# Supplementary figures and images for: Metabolomics of primary cutaneous melanoma and matched adjacent extratumoral microenvironment
Source: PLoS One. 2020 Oct 27;15(10):e0240849. doi: 10.1371/journal.pone.0240849 (PMC7591037; doi:10.1371/journal.pone.0240849)

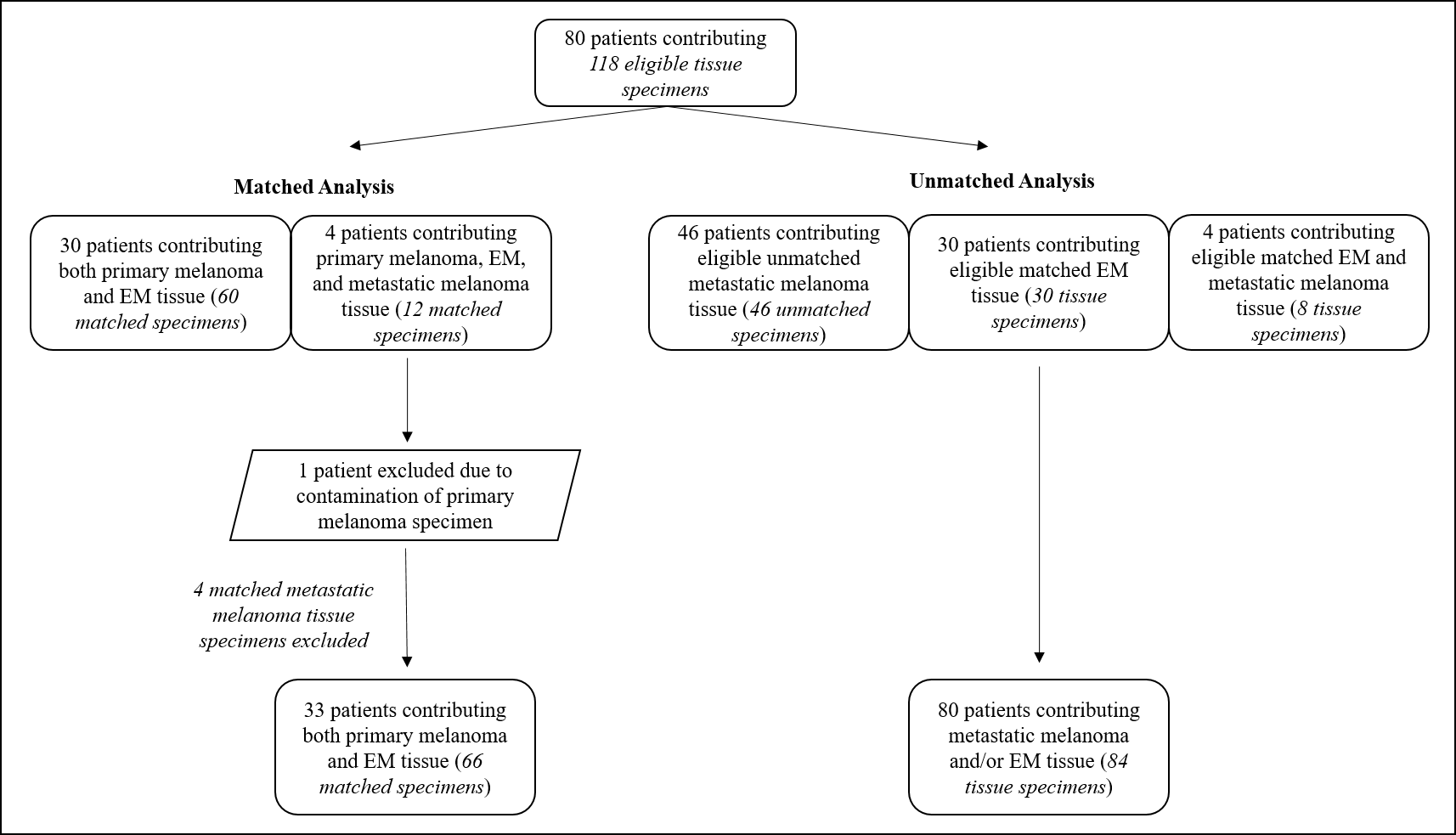

Supplement: S1 Fig — Flow chart illustrating the number of eligible study participants contributing eligible tissue to the study according to matched and unmatched analysis. (TIF) [file pone.0240849.s001.tif]

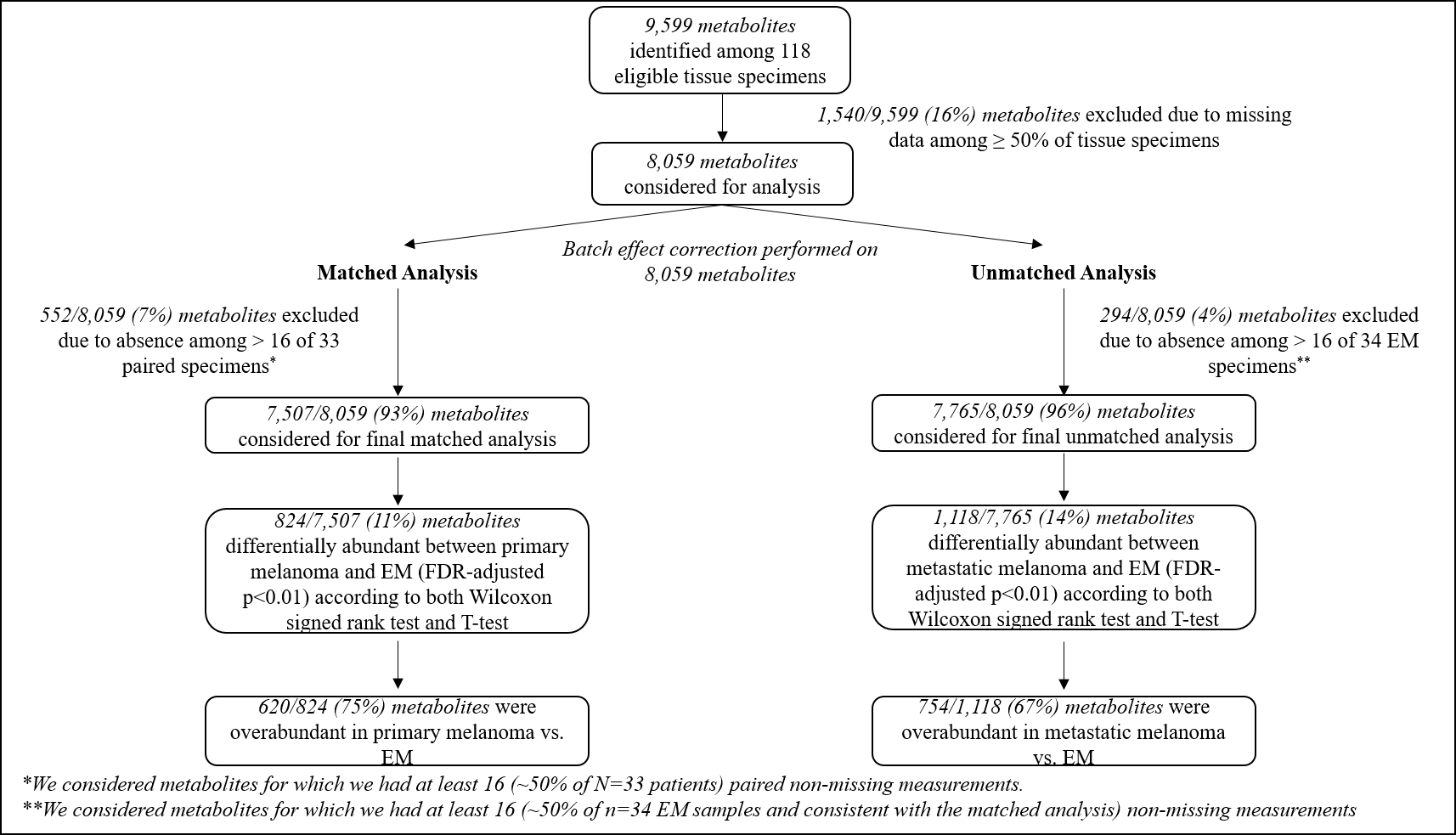

Supplement: S2 Fig — Flow chart illustrating the number of metabolites (MS peaks) identified among eligible tissue specimens and the final number of metabolites (MS peaks) considered for matched and unmatched analyses after batch correction and quality control measures. (TIF) [file pone.0240849.s002.tif]

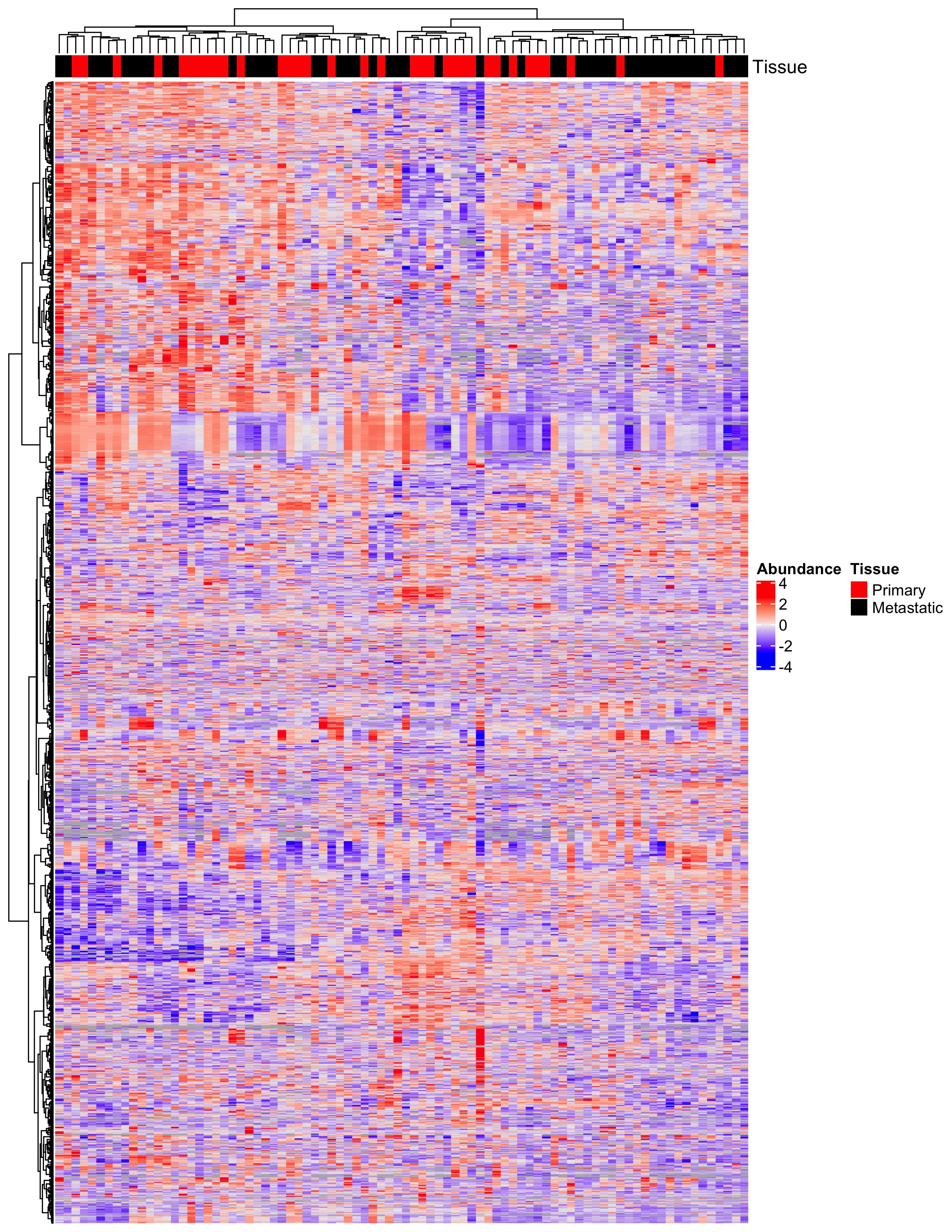

Supplement: S7 Fig — Heatmap of all metabolites with tissue type (primary melanoma: Red or metastatic melanoma: Black) noted in the top color bar. (TIF) [file pone.0240849.s007.tif]

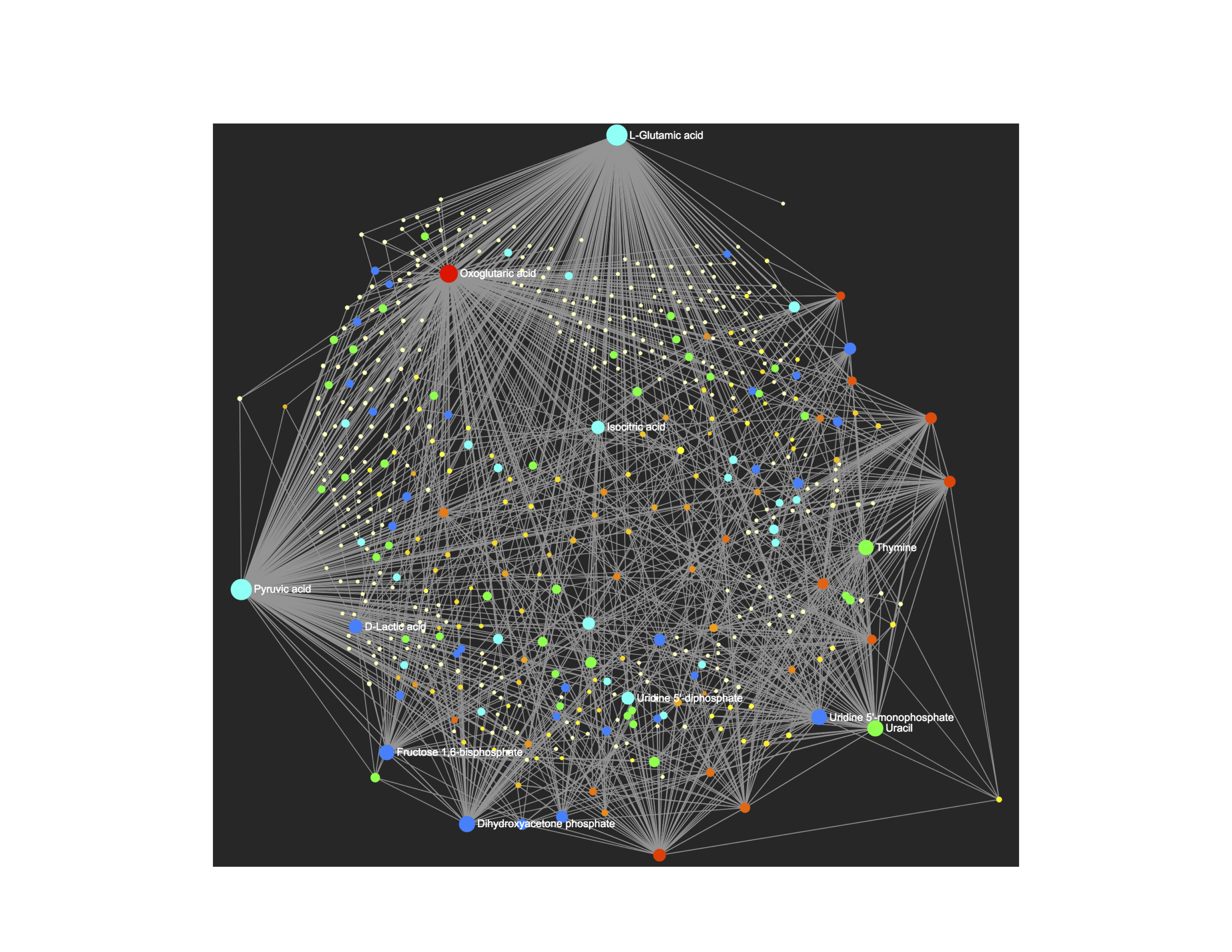

Supplement: S8 Fig — Network of metabolite-metabolite interactions is shown among metabolites that were significantly overabundant in primary melanoma vs. EM. (TIFF) [file pone.0240849.s008.tiff]

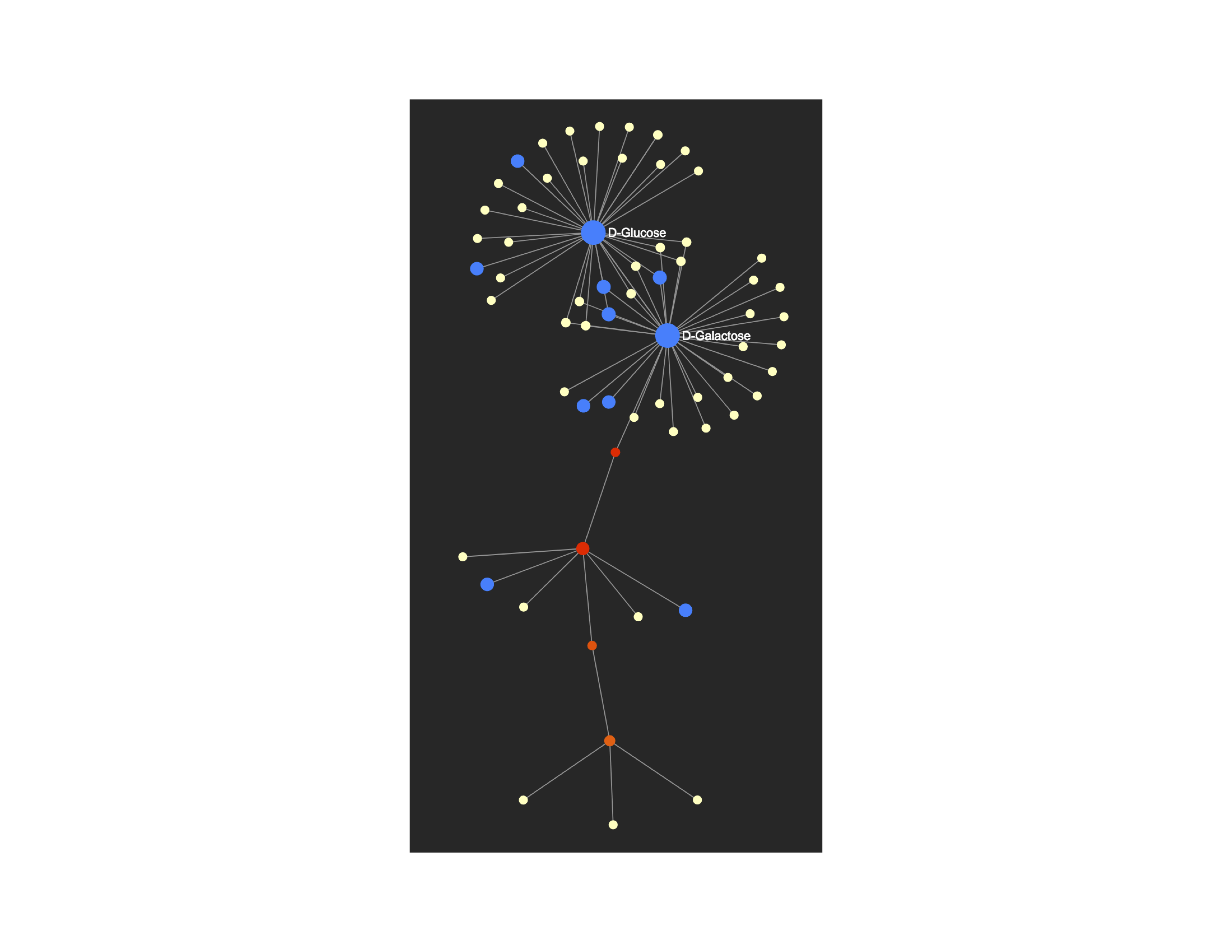

Supplement: S9 Fig — Network of metabolite-metabolite interactions is shown among metabolites that were significantly less abundant in primary melanoma vs. EM. (TIFF) [file pone.0240849.s009.tiff]

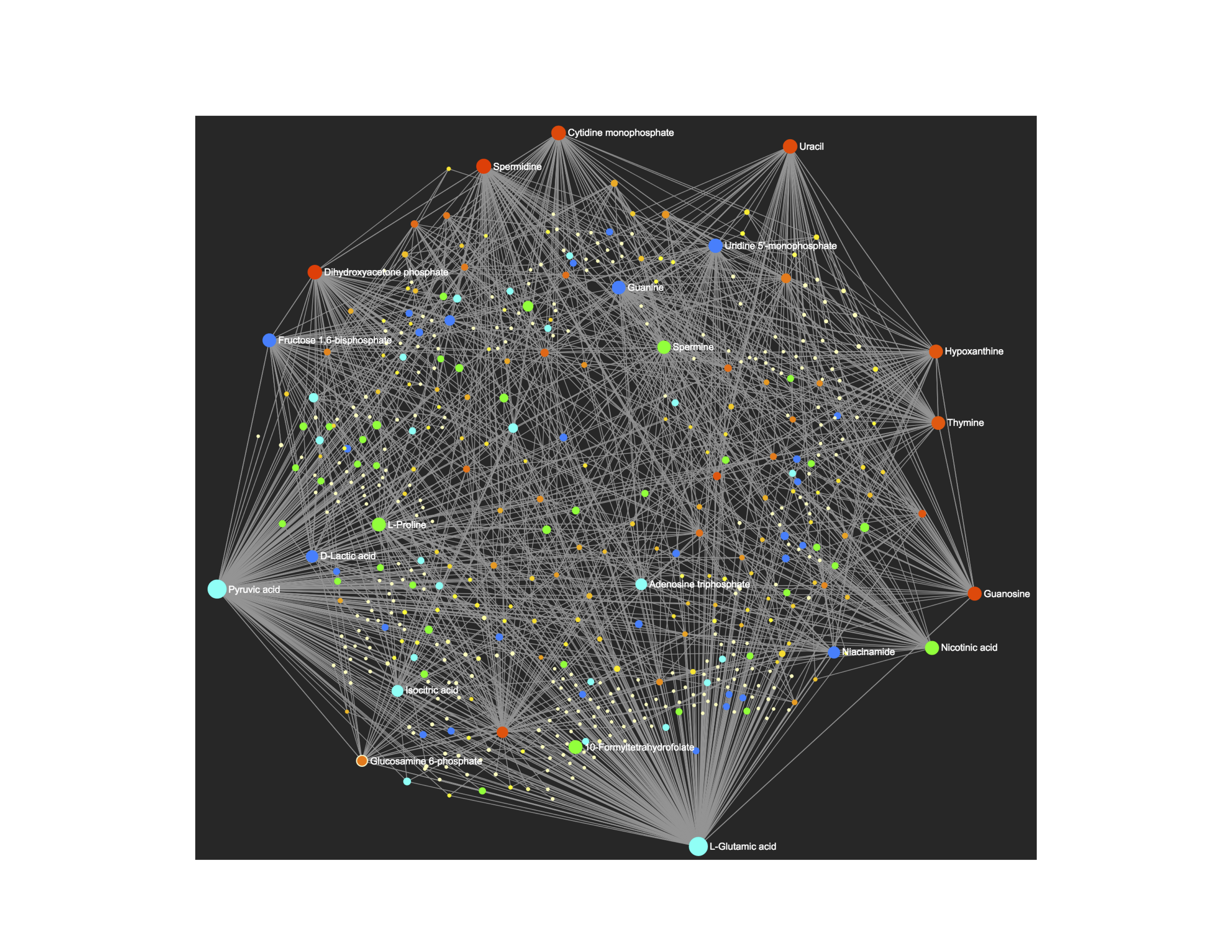

Supplement: S10 Fig — Network of metabolite-metabolite interactions is shown among metabolites that were significantly overabundant in metastatic melanoma vs. EM. (TIFF) [file pone.0240849.s010.tiff]

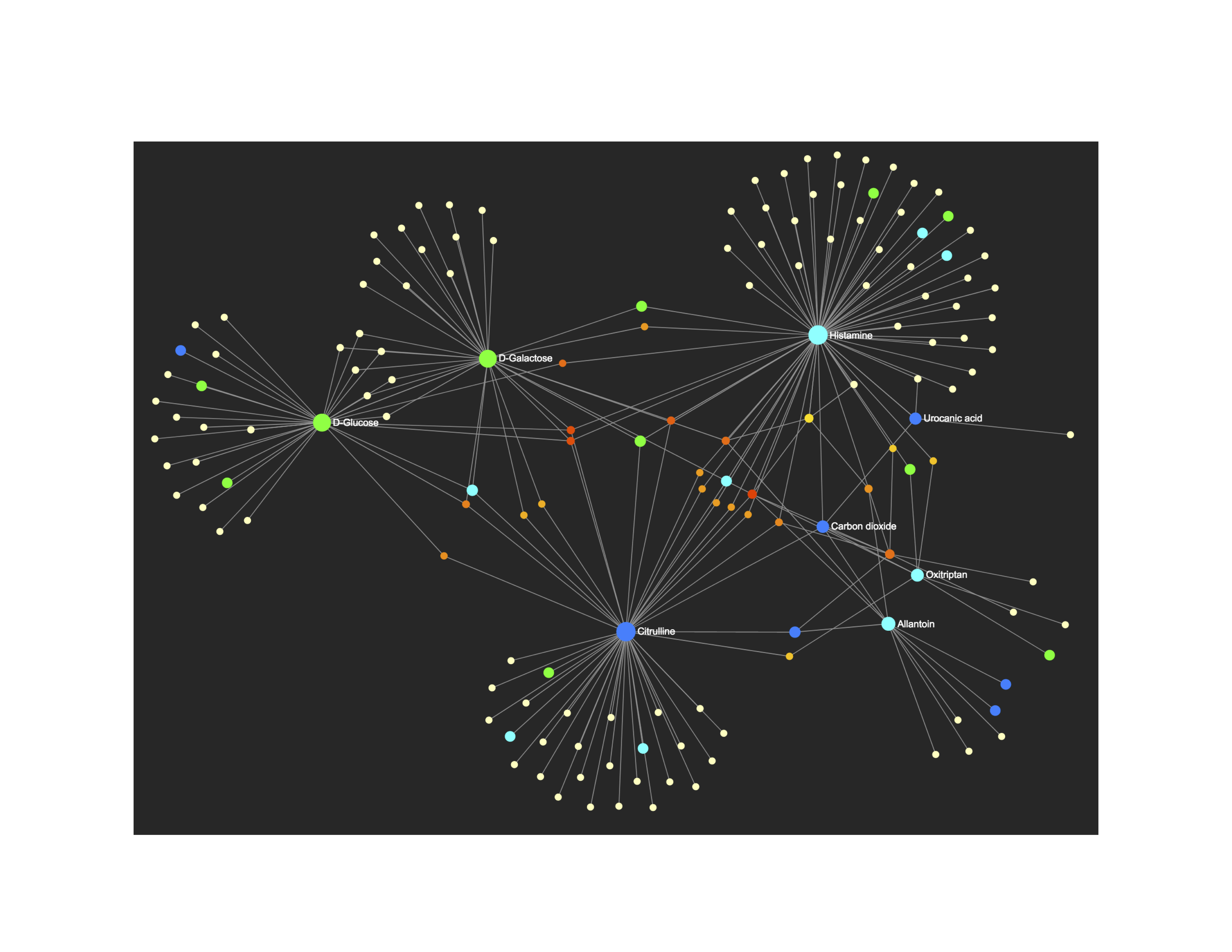

Supplement: S11 Fig — Network of metabolite-metabolite interactions is shown among metabolites that were significantly less abundant in metastatic melanoma vs. EM. (TIFF) [file pone.0240849.s011.tiff]
