# Supplementary material for: Metabolomics of primary cutaneous melanoma and matched adjacent extratumoral microenvironment
Source: PLoS One. 2020 Oct 27;15(10):e0240849. doi: 10.1371/journal.pone.0240849 (PMC7591037; doi:10.1371/journal.pone.0240849)
Supplement: S3 Fig — Bar charts reporting noteworthy differentially abundant metabolites between primary melanoma and matched EM. (PDF) [file pone.0240849.s003.pdf]

S3 Fig

Selected differentially abundant metabolites

primary vs. EM

Supplement to

"Metabolomics of primary cutaneous melanoma

and matched adjacent extratumoral microenvironment"

Nicholas J. Taylor, Irina Gaynanova, Eric A. Welsh, Timothy J. Garrett, Chris Beecher,
Ritin Sharma, John Koomen, Keiran S.M. Smalley, Steven A. Eschrich, Jane L. Messina, Peter A. Kanetsky

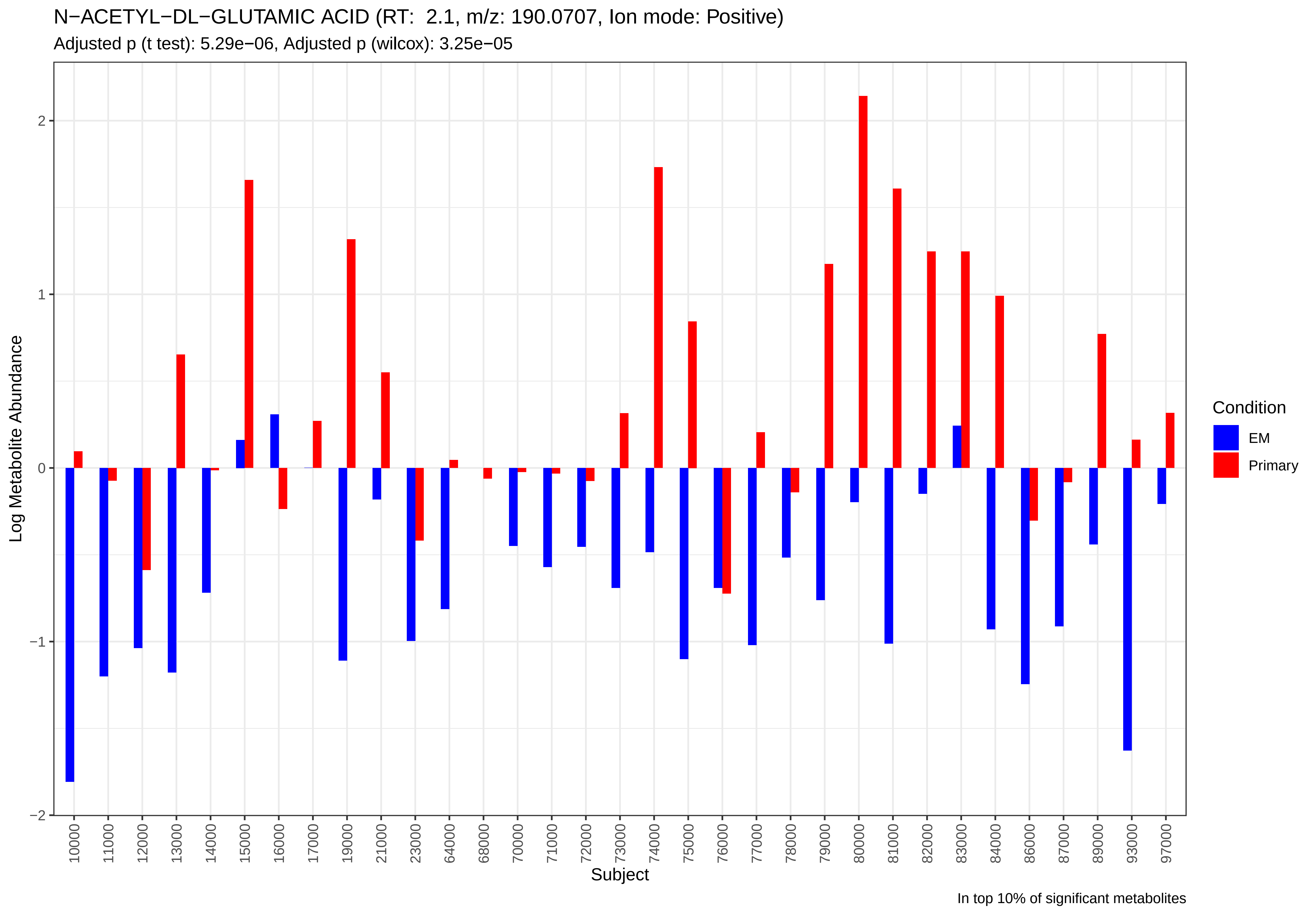
N−ACETYL−DL−GLUTAMIC ACID (RT: 2.1, m/z: 190.0707, Ion mode: Positive)
Adjusted p (t test): 5.29e−06, Adjusted p (wilcox): 3.25e−05
Log Metabolite Abundance
2
1
0
−1
−2
10000
11000
12000
13000
14000
15000
16000
17000
19000
21000
23000
64000
68000
70000
71000
72000
73000
74000
75000
76000
77000
78000
79000
80000
81000
82000
83000
84000
86000
87000
89000
93000
97000
Subject
Condition
EM
Primary
In top 10% of significant metabolites

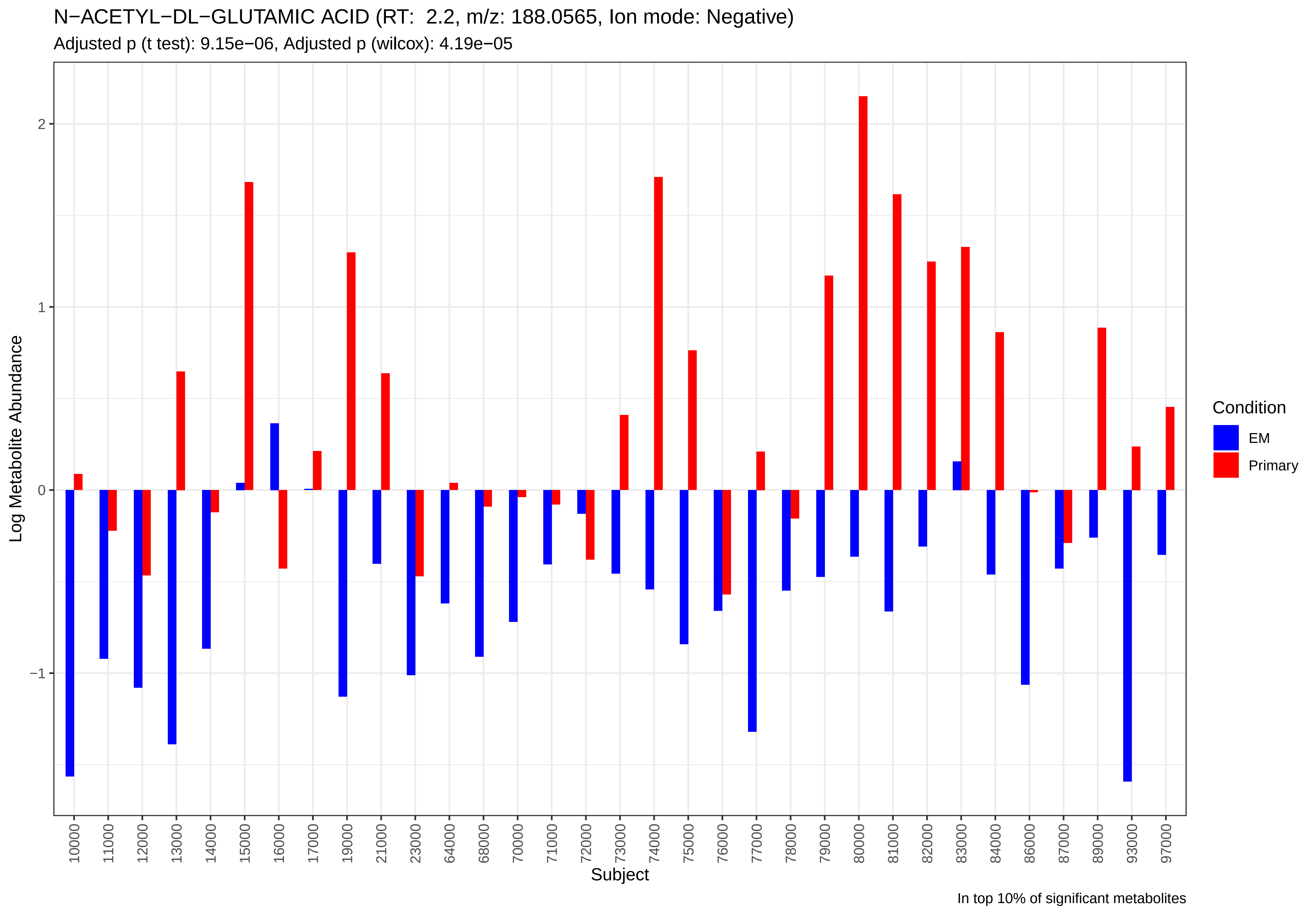
N−ACETYL−DL−GLUTAMIC ACID (RT: 2.2, m/z: 188.0565, Ion mode: Negative)
Adjusted p (t test): 9.15e−06, Adjusted p (wilcox): 4.19e−05
Log Metabolite Abundance
2
1
0
−1
10000
11000
12000
13000
14000
15000
16000
17000
19000
21000
23000
64000
68000
70000
71000
72000
73000
74000
75000
76000
77000
78000
79000
80000
81000
82000
83000
84000
86000
87000
89000
93000
97000
Subject
Condition
EM
Primary
In top 10% of significant metabolites

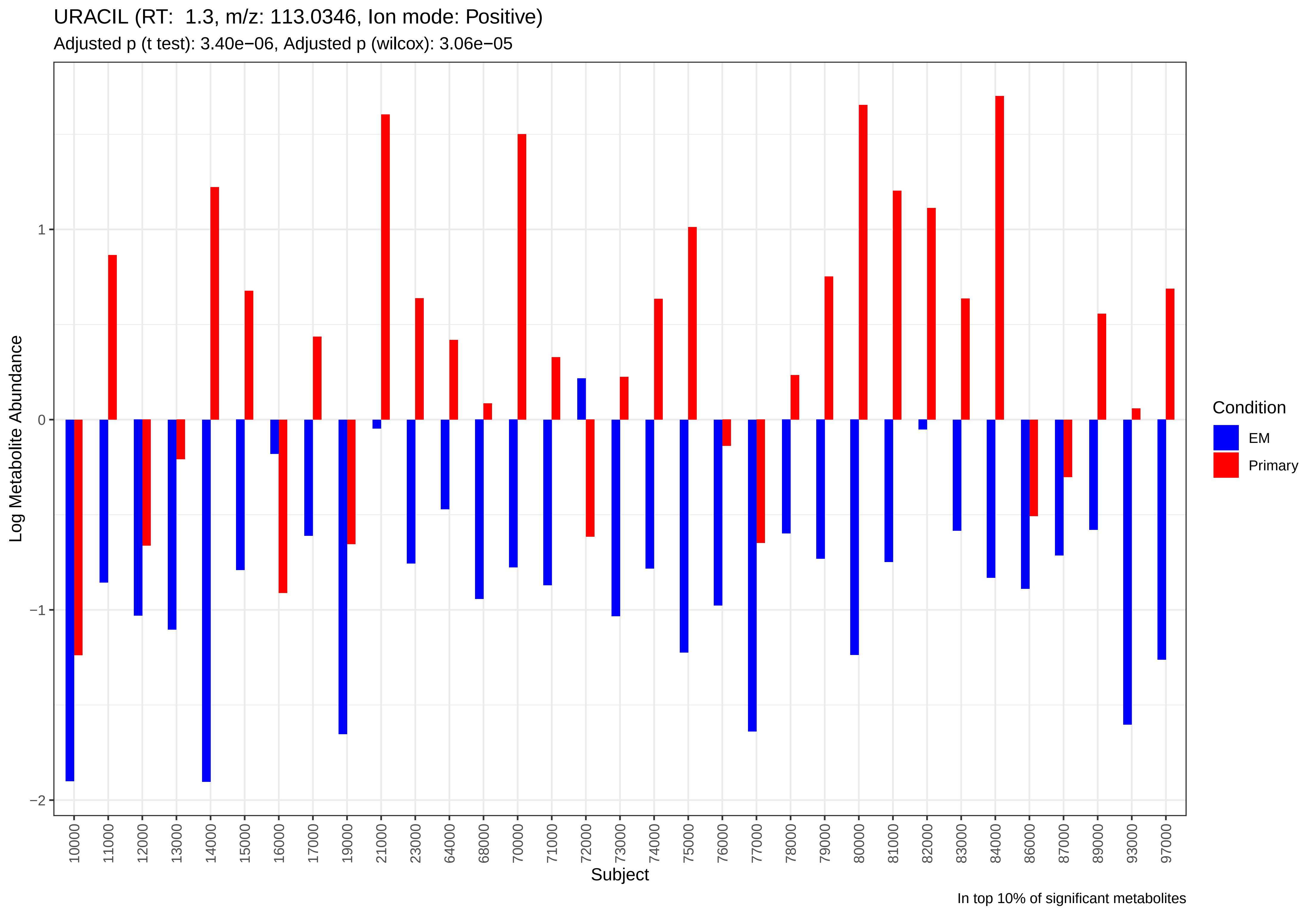
URACIL (RT: 1.3, m/z: 113.0346, Ion mode: Positive)
Adjusted p (t test): 3.40e−06, Adjusted p (wilcox): 3.06e−05
Log Metabolite Abundance
1
0
−1
−2
10000
11000
12000
13000
14000
15000
16000
17000
19000
21000
23000
64000
68000
70000
71000
72000
73000
74000
75000
76000
77000
78000
79000
80000
81000
82000
83000
84000
86000
87000
89000
93000
97000
Subject
Condition
EM
Primary
In top 10% of significant metabolites

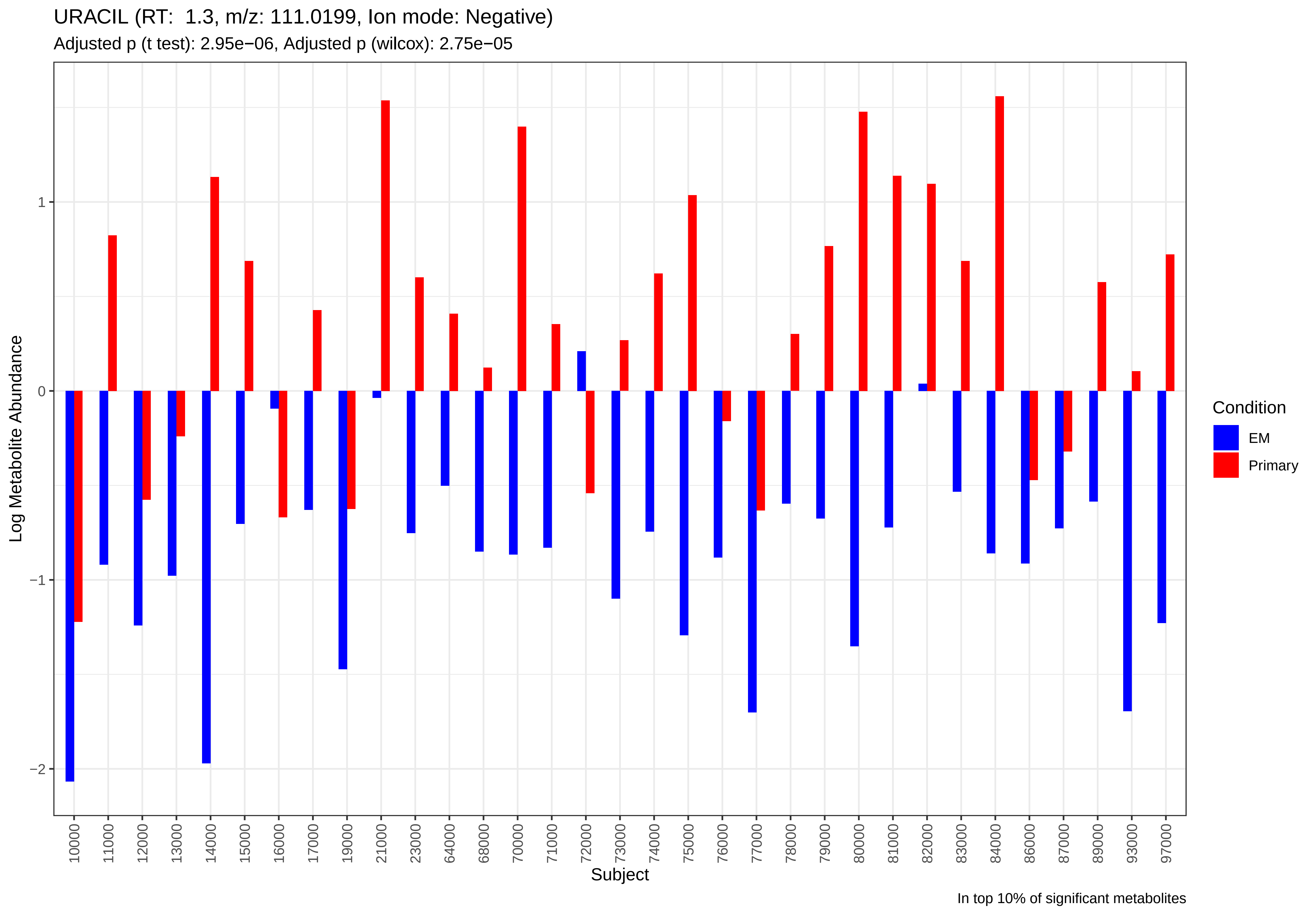

URACIL (RT: 1.3, m/z: 111.0199, Ion mode: Negative)
Adjusted p (t test): 2.95e−06, Adjusted p (wilcox): 2.75e−05
Log Metabolite Abundance
1
0
−1
−2
10000
11000
12000
13000
14000
15000
16000
17000
19000
21000
23000
64000
68000
70000
71000
72000
73000
74000
75000
76000
77000
78000
79000
80000
81000
82000
83000
84000
86000
87000
89000
93000
97000
Subject
Condition
EM
Primary
In top 10% of significant metabolites

DIHYDROXYACETONE PHOSPHATE (RT: 0.7, m/z: 171.0051, Ion mode: Positive)

Adjusted p (t test): 1.78e−05, Adjusted p (wilcox): 7.74e−05

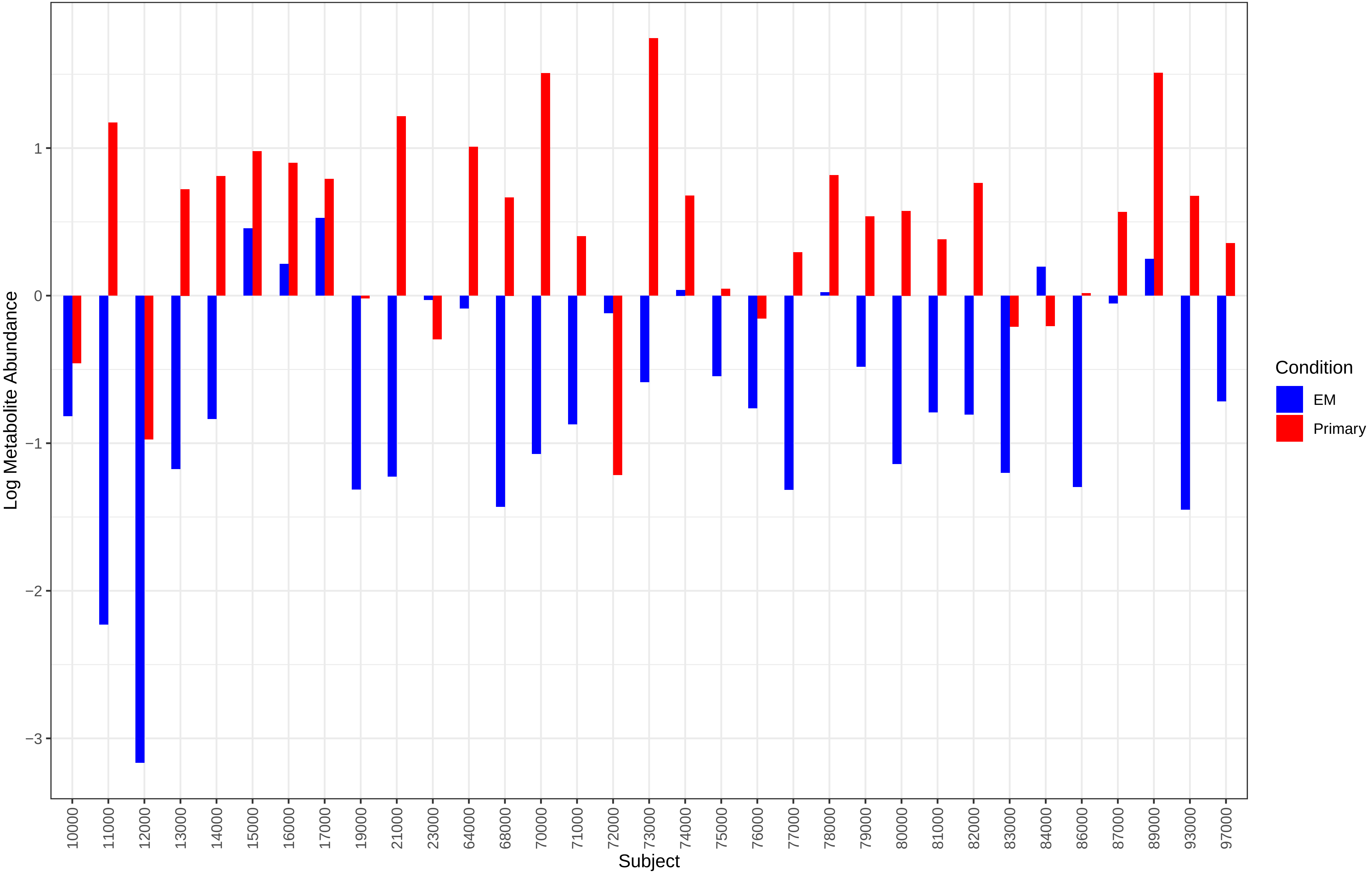

In top 10% of significant metabolites

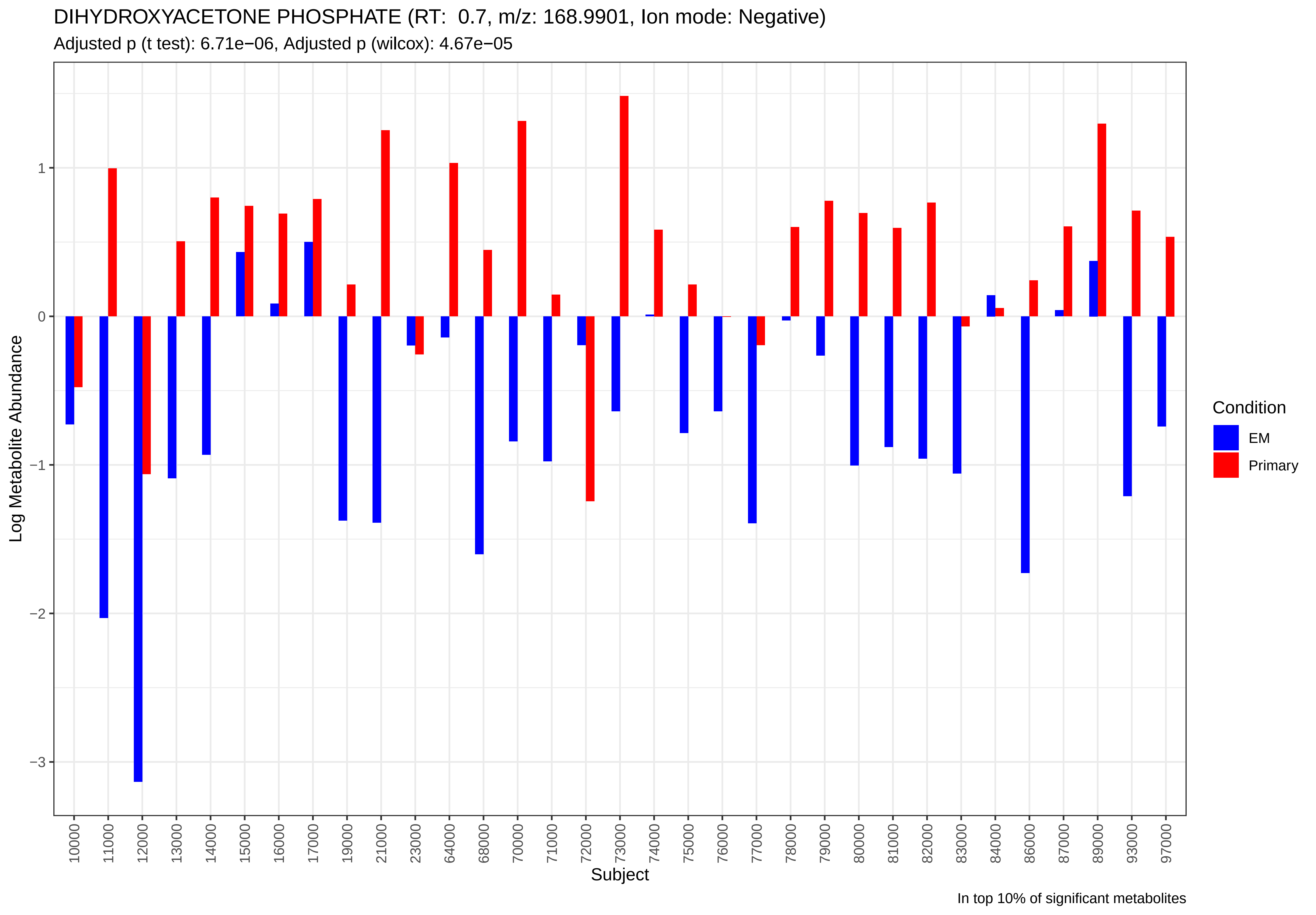

DIHYDROXYACETONE PHOSPHATE (RT: 0.7, m/z: 168.9901, Ion mode: Negative)
Adjusted p (t test): 6.71e−06, Adjusted p (wilcox): 4.67e−05
Log Metabolite Abundance
1
0
−1
−2
−3
10000
11000
12000
13000
14000
15000
16000
17000
19000
21000
23000
64000
68000
70000
71000
72000
73000
74000
75000
76000
77000
78000
79000
80000
81000
82000
83000
84000
86000
87000
89000
93000
97000
Subject
Condition
EM
Primary
In top 10% of significant metabolites

N−METHYL−D−ASPARTIC ACID (RT: 0.7, m/z: 148.0603, Ion mode: Positive)

Adjusted p (t test): 6.63e−06, Adjusted p (wilcox): 3.45e−05

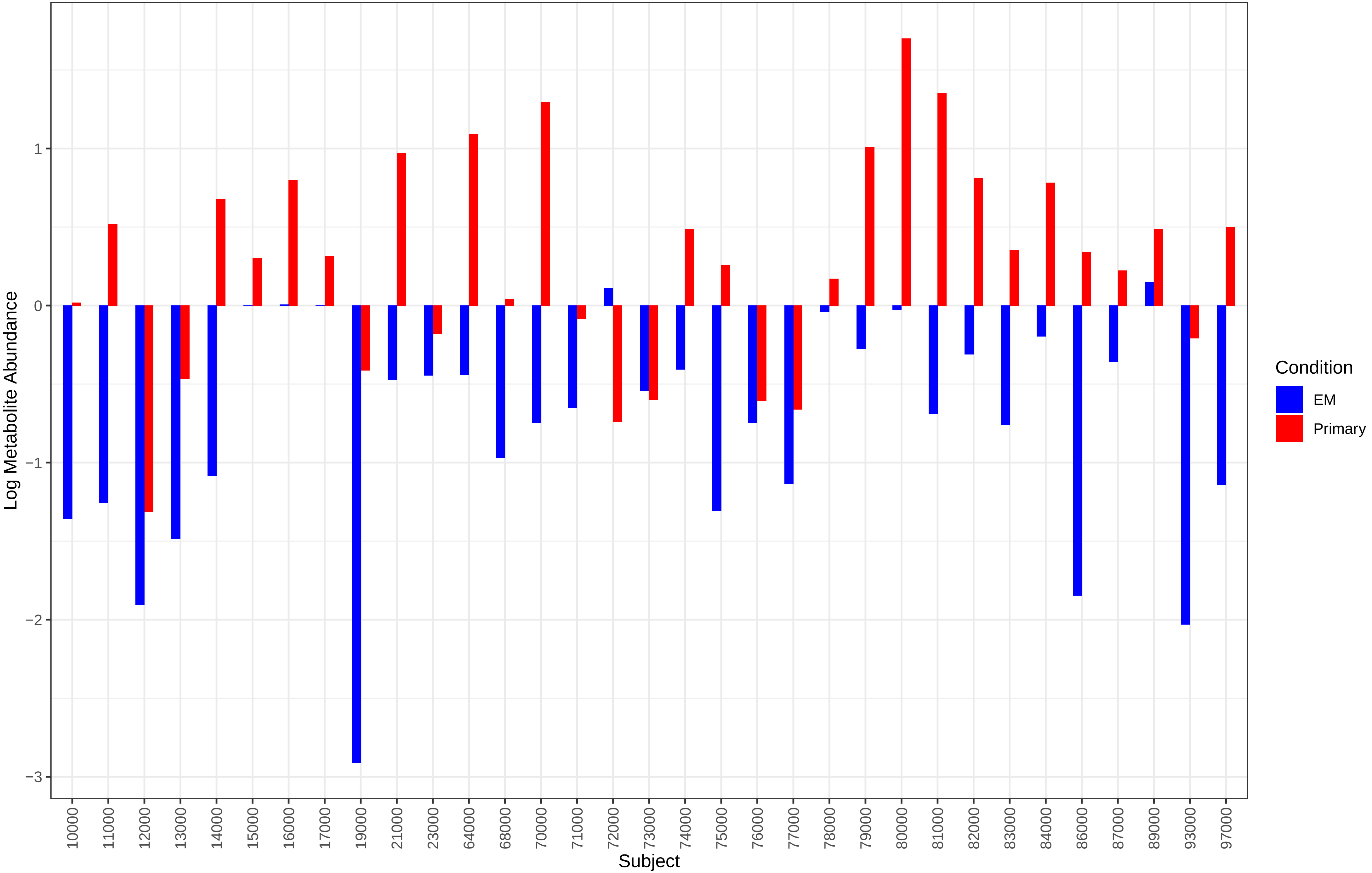

In top 10% of significant metabolites

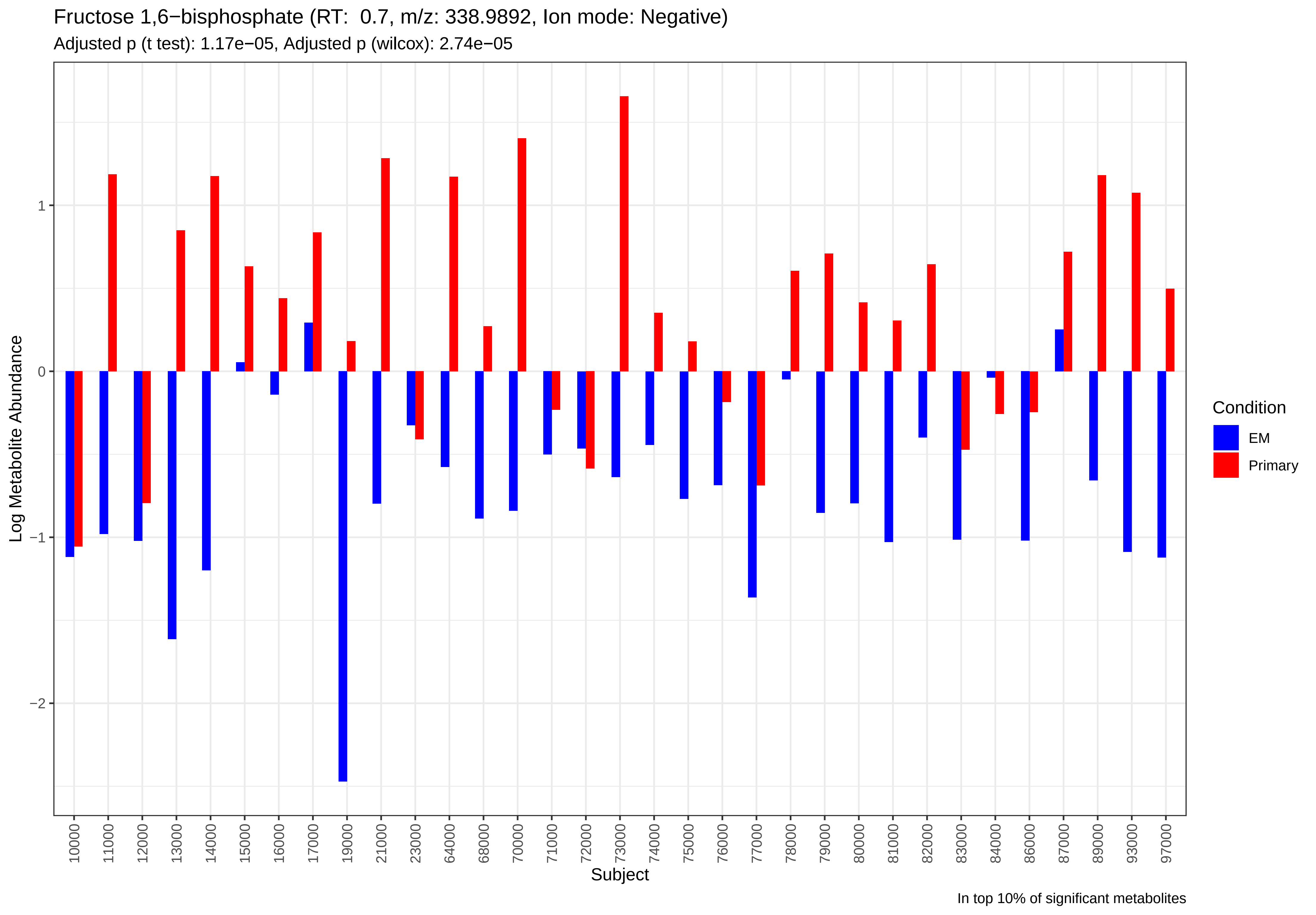
Fructose 1,6−bisphosphate (RT: 0.7, m/z: 338.9892, Ion mode: Negative)
Adjusted p (t test): 1.17e−05, Adjusted p (wilcox): 2.74e−05
Log Metabolite Abundance
1
0
−1
−2
Subject
10000
11000
12000
13000
14000
15000
16000
17000
19000
21000
23000
64000
68000
70000
71000
72000
73000
74000
75000
76000
77000
78000
79000
80000
81000
82000
83000
84000
86000
87000
89000
93000
97000
Condition
EM
Primary
In top 10% of significant metabolites
