# Supplementary material for: Metabolomics of primary cutaneous melanoma and matched adjacent extratumoral microenvironment
Source: PLoS One. 2020 Oct 27;15(10):e0240849. doi: 10.1371/journal.pone.0240849 (PMC7591037; doi:10.1371/journal.pone.0240849)
Supplement: S5 Fig — Bar charts reporting noteworthy differentially abundant metabolites between metastatic melanoma and EM. (PDF) [file pone.0240849.s005.pdf]

S5 Fig
Selected differentially abundant metabolites
EM vs. Metastasis

Supplement to

"Metabolomics of primary cutaneous melanoma and matched adjacent extratumoral microenvironment"

Nicholas J. Taylor, Irina Gaynanova, Eric A. Welsh, Timothy J. Garrett, Chris Beecher,
Ritin Sharma, John Koomen, Keiran S.M. Smalley, Steven A. Eschrich, Jane L. Messina, Peter A. Kanetsky

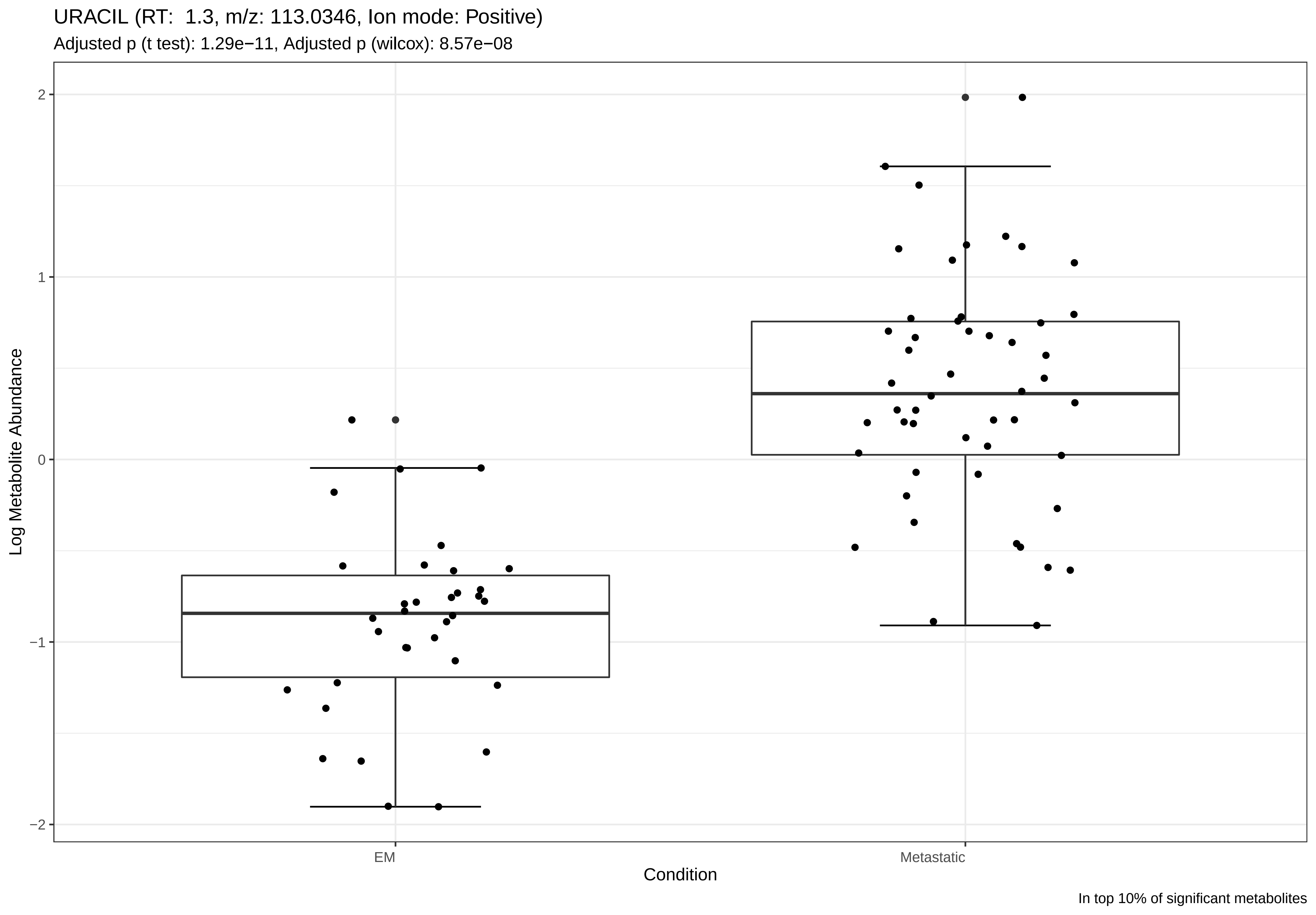
URACIL (RT: 1.3, m/z: 113.0346, Ion mode: Positive)
Adjusted p (t test): 1.29e−11, Adjusted p (wilcox): 8.57e−08
Log Metabolite Abundance
2
1
0
−1
−2
EM
Metastatic
Condition
In top 10% of significant metabolites

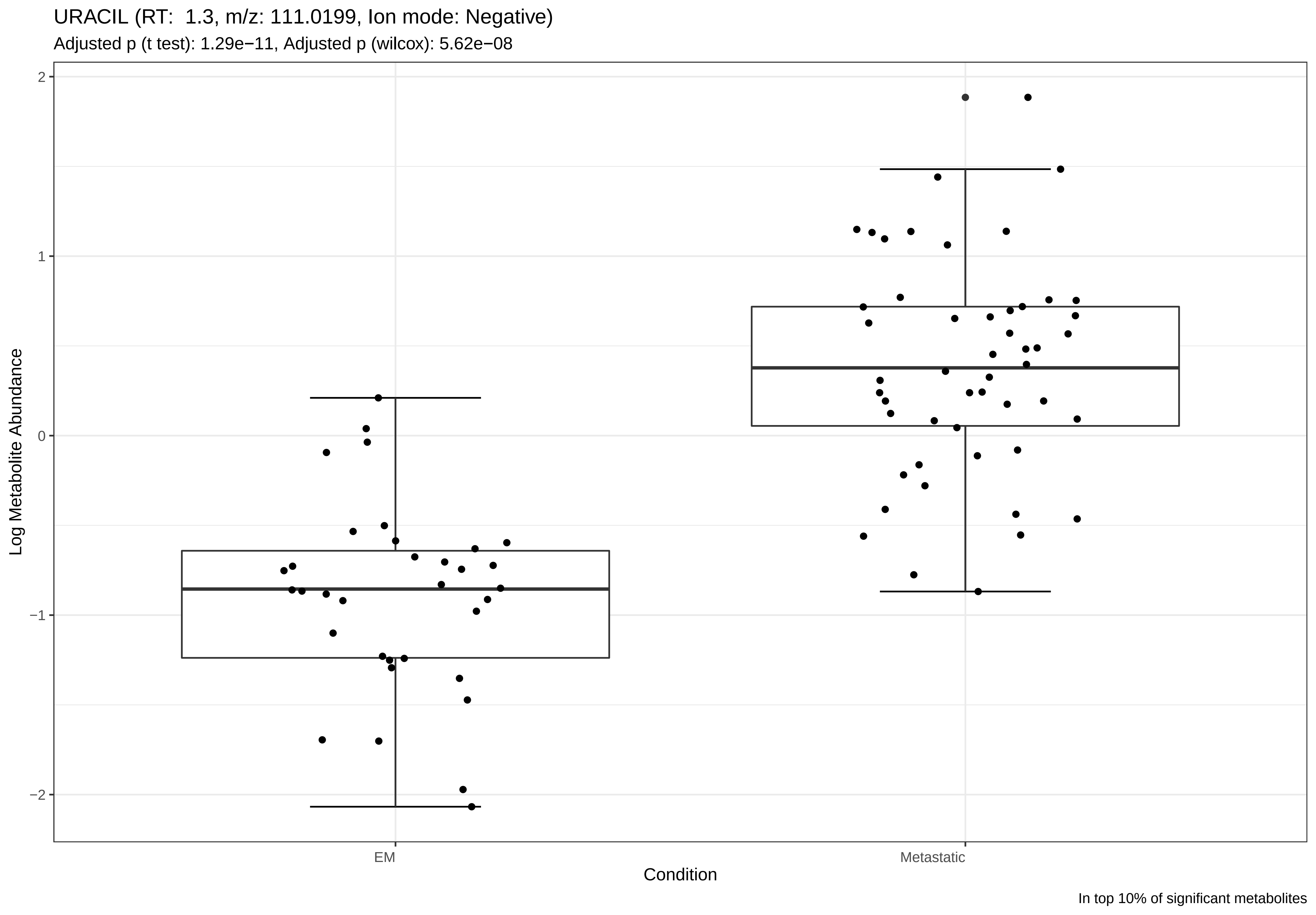
URACIL (RT: 1.3, m/z: 111.0199, Ion mode: Negative)
Adjusted p (t test): 1.29e−11, Adjusted p (wilcox): 5.62e−08
Log Metabolite Abundance
2
1
0
−1
−2
EM
Metastatic
Condition
In top 10% of significant metabolites

CYTIDINE 5'−DIPHOSPHOCHOLINE (RT: 0.9, m/z: 489.1135, Ion mode: Positive)

Adjusted p (t test): 1.98e−08, Adjusted p (wilcox): 1.10e−07

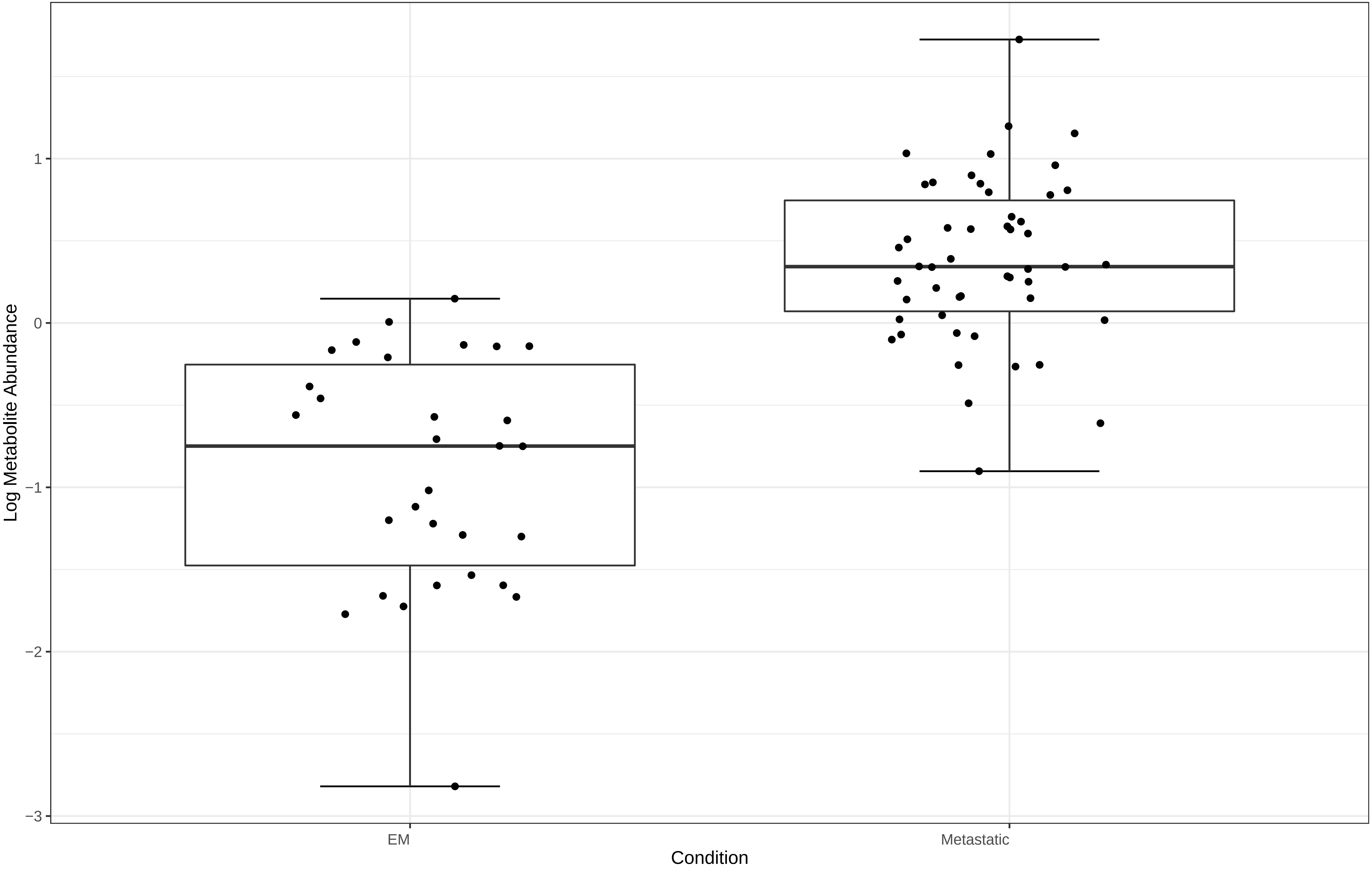

In top 10% of significant metabolites

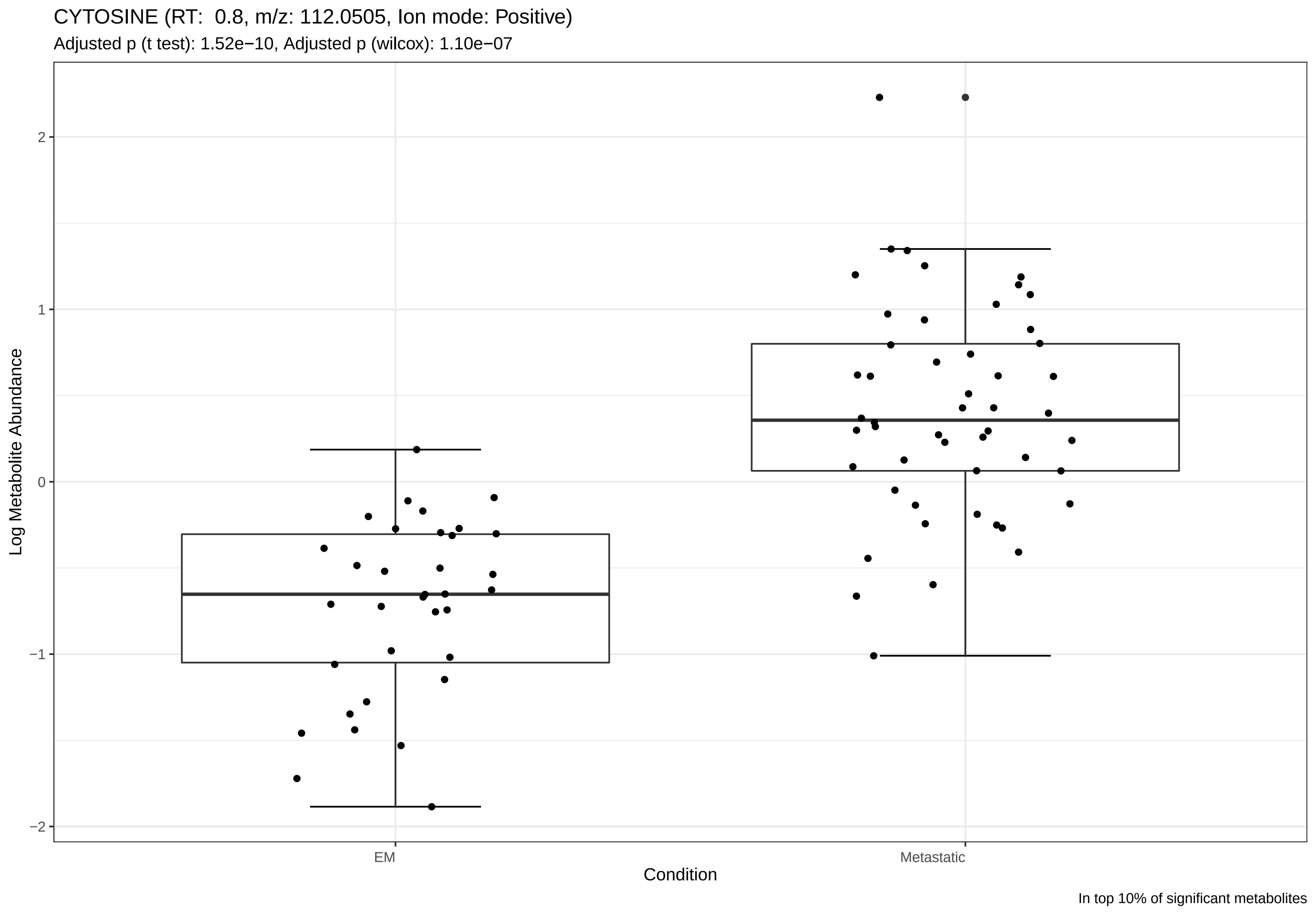
CYTOSINE (RT: 0.8, m/z: 112.0505, Ion mode: Positive)
Adjusted p (t test): 1.52e−10, Adjusted p (wilcox): 1.10e−07
Log Metabolite Abundance
2
1
0
−1
−2
EM
Metastatic
Condition
In top 10% of significant metabolites

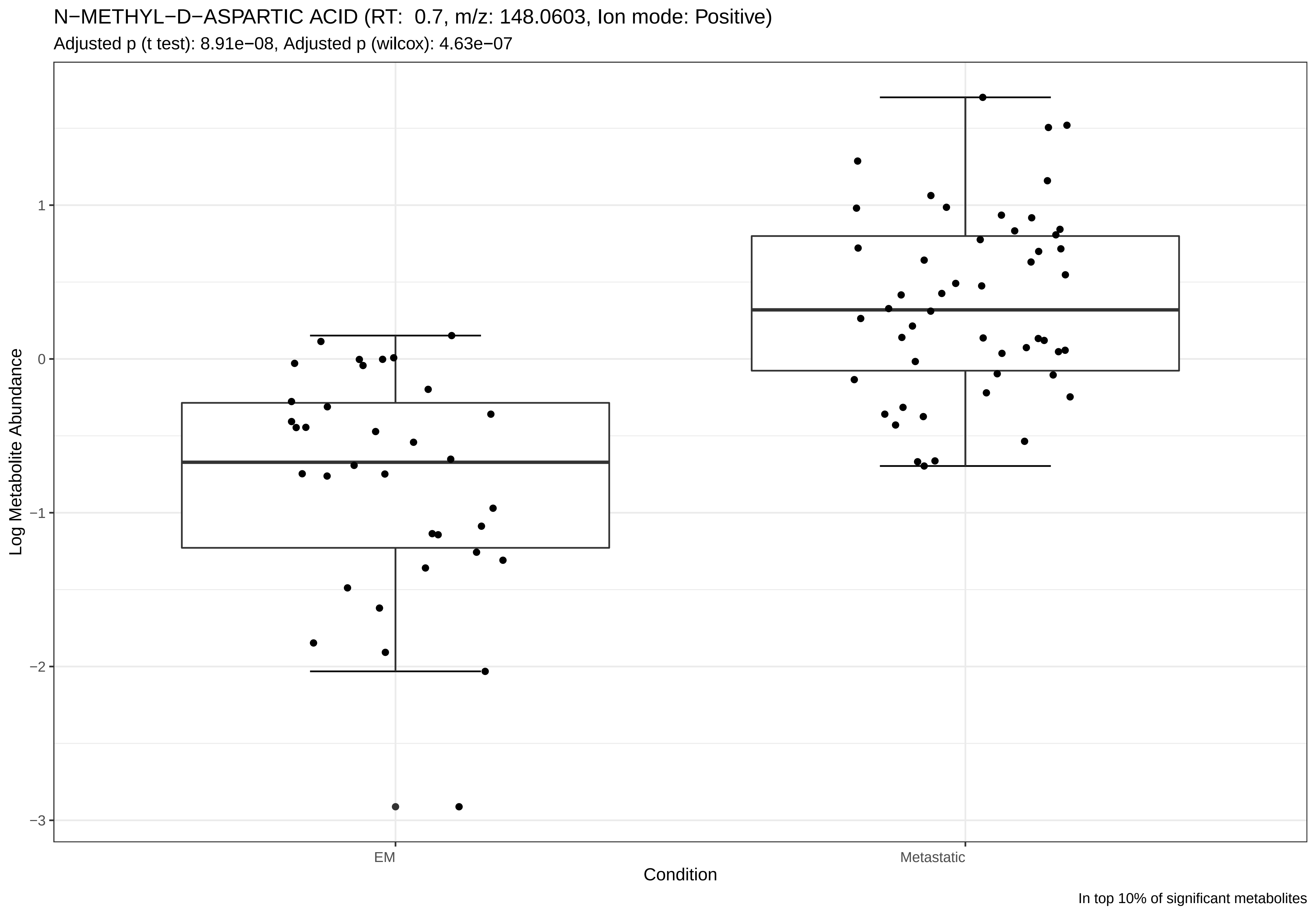
N−METHYL−D−ASPARTIC ACID (RT: 0.7, m/z: 148.0603, Ion mode: Positive)
Adjusted p (t test): 8.91e−08, Adjusted p (wilcox): 4.63e−07
Log Metabolite Abundance
1
0
−1
−2
−3
EM
Metastatic
Condition
In top 10% of significant metabolites
