# Supplementary material for: Metabolomics of primary cutaneous melanoma and matched adjacent extratumoral microenvironment
Source: PLoS One. 2020 Oct 27;15(10):e0240849. doi: 10.1371/journal.pone.0240849 (PMC7591037; doi:10.1371/journal.pone.0240849)

S4 Fig

All differentially abundant metabolites

primary vs. EM

Supplement to

"Metabolomics of primary cutaneous melanoma

and matched adjacent extratumoral microenvironment"

Nicholas J. Taylor, Irina Gaynanova, Eric A. Welsh, Timothy J. Garrett, Chris Beecher,

Ritin Sharma, John Koomen, Keiran S.M. Smalley, Steven A. Eschrich, Jane L. Messina, Peter A. Kanetsky

URACIL (RT: 1.3, m/z: 111.0199, Ion mode: Negative)

Adjusted p (t test): 2.95e−06, Adjusted p (wilcox): 2.75e−05

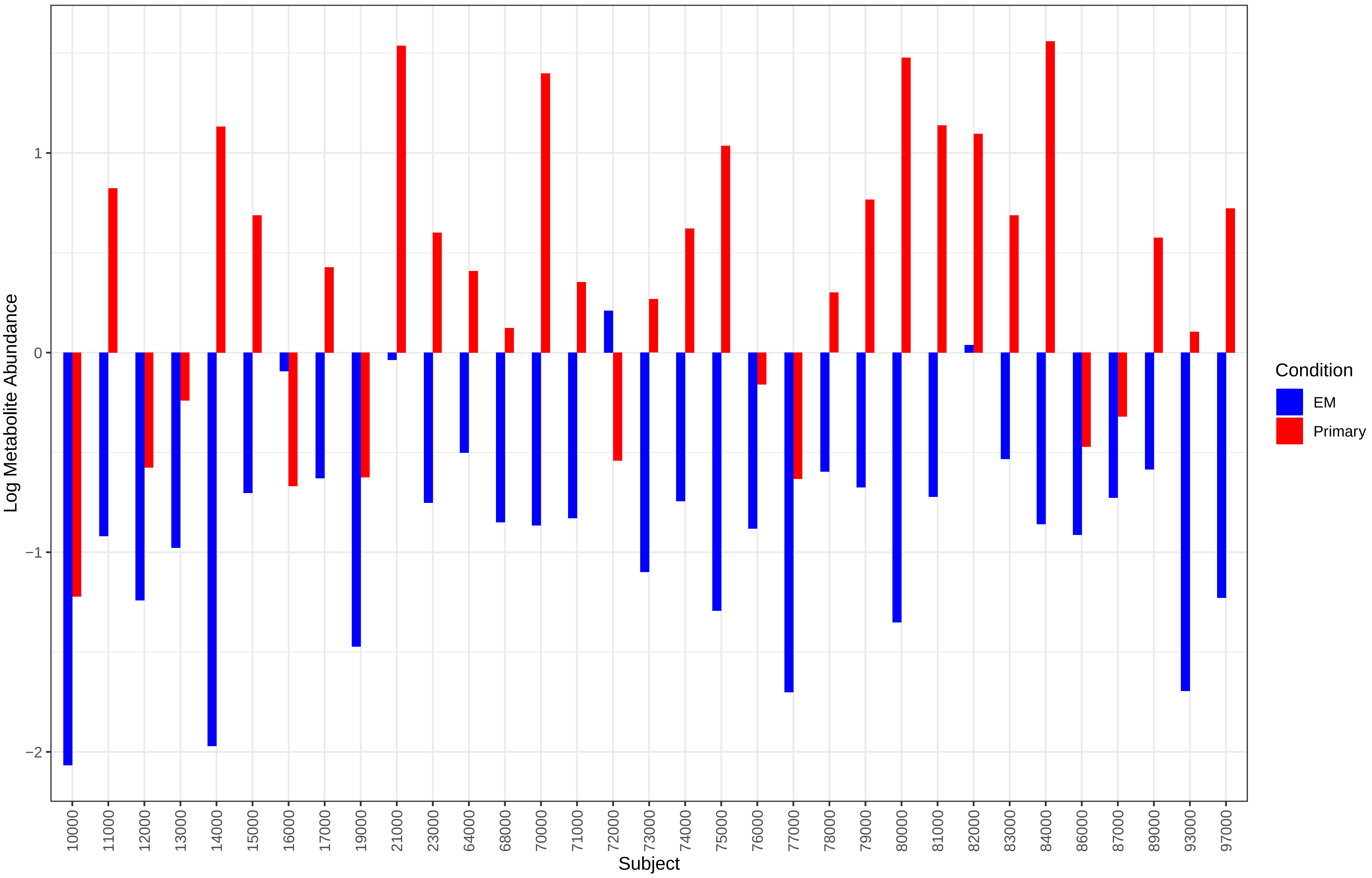

In top 10% of significant metabolites

RT: 0.7, m/z: 89.0239, Ion mode: Negative

Adjusted p (t test): 2.95e−06, Adjusted p (wilcox): 2.75e−05

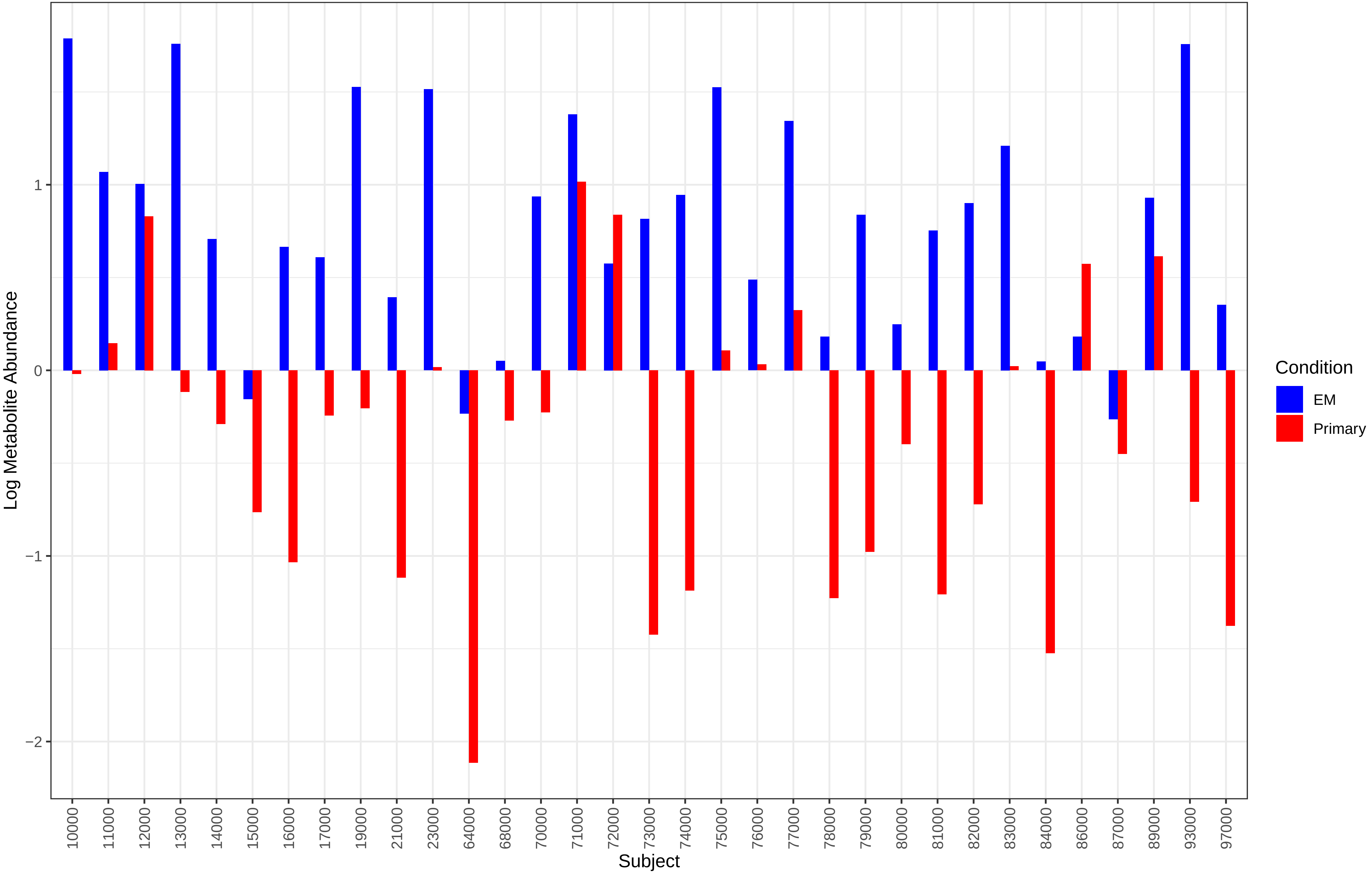

In top 10% of significant metabolites

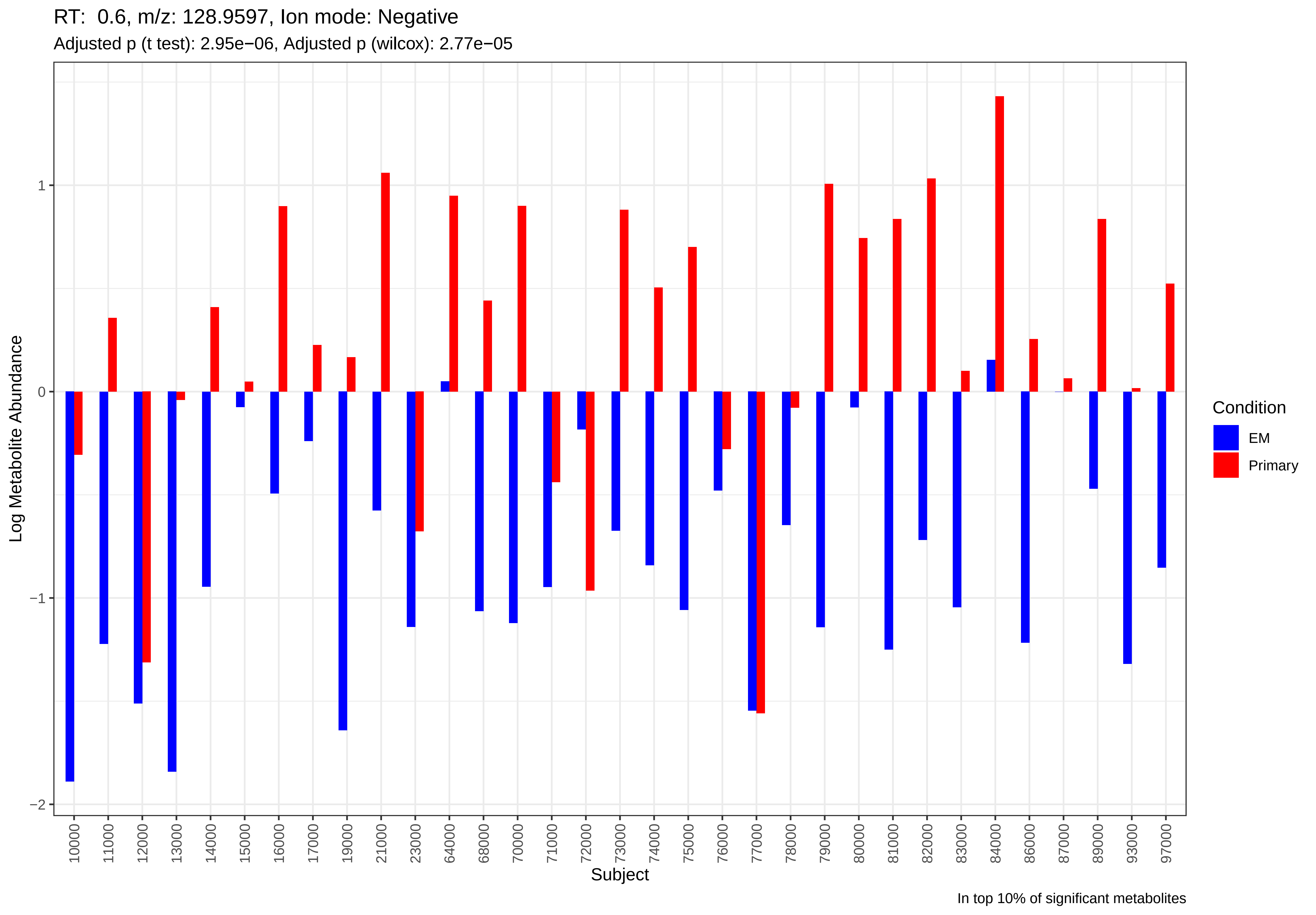

RT: 0.6, m/z: 128.9597, Ion mode: Negative
Adjusted p (t test): 2.95e−06, Adjusted p (wilcox): 2.77e−05
Log Metabolite Abundance
1
0
−1
−2
10000
11000
12000
13000
14000
15000
16000
17000
19000
21000
23000
64000
68000
70000
71000
72000
73000
74000
75000
76000
77000
78000
79000
80000
81000
82000
83000
84000
86000
87000
89000
93000
97000
Subject
Condition
EM
Primary
In top 10% of significant metabolites

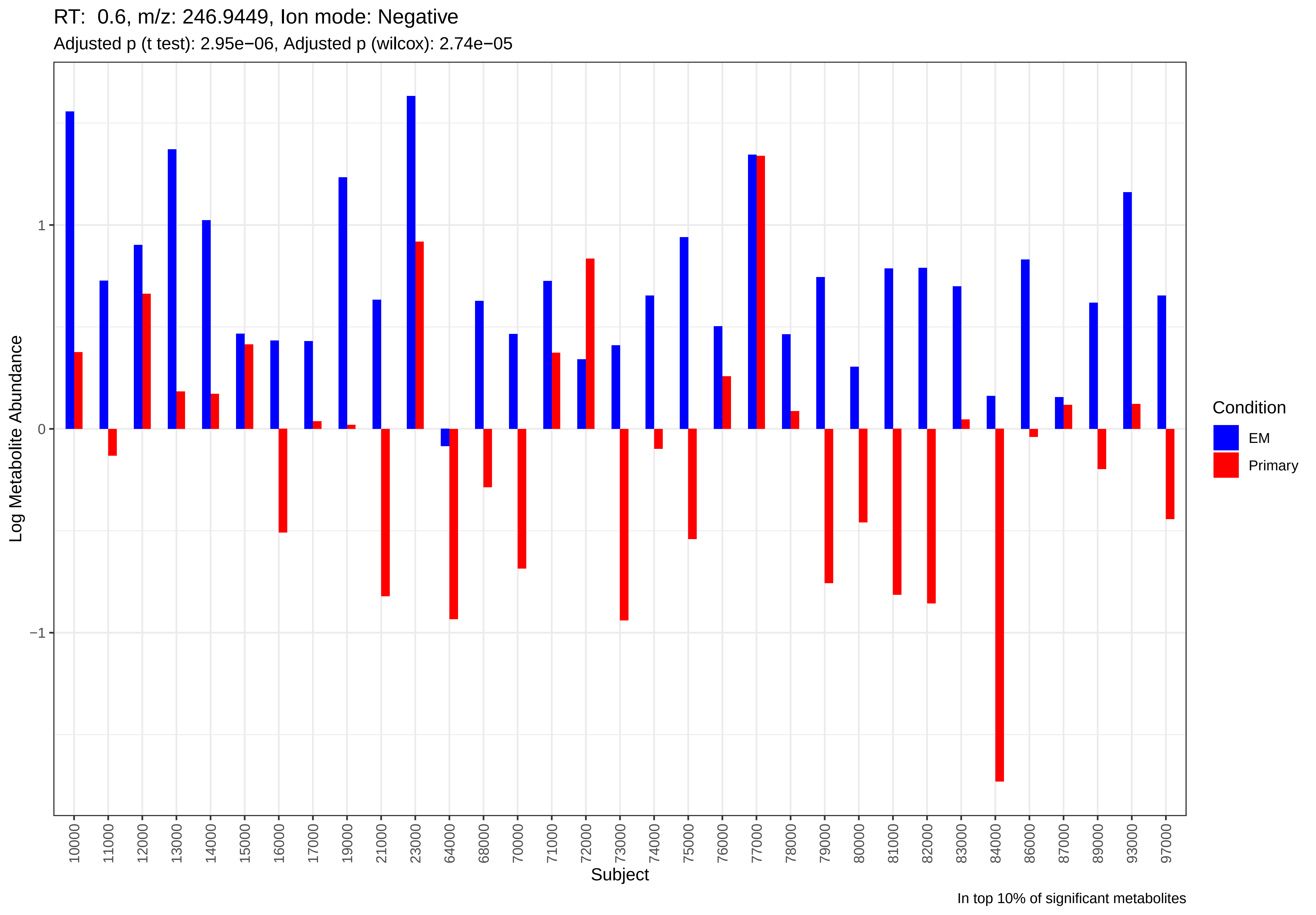

RT: 0.6, m/z: 246.9449, Ion mode: Negative
Adjusted p (t test): 2.95e−06, Adjusted p (wilcox): 2.74e−05
Log Metabolite Abundance
1
0
−1
10000
11000
12000
13000
14000
15000
16000
17000
19000
21000
23000
64000
68000
70000
71000
72000
73000
74000
75000
76000
77000
78000
79000
80000
81000
82000
83000
84000
86000
87000
89000
93000
97000
Subject
Condition
EM
Primary
In top 10% of significant metabolites

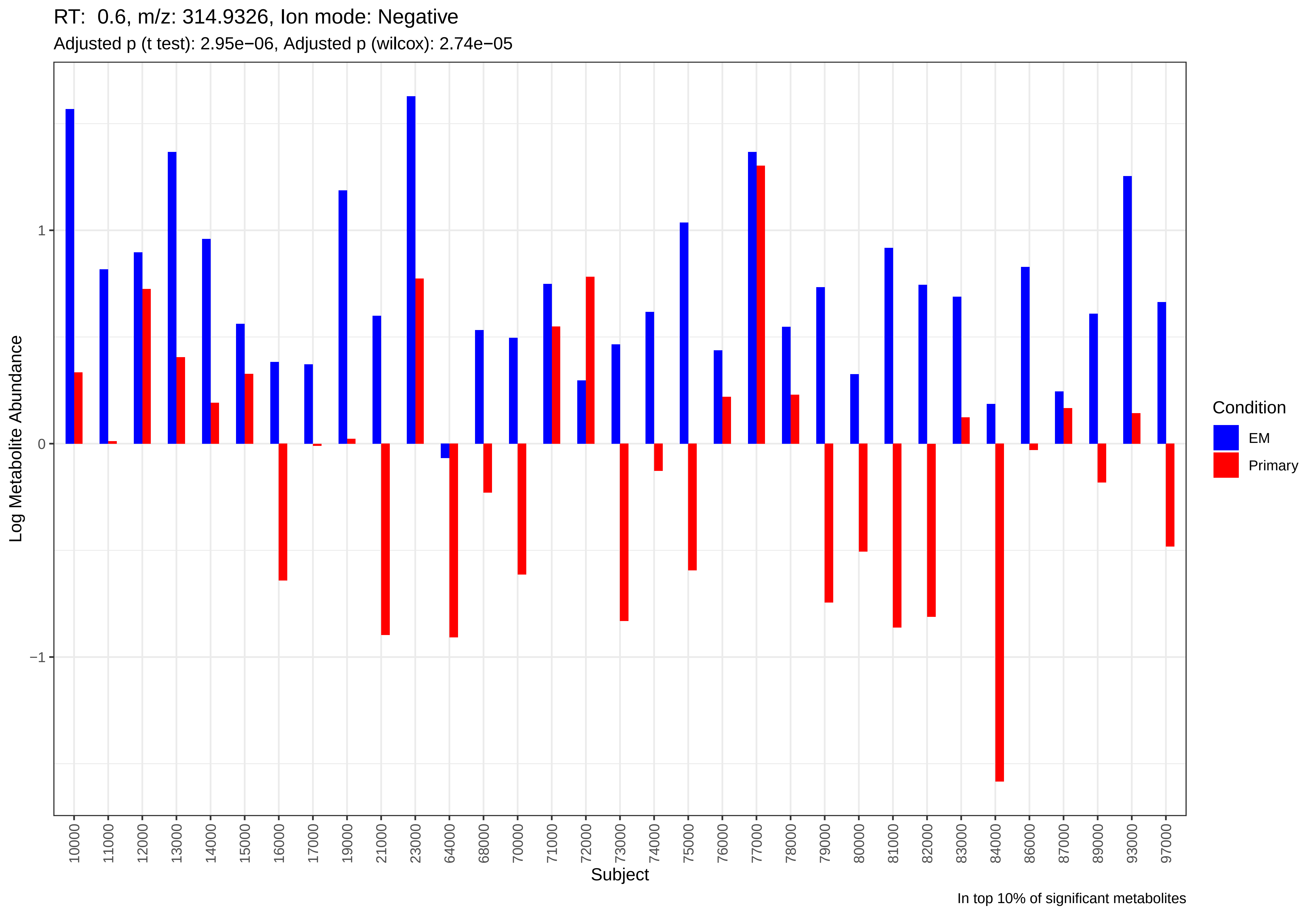

RT: 0.6, m/z: 314.9326, Ion mode: Negative
Adjusted p (t test): 2.95e−06, Adjusted p (wilcox): 2.74e−05
Log Metabolite Abundance
1
0
−1
10000
11000
12000
13000
14000
15000
16000
17000
19000
21000
23000
64000
68000
70000
71000
72000
73000
74000
75000
76000
77000
78000
79000
80000
81000
82000
83000
84000
86000
87000
89000
93000
97000
Subject
Condition
EM
Primary
In top 10% of significant metabolites

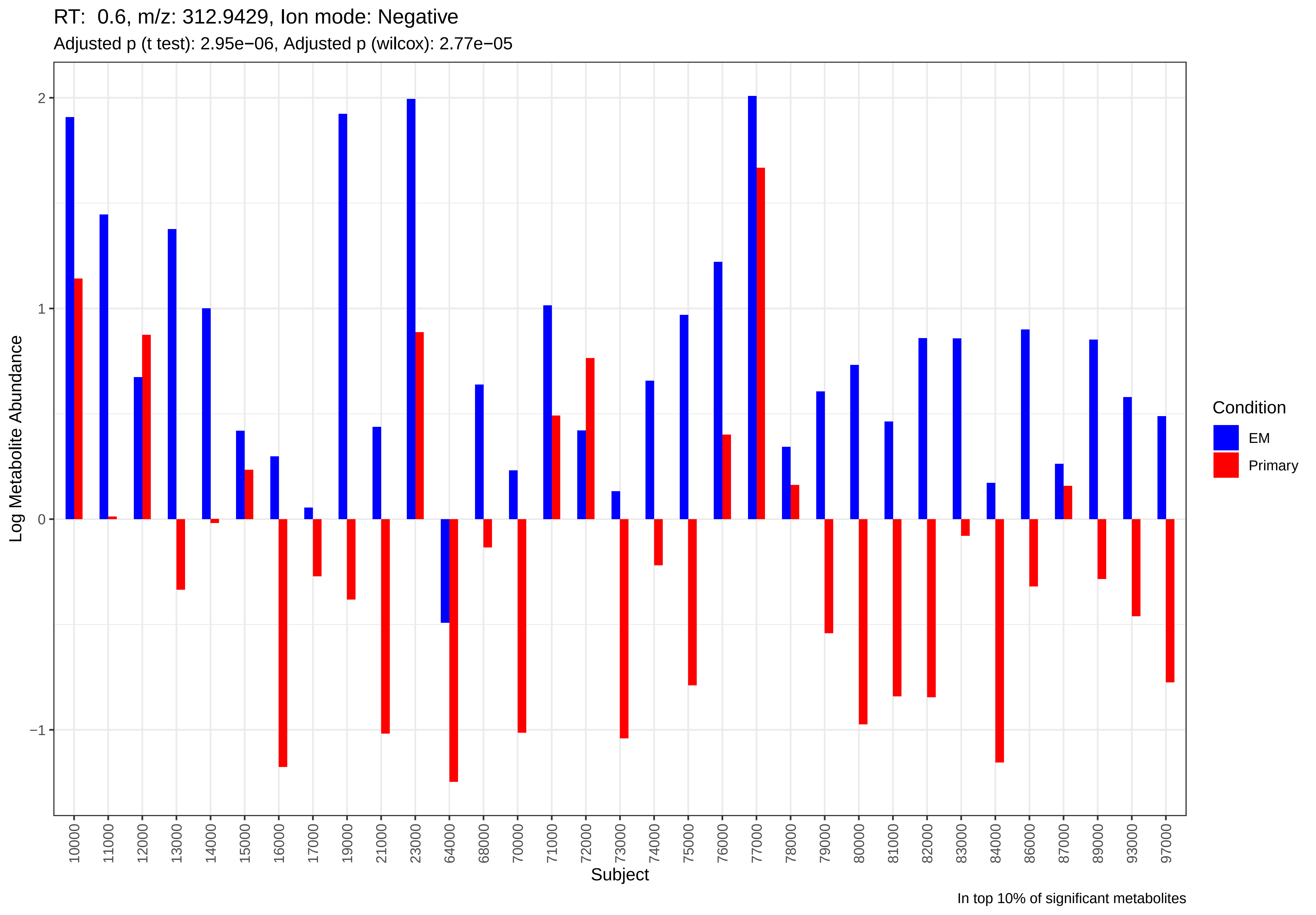
RT: 0.6, m/z: 312.9429, Ion mode: Negative
Adjusted p (t test): 2.95e−06, Adjusted p (wilcox): 2.77e−05
Log Metabolite Abundance
2
1
0
−1
10000
11000
12000
13000
14000
15000
16000
17000
19000
21000
23000
64000
68000
70000
71000
72000
73000
74000
75000
76000
77000
78000
79000
80000
81000
82000
83000
84000
86000
87000
89000
93000
97000
Subject
Condition
EM
Primary
In top 10% of significant metabolites

GLUCOSE/FRUCTOSE (RT: 0.7, m/z: 215.0327, Ion mode: Negative)

Adjusted p (t test): 2.95e−06, Adjusted p (wilcox): 3.55e−05

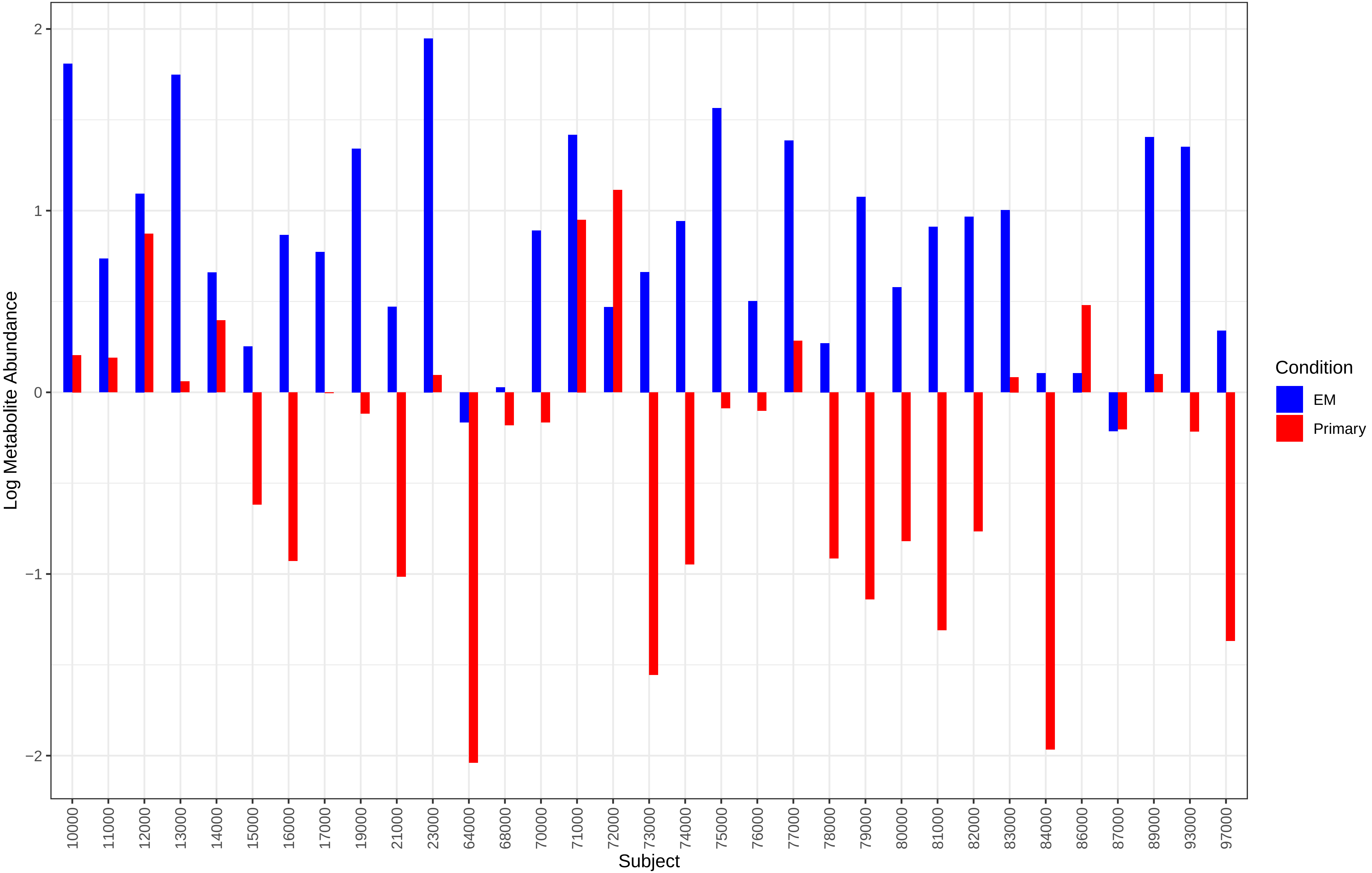

In top 10% of significant metabolites

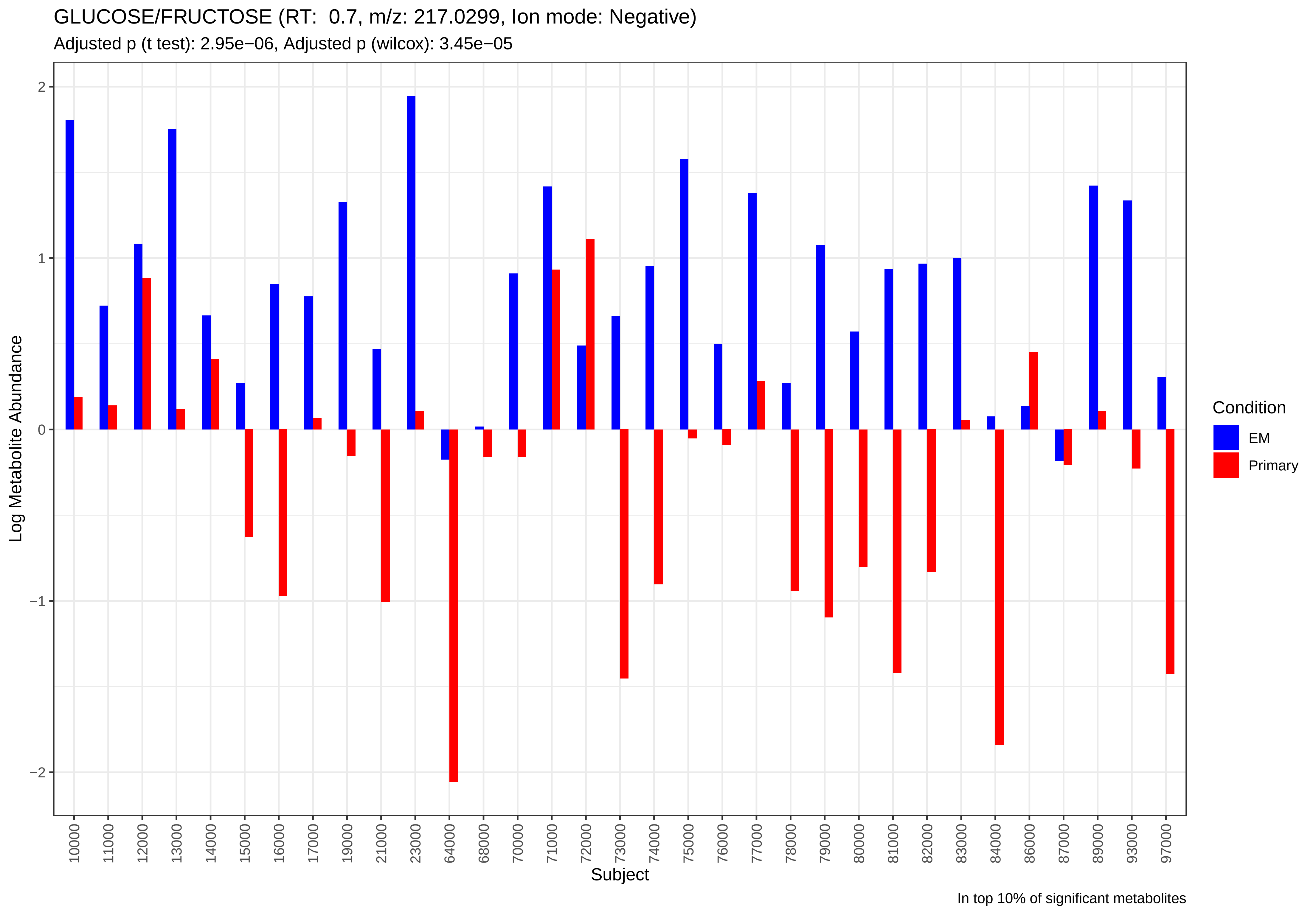
GLUCOSE/FRUCTOSE (RT: 0.7, m/z: 217.0299, Ion mode: Negative)
Adjusted p (t test): 2.95e−06, Adjusted p (wilcox): 3.45e−05
Log Metabolite Abundance
2
1
0
−1
−2
10000
11000
12000
13000
14000
15000
16000
17000
19000
21000
23000
64000
68000
70000
71000
72000
73000
74000
75000
76000
77000
78000
79000
80000
81000
82000
83000
84000
86000
87000
89000
93000
97000
Subject
Condition
EM
Primary
In top 10% of significant metabolites

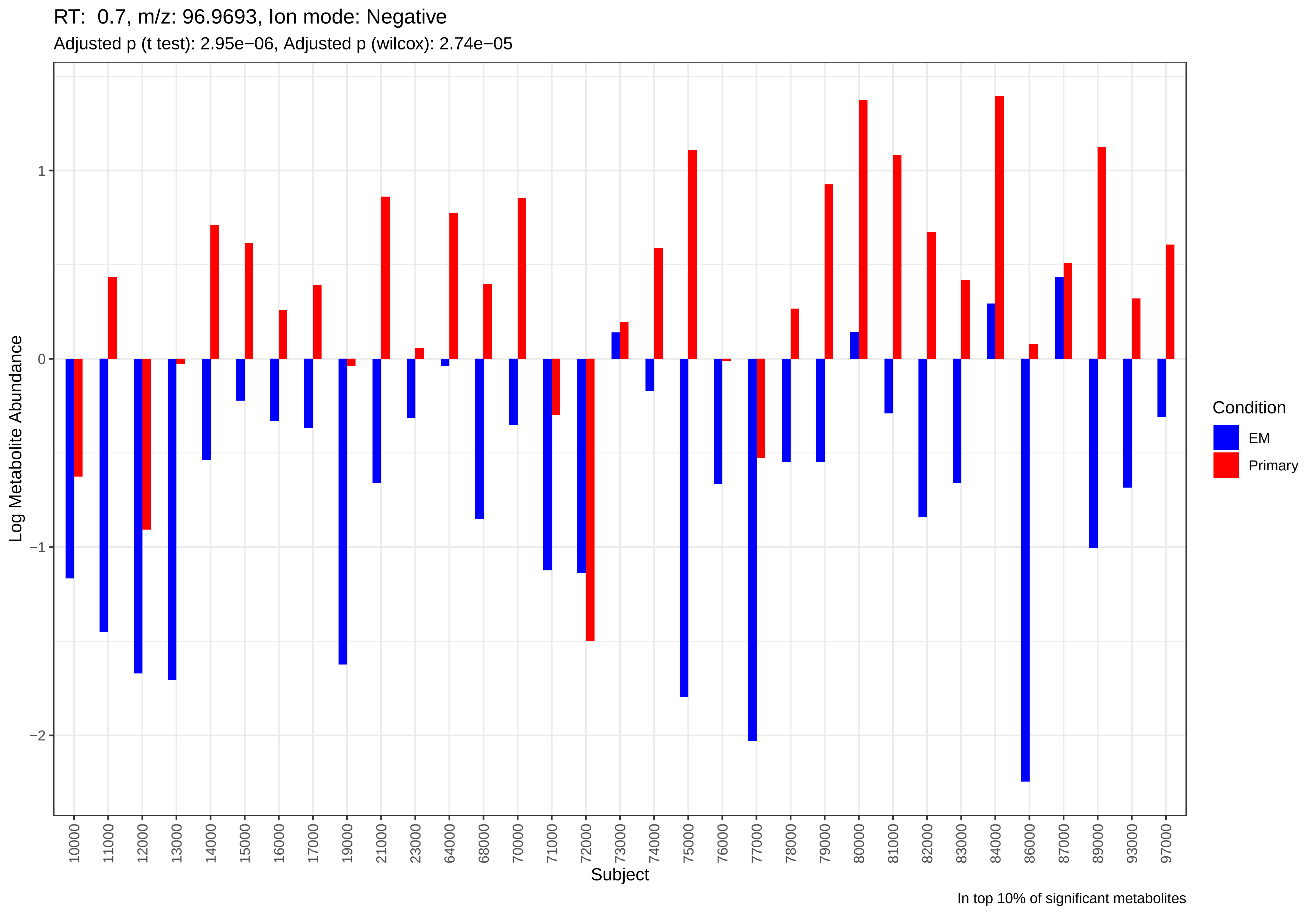
RT: 0.7, m/z: 96.9693, Ion mode: Negative
Adjusted p (t test): 2.95e−06, Adjusted p (wilcox): 2.74e−05
Log Metabolite Abundance
1
0
−1
−2
10000
11000
12000
13000
14000
15000
16000
17000
19000
21000
23000
64000
68000
70000
71000
72000
73000
74000
75000
76000
77000
78000
79000
80000
81000
82000
83000
84000
86000
87000
89000
93000
97000
Subject
Condition
EM
Primary
In top 10% of significant metabolites

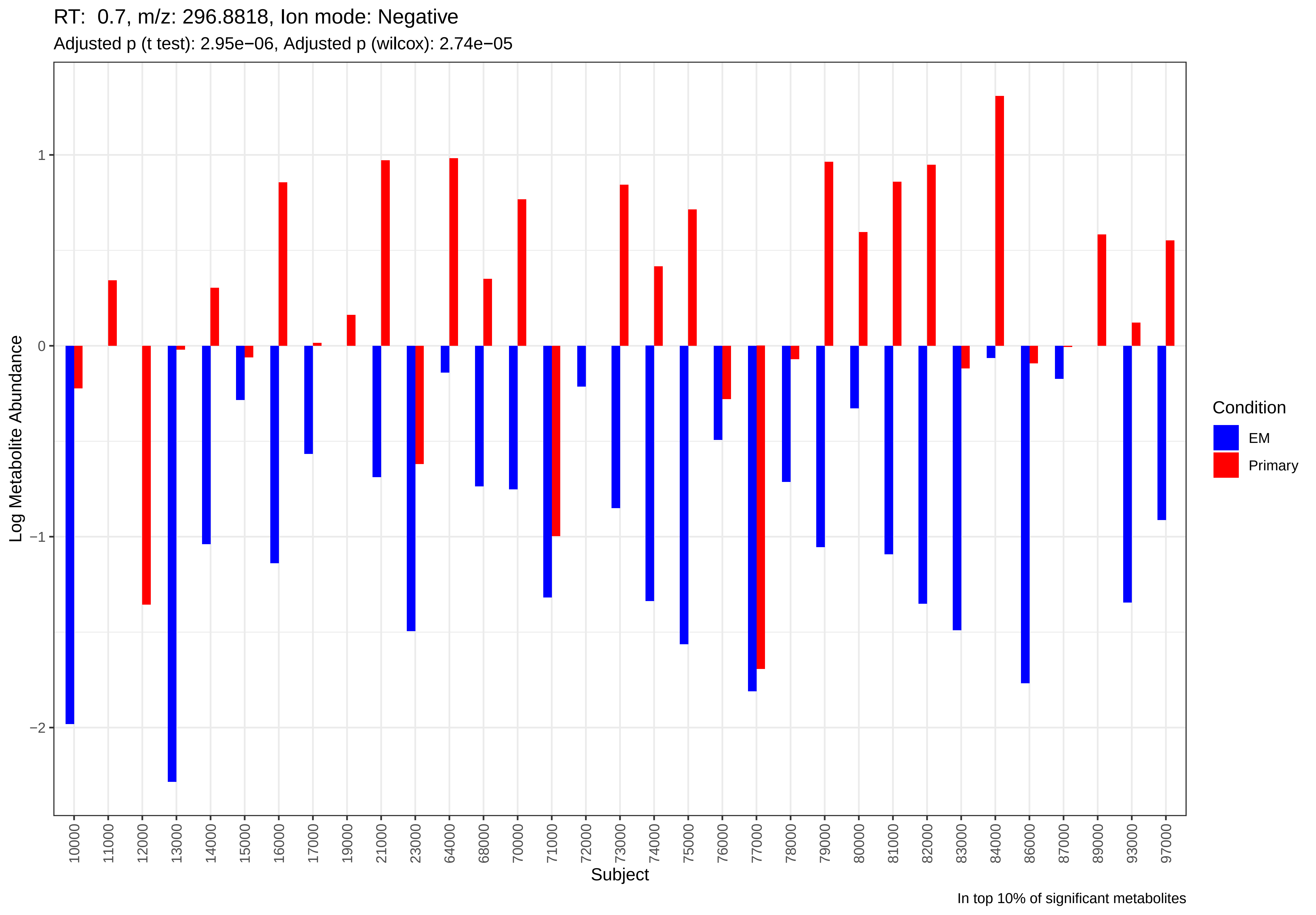
RT: 0.7, m/z: 296.8818, Ion mode: Negative
Adjusted p (t test): 2.95e−06, Adjusted p (wilcox): 2.74e−05
Log Metabolite Abundance
1
0
−1
−2
Subject
10000
11000
12000
13000
14000
15000
16000
17000
19000
21000
23000
64000
68000
70000
71000
72000
73000
74000
75000
76000
77000
78000
79000
80000
81000
82000
83000
84000
86000
87000
89000
93000
97000
Condition
EM
Primary
In top 10% of significant metabolites

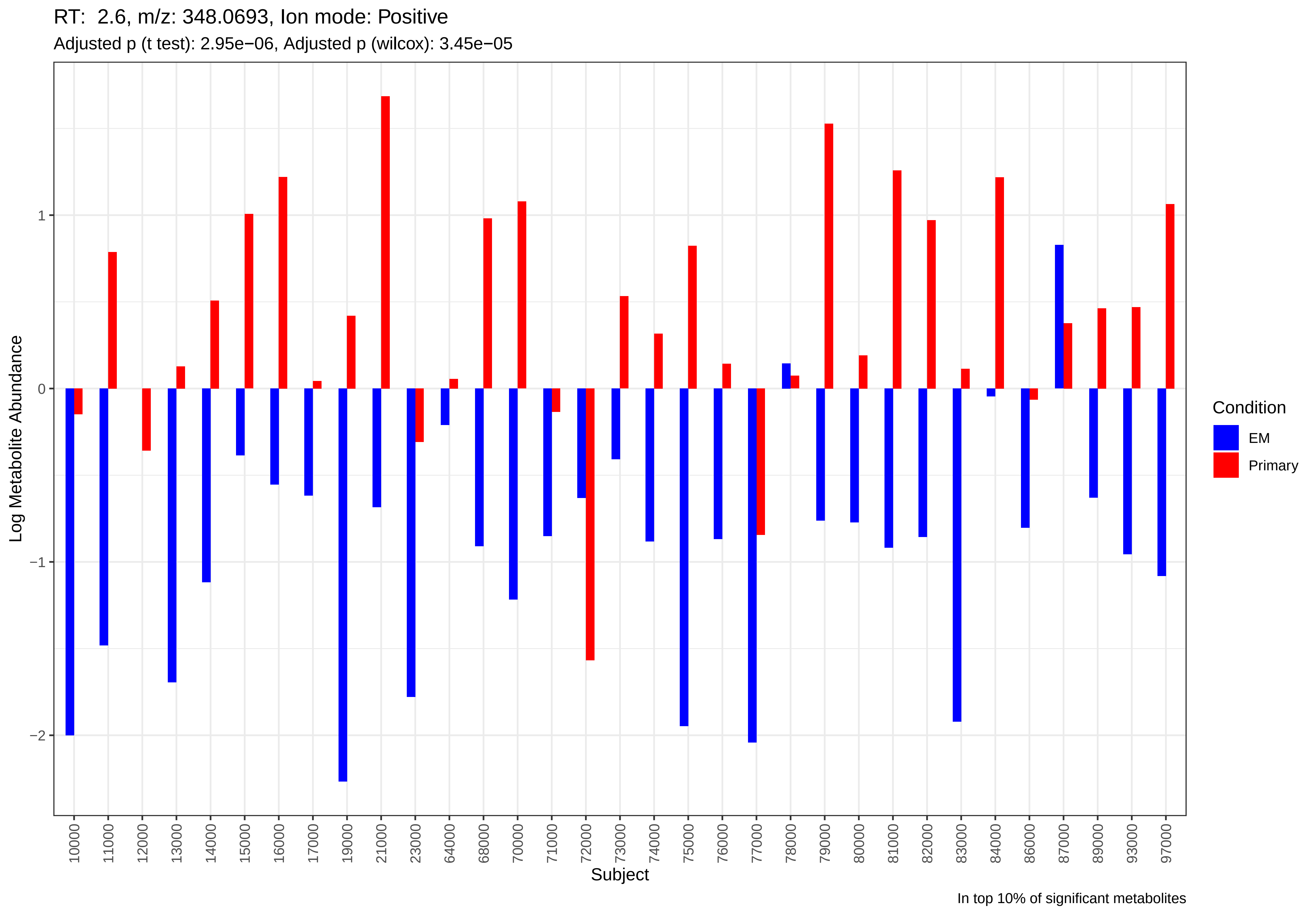

RT: 2.6, m/z: 348.0693, Ion mode: Positive
Adjusted p (t test): 2.95e−06, Adjusted p (wilcox): 3.45e−05
Log Metabolite Abundance
1
0
−1
−2
10000
11000
12000
13000
14000
15000
16000
17000
19000
21000
23000
64000
68000
70000
71000
72000
73000
74000
75000
76000
77000
78000
79000
80000
81000
82000
83000
84000
86000
87000
89000
93000
97000
Subject
Condition
EM
Primary
In top 10% of significant metabolites

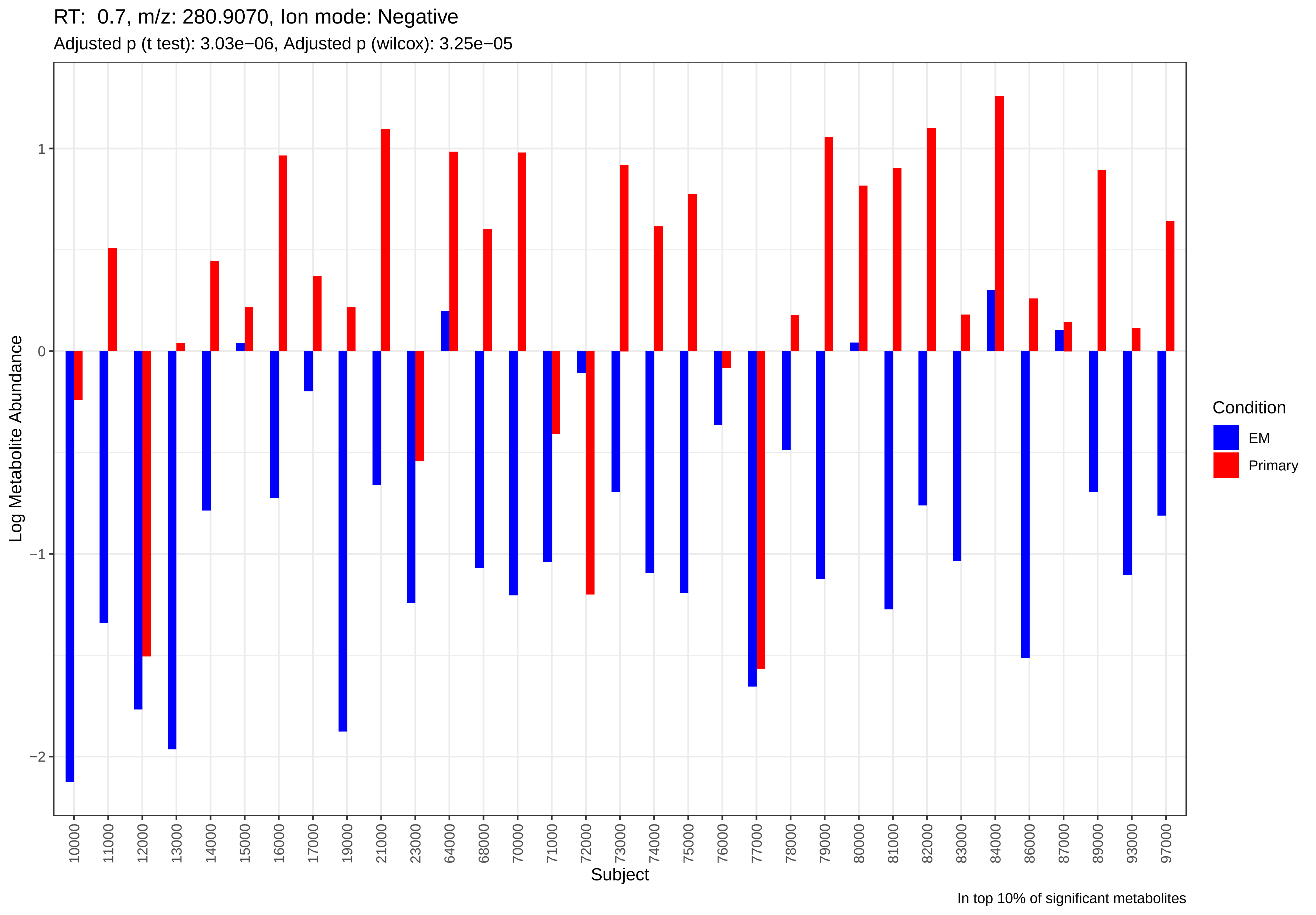
RT: 0.7, m/z: 280.9070, Ion mode: Negative
Adjusted p (t test): 3.03e−06, Adjusted p (wilcox): 3.25e−05
Log Metabolite Abundance
1
0
−1
−2
10000
11000
12000
13000
14000
15000
16000
17000
19000
21000
23000
64000
68000
70000
71000
72000
73000
74000
75000
76000
77000
78000
79000
80000
81000
82000
83000
84000
86000
87000
89000
93000
97000
Subject
Condition
EM
Primary
In top 10% of significant metabolites

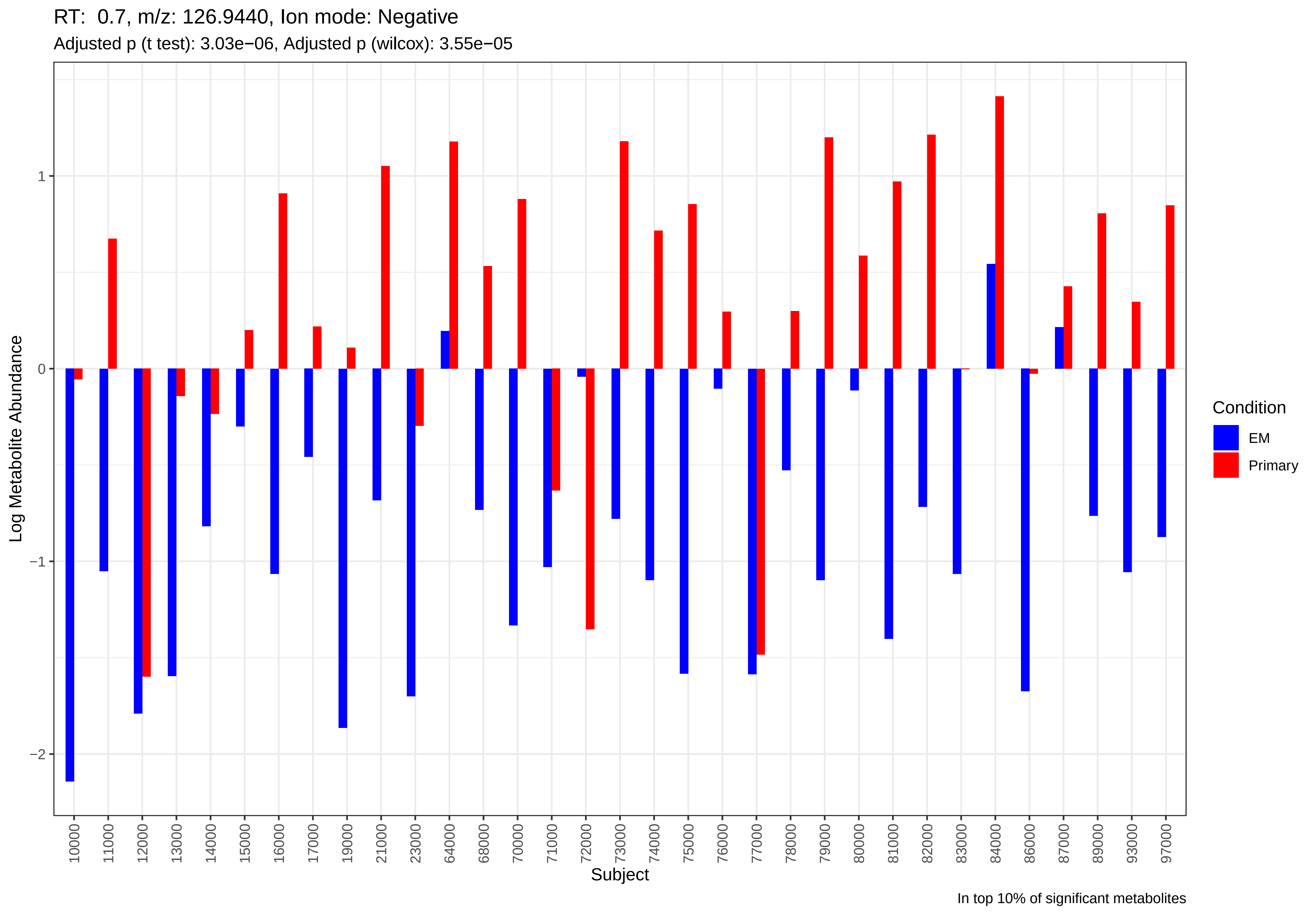
RT: 0.7, m/z: 126.9440, Ion mode: Negative
Adjusted p (t test): 3.03e−06, Adjusted p (wilcox): 3.55e−05
Log Metabolite Abundance
1
0
−1
−2
Subject
10000
11000
12000
13000
14000
15000
16000
17000
19000
21000
23000
64000
68000
70000
71000
72000
73000
74000
75000
76000
77000
78000
79000
80000
81000
82000
83000
84000
86000
87000
89000
93000
97000
Condition
EM
Primary
In top 10% of significant metabolites

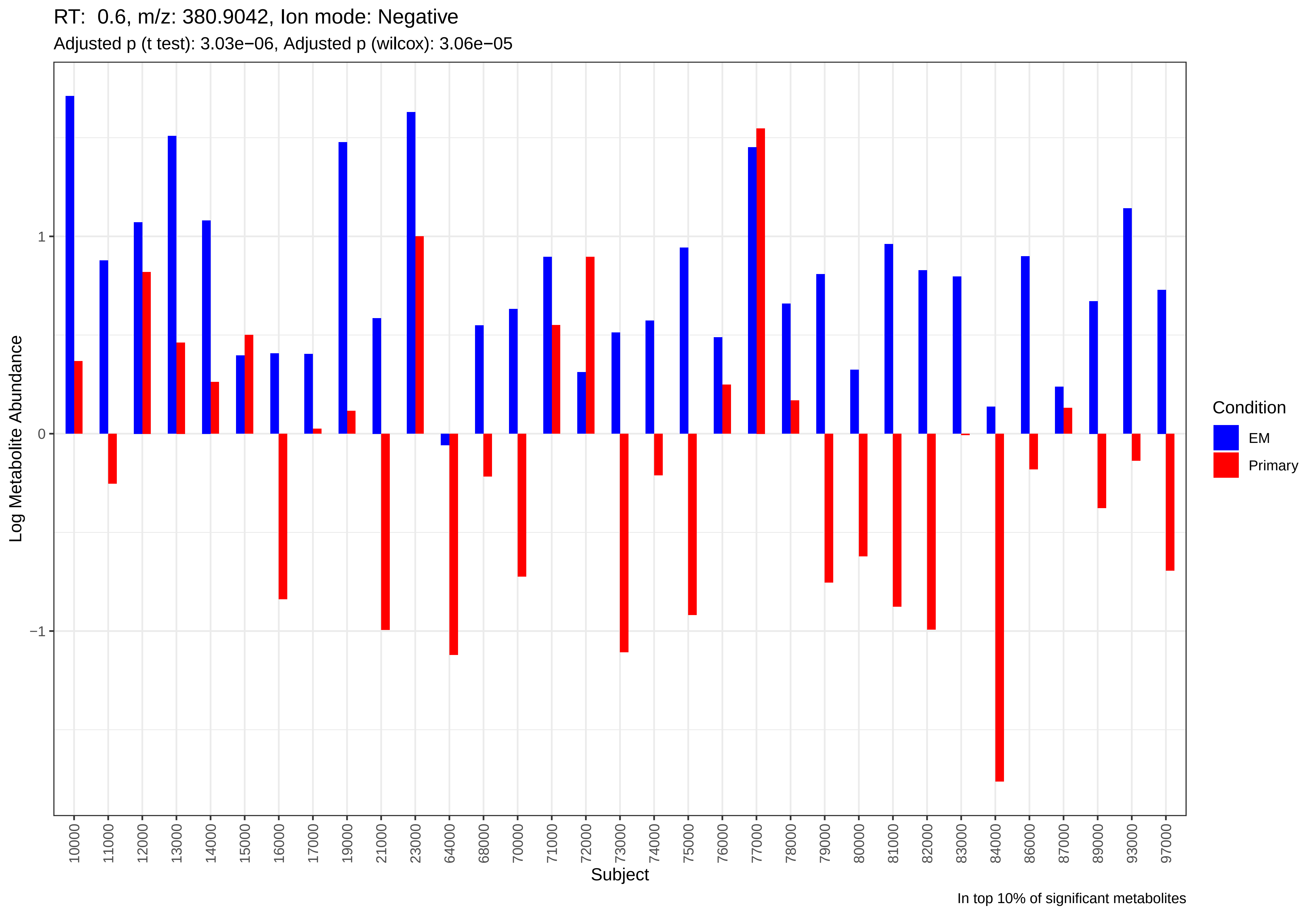
RT: 0.6, m/z: 380.9042, Ion mode: Negative
Adjusted p (t test): 3.03e−06, Adjusted p (wilcox): 3.06e−05
Log Metabolite Abundance
1
0
−1
10000
11000
12000
13000
14000
15000
16000
17000
19000
21000
23000
64000
68000
70000
71000
72000
73000
74000
75000
76000
77000
78000
79000
80000
81000
82000
83000
84000
86000
87000
89000
93000
97000
Subject
Condition
EM
Primary
In top 10% of significant metabolites

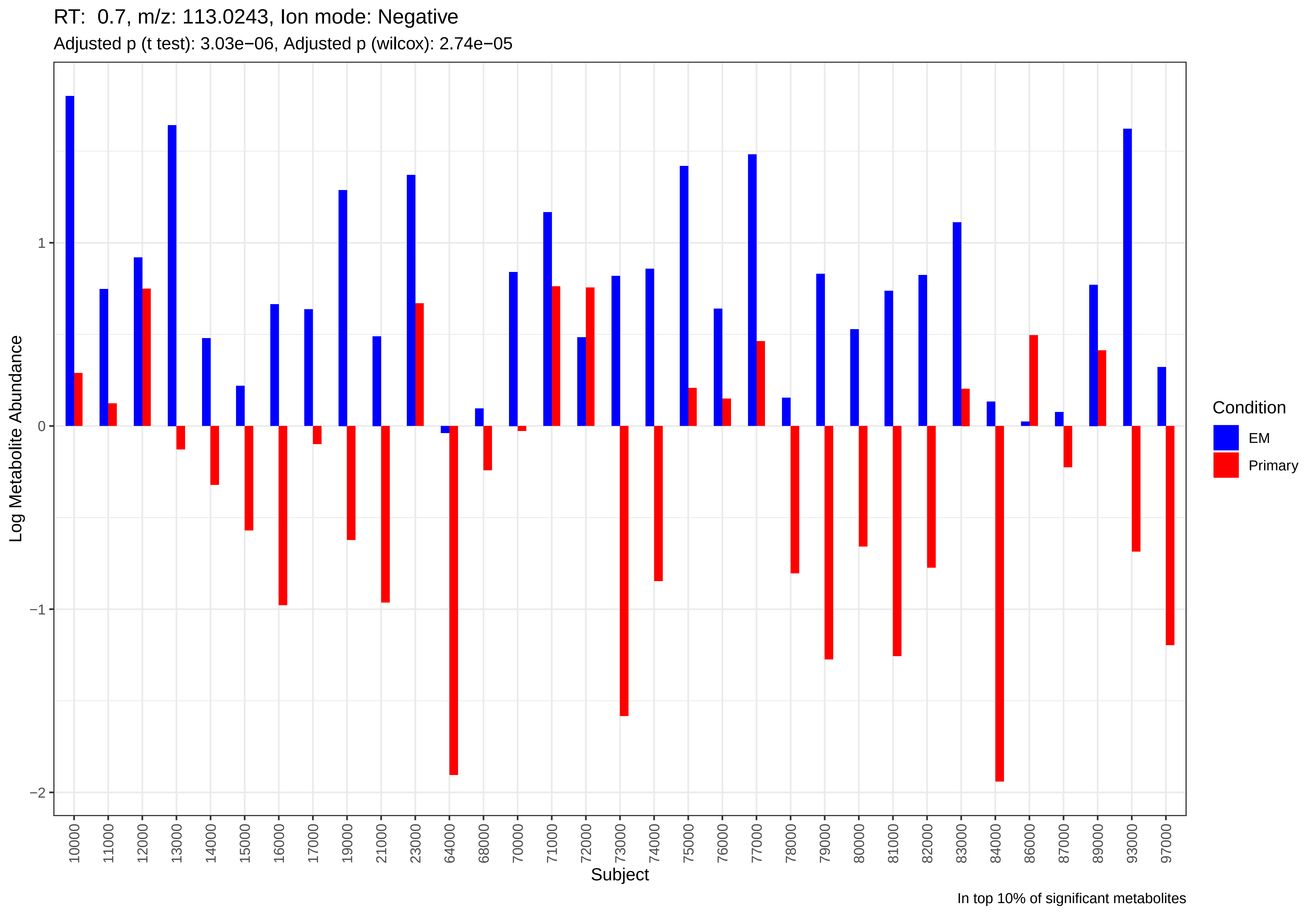
RT: 0.7, m/z: 113.0243, Ion mode: Negative
Adjusted p (t test): 3.03e−06, Adjusted p (wilcox): 2.74e−05
Log Metabolite Abundance
Subject
Condition
EM
Primary
1
0
−1
−2
10000
11000
12000
13000
14000
15000
16000
17000
19000
21000
23000
64000
68000
70000
71000
72000
73000
74000
75000
76000
77000
78000
79000
80000
81000
82000
83000
84000
86000
87000
89000
93000
97000
In top 10% of significant metabolites

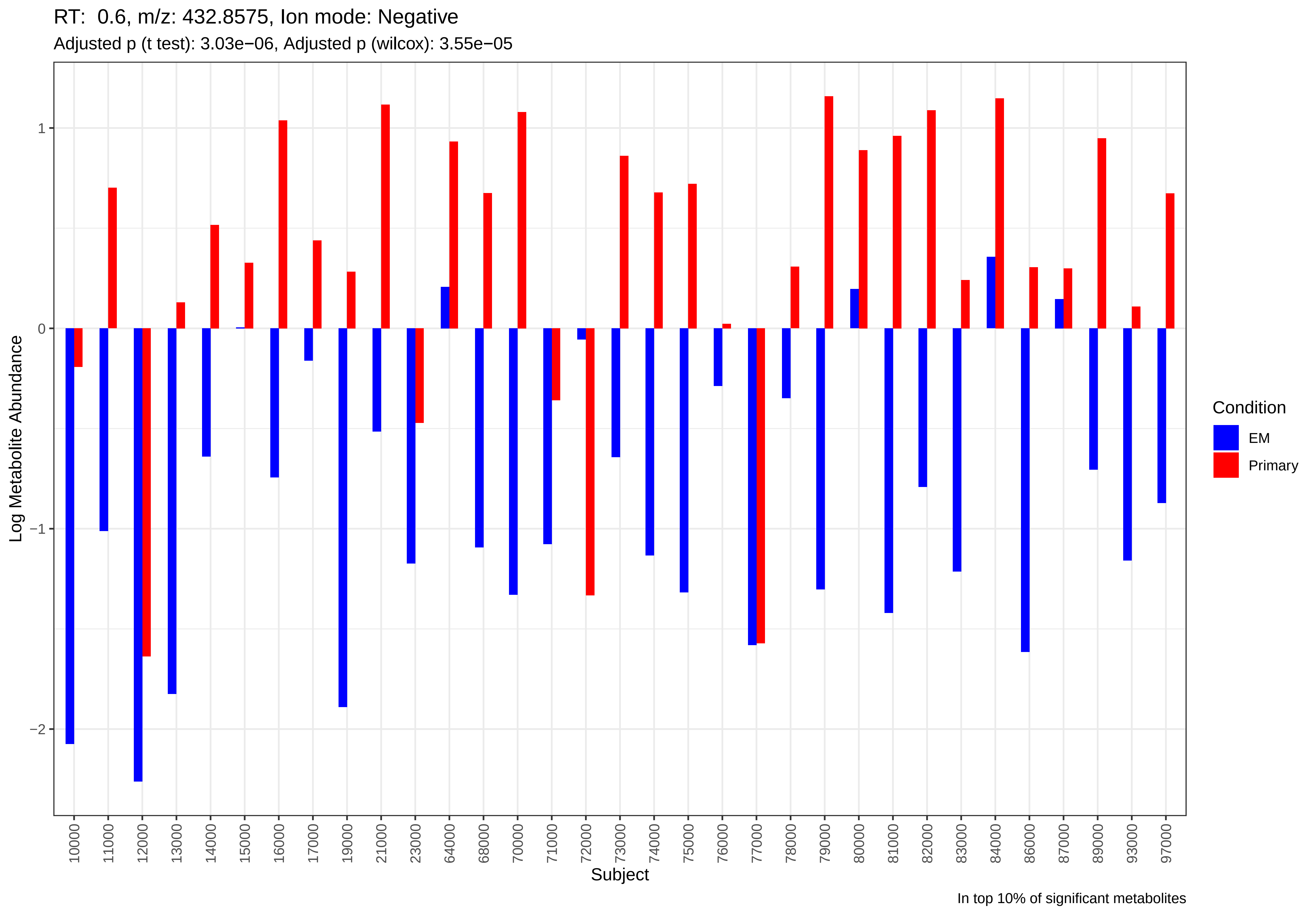
RT: 0.6, m/z: 432.8575, Ion mode: Negative
Adjusted p (t test): 3.03e−06, Adjusted p (wilcox): 3.55e−05
Log Metabolite Abundance
1
0
−1
−2
Subject
10000
11000
12000
13000
14000
15000
16000
17000
19000
21000
23000
64000
68000
70000
71000
72000
73000
74000
75000
76000
77000
78000
79000
80000
81000
82000
83000
84000
86000
87000
89000
93000
97000
Condition
EM
Primary
In top 10% of significant metabolites

RT: 0.7, m/z: 278.8926, Ion mode: Negative

Adjusted p (t test): 3.03e−06, Adjusted p (wilcox): 3.79e−05

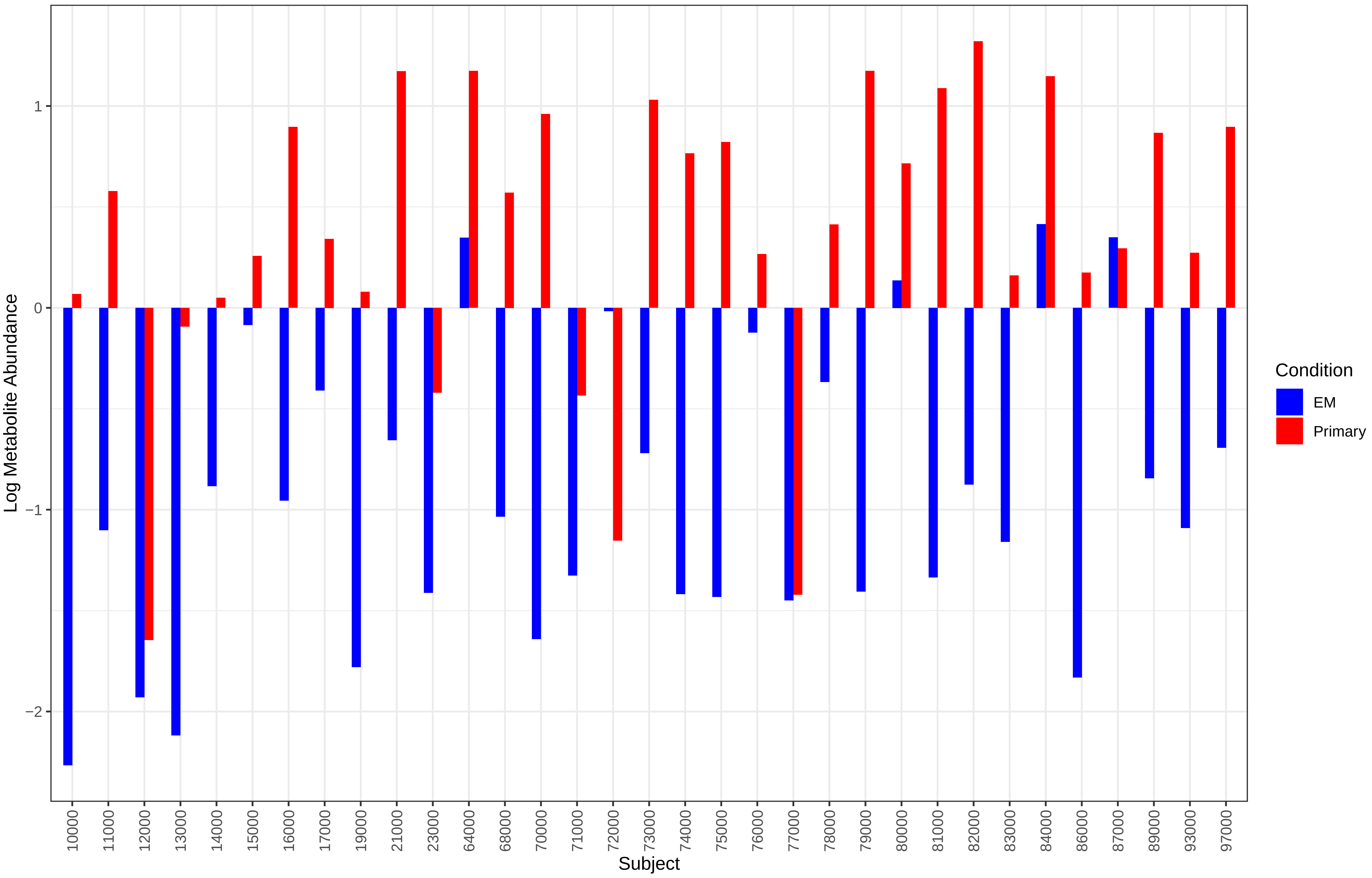

In top 10% of significant metabolites

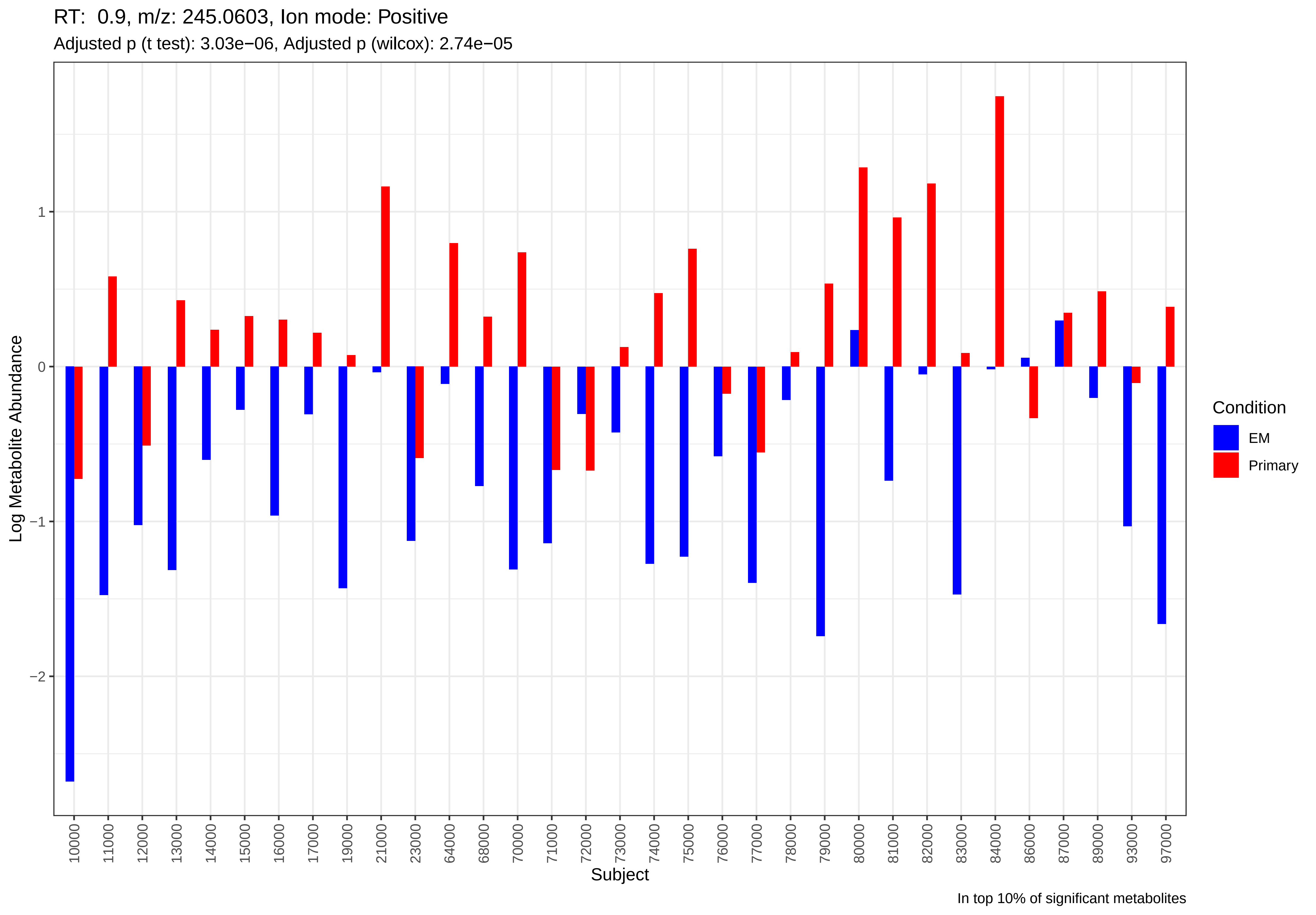
RT: 0.9, m/z: 245.0603, Ion mode: Positive
Adjusted p (t test): 3.03e−06, Adjusted p (wilcox): 2.74e−05
Log Metabolite Abundance
1
0
−1
−2
10000
11000
12000
13000
14000
15000
16000
17000
19000
21000
23000
64000
68000
70000
71000
72000
73000
74000
75000
76000
77000
78000
79000
80000
81000
82000
83000
84000
86000
87000
89000
93000
97000
Subject
Condition
EM
Primary
In top 10% of significant metabolites

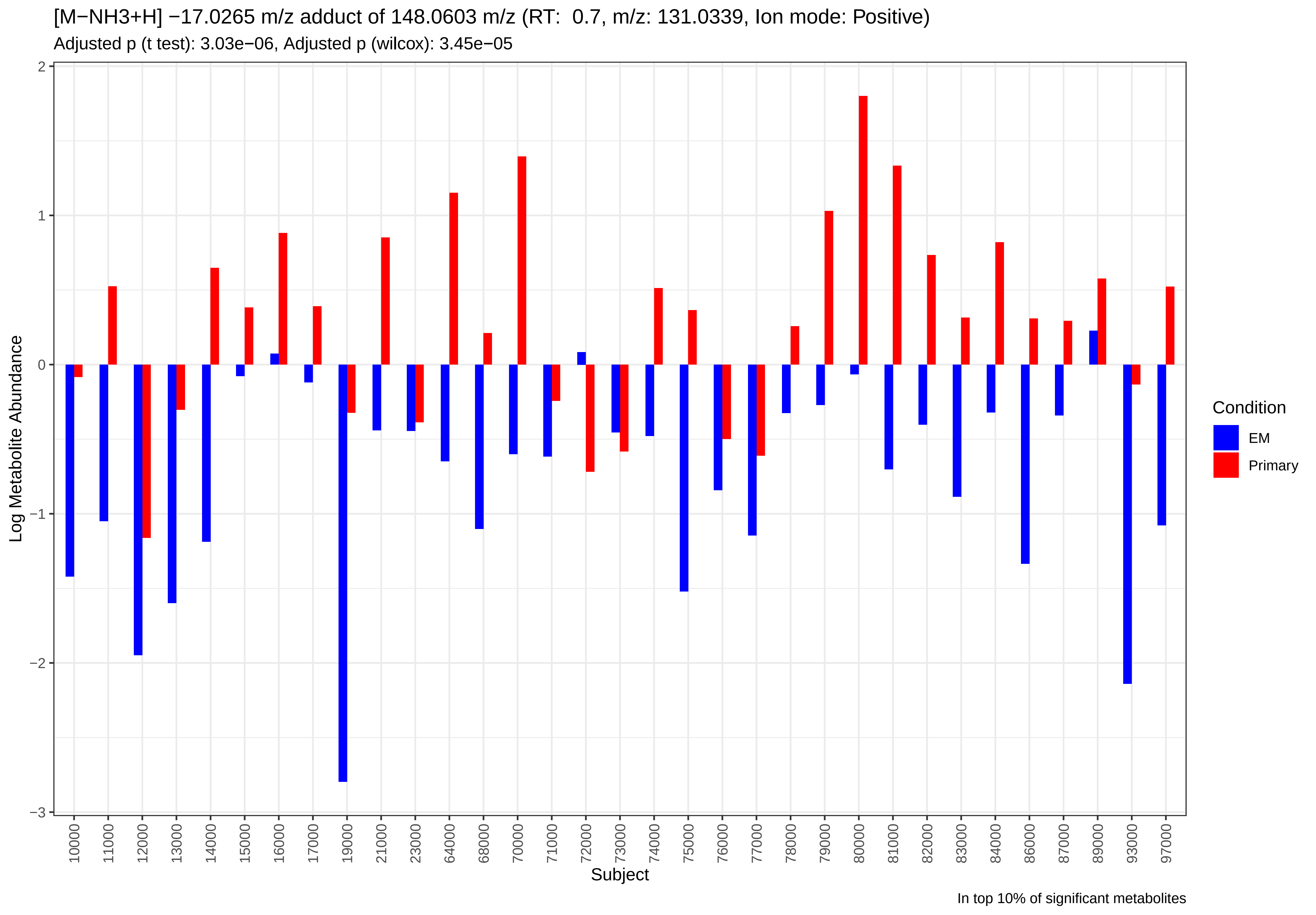

[M−NH3+H] −17.0265 m/z adduct of 148.0603 m/z (RT: 0.7, m/z: 131.0339, Ion mode: Positive)
Adjusted p (t test): 3.03e−06, Adjusted p (wilcox): 3.45e−05
Log Metabolite Abundance
2
1
0
−1
−2
−3
10000
11000
12000
13000
14000
15000
16000
17000
19000
21000
23000
64000
68000
70000
71000
72000
73000
74000
75000
76000
77000
78000
79000
80000
81000
82000
83000
84000
86000
87000
89000
93000
97000
Subject
Condition
EM
Primary
In top 10% of significant metabolites

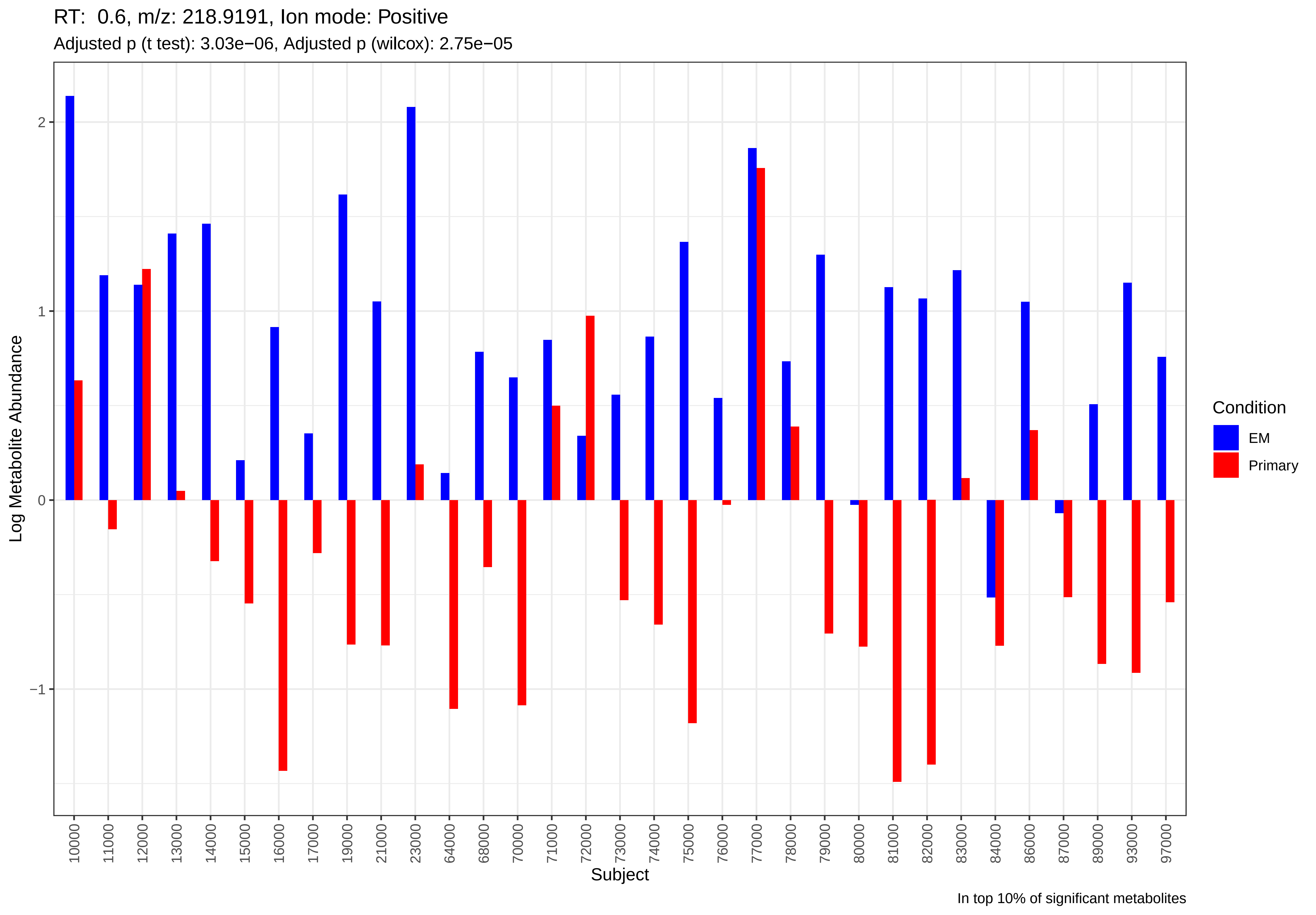
RT: 0.6, m/z: 218.9191, Ion mode: Positive
Adjusted p (t test): 3.03e−06, Adjusted p (wilcox): 2.75e−05
Log Metabolite Abundance
2
1
0
−1
10000
11000
12000
13000
14000
15000
16000
17000
19000
21000
23000
64000
68000
70000
71000
72000
73000
74000
75000
76000
77000
78000
79000
80000
81000
82000
83000
84000
86000
87000
89000
93000
97000
Subject
Condition
EM
Primary
In top 10% of significant metabolites

RT: 0.7, m/z: 216.9390, Ion mode: Negative

Adjusted p (t test): 3.40e−06, Adjusted p (wilcox): 3.55e−05

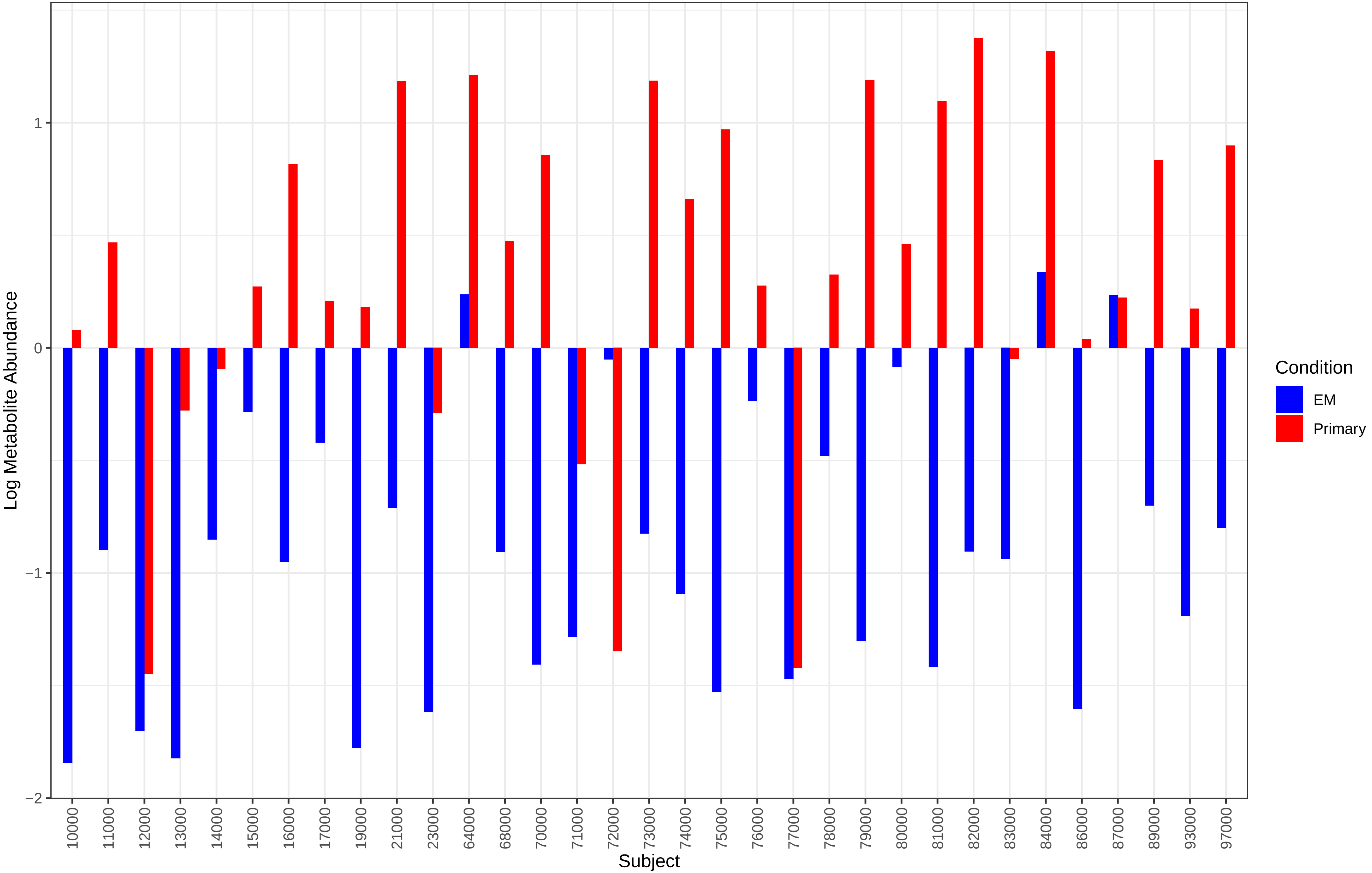

In top 10% of significant metabolites

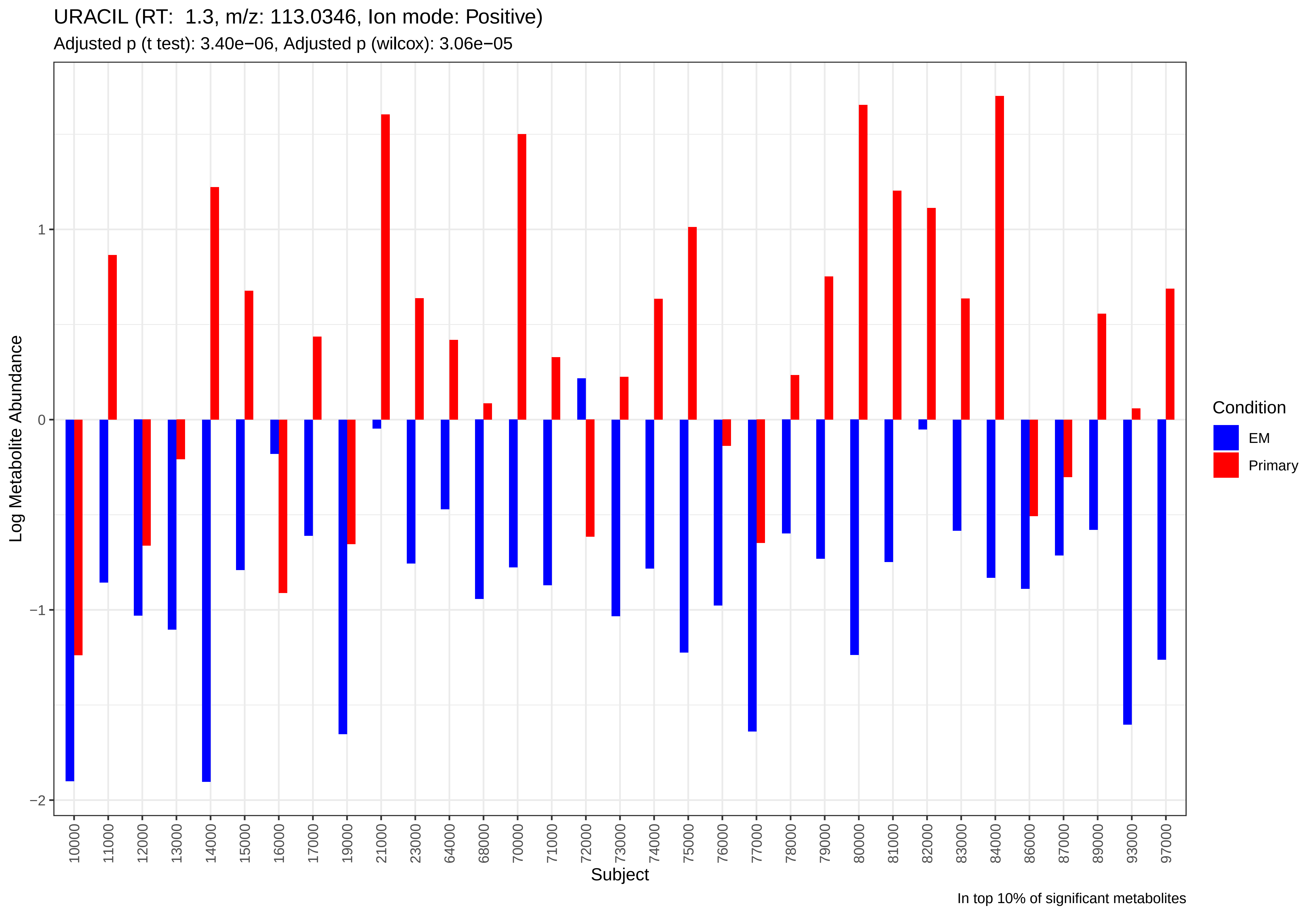
URACIL (RT: 1.3, m/z: 113.0346, Ion mode: Positive)
Adjusted p (t test): 3.40e−06, Adjusted p (wilcox): 3.06e−05
Log Metabolite Abundance
1
0
−1
−2
Subject
10000
11000
12000
13000
14000
15000
16000
17000
19000
21000
23000
64000
68000
70000
71000
72000
73000
74000
75000
76000
77000
78000
79000
80000
81000
82000
83000
84000
86000
87000
89000
93000
97000
Condition
EM
Primary
In top 10% of significant metabolites

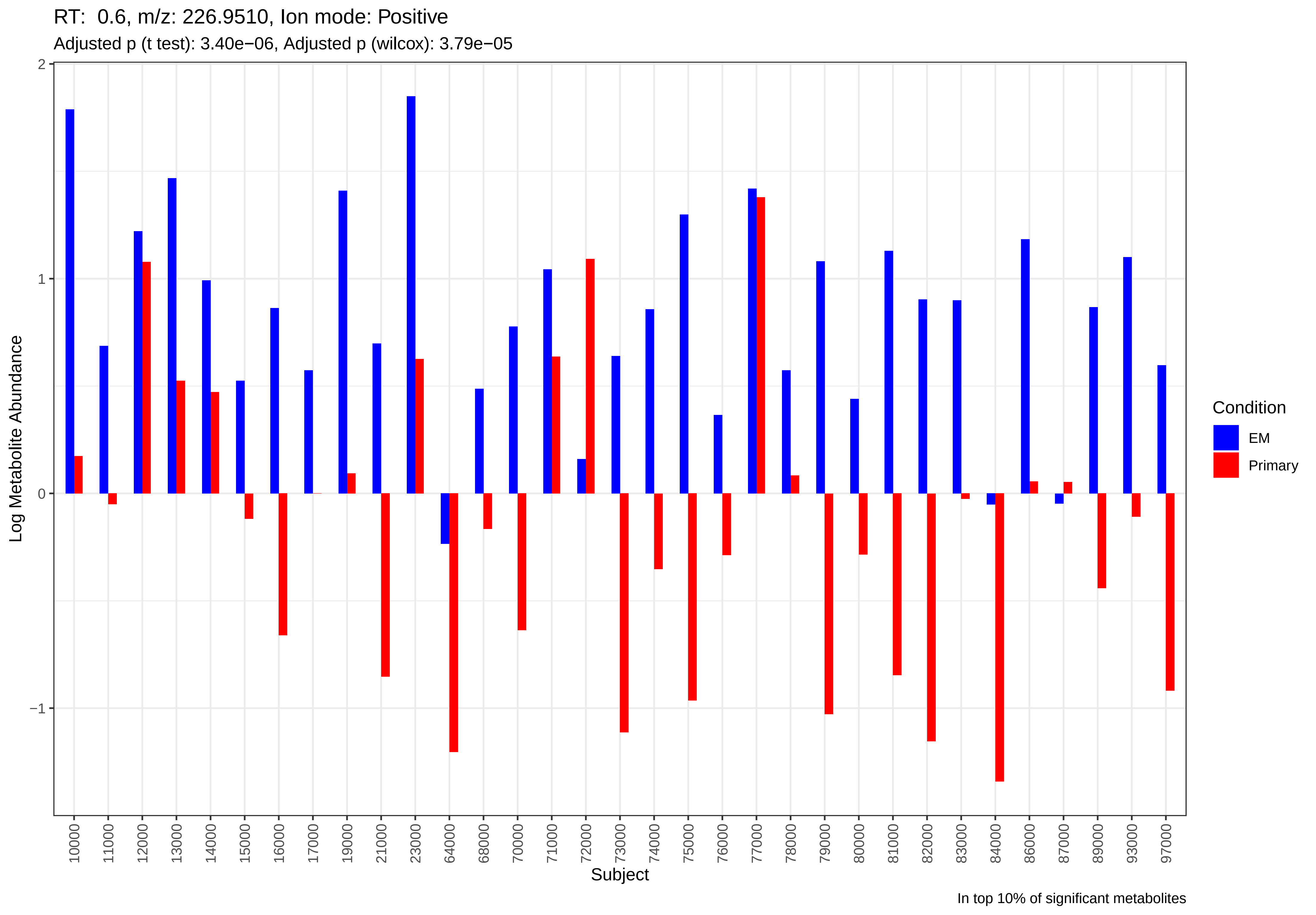
RT: 0.6, m/z: 226.9510, Ion mode: Positive
Adjusted p (t test): 3.40e−06, Adjusted p (wilcox): 3.79e−05
Log Metabolite Abundance
2
1
0
−1
10000
11000
12000
13000
14000
15000
16000
17000
19000
21000
23000
64000
68000
70000
71000
72000
73000
74000
75000
76000
77000
78000
79000
80000
81000
82000
83000
84000
86000
87000
89000
93000
97000
Subject
Condition
EM
Primary
In top 10% of significant metabolites

RT: 0.7, m/z: 192.9872, Ion mode: Positive

Adjusted p (t test): 3.43e−06, Adjusted p (wilcox): 4.19e−05

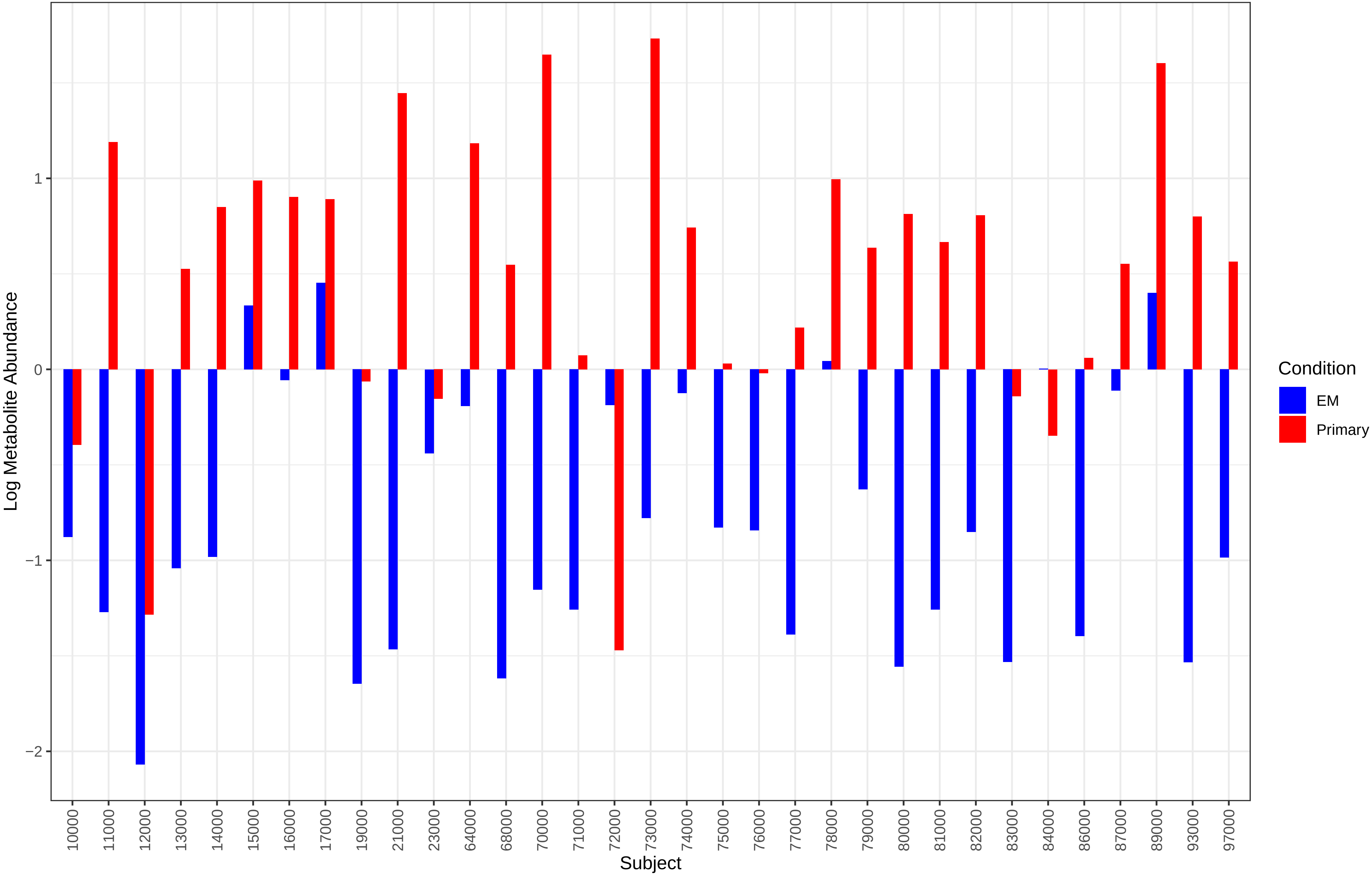

In top 10% of significant metabolites

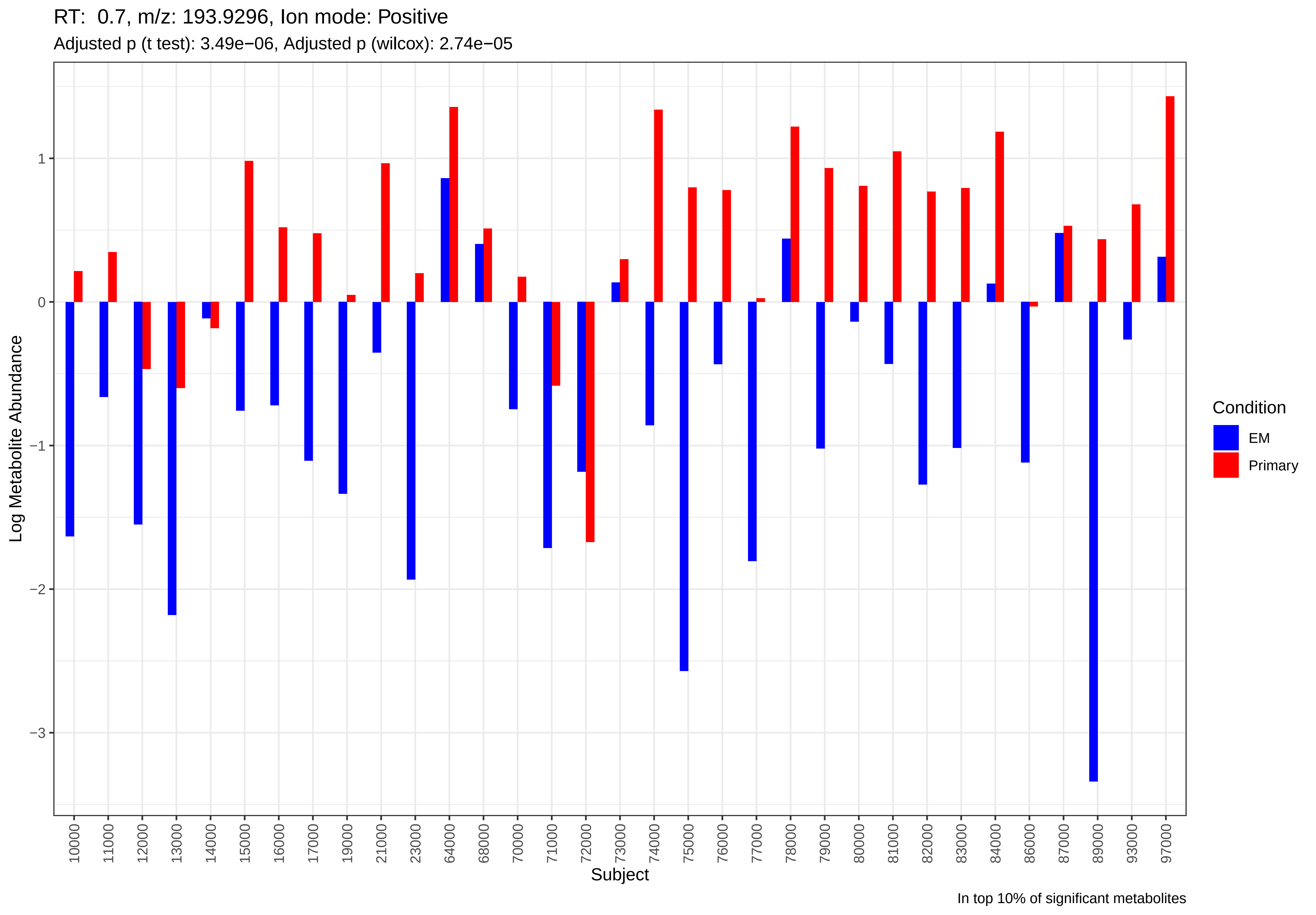
RT: 0.7, m/z: 193.9296, Ion mode: Positive
Adjusted p (t test): 3.49e−06, Adjusted p (wilcox): 2.74e−05
Log Metabolite Abundance
1
0
−1
−2
−3
10000
11000
12000
13000
14000
15000
16000
17000
19000
21000
23000
64000
68000
70000
71000
72000
73000
74000
75000
76000
77000
78000
79000
80000
81000
82000
83000
84000
86000
87000
89000
93000
97000
Subject
Condition
EM
Primary
In top 10% of significant metabolites

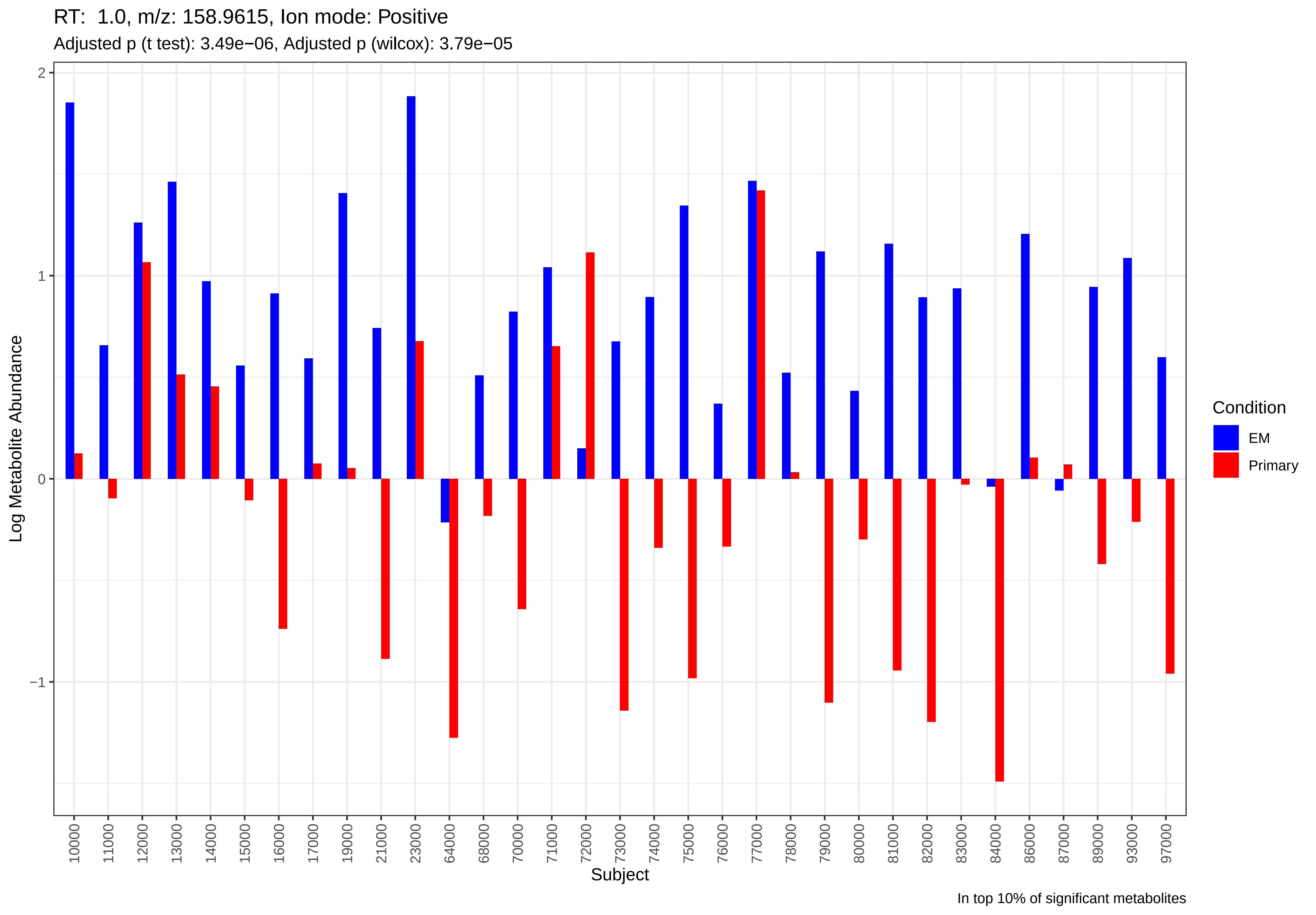
RT: 1.0, m/z: 158.9615, Ion mode: Positive
Adjusted p (t test): 3.49e−06, Adjusted p (wilcox): 3.79e−05
Log Metabolite Abundance
2
1
0
−1
10000
11000
12000
13000
14000
15000
16000
17000
19000
21000
23000
64000
68000
70000
71000
72000
73000
74000
75000
76000
77000
78000
79000
80000
81000
82000
83000
84000
86000
87000
89000
93000
97000
Subject
Condition
EM
Primary
In top 10% of significant metabolites

RT: 3.6, m/z: 152.0566, Ion mode: Positive

Adjusted p (t test): 3.49e−06, Adjusted p (wilcox): 5.05e−05

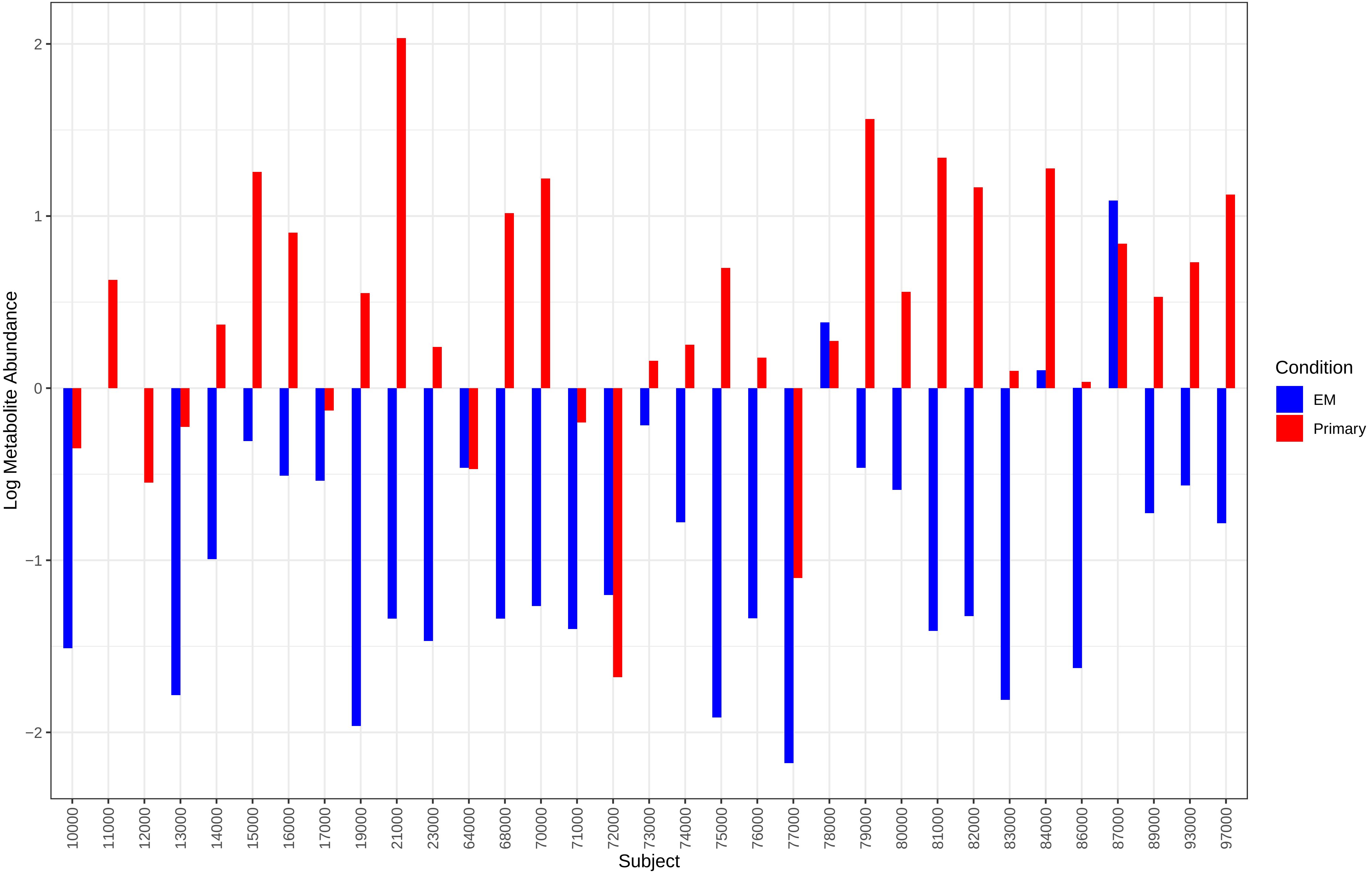

In top 10% of significant metabolites

RT: 0.6, m/z: 346.8808, Ion mode: Negative

Adjusted p (t test): 3.84e−06, Adjusted p (wilcox): 4.19e−05

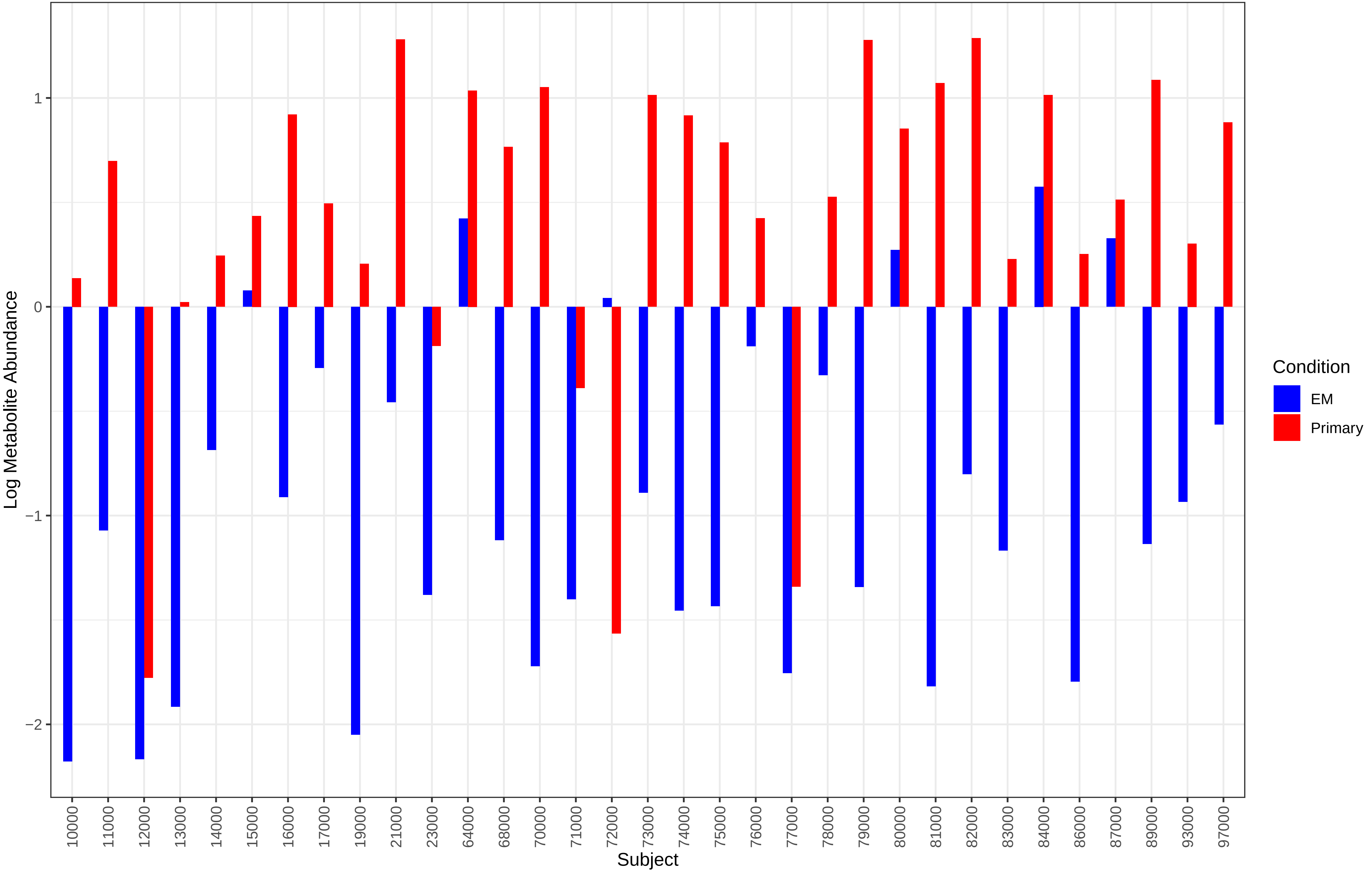

In top 10% of significant metabolites

RT: 0.6, m/z: 316.9480, Ion mode: Negative

Adjusted p (t test): 3.84e−06, Adjusted p (wilcox): 3.55e−05

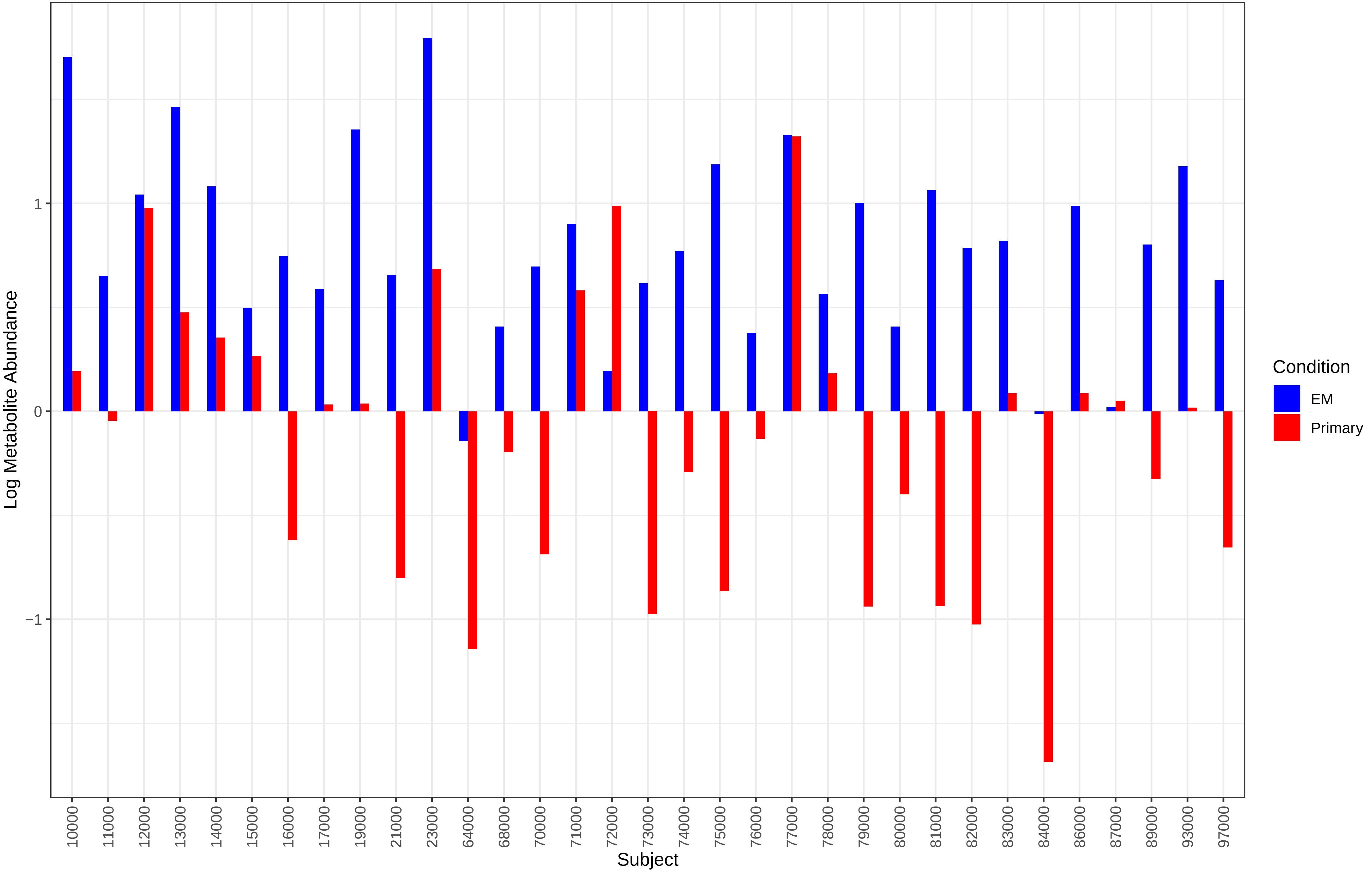

In top 10% of significant metabolites

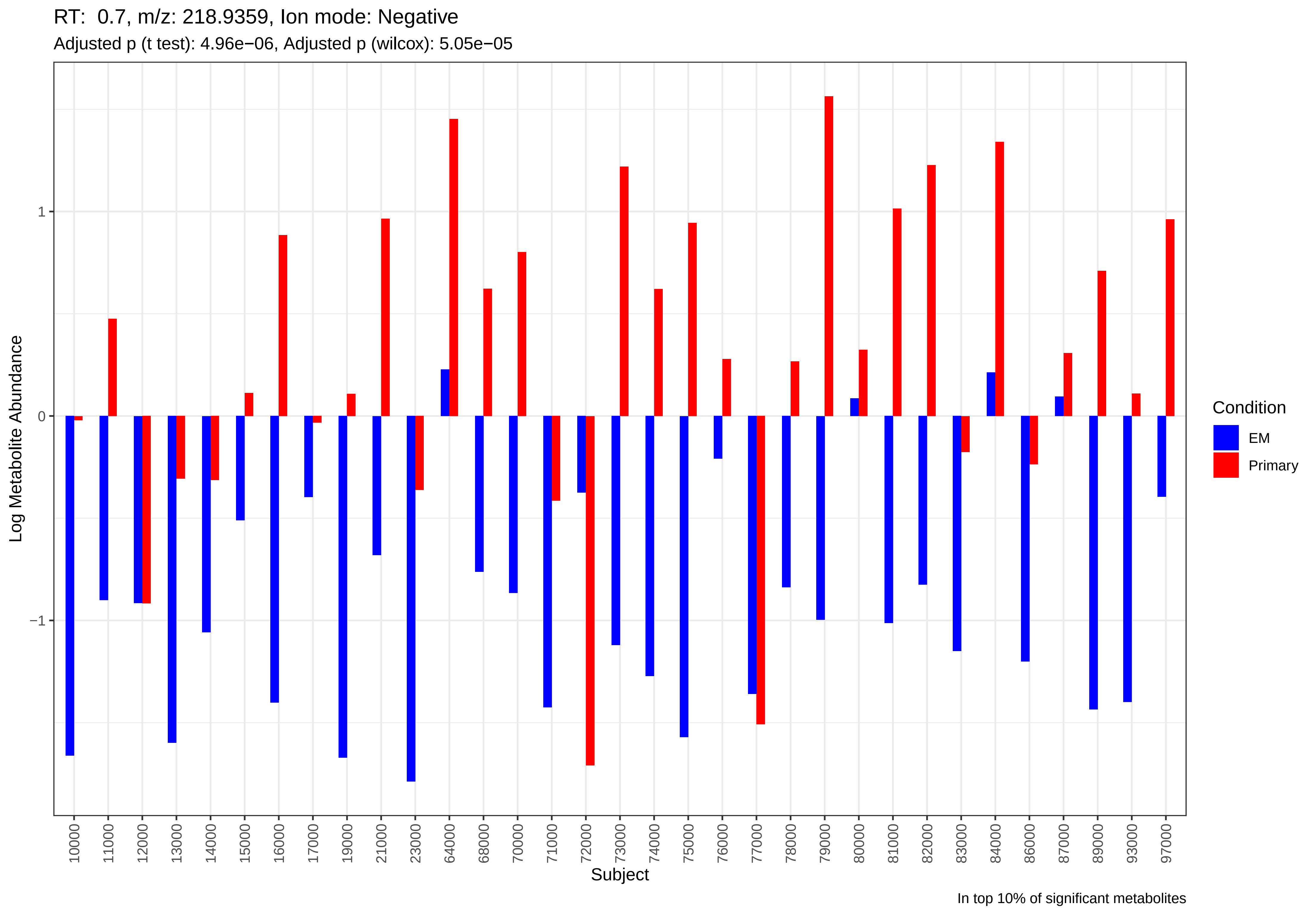
RT: 0.7, m/z: 218.9359, Ion mode: Negative
Adjusted p (t test): 4.96e−06, Adjusted p (wilcox): 5.05e−05
Log Metabolite Abundance
1
0
−1
10000
11000
12000
13000
14000
15000
16000
17000
19000
21000
23000
64000
68000
70000
71000
72000
73000
74000
75000
76000
77000
78000
79000
80000
81000
82000
83000
84000
86000
87000
89000
93000
97000
Subject
Condition
EM
Primary
In top 10% of significant metabolites

RT: 0.7, m/z: 90.9766, Ion mode: Positive

Adjusted p (t test): 4.96e−06, Adjusted p (wilcox): 4.19e−05

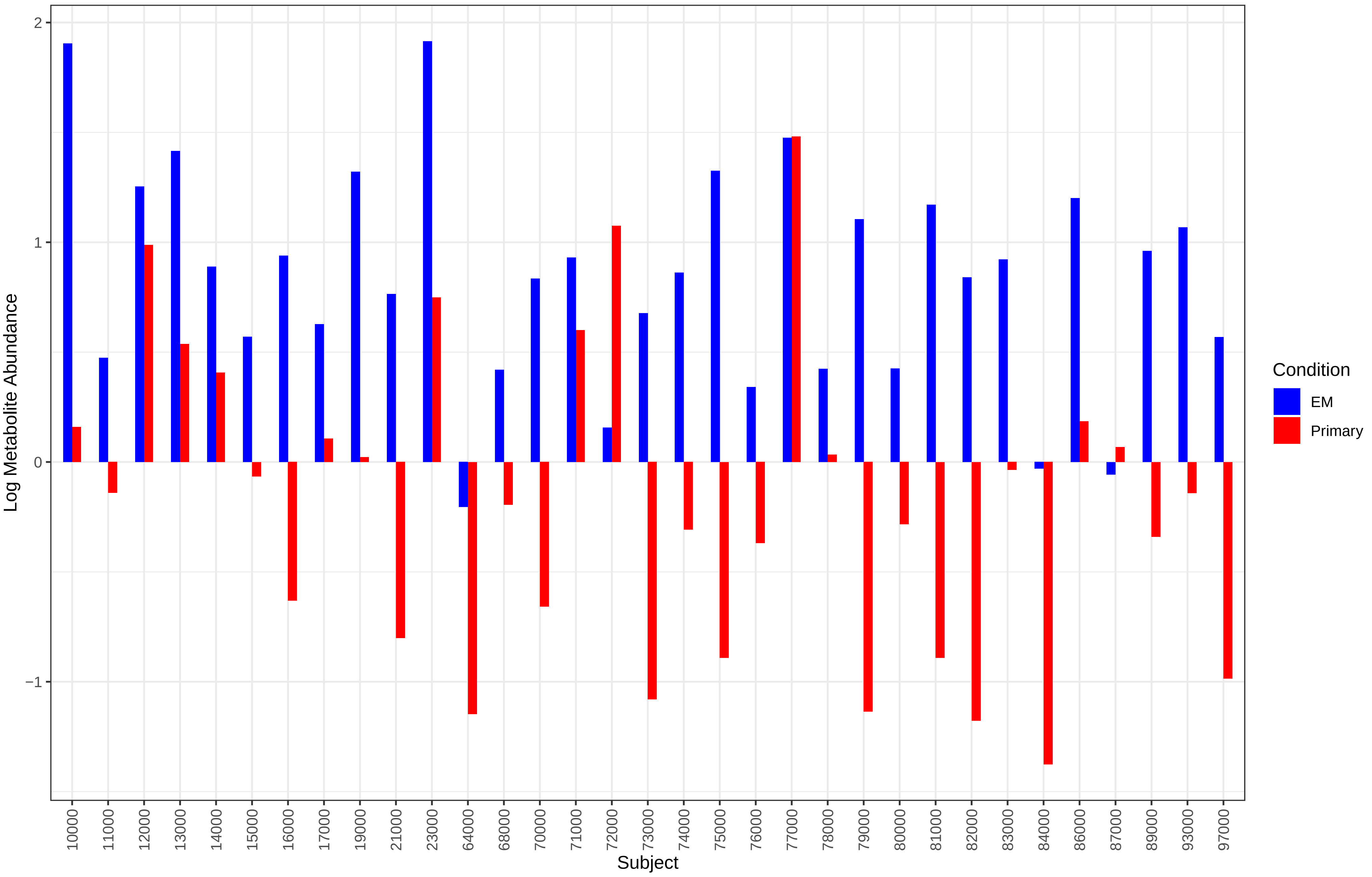

In top 10% of significant metabolites

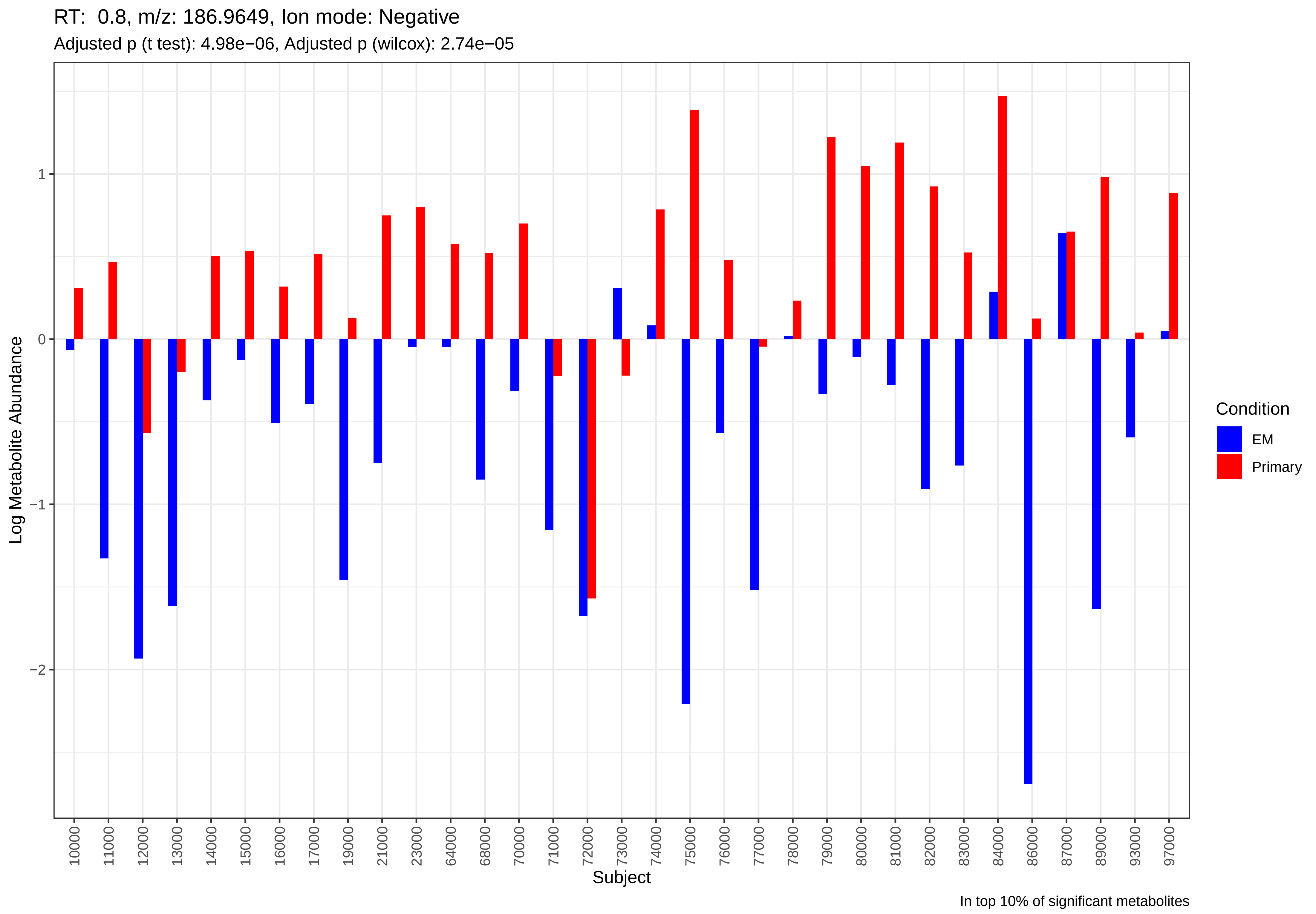
RT: 0.8, m/z: 186.9649, Ion mode: Negative
Adjusted p (t test): 4.98e−06, Adjusted p (wilcox): 2.74e−05
Log Metabolite Abundance
1
0
−1
−2
Subject
10000
11000
12000
13000
14000
15000
16000
17000
19000
21000
23000
64000
68000
70000
71000
72000
73000
74000
75000
76000
77000
78000
79000
80000
81000
82000
83000
84000
86000
87000
89000
93000
97000
Condition
EM
Primary
In top 10% of significant metabolites

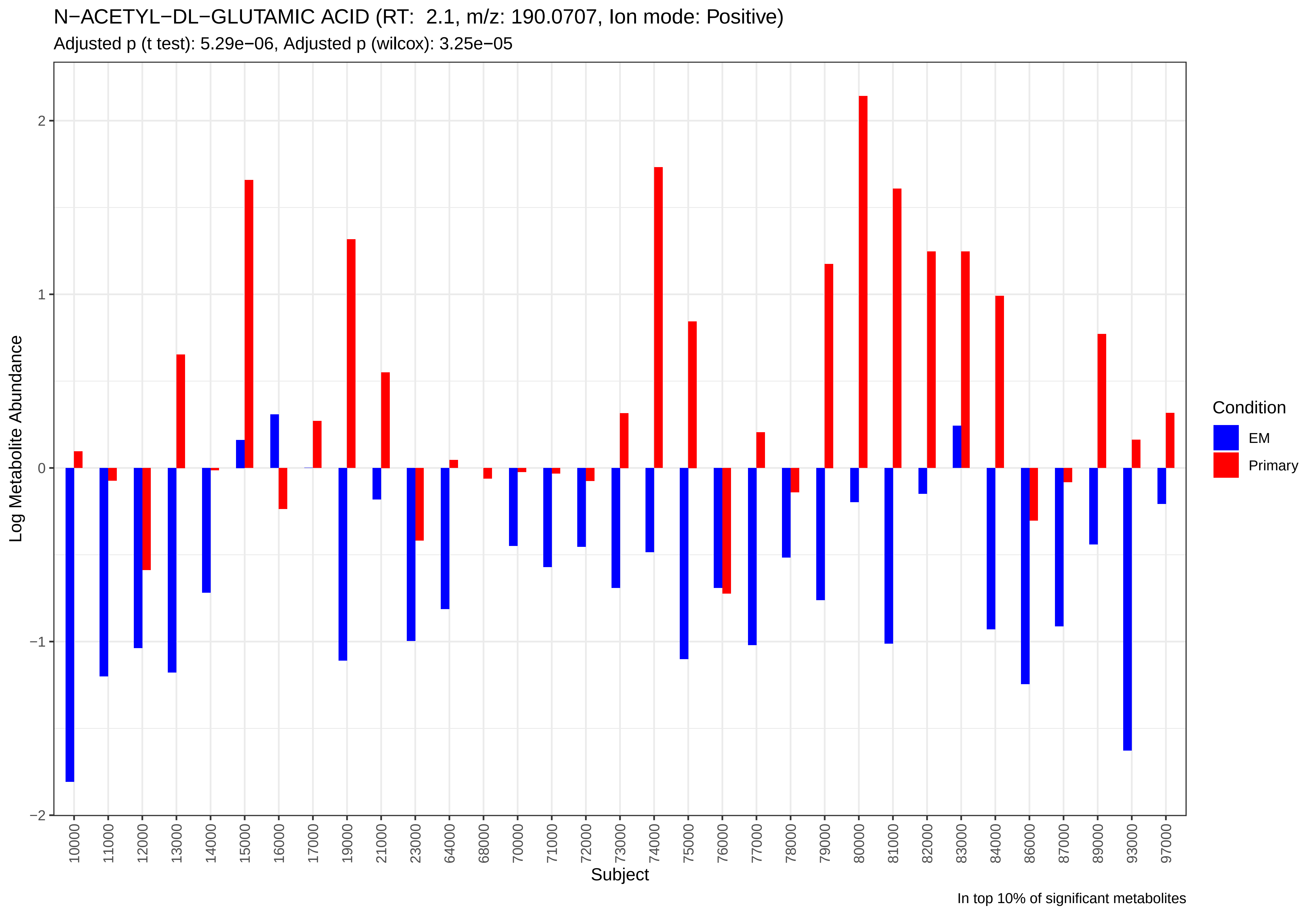
N−ACETYL−DL−GLUTAMIC ACID (RT: 2.1, m/z: 190.0707, Ion mode: Positive)
Adjusted p (t test): 5.29e−06, Adjusted p (wilcox): 3.25e−05
Log Metabolite Abundance
2
1
0
−1
−2
10000
11000
12000
13000
14000
15000
16000
17000
19000
21000
23000
64000
68000
70000
71000
72000
73000
74000
75000
76000
77000
78000
79000
80000
81000
82000
83000
84000
86000
87000
89000
93000
97000
Subject
Condition
EM
Primary
In top 10% of significant metabolites

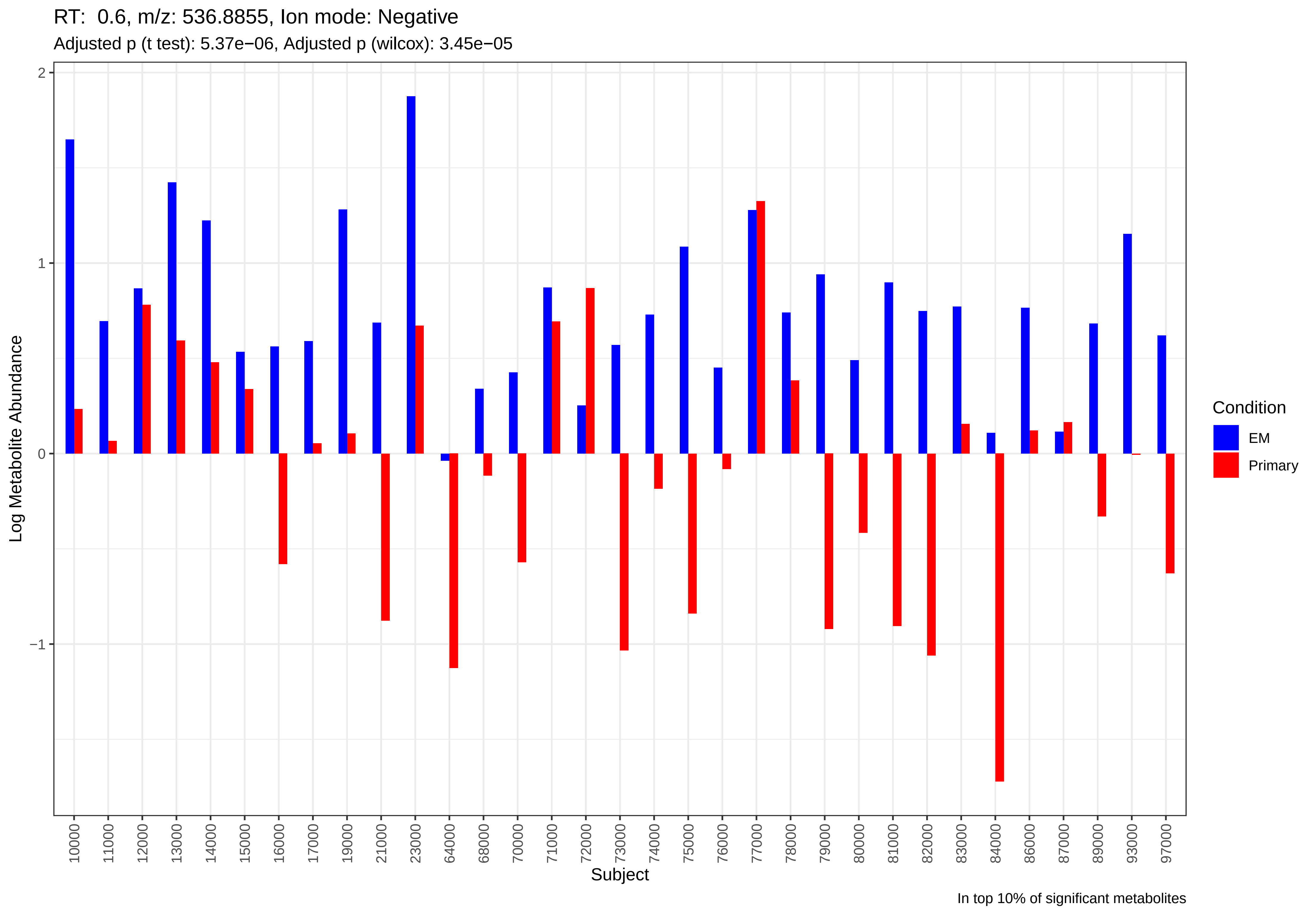

RT: 0.6, m/z: 536.8855, Ion mode: Negative
Adjusted p (t test): 5.37e−06, Adjusted p (wilcox): 3.45e−05
Log Metabolite Abundance
2
1
0
−1
Subject
10000
11000
12000
13000
14000
15000
16000
17000
19000
21000
23000
64000
68000
70000
71000
72000
73000
74000
75000
76000
77000
78000
79000
80000
81000
82000
83000
84000
86000
87000
89000
93000
97000
Condition
EM
Primary
In top 10% of significant metabolites

Complex of 91.0576 and 177.0067 m/z (RT: 0.8, m/z: 267.0562, Ion mode: Positive)
Adjusted p (t test): 5.37e−06, Adjusted p (wilcox): 2.74e−05

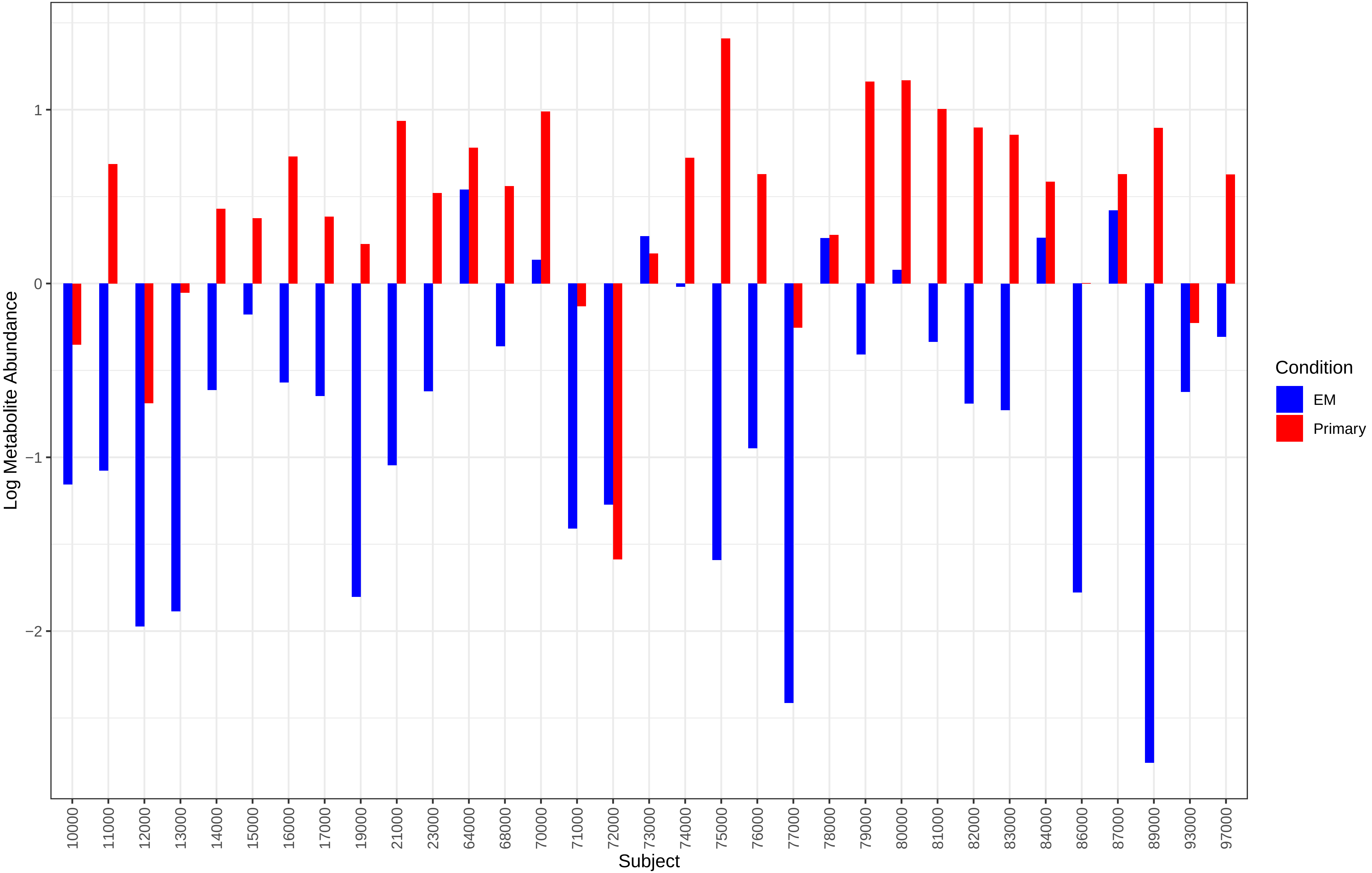

In top 10% of significant metabolites

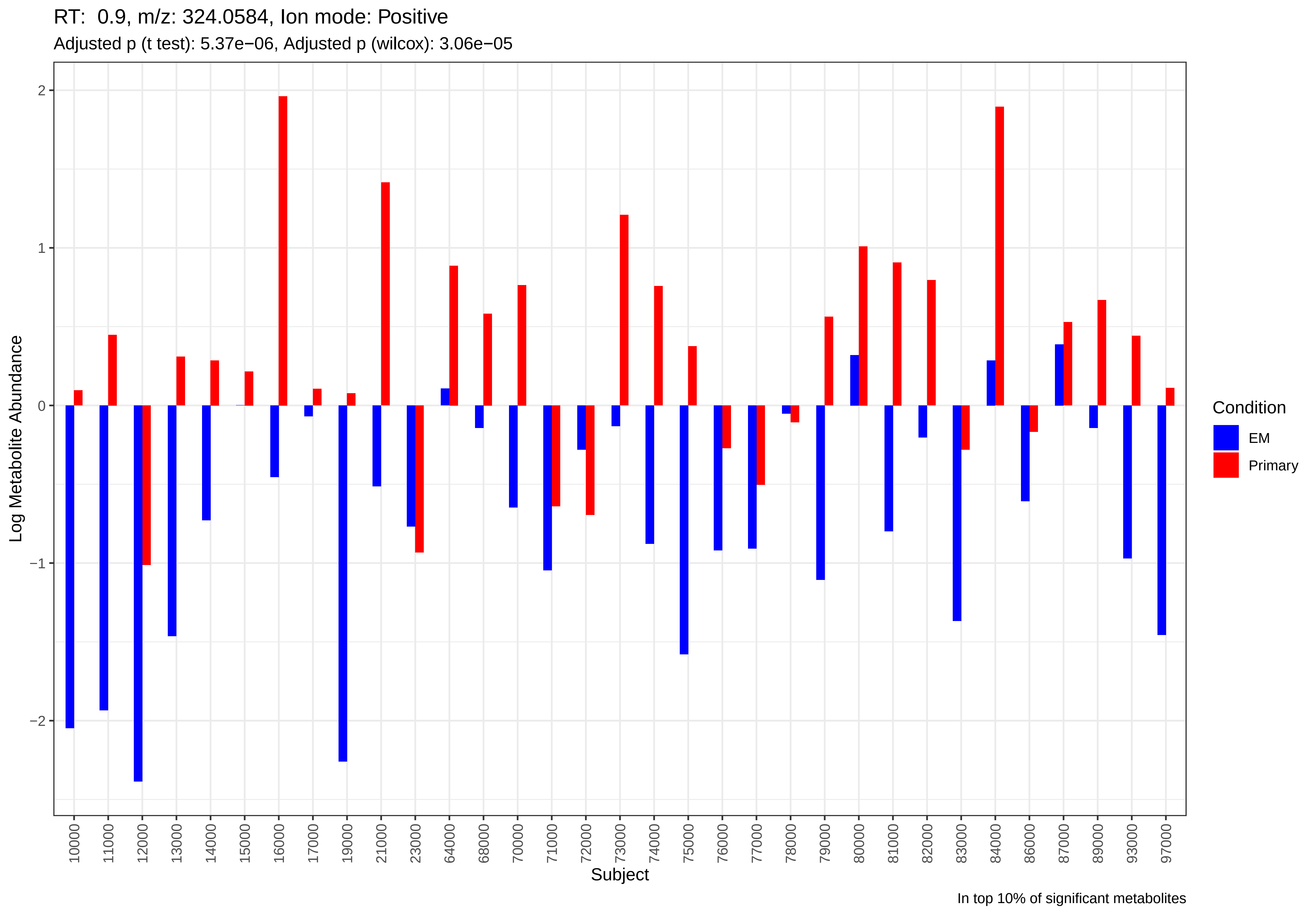
RT: 0.9, m/z: 324.0584, Ion mode: Positive
Adjusted p (t test): 5.37e−06, Adjusted p (wilcox): 3.06e−05
Log Metabolite Abundance
2
1
0
−1
−2
Subject
10000
11000
12000
13000
14000
15000
16000
17000
19000
21000
23000
64000
68000
70000
71000
72000
73000
74000
75000
76000
77000
78000
79000
80000
81000
82000
83000
84000
86000
87000
89000
93000
97000
Condition
EM
Primary
In top 10% of significant metabolites

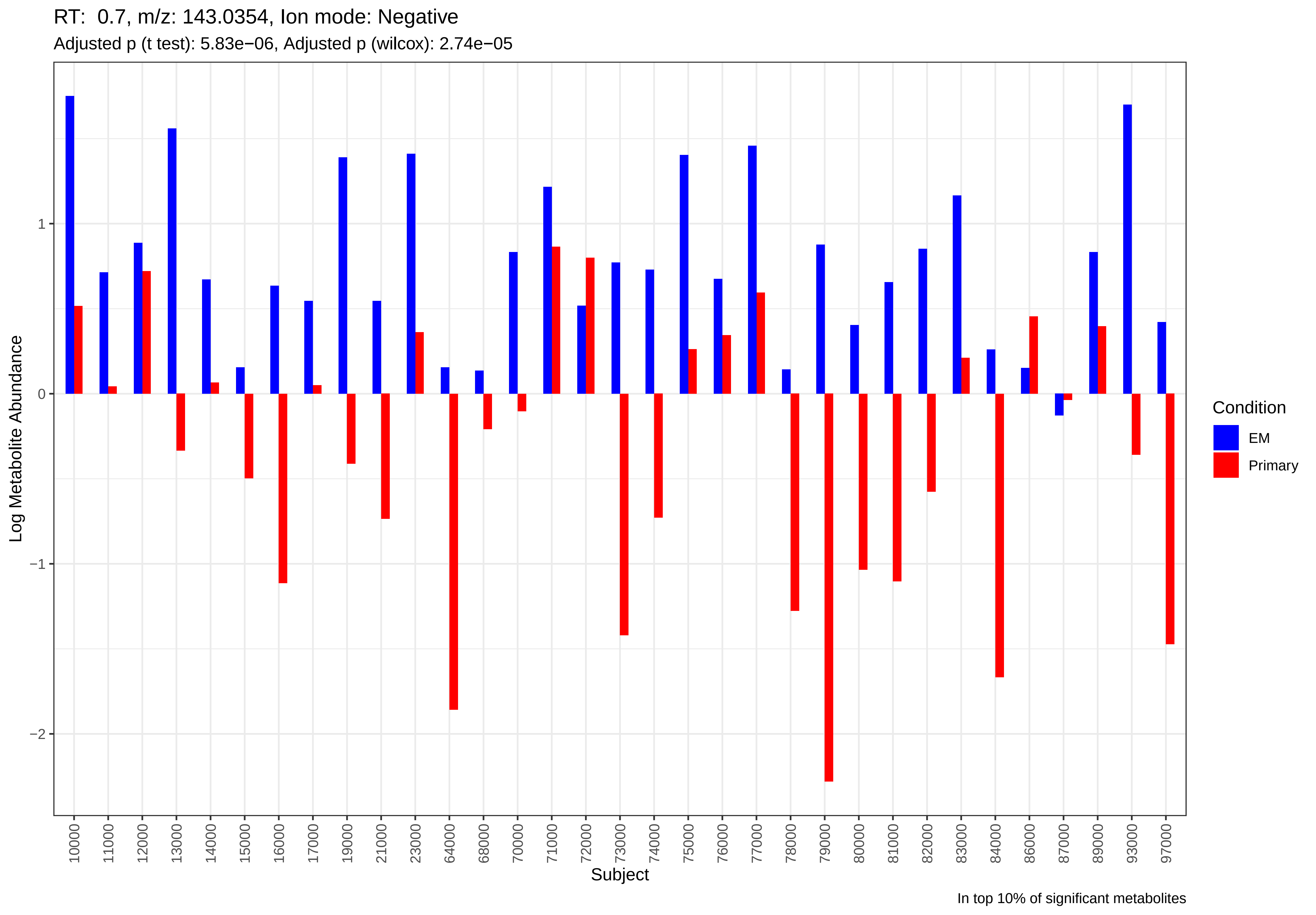

RT: 0.7, m/z: 143.0354, Ion mode: Negative
Adjusted p (t test): 5.83e−06, Adjusted p (wilcox): 2.74e−05
Log Metabolite Abundance
1
0
−1
−2
10000
11000
12000
13000
14000
15000
16000
17000
19000
21000
23000
64000
68000
70000
71000
72000
73000
74000
75000
76000
77000
78000
79000
80000
81000
82000
83000
84000
86000
87000
89000
93000
97000
Subject
Condition
EM
Primary
In top 10% of significant metabolites

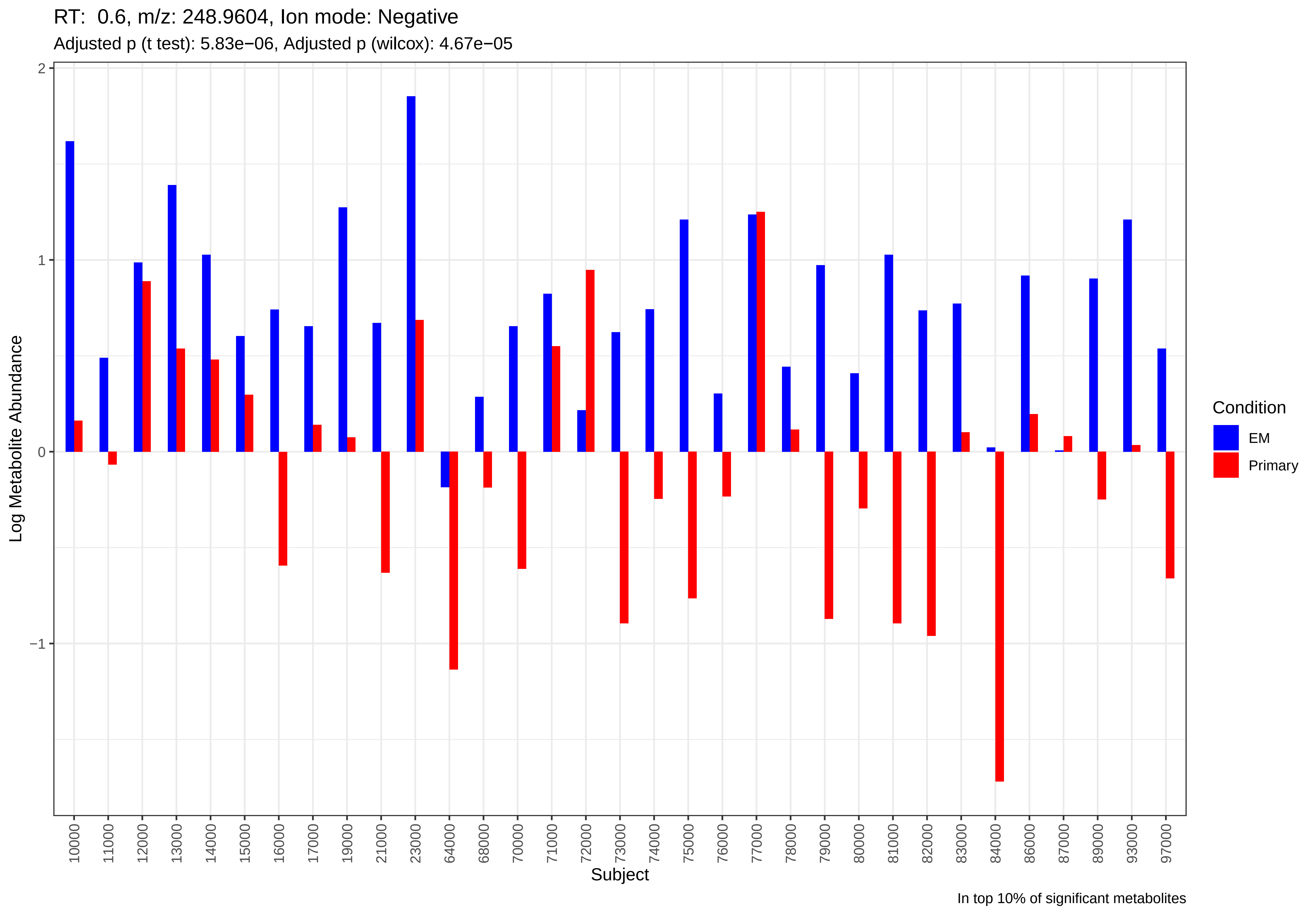

RT: 0.6, m/z: 248.9604, Ion mode: Negative
Adjusted p (t test): 5.83e−06, Adjusted p (wilcox): 4.67e−05
Log Metabolite Abundance
2
1
0
−1
10000
11000
12000
13000
14000
15000
16000
17000
19000
21000
23000
64000
68000
70000
71000
72000
73000
74000
75000
76000
77000
78000
79000
80000
81000
82000
83000
84000
86000
87000
89000
93000
97000
Subject
Condition
EM
Primary
In top 10% of significant metabolites

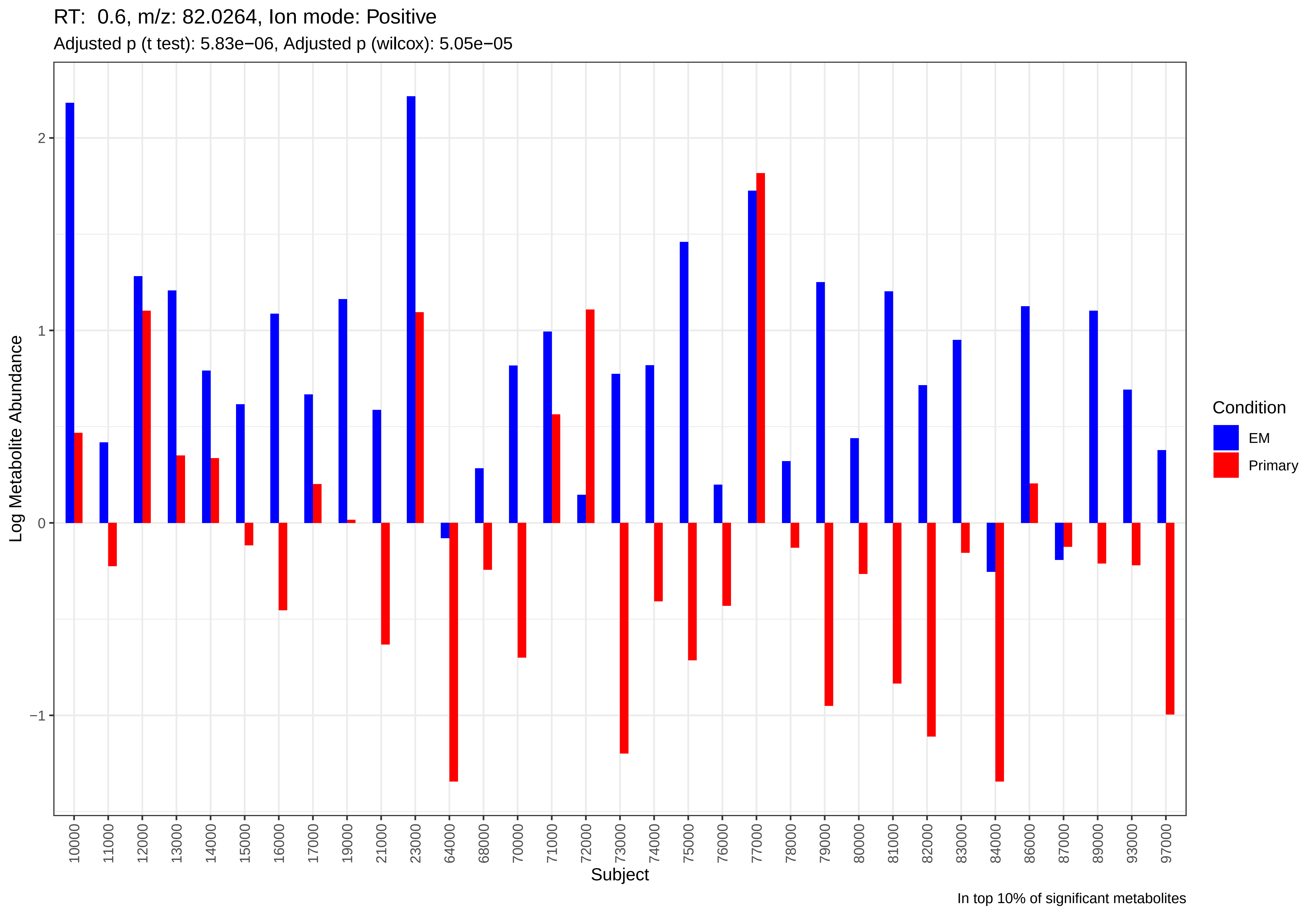
RT: 0.6, m/z: 82.0264, Ion mode: Positive
Adjusted p (t test): 5.83e−06, Adjusted p (wilcox): 5.05e−05
Log Metabolite Abundance
2
1
0
−1
10000
11000
12000
13000
14000
15000
16000
17000
19000
21000
23000
64000
68000
70000
71000
72000
73000
74000
75000
76000
77000
78000
79000
80000
81000
82000
83000
84000
86000
87000
89000
93000
97000
Subject
Condition
EM
Primary
In top 10% of significant metabolites

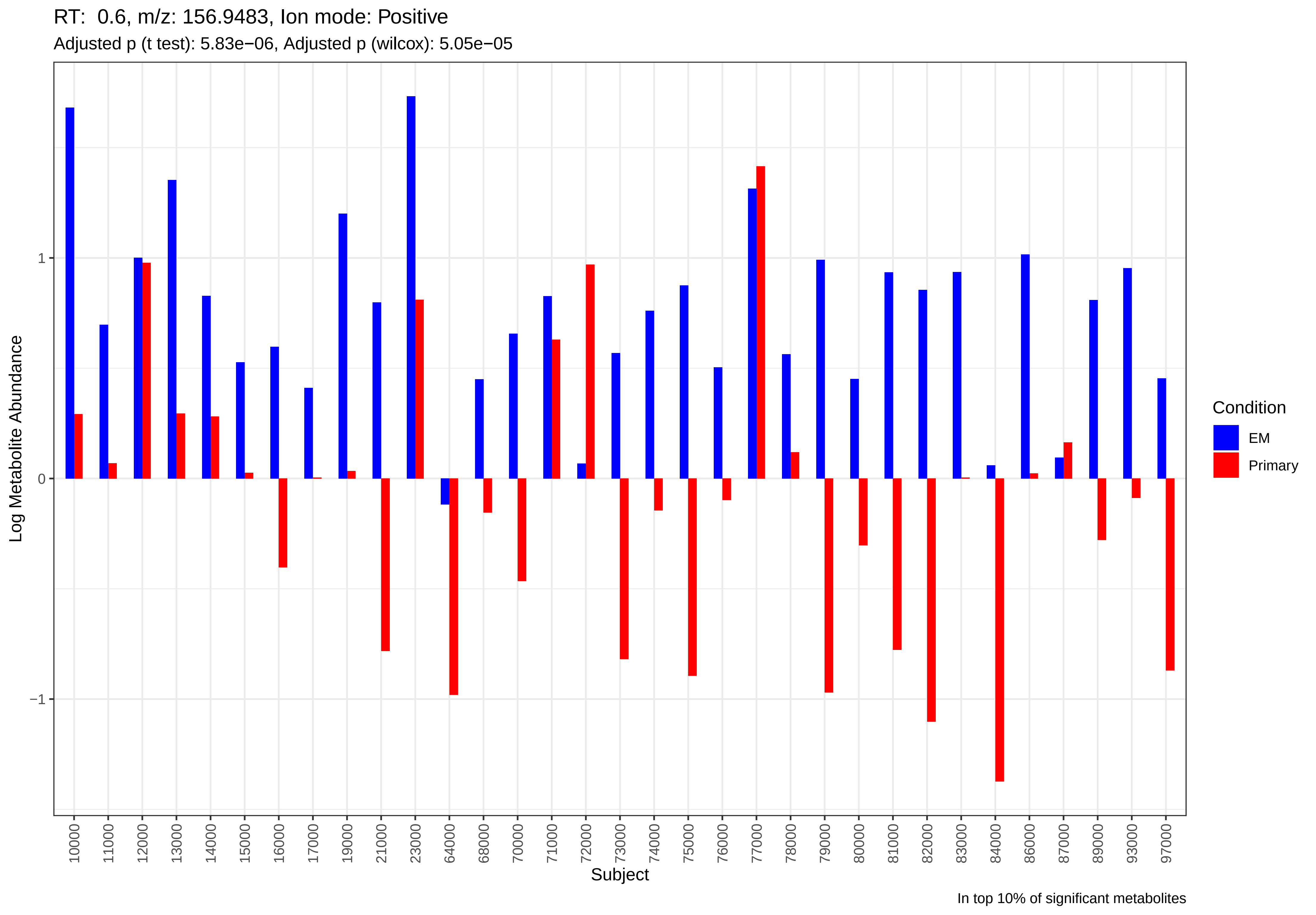

RT: 0.6, m/z: 156.9483, Ion mode: Positive
Adjusted p (t test): 5.83e−06, Adjusted p (wilcox): 5.05e−05
Log Metabolite Abundance
1
0
−1
Subject
10000
11000
12000
13000
14000
15000
16000
17000
19000
21000
23000
64000
68000
70000
71000
72000
73000
74000
75000
76000
77000
78000
79000
80000
81000
82000
83000
84000
86000
87000
89000
93000
97000
Condition
EM
Primary
In top 10% of significant metabolites

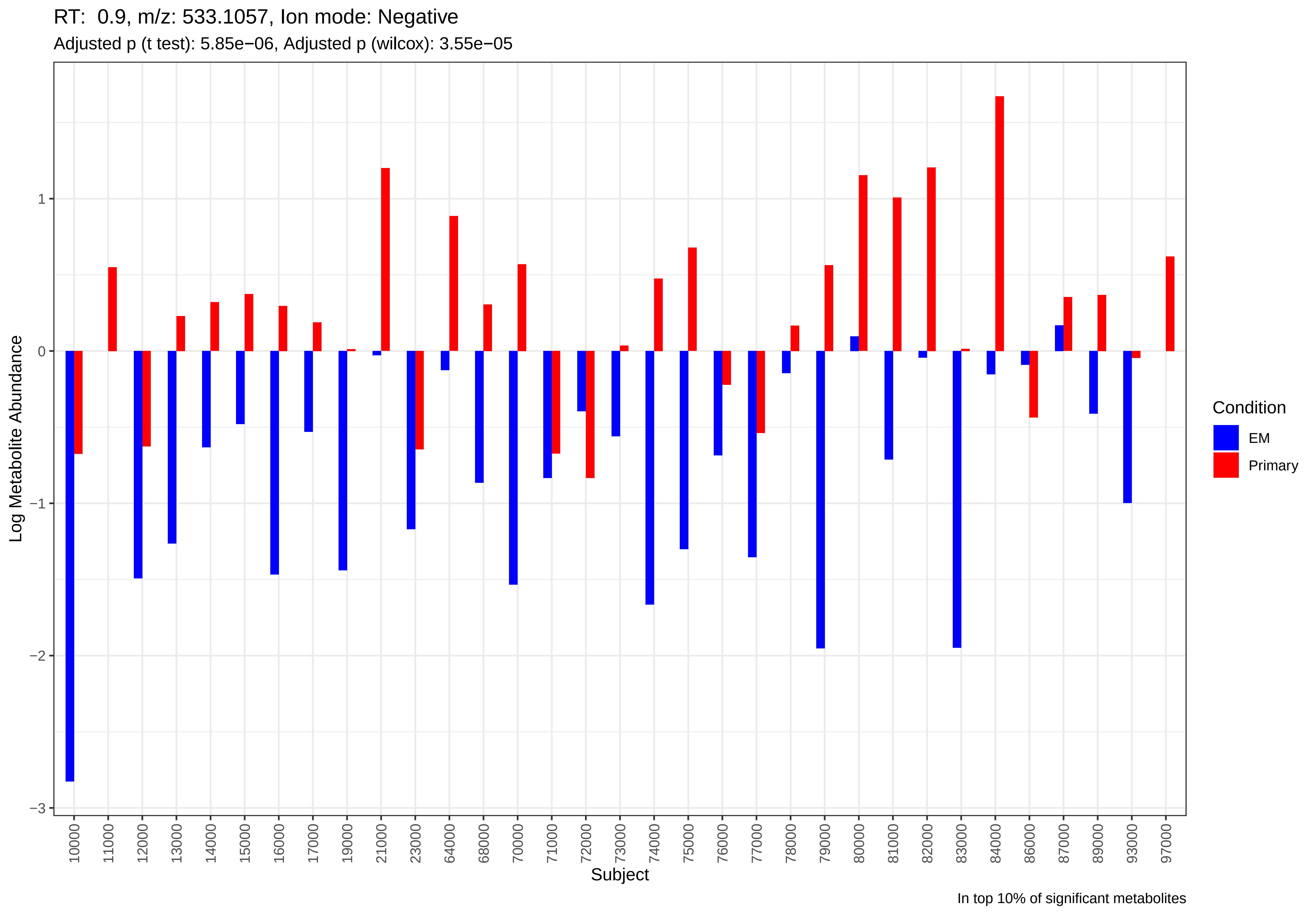
RT: 0.9, m/z: 533.1057, Ion mode: Negative
Adjusted p (t test): 5.85e−06, Adjusted p (wilcox): 3.55e−05
Log Metabolite Abundance
1
0
−1
−2
−3
10000
11000
12000
13000
14000
15000
16000
17000
19000
21000
23000
64000
68000
70000
71000
72000
73000
74000
75000
76000
77000
78000
79000
80000
81000
82000
83000
84000
86000
87000
89000
93000
97000
Subject
Condition
EM
Primary
In top 10% of significant metabolites

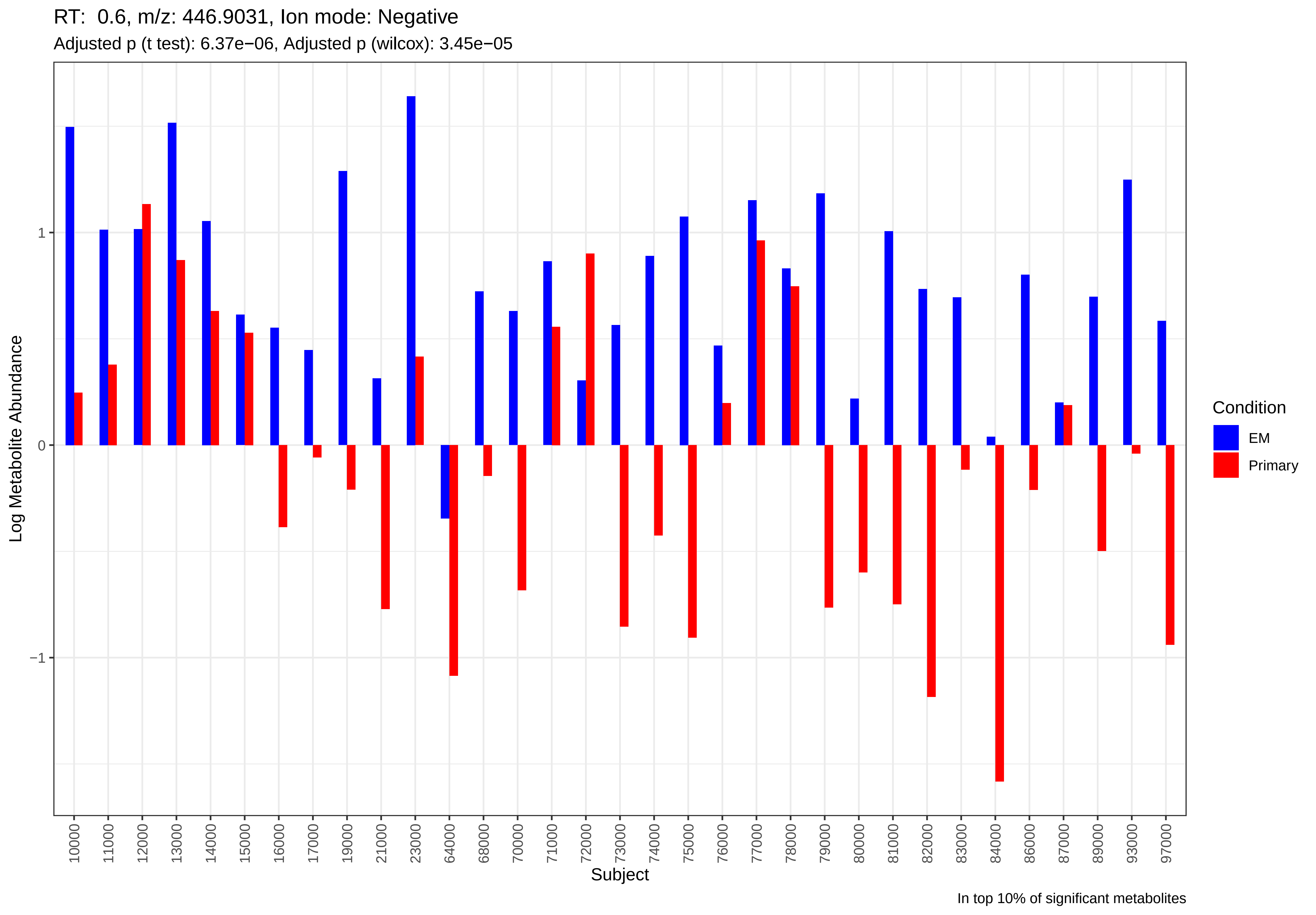
RT: 0.6, m/z: 446.9031, Ion mode: Negative
Adjusted p (t test): 6.37e−06, Adjusted p (wilcox): 3.45e−05
Log Metabolite Abundance
1
0
−1
10000
11000
12000
13000
14000
15000
16000
17000
19000
21000
23000
64000
68000
70000
71000
72000
73000
74000
75000
76000
77000
78000
79000
80000
81000
82000
83000
84000
86000
87000
89000
93000
97000
Subject
Condition
EM
Primary
In top 10% of significant metabolites

RT: 0.7, m/z: 181.9661, Ion mode: Negative

Adjusted p (t test): 6.37e−06, Adjusted p (wilcox): 2.75e−05

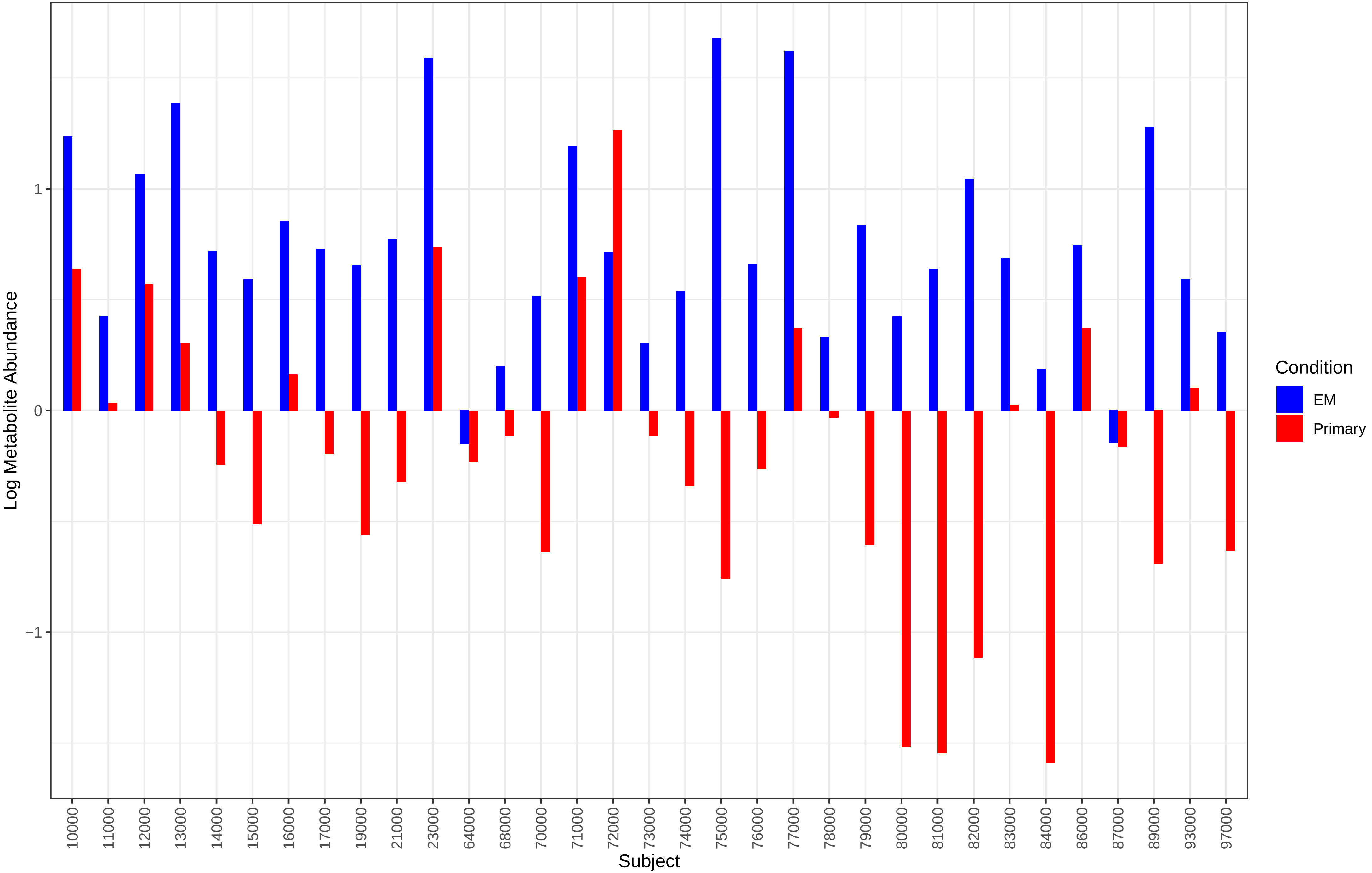

In top 10% of significant metabolites

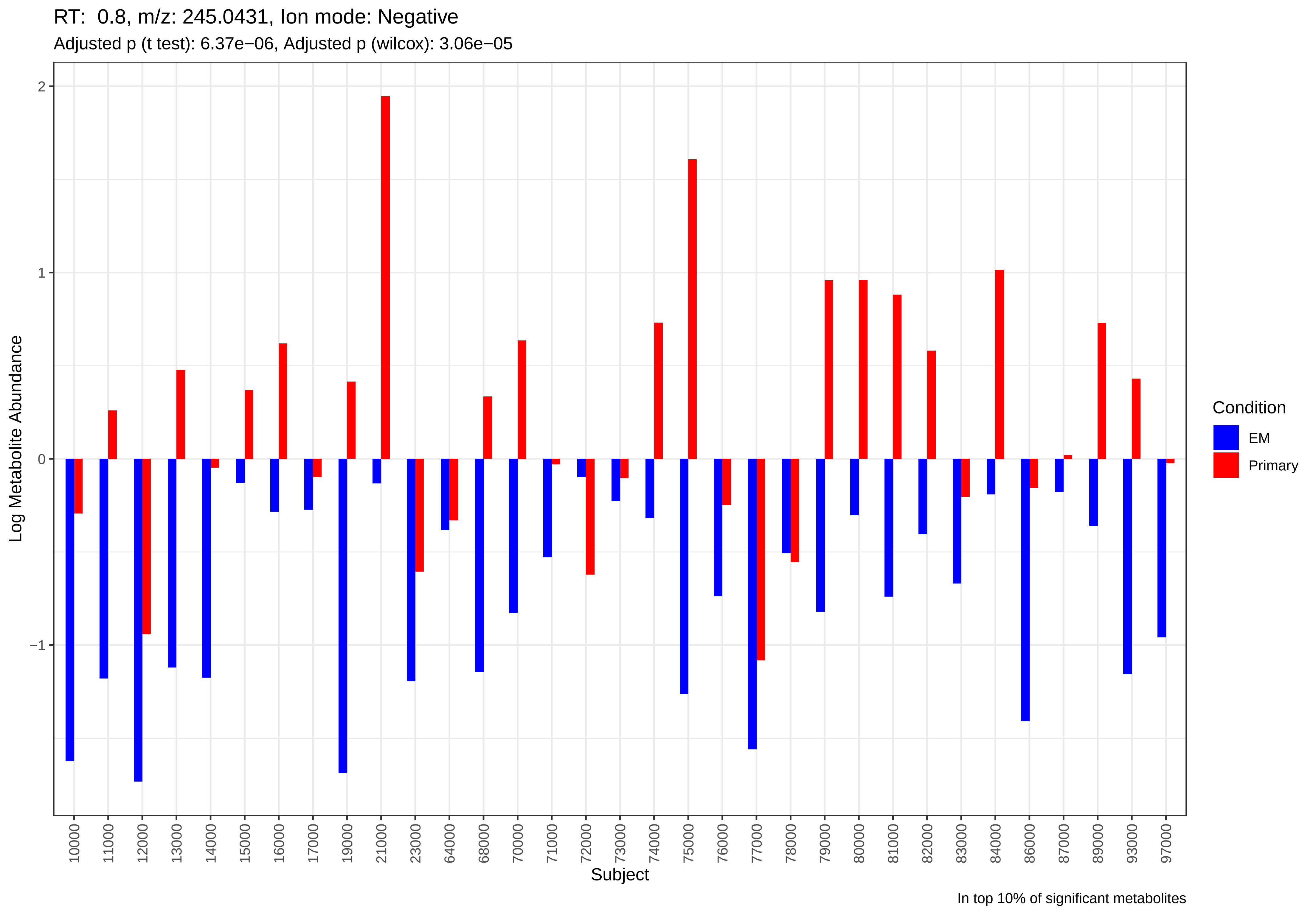

RT: 0.8, m/z: 245.0431, Ion mode: Negative
Adjusted p (t test): 6.37e−06, Adjusted p (wilcox): 3.06e−05
Log Metabolite Abundance
2
1
0
−1
10000
11000
12000
13000
14000
15000
16000
17000
19000
21000
23000
64000
68000
70000
71000
72000
73000
74000
75000
76000
77000
78000
79000
80000
81000
82000
83000
84000
86000
87000
89000
93000
97000
Subject
Condition
EM
Primary
In top 10% of significant metabolites

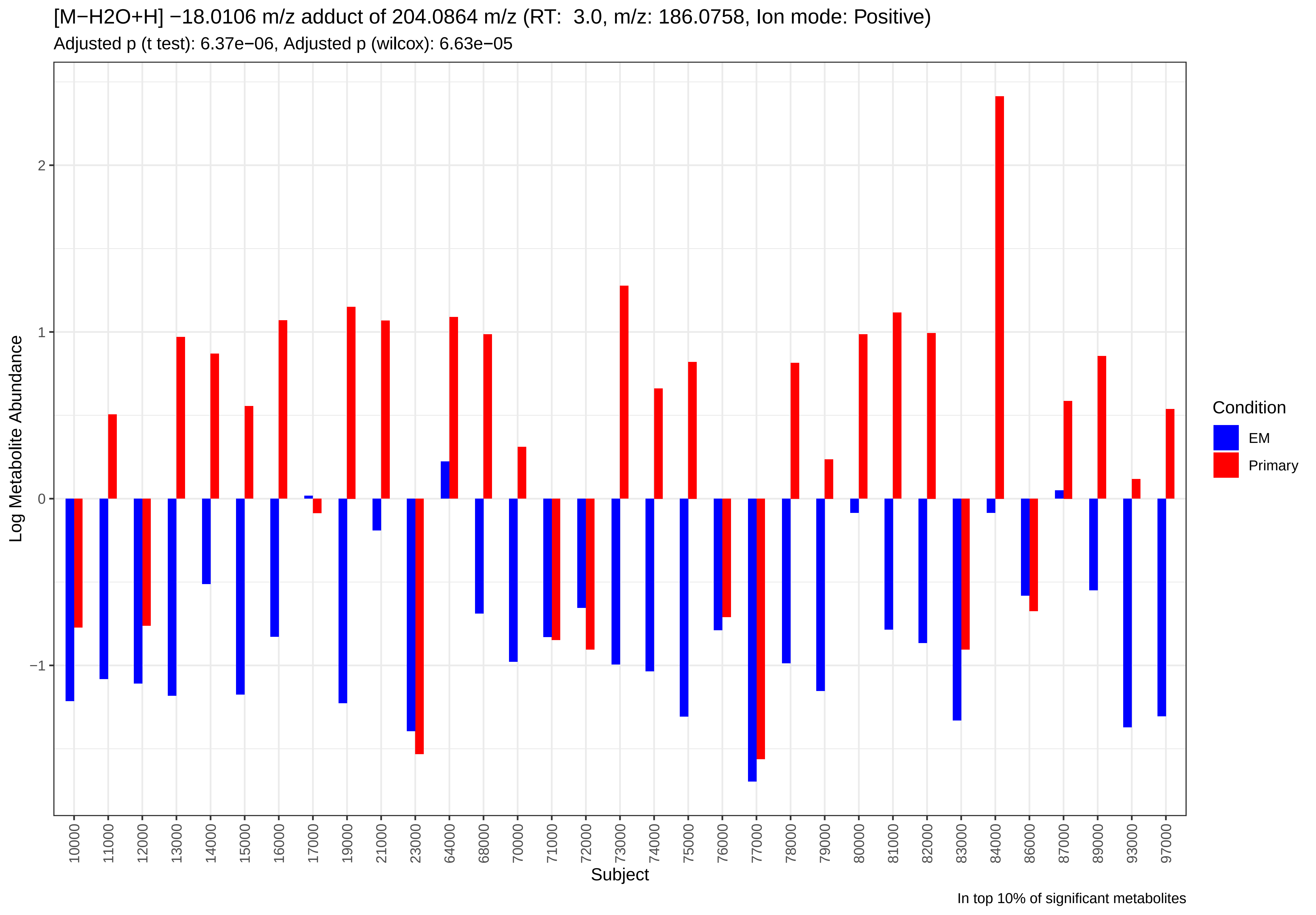

[M−H2O+H] −18.0106 m/z adduct of 204.0864 m/z (RT: 3.0, m/z: 186.0758, Ion mode: Positive)
Adjusted p (t test): 6.37e−06, Adjusted p (wilcox): 6.63e−05
Log Metabolite Abundance
2
1
0
−1
10000
11000
12000
13000
14000
15000
16000
17000
19000
21000
23000
64000
68000
70000
71000
72000
73000
74000
75000
76000
77000
78000
79000
80000
81000
82000
83000
84000
86000
87000
89000
93000
97000
Subject
Condition
EM
Primary
In top 10% of significant metabolites

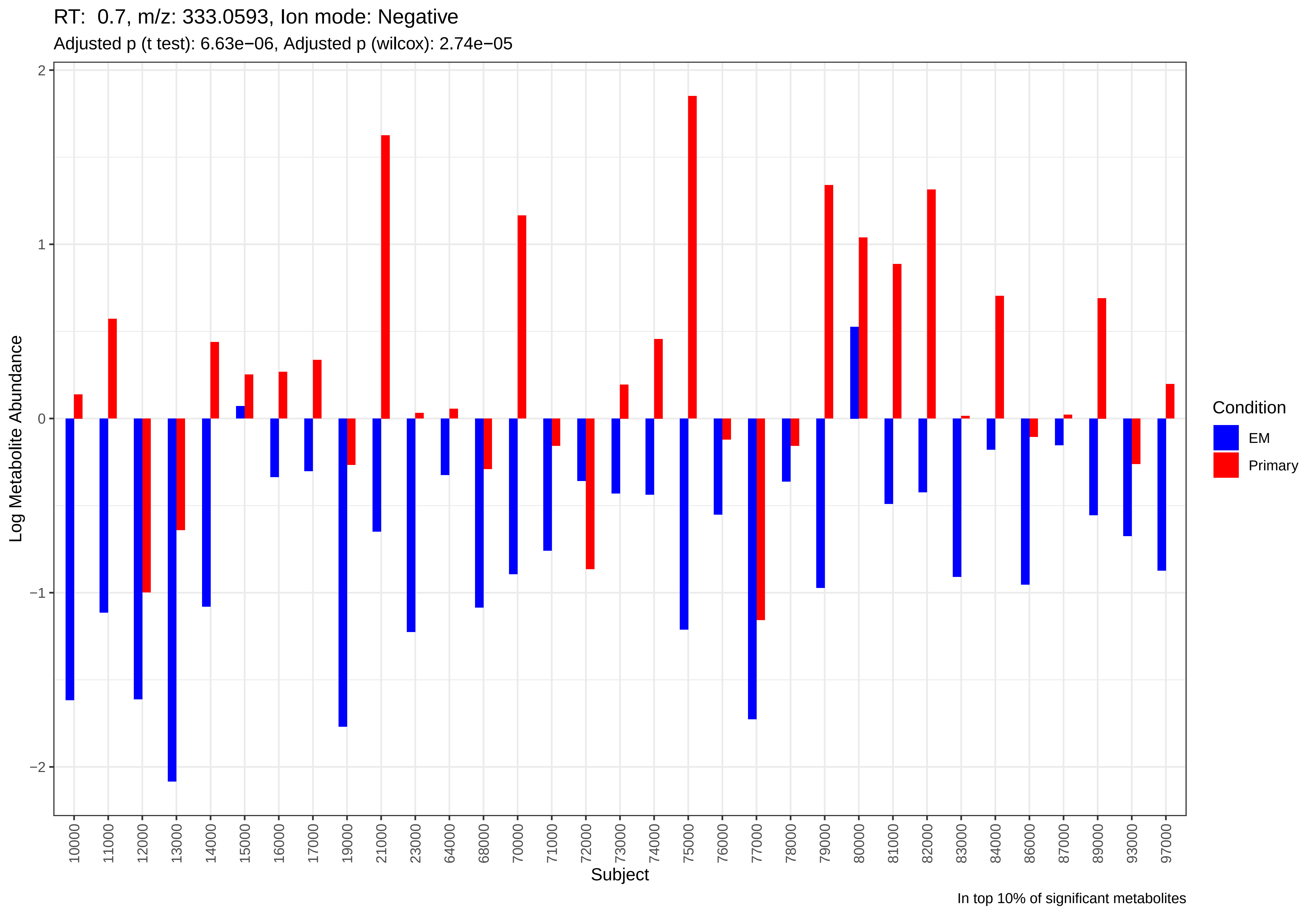
RT: 0.7, m/z: 333.0593, Ion mode: Negative
Adjusted p (t test): 6.63e−06, Adjusted p (wilcox): 2.74e−05
Log Metabolite Abundance
Subject
Condition
EM
Primary
2
1
0
−1
−2
10000
11000
12000
13000
14000
15000
16000
17000
19000
21000
23000
64000
68000
70000
71000
72000
73000
74000
75000
76000
77000
78000
79000
80000
81000
82000
83000
84000
86000
87000
89000
93000
97000
In top 10% of significant metabolites

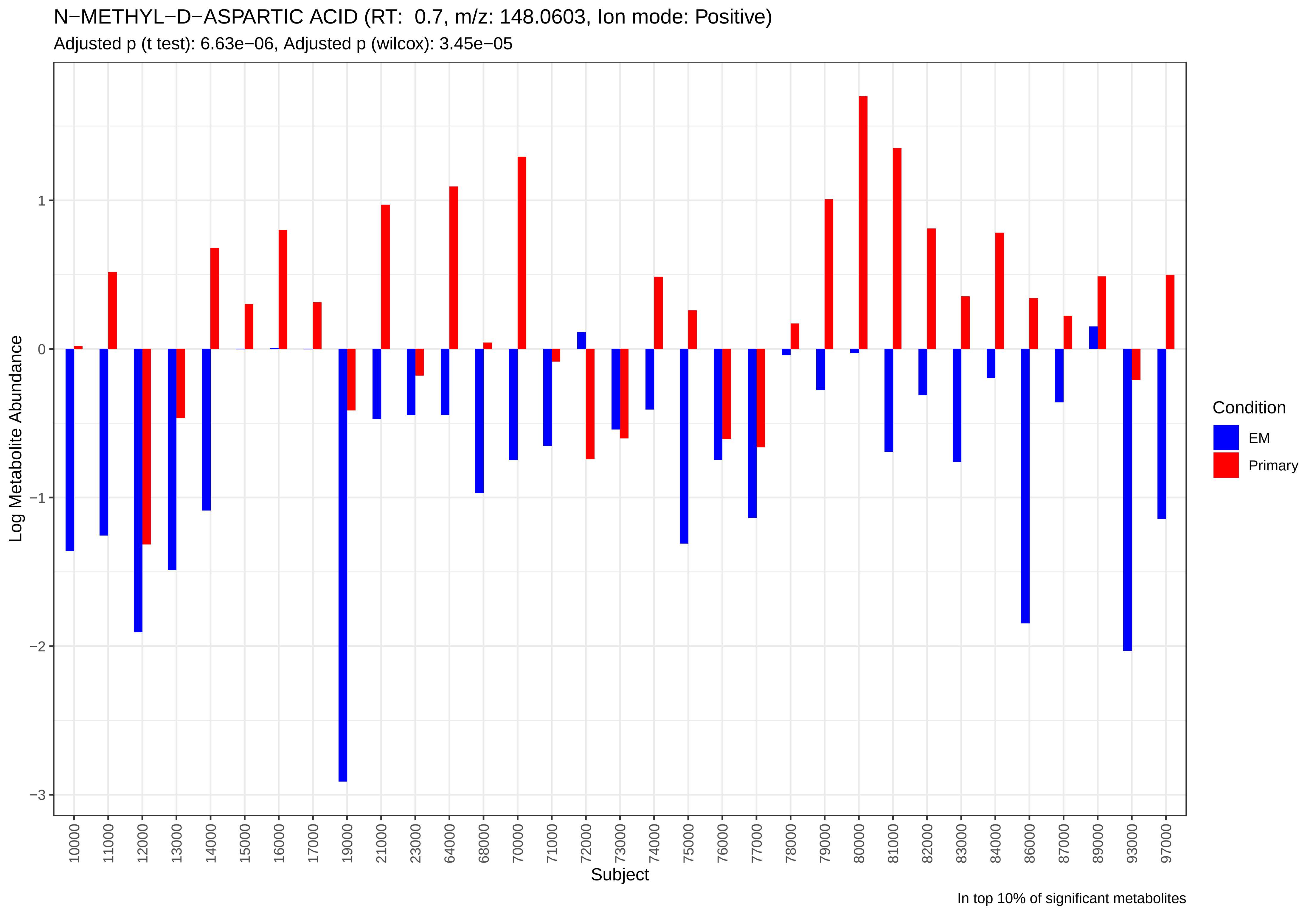
N−METHYL−D−ASPARTIC ACID (RT: 0.7, m/z: 148.0603, Ion mode: Positive)
Adjusted p (t test): 6.63e−06, Adjusted p (wilcox): 3.45e−05
Log Metabolite Abundance
1
0
−1
−2
−3
10000
11000
12000
13000
14000
15000
16000
17000
19000
21000
23000
64000
68000
70000
71000
72000
73000
74000
75000
76000
77000
78000
79000
80000
81000
82000
83000
84000
86000
87000
89000
93000
97000
Subject
Condition
EM
Primary
In top 10% of significant metabolites

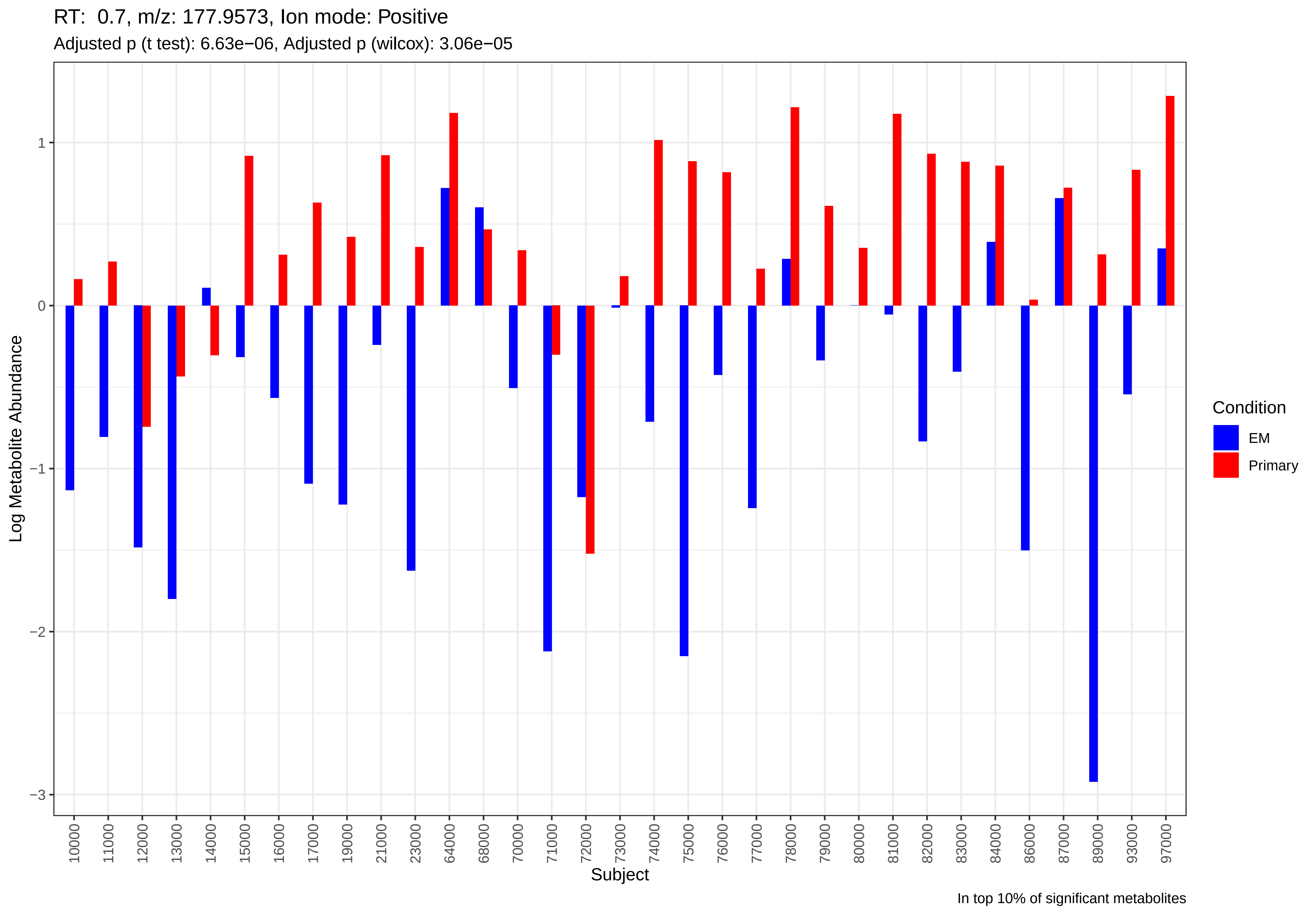
RT: 0.7, m/z: 177.9573, Ion mode: Positive
Adjusted p (t test): 6.63e−06, Adjusted p (wilcox): 3.06e−05
Log Metabolite Abundance
1
0
−1
−2
−3
10000
11000
12000
13000
14000
15000
16000
17000
19000
21000
23000
64000
68000
70000
71000
72000
73000
74000
75000
76000
77000
78000
79000
80000
81000
82000
83000
84000
86000
87000
89000
93000
97000
Subject
Condition
EM
Primary
In top 10% of significant metabolites

RT: 0.7, m/z: 150.0651, Ion mode: Positive

Adjusted p (t test): 6.63e−06, Adjusted p (wilcox): 3.25e−05

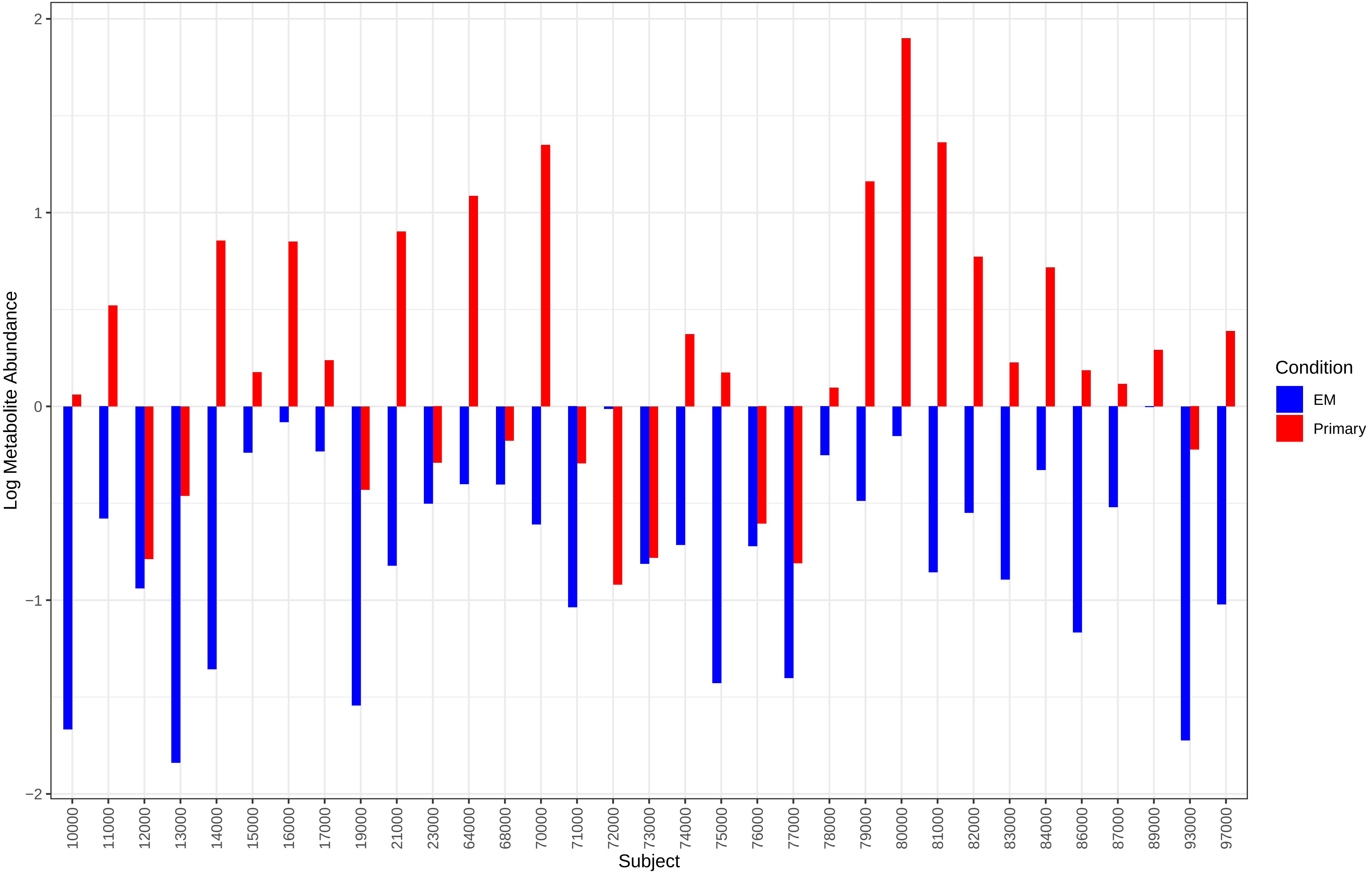

In top 10% of significant metabolites

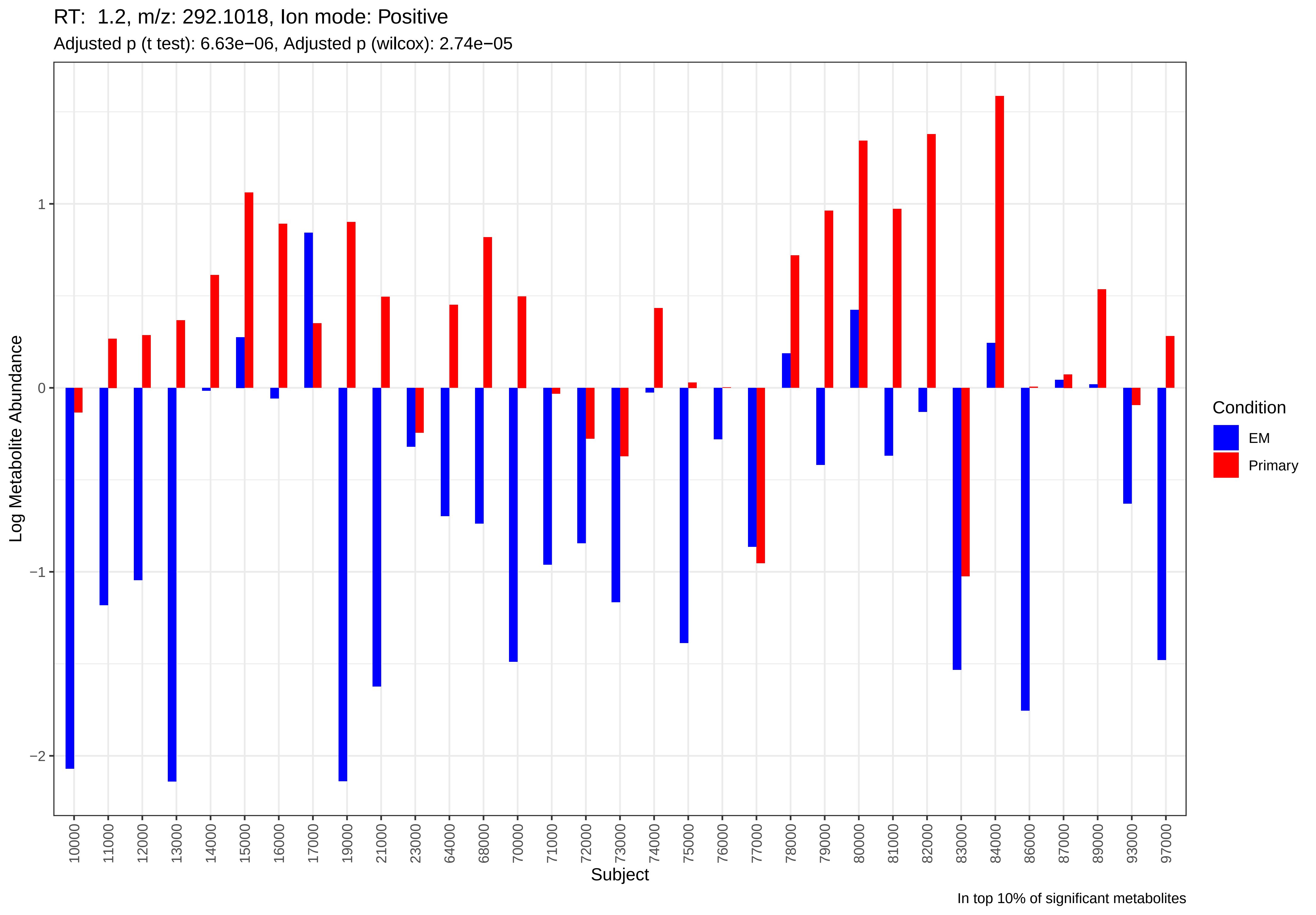
RT: 1.2, m/z: 292.1018, Ion mode: Positive
Adjusted p (t test): 6.63e−06, Adjusted p (wilcox): 2.74e−05
Log Metabolite Abundance
1
0
−1
−2
10000
11000
12000
13000
14000
15000
16000
17000
19000
21000
23000
64000
68000
70000
71000
72000
73000
74000
75000
76000
77000
78000
79000
80000
81000
82000
83000
84000
86000
87000
89000
93000
97000
Subject
Condition
EM
Primary
In top 10% of significant metabolites

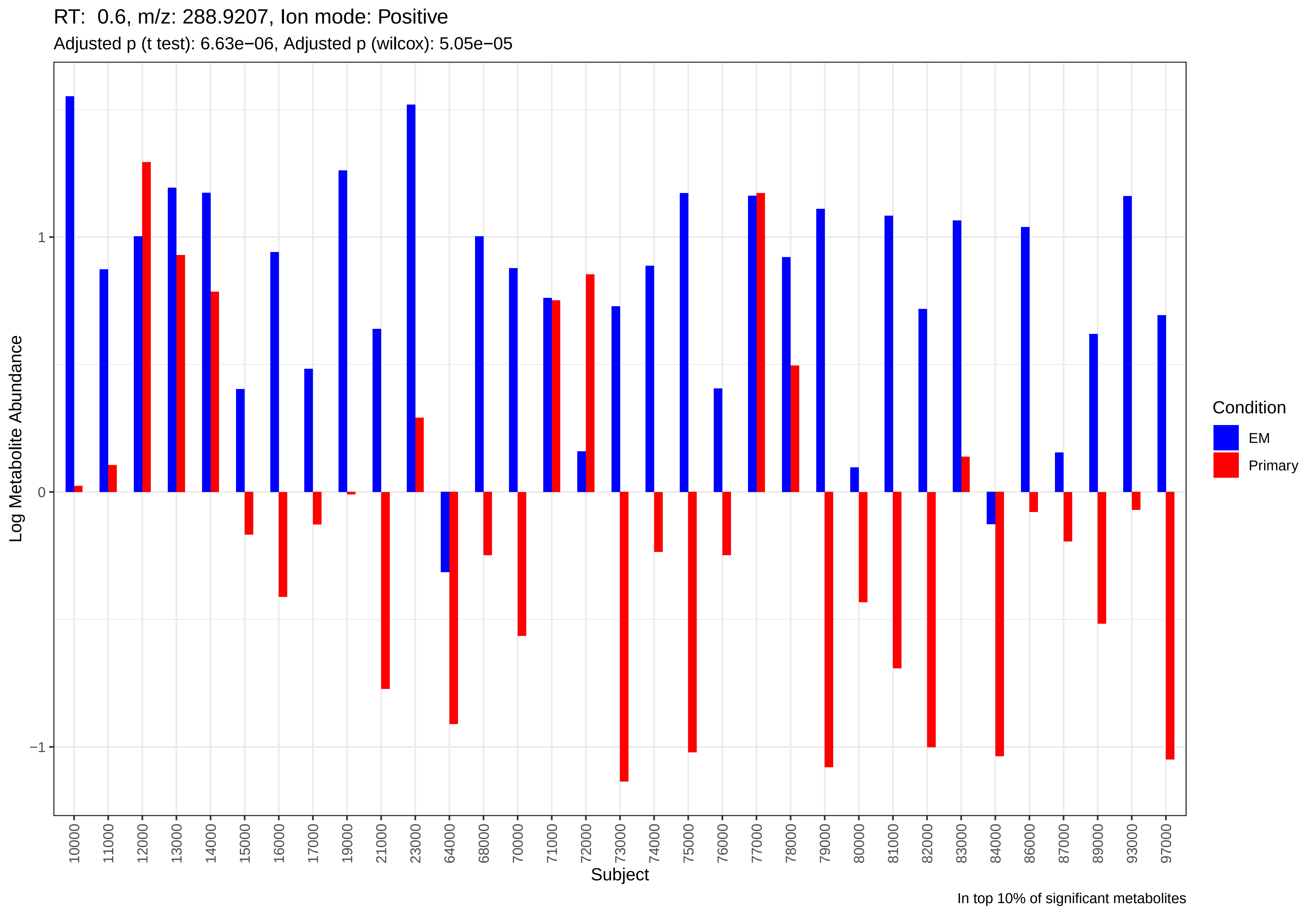
RT: 0.6, m/z: 288.9207, Ion mode: Positive
Adjusted p (t test): 6.63e−06, Adjusted p (wilcox): 5.05e−05
Log Metabolite Abundance
1
0
−1
10000
11000
12000
13000
14000
15000
16000
17000
19000
21000
23000
64000
68000
70000
71000
72000
73000
74000
75000
76000
77000
78000
79000
80000
81000
82000
83000
84000
86000
87000
89000
93000
97000
Subject
Condition
EM
Primary
In top 10% of significant metabolites

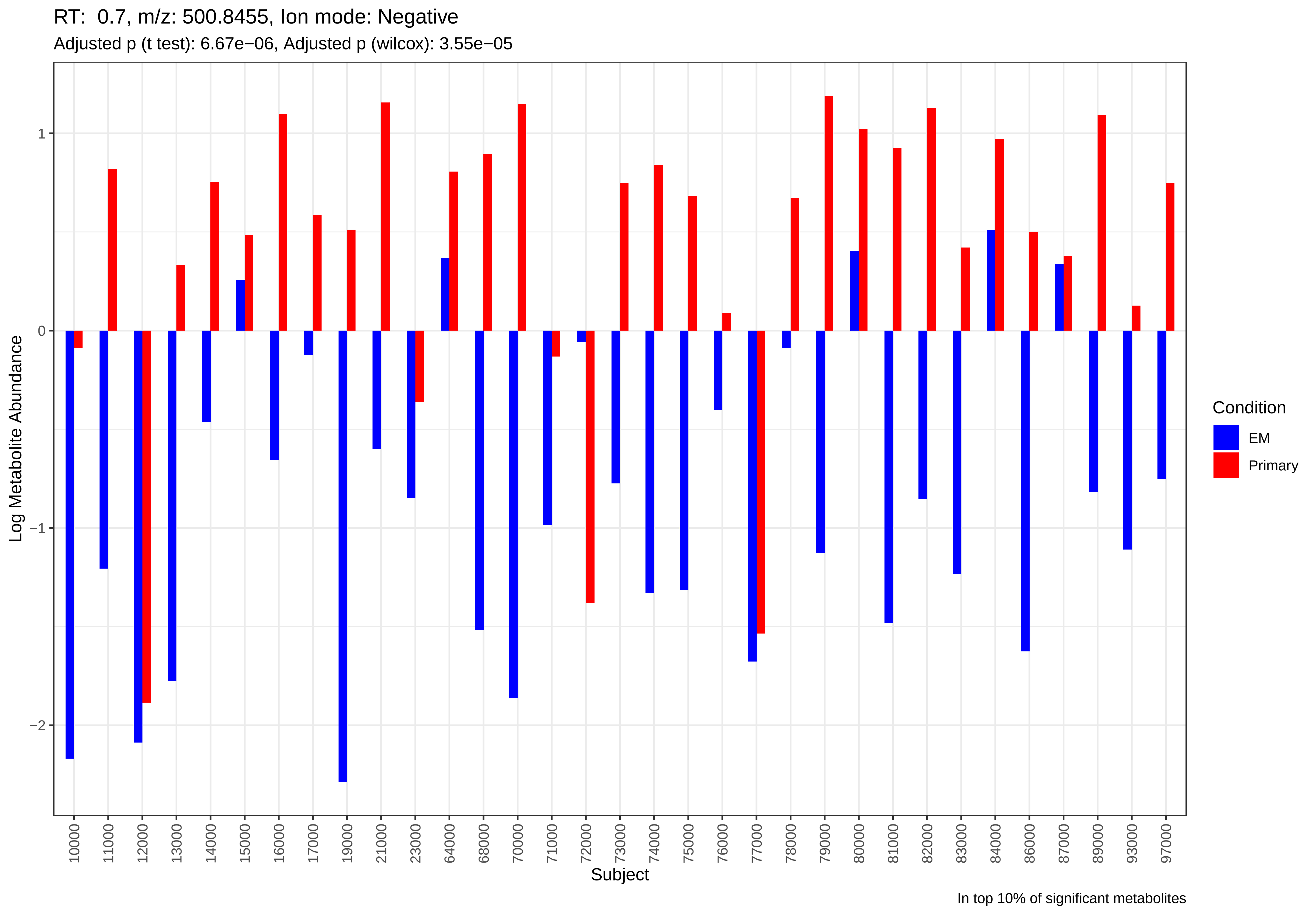
RT: 0.7, m/z: 500.8455, Ion mode: Negative
Adjusted p (t test): 6.67e−06, Adjusted p (wilcox): 3.55e−05
Log Metabolite Abundance
1
0
−1
−2
Subject
10000
11000
12000
13000
14000
15000
16000
17000
19000
21000
23000
64000
68000
70000
71000
72000
73000
74000
75000
76000
77000
78000
79000
80000
81000
82000
83000
84000
86000
87000
89000
93000
97000
Condition
EM
Primary
In top 10% of significant metabolites

DIHYDROXYACETONE PHOSPHATE (RT: 0.7, m/z: 168.9901, Ion mode: Negative)

Adjusted p (t test): 6.71e−06, Adjusted p (wilcox): 4.67e−05

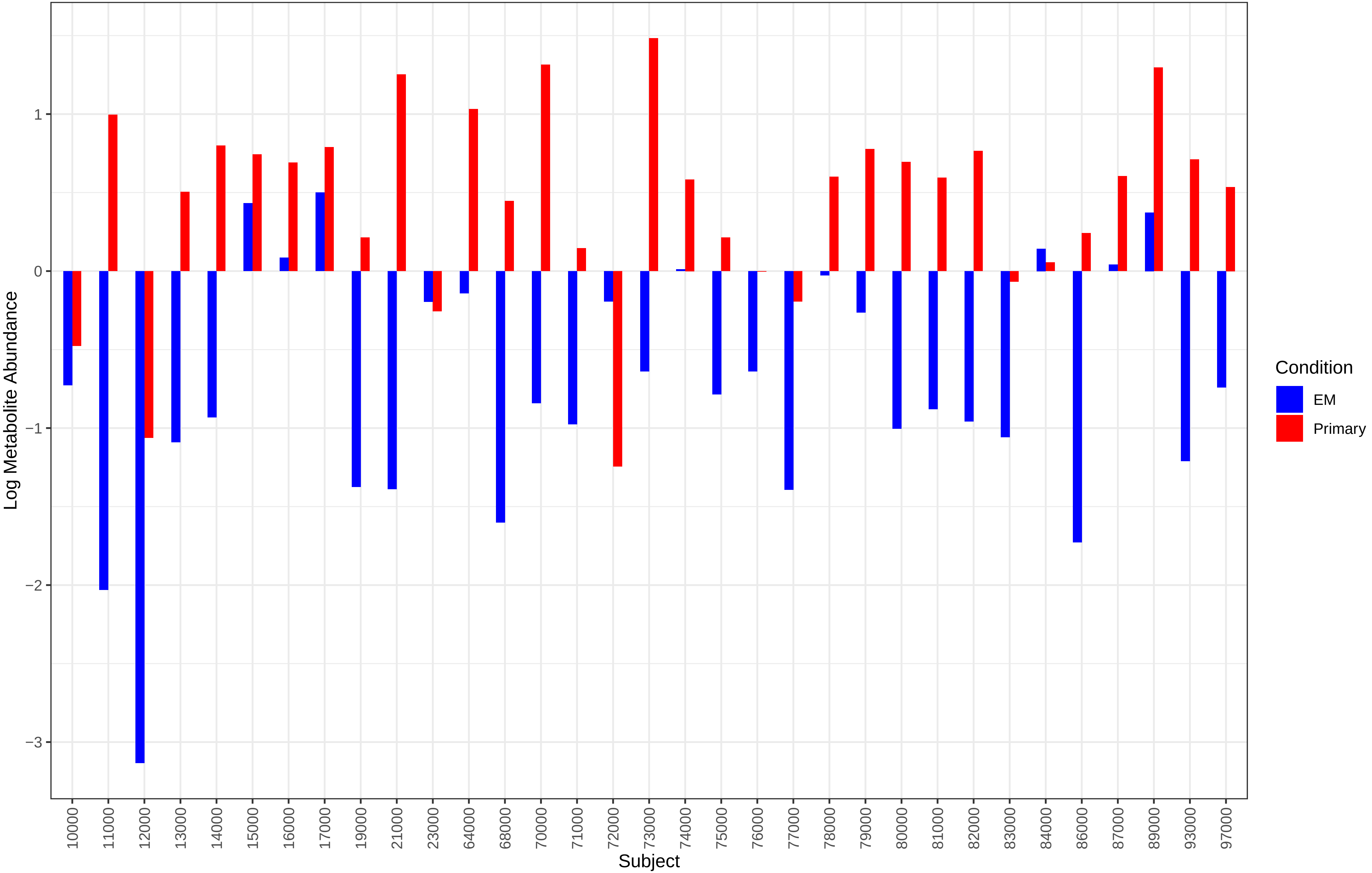

In top 10% of significant metabolites

RT: 0.6, m/z: 358.9227, Ion mode: Negative

Adjusted p (t test): 6.71e−06, Adjusted p (wilcox): 2.77e−05

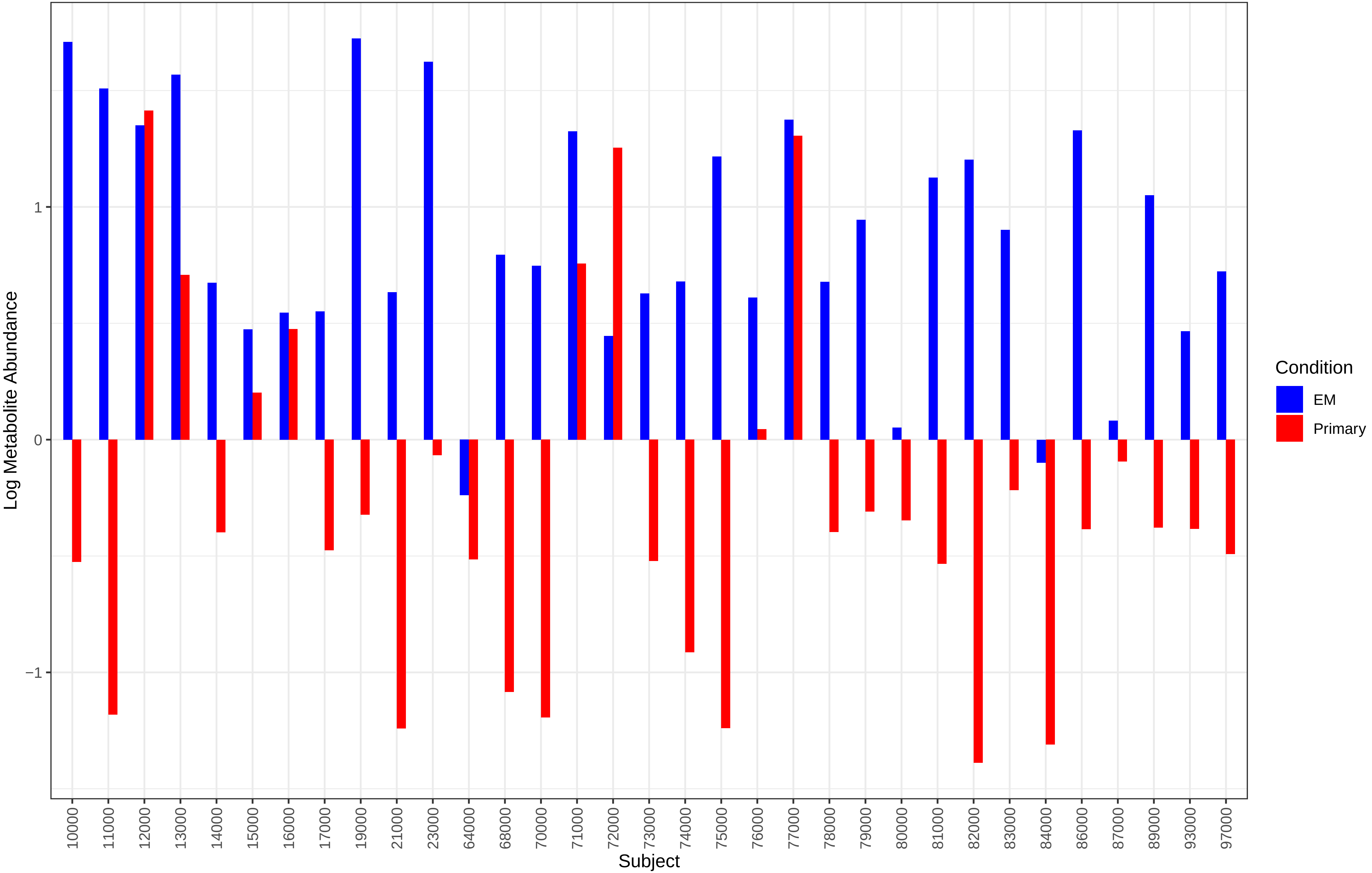

In top 10% of significant metabolites

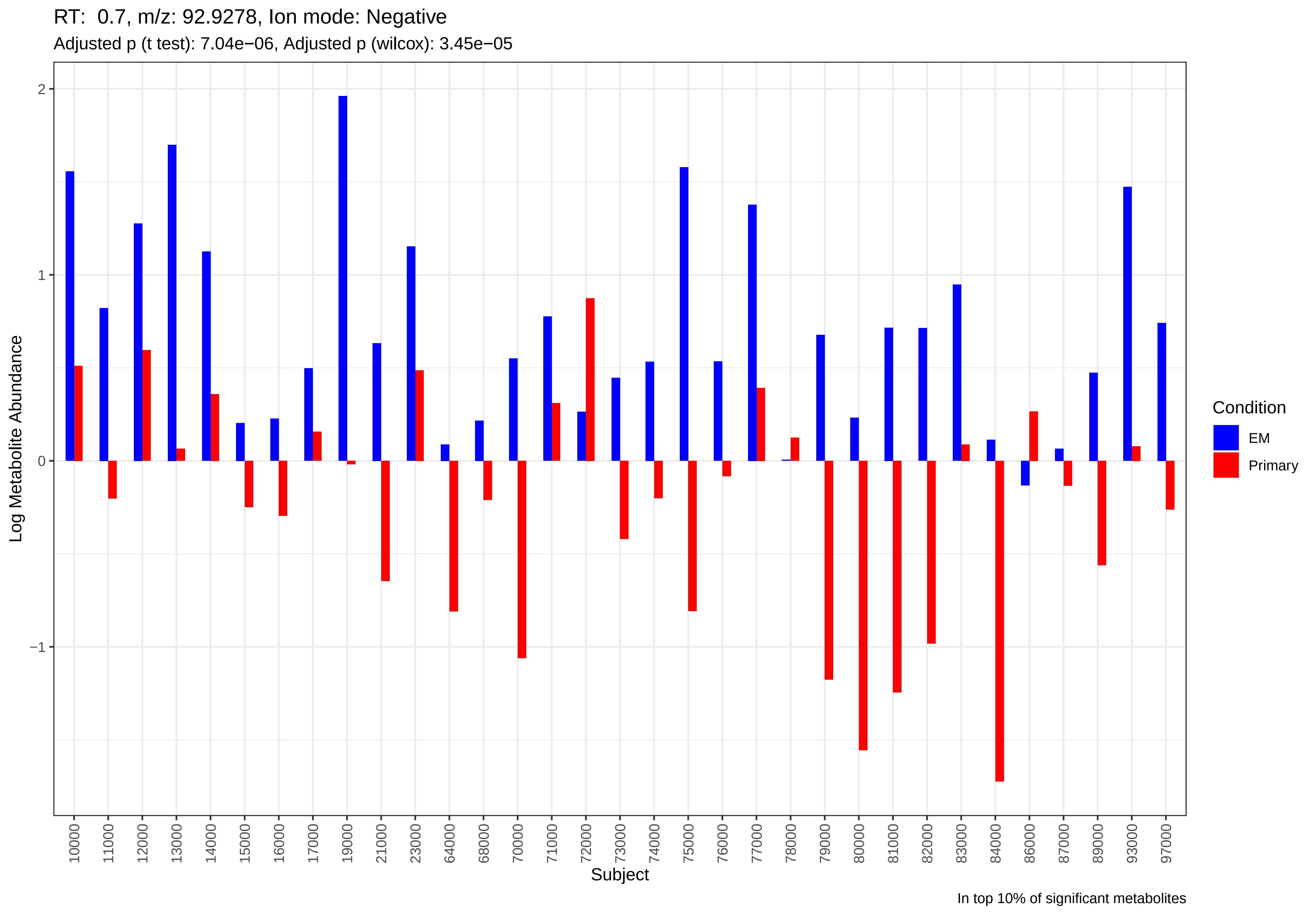
RT: 0.7, m/z: 92.9278, Ion mode: Negative
Adjusted p (t test): 7.04e−06, Adjusted p (wilcox): 3.45e−05
Log Metabolite Abundance
2
1
0
−1
Condition
EM
Primary
10000
11000
12000
13000
14000
15000
16000
17000
19000
21000
23000
64000
68000
70000
71000
72000
73000
74000
75000
76000
77000
78000
79000
80000
81000
82000
83000
84000
86000
87000
89000
93000
97000
Subject
In top 10% of significant metabolites

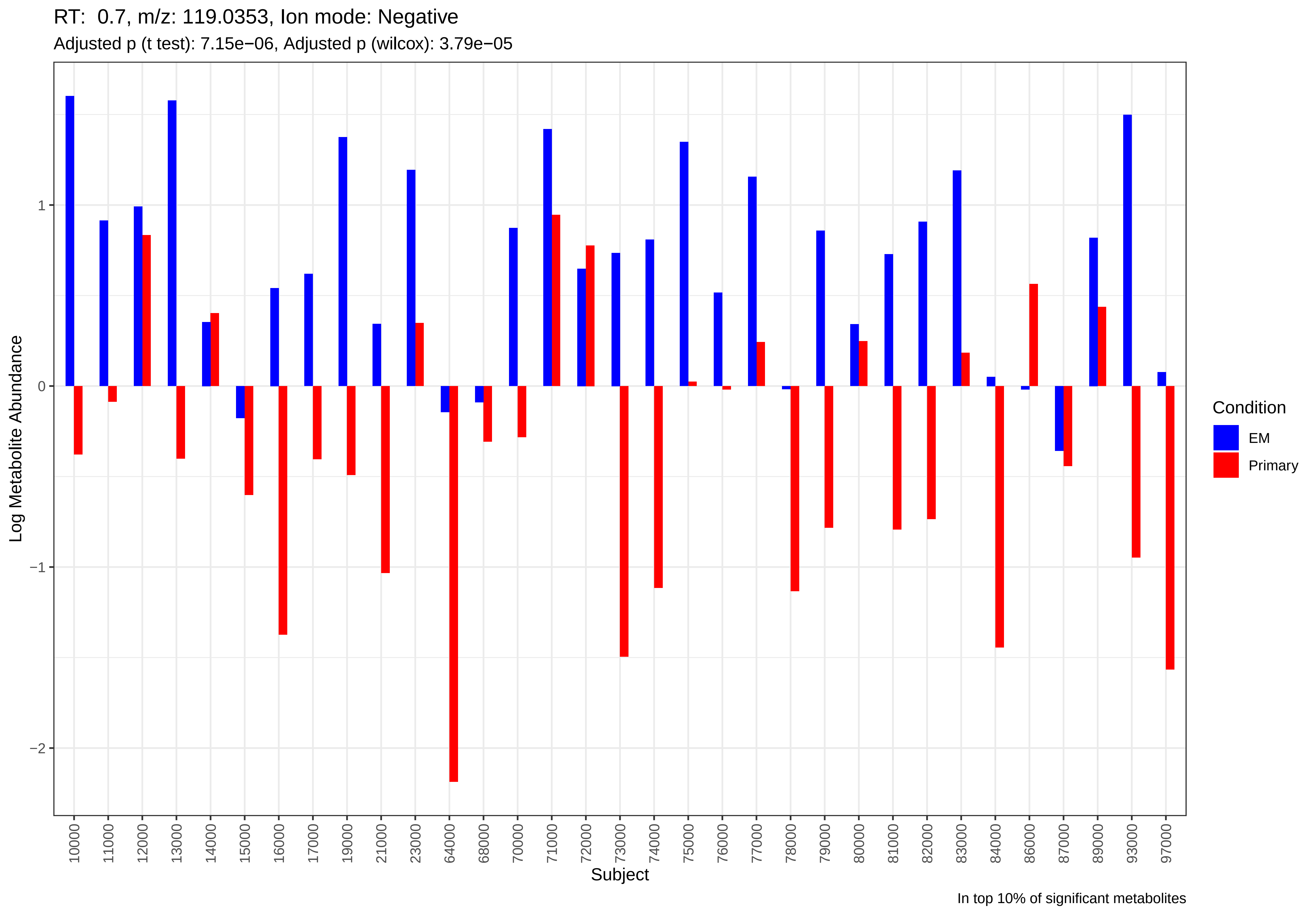
RT: 0.7, m/z: 119.0353, Ion mode: Negative
Adjusted p (t test): 7.15e−06, Adjusted p (wilcox): 3.79e−05
Log Metabolite Abundance
1
0
−1
−2
Subject
10000
11000
12000
13000
14000
15000
16000
17000
19000
21000
23000
64000
68000
70000
71000
72000
73000
74000
75000
76000
77000
78000
79000
80000
81000
82000
83000
84000
86000
87000
89000
93000
97000
Condition
EM
Primary
In top 10% of significant metabolites

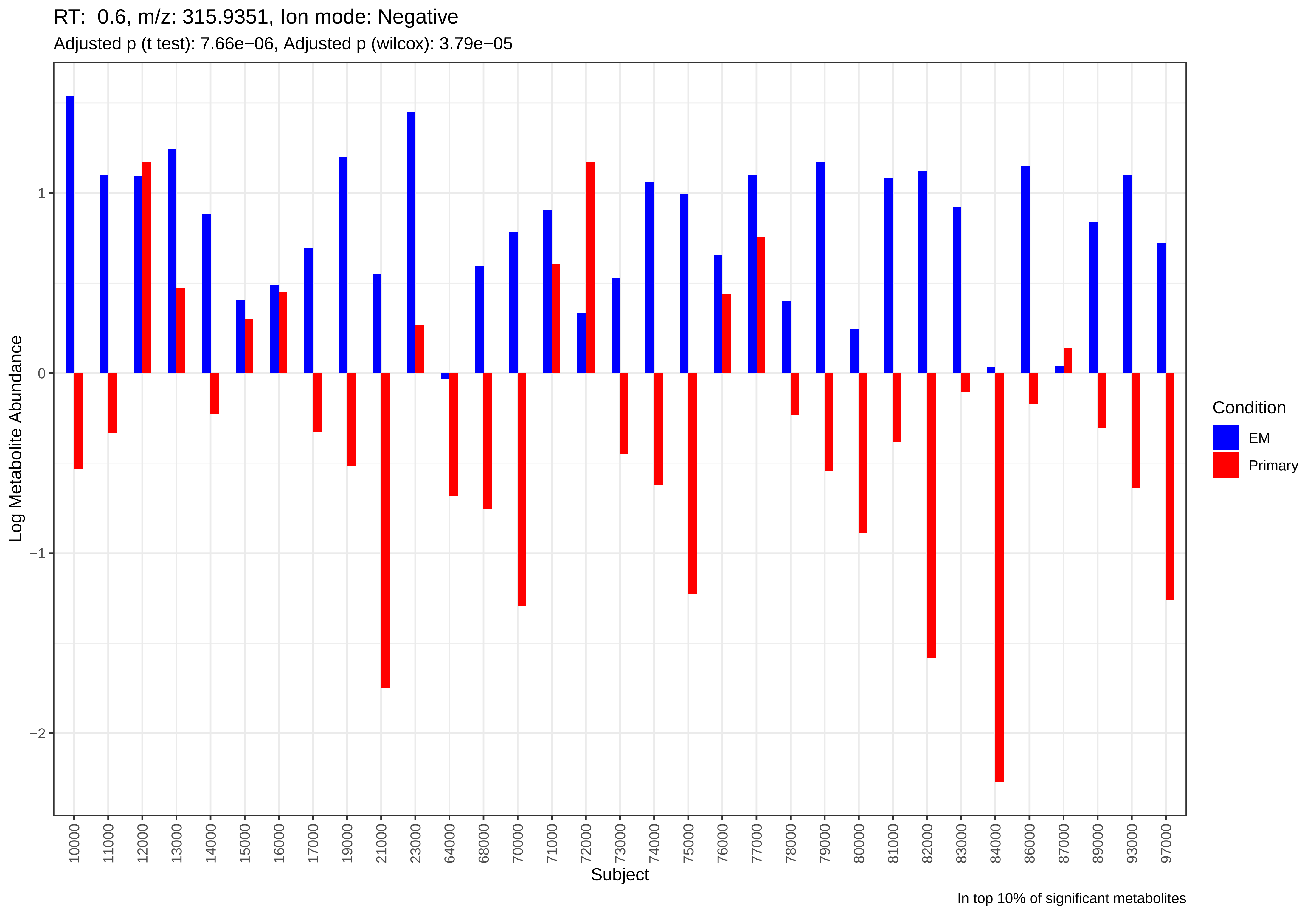
RT: 0.6, m/z: 315.9351, Ion mode: Negative
Adjusted p (t test): 7.66e−06, Adjusted p (wilcox): 3.79e−05
Log Metabolite Abundance
1
0
−1
−2
Subject
10000
11000
12000
13000
14000
15000
16000
17000
19000
21000
23000
64000
68000
70000
71000
72000
73000
74000
75000
76000
77000
78000
79000
80000
81000
82000
83000
84000
86000
87000
89000
93000
97000
Condition
EM
Primary
In top 10% of significant metabolites

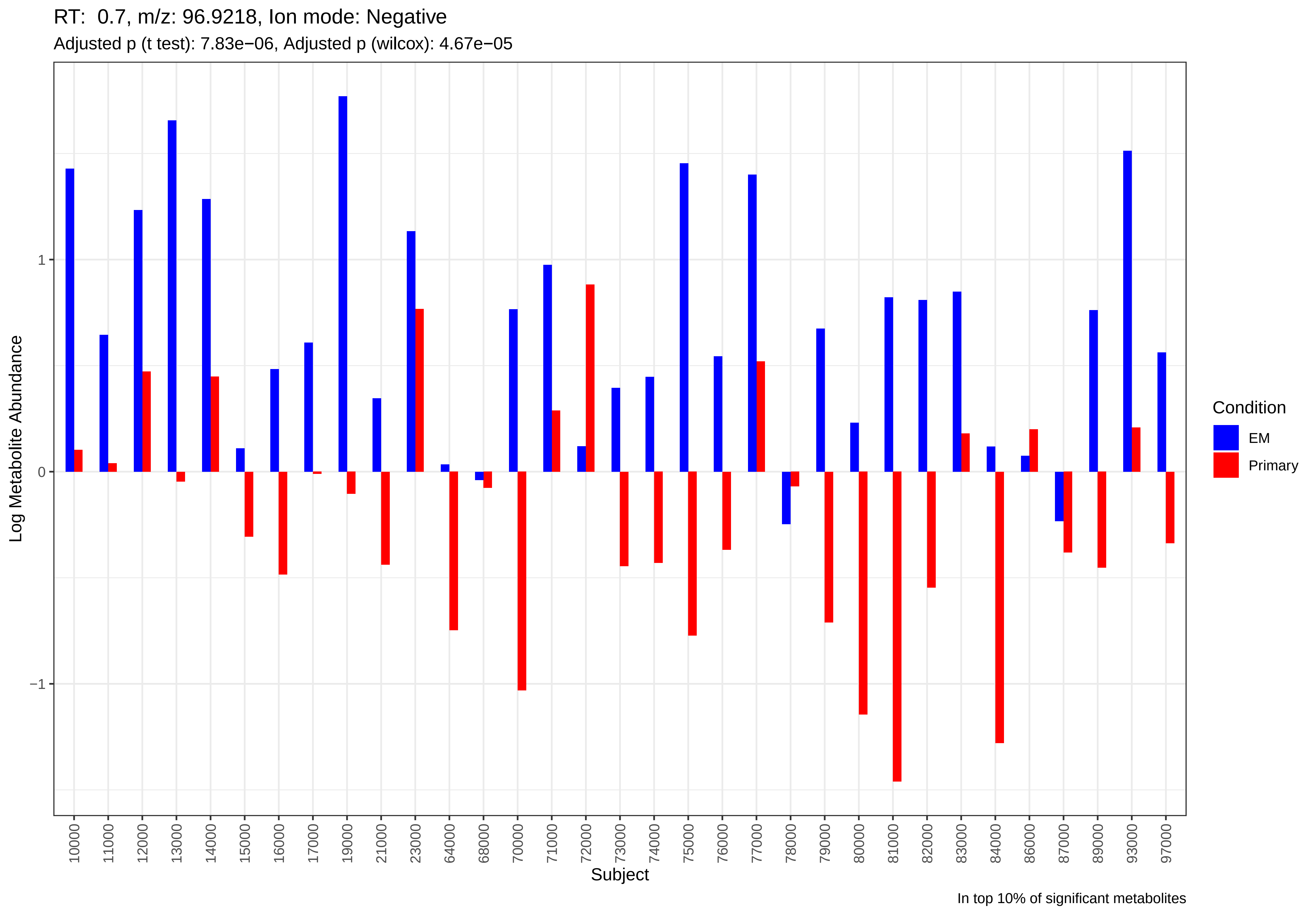

RT: 0.7, m/z: 96.9218, Ion mode: Negative
Adjusted p (t test): 7.83e−06, Adjusted p (wilcox): 4.67e−05
Log Metabolite Abundance
1
0
−1
10000
11000
12000
13000
14000
15000
16000
17000
19000
21000
23000
64000
68000
70000
71000
72000
73000
74000
75000
76000
77000
78000
79000
80000
81000
82000
83000
84000
86000
87000
89000
93000
97000
Subject
Condition
EM
Primary
In top 10% of significant metabolites

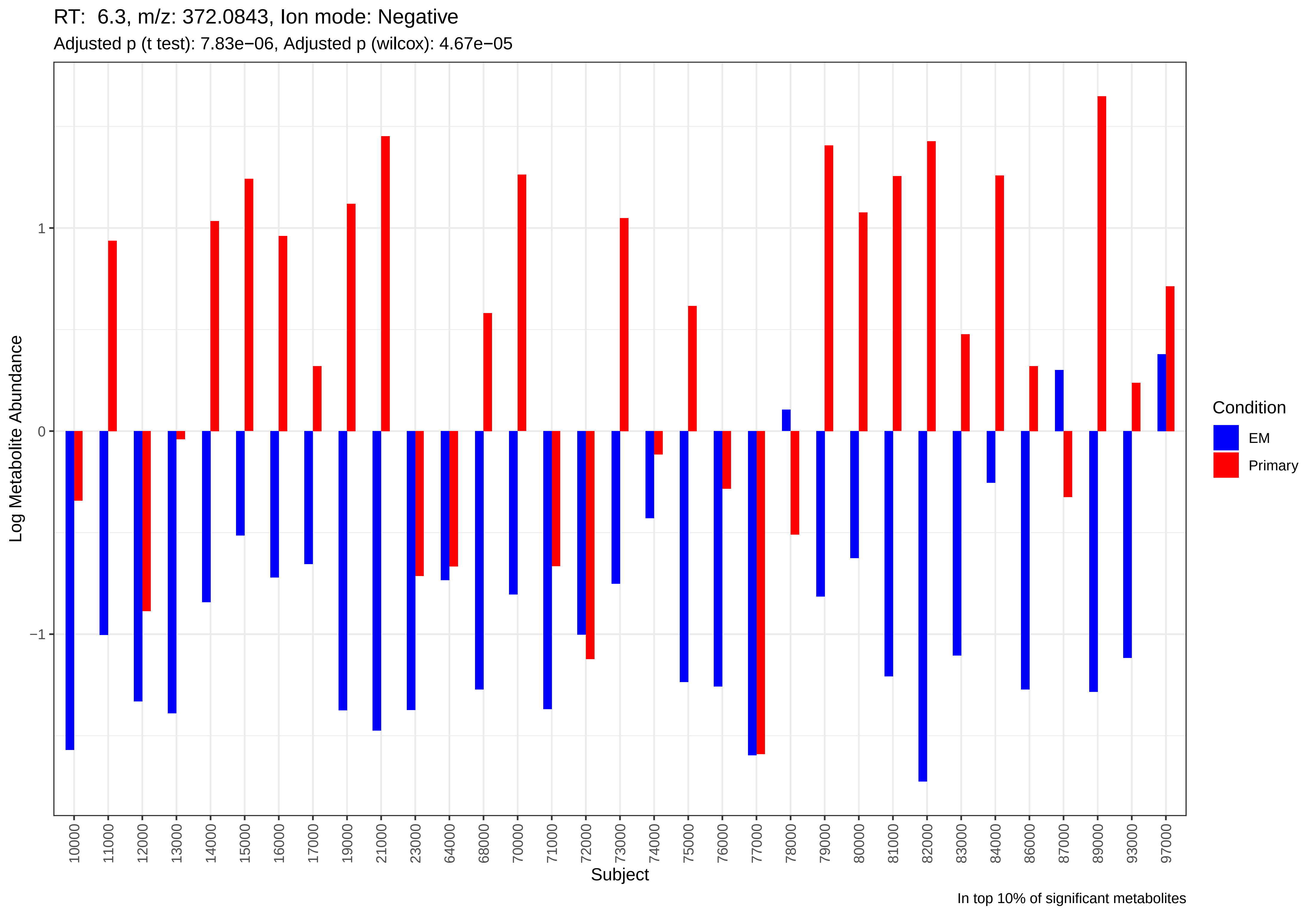
RT: 6.3, m/z: 372.0843, Ion mode: Negative
Adjusted p (t test): 7.83e−06, Adjusted p (wilcox): 4.67e−05
Log Metabolite Abundance
1
0
−1
10000
11000
12000
13000
14000
15000
16000
17000
19000
21000
23000
64000
68000
70000
71000
72000
73000
74000
75000
76000
77000
78000
79000
80000
81000
82000
83000
84000
86000
87000
89000
93000
97000
Subject
Condition
EM
Primary
In top 10% of significant metabolites

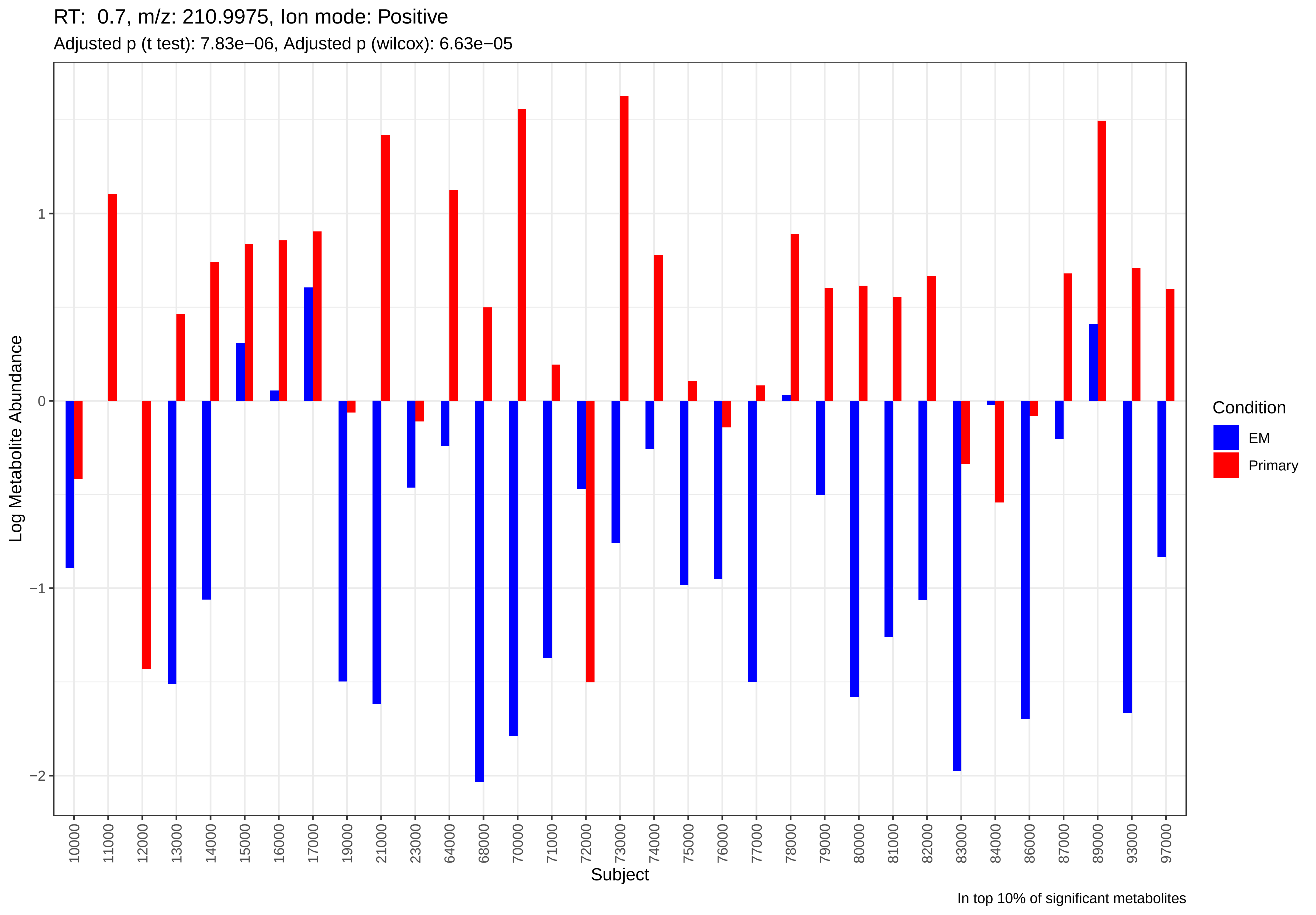
RT: 0.7, m/z: 210.9975, Ion mode: Positive
Adjusted p (t test): 7.83e−06, Adjusted p (wilcox): 6.63e−05
Log Metabolite Abundance
1
0
−1
−2
Subject
10000
11000
12000
13000
14000
15000
16000
17000
19000
21000
23000
64000
68000
70000
71000
72000
73000
74000
75000
76000
77000
78000
79000
80000
81000
82000
83000
84000
86000
87000
89000
93000
97000
Condition
EM
Primary
In top 10% of significant metabolites

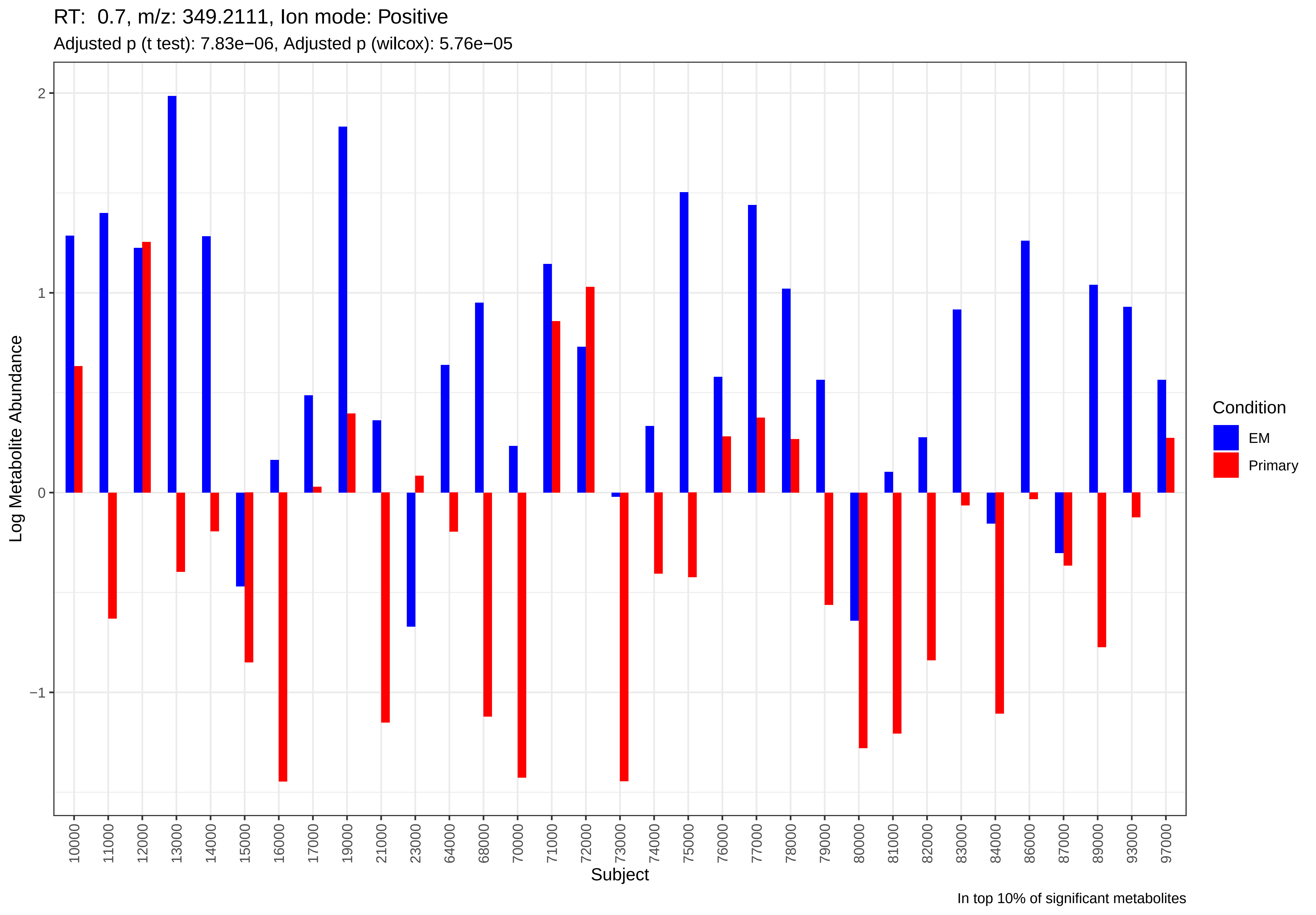

RT: 0.7, m/z: 349.2111, Ion mode: Positive
Adjusted p (t test): 7.83e−06, Adjusted p (wilcox): 5.76e−05
Log Metabolite Abundance
2
1
0
−1
Subject
10000
11000
12000
13000
14000
15000
16000
17000
19000
21000
23000
64000
68000
70000
71000
72000
73000
74000
75000
76000
77000
78000
79000
80000
81000
82000
83000
84000
86000
87000
89000
93000
97000
Condition
EM
Primary
In top 10% of significant metabolites

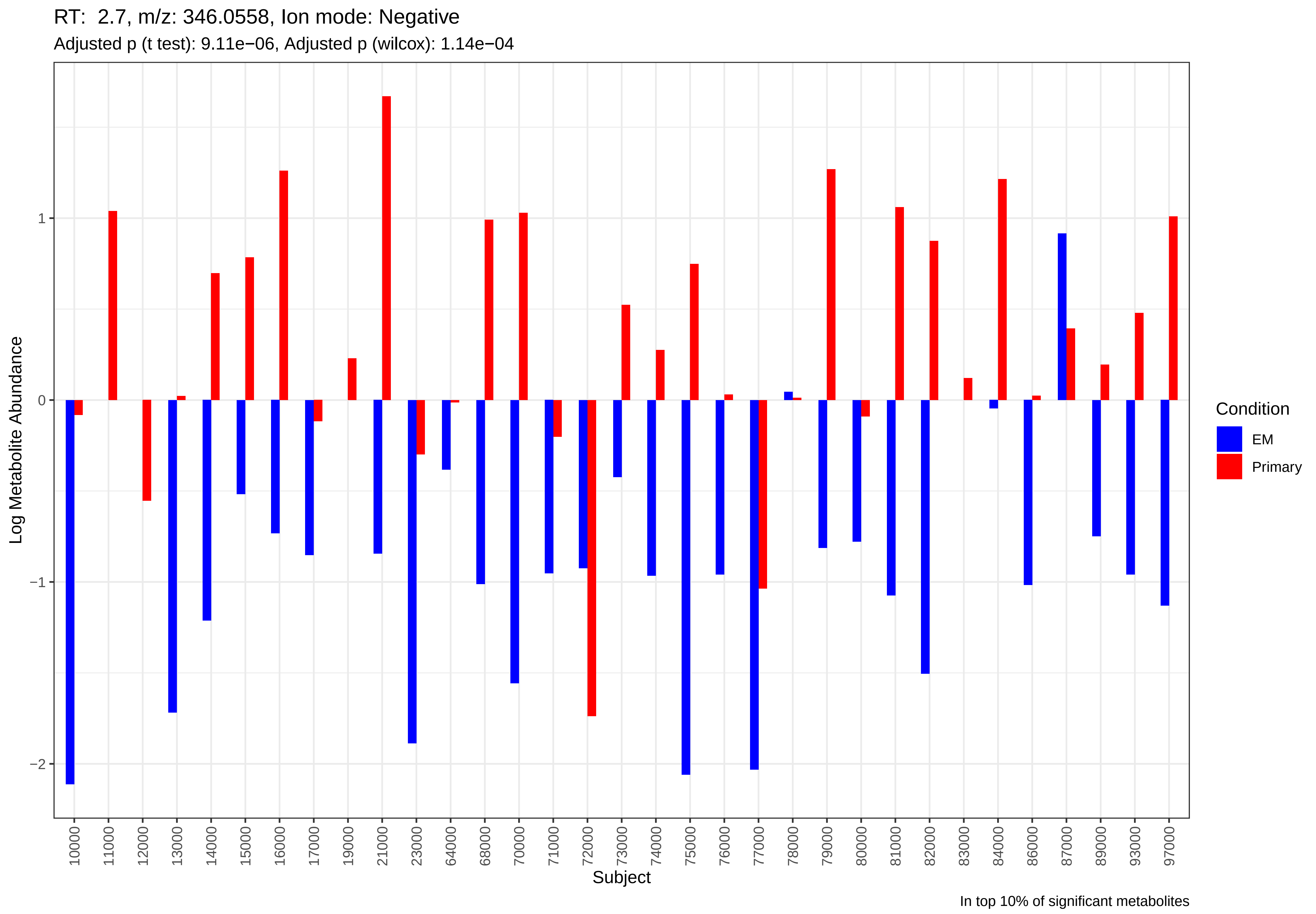

RT: 2.7, m/z: 346.0558, Ion mode: Negative
Adjusted p (t test): 9.11e−06, Adjusted p (wilcox): 1.14e−04
Log Metabolite Abundance
1
0
−1
−2
10000
11000
12000
13000
14000
15000
16000
17000
19000
21000
23000
64000
68000
70000
71000
72000
73000
74000
75000
76000
77000
78000
79000
80000
81000
82000
83000
84000
86000
87000
89000
93000
97000
Subject
Condition
EM
Primary
In top 10% of significant metabolites

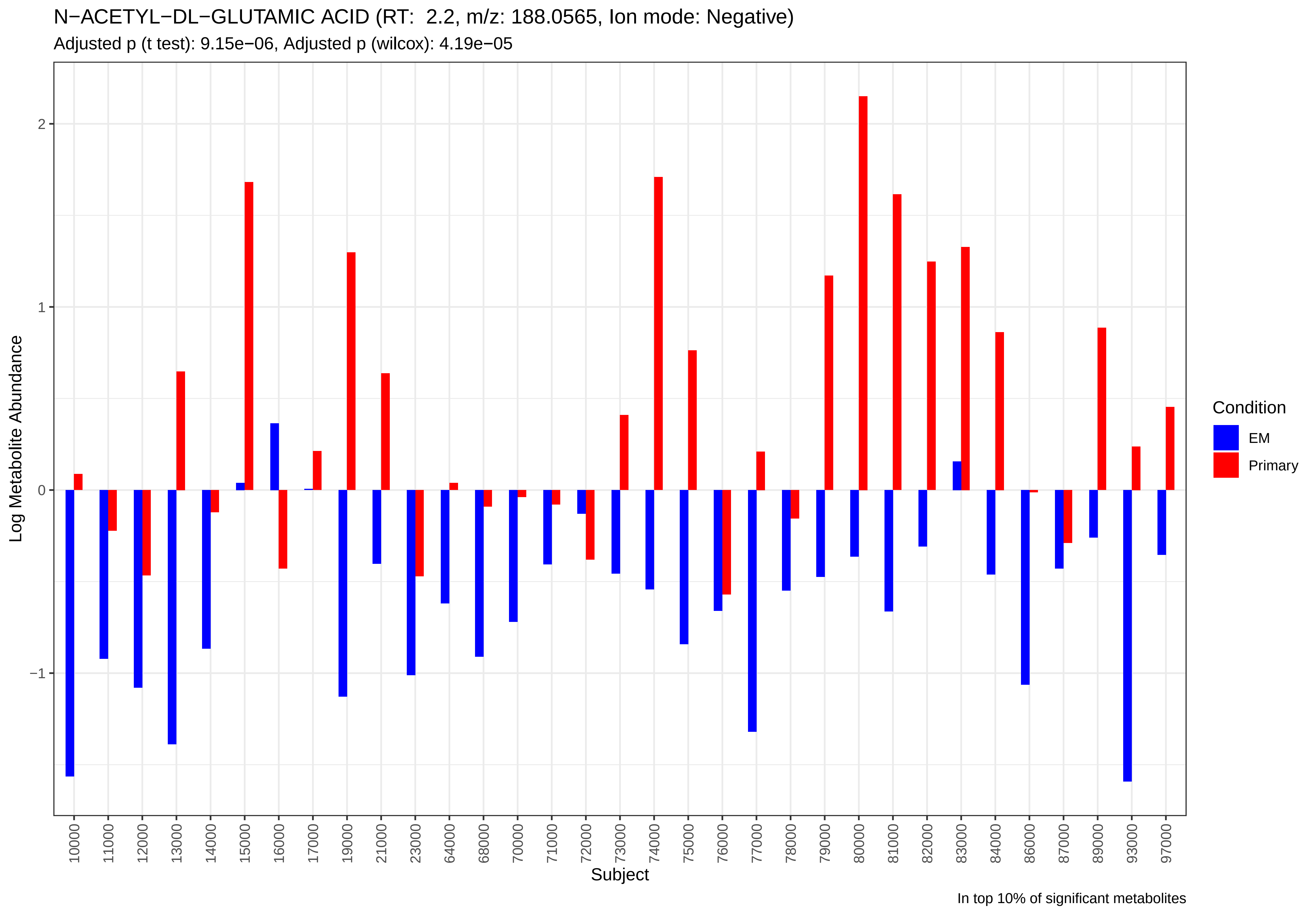
N−ACETYL−DL−GLUTAMIC ACID (RT: 2.2, m/z: 188.0565, Ion mode: Negative)
Adjusted p (t test): 9.15e−06, Adjusted p (wilcox): 4.19e−05
Log Metabolite Abundance
2
1
0
−1
Subject
10000
11000
12000
13000
14000
15000
16000
17000
19000
21000
23000
64000
68000
70000
71000
72000
73000
74000
75000
76000
77000
78000
79000
80000
81000
82000
83000
84000
86000
87000
89000
93000
97000
Condition
EM
Primary
In top 10% of significant metabolites

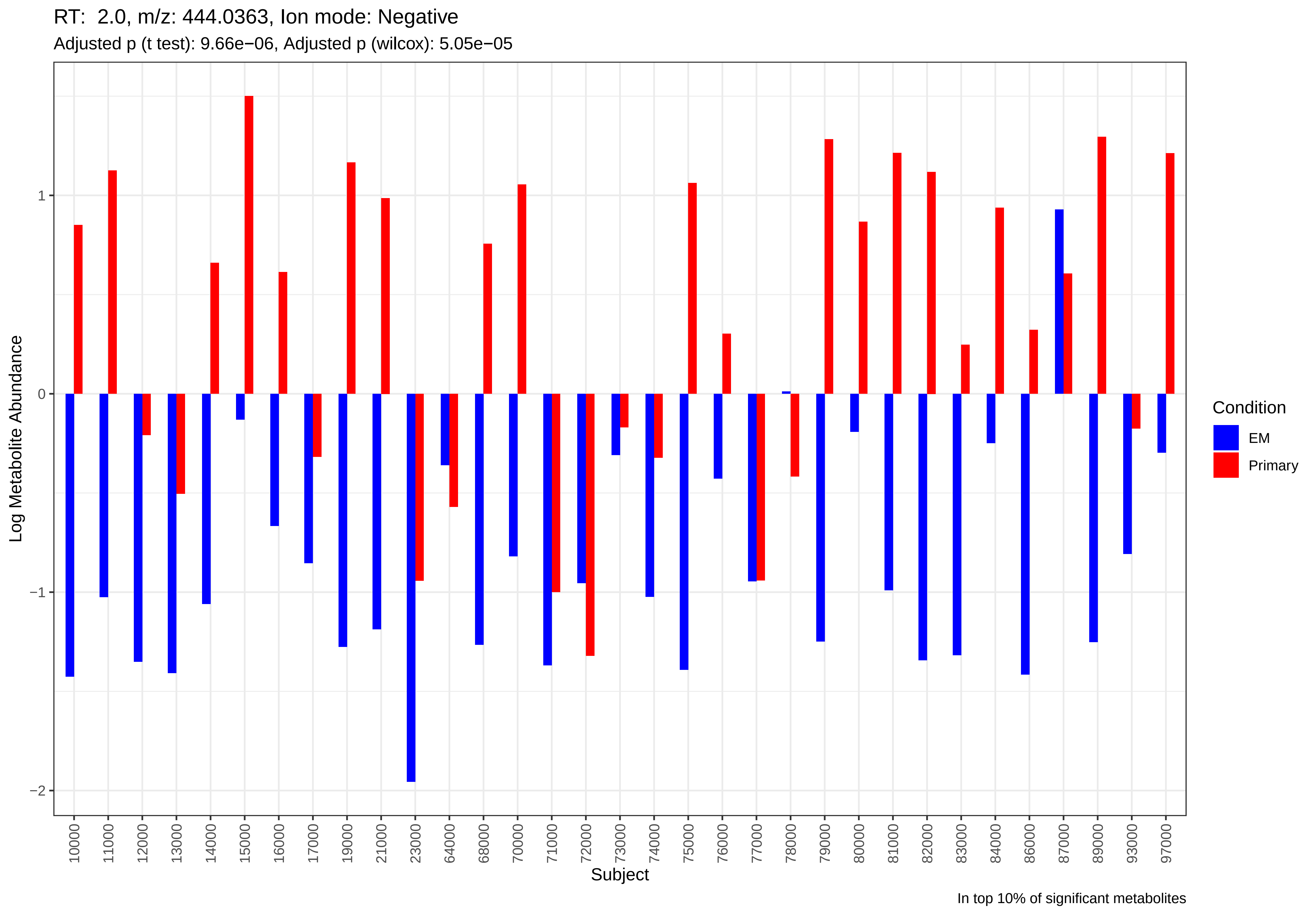

RT: 2.0, m/z: 444.0363, Ion mode: Negative
Adjusted p (t test): 9.66e−06, Adjusted p (wilcox): 5.05e−05
Log Metabolite Abundance
1
0
−1
−2
10000
11000
12000
13000
14000
15000
16000
17000
19000
21000
23000
64000
68000
70000
71000
72000
73000
74000
75000
76000
77000
78000
79000
80000
81000
82000
83000
84000
86000
87000
89000
93000
97000
Subject
Condition
EM
Primary
In top 10% of significant metabolites

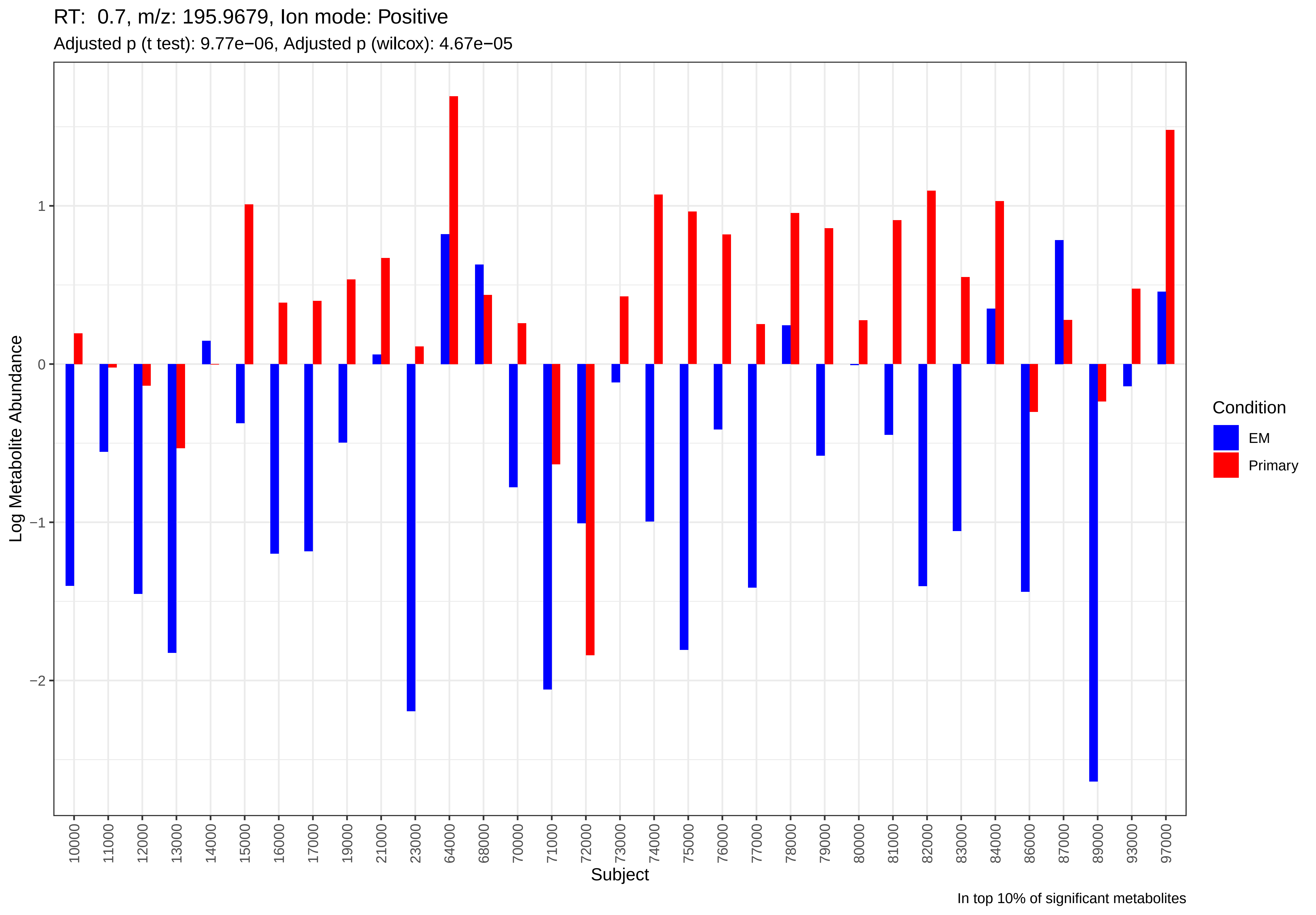

RT: 0.7, m/z: 195.9679, Ion mode: Positive
Adjusted p (t test): 9.77e−06, Adjusted p (wilcox): 4.67e−05
Log Metabolite Abundance
1
0
−1
−2
10000
11000
12000
13000
14000
15000
16000
17000
19000
21000
23000
64000
68000
70000
71000
72000
73000
74000
75000
76000
77000
78000
79000
80000
81000
82000
83000
84000
86000
87000
89000
93000
97000
Subject
Condition
EM
Primary
In top 10% of significant metabolites

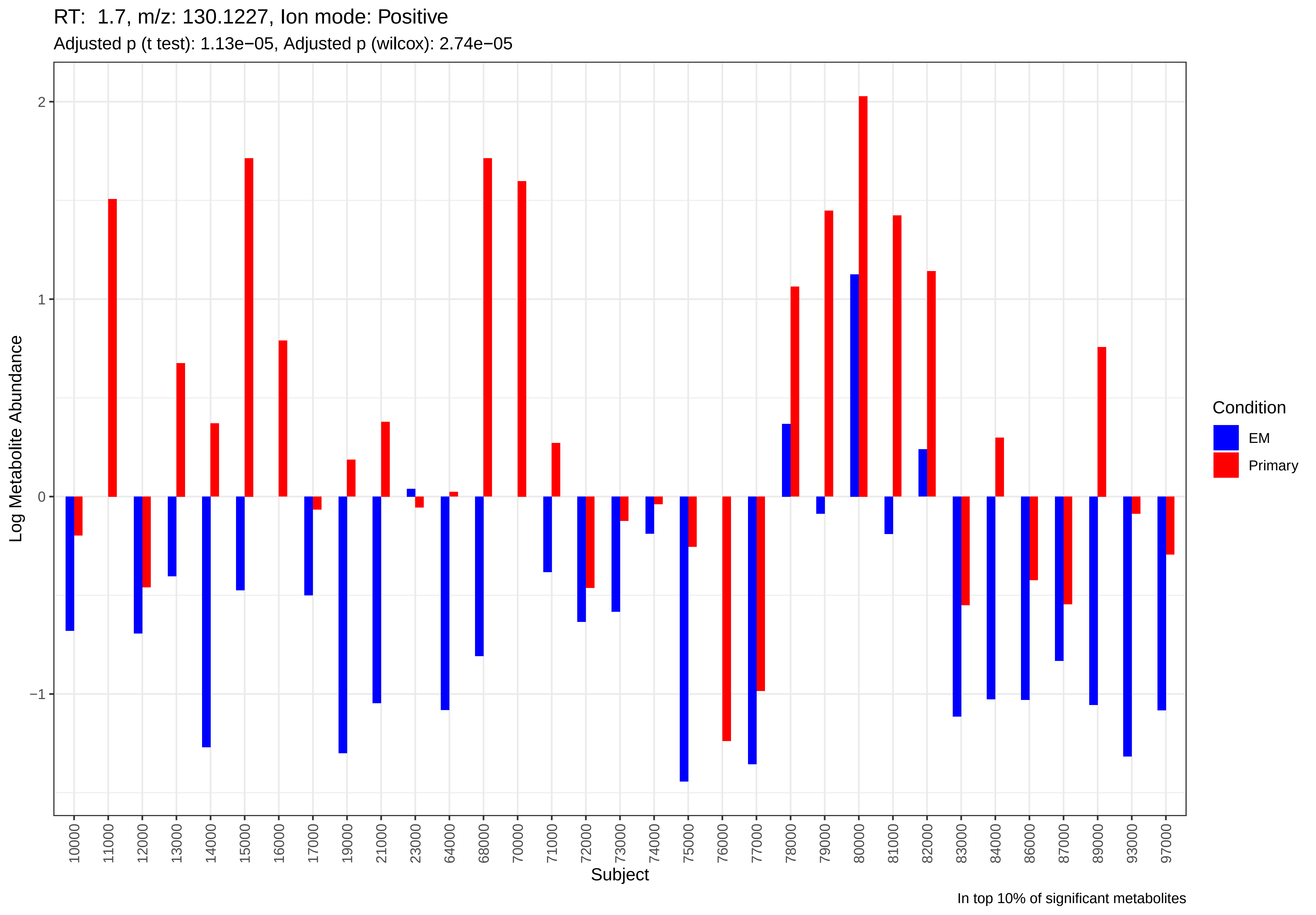
RT: 1.7, m/z: 130.1227, Ion mode: Positive
Adjusted p (t test): 1.13e−05, Adjusted p (wilcox): 2.74e−05
Log Metabolite Abundance
2
1
0
−1
10000
11000
12000
13000
14000
15000
16000
17000
19000
21000
23000
64000
68000
70000
71000
72000
73000
74000
75000
76000
77000
78000
79000
80000
81000
82000
83000
84000
86000
87000
89000
93000
97000
Subject
Condition
EM
Primary
In top 10% of significant metabolites

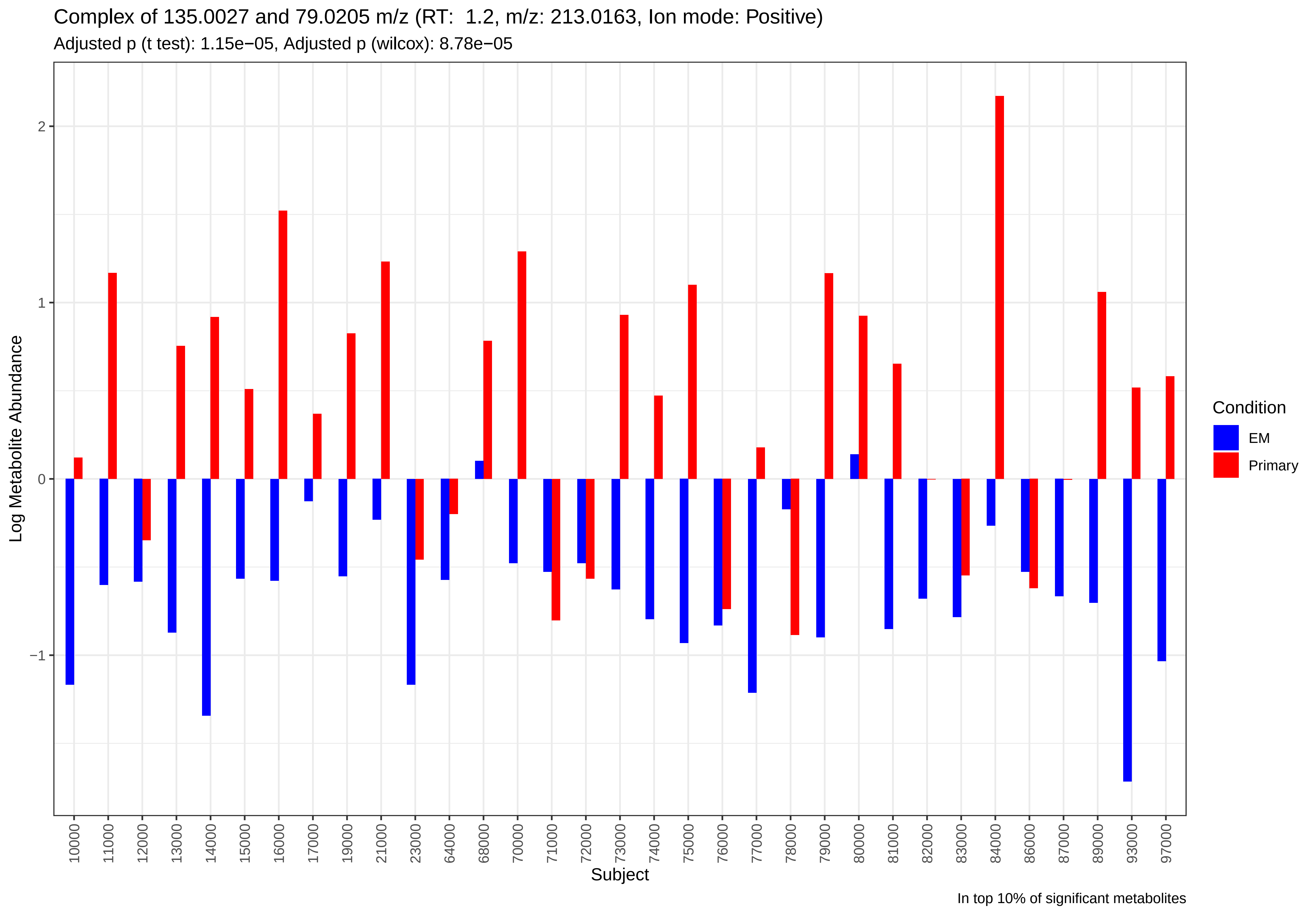

Complex of 135.0027 and 79.0205 m/z (RT: 1.2, m/z: 213.0163, Ion mode: Positive)
Adjusted p (t test): 1.15e−05, Adjusted p (wilcox): 8.78e−05
Log Metabolite Abundance
2
1
0
−1
10000
11000
12000
13000
14000
15000
16000
17000
19000
21000
23000
64000
68000
70000
71000
72000
73000
74000
75000
76000
77000
78000
79000
80000
81000
82000
83000
84000
86000
87000
89000
93000
97000
Subject
Condition
EM
Primary
In top 10% of significant metabolites

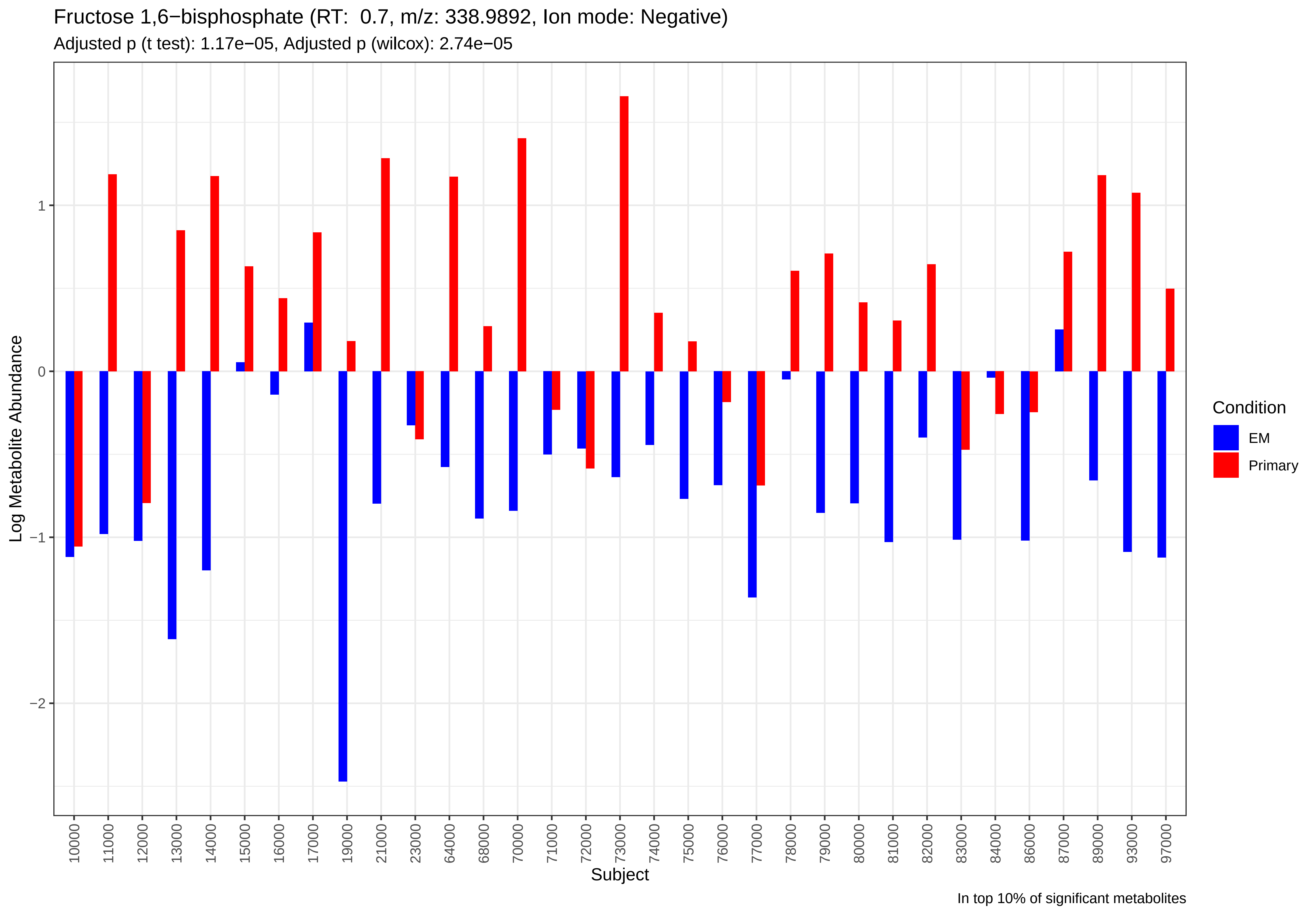

Fructose 1,6−bisphosphate (RT: 0.7, m/z: 338.9892, Ion mode: Negative)
Adjusted p (t test): 1.17e−05, Adjusted p (wilcox): 2.74e−05
Log Metabolite Abundance
1
0
−1
−2
10000
11000
12000
13000
14000
15000
16000
17000
19000
21000
23000
64000
68000
70000
71000
72000
73000
74000
75000
76000
77000
78000
79000
80000
81000
82000
83000
84000
86000
87000
89000
93000
97000
Subject
Condition
EM
Primary
In top 10% of significant metabolites

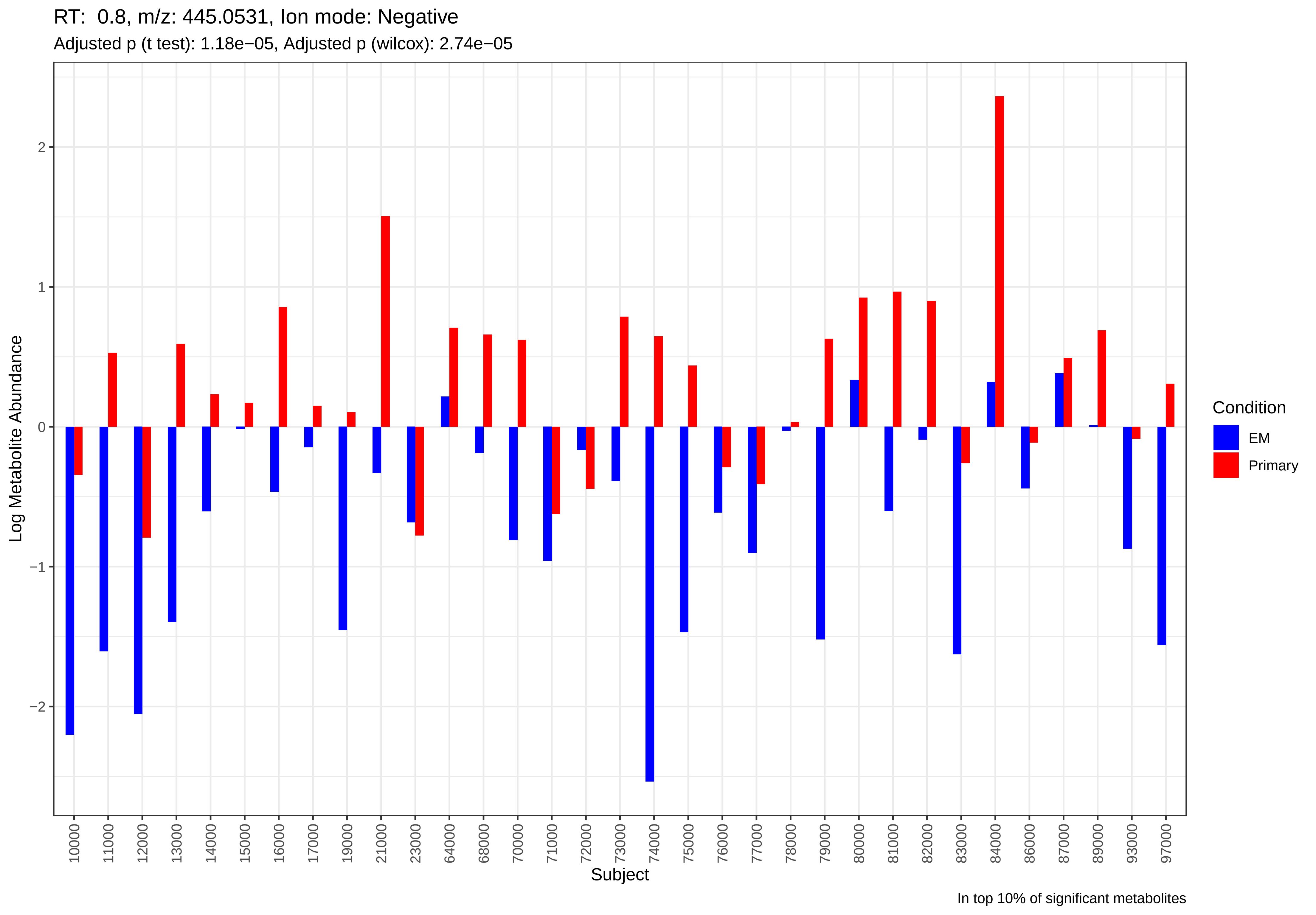
RT: 0.8, m/z: 445.0531, Ion mode: Negative
Adjusted p (t test): 1.18e−05, Adjusted p (wilcox): 2.74e−05
Log Metabolite Abundance
2
1
0
−1
−2
10000
11000
12000
13000
14000
15000
16000
17000
19000
21000
23000
64000
68000
70000
71000
72000
73000
74000
75000
76000
77000
78000
79000
80000
81000
82000
83000
84000
86000
87000
89000
93000
97000
Subject
Condition
EM
Primary
In top 10% of significant metabolites

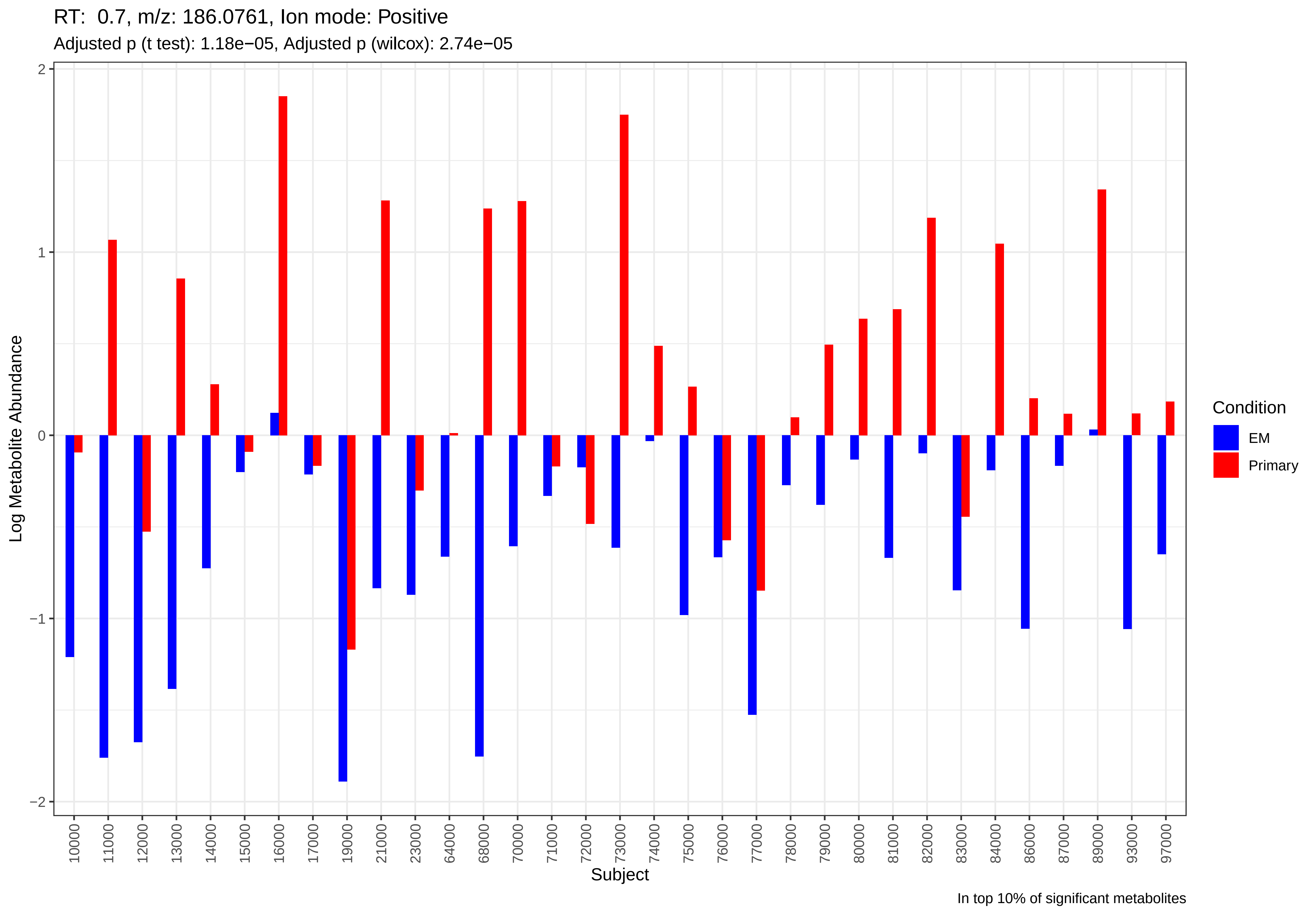
RT: 0.7, m/z: 186.0761, Ion mode: Positive
Adjusted p (t test): 1.18e−05, Adjusted p (wilcox): 2.74e−05
Log Metabolite Abundance
Subject
Condition
EM
Primary
In top 10% of significant metabolites

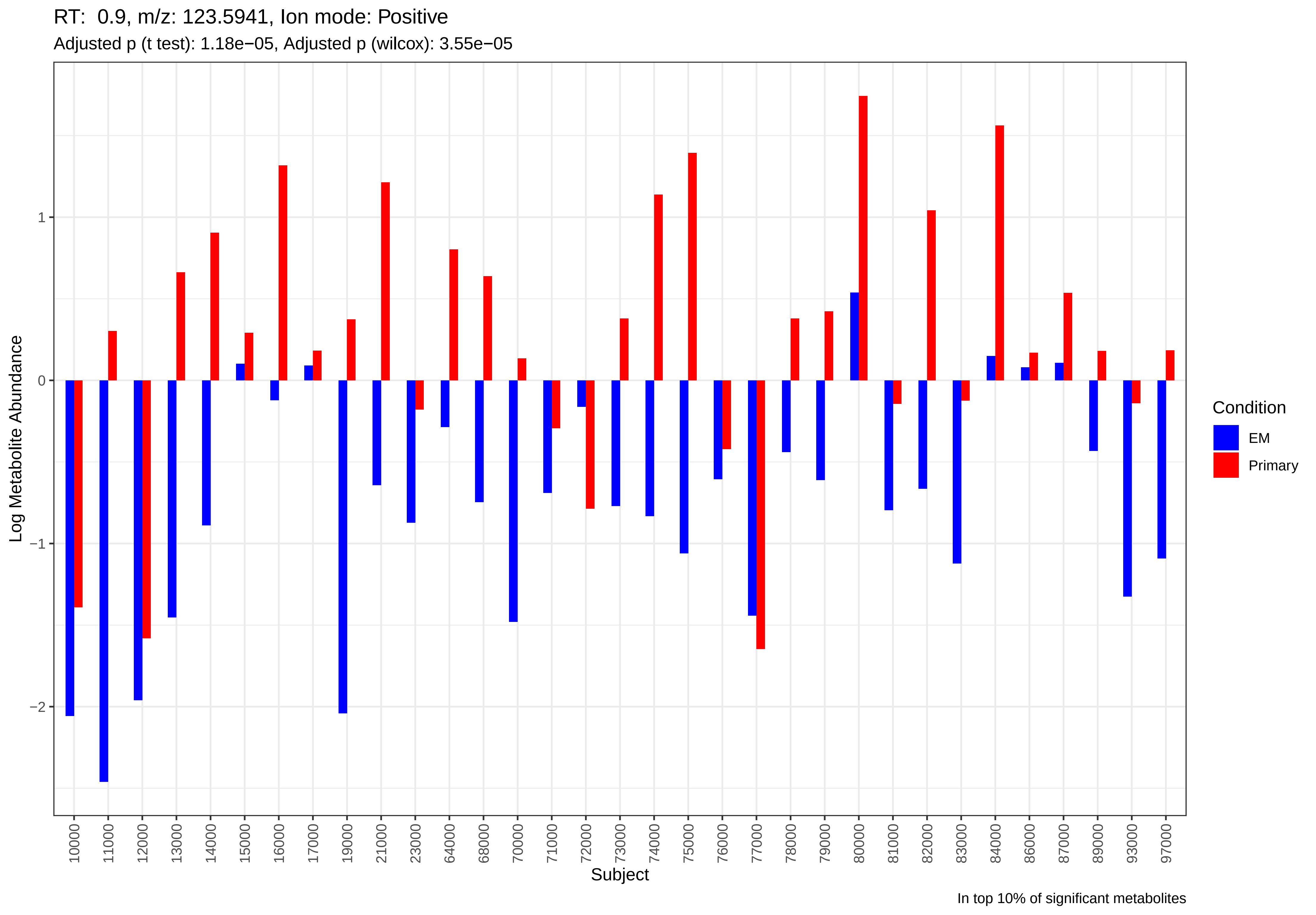

RT: 0.9, m/z: 123.5941, Ion mode: Positive
Adjusted p (t test): 1.18e−05, Adjusted p (wilcox): 3.55e−05
Log Metabolite Abundance
1
0
−1
−2
Subject
10000
11000
12000
13000
14000
15000
16000
17000
19000
21000
23000
64000
68000
70000
71000
72000
73000
74000
75000
76000
77000
78000
79000
80000
81000
82000
83000
84000
86000
87000
89000
93000
97000
Condition
EM
Primary
In top 10% of significant metabolites

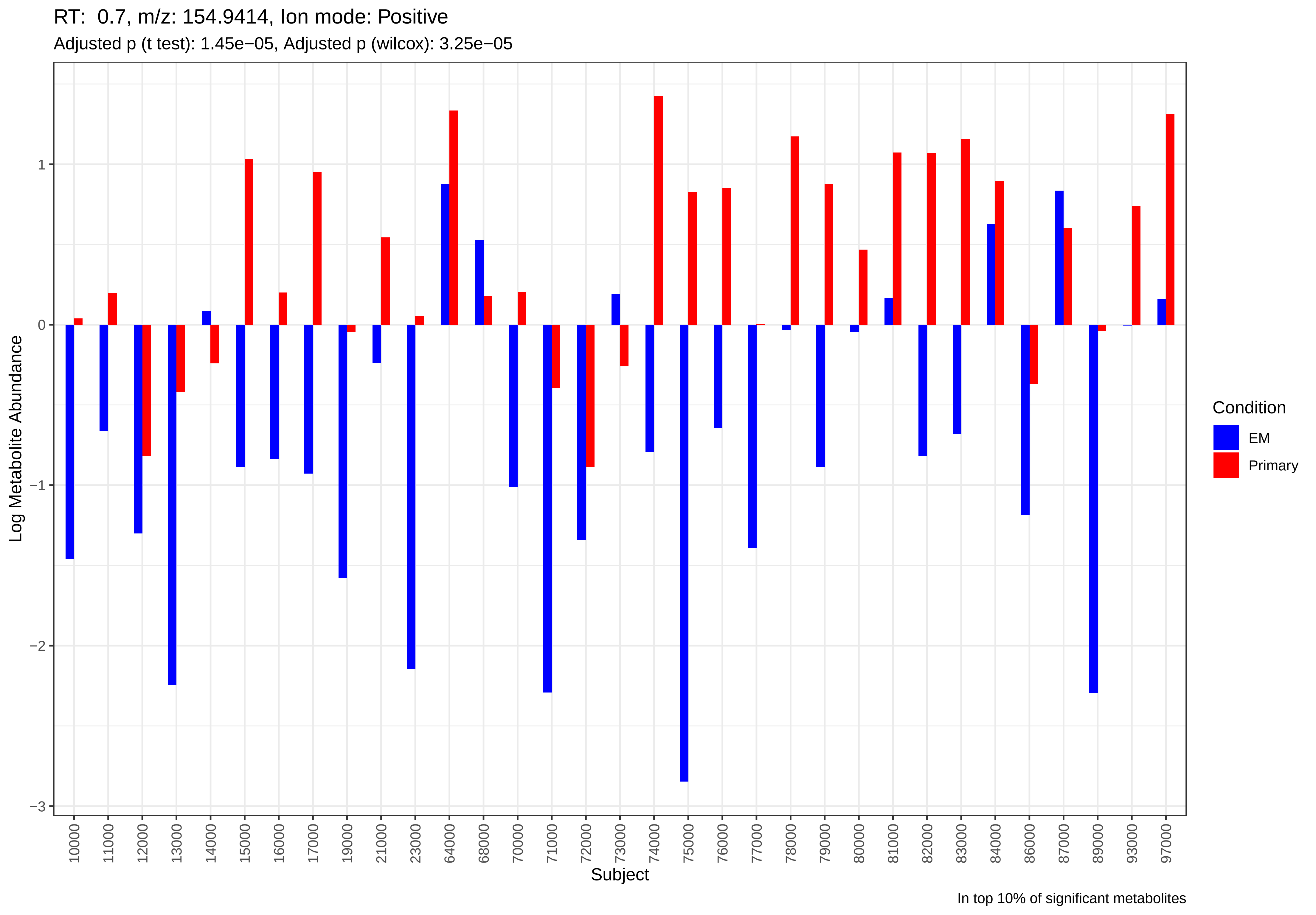
RT: 0.7, m/z: 154.9414, Ion mode: Positive
Adjusted p (t test): 1.45e−05, Adjusted p (wilcox): 3.25e−05
Log Metabolite Abundance
Subject
Condition
EM
Primary
1
0
−1
−2
−3
10000
11000
12000
13000
14000
15000
16000
17000
19000
21000
23000
64000
68000
70000
71000
72000
73000
74000
75000
76000
77000
78000
79000
80000
81000
82000
83000
84000
86000
87000
89000
93000
97000
In top 10% of significant metabolites

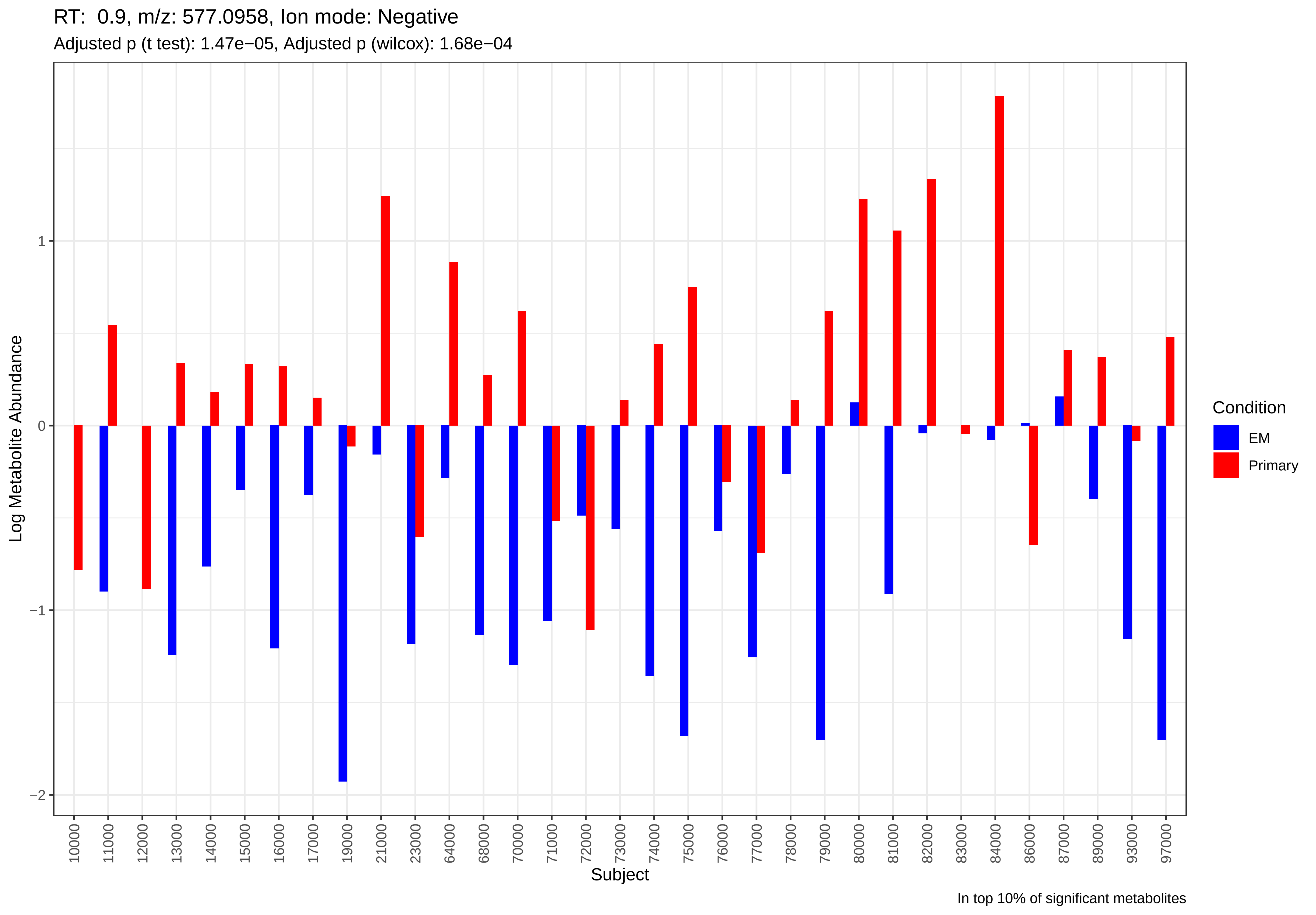
RT: 0.9, m/z: 577.0958, Ion mode: Negative
Adjusted p (t test): 1.47e−05, Adjusted p (wilcox): 1.68e−04
Log Metabolite Abundance
1
0
−1
−2
10000
11000
12000
13000
14000
15000
16000
17000
19000
21000
23000
64000
68000
70000
71000
72000
73000
74000
75000
76000
77000
78000
79000
80000
81000
82000
83000
84000
86000
87000
89000
93000
97000
Subject
Condition
EM
Primary
In top 10% of significant metabolites

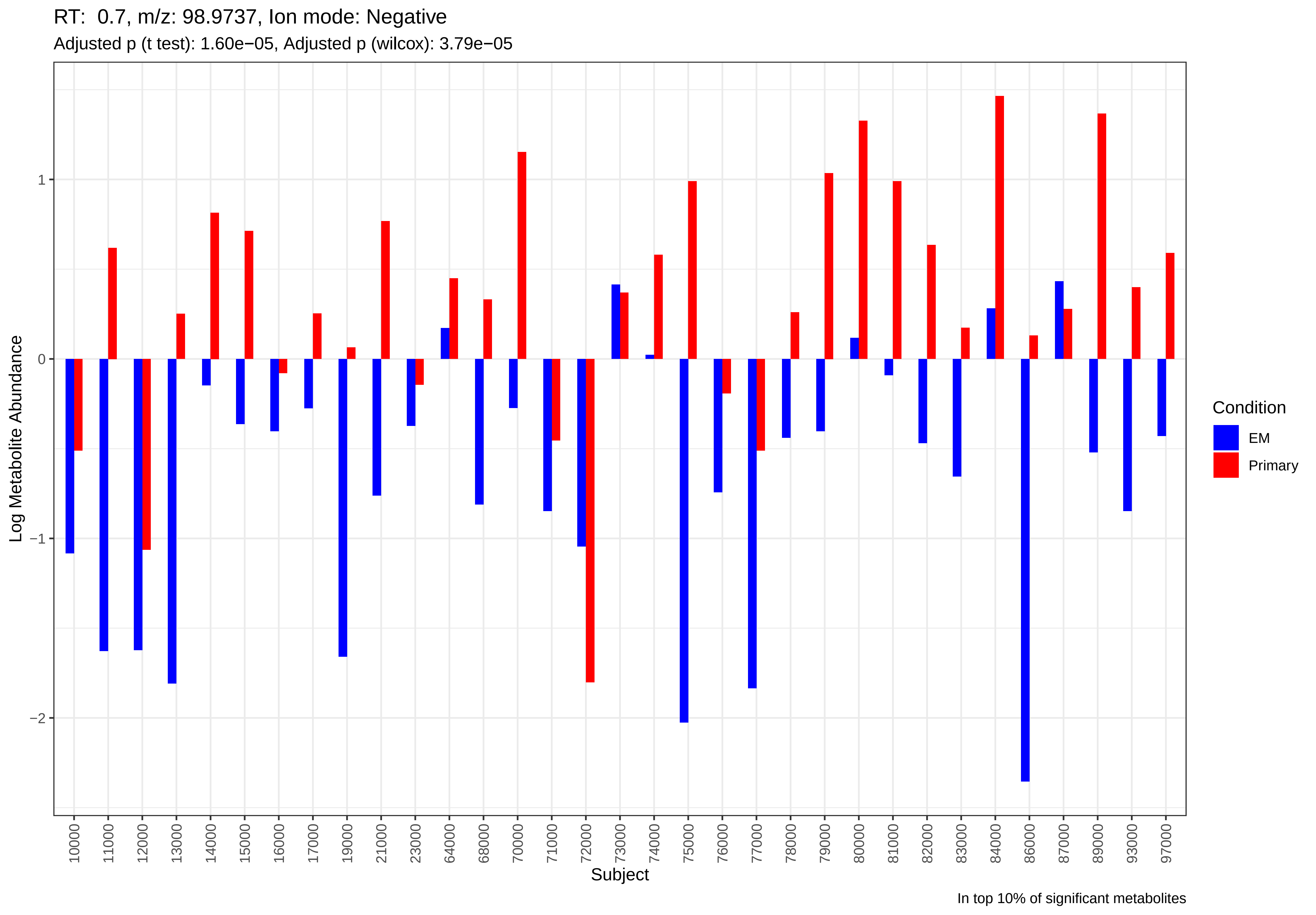

RT: 0.7, m/z: 98.9737, Ion mode: Negative
Adjusted p (t test): 1.60e−05, Adjusted p (wilcox): 3.79e−05
Log Metabolite Abundance
1
0
−1
−2
10000
11000
12000
13000
14000
15000
16000
17000
19000
21000
23000
64000
68000
70000
71000
72000
73000
74000
75000
76000
77000
78000
79000
80000
81000
82000
83000
84000
86000
87000
89000
93000
97000
Subject
Condition
EM
Primary
In top 10% of significant metabolites

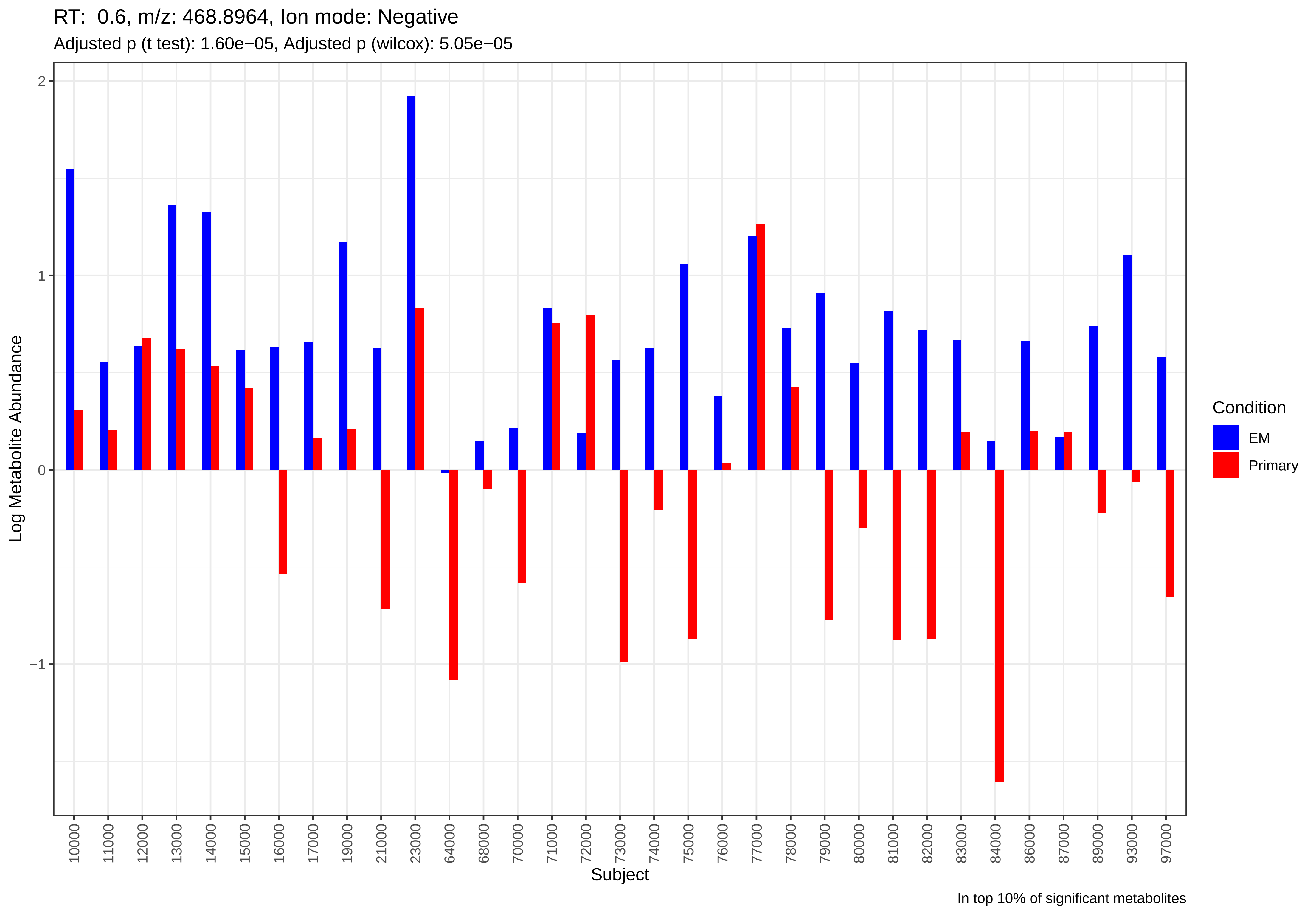

RT: 0.6, m/z: 468.8964, Ion mode: Negative
Adjusted p (t test): 1.60e−05, Adjusted p (wilcox): 5.05e−05
Log Metabolite Abundance
2
1
0
−1
10000
11000
12000
13000
14000
15000
16000
17000
19000
21000
23000
64000
68000
70000
71000
72000
73000
74000
75000
76000
77000
78000
79000
80000
81000
82000
83000
84000
86000
87000
89000
93000
97000
Subject
Condition
EM
Primary
In top 10% of significant metabolites

RT: 0.8, m/z: 154.8986, Ion mode: Negative

Adjusted p (t test): 1.66e−05, Adjusted p (wilcox): 2.74e−05

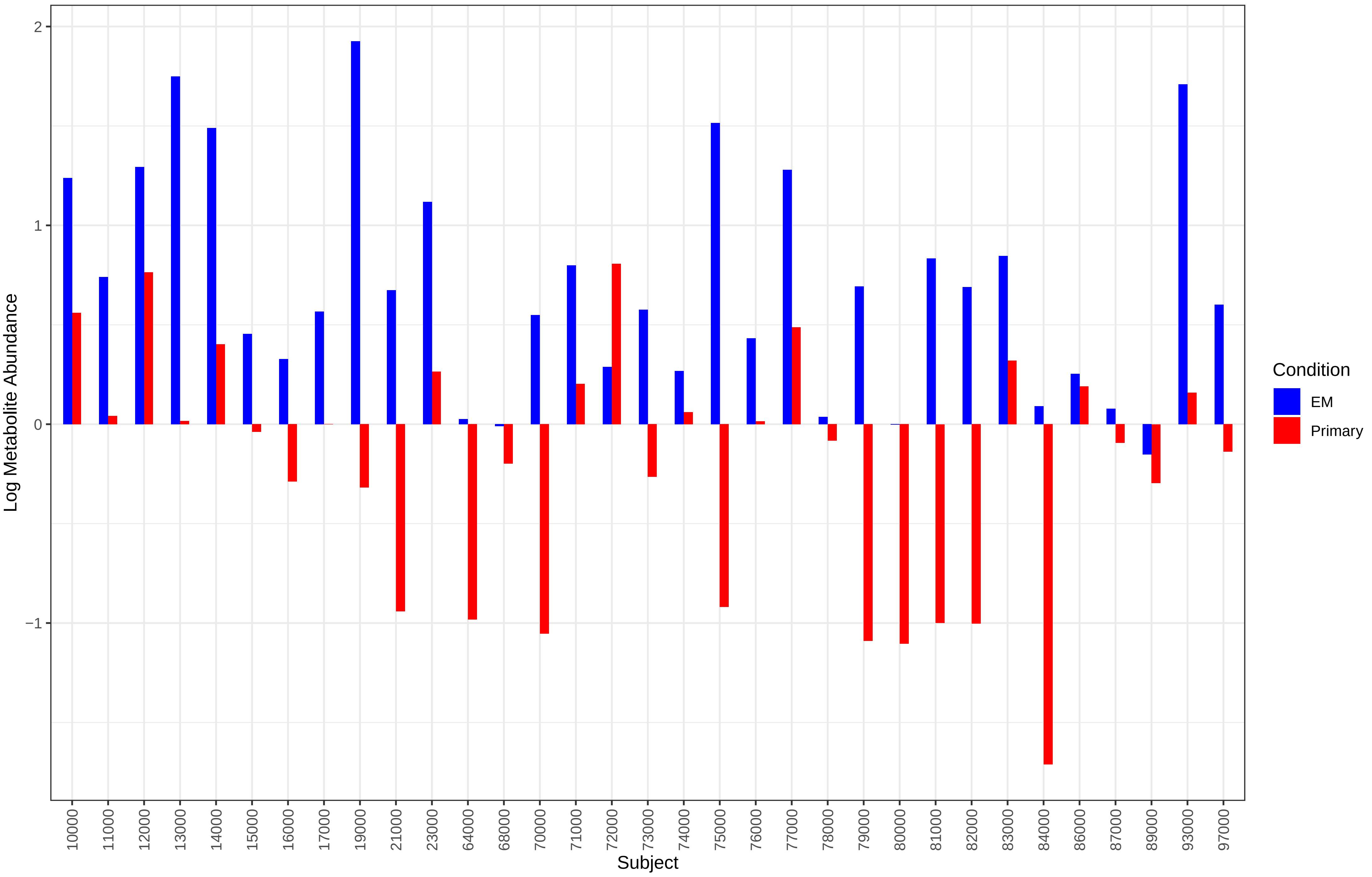

In top 10% of significant metabolites

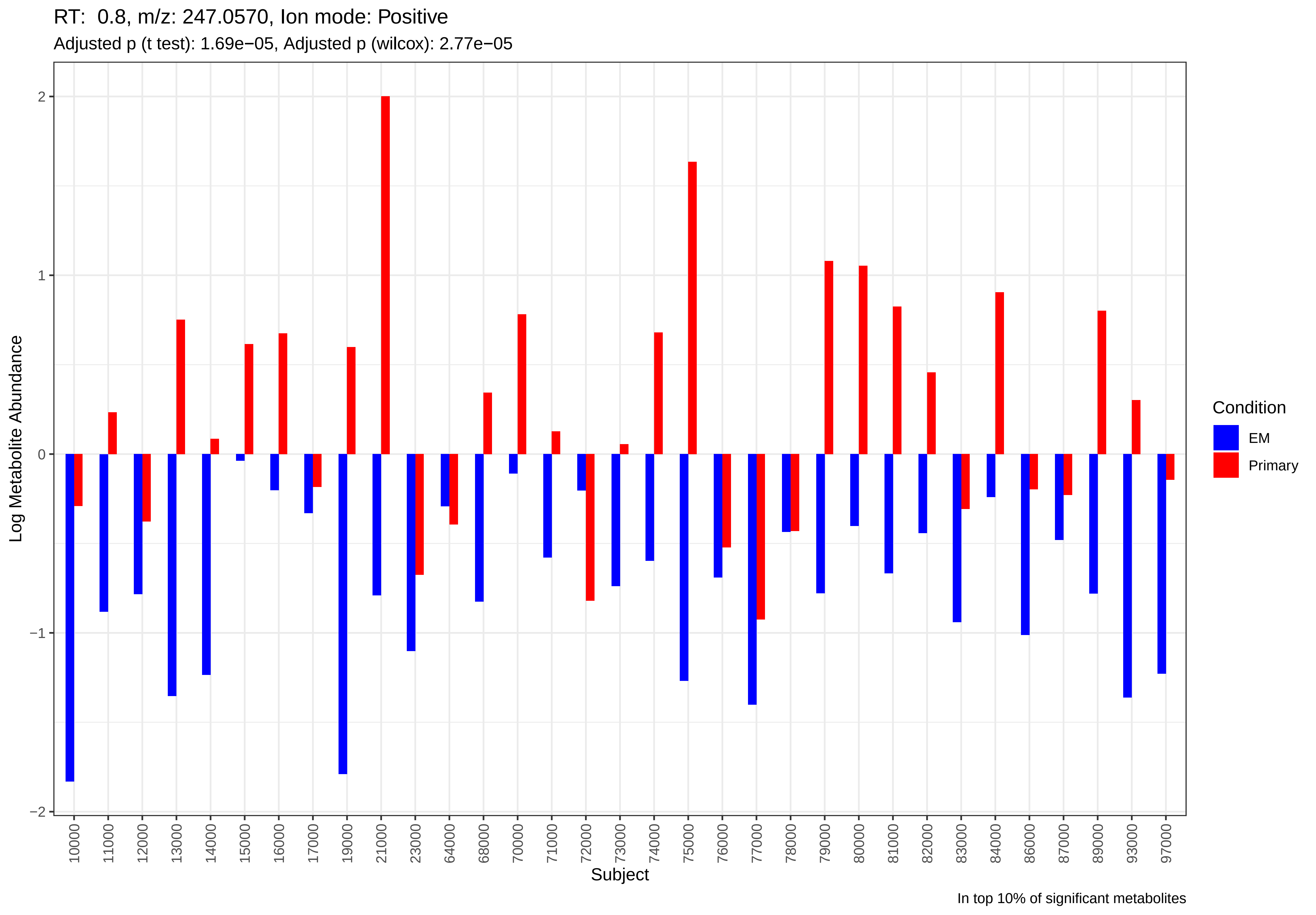

RT: 0.8, m/z: 247.0570, Ion mode: Positive
Adjusted p (t test): 1.69e−05, Adjusted p (wilcox): 2.77e−05
Log Metabolite Abundance
2
1
0
−1
−2
10000
11000
12000
13000
14000
15000
16000
17000
19000
21000
23000
64000
68000
70000
71000
72000
73000
74000
75000
76000
77000
78000
79000
80000
81000
82000
83000
84000
86000
87000
89000
93000
97000
Subject
Condition
EM
Primary
In top 10% of significant metabolites

RT: 0.8, m/z: 451.1265, Ion mode: Negative

Adjusted p (t test): 1.72e−05, Adjusted p (wilcox): 3.25e−05

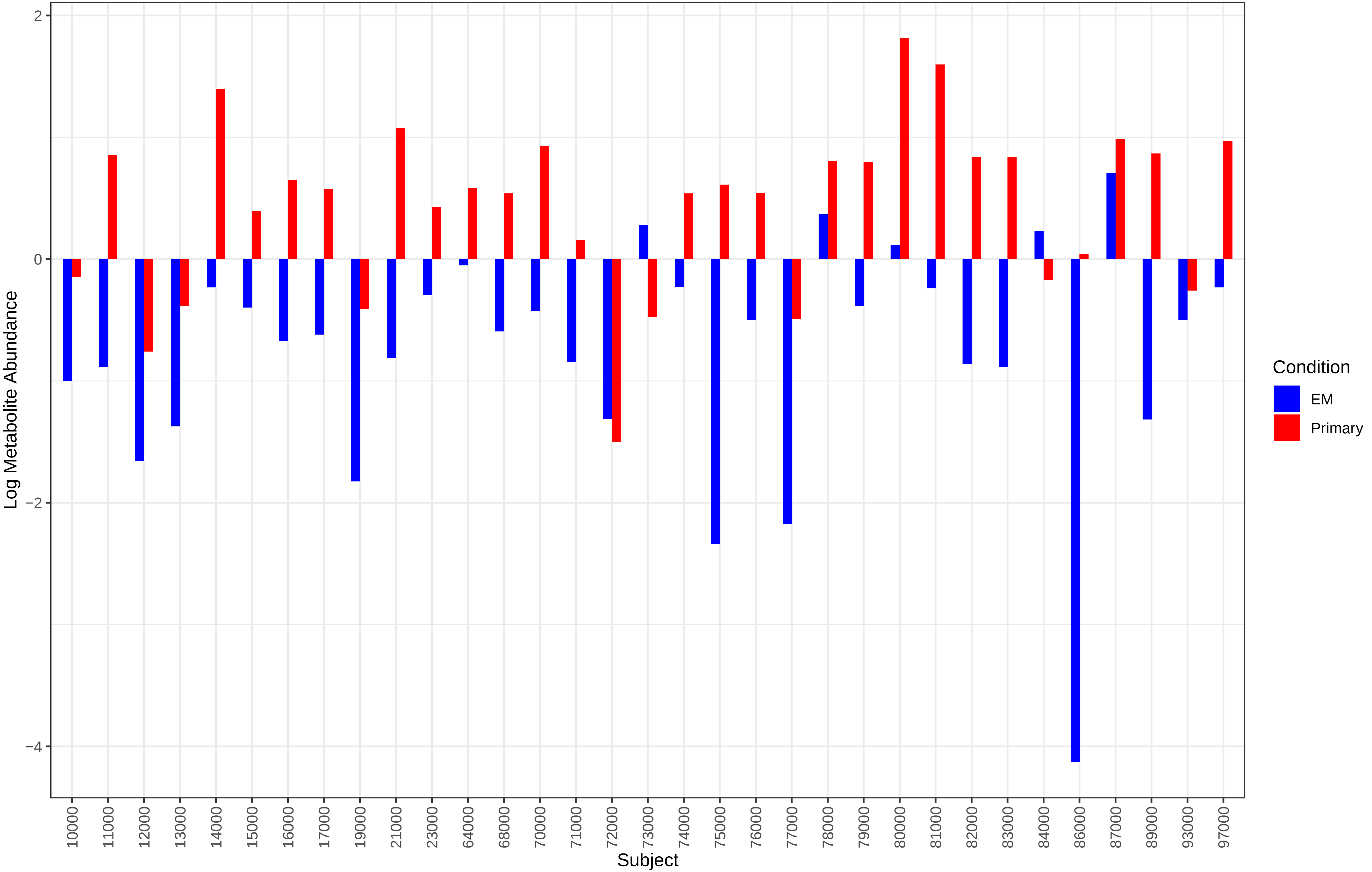

In top 10% of significant metabolites

DIHYDROXYACETONE PHOSPHATE (RT: 0.7, m/z: 171.0051, Ion mode: Positive)

Adjusted p (t test): 1.78e−05, Adjusted p (wilcox): 7.74e−05

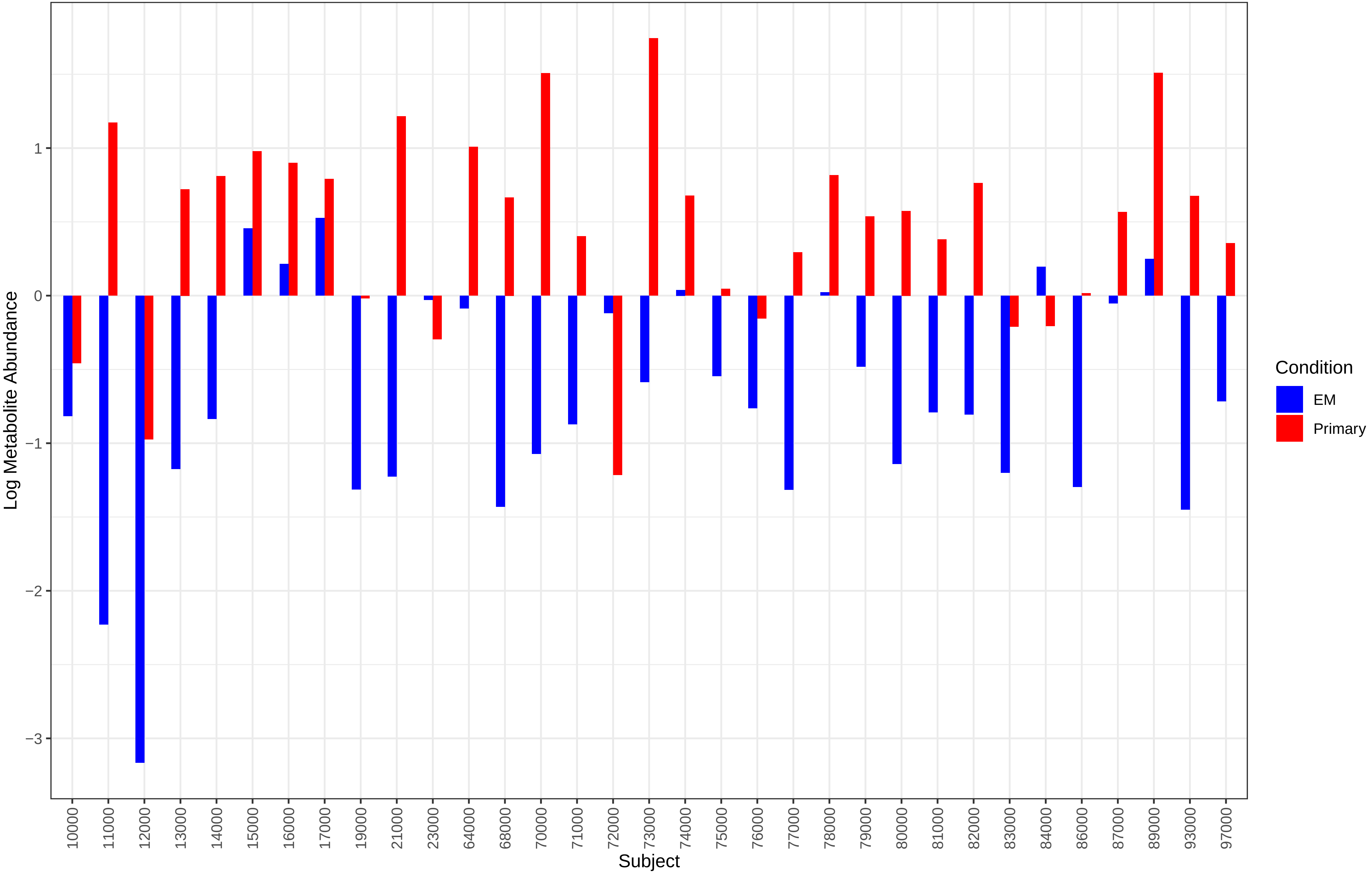

In top 10% of significant metabolites

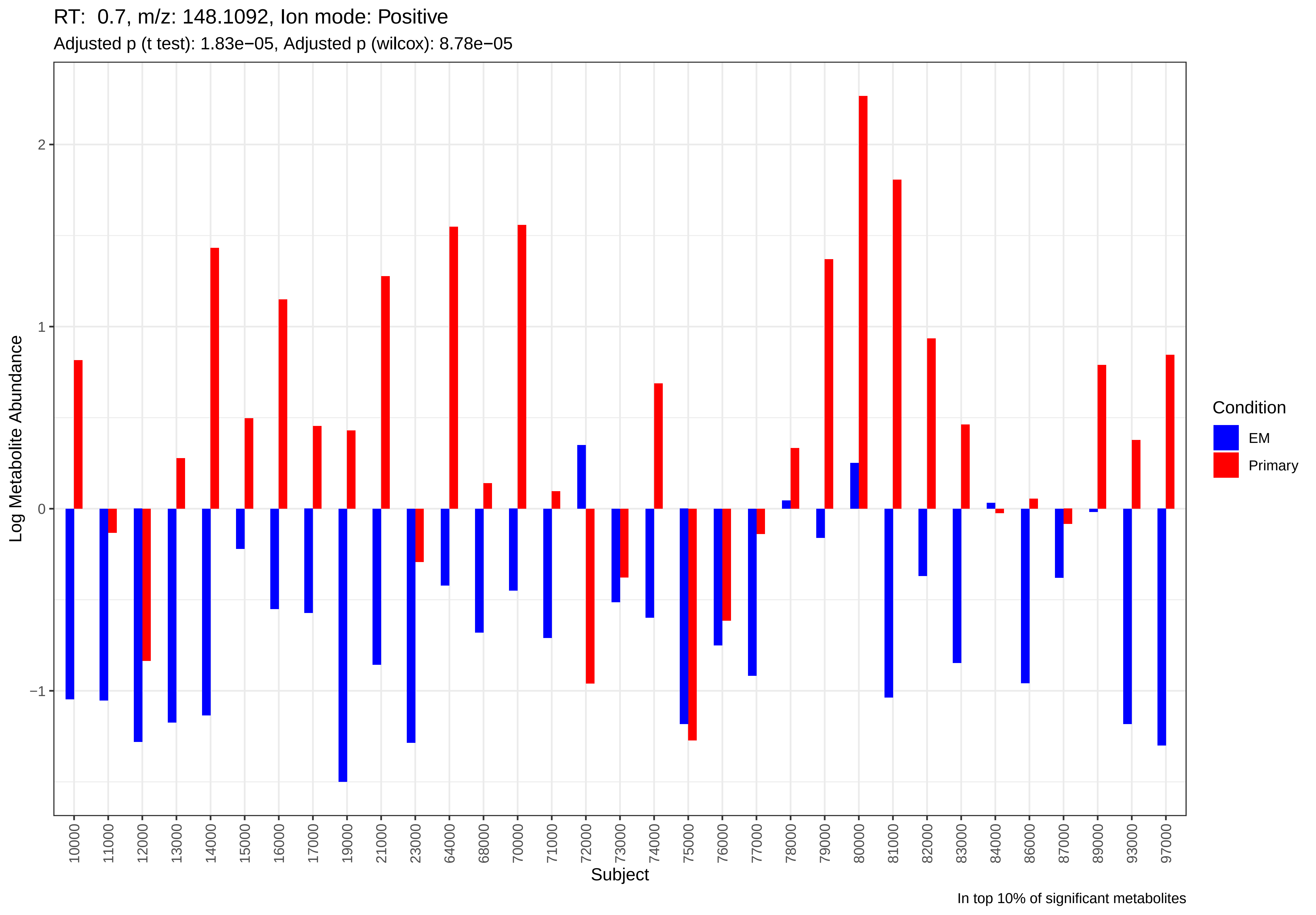
RT: 0.7, m/z: 148.1092, Ion mode: Positive
Adjusted p (t test): 1.83e−05, Adjusted p (wilcox): 8.78e−05
Log Metabolite Abundance
2
1
0
−1
10000
11000
12000
13000
14000
15000
16000
17000
19000
21000
23000
64000
68000
70000
71000
72000
73000
74000
75000
76000
77000
78000
79000
80000
81000
82000
83000
84000
86000
87000
89000
93000
97000
Subject
Condition
EM
Primary
In top 10% of significant metabolites

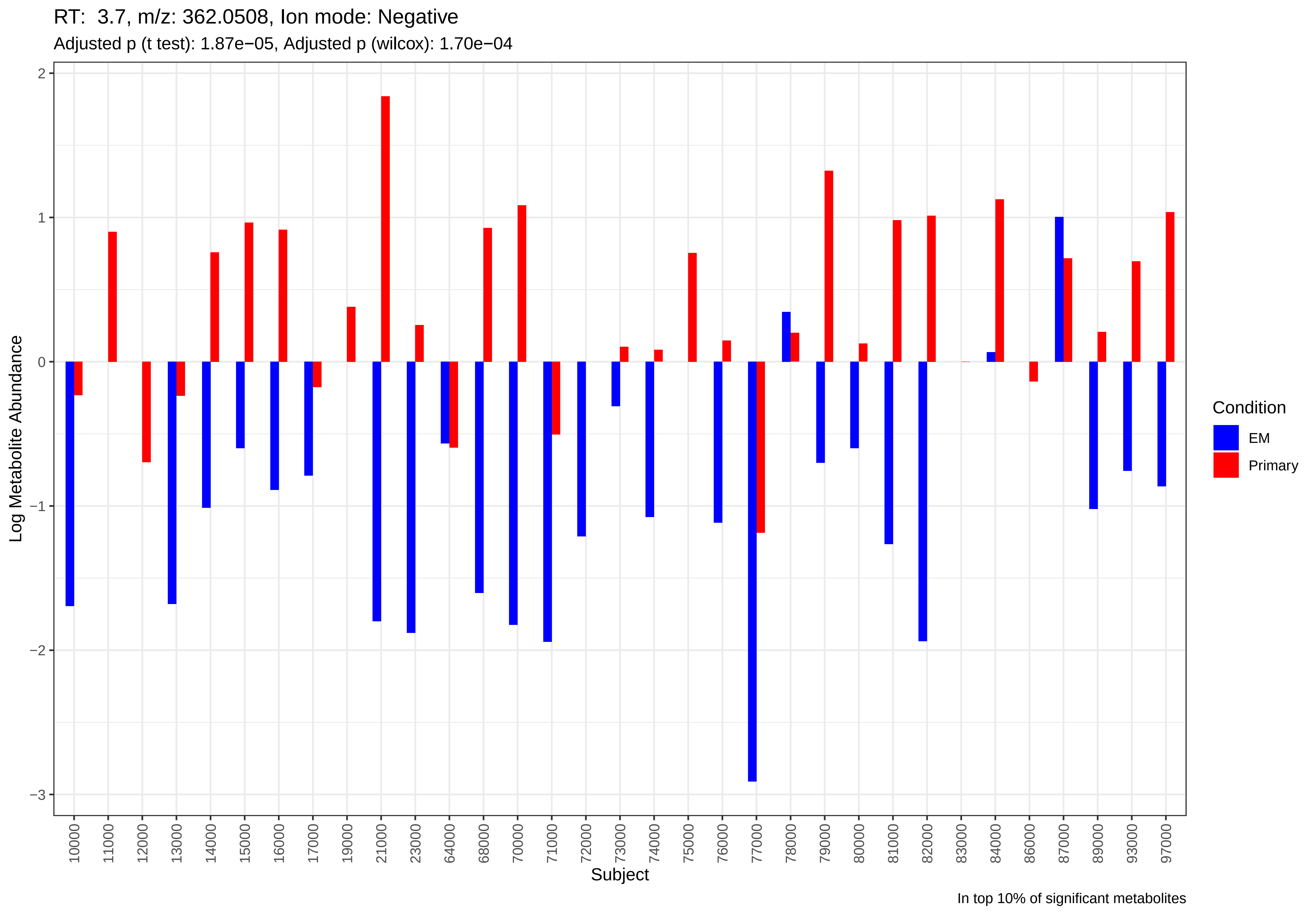

RT: 3.7, m/z: 362.0508, Ion mode: Negative
Adjusted p (t test): 1.87e−05, Adjusted p (wilcox): 1.70e−04
Log Metabolite Abundance
2
1
0
−1
−2
−3
Subject
10000
11000
12000
13000
14000
15000
16000
17000
19000
21000
23000
64000
68000
70000
71000
72000
73000
74000
75000
76000
77000
78000
79000
80000
81000
82000
83000
84000
86000
87000
89000
93000
97000
Condition
EM
Primary
In top 10% of significant metabolites

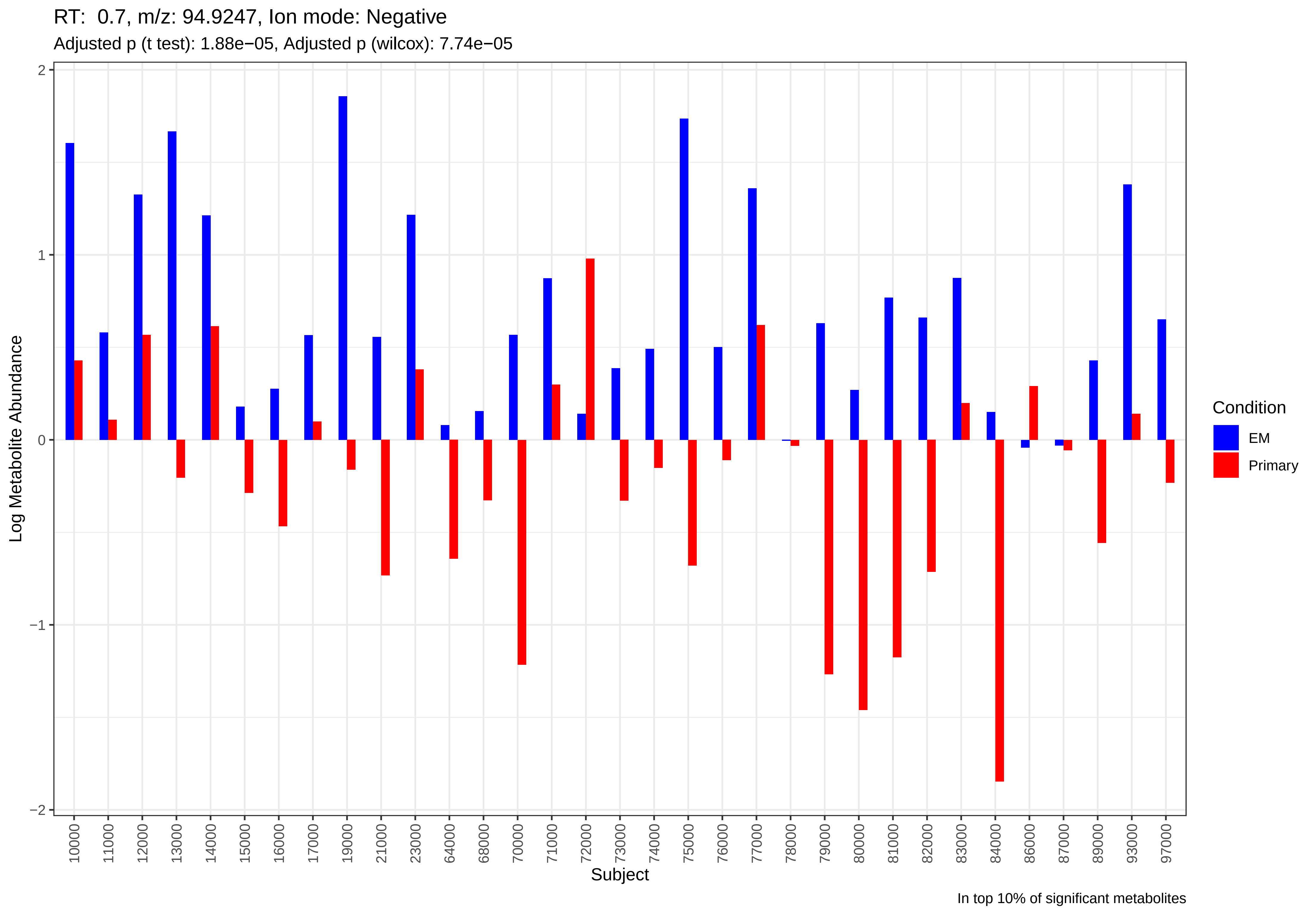

RT: 0.7, m/z: 94.9247, Ion mode: Negative
Adjusted p (t test): 1.88e−05, Adjusted p (wilcox): 7.74e−05
Log Metabolite Abundance
2
1
0
−1
−2
10000
11000
12000
13000
14000
15000
16000
17000
19000
21000
23000
64000
68000
70000
71000
72000
73000
74000
75000
76000
77000
78000
79000
80000
81000
82000
83000
84000
86000
87000
89000
93000
97000
Subject
Condition
EM
Primary
In top 10% of significant metabolites

RT: 2.1, m/z: 323.0286, Ion mode: Negative
Adjusted p (t test): 1.88e−05, Adjusted p (wilcox): 6.63e−05

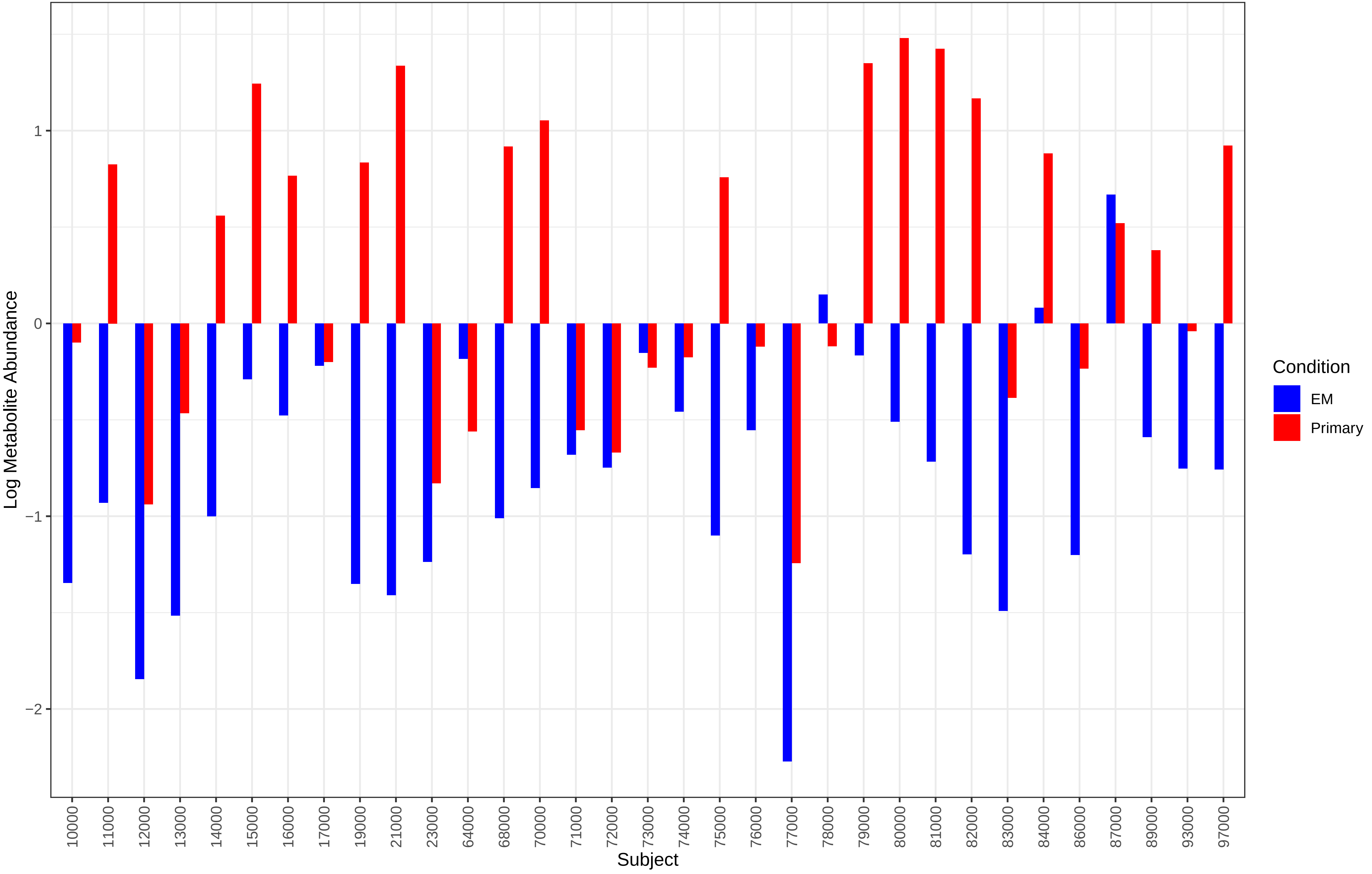

[M+ACN+H] 42.0338 m/z adduct of 138.0529 m/z (RT: 0.7, m/z: 180.0865, Ion mode: Positive)

Adjusted p (t test): 1.93e−05, Adjusted p (wilcox): 3.25e−05

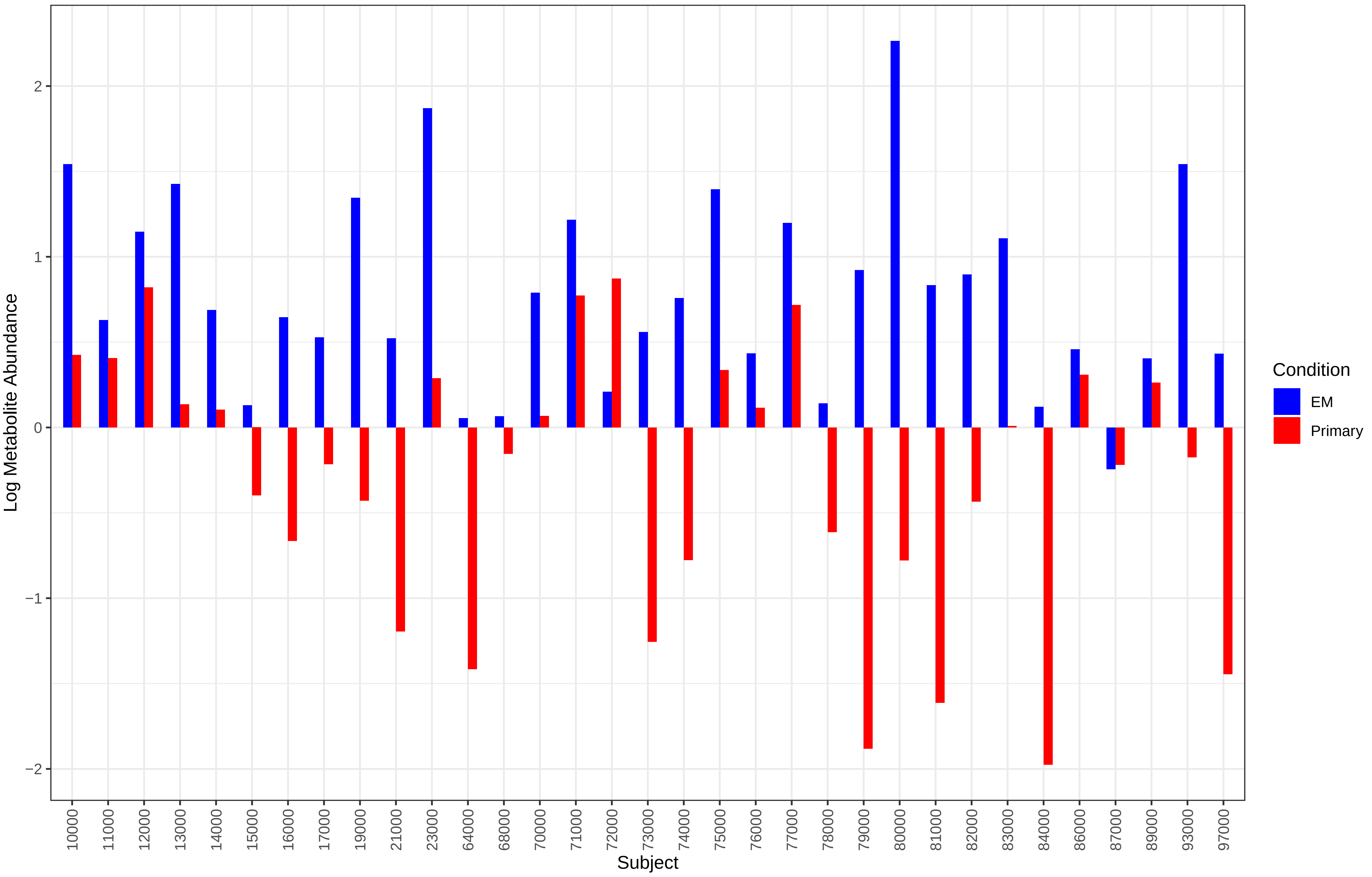

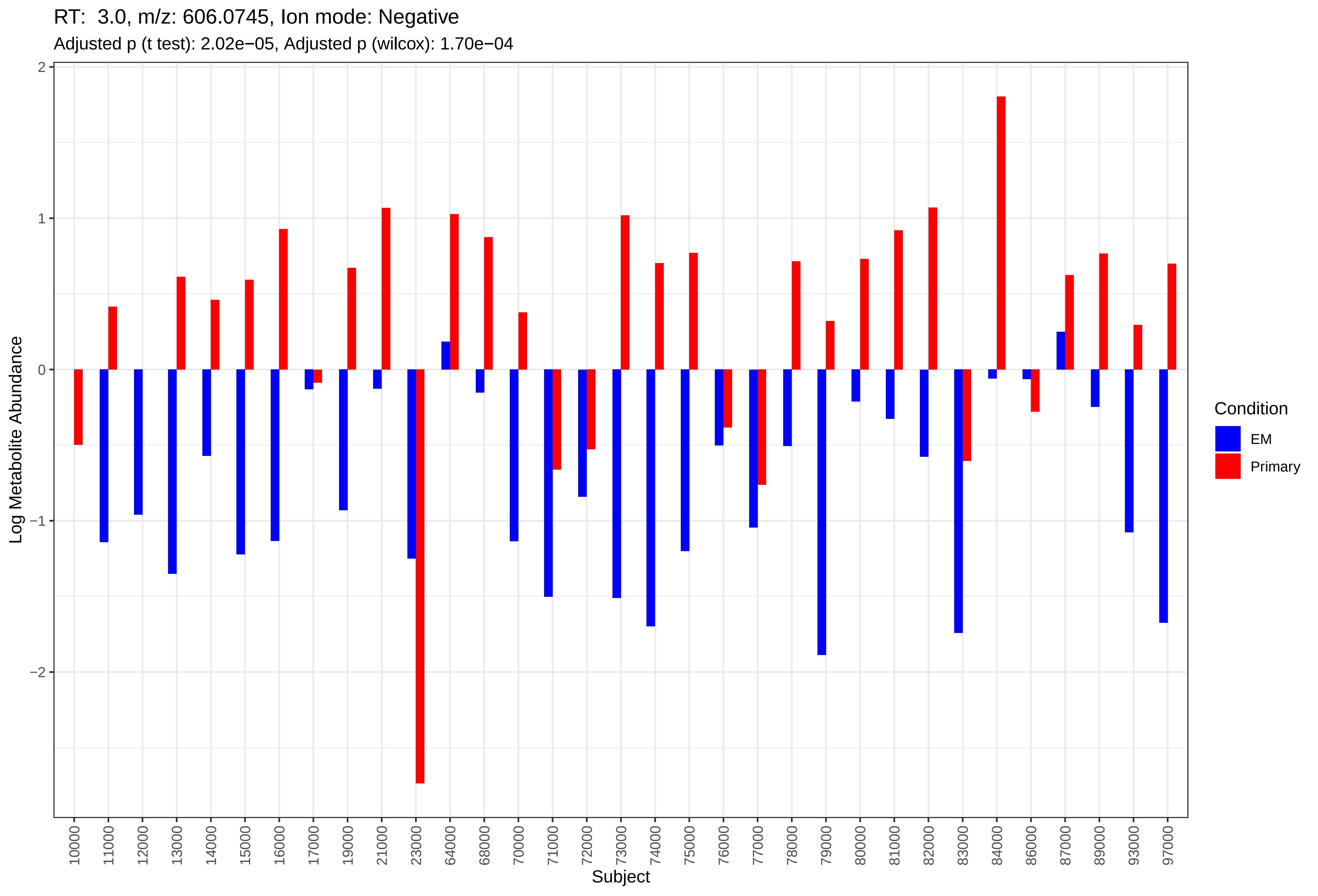
RT: 3.0, m/z: 606.0745, Ion mode: Negative
Adjusted p (t test): 2.02e−05, Adjusted p (wilcox): 1.70e−04
Log Metabolite Abundance
2
1
0
−1
−2
Subject
10000
11000
12000
13000
14000
15000
16000
17000
19000
21000
23000
64000
68000
70000
71000
72000
73000
74000
75000
76000
77000
78000
79000
80000
81000
82000
83000
84000
86000
87000
89000
93000
97000
Condition
EM
Primary

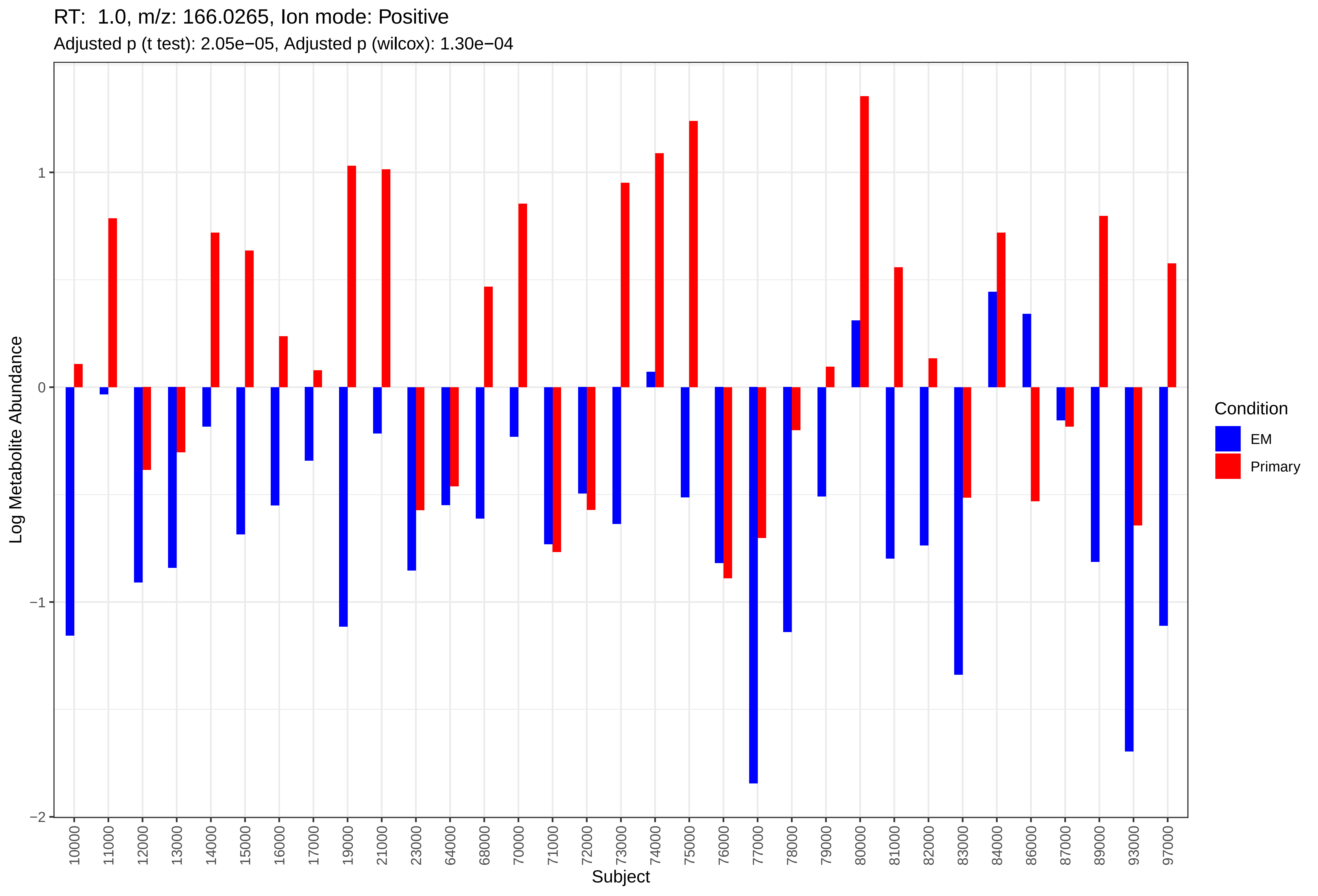
RT: 1.0, m/z: 166.0265, Ion mode: Positive
Adjusted p (t test): 2.05e−05, Adjusted p (wilcox): 1.30e−04
Log Metabolite Abundance
1
0
−1
−2
10000
11000
12000
13000
14000
15000
16000
17000
19000
21000
23000
64000
68000
70000
71000
72000
73000
74000
75000
76000
77000
78000
79000
80000
81000
82000
83000
84000
86000
87000
89000
93000
97000
Subject
Condition
EM
Primary

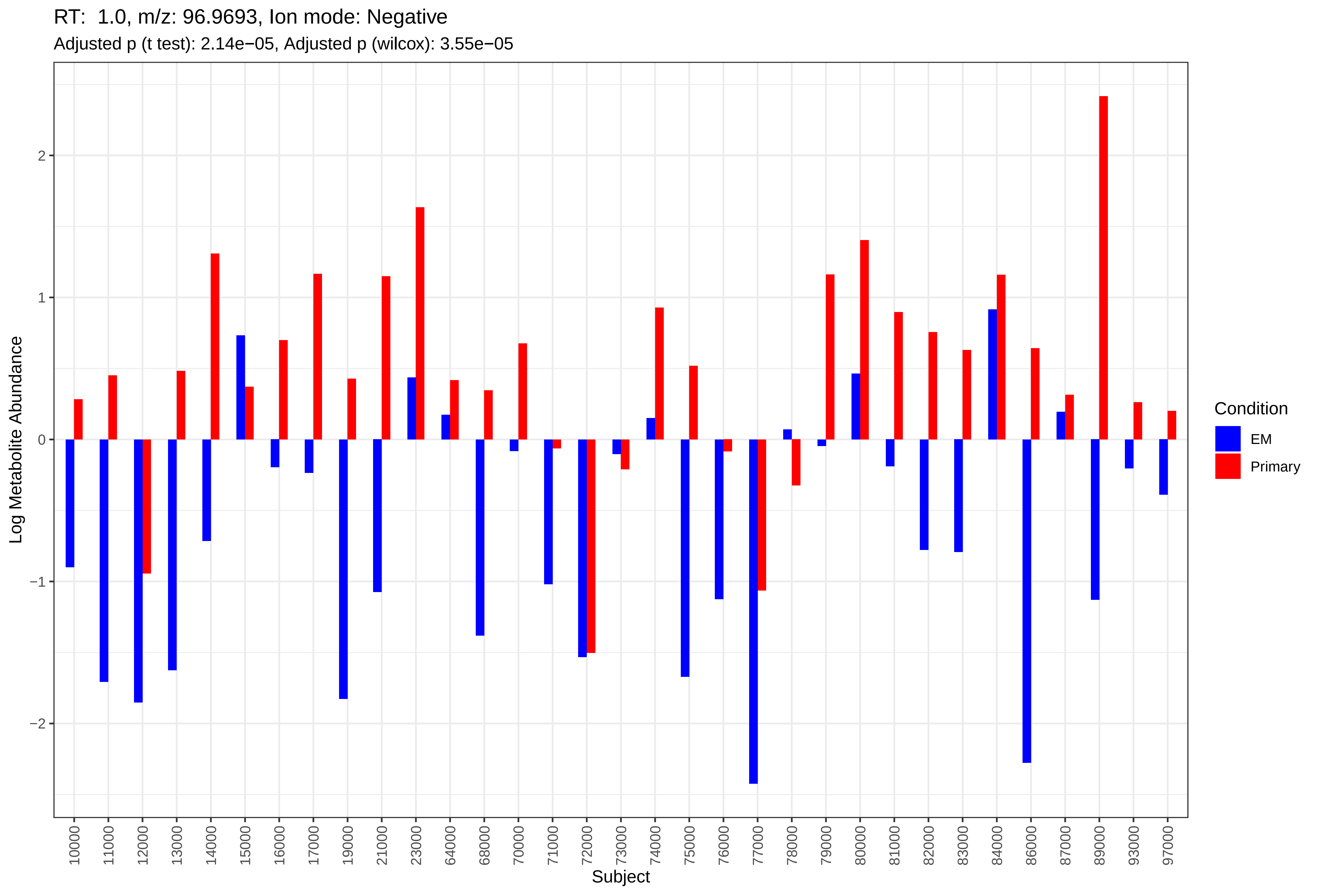
RT: 1.0, m/z: 96.9693, Ion mode: Negative
Adjusted p (t test): 2.14e−05, Adjusted p (wilcox): 3.55e−05
Log Metabolite Abundance
2
1
0
−1
−2
10000
11000
12000
13000
14000
15000
16000
17000
19000
21000
23000
64000
68000
70000
71000
72000
73000
74000
75000
76000
77000
78000
79000
80000
81000
82000
83000
84000
86000
87000
89000
93000
97000
Subject
Condition
EM
Primary

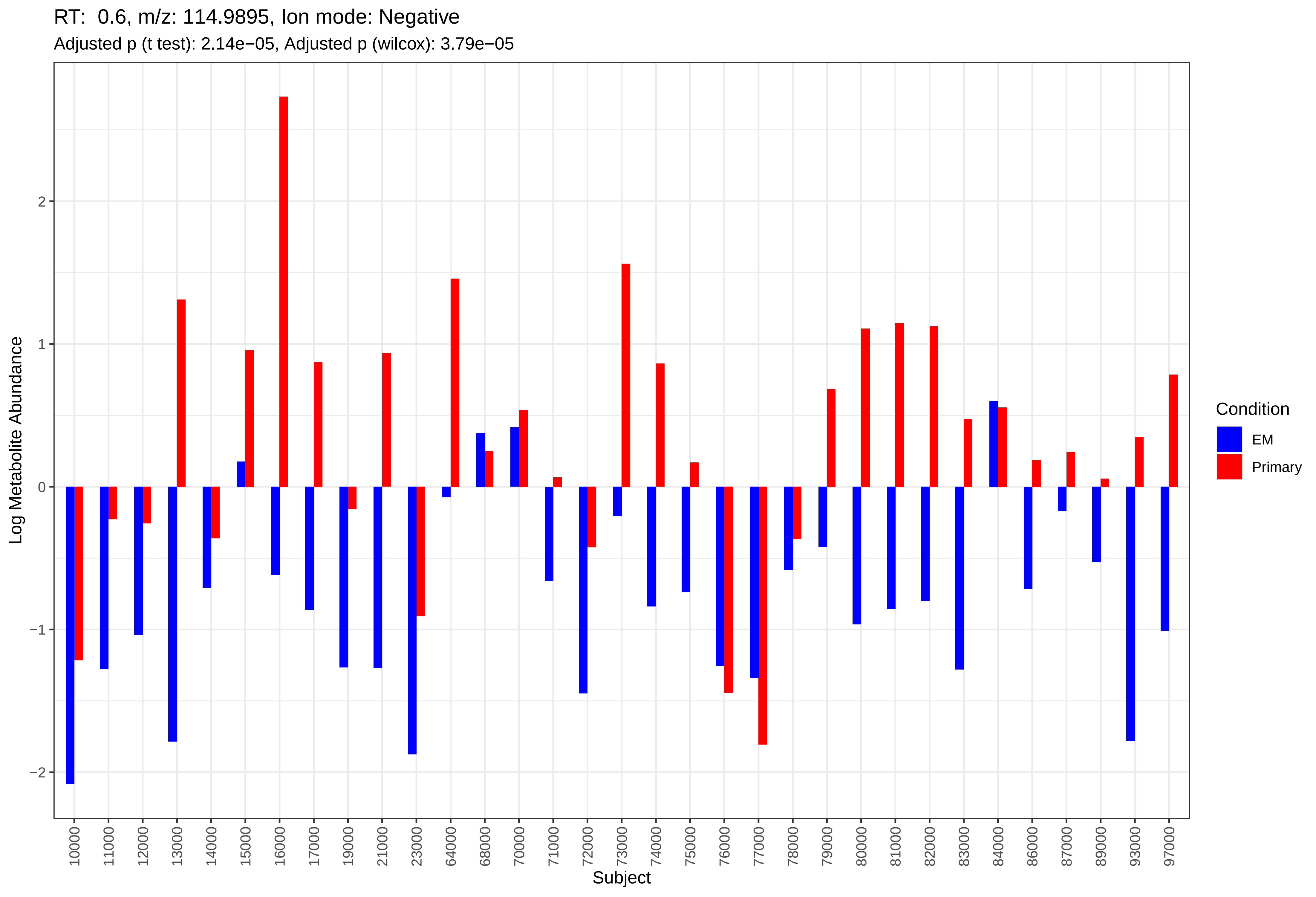
RT: 0.6, m/z: 114.9895, Ion mode: Negative
Adjusted p (t test): 2.14e−05, Adjusted p (wilcox): 3.79e−05
Log Metabolite Abundance
2
1
0
−1
−2
10000
11000
12000
13000
14000
15000
16000
17000
19000
21000
23000
64000
68000
70000
71000
72000
73000
74000
75000
76000
77000
78000
79000
80000
81000
82000
83000
84000
86000
87000
89000
93000
97000
Subject
Condition
EM
Primary

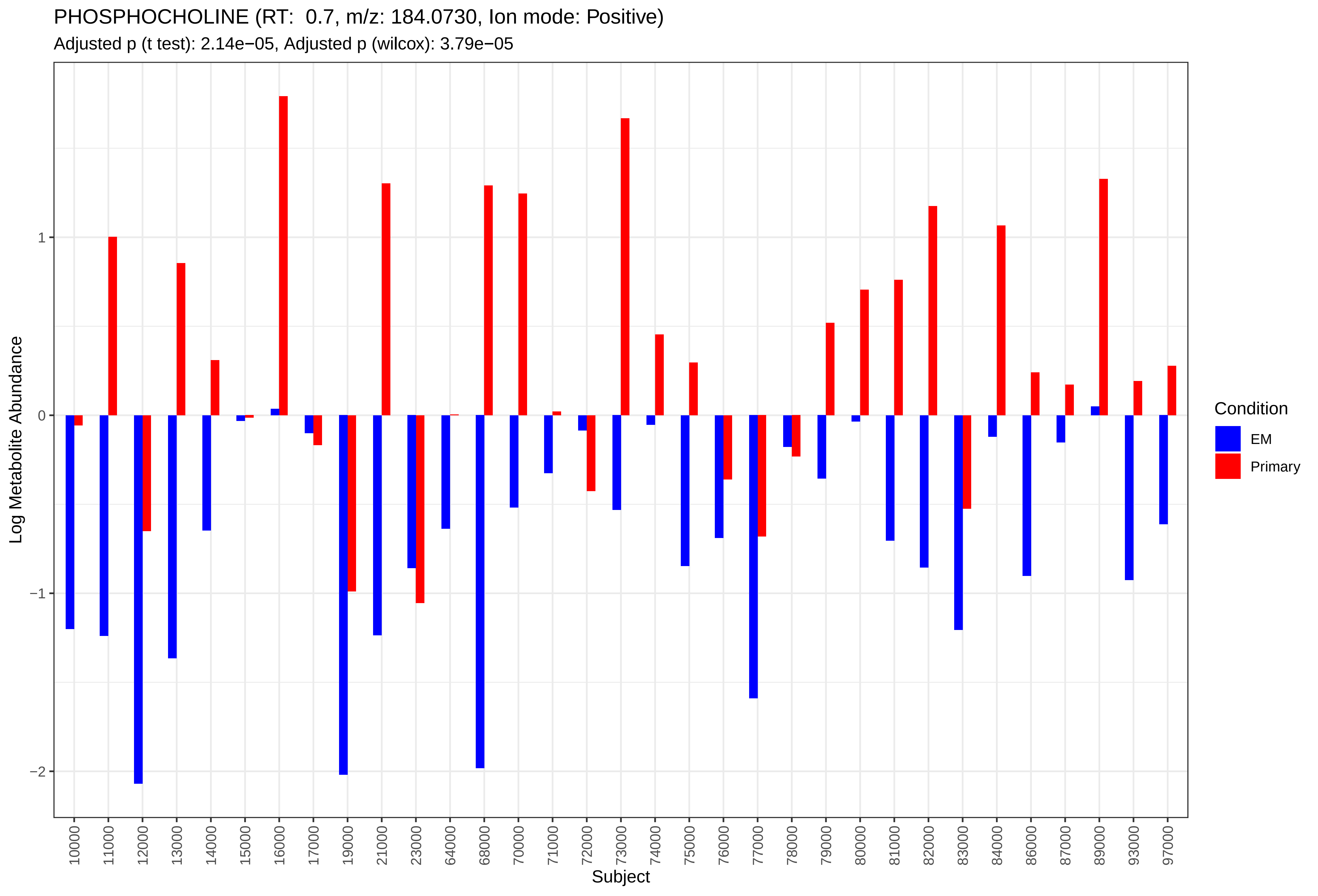

PHOSPHOCHOLINE (RT: 0.7, m/z: 184.0730, Ion mode: Positive)
Adjusted p (t test): 2.14e−05, Adjusted p (wilcox): 3.79e−05
Log Metabolite Abundance
1
0
−1
−2
10000
11000
12000
13000
14000
15000
16000
17000
19000
21000
23000
64000
68000
70000
71000
72000
73000
74000
75000
76000
77000
78000
79000
80000
81000
82000
83000
84000
86000
87000
89000
93000
97000
Subject
Condition
EM
Primary

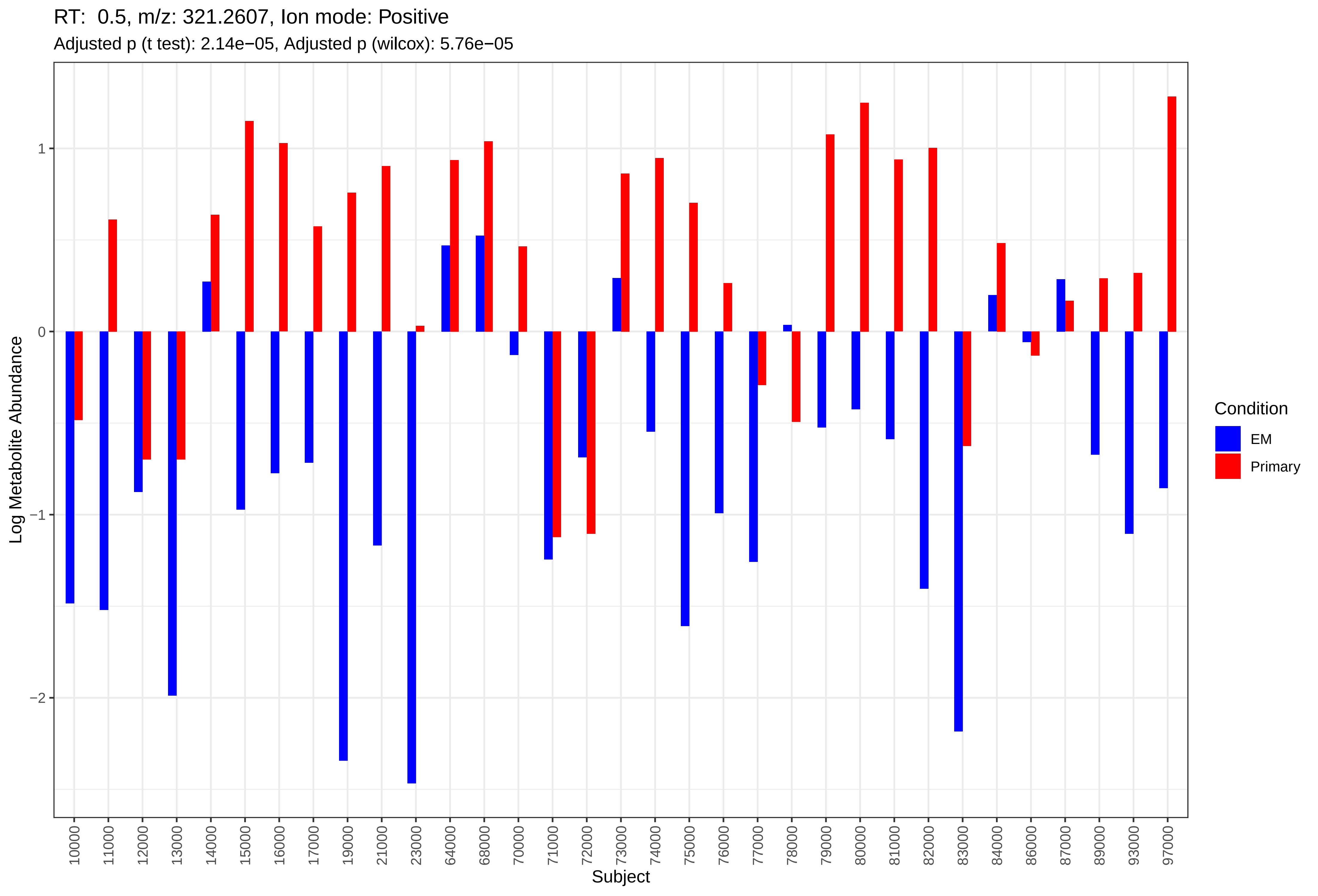

RT: 0.5, m/z: 321.2607, Ion mode: Positive
Adjusted p (t test): 2.14e−05, Adjusted p (wilcox): 5.76e−05
Log Metabolite Abundance
1
0
−1
−2
Subject
10000
11000
12000
13000
14000
15000
16000
17000
19000
21000
23000
64000
68000
70000
71000
72000
73000
74000
75000
76000
77000
78000
79000
80000
81000
82000
83000
84000
86000
87000
89000
93000
97000
Condition
EM
Primary

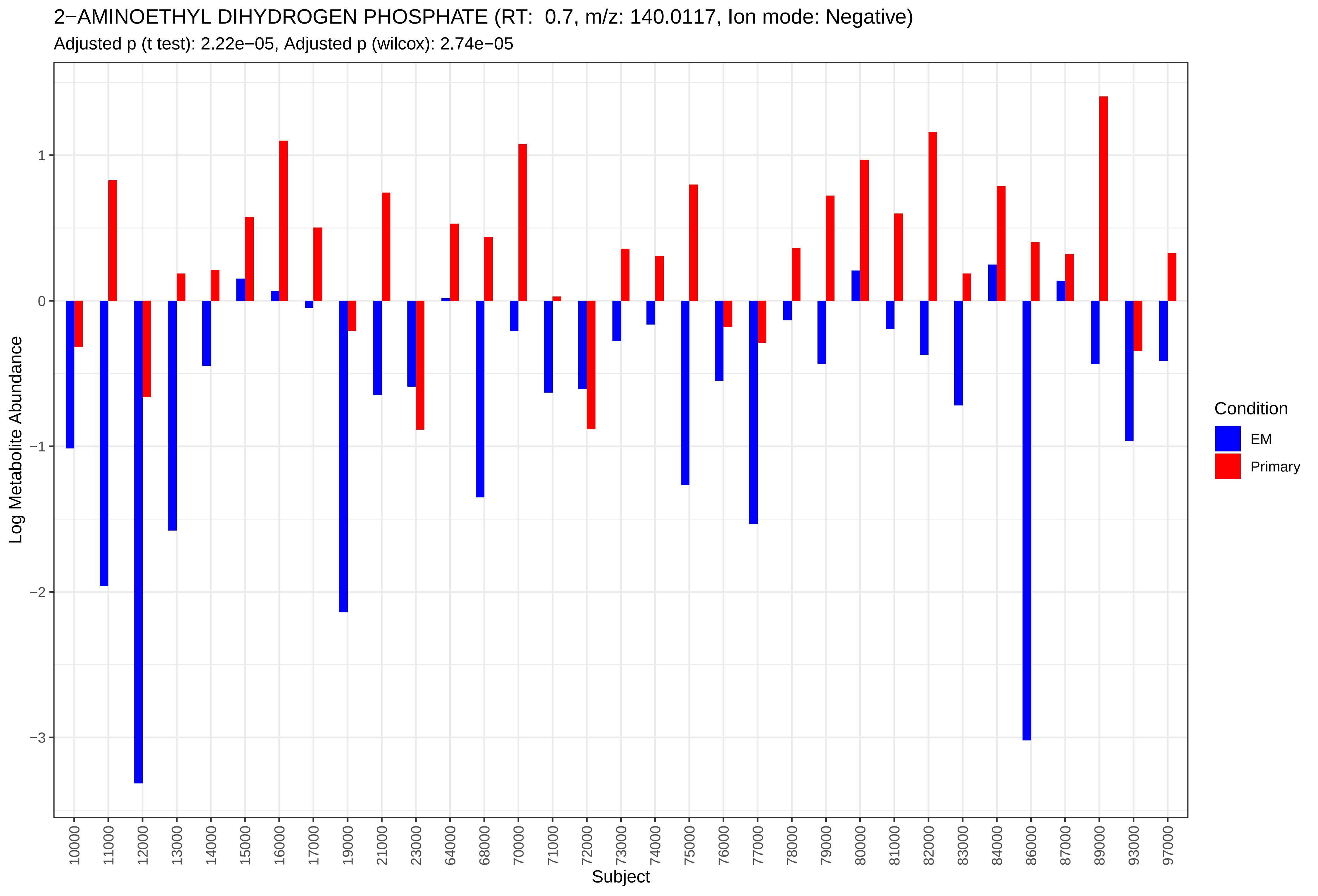

2−AMINOETHYL DIHYDROGEN PHOSPHATE (RT: 0.7, m/z: 140.0117, Ion mode: Negative)
Adjusted p (t test): 2.22e−05, Adjusted p (wilcox): 2.74e−05
Log Metabolite Abundance
1
0
−1
−2
−3
Subject
10000
11000
12000
13000
14000
15000
16000
17000
19000
21000
23000
64000
68000
70000
71000
72000
73000
74000
75000
76000
77000
78000
79000
80000
81000
82000
83000
84000
86000
87000
89000
93000
97000
Condition
EM
Primary

RT: 0.7, m/z: 142.9750, Ion mode: Negative

Adjusted p (t test): 2.27e−05, Adjusted p (wilcox): 6.63e−05

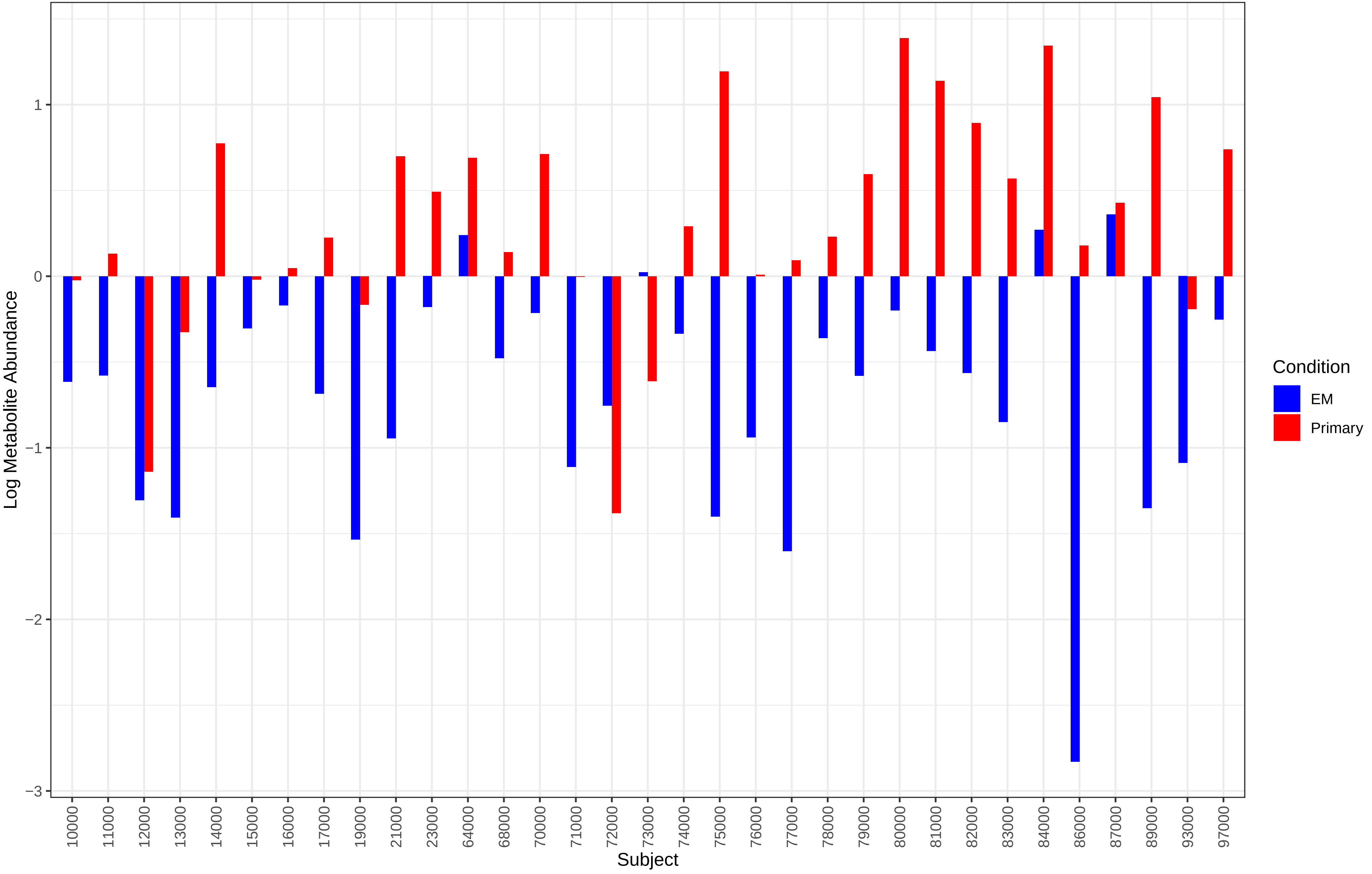

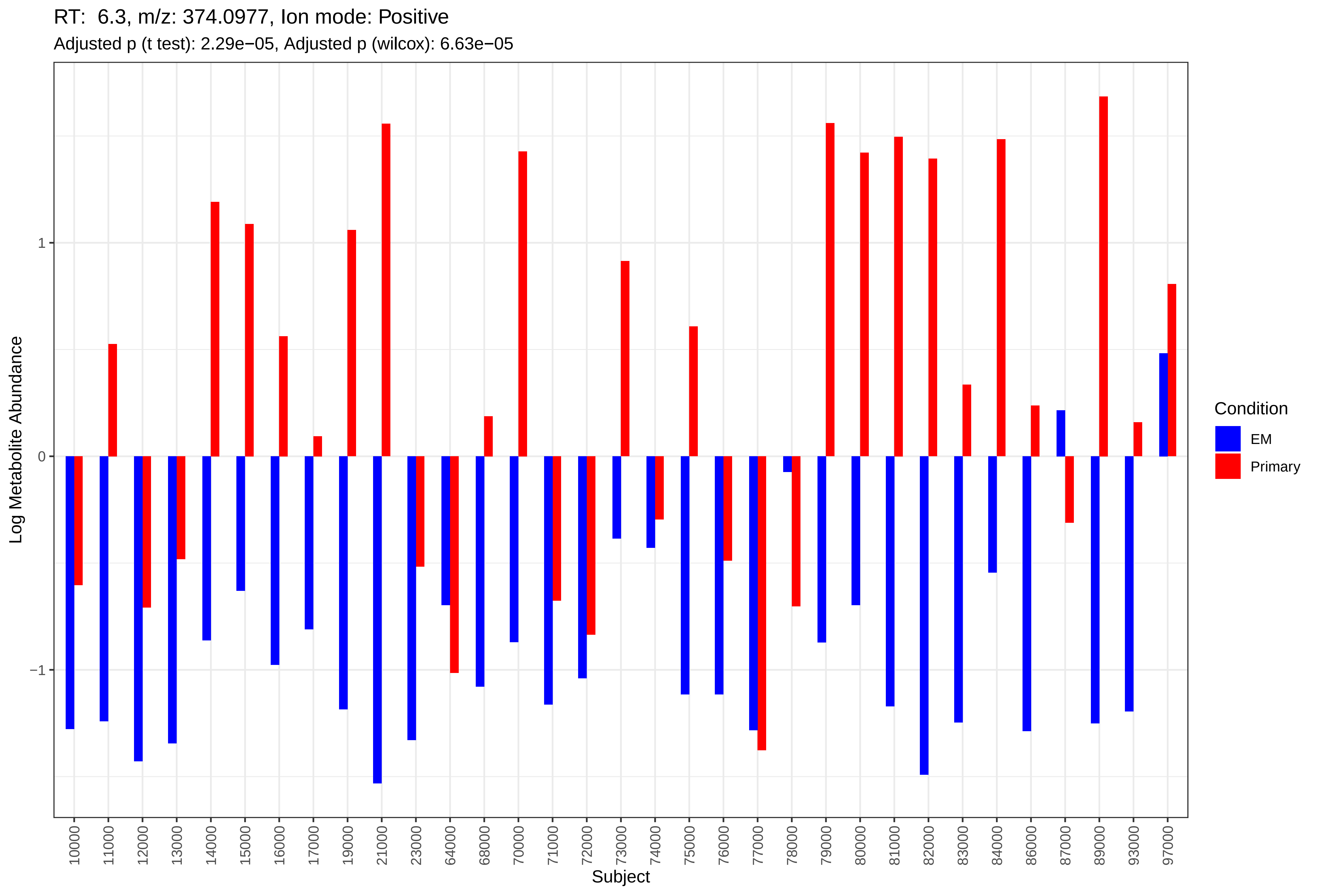

RT: 6.3, m/z: 374.0977, Ion mode: Positive
Adjusted p (t test): 2.29e−05, Adjusted p (wilcox): 6.63e−05
Log Metabolite Abundance
1
0
−1
10000
11000
12000
13000
14000
15000
16000
17000
19000
21000
23000
64000
68000
70000
71000
72000
73000
74000
75000
76000
77000
78000
79000
80000
81000
82000
83000
84000
86000
87000
89000
93000
97000
Subject
Condition
EM
Primary

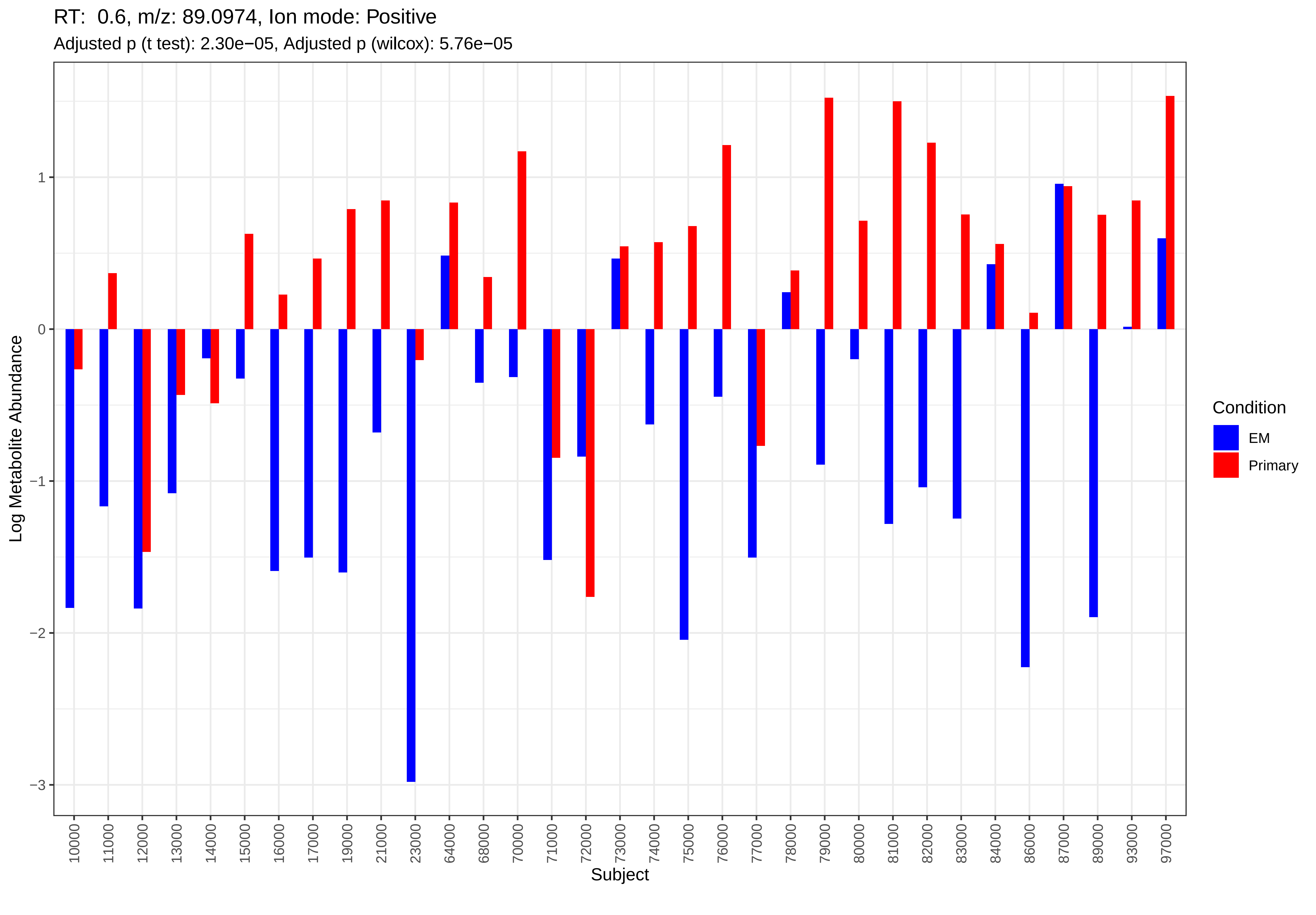
RT: 0.6, m/z: 89.0974, Ion mode: Positive
Adjusted p (t test): 2.30e−05, Adjusted p (wilcox): 5.76e−05
Log Metabolite Abundance
1
0
−1
−2
−3
10000
11000
12000
13000
14000
15000
16000
17000
19000
21000
23000
64000
68000
70000
71000
72000
73000
74000
75000
76000
77000
78000
79000
80000
81000
82000
83000
84000
86000
87000
89000
93000
97000
Subject
Condition
EM
Primary

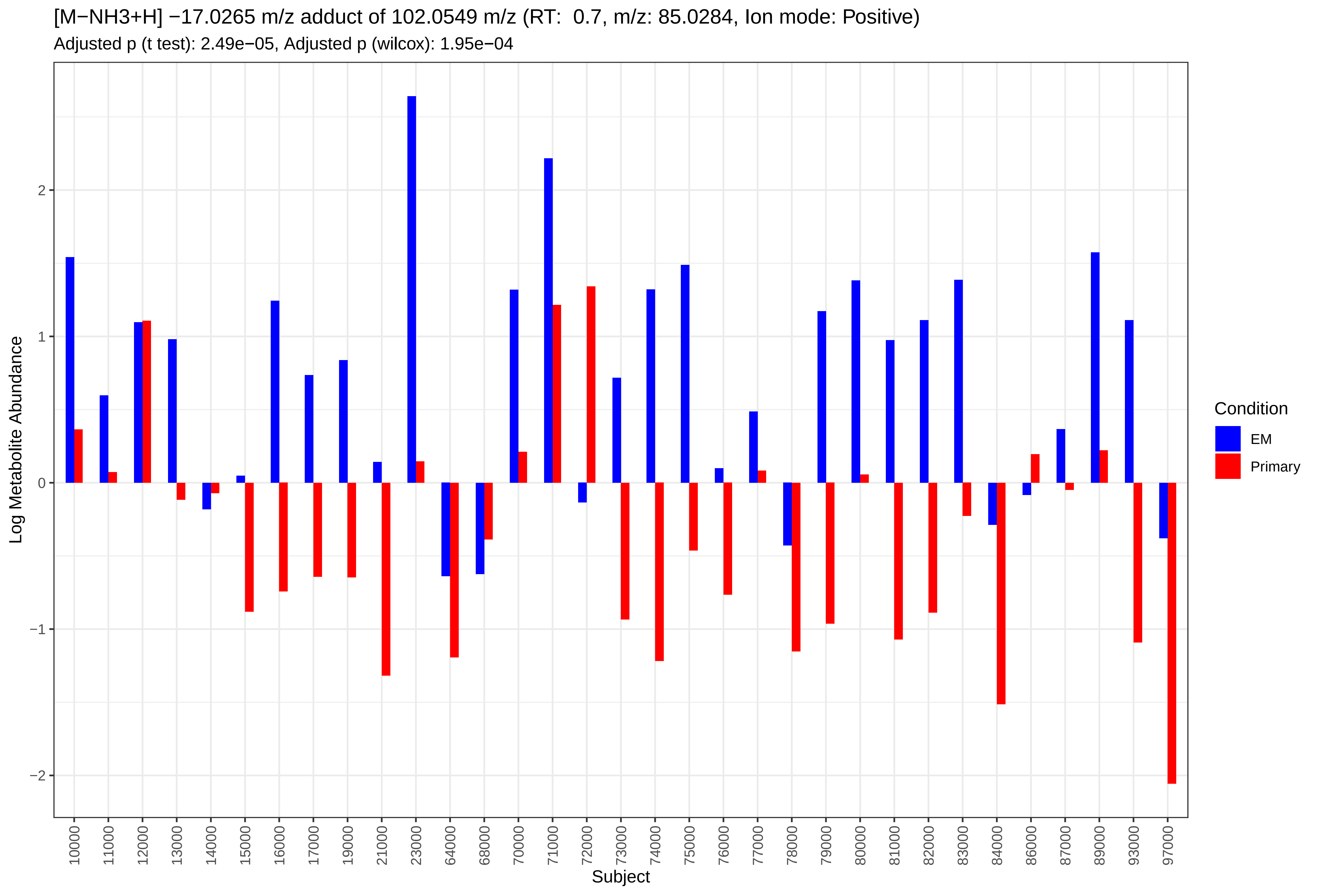
[M−NH3+H] −17.0265 m/z adduct of 102.0549 m/z (RT: 0.7, m/z: 85.0284, Ion mode: Positive)
Adjusted p (t test): 2.49e−05, Adjusted p (wilcox): 1.95e−04
Log Metabolite Abundance
Subject
Condition
EM
Primary
2
1
0
−1
−2
10000
11000
12000
13000
14000
15000
16000
17000
19000
21000
23000
64000
68000
70000
71000
72000
73000
74000
75000
76000
77000
78000
79000
80000
81000
82000
83000
84000
86000
87000
89000
93000
97000

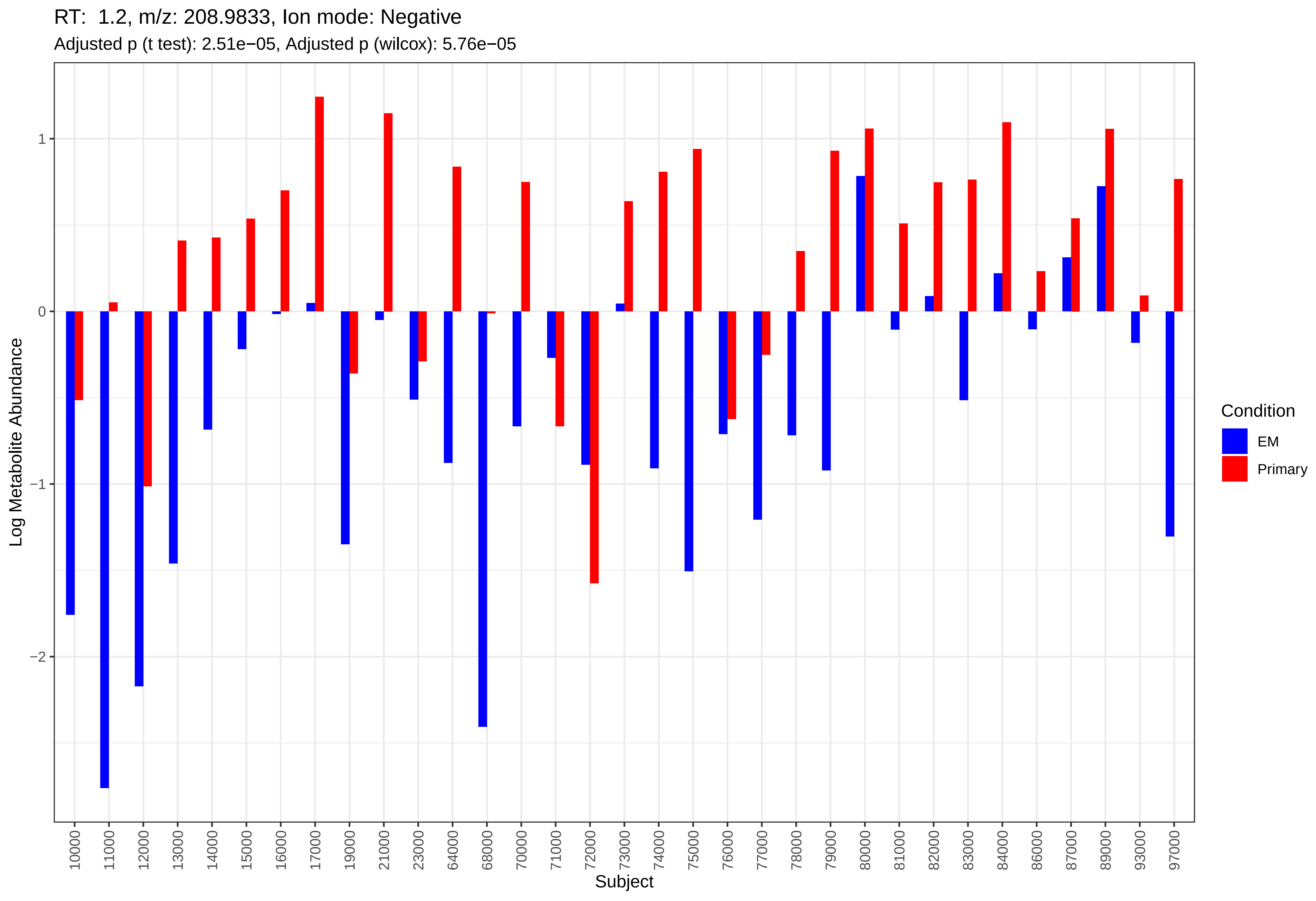

RT: 1.2, m/z: 208.9833, Ion mode: Negative
Adjusted p (t test): 2.51e−05, Adjusted p (wilcox): 5.76e−05
Log Metabolite Abundance
1
0
−1
−2
10000
11000
12000
13000
14000
15000
16000
17000
19000
21000
23000
64000
68000
70000
71000
72000
73000
74000
75000
76000
77000
78000
79000
80000
81000
82000
83000
84000
86000
87000
89000
93000
97000
Subject
Condition
EM
Primary

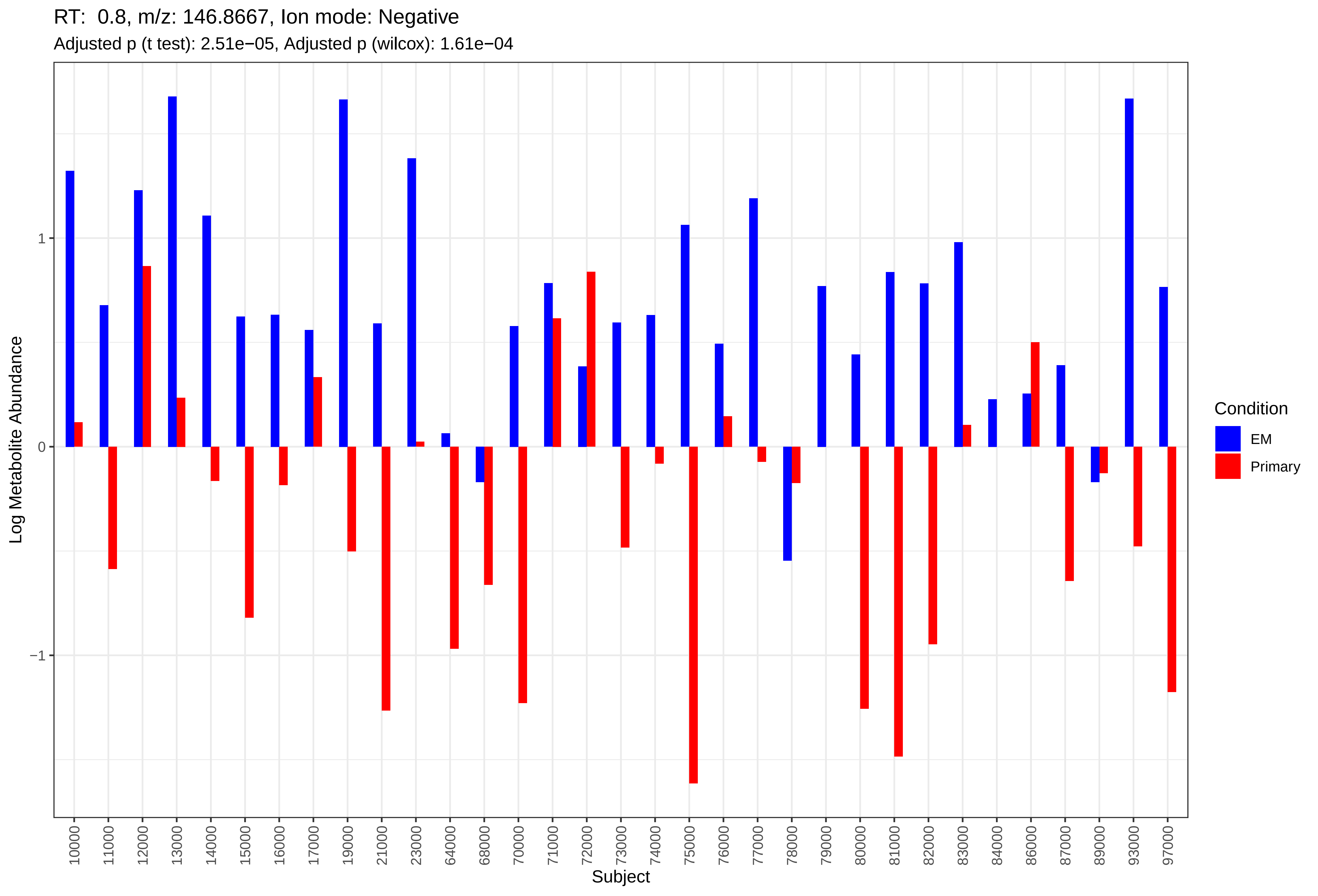

RT: 0.8, m/z: 146.8667, Ion mode: Negative
Adjusted p (t test): 2.51e−05, Adjusted p (wilcox): 1.61e−04
Log Metabolite Abundance
1
0
−1
10000
11000
12000
13000
14000
15000
16000
17000
19000
21000
23000
64000
68000
70000
71000
72000
73000
74000
75000
76000
77000
78000
79000
80000
81000
82000
83000
84000
86000
87000
89000
93000
97000
Subject
Condition
EM
Primary

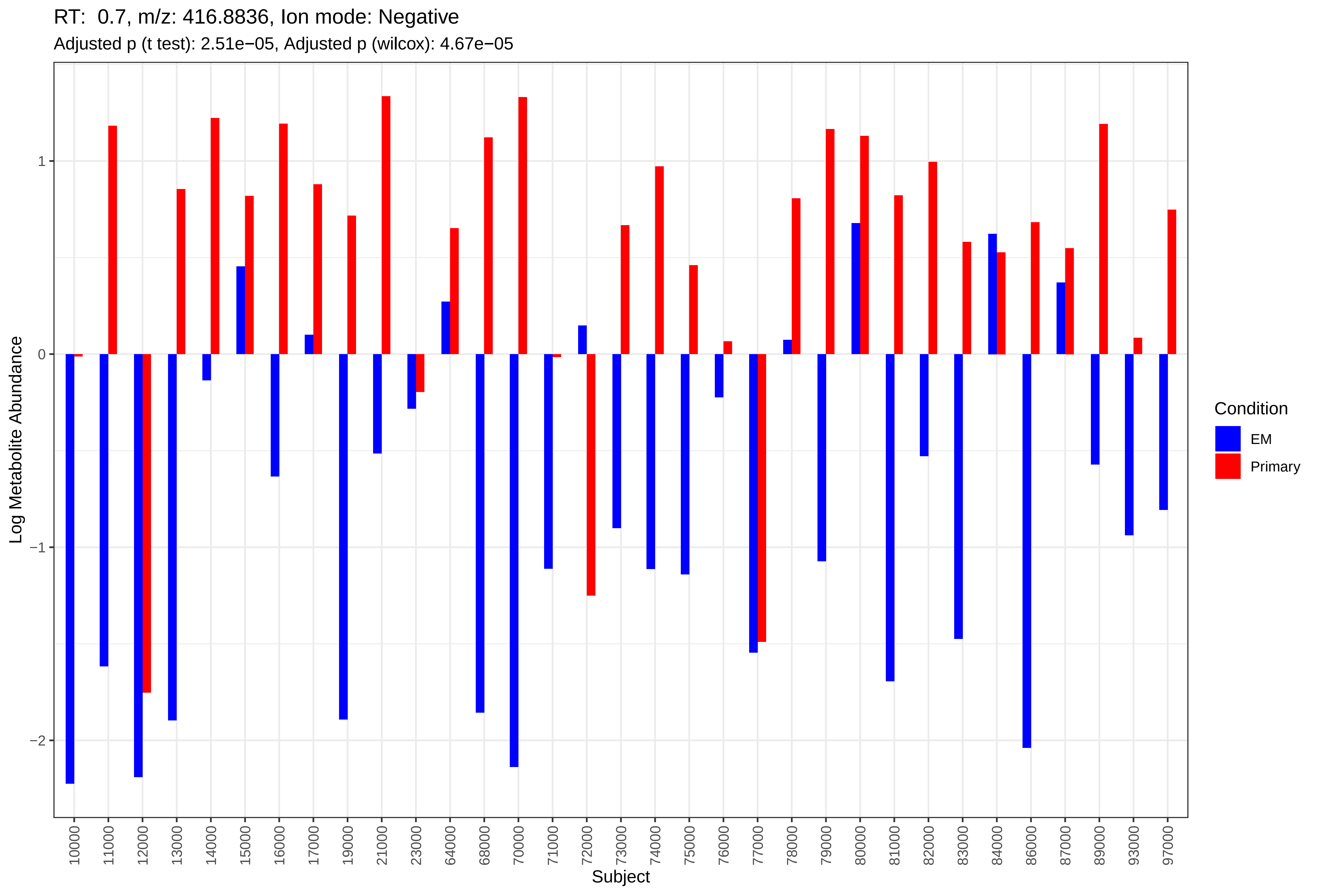
RT: 0.7, m/z: 416.8836, Ion mode: Negative
Adjusted p (t test): 2.51e−05, Adjusted p (wilcox): 4.67e−05
Log Metabolite Abundance
1
0
−1
−2
Subject
10000
11000
12000
13000
14000
15000
16000
17000
19000
21000
23000
64000
68000
70000
71000
72000
73000
74000
75000
76000
77000
78000
79000
80000
81000
82000
83000
84000
86000
87000
89000
93000
97000
Condition
EM
Primary

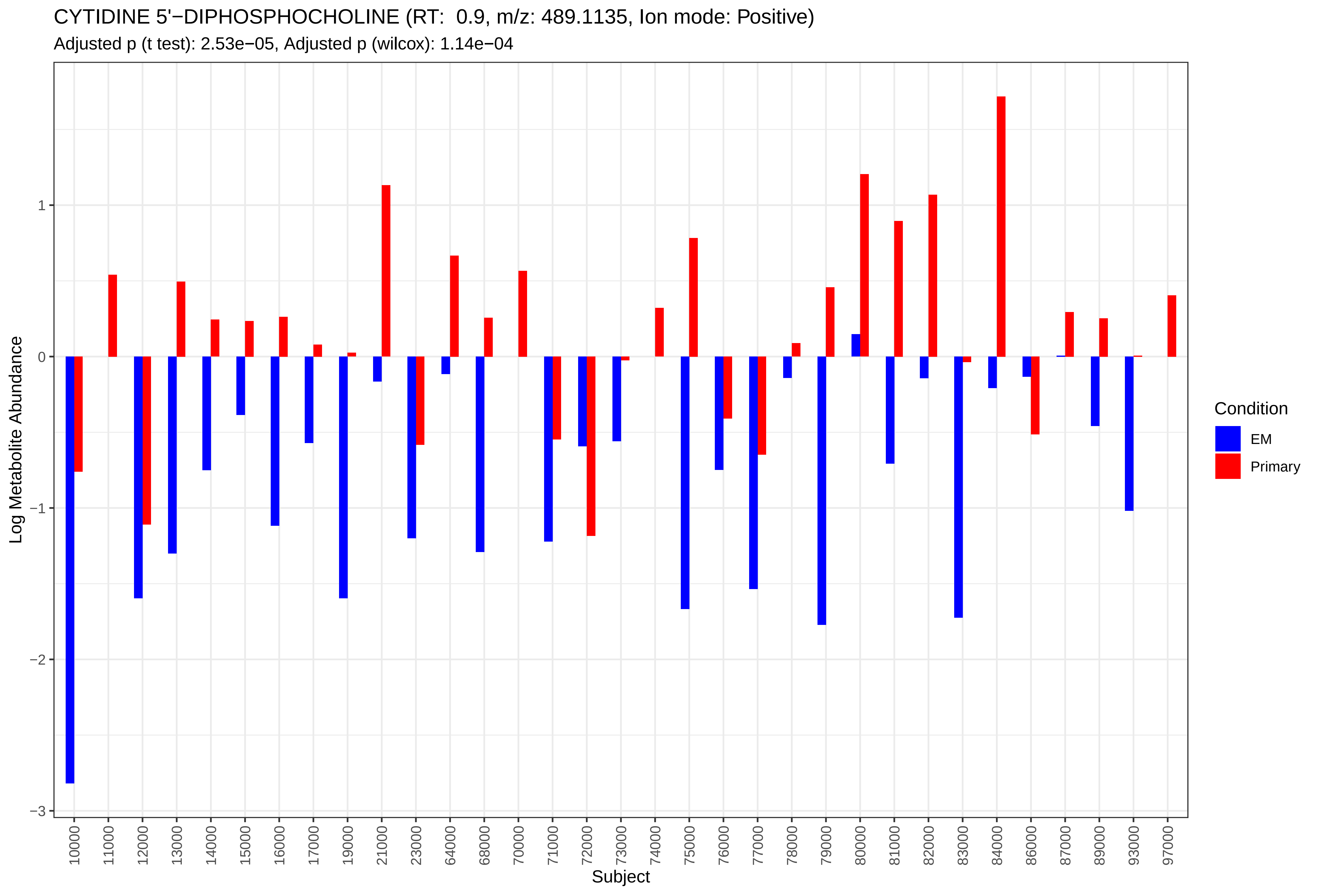
CYTIDINE 5'−DIPHOSPHOCHOLINE (RT: 0.9, m/z: 489.1135, Ion mode: Positive)
Adjusted p (t test): 2.53e−05, Adjusted p (wilcox): 1.14e−04
Log Metabolite Abundance
1
0
−1
−2
−3
10000
11000
12000
13000
14000
15000
16000
17000
19000
21000
23000
64000
68000
70000
71000
72000
73000
74000
75000
76000
77000
78000
79000
80000
81000
82000
83000
84000
86000
87000
89000
93000
97000
Subject
Condition
EM
Primary

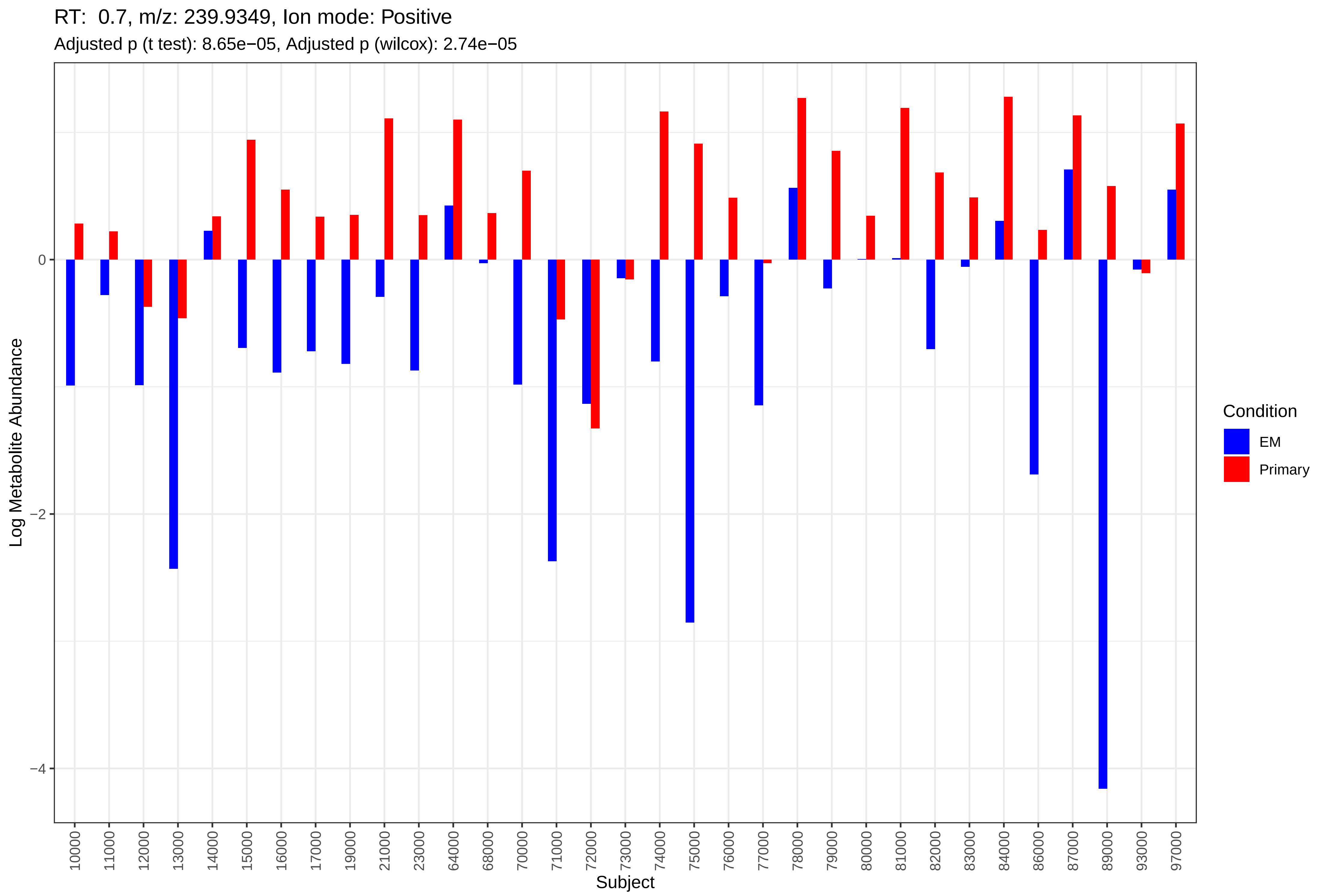

RT: 0.7, m/z: 239.9349, Ion mode: Positive
Adjusted p (t test): 8.65e−05, Adjusted p (wilcox): 2.74e−05
Log Metabolite Abundance
0
−2
−4
10000
11000
12000
13000
14000
15000
16000
17000
19000
21000
23000
64000
68000
70000
71000
72000
73000
74000
75000
76000
77000
78000
79000
80000
81000
82000
83000
84000
86000
87000
89000
93000
97000
Subject
Condition
EM
Primary

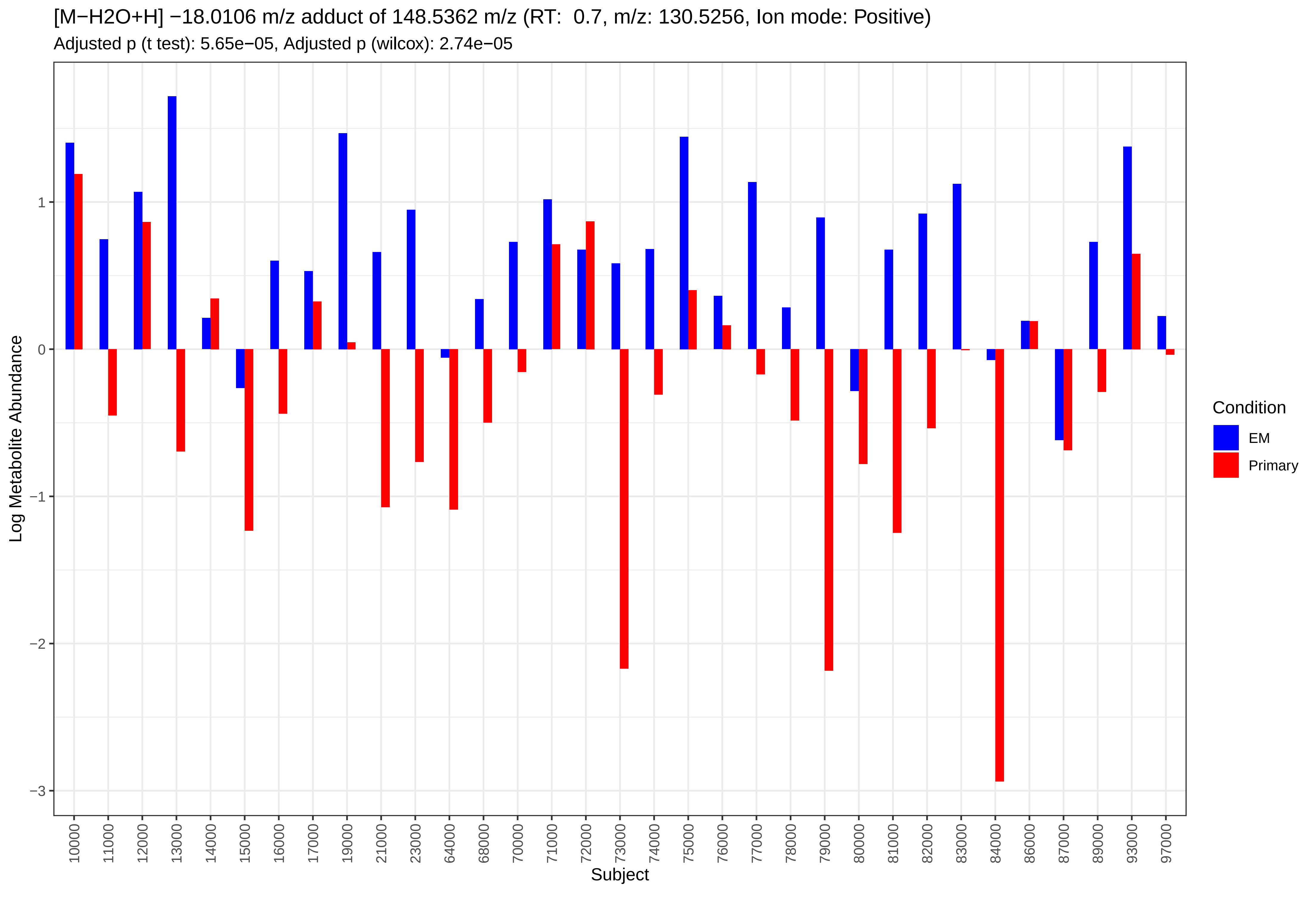
[M−H2O+H] −18.0106 m/z adduct of 148.5362 m/z (RT: 0.7, m/z: 130.5256, Ion mode: Positive)
Adjusted p (t test): 5.65e−05, Adjusted p (wilcox): 2.74e−05
Log Metabolite Abundance
1
0
−1
−2
−3
Subject
10000
11000
12000
13000
14000
15000
16000
17000
19000
21000
23000
64000
68000
70000
71000
72000
73000
74000
75000
76000
77000
78000
79000
80000
81000
82000
83000
84000
86000
87000
89000
93000
97000
Condition
EM
Primary

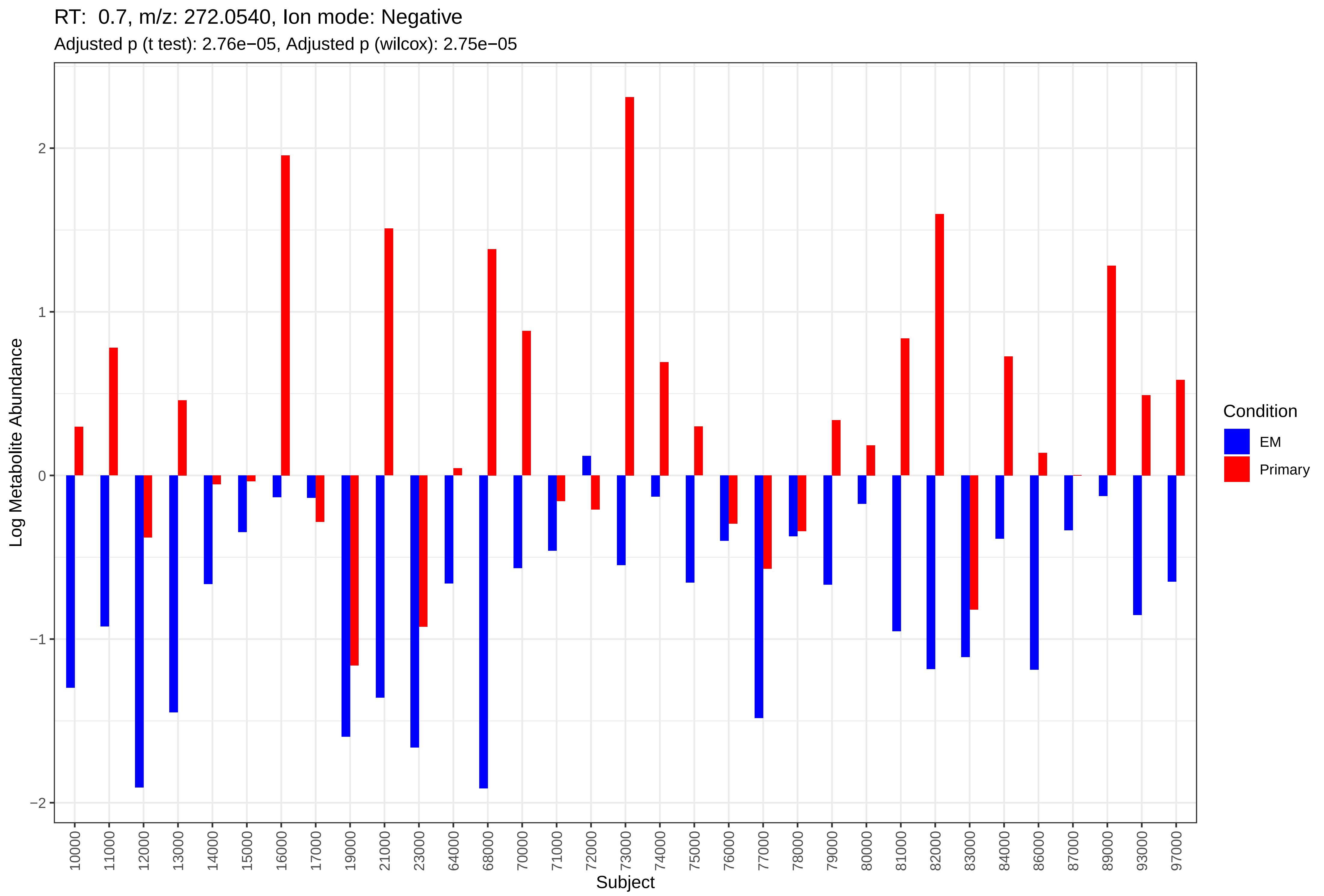
RT: 0.7, m/z: 272.0540, Ion mode: Negative
Adjusted p (t test): 2.76e−05, Adjusted p (wilcox): 2.75e−05
Log Metabolite Abundance
Subject
2
1
0
−1
−2
10000
11000
12000
13000
14000
15000
16000
17000
19000
21000
23000
64000
68000
70000
71000
72000
73000
74000
75000
76000
77000
78000
79000
80000
81000
82000
83000
84000
86000
87000
89000
93000
97000
Condition
EM
Primary

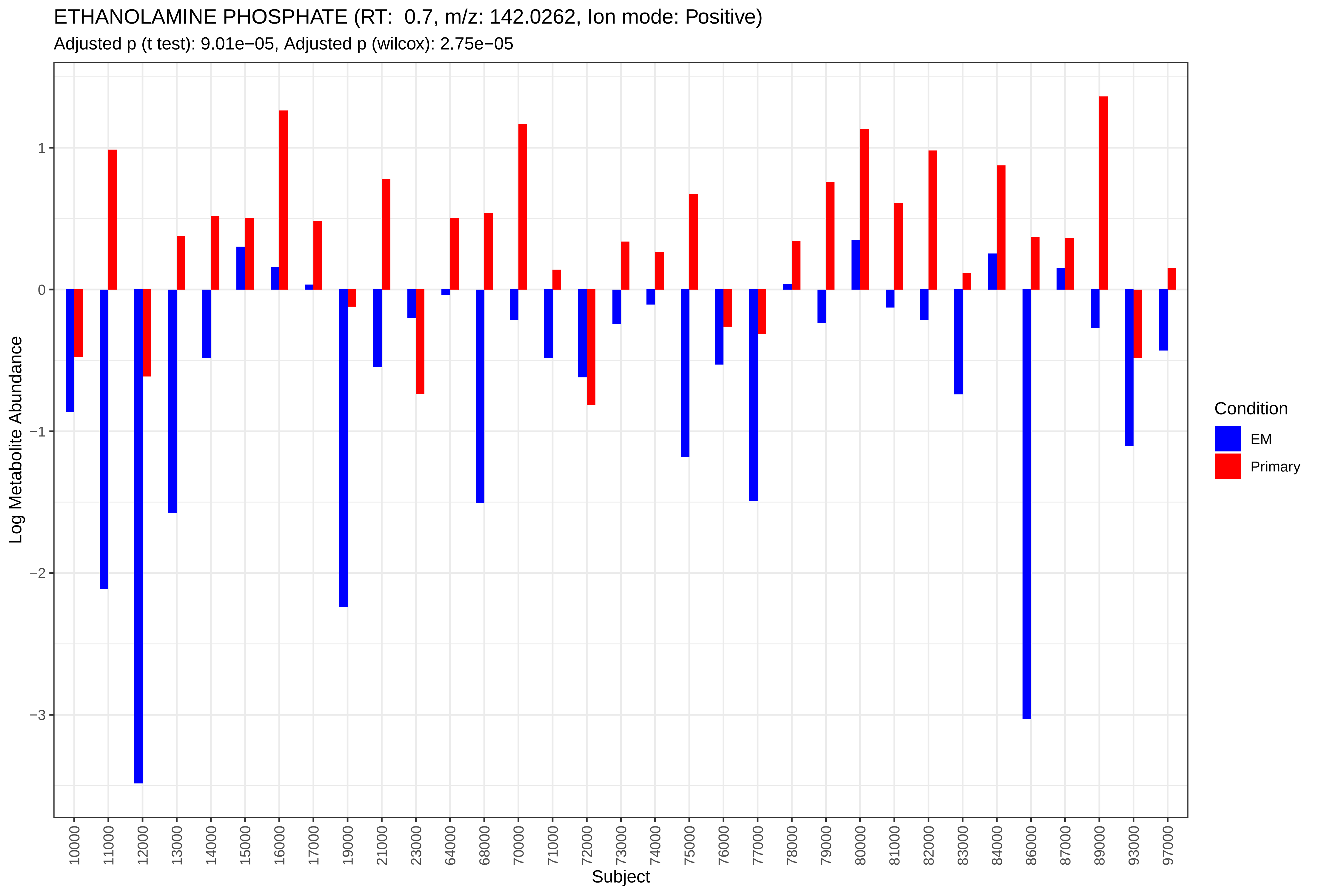

ETHANOLAMINE PHOSPHATE (RT: 0.7, m/z: 142.0262, Ion mode: Positive)
Adjusted p (t test): 9.01e−05, Adjusted p (wilcox): 2.75e−05
Log Metabolite Abundance
1
0
−1
−2
−3
Subject
10000
11000
12000
13000
14000
15000
16000
17000
19000
21000
23000
64000
68000
70000
71000
72000
73000
74000
75000
76000
77000
78000
79000
80000
81000
82000
83000
84000
86000
87000
89000
93000
97000
Condition
EM
Primary

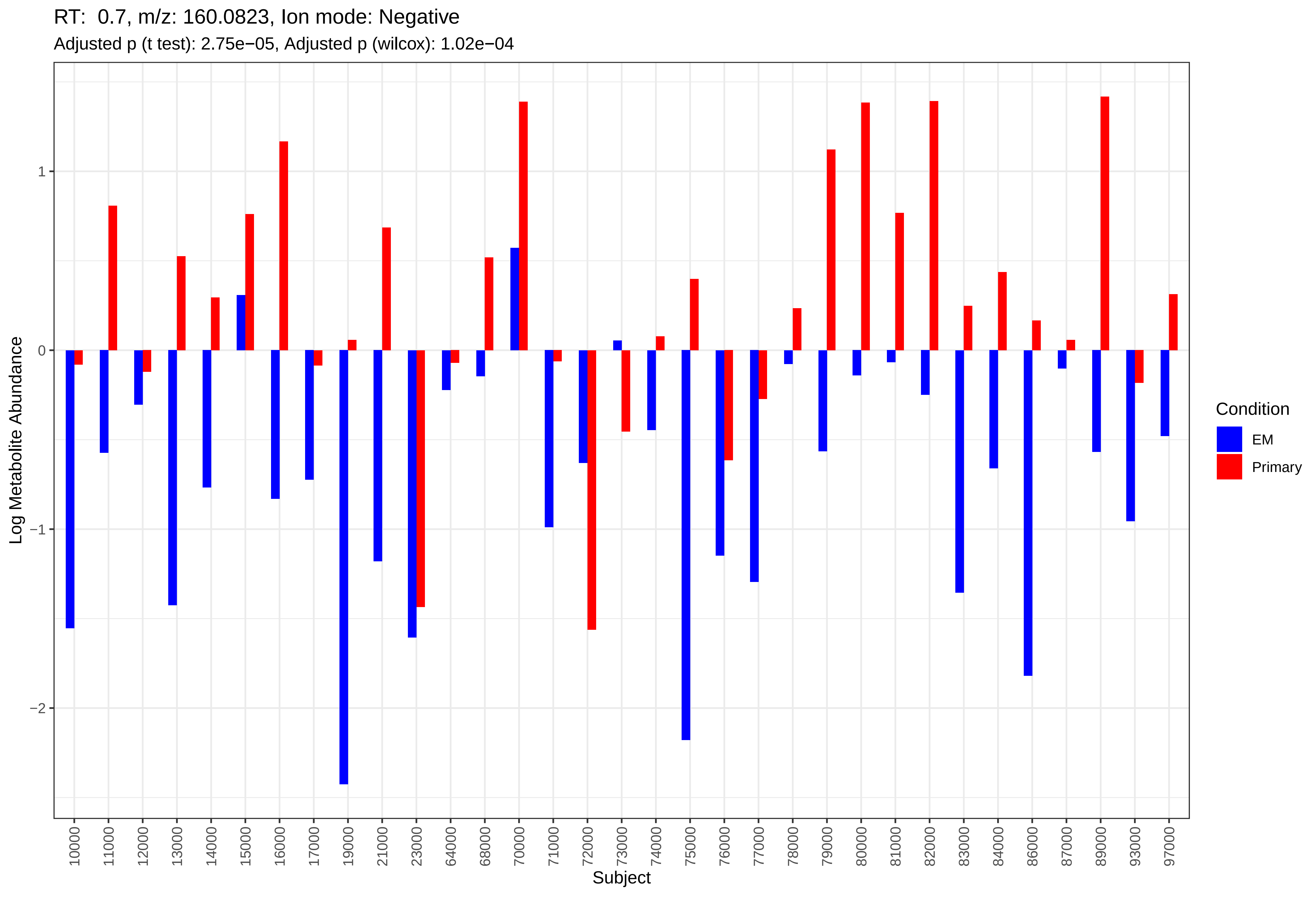
RT: 0.7, m/z: 160.0823, Ion mode: Negative
Adjusted p (t test): 2.75e−05, Adjusted p (wilcox): 1.02e−04
Log Metabolite Abundance
1
0
−1
−2
10000
11000
12000
13000
14000
15000
16000
17000
19000
21000
23000
64000
68000
70000
71000
72000
73000
74000
75000
76000
77000
78000
79000
80000
81000
82000
83000
84000
86000
87000
89000
93000
97000
Subject
Condition
EM
Primary

RT: 10.5, m/z: 449.2761, Ion mode: Negative

Adjusted p (t test): 2.76e−05, Adjusted p (wilcox): 2.77e−05

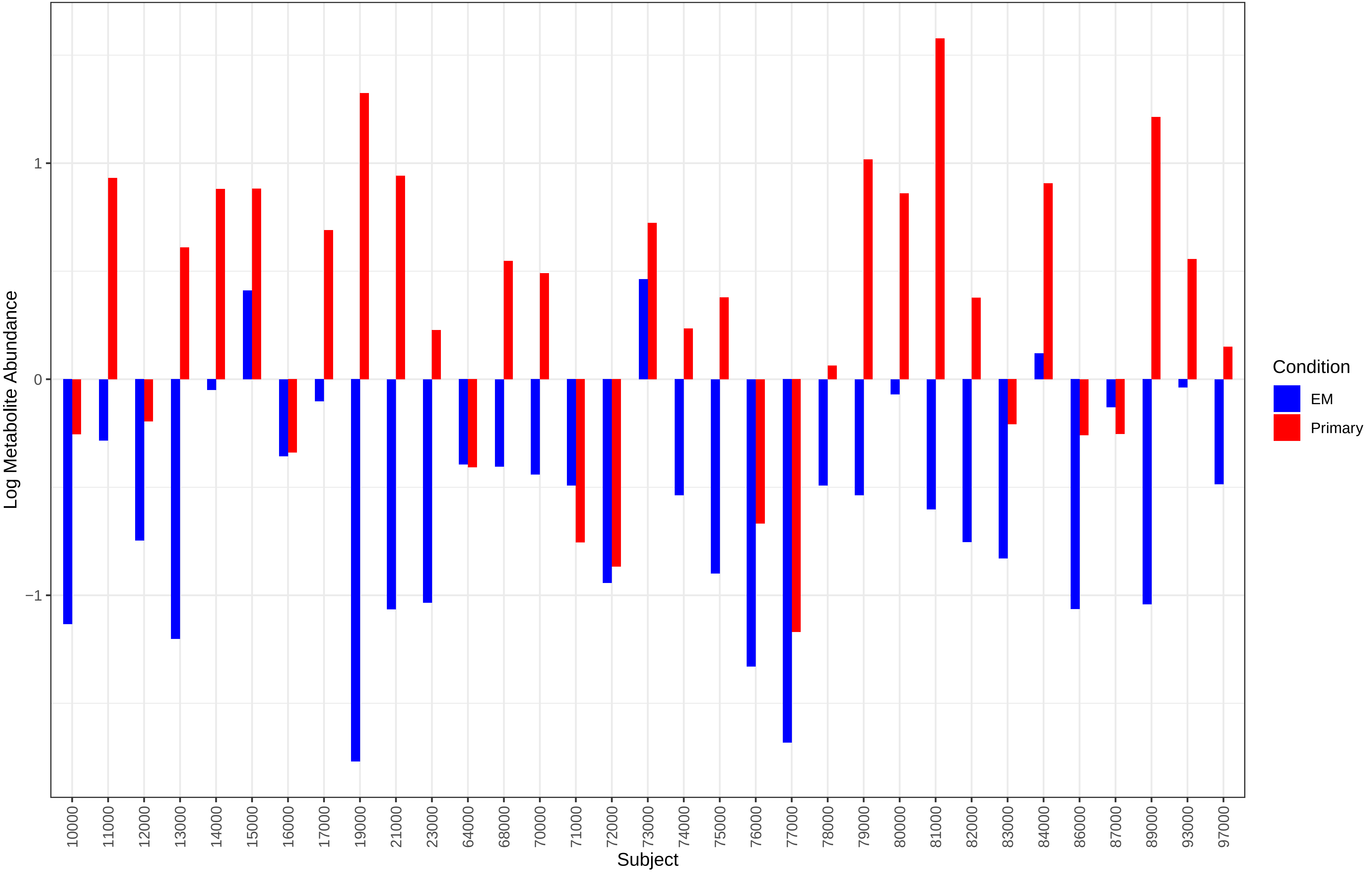

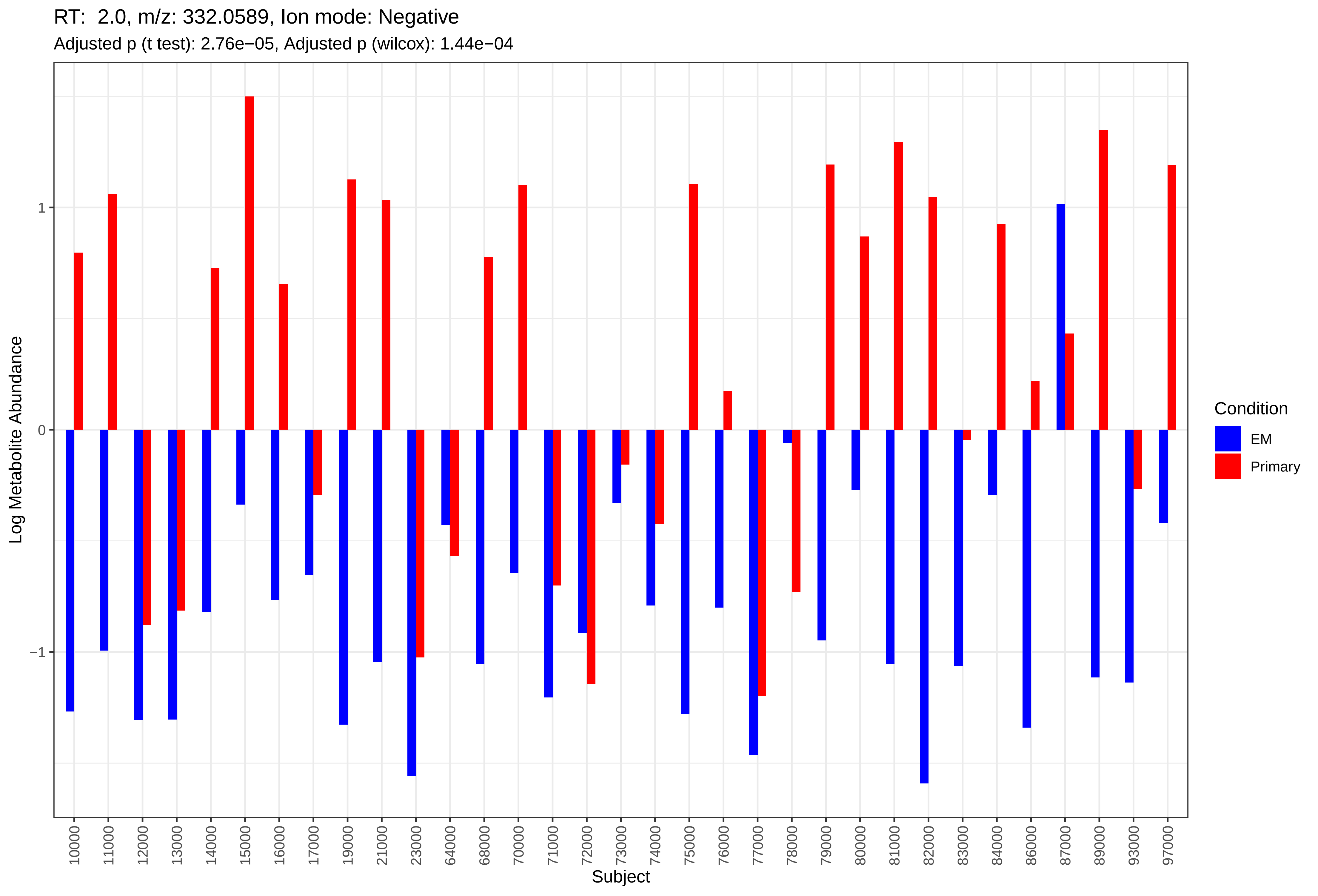

RT: 2.0, m/z: 332.0589, Ion mode: Negative
Adjusted p (t test): 2.76e−05, Adjusted p (wilcox): 1.44e−04
Log Metabolite Abundance
1
0
−1
10000
11000
12000
13000
14000
15000
16000
17000
19000
21000
23000
64000
68000
70000
71000
72000
73000
74000
75000
76000
77000
78000
79000
80000
81000
82000
83000
84000
86000
87000
89000
93000
97000
Subject
Condition
EM
Primary

Complex of 114.0661 and 158.9615 m/z (RT: 0.9, m/z: 272.0199, Ion mode: Positive)

Adjusted p (t test): 2.76e−05, Adjusted p (wilcox): 1.60e−04

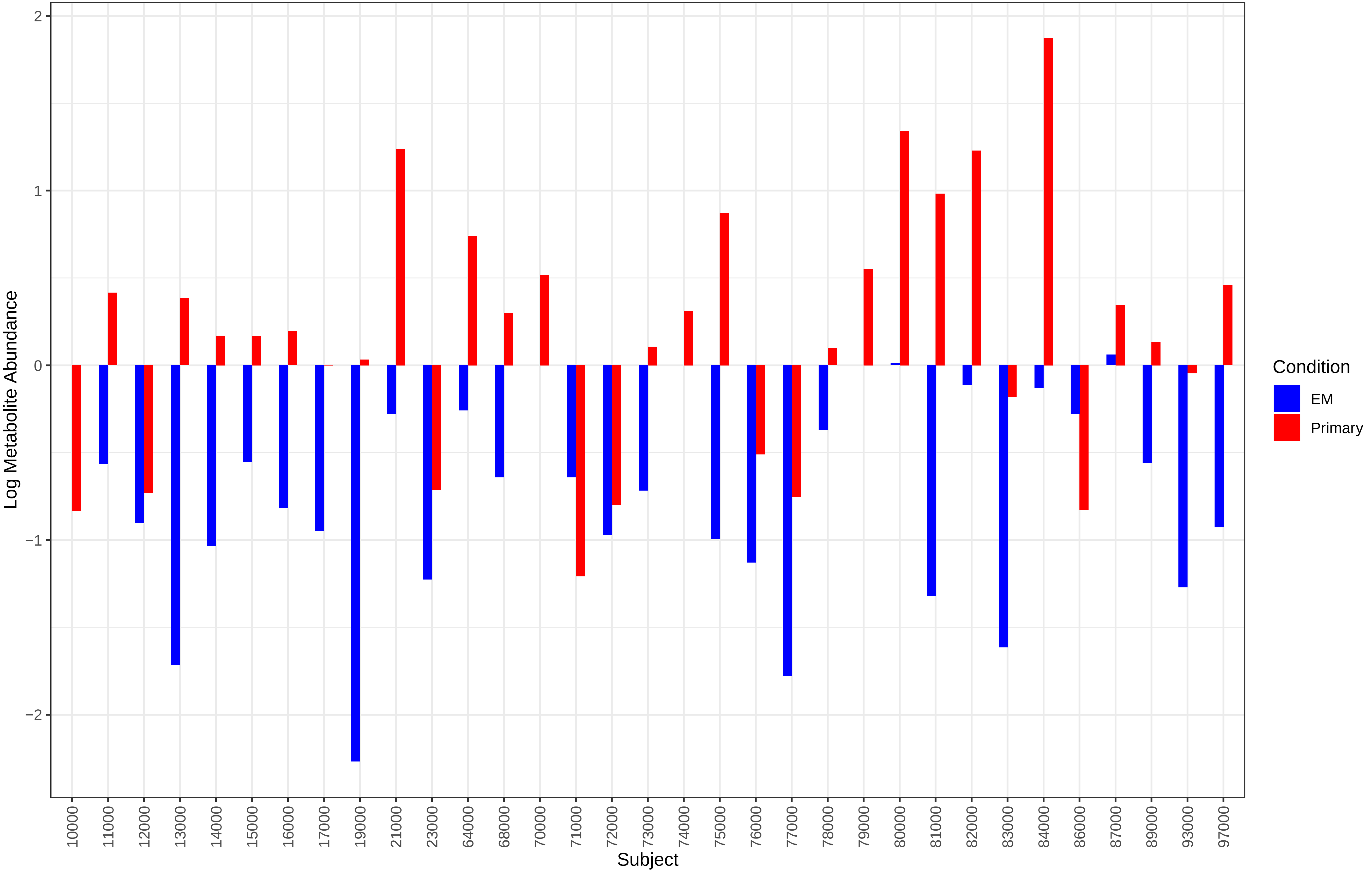

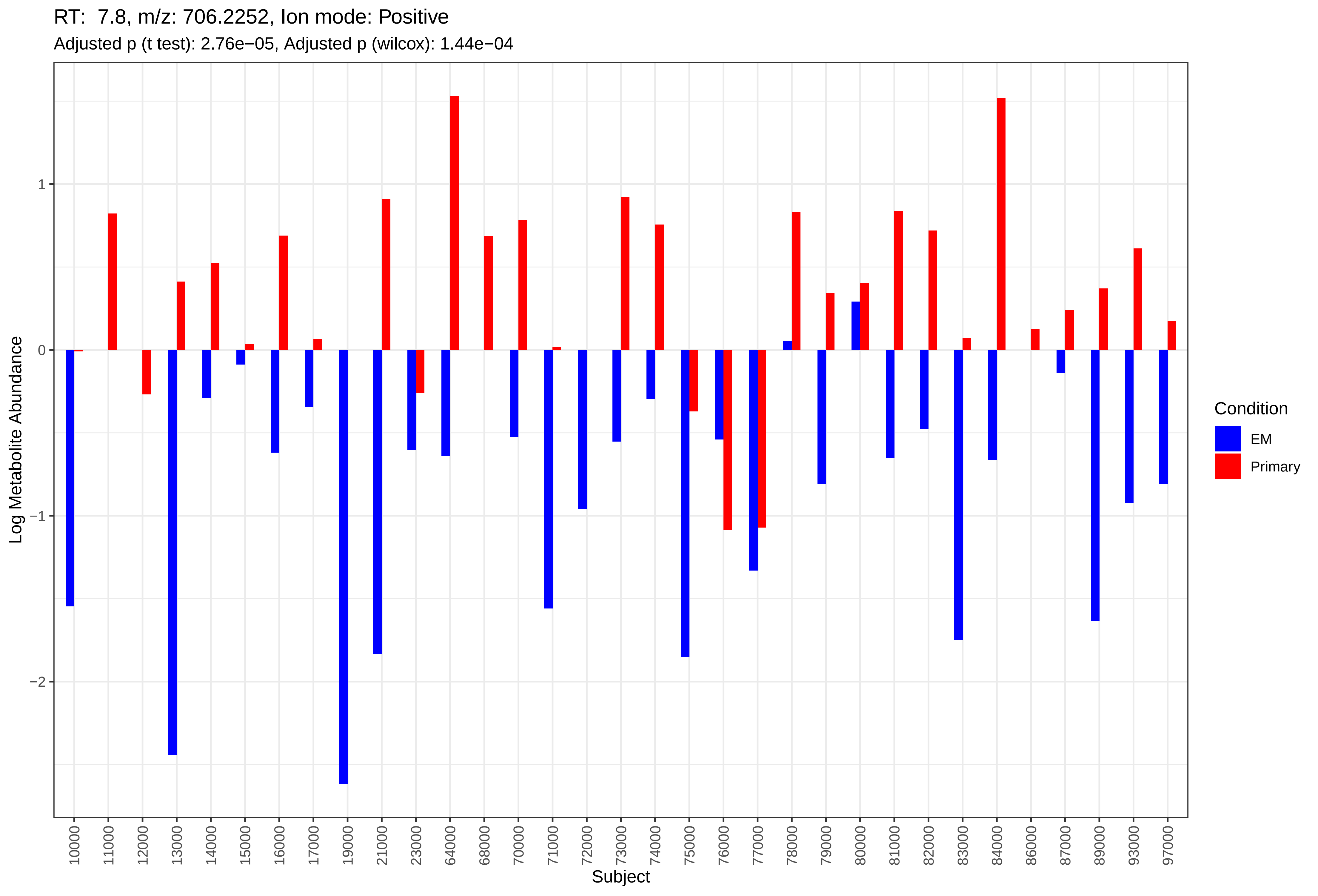
RT: 7.8, m/z: 706.2252, Ion mode: Positive
Adjusted p (t test): 2.76e−05, Adjusted p (wilcox): 1.44e−04
Log Metabolite Abundance
Subject
Condition
EM
Primary
1
0
−1
−2
10000
11000
12000
13000
14000
15000
16000
17000
19000
21000
23000
64000
68000
70000
71000
72000
73000
74000
75000
76000
77000
78000
79000
80000
81000
82000
83000
84000
86000
87000
89000
93000
97000

RT: 3.0, m/z: 204.0864, Ion mode: Positive

Adjusted p (t test): 2.76e−05, Adjusted p (wilcox): 8.71e−05

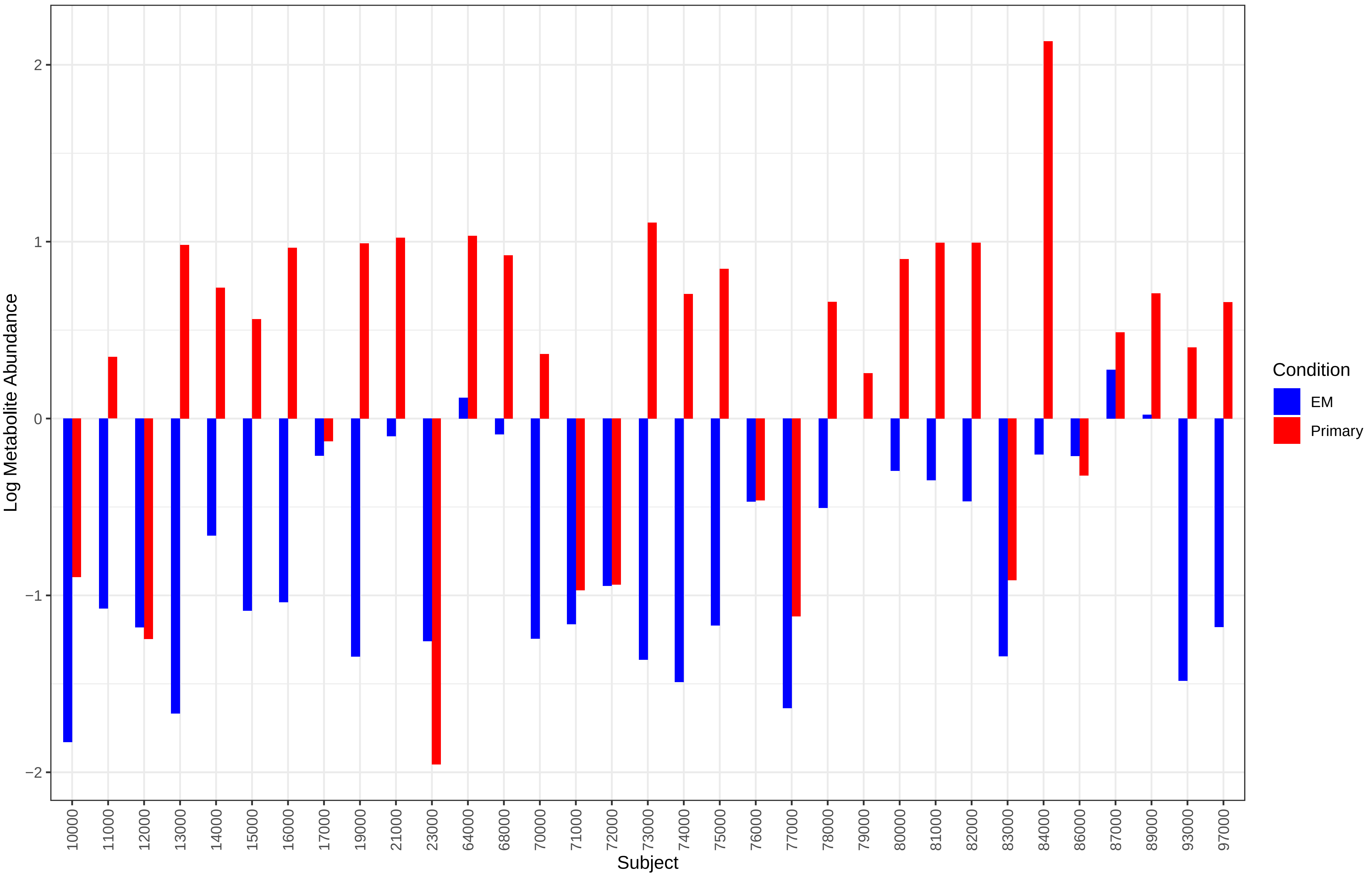

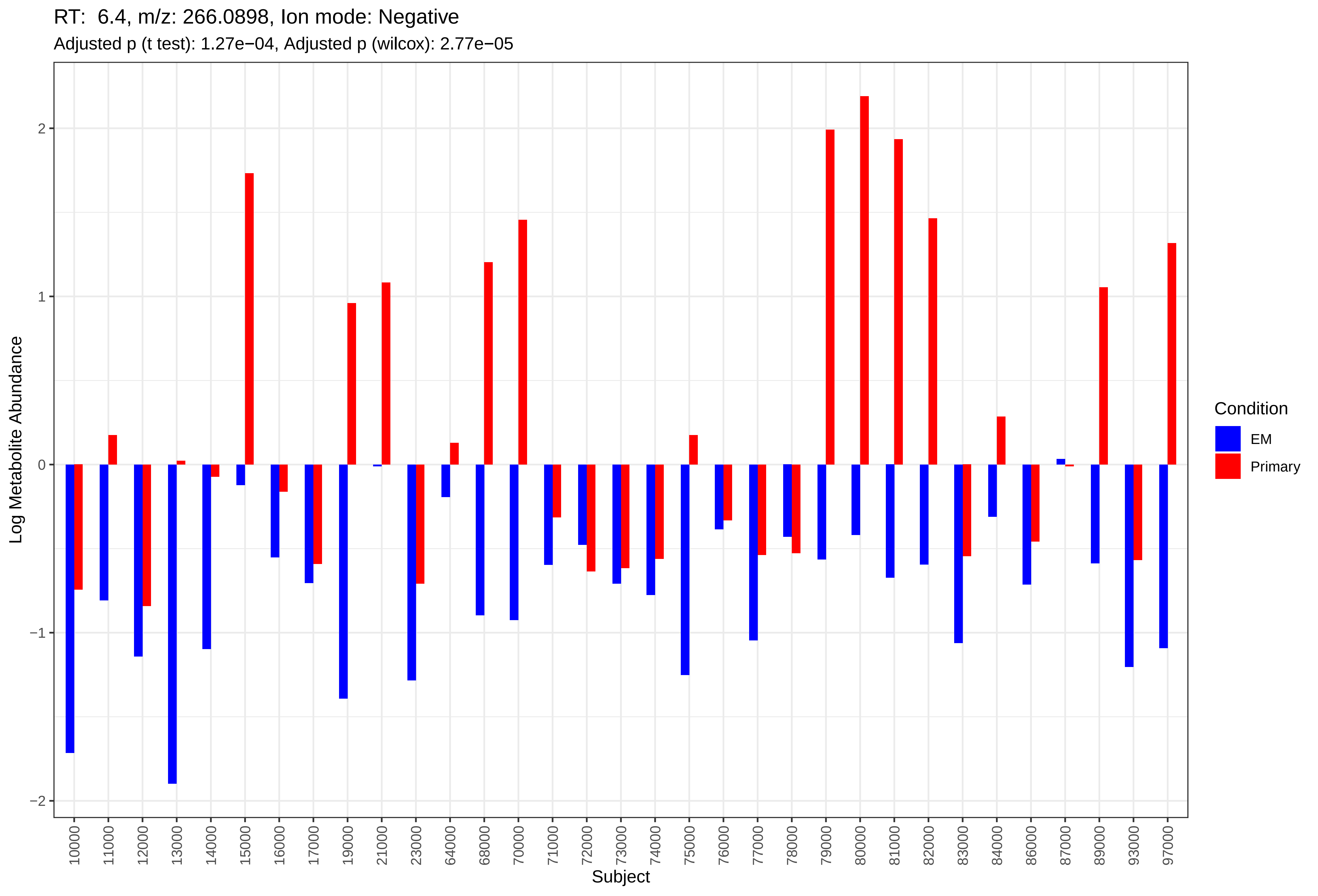
RT: 6.4, m/z: 266.0898, Ion mode: Negative
Adjusted p (t test): 1.27e−04, Adjusted p (wilcox): 2.77e−05
Log Metabolite Abundance
2
1
0
−1
−2
Subject
10000
11000
12000
13000
14000
15000
16000
17000
19000
21000
23000
64000
68000
70000
71000
72000
73000
74000
75000
76000
77000
78000
79000
80000
81000
82000
83000
84000
86000
87000
89000
93000
97000
Condition
EM
Primary

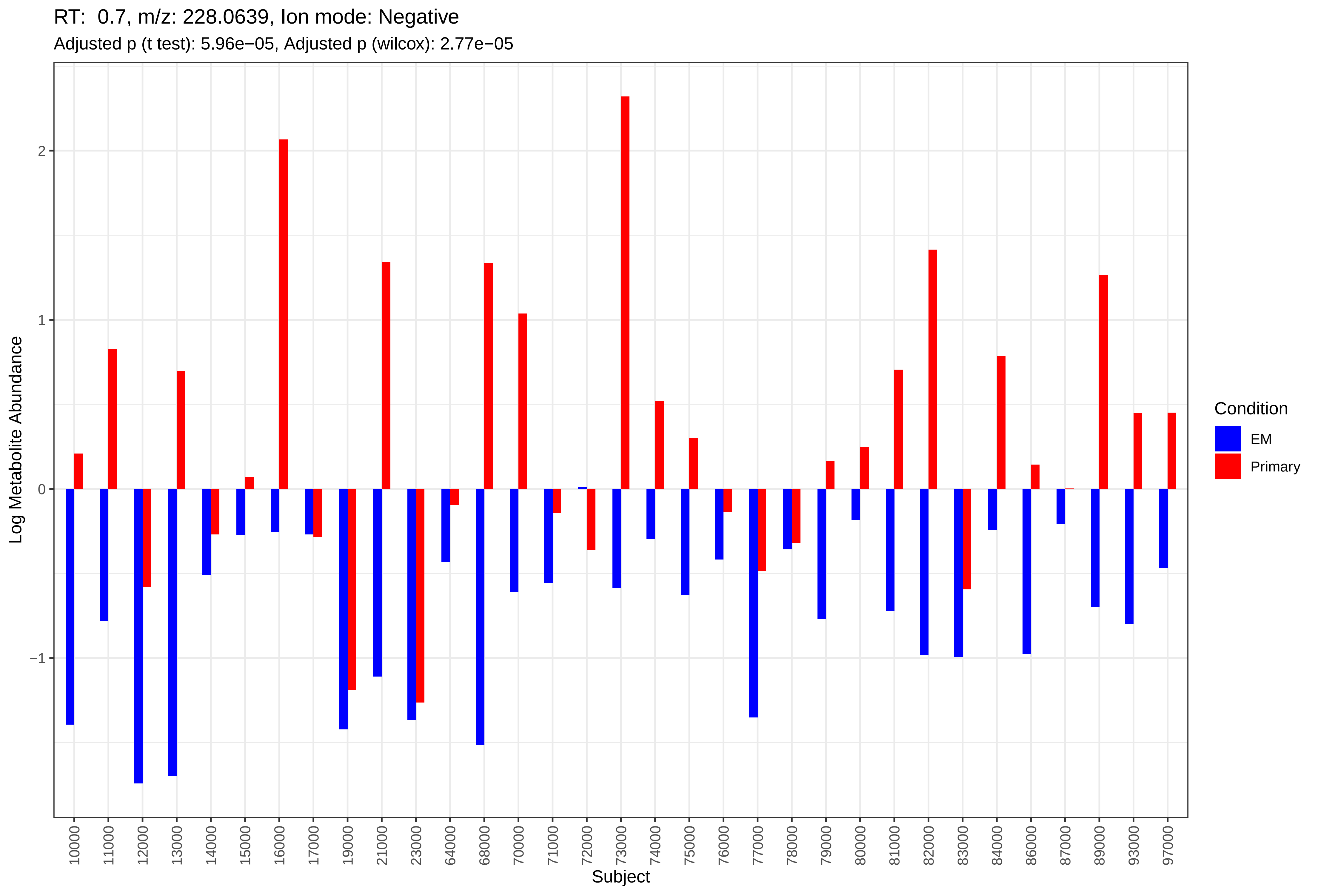

RT: 0.7, m/z: 228.0639, Ion mode: Negative
Adjusted p (t test): 5.96e−05, Adjusted p (wilcox): 2.77e−05
Log Metabolite Abundance
2
1
0
−1
10000
11000
12000
13000
14000
15000
16000
17000
19000
21000
23000
64000
68000
70000
71000
72000
73000
74000
75000
76000
77000
78000
79000
80000
81000
82000
83000
84000
86000
87000
89000
93000
97000
Subject
Condition
EM
Primary

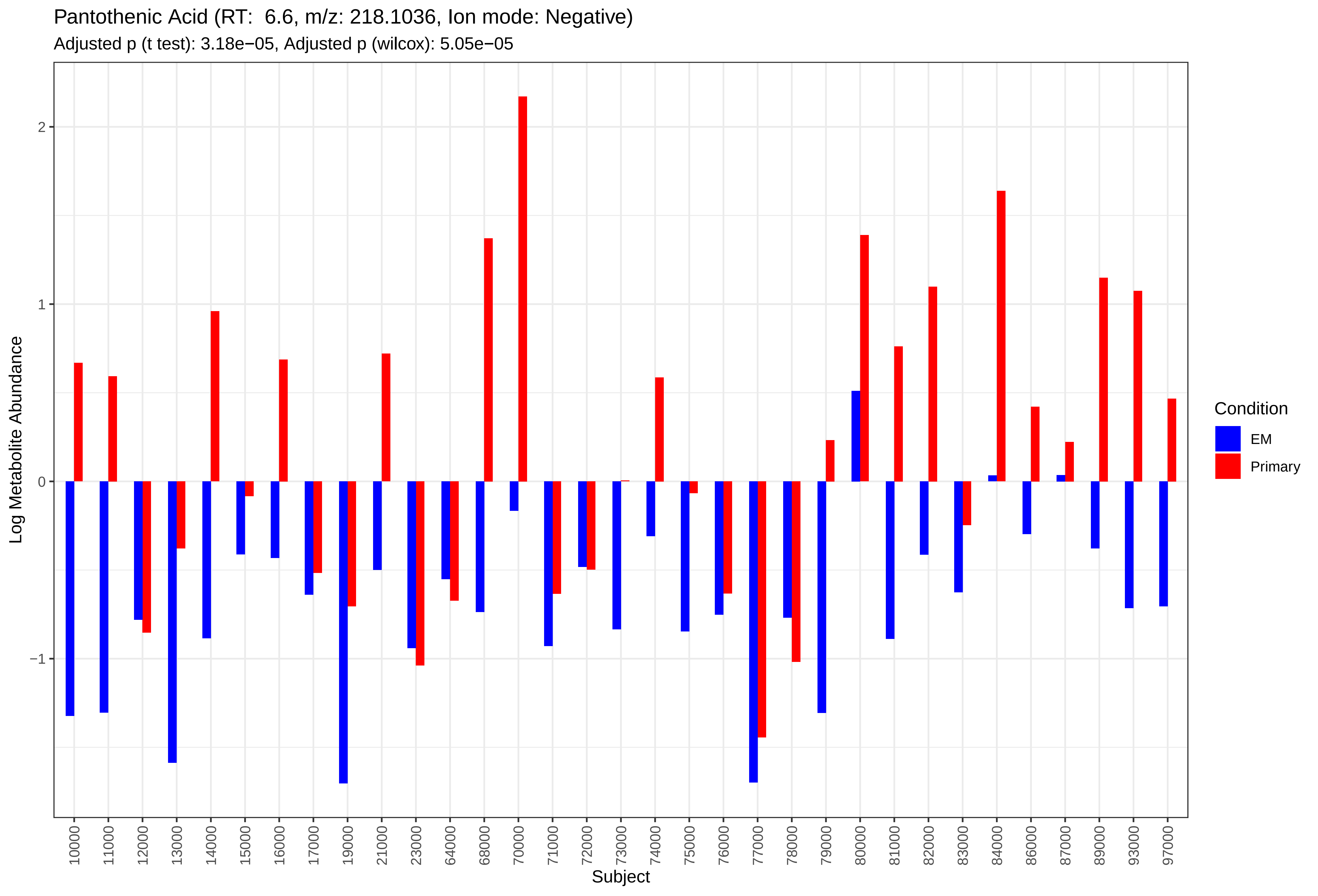

Pantothenic Acid (RT: 6.6, m/z: 218.1036, Ion mode: Negative)
Adjusted p (t test): 3.18e−05, Adjusted p (wilcox): 5.05e−05
Log Metabolite Abundance
2
1
0
−1
10000
11000
12000
13000
14000
15000
16000
17000
19000
21000
23000
64000
68000
70000
71000
72000
73000
74000
75000
76000
77000
78000
79000
80000
81000
82000
83000
84000
86000
87000
89000
93000
97000
Subject
Condition
EM
Primary

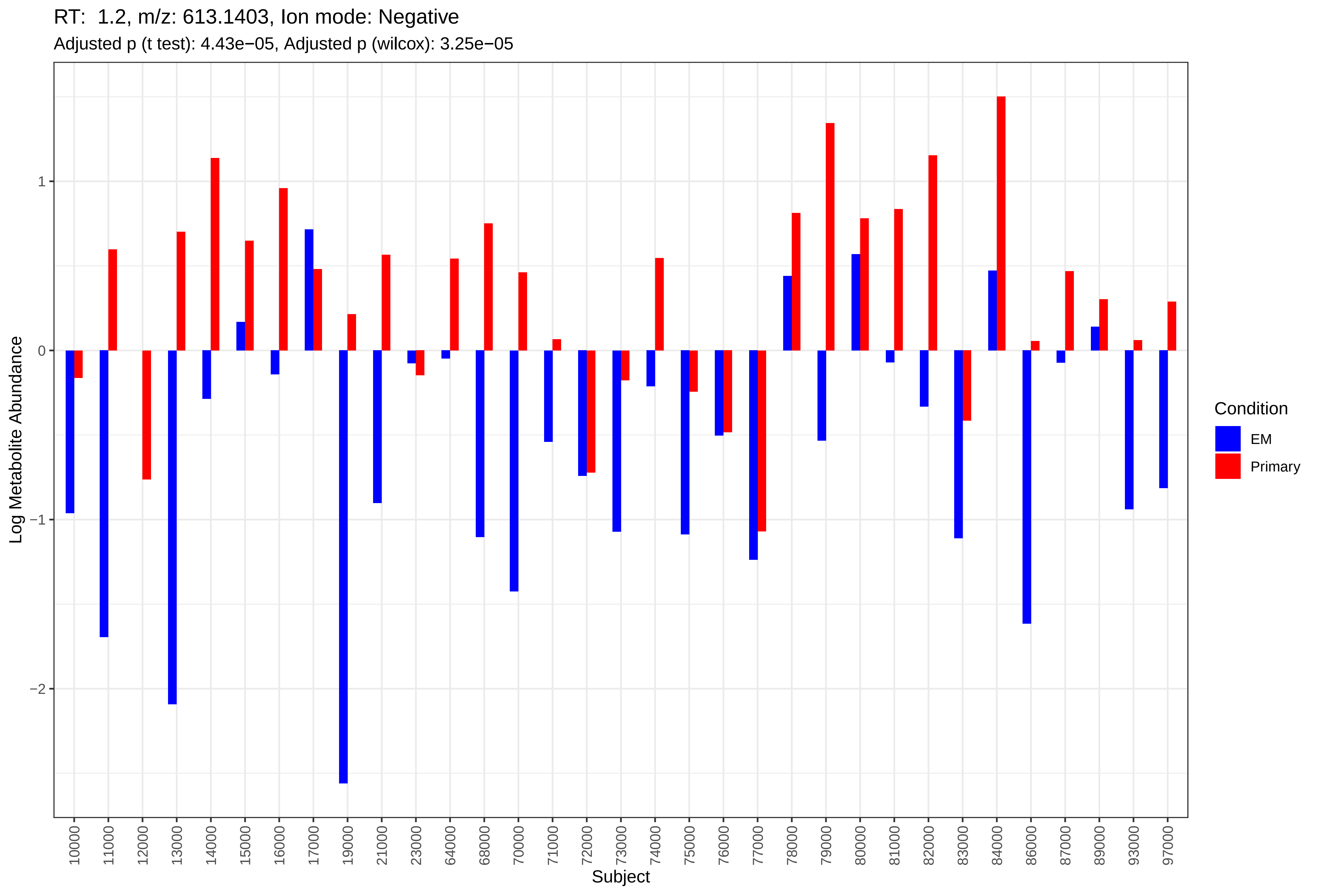
RT: 1.2, m/z: 613.1403, Ion mode: Negative
Adjusted p (t test): 4.43e−05, Adjusted p (wilcox): 3.25e−05
Log Metabolite Abundance
1
0
−1
−2
10000
11000
12000
13000
14000
15000
16000
17000
19000
21000
23000
64000
68000
70000
71000
72000
73000
74000
75000
76000
77000
78000
79000
80000
81000
82000
83000
84000
86000
87000
89000
93000
97000
Subject
Condition
EM
Primary

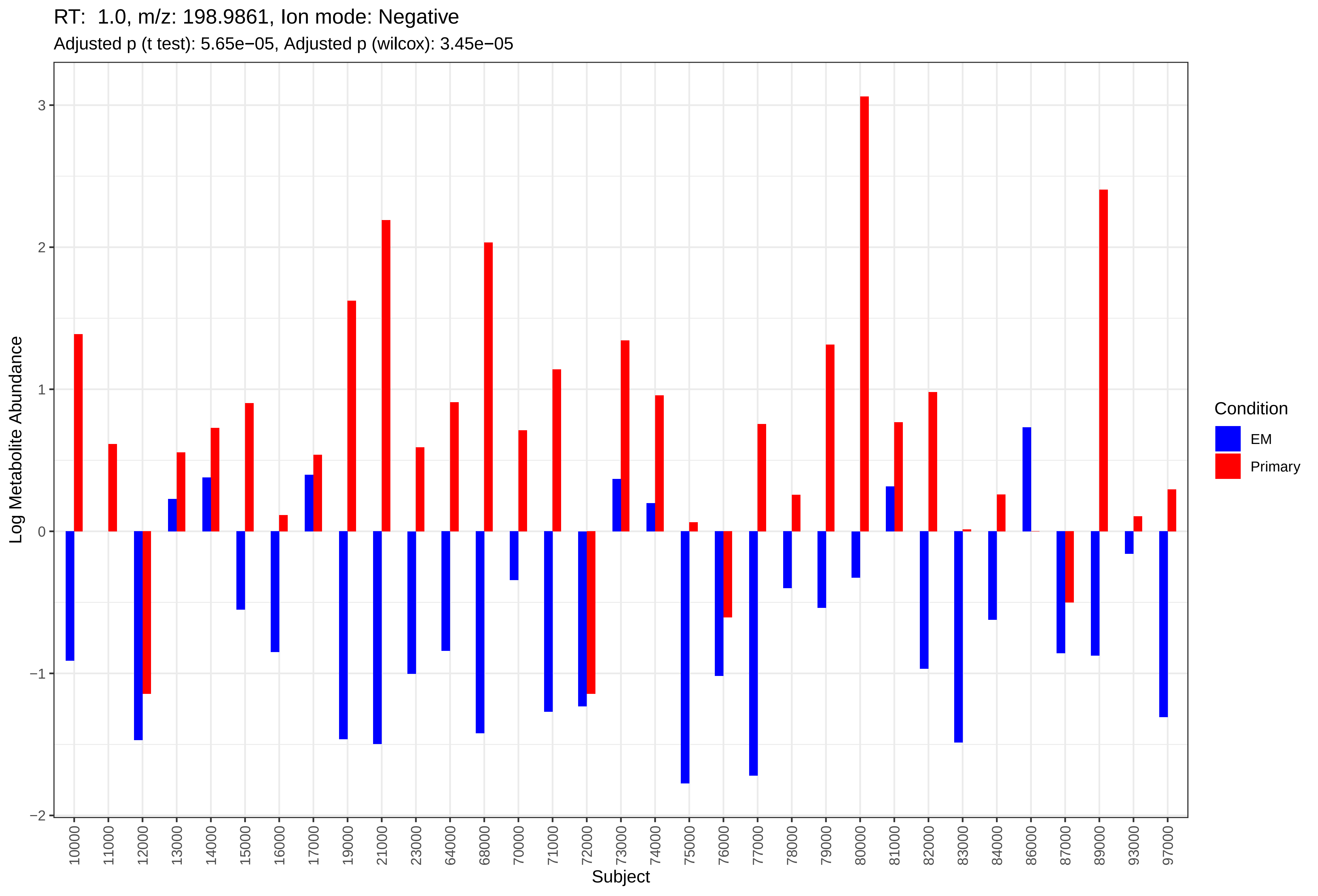

RT: 1.0, m/z: 198.9861, Ion mode: Negative
Adjusted p (t test): 5.65e−05, Adjusted p (wilcox): 3.45e−05
Log Metabolite Abundance
3
2
1
0
−1
−2
10000
11000
12000
13000
14000
15000
16000
17000
19000
21000
23000
64000
68000
70000
71000
72000
73000
74000
75000
76000
77000
78000
79000
80000
81000
82000
83000
84000
86000
87000
89000
93000
97000
Subject
Condition
EM
Primary

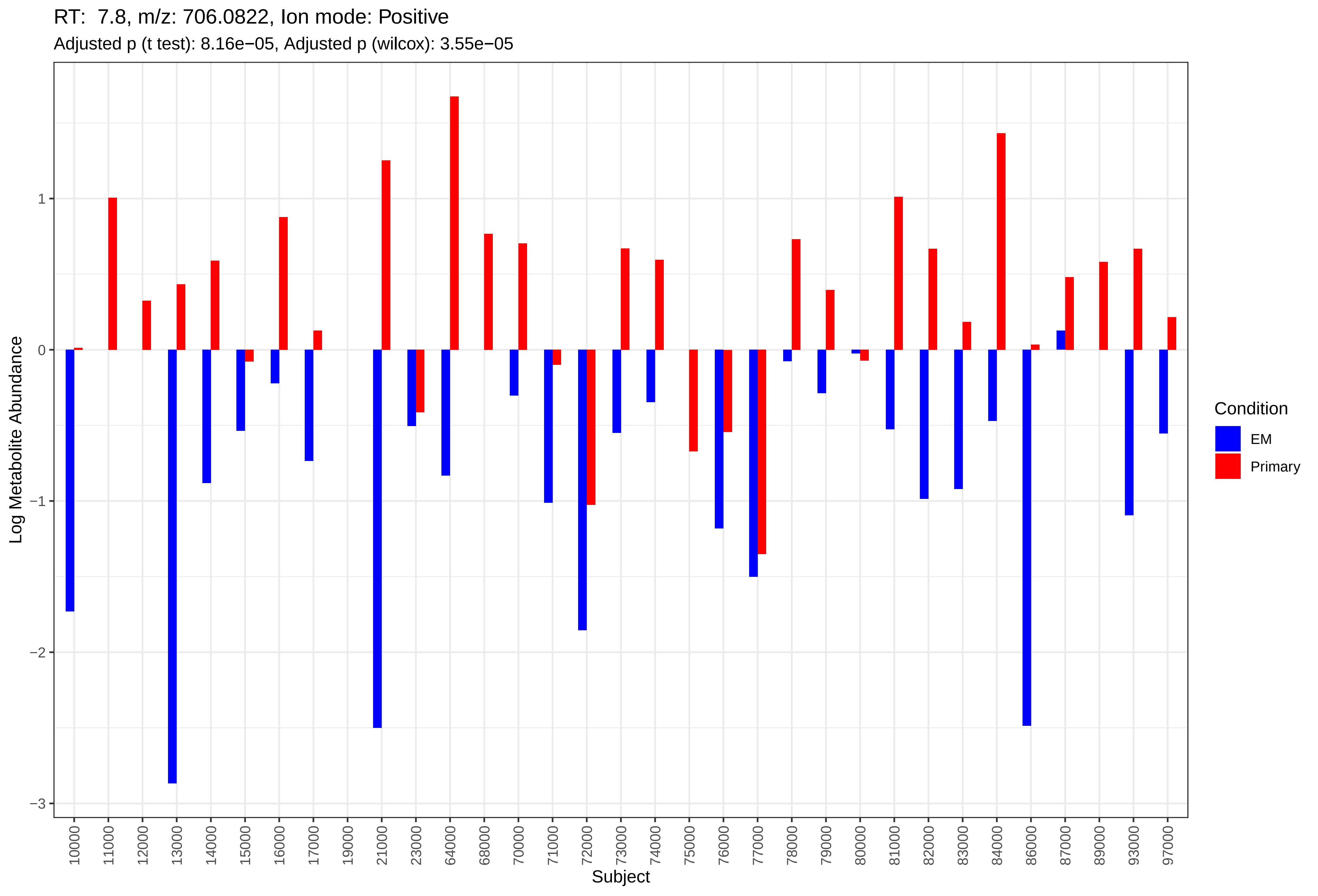
RT: 7.8, m/z: 706.0822, Ion mode: Positive
Adjusted p (t test): 8.16e−05, Adjusted p (wilcox): 3.55e−05
Log Metabolite Abundance
1
0
−1
−2
−3
10000
11000
12000
13000
14000
15000
16000
17000
19000
21000
23000
64000
68000
70000
71000
72000
73000
74000
75000
76000
77000
78000
79000
80000
81000
82000
83000
84000
86000
87000
89000
93000
97000
Subject
Condition
EM
Primary

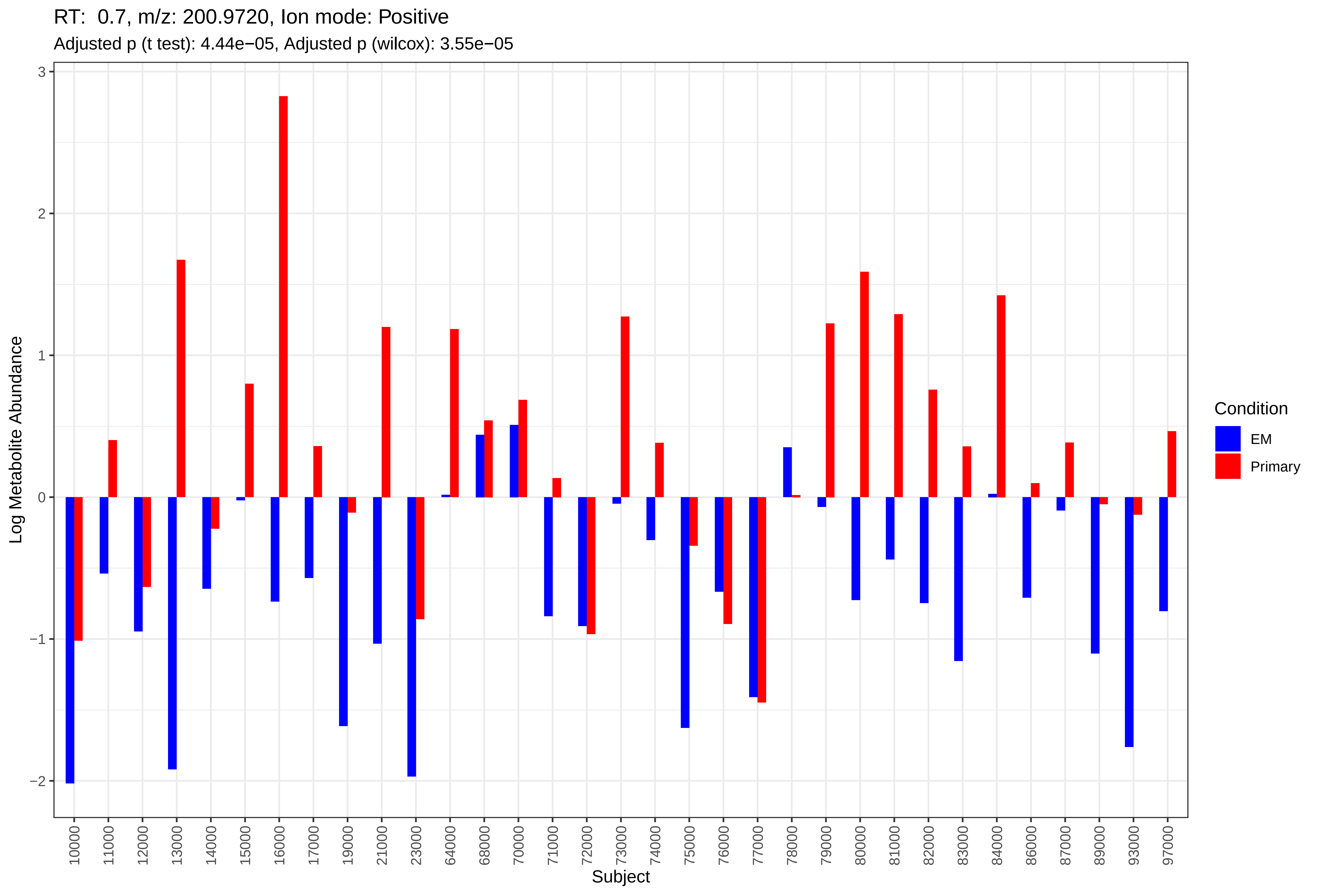

RT: 0.7, m/z: 200.9720, Ion mode: Positive
Adjusted p (t test): 4.44e−05, Adjusted p (wilcox): 3.55e−05
Log Metabolite Abundance
3
2
1
0
−1
−2
Subject
10000
11000
12000
13000
14000
15000
16000
17000
19000
21000
23000
64000
68000
70000
71000
72000
73000
74000
75000
76000
77000
78000
79000
80000
81000
82000
83000
84000
86000
87000
89000
93000
97000
Condition
EM
Primary

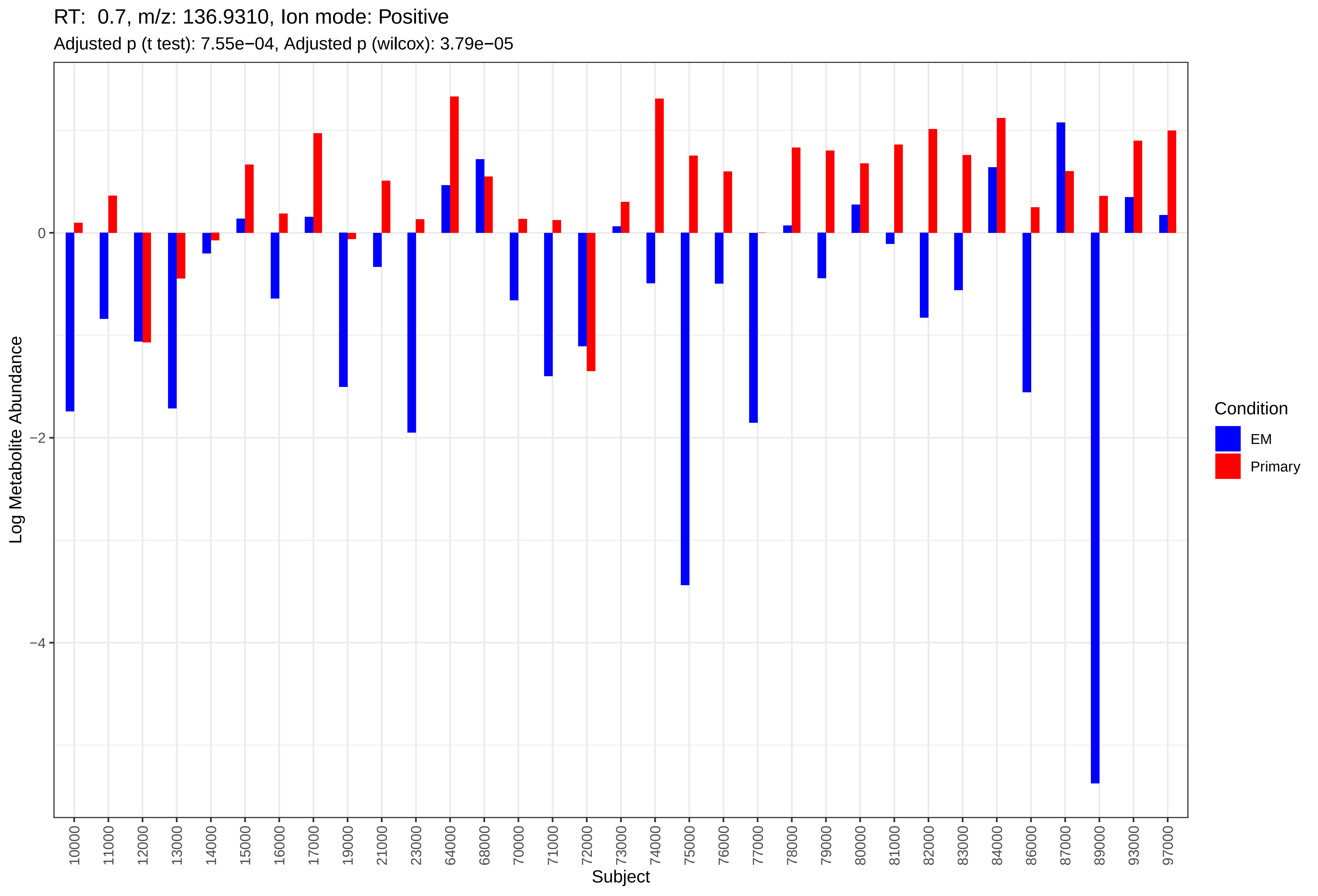
RT: 0.7, m/z: 136.9310, Ion mode: Positive
Adjusted p (t test): 7.55e−04, Adjusted p (wilcox): 3.79e−05
Log Metabolite Abundance
0
−2
−4
Subject
10000
11000
12000
13000
14000
15000
16000
17000
19000
21000
23000
64000
68000
70000
71000
72000
73000
74000
75000
76000
77000
78000
79000
80000
81000
82000
83000
84000
86000
87000
89000
93000
97000
Condition
EM
Primary

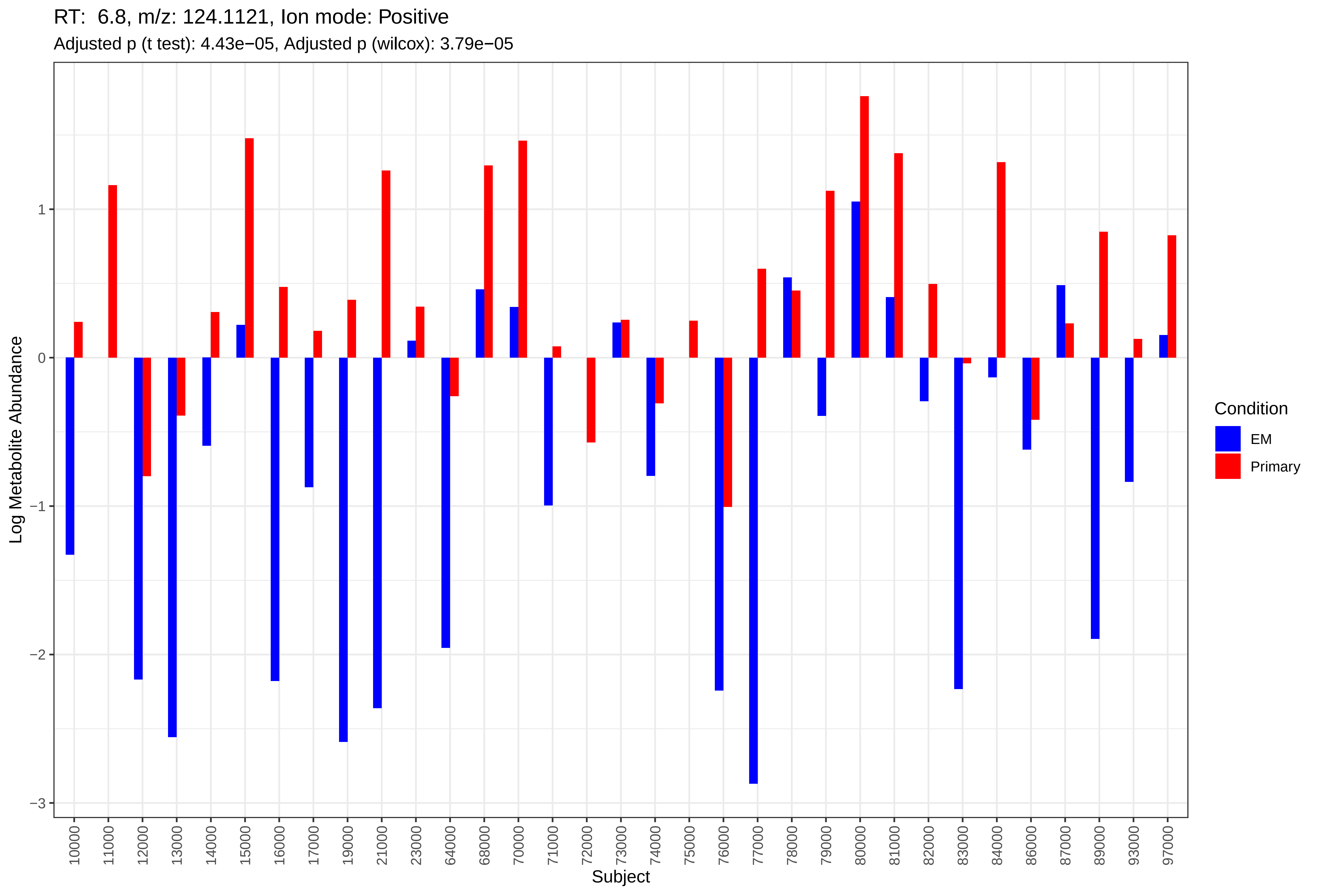
RT: 6.8, m/z: 124.1121, Ion mode: Positive
Adjusted p (t test): 4.43e−05, Adjusted p (wilcox): 3.79e−05
Log Metabolite Abundance
1
0
−1
−2
−3
10000
11000
12000
13000
14000
15000
16000
17000
19000
21000
23000
64000
68000
70000
71000
72000
73000
74000
75000
76000
77000
78000
79000
80000
81000
82000
83000
84000
86000
87000
89000
93000
97000
Subject
Condition
EM
Primary

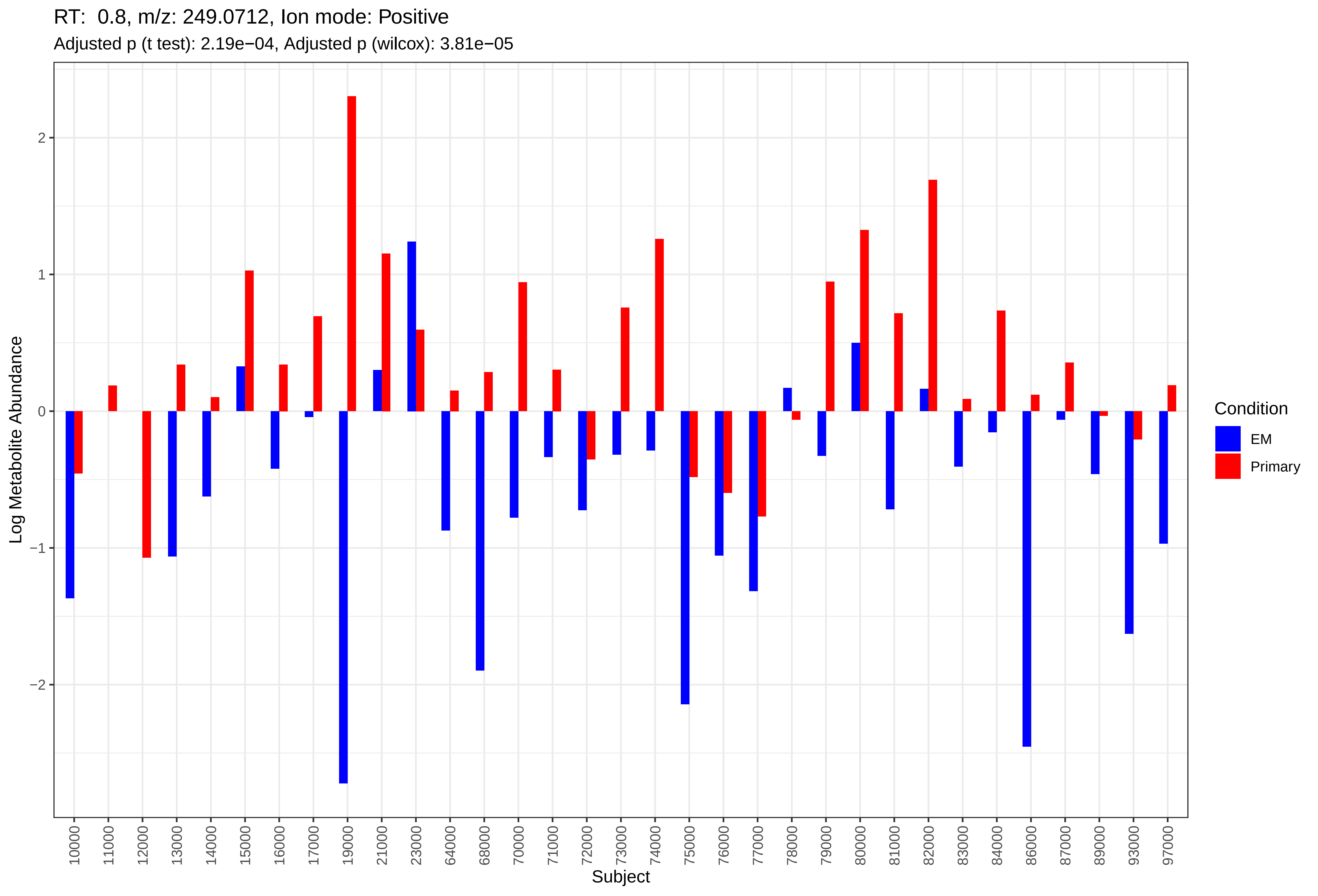

RT: 0.8, m/z: 249.0712, Ion mode: Positive
Adjusted p (t test): 2.19e−04, Adjusted p (wilcox): 3.81e−05
Log Metabolite Abundance
2
1
0
−1
−2
10000
11000
12000
13000
14000
15000
16000
17000
19000
21000
23000
64000
68000
70000
71000
72000
73000
74000
75000
76000
77000
78000
79000
80000
81000
82000
83000
84000
86000
87000
89000
93000
97000
Subject
Condition
EM
Primary

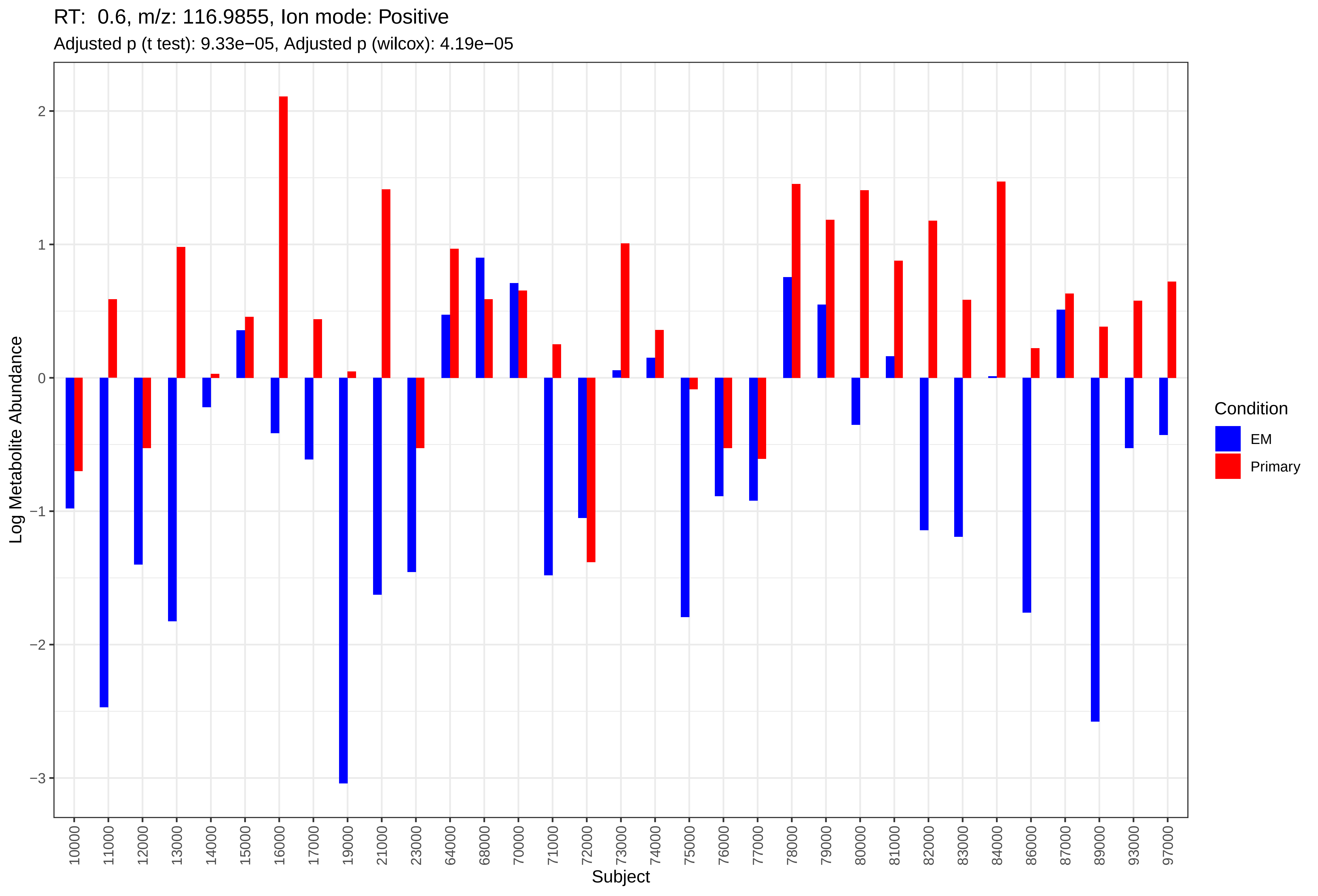
RT: 0.6, m/z: 116.9855, Ion mode: Positive
Adjusted p (t test): 9.33e−05, Adjusted p (wilcox): 4.19e−05
Log Metabolite Abundance
2
1
0
−1
−2
−3
10000
11000
12000
13000
14000
15000
16000
17000
19000
21000
23000
64000
68000
70000
71000
72000
73000
74000
75000
76000
77000
78000
79000
80000
81000
82000
83000
84000
86000
87000
89000
93000
97000
Subject
Condition
EM
Primary

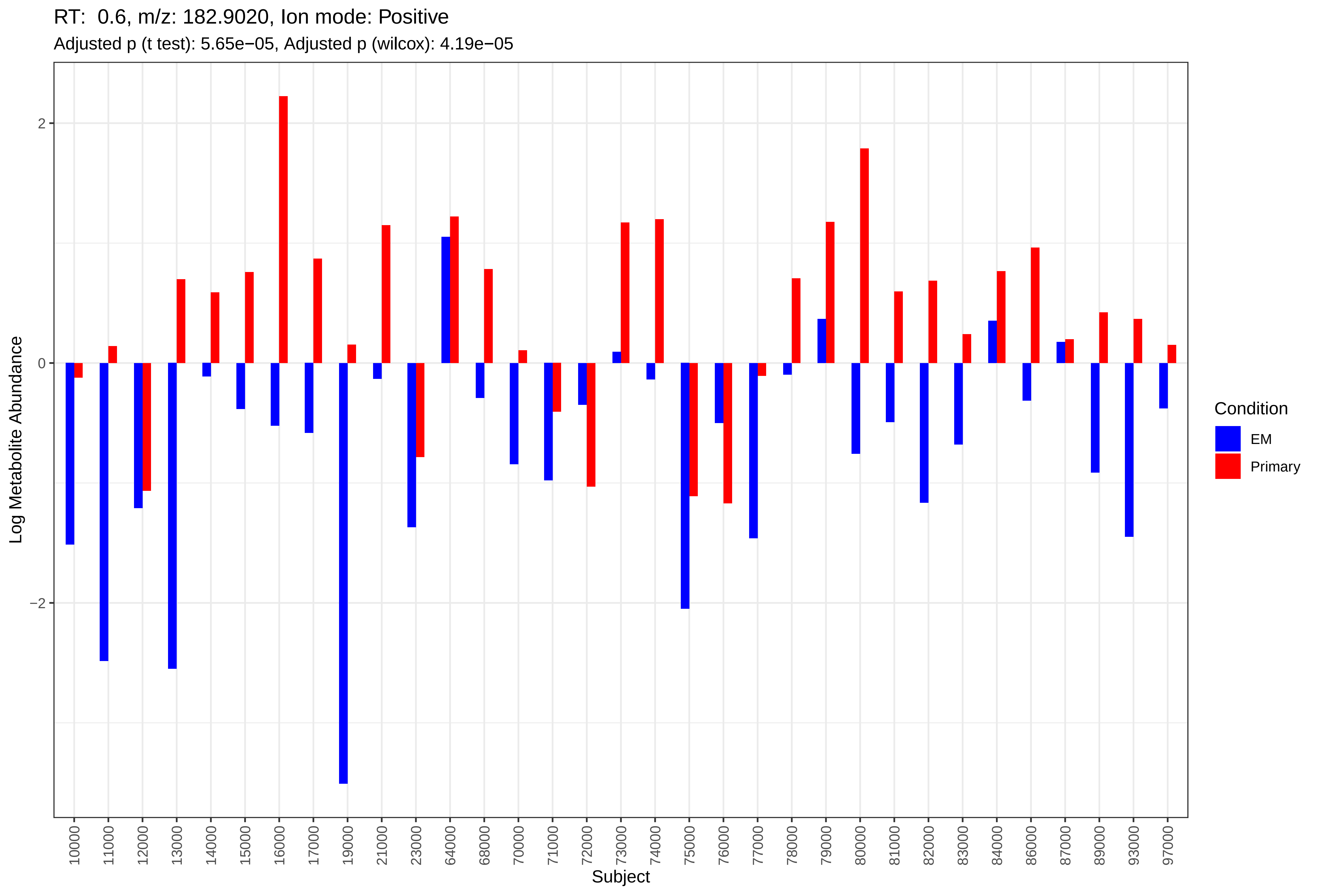
RT: 0.6, m/z: 182.9020, Ion mode: Positive
Adjusted p (t test): 5.65e−05, Adjusted p (wilcox): 4.19e−05
Log Metabolite Abundance
2
0
−2
10000
11000
12000
13000
14000
15000
16000
17000
19000
21000
23000
64000
68000
70000
71000
72000
73000
74000
75000
76000
77000
78000
79000
80000
81000
82000
83000
84000
86000
87000
89000
93000
97000
Subject
Condition
EM
Primary

RT: 0.6, m/z: 98.4707, Ion mode: Positive

Adjusted p (t test): 1.72e−04, Adjusted p (wilcox): 4.19e−05

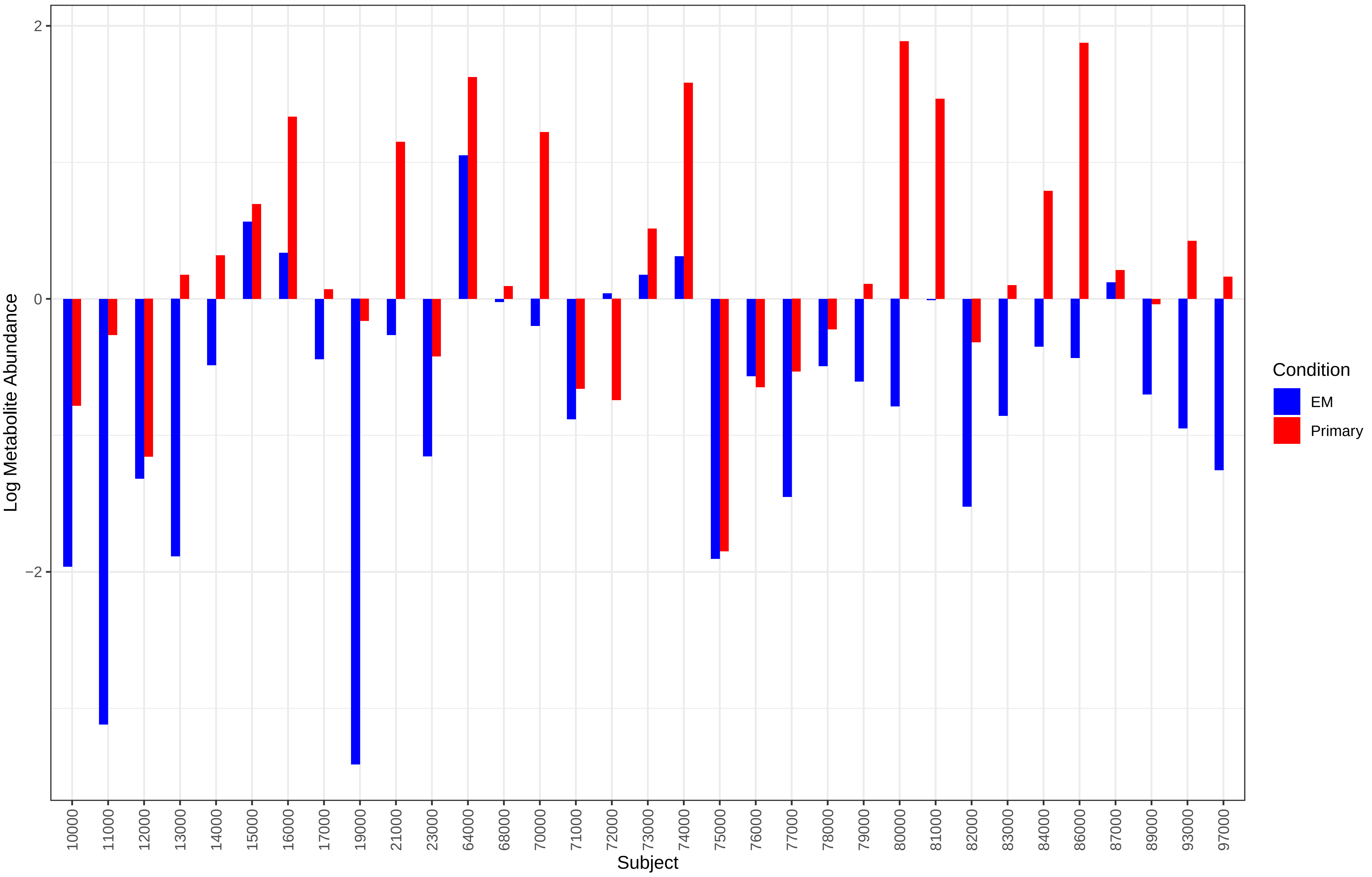

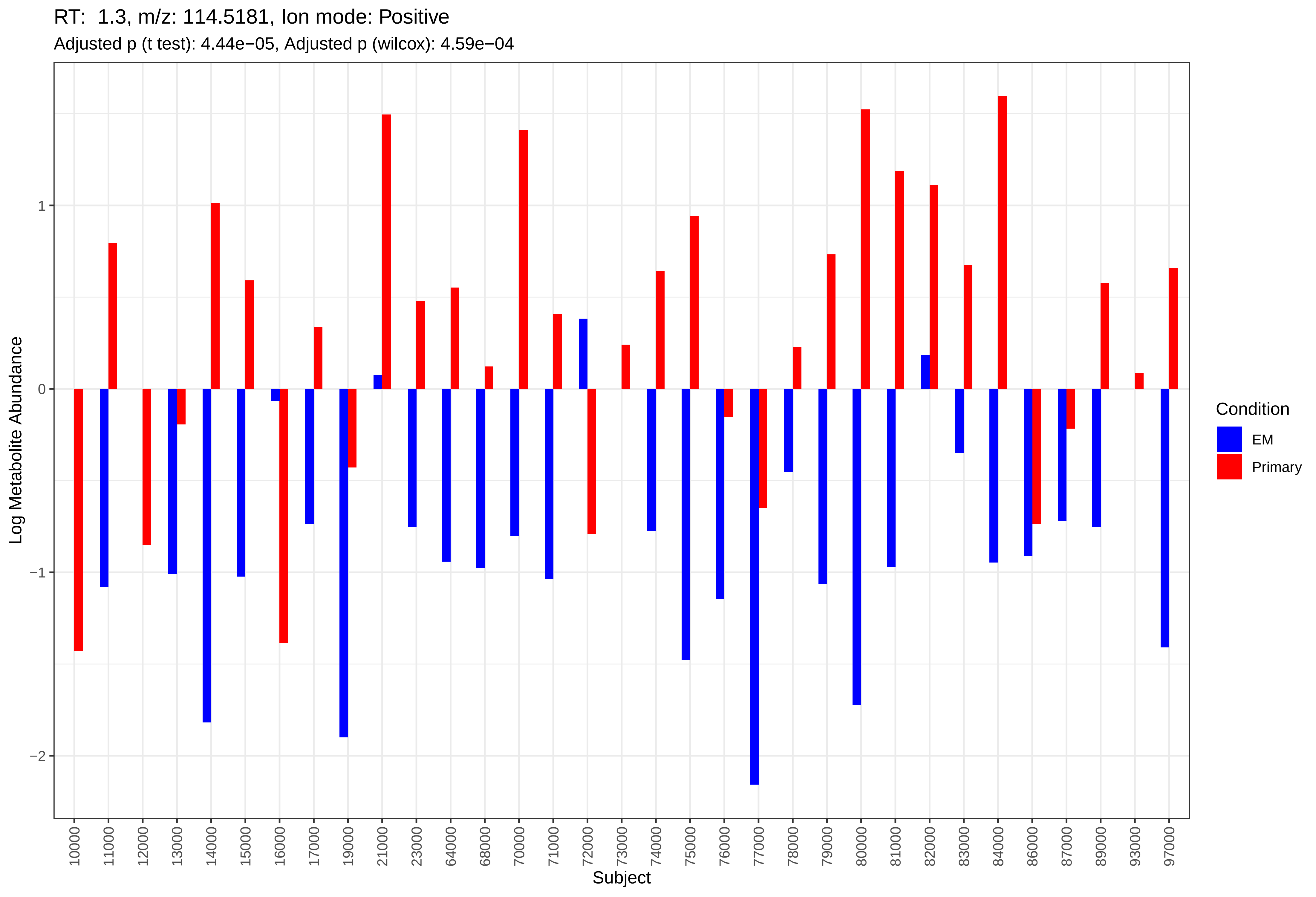

RT: 1.3, m/z: 114.5181, Ion mode: Positive
Adjusted p (t test): 4.44e−05, Adjusted p (wilcox): 4.59e−04
Log Metabolite Abundance
1
0
−1
−2
Subject
10000
11000
12000
13000
14000
15000
16000
17000
19000
21000
23000
64000
68000
70000
71000
72000
73000
74000
75000
76000
77000
78000
79000
80000
81000
82000
83000
84000
86000
87000
89000
93000
97000
Condition
EM
Primary

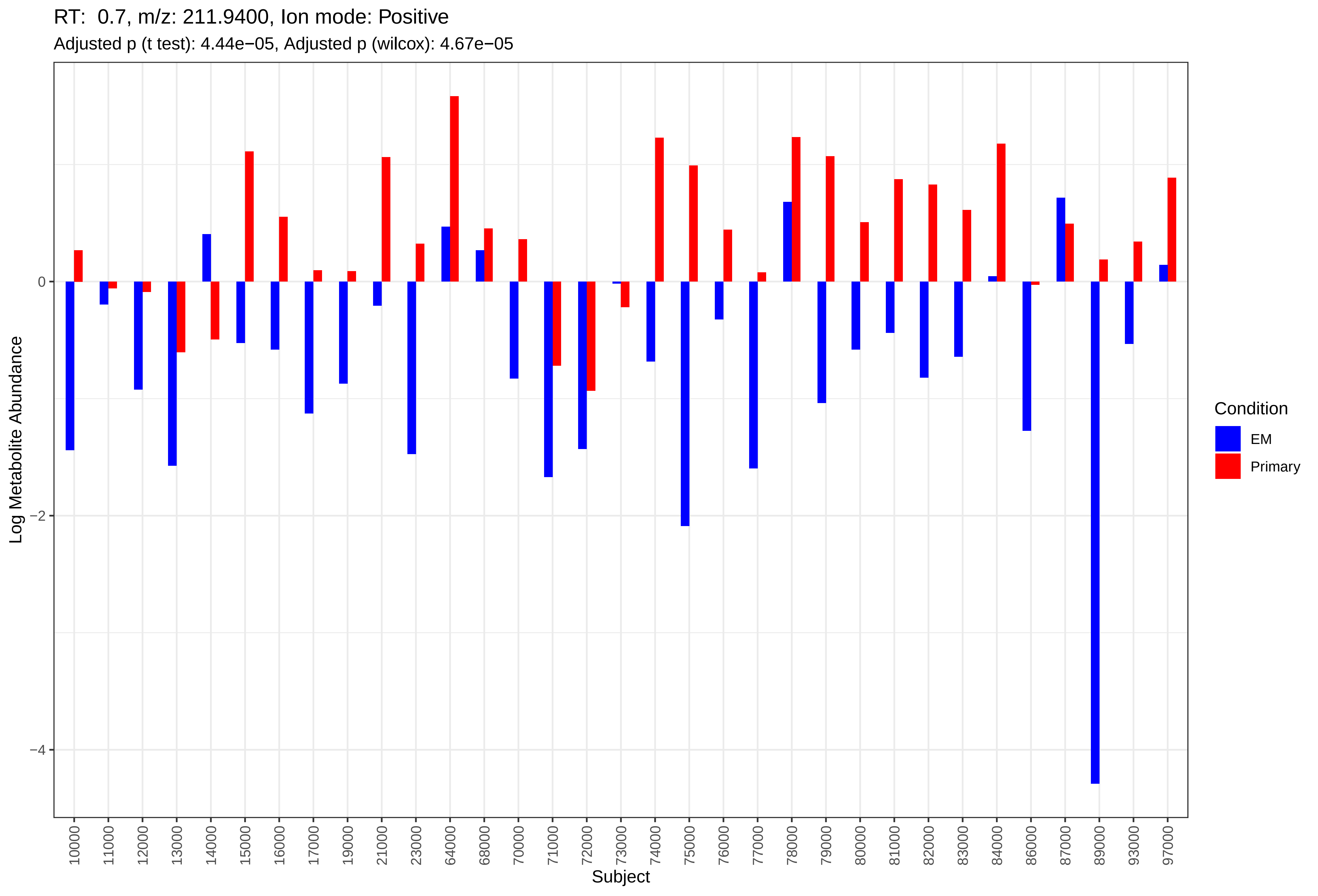
RT: 0.7, m/z: 211.9400, Ion mode: Positive
Adjusted p (t test): 4.44e−05, Adjusted p (wilcox): 4.67e−05
Log Metabolite Abundance
0
−2
−4
Subject
10000
11000
12000
13000
14000
15000
16000
17000
19000
21000
23000
64000
68000
70000
71000
72000
73000
74000
75000
76000
77000
78000
79000
80000
81000
82000
83000
84000
86000
87000
89000
93000
97000
Condition
EM
Primary

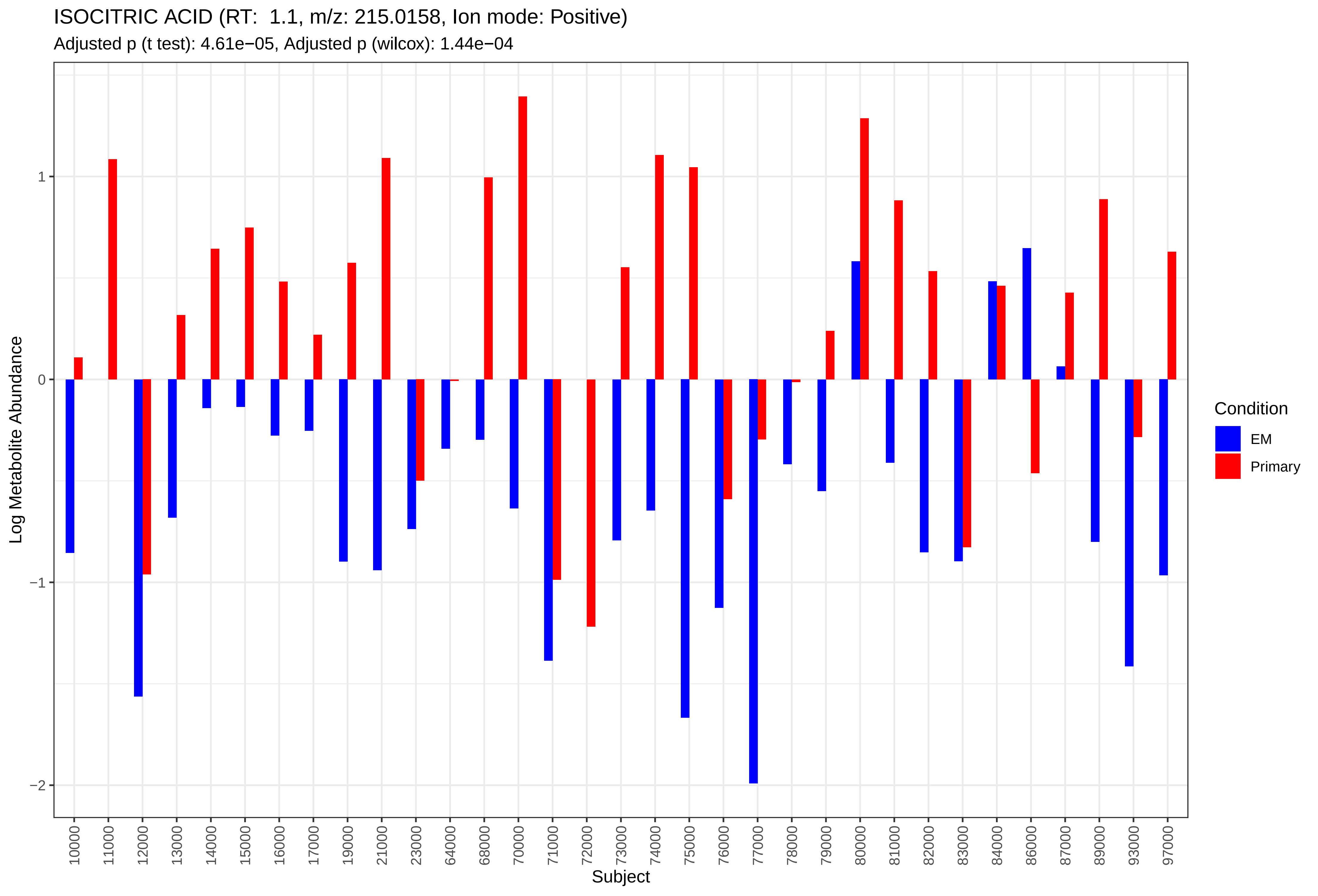

ISOCITRIC ACID (RT: 1.1, m/z: 215.0158, Ion mode: Positive)
Adjusted p (t test): 4.61e−05, Adjusted p (wilcox): 1.44e−04
Log Metabolite Abundance
1
0
−1
−2
Subject
10000
11000
12000
13000
14000
15000
16000
17000
19000
21000
23000
64000
68000
70000
71000
72000
73000
74000
75000
76000
77000
78000
79000
80000
81000
82000
83000
84000
86000
87000
89000
93000
97000
Condition
EM
Primary

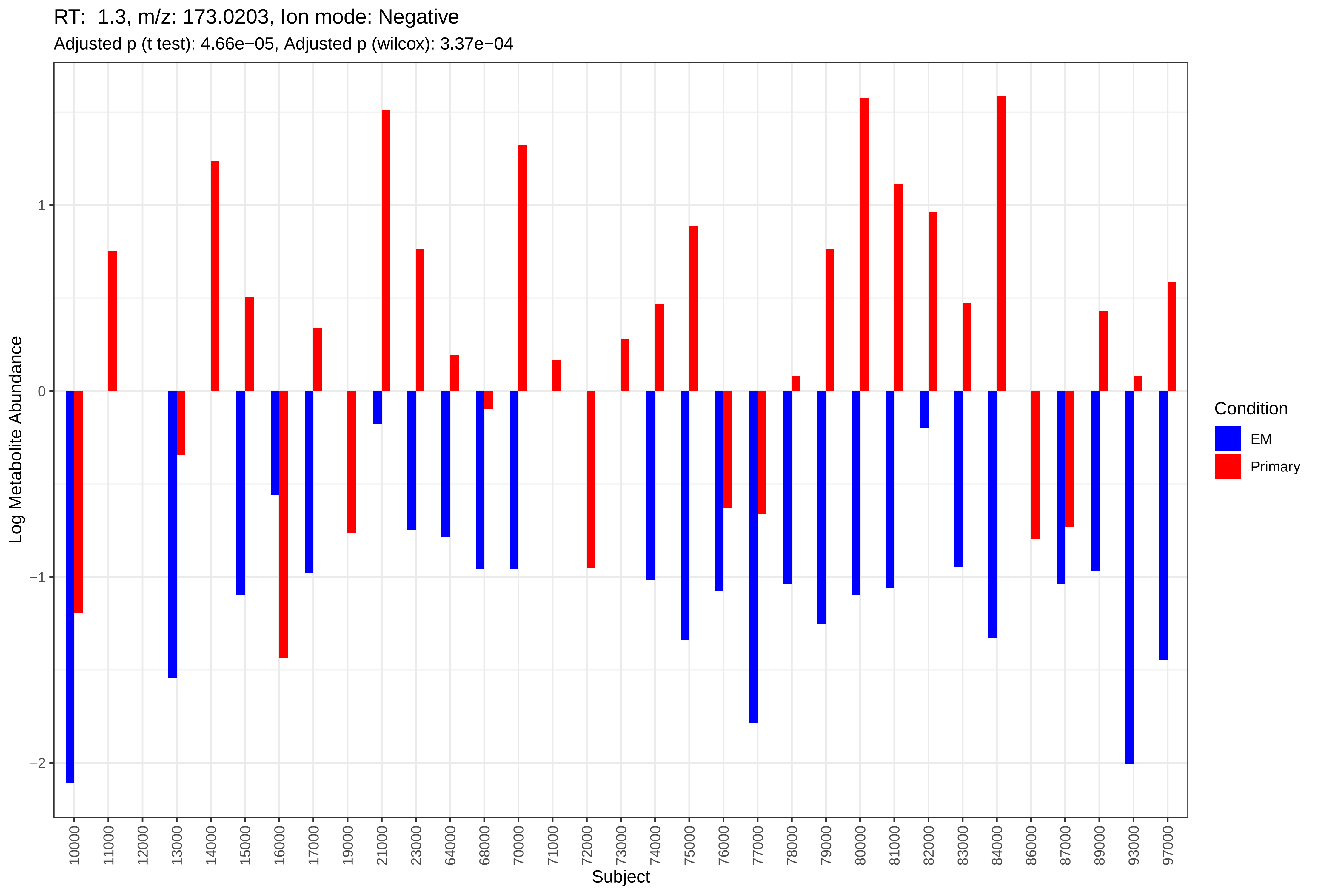
RT: 1.3, m/z: 173.0203, Ion mode: Negative
Adjusted p (t test): 4.66e−05, Adjusted p (wilcox): 3.37e−04
Log Metabolite Abundance
1
0
−1
−2
10000
11000
12000
13000
14000
15000
16000
17000
19000
21000
23000
64000
68000
70000
71000
72000
73000
74000
75000
76000
77000
78000
79000
80000
81000
82000
83000
84000
86000
87000
89000
93000
97000
Subject
Condition
EM
Primary

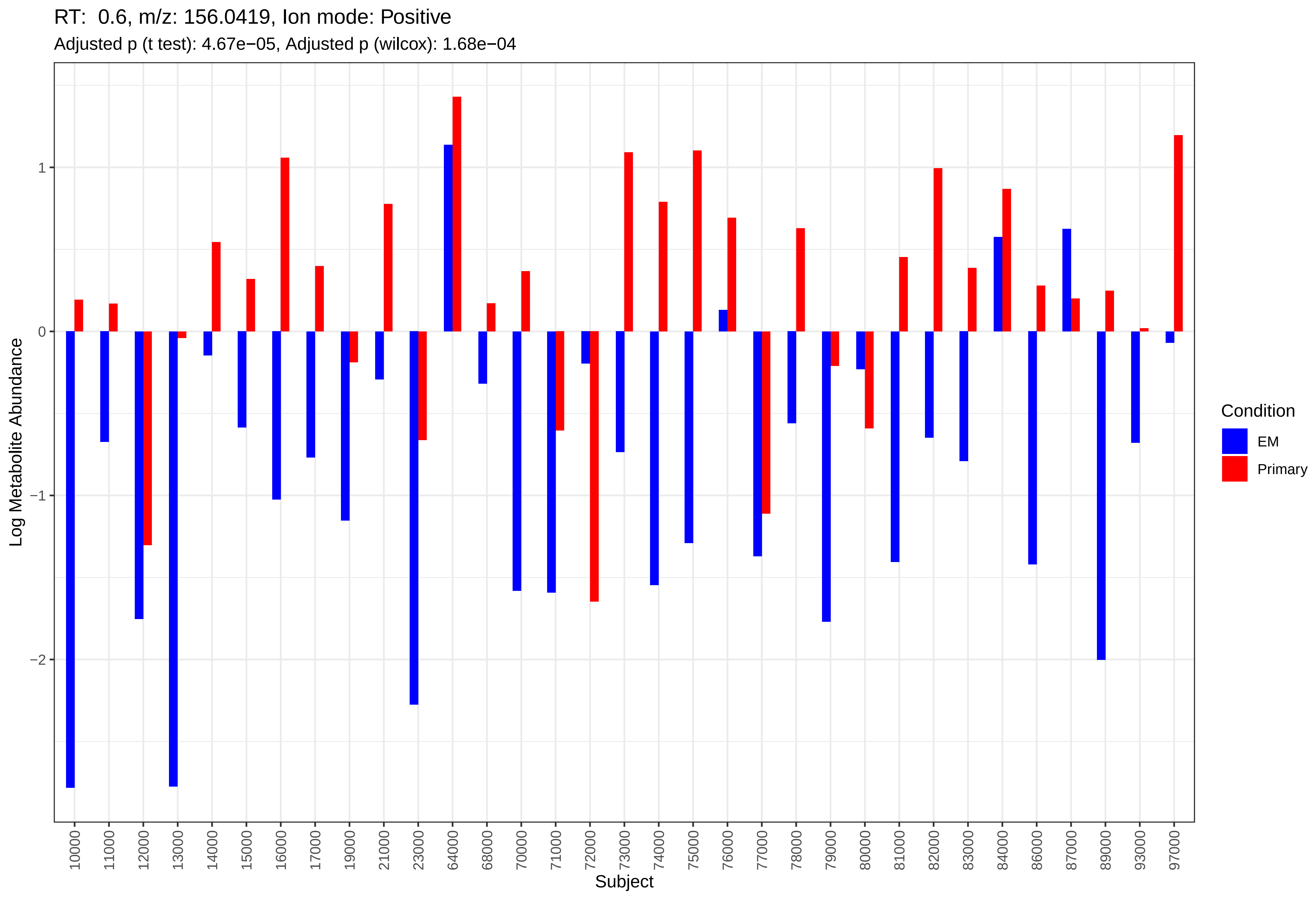

RT: 0.6, m/z: 156.0419, Ion mode: Positive
Adjusted p (t test): 4.67e−05, Adjusted p (wilcox): 1.68e−04
Log Metabolite Abundance
1
0
−1
−2
Subject
10000
11000
12000
13000
14000
15000
16000
17000
19000
21000
23000
64000
68000
70000
71000
72000
73000
74000
75000
76000
77000
78000
79000
80000
81000
82000
83000
84000
86000
87000
89000
93000
97000
Condition
EM
Primary

CYTOSINE (RT: 0.8, m/z: 112.0505, Ion mode: Positive)

Adjusted p (t test): 5.96e−05, Adjusted p (wilcox): 4.67e−05

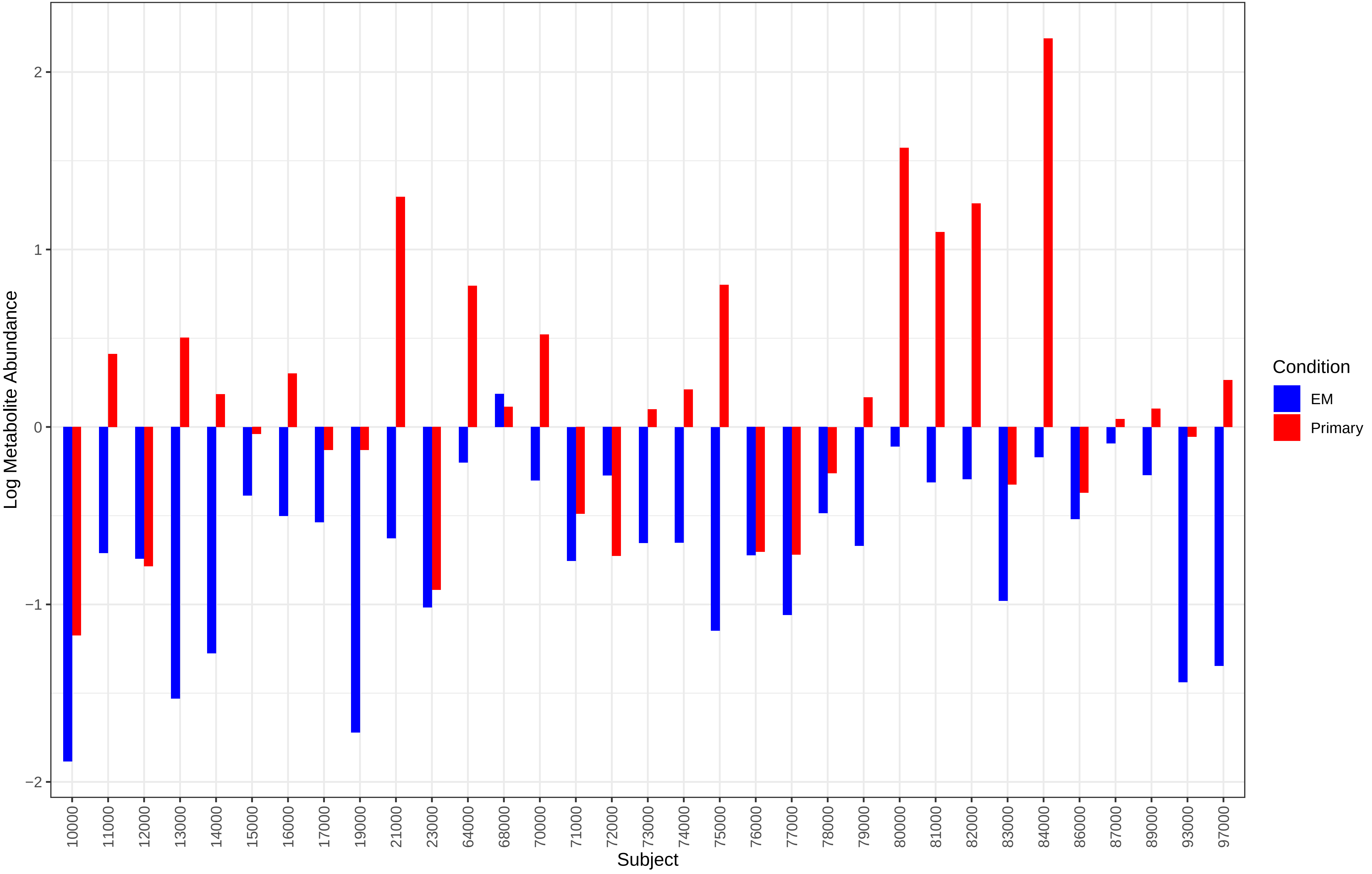

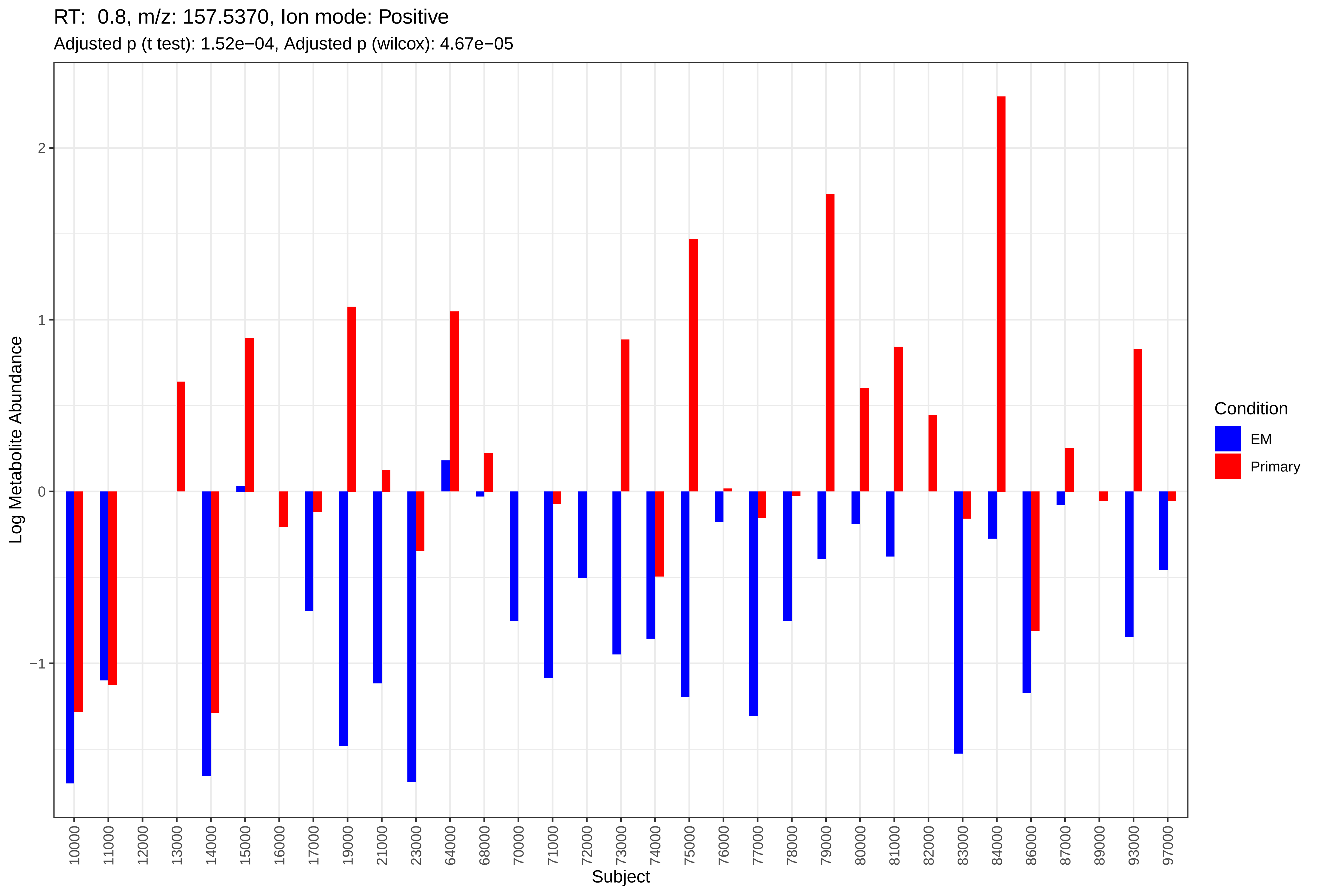
RT: 0.8, m/z: 157.5370, Ion mode: Positive
Adjusted p (t test): 1.52e−04, Adjusted p (wilcox): 4.67e−05
Log Metabolite Abundance
2
1
0
−1
10000
11000
12000
13000
14000
15000
16000
17000
19000
21000
23000
64000
68000
70000
71000
72000
73000
74000
75000
76000
77000
78000
79000
80000
81000
82000
83000
84000
86000
87000
89000
93000
97000
Subject
Condition
EM
Primary

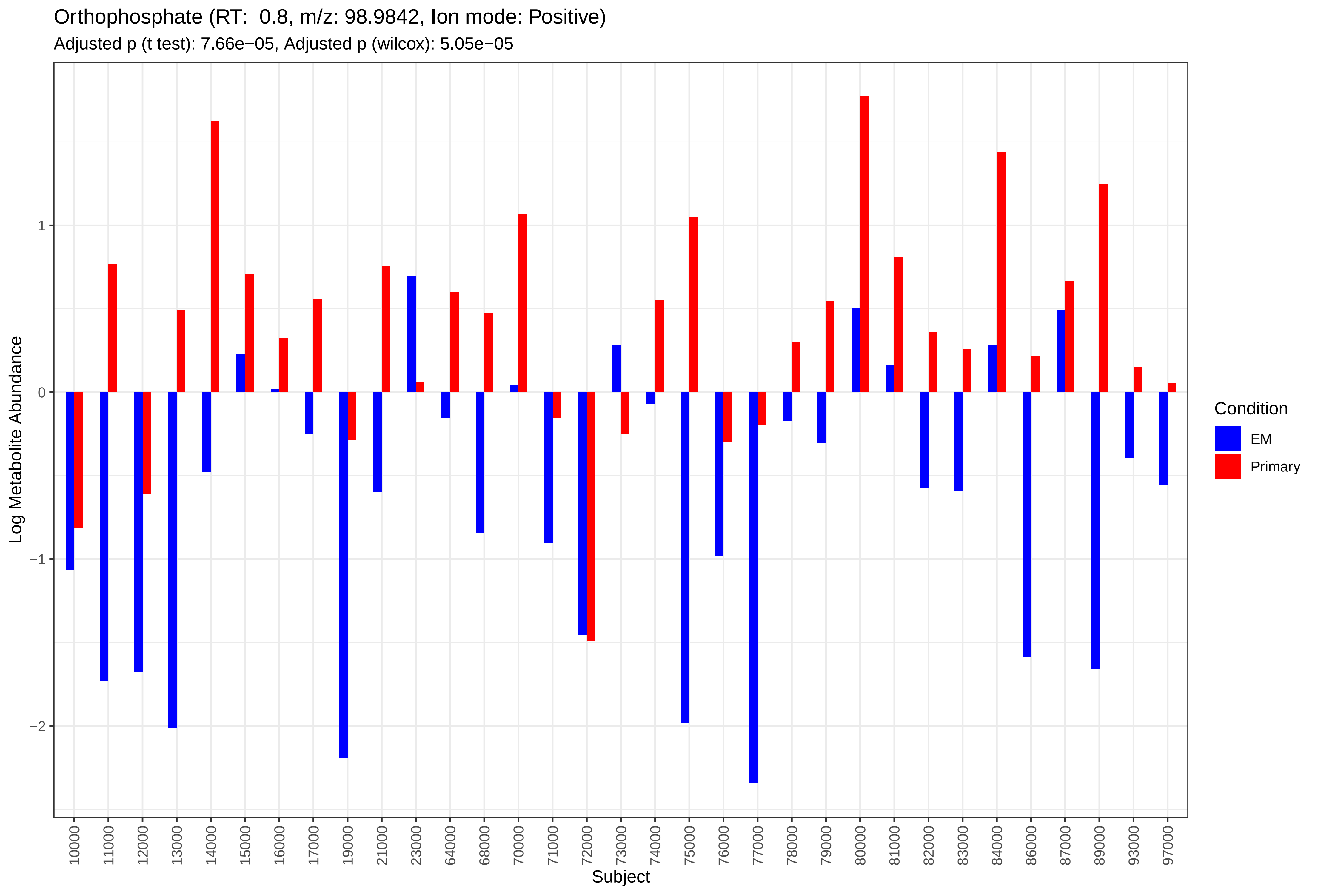
Orthophosphate (RT: 0.8, m/z: 98.9842, Ion mode: Positive)
Adjusted p (t test): 7.66e−05, Adjusted p (wilcox): 5.05e−05
Log Metabolite Abundance
1
0
−1
−2
10000
11000
12000
13000
14000
15000
16000
17000
19000
21000
23000
64000
68000
70000
71000
72000
73000
74000
75000
76000
77000
78000
79000
80000
81000
82000
83000
84000
86000
87000
89000
93000
97000
Subject
Condition
EM
Primary

RT: 6.5, m/z: 225.1716, Ion mode: Positive

Adjusted p (t test): 4.85e−03, Adjusted p (wilcox): 5.05e−05

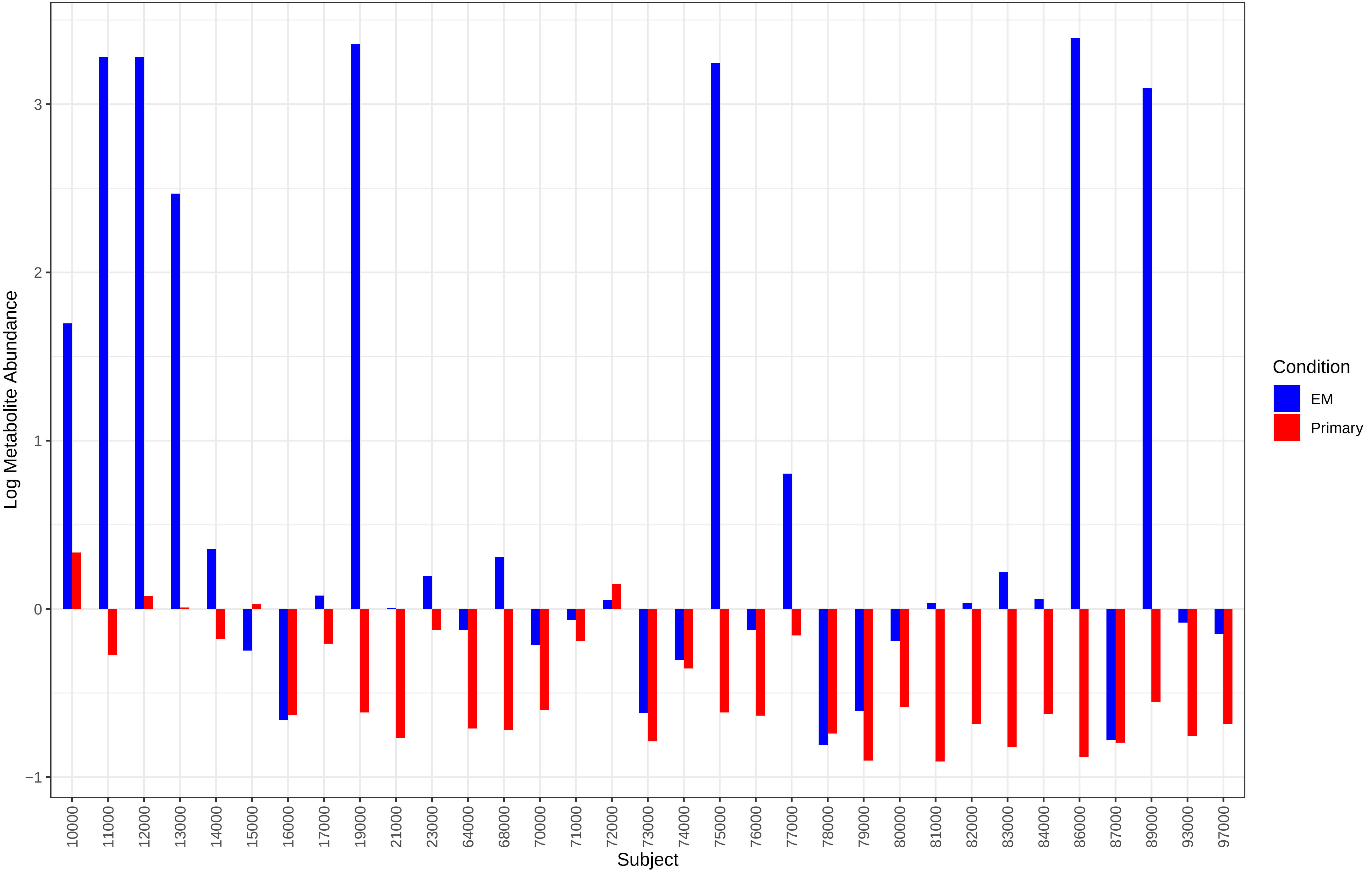

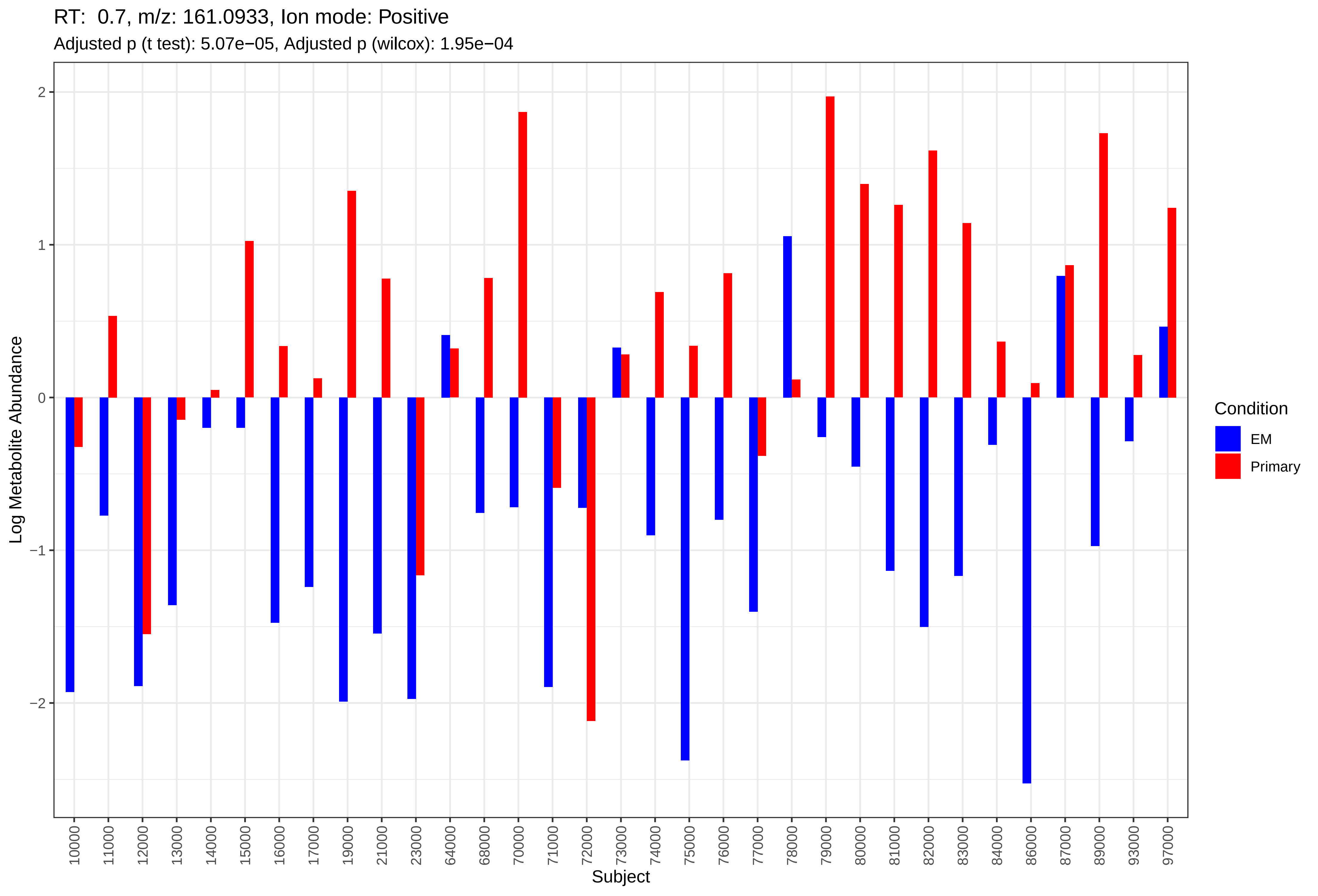

RT: 0.7, m/z: 161.0933, Ion mode: Positive
Adjusted p (t test): 5.07e−05, Adjusted p (wilcox): 1.95e−04
Log Metabolite Abundance
2
1
0
−1
−2
10000
11000
12000
13000
14000
15000
16000
17000
19000
21000
23000
64000
68000
70000
71000
72000
73000
74000
75000
76000
77000
78000
79000
80000
81000
82000
83000
84000
86000
87000
89000
93000
97000
Subject
Condition
EM
Primary

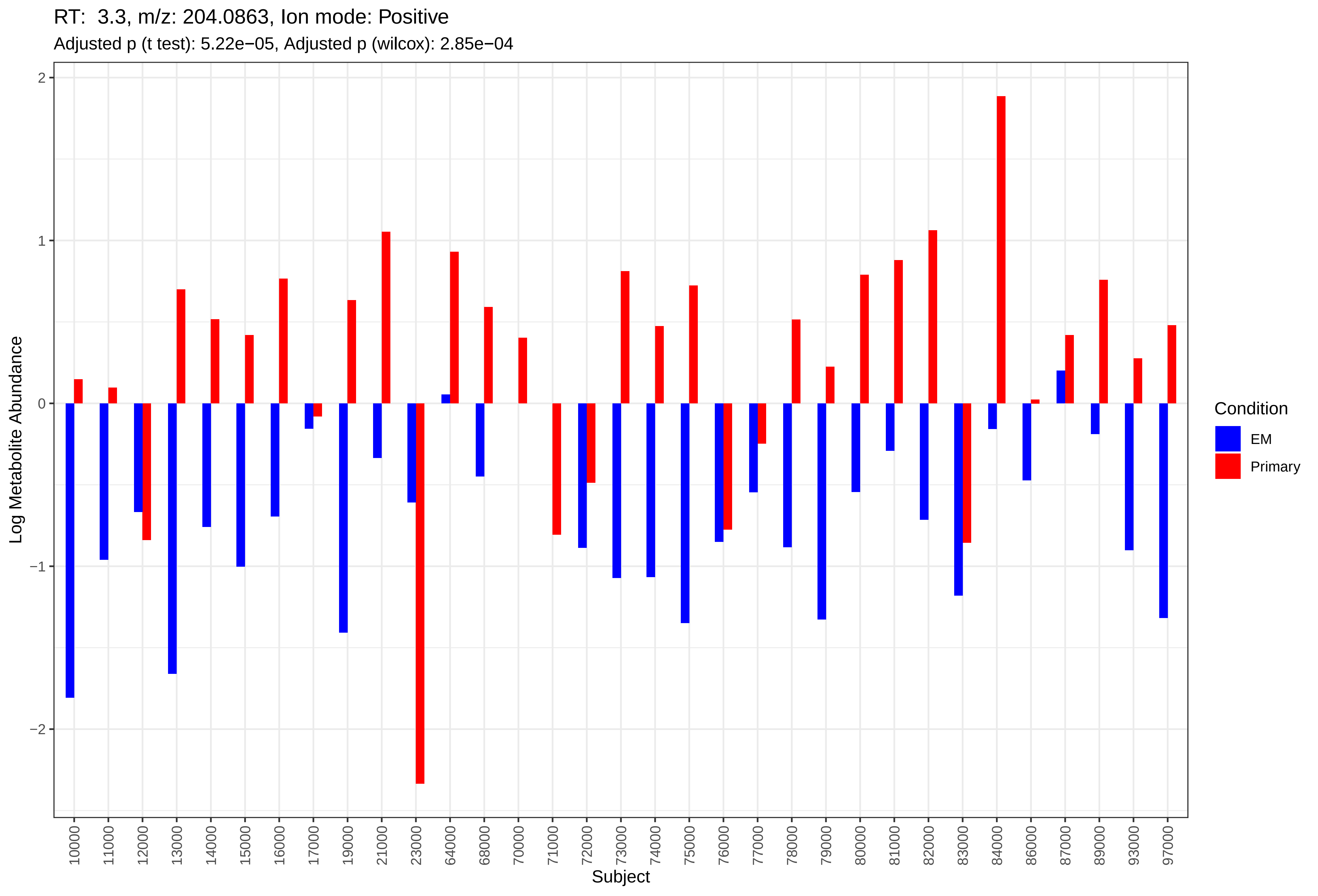
RT: 3.3, m/z: 204.0863, Ion mode: Positive
Adjusted p (t test): 5.22e−05, Adjusted p (wilcox): 2.85e−04
Log Metabolite Abundance
2
1
0
−1
−2
Subject
10000
11000
12000
13000
14000
15000
16000
17000
19000
21000
23000
64000
68000
70000
71000
72000
73000
74000
75000
76000
77000
78000
79000
80000
81000
82000
83000
84000
86000
87000
89000
93000
97000
Condition
EM
Primary

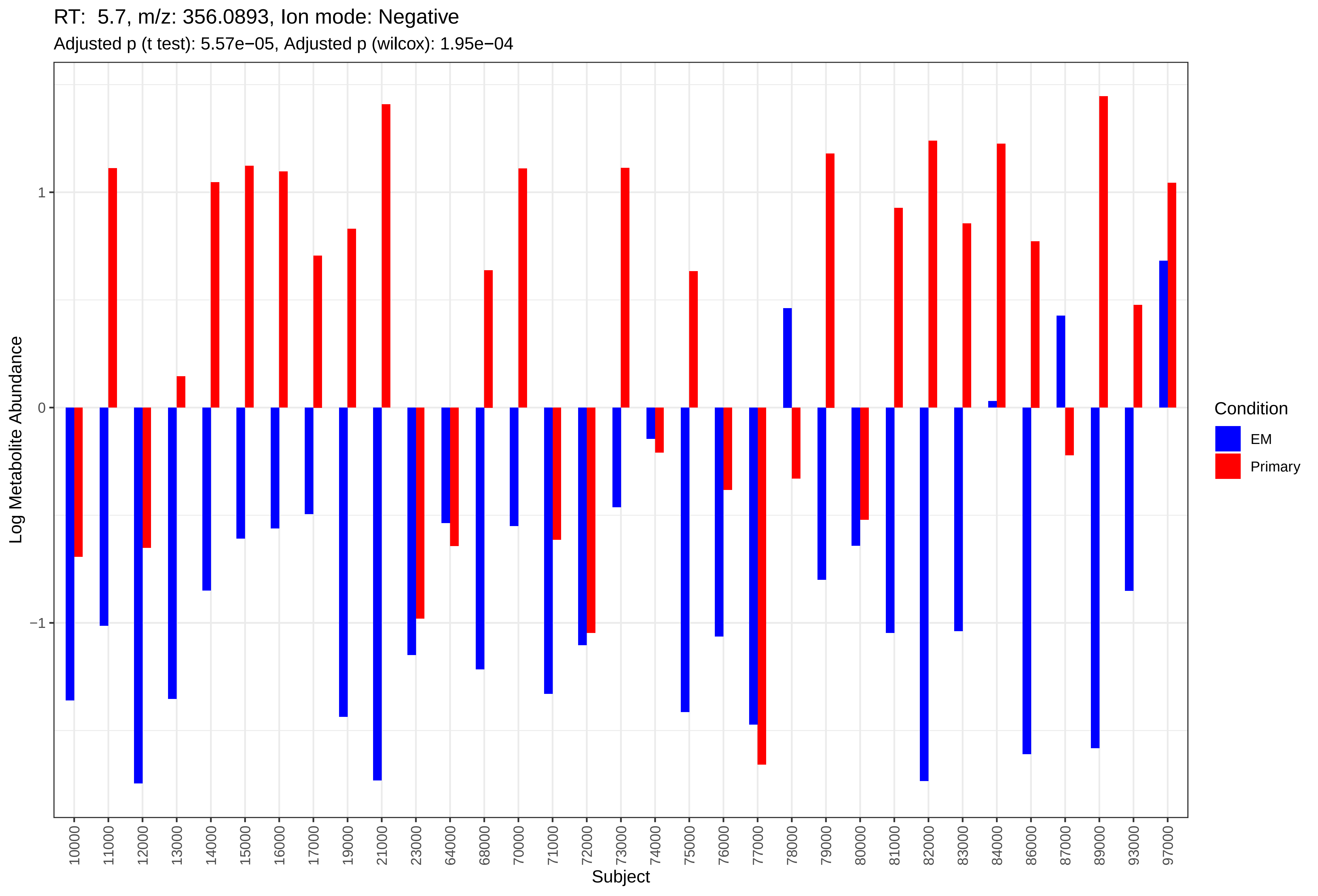
RT: 5.7, m/z: 356.0893, Ion mode: Negative
Adjusted p (t test): 5.57e−05, Adjusted p (wilcox): 1.95e−04
Log Metabolite Abundance
1
0
−1
Subject
10000
11000
12000
13000
14000
15000
16000
17000
19000
21000
23000
64000
68000
70000
71000
72000
73000
74000
75000
76000
77000
78000
79000
80000
81000
82000
83000
84000
86000
87000
89000
93000
97000
Condition
EM
Primary

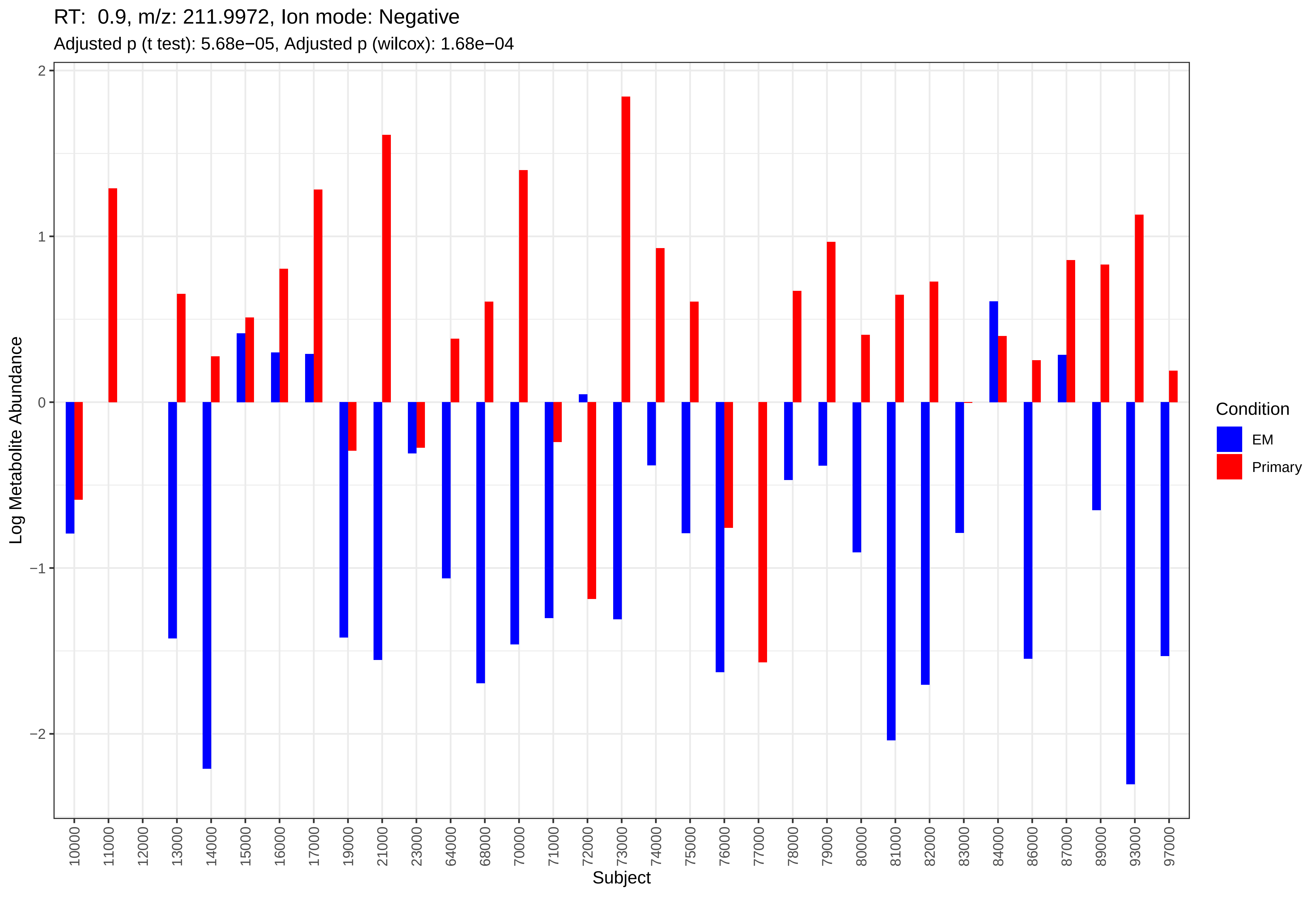
RT: 0.9, m/z: 211.9972, Ion mode: Negative
Adjusted p (t test): 5.68e−05, Adjusted p (wilcox): 1.68e−04
Log Metabolite Abundance
2
1
0
−1
−2
Subject
10000
11000
12000
13000
14000
15000
16000
17000
19000
21000
23000
64000
68000
70000
71000
72000
73000
74000
75000
76000
77000
78000
79000
80000
81000
82000
83000
84000
86000
87000
89000
93000
97000
Condition
EM
Primary

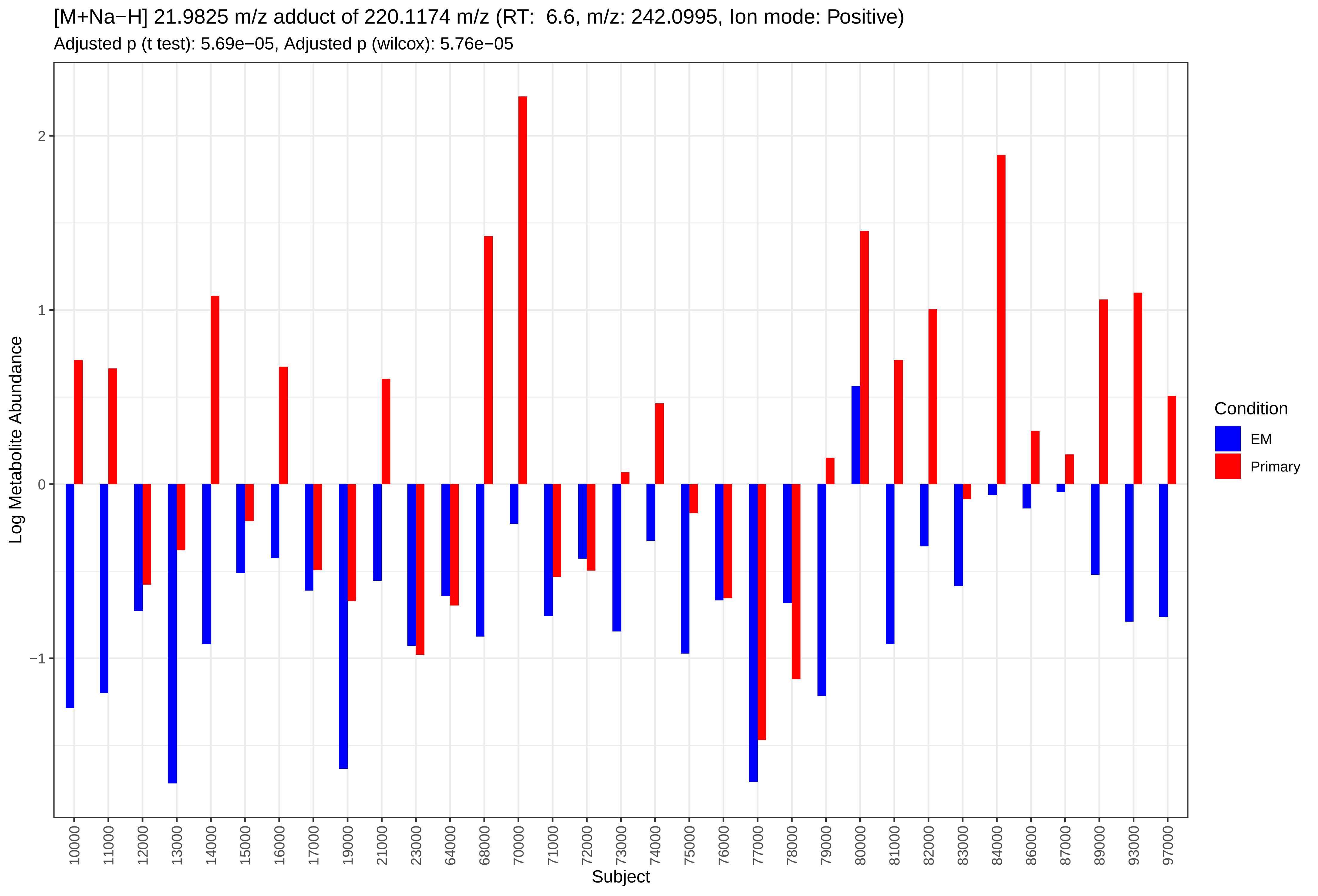

[M+Na−H] 21.9825 m/z adduct of 220.1174 m/z (RT: 6.6, m/z: 242.0995, Ion mode: Positive)
Adjusted p (t test): 5.69e−05, Adjusted p (wilcox): 5.76e−05
Log Metabolite Abundance
2
1
0
−1
10000
11000
12000
13000
14000
15000
16000
17000
19000
21000
23000
64000
68000
70000
71000
72000
73000
74000
75000
76000
77000
78000
79000
80000
81000
82000
83000
84000
86000
87000
89000
93000
97000
Subject
Condition
EM
Primary

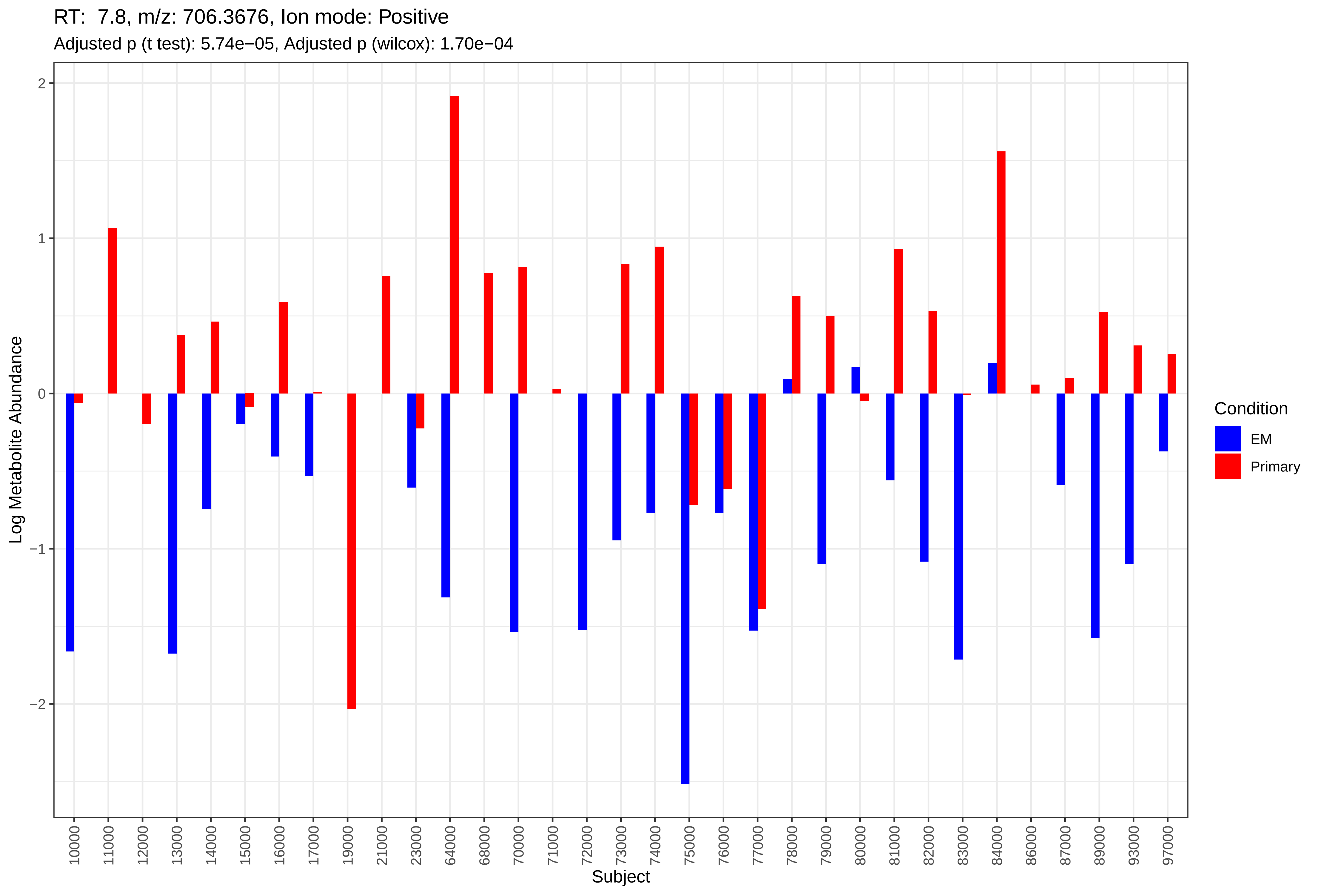
RT: 7.8, m/z: 706.3676, Ion mode: Positive
Adjusted p (t test): 5.74e−05, Adjusted p (wilcox): 1.70e−04
Log Metabolite Abundance
2
1
0
−1
−2
Subject
10000
11000
12000
13000
14000
15000
16000
17000
19000
21000
23000
64000
68000
70000
71000
72000
73000
74000
75000
76000
77000
78000
79000
80000
81000
82000
83000
84000
86000
87000
89000
93000
97000
Condition
EM
Primary

[M+Na−H] 21.9825 m/z adduct of 184.0730 m/z (RT: 0.7, m/z: 206.0548, Ion mode: Positive)

Adjusted p (t test): 1.46e−04, Adjusted p (wilcox): 5.76e−05

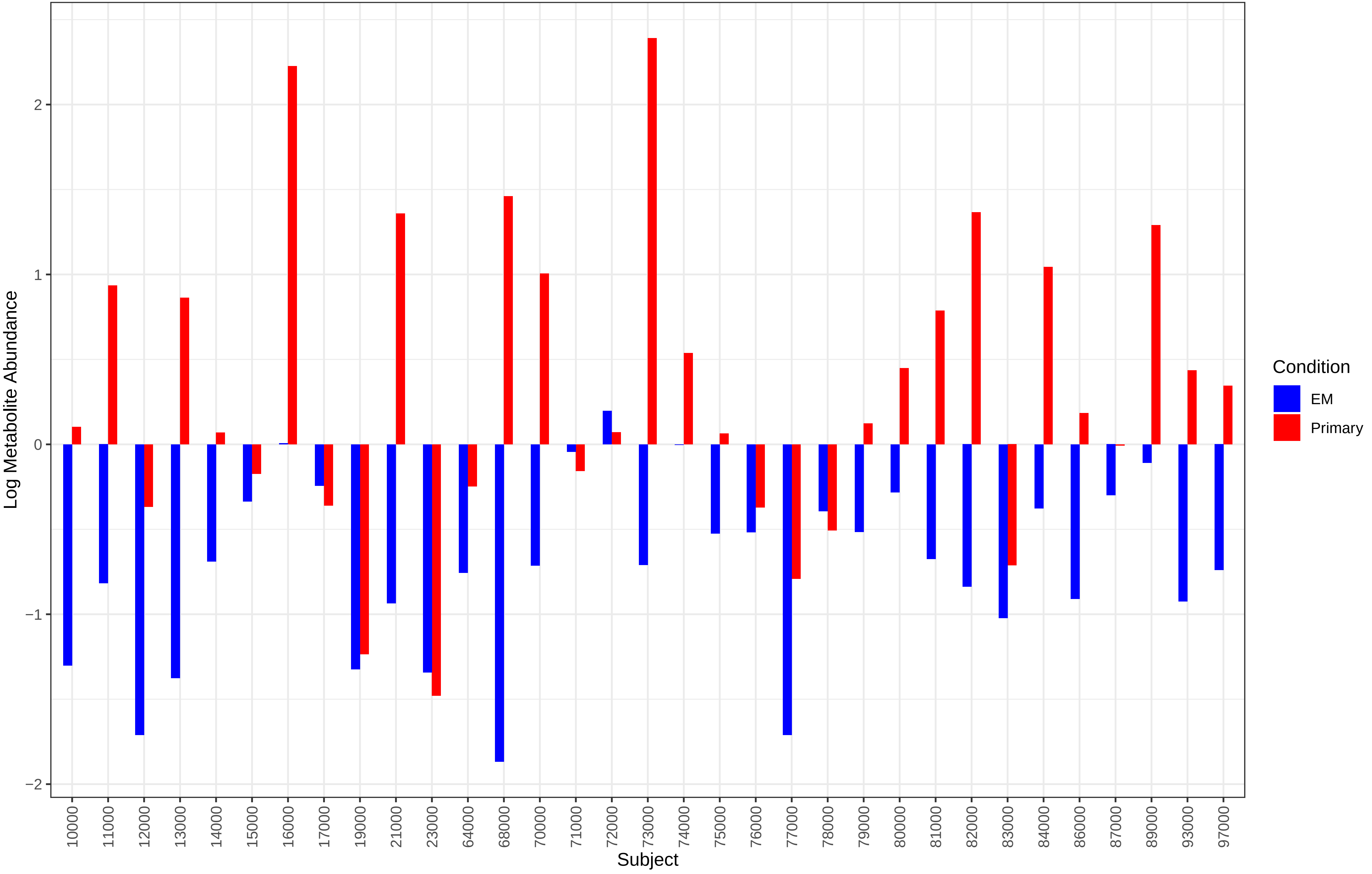

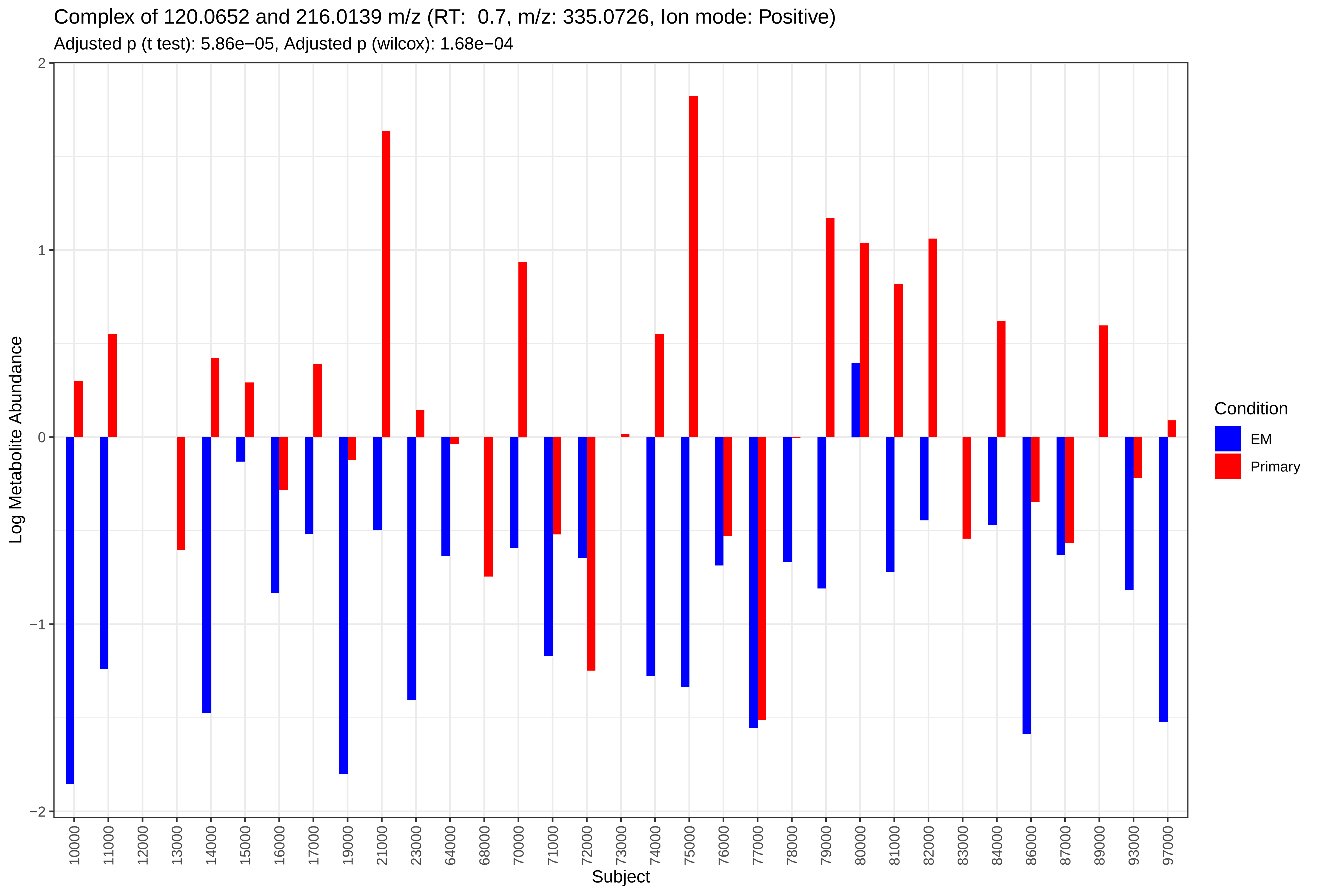
Complex of 120.0652 and 216.0139 m/z (RT: 0.7, m/z: 335.0726, Ion mode: Positive)
Adjusted p (t test): 5.86e−05, Adjusted p (wilcox): 1.68e−04
Log Metabolite Abundance
2
1
0
−1
−2
10000
11000
12000
13000
14000
15000
16000
17000
19000
21000
23000
64000
68000
70000
71000
72000
73000
74000
75000
76000
77000
78000
79000
80000
81000
82000
83000
84000
86000
87000
89000
93000
97000
Subject
Condition
EM
Primary

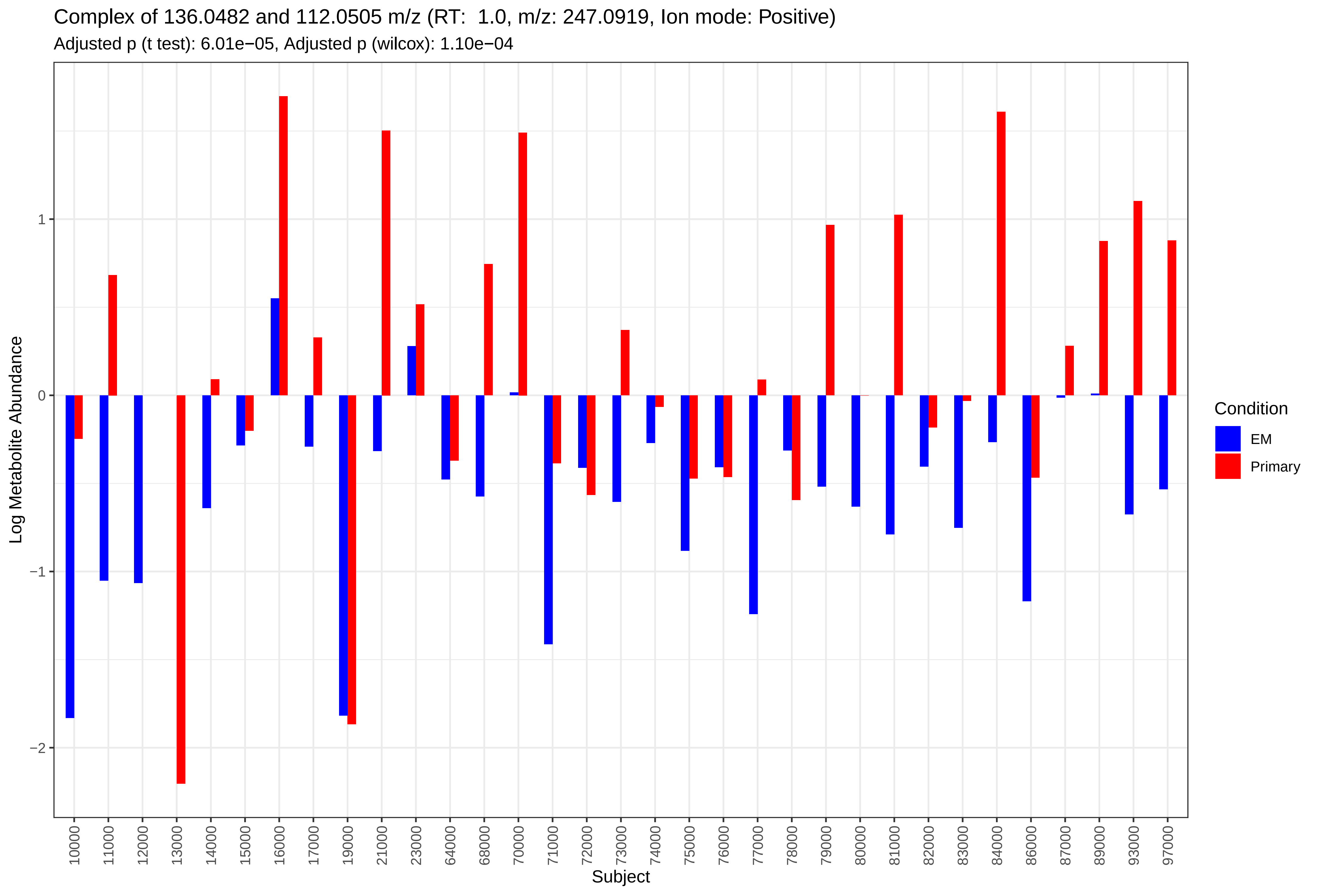

Complex of 136.0482 and 112.0505 m/z (RT: 1.0, m/z: 247.0919, Ion mode: Positive)
Adjusted p (t test): 6.01e−05, Adjusted p (wilcox): 1.10e−04
Log Metabolite Abundance
1
0
−1
−2
10000
11000
12000
13000
14000
15000
16000
17000
19000
21000
23000
64000
68000
70000
71000
72000
73000
74000
75000
76000
77000
78000
79000
80000
81000
82000
83000
84000
86000
87000
89000
93000
97000
Subject
Condition
EM
Primary

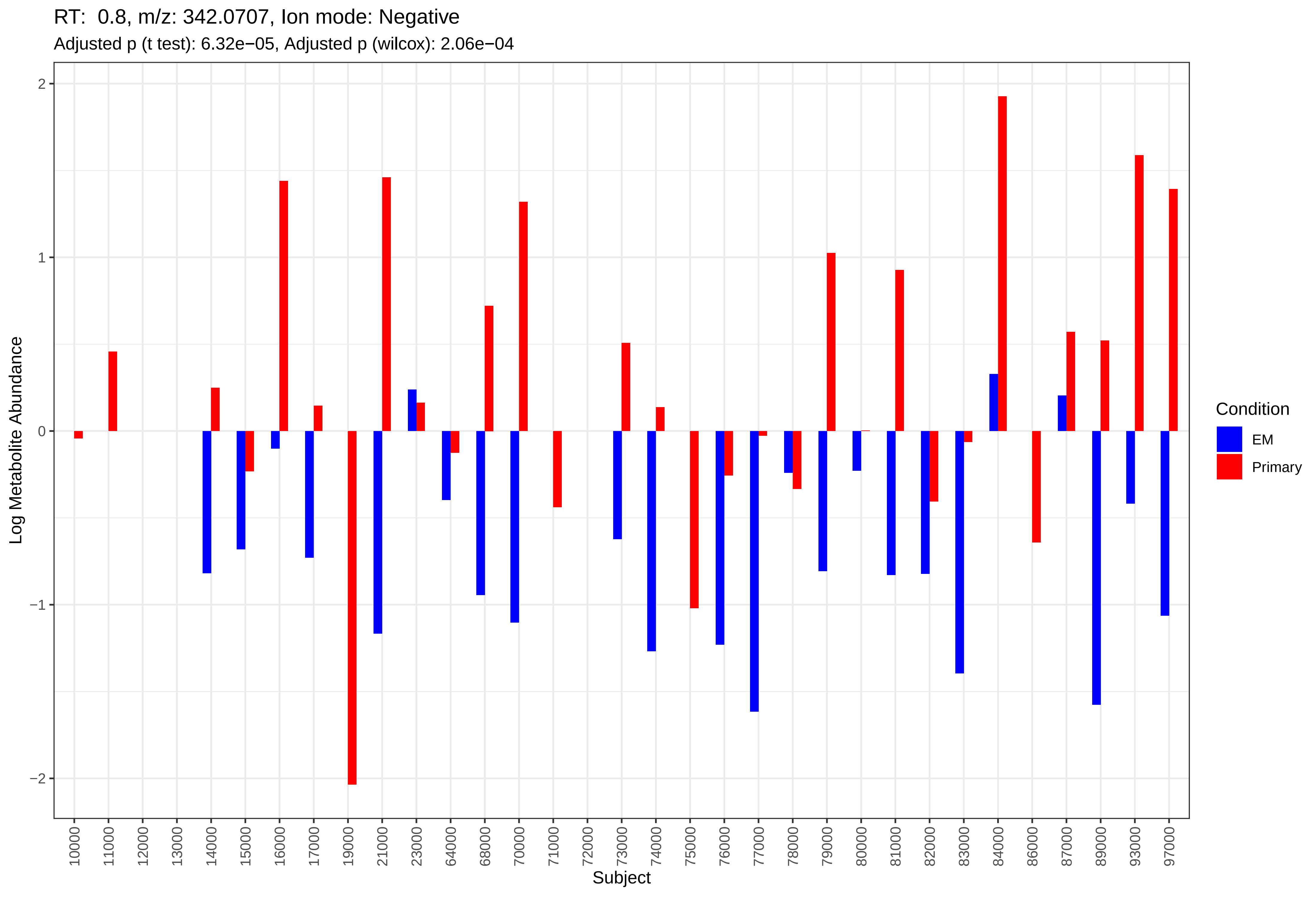

RT: 0.8, m/z: 342.0707, Ion mode: Negative
Adjusted p (t test): 6.32e−05, Adjusted p (wilcox): 2.06e−04
Log Metabolite Abundance
2
1
0
−1
−2
10000
11000
12000
13000
14000
15000
16000
17000
19000
21000
23000
64000
68000
70000
71000
72000
73000
74000
75000
76000
77000
78000
79000
80000
81000
82000
83000
84000
86000
87000
89000
93000
97000
Subject
Condition
EM
Primary

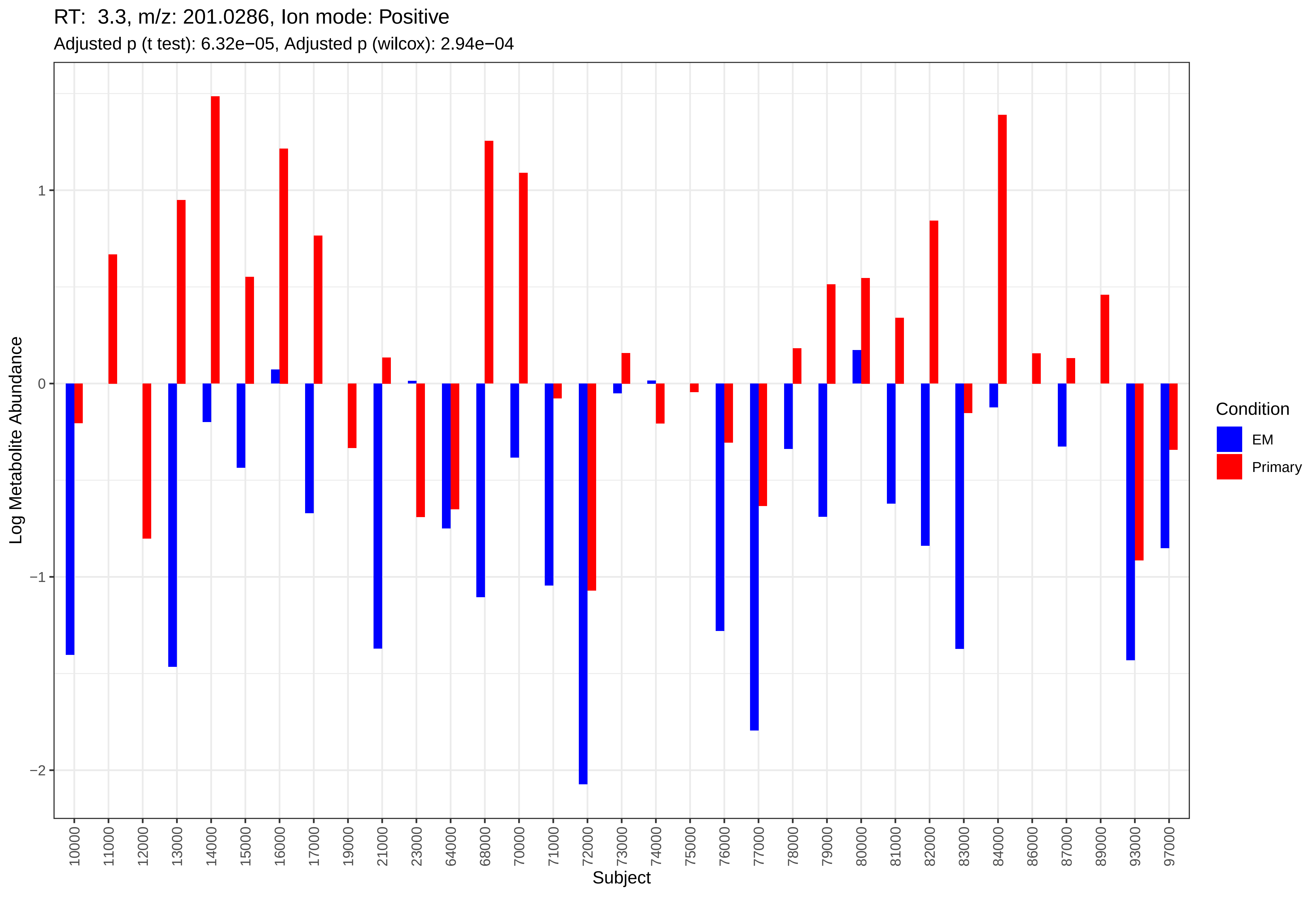
RT: 3.3, m/z: 201.0286, Ion mode: Positive
Adjusted p (t test): 6.32e−05, Adjusted p (wilcox): 2.94e−04
Log Metabolite Abundance
1
0
−1
−2
Subject
10000
11000
12000
13000
14000
15000
16000
17000
19000
21000
23000
64000
68000
70000
71000
72000
73000
74000
75000
76000
77000
78000
79000
80000
81000
82000
83000
84000
86000
87000
89000
93000
97000
Condition
EM
Primary

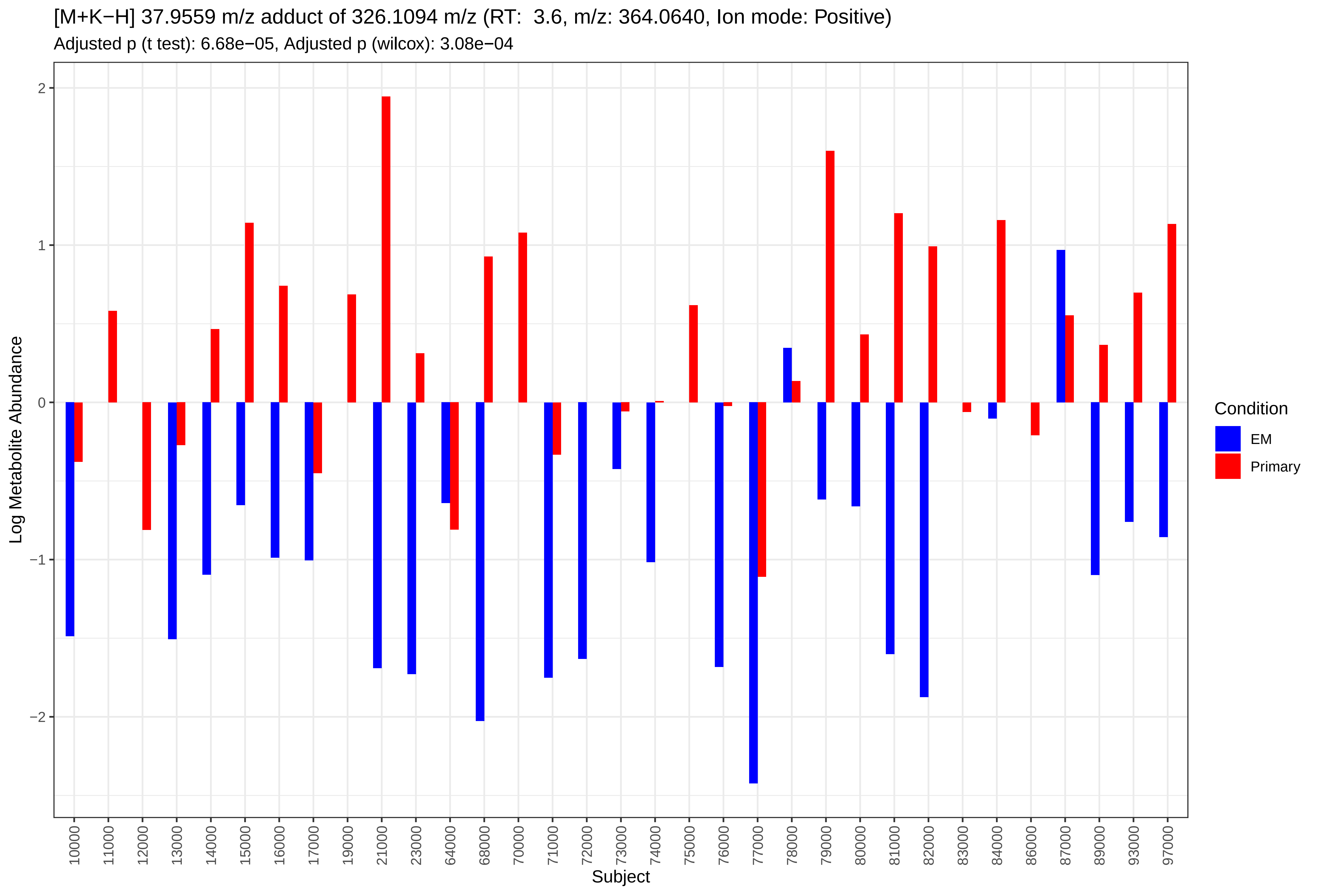
[M+K−H] 37.9559 m/z adduct of 326.1094 m/z (RT: 3.6, m/z: 364.0640, Ion mode: Positive)
Adjusted p (t test): 6.68e−05, Adjusted p (wilcox): 3.08e−04
Log Metabolite Abundance
2
1
0
−1
−2
Subject
10000
11000
12000
13000
14000
15000
16000
17000
19000
21000
23000
64000
68000
70000
71000
72000
73000
74000
75000
76000
77000
78000
79000
80000
81000
82000
83000
84000
86000
87000
89000
93000
97000
Condition
EM
Primary

RT: 2.0, m/z: 213.0159, Ion mode: Positive

Adjusted p (t test): 6.90e−05, Adjusted p (wilcox): 1.30e−04

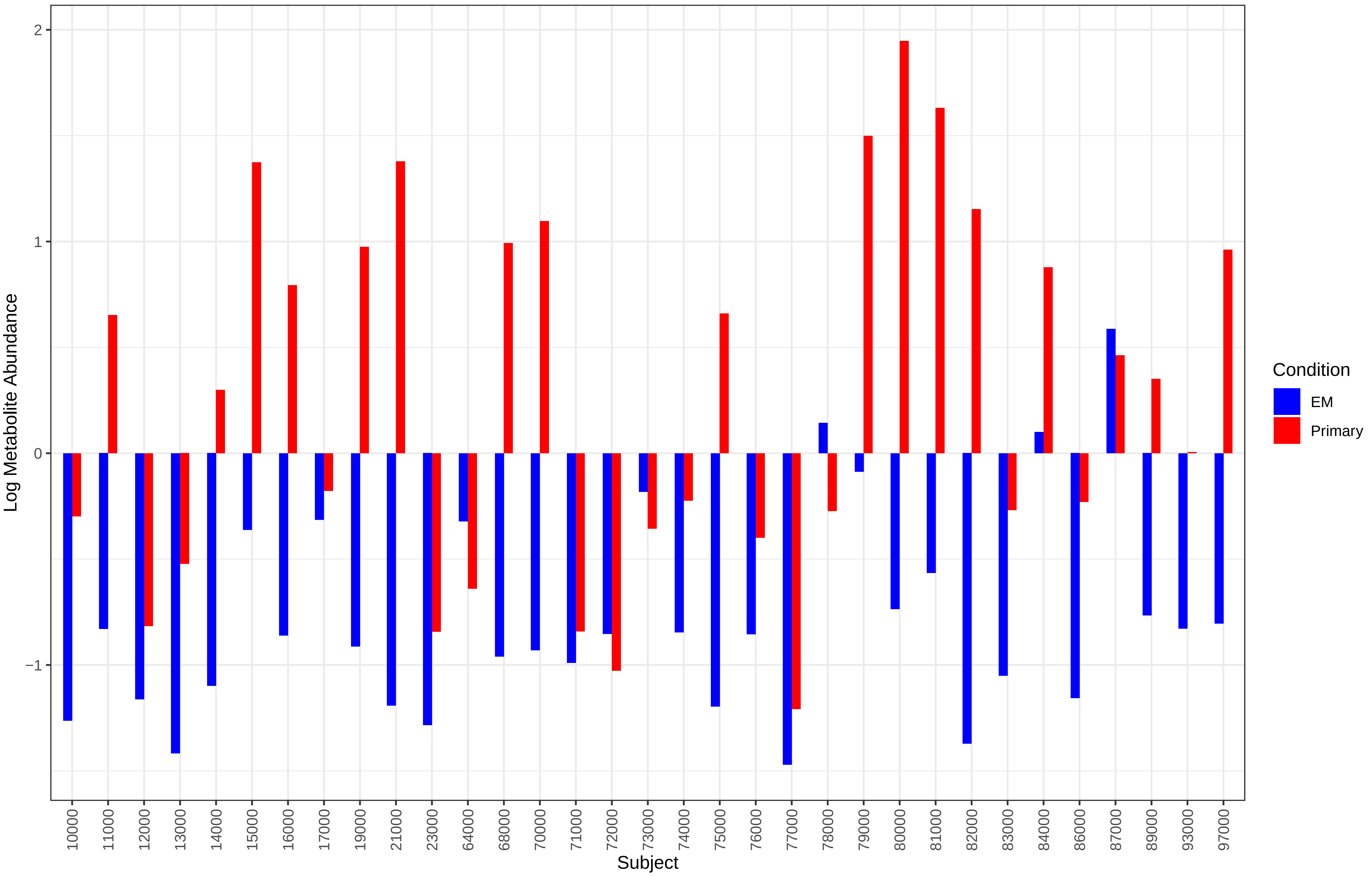

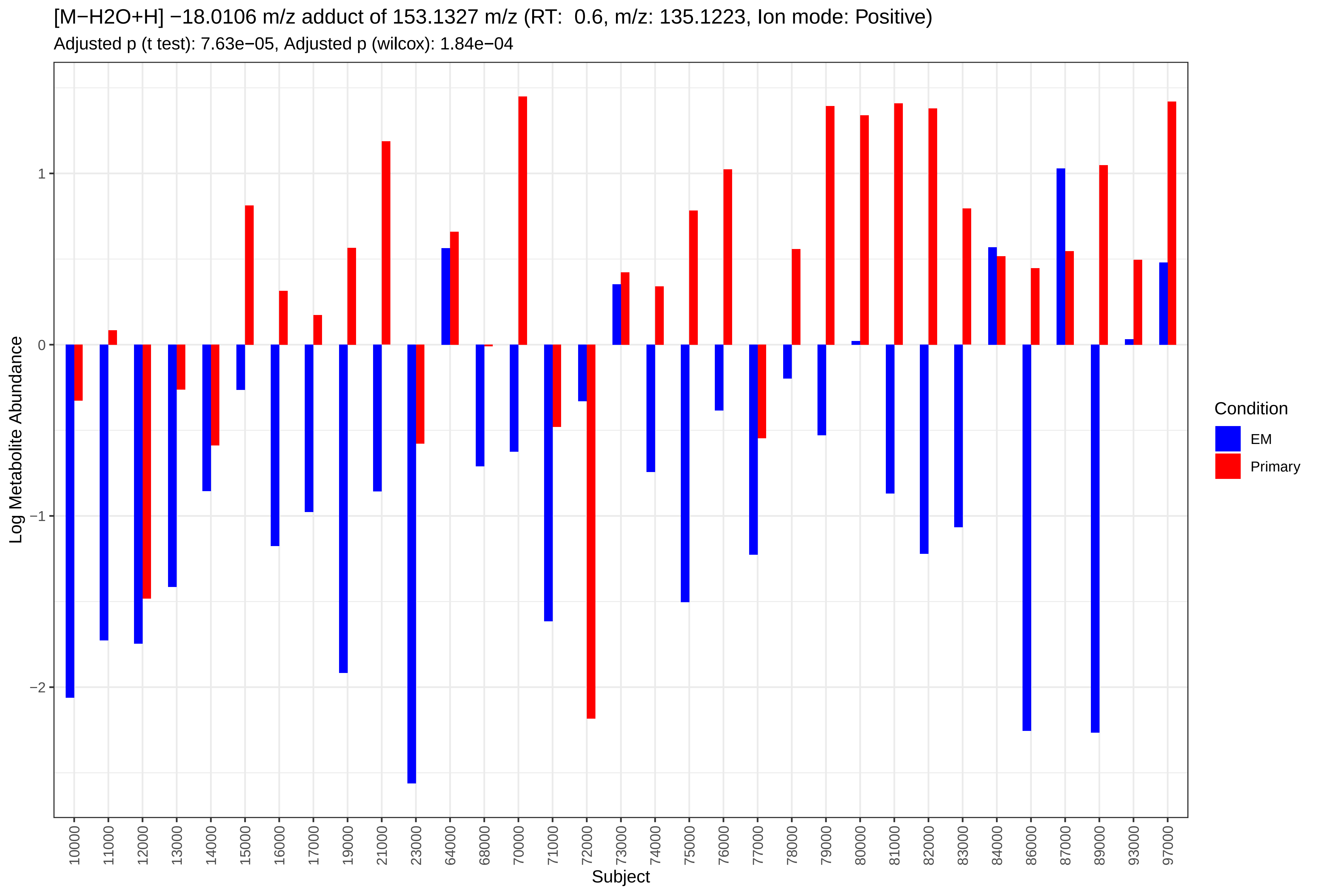
[M−H2O+H] −18.0106 m/z adduct of 153.1327 m/z (RT: 0.6, m/z: 135.1223, Ion mode: Positive)
Adjusted p (t test): 7.63e−05, Adjusted p (wilcox): 1.84e−04
Log Metabolite Abundance
1
0
−1
−2
Subject
10000
11000
12000
13000
14000
15000
16000
17000
19000
21000
23000
64000
68000
70000
71000
72000
73000
74000
75000
76000
77000
78000
79000
80000
81000
82000
83000
84000
86000
87000
89000
93000
97000
Condition
EM
Primary

RT: 1.5, m/z: 206.0478, Ion mode: Positive

Adjusted p (t test): 7.66e−05, Adjusted p (wilcox): 7.68e−04

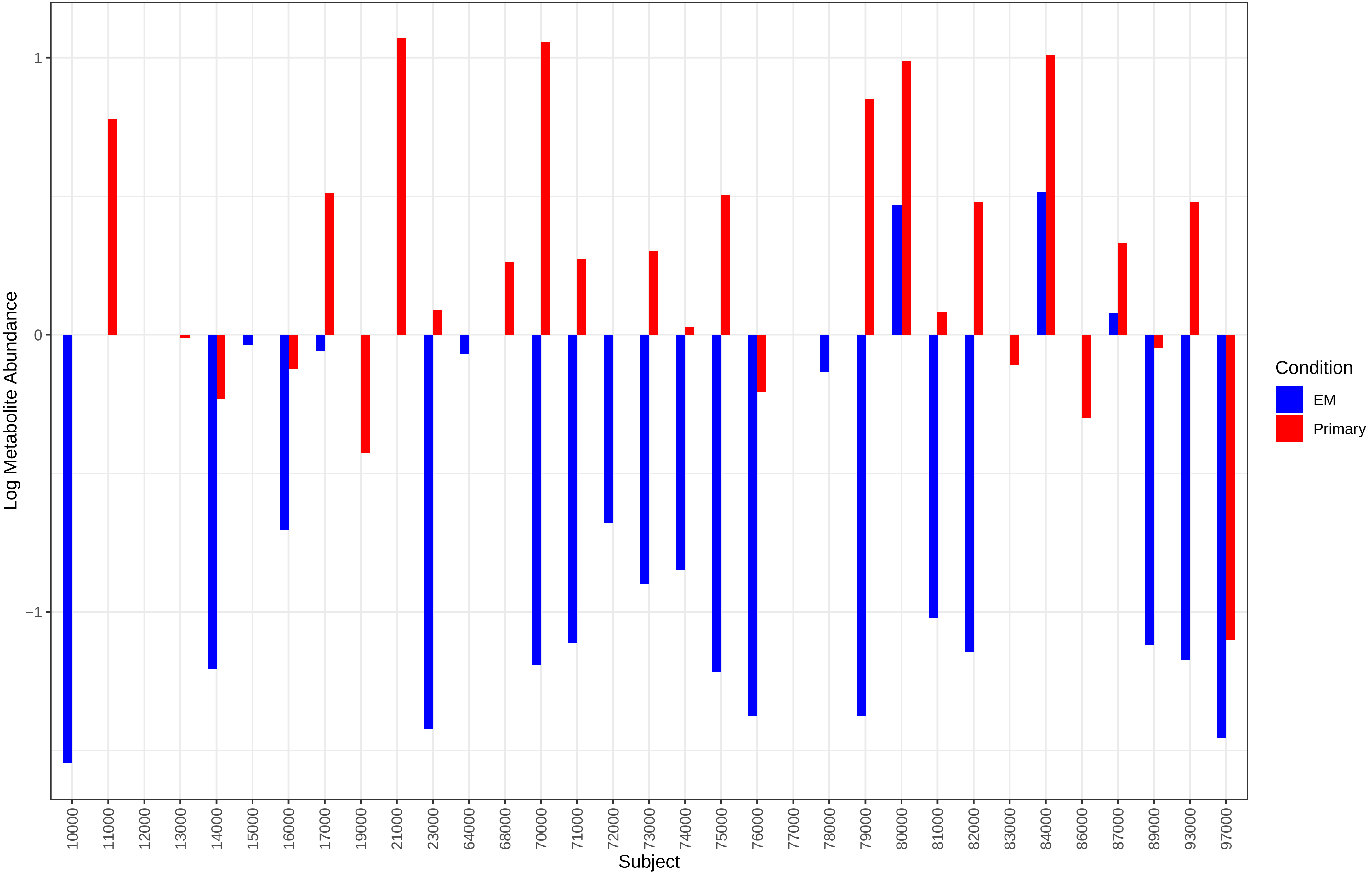

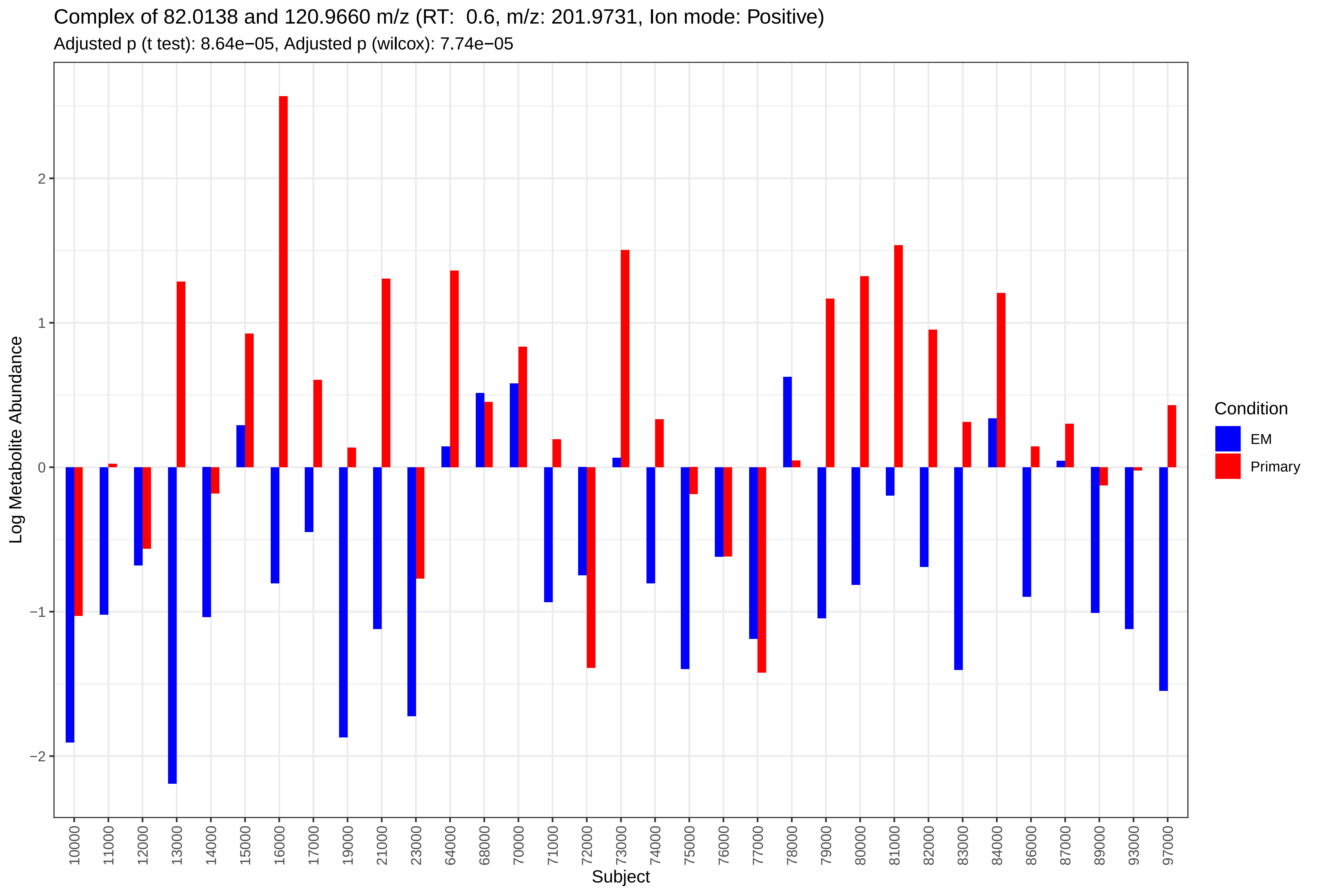

Complex of 82.0138 and 120.9660 m/z (RT: 0.6, m/z: 201.9731, Ion mode: Positive)
Adjusted p (t test): 8.64e−05, Adjusted p (wilcox): 7.74e−05
Log Metabolite Abundance
2
1
0
−1
−2
10000
11000
12000
13000
14000
15000
16000
17000
19000
21000
23000
64000
68000
70000
71000
72000
73000
74000
75000
76000
77000
78000
79000
80000
81000
82000
83000
84000
86000
87000
89000
93000
97000
Subject
Condition
EM
Primary

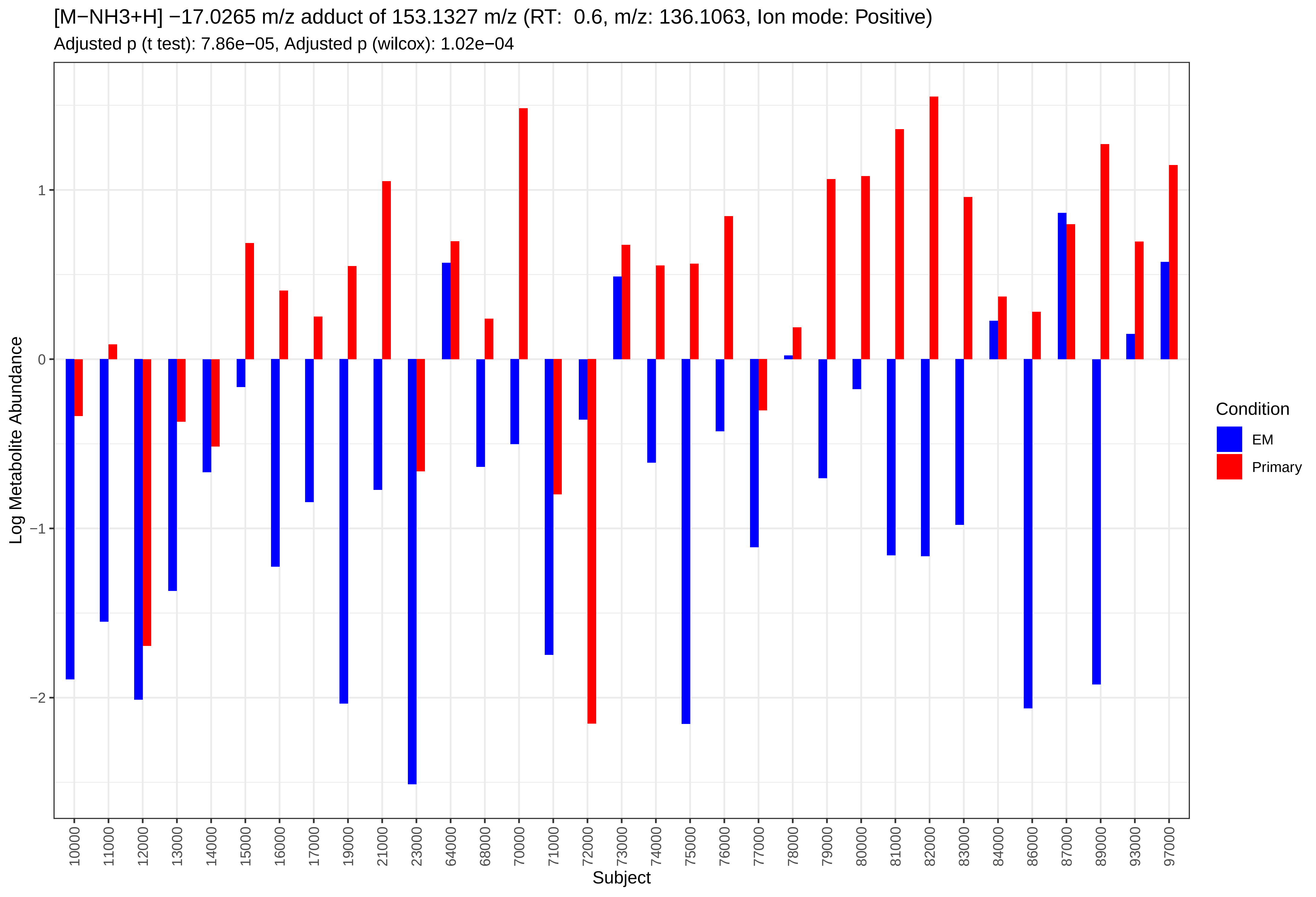

[M−NH3+H] −17.0265 m/z adduct of 153.1327 m/z (RT: 0.6, m/z: 136.1063, Ion mode: Positive)
Adjusted p (t test): 7.86e−05, Adjusted p (wilcox): 1.02e−04
Log Metabolite Abundance
1
0
−1
−2
Subject
10000
11000
12000
13000
14000
15000
16000
17000
19000
21000
23000
64000
68000
70000
71000
72000
73000
74000
75000
76000
77000
78000
79000
80000
81000
82000
83000
84000
86000
87000
89000
93000
97000
Condition
EM
Primary

RT: 0.6, m/z: 153.1327, Ion mode: Positive

Adjusted p (t test): 8.03e−05, Adjusted p (wilcox): 1.44e−04

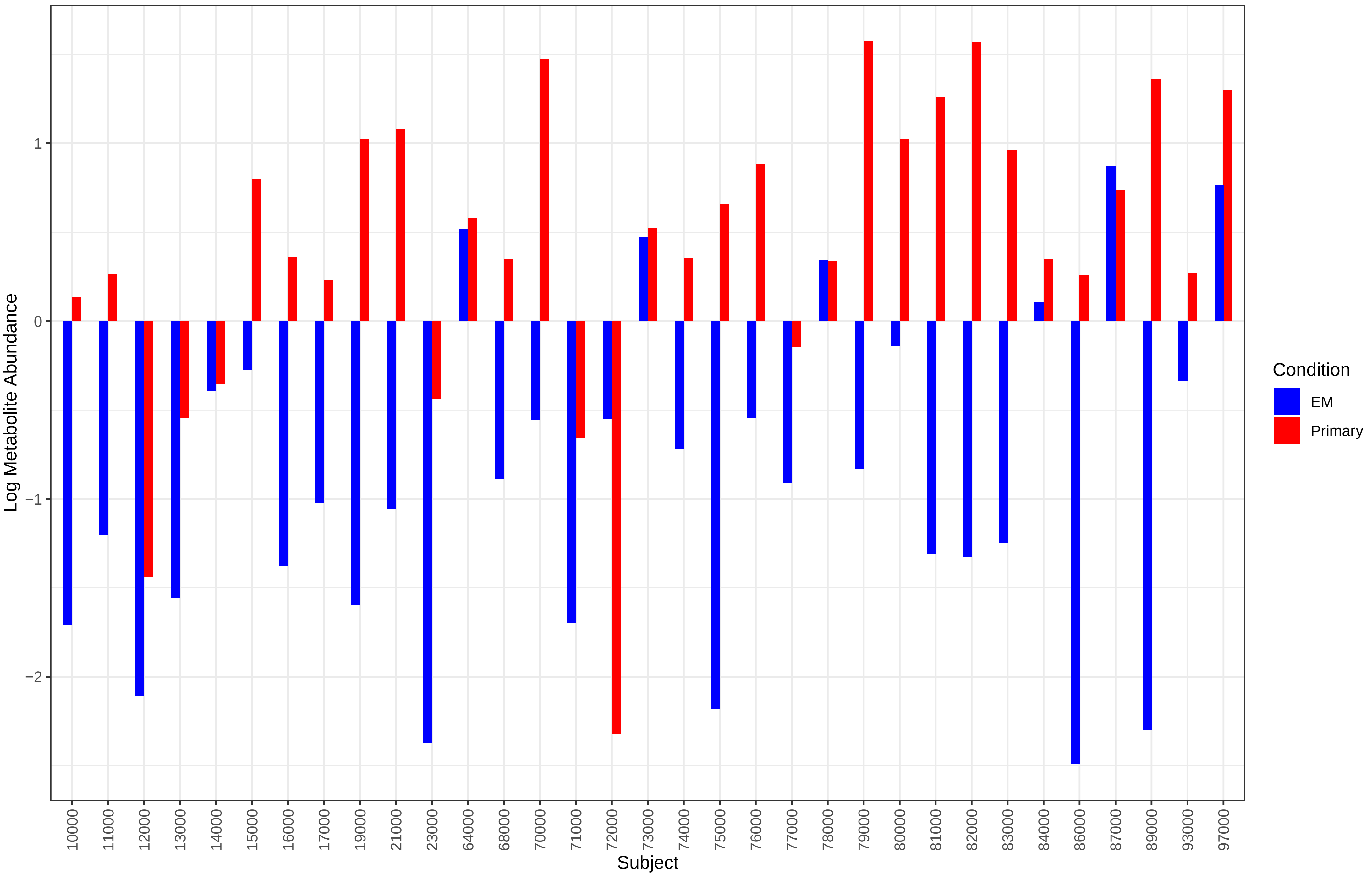

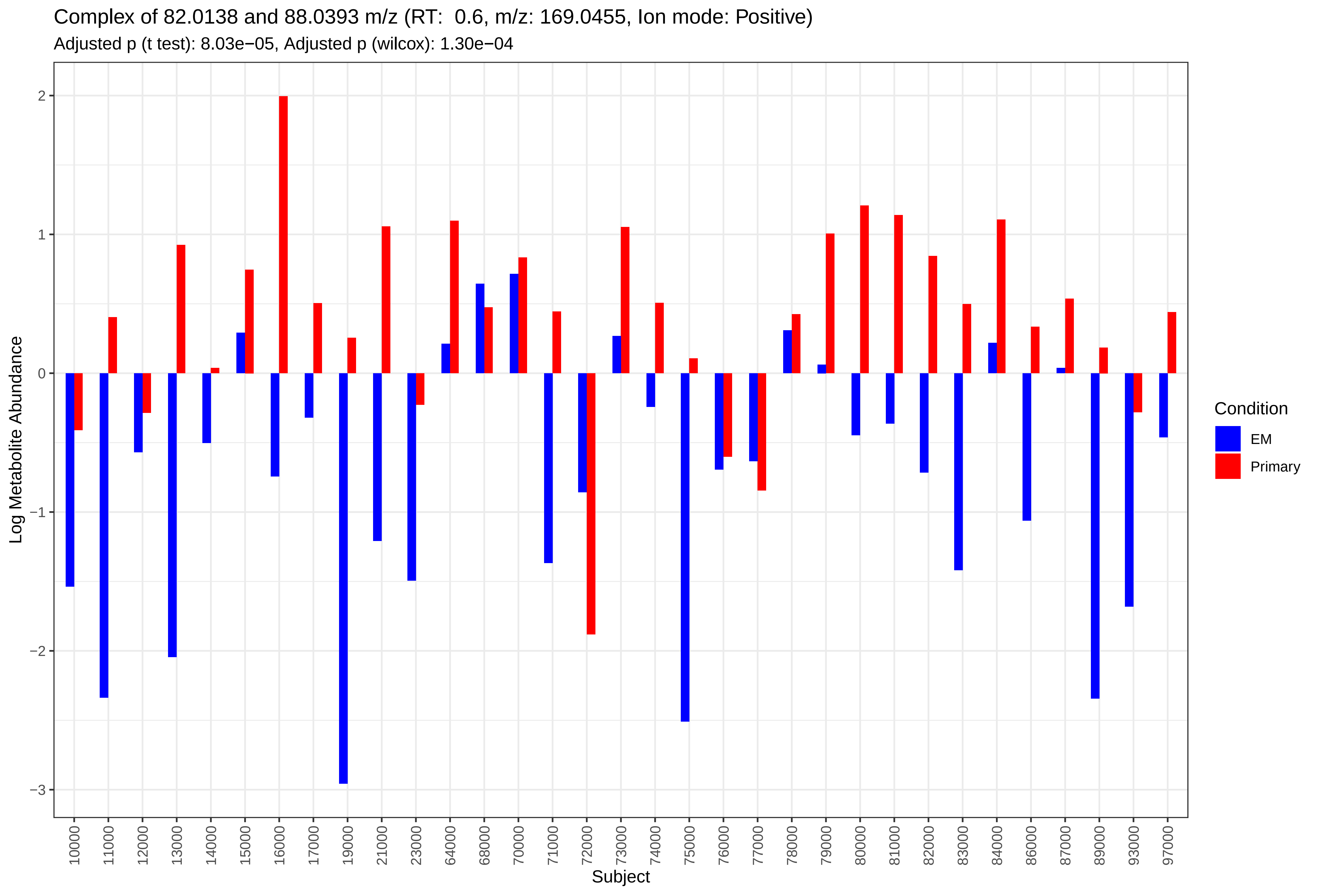

Complex of 82.0138 and 88.0393 m/z (RT: 0.6, m/z: 169.0455, Ion mode: Positive)
Adjusted p (t test): 8.03e−05, Adjusted p (wilcox): 1.30e−04
Log Metabolite Abundance
2
1
0
−1
−2
−3
Subject
10000
11000
12000
13000
14000
15000
16000
17000
19000
21000
23000
64000
68000
70000
71000
72000
73000
74000
75000
76000
77000
78000
79000
80000
81000
82000
83000
84000
86000
87000
89000
93000
97000
Condition
EM
Primary

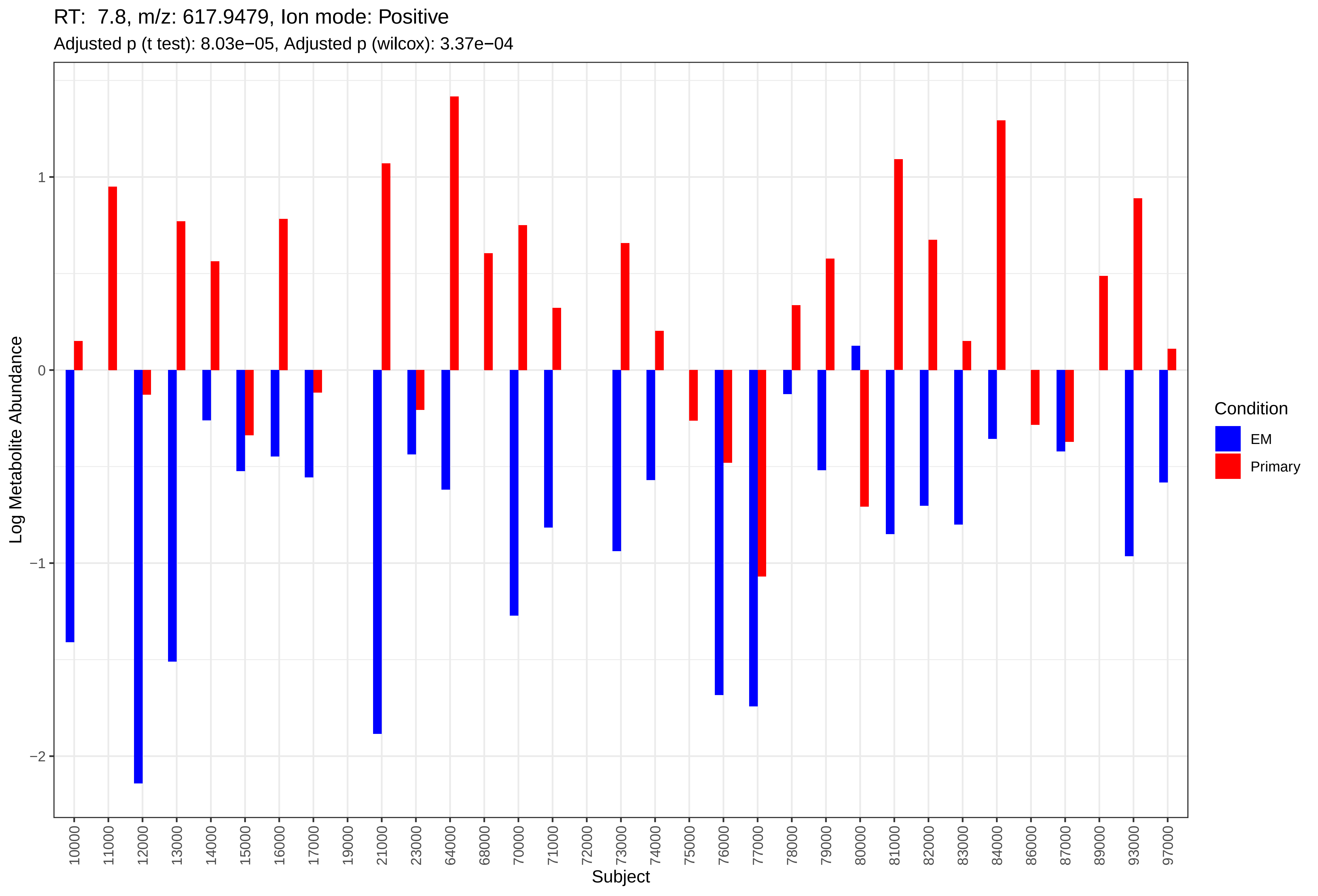
RT: 7.8, m/z: 617.9479, Ion mode: Positive
Adjusted p (t test): 8.03e−05, Adjusted p (wilcox): 3.37e−04
Log Metabolite Abundance
1
0
−1
−2
10000
11000
12000
13000
14000
15000
16000
17000
19000
21000
23000
64000
68000
70000
71000
72000
73000
74000
75000
76000
77000
78000
79000
80000
81000
82000
83000
84000
86000
87000
89000
93000
97000
Subject
Condition
EM
Primary

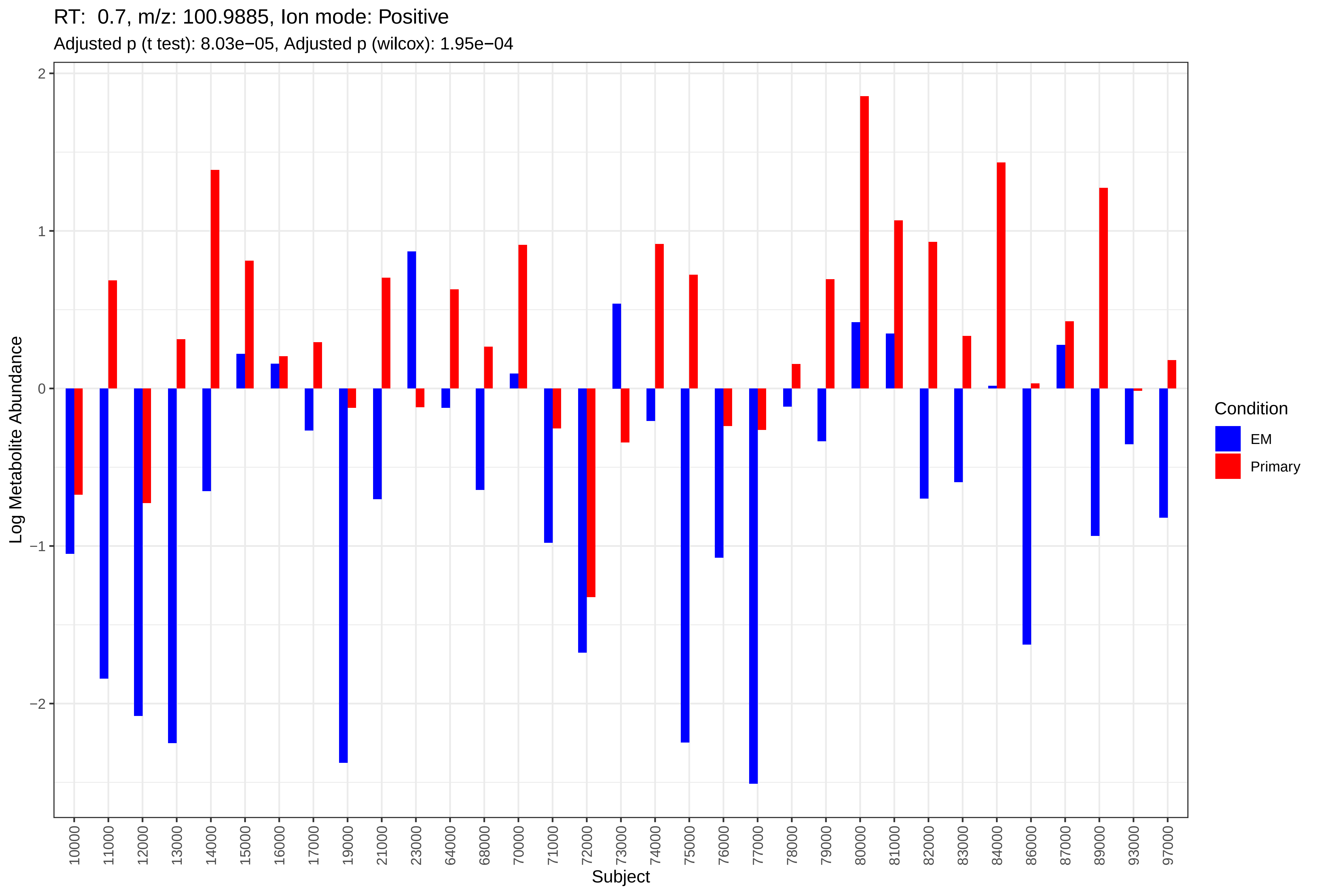
RT: 0.7, m/z: 100.9885, Ion mode: Positive
Adjusted p (t test): 8.03e−05, Adjusted p (wilcox): 1.95e−04
Log Metabolite Abundance
2
1
0
−1
−2
Subject
10000
11000
12000
13000
14000
15000
16000
17000
19000
21000
23000
64000
68000
70000
71000
72000
73000
74000
75000
76000
77000
78000
79000
80000
81000
82000
83000
84000
86000
87000
89000
93000
97000
Condition
EM
Primary

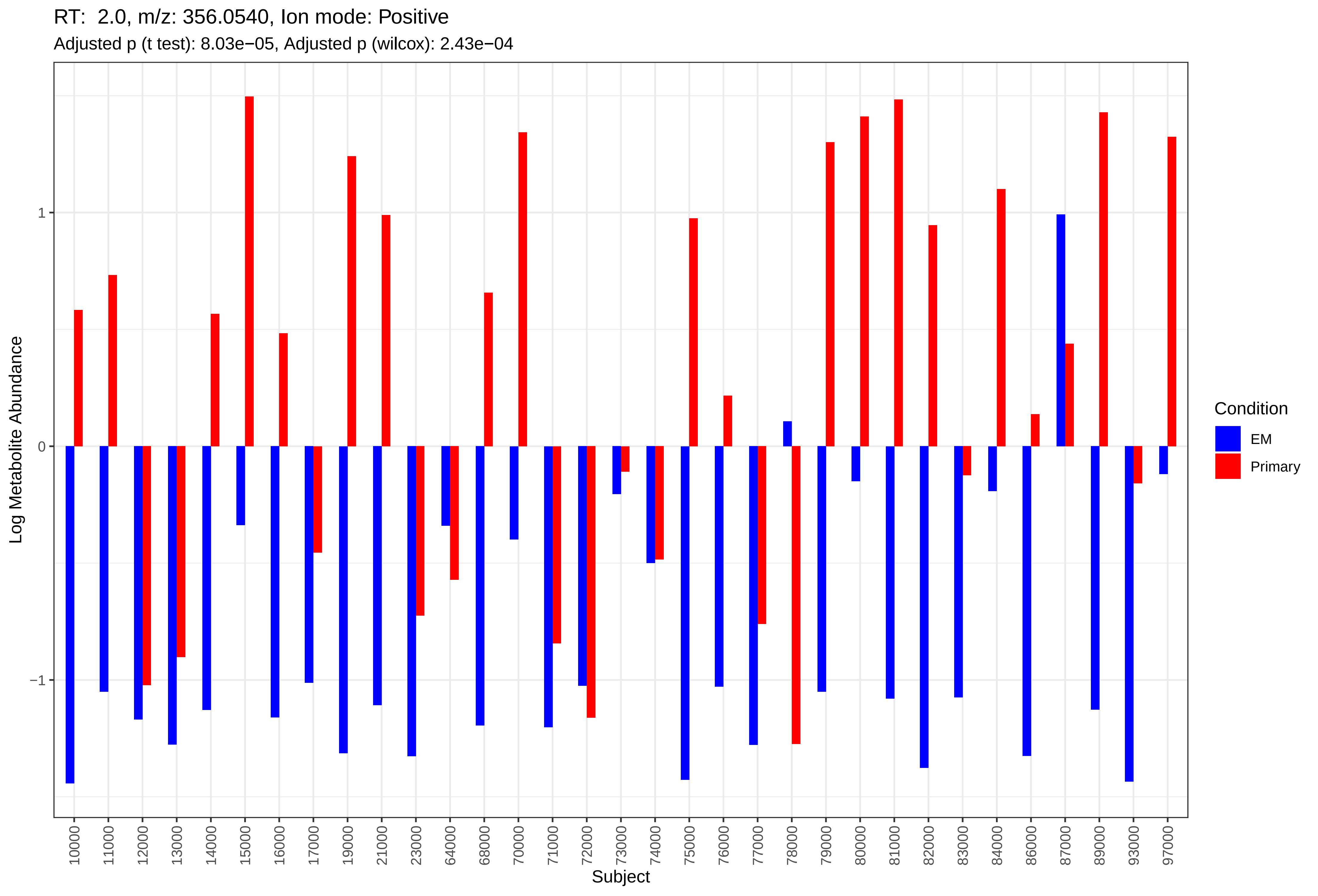
RT: 2.0, m/z: 356.0540, Ion mode: Positive
Adjusted p (t test): 8.03e−05, Adjusted p (wilcox): 2.43e−04
Log Metabolite Abundance
1
0
−1
10000
11000
12000
13000
14000
15000
16000
17000
19000
21000
23000
64000
68000
70000
71000
72000
73000
74000
75000
76000
77000
78000
79000
80000
81000
82000
83000
84000
86000
87000
89000
93000
97000
Subject
Condition
EM
Primary

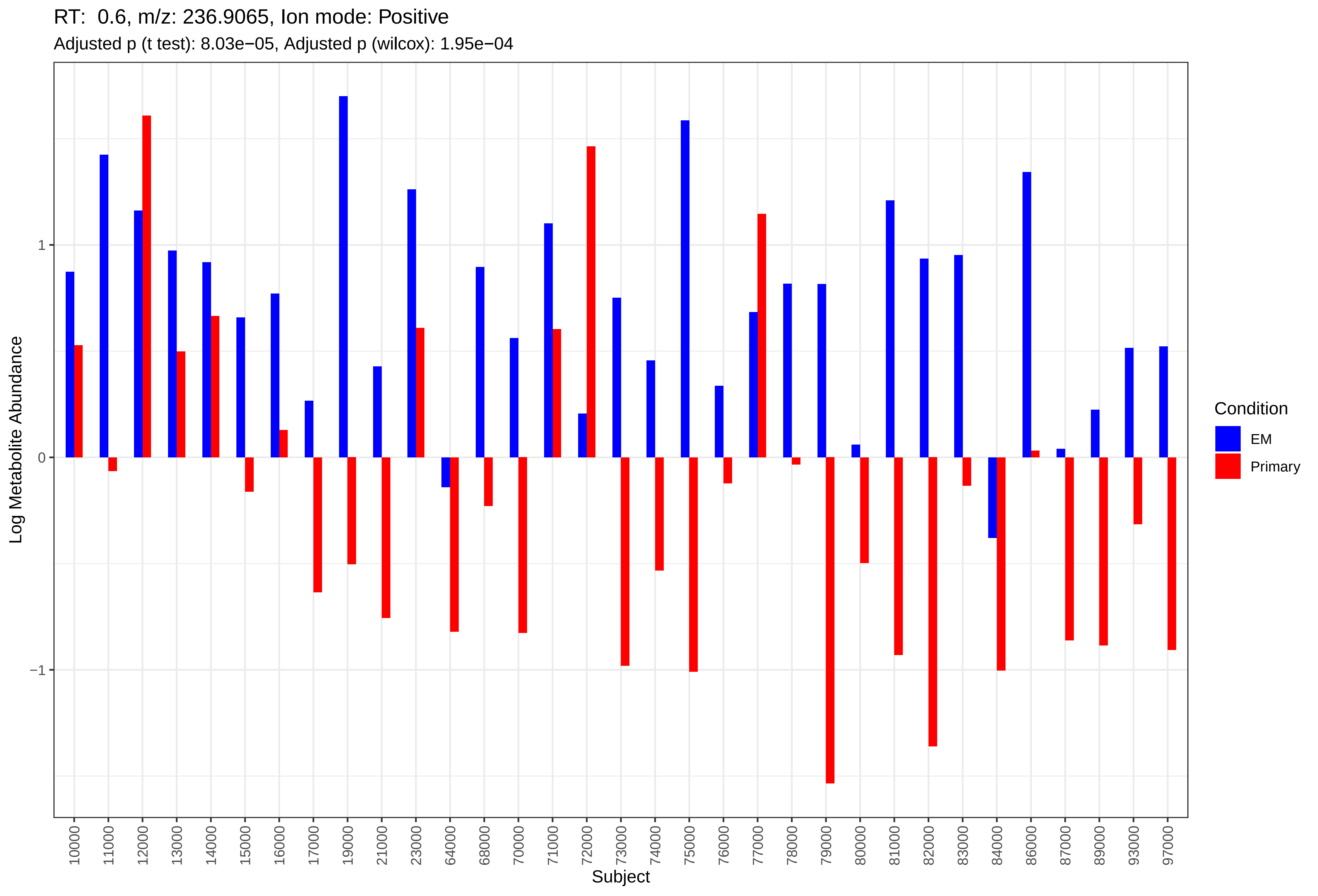
RT: 0.6, m/z: 236.9065, Ion mode: Positive
Adjusted p (t test): 8.03e−05, Adjusted p (wilcox): 1.95e−04
Log Metabolite Abundance
1
0
−1
10000
11000
12000
13000
14000
15000
16000
17000
19000
21000
23000
64000
68000
70000
71000
72000
73000
74000
75000
76000
77000
78000
79000
80000
81000
82000
83000
84000
86000
87000
89000
93000
97000
Subject
Condition
EM
Primary

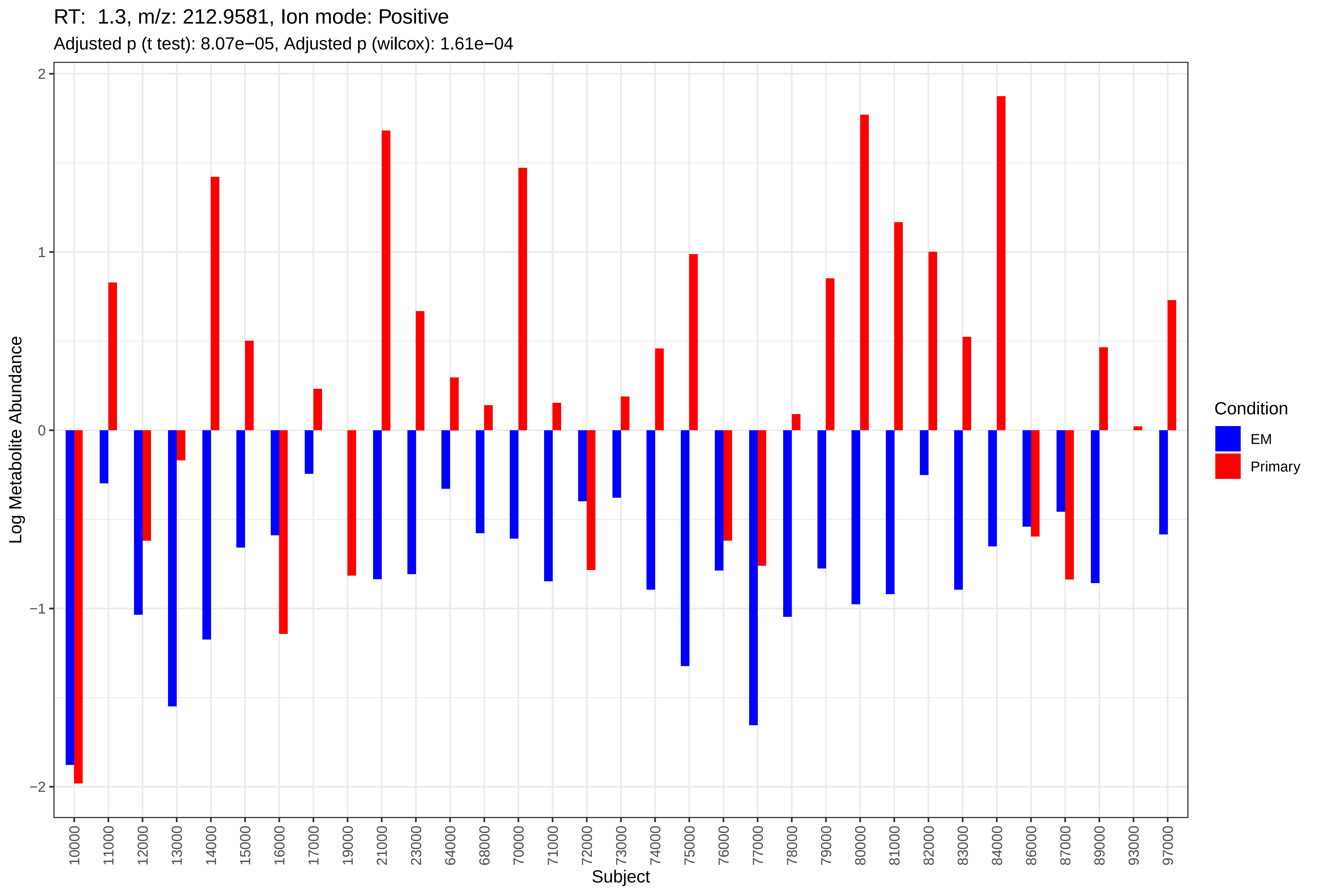

RT: 1.3, m/z: 212.9581, Ion mode: Positive
Adjusted p (t test): 8.07e−05, Adjusted p (wilcox): 1.61e−04
Log Metabolite Abundance
2
1
0
−1
−2
Subject
10000
11000
12000
13000
14000
15000
16000
17000
19000
21000
23000
64000
68000
70000
71000
72000
73000
74000
75000
76000
77000
78000
79000
80000
81000
82000
83000
84000
86000
87000
89000
93000
97000
Condition
EM
Primary

RT: 0.7, m/z: 194.9464, Ion mode: Negative

Adjusted p (t test): 8.16e−05, Adjusted p (wilcox): 1.61e−04

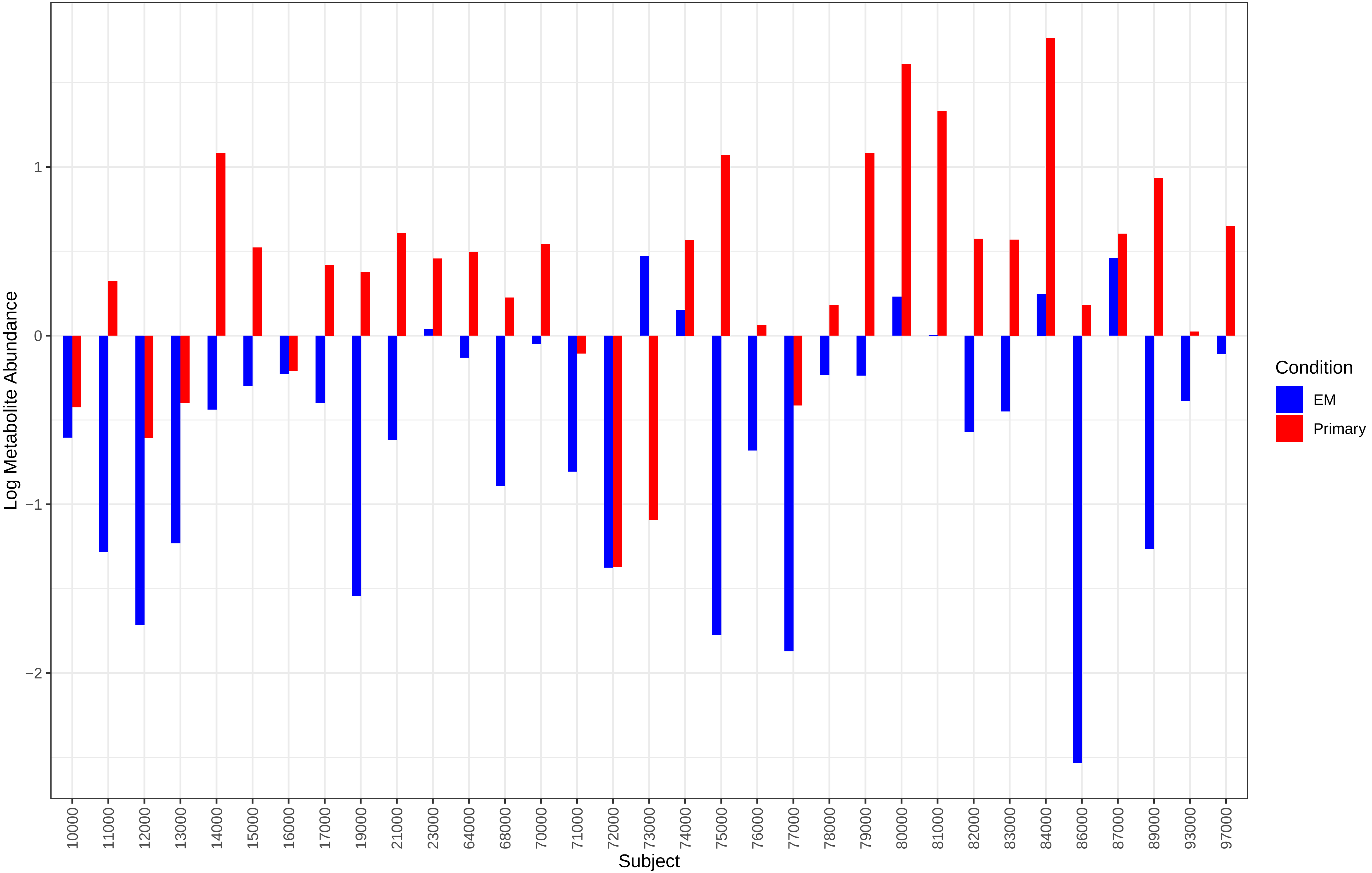

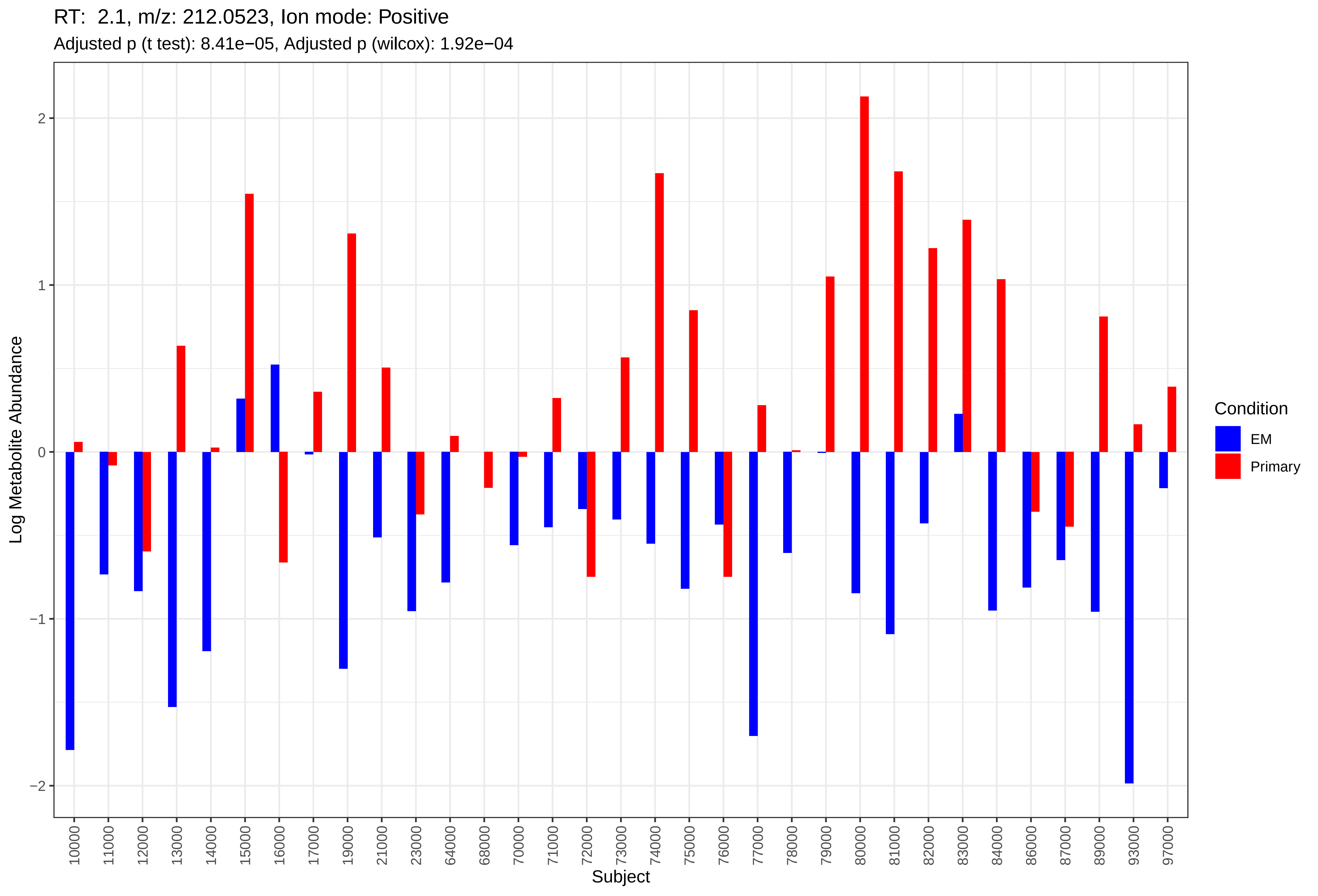

RT: 2.1, m/z: 212.0523, Ion mode: Positive
Adjusted p (t test): 8.41e−05, Adjusted p (wilcox): 1.92e−04
Log Metabolite Abundance
2
1
0
−1
−2
10000
11000
12000
13000
14000
15000
16000
17000
19000
21000
23000
64000
68000
70000
71000
72000
73000
74000
75000
76000
77000
78000
79000
80000
81000
82000
83000
84000
86000
87000
89000
93000
97000
Subject
Condition
EM
Primary

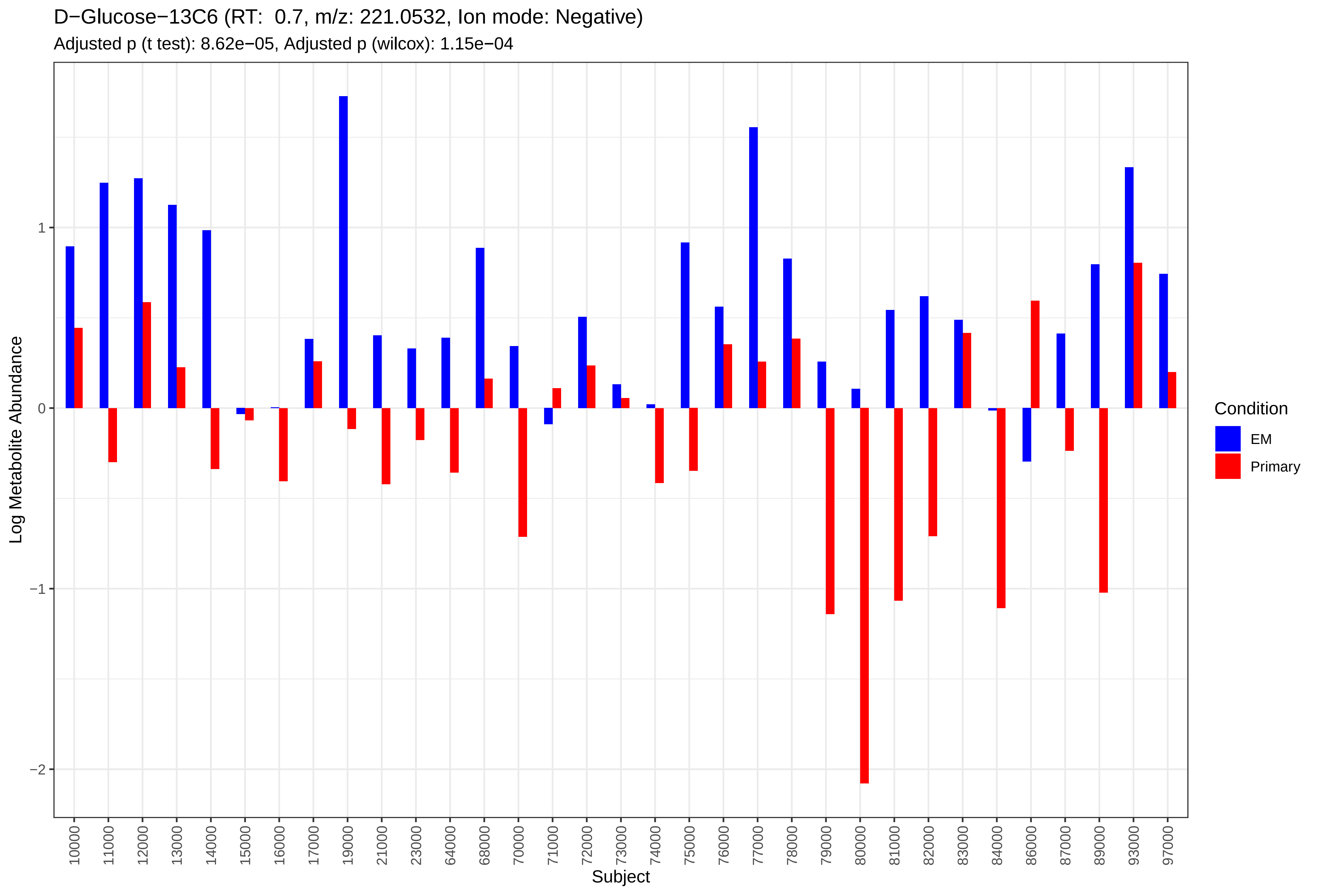
D−Glucose−13C6 (RT: 0.7, m/z: 221.0532, Ion mode: Negative)
Adjusted p (t test): 8.62e−05, Adjusted p (wilcox): 1.15e−04
Log Metabolite Abundance
1
0
−1
−2
10000
11000
12000
13000
14000
15000
16000
17000
19000
21000
23000
64000
68000
70000
71000
72000
73000
74000
75000
76000
77000
78000
79000
80000
81000
82000
83000
84000
86000
87000
89000
93000
97000
Subject
Condition
EM
Primary

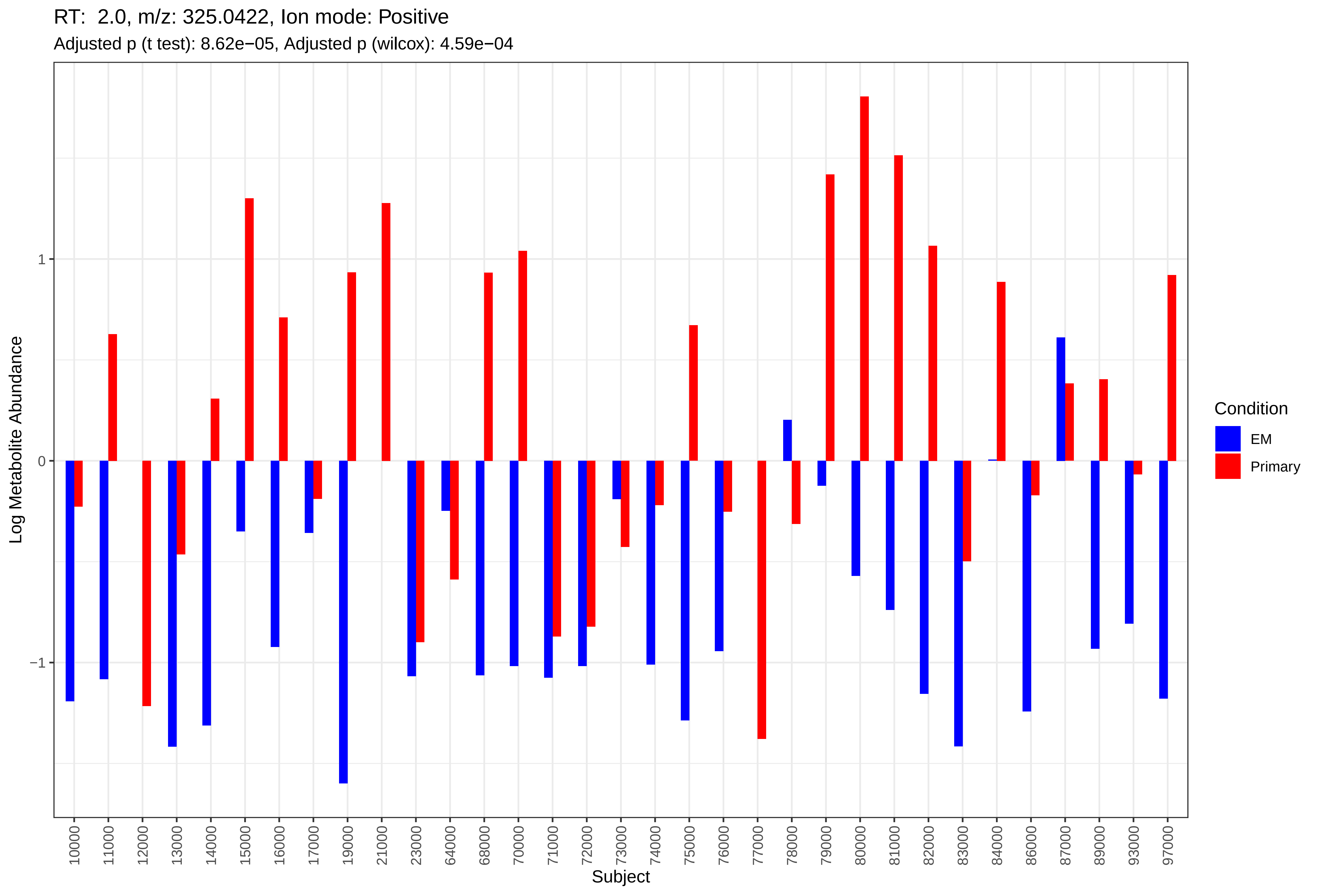

RT: 2.0, m/z: 325.0422, Ion mode: Positive
Adjusted p (t test): 8.62e−05, Adjusted p (wilcox): 4.59e−04
Log Metabolite Abundance
1
0
−1
10000
11000
12000
13000
14000
15000
16000
17000
19000
21000
23000
64000
68000
70000
71000
72000
73000
74000
75000
76000
77000
78000
79000
80000
81000
82000
83000
84000
86000
87000
89000
93000
97000
Subject
Condition
EM
Primary

RT: 0.7, m/z: 216.0626, Ion mode: Positive

Adjusted p (t test): 8.62e−05, Adjusted p (wilcox): 1.44e−04

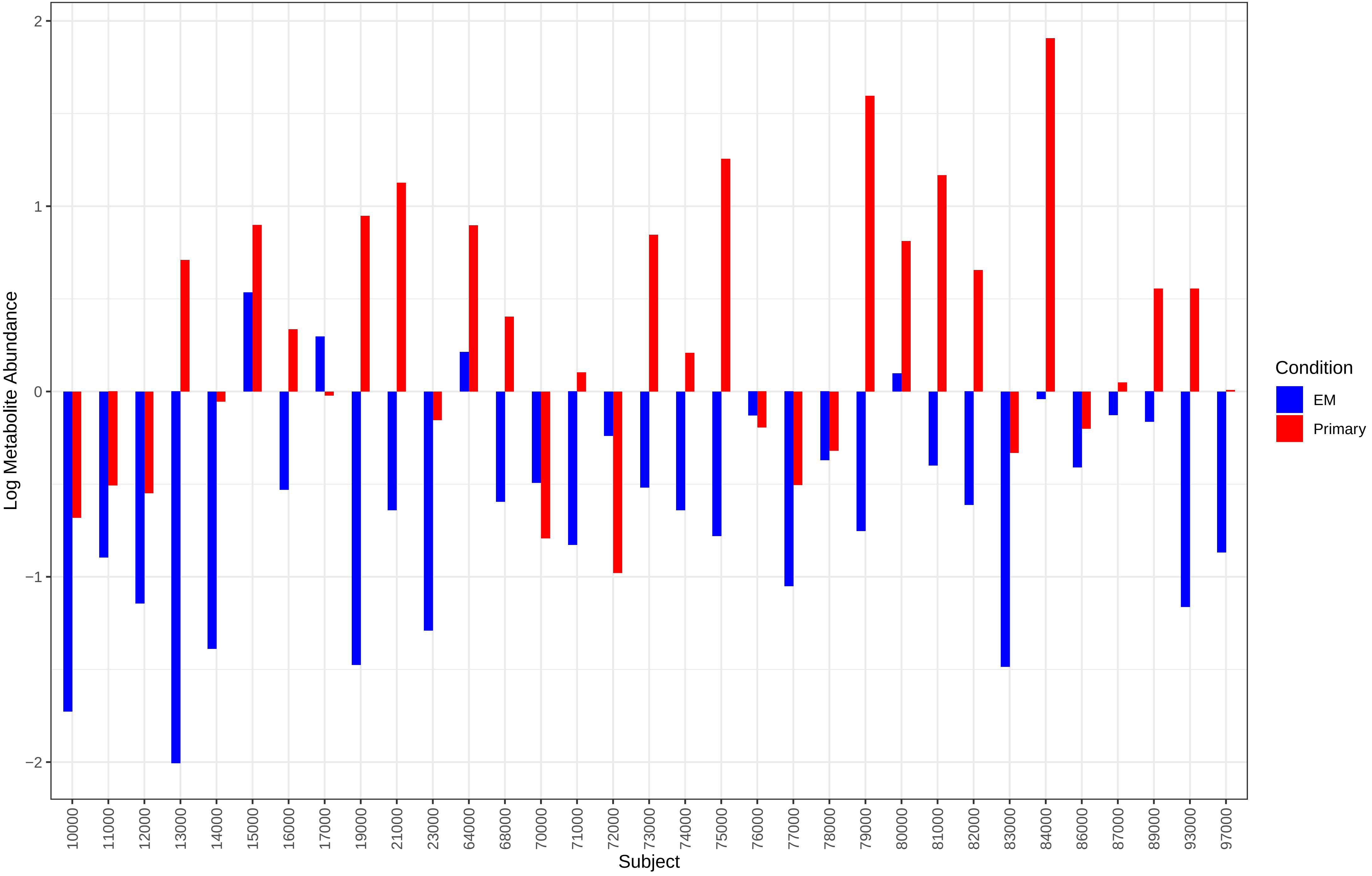

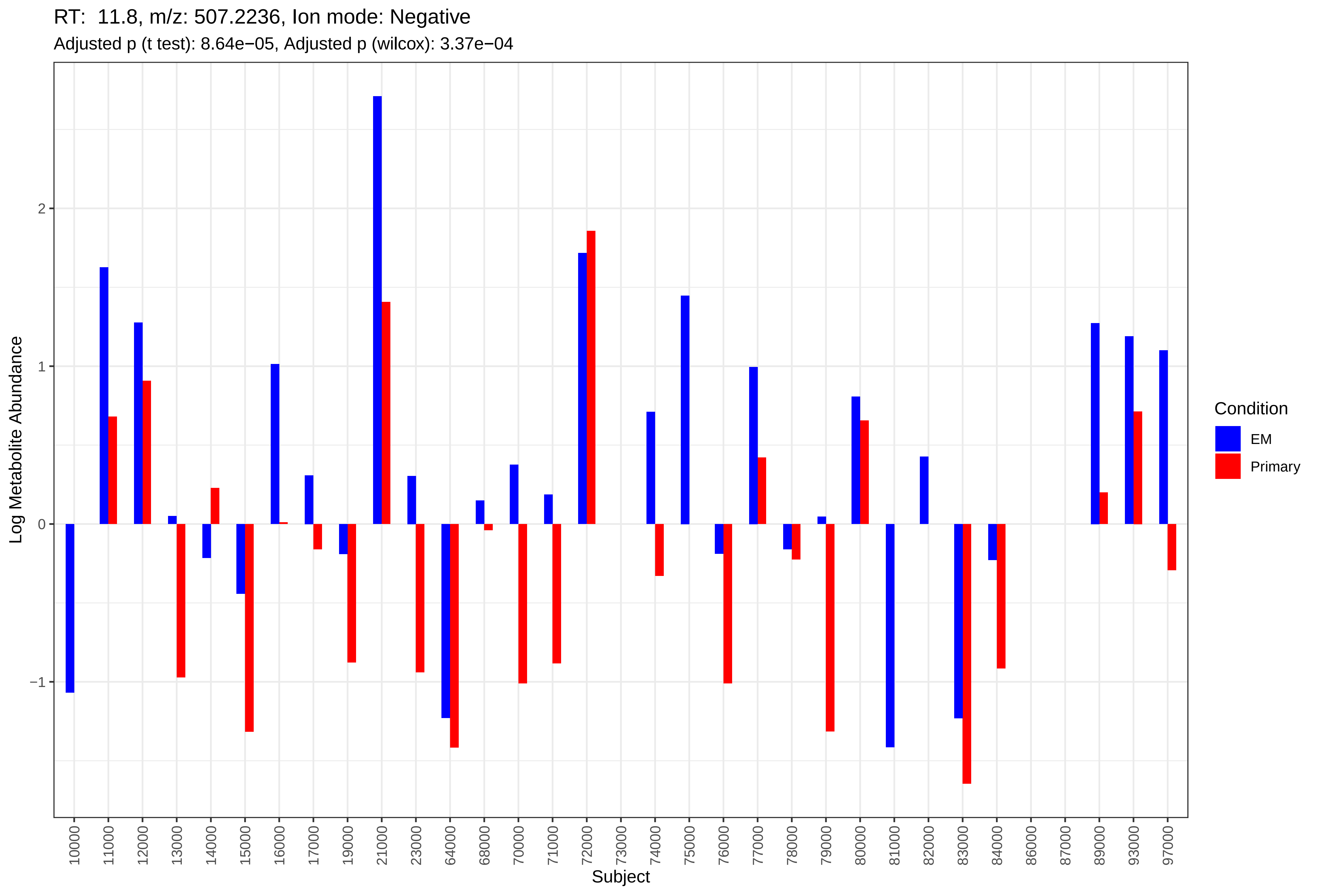
RT: 11.8, m/z: 507.2236, Ion mode: Negative
Adjusted p (t test): 8.64e−05, Adjusted p (wilcox): 3.37e−04
Log Metabolite Abundance
2
1
0
−1
10000
11000
12000
13000
14000
15000
16000
17000
19000
21000
23000
64000
68000
70000
71000
72000
73000
74000
75000
76000
77000
78000
79000
80000
81000
82000
83000
84000
86000
87000
89000
93000
97000
Subject
Condition
EM
Primary

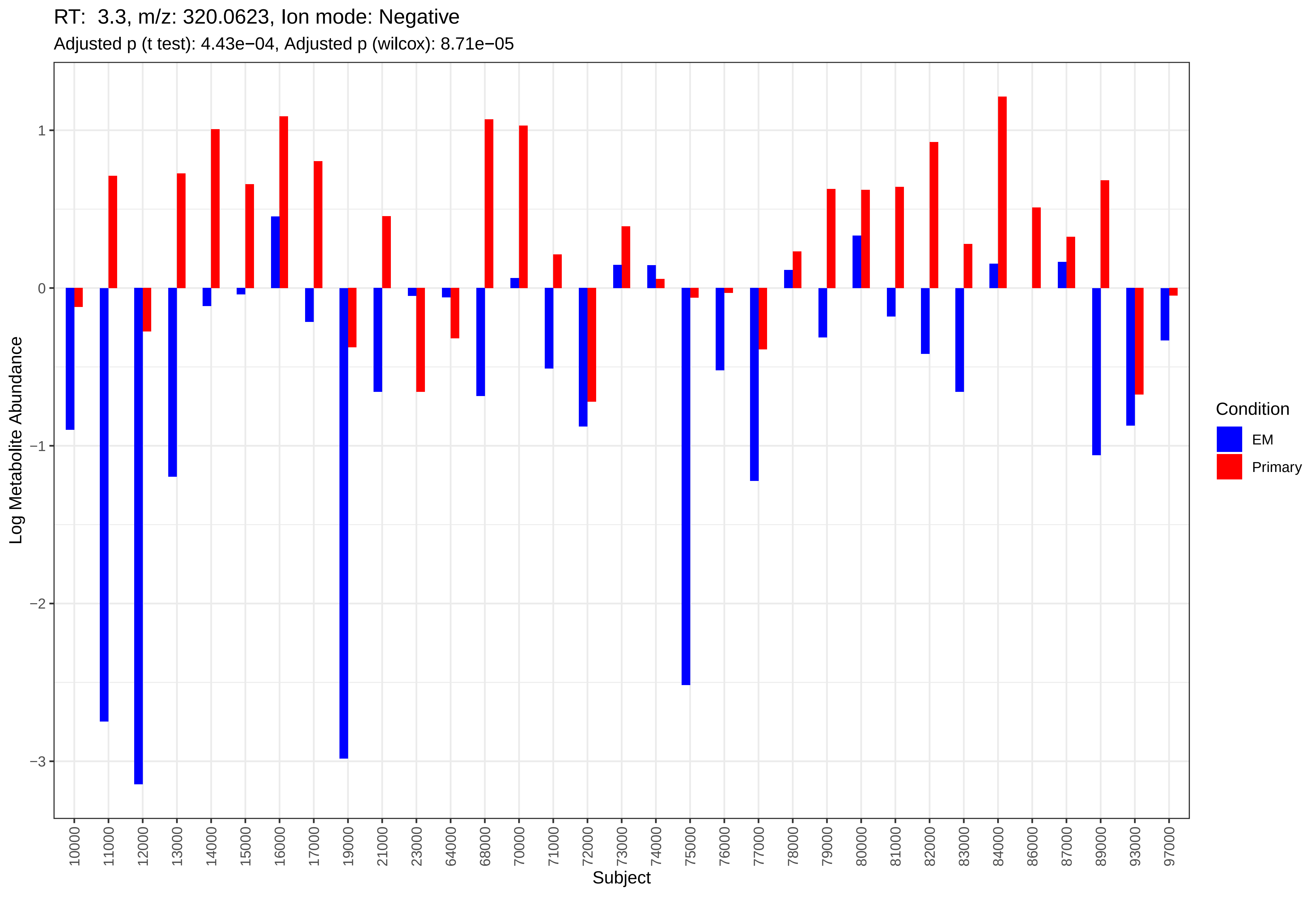

RT: 3.3, m/z: 320.0623, Ion mode: Negative
Adjusted p (t test): 4.43e−04, Adjusted p (wilcox): 8.71e−05
Log Metabolite Abundance
1
0
−1
−2
−3
10000
11000
12000
13000
14000
15000
16000
17000
19000
21000
23000
64000
68000
70000
71000
72000
73000
74000
75000
76000
77000
78000
79000
80000
81000
82000
83000
84000
86000
87000
89000
93000
97000
Subject
Condition
EM
Primary

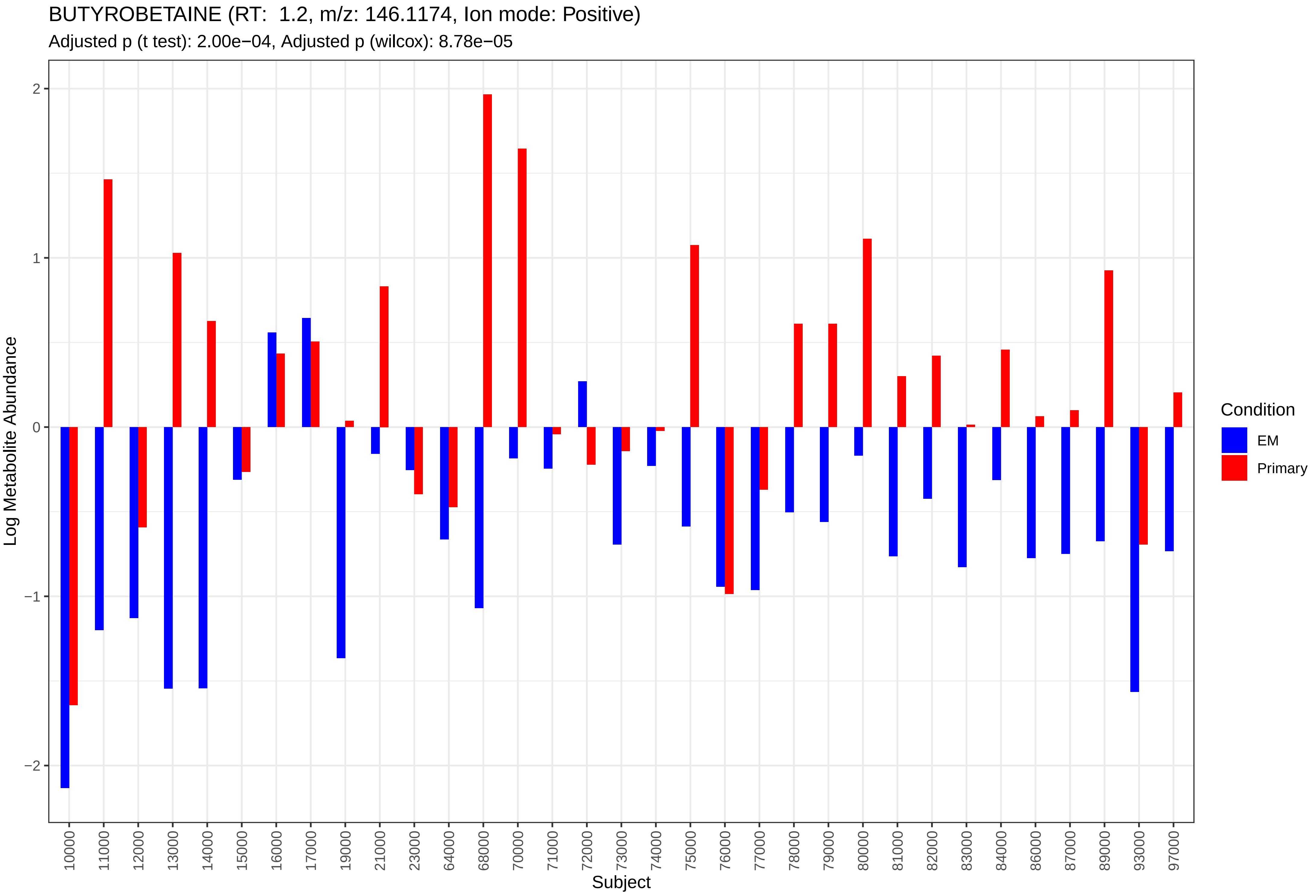
BUTYROBETAINE (RT: 1.2, m/z: 146.1174, Ion mode: Positive)
Adjusted p (t test): 2.00e−04, Adjusted p (wilcox): 8.78e−05
Log Metabolite Abundance
2
1
0
−1
−2
10000
11000
12000
13000
14000
15000
16000
17000
19000
21000
23000
64000
68000
70000
71000
72000
73000
74000
75000
76000
77000
78000
79000
80000
81000
82000
83000
84000
86000
87000
89000
93000
97000
Subject
Condition
EM
Primary

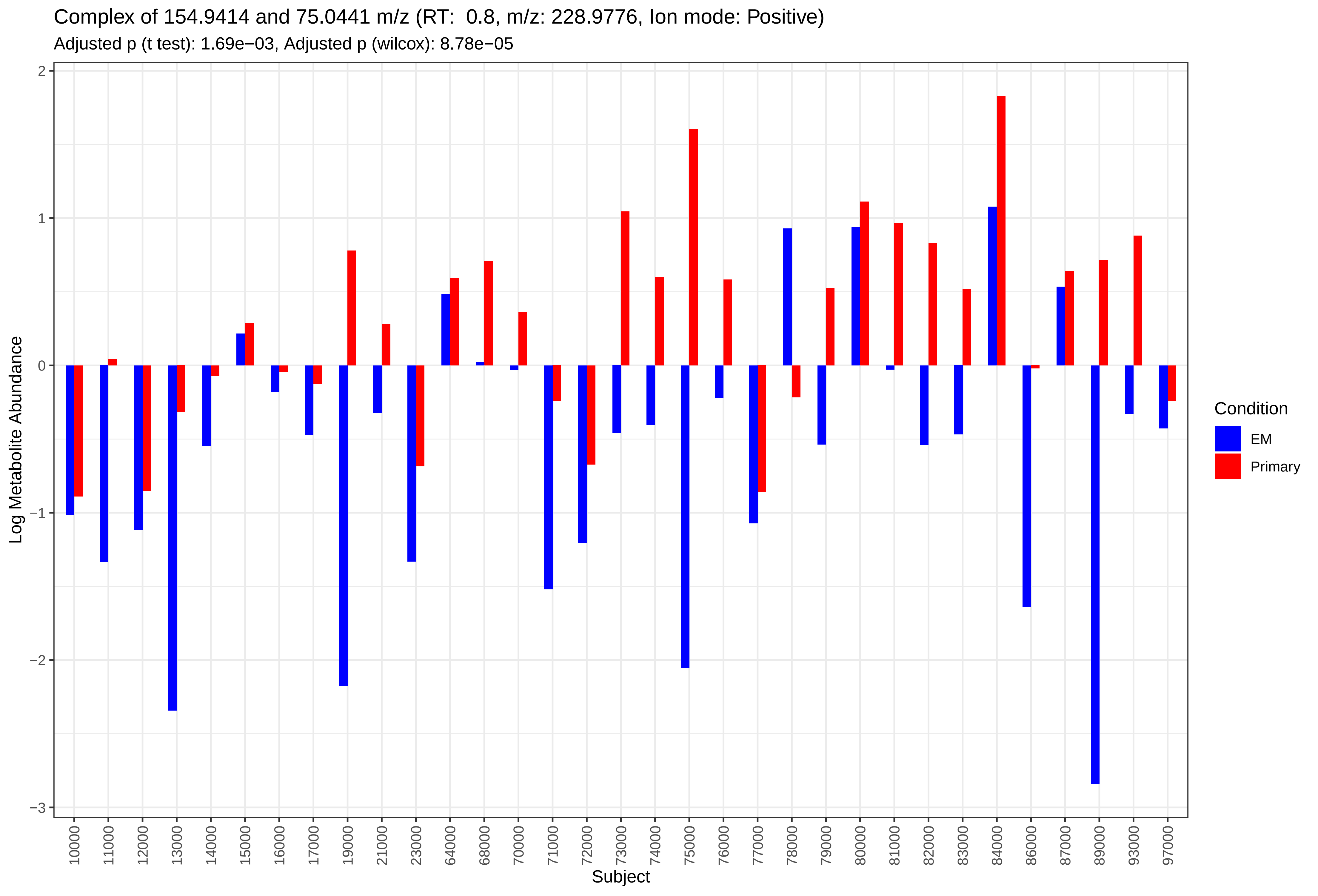
Complex of 154.9414 and 75.0441 m/z (RT: 0.8, m/z: 228.9776, Ion mode: Positive)
Adjusted p (t test): 1.69e−03, Adjusted p (wilcox): 8.78e−05
Log Metabolite Abundance
2
1
0
−1
−2
−3
10000
11000
12000
13000
14000
15000
16000
17000
19000
21000
23000
64000
68000
70000
71000
72000
73000
74000
75000
76000
77000
78000
79000
80000
81000
82000
83000
84000
86000
87000
89000
93000
97000
Subject
Condition
EM
Primary

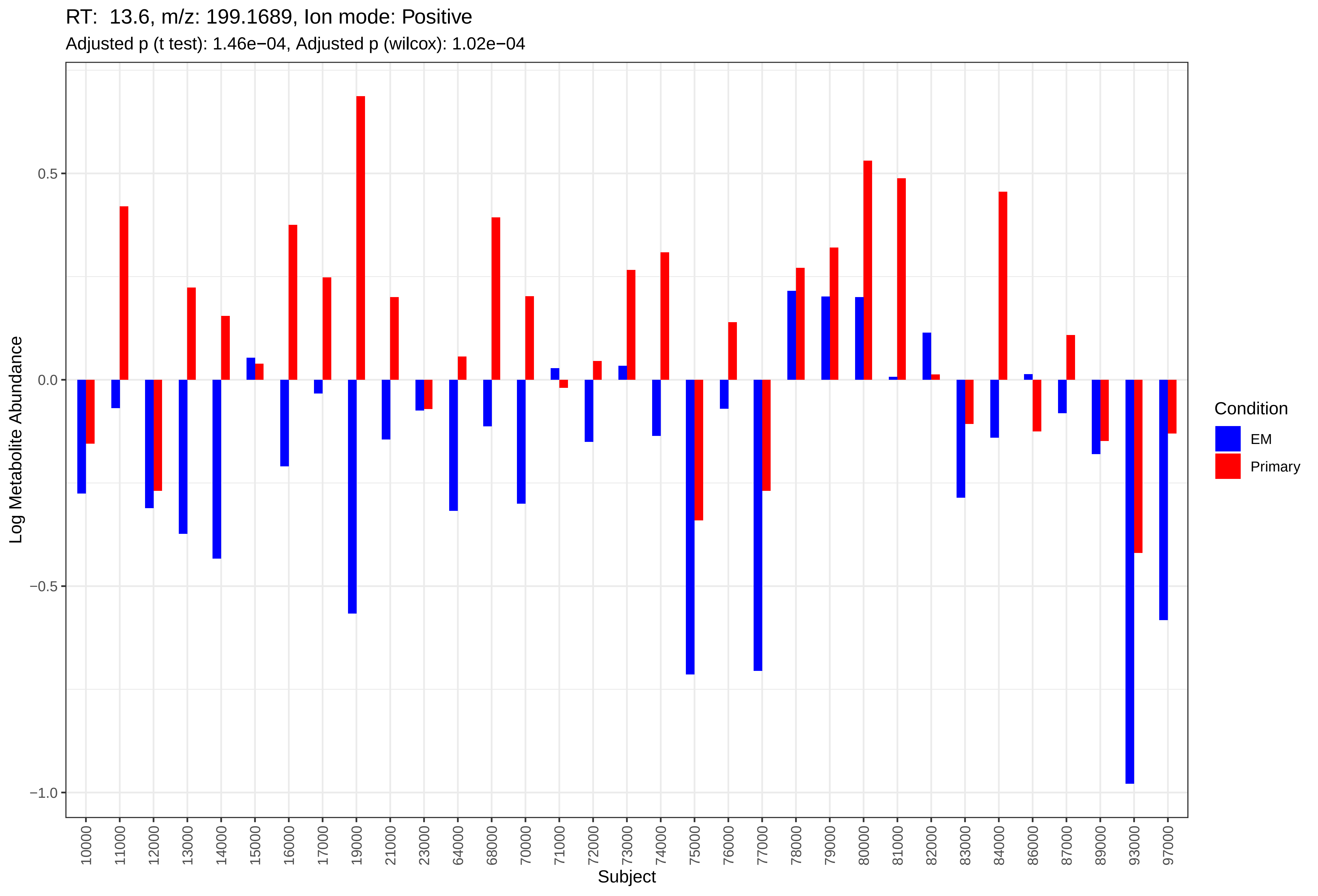
RT: 13.6, m/z: 199.1689, Ion mode: Positive
Adjusted p (t test): 1.46e−04, Adjusted p (wilcox): 1.02e−04
Log Metabolite Abundance
0.5
0.0
−0.5
−1.0
Subject
10000
11000
12000
13000
14000
15000
16000
17000
19000
21000
23000
64000
68000
70000
71000
72000
73000
74000
75000
76000
77000
78000
79000
80000
81000
82000
83000
84000
86000
87000
89000
93000
97000
Condition
EM
Primary

Pantothenic Acid (RT: 6.6, m/z: 220.1174, Ion mode: Positive)

Adjusted p (t test): 1.06e−04, Adjusted p (wilcox): 1.84e−04

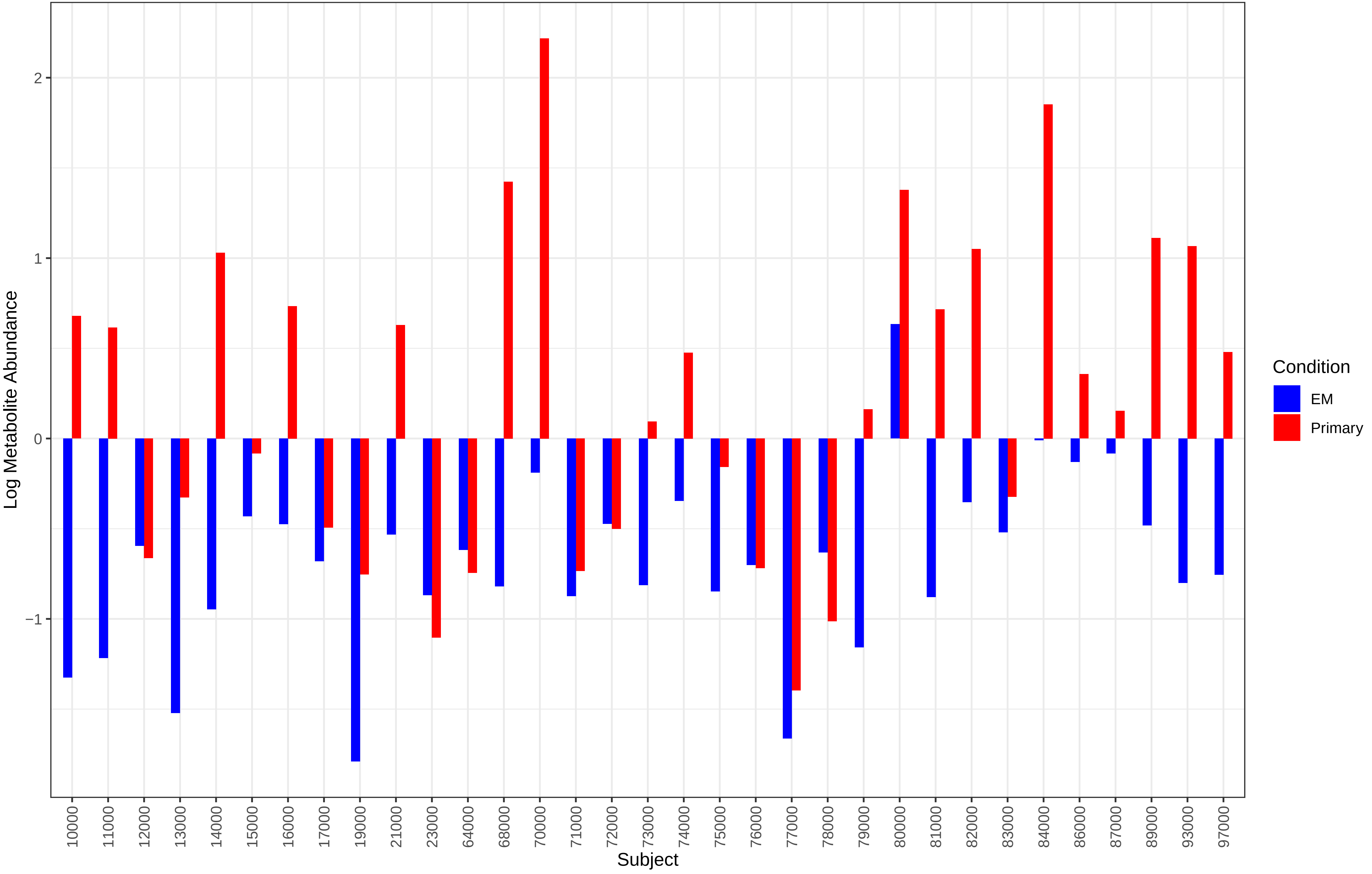

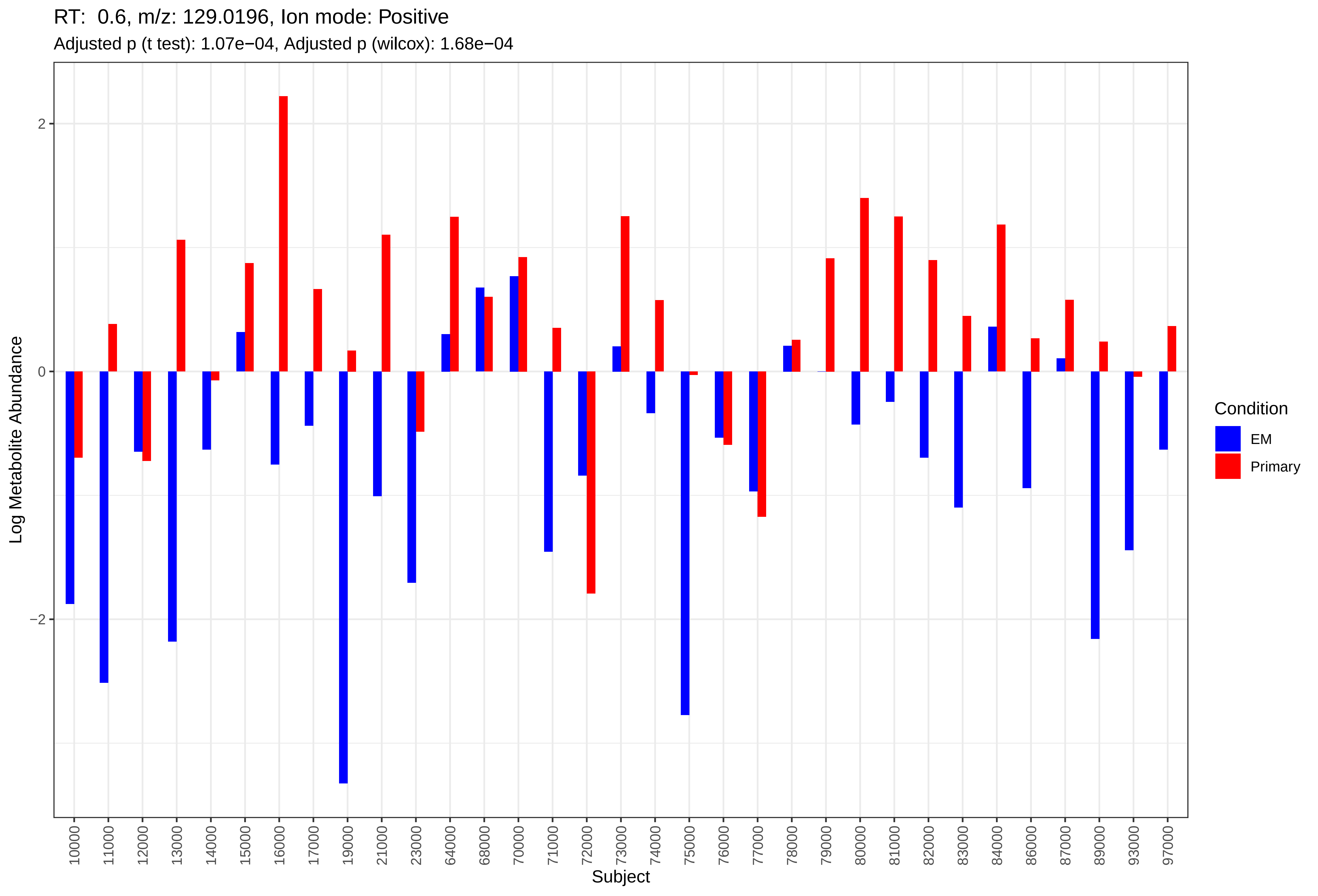

RT: 0.6, m/z: 129.0196, Ion mode: Positive
Adjusted p (t test): 1.07e−04, Adjusted p (wilcox): 1.68e−04
Log Metabolite Abundance
2
0
−2
Subject
10000
11000
12000
13000
14000
15000
16000
17000
19000
21000
23000
64000
68000
70000
71000
72000
73000
74000
75000
76000
77000
78000
79000
80000
81000
82000
83000
84000
86000
87000
89000
93000
97000
Condition
EM
Primary

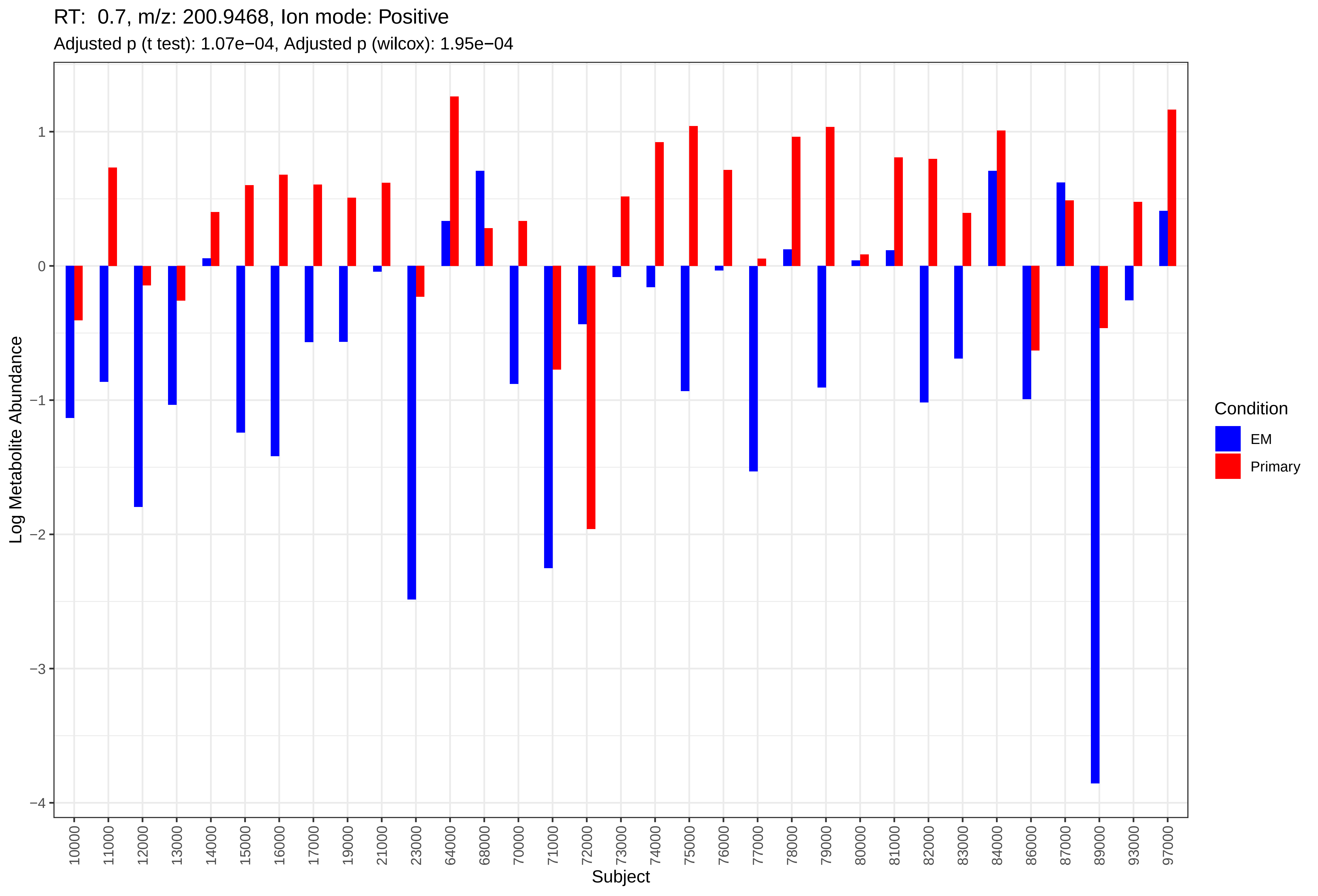

RT: 0.7, m/z: 200.9468, Ion mode: Positive
Adjusted p (t test): 1.07e−04, Adjusted p (wilcox): 1.95e−04
Log Metabolite Abundance
1
0
−1
−2
−3
−4
10000
11000
12000
13000
14000
15000
16000
17000
19000
21000
23000
64000
68000
70000
71000
72000
73000
74000
75000
76000
77000
78000
79000
80000
81000
82000
83000
84000
86000
87000
89000
93000
97000
Subject
Condition
EM
Primary

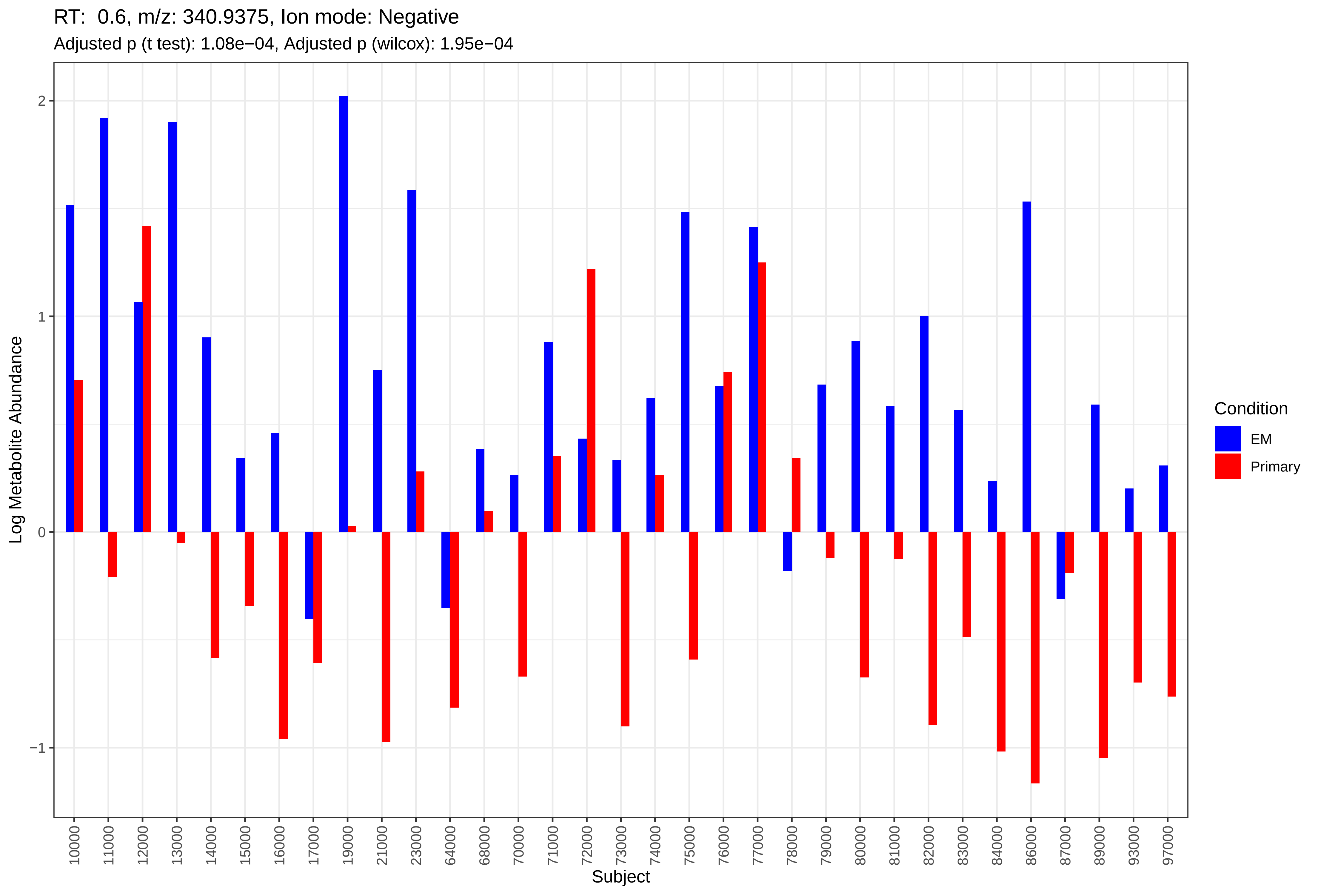
RT: 0.6, m/z: 340.9375, Ion mode: Negative
Adjusted p (t test): 1.08e−04, Adjusted p (wilcox): 1.95e−04
Log Metabolite Abundance
2
1
0
−1
Subject
10000
11000
12000
13000
14000
15000
16000
17000
19000
21000
23000
64000
68000
70000
71000
72000
73000
74000
75000
76000
77000
78000
79000
80000
81000
82000
83000
84000
86000
87000
89000
93000
97000
Condition
EM
Primary

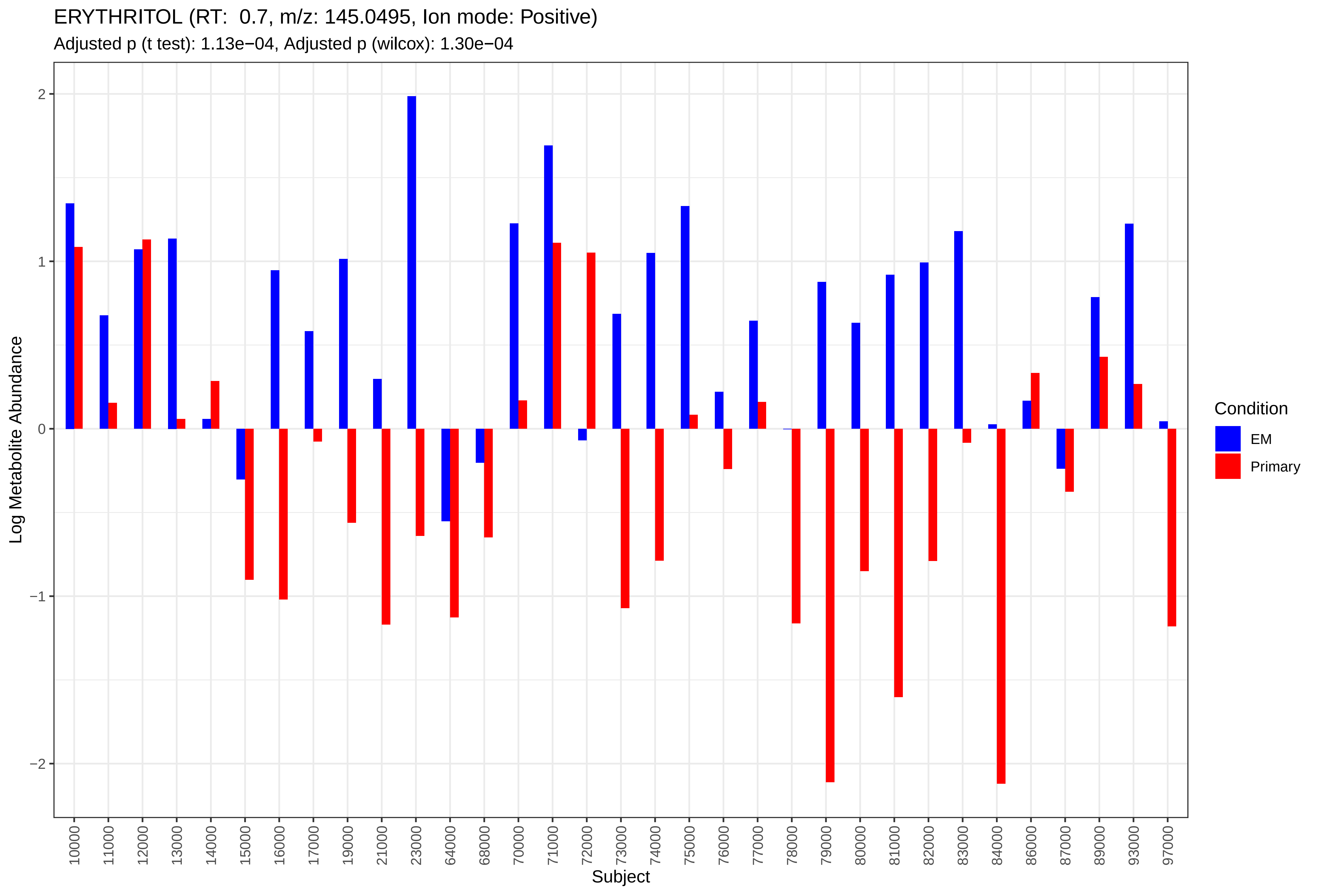

ERYTHRITOL (RT: 0.7, m/z: 145.0495, Ion mode: Positive)
Adjusted p (t test): 1.13e−04, Adjusted p (wilcox): 1.30e−04
Log Metabolite Abundance
2
1
0
−1
−2
10000
11000
12000
13000
14000
15000
16000
17000
19000
21000
23000
64000
68000
70000
71000
72000
73000
74000
75000
76000
77000
78000
79000
80000
81000
82000
83000
84000
86000
87000
89000
93000
97000
Subject
Condition
EM
Primary

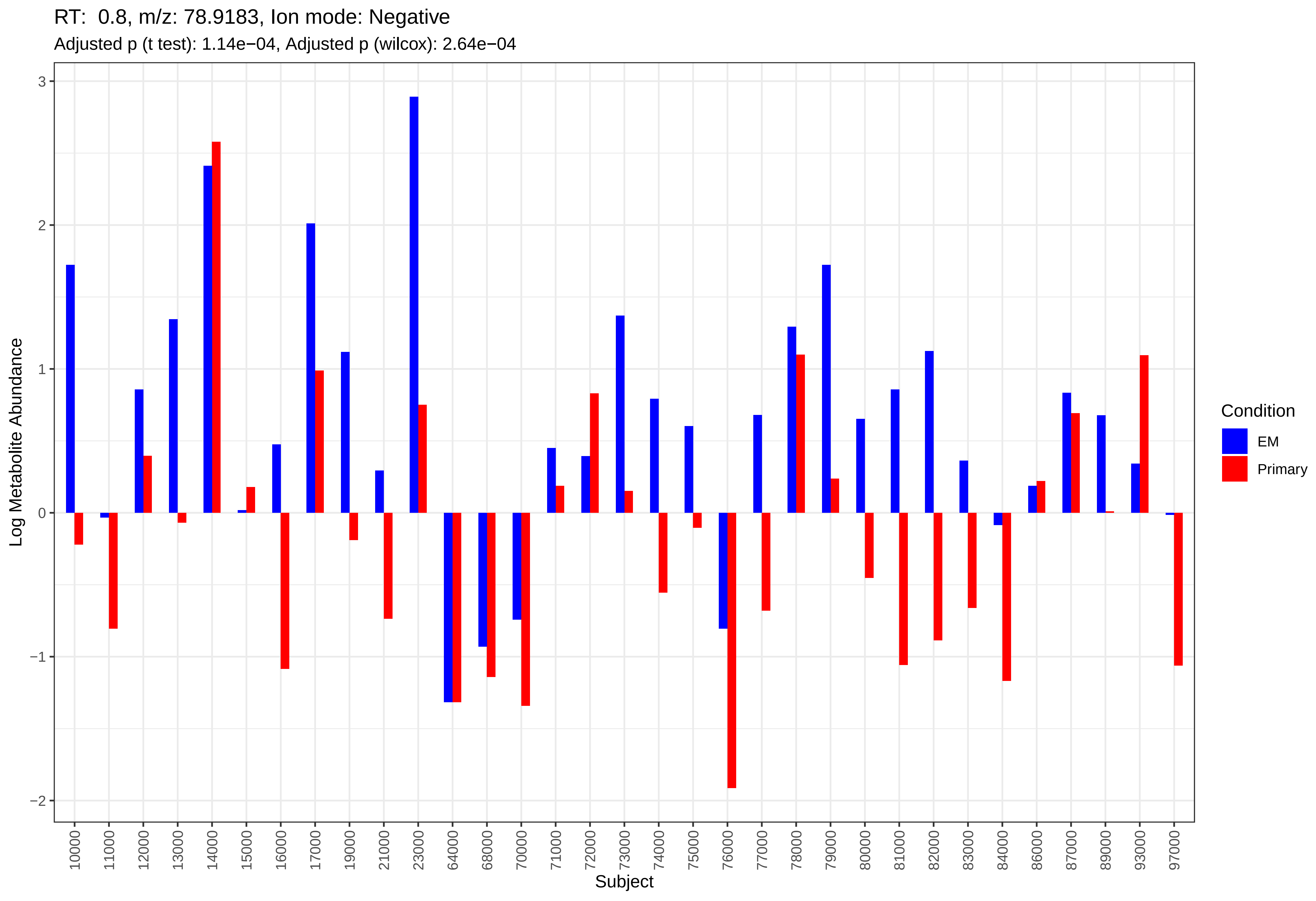
RT: 0.8, m/z: 78.9183, Ion mode: Negative
Adjusted p (t test): 1.14e−04, Adjusted p (wilcox): 2.64e−04
Log Metabolite Abundance
3
2
1
0
−1
−2
Subject
10000
11000
12000
13000
14000
15000
16000
17000
19000
21000
23000
64000
68000
70000
71000
72000
73000
74000
75000
76000
77000
78000
79000
80000
81000
82000
83000
84000
86000
87000
89000
93000
97000
Condition
EM
Primary

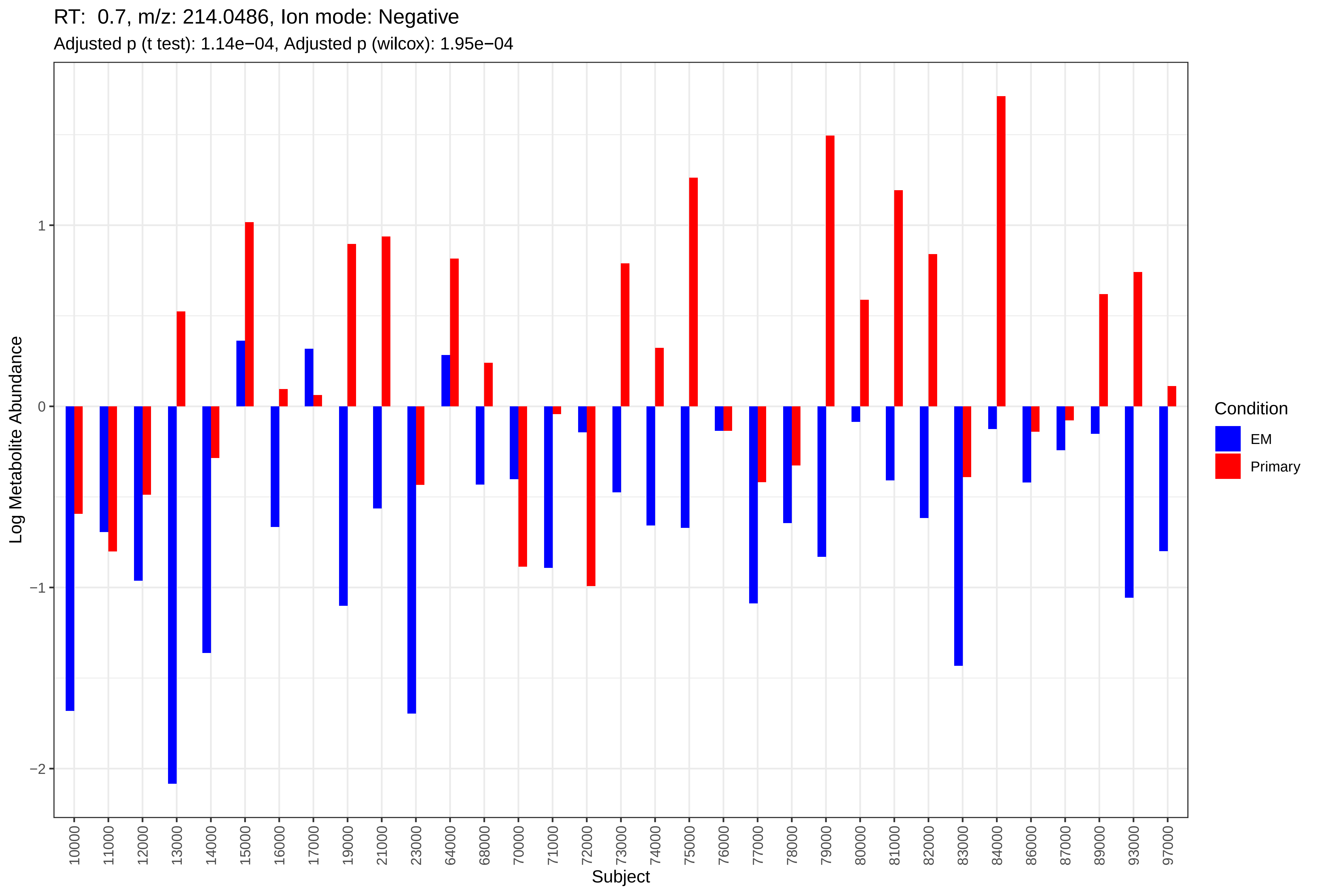
RT: 0.7, m/z: 214.0486, Ion mode: Negative
Adjusted p (t test): 1.14e−04, Adjusted p (wilcox): 1.95e−04
Log Metabolite Abundance
1
0
−1
−2
10000
11000
12000
13000
14000
15000
16000
17000
19000
21000
23000
64000
68000
70000
71000
72000
73000
74000
75000
76000
77000
78000
79000
80000
81000
82000
83000
84000
86000
87000
89000
93000
97000
Subject
Condition
EM
Primary

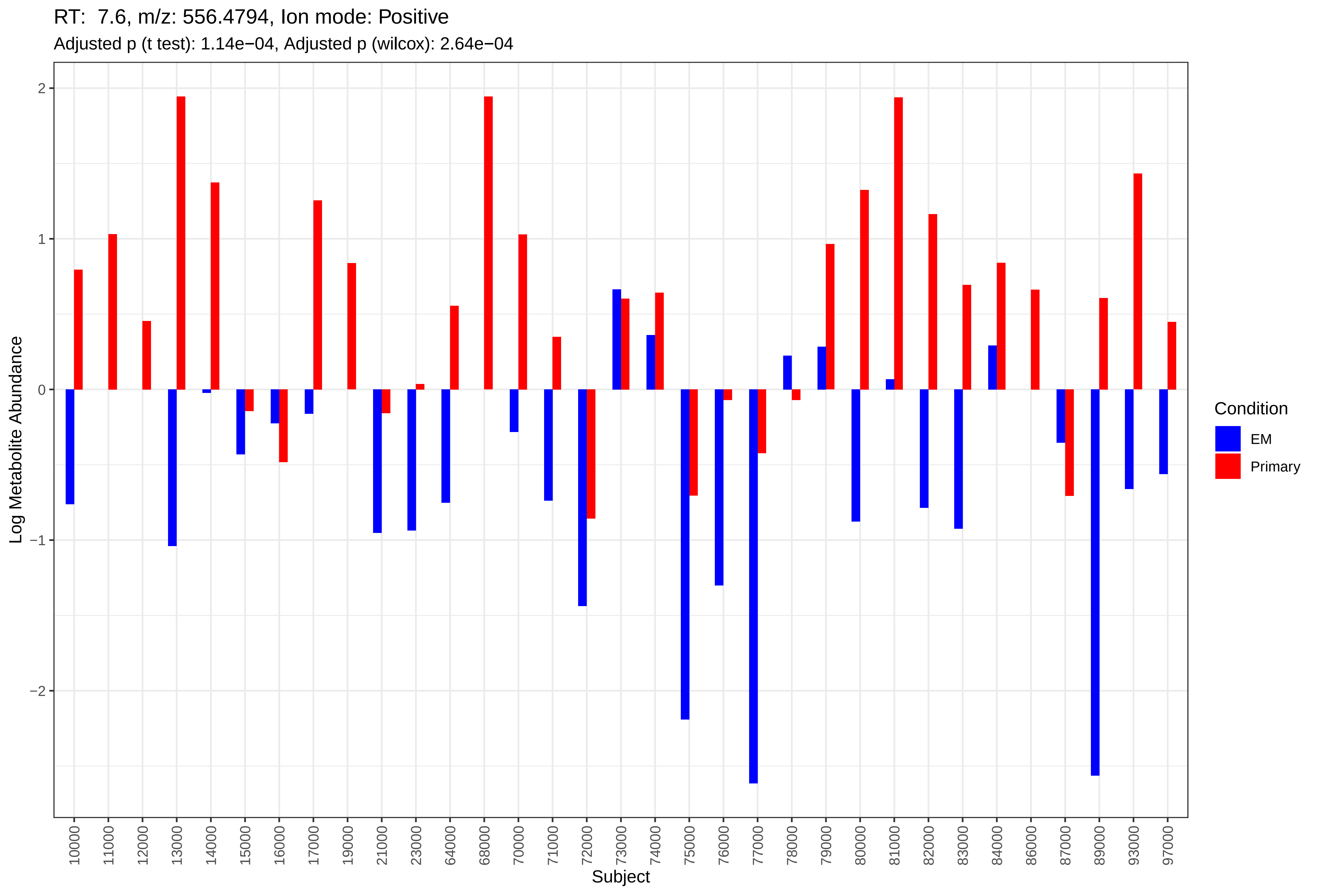

RT: 7.6, m/z: 556.4794, Ion mode: Positive
Adjusted p (t test): 1.14e−04, Adjusted p (wilcox): 2.64e−04
Log Metabolite Abundance
2
1
0
−1
−2
Subject
10000
11000
12000
13000
14000
15000
16000
17000
19000
21000
23000
64000
68000
70000
71000
72000
73000
74000
75000
76000
77000
78000
79000
80000
81000
82000
83000
84000
86000
87000
89000
93000
97000
Condition
EM
Primary

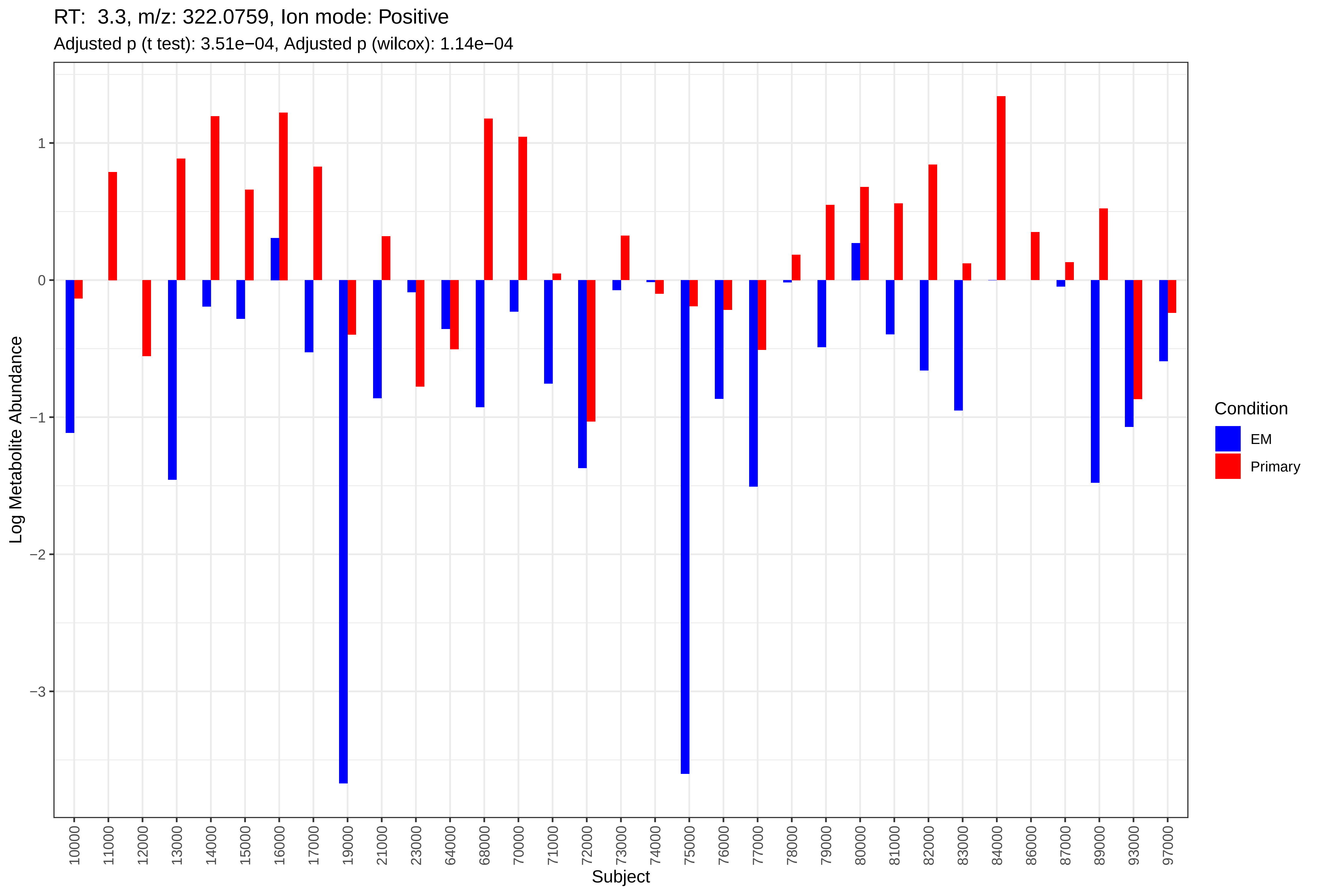

RT: 3.3, m/z: 322.0759, Ion mode: Positive
Adjusted p (t test): 3.51e−04, Adjusted p (wilcox): 1.14e−04
Log Metabolite Abundance
1
0
−1
−2
−3
10000
11000
12000
13000
14000
15000
16000
17000
19000
21000
23000
64000
68000
70000
71000
72000
73000
74000
75000
76000
77000
78000
79000
80000
81000
82000
83000
84000
86000
87000
89000
93000
97000
Subject
Condition
EM
Primary

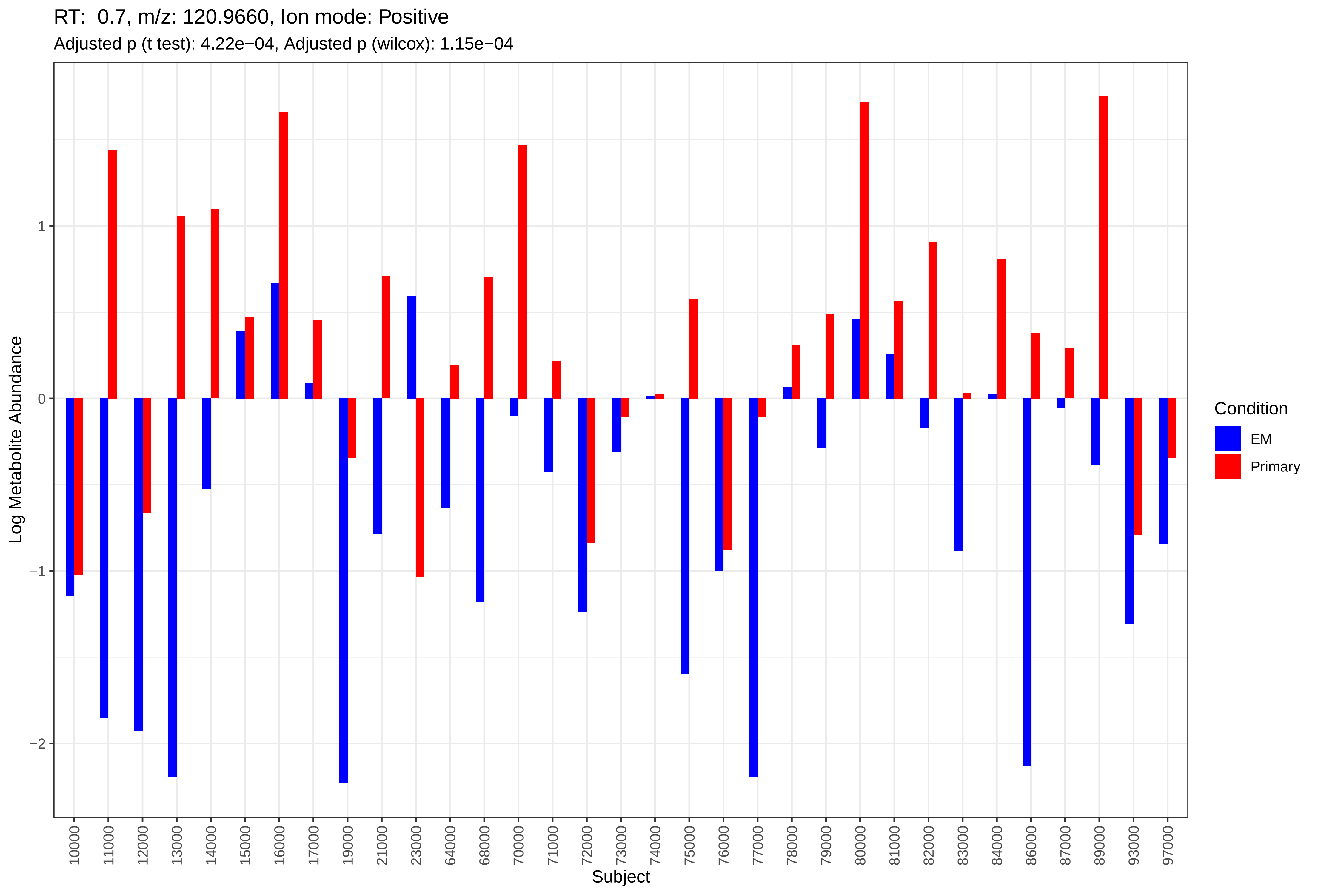

RT: 0.7, m/z: 120.9660, Ion mode: Positive
Adjusted p (t test): 4.22e−04, Adjusted p (wilcox): 1.15e−04
Log Metabolite Abundance
1
0
−1
−2
Subject
10000
11000
12000
13000
14000
15000
16000
17000
19000
21000
23000
64000
68000
70000
71000
72000
73000
74000
75000
76000
77000
78000
79000
80000
81000
82000
83000
84000
86000
87000
89000
93000
97000
Condition
EM
Primary

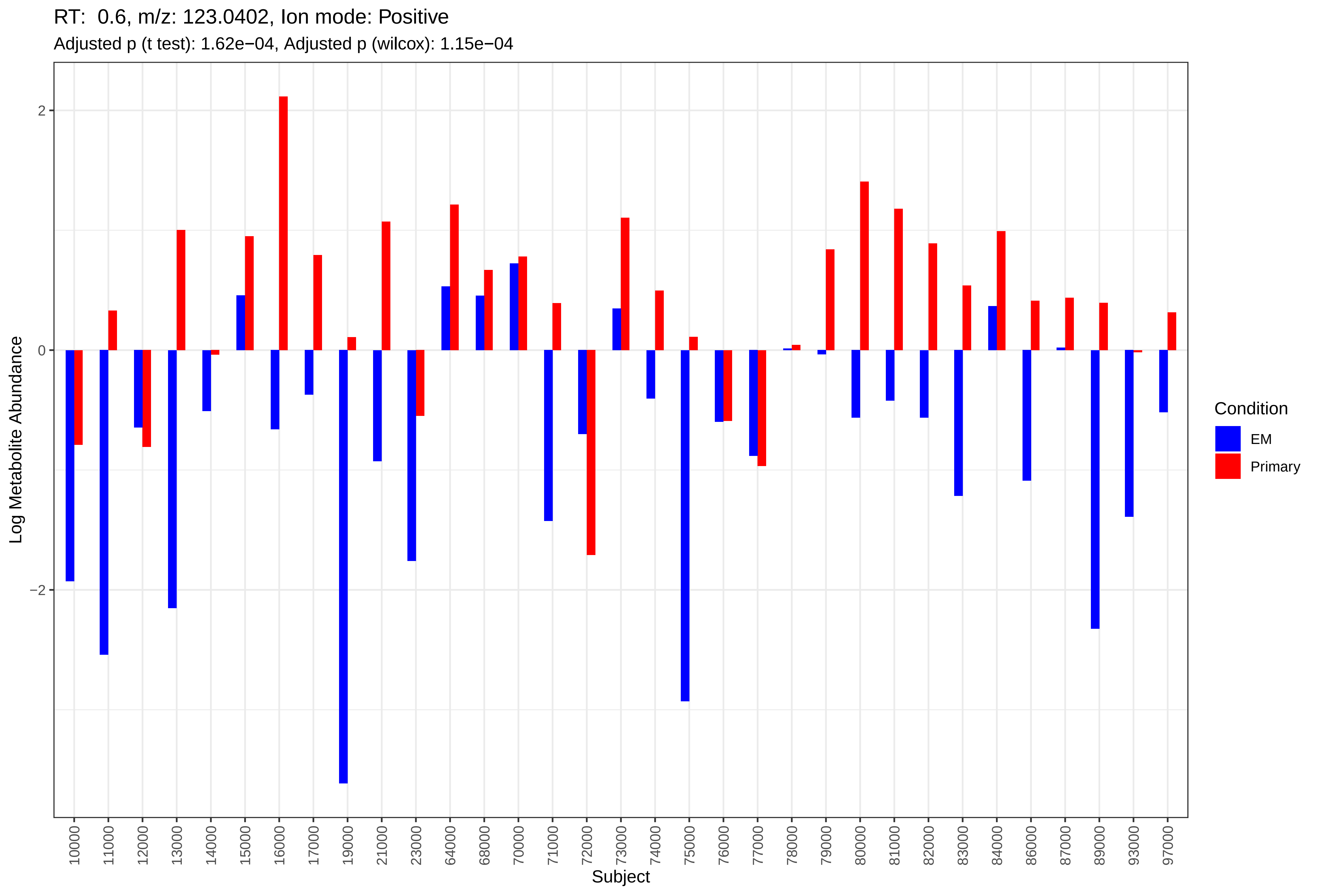

RT: 0.6, m/z: 123.0402, Ion mode: Positive
Adjusted p (t test): 1.62e−04, Adjusted p (wilcox): 1.15e−04
Log Metabolite Abundance
2
0
−2
Subject
10000
11000
12000
13000
14000
15000
16000
17000
19000
21000
23000
64000
68000
70000
71000
72000
73000
74000
75000
76000
77000
78000
79000
80000
81000
82000
83000
84000
86000
87000
89000
93000
97000
Condition
EM
Primary

RT: 0.8, m/z: 148.9439, Ion mode: Negative

Adjusted p (t test): 1.15e−04, Adjusted p (wilcox): 3.08e−04

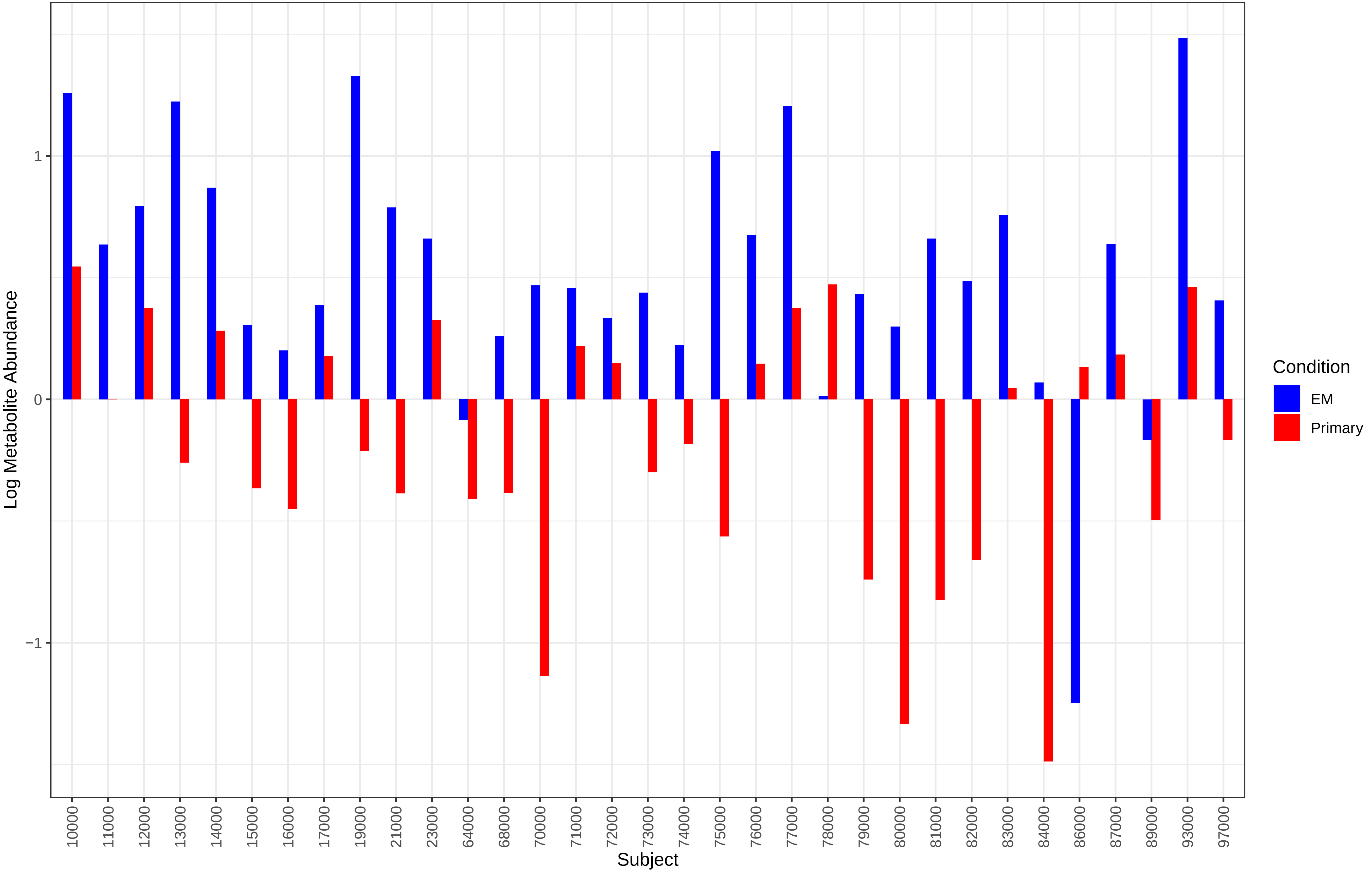

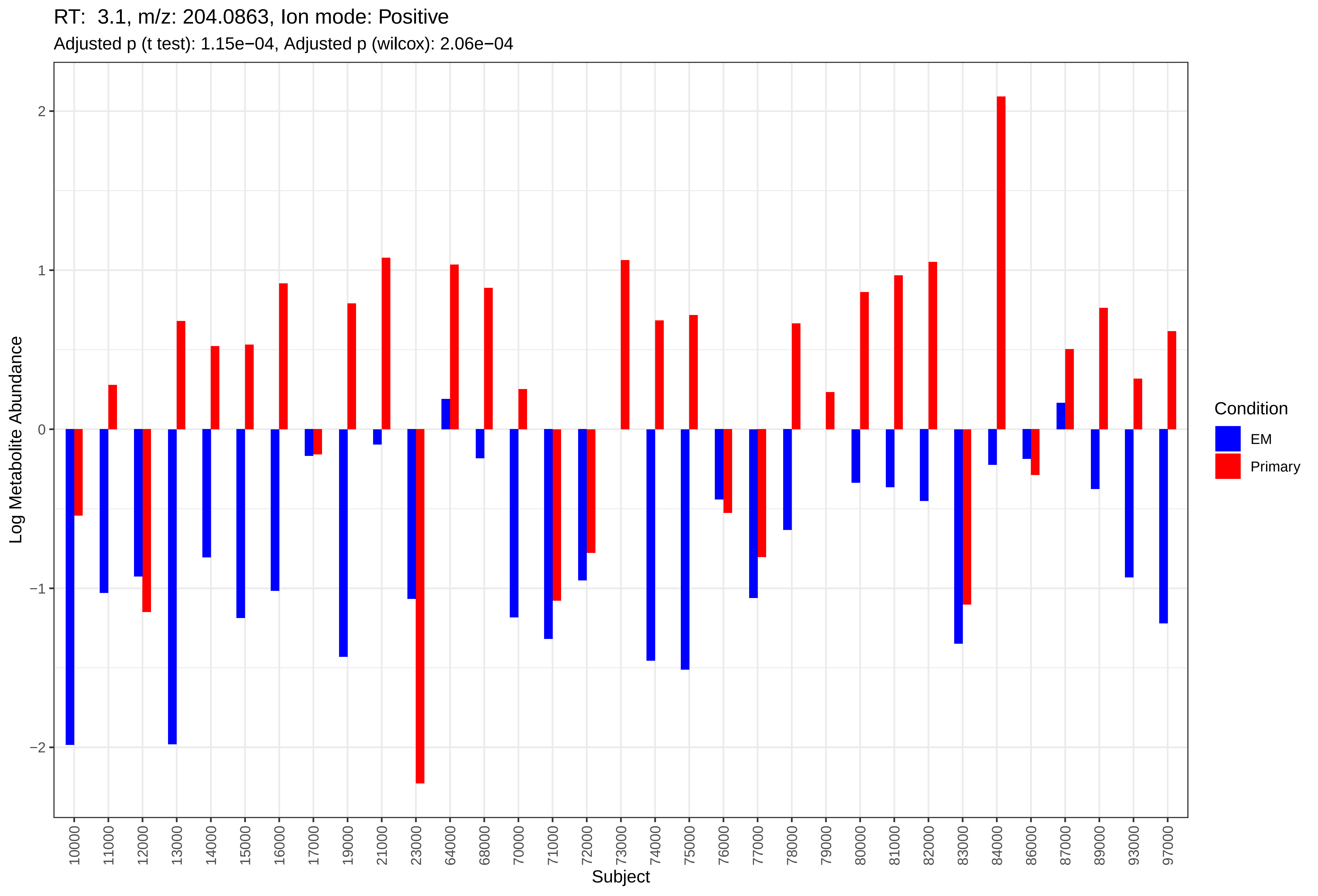

RT: 3.1, m/z: 204.0863, Ion mode: Positive
Adjusted p (t test): 1.15e−04, Adjusted p (wilcox): 2.06e−04
Log Metabolite Abundance
2
1
0
−1
−2
Subject
10000
11000
12000
13000
14000
15000
16000
17000
19000
21000
23000
64000
68000
70000
71000
72000
73000
74000
75000
76000
77000
78000
79000
80000
81000
82000
83000
84000
86000
87000
89000
93000
97000
Condition
EM
Primary

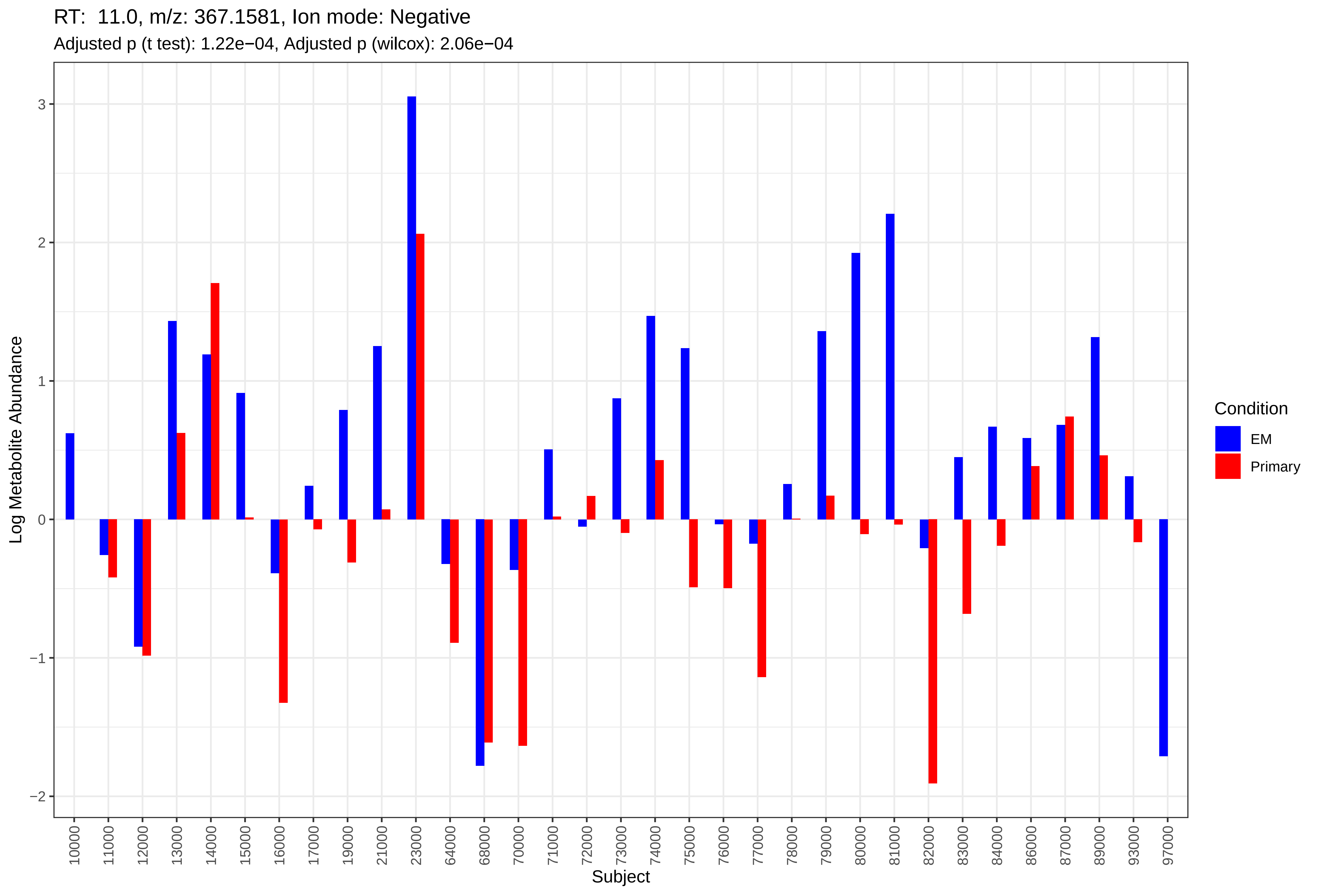
RT: 11.0, m/z: 367.1581, Ion mode: Negative
Adjusted p (t test): 1.22e−04, Adjusted p (wilcox): 2.06e−04
Log Metabolite Abundance
3
2
1
0
−1
−2
10000
11000
12000
13000
14000
15000
16000
17000
19000
21000
23000
64000
68000
70000
71000
72000
73000
74000
75000
76000
77000
78000
79000
80000
81000
82000
83000
84000
86000
87000
89000
93000
97000
Subject
Condition
EM
Primary

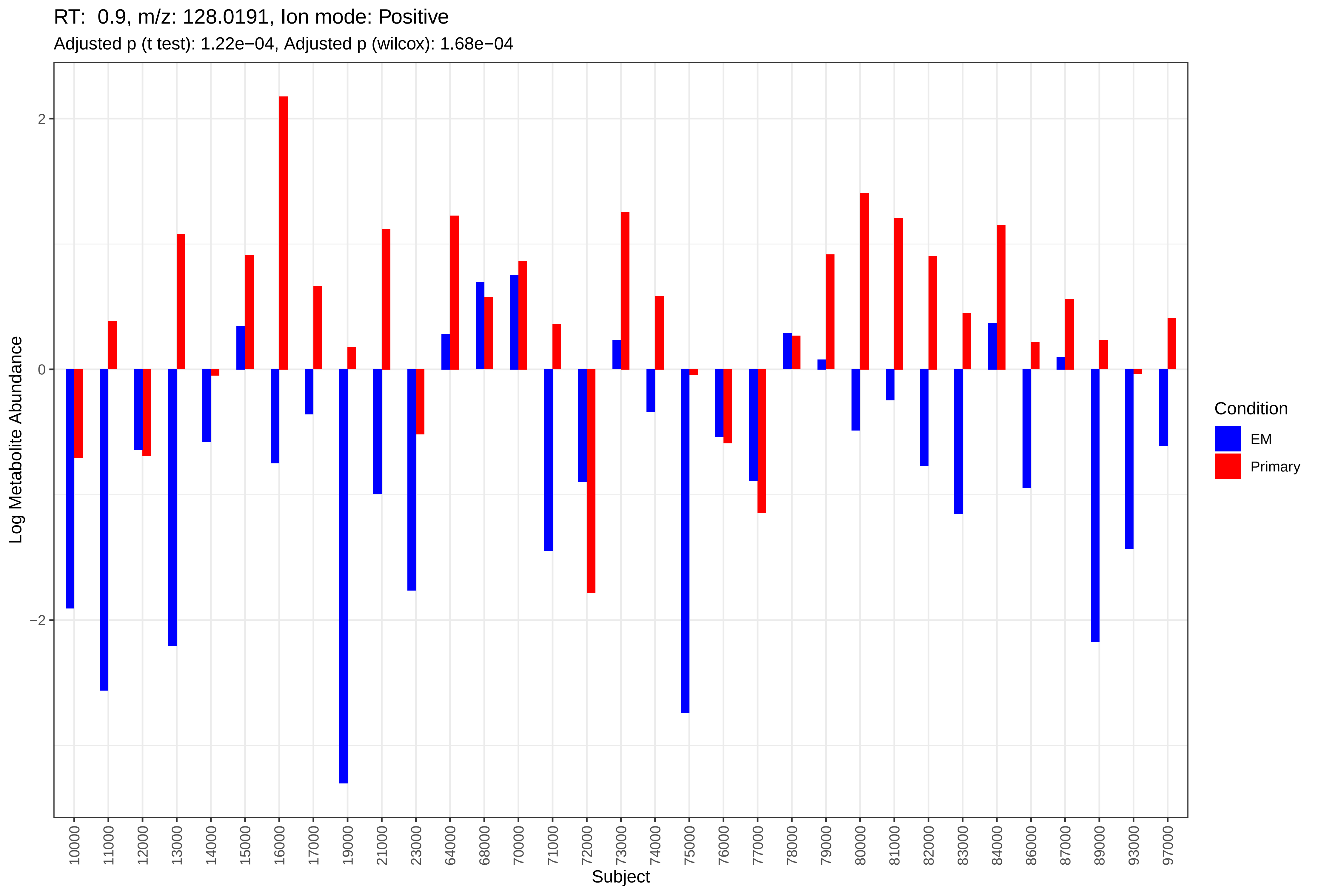

RT: 0.9, m/z: 128.0191, Ion mode: Positive
Adjusted p (t test): 1.22e−04, Adjusted p (wilcox): 1.68e−04
Log Metabolite Abundance
2
0
−2
10000
11000
12000
13000
14000
15000
16000
17000
19000
21000
23000
64000
68000
70000
71000
72000
73000
74000
75000
76000
77000
78000
79000
80000
81000
82000
83000
84000
86000
87000
89000
93000
97000
Subject
Condition
EM
Primary

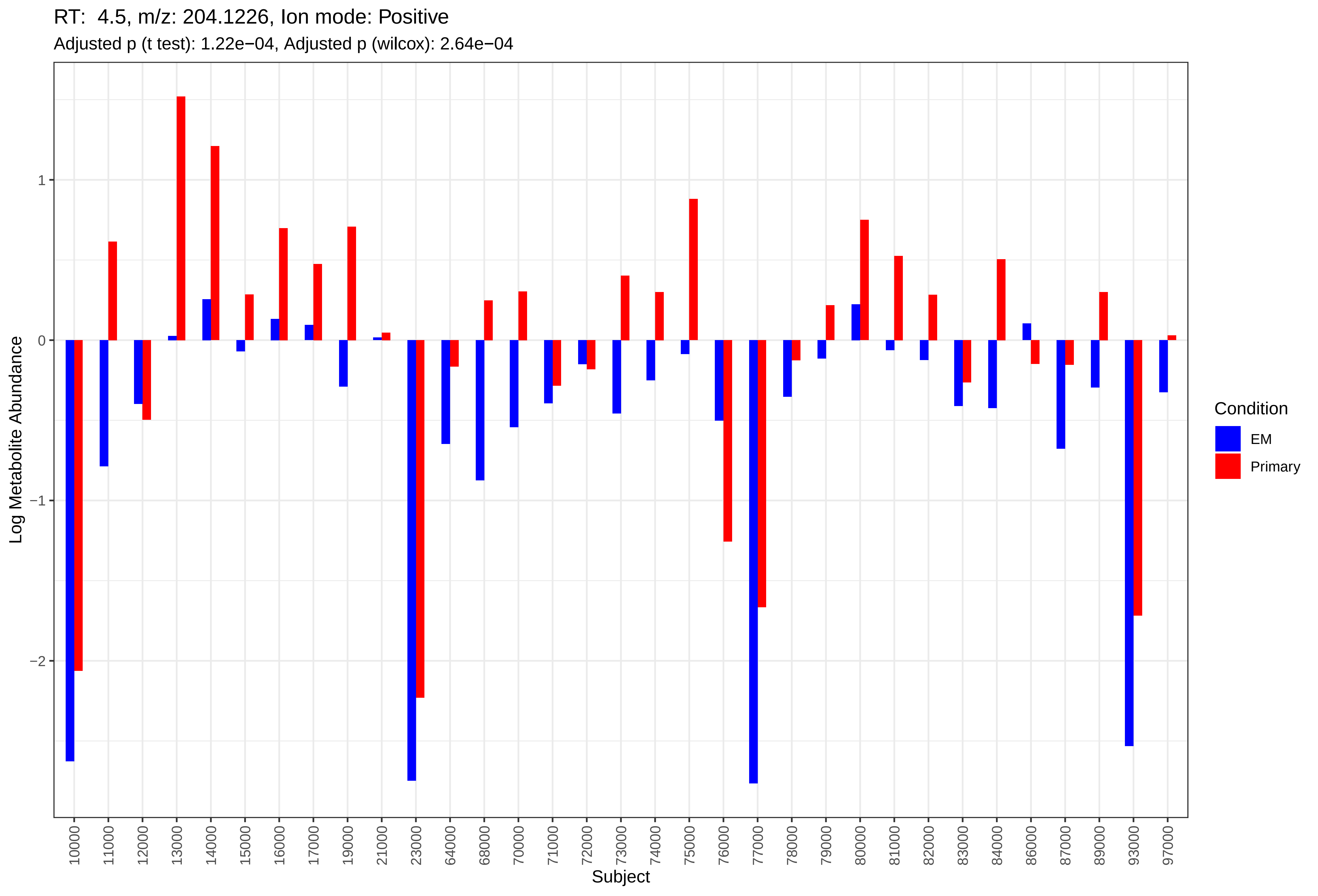
RT: 4.5, m/z: 204.1226, Ion mode: Positive
Adjusted p (t test): 1.22e−04, Adjusted p (wilcox): 2.64e−04
Log Metabolite Abundance
1
0
−1
−2
10000
11000
12000
13000
14000
15000
16000
17000
19000
21000
23000
64000
68000
70000
71000
72000
73000
74000
75000
76000
77000
78000
79000
80000
81000
82000
83000
84000
86000
87000
89000
93000
97000
Subject
Condition
EM
Primary

RT: 0.6, m/z: 146.0295, Ion mode: Positive

Adjusted p (t test): 1.26e−04, Adjusted p (wilcox): 1.68e−04

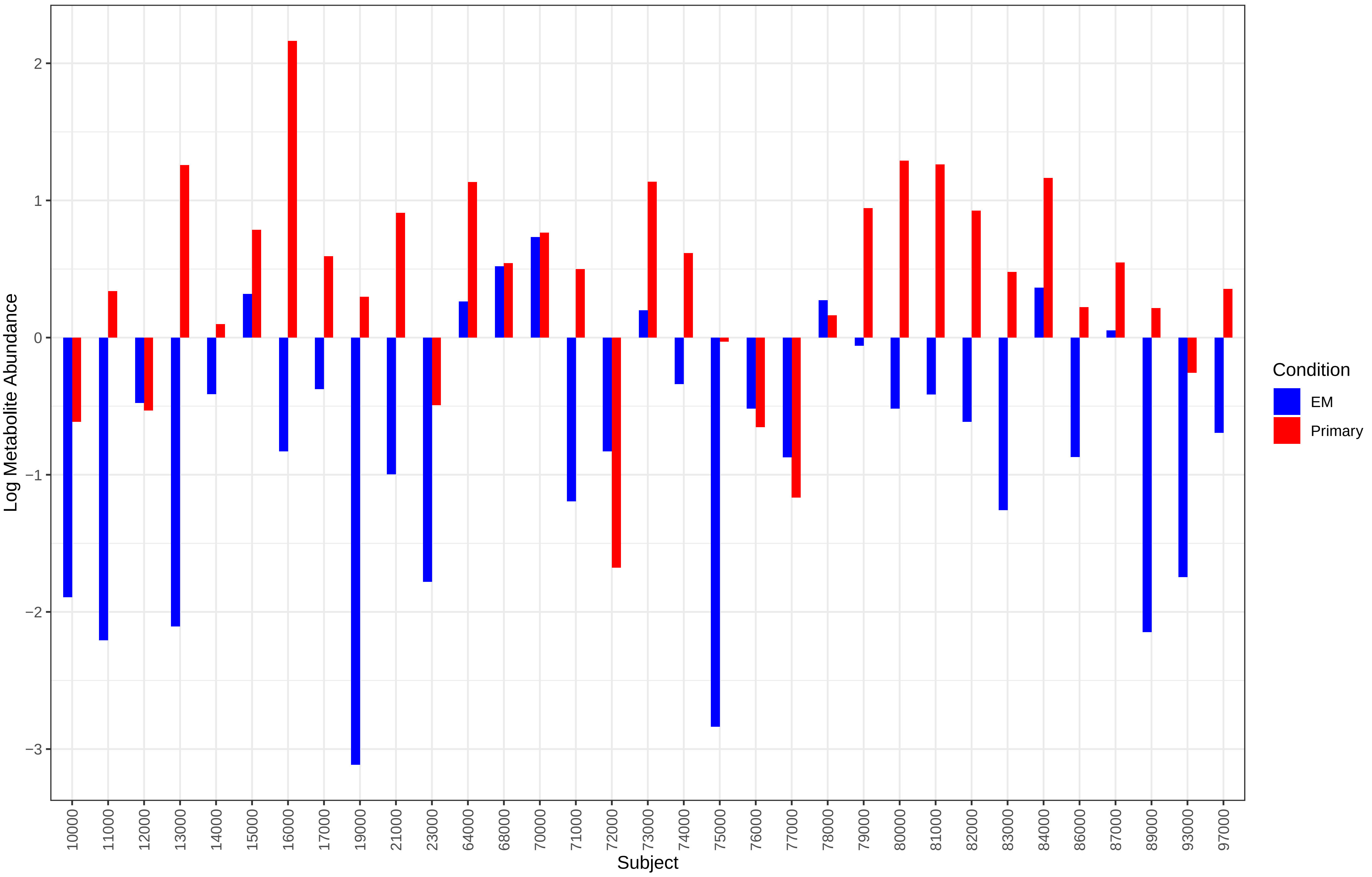

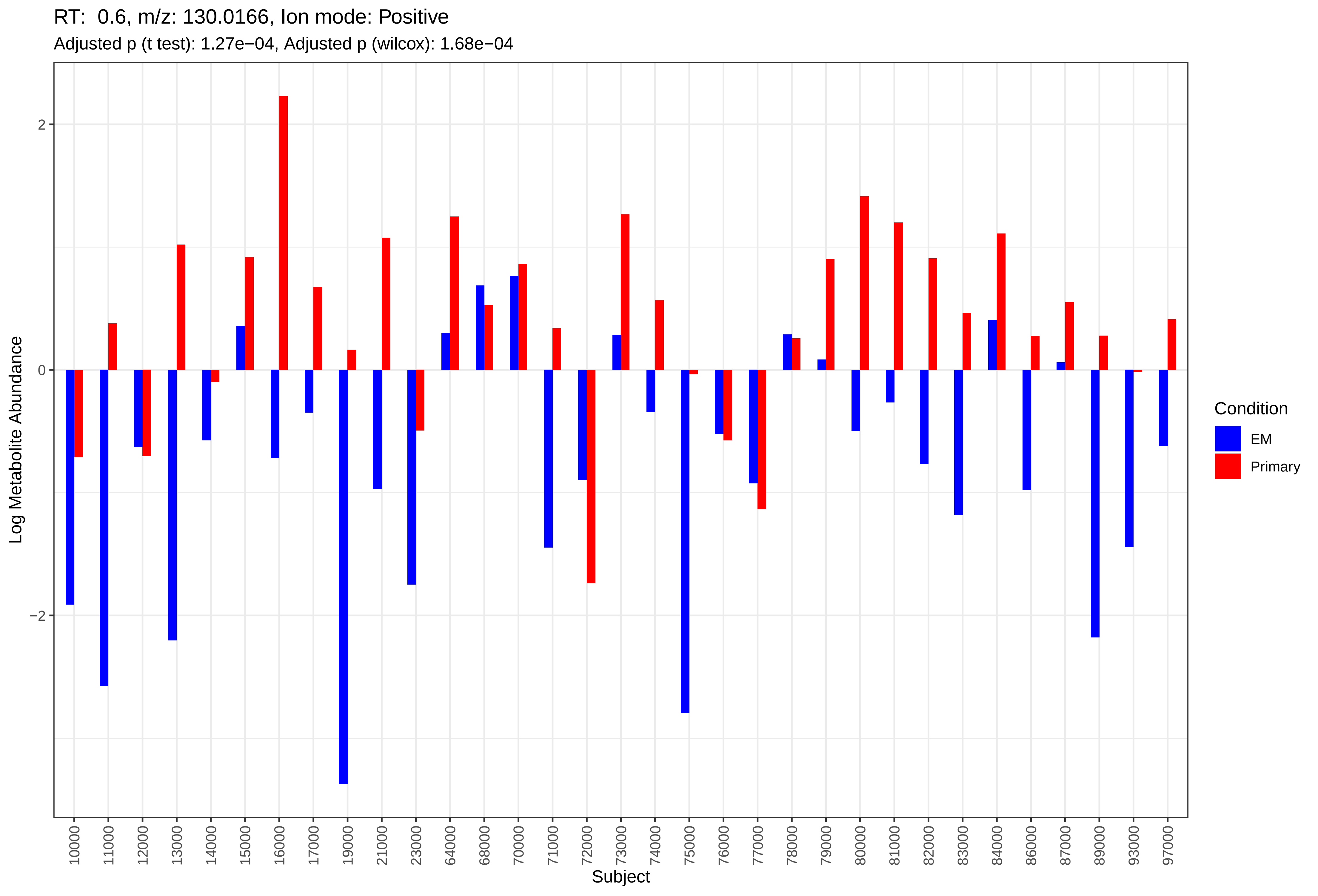

RT: 0.6, m/z: 130.0166, Ion mode: Positive
Adjusted p (t test): 1.27e−04, Adjusted p (wilcox): 1.68e−04
Log Metabolite Abundance
2
0
−2
Subject
10000
11000
12000
13000
14000
15000
16000
17000
19000
21000
23000
64000
68000
70000
71000
72000
73000
74000
75000
76000
77000
78000
79000
80000
81000
82000
83000
84000
86000
87000
89000
93000
97000
Condition
EM
Primary

RT: 0.6, m/z: 123.0138, Ion mode: Positive

Adjusted p (t test): 1.56e−04, Adjusted p (wilcox): 1.30e−04

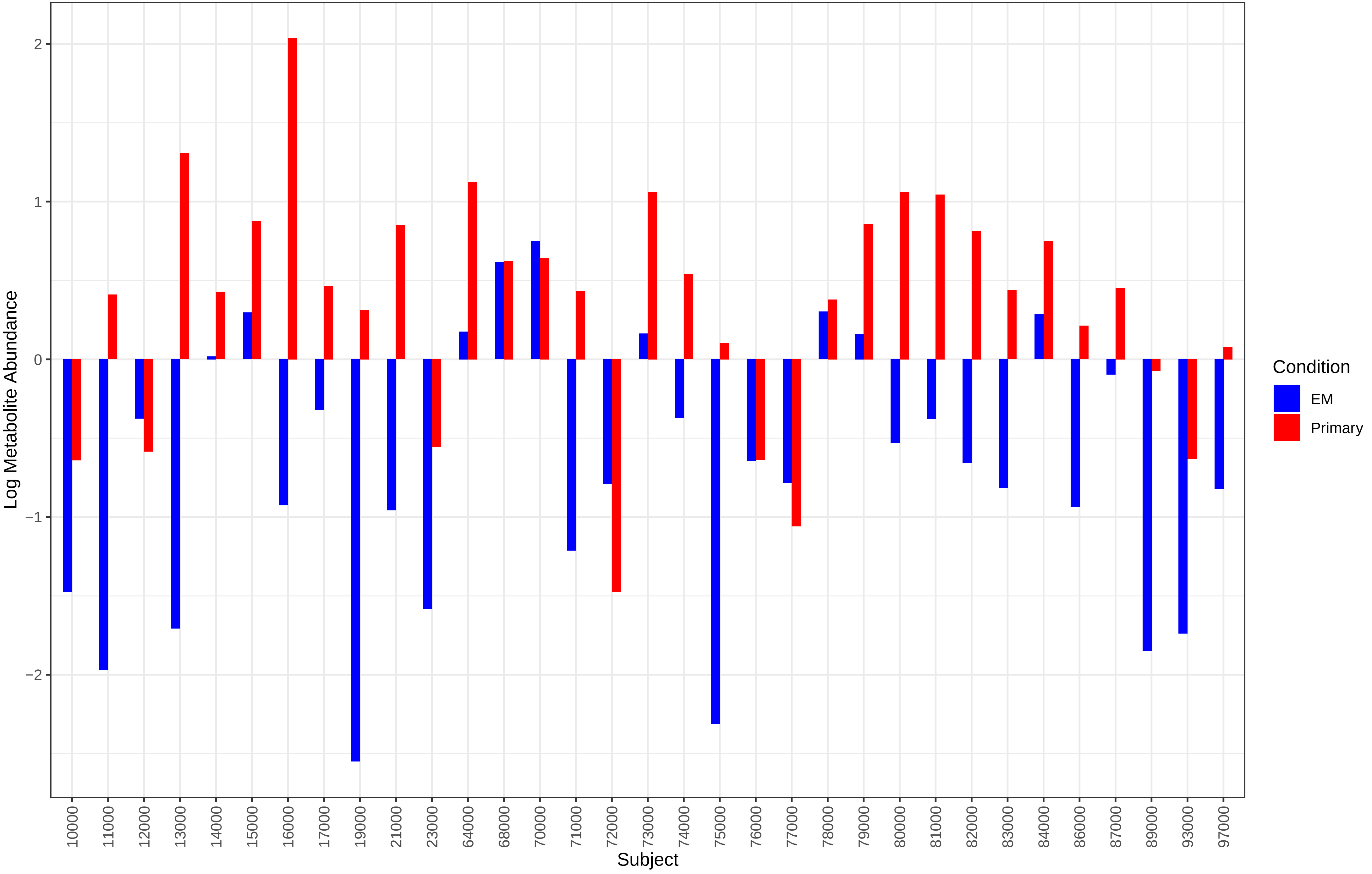

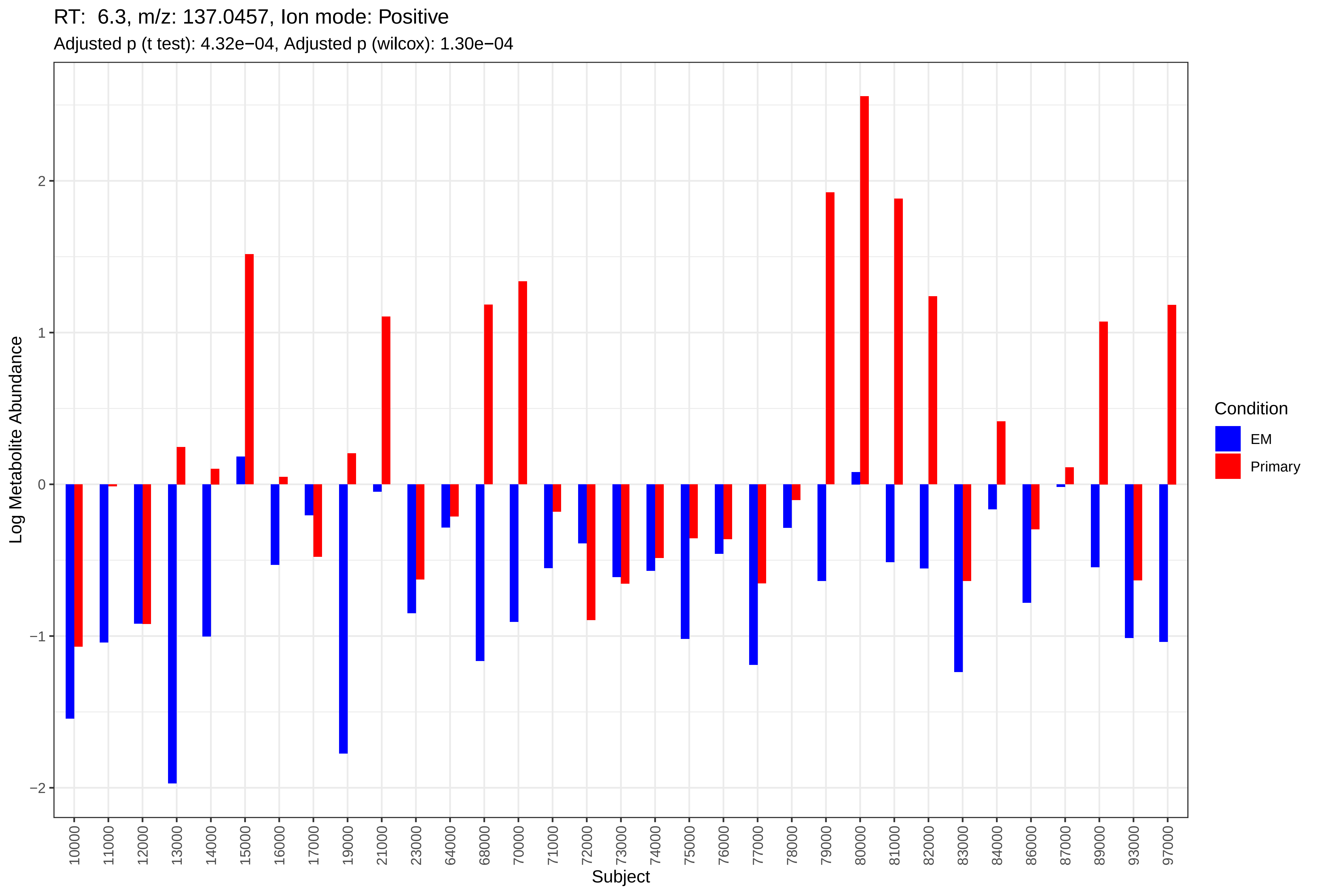
RT: 6.3, m/z: 137.0457, Ion mode: Positive
Adjusted p (t test): 4.32e−04, Adjusted p (wilcox): 1.30e−04
Log Metabolite Abundance
2
1
0
−1
−2
Subject
10000
11000
12000
13000
14000
15000
16000
17000
19000
21000
23000
64000
68000
70000
71000
72000
73000
74000
75000
76000
77000
78000
79000
80000
81000
82000
83000
84000
86000
87000
89000
93000
97000
Condition
EM
Primary

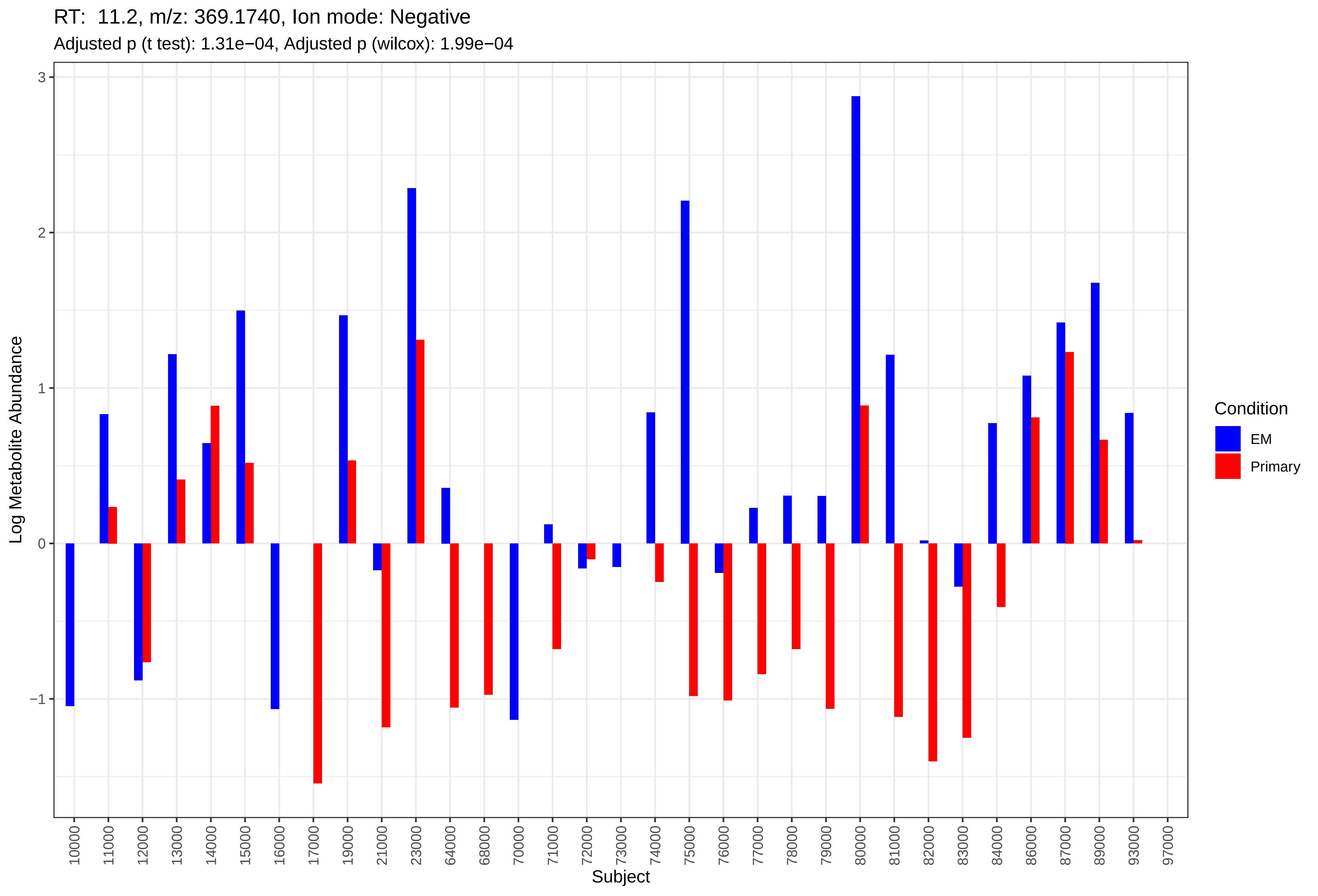
RT: 11.2, m/z: 369.1740, Ion mode: Negative
Adjusted p (t test): 1.31e−04, Adjusted p (wilcox): 1.99e−04
Log Metabolite Abundance
3
2
1
0
−1
Subject
10000
11000
12000
13000
14000
15000
16000
17000
19000
21000
23000
64000
68000
70000
71000
72000
73000
74000
75000
76000
77000
78000
79000
80000
81000
82000
83000
84000
86000
87000
89000
93000
97000
Condition
EM
Primary

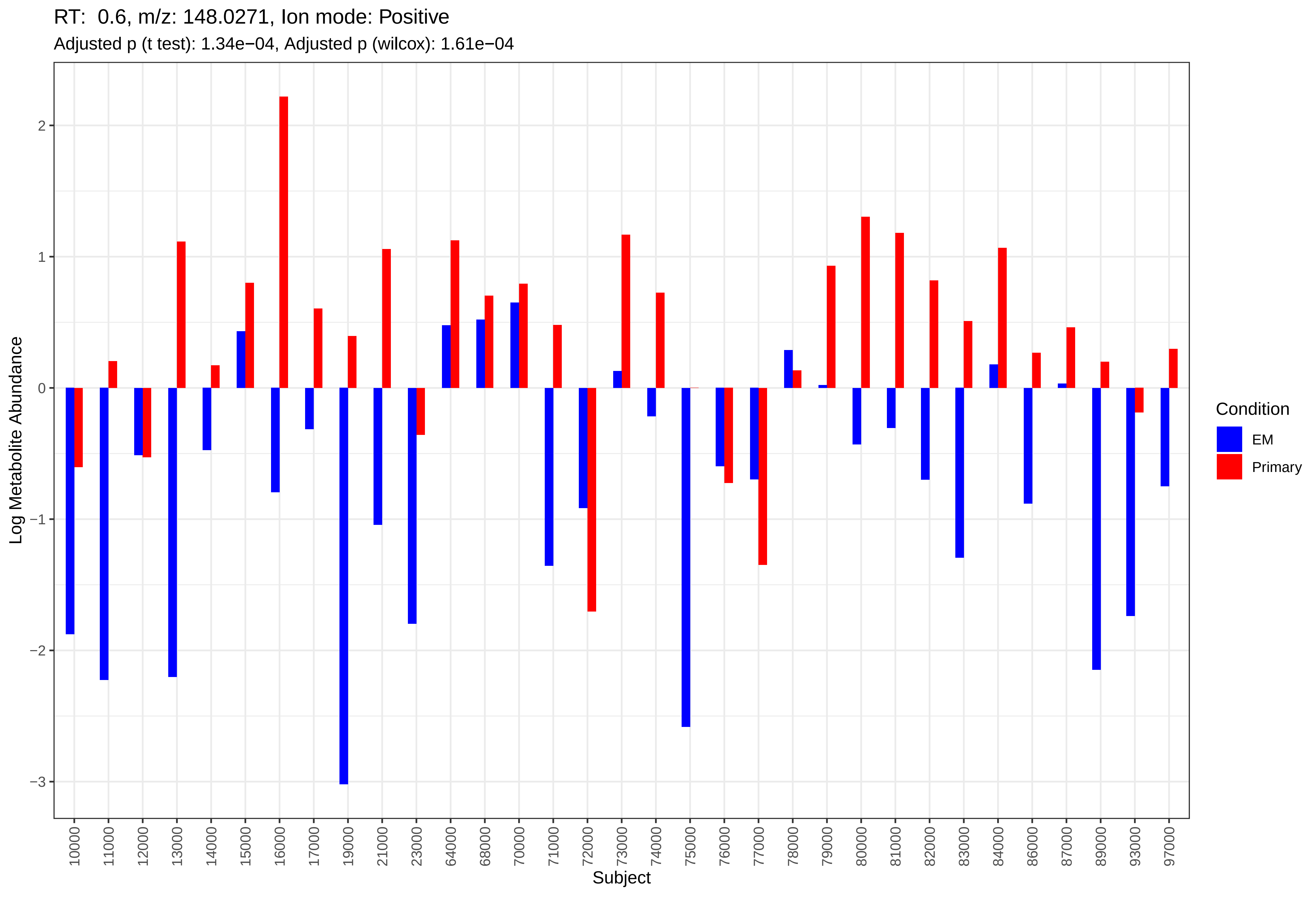
RT: 0.6, m/z: 148.0271, Ion mode: Positive
Adjusted p (t test): 1.34e−04, Adjusted p (wilcox): 1.61e−04
Log Metabolite Abundance
2
1
0
−1
−2
−3
Subject
10000
11000
12000
13000
14000
15000
16000
17000
19000
21000
23000
64000
68000
70000
71000
72000
73000
74000
75000
76000
77000
78000
79000
80000
81000
82000
83000
84000
86000
87000
89000
93000
97000
Condition
EM
Primary

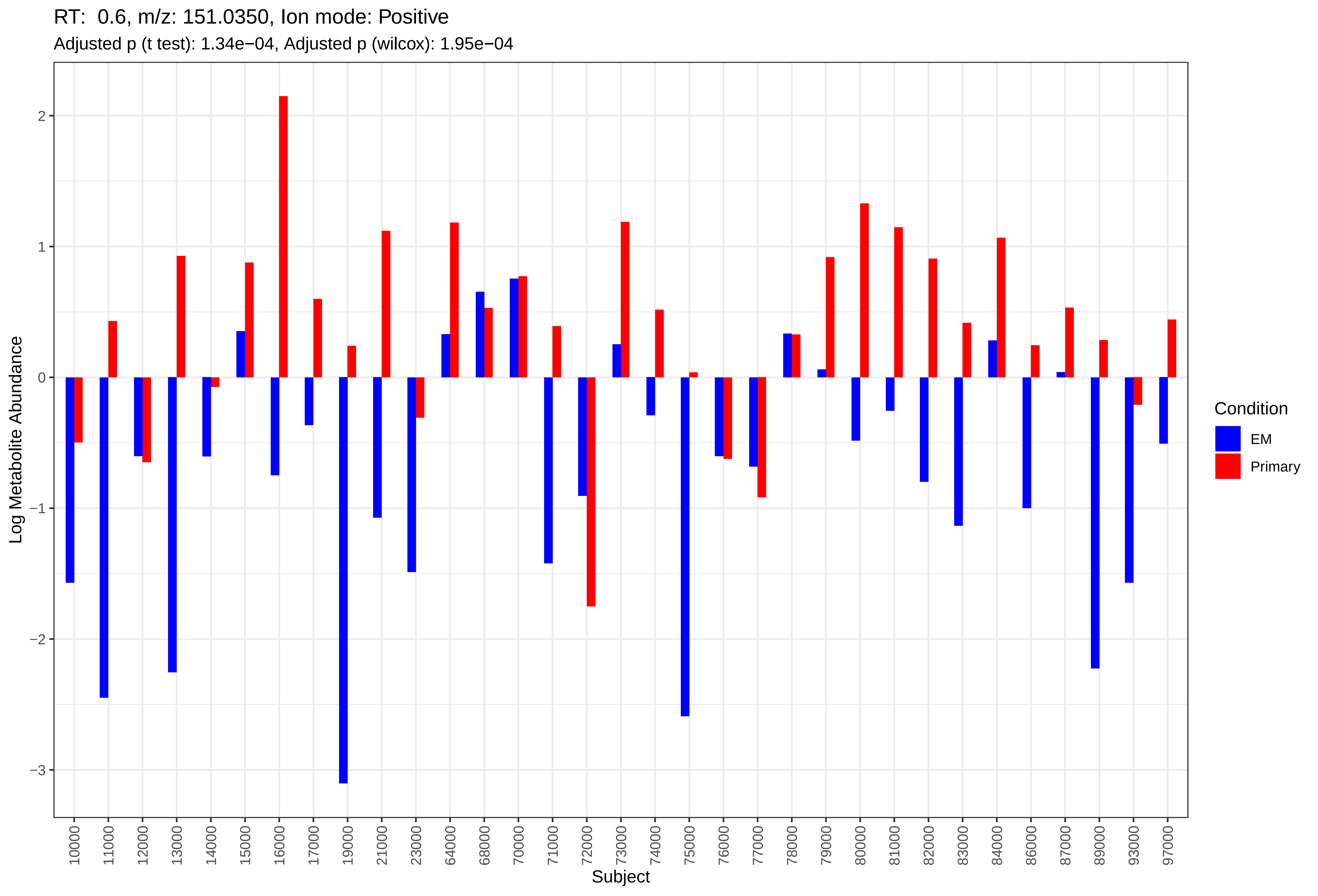

RT: 0.6, m/z: 151.0350, Ion mode: Positive
Adjusted p (t test): 1.34e−04, Adjusted p (wilcox): 1.95e−04
Log Metabolite Abundance
2
1
0
−1
−2
−3
10000
11000
12000
13000
14000
15000
16000
17000
19000
21000
23000
64000
68000
70000
71000
72000
73000
74000
75000
76000
77000
78000
79000
80000
81000
82000
83000
84000
86000
87000
89000
93000
97000
Subject
Condition
EM
Primary

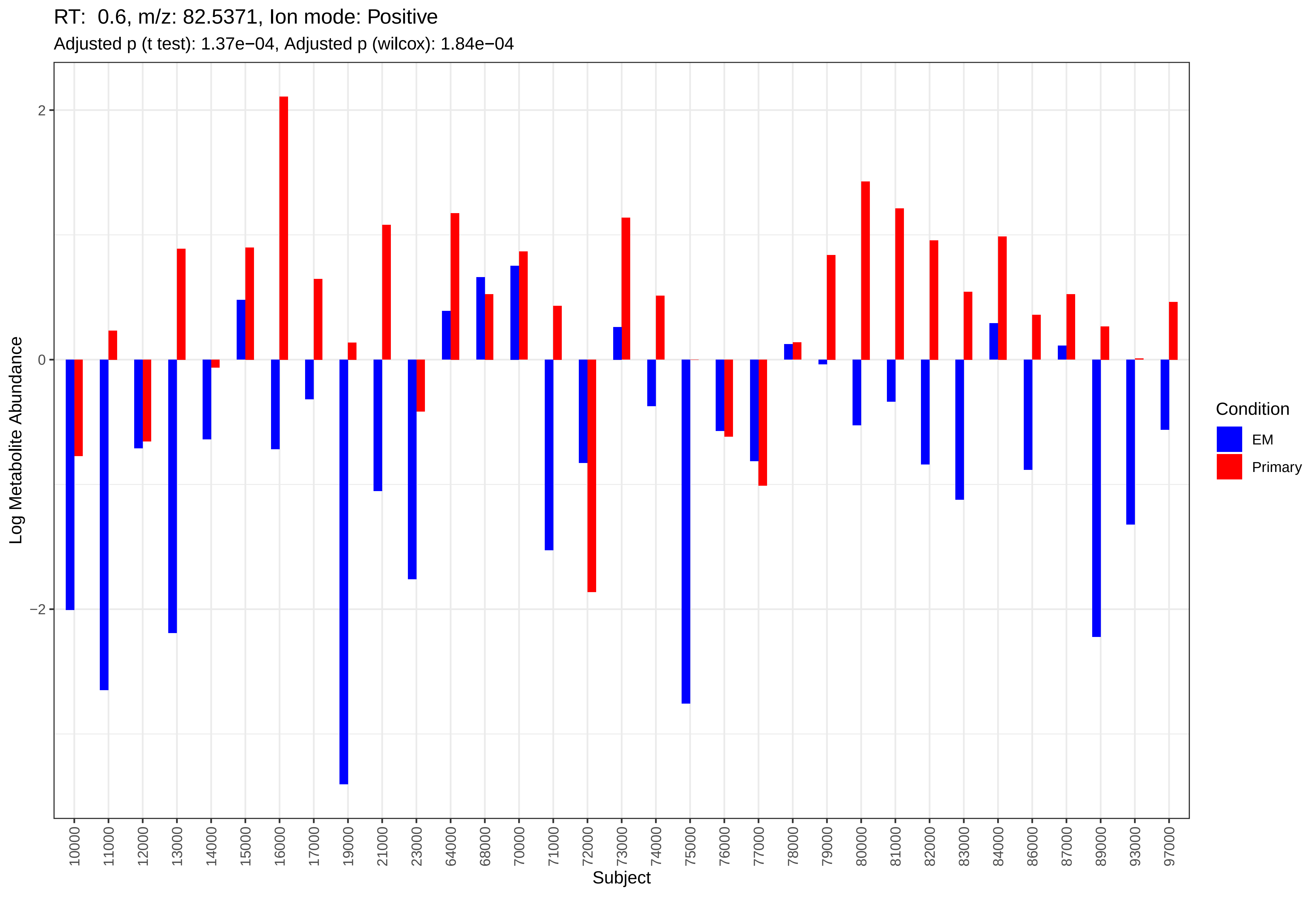

RT: 0.6, m/z: 82.5371, Ion mode: Positive
Adjusted p (t test): 1.37e−04, Adjusted p (wilcox): 1.84e−04
Log Metabolite Abundance
2
0
−2
10000
11000
12000
13000
14000
15000
16000
17000
19000
21000
23000
64000
68000
70000
71000
72000
73000
74000
75000
76000
77000
78000
79000
80000
81000
82000
83000
84000
86000
87000
89000
93000
97000
Subject
Condition
EM
Primary

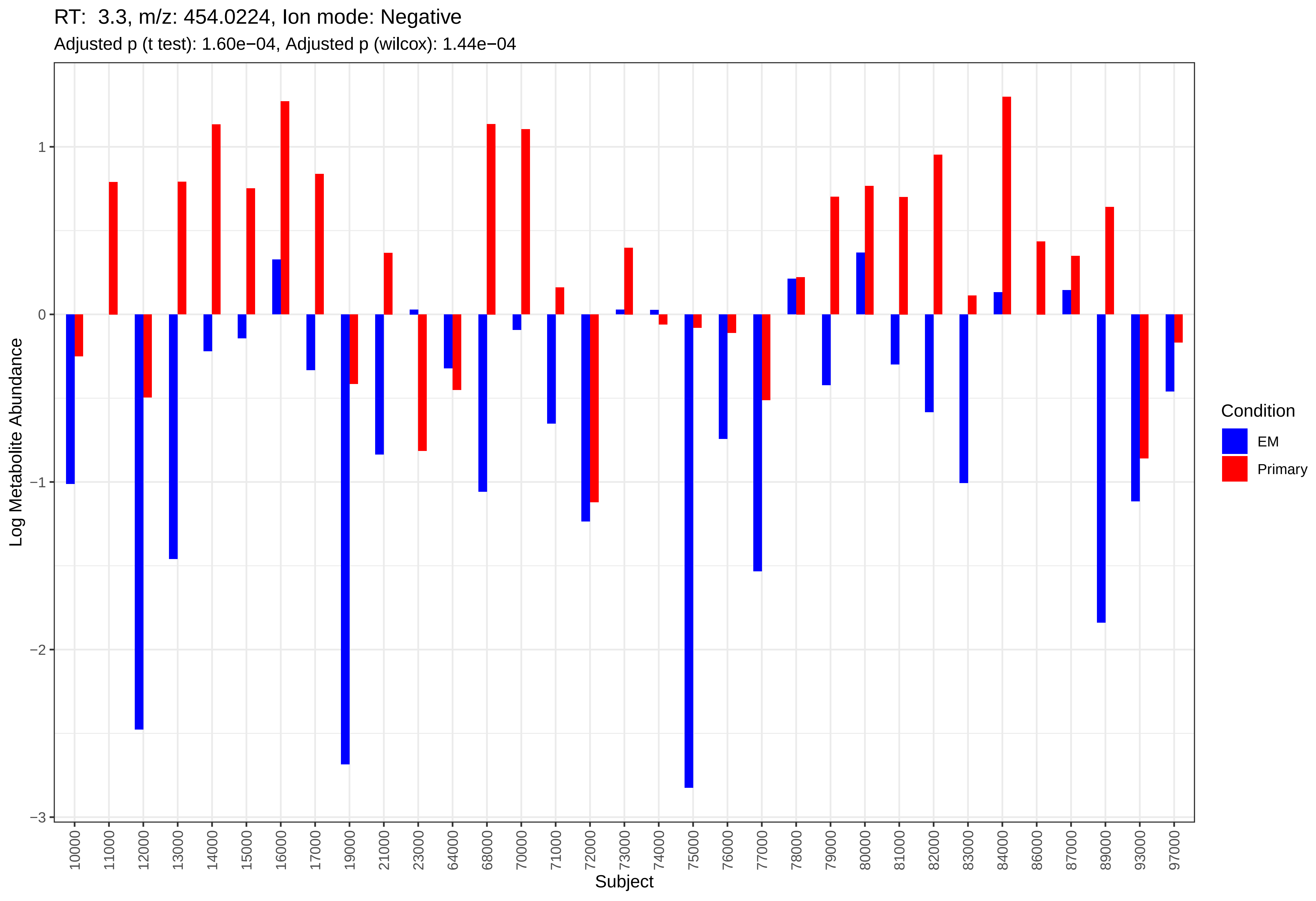

RT: 3.3, m/z: 454.0224, Ion mode: Negative
Adjusted p (t test): 1.60e−04, Adjusted p (wilcox): 1.44e−04
Log Metabolite Abundance
1
0
−1
−2
−3
10000
11000
12000
13000
14000
15000
16000
17000
19000
21000
23000
64000
68000
70000
71000
72000
73000
74000
75000
76000
77000
78000
79000
80000
81000
82000
83000
84000
86000
87000
89000
93000
97000
Subject
Condition
EM
Primary

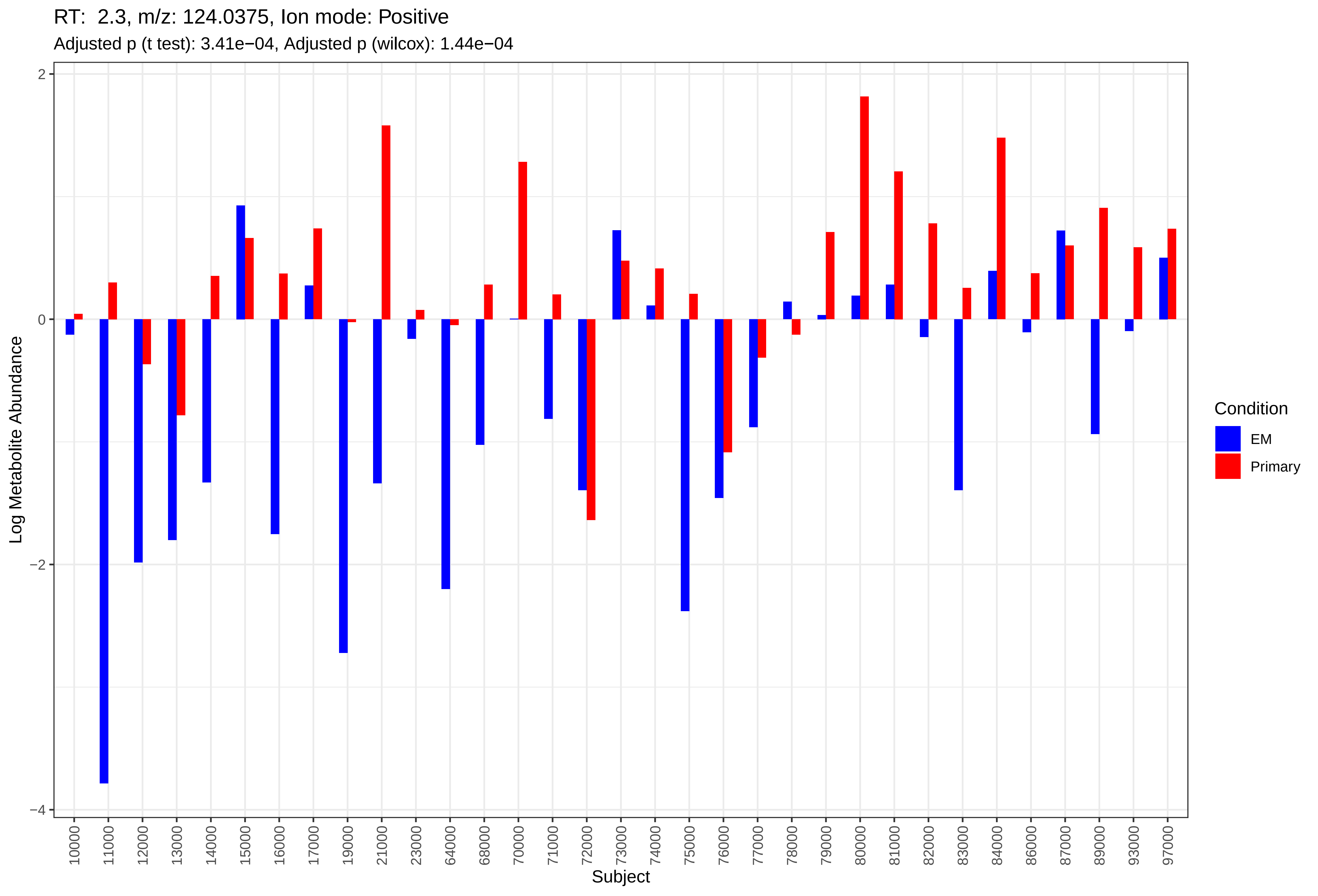

RT: 2.3, m/z: 124.0375, Ion mode: Positive
Adjusted p (t test): 3.41e−04, Adjusted p (wilcox): 1.44e−04
Log Metabolite Abundance
2
0
−2
−4
Subject
10000
11000
12000
13000
14000
15000
16000
17000
19000
21000
23000
64000
68000
70000
71000
72000
73000
74000
75000
76000
77000
78000
79000
80000
81000
82000
83000
84000
86000
87000
89000
93000
97000
Condition
EM
Primary

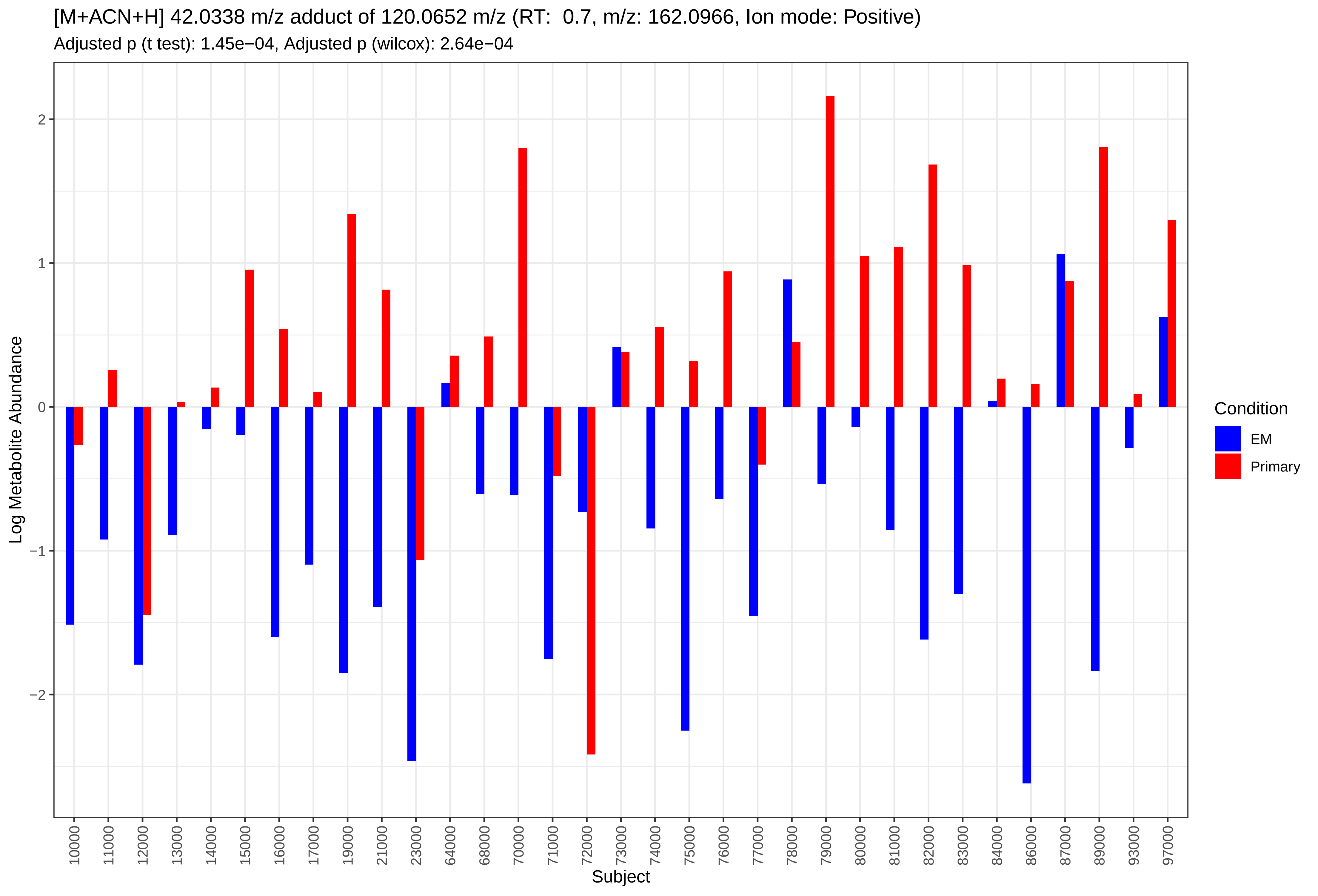

[M+ACN+H] 42.0338 m/z adduct of 120.0652 m/z (RT: 0.7, m/z: 162.0966, Ion mode: Positive)
Adjusted p (t test): 1.45e−04, Adjusted p (wilcox): 2.64e−04
Log Metabolite Abundance
2
1
0
−1
−2
Subject
10000
11000
12000
13000
14000
15000
16000
17000
19000
21000
23000
64000
68000
70000
71000
72000
73000
74000
75000
76000
77000
78000
79000
80000
81000
82000
83000
84000
86000
87000
89000
93000
97000
Condition
EM
Primary

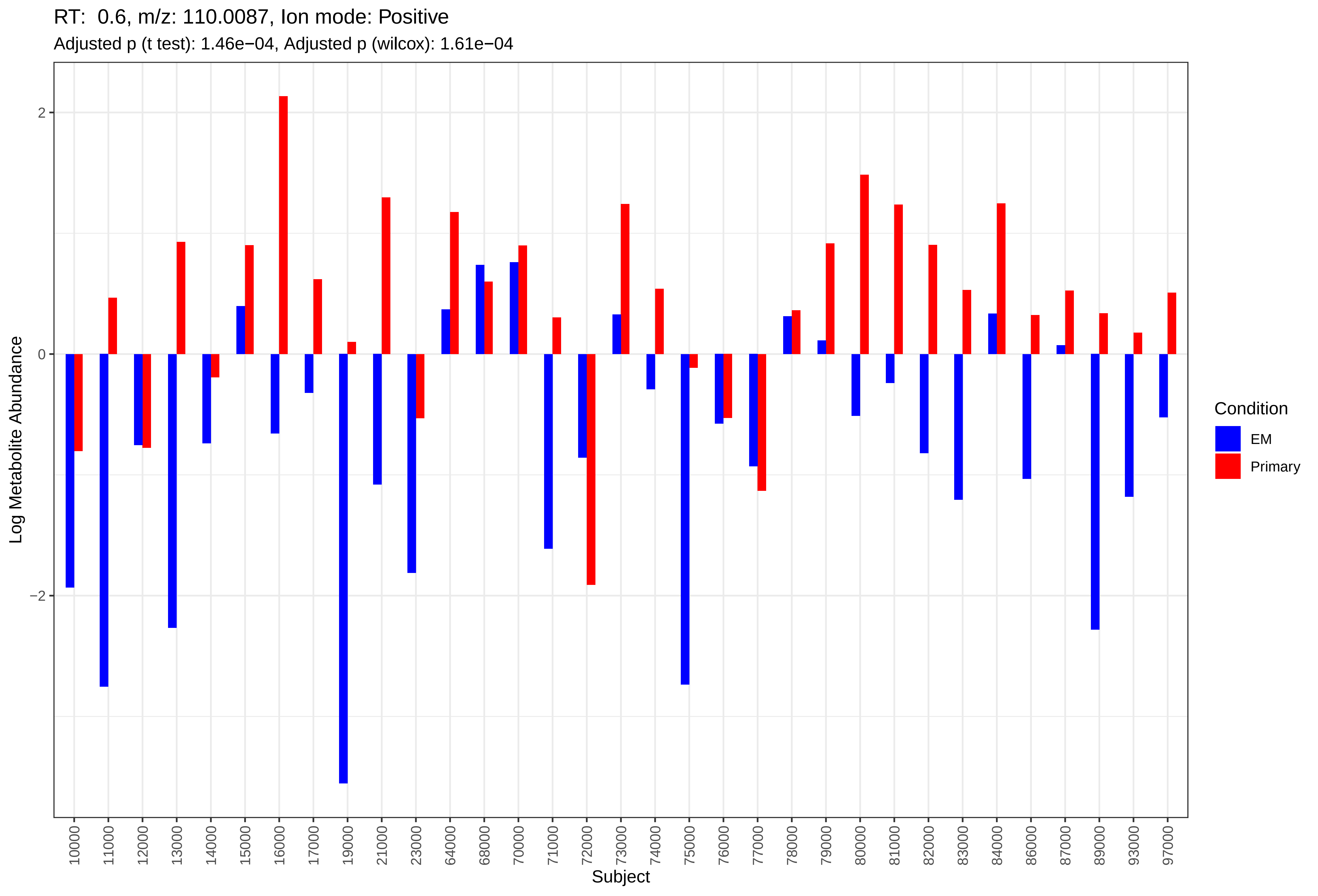

RT: 0.6, m/z: 110.0087, Ion mode: Positive
Adjusted p (t test): 1.46e−04, Adjusted p (wilcox): 1.61e−04
Log Metabolite Abundance
2
0
−2
10000
11000
12000
13000
14000
15000
16000
17000
19000
21000
23000
64000
68000
70000
71000
72000
73000
74000
75000
76000
77000
78000
79000
80000
81000
82000
83000
84000
86000
87000
89000
93000
97000
Subject
Condition
EM
Primary

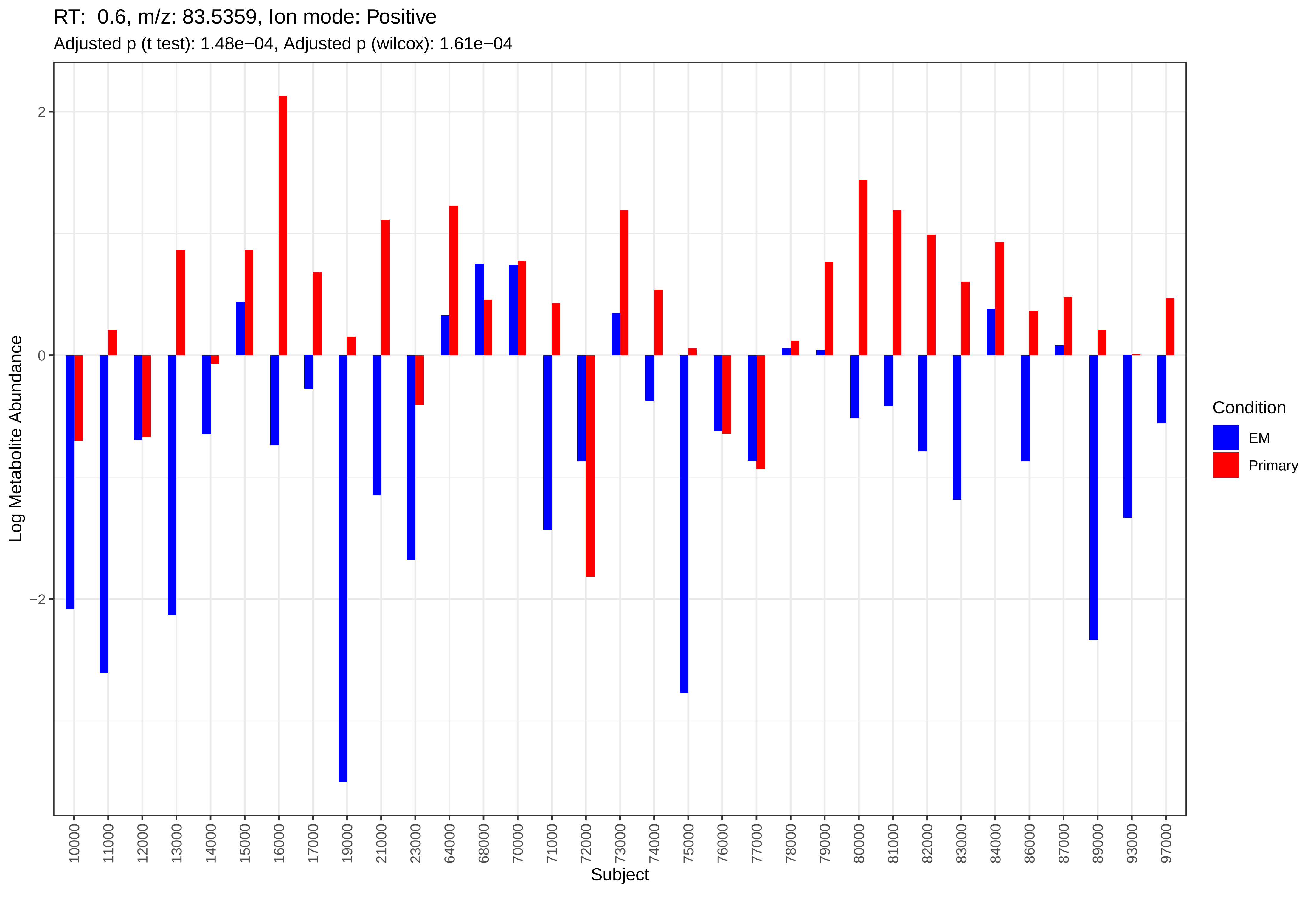
RT: 0.6, m/z: 83.5359, Ion mode: Positive
Adjusted p (t test): 1.48e−04, Adjusted p (wilcox): 1.61e−04
Log Metabolite Abundance
2
0
−2
Subject
10000
11000
12000
13000
14000
15000
16000
17000
19000
21000
23000
64000
68000
70000
71000
72000
73000
74000
75000
76000
77000
78000
79000
80000
81000
82000
83000
84000
86000
87000
89000
93000
97000
Condition
EM
Primary

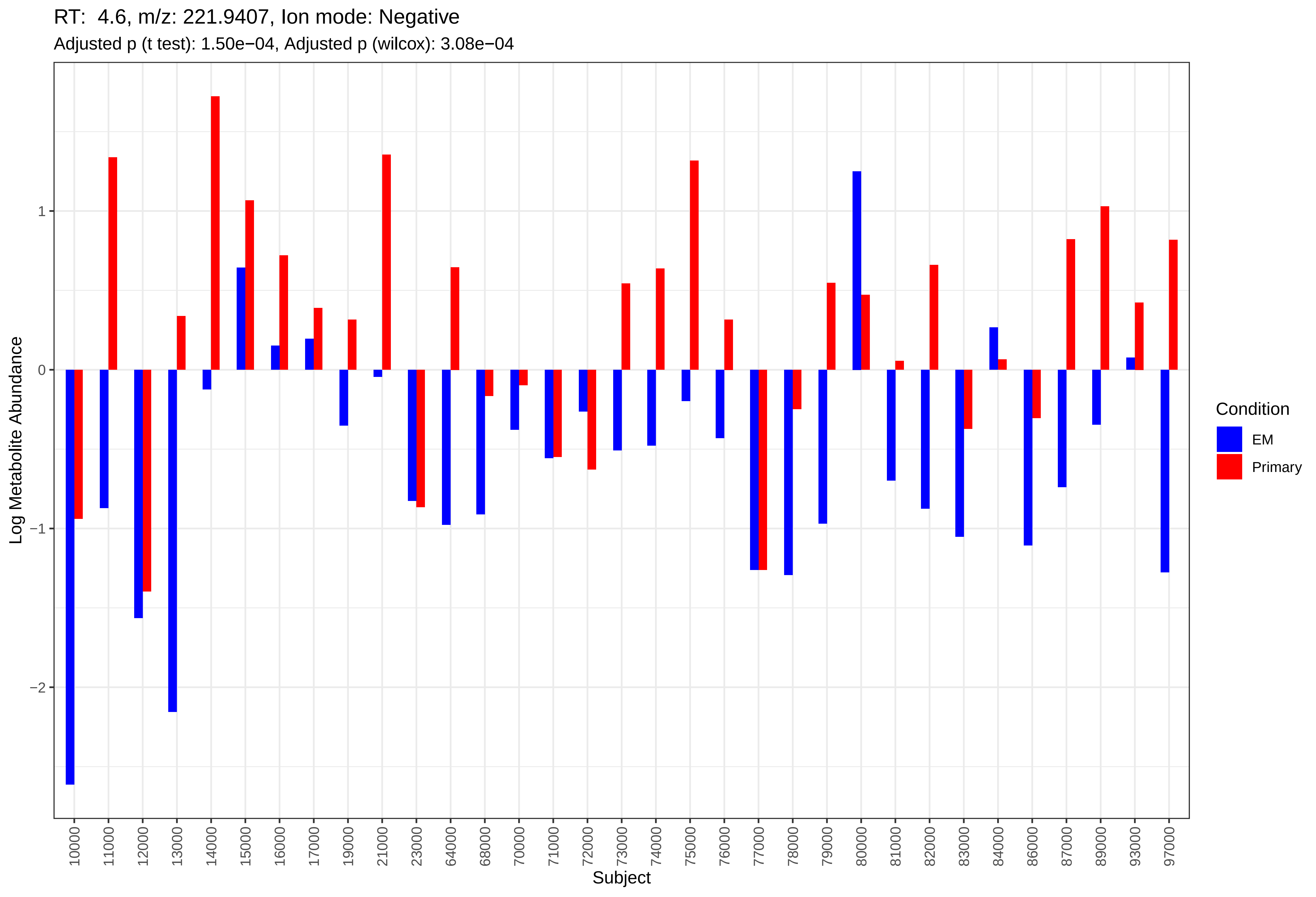
RT: 4.6, m/z: 221.9407, Ion mode: Negative
Adjusted p (t test): 1.50e−04, Adjusted p (wilcox): 3.08e−04
Log Metabolite Abundance
1
0
−1
−2
Subject
10000
11000
12000
13000
14000
15000
16000
17000
19000
21000
23000
64000
68000
70000
71000
72000
73000
74000
75000
76000
77000
78000
79000
80000
81000
82000
83000
84000
86000
87000
89000
93000
97000
Condition
EM
Primary

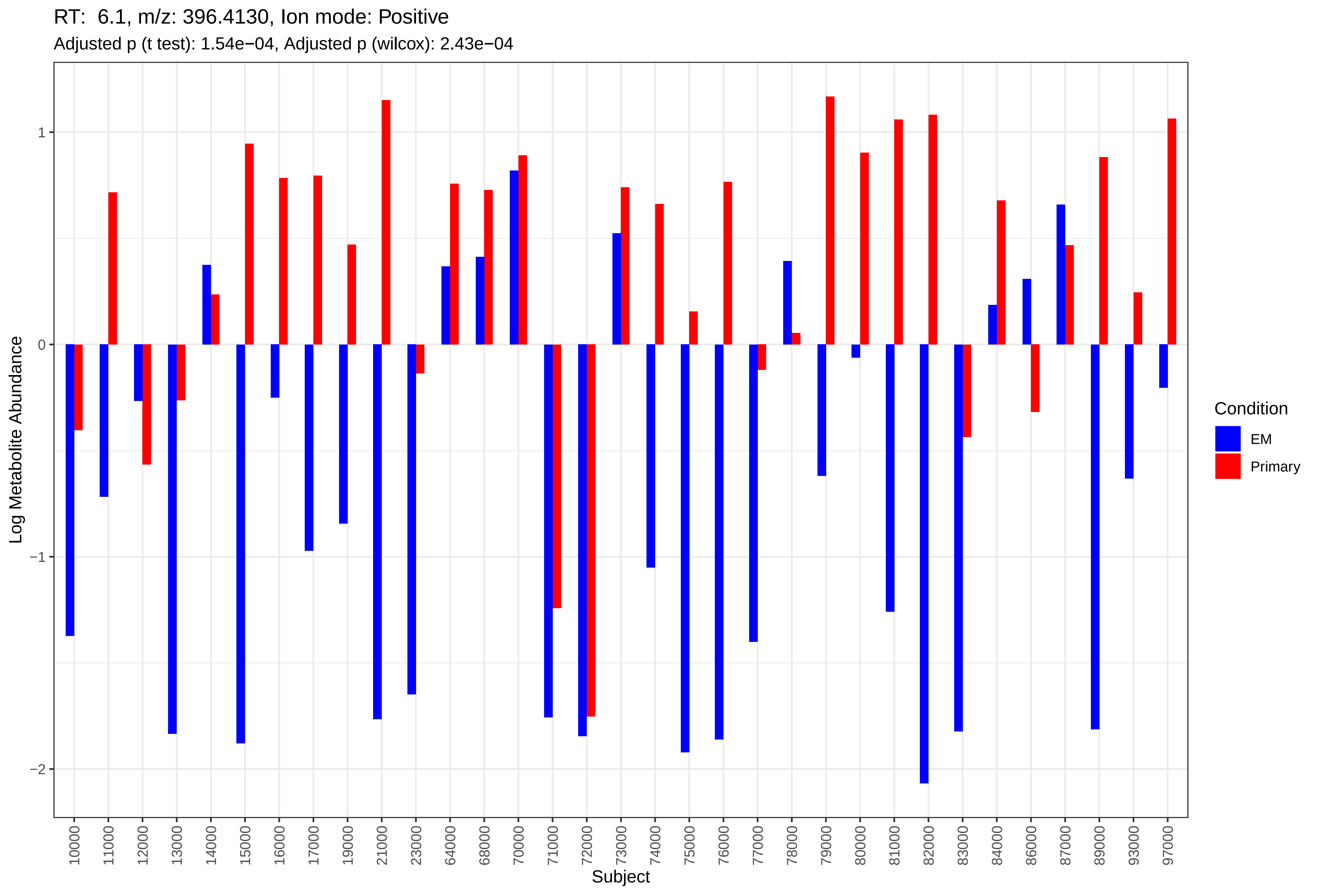
RT: 6.1, m/z: 396.4130, Ion mode: Positive
Adjusted p (t test): 1.54e−04, Adjusted p (wilcox): 2.43e−04
Log Metabolite Abundance
1
0
−1
−2
Subject
10000
11000
12000
13000
14000
15000
16000
17000
19000
21000
23000
64000
68000
70000
71000
72000
73000
74000
75000
76000
77000
78000
79000
80000
81000
82000
83000
84000
86000
87000
89000
93000
97000
Condition
EM
Primary

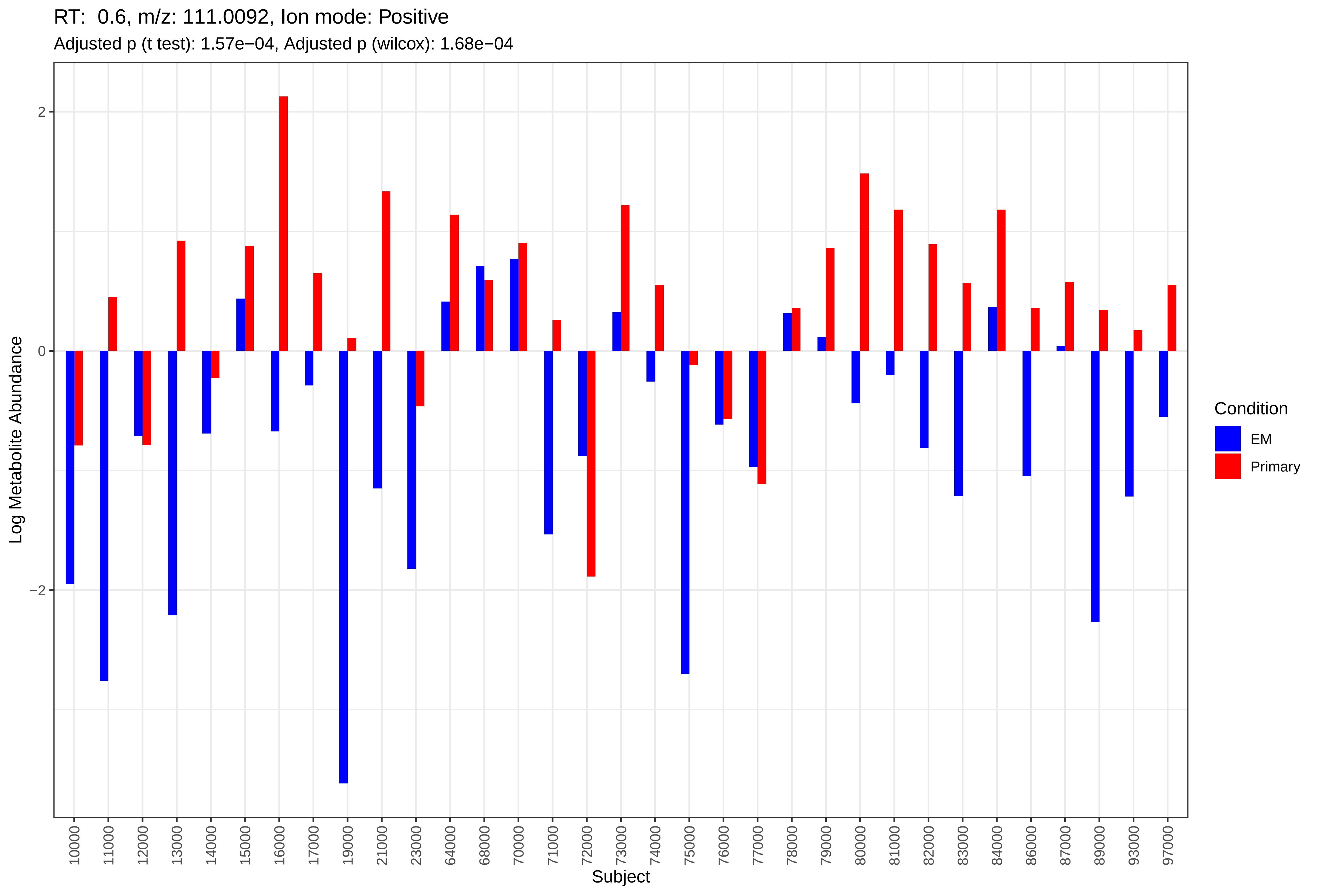
RT: 0.6, m/z: 111.0092, Ion mode: Positive
Adjusted p (t test): 1.57e−04, Adjusted p (wilcox): 1.68e−04
Log Metabolite Abundance
2
0
−2
Subject
10000
11000
12000
13000
14000
15000
16000
17000
19000
21000
23000
64000
68000
70000
71000
72000
73000
74000
75000
76000
77000
78000
79000
80000
81000
82000
83000
84000
86000
87000
89000
93000
97000
Condition
EM
Primary

RT: 7.9, m/z: 331.9877, Ion mode: Positive

Adjusted p (t test): 1.60e−04, Adjusted p (wilcox): 2.64e−04

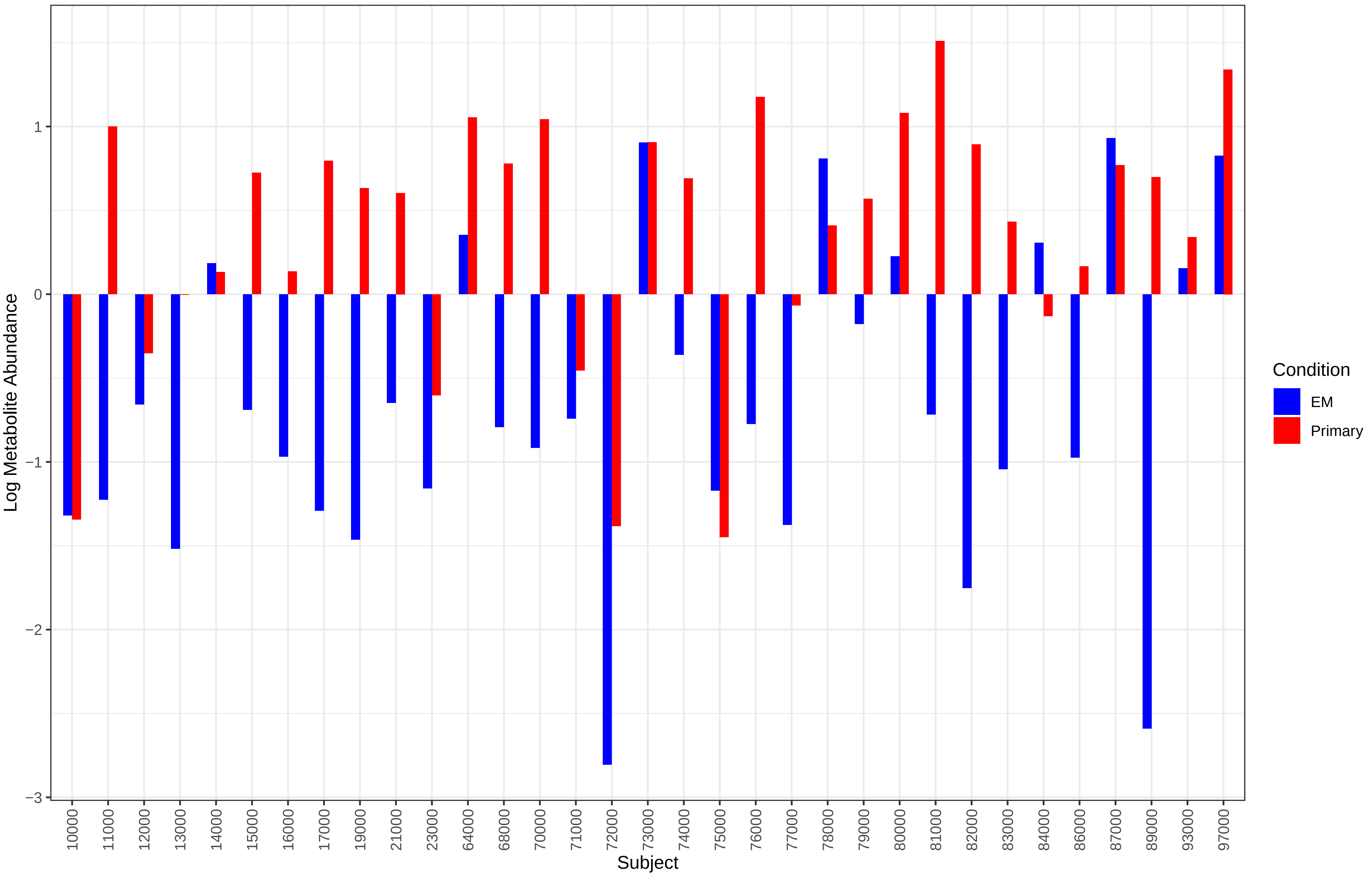

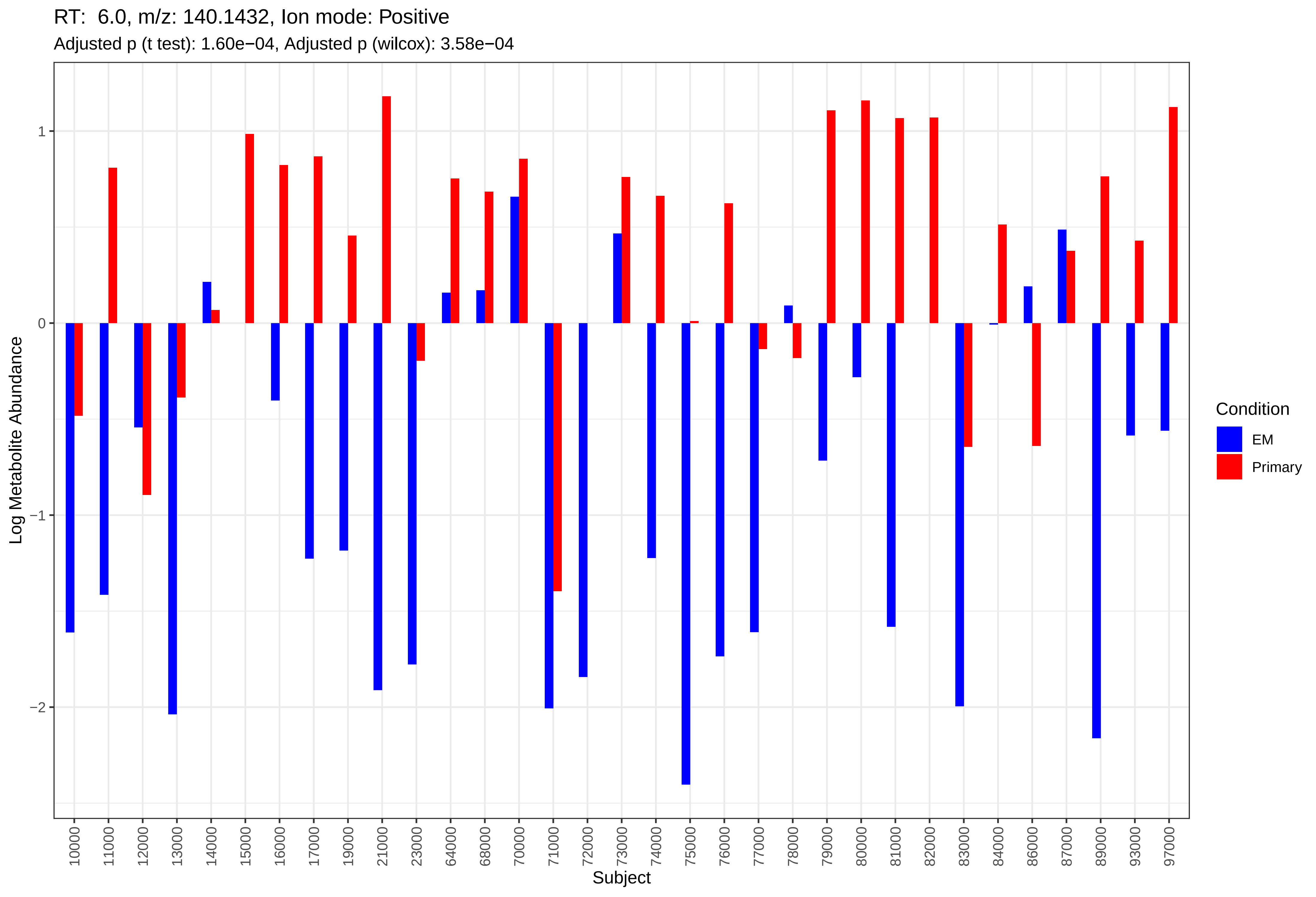

RT: 6.0, m/z: 140.1432, Ion mode: Positive
Adjusted p (t test): 1.60e−04, Adjusted p (wilcox): 3.58e−04
Log Metabolite Abundance
1
0
−1
−2
10000
11000
12000
13000
14000
15000
16000
17000
19000
21000
23000
64000
68000
70000
71000
72000
73000
74000
75000
76000
77000
78000
79000
80000
81000
82000
83000
84000
86000
87000
89000
93000
97000
Subject
Condition
EM
Primary

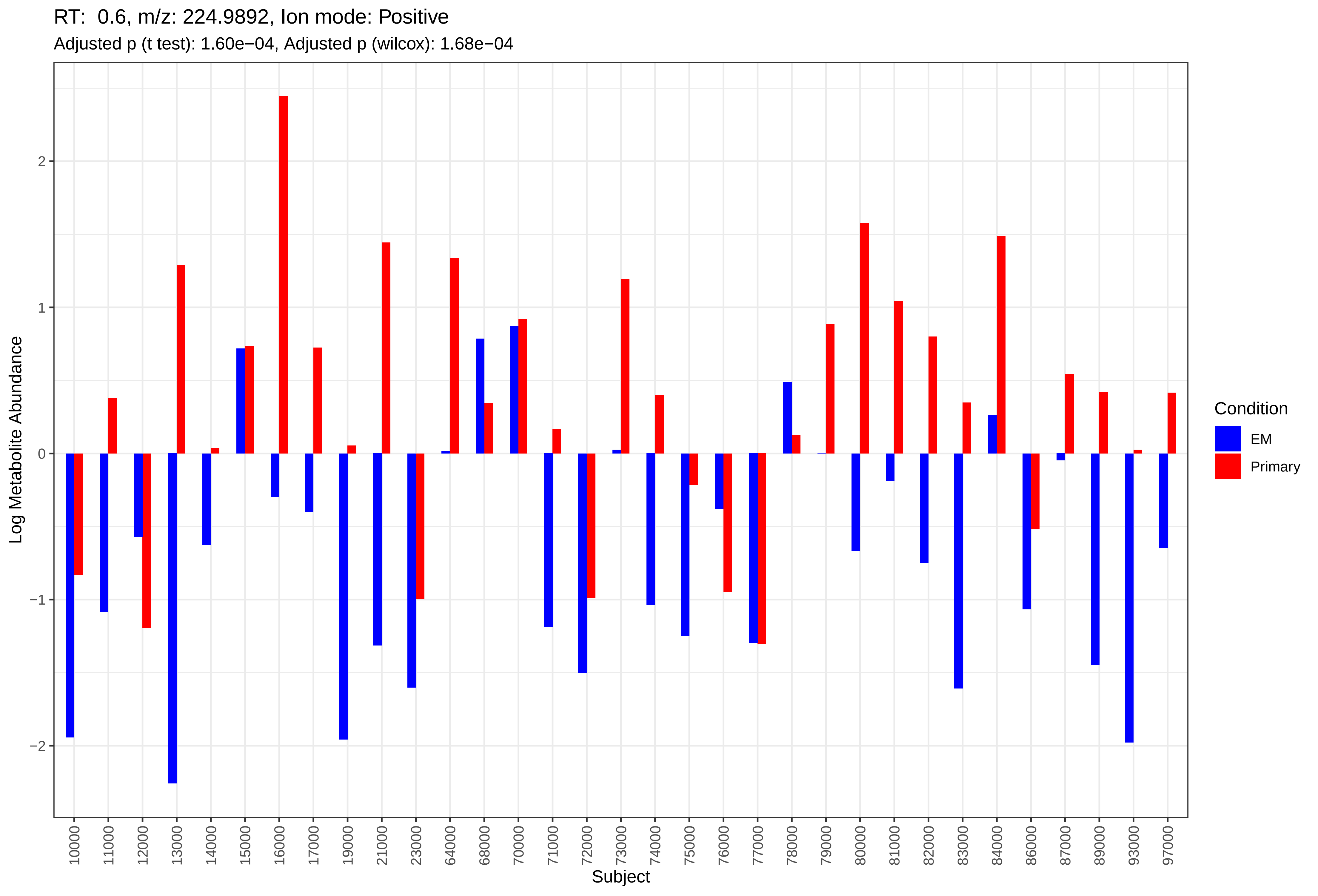
RT: 0.6, m/z: 224.9892, Ion mode: Positive
Adjusted p (t test): 1.60e−04, Adjusted p (wilcox): 1.68e−04
Log Metabolite Abundance
2
1
0
−1
−2
10000
11000
12000
13000
14000
15000
16000
17000
19000
21000
23000
64000
68000
70000
71000
72000
73000
74000
75000
76000
77000
78000
79000
80000
81000
82000
83000
84000
86000
87000
89000
93000
97000
Subject
Condition
EM
Primary

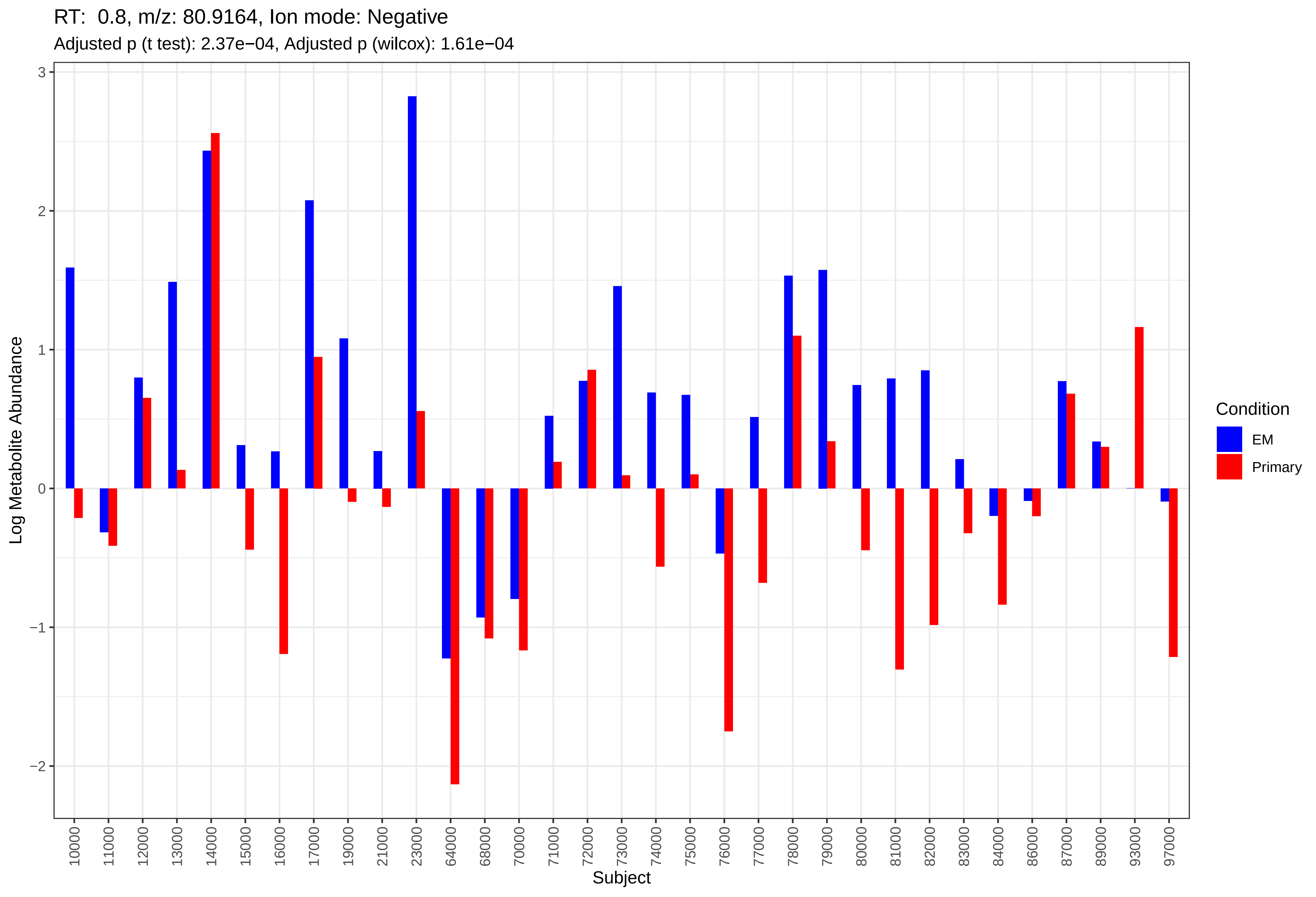

RT: 0.8, m/z: 80.9164, Ion mode: Negative
Adjusted p (t test): 2.37e−04, Adjusted p (wilcox): 1.61e−04
Log Metabolite Abundance
3
2
1
0
−1
−2
Subject
10000
11000
12000
13000
14000
15000
16000
17000
19000
21000
23000
64000
68000
70000
71000
72000
73000
74000
75000
76000
77000
78000
79000
80000
81000
82000
83000
84000
86000
87000
89000
93000
97000
Condition
EM
Primary

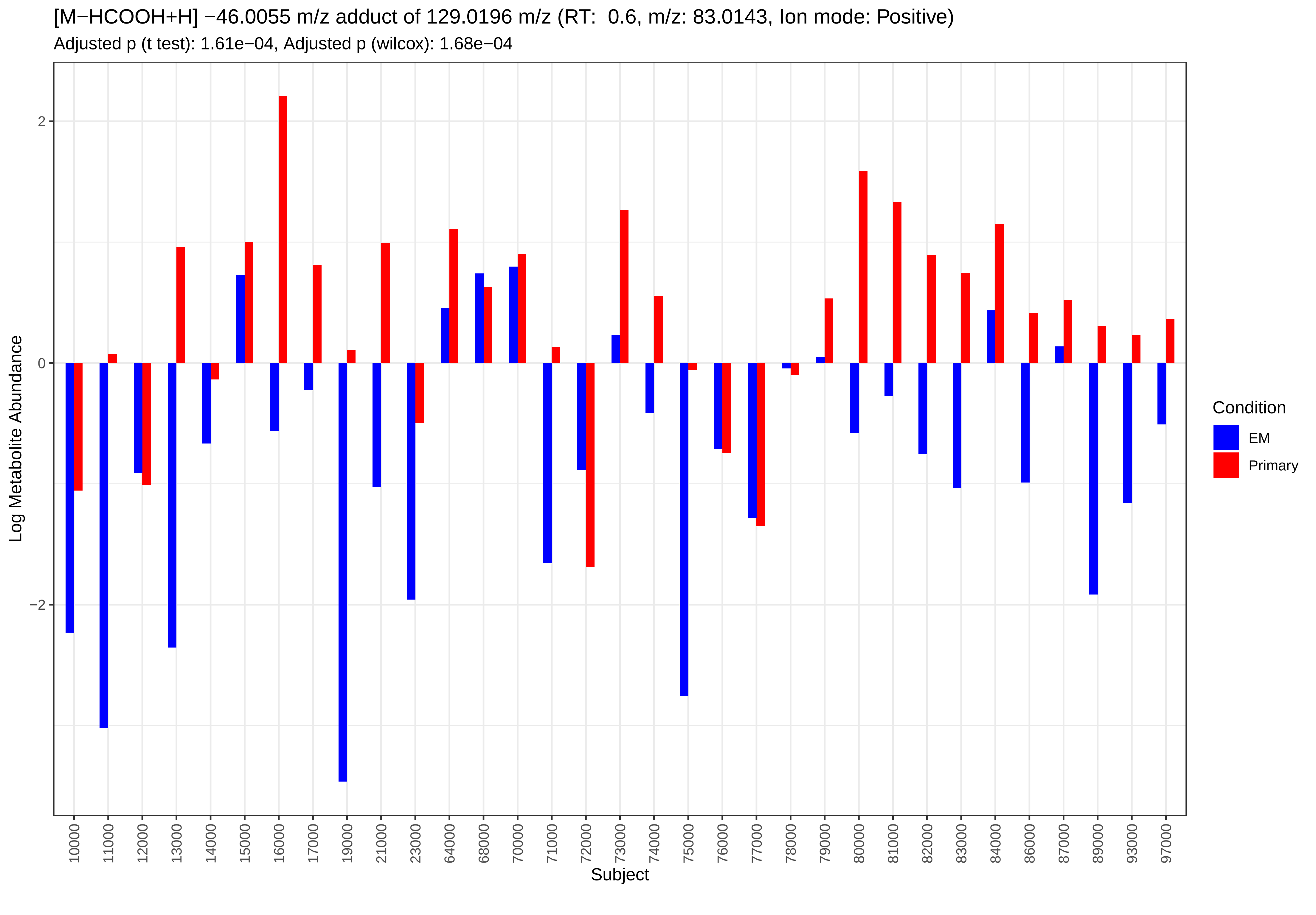

[M−HCOOH+H] −46.0055 m/z adduct of 129.0196 m/z (RT: 0.6, m/z: 83.0143, Ion mode: Positive)
Adjusted p (t test): 1.61e−04, Adjusted p (wilcox): 1.68e−04
Log Metabolite Abundance
2
0
−2
Subject
10000
11000
12000
13000
14000
15000
16000
17000
19000
21000
23000
64000
68000
70000
71000
72000
73000
74000
75000
76000
77000
78000
79000
80000
81000
82000
83000
84000
86000
87000
89000
93000
97000
Condition
EM
Primary

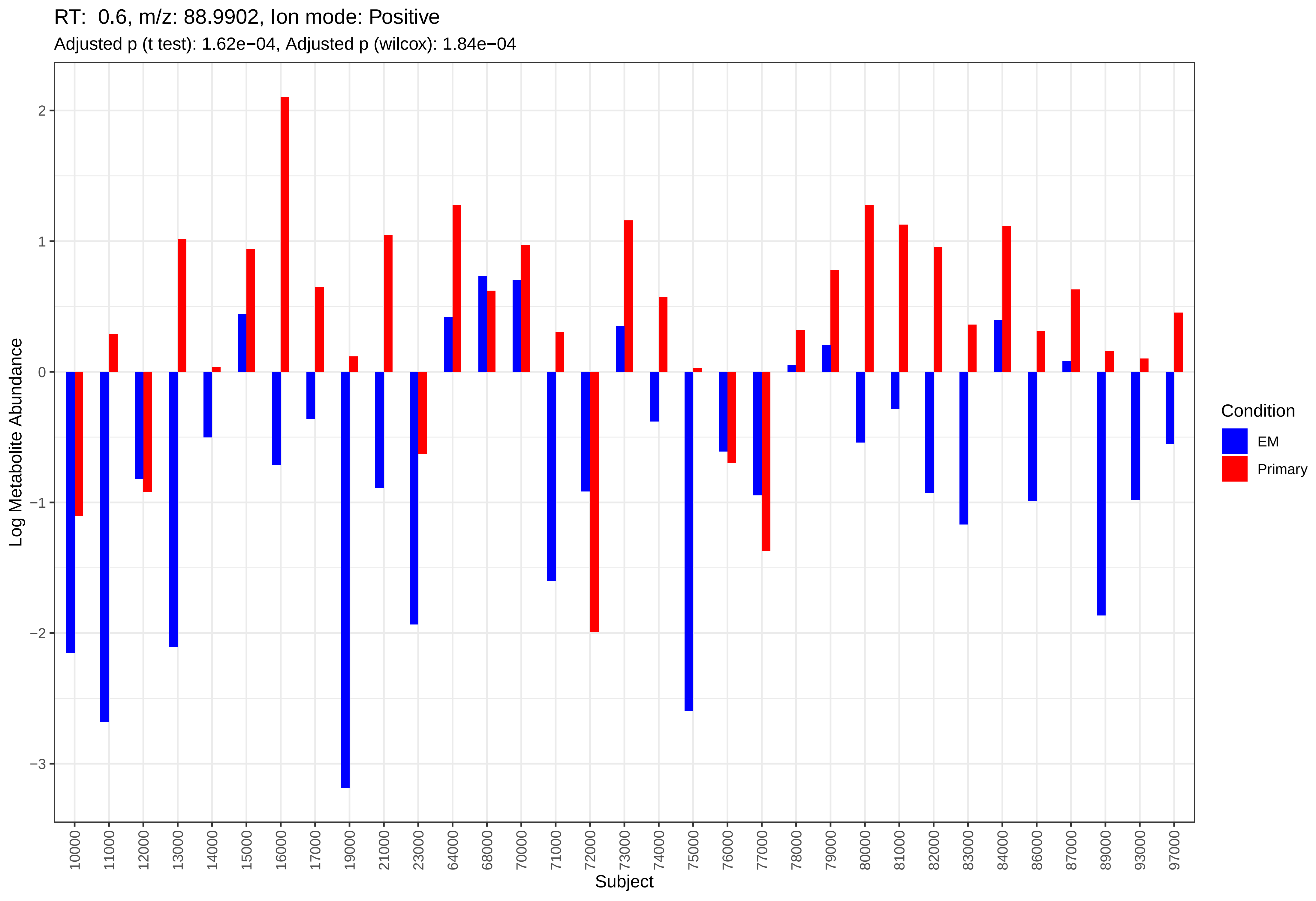

RT: 0.6, m/z: 88.9902, Ion mode: Positive
Adjusted p (t test): 1.62e−04, Adjusted p (wilcox): 1.84e−04
Log Metabolite Abundance
2
1
0
−1
−2
−3
10000
11000
12000
13000
14000
15000
16000
17000
19000
21000
23000
64000
68000
70000
71000
72000
73000
74000
75000
76000
77000
78000
79000
80000
81000
82000
83000
84000
86000
87000
89000
93000
97000
Subject
Condition
EM
Primary

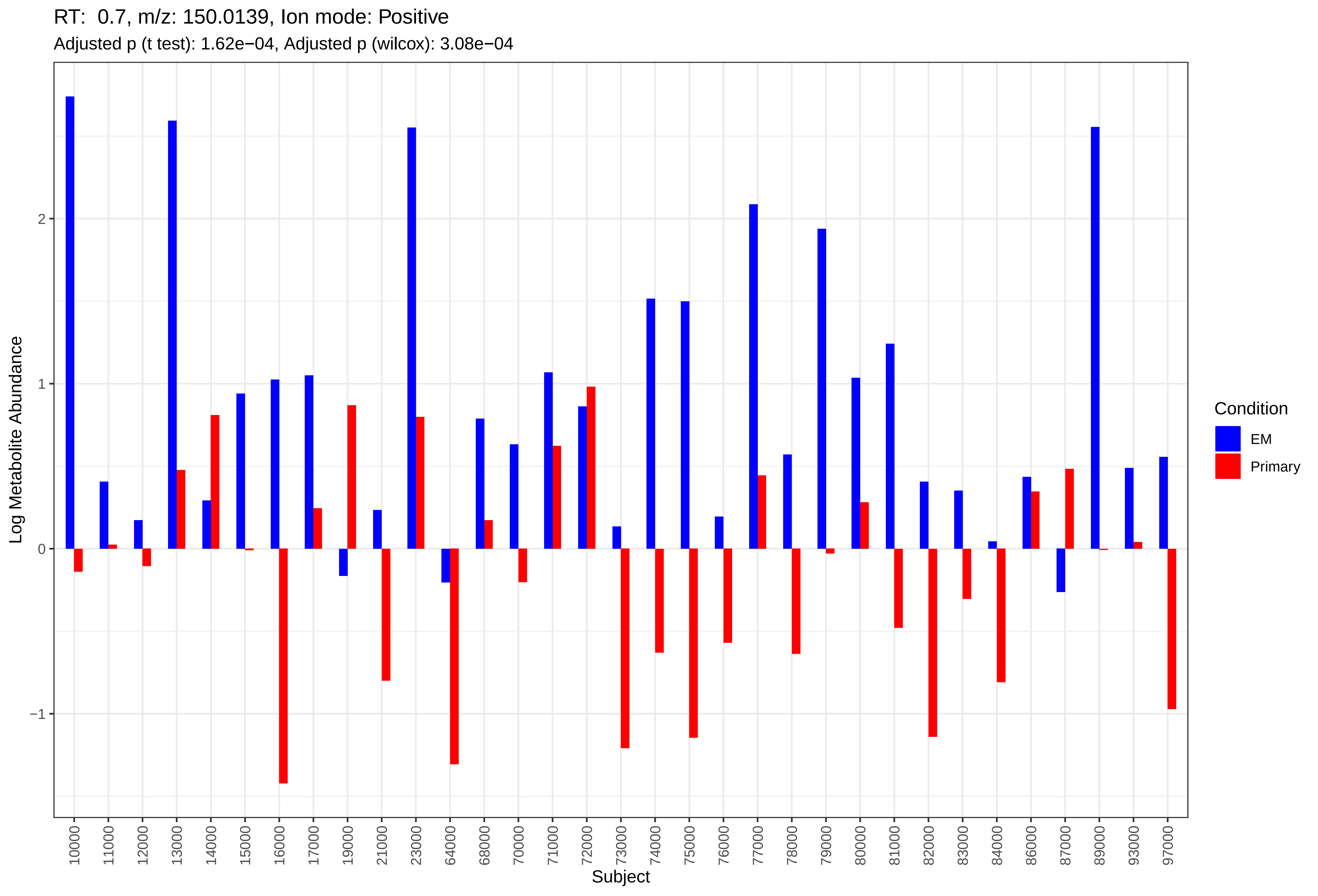
RT: 0.7, m/z: 150.0139, Ion mode: Positive
Adjusted p (t test): 1.62e−04, Adjusted p (wilcox): 3.08e−04
Log Metabolite Abundance
2
1
0
−1
10000
11000
12000
13000
14000
15000
16000
17000
19000
21000
23000
64000
68000
70000
71000
72000
73000
74000
75000
76000
77000
78000
79000
80000
81000
82000
83000
84000
86000
87000
89000
93000
97000
Subject
Condition
EM
Primary

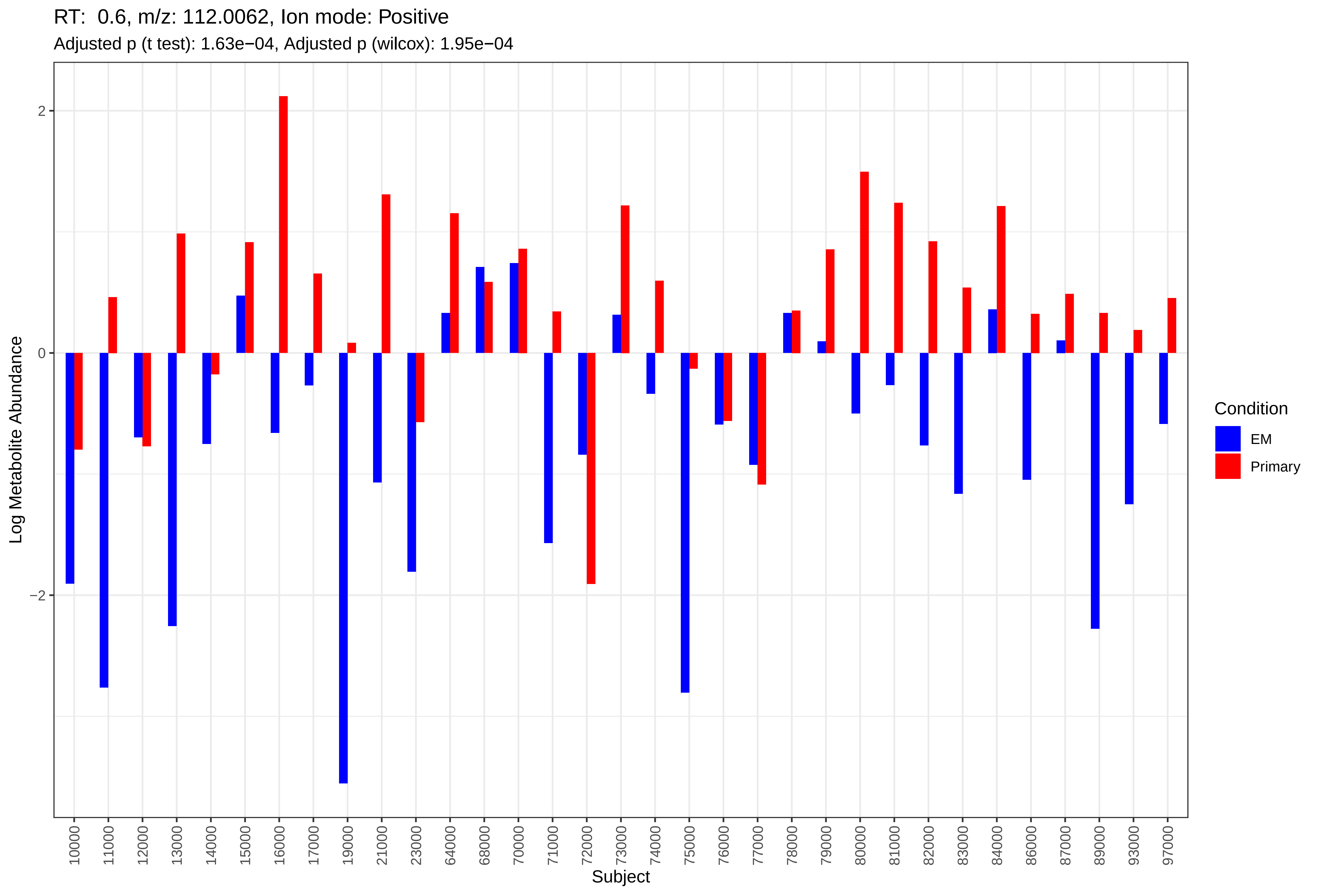

RT: 0.6, m/z: 112.0062, Ion mode: Positive
Adjusted p (t test): 1.63e−04, Adjusted p (wilcox): 1.95e−04
Log Metabolite Abundance
2
0
−2
10000
11000
12000
13000
14000
15000
16000
17000
19000
21000
23000
64000
68000
70000
71000
72000
73000
74000
75000
76000
77000
78000
79000
80000
81000
82000
83000
84000
86000
87000
89000
93000
97000
Subject
Condition
EM
Primary

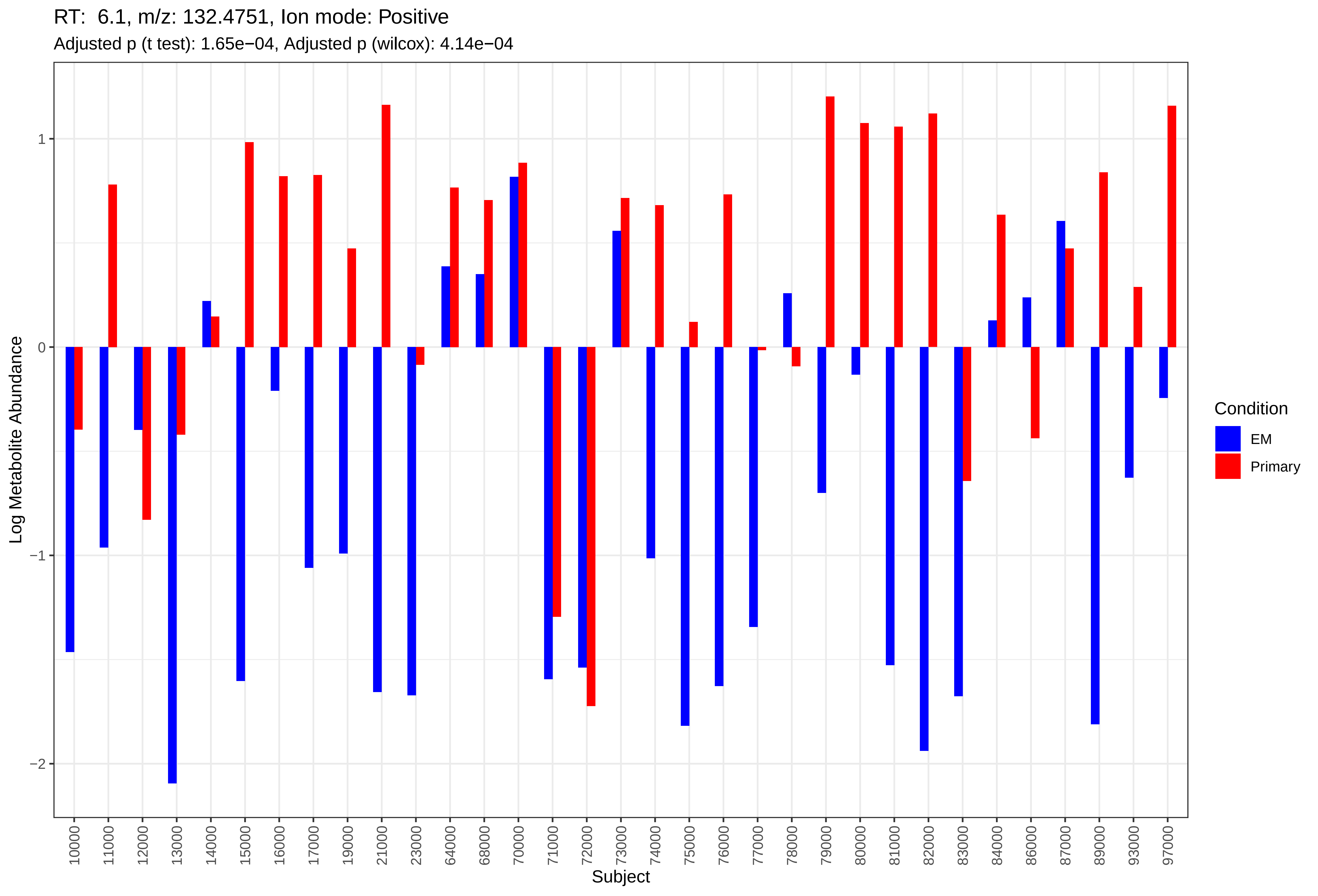
RT: 6.1, m/z: 132.4751, Ion mode: Positive
Adjusted p (t test): 1.65e−04, Adjusted p (wilcox): 4.14e−04
Log Metabolite Abundance
1
0
−1
−2
10000
11000
12000
13000
14000
15000
16000
17000
19000
21000
23000
64000
68000
70000
71000
72000
73000
74000
75000
76000
77000
78000
79000
80000
81000
82000
83000
84000
86000
87000
89000
93000
97000
Subject
Condition
EM
Primary

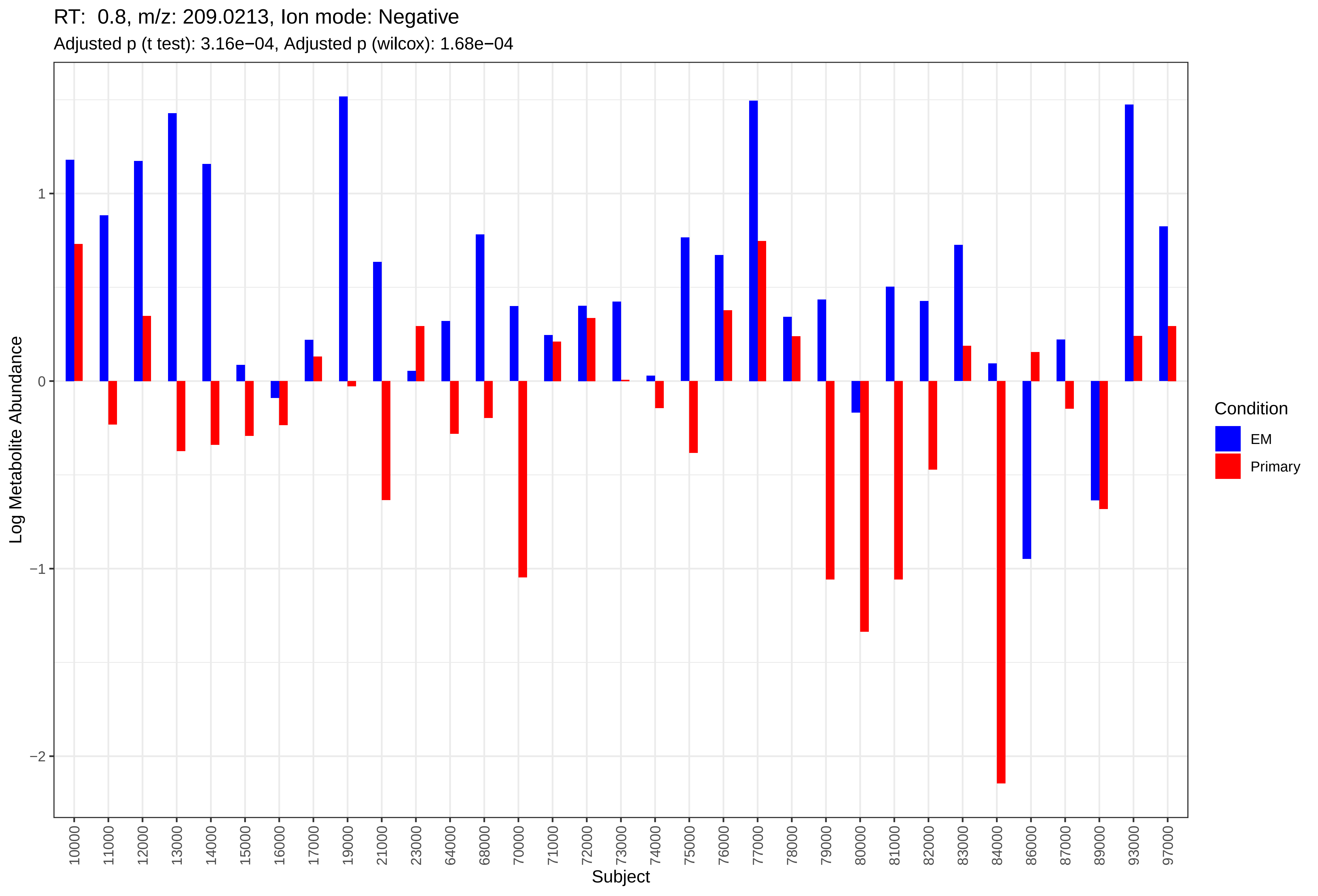

RT: 0.8, m/z: 209.0213, Ion mode: Negative
Adjusted p (t test): 3.16e−04, Adjusted p (wilcox): 1.68e−04
Log Metabolite Abundance
1
0
−1
−2
Subject
10000
11000
12000
13000
14000
15000
16000
17000
19000
21000
23000
64000
68000
70000
71000
72000
73000
74000
75000
76000
77000
78000
79000
80000
81000
82000
83000
84000
86000
87000
89000
93000
97000
Condition
EM
Primary

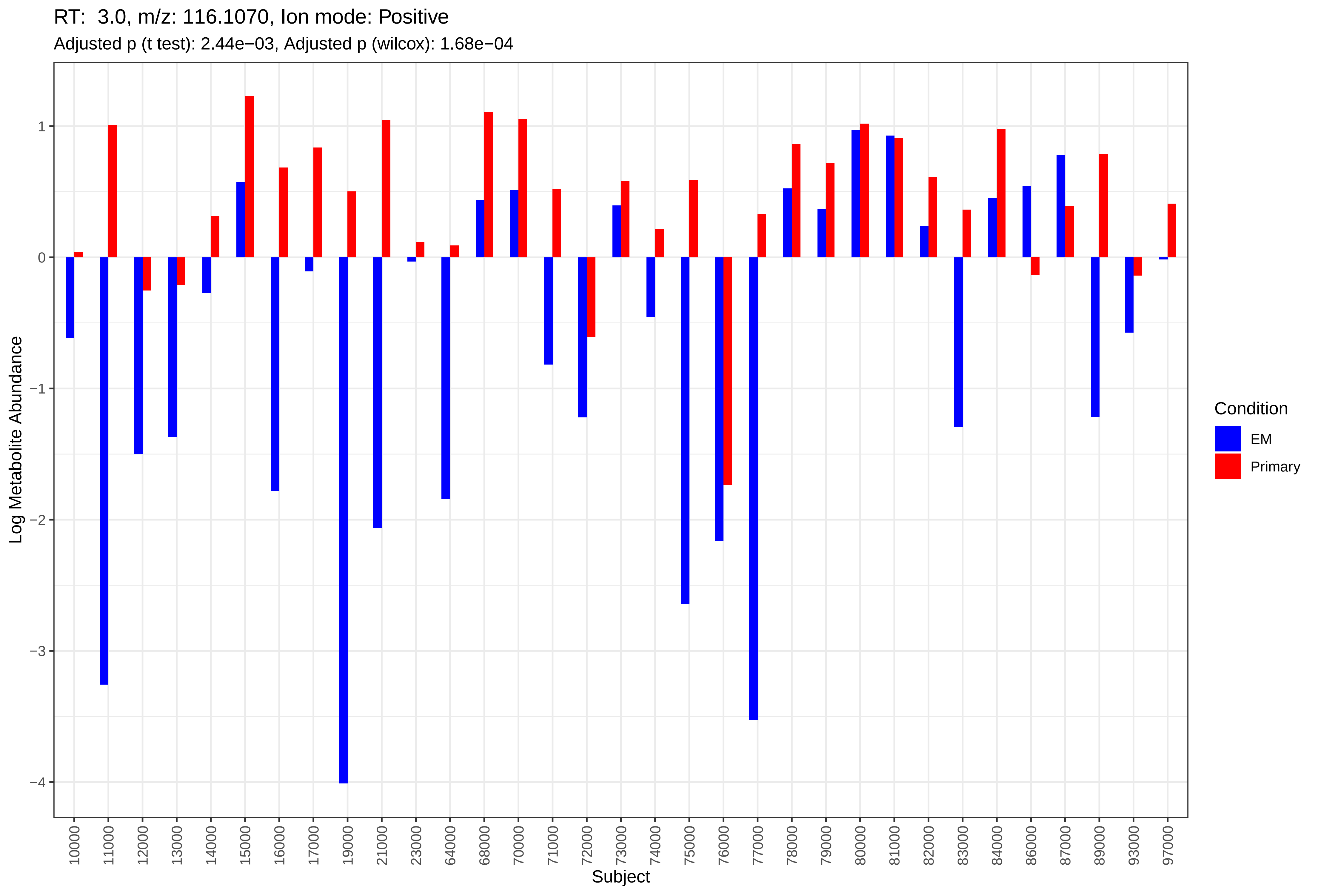
RT: 3.0, m/z: 116.1070, Ion mode: Positive
Adjusted p (t test): 2.44e−03, Adjusted p (wilcox): 1.68e−04
Log Metabolite Abundance
1
0
−1
−2
−3
−4
Subject
10000
11000
12000
13000
14000
15000
16000
17000
19000
21000
23000
64000
68000
70000
71000
72000
73000
74000
75000
76000
77000
78000
79000
80000
81000
82000
83000
84000
86000
87000
89000
93000
97000
Condition
EM
Primary

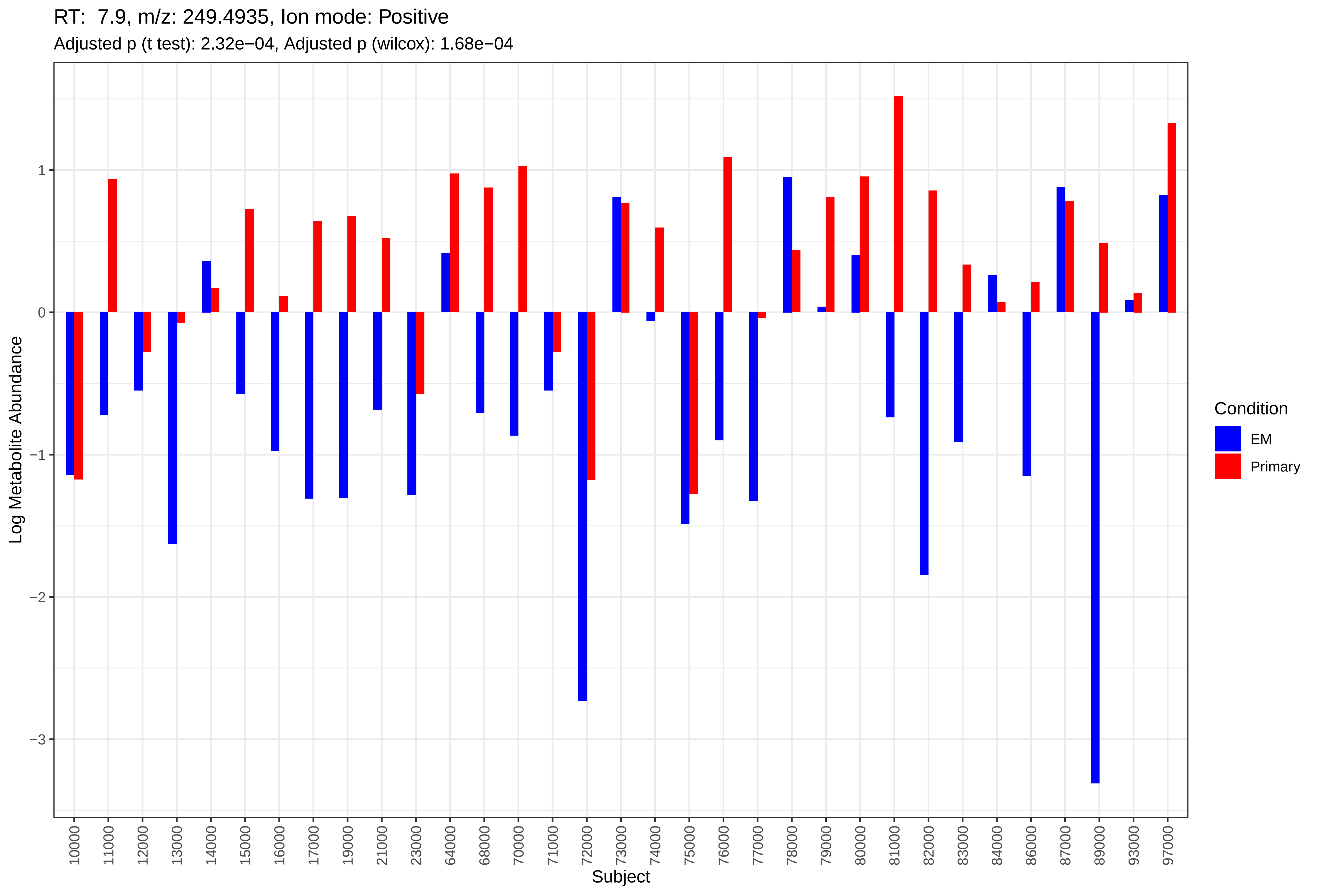
RT: 7.9, m/z: 249.4935, Ion mode: Positive
Adjusted p (t test): 2.32e−04, Adjusted p (wilcox): 1.68e−04
Log Metabolite Abundance
1
0
−1
−2
−3
10000
11000
12000
13000
14000
15000
16000
17000
19000
21000
23000
64000
68000
70000
71000
72000
73000
74000
75000
76000
77000
78000
79000
80000
81000
82000
83000
84000
86000
87000
89000
93000
97000
Subject
Condition
EM
Primary

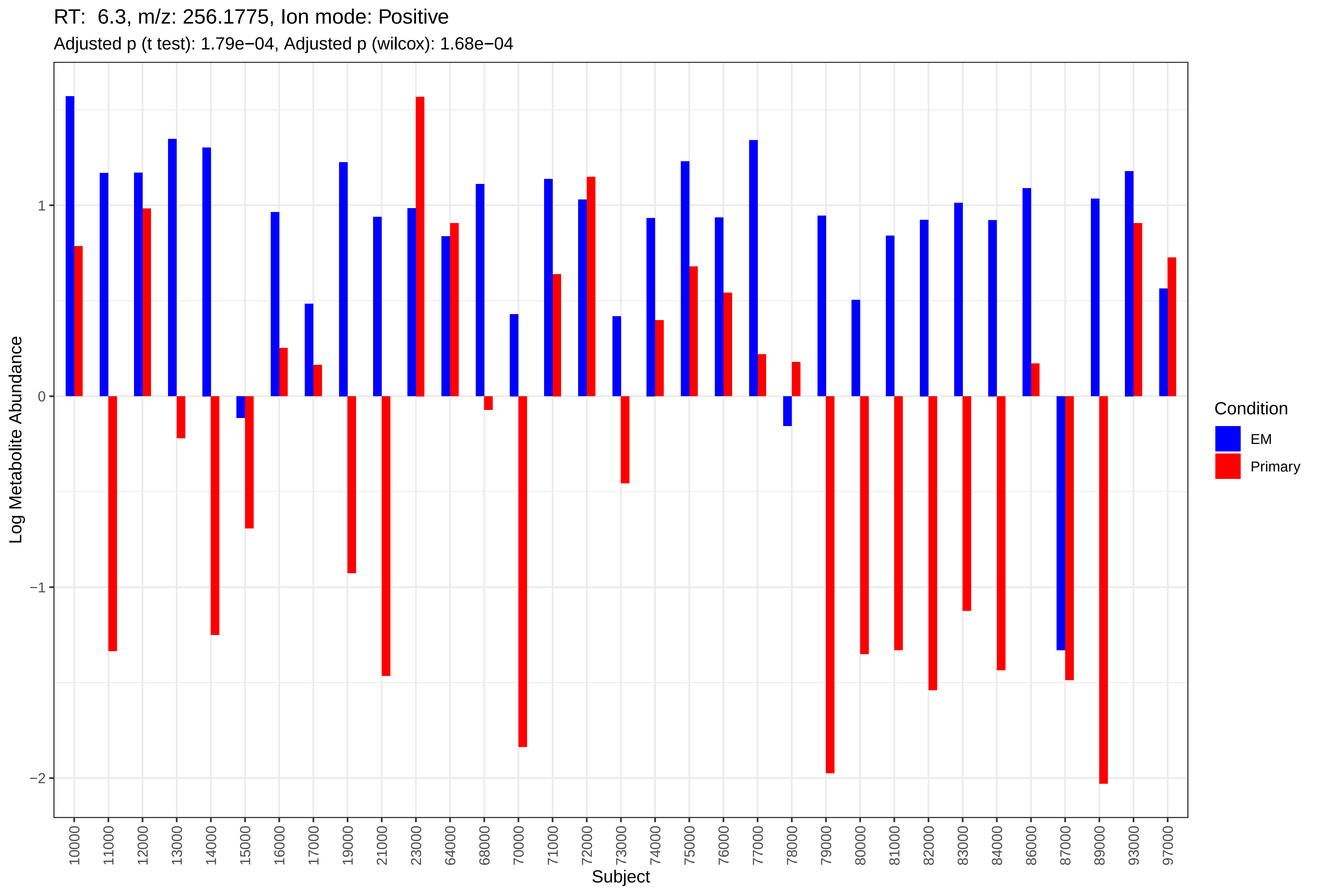

RT: 6.3, m/z: 256.1775, Ion mode: Positive
Adjusted p (t test): 1.79e−04, Adjusted p (wilcox): 1.68e−04
Log Metabolite Abundance
1
0
−1
−2
Subject
10000
11000
12000
13000
14000
15000
16000
17000
19000
21000
23000
64000
68000
70000
71000
72000
73000
74000
75000
76000
77000
78000
79000
80000
81000
82000
83000
84000
86000
87000
89000
93000
97000
Condition
EM
Primary

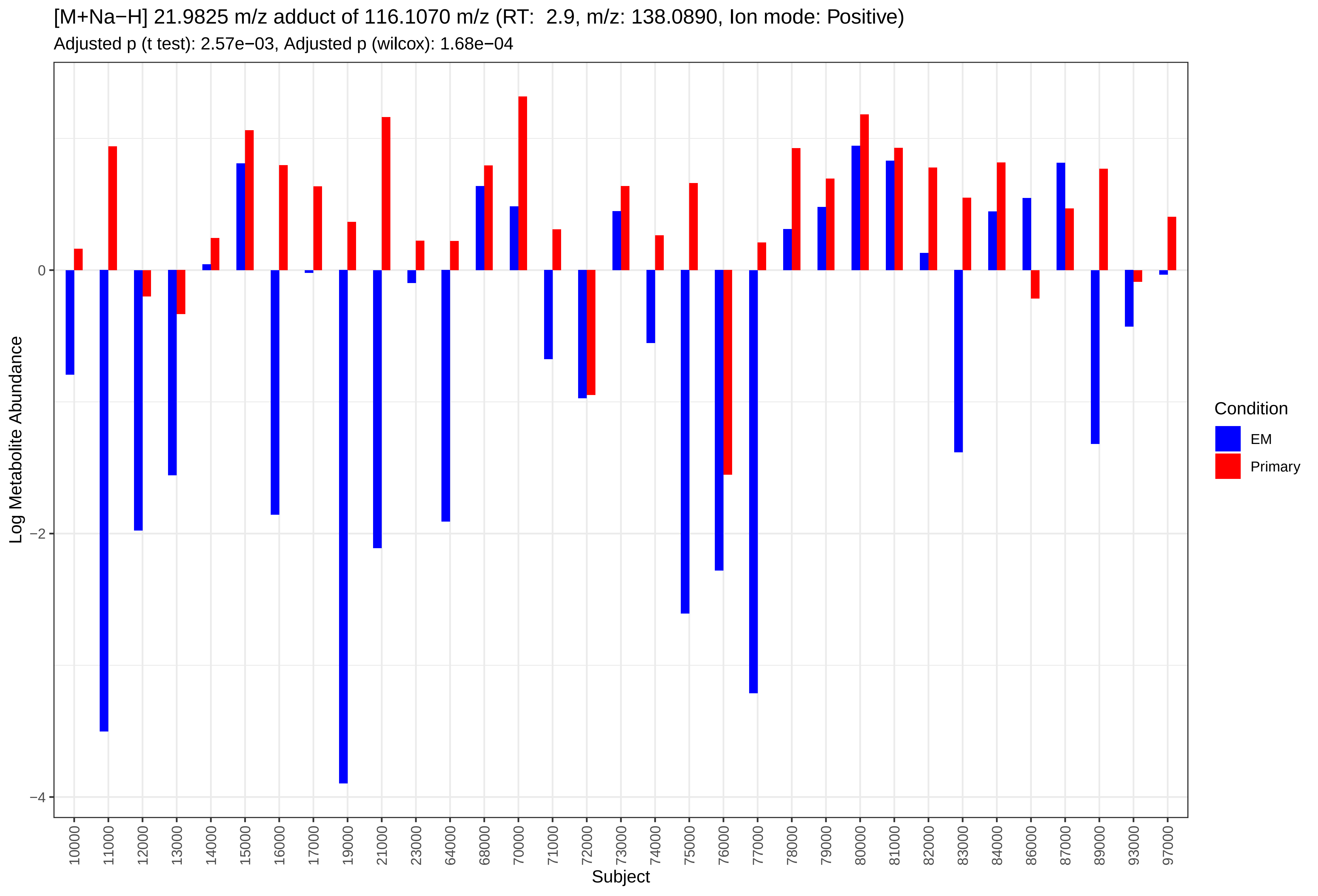
[M+Na−H] 21.9825 m/z adduct of 116.1070 m/z (RT: 2.9, m/z: 138.0890, Ion mode: Positive)
Adjusted p (t test): 2.57e−03, Adjusted p (wilcox): 1.68e−04
Log Metabolite Abundance
0
−2
−4
Subject
10000
11000
12000
13000
14000
15000
16000
17000
19000
21000
23000
64000
68000
70000
71000
72000
73000
74000
75000
76000
77000
78000
79000
80000
81000
82000
83000
84000
86000
87000
89000
93000
97000
Condition
EM
Primary

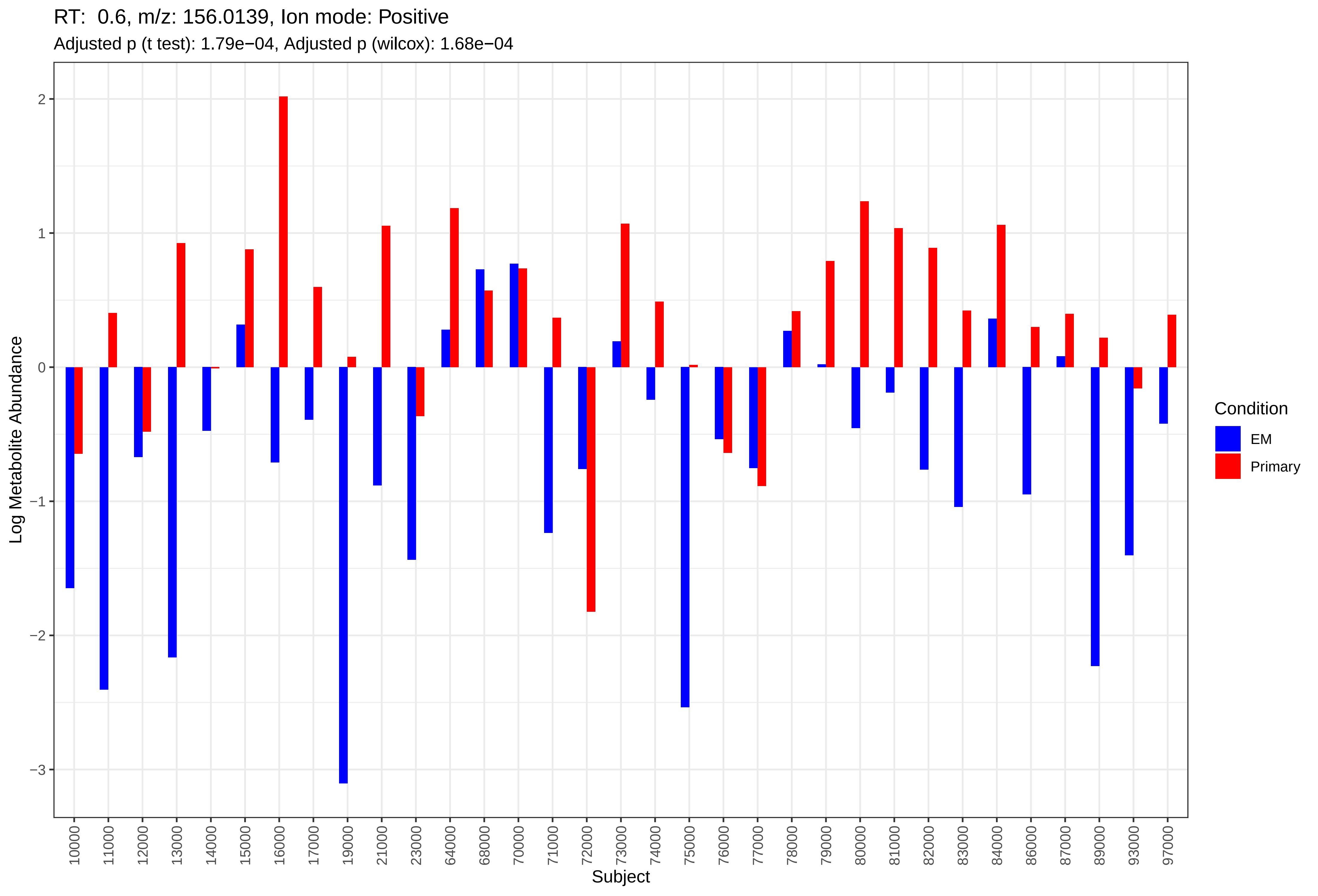

RT: 0.6, m/z: 156.0139, Ion mode: Positive
Adjusted p (t test): 1.79e−04, Adjusted p (wilcox): 1.68e−04
Log Metabolite Abundance
2
1
0
−1
−2
−3
Subject
10000
11000
12000
13000
14000
15000
16000
17000
19000
21000
23000
64000
68000
70000
71000
72000
73000
74000
75000
76000
77000
78000
79000
80000
81000
82000
83000
84000
86000
87000
89000
93000
97000
Condition
EM
Primary

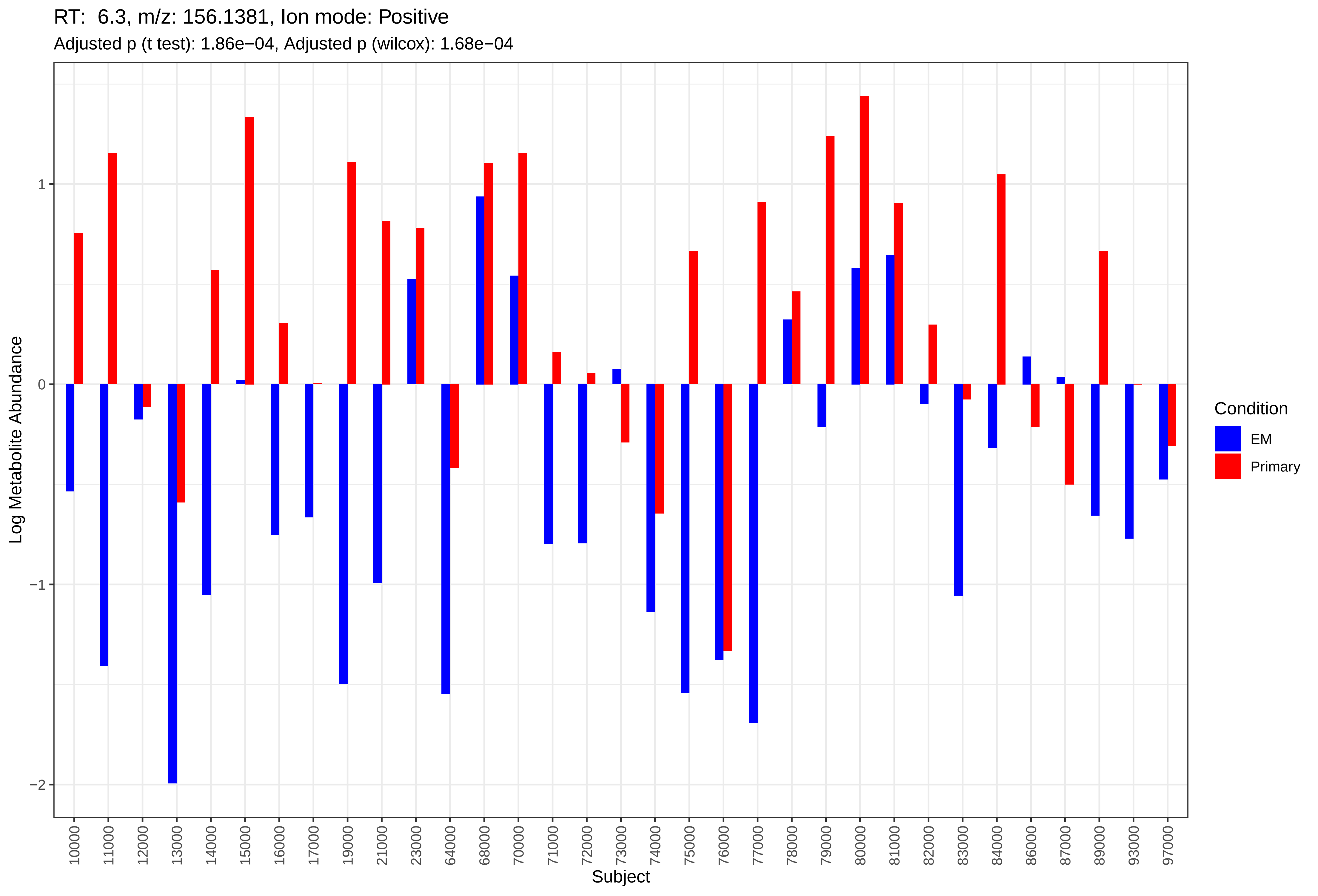

RT: 6.3, m/z: 156.1381, Ion mode: Positive
Adjusted p (t test): 1.86e−04, Adjusted p (wilcox): 1.68e−04
Log Metabolite Abundance
1
0
−1
−2
10000
11000
12000
13000
14000
15000
16000
17000
19000
21000
23000
64000
68000
70000
71000
72000
73000
74000
75000
76000
77000
78000
79000
80000
81000
82000
83000
84000
86000
87000
89000
93000
97000
Subject
Condition
EM
Primary

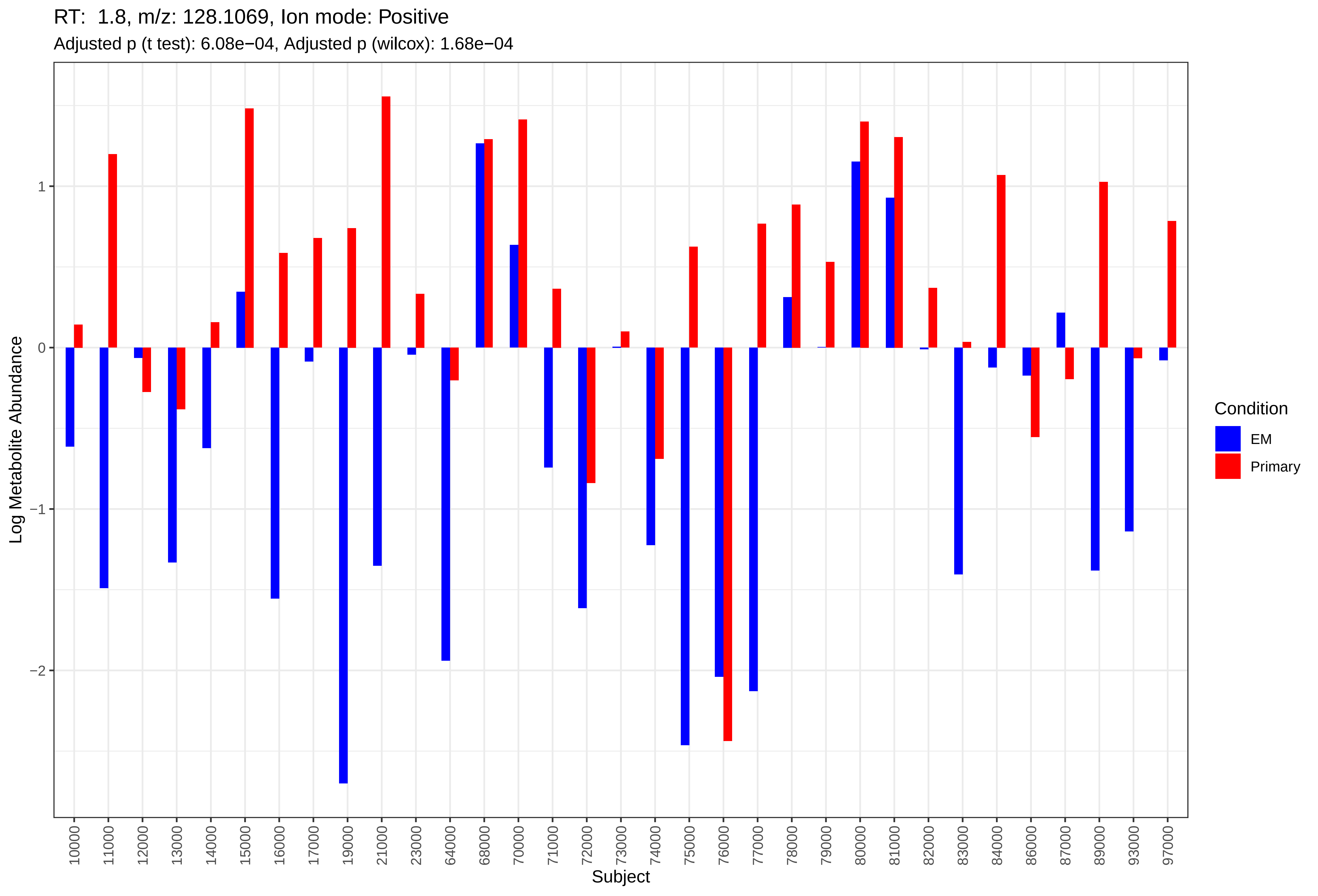

RT: 1.8, m/z: 128.1069, Ion mode: Positive
Adjusted p (t test): 6.08e−04, Adjusted p (wilcox): 1.68e−04
Log Metabolite Abundance
1
0
−1
−2
10000
11000
12000
13000
14000
15000
16000
17000
19000
21000
23000
64000
68000
70000
71000
72000
73000
74000
75000
76000
77000
78000
79000
80000
81000
82000
83000
84000
86000
87000
89000
93000
97000
Subject
Condition
EM
Primary

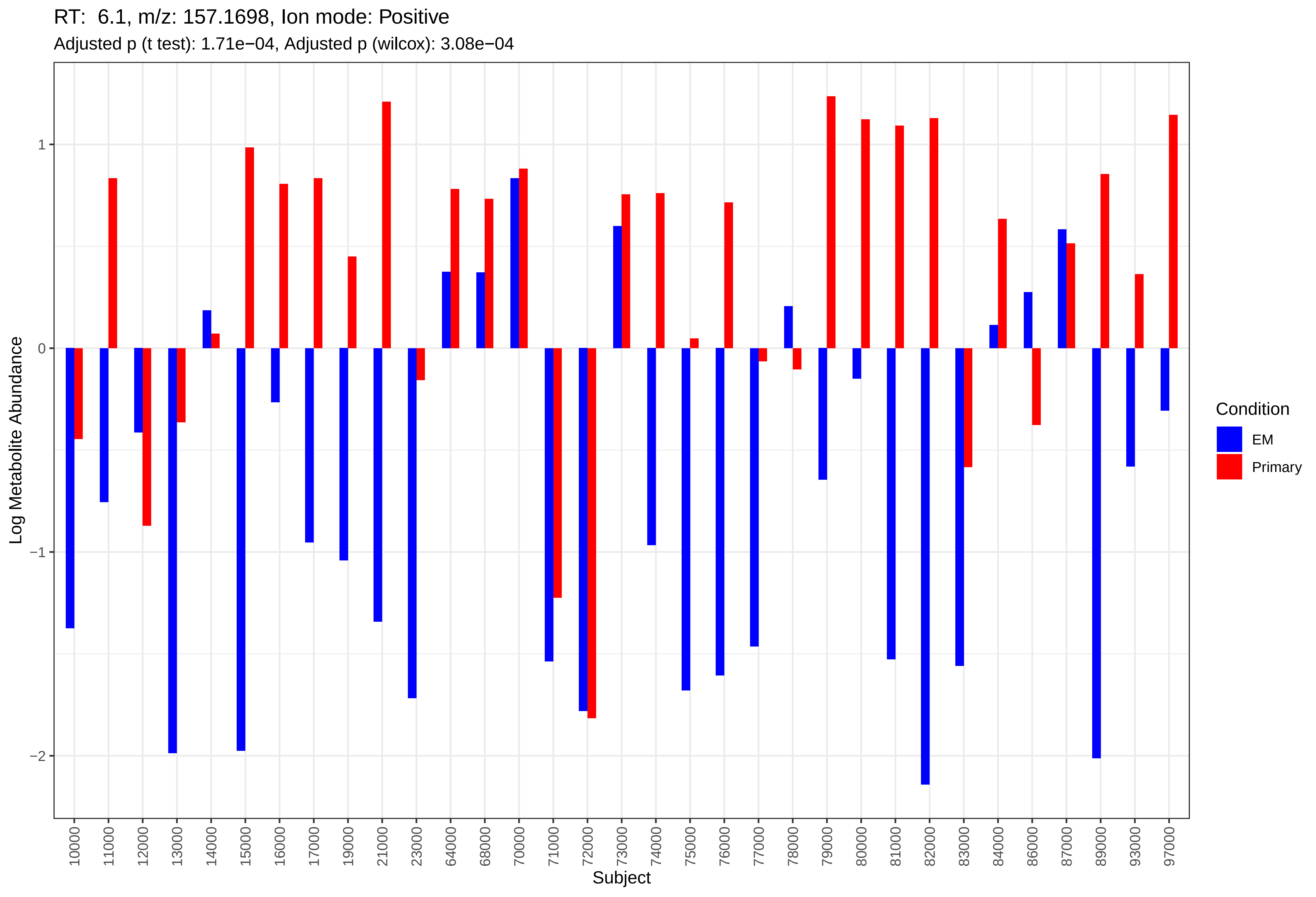

RT: 6.1, m/z: 157.1698, Ion mode: Positive
Adjusted p (t test): 1.71e−04, Adjusted p (wilcox): 3.08e−04
Log Metabolite Abundance
1
0
−1
−2
10000
11000
12000
13000
14000
15000
16000
17000
19000
21000
23000
64000
68000
70000
71000
72000
73000
74000
75000
76000
77000
78000
79000
80000
81000
82000
83000
84000
86000
87000
89000
93000
97000
Subject
Condition
EM
Primary

[M+Na−H] 21.9825 m/z adduct of 148.0603 m/z (RT: 0.7, m/z: 170.0421, Ion mode: Positive)

Adjusted p (t test): 1.71e−04, Adjusted p (wilcox): 1.84e−04

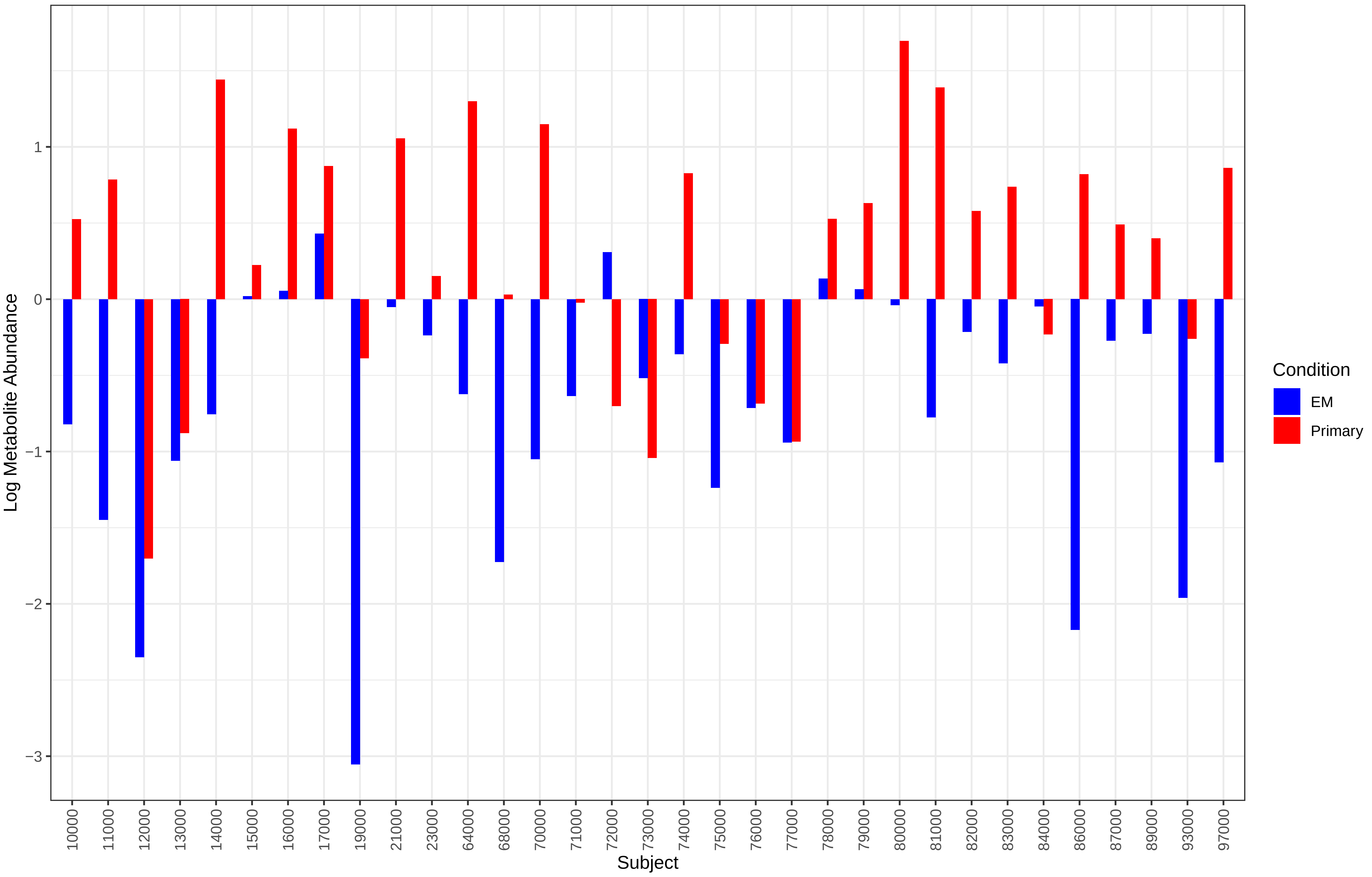

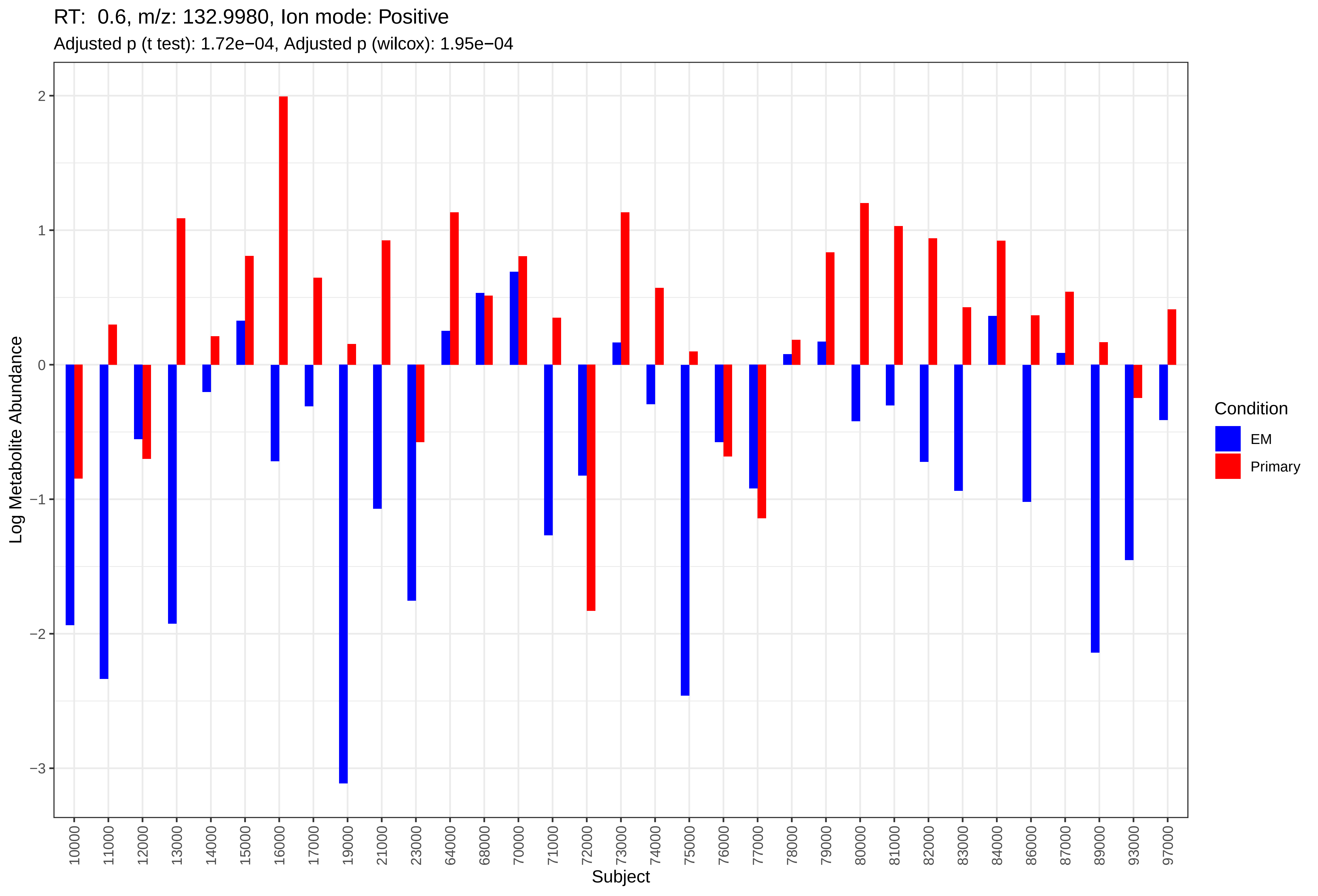
RT: 0.6, m/z: 132.9980, Ion mode: Positive
Adjusted p (t test): 1.72e−04, Adjusted p (wilcox): 1.95e−04
Log Metabolite Abundance
2
1
0
−1
−2
−3
Subject
10000
11000
12000
13000
14000
15000
16000
17000
19000
21000
23000
64000
68000
70000
71000
72000
73000
74000
75000
76000
77000
78000
79000
80000
81000
82000
83000
84000
86000
87000
89000
93000
97000
Condition
EM
Primary

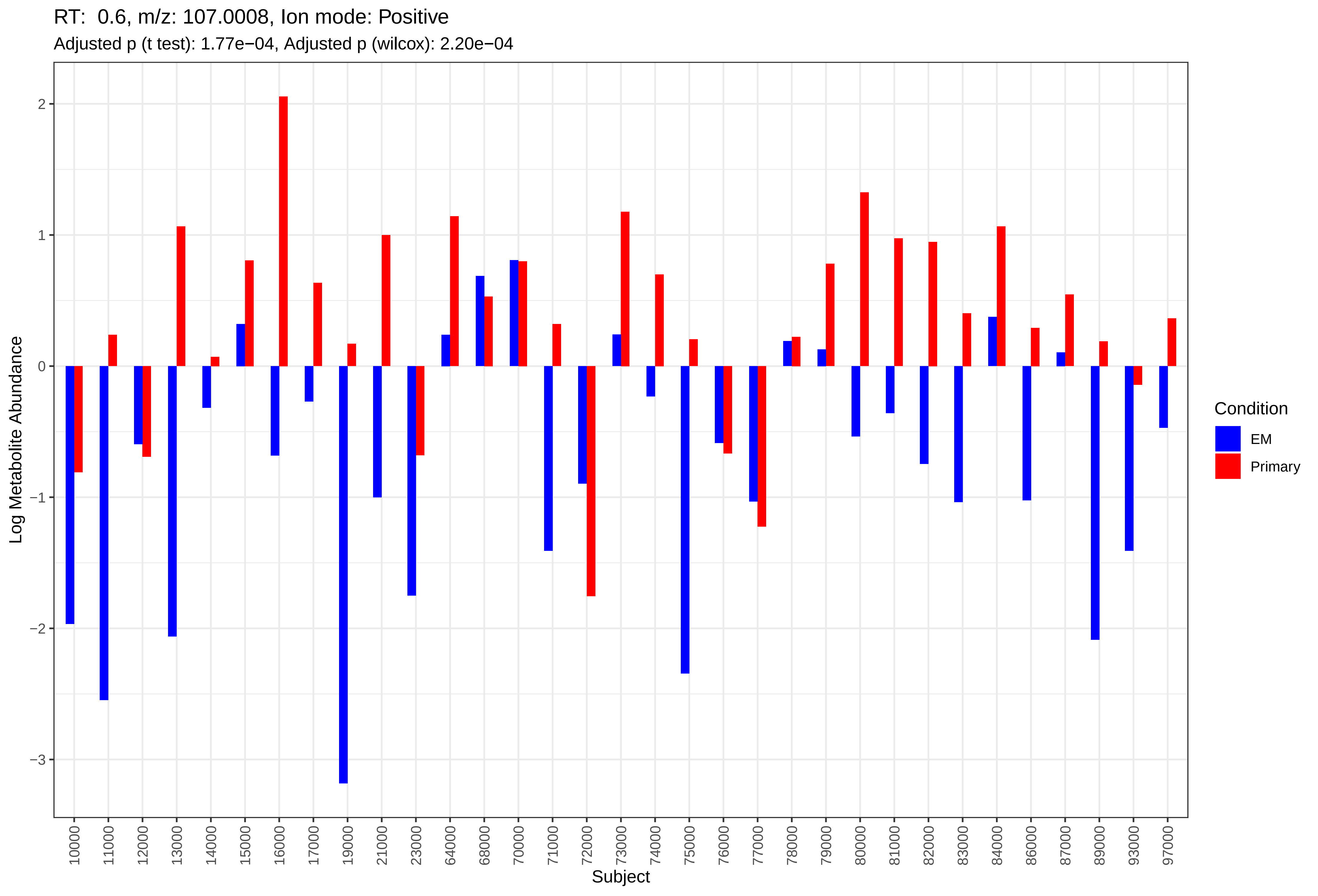

RT: 0.6, m/z: 107.0008, Ion mode: Positive
Adjusted p (t test): 1.77e−04, Adjusted p (wilcox): 2.20e−04
Log Metabolite Abundance
2
1
0
−1
−2
−3
10000
11000
12000
13000
14000
15000
16000
17000
19000
21000
23000
64000
68000
70000
71000
72000
73000
74000
75000
76000
77000
78000
79000
80000
81000
82000
83000
84000
86000
87000
89000
93000
97000
Subject
Condition
EM
Primary

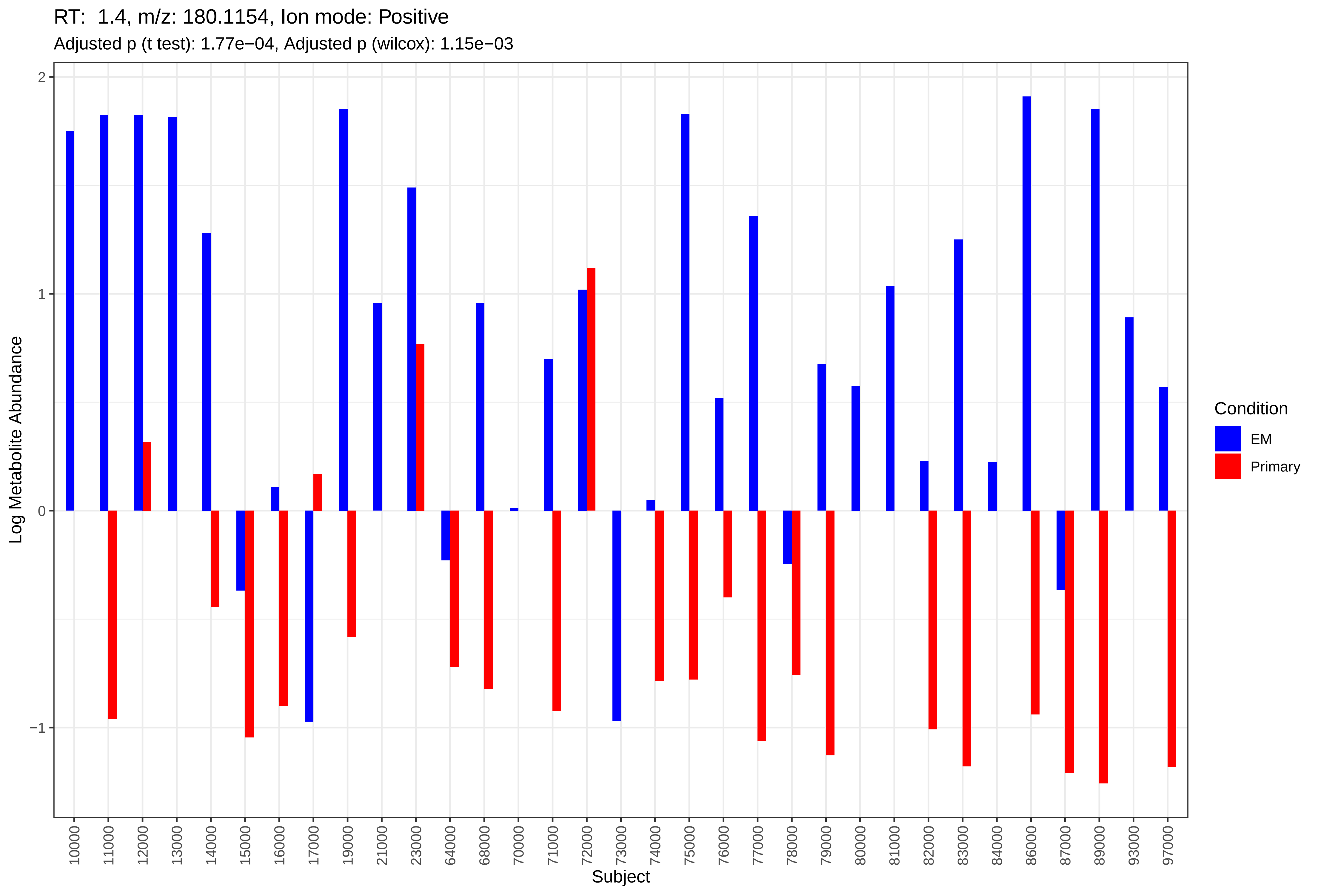
RT: 1.4, m/z: 180.1154, Ion mode: Positive
Adjusted p (t test): 1.77e−04, Adjusted p (wilcox): 1.15e−03
Log Metabolite Abundance
2
1
0
−1
Subject
10000
11000
12000
13000
14000
15000
16000
17000
19000
21000
23000
64000
68000
70000
71000
72000
73000
74000
75000
76000
77000
78000
79000
80000
81000
82000
83000
84000
86000
87000
89000
93000
97000
Condition
EM
Primary

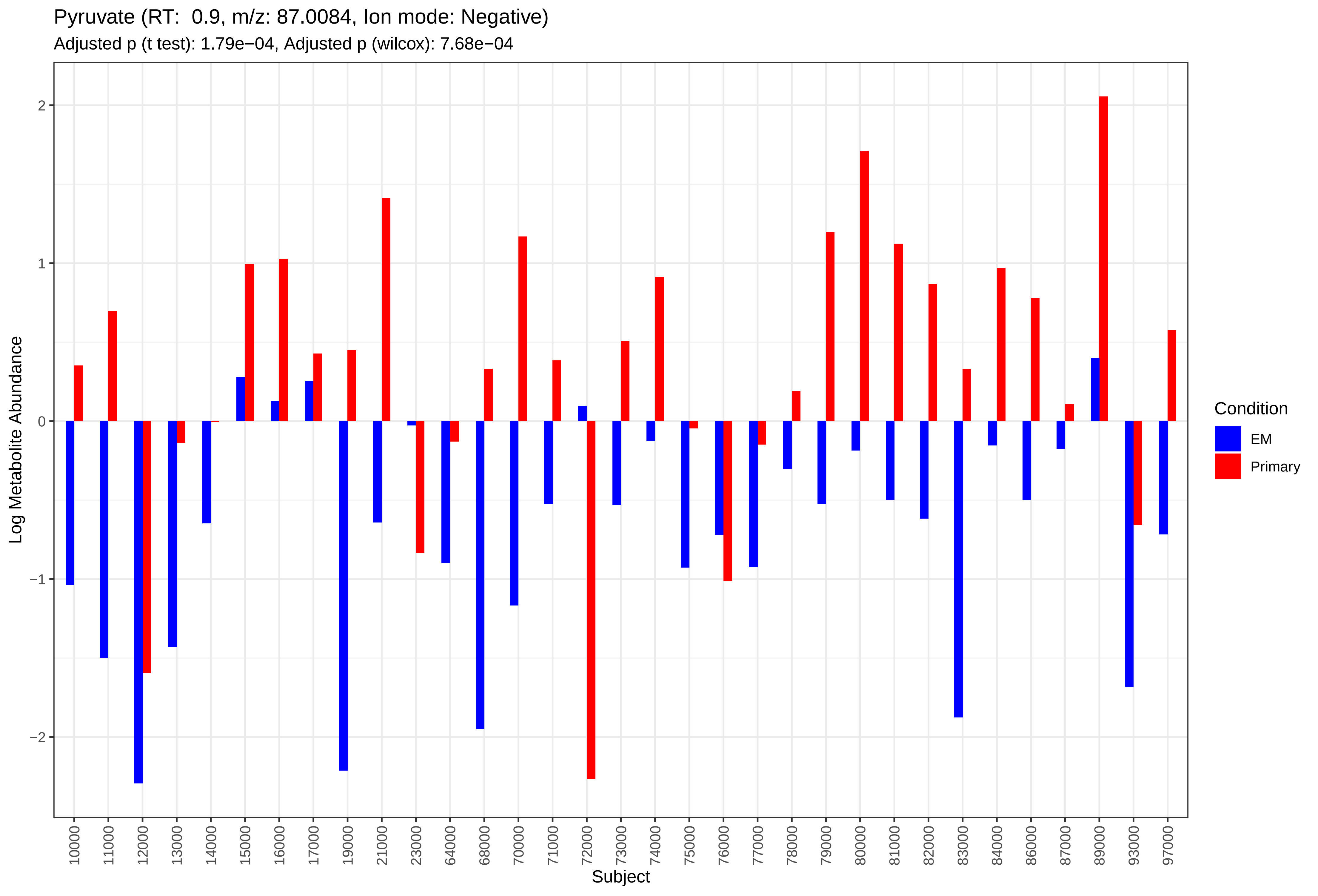

Pyruvate (RT: 0.9, m/z: 87.0084, Ion mode: Negative)
Adjusted p (t test): 1.79e−04, Adjusted p (wilcox): 7.68e−04
Log Metabolite Abundance
2
1
0
−1
−2
Subject
10000
11000
12000
13000
14000
15000
16000
17000
19000
21000
23000
64000
68000
70000
71000
72000
73000
74000
75000
76000
77000
78000
79000
80000
81000
82000
83000
84000
86000
87000
89000
93000
97000
Condition
EM
Primary

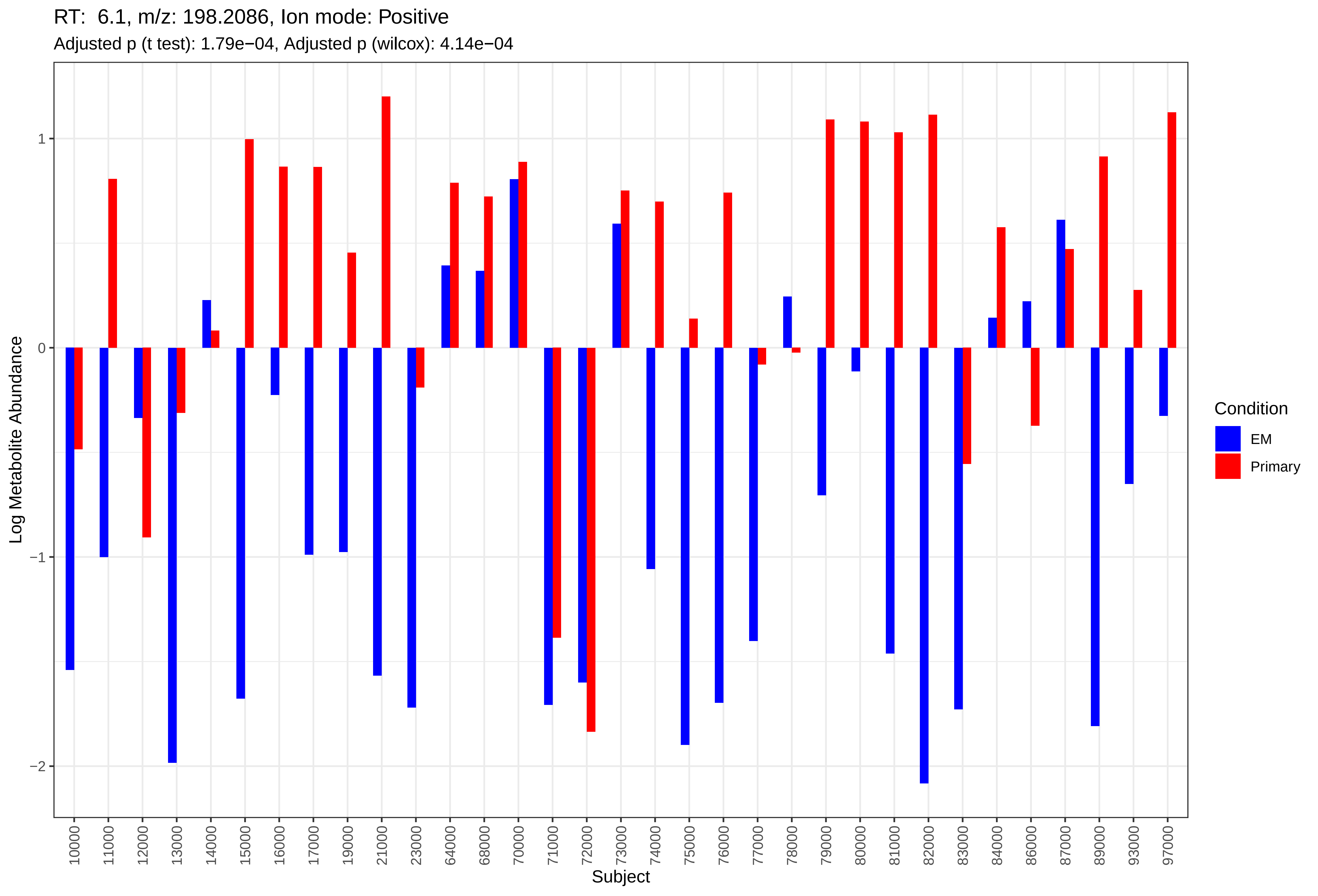

RT: 6.1, m/z: 198.2086, Ion mode: Positive
Adjusted p (t test): 1.79e−04, Adjusted p (wilcox): 4.14e−04
Log Metabolite Abundance
1
0
−1
−2
10000
11000
12000
13000
14000
15000
16000
17000
19000
21000
23000
64000
68000
70000
71000
72000
73000
74000
75000
76000
77000
78000
79000
80000
81000
82000
83000
84000
86000
87000
89000
93000
97000
Subject
Condition
EM
Primary

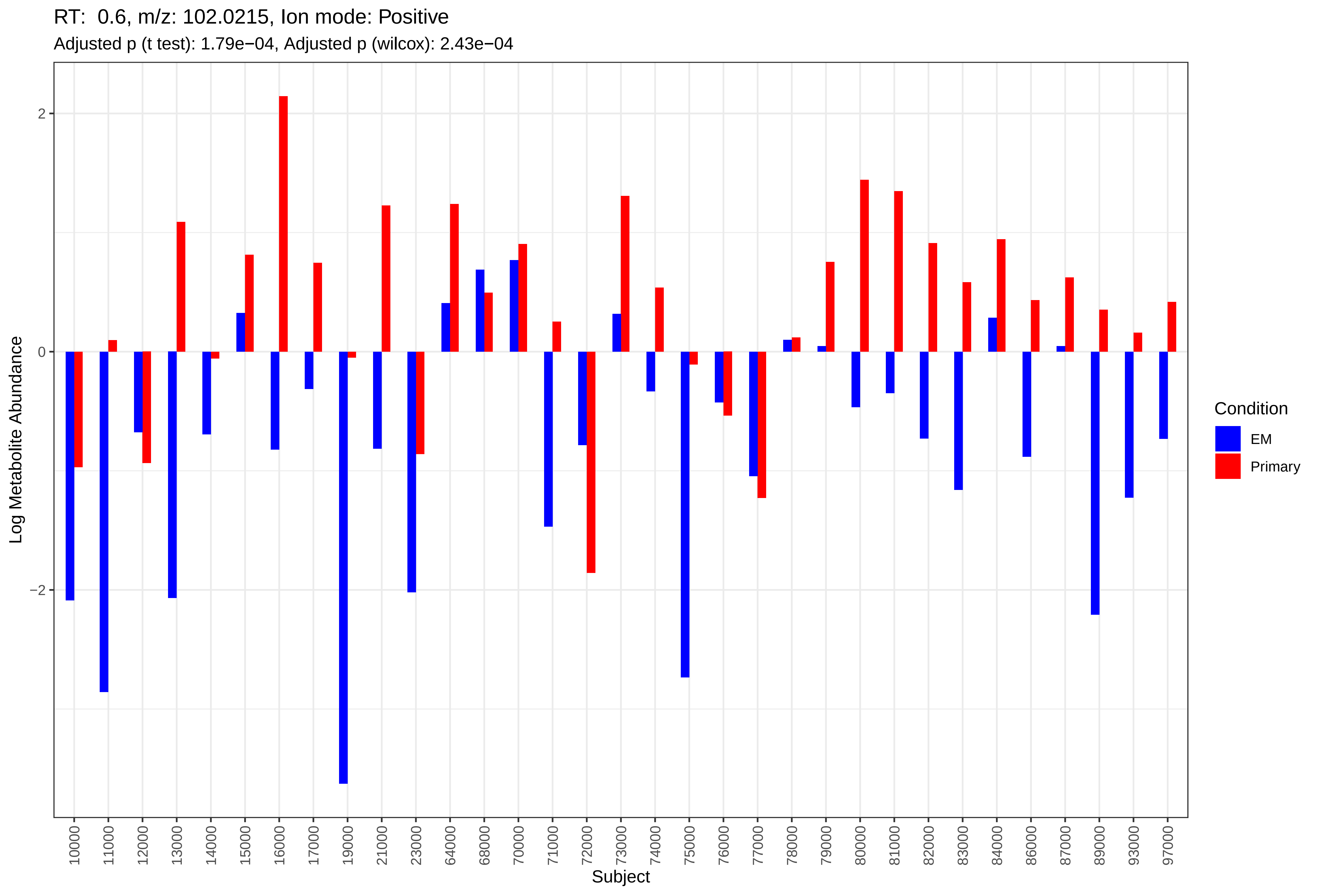
RT: 0.6, m/z: 102.0215, Ion mode: Positive
Adjusted p (t test): 1.79e−04, Adjusted p (wilcox): 2.43e−04
Log Metabolite Abundance
2
0
−2
Subject
10000
11000
12000
13000
14000
15000
16000
17000
19000
21000
23000
64000
68000
70000
71000
72000
73000
74000
75000
76000
77000
78000
79000
80000
81000
82000
83000
84000
86000
87000
89000
93000
97000
Condition
EM
Primary

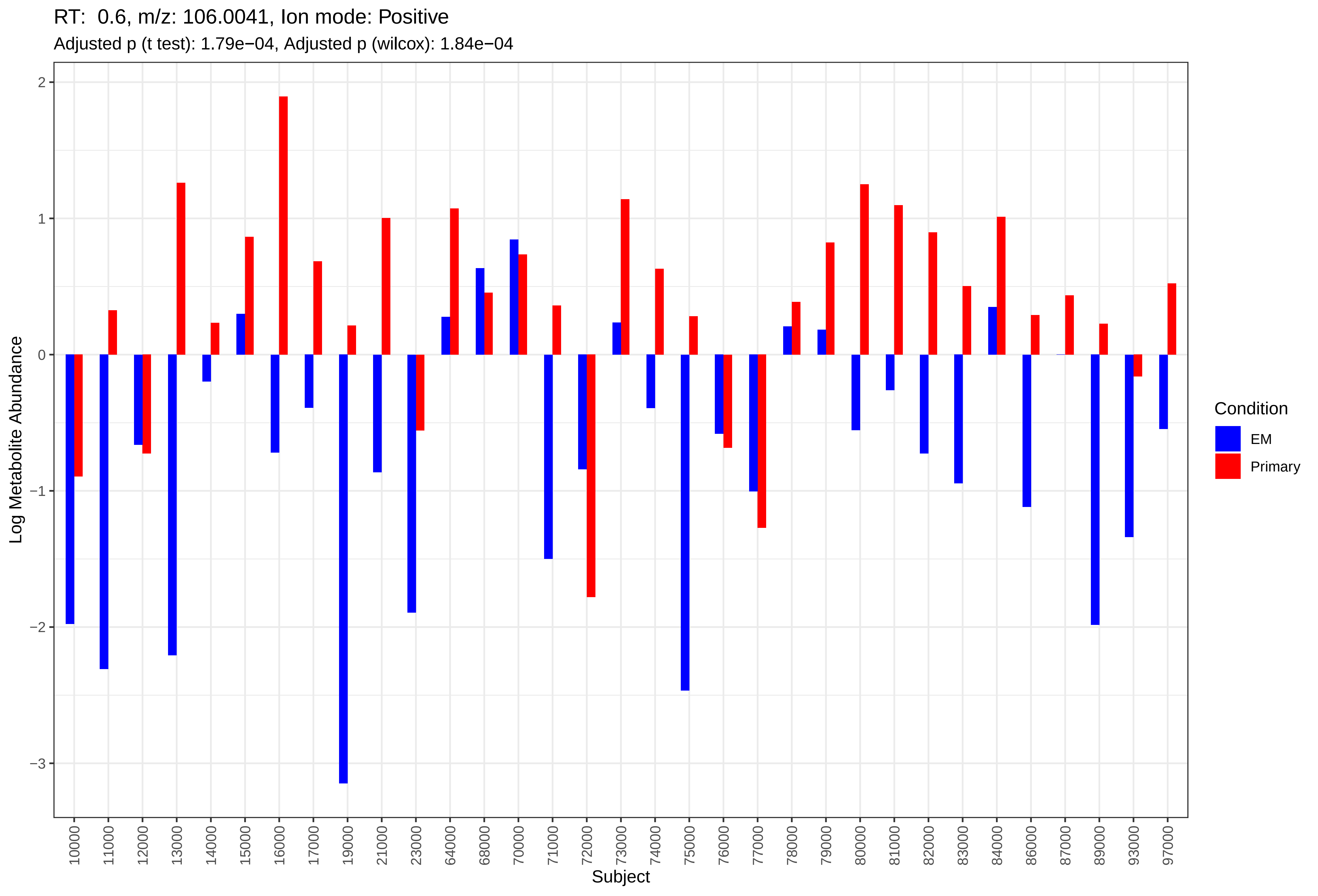
RT: 0.6, m/z: 106.0041, Ion mode: Positive
Adjusted p (t test): 1.79e−04, Adjusted p (wilcox): 1.84e−04
Log Metabolite Abundance
2
1
0
−1
−2
−3
Subject
10000
11000
12000
13000
14000
15000
16000
17000
19000
21000
23000
64000
68000
70000
71000
72000
73000
74000
75000
76000
77000
78000
79000
80000
81000
82000
83000
84000
86000
87000
89000
93000
97000
Condition
EM
Primary

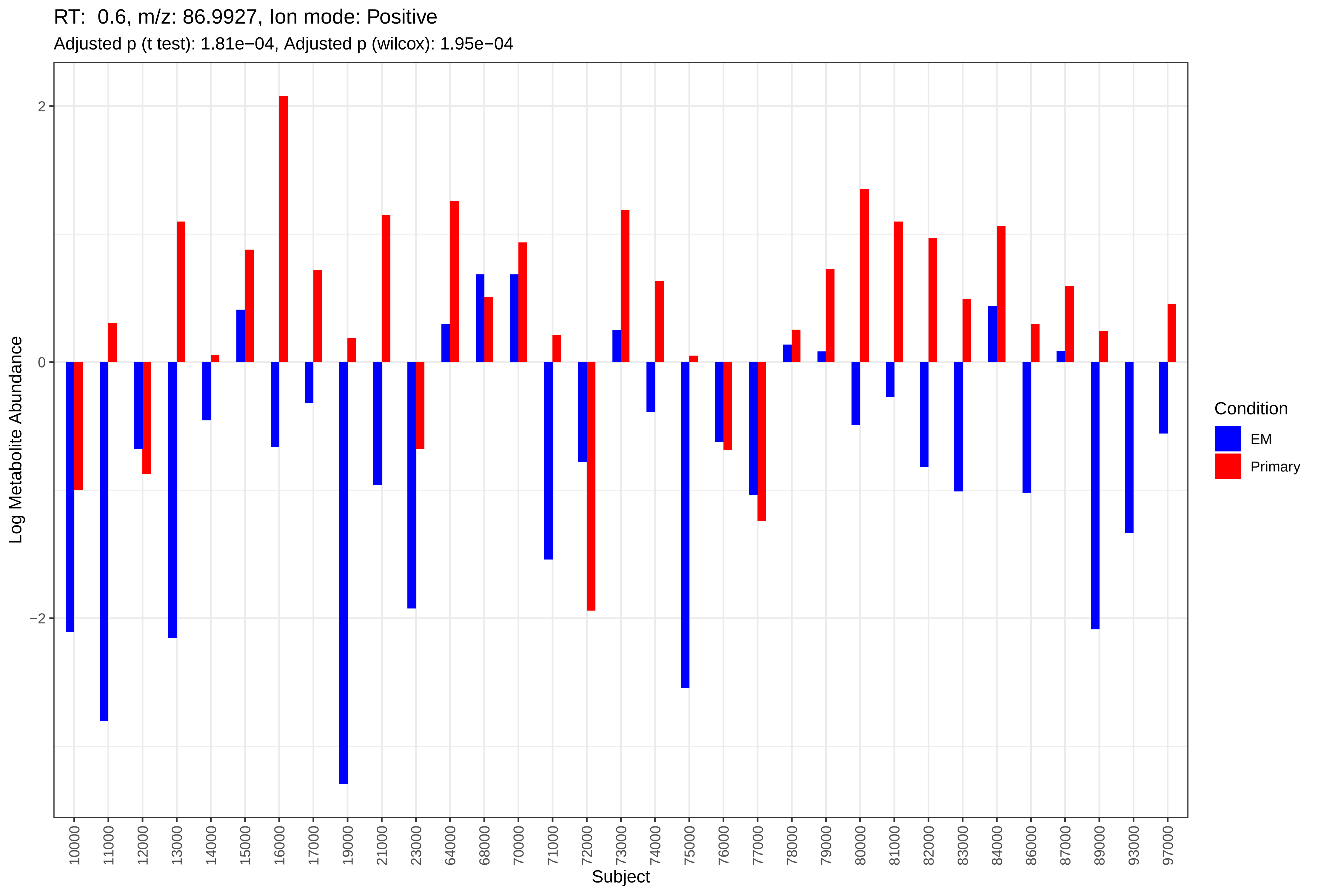

RT: 0.6, m/z: 86.9927, Ion mode: Positive
Adjusted p (t test): 1.81e−04, Adjusted p (wilcox): 1.95e−04
Log Metabolite Abundance
2
0
−2
10000
11000
12000
13000
14000
15000
16000
17000
19000
21000
23000
64000
68000
70000
71000
72000
73000
74000
75000
76000
77000
78000
79000
80000
81000
82000
83000
84000
86000
87000
89000
93000
97000
Subject
Condition
EM
Primary

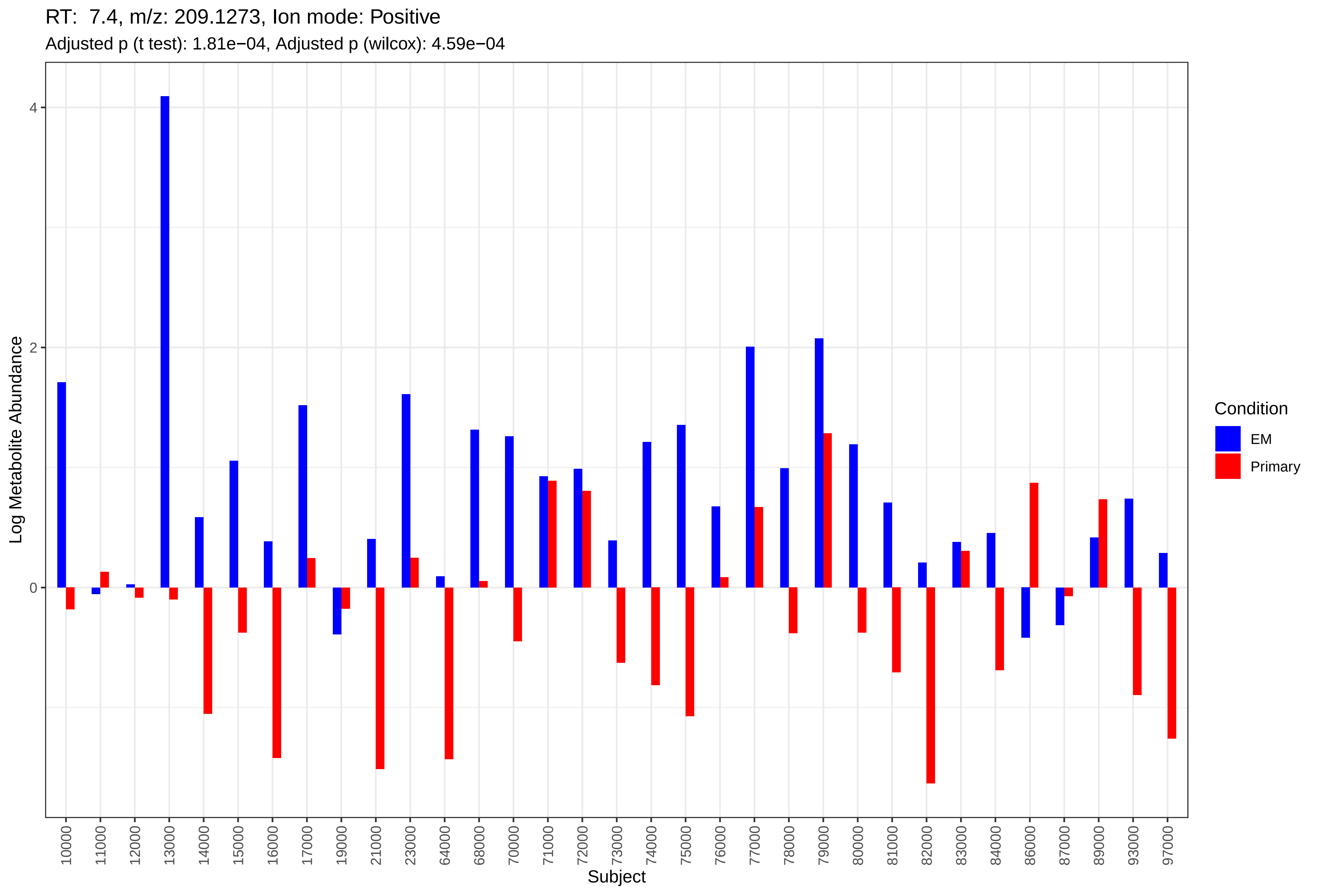
RT: 7.4, m/z: 209.1273, Ion mode: Positive
Adjusted p (t test): 1.81e−04, Adjusted p (wilcox): 4.59e−04
Log Metabolite Abundance
4
2
0
Subject
10000
11000
12000
13000
14000
15000
16000
17000
19000
21000
23000
64000
68000
70000
71000
72000
73000
74000
75000
76000
77000
78000
79000
80000
81000
82000
83000
84000
86000
87000
89000
93000
97000
Condition
EM
Primary

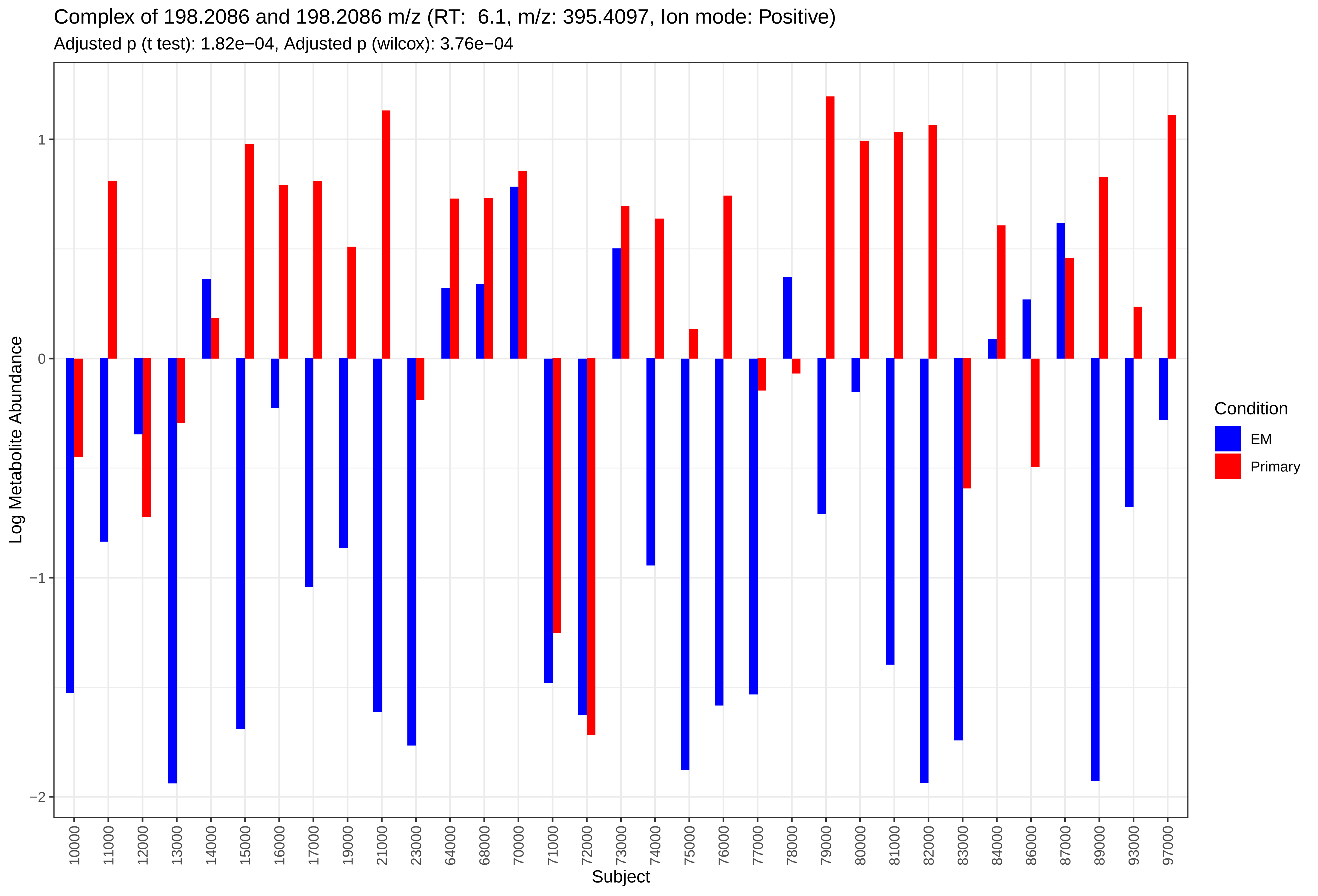

Complex of 198.2086 and 198.2086 m/z (RT: 6.1, m/z: 395.4097, Ion mode: Positive)
Adjusted p (t test): 1.82e−04, Adjusted p (wilcox): 3.76e−04
Log Metabolite Abundance
1
0
−1
−2
10000 11000 12000 13000 14000 15000 16000 17000 19000 21000 23000 64000 68000 70000 71000 72000 73000 74000 75000 76000 77000 78000 79000 80000 81000 82000 83000 84000 86000 87000 89000 93000 97000
Subject
Condition
EM
Primary

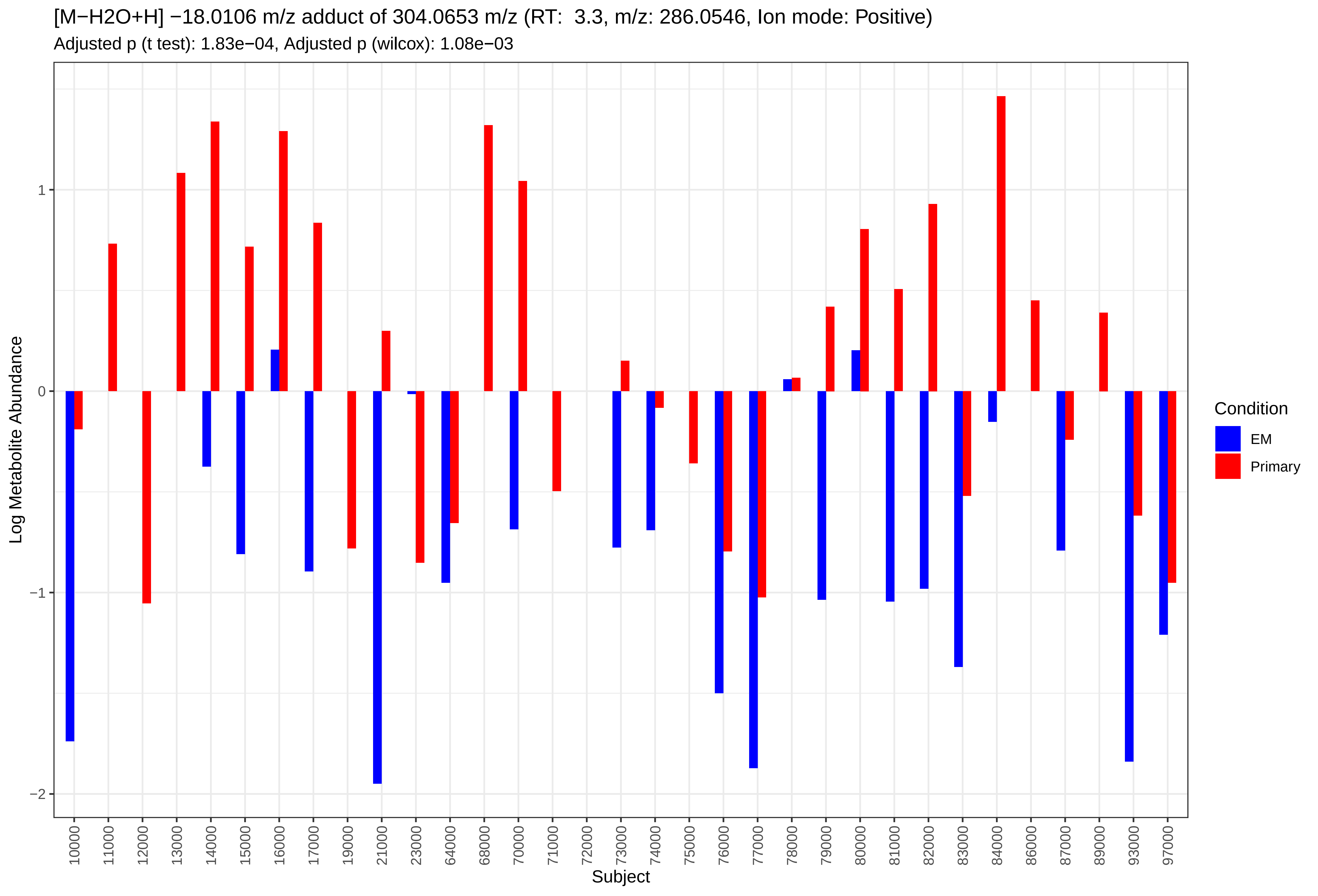

[M−H2O+H] −18.0106 m/z adduct of 304.0653 m/z (RT: 3.3, m/z: 286.0546, Ion mode: Positive)
Adjusted p (t test): 1.83e−04, Adjusted p (wilcox): 1.08e−03
Log Metabolite Abundance
1
0
−1
−2
Subject
10000
11000
12000
13000
14000
15000
16000
17000
19000
21000
23000
64000
68000
70000
71000
72000
73000
74000
75000
76000
77000
78000
79000
80000
81000
82000
83000
84000
86000
87000
89000
93000
97000
Condition
EM
Primary

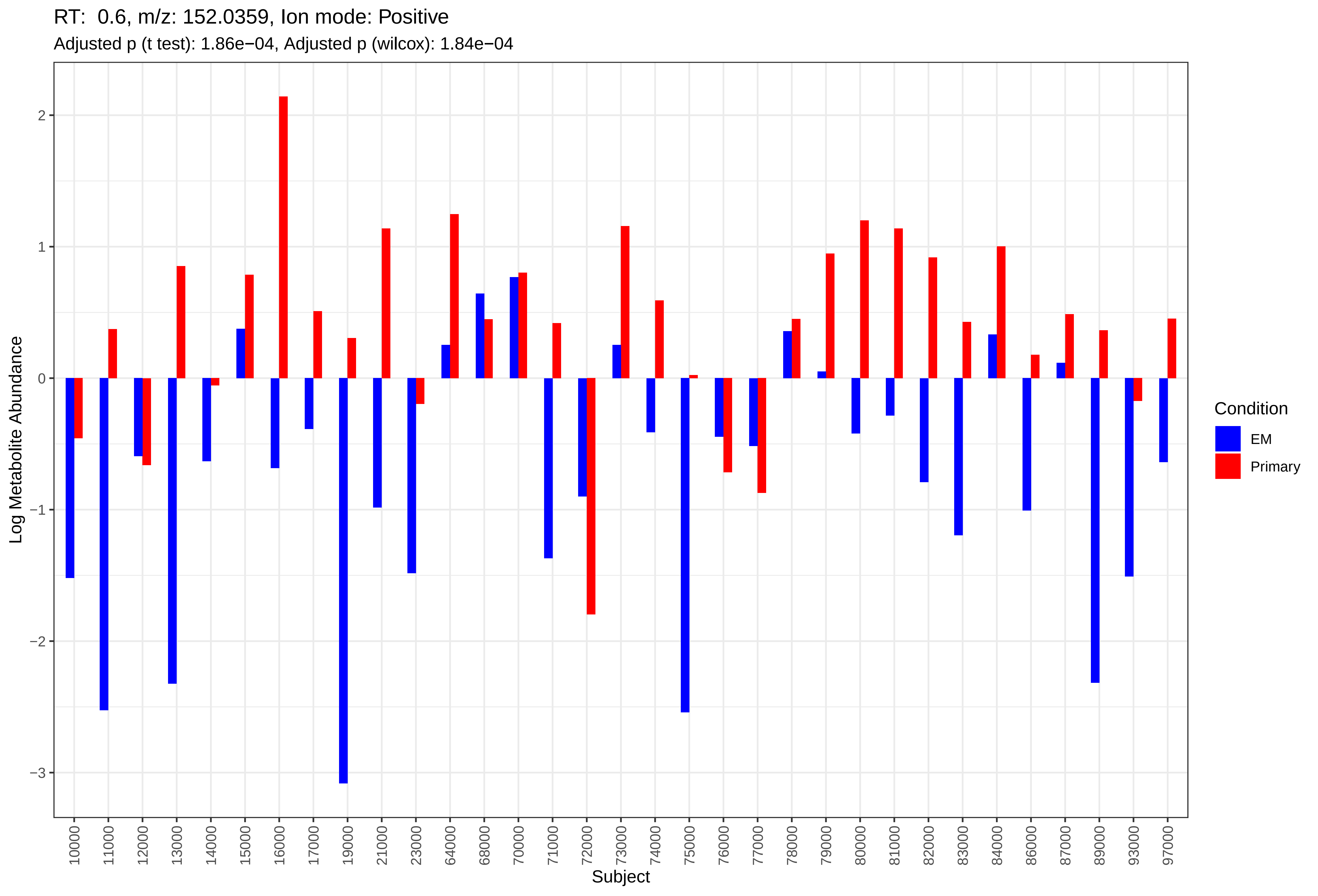
RT: 0.6, m/z: 152.0359, Ion mode: Positive
Adjusted p (t test): 1.86e−04, Adjusted p (wilcox): 1.84e−04
Log Metabolite Abundance
2
1
0
−1
−2
−3
10000
11000
12000
13000
14000
15000
16000
17000
19000
21000
23000
64000
68000
70000
71000
72000
73000
74000
75000
76000
77000
78000
79000
80000
81000
82000
83000
84000
86000
87000
89000
93000
97000
Subject
Condition
EM
Primary

RT: 7.9, m/z: 285.6094, Ion mode: Positive

Adjusted p (t test): 2.62e−04, Adjusted p (wilcox): 1.84e−04

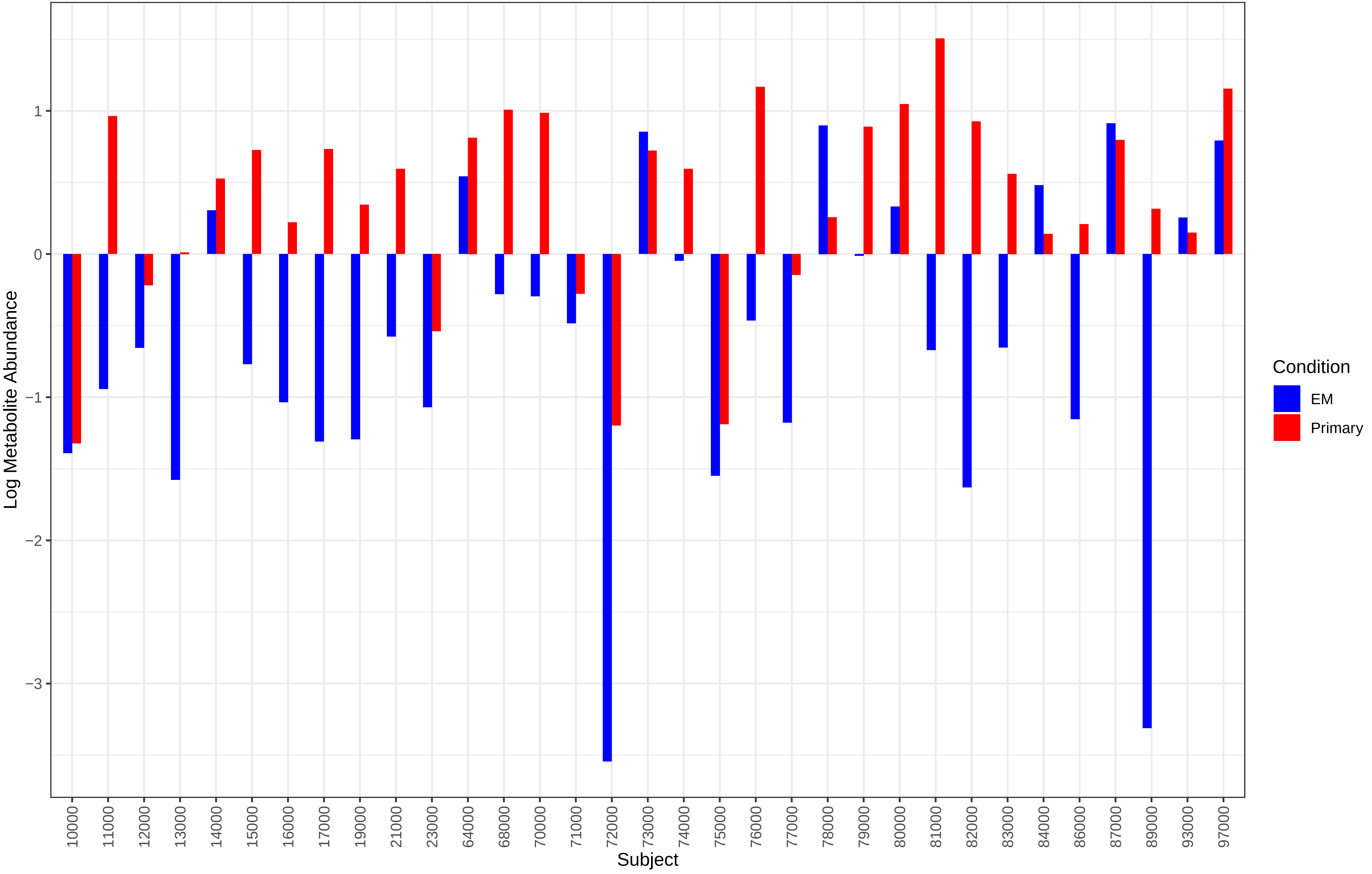

RT: 0.6, m/z: 73.5318, Ion mode: Positive

Adjusted p (t test): 2.05e−04, Adjusted p (wilcox): 1.84e−04

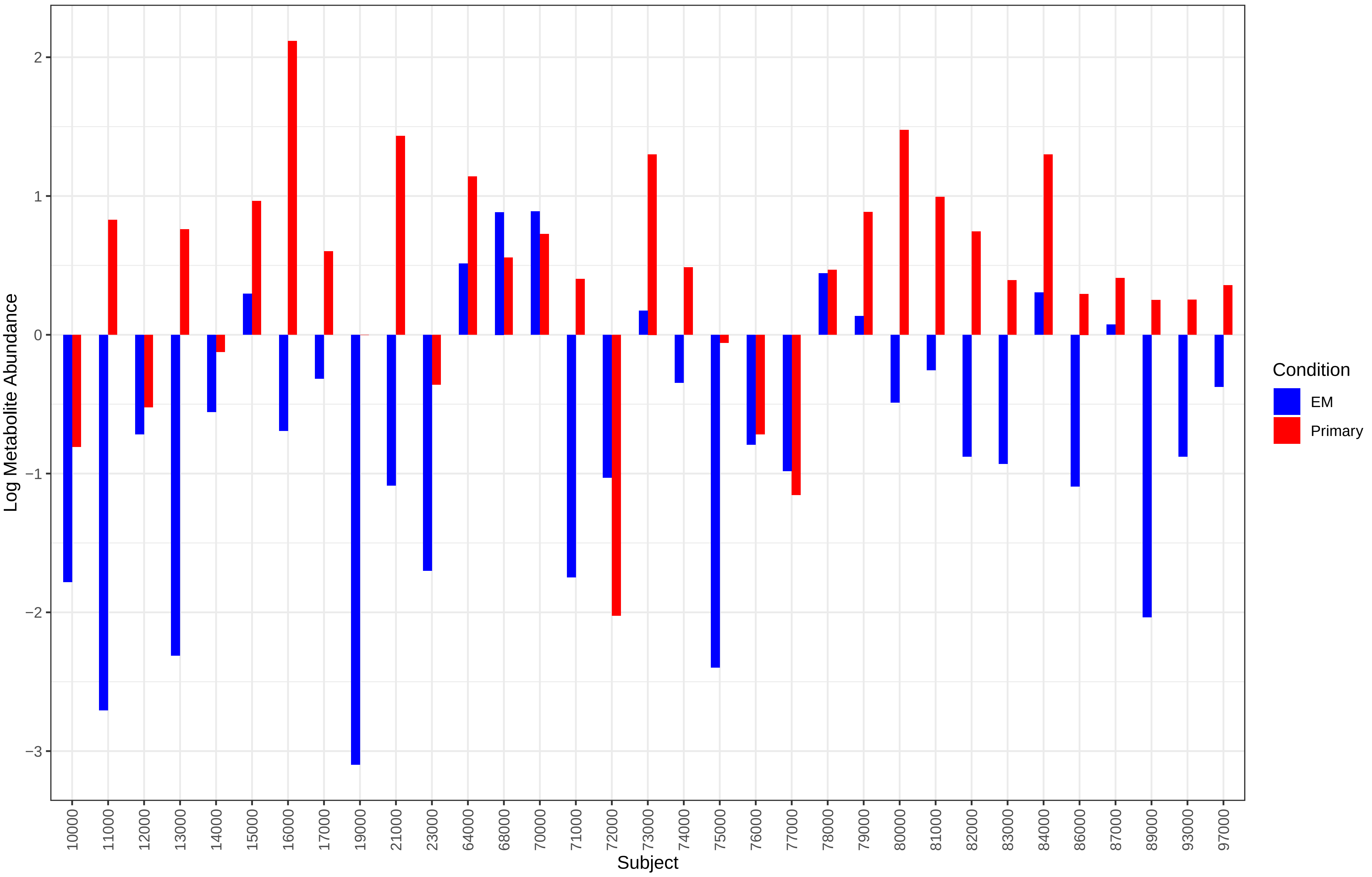

RT: 5.7, m/z: 358.1028, Ion mode: Positive

Adjusted p (t test): 1.90e−04, Adjusted p (wilcox): 6.27e−04

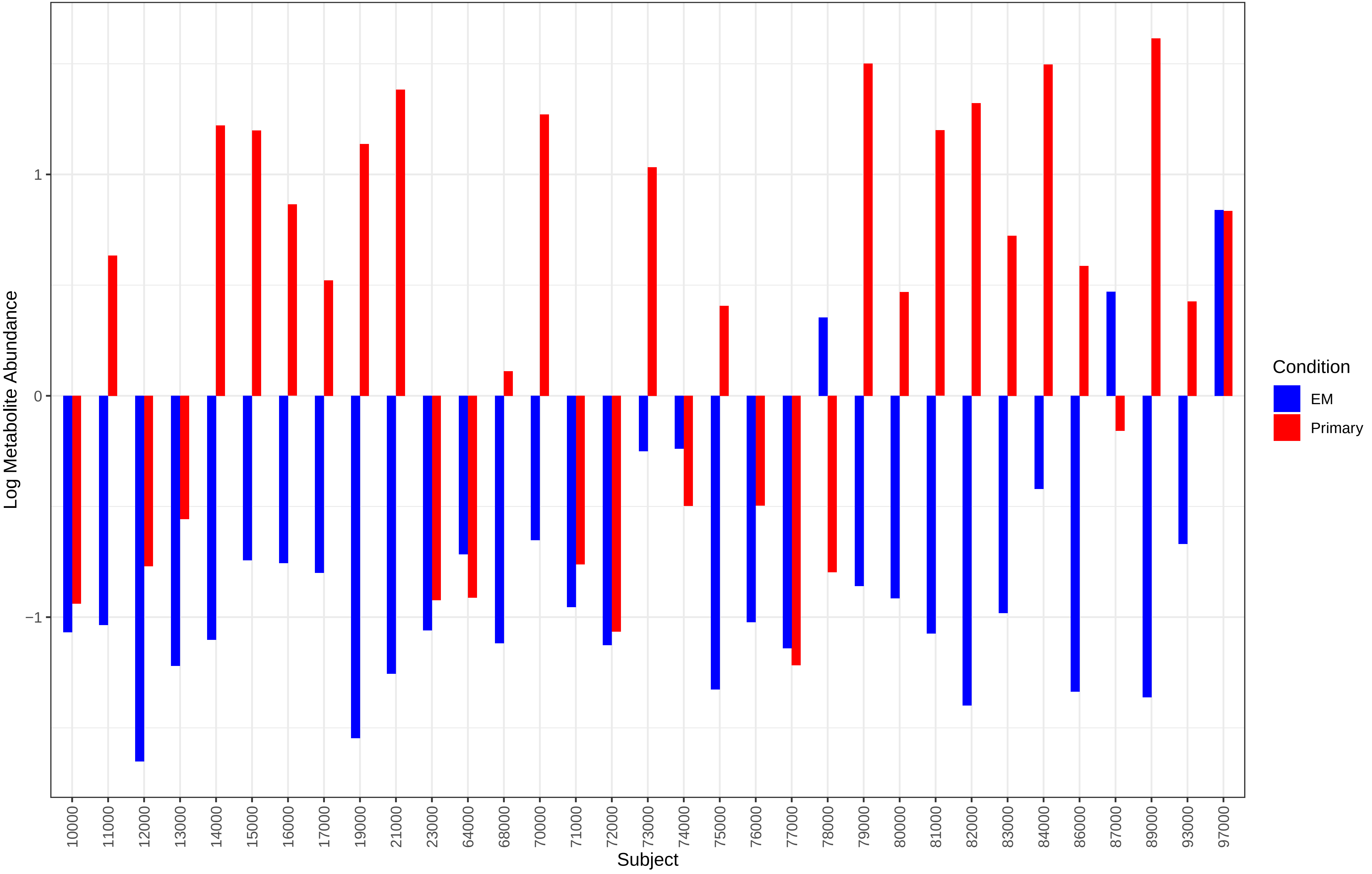

L−KYNURENINE (RT: 6.4, m/z: 209.0916, Ion mode: Positive)

Adjusted p (t test): 1.90e−04, Adjusted p (wilcox): 3.37e−04

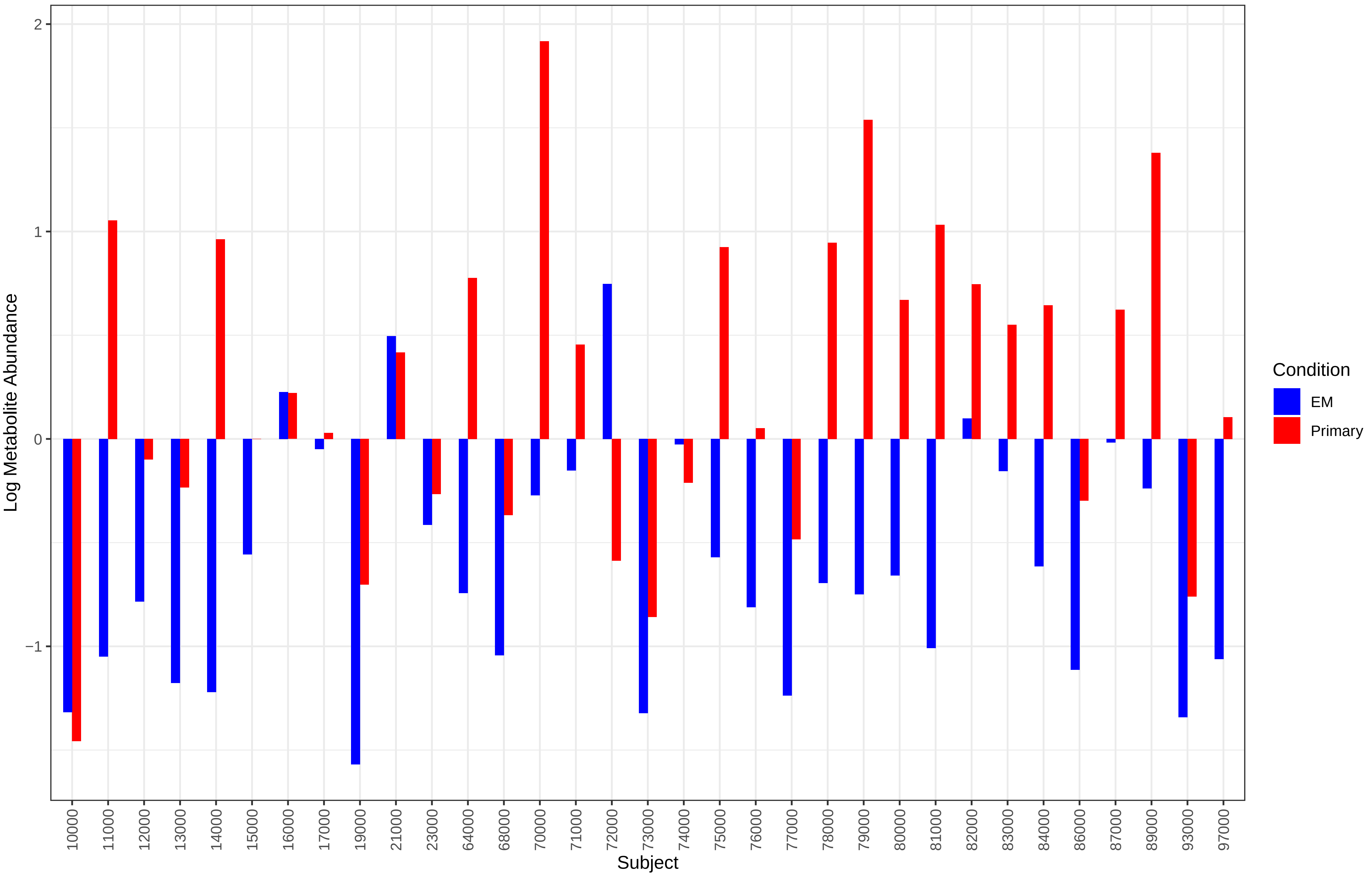

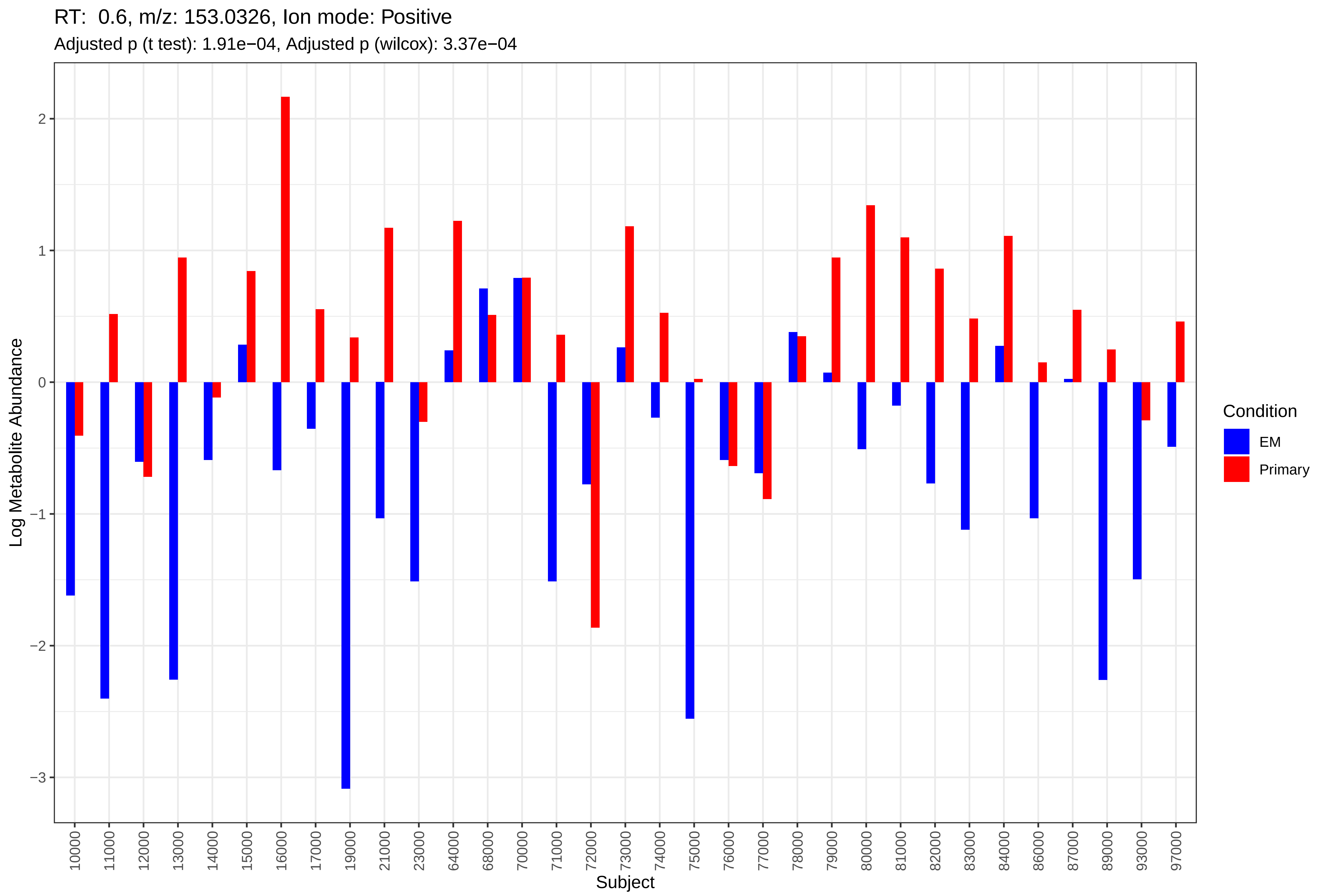

RT: 0.6, m/z: 153.0326, Ion mode: Positive
Adjusted p (t test): 1.91e−04, Adjusted p (wilcox): 3.37e−04
Log Metabolite Abundance
2
1
0
−1
−2
−3
Subject
10000
11000
12000
13000
14000
15000
16000
17000
19000
21000
23000
64000
68000
70000
71000
72000
73000
74000
75000
76000
77000
78000
79000
80000
81000
82000
83000
84000
86000
87000
89000
93000
97000
Condition
EM
Primary

RT: 0.6, m/z: 100.0243, Ion mode: Positive

Adjusted p (t test): 1.93e−04, Adjusted p (wilcox): 1.95e−04

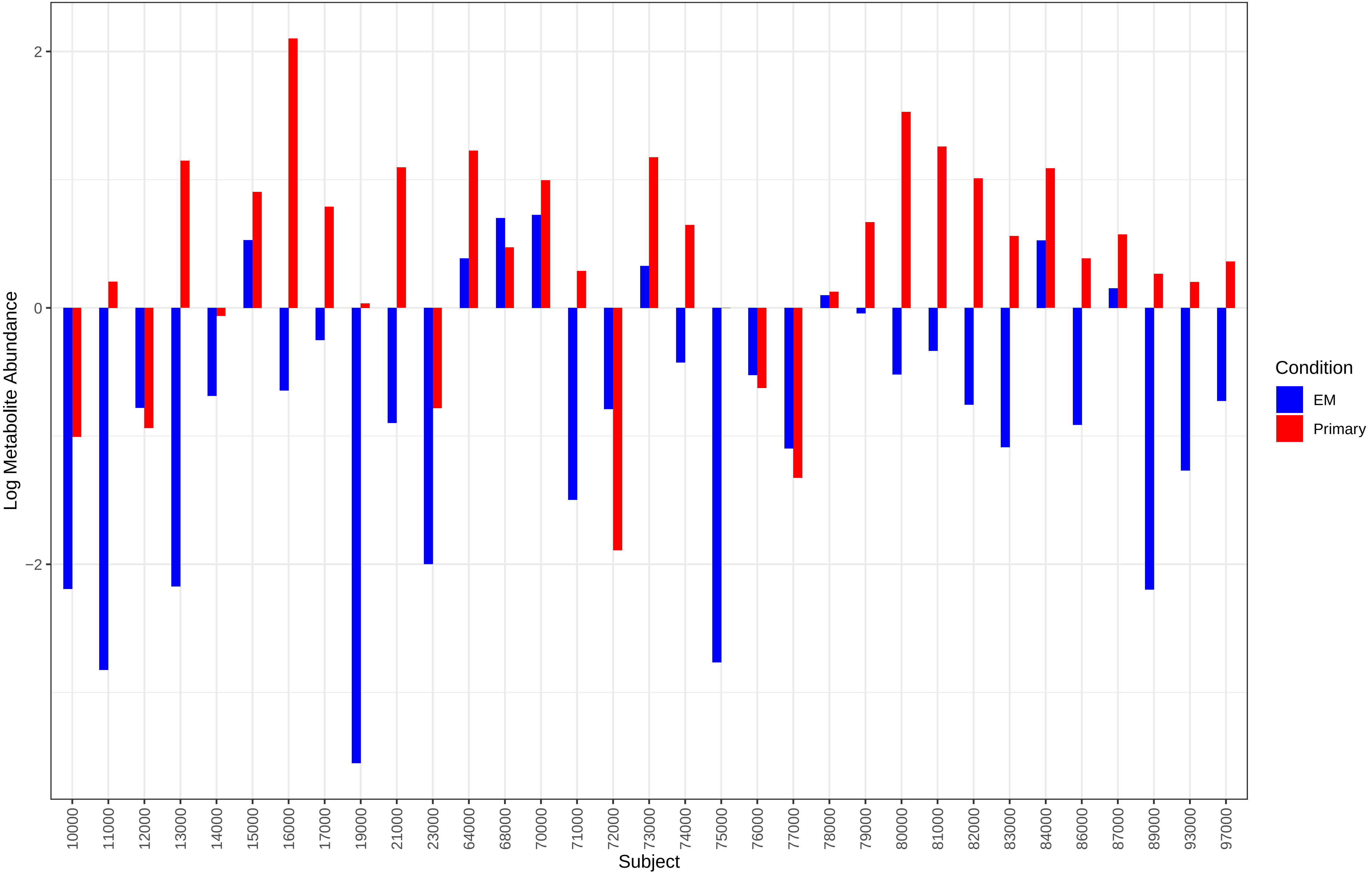

RT: 0.6, m/z: 82.0138, Ion mode: Positive

Adjusted p (t test): 1.94e−04, Adjusted p (wilcox): 2.43e−04

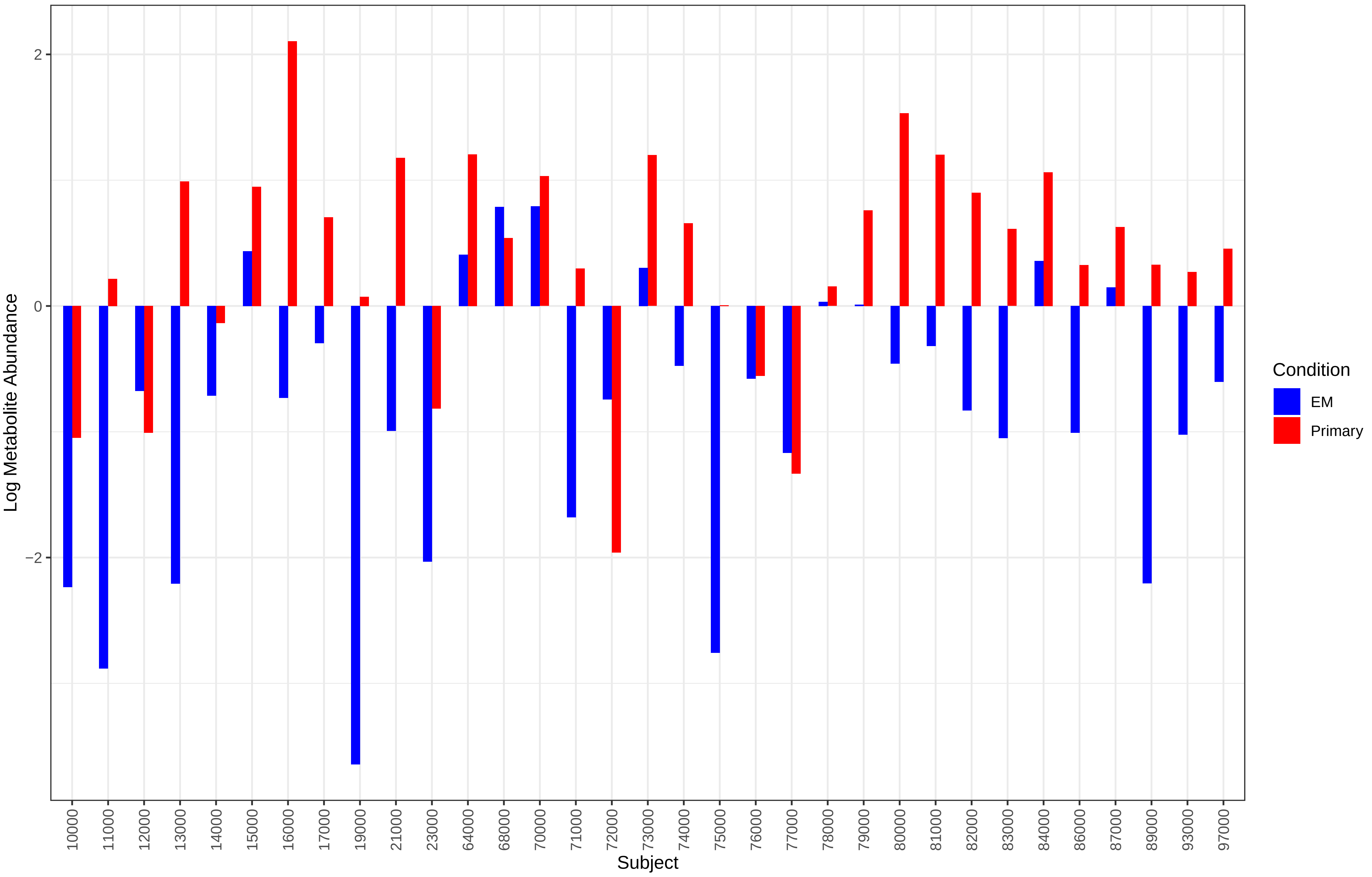

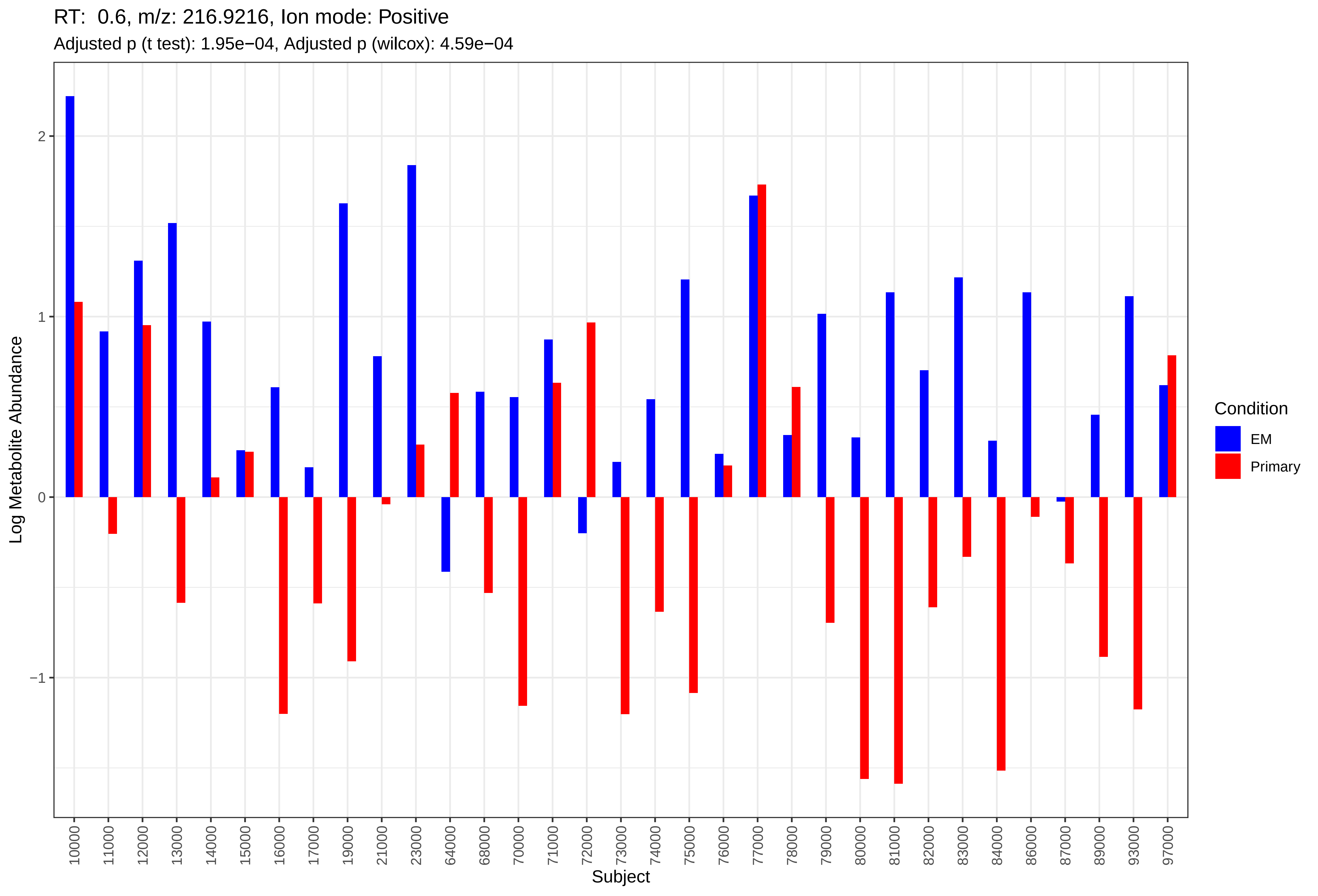
RT: 0.6, m/z: 216.9216, Ion mode: Positive
Adjusted p (t test): 1.95e−04, Adjusted p (wilcox): 4.59e−04
Log Metabolite Abundance
2
1
0
−1
10000
11000
12000
13000
14000
15000
16000
17000
19000
21000
23000
64000
68000
70000
71000
72000
73000
74000
75000
76000
77000
78000
79000
80000
81000
82000
83000
84000
86000
87000
89000
93000
97000
Subject
Condition
EM
Primary

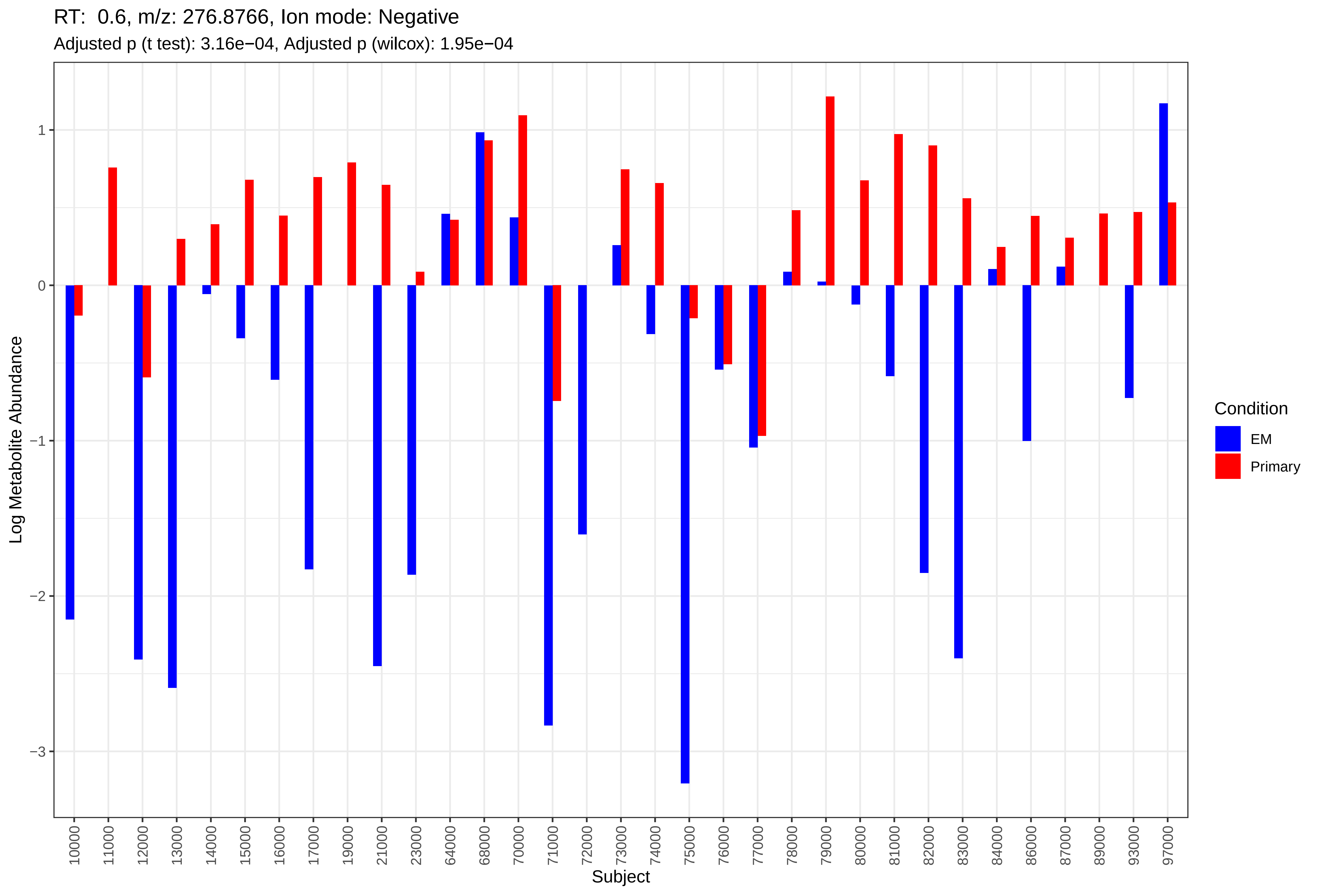

RT: 0.6, m/z: 276.8766, Ion mode: Negative
Adjusted p (t test): 3.16e−04, Adjusted p (wilcox): 1.95e−04
Log Metabolite Abundance
1
0
−1
−2
−3
10000
11000
12000
13000
14000
15000
16000
17000
19000
21000
23000
64000
68000
70000
71000
72000
73000
74000
75000
76000
77000
78000
79000
80000
81000
82000
83000
84000
86000
87000
89000
93000
97000
Subject
Condition
EM
Primary

RT: 1.5, m/z: 96.9693, Ion mode: Negative

Adjusted p (t test): 1.46e−03, Adjusted p (wilcox): 1.95e−04

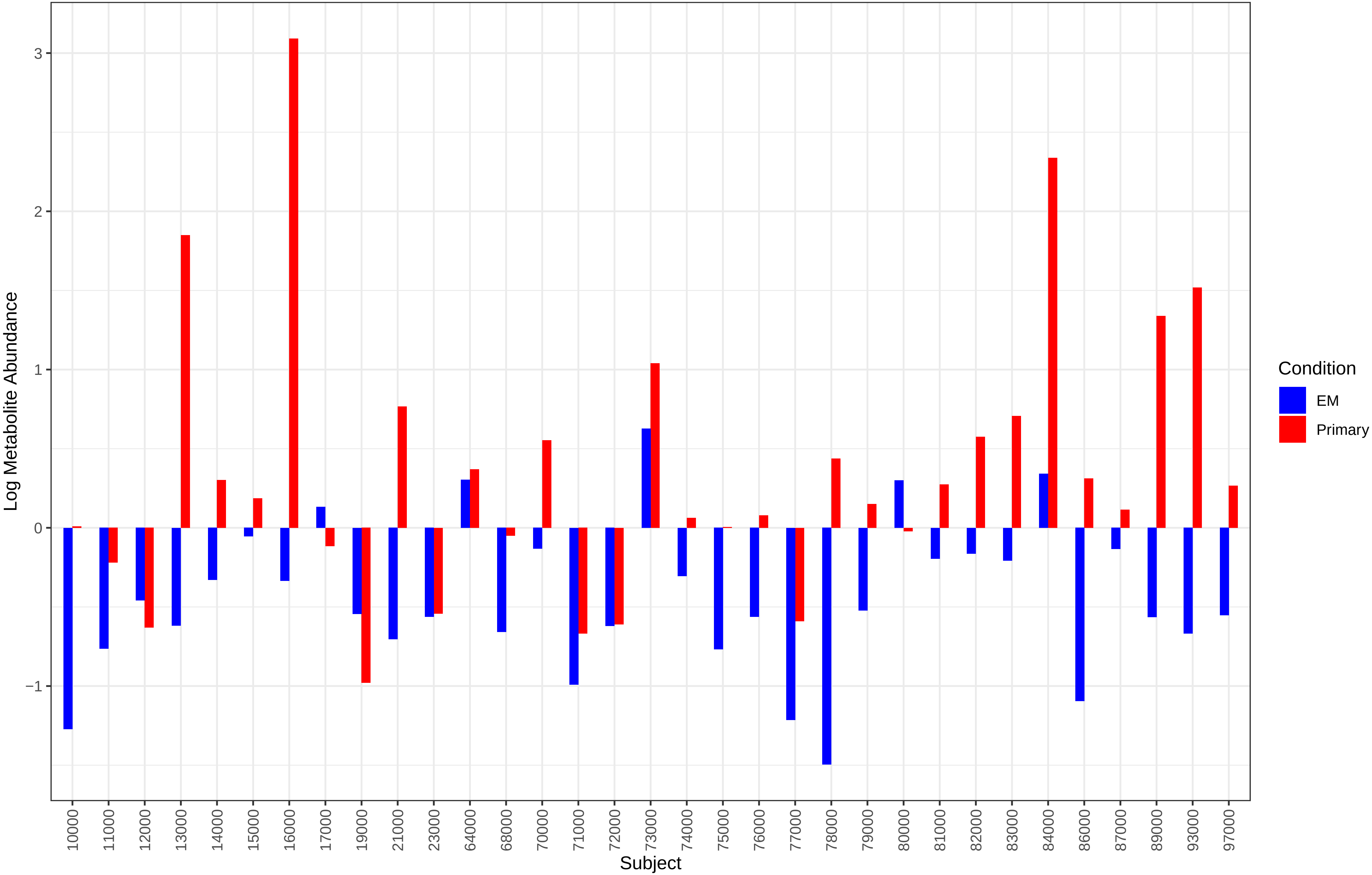

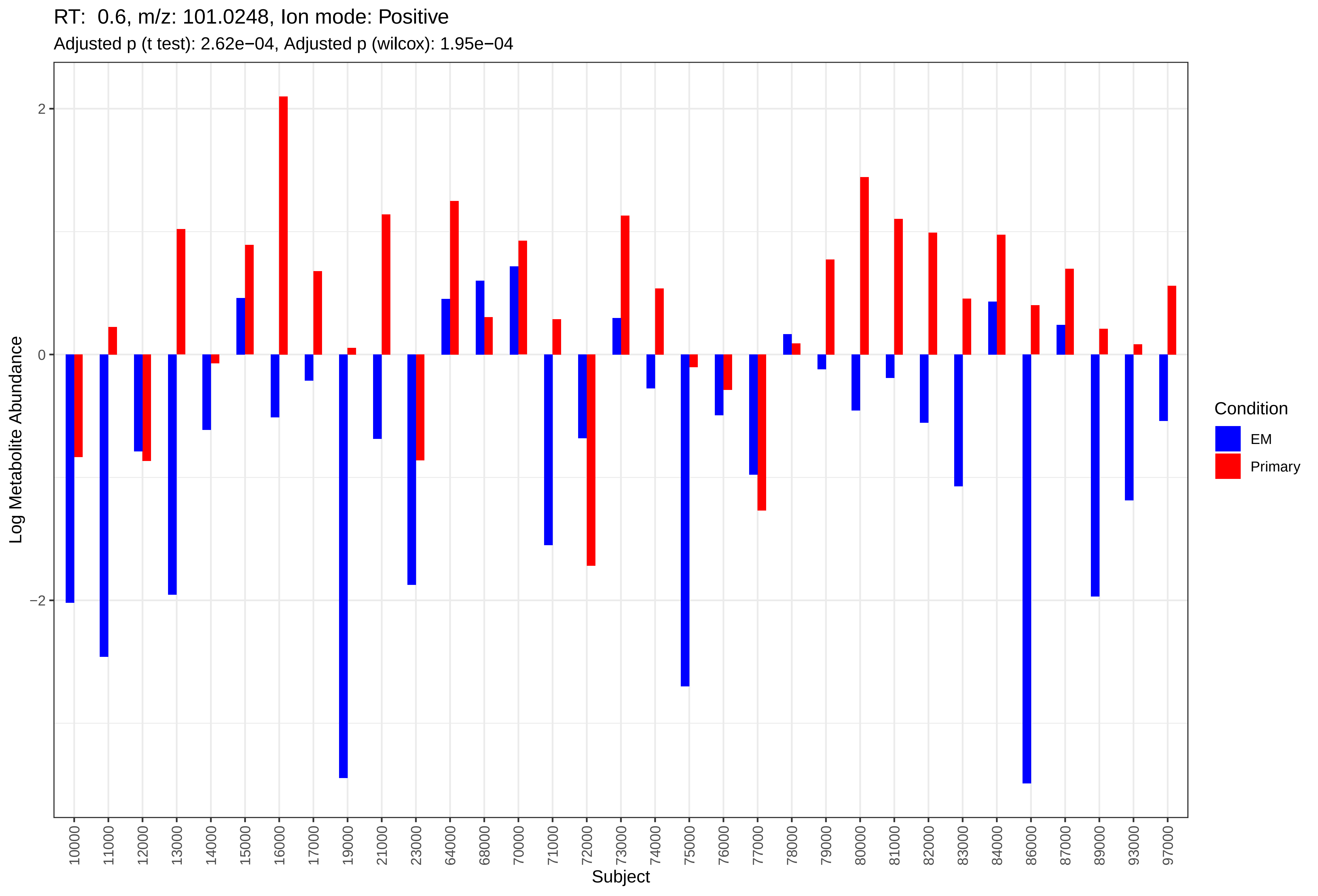

RT: 0.6, m/z: 101.0248, Ion mode: Positive
Adjusted p (t test): 2.62e−04, Adjusted p (wilcox): 1.95e−04
Log Metabolite Abundance
2
0
−2
10000
11000
12000
13000
14000
15000
16000
17000
19000
21000
23000
64000
68000
70000
71000
72000
73000
74000
75000
76000
77000
78000
79000
80000
81000
82000
83000
84000
86000
87000
89000
93000
97000
Subject
Condition
EM
Primary

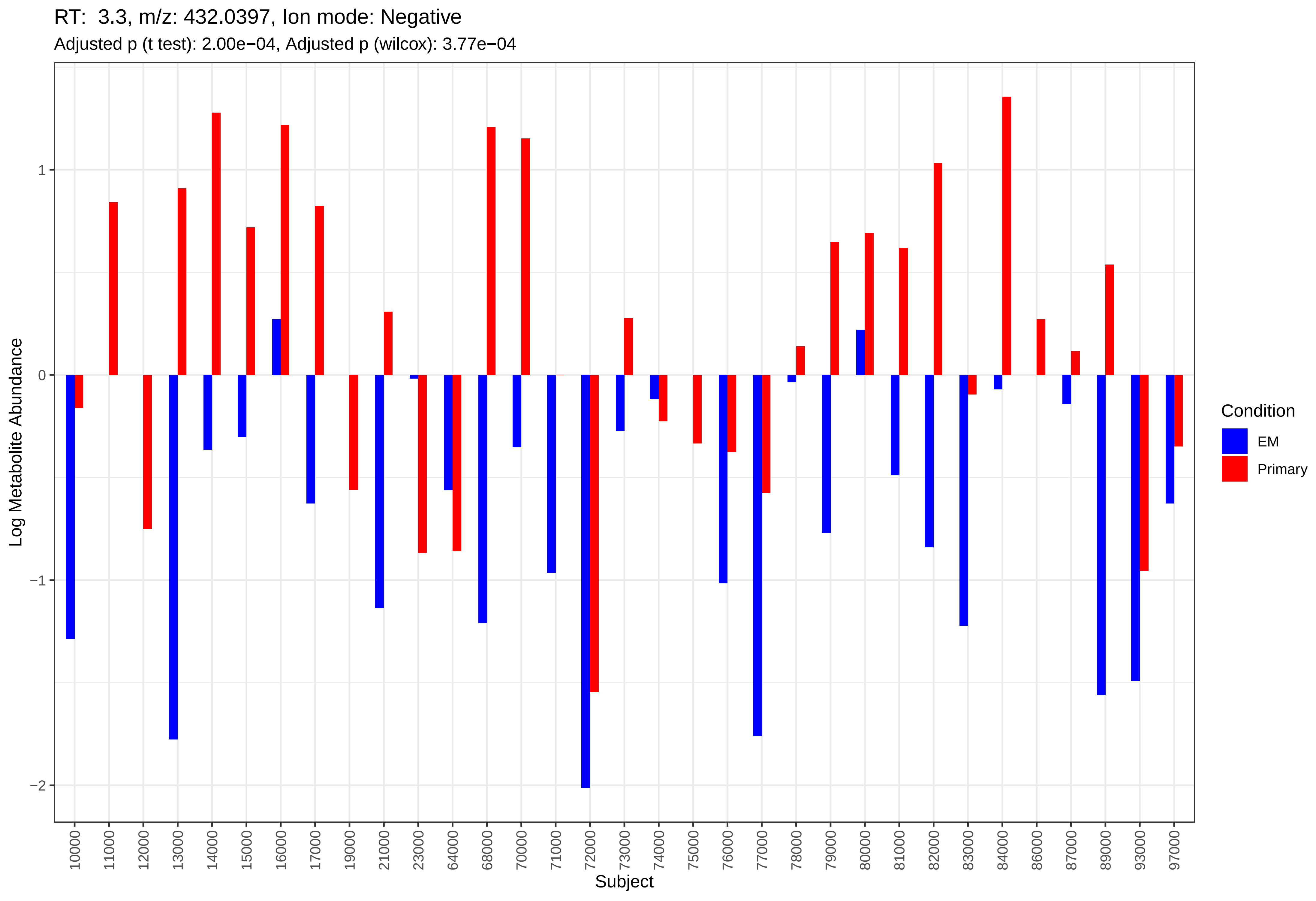

RT: 3.3, m/z: 432.0397, Ion mode: Negative
Adjusted p (t test): 2.00e−04, Adjusted p (wilcox): 3.77e−04
Log Metabolite Abundance
1
0
−1
−2
10000
11000
12000
13000
14000
15000
16000
17000
19000
21000
23000
64000
68000
70000
71000
72000
73000
74000
75000
76000
77000
78000
79000
80000
81000
82000
83000
84000
86000
87000
89000
93000
97000
Subject
Condition
EM
Primary

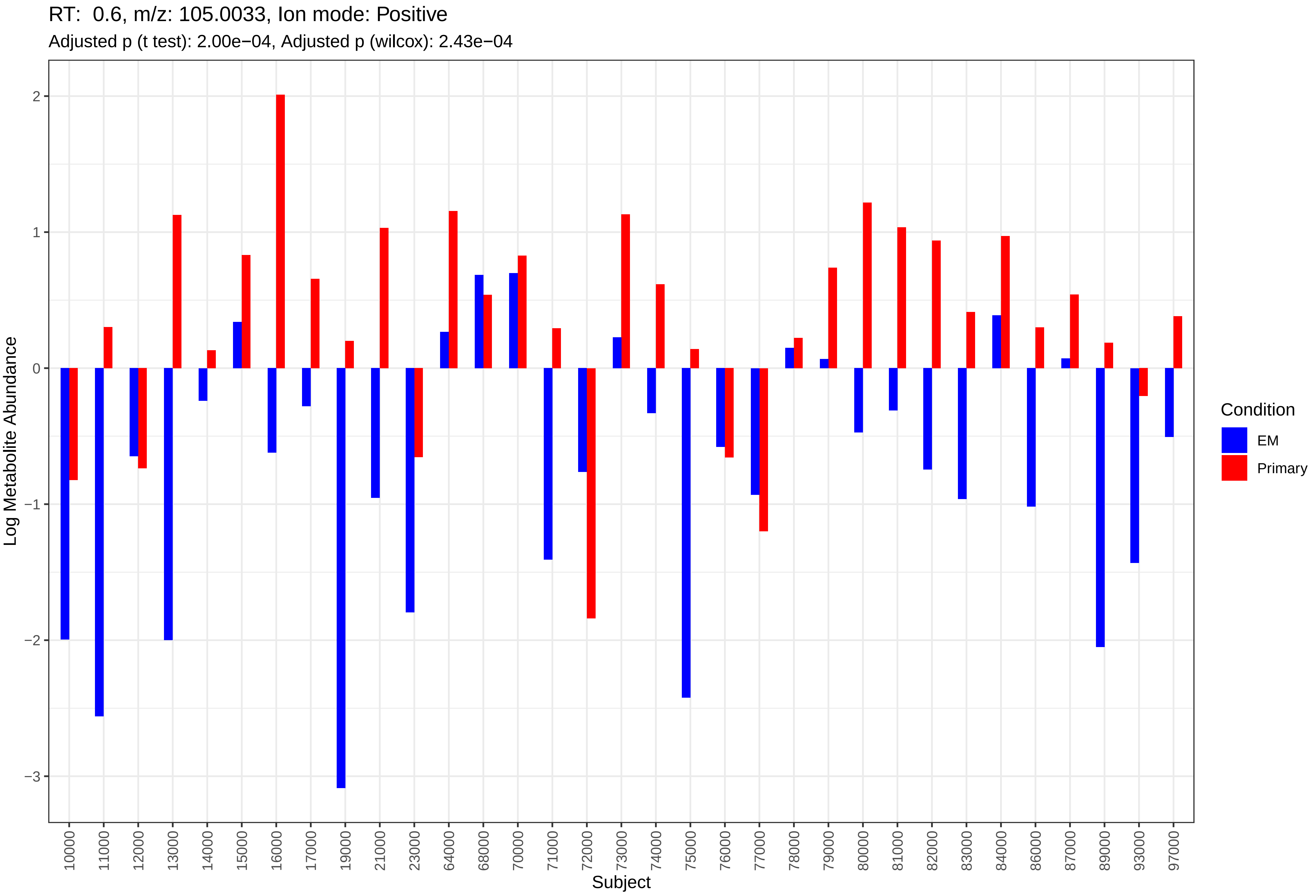
RT: 0.6, m/z: 105.0033, Ion mode: Positive
Adjusted p (t test): 2.00e−04, Adjusted p (wilcox): 2.43e−04
Log Metabolite Abundance
2
1
0
−1
−2
−3
10000
11000
12000
13000
14000
15000
16000
17000
19000
21000
23000
64000
68000
70000
71000
72000
73000
74000
75000
76000
77000
78000
79000
80000
81000
82000
83000
84000
86000
87000
89000
93000
97000
Subject
Condition
EM
Primary

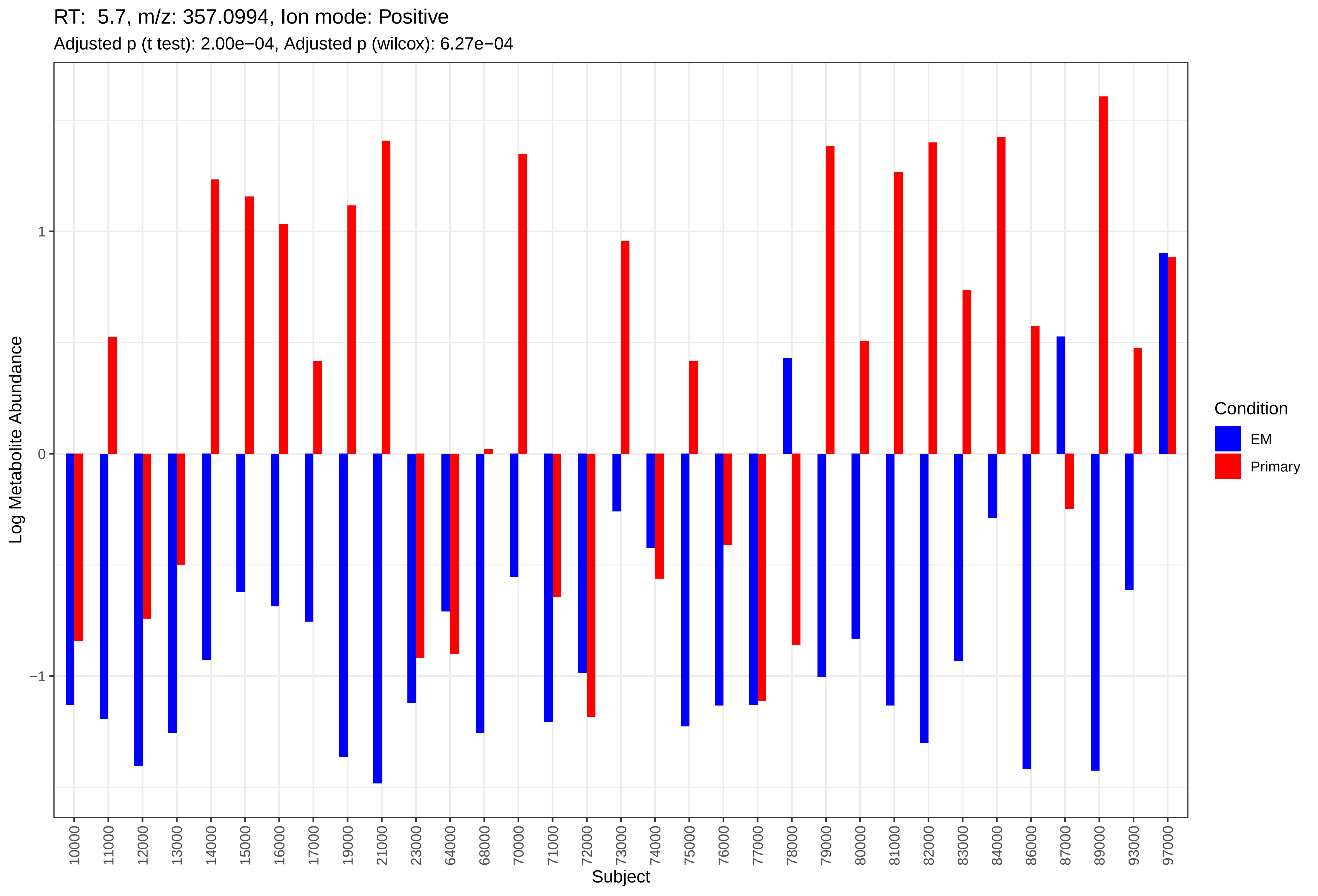

RT: 5.7, m/z: 357.0994, Ion mode: Positive
Adjusted p (t test): 2.00e−04, Adjusted p (wilcox): 6.27e−04
Log Metabolite Abundance
1
0
−1
10000
11000
12000
13000
14000
15000
16000
17000
19000
21000
23000
64000
68000
70000
71000
72000
73000
74000
75000
76000
77000
78000
79000
80000
81000
82000
83000
84000
86000
87000
89000
93000
97000
Subject
Condition
EM
Primary

RT: 3.9, m/z: 257.1003, Ion mode: Positive

Adjusted p (t test): 2.05e−04, Adjusted p (wilcox): 4.59e−04

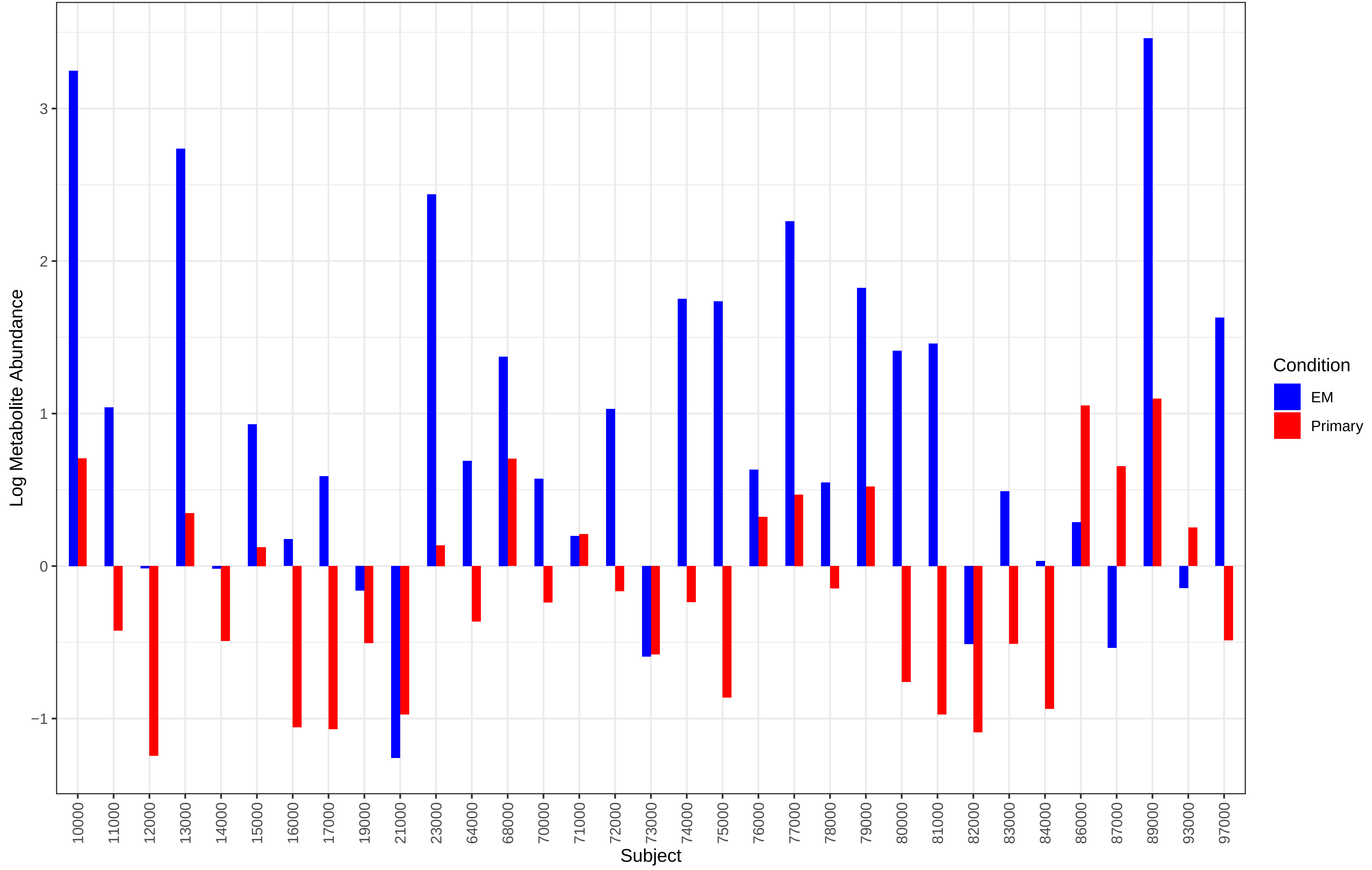

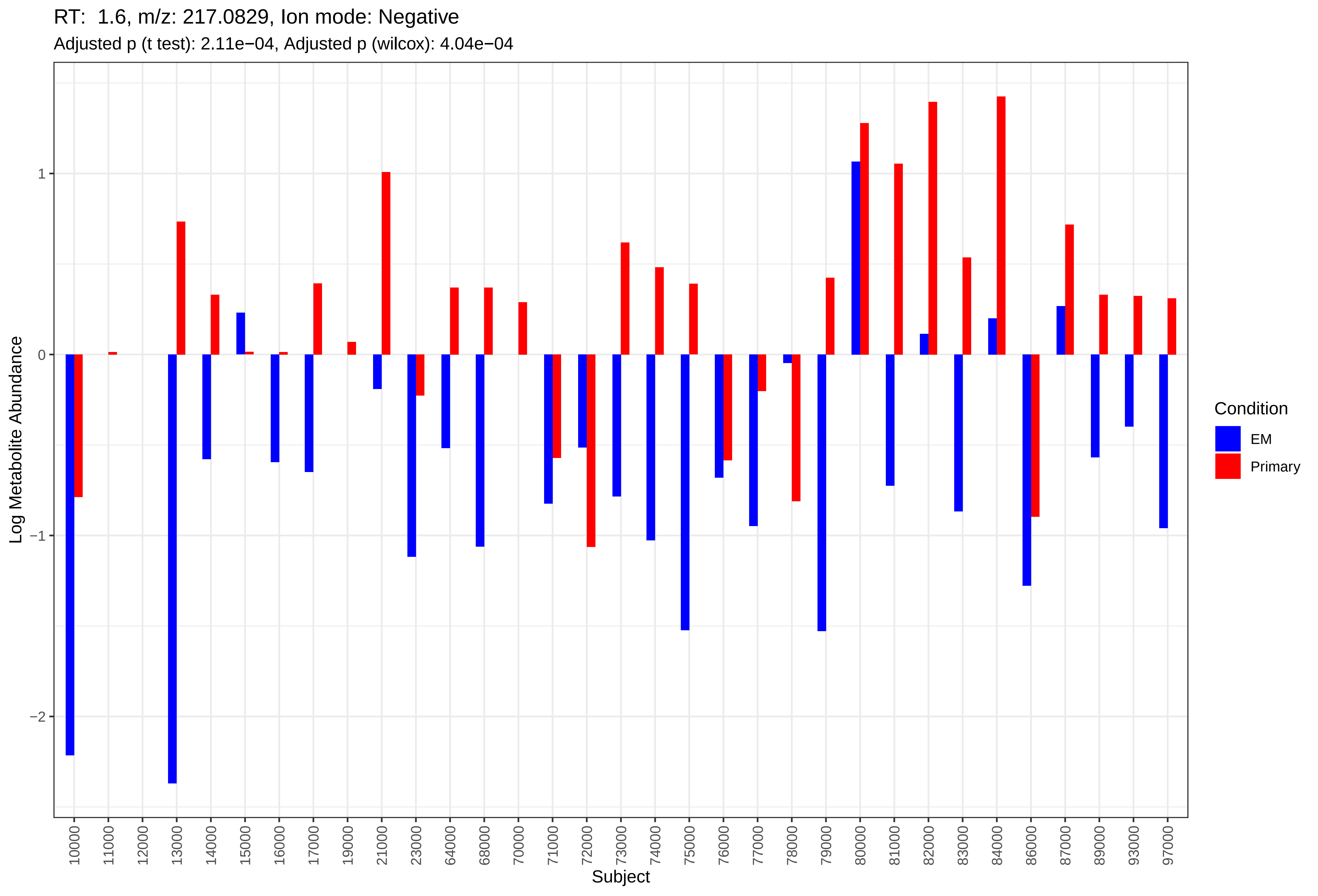
RT: 1.6, m/z: 217.0829, Ion mode: Negative
Adjusted p (t test): 2.11e−04, Adjusted p (wilcox): 4.04e−04
Log Metabolite Abundance
1
0
−1
−2
Subject
10000
11000
12000
13000
14000
15000
16000
17000
19000
21000
23000
64000
68000
70000
71000
72000
73000
74000
75000
76000
77000
78000
79000
80000
81000
82000
83000
84000
86000
87000
89000
93000
97000
Condition
EM
Primary

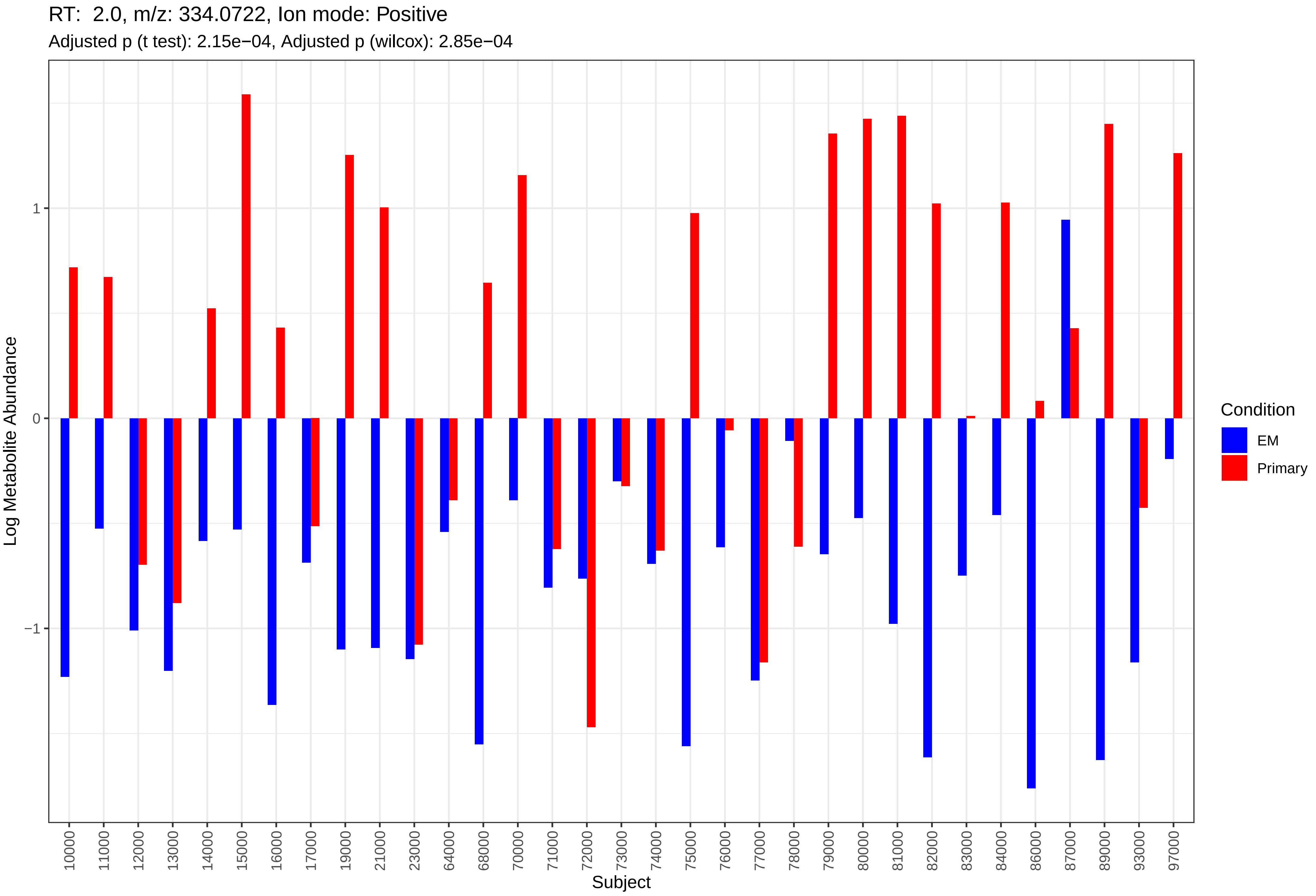
RT: 2.0, m/z: 334.0722, Ion mode: Positive
Adjusted p (t test): 2.15e−04, Adjusted p (wilcox): 2.85e−04
Log Metabolite Abundance
1
0
−1
10000
11000
12000
13000
14000
15000
16000
17000
19000
21000
23000
64000
68000
70000
71000
72000
73000
74000
75000
76000
77000
78000
79000
80000
81000
82000
83000
84000
86000
87000
89000
93000
97000
Subject
Condition
EM
Primary

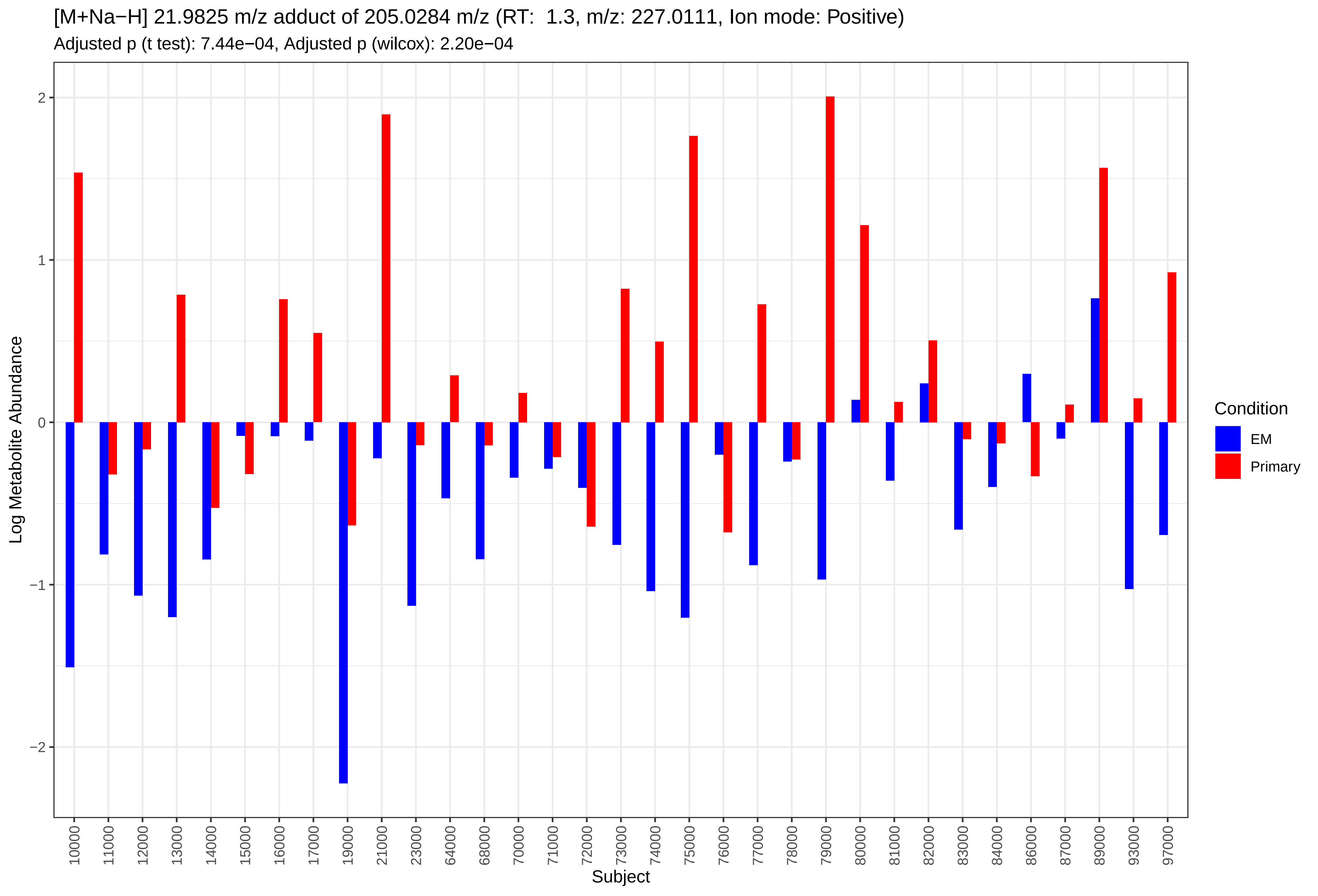

[M+Na−H] 21.9825 m/z adduct of 205.0284 m/z (RT: 1.3, m/z: 227.0111, Ion mode: Positive)
Adjusted p (t test): 7.44e−04, Adjusted p (wilcox): 2.20e−04
Log Metabolite Abundance
2
1
0
−1
−2
Subject
10000
11000
12000
13000
14000
15000
16000
17000
19000
21000
23000
64000
68000
70000
71000
72000
73000
74000
75000
76000
77000
78000
79000
80000
81000
82000
83000
84000
86000
87000
89000
93000
97000
Condition
EM
Primary

RT: 8.7, m/z: 266.2540, Ion mode: Positive

Adjusted p (t test): 1.31e−03, Adjusted p (wilcox): 2.20e−04

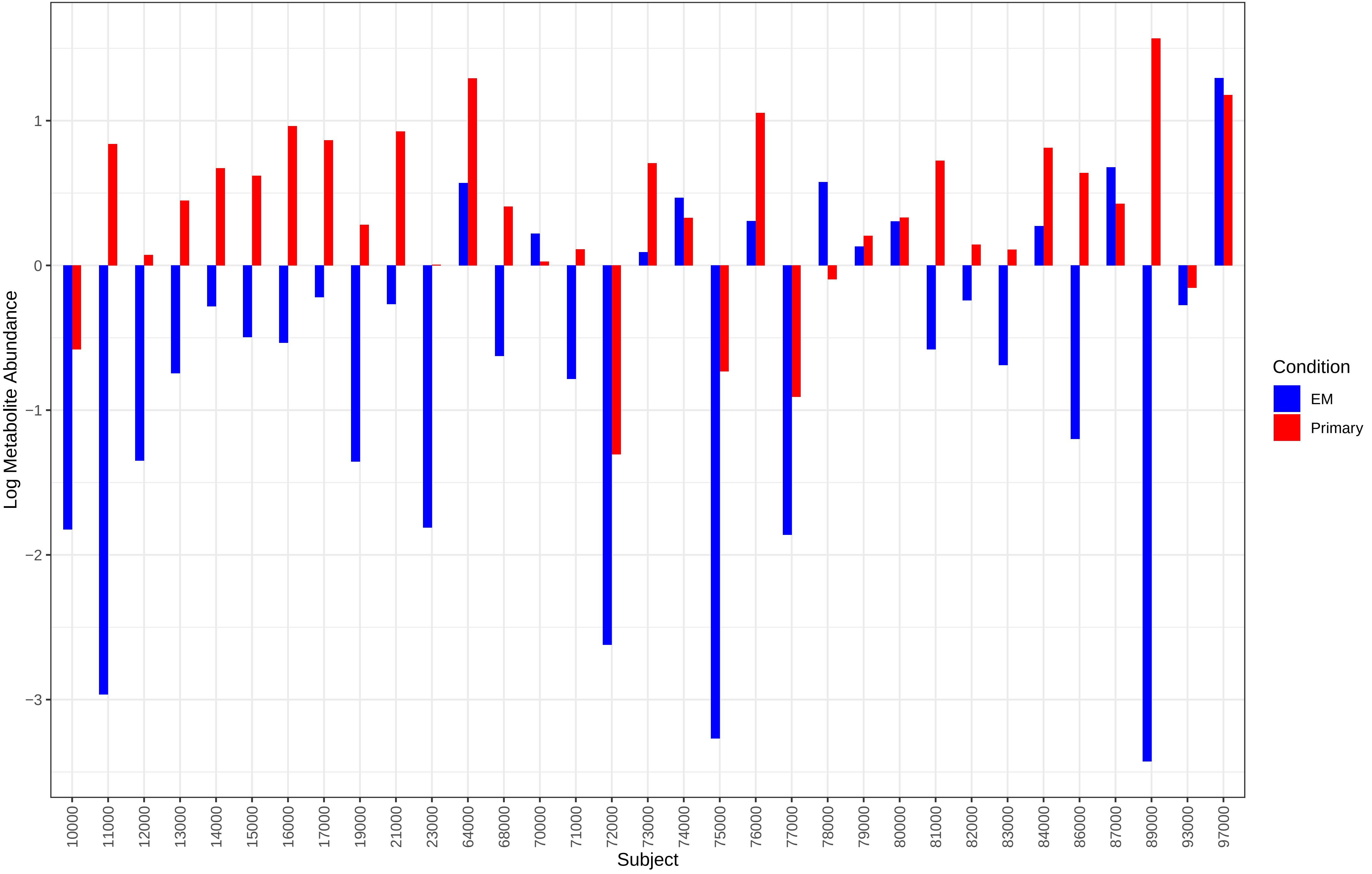

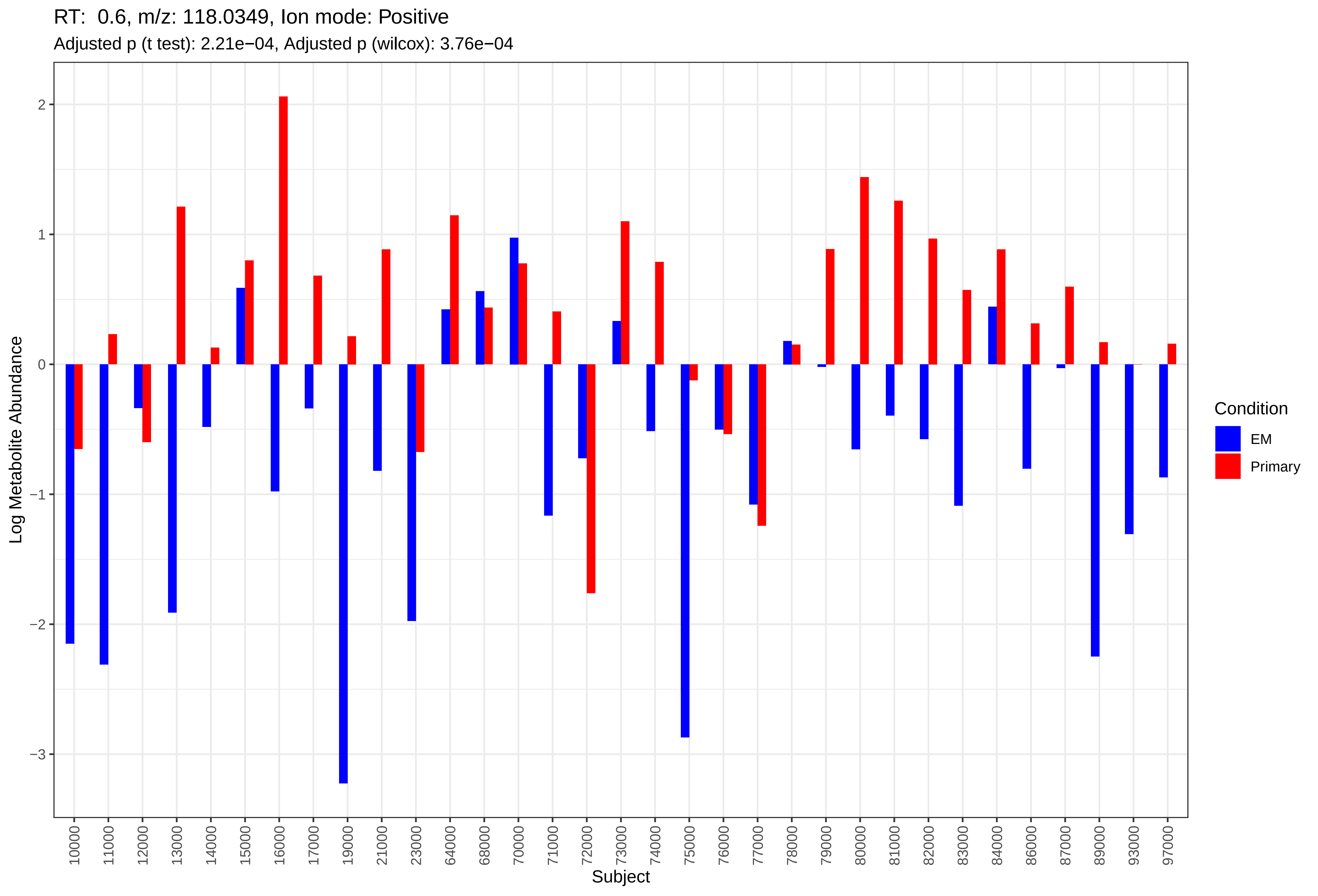
RT: 0.6, m/z: 118.0349, Ion mode: Positive
Adjusted p (t test): 2.21e−04, Adjusted p (wilcox): 3.76e−04
Log Metabolite Abundance
2
1
0
−1
−2
−3
10000
11000
12000
13000
14000
15000
16000
17000
19000
21000
23000
64000
68000
70000
71000
72000
73000
74000
75000
76000
77000
78000
79000
80000
81000
82000
83000
84000
86000
87000
89000
93000
97000
Subject
Condition
EM
Primary

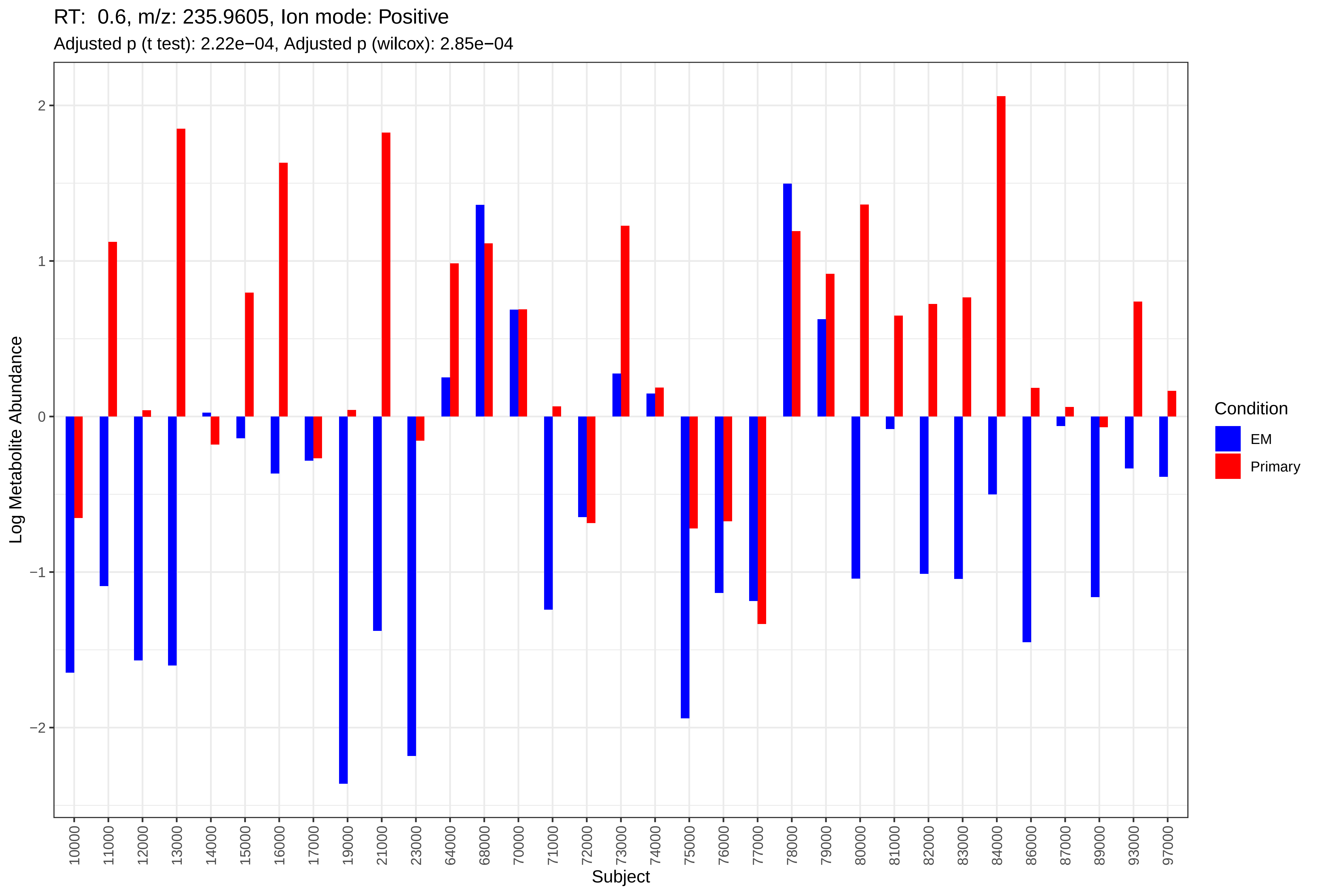
RT: 0.6, m/z: 235.9605, Ion mode: Positive
Adjusted p (t test): 2.22e−04, Adjusted p (wilcox): 2.85e−04
Log Metabolite Abundance
2
1
0
−1
−2
10000
11000
12000
13000
14000
15000
16000
17000
19000
21000
23000
64000
68000
70000
71000
72000
73000
74000
75000
76000
77000
78000
79000
80000
81000
82000
83000
84000
86000
87000
89000
93000
97000
Subject
Condition
EM
Primary

Sphingosine (d18:1) (RT: 13.6, m/z: 300.2890, Ion mode: Positive)

Adjusted p (t test): 2.26e−04, Adjusted p (wilcox): 2.64e−04

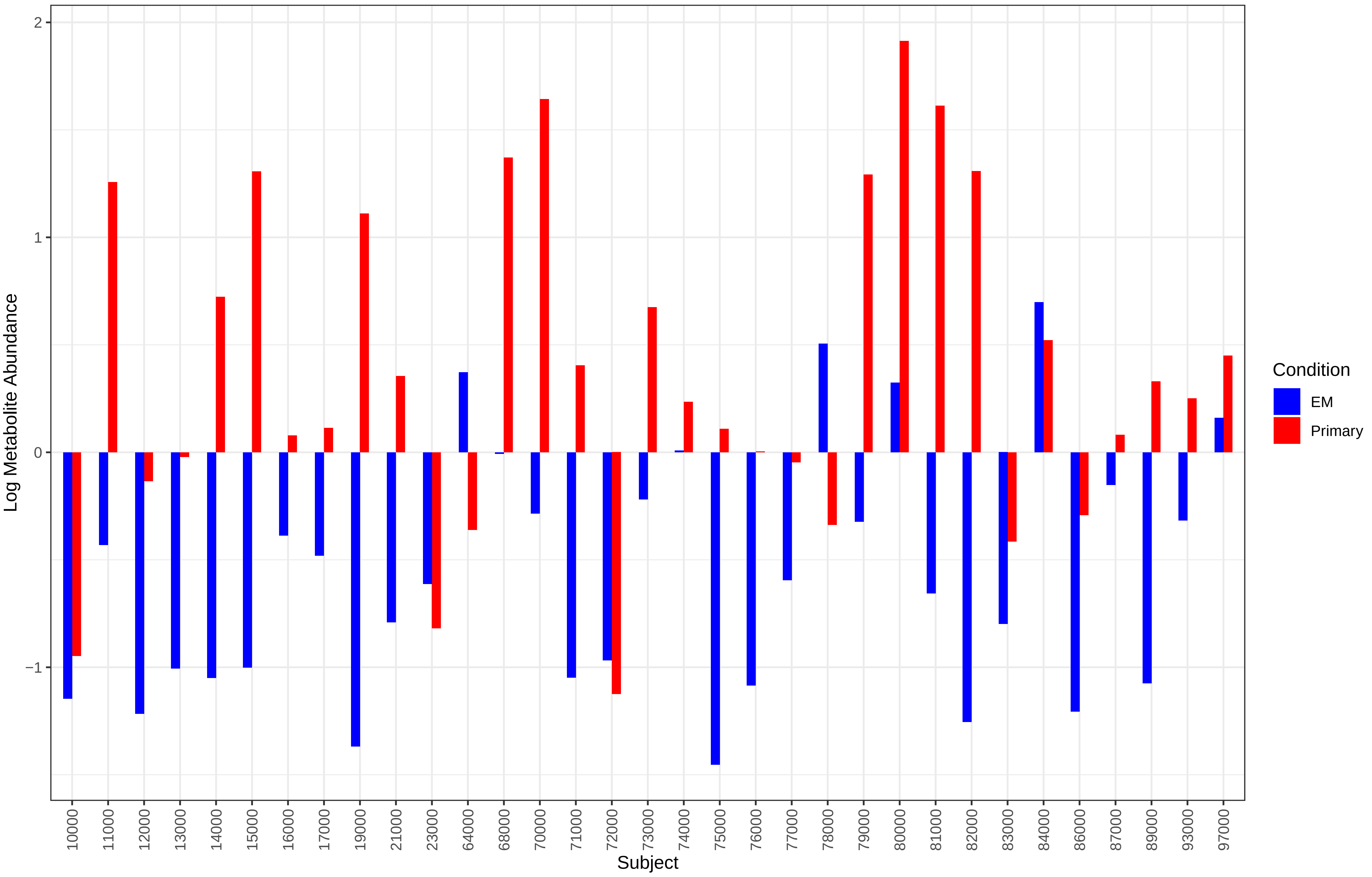

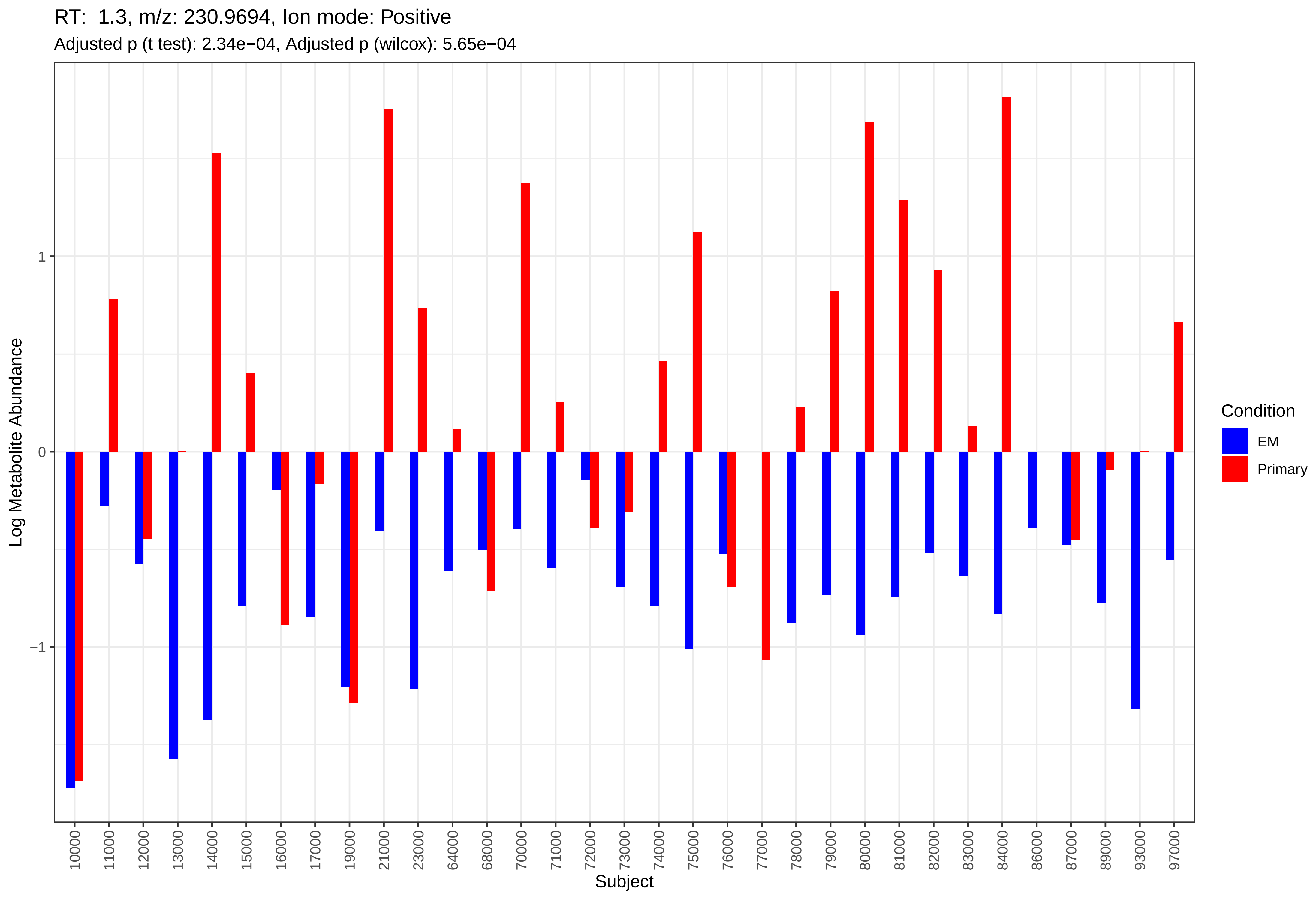
RT: 1.3, m/z: 230.9694, Ion mode: Positive
Adjusted p (t test): 2.34e−04, Adjusted p (wilcox): 5.65e−04
Log Metabolite Abundance
1
0
−1
Subject
10000
11000
12000
13000
14000
15000
16000
17000
19000
21000
23000
64000
68000
70000
71000
72000
73000
74000
75000
76000
77000
78000
79000
80000
81000
82000
83000
84000
86000
87000
89000
93000
97000
Condition
EM
Primary

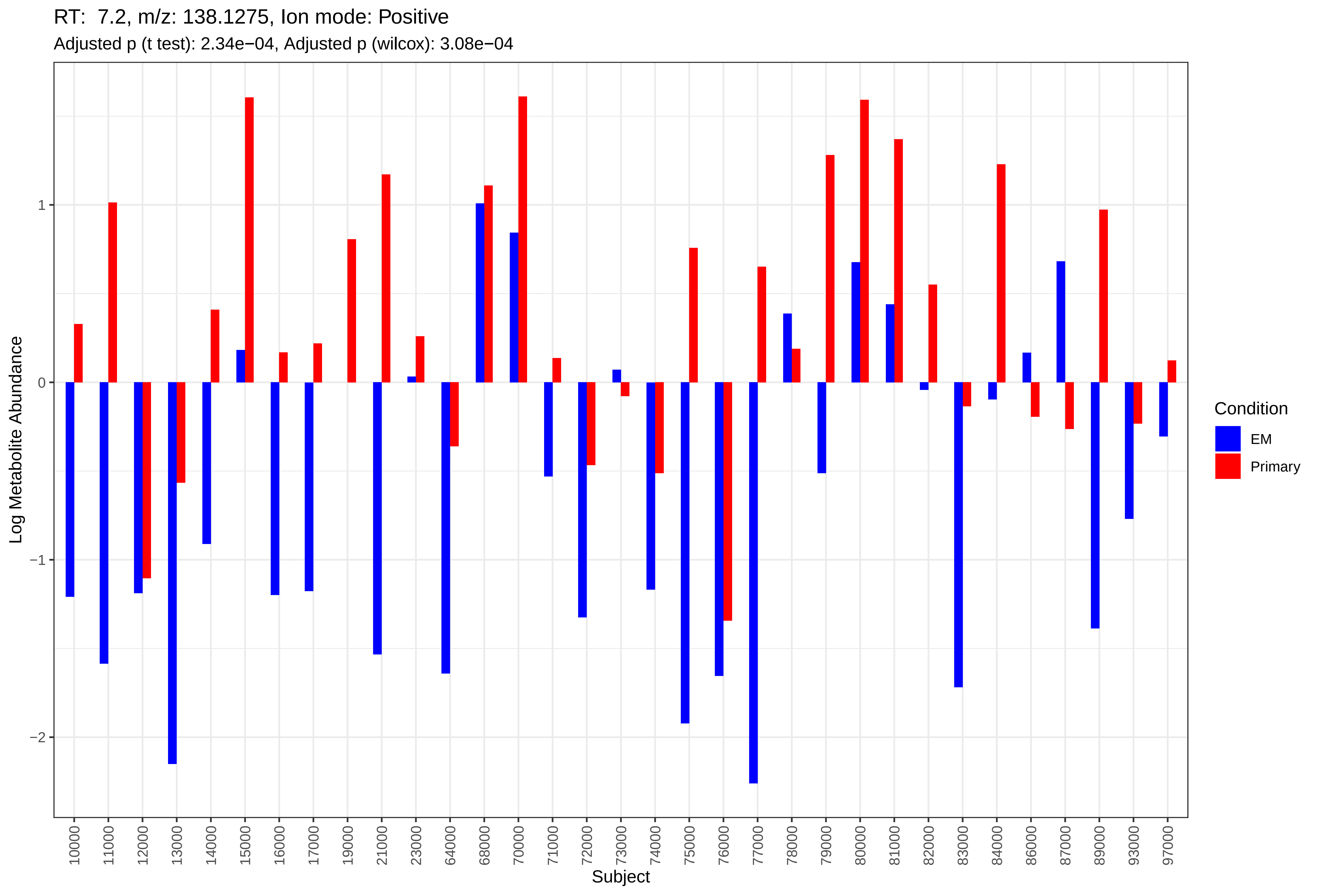
RT: 7.2, m/z: 138.1275, Ion mode: Positive
Adjusted p (t test): 2.34e−04, Adjusted p (wilcox): 3.08e−04
Log Metabolite Abundance
1
0
−1
−2
Subject
10000
11000
12000
13000
14000
15000
16000
17000
19000
21000
23000
64000
68000
70000
71000
72000
73000
74000
75000
76000
77000
78000
79000
80000
81000
82000
83000
84000
86000
87000
89000
93000
97000
Condition
EM
Primary

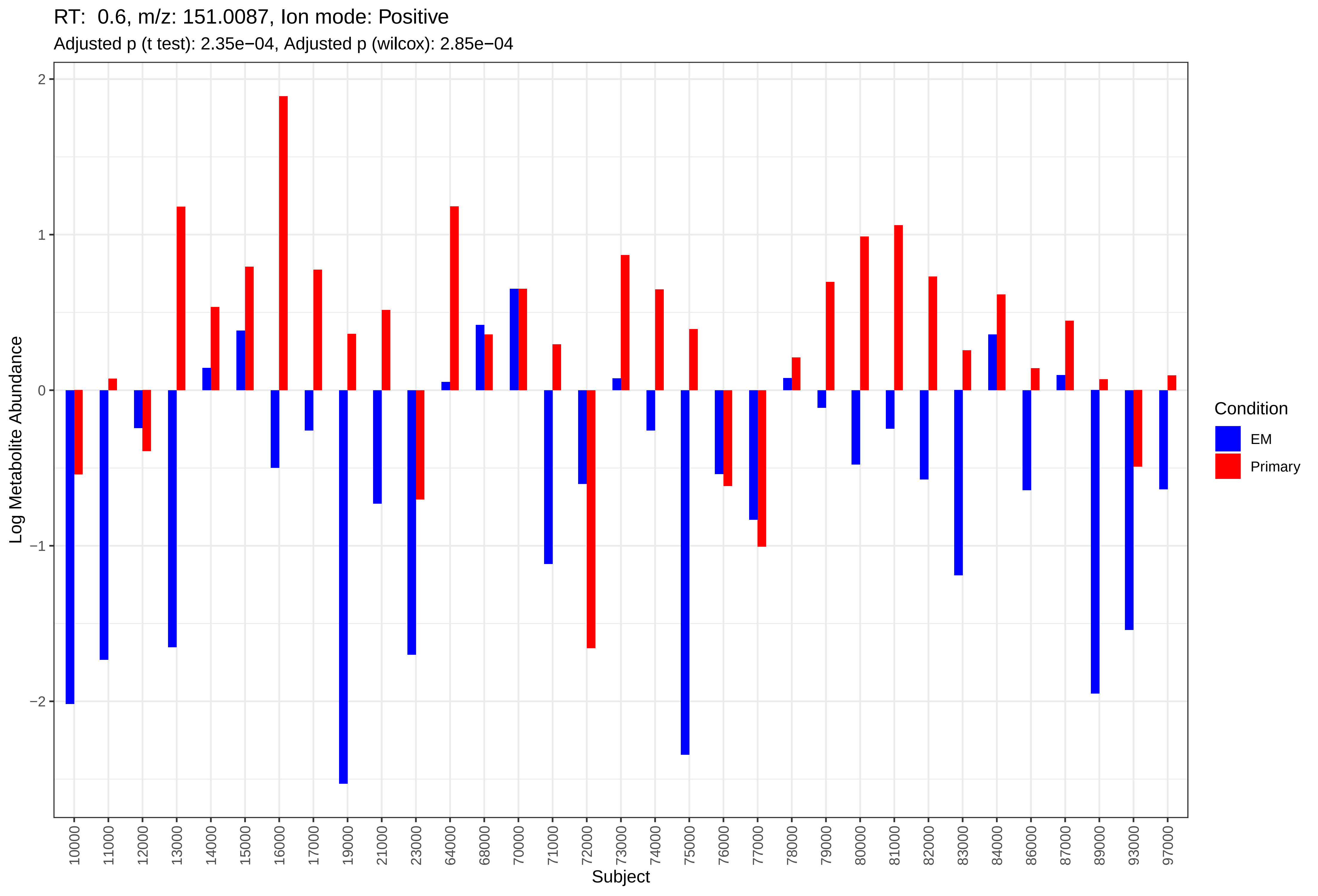
RT: 0.6, m/z: 151.0087, Ion mode: Positive
Adjusted p (t test): 2.35e−04, Adjusted p (wilcox): 2.85e−04
Log Metabolite Abundance
2
1
0
−1
−2
Subject
10000
11000
12000
13000
14000
15000
16000
17000
19000
21000
23000
64000
68000
70000
71000
72000
73000
74000
75000
76000
77000
78000
79000
80000
81000
82000
83000
84000
86000
87000
89000
93000
97000
Condition
EM
Primary

RT: 2.0, m/z: 218.0323, Ion mode: Positive

Adjusted p (t test): 2.36e−04, Adjusted p (wilcox): 3.76e−04

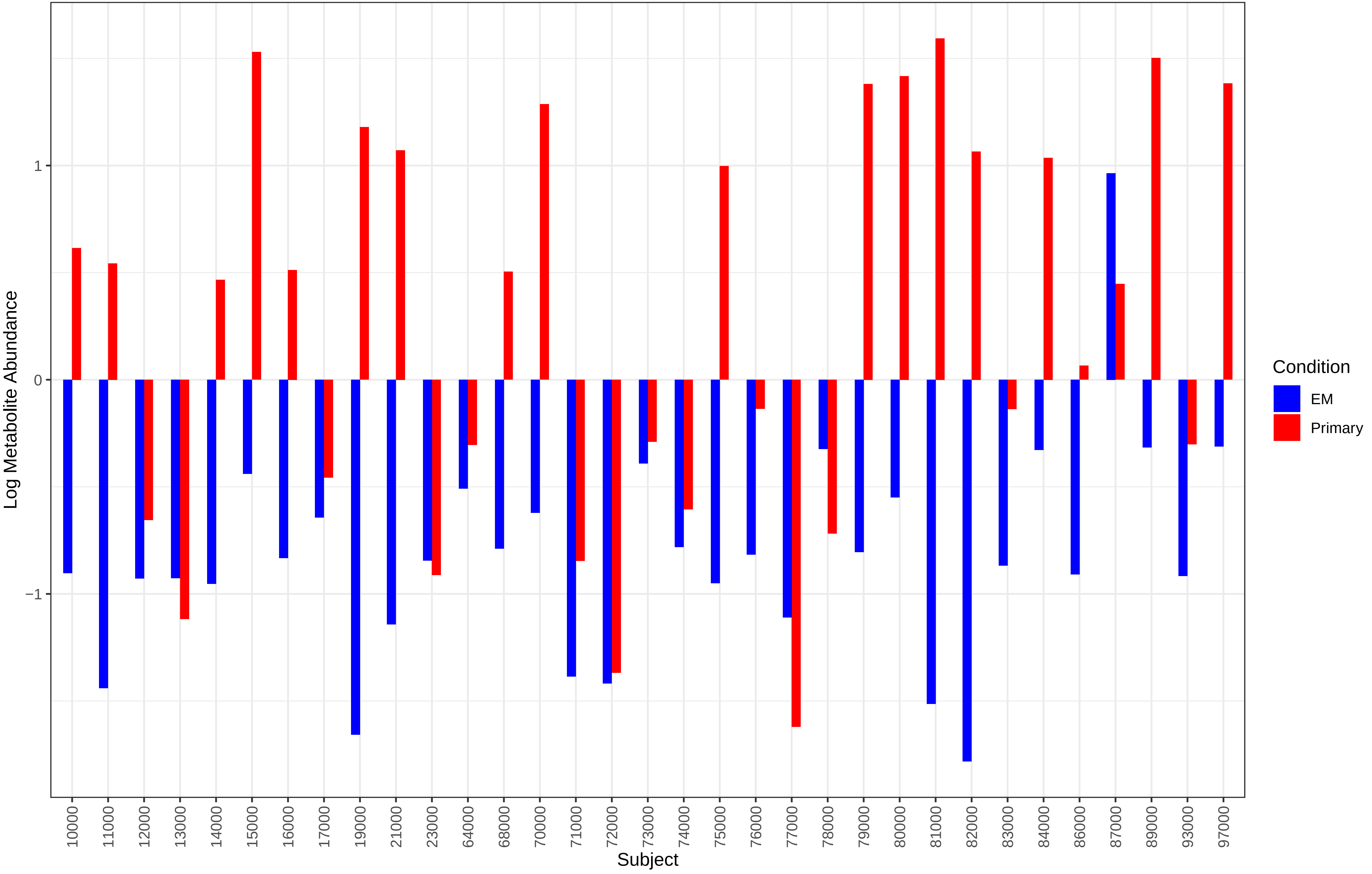

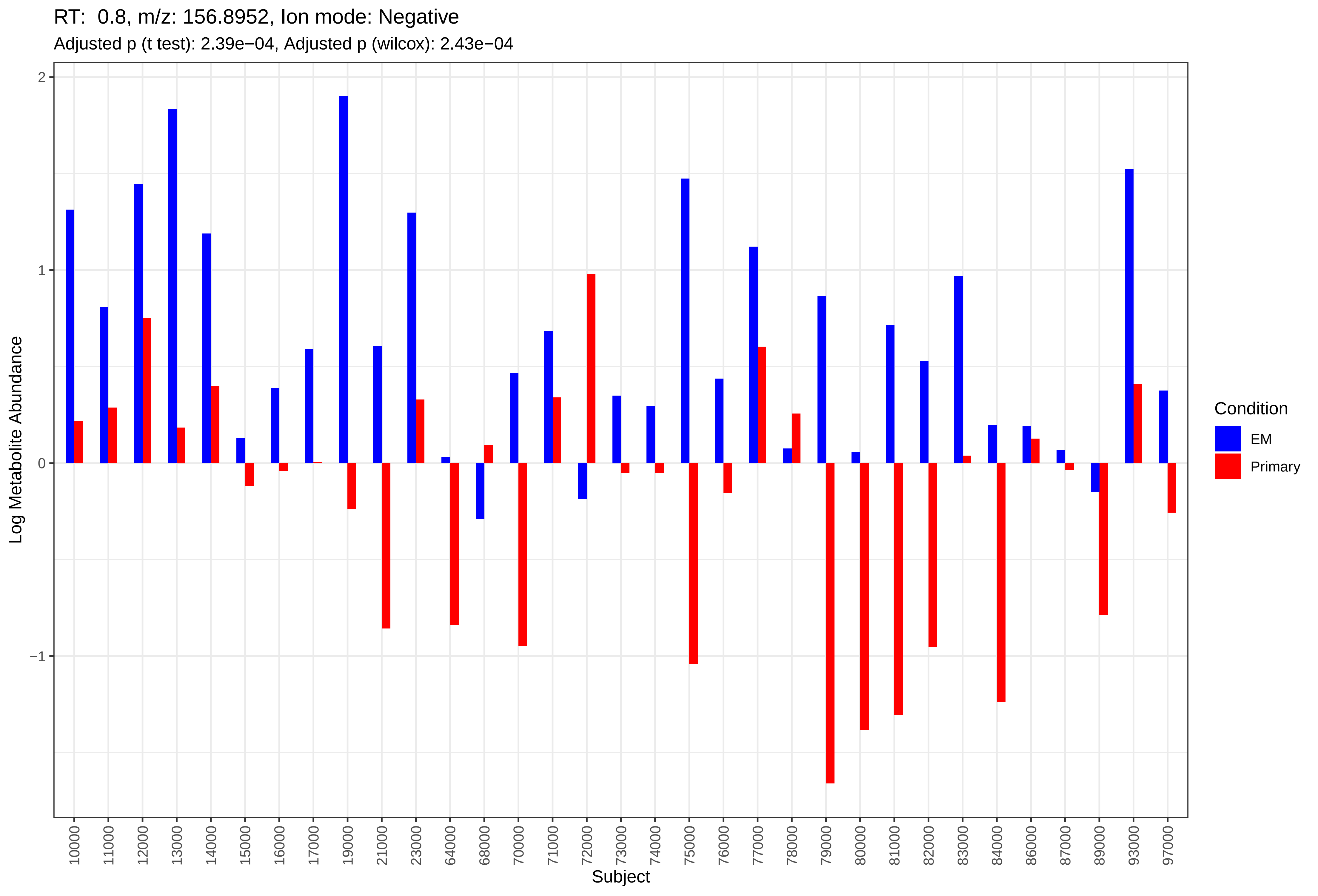

RT: 0.8, m/z: 156.8952, Ion mode: Negative
Adjusted p (t test): 2.39e−04, Adjusted p (wilcox): 2.43e−04
Log Metabolite Abundance
2
1
0
−1
Subject
10000
11000
12000
13000
14000
15000
16000
17000
19000
21000
23000
64000
68000
70000
71000
72000
73000
74000
75000
76000
77000
78000
79000
80000
81000
82000
83000
84000
86000
87000
89000
93000
97000
Condition
EM
Primary

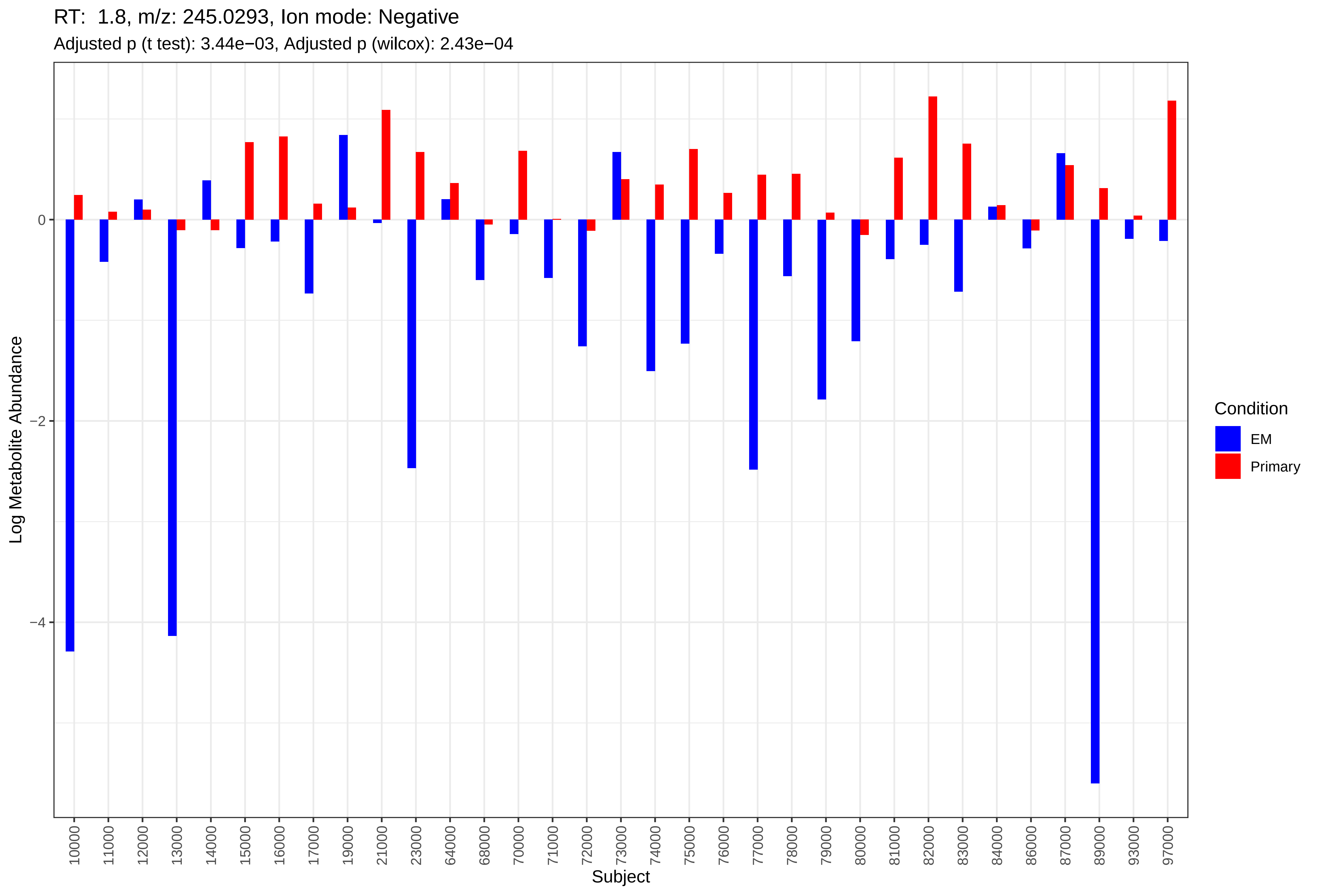

RT: 1.8, m/z: 245.0293, Ion mode: Negative
Adjusted p (t test): 3.44e−03, Adjusted p (wilcox): 2.43e−04
Log Metabolite Abundance
0
−2
−4
10000
11000
12000
13000
14000
15000
16000
17000
19000
21000
23000
64000
68000
70000
71000
72000
73000
74000
75000
76000
77000
78000
79000
80000
81000
82000
83000
84000
86000
87000
89000
93000
97000
Subject
Condition
EM
Primary

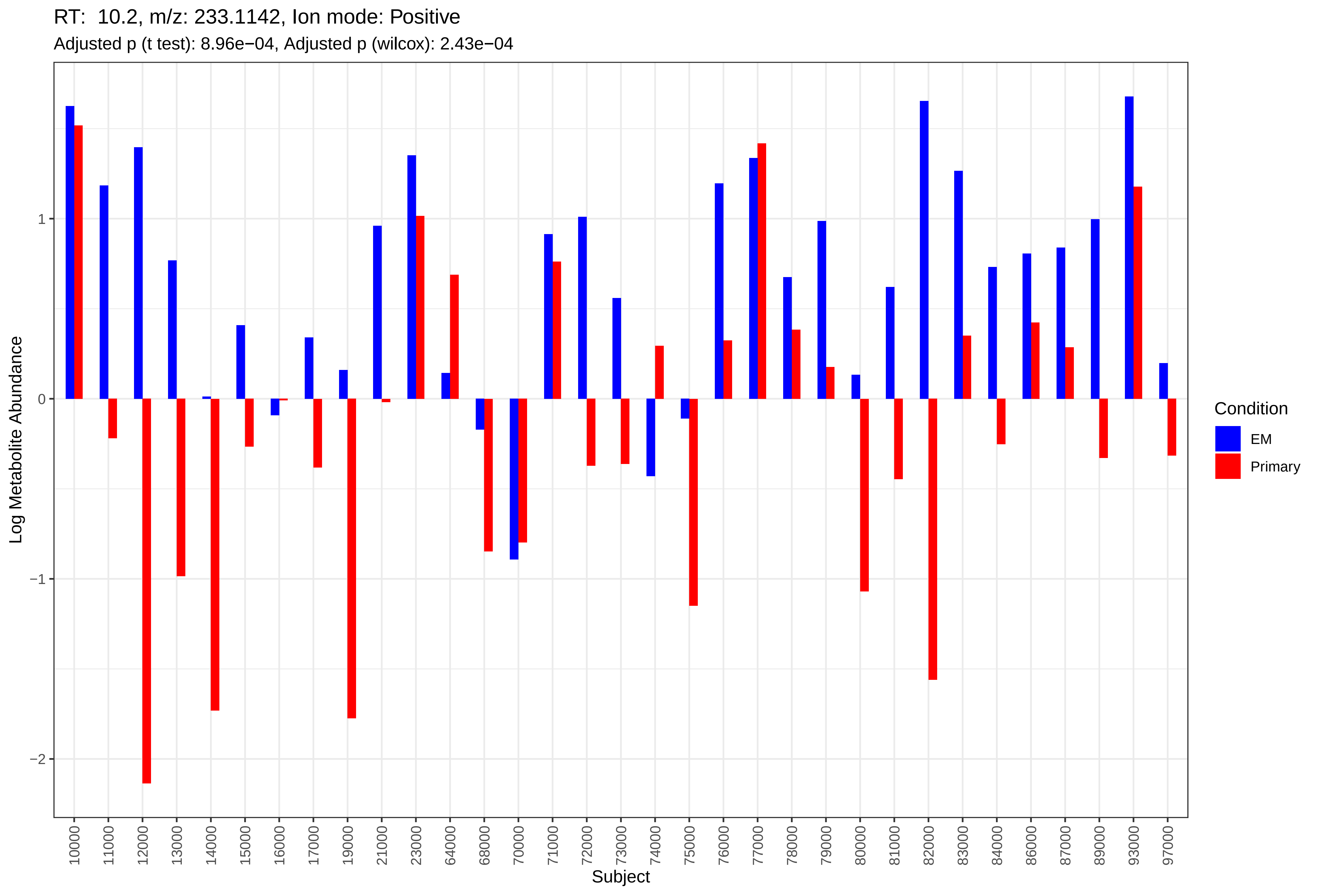

RT: 10.2, m/z: 233.1142, Ion mode: Positive
Adjusted p (t test): 8.96e−04, Adjusted p (wilcox): 2.43e−04
Log Metabolite Abundance
1
0
−1
−2
10000
11000
12000
13000
14000
15000
16000
17000
19000
21000
23000
64000
68000
70000
71000
72000
73000
74000
75000
76000
77000
78000
79000
80000
81000
82000
83000
84000
86000
87000
89000
93000
97000
Subject
Condition
EM
Primary

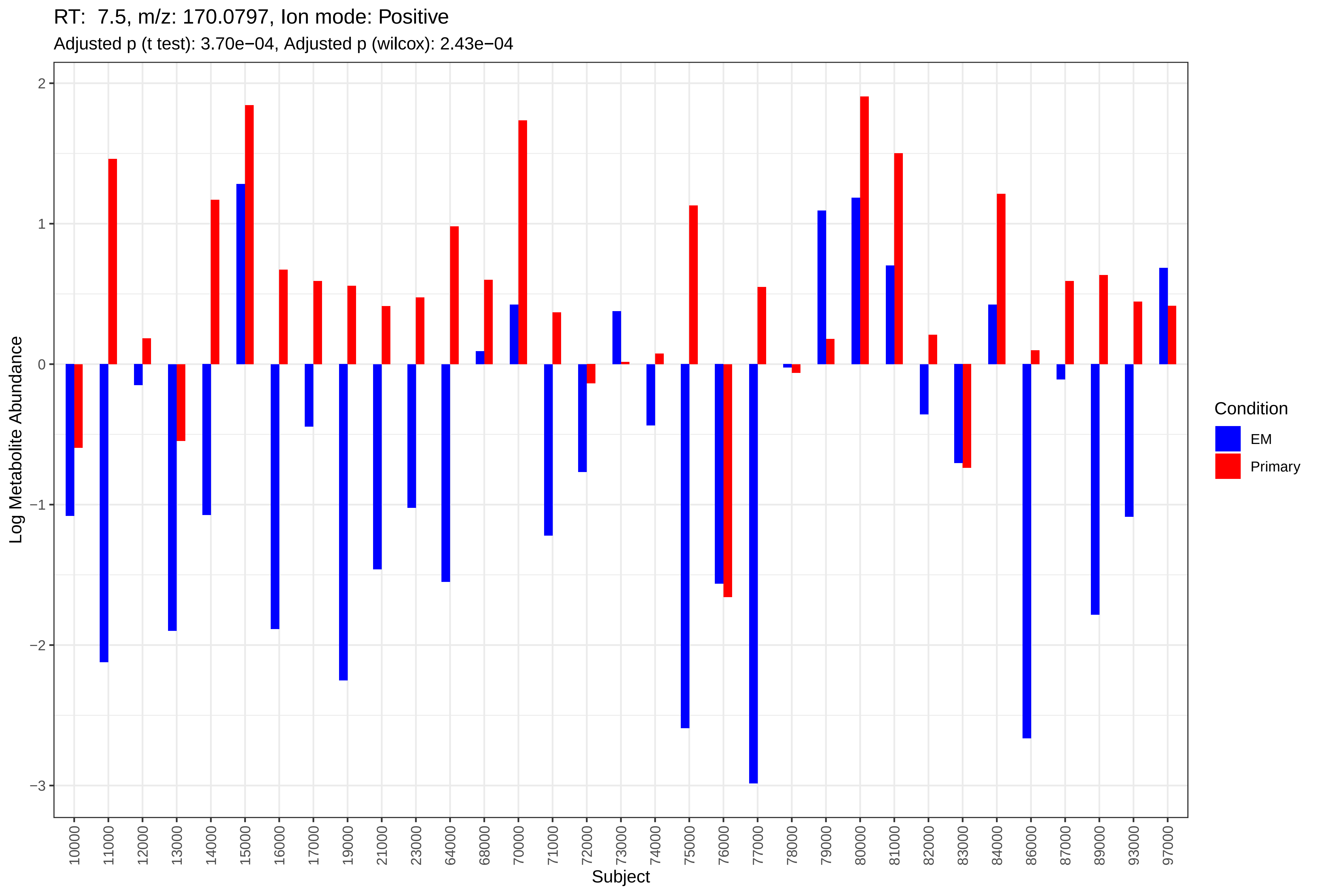

RT: 7.5, m/z: 170.0797, Ion mode: Positive
Adjusted p (t test): 3.70e−04, Adjusted p (wilcox): 2.43e−04
Log Metabolite Abundance
2
1
0
−1
−2
−3
10000
11000
12000
13000
14000
15000
16000
17000
19000
21000
23000
64000
68000
70000
71000
72000
73000
74000
75000
76000
77000
78000
79000
80000
81000
82000
83000
84000
86000
87000
89000
93000
97000
Subject
Condition
EM
Primary

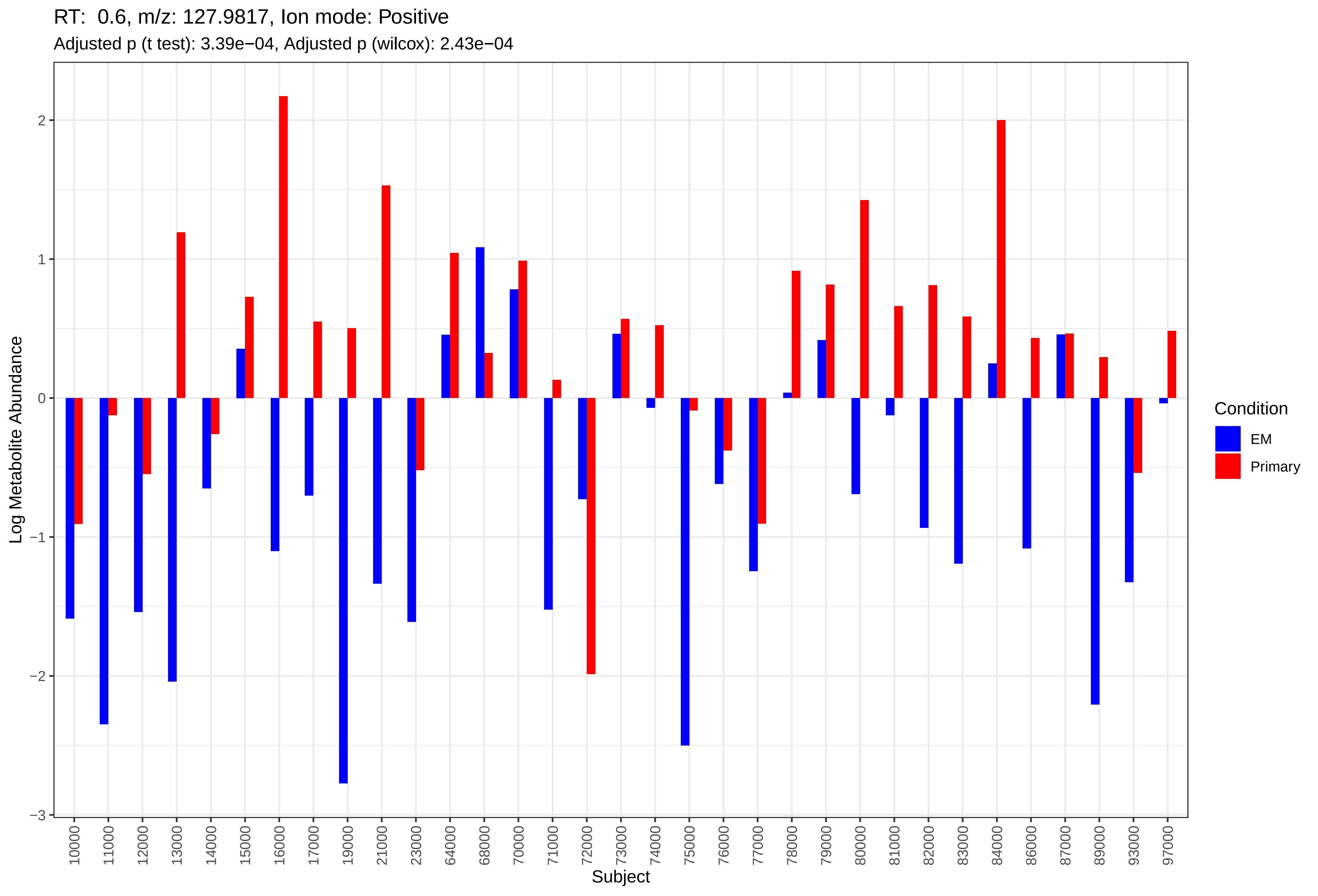

RT: 0.6, m/z: 127.9817, Ion mode: Positive
Adjusted p (t test): 3.39e−04, Adjusted p (wilcox): 2.43e−04
Log Metabolite Abundance
2
1
0
−1
−2
−3
10000
11000
12000
13000
14000
15000
16000
17000
19000
21000
23000
64000
68000
70000
71000
72000
73000
74000
75000
76000
77000
78000
79000
80000
81000
82000
83000
84000
86000
87000
89000
93000
97000
Subject
Condition
EM
Primary

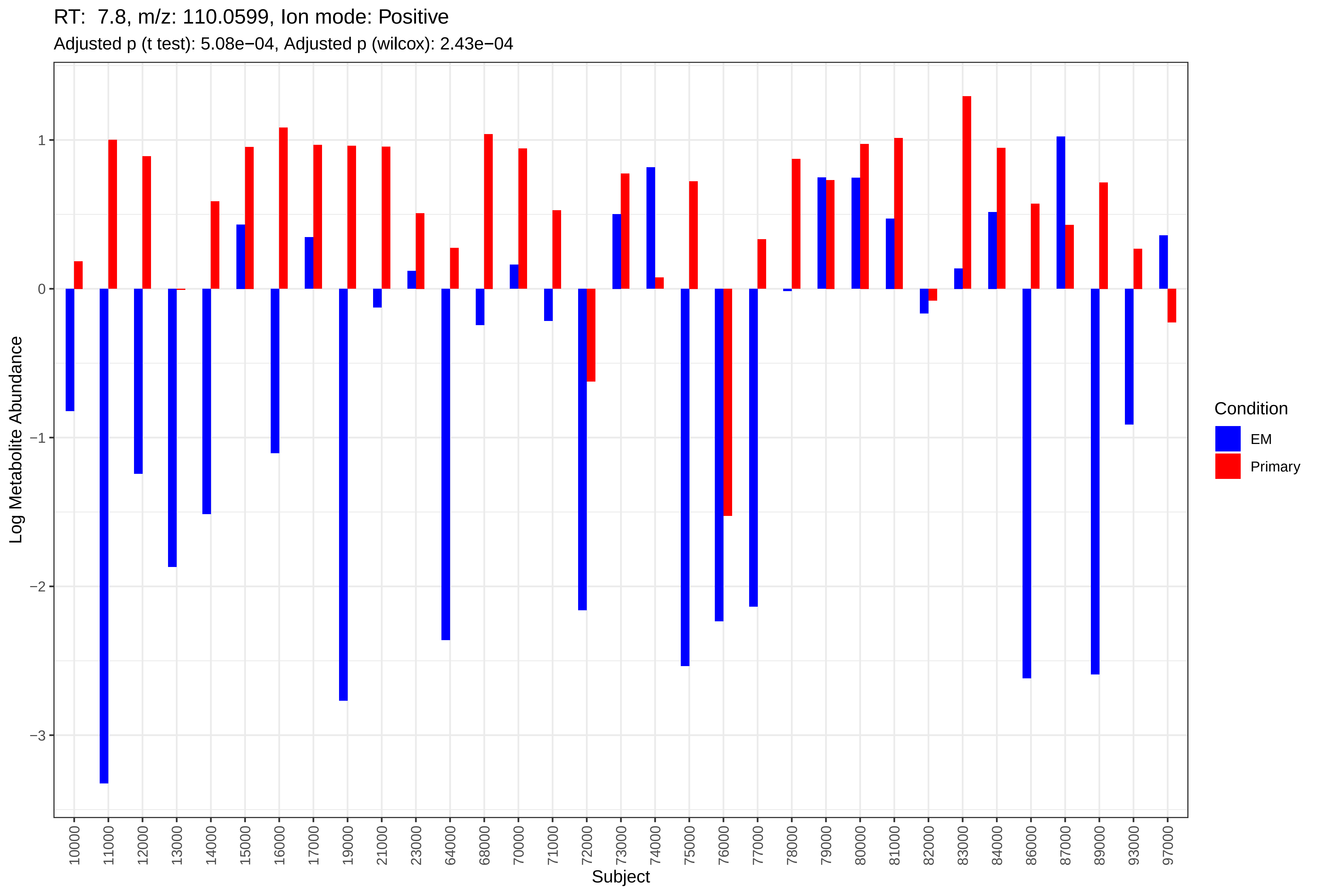
RT: 7.8, m/z: 110.0599, Ion mode: Positive
Adjusted p (t test): 5.08e−04, Adjusted p (wilcox): 2.43e−04
Log Metabolite Abundance
1
0
−1
−2
−3
Subject
10000
11000
12000
13000
14000
15000
16000
17000
19000
21000
23000
64000
68000
70000
71000
72000
73000
74000
75000
76000
77000
78000
79000
80000
81000
82000
83000
84000
86000
87000
89000
93000
97000
Condition
EM
Primary

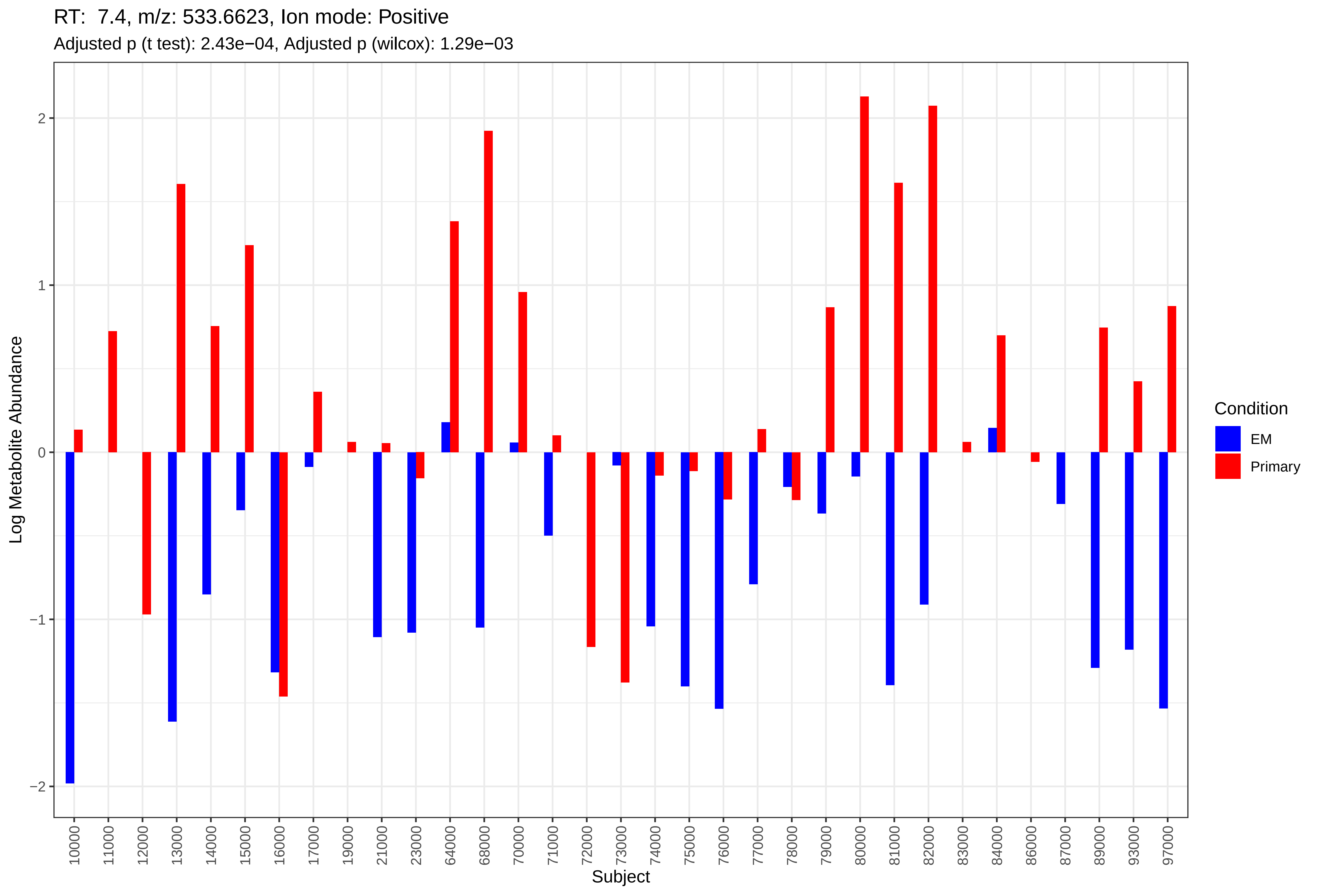
RT: 7.4, m/z: 533.6623, Ion mode: Positive
Adjusted p (t test): 2.43e−04, Adjusted p (wilcox): 1.29e−03
Log Metabolite Abundance
2
1
0
−1
−2
Subject
10000
11000
12000
13000
14000
15000
16000
17000
19000
21000
23000
64000
68000
70000
71000
72000
73000
74000
75000
76000
77000
78000
79000
80000
81000
82000
83000
84000
86000
87000
89000
93000
97000
Condition
EM
Primary

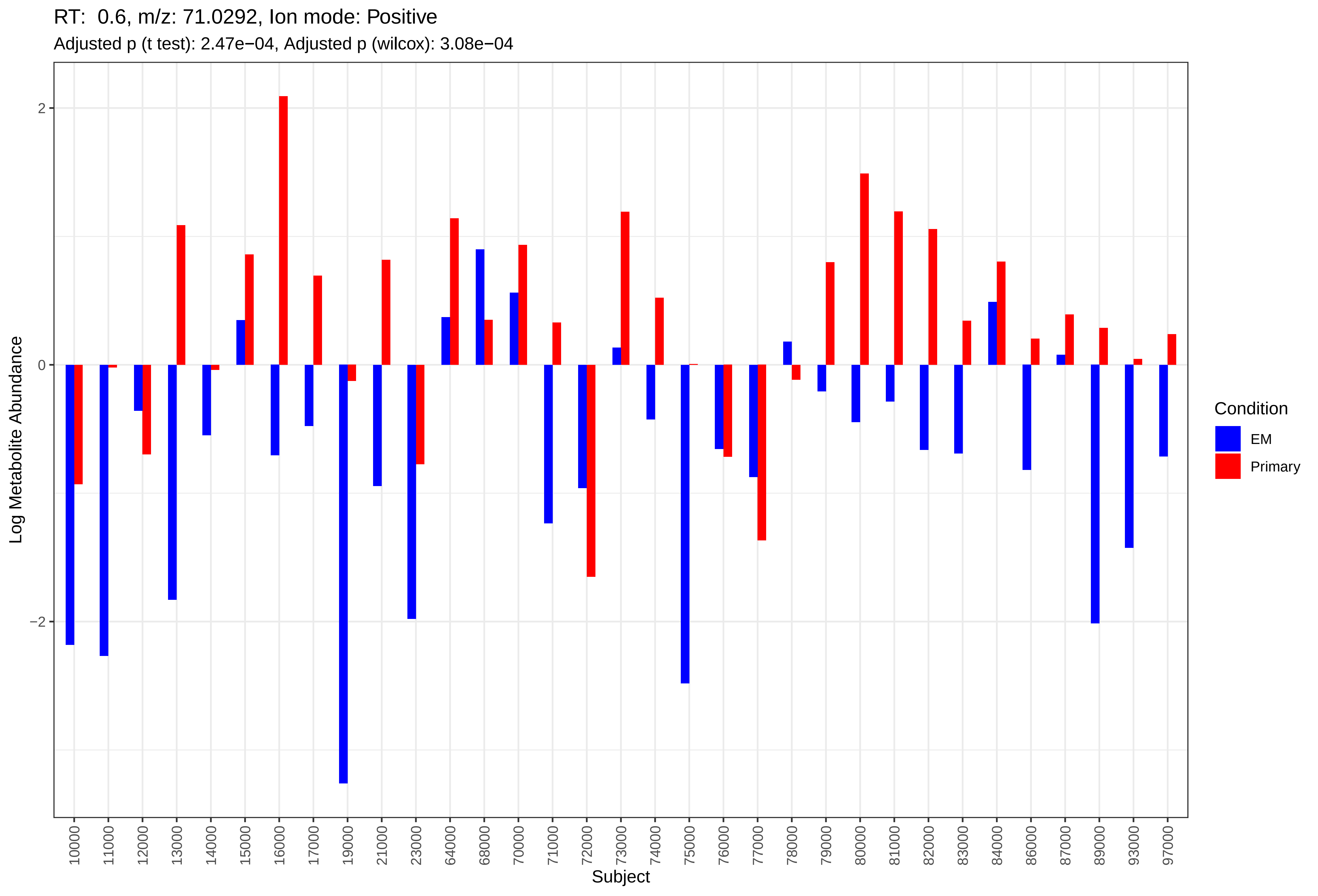

RT: 0.6, m/z: 71.0292, Ion mode: Positive
Adjusted p (t test): 2.47e−04, Adjusted p (wilcox): 3.08e−04
Log Metabolite Abundance
2
0
−2
Subject
10000
11000
12000
13000
14000
15000
16000
17000
19000
21000
23000
64000
68000
70000
71000
72000
73000
74000
75000
76000
77000
78000
79000
80000
81000
82000
83000
84000
86000
87000
89000
93000
97000
Condition
EM
Primary

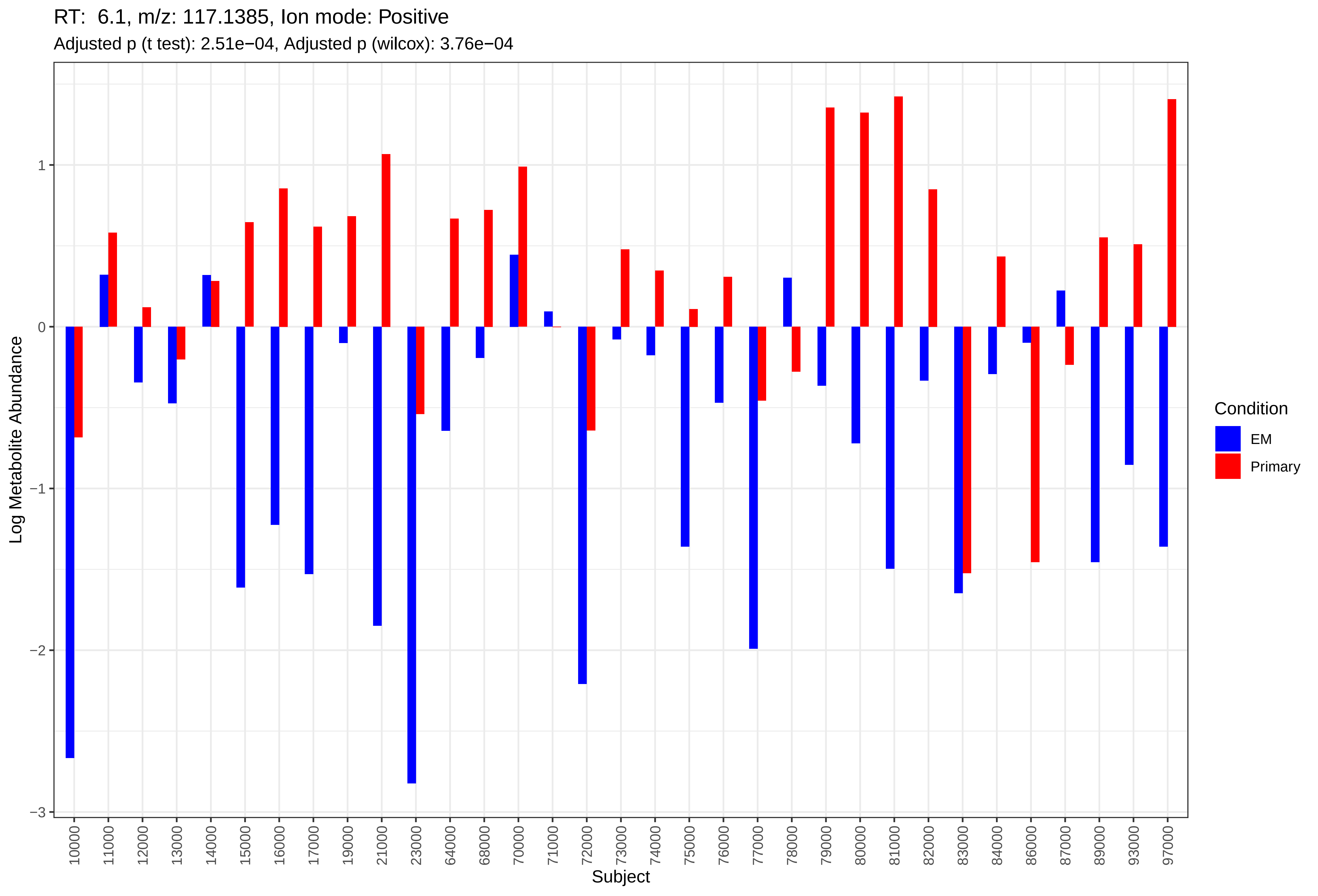

RT: 6.1, m/z: 117.1385, Ion mode: Positive
Adjusted p (t test): 2.51e−04, Adjusted p (wilcox): 3.76e−04
Log Metabolite Abundance
1
0
−1
−2
−3
10000
11000
12000
13000
14000
15000
16000
17000
19000
21000
23000
64000
68000
70000
71000
72000
73000
74000
75000
76000
77000
78000
79000
80000
81000
82000
83000
84000
86000
87000
89000
93000
97000
Subject
Condition
EM
Primary

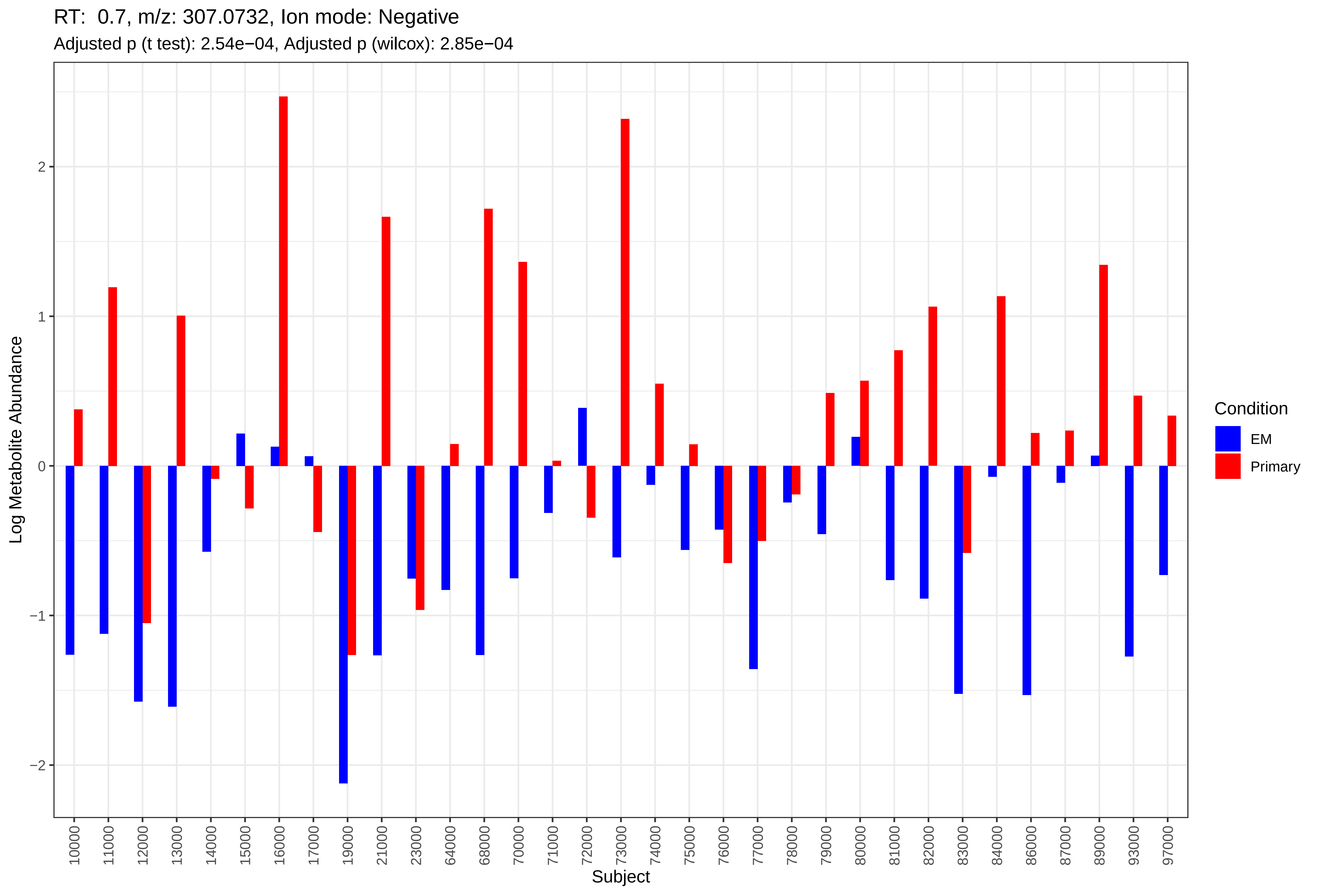

RT: 0.7, m/z: 307.0732, Ion mode: Negative
Adjusted p (t test): 2.54e−04, Adjusted p (wilcox): 2.85e−04
Log Metabolite Abundance
2
1
0
−1
−2
10000
11000
12000
13000
14000
15000
16000
17000
19000
21000
23000
64000
68000
70000
71000
72000
73000
74000
75000
76000
77000
78000
79000
80000
81000
82000
83000
84000
86000
87000
89000
93000
97000
Subject
Condition
EM
Primary

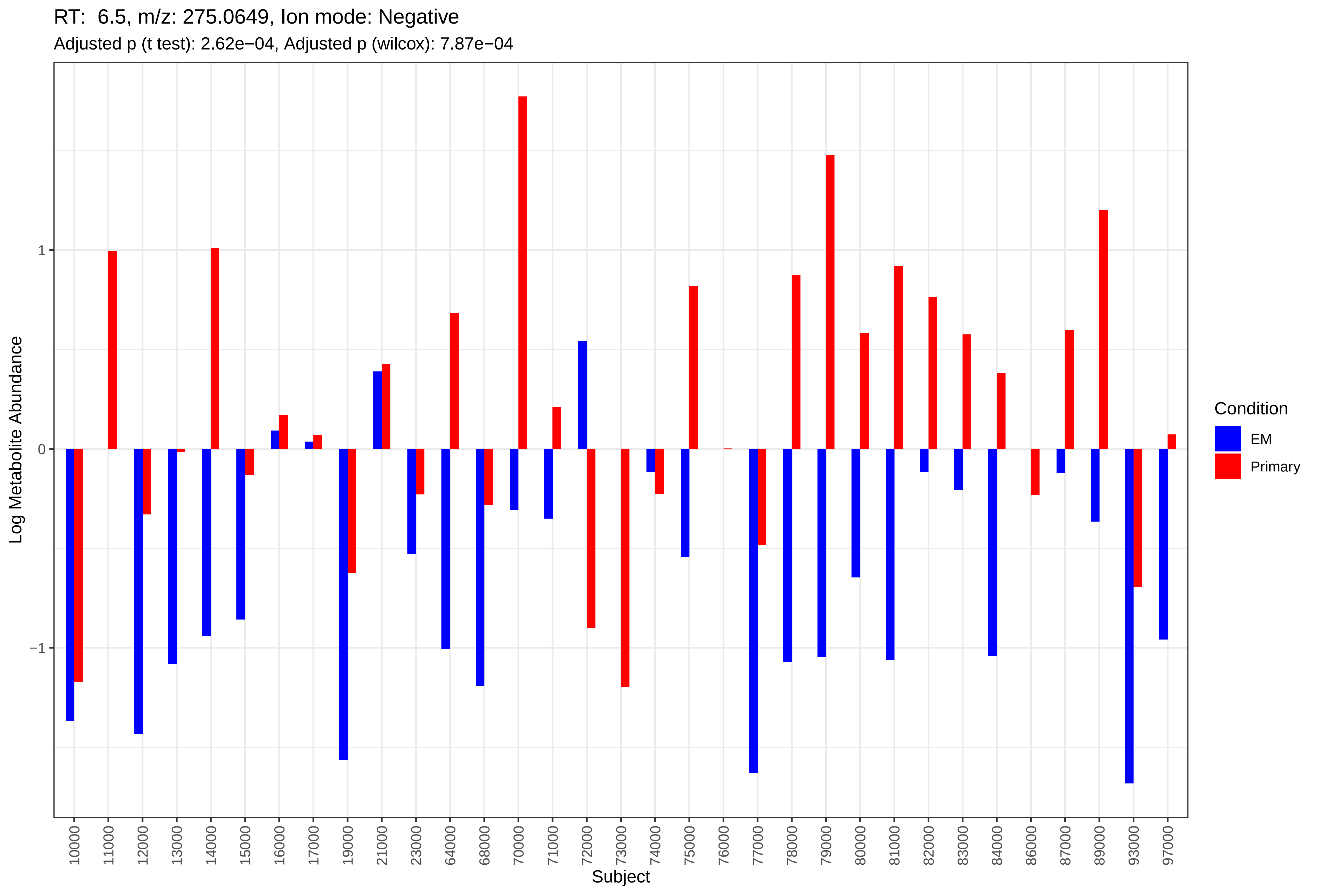

RT: 6.5, m/z: 275.0649, Ion mode: Negative
Adjusted p (t test): 2.62e−04, Adjusted p (wilcox): 7.87e−04
Log Metabolite Abundance
1
0
−1
10000
11000
12000
13000
14000
15000
16000
17000
19000
21000
23000
64000
68000
70000
71000
72000
73000
74000
75000
76000
77000
78000
79000
80000
81000
82000
83000
84000
86000
87000
89000
93000
97000
Subject
Condition
EM
Primary

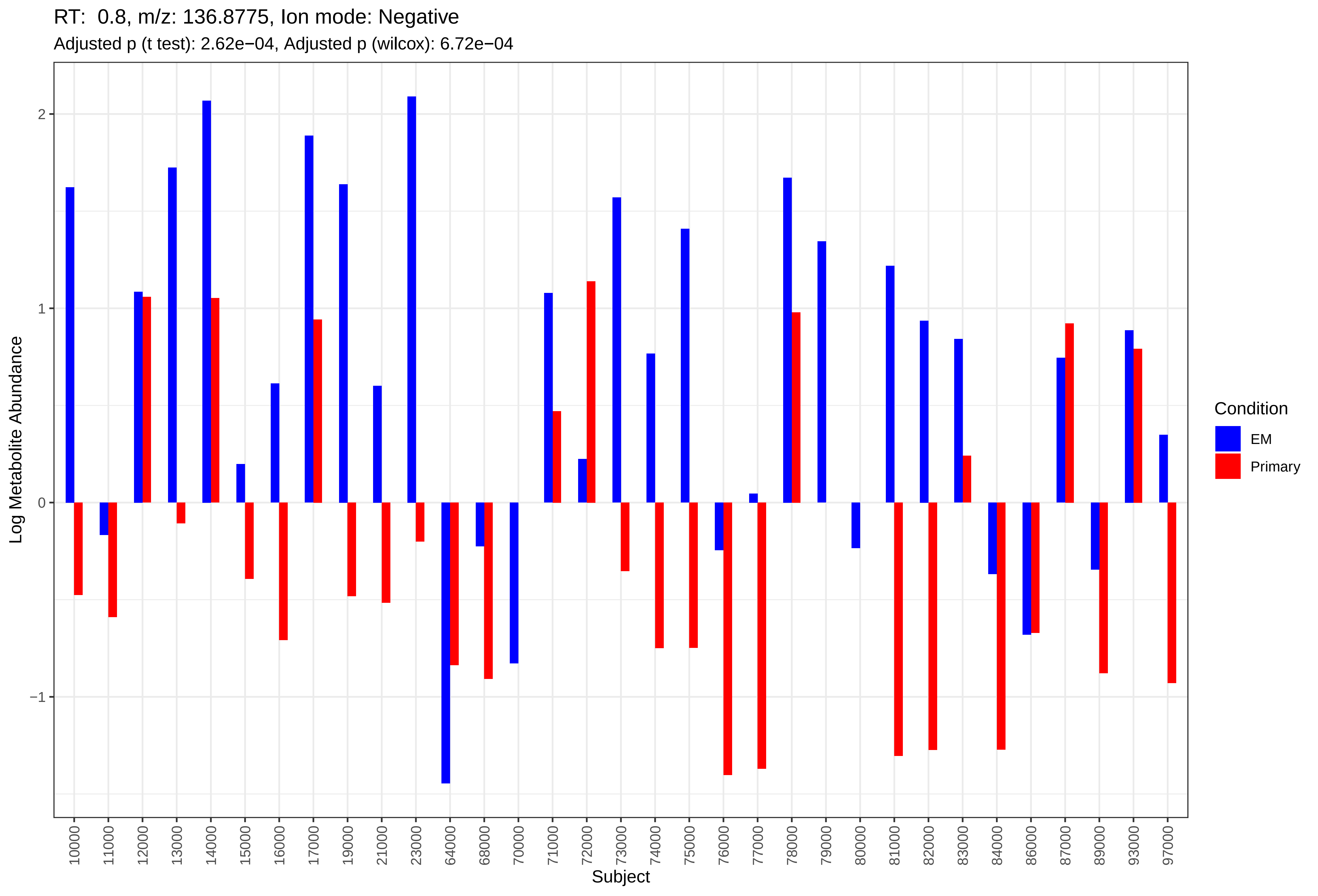
RT: 0.8, m/z: 136.8775, Ion mode: Negative
Adjusted p (t test): 2.62e−04, Adjusted p (wilcox): 6.72e−04
Log Metabolite Abundance
2
1
0
−1
10000
11000
12000
13000
14000
15000
16000
17000
19000
21000
23000
64000
68000
70000
71000
72000
73000
74000
75000
76000
77000
78000
79000
80000
81000
82000
83000
84000
86000
87000
89000
93000
97000
Subject
Condition
EM
Primary

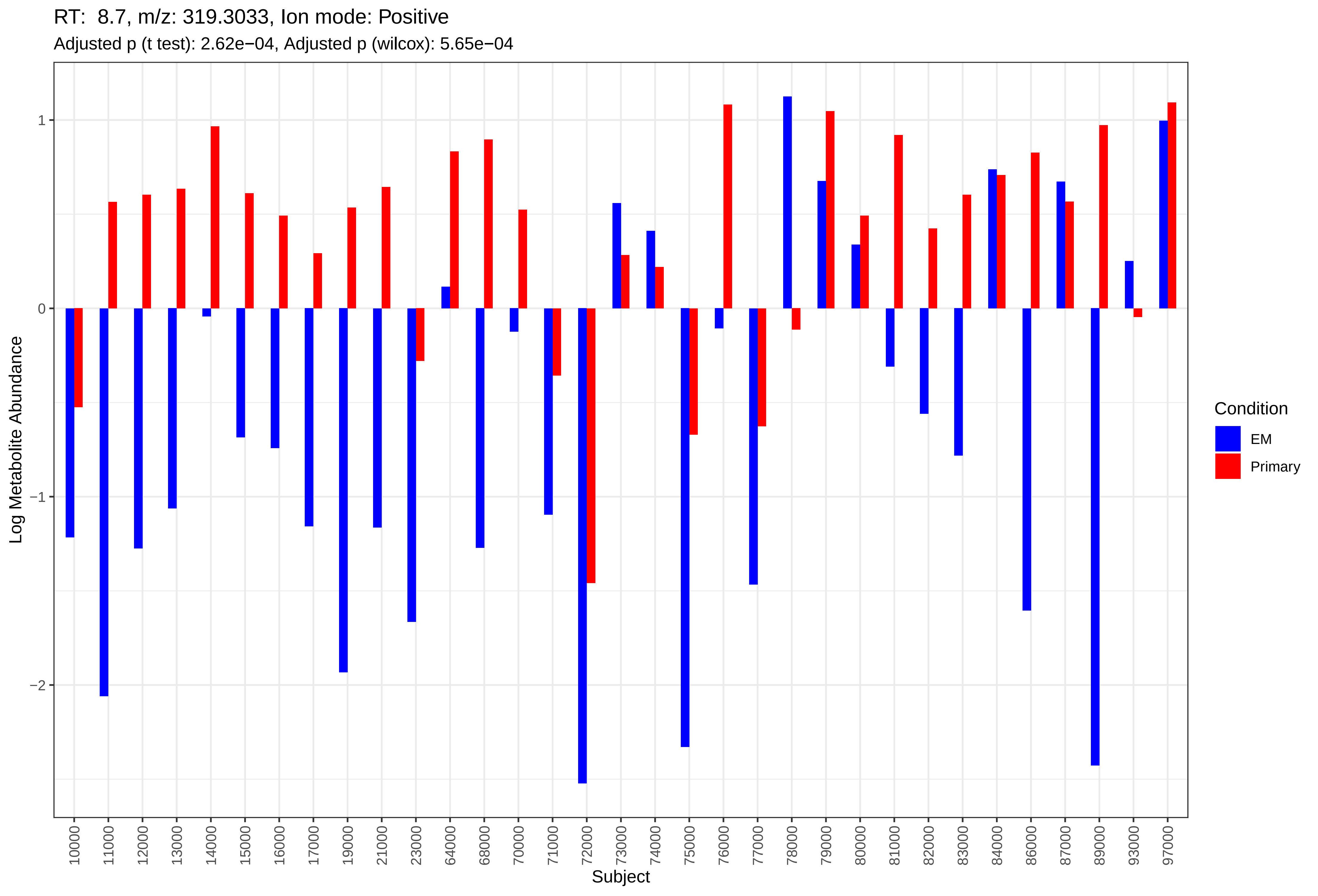

RT: 8.7, m/z: 319.3033, Ion mode: Positive
Adjusted p (t test): 2.62e−04, Adjusted p (wilcox): 5.65e−04
Log Metabolite Abundance
1
0
−1
−2
10000
11000
12000
13000
14000
15000
16000
17000
19000
21000
23000
64000
68000
70000
71000
72000
73000
74000
75000
76000
77000
78000
79000
80000
81000
82000
83000
84000
86000
87000
89000
93000
97000
Subject
Condition
EM
Primary

RT: 12.4, m/z: 191.1427, Ion mode: Positive

Adjusted p (t test): 2.63e−04, Adjusted p (wilcox): 2.85e−04

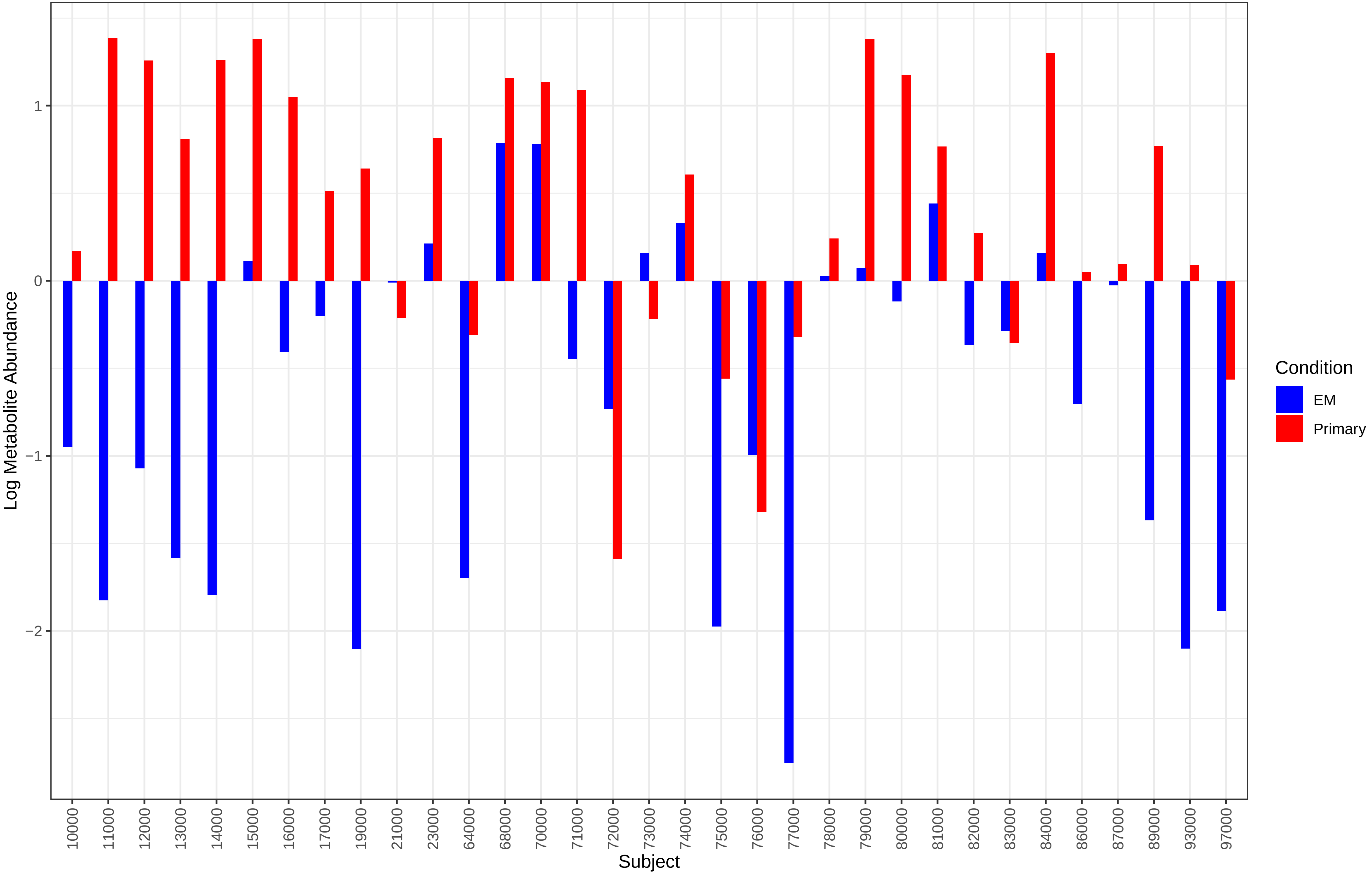

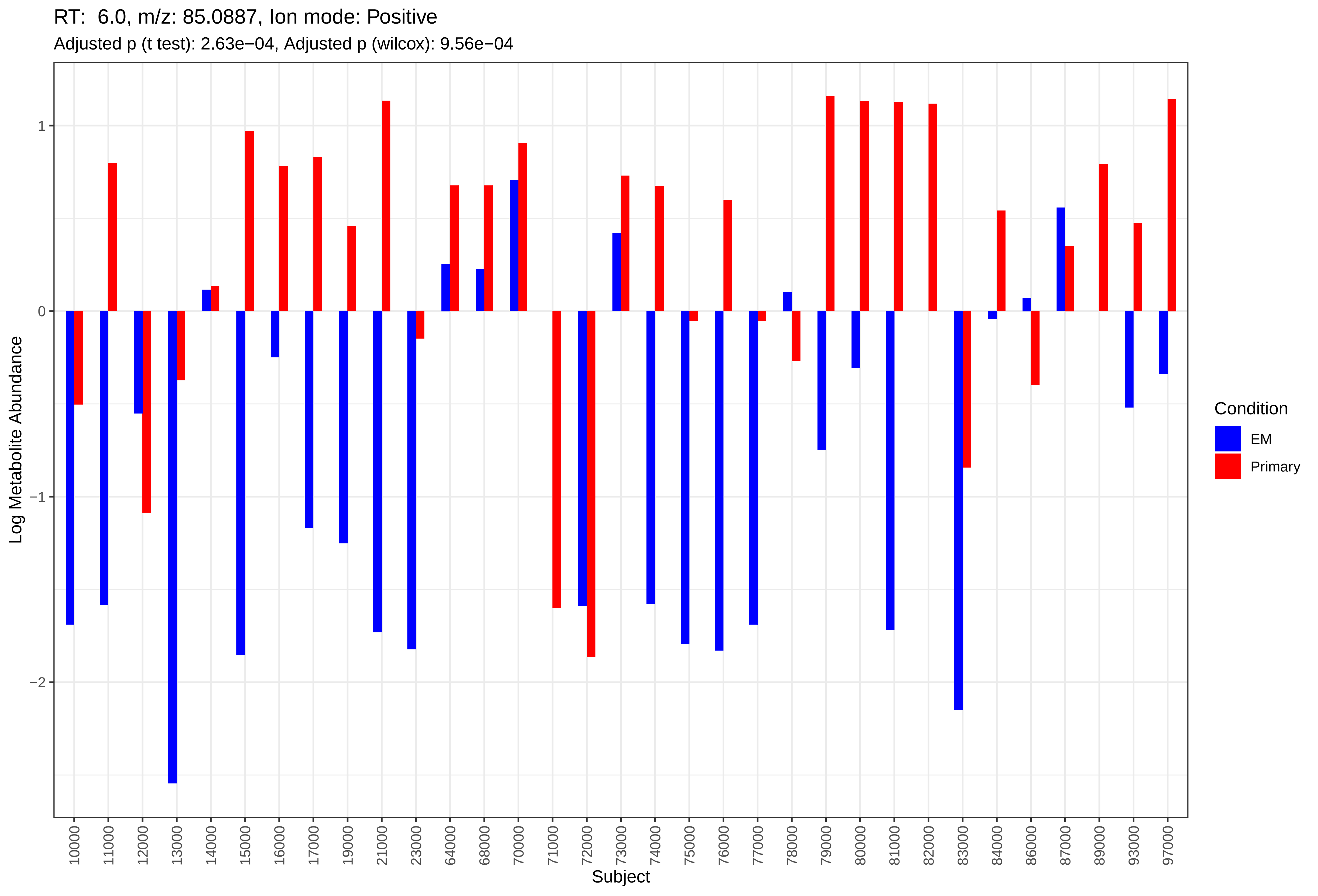

RT: 6.0, m/z: 85.0887, Ion mode: Positive
Adjusted p (t test): 2.63e−04, Adjusted p (wilcox): 9.56e−04
Log Metabolite Abundance
1
0
−1
−2
Subject
10000
11000
12000
13000
14000
15000
16000
17000
19000
21000
23000
64000
68000
70000
71000
72000
73000
74000
75000
76000
77000
78000
79000
80000
81000
82000
83000
84000
86000
87000
89000
93000
97000
Condition
EM
Primary

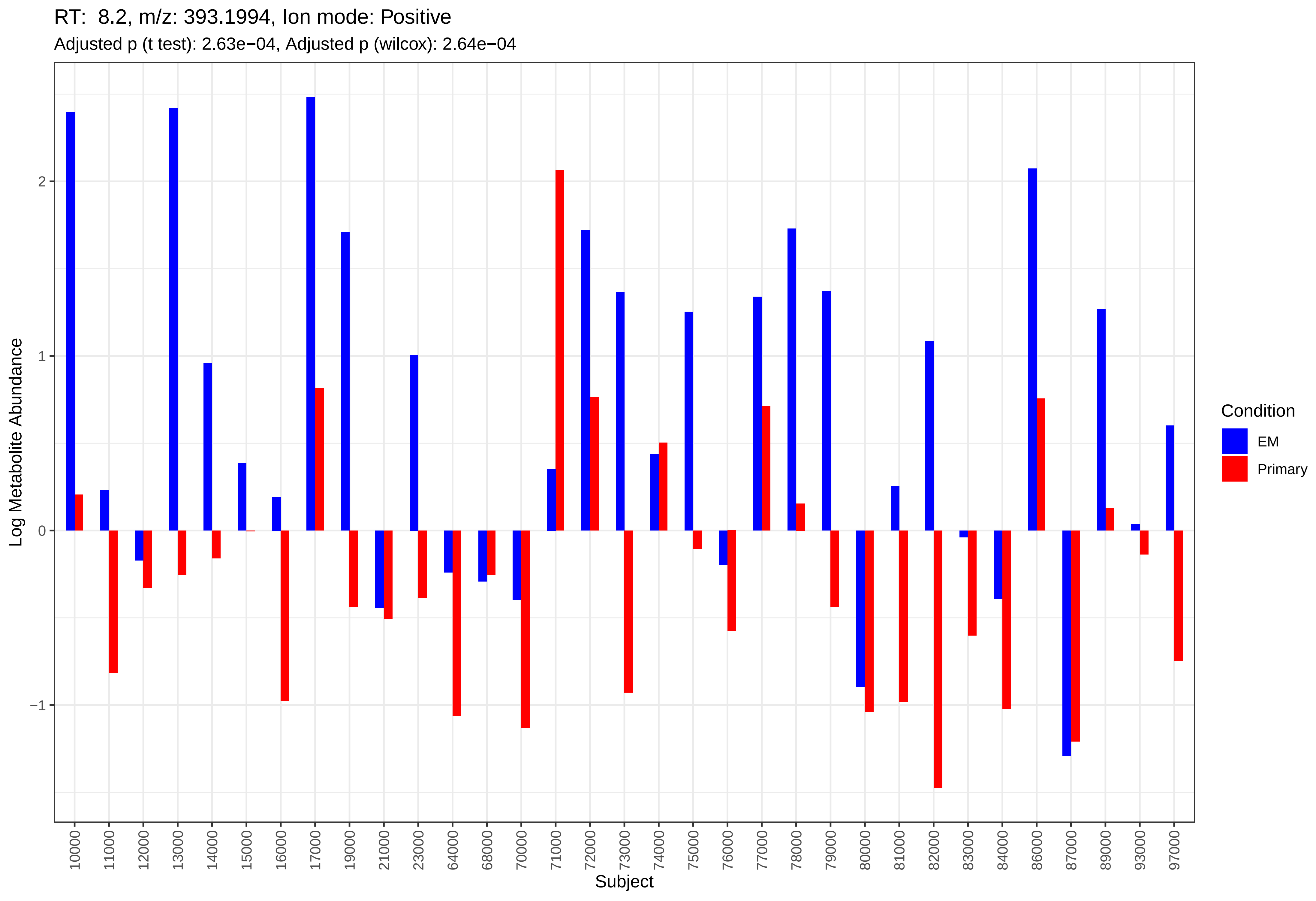
RT: 8.2, m/z: 393.1994, Ion mode: Positive
Adjusted p (t test): 2.63e−04, Adjusted p (wilcox): 2.64e−04
Log Metabolite Abundance
2
1
0
−1
Subject
10000
11000
12000
13000
14000
15000
16000
17000
19000
21000
23000
64000
68000
70000
71000
72000
73000
74000
75000
76000
77000
78000
79000
80000
81000
82000
83000
84000
86000
87000
89000
93000
97000
Condition
EM
Primary

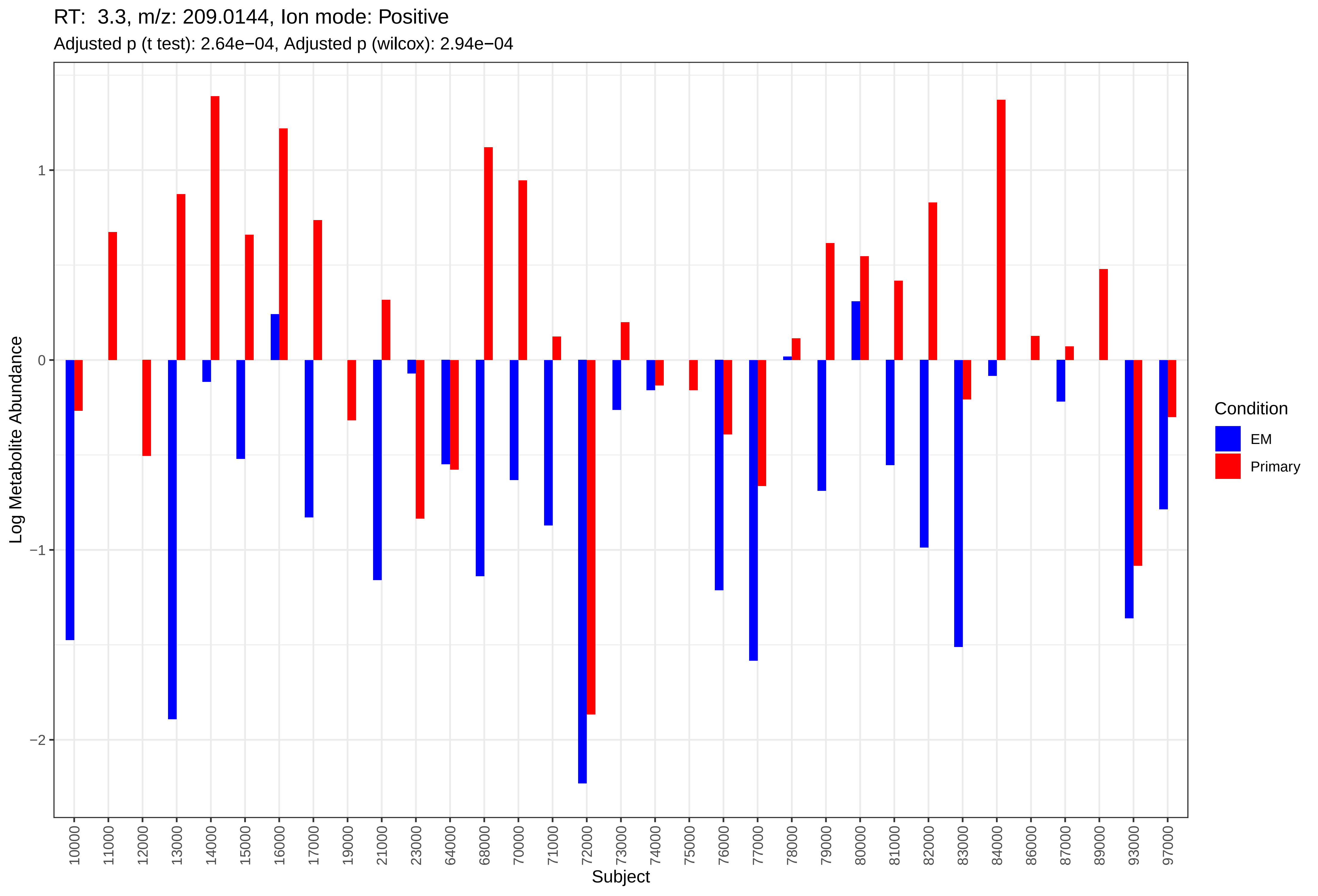

RT: 3.3, m/z: 209.0144, Ion mode: Positive
Adjusted p (t test): 2.64e−04, Adjusted p (wilcox): 2.94e−04
Log Metabolite Abundance
1
0
−1
−2
10000
11000
12000
13000
14000
15000
16000
17000
19000
21000
23000
64000
68000
70000
71000
72000
73000
74000
75000
76000
77000
78000
79000
80000
81000
82000
83000
84000
86000
87000
89000
93000
97000
Subject
Condition
EM
Primary

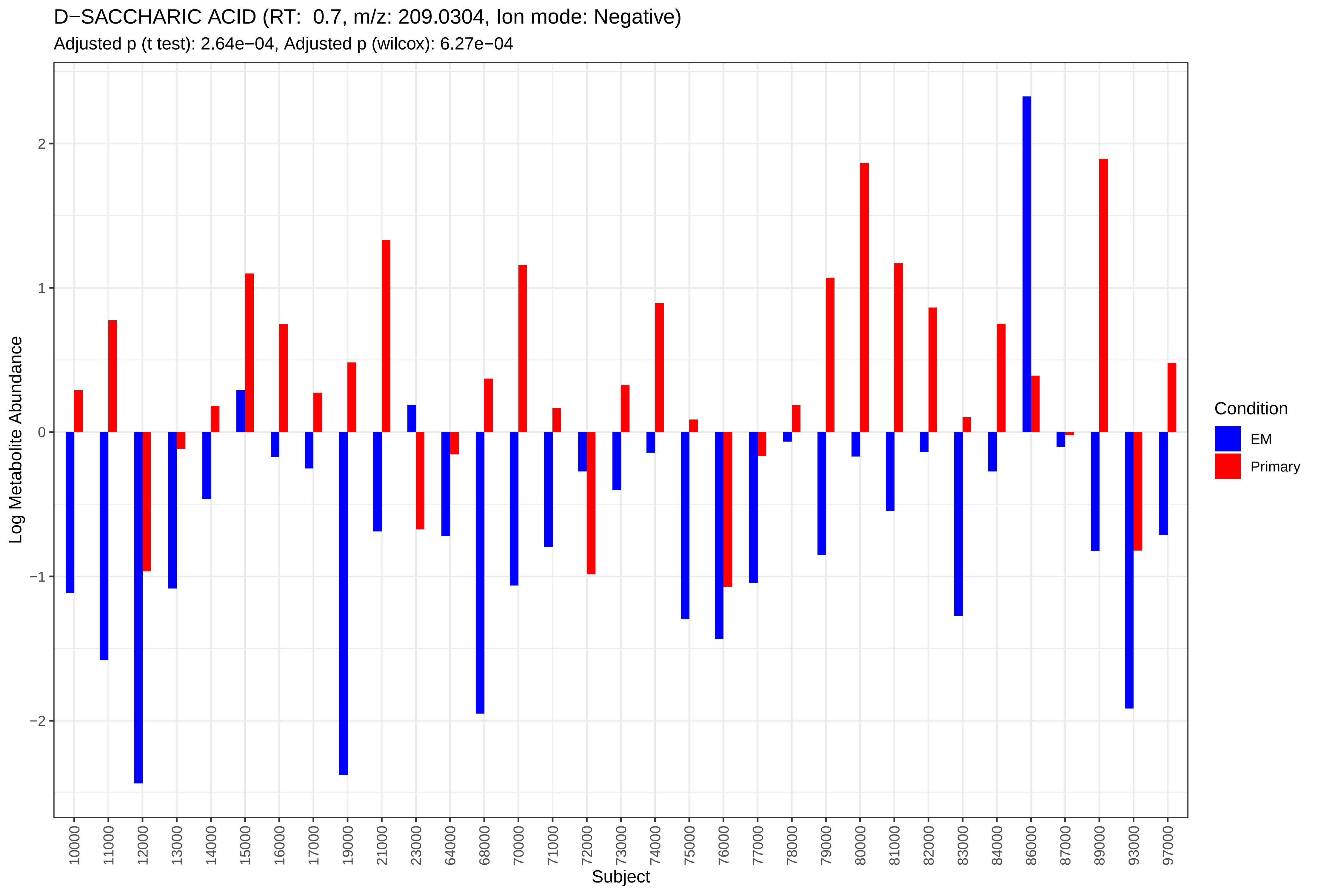
D−SACCHARIC ACID (RT: 0.7, m/z: 209.0304, Ion mode: Negative)
Adjusted p (t test): 2.64e−04, Adjusted p (wilcox): 6.27e−04
Log Metabolite Abundance
2
1
0
−1
−2
10000
11000
12000
13000
14000
15000
16000
17000
19000
21000
23000
64000
68000
70000
71000
72000
73000
74000
75000
76000
77000
78000
79000
80000
81000
82000
83000
84000
86000
87000
89000
93000
97000
Subject
Condition
EM
Primary

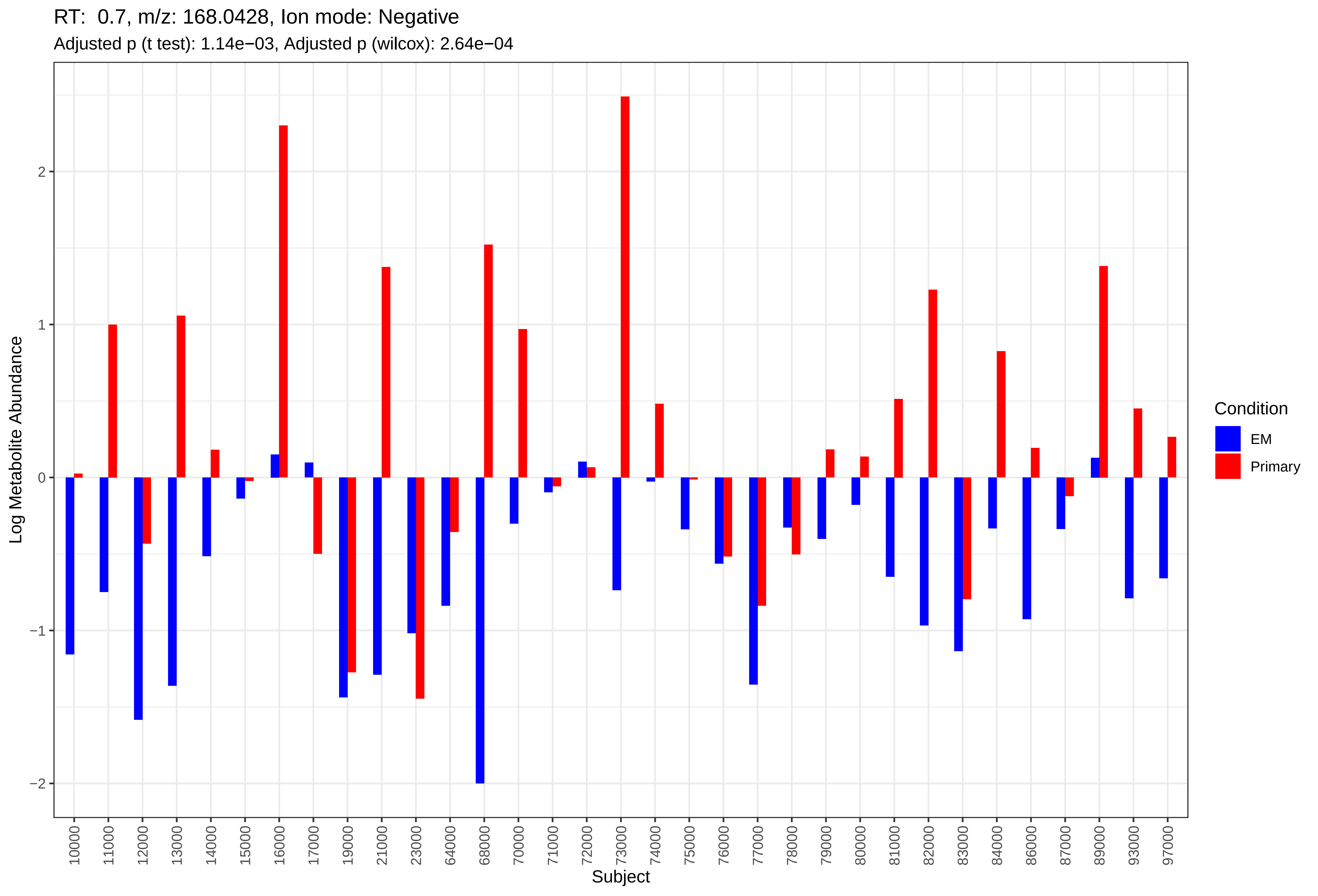
RT: 0.7, m/z: 168.0428, Ion mode: Negative
Adjusted p (t test): 1.14e−03, Adjusted p (wilcox): 2.64e−04
Log Metabolite Abundance
2
1
0
−1
−2
10000
11000
12000
13000
14000
15000
16000
17000
19000
21000
23000
64000
68000
70000
71000
72000
73000
74000
75000
76000
77000
78000
79000
80000
81000
82000
83000
84000
86000
87000
89000
93000
97000
Subject
Condition
EM
Primary

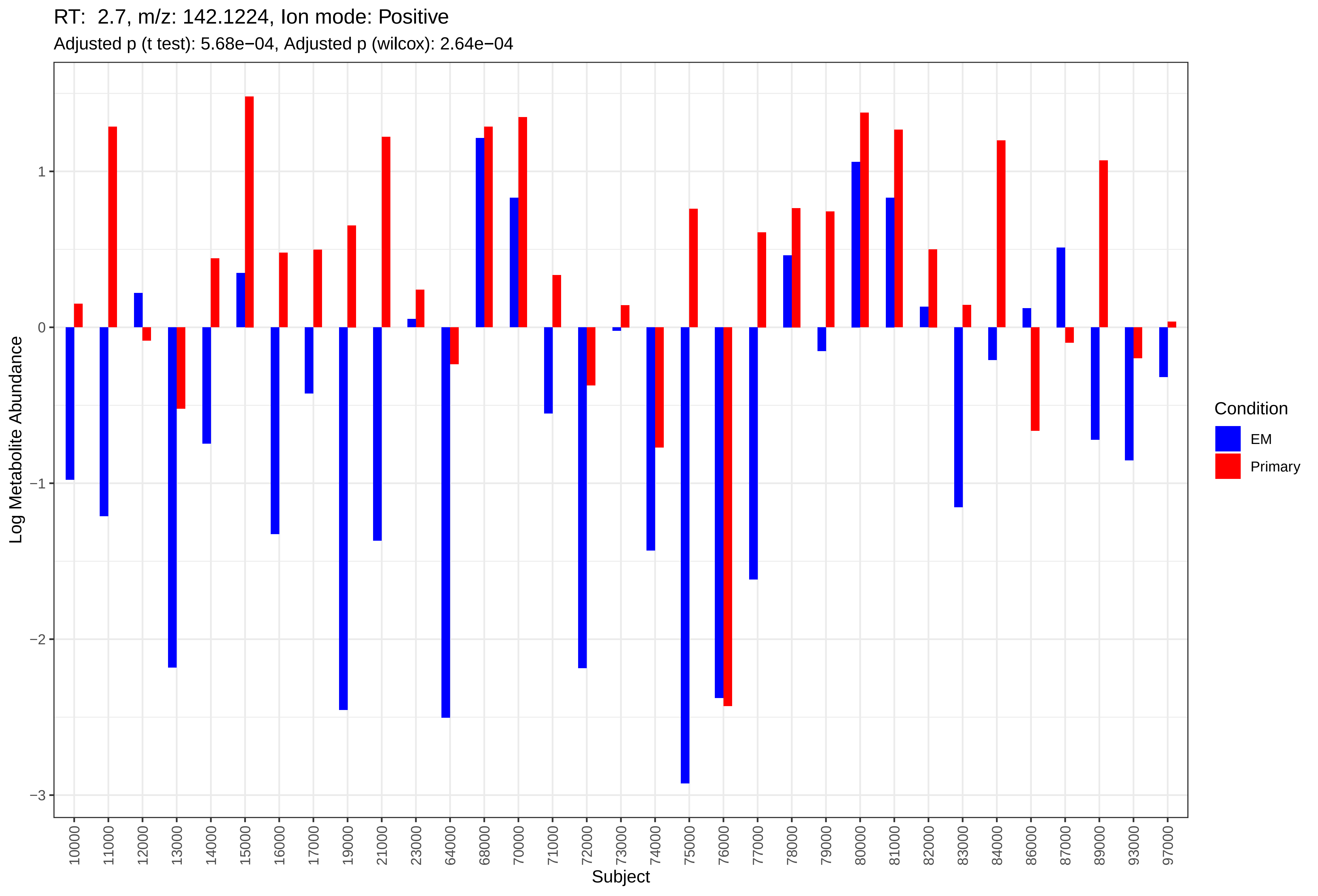

RT: 2.7, m/z: 142.1224, Ion mode: Positive
Adjusted p (t test): 5.68e−04, Adjusted p (wilcox): 2.64e−04
Log Metabolite Abundance
1
0
−1
−2
−3
10000
11000
12000
13000
14000
15000
16000
17000
19000
21000
23000
64000
68000
70000
71000
72000
73000
74000
75000
76000
77000
78000
79000
80000
81000
82000
83000
84000
86000
87000
89000
93000
97000
Subject
Condition
EM
Primary

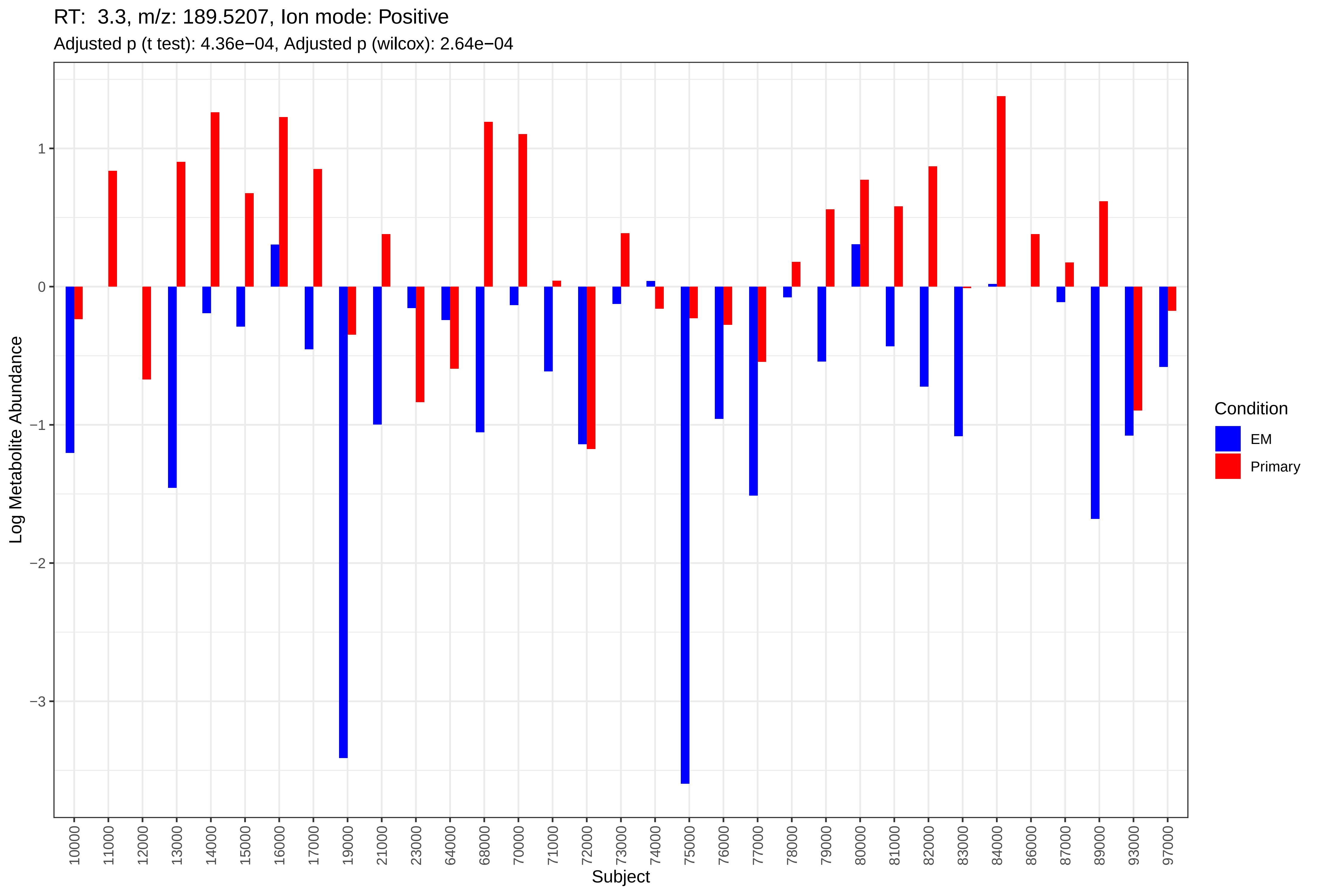

RT: 3.3, m/z: 189.5207, Ion mode: Positive
Adjusted p (t test): 4.36e−04, Adjusted p (wilcox): 2.64e−04
Log Metabolite Abundance
1
0
−1
−2
−3
10000
11000
12000
13000
14000
15000
16000
17000
19000
21000
23000
64000
68000
70000
71000
72000
73000
74000
75000
76000
77000
78000
79000
80000
81000
82000
83000
84000
86000
87000
89000
93000
97000
Subject
Condition
EM
Primary

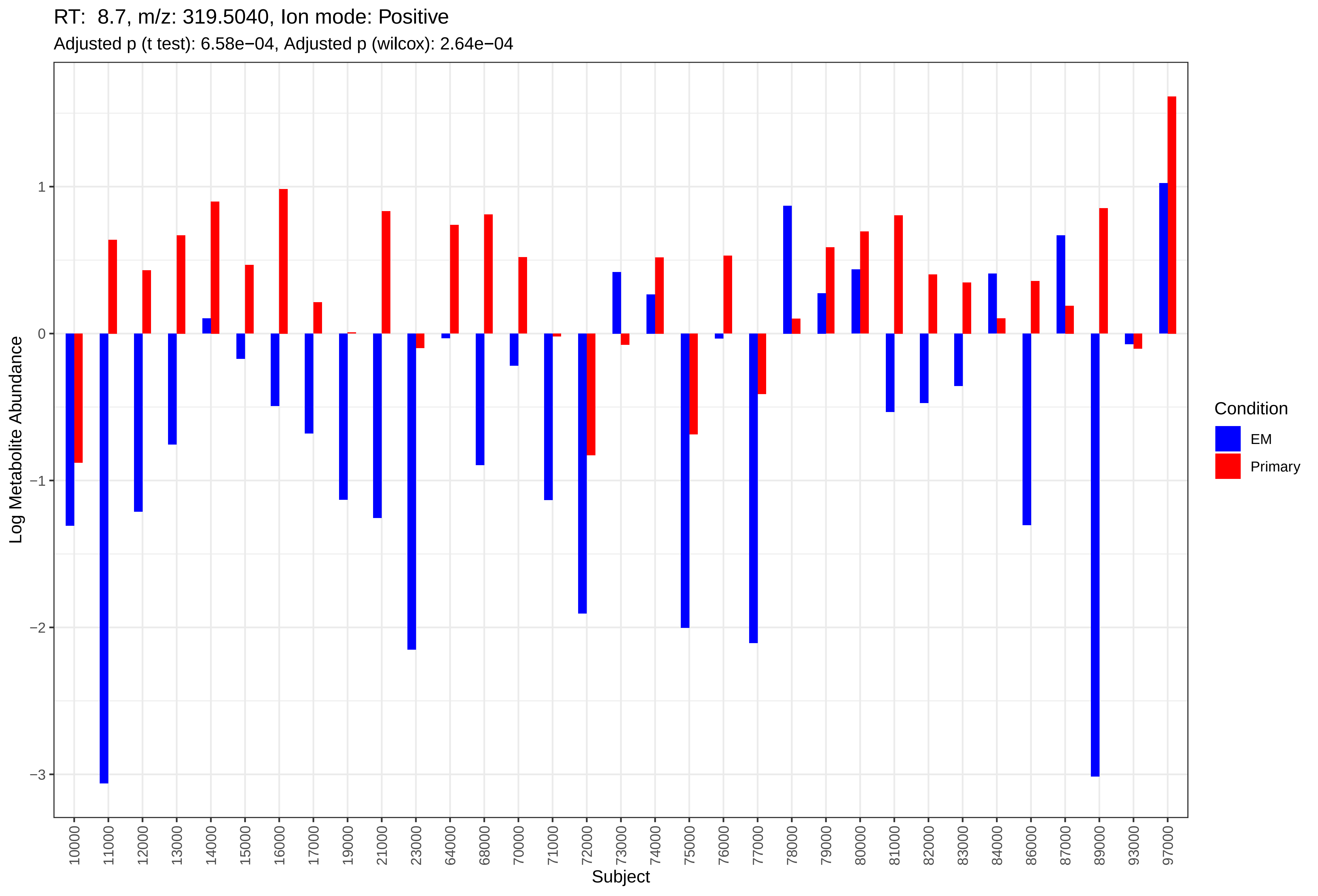
RT: 8.7, m/z: 319.5040, Ion mode: Positive
Adjusted p (t test): 6.58e−04, Adjusted p (wilcox): 2.64e−04
Log Metabolite Abundance
1
0
−1
−2
−3
Subject
10000
11000
12000
13000
14000
15000
16000
17000
19000
21000
23000
64000
68000
70000
71000
72000
73000
74000
75000
76000
77000
78000
79000
80000
81000
82000
83000
84000
86000
87000
89000
93000
97000
Condition
EM
Primary

[M+Na−H] 21.9825 m/z adduct of 142.0262 m/z (RT: 0.7, m/z: 164.0081, Ion mode: Positive)

Adjusted p (t test): 3.38e−03, Adjusted p (wilcox): 2.64e−04

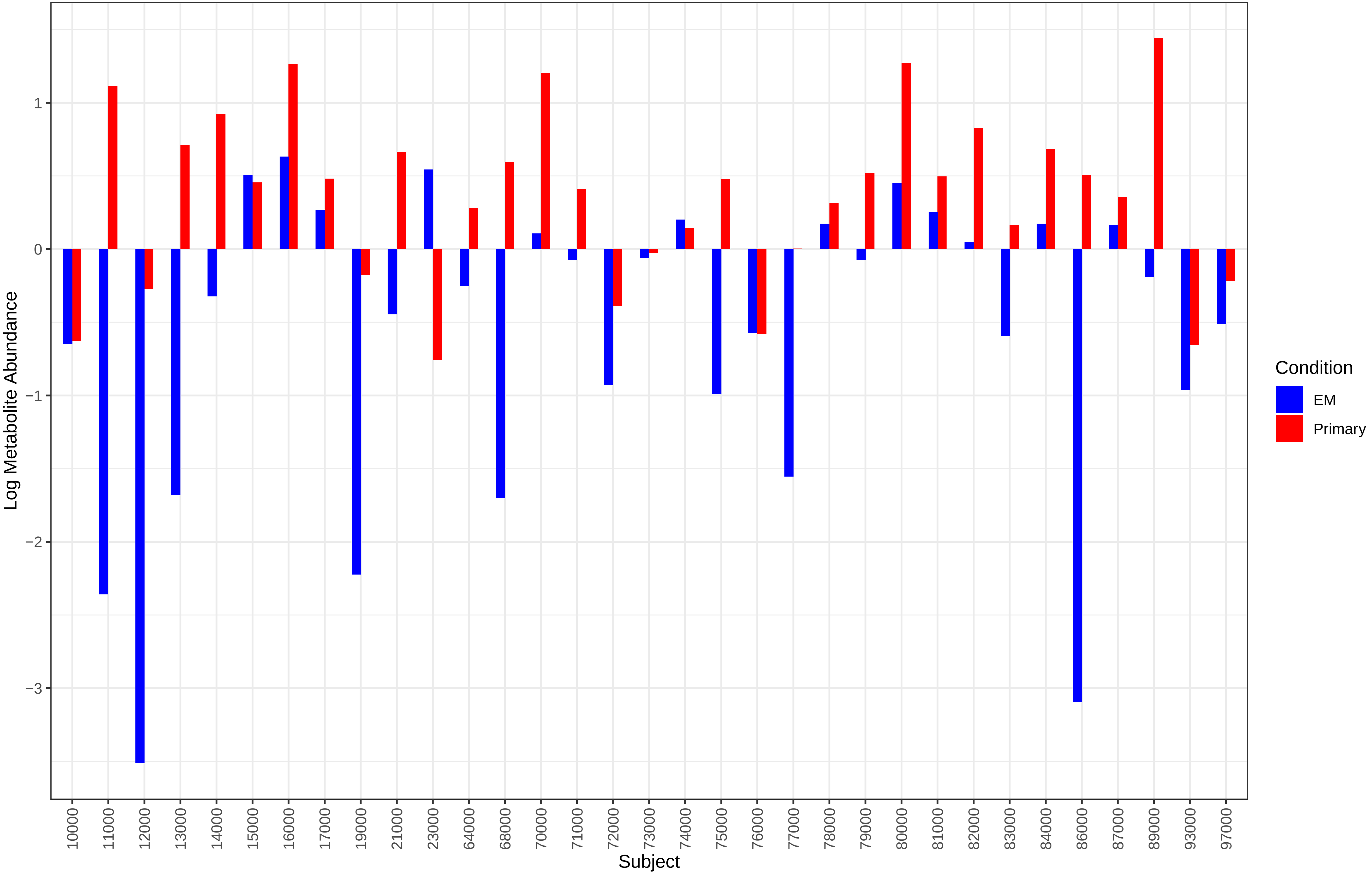

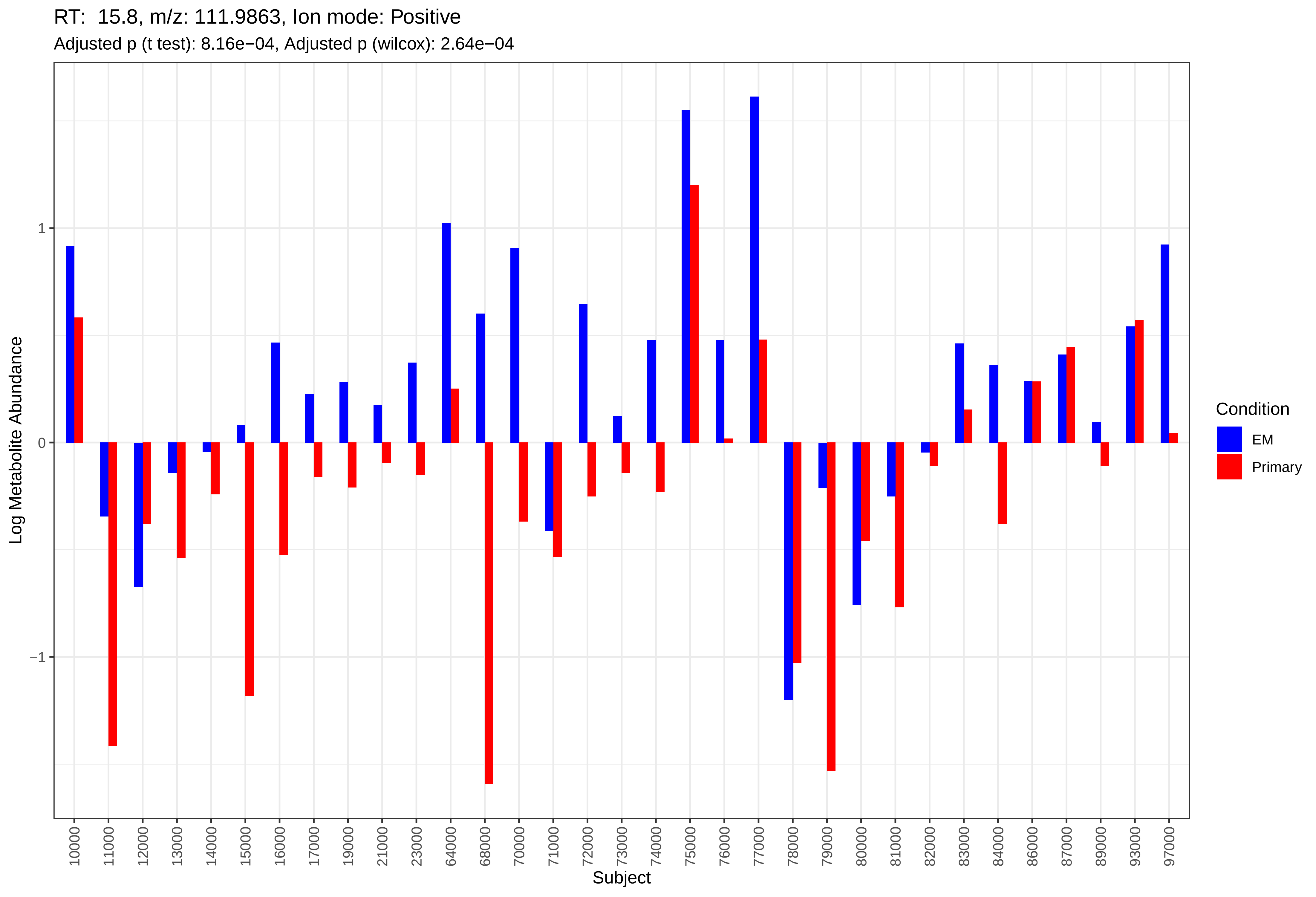

RT: 15.8, m/z: 111.9863, Ion mode: Positive
Adjusted p (t test): 8.16e−04, Adjusted p (wilcox): 2.64e−04
Log Metabolite Abundance
1
0
−1
10000
11000
12000
13000
14000
15000
16000
17000
19000
21000
23000
64000
68000
70000
71000
72000
73000
74000
75000
76000
77000
78000
79000
80000
81000
82000
83000
84000
86000
87000
89000
93000
97000
Subject
Condition
EM
Primary

RT: 0.9, m/z: 211.1291, Ion mode: Positive

Adjusted p (t test): 2.97e−03, Adjusted p (wilcox): 2.64e−04

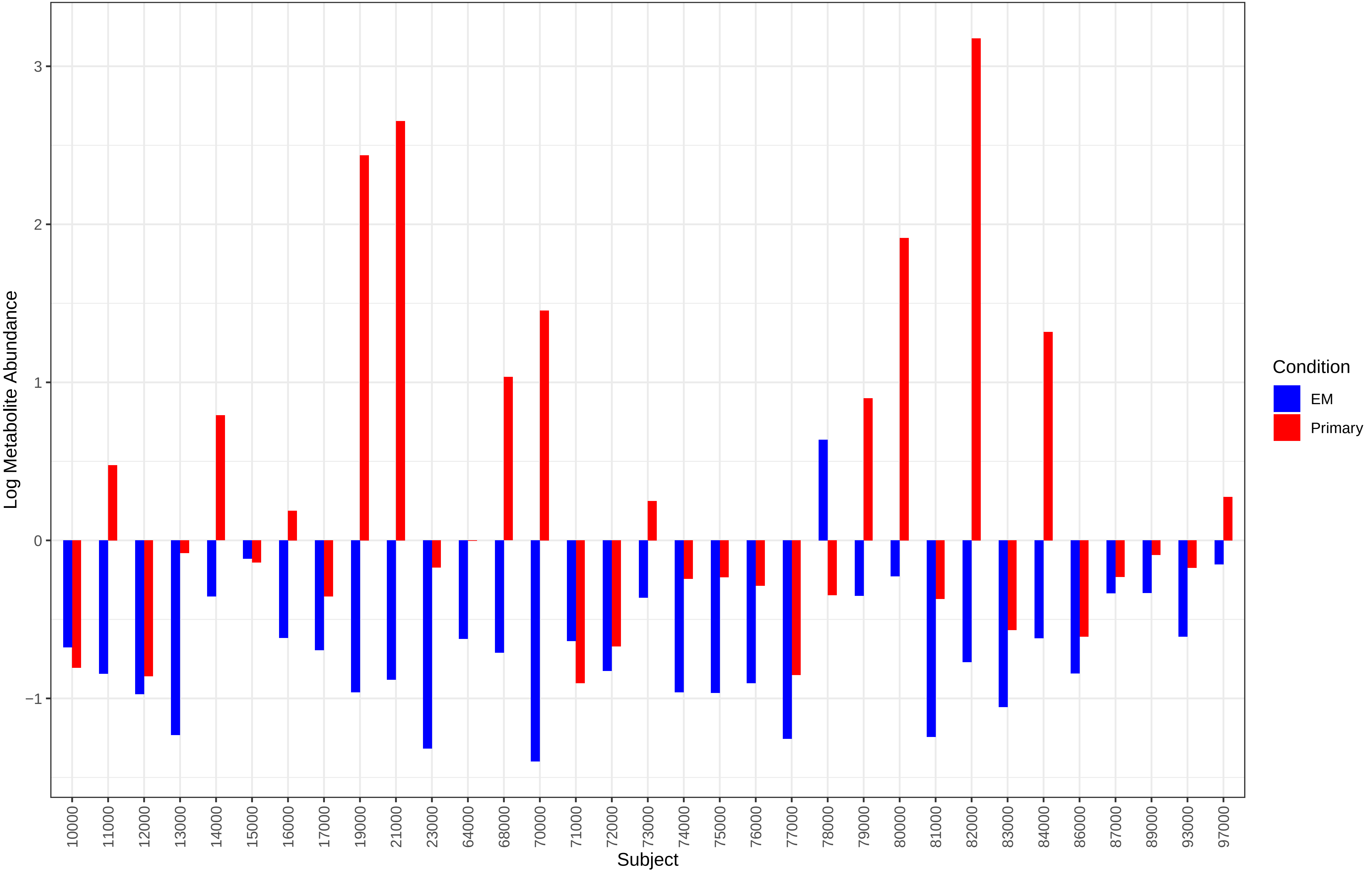

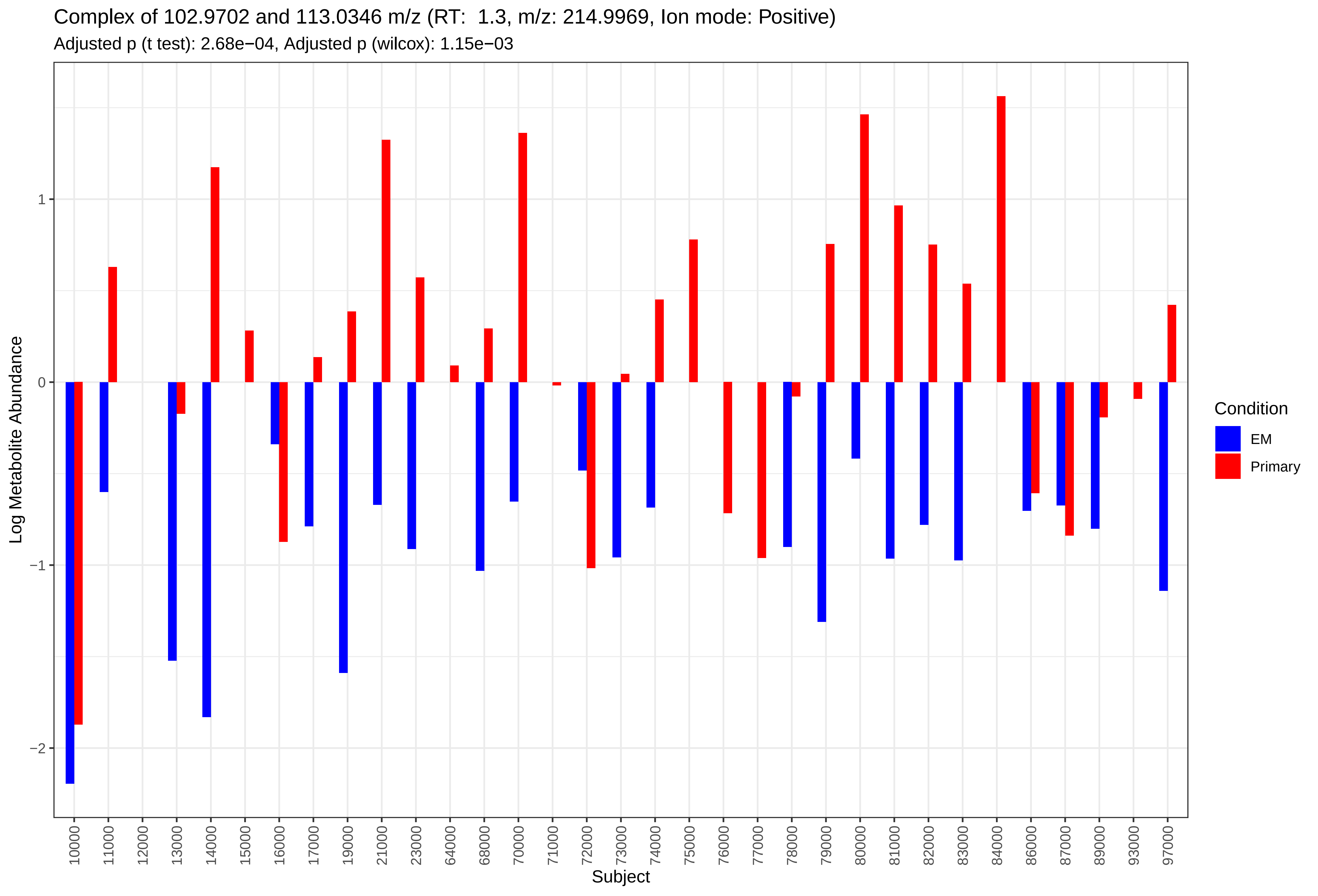
Complex of 102.9702 and 113.0346 m/z (RT: 1.3, m/z: 214.9969, Ion mode: Positive)
Adjusted p (t test): 2.68e−04, Adjusted p (wilcox): 1.15e−03
Log Metabolite Abundance
1
0
−1
−2
Subject
10000
11000
12000
13000
14000
15000
16000
17000
19000
21000
23000
64000
68000
70000
71000
72000
73000
74000
75000
76000
77000
78000
79000
80000
81000
82000
83000
84000
86000
87000
89000
93000
97000
Condition
EM
Primary

RT: 0.6, m/z: 91.5423, Ion mode: Positive

Adjusted p (t test): 2.73e−04, Adjusted p (wilcox): 2.85e−04

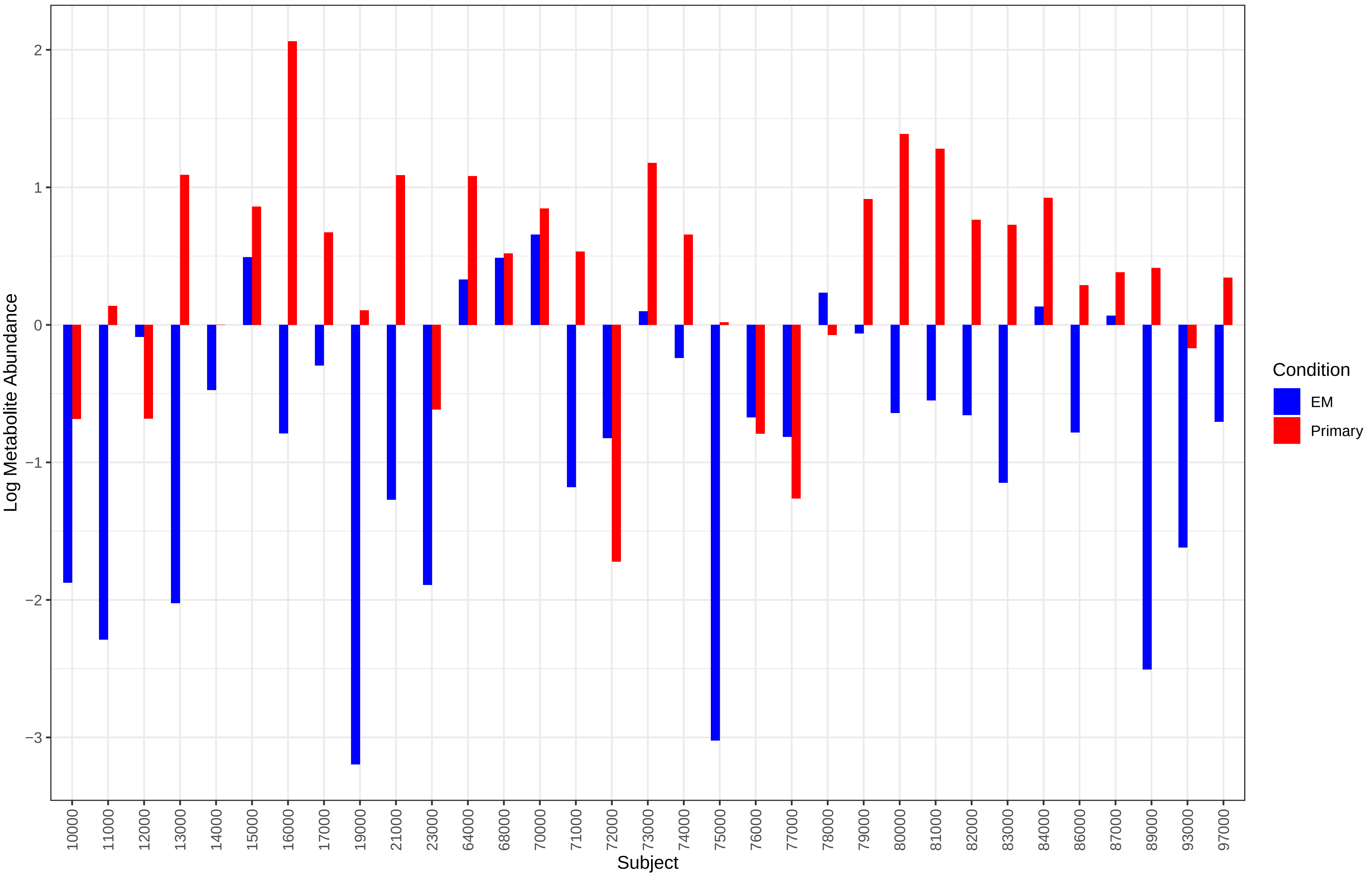

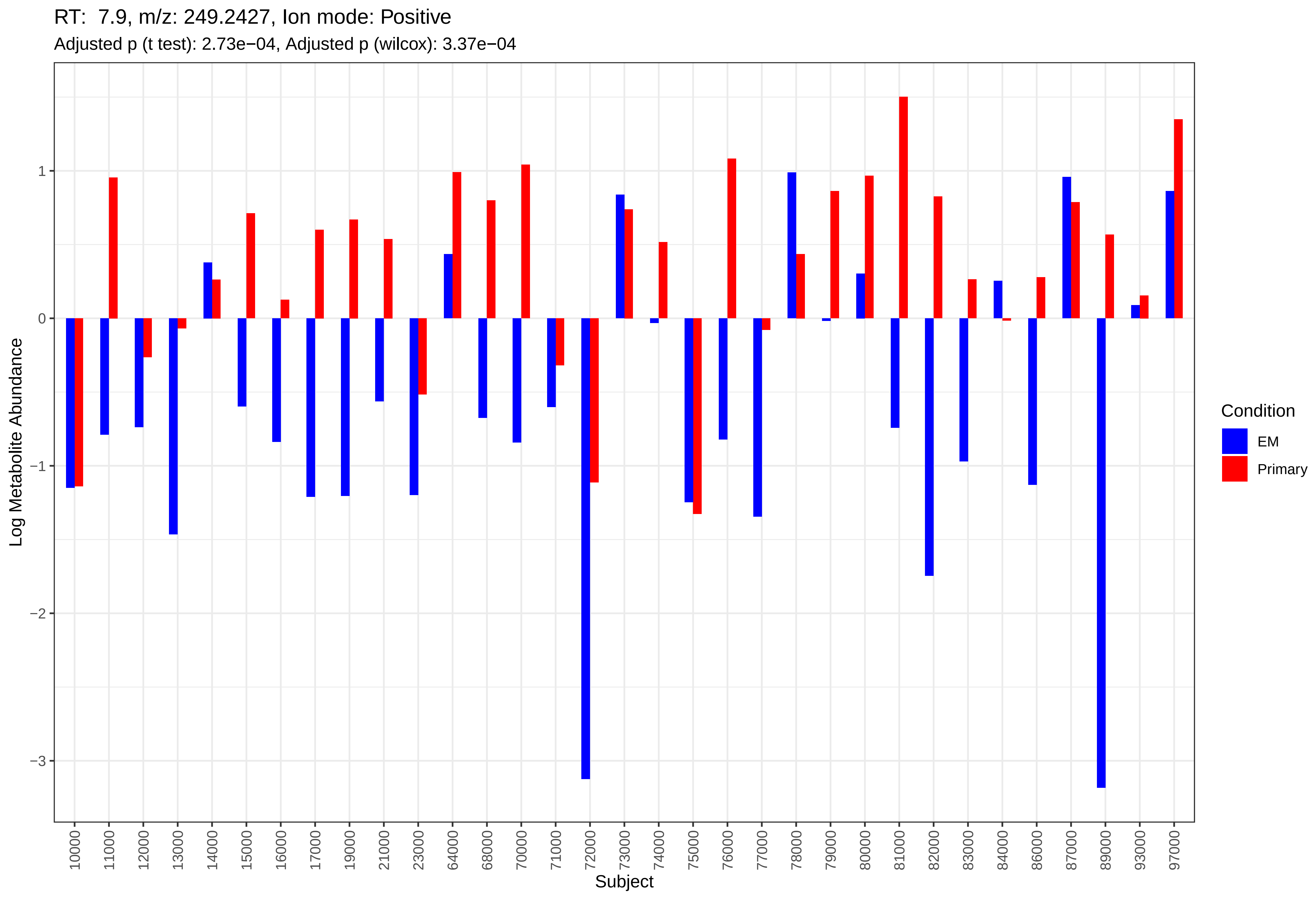

RT: 7.9, m/z: 249.2427, Ion mode: Positive
Adjusted p (t test): 2.73e−04, Adjusted p (wilcox): 3.37e−04
Log Metabolite Abundance
1
0
−1
−2
−3
Subject
10000
11000
12000
13000
14000
15000
16000
17000
19000
21000
23000
64000
68000
70000
71000
72000
73000
74000
75000
76000
77000
78000
79000
80000
81000
82000
83000
84000
86000
87000
89000
93000
97000
Condition
EM
Primary

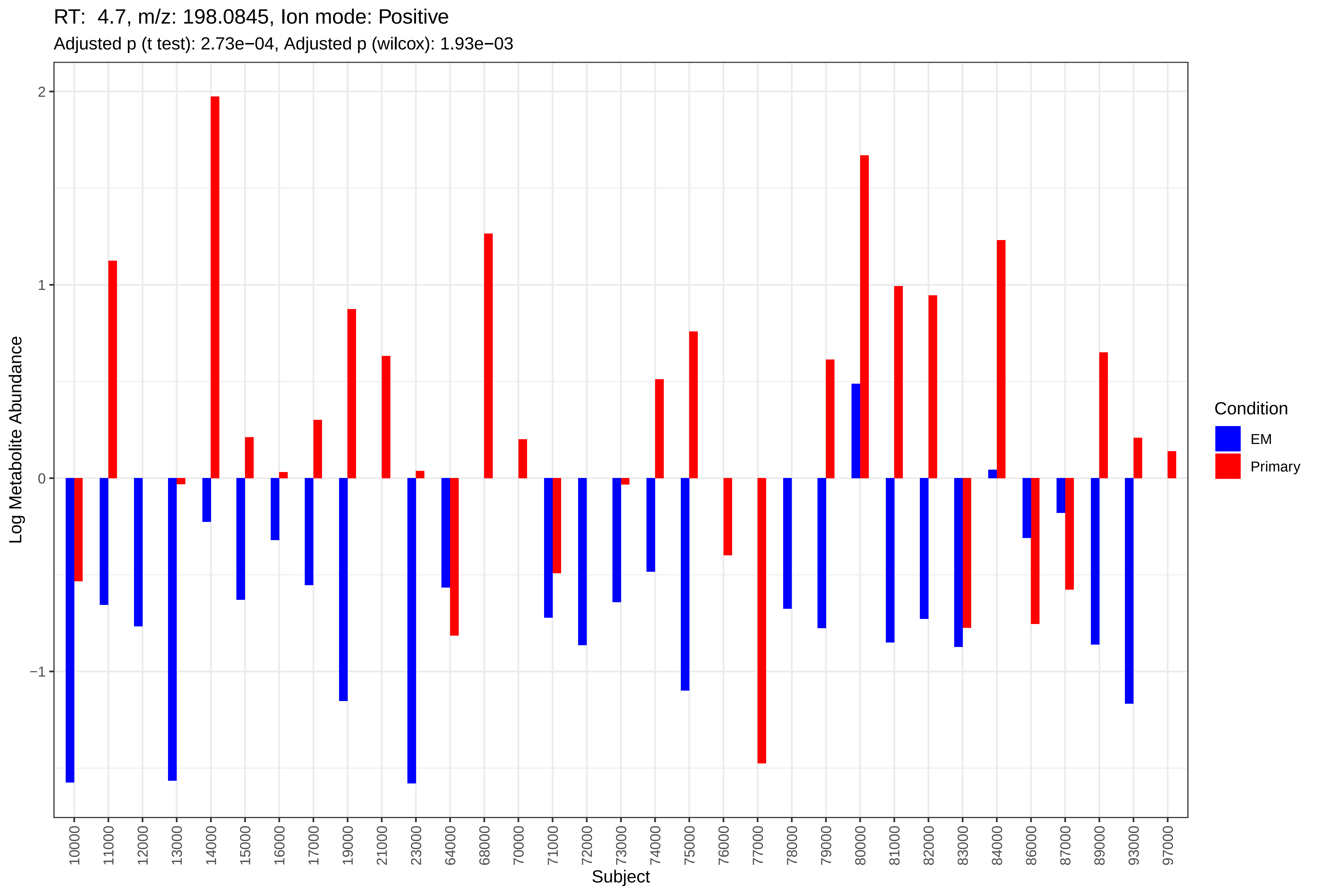
RT: 4.7, m/z: 198.0845, Ion mode: Positive
Adjusted p (t test): 2.73e−04, Adjusted p (wilcox): 1.93e−03
Log Metabolite Abundance
2
1
0
−1
10000
11000
12000
13000
14000
15000
16000
17000
19000
21000
23000
64000
68000
70000
71000
72000
73000
74000
75000
76000
77000
78000
79000
80000
81000
82000
83000
84000
86000
87000
89000
93000
97000
Subject
Condition
EM
Primary

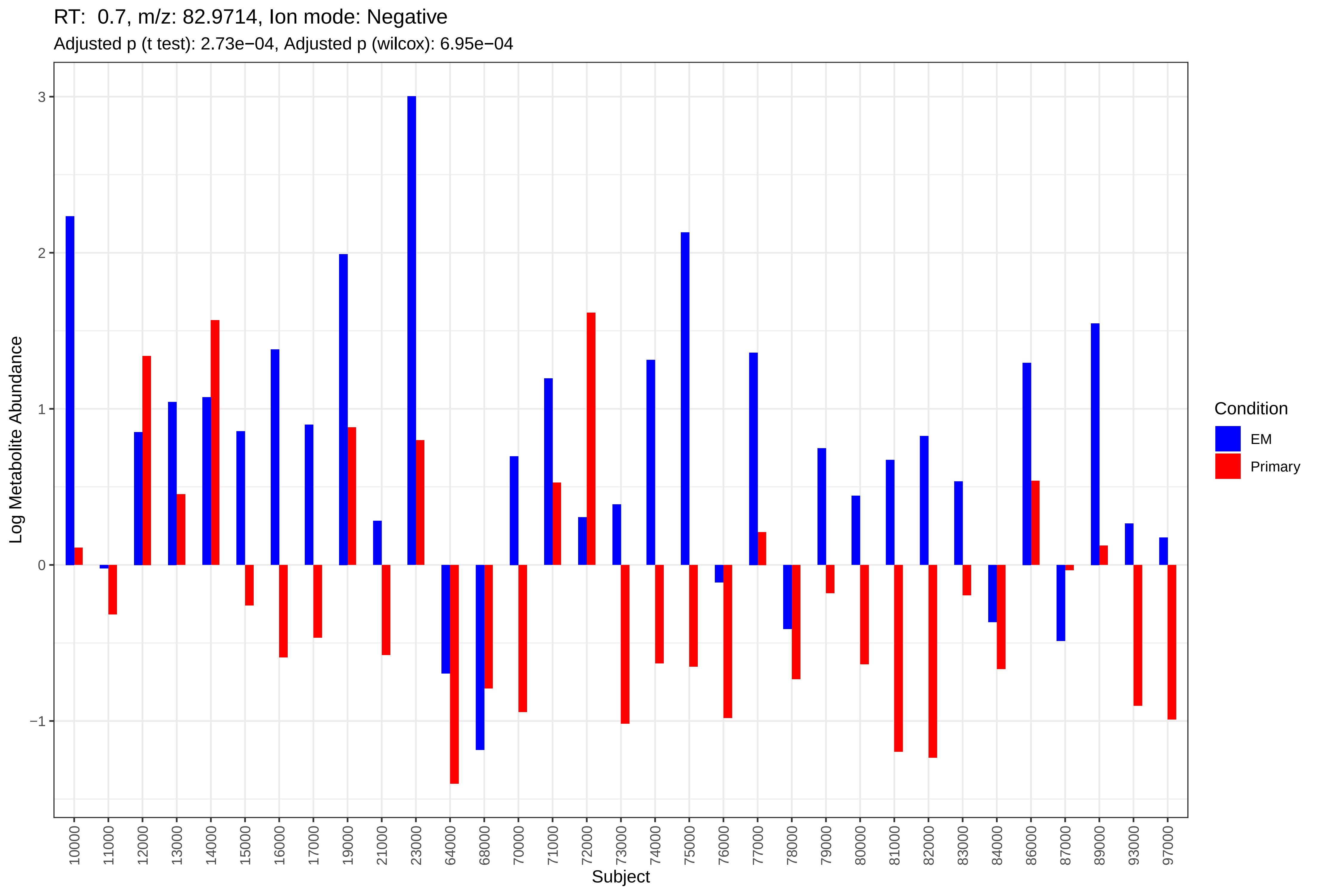
RT: 0.7, m/z: 82.9714, Ion mode: Negative
Adjusted p (t test): 2.73e−04, Adjusted p (wilcox): 6.95e−04
Log Metabolite Abundance
3
2
1
0
−1
Subject
10000
11000
12000
13000
14000
15000
16000
17000
19000
21000
23000
64000
68000
70000
71000
72000
73000
74000
75000
76000
77000
78000
79000
80000
81000
82000
83000
84000
86000
87000
89000
93000
97000
Condition
EM
Primary

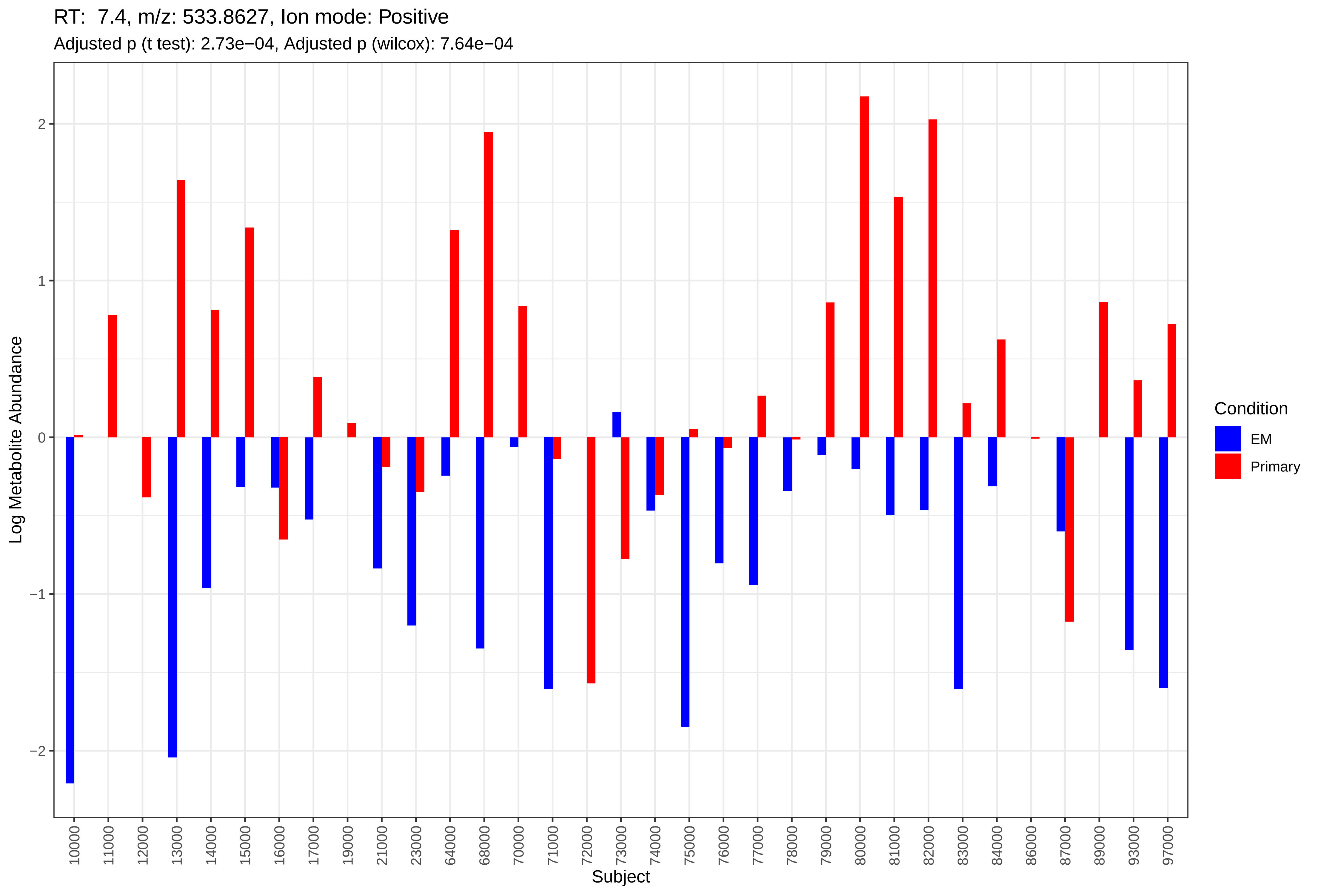
RT: 7.4, m/z: 533.8627, Ion mode: Positive
Adjusted p (t test): 2.73e−04, Adjusted p (wilcox): 7.64e−04
Log Metabolite Abundance
2
1
0
−1
−2
10000
11000
12000
13000
14000
15000
16000
17000
19000
21000
23000
64000
68000
70000
71000
72000
73000
74000
75000
76000
77000
78000
79000
80000
81000
82000
83000
84000
86000
87000
89000
93000
97000
Subject
Condition
EM
Primary

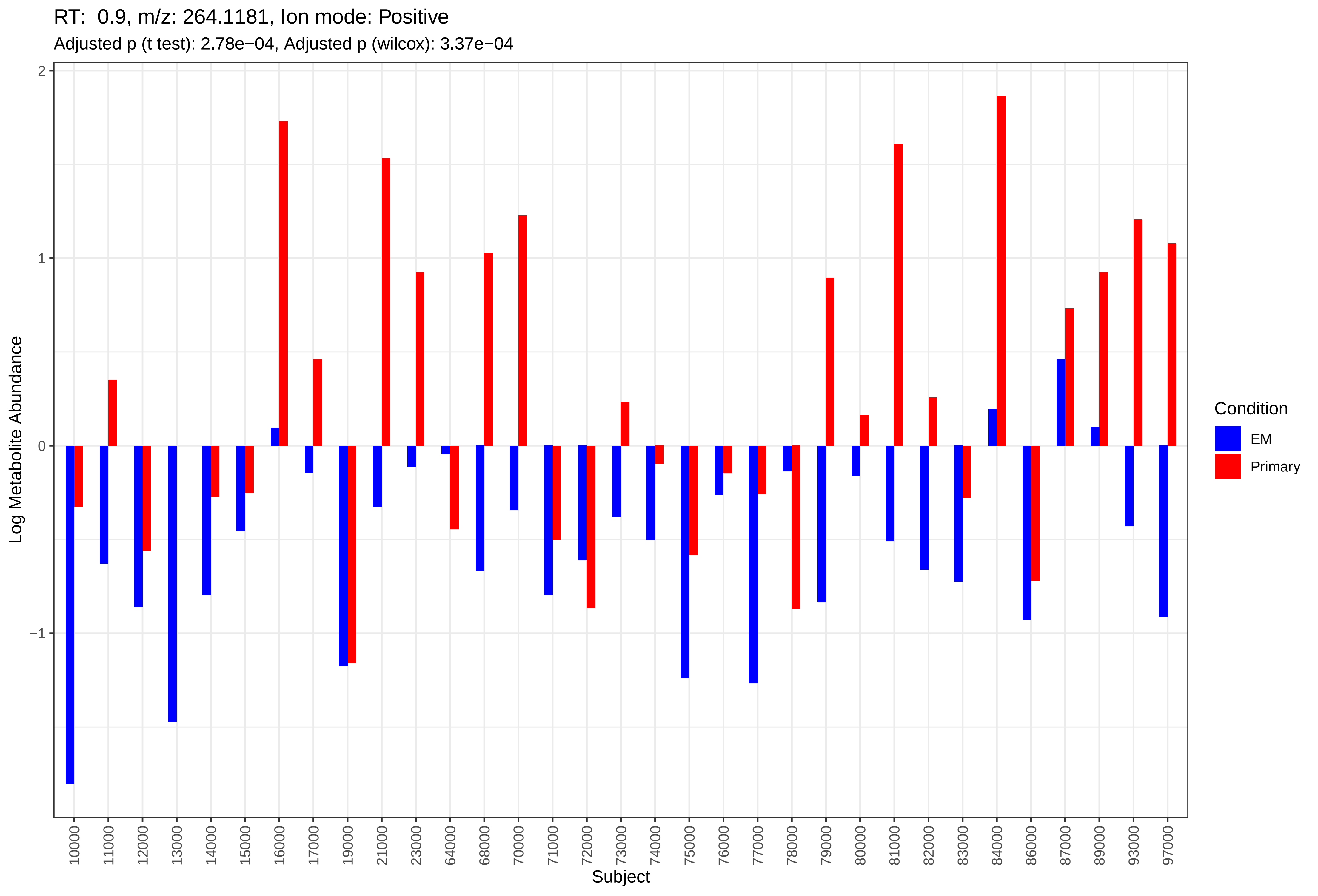

RT: 0.9, m/z: 264.1181, Ion mode: Positive
Adjusted p (t test): 2.78e−04, Adjusted p (wilcox): 3.37e−04
Log Metabolite Abundance
2
1
0
−1
10000
11000
12000
13000
14000
15000
16000
17000
19000
21000
23000
64000
68000
70000
71000
72000
73000
74000
75000
76000
77000
78000
79000
80000
81000
82000
83000
84000
86000
87000
89000
93000
97000
Subject
Condition
EM
Primary

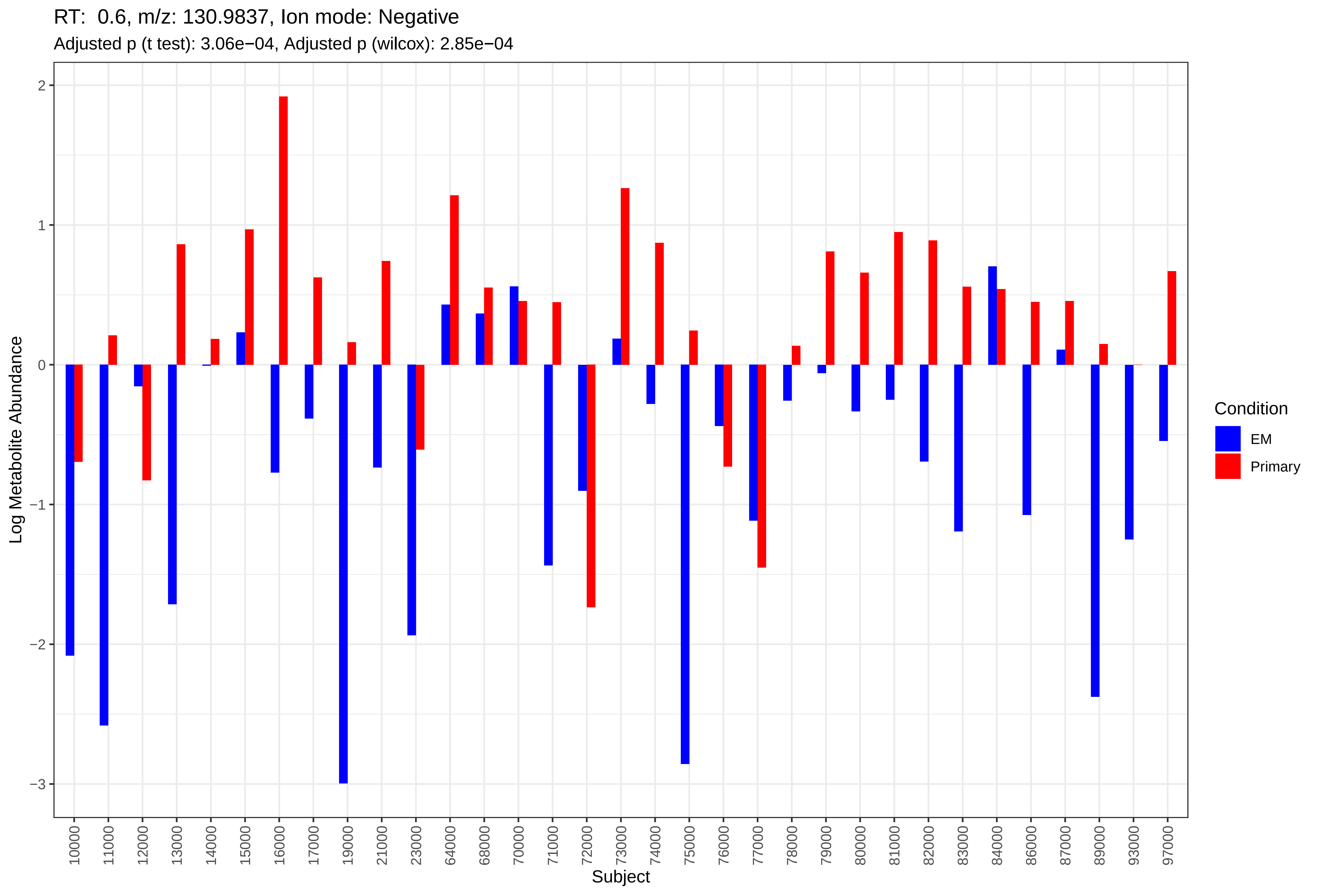

RT: 0.6, m/z: 130.9837, Ion mode: Negative
Adjusted p (t test): 3.06e−04, Adjusted p (wilcox): 2.85e−04
Log Metabolite Abundance
Subject
Condition
EM
Primary
2
1
0
−1
−2
−3
10000
11000
12000
13000
14000
15000
16000
17000
19000
21000
23000
64000
68000
70000
71000
72000
73000
74000
75000
76000
77000
78000
79000
80000
81000
82000
83000
84000
86000
87000
89000
93000
97000

RT: 3.3, m/z: 240.0513, Ion mode: Negative

Adjusted p (t test): 3.70e−04, Adjusted p (wilcox): 2.85e−04

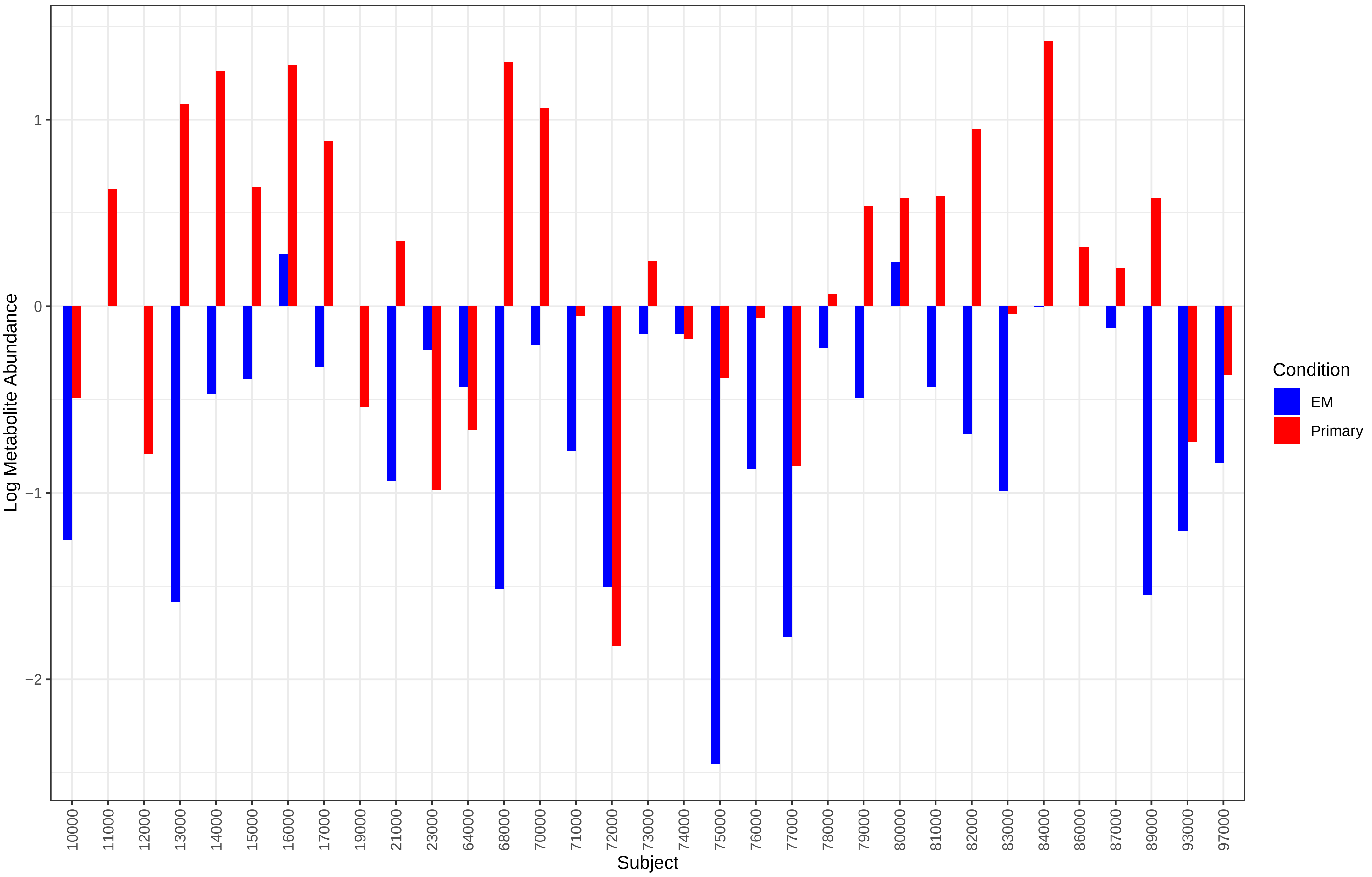

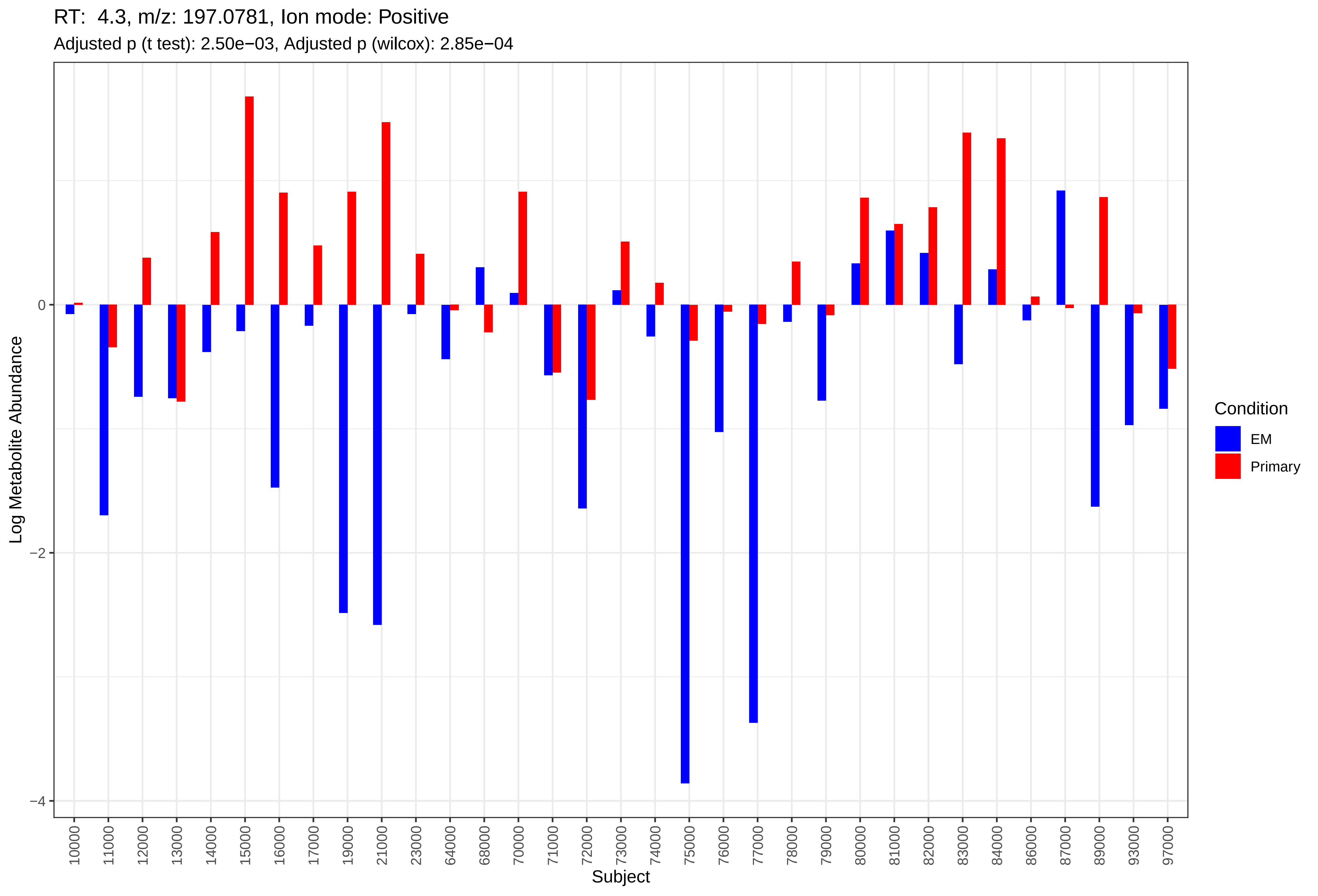

RT: 4.3, m/z: 197.0781, Ion mode: Positive
Adjusted p (t test): 2.50e−03, Adjusted p (wilcox): 2.85e−04
Log Metabolite Abundance
0
−2
−4
10000
11000
12000
13000
14000
15000
16000
17000
19000
21000
23000
64000
68000
70000
71000
72000
73000
74000
75000
76000
77000
78000
79000
80000
81000
82000
83000
84000
86000
87000
89000
93000
97000
Subject
Condition
EM
Primary

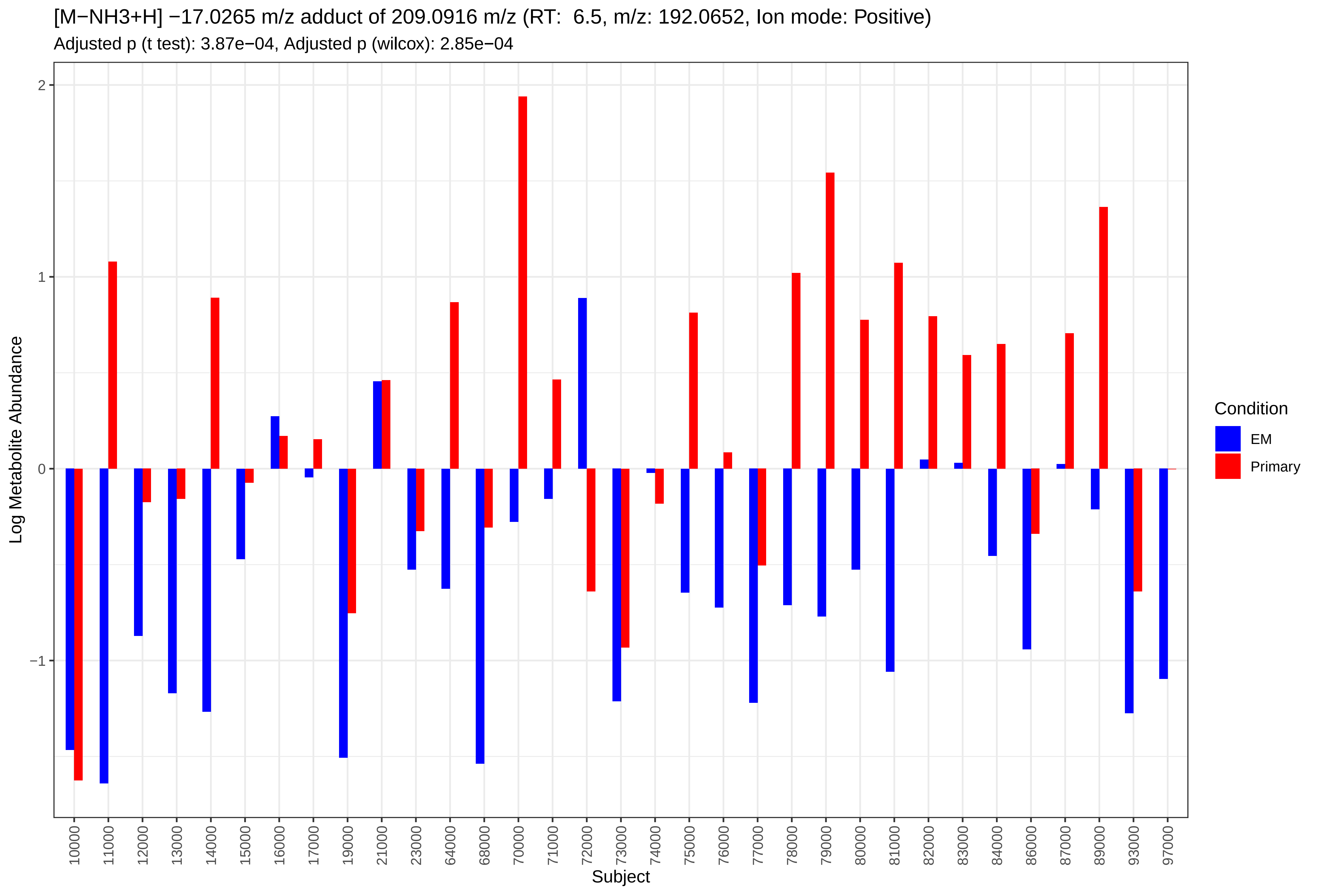
[M−NH3+H] −17.0265 m/z adduct of 209.0916 m/z (RT: 6.5, m/z: 192.0652, Ion mode: Positive)
Adjusted p (t test): 3.87e−04, Adjusted p (wilcox): 2.85e−04
Log Metabolite Abundance
2
1
0
−1
10000
11000
12000
13000
14000
15000
16000
17000
19000
21000
23000
64000
68000
70000
71000
72000
73000
74000
75000
76000
77000
78000
79000
80000
81000
82000
83000
84000
86000
87000
89000
93000
97000
Subject
Condition
EM
Primary

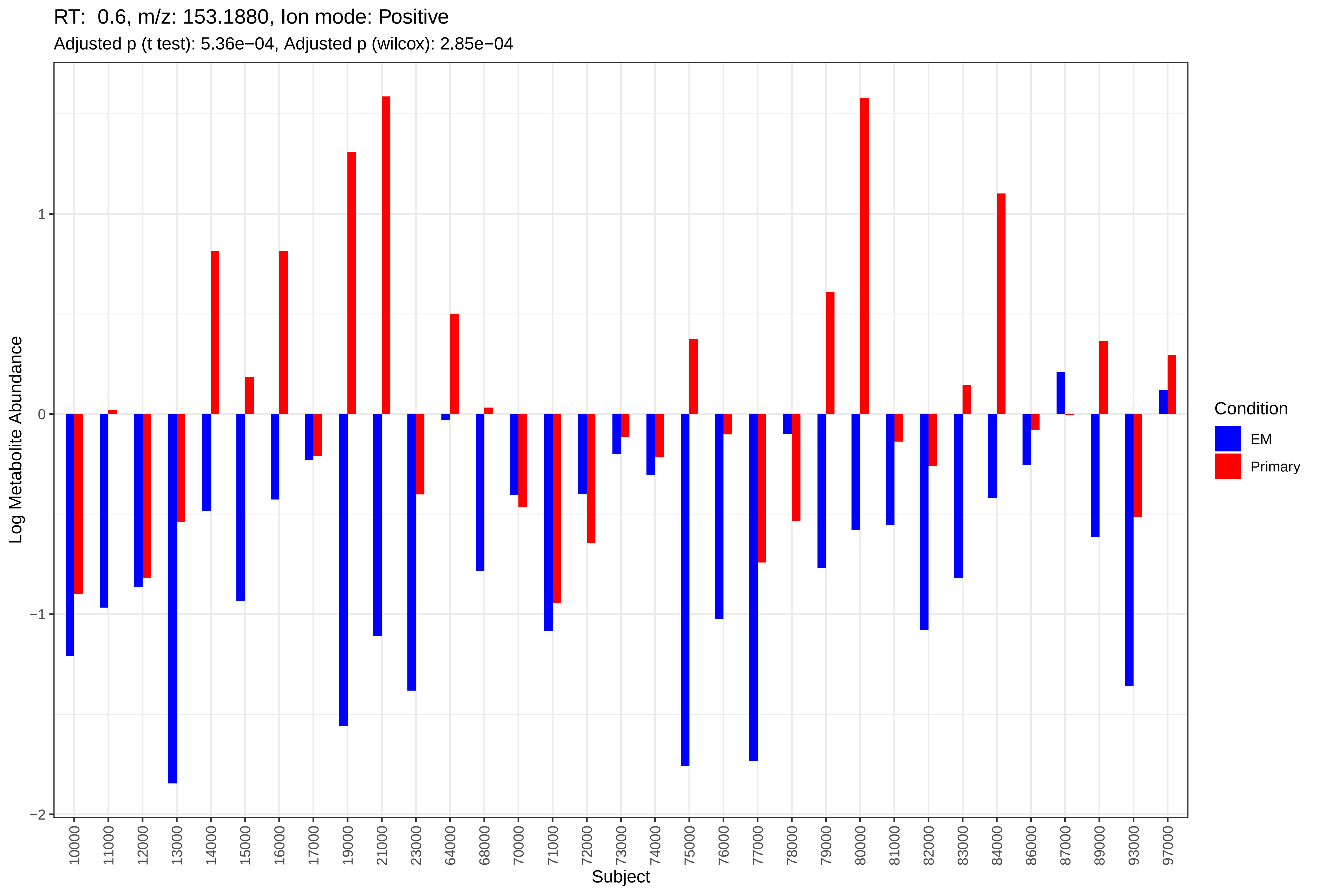
RT: 0.6, m/z: 153.1880, Ion mode: Positive
Adjusted p (t test): 5.36e−04, Adjusted p (wilcox): 2.85e−04
Log Metabolite Abundance
1
0
−1
−2
10000
11000
12000
13000
14000
15000
16000
17000
19000
21000
23000
64000
68000
70000
71000
72000
73000
74000
75000
76000
77000
78000
79000
80000
81000
82000
83000
84000
86000
87000
89000
93000
97000
Subject
Condition
EM
Primary

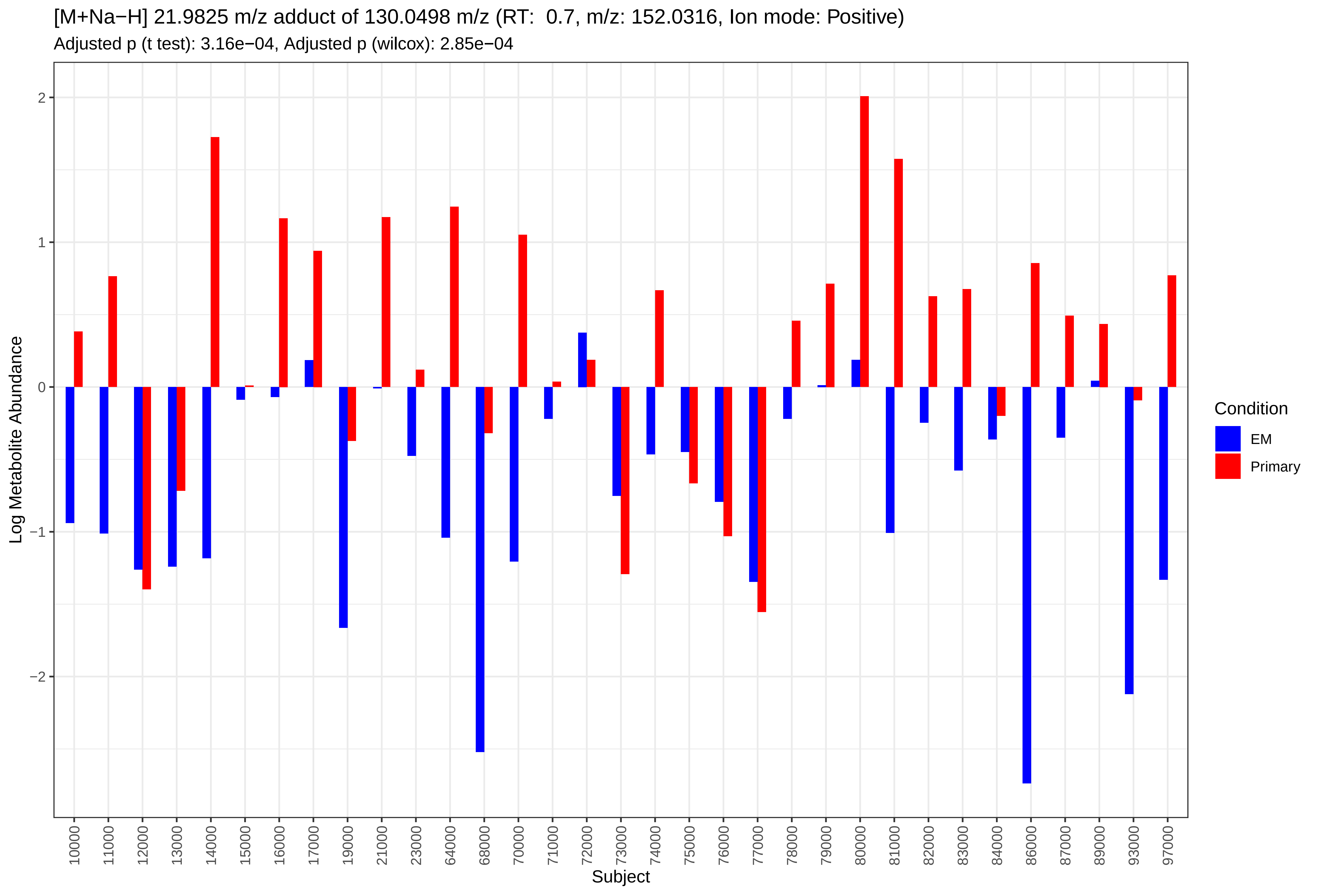
[M+Na−H] 21.9825 m/z adduct of 130.0498 m/z (RT: 0.7, m/z: 152.0316, Ion mode: Positive)
Adjusted p (t test): 3.16e−04, Adjusted p (wilcox): 2.85e−04
Log Metabolite Abundance
2
1
0
−1
−2
Subject
10000
11000
12000
13000
14000
15000
16000
17000
19000
21000
23000
64000
68000
70000
71000
72000
73000
74000
75000
76000
77000
78000
79000
80000
81000
82000
83000
84000
86000
87000
89000
93000
97000
Condition
EM
Primary

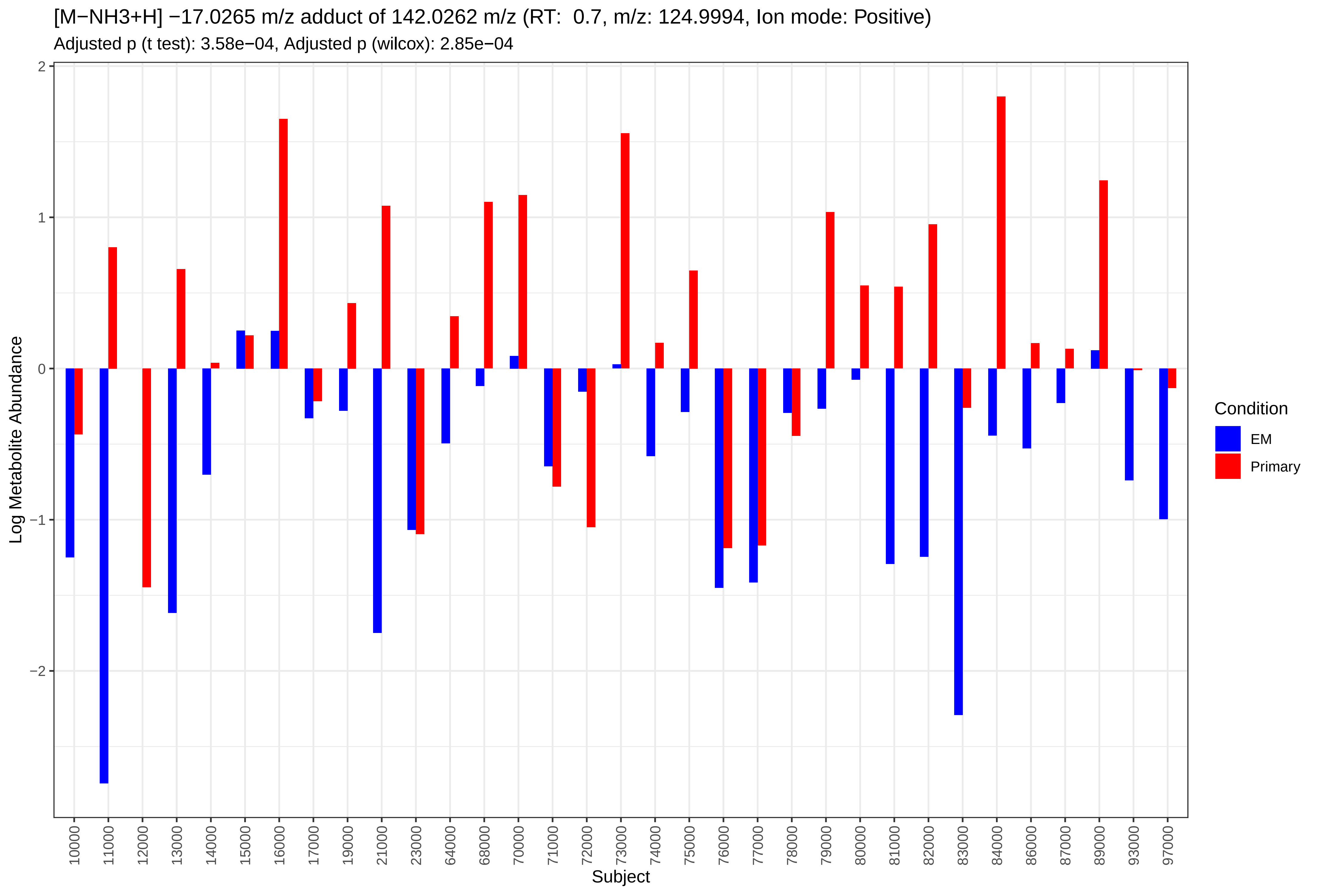
[M−NH3+H] −17.0265 m/z adduct of 142.0262 m/z (RT: 0.7, m/z: 124.9994, Ion mode: Positive)
Adjusted p (t test): 3.58e−04, Adjusted p (wilcox): 2.85e−04
Log Metabolite Abundance
Subject
Condition
EM
Primary
10000
11000
12000
13000
14000
15000
16000
17000
19000
21000
23000
64000
68000
70000
71000
72000
73000
74000
75000
76000
77000
78000
79000
80000
81000
82000
83000
84000
86000
87000
89000
93000
97000

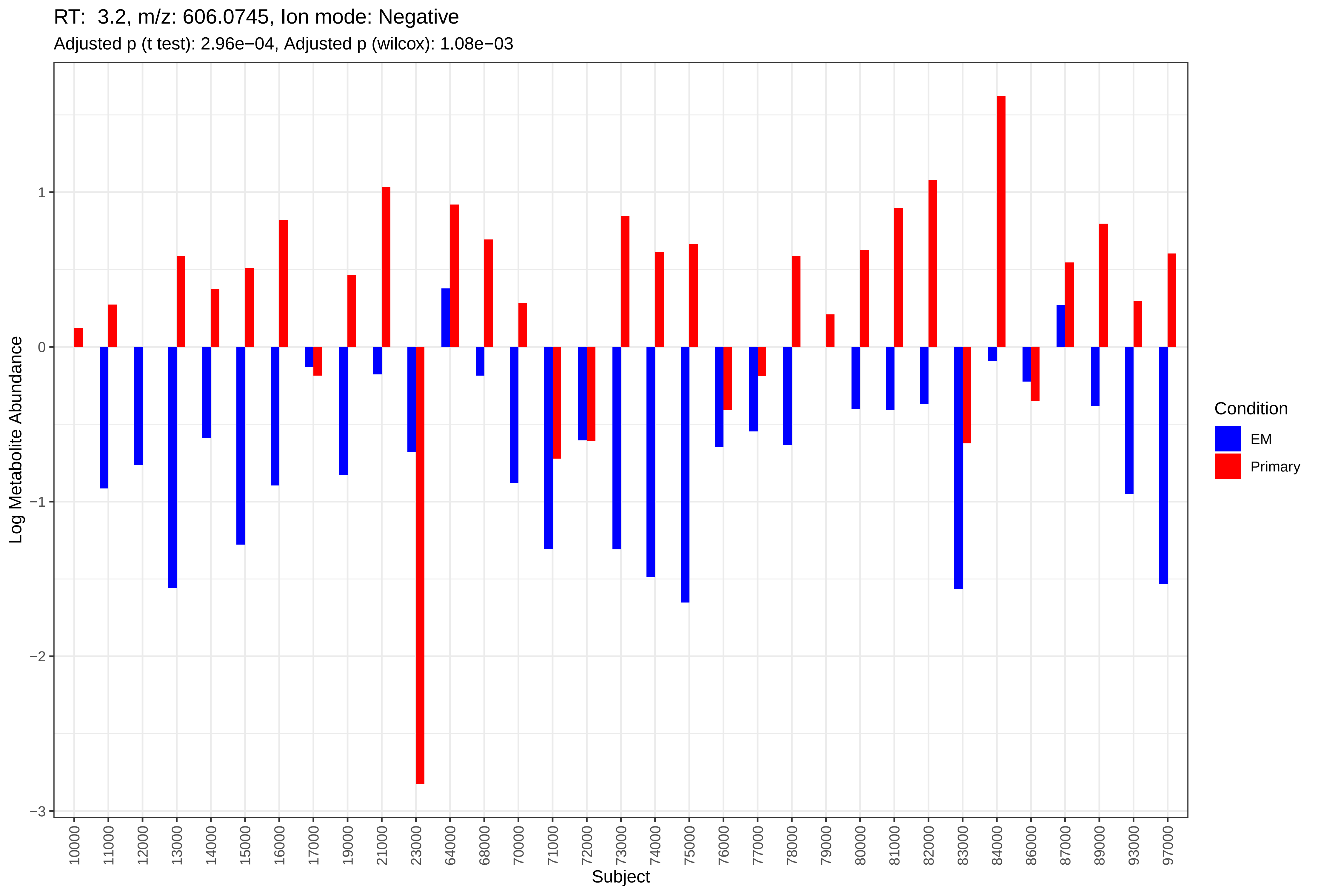

RT: 3.2, m/z: 606.0745, Ion mode: Negative
Adjusted p (t test): 2.96e−04, Adjusted p (wilcox): 1.08e−03
Log Metabolite Abundance
1
0
−1
−2
−3
Subject
10000 11000 12000 13000 14000 15000 16000 17000 19000 21000 23000 64000 68000 70000 71000 72000 73000 74000 75000 76000 77000 78000 79000 80000 81000 82000 83000 84000 86000 87000 89000 93000 97000
Condition
EM
Primary

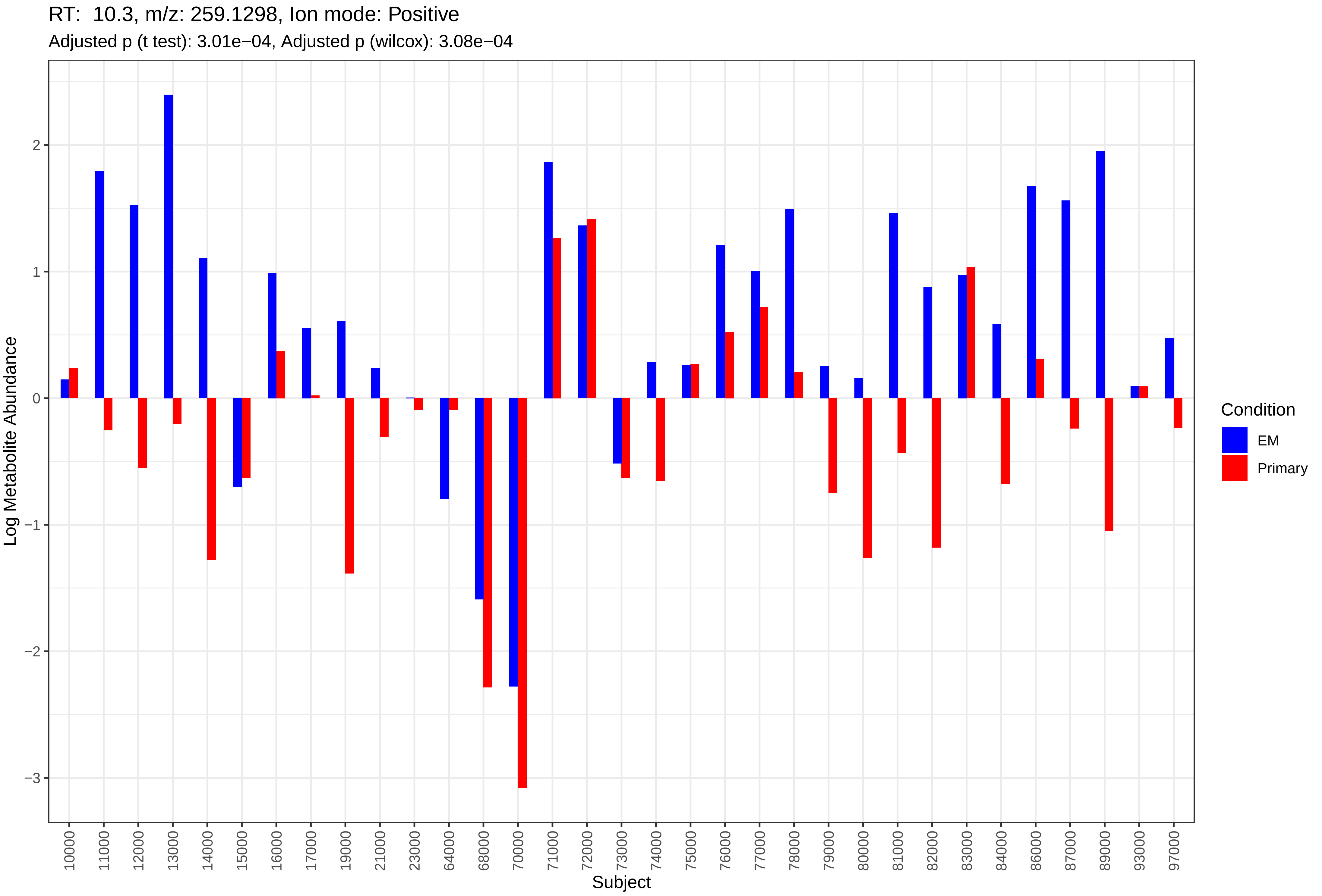
RT: 10.3, m/z: 259.1298, Ion mode: Positive
Adjusted p (t test): 3.01e−04, Adjusted p (wilcox): 3.08e−04
Log Metabolite Abundance
Subject
Condition
EM
Primary
2
1
0
−1
−2
−3
10000
11000
12000
13000
14000
15000
16000
17000
19000
21000
23000
64000
68000
70000
71000
72000
73000
74000
75000
76000
77000
78000
79000
80000
81000
82000
83000
84000
86000
87000
89000
93000
97000

RT: 0.7, m/z: 164.8361, Ion mode: Negative

Adjusted p (t test): 4.14e−04, Adjusted p (wilcox): 3.08e−04

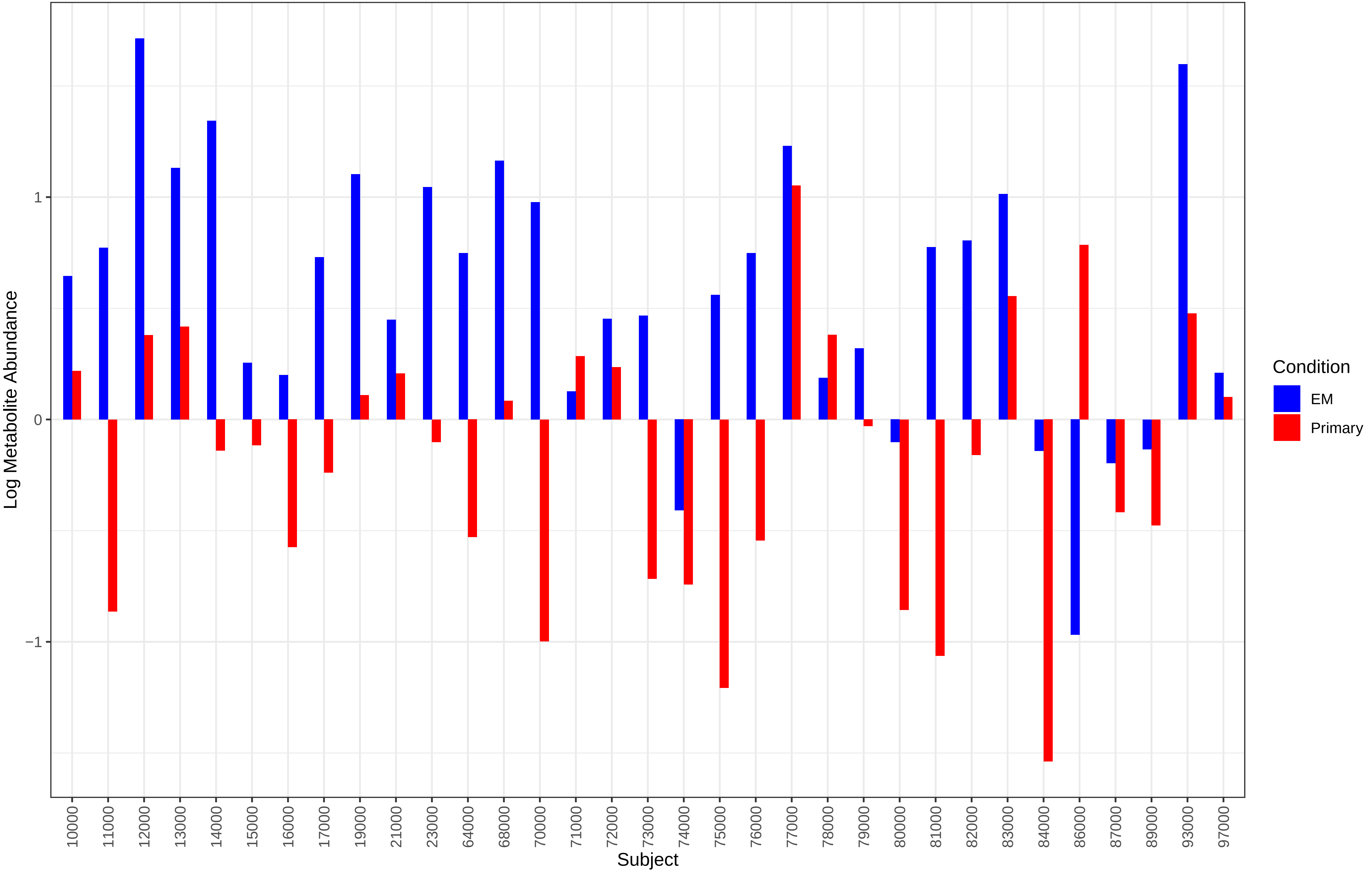

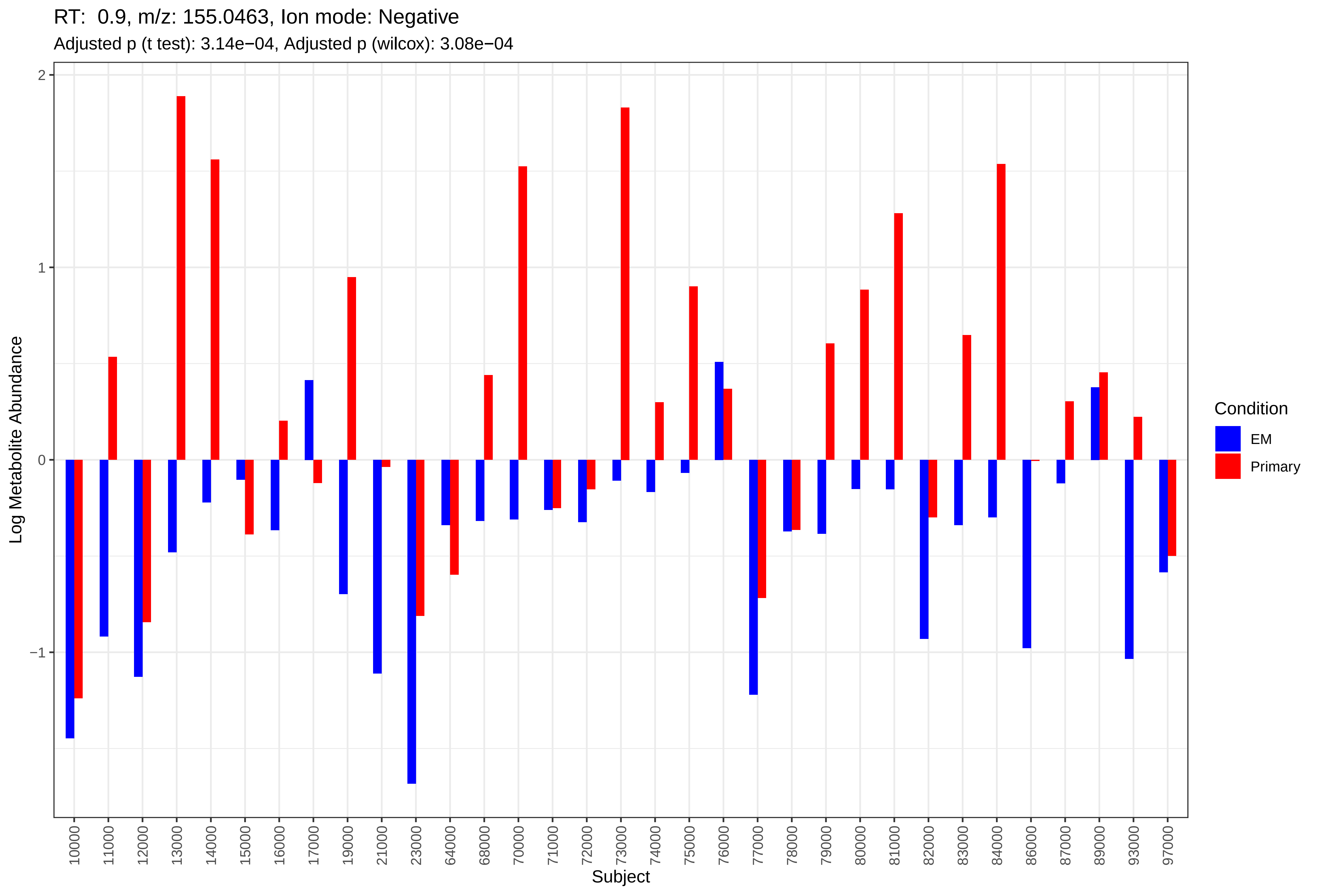
RT: 0.9, m/z: 155.0463, Ion mode: Negative
Adjusted p (t test): 3.14e−04, Adjusted p (wilcox): 3.08e−04
Log Metabolite Abundance
2
1
0
−1
10000
11000
12000
13000
14000
15000
16000
17000
19000
21000
23000
64000
68000
70000
71000
72000
73000
74000
75000
76000
77000
78000
79000
80000
81000
82000
83000
84000
86000
87000
89000
93000
97000
Subject
Condition
EM
Primary

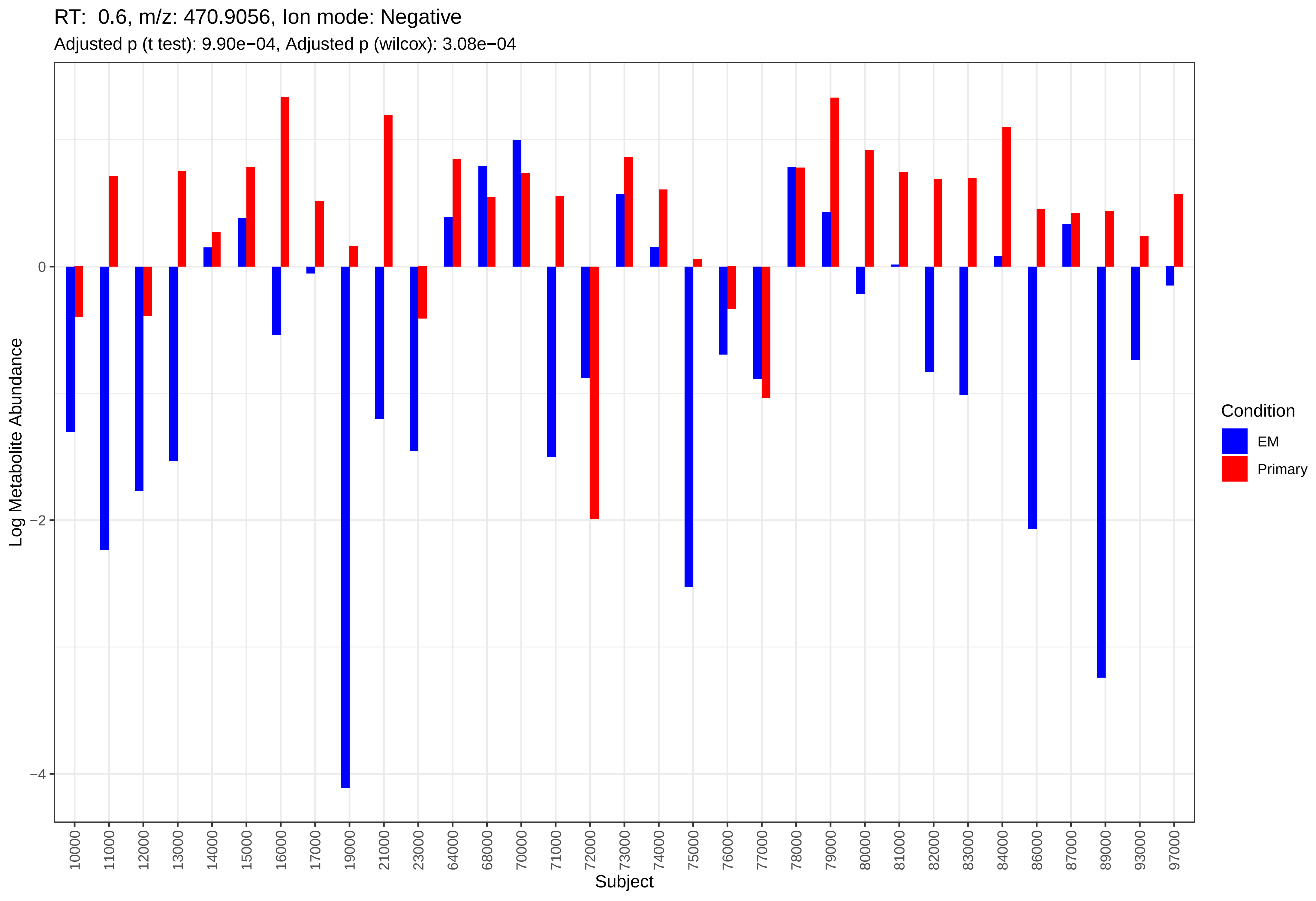

RT: 0.6, m/z: 470.9056, Ion mode: Negative
Adjusted p (t test): 9.90e−04, Adjusted p (wilcox): 3.08e−04
Log Metabolite Abundance
0
−2
−4
Subject
10000
11000
12000
13000
14000
15000
16000
17000
19000
21000
23000
64000
68000
70000
71000
72000
73000
74000
75000
76000
77000
78000
79000
80000
81000
82000
83000
84000
86000
87000
89000
93000
97000
Condition
EM
Primary

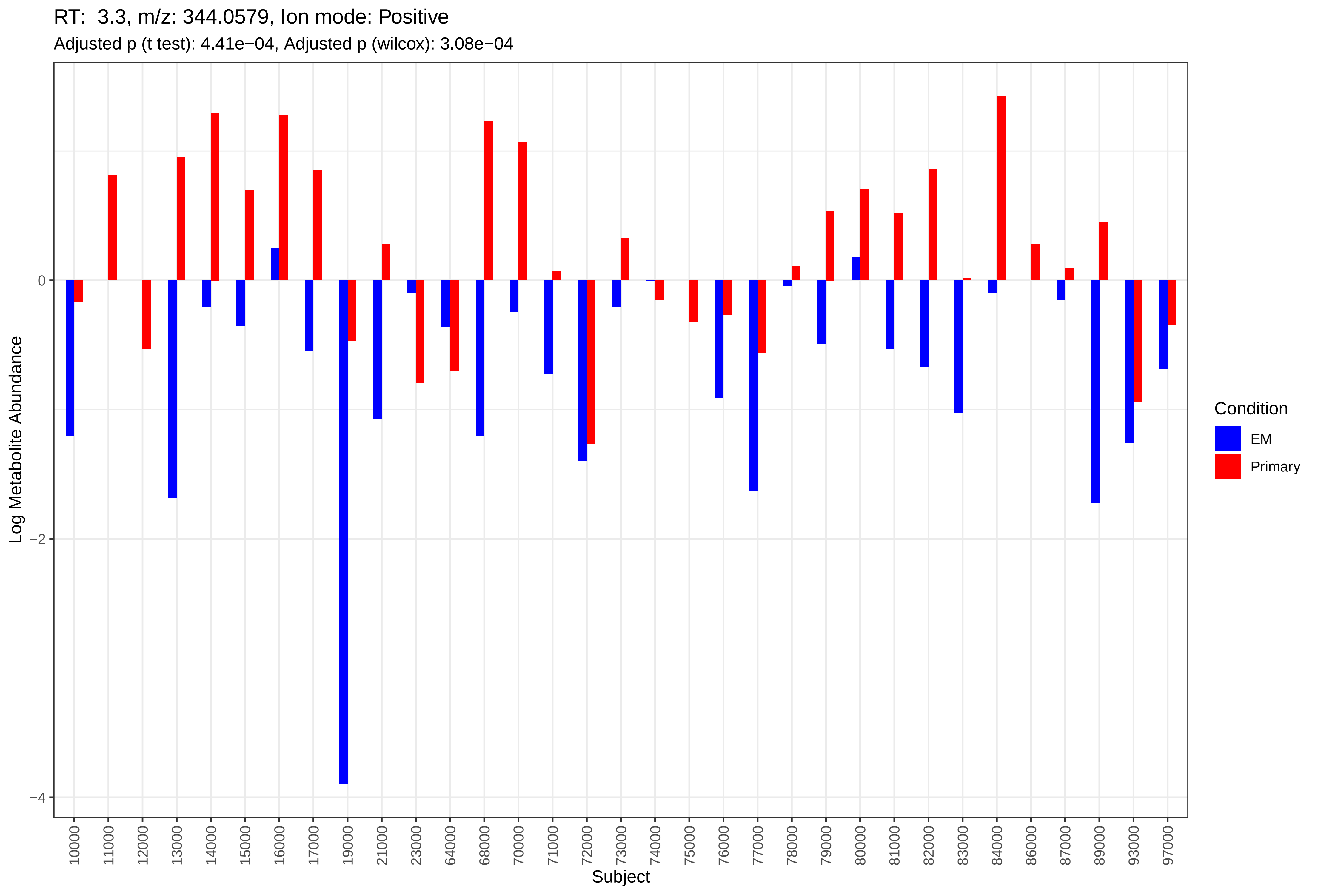
RT: 3.3, m/z: 344.0579, Ion mode: Positive
Adjusted p (t test): 4.41e−04, Adjusted p (wilcox): 3.08e−04
Log Metabolite Abundance
0
−2
−4
10000
11000
12000
13000
14000
15000
16000
17000
19000
21000
23000
64000
68000
70000
71000
72000
73000
74000
75000
76000
77000
78000
79000
80000
81000
82000
83000
84000
86000
87000
89000
93000
97000
Subject
Condition
EM
Primary

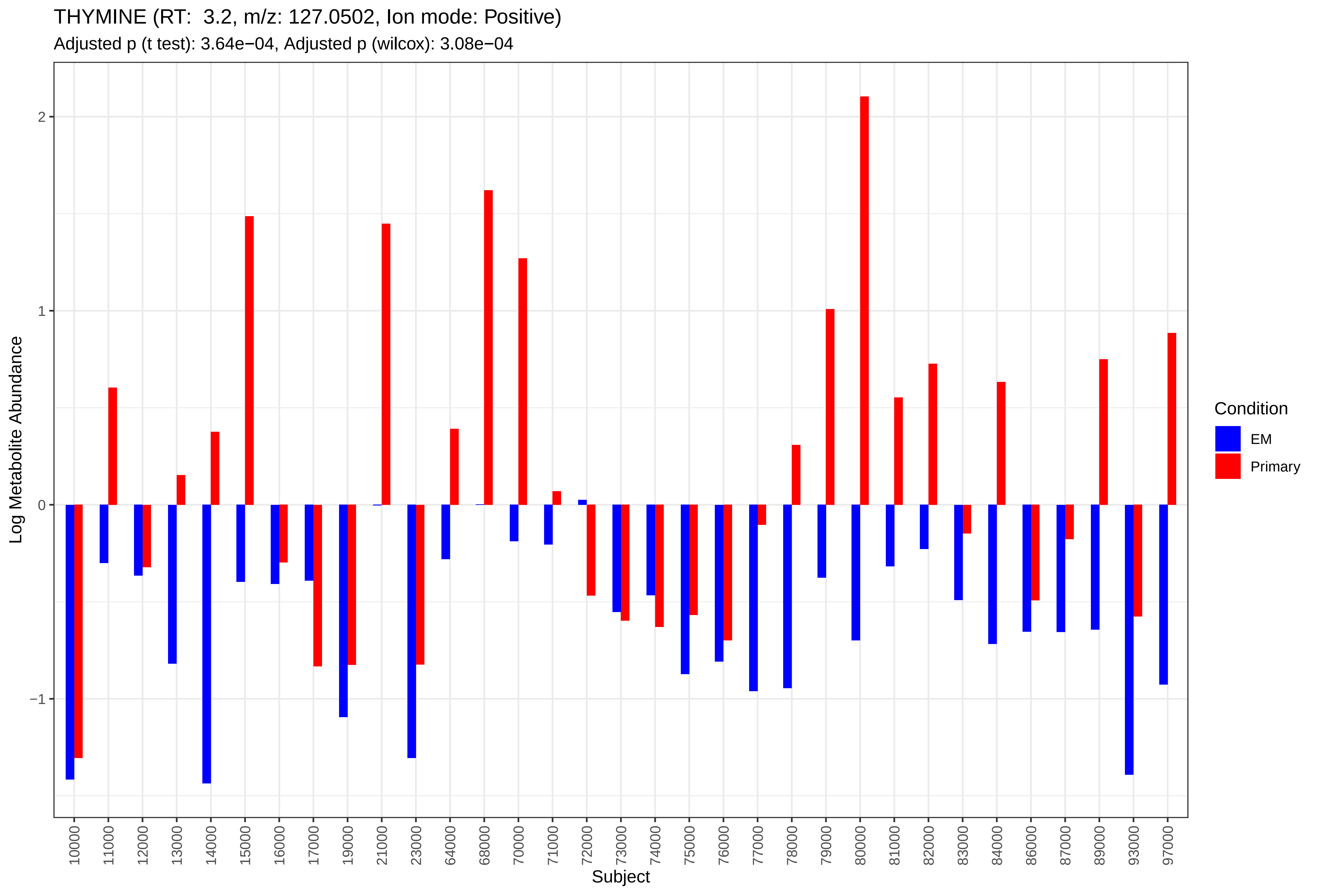
THYMINE (RT: 3.2, m/z: 127.0502, Ion mode: Positive)
Adjusted p (t test): 3.64e−04, Adjusted p (wilcox): 3.08e−04
Log Metabolite Abundance
2
1
0
−1
Subject
10000
11000
12000
13000
14000
15000
16000
17000
19000
21000
23000
64000
68000
70000
71000
72000
73000
74000
75000
76000
77000
78000
79000
80000
81000
82000
83000
84000
86000
87000
89000
93000
97000
Condition
EM
Primary

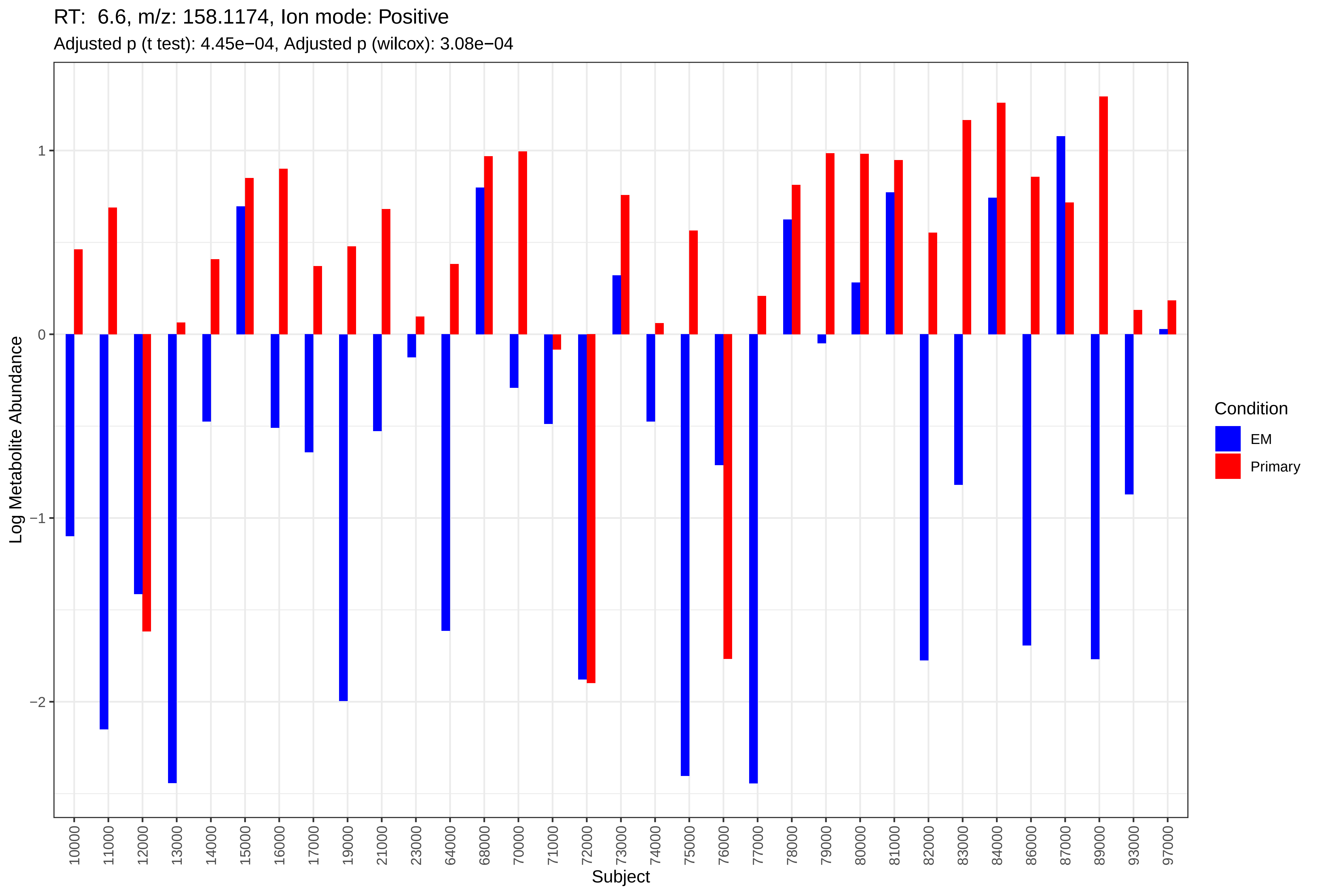
RT: 6.6, m/z: 158.1174, Ion mode: Positive
Adjusted p (t test): 4.45e−04, Adjusted p (wilcox): 3.08e−04
Log Metabolite Abundance
1
0
−1
−2
10000
11000
12000
13000
14000
15000
16000
17000
19000
21000
23000
64000
68000
70000
71000
72000
73000
74000
75000
76000
77000
78000
79000
80000
81000
82000
83000
84000
86000
87000
89000
93000
97000
Subject
Condition
EM
Primary

[M−H2O+H] −18.0106 m/z adduct of 216.0626 m/z (RT: 0.7, m/z: 198.0520, Ion mode: Positive)

Adjusted p (t test): 4.64e−04, Adjusted p (wilcox): 3.08e−04

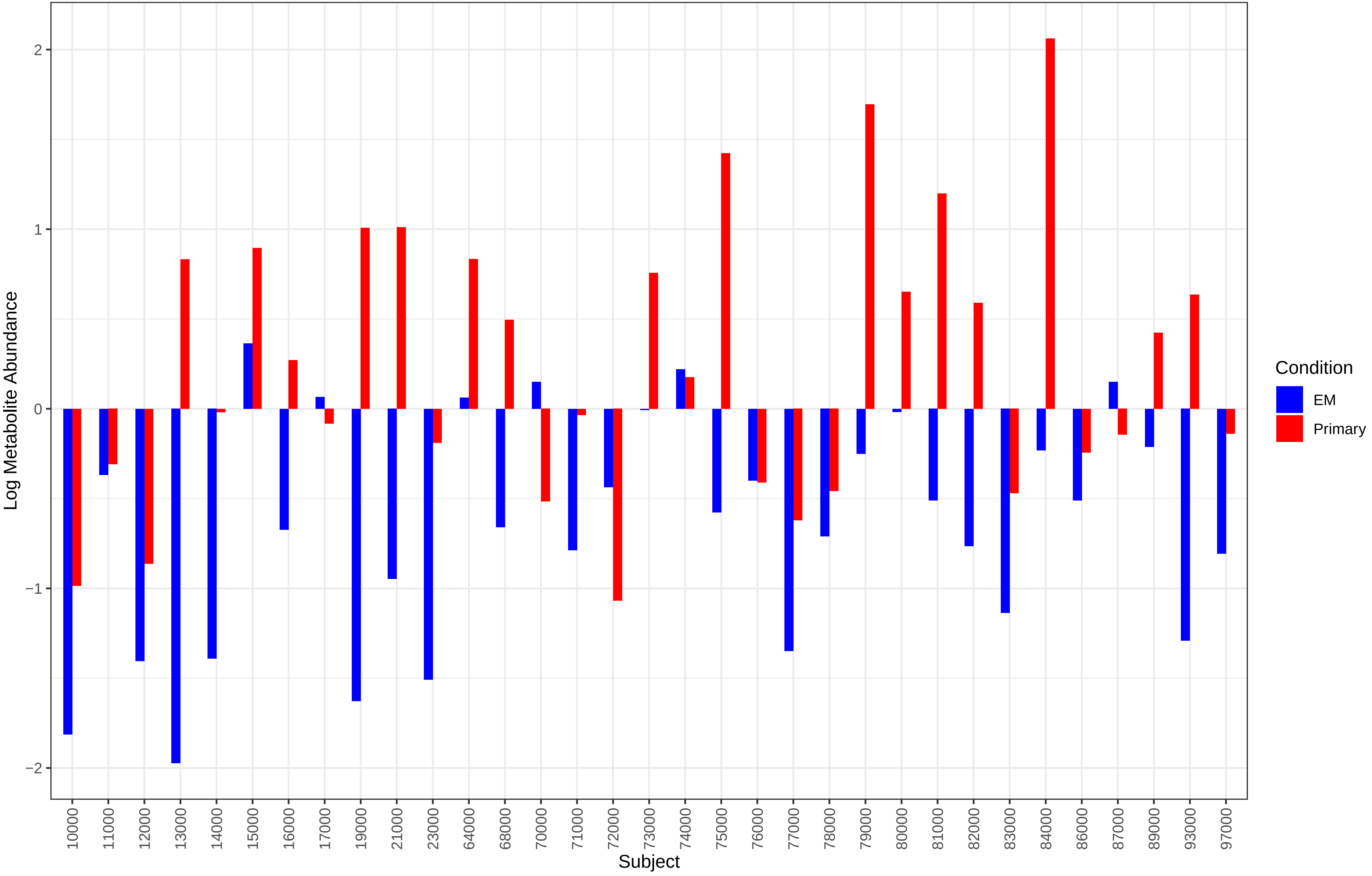

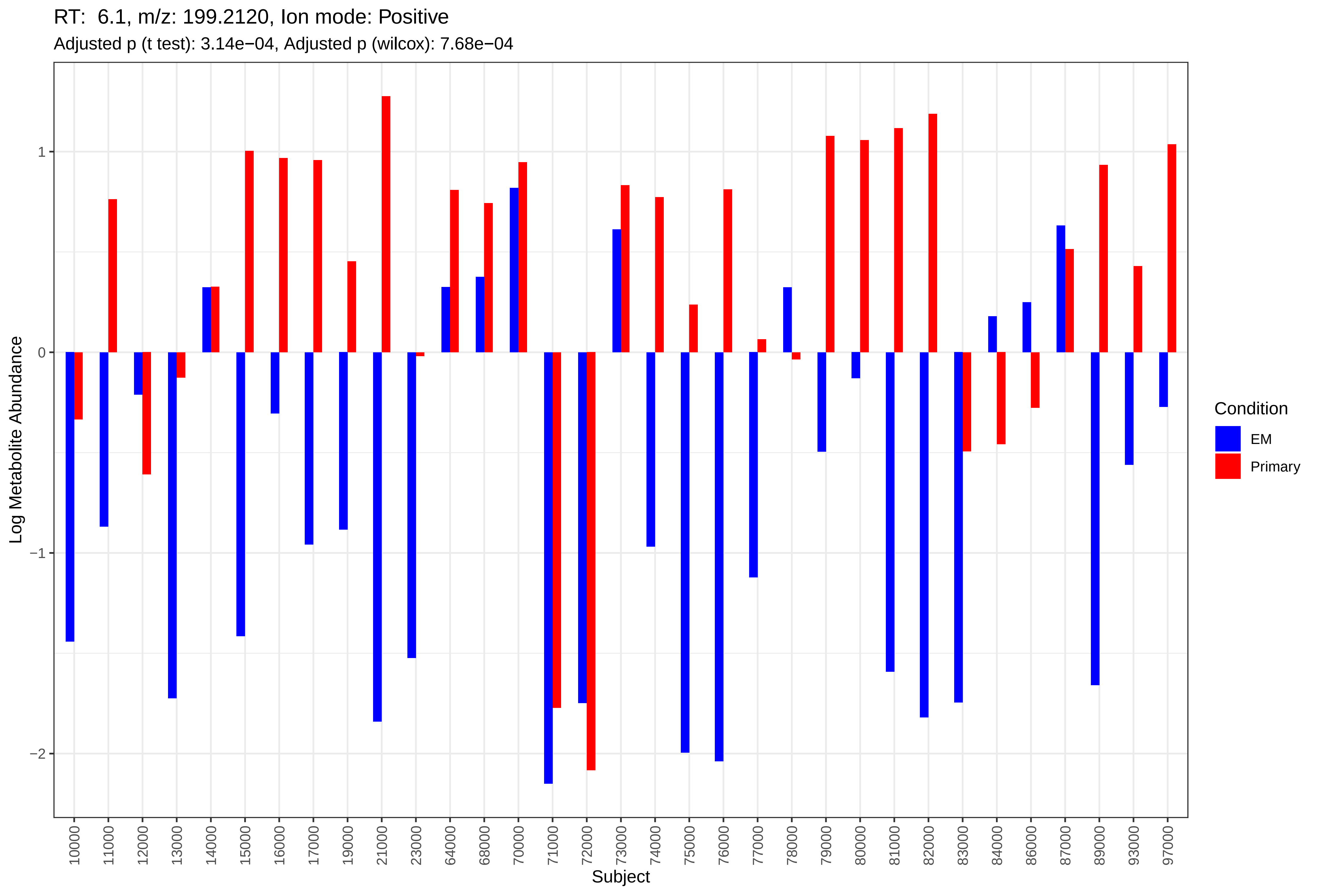
RT: 6.1, m/z: 199.2120, Ion mode: Positive
Adjusted p (t test): 3.14e−04, Adjusted p (wilcox): 7.68e−04
Log Metabolite Abundance
1
0
−1
−2
Subject
10000
11000
12000
13000
14000
15000
16000
17000
19000
21000
23000
64000
68000
70000
71000
72000
73000
74000
75000
76000
77000
78000
79000
80000
81000
82000
83000
84000
86000
87000
89000
93000
97000
Condition
EM
Primary

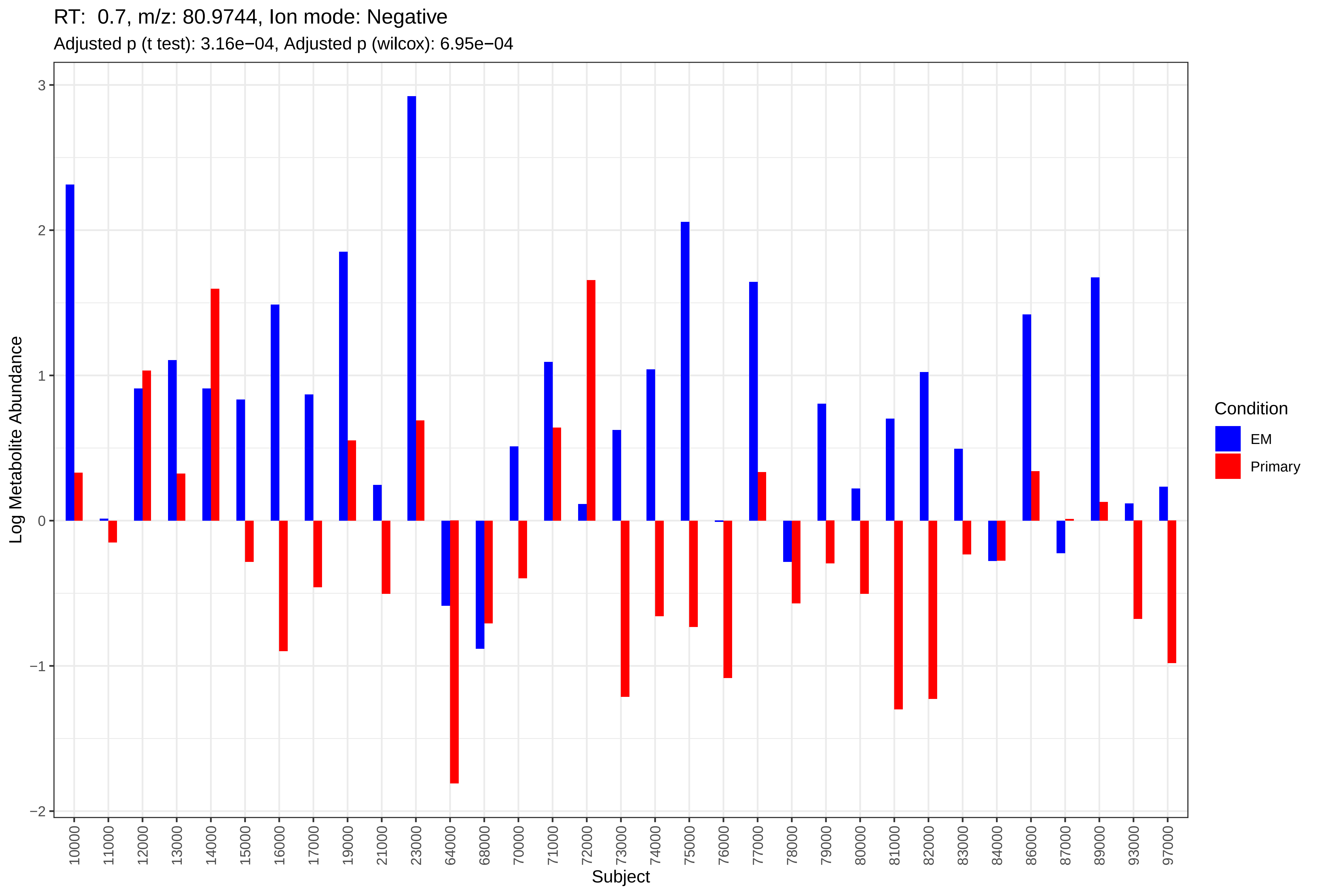
RT: 0.7, m/z: 80.9744, Ion mode: Negative
Adjusted p (t test): 3.16e−04, Adjusted p (wilcox): 6.95e−04
Log Metabolite Abundance
3
2
1
0
−1
−2
Subject
10000
11000
12000
13000
14000
15000
16000
17000
19000
21000
23000
64000
68000
70000
71000
72000
73000
74000
75000
76000
77000
78000
79000
80000
81000
82000
83000
84000
86000
87000
89000
93000
97000
Condition
EM
Primary

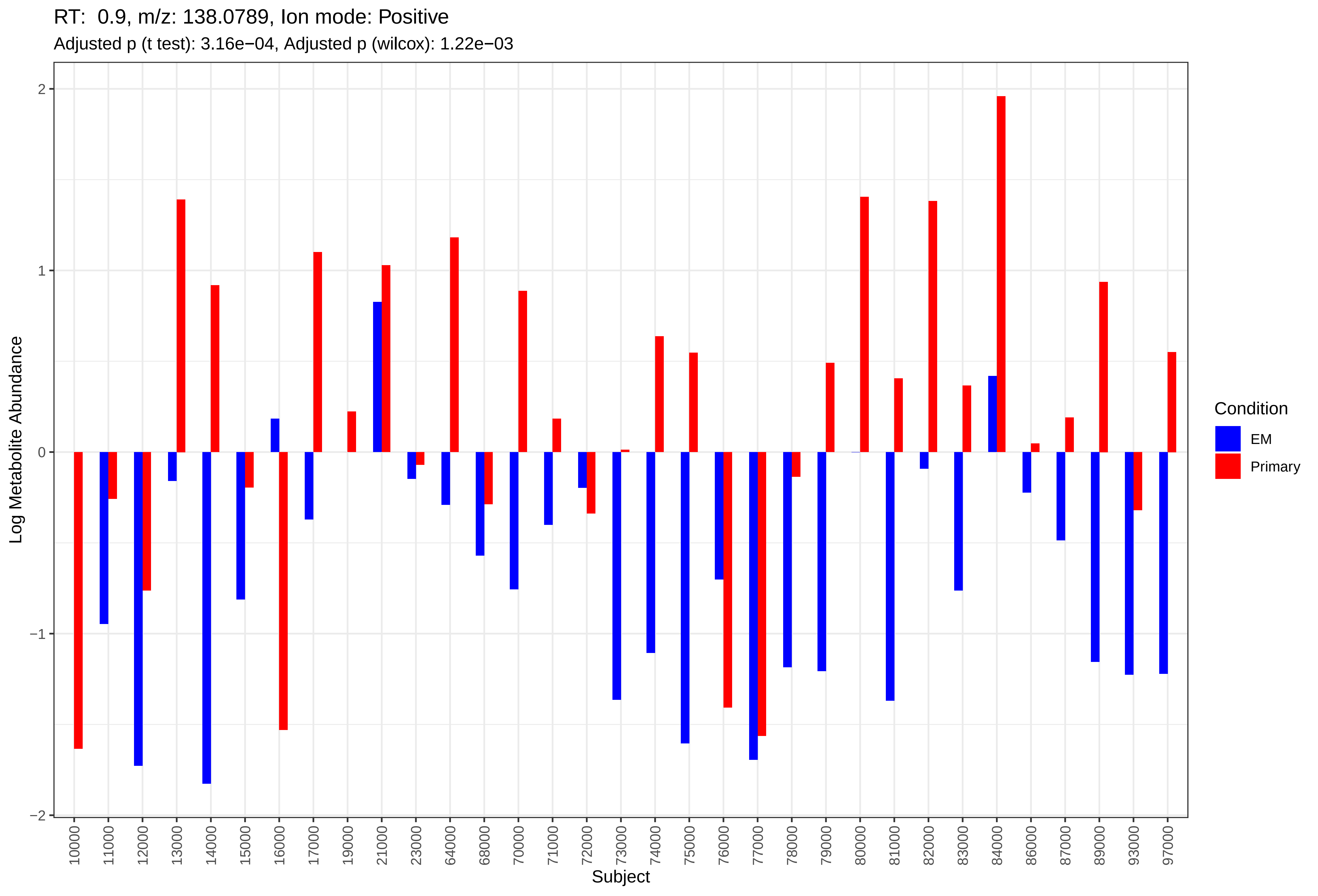

RT: 0.9, m/z: 138.0789, Ion mode: Positive
Adjusted p (t test): 3.16e−04, Adjusted p (wilcox): 1.22e−03
Log Metabolite Abundance
2
1
0
−1
−2
Subject
10000
11000
12000
13000
14000
15000
16000
17000
19000
21000
23000
64000
68000
70000
71000
72000
73000
74000
75000
76000
77000
78000
79000
80000
81000
82000
83000
84000
86000
87000
89000
93000
97000
Condition
EM
Primary

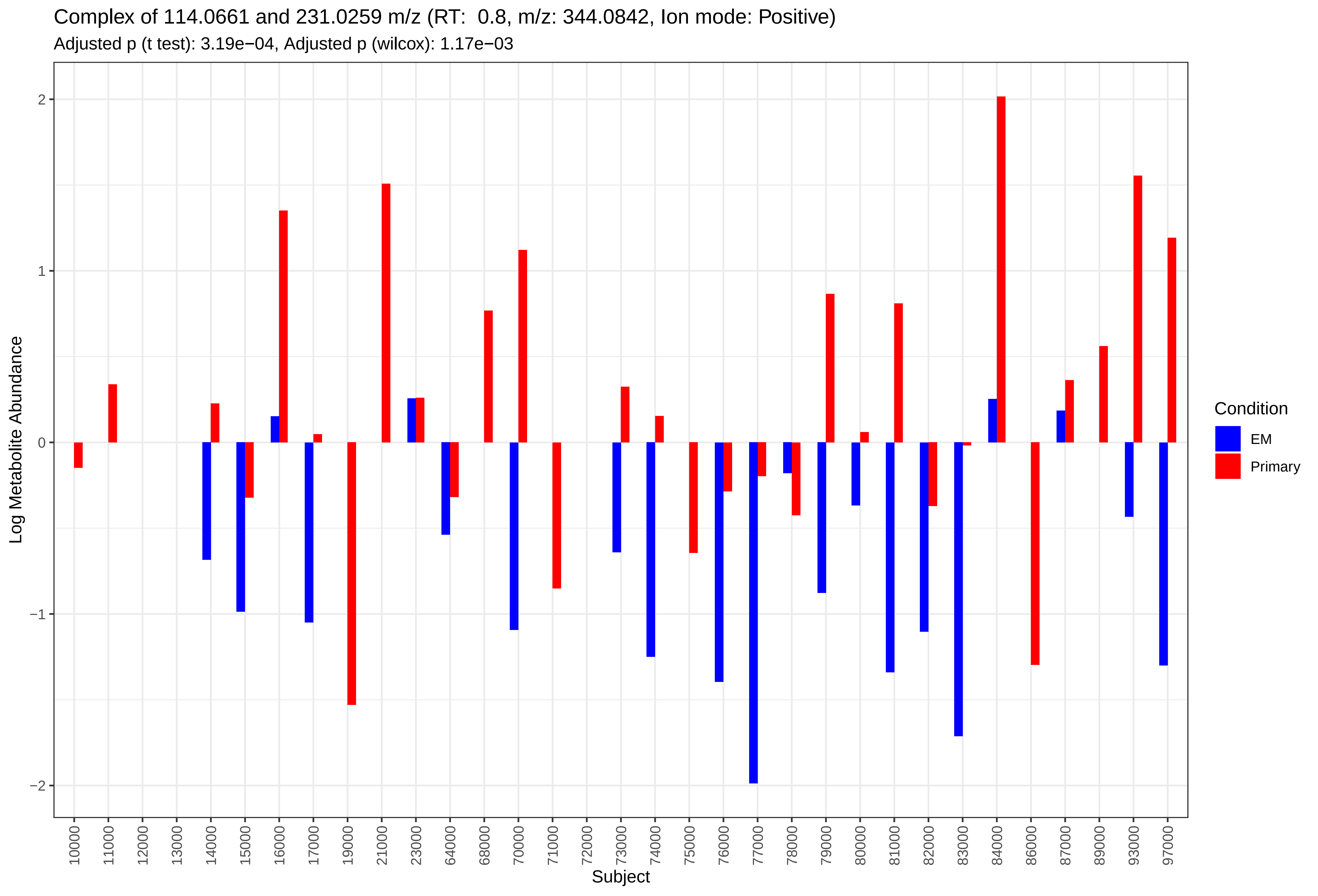

Complex of 114.0661 and 231.0259 m/z (RT: 0.8, m/z: 344.0842, Ion mode: Positive)
Adjusted p (t test): 3.19e−04, Adjusted p (wilcox): 1.17e−03
Log Metabolite Abundance
2
1
0
−1
−2
10000
11000
12000
13000
14000
15000
16000
17000
19000
21000
23000
64000
68000
70000
71000
72000
73000
74000
75000
76000
77000
78000
79000
80000
81000
82000
83000
84000
86000
87000
89000
93000
97000
Subject
Condition
EM
Primary

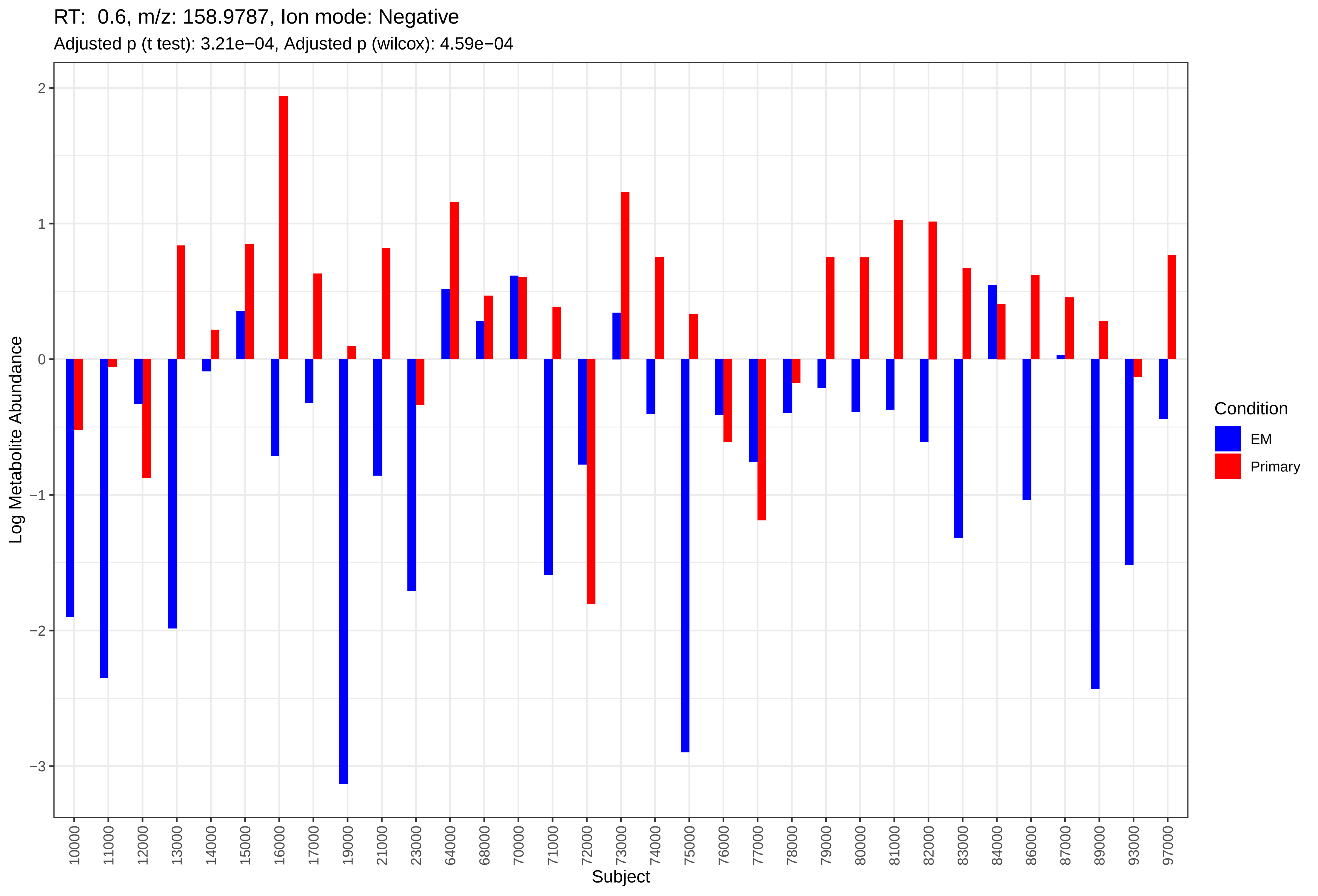

RT: 0.6, m/z: 158.9787, Ion mode: Negative
Adjusted p (t test): 3.21e−04, Adjusted p (wilcox): 4.59e−04
Log Metabolite Abundance
2
1
0
−1
−2
−3
10000
11000
12000
13000
14000
15000
16000
17000
19000
21000
23000
64000
68000
70000
71000
72000
73000
74000
75000
76000
77000
78000
79000
80000
81000
82000
83000
84000
86000
87000
89000
93000
97000
Subject
Condition
EM
Primary

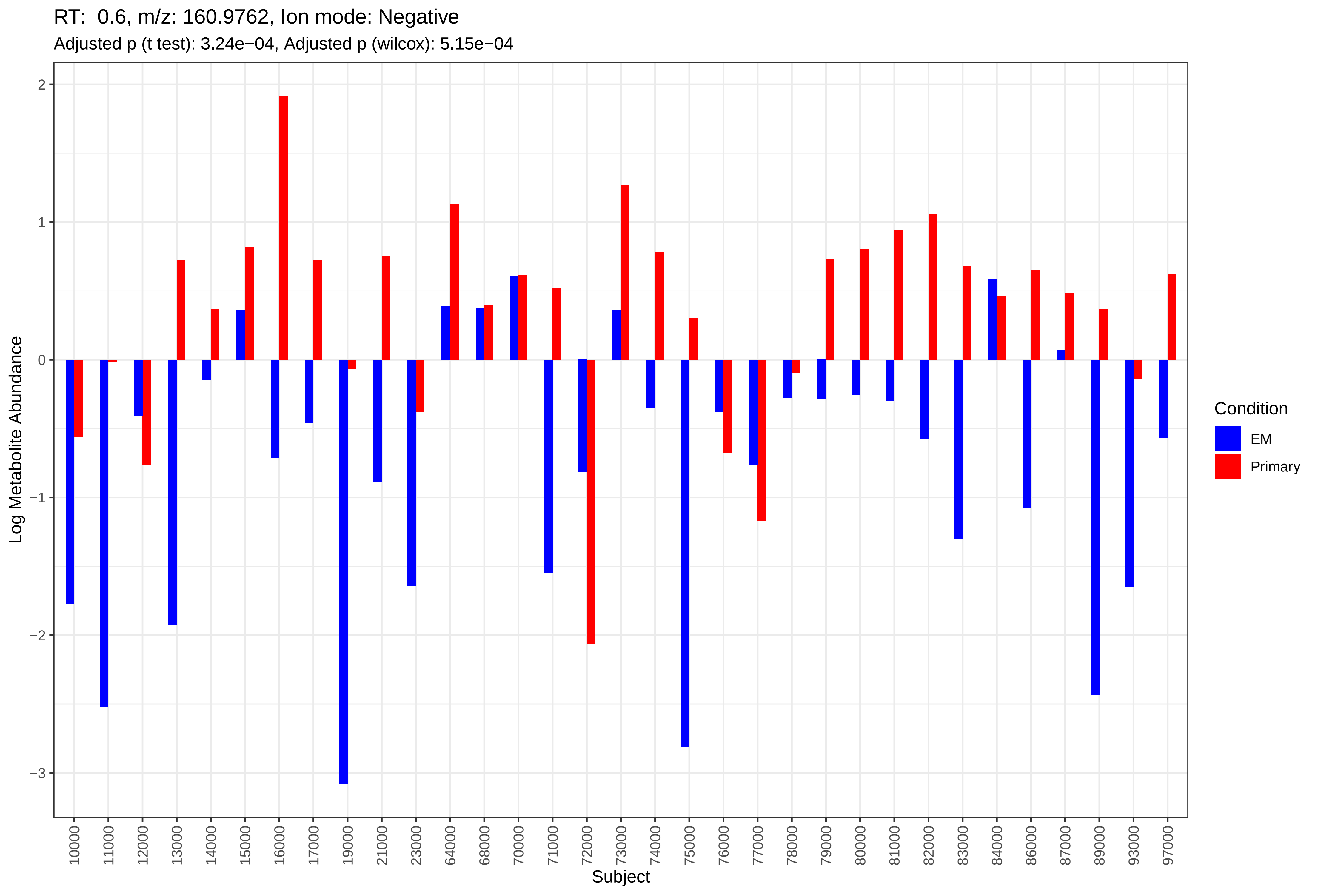

RT: 0.6, m/z: 160.9762, Ion mode: Negative
Adjusted p (t test): 3.24e−04, Adjusted p (wilcox): 5.15e−04
Log Metabolite Abundance
2
1
0
−1
−2
−3
10000
11000
12000
13000
14000
15000
16000
17000
19000
21000
23000
64000
68000
70000
71000
72000
73000
74000
75000
76000
77000
78000
79000
80000
81000
82000
83000
84000
86000
87000
89000
93000
97000
Subject
Condition
EM
Primary

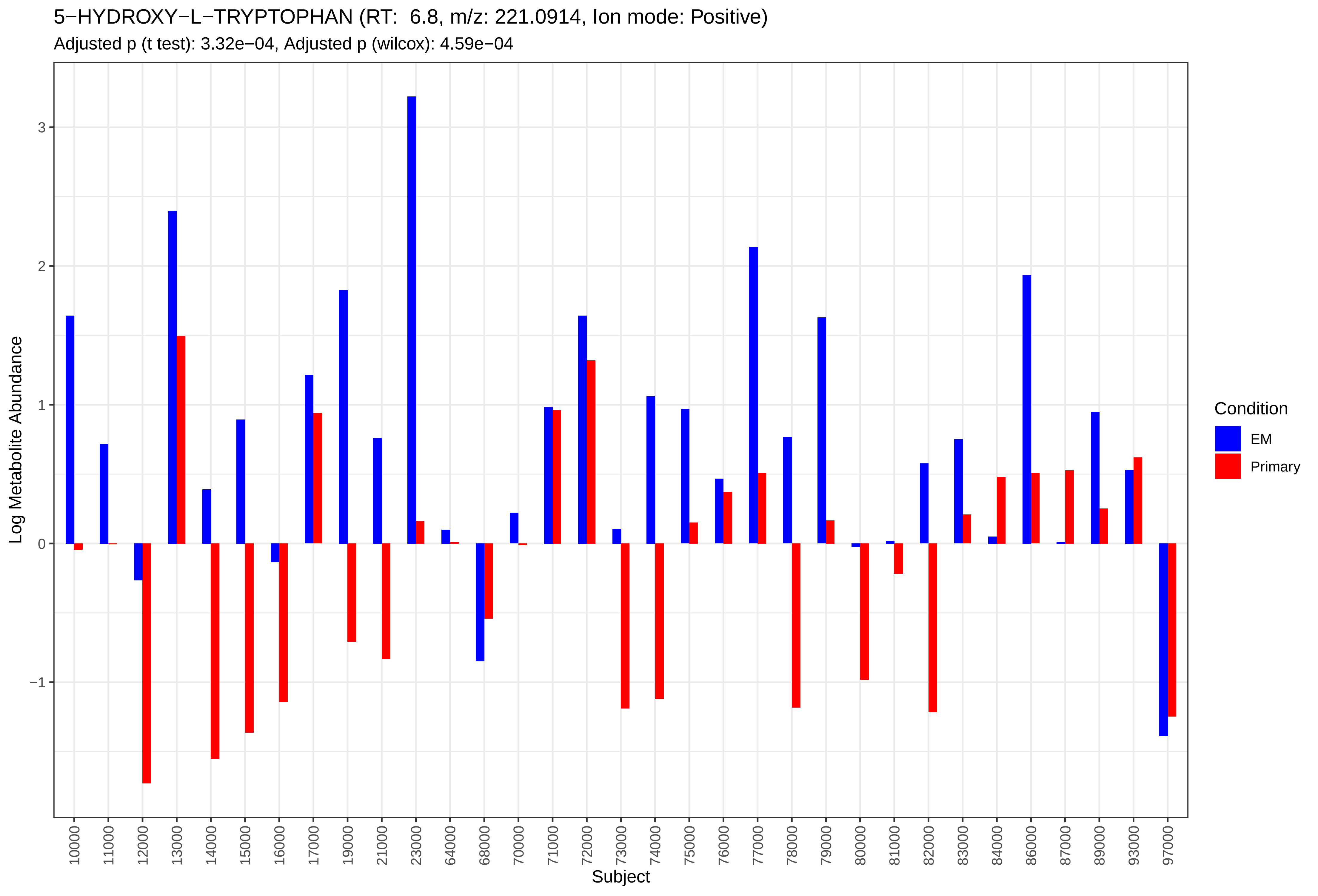
5−HYDROXY−L−TRYPTOPHAN (RT: 6.8, m/z: 221.0914, Ion mode: Positive)
Adjusted p (t test): 3.32e−04, Adjusted p (wilcox): 4.59e−04
Log Metabolite Abundance
3
2
1
0
−1
Subject
10000
11000
12000
13000
14000
15000
16000
17000
19000
21000
23000
64000
68000
70000
71000
72000
73000
74000
75000
76000
77000
78000
79000
80000
81000
82000
83000
84000
86000
87000
89000
93000
97000
Condition
EM
Primary

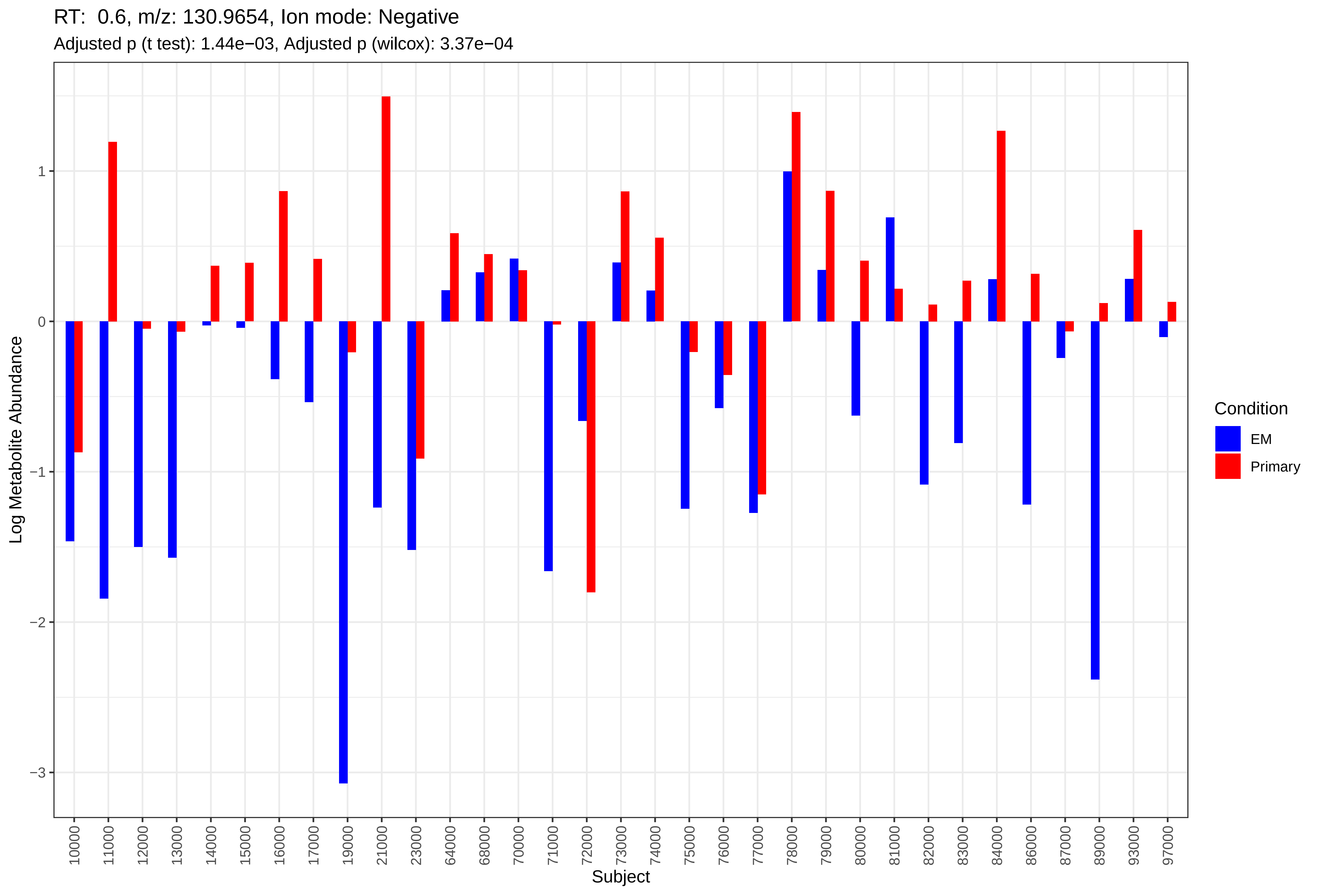
RT: 0.6, m/z: 130.9654, Ion mode: Negative
Adjusted p (t test): 1.44e−03, Adjusted p (wilcox): 3.37e−04
Log Metabolite Abundance
1
0
−1
−2
−3
10000
11000
12000
13000
14000
15000
16000
17000
19000
21000
23000
64000
68000
70000
71000
72000
73000
74000
75000
76000
77000
78000
79000
80000
81000
82000
83000
84000
86000
87000
89000
93000
97000
Subject
Condition
EM
Primary

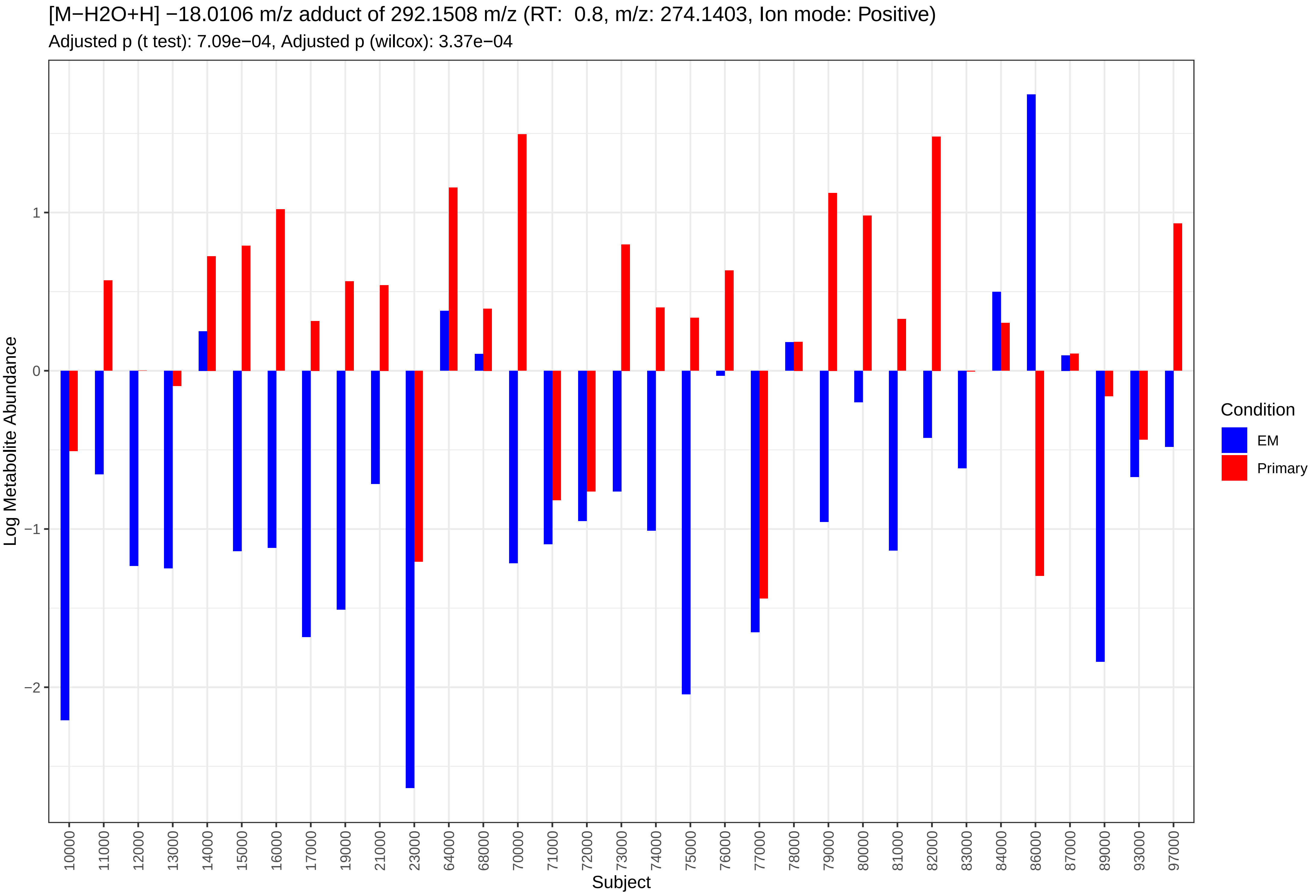
[M−H2O+H] −18.0106 m/z adduct of 292.1508 m/z (RT: 0.8, m/z: 274.1403, Ion mode: Positive)
Adjusted p (t test): 7.09e−04, Adjusted p (wilcox): 3.37e−04
Log Metabolite Abundance
1
0
−1
−2
Subject
10000
11000
12000
13000
14000
15000
16000
17000
19000
21000
23000
64000
68000
70000
71000
72000
73000
74000
75000
76000
77000
78000
79000
80000
81000
82000
83000
84000
86000
87000
89000
93000
97000
Condition
EM
Primary

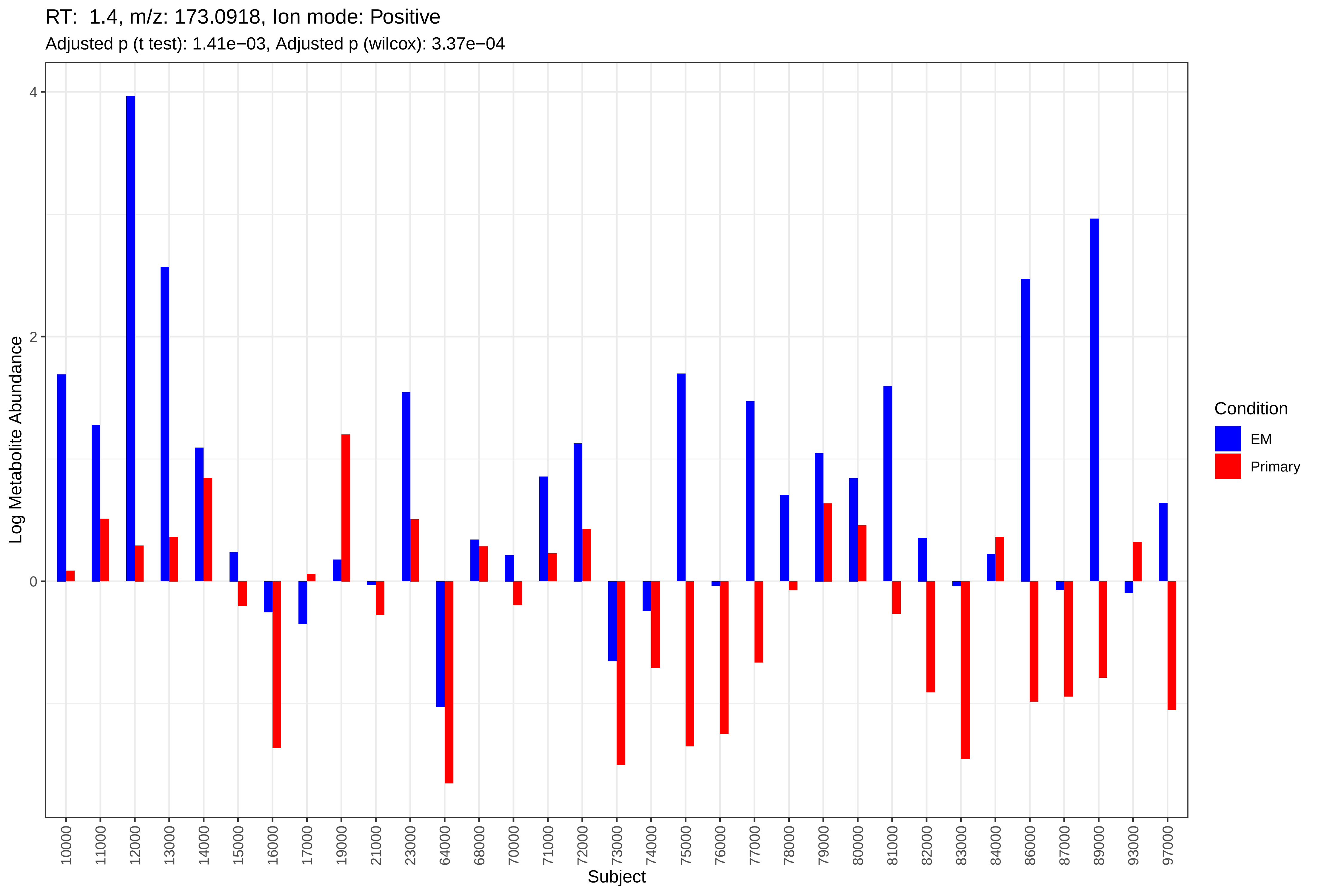
RT: 1.4, m/z: 173.0918, Ion mode: Positive
Adjusted p (t test): 1.41e−03, Adjusted p (wilcox): 3.37e−04
Log Metabolite Abundance
4
2
0
Subject
10000
11000
12000
13000
14000
15000
16000
17000
19000
21000
23000
64000
68000
70000
71000
72000
73000
74000
75000
76000
77000
78000
79000
80000
81000
82000
83000
84000
86000
87000
89000
93000
97000
Condition
EM
Primary

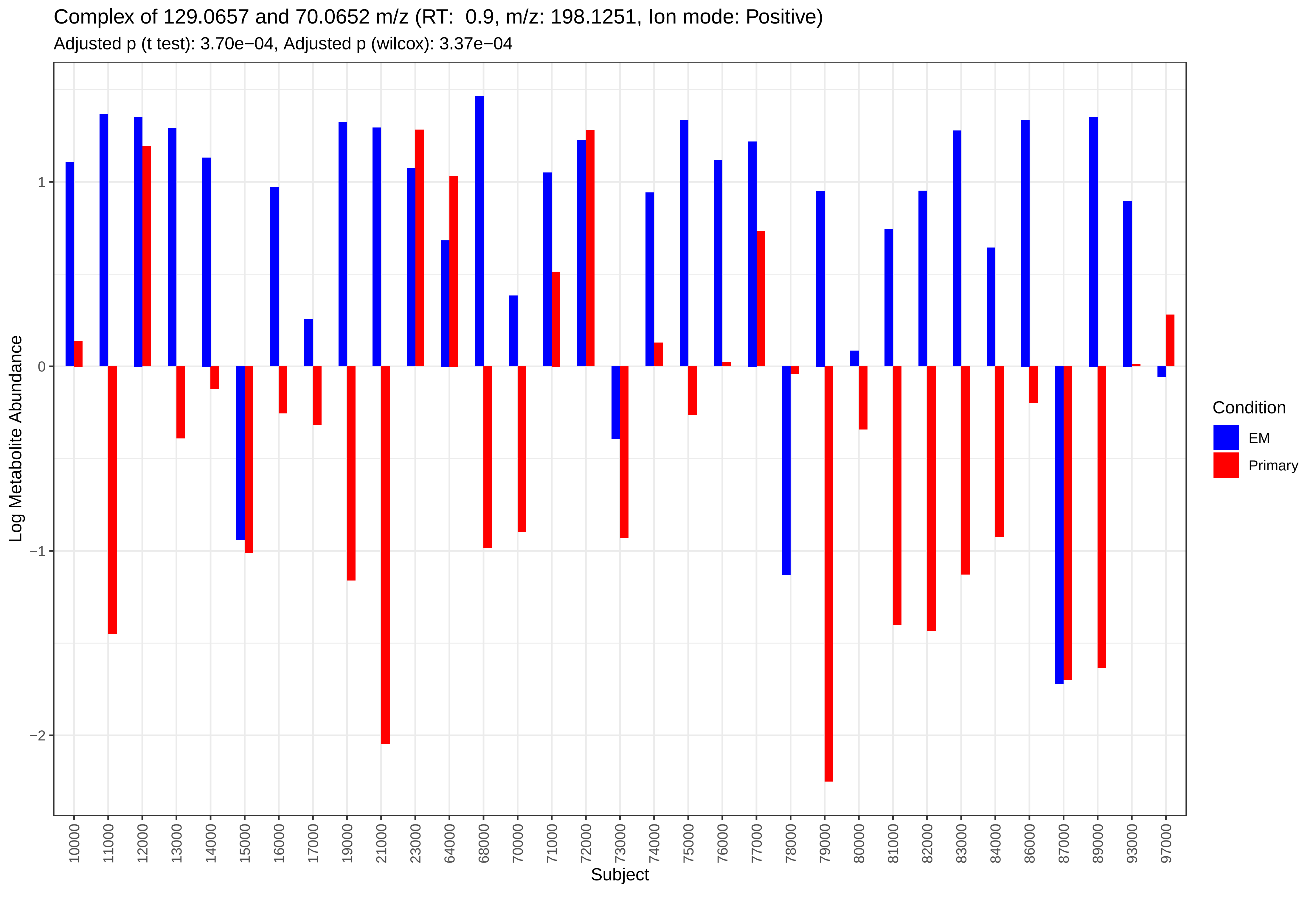

Complex of 129.0657 and 70.0652 m/z (RT: 0.9, m/z: 198.1251, Ion mode: Positive)
Adjusted p (t test): 3.70e−04, Adjusted p (wilcox): 3.37e−04
Log Metabolite Abundance
1
0
−1
−2
Subject
10000
11000
12000
13000
14000
15000
16000
17000
19000
21000
23000
64000
68000
70000
71000
72000
73000
74000
75000
76000
77000
78000
79000
80000
81000
82000
83000
84000
86000
87000
89000
93000
97000
Condition
EM
Primary

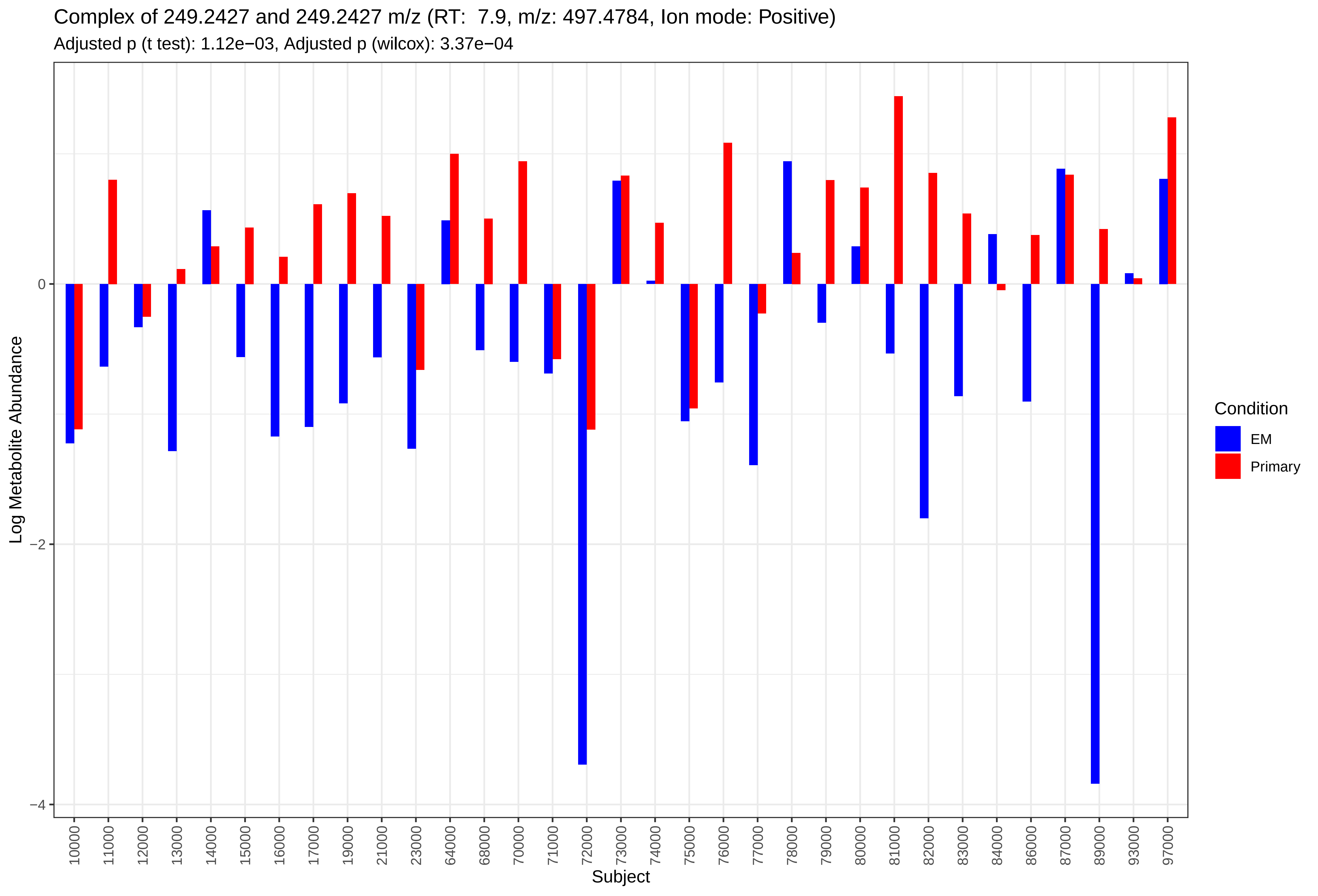

Complex of 249.2427 and 249.2427 m/z (RT: 7.9, m/z: 497.4784, Ion mode: Positive)
Adjusted p (t test): 1.12e−03, Adjusted p (wilcox): 3.37e−04
Log Metabolite Abundance
0
−2
−4
Subject
10000
11000
12000
13000
14000
15000
16000
17000
19000
21000
23000
64000
68000
70000
71000
72000
73000
74000
75000
76000
77000
78000
79000
80000
81000
82000
83000
84000
86000
87000
89000
93000
97000
Condition
EM
Primary

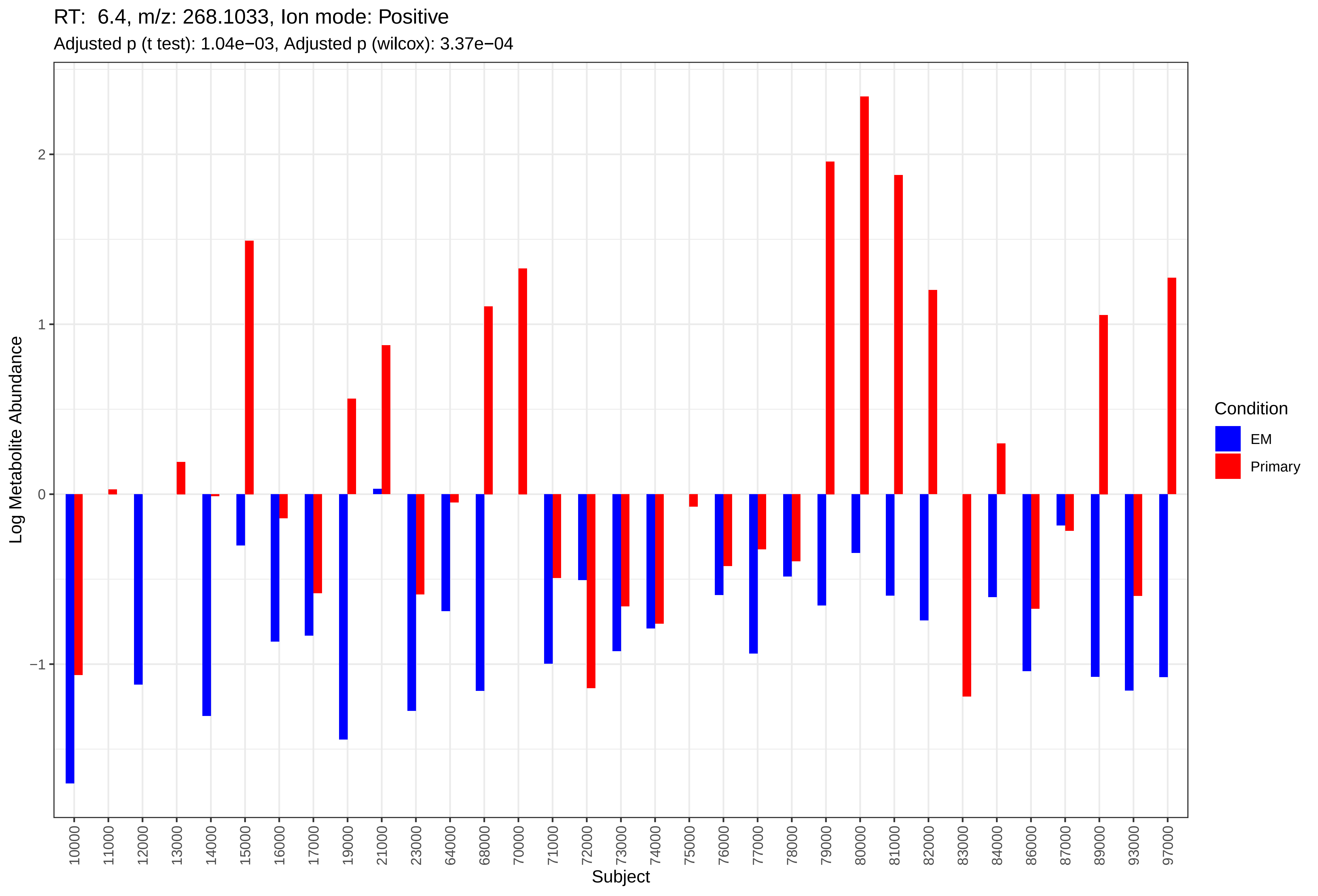

RT: 6.4, m/z: 268.1033, Ion mode: Positive
Adjusted p (t test): 1.04e−03, Adjusted p (wilcox): 3.37e−04
Log Metabolite Abundance
2
1
0
−1
Subject
10000
11000
12000
13000
14000
15000
16000
17000
19000
21000
23000
64000
68000
70000
71000
72000
73000
74000
75000
76000
77000
78000
79000
80000
81000
82000
83000
84000
86000
87000
89000
93000
97000
Condition
EM
Primary

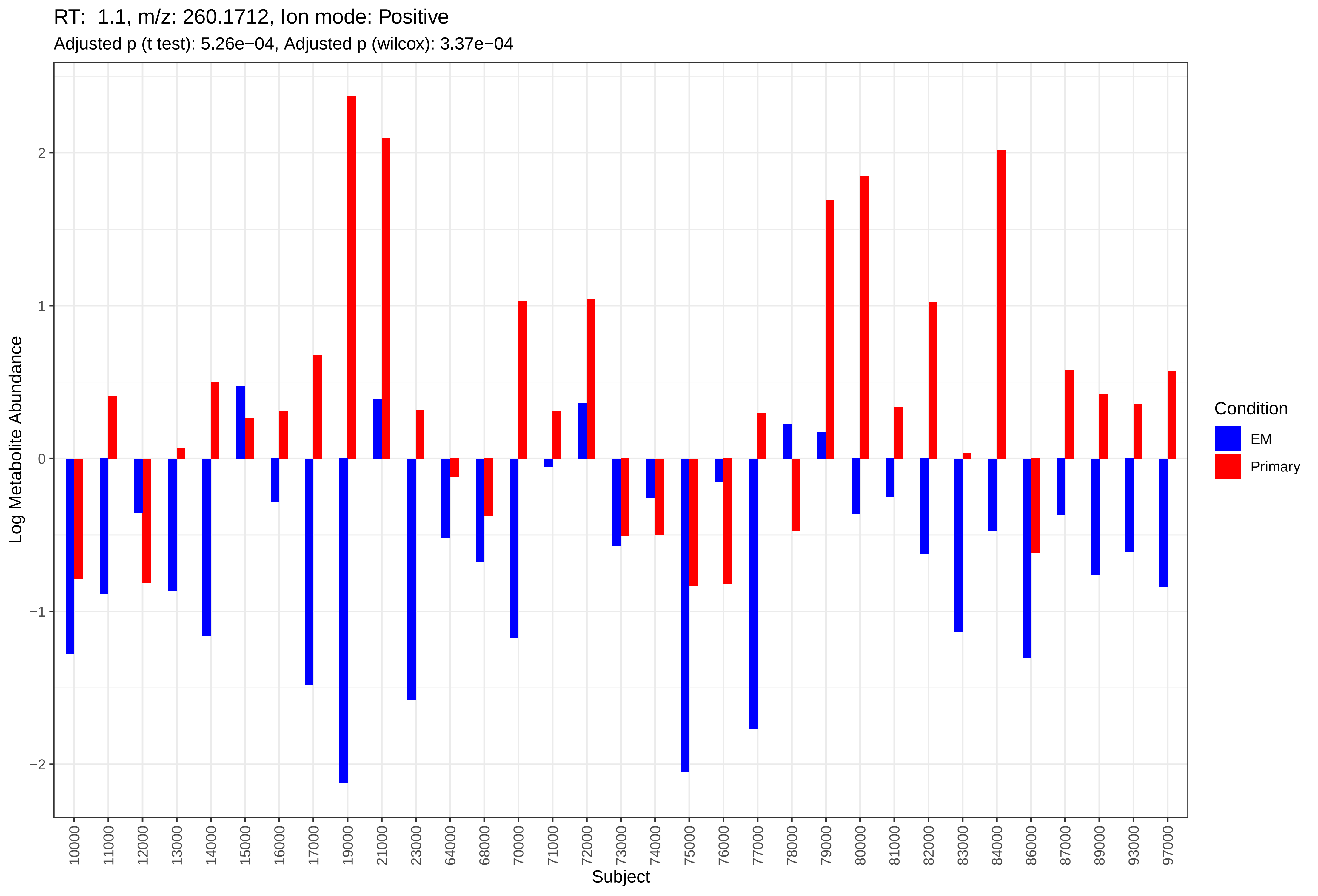
RT: 1.1, m/z: 260.1712, Ion mode: Positive
Adjusted p (t test): 5.26e−04, Adjusted p (wilcox): 3.37e−04
Log Metabolite Abundance
2
1
0
−1
−2
Subject
10000
11000
12000
13000
14000
15000
16000
17000
19000
21000
23000
64000
68000
70000
71000
72000
73000
74000
75000
76000
77000
78000
79000
80000
81000
82000
83000
84000
86000
87000
89000
93000
97000
Condition
EM
Primary

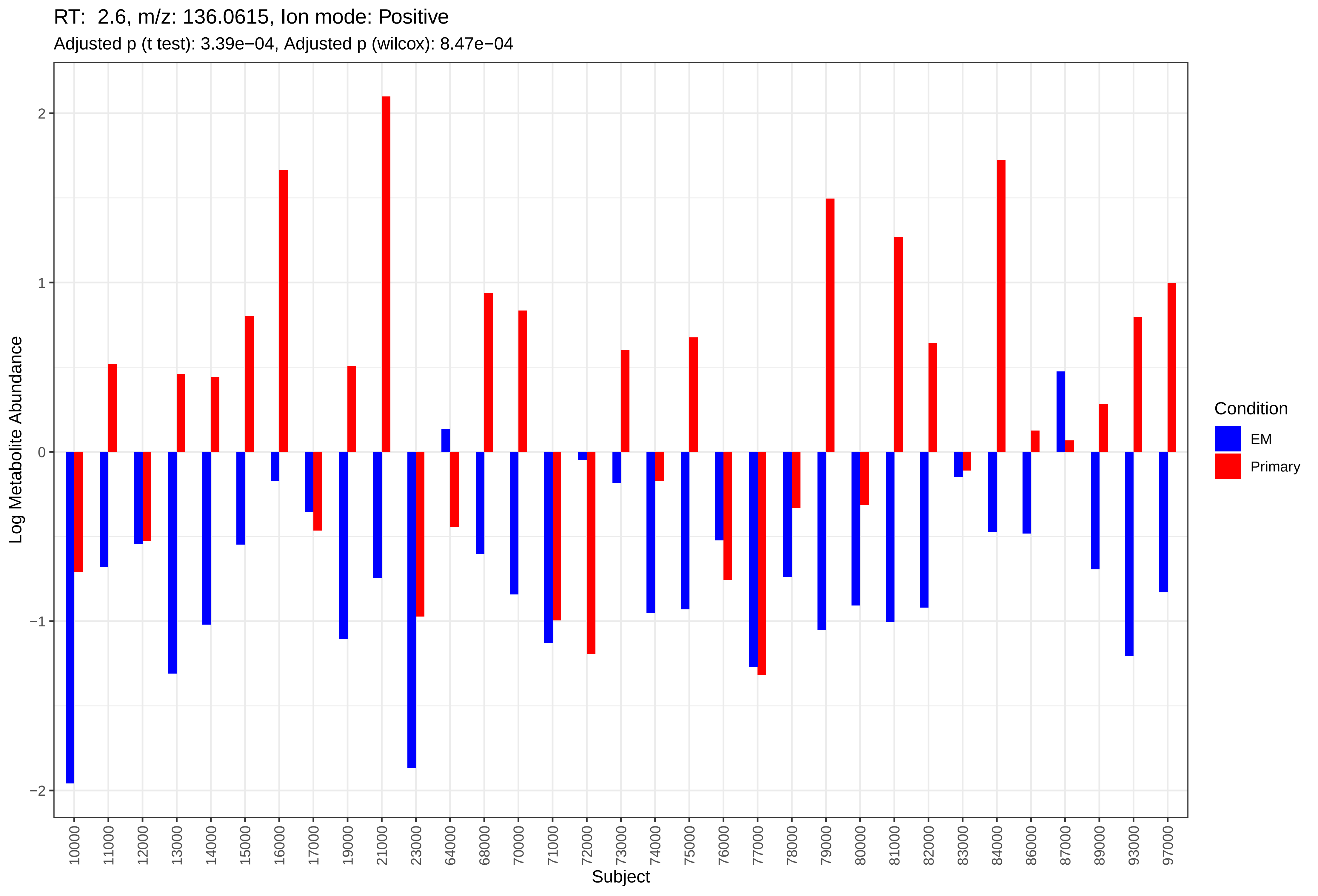
RT: 2.6, m/z: 136.0615, Ion mode: Positive
Adjusted p (t test): 3.39e−04, Adjusted p (wilcox): 8.47e−04
Log Metabolite Abundance
2
1
0
−1
−2
10000
11000
12000
13000
14000
15000
16000
17000
19000
21000
23000
64000
68000
70000
71000
72000
73000
74000
75000
76000
77000
78000
79000
80000
81000
82000
83000
84000
86000
87000
89000
93000
97000
Subject
Condition
EM
Primary

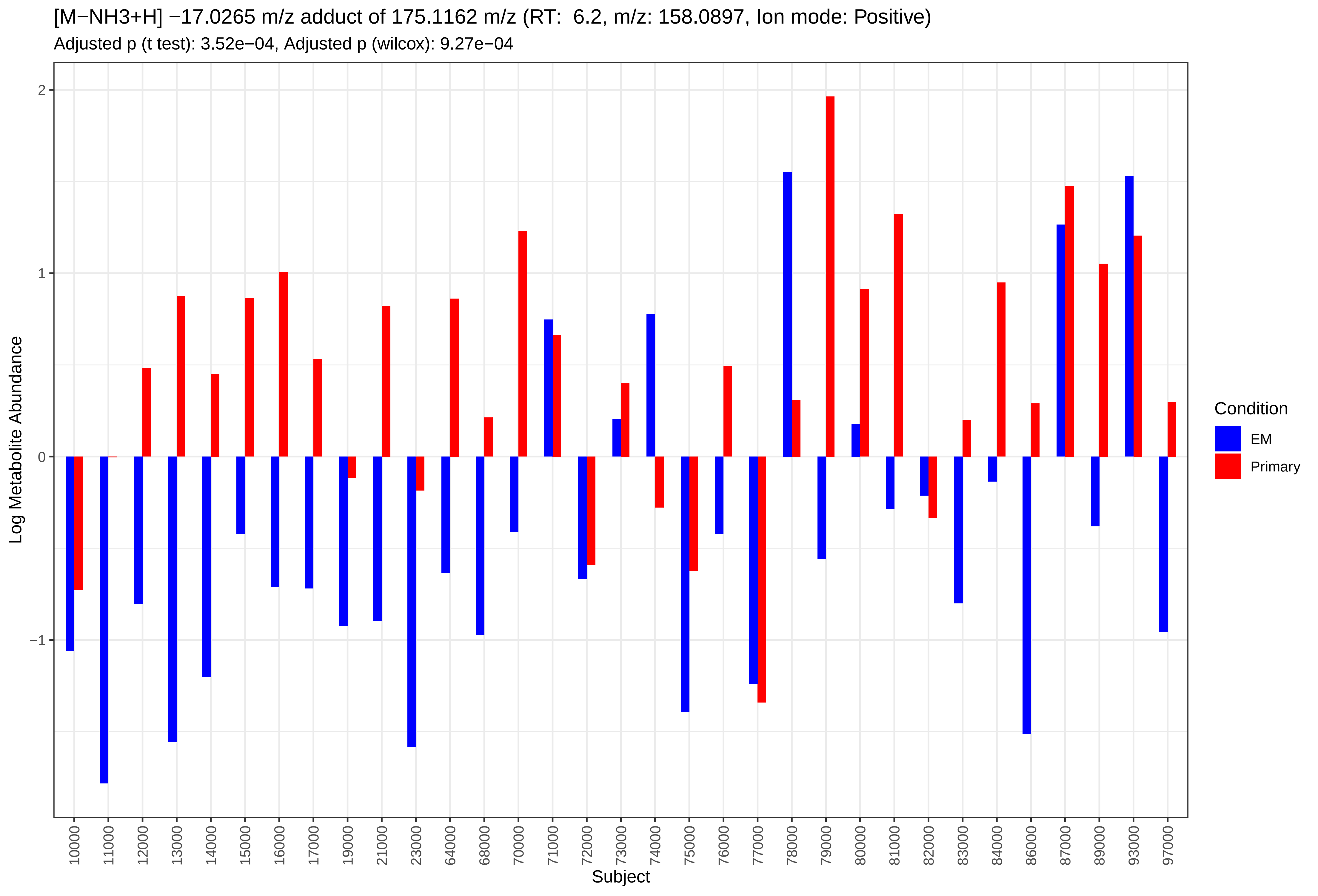

[M−NH3+H] −17.0265 m/z adduct of 175.1162 m/z (RT: 6.2, m/z: 158.0897, Ion mode: Positive)
Adjusted p (t test): 3.52e−04, Adjusted p (wilcox): 9.27e−04
Log Metabolite Abundance
Subject
Condition
EM
Primary
10000
11000
12000
13000
14000
15000
16000
17000
19000
21000
23000
64000
68000
70000
71000
72000
73000
74000
75000
76000
77000
78000
79000
80000
81000
82000
83000
84000
86000
87000
89000
93000
97000

RT: 11.3, m/z: 228.1952, Ion mode: Positive

Adjusted p (t test): 3.64e−04, Adjusted p (wilcox): 4.14e−04

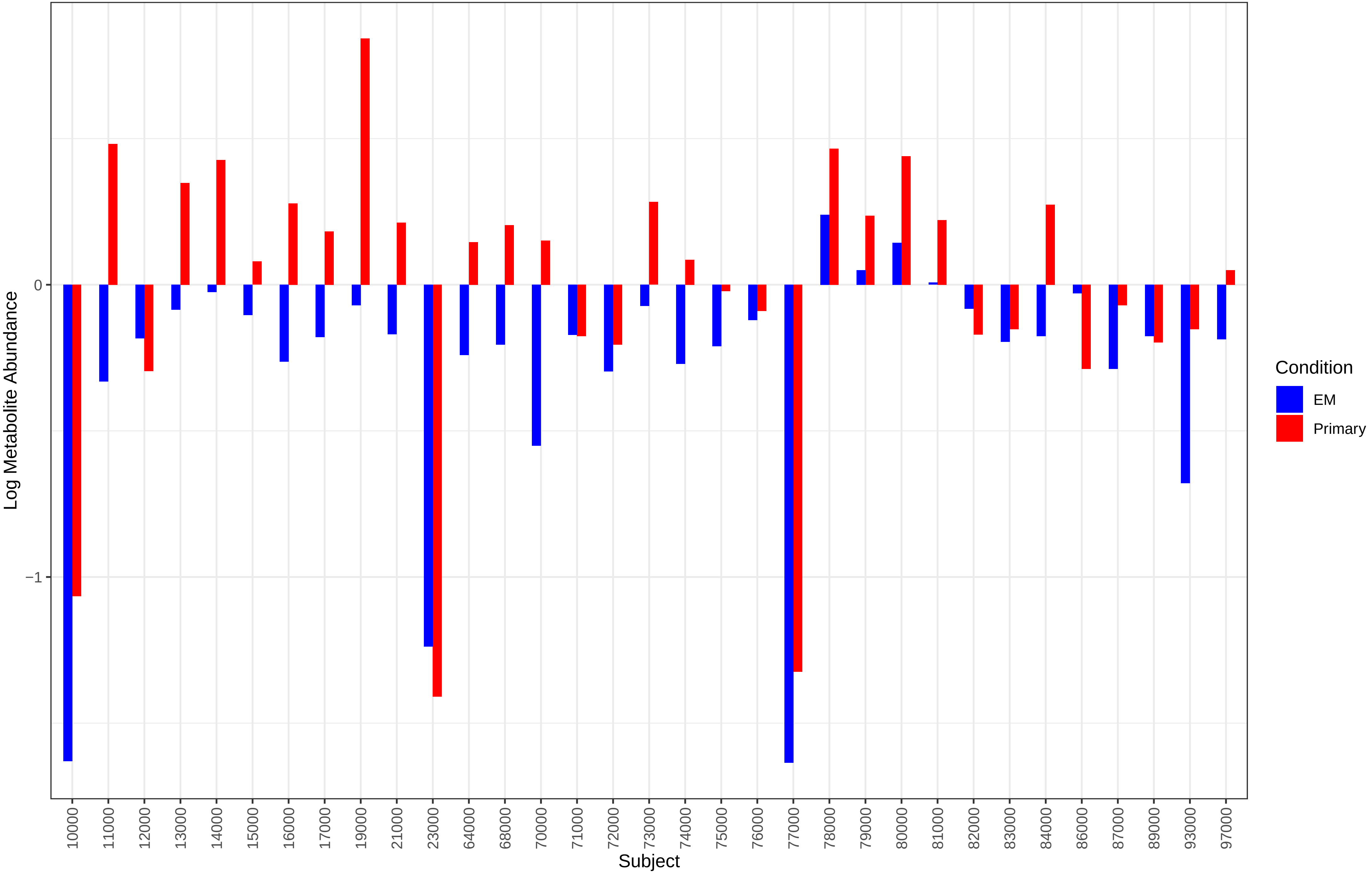

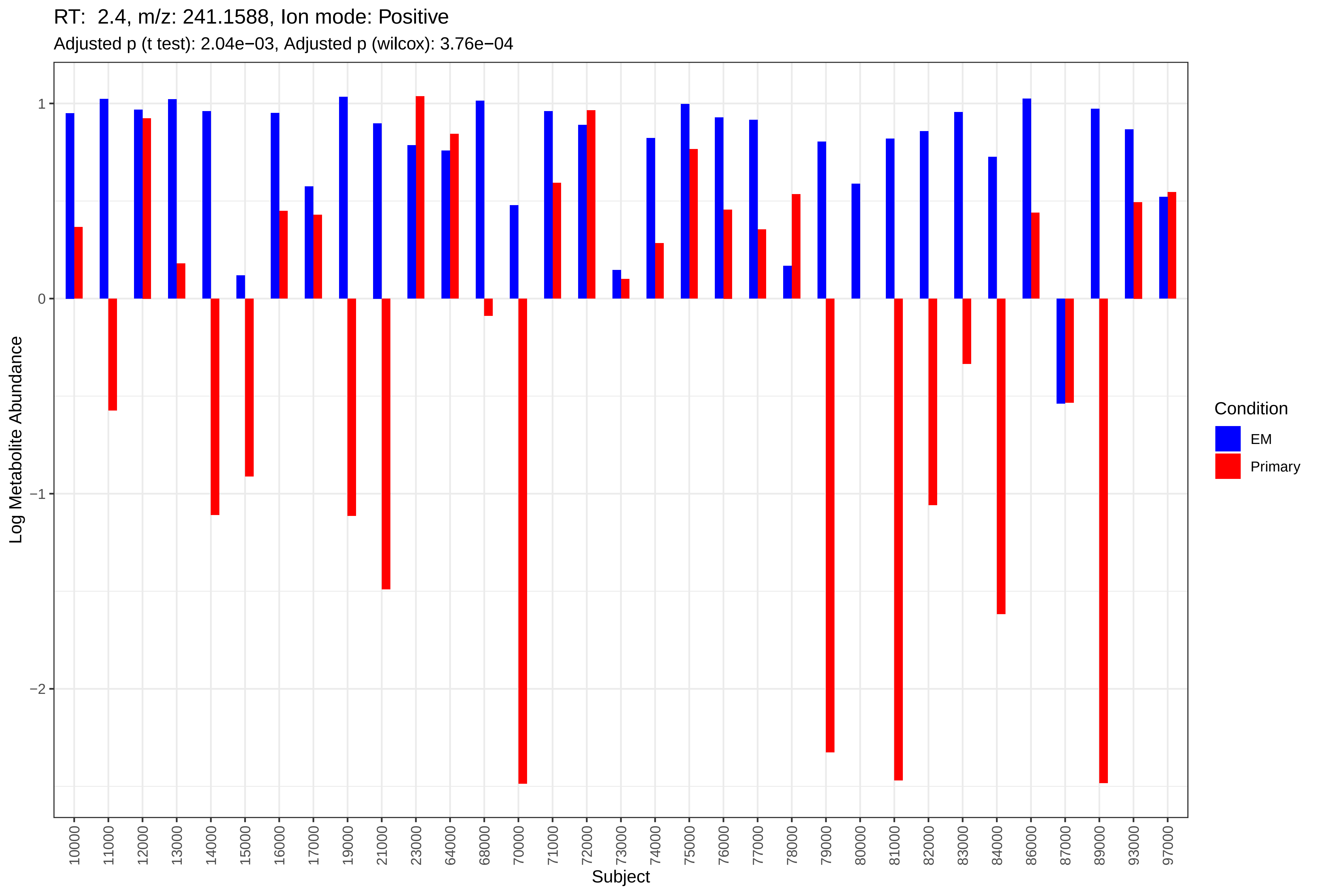
RT: 2.4, m/z: 241.1588, Ion mode: Positive
Adjusted p (t test): 2.04e−03, Adjusted p (wilcox): 3.76e−04
Log Metabolite Abundance
1
0
−1
−2
Subject
10000
11000
12000
13000
14000
15000
16000
17000
19000
21000
23000
64000
68000
70000
71000
72000
73000
74000
75000
76000
77000
78000
79000
80000
81000
82000
83000
84000
86000
87000
89000
93000
97000
Condition
EM
Primary

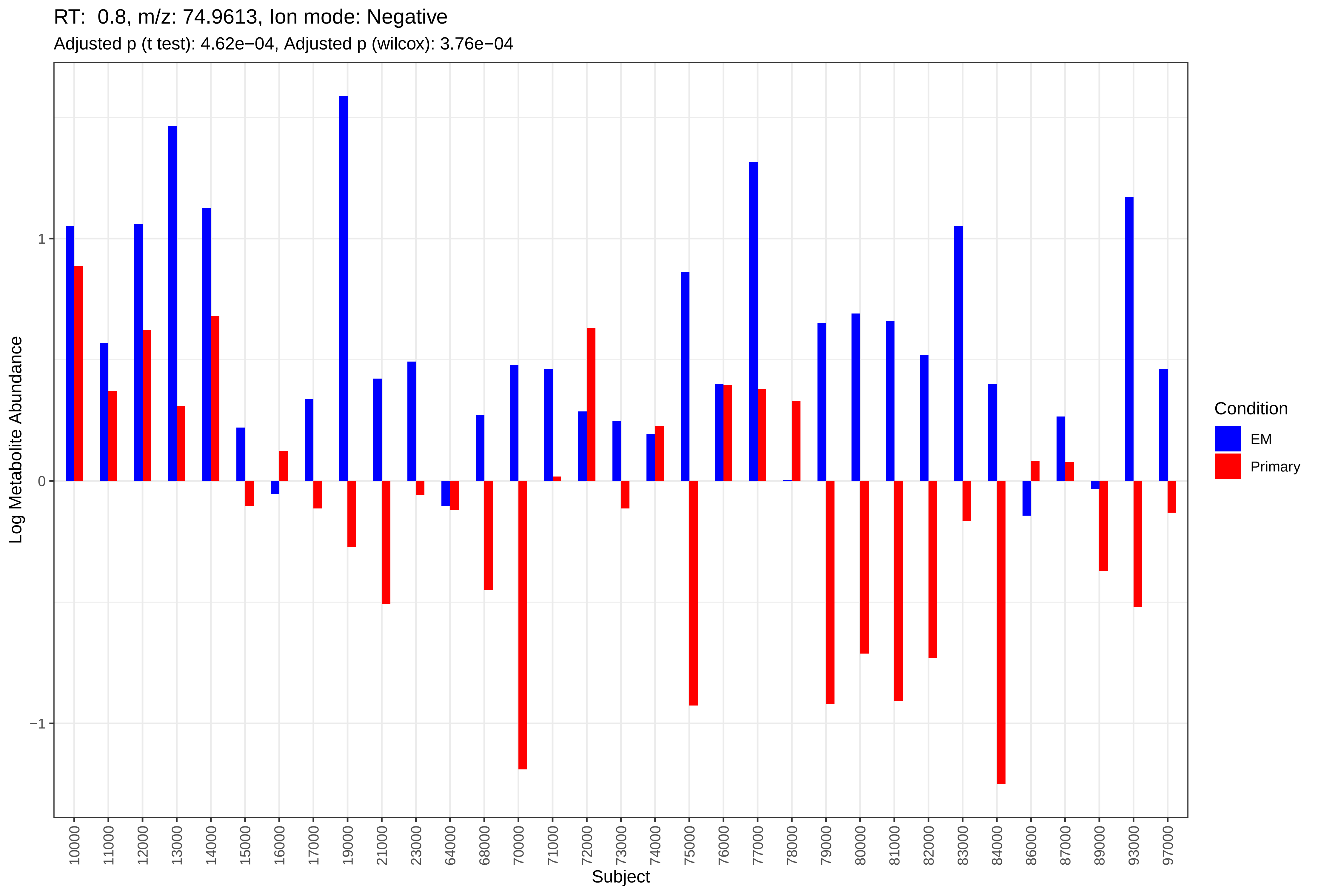

RT: 0.8, m/z: 74.9613, Ion mode: Negative
Adjusted p (t test): 4.62e−04, Adjusted p (wilcox): 3.76e−04
Log Metabolite Abundance
1
0
−1
10000
11000
12000
13000
14000
15000
16000
17000
19000
21000
23000
64000
68000
70000
71000
72000
73000
74000
75000
76000
77000
78000
79000
80000
81000
82000
83000
84000
86000
87000
89000
93000
97000
Subject
Condition
EM
Primary

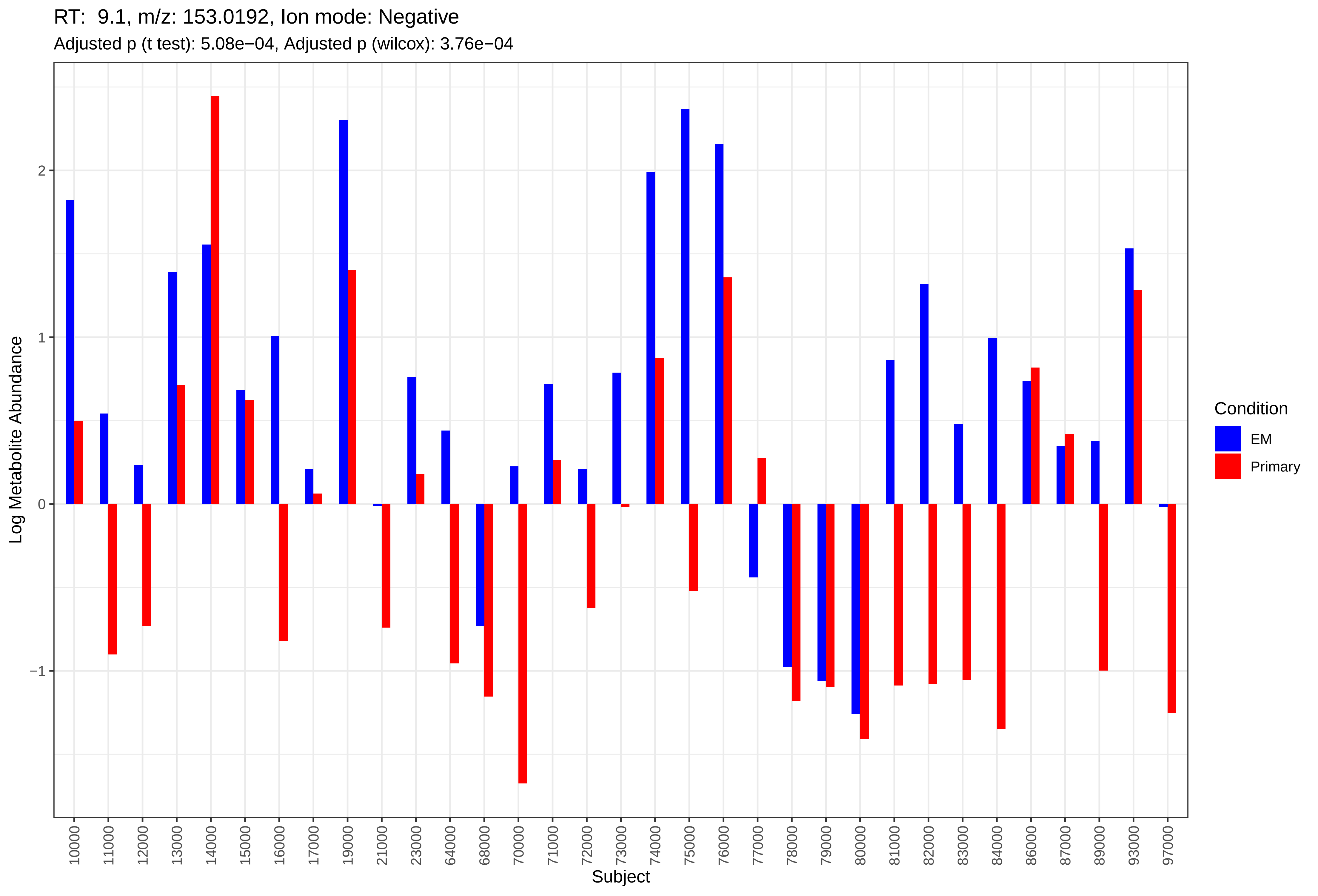

RT: 9.1, m/z: 153.0192, Ion mode: Negative
Adjusted p (t test): 5.08e−04, Adjusted p (wilcox): 3.76e−04
Log Metabolite Abundance
2
1
0
−1
Subject
10000
11000
12000
13000
14000
15000
16000
17000
19000
21000
23000
64000
68000
70000
71000
72000
73000
74000
75000
76000
77000
78000
79000
80000
81000
82000
83000
84000
86000
87000
89000
93000
97000
Condition
EM
Primary

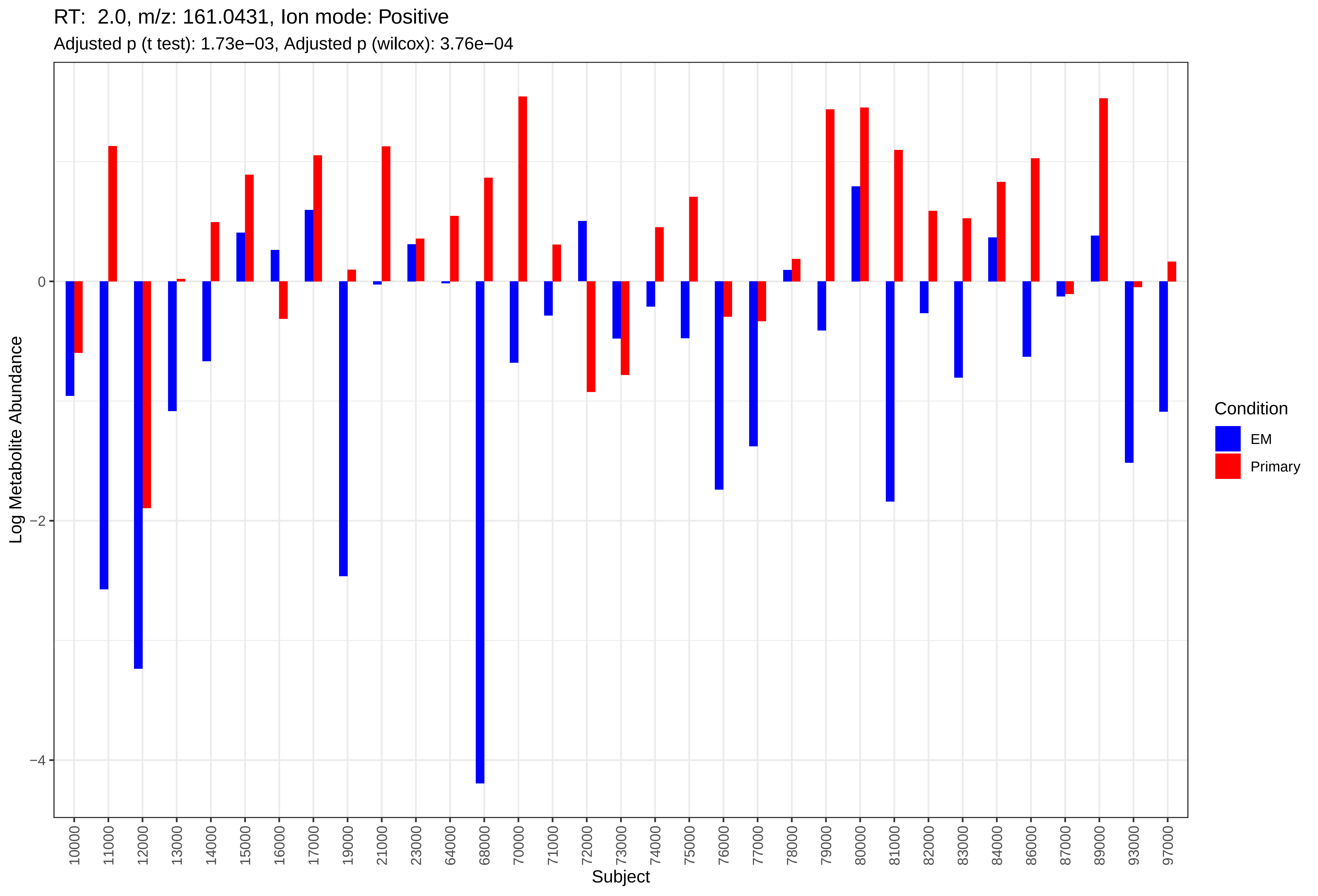
RT: 2.0, m/z: 161.0431, Ion mode: Positive
Adjusted p (t test): 1.73e−03, Adjusted p (wilcox): 3.76e−04
Log Metabolite Abundance
0
−2
−4
Subject
10000
11000
12000
13000
14000
15000
16000
17000
19000
21000
23000
64000
68000
70000
71000
72000
73000
74000
75000
76000
77000
78000
79000
80000
81000
82000
83000
84000
86000
87000
89000
93000
97000
Condition
EM
Primary

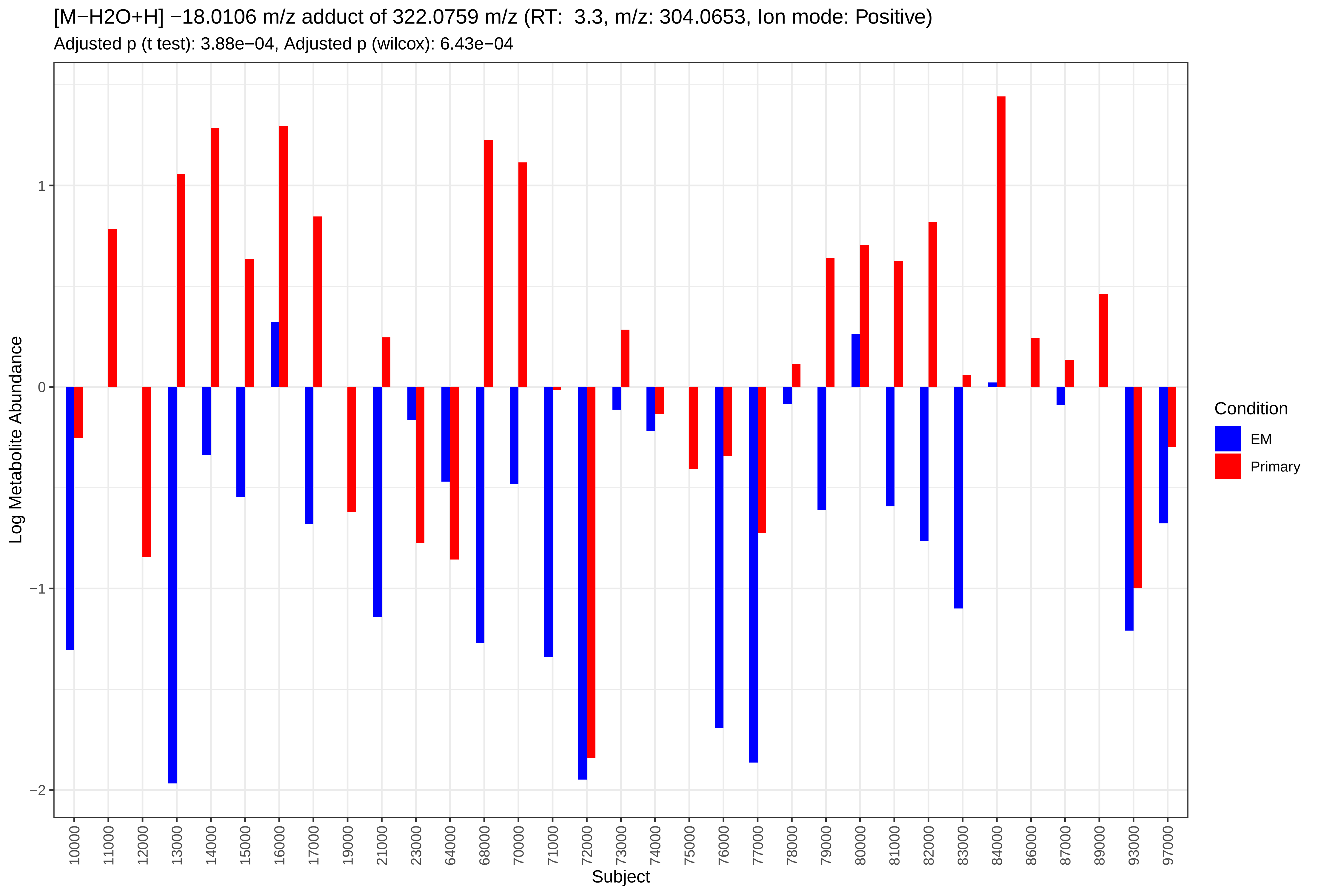
[M−H2O+H] −18.0106 m/z adduct of 322.0759 m/z (RT: 3.3, m/z: 304.0653, Ion mode: Positive)
Adjusted p (t test): 3.88e−04, Adjusted p (wilcox): 6.43e−04
Log Metabolite Abundance
1
0
−1
−2
Subject
10000
11000
12000
13000
14000
15000
16000
17000
19000
21000
23000
64000
68000
70000
71000
72000
73000
74000
75000
76000
77000
78000
79000
80000
81000
82000
83000
84000
86000
87000
89000
93000
97000
Condition
EM
Primary

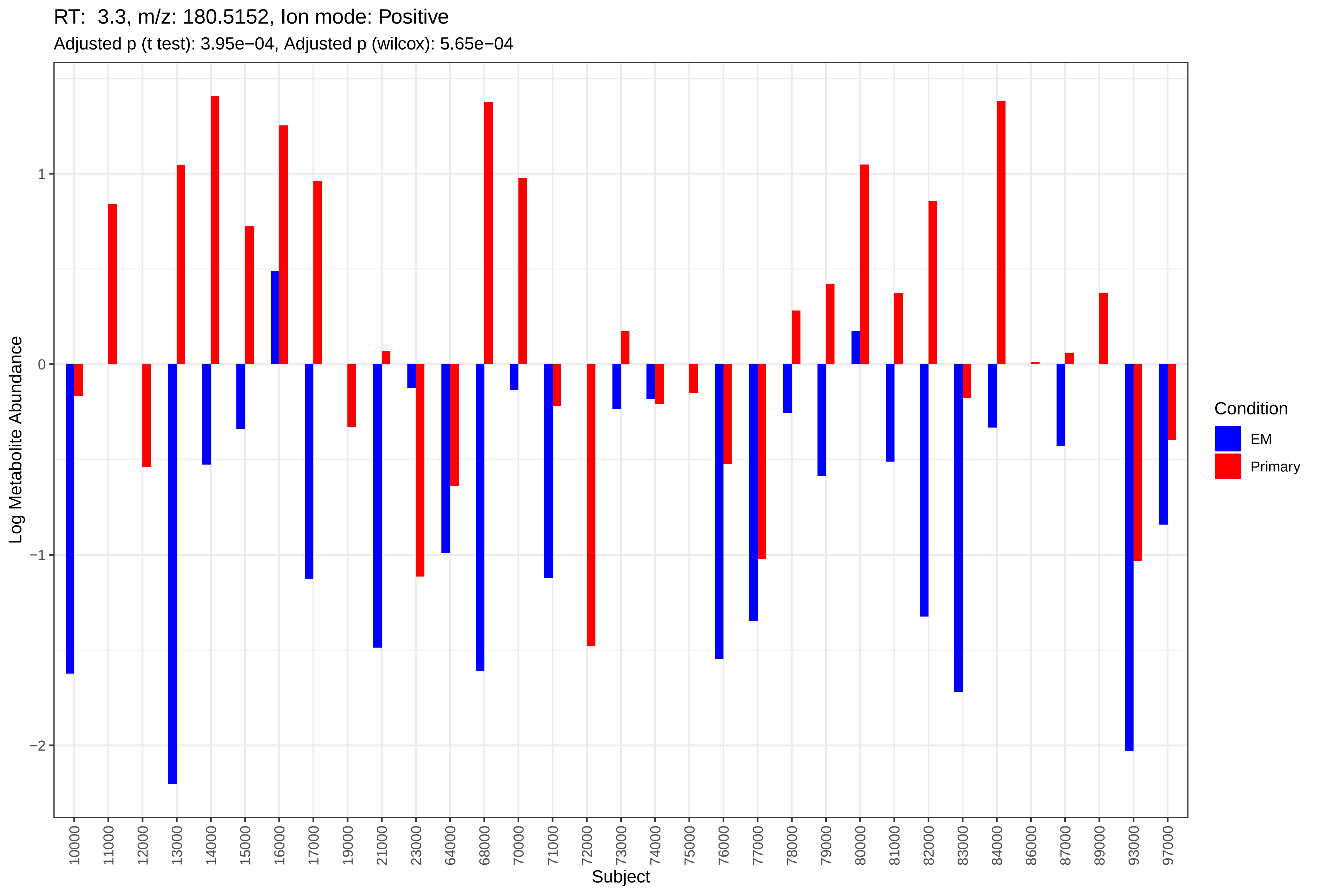

RT: 3.3, m/z: 180.5152, Ion mode: Positive
Adjusted p (t test): 3.95e−04, Adjusted p (wilcox): 5.65e−04
Log Metabolite Abundance
1
0
−1
−2
10000
11000
12000
13000
14000
15000
16000
17000
19000
21000
23000
64000
68000
70000
71000
72000
73000
74000
75000
76000
77000
78000
79000
80000
81000
82000
83000
84000
86000
87000
89000
93000
97000
Subject
Condition
EM
Primary

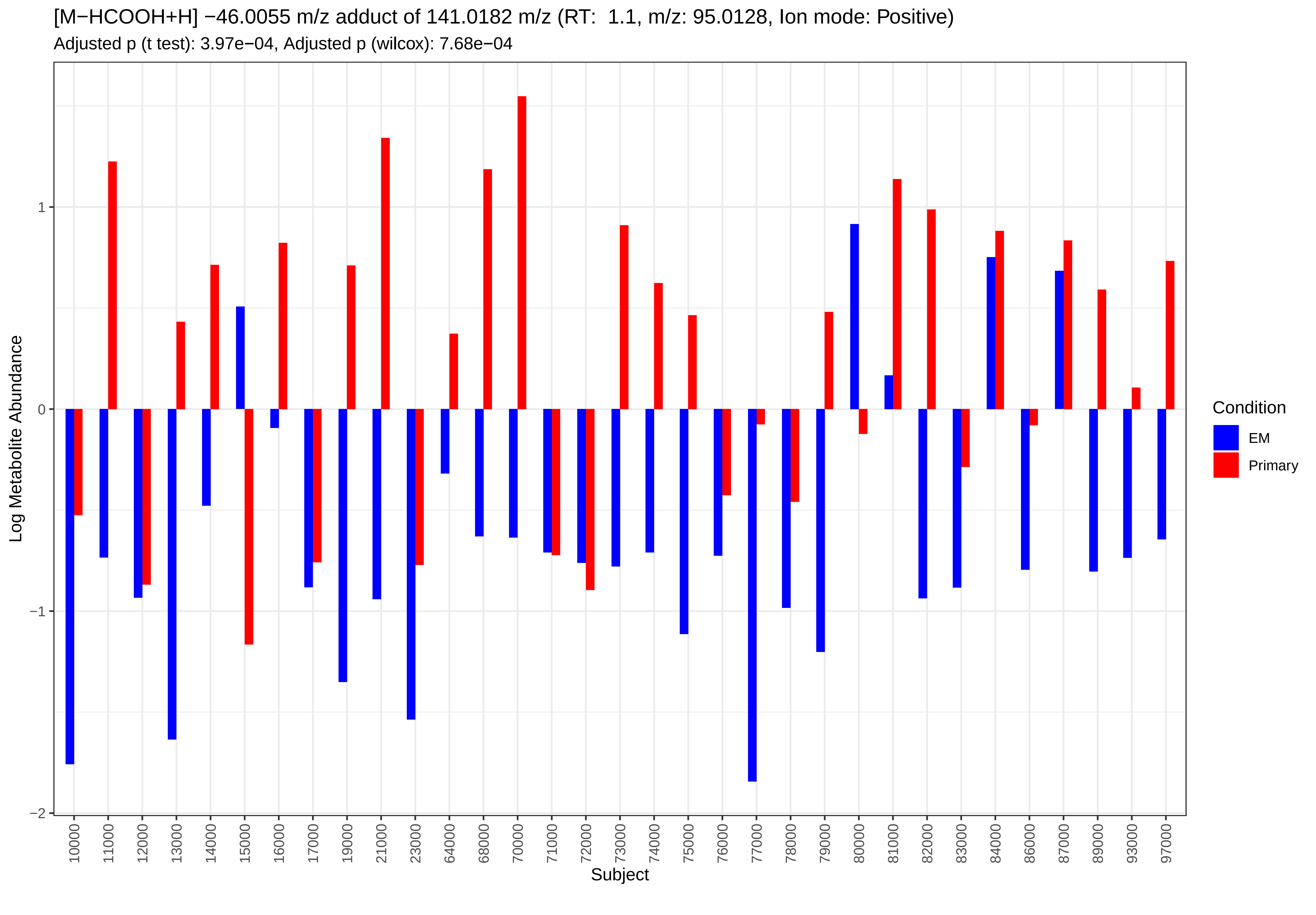

[M−HCOOH+H] −46.0055 m/z adduct of 141.0182 m/z (RT: 1.1, m/z: 95.0128, Ion mode: Positive)
Adjusted p (t test): 3.97e−04, Adjusted p (wilcox): 7.68e−04
Log Metabolite Abundance
1
0
−1
−2
10000
11000
12000
13000
14000
15000
16000
17000
19000
21000
23000
64000
68000
70000
71000
72000
73000
74000
75000
76000
77000
78000
79000
80000
81000
82000
83000
84000
86000
87000
89000
93000
97000
Subject
Condition
EM
Primary

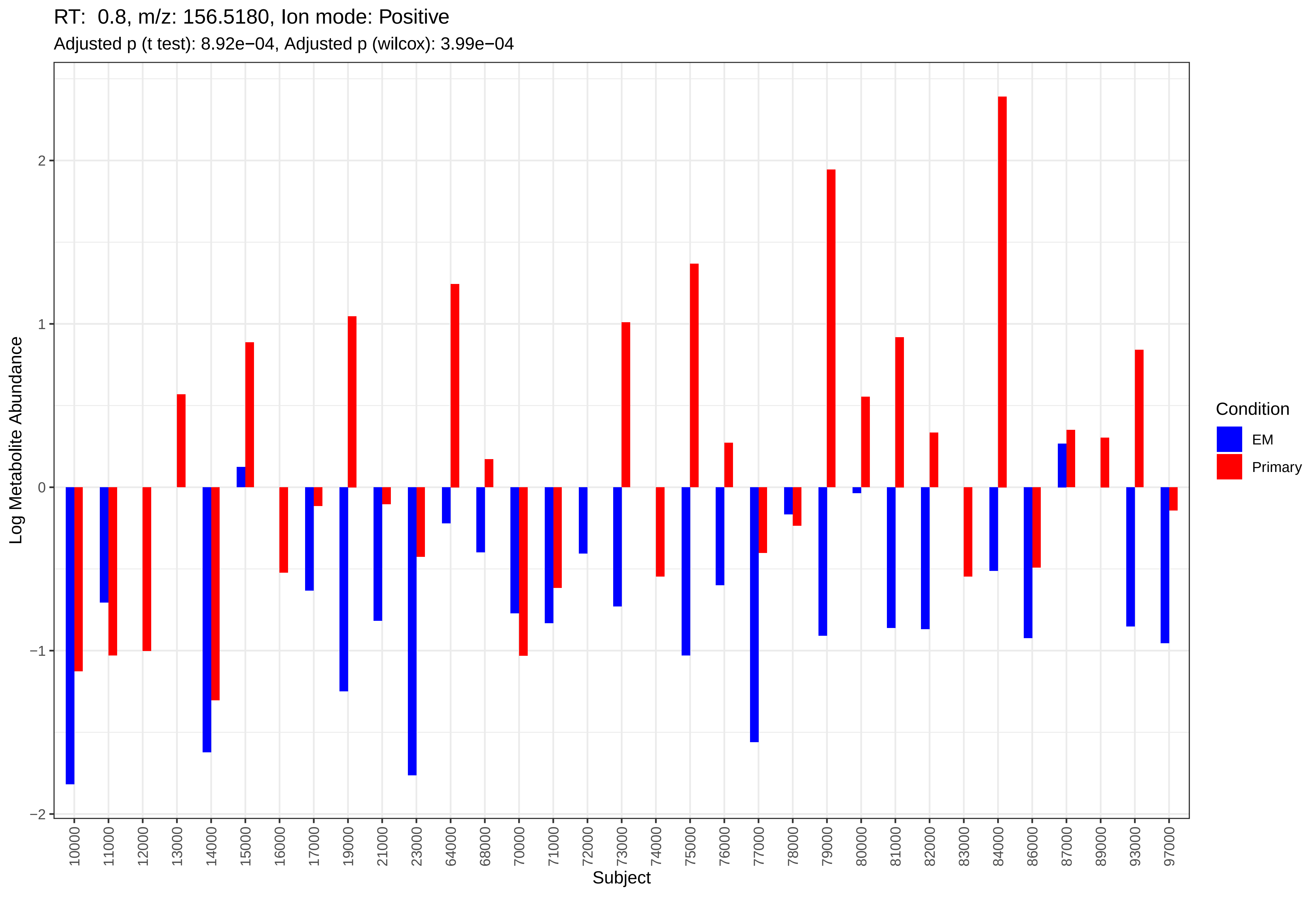
RT: 0.8, m/z: 156.5180, Ion mode: Positive
Adjusted p (t test): 8.92e−04, Adjusted p (wilcox): 3.99e−04
Log Metabolite Abundance
2
1
0
−1
−2
10000
11000
12000
13000
14000
15000
16000
17000
19000
21000
23000
64000
68000
70000
71000
72000
73000
74000
75000
76000
77000
78000
79000
80000
81000
82000
83000
84000
86000
87000
89000
93000
97000
Subject
Condition
EM
Primary

[M+Na−H] 21.9825 m/z adduct of 344.0579 m/z (RT: 3.3, m/z: 366.0399, Ion mode: Positive)

Adjusted p (t test): 4.00e−04, Adjusted p (wilcox): 8.22e−04

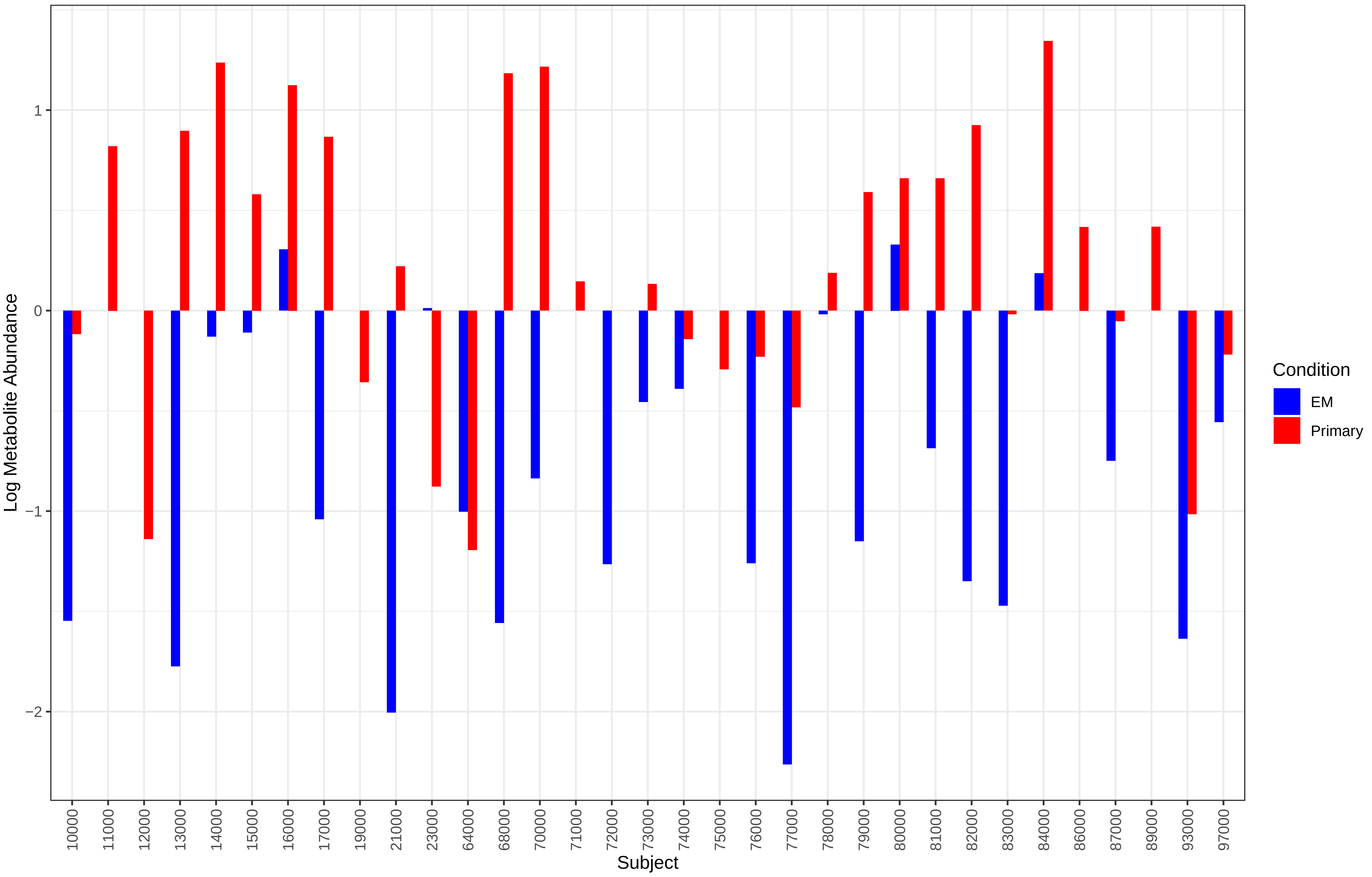

RT: 7.8, m/z: 823.7619, Ion mode: Positive

Adjusted p (t test): 4.27e−04, Adjusted p (wilcox): 4.04e−04

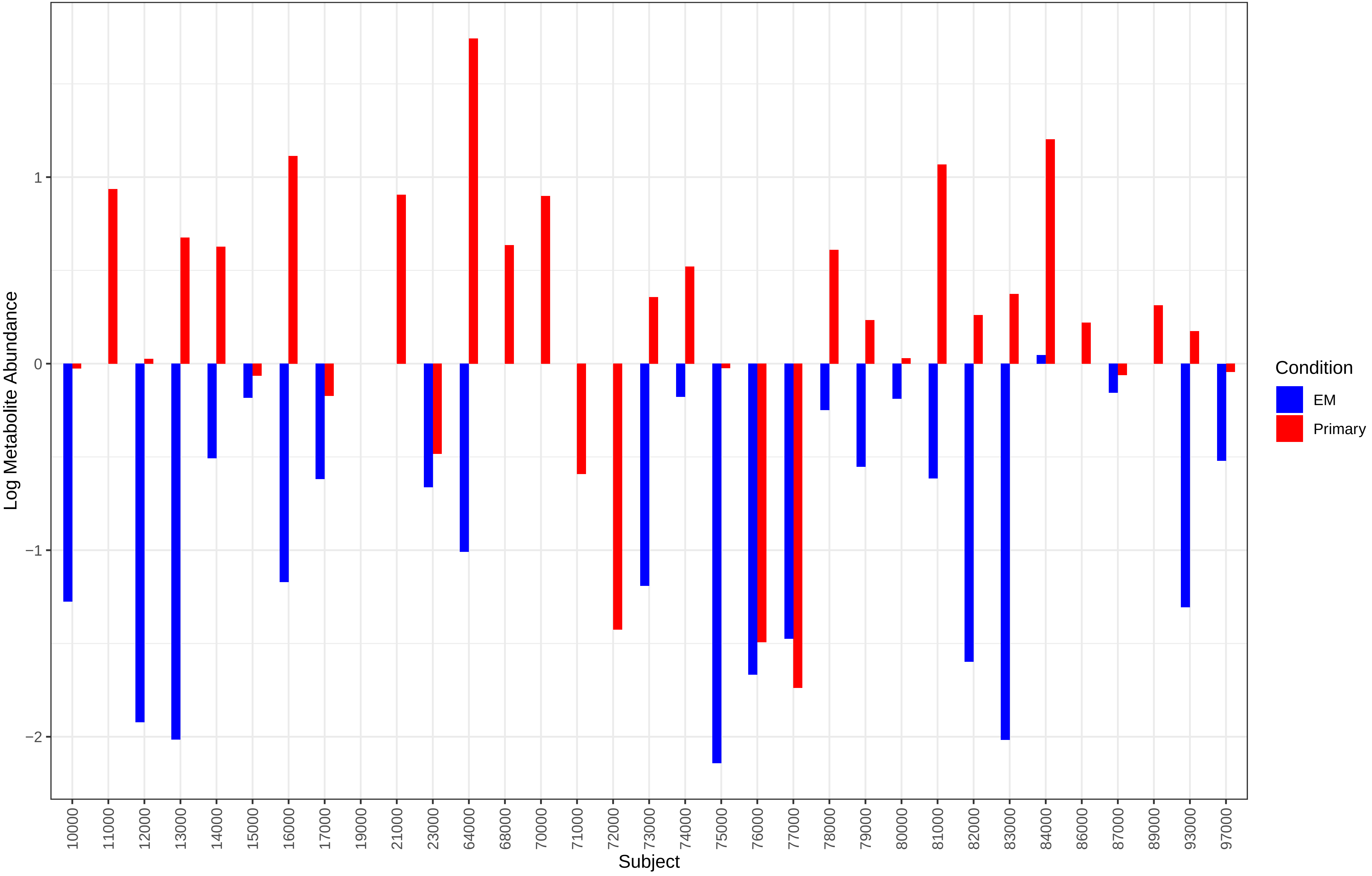

RT: 6.4, m/z: 251.0786, Ion mode: Negative

Adjusted p (t test): 8.26e−04, Adjusted p (wilcox): 4.14e−04

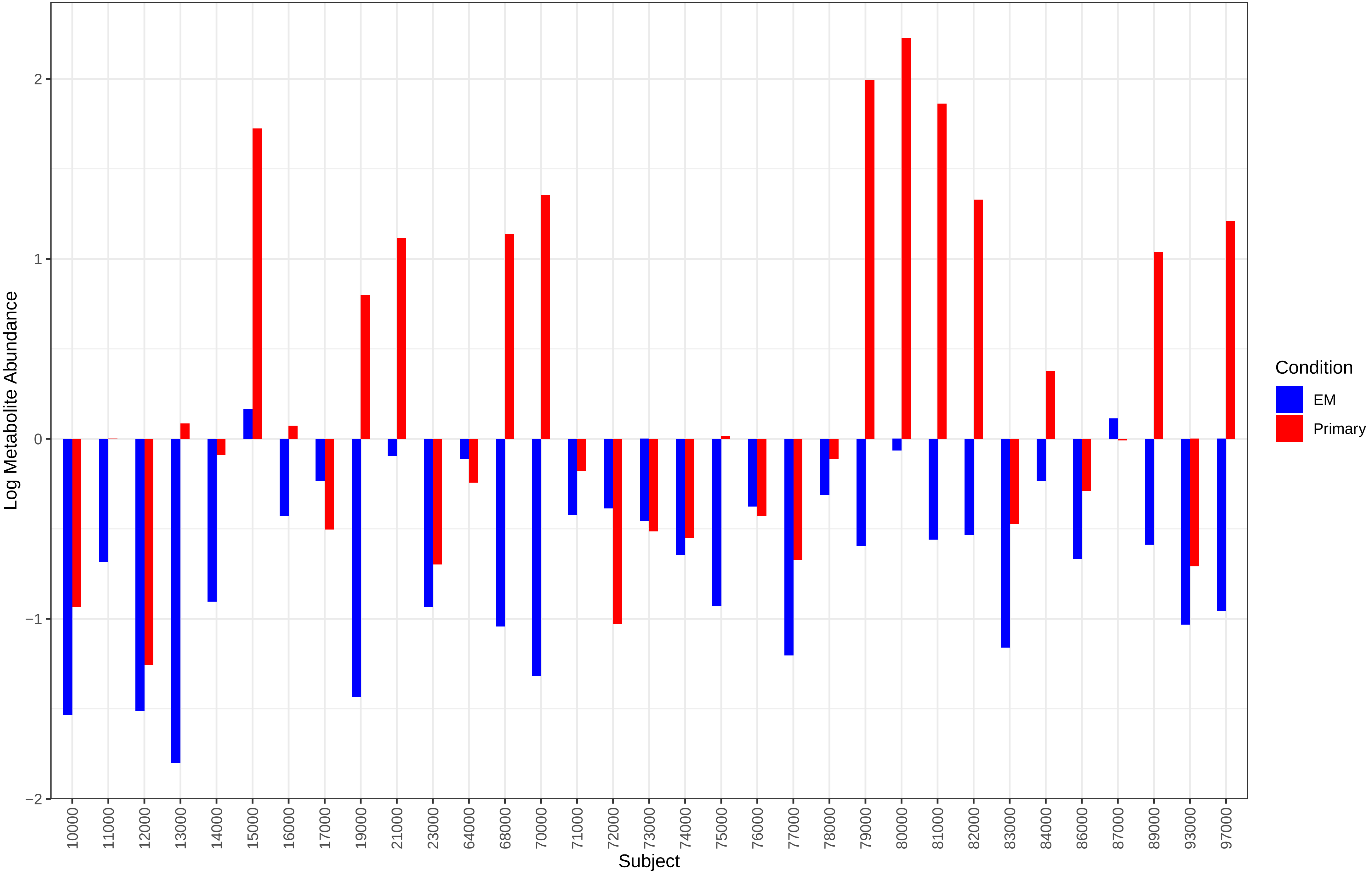

RT: 8.5, m/z: 218.9998, Ion mode: Negative

Adjusted p (t test): 6.19e−04, Adjusted p (wilcox): 4.14e−04

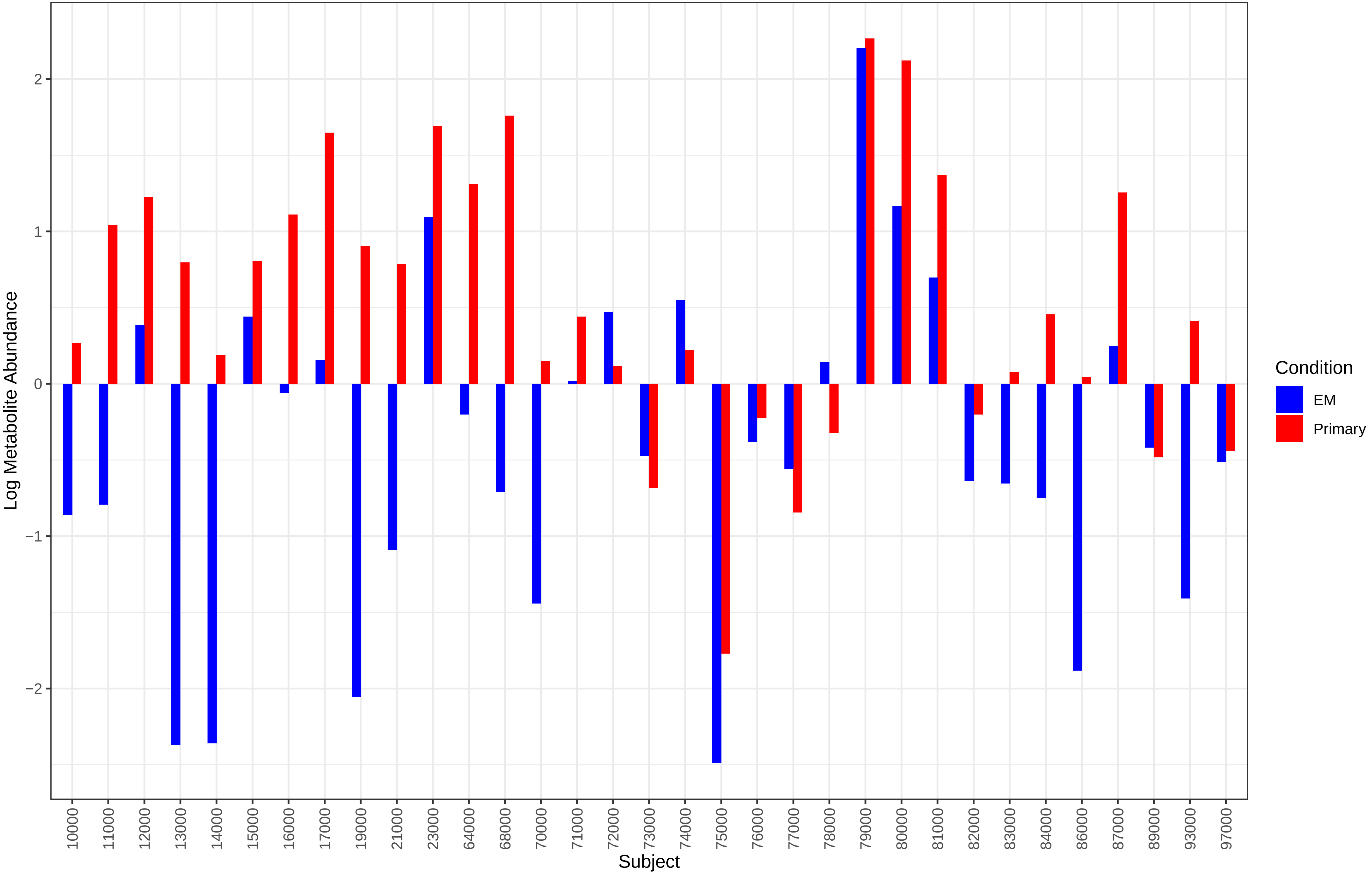

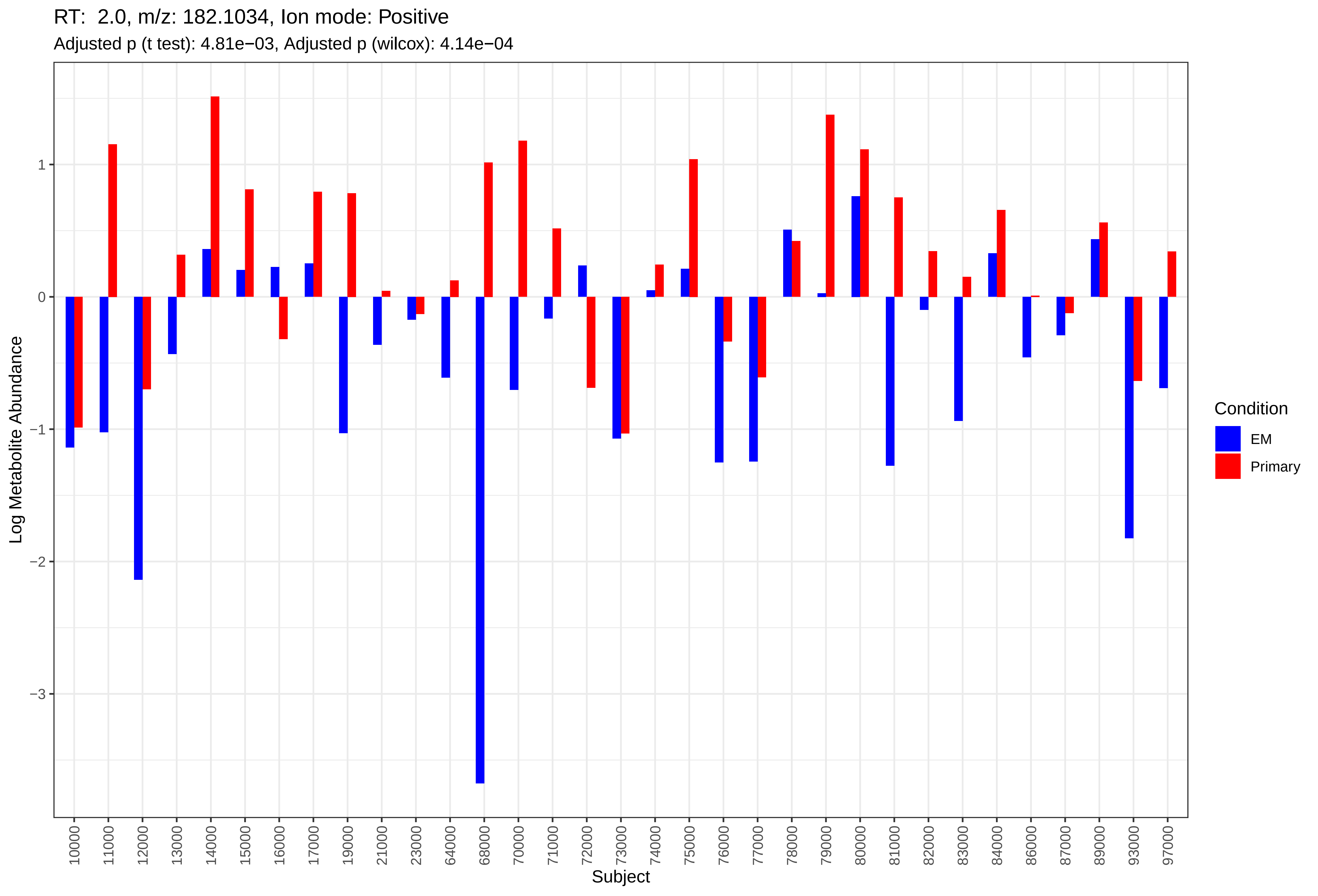

RT: 2.0, m/z: 182.1034, Ion mode: Positive
Adjusted p (t test): 4.81e−03, Adjusted p (wilcox): 4.14e−04
Log Metabolite Abundance
1
0
−1
−2
−3
10000
11000
12000
13000
14000
15000
16000
17000
19000
21000
23000
64000
68000
70000
71000
72000
73000
74000
75000
76000
77000
78000
79000
80000
81000
82000
83000
84000
86000
87000
89000
93000
97000
Subject
Condition
EM
Primary

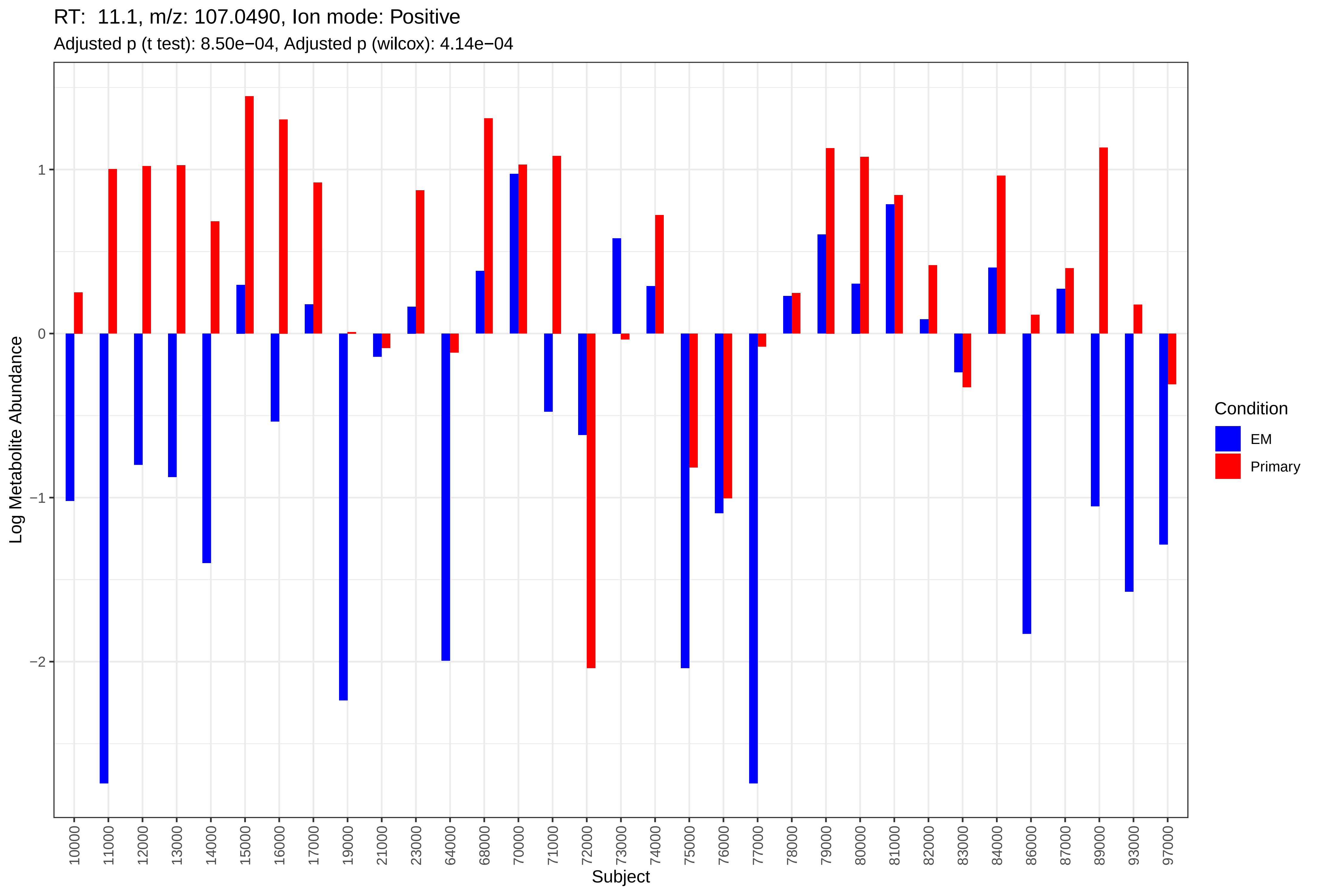
RT: 11.1, m/z: 107.0490, Ion mode: Positive
Adjusted p (t test): 8.50e−04, Adjusted p (wilcox): 4.14e−04
Log Metabolite Abundance
1
0
−1
−2
Subject
10000
11000
12000
13000
14000
15000
16000
17000
19000
21000
23000
64000
68000
70000
71000
72000
73000
74000
75000
76000
77000
78000
79000
80000
81000
82000
83000
84000
86000
87000
89000
93000
97000
Condition
EM
Primary

RT: 3.6, m/z: 222.0231, Ion mode: Positive

Adjusted p (t test): 2.59e−03, Adjusted p (wilcox): 4.14e−04

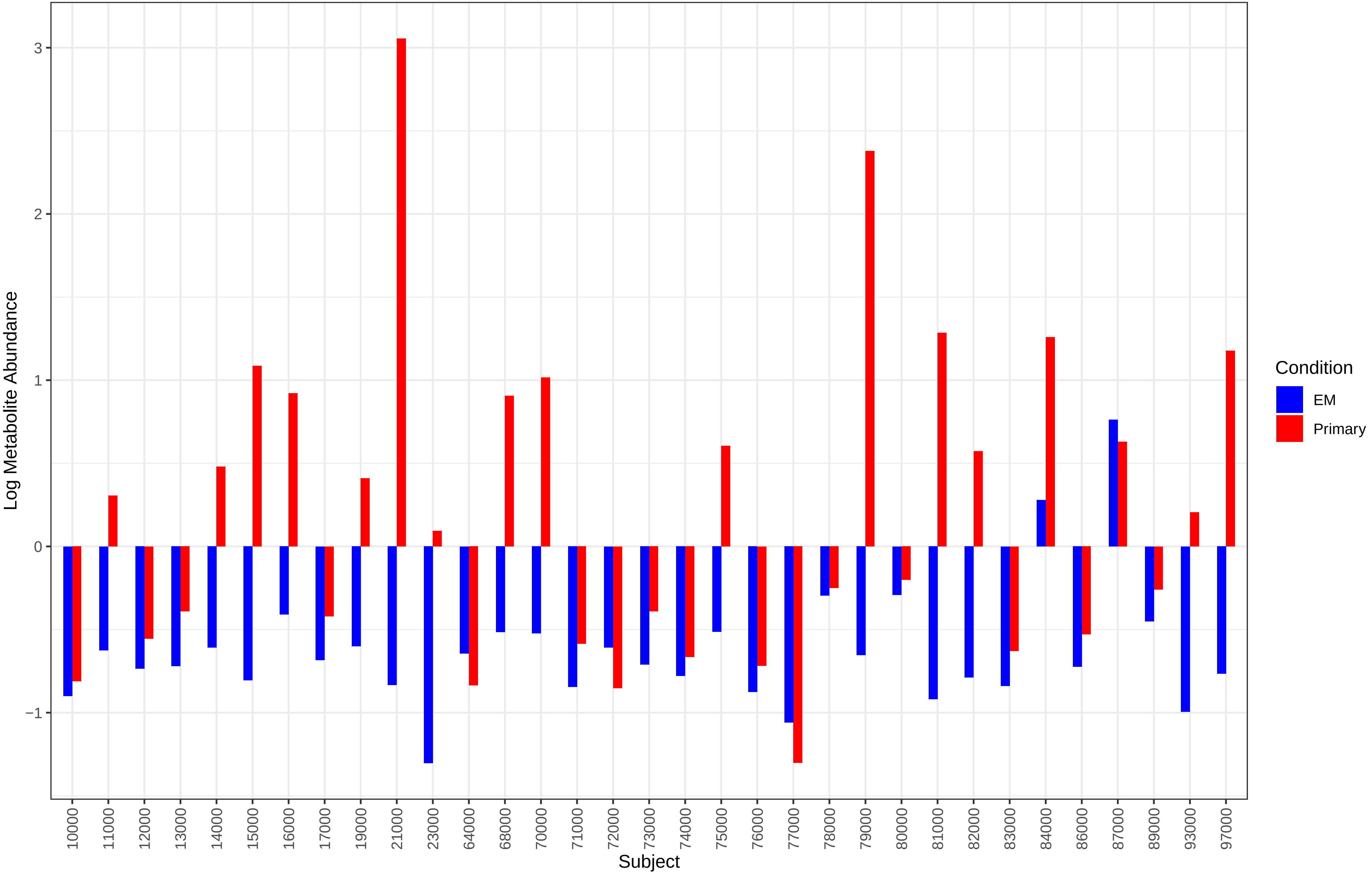

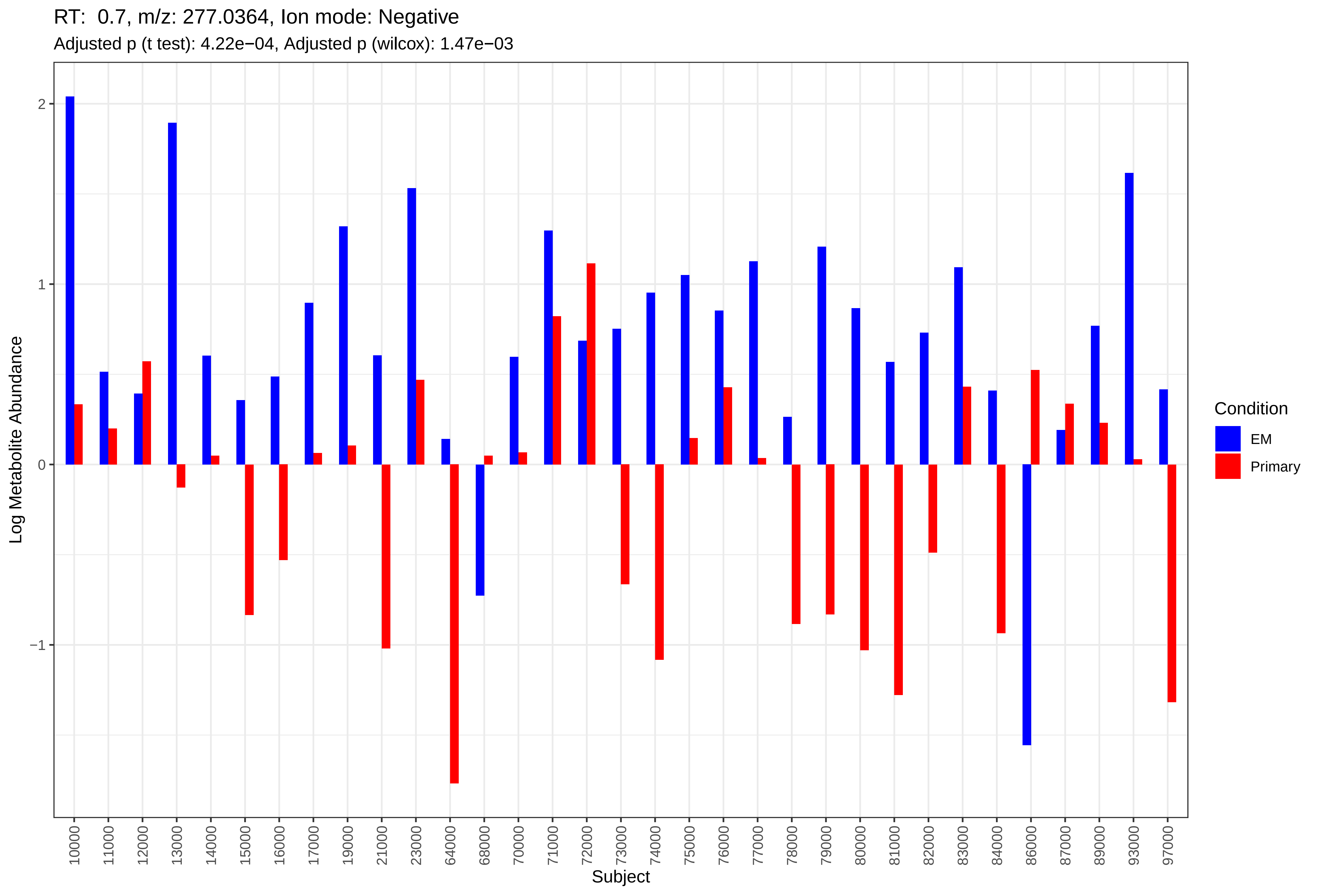
RT: 0.7, m/z: 277.0364, Ion mode: Negative
Adjusted p (t test): 4.22e−04, Adjusted p (wilcox): 1.47e−03
Log Metabolite Abundance
2
1
0
−1
10000
11000
12000
13000
14000
15000
16000
17000
19000
21000
23000
64000
68000
70000
71000
72000
73000
74000
75000
76000
77000
78000
79000
80000
81000
82000
83000
84000
86000
87000
89000
93000
97000
Subject
Condition
EM
Primary

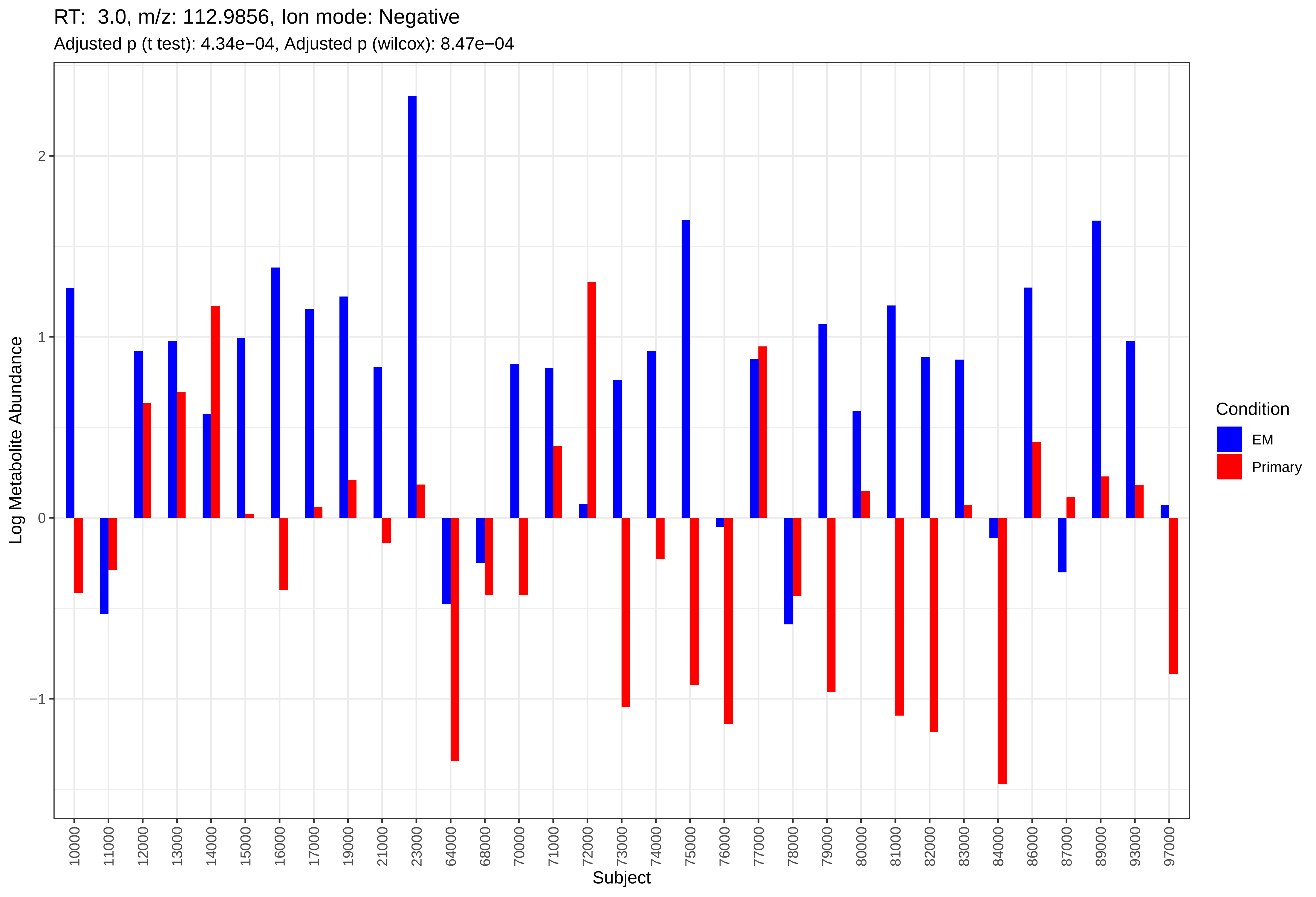
RT: 3.0, m/z: 112.9856, Ion mode: Negative
Adjusted p (t test): 4.34e−04, Adjusted p (wilcox): 8.47e−04
Log Metabolite Abundance
2
1
0
−1
10000
11000
12000
13000
14000
15000
16000
17000
19000
21000
23000
64000
68000
70000
71000
72000
73000
74000
75000
76000
77000
78000
79000
80000
81000
82000
83000
84000
86000
87000
89000
93000
97000
Subject
Condition
EM
Primary

RT: 1.5, m/z: 219.0972, Ion mode: Positive

Adjusted p (t test): 6.58e−04, Adjusted p (wilcox): 4.42e−04

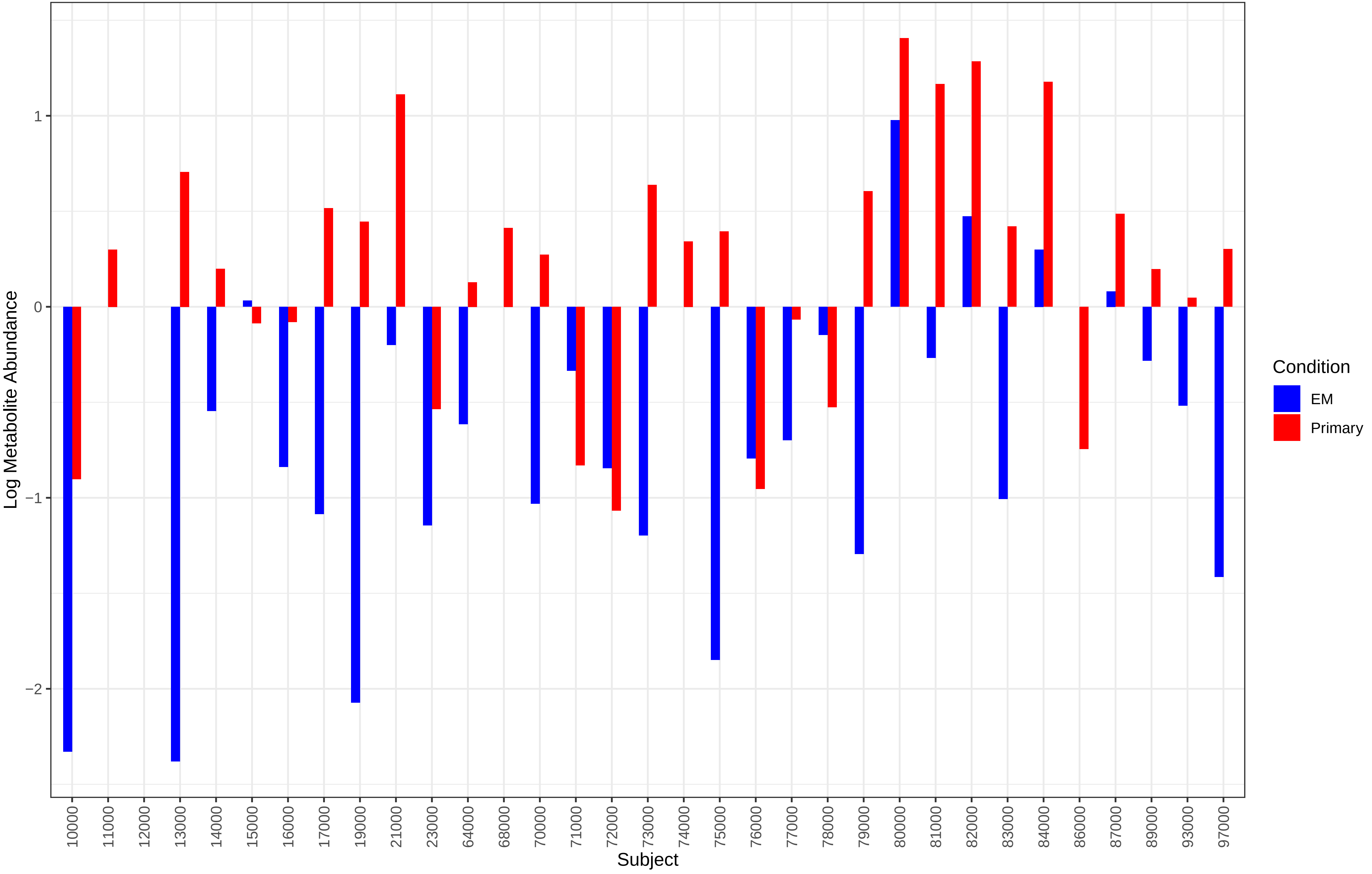

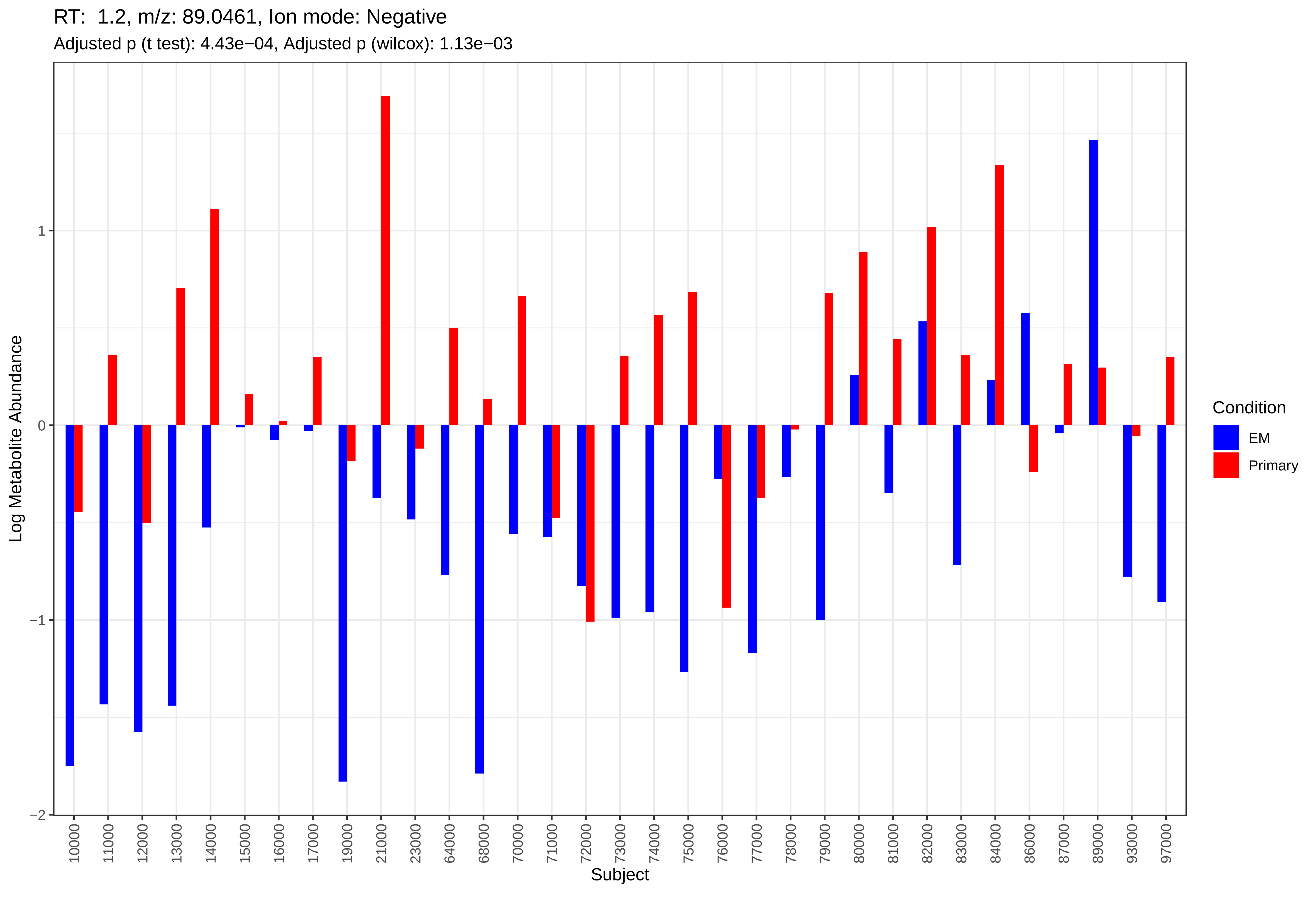
RT: 1.2, m/z: 89.0461, Ion mode: Negative
Adjusted p (t test): 4.43e−04, Adjusted p (wilcox): 1.13e−03
Log Metabolite Abundance
1
0
−1
−2
Subject
10000
11000
12000
13000
14000
15000
16000
17000
19000
21000
23000
64000
68000
70000
71000
72000
73000
74000
75000
76000
77000
78000
79000
80000
81000
82000
83000
84000
86000
87000
89000
93000
97000
Condition
EM
Primary

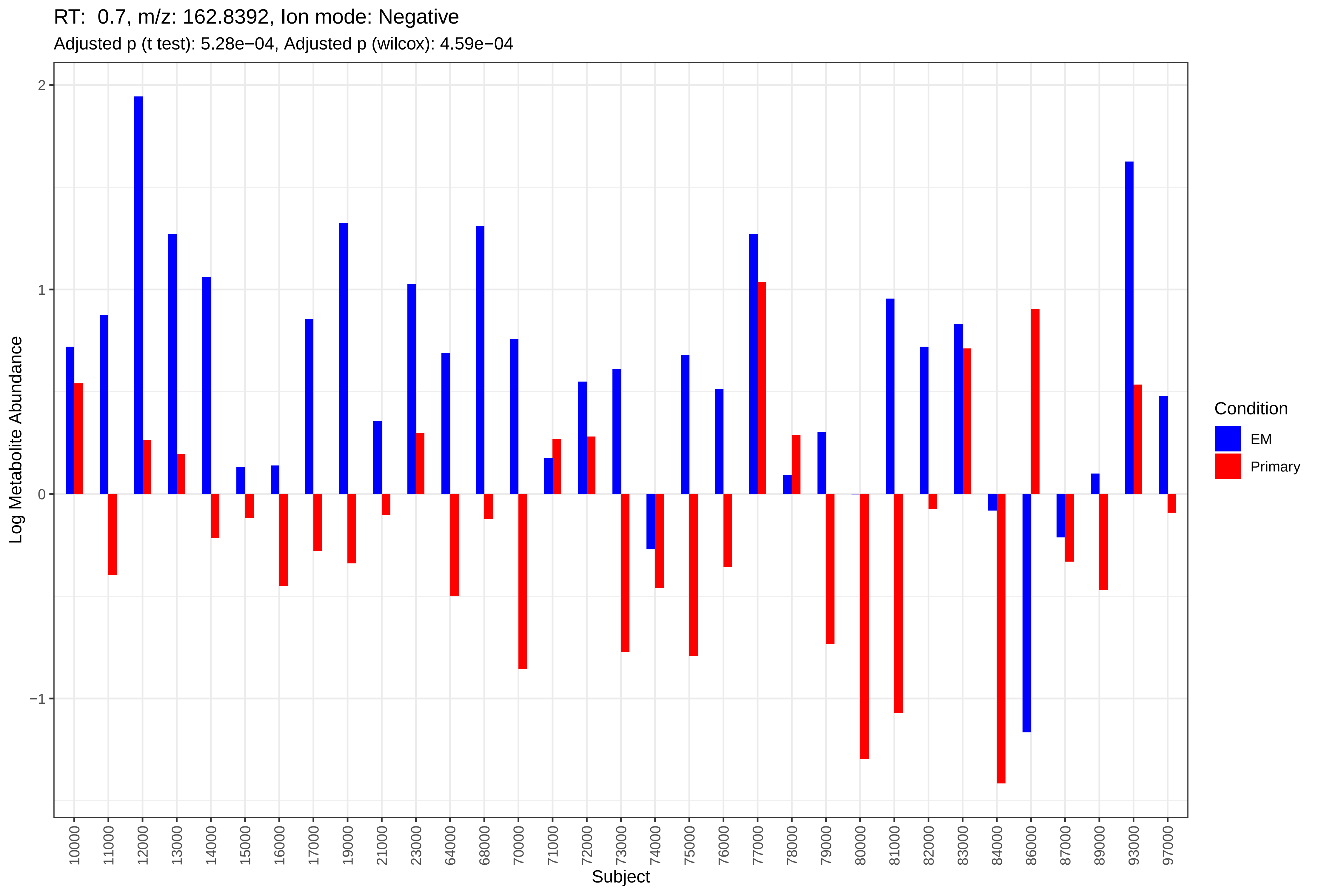
RT: 0.7, m/z: 162.8392, Ion mode: Negative
Adjusted p (t test): 5.28e−04, Adjusted p (wilcox): 4.59e−04
Log Metabolite Abundance
2
1
0
−1
Subject
10000
11000
12000
13000
14000
15000
16000
17000
19000
21000
23000
64000
68000
70000
71000
72000
73000
74000
75000
76000
77000
78000
79000
80000
81000
82000
83000
84000
86000
87000
89000
93000
97000
Condition
EM
Primary

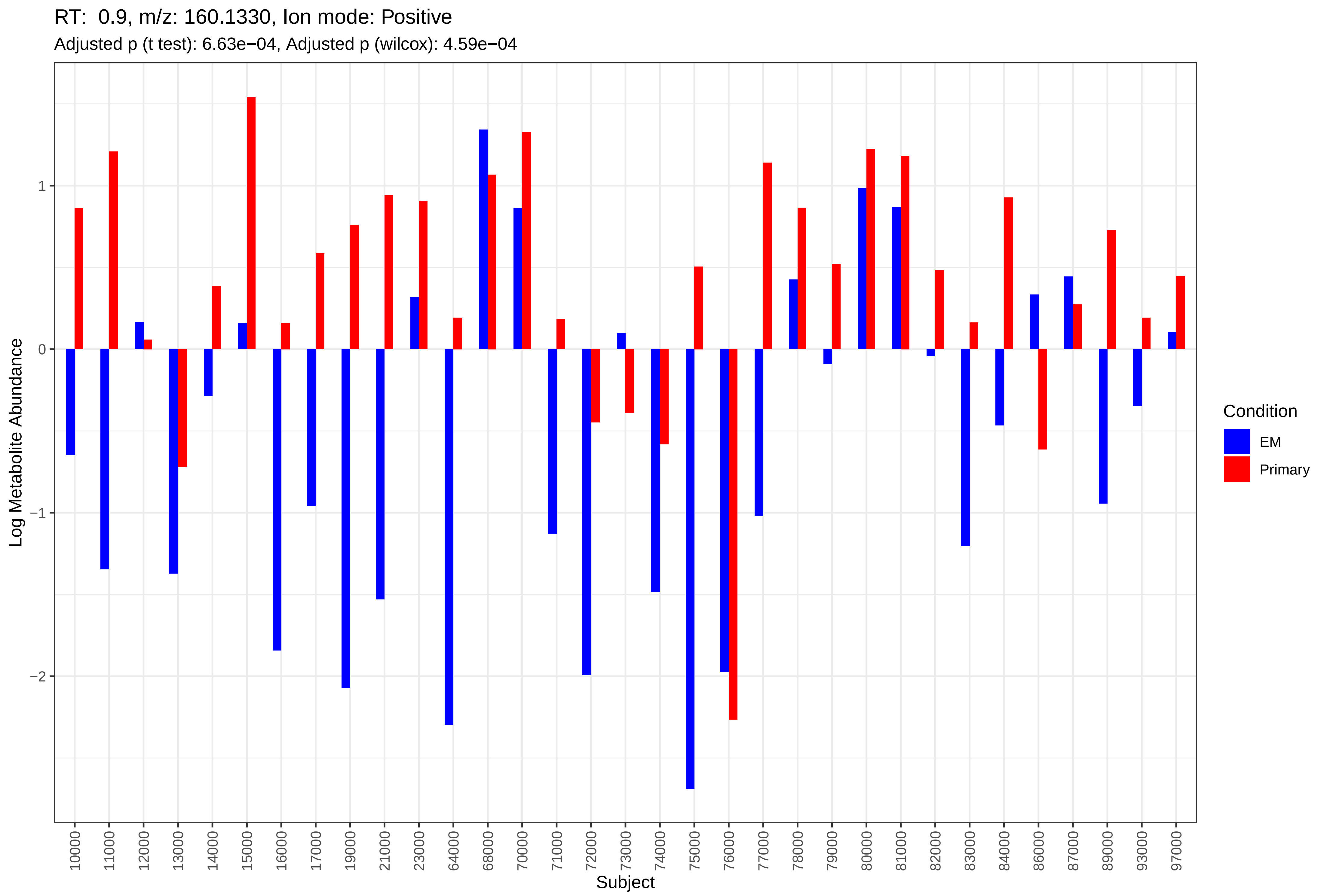
RT: 0.9, m/z: 160.1330, Ion mode: Positive
Adjusted p (t test): 6.63e−04, Adjusted p (wilcox): 4.59e−04
Log Metabolite Abundance
1
0
−1
−2
10000
11000
12000
13000
14000
15000
16000
17000
19000
21000
23000
64000
68000
70000
71000
72000
73000
74000
75000
76000
77000
78000
79000
80000
81000
82000
83000
84000
86000
87000
89000
93000
97000
Subject
Condition
EM
Primary

RT: 6.6, m/z: 256.1774, Ion mode: Positive

Adjusted p (t test): 4.66e−04, Adjusted p (wilcox): 1.89e−03

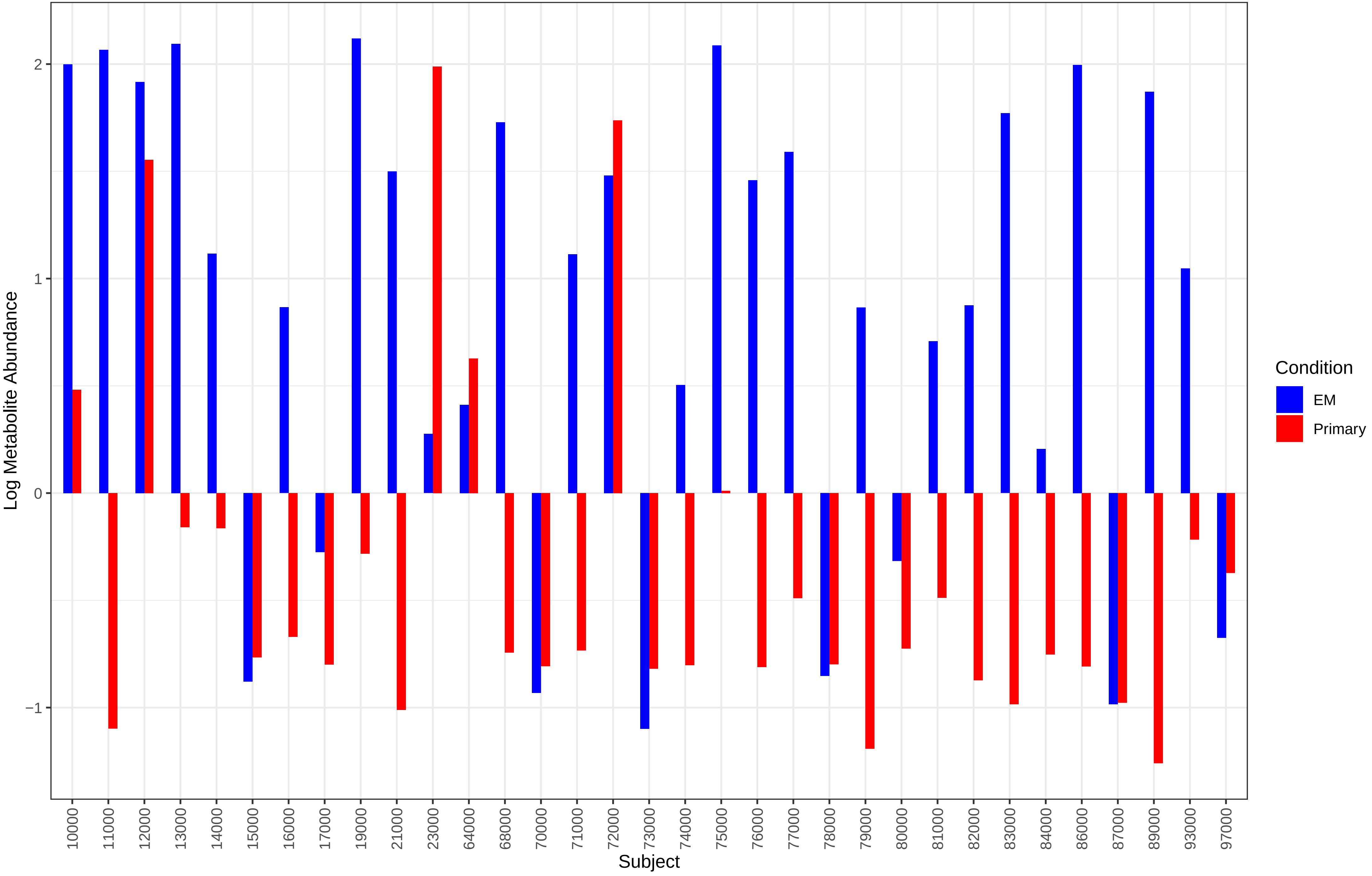

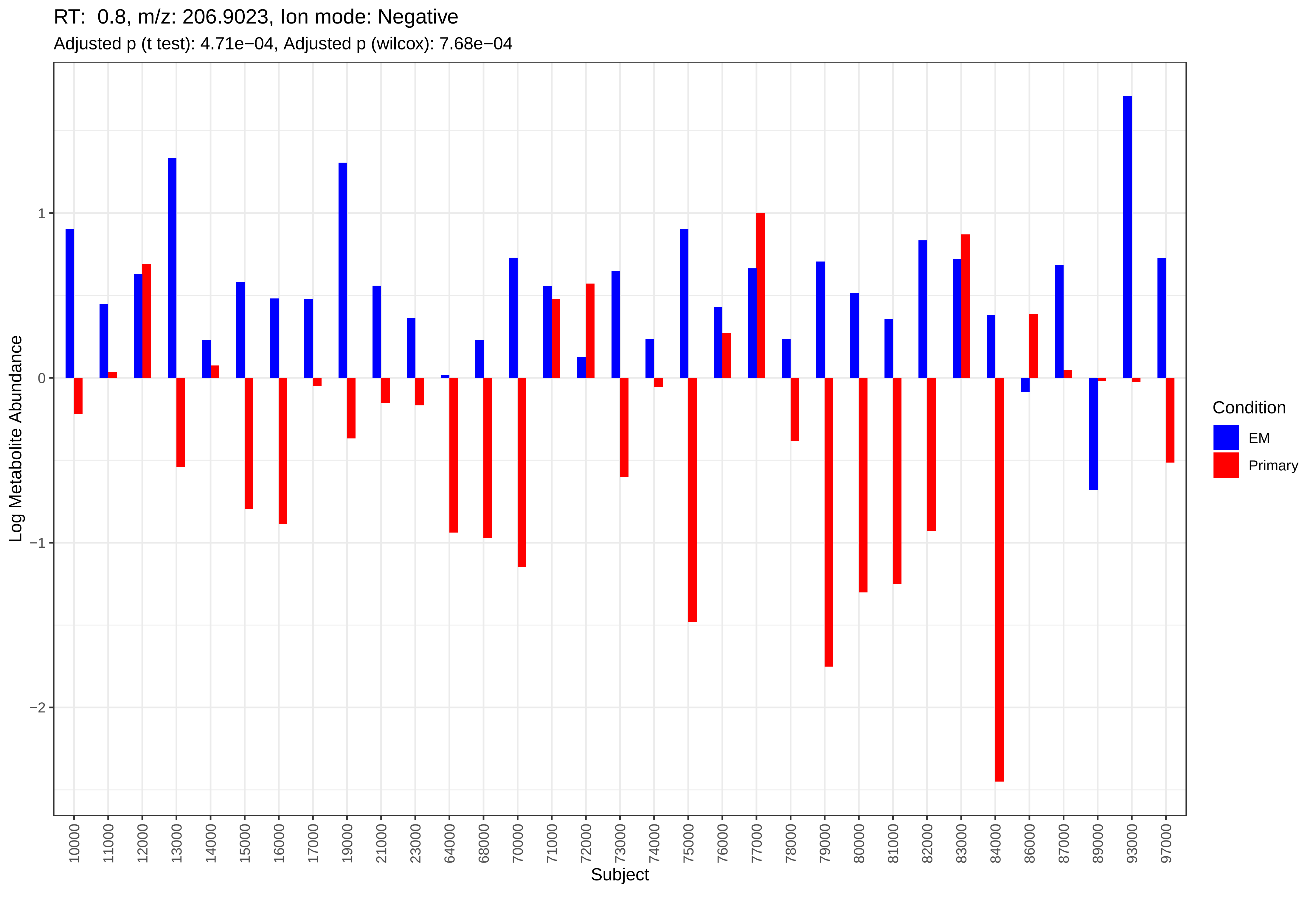
RT: 0.8, m/z: 206.9023, Ion mode: Negative
Adjusted p (t test): 4.71e−04, Adjusted p (wilcox): 7.68e−04
Log Metabolite Abundance
1
0
−1
−2
Subject
10000
11000
12000
13000
14000
15000
16000
17000
19000
21000
23000
64000
68000
70000
71000
72000
73000
74000
75000
76000
77000
78000
79000
80000
81000
82000
83000
84000
86000
87000
89000
93000
97000
Condition
EM
Primary

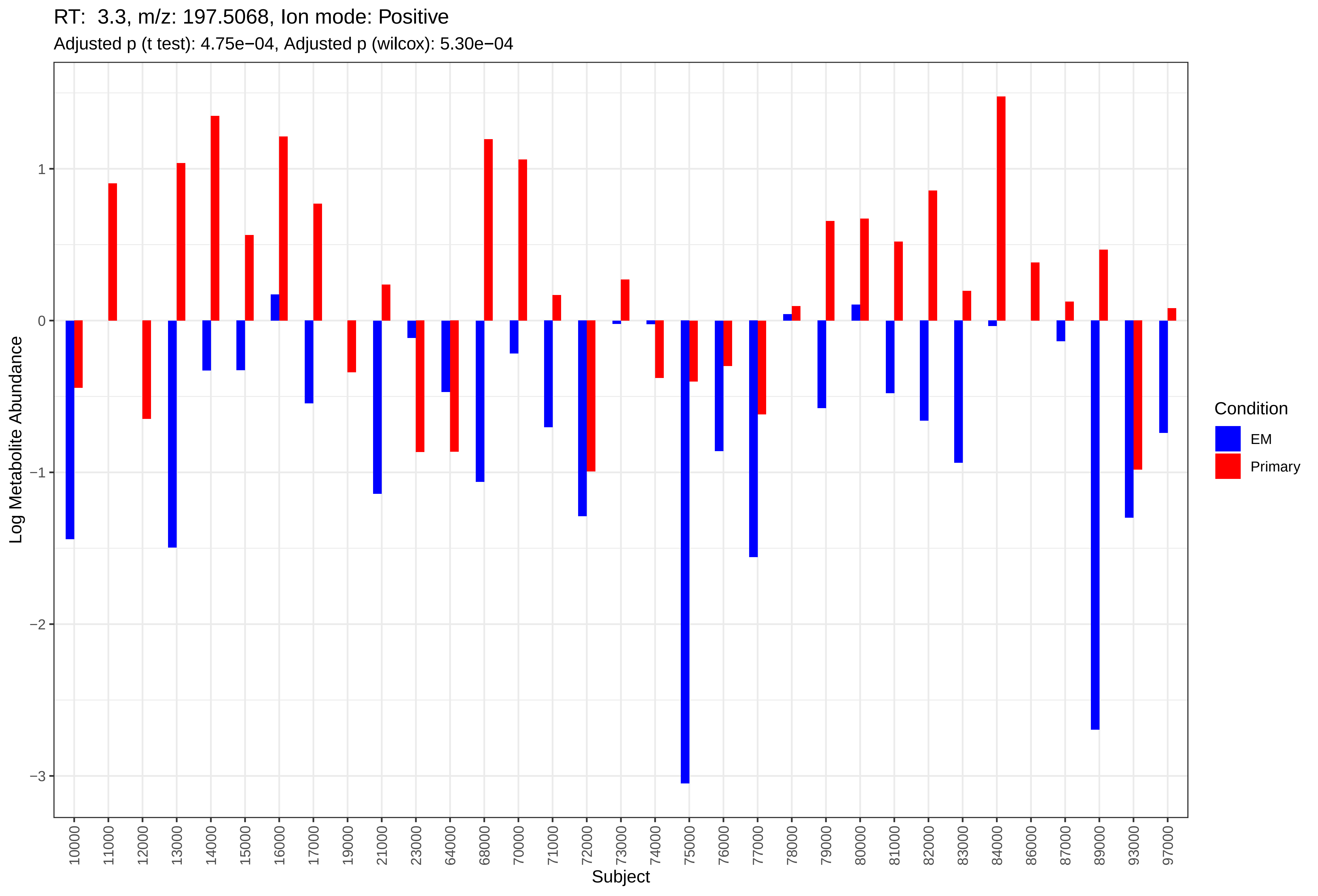
RT: 3.3, m/z: 197.5068, Ion mode: Positive
Adjusted p (t test): 4.75e−04, Adjusted p (wilcox): 5.30e−04
Log Metabolite Abundance
Subject
Condition
EM
Primary
1
0
−1
−2
−3
10000
11000
12000
13000
14000
15000
16000
17000
19000
21000
23000
64000
68000
70000
71000
72000
73000
74000
75000
76000
77000
78000
79000
80000
81000
82000
83000
84000
86000
87000
89000
93000
97000

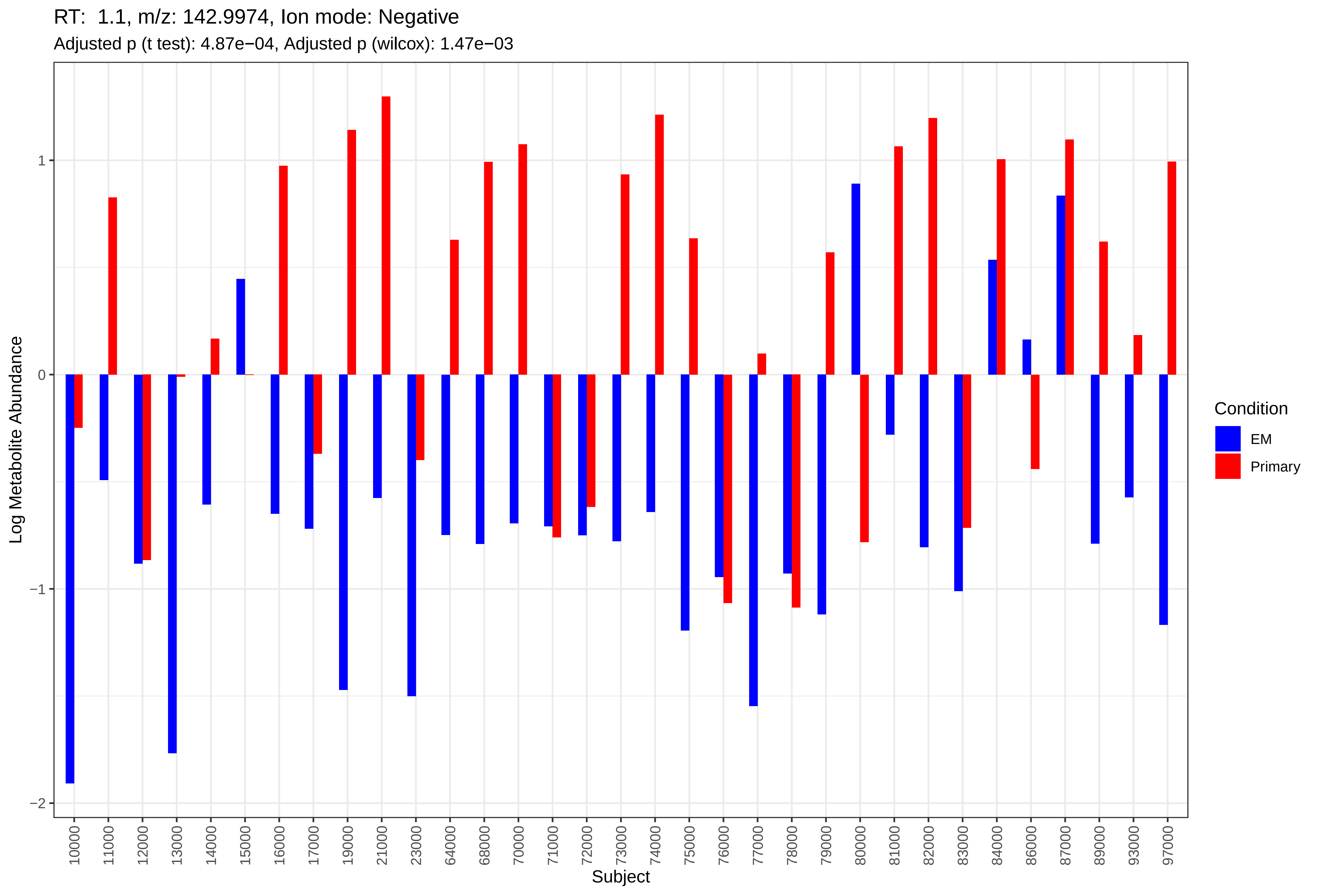

RT: 1.1, m/z: 142.9974, Ion mode: Negative
Adjusted p (t test): 4.87e−04, Adjusted p (wilcox): 1.47e−03
Log Metabolite Abundance
1
0
−1
−2
Subject
10000
11000
12000
13000
14000
15000
16000
17000
19000
21000
23000
64000
68000
70000
71000
72000
73000
74000
75000
76000
77000
78000
79000
80000
81000
82000
83000
84000
86000
87000
89000
93000
97000
Condition
EM
Primary

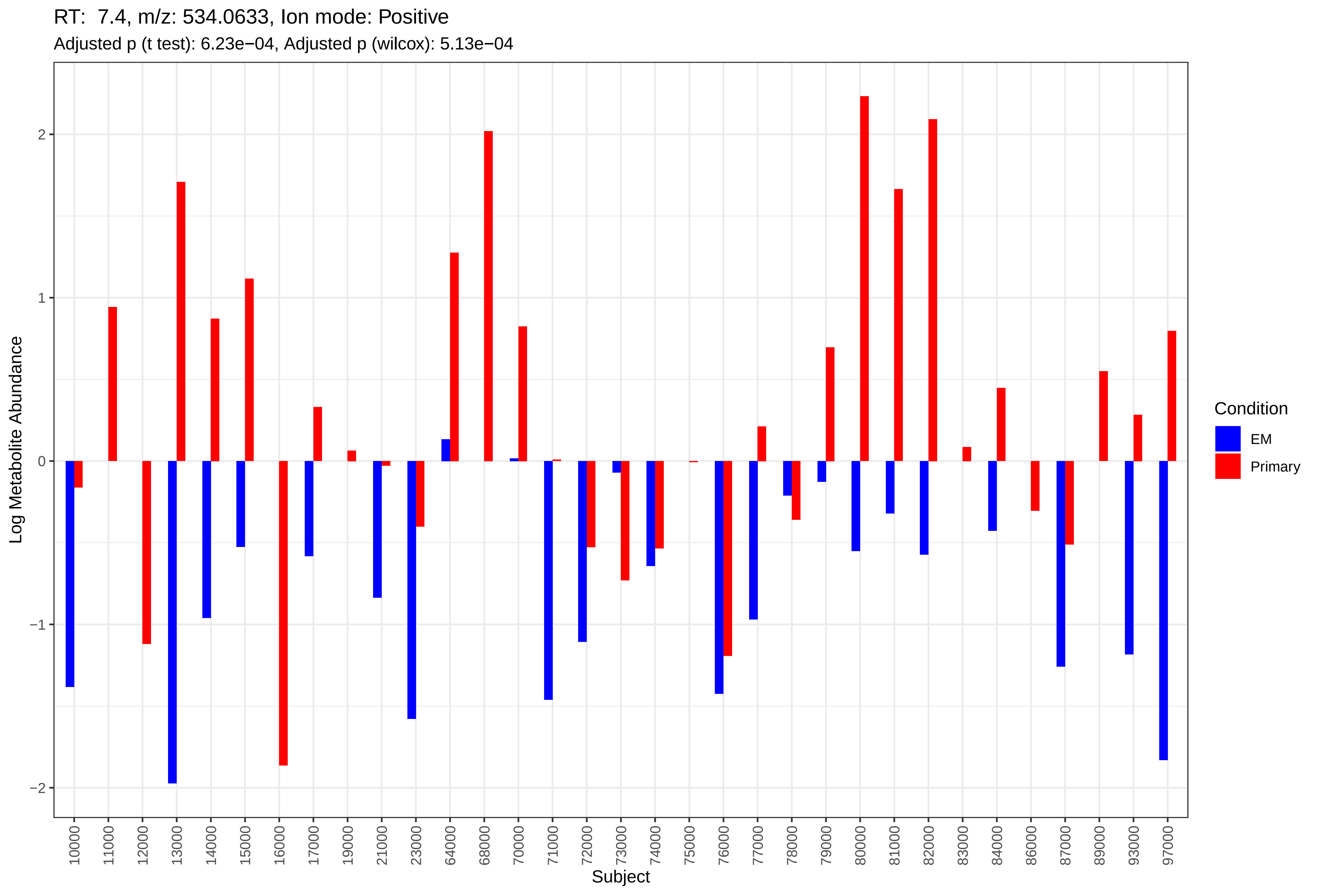
RT: 7.4, m/z: 534.0633, Ion mode: Positive
Adjusted p (t test): 6.23e−04, Adjusted p (wilcox): 5.13e−04
Log Metabolite Abundance
Subject
Condition
EM
Primary
2
1
0
−1
−2
10000
11000
12000
13000
14000
15000
16000
17000
19000
21000
23000
64000
68000
70000
71000
72000
73000
74000
75000
76000
77000
78000
79000
80000
81000
82000
83000
84000
86000
87000
89000
93000
97000

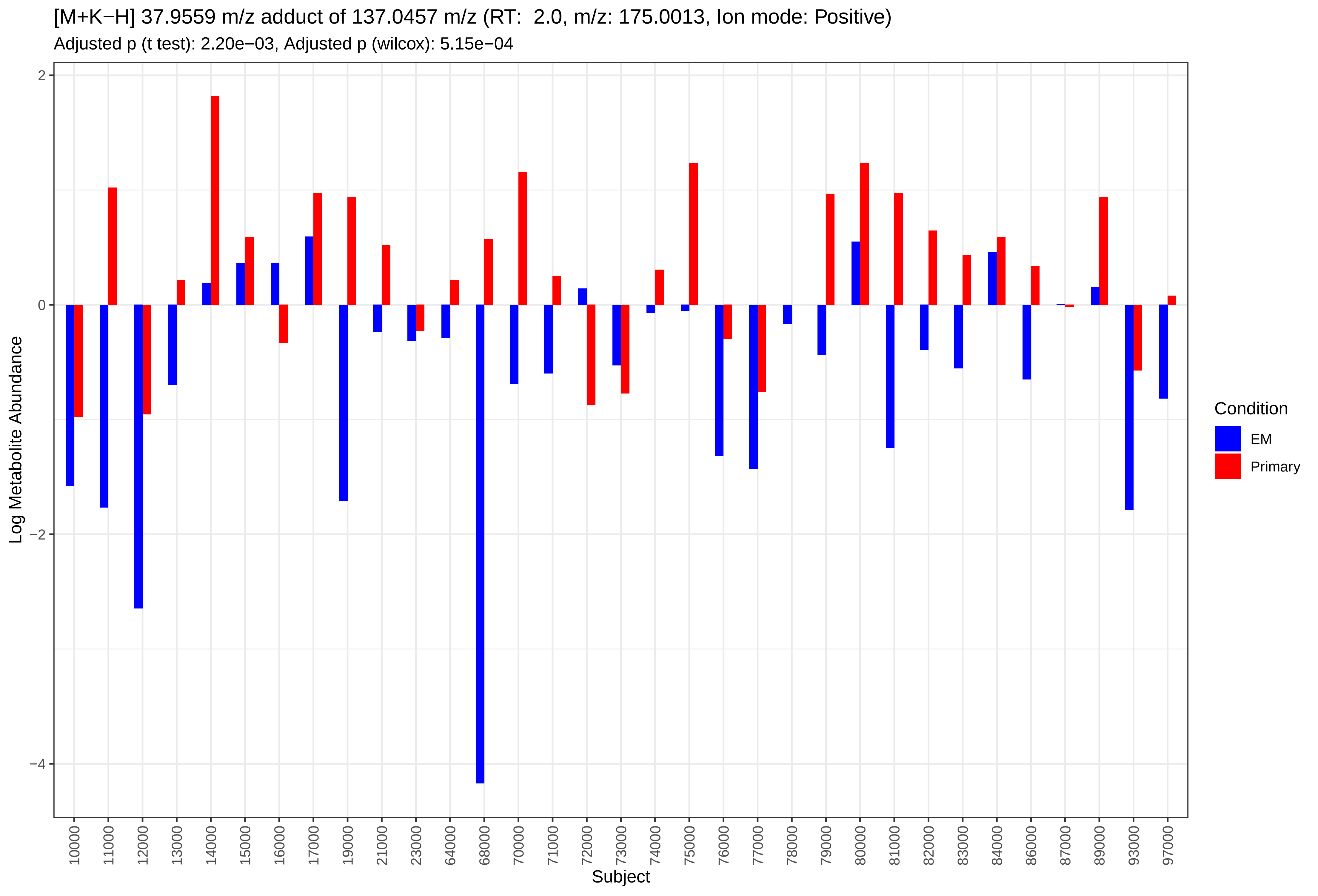

[M+K−H] 37.9559 m/z adduct of 137.0457 m/z (RT: 2.0, m/z: 175.0013, Ion mode: Positive)
Adjusted p (t test): 2.20e−03, Adjusted p (wilcox): 5.15e−04
Log Metabolite Abundance
2
0
−2
−4
Subject
10000
11000
12000
13000
14000
15000
16000
17000
19000
21000
23000
64000
68000
70000
71000
72000
73000
74000
75000
76000
77000
78000
79000
80000
81000
82000
83000
84000
86000
87000
89000
93000
97000
Condition
EM
Primary

RT: 2.9, m/z: 272.2364, Ion mode: Positive

Adjusted p (t test): 5.46e−03, Adjusted p (wilcox): 5.15e−04

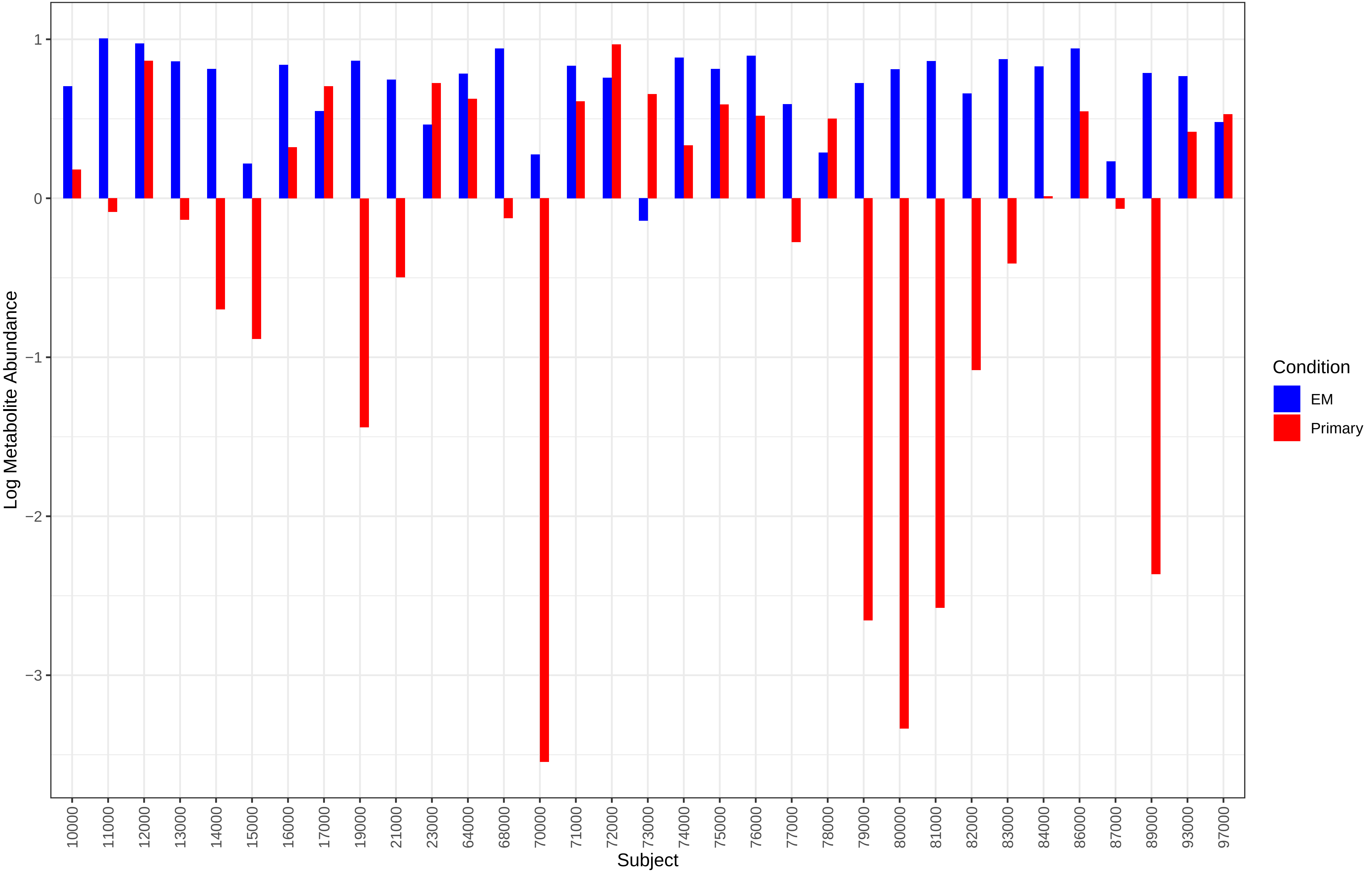

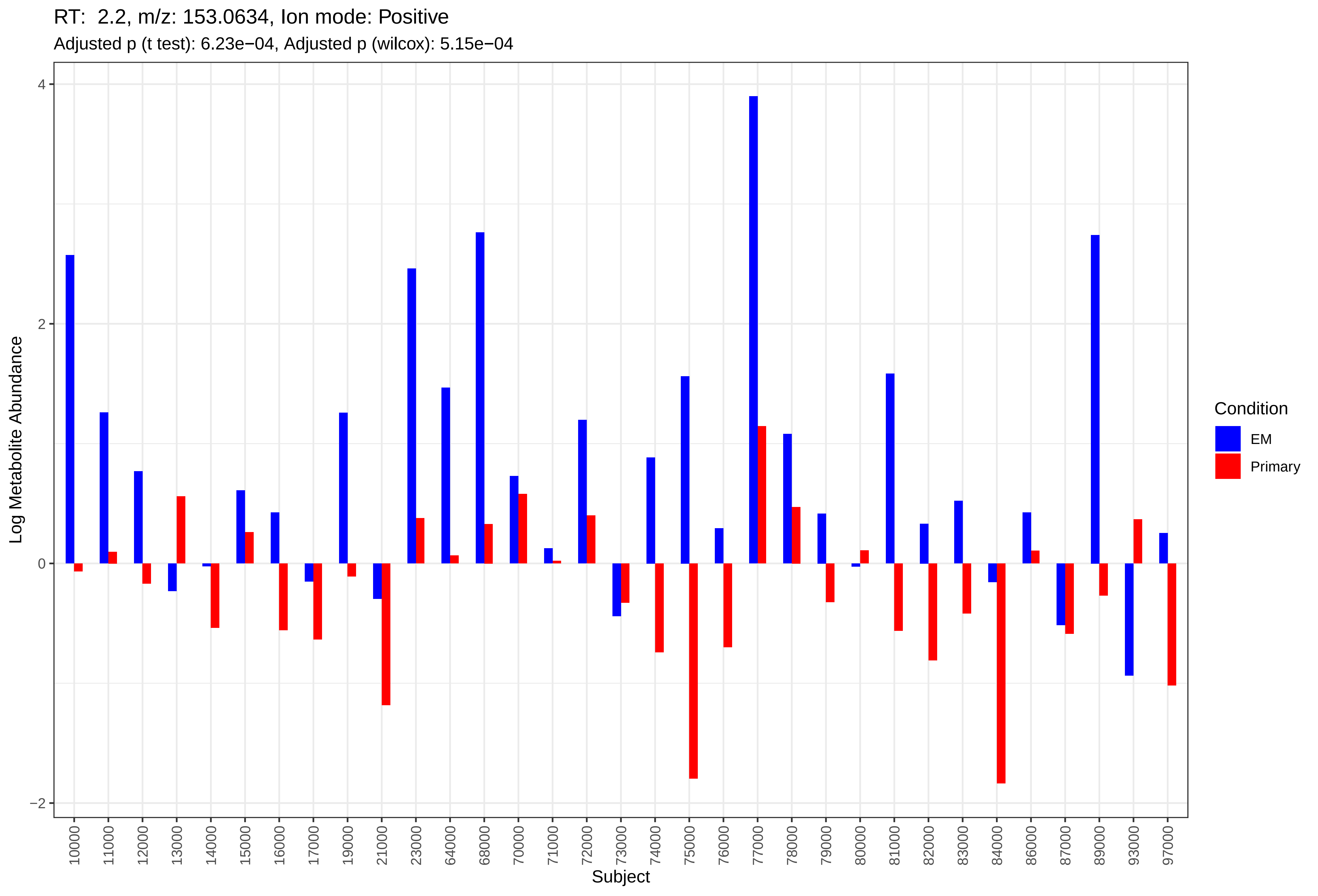
RT: 2.2, m/z: 153.0634, Ion mode: Positive
Adjusted p (t test): 6.23e−04, Adjusted p (wilcox): 5.15e−04
Log Metabolite Abundance
4
2
0
−2
Subject
10000
11000
12000
13000
14000
15000
16000
17000
19000
21000
23000
64000
68000
70000
71000
72000
73000
74000
75000
76000
77000
78000
79000
80000
81000
82000
83000
84000
86000
87000
89000
93000
97000
Condition
EM
Primary

RT: 15.8, m/z: 105.0016, Ion mode: Negative

Adjusted p (t test): 5.90e−04, Adjusted p (wilcox): 5.16e−04

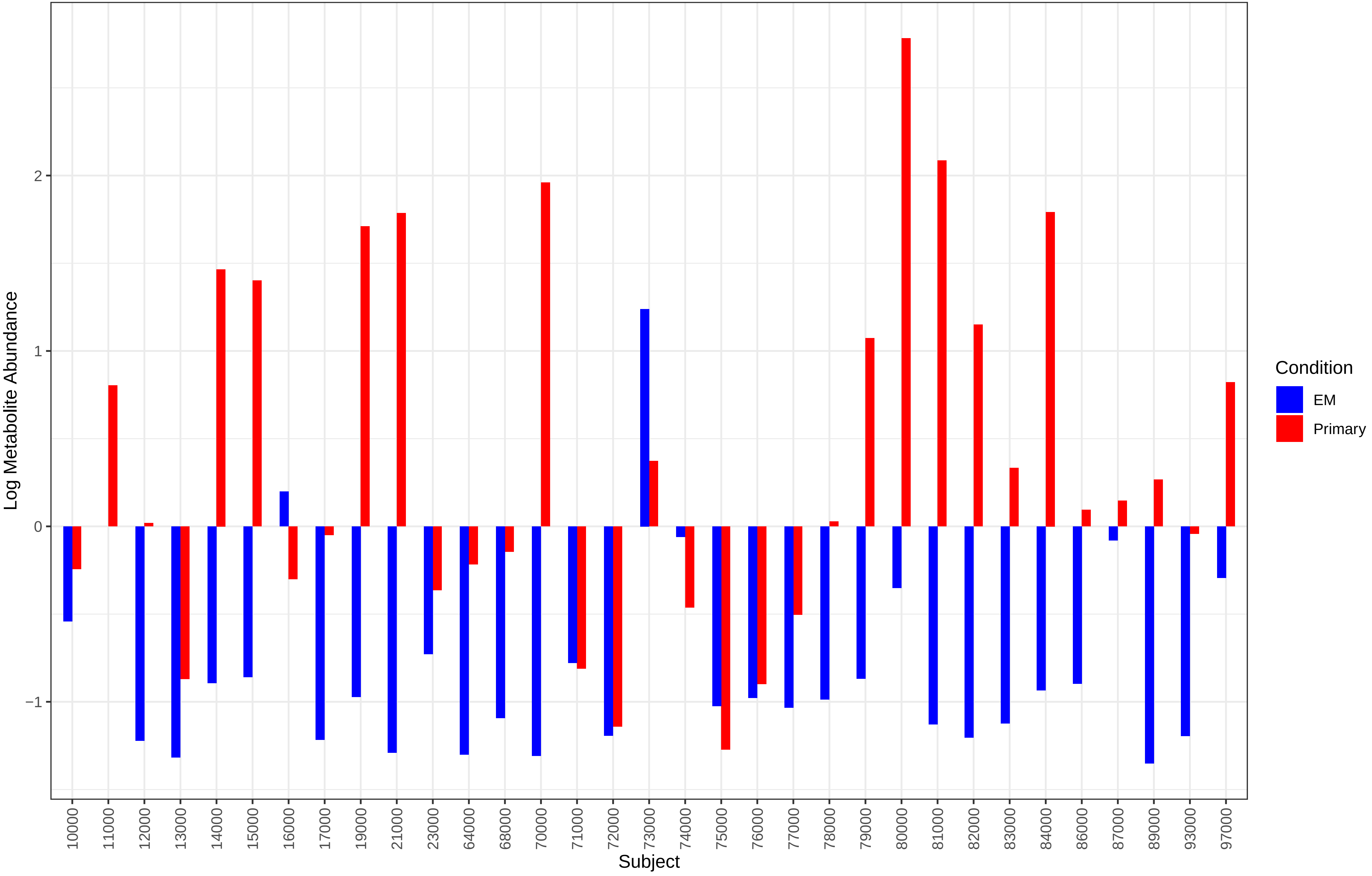

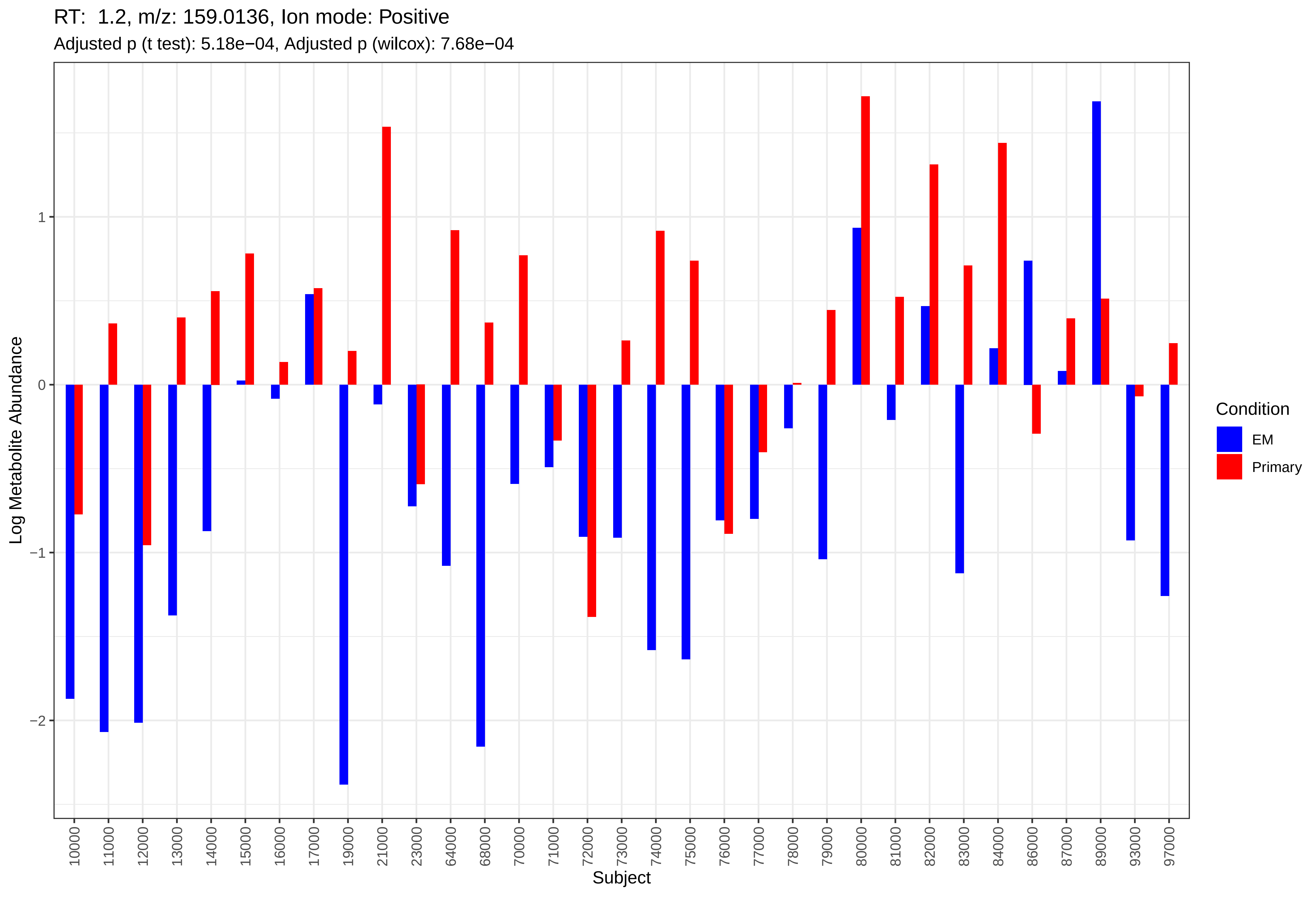

RT: 1.2, m/z: 159.0136, Ion mode: Positive
Adjusted p (t test): 5.18e−04, Adjusted p (wilcox): 7.68e−04
Log Metabolite Abundance
1
0
−1
−2
10000
11000
12000
13000
14000
15000
16000
17000
19000
21000
23000
64000
68000
70000
71000
72000
73000
74000
75000
76000
77000
78000
79000
80000
81000
82000
83000
84000
86000
87000
89000
93000
97000
Subject
Condition
EM
Primary

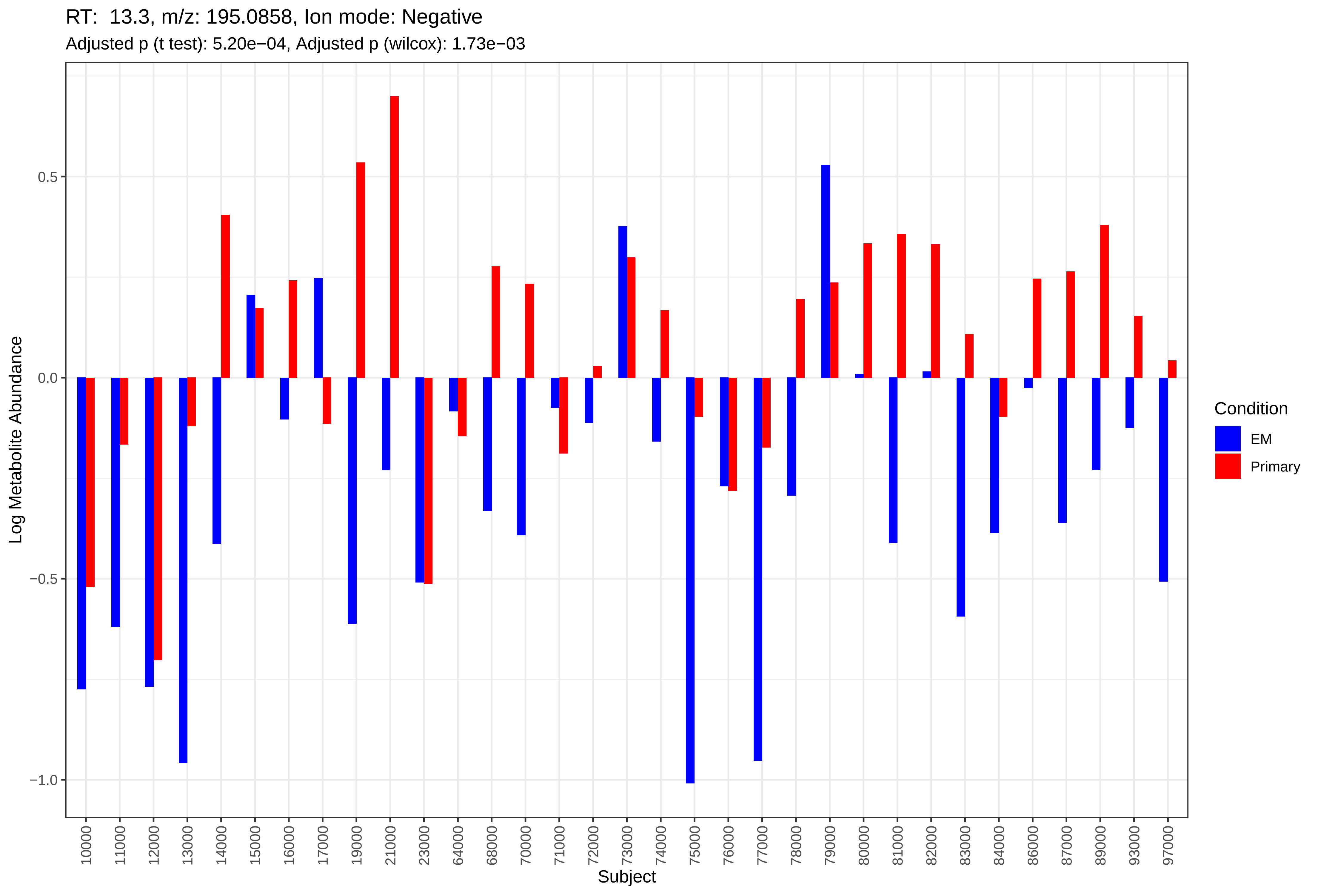

RT: 13.3, m/z: 195.0858, Ion mode: Negative
Adjusted p (t test): 5.20e−04, Adjusted p (wilcox): 1.73e−03
Log Metabolite Abundance
0.5
0.0
−0.5
−1.0
Subject
10000
11000
12000
13000
14000
15000
16000
17000
19000
21000
23000
64000
68000
70000
71000
72000
73000
74000
75000
76000
77000
78000
79000
80000
81000
82000
83000
84000
86000
87000
89000
93000
97000
Condition
EM
Primary

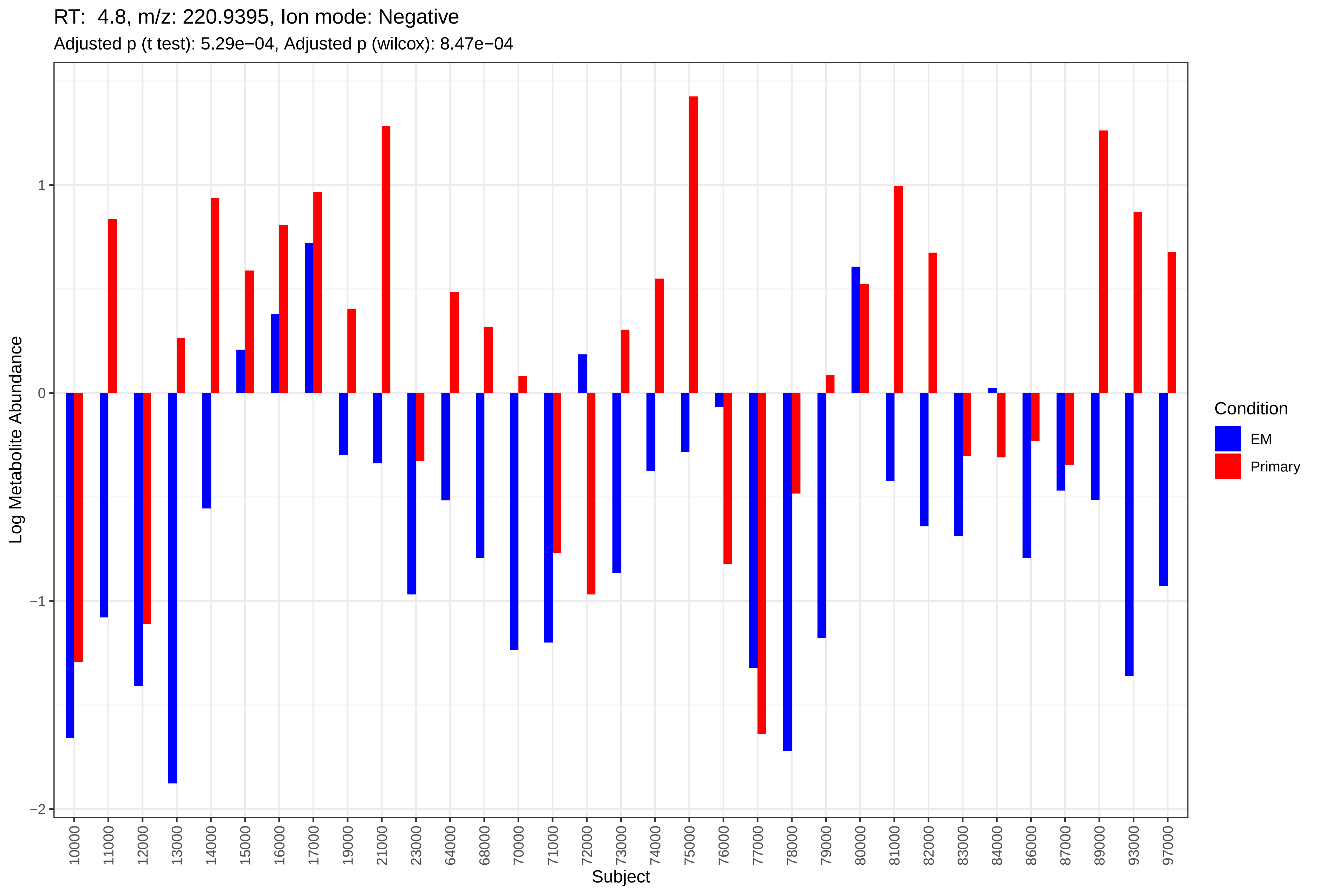
RT: 4.8, m/z: 220.9395, Ion mode: Negative
Adjusted p (t test): 5.29e−04, Adjusted p (wilcox): 8.47e−04
Log Metabolite Abundance
1
0
−1
−2
Subject
10000
11000
12000
13000
14000
15000
16000
17000
19000
21000
23000
64000
68000
70000
71000
72000
73000
74000
75000
76000
77000
78000
79000
80000
81000
82000
83000
84000
86000
87000
89000
93000
97000
Condition
EM
Primary

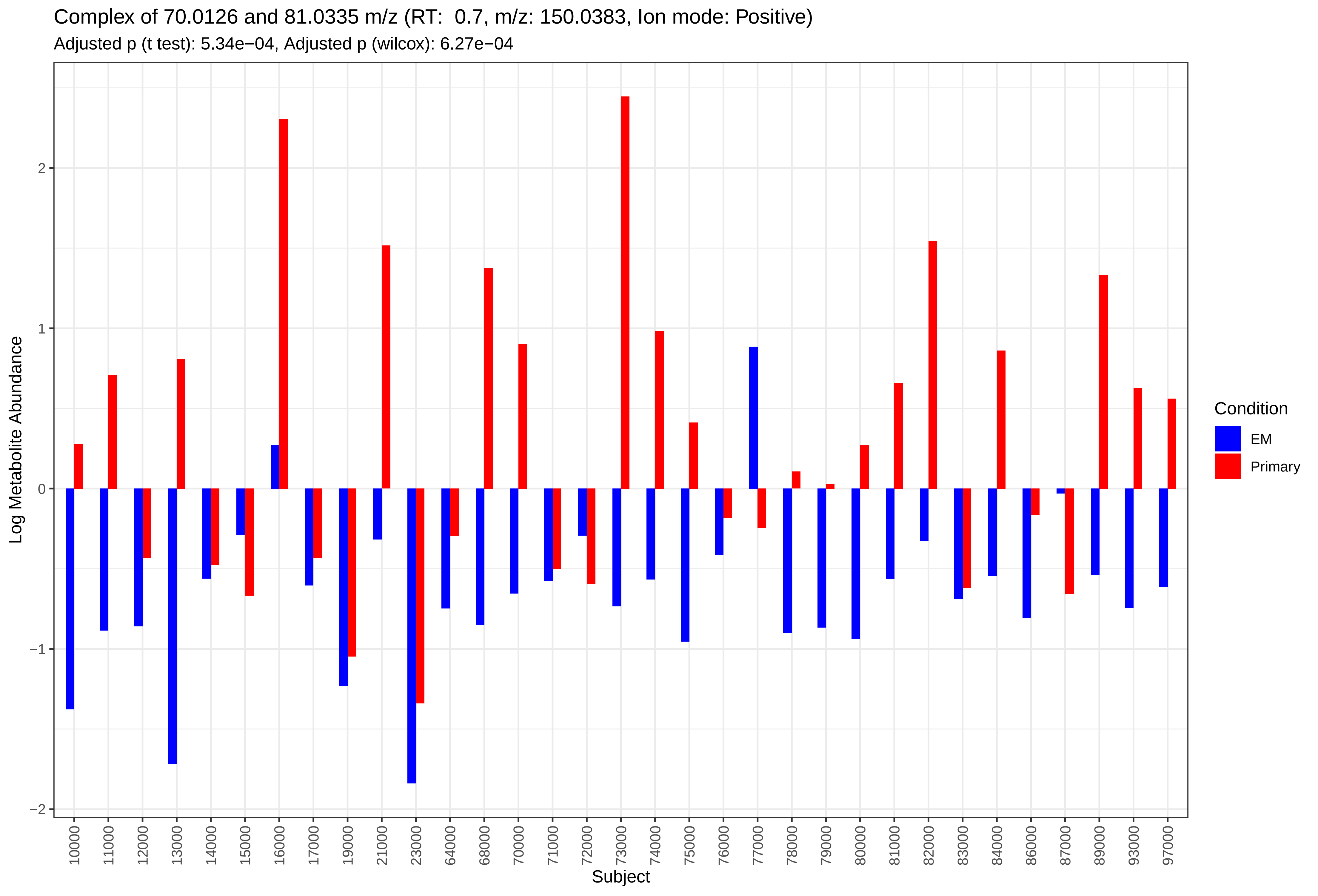

Complex of 70.0126 and 81.0335 m/z (RT: 0.7, m/z: 150.0383, Ion mode: Positive)
Adjusted p (t test): 5.34e−04, Adjusted p (wilcox): 6.27e−04
Log Metabolite Abundance
Subject
Condition
EM
Primary
2
1
0
−1
−2
10000
11000
12000
13000
14000
15000
16000
17000
19000
21000
23000
64000
68000
70000
71000
72000
73000
74000
75000
76000
77000
78000
79000
80000
81000
82000
83000
84000
86000
87000
89000
93000
97000

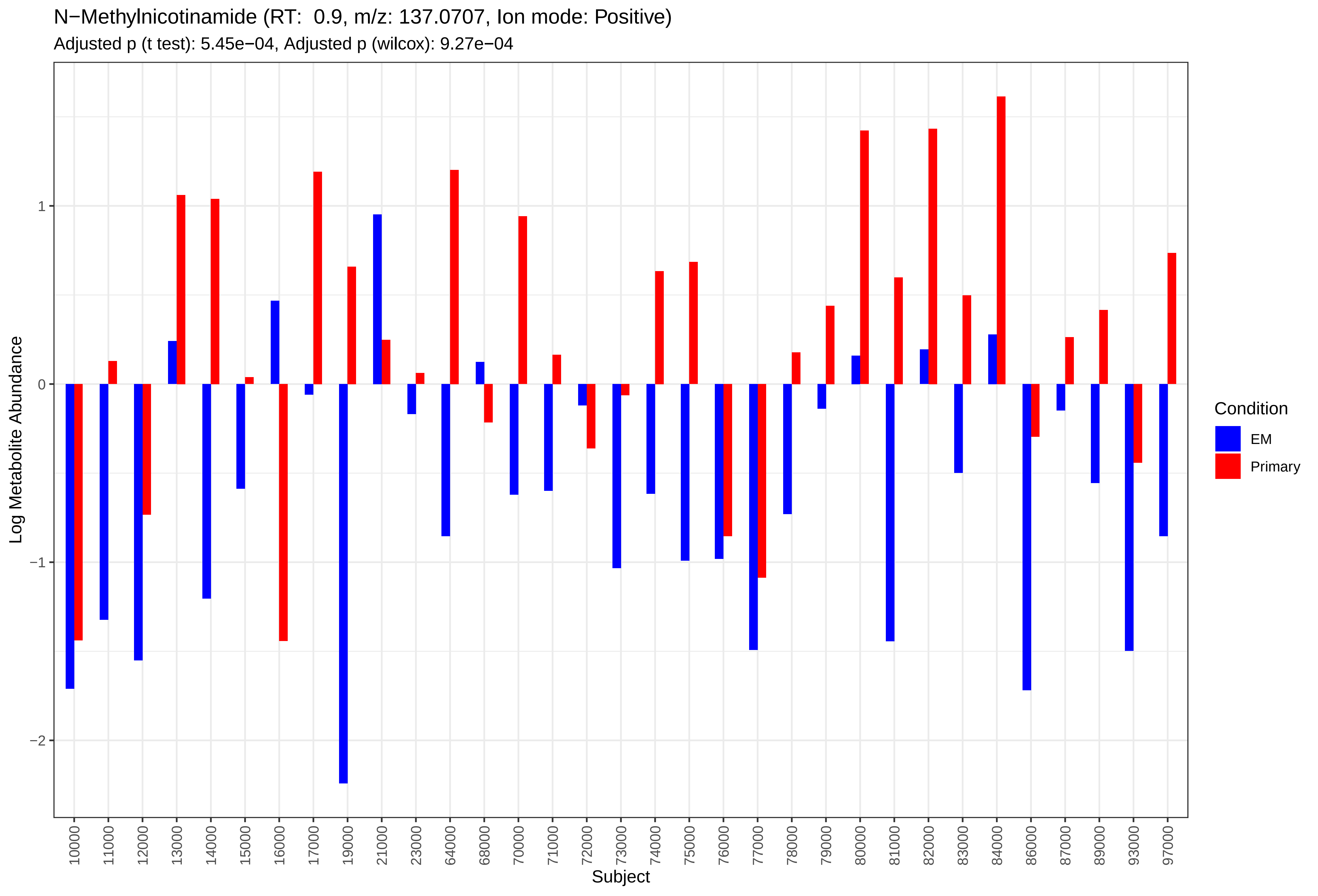

N−Methylnicotinamide (RT: 0.9, m/z: 137.0707, Ion mode: Positive)
Adjusted p (t test): 5.45e−04, Adjusted p (wilcox): 9.27e−04
Log Metabolite Abundance
1
0
−1
−2
Subject
10000
11000
12000
13000
14000
15000
16000
17000
19000
21000
23000
64000
68000
70000
71000
72000
73000
74000
75000
76000
77000
78000
79000
80000
81000
82000
83000
84000
86000
87000
89000
93000
97000
Condition
EM
Primary

RT: 2.1, m/z: 138.1220, Ion mode: Positive

Adjusted p (t test): 1.55e−03, Adjusted p (wilcox): 5.65e−04

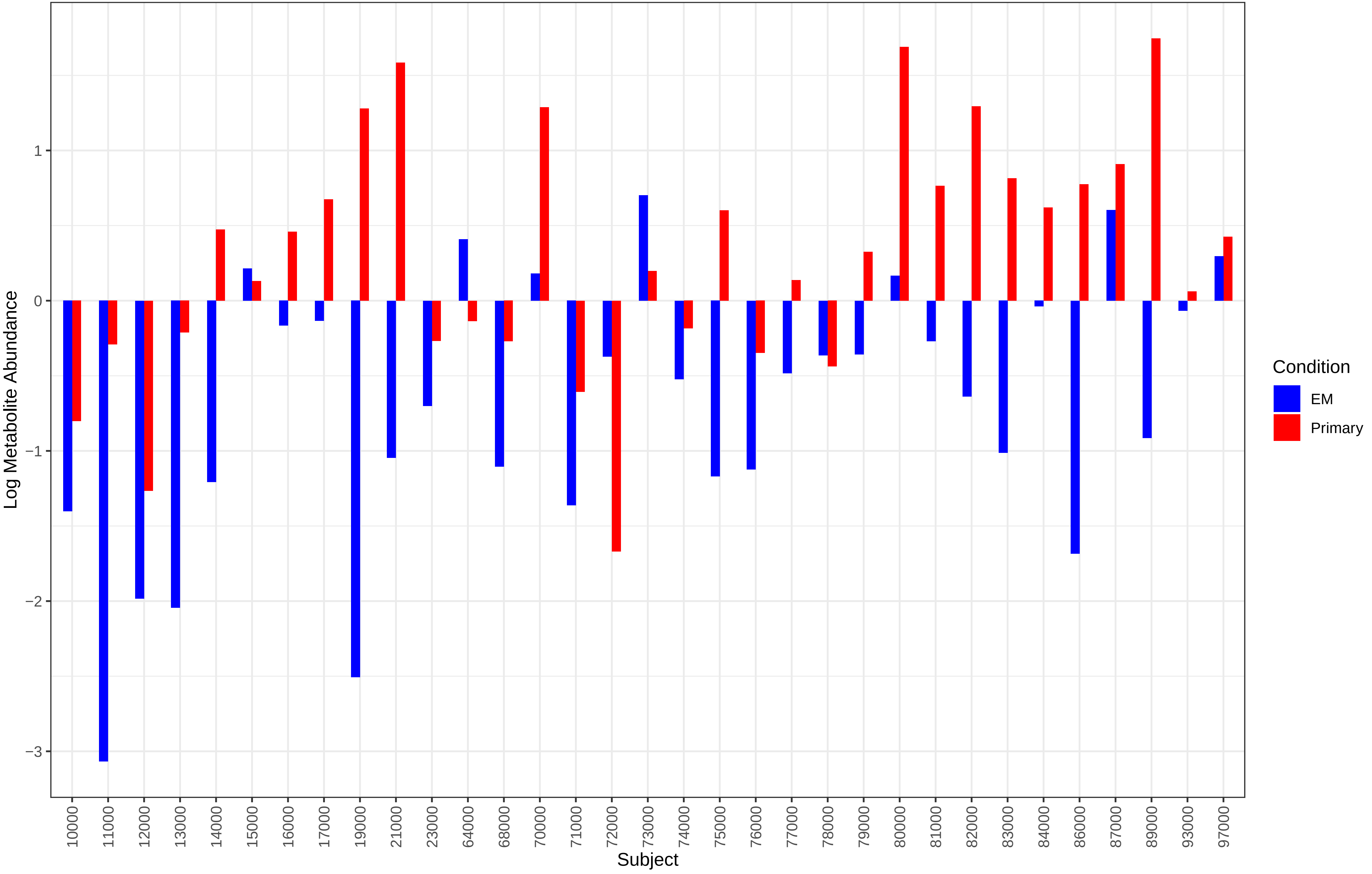

RT: 2.1, m/z: 91.1132, Ion mode: Positive

Adjusted p (t test): 8.65e−04, Adjusted p (wilcox): 5.65e−04

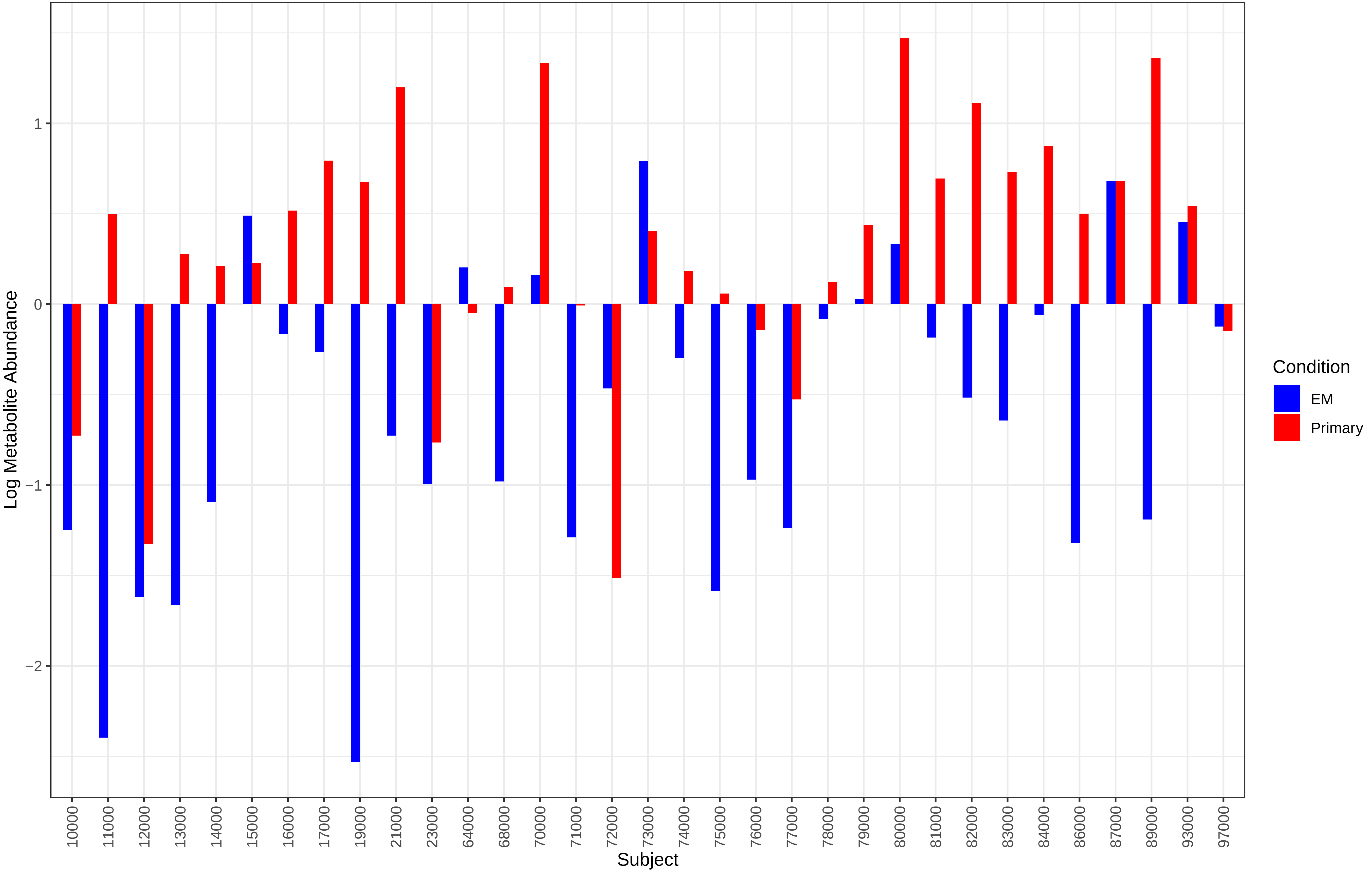

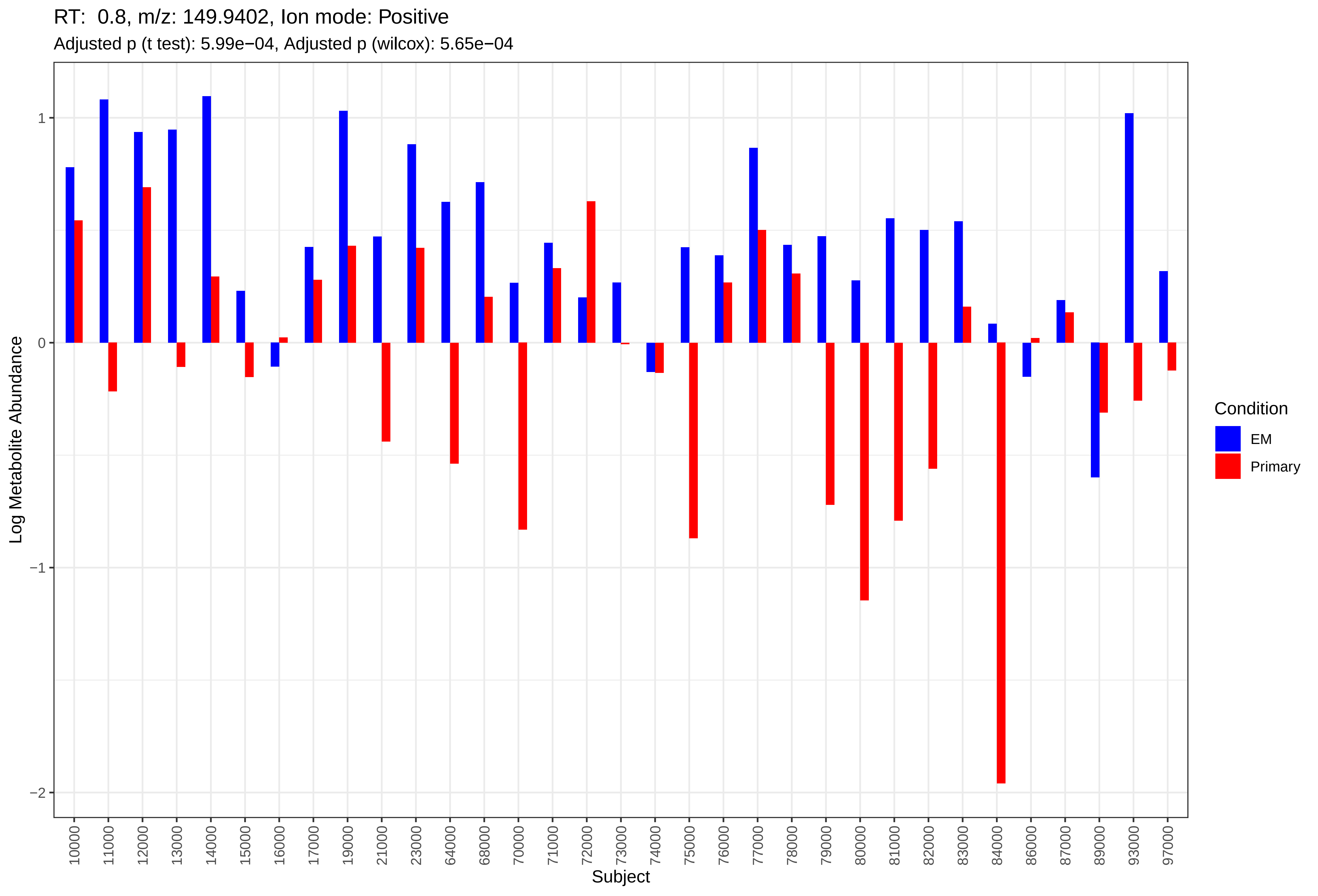

RT: 0.8, m/z: 149.9402, Ion mode: Positive
Adjusted p (t test): 5.99e−04, Adjusted p (wilcox): 5.65e−04
Log Metabolite Abundance
1
0
−1
−2
10000
11000
12000
13000
14000
15000
16000
17000
19000
21000
23000
64000
68000
70000
71000
72000
73000
74000
75000
76000
77000
78000
79000
80000
81000
82000
83000
84000
86000
87000
89000
93000
97000
Subject
Condition
EM
Primary

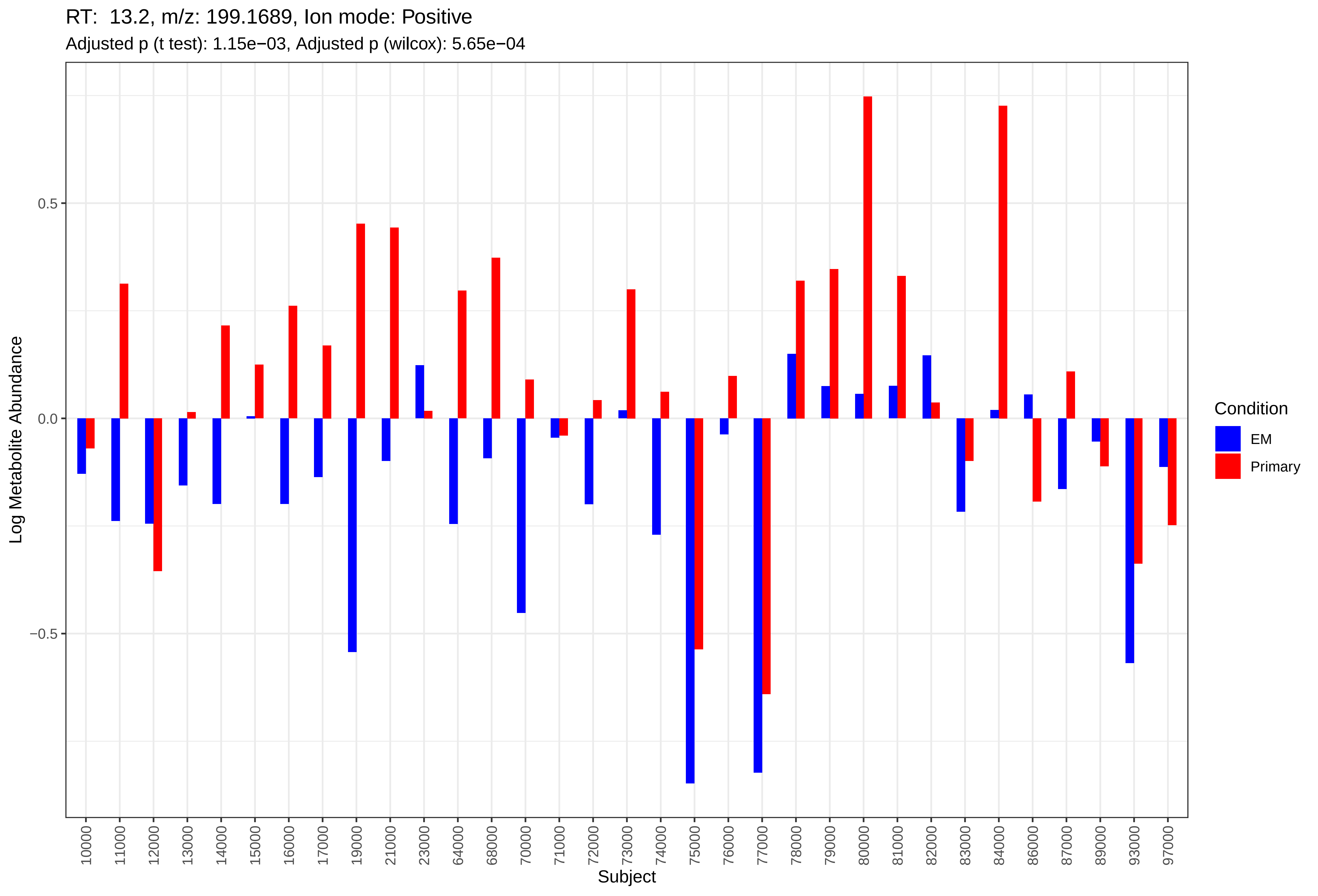

RT: 13.2, m/z: 199.1689, Ion mode: Positive
Adjusted p (t test): 1.15e−03, Adjusted p (wilcox): 5.65e−04
Log Metabolite Abundance
0.5
0.0
−0.5
10000
11000
12000
13000
14000
15000
16000
17000
19000
21000
23000
64000
68000
70000
71000
72000
73000
74000
75000
76000
77000
78000
79000
80000
81000
82000
83000
84000
86000
87000
89000
93000
97000
Subject
Condition
EM
Primary

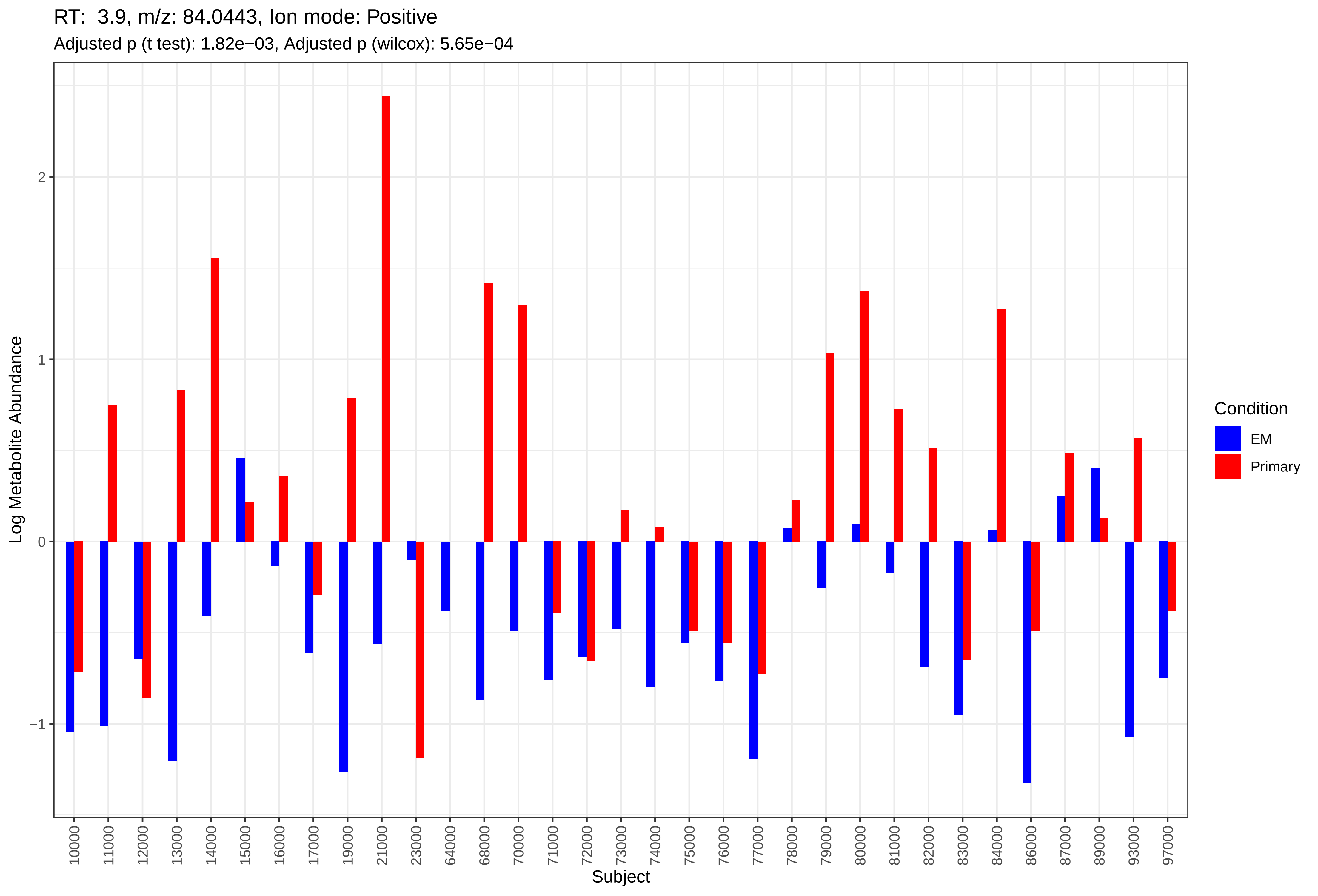

RT: 3.9, m/z: 84.0443, Ion mode: Positive
Adjusted p (t test): 1.82e−03, Adjusted p (wilcox): 5.65e−04
Log Metabolite Abundance
2
1
0
−1
10000
11000
12000
13000
14000
15000
16000
17000
19000
21000
23000
64000
68000
70000
71000
72000
73000
74000
75000
76000
77000
78000
79000
80000
81000
82000
83000
84000
86000
87000
89000
93000
97000
Subject
Condition
EM
Primary

RT: 6.6, m/z: 166.0973, Ion mode: Positive

Adjusted p (t test): 6.20e−03, Adjusted p (wilcox): 5.65e−04

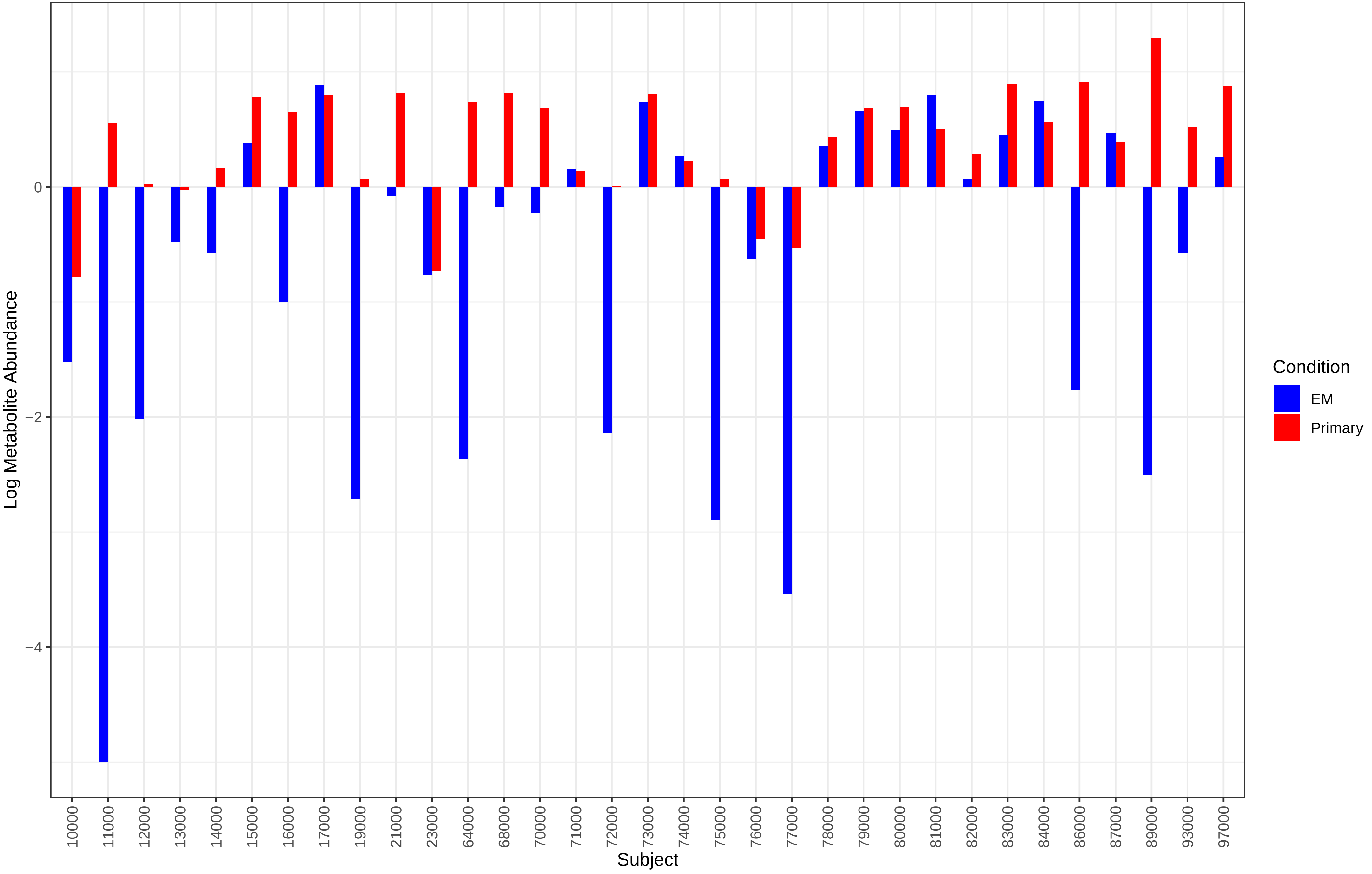

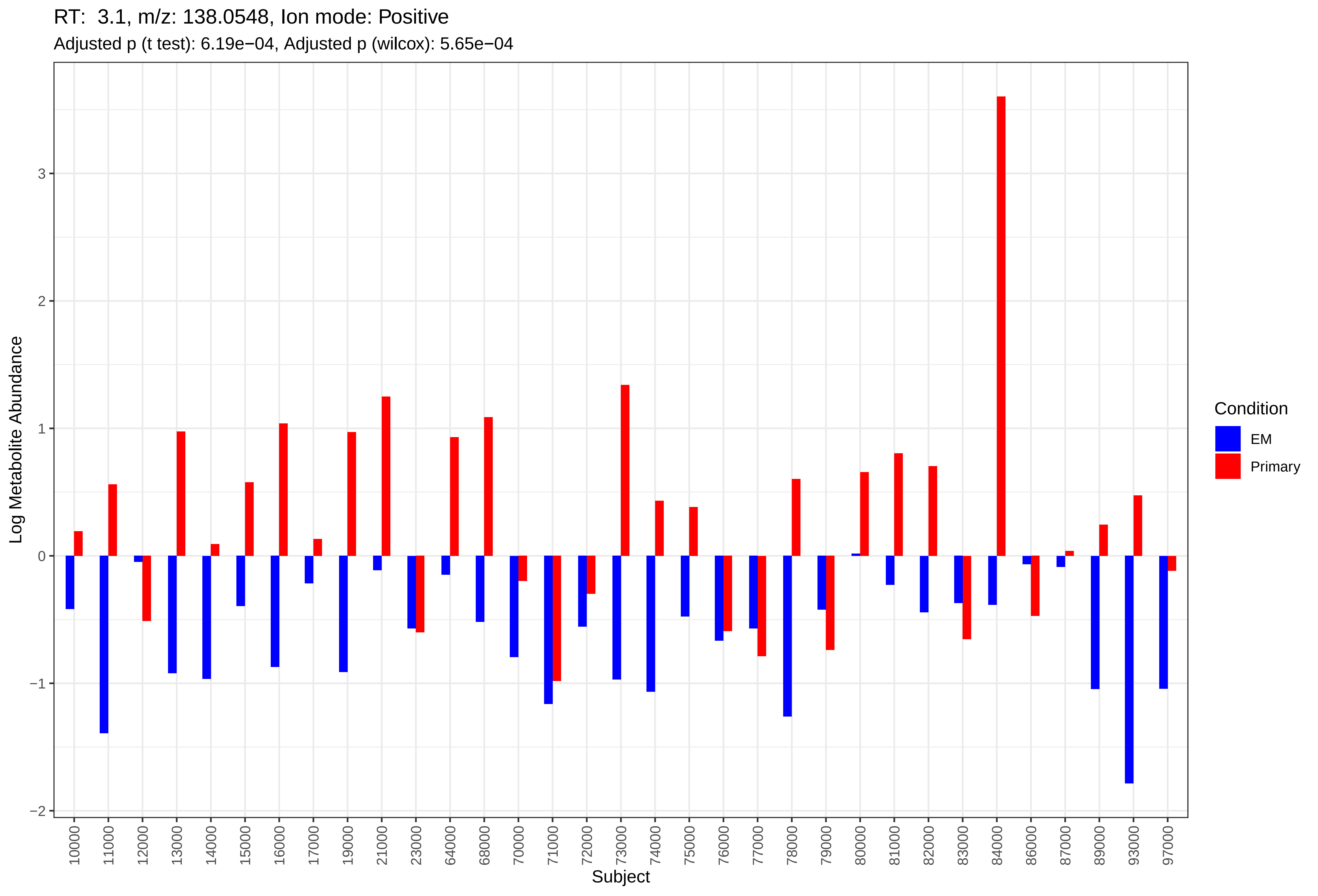
RT: 3.1, m/z: 138.0548, Ion mode: Positive
Adjusted p (t test): 6.19e−04, Adjusted p (wilcox): 5.65e−04
Log Metabolite Abundance
3
2
1
0
−1
−2
Subject
10000
11000
12000
13000
14000
15000
16000
17000
19000
21000
23000
64000
68000
70000
71000
72000
73000
74000
75000
76000
77000
78000
79000
80000
81000
82000
83000
84000
86000
87000
89000
93000
97000
Condition
EM
Primary

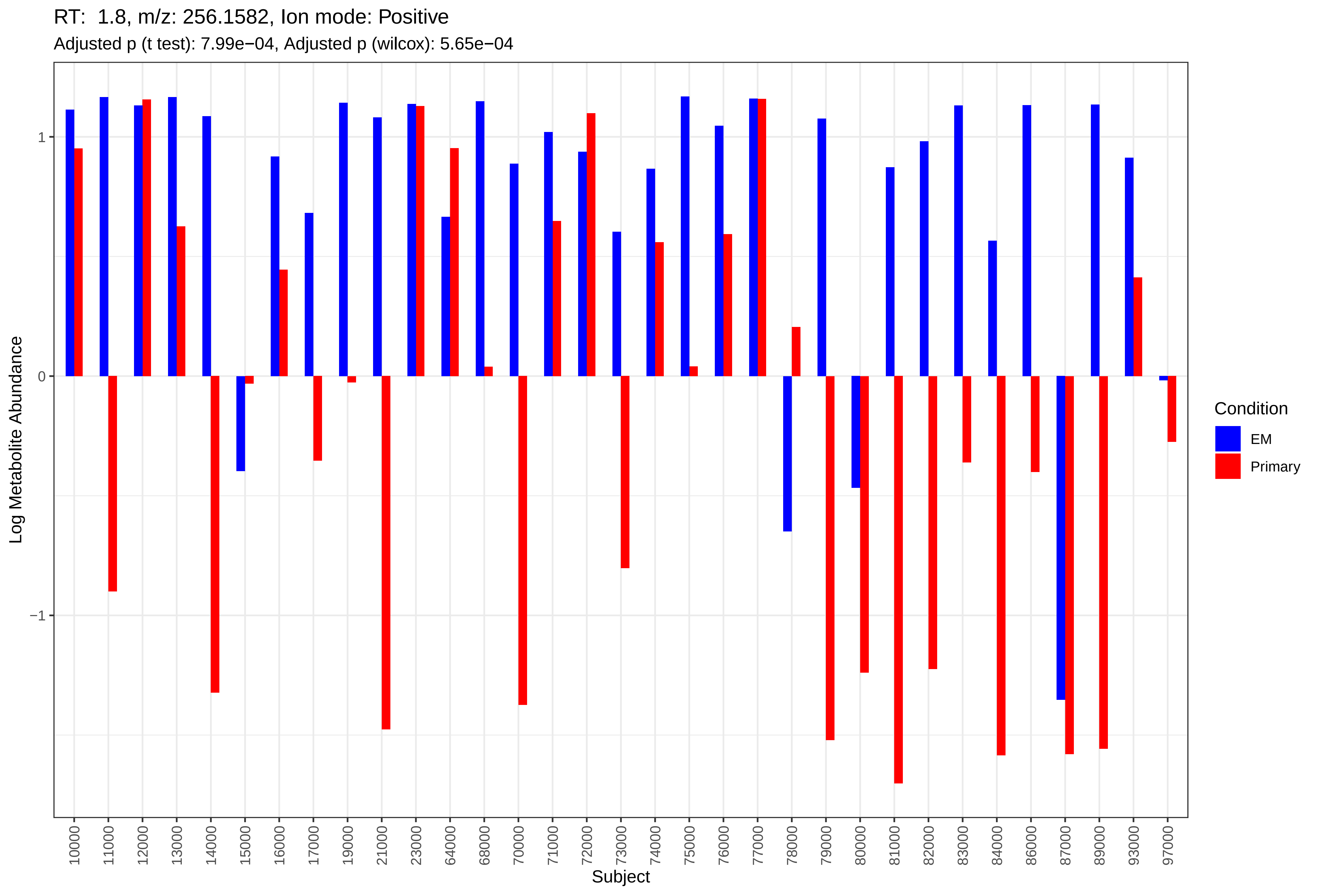

RT: 1.8, m/z: 256.1582, Ion mode: Positive
Adjusted p (t test): 7.99e−04, Adjusted p (wilcox): 5.65e−04
Log Metabolite Abundance
1
0
−1
Subject
10000
11000
12000
13000
14000
15000
16000
17000
19000
21000
23000
64000
68000
70000
71000
72000
73000
74000
75000
76000
77000
78000
79000
80000
81000
82000
83000
84000
86000
87000
89000
93000
97000
Condition
EM
Primary

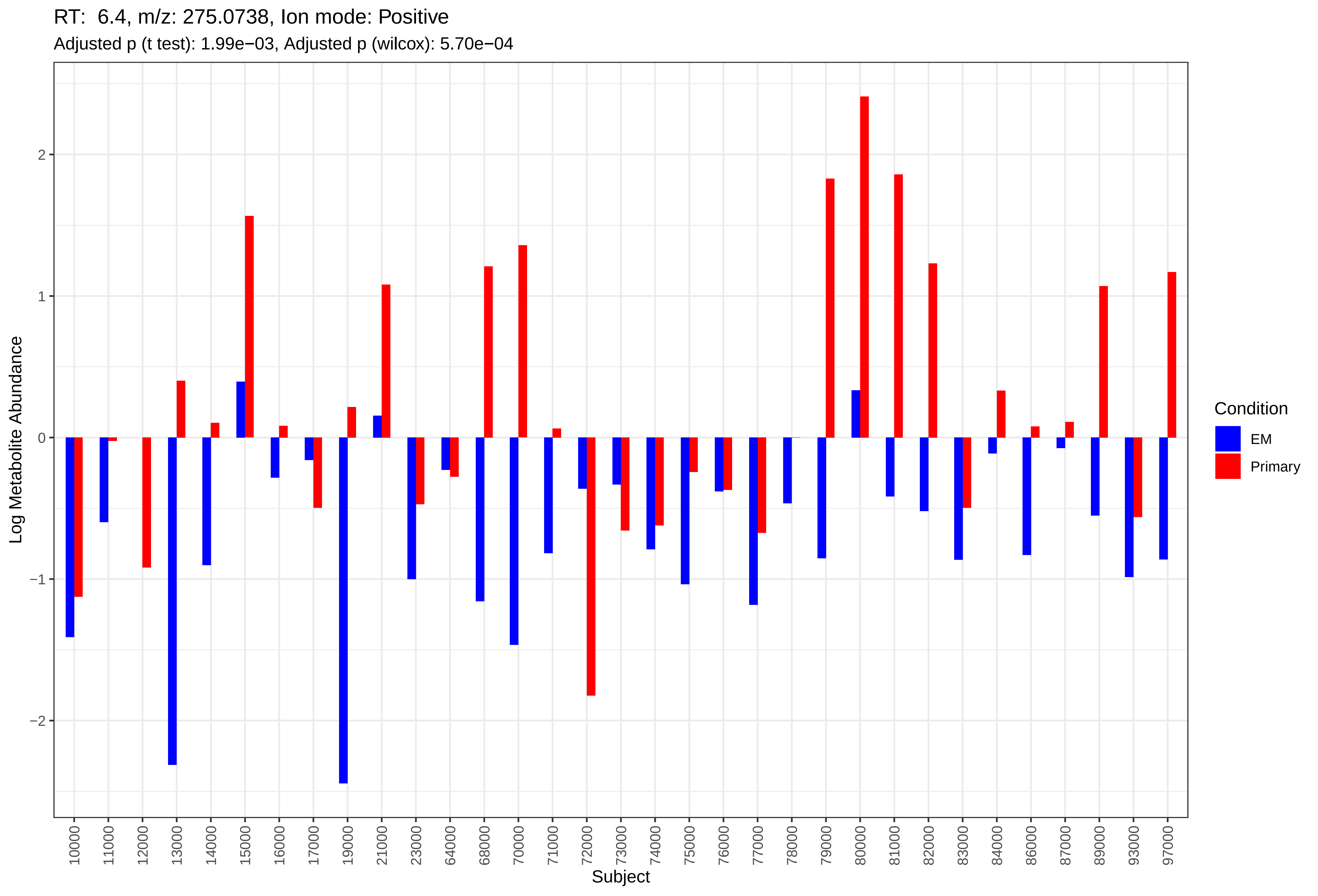
RT: 6.4, m/z: 275.0738, Ion mode: Positive
Adjusted p (t test): 1.99e−03, Adjusted p (wilcox): 5.70e−04
Log Metabolite Abundance
2
1
0
−1
−2
Subject
10000
11000
12000
13000
14000
15000
16000
17000
19000
21000
23000
64000
68000
70000
71000
72000
73000
74000
75000
76000
77000
78000
79000
80000
81000
82000
83000
84000
86000
87000
89000
93000
97000
Condition
EM
Primary

Complex of 86.9927 and 71.0292 m/z (RT: 0.6, m/z: 157.0146, Ion mode: Positive)

Adjusted p (t test): 5.80e−04, Adjusted p (wilcox): 6.27e−04

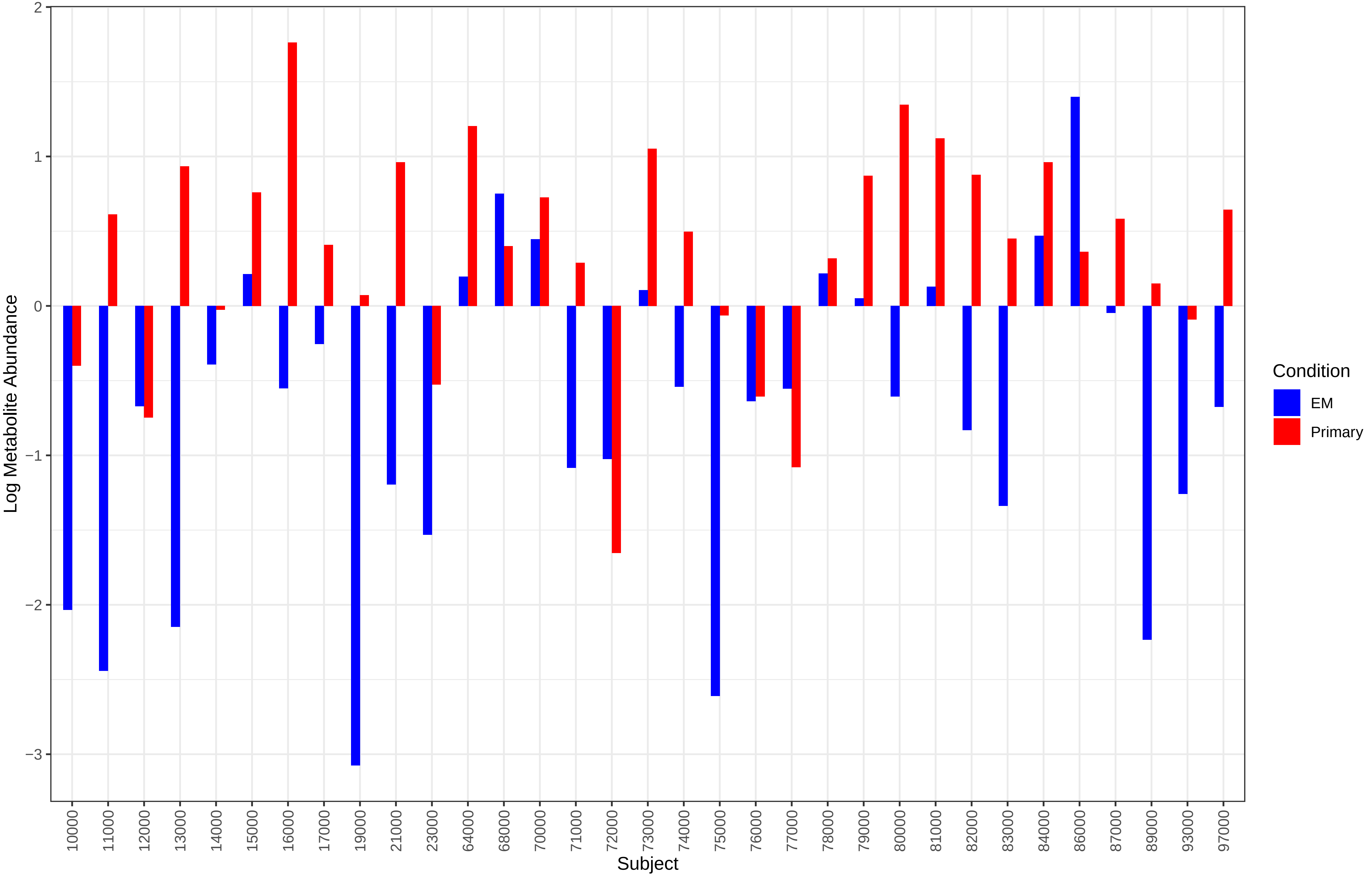

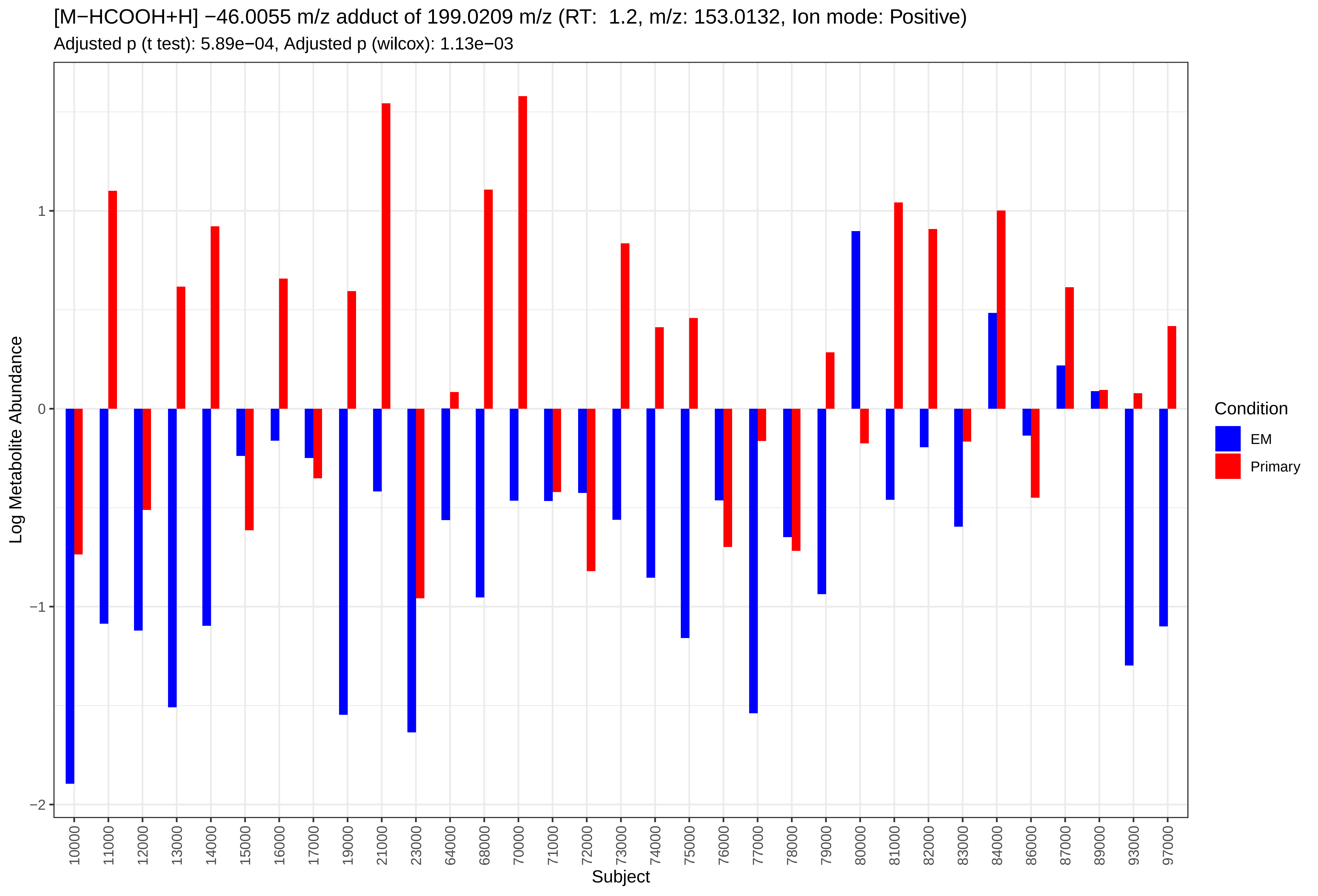

[M−HCOOH+H] −46.0055 m/z adduct of 199.0209 m/z (RT: 1.2, m/z: 153.0132, Ion mode: Positive)
Adjusted p (t test): 5.89e−04, Adjusted p (wilcox): 1.13e−03
Log Metabolite Abundance
1
0
−1
−2
Subject
10000
11000
12000
13000
14000
15000
16000
17000
19000
21000
23000
64000
68000
70000
71000
72000
73000
74000
75000
76000
77000
78000
79000
80000
81000
82000
83000
84000
86000
87000
89000
93000
97000
Condition
EM
Primary

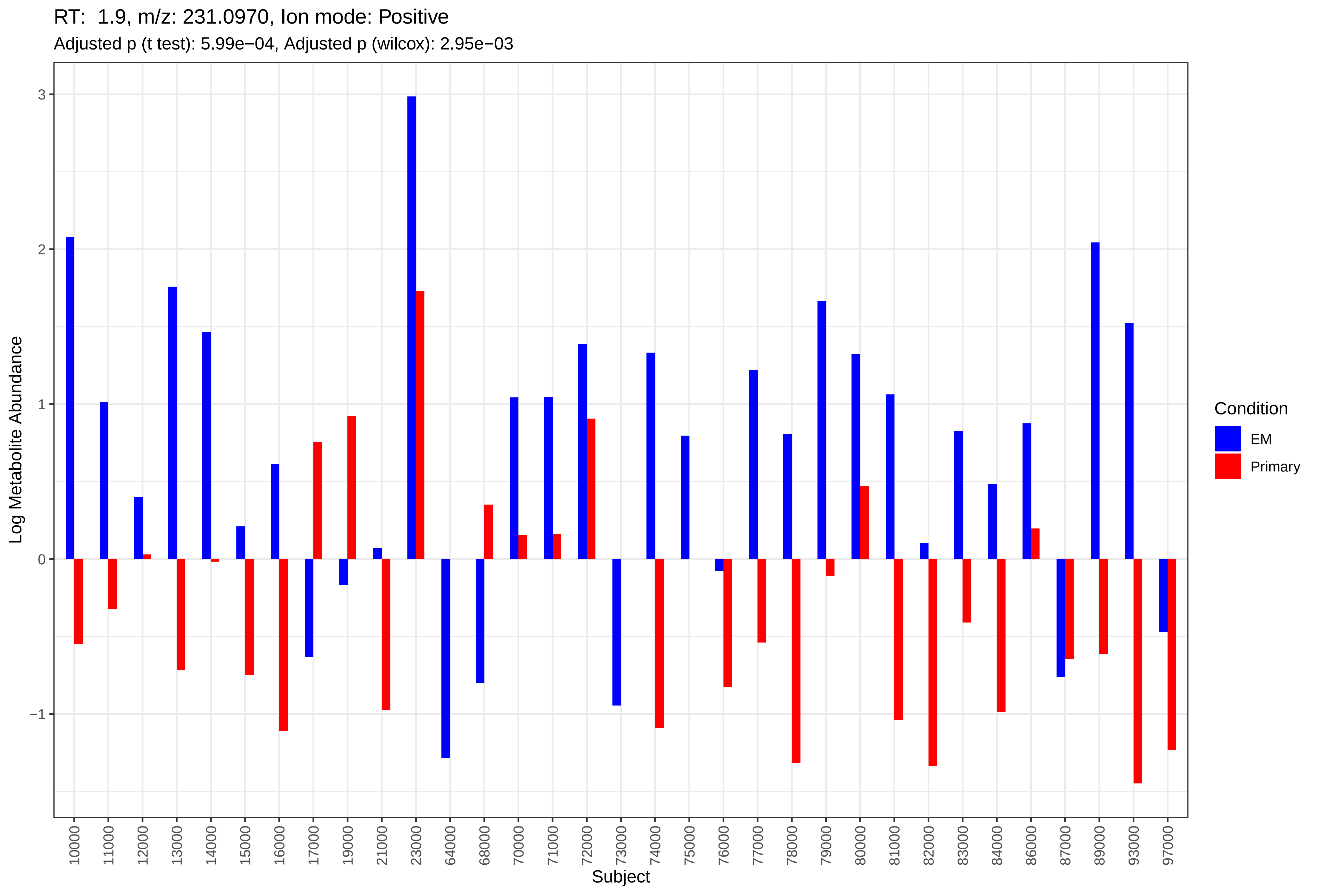

RT: 1.9, m/z: 231.0970, Ion mode: Positive
Adjusted p (t test): 5.99e−04, Adjusted p (wilcox): 2.95e−03
Log Metabolite Abundance
3
2
1
0
−1
Subject
10000
11000
12000
13000
14000
15000
16000
17000
19000
21000
23000
64000
68000
70000
71000
72000
73000
74000
75000
76000
77000
78000
79000
80000
81000
82000
83000
84000
86000
87000
89000
93000
97000
Condition
EM
Primary

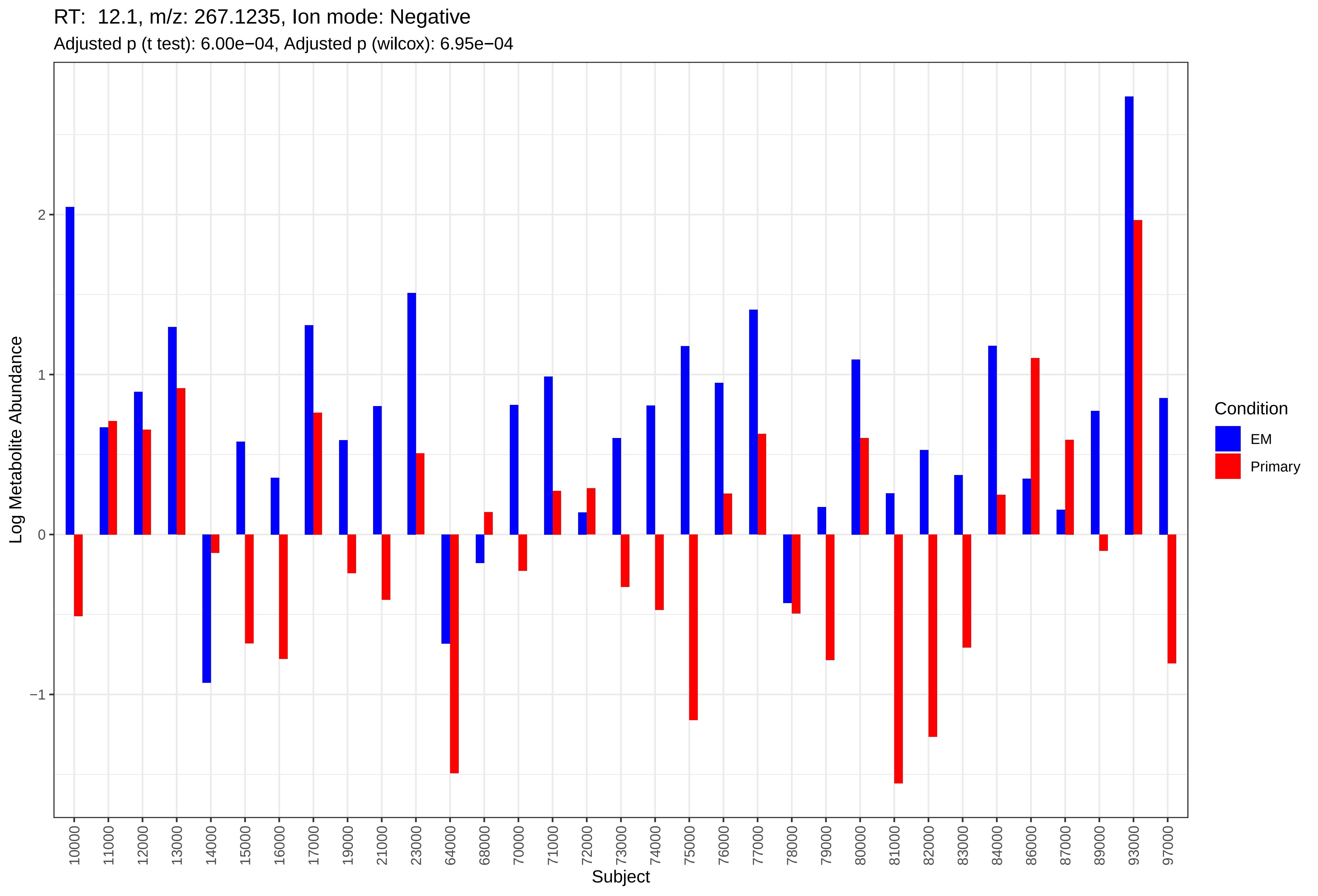
RT: 12.1, m/z: 267.1235, Ion mode: Negative
Adjusted p (t test): 6.00e−04, Adjusted p (wilcox): 6.95e−04
Log Metabolite Abundance
2
1
0
−1
10000
11000
12000
13000
14000
15000
16000
17000
19000
21000
23000
64000
68000
70000
71000
72000
73000
74000
75000
76000
77000
78000
79000
80000
81000
82000
83000
84000
86000
87000
89000
93000
97000
Subject
Condition
EM
Primary

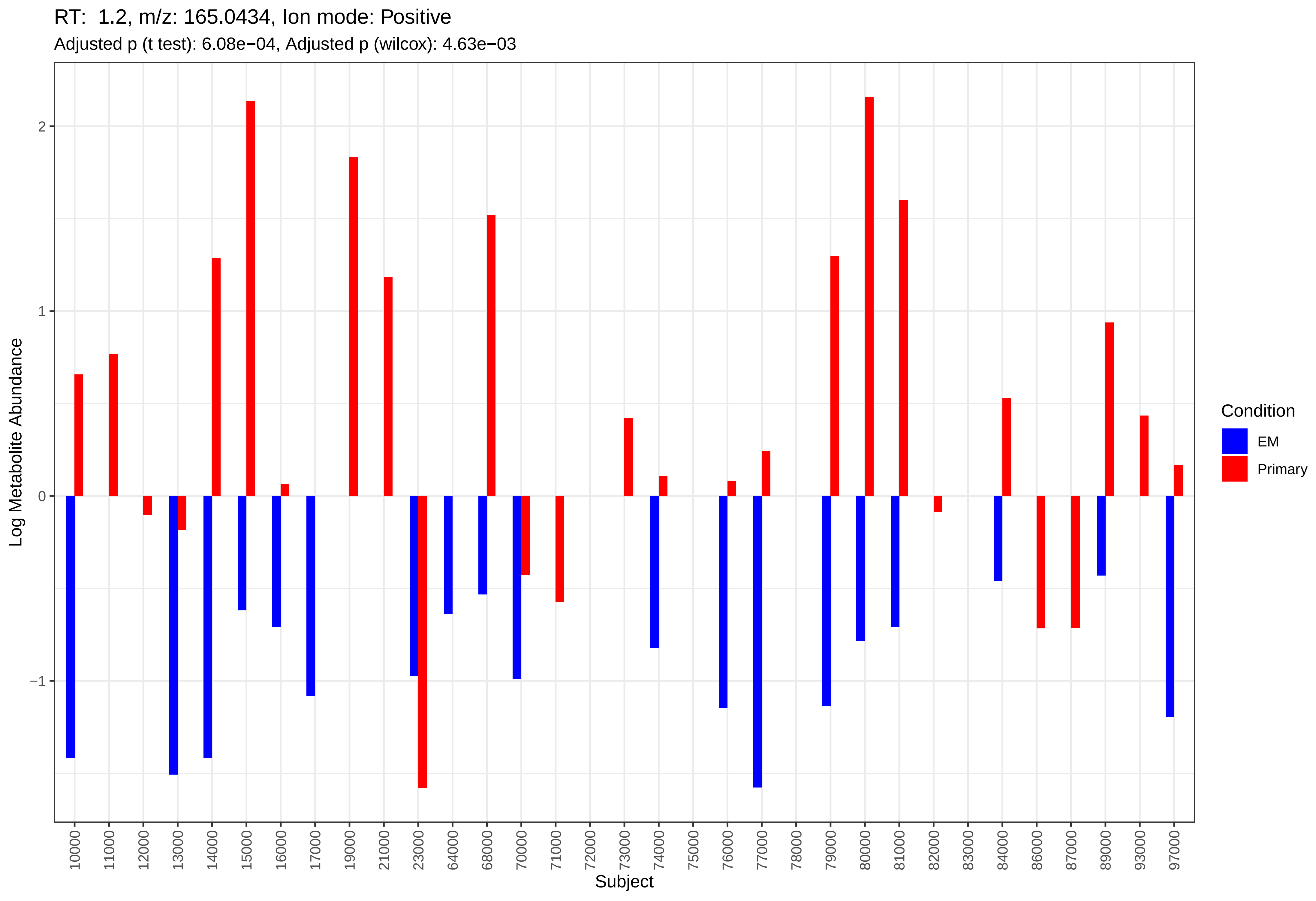

RT: 1.2, m/z: 165.0434, Ion mode: Positive
Adjusted p (t test): 6.08e−04, Adjusted p (wilcox): 4.63e−03
Log Metabolite Abundance
2
1
0
−1
10000
11000
12000
13000
14000
15000
16000
17000
19000
21000
23000
64000
68000
70000
71000
72000
73000
74000
75000
76000
77000
78000
79000
80000
81000
82000
83000
84000
86000
87000
89000
93000
97000
Subject
Condition
EM
Primary

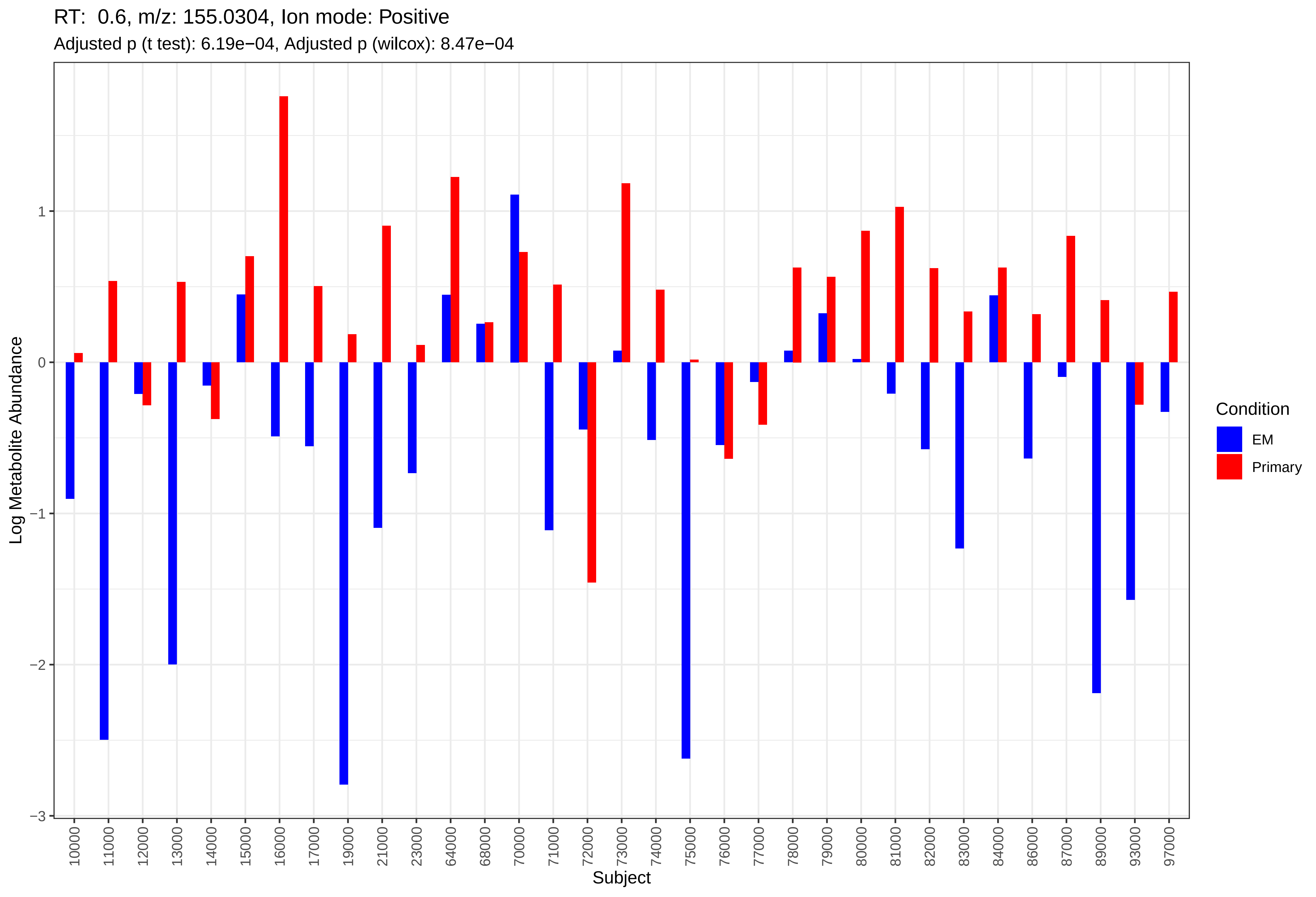

RT: 0.6, m/z: 155.0304, Ion mode: Positive
Adjusted p (t test): 6.19e−04, Adjusted p (wilcox): 8.47e−04
Log Metabolite Abundance
1
0
−1
−2
−3
10000
11000
12000
13000
14000
15000
16000
17000
19000
21000
23000
64000
68000
70000
71000
72000
73000
74000
75000
76000
77000
78000
79000
80000
81000
82000
83000
84000
86000
87000
89000
93000
97000
Subject
Condition
EM
Primary

RT: 1.6, m/z: 285.2236, Ion mode: Positive

Adjusted p (t test): 2.68e−03, Adjusted p (wilcox): 6.27e−04

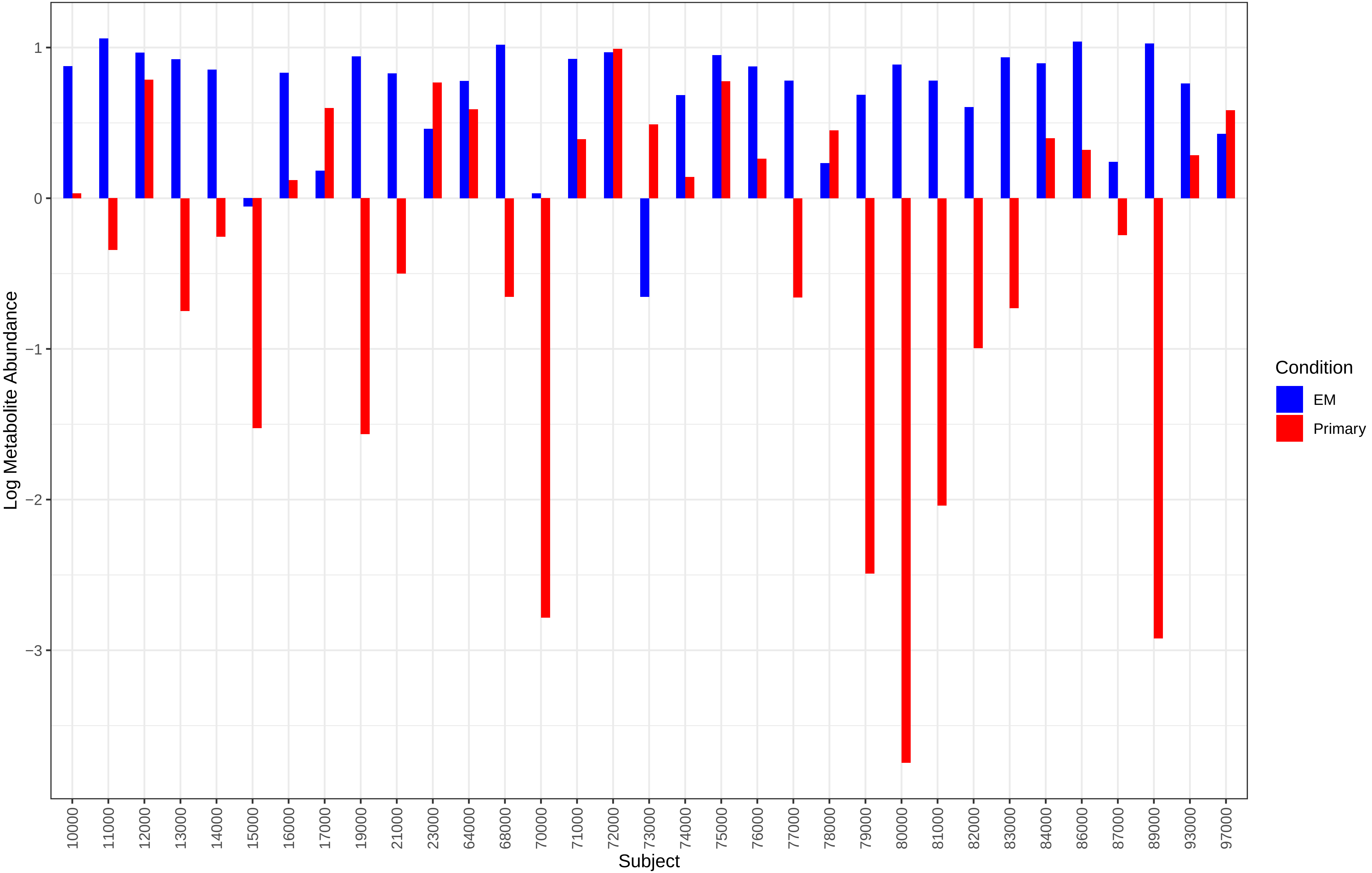

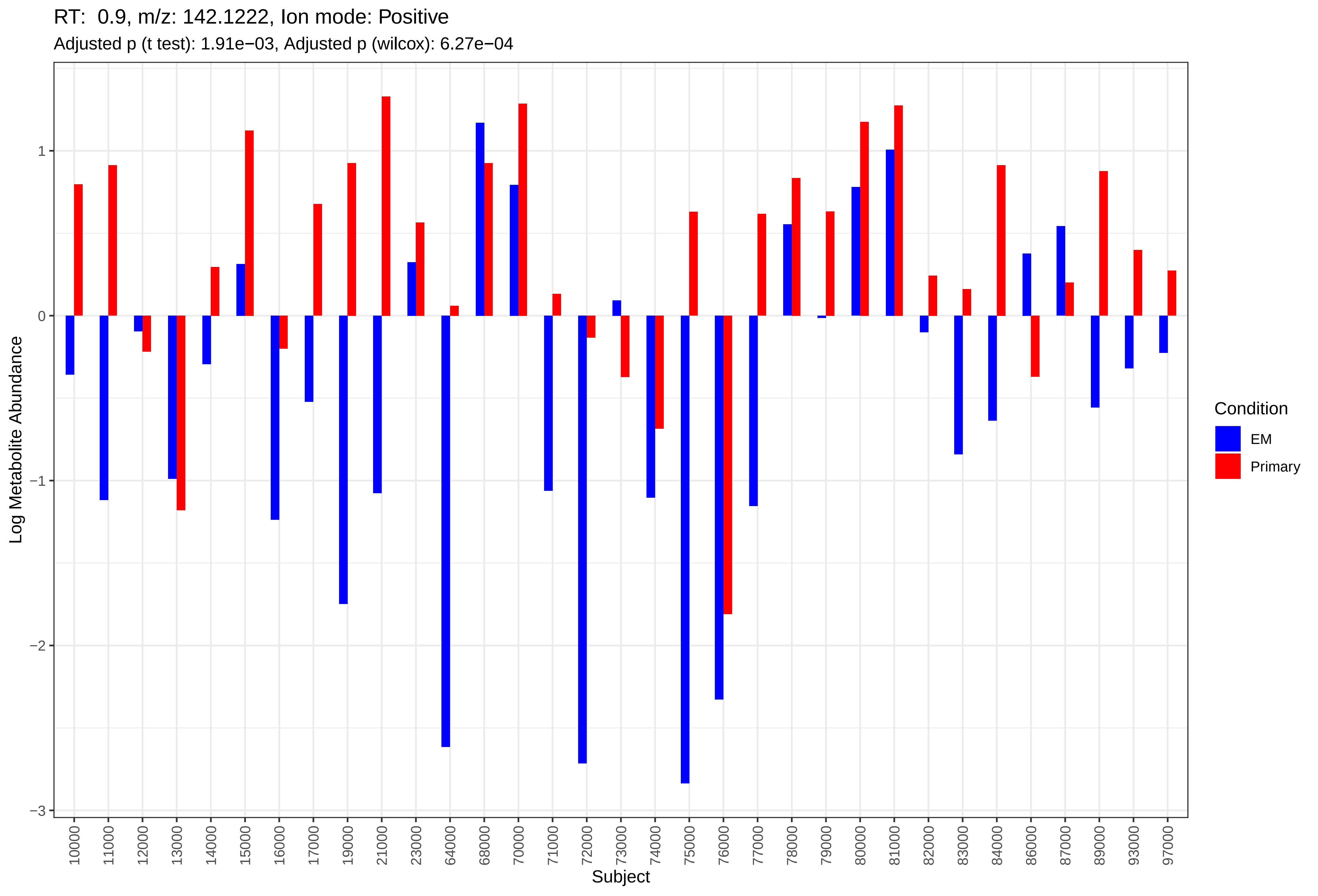
RT: 0.9, m/z: 142.1222, Ion mode: Positive
Adjusted p (t test): 1.91e−03, Adjusted p (wilcox): 6.27e−04
Log Metabolite Abundance
1
0
−1
−2
−3
Subject
10000
11000
12000
13000
14000
15000
16000
17000
19000
21000
23000
64000
68000
70000
71000
72000
73000
74000
75000
76000
77000
78000
79000
80000
81000
82000
83000
84000
86000
87000
89000
93000
97000
Condition
EM
Primary

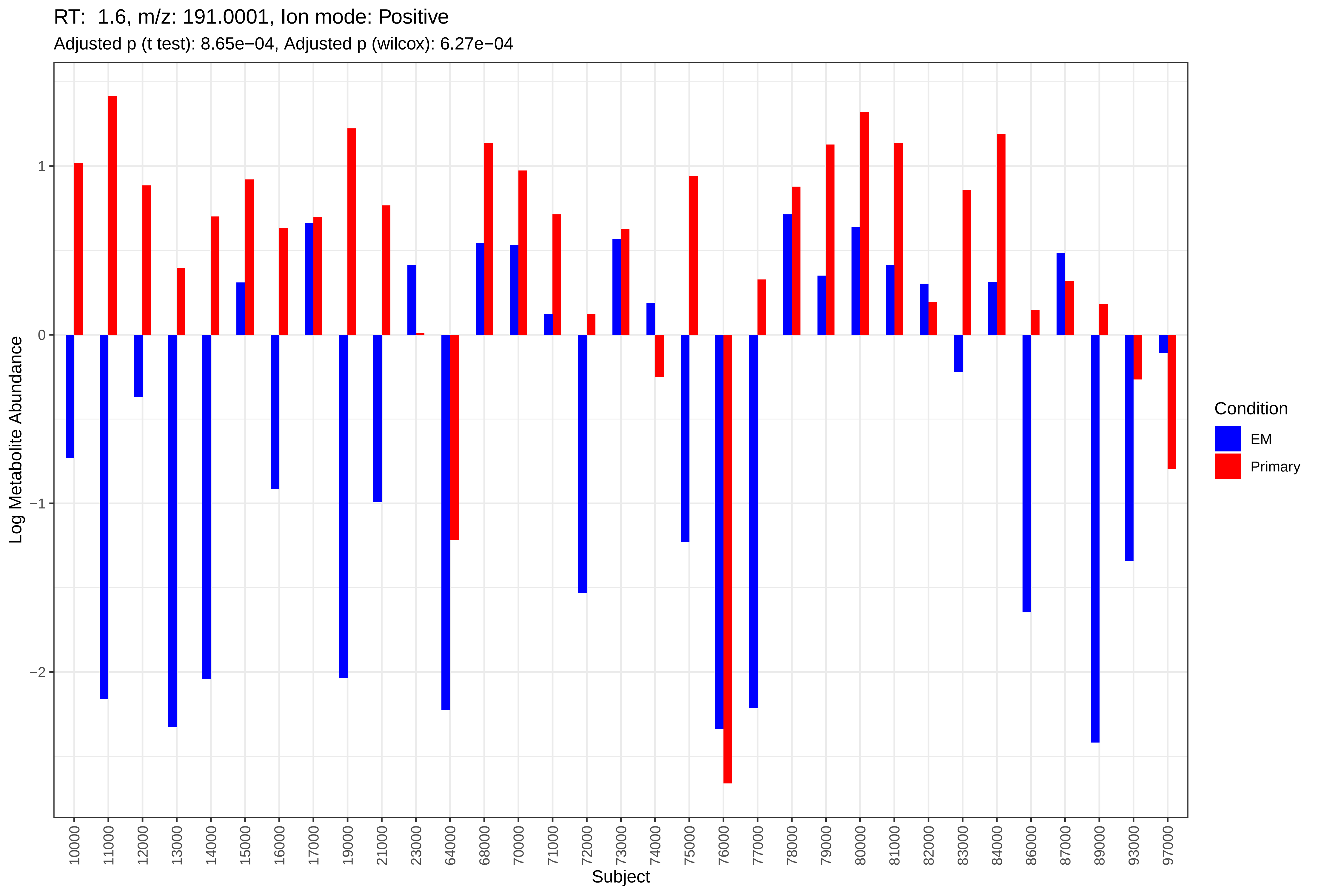

RT: 1.6, m/z: 191.0001, Ion mode: Positive
Adjusted p (t test): 8.65e−04, Adjusted p (wilcox): 6.27e−04
Log Metabolite Abundance
1
0
−1
−2
Subject
10000
11000
12000
13000
14000
15000
16000
17000
19000
21000
23000
64000
68000
70000
71000
72000
73000
74000
75000
76000
77000
78000
79000
80000
81000
82000
83000
84000
86000
87000
89000
93000
97000
Condition
EM
Primary

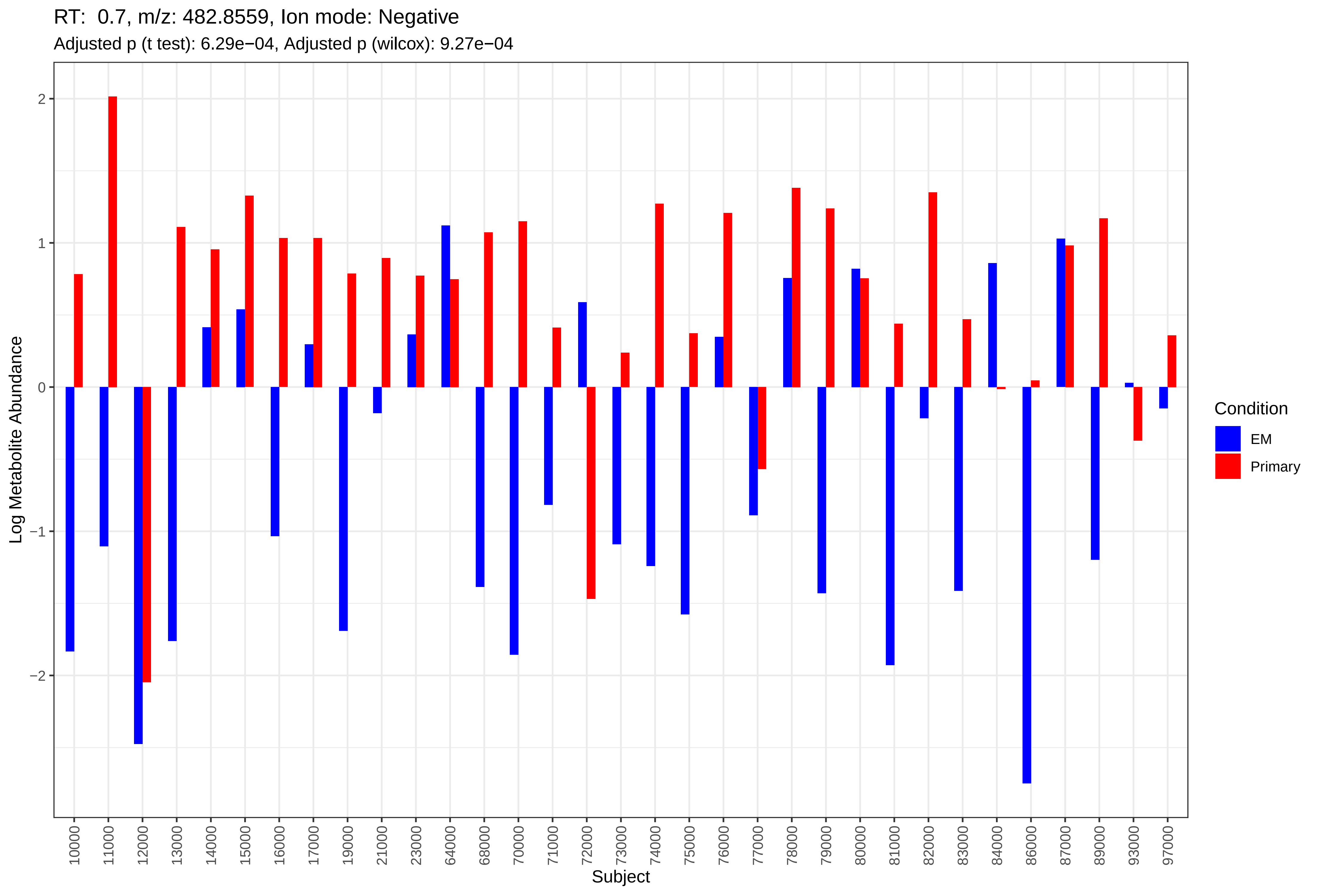
RT: 0.7, m/z: 482.8559, Ion mode: Negative
Adjusted p (t test): 6.29e−04, Adjusted p (wilcox): 9.27e−04
Log Metabolite Abundance
2
1
0
−1
−2
Subject
10000
11000
12000
13000
14000
15000
16000
17000
19000
21000
23000
64000
68000
70000
71000
72000
73000
74000
75000
76000
77000
78000
79000
80000
81000
82000
83000
84000
86000
87000
89000
93000
97000
Condition
EM
Primary

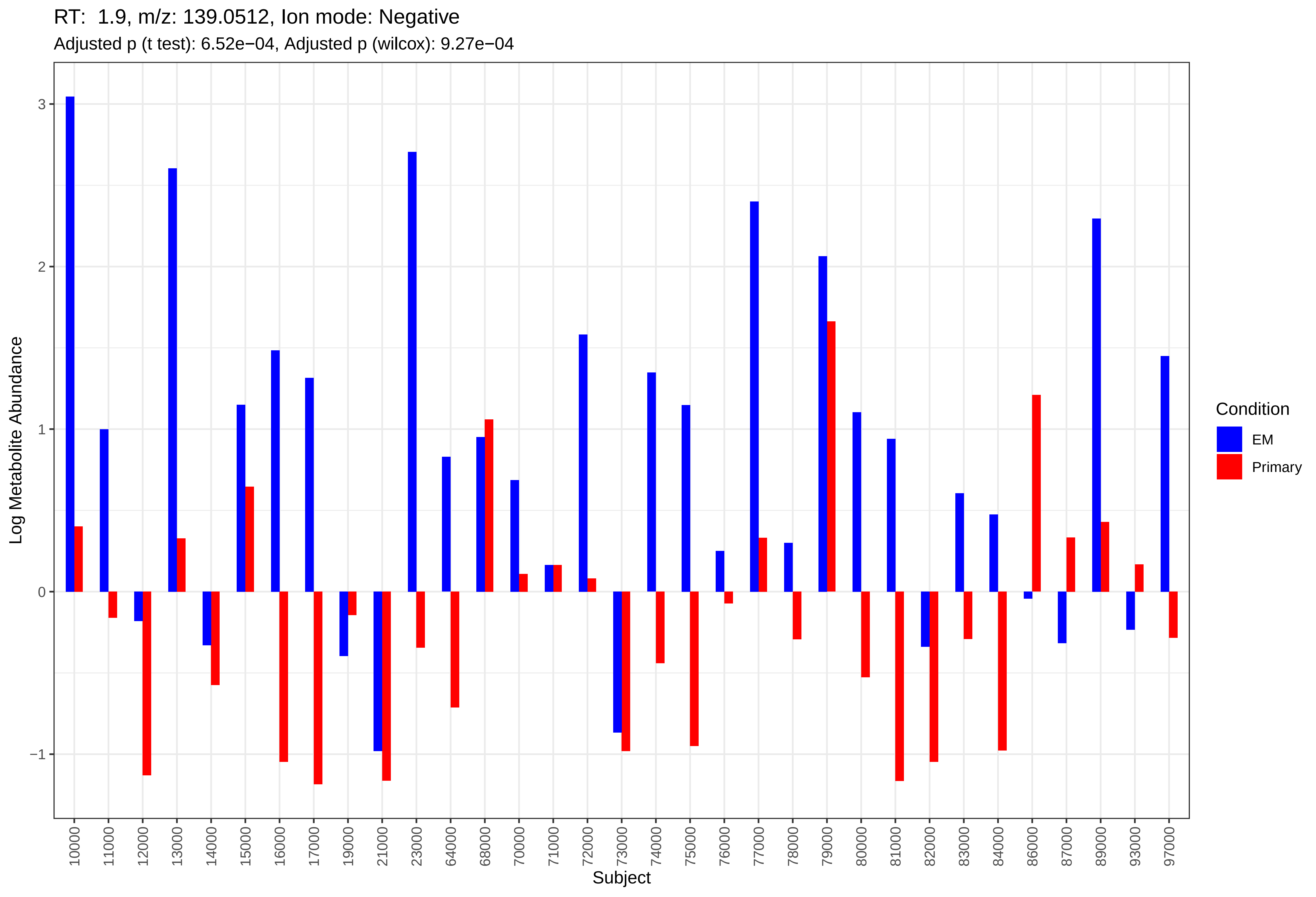
RT: 1.9, m/z: 139.0512, Ion mode: Negative
Adjusted p (t test): 6.52e−04, Adjusted p (wilcox): 9.27e−04
Log Metabolite Abundance
3
2
1
0
−1
10000
11000
12000
13000
14000
15000
16000
17000
19000
21000
23000
64000
68000
70000
71000
72000
73000
74000
75000
76000
77000
78000
79000
80000
81000
82000
83000
84000
86000
87000
89000
93000
97000
Subject
Condition
EM
Primary

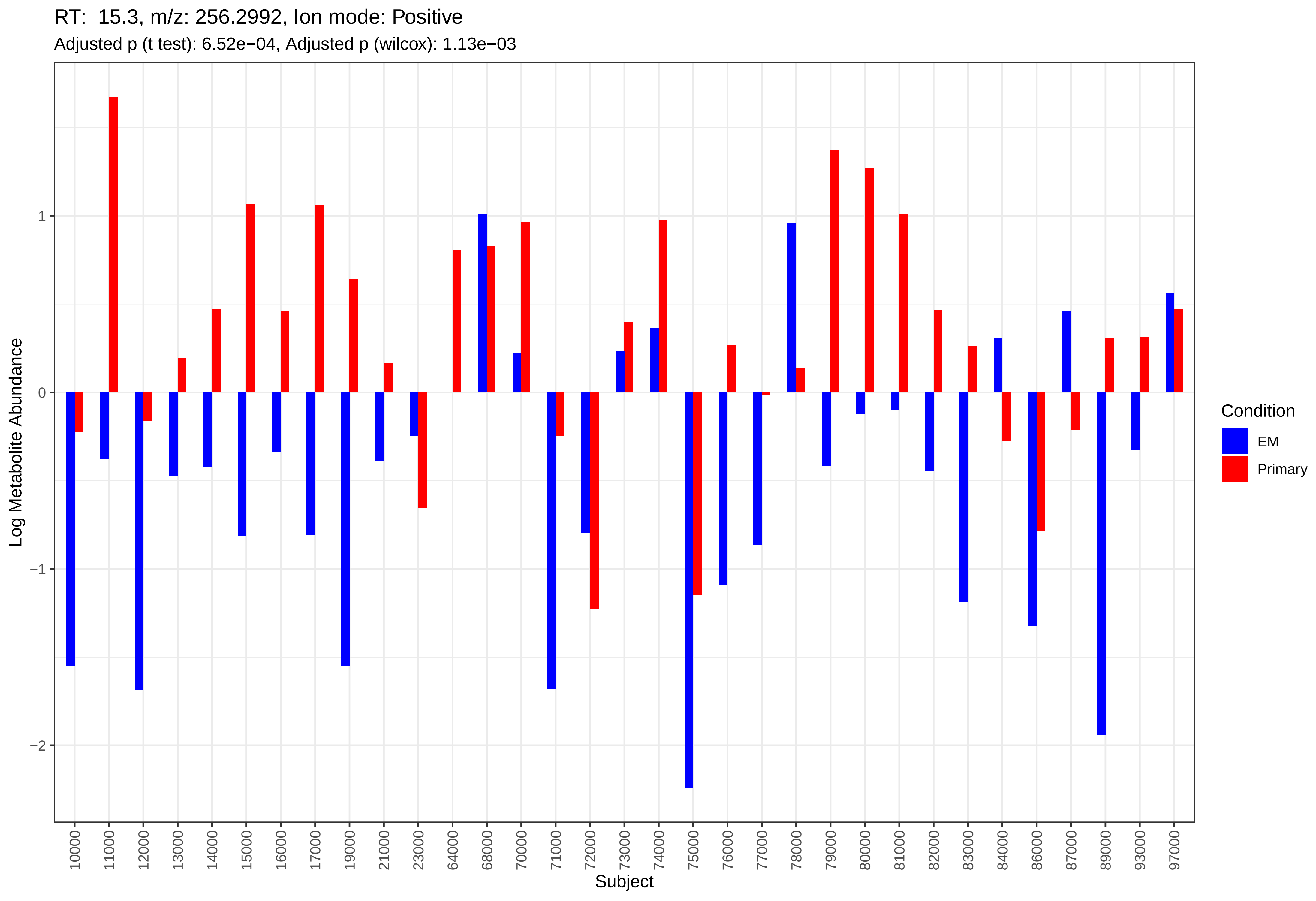
RT: 15.3, m/z: 256.2992, Ion mode: Positive
Adjusted p (t test): 6.52e−04, Adjusted p (wilcox): 1.13e−03
Log Metabolite Abundance
1
0
−1
−2
10000
11000
12000
13000
14000
15000
16000
17000
19000
21000
23000
64000
68000
70000
71000
72000
73000
74000
75000
76000
77000
78000
79000
80000
81000
82000
83000
84000
86000
87000
89000
93000
97000
Subject
Condition
EM
Primary

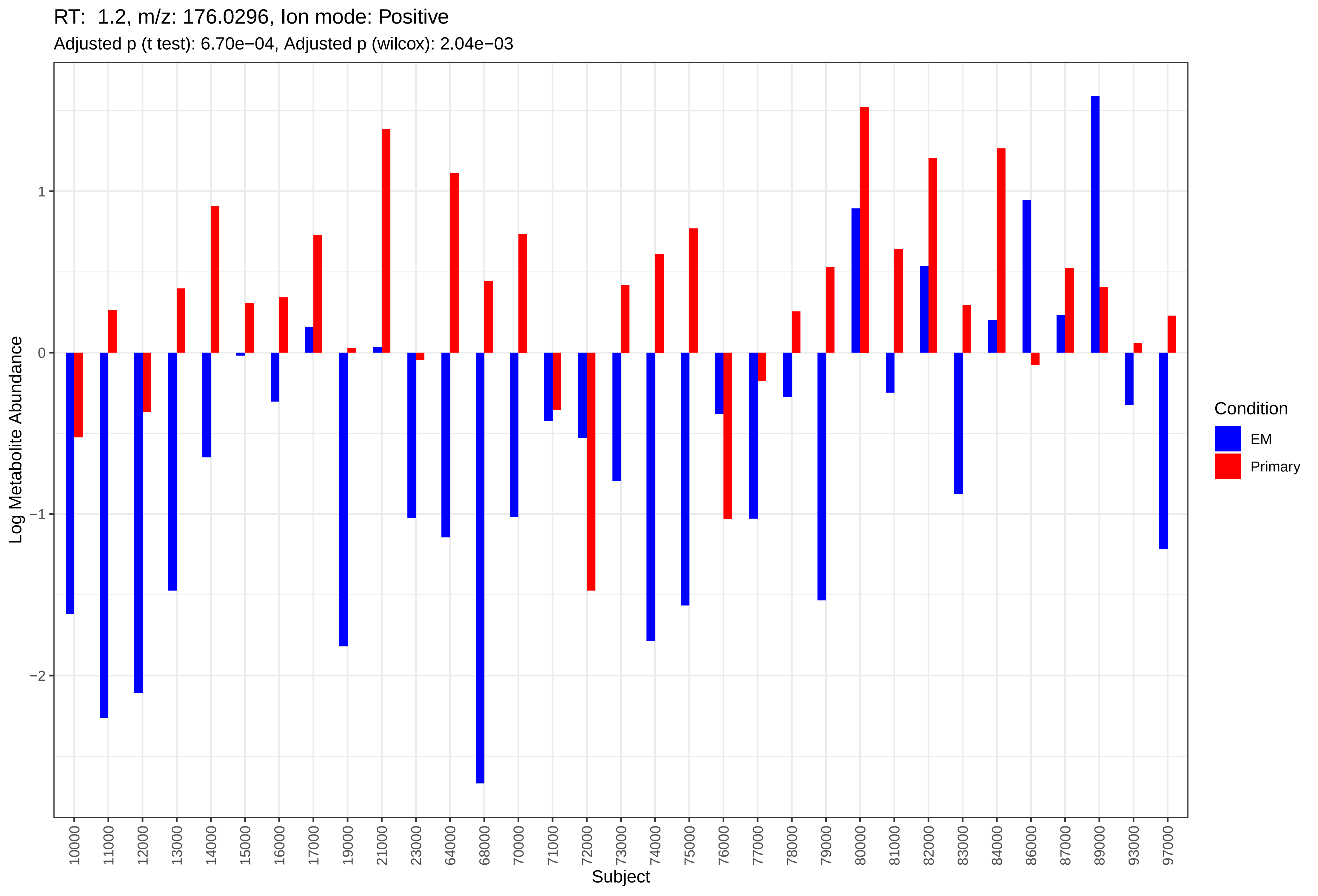
RT: 1.2, m/z: 176.0296, Ion mode: Positive
Adjusted p (t test): 6.70e−04, Adjusted p (wilcox): 2.04e−03
Log Metabolite Abundance
1
0
−1
−2
10000
11000
12000
13000
14000
15000
16000
17000
19000
21000
23000
64000
68000
70000
71000
72000
73000
74000
75000
76000
77000
78000
79000
80000
81000
82000
83000
84000
86000
87000
89000
93000
97000
Subject
Condition
EM
Primary

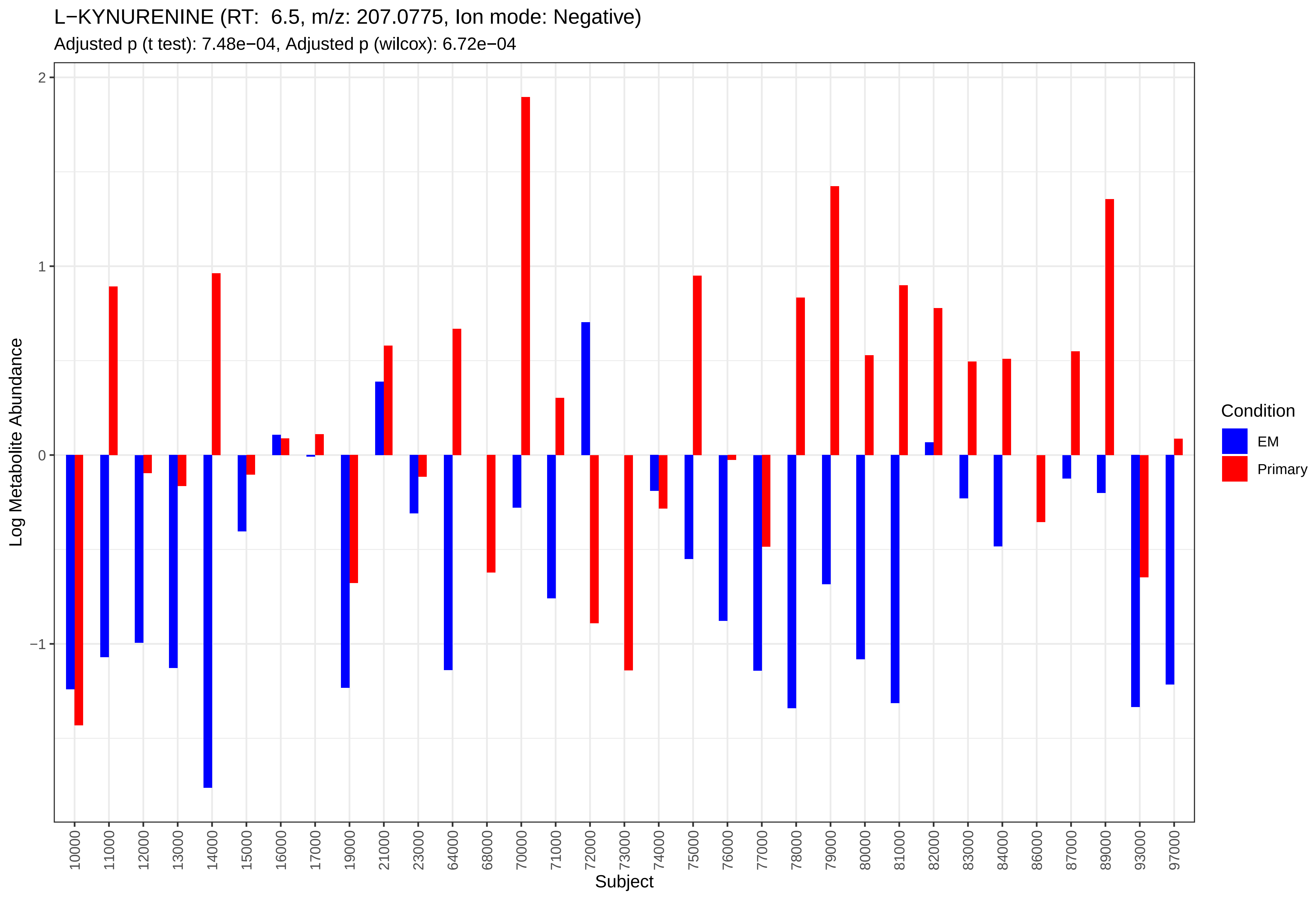
L−KYNURENINE (RT: 6.5, m/z: 207.0775, Ion mode: Negative)
Adjusted p (t test): 7.48e−04, Adjusted p (wilcox): 6.72e−04
Log Metabolite Abundance
2
1
0
−1
10000
11000
12000
13000
14000
15000
16000
17000
19000
21000
23000
64000
68000
70000
71000
72000
73000
74000
75000
76000
77000
78000
79000
80000
81000
82000
83000
84000
86000
87000
89000
93000
97000
Subject
Condition
EM
Primary

RT: 0.8, m/z: 127.9986, Ion mode: Negative

Adjusted p (t test): 9.99e−04, Adjusted p (wilcox): 6.95e−04

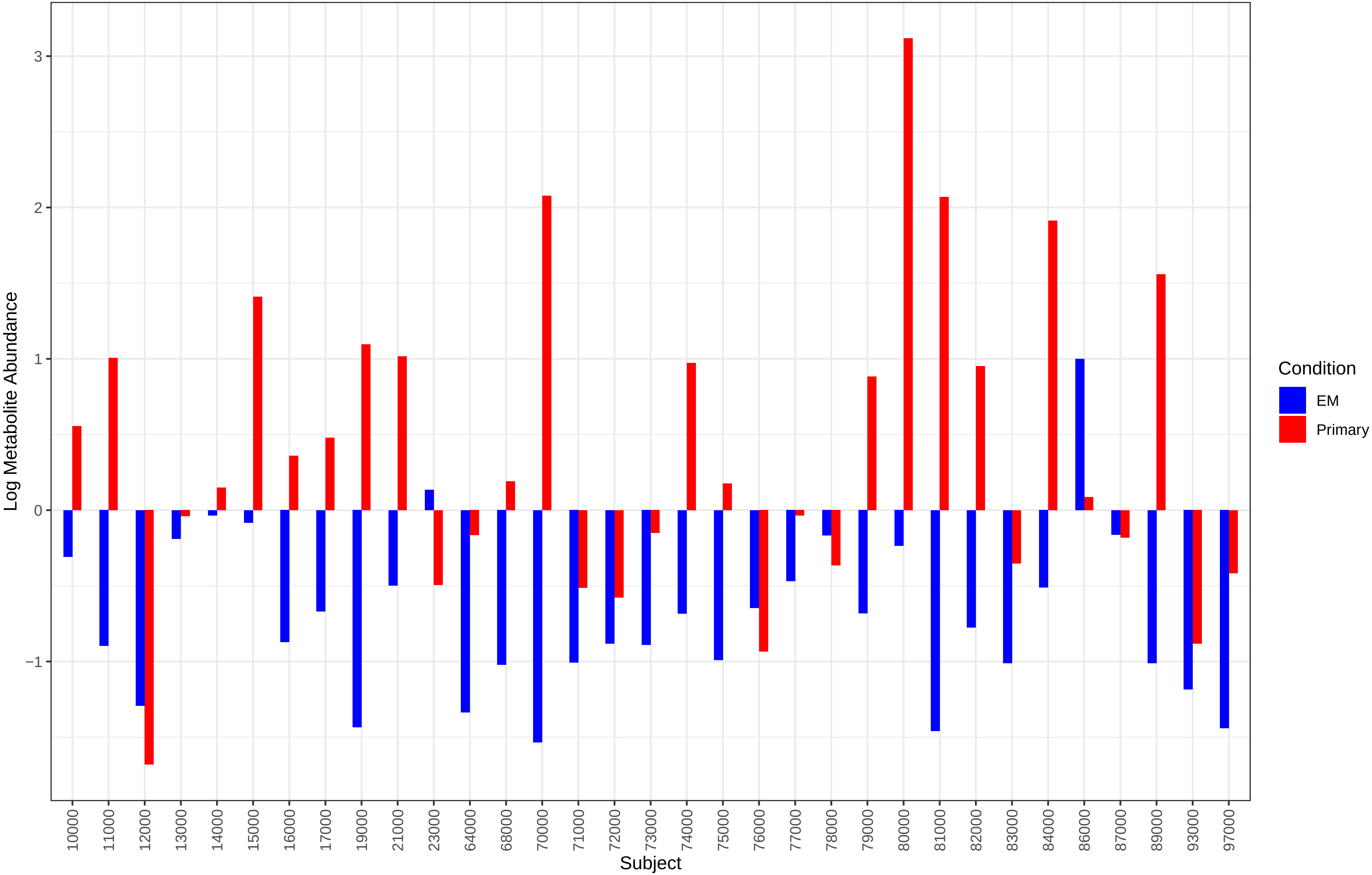

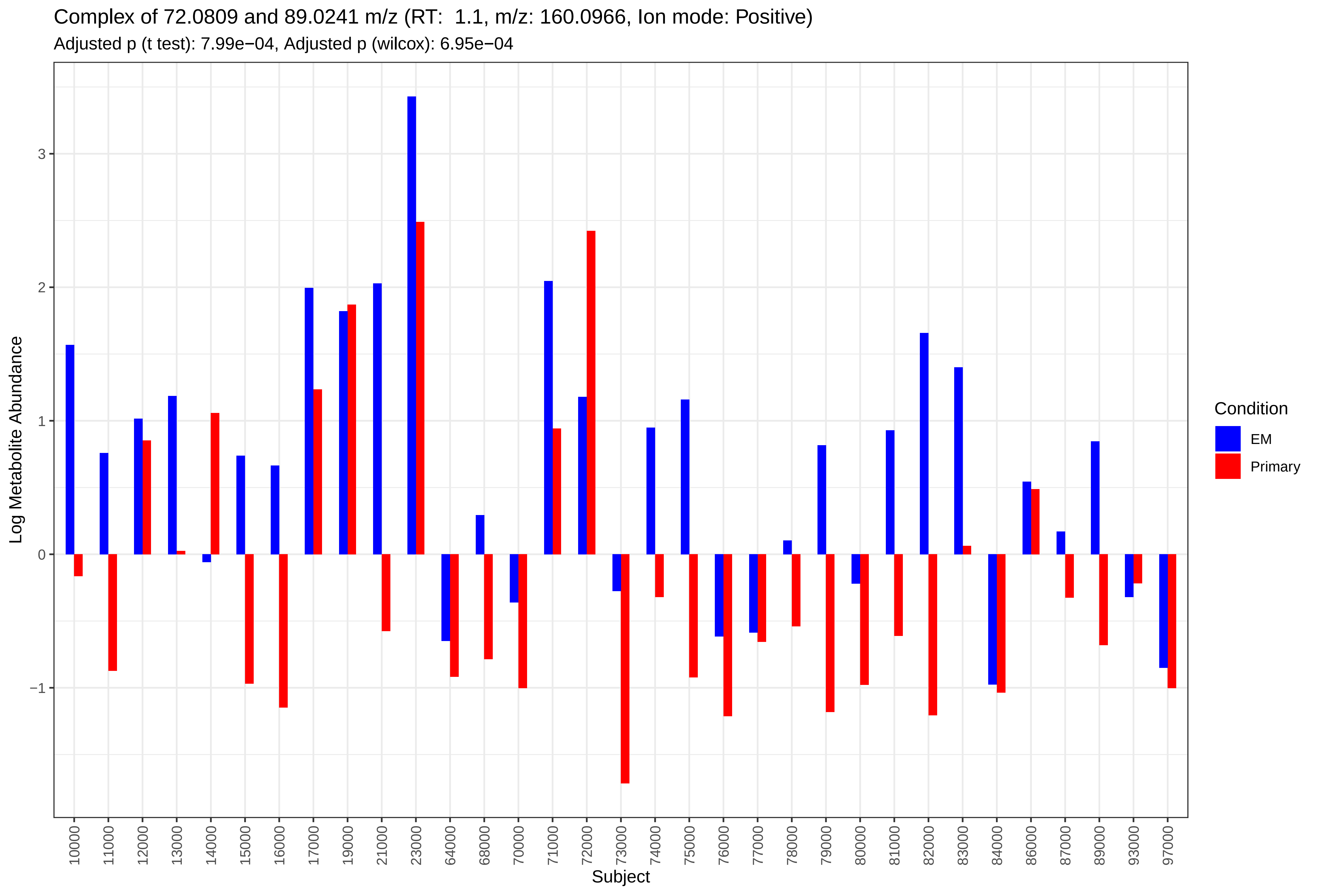
Complex of 72.0809 and 89.0241 m/z (RT: 1.1, m/z: 160.0966, Ion mode: Positive)
Adjusted p (t test): 7.99e−04, Adjusted p (wilcox): 6.95e−04
Log Metabolite Abundance
3
2
1
0
−1
Subject
10000
11000
12000
13000
14000
15000
16000
17000
19000
21000
23000
64000
68000
70000
71000
72000
73000
74000
75000
76000
77000
78000
79000
80000
81000
82000
83000
84000
86000
87000
89000
93000
97000
Condition
EM
Primary

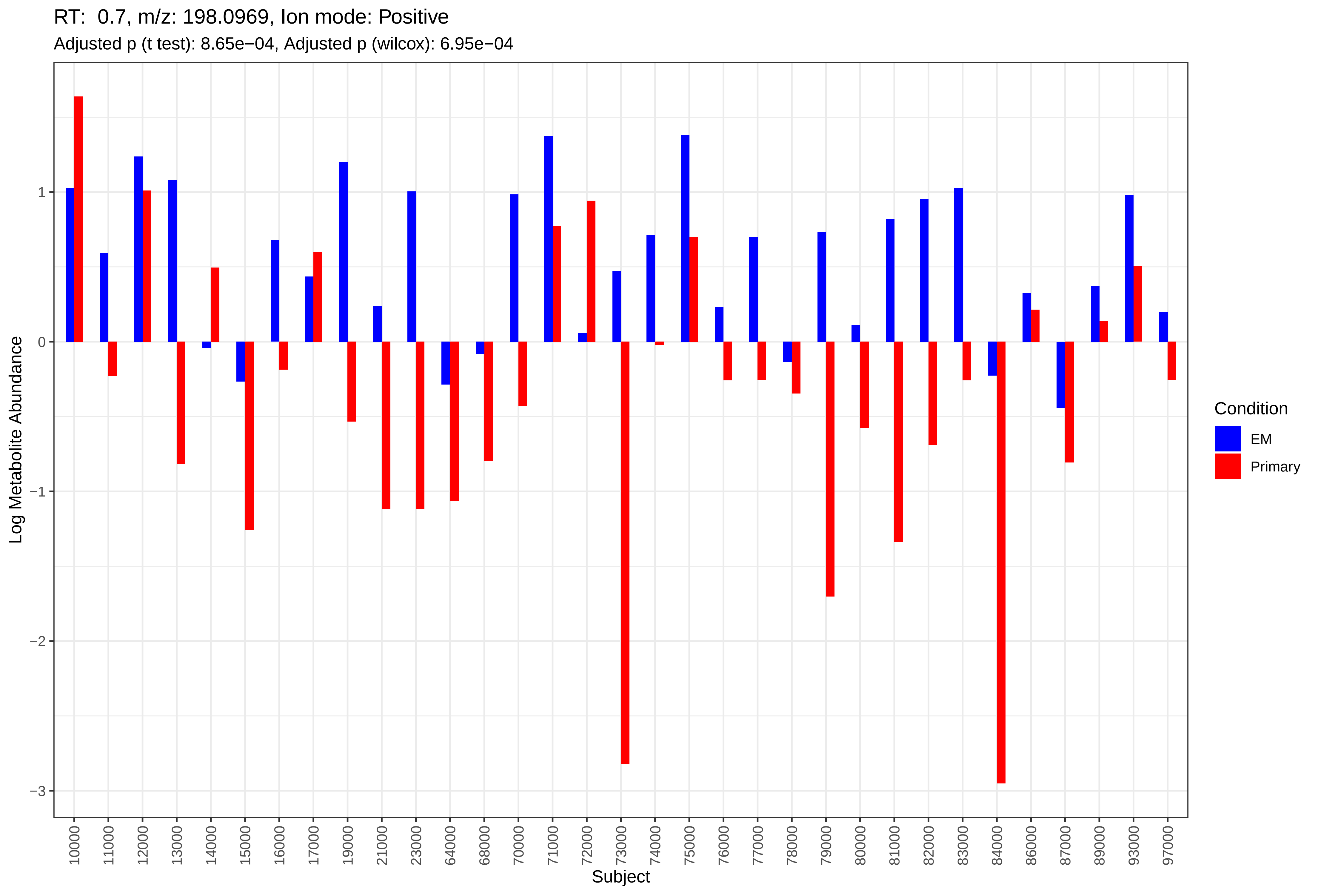
RT: 0.7, m/z: 198.0969, Ion mode: Positive
Adjusted p (t test): 8.65e−04, Adjusted p (wilcox): 6.95e−04
Log Metabolite Abundance
1
0
−1
−2
−3
Subject
10000
11000
12000
13000
14000
15000
16000
17000
19000
21000
23000
64000
68000
70000
71000
72000
73000
74000
75000
76000
77000
78000
79000
80000
81000
82000
83000
84000
86000
87000
89000
93000
97000
Condition
EM
Primary

RT: 0.7, m/z: 260.0676, Ion mode: Positive
Adjusted p (t test): 7.00e−04, Adjusted p (wilcox): 1.89e−03

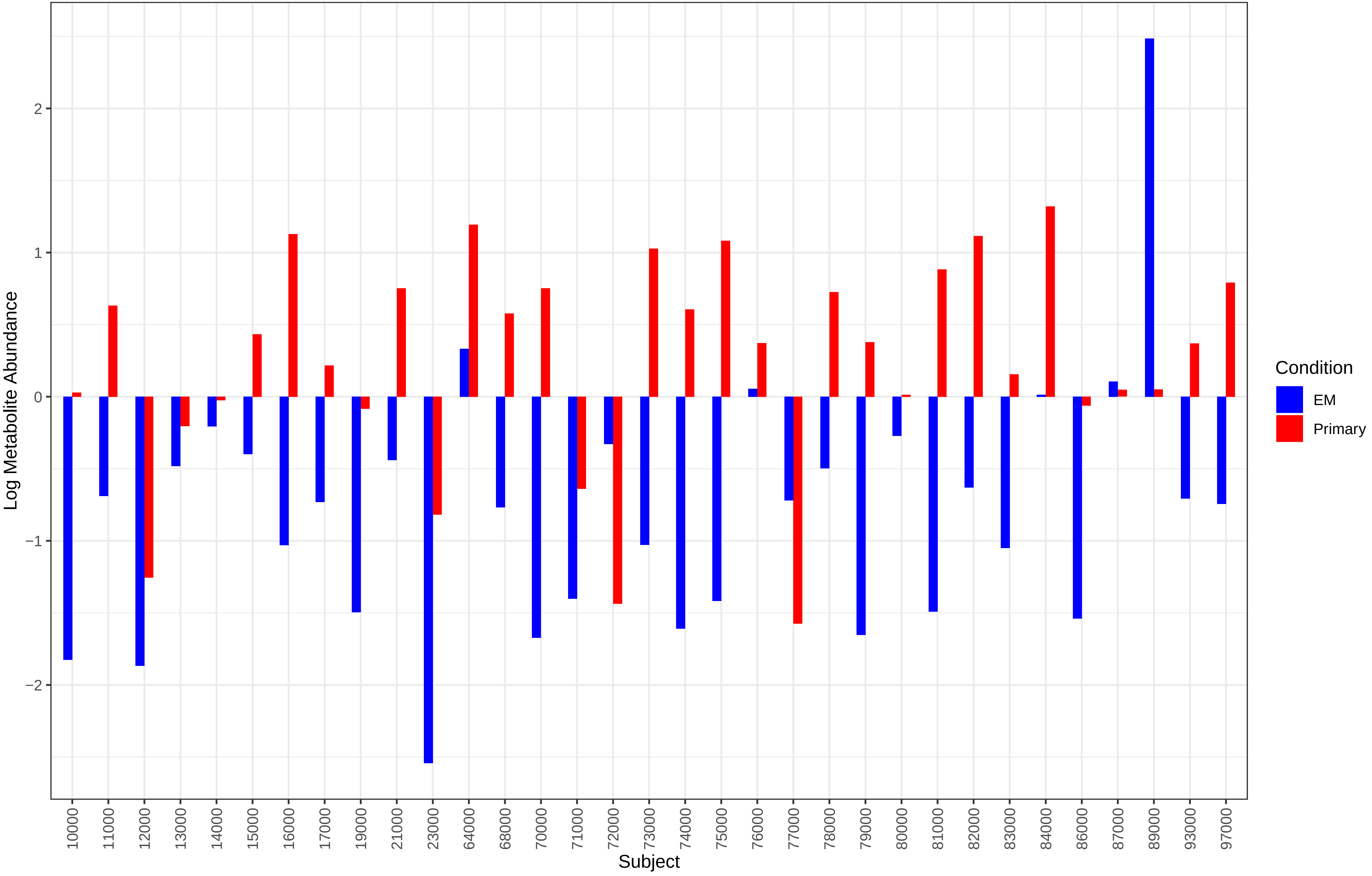

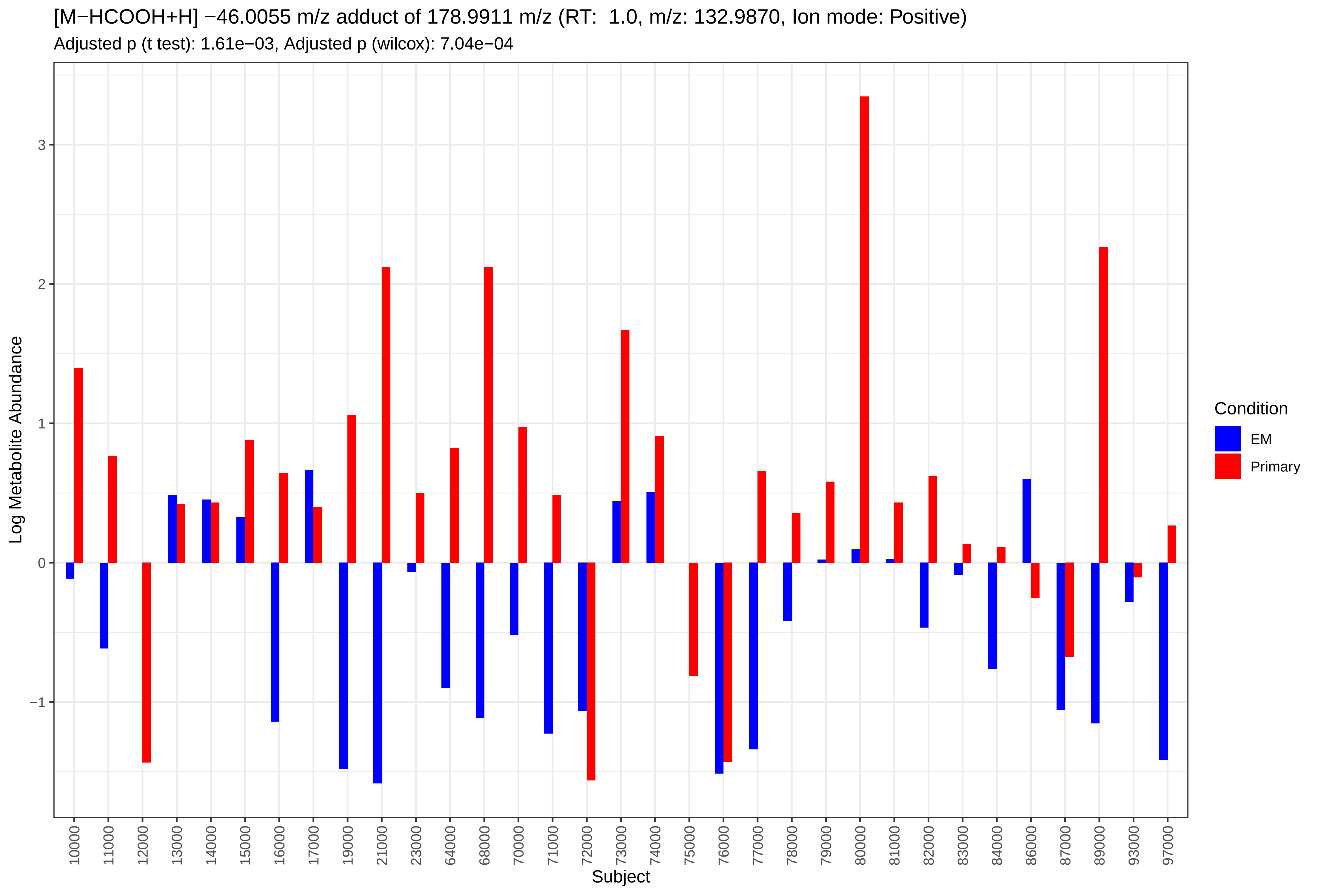

[M−HCOOH+H] −46.0055 m/z adduct of 178.9911 m/z (RT: 1.0, m/z: 132.9870, Ion mode: Positive)
Adjusted p (t test): 1.61e−03, Adjusted p (wilcox): 7.04e−04
Log Metabolite Abundance
3
2
1
0
−1
10000
11000
12000
13000
14000
15000
16000
17000
19000
21000
23000
64000
68000
70000
71000
72000
73000
74000
75000
76000
77000
78000
79000
80000
81000
82000
83000
84000
86000
87000
89000
93000
97000
Subject
Condition
EM
Primary

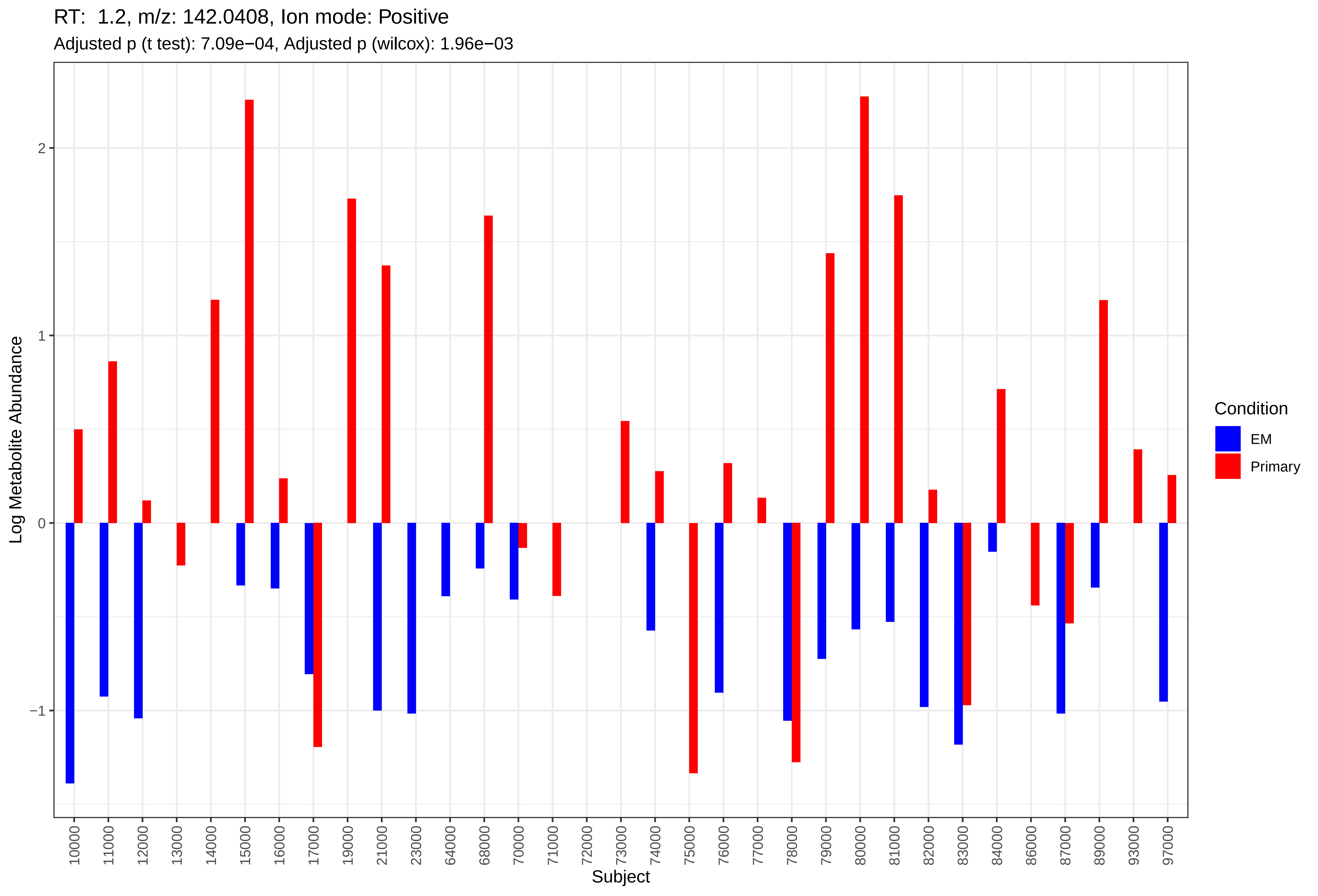
RT: 1.2, m/z: 142.0408, Ion mode: Positive
Adjusted p (t test): 7.09e−04, Adjusted p (wilcox): 1.96e−03
Log Metabolite Abundance
2
1
0
−1
10000
11000
12000
13000
14000
15000
16000
17000
19000
21000
23000
64000
68000
70000
71000
72000
73000
74000
75000
76000
77000
78000
79000
80000
81000
82000
83000
84000
86000
87000
89000
93000
97000
Subject
Condition
EM
Primary

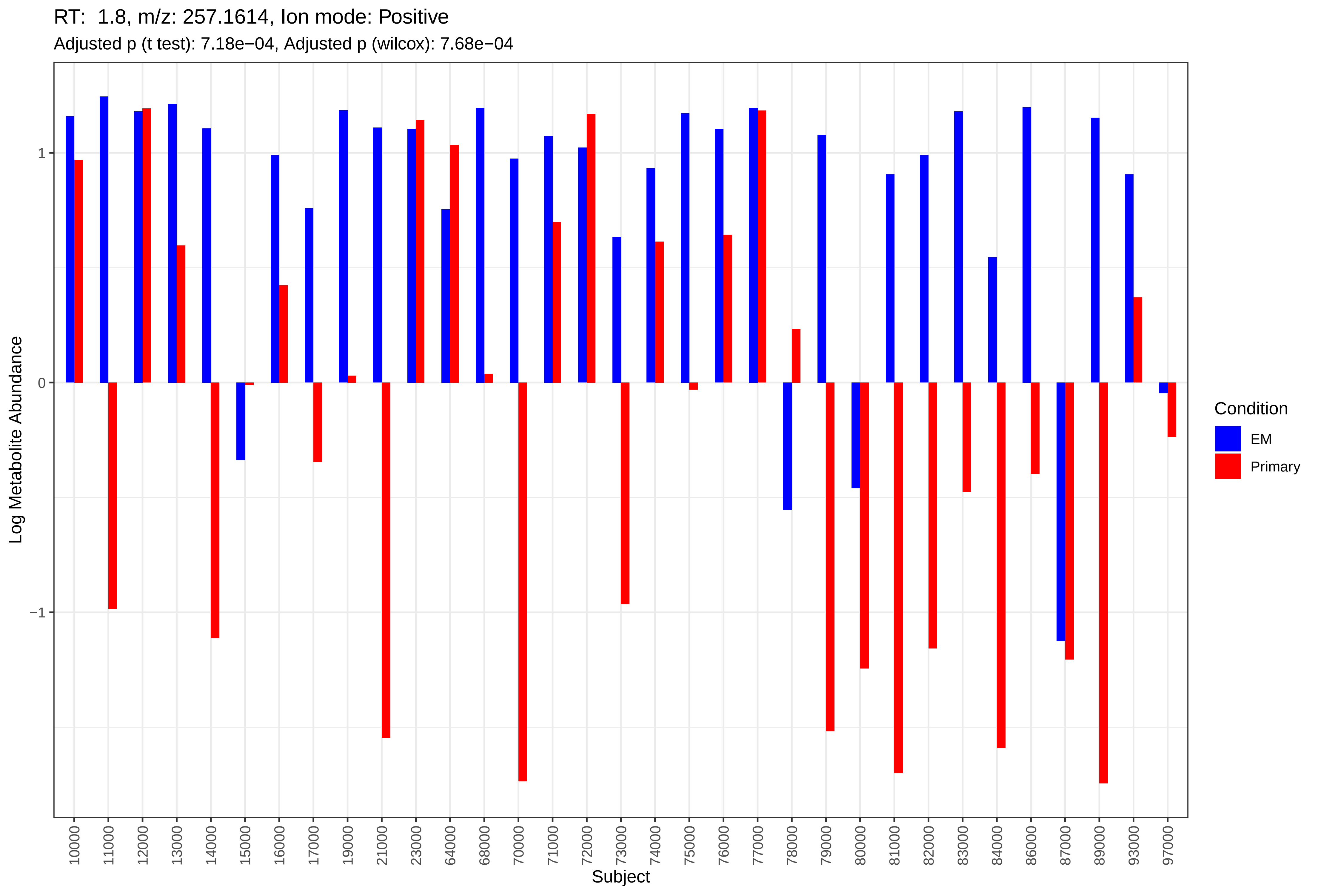

RT: 1.8, m/z: 257.1614, Ion mode: Positive
Adjusted p (t test): 7.18e−04, Adjusted p (wilcox): 7.68e−04
Log Metabolite Abundance
1
0
−1
Subject
10000
11000
12000
13000
14000
15000
16000
17000
19000
21000
23000
64000
68000
70000
71000
72000
73000
74000
75000
76000
77000
78000
79000
80000
81000
82000
83000
84000
86000
87000
89000
93000
97000
Condition
EM
Primary

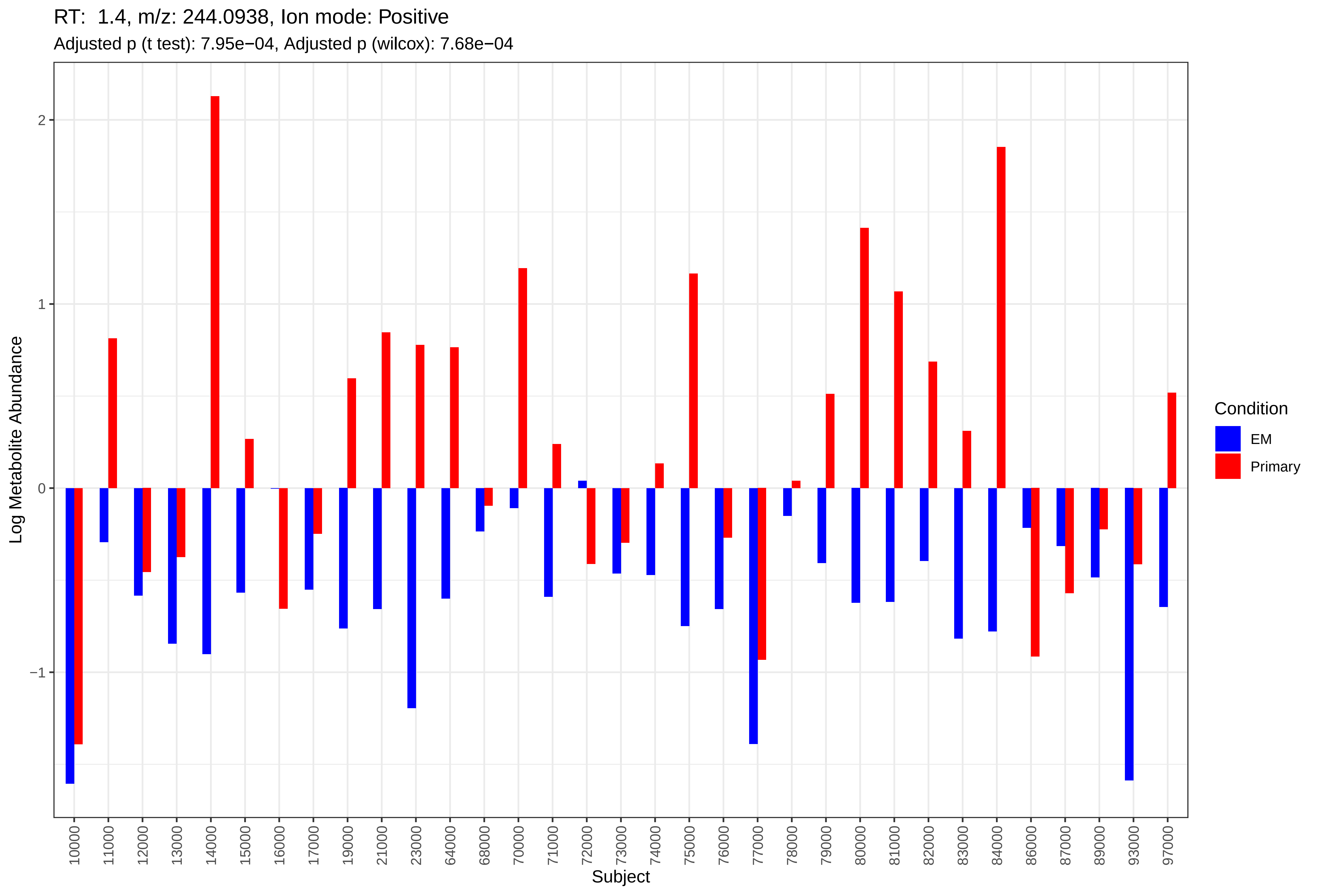
RT: 1.4, m/z: 244.0938, Ion mode: Positive
Adjusted p (t test): 7.95e−04, Adjusted p (wilcox): 7.68e−04
Log Metabolite Abundance
2
1
0
−1
Subject
10000
11000
12000
13000
14000
15000
16000
17000
19000
21000
23000
64000
68000
70000
71000
72000
73000
74000
75000
76000
77000
78000
79000
80000
81000
82000
83000
84000
86000
87000
89000
93000
97000
Condition
EM
Primary

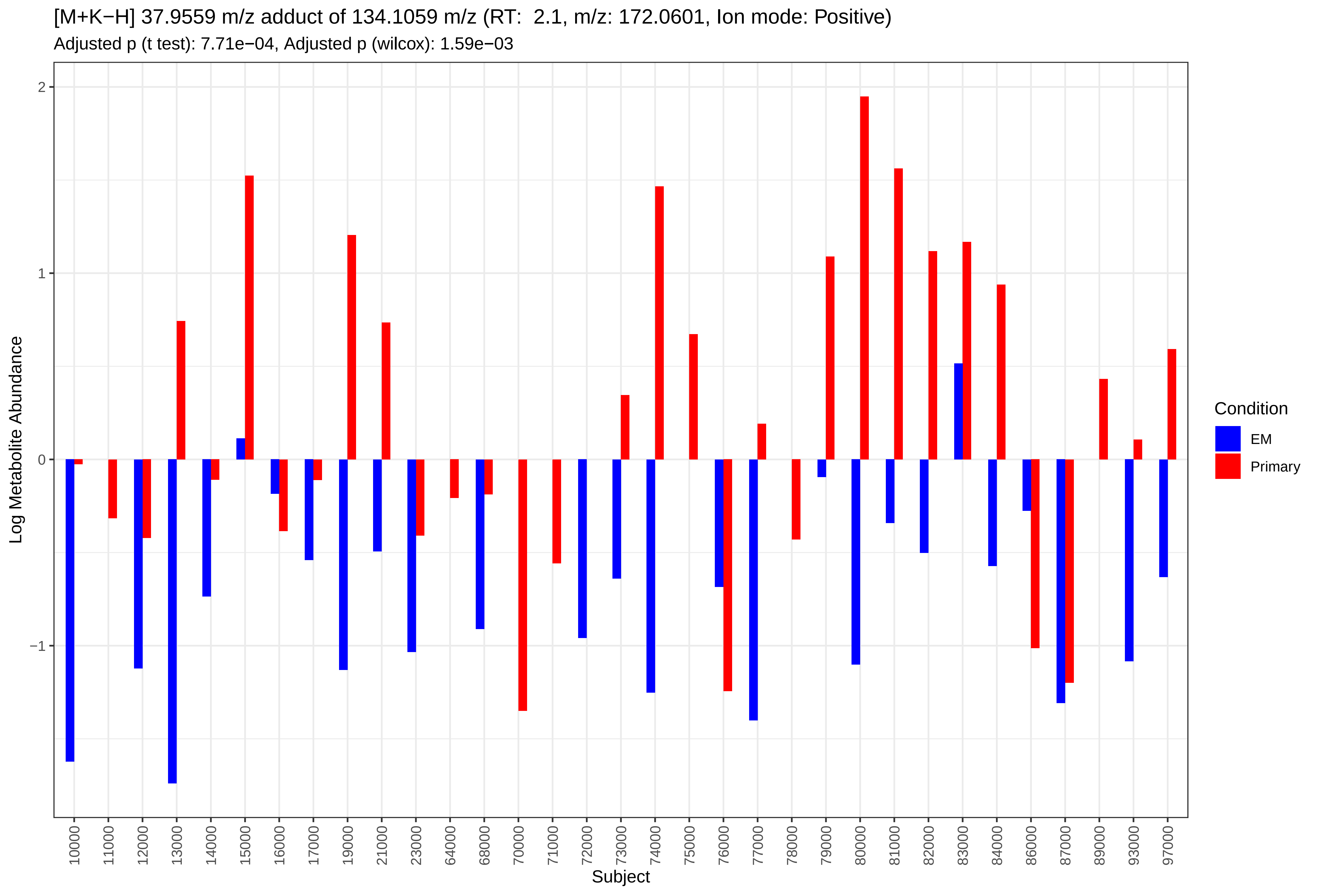
[M+K−H] 37.9559 m/z adduct of 134.1059 m/z (RT: 2.1, m/z: 172.0601, Ion mode: Positive)
Adjusted p (t test): 7.71e−04, Adjusted p (wilcox): 1.59e−03
Log Metabolite Abundance
2
1
0
−1
Subject
10000
11000
12000
13000
14000
15000
16000
17000
19000
21000
23000
64000
68000
70000
71000
72000
73000
74000
75000
76000
77000
78000
79000
80000
81000
82000
83000
84000
86000
87000
89000
93000
97000
Condition
EM
Primary

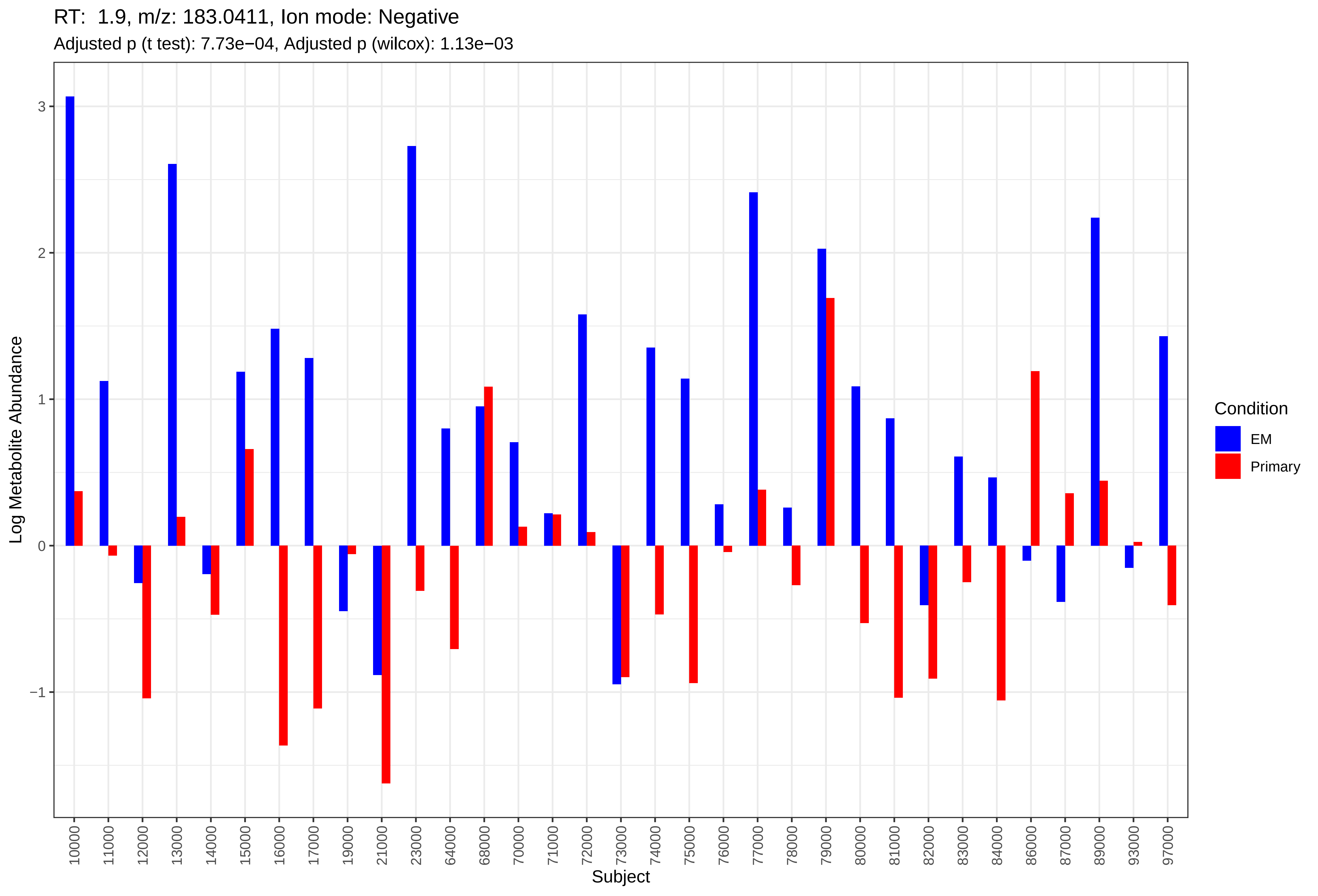

RT: 1.9, m/z: 183.0411, Ion mode: Negative
Adjusted p (t test): 7.73e−04, Adjusted p (wilcox): 1.13e−03
Log Metabolite Abundance
3
2
1
0
−1
Subject
10000
11000
12000
13000
14000
15000
16000
17000
19000
21000
23000
64000
68000
70000
71000
72000
73000
74000
75000
76000
77000
78000
79000
80000
81000
82000
83000
84000
86000
87000
89000
93000
97000
Condition
EM
Primary

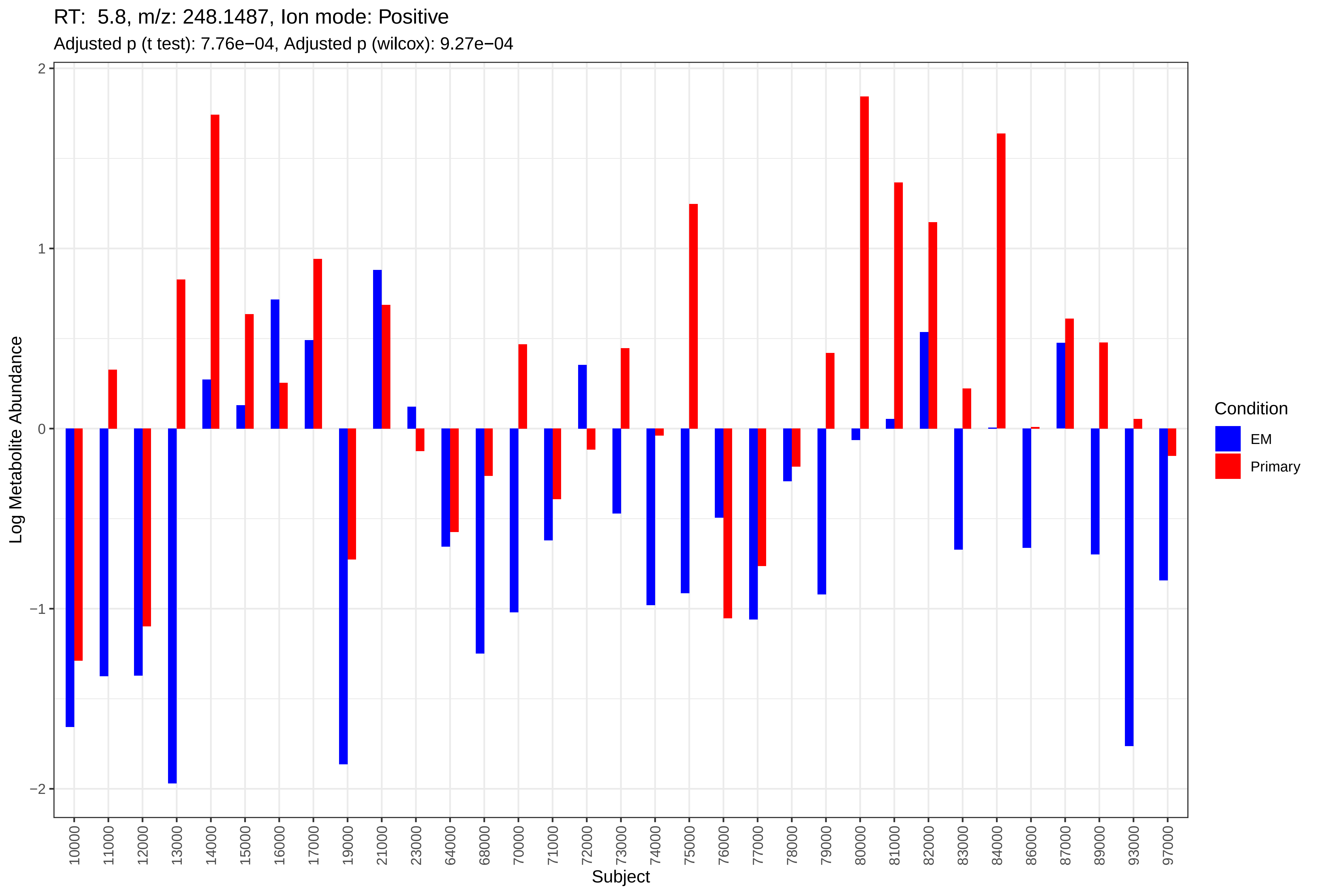
RT: 5.8, m/z: 248.1487, Ion mode: Positive
Adjusted p (t test): 7.76e−04, Adjusted p (wilcox): 9.27e−04
Log Metabolite Abundance
2
1
0
−1
−2
Subject
10000
11000
12000
13000
14000
15000
16000
17000
19000
21000
23000
64000
68000
70000
71000
72000
73000
74000
75000
76000
77000
78000
79000
80000
81000
82000
83000
84000
86000
87000
89000
93000
97000
Condition
EM
Primary

RT: 1.9, m/z: 181.0254, Ion mode: Negative

Adjusted p (t test): 7.78e−04, Adjusted p (wilcox): 1.03e−03

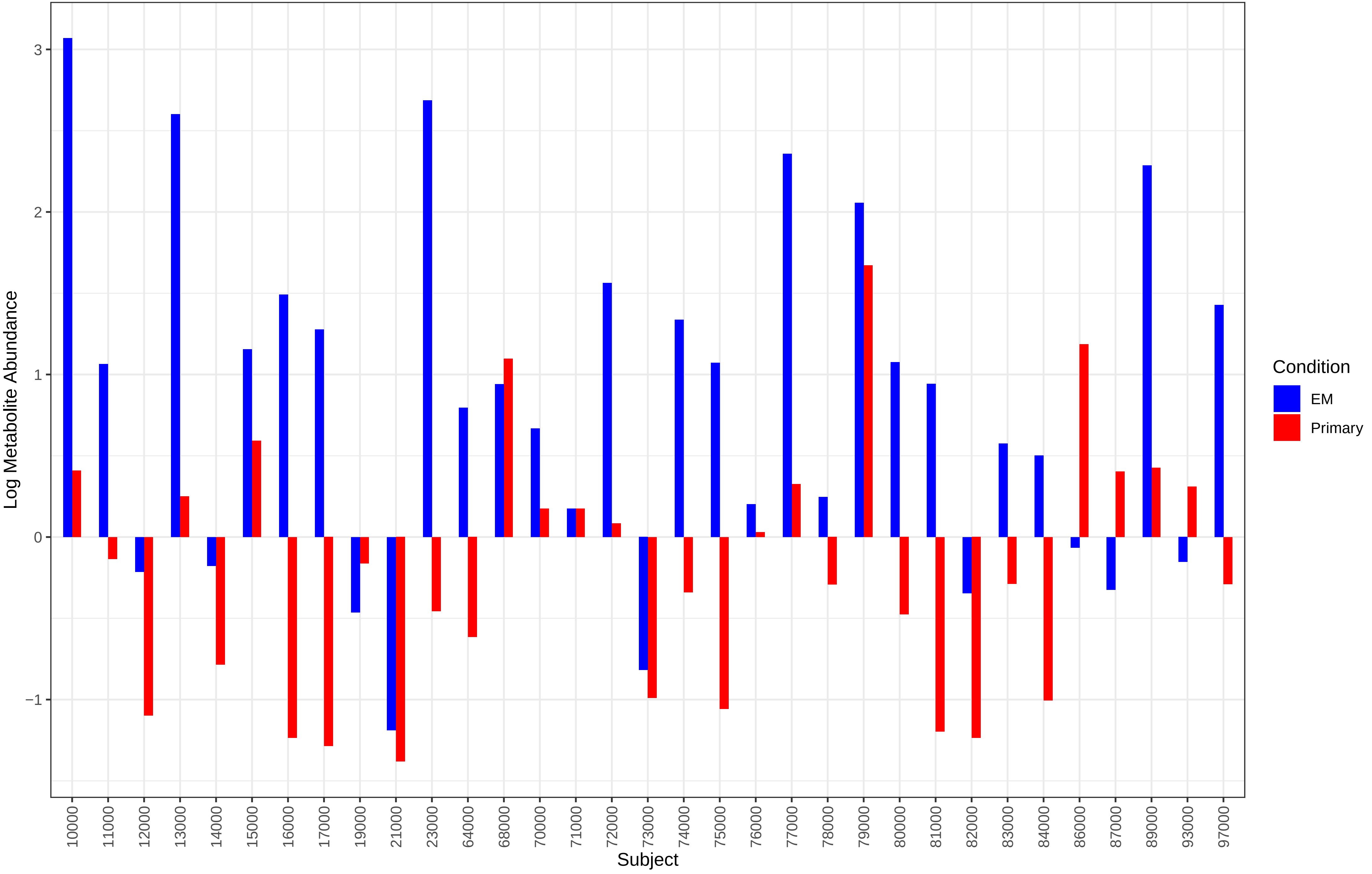

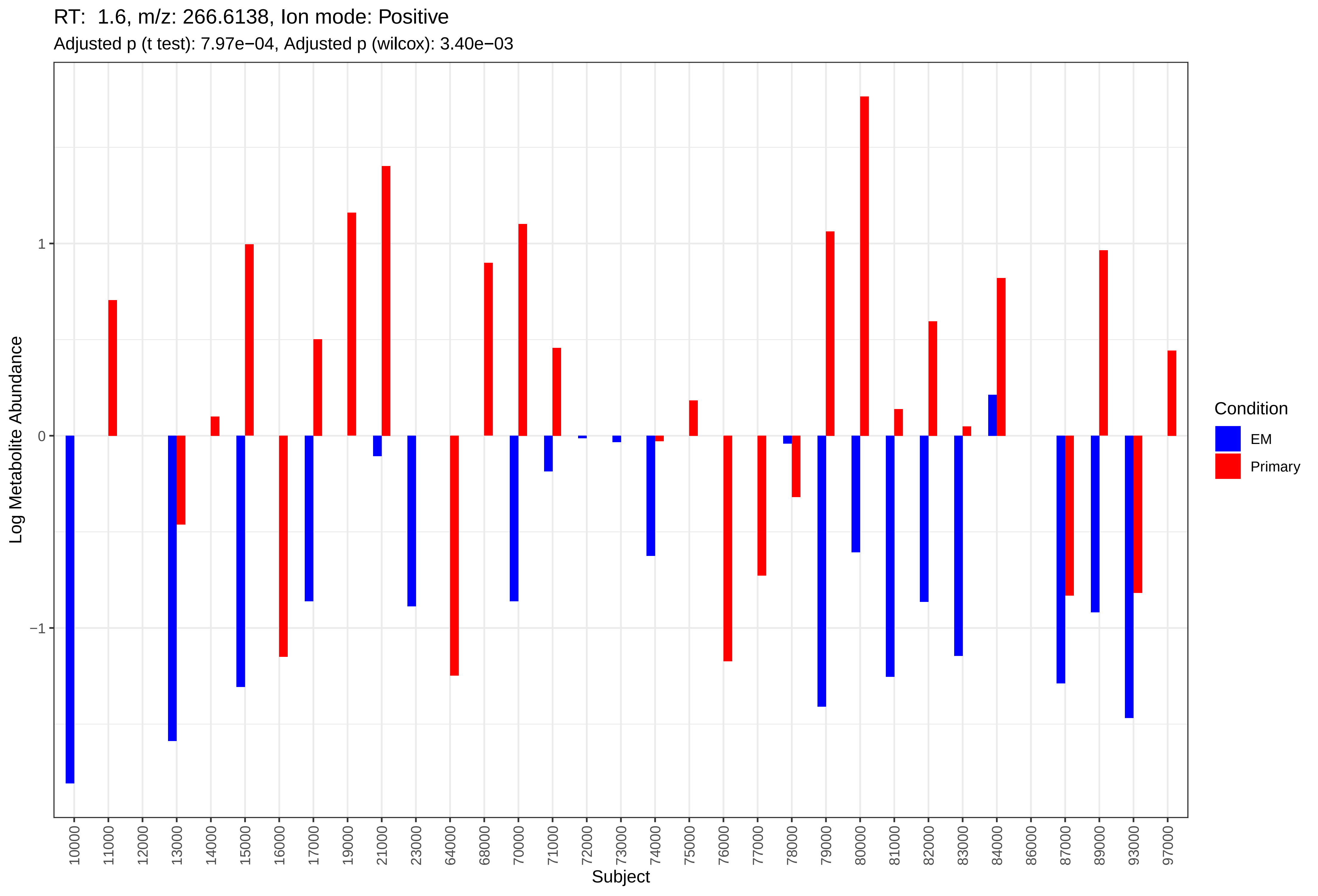

RT: 1.6, m/z: 266.6138, Ion mode: Positive
Adjusted p (t test): 7.97e−04, Adjusted p (wilcox): 3.40e−03
Log Metabolite Abundance
1
0
−1
10000
11000
12000
13000
14000
15000
16000
17000
19000
21000
23000
64000
68000
70000
71000
72000
73000
74000
75000
76000
77000
78000
79000
80000
81000
82000
83000
84000
86000
87000
89000
93000
97000
Subject
Condition
EM
Primary

UROCANATE (RT: 1.9, m/z: 139.0499, Ion mode: Positive)

Adjusted p (t test): 8.16e−04, Adjusted p (wilcox): 1.47e−03

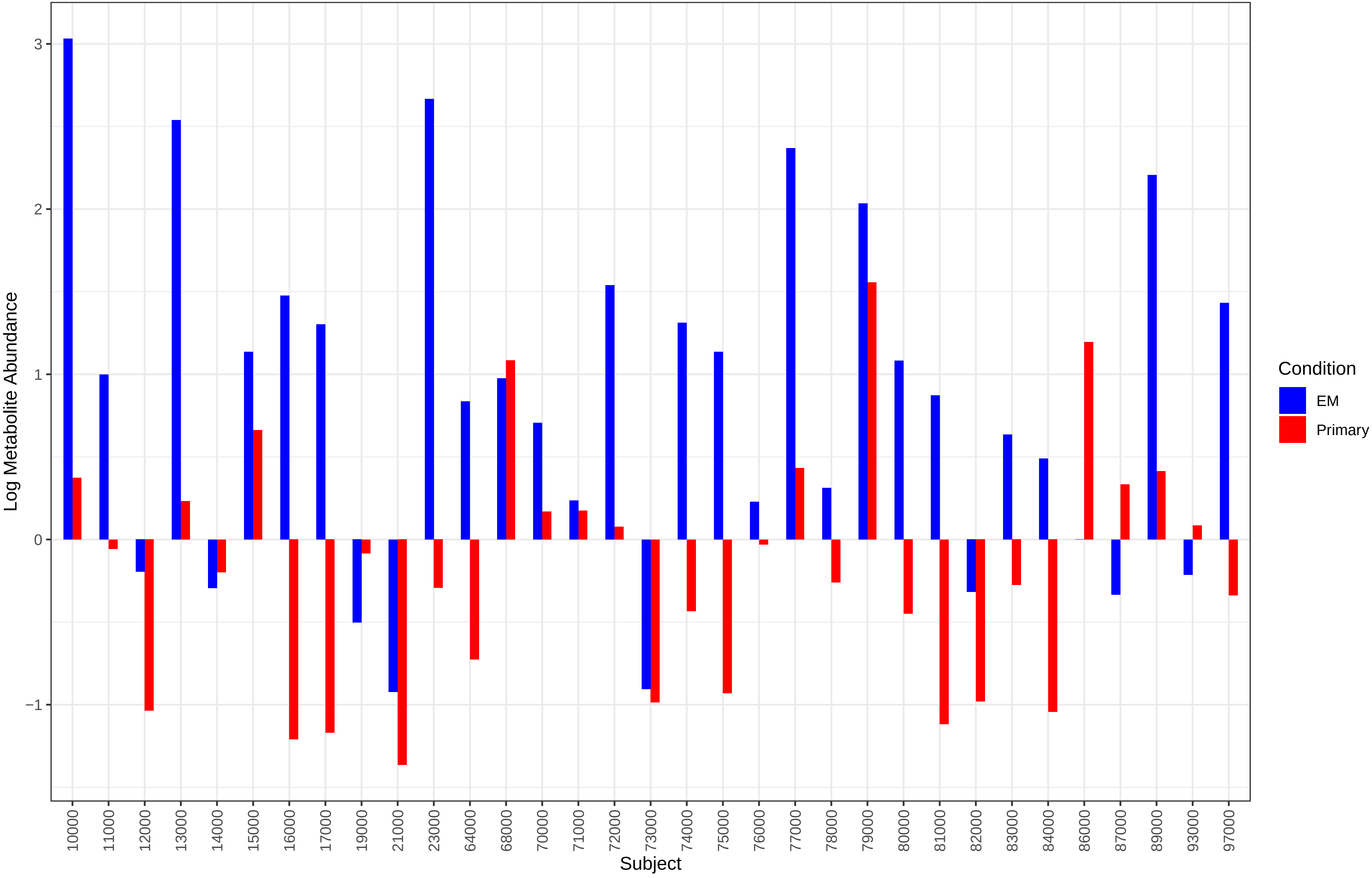

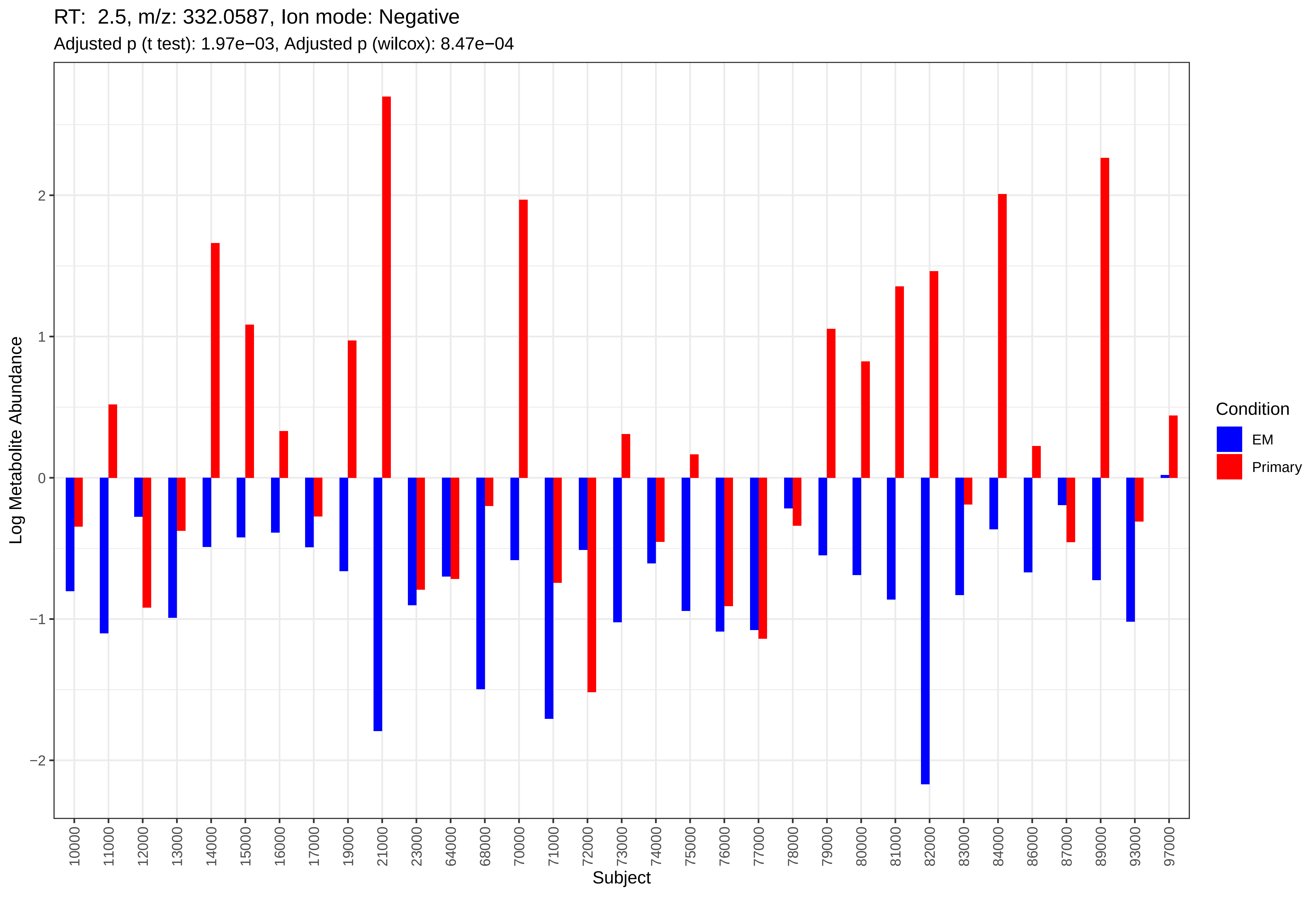
RT: 2.5, m/z: 332.0587, Ion mode: Negative
Adjusted p (t test): 1.97e−03, Adjusted p (wilcox): 8.47e−04
Log Metabolite Abundance
2
1
0
−1
−2
Subject
10000
11000
12000
13000
14000
15000
16000
17000
19000
21000
23000
64000
68000
70000
71000
72000
73000
74000
75000
76000
77000
78000
79000
80000
81000
82000
83000
84000
86000
87000
89000
93000
97000
Condition
EM
Primary

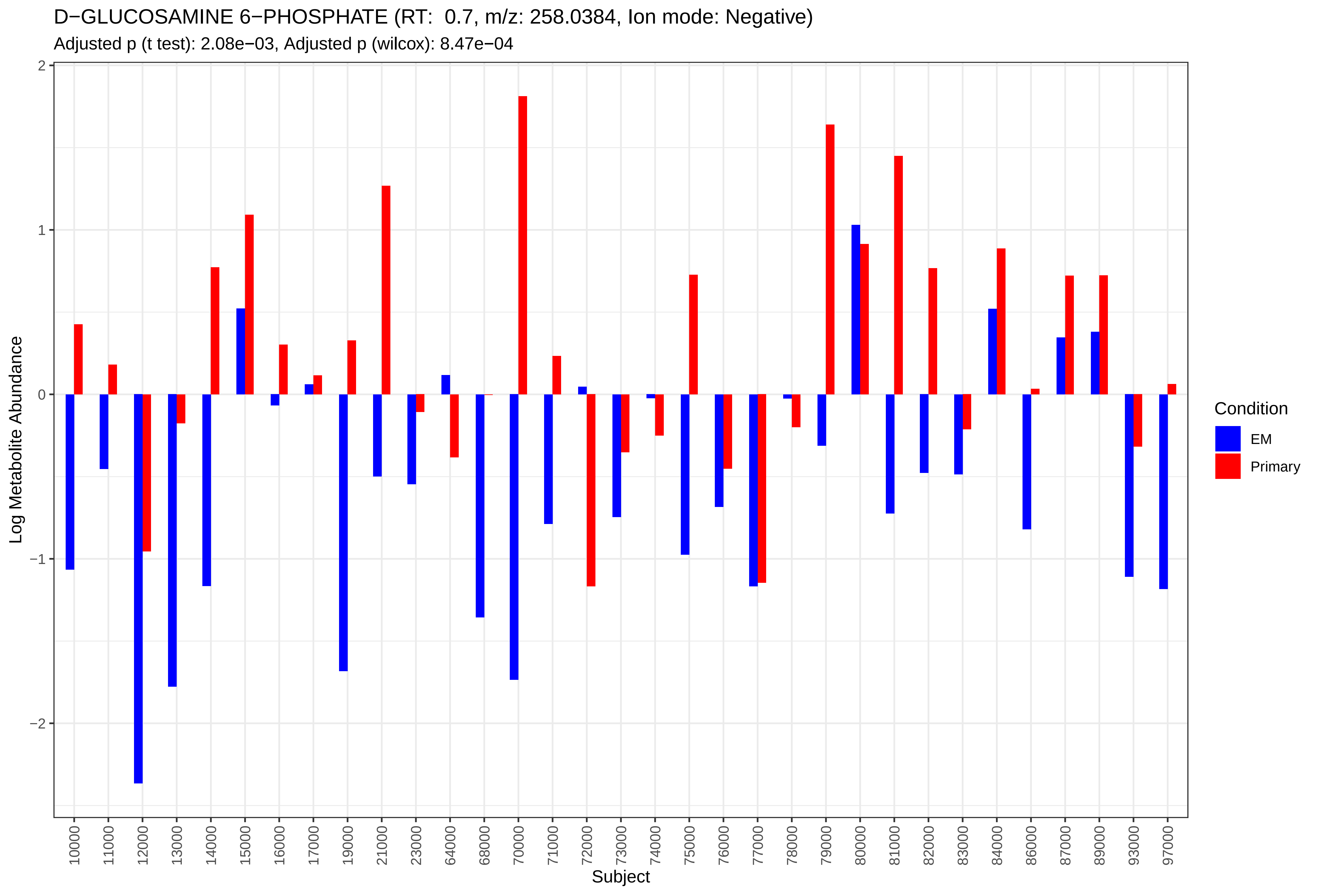
D−GLUCOSAMINE 6−PHOSPHATE (RT: 0.7, m/z: 258.0384, Ion mode: Negative)
Adjusted p (t test): 2.08e−03, Adjusted p (wilcox): 8.47e−04
Log Metabolite Abundance
2
1
0
−1
−2
10000
11000
12000
13000
14000
15000
16000
17000
19000
21000
23000
64000
68000
70000
71000
72000
73000
74000
75000
76000
77000
78000
79000
80000
81000
82000
83000
84000
86000
87000
89000
93000
97000
Subject
Condition
EM
Primary

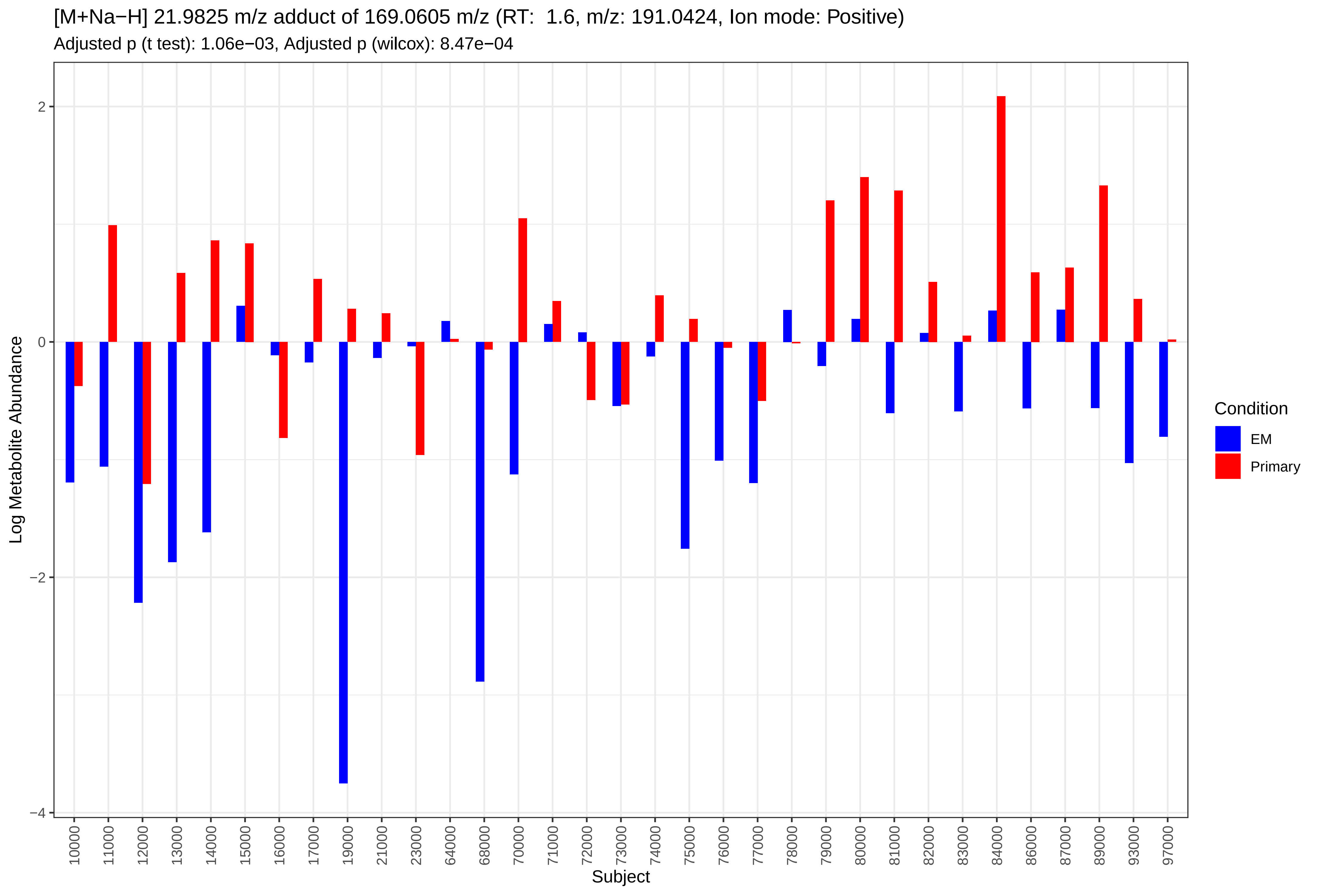

[M+Na−H] 21.9825 m/z adduct of 169.0605 m/z (RT: 1.6, m/z: 191.0424, Ion mode: Positive)
Adjusted p (t test): 1.06e−03, Adjusted p (wilcox): 8.47e−04
Log Metabolite Abundance
2
0
−2
−4
10000 11000 12000 13000 14000 15000 16000 17000 19000 21000 23000 64000 68000 70000 71000 72000 73000 74000 75000 76000 77000 78000 79000 80000 81000 82000 83000 84000 86000 87000 89000 93000 97000
Subject
Condition
EM
Primary

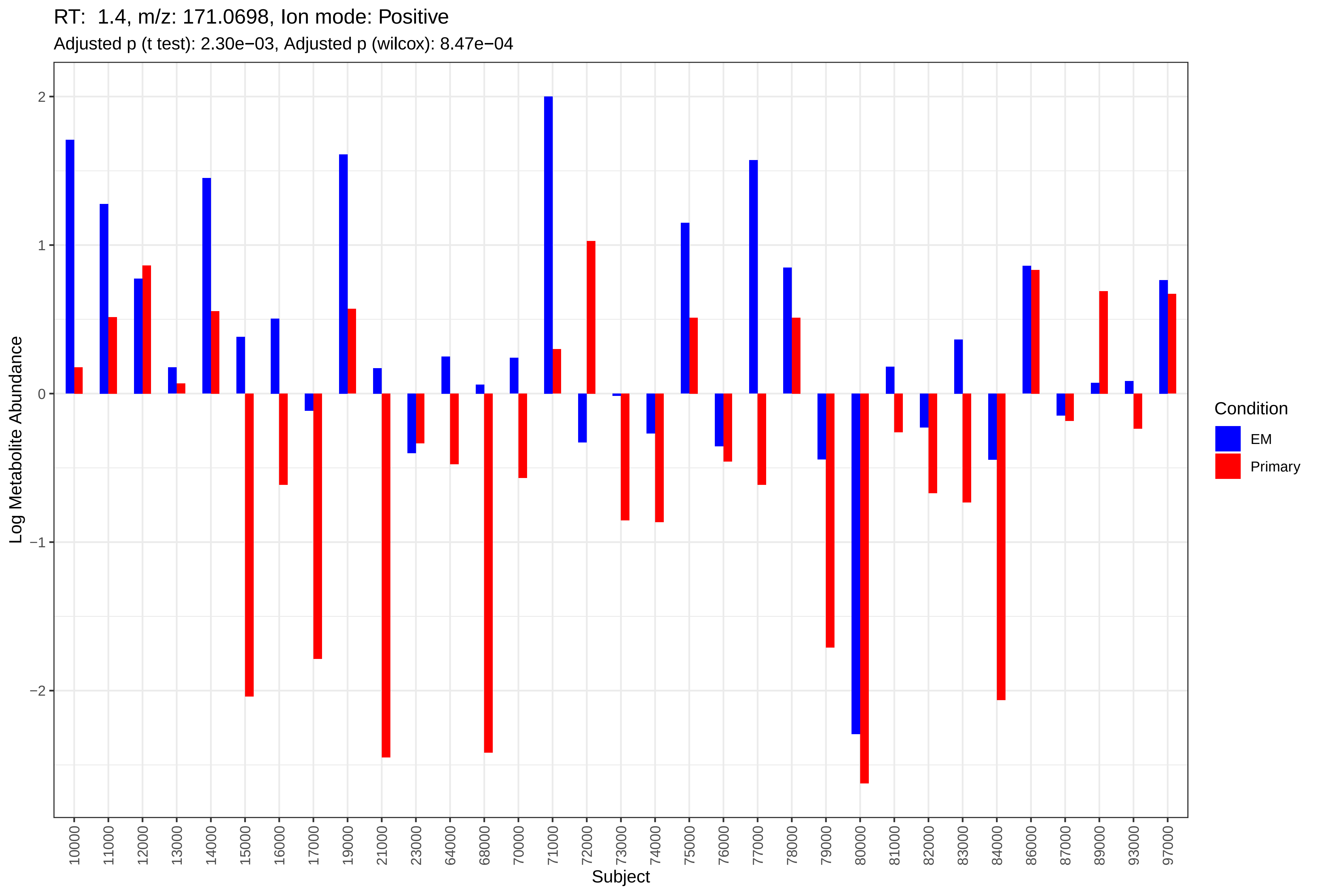

RT: 1.4, m/z: 171.0698, Ion mode: Positive
Adjusted p (t test): 2.30e−03, Adjusted p (wilcox): 8.47e−04
Log Metabolite Abundance
2
1
0
−1
−2
Subject
10000
11000
12000
13000
14000
15000
16000
17000
19000
21000
23000
64000
68000
70000
71000
72000
73000
74000
75000
76000
77000
78000
79000
80000
81000
82000
83000
84000
86000
87000
89000
93000
97000
Condition
EM
Primary

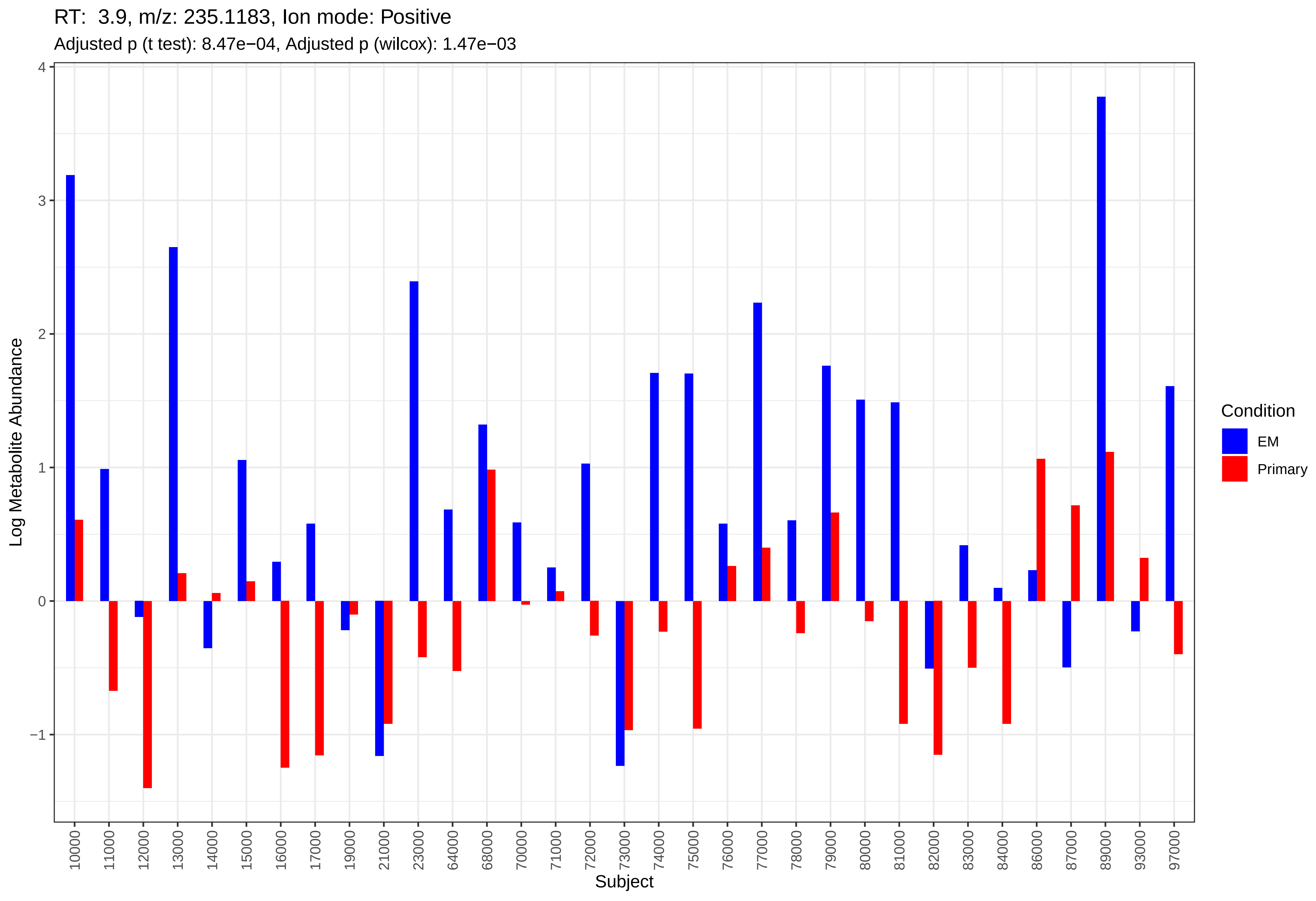
RT: 3.9, m/z: 235.1183, Ion mode: Positive
Adjusted p (t test): 8.47e−04, Adjusted p (wilcox): 1.47e−03
Log Metabolite Abundance
Subject
Condition
EM
Primary
4
3
2
1
0
−1
10000
11000
12000
13000
14000
15000
16000
17000
19000
21000
23000
64000
68000
70000
71000
72000
73000
74000
75000
76000
77000
78000
79000
80000
81000
82000
83000
84000
86000
87000
89000
93000
97000

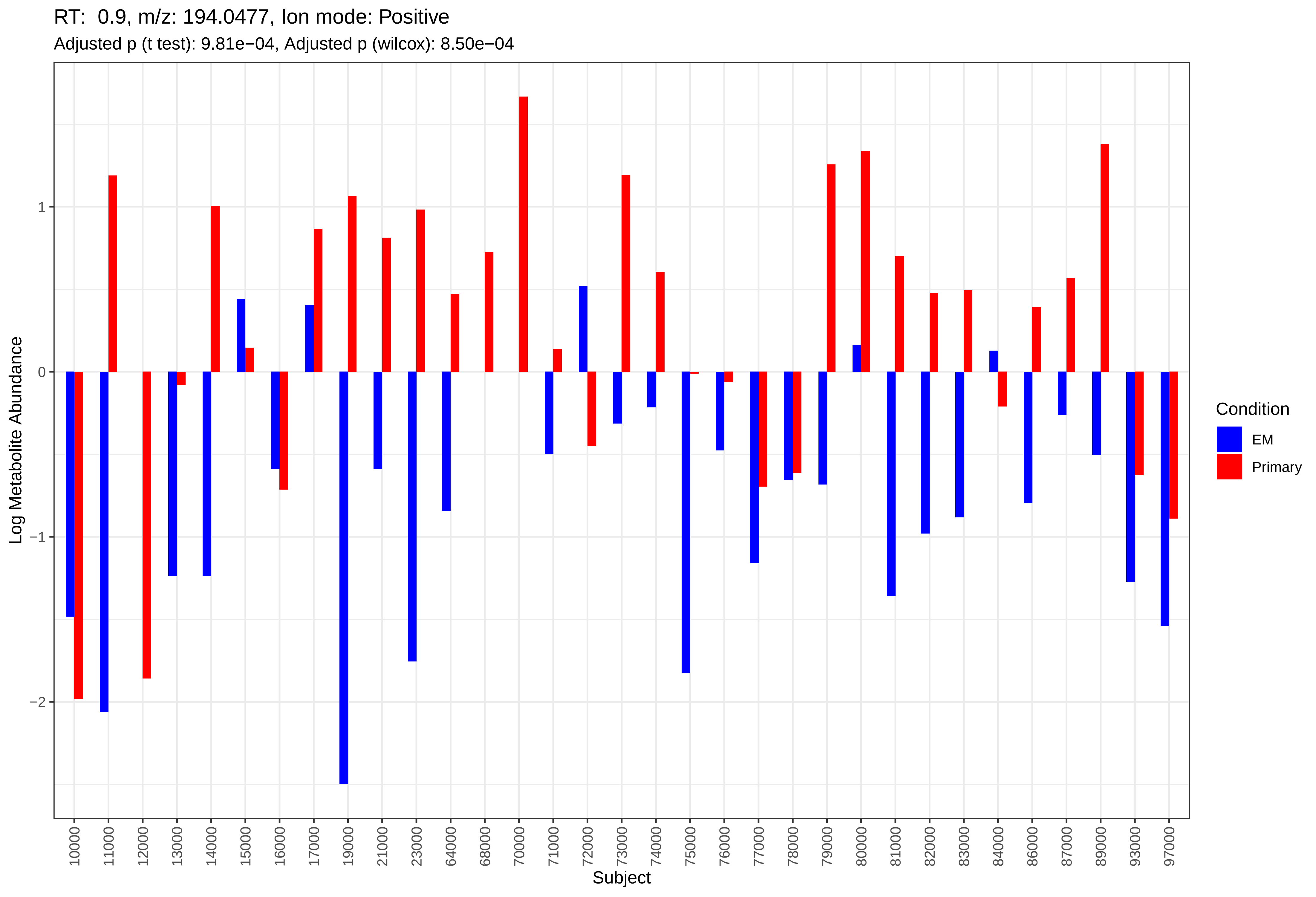
RT: 0.9, m/z: 194.0477, Ion mode: Positive
Adjusted p (t test): 9.81e−04, Adjusted p (wilcox): 8.50e−04
Log Metabolite Abundance
1
0
−1
−2
Subject
10000
11000
12000
13000
14000
15000
16000
17000
19000
21000
23000
64000
68000
70000
71000
72000
73000
74000
75000
76000
77000
78000
79000
80000
81000
82000
83000
84000
86000
87000
89000
93000
97000
Condition
EM
Primary

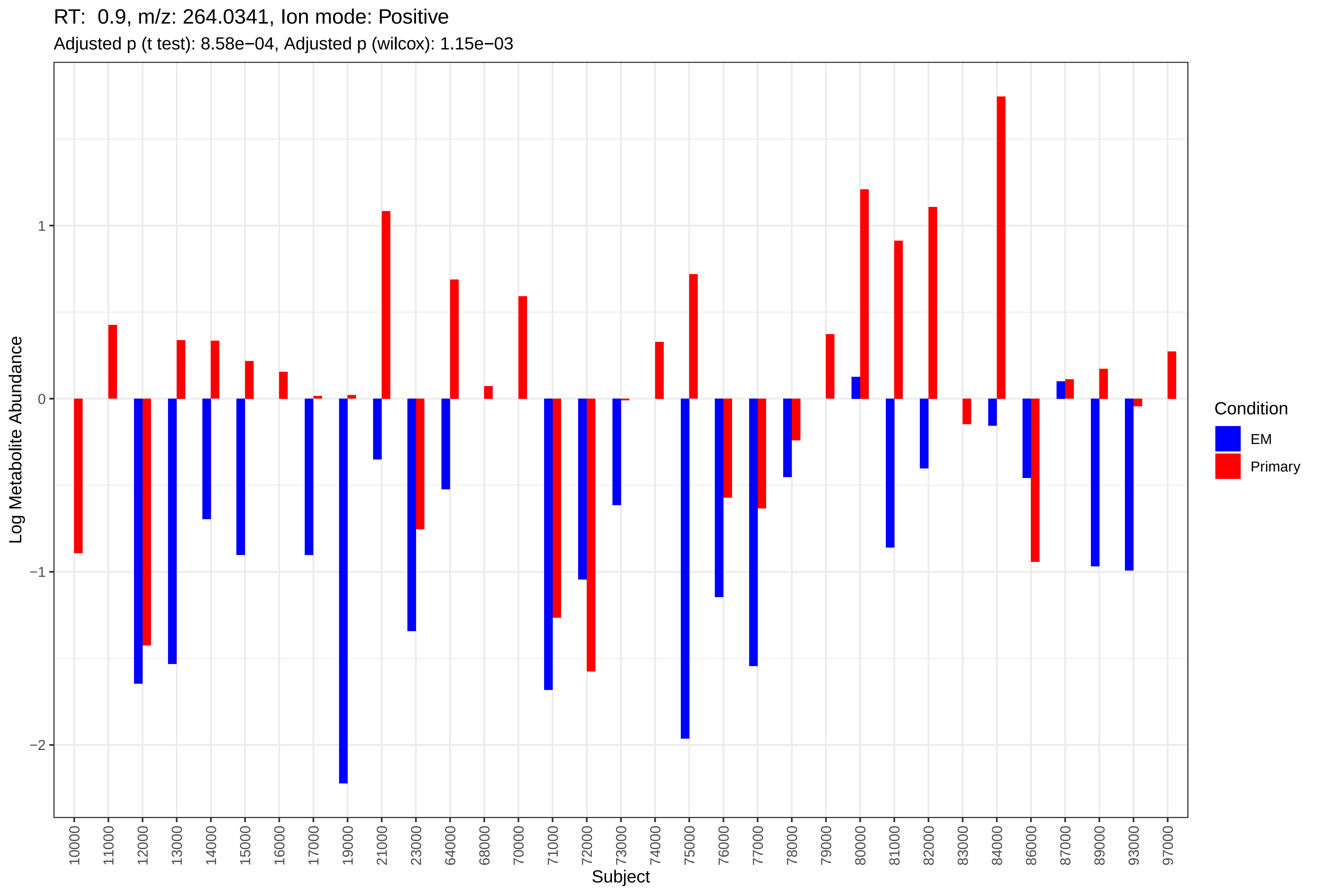
RT: 0.9, m/z: 264.0341, Ion mode: Positive
Adjusted p (t test): 8.58e−04, Adjusted p (wilcox): 1.15e−03
Log Metabolite Abundance
1
0
−1
−2
Subject
10000
11000
12000
13000
14000
15000
16000
17000
19000
21000
23000
64000
68000
70000
71000
72000
73000
74000
75000
76000
77000
78000
79000
80000
81000
82000
83000
84000
86000
87000
89000
93000
97000
Condition
EM
Primary

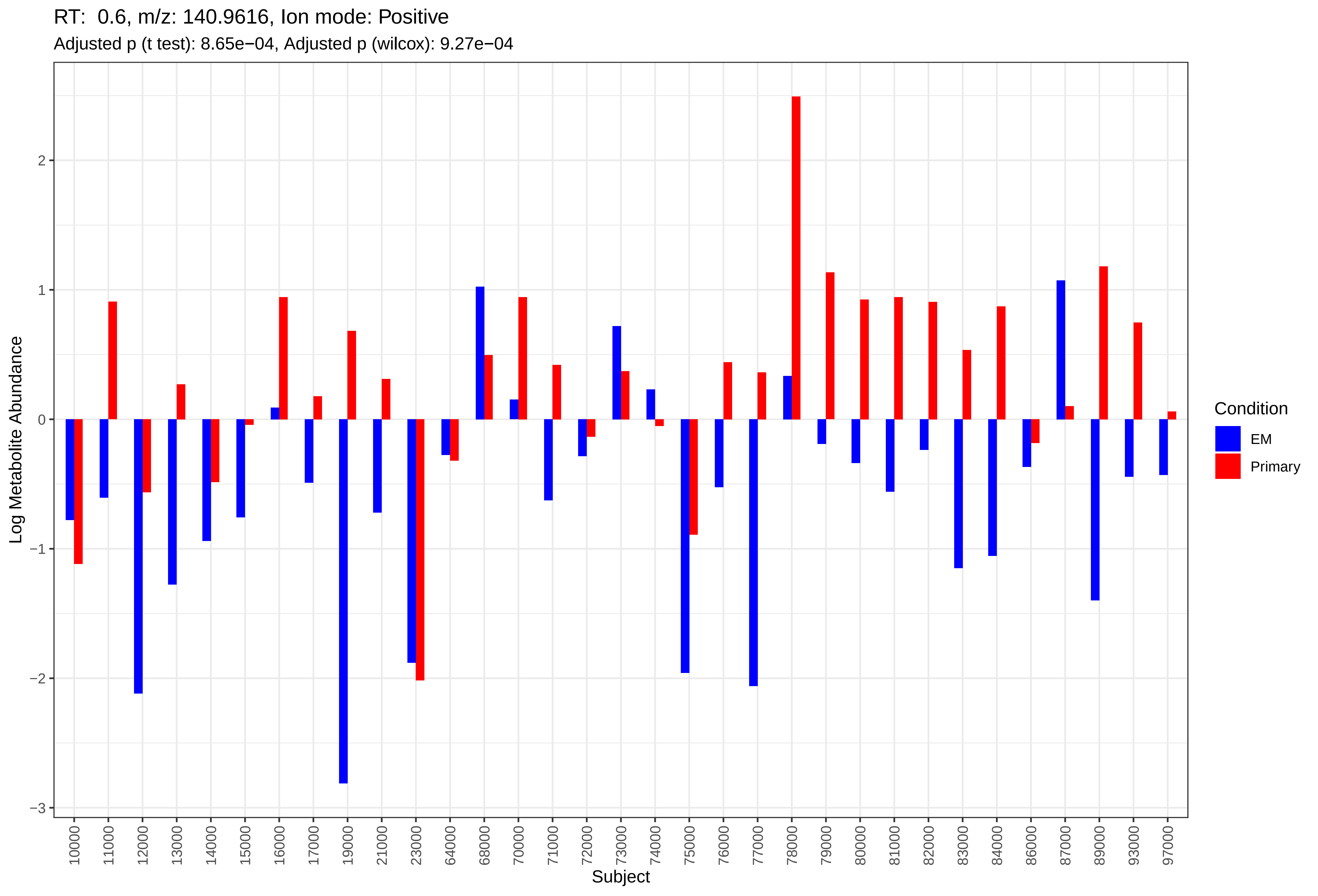
RT: 0.6, m/z: 140.9616, Ion mode: Positive
Adjusted p (t test): 8.65e−04, Adjusted p (wilcox): 9.27e−04
Log Metabolite Abundance
2
1
0
−1
−2
−3
Subject
10000
11000
12000
13000
14000
15000
16000
17000
19000
21000
23000
64000
68000
70000
71000
72000
73000
74000
75000
76000
77000
78000
79000
80000
81000
82000
83000
84000
86000
87000
89000
93000
97000
Condition
EM
Primary

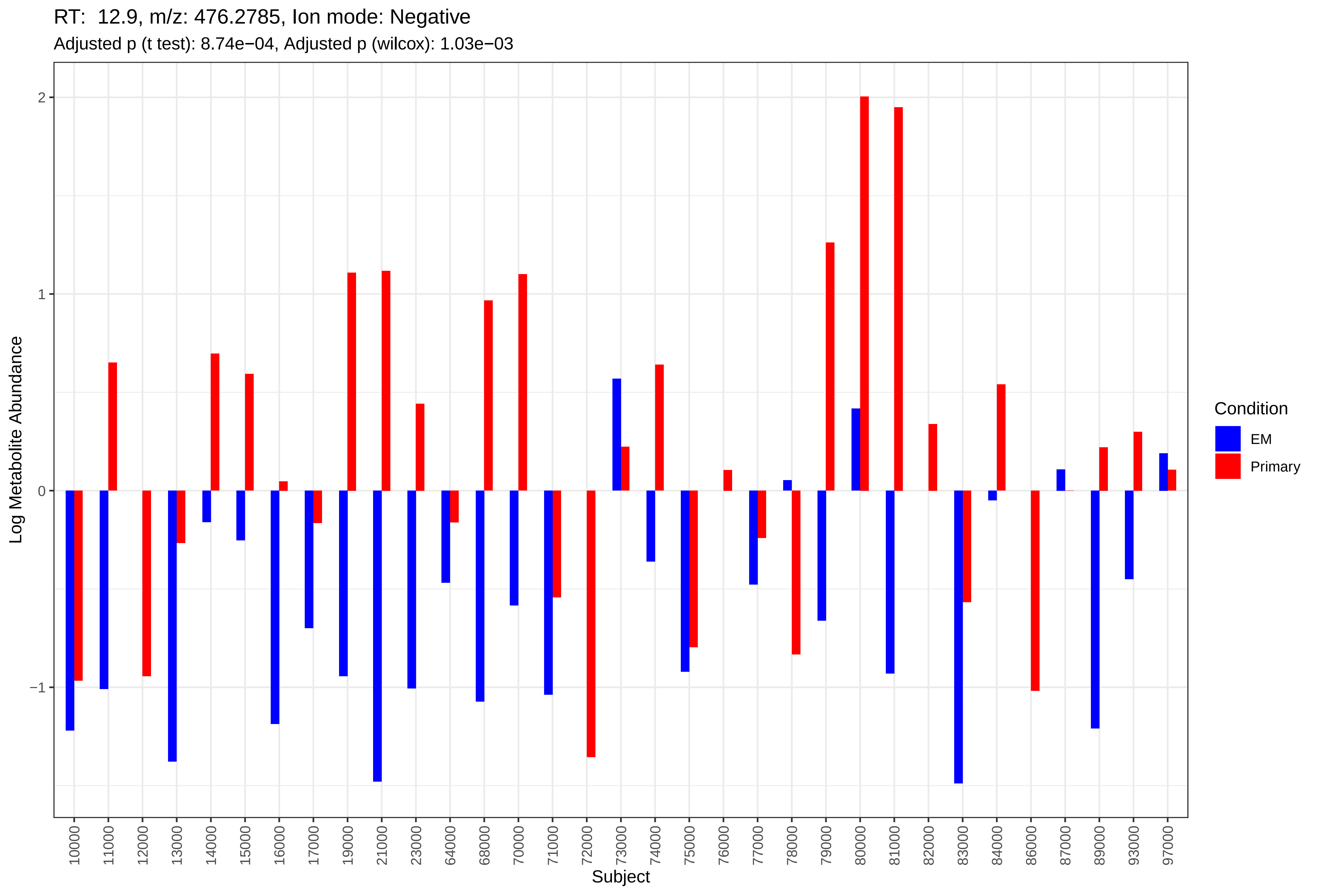

RT: 12.9, m/z: 476.2785, Ion mode: Negative
Adjusted p (t test): 8.74e−04, Adjusted p (wilcox): 1.03e−03
Log Metabolite Abundance
2
1
0
−1
Subject
10000
11000
12000
13000
14000
15000
16000
17000
19000
21000
23000
64000
68000
70000
71000
72000
73000
74000
75000
76000
77000
78000
79000
80000
81000
82000
83000
84000
86000
87000
89000
93000
97000
Condition
EM
Primary

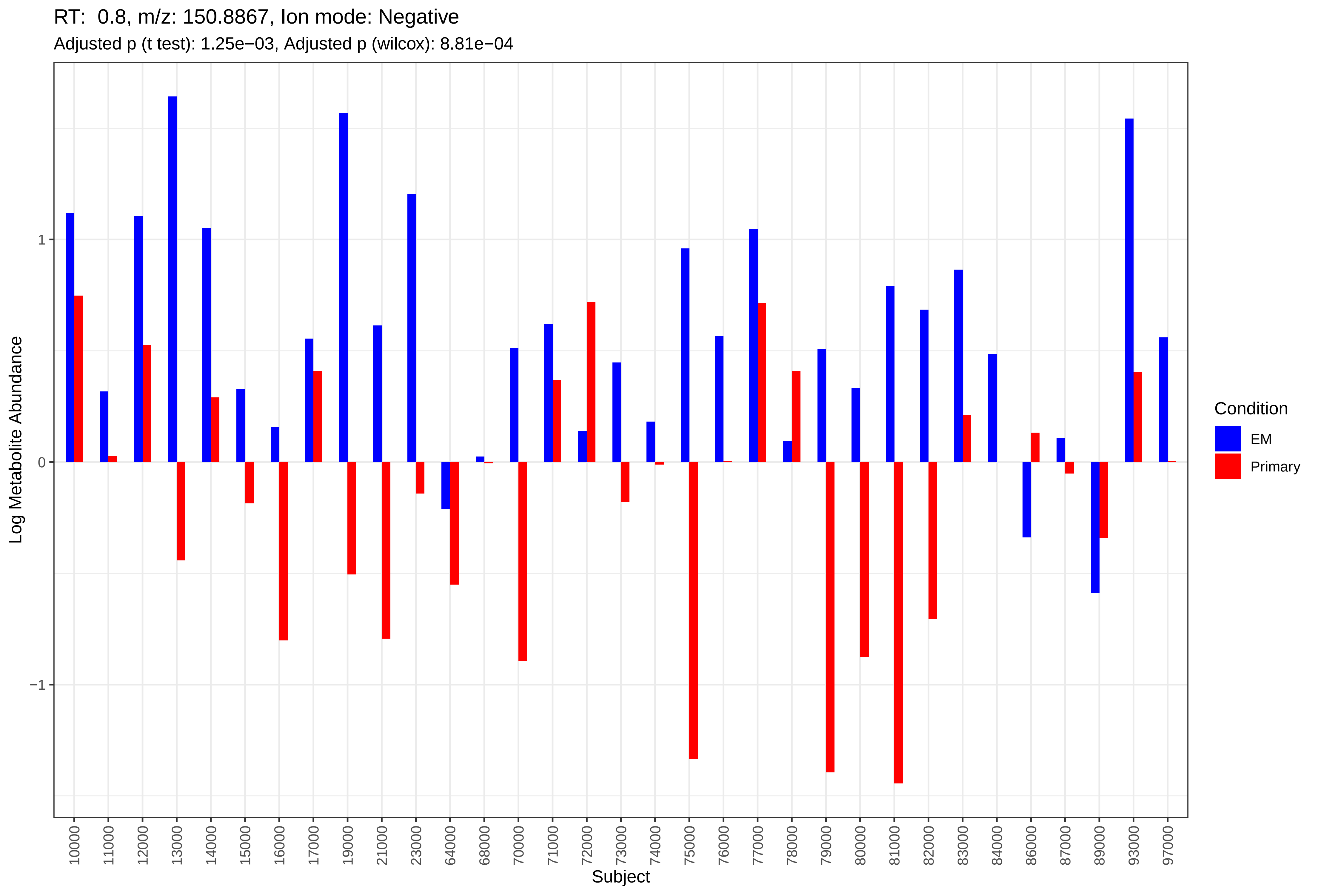

RT: 0.8, m/z: 150.8867, Ion mode: Negative
Adjusted p (t test): 1.25e−03, Adjusted p (wilcox): 8.81e−04
Log Metabolite Abundance
1
0
−1
10000
11000
12000
13000
14000
15000
16000
17000
19000
21000
23000
64000
68000
70000
71000
72000
73000
74000
75000
76000
77000
78000
79000
80000
81000
82000
83000
84000
86000
87000
89000
93000
97000
Subject
Condition
EM
Primary

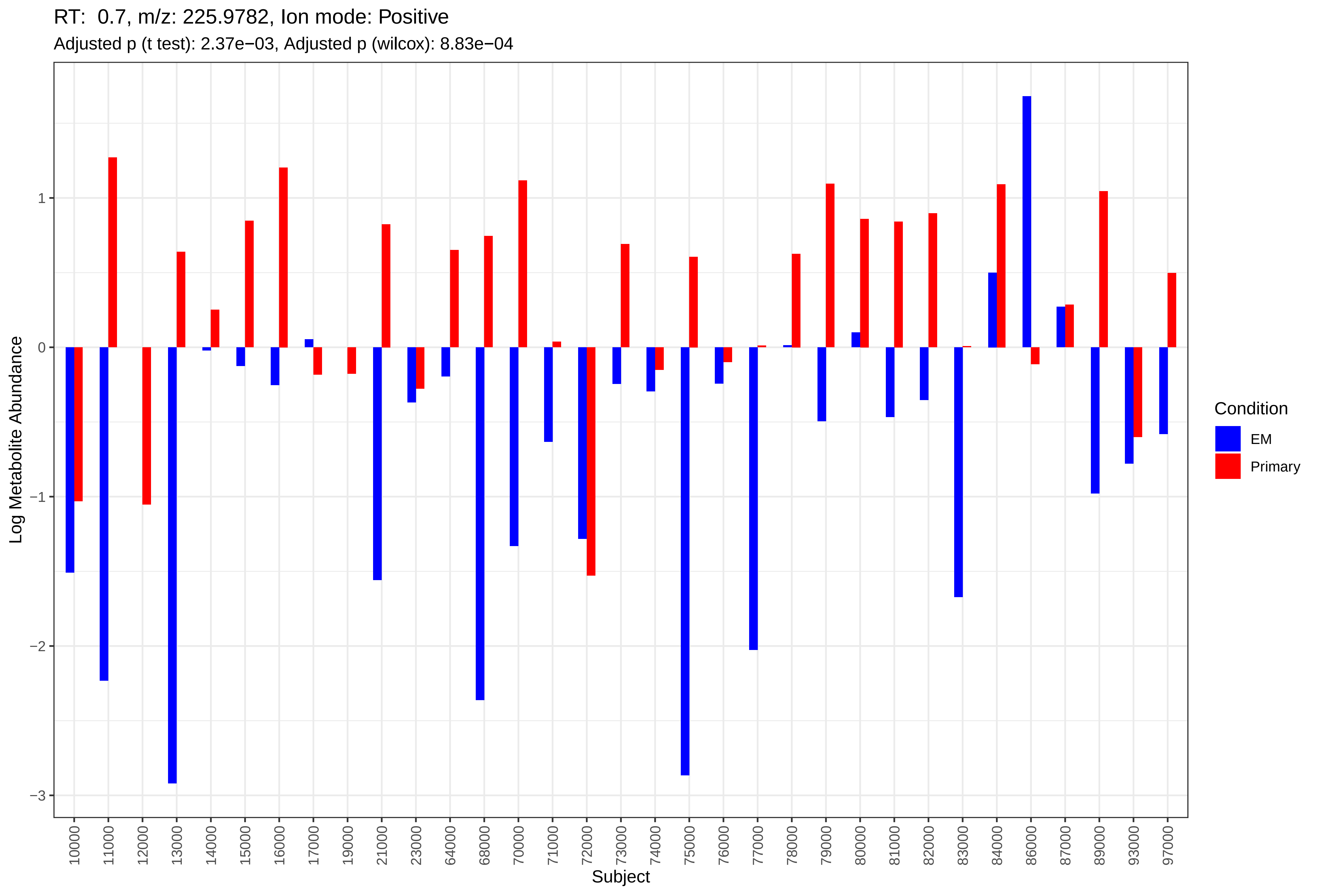

RT: 0.7, m/z: 225.9782, Ion mode: Positive
Adjusted p (t test): 2.37e−03, Adjusted p (wilcox): 8.83e−04
Log Metabolite Abundance
1
0
−1
−2
−3
10000
11000
12000
13000
14000
15000
16000
17000
19000
21000
23000
64000
68000
70000
71000
72000
73000
74000
75000
76000
77000
78000
79000
80000
81000
82000
83000
84000
86000
87000
89000
93000
97000
Subject
Condition
EM
Primary

RT: 1.0, m/z: 176.0914, Ion mode: Positive

Adjusted p (t test): 9.24e−04, Adjusted p (wilcox): 1.23e−03

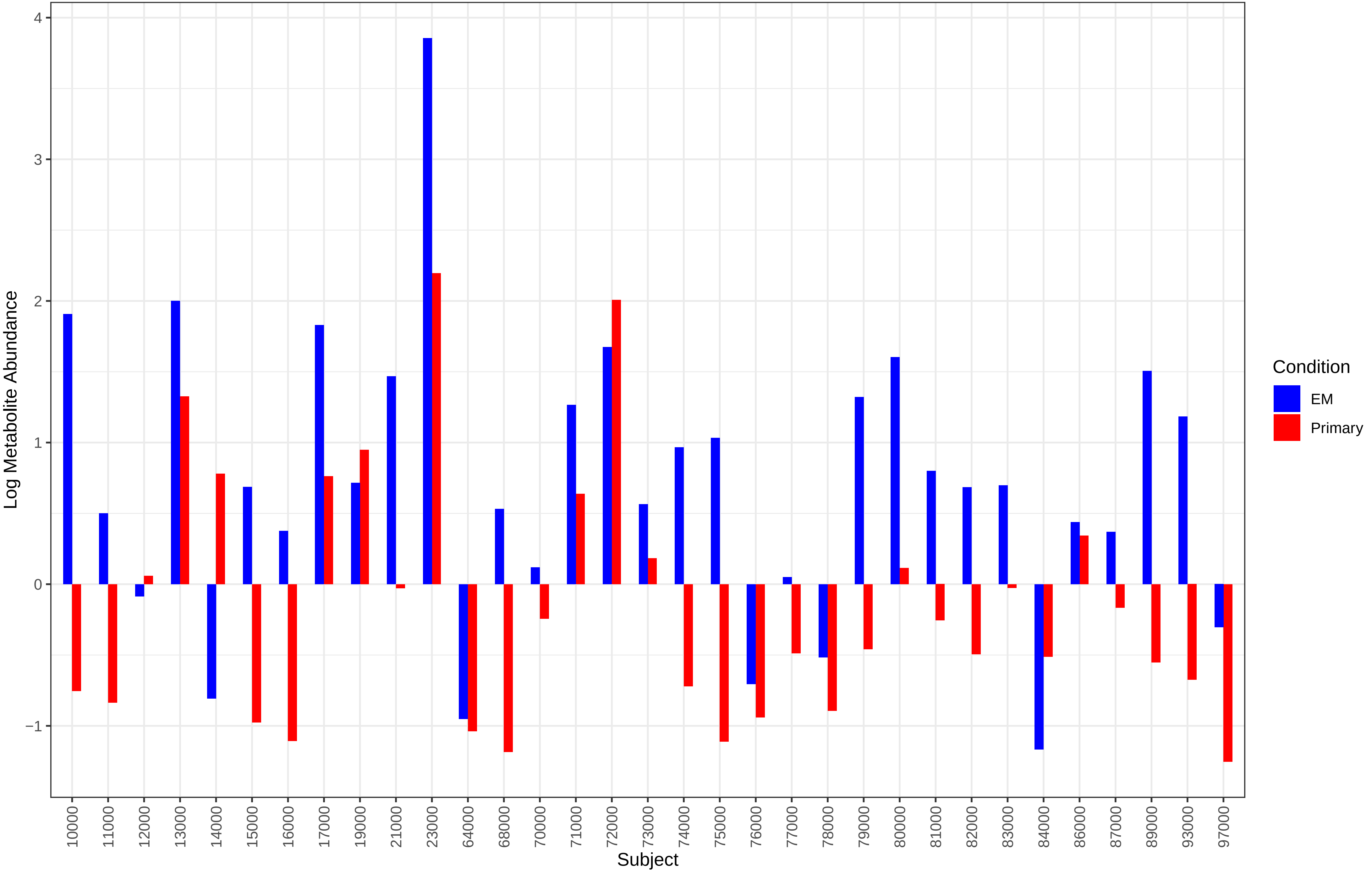

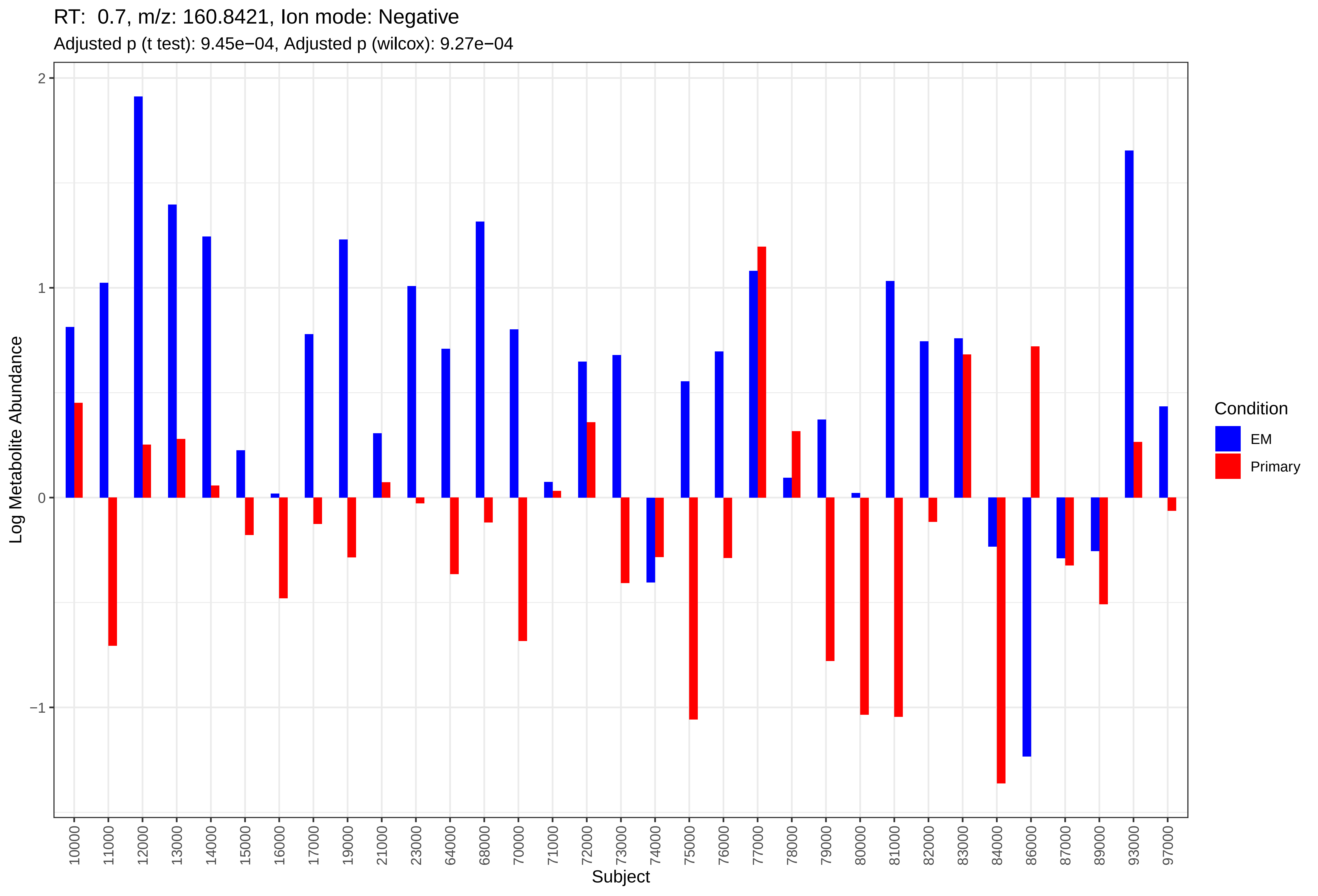

RT: 0.7, m/z: 160.8421, Ion mode: Negative
Adjusted p (t test): 9.45e−04, Adjusted p (wilcox): 9.27e−04
Log Metabolite Abundance
2
1
0
−1
10000
11000
12000
13000
14000
15000
16000
17000
19000
21000
23000
64000
68000
70000
71000
72000
73000
74000
75000
76000
77000
78000
79000
80000
81000
82000
83000
84000
86000
87000
89000
93000
97000
Subject
Condition
EM
Primary

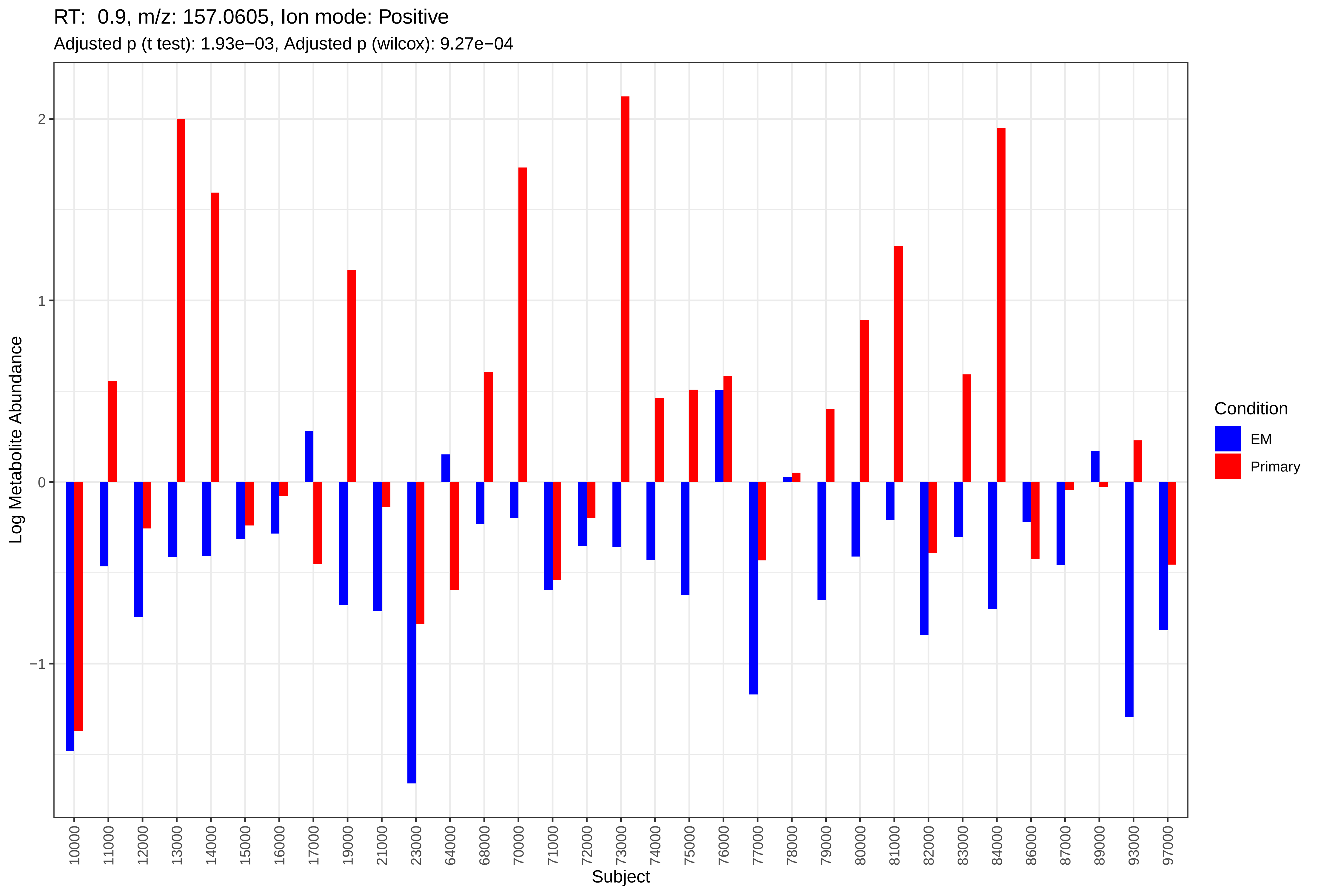
RT: 0.9, m/z: 157.0605, Ion mode: Positive
Adjusted p (t test): 1.93e−03, Adjusted p (wilcox): 9.27e−04
Log Metabolite Abundance
Subject
Condition
EM
Primary
2
1
0
−1
10000
11000
12000
13000
14000
15000
16000
17000
19000
21000
23000
64000
68000
70000
71000
72000
73000
74000
75000
76000
77000
78000
79000
80000
81000
82000
83000
84000
86000
87000
89000
93000
97000

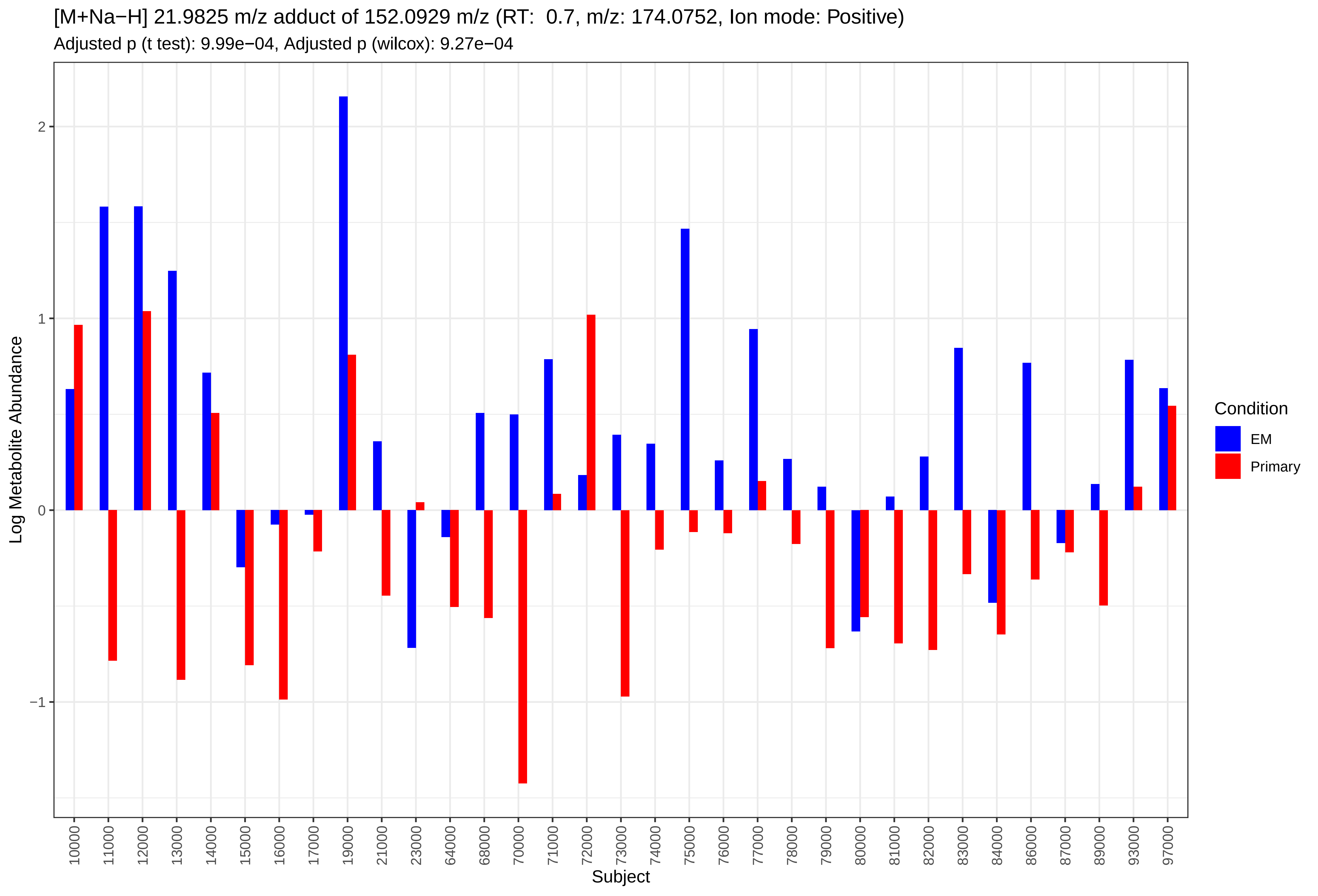
[M+Na−H] 21.9825 m/z adduct of 152.0929 m/z (RT: 0.7, m/z: 174.0752, Ion mode: Positive)
Adjusted p (t test): 9.99e−04, Adjusted p (wilcox): 9.27e−04
Log Metabolite Abundance
2
1
0
−1
Subject
10000
11000
12000
13000
14000
15000
16000
17000
19000
21000
23000
64000
68000
70000
71000
72000
73000
74000
75000
76000
77000
78000
79000
80000
81000
82000
83000
84000
86000
87000
89000
93000
97000
Condition
EM
Primary

RT: 3.6, m/z: 156.0700, Ion mode: Positive

Adjusted p (t test): 9.36e−04, Adjusted p (wilcox): 1.89e−03

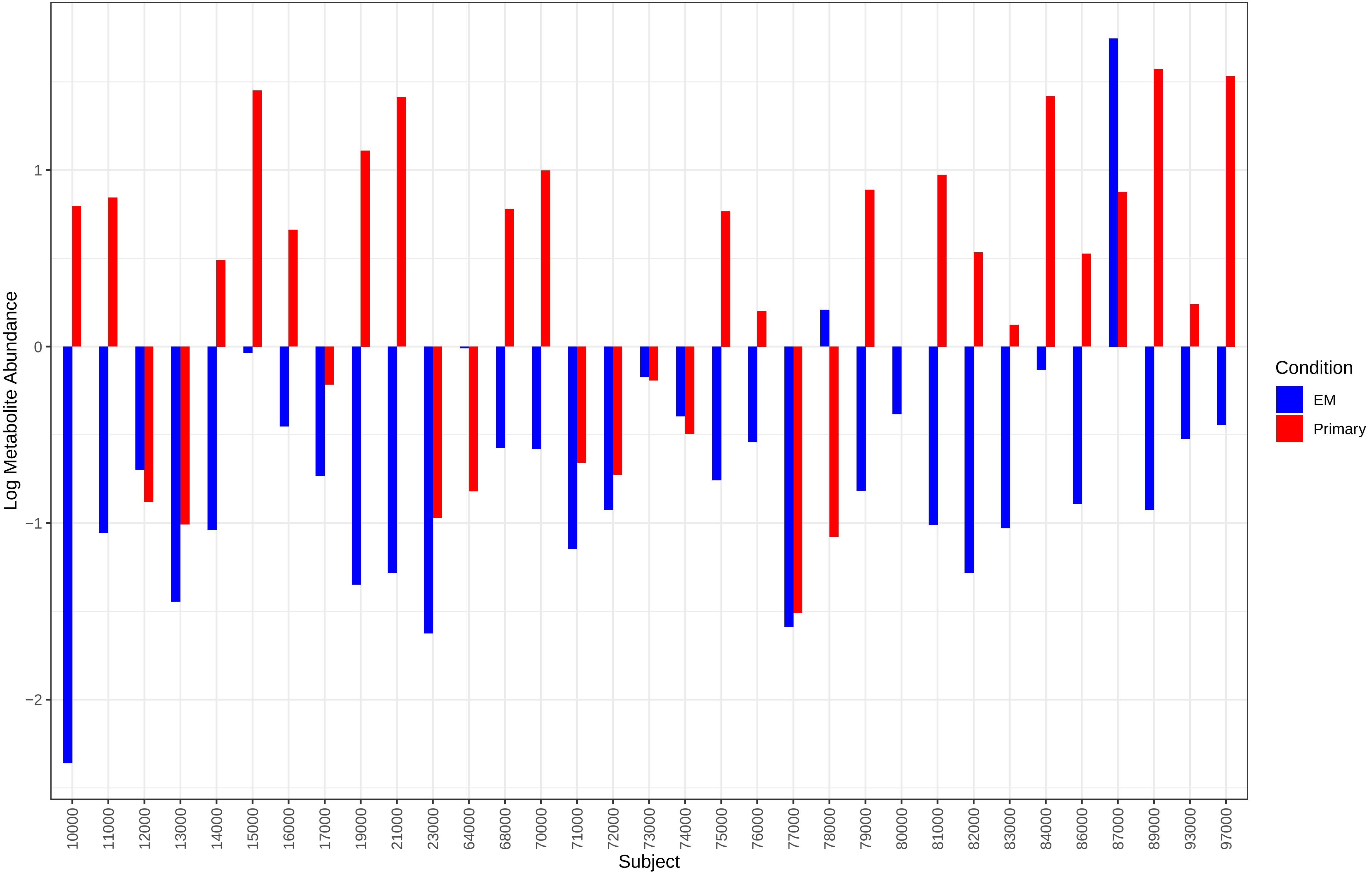

LACTATE (RT: 1.2, m/z: 89.0239, Ion mode: Negative)

Adjusted p (t test): 9.48e−04, Adjusted p (wilcox): 2.39e−03

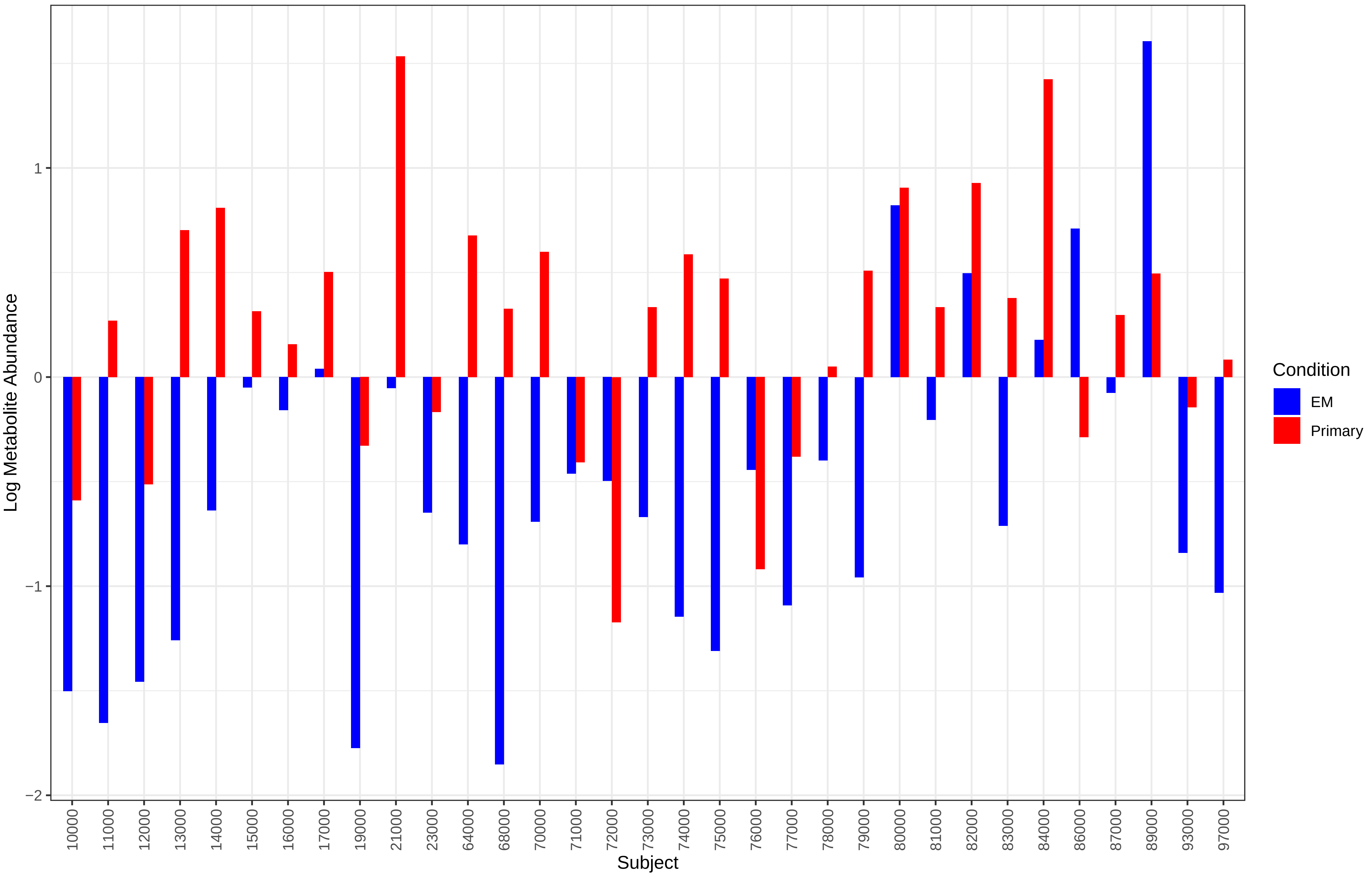

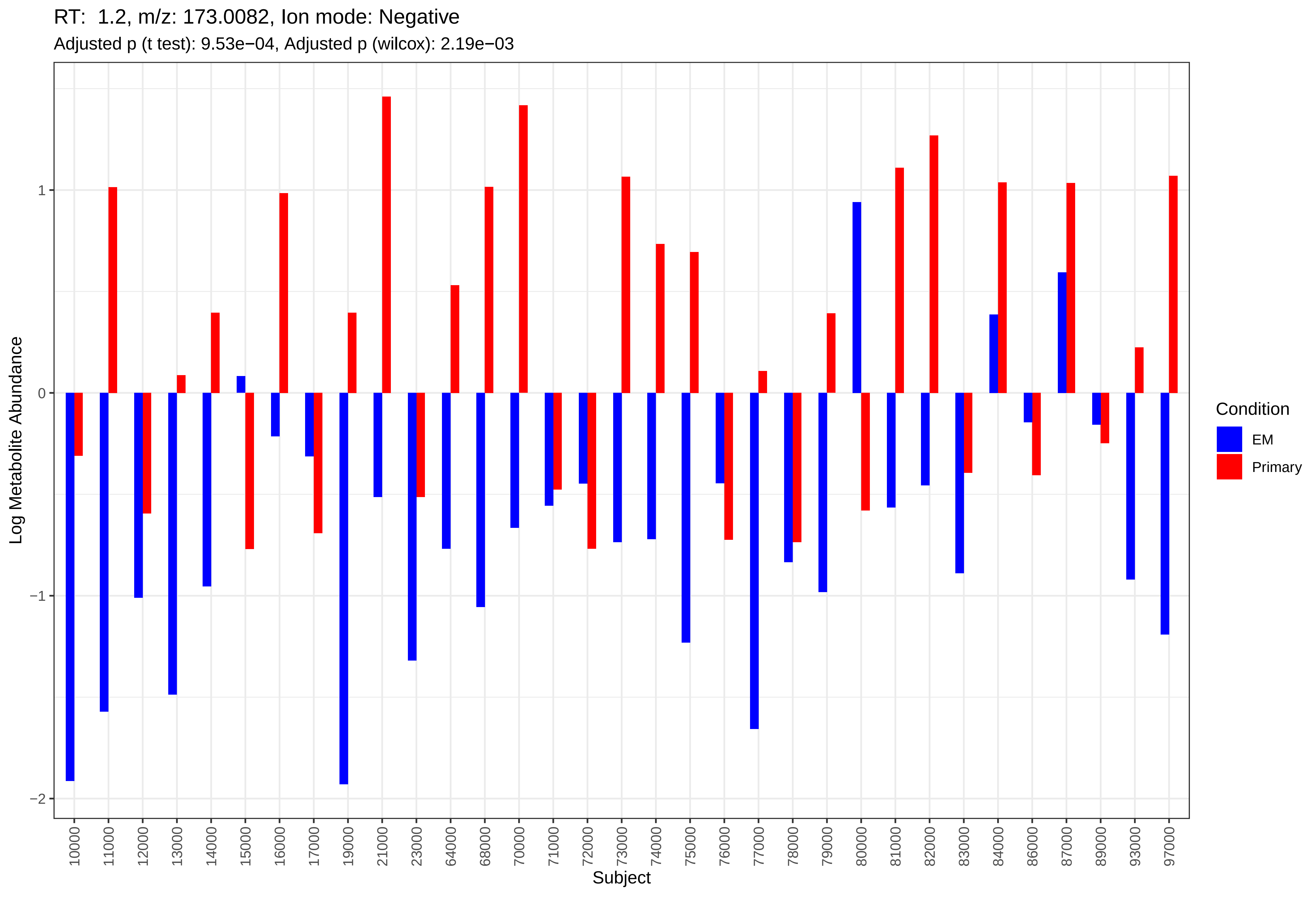
RT: 1.2, m/z: 173.0082, Ion mode: Negative
Adjusted p (t test): 9.53e−04, Adjusted p (wilcox): 2.19e−03
Log Metabolite Abundance
1
0
−1
−2
10000
11000
12000
13000
14000
15000
16000
17000
19000
21000
23000
64000
68000
70000
71000
72000
73000
74000
75000
76000
77000
78000
79000
80000
81000
82000
83000
84000
86000
87000
89000
93000
97000
Subject
Condition
EM
Primary

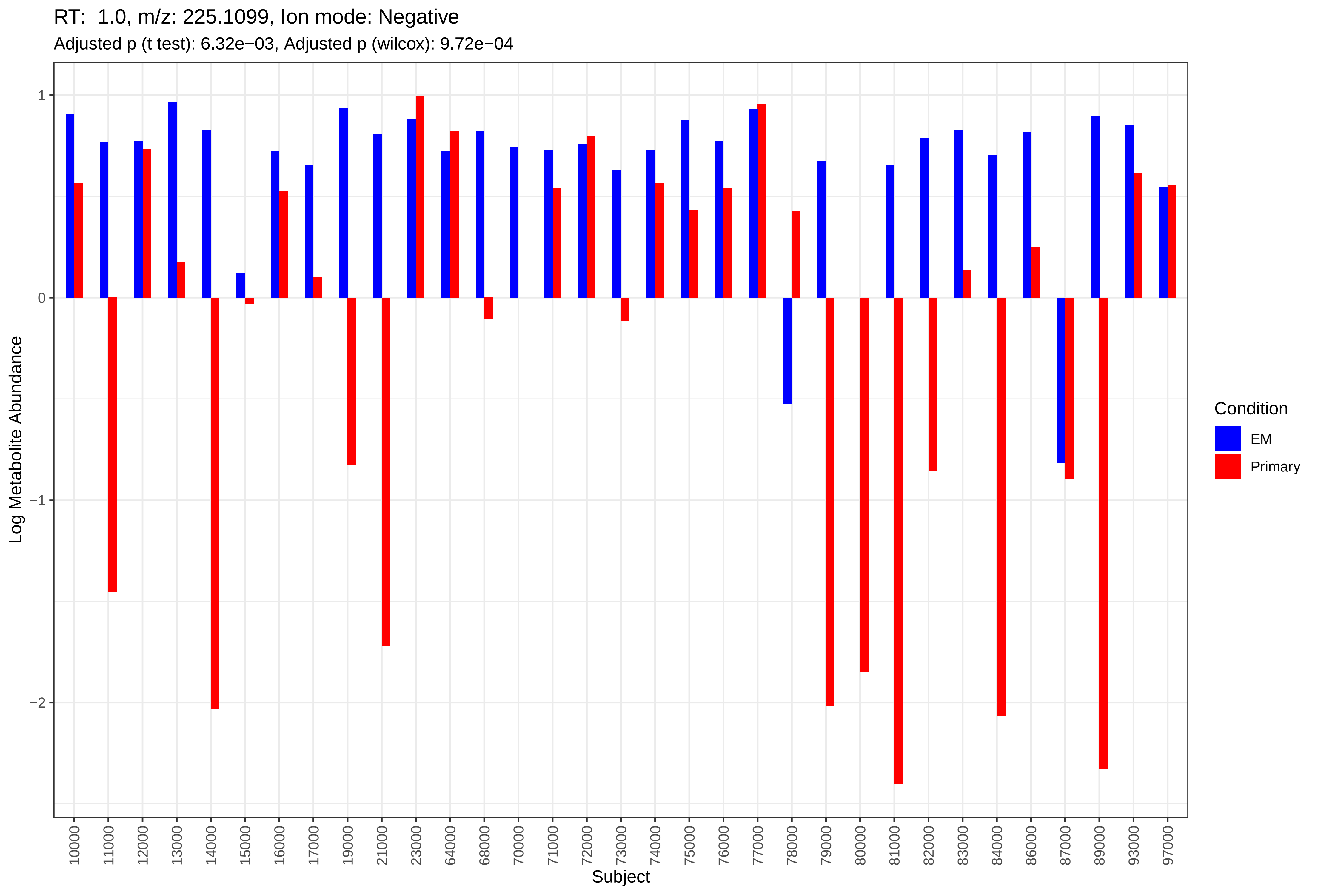
RT: 1.0, m/z: 225.1099, Ion mode: Negative
Adjusted p (t test): 6.32e−03, Adjusted p (wilcox): 9.72e−04
Log Metabolite Abundance
1
0
−1
−2
Subject
10000
11000
12000
13000
14000
15000
16000
17000
19000
21000
23000
64000
68000
70000
71000
72000
73000
74000
75000
76000
77000
78000
79000
80000
81000
82000
83000
84000
86000
87000
89000
93000
97000
Condition
EM
Primary

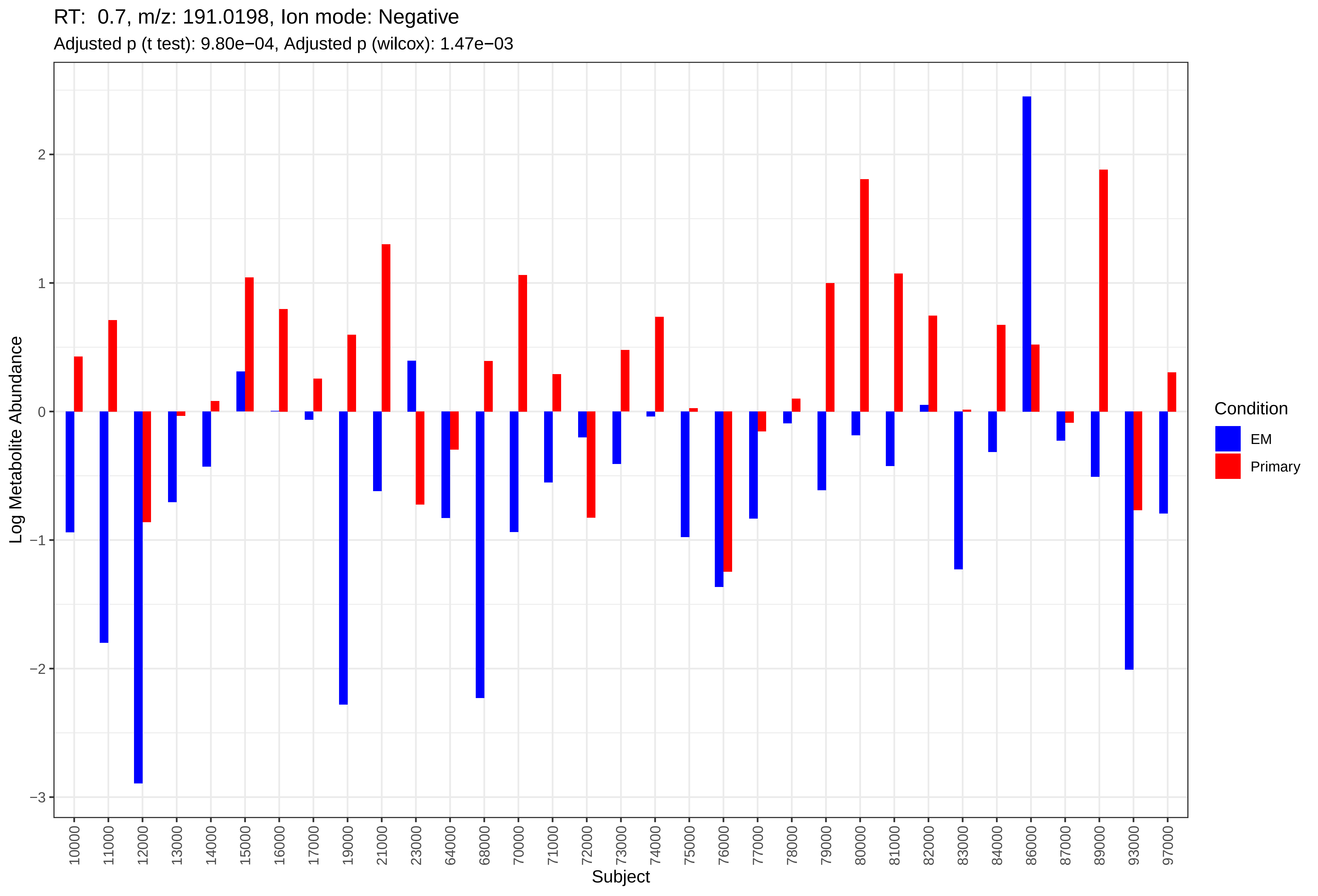

RT: 0.7, m/z: 191.0198, Ion mode: Negative
Adjusted p (t test): 9.80e−04, Adjusted p (wilcox): 1.47e−03
Log Metabolite Abundance
2
1
0
−1
−2
−3
10000
11000
12000
13000
14000
15000
16000
17000
19000
21000
23000
64000
68000
70000
71000
72000
73000
74000
75000
76000
77000
78000
79000
80000
81000
82000
83000
84000
86000
87000
89000
93000
97000
Subject
Condition
EM
Primary

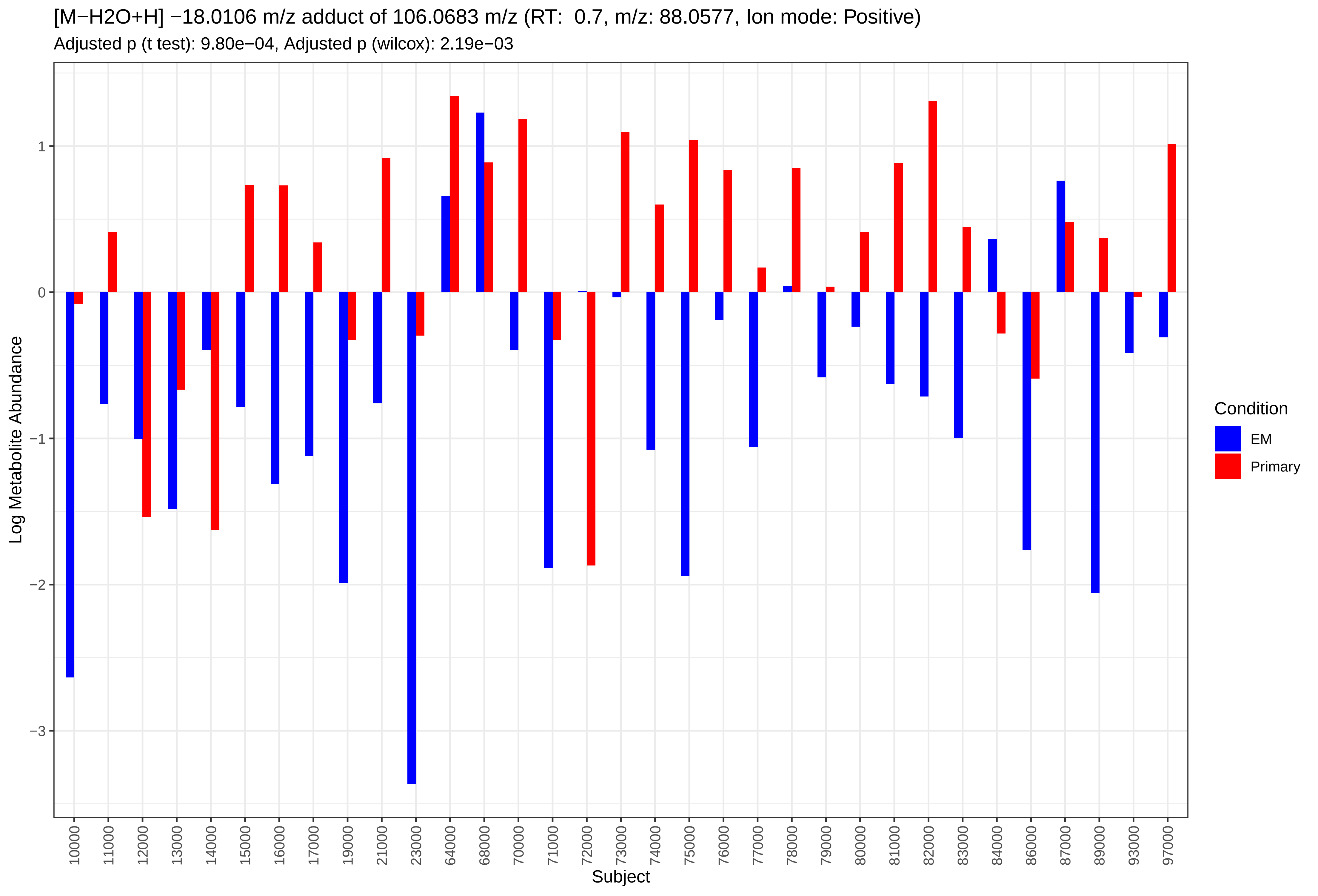

[M−H2O+H] −18.0106 m/z adduct of 106.0683 m/z (RT: 0.7, m/z: 88.0577, Ion mode: Positive)
Adjusted p (t test): 9.80e−04, Adjusted p (wilcox): 2.19e−03
Log Metabolite Abundance
1
0
−1
−2
−3
Subject
10000
11000
12000
13000
14000
15000
16000
17000
19000
21000
23000
64000
68000
70000
71000
72000
73000
74000
75000
76000
77000
78000
79000
80000
81000
82000
83000
84000
86000
87000
89000
93000
97000
Condition
EM
Primary

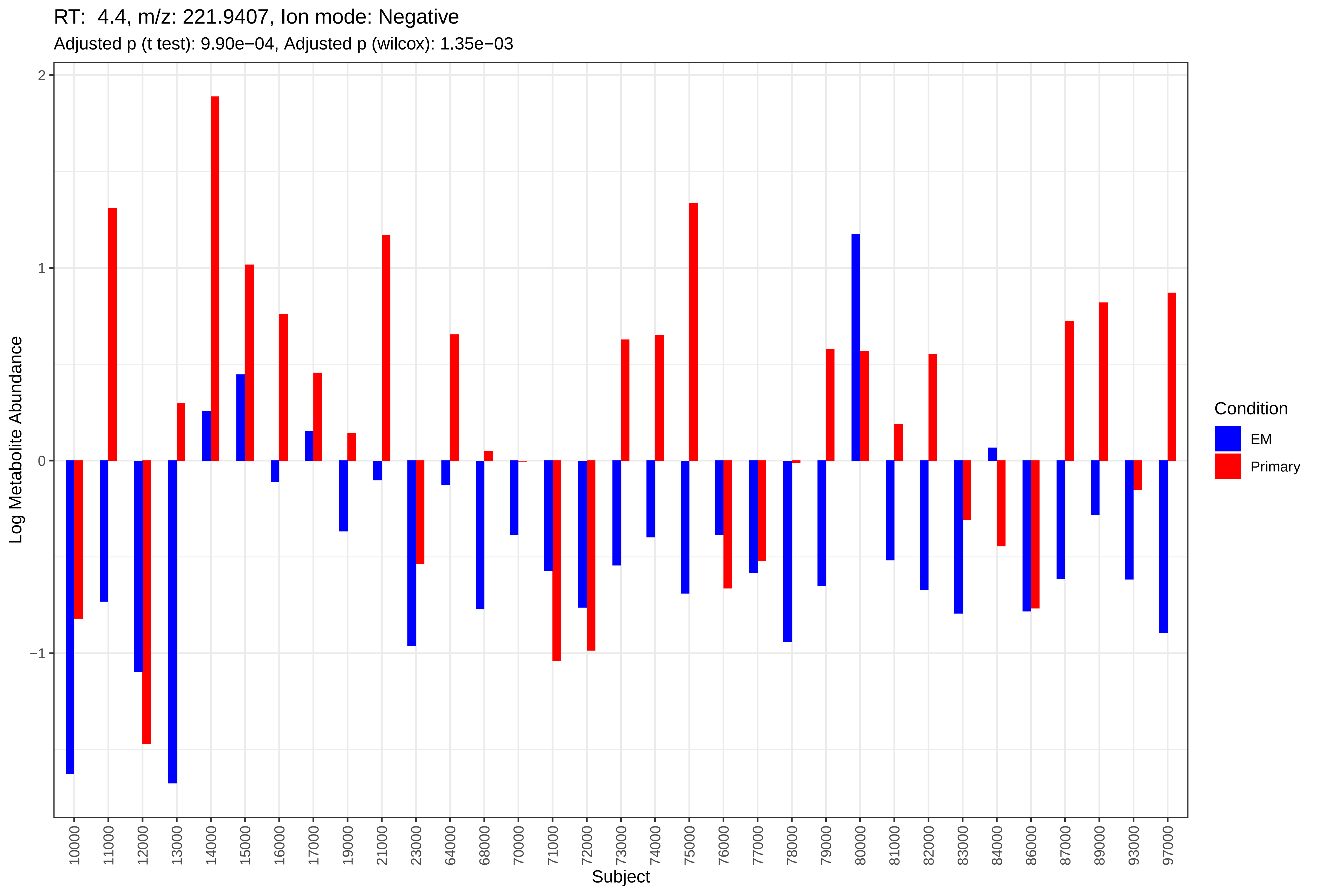
RT: 4.4, m/z: 221.9407, Ion mode: Negative
Adjusted p (t test): 9.90e−04, Adjusted p (wilcox): 1.35e−03
Log Metabolite Abundance
2
1
0
−1
10000
11000
12000
13000
14000
15000
16000
17000
19000
21000
23000
64000
68000
70000
71000
72000
73000
74000
75000
76000
77000
78000
79000
80000
81000
82000
83000
84000
86000
87000
89000
93000
97000
Subject
Condition
EM
Primary

UROCANATE (RT: 1.9, m/z: 137.0358, Ion mode: Negative)

Adjusted p (t test): 9.99e−04, Adjusted p (wilcox): 1.47e−03

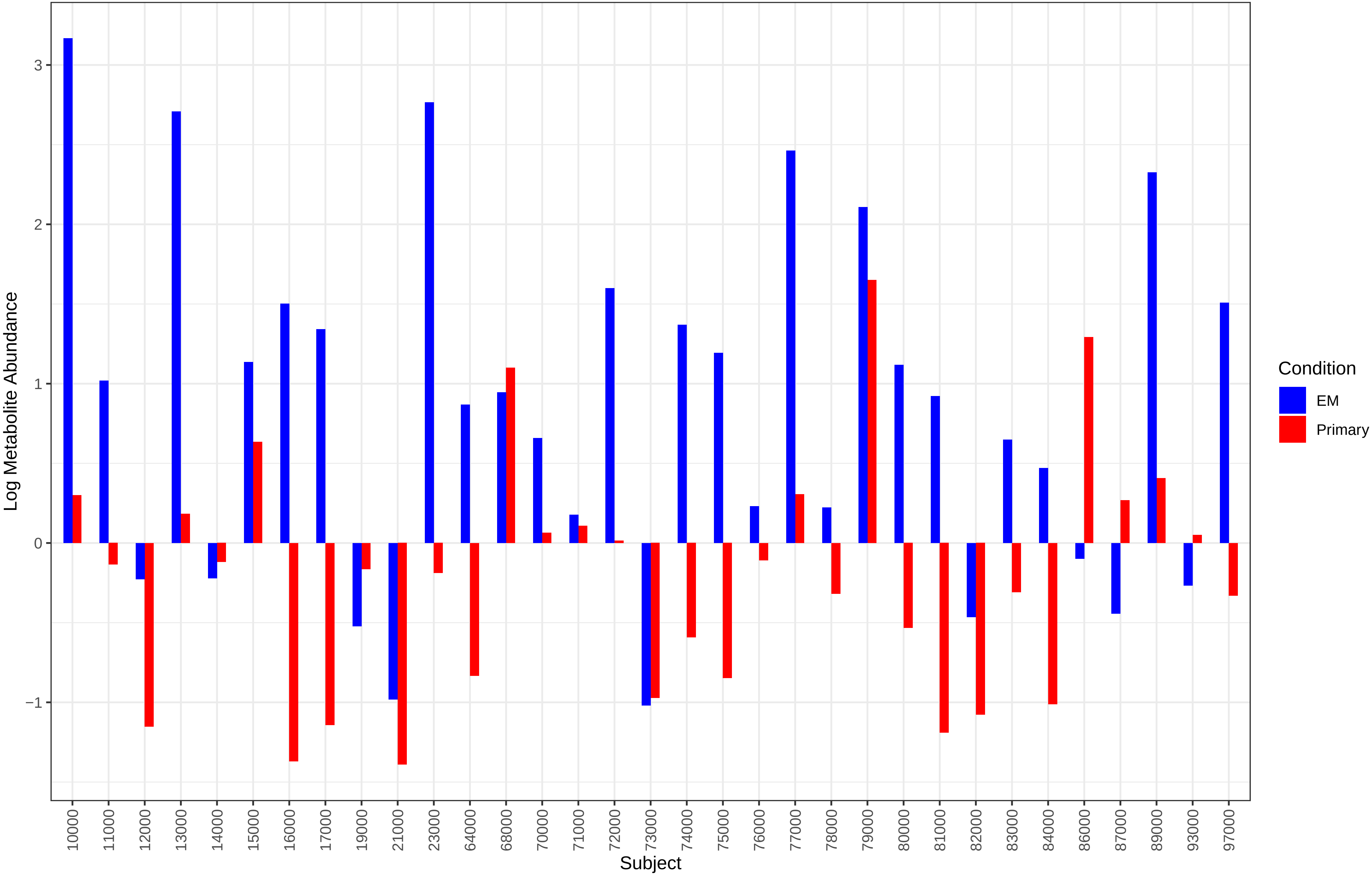

RT: 1.2, m/z: 188.0227, Ion mode: Positive

Adjusted p (t test): 9.99e−04, Adjusted p (wilcox): 1.89e−03

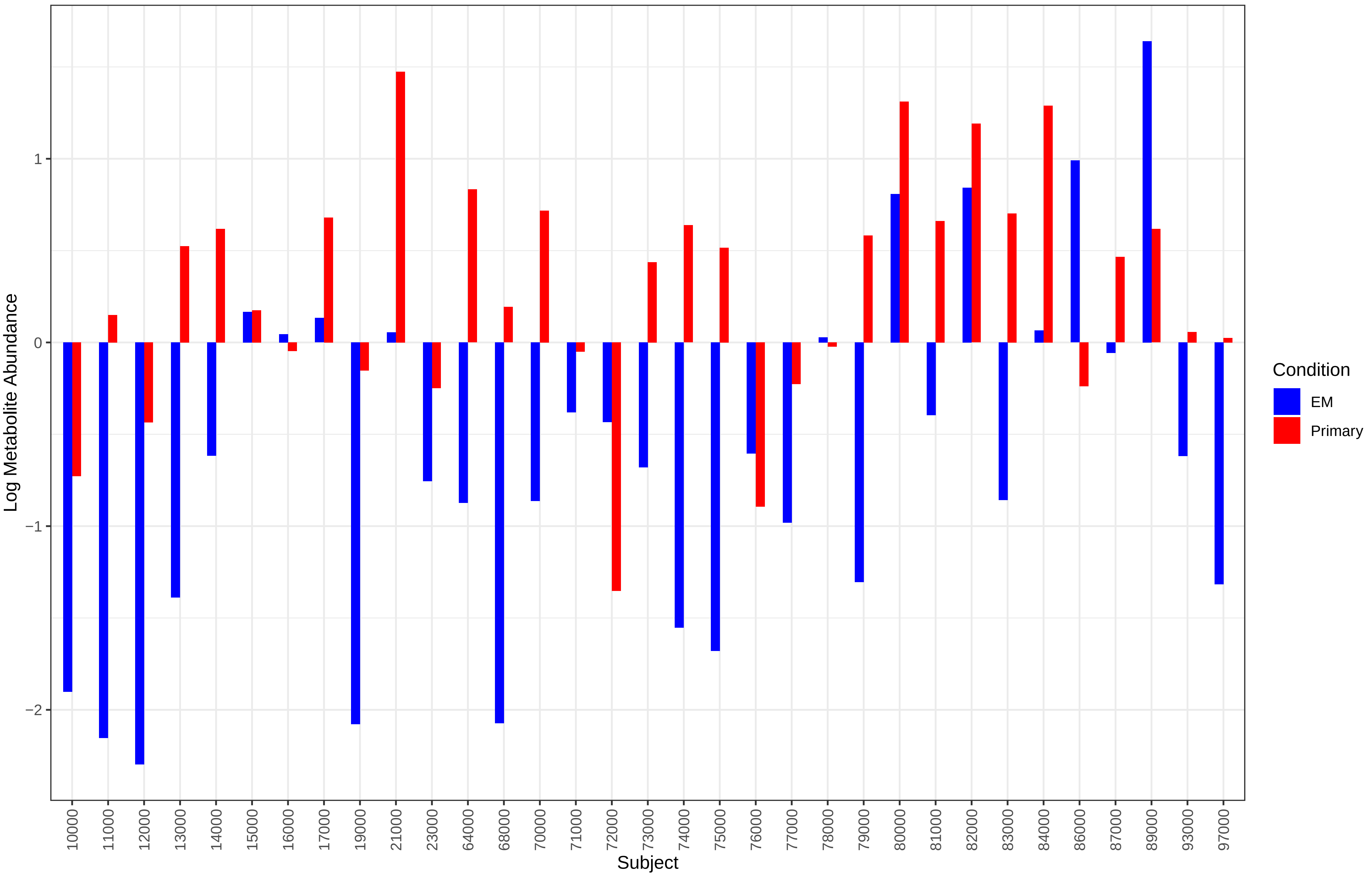

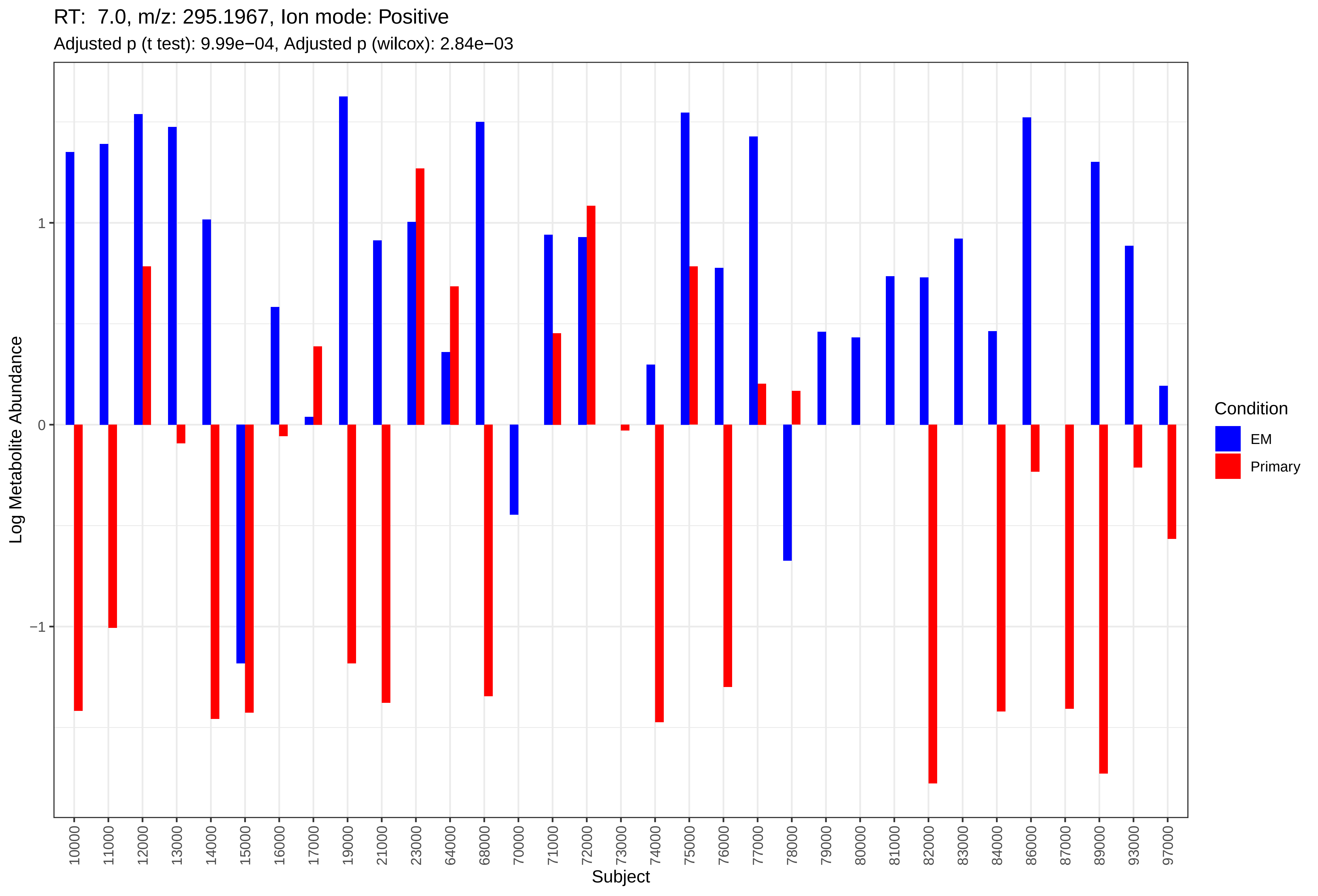
RT: 7.0, m/z: 295.1967, Ion mode: Positive
Adjusted p (t test): 9.99e−04, Adjusted p (wilcox): 2.84e−03
Log Metabolite Abundance
1
0
−1
10000
11000
12000
13000
14000
15000
16000
17000
19000
21000
23000
64000
68000
70000
71000
72000
73000
74000
75000
76000
77000
78000
79000
80000
81000
82000
83000
84000
86000
87000
89000
93000
97000
Subject
Condition
EM
Primary

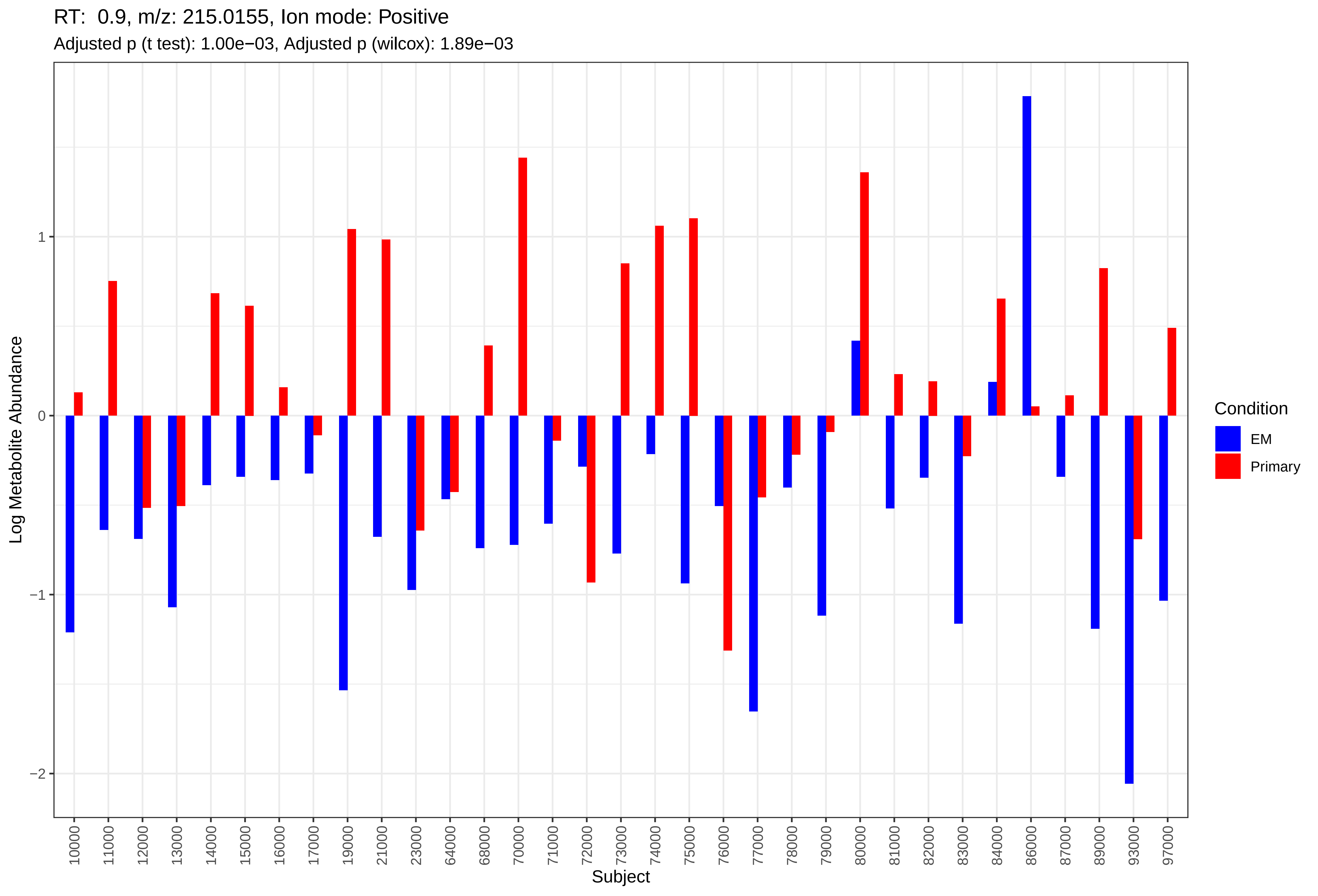

RT: 0.9, m/z: 215.0155, Ion mode: Positive
Adjusted p (t test): 1.00e−03, Adjusted p (wilcox): 1.89e−03
Log Metabolite Abundance
1
0
−1
−2
Subject
10000
11000
12000
13000
14000
15000
16000
17000
19000
21000
23000
64000
68000
70000
71000
72000
73000
74000
75000
76000
77000
78000
79000
80000
81000
82000
83000
84000
86000
87000
89000
93000
97000
Condition
EM
Primary

RT: 0.6, m/z: 400.9093, Ion mode: Negative

Adjusted p (t test): 1.04e−03, Adjusted p (wilcox): 1.03e−03

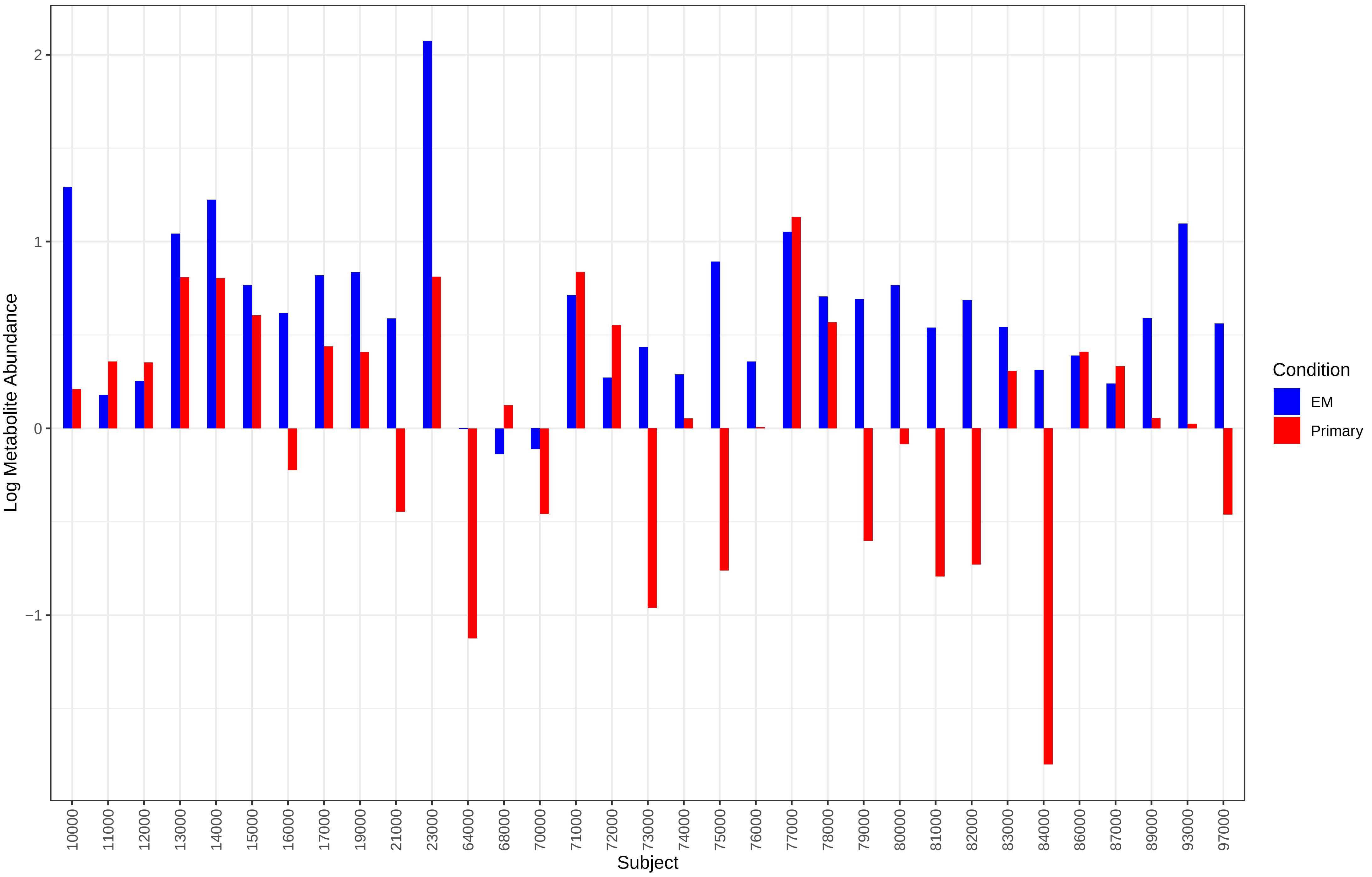

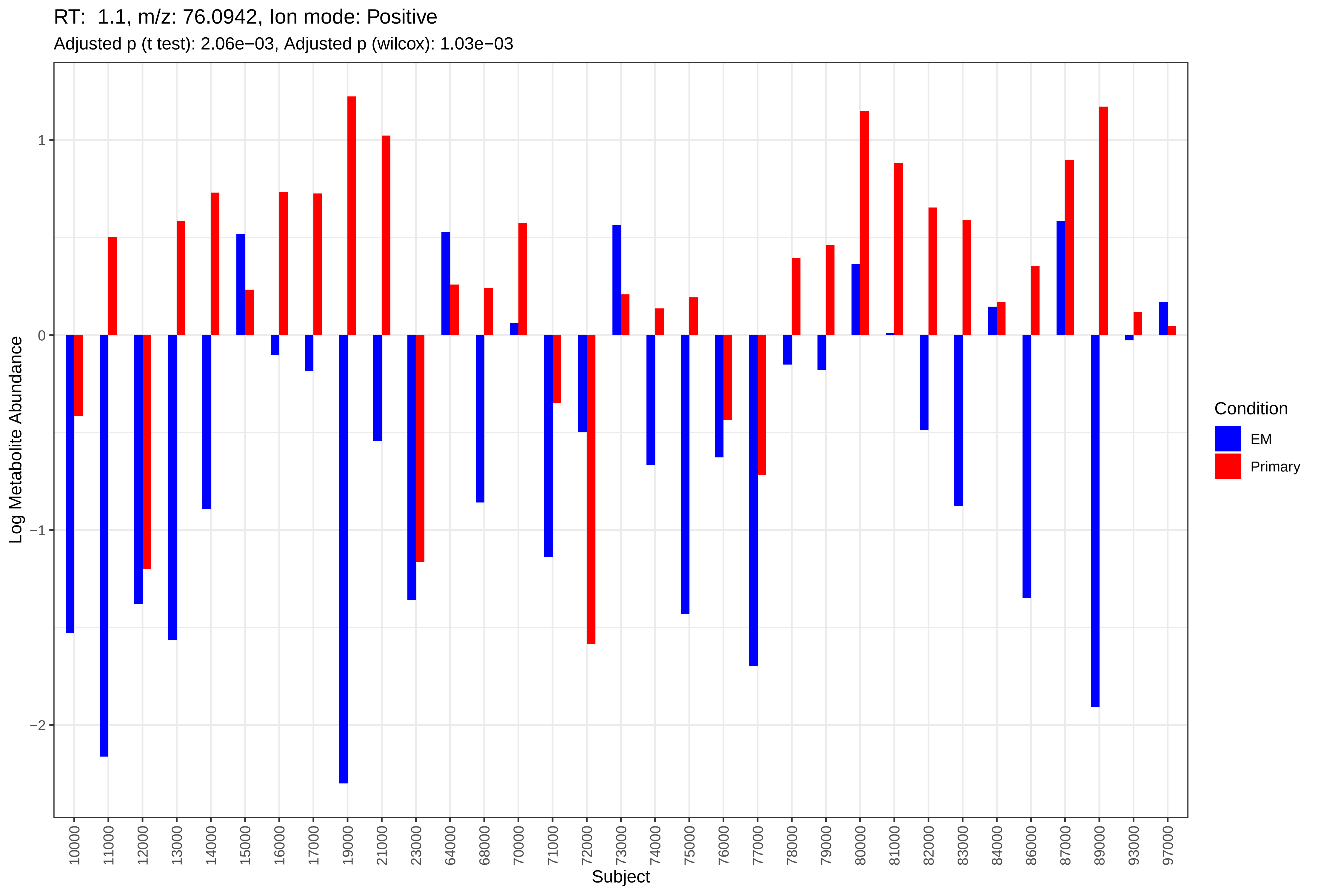

RT: 1.1, m/z: 76.0942, Ion mode: Positive
Adjusted p (t test): 2.06e−03, Adjusted p (wilcox): 1.03e−03
Log Metabolite Abundance
1
0
−1
−2
10000
11000
12000
13000
14000
15000
16000
17000
19000
21000
23000
64000
68000
70000
71000
72000
73000
74000
75000
76000
77000
78000
79000
80000
81000
82000
83000
84000
86000
87000
89000
93000
97000
Subject
Condition
EM
Primary

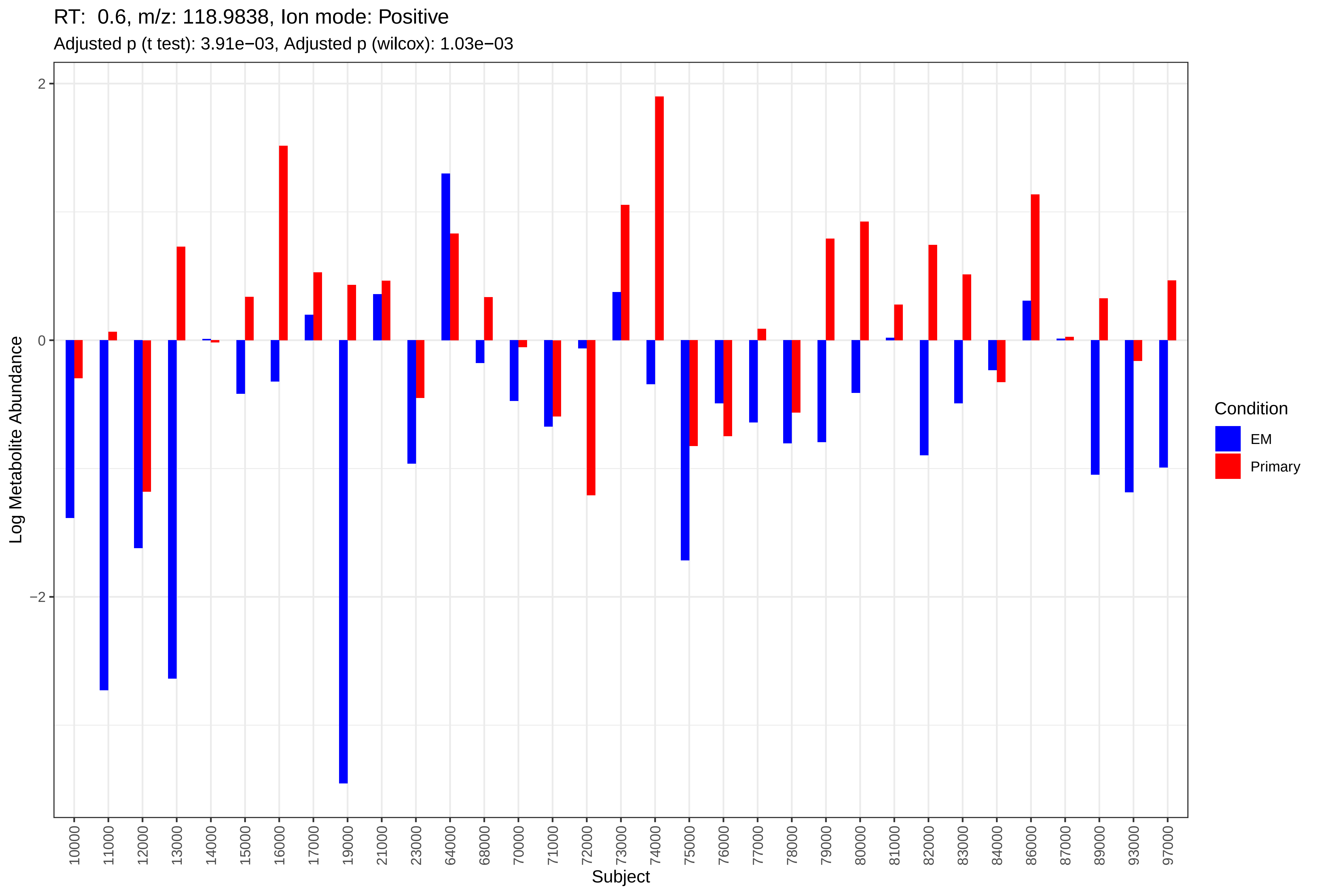

RT: 0.6, m/z: 118.9838, Ion mode: Positive
Adjusted p (t test): 3.91e−03, Adjusted p (wilcox): 1.03e−03
Log Metabolite Abundance
2
0
−2
10000
11000
12000
13000
14000
15000
16000
17000
19000
21000
23000
64000
68000
70000
71000
72000
73000
74000
75000
76000
77000
78000
79000
80000
81000
82000
83000
84000
86000
87000
89000
93000
97000
Subject
Condition
EM
Primary

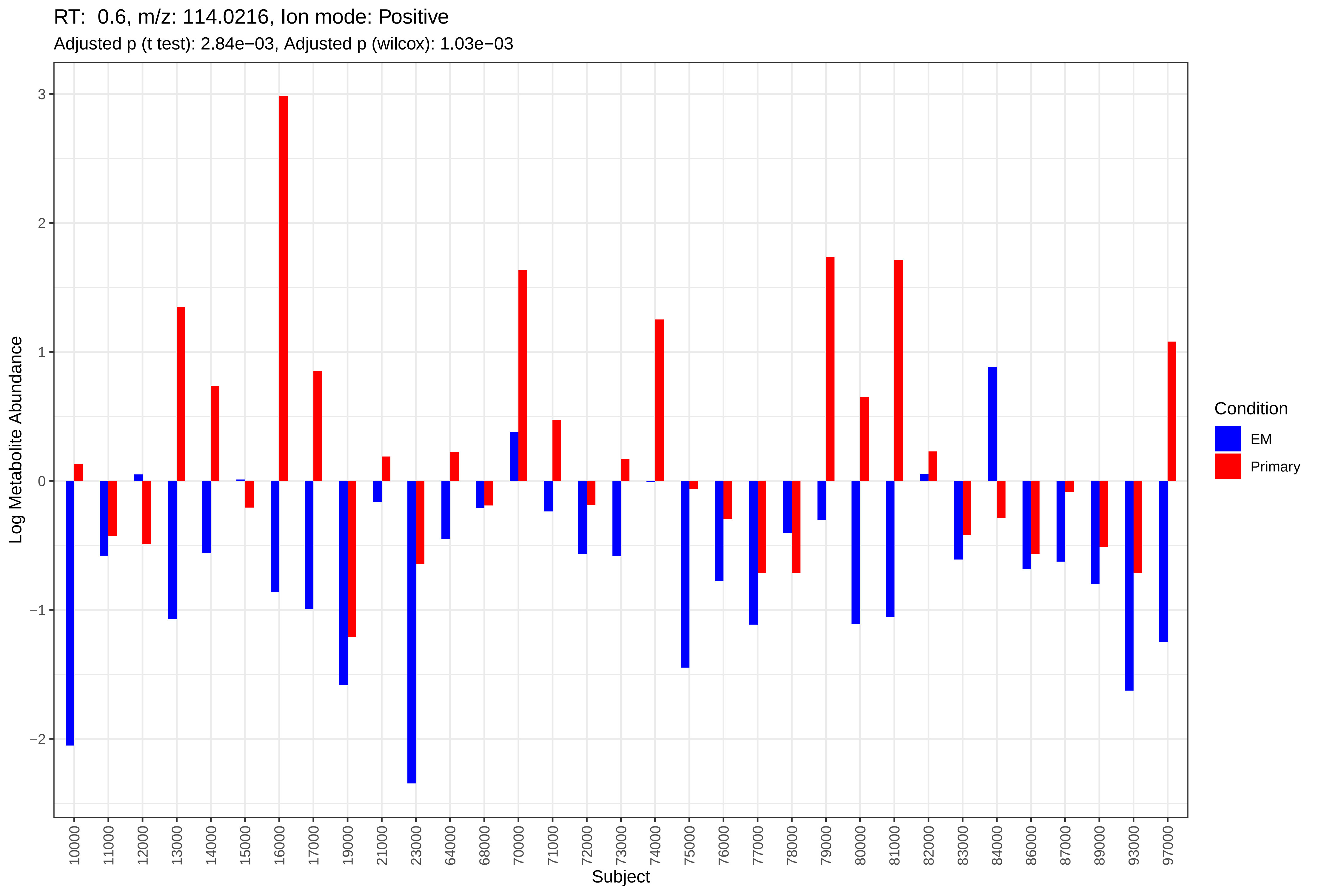

RT: 0.6, m/z: 114.0216, Ion mode: Positive
Adjusted p (t test): 2.84e−03, Adjusted p (wilcox): 1.03e−03
Log Metabolite Abundance
3
2
1
0
−1
−2
10000
11000
12000
13000
14000
15000
16000
17000
19000
21000
23000
64000
68000
70000
71000
72000
73000
74000
75000
76000
77000
78000
79000
80000
81000
82000
83000
84000
86000
87000
89000
93000
97000
Subject
Condition
EM
Primary

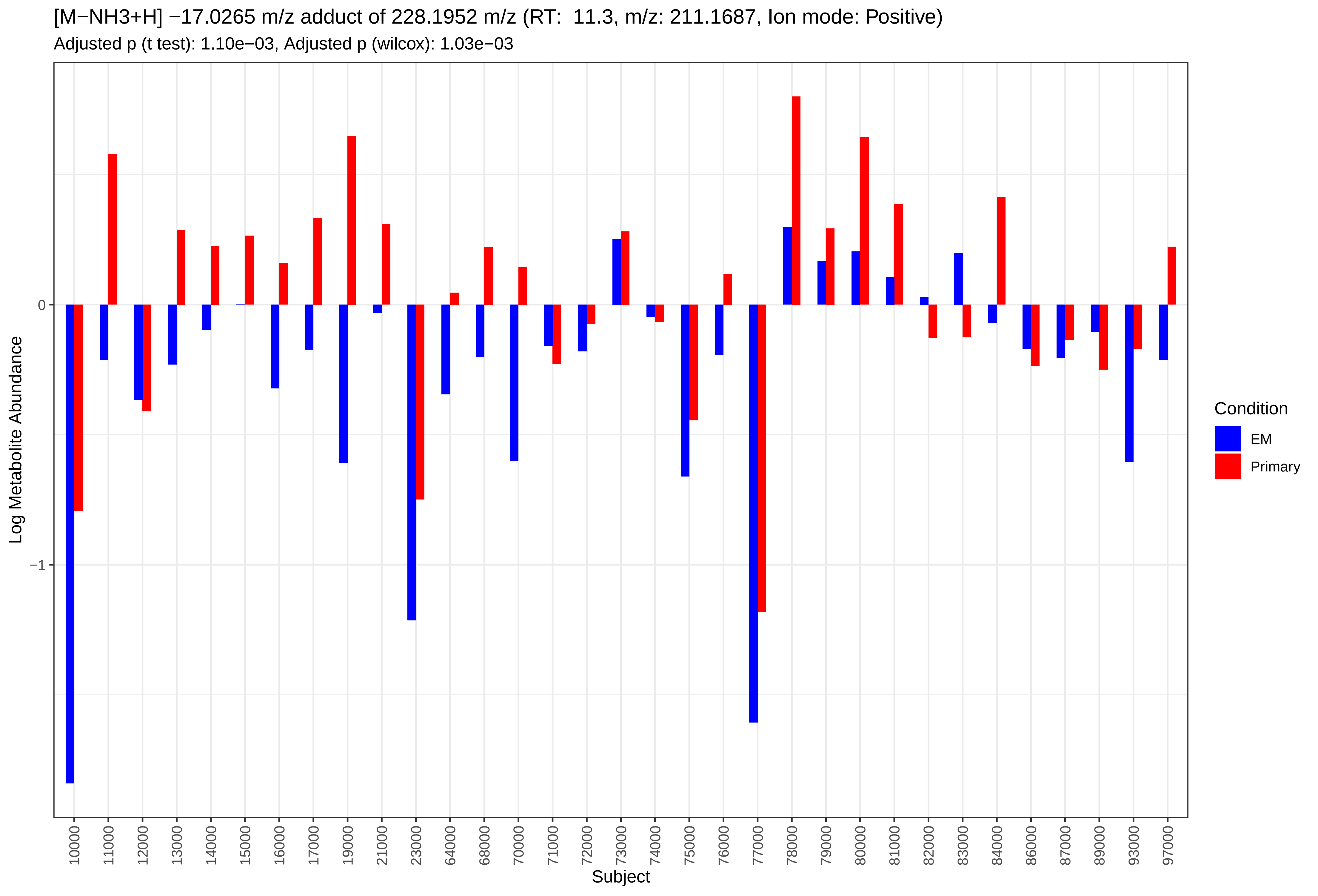

[M−NH3+H] −17.0265 m/z adduct of 228.1952 m/z (RT: 11.3, m/z: 211.1687, Ion mode: Positive)
Adjusted p (t test): 1.10e−03, Adjusted p (wilcox): 1.03e−03
Log Metabolite Abundance
0
−1
Subject
10000
11000
12000
13000
14000
15000
16000
17000
19000
21000
23000
64000
68000
70000
71000
72000
73000
74000
75000
76000
77000
78000
79000
80000
81000
82000
83000
84000
86000
87000
89000
93000
97000
Condition
EM
Primary

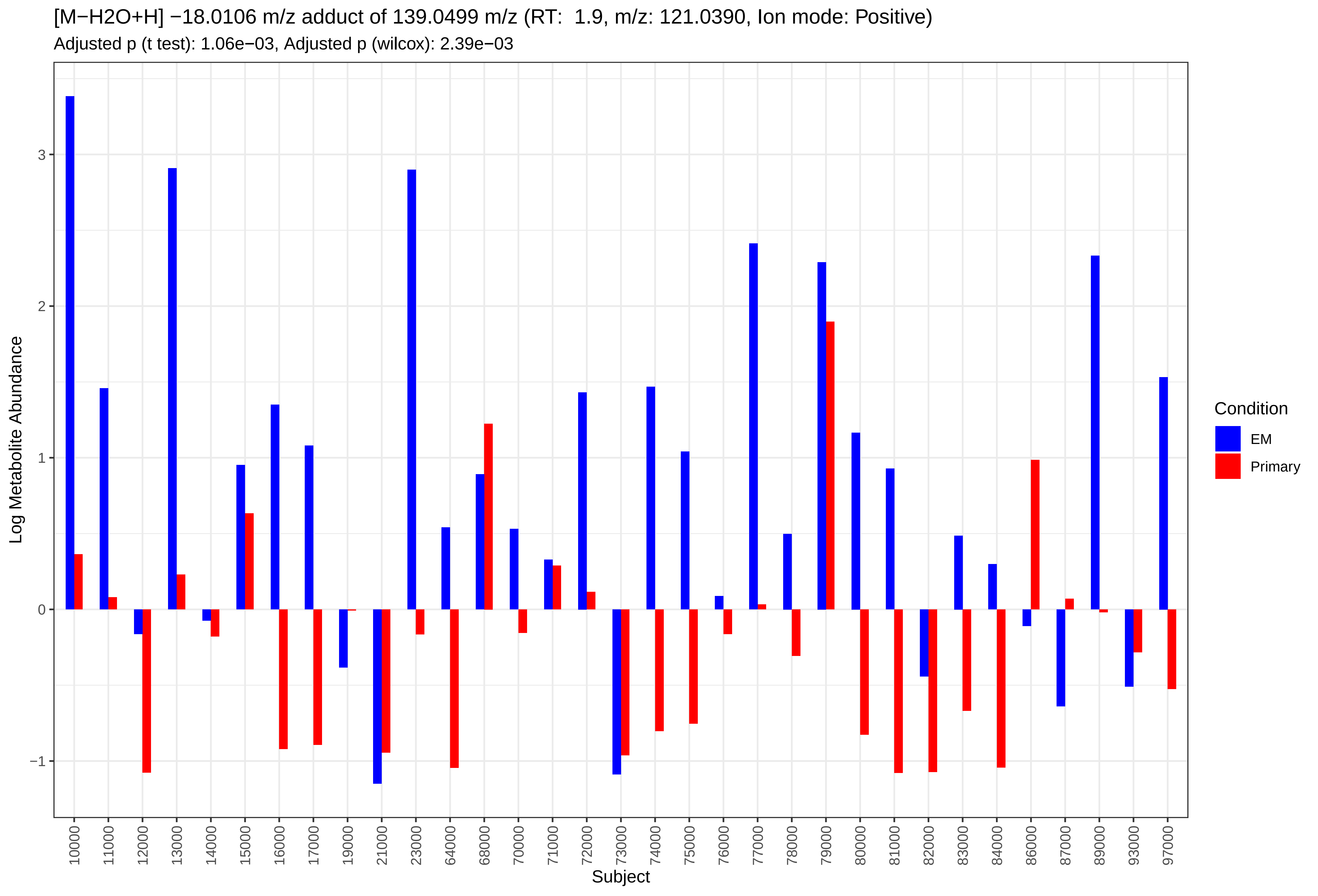

[M−H2O+H] −18.0106 m/z adduct of 139.0499 m/z (RT: 1.9, m/z: 121.0390, Ion mode: Positive)
Adjusted p (t test): 1.06e−03, Adjusted p (wilcox): 2.39e−03
Log Metabolite Abundance
Subject
Condition
EM
Primary
10000
11000
12000
13000
14000
15000
16000
17000
19000
21000
23000
64000
68000
70000
71000
72000
73000
74000
75000
76000
77000
78000
79000
80000
81000
82000
83000
84000
86000
87000
89000
93000
97000

RT: 0.7, m/z: 129.5759, Ion mode: Positive

Adjusted p (t test): 1.69e−03, Adjusted p (wilcox): 1.08e−03

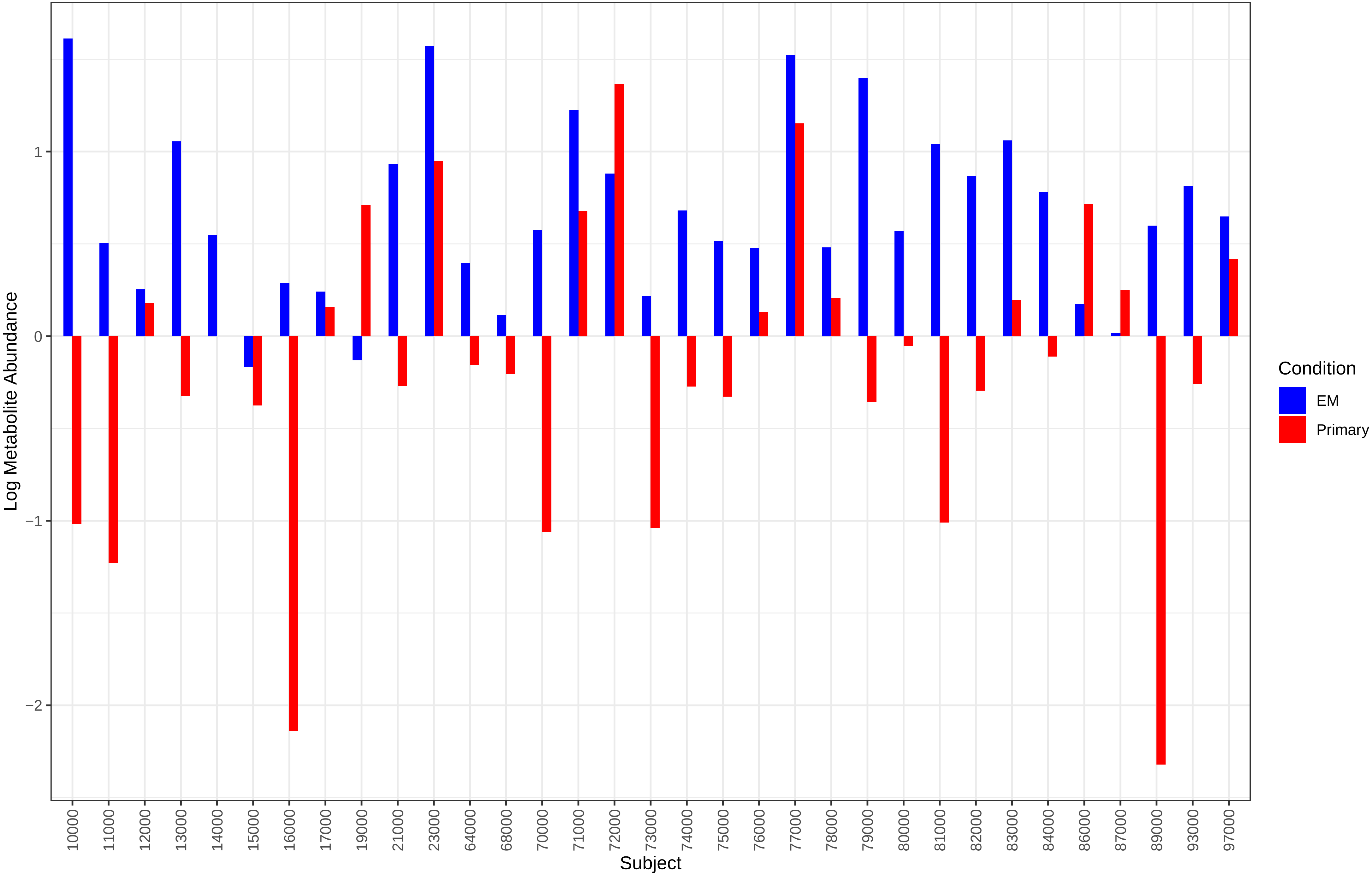

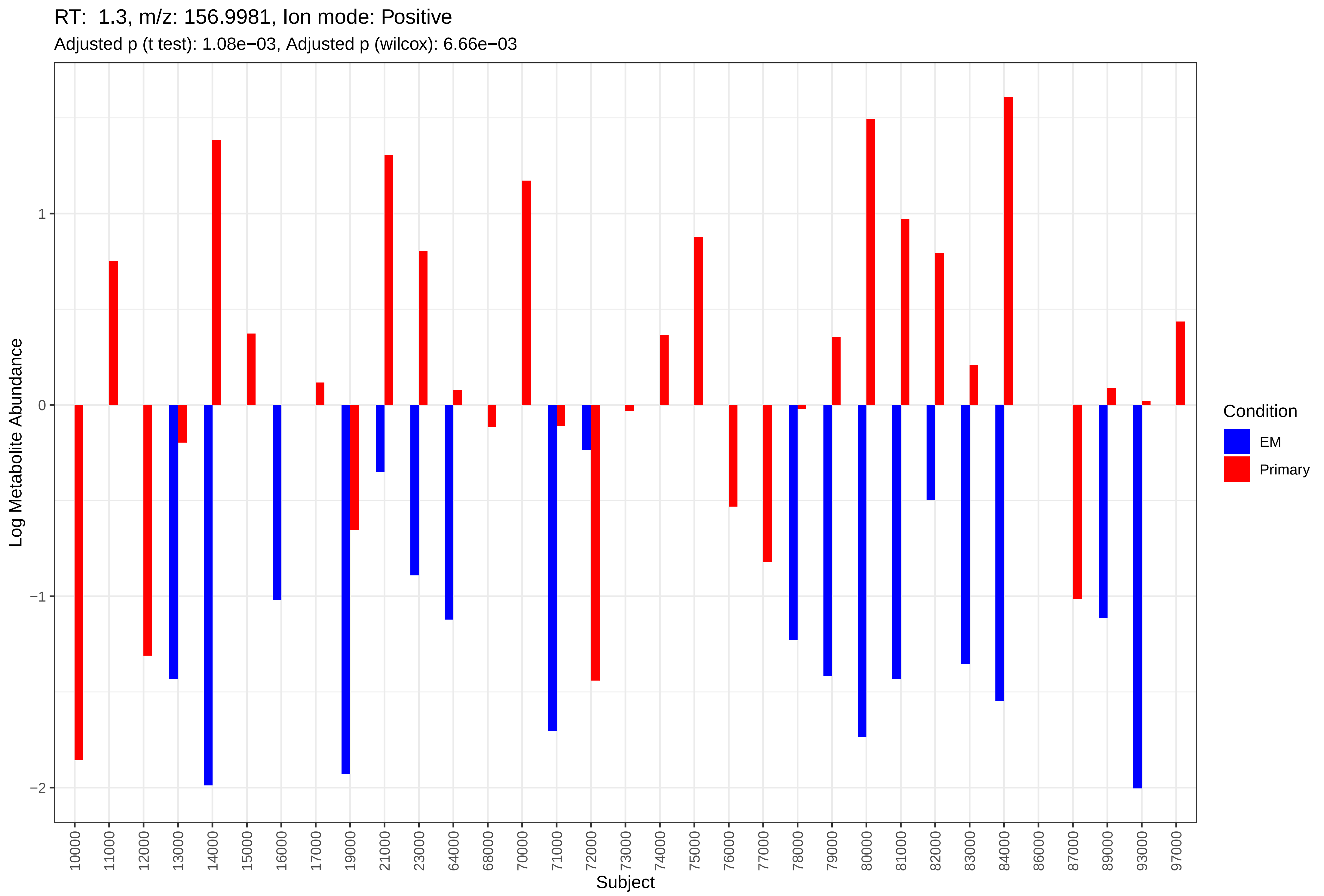
RT: 1.3, m/z: 156.9981, Ion mode: Positive
Adjusted p (t test): 1.08e−03, Adjusted p (wilcox): 6.66e−03
Log Metabolite Abundance
1
0
−1
−2
10000
11000
12000
13000
14000
15000
16000
17000
19000
21000
23000
64000
68000
70000
71000
72000
73000
74000
75000
76000
77000
78000
79000
80000
81000
82000
83000
84000
86000
87000
89000
93000
97000
Subject
Condition
EM
Primary

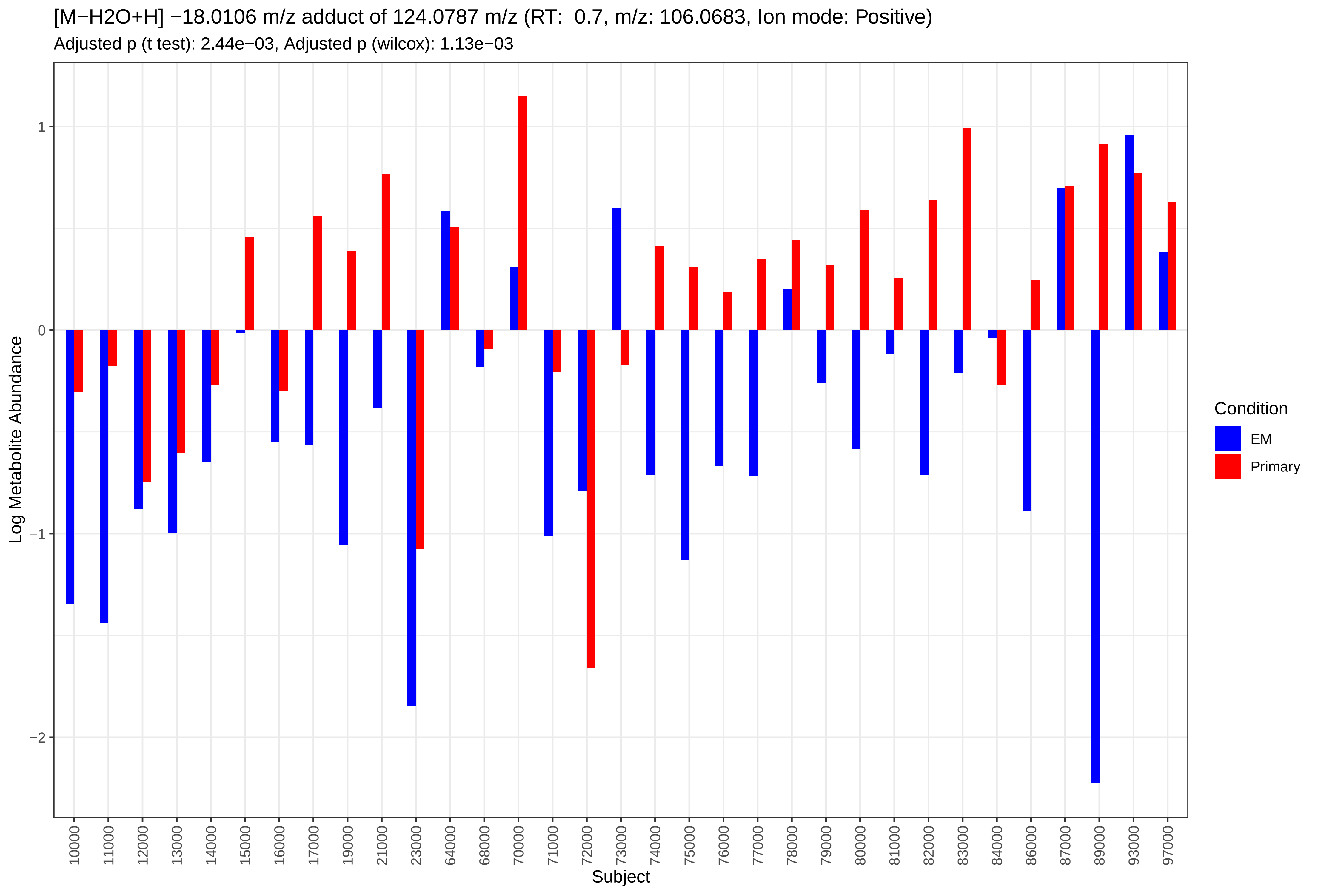

[M−H2O+H] −18.0106 m/z adduct of 124.0787 m/z (RT: 0.7, m/z: 106.0683, Ion mode: Positive)
Adjusted p (t test): 2.44e−03, Adjusted p (wilcox): 1.13e−03
Log Metabolite Abundance
1
0
−1
−2
Subject
10000
11000
12000
13000
14000
15000
16000
17000
19000
21000
23000
64000
68000
70000
71000
72000
73000
74000
75000
76000
77000
78000
79000
80000
81000
82000
83000
84000
86000
87000
89000
93000
97000
Condition
EM
Primary

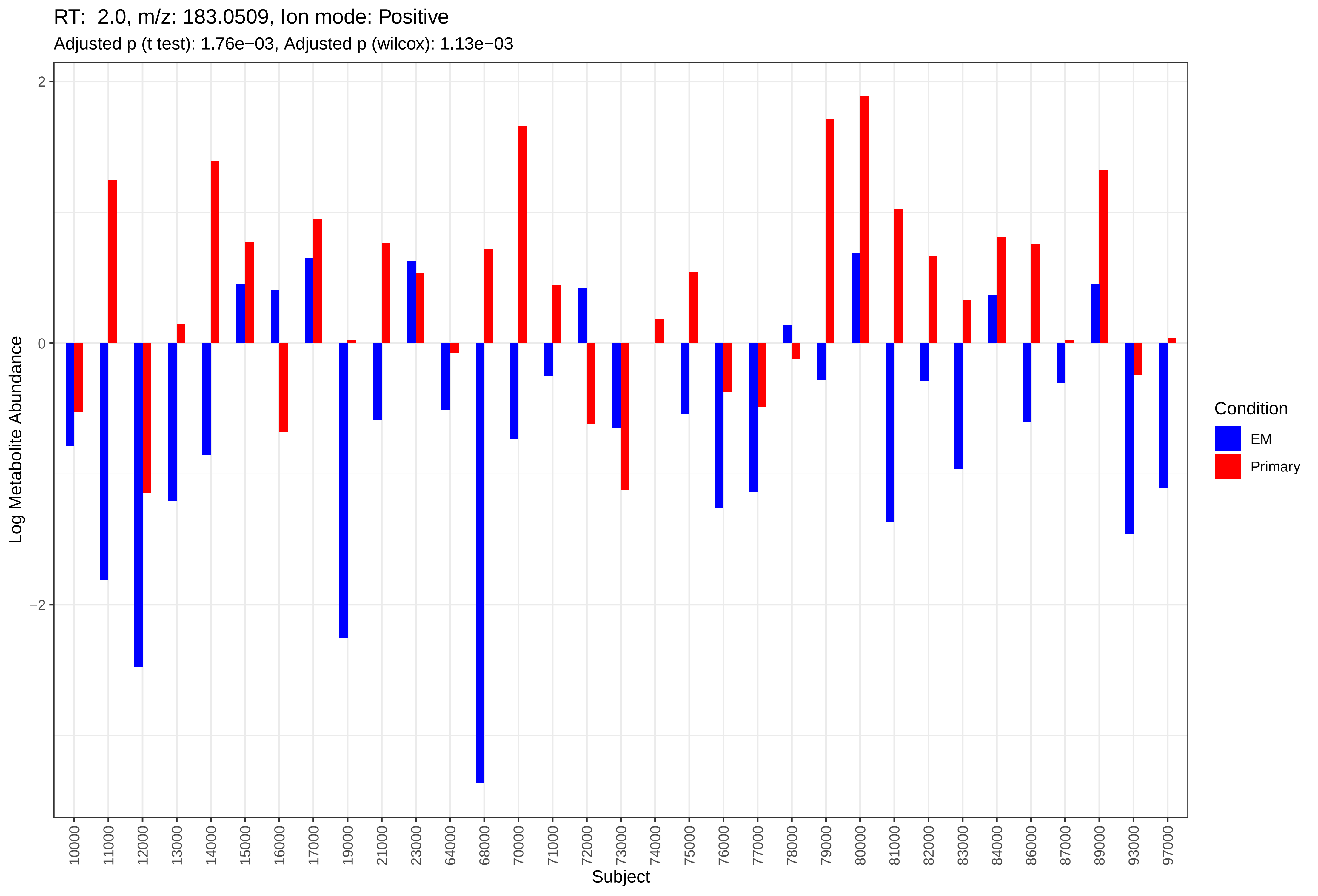

RT: 2.0, m/z: 183.0509, Ion mode: Positive
Adjusted p (t test): 1.76e−03, Adjusted p (wilcox): 1.13e−03
Log Metabolite Abundance
2
0
−2
Subject
10000
11000
12000
13000
14000
15000
16000
17000
19000
21000
23000
64000
68000
70000
71000
72000
73000
74000
75000
76000
77000
78000
79000
80000
81000
82000
83000
84000
86000
87000
89000
93000
97000
Condition
EM
Primary

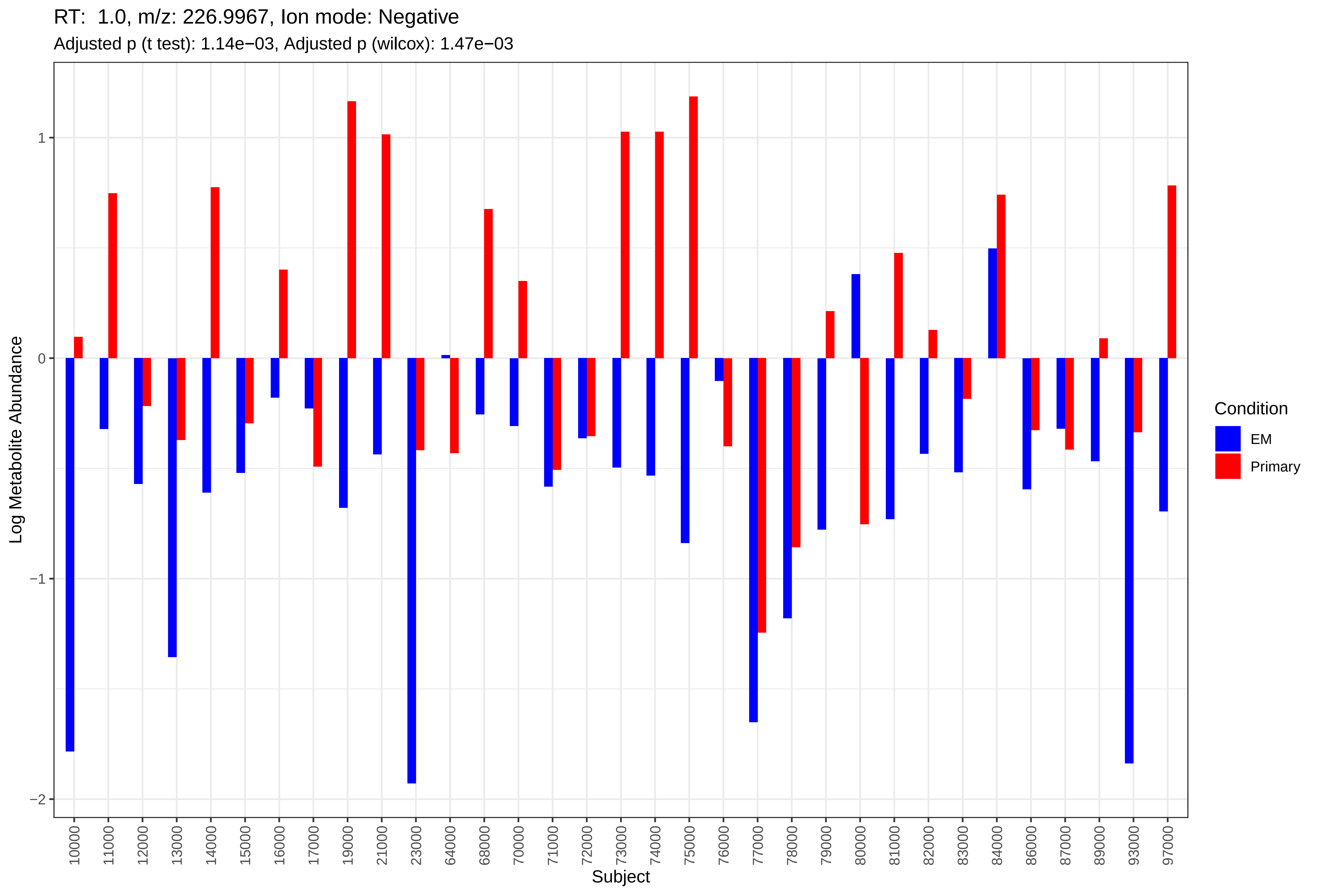

RT: 1.0, m/z: 226.9967, Ion mode: Negative
Adjusted p (t test): 1.14e−03, Adjusted p (wilcox): 1.47e−03
Log Metabolite Abundance
1
0
−1
−2
Subject
10000
11000
12000
13000
14000
15000
16000
17000
19000
21000
23000
64000
68000
70000
71000
72000
73000
74000
75000
76000
77000
78000
79000
80000
81000
82000
83000
84000
86000
87000
89000
93000
97000
Condition
EM
Primary

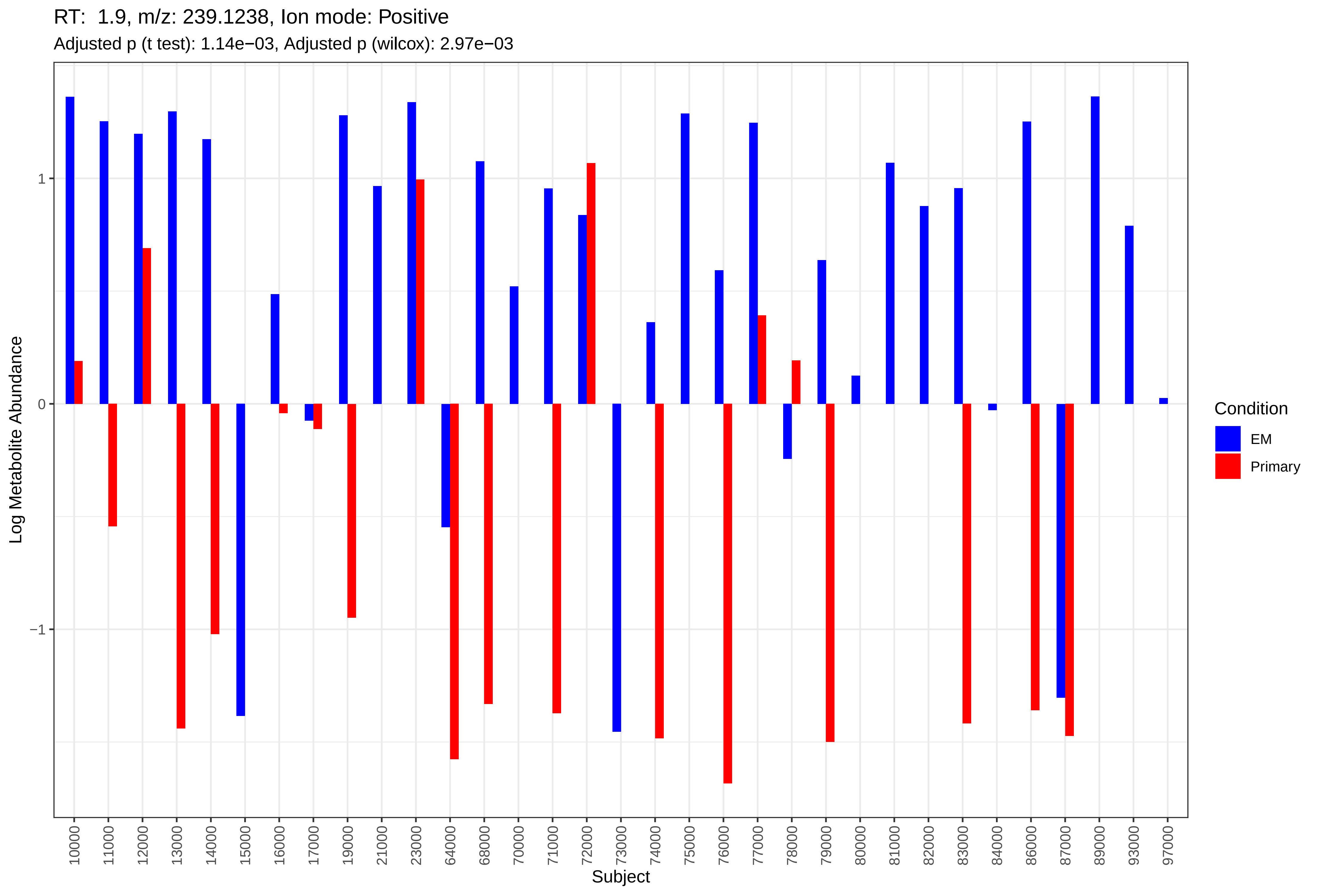
RT: 1.9, m/z: 239.1238, Ion mode: Positive
Adjusted p (t test): 1.14e−03, Adjusted p (wilcox): 2.97e−03
Log Metabolite Abundance
1
0
−1
10000
11000
12000
13000
14000
15000
16000
17000
19000
21000
23000
64000
68000
70000
71000
72000
73000
74000
75000
76000
77000
78000
79000
80000
81000
82000
83000
84000
86000
87000
89000
93000
97000
Subject
Condition
EM
Primary

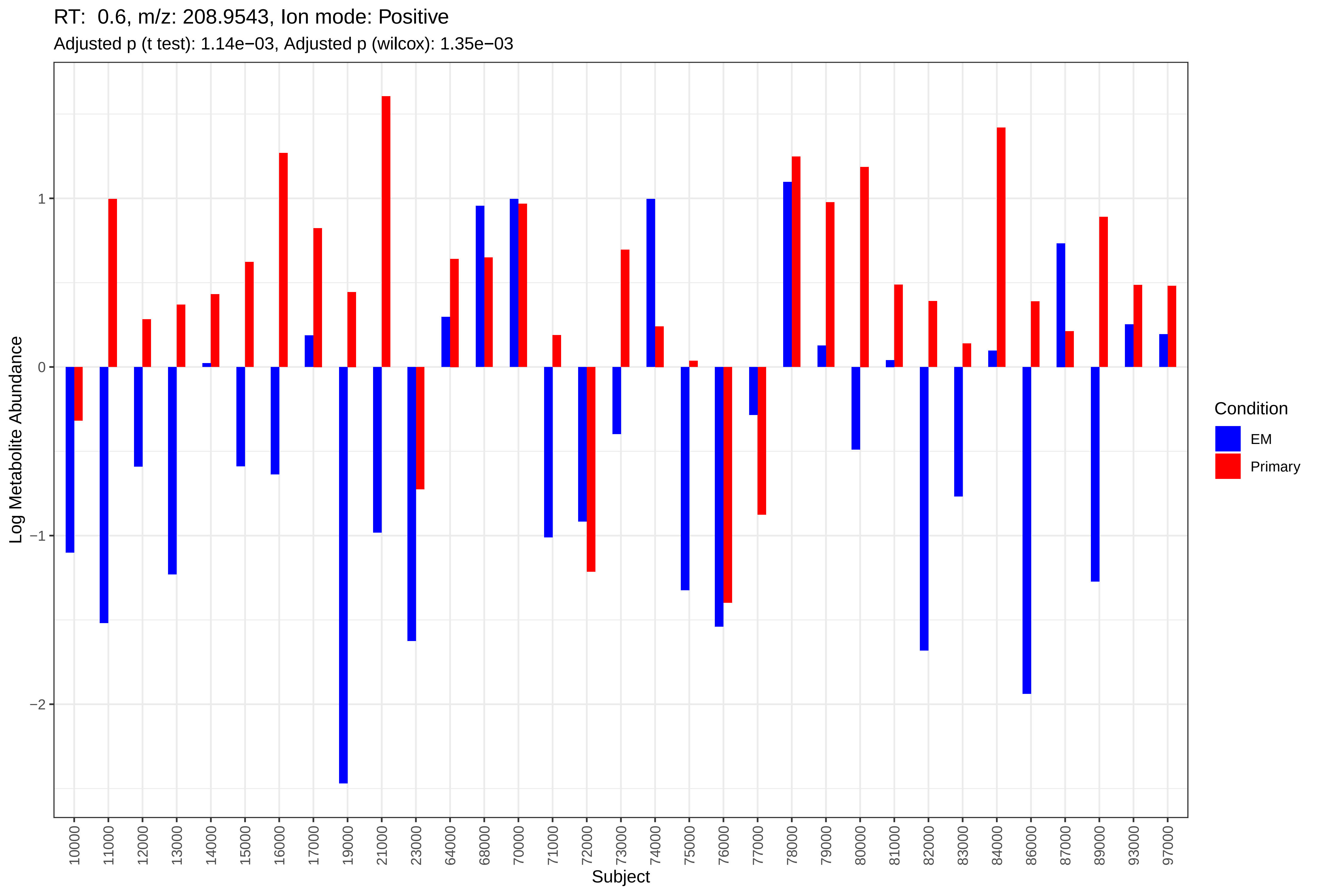

RT: 0.6, m/z: 208.9543, Ion mode: Positive
Adjusted p (t test): 1.14e−03, Adjusted p (wilcox): 1.35e−03
Log Metabolite Abundance
1
0
−1
−2
Subject
10000
11000
12000
13000
14000
15000
16000
17000
19000
21000
23000
64000
68000
70000
71000
72000
73000
74000
75000
76000
77000
78000
79000
80000
81000
82000
83000
84000
86000
87000
89000
93000
97000
Condition
EM
Primary

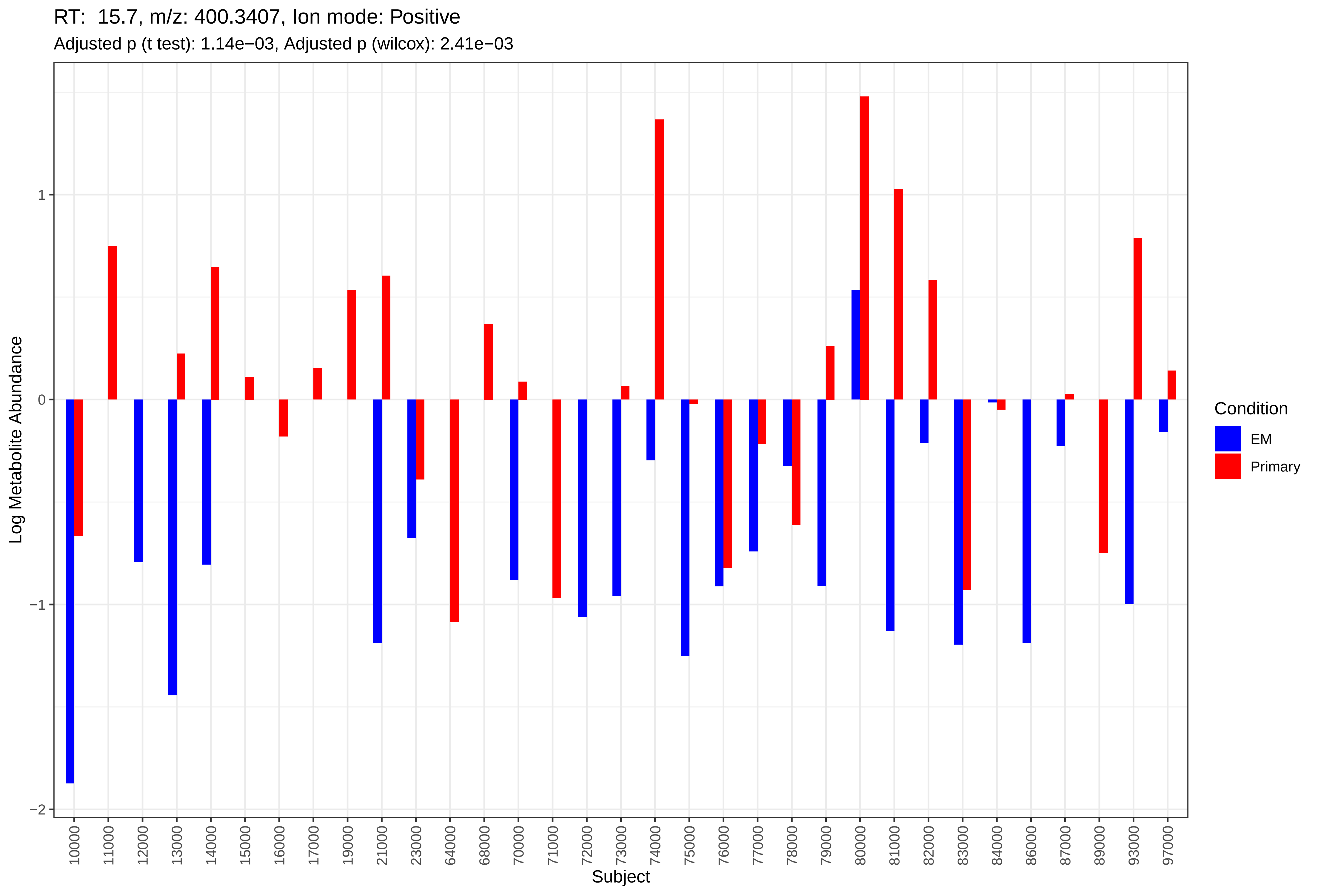
RT: 15.7, m/z: 400.3407, Ion mode: Positive
Adjusted p (t test): 1.14e−03, Adjusted p (wilcox): 2.41e−03
Log Metabolite Abundance
1
0
−1
−2
Subject
10000
11000
12000
13000
14000
15000
16000
17000
19000
21000
23000
64000
68000
70000
71000
72000
73000
74000
75000
76000
77000
78000
79000
80000
81000
82000
83000
84000
86000
87000
89000
93000
97000
Condition
EM
Primary

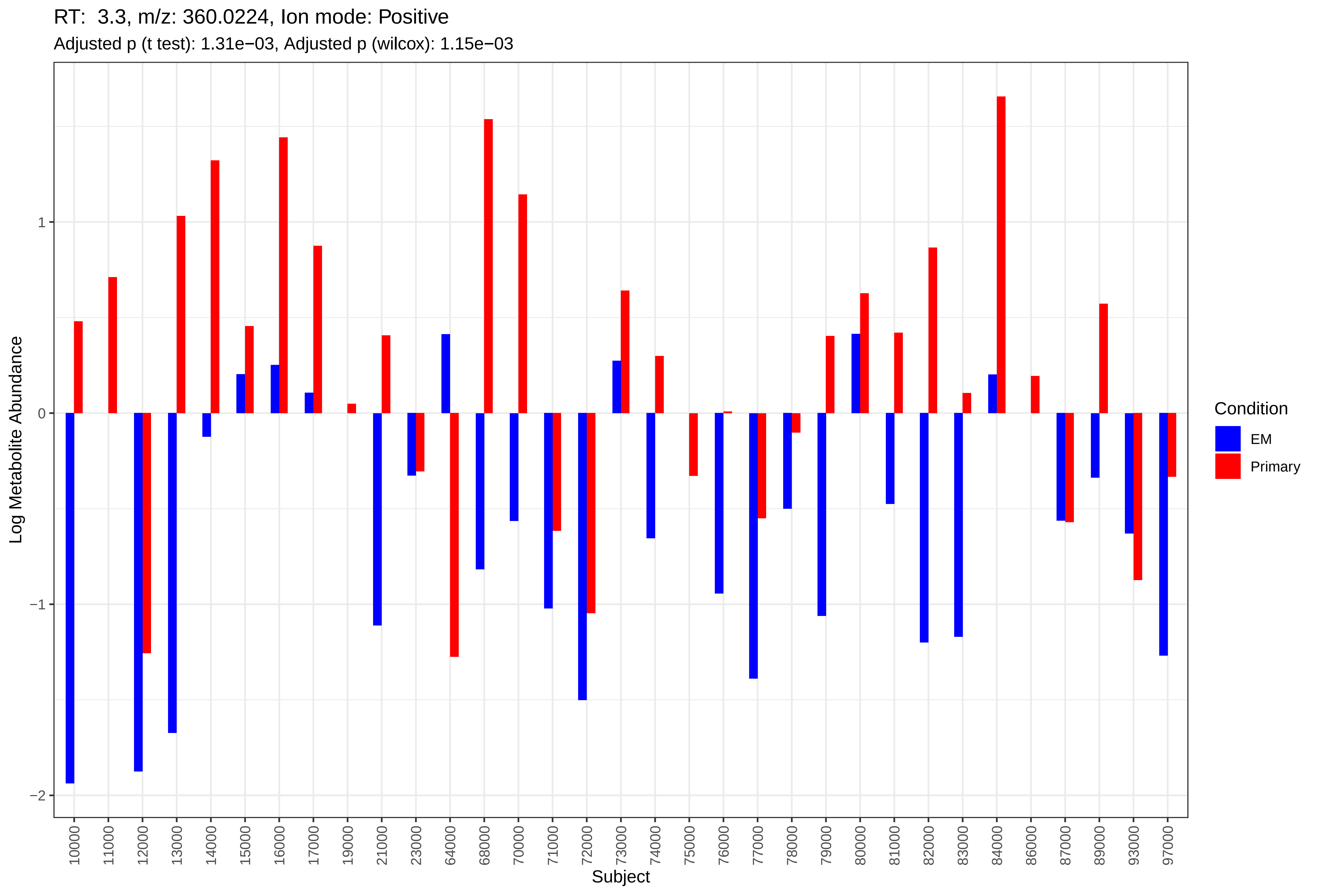
RT: 3.3, m/z: 360.0224, Ion mode: Positive
Adjusted p (t test): 1.31e−03, Adjusted p (wilcox): 1.15e−03
Log Metabolite Abundance
1
0
−1
−2
Subject
10000
11000
12000
13000
14000
15000
16000
17000
19000
21000
23000
64000
68000
70000
71000
72000
73000
74000
75000
76000
77000
78000
79000
80000
81000
82000
83000
84000
86000
87000
89000
93000
97000
Condition
EM
Primary

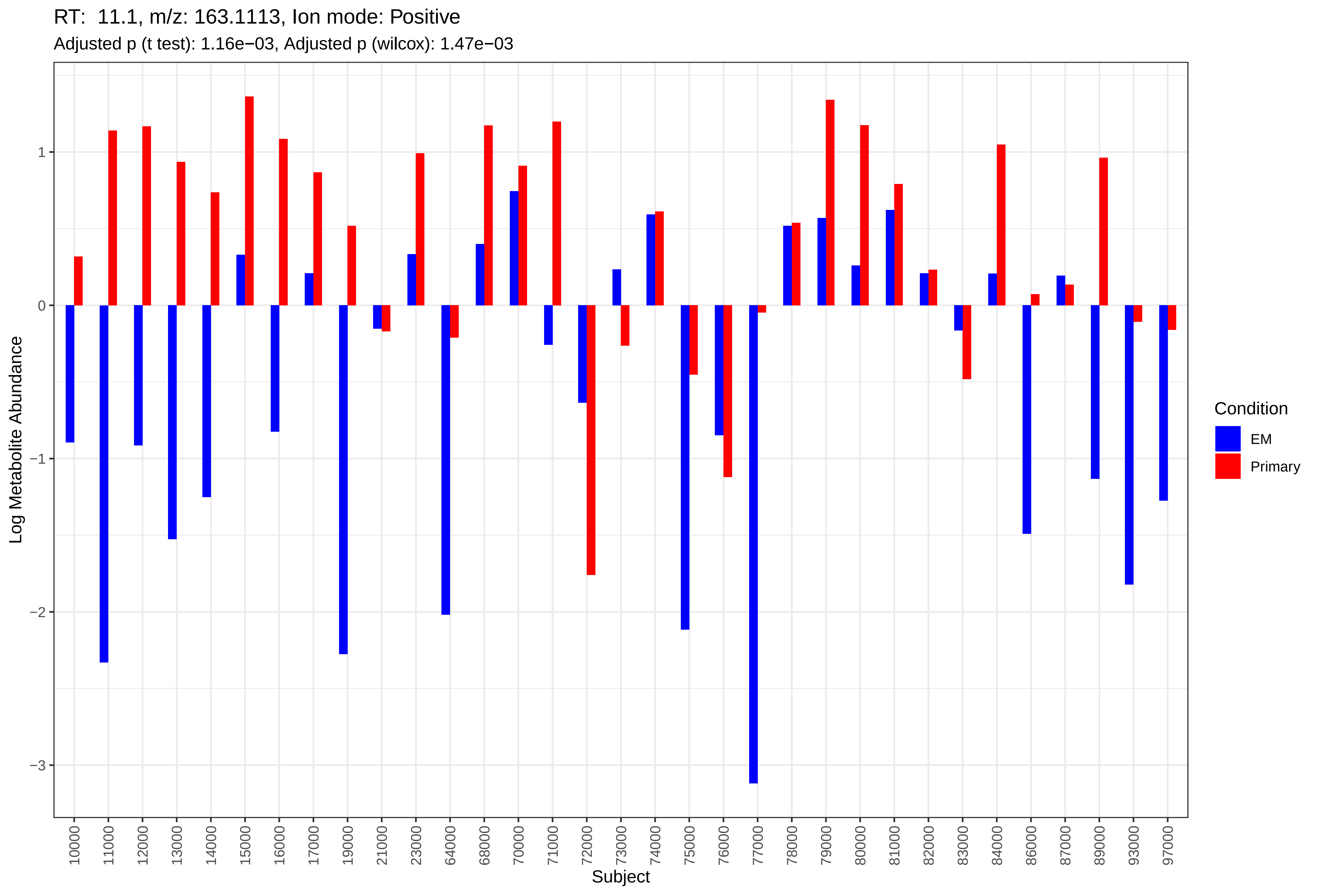

RT: 11.1, m/z: 163.1113, Ion mode: Positive
Adjusted p (t test): 1.16e−03, Adjusted p (wilcox): 1.47e−03
Log Metabolite Abundance
1
0
−1
−2
−3
10000
11000
12000
13000
14000
15000
16000
17000
19000
21000
23000
64000
68000
70000
71000
72000
73000
74000
75000
76000
77000
78000
79000
80000
81000
82000
83000
84000
86000
87000
89000
93000
97000
Subject
Condition
EM
Primary

RT: 4.9, m/z: 220.9395, Ion mode: Negative

Adjusted p (t test): 1.18e−03, Adjusted p (wilcox): 1.59e−03

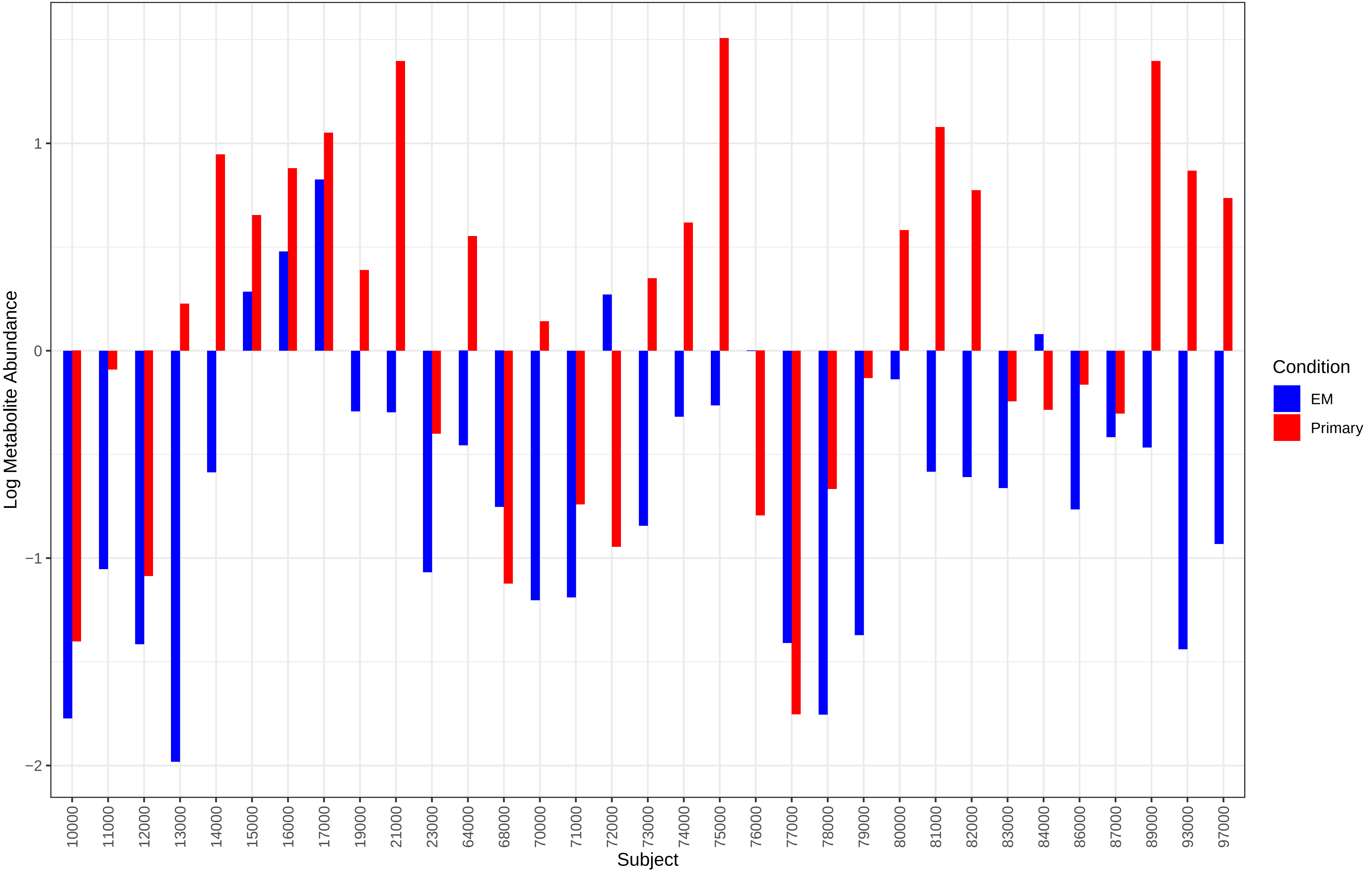

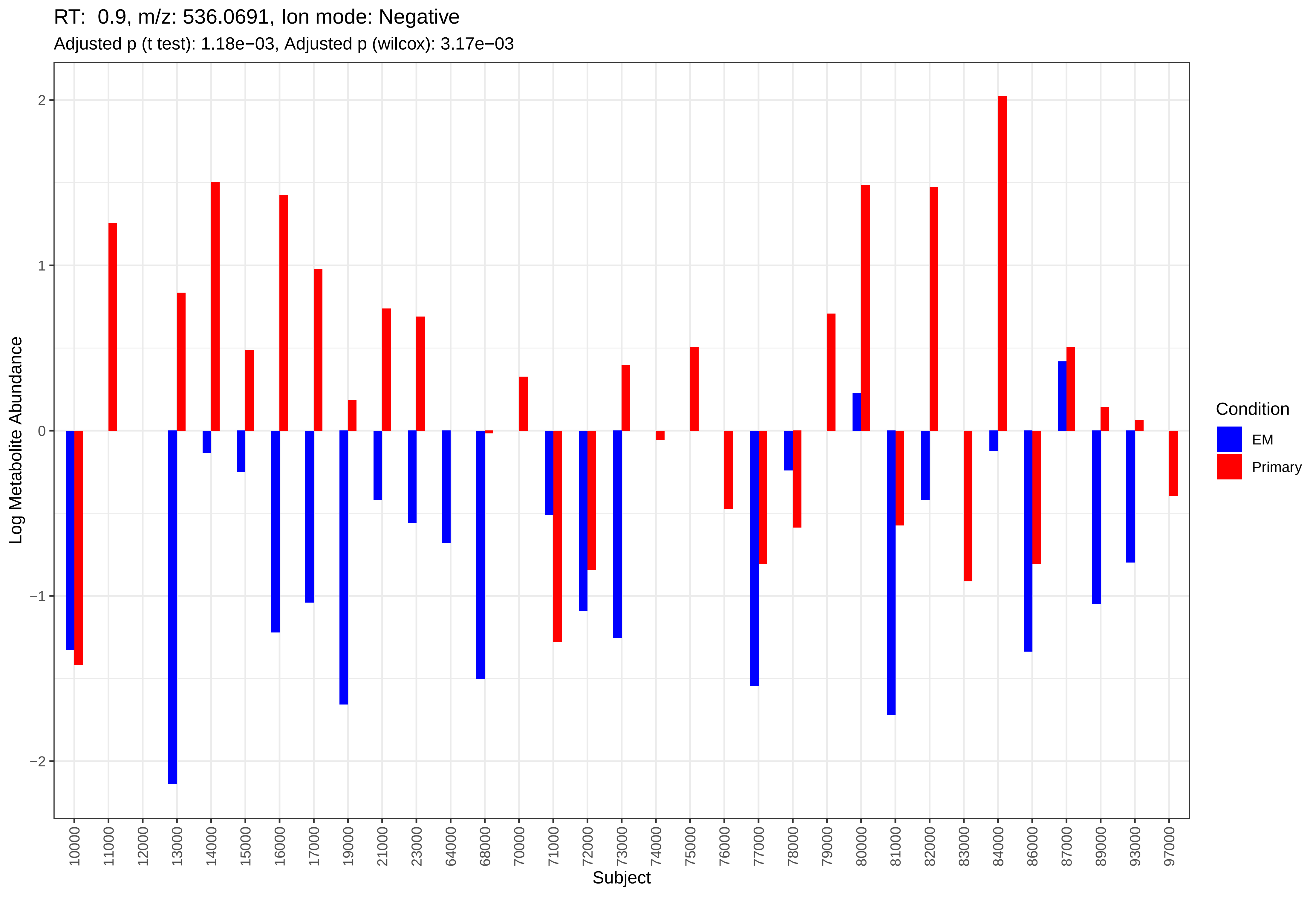

RT: 0.9, m/z: 536.0691, Ion mode: Negative
Adjusted p (t test): 1.18e−03, Adjusted p (wilcox): 3.17e−03
Log Metabolite Abundance
2
1
0
−1
−2
Subject
10000
11000
12000
13000
14000
15000
16000
17000
19000
21000
23000
64000
68000
70000
71000
72000
73000
74000
75000
76000
77000
78000
79000
80000
81000
82000
83000
84000
86000
87000
89000
93000
97000
Condition
EM
Primary

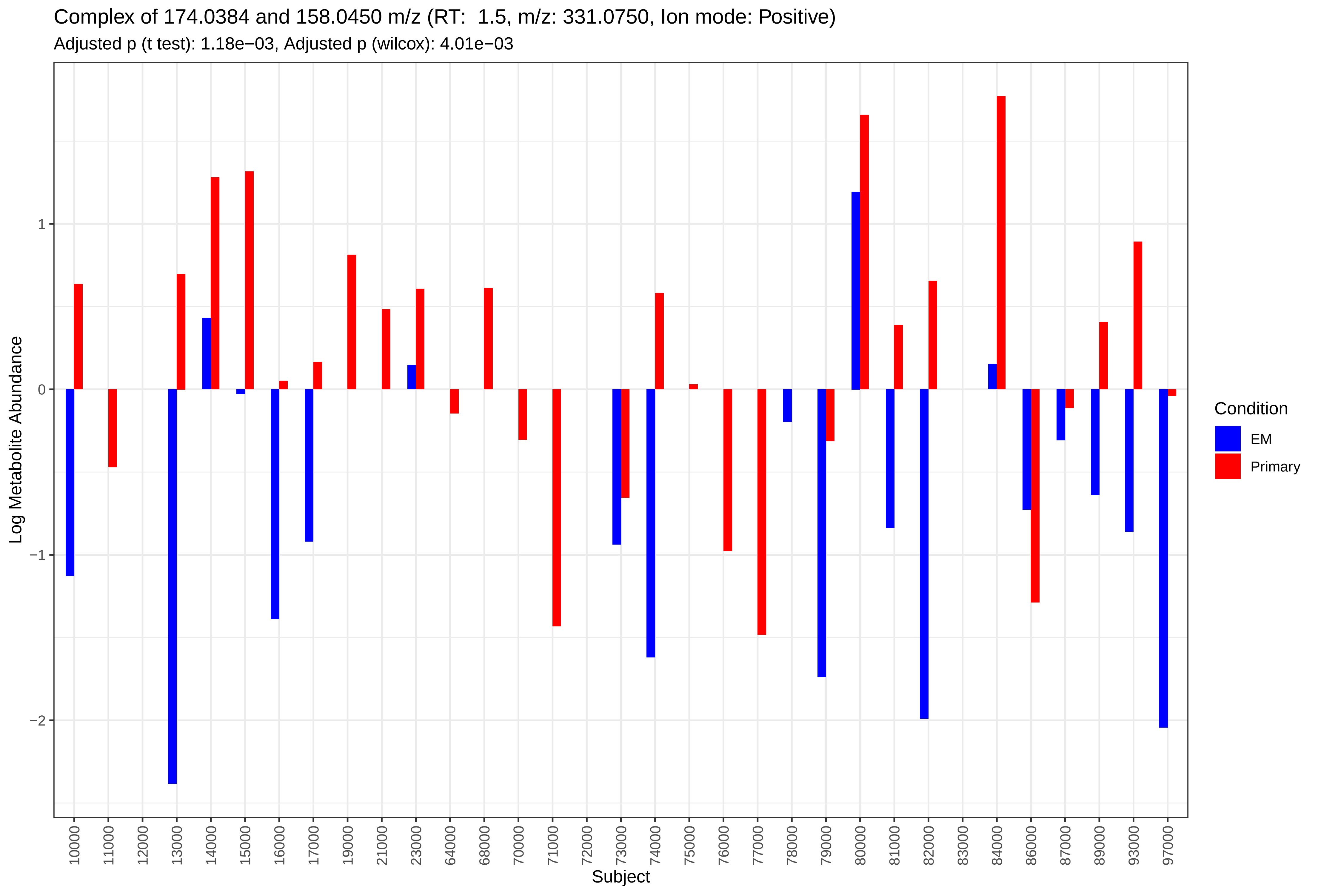

Complex of 174.0384 and 158.0450 m/z (RT: 1.5, m/z: 331.0750, Ion mode: Positive)
Adjusted p (t test): 1.18e−03, Adjusted p (wilcox): 4.01e−03
Log Metabolite Abundance
1
0
−1
−2
10000
11000
12000
13000
14000
15000
16000
17000
19000
21000
23000
64000
68000
70000
71000
72000
73000
74000
75000
76000
77000
78000
79000
80000
81000
82000
83000
84000
86000
87000
89000
93000
97000
Subject
Condition
EM
Primary

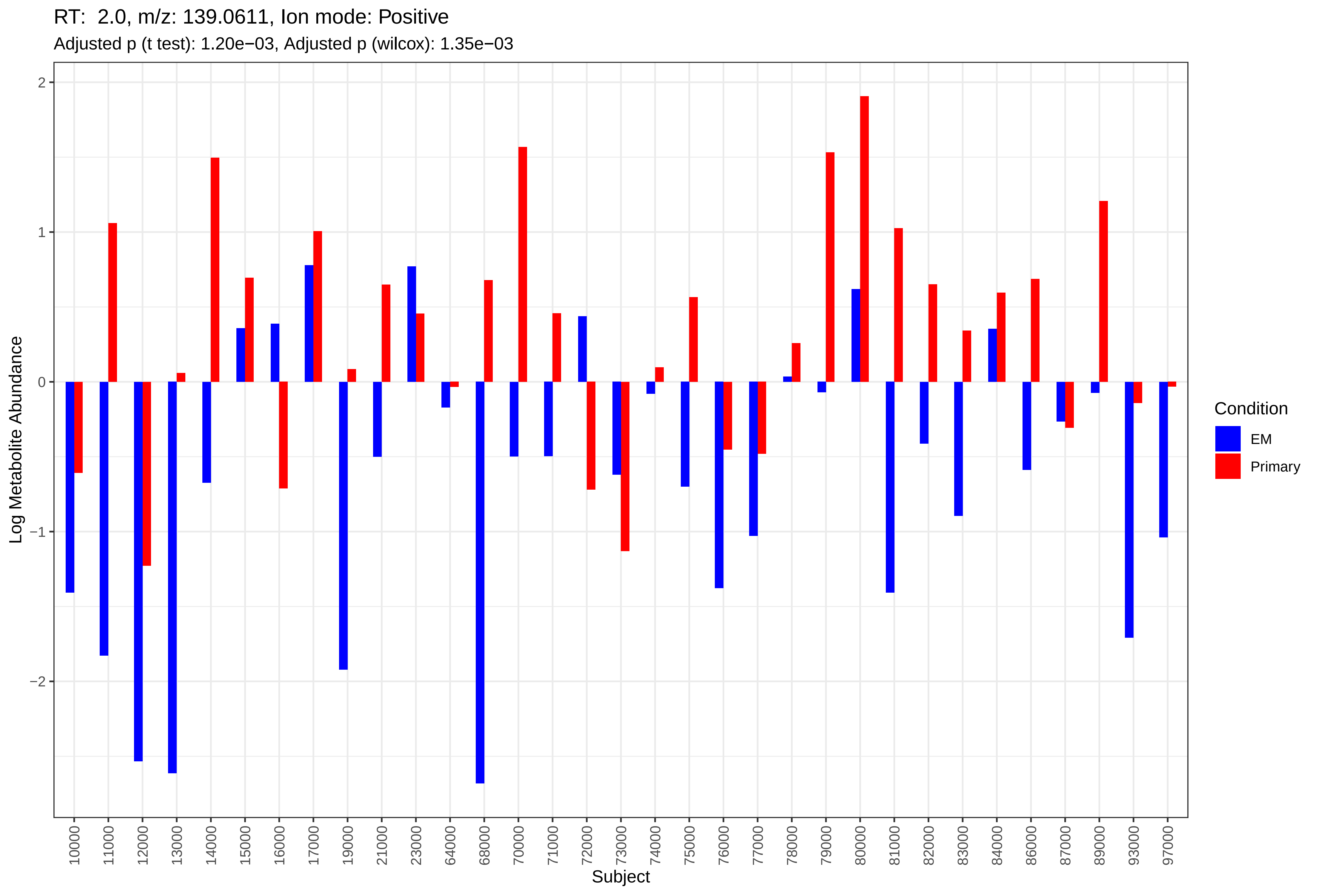
RT: 2.0, m/z: 139.0611, Ion mode: Positive
Adjusted p (t test): 1.20e−03, Adjusted p (wilcox): 1.35e−03
Log Metabolite Abundance
2
1
0
−1
−2
Subject
10000
11000
12000
13000
14000
15000
16000
17000
19000
21000
23000
64000
68000
70000
71000
72000
73000
74000
75000
76000
77000
78000
79000
80000
81000
82000
83000
84000
86000
87000
89000
93000
97000
Condition
EM
Primary

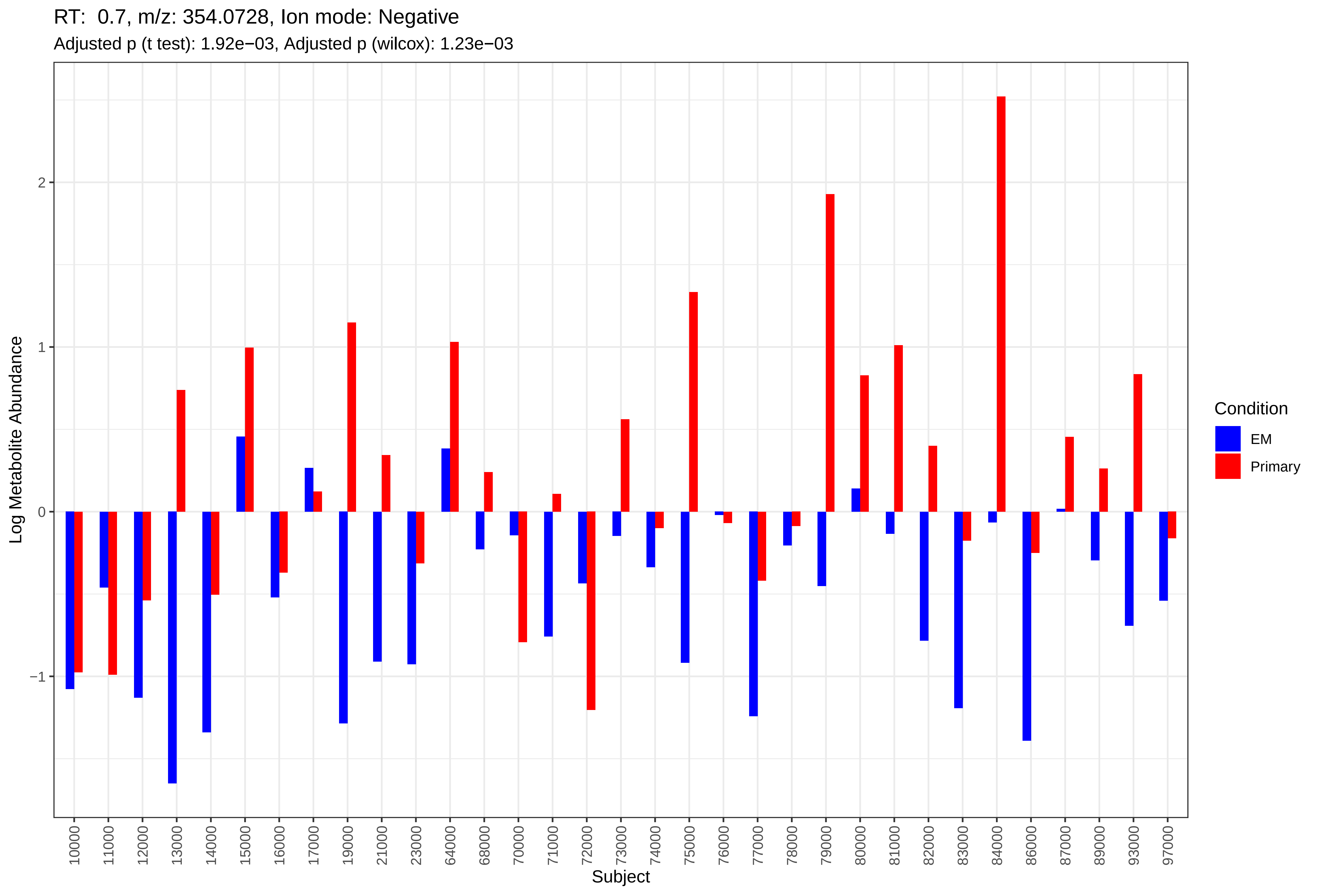
RT: 0.7, m/z: 354.0728, Ion mode: Negative
Adjusted p (t test): 1.92e−03, Adjusted p (wilcox): 1.23e−03
Log Metabolite Abundance
2
1
0
−1
Subject
10000
11000
12000
13000
14000
15000
16000
17000
19000
21000
23000
64000
68000
70000
71000
72000
73000
74000
75000
76000
77000
78000
79000
80000
81000
82000
83000
84000
86000
87000
89000
93000
97000
Condition
EM
Primary

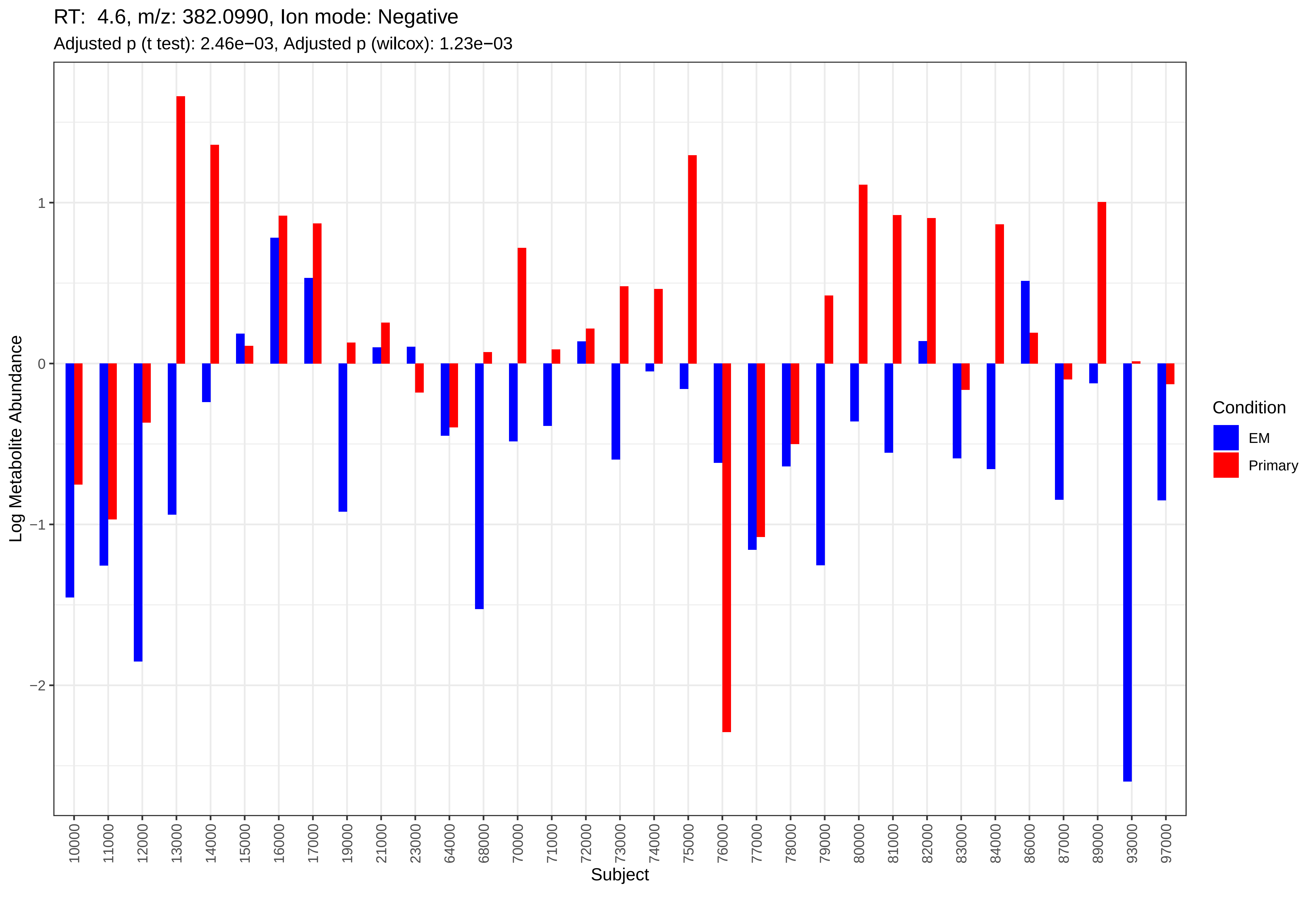

RT: 4.6, m/z: 382.0990, Ion mode: Negative
Adjusted p (t test): 2.46e−03, Adjusted p (wilcox): 1.23e−03
Log Metabolite Abundance
1
0
−1
−2
Subject
10000
11000
12000
13000
14000
15000
16000
17000
19000
21000
23000
64000
68000
70000
71000
72000
73000
74000
75000
76000
77000
78000
79000
80000
81000
82000
83000
84000
86000
87000
89000
93000
97000
Condition
EM
Primary

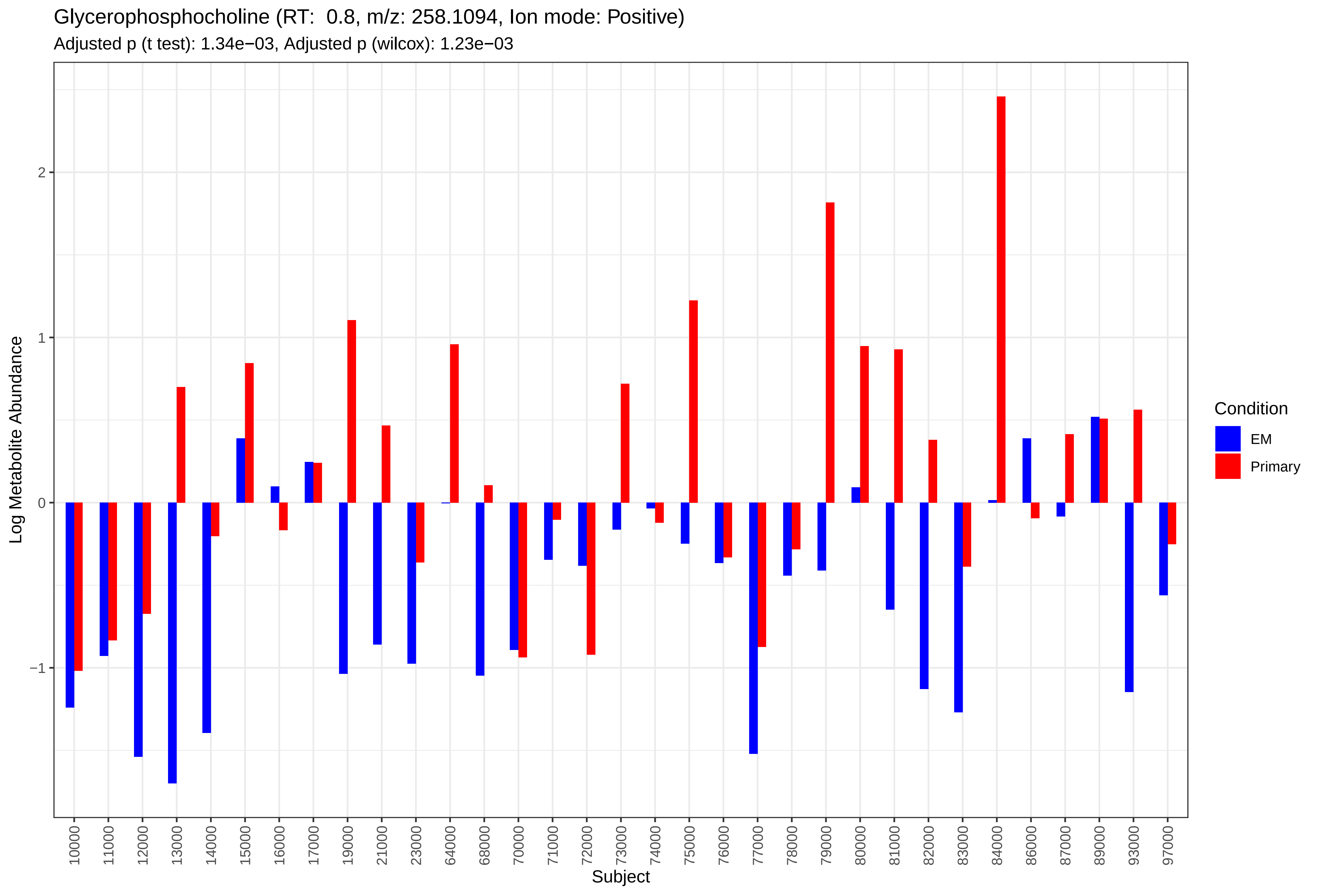

Glycerophosphocholine (RT: 0.8, m/z: 258.1094, Ion mode: Positive)
Adjusted p (t test): 1.34e−03, Adjusted p (wilcox): 1.23e−03
Log Metabolite Abundance
2
1
0
−1
10000
11000
12000
13000
14000
15000
16000
17000
19000
21000
23000
64000
68000
70000
71000
72000
73000
74000
75000
76000
77000
78000
79000
80000
81000
82000
83000
84000
86000
87000
89000
93000
97000
Subject
Condition
EM
Primary

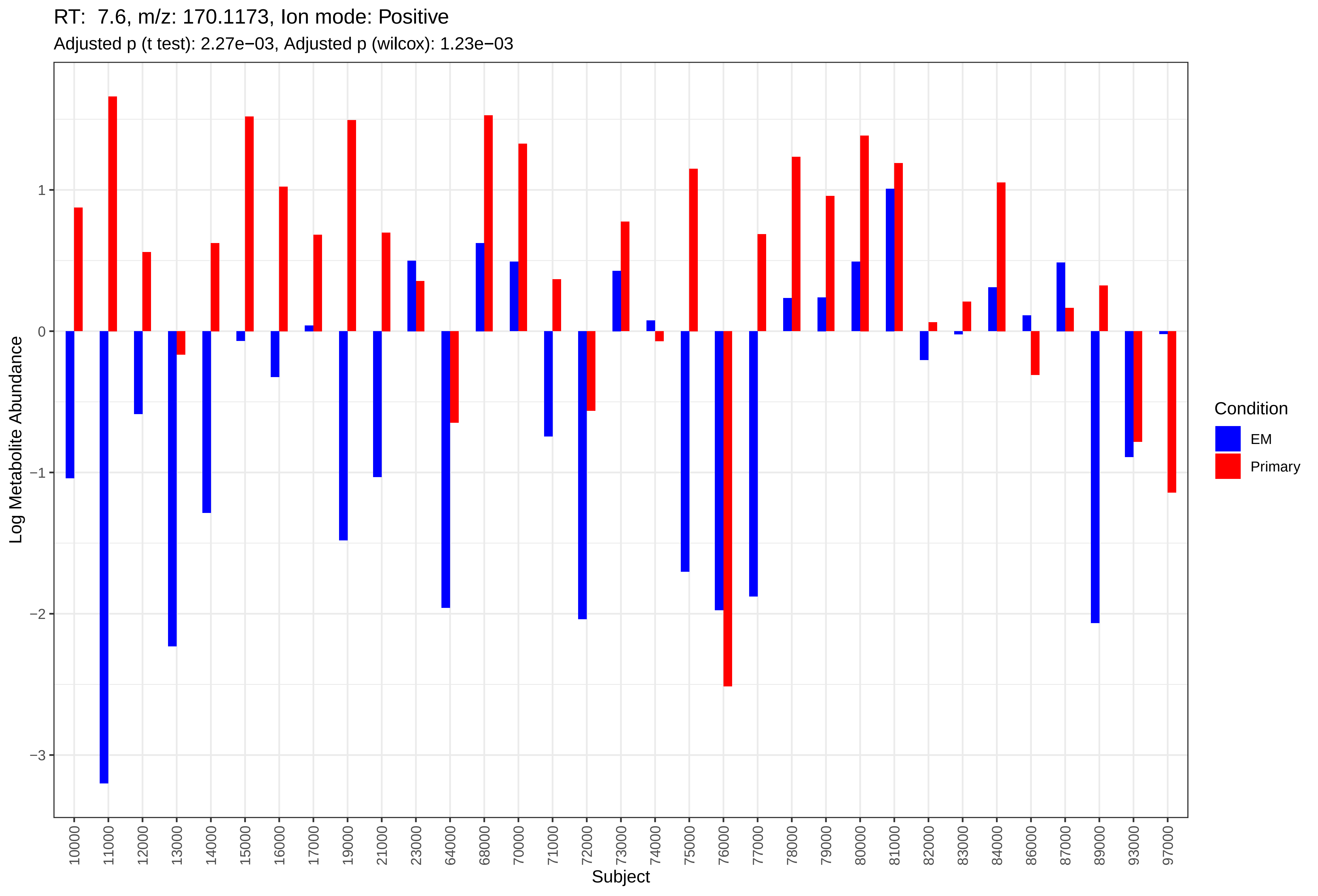
RT: 7.6, m/z: 170.1173, Ion mode: Positive
Adjusted p (t test): 2.27e−03, Adjusted p (wilcox): 1.23e−03
Log Metabolite Abundance
1
0
−1
−2
−3
10000
11000
12000
13000
14000
15000
16000
17000
19000
21000
23000
64000
68000
70000
71000
72000
73000
74000
75000
76000
77000
78000
79000
80000
81000
82000
83000
84000
86000
87000
89000
93000
97000
Subject
Condition
EM
Primary

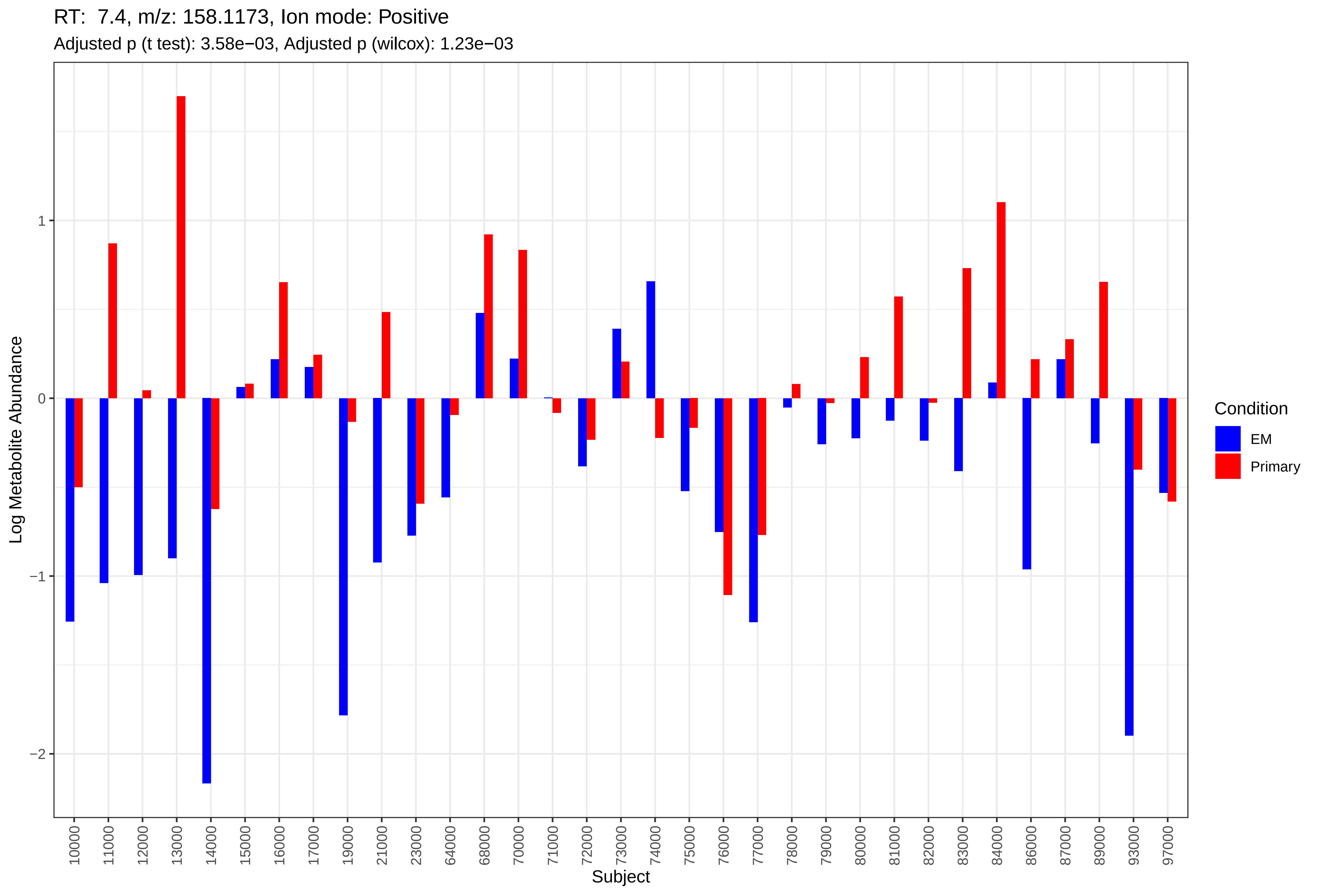

RT: 7.4, m/z: 158.1173, Ion mode: Positive
Adjusted p (t test): 3.58e−03, Adjusted p (wilcox): 1.23e−03
Log Metabolite Abundance
1
0
−1
−2
10000
11000
12000
13000
14000
15000
16000
17000
19000
21000
23000
64000
68000
70000
71000
72000
73000
74000
75000
76000
77000
78000
79000
80000
81000
82000
83000
84000
86000
87000
89000
93000
97000
Subject
Condition
EM
Primary

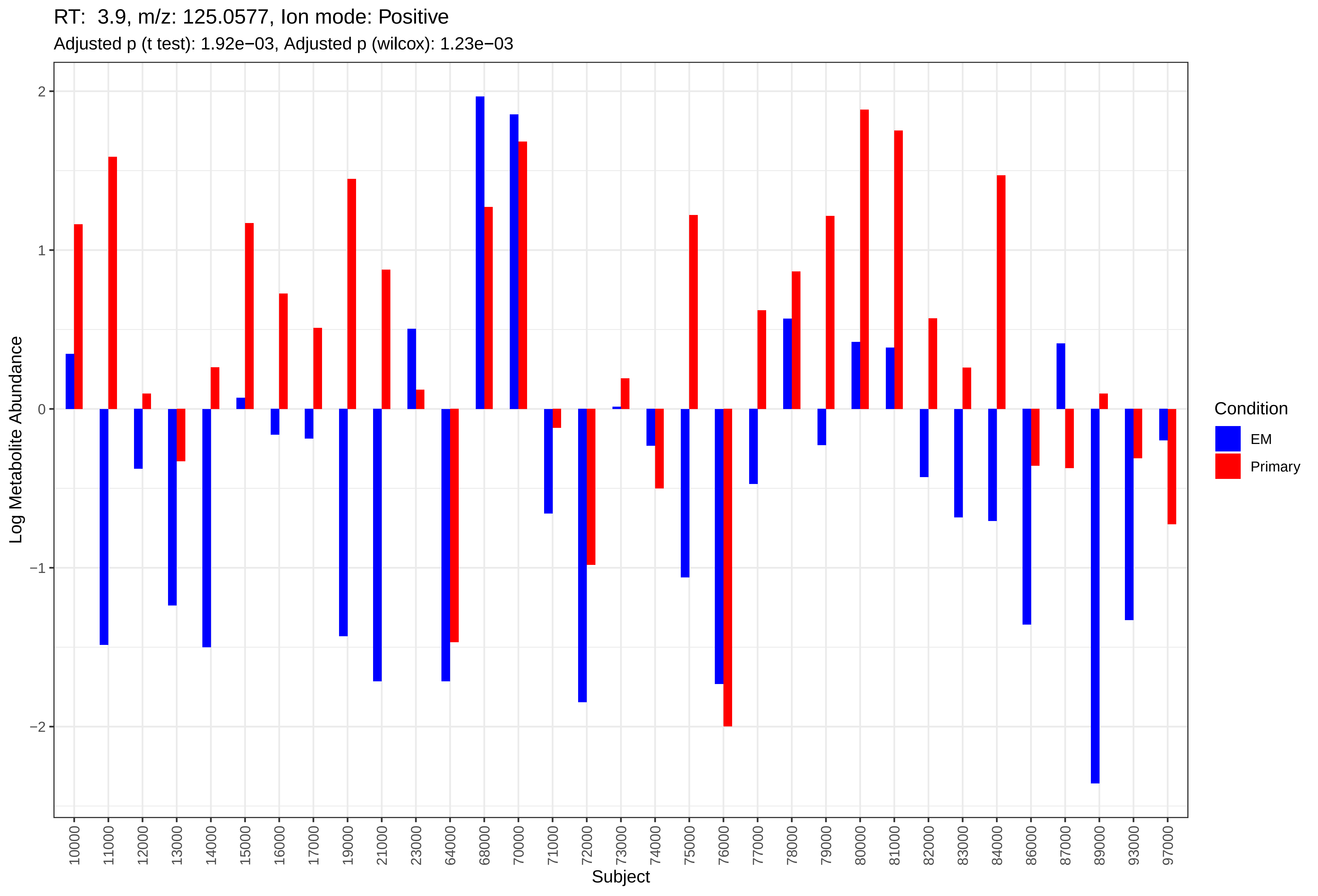
RT: 3.9, m/z: 125.0577, Ion mode: Positive
Adjusted p (t test): 1.92e−03, Adjusted p (wilcox): 1.23e−03
Log Metabolite Abundance
2
1
0
−1
−2
10000
11000
12000
13000
14000
15000
16000
17000
19000
21000
23000
64000
68000
70000
71000
72000
73000
74000
75000
76000
77000
78000
79000
80000
81000
82000
83000
84000
86000
87000
89000
93000
97000
Subject
Condition
EM
Primary

RT: 8.0, m/z: 249.4935, Ion mode: Positive

Adjusted p (t test): 3.01e−03, Adjusted p (wilcox): 1.23e−03

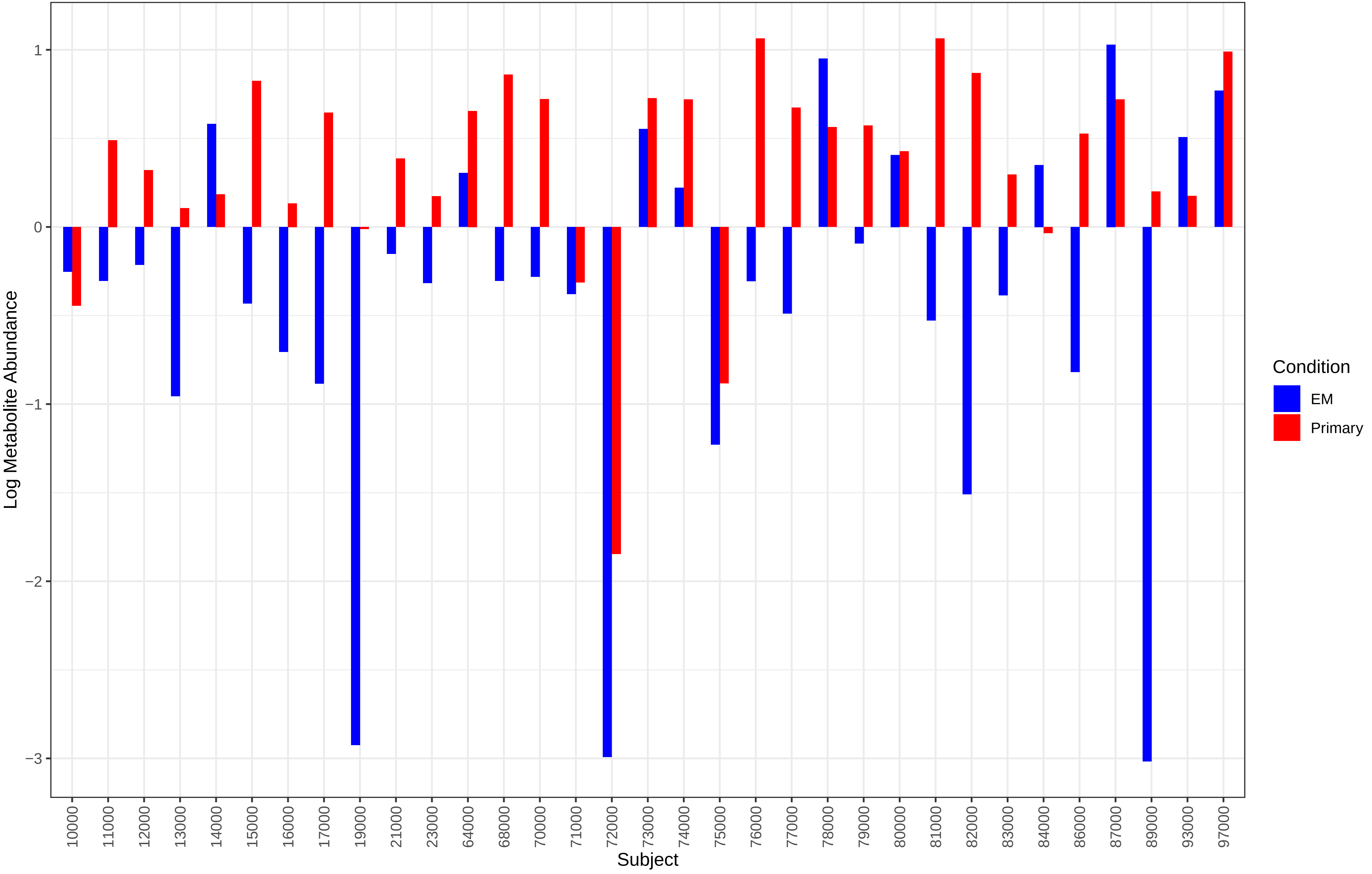

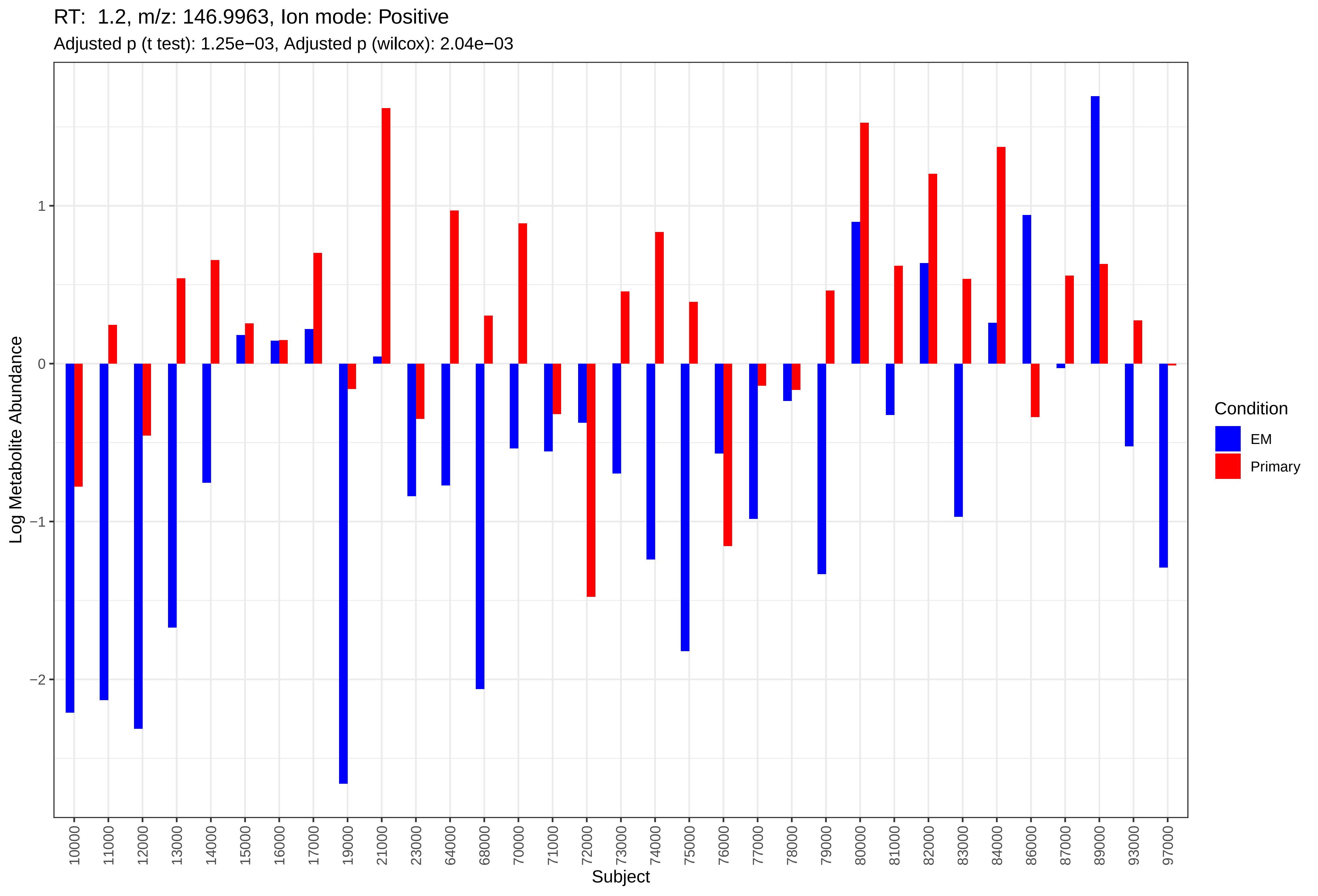

RT: 1.2, m/z: 146.9963, Ion mode: Positive
Adjusted p (t test): 1.25e−03, Adjusted p (wilcox): 2.04e−03
Log Metabolite Abundance
1
0
−1
−2
Subject
10000
11000
12000
13000
14000
15000
16000
17000
19000
21000
23000
64000
68000
70000
71000
72000
73000
74000
75000
76000
77000
78000
79000
80000
81000
82000
83000
84000
86000
87000
89000
93000
97000
Condition
EM
Primary

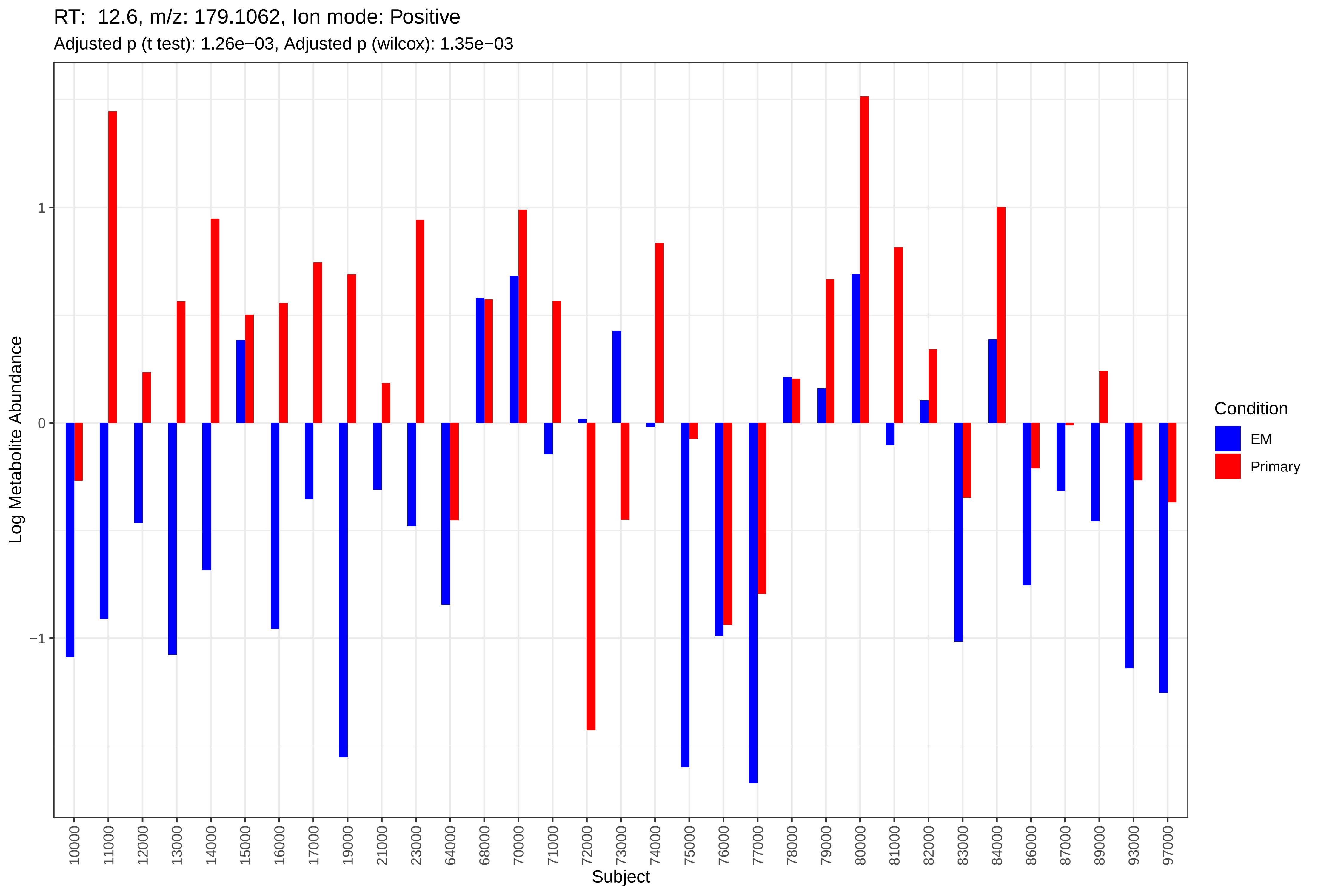
RT: 12.6, m/z: 179.1062, Ion mode: Positive
Adjusted p (t test): 1.26e−03, Adjusted p (wilcox): 1.35e−03
Log Metabolite Abundance
1
0
−1
10000
11000
12000
13000
14000
15000
16000
17000
19000
21000
23000
64000
68000
70000
71000
72000
73000
74000
75000
76000
77000
78000
79000
80000
81000
82000
83000
84000
86000
87000
89000
93000
97000
Subject
Condition
EM
Primary

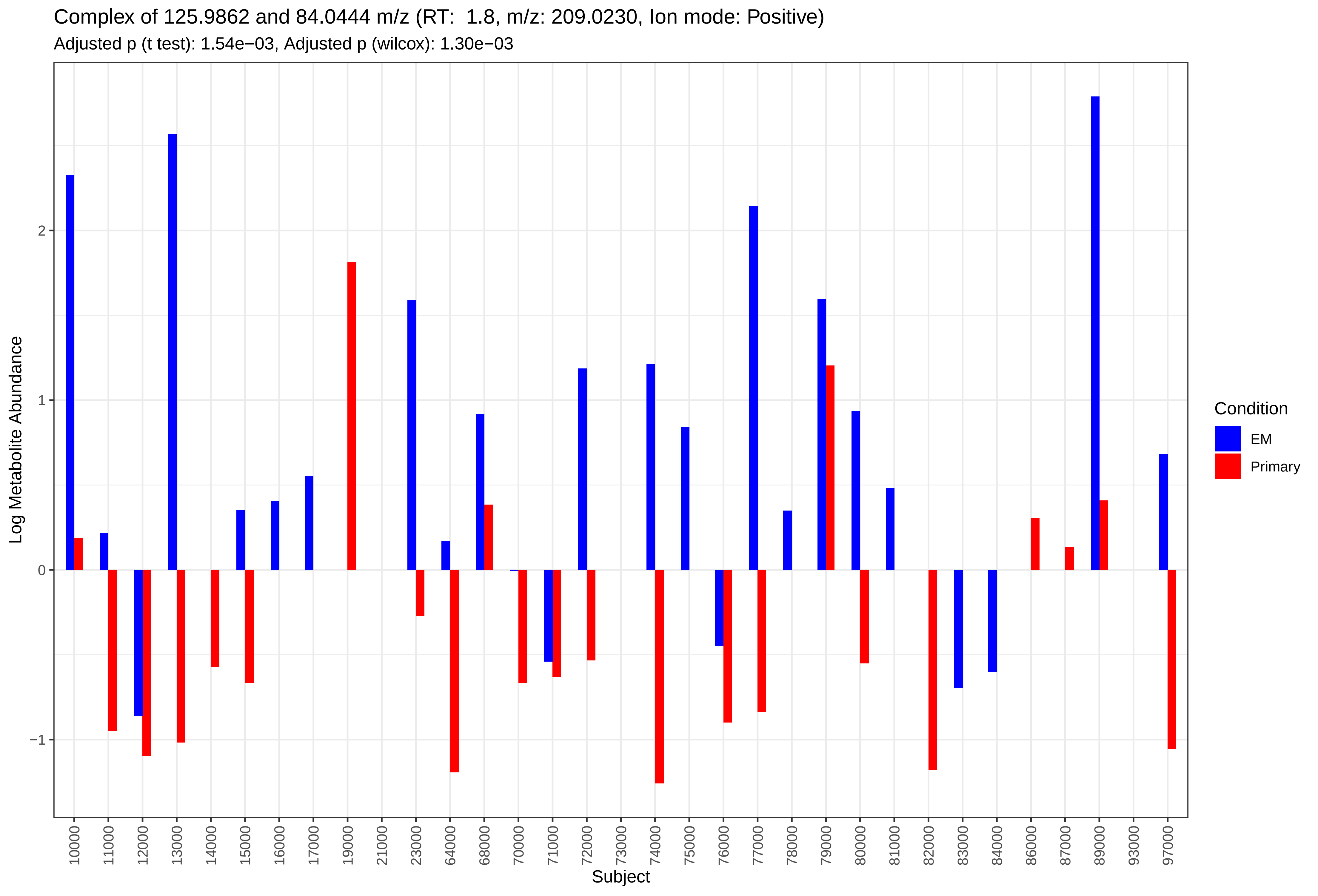

Complex of 125.9862 and 84.0444 m/z (RT: 1.8, m/z: 209.0230, Ion mode: Positive)
Adjusted p (t test): 1.54e−03, Adjusted p (wilcox): 1.30e−03
Log Metabolite Abundance
2
1
0
−1
10000
11000
12000
13000
14000
15000
16000
17000
19000
21000
23000
64000
68000
70000
71000
72000
73000
74000
75000
76000
77000
78000
79000
80000
81000
82000
83000
84000
86000
87000
89000
93000
97000
Subject
Condition
EM
Primary

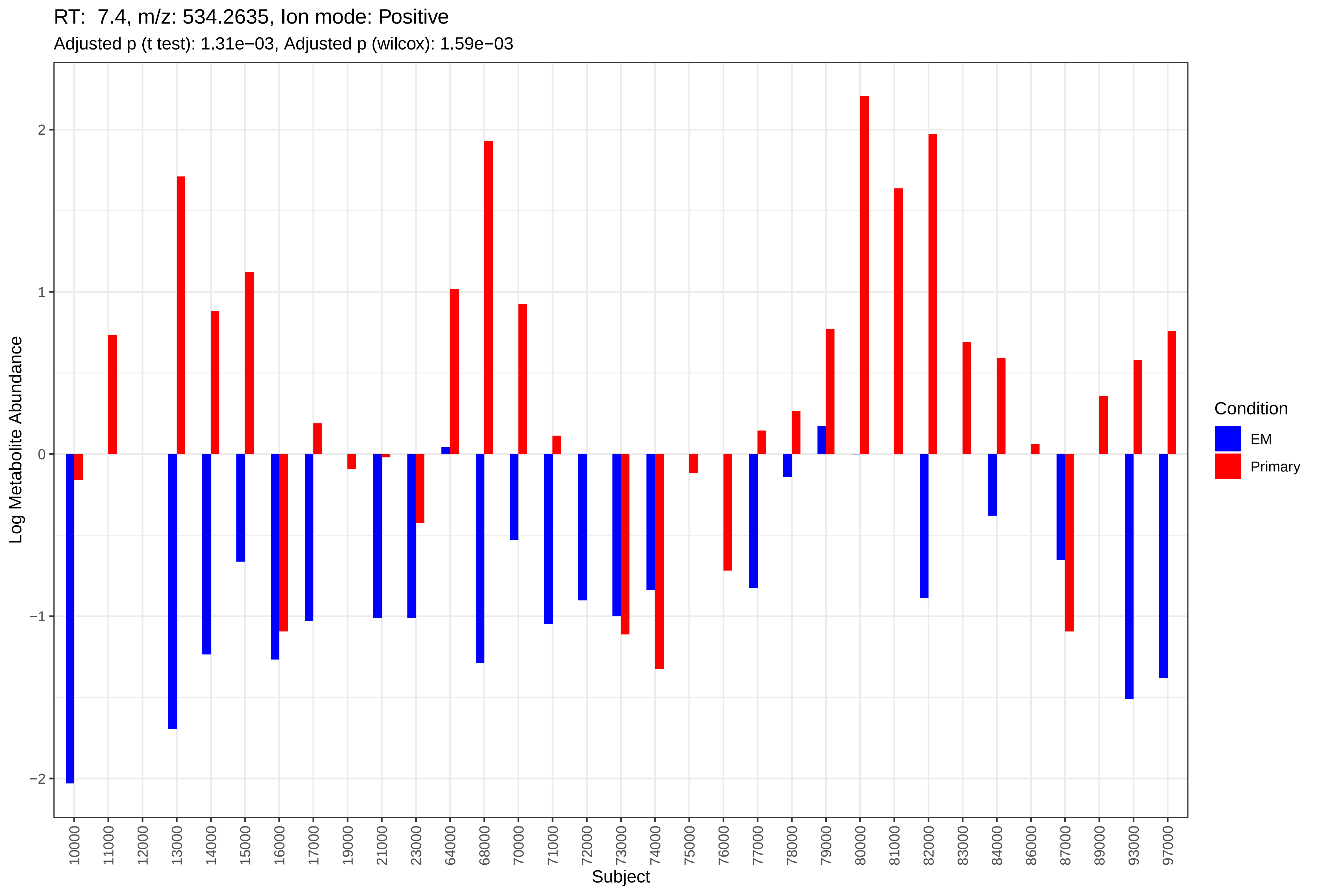
RT: 7.4, m/z: 534.2635, Ion mode: Positive
Adjusted p (t test): 1.31e−03, Adjusted p (wilcox): 1.59e−03
Log Metabolite Abundance
2
1
0
−1
−2
Subject
10000
11000
12000
13000
14000
15000
16000
17000
19000
21000
23000
64000
68000
70000
71000
72000
73000
74000
75000
76000
77000
78000
79000
80000
81000
82000
83000
84000
86000
87000
89000
93000
97000
Condition
EM
Primary

RT: 0.7, m/z: 221.0301, Ion mode: Negative

Adjusted p (t test): 1.32e−03, Adjusted p (wilcox): 1.46e−03

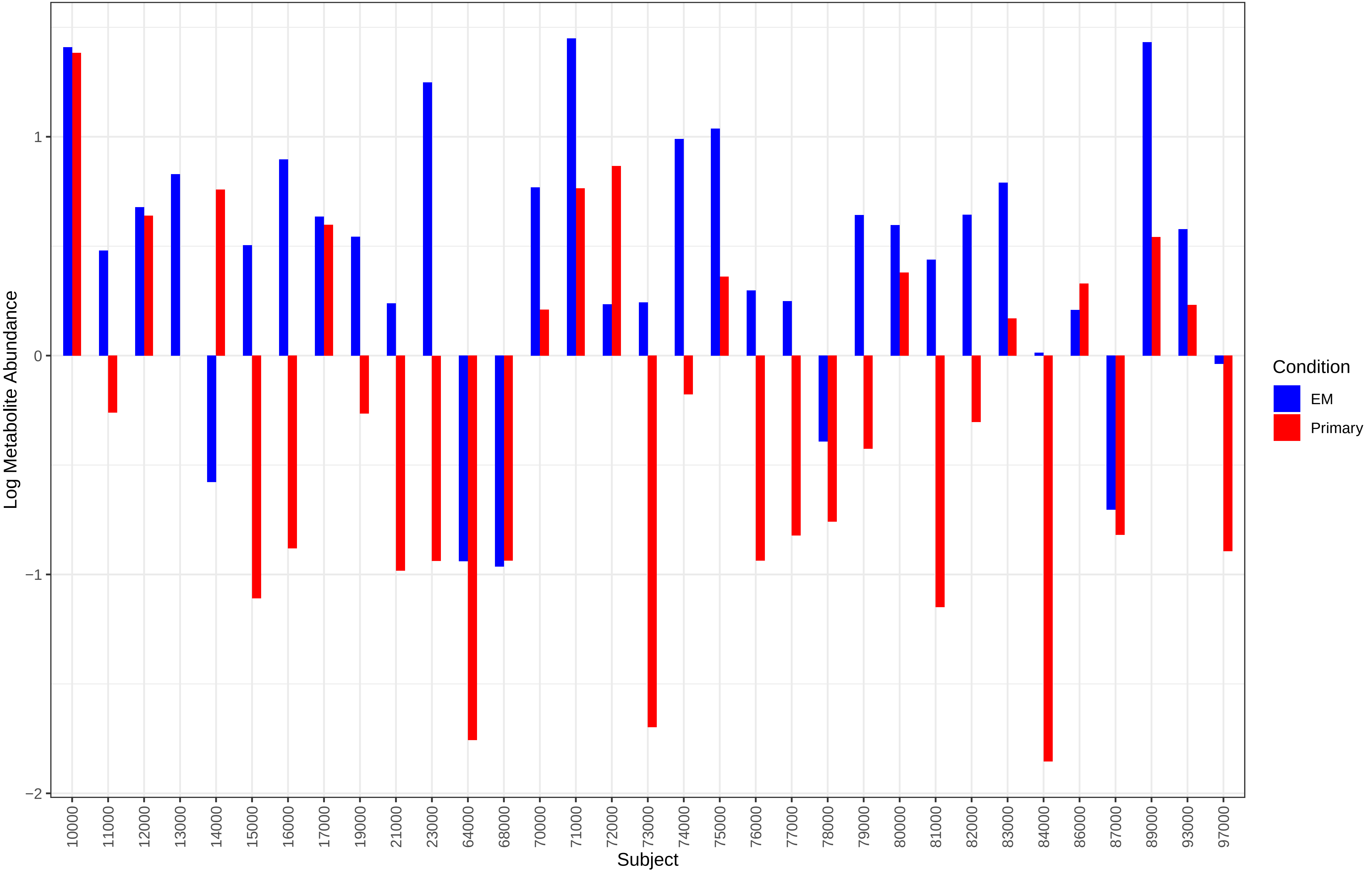

[M−H2O+H] −18.0106 m/z adduct of 212.1026 m/z (RT: 1.2, m/z: 194.0895, Ion mode: Positive)

Adjusted p (t test): 1.33e−03, Adjusted p (wilcox): 1.72e−03

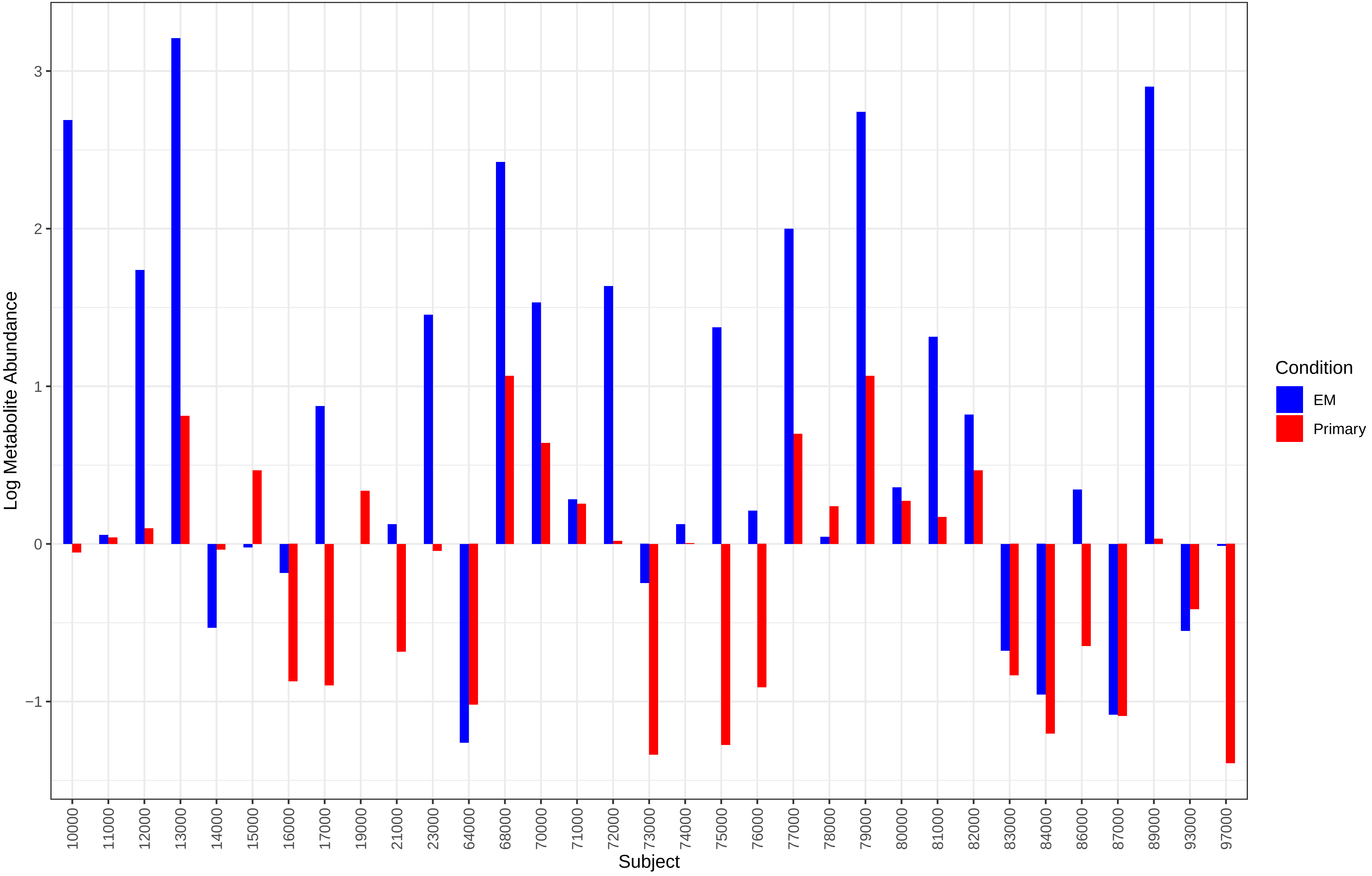

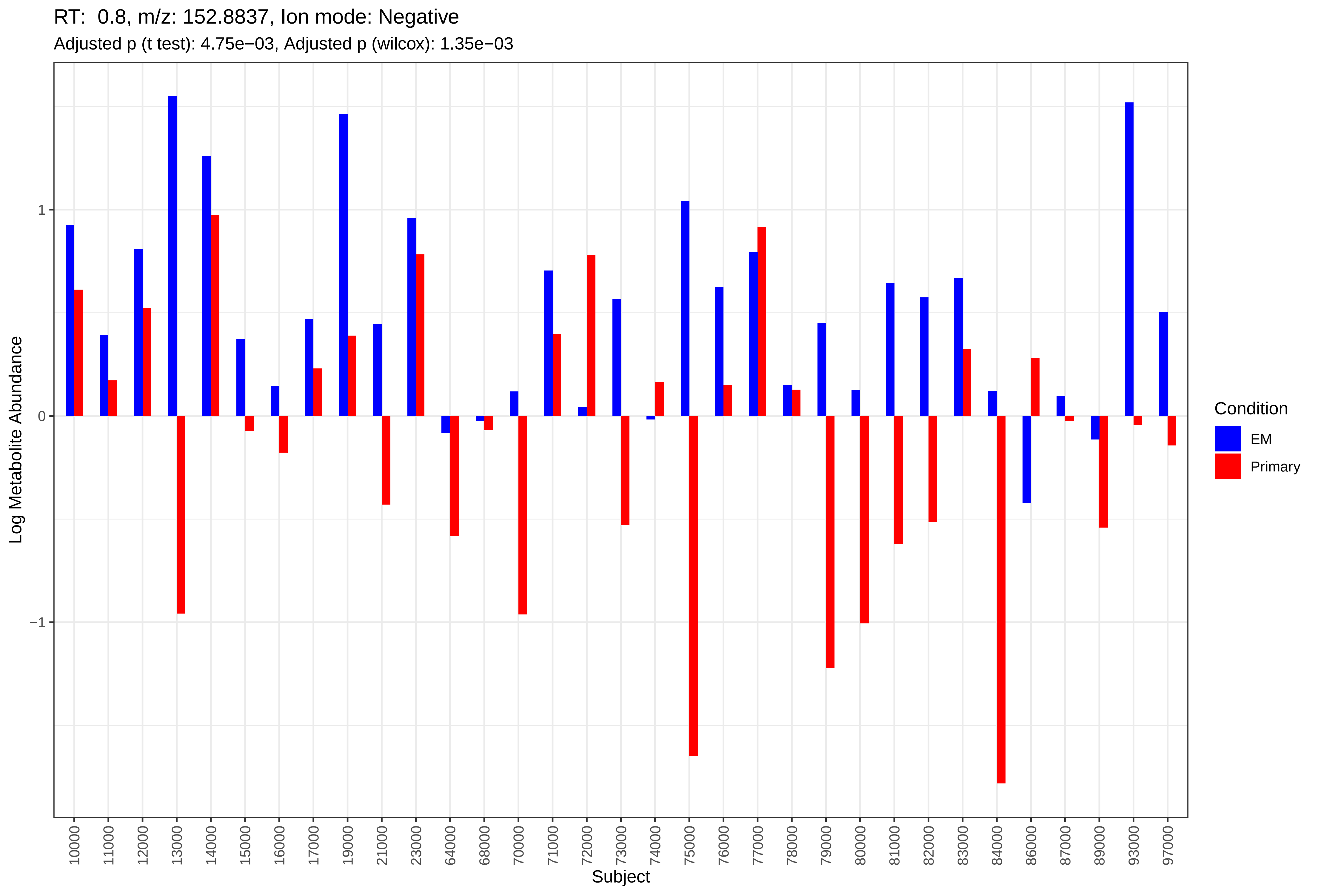
RT: 0.8, m/z: 152.8837, Ion mode: Negative
Adjusted p (t test): 4.75e−03, Adjusted p (wilcox): 1.35e−03
Log Metabolite Abundance
1
0
−1
10000
11000
12000
13000
14000
15000
16000
17000
19000
21000
23000
64000
68000
70000
71000
72000
73000
74000
75000
76000
77000
78000
79000
80000
81000
82000
83000
84000
86000
87000
89000
93000
97000
Subject
Condition
EM
Primary

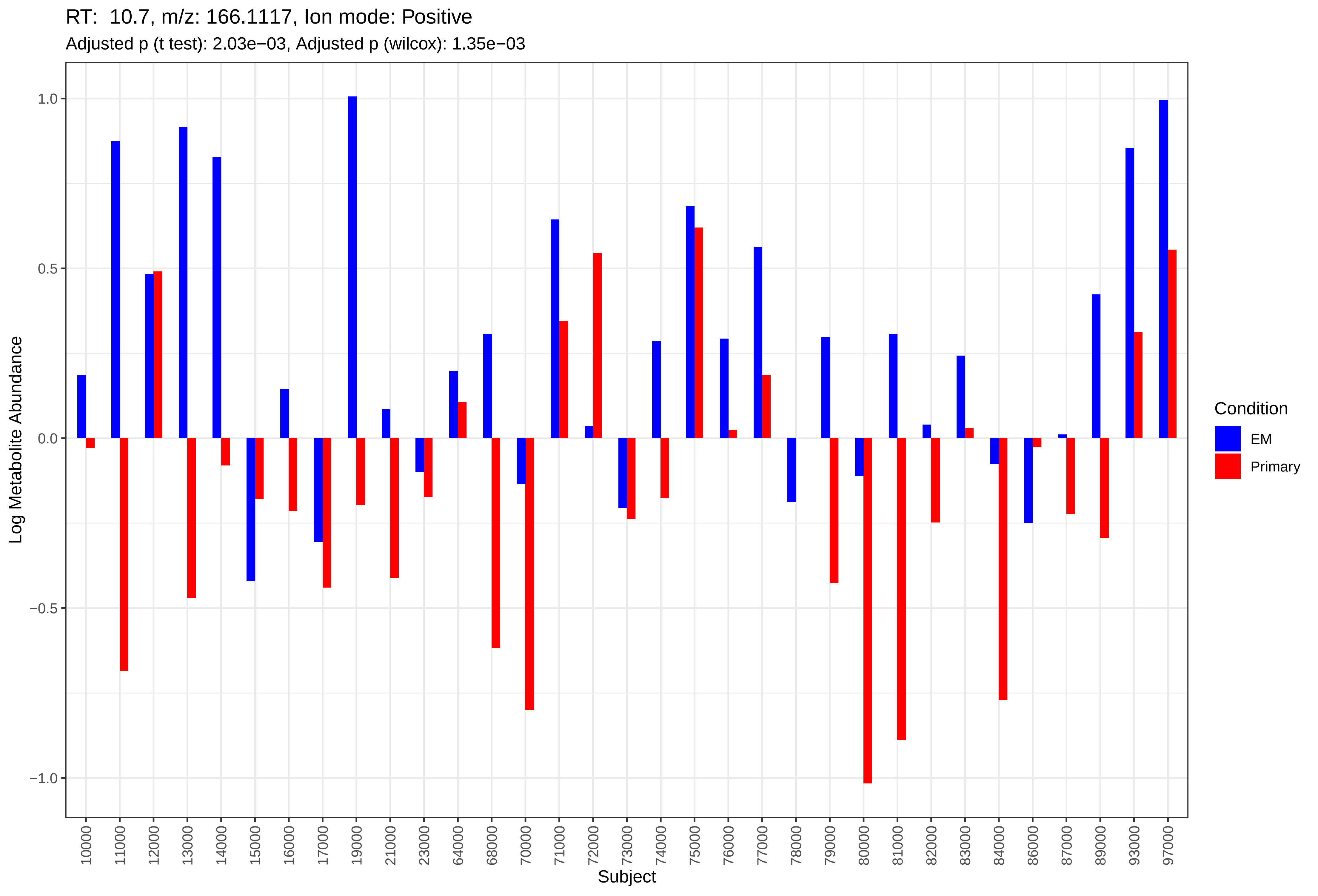
RT: 10.7, m/z: 166.1117, Ion mode: Positive
Adjusted p (t test): 2.03e−03, Adjusted p (wilcox): 1.35e−03
Log Metabolite Abundance
1.0
0.5
0.0
−0.5
−1.0
Subject
10000
11000
12000
13000
14000
15000
16000
17000
19000
21000
23000
64000
68000
70000
71000
72000
73000
74000
75000
76000
77000
78000
79000
80000
81000
82000
83000
84000
86000
87000
89000
93000
97000
Condition
EM
Primary

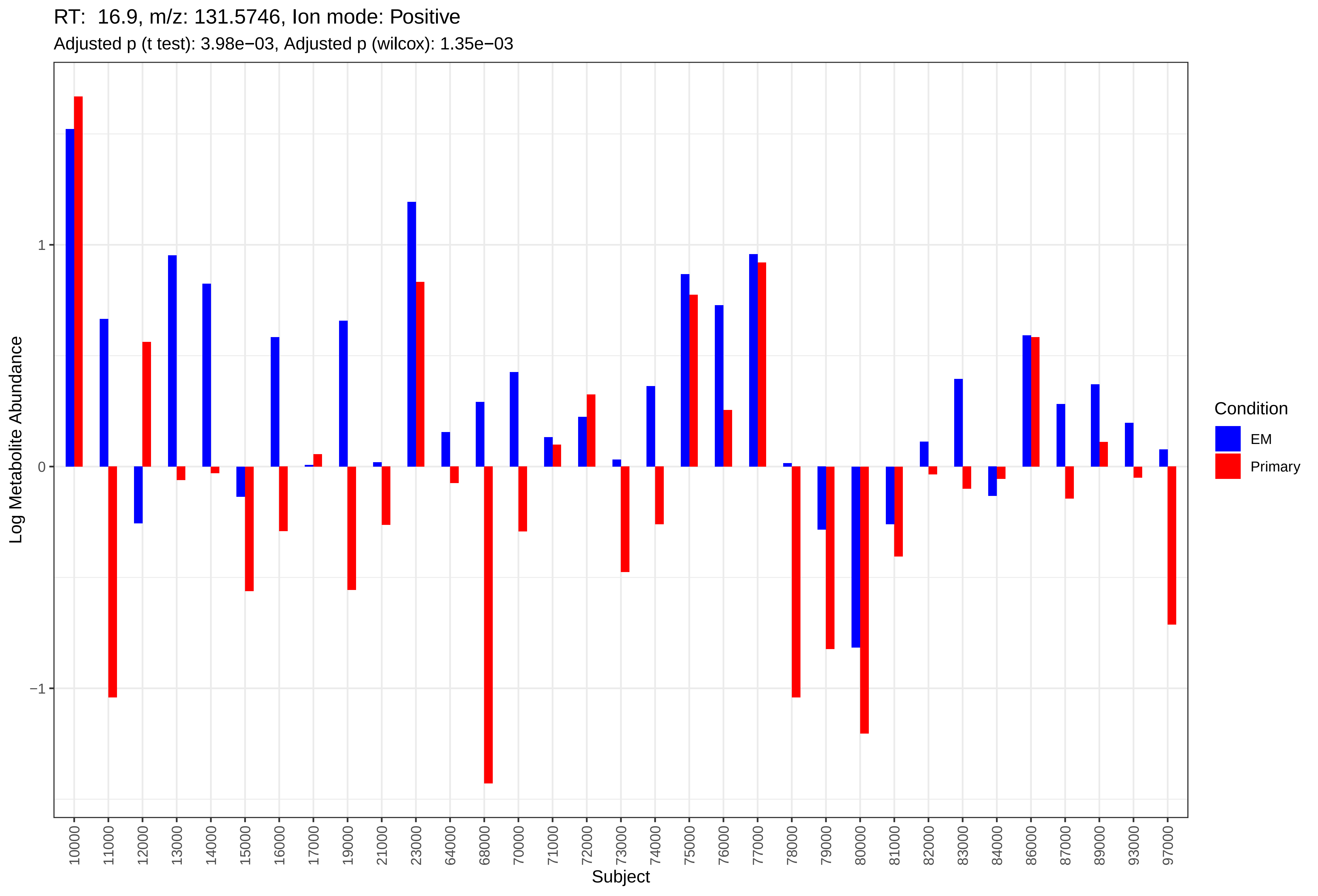

RT: 16.9, m/z: 131.5746, Ion mode: Positive
Adjusted p (t test): 3.98e−03, Adjusted p (wilcox): 1.35e−03
Log Metabolite Abundance
1
0
−1
Subject
10000
11000
12000
13000
14000
15000
16000
17000
19000
21000
23000
64000
68000
70000
71000
72000
73000
74000
75000
76000
77000
78000
79000
80000
81000
82000
83000
84000
86000
87000
89000
93000
97000
Condition
EM
Primary

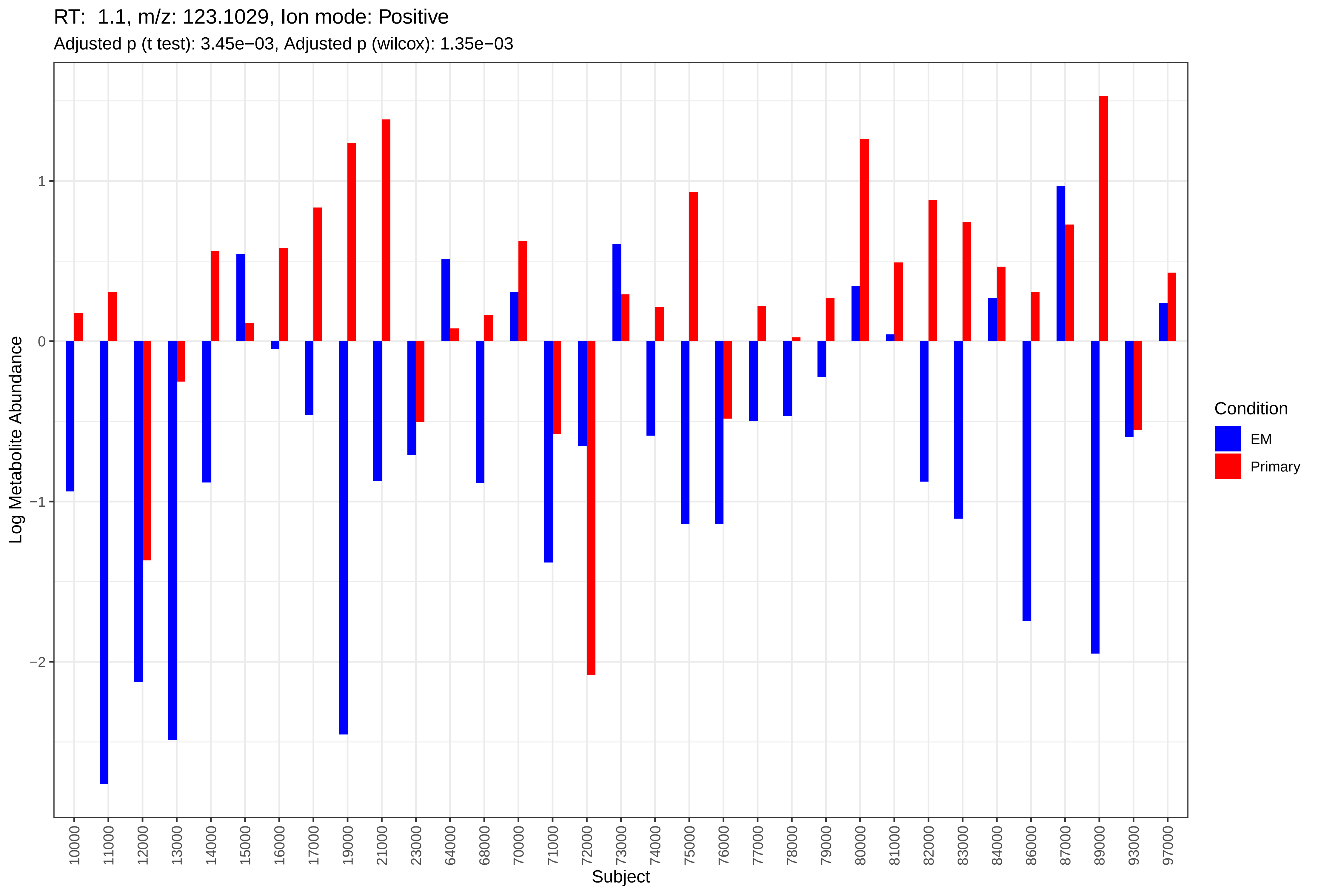

RT: 1.1, m/z: 123.1029, Ion mode: Positive
Adjusted p (t test): 3.45e−03, Adjusted p (wilcox): 1.35e−03
Log Metabolite Abundance
1
0
−1
−2
10000
11000
12000
13000
14000
15000
16000
17000
19000
21000
23000
64000
68000
70000
71000
72000
73000
74000
75000
76000
77000
78000
79000
80000
81000
82000
83000
84000
86000
87000
89000
93000
97000
Subject
Condition
EM
Primary

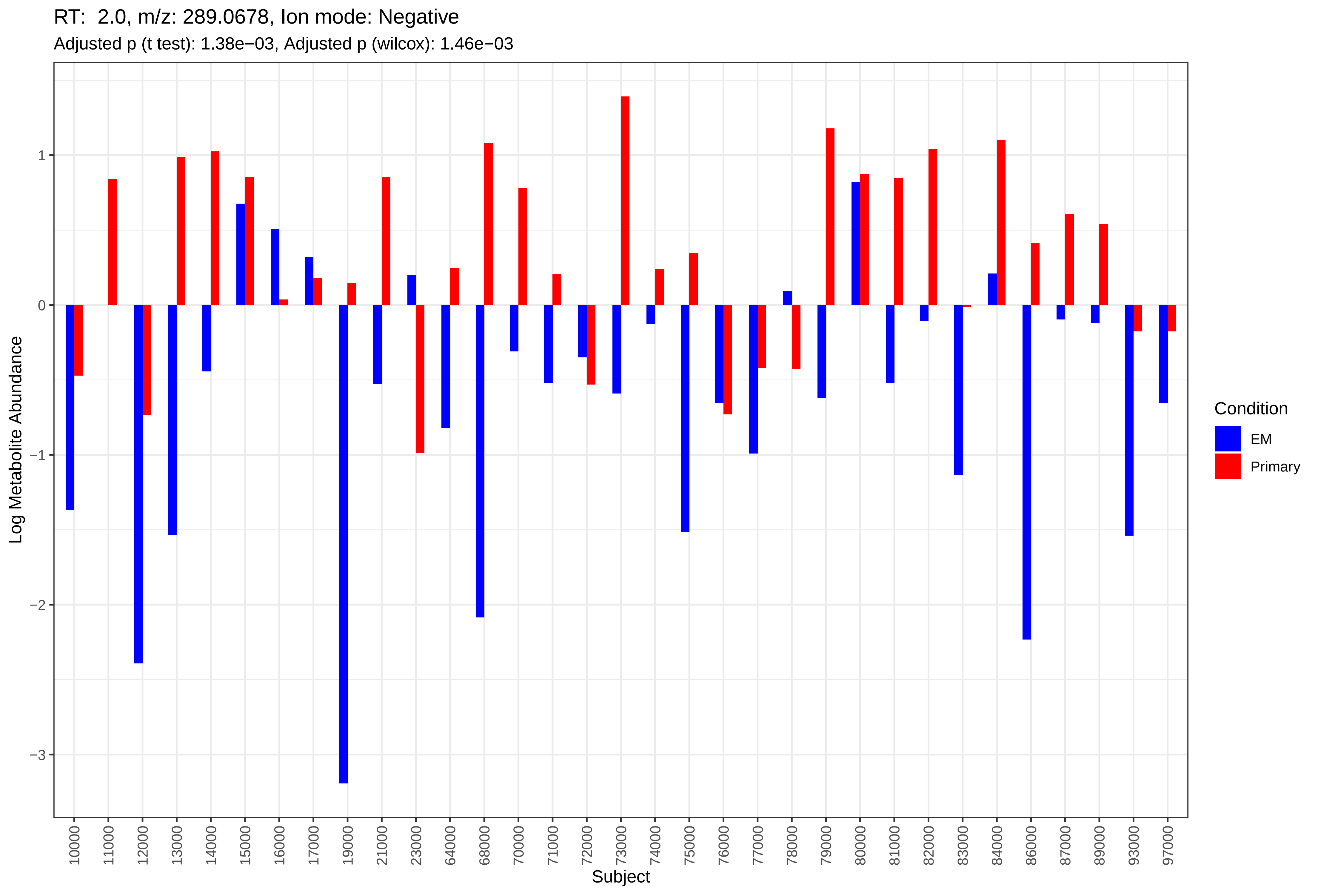
RT: 2.0, m/z: 289.0678, Ion mode: Negative
Adjusted p (t test): 1.38e−03, Adjusted p (wilcox): 1.46e−03
Log Metabolite Abundance
1
0
−1
−2
−3
Subject
10000
11000
12000
13000
14000
15000
16000
17000
19000
21000
23000
64000
68000
70000
71000
72000
73000
74000
75000
76000
77000
78000
79000
80000
81000
82000
83000
84000
86000
87000
89000
93000
97000
Condition
EM
Primary

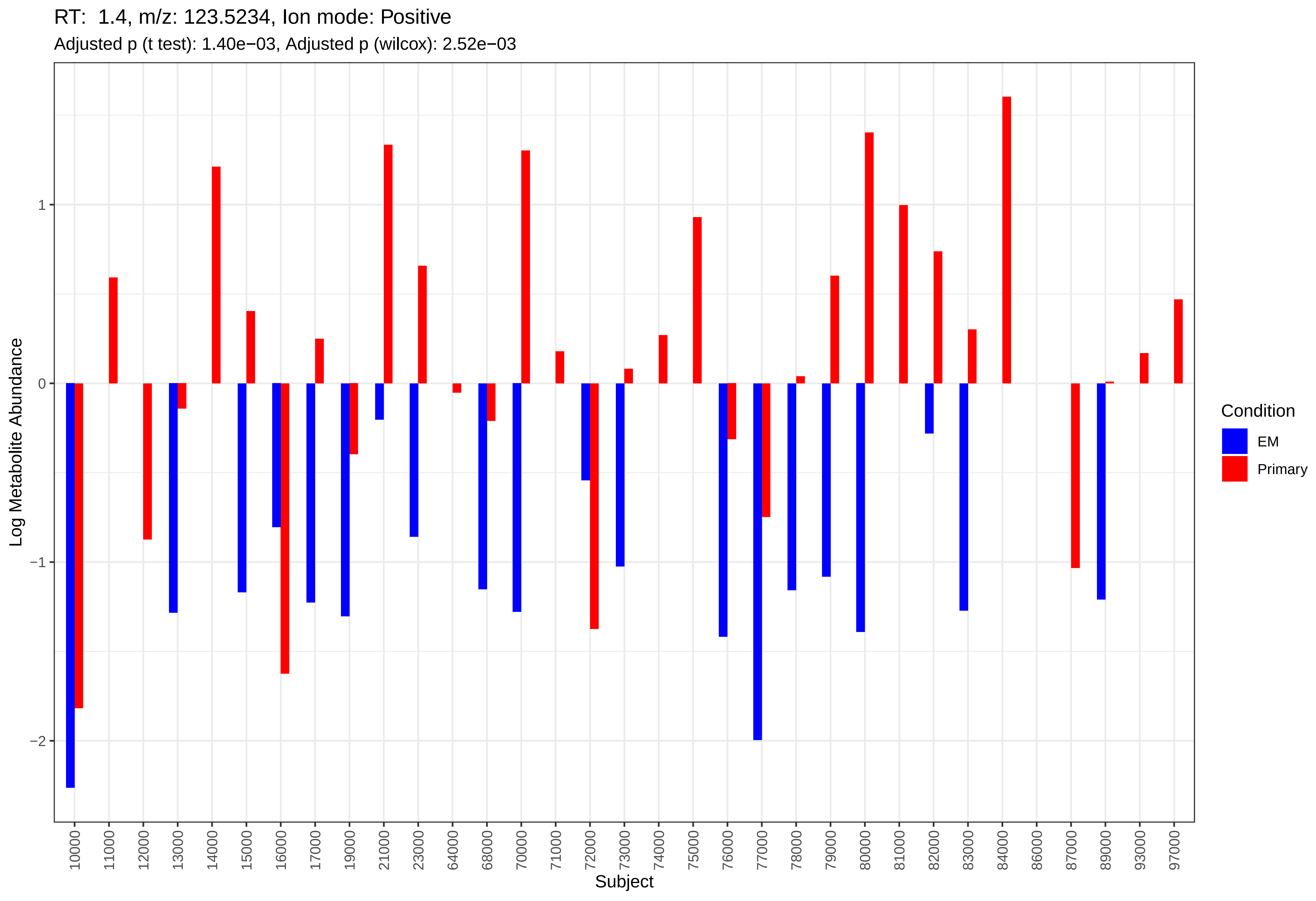
RT: 1.4, m/z: 123.5234, Ion mode: Positive
Adjusted p (t test): 1.40e−03, Adjusted p (wilcox): 2.52e−03
Log Metabolite Abundance
1
0
−1
−2
Subject
10000
11000
12000
13000
14000
15000
16000
17000
19000
21000
23000
64000
68000
70000
71000
72000
73000
74000
75000
76000
77000
78000
79000
80000
81000
82000
83000
84000
86000
87000
89000
93000
97000
Condition
EM
Primary

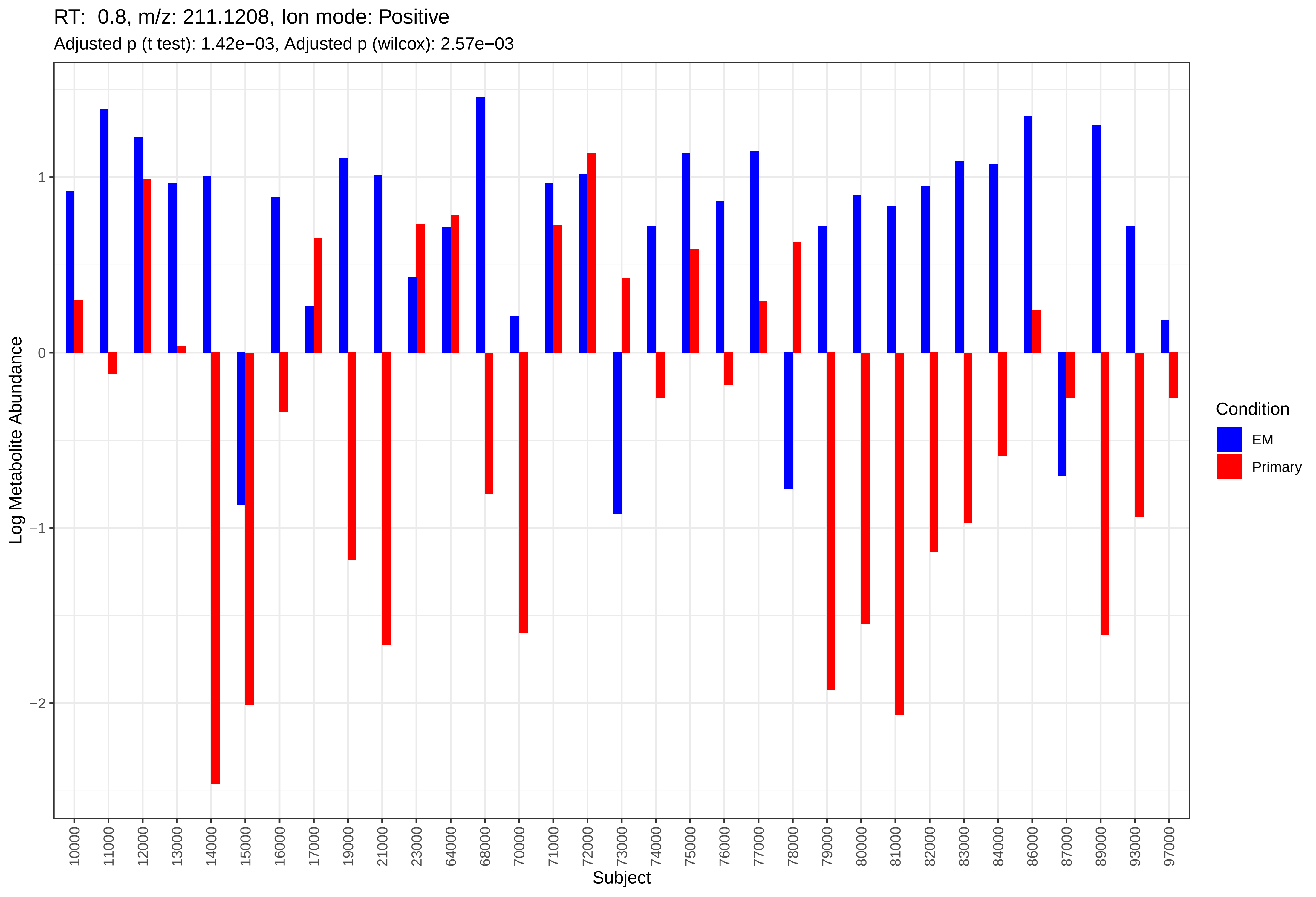

RT: 0.8, m/z: 211.1208, Ion mode: Positive
Adjusted p (t test): 1.42e−03, Adjusted p (wilcox): 2.57e−03
Log Metabolite Abundance
1
0
−1
−2
10000
11000
12000
13000
14000
15000
16000
17000
19000
21000
23000
64000
68000
70000
71000
72000
73000
74000
75000
76000
77000
78000
79000
80000
81000
82000
83000
84000
86000
87000
89000
93000
97000
Subject
Condition
EM
Primary

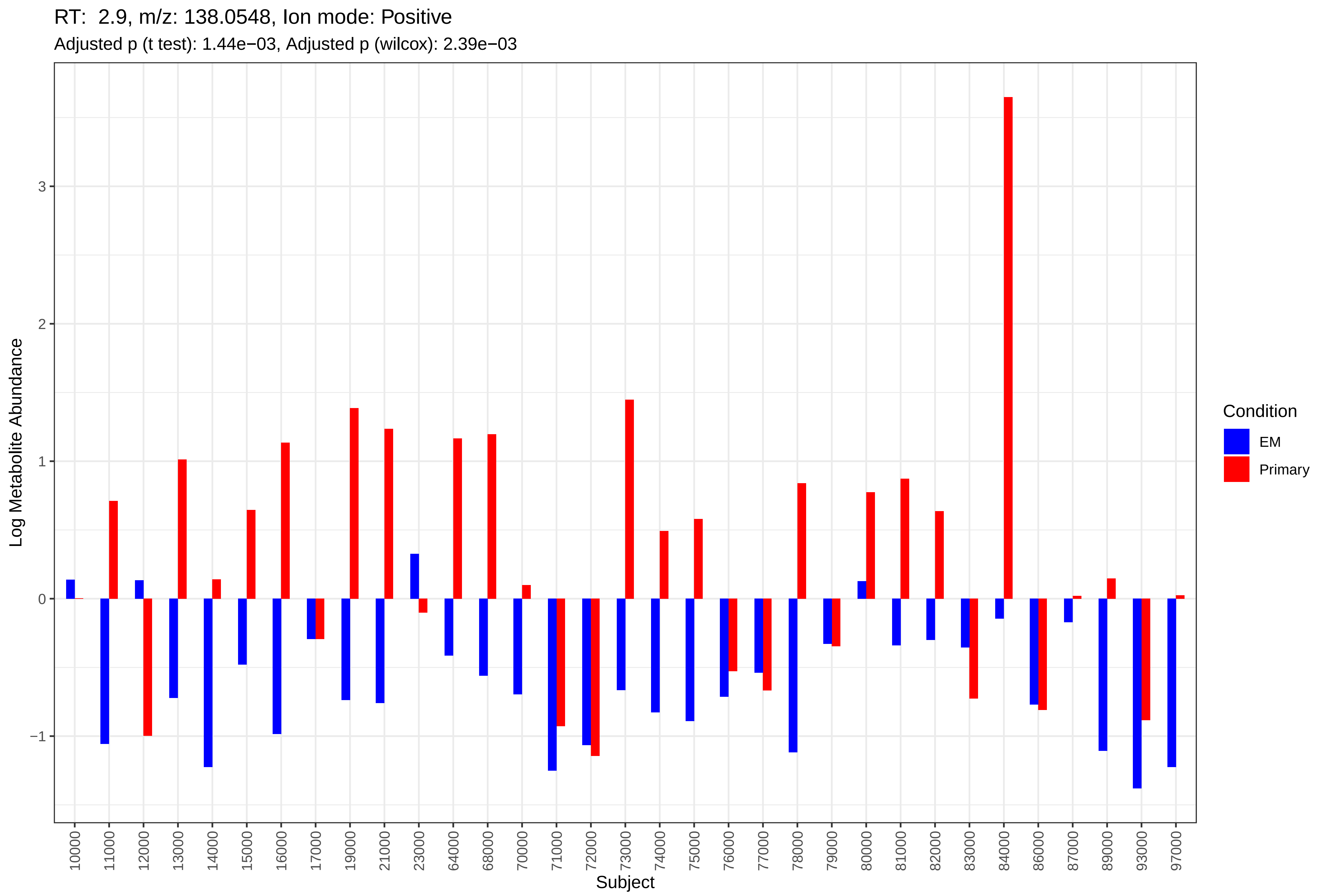

RT: 2.9, m/z: 138.0548, Ion mode: Positive
Adjusted p (t test): 1.44e−03, Adjusted p (wilcox): 2.39e−03
Log Metabolite Abundance
3
2
1
0
−1
10000
11000
12000
13000
14000
15000
16000
17000
19000
21000
23000
64000
68000
70000
71000
72000
73000
74000
75000
76000
77000
78000
79000
80000
81000
82000
83000
84000
86000
87000
89000
93000
97000
Subject
Condition
EM
Primary

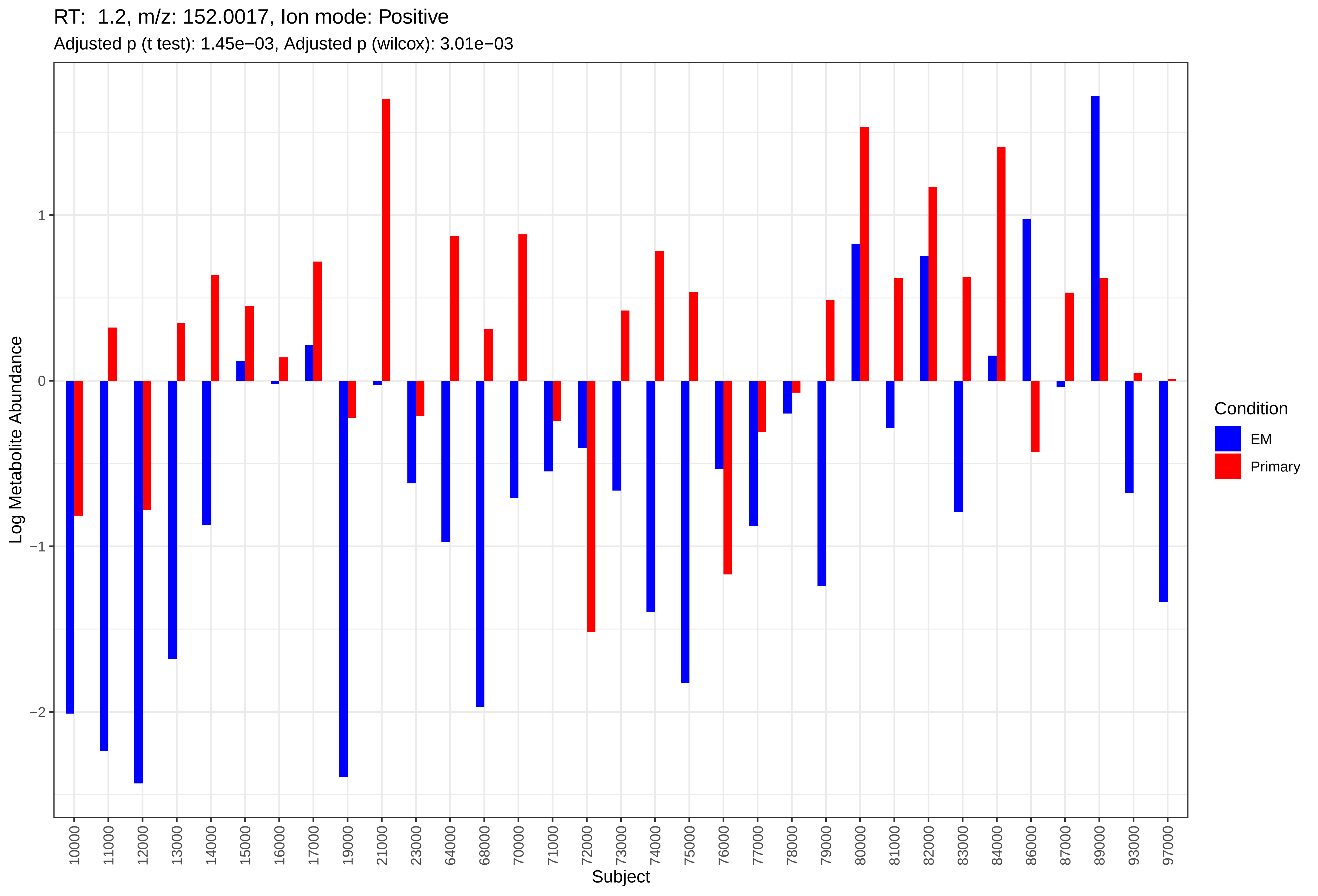

RT: 1.2, m/z: 152.0017, Ion mode: Positive
Adjusted p (t test): 1.45e−03, Adjusted p (wilcox): 3.01e−03
Log Metabolite Abundance
1
0
−1
−2
10000
11000
12000
13000
14000
15000
16000
17000
19000
21000
23000
64000
68000
70000
71000
72000
73000
74000
75000
76000
77000
78000
79000
80000
81000
82000
83000
84000
86000
87000
89000
93000
97000
Subject
Condition
EM
Primary

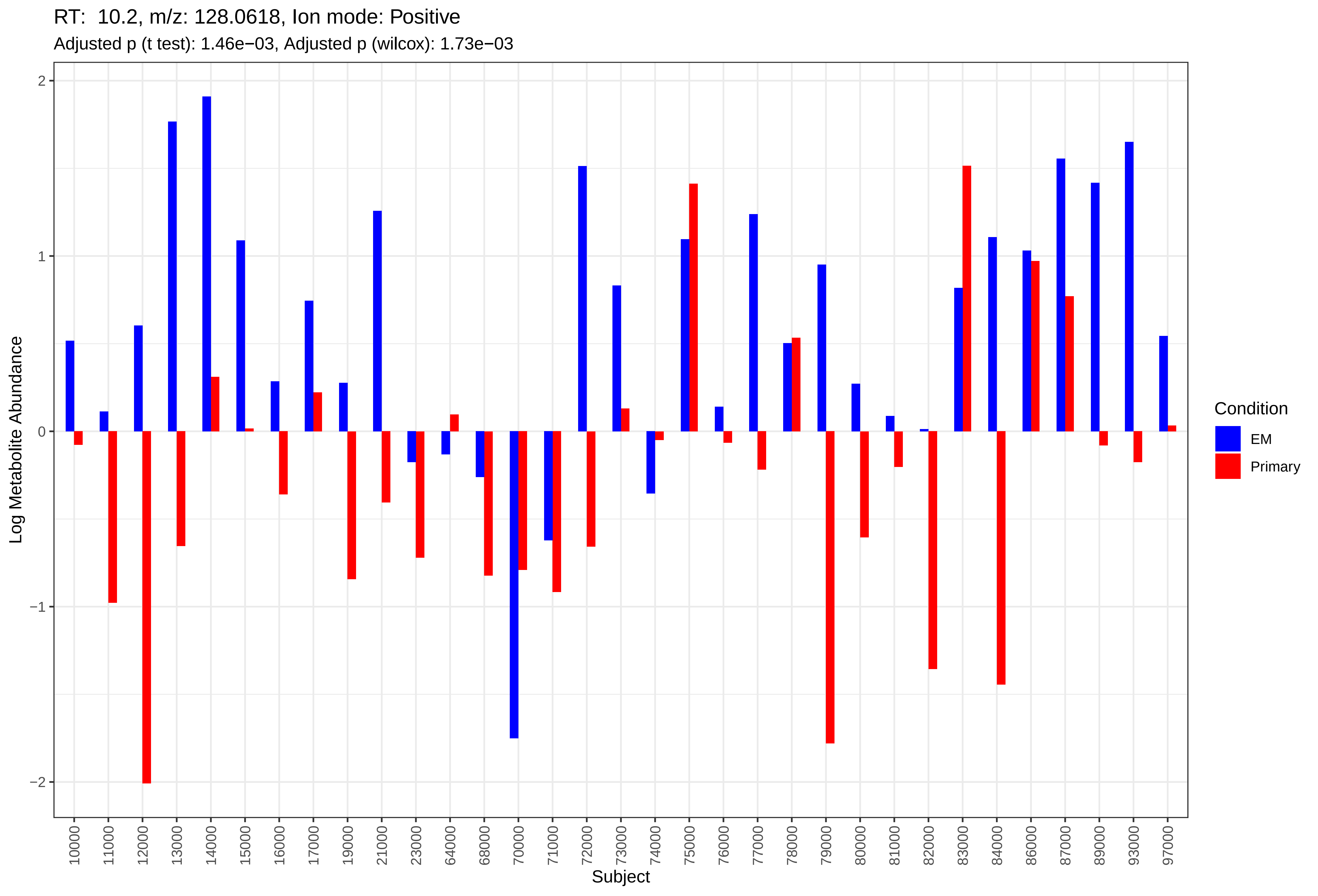
RT: 10.2, m/z: 128.0618, Ion mode: Positive
Adjusted p (t test): 1.46e−03, Adjusted p (wilcox): 1.73e−03
Log Metabolite Abundance
2
1
0
−1
−2
Subject
10000
11000
12000
13000
14000
15000
16000
17000
19000
21000
23000
64000
68000
70000
71000
72000
73000
74000
75000
76000
77000
78000
79000
80000
81000
82000
83000
84000
86000
87000
89000
93000
97000
Condition
EM
Primary

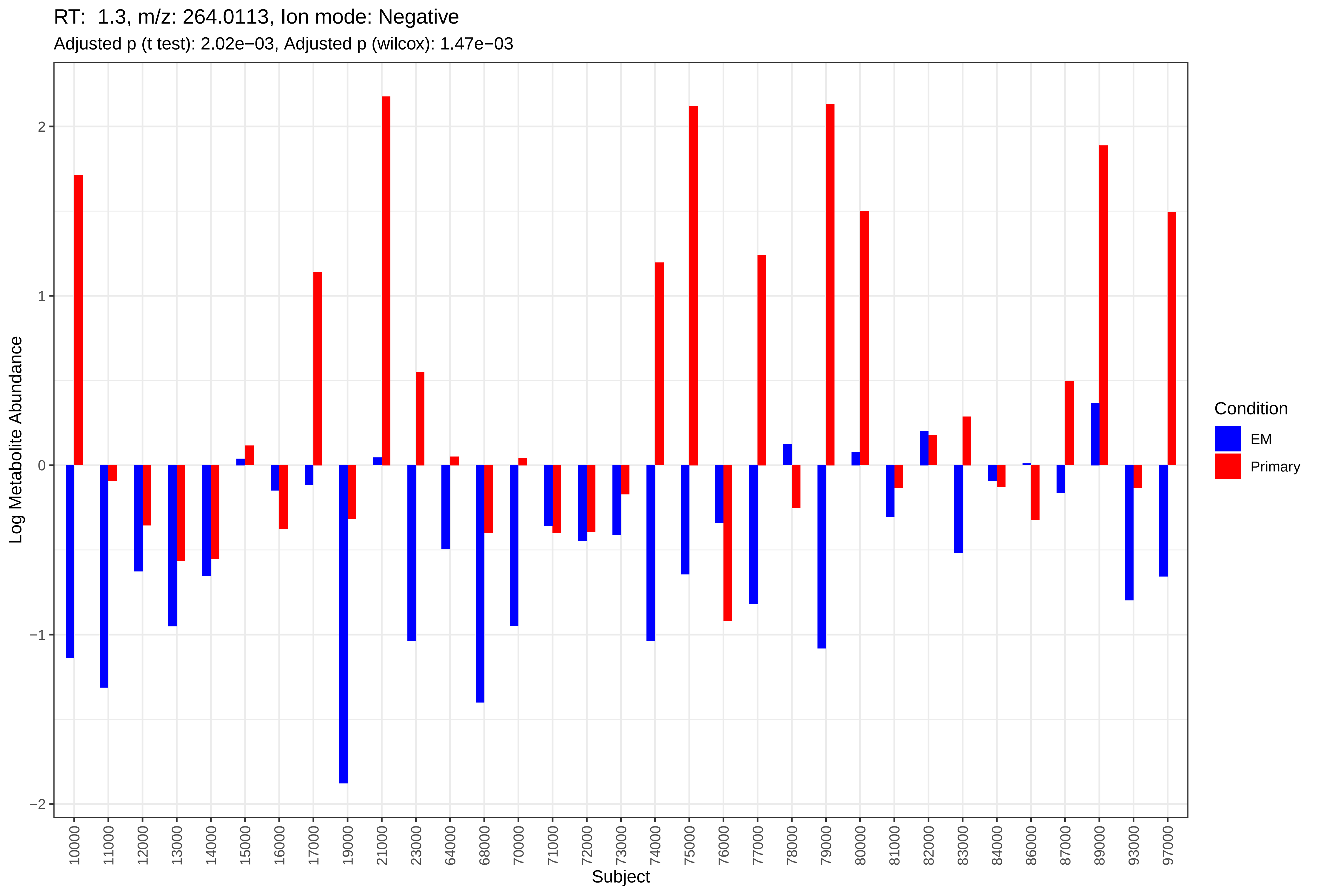

RT: 1.3, m/z: 264.0113, Ion mode: Negative
Adjusted p (t test): 2.02e−03, Adjusted p (wilcox): 1.47e−03
Log Metabolite Abundance
2
1
0
−1
−2
Subject
10000
11000
12000
13000
14000
15000
16000
17000
19000
21000
23000
64000
68000
70000
71000
72000
73000
74000
75000
76000
77000
78000
79000
80000
81000
82000
83000
84000
86000
87000
89000
93000
97000
Condition
EM
Primary

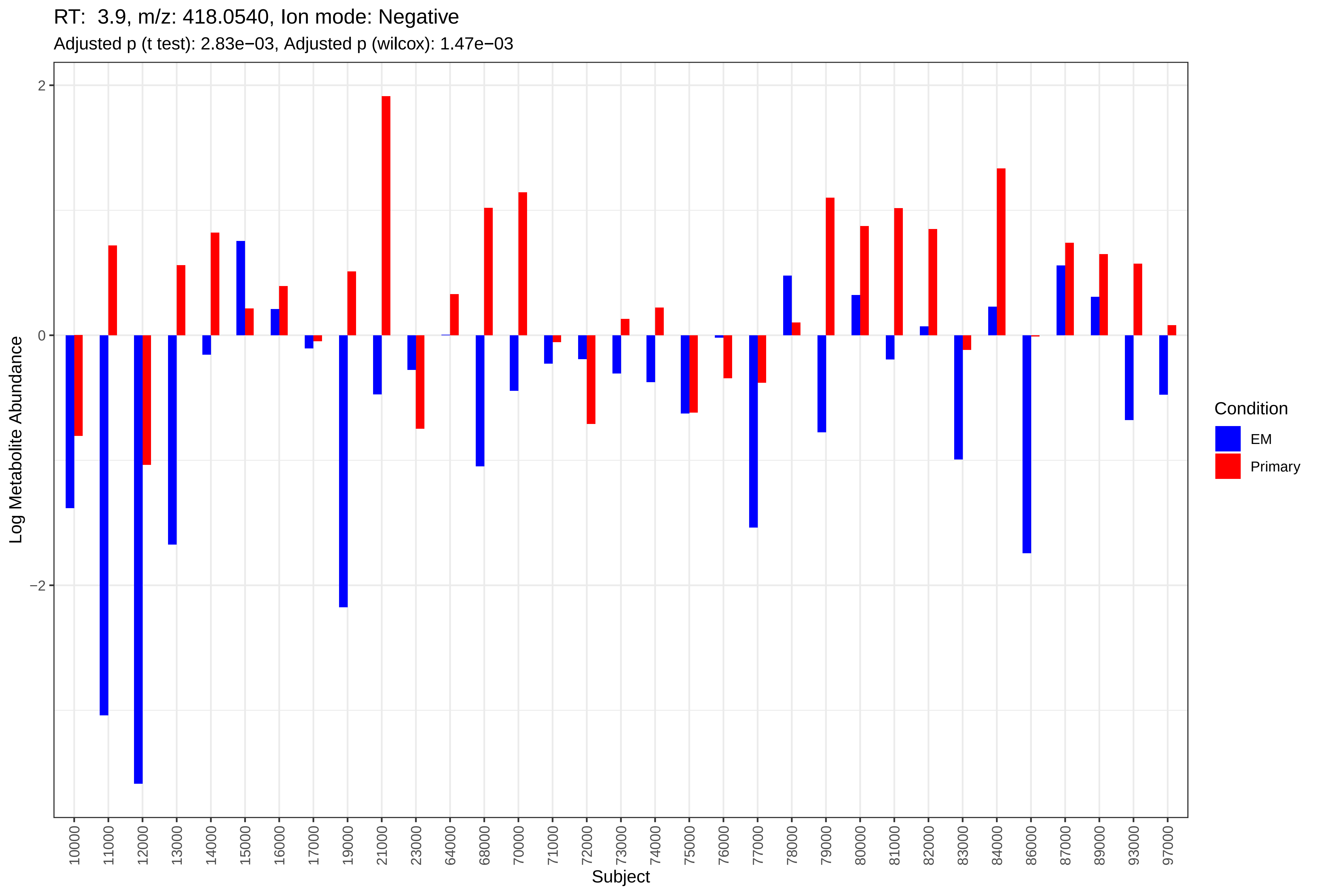
RT: 3.9, m/z: 418.0540, Ion mode: Negative
Adjusted p (t test): 2.83e−03, Adjusted p (wilcox): 1.47e−03
Log Metabolite Abundance
2
0
−2
10000
11000
12000
13000
14000
15000
16000
17000
19000
21000
23000
64000
68000
70000
71000
72000
73000
74000
75000
76000
77000
78000
79000
80000
81000
82000
83000
84000
86000
87000
89000
93000
97000
Subject
Condition
EM
Primary

RT: 1.9, m/z: 96.9693, Ion mode: Negative

Adjusted p (t test): 3.45e−03, Adjusted p (wilcox): 1.47e−03

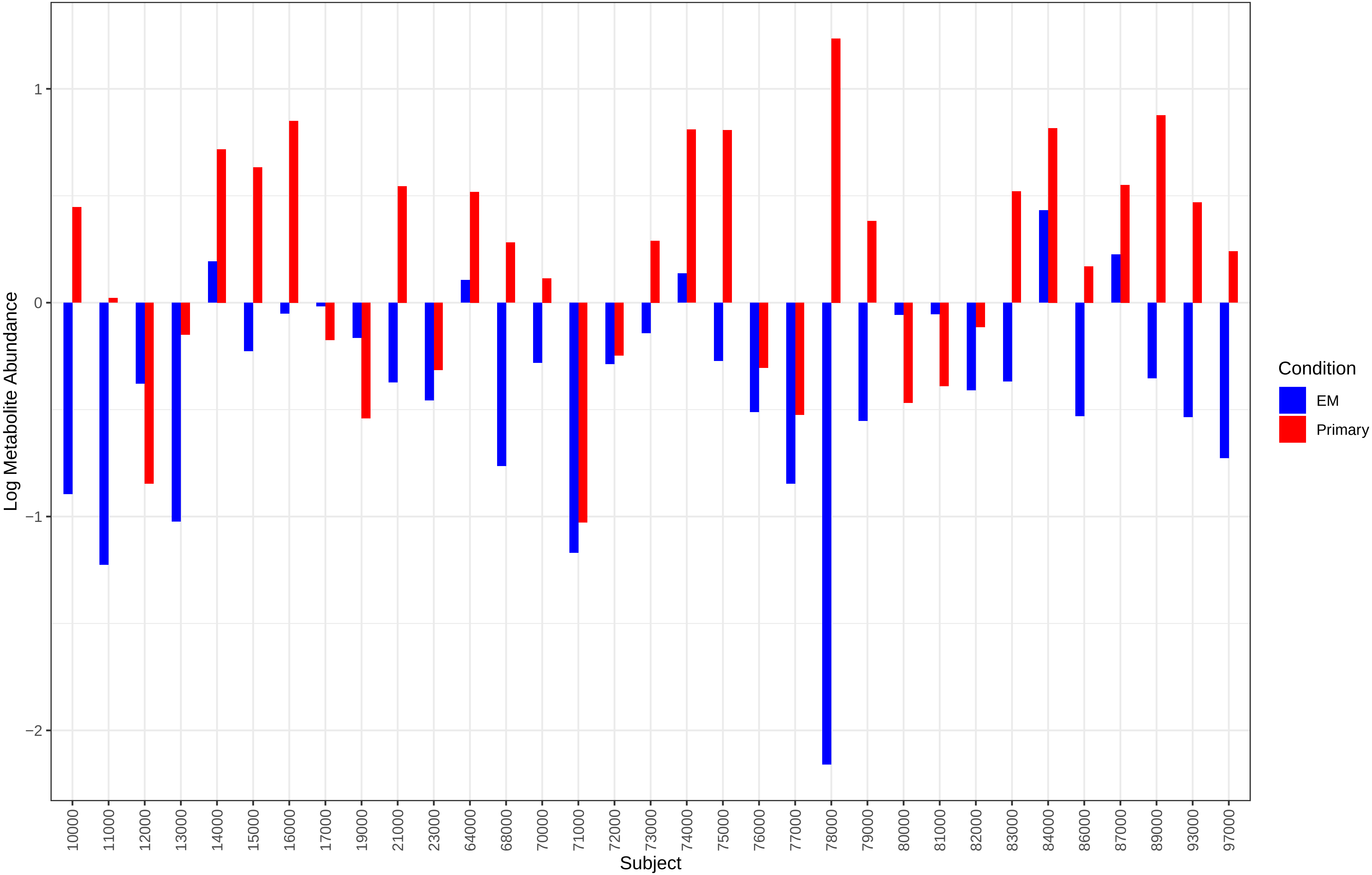

RT: 1.3, m/z: 241.1395, Ion mode: Positive

Adjusted p (t test): 3.44e−03, Adjusted p (wilcox): 1.47e−03

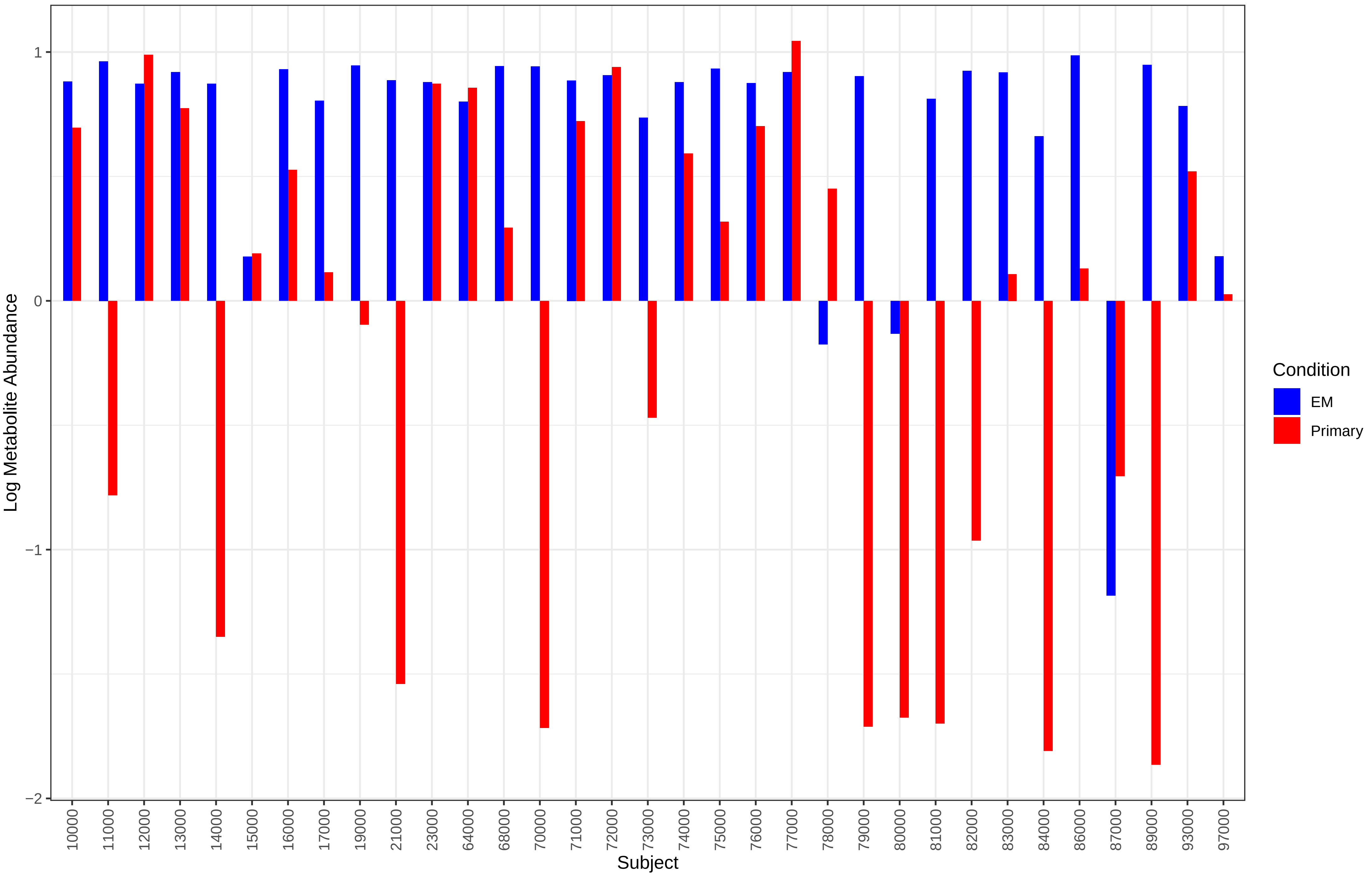

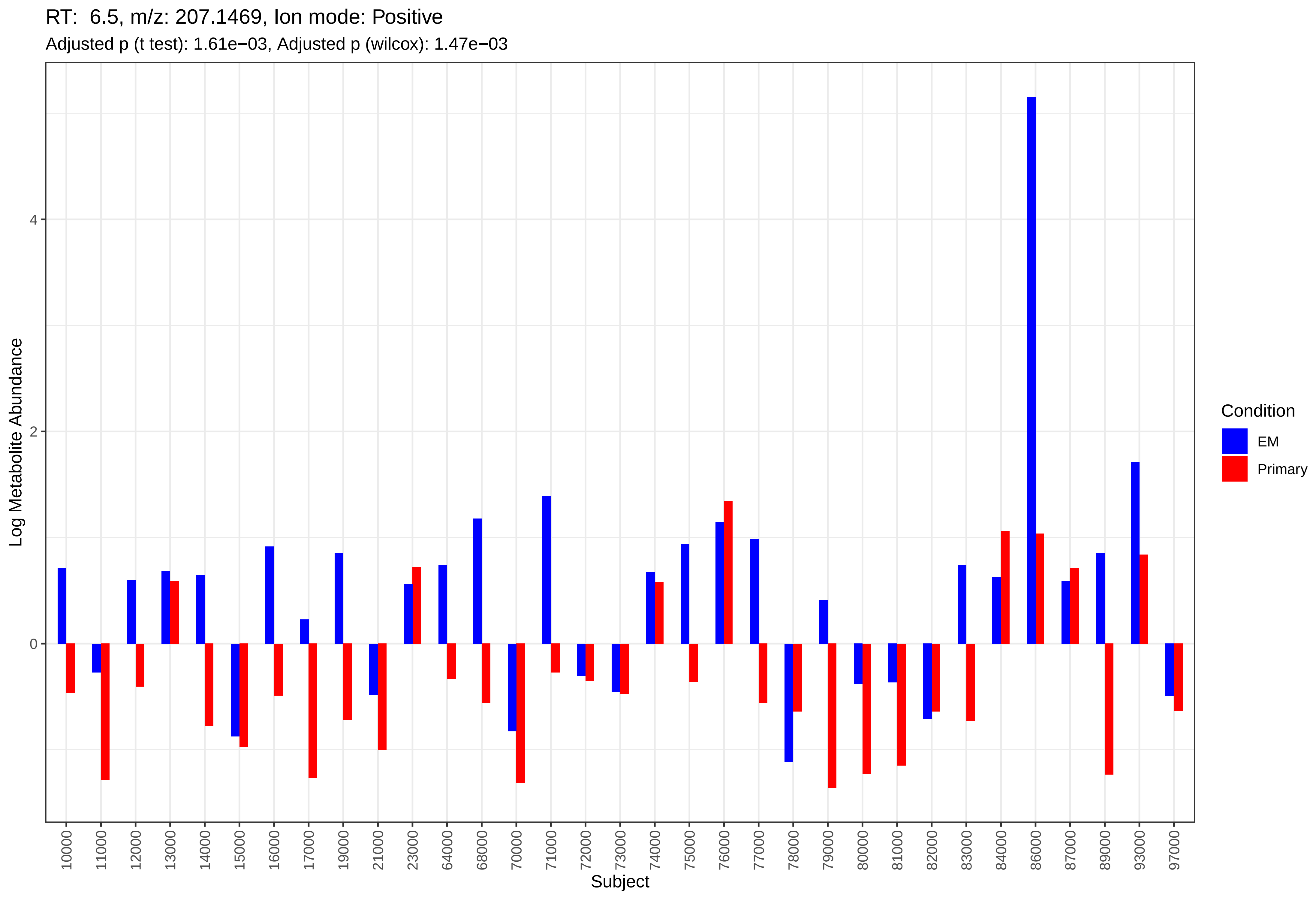

RT: 6.5, m/z: 207.1469, Ion mode: Positive
Adjusted p (t test): 1.61e−03, Adjusted p (wilcox): 1.47e−03
Log Metabolite Abundance
4
2
0
10000
11000
12000
13000
14000
15000
16000
17000
19000
21000
23000
64000
68000
70000
71000
72000
73000
74000
75000
76000
77000
78000
79000
80000
81000
82000
83000
84000
86000
87000
89000
93000
97000
Subject
Condition
EM
Primary

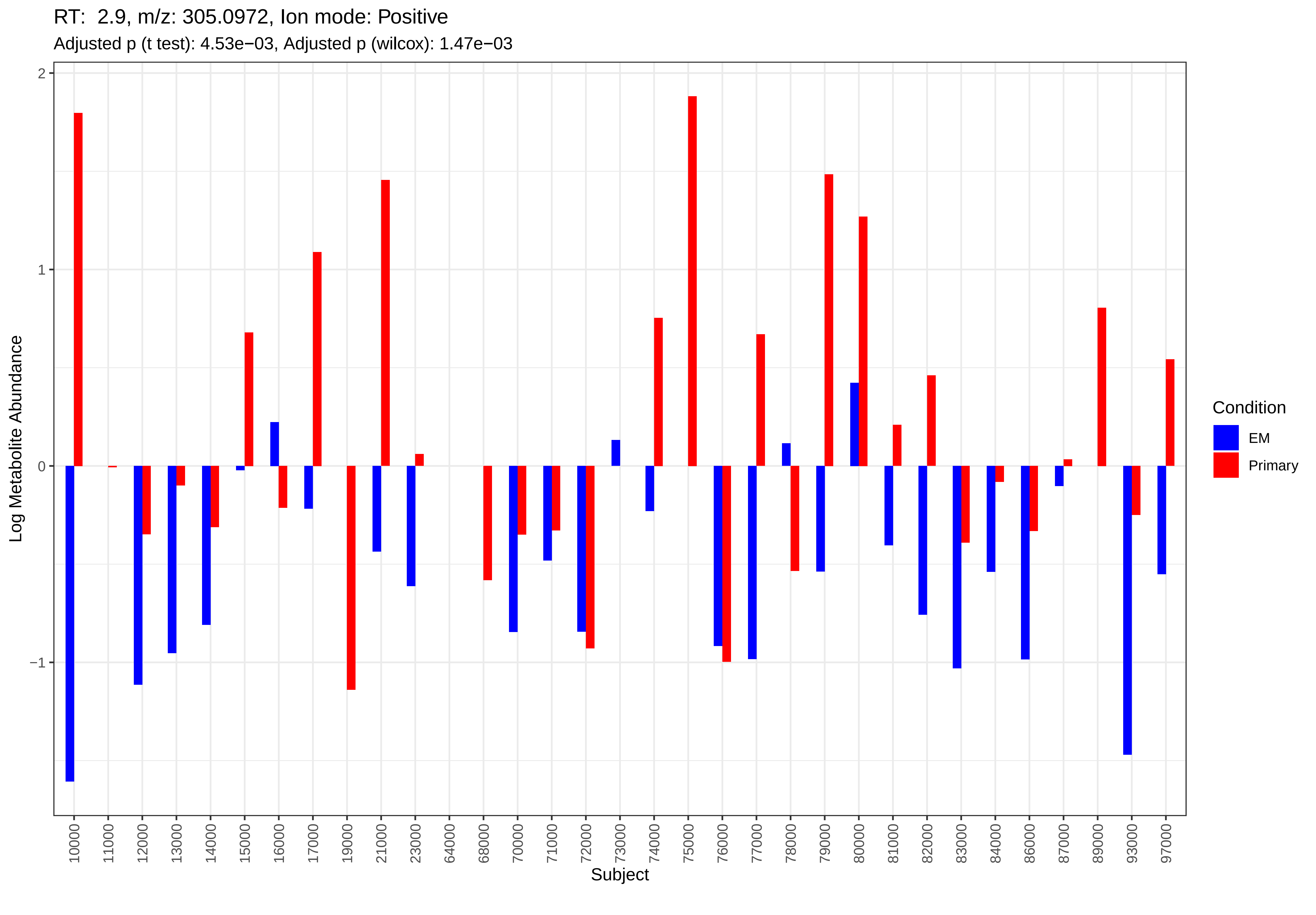

RT: 2.9, m/z: 305.0972, Ion mode: Positive
Adjusted p (t test): 4.53e−03, Adjusted p (wilcox): 1.47e−03
Log Metabolite Abundance
2
1
0
−1
Subject
10000
11000
12000
13000
14000
15000
16000
17000
19000
21000
23000
64000
68000
70000
71000
72000
73000
74000
75000
76000
77000
78000
79000
80000
81000
82000
83000
84000
86000
87000
89000
93000
97000
Condition
EM
Primary

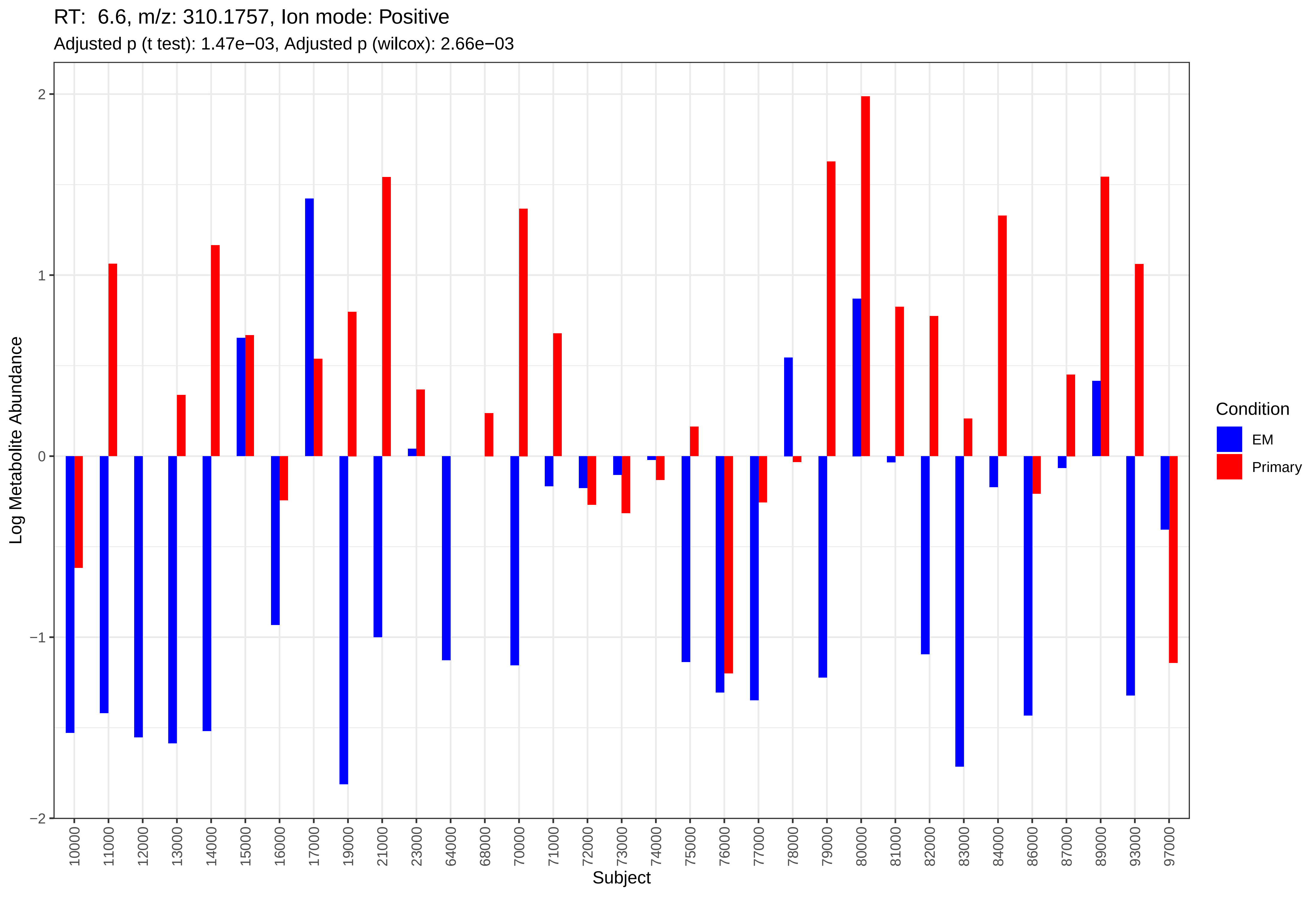
RT: 6.6, m/z: 310.1757, Ion mode: Positive
Adjusted p (t test): 1.47e−03, Adjusted p (wilcox): 2.66e−03
Log Metabolite Abundance
2
1
0
−1
−2
Subject
10000
11000
12000
13000
14000
15000
16000
17000
19000
21000
23000
64000
68000
70000
71000
72000
73000
74000
75000
76000
77000
78000
79000
80000
81000
82000
83000
84000
86000
87000
89000
93000
97000
Condition
EM
Primary

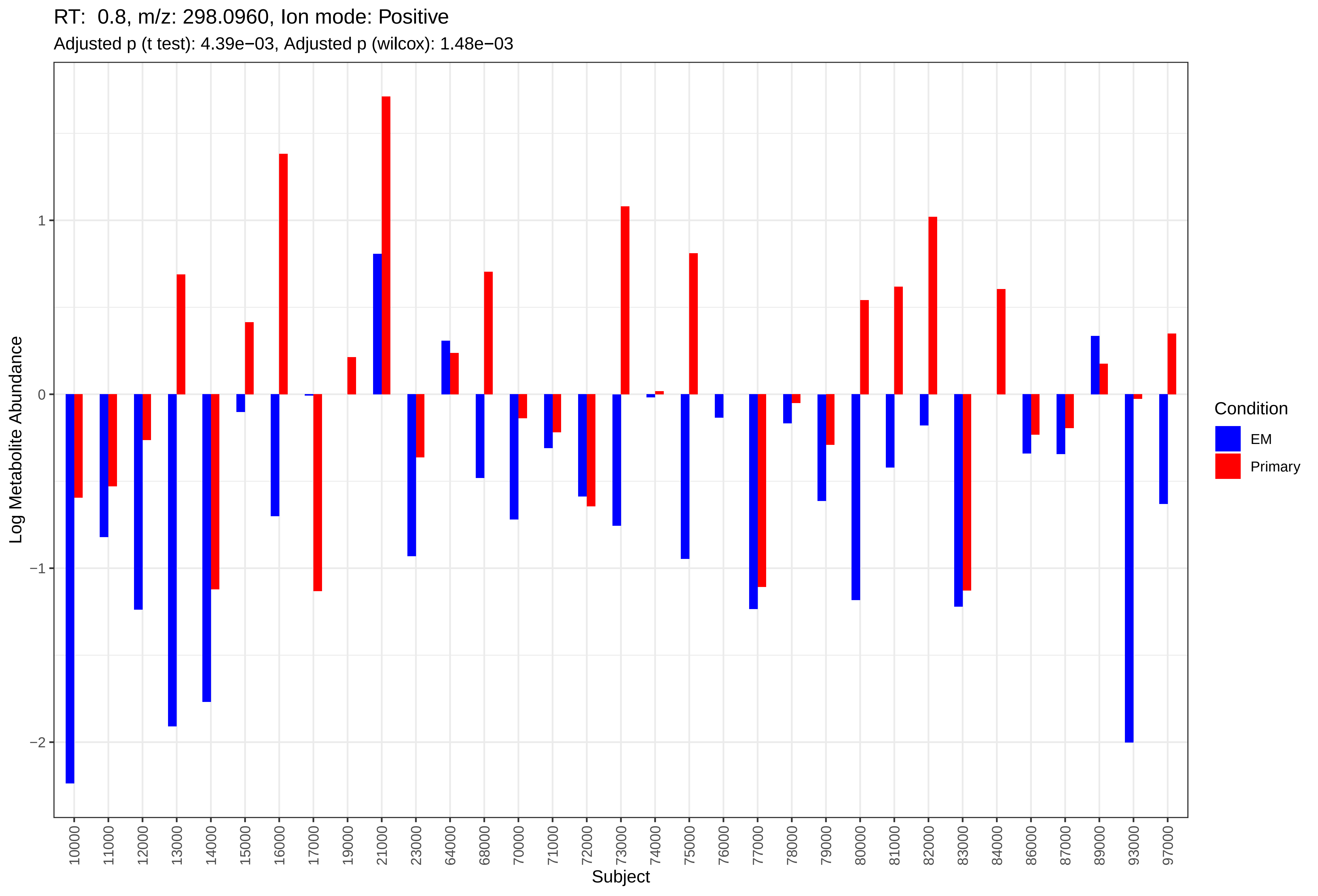
RT: 0.8, m/z: 298.0960, Ion mode: Positive
Adjusted p (t test): 4.39e−03, Adjusted p (wilcox): 1.48e−03
Log Metabolite Abundance
1
0
−1
−2
Subject
10000
11000
12000
13000
14000
15000
16000
17000
19000
21000
23000
64000
68000
70000
71000
72000
73000
74000
75000
76000
77000
78000
79000
80000
81000
82000
83000
84000
86000
87000
89000
93000
97000
Condition
EM
Primary

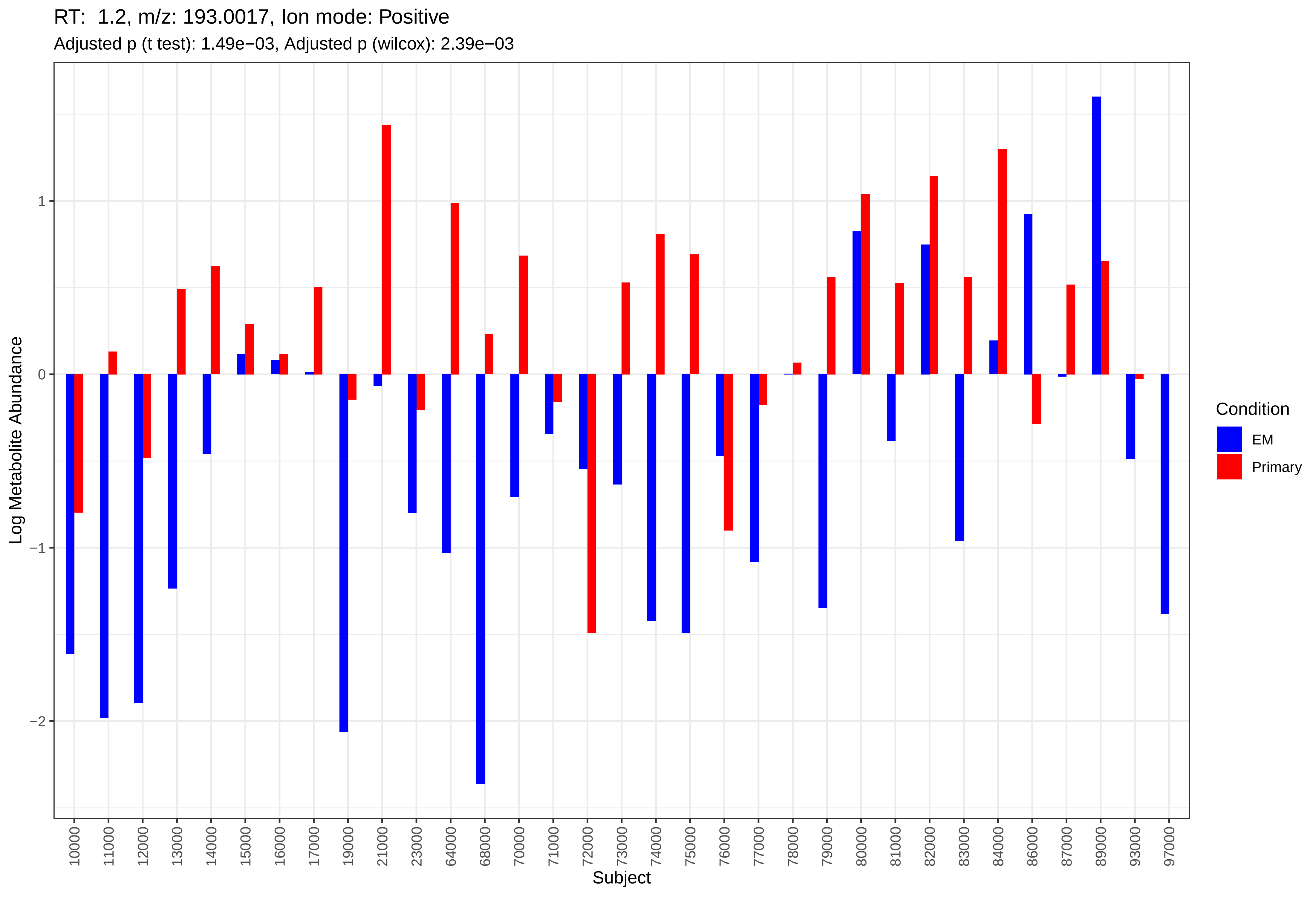

RT: 1.2, m/z: 193.0017, Ion mode: Positive
Adjusted p (t test): 1.49e−03, Adjusted p (wilcox): 2.39e−03
Log Metabolite Abundance
1
0
−1
−2
10000
11000
12000
13000
14000
15000
16000
17000
19000
21000
23000
64000
68000
70000
71000
72000
73000
74000
75000
76000
77000
78000
79000
80000
81000
82000
83000
84000
86000
87000
89000
93000
97000
Subject
Condition
EM
Primary

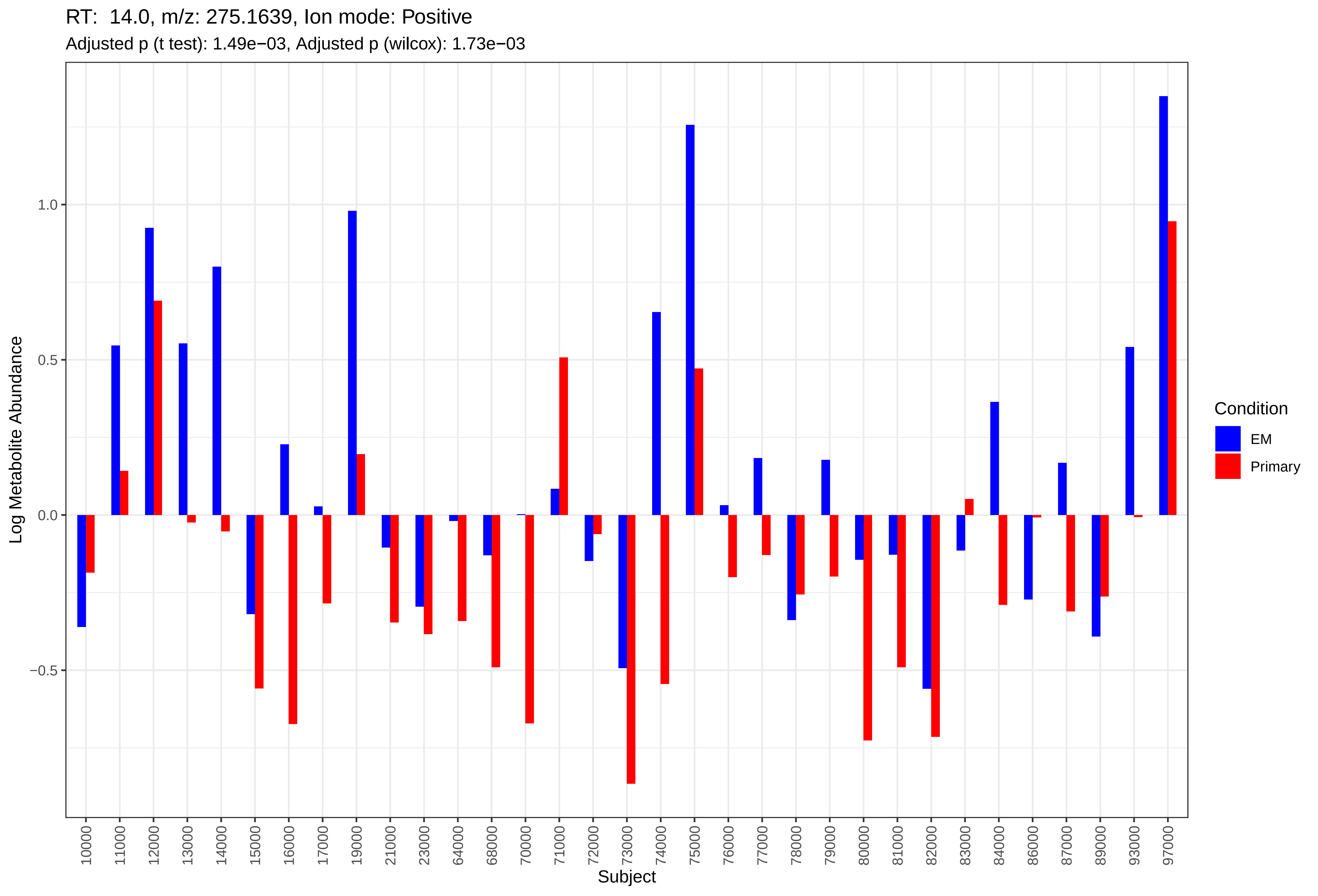
RT: 14.0, m/z: 275.1639, Ion mode: Positive
Adjusted p (t test): 1.49e−03, Adjusted p (wilcox): 1.73e−03
Log Metabolite Abundance
1.0
0.5
0.0
−0.5
Subject
10000
11000
12000
13000
14000
15000
16000
17000
19000
21000
23000
64000
68000
70000
71000
72000
73000
74000
75000
76000
77000
78000
79000
80000
81000
82000
83000
84000
86000
87000
89000
93000
97000
Condition
EM
Primary

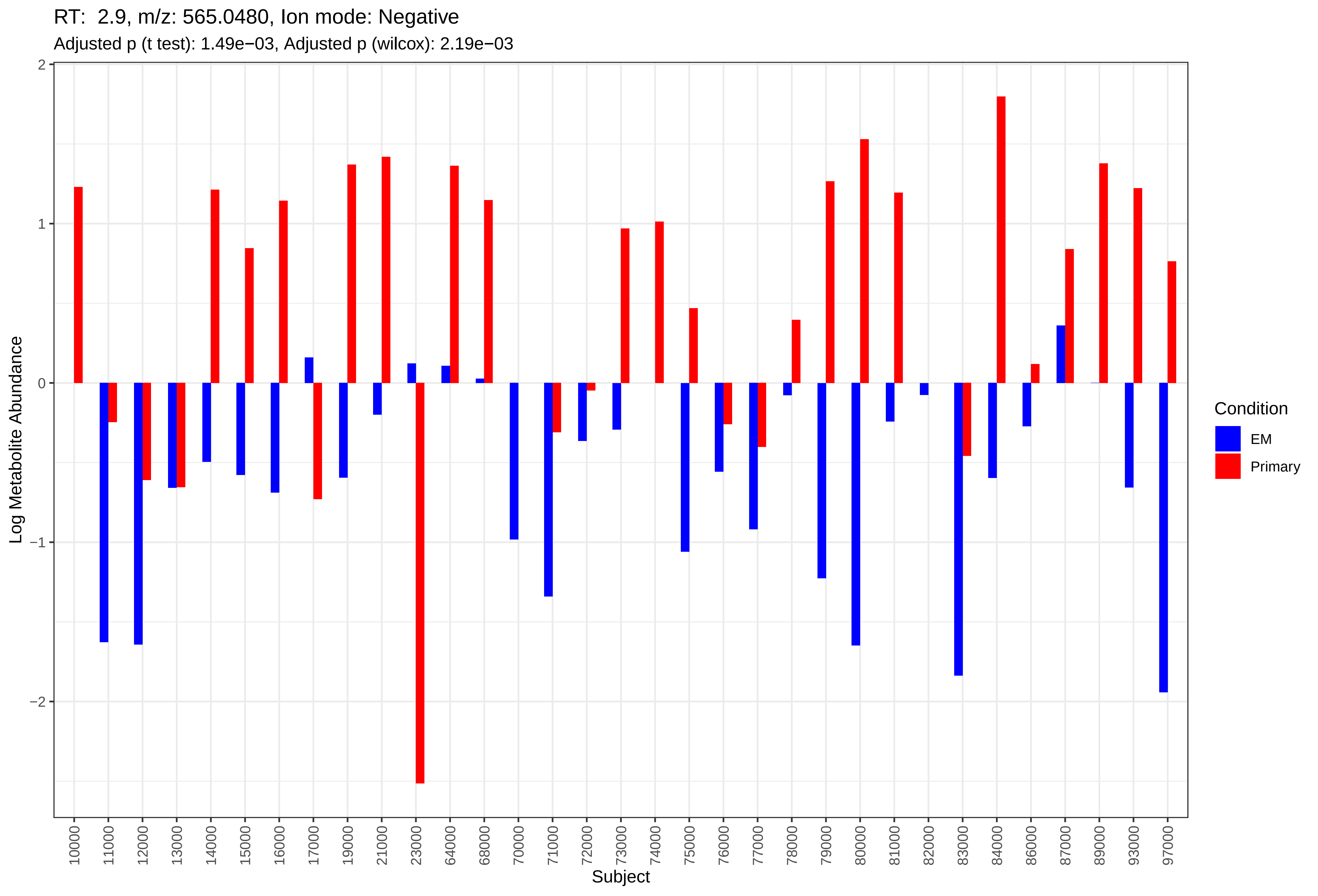

RT: 2.9, m/z: 565.0480, Ion mode: Negative
Adjusted p (t test): 1.49e−03, Adjusted p (wilcox): 2.19e−03
Log Metabolite Abundance
2
1
0
−1
−2
10000
11000
12000
13000
14000
15000
16000
17000
19000
21000
23000
64000
68000
70000
71000
72000
73000
74000
75000
76000
77000
78000
79000
80000
81000
82000
83000
84000
86000
87000
89000
93000
97000
Subject
Condition
EM
Primary

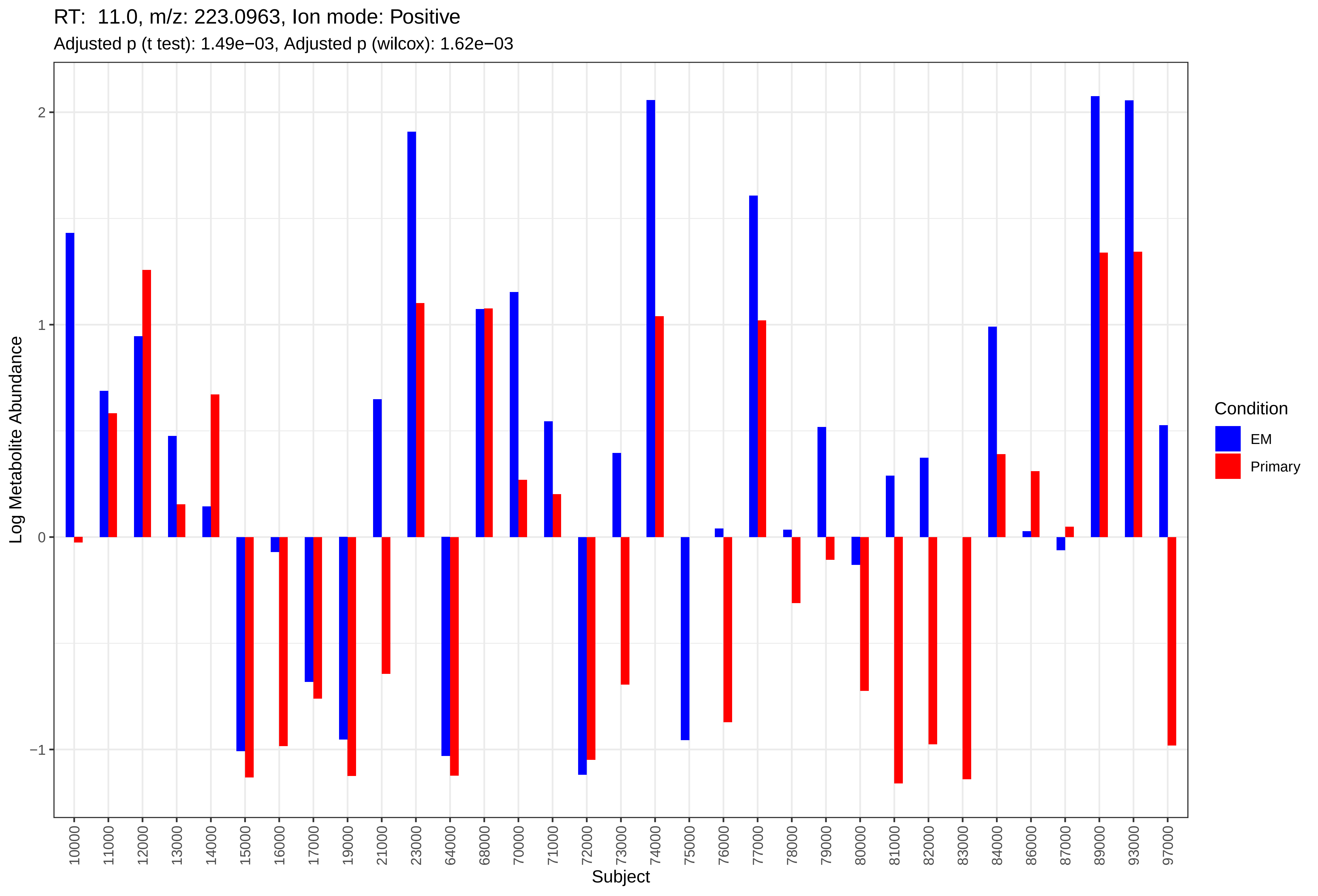

RT: 11.0, m/z: 223.0963, Ion mode: Positive
Adjusted p (t test): 1.49e−03, Adjusted p (wilcox): 1.62e−03
Log Metabolite Abundance
2
1
0
−1
Subject
10000
11000
12000
13000
14000
15000
16000
17000
19000
21000
23000
64000
68000
70000
71000
72000
73000
74000
75000
76000
77000
78000
79000
80000
81000
82000
83000
84000
86000
87000
89000
93000
97000
Condition
EM
Primary

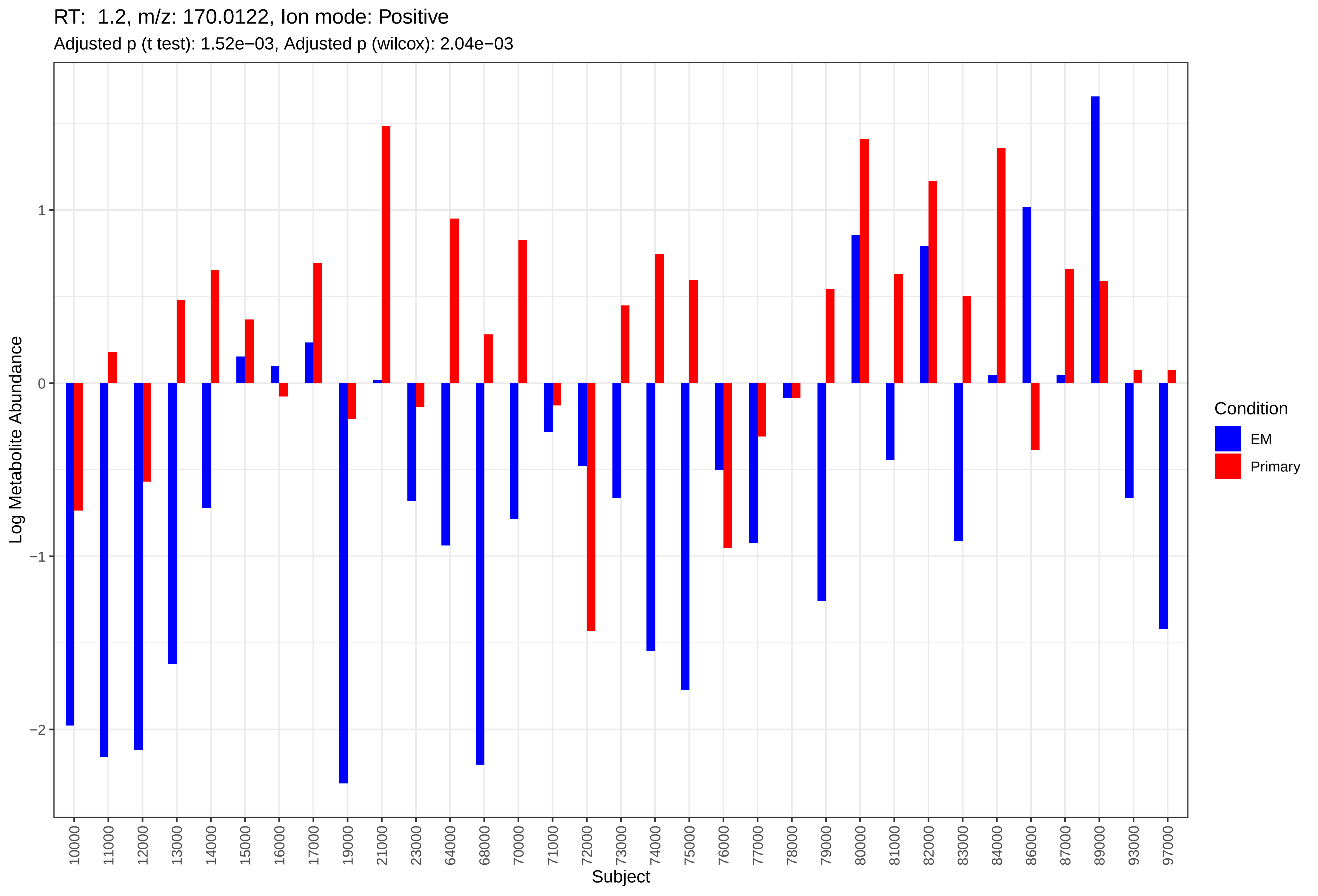
RT: 1.2, m/z: 170.0122, Ion mode: Positive
Adjusted p (t test): 1.52e−03, Adjusted p (wilcox): 2.04e−03
Log Metabolite Abundance
1
0
−1
−2
10000
11000
12000
13000
14000
15000
16000
17000
19000
21000
23000
64000
68000
70000
71000
72000
73000
74000
75000
76000
77000
78000
79000
80000
81000
82000
83000
84000
86000
87000
89000
93000
97000
Subject
Condition
EM
Primary

RT: 0.9, m/z: 226.9962, Ion mode: Negative

Adjusted p (t test): 5.54e−03, Adjusted p (wilcox): 1.53e−03

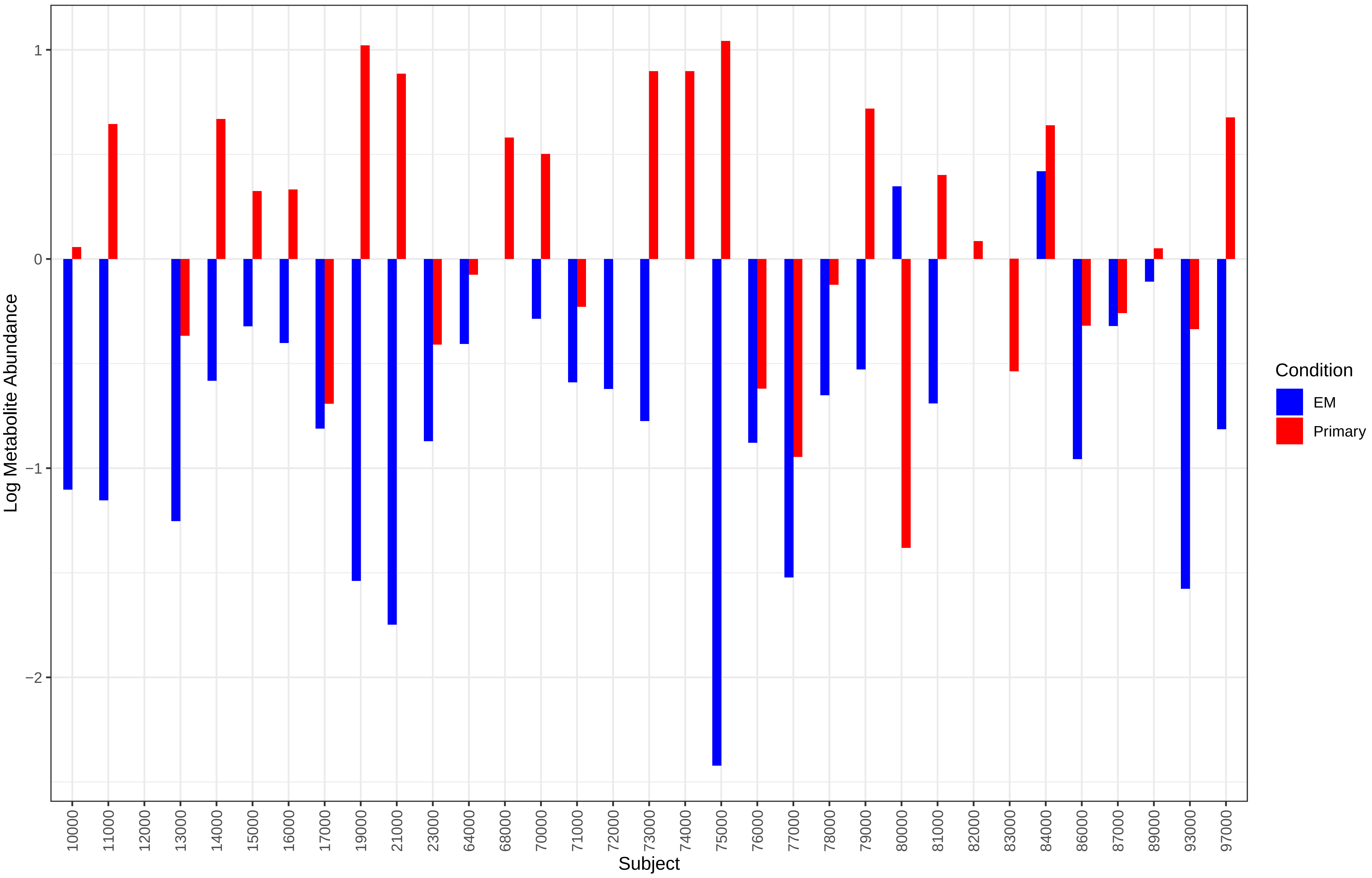

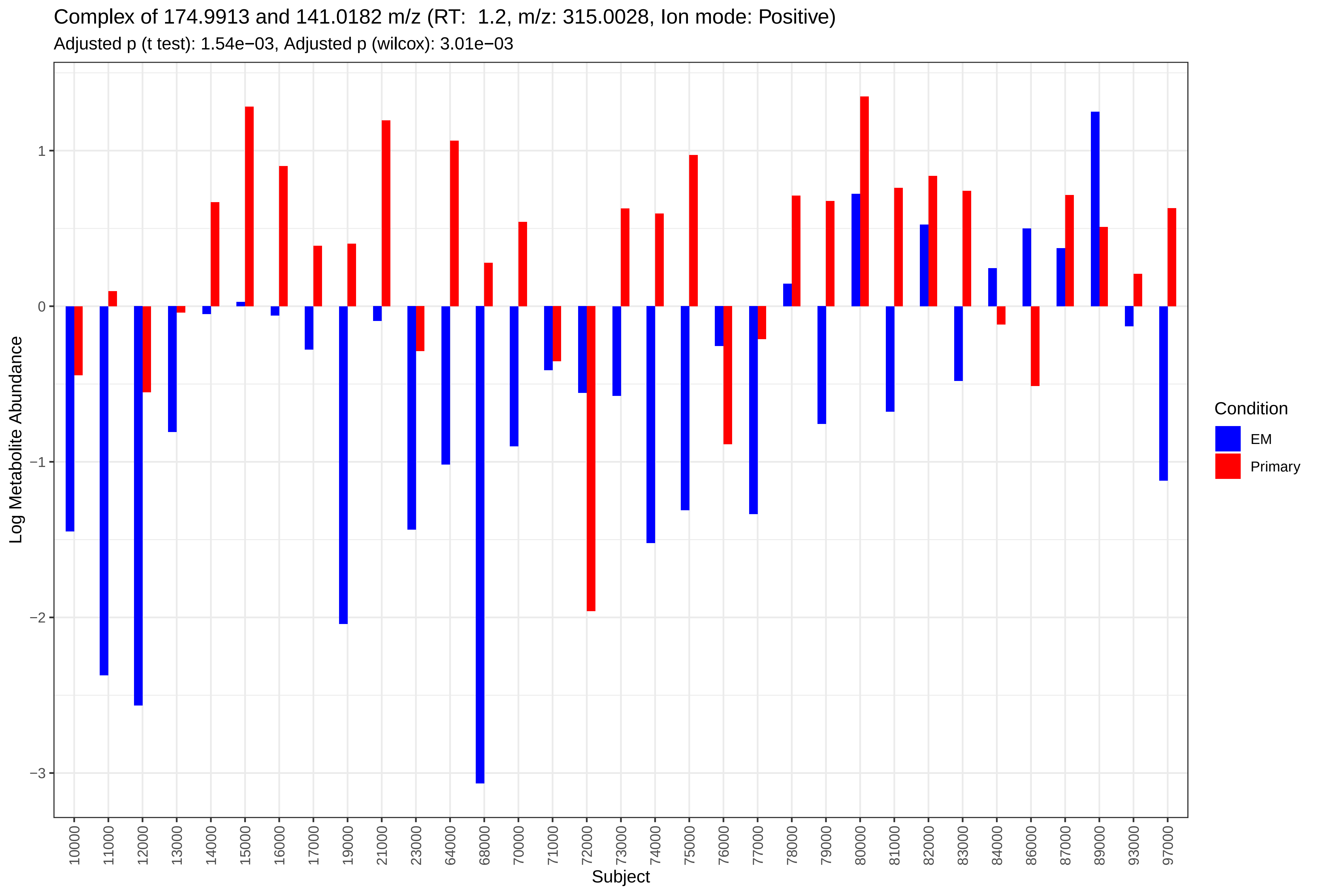

Complex of 174.9913 and 141.0182 m/z (RT: 1.2, m/z: 315.0028, Ion mode: Positive)
Adjusted p (t test): 1.54e−03, Adjusted p (wilcox): 3.01e−03
Log Metabolite Abundance
1
0
−1
−2
−3
Subject
10000
11000
12000
13000
14000
15000
16000
17000
19000
21000
23000
64000
68000
70000
71000
72000
73000
74000
75000
76000
77000
78000
79000
80000
81000
82000
83000
84000
86000
87000
89000
93000
97000
Condition
EM
Primary

RT: 1.5, m/z: 153.1329, Ion mode: Positive

Adjusted p (t test): 1.55e−03, Adjusted p (wilcox): 3.26e−03

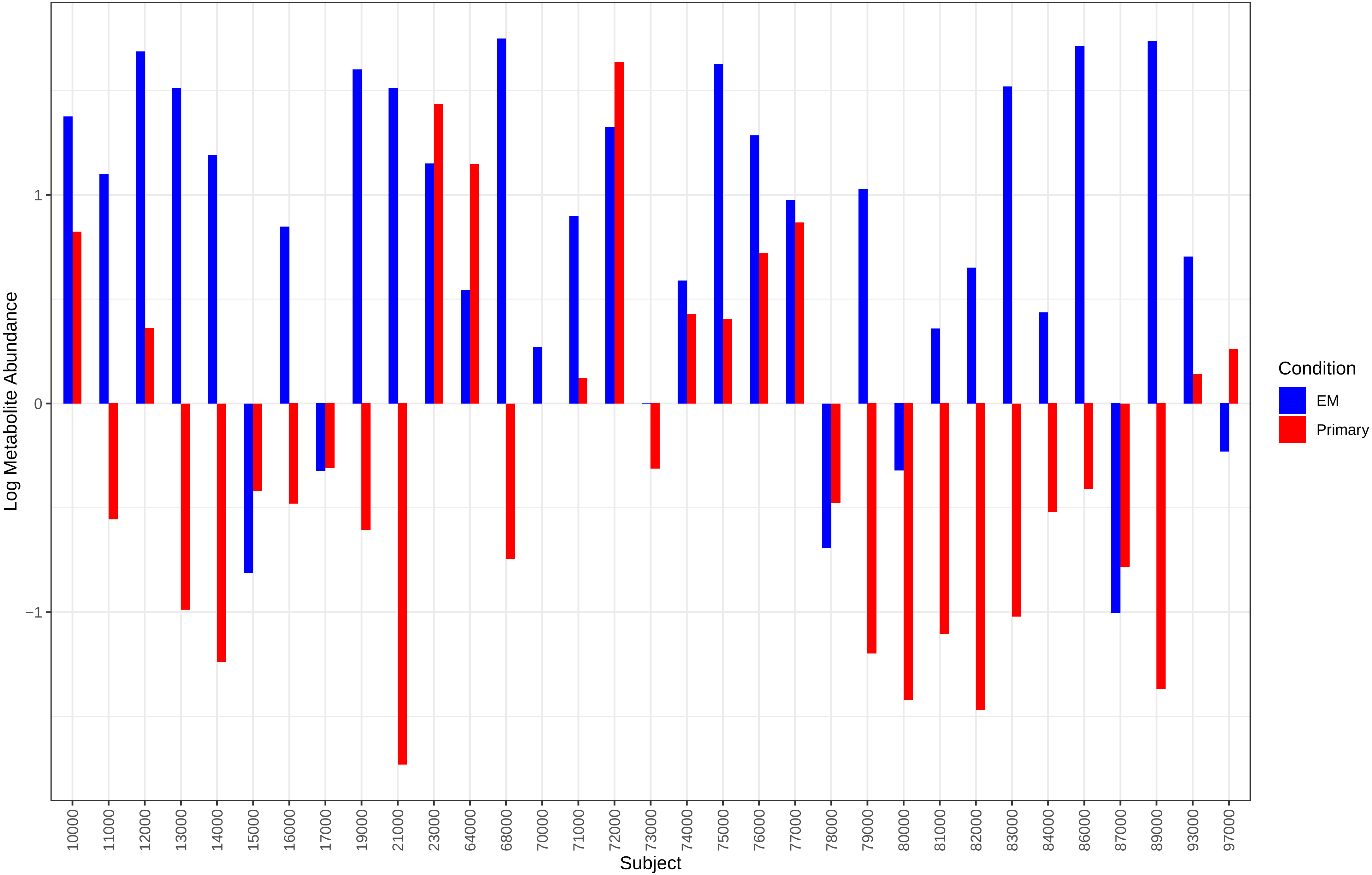

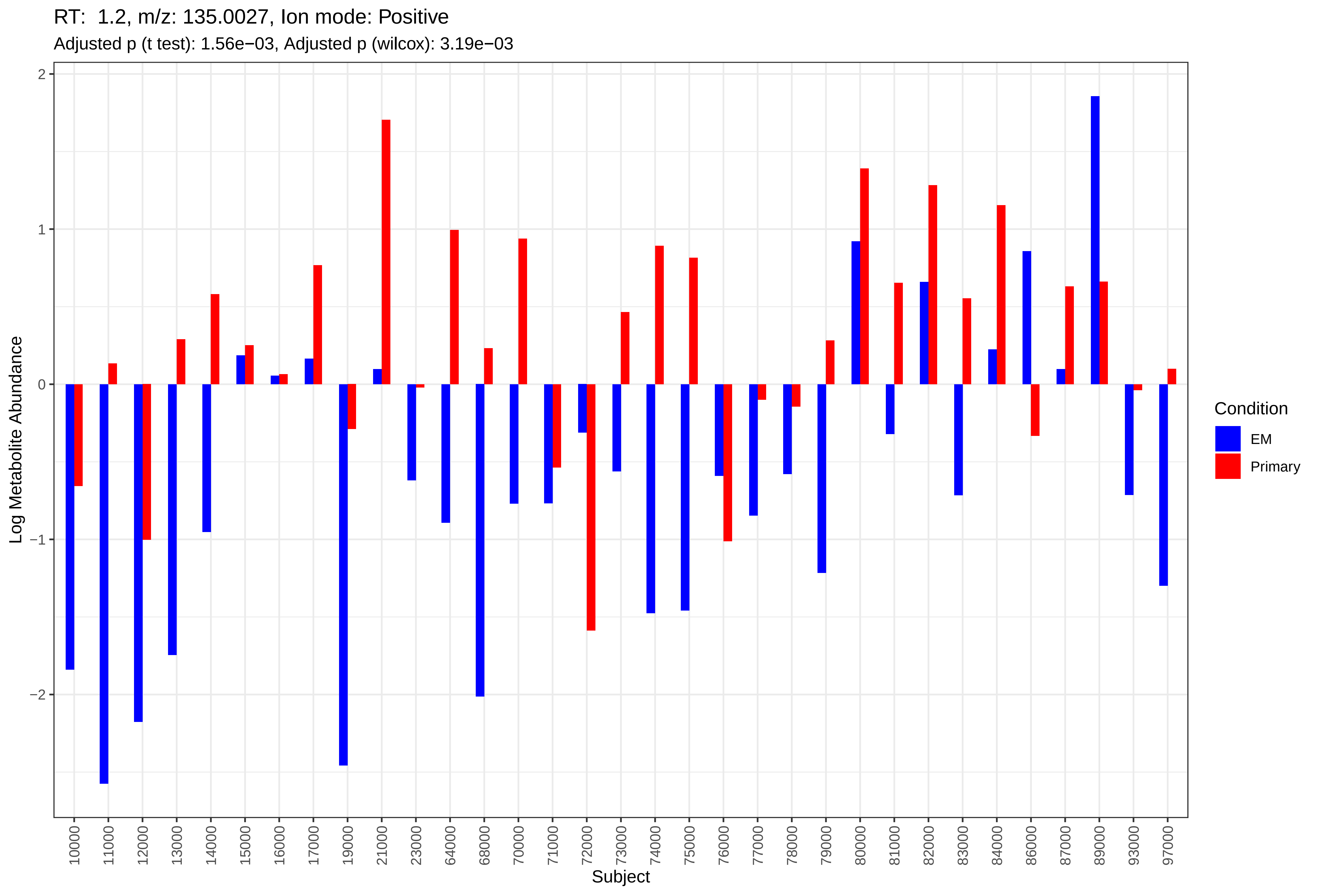

RT: 1.2, m/z: 135.0027, Ion mode: Positive
Adjusted p (t test): 1.56e−03, Adjusted p (wilcox): 3.19e−03
Log Metabolite Abundance
2
1
0
−1
−2
10000
11000
12000
13000
14000
15000
16000
17000
19000
21000
23000
64000
68000
70000
71000
72000
73000
74000
75000
76000
77000
78000
79000
80000
81000
82000
83000
84000
86000
87000
89000
93000
97000
Subject
Condition
EM
Primary

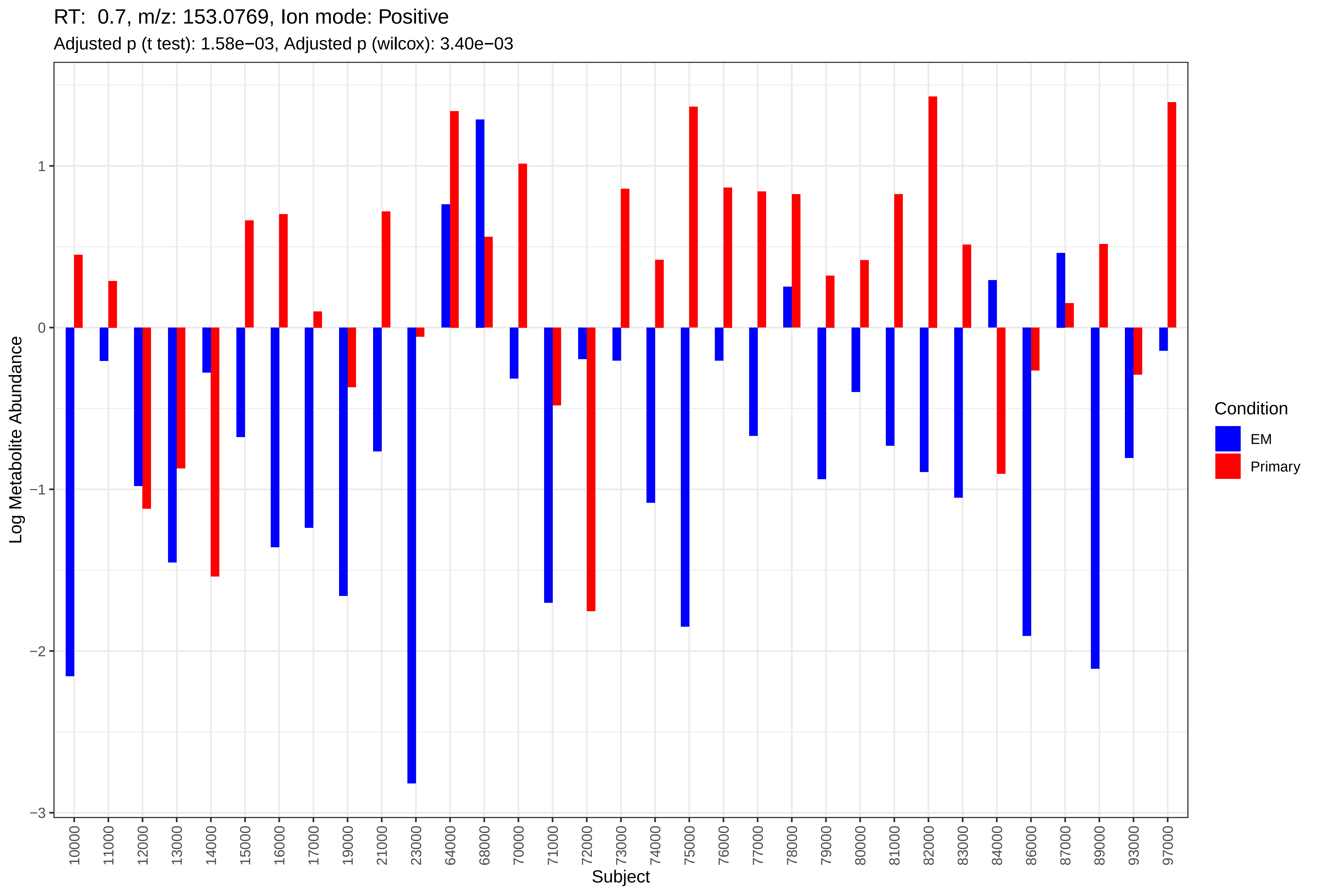

RT: 0.7, m/z: 153.0769, Ion mode: Positive
Adjusted p (t test): 1.58e−03, Adjusted p (wilcox): 3.40e−03
Log Metabolite Abundance
1
0
−1
−2
−3
10000
11000
12000
13000
14000
15000
16000
17000
19000
21000
23000
64000
68000
70000
71000
72000
73000
74000
75000
76000
77000
78000
79000
80000
81000
82000
83000
84000
86000
87000
89000
93000
97000
Subject
Condition
EM
Primary

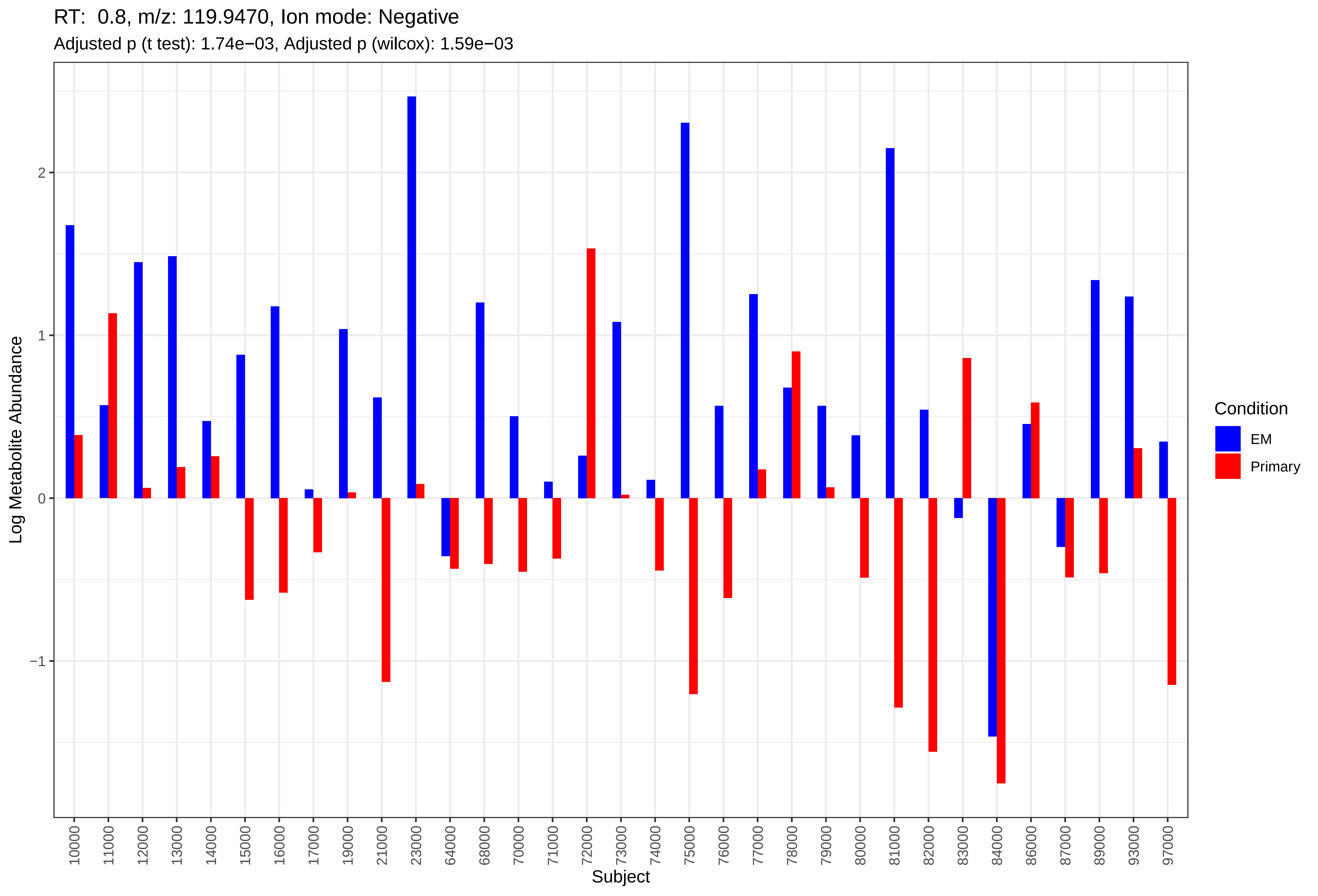
RT: 0.8, m/z: 119.9470, Ion mode: Negative
Adjusted p (t test): 1.74e−03, Adjusted p (wilcox): 1.59e−03
Log Metabolite Abundance
2
1
0
−1
Subject
10000
11000
12000
13000
14000
15000
16000
17000
19000
21000
23000
64000
68000
70000
71000
72000
73000
74000
75000
76000
77000
78000
79000
80000
81000
82000
83000
84000
86000
87000
89000
93000
97000
Condition
EM
Primary

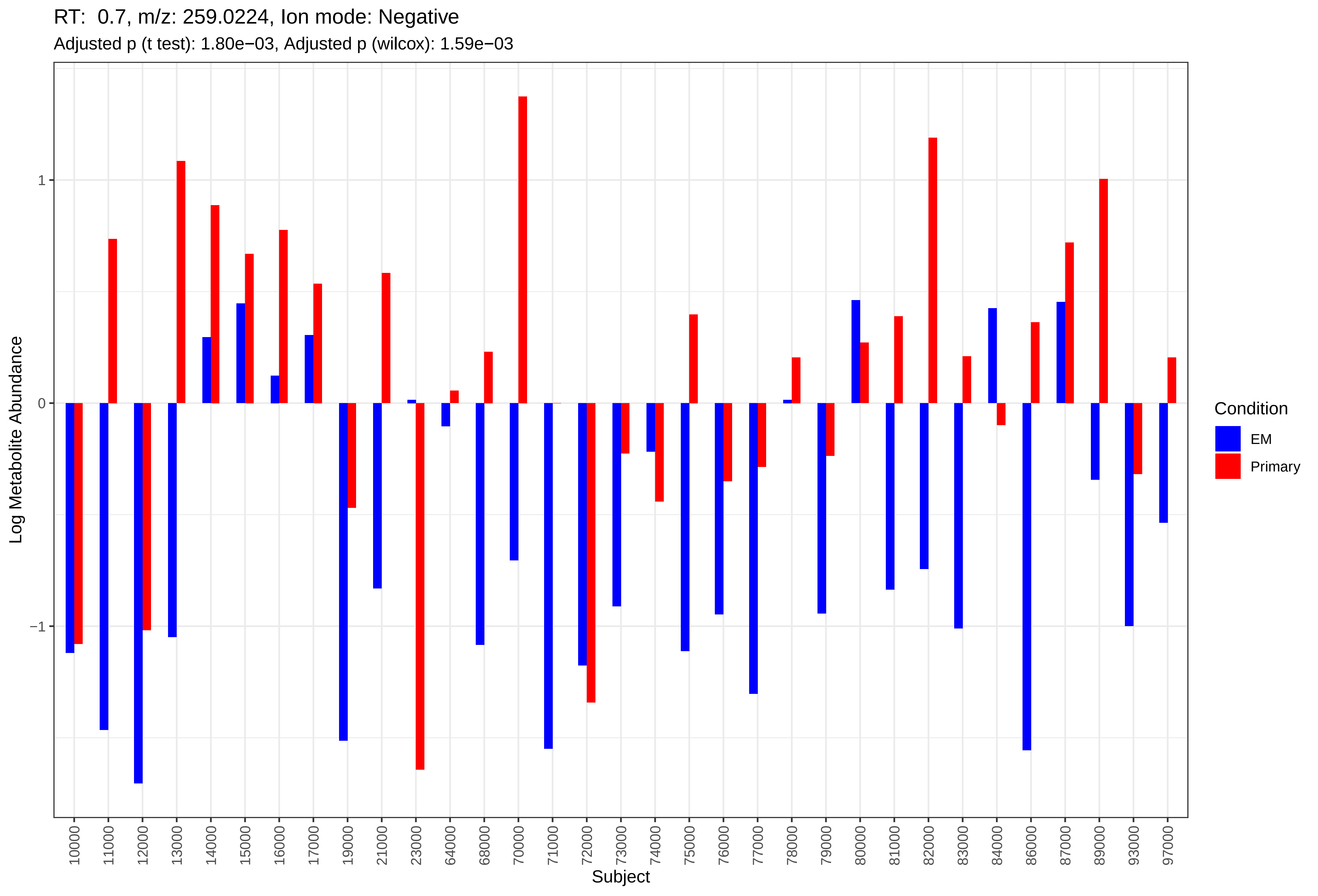
RT: 0.7, m/z: 259.0224, Ion mode: Negative
Adjusted p (t test): 1.80e−03, Adjusted p (wilcox): 1.59e−03
Log Metabolite Abundance
1
0
−1
10000
11000
12000
13000
14000
15000
16000
17000
19000
21000
23000
64000
68000
70000
71000
72000
73000
74000
75000
76000
77000
78000
79000
80000
81000
82000
83000
84000
86000
87000
89000
93000
97000
Subject
Condition
EM
Primary

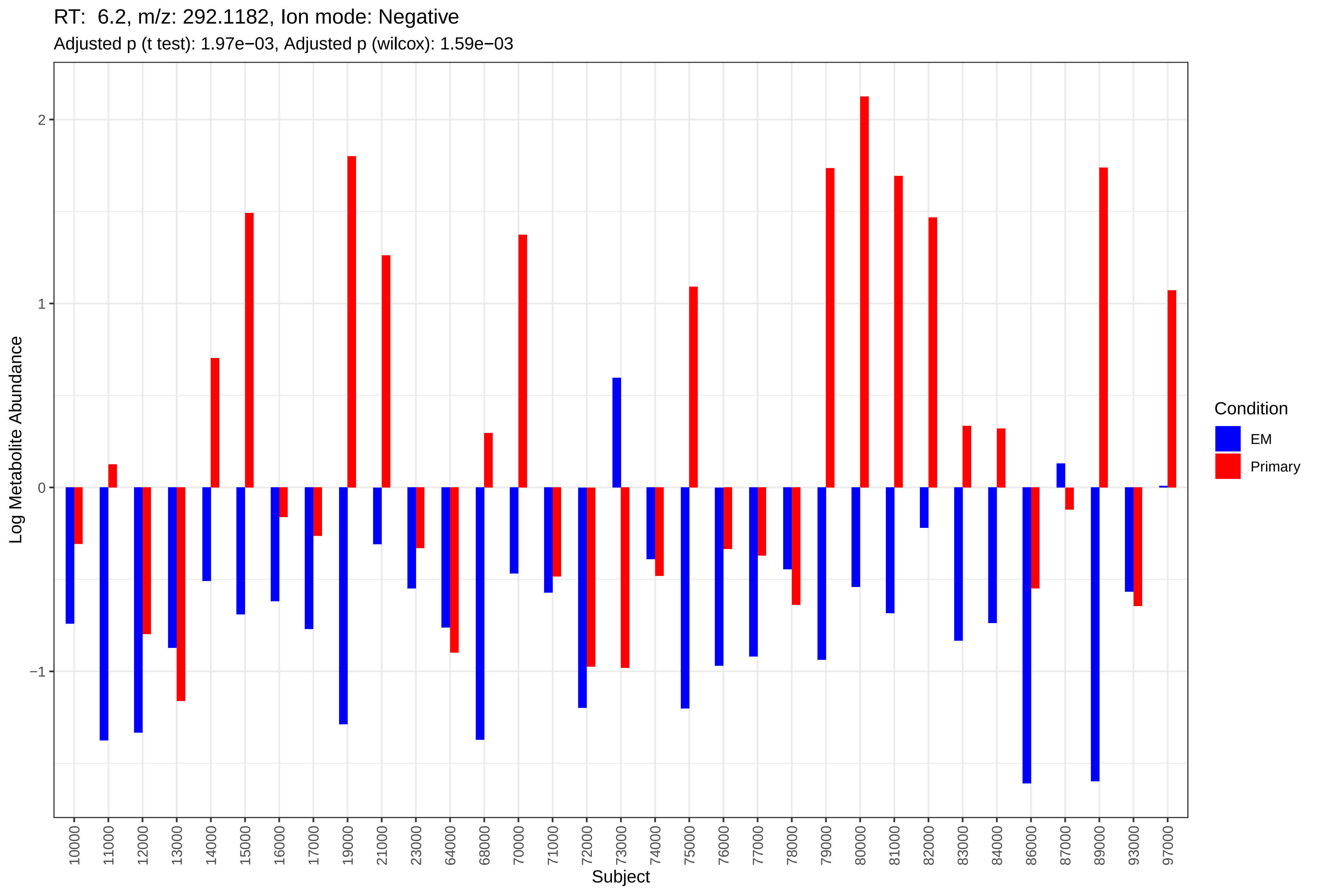
RT: 6.2, m/z: 292.1182, Ion mode: Negative
Adjusted p (t test): 1.97e−03, Adjusted p (wilcox): 1.59e−03
Log Metabolite Abundance
2
1
0
−1
Subject
10000
11000
12000
13000
14000
15000
16000
17000
19000
21000
23000
64000
68000
70000
71000
72000
73000
74000
75000
76000
77000
78000
79000
80000
81000
82000
83000
84000
86000
87000
89000
93000
97000
Condition
EM
Primary

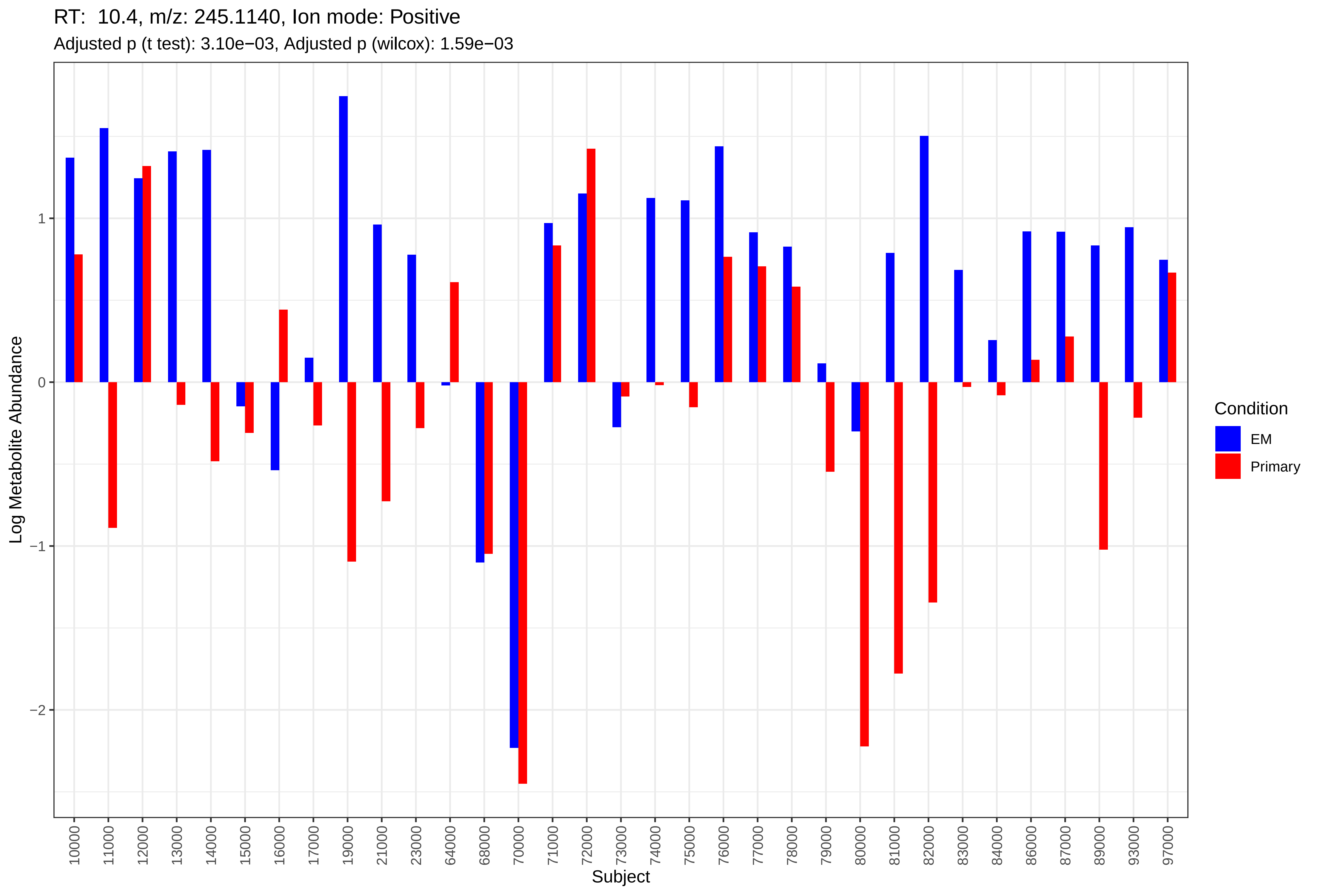

RT: 10.4, m/z: 245.1140, Ion mode: Positive
Adjusted p (t test): 3.10e−03, Adjusted p (wilcox): 1.59e−03
Log Metabolite Abundance
1
0
−1
−2
10000
11000
12000
13000
14000
15000
16000
17000
19000
21000
23000
64000
68000
70000
71000
72000
73000
74000
75000
76000
77000
78000
79000
80000
81000
82000
83000
84000
86000
87000
89000
93000
97000
Subject
Condition
EM
Primary

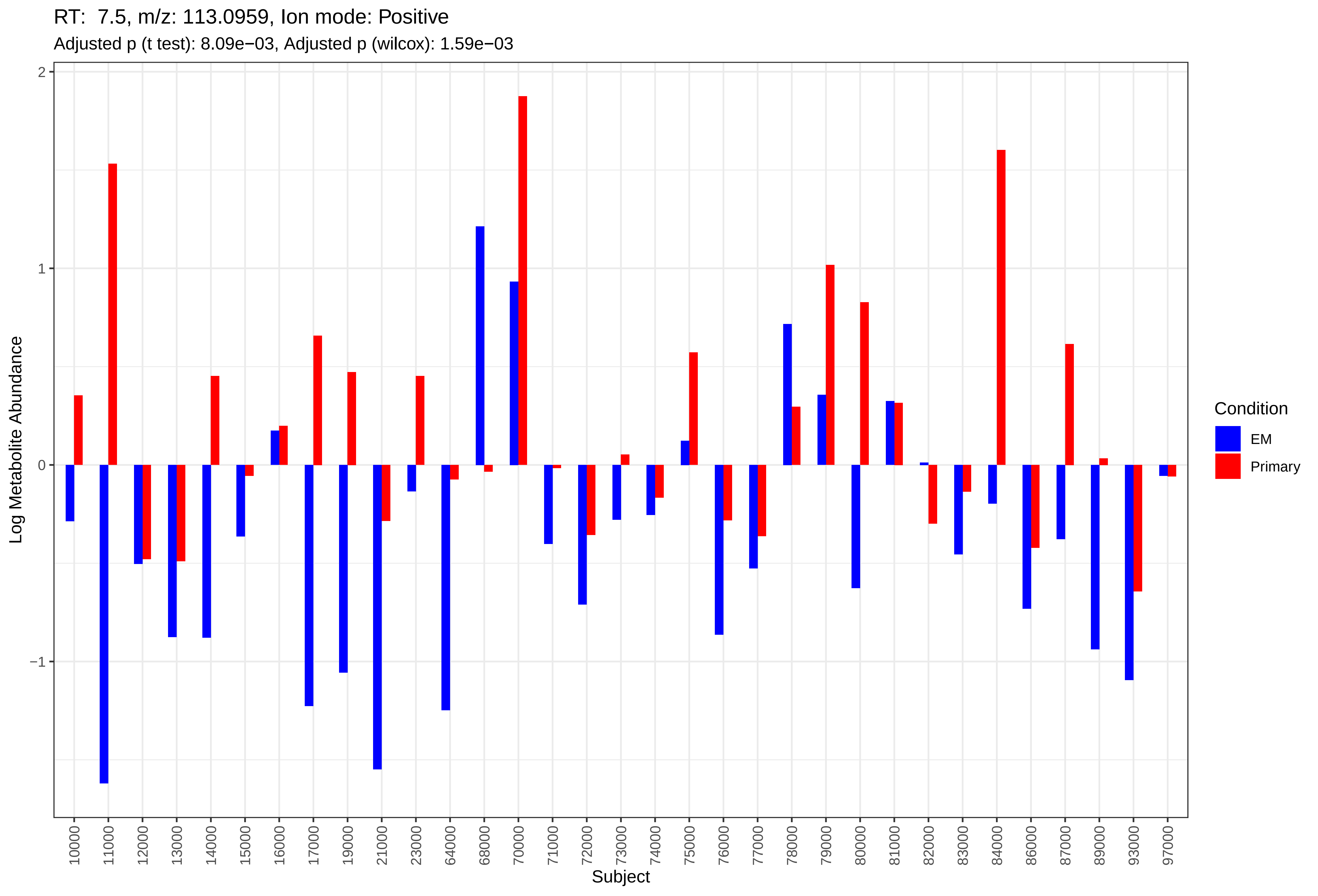
RT: 7.5, m/z: 113.0959, Ion mode: Positive
Adjusted p (t test): 8.09e−03, Adjusted p (wilcox): 1.59e−03
Log Metabolite Abundance
2
1
0
−1
Subject
10000
11000
12000
13000
14000
15000
16000
17000
19000
21000
23000
64000
68000
70000
71000
72000
73000
74000
75000
76000
77000
78000
79000
80000
81000
82000
83000
84000
86000
87000
89000
93000
97000
Condition
EM
Primary

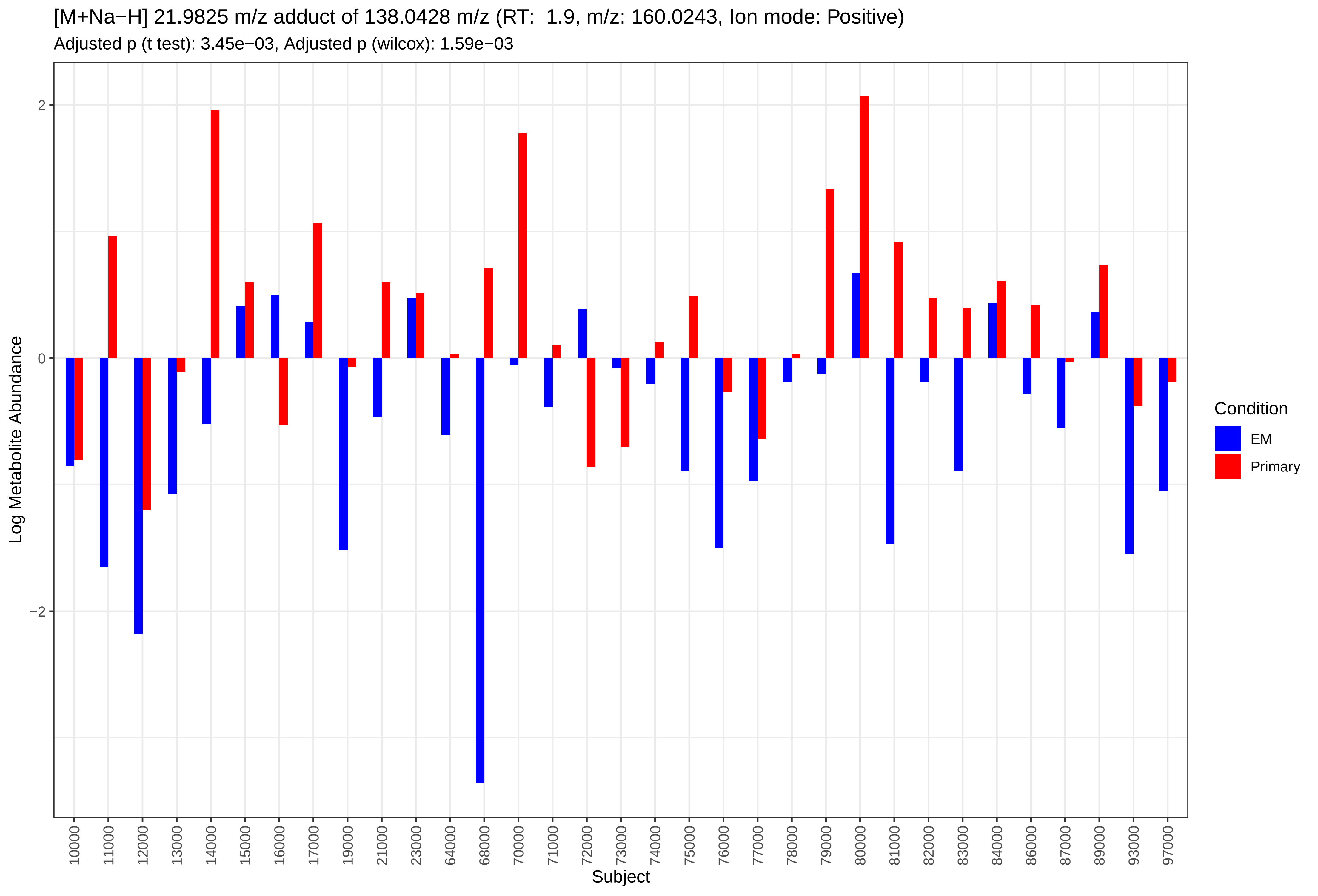

[M+Na−H] 21.9825 m/z adduct of 138.0428 m/z (RT: 1.9, m/z: 160.0243, Ion mode: Positive)
Adjusted p (t test): 3.45e−03, Adjusted p (wilcox): 1.59e−03
Log Metabolite Abundance
2
0
−2
Subject
10000
11000
12000
13000
14000
15000
16000
17000
19000
21000
23000
64000
68000
70000
71000
72000
73000
74000
75000
76000
77000
78000
79000
80000
81000
82000
83000
84000
86000
87000
89000
93000
97000
Condition
EM
Primary

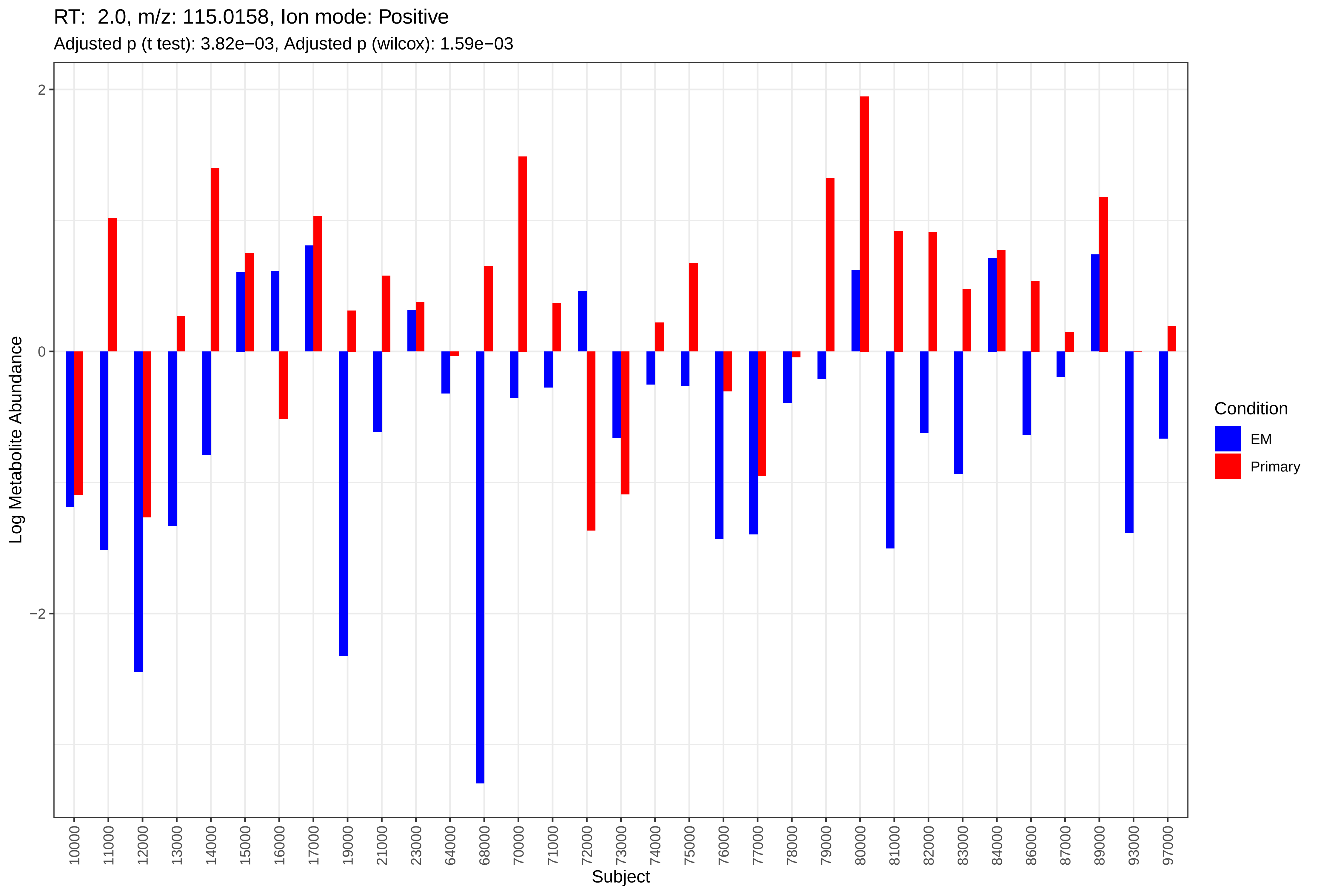

RT: 2.0, m/z: 115.0158, Ion mode: Positive
Adjusted p (t test): 3.82e−03, Adjusted p (wilcox): 1.59e−03
Log Metabolite Abundance
2
0
−2
Subject
10000
11000
12000
13000
14000
15000
16000
17000
19000
21000
23000
64000
68000
70000
71000
72000
73000
74000
75000
76000
77000
78000
79000
80000
81000
82000
83000
84000
86000
87000
89000
93000
97000
Condition
EM
Primary

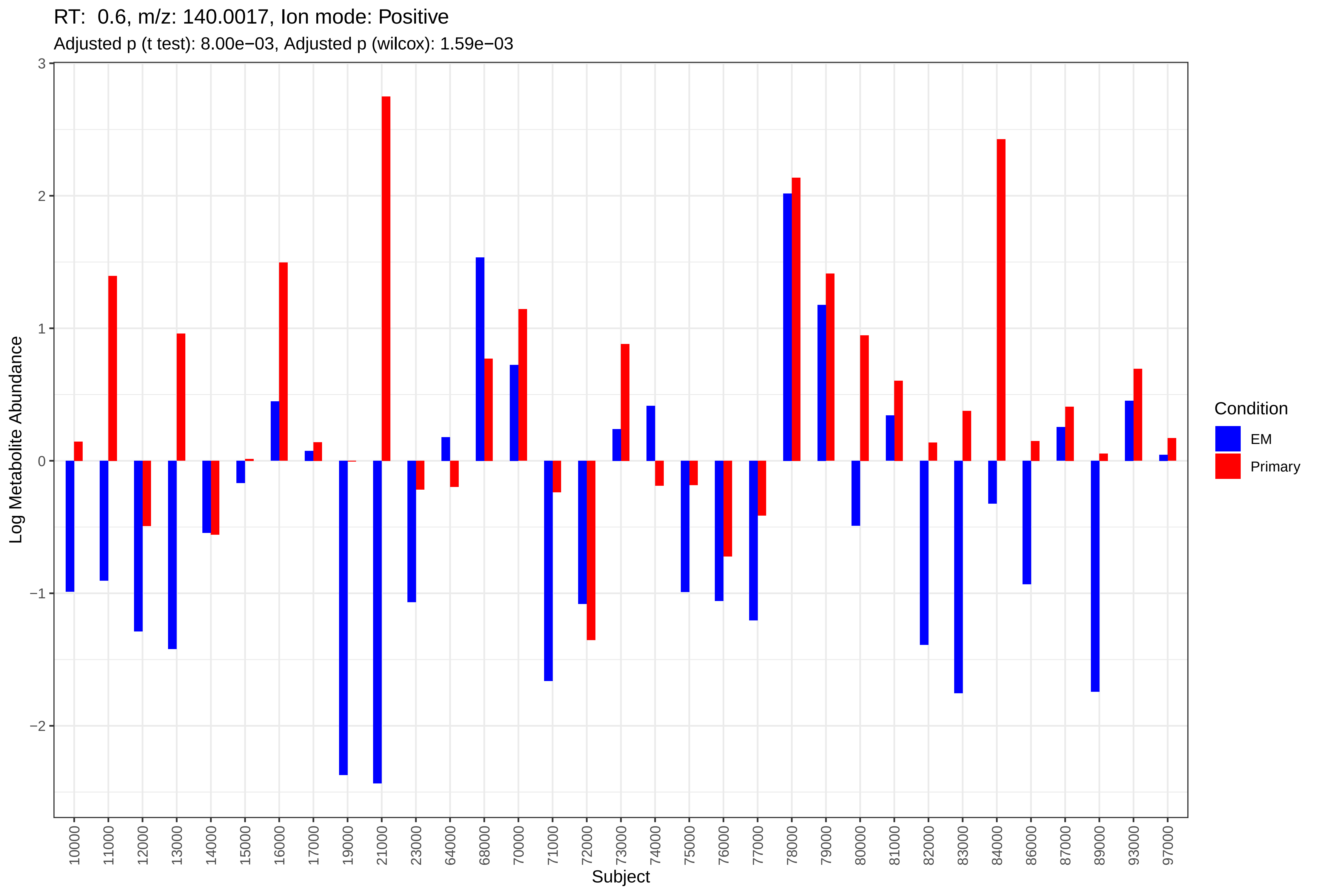

RT: 0.6, m/z: 140.0017, Ion mode: Positive
Adjusted p (t test): 8.00e−03, Adjusted p (wilcox): 1.59e−03
Log Metabolite Abundance
3
2
1
0
−1
−2
10000
11000
12000
13000
14000
15000
16000
17000
19000
21000
23000
64000
68000
70000
71000
72000
73000
74000
75000
76000
77000
78000
79000
80000
81000
82000
83000
84000
86000
87000
89000
93000
97000
Subject
Condition
EM
Primary

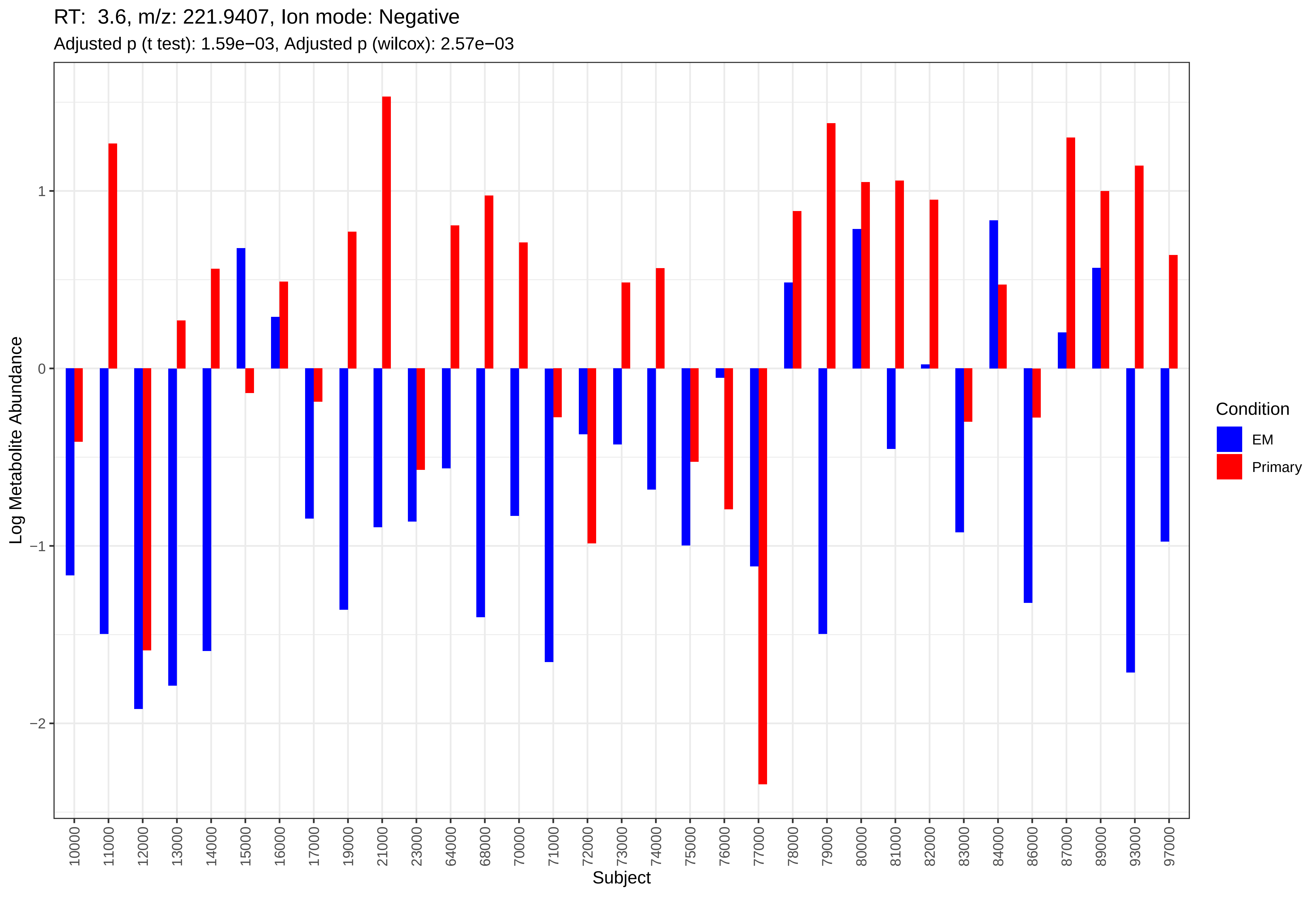
RT: 3.6, m/z: 221.9407, Ion mode: Negative
Adjusted p (t test): 1.59e−03, Adjusted p (wilcox): 2.57e−03
Log Metabolite Abundance
1
0
−1
−2
Subject
10000
11000
12000
13000
14000
15000
16000
17000
19000
21000
23000
64000
68000
70000
71000
72000
73000
74000
75000
76000
77000
78000
79000
80000
81000
82000
83000
84000
86000
87000
89000
93000
97000
Condition
EM
Primary

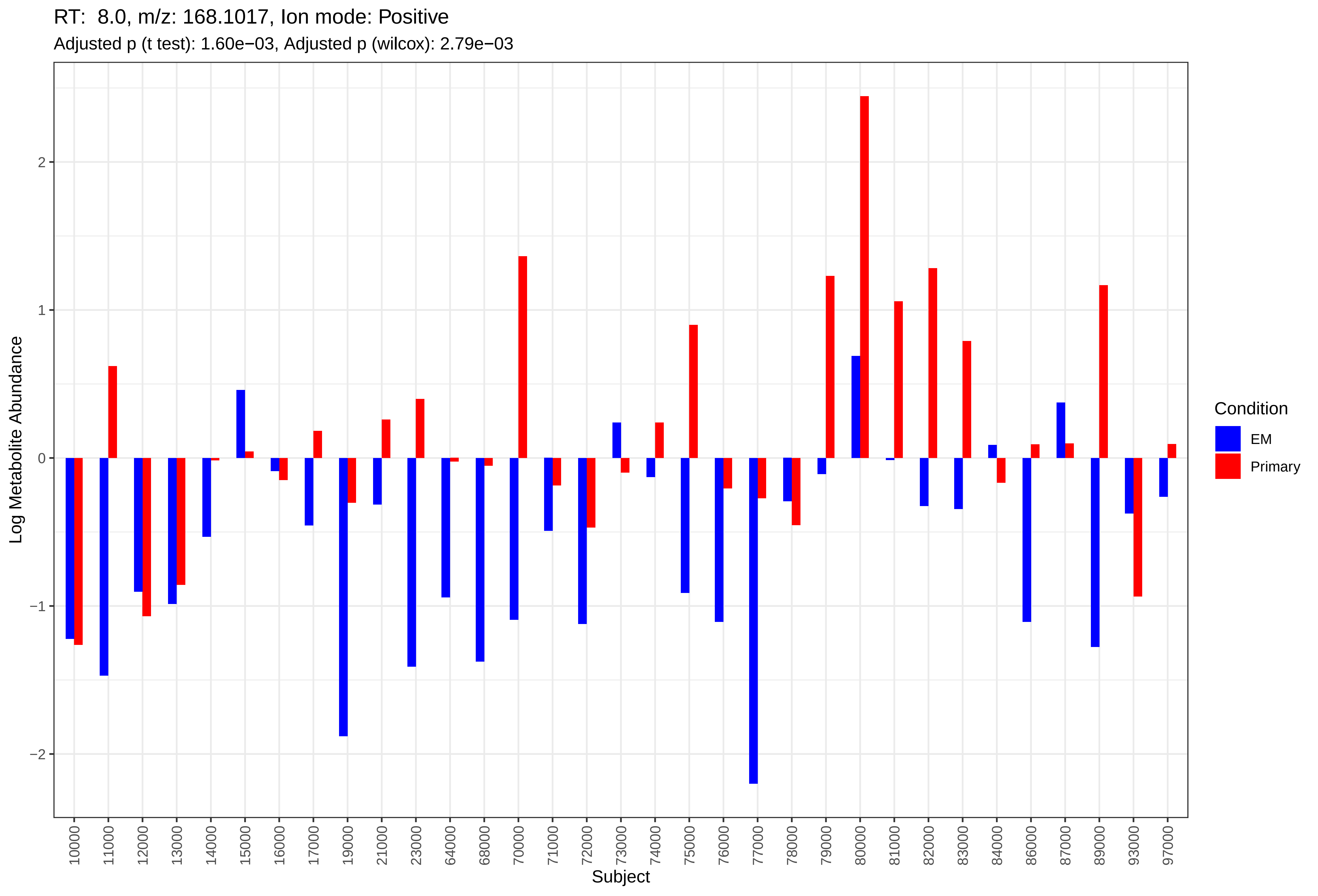
RT: 8.0, m/z: 168.1017, Ion mode: Positive
Adjusted p (t test): 1.60e−03, Adjusted p (wilcox): 2.79e−03
Log Metabolite Abundance
2
1
0
−1
−2
10000
11000
12000
13000
14000
15000
16000
17000
19000
21000
23000
64000
68000
70000
71000
72000
73000
74000
75000
76000
77000
78000
79000
80000
81000
82000
83000
84000
86000
87000
89000
93000
97000
Subject
Condition
EM
Primary

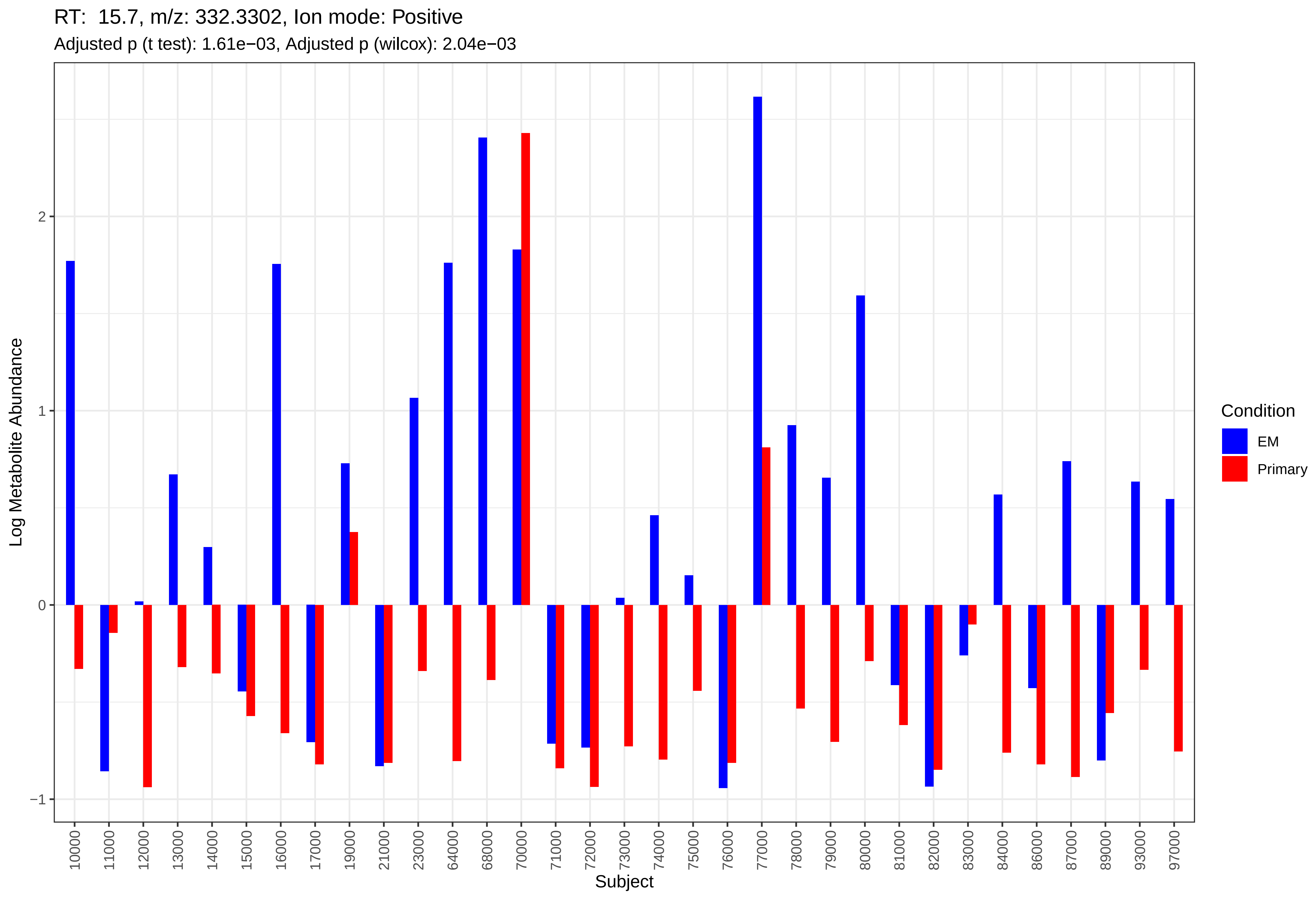

RT: 15.7, m/z: 332.3302, Ion mode: Positive
Adjusted p (t test): 1.61e−03, Adjusted p (wilcox): 2.04e−03
Log Metabolite Abundance
2
1
0
−1
10000
11000
12000
13000
14000
15000
16000
17000
19000
21000
23000
64000
68000
70000
71000
72000
73000
74000
75000
76000
77000
78000
79000
80000
81000
82000
83000
84000
86000
87000
89000
93000
97000
Subject
Condition
EM
Primary

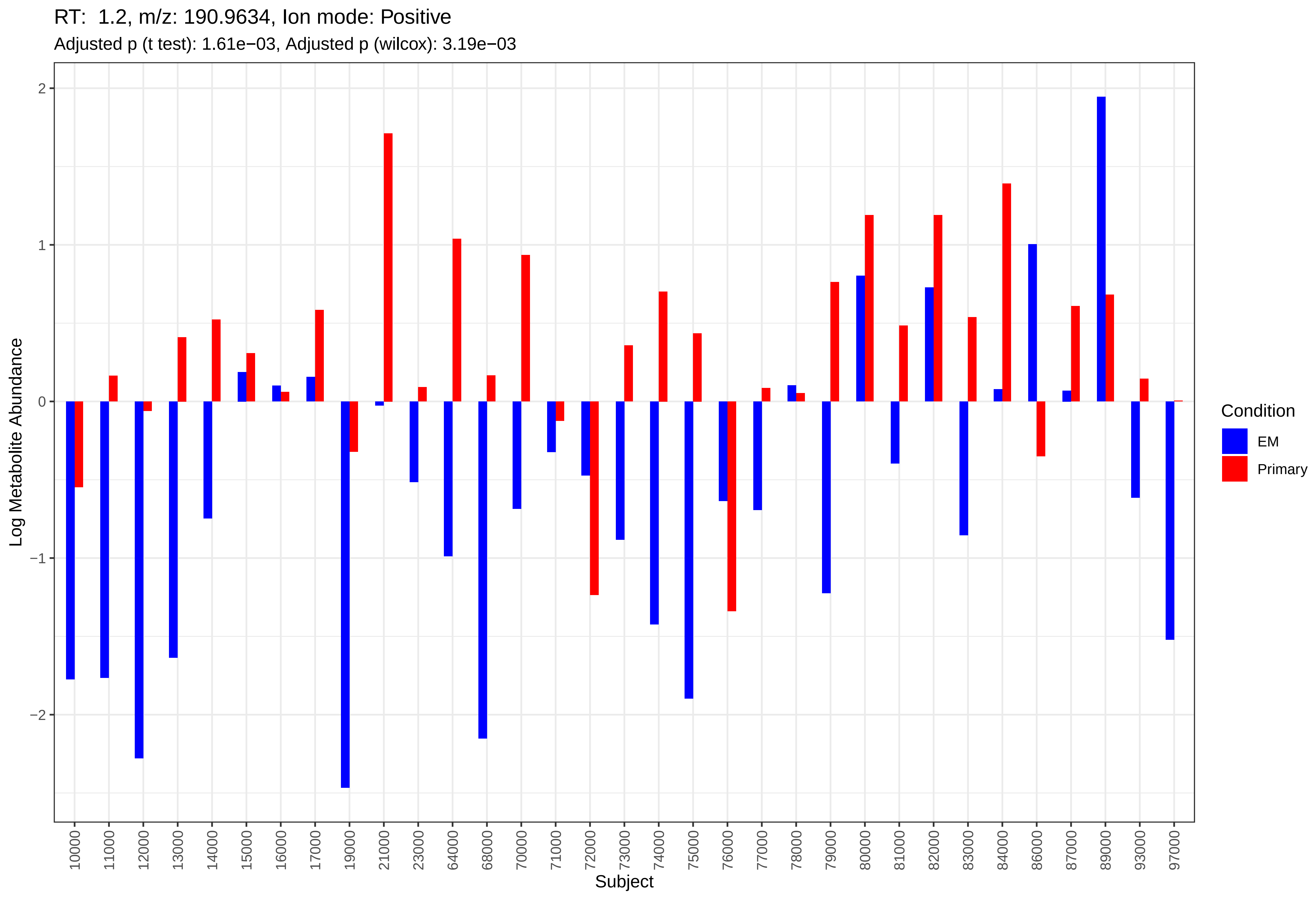

RT: 1.2, m/z: 190.9634, Ion mode: Positive
Adjusted p (t test): 1.61e−03, Adjusted p (wilcox): 3.19e−03
Log Metabolite Abundance
2
1
0
−1
−2
10000
11000
12000
13000
14000
15000
16000
17000
19000
21000
23000
64000
68000
70000
71000
72000
73000
74000
75000
76000
77000
78000
79000
80000
81000
82000
83000
84000
86000
87000
89000
93000
97000
Subject
Condition
EM
Primary

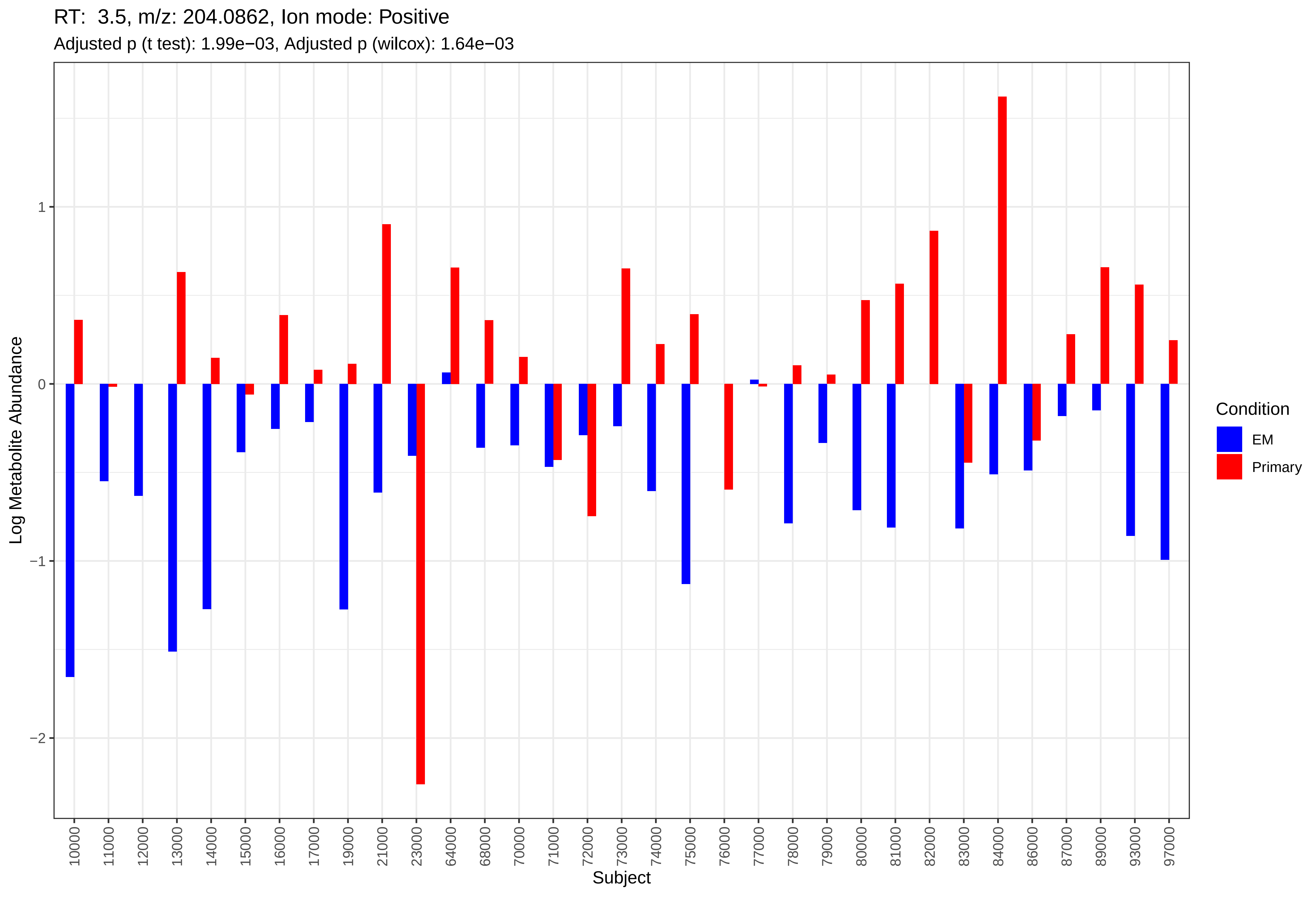
RT: 3.5, m/z: 204.0862, Ion mode: Positive
Adjusted p (t test): 1.99e−03, Adjusted p (wilcox): 1.64e−03
Log Metabolite Abundance
1
0
−1
−2
Subject
10000
11000
12000
13000
14000
15000
16000
17000
19000
21000
23000
64000
68000
70000
71000
72000
73000
74000
75000
76000
77000
78000
79000
80000
81000
82000
83000
84000
86000
87000
89000
93000
97000
Condition
EM
Primary

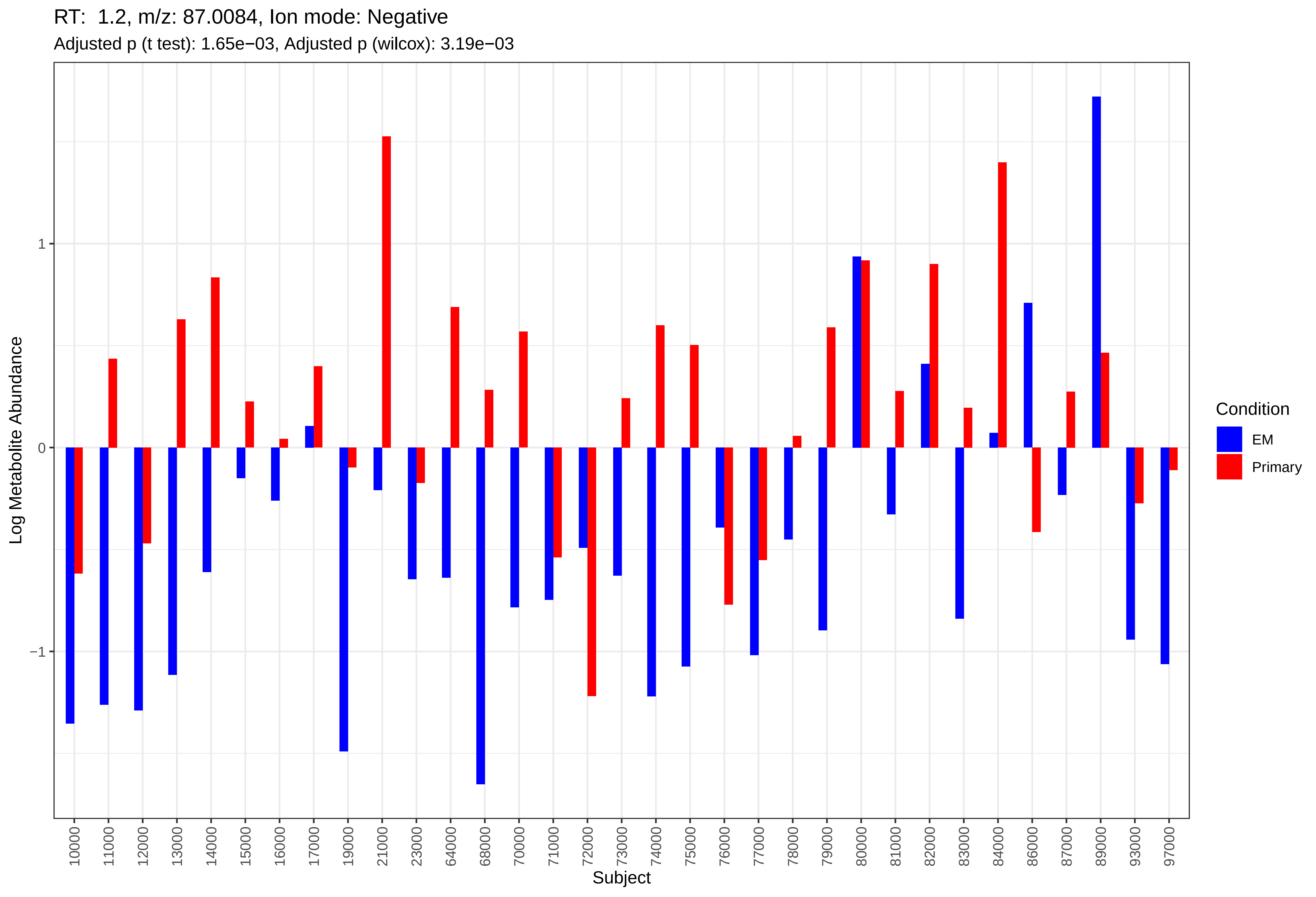

RT: 1.2, m/z: 87.0084, Ion mode: Negative
Adjusted p (t test): 1.65e−03, Adjusted p (wilcox): 3.19e−03
Log Metabolite Abundance
1
0
−1
10000
11000
12000
13000
14000
15000
16000
17000
19000
21000
23000
64000
68000
70000
71000
72000
73000
74000
75000
76000
77000
78000
79000
80000
81000
82000
83000
84000
86000
87000
89000
93000
97000
Subject
Condition
EM
Primary

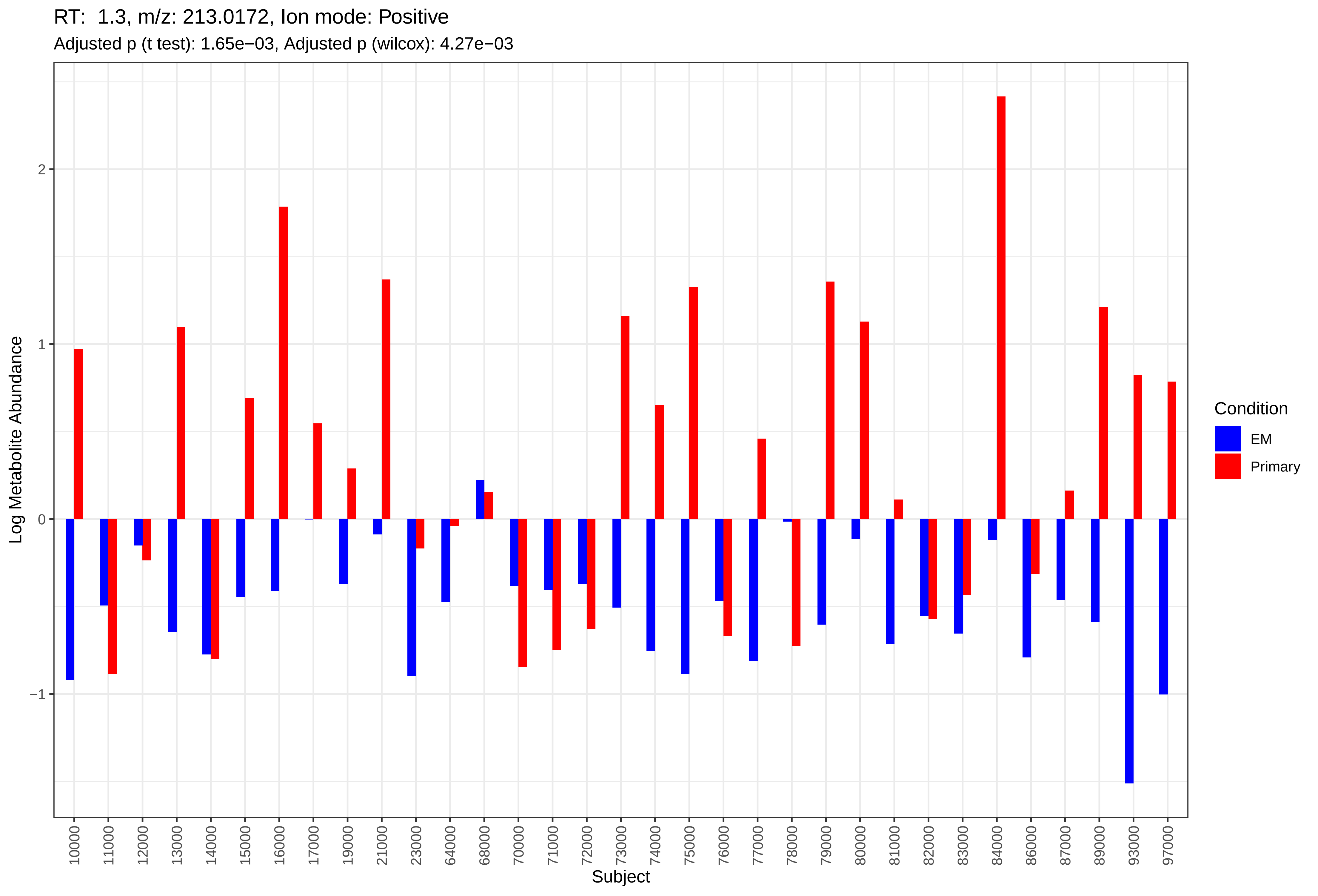
RT: 1.3, m/z: 213.0172, Ion mode: Positive
Adjusted p (t test): 1.65e−03, Adjusted p (wilcox): 4.27e−03
Log Metabolite Abundance
2
1
0
−1
10000
11000
12000
13000
14000
15000
16000
17000
19000
21000
23000
64000
68000
70000
71000
72000
73000
74000
75000
76000
77000
78000
79000
80000
81000
82000
83000
84000
86000
87000
89000
93000
97000
Subject
Condition
EM
Primary

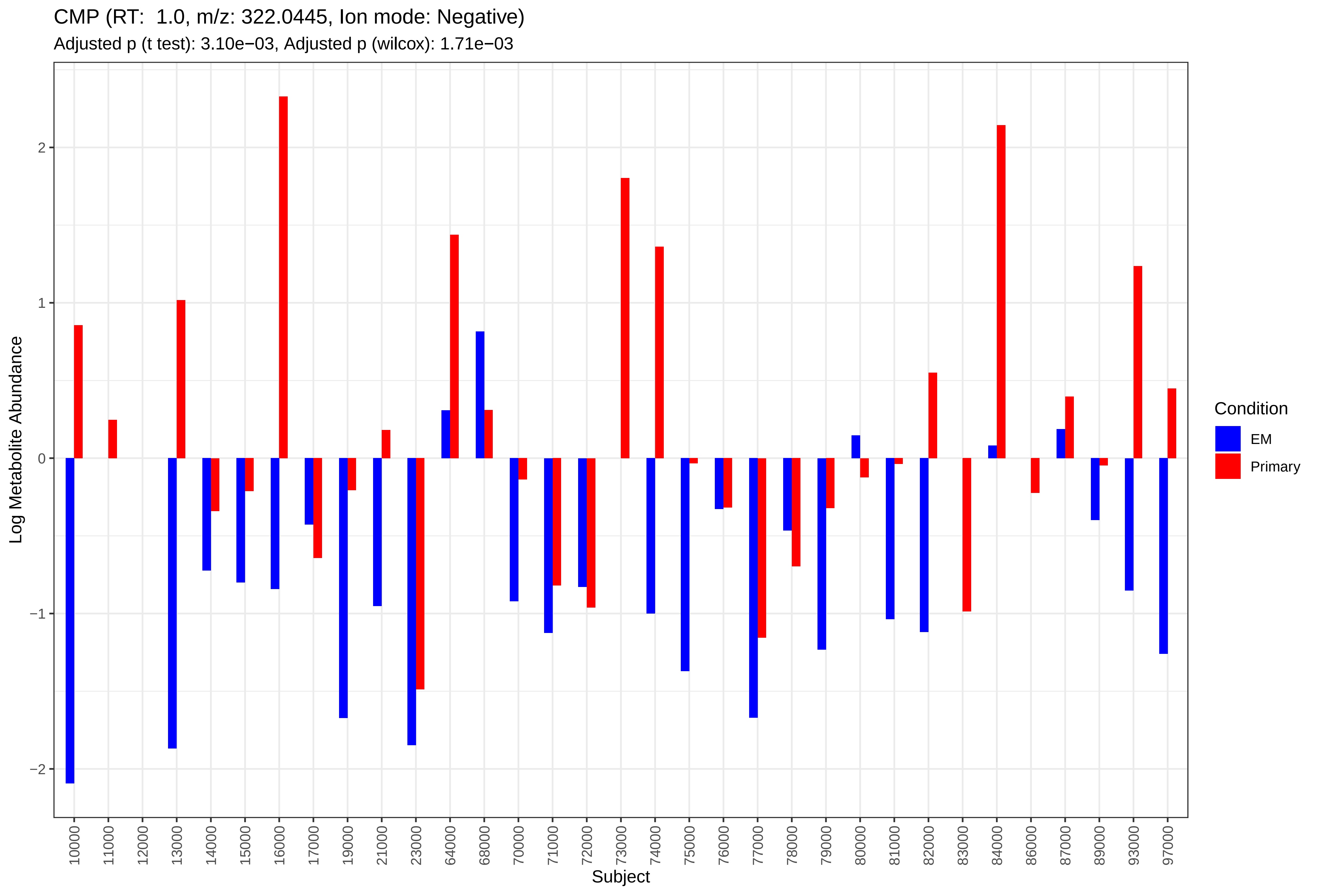
CMP (RT: 1.0, m/z: 322.0445, Ion mode: Negative)
Adjusted p (t test): 3.10e−03, Adjusted p (wilcox): 1.71e−03
Log Metabolite Abundance
2
1
0
−1
−2
10000
11000
12000
13000
14000
15000
16000
17000
19000
21000
23000
64000
68000
70000
71000
72000
73000
74000
75000
76000
77000
78000
79000
80000
81000
82000
83000
84000
86000
87000
89000
93000
97000
Subject
Condition
EM
Primary

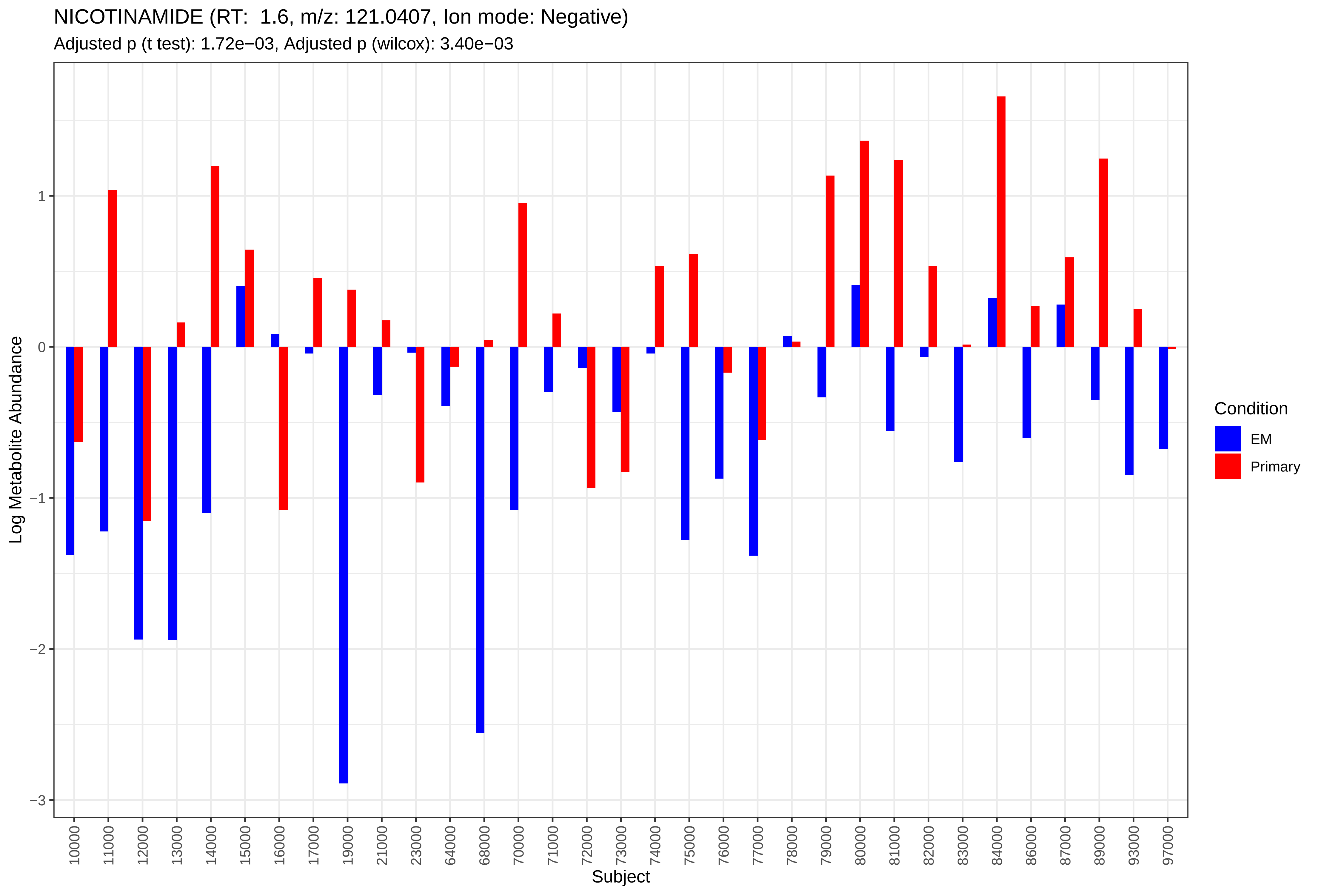
NICOTINAMIDE (RT: 1.6, m/z: 121.0407, Ion mode: Negative)
Adjusted p (t test): 1.72e−03, Adjusted p (wilcox): 3.40e−03
Log Metabolite Abundance
1
0
−1
−2
−3
Subject
10000
11000
12000
13000
14000
15000
16000
17000
19000
21000
23000
64000
68000
70000
71000
72000
73000
74000
75000
76000
77000
78000
79000
80000
81000
82000
83000
84000
86000
87000
89000
93000
97000
Condition
EM
Primary

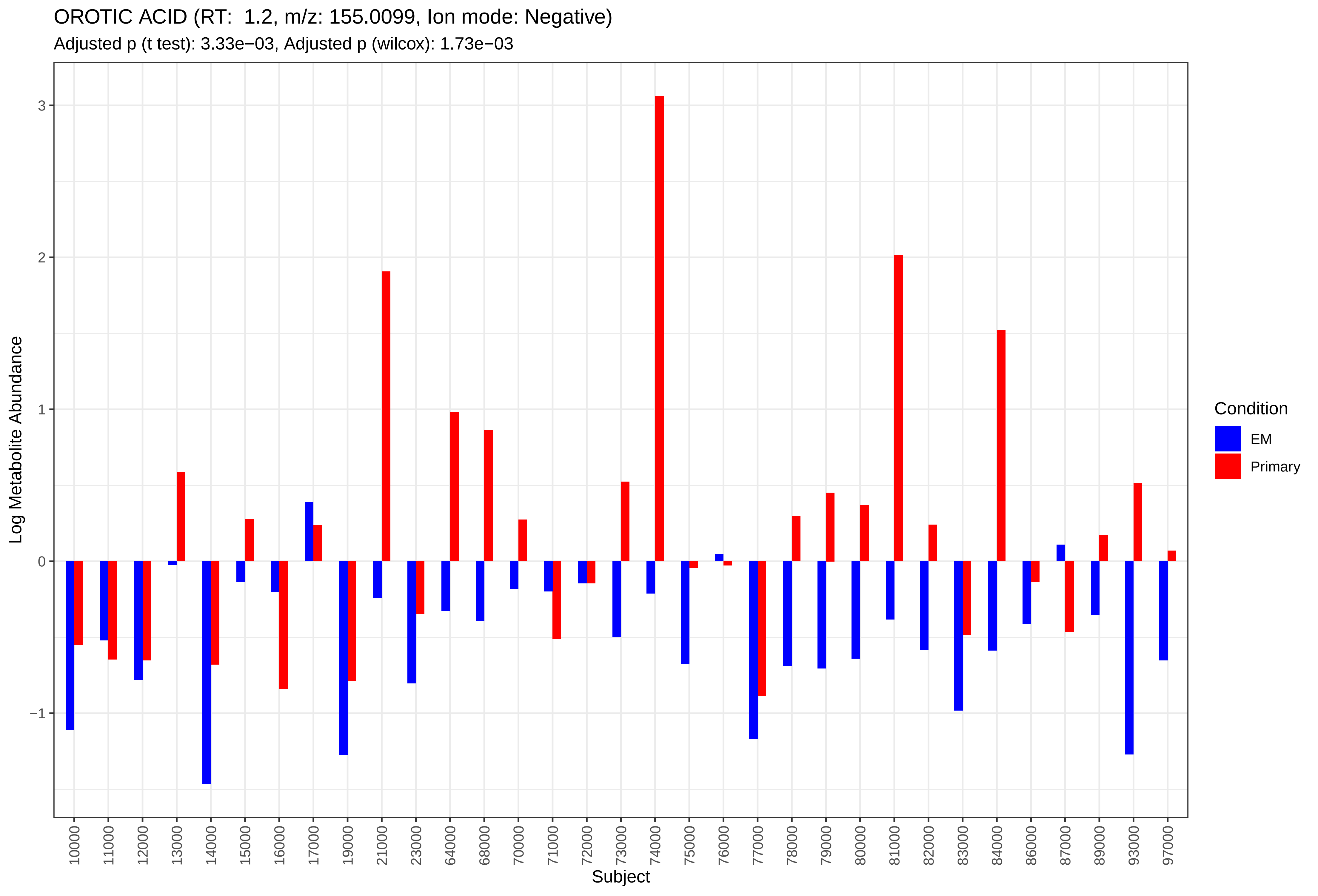
OROTIC ACID (RT: 1.2, m/z: 155.0099, Ion mode: Negative)
Adjusted p (t test): 3.33e−03, Adjusted p (wilcox): 1.73e−03
Log Metabolite Abundance
3
2
1
0
−1
Subject
10000
11000
12000
13000
14000
15000
16000
17000
19000
21000
23000
64000
68000
70000
71000
72000
73000
74000
75000
76000
77000
78000
79000
80000
81000
82000
83000
84000
86000
87000
89000
93000
97000
Condition
EM
Primary

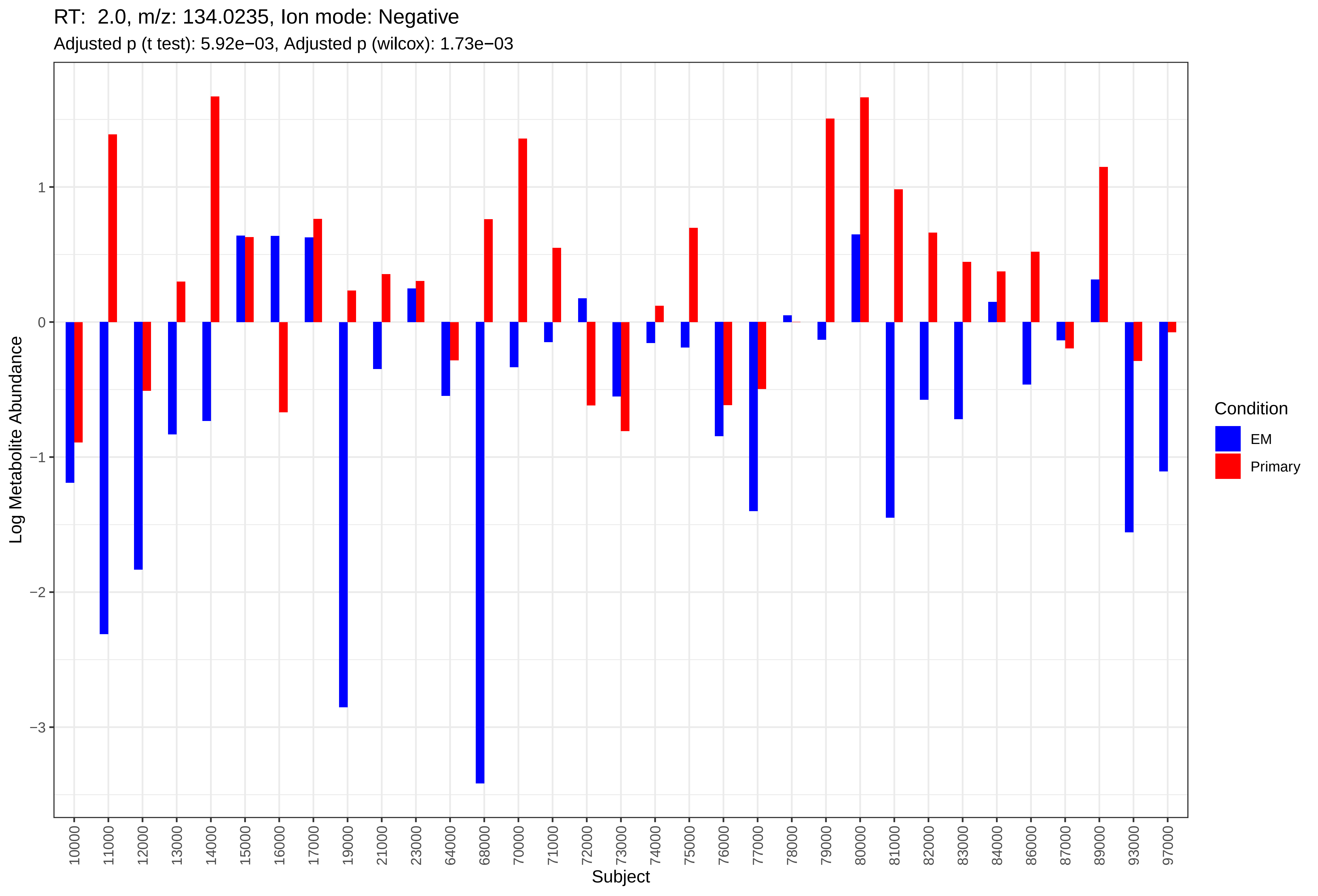
RT: 2.0, m/z: 134.0235, Ion mode: Negative
Adjusted p (t test): 5.92e−03, Adjusted p (wilcox): 1.73e−03
Log Metabolite Abundance
Subject
Condition
EM
Primary
1
0
−1
−2
−3
10000
11000
12000
13000
14000
15000
16000
17000
19000
21000
23000
64000
68000
70000
71000
72000
73000
74000
75000
76000
77000
78000
79000
80000
81000
82000
83000
84000
86000
87000
89000
93000
97000

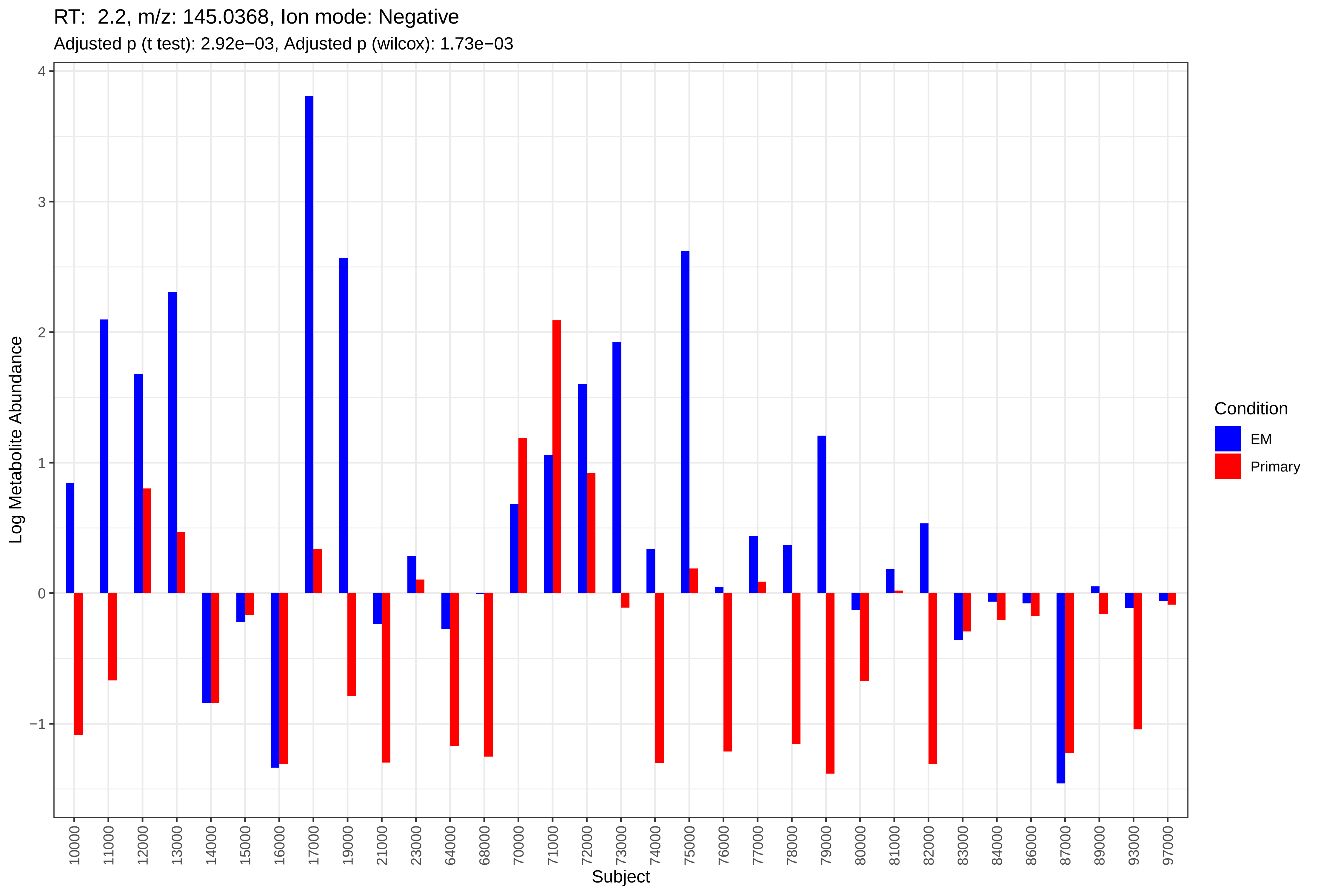

RT: 2.2, m/z: 145.0368, Ion mode: Negative
Adjusted p (t test): 2.92e−03, Adjusted p (wilcox): 1.73e−03
Log Metabolite Abundance
4
3
2
1
0
−1
10000
11000
12000
13000
14000
15000
16000
17000
19000
21000
23000
64000
68000
70000
71000
72000
73000
74000
75000
76000
77000
78000
79000
80000
81000
82000
83000
84000
86000
87000
89000
93000
97000
Subject
Condition
EM
Primary

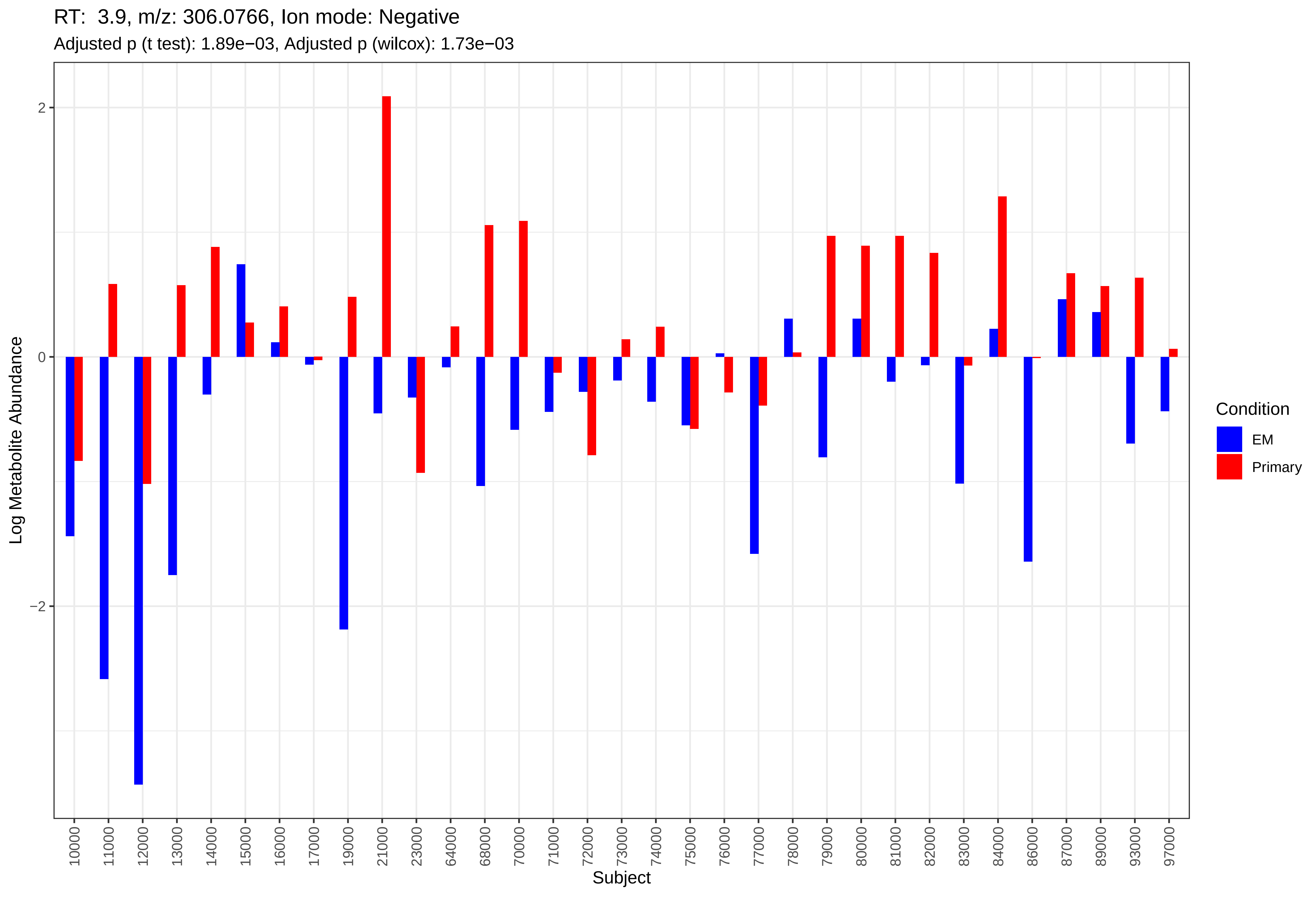
RT: 3.9, m/z: 306.0766, Ion mode: Negative
Adjusted p (t test): 1.89e−03, Adjusted p (wilcox): 1.73e−03
Log Metabolite Abundance
2
0
−2
Subject
10000
11000
12000
13000
14000
15000
16000
17000
19000
21000
23000
64000
68000
70000
71000
72000
73000
74000
75000
76000
77000
78000
79000
80000
81000
82000
83000
84000
86000
87000
89000
93000
97000
Condition
EM
Primary

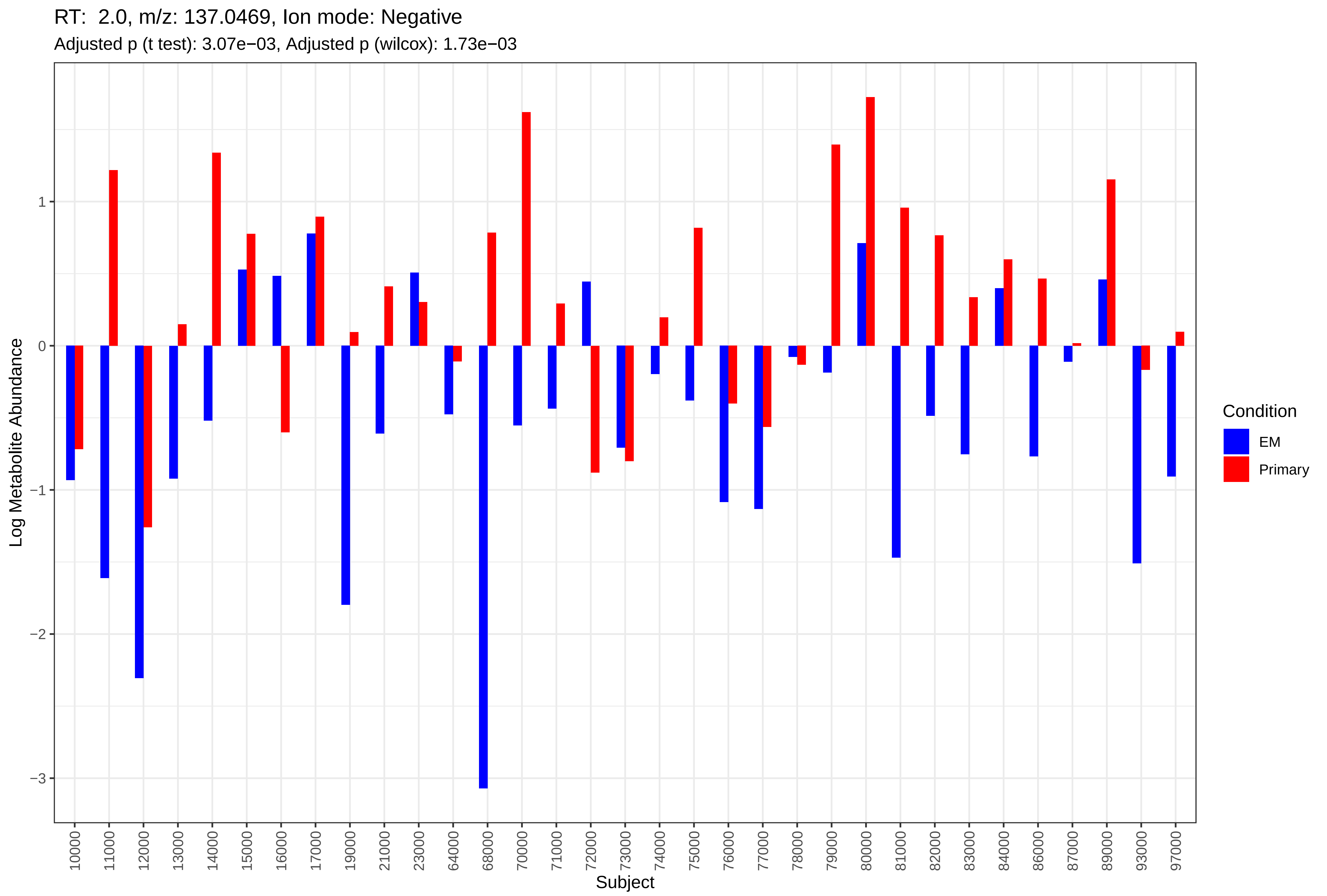
RT: 2.0, m/z: 137.0469, Ion mode: Negative
Adjusted p (t test): 3.07e−03, Adjusted p (wilcox): 1.73e−03
Log Metabolite Abundance
1
0
−1
−2
−3
10000
11000
12000
13000
14000
15000
16000
17000
19000
21000
23000
64000
68000
70000
71000
72000
73000
74000
75000
76000
77000
78000
79000
80000
81000
82000
83000
84000
86000
87000
89000
93000
97000
Subject
Condition
EM
Primary

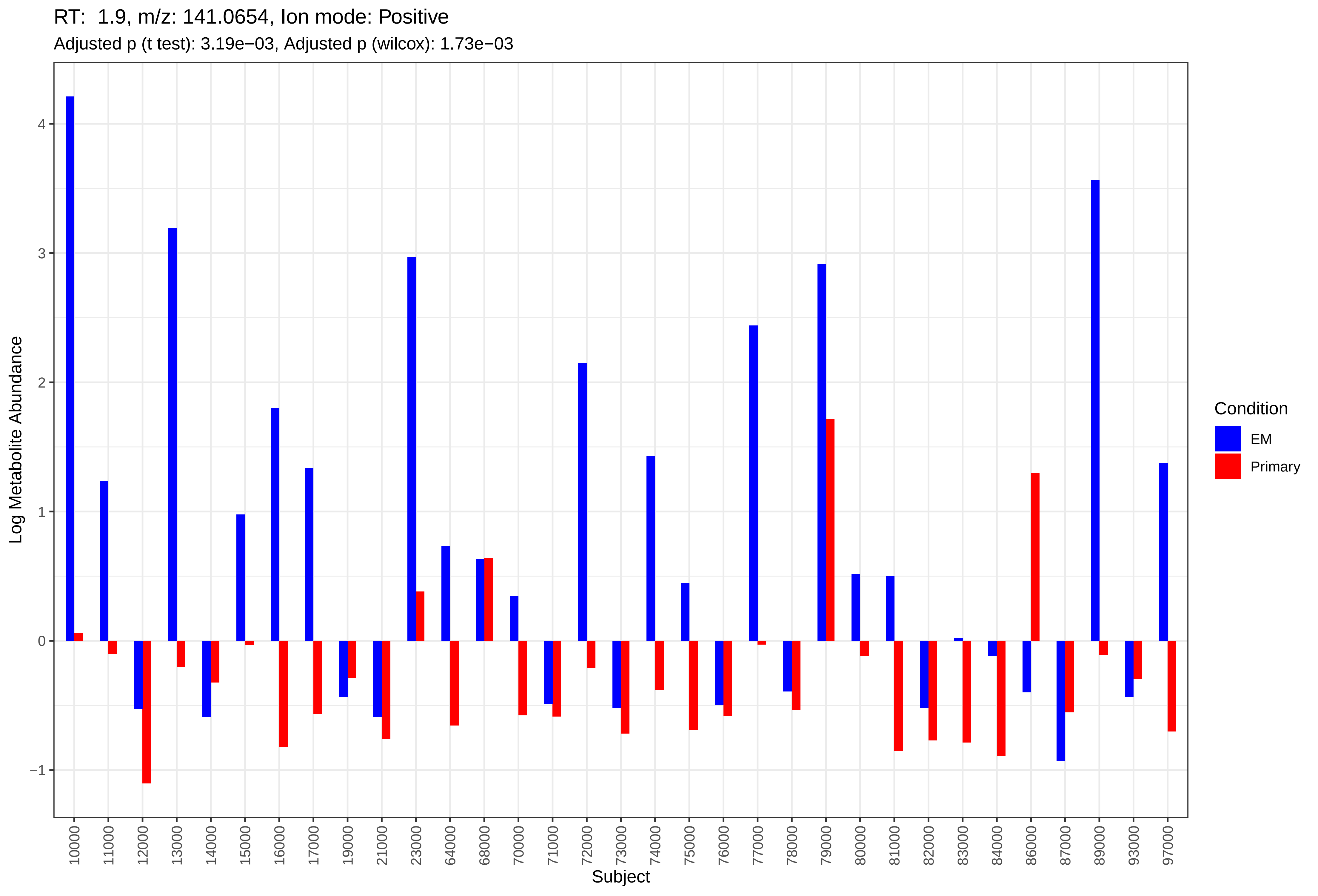
RT: 1.9, m/z: 141.0654, Ion mode: Positive
Adjusted p (t test): 3.19e−03, Adjusted p (wilcox): 1.73e−03
Log Metabolite Abundance
4
3
2
1
0
−1
10000
11000
12000
13000
14000
15000
16000
17000
19000
21000
23000
64000
68000
70000
71000
72000
73000
74000
75000
76000
77000
78000
79000
80000
81000
82000
83000
84000
86000
87000
89000
93000
97000
Subject
Condition
EM
Primary

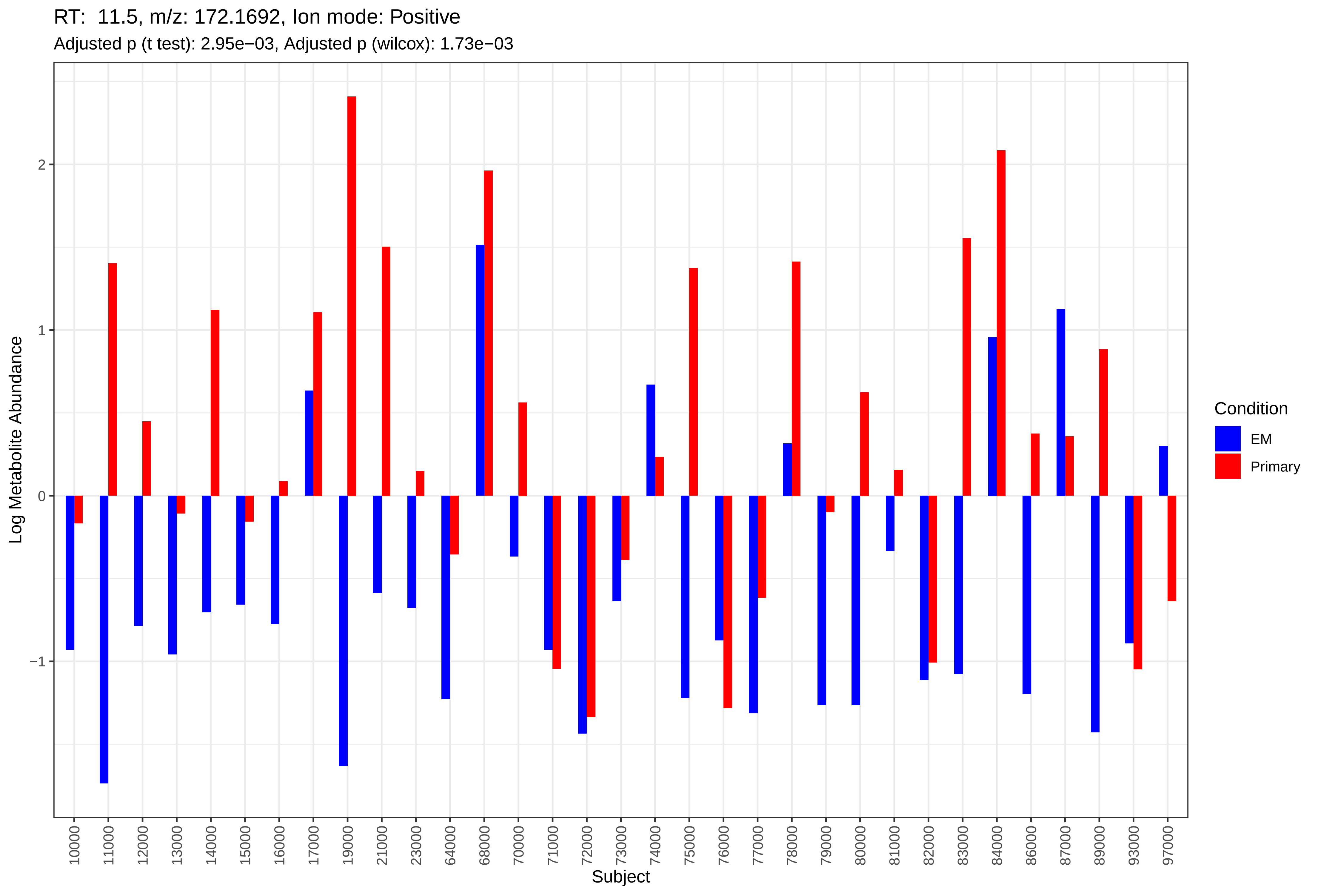

RT: 11.5, m/z: 172.1692, Ion mode: Positive
Adjusted p (t test): 2.95e−03, Adjusted p (wilcox): 1.73e−03
Log Metabolite Abundance
Subject
Condition
EM
Primary
10000
11000
12000
13000
14000
15000
16000
17000
19000
21000
23000
64000
68000
70000
71000
72000
73000
74000
75000
76000
77000
78000
79000
80000
81000
82000
83000
84000
86000
87000
89000
93000
97000

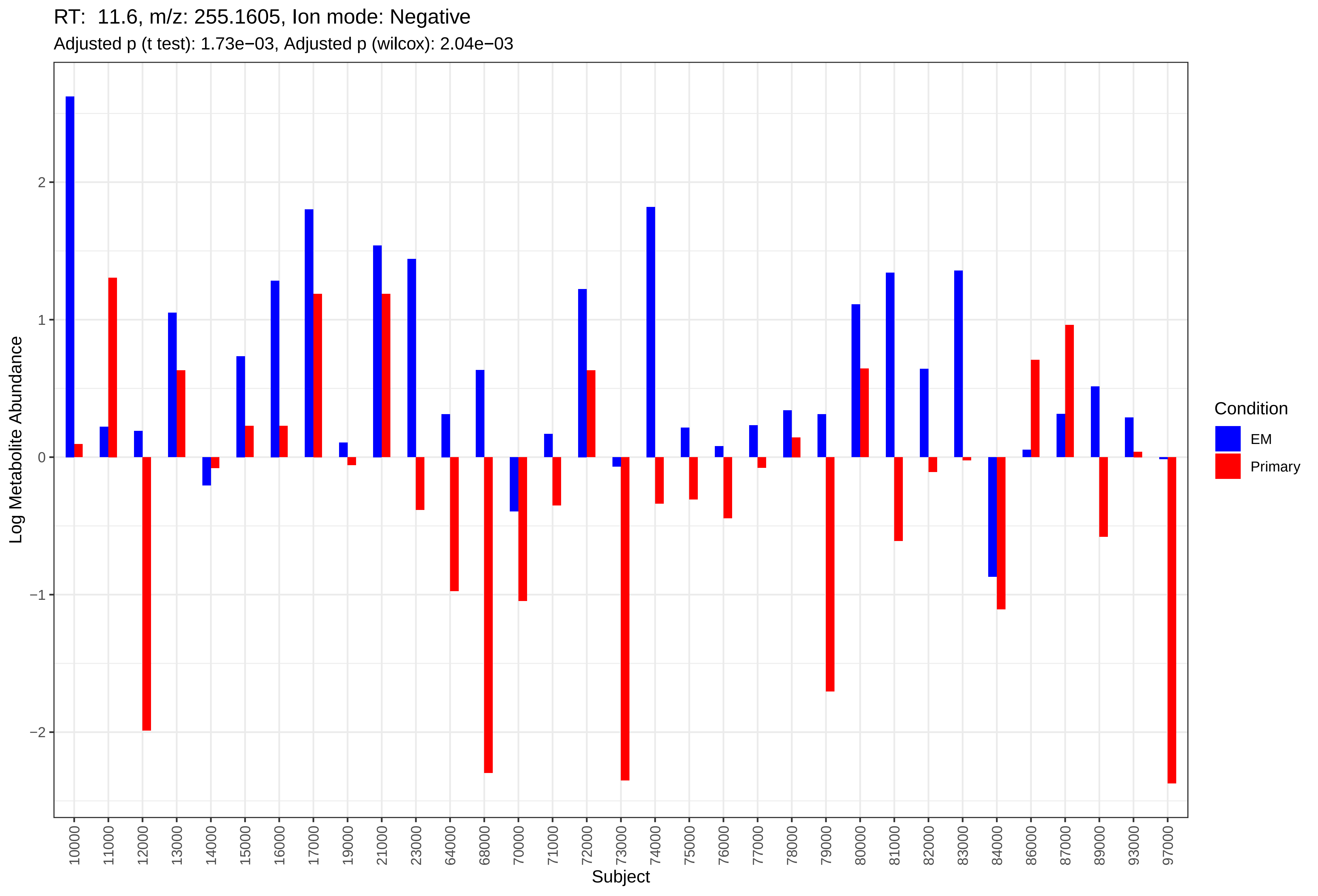
RT: 11.6, m/z: 255.1605, Ion mode: Negative
Adjusted p (t test): 1.73e−03, Adjusted p (wilcox): 2.04e−03
Log Metabolite Abundance
2
1
0
−1
−2
10000
11000
12000
13000
14000
15000
16000
17000
19000
21000
23000
64000
68000
70000
71000
72000
73000
74000
75000
76000
77000
78000
79000
80000
81000
82000
83000
84000
86000
87000
89000
93000
97000
Subject
Condition
EM
Primary

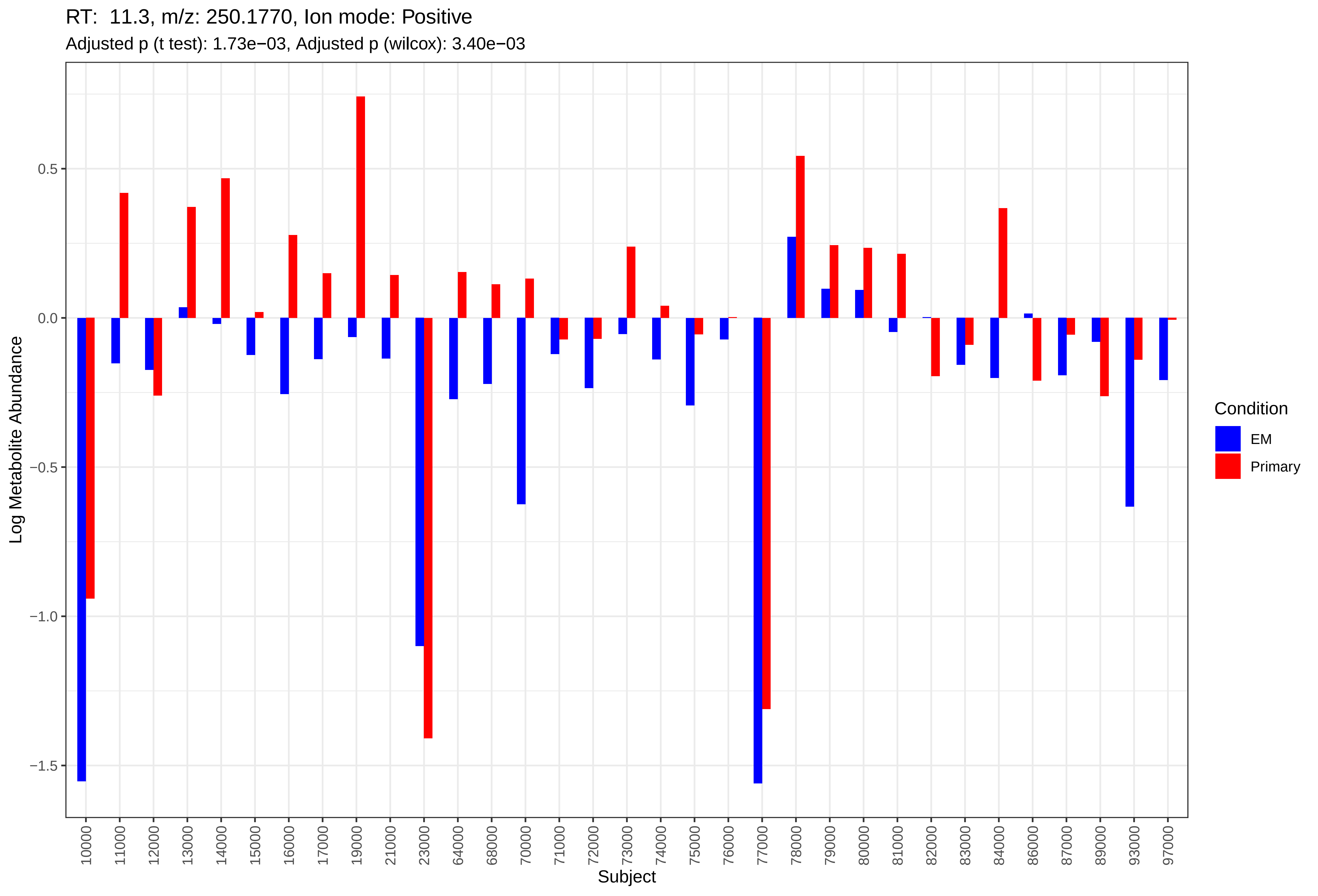
RT: 11.3, m/z: 250.1770, Ion mode: Positive
Adjusted p (t test): 1.73e−03, Adjusted p (wilcox): 3.40e−03
Log Metabolite Abundance
0.5
0.0
−0.5
−1.0
−1.5
10000
11000
12000
13000
14000
15000
16000
17000
19000
21000
23000
64000
68000
70000
71000
72000
73000
74000
75000
76000
77000
78000
79000
80000
81000
82000
83000
84000
86000
87000
89000
93000
97000
Subject
Condition
EM
Primary

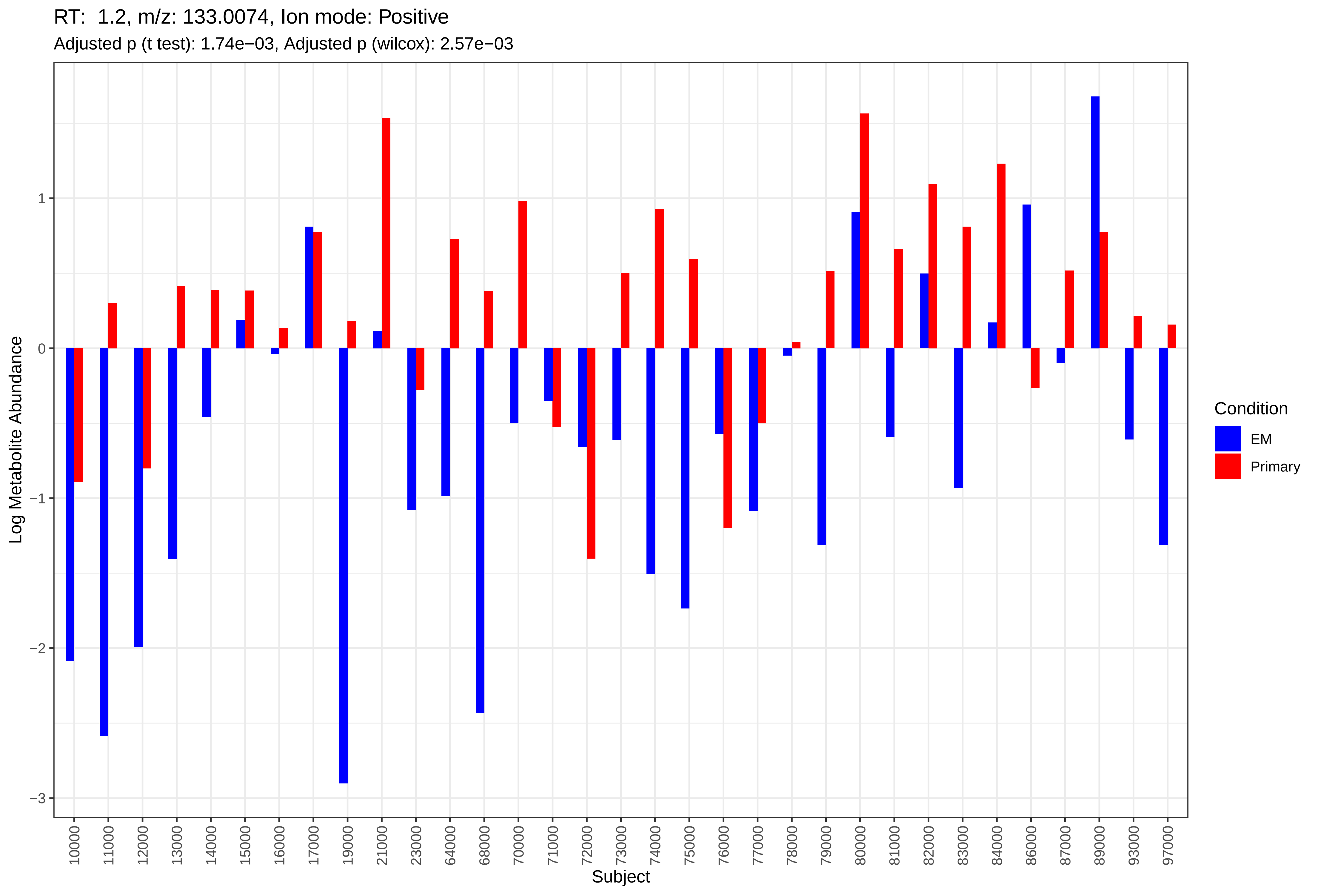
RT: 1.2, m/z: 133.0074, Ion mode: Positive
Adjusted p (t test): 1.74e−03, Adjusted p (wilcox): 2.57e−03
Log Metabolite Abundance
1
0
−1
−2
−3
10000
11000
12000
13000
14000
15000
16000
17000
19000
21000
23000
64000
68000
70000
71000
72000
73000
74000
75000
76000
77000
78000
79000
80000
81000
82000
83000
84000
86000
87000
89000
93000
97000
Subject
Condition
EM
Primary

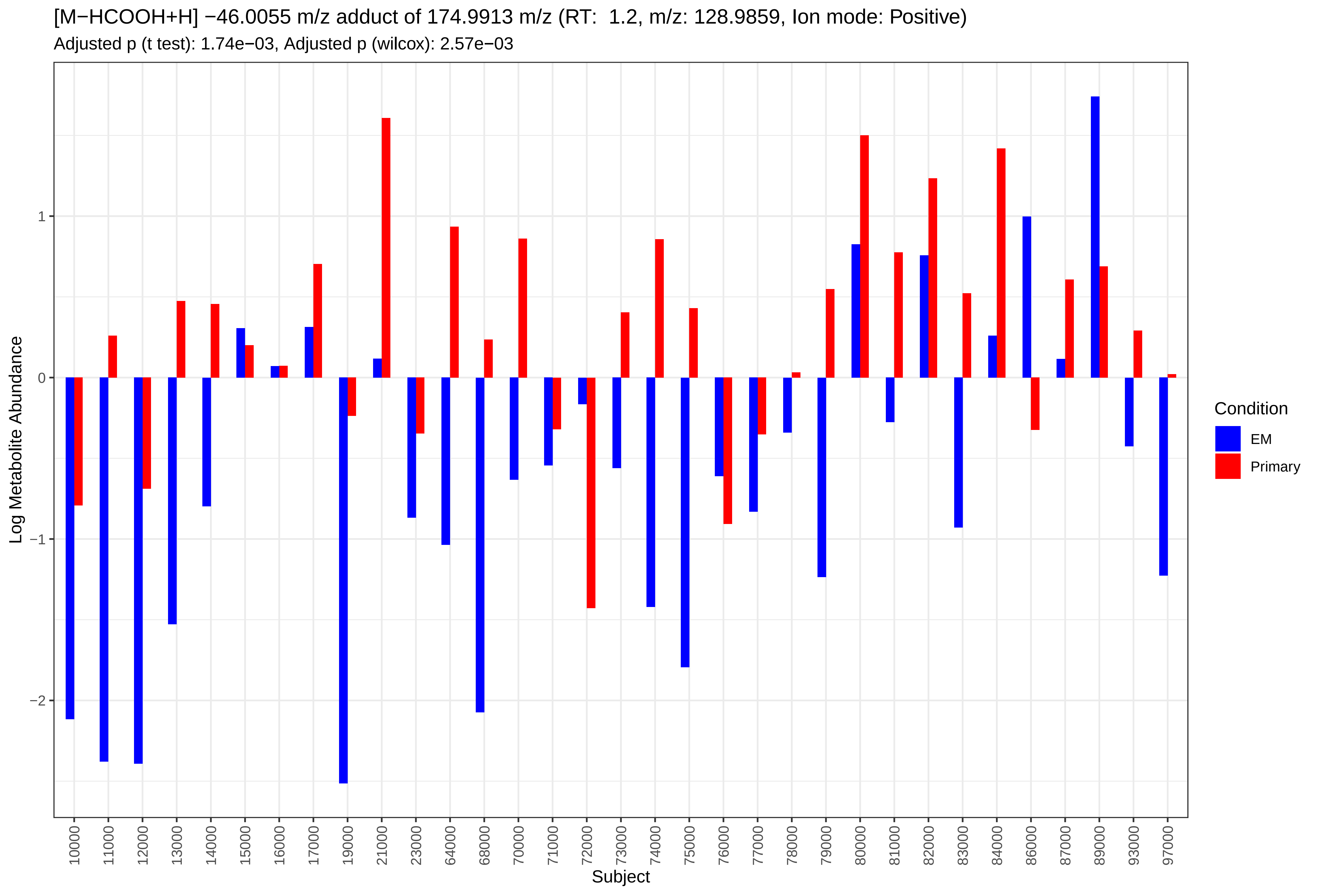

[M−HCOOH+H] −46.0055 m/z adduct of 174.9913 m/z (RT: 1.2, m/z: 128.9859, Ion mode: Positive)
Adjusted p (t test): 1.74e−03, Adjusted p (wilcox): 2.57e−03
Log Metabolite Abundance
1
0
−1
−2
Subject
10000
11000
12000
13000
14000
15000
16000
17000
19000
21000
23000
64000
68000
70000
71000
72000
73000
74000
75000
76000
77000
78000
79000
80000
81000
82000
83000
84000
86000
87000
89000
93000
97000
Condition
EM
Primary

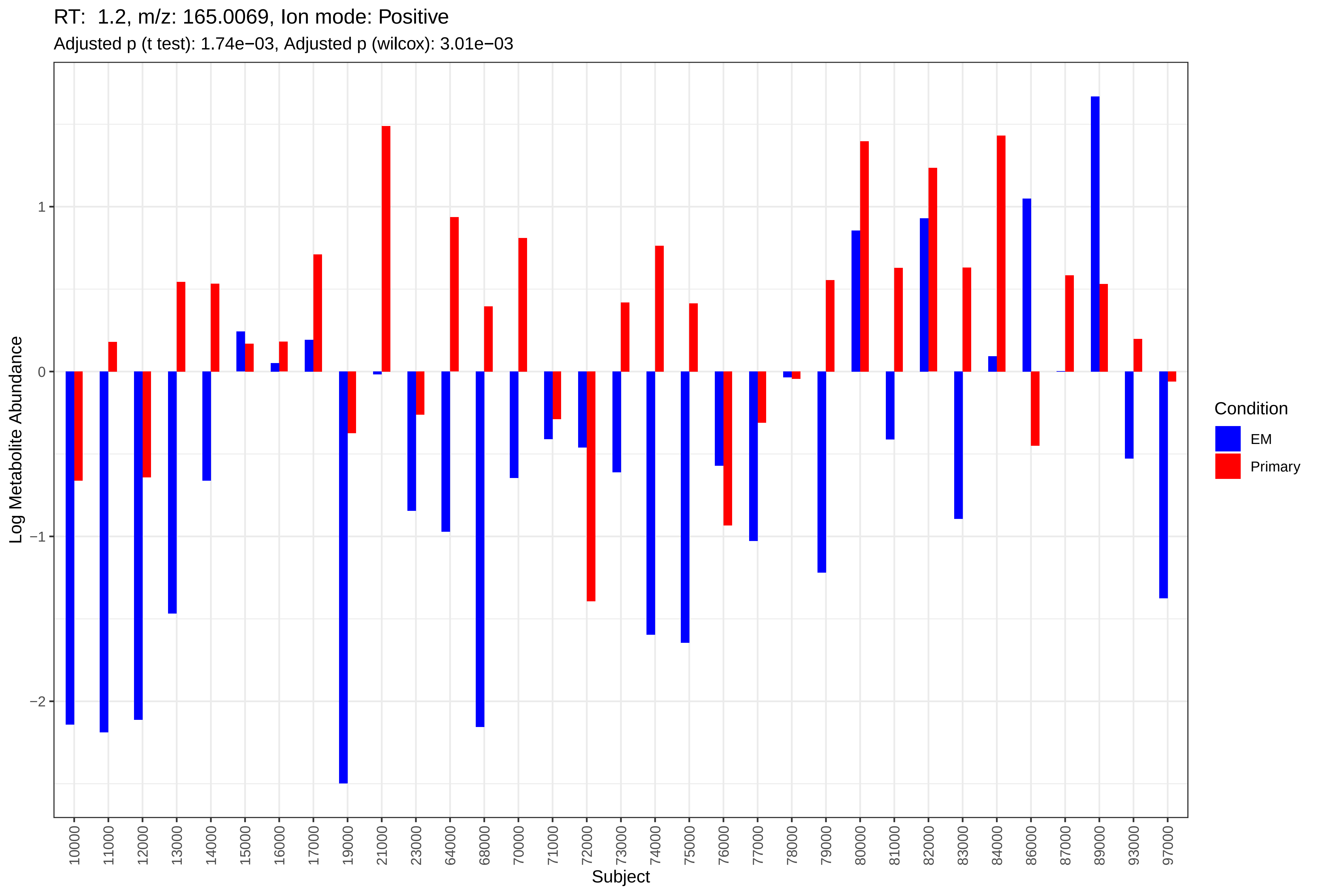

RT: 1.2, m/z: 165.0069, Ion mode: Positive
Adjusted p (t test): 1.74e−03, Adjusted p (wilcox): 3.01e−03
Log Metabolite Abundance
1
0
−1
−2
Subject
10000
11000
12000
13000
14000
15000
16000
17000
19000
21000
23000
64000
68000
70000
71000
72000
73000
74000
75000
76000
77000
78000
79000
80000
81000
82000
83000
84000
86000
87000
89000
93000
97000
Condition
EM
Primary

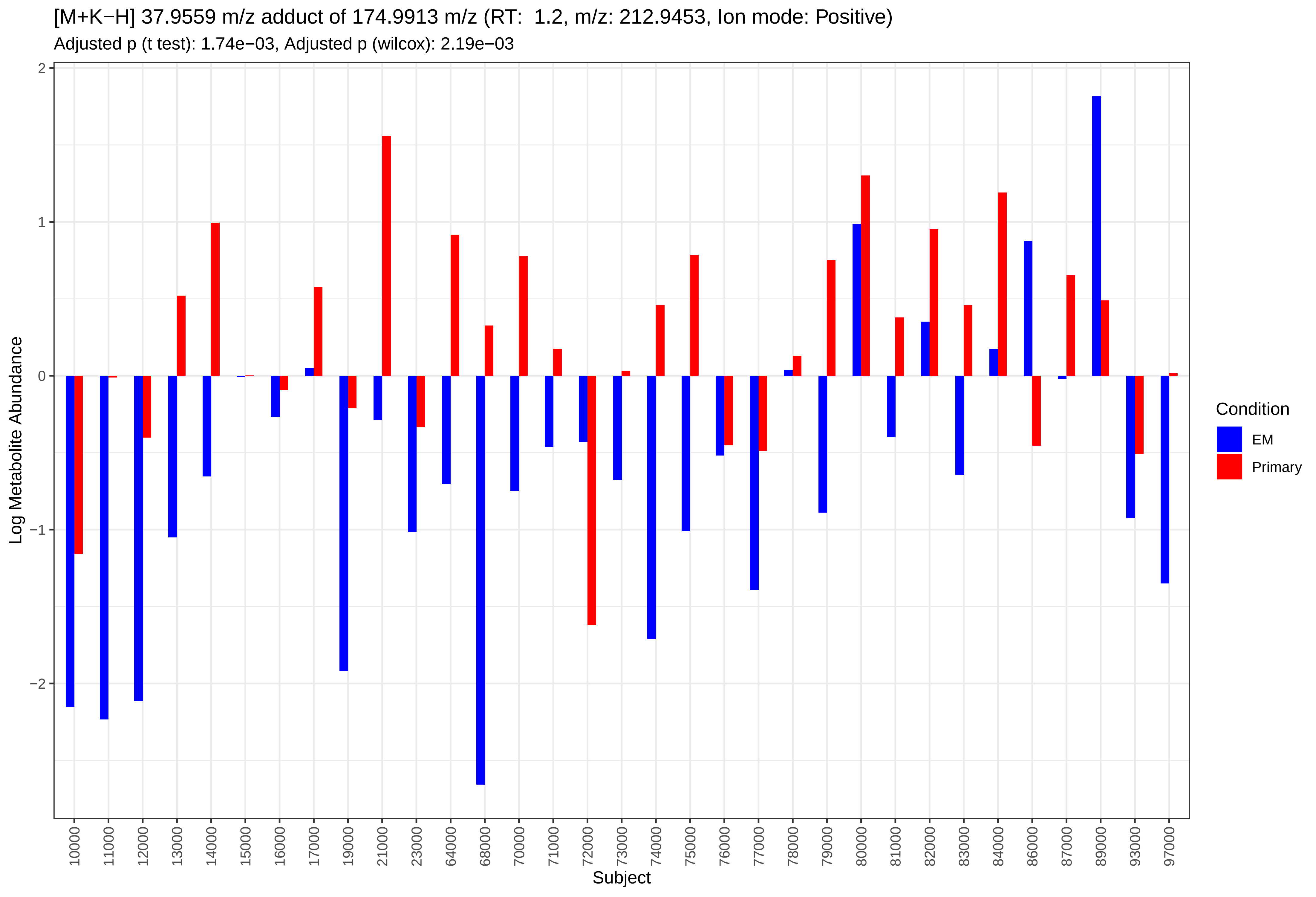
[M+K−H] 37.9559 m/z adduct of 174.9913 m/z (RT: 1.2, m/z: 212.9453, Ion mode: Positive)
Adjusted p (t test): 1.74e−03, Adjusted p (wilcox): 2.19e−03
Log Metabolite Abundance
2
1
0
−1
−2
Subject
10000
11000
12000
13000
14000
15000
16000
17000
19000
21000
23000
64000
68000
70000
71000
72000
73000
74000
75000
76000
77000
78000
79000
80000
81000
82000
83000
84000
86000
87000
89000
93000
97000
Condition
EM
Primary

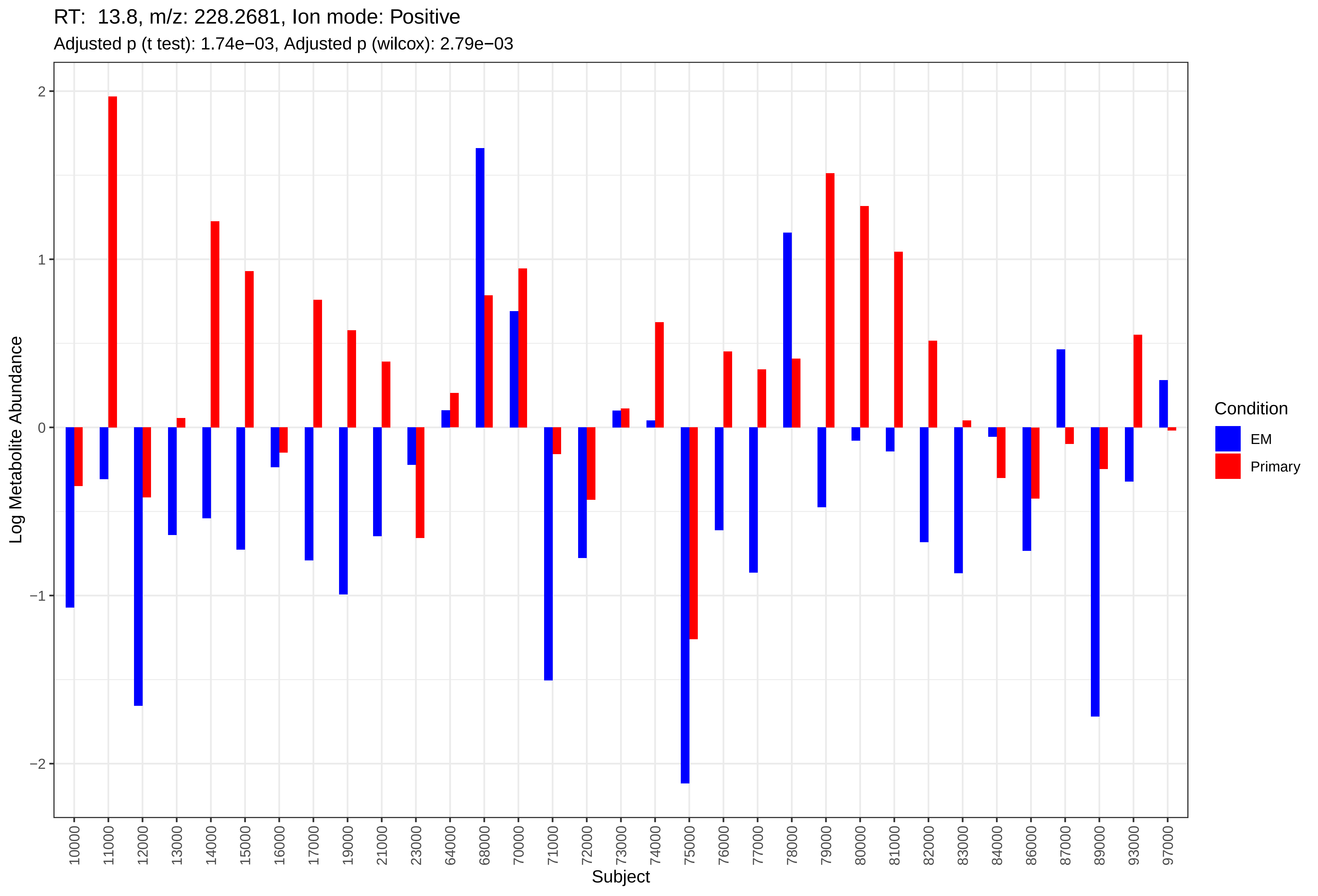
RT: 13.8, m/z: 228.2681, Ion mode: Positive
Adjusted p (t test): 1.74e−03, Adjusted p (wilcox): 2.79e−03
Log Metabolite Abundance
2
1
0
−1
−2
Subject
10000
11000
12000
13000
14000
15000
16000
17000
19000
21000
23000
64000
68000
70000
71000
72000
73000
74000
75000
76000
77000
78000
79000
80000
81000
82000
83000
84000
86000
87000
89000
93000
97000
Condition
EM
Primary

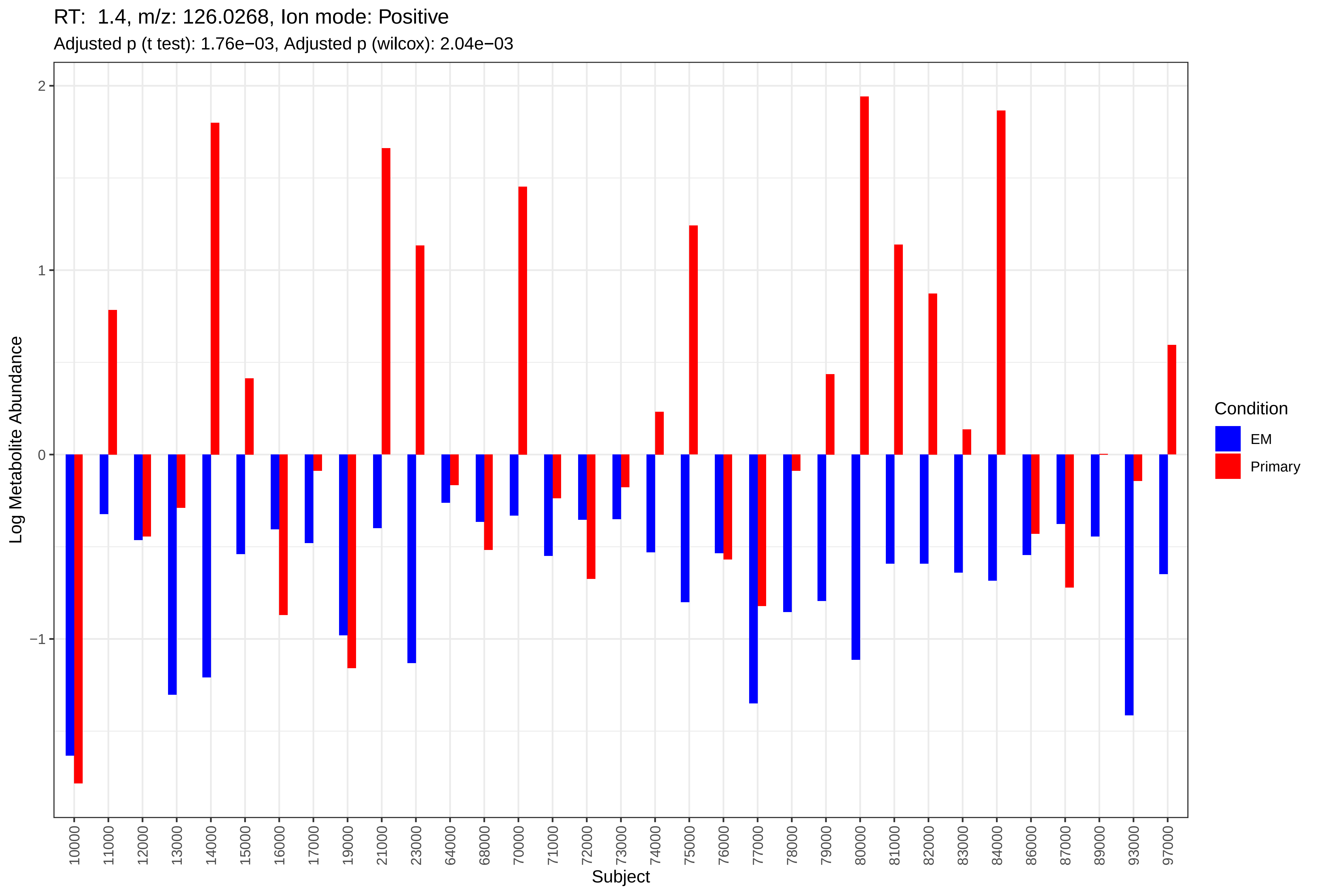

RT: 1.4, m/z: 126.0268, Ion mode: Positive
Adjusted p (t test): 1.76e−03, Adjusted p (wilcox): 2.04e−03
Log Metabolite Abundance
2
1
0
−1
Subject
10000
11000
12000
13000
14000
15000
16000
17000
19000
21000
23000
64000
68000
70000
71000
72000
73000
74000
75000
76000
77000
78000
79000
80000
81000
82000
83000
84000
86000
87000
89000
93000
97000
Condition
EM
Primary

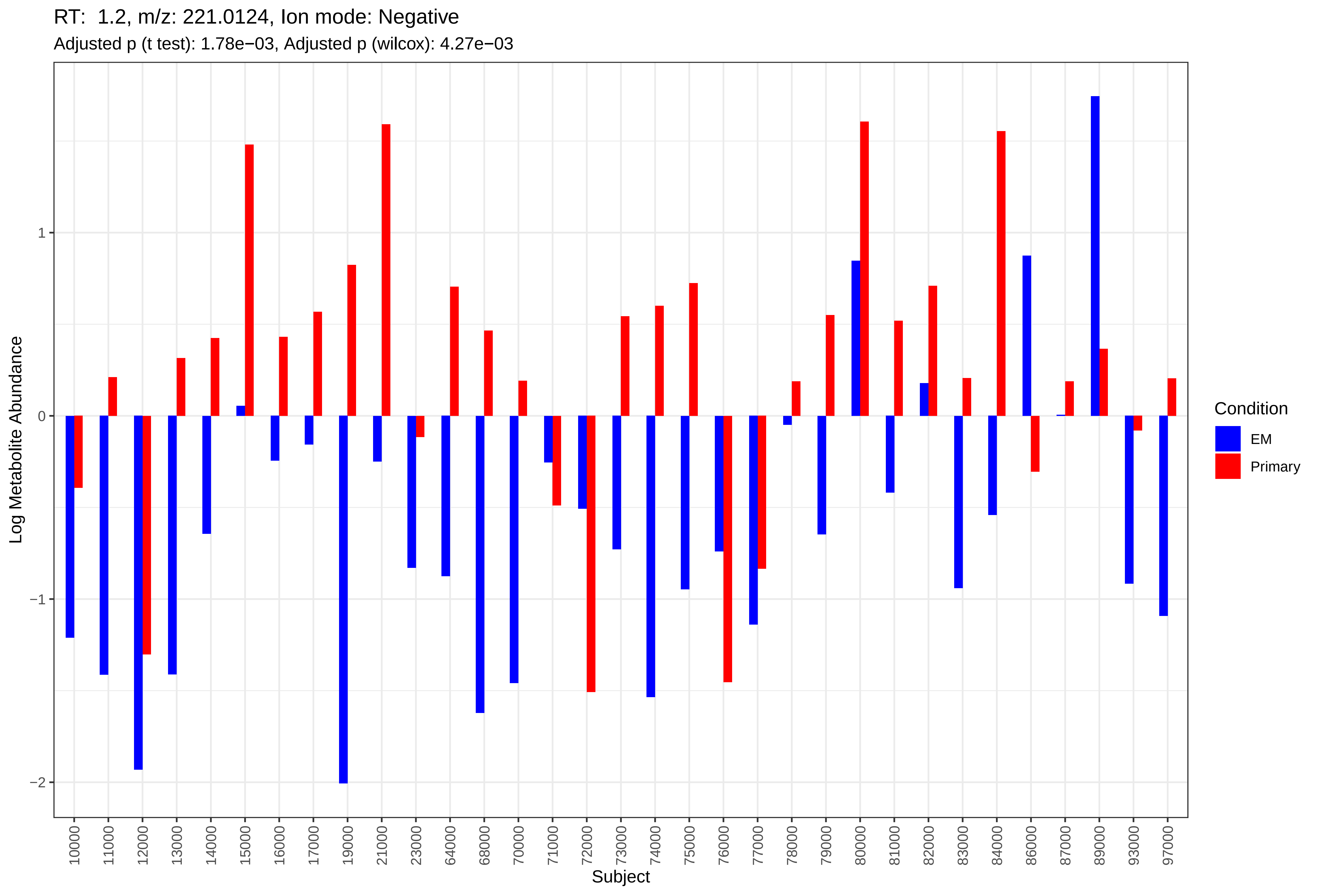
RT: 1.2, m/z: 221.0124, Ion mode: Negative
Adjusted p (t test): 1.78e−03, Adjusted p (wilcox): 4.27e−03
Log Metabolite Abundance
1
0
−1
−2
Subject
10000
11000
12000
13000
14000
15000
16000
17000
19000
21000
23000
64000
68000
70000
71000
72000
73000
74000
75000
76000
77000
78000
79000
80000
81000
82000
83000
84000
86000
87000
89000
93000
97000
Condition
EM
Primary

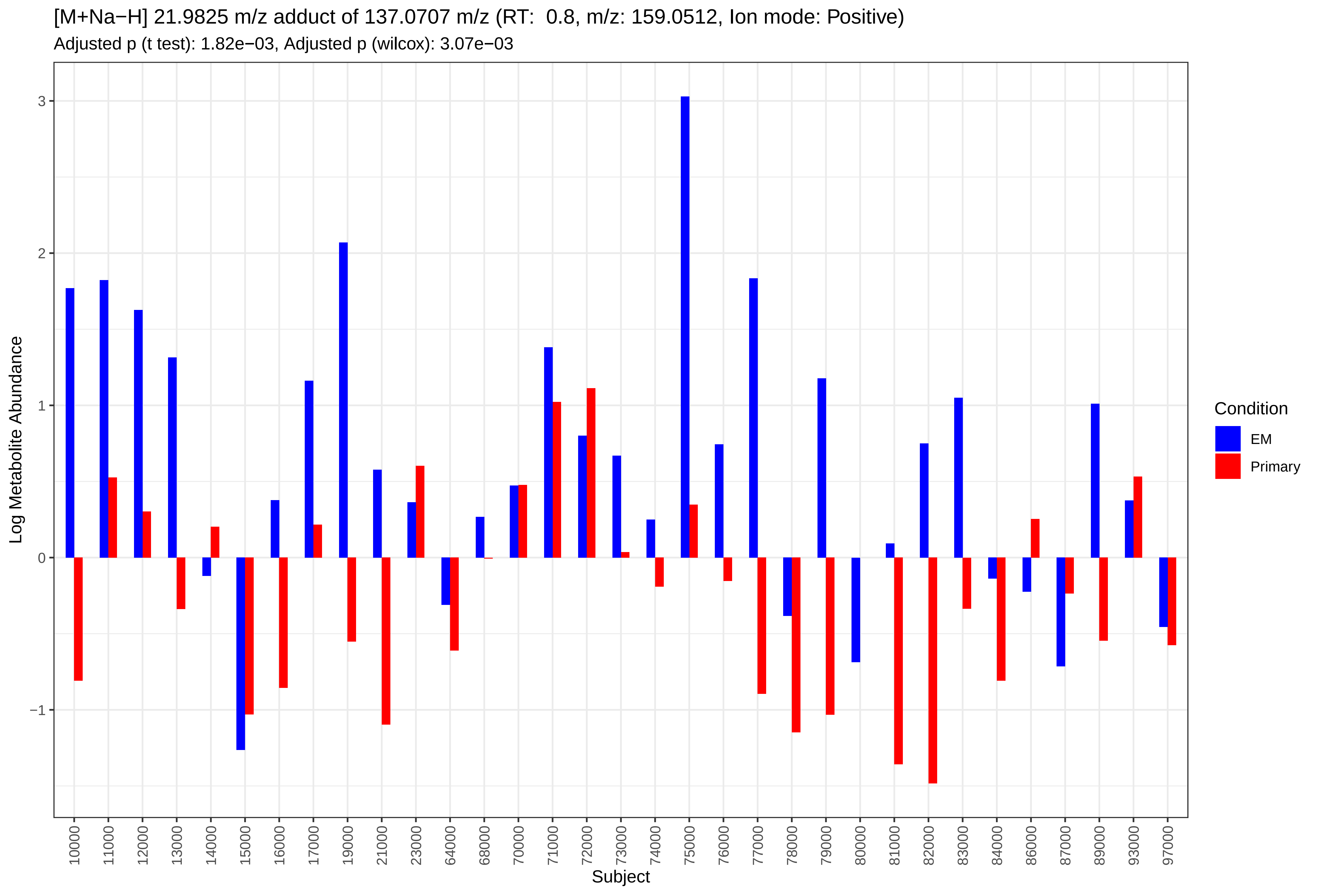
[M+Na−H] 21.9825 m/z adduct of 137.0707 m/z (RT: 0.8, m/z: 159.0512, Ion mode: Positive)
Adjusted p (t test): 1.82e−03, Adjusted p (wilcox): 3.07e−03
Log Metabolite Abundance
3
2
1
0
−1
Subject
10000
11000
12000
13000
14000
15000
16000
17000
19000
21000
23000
64000
68000
70000
71000
72000
73000
74000
75000
76000
77000
78000
79000
80000
81000
82000
83000
84000
86000
87000
89000
93000
97000
Condition
EM
Primary

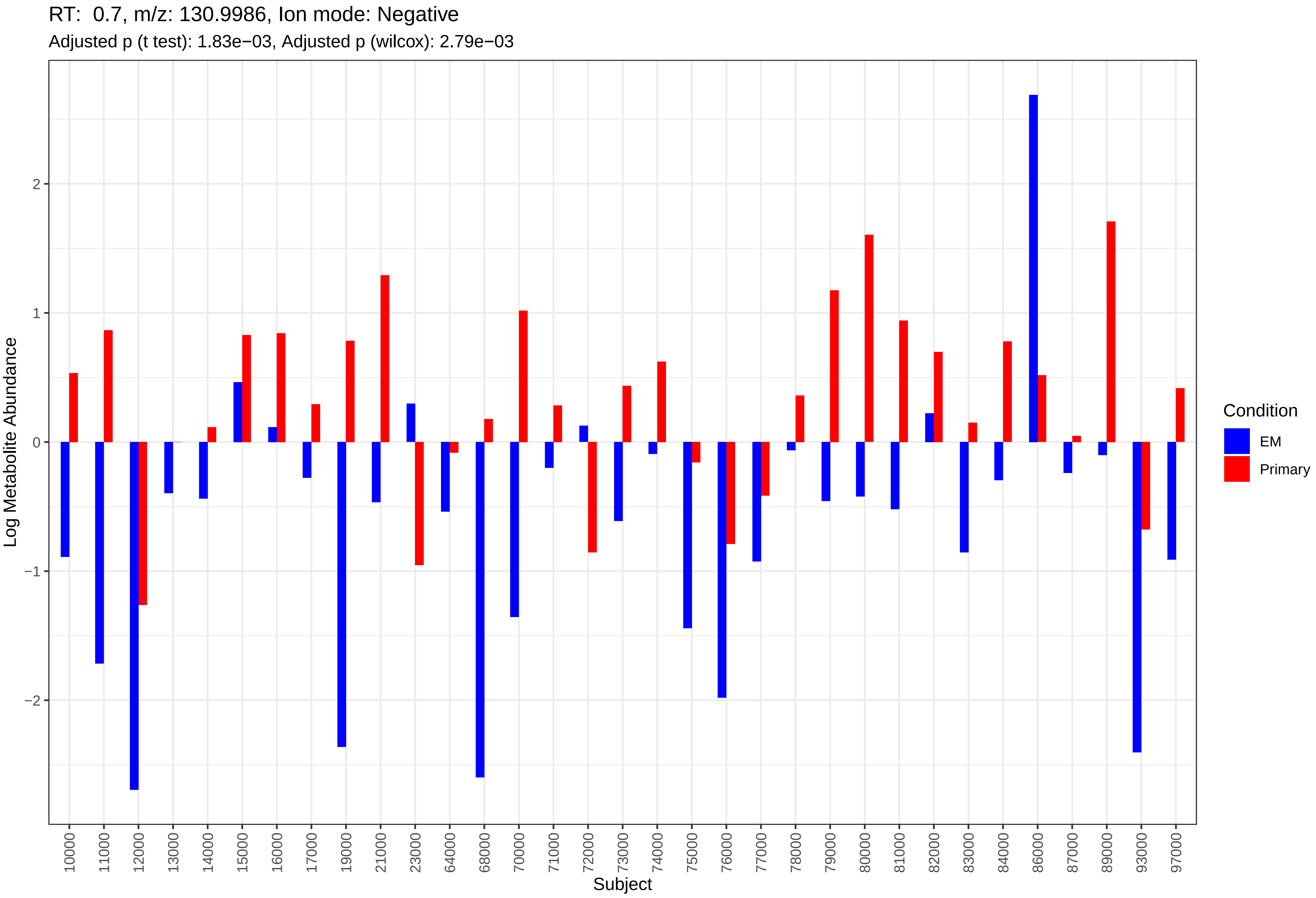
RT: 0.7, m/z: 130.9986, Ion mode: Negative
Adjusted p (t test): 1.83e−03, Adjusted p (wilcox): 2.79e−03
Log Metabolite Abundance
2
1
0
−1
−2
10000
11000
12000
13000
14000
15000
16000
17000
19000
21000
23000
64000
68000
70000
71000
72000
73000
74000
75000
76000
77000
78000
79000
80000
81000
82000
83000
84000
86000
87000
89000
93000
97000
Subject
Condition
EM
Primary

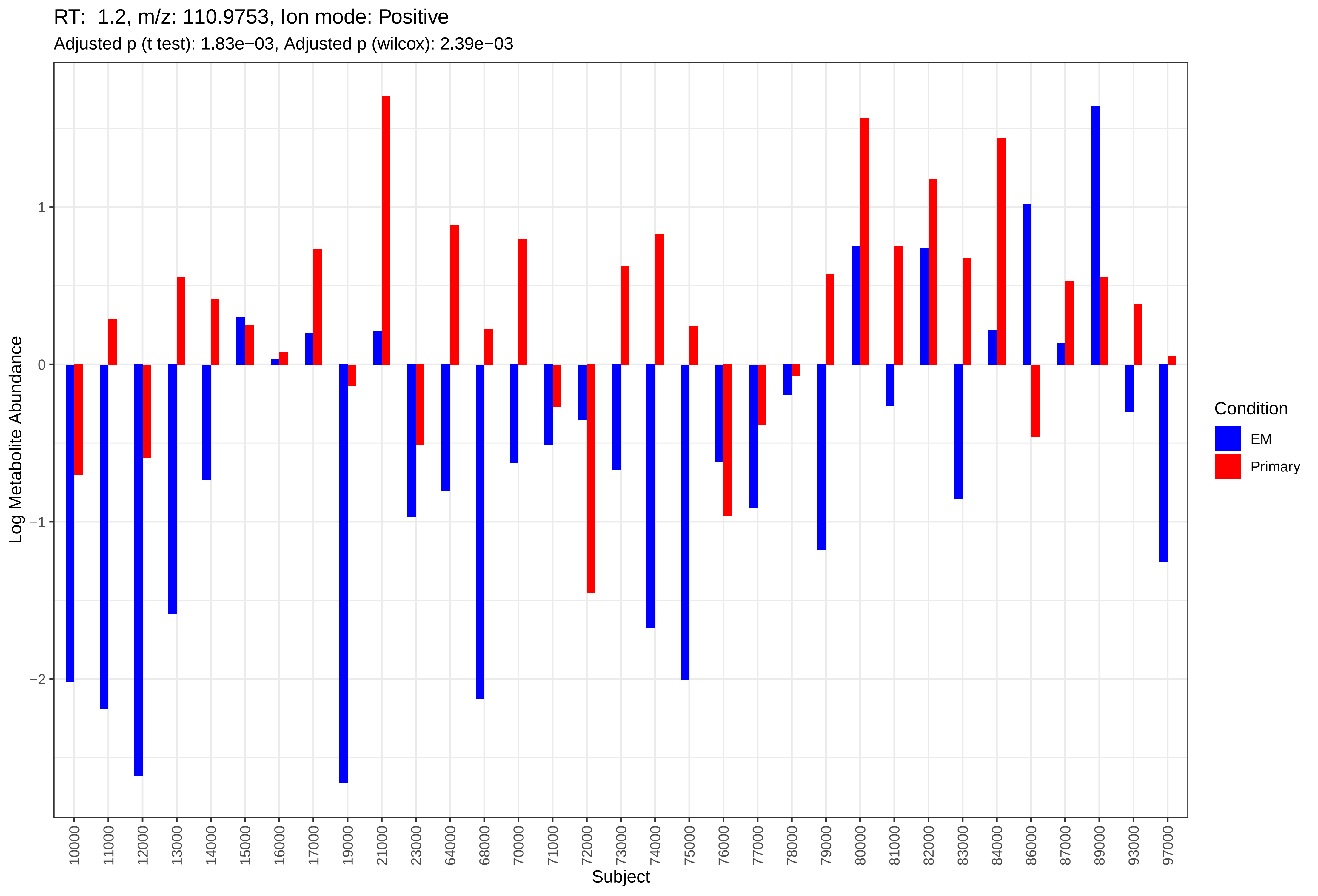
RT: 1.2, m/z: 110.9753, Ion mode: Positive
Adjusted p (t test): 1.83e−03, Adjusted p (wilcox): 2.39e−03
Log Metabolite Abundance
1
0
−1
−2
Subject
10000
11000
12000
13000
14000
15000
16000
17000
19000
21000
23000
64000
68000
70000
71000
72000
73000
74000
75000
76000
77000
78000
79000
80000
81000
82000
83000
84000
86000
87000
89000
93000
97000
Condition
EM
Primary

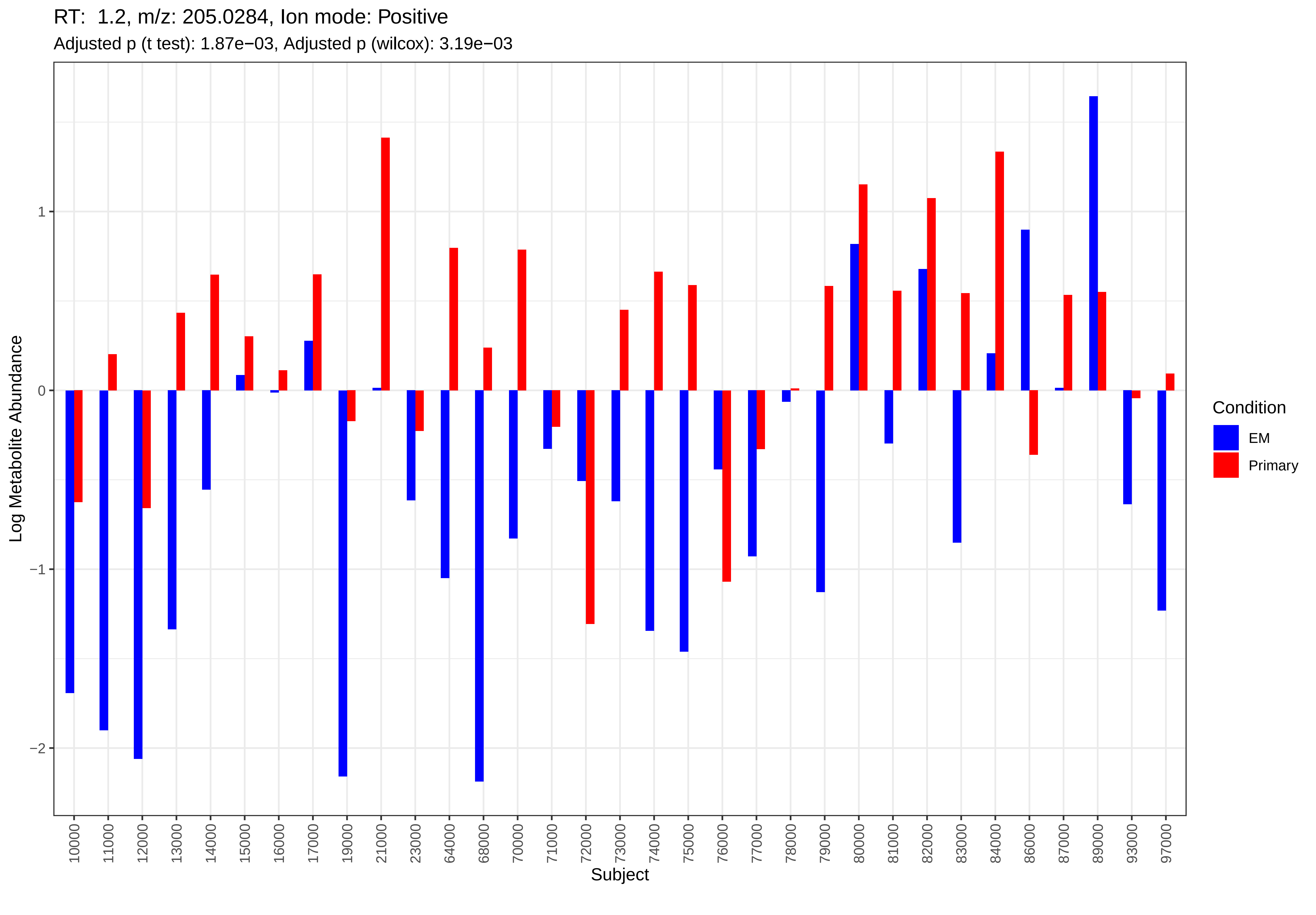
RT: 1.2, m/z: 205.0284, Ion mode: Positive
Adjusted p (t test): 1.87e−03, Adjusted p (wilcox): 3.19e−03
Log Metabolite Abundance
1
0
−1
−2
Subject
10000
11000
12000
13000
14000
15000
16000
17000
19000
21000
23000
64000
68000
70000
71000
72000
73000
74000
75000
76000
77000
78000
79000
80000
81000
82000
83000
84000
86000
87000
89000
93000
97000
Condition
EM
Primary

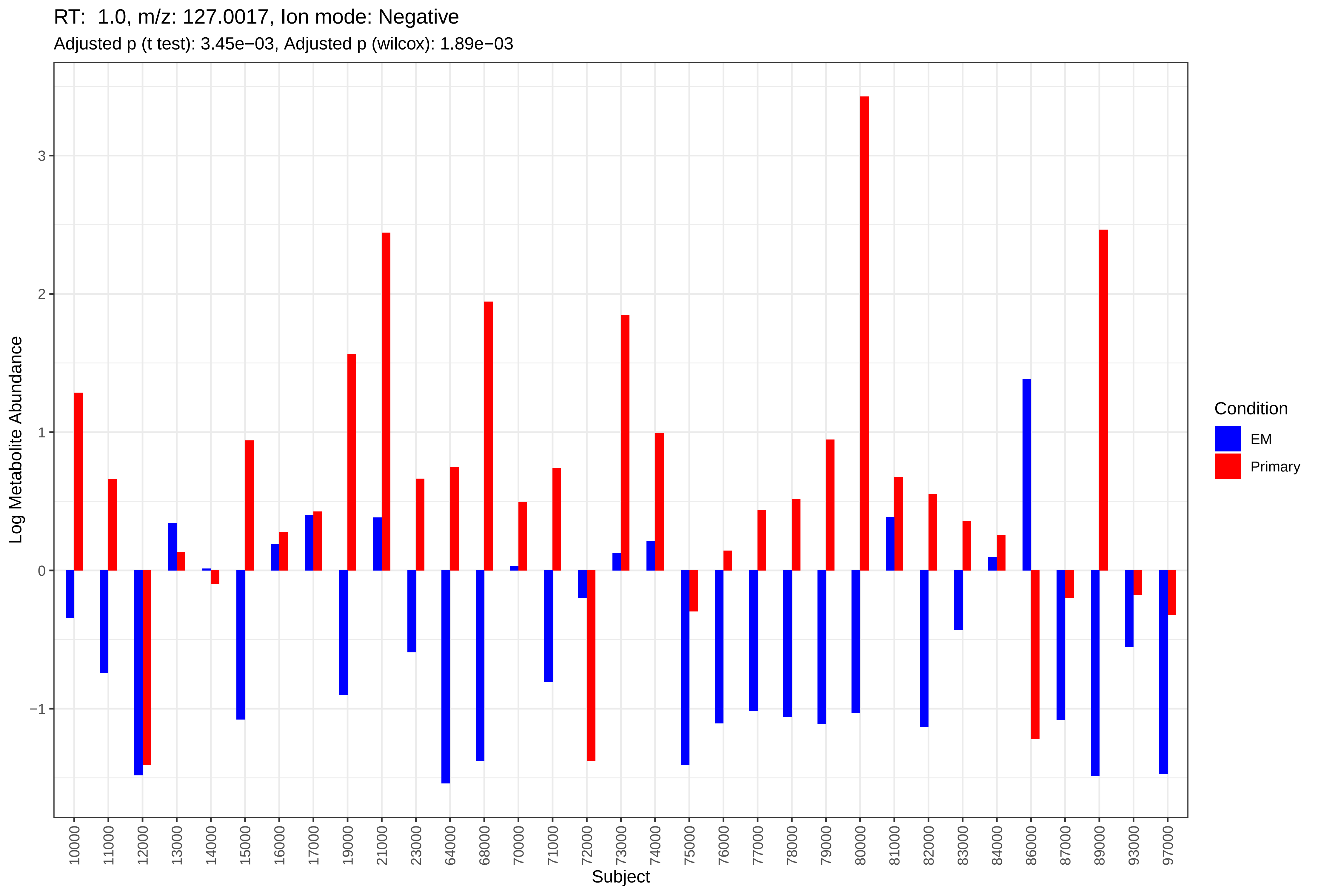
RT: 1.0, m/z: 127.0017, Ion mode: Negative
Adjusted p (t test): 3.45e−03, Adjusted p (wilcox): 1.89e−03
Log Metabolite Abundance
3
2
1
0
−1
Subject
10000
11000
12000
13000
14000
15000
16000
17000
19000
21000
23000
64000
68000
70000
71000
72000
73000
74000
75000
76000
77000
78000
79000
80000
81000
82000
83000
84000
86000
87000
89000
93000
97000
Condition
EM
Primary

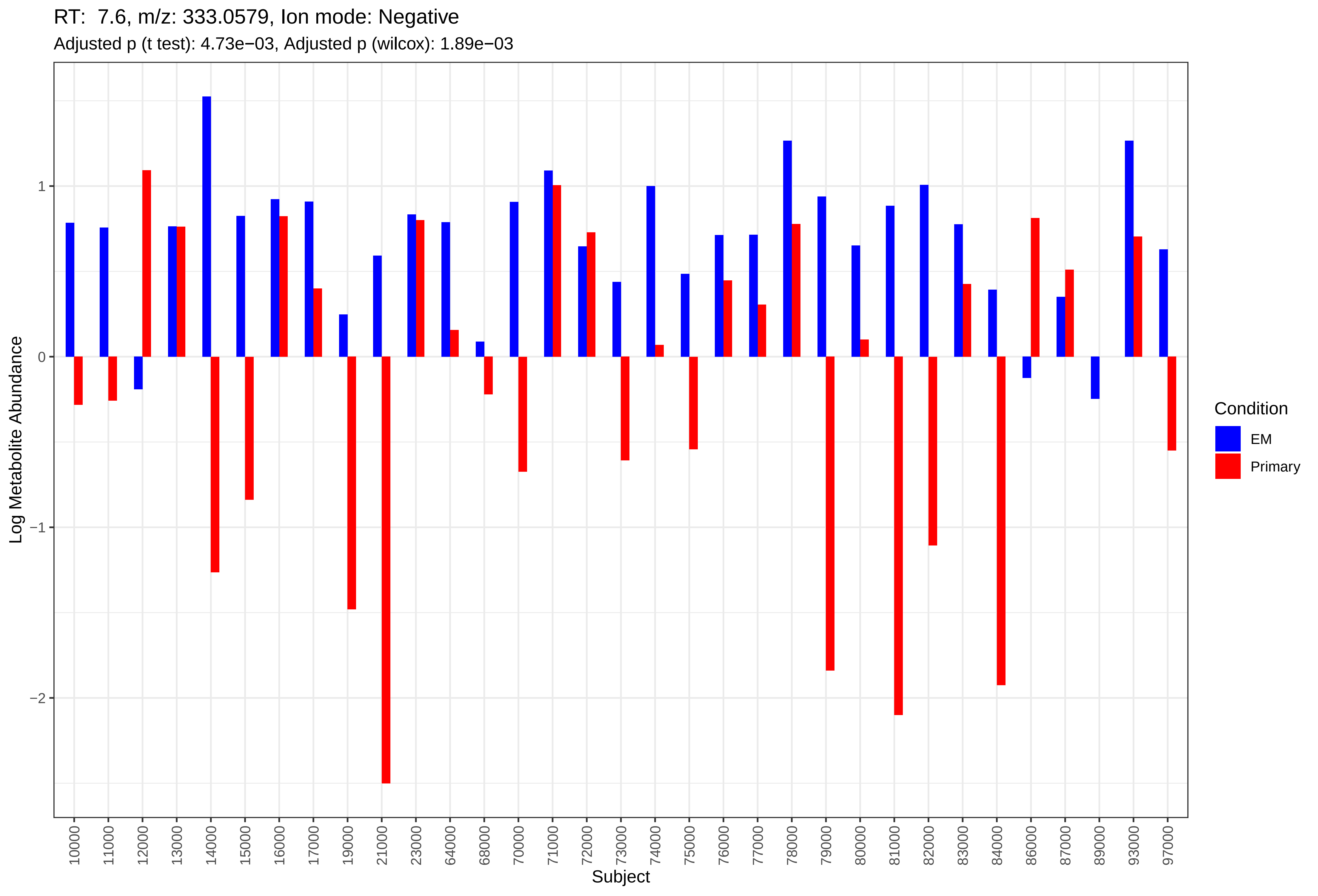

RT: 7.6, m/z: 333.0579, Ion mode: Negative
Adjusted p (t test): 4.73e−03, Adjusted p (wilcox): 1.89e−03
Log Metabolite Abundance
1
0
−1
−2
10000
11000
12000
13000
14000
15000
16000
17000
19000
21000
23000
64000
68000
70000
71000
72000
73000
74000
75000
76000
77000
78000
79000
80000
81000
82000
83000
84000
86000
87000
89000
93000
97000
Subject
Condition
EM
Primary

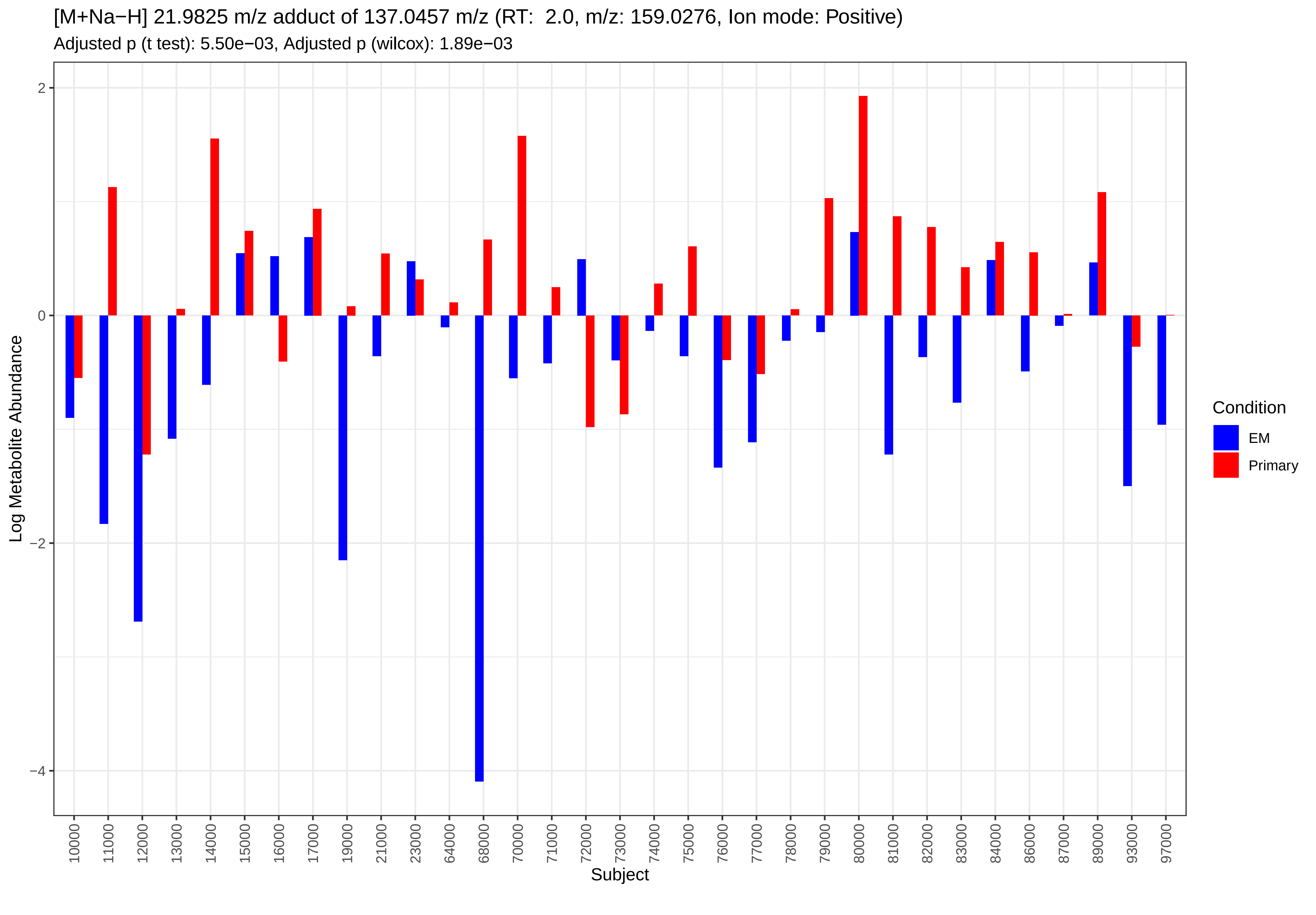
[M+Na−H] 21.9825 m/z adduct of 137.0457 m/z (RT: 2.0, m/z: 159.0276, Ion mode: Positive)
Adjusted p (t test): 5.50e−03, Adjusted p (wilcox): 1.89e−03
Log Metabolite Abundance
2
0
−2
−4
10000
11000
12000
13000
14000
15000
16000
17000
19000
21000
23000
64000
68000
70000
71000
72000
73000
74000
75000
76000
77000
78000
79000
80000
81000
82000
83000
84000
86000
87000
89000
93000
97000
Subject
Condition
EM
Primary

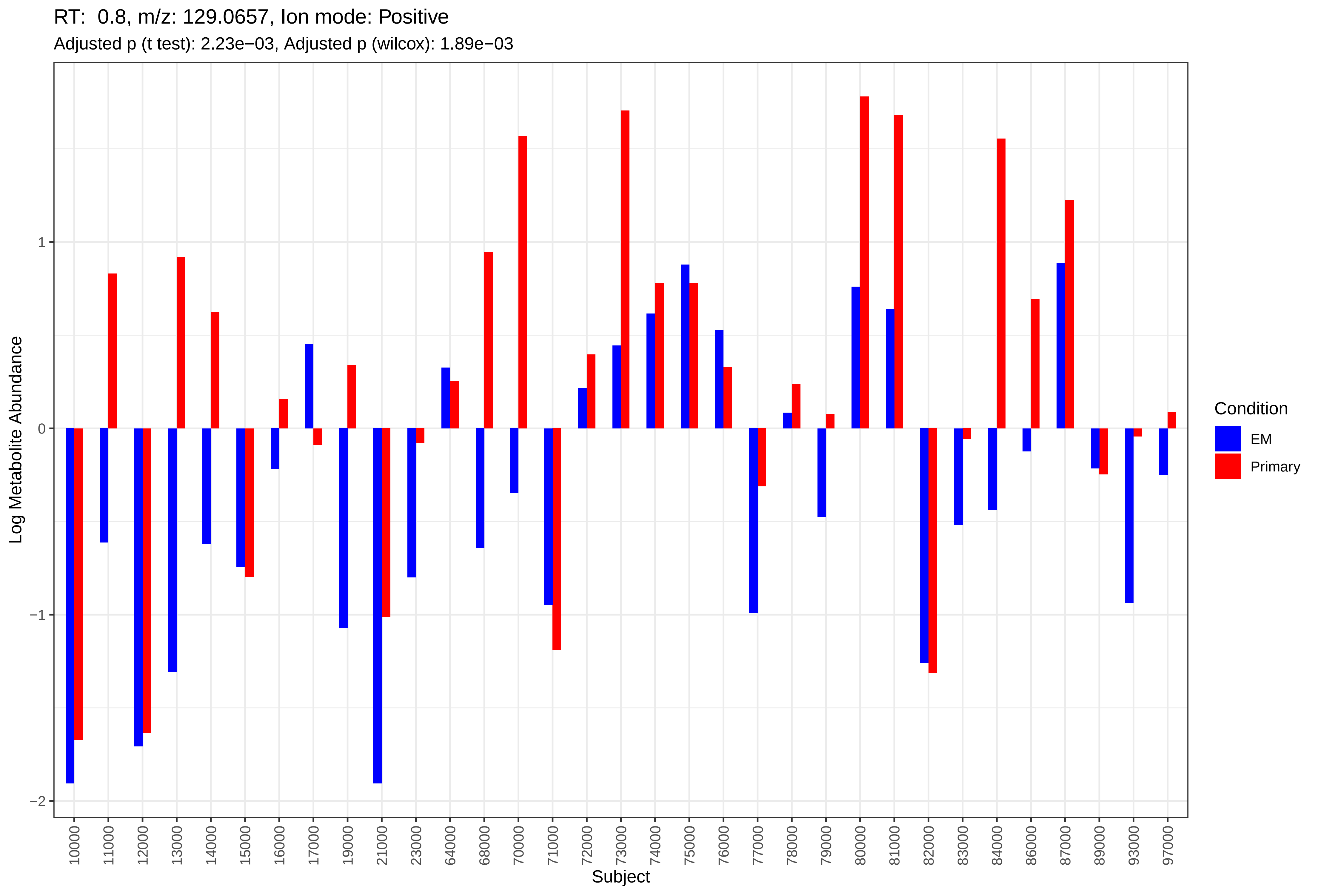
RT: 0.8, m/z: 129.0657, Ion mode: Positive
Adjusted p (t test): 2.23e−03, Adjusted p (wilcox): 1.89e−03
Log Metabolite Abundance
1
0
−1
−2
10000
11000
12000
13000
14000
15000
16000
17000
19000
21000
23000
64000
68000
70000
71000
72000
73000
74000
75000
76000
77000
78000
79000
80000
81000
82000
83000
84000
86000
87000
89000
93000
97000
Subject
Condition
EM
Primary

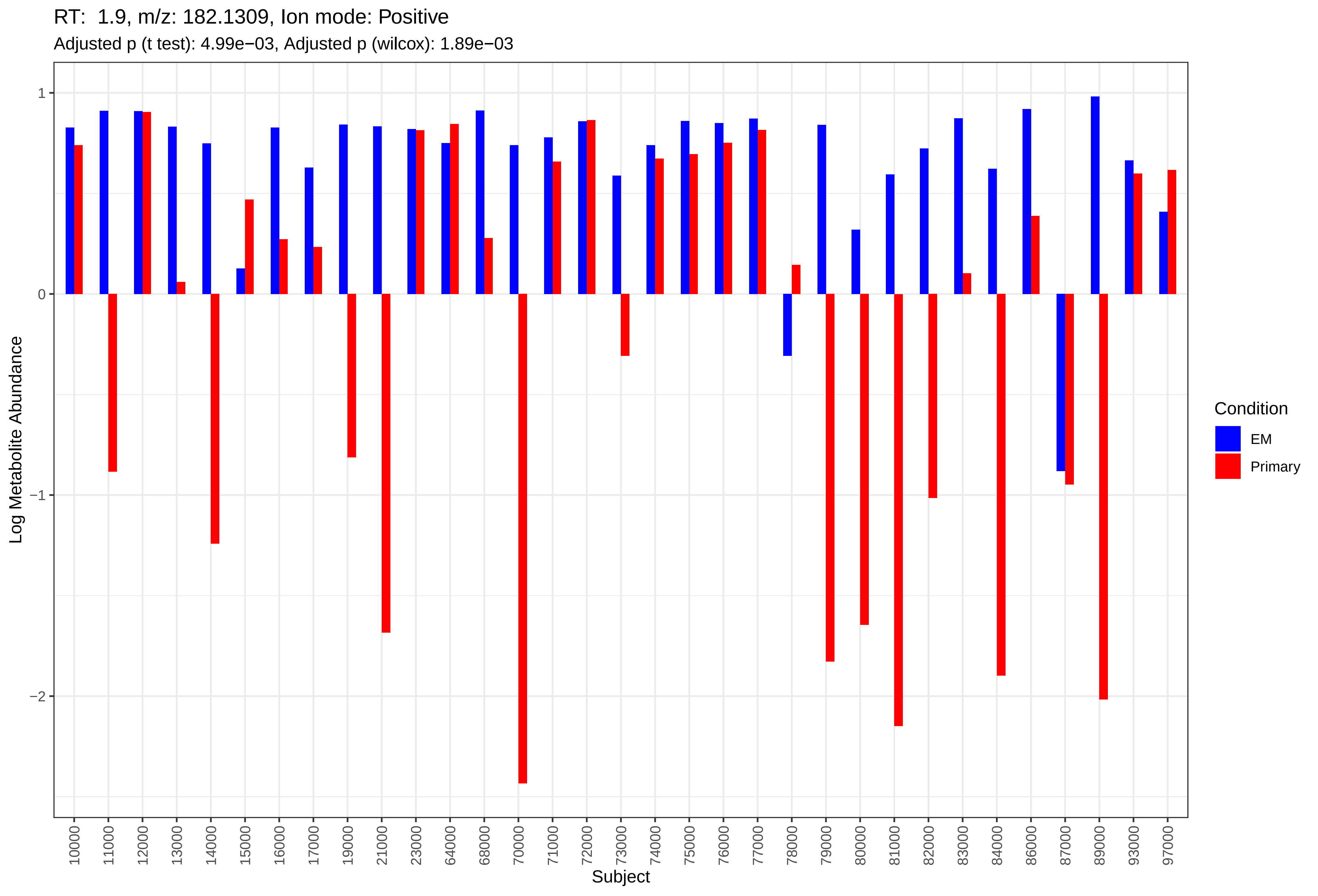

RT: 1.9, m/z: 182.1309, Ion mode: Positive
Adjusted p (t test): 4.99e−03, Adjusted p (wilcox): 1.89e−03
Log Metabolite Abundance
1
0
−1
−2
10000
11000
12000
13000
14000
15000
16000
17000
19000
21000
23000
64000
68000
70000
71000
72000
73000
74000
75000
76000
77000
78000
79000
80000
81000
82000
83000
84000
86000
87000
89000
93000
97000
Subject
Condition
EM
Primary

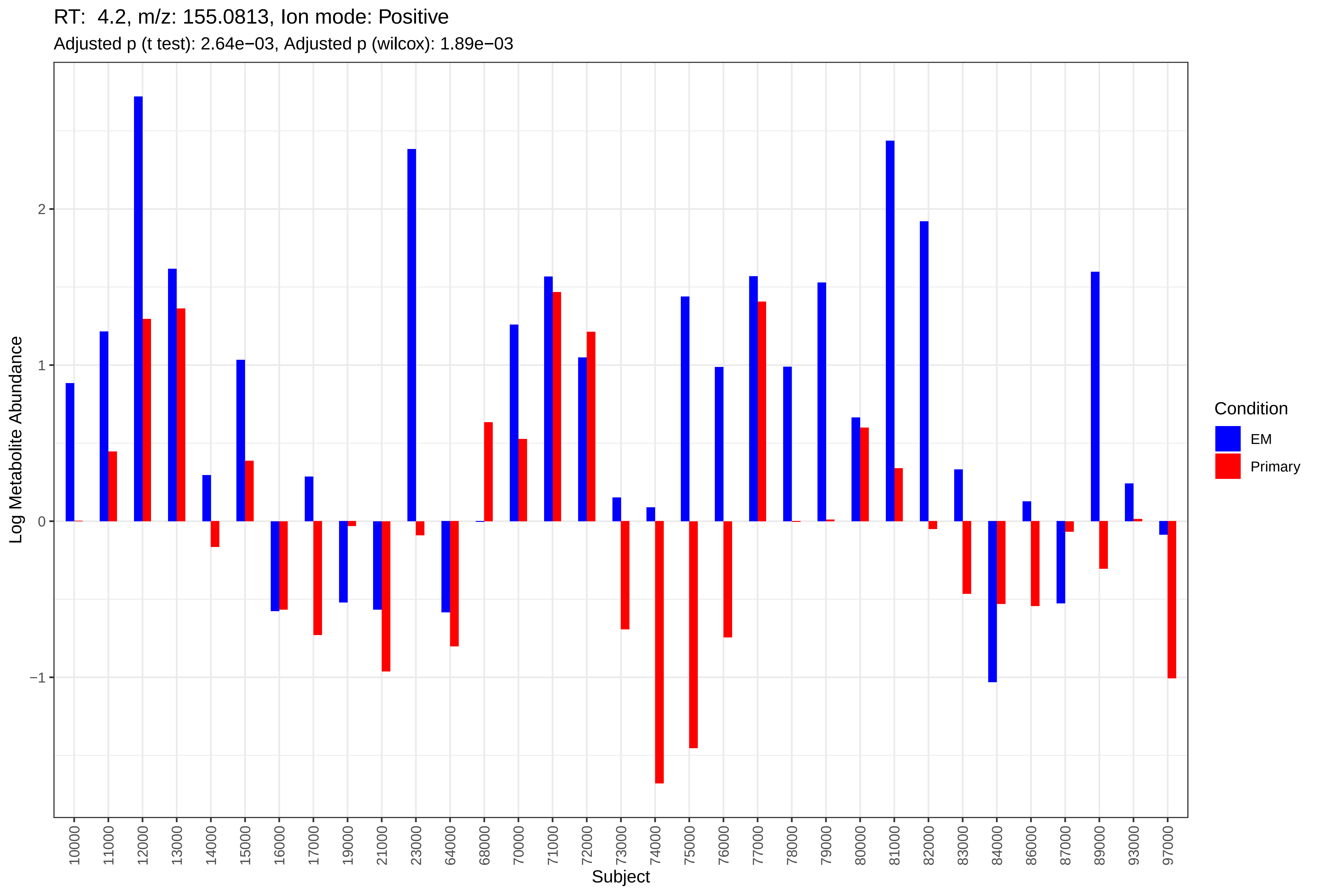
RT: 4.2, m/z: 155.0813, Ion mode: Positive
Adjusted p (t test): 2.64e−03, Adjusted p (wilcox): 1.89e−03
Log Metabolite Abundance
2
1
0
−1
Subject
10000
11000
12000
13000
14000
15000
16000
17000
19000
21000
23000
64000
68000
70000
71000
72000
73000
74000
75000
76000
77000
78000
79000
80000
81000
82000
83000
84000
86000
87000
89000
93000
97000
Condition
EM
Primary

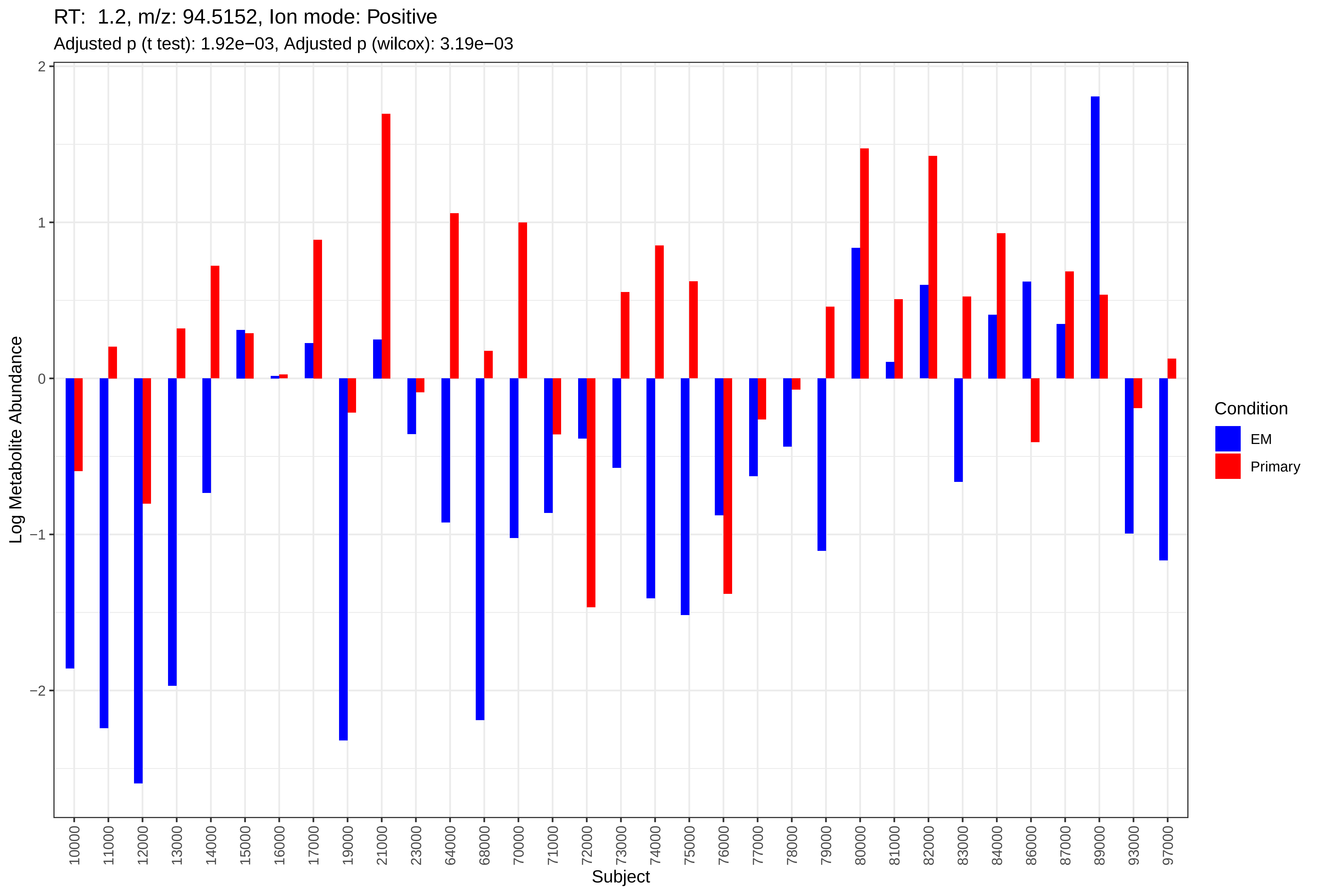

RT: 1.2, m/z: 94.5152, Ion mode: Positive
Adjusted p (t test): 1.92e−03, Adjusted p (wilcox): 3.19e−03
Log Metabolite Abundance
2
1
0
−1
−2
10000
11000
12000
13000
14000
15000
16000
17000
19000
21000
23000
64000
68000
70000
71000
72000
73000
74000
75000
76000
77000
78000
79000
80000
81000
82000
83000
84000
86000
87000
89000
93000
97000
Subject
Condition
EM
Primary

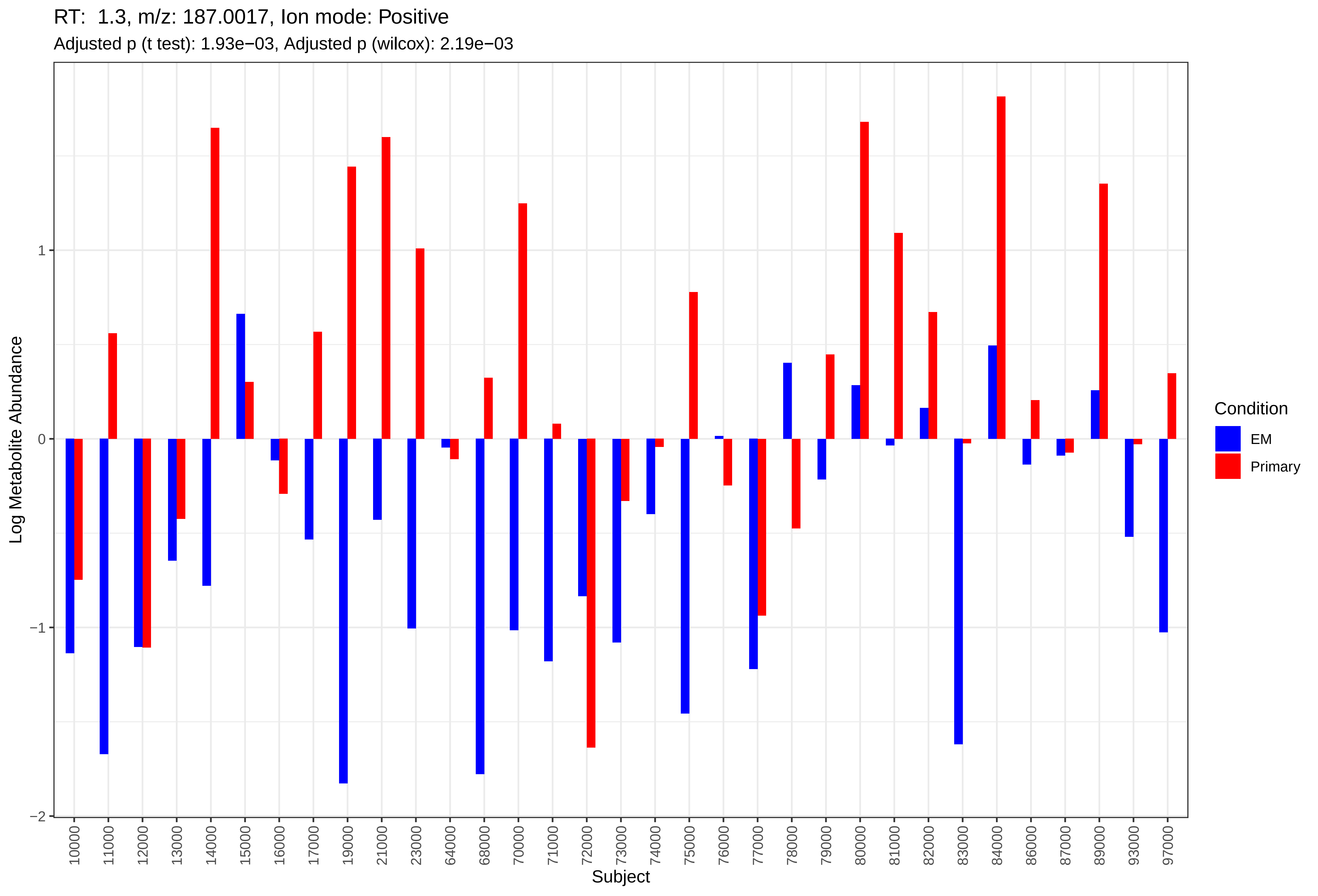

RT: 1.3, m/z: 187.0017, Ion mode: Positive
Adjusted p (t test): 1.93e−03, Adjusted p (wilcox): 2.19e−03
Log Metabolite Abundance
1
0
−1
−2
10000
11000
12000
13000
14000
15000
16000
17000
19000
21000
23000
64000
68000
70000
71000
72000
73000
74000
75000
76000
77000
78000
79000
80000
81000
82000
83000
84000
86000
87000
89000
93000
97000
Subject
Condition
EM
Primary

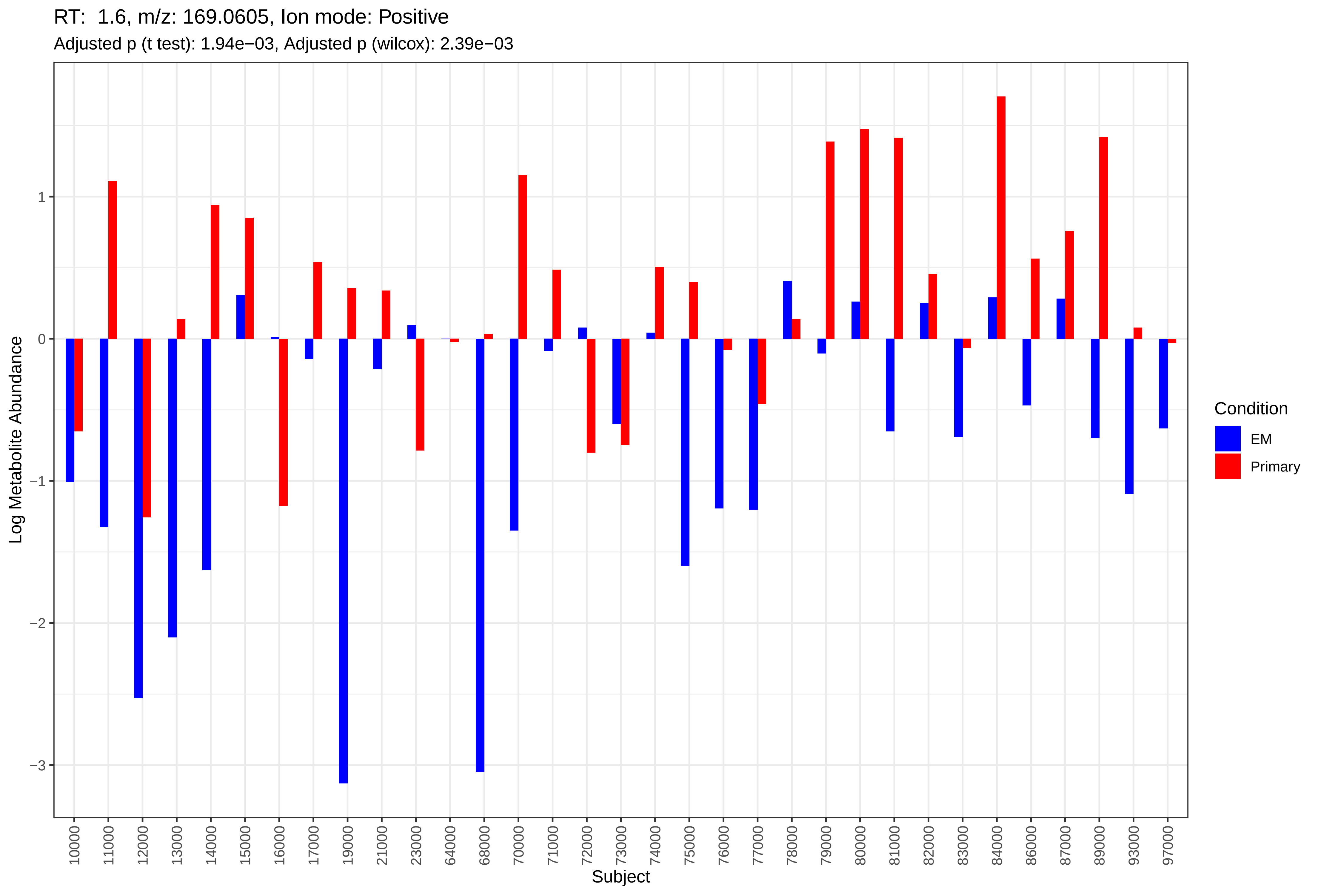

RT: 1.6, m/z: 169.0605, Ion mode: Positive
Adjusted p (t test): 1.94e−03, Adjusted p (wilcox): 2.39e−03
Log Metabolite Abundance
1
0
−1
−2
−3
10000
11000
12000
13000
14000
15000
16000
17000
19000
21000
23000
64000
68000
70000
71000
72000
73000
74000
75000
76000
77000
78000
79000
80000
81000
82000
83000
84000
86000
87000
89000
93000
97000
Subject
Condition
EM
Primary

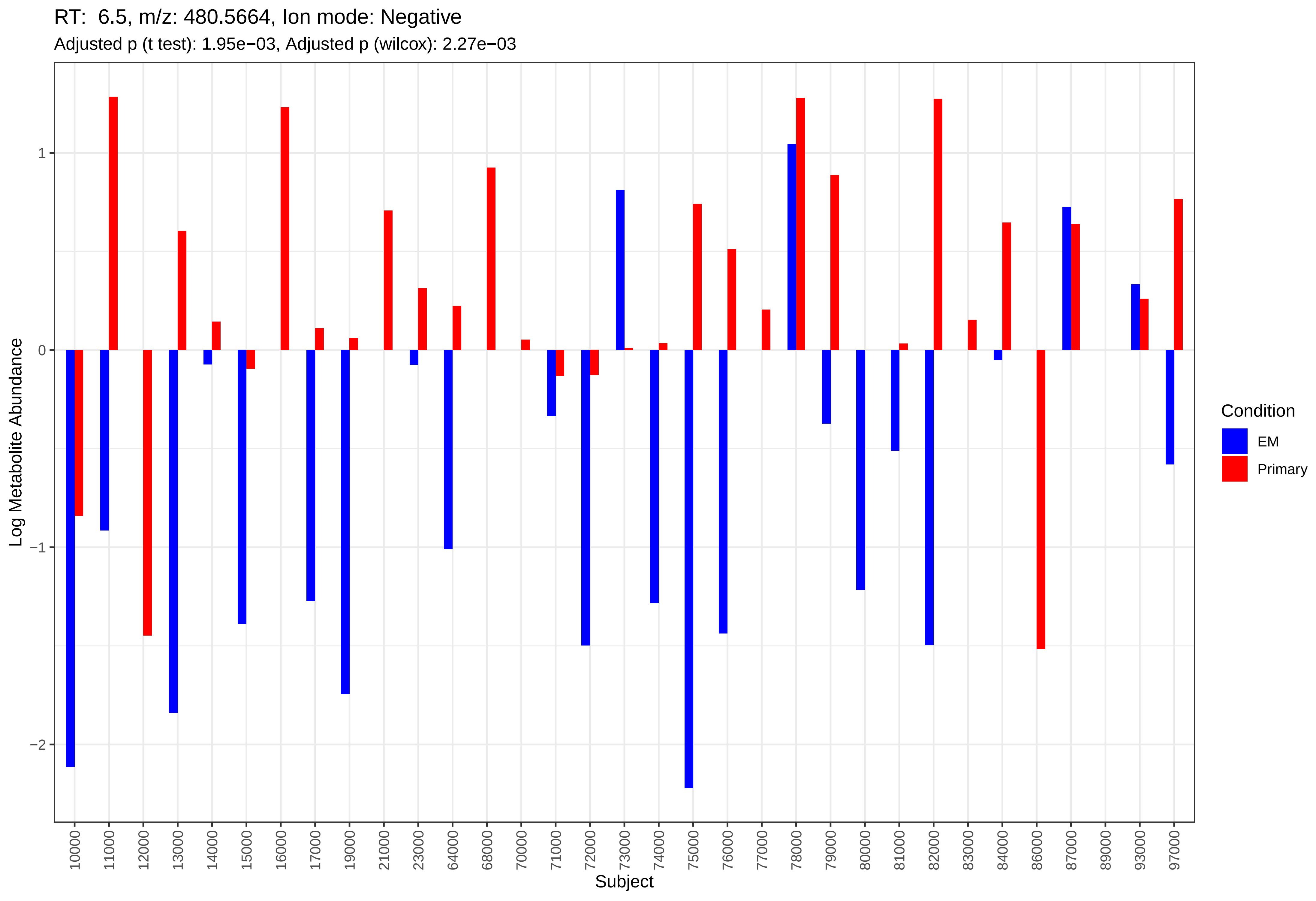

RT: 6.5, m/z: 480.5664, Ion mode: Negative
Adjusted p (t test): 1.95e−03, Adjusted p (wilcox): 2.27e−03
Log Metabolite Abundance
1
0
−1
−2
Subject
10000
11000
12000
13000
14000
15000
16000
17000
19000
21000
23000
64000
68000
70000
71000
72000
73000
74000
75000
76000
77000
78000
79000
80000
81000
82000
83000
84000
86000
87000
89000
93000
97000
Condition
EM
Primary

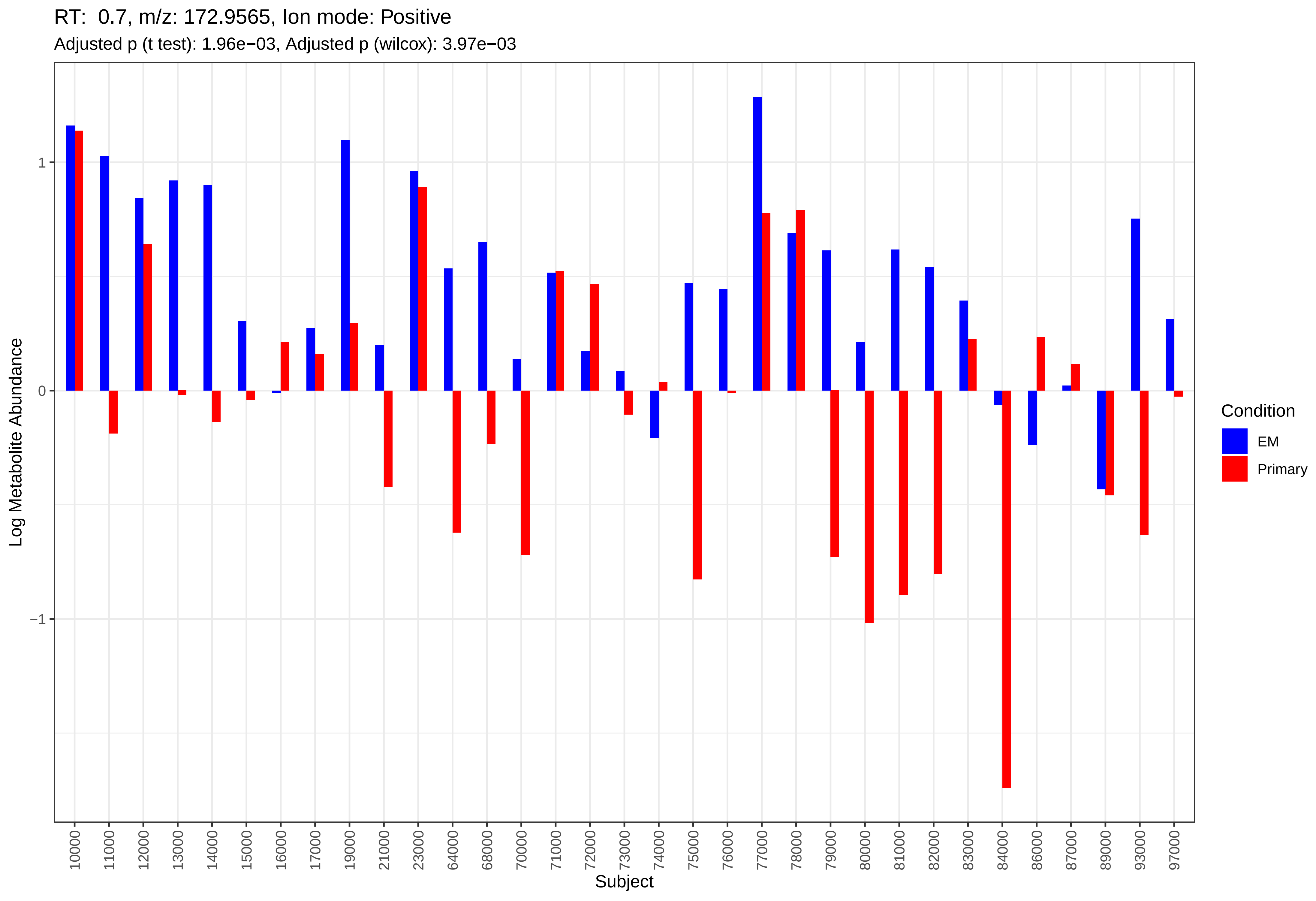

RT: 0.7, m/z: 172.9565, Ion mode: Positive
Adjusted p (t test): 1.96e−03, Adjusted p (wilcox): 3.97e−03
Log Metabolite Abundance
1
0
−1
Subject
10000
11000
12000
13000
14000
15000
16000
17000
19000
21000
23000
64000
68000
70000
71000
72000
73000
74000
75000
76000
77000
78000
79000
80000
81000
82000
83000
84000
86000
87000
89000
93000
97000
Condition
EM
Primary

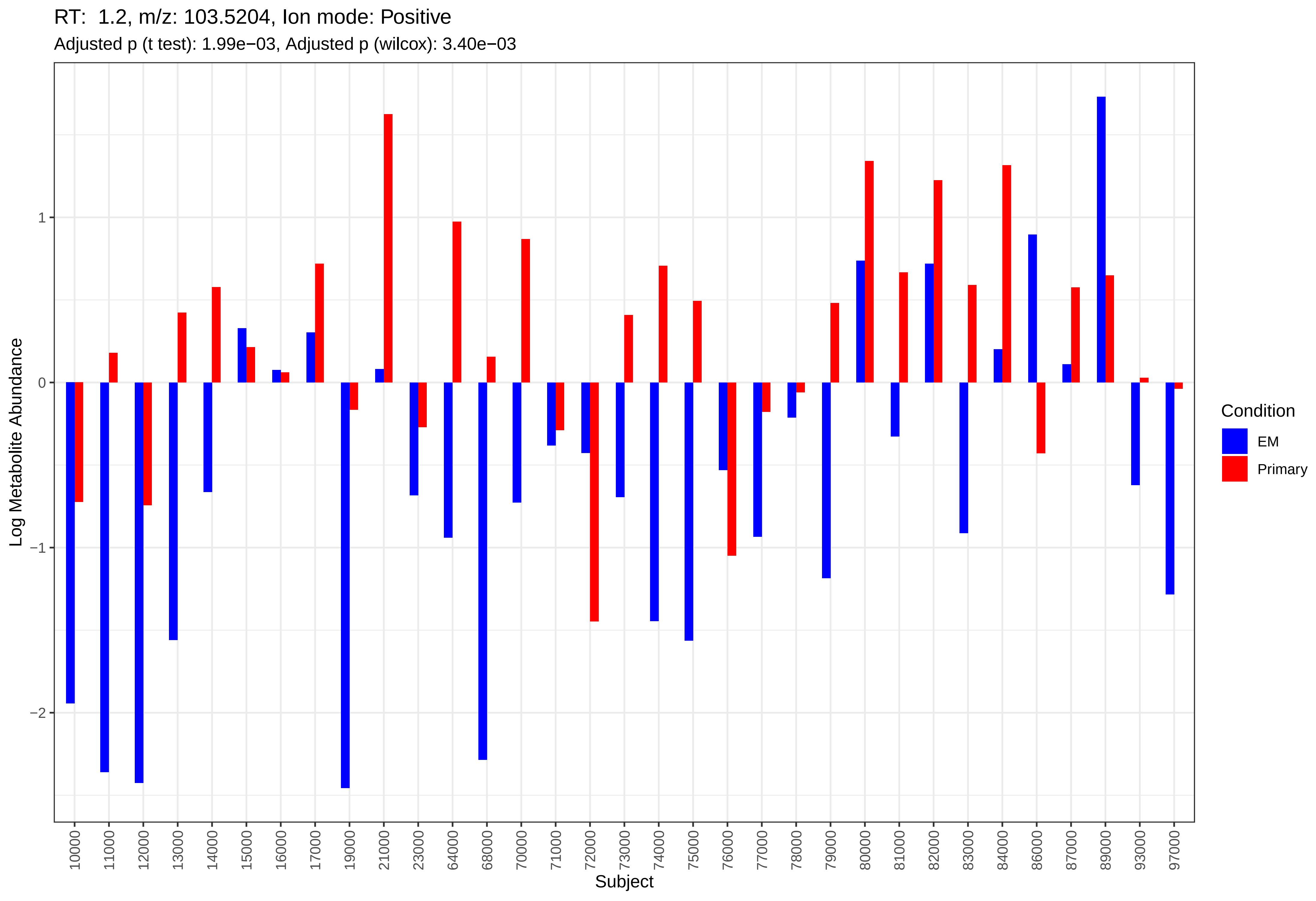

RT: 1.2, m/z: 103.5204, Ion mode: Positive
Adjusted p (t test): 1.99e−03, Adjusted p (wilcox): 3.40e−03
Log Metabolite Abundance
1
0
−1
−2
Subject
10000
11000
12000
13000
14000
15000
16000
17000
19000
21000
23000
64000
68000
70000
71000
72000
73000
74000
75000
76000
77000
78000
79000
80000
81000
82000
83000
84000
86000
87000
89000
93000
97000
Condition
EM
Primary

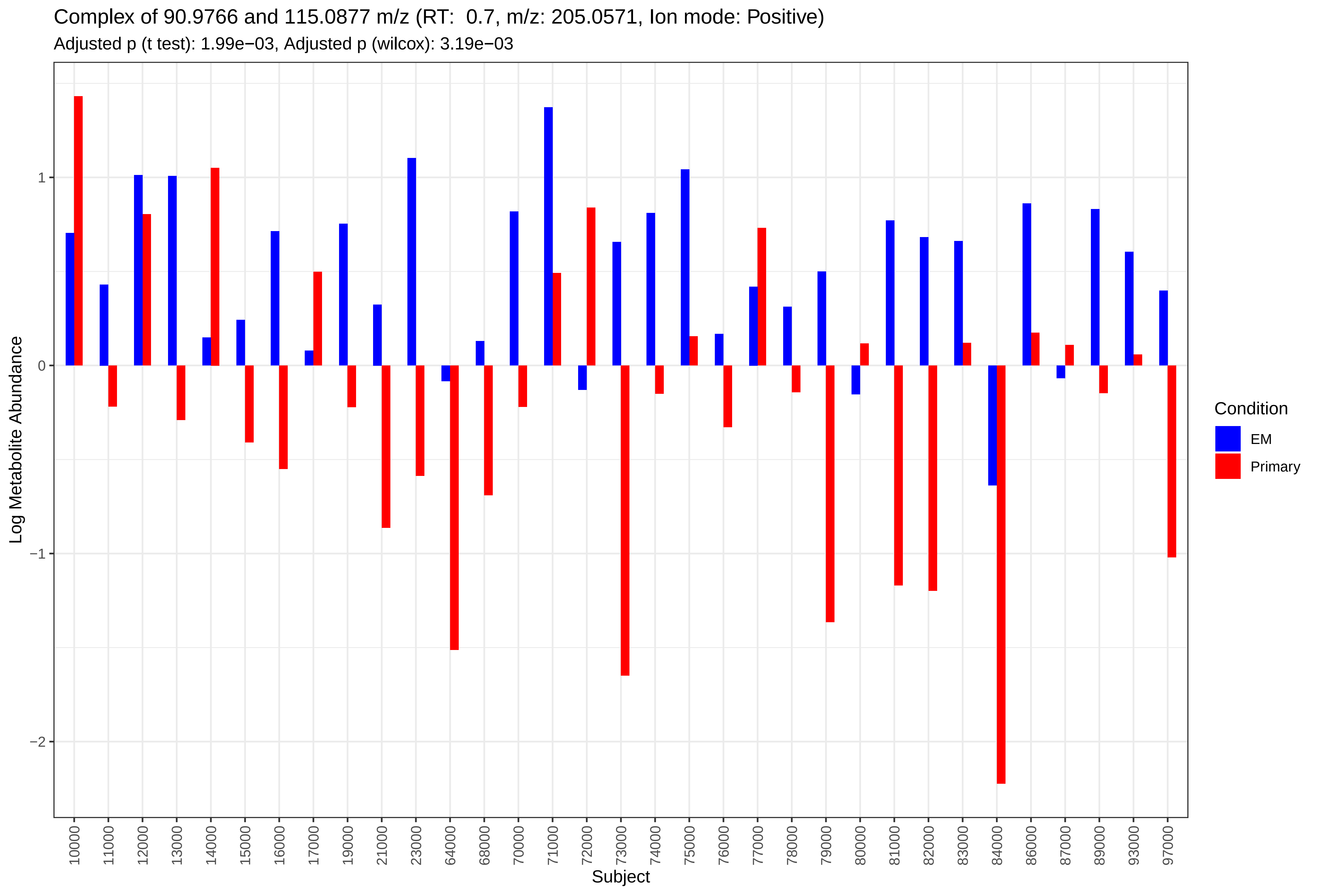

Complex of 90.9766 and 115.0877 m/z (RT: 0.7, m/z: 205.0571, Ion mode: Positive)
Adjusted p (t test): 1.99e−03, Adjusted p (wilcox): 3.19e−03
Log Metabolite Abundance
1
0
−1
−2
Subject
10000
11000
12000
13000
14000
15000
16000
17000
19000
21000
23000
64000
68000
70000
71000
72000
73000
74000
75000
76000
77000
78000
79000
80000
81000
82000
83000
84000
86000
87000
89000
93000
97000
Condition
EM
Primary

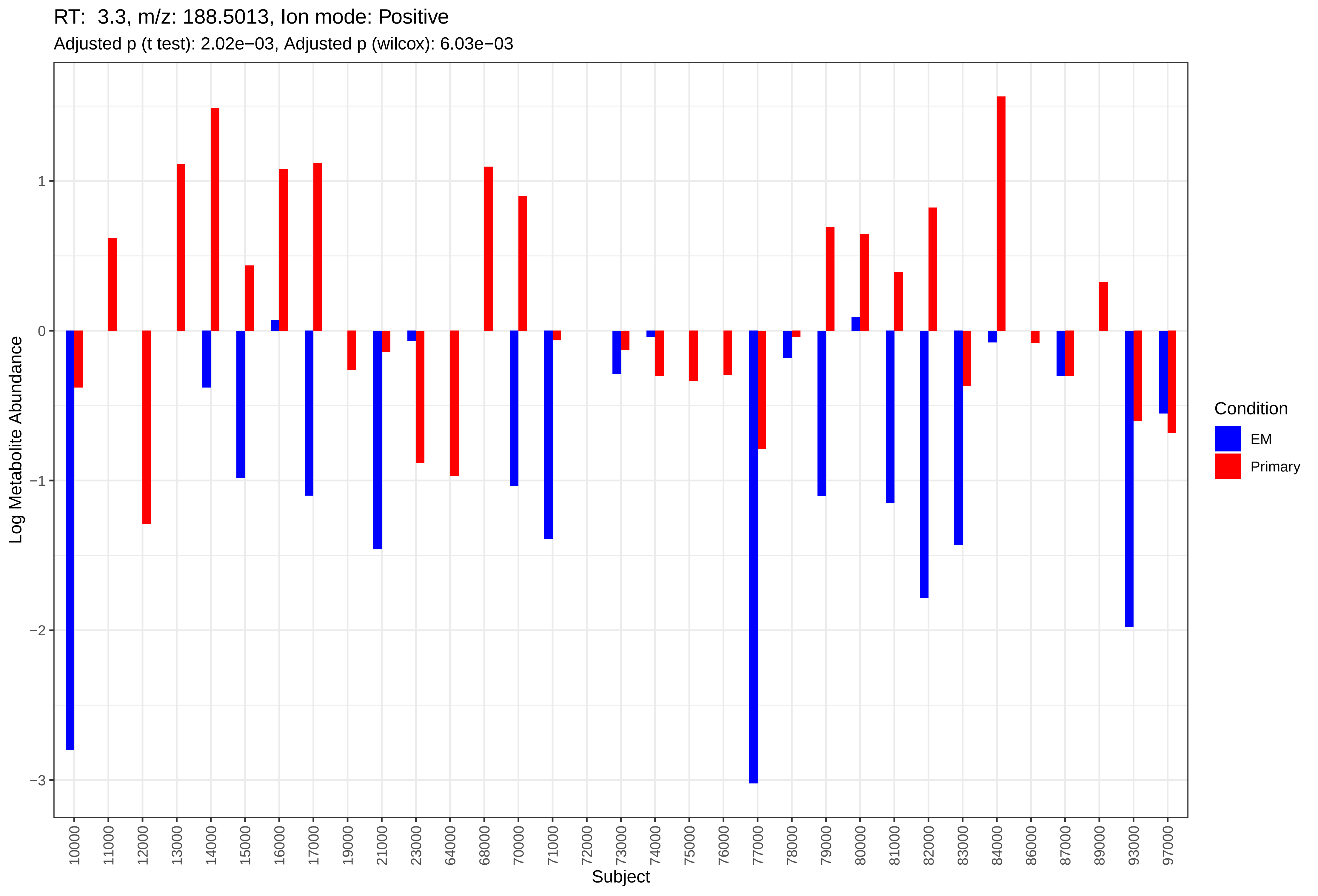

RT: 3.3, m/z: 188.5013, Ion mode: Positive
Adjusted p (t test): 2.02e−03, Adjusted p (wilcox): 6.03e−03
Log Metabolite Abundance
1
0
−1
−2
−3
10000
11000
12000
13000
14000
15000
16000
17000
19000
21000
23000
64000
68000
70000
71000
72000
73000
74000
75000
76000
77000
78000
79000
80000
81000
82000
83000
84000
86000
87000
89000
93000
97000
Subject
Condition
EM
Primary

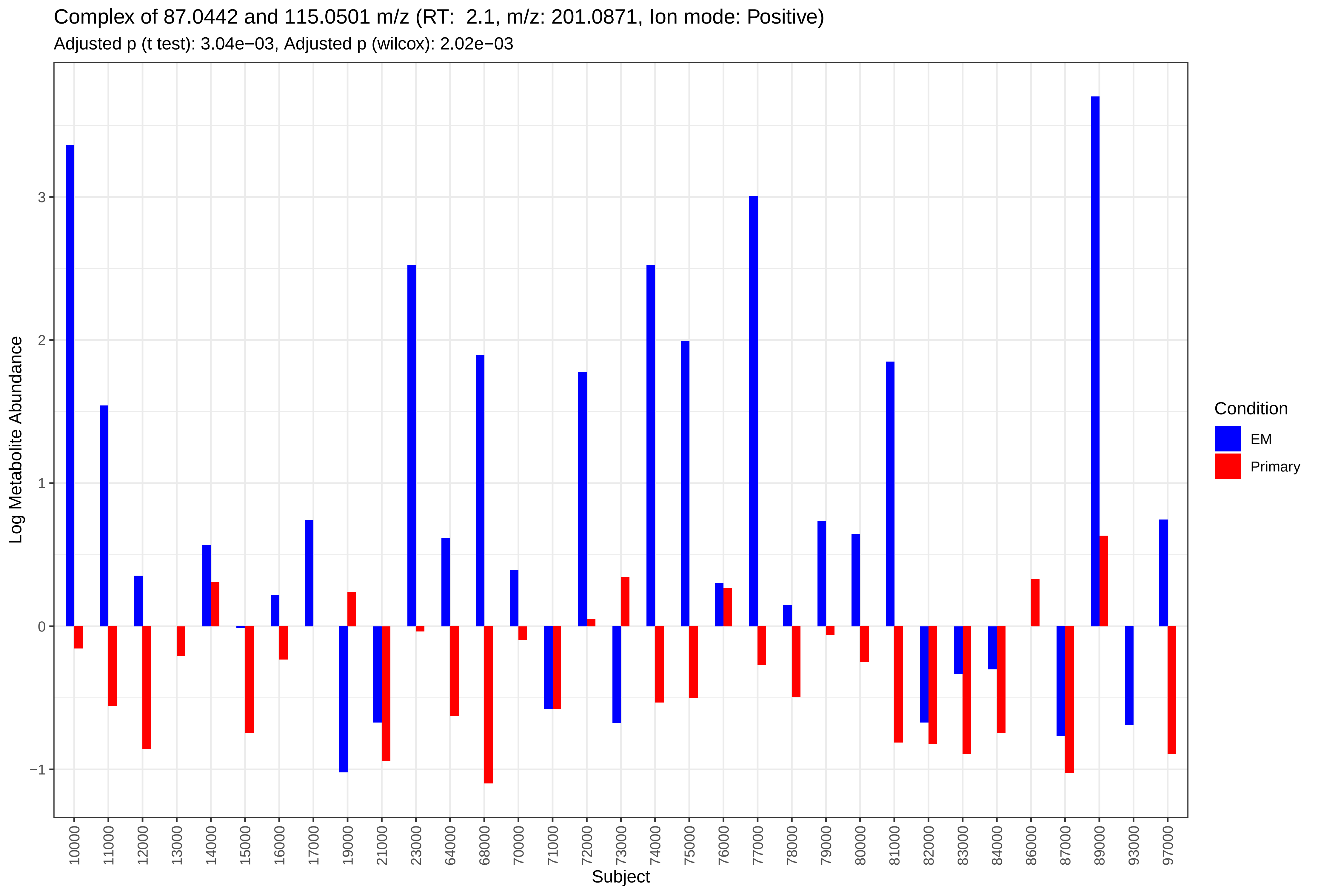

Complex of 87.0442 and 115.0501 m/z (RT: 2.1, m/z: 201.0871, Ion mode: Positive)
Adjusted p (t test): 3.04e−03, Adjusted p (wilcox): 2.02e−03
Log Metabolite Abundance
3
2
1
0
−1
10000
11000
12000
13000
14000
15000
16000
17000
19000
21000
23000
64000
68000
70000
71000
72000
73000
74000
75000
76000
77000
78000
79000
80000
81000
82000
83000
84000
86000
87000
89000
93000
97000
Subject
Condition
EM
Primary

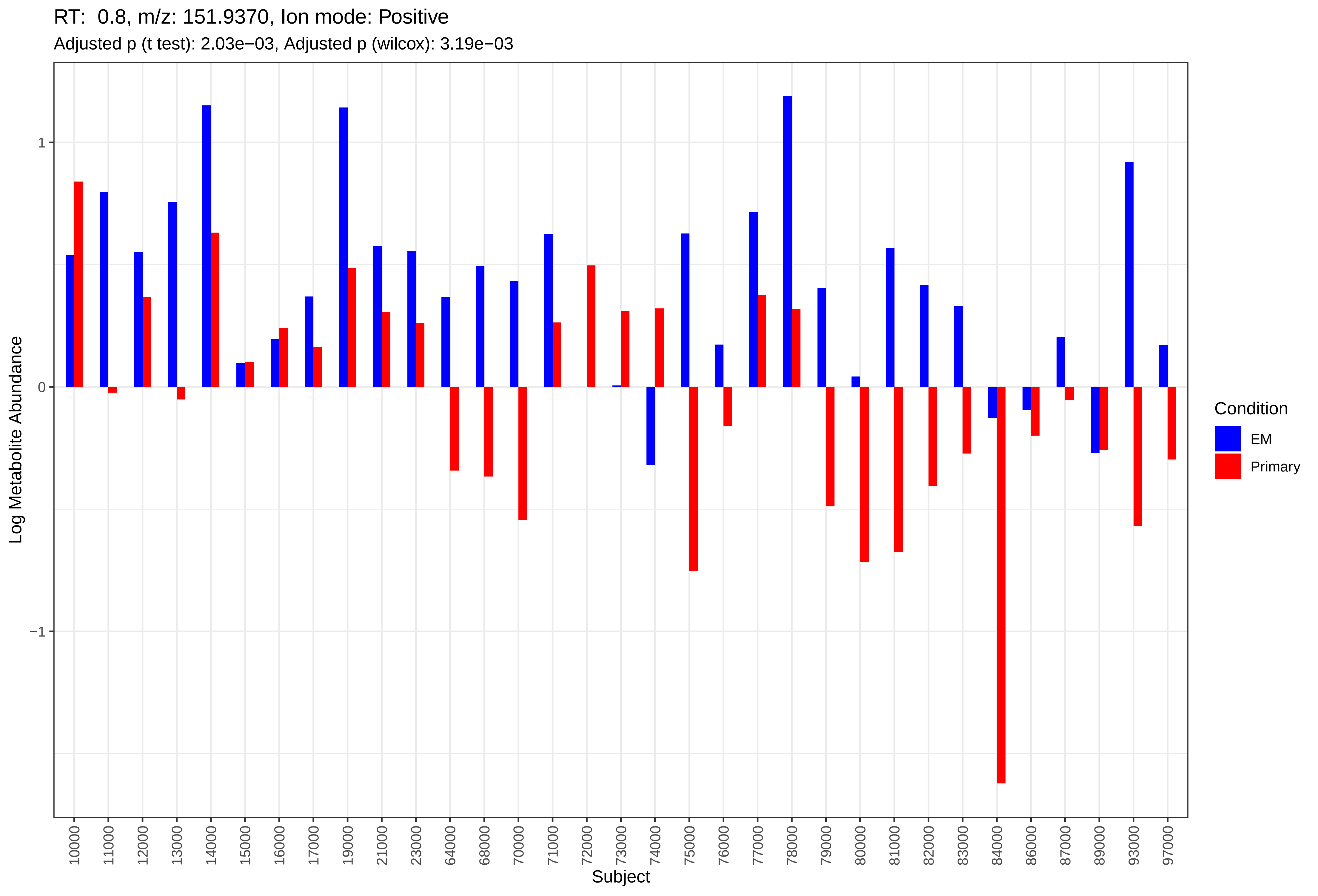
RT: 0.8, m/z: 151.9370, Ion mode: Positive
Adjusted p (t test): 2.03e−03, Adjusted p (wilcox): 3.19e−03
Log Metabolite Abundance
1
0
−1
Subject
10000
11000
12000
13000
14000
15000
16000
17000
19000
21000
23000
64000
68000
70000
71000
72000
73000
74000
75000
76000
77000
78000
79000
80000
81000
82000
83000
84000
86000
87000
89000
93000
97000
Condition
EM
Primary

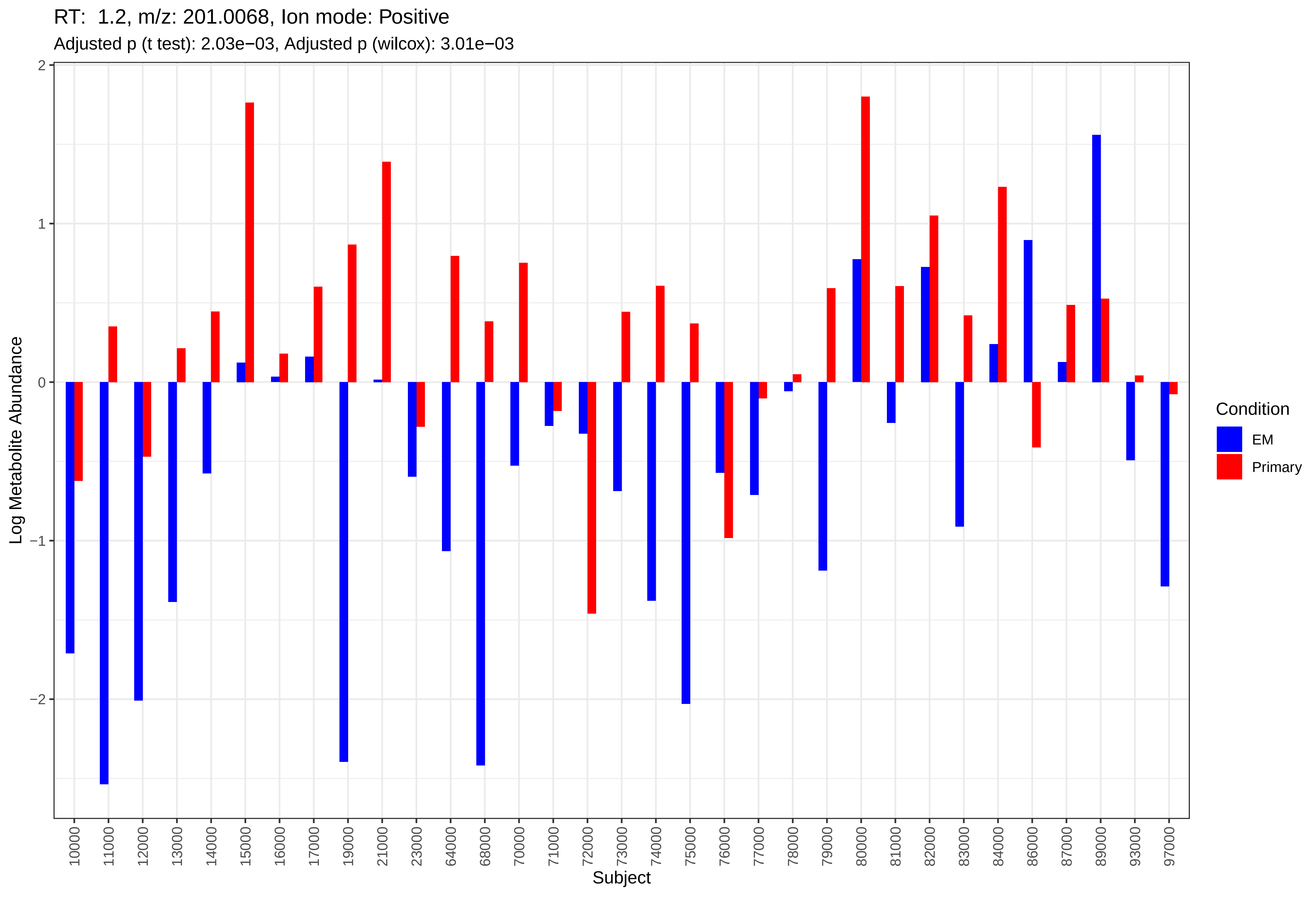

RT: 1.2, m/z: 201.0068, Ion mode: Positive
Adjusted p (t test): 2.03e−03, Adjusted p (wilcox): 3.01e−03
Log Metabolite Abundance
2
1
0
−1
−2
10000
11000
12000
13000
14000
15000
16000
17000
19000
21000
23000
64000
68000
70000
71000
72000
73000
74000
75000
76000
77000
78000
79000
80000
81000
82000
83000
84000
86000
87000
89000
93000
97000
Subject
Condition
EM
Primary

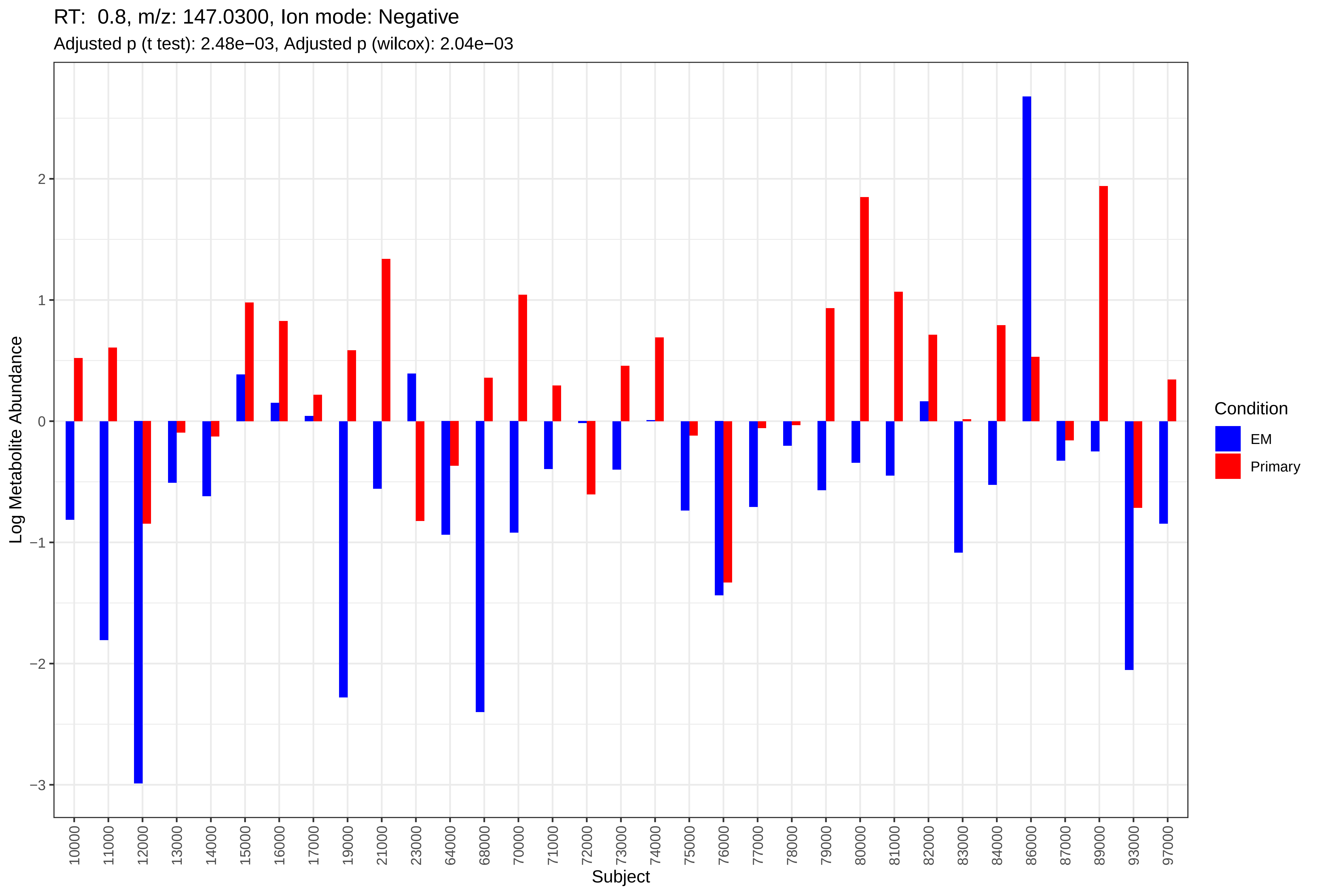
RT: 0.8, m/z: 147.0300, Ion mode: Negative
Adjusted p (t test): 2.48e−03, Adjusted p (wilcox): 2.04e−03
Log Metabolite Abundance
2
1
0
−1
−2
−3
Subject
10000
11000
12000
13000
14000
15000
16000
17000
19000
21000
23000
64000
68000
70000
71000
72000
73000
74000
75000
76000
77000
78000
79000
80000
81000
82000
83000
84000
86000
87000
89000
93000
97000
Condition
EM
Primary

RT: 0.8, m/z: 447.0847, Ion mode: Negative

Adjusted p (t test): 5.32e−03, Adjusted p (wilcox): 2.04e−03

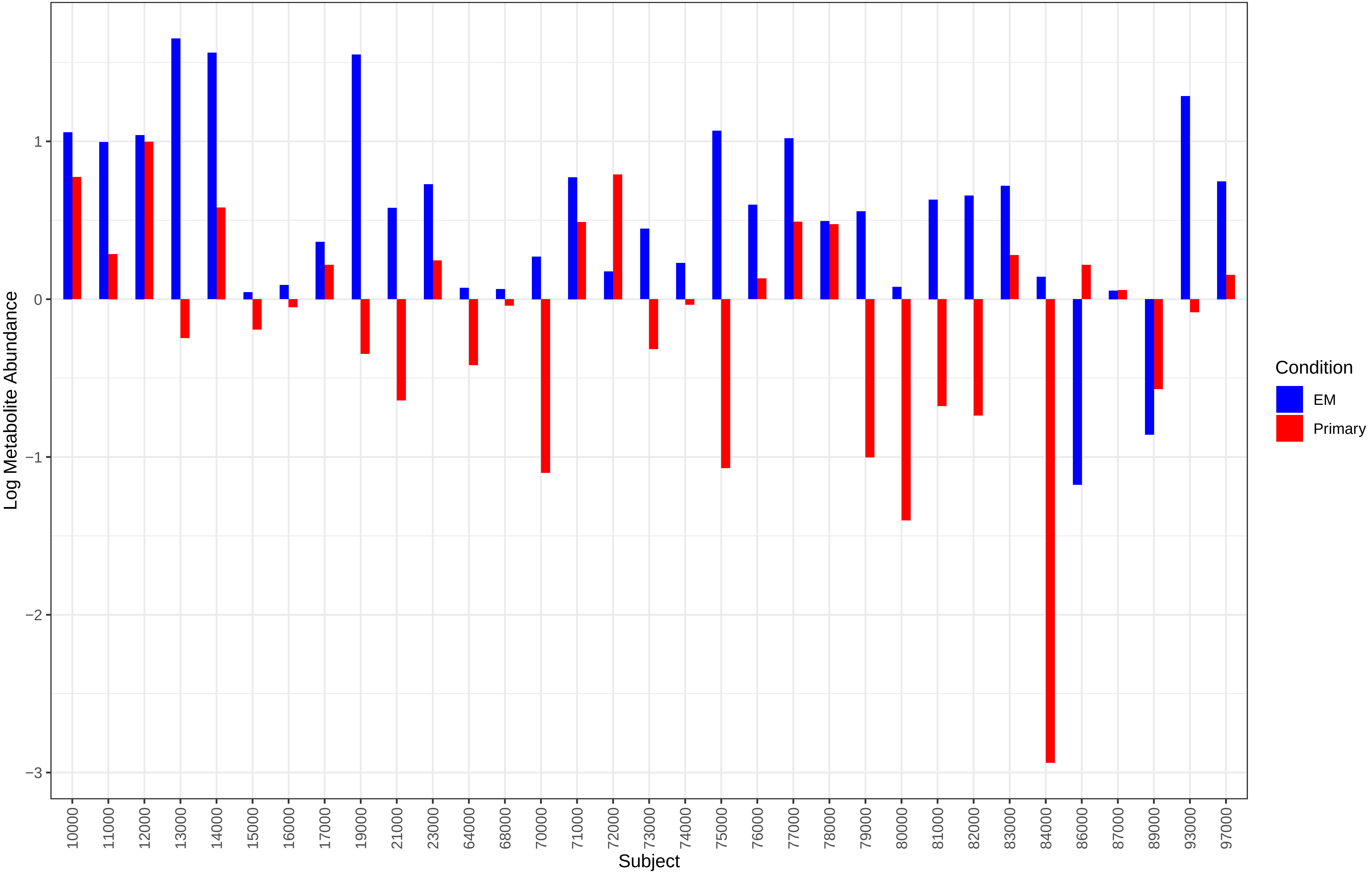

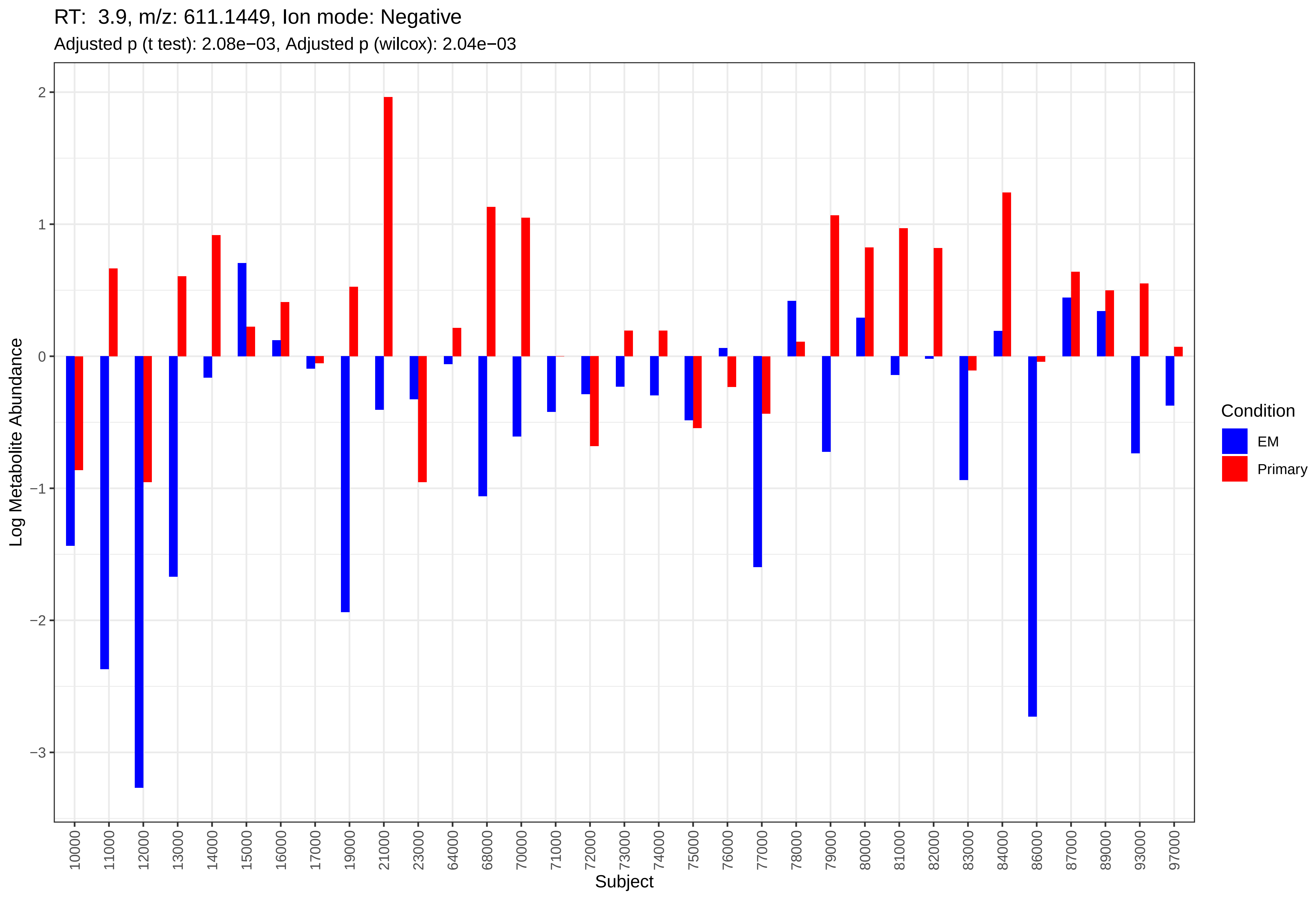

RT: 3.9, m/z: 611.1449, Ion mode: Negative
Adjusted p (t test): 2.08e−03, Adjusted p (wilcox): 2.04e−03
Log Metabolite Abundance
2
1
0
−1
−2
−3
10000
11000
12000
13000
14000
15000
16000
17000
19000
21000
23000
64000
68000
70000
71000
72000
73000
74000
75000
76000
77000
78000
79000
80000
81000
82000
83000
84000
86000
87000
89000
93000
97000
Subject
Condition
EM
Primary

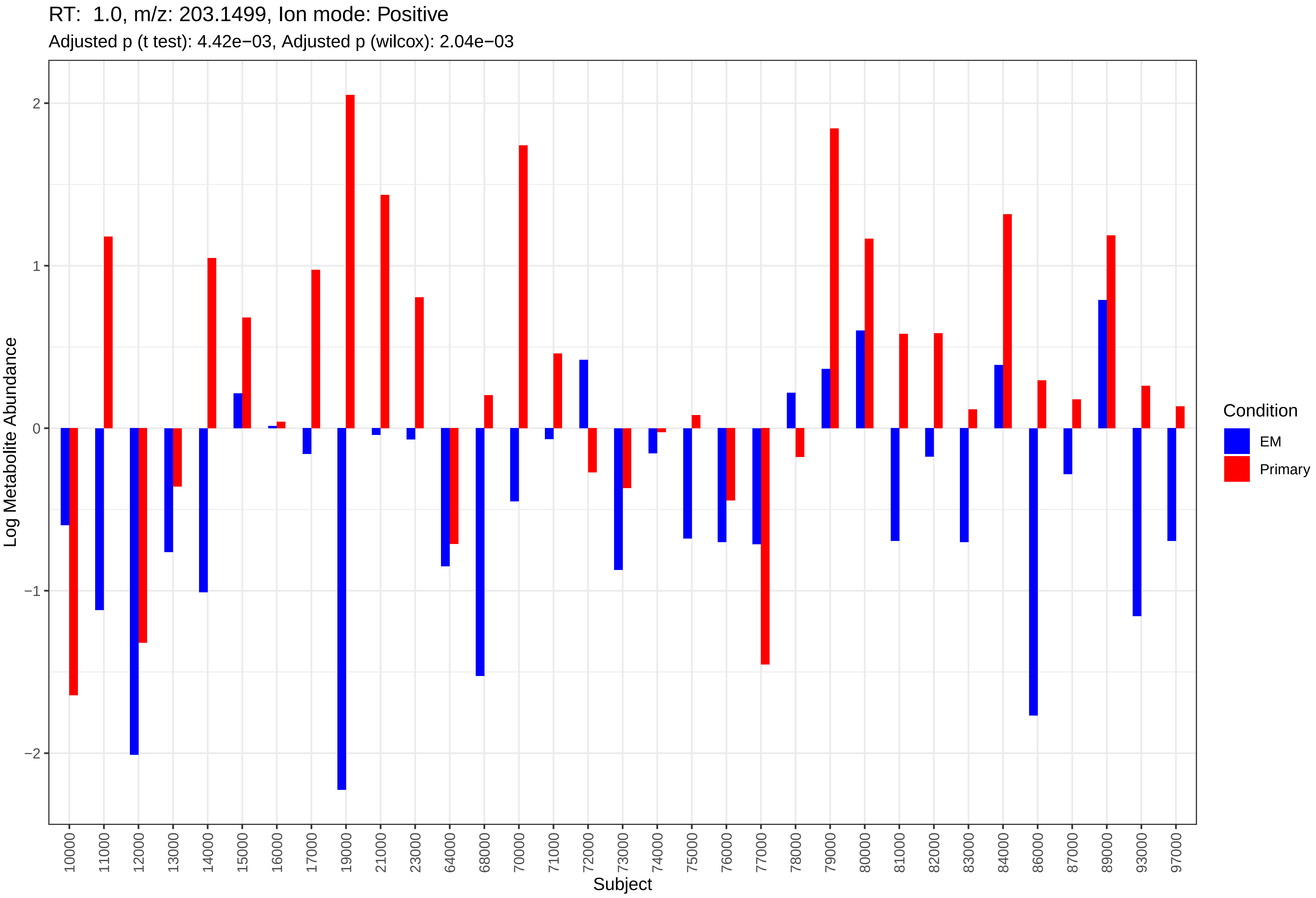
RT: 1.0, m/z: 203.1499, Ion mode: Positive
Adjusted p (t test): 4.42e−03, Adjusted p (wilcox): 2.04e−03
Log Metabolite Abundance
2
1
0
−1
−2
10000
11000
12000
13000
14000
15000
16000
17000
19000
21000
23000
64000
68000
70000
71000
72000
73000
74000
75000
76000
77000
78000
79000
80000
81000
82000
83000
84000
86000
87000
89000
93000
97000
Subject
Condition
EM
Primary

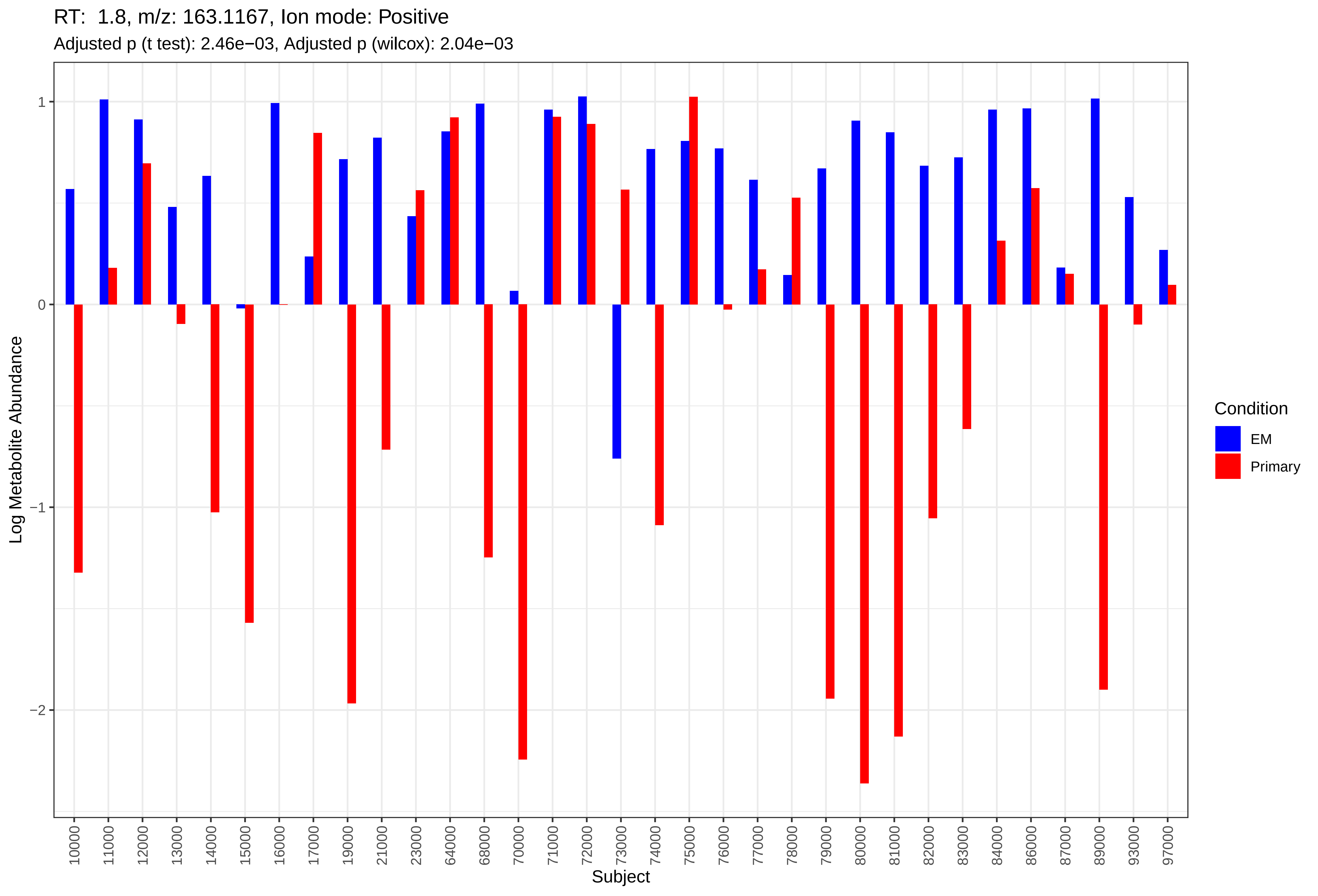
RT: 1.8, m/z: 163.1167, Ion mode: Positive
Adjusted p (t test): 2.46e−03, Adjusted p (wilcox): 2.04e−03
Log Metabolite Abundance
1
0
−1
−2
Subject
10000
11000
12000
13000
14000
15000
16000
17000
19000
21000
23000
64000
68000
70000
71000
72000
73000
74000
75000
76000
77000
78000
79000
80000
81000
82000
83000
84000
86000
87000
89000
93000
97000
Condition
EM
Primary

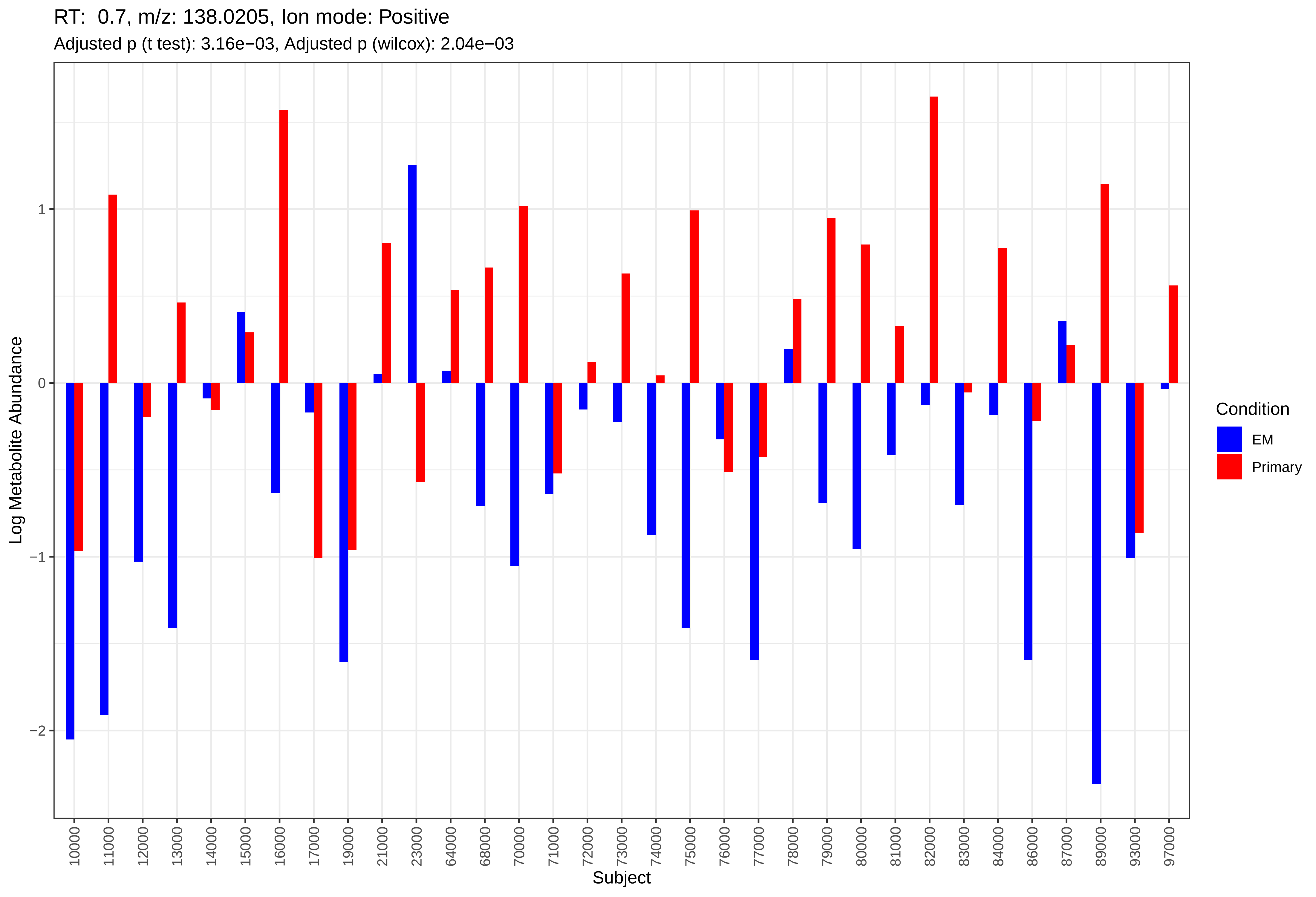

RT: 0.7, m/z: 138.0205, Ion mode: Positive
Adjusted p (t test): 3.16e−03, Adjusted p (wilcox): 2.04e−03
Log Metabolite Abundance
1
0
−1
−2
Subject
10000
11000
12000
13000
14000
15000
16000
17000
19000
21000
23000
64000
68000
70000
71000
72000
73000
74000
75000
76000
77000
78000
79000
80000
81000
82000
83000
84000
86000
87000
89000
93000
97000
Condition
EM
Primary

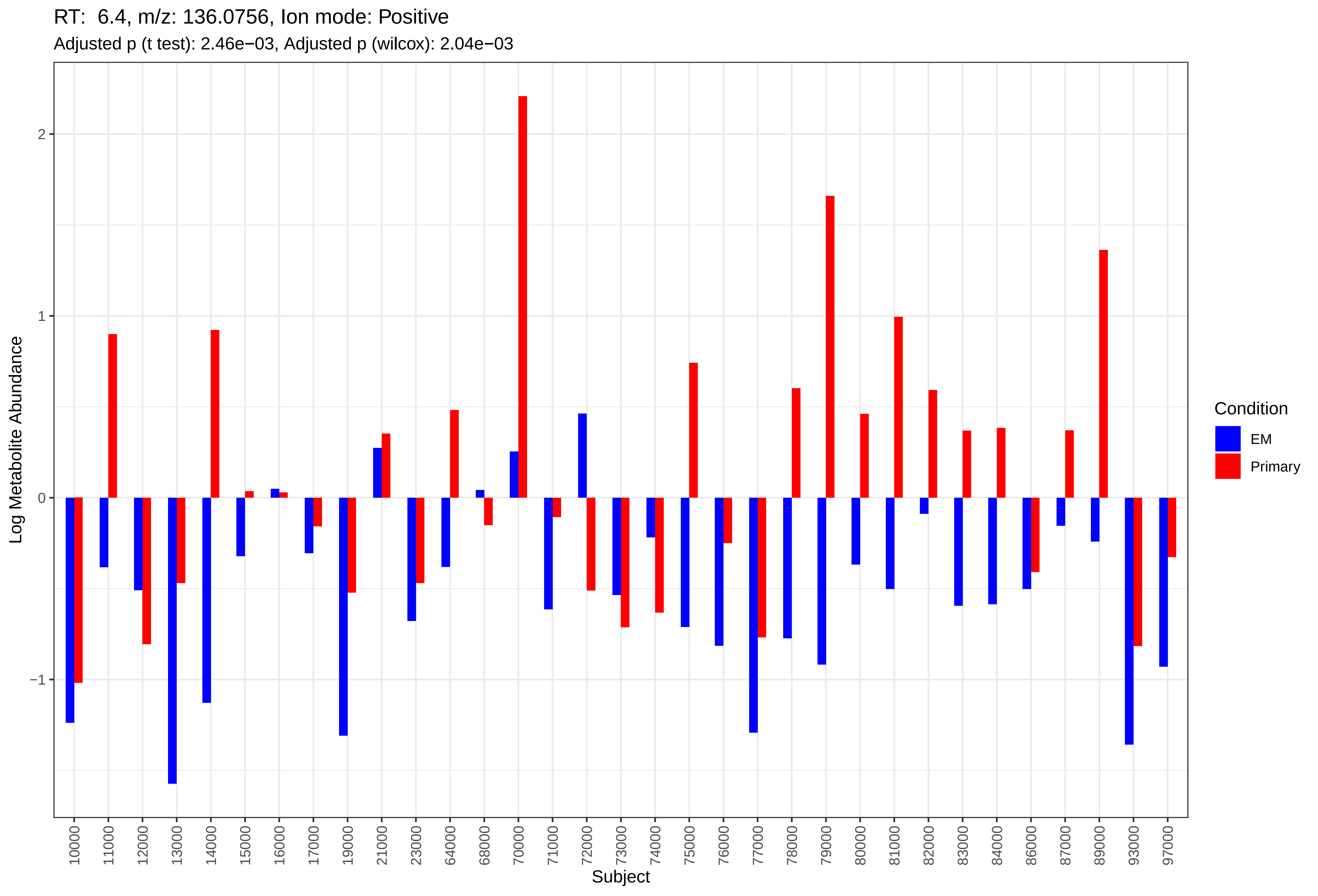

RT: 6.4, m/z: 136.0756, Ion mode: Positive
Adjusted p (t test): 2.46e−03, Adjusted p (wilcox): 2.04e−03
Log Metabolite Abundance
2
1
0
−1
10000
11000
12000
13000
14000
15000
16000
17000
19000
21000
23000
64000
68000
70000
71000
72000
73000
74000
75000
76000
77000
78000
79000
80000
81000
82000
83000
84000
86000
87000
89000
93000
97000
Subject
Condition
EM
Primary

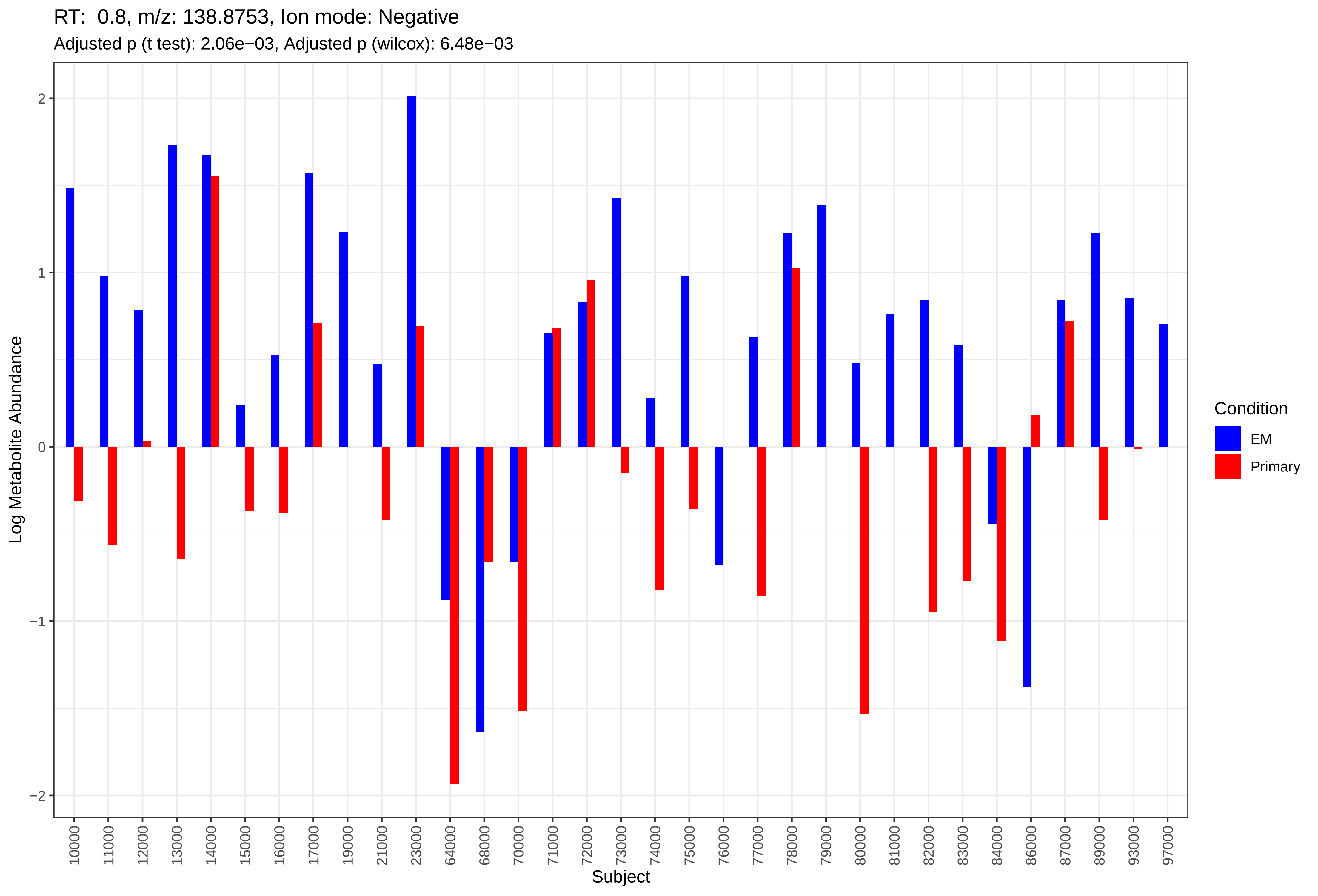
RT: 0.8, m/z: 138.8753, Ion mode: Negative
Adjusted p (t test): 2.06e−03, Adjusted p (wilcox): 6.48e−03
Log Metabolite Abundance
2
1
0
−1
−2
10000
11000
12000
13000
14000
15000
16000
17000
19000
21000
23000
64000
68000
70000
71000
72000
73000
74000
75000
76000
77000
78000
79000
80000
81000
82000
83000
84000
86000
87000
89000
93000
97000
Subject
Condition
EM
Primary

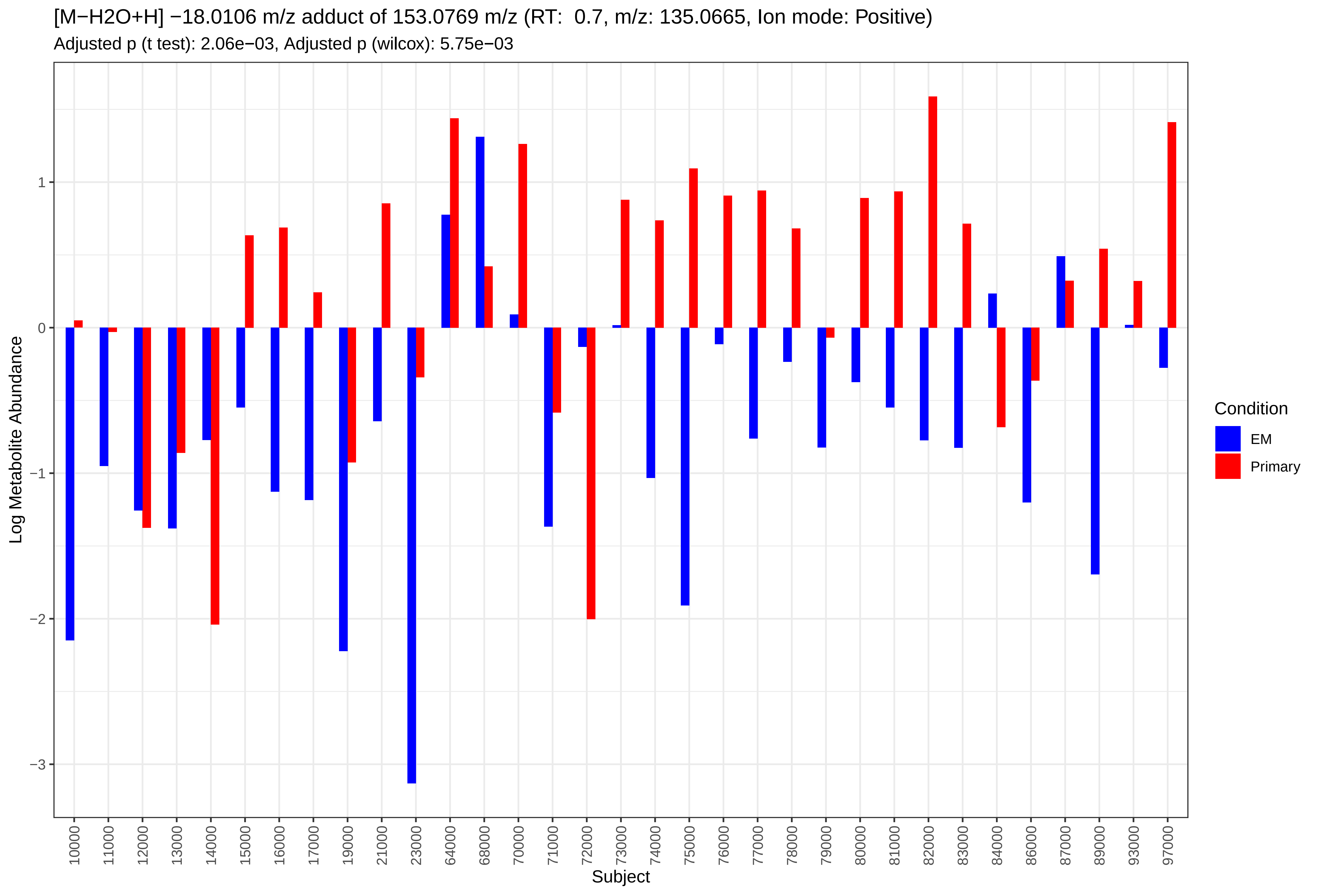
[M−H2O+H] −18.0106 m/z adduct of 153.0769 m/z (RT: 0.7, m/z: 135.0665, Ion mode: Positive)
Adjusted p (t test): 2.06e−03, Adjusted p (wilcox): 5.75e−03
Log Metabolite Abundance
1
0
−1
−2
−3
10000
11000
12000
13000
14000
15000
16000
17000
19000
21000
23000
64000
68000
70000
71000
72000
73000
74000
75000
76000
77000
78000
79000
80000
81000
82000
83000
84000
86000
87000
89000
93000
97000
Subject
Condition
EM
Primary

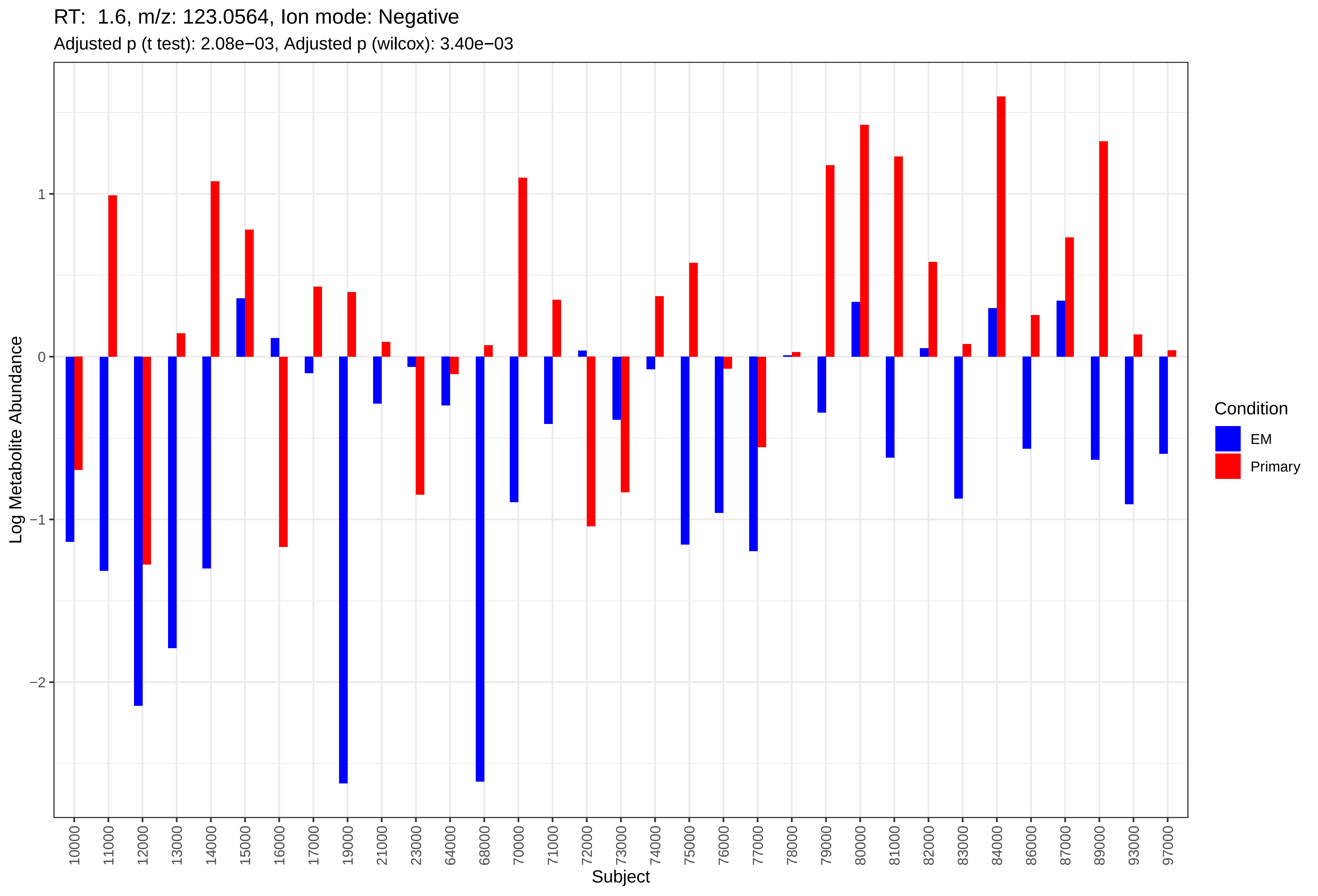

RT: 1.6, m/z: 123.0564, Ion mode: Negative
Adjusted p (t test): 2.08e−03, Adjusted p (wilcox): 3.40e−03
Log Metabolite Abundance
1
0
−1
−2
Subject
10000
11000
12000
13000
14000
15000
16000
17000
19000
21000
23000
64000
68000
70000
71000
72000
73000
74000
75000
76000
77000
78000
79000
80000
81000
82000
83000
84000
86000
87000
89000
93000
97000
Condition
EM
Primary

Complex of 114.0661 and 114.0661 m/z (RT: 0.9, m/z: 227.1241, Ion mode: Positive)

Adjusted p (t test): 2.08e−03, Adjusted p (wilcox): 2.19e−03

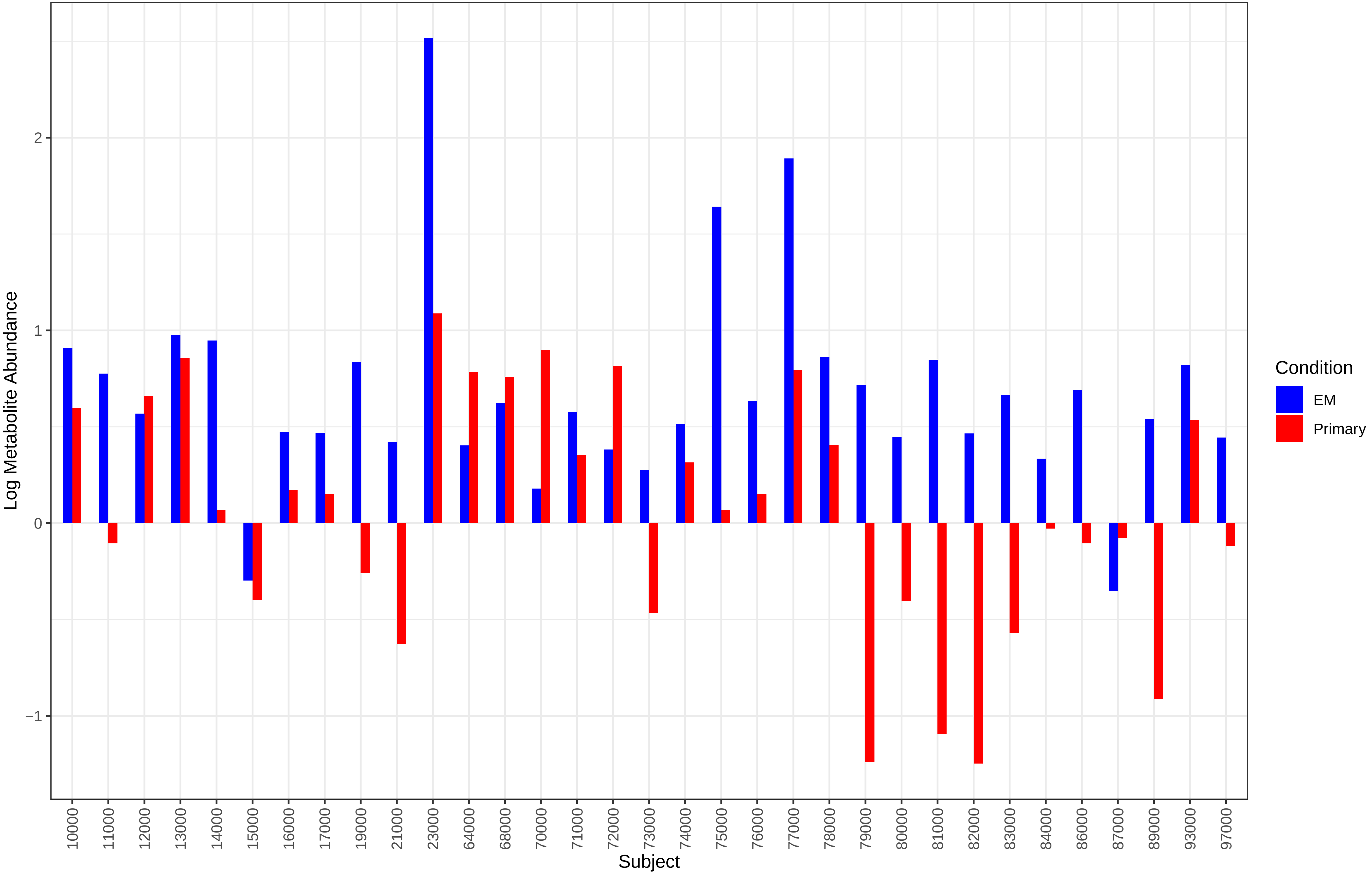

RT: 1.9, m/z: 185.0552, Ion mode: Positive

Adjusted p (t test): 2.08e−03, Adjusted p (wilcox): 3.19e−03

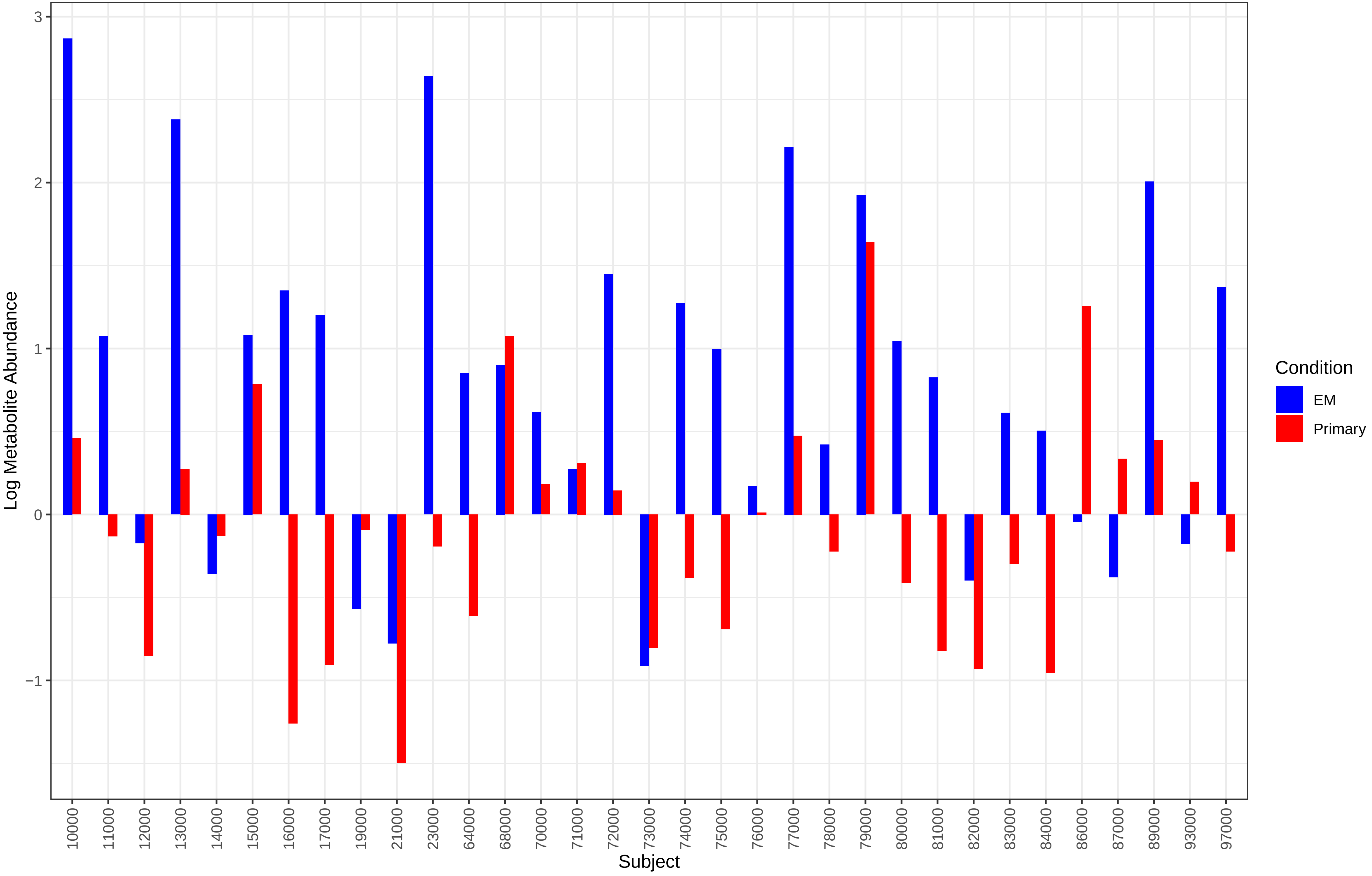

RT: 2.2, m/z: 221.0917, Ion mode: Positive

Adjusted p (t test): 2.08e−03, Adjusted p (wilcox): 2.52e−03

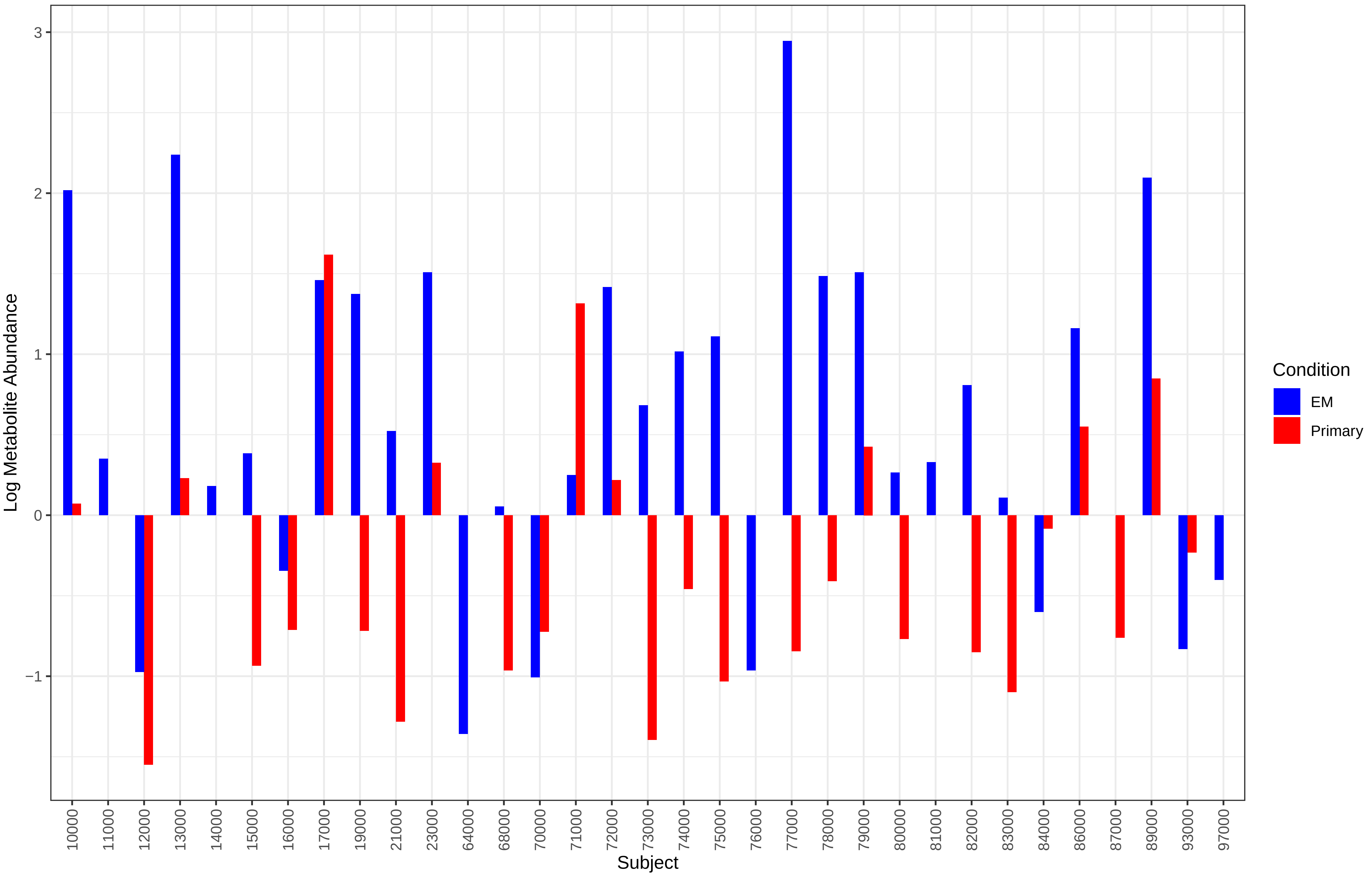

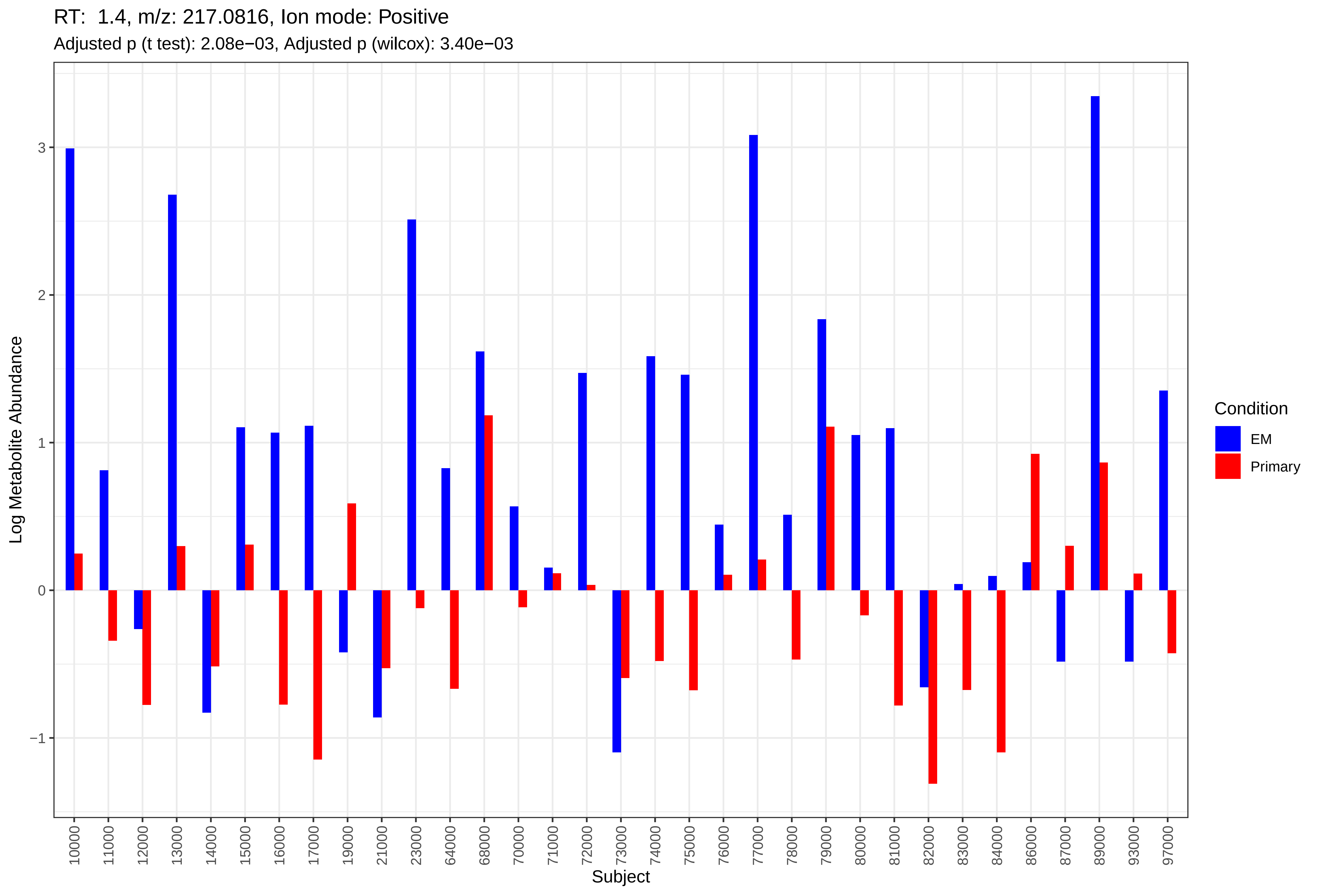
RT: 1.4, m/z: 217.0816, Ion mode: Positive
Adjusted p (t test): 2.08e−03, Adjusted p (wilcox): 3.40e−03
Log Metabolite Abundance
Subject
Condition
EM
Primary
3
2
1
0
−1
10000
11000
12000
13000
14000
15000
16000
17000
19000
21000
23000
64000
68000
70000
71000
72000
73000
74000
75000
76000
77000
78000
79000
80000
81000
82000
83000
84000
86000
87000
89000
93000
97000

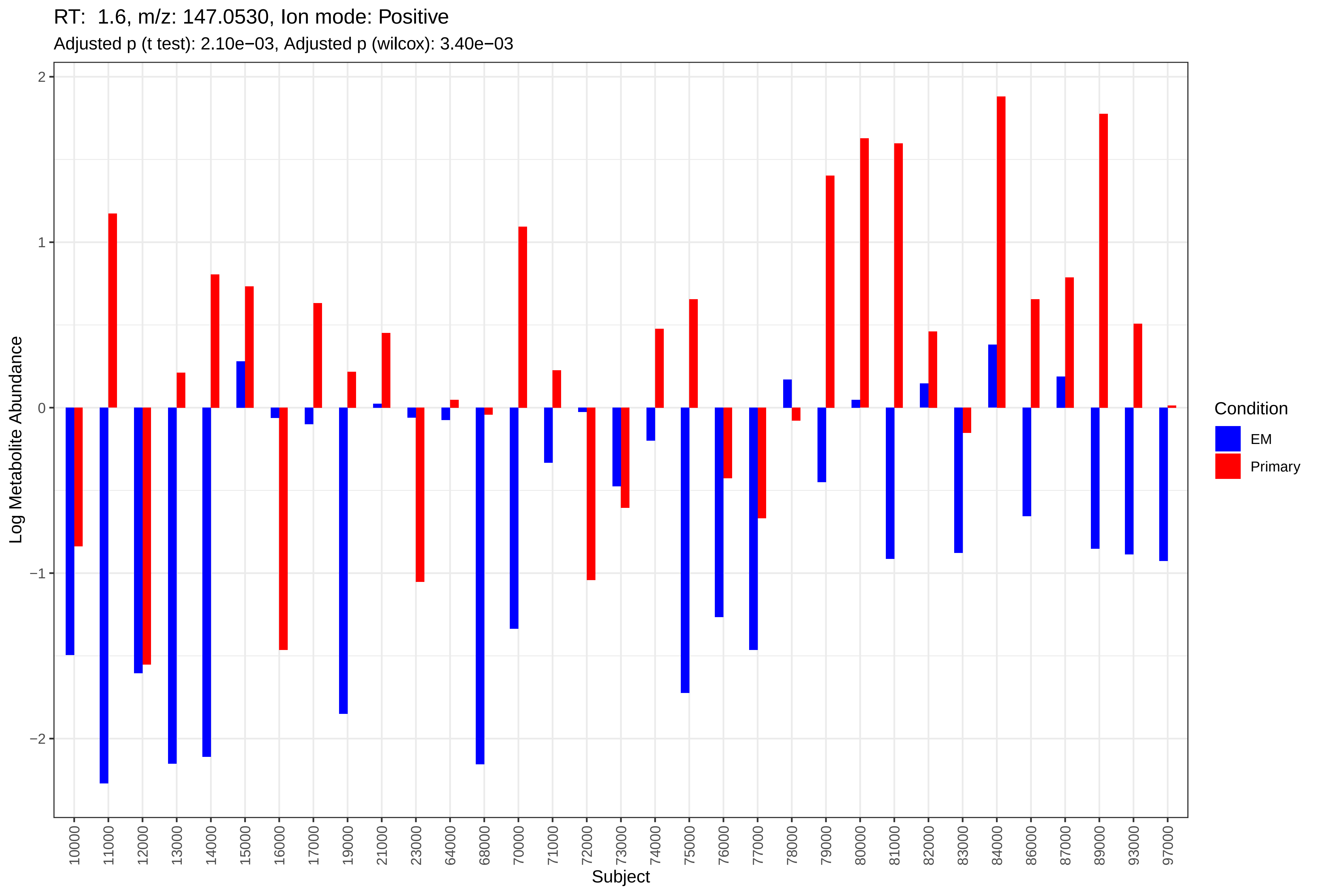
RT: 1.6, m/z: 147.0530, Ion mode: Positive
Adjusted p (t test): 2.10e−03, Adjusted p (wilcox): 3.40e−03
Log Metabolite Abundance
2
1
0
−1
−2
Subject
10000
11000
12000
13000
14000
15000
16000
17000
19000
21000
23000
64000
68000
70000
71000
72000
73000
74000
75000
76000
77000
78000
79000
80000
81000
82000
83000
84000
86000
87000
89000
93000
97000
Condition
EM
Primary

RT: 1.6, m/z: 124.0631, Ion mode: Positive

Adjusted p (t test): 2.11e−03, Adjusted p (wilcox): 2.19e−03

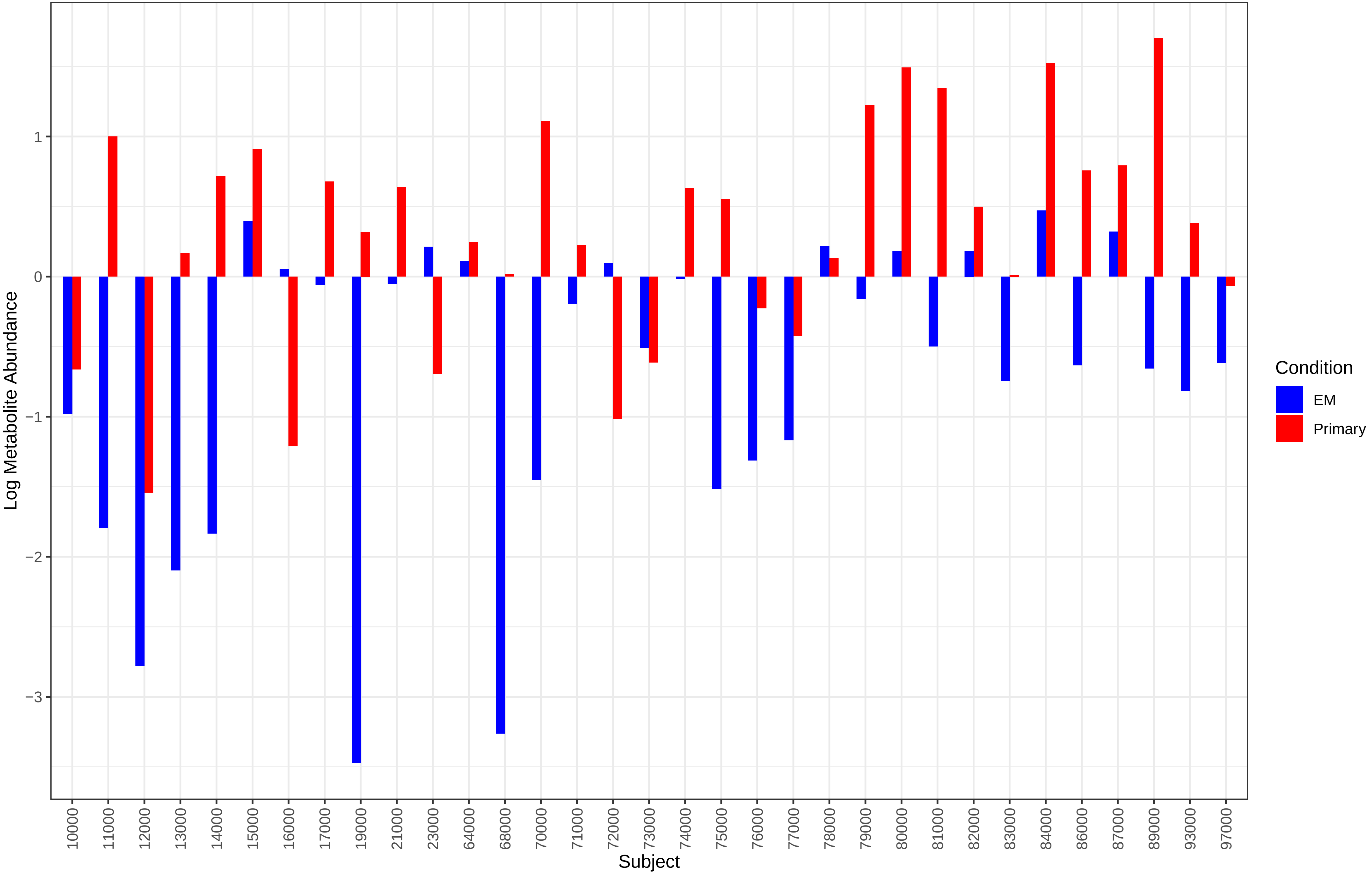

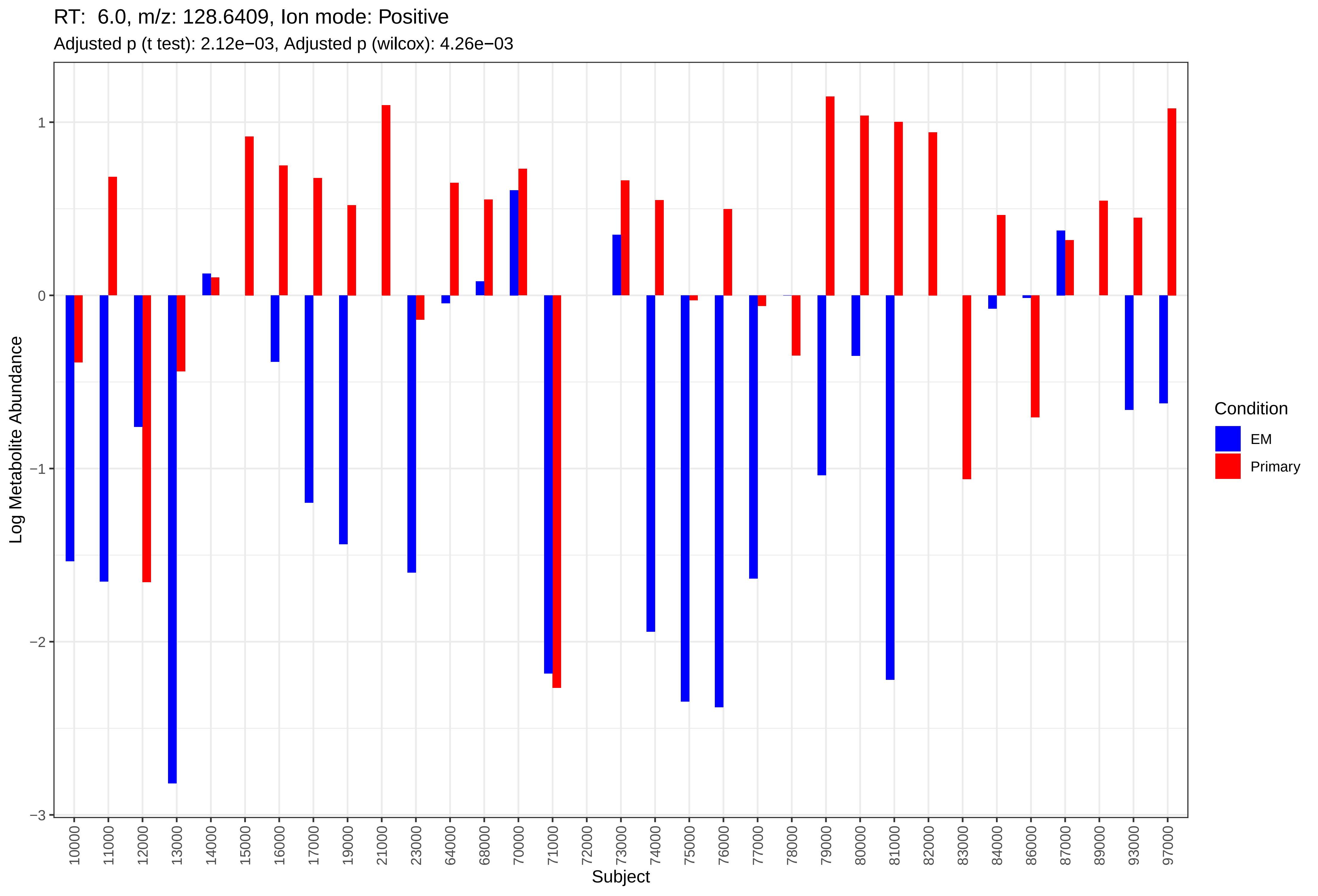
RT: 6.0, m/z: 128.6409, Ion mode: Positive
Adjusted p (t test): 2.12e−03, Adjusted p (wilcox): 4.26e−03
Log Metabolite Abundance
1
0
−1
−2
−3
10000
11000
12000
13000
14000
15000
16000
17000
19000
21000
23000
64000
68000
70000
71000
72000
73000
74000
75000
76000
77000
78000
79000
80000
81000
82000
83000
84000
86000
87000
89000
93000
97000
Subject
Condition
EM
Primary

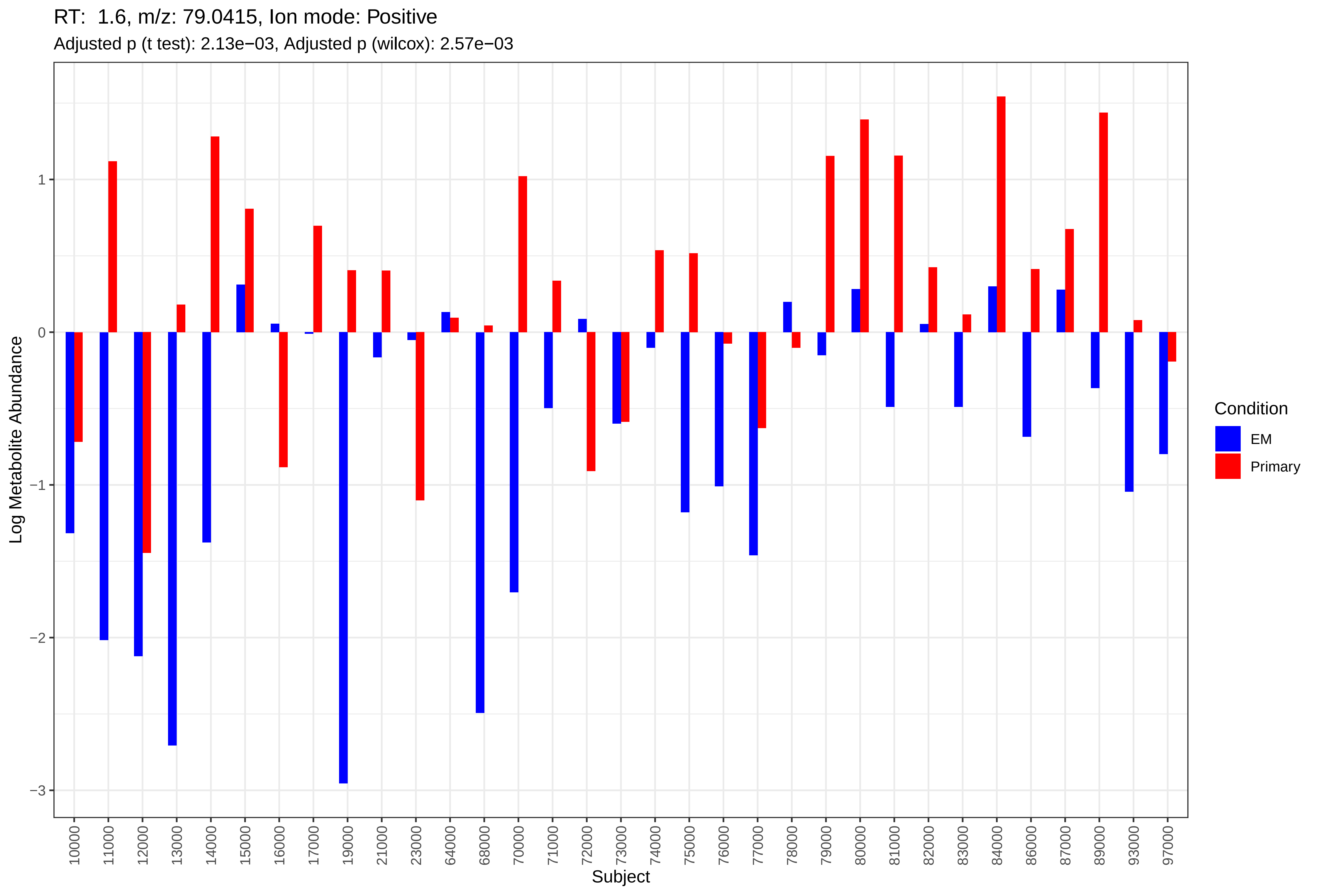

RT: 1.6, m/z: 79.0415, Ion mode: Positive
Adjusted p (t test): 2.13e−03, Adjusted p (wilcox): 2.57e−03
Log Metabolite Abundance
1
0
−1
−2
−3
10000
11000
12000
13000
14000
15000
16000
17000
19000
21000
23000
64000
68000
70000
71000
72000
73000
74000
75000
76000
77000
78000
79000
80000
81000
82000
83000
84000
86000
87000
89000
93000
97000
Subject
Condition
EM
Primary

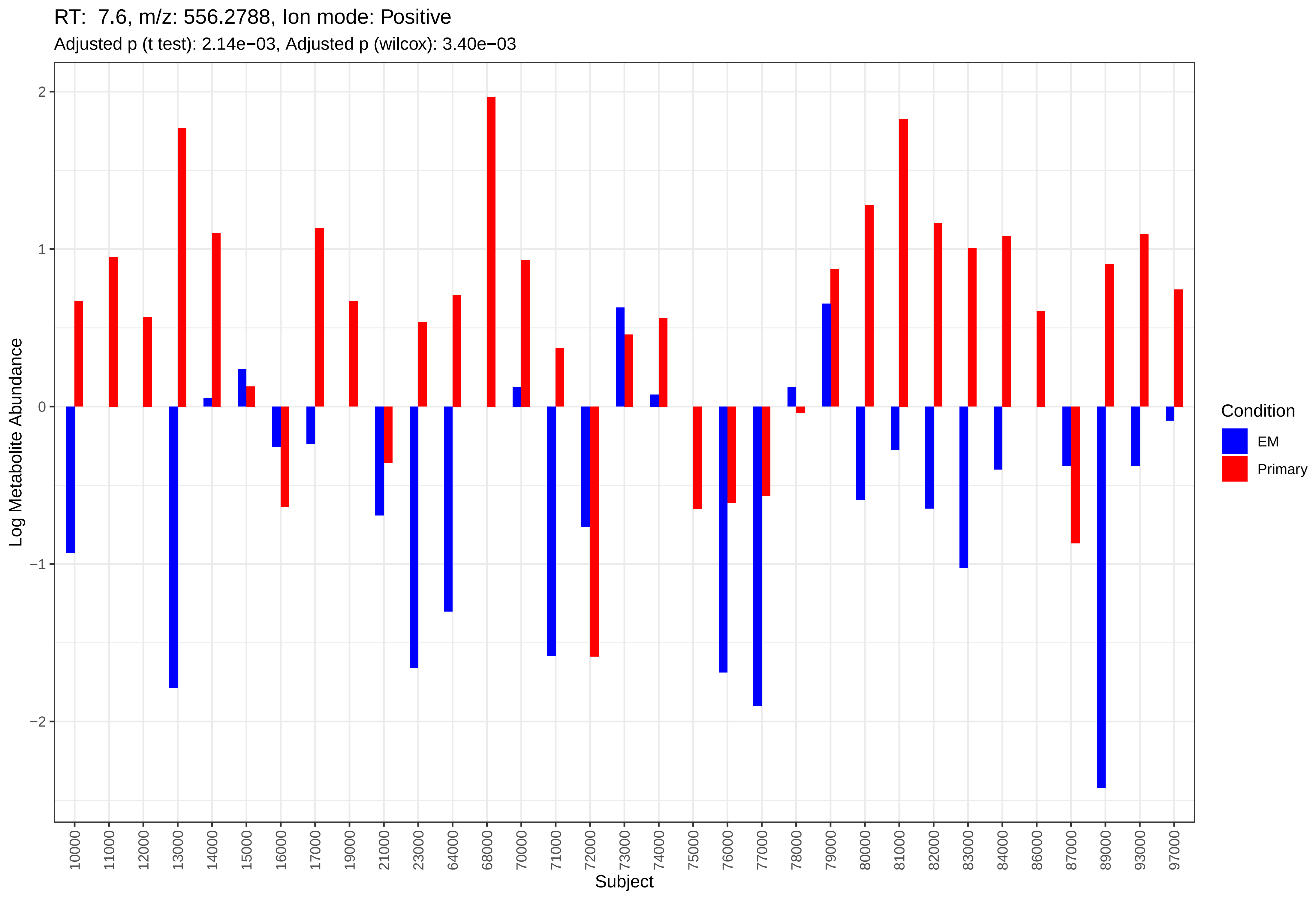
RT: 7.6, m/z: 556.2788, Ion mode: Positive
Adjusted p (t test): 2.14e−03, Adjusted p (wilcox): 3.40e−03
Log Metabolite Abundance
Subject
Condition
EM
Primary
2
1
0
−1
−2
10000
11000
12000
13000
14000
15000
16000
17000
19000
21000
23000
64000
68000
70000
71000
72000
73000
74000
75000
76000
77000
78000
79000
80000
81000
82000
83000
84000
86000
87000
89000
93000
97000

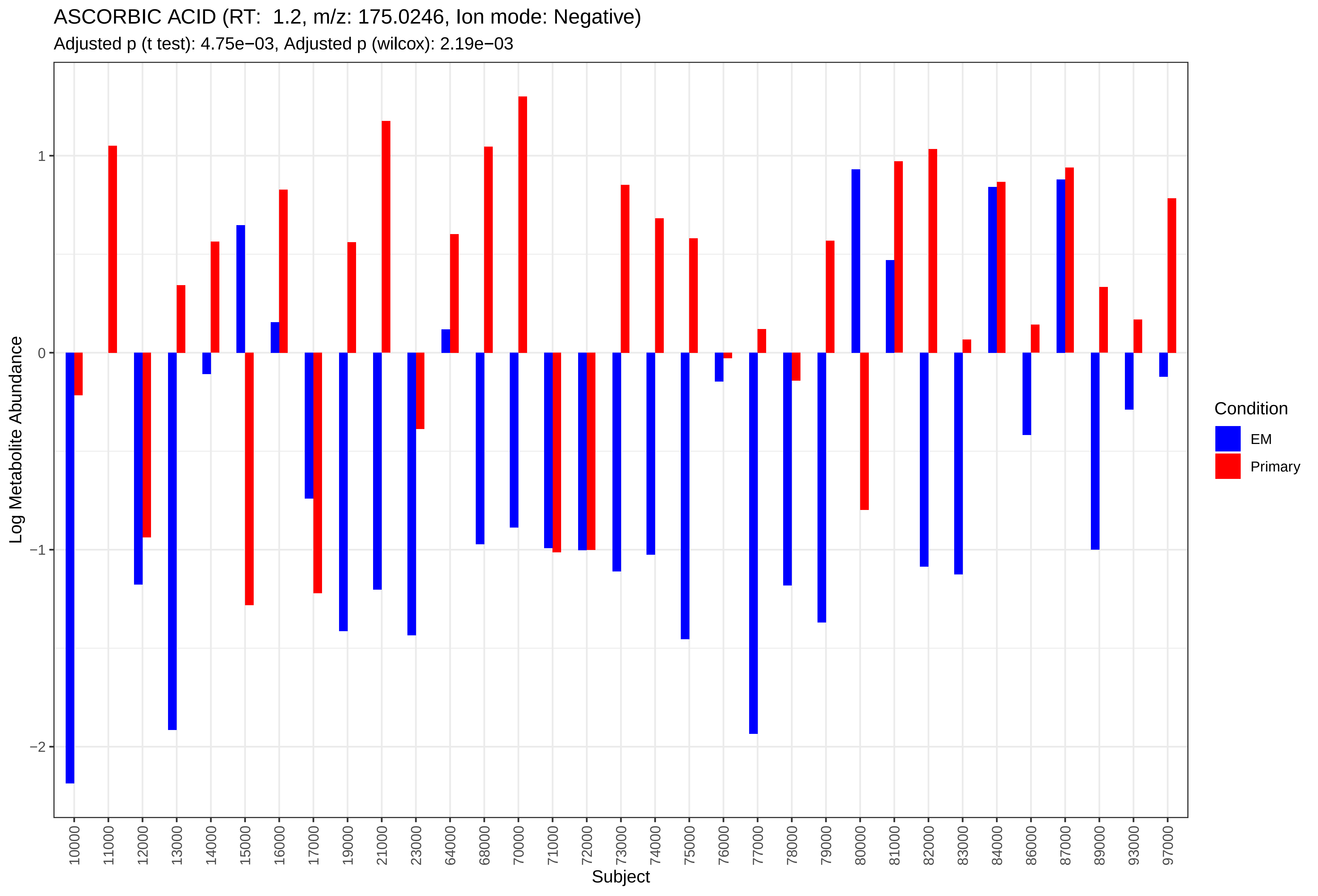
ASCORBIC ACID (RT: 1.2, m/z: 175.0246, Ion mode: Negative)
Adjusted p (t test): 4.75e−03, Adjusted p (wilcox): 2.19e−03
Log Metabolite Abundance
1
0
−1
−2
Subject
10000
11000
12000
13000
14000
15000
16000
17000
19000
21000
23000
64000
68000
70000
71000
72000
73000
74000
75000
76000
77000
78000
79000
80000
81000
82000
83000
84000
86000
87000
89000
93000
97000
Condition
EM
Primary

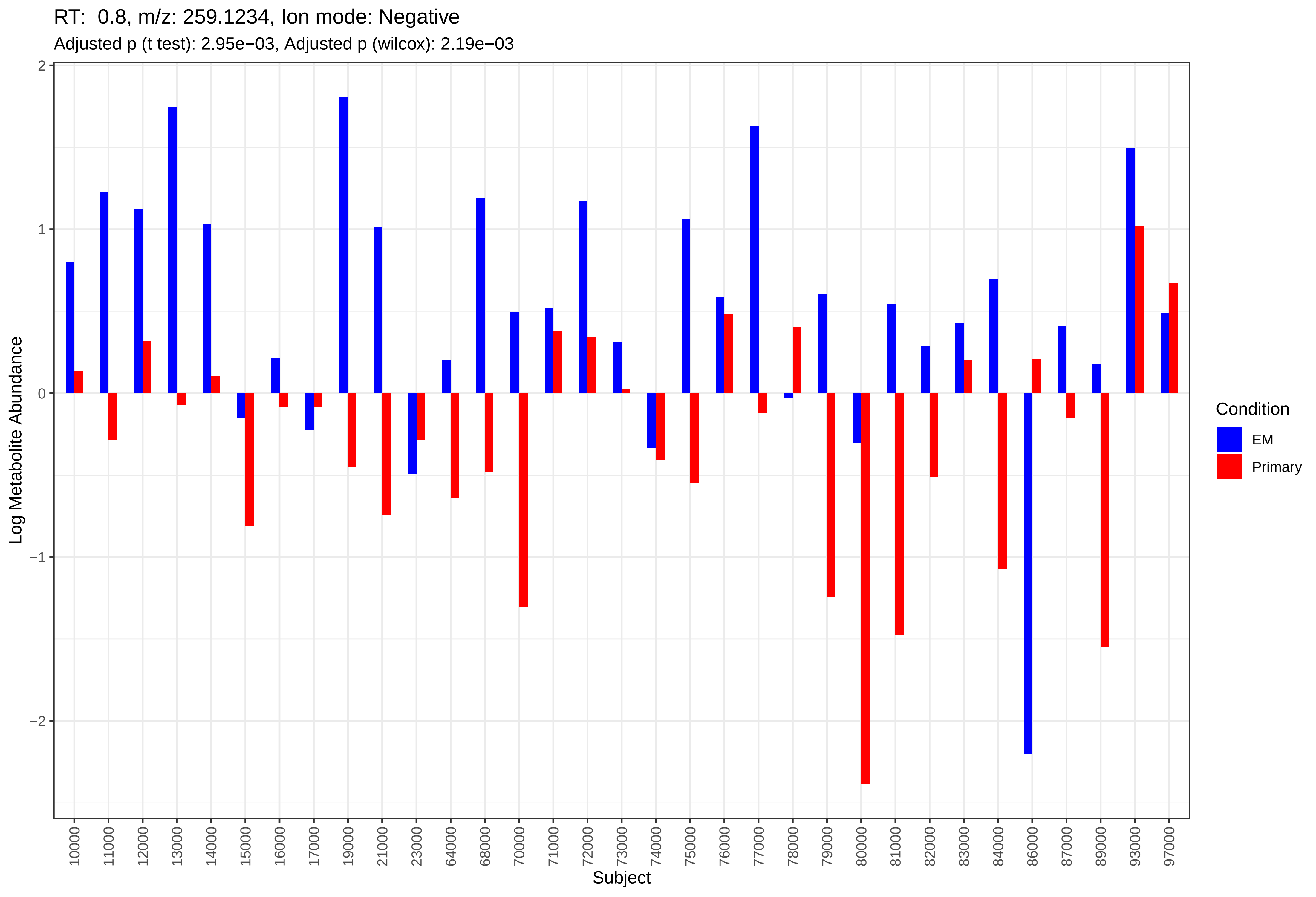
RT: 0.8, m/z: 259.1234, Ion mode: Negative
Adjusted p (t test): 2.95e−03, Adjusted p (wilcox): 2.19e−03
Log Metabolite Abundance
2
1
0
−1
−2
10000
11000
12000
13000
14000
15000
16000
17000
19000
21000
23000
64000
68000
70000
71000
72000
73000
74000
75000
76000
77000
78000
79000
80000
81000
82000
83000
84000
86000
87000
89000
93000
97000
Subject
Condition
EM
Primary

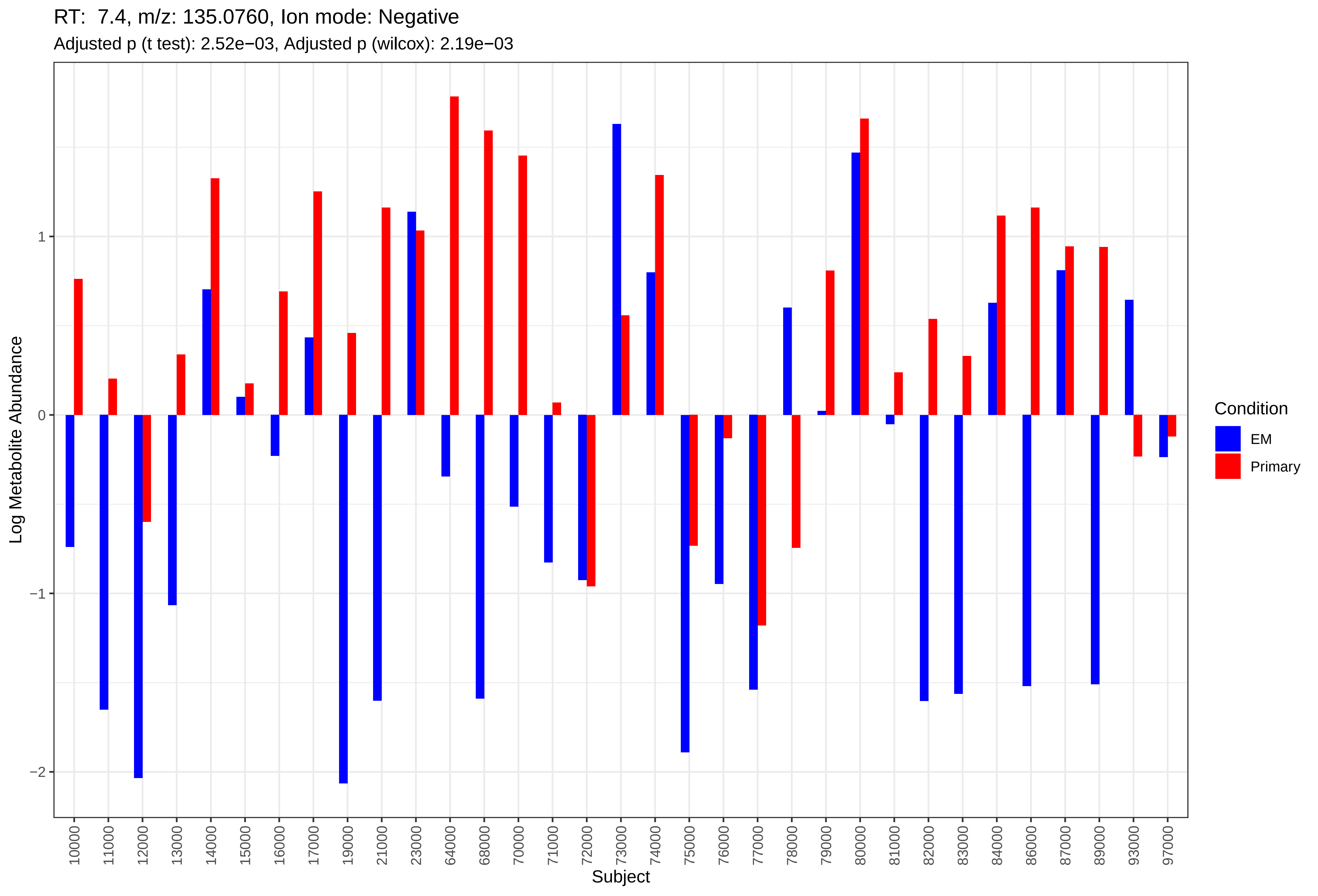
RT: 7.4, m/z: 135.0760, Ion mode: Negative
Adjusted p (t test): 2.52e−03, Adjusted p (wilcox): 2.19e−03
Log Metabolite Abundance
1
0
−1
−2
10000
11000
12000
13000
14000
15000
16000
17000
19000
21000
23000
64000
68000
70000
71000
72000
73000
74000
75000
76000
77000
78000
79000
80000
81000
82000
83000
84000
86000
87000
89000
93000
97000
Subject
Condition
EM
Primary

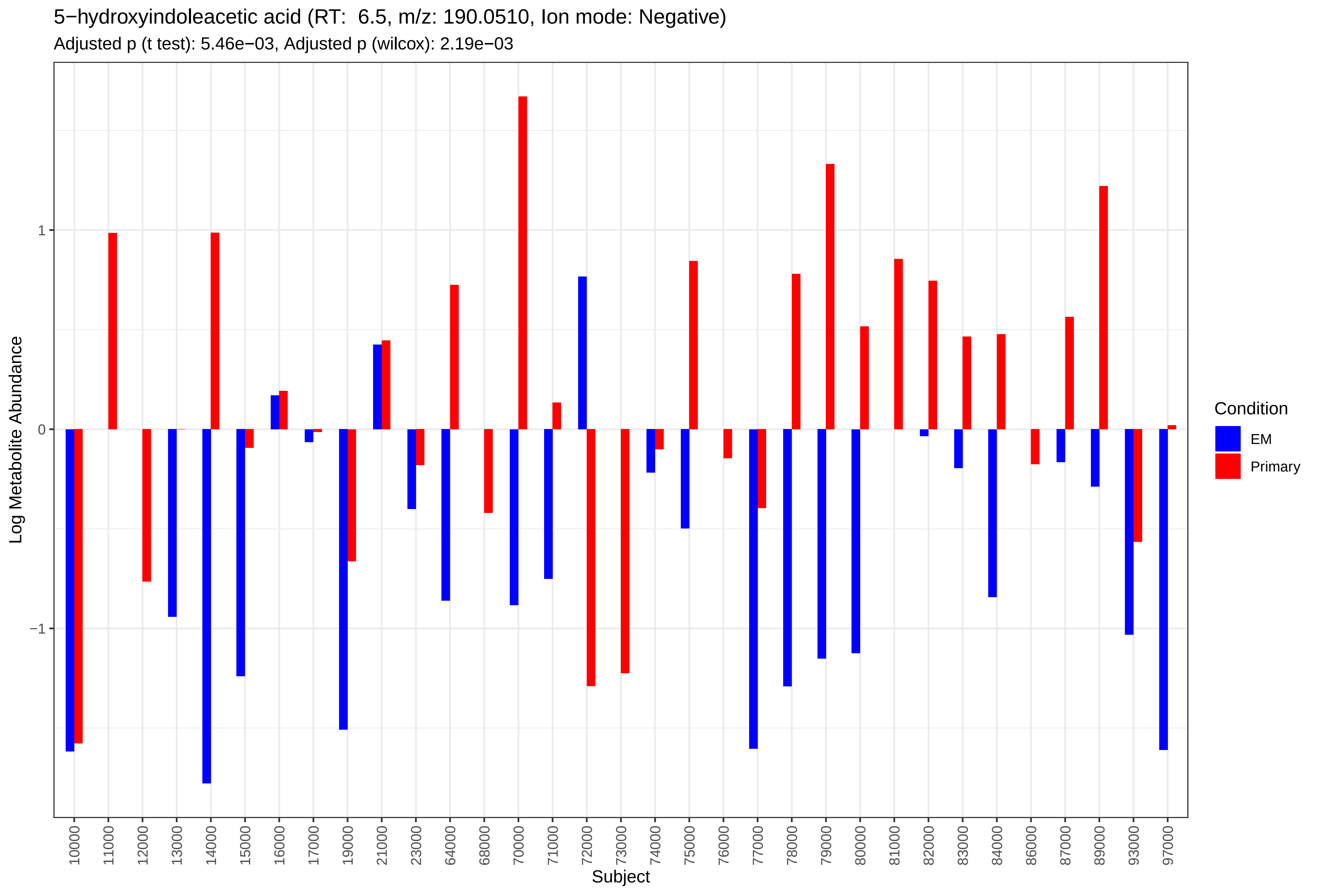

5−hydroxyindoleacetic acid (RT: 6.5, m/z: 190.0510, Ion mode: Negative)
Adjusted p (t test): 5.46e−03, Adjusted p (wilcox): 2.19e−03
Log Metabolite Abundance
1
0
−1
10000
11000
12000
13000
14000
15000
16000
17000
19000
21000
23000
64000
68000
70000
71000
72000
73000
74000
75000
76000
77000
78000
79000
80000
81000
82000
83000
84000
86000
87000
89000
93000
97000
Subject
Condition
EM
Primary

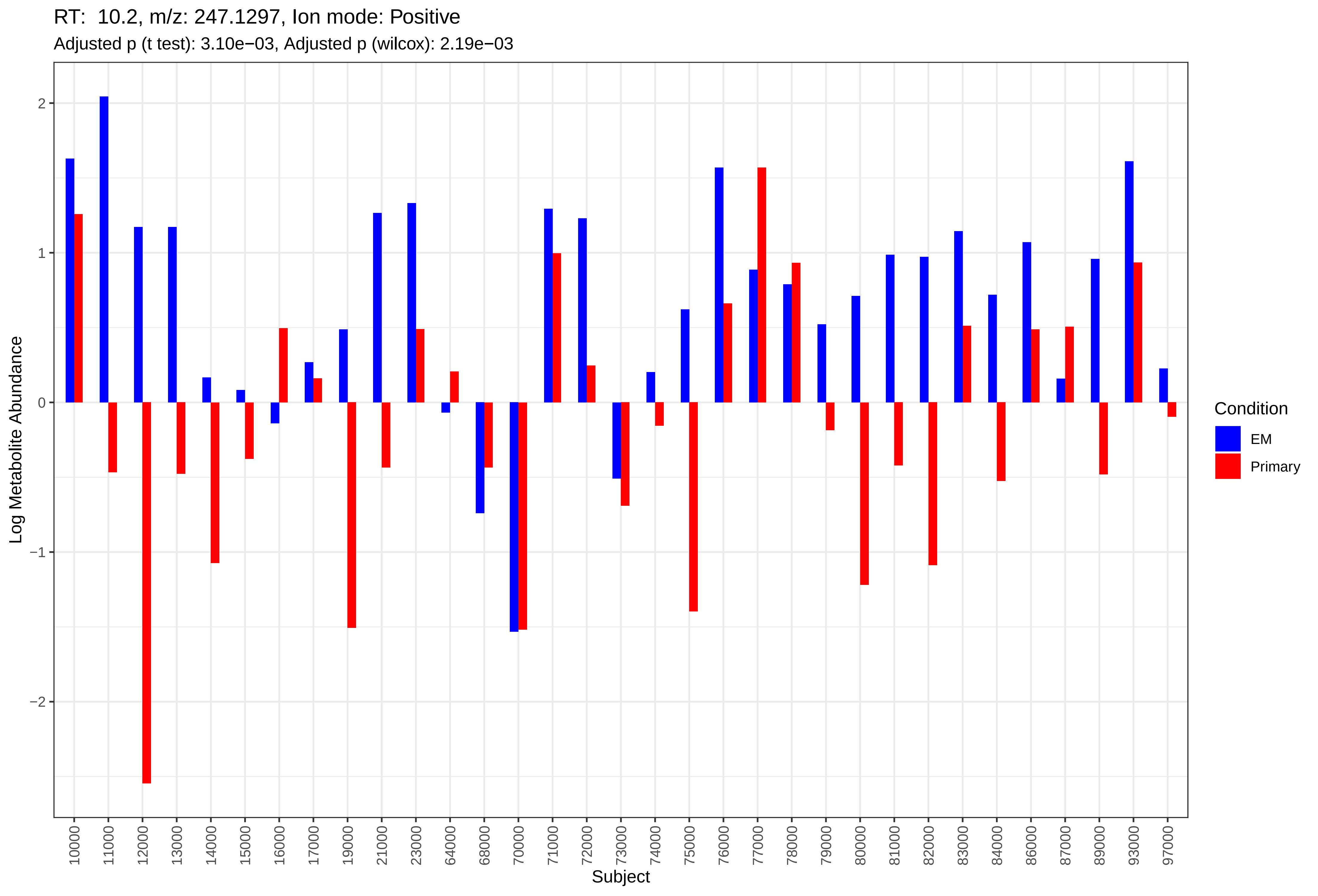
RT: 10.2, m/z: 247.1297, Ion mode: Positive
Adjusted p (t test): 3.10e−03, Adjusted p (wilcox): 2.19e−03
Log Metabolite Abundance
2
1
0
−1
−2
10000
11000
12000
13000
14000
15000
16000
17000
19000
21000
23000
64000
68000
70000
71000
72000
73000
74000
75000
76000
77000
78000
79000
80000
81000
82000
83000
84000
86000
87000
89000
93000
97000
Subject
Condition
EM
Primary

RT: 1.3, m/z: 242.1428, Ion mode: Positive

Adjusted p (t test): 3.07e−03, Adjusted p (wilcox): 2.19e−03

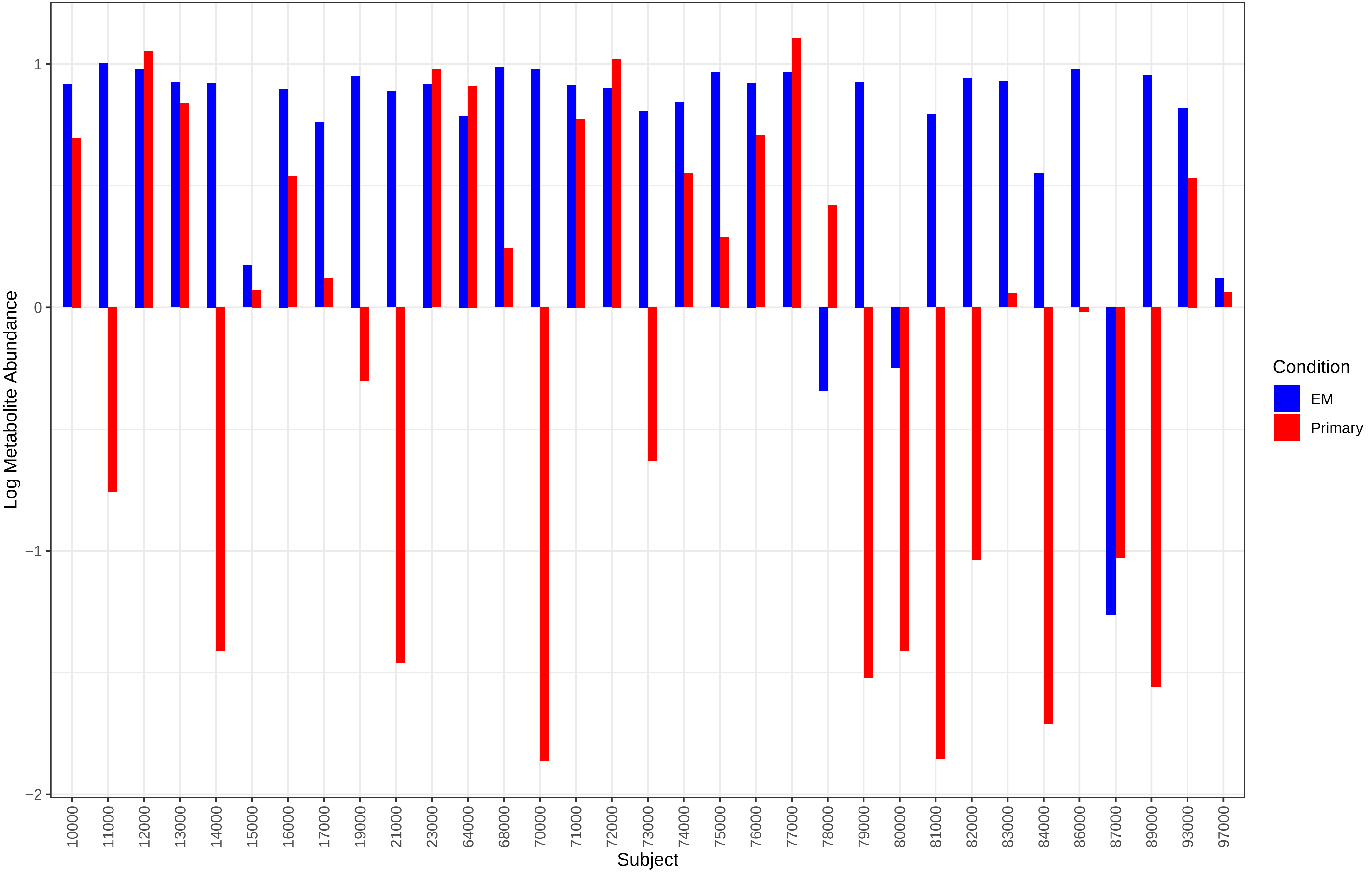

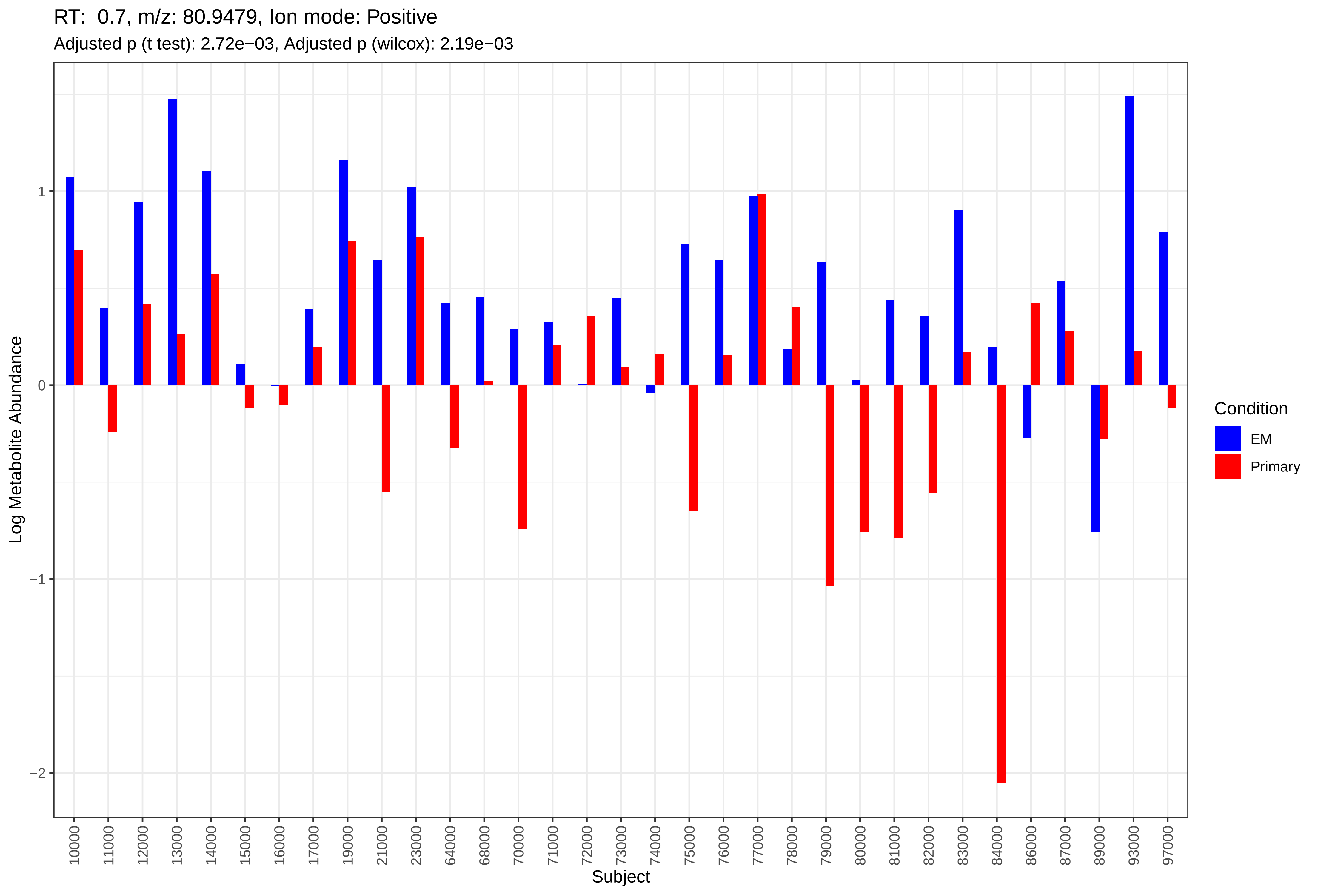

RT: 0.7, m/z: 80.9479, Ion mode: Positive
Adjusted p (t test): 2.72e−03, Adjusted p (wilcox): 2.19e−03
Log Metabolite Abundance
1
0
−1
−2
Subject
10000
11000
12000
13000
14000
15000
16000
17000
19000
21000
23000
64000
68000
70000
71000
72000
73000
74000
75000
76000
77000
78000
79000
80000
81000
82000
83000
84000
86000
87000
89000
93000
97000
Condition
EM
Primary

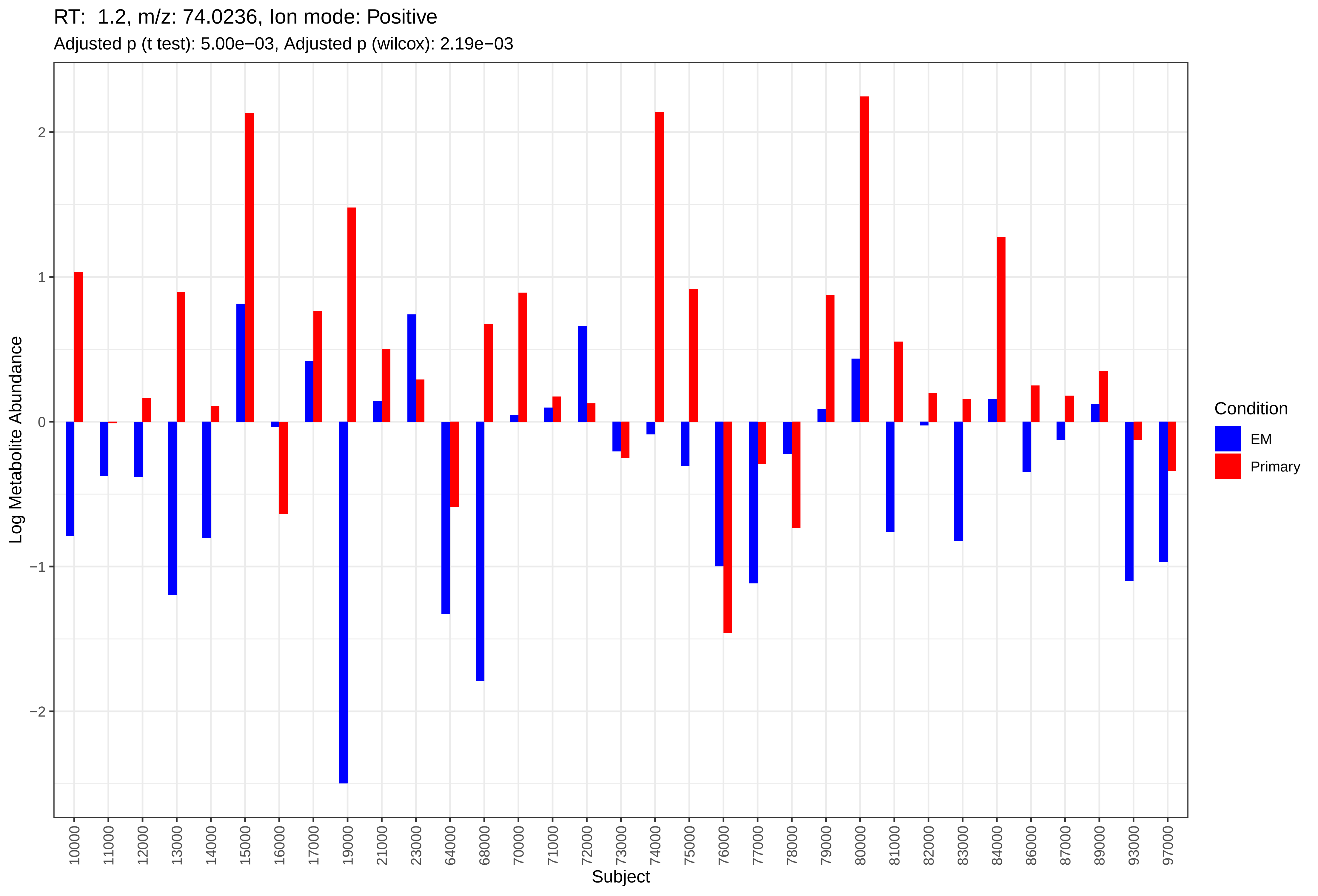

RT: 1.2, m/z: 74.0236, Ion mode: Positive
Adjusted p (t test): 5.00e−03, Adjusted p (wilcox): 2.19e−03
Log Metabolite Abundance
2
1
0
−1
−2
10000
11000
12000
13000
14000
15000
16000
17000
19000
21000
23000
64000
68000
70000
71000
72000
73000
74000
75000
76000
77000
78000
79000
80000
81000
82000
83000
84000
86000
87000
89000
93000
97000
Subject
Condition
EM
Primary

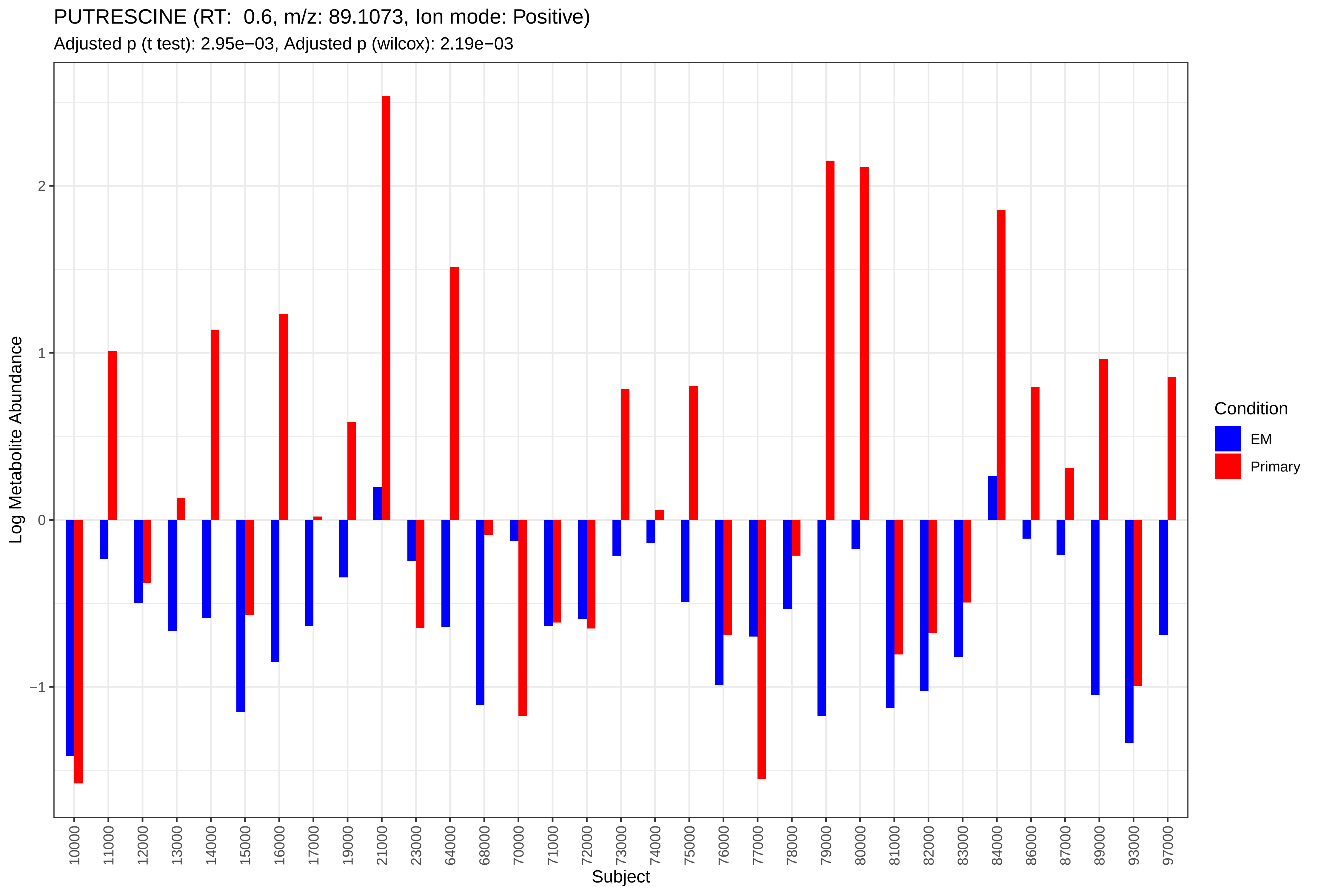
PUTRESCINE (RT: 0.6, m/z: 89.1073, Ion mode: Positive)
Adjusted p (t test): 2.95e−03, Adjusted p (wilcox): 2.19e−03
Log Metabolite Abundance
2
1
0
−1
10000
11000
12000
13000
14000
15000
16000
17000
19000
21000
23000
64000
68000
70000
71000
72000
73000
74000
75000
76000
77000
78000
79000
80000
81000
82000
83000
84000
86000
87000
89000
93000
97000
Subject
Condition
EM
Primary

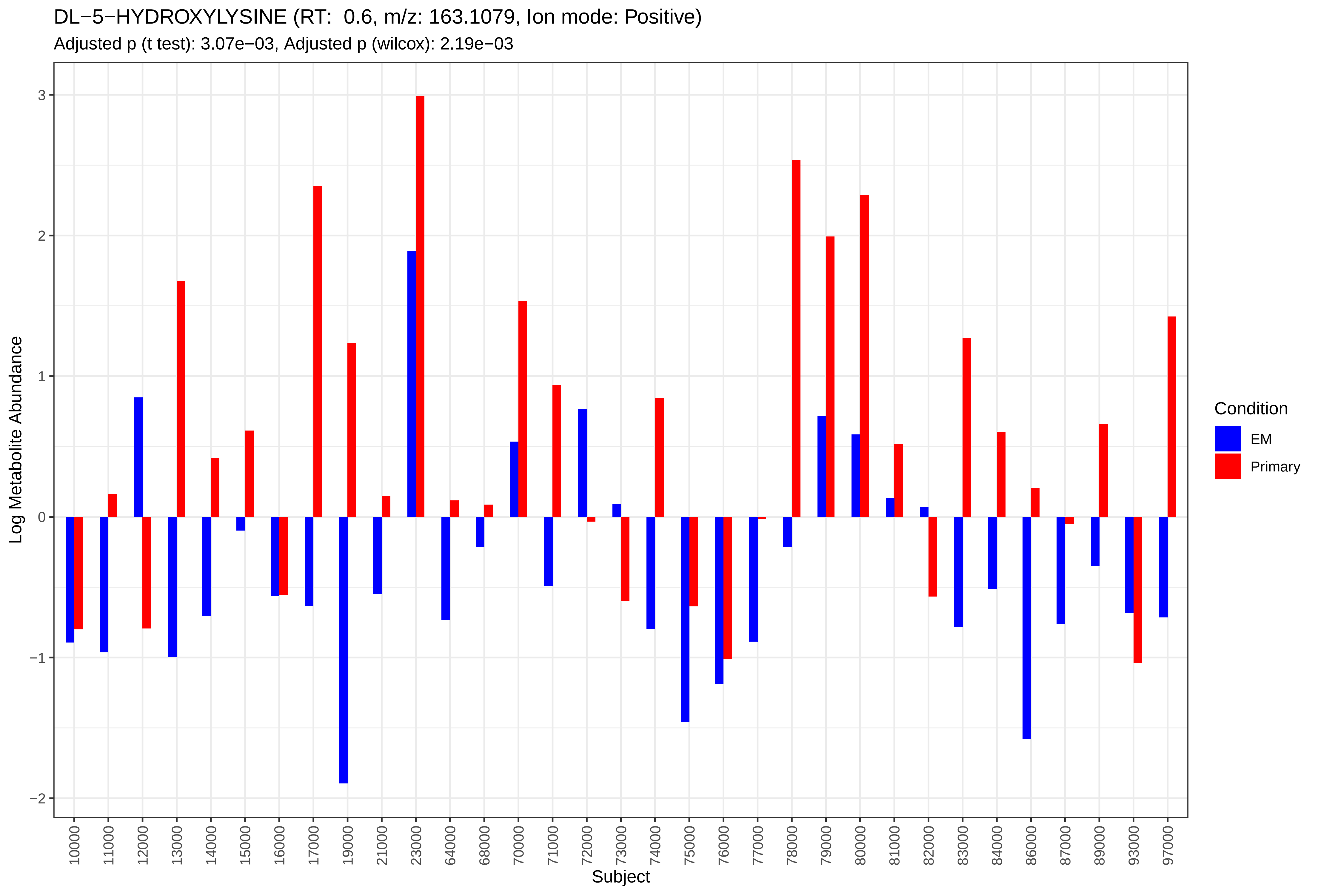
DL−5−HYDROXYLYSINE (RT: 0.6, m/z: 163.1079, Ion mode: Positive)
Adjusted p (t test): 3.07e−03, Adjusted p (wilcox): 2.19e−03
Log Metabolite Abundance
3
2
1
0
−1
−2
Subject
10000
11000
12000
13000
14000
15000
16000
17000
19000
21000
23000
64000
68000
70000
71000
72000
73000
74000
75000
76000
77000
78000
79000
80000
81000
82000
83000
84000
86000
87000
89000
93000
97000
Condition
EM
Primary

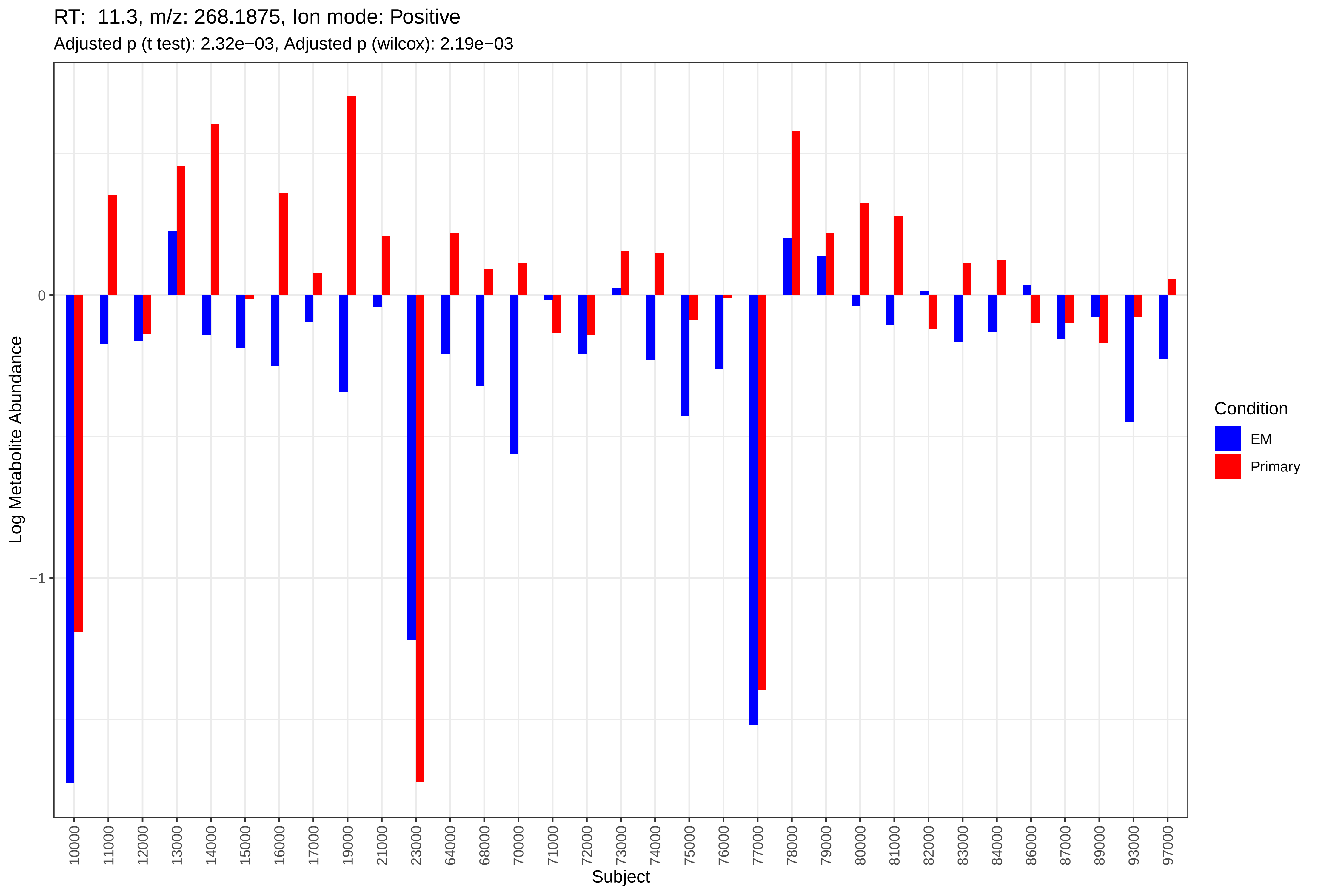

RT: 11.3, m/z: 268.1875, Ion mode: Positive
Adjusted p (t test): 2.32e−03, Adjusted p (wilcox): 2.19e−03
Log Metabolite Abundance
0
−1
Subject
10000
11000
12000
13000
14000
15000
16000
17000
19000
21000
23000
64000
68000
70000
71000
72000
73000
74000
75000
76000
77000
78000
79000
80000
81000
82000
83000
84000
86000
87000
89000
93000
97000
Condition
EM
Primary

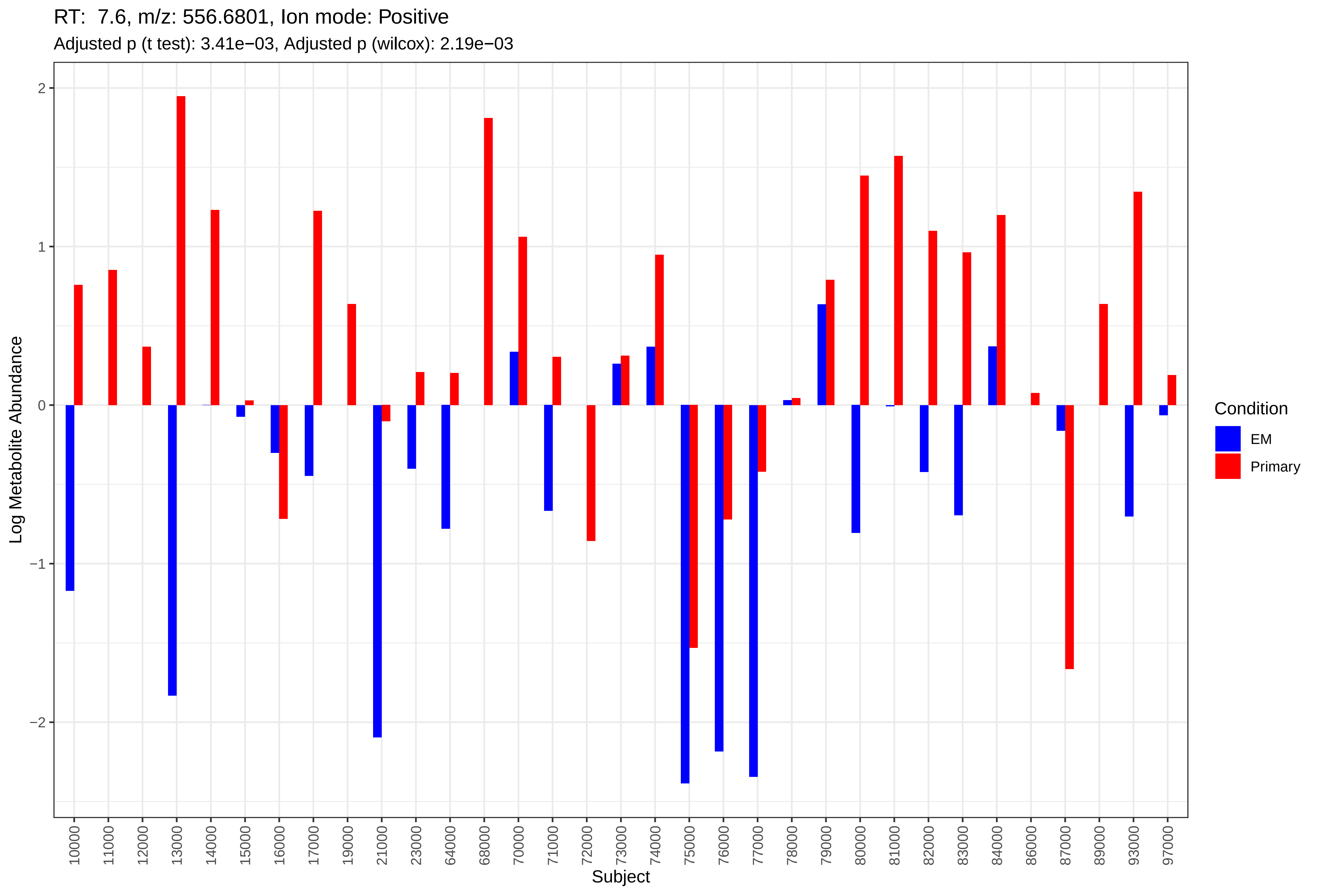
RT: 7.6, m/z: 556.6801, Ion mode: Positive
Adjusted p (t test): 3.41e−03, Adjusted p (wilcox): 2.19e−03
Log Metabolite Abundance
2
1
0
−1
−2
Subject
10000
11000
12000
13000
14000
15000
16000
17000
19000
21000
23000
64000
68000
70000
71000
72000
73000
74000
75000
76000
77000
78000
79000
80000
81000
82000
83000
84000
86000
87000
89000
93000
97000
Condition
EM
Primary

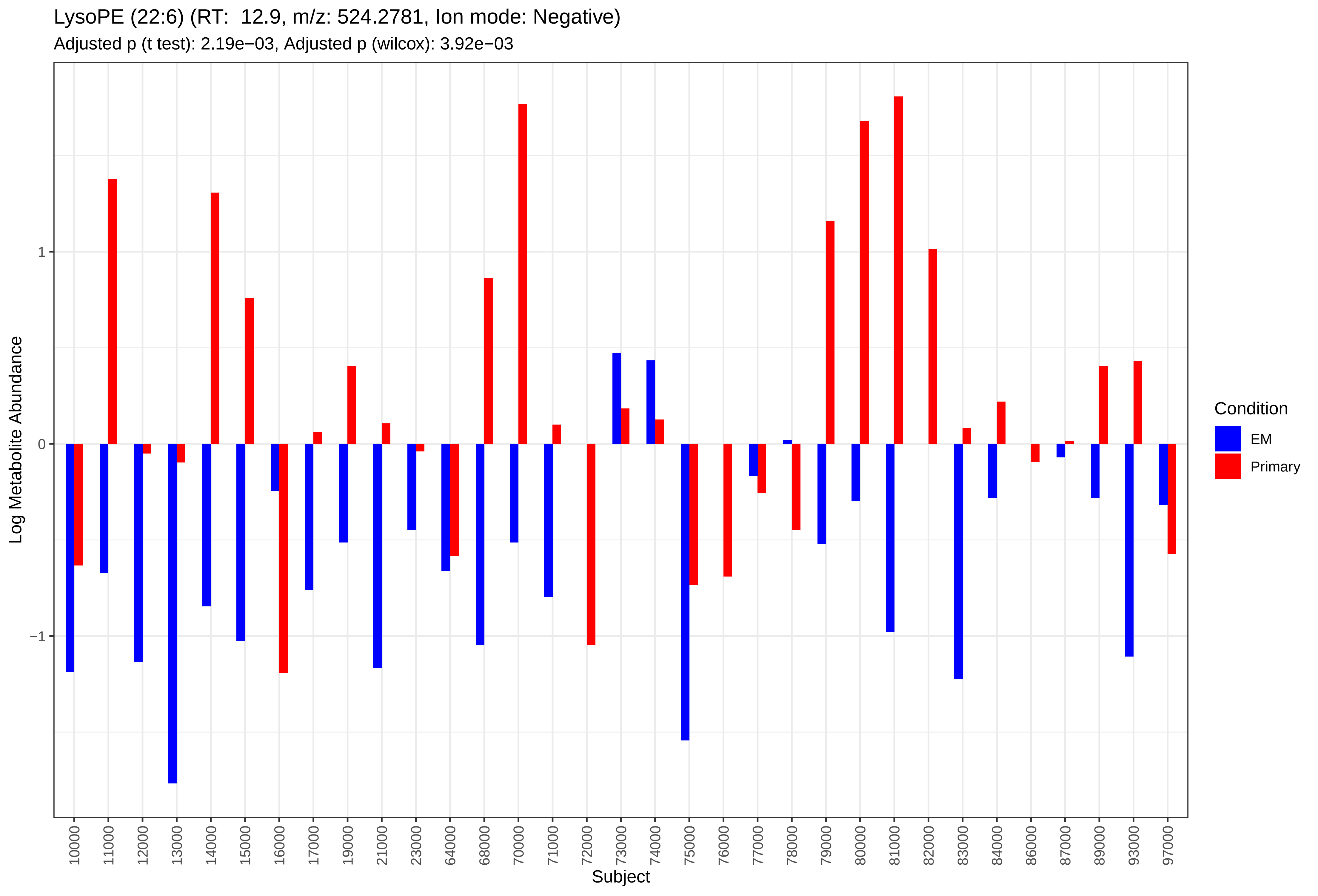
LysoPE (22:6) (RT: 12.9, m/z: 524.2781, Ion mode: Negative)
Adjusted p (t test): 2.19e−03, Adjusted p (wilcox): 3.92e−03
Log Metabolite Abundance
1
0
−1
10000
11000
12000
13000
14000
15000
16000
17000
19000
21000
23000
64000
68000
70000
71000
72000
73000
74000
75000
76000
77000
78000
79000
80000
81000
82000
83000
84000
86000
87000
89000
93000
97000
Subject
Condition
EM
Primary

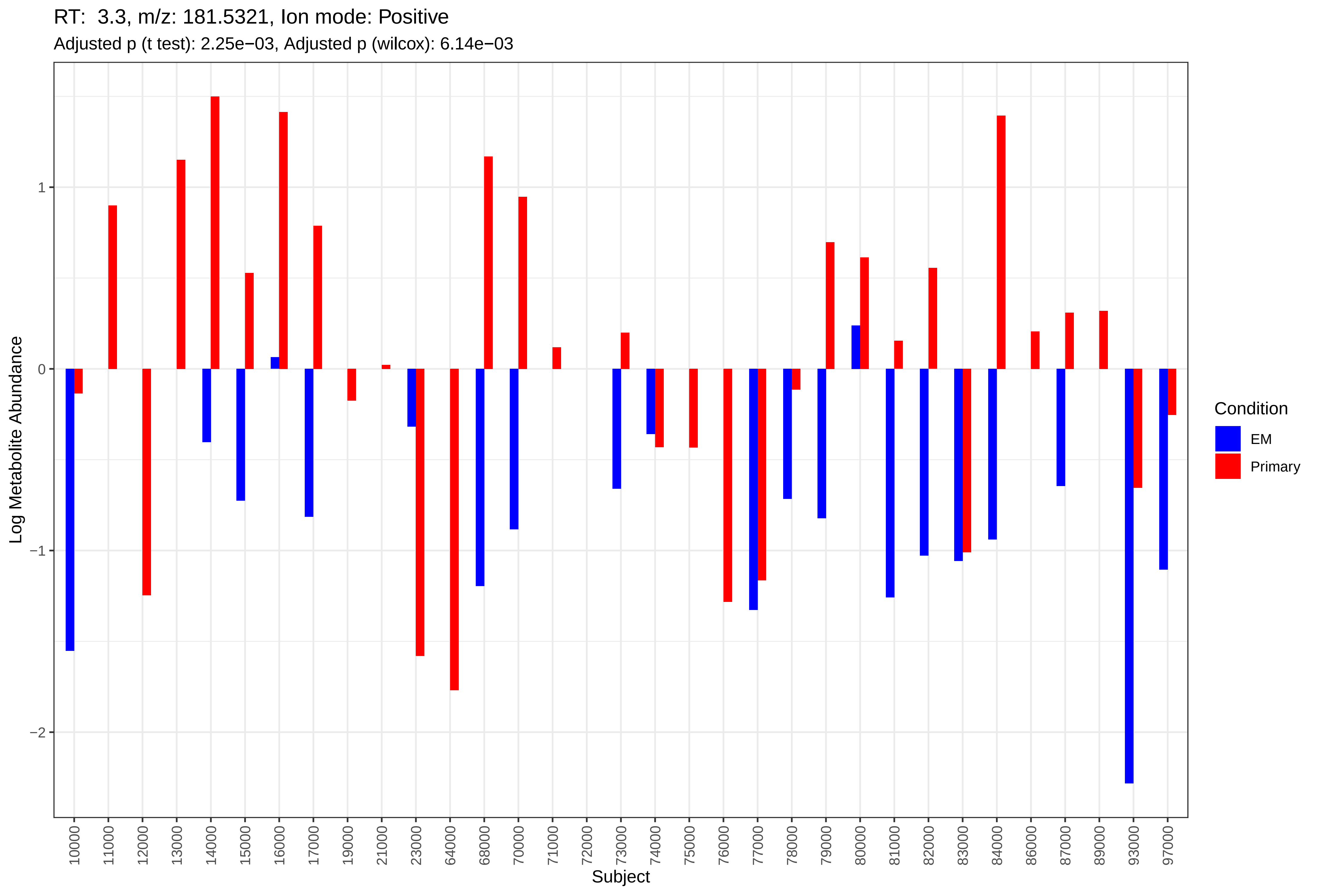

RT: 3.3, m/z: 181.5321, Ion mode: Positive
Adjusted p (t test): 2.25e−03, Adjusted p (wilcox): 6.14e−03
Log Metabolite Abundance
1
0
−1
−2
10000
11000
12000
13000
14000
15000
16000
17000
19000
21000
23000
64000
68000
70000
71000
72000
73000
74000
75000
76000
77000
78000
79000
80000
81000
82000
83000
84000
86000
87000
89000
93000
97000
Subject
Condition
EM
Primary

RT: 2.2, m/z: 140.1261, Ion mode: Positive

Adjusted p (t test): 2.25e−03, Adjusted p (wilcox): 2.57e−03

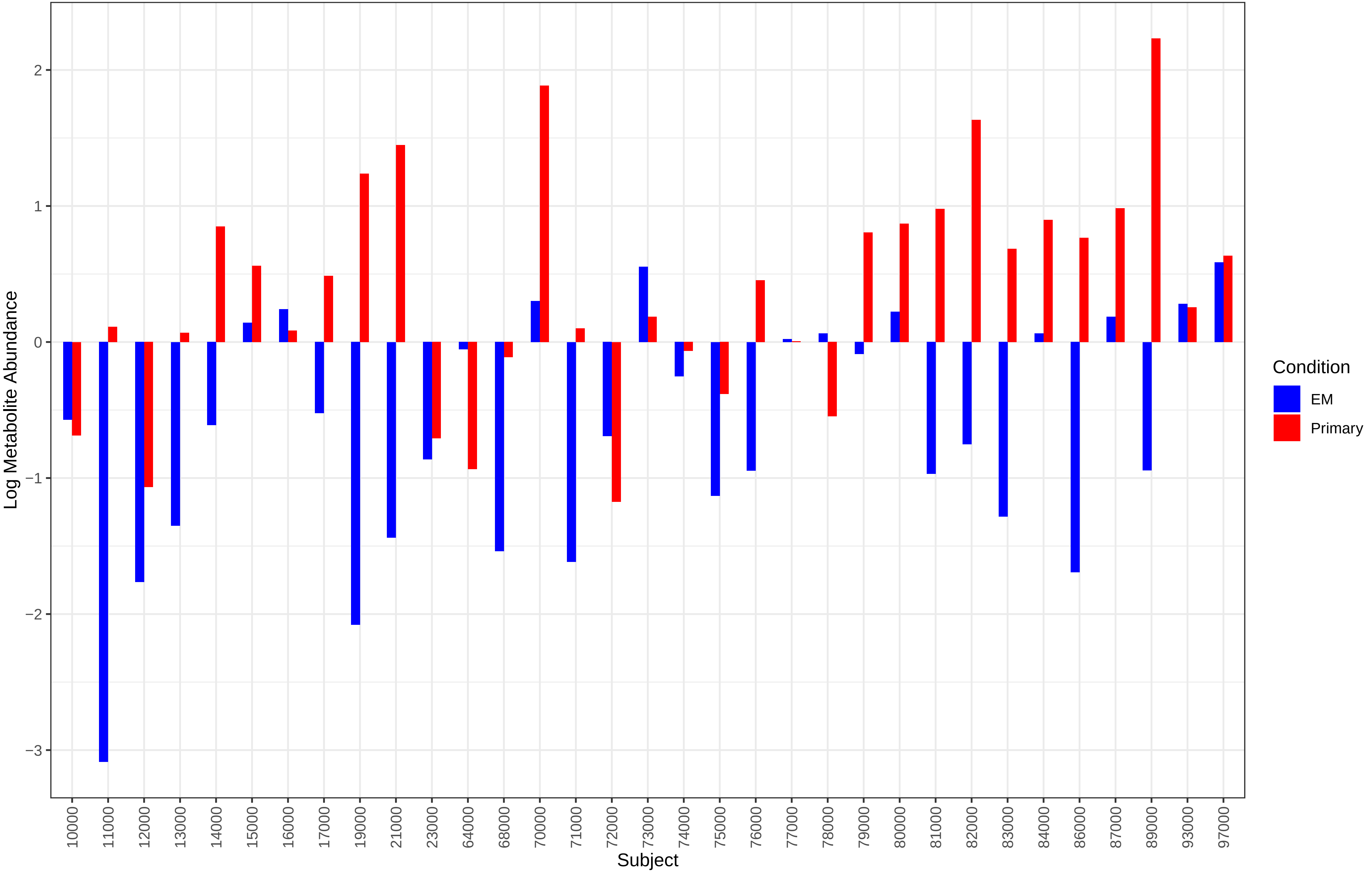

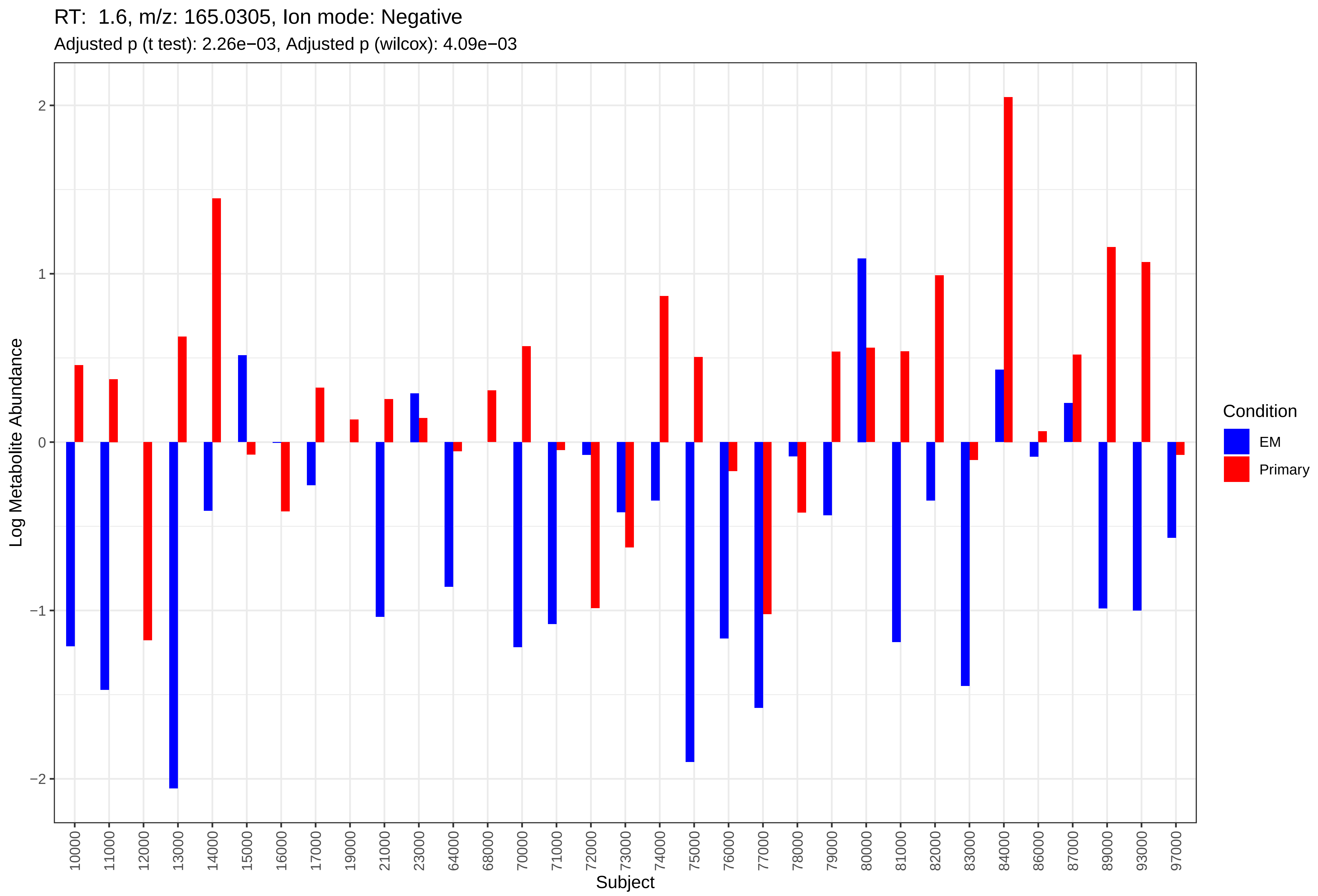

RT: 1.6, m/z: 165.0305, Ion mode: Negative
Adjusted p (t test): 2.26e−03, Adjusted p (wilcox): 4.09e−03
Log Metabolite Abundance
2
1
0
−1
−2
10000
11000
12000
13000
14000
15000
16000
17000
19000
21000
23000
64000
68000
70000
71000
72000
73000
74000
75000
76000
77000
78000
79000
80000
81000
82000
83000
84000
86000
87000
89000
93000
97000
Subject
Condition
EM
Primary

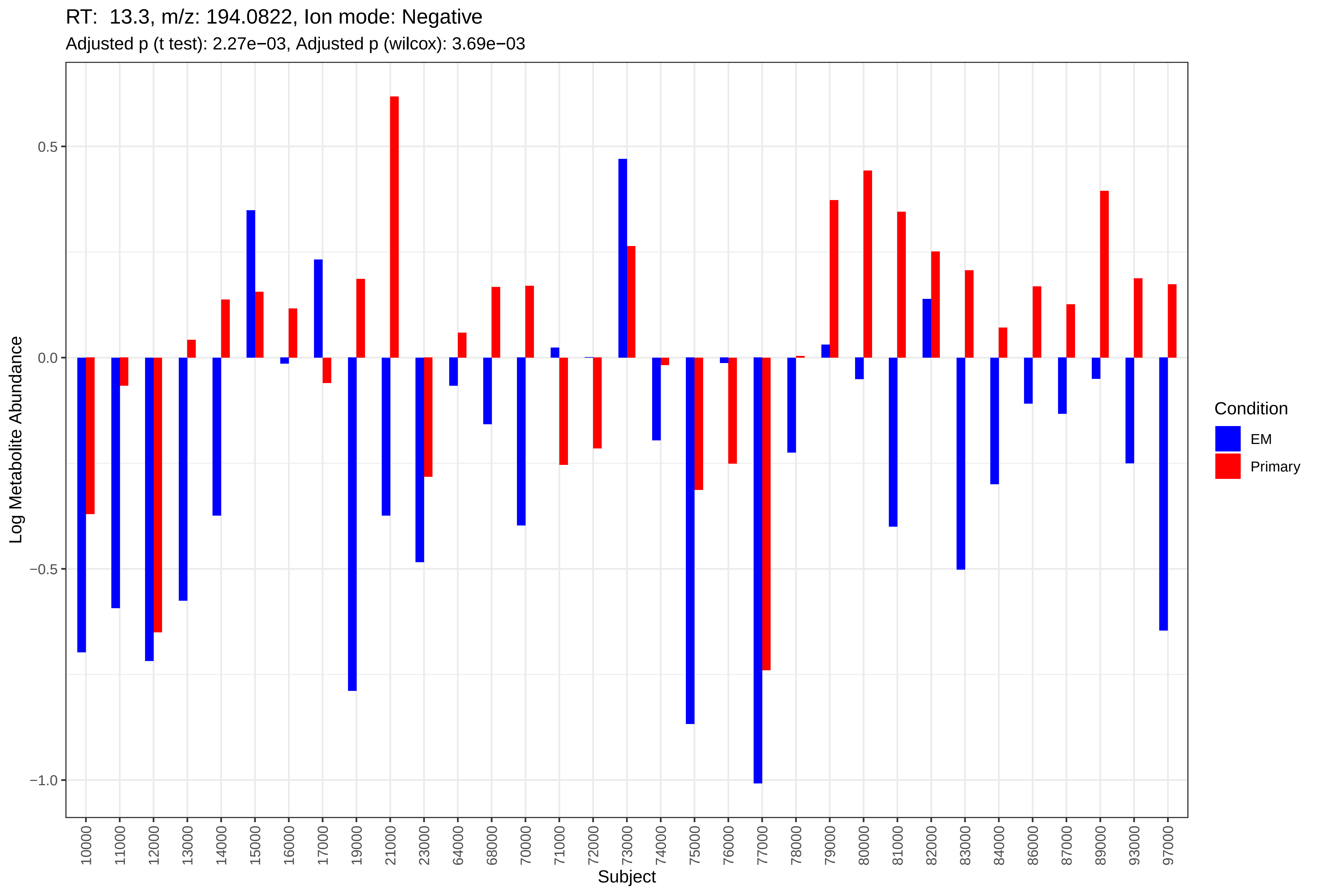

RT: 13.3, m/z: 194.0822, Ion mode: Negative
Adjusted p (t test): 2.27e−03, Adjusted p (wilcox): 3.69e−03
Log Metabolite Abundance
0.5
0.0
−0.5
−1.0
Subject
10000
11000
12000
13000
14000
15000
16000
17000
19000
21000
23000
64000
68000
70000
71000
72000
73000
74000
75000
76000
77000
78000
79000
80000
81000
82000
83000
84000
86000
87000
89000
93000
97000
Condition
EM
Primary

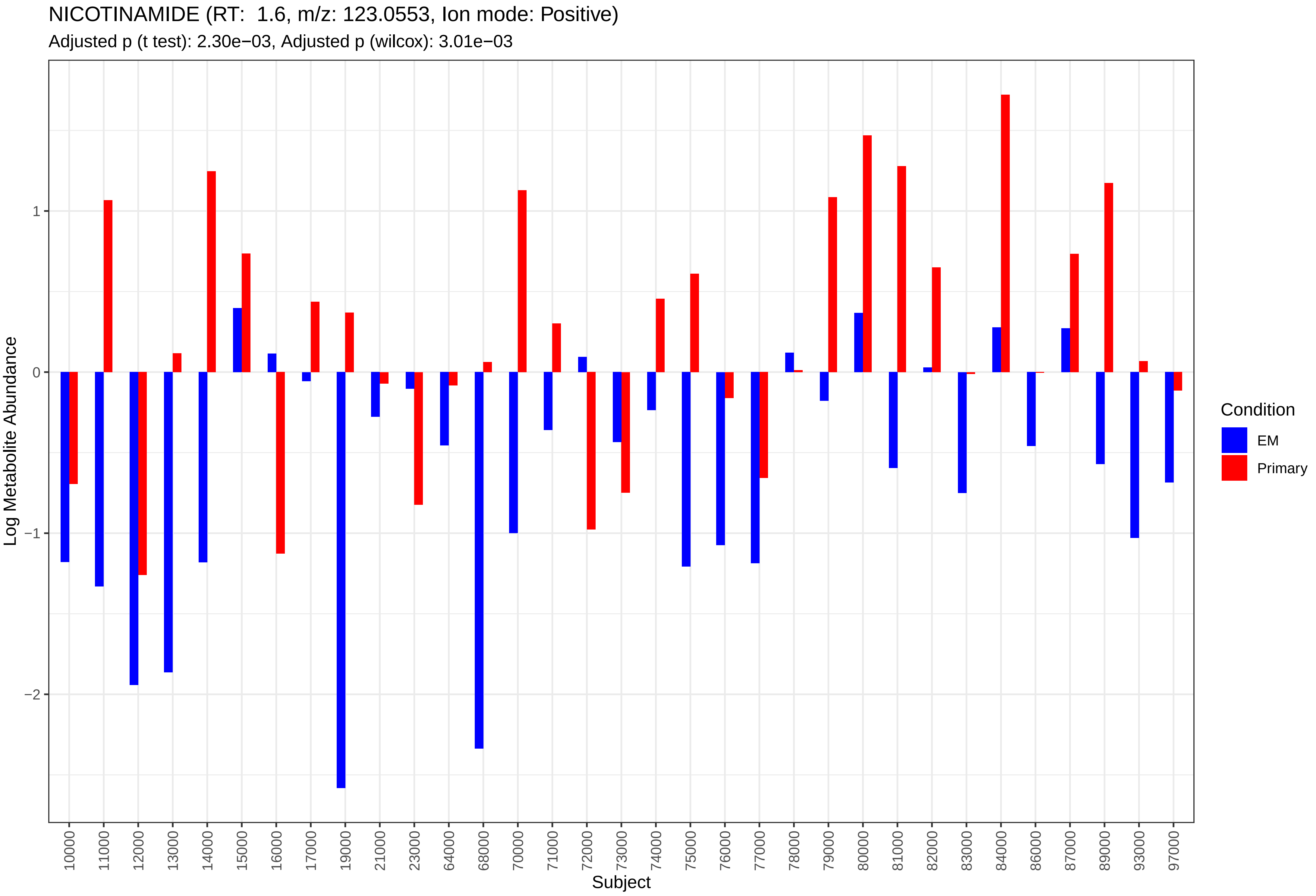

NICOTINAMIDE (RT: 1.6, m/z: 123.0553, Ion mode: Positive)
Adjusted p (t test): 2.30e−03, Adjusted p (wilcox): 3.01e−03
Log Metabolite Abundance
1
0
−1
−2
10000
11000
12000
13000
14000
15000
16000
17000
19000
21000
23000
64000
68000
70000
71000
72000
73000
74000
75000
76000
77000
78000
79000
80000
81000
82000
83000
84000
86000
87000
89000
93000
97000
Subject
Condition
EM
Primary

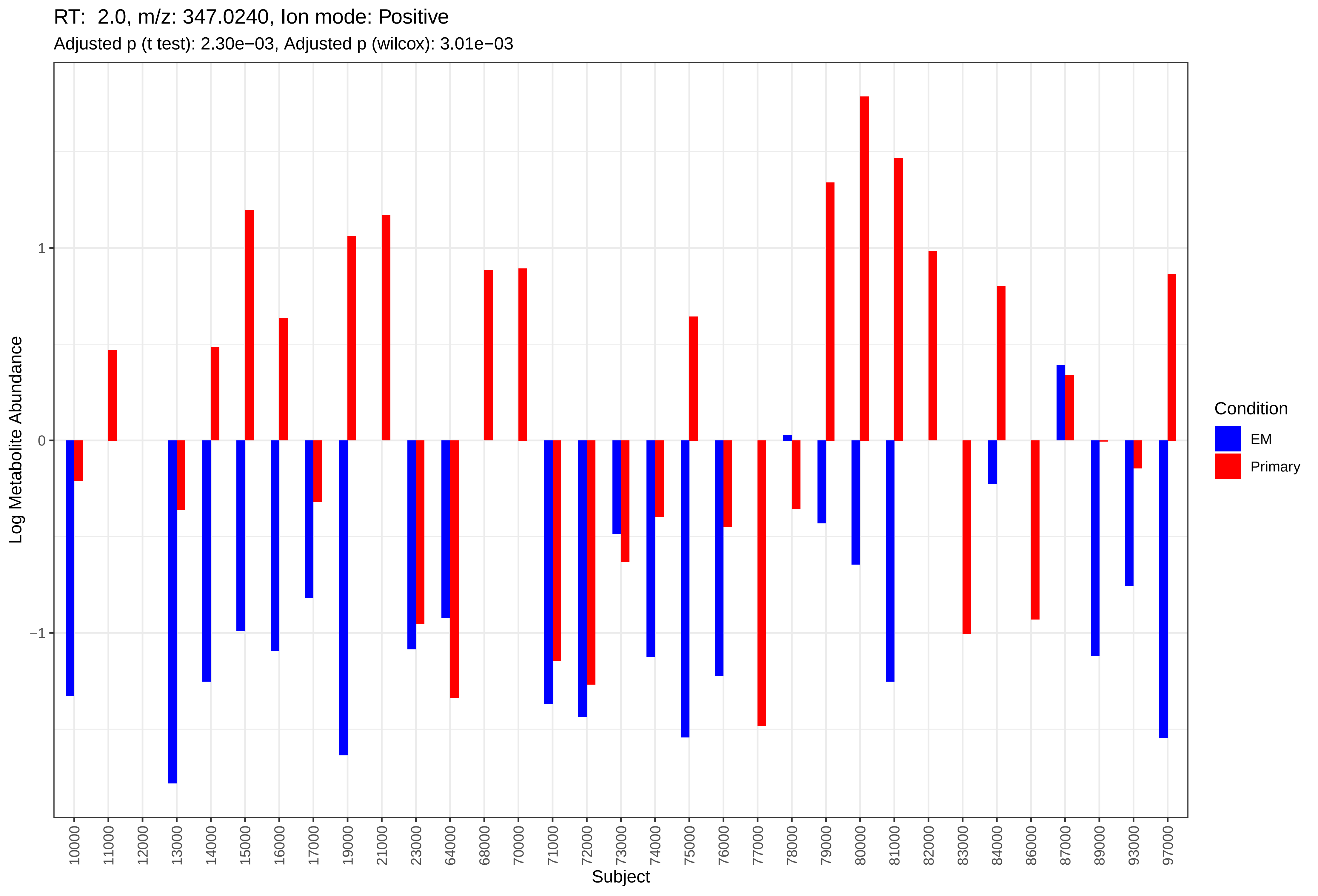
RT: 2.0, m/z: 347.0240, Ion mode: Positive
Adjusted p (t test): 2.30e−03, Adjusted p (wilcox): 3.01e−03
Log Metabolite Abundance
1
0
−1
Subject
10000
11000
12000
13000
14000
15000
16000
17000
19000
21000
23000
64000
68000
70000
71000
72000
73000
74000
75000
76000
77000
78000
79000
80000
81000
82000
83000
84000
86000
87000
89000
93000
97000
Condition
EM
Primary

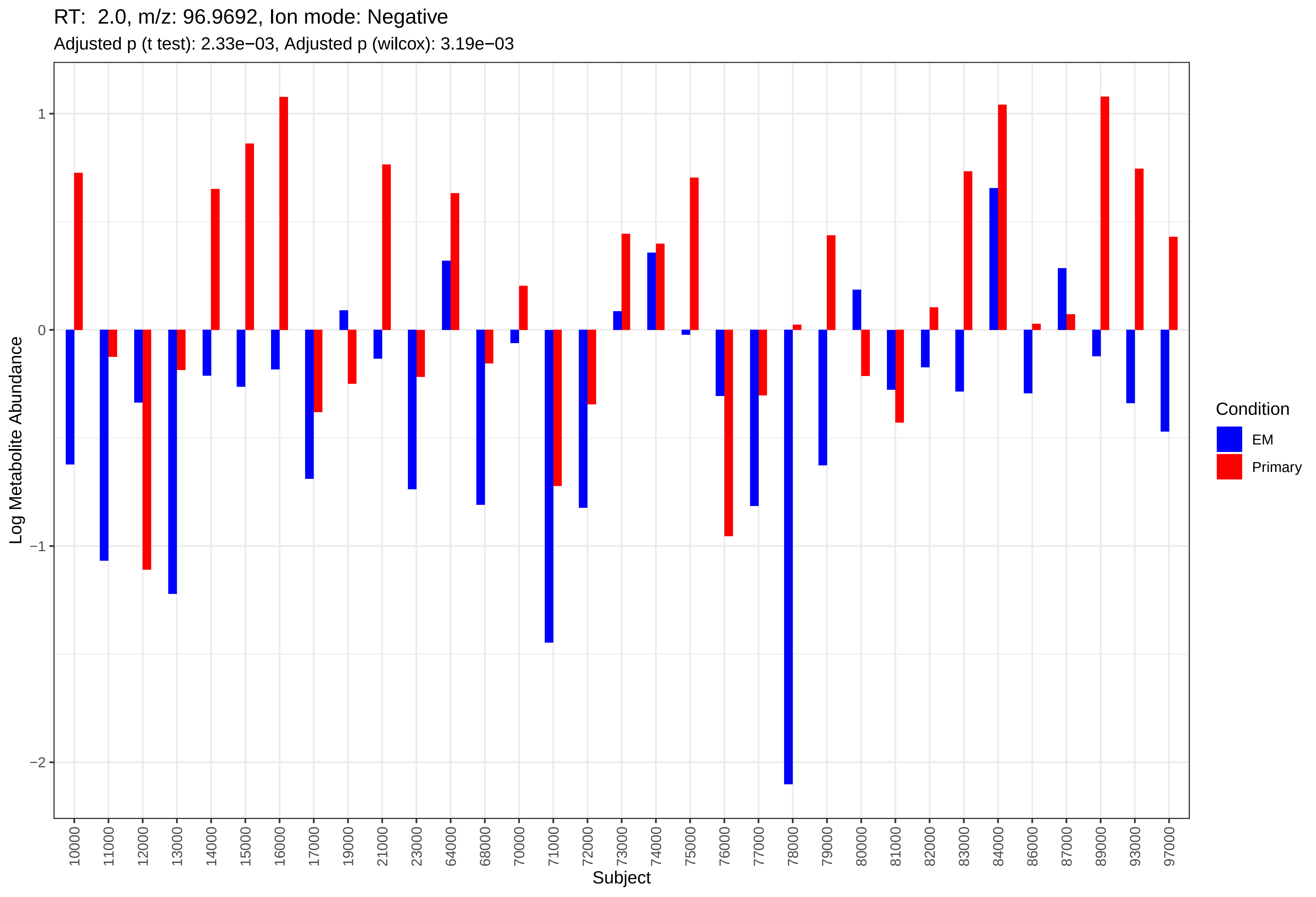

RT: 2.0, m/z: 96.9692, Ion mode: Negative
Adjusted p (t test): 2.33e−03, Adjusted p (wilcox): 3.19e−03
Log Metabolite Abundance
1
0
−1
−2
10000
11000
12000
13000
14000
15000
16000
17000
19000
21000
23000
64000
68000
70000
71000
72000
73000
74000
75000
76000
77000
78000
79000
80000
81000
82000
83000
84000
86000
87000
89000
93000
97000
Subject
Condition
EM
Primary

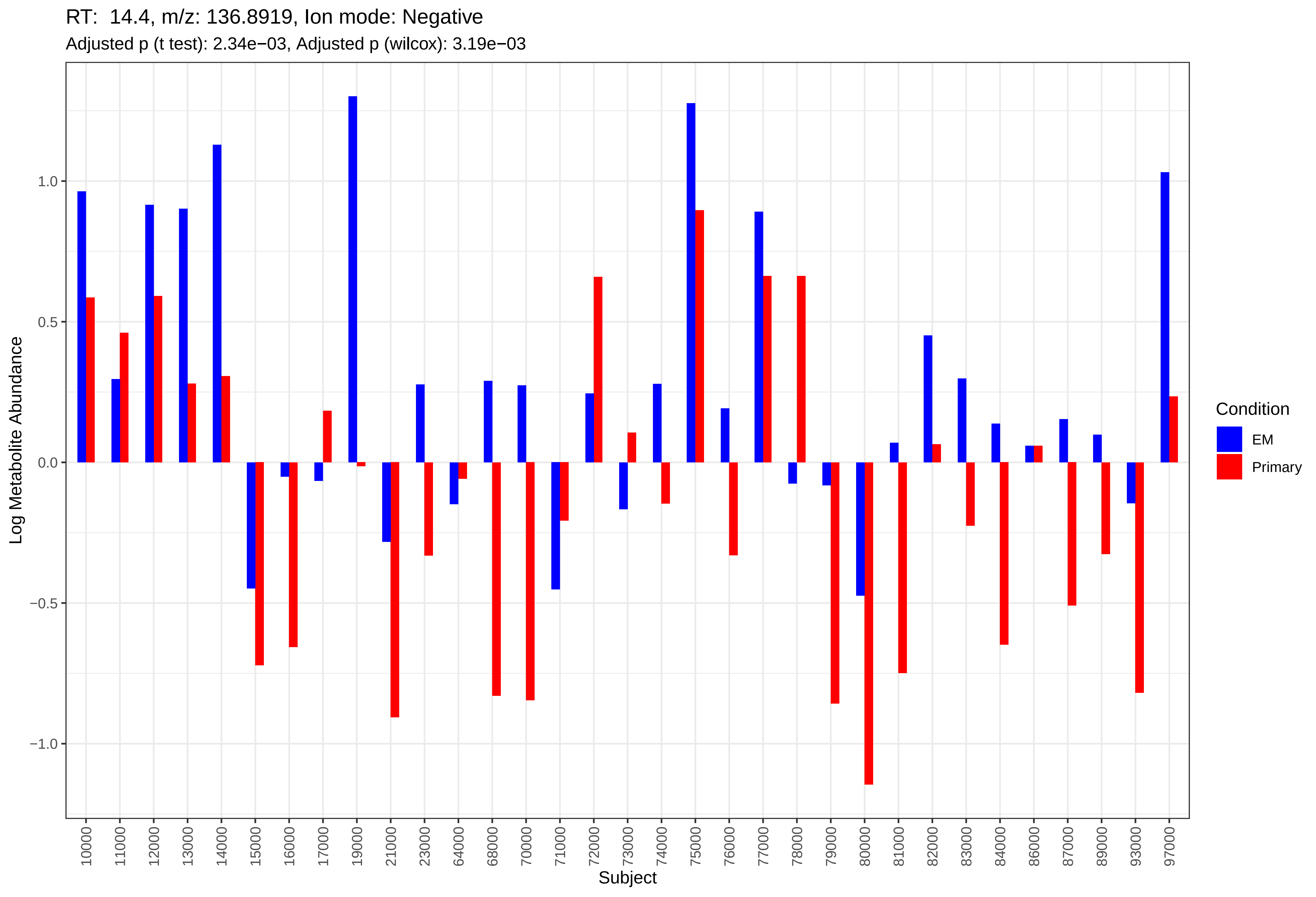

RT: 14.4, m/z: 136.8919, Ion mode: Negative
Adjusted p (t test): 2.34e−03, Adjusted p (wilcox): 3.19e−03
Log Metabolite Abundance
1.0
0.5
0.0
−0.5
−1.0
Subject
10000
11000
12000
13000
14000
15000
16000
17000
19000
21000
23000
64000
68000
70000
71000
72000
73000
74000
75000
76000
77000
78000
79000
80000
81000
82000
83000
84000
86000
87000
89000
93000
97000
Condition
EM
Primary

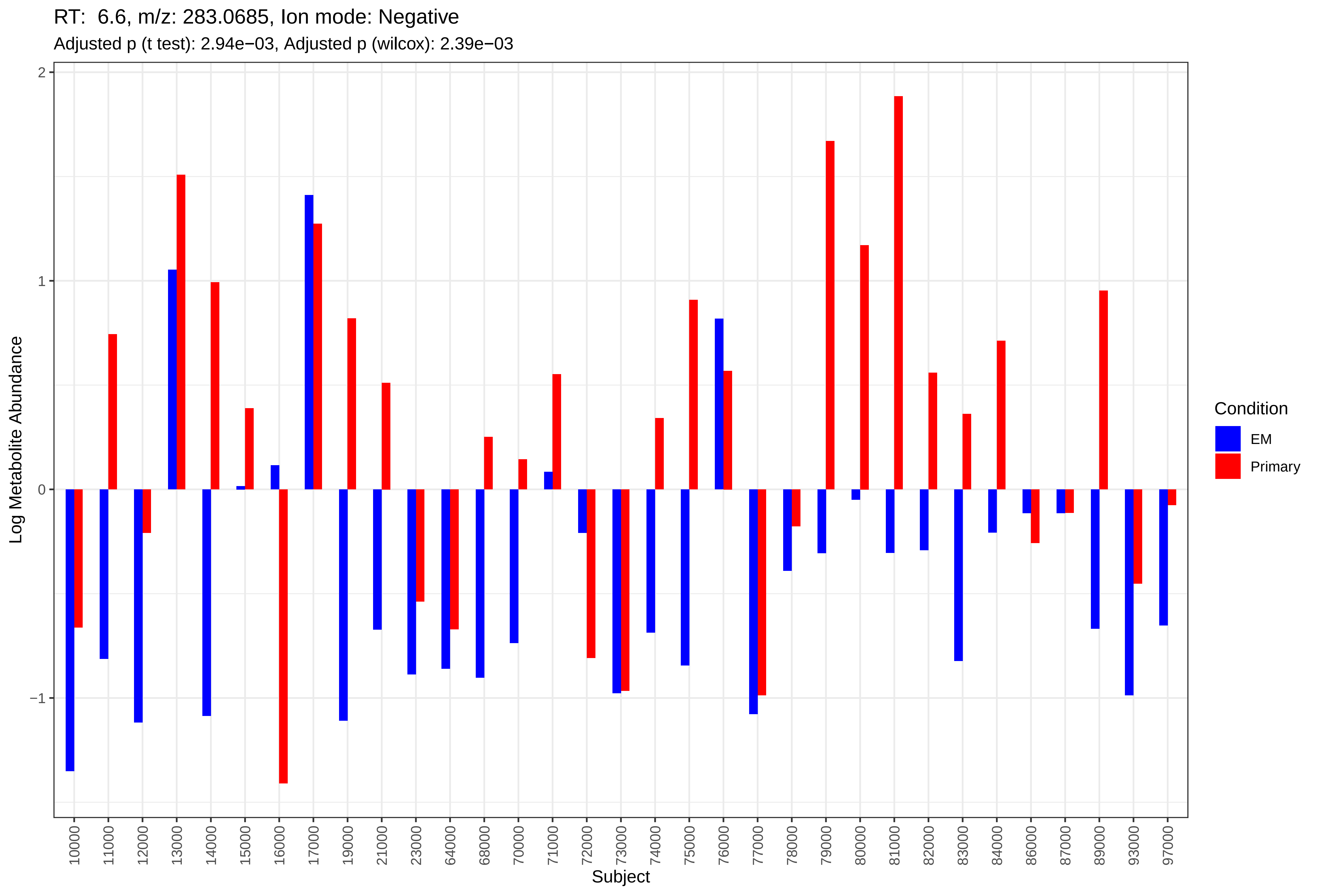
RT: 6.6, m/z: 283.0685, Ion mode: Negative
Adjusted p (t test): 2.94e−03, Adjusted p (wilcox): 2.39e−03
Log Metabolite Abundance
2
1
0
−1
Subject
10000
11000
12000
13000
14000
15000
16000
17000
19000
21000
23000
64000
68000
70000
71000
72000
73000
74000
75000
76000
77000
78000
79000
80000
81000
82000
83000
84000
86000
87000
89000
93000
97000
Condition
EM
Primary

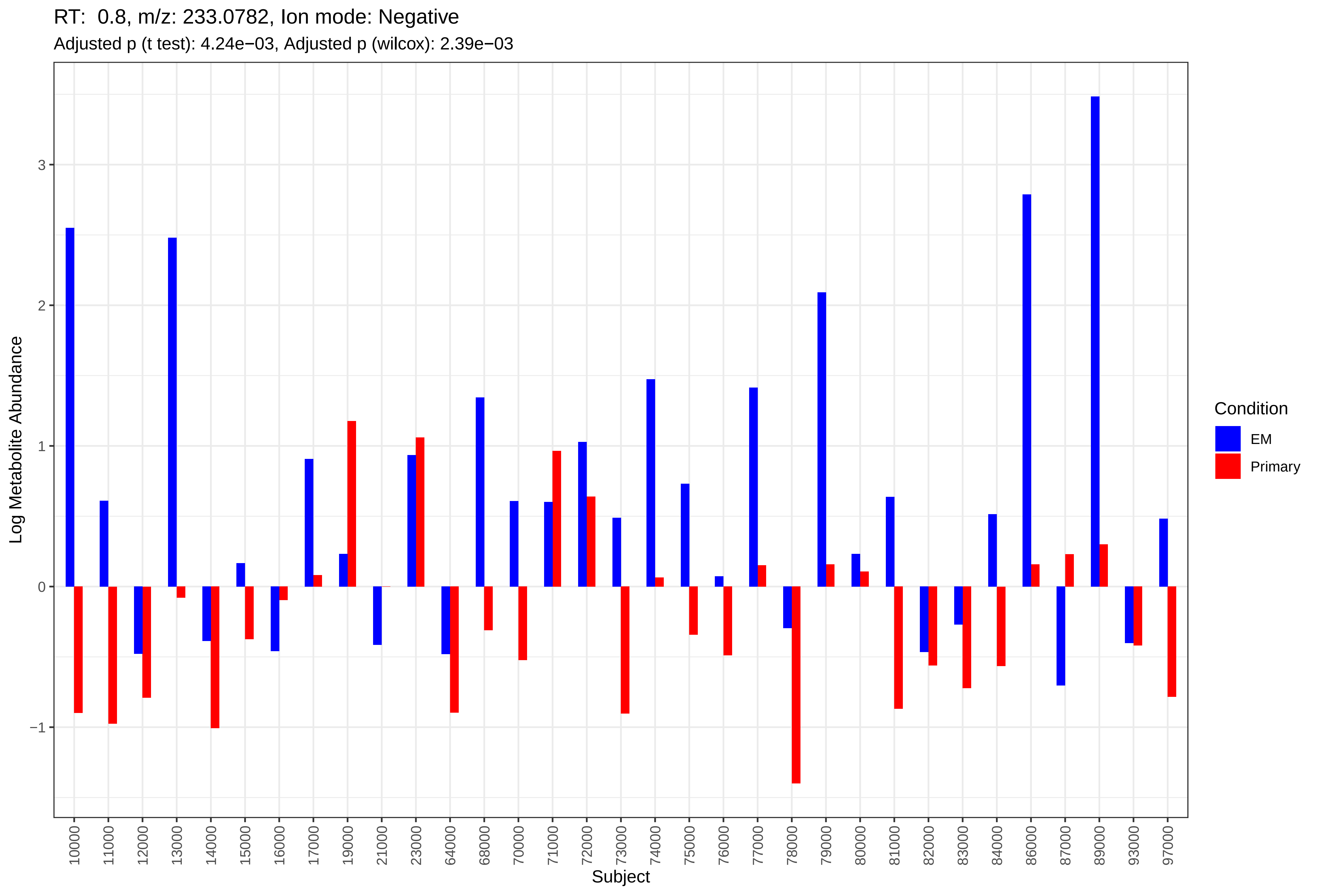

RT: 0.8, m/z: 233.0782, Ion mode: Negative
Adjusted p (t test): 4.24e−03, Adjusted p (wilcox): 2.39e−03
Log Metabolite Abundance
3
2
1
0
−1
10000
11000
12000
13000
14000
15000
16000
17000
19000
21000
23000
64000
68000
70000
71000
72000
73000
74000
75000
76000
77000
78000
79000
80000
81000
82000
83000
84000
86000
87000
89000
93000
97000
Subject
Condition
EM
Primary

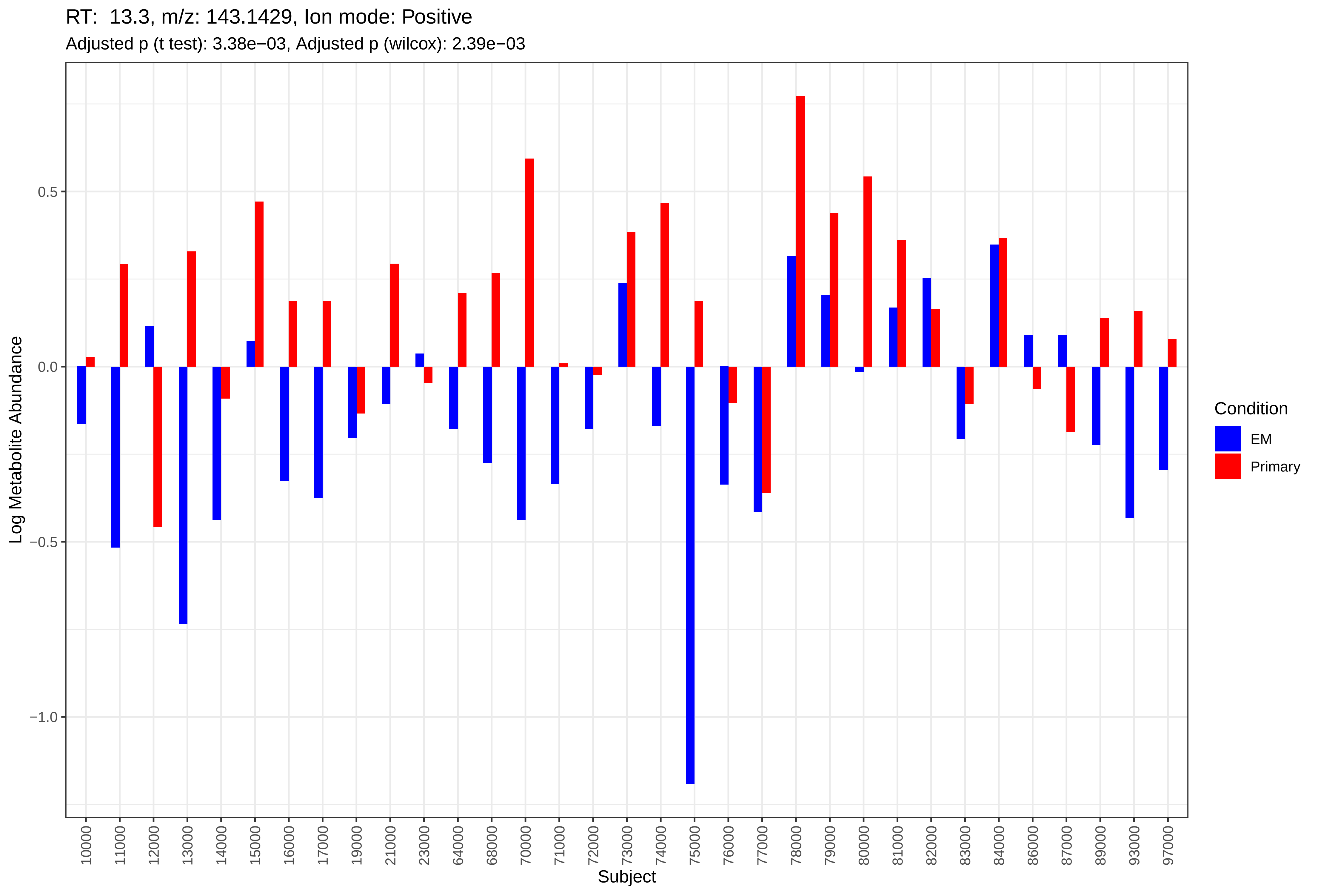
RT: 13.3, m/z: 143.1429, Ion mode: Positive
Adjusted p (t test): 3.38e−03, Adjusted p (wilcox): 2.39e−03
Log Metabolite Abundance
0.5
0.0
−0.5
−1.0
Subject
10000
11000
12000
13000
14000
15000
16000
17000
19000
21000
23000
64000
68000
70000
71000
72000
73000
74000
75000
76000
77000
78000
79000
80000
81000
82000
83000
84000
86000
87000
89000
93000
97000
Condition
EM
Primary

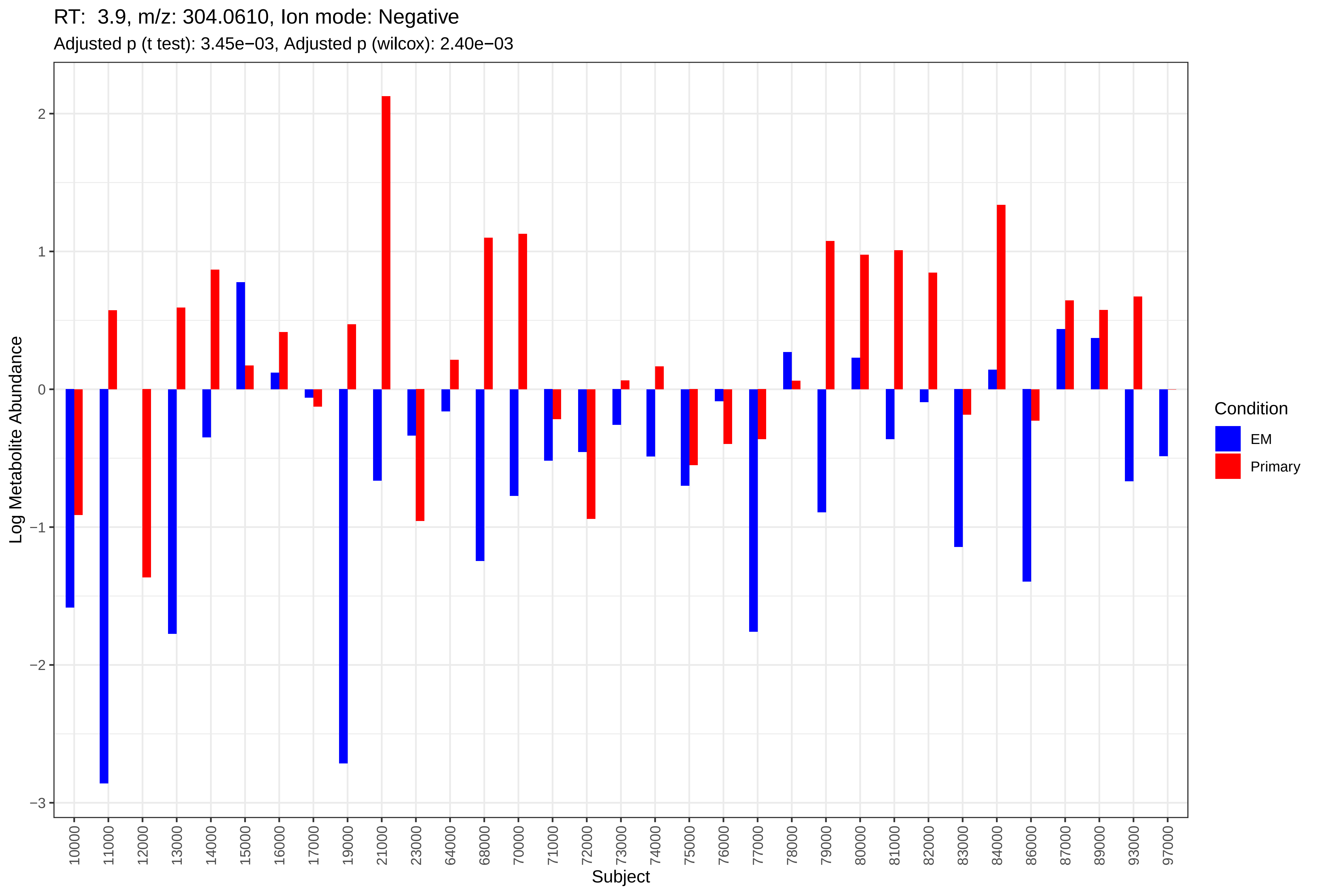

RT: 3.9, m/z: 304.0610, Ion mode: Negative
Adjusted p (t test): 3.45e−03, Adjusted p (wilcox): 2.40e−03
Log Metabolite Abundance
2
1
0
−1
−2
−3
10000
11000
12000
13000
14000
15000
16000
17000
19000
21000
23000
64000
68000
70000
71000
72000
73000
74000
75000
76000
77000
78000
79000
80000
81000
82000
83000
84000
86000
87000
89000
93000
97000
Subject
Condition
EM
Primary

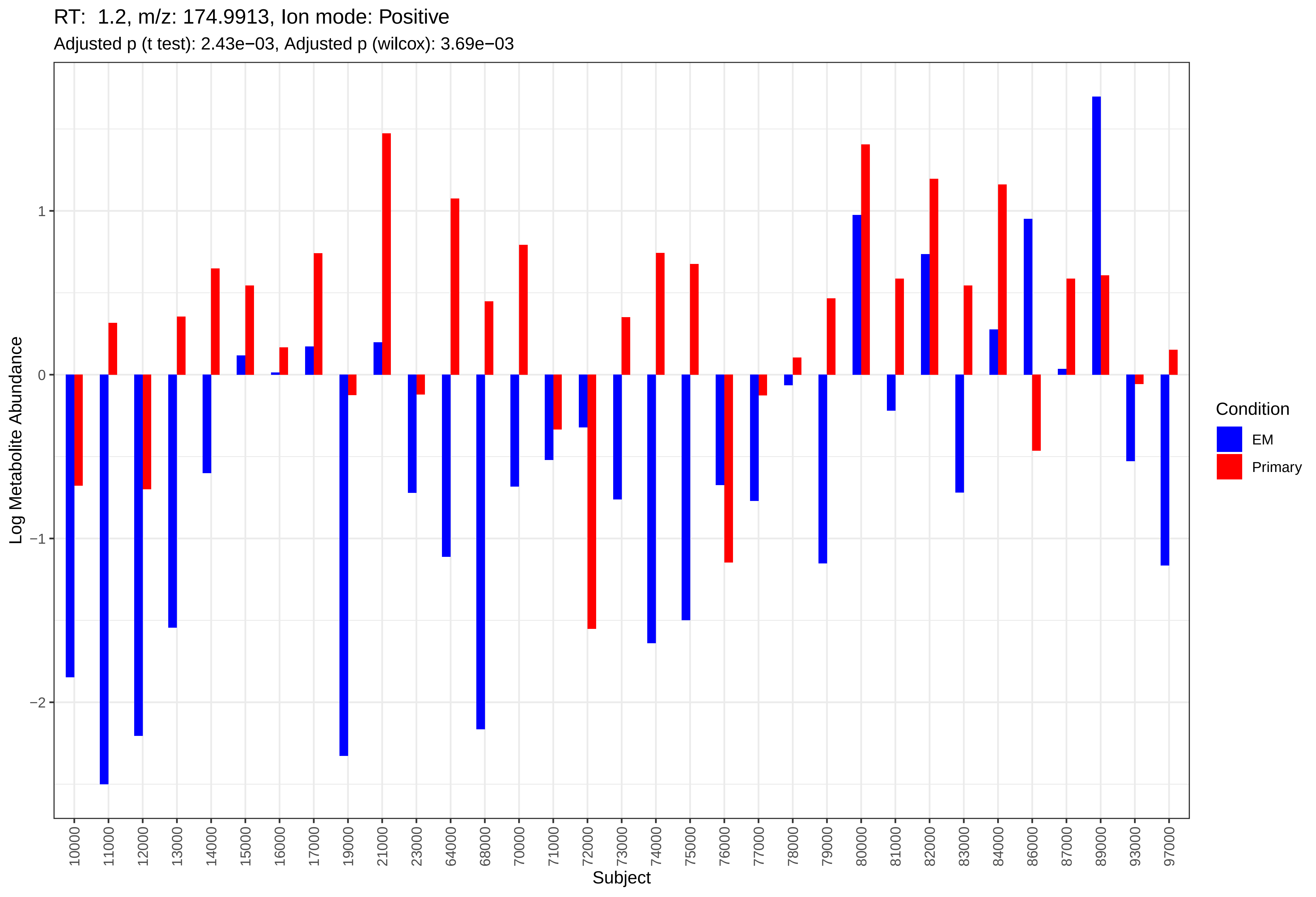

RT: 1.2, m/z: 174.9913, Ion mode: Positive
Adjusted p (t test): 2.43e−03, Adjusted p (wilcox): 3.69e−03
Log Metabolite Abundance
1
0
−1
−2
Subject
10000
11000
12000
13000
14000
15000
16000
17000
19000
21000
23000
64000
68000
70000
71000
72000
73000
74000
75000
76000
77000
78000
79000
80000
81000
82000
83000
84000
86000
87000
89000
93000
97000
Condition
EM
Primary

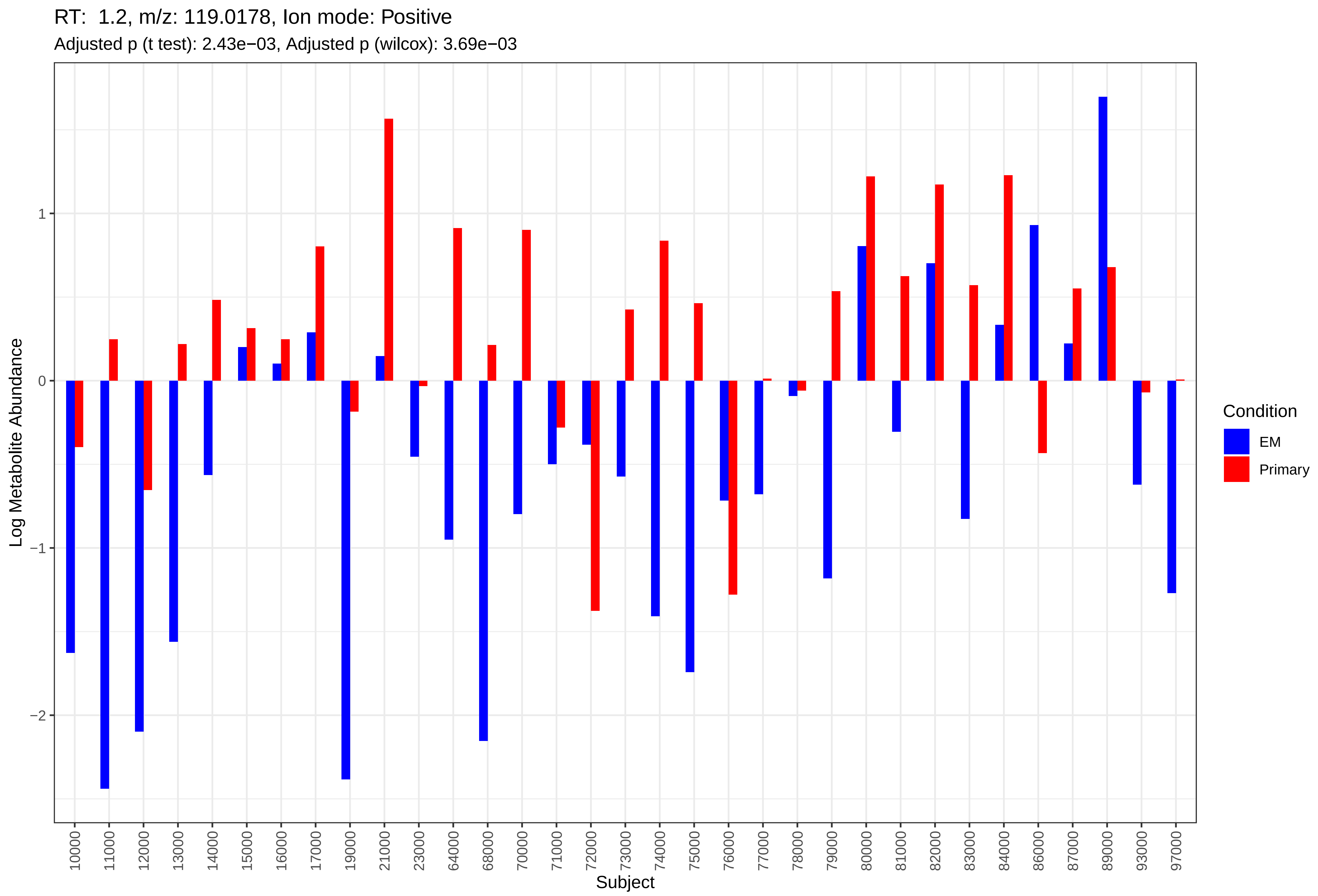
RT: 1.2, m/z: 119.0178, Ion mode: Positive
Adjusted p (t test): 2.43e−03, Adjusted p (wilcox): 3.69e−03
Log Metabolite Abundance
1
0
−1
−2
Subject
10000
11000
12000
13000
14000
15000
16000
17000
19000
21000
23000
64000
68000
70000
71000
72000
73000
74000
75000
76000
77000
78000
79000
80000
81000
82000
83000
84000
86000
87000
89000
93000
97000
Condition
EM
Primary

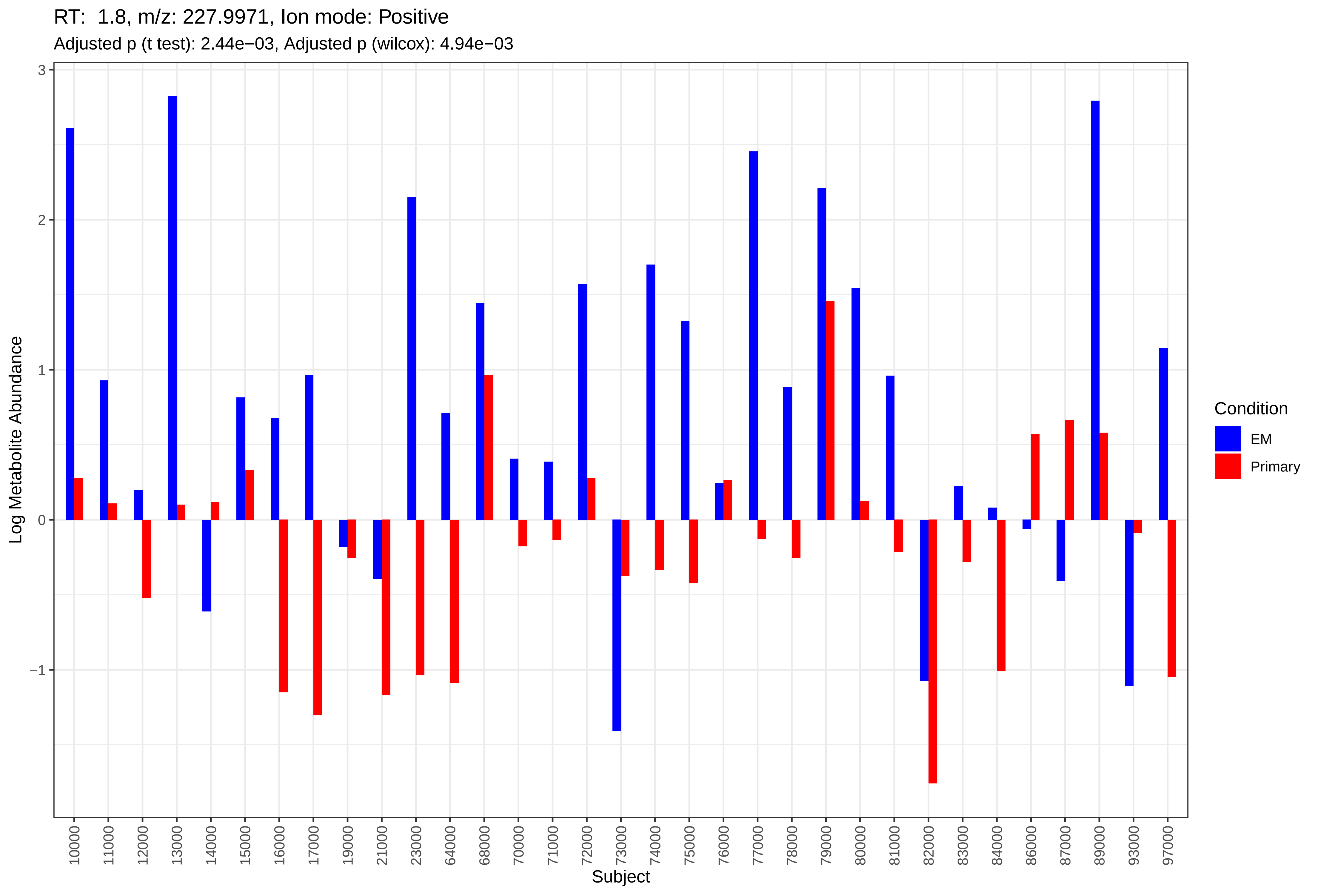
RT: 1.8, m/z: 227.9971, Ion mode: Positive
Adjusted p (t test): 2.44e−03, Adjusted p (wilcox): 4.94e−03
Log Metabolite Abundance
3
2
1
0
−1
Subject
10000
11000
12000
13000
14000
15000
16000
17000
19000
21000
23000
64000
68000
70000
71000
72000
73000
74000
75000
76000
77000
78000
79000
80000
81000
82000
83000
84000
86000
87000
89000
93000
97000
Condition
EM
Primary

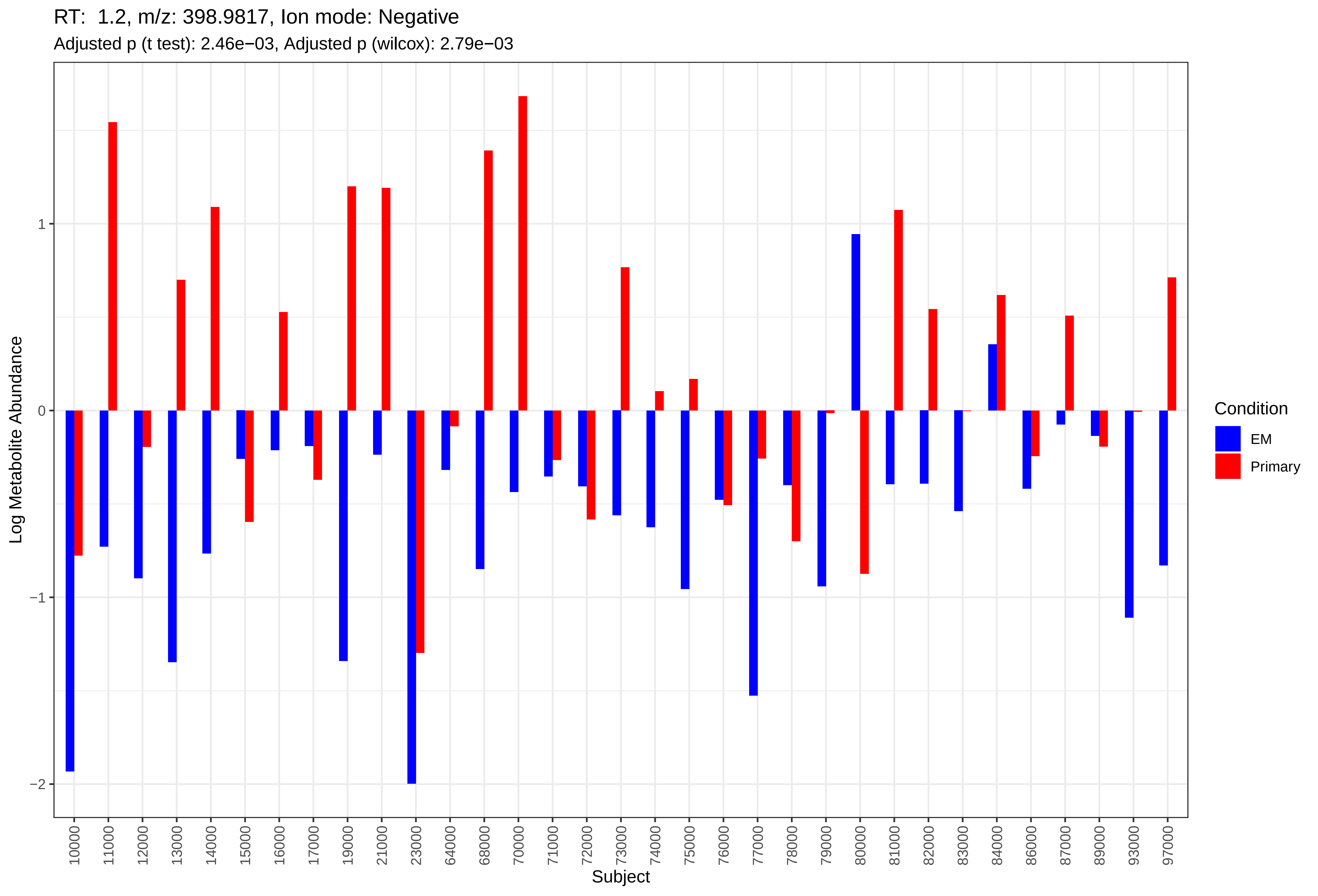

RT: 1.2, m/z: 398.9817, Ion mode: Negative
Adjusted p (t test): 2.46e−03, Adjusted p (wilcox): 2.79e−03
Log Metabolite Abundance
1
0
−1
−2
10000
11000
12000
13000
14000
15000
16000
17000
19000
21000
23000
64000
68000
70000
71000
72000
73000
74000
75000
76000
77000
78000
79000
80000
81000
82000
83000
84000
86000
87000
89000
93000
97000
Subject
Condition
EM
Primary

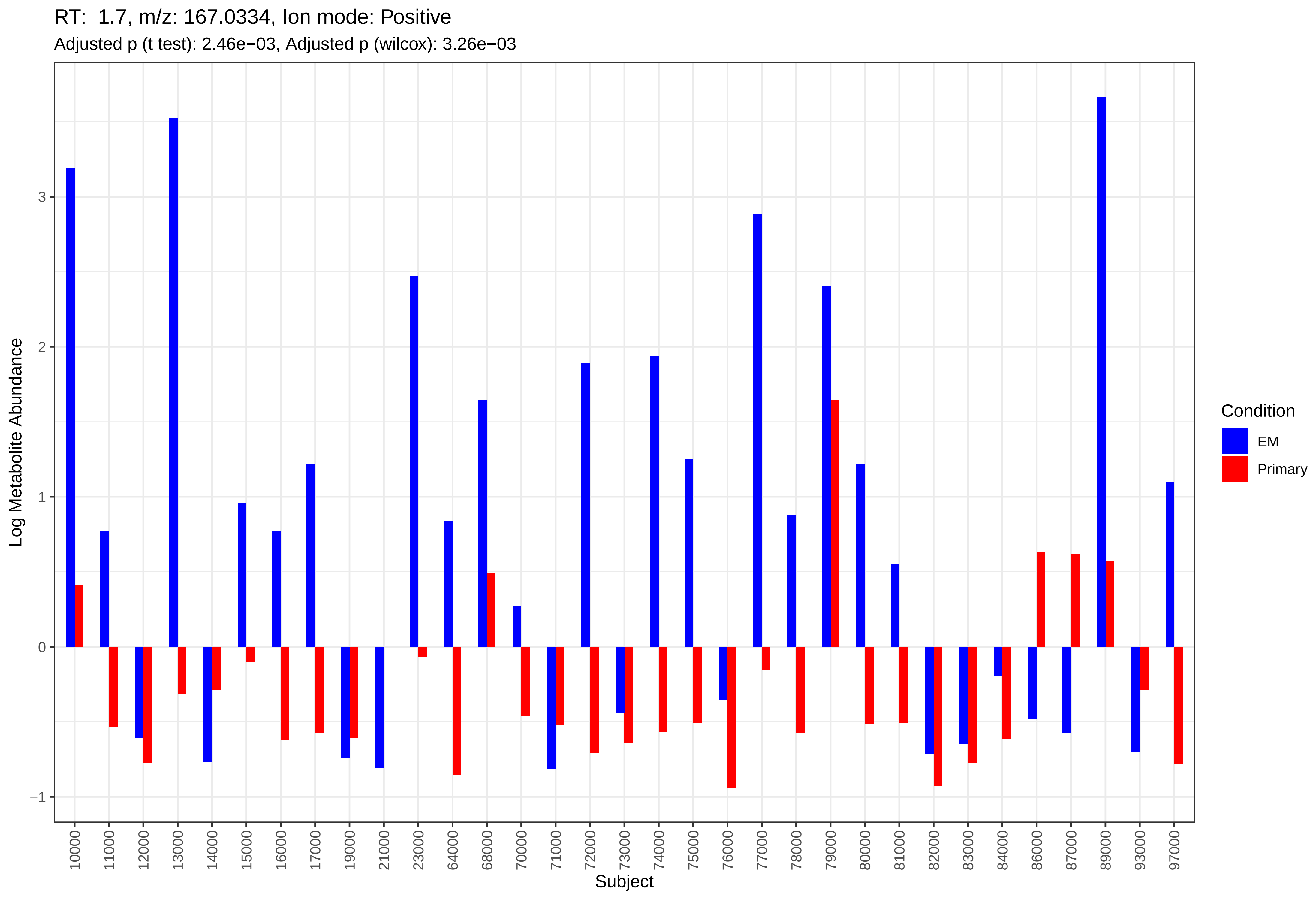
RT: 1.7, m/z: 167.0334, Ion mode: Positive
Adjusted p (t test): 2.46e−03, Adjusted p (wilcox): 3.26e−03
Log Metabolite Abundance
3
2
1
0
−1
10000
11000
12000
13000
14000
15000
16000
17000
19000
21000
23000
64000
68000
70000
71000
72000
73000
74000
75000
76000
77000
78000
79000
80000
81000
82000
83000
84000
86000
87000
89000
93000
97000
Subject
Condition
EM
Primary

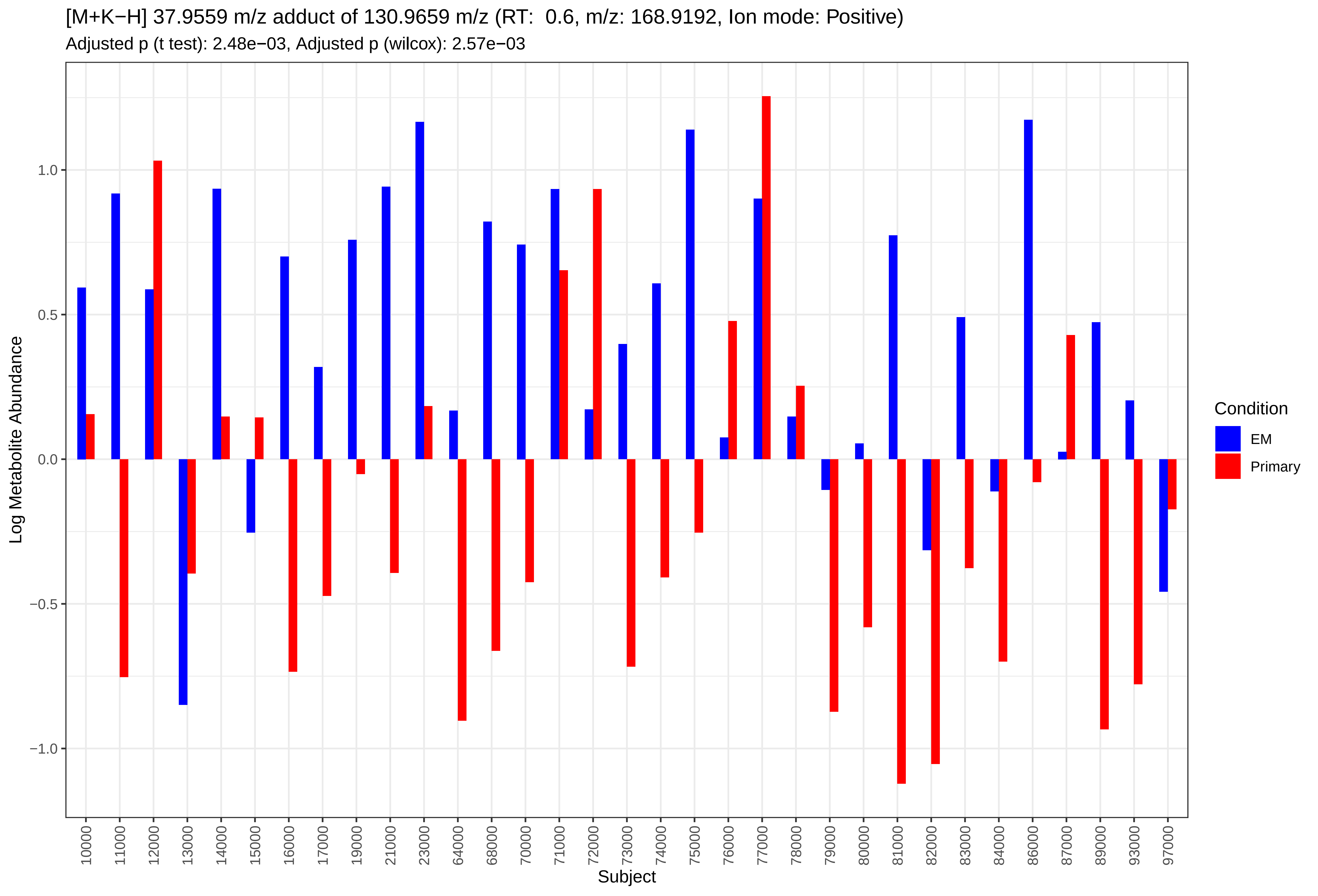

[M+K−H] 37.9559 m/z adduct of 130.9659 m/z (RT: 0.6, m/z: 168.9192, Ion mode: Positive)
Adjusted p (t test): 2.48e−03, Adjusted p (wilcox): 2.57e−03
Log Metabolite Abundance
1.0
0.5
0.0
−0.5
−1.0
10000
11000
12000
13000
14000
15000
16000
17000
19000
21000
23000
64000
68000
70000
71000
72000
73000
74000
75000
76000
77000
78000
79000
80000
81000
82000
83000
84000
86000
87000
89000
93000
97000
Subject
Condition
EM
Primary

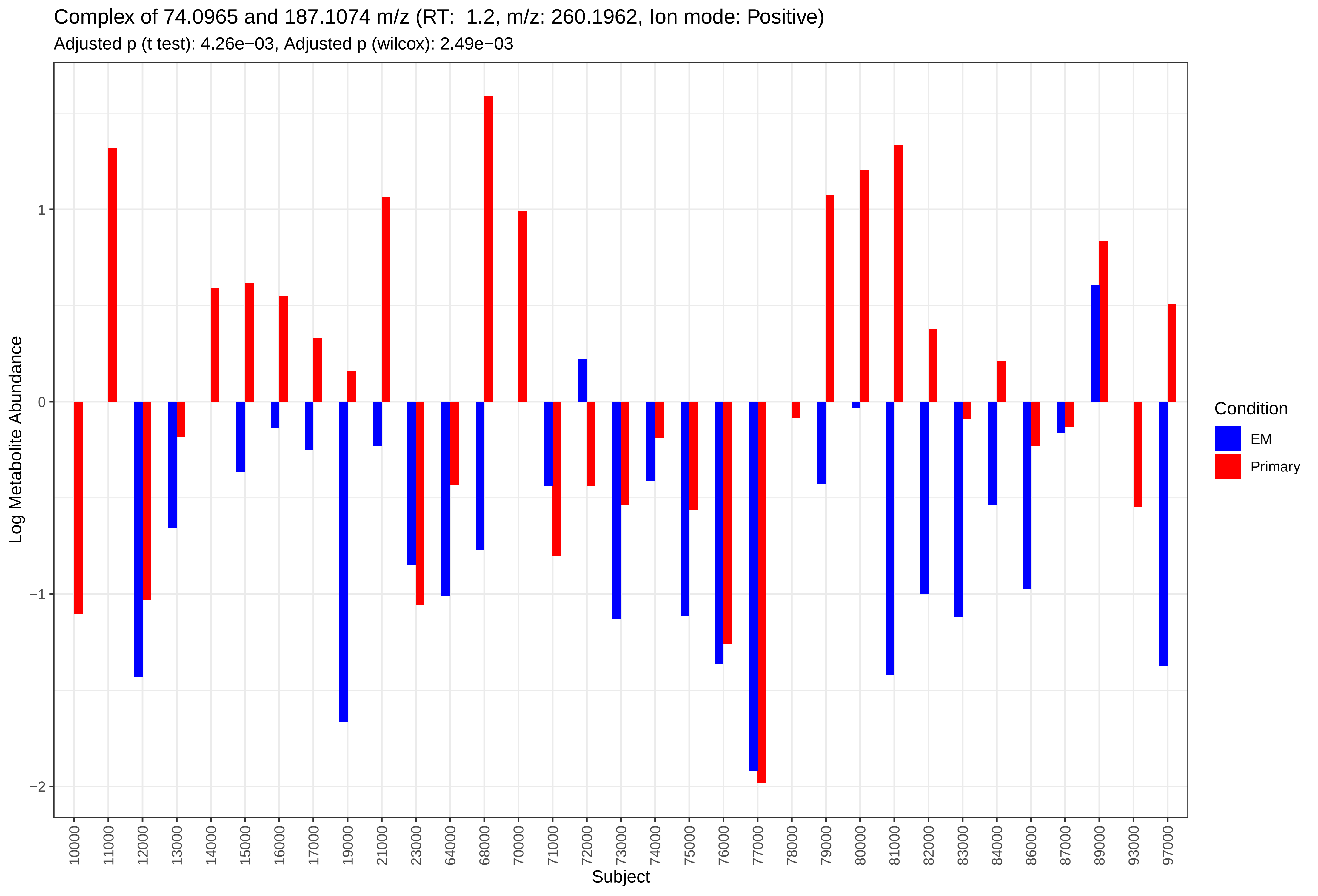

Complex of 74.0965 and 187.1074 m/z (RT: 1.2, m/z: 260.1962, Ion mode: Positive)
Adjusted p (t test): 4.26e−03, Adjusted p (wilcox): 2.49e−03
Log Metabolite Abundance
1
0
−1
−2
10000
11000
12000
13000
14000
15000
16000
17000
19000
21000
23000
64000
68000
70000
71000
72000
73000
74000
75000
76000
77000
78000
79000
80000
81000
82000
83000
84000
86000
87000
89000
93000
97000
Subject
Condition
EM
Primary

RT: 1.4, m/z: 215.0675, Ion mode: Negative

Adjusted p (t test): 2.50e−03, Adjusted p (wilcox): 4.94e−03

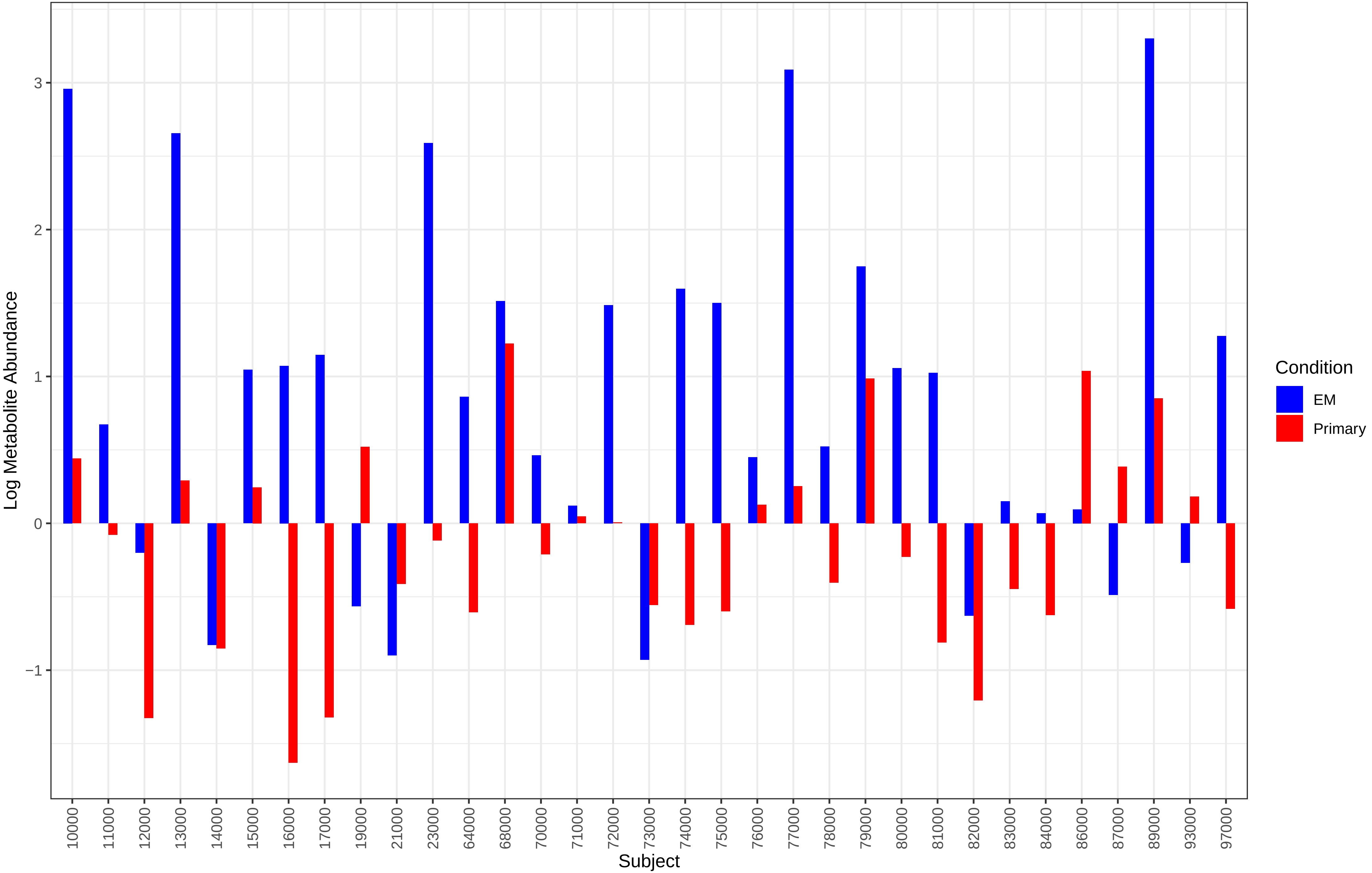

RT: 0.7, m/z: 119.0011, Ion mode: Positive

Adjusted p (t test): 2.50e−03, Adjusted p (wilcox): 4.63e−03

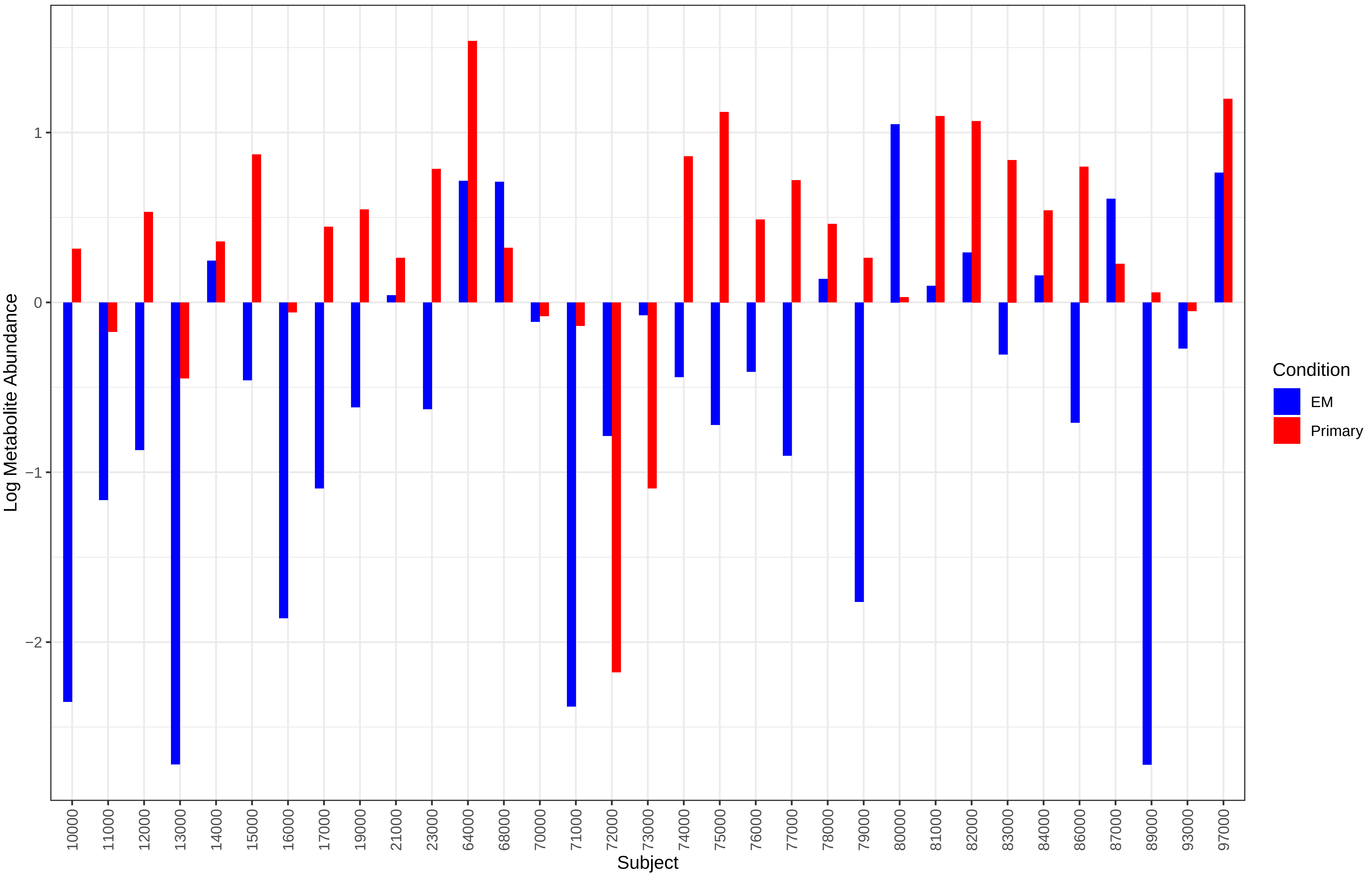

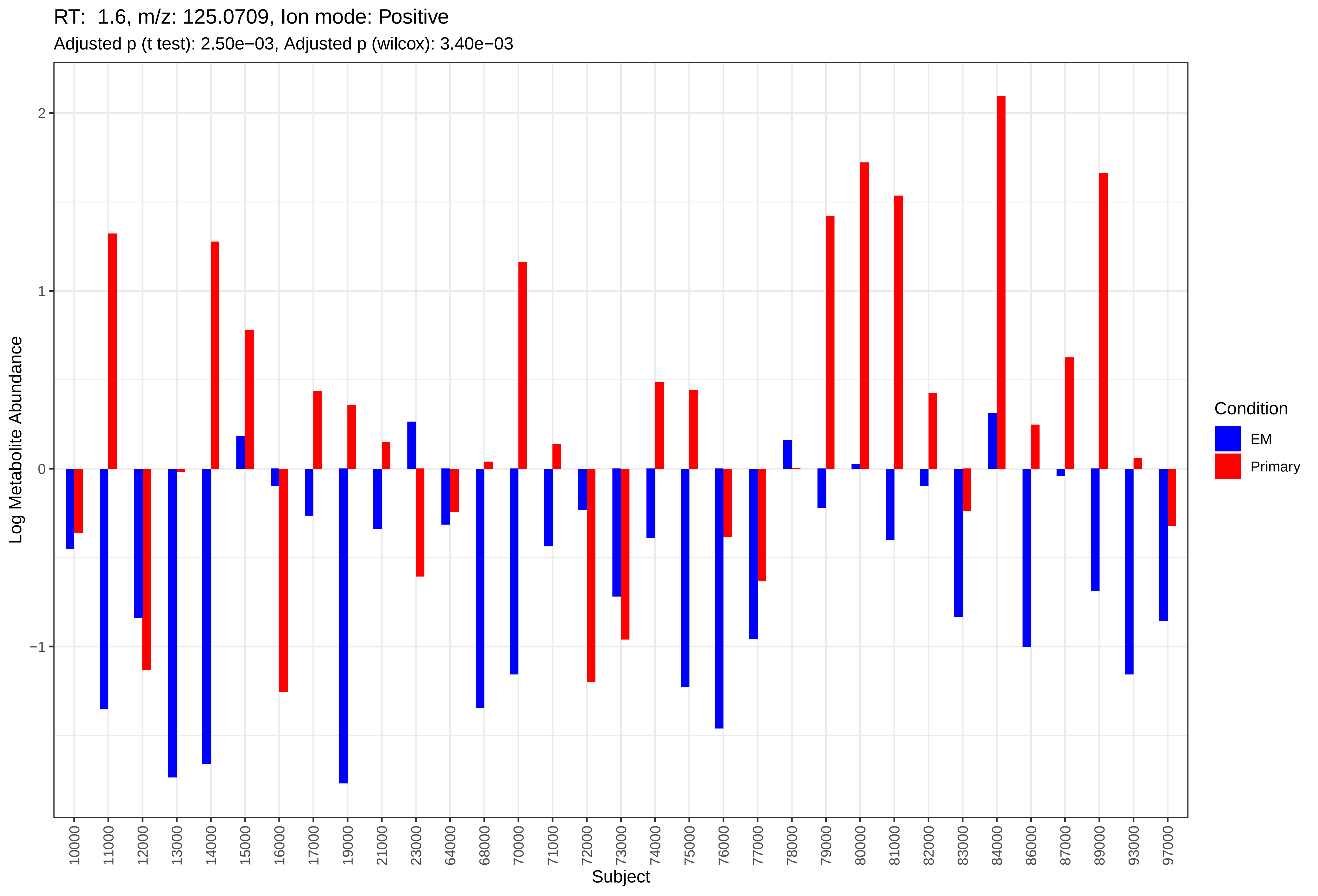
RT: 1.6, m/z: 125.0709, Ion mode: Positive
Adjusted p (t test): 2.50e−03, Adjusted p (wilcox): 3.40e−03
Log Metabolite Abundance
2
1
0
−1
Subject
10000
11000
12000
13000
14000
15000
16000
17000
19000
21000
23000
64000
68000
70000
71000
72000
73000
74000
75000
76000
77000
78000
79000
80000
81000
82000
83000
84000
86000
87000
89000
93000
97000
Condition
EM
Primary

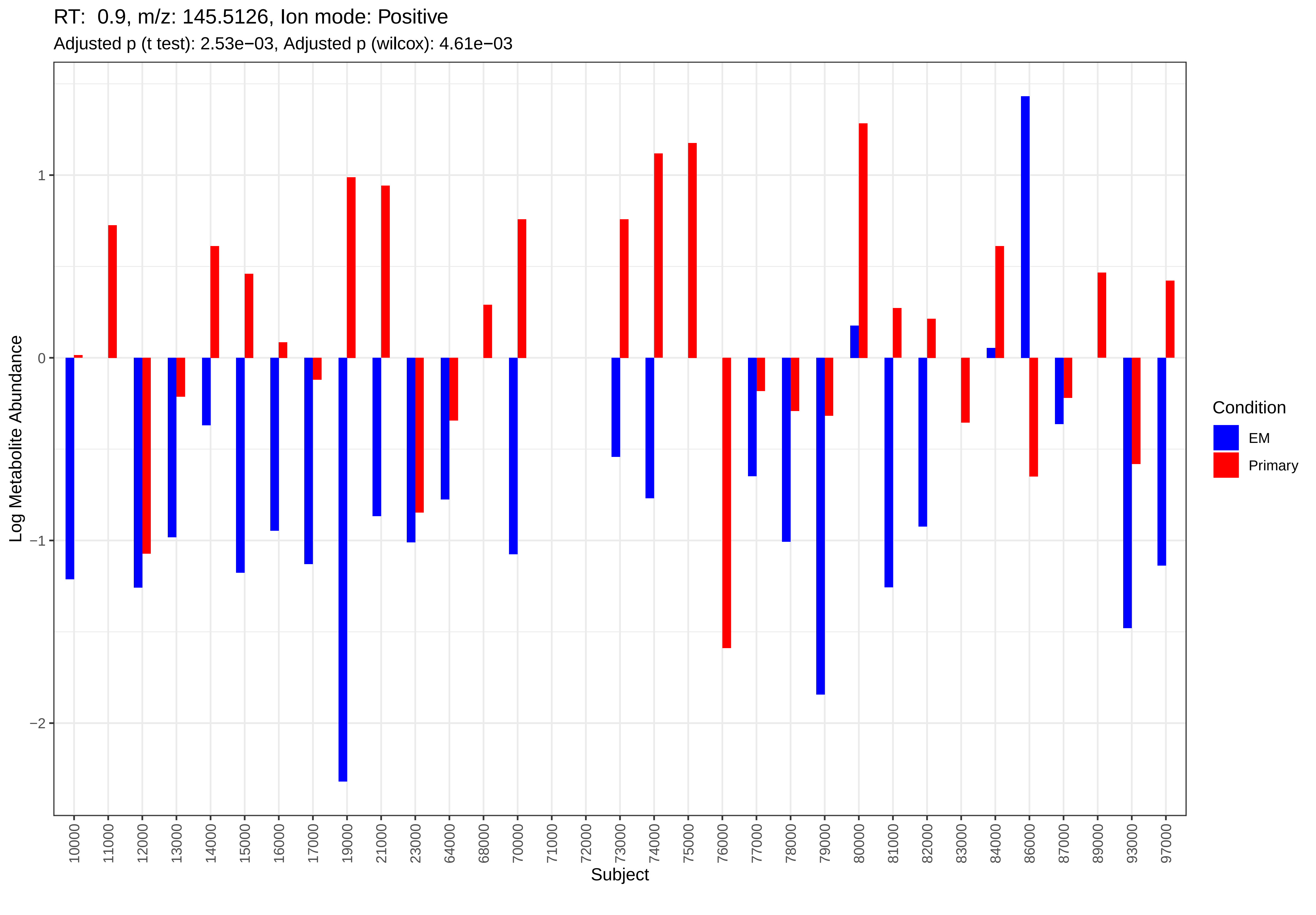

RT: 0.9, m/z: 145.5126, Ion mode: Positive
Adjusted p (t test): 2.53e−03, Adjusted p (wilcox): 4.61e−03
Log Metabolite Abundance
1
0
−1
−2
Subject
10000
11000
12000
13000
14000
15000
16000
17000
19000
21000
23000
64000
68000
70000
71000
72000
73000
74000
75000
76000
77000
78000
79000
80000
81000
82000
83000
84000
86000
87000
89000
93000
97000
Condition
EM
Primary

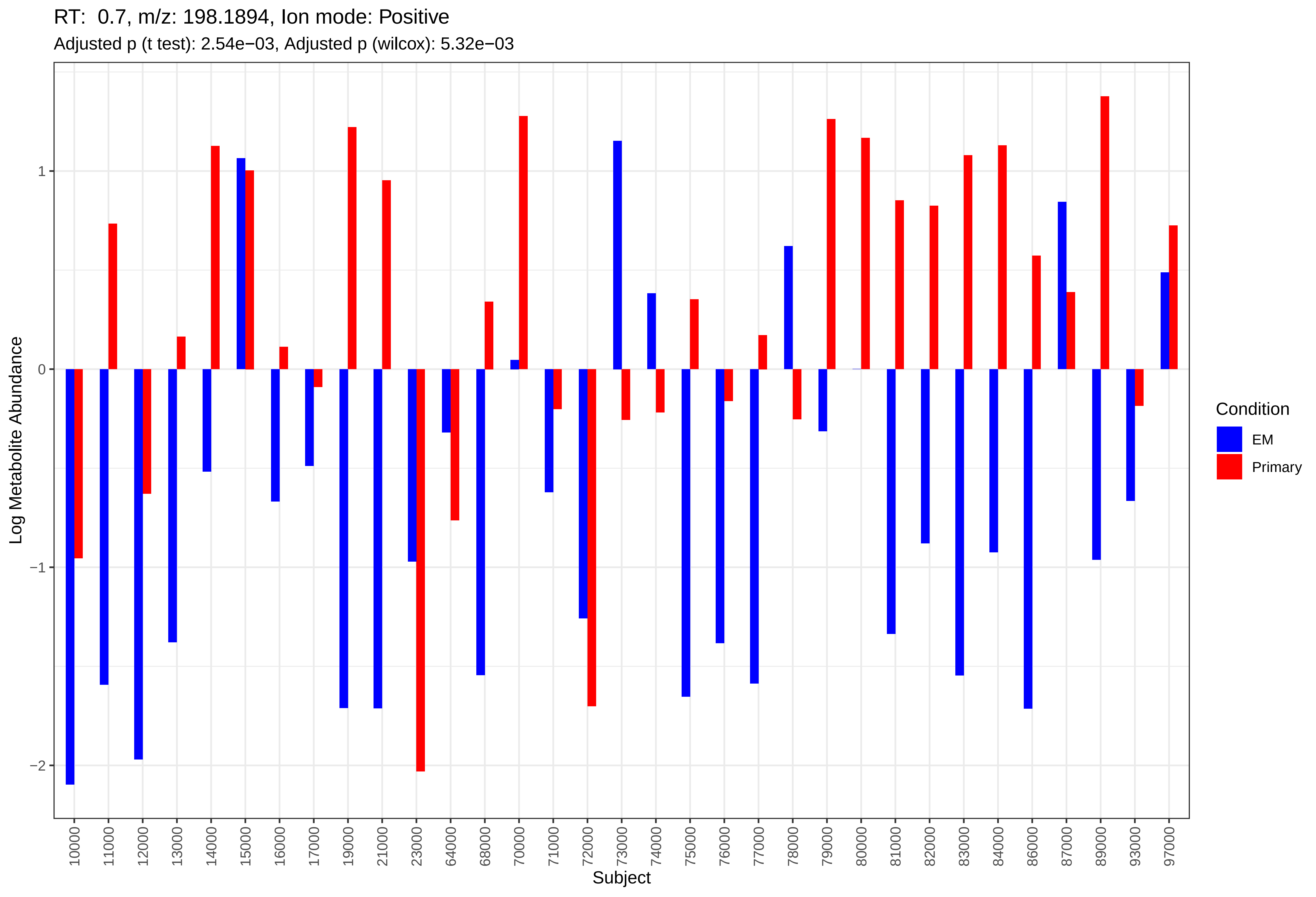

RT: 0.7, m/z: 198.1894, Ion mode: Positive
Adjusted p (t test): 2.54e−03, Adjusted p (wilcox): 5.32e−03
Log Metabolite Abundance
1
0
−1
−2
10000
11000
12000
13000
14000
15000
16000
17000
19000
21000
23000
64000
68000
70000
71000
72000
73000
74000
75000
76000
77000
78000
79000
80000
81000
82000
83000
84000
86000
87000
89000
93000
97000
Subject
Condition
EM
Primary

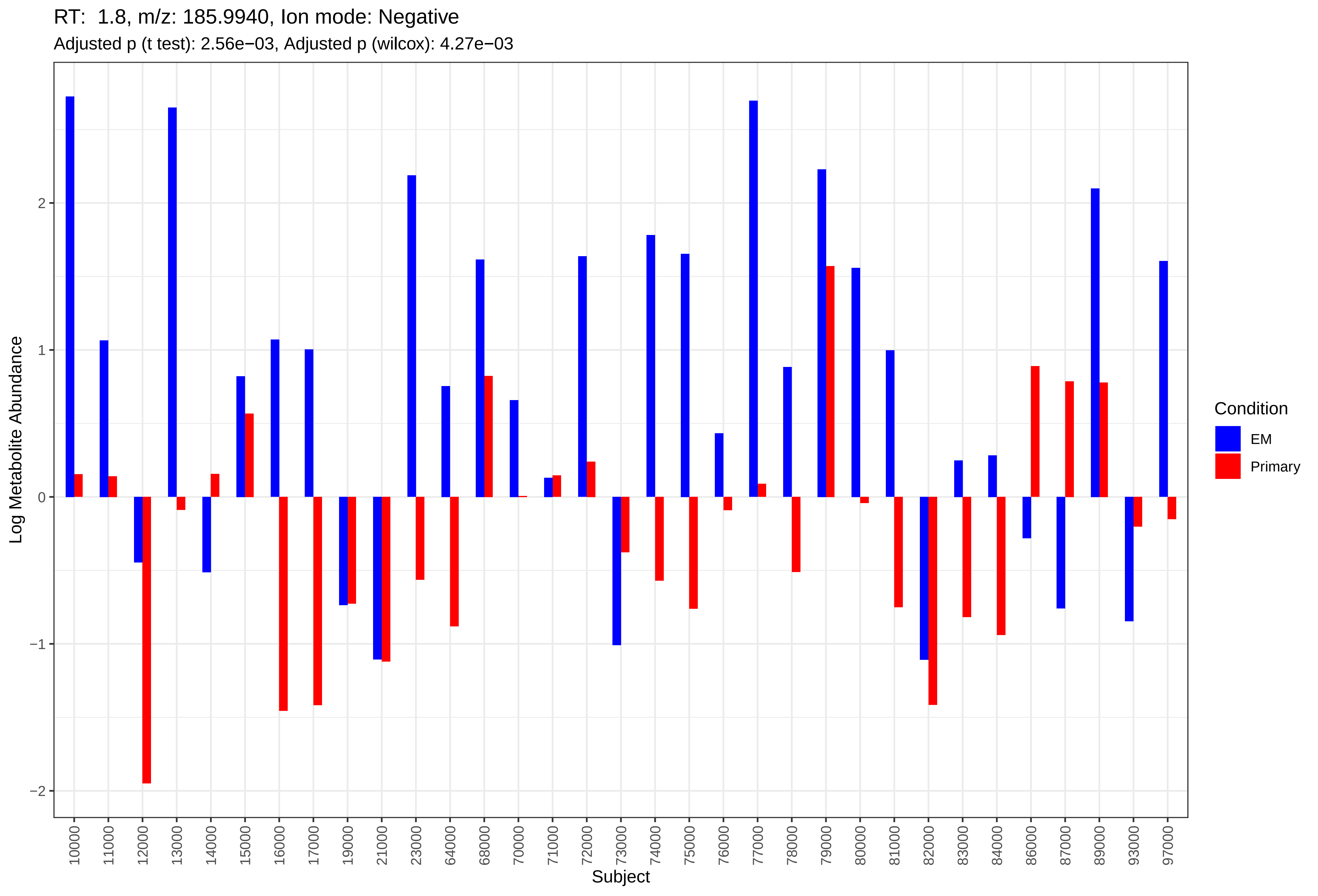

RT: 1.8, m/z: 185.9940, Ion mode: Negative
Adjusted p (t test): 2.56e−03, Adjusted p (wilcox): 4.27e−03
Log Metabolite Abundance
2
1
0
−1
−2
10000
11000
12000
13000
14000
15000
16000
17000
19000
21000
23000
64000
68000
70000
71000
72000
73000
74000
75000
76000
77000
78000
79000
80000
81000
82000
83000
84000
86000
87000
89000
93000
97000
Subject
Condition
EM
Primary

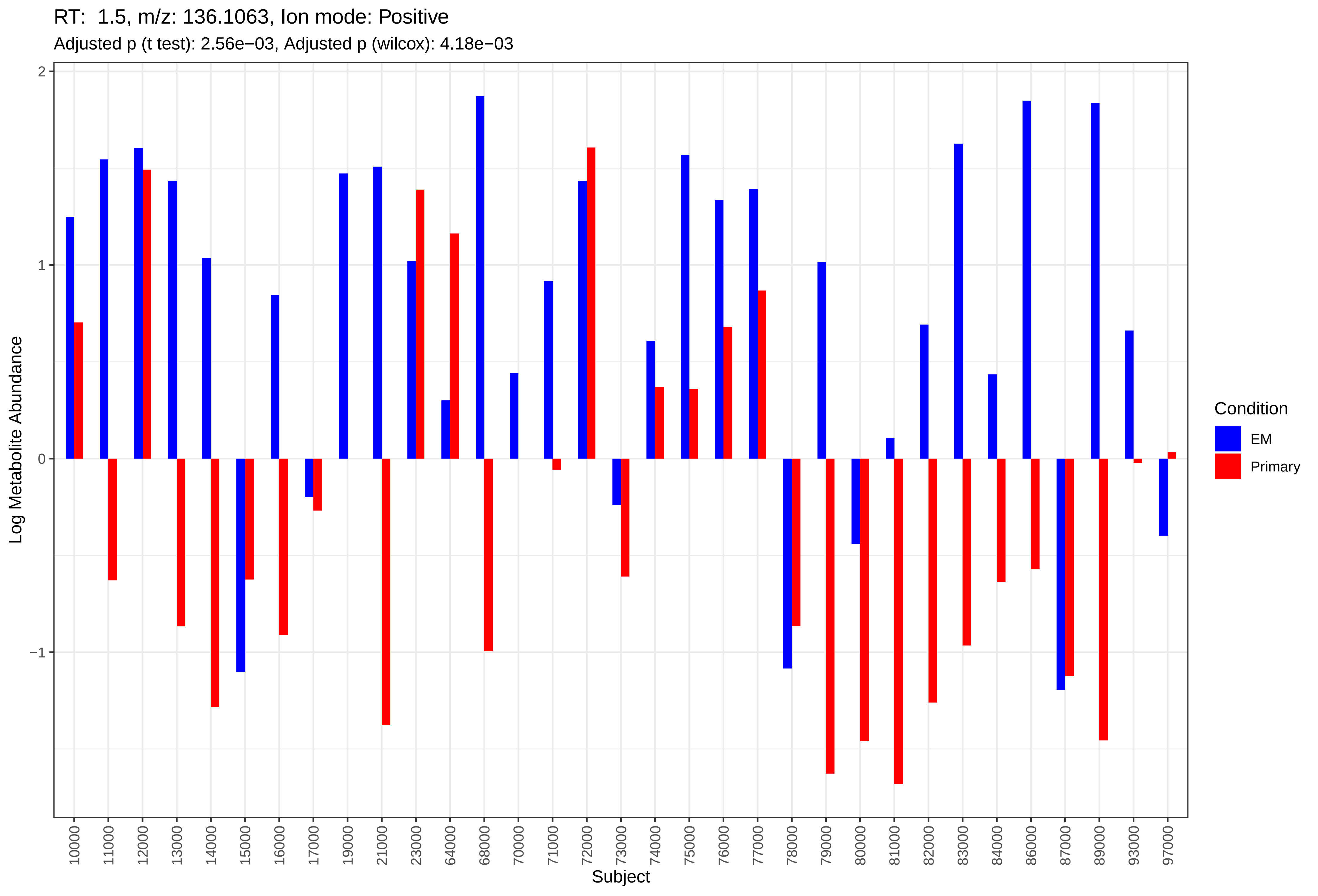
RT: 1.5, m/z: 136.1063, Ion mode: Positive
Adjusted p (t test): 2.56e−03, Adjusted p (wilcox): 4.18e−03
Log Metabolite Abundance
2
1
0
−1
Subject
10000
11000
12000
13000
14000
15000
16000
17000
19000
21000
23000
64000
68000
70000
71000
72000
73000
74000
75000
76000
77000
78000
79000
80000
81000
82000
83000
84000
86000
87000
89000
93000
97000
Condition
EM
Primary

RT: 3.9, m/z: 352.0799, Ion mode: Positive

Adjusted p (t test): 2.57e−03, Adjusted p (wilcox): 2.57e−03

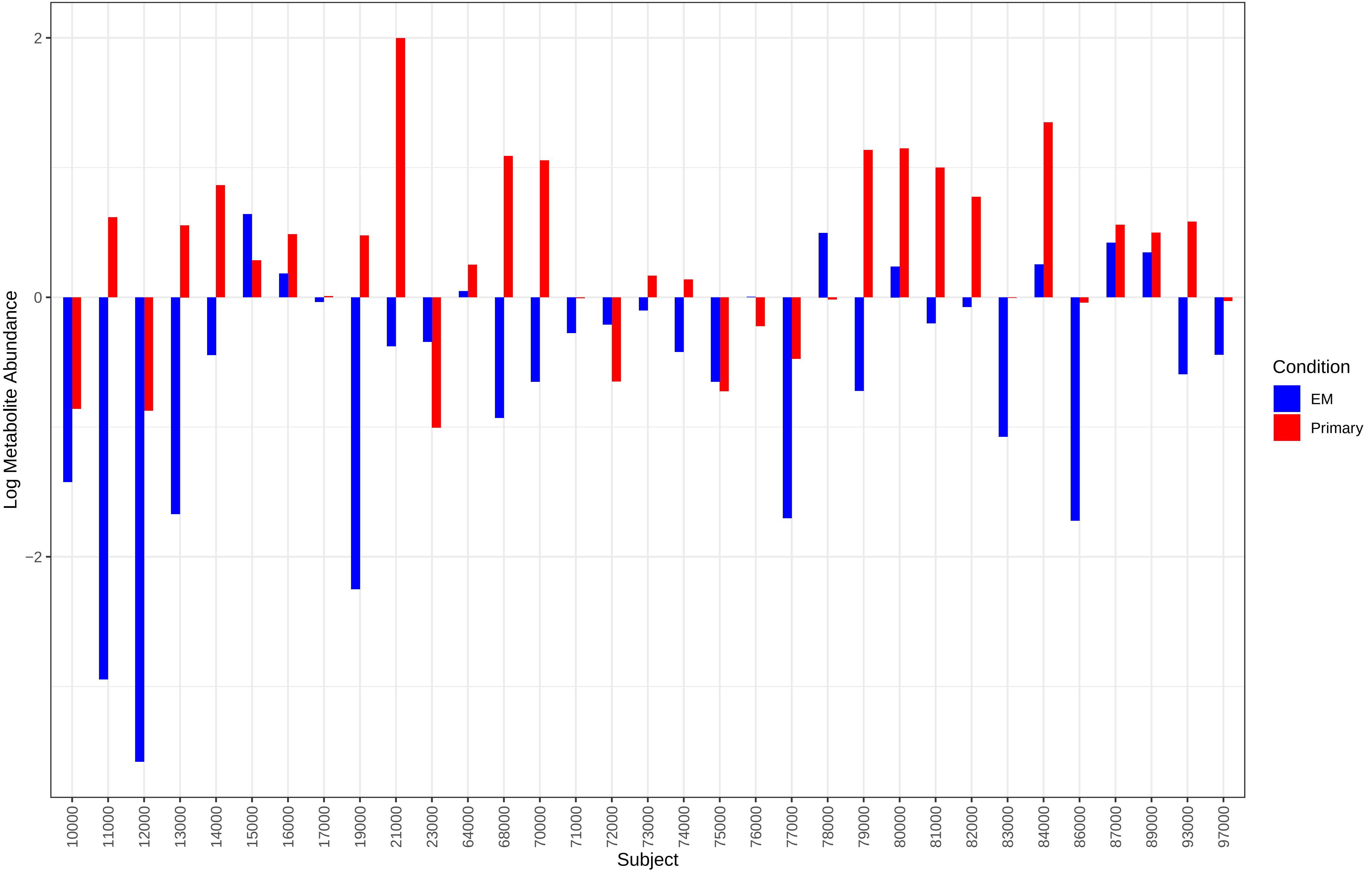

RT: 3.9, m/z: 308.5825, Ion mode: Positive

Adjusted p (t test): 2.57e−03, Adjusted p (wilcox): 2.79e−03

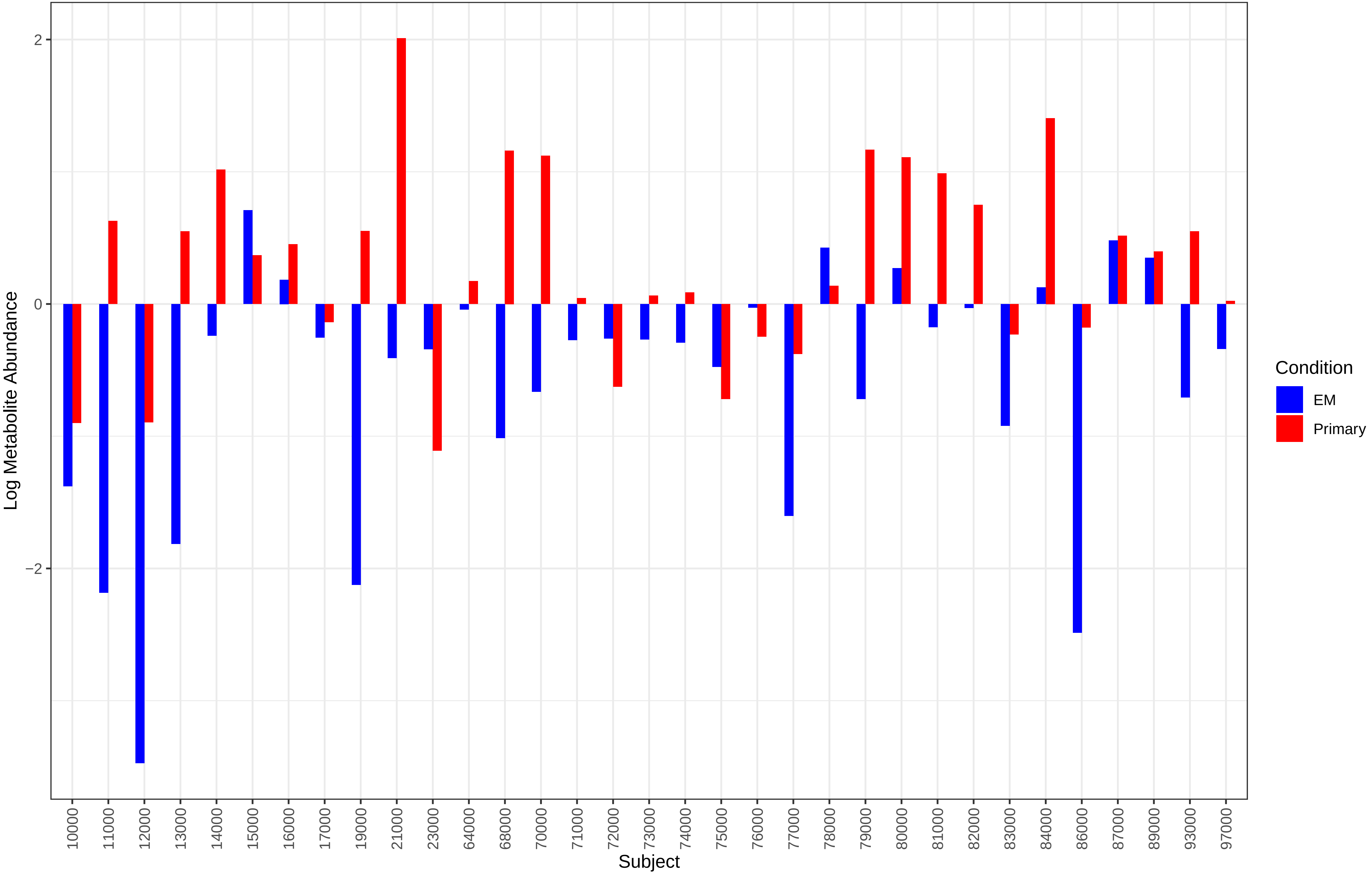

RT: 14.2, m/z: 136.8920, Ion mode: Negative

Adjusted p (t test): 3.43e−03, Adjusted p (wilcox): 2.57e−03

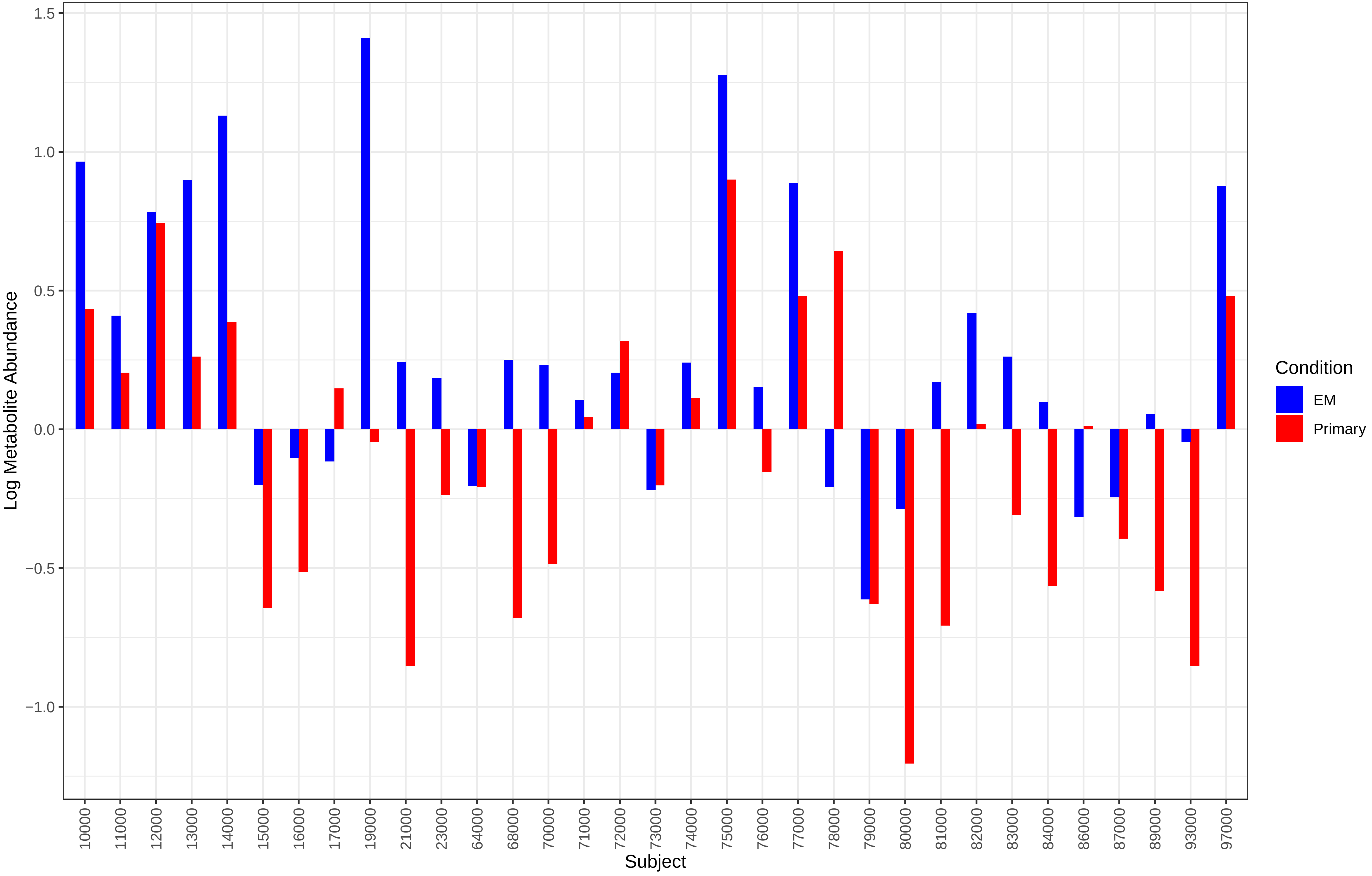

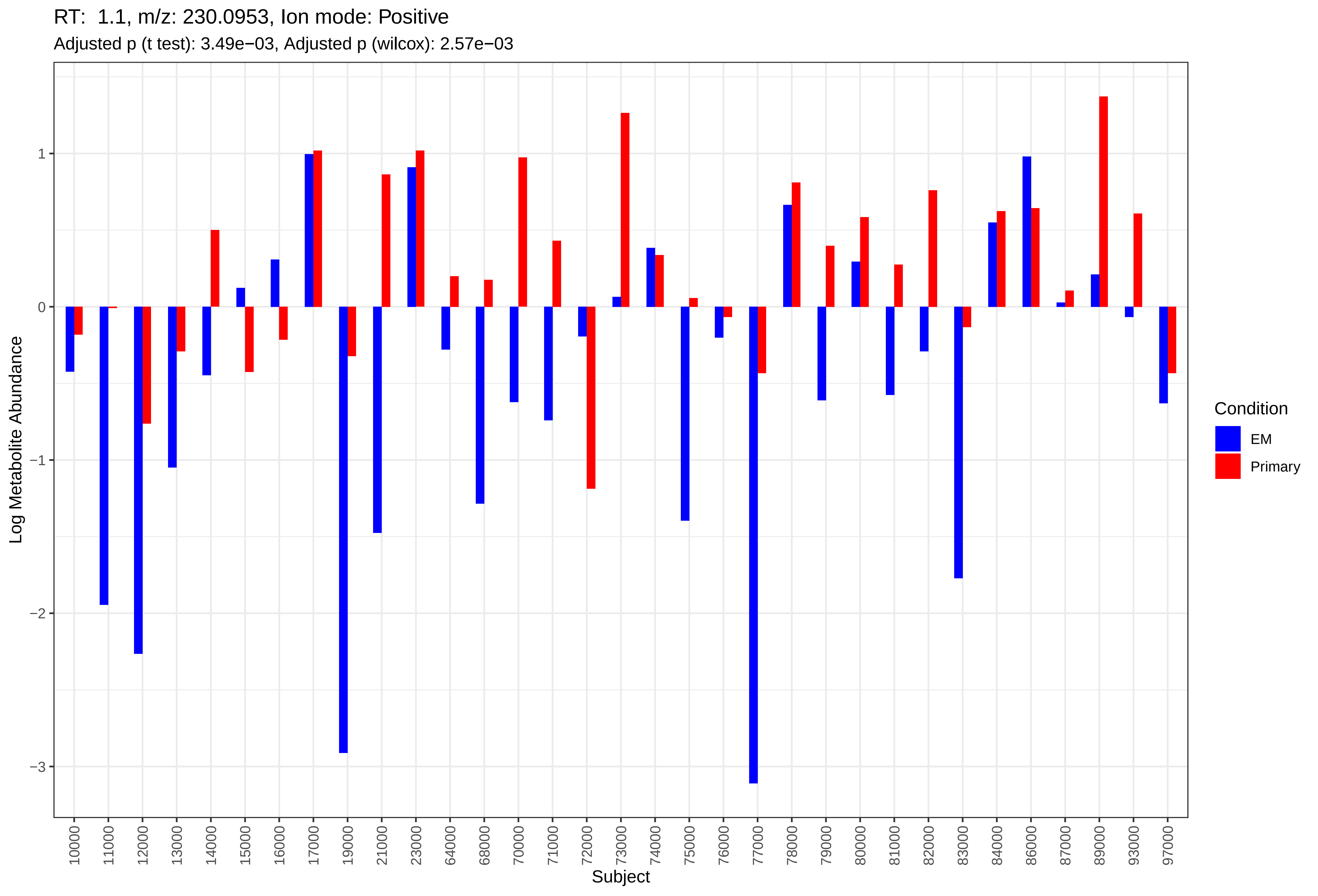

RT: 1.1, m/z: 230.0953, Ion mode: Positive
Adjusted p (t test): 3.49e−03, Adjusted p (wilcox): 2.57e−03
Log Metabolite Abundance
1
0
−1
−2
−3
10000
11000
12000
13000
14000
15000
16000
17000
19000
21000
23000
64000
68000
70000
71000
72000
73000
74000
75000
76000
77000
78000
79000
80000
81000
82000
83000
84000
86000
87000
89000
93000
97000
Subject
Condition
EM
Primary

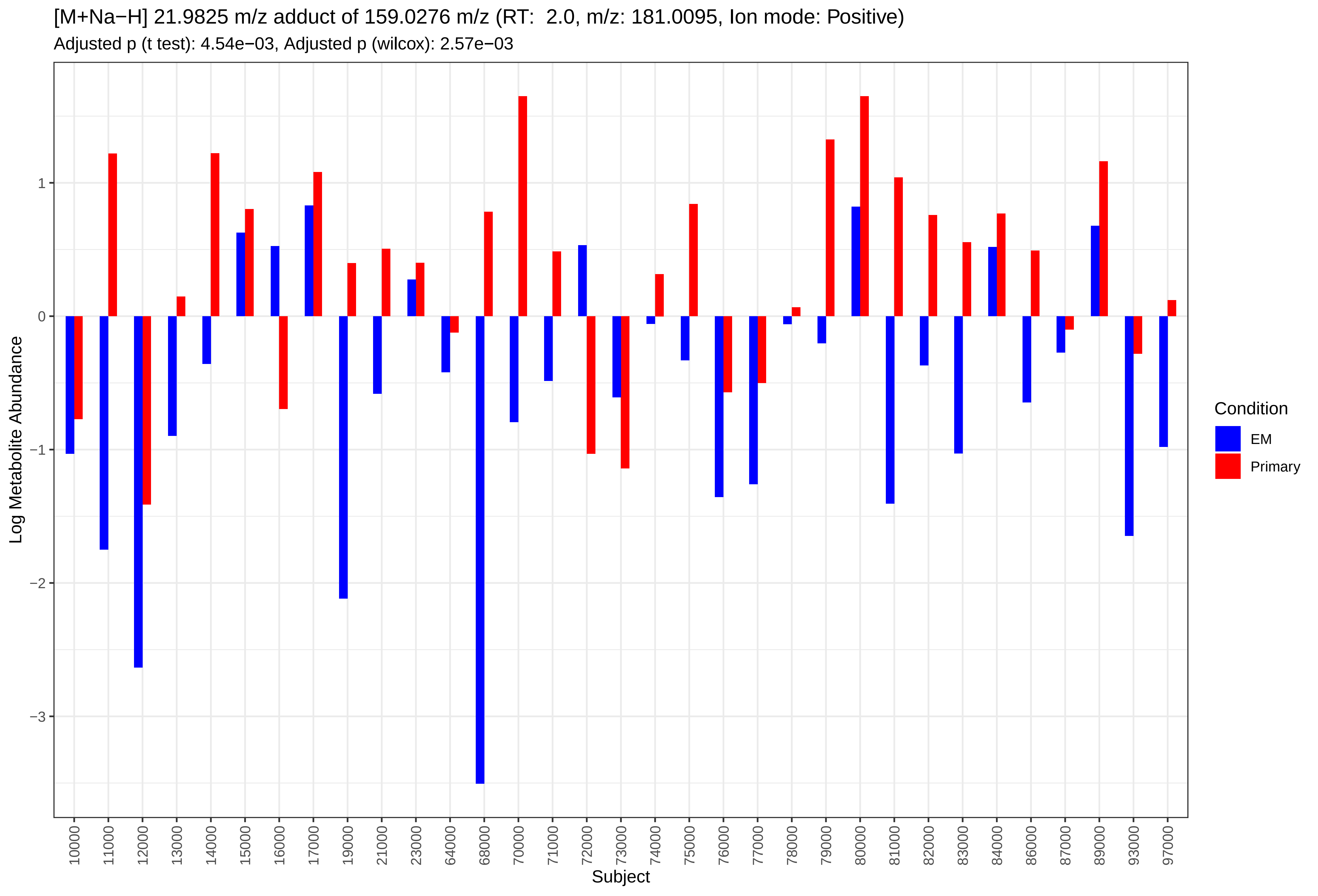
[M+Na−H] 21.9825 m/z adduct of 159.0276 m/z (RT: 2.0, m/z: 181.0095, Ion mode: Positive)
Adjusted p (t test): 4.54e−03, Adjusted p (wilcox): 2.57e−03
Log Metabolite Abundance
1
0
−1
−2
−3
10000
11000
12000
13000
14000
15000
16000
17000
19000
21000
23000
64000
68000
70000
71000
72000
73000
74000
75000
76000
77000
78000
79000
80000
81000
82000
83000
84000
86000
87000
89000
93000
97000
Subject
Condition
EM
Primary

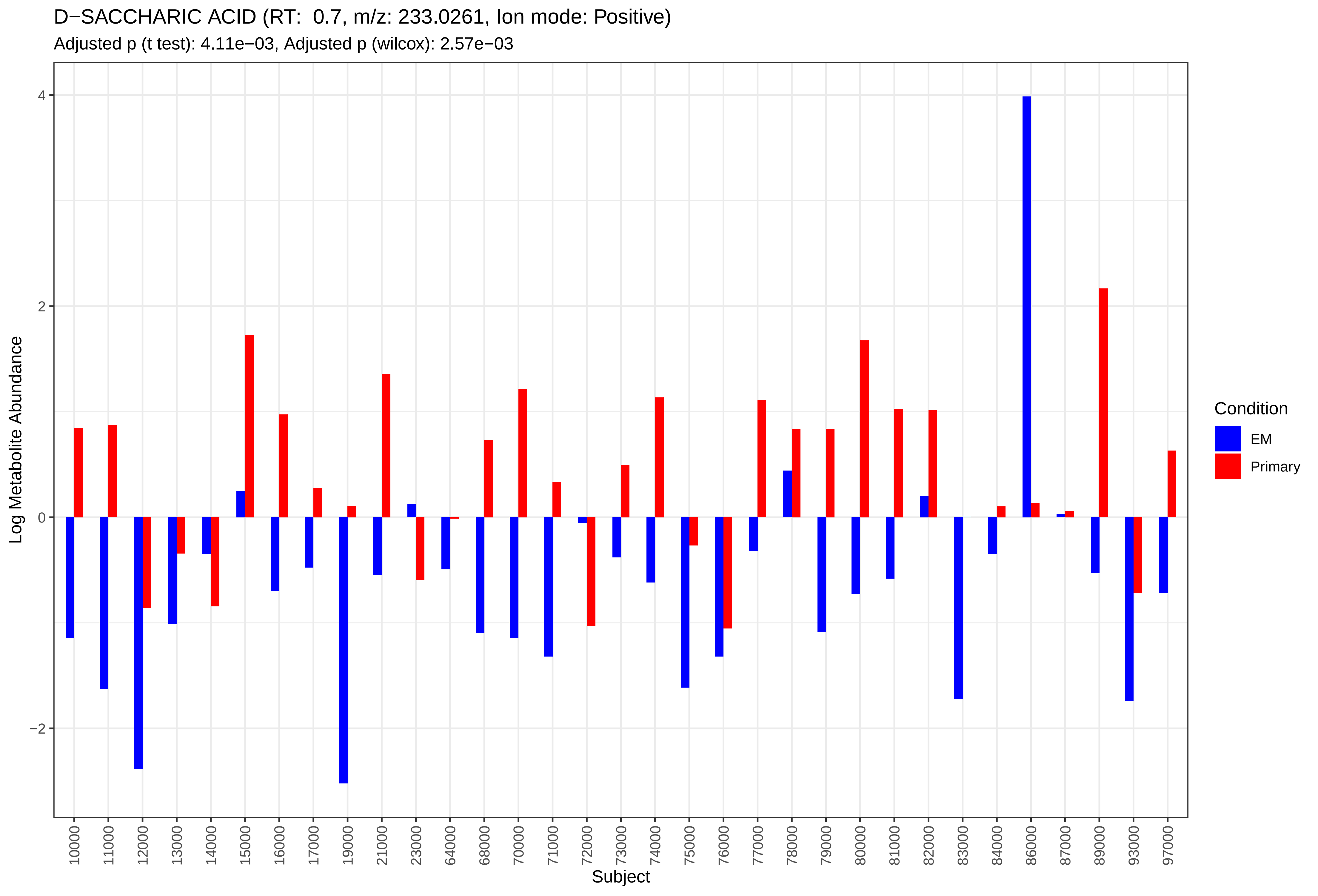

D−SACCHARIC ACID (RT: 0.7, m/z: 233.0261, Ion mode: Positive)
Adjusted p (t test): 4.11e−03, Adjusted p (wilcox): 2.57e−03
Log Metabolite Abundance
4
2
0
−2
Subject
10000
11000
12000
13000
14000
15000
16000
17000
19000
21000
23000
64000
68000
70000
71000
72000
73000
74000
75000
76000
77000
78000
79000
80000
81000
82000
83000
84000
86000
87000
89000
93000
97000
Condition
EM
Primary

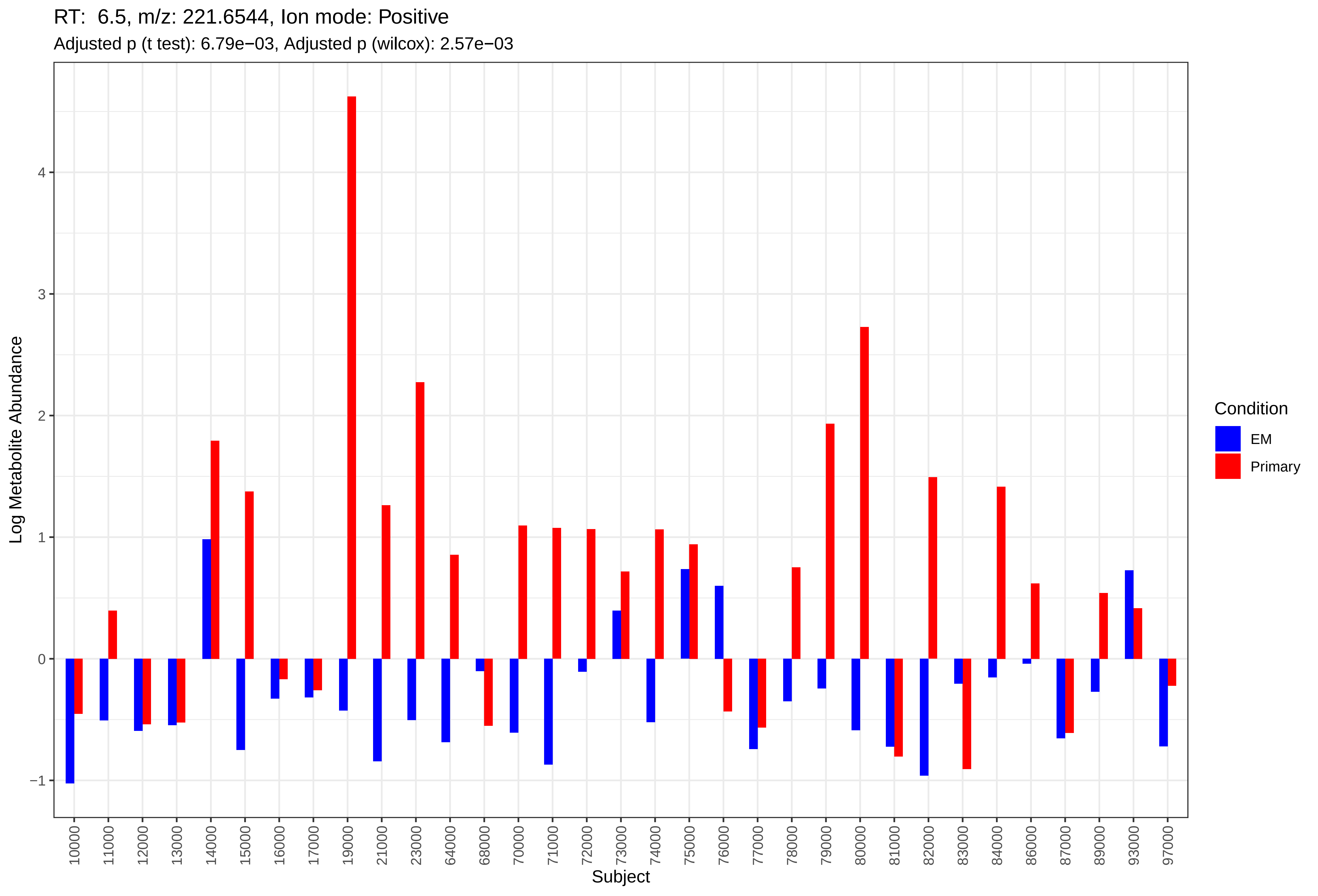
RT: 6.5, m/z: 221.6544, Ion mode: Positive
Adjusted p (t test): 6.79e−03, Adjusted p (wilcox): 2.57e−03
Log Metabolite Abundance
4
3
2
1
0
−1
Subject
10000
11000
12000
13000
14000
15000
16000
17000
19000
21000
23000
64000
68000
70000
71000
72000
73000
74000
75000
76000
77000
78000
79000
80000
81000
82000
83000
84000
86000
87000
89000
93000
97000
Condition
EM
Primary

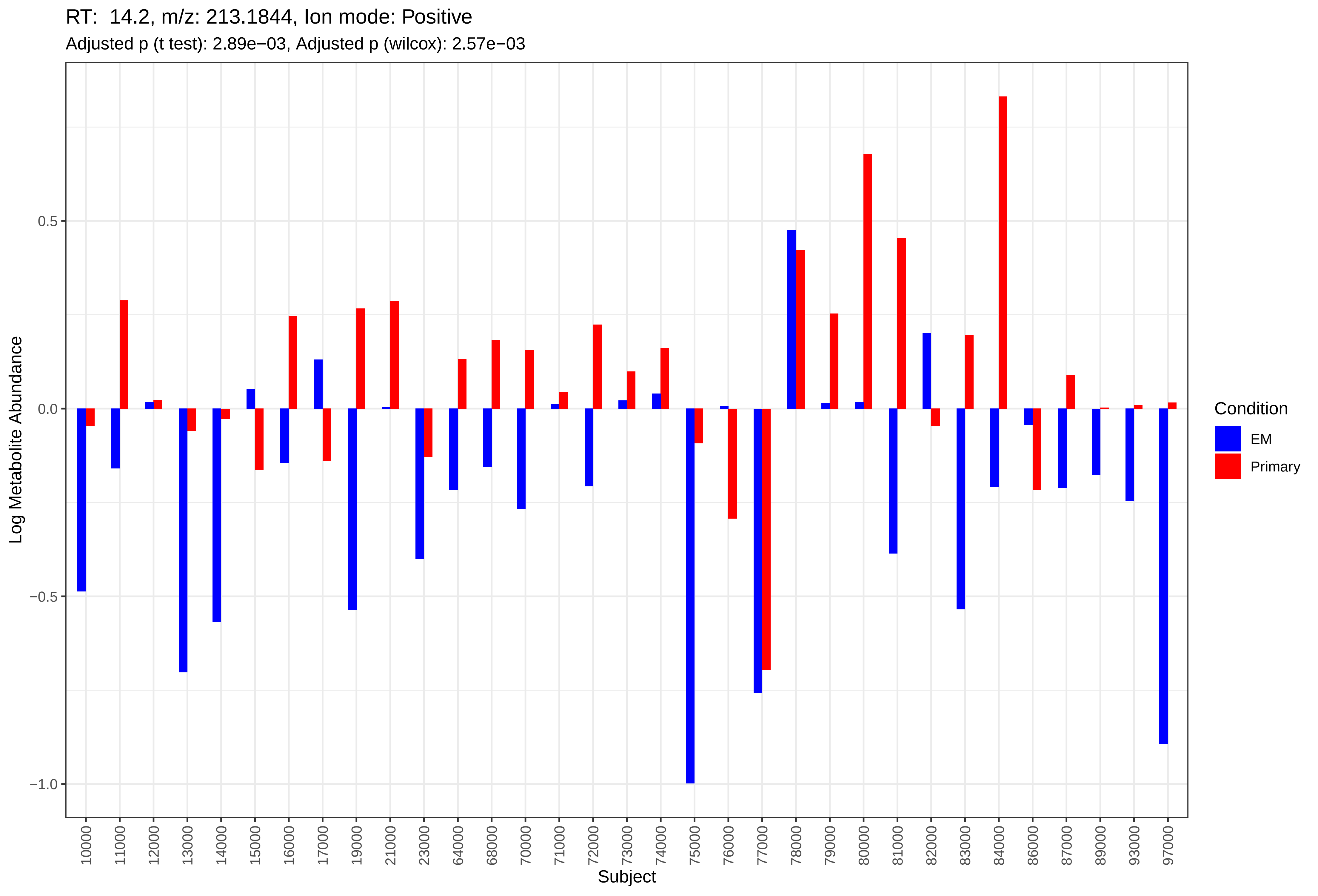

RT: 14.2, m/z: 213.1844, Ion mode: Positive
Adjusted p (t test): 2.89e−03, Adjusted p (wilcox): 2.57e−03
Log Metabolite Abundance
0.5
0.0
−0.5
−1.0
Subject
10000
11000
12000
13000
14000
15000
16000
17000
19000
21000
23000
64000
68000
70000
71000
72000
73000
74000
75000
76000
77000
78000
79000
80000
81000
82000
83000
84000
86000
87000
89000
93000
97000
Condition
EM
Primary

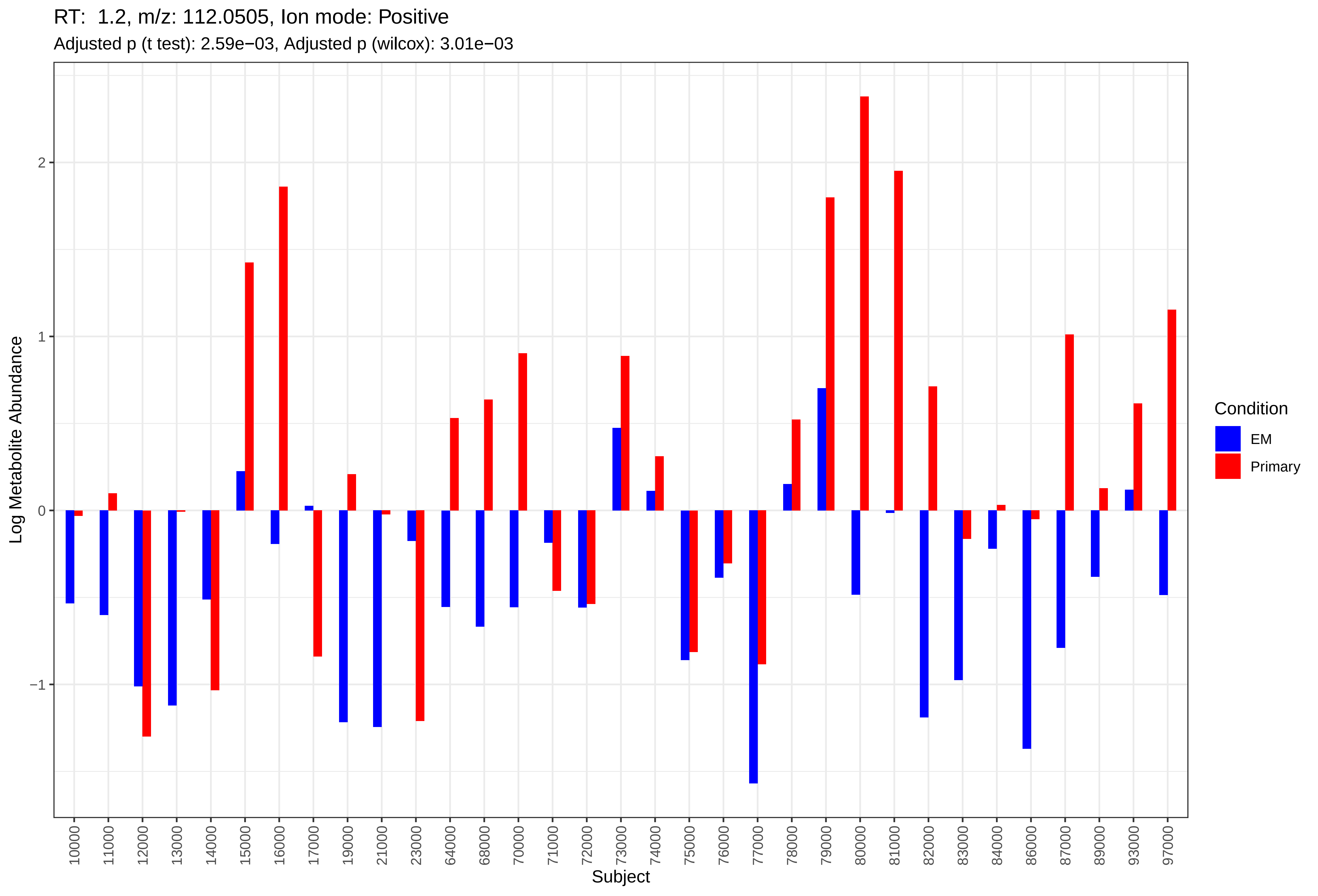
RT: 1.2, m/z: 112.0505, Ion mode: Positive
Adjusted p (t test): 2.59e−03, Adjusted p (wilcox): 3.01e−03
Log Metabolite Abundance
2
1
0
−1
10000
11000
12000
13000
14000
15000
16000
17000
19000
21000
23000
64000
68000
70000
71000
72000
73000
74000
75000
76000
77000
78000
79000
80000
81000
82000
83000
84000
86000
87000
89000
93000
97000
Subject
Condition
EM
Primary

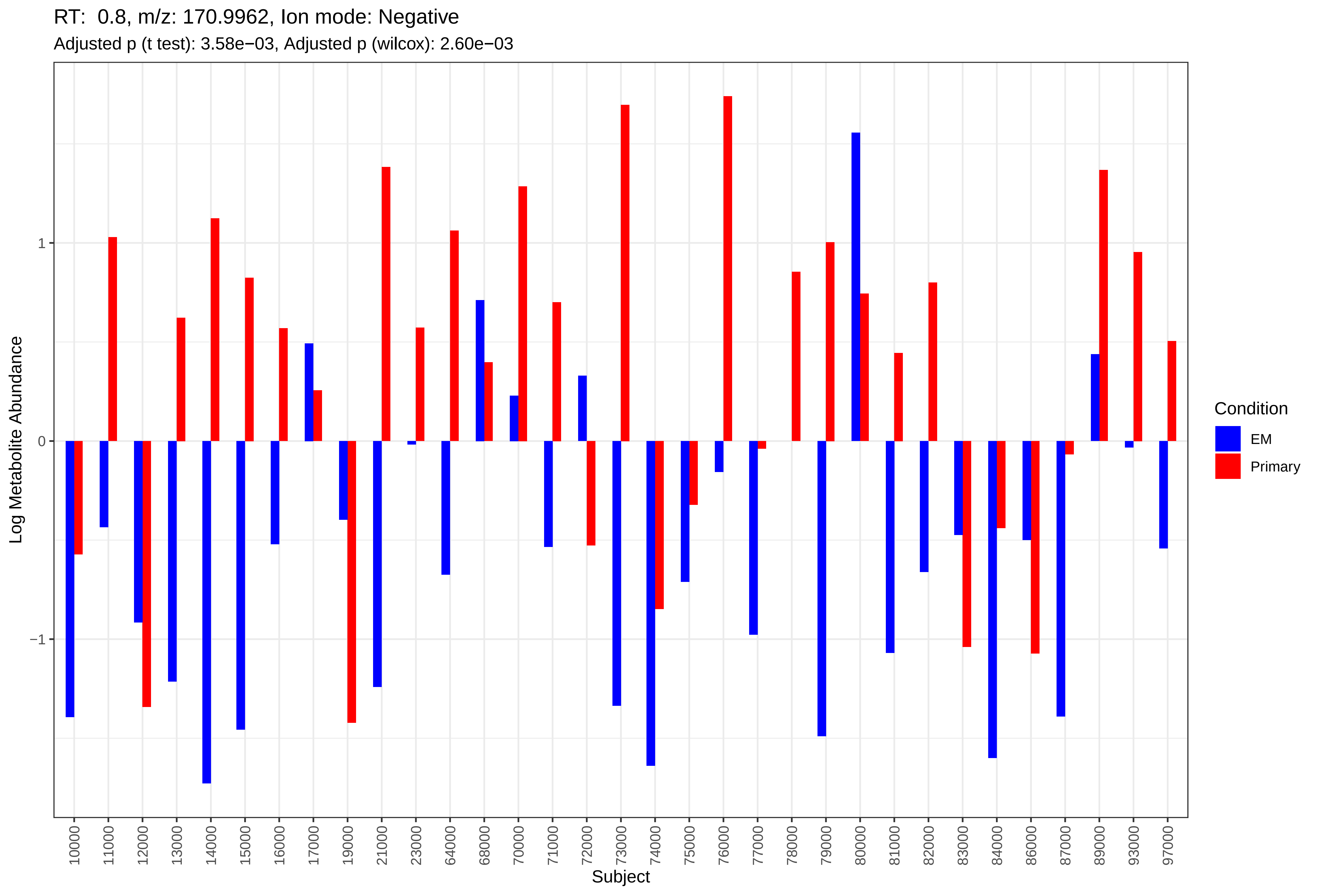

RT: 0.8, m/z: 170.9962, Ion mode: Negative
Adjusted p (t test): 3.58e−03, Adjusted p (wilcox): 2.60e−03
Log Metabolite Abundance
1
0
−1
Subject
10000
11000
12000
13000
14000
15000
16000
17000
19000
21000
23000
64000
68000
70000
71000
72000
73000
74000
75000
76000
77000
78000
79000
80000
81000
82000
83000
84000
86000
87000
89000
93000
97000
Condition
EM
Primary

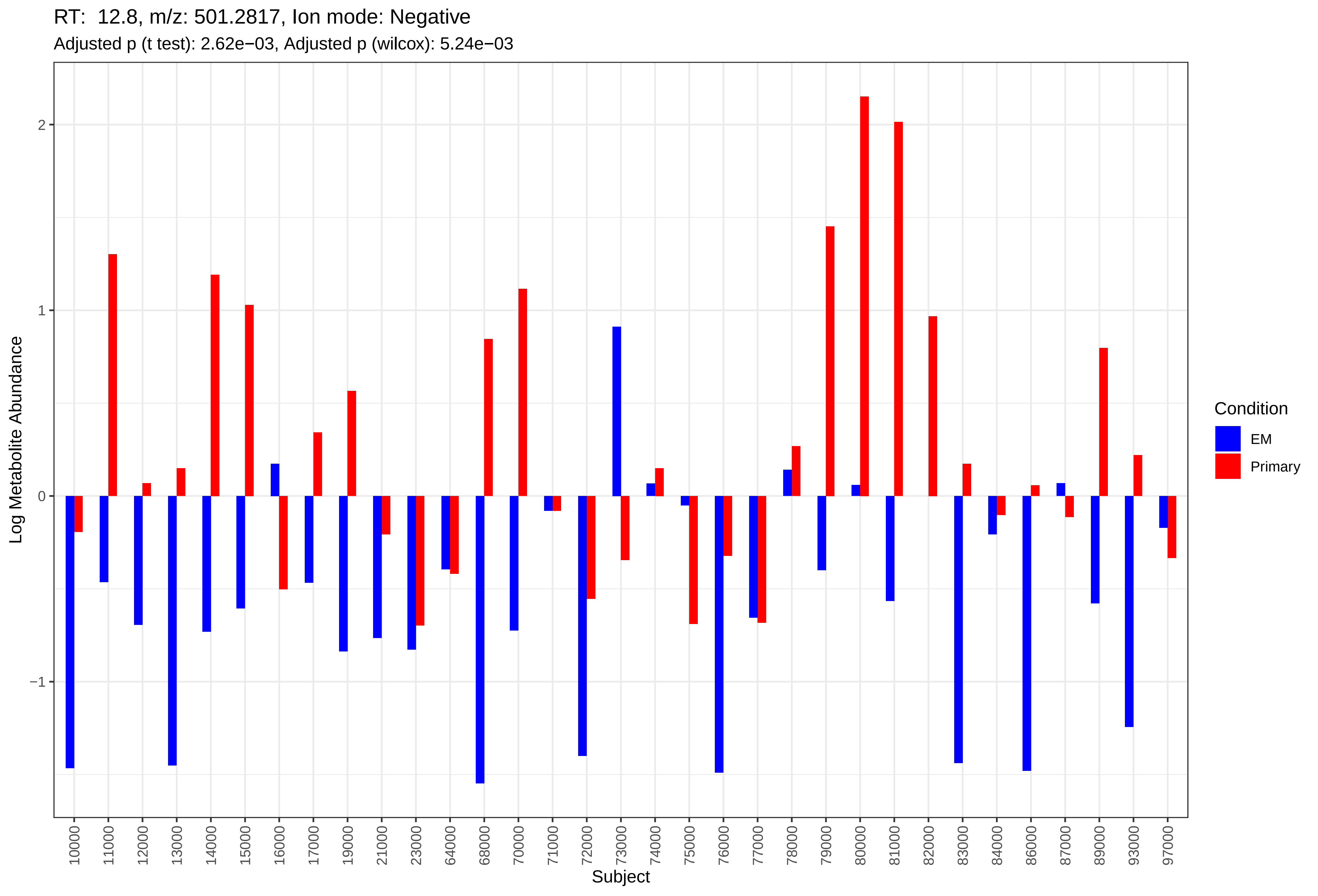
RT: 12.8, m/z: 501.2817, Ion mode: Negative
Adjusted p (t test): 2.62e−03, Adjusted p (wilcox): 5.24e−03
Log Metabolite Abundance
2
1
0
−1
Subject
10000
11000
12000
13000
14000
15000
16000
17000
19000
21000
23000
64000
68000
70000
71000
72000
73000
74000
75000
76000
77000
78000
79000
80000
81000
82000
83000
84000
86000
87000
89000
93000
97000
Condition
EM
Primary

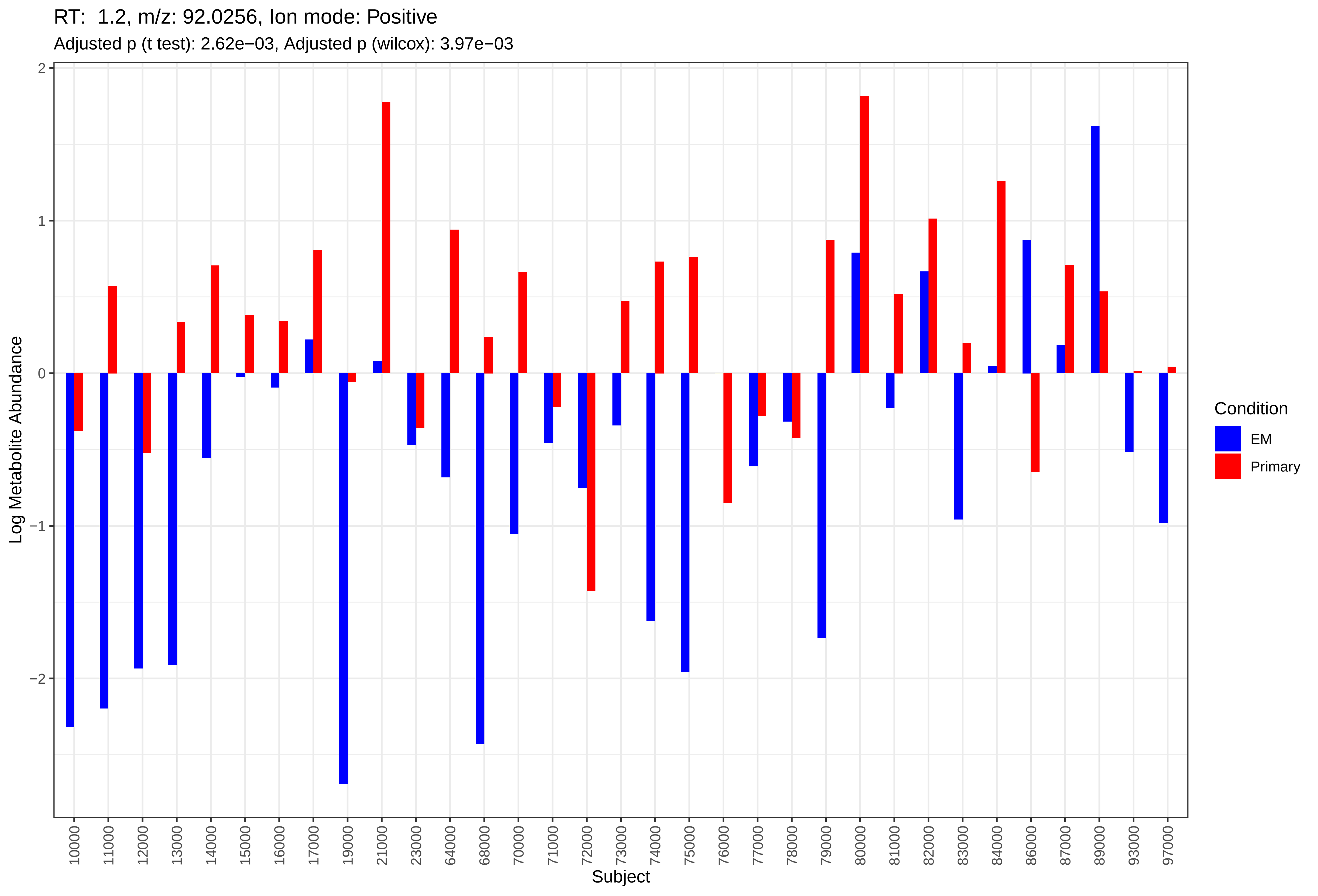
RT: 1.2, m/z: 92.0256, Ion mode: Positive
Adjusted p (t test): 2.62e−03, Adjusted p (wilcox): 3.97e−03
Log Metabolite Abundance
2
1
0
−1
−2
10000
11000
12000
13000
14000
15000
16000
17000
19000
21000
23000
64000
68000
70000
71000
72000
73000
74000
75000
76000
77000
78000
79000
80000
81000
82000
83000
84000
86000
87000
89000
93000
97000
Subject
Condition
EM
Primary

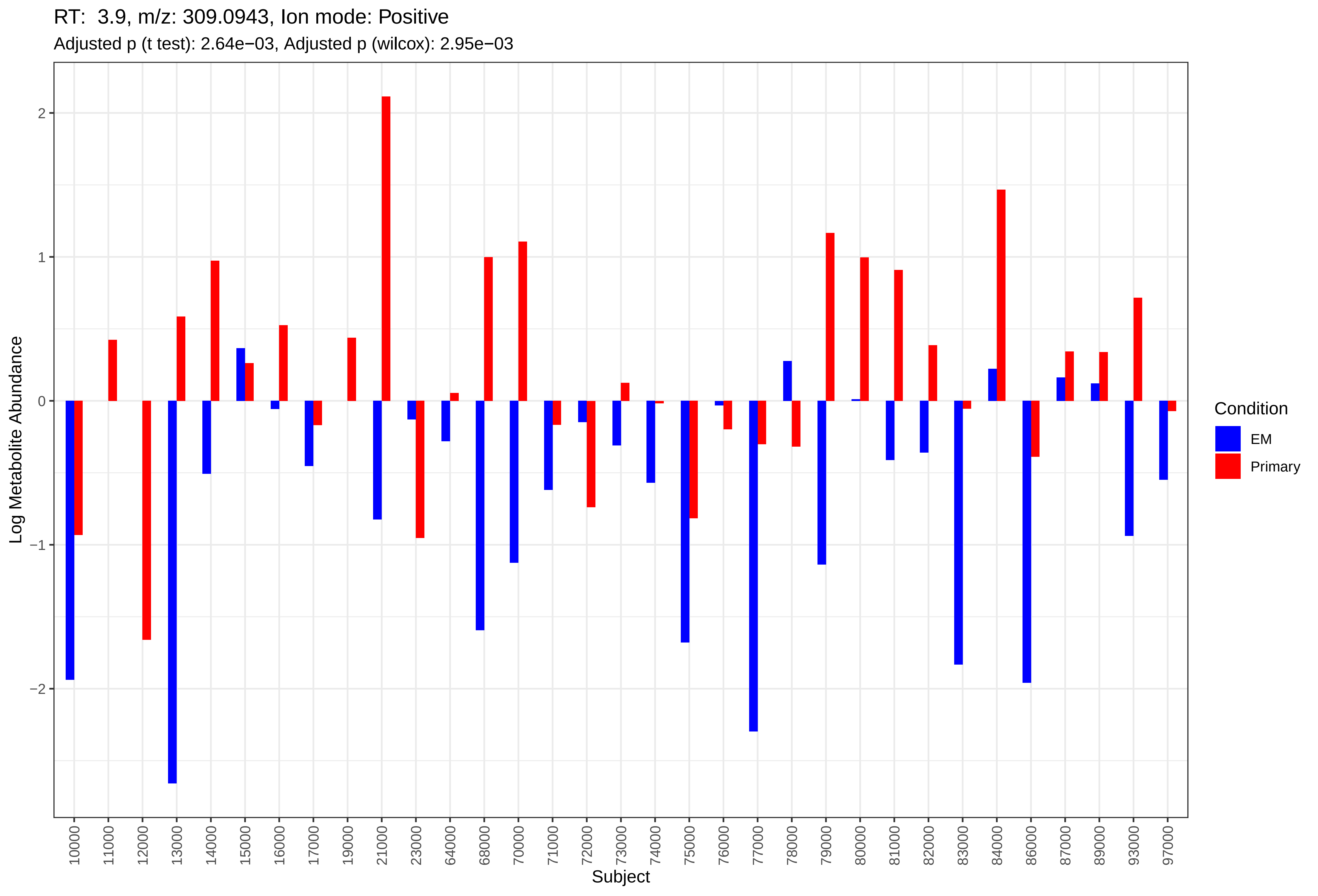

RT: 3.9, m/z: 309.0943, Ion mode: Positive
Adjusted p (t test): 2.64e−03, Adjusted p (wilcox): 2.95e−03
Log Metabolite Abundance
2
1
0
−1
−2
10000
11000
12000
13000
14000
15000
16000
17000
19000
21000
23000
64000
68000
70000
71000
72000
73000
74000
75000
76000
77000
78000
79000
80000
81000
82000
83000
84000
86000
87000
89000
93000
97000
Subject
Condition
EM
Primary

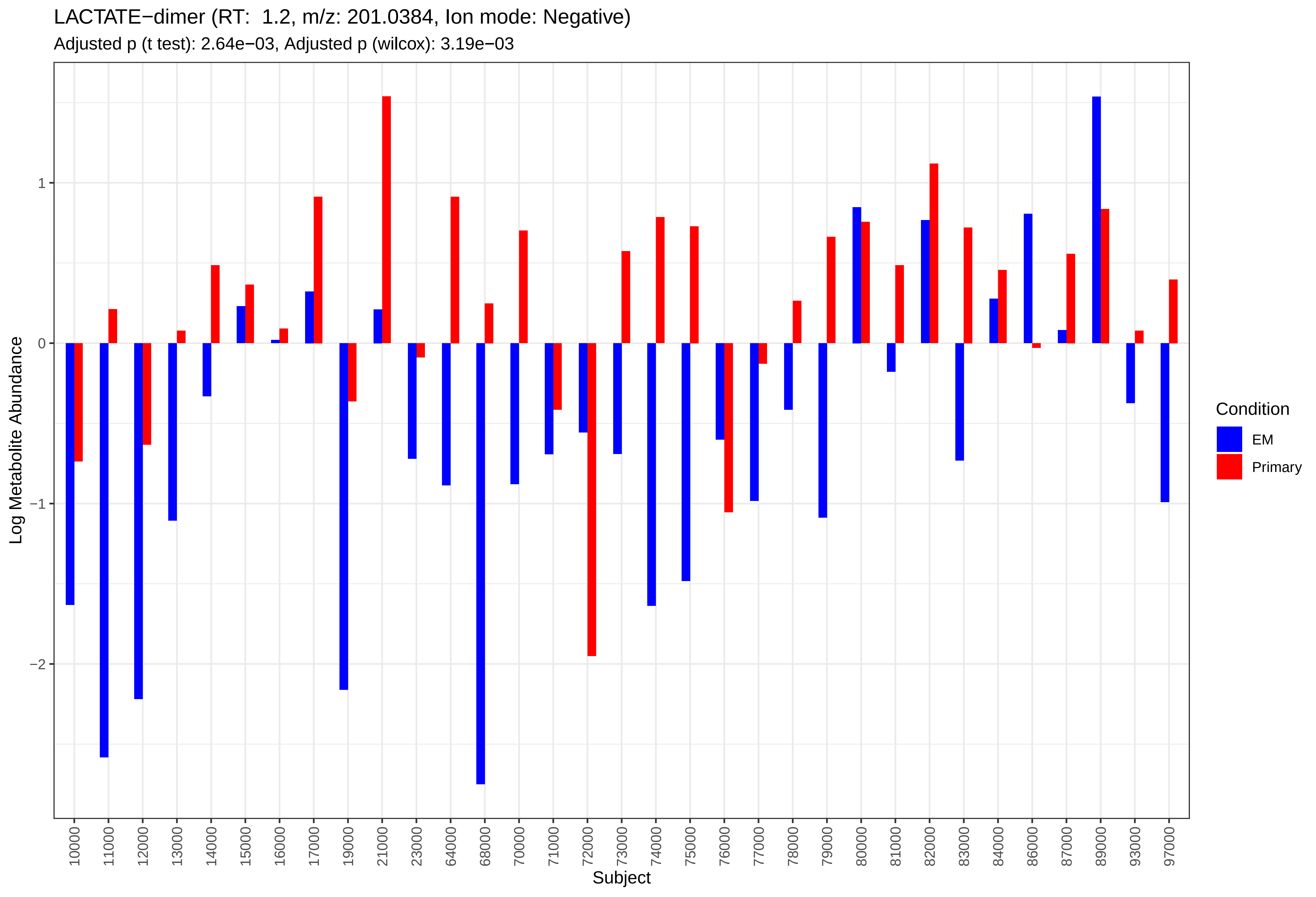

LACTATE−dimer (RT: 1.2, m/z: 201.0384, Ion mode: Negative)
Adjusted p (t test): 2.64e−03, Adjusted p (wilcox): 3.19e−03
Log Metabolite Abundance
1
0
−1
−2
Subject
10000
11000
12000
13000
14000
15000
16000
17000
19000
21000
23000
64000
68000
70000
71000
72000
73000
74000
75000
76000
77000
78000
79000
80000
81000
82000
83000
84000
86000
87000
89000
93000
97000
Condition
EM
Primary

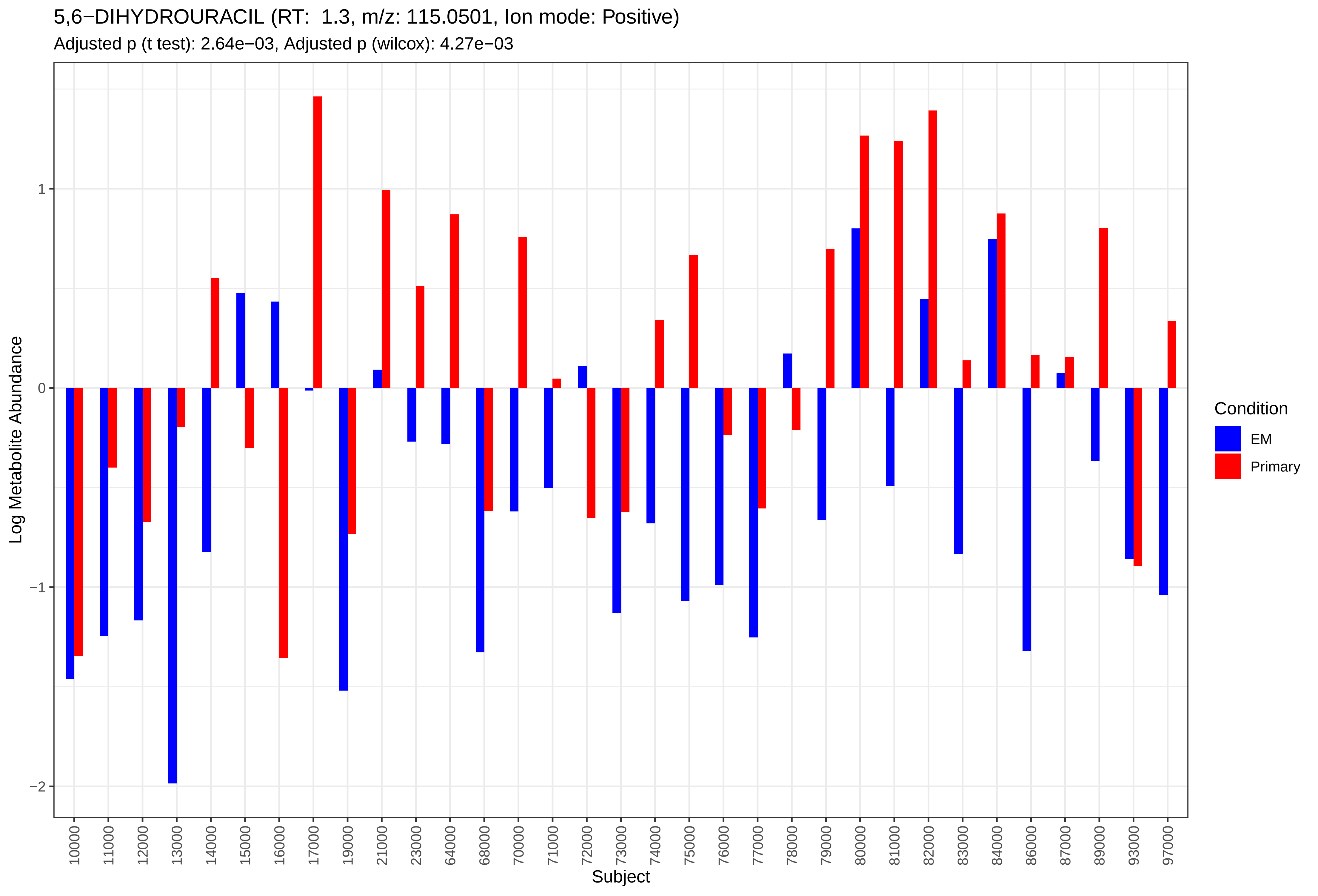

5,6−DIHYDROURACIL (RT: 1.3, m/z: 115.0501, Ion mode: Positive)
Adjusted p (t test): 2.64e−03, Adjusted p (wilcox): 4.27e−03
Log Metabolite Abundance
1
0
−1
−2
10000
11000
12000
13000
14000
15000
16000
17000
19000
21000
23000
64000
68000
70000
71000
72000
73000
74000
75000
76000
77000
78000
79000
80000
81000
82000
83000
84000
86000
87000
89000
93000
97000
Subject
Condition
EM
Primary

RT: 3.1, m/z: 565.0480, Ion mode: Negative

Adjusted p (t test): 3.71e−03, Adjusted p (wilcox): 2.66e−03

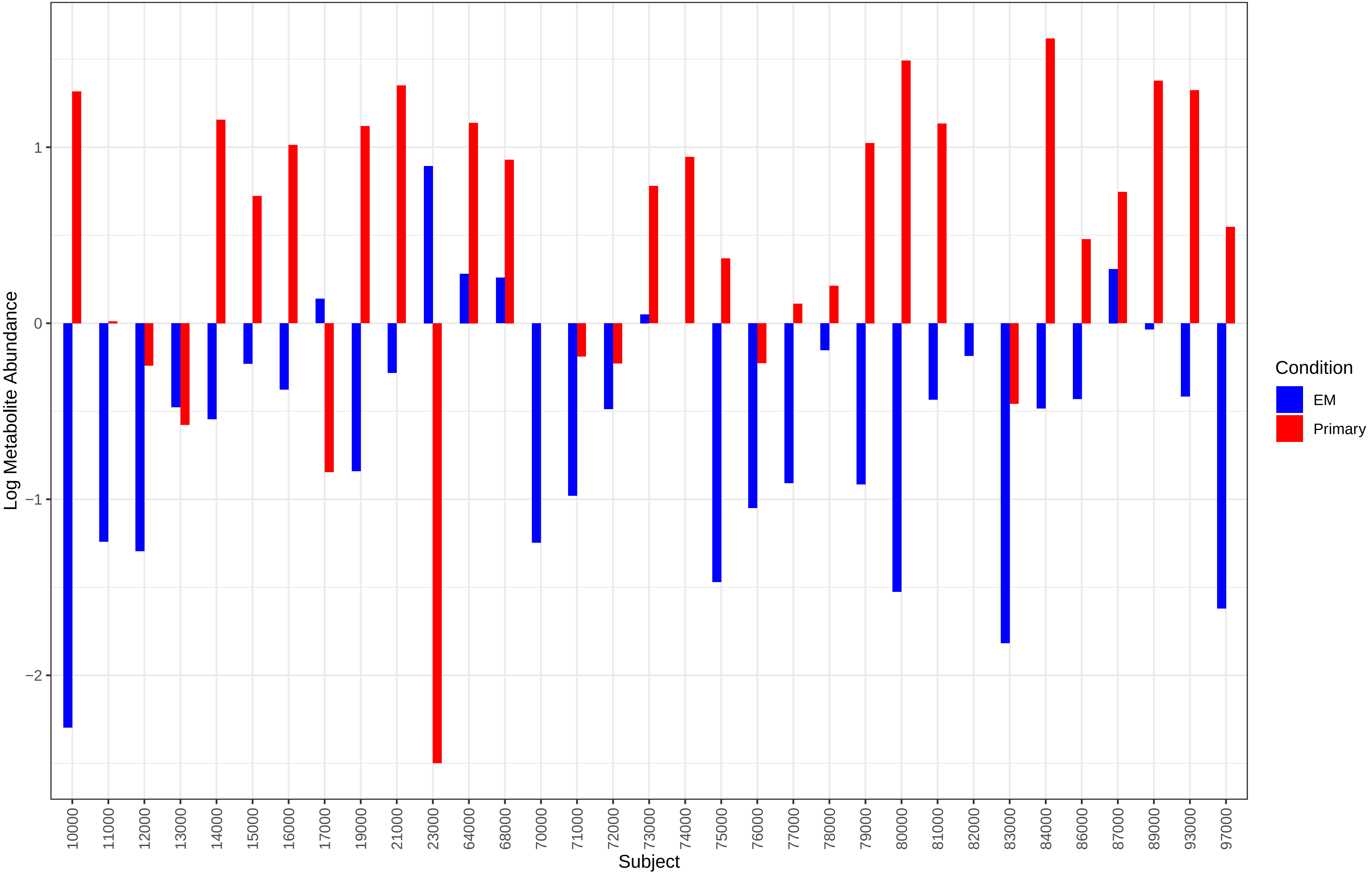

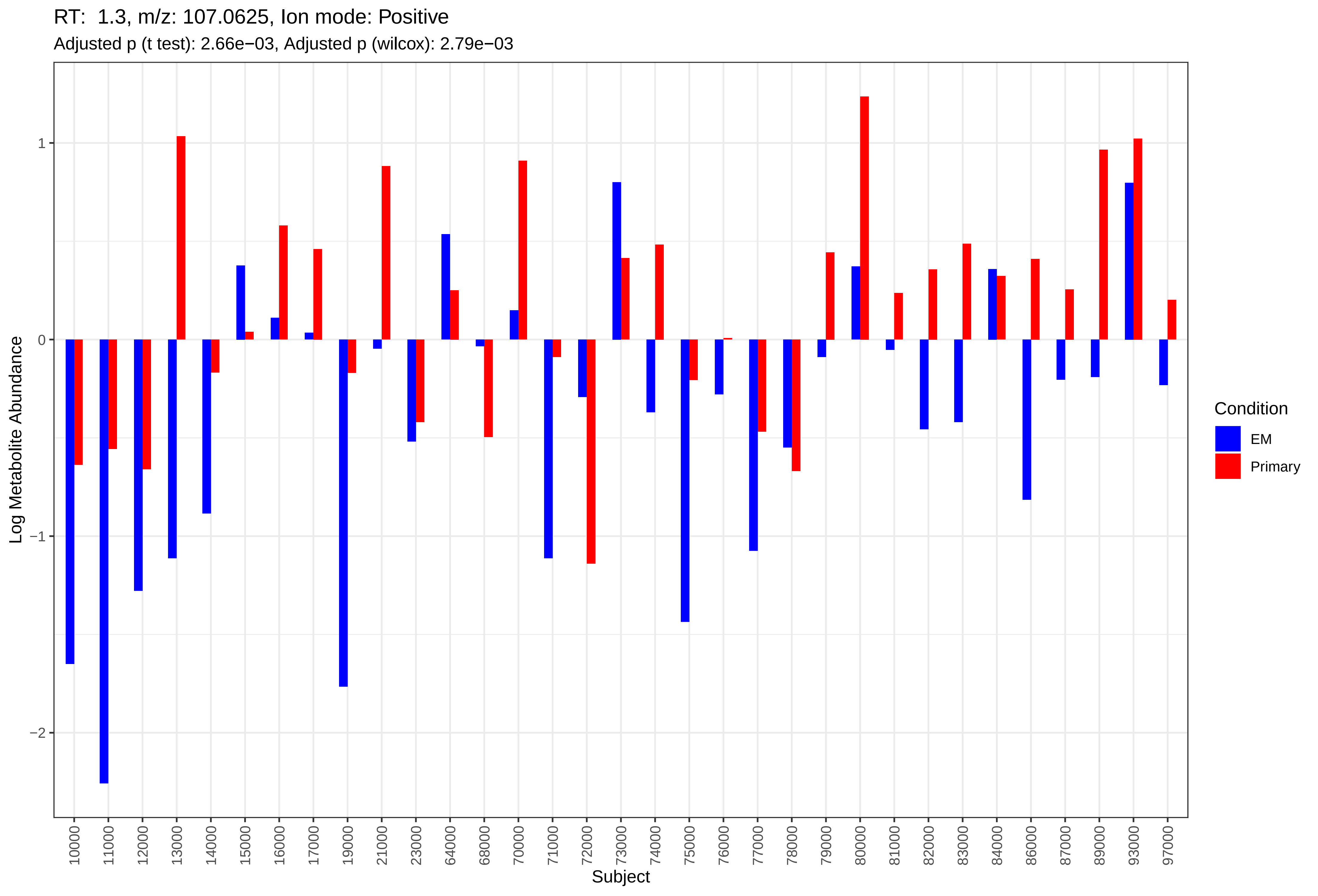
RT: 1.3, m/z: 107.0625, Ion mode: Positive
Adjusted p (t test): 2.66e−03, Adjusted p (wilcox): 2.79e−03
Log Metabolite Abundance
1
0
−1
−2
Subject
10000
11000
12000
13000
14000
15000
16000
17000
19000
21000
23000
64000
68000
70000
71000
72000
73000
74000
75000
76000
77000
78000
79000
80000
81000
82000
83000
84000
86000
87000
89000
93000
97000
Condition
EM
Primary

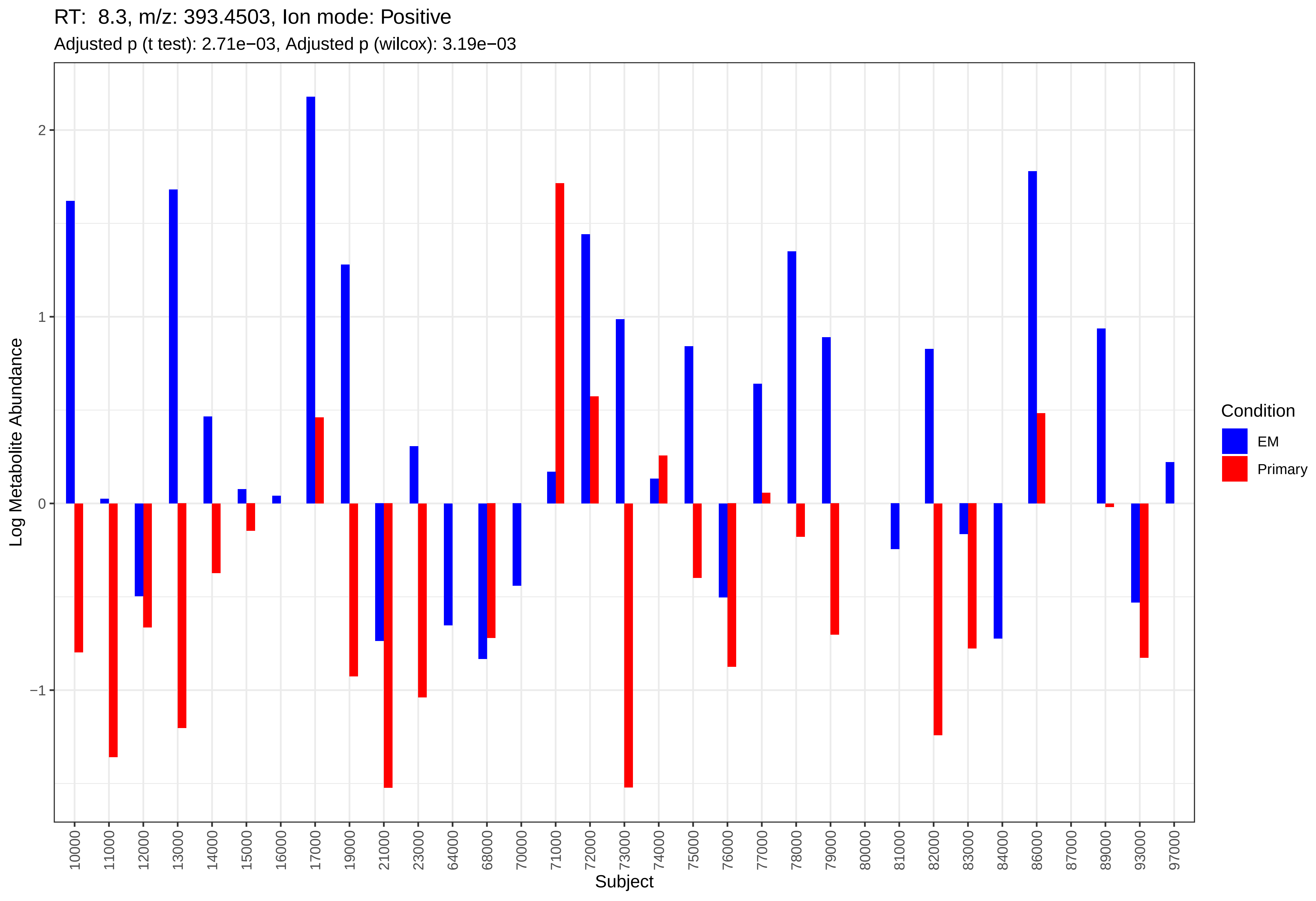
RT: 8.3, m/z: 393.4503, Ion mode: Positive
Adjusted p (t test): 2.71e−03, Adjusted p (wilcox): 3.19e−03
Log Metabolite Abundance
2
1
0
−1
10000
11000
12000
13000
14000
15000
16000
17000
19000
21000
23000
64000
68000
70000
71000
72000
73000
74000
75000
76000
77000
78000
79000
80000
81000
82000
83000
84000
86000
87000
89000
93000
97000
Subject
Condition
EM
Primary

RT: 0.7, m/z: 217.0163, Ion mode: Negative

Adjusted p (t test): 2.72e−03, Adjusted p (wilcox): 3.01e−03

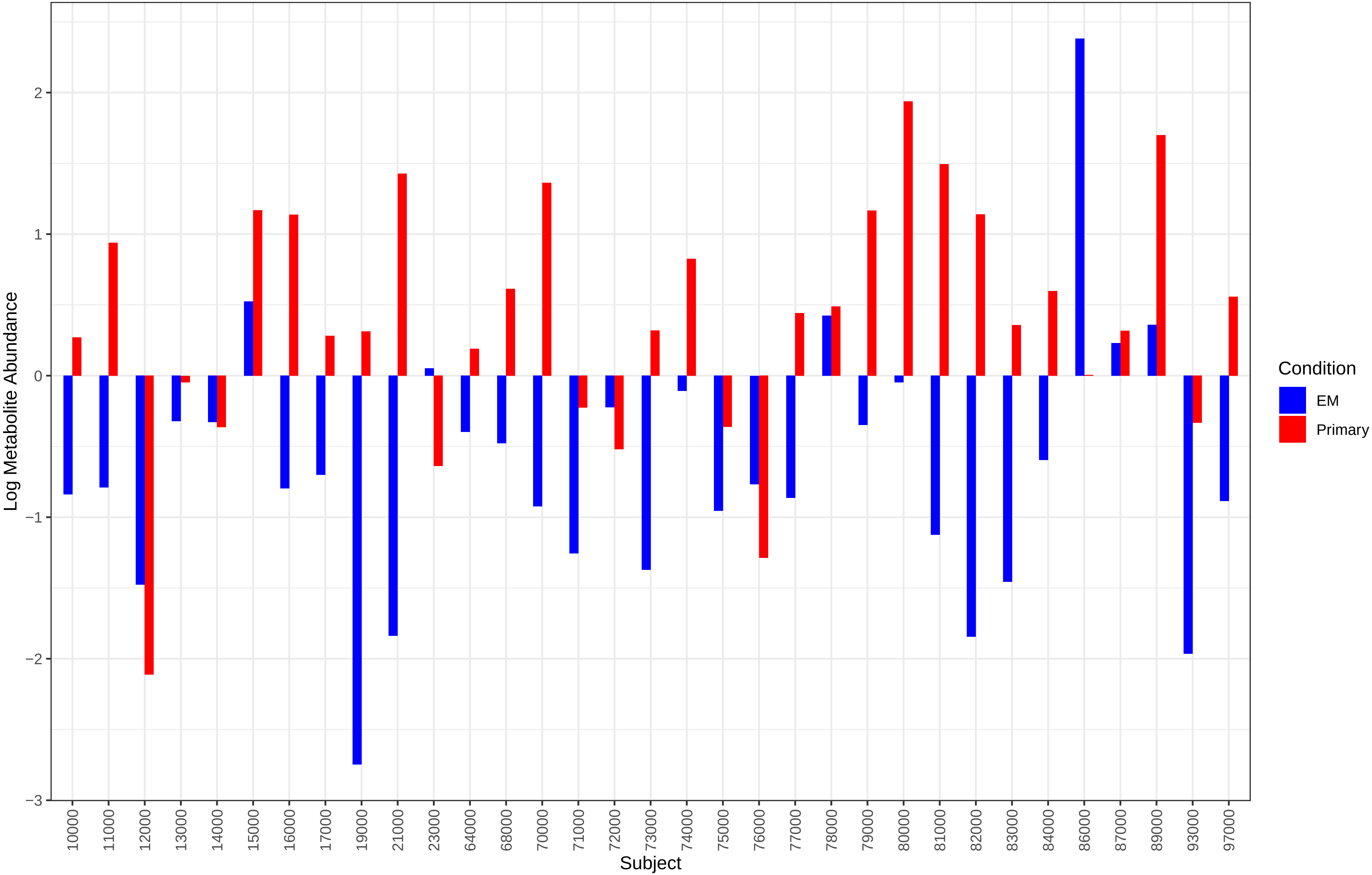

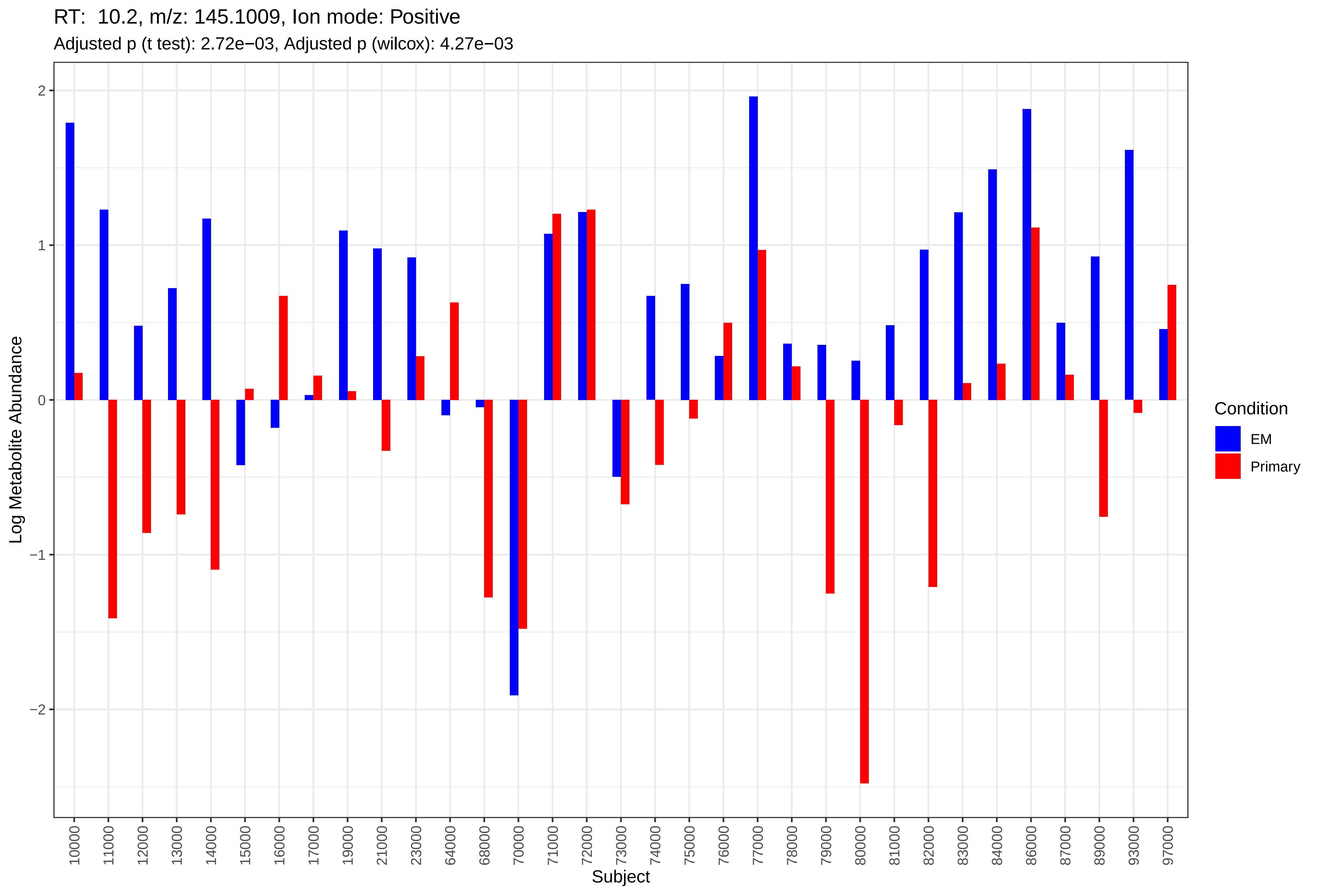
RT: 10.2, m/z: 145.1009, Ion mode: Positive
Adjusted p (t test): 2.72e−03, Adjusted p (wilcox): 4.27e−03
Log Metabolite Abundance
2
1
0
−1
−2
Subject
10000
11000
12000
13000
14000
15000
16000
17000
19000
21000
23000
64000
68000
70000
71000
72000
73000
74000
75000
76000
77000
78000
79000
80000
81000
82000
83000
84000
86000
87000
89000
93000
97000
Condition
EM
Primary

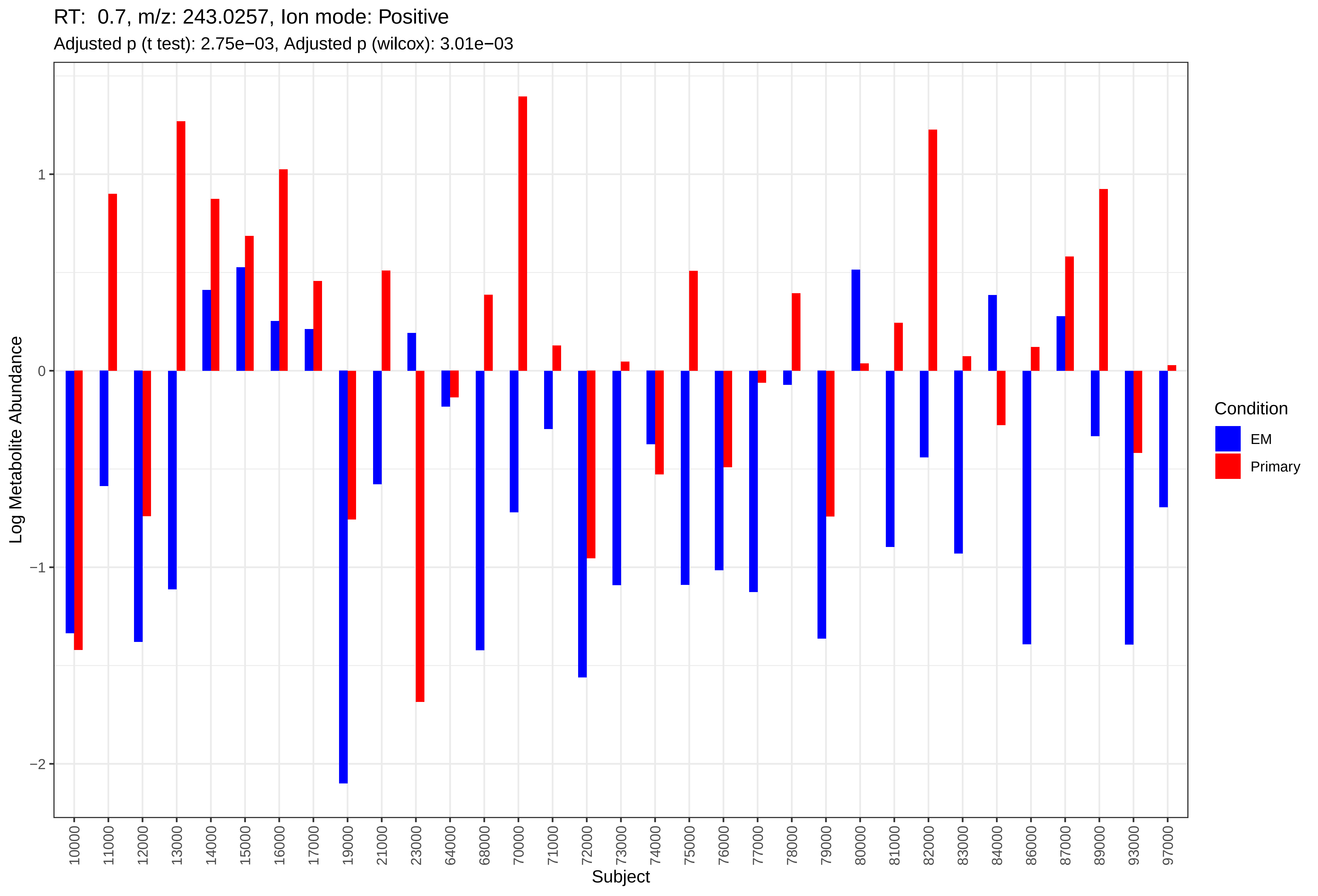

RT: 0.7, m/z: 243.0257, Ion mode: Positive
Adjusted p (t test): 2.75e−03, Adjusted p (wilcox): 3.01e−03
Log Metabolite Abundance
1
0
−1
−2
10000
11000
12000
13000
14000
15000
16000
17000
19000
21000
23000
64000
68000
70000
71000
72000
73000
74000
75000
76000
77000
78000
79000
80000
81000
82000
83000
84000
86000
87000
89000
93000
97000
Subject
Condition
EM
Primary

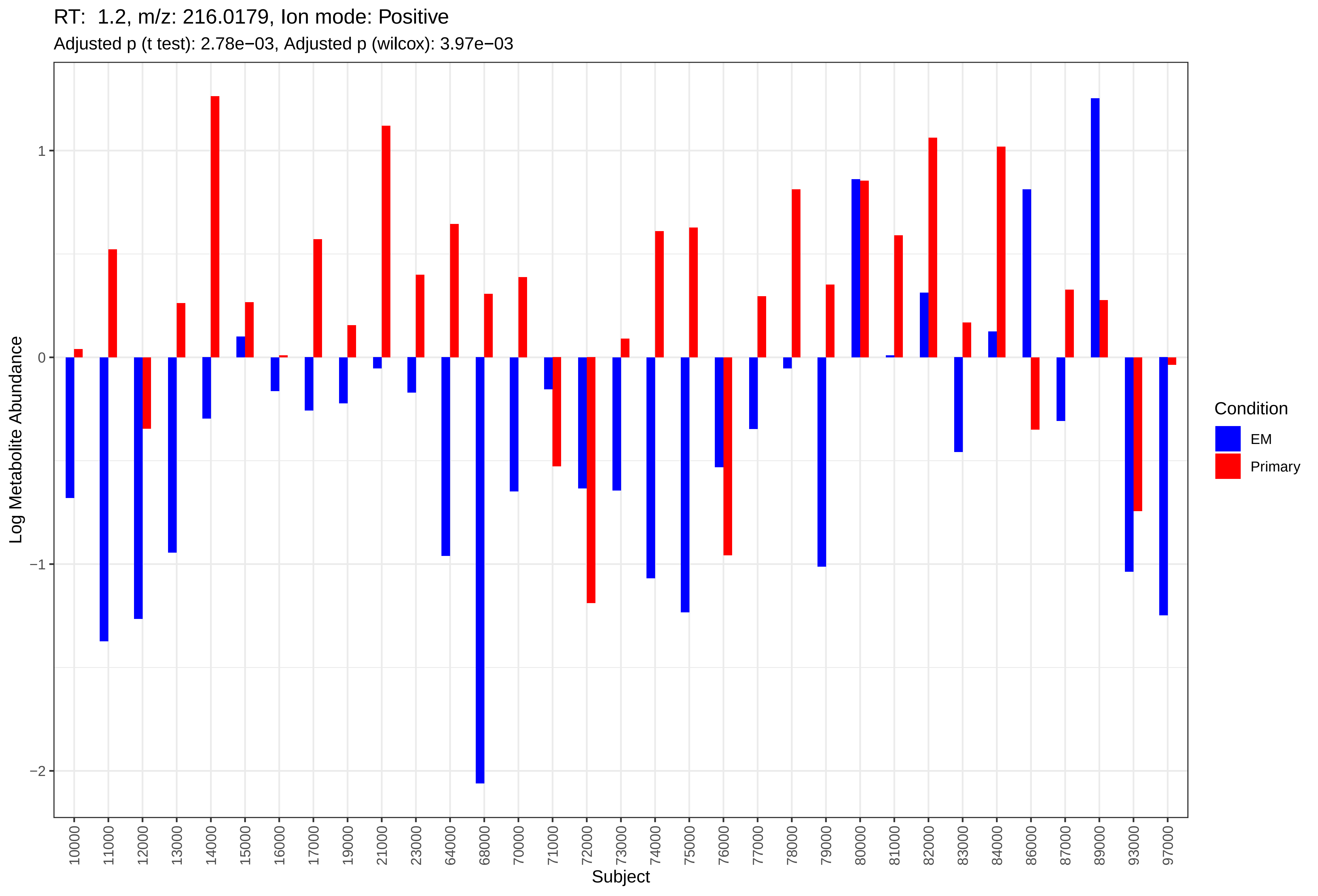
RT: 1.2, m/z: 216.0179, Ion mode: Positive
Adjusted p (t test): 2.78e−03, Adjusted p (wilcox): 3.97e−03
Log Metabolite Abundance
1
0
−1
−2
Subject
10000
11000
12000
13000
14000
15000
16000
17000
19000
21000
23000
64000
68000
70000
71000
72000
73000
74000
75000
76000
77000
78000
79000
80000
81000
82000
83000
84000
86000
87000
89000
93000
97000
Condition
EM
Primary

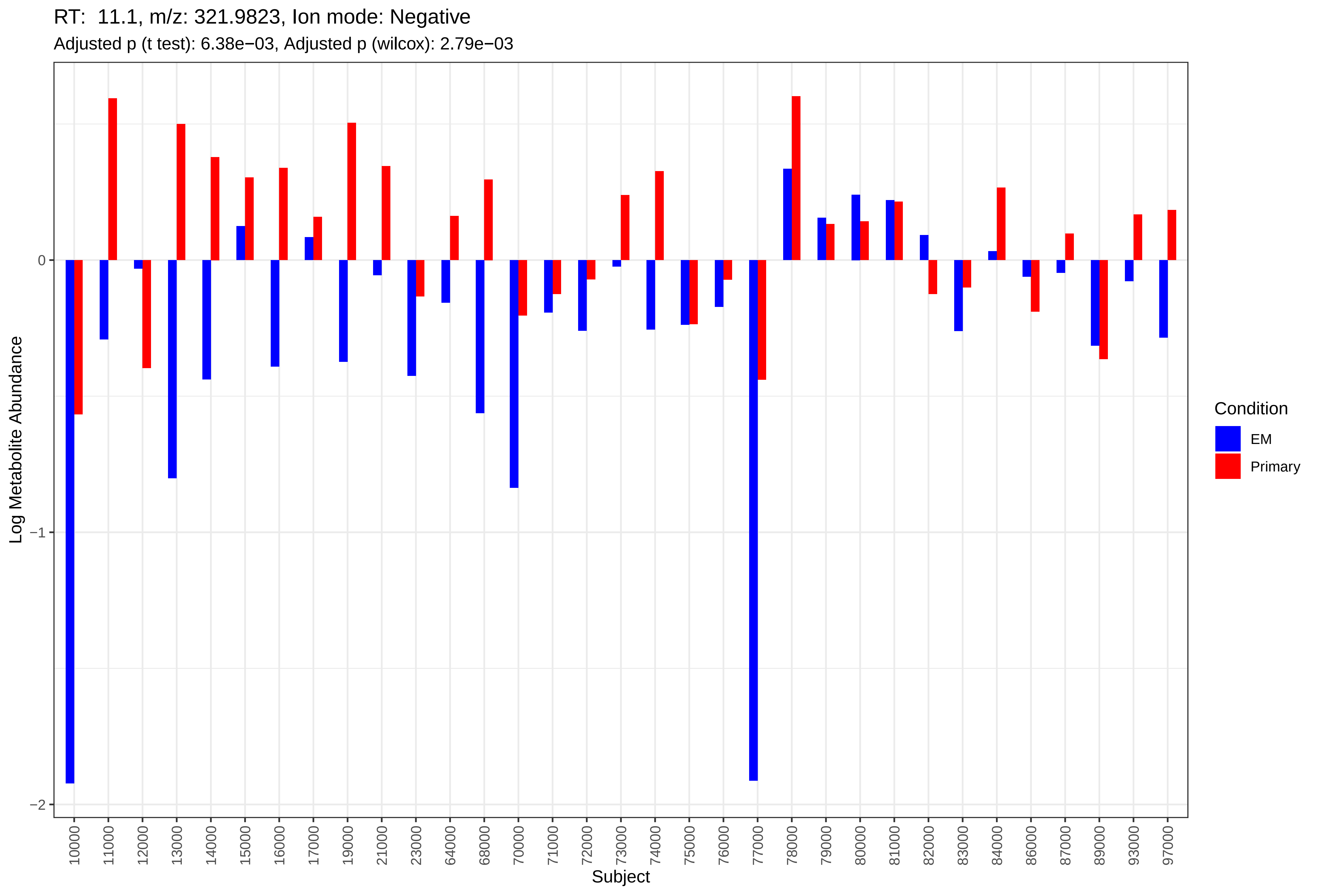
RT: 11.1, m/z: 321.9823, Ion mode: Negative
Adjusted p (t test): 6.38e−03, Adjusted p (wilcox): 2.79e−03
Log Metabolite Abundance
0
−1
−2
Subject
10000
11000
12000
13000
14000
15000
16000
17000
19000
21000
23000
64000
68000
70000
71000
72000
73000
74000
75000
76000
77000
78000
79000
80000
81000
82000
83000
84000
86000
87000
89000
93000
97000
Condition
EM
Primary

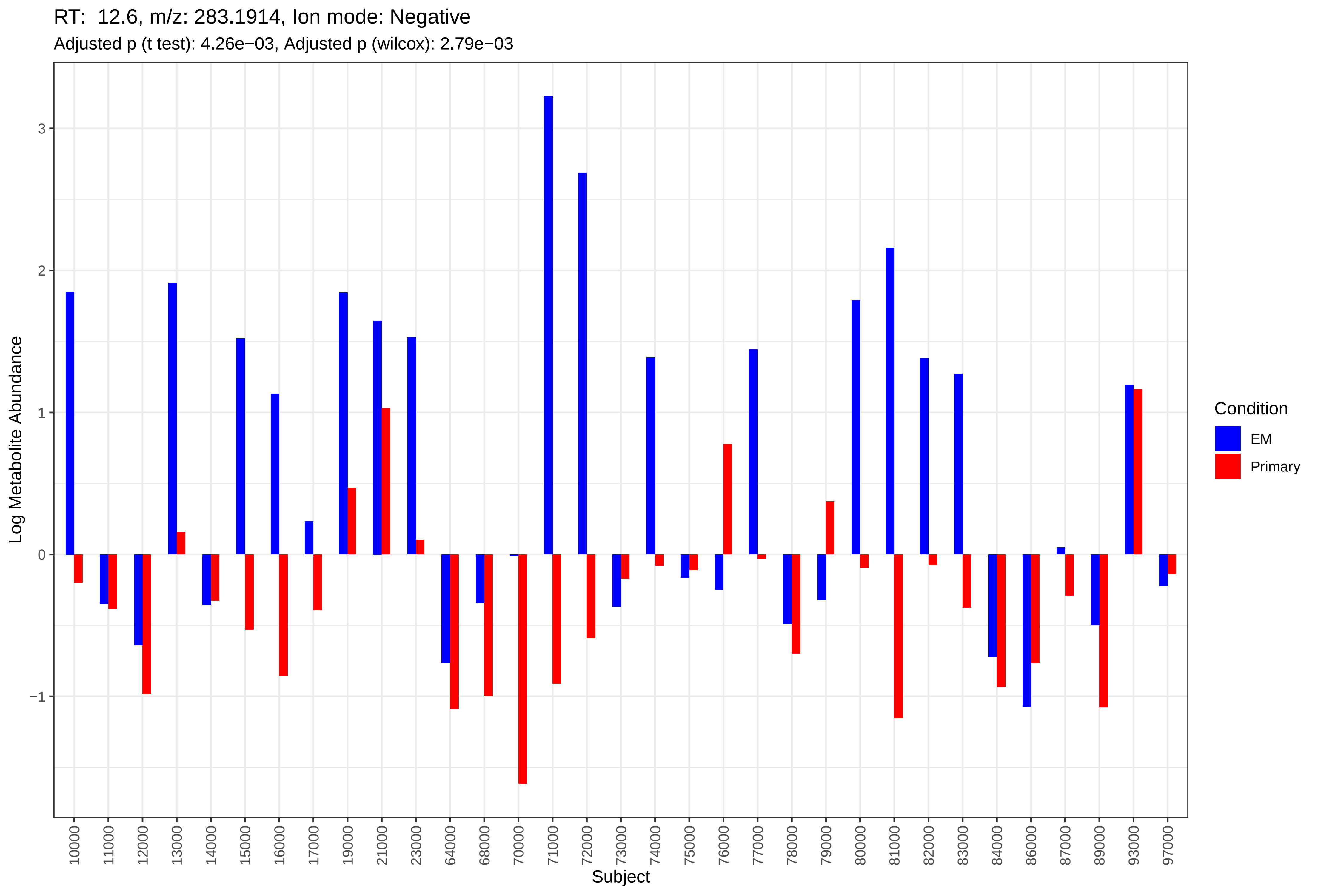

RT: 12.6, m/z: 283.1914, Ion mode: Negative
Adjusted p (t test): 4.26e−03, Adjusted p (wilcox): 2.79e−03
Log Metabolite Abundance
3
2
1
0
−1
10000
11000
12000
13000
14000
15000
16000
17000
19000
21000
23000
64000
68000
70000
71000
72000
73000
74000
75000
76000
77000
78000
79000
80000
81000
82000
83000
84000
86000
87000
89000
93000
97000
Subject
Condition
EM
Primary

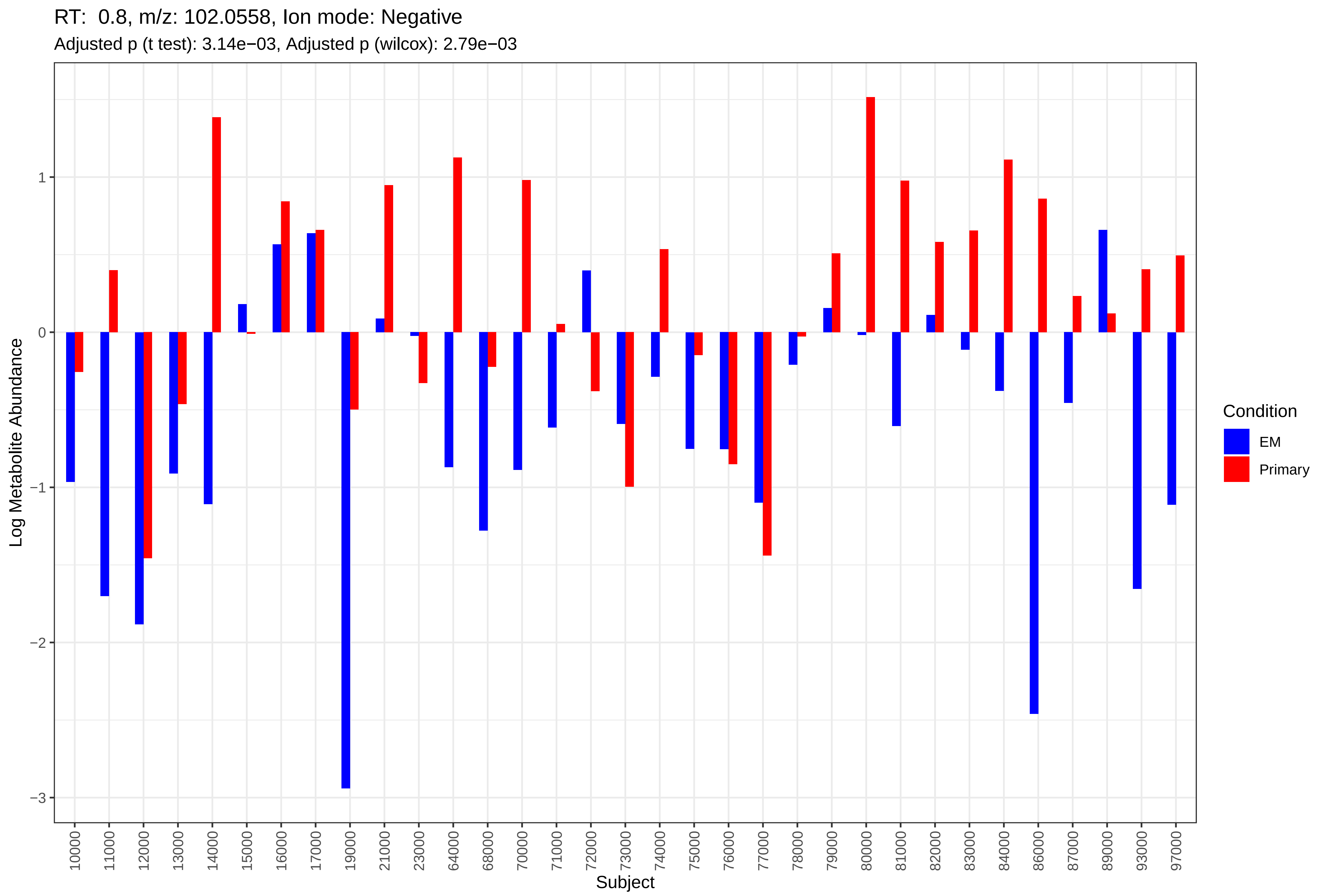
RT: 0.8, m/z: 102.0558, Ion mode: Negative
Adjusted p (t test): 3.14e−03, Adjusted p (wilcox): 2.79e−03
Log Metabolite Abundance
1
0
−1
−2
−3
10000
11000
12000
13000
14000
15000
16000
17000
19000
21000
23000
64000
68000
70000
71000
72000
73000
74000
75000
76000
77000
78000
79000
80000
81000
82000
83000
84000
86000
87000
89000
93000
97000
Subject
Condition
EM
Primary

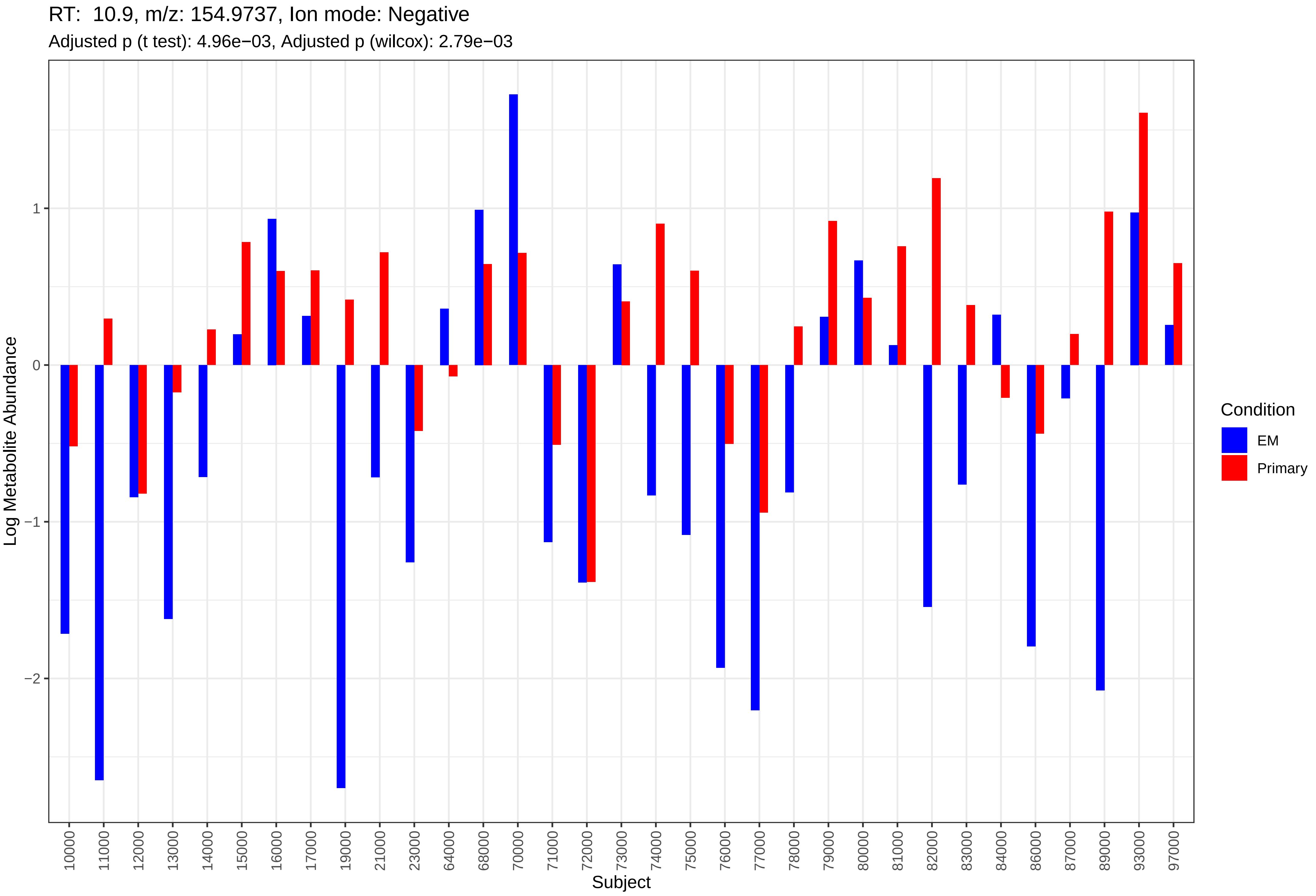
RT: 10.9, m/z: 154.9737, Ion mode: Negative
Adjusted p (t test): 4.96e−03, Adjusted p (wilcox): 2.79e−03
Log Metabolite Abundance
1
0
−1
−2
10000
11000
12000
13000
14000
15000
16000
17000
19000
21000
23000
64000
68000
70000
71000
72000
73000
74000
75000
76000
77000
78000
79000
80000
81000
82000
83000
84000
86000
87000
89000
93000
97000
Subject
Condition
EM
Primary

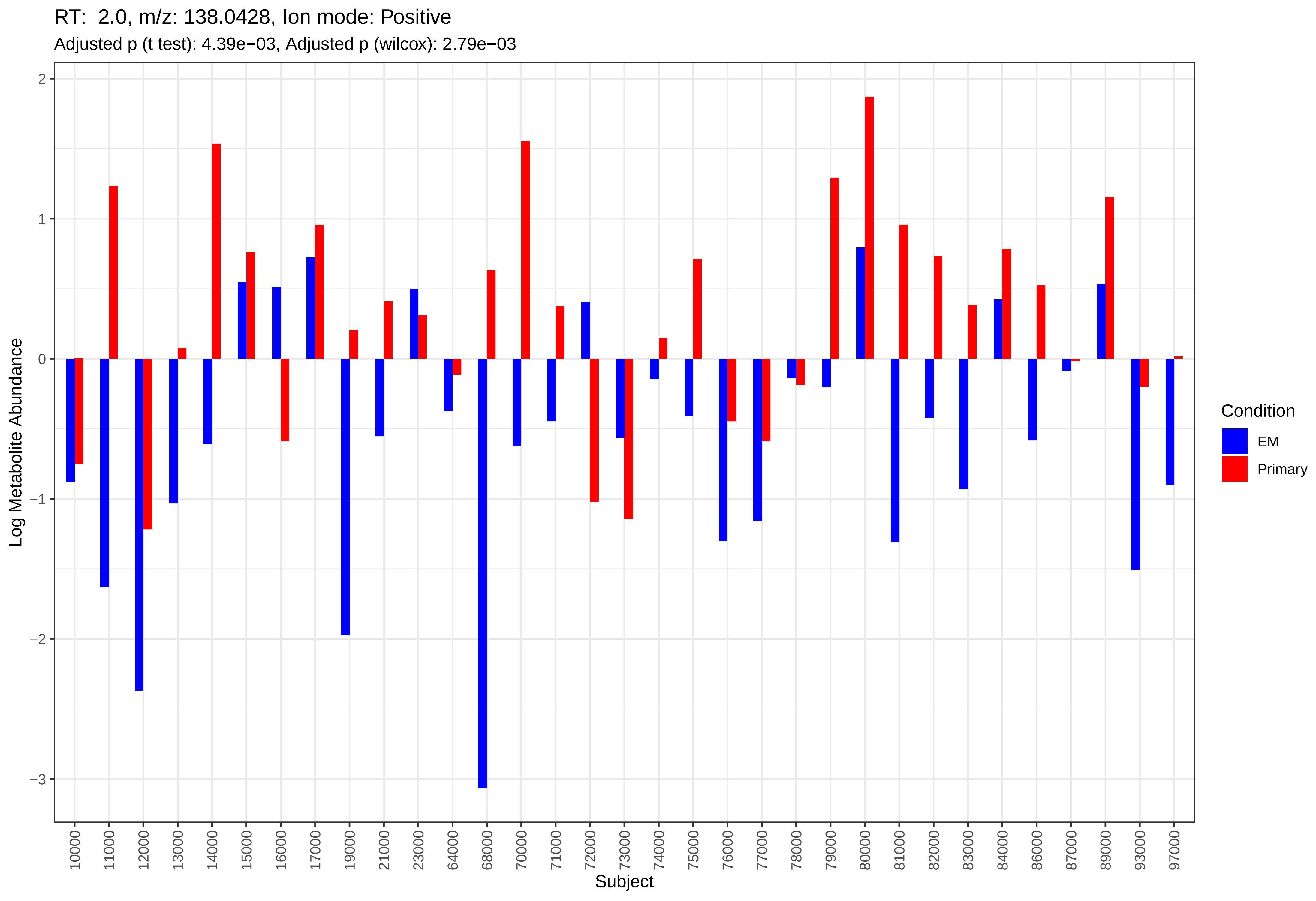

RT: 2.0, m/z: 138.0428, Ion mode: Positive
Adjusted p (t test): 4.39e−03, Adjusted p (wilcox): 2.79e−03
Log Metabolite Abundance
2
1
0
−1
−2
−3
Subject
10000
11000
12000
13000
14000
15000
16000
17000
19000
21000
23000
64000
68000
70000
71000
72000
73000
74000
75000
76000
77000
78000
79000
80000
81000
82000
83000
84000
86000
87000
89000
93000
97000
Condition
EM
Primary

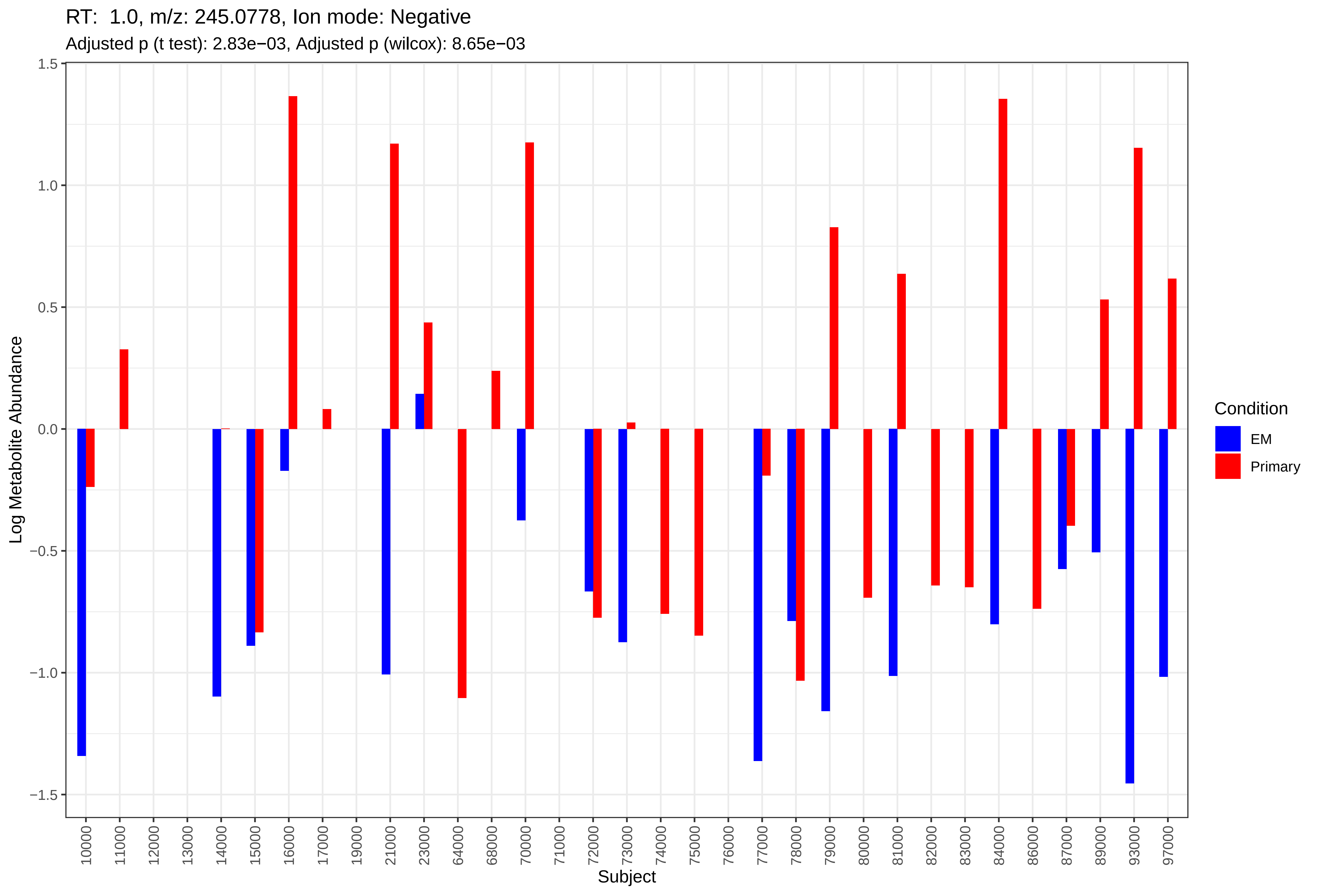
RT: 1.0, m/z: 245.0778, Ion mode: Negative
Adjusted p (t test): 2.83e−03, Adjusted p (wilcox): 8.65e−03
Log Metabolite Abundance
1.5
1.0
0.5
0.0
−0.5
−1.0
−1.5
10000
11000
12000
13000
14000
15000
16000
17000
19000
21000
23000
64000
68000
70000
71000
72000
73000
74000
75000
76000
77000
78000
79000
80000
81000
82000
83000
84000
86000
87000
89000
93000
97000
Subject
Condition
EM
Primary

Complex of 152.0929 and 109.0284 m/z (RT: 0.7, m/z: 260.1138, Ion mode: Positive)

Adjusted p (t test): 2.83e−03, Adjusted p (wilcox): 3.69e−03

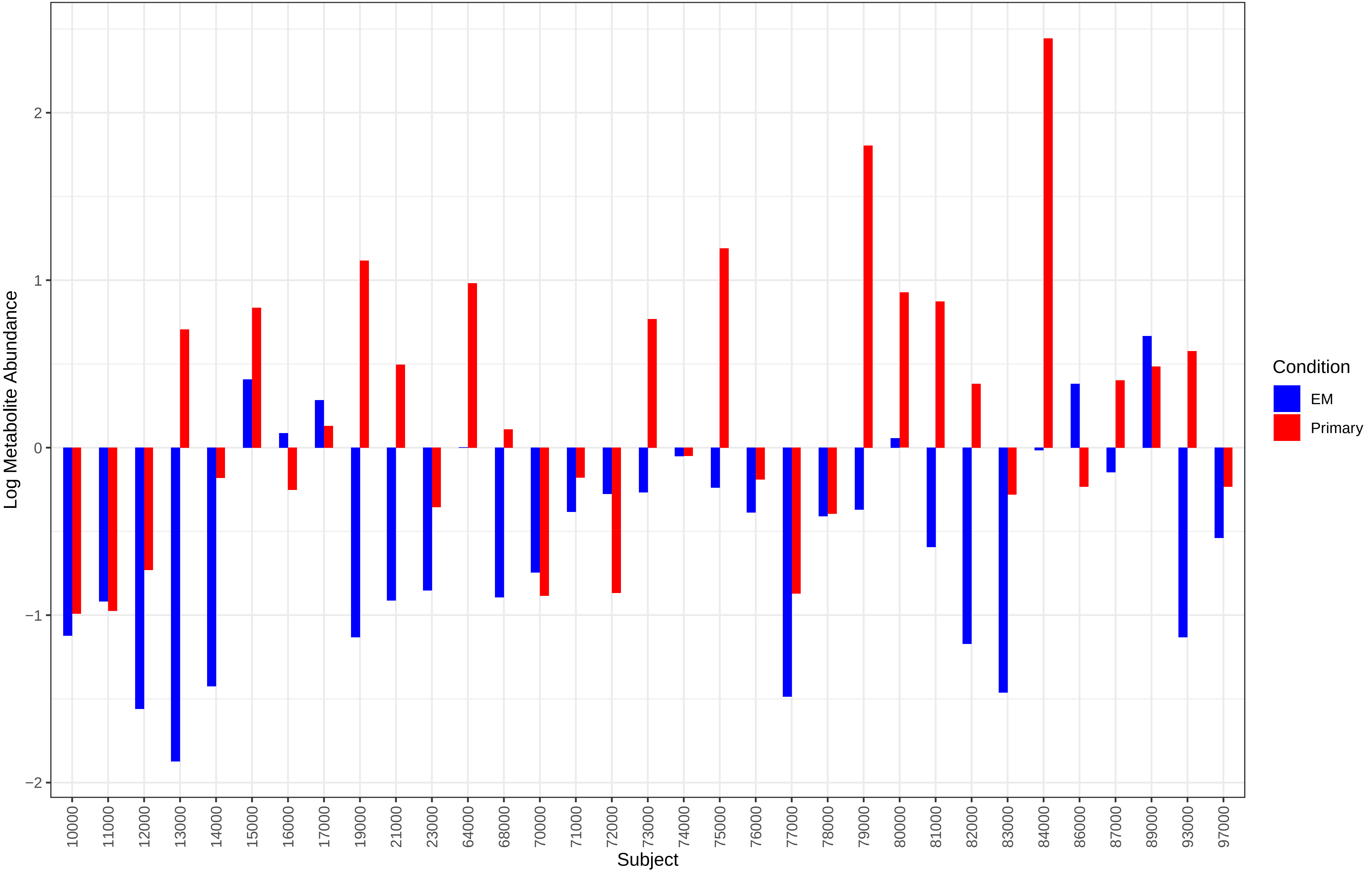

RT: 6.2, m/z: 144.1020, Ion mode: Positive

Adjusted p (t test): 2.88e−03, Adjusted p (wilcox): 4.94e−03

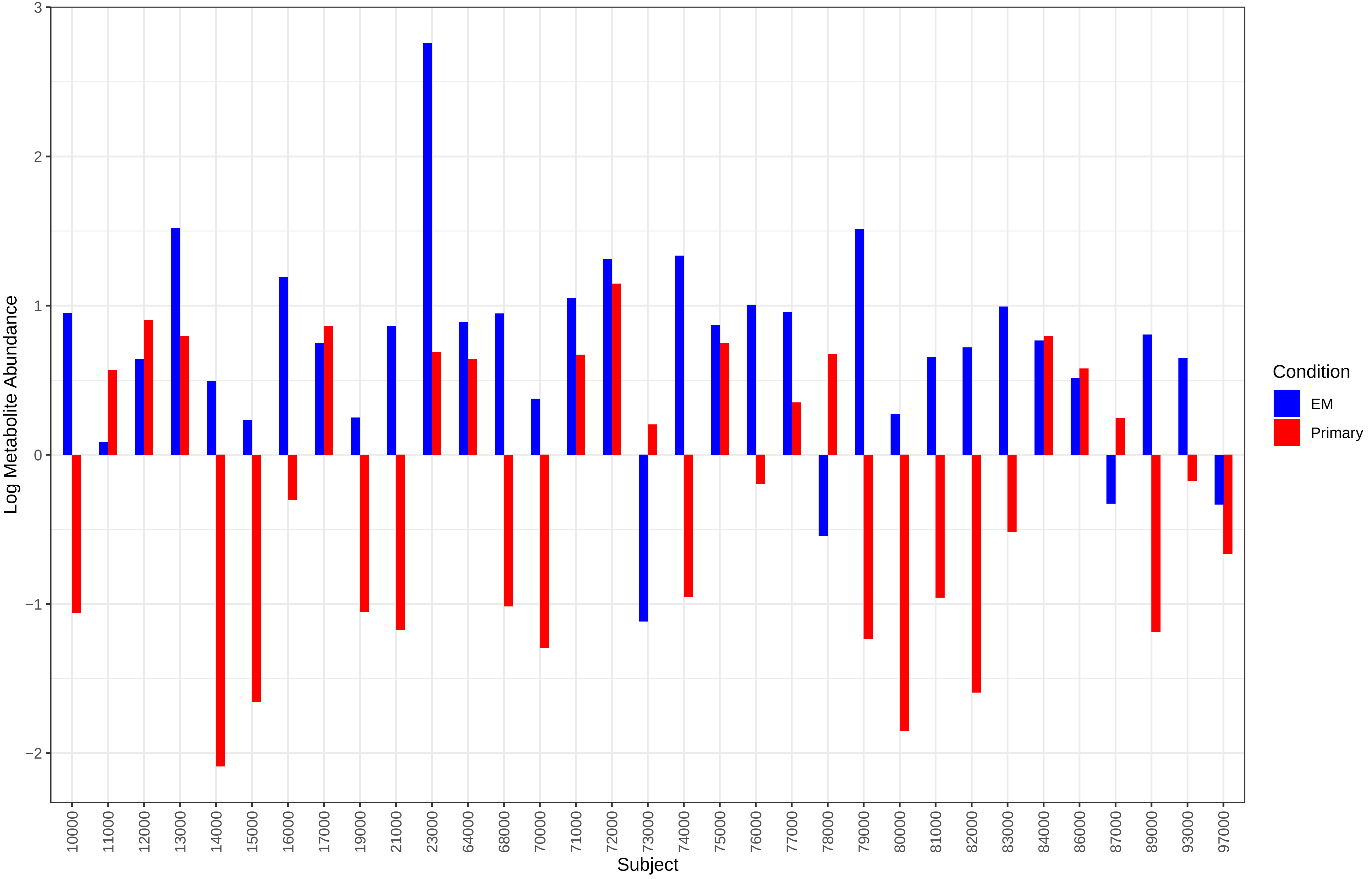

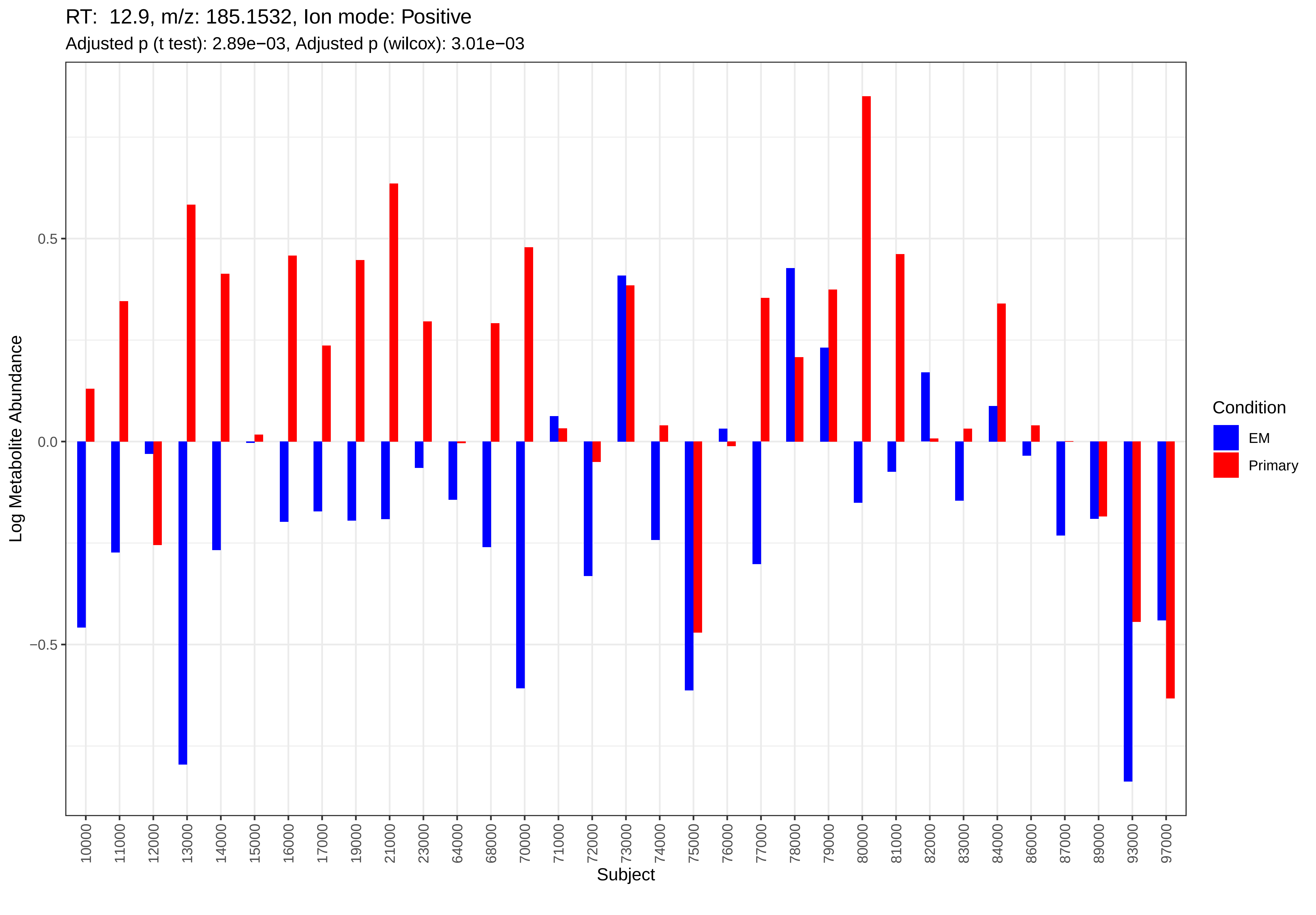
RT: 12.9, m/z: 185.1532, Ion mode: Positive
Adjusted p (t test): 2.89e−03, Adjusted p (wilcox): 3.01e−03
Log Metabolite Abundance
0.5
0.0
−0.5
Subject
10000
11000
12000
13000
14000
15000
16000
17000
19000
21000
23000
64000
68000
70000
71000
72000
73000
74000
75000
76000
77000
78000
79000
80000
81000
82000
83000
84000
86000
87000
89000
93000
97000
Condition
EM
Primary

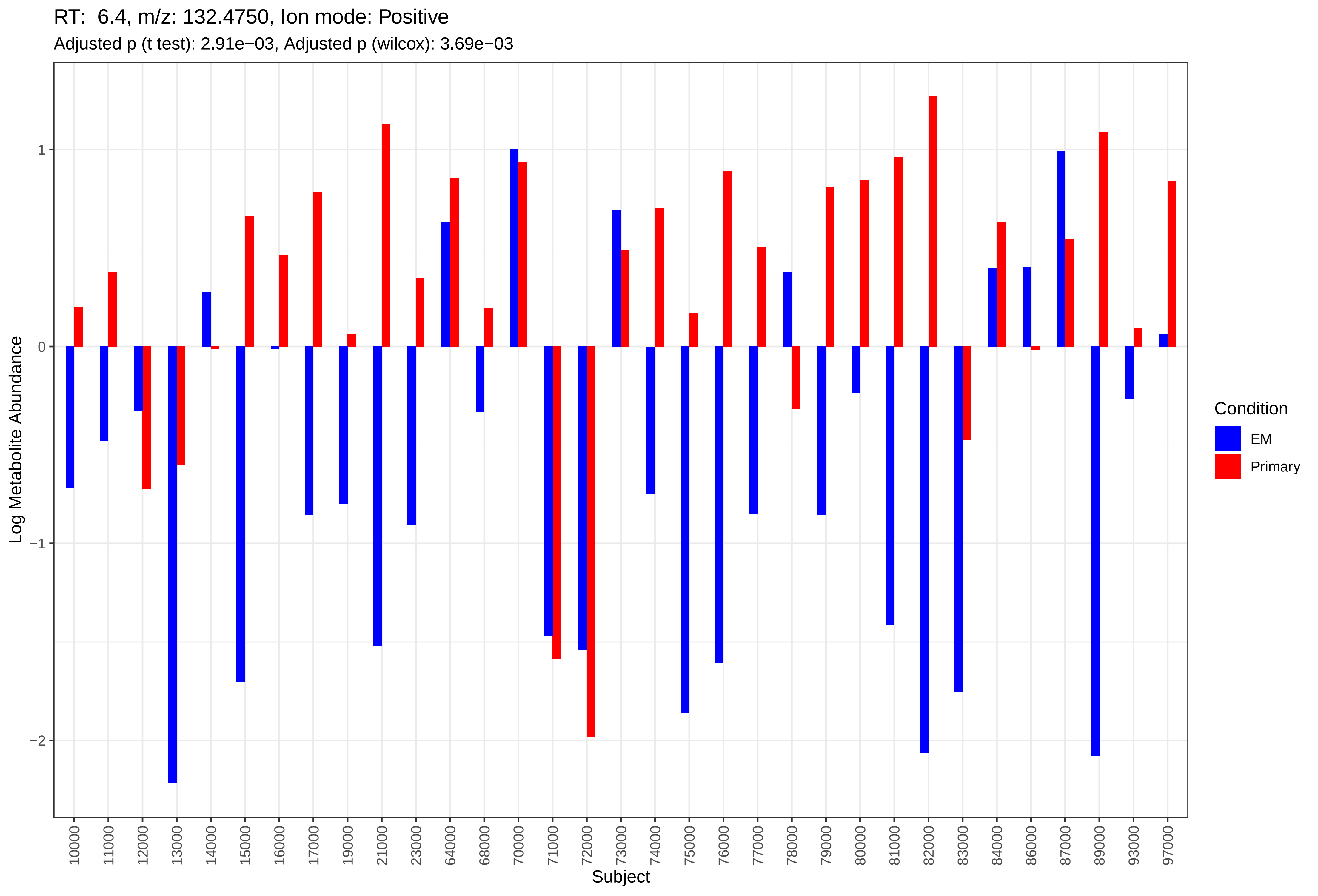

RT: 6.4, m/z: 132.4750, Ion mode: Positive
Adjusted p (t test): 2.91e−03, Adjusted p (wilcox): 3.69e−03
Log Metabolite Abundance
1
0
−1
−2
Subject
10000
11000
12000
13000
14000
15000
16000
17000
19000
21000
23000
64000
68000
70000
71000
72000
73000
74000
75000
76000
77000
78000
79000
80000
81000
82000
83000
84000
86000
87000
89000
93000
97000
Condition
EM
Primary

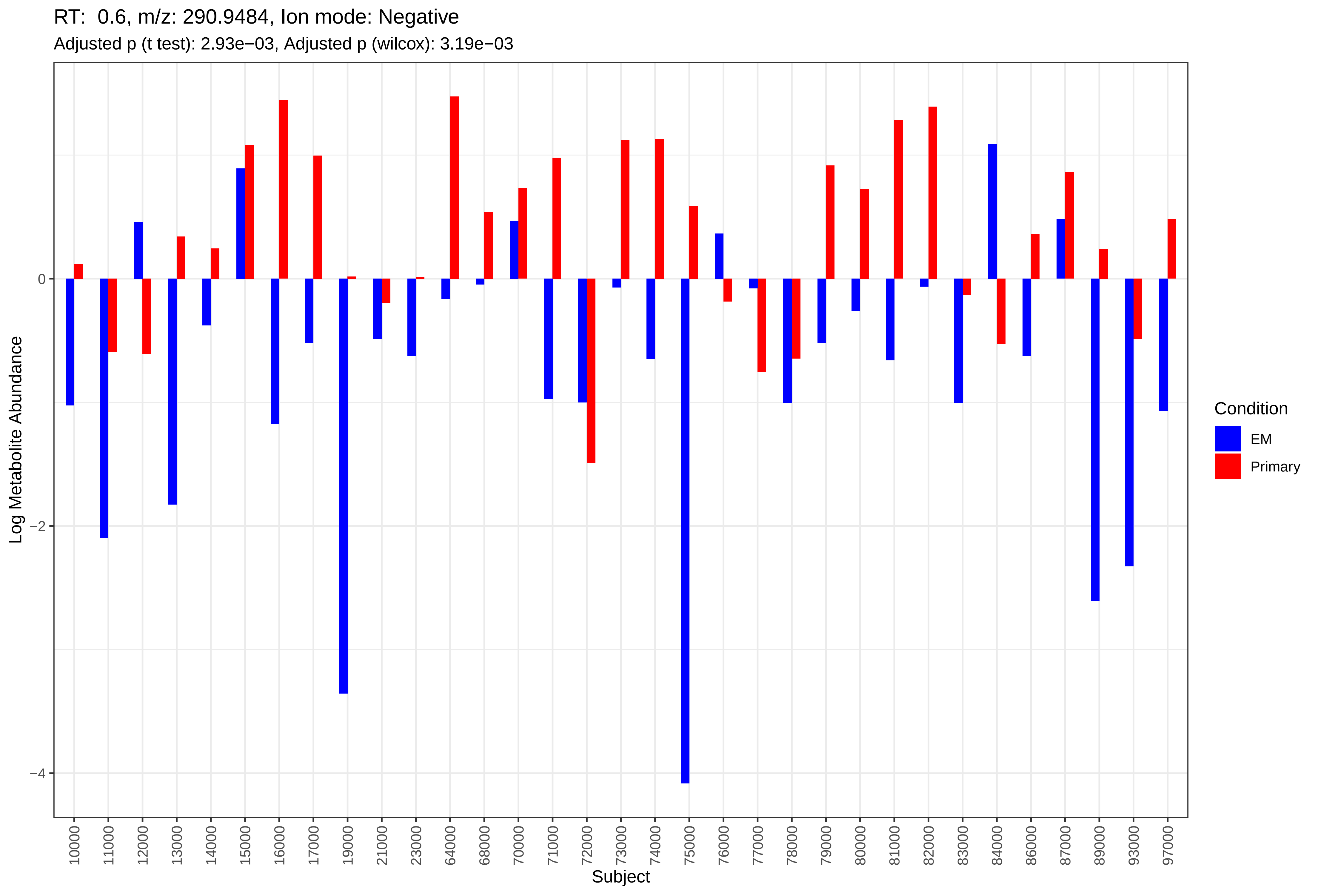
RT: 0.6, m/z: 290.9484, Ion mode: Negative
Adjusted p (t test): 2.93e−03, Adjusted p (wilcox): 3.19e−03
Log Metabolite Abundance
0
−2
−4
Subject
10000
11000
12000
13000
14000
15000
16000
17000
19000
21000
23000
64000
68000
70000
71000
72000
73000
74000
75000
76000
77000
78000
79000
80000
81000
82000
83000
84000
86000
87000
89000
93000
97000
Condition
EM
Primary

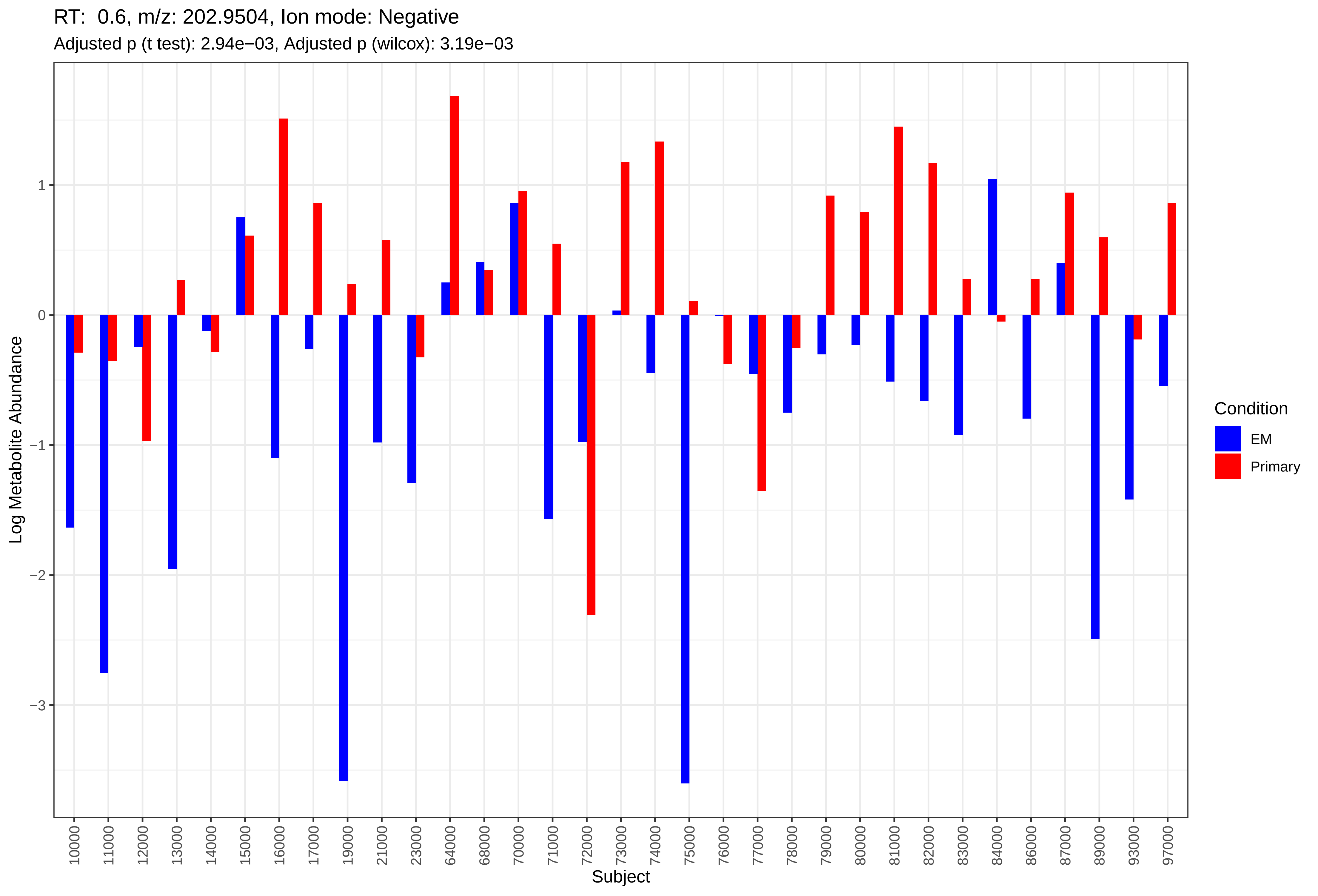

RT: 0.6, m/z: 202.9504, Ion mode: Negative
Adjusted p (t test): 2.94e−03, Adjusted p (wilcox): 3.19e−03
Log Metabolite Abundance
1
0
−1
−2
−3
10000
11000
12000
13000
14000
15000
16000
17000
19000
21000
23000
64000
68000
70000
71000
72000
73000
74000
75000
76000
77000
78000
79000
80000
81000
82000
83000
84000
86000
87000
89000
93000
97000
Subject
Condition
EM
Primary

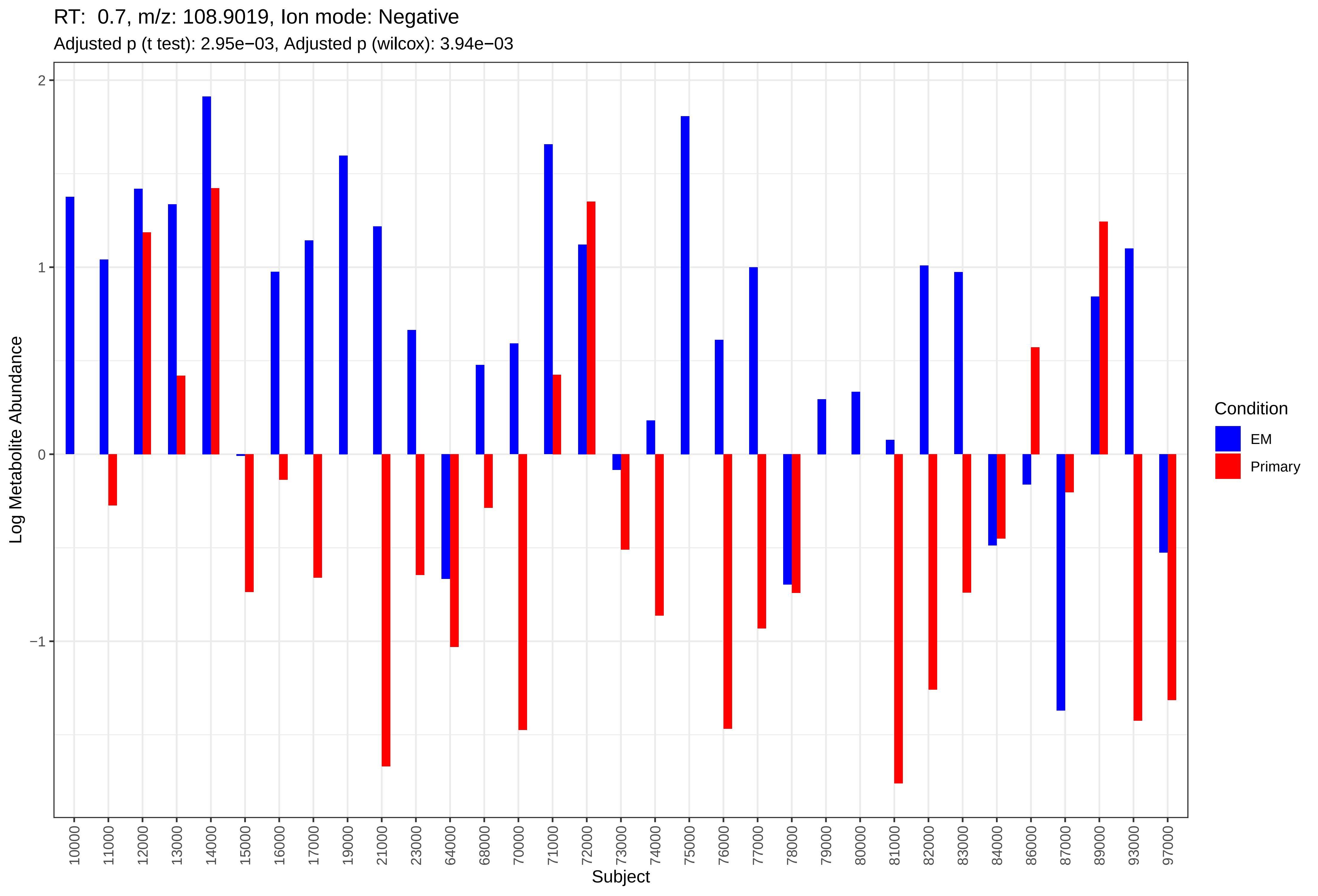

RT: 0.7, m/z: 108.9019, Ion mode: Negative
Adjusted p (t test): 2.95e−03, Adjusted p (wilcox): 3.94e−03
Log Metabolite Abundance
2
1
0
−1
Subject
10000
11000
12000
13000
14000
15000
16000
17000
19000
21000
23000
64000
68000
70000
71000
72000
73000
74000
75000
76000
77000
78000
79000
80000
81000
82000
83000
84000
86000
87000
89000
93000
97000
Condition
EM
Primary

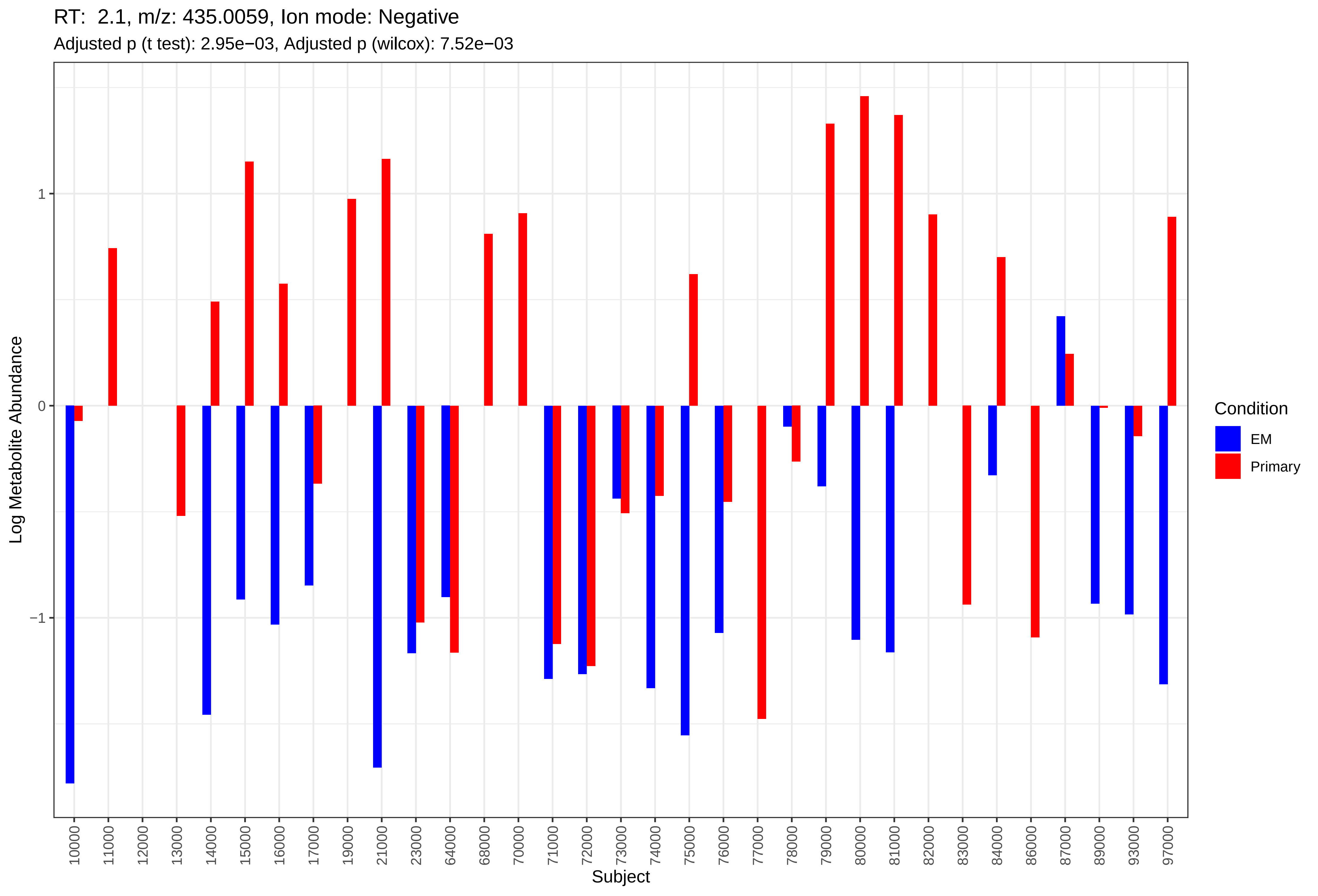
RT: 2.1, m/z: 435.0059, Ion mode: Negative
Adjusted p (t test): 2.95e−03, Adjusted p (wilcox): 7.52e−03
Log Metabolite Abundance
1
0
−1
10000
11000
12000
13000
14000
15000
16000
17000
19000
21000
23000
64000
68000
70000
71000
72000
73000
74000
75000
76000
77000
78000
79000
80000
81000
82000
83000
84000
86000
87000
89000
93000
97000
Subject
Condition
EM
Primary

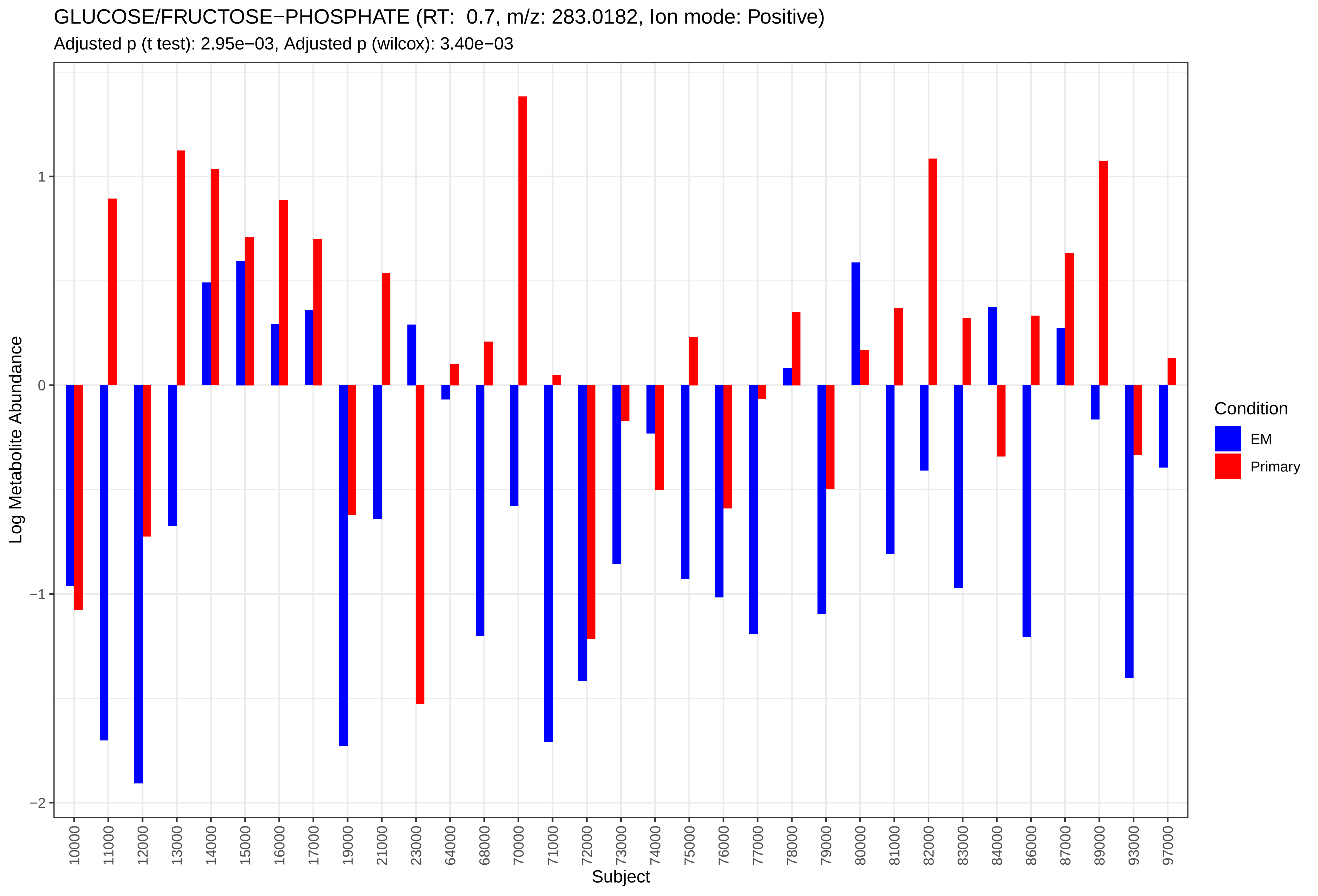
GLUCOSE/FRUCTOSE−PHOSPHATE (RT: 0.7, m/z: 283.0182, Ion mode: Positive)
Adjusted p (t test): 2.95e−03, Adjusted p (wilcox): 3.40e−03
Log Metabolite Abundance
1
0
−1
−2
10000
11000
12000
13000
14000
15000
16000
17000
19000
21000
23000
64000
68000
70000
71000
72000
73000
74000
75000
76000
77000
78000
79000
80000
81000
82000
83000
84000
86000
87000
89000
93000
97000
Subject
Condition
EM
Primary

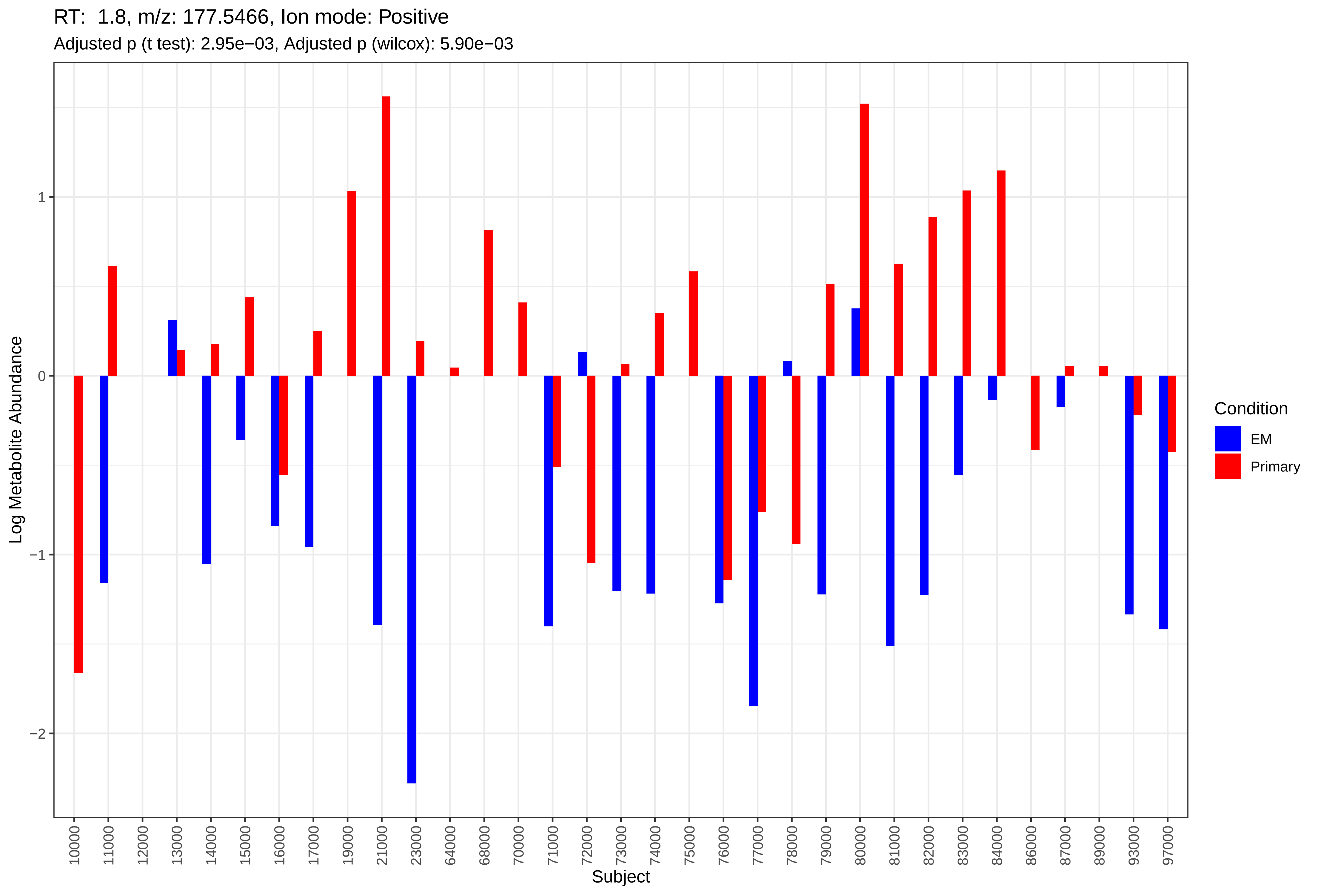
RT: 1.8, m/z: 177.5466, Ion mode: Positive
Adjusted p (t test): 2.95e−03, Adjusted p (wilcox): 5.90e−03
Log Metabolite Abundance
1
0
−1
−2
10000
11000
12000
13000
14000
15000
16000
17000
19000
21000
23000
64000
68000
70000
71000
72000
73000
74000
75000
76000
77000
78000
79000
80000
81000
82000
83000
84000
86000
87000
89000
93000
97000
Subject
Condition
EM
Primary

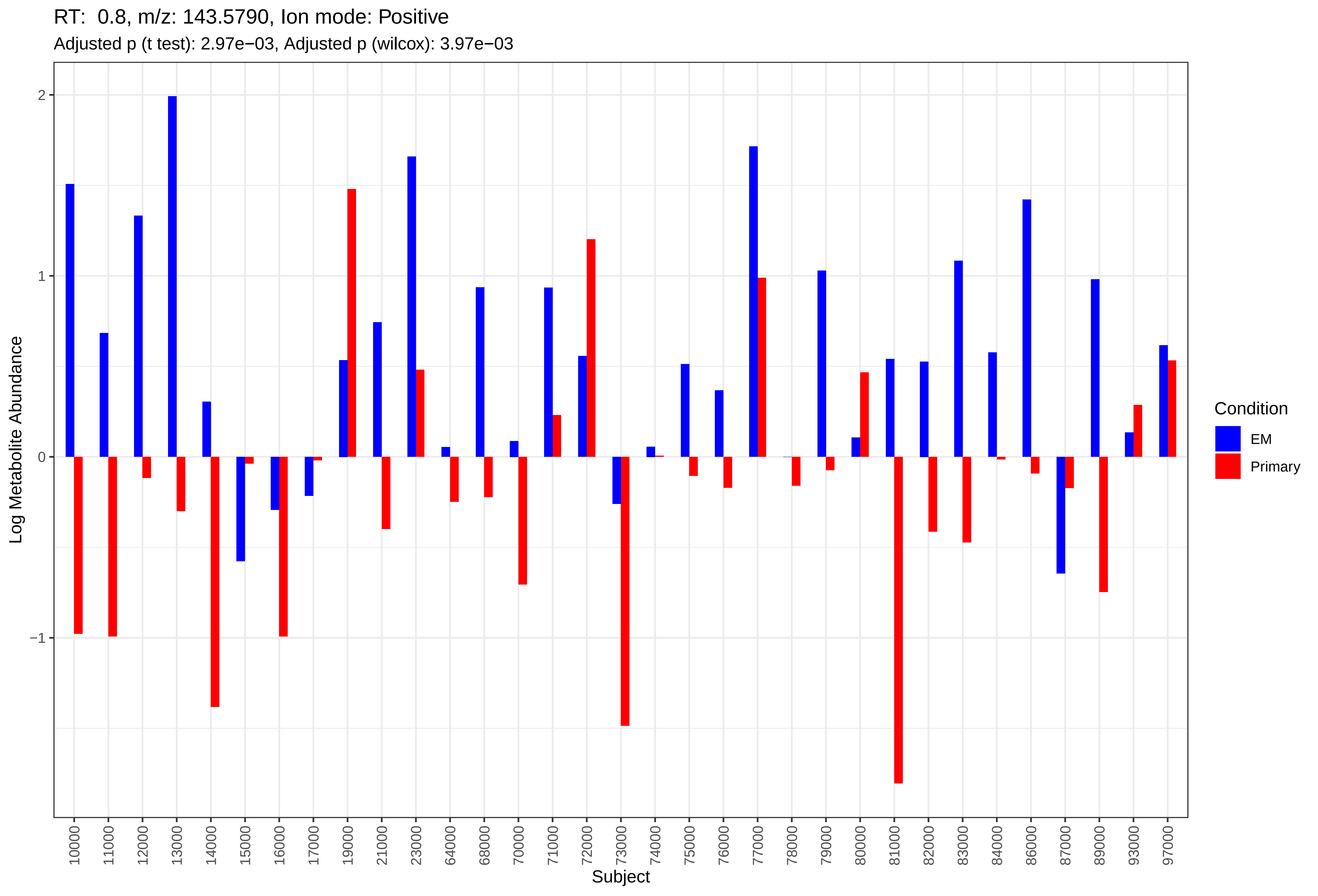

RT: 0.8, m/z: 143.5790, Ion mode: Positive
Adjusted p (t test): 2.97e−03, Adjusted p (wilcox): 3.97e−03
Log Metabolite Abundance
2
1
0
−1
10000
11000
12000
13000
14000
15000
16000
17000
19000
21000
23000
64000
68000
70000
71000
72000
73000
74000
75000
76000
77000
78000
79000
80000
81000
82000
83000
84000
86000
87000
89000
93000
97000
Subject
Condition
EM
Primary

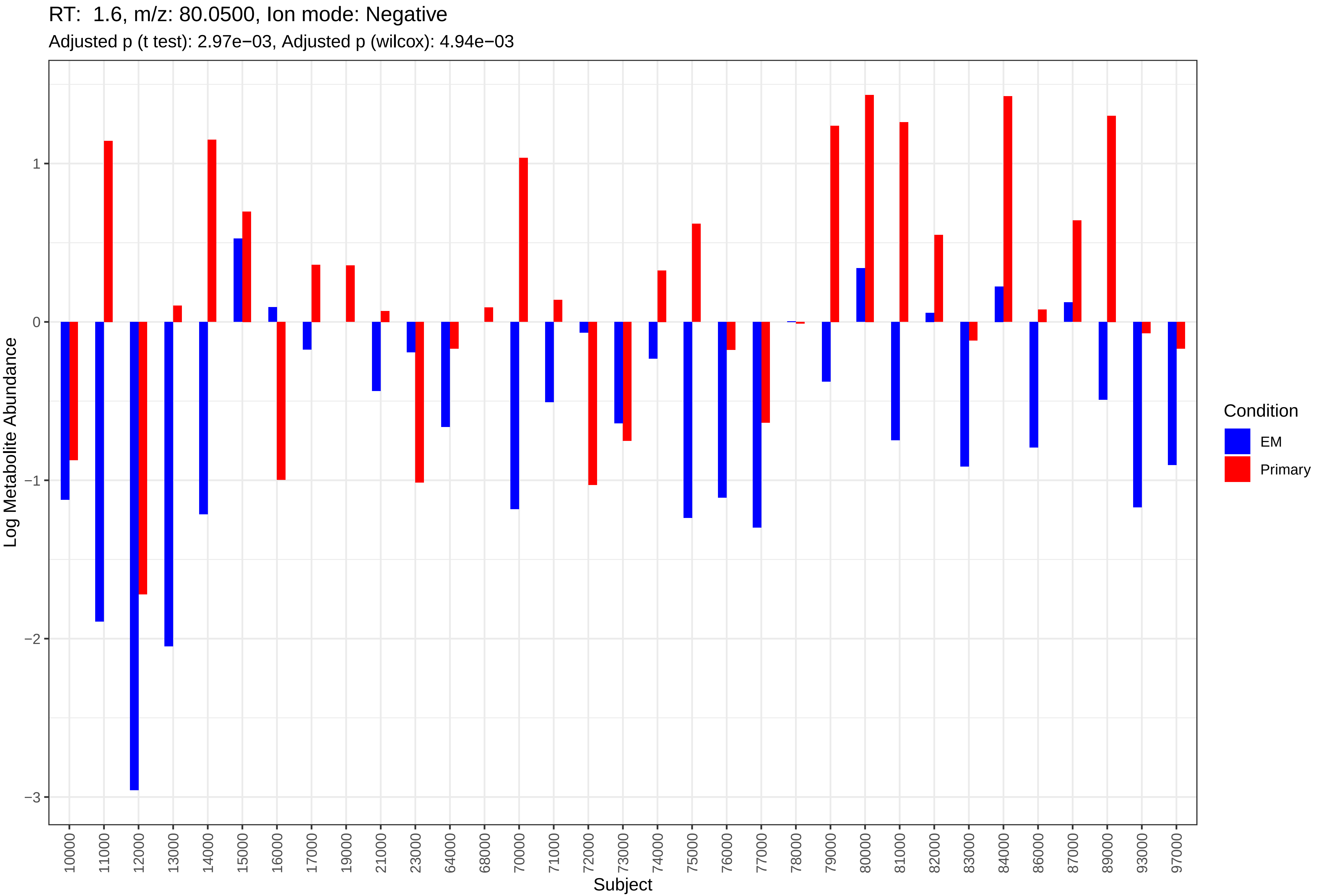
RT: 1.6, m/z: 80.0500, Ion mode: Negative
Adjusted p (t test): 2.97e−03, Adjusted p (wilcox): 4.94e−03
Log Metabolite Abundance
1
0
−1
−2
−3
Subject
10000
11000
12000
13000
14000
15000
16000
17000
19000
21000
23000
64000
68000
70000
71000
72000
73000
74000
75000
76000
77000
78000
79000
80000
81000
82000
83000
84000
86000
87000
89000
93000
97000
Condition
EM
Primary

L−GLUTAMIC ACID (RT: 0.8, m/z: 146.0460, Ion mode: Negative)

Adjusted p (t test): 3.69e−03, Adjusted p (wilcox): 3.01e−03

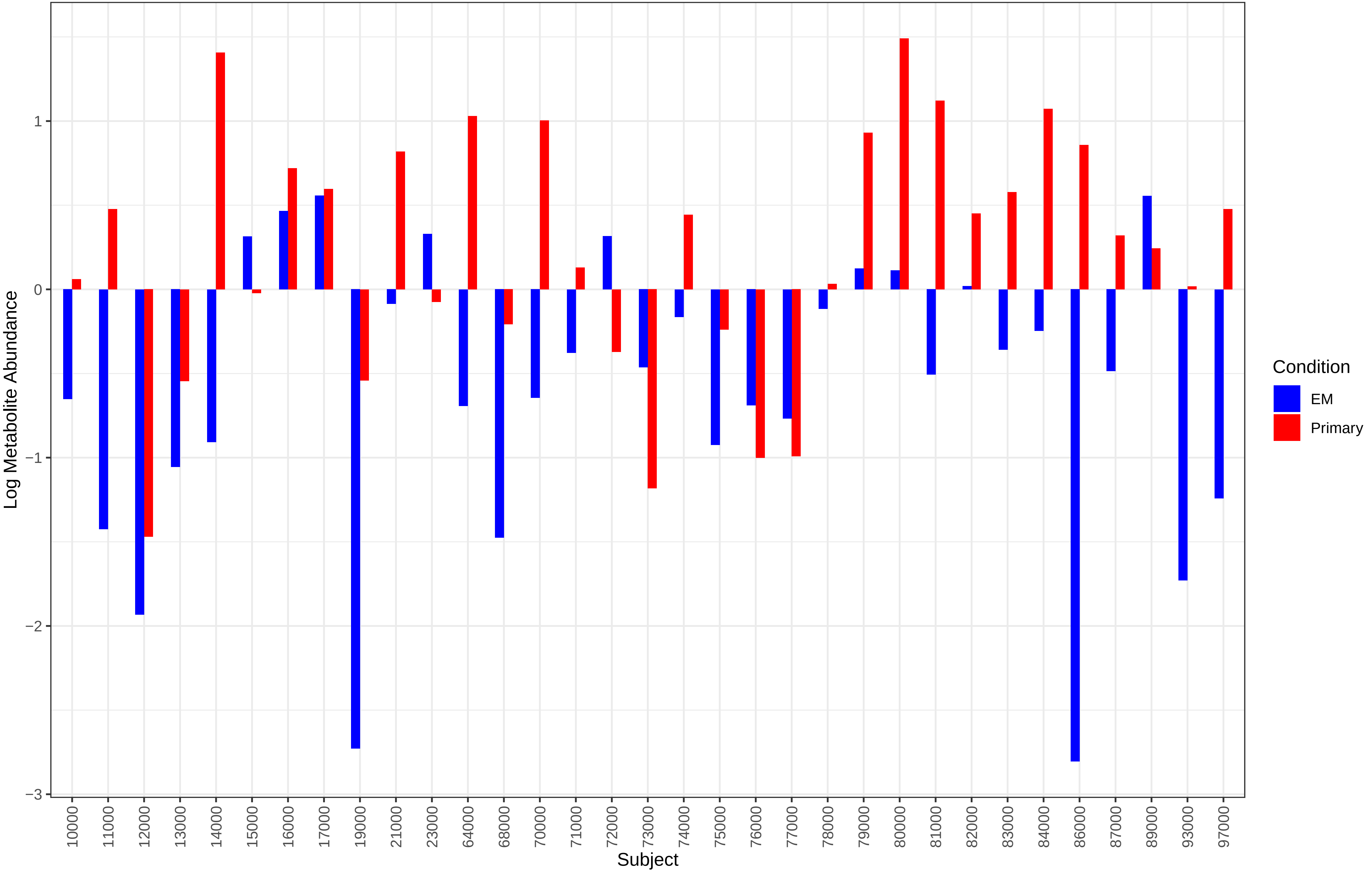

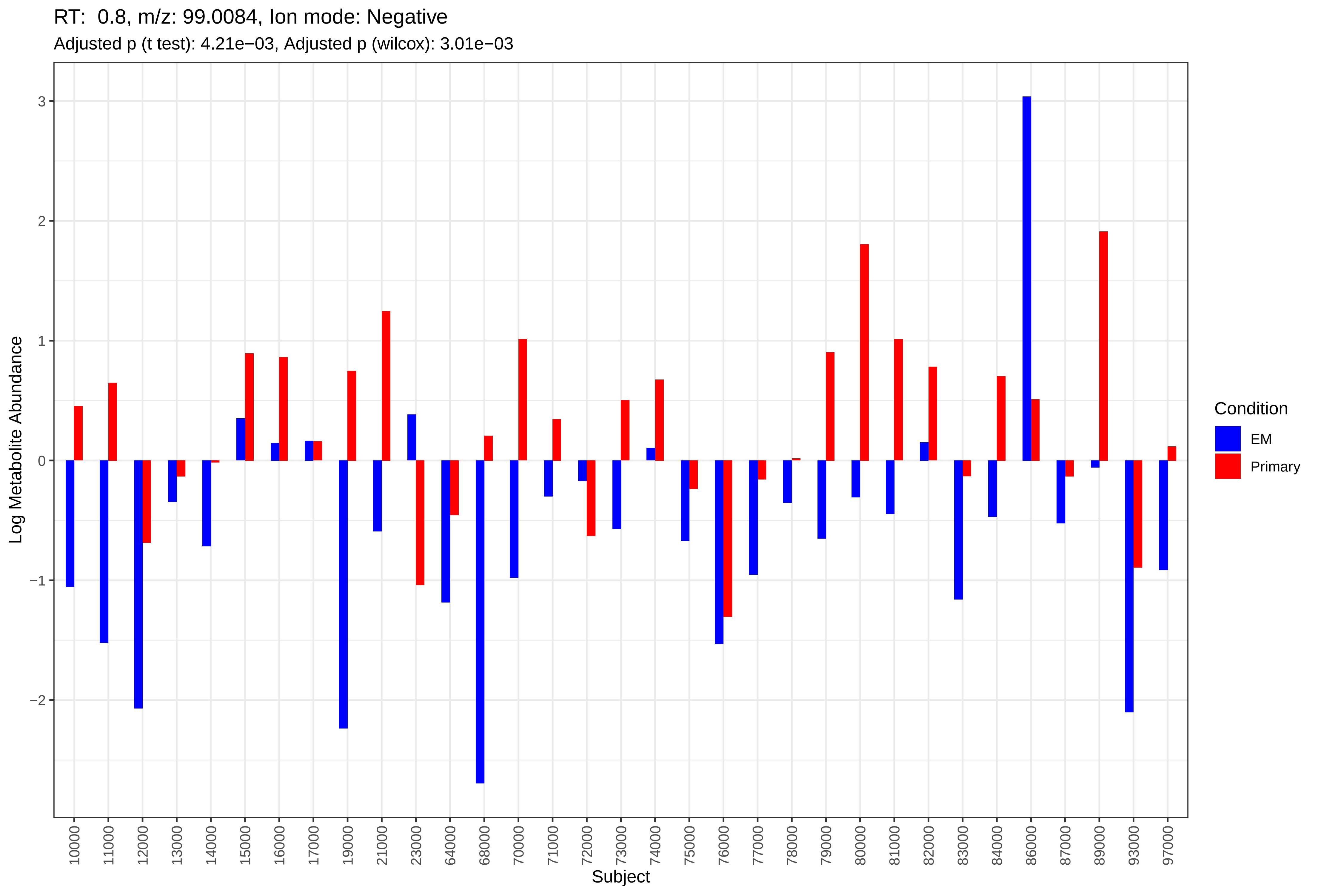
RT: 0.8, m/z: 99.0084, Ion mode: Negative
Adjusted p (t test): 4.21e−03, Adjusted p (wilcox): 3.01e−03
Log Metabolite Abundance
Subject
Condition
EM
Primary
3
2
1
0
−1
−2
10000
11000
12000
13000
14000
15000
16000
17000
19000
21000
23000
64000
68000
70000
71000
72000
73000
74000
75000
76000
77000
78000
79000
80000
81000
82000
83000
84000
86000
87000
89000
93000
97000

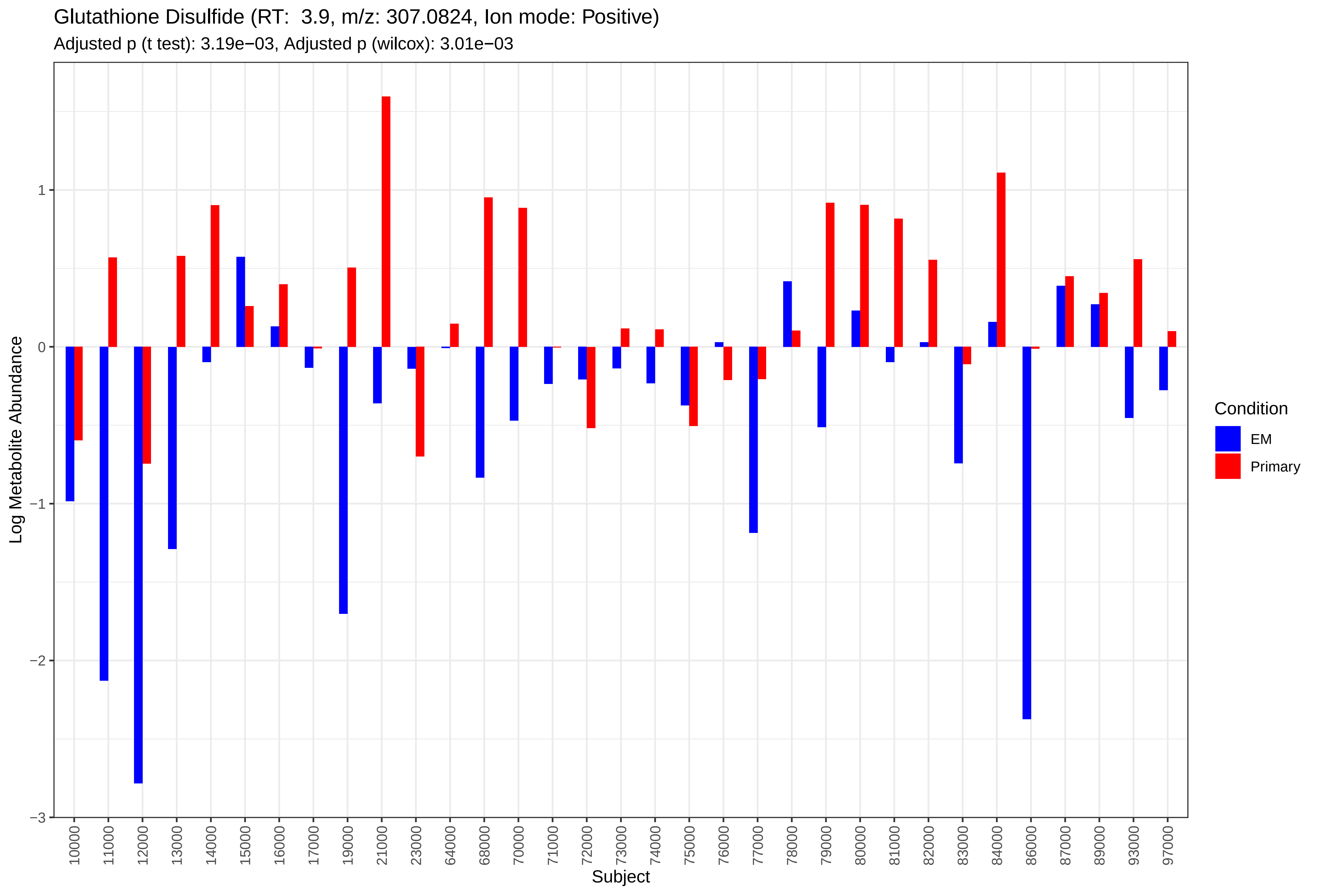

Glutathione Disulfide (RT: 3.9, m/z: 307.0824, Ion mode: Positive)
Adjusted p (t test): 3.19e−03, Adjusted p (wilcox): 3.01e−03
Log Metabolite Abundance
1
0
−1
−2
−3
10000
11000
12000
13000
14000
15000
16000
17000
19000
21000
23000
64000
68000
70000
71000
72000
73000
74000
75000
76000
77000
78000
79000
80000
81000
82000
83000
84000
86000
87000
89000
93000
97000
Subject
Condition
EM
Primary

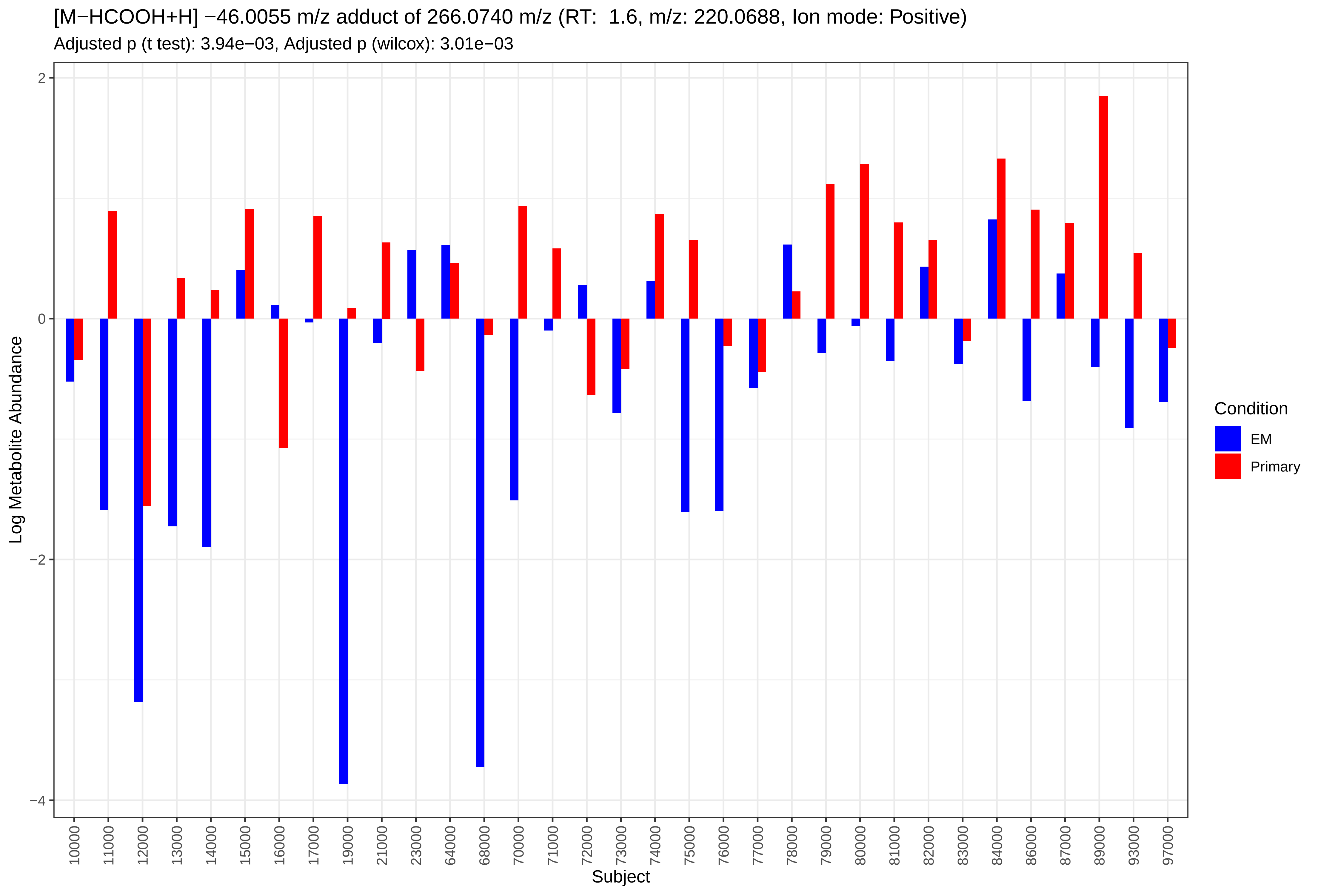

[M−HCOOH+H] −46.0055 m/z adduct of 266.0740 m/z (RT: 1.6, m/z: 220.0688, Ion mode: Positive)
Adjusted p (t test): 3.94e−03, Adjusted p (wilcox): 3.01e−03
Log Metabolite Abundance
2
0
−2
−4
Subject
10000
11000
12000
13000
14000
15000
16000
17000
19000
21000
23000
64000
68000
70000
71000
72000
73000
74000
75000
76000
77000
78000
79000
80000
81000
82000
83000
84000
86000
87000
89000
93000
97000
Condition
EM
Primary

RT: 6.1, m/z: 242.6414, Ion mode: Positive

Adjusted p (t test): 3.01e−03, Adjusted p (wilcox): 4.48e−03

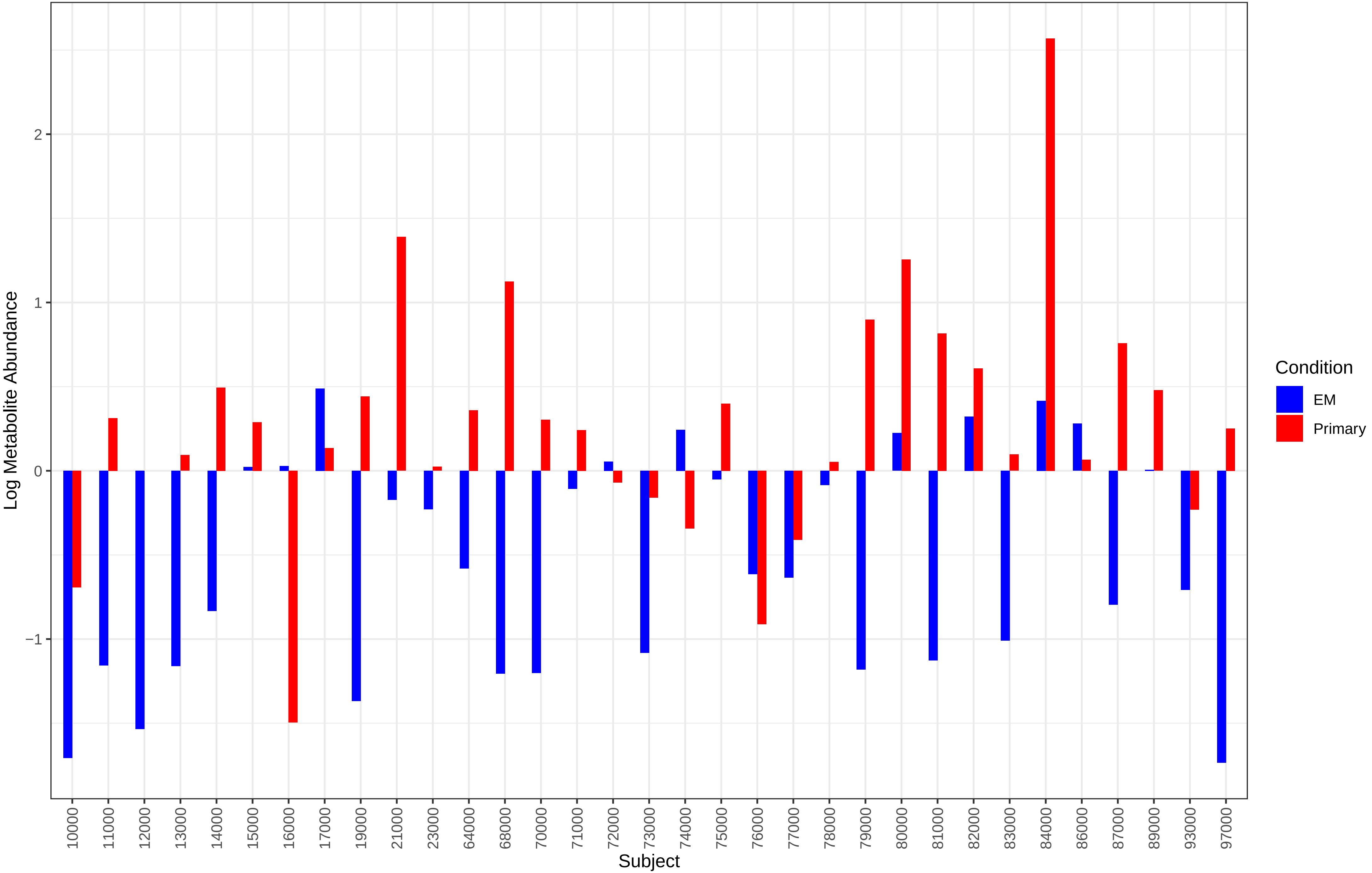

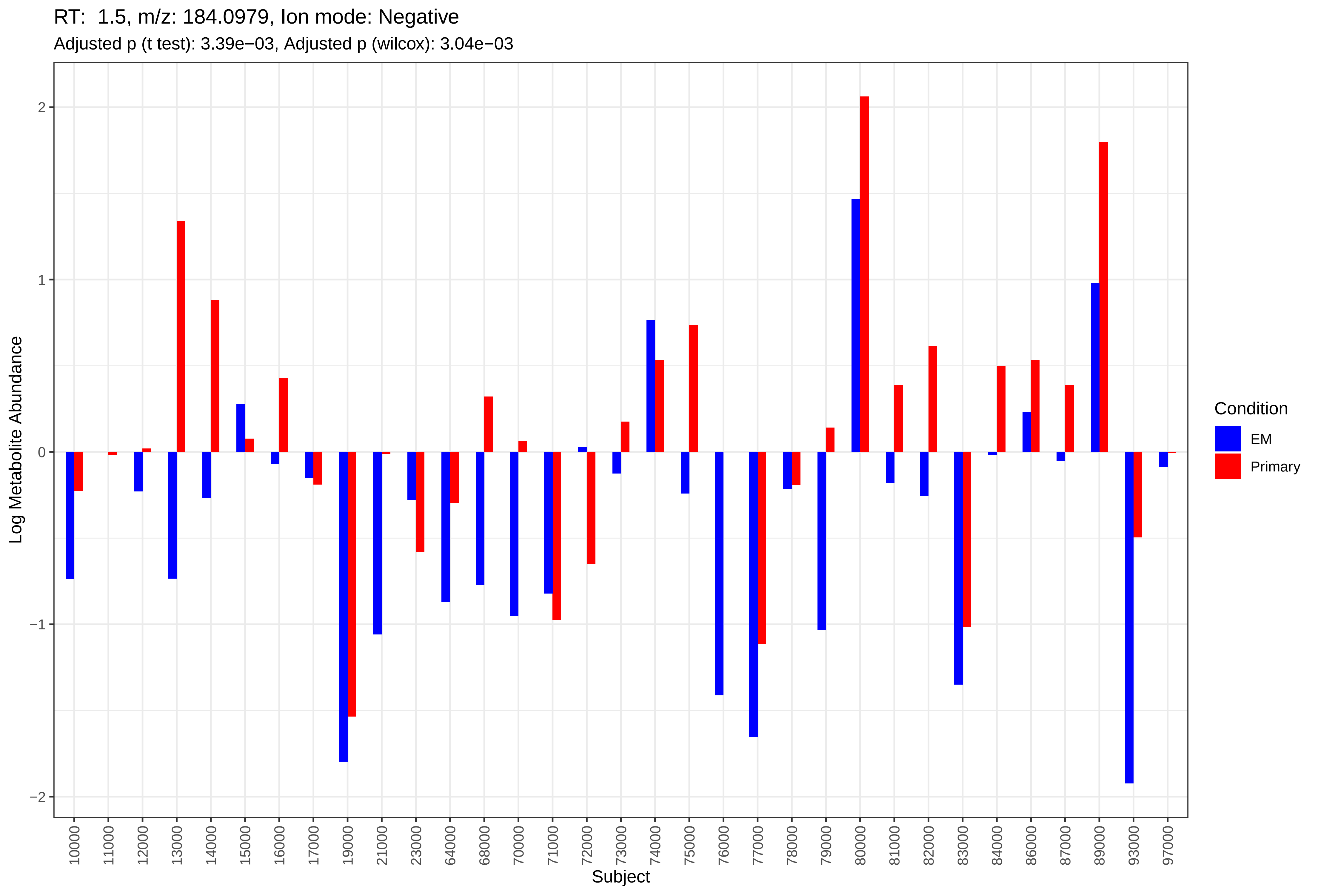

RT: 1.5, m/z: 184.0979, Ion mode: Negative
Adjusted p (t test): 3.39e−03, Adjusted p (wilcox): 3.04e−03
Log Metabolite Abundance
2
1
0
−1
−2
10000
11000
12000
13000
14000
15000
16000
17000
19000
21000
23000
64000
68000
70000
71000
72000
73000
74000
75000
76000
77000
78000
79000
80000
81000
82000
83000
84000
86000
87000
89000
93000
97000
Subject
Condition
EM
Primary

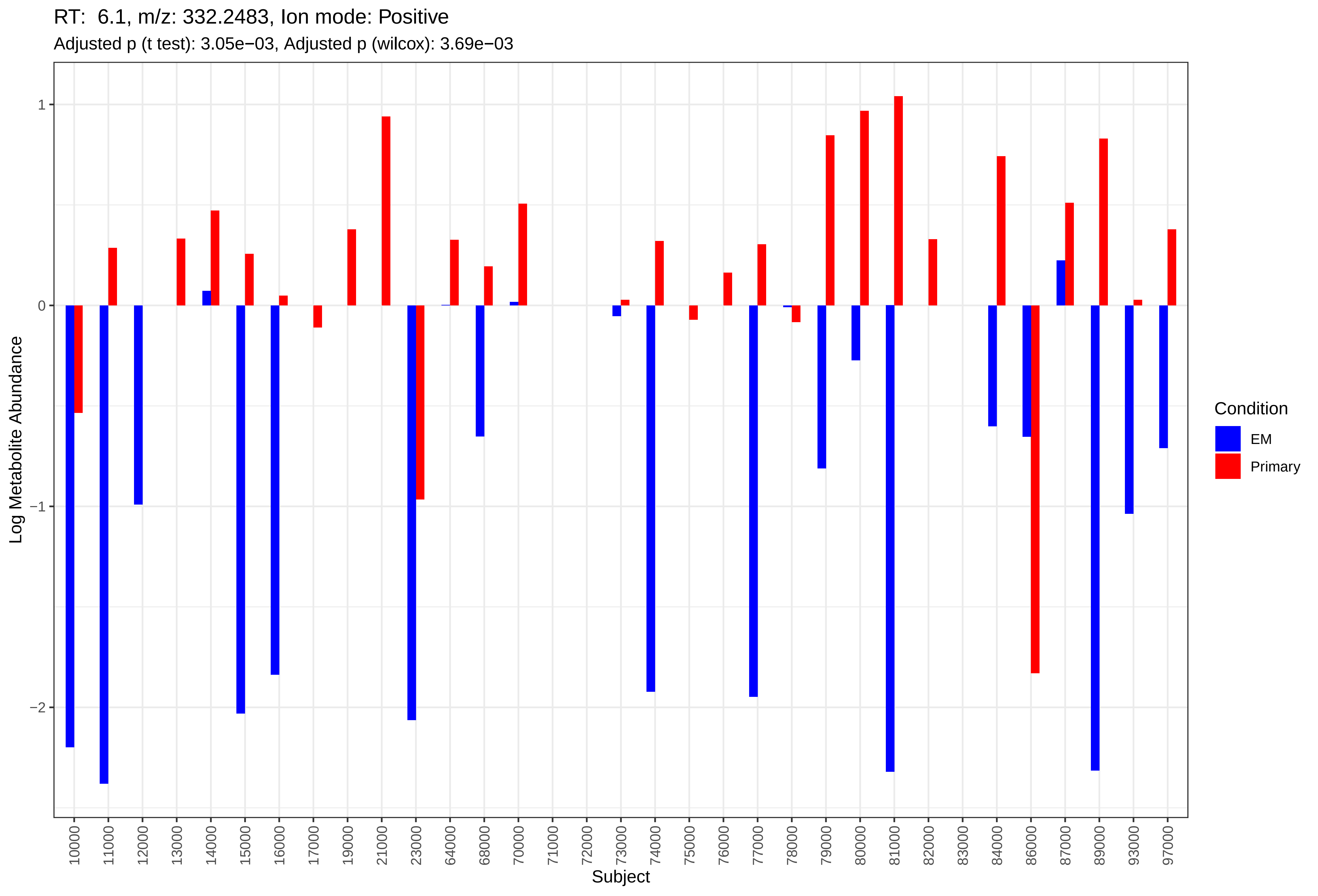

RT: 6.1, m/z: 332.2483, Ion mode: Positive
Adjusted p (t test): 3.05e−03, Adjusted p (wilcox): 3.69e−03
Log Metabolite Abundance
1
0
−1
−2
Subject
10000
11000
12000
13000
14000
15000
16000
17000
19000
21000
23000
64000
68000
70000
71000
72000
73000
74000
75000
76000
77000
78000
79000
80000
81000
82000
83000
84000
86000
87000
89000
93000
97000
Condition
EM
Primary

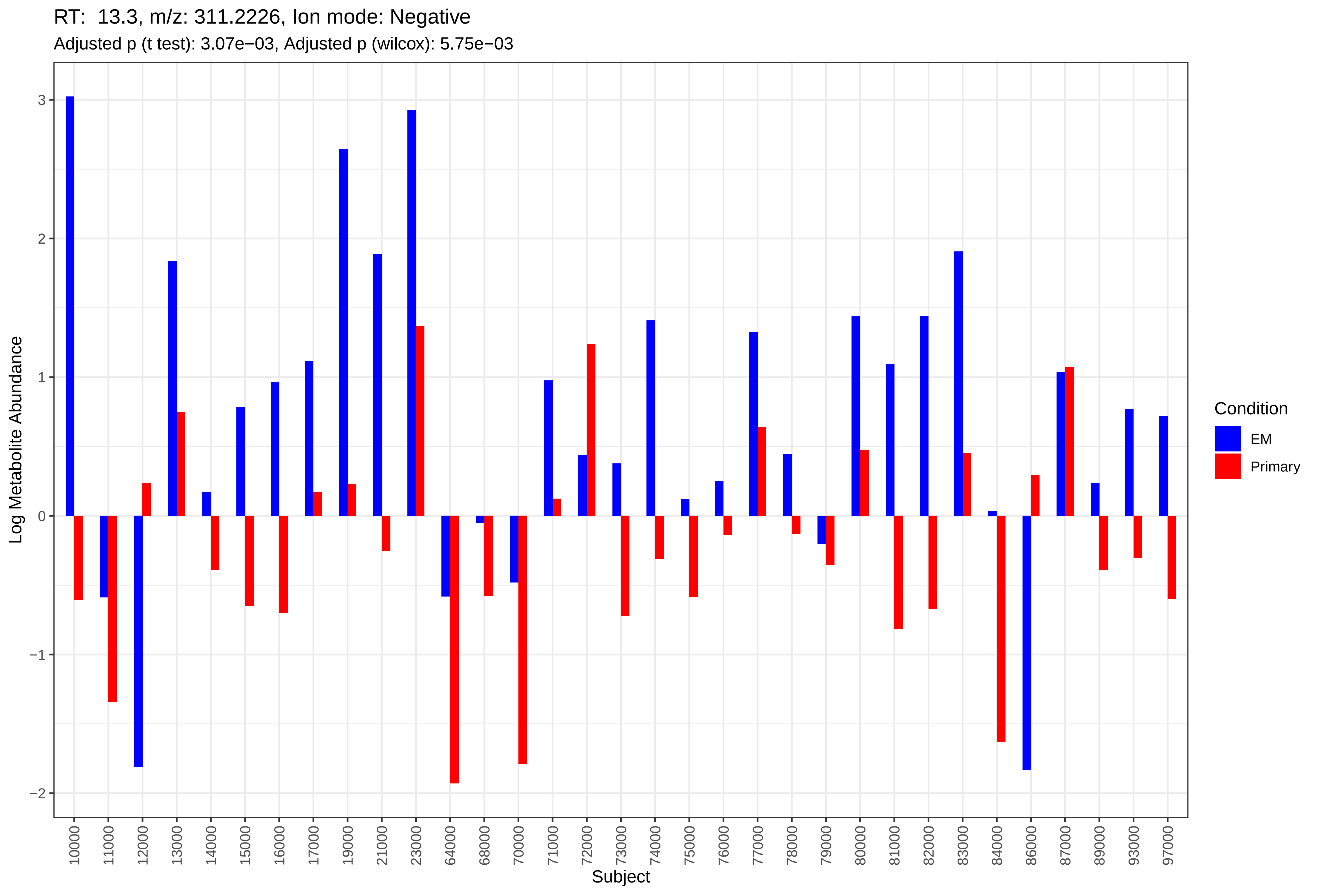
RT: 13.3, m/z: 311.2226, Ion mode: Negative
Adjusted p (t test): 3.07e−03, Adjusted p (wilcox): 5.75e−03
Log Metabolite Abundance
3
2
1
0
−1
−2
10000
11000
12000
13000
14000
15000
16000
17000
19000
21000
23000
64000
68000
70000
71000
72000
73000
74000
75000
76000
77000
78000
79000
80000
81000
82000
83000
84000
86000
87000
89000
93000
97000
Subject
Condition
EM
Primary

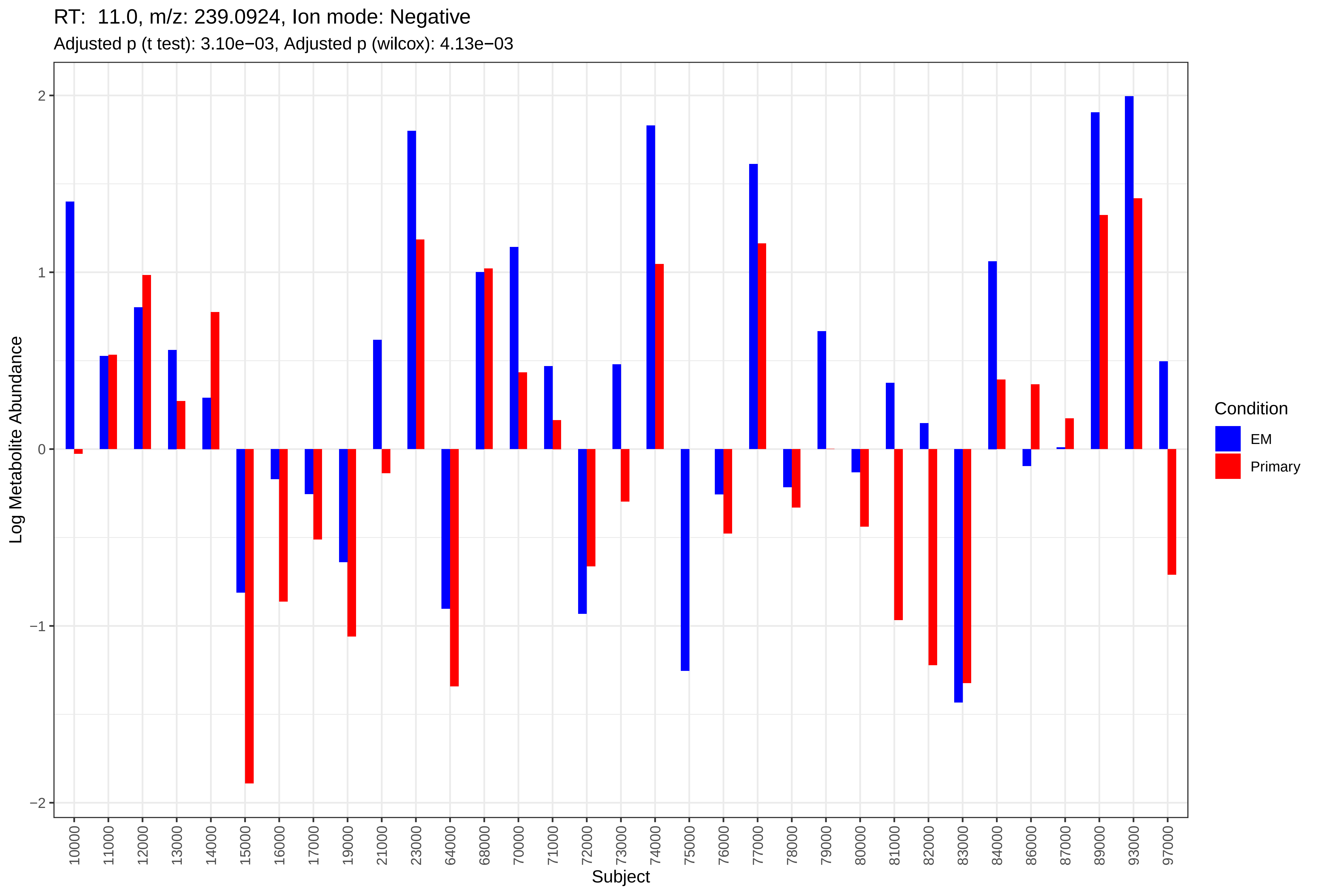
RT: 11.0, m/z: 239.0924, Ion mode: Negative
Adjusted p (t test): 3.10e−03, Adjusted p (wilcox): 4.13e−03
Log Metabolite Abundance
2
1
0
−1
−2
Subject
10000
11000
12000
13000
14000
15000
16000
17000
19000
21000
23000
64000
68000
70000
71000
72000
73000
74000
75000
76000
77000
78000
79000
80000
81000
82000
83000
84000
86000
87000
89000
93000
97000
Condition
EM
Primary

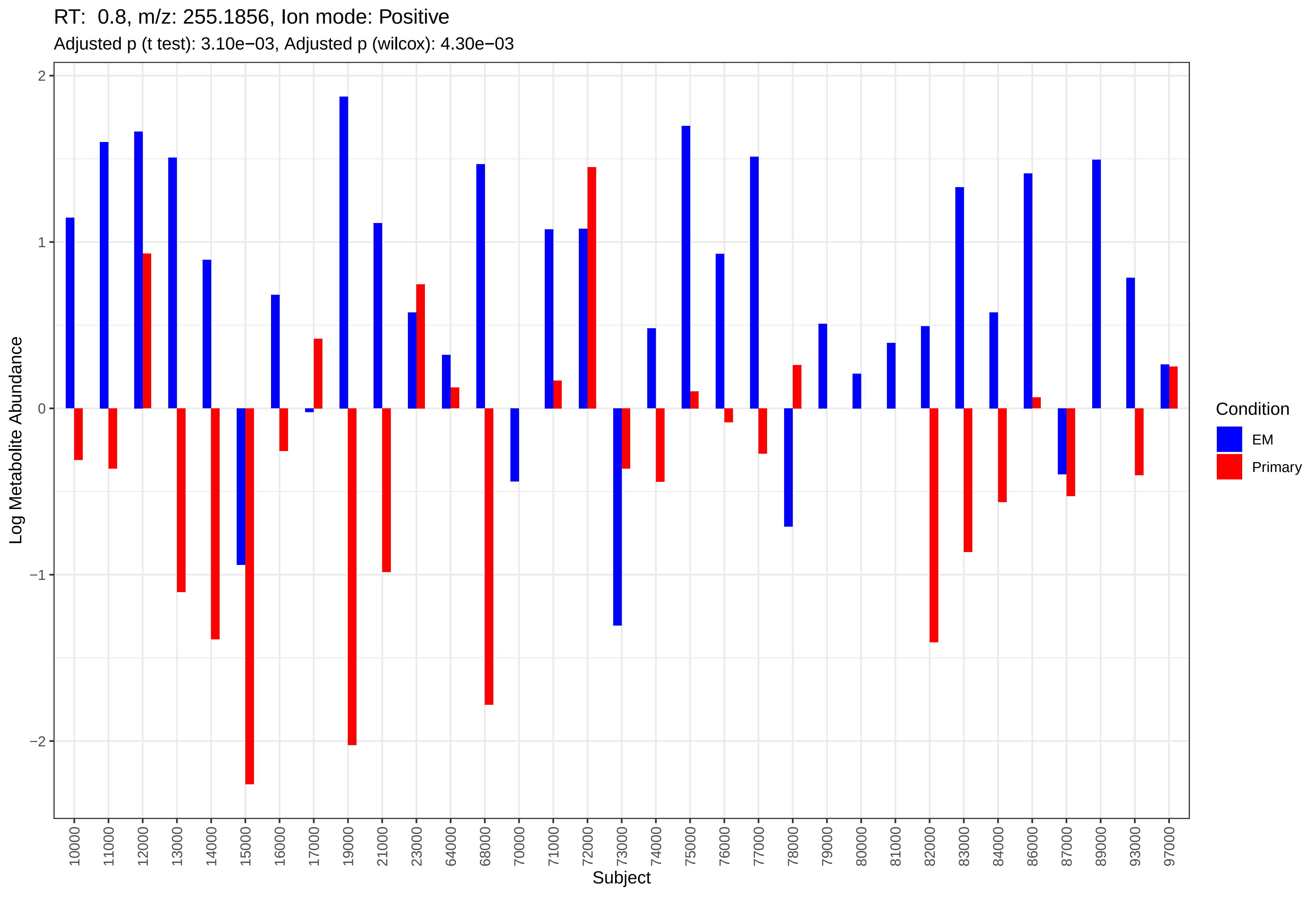

RT: 0.8, m/z: 255.1856, Ion mode: Positive
Adjusted p (t test): 3.10e−03, Adjusted p (wilcox): 4.30e−03
Log Metabolite Abundance
2
1
0
−1
−2
10000
11000
12000
13000
14000
15000
16000
17000
19000
21000
23000
64000
68000
70000
71000
72000
73000
74000
75000
76000
77000
78000
79000
80000
81000
82000
83000
84000
86000
87000
89000
93000
97000
Subject
Condition
EM
Primary

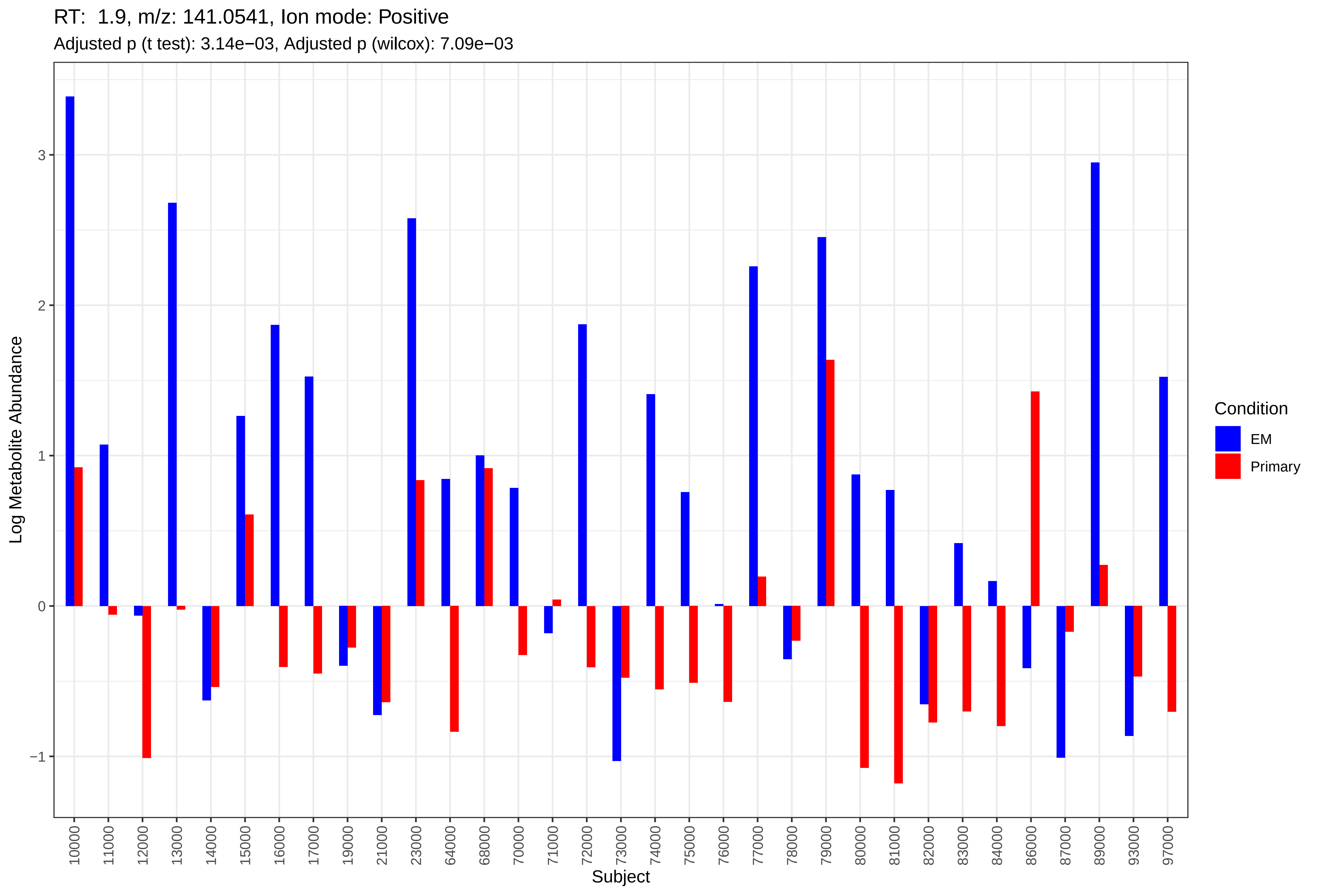

RT: 1.9, m/z: 141.0541, Ion mode: Positive
Adjusted p (t test): 3.14e−03, Adjusted p (wilcox): 7.09e−03
Log Metabolite Abundance
3
2
1
0
−1
10000
11000
12000
13000
14000
15000
16000
17000
19000
21000
23000
64000
68000
70000
71000
72000
73000
74000
75000
76000
77000
78000
79000
80000
81000
82000
83000
84000
86000
87000
89000
93000
97000
Subject
Condition
EM
Primary

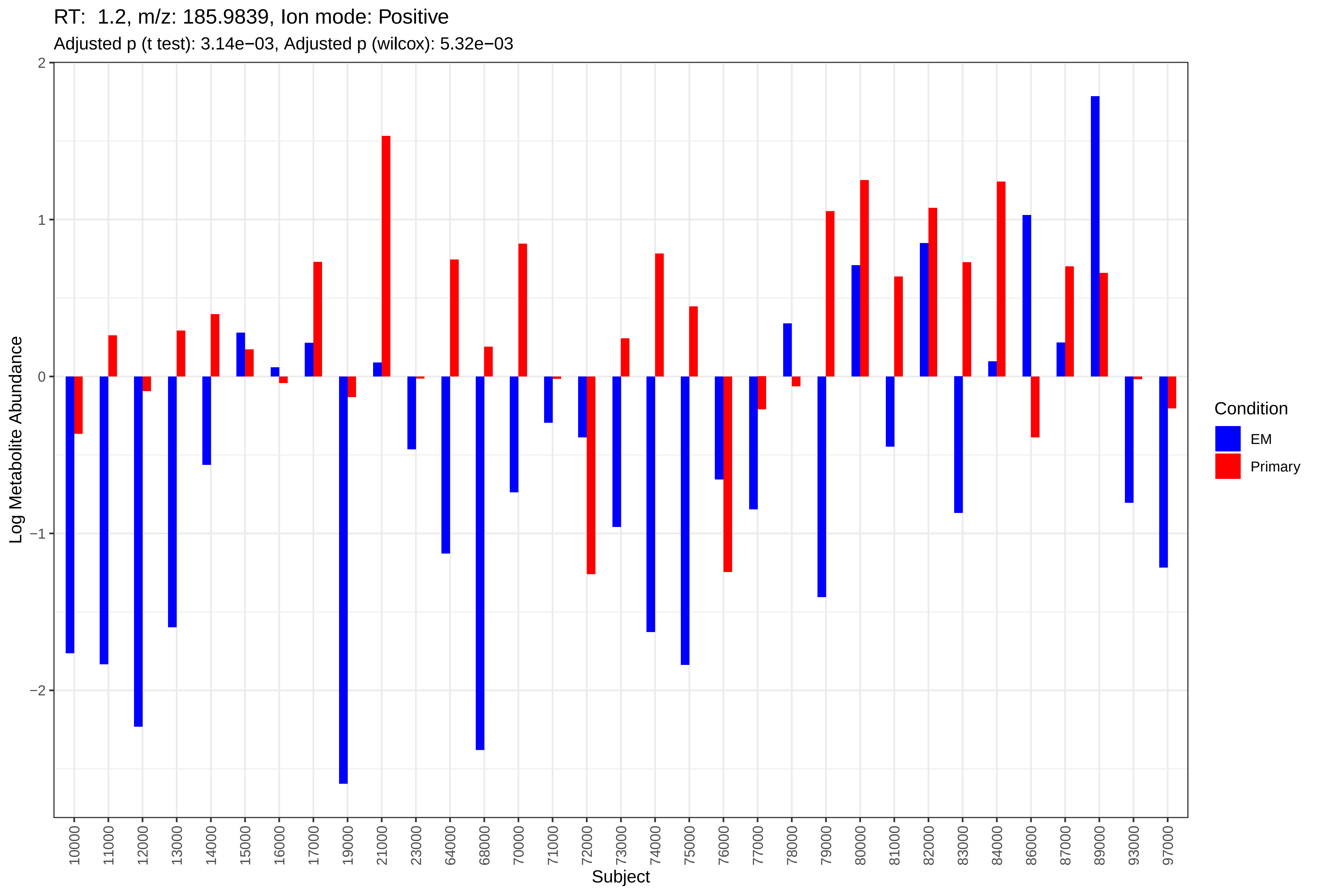
RT: 1.2, m/z: 185.9839, Ion mode: Positive
Adjusted p (t test): 3.14e−03, Adjusted p (wilcox): 5.32e−03
Log Metabolite Abundance
2
1
0
−1
−2
10000
11000
12000
13000
14000
15000
16000
17000
19000
21000
23000
64000
68000
70000
71000
72000
73000
74000
75000
76000
77000
78000
79000
80000
81000
82000
83000
84000
86000
87000
89000
93000
97000
Subject
Condition
EM
Primary

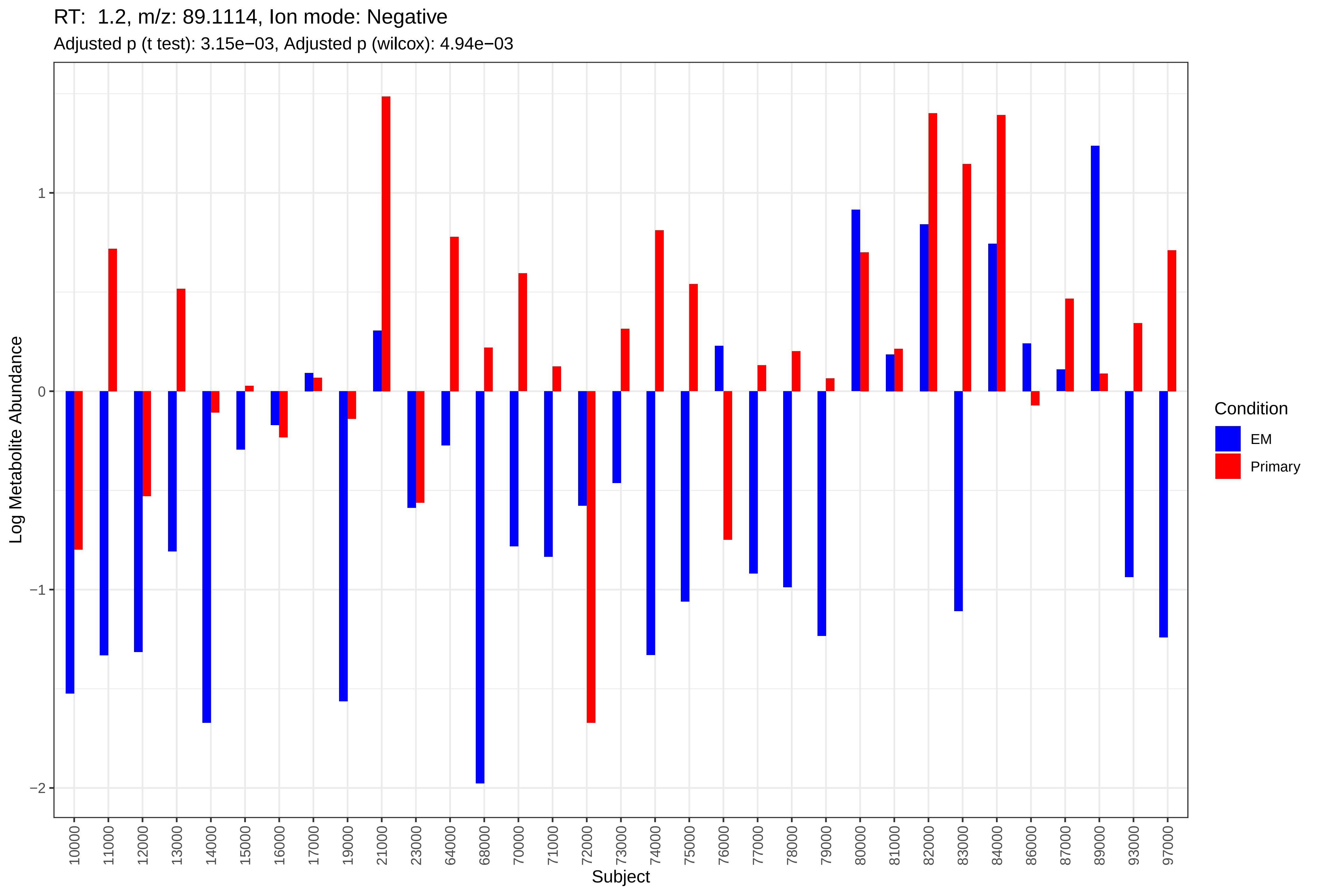

RT: 1.2, m/z: 89.1114, Ion mode: Negative
Adjusted p (t test): 3.15e−03, Adjusted p (wilcox): 4.94e−03
Log Metabolite Abundance
1
0
−1
−2
10000
11000
12000
13000
14000
15000
16000
17000
19000
21000
23000
64000
68000
70000
71000
72000
73000
74000
75000
76000
77000
78000
79000
80000
81000
82000
83000
84000
86000
87000
89000
93000
97000
Subject
Condition
EM
Primary

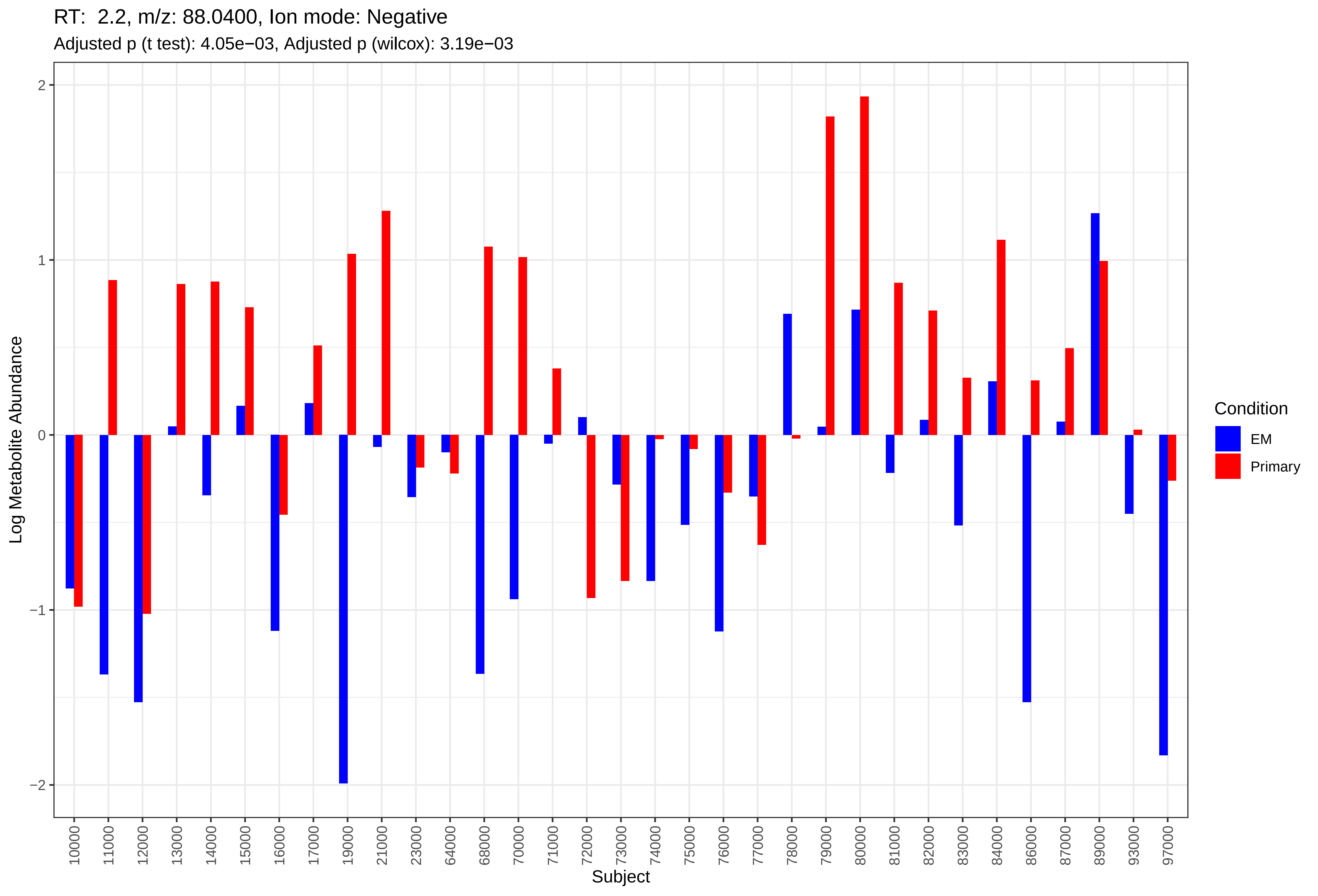

RT: 2.2, m/z: 88.0400, Ion mode: Negative
Adjusted p (t test): 4.05e−03, Adjusted p (wilcox): 3.19e−03
Log Metabolite Abundance
2
1
0
−1
−2
10000
11000
12000
13000
14000
15000
16000
17000
19000
21000
23000
64000
68000
70000
71000
72000
73000
74000
75000
76000
77000
78000
79000
80000
81000
82000
83000
84000
86000
87000
89000
93000
97000
Subject
Condition
EM
Primary

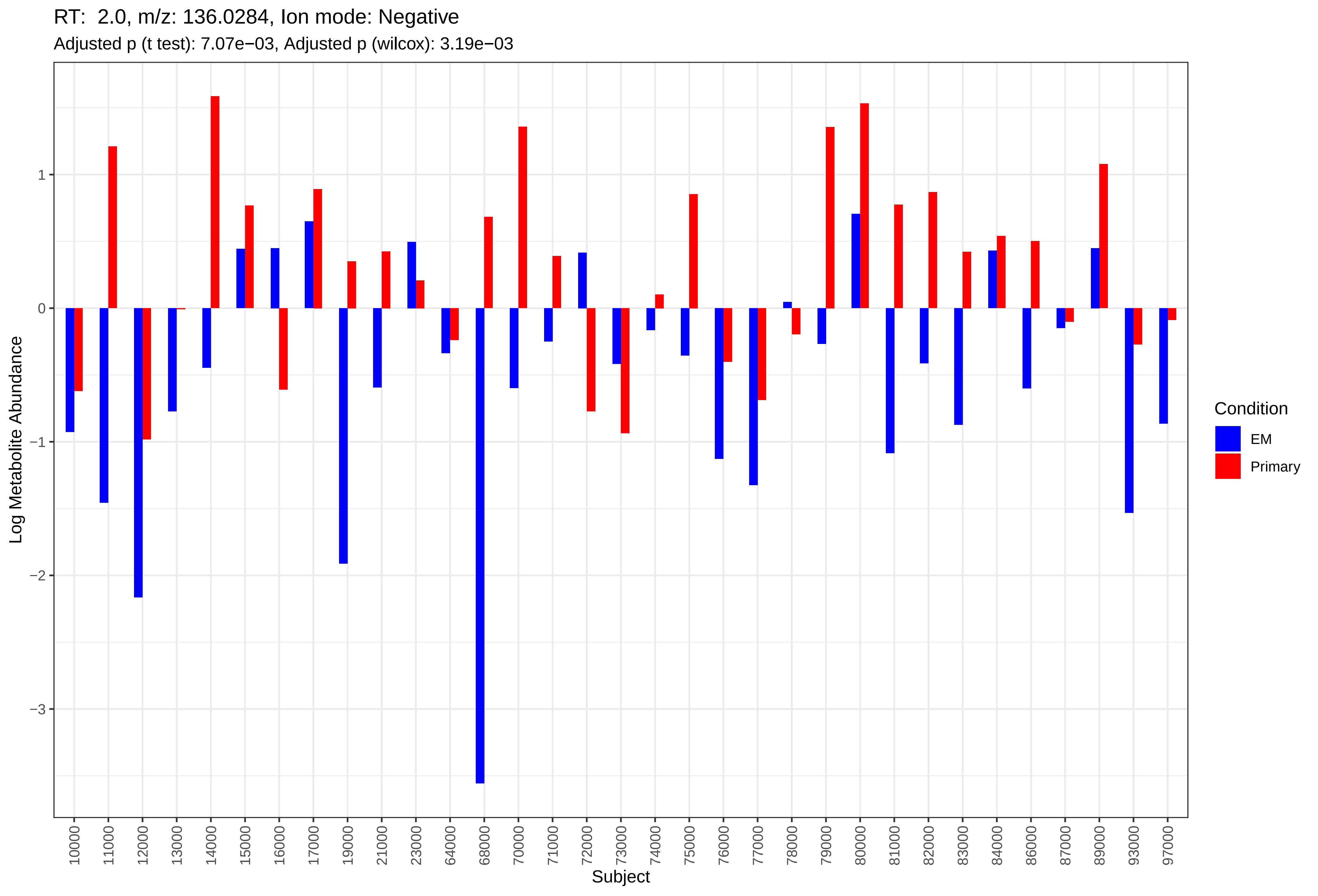

RT: 2.0, m/z: 136.0284, Ion mode: Negative
Adjusted p (t test): 7.07e−03, Adjusted p (wilcox): 3.19e−03
Log Metabolite Abundance
1
0
−1
−2
−3
Subject
10000
11000
12000
13000
14000
15000
16000
17000
19000
21000
23000
64000
68000
70000
71000
72000
73000
74000
75000
76000
77000
78000
79000
80000
81000
82000
83000
84000
86000
87000
89000
93000
97000
Condition
EM
Primary

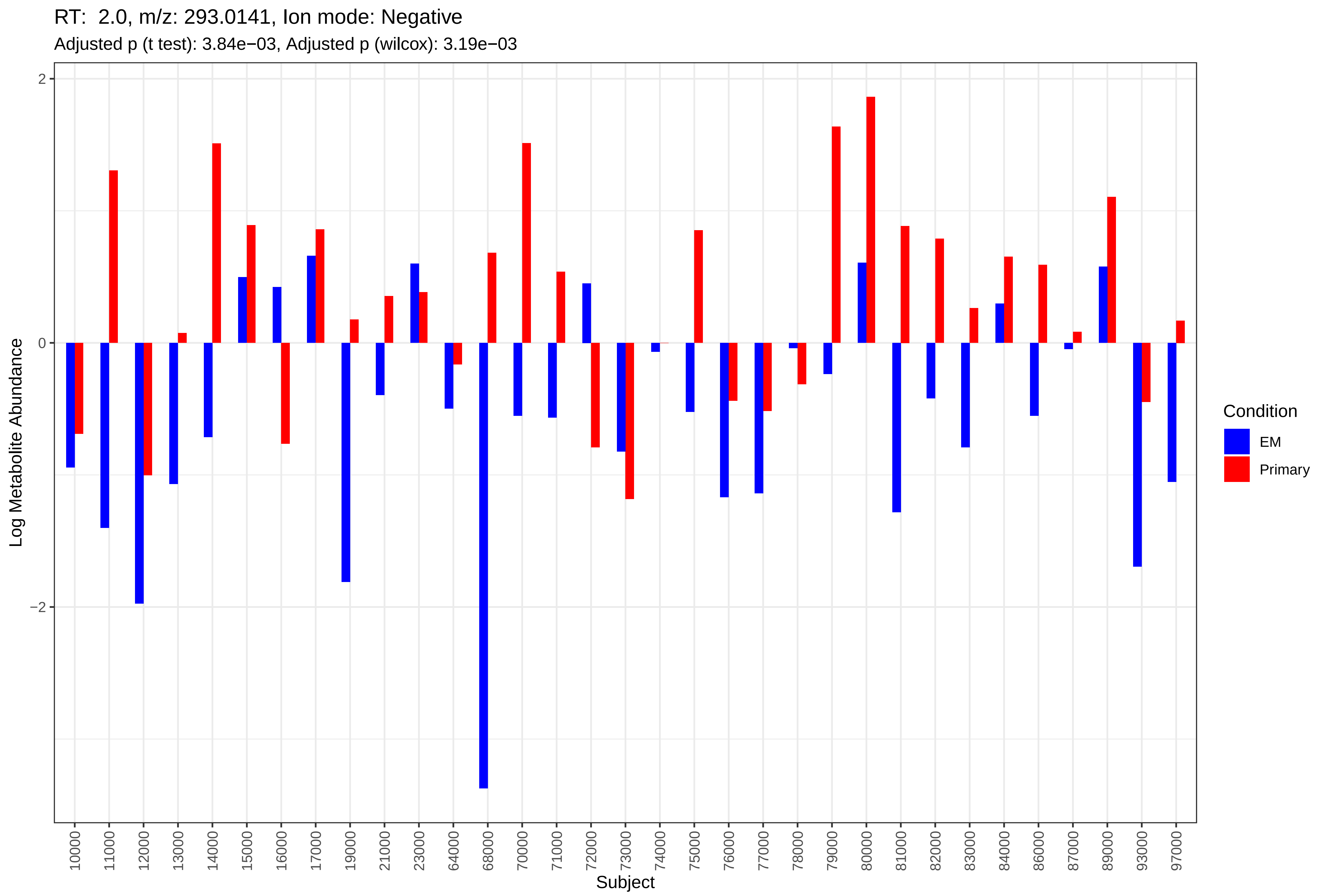

RT: 2.0, m/z: 293.0141, Ion mode: Negative
Adjusted p (t test): 3.84e−03, Adjusted p (wilcox): 3.19e−03
Log Metabolite Abundance
2
0
−2
10000
11000
12000
13000
14000
15000
16000
17000
19000
21000
23000
64000
68000
70000
71000
72000
73000
74000
75000
76000
77000
78000
79000
80000
81000
82000
83000
84000
86000
87000
89000
93000
97000
Subject
Condition
EM
Primary

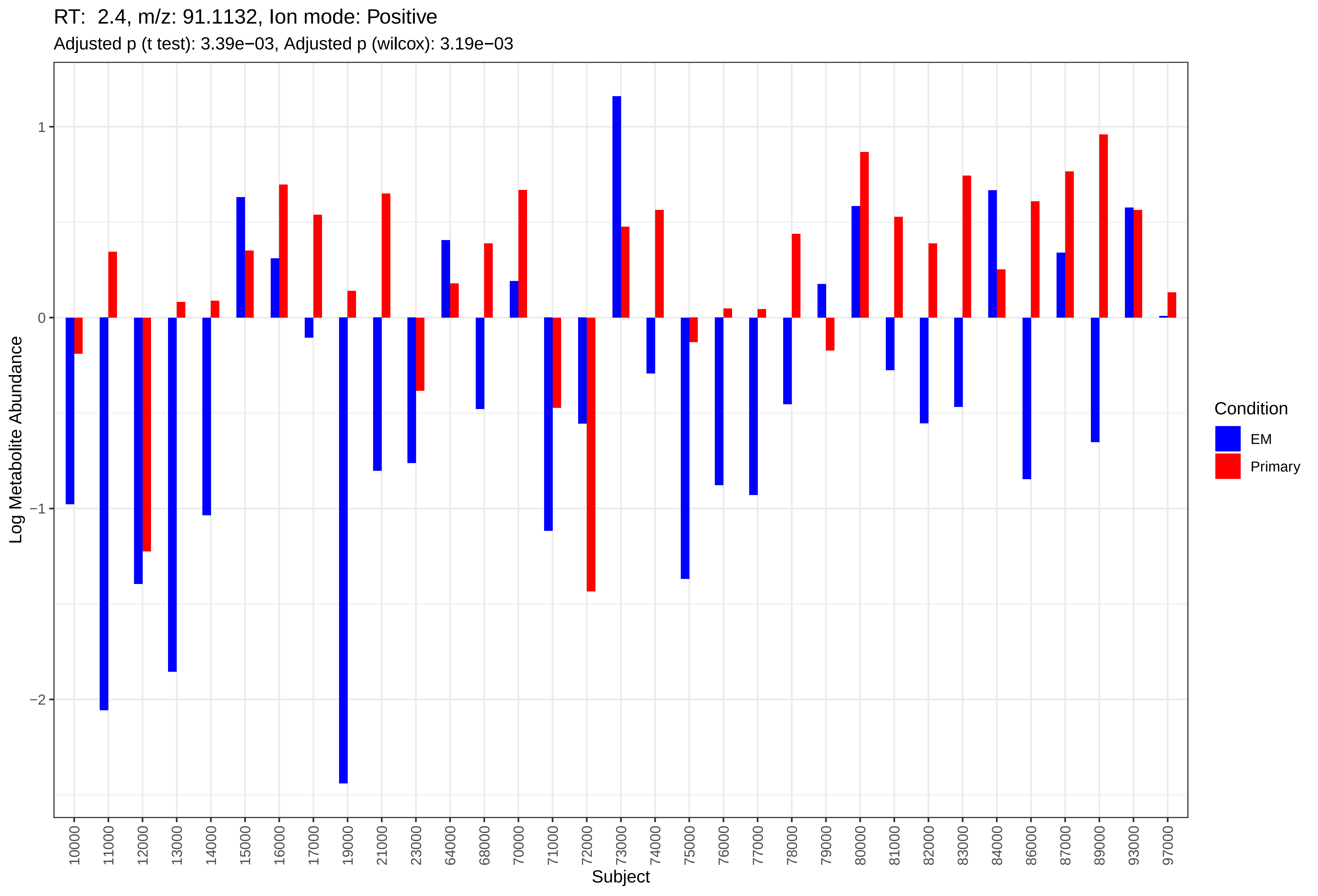
RT: 2.4, m/z: 91.1132, Ion mode: Positive
Adjusted p (t test): 3.39e−03, Adjusted p (wilcox): 3.19e−03
Log Metabolite Abundance
1
0
−1
−2
Subject
10000
11000
12000
13000
14000
15000
16000
17000
19000
21000
23000
64000
68000
70000
71000
72000
73000
74000
75000
76000
77000
78000
79000
80000
81000
82000
83000
84000
86000
87000
89000
93000
97000
Condition
EM
Primary

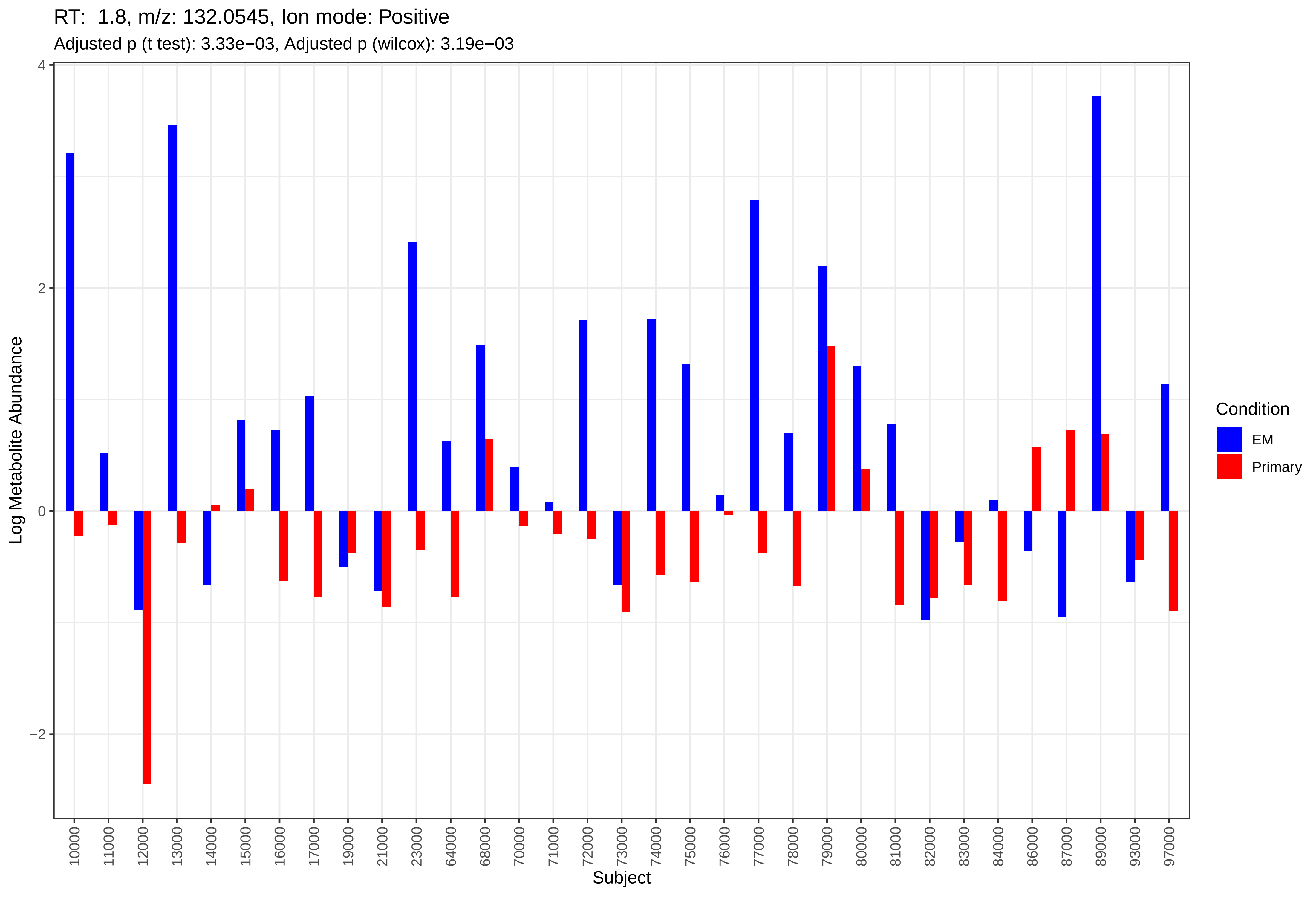

RT: 1.8, m/z: 132.0545, Ion mode: Positive
Adjusted p (t test): 3.33e−03, Adjusted p (wilcox): 3.19e−03
Log Metabolite Abundance
4
2
0
−2
10000
11000
12000
13000
14000
15000
16000
17000
19000
21000
23000
64000
68000
70000
71000
72000
73000
74000
75000
76000
77000
78000
79000
80000
81000
82000
83000
84000
86000
87000
89000
93000
97000
Subject
Condition
EM
Primary

RT: 0.7, m/z: 82.9449, Ion mode: Positive

Adjusted p (t test): 3.66e−03, Adjusted p (wilcox): 3.19e−03

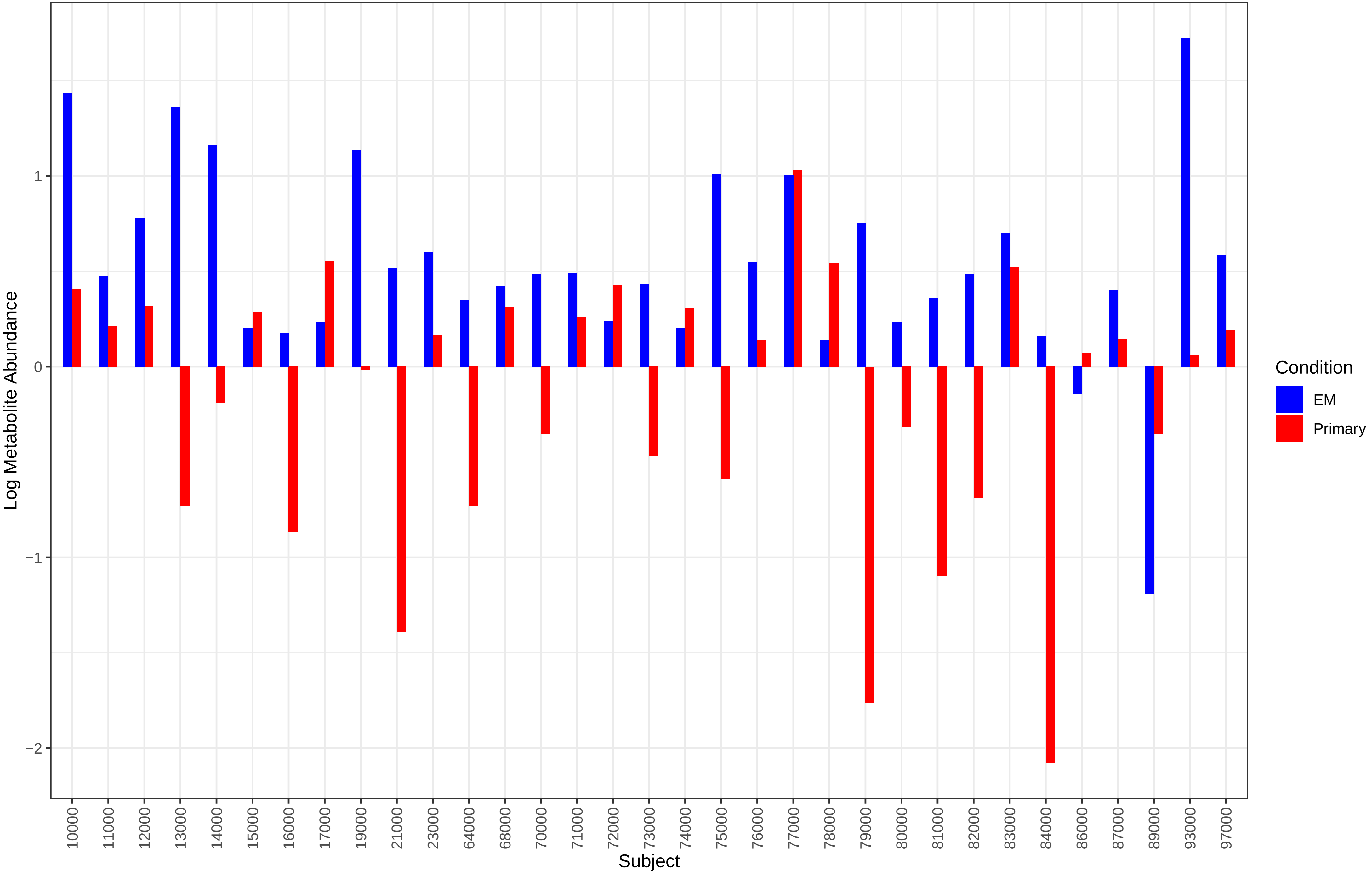

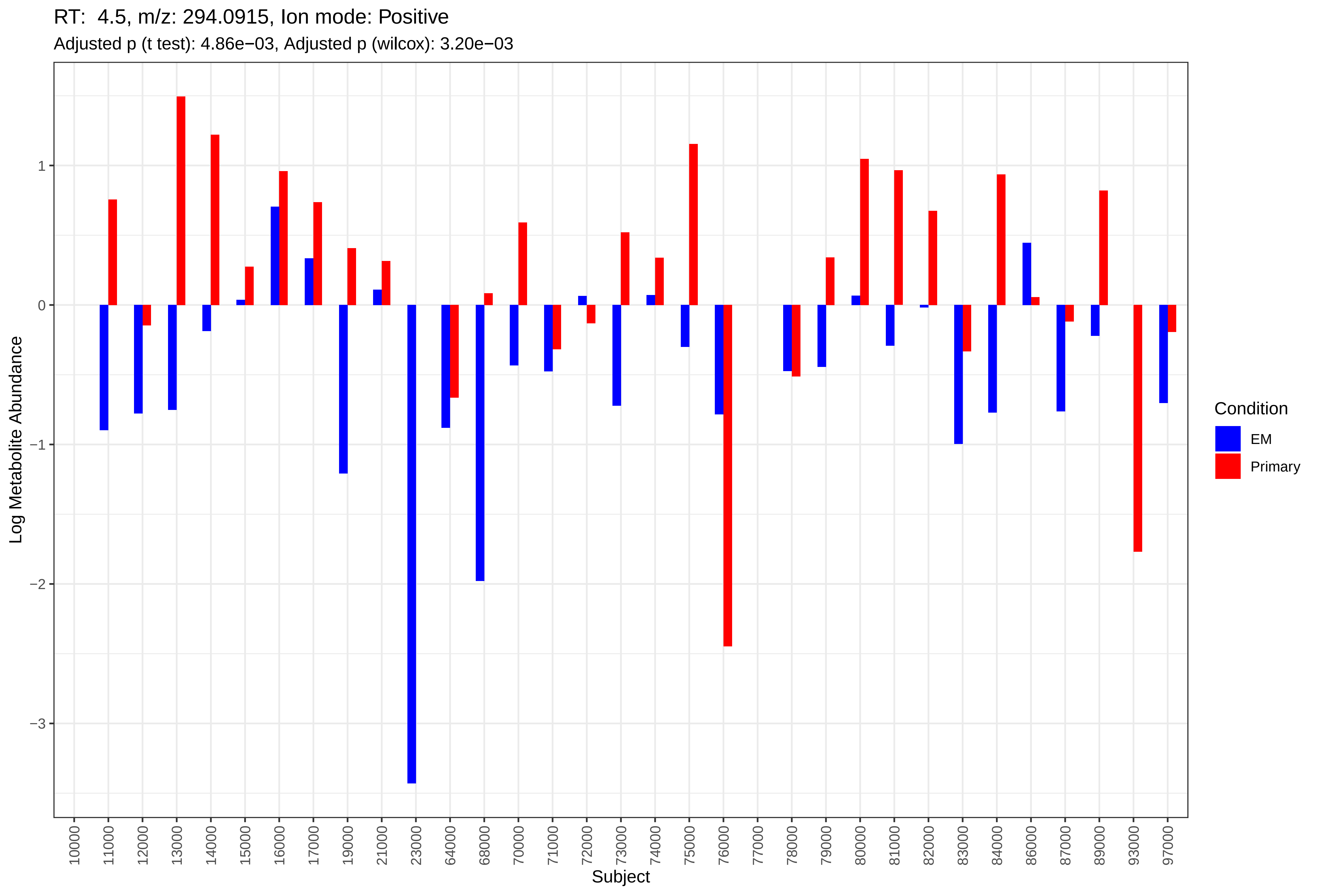

RT: 4.5, m/z: 294.0915, Ion mode: Positive
Adjusted p (t test): 4.86e−03, Adjusted p (wilcox): 3.20e−03
Log Metabolite Abundance
1
0
−1
−2
−3
10000
11000
12000
13000
14000
15000
16000
17000
19000
21000
23000
64000
68000
70000
71000
72000
73000
74000
75000
76000
77000
78000
79000
80000
81000
82000
83000
84000
86000
87000
89000
93000
97000
Subject
Condition
EM
Primary

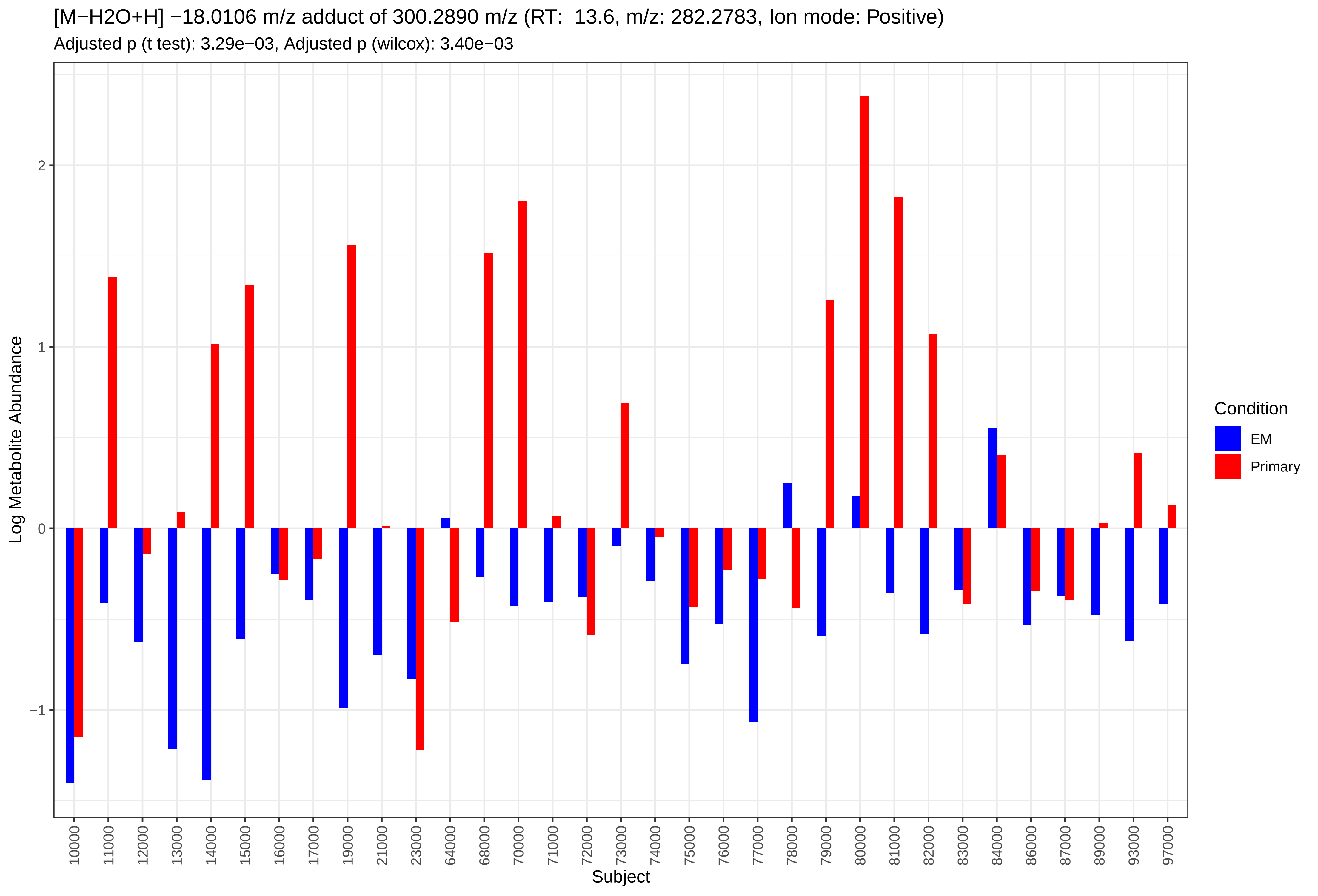

[M−H2O+H] −18.0106 m/z adduct of 300.2890 m/z (RT: 13.6, m/z: 282.2783, Ion mode: Positive)
Adjusted p (t test): 3.29e−03, Adjusted p (wilcox): 3.40e−03
Log Metabolite Abundance
2
1
0
−1
10000
11000
12000
13000
14000
15000
16000
17000
19000
21000
23000
64000
68000
70000
71000
72000
73000
74000
75000
76000
77000
78000
79000
80000
81000
82000
83000
84000
86000
87000
89000
93000
97000
Subject
Condition
EM
Primary

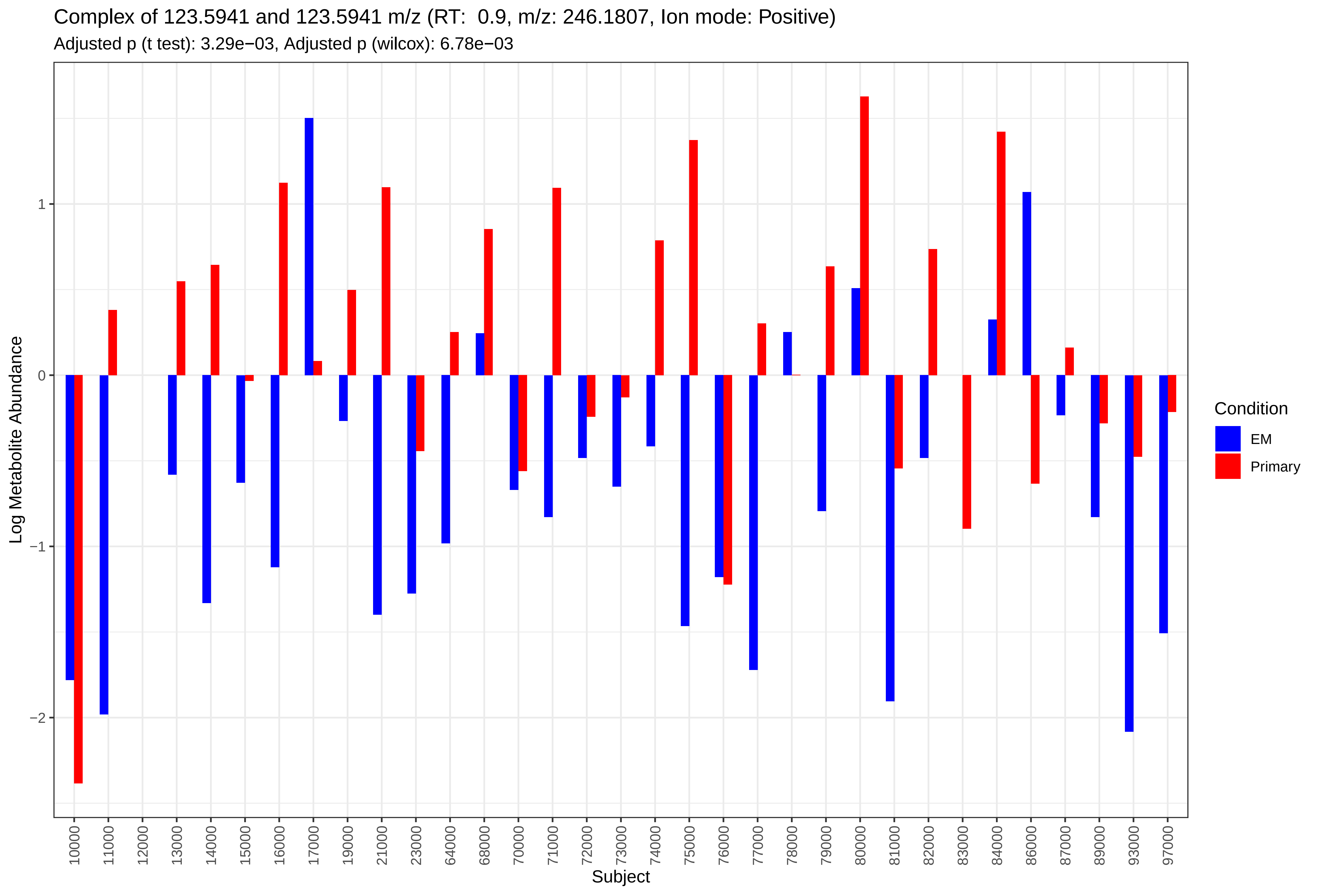

Complex of 123.5941 and 123.5941 m/z (RT: 0.9, m/z: 246.1807, Ion mode: Positive)
Adjusted p (t test): 3.29e−03, Adjusted p (wilcox): 6.78e−03
Log Metabolite Abundance
1
0
−1
−2
Subject
10000
11000
12000
13000
14000
15000
16000
17000
19000
21000
23000
64000
68000
70000
71000
72000
73000
74000
75000
76000
77000
78000
79000
80000
81000
82000
83000
84000
86000
87000
89000
93000
97000
Condition
EM
Primary

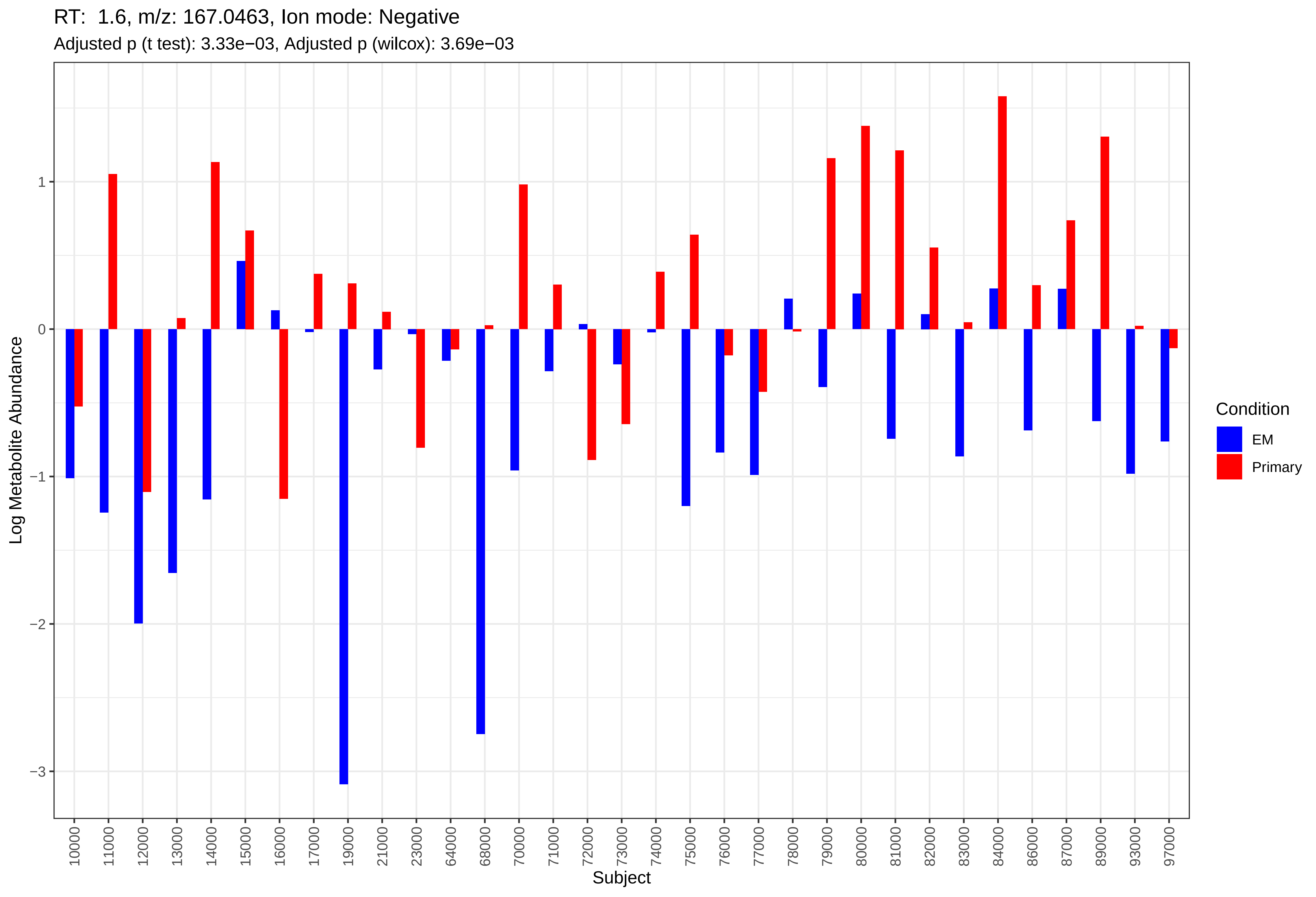

RT: 1.6, m/z: 167.0463, Ion mode: Negative
Adjusted p (t test): 3.33e−03, Adjusted p (wilcox): 3.69e−03
Log Metabolite Abundance
1
0
−1
−2
−3
10000
11000
12000
13000
14000
15000
16000
17000
19000
21000
23000
64000
68000
70000
71000
72000
73000
74000
75000
76000
77000
78000
79000
80000
81000
82000
83000
84000
86000
87000
89000
93000
97000
Subject
Condition
EM
Primary

RT: 7.2, m/z: 122.0962, Ion mode: Positive

Adjusted p (t test): 3.33e−03, Adjusted p (wilcox): 4.94e−03

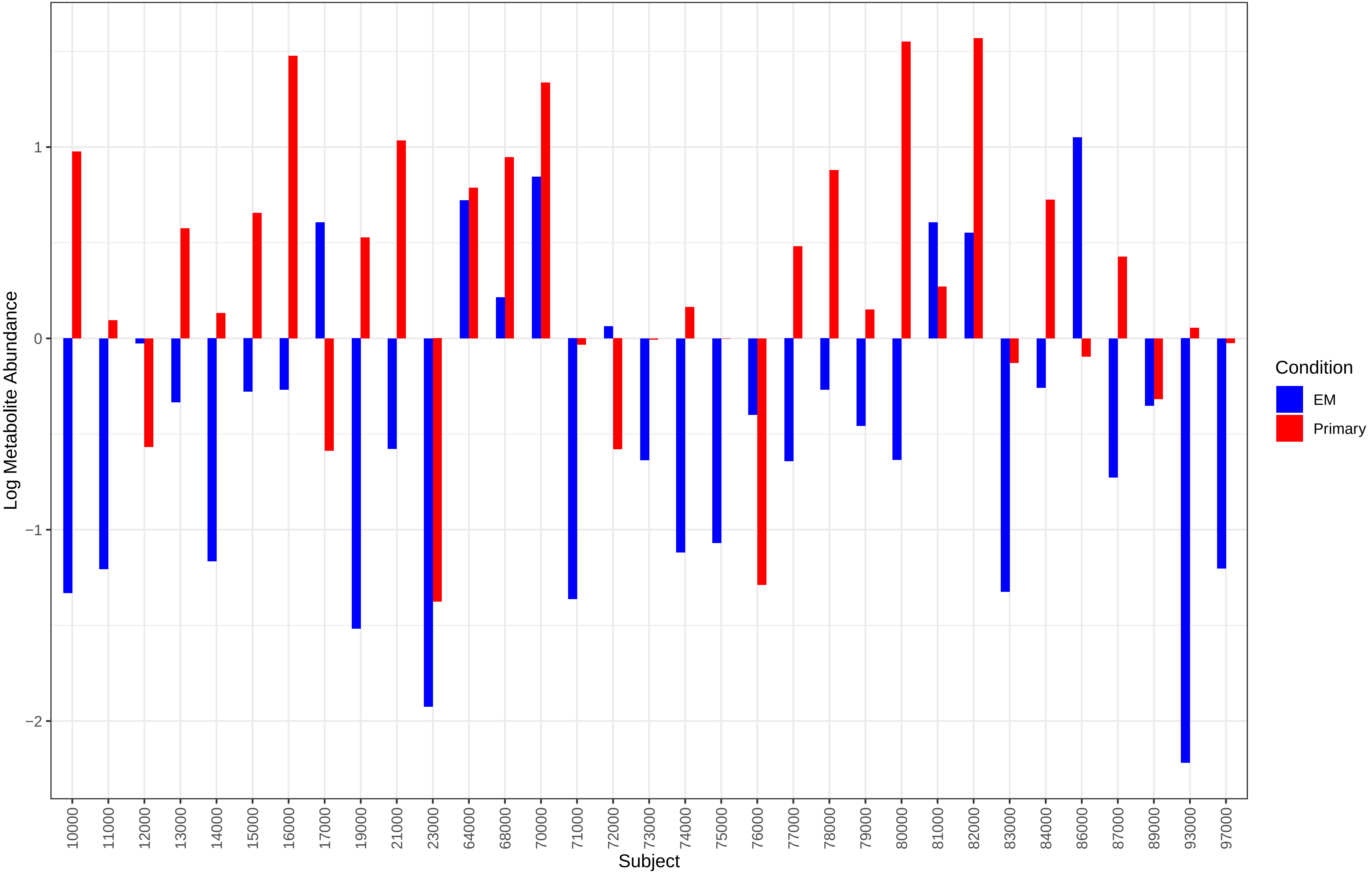

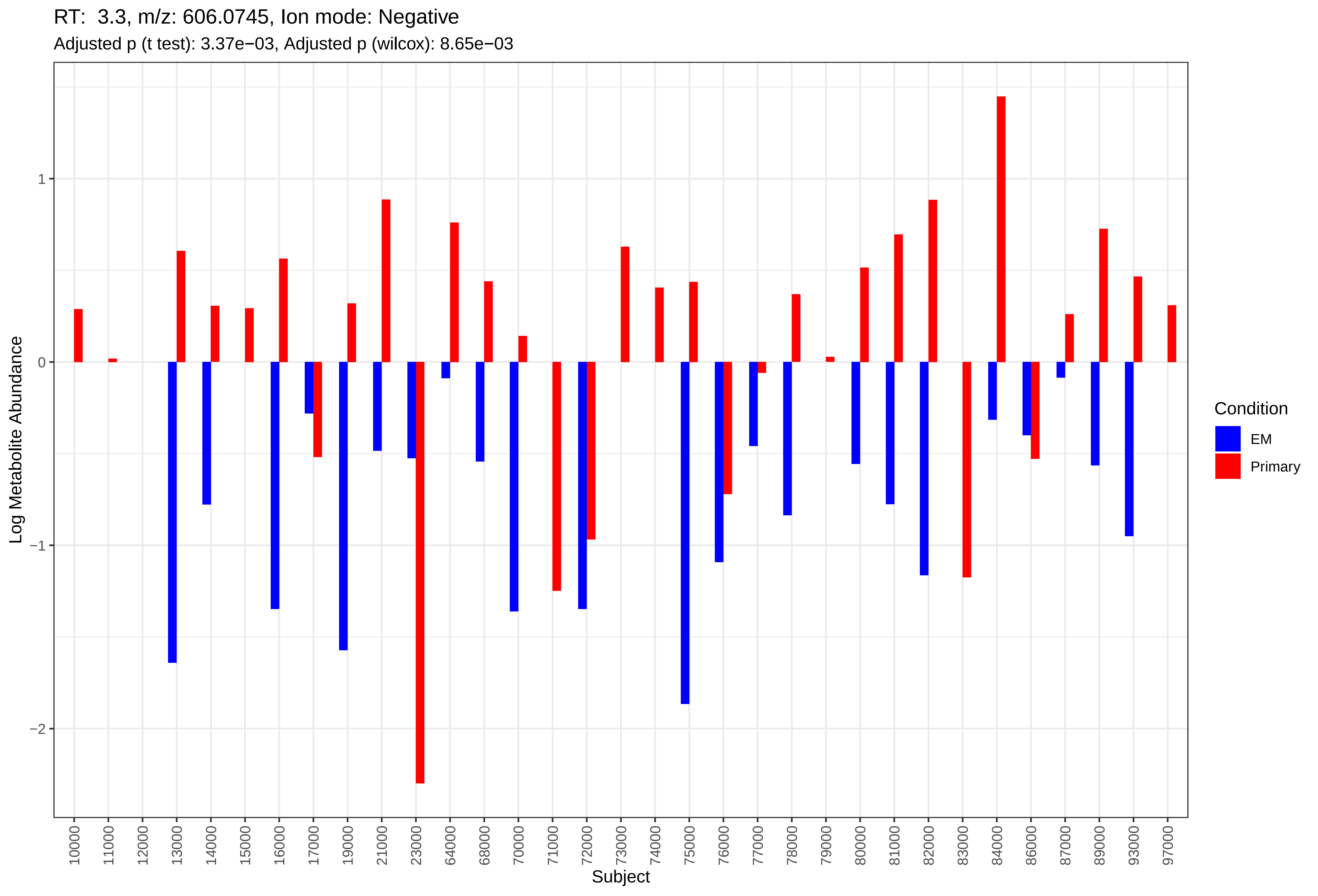

RT: 3.3, m/z: 606.0745, Ion mode: Negative
Adjusted p (t test): 3.37e−03, Adjusted p (wilcox): 8.65e−03
Log Metabolite Abundance
1
0
−1
−2
Subject
10000
11000
12000
13000
14000
15000
16000
17000
19000
21000
23000
64000
68000
70000
71000
72000
73000
74000
75000
76000
77000
78000
79000
80000
81000
82000
83000
84000
86000
87000
89000
93000
97000
Condition
EM
Primary

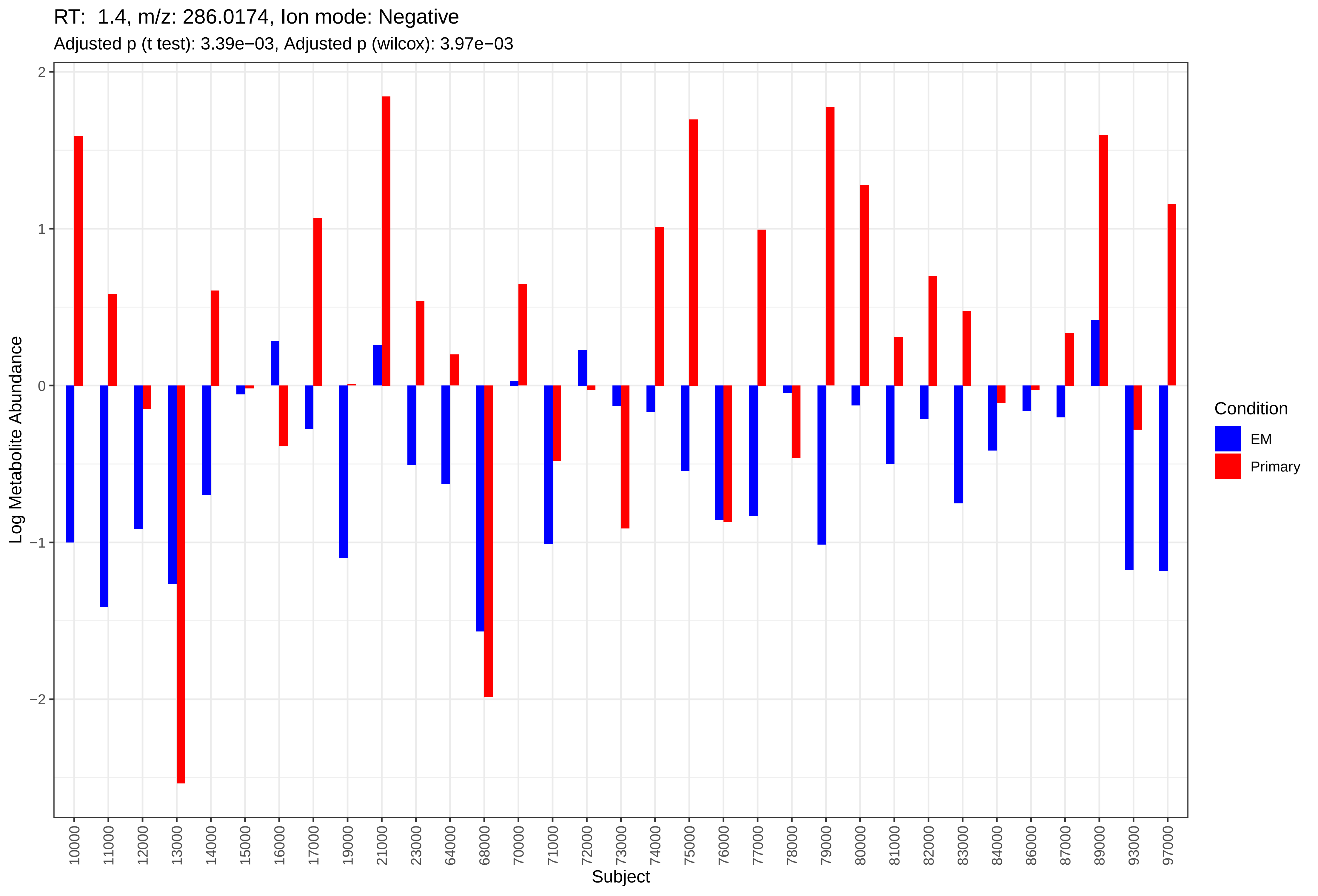

RT: 1.4, m/z: 286.0174, Ion mode: Negative
Adjusted p (t test): 3.39e−03, Adjusted p (wilcox): 3.97e−03
Log Metabolite Abundance
2
1
0
−1
−2
Subject
10000
11000
12000
13000
14000
15000
16000
17000
19000
21000
23000
64000
68000
70000
71000
72000
73000
74000
75000
76000
77000
78000
79000
80000
81000
82000
83000
84000
86000
87000
89000
93000
97000
Condition
EM
Primary

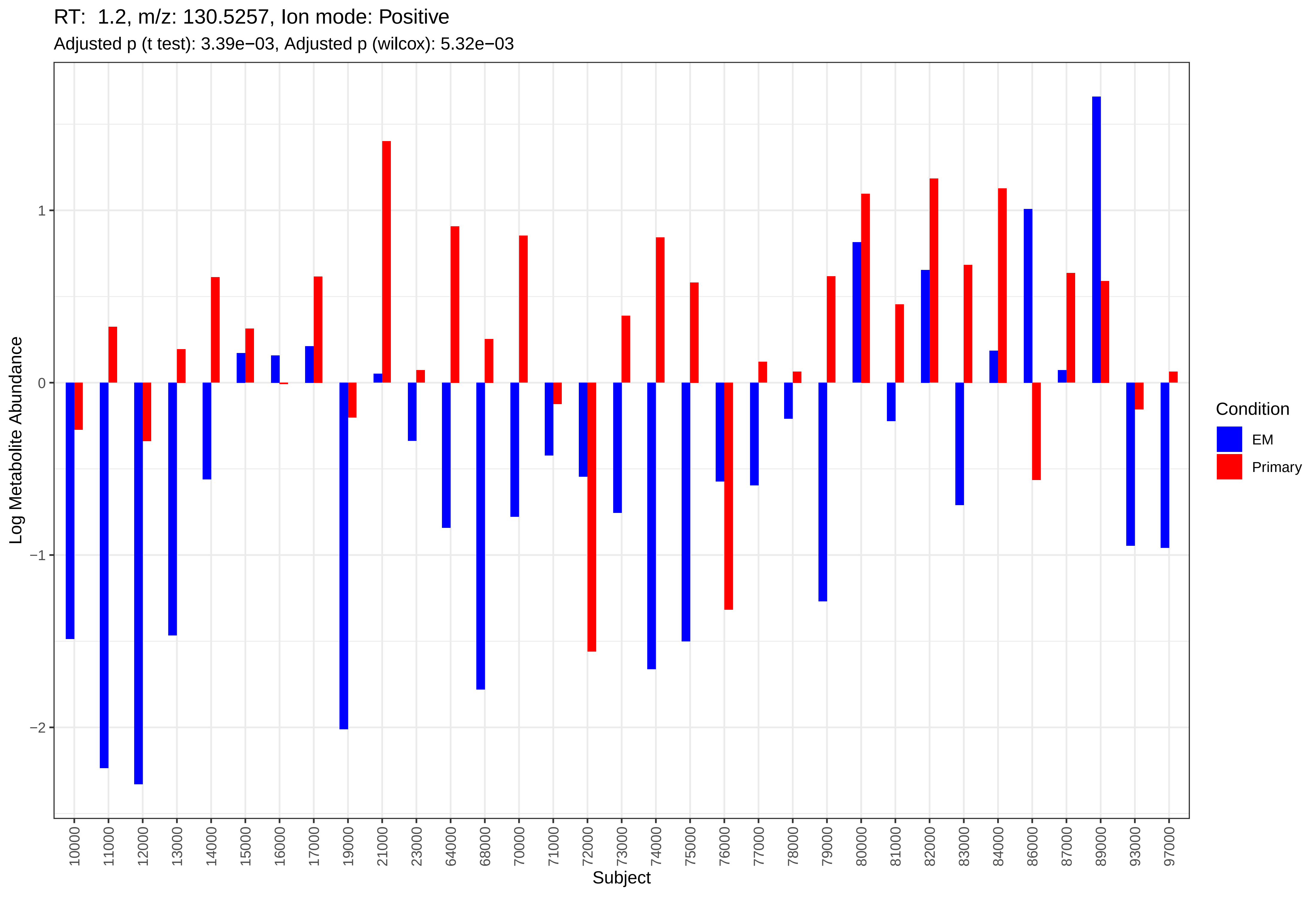
RT: 1.2, m/z: 130.5257, Ion mode: Positive
Adjusted p (t test): 3.39e−03, Adjusted p (wilcox): 5.32e−03
Log Metabolite Abundance
1
0
−1
−2
10000
11000
12000
13000
14000
15000
16000
17000
19000
21000
23000
64000
68000
70000
71000
72000
73000
74000
75000
76000
77000
78000
79000
80000
81000
82000
83000
84000
86000
87000
89000
93000
97000
Subject
Condition
EM
Primary

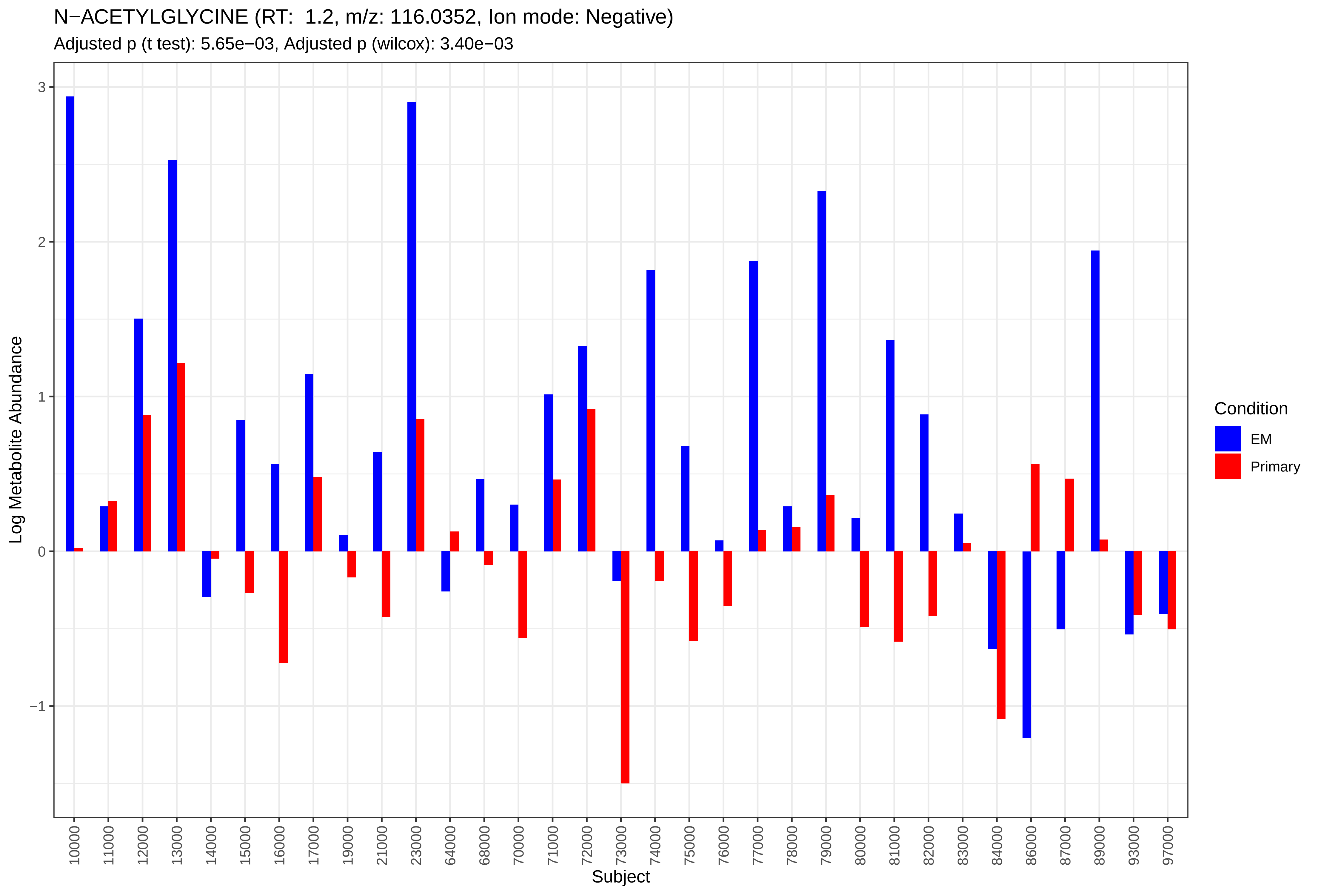

N−ACETYLGLYCINE (RT: 1.2, m/z: 116.0352, Ion mode: Negative)
Adjusted p (t test): 5.65e−03, Adjusted p (wilcox): 3.40e−03
Log Metabolite Abundance
3
2
1
0
−1
10000
11000
12000
13000
14000
15000
16000
17000
19000
21000
23000
64000
68000
70000
71000
72000
73000
74000
75000
76000
77000
78000
79000
80000
81000
82000
83000
84000
86000
87000
89000
93000
97000
Subject
Condition
EM
Primary

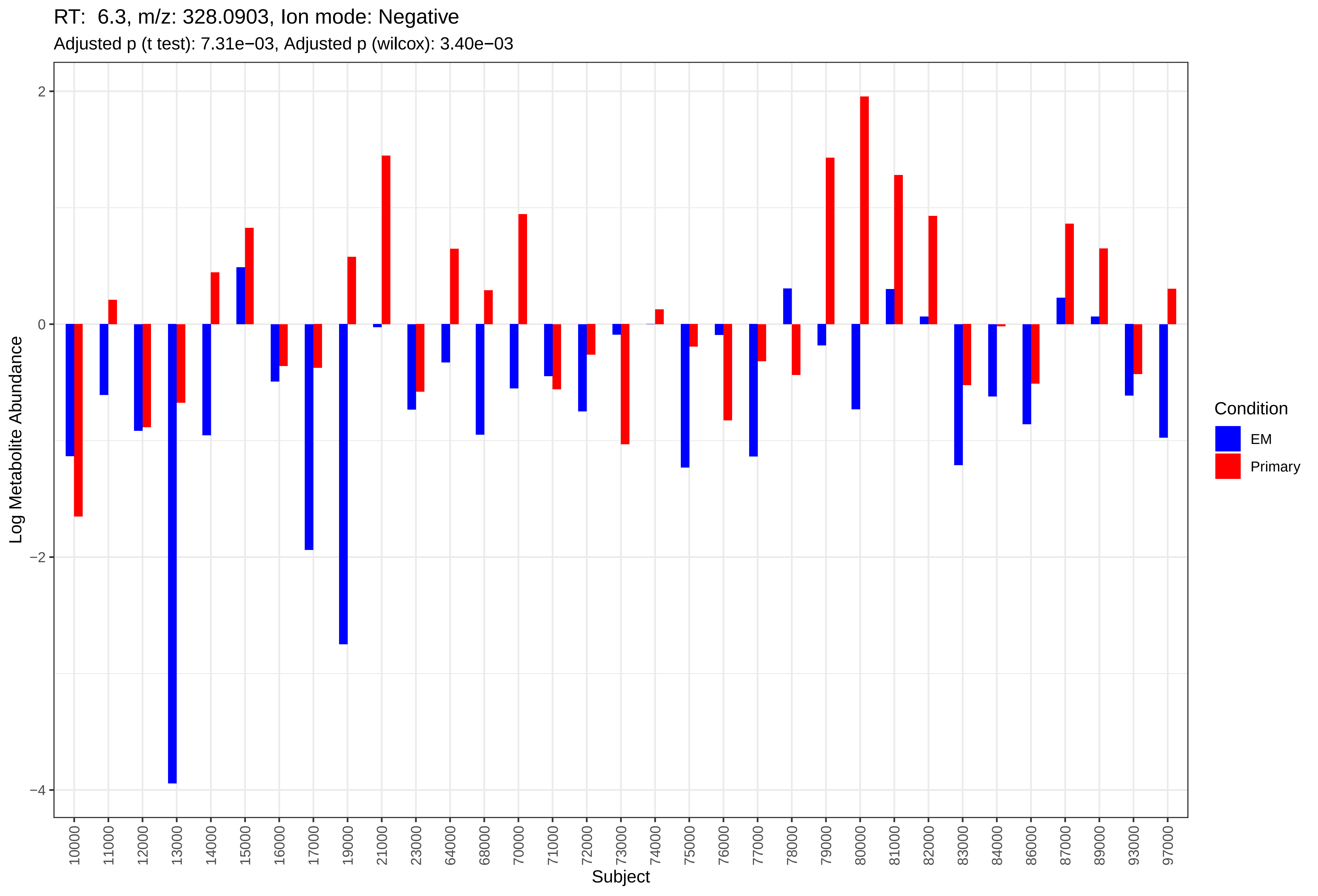
RT: 6.3, m/z: 328.0903, Ion mode: Negative
Adjusted p (t test): 7.31e−03, Adjusted p (wilcox): 3.40e−03
Log Metabolite Abundance
2
0
−2
−4
Subject
10000
11000
12000
13000
14000
15000
16000
17000
19000
21000
23000
64000
68000
70000
71000
72000
73000
74000
75000
76000
77000
78000
79000
80000
81000
82000
83000
84000
86000
87000
89000
93000
97000
Condition
EM
Primary

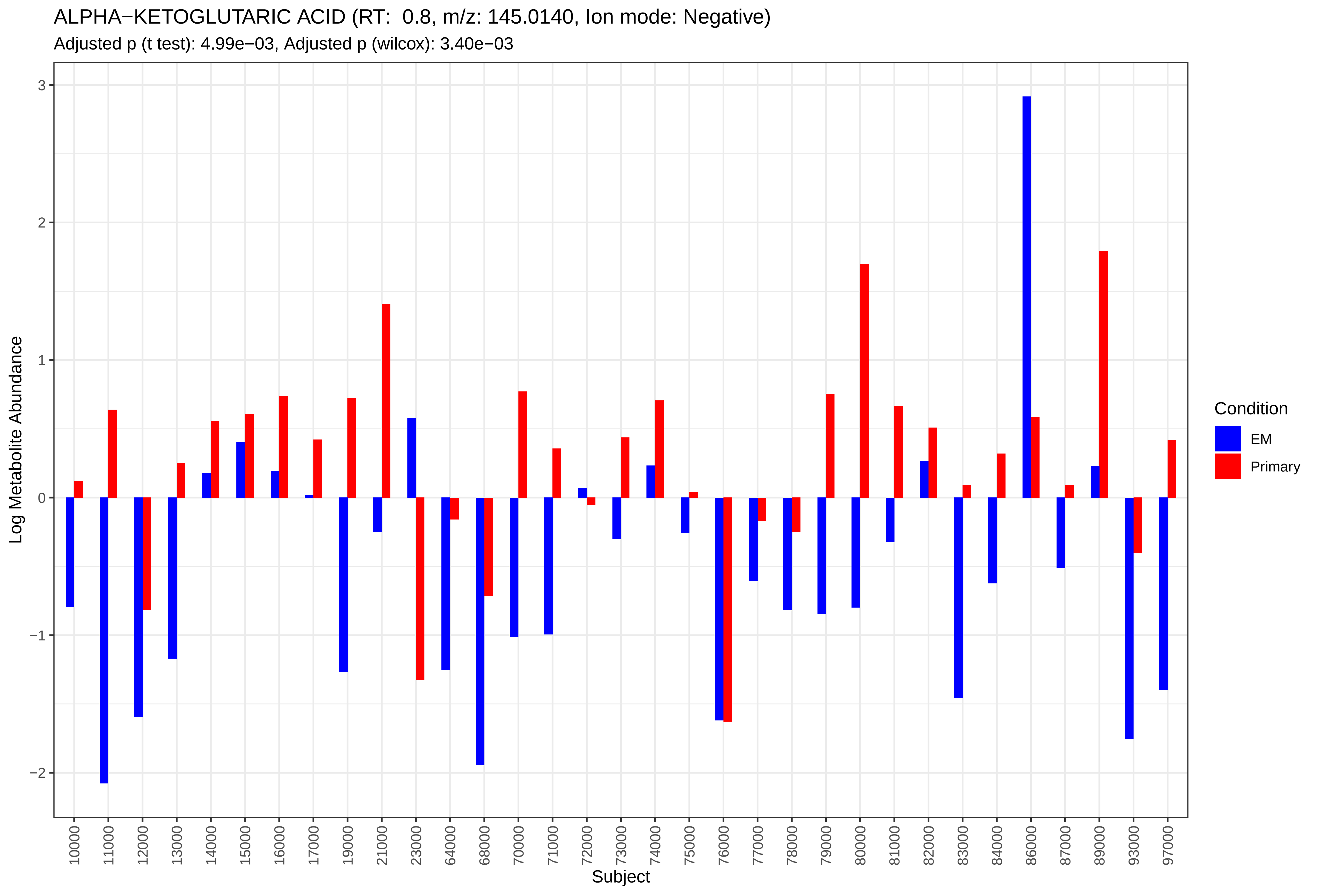

ALPHA−KETOGLUTARIC ACID (RT: 0.8, m/z: 145.0140, Ion mode: Negative)
Adjusted p (t test): 4.99e−03, Adjusted p (wilcox): 3.40e−03
Log Metabolite Abundance
3
2
1
0
−1
−2
Subject
10000
11000
12000
13000
14000
15000
16000
17000
19000
21000
23000
64000
68000
70000
71000
72000
73000
74000
75000
76000
77000
78000
79000
80000
81000
82000
83000
84000
86000
87000
89000
93000
97000
Condition
EM
Primary

Complex of 118.0862 and 159.0276 m/z (RT: 2.0, m/z: 276.1060, Ion mode: Positive)

Adjusted p (t test): 7.68e−03, Adjusted p (wilcox): 3.40e−03

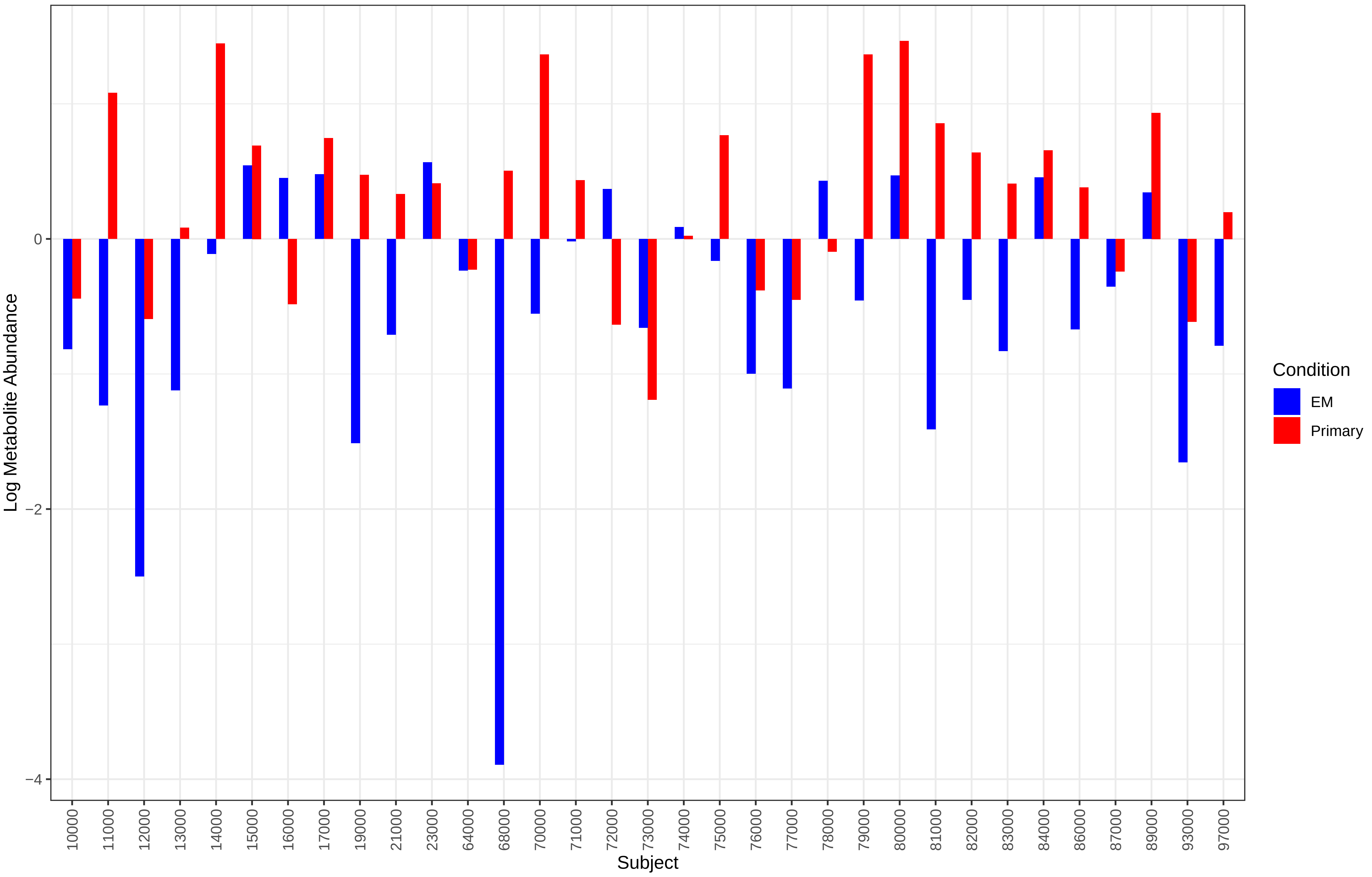

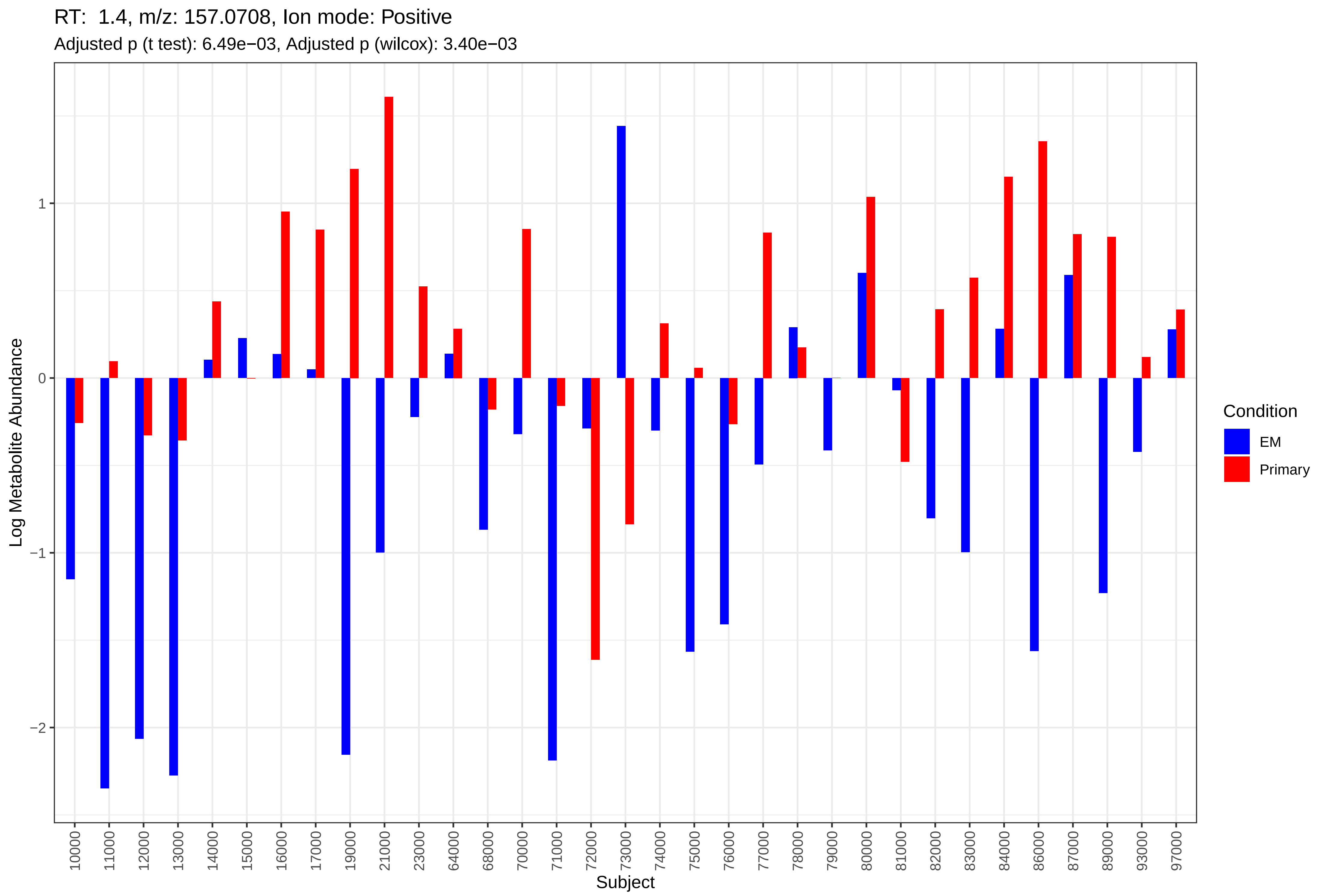
RT: 1.4, m/z: 157.0708, Ion mode: Positive
Adjusted p (t test): 6.49e−03, Adjusted p (wilcox): 3.40e−03
Log Metabolite Abundance
1
0
−1
−2
10000
11000
12000
13000
14000
15000
16000
17000
19000
21000
23000
64000
68000
70000
71000
72000
73000
74000
75000
76000
77000
78000
79000
80000
81000
82000
83000
84000
86000
87000
89000
93000
97000
Subject
Condition
EM
Primary

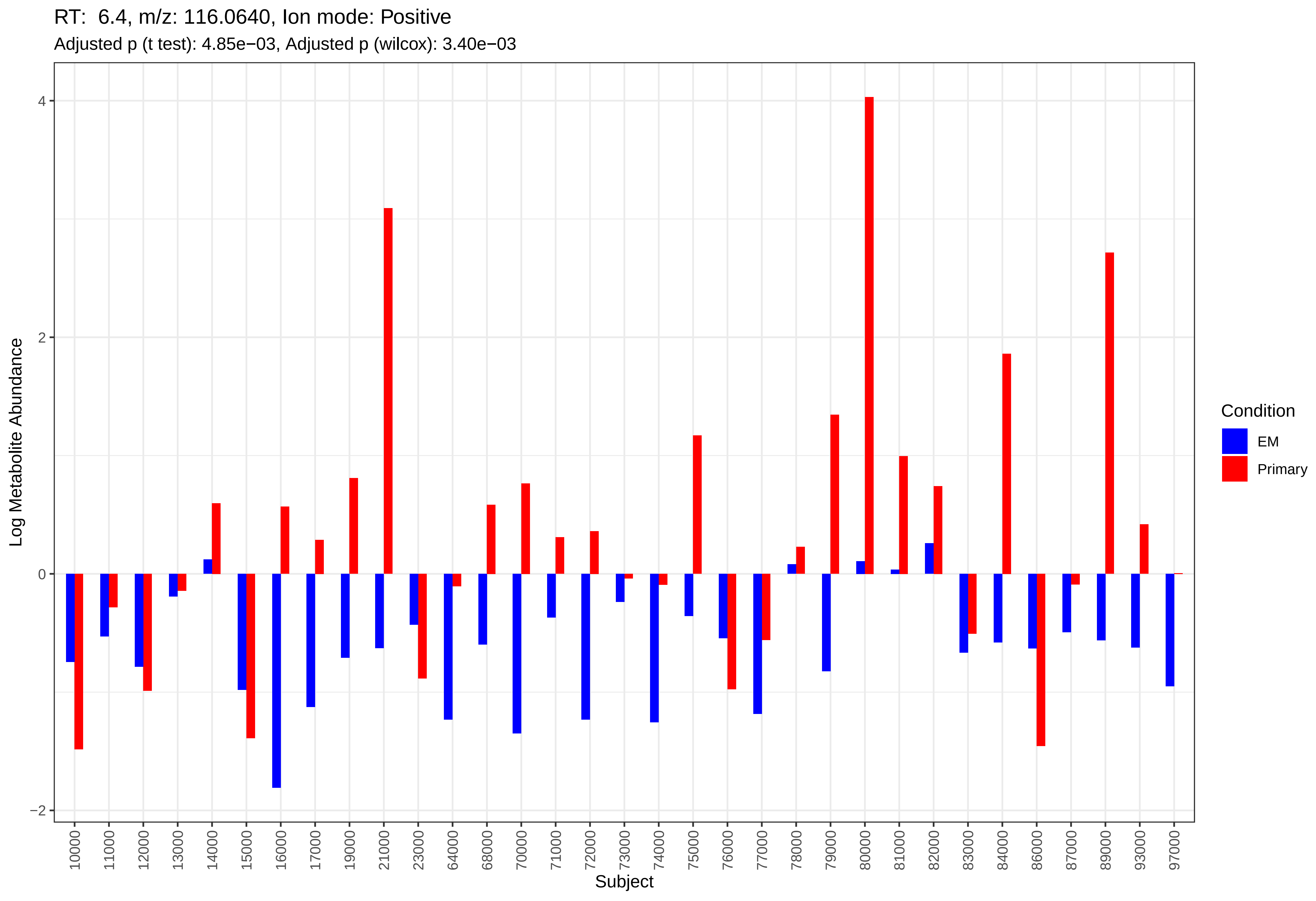
RT: 6.4, m/z: 116.0640, Ion mode: Positive
Adjusted p (t test): 4.85e−03, Adjusted p (wilcox): 3.40e−03
Log Metabolite Abundance
4
2
0
−2
Subject
10000
11000
12000
13000
14000
15000
16000
17000
19000
21000
23000
64000
68000
70000
71000
72000
73000
74000
75000
76000
77000
78000
79000
80000
81000
82000
83000
84000
86000
87000
89000
93000
97000
Condition
EM
Primary

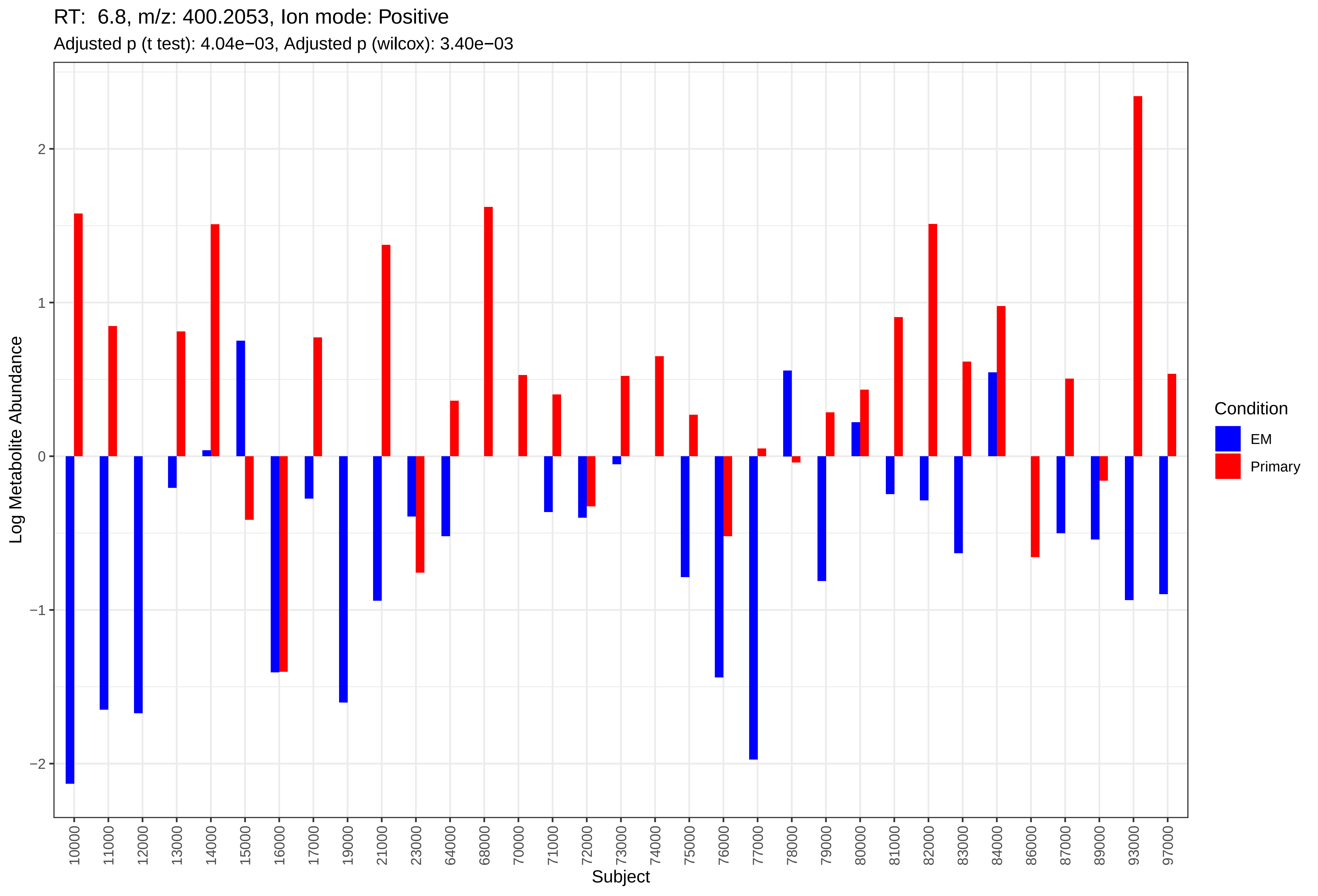
RT: 6.8, m/z: 400.2053, Ion mode: Positive
Adjusted p (t test): 4.04e−03, Adjusted p (wilcox): 3.40e−03
Log Metabolite Abundance
2
1
0
−1
−2
10000
11000
12000
13000
14000
15000
16000
17000
19000
21000
23000
64000
68000
70000
71000
72000
73000
74000
75000
76000
77000
78000
79000
80000
81000
82000
83000
84000
86000
87000
89000
93000
97000
Subject
Condition
EM
Primary

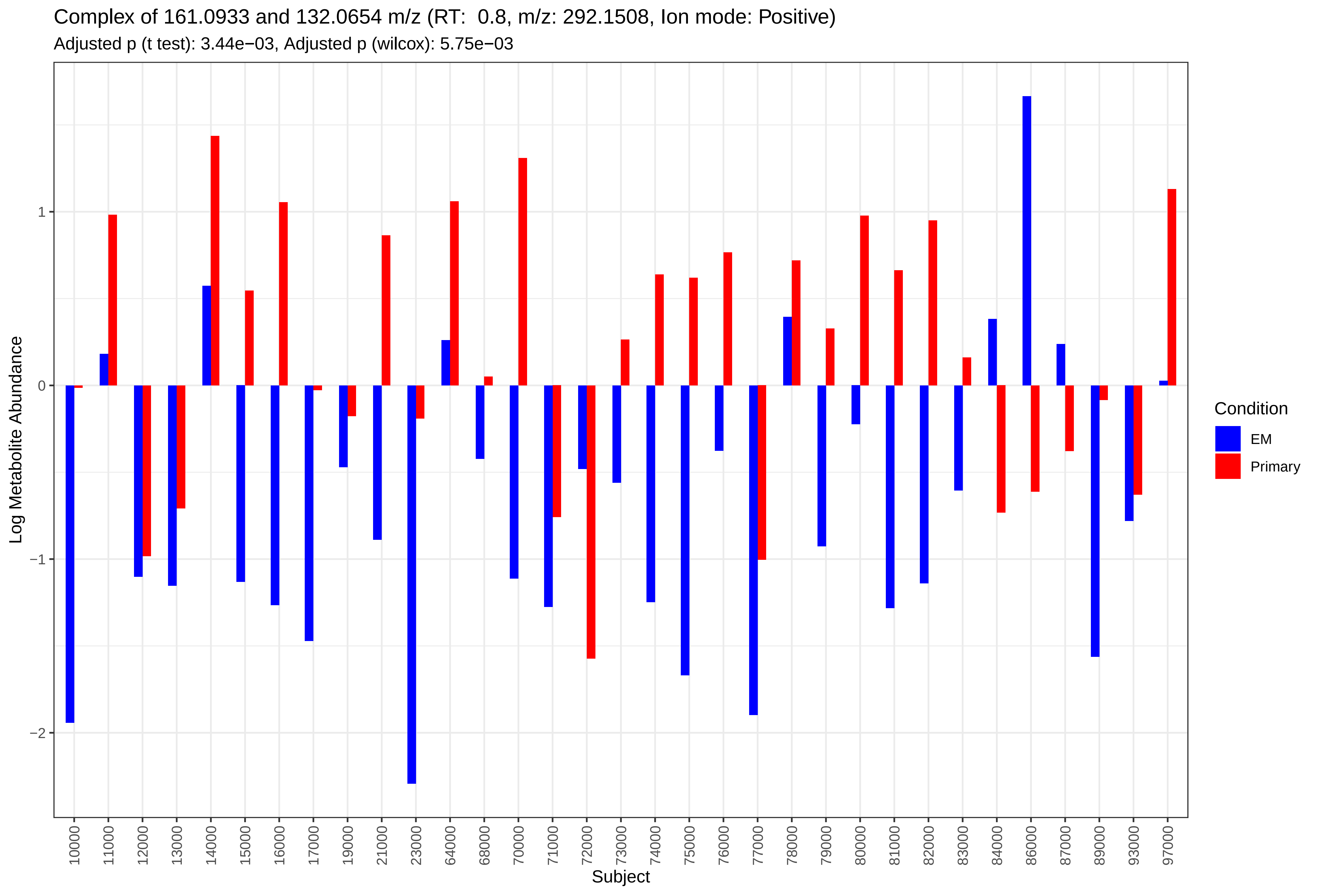

Complex of 161.0933 and 132.0654 m/z (RT: 0.8, m/z: 292.1508, Ion mode: Positive)
Adjusted p (t test): 3.44e−03, Adjusted p (wilcox): 5.75e−03
Log Metabolite Abundance
1
0
−1
−2
10000
11000
12000
13000
14000
15000
16000
17000
19000
21000
23000
64000
68000
70000
71000
72000
73000
74000
75000
76000
77000
78000
79000
80000
81000
82000
83000
84000
86000
87000
89000
93000
97000
Subject
Condition
EM
Primary

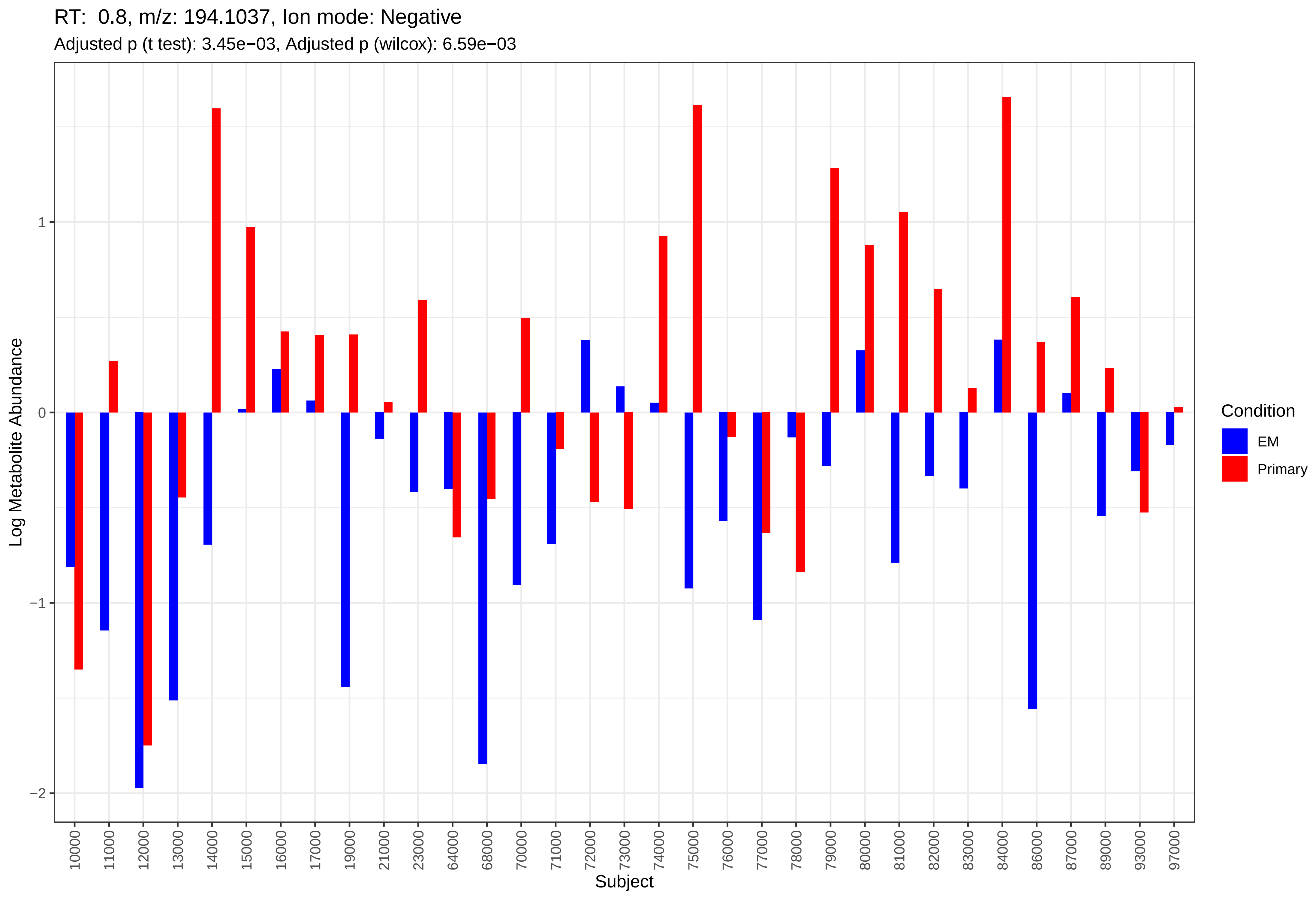
RT: 0.8, m/z: 194.1037, Ion mode: Negative
Adjusted p (t test): 3.45e−03, Adjusted p (wilcox): 6.59e−03
Log Metabolite Abundance
1
0
−1
−2
10000
11000
12000
13000
14000
15000
16000
17000
19000
21000
23000
64000
68000
70000
71000
72000
73000
74000
75000
76000
77000
78000
79000
80000
81000
82000
83000
84000
86000
87000
89000
93000
97000
Subject
Condition
EM
Primary

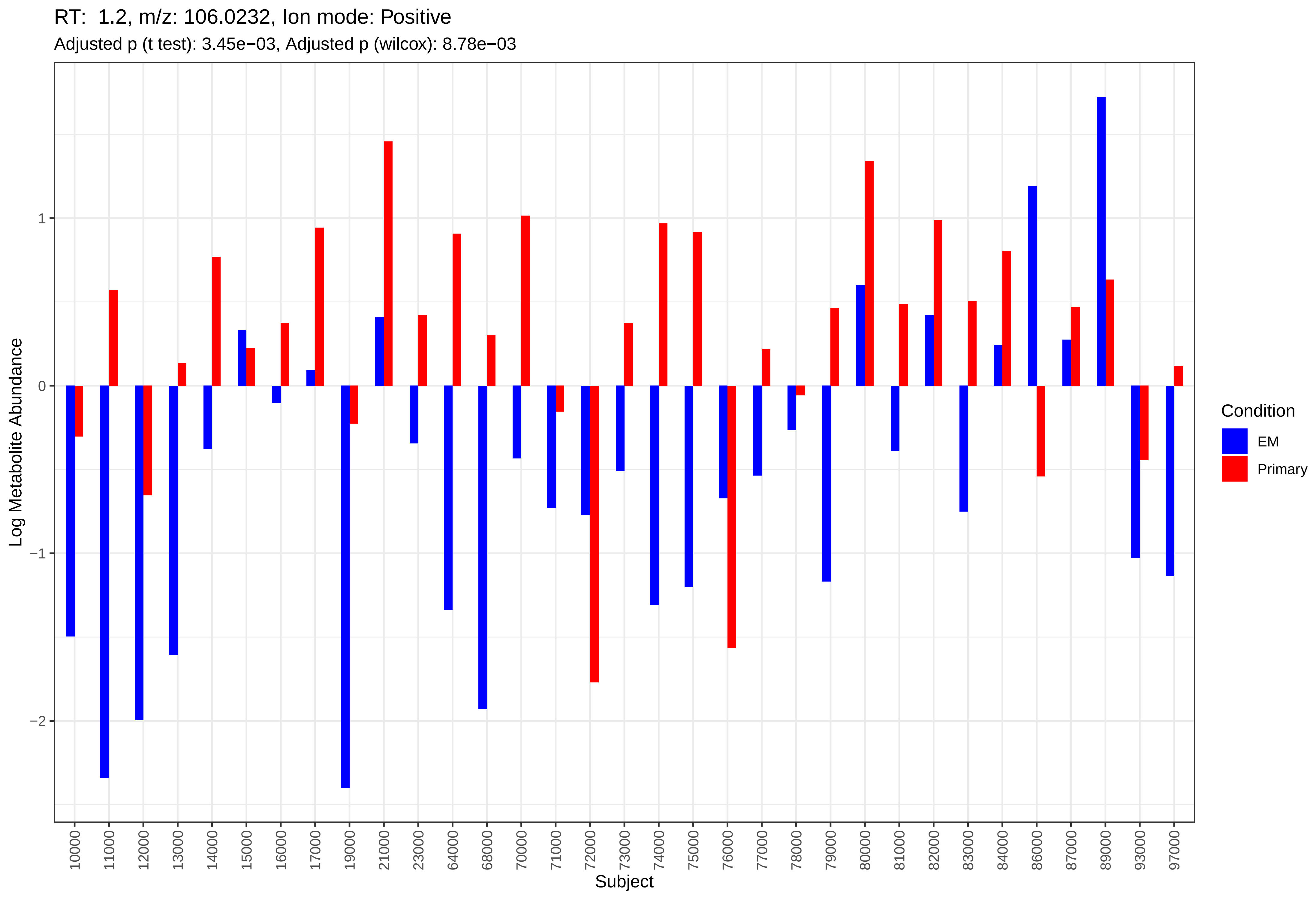
RT: 1.2, m/z: 106.0232, Ion mode: Positive
Adjusted p (t test): 3.45e−03, Adjusted p (wilcox): 8.78e−03
Log Metabolite Abundance
1
0
−1
−2
10000
11000
12000
13000
14000
15000
16000
17000
19000
21000
23000
64000
68000
70000
71000
72000
73000
74000
75000
76000
77000
78000
79000
80000
81000
82000
83000
84000
86000
87000
89000
93000
97000
Subject
Condition
EM
Primary

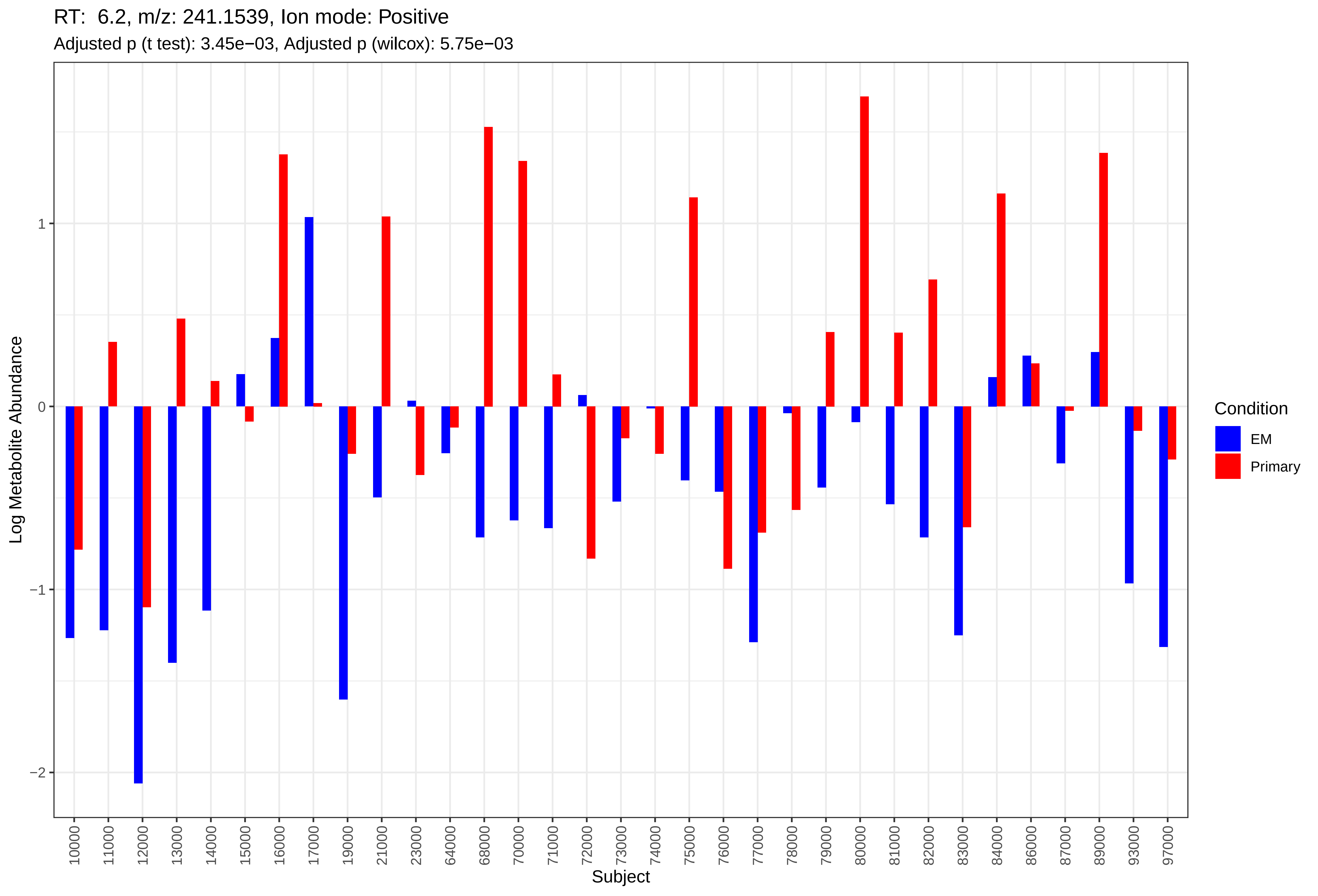
RT: 6.2, m/z: 241.1539, Ion mode: Positive
Adjusted p (t test): 3.45e−03, Adjusted p (wilcox): 5.75e−03
Log Metabolite Abundance
1
0
−1
−2
10000
11000
12000
13000
14000
15000
16000
17000
19000
21000
23000
64000
68000
70000
71000
72000
73000
74000
75000
76000
77000
78000
79000
80000
81000
82000
83000
84000
86000
87000
89000
93000
97000
Subject
Condition
EM
Primary

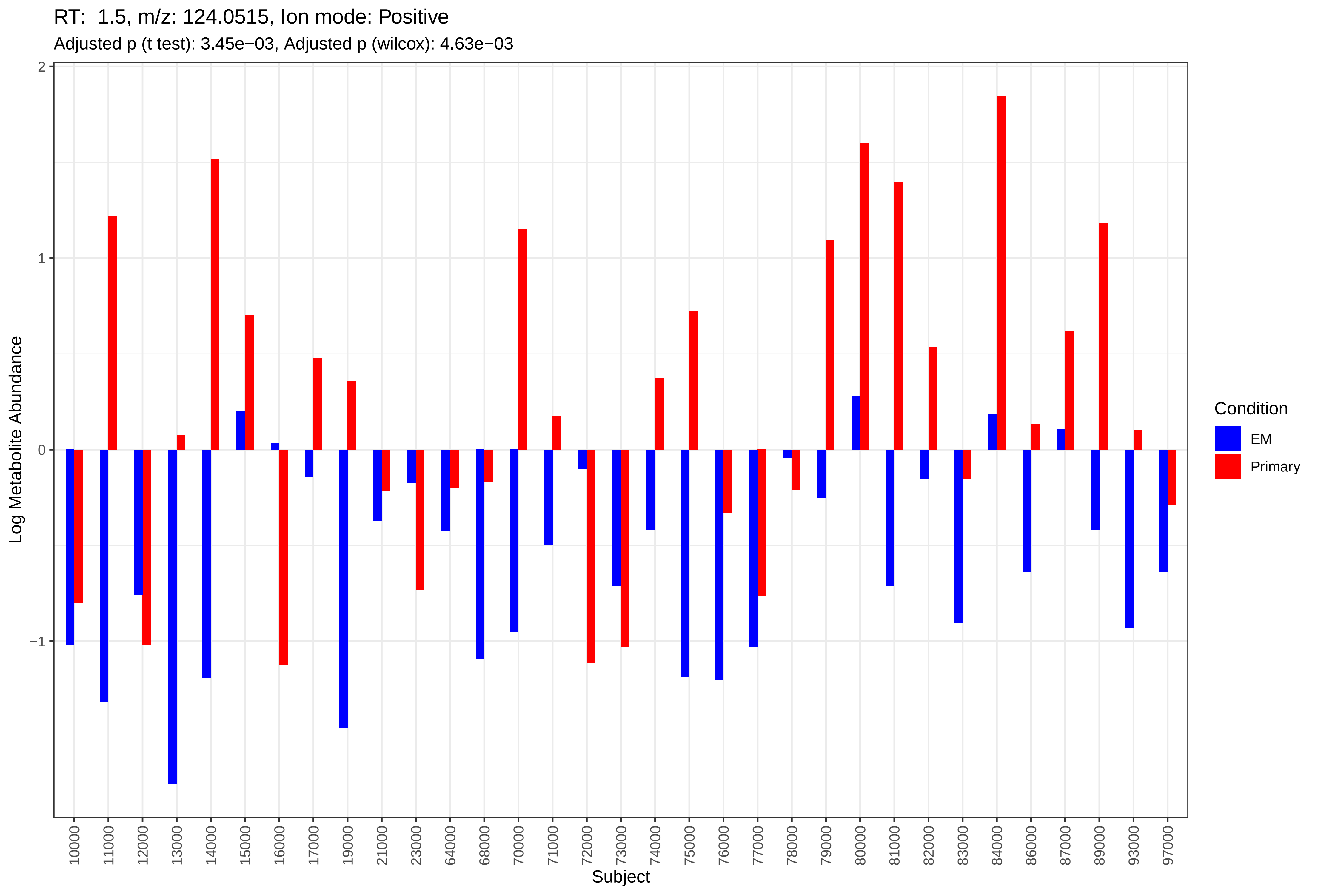

RT: 1.5, m/z: 124.0515, Ion mode: Positive
Adjusted p (t test): 3.45e−03, Adjusted p (wilcox): 4.63e−03
Log Metabolite Abundance
2
1
0
−1
Subject
10000
11000
12000
13000
14000
15000
16000
17000
19000
21000
23000
64000
68000
70000
71000
72000
73000
74000
75000
76000
77000
78000
79000
80000
81000
82000
83000
84000
86000
87000
89000
93000
97000
Condition
EM
Primary

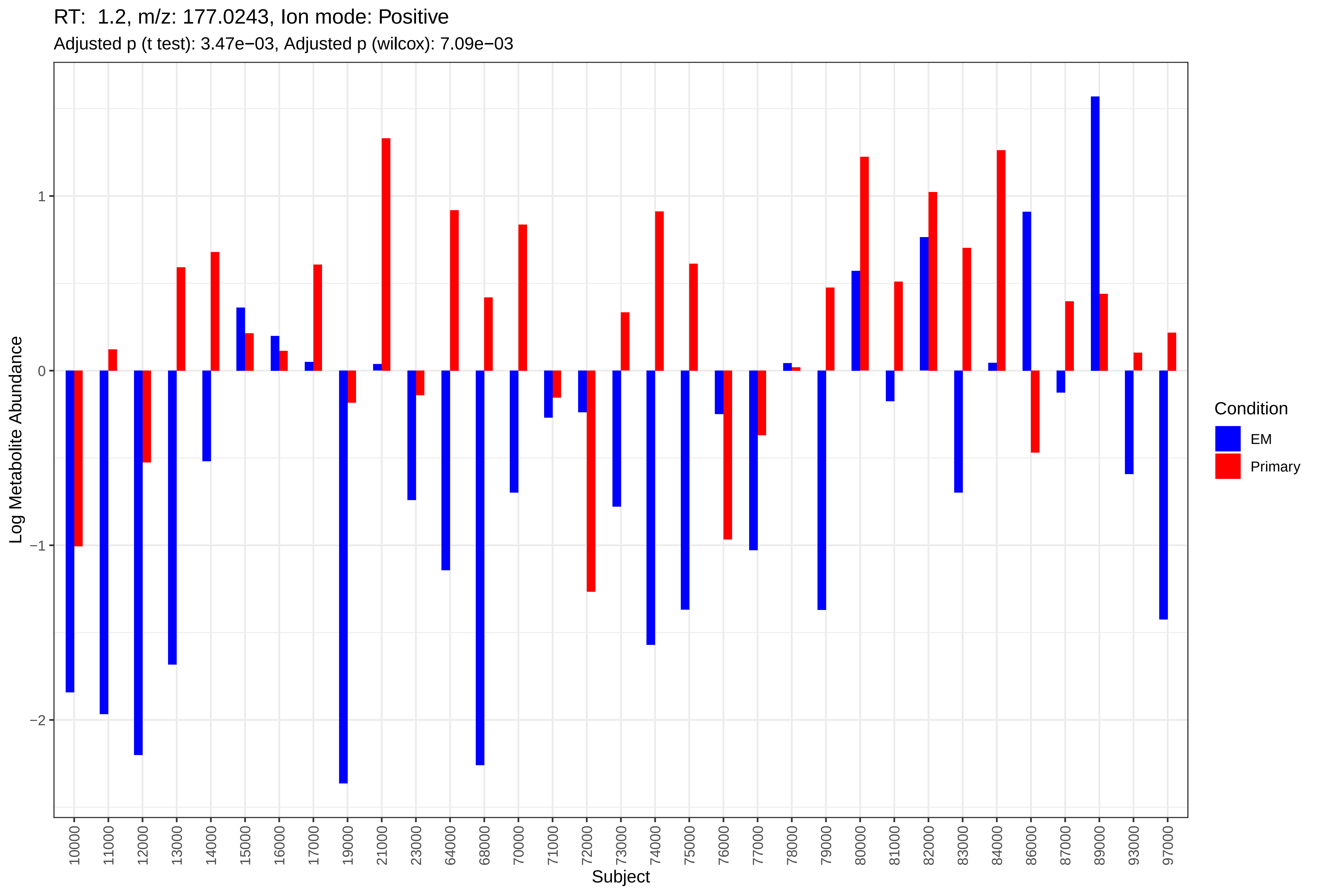
RT: 1.2, m/z: 177.0243, Ion mode: Positive
Adjusted p (t test): 3.47e−03, Adjusted p (wilcox): 7.09e−03
Log Metabolite Abundance
1
0
−1
−2
Subject
10000
11000
12000
13000
14000
15000
16000
17000
19000
21000
23000
64000
68000
70000
71000
72000
73000
74000
75000
76000
77000
78000
79000
80000
81000
82000
83000
84000
86000
87000
89000
93000
97000
Condition
EM
Primary

RT: 9.3, m/z: 541.2655, Ion mode: Negative

Adjusted p (t test): 3.58e−03, Adjusted p (wilcox): 4.48e−03

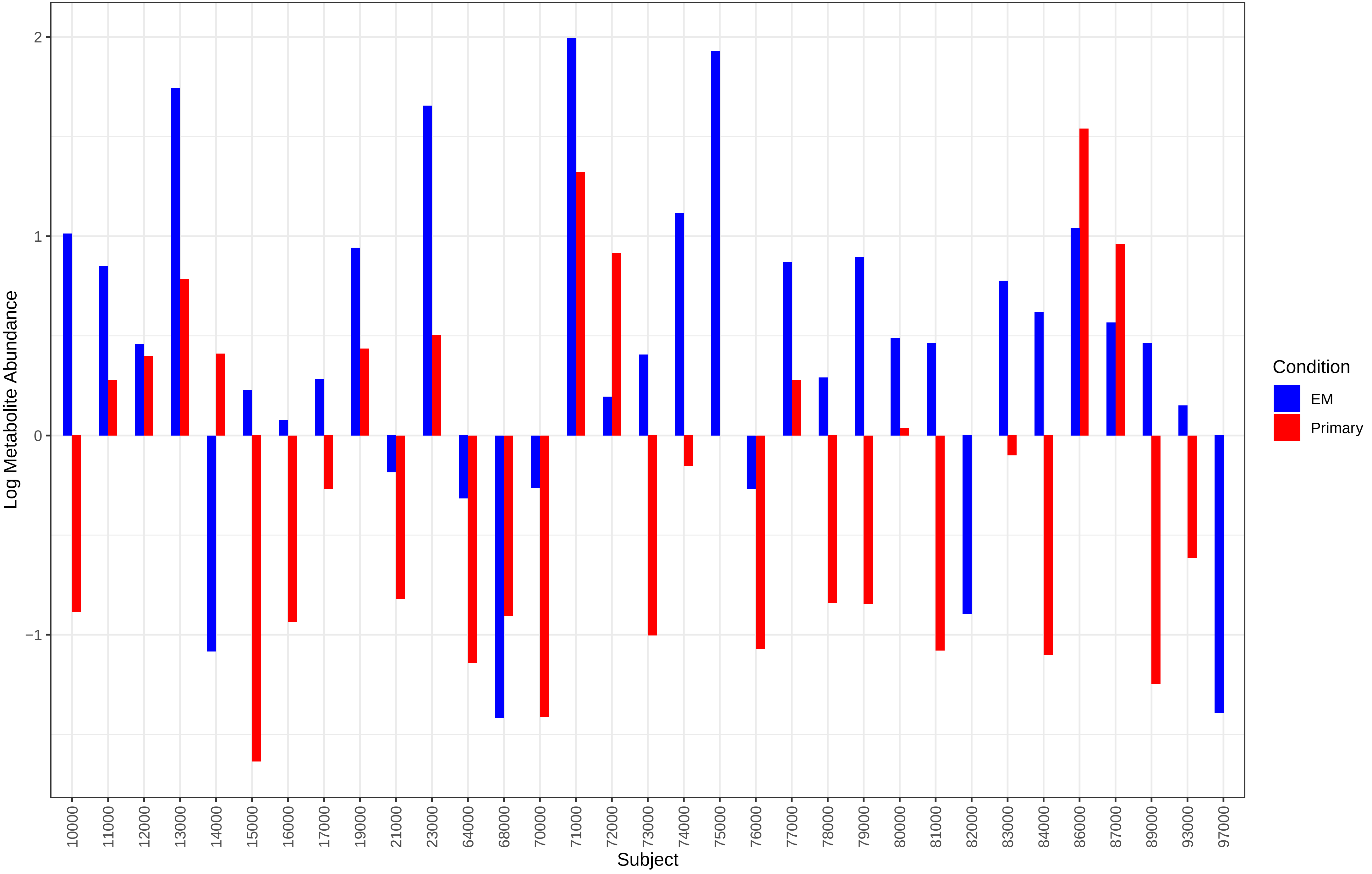

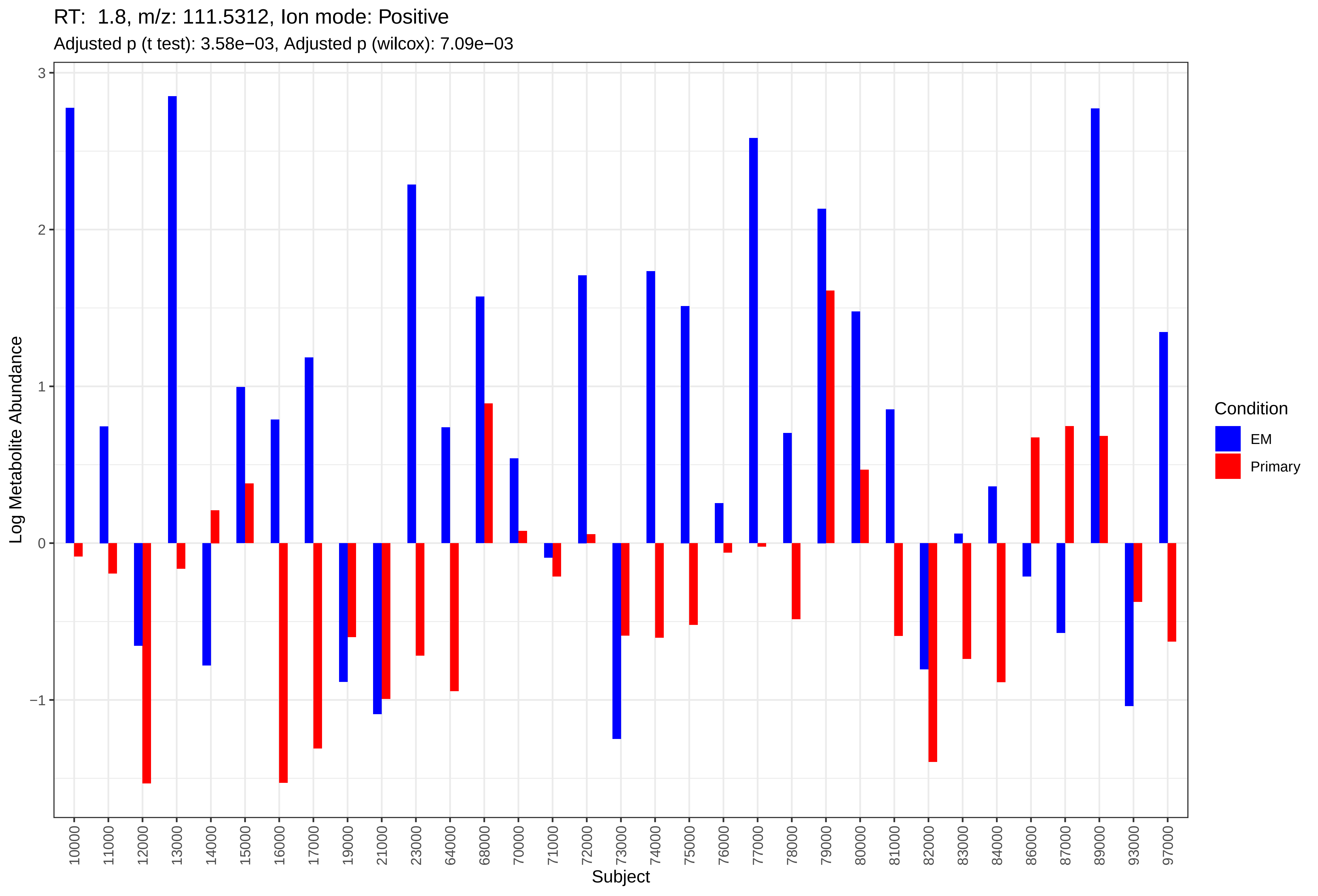

RT: 1.8, m/z: 111.5312, Ion mode: Positive
Adjusted p (t test): 3.58e−03, Adjusted p (wilcox): 7.09e−03
Log Metabolite Abundance
3
2
1
0
−1
Subject
10000
11000
12000
13000
14000
15000
16000
17000
19000
21000
23000
64000
68000
70000
71000
72000
73000
74000
75000
76000
77000
78000
79000
80000
81000
82000
83000
84000
86000
87000
89000
93000
97000
Condition
EM
Primary

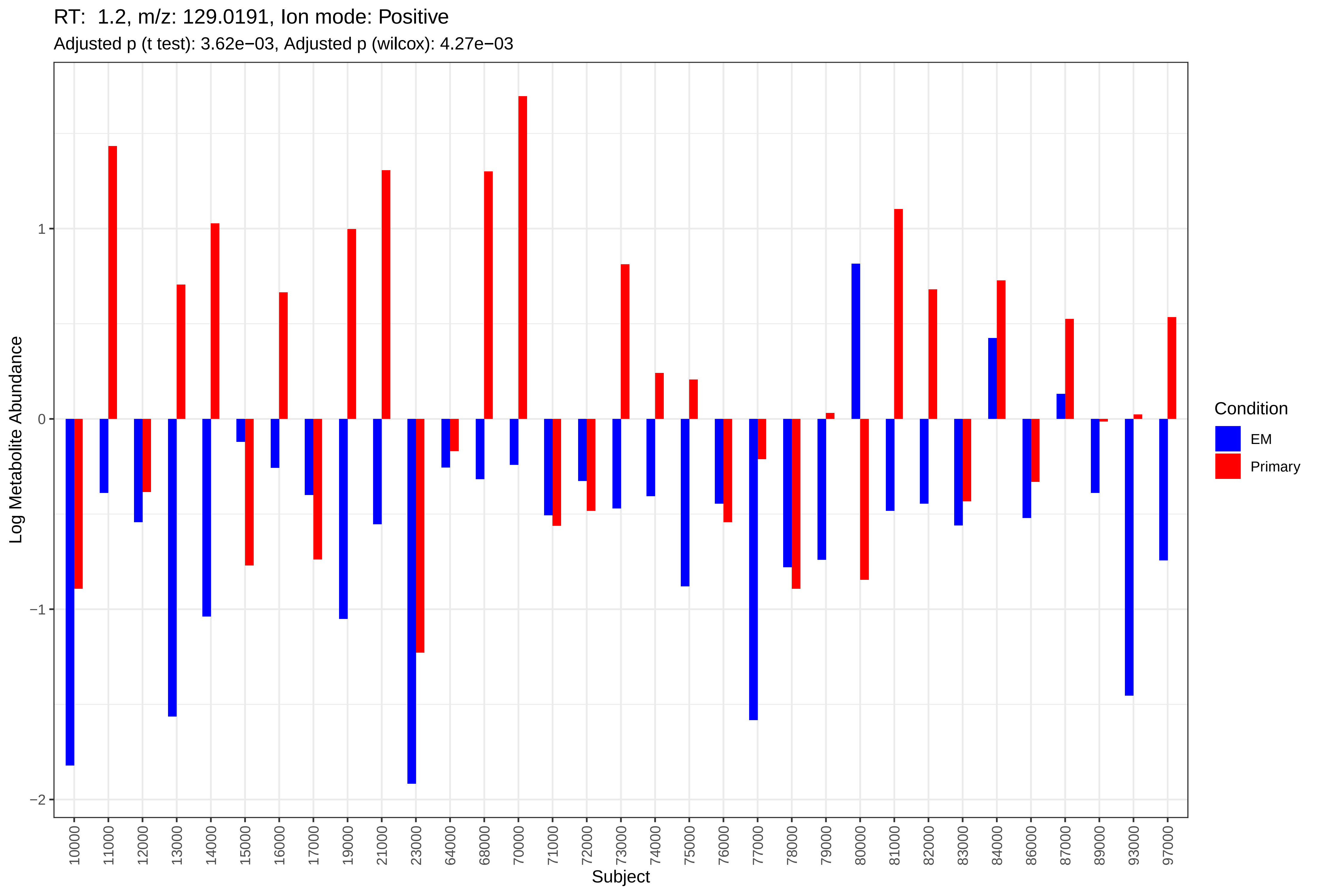

RT: 1.2, m/z: 129.0191, Ion mode: Positive
Adjusted p (t test): 3.62e−03, Adjusted p (wilcox): 4.27e−03
Log Metabolite Abundance
1
0
−1
−2
10000
11000
12000
13000
14000
15000
16000
17000
19000
21000
23000
64000
68000
70000
71000
72000
73000
74000
75000
76000
77000
78000
79000
80000
81000
82000
83000
84000
86000
87000
89000
93000
97000
Subject
Condition
EM
Primary

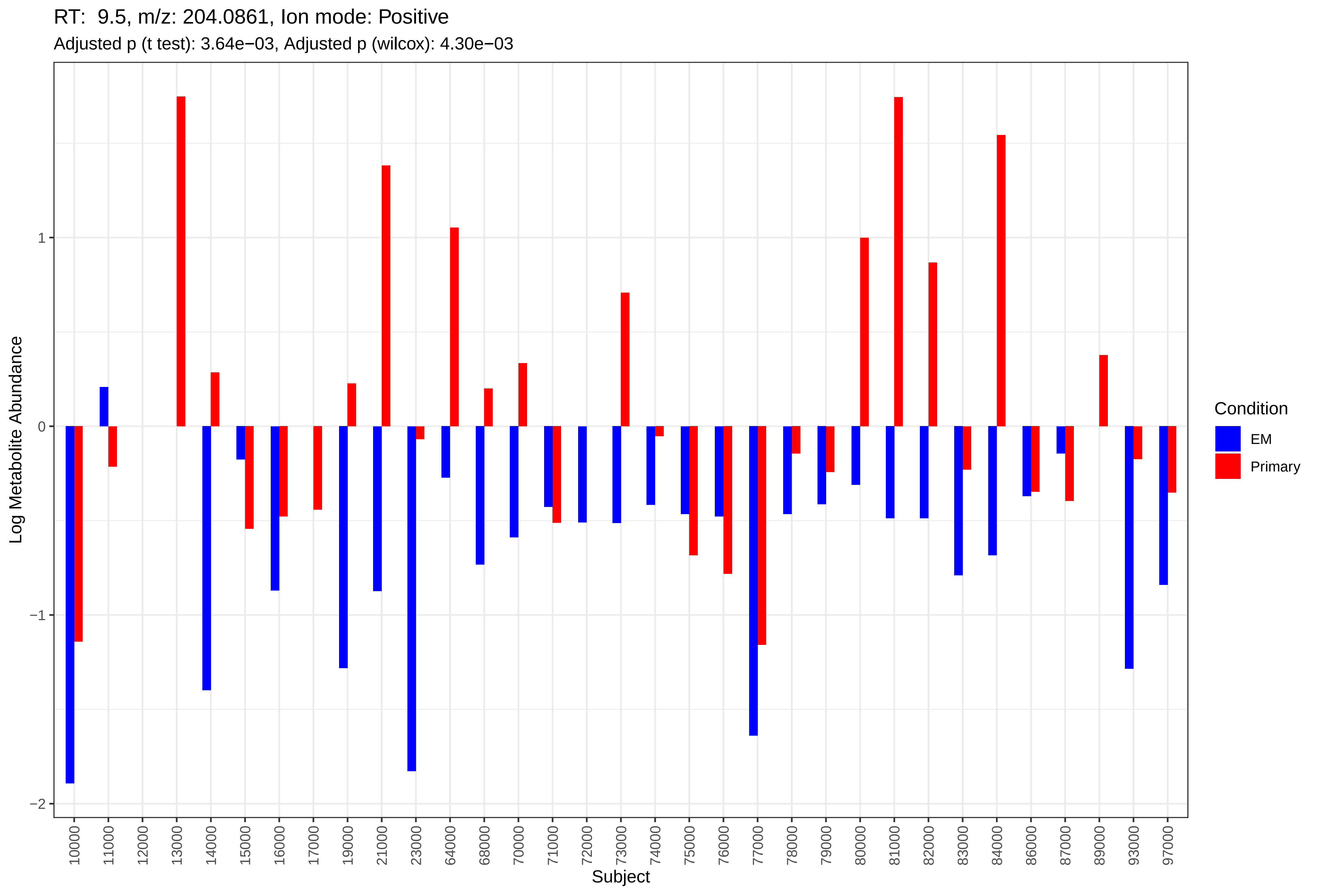
RT: 9.5, m/z: 204.0861, Ion mode: Positive
Adjusted p (t test): 3.64e−03, Adjusted p (wilcox): 4.30e−03
Log Metabolite Abundance
1
0
−1
−2
10000
11000
12000
13000
14000
15000
16000
17000
19000
21000
23000
64000
68000
70000
71000
72000
73000
74000
75000
76000
77000
78000
79000
80000
81000
82000
83000
84000
86000
87000
89000
93000
97000
Subject
Condition
EM
Primary

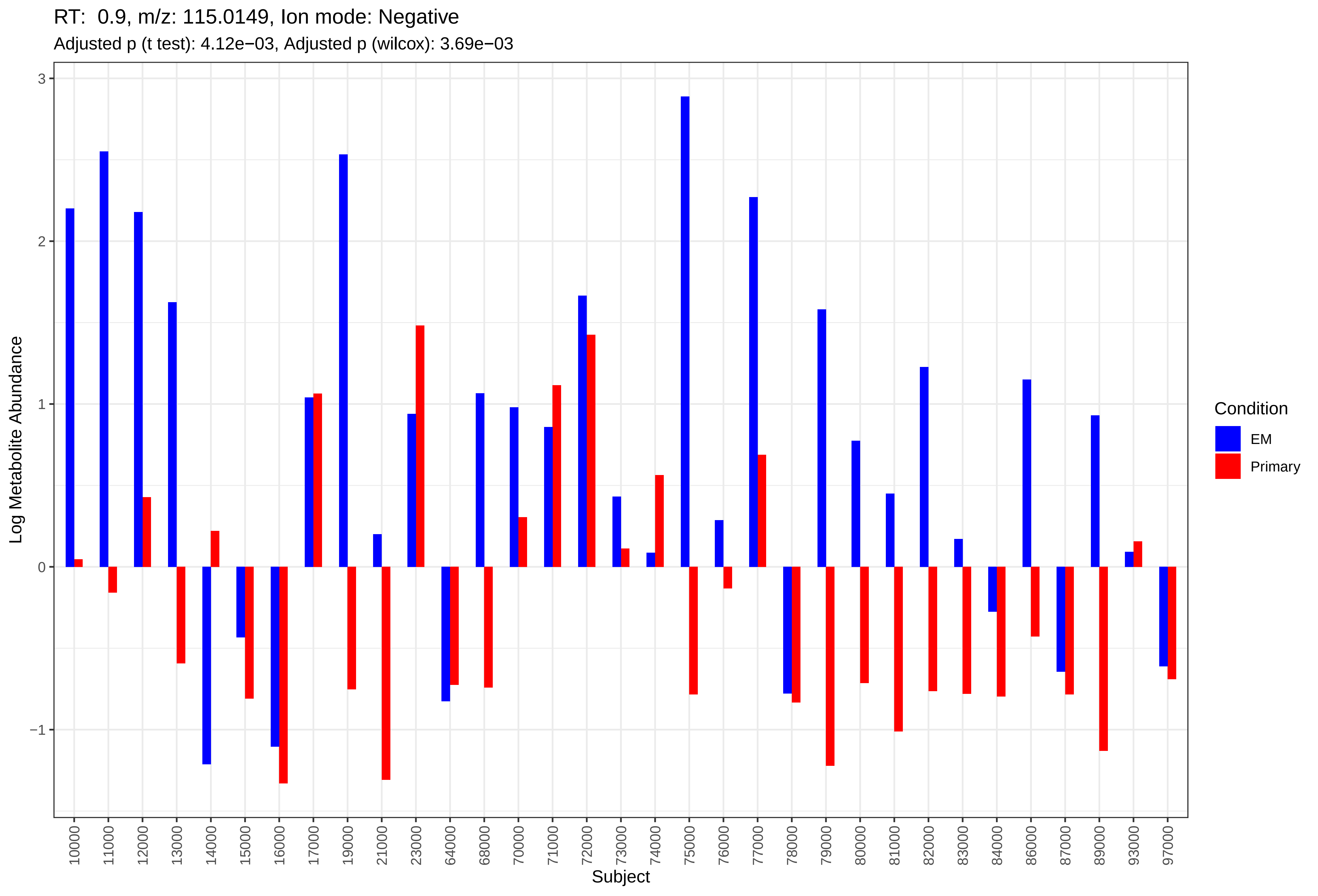

RT: 0.9, m/z: 115.0149, Ion mode: Negative
Adjusted p (t test): 4.12e−03, Adjusted p (wilcox): 3.69e−03
Log Metabolite Abundance
3
2
1
0
−1
10000
11000
12000
13000
14000
15000
16000
17000
19000
21000
23000
64000
68000
70000
71000
72000
73000
74000
75000
76000
77000
78000
79000
80000
81000
82000
83000
84000
86000
87000
89000
93000
97000
Subject
Condition
EM
Primary

RT: 2.0, m/z: 271.0062, Ion mode: Negative

Adjusted p (t test): 5.66e−03, Adjusted p (wilcox): 3.69e−03

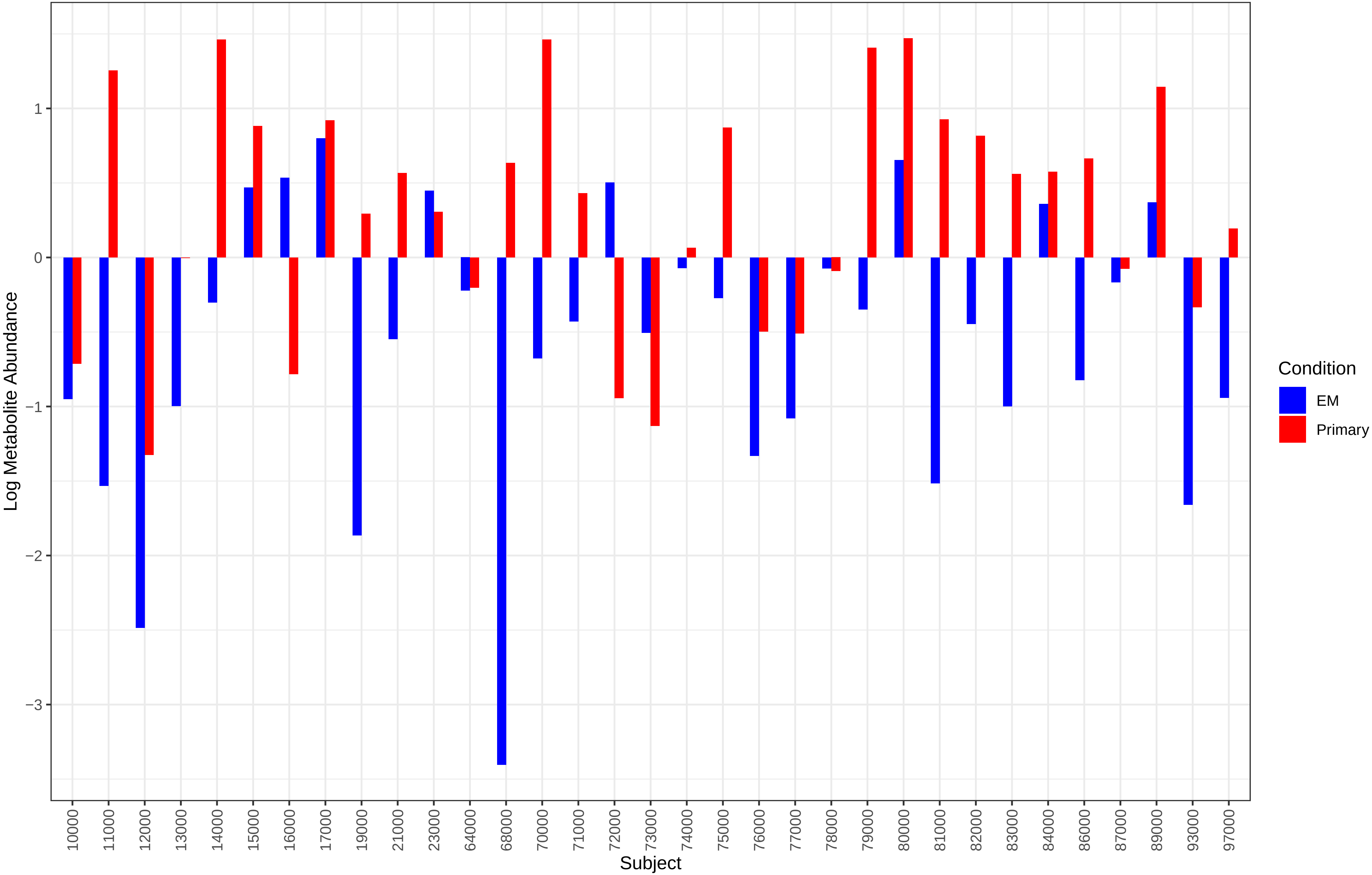

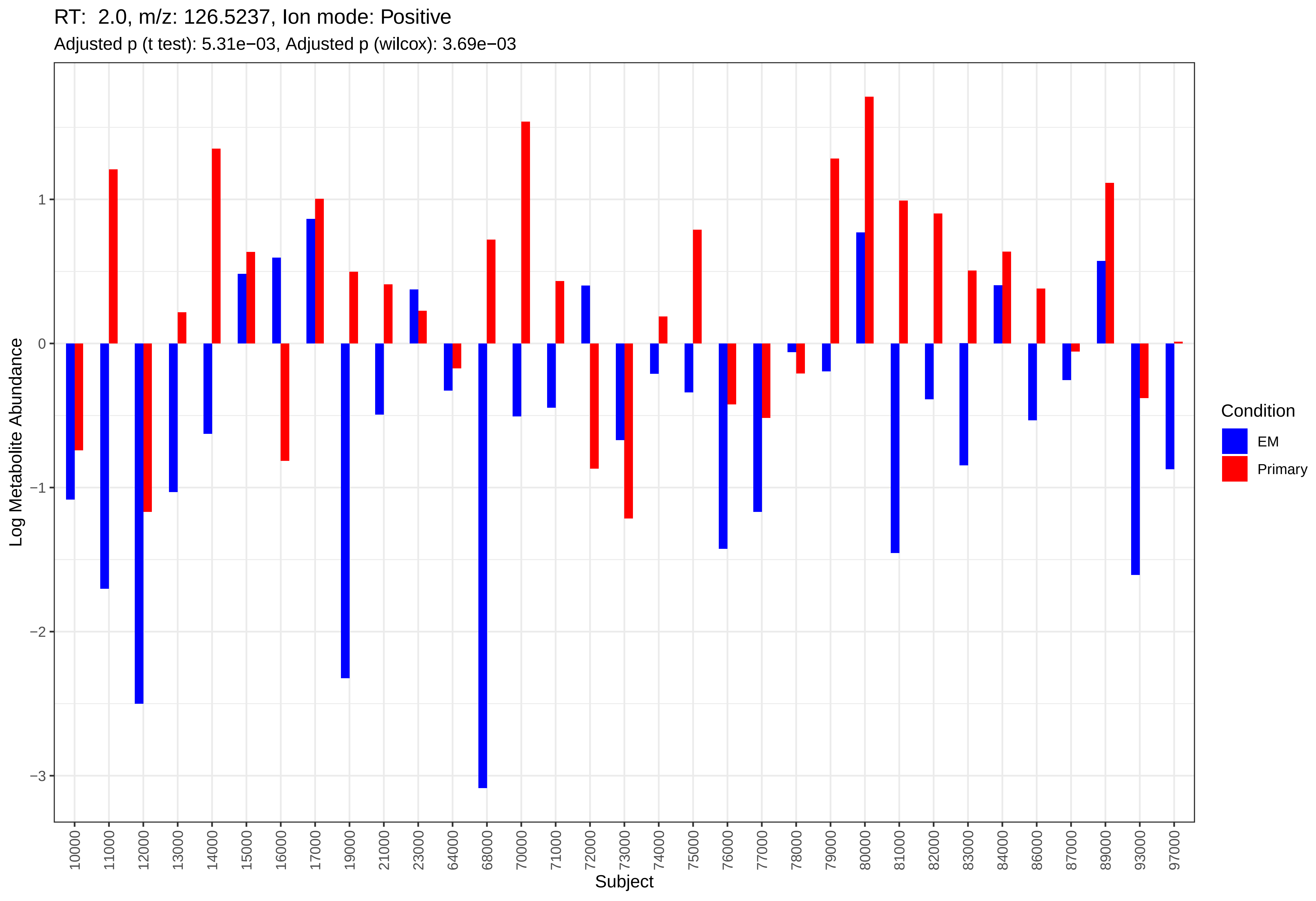

RT: 2.0, m/z: 126.5237, Ion mode: Positive
Adjusted p (t test): 5.31e−03, Adjusted p (wilcox): 3.69e−03
Log Metabolite Abundance
1
0
−1
−2
−3
Subject
10000
11000
12000
13000
14000
15000
16000
17000
19000
21000
23000
64000
68000
70000
71000
72000
73000
74000
75000
76000
77000
78000
79000
80000
81000
82000
83000
84000
86000
87000
89000
93000
97000
Condition
EM
Primary

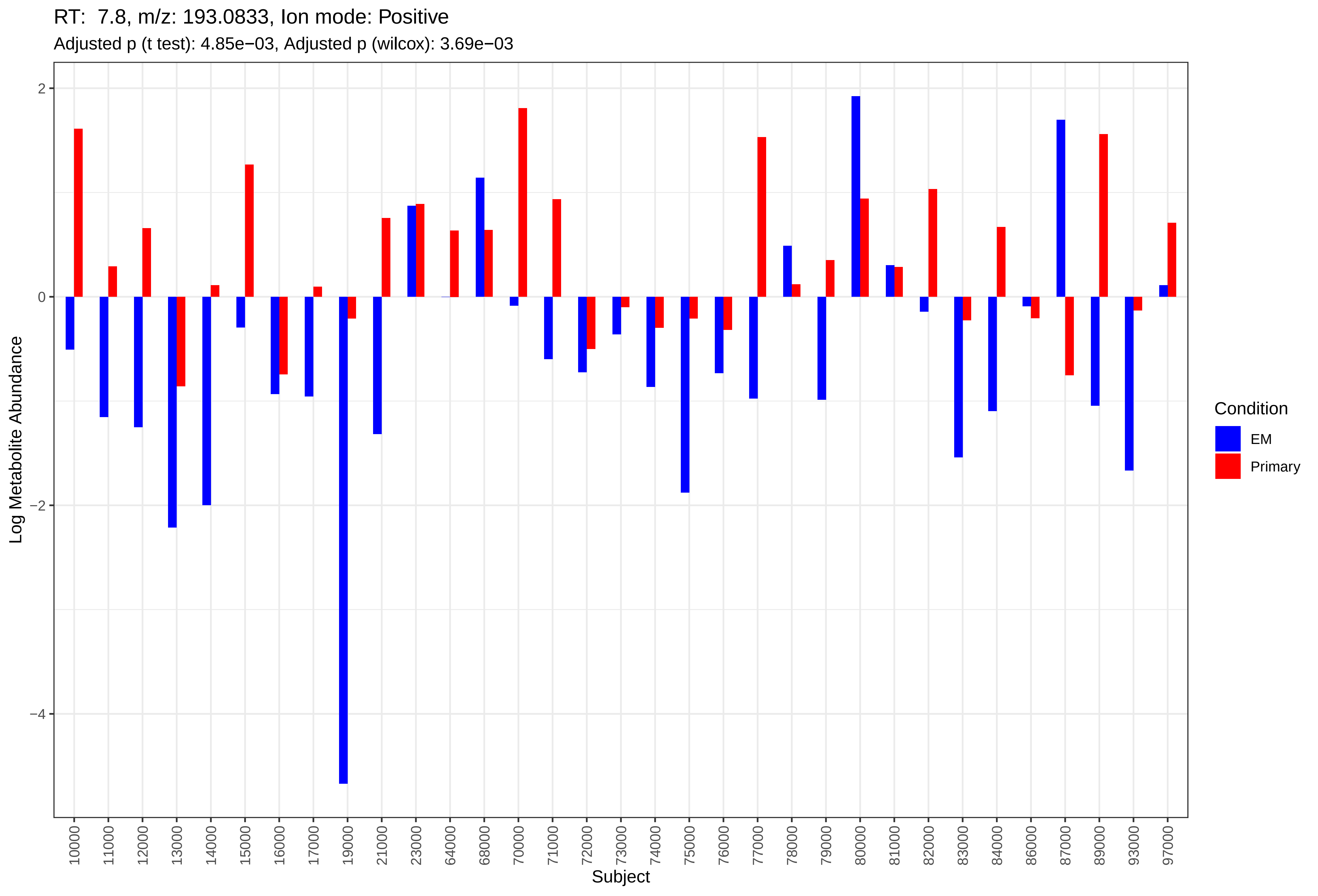
RT: 7.8, m/z: 193.0833, Ion mode: Positive
Adjusted p (t test): 4.85e−03, Adjusted p (wilcox): 3.69e−03
Log Metabolite Abundance
2
0
−2
−4
Subject
10000
11000
12000
13000
14000
15000
16000
17000
19000
21000
23000
64000
68000
70000
71000
72000
73000
74000
75000
76000
77000
78000
79000
80000
81000
82000
83000
84000
86000
87000
89000
93000
97000
Condition
EM
Primary

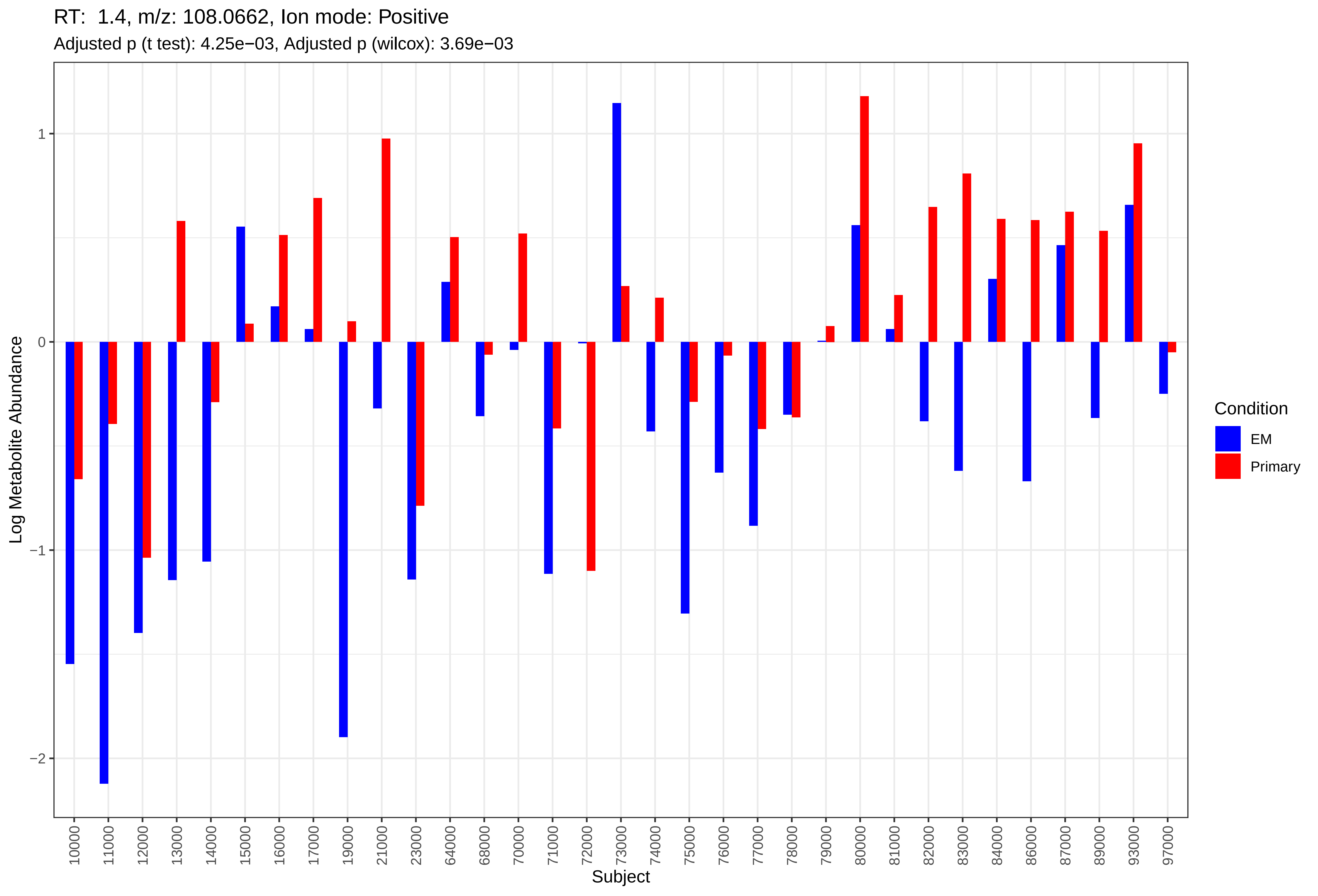

RT: 1.4, m/z: 108.0662, Ion mode: Positive
Adjusted p (t test): 4.25e−03, Adjusted p (wilcox): 3.69e−03
Log Metabolite Abundance
1
0
−1
−2
10000
11000
12000
13000
14000
15000
16000
17000
19000
21000
23000
64000
68000
70000
71000
72000
73000
74000
75000
76000
77000
78000
79000
80000
81000
82000
83000
84000
86000
87000
89000
93000
97000
Subject
Condition
EM
Primary

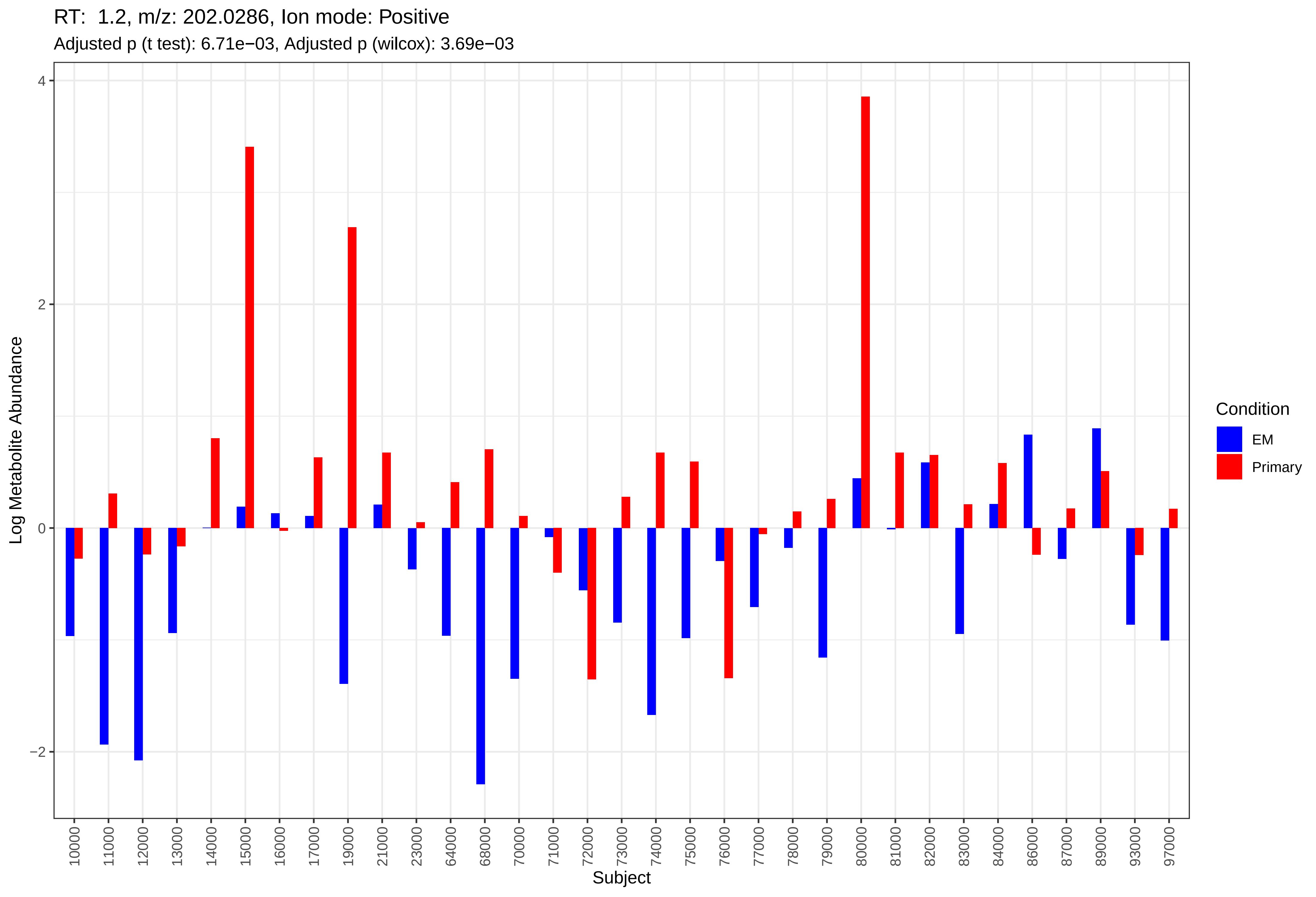

RT: 1.2, m/z: 202.0286, Ion mode: Positive
Adjusted p (t test): 6.71e−03, Adjusted p (wilcox): 3.69e−03
Log Metabolite Abundance
4
2
0
−2
Subject
10000
11000
12000
13000
14000
15000
16000
17000
19000
21000
23000
64000
68000
70000
71000
72000
73000
74000
75000
76000
77000
78000
79000
80000
81000
82000
83000
84000
86000
87000
89000
93000
97000
Condition
EM
Primary

RT: 2.0, m/z: 106.0098, Ion mode: Positive

Adjusted p (t test): 4.24e−03, Adjusted p (wilcox): 3.69e−03

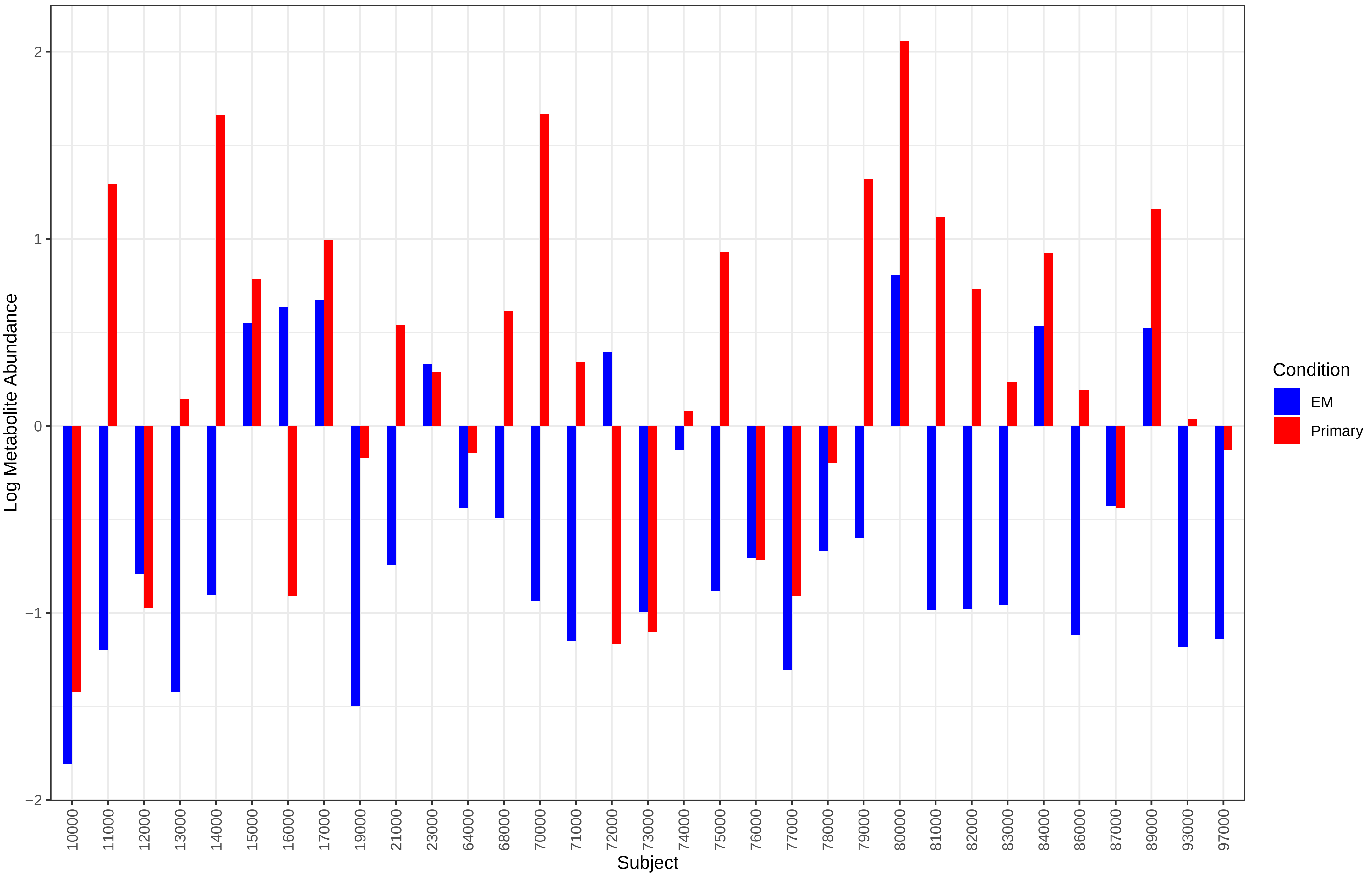

RT: 3.9, m/z: 308.0810, Ion mode: Positive

Adjusted p (t test): 3.70e−03, Adjusted p (wilcox): 5.75e−03

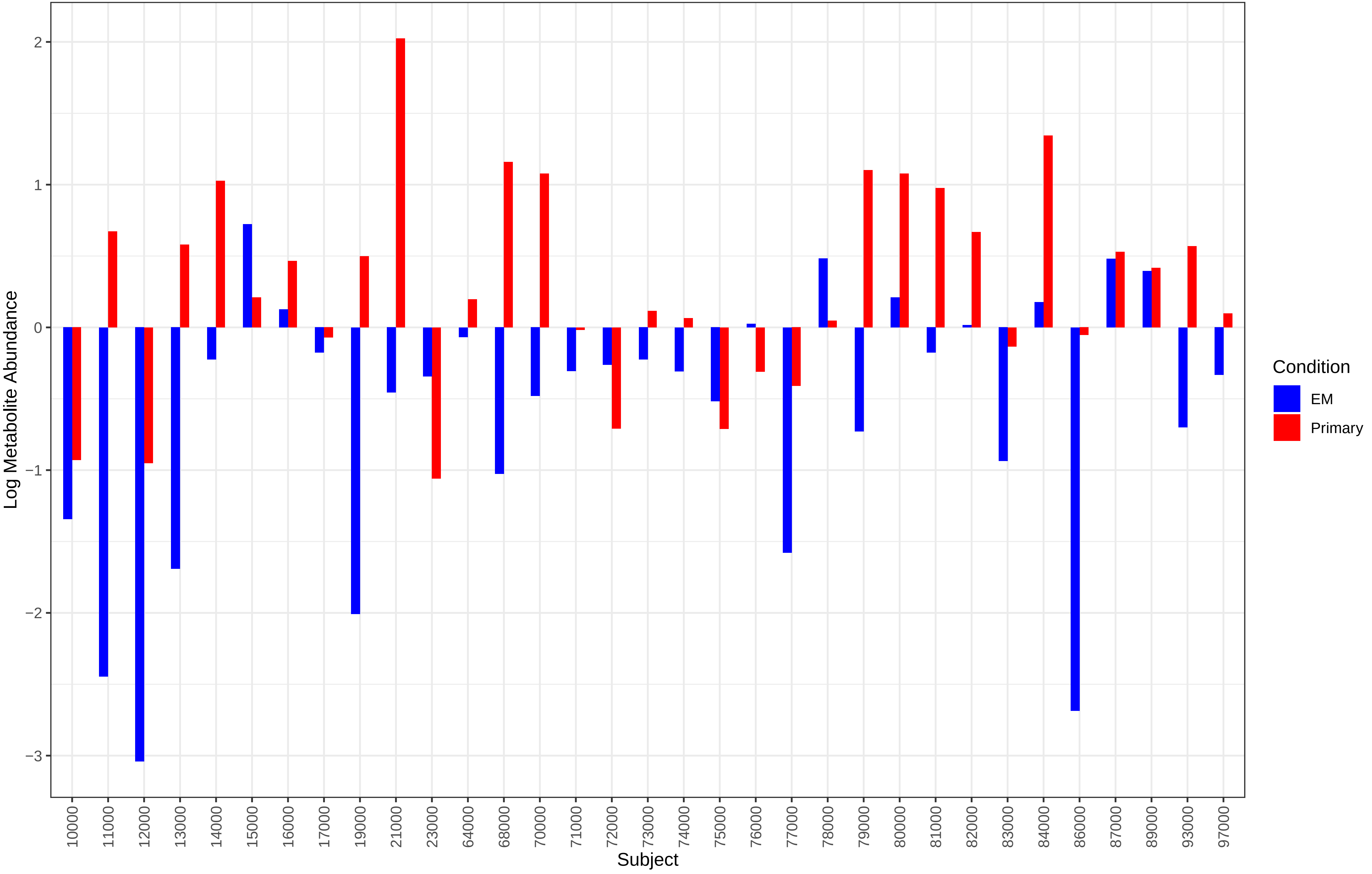

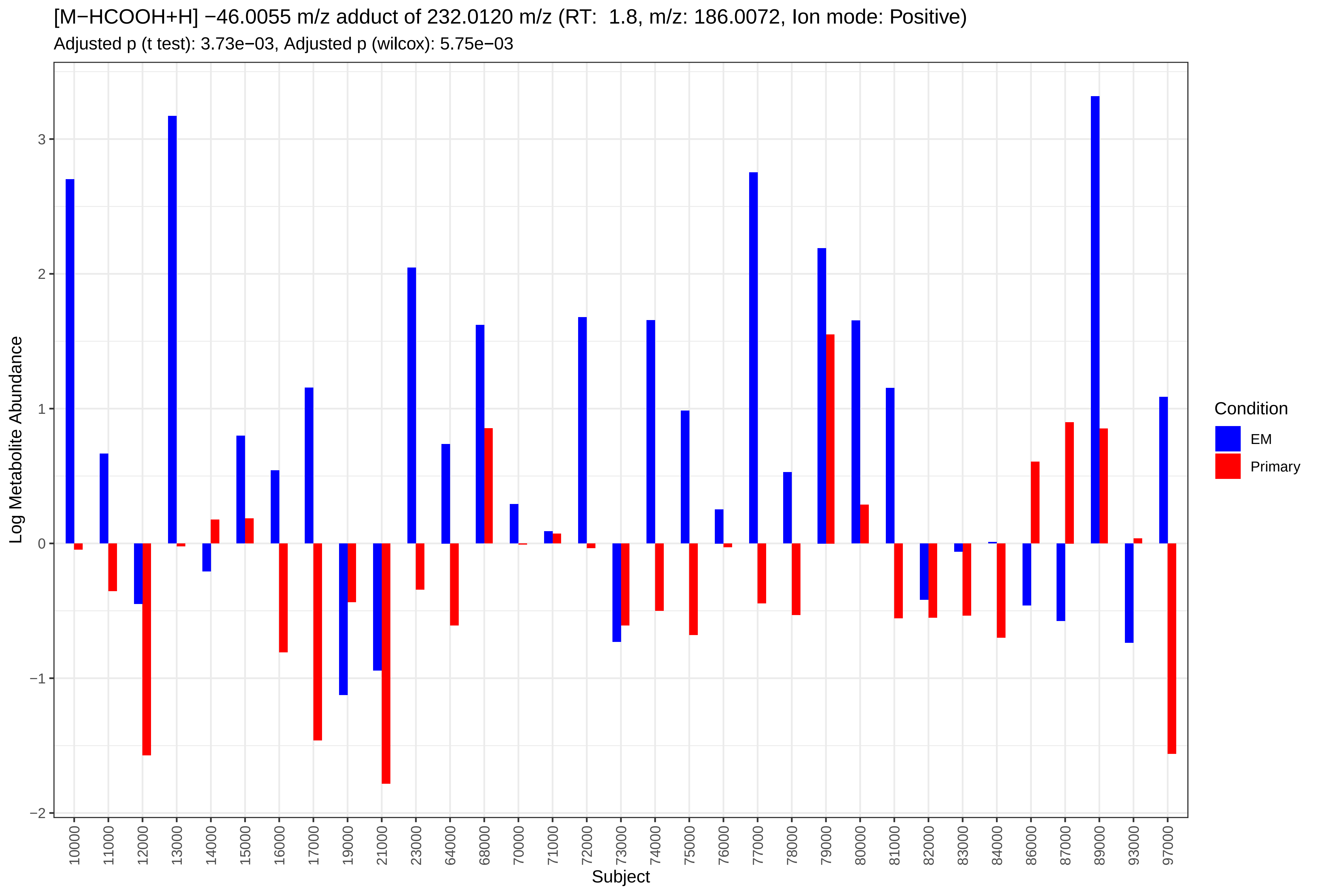

[M−HCOOH+H] −46.0055 m/z adduct of 232.0120 m/z (RT: 1.8, m/z: 186.0072, Ion mode: Positive)
Adjusted p (t test): 3.73e−03, Adjusted p (wilcox): 5.75e−03
Log Metabolite Abundance
3
2
1
0
−1
−2
10000
11000
12000
13000
14000
15000
16000
17000
19000
21000
23000
64000
68000
70000
71000
72000
73000
74000
75000
76000
77000
78000
79000
80000
81000
82000
83000
84000
86000
87000
89000
93000
97000
Subject
Condition
EM
Primary

RT: 1.1, m/z: 146.1650, Ion mode: Positive

Adjusted p (t test): 5.15e−03, Adjusted p (wilcox): 3.74e−03

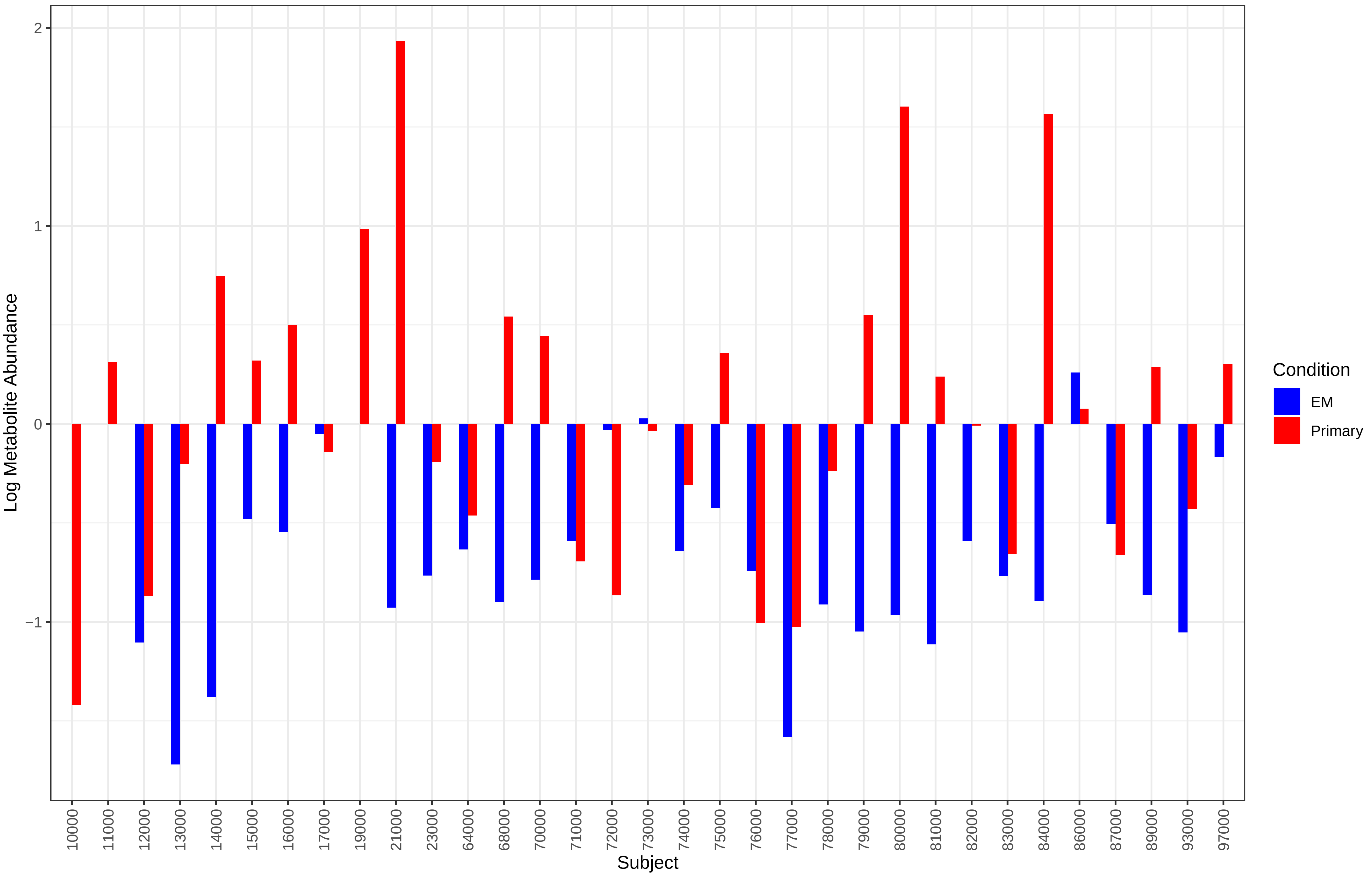

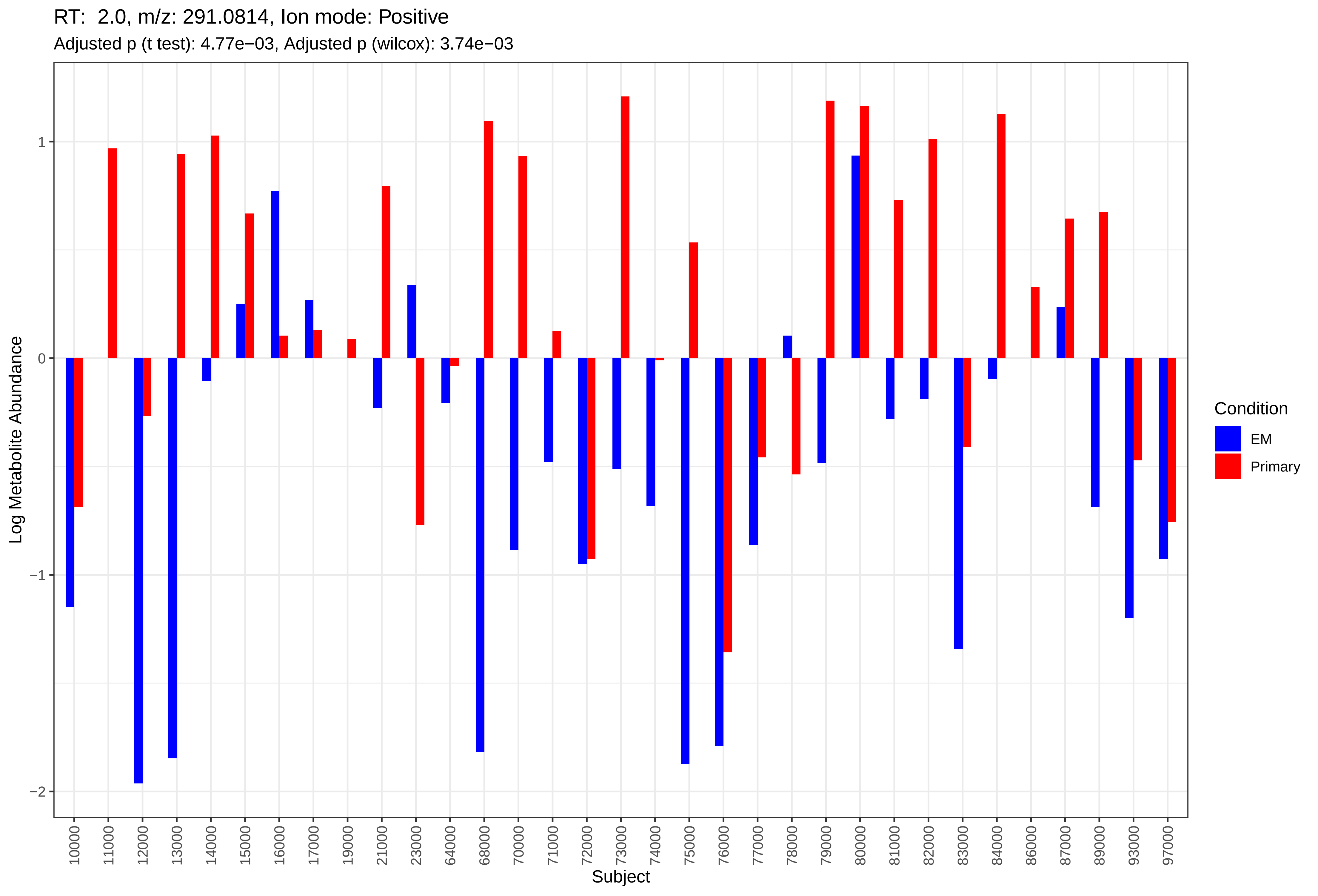

RT: 2.0, m/z: 291.0814, Ion mode: Positive
Adjusted p (t test): 4.77e−03, Adjusted p (wilcox): 3.74e−03
Log Metabolite Abundance
1
0
−1
−2
Subject
10000
11000
12000
13000
14000
15000
16000
17000
19000
21000
23000
64000
68000
70000
71000
72000
73000
74000
75000
76000
77000
78000
79000
80000
81000
82000
83000
84000
86000
87000
89000
93000
97000
Condition
EM
Primary

RT: 1.5, m/z: 125.0596, Ion mode: Positive

Adjusted p (t test): 3.77e−03, Adjusted p (wilcox): 5.32e−03

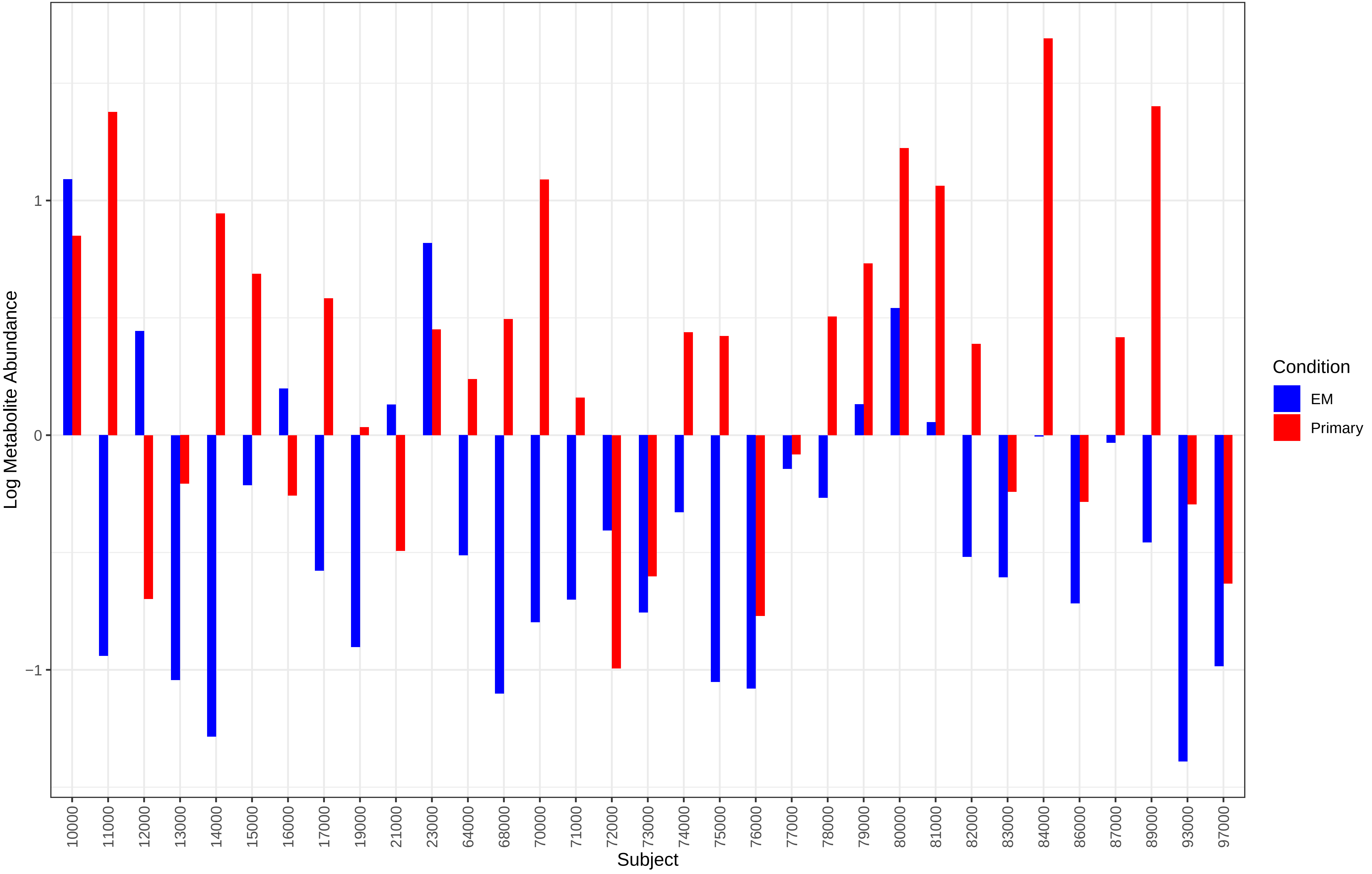

URIDINE−5−MONOPHOSPHATE (RT: 1.2, m/z: 323.0286, Ion mode: Negative)

Adjusted p (t test): 3.79e−03, Adjusted p (wilcox): 3.92e−03

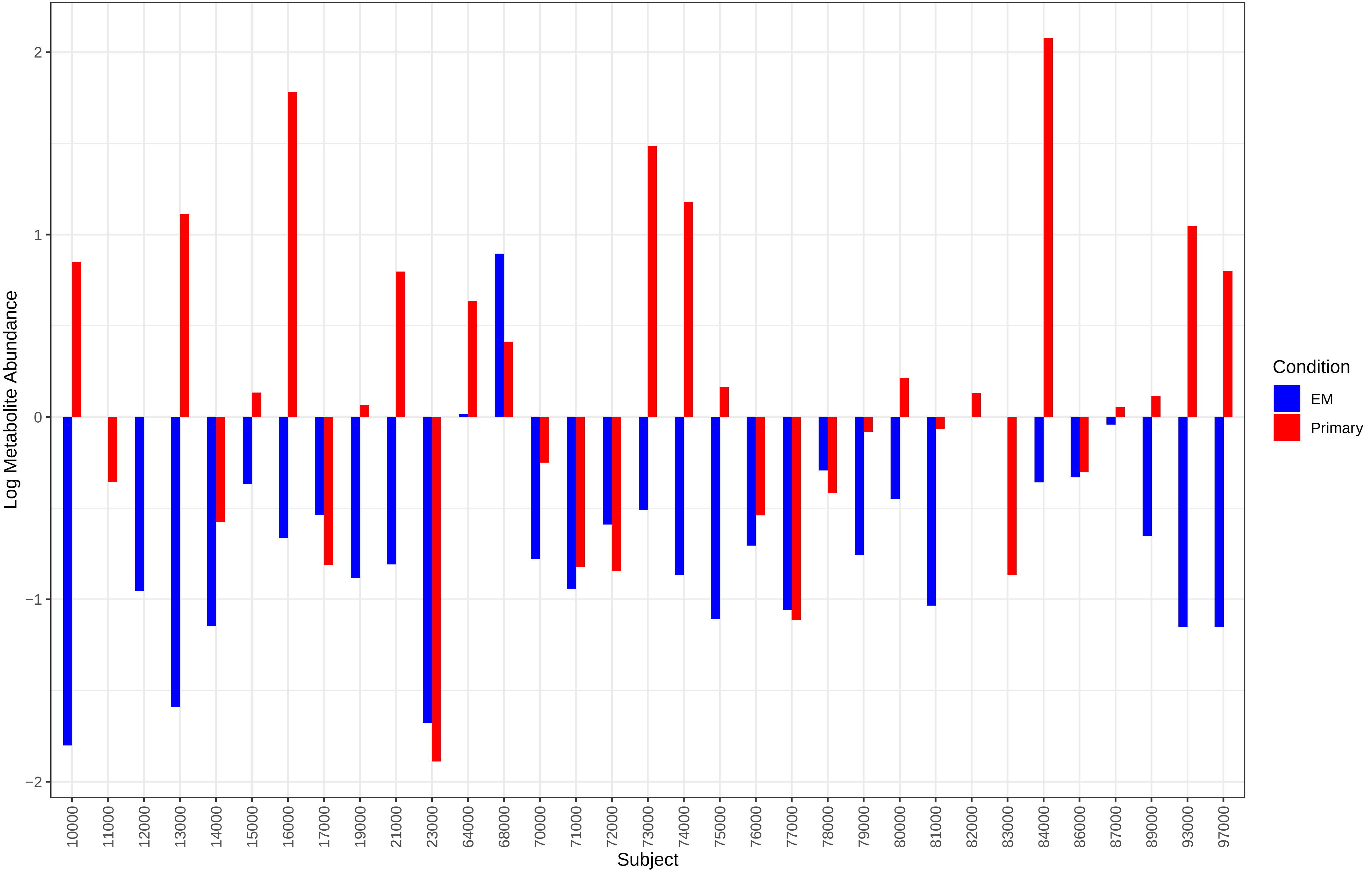

RT: 1.2, m/z: 115.9642, Ion mode: Positive

Adjusted p (t test): 3.84e−03, Adjusted p (wilcox): 4.94e−03

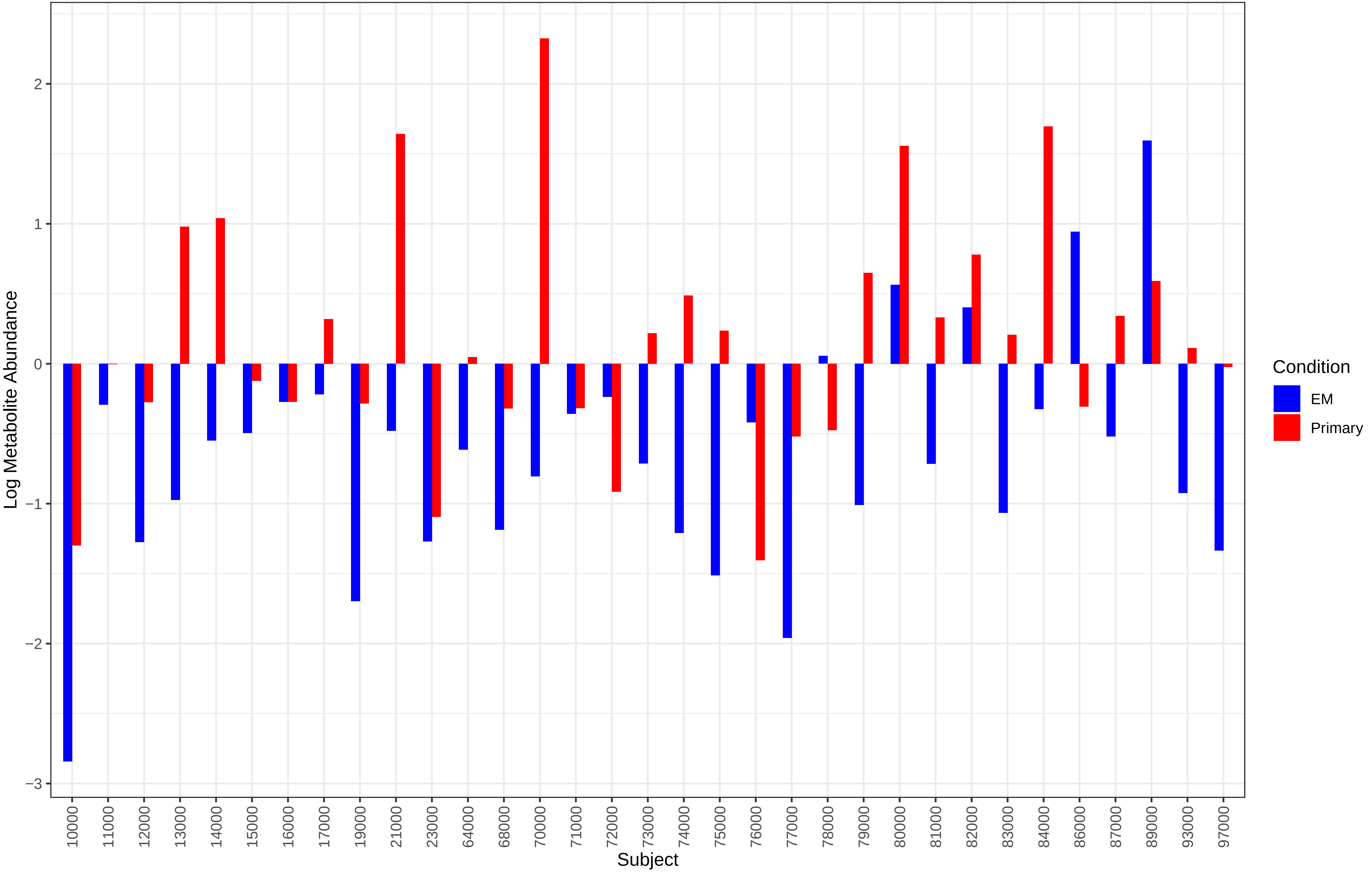

RT: 4.2, m/z: 220.9395, Ion mode: Negative

Adjusted p (t test): 3.85e−03, Adjusted p (wilcox): 5.32e−03

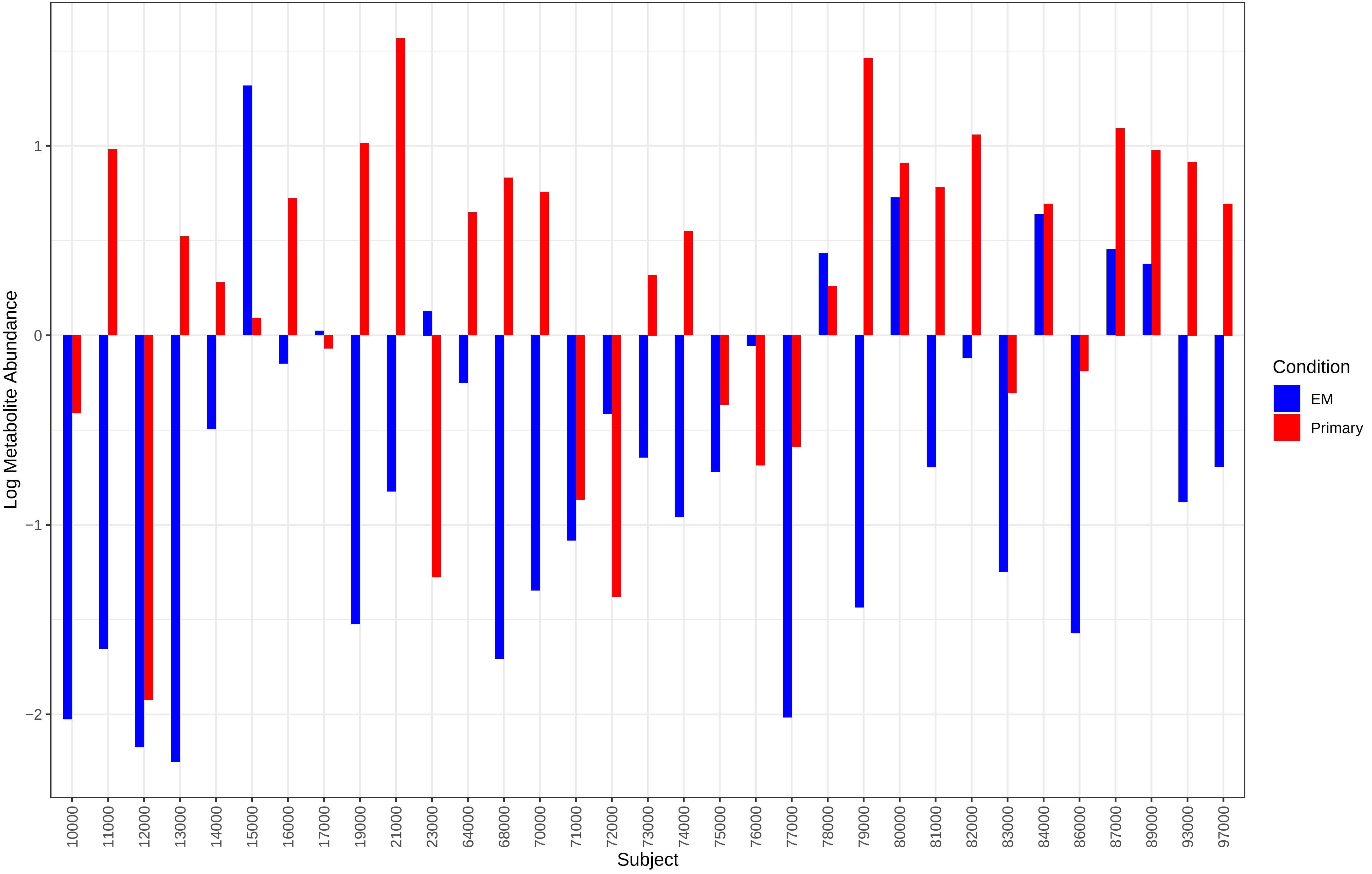

RT: 13.4, m/z: 614.4818, Ion mode: Positive

Adjusted p (t test): 3.89e−03, Adjusted p (wilcox): 3.97e−03

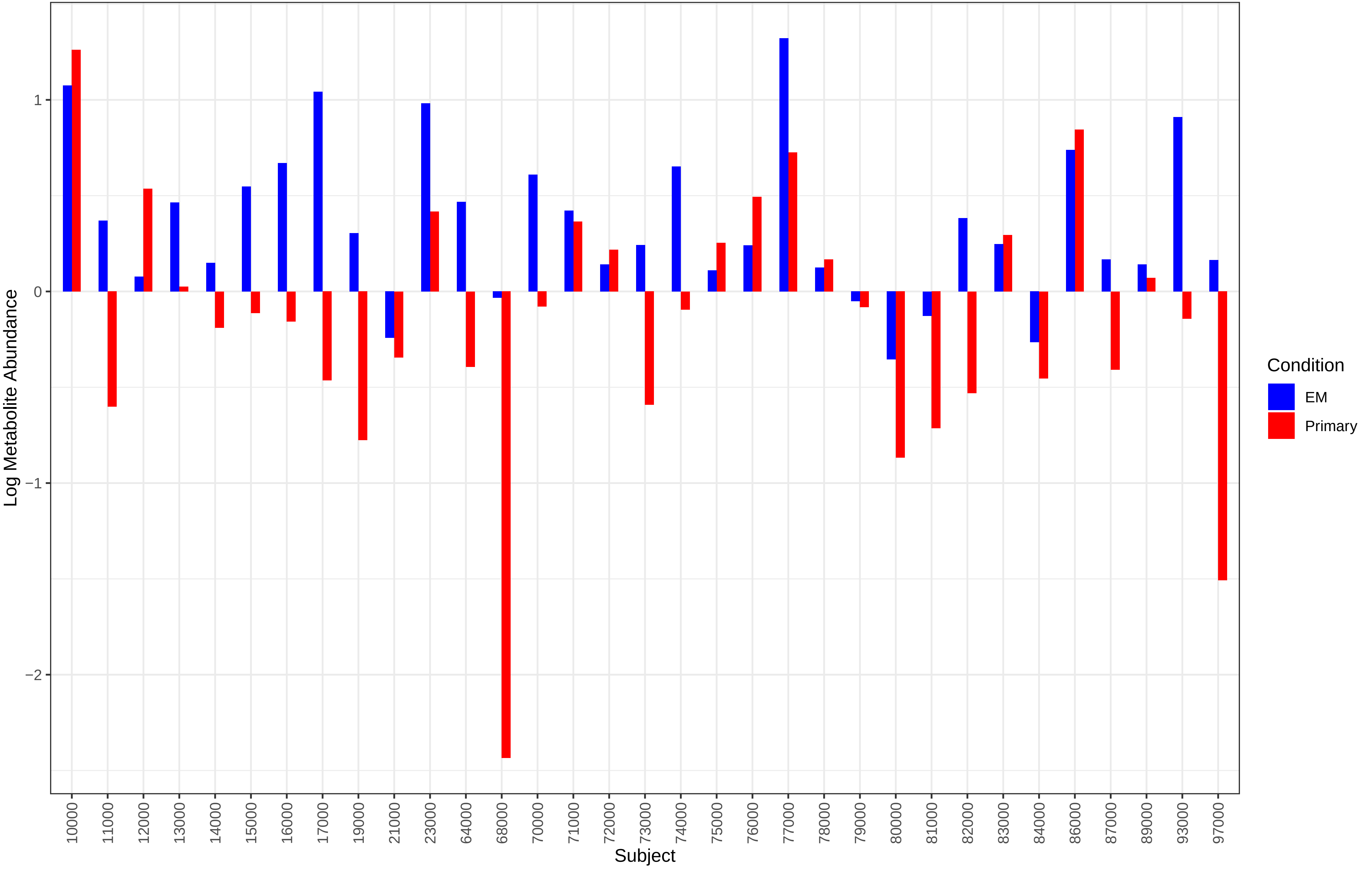

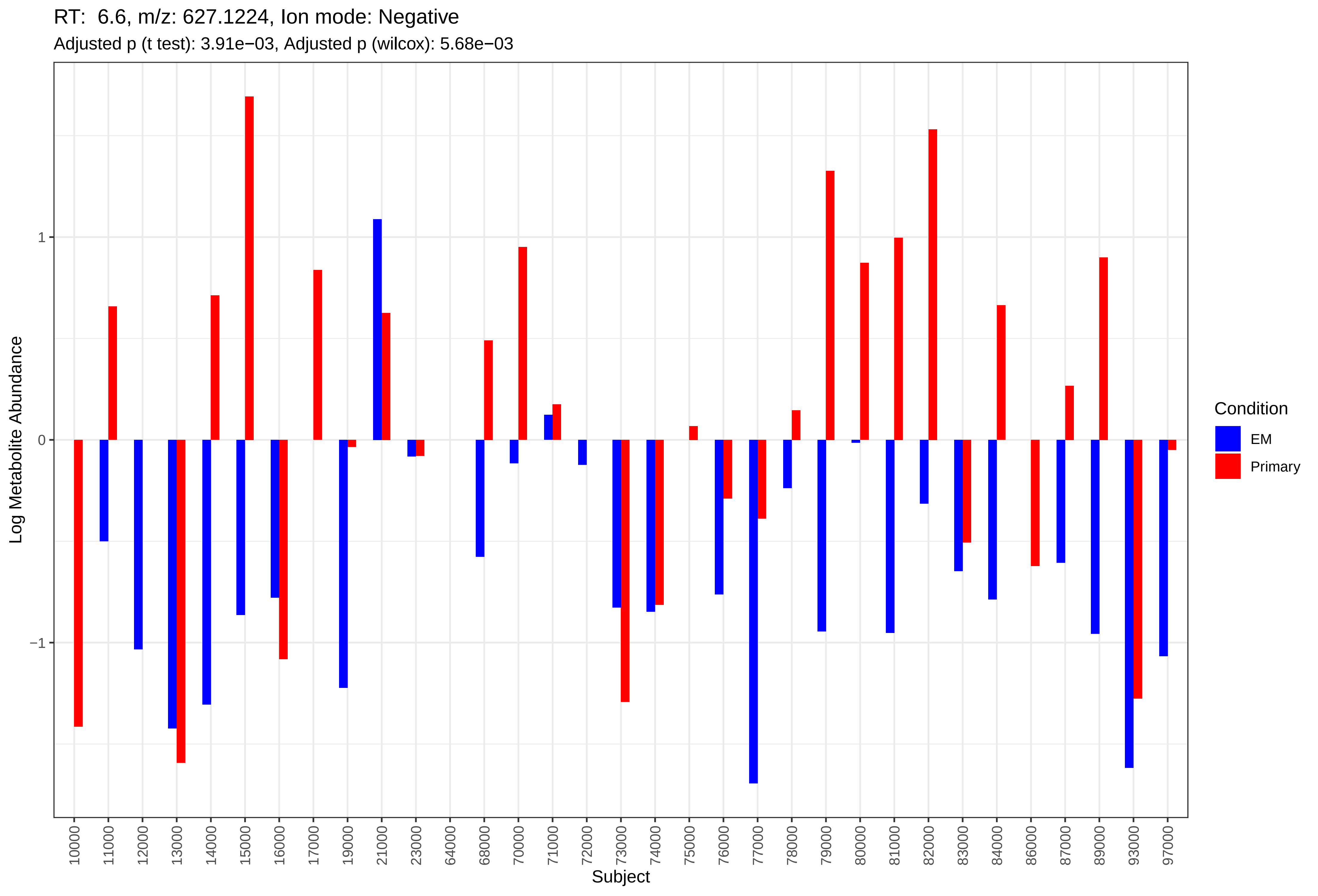
RT: 6.6, m/z: 627.1224, Ion mode: Negative
Adjusted p (t test): 3.91e−03, Adjusted p (wilcox): 5.68e−03
Log Metabolite Abundance
1
0
−1
Subject
10000
11000
12000
13000
14000
15000
16000
17000
19000
21000
23000
64000
68000
70000
71000
72000
73000
74000
75000
76000
77000
78000
79000
80000
81000
82000
83000
84000
86000
87000
89000
93000
97000
Condition
EM
Primary

Glycodeoxycholic acid (GDCA) (RT: 11.3, m/z: 448.3071, Ion mode: Negative)

Adjusted p (t test): 5.49e−03, Adjusted p (wilcox): 3.94e−03

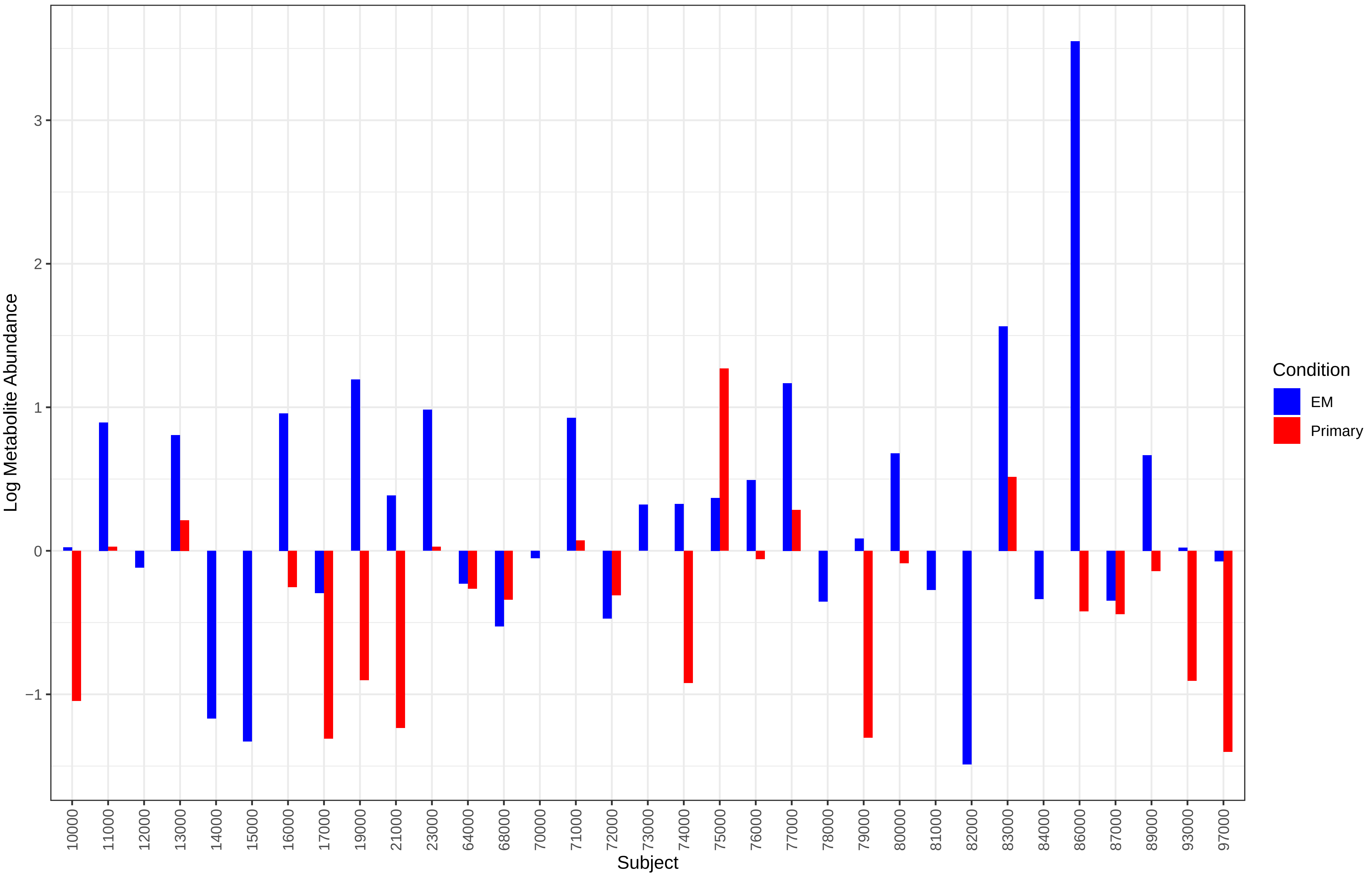

RT: 10.3, m/z: 419.1714, Ion mode: Negative

Adjusted p (t test): 3.96e−03, Adjusted p (wilcox): 5.75e−03

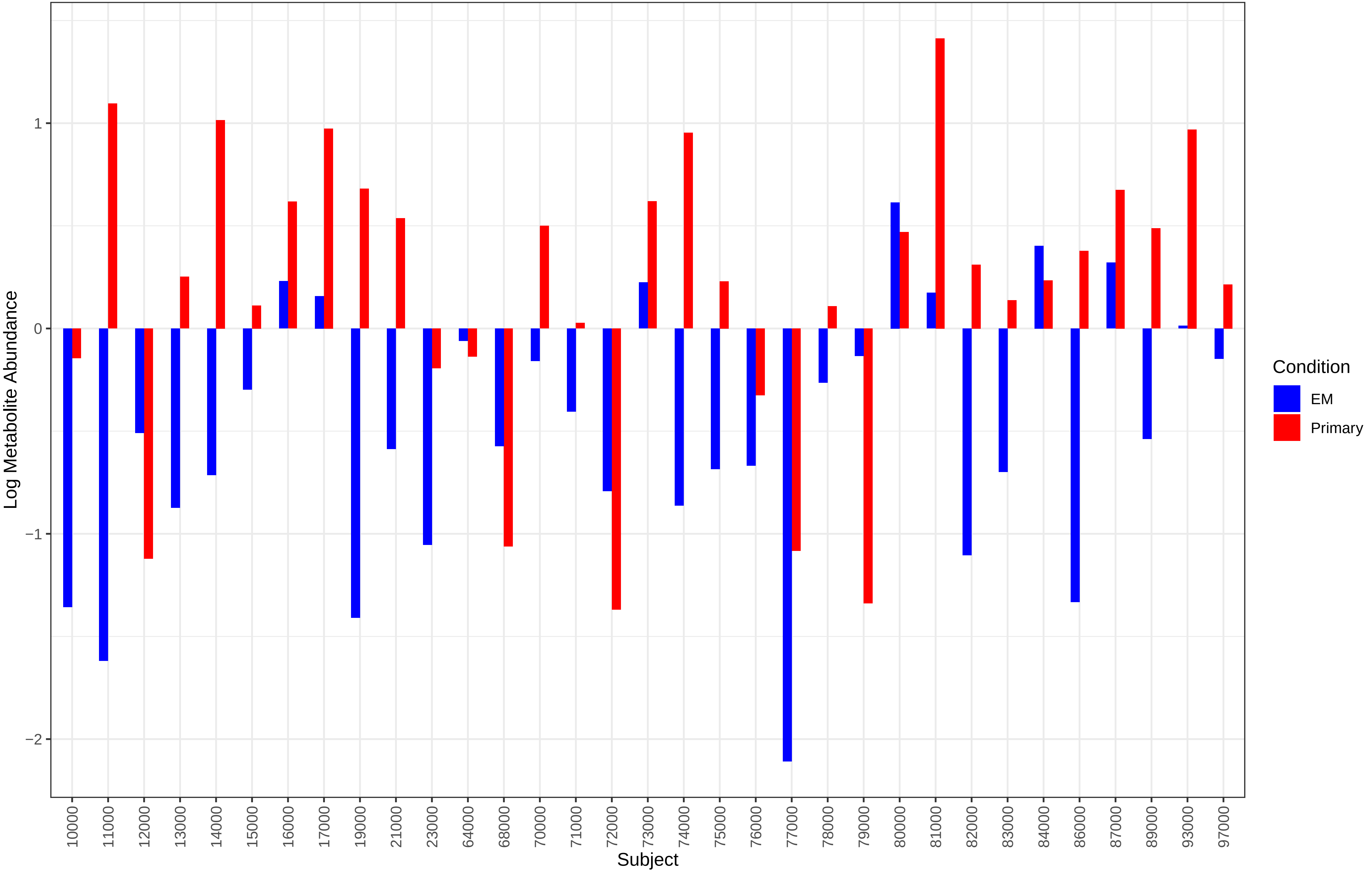

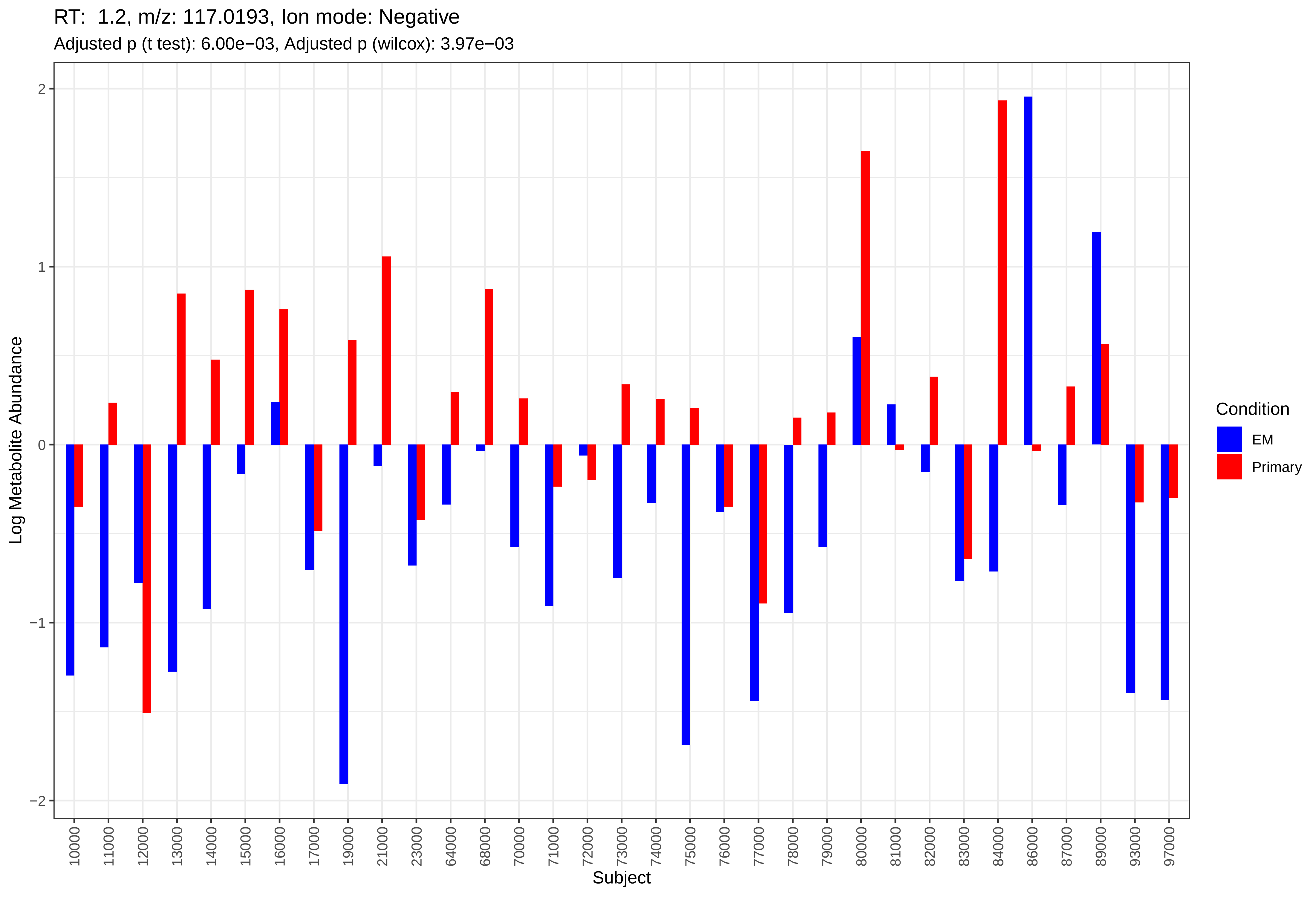

RT: 1.2, m/z: 117.0193, Ion mode: Negative
Adjusted p (t test): 6.00e−03, Adjusted p (wilcox): 3.97e−03
Log Metabolite Abundance
2
1
0
−1
−2
Subject
10000
11000
12000
13000
14000
15000
16000
17000
19000
21000
23000
64000
68000
70000
71000
72000
73000
74000
75000
76000
77000
78000
79000
80000
81000
82000
83000
84000
86000
87000
89000
93000
97000
Condition
EM
Primary

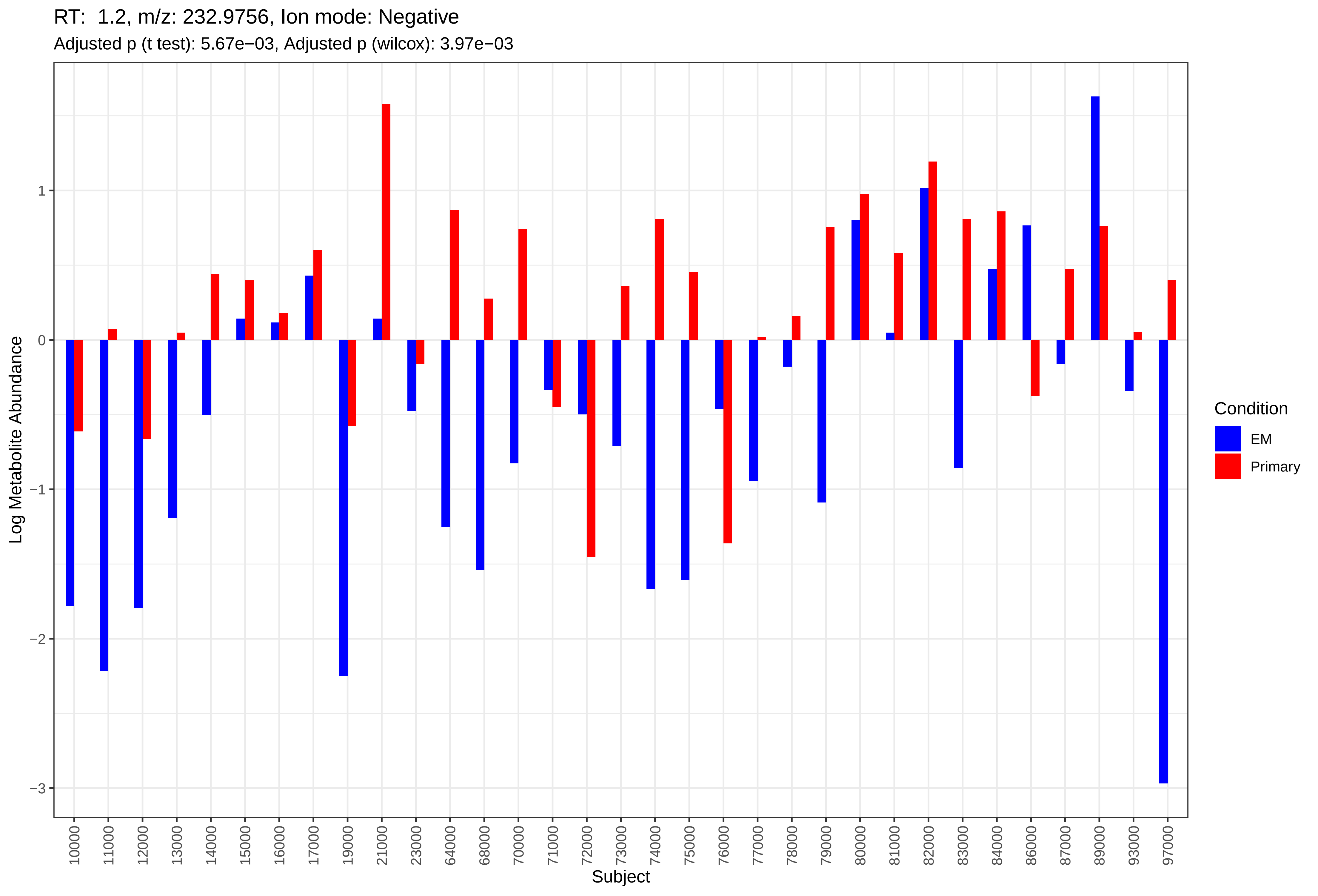

RT: 1.2, m/z: 232.9756, Ion mode: Negative
Adjusted p (t test): 5.67e−03, Adjusted p (wilcox): 3.97e−03
Log Metabolite Abundance
1
0
−1
−2
−3
10000
11000
12000
13000
14000
15000
16000
17000
19000
21000
23000
64000
68000
70000
71000
72000
73000
74000
75000
76000
77000
78000
79000
80000
81000
82000
83000
84000
86000
87000
89000
93000
97000
Subject
Condition
EM
Primary

HYPOXANTHINE (RT: 2.0, m/z: 135.0311, Ion mode: Negative)

Adjusted p (t test): 4.89e−03, Adjusted p (wilcox): 3.97e−03

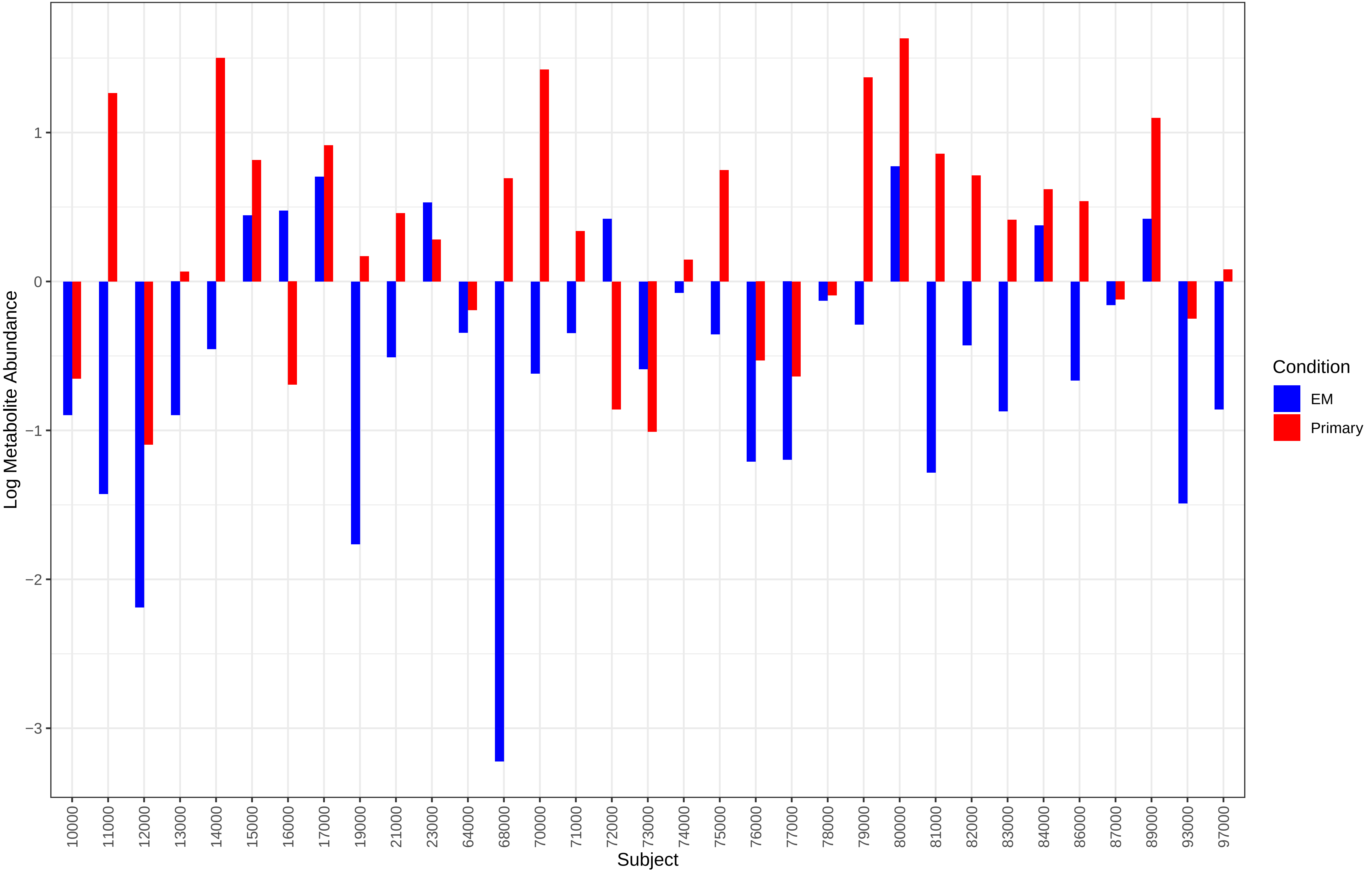

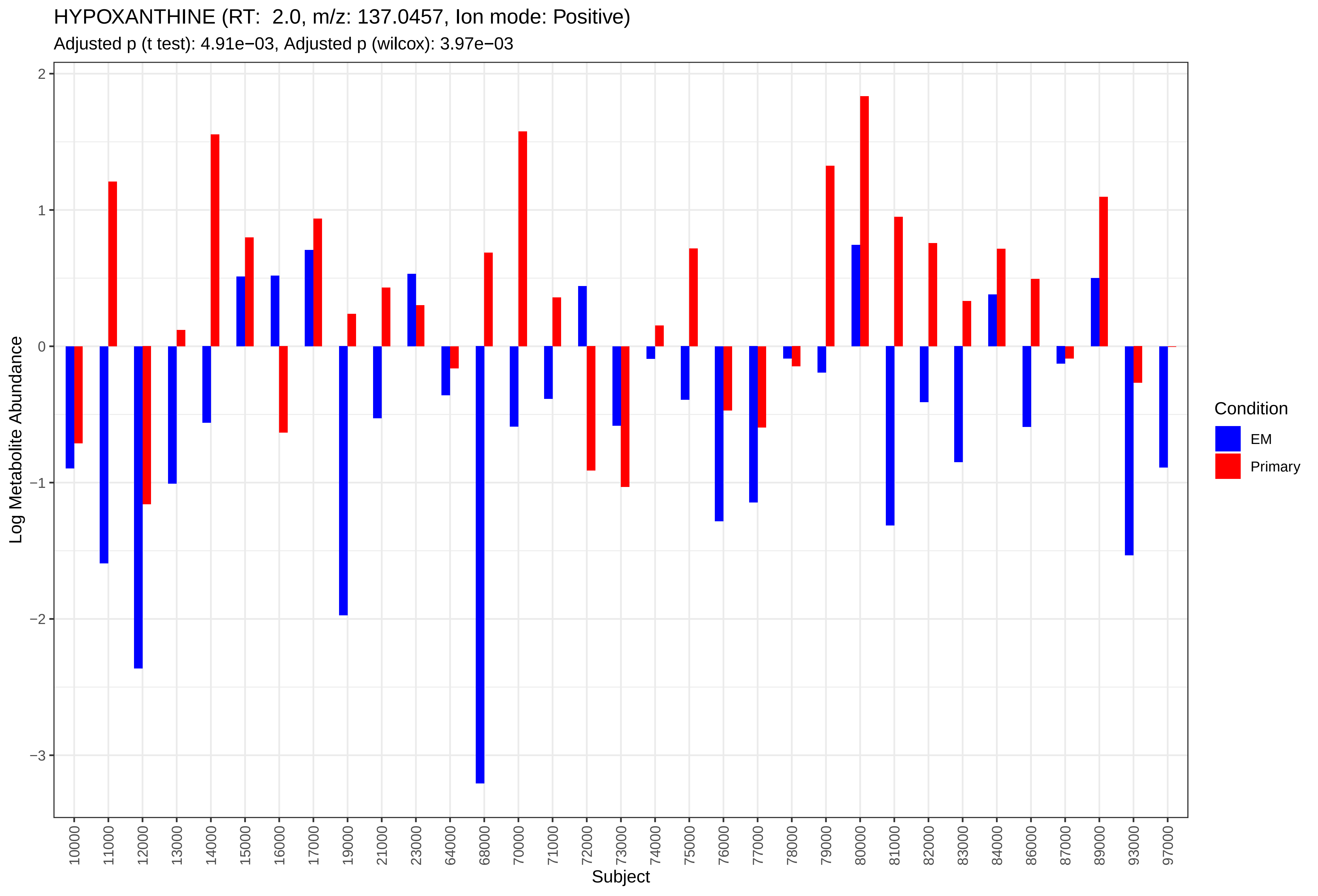
HYPOXANTHINE (RT: 2.0, m/z: 137.0457, Ion mode: Positive)
Adjusted p (t test): 4.91e−03, Adjusted p (wilcox): 3.97e−03
Log Metabolite Abundance
2
1
0
−1
−2
−3
10000
11000
12000
13000
14000
15000
16000
17000
19000
21000
23000
64000
68000
70000
71000
72000
73000
74000
75000
76000
77000
78000
79000
80000
81000
82000
83000
84000
86000
87000
89000
93000
97000
Subject
Condition
EM
Primary

ALDO/KETO−HEXOSE (RT: 0.7, m/z: 203.0522, Ion mode: Positive)

Adjusted p (t test): 5.29e−03, Adjusted p (wilcox): 3.97e−03

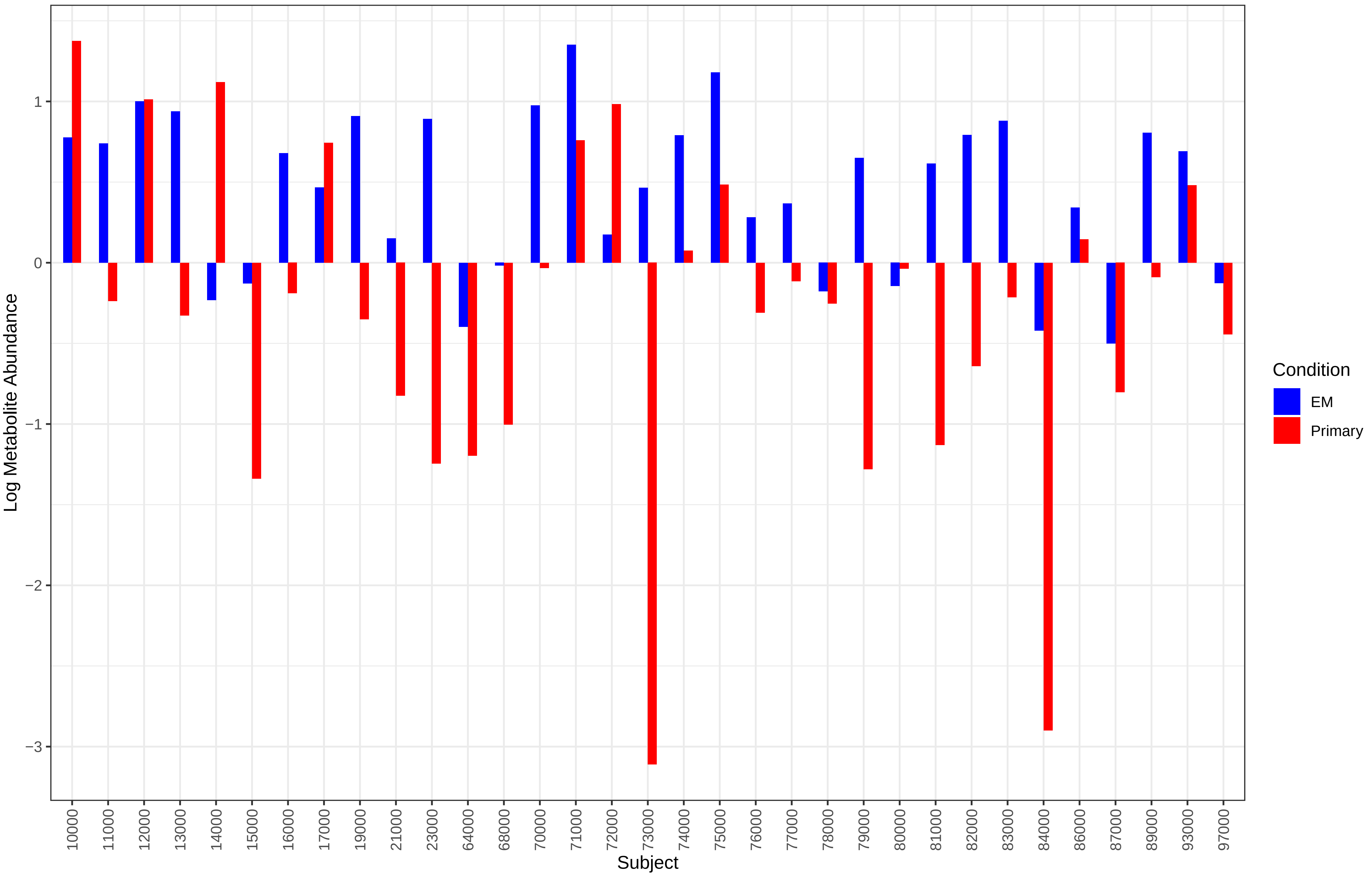

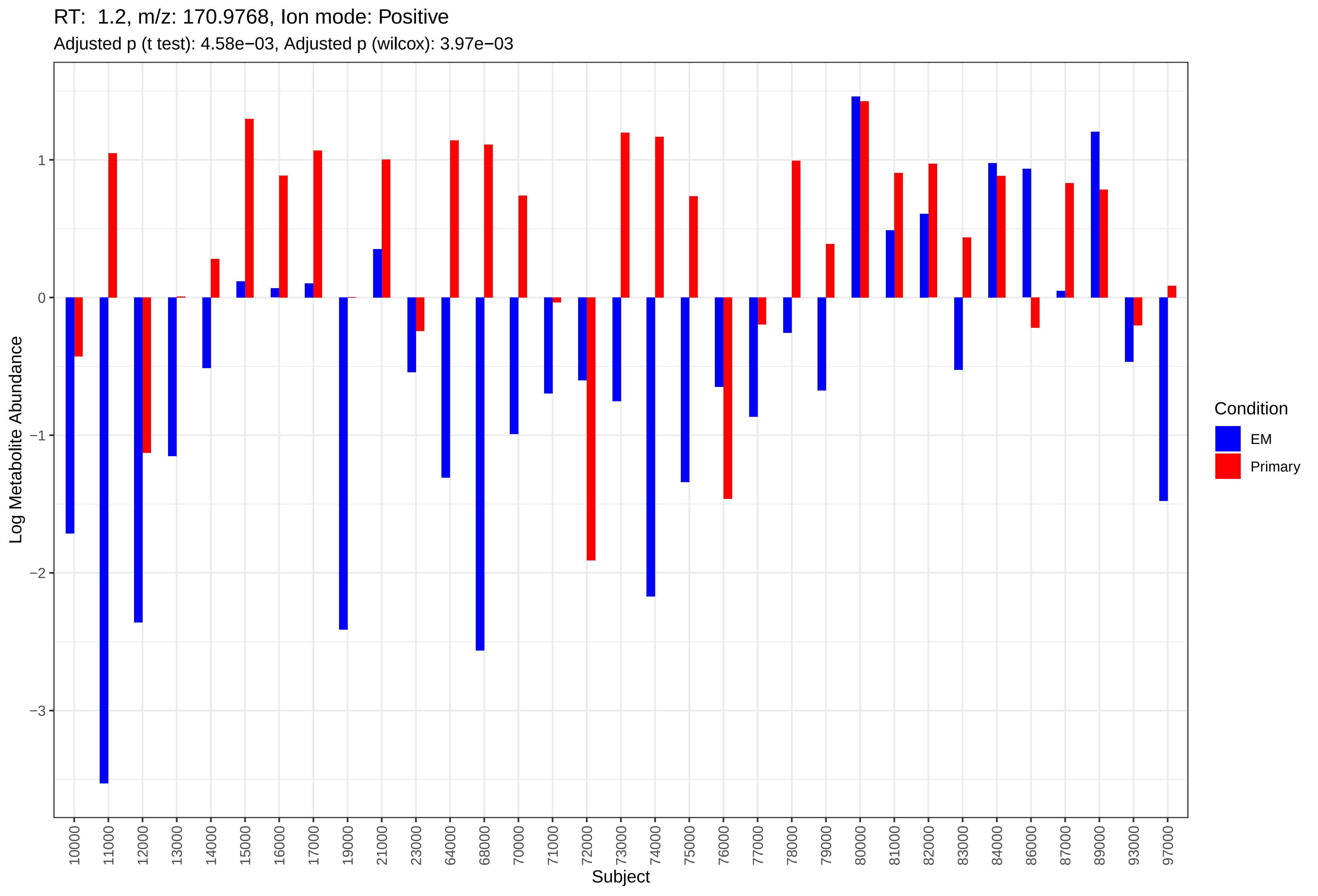
RT: 1.2, m/z: 170.9768, Ion mode: Positive
Adjusted p (t test): 4.58e−03, Adjusted p (wilcox): 3.97e−03
Log Metabolite Abundance
1
0
−1
−2
−3
10000
11000
12000
13000
14000
15000
16000
17000
19000
21000
23000
64000
68000
70000
71000
72000
73000
74000
75000
76000
77000
78000
79000
80000
81000
82000
83000
84000
86000
87000
89000
93000
97000
Subject
Condition
EM
Primary

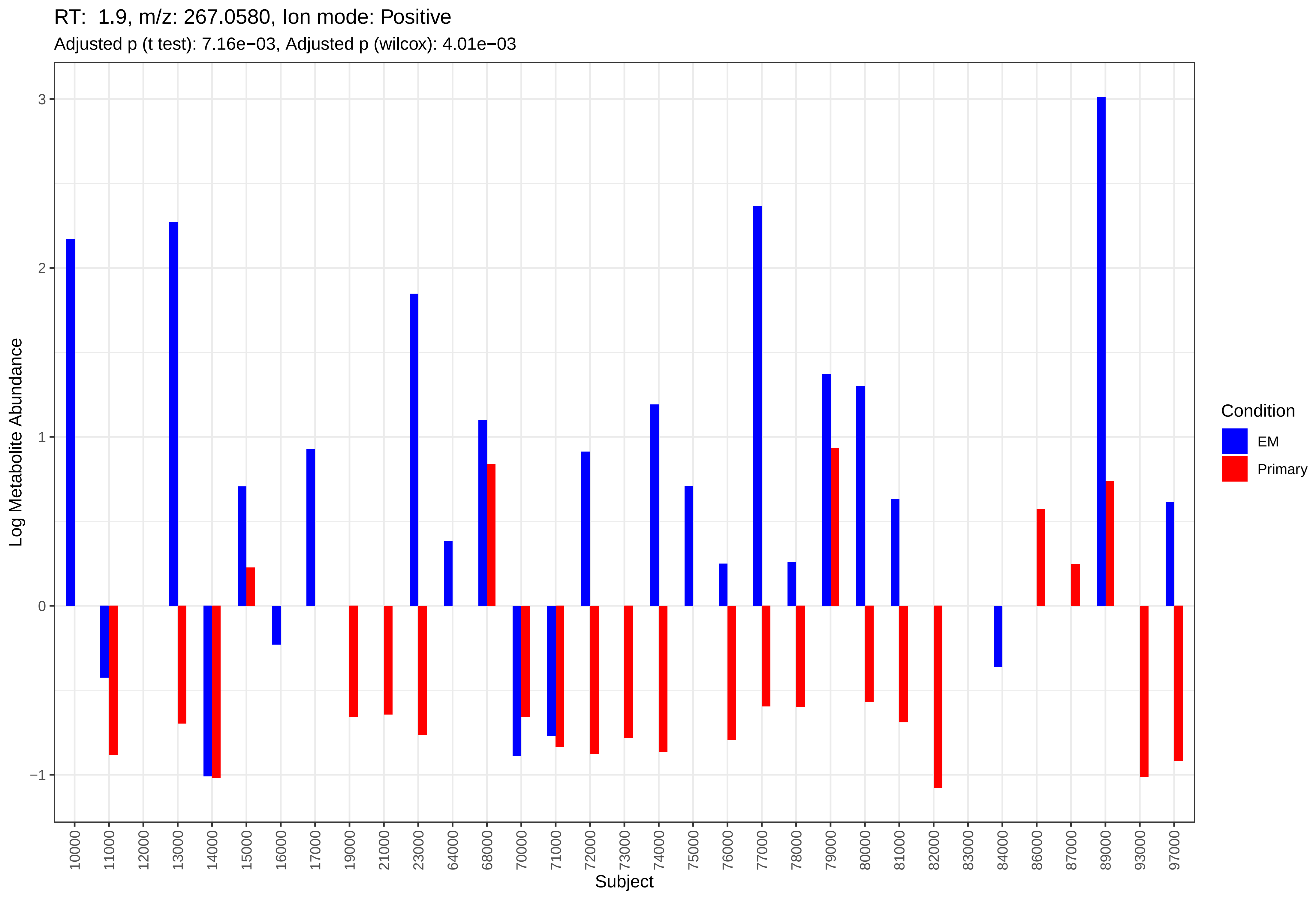

RT: 1.9, m/z: 267.0580, Ion mode: Positive
Adjusted p (t test): 7.16e−03, Adjusted p (wilcox): 4.01e−03
Log Metabolite Abundance
3
2
1
0
−1
10000
11000
12000
13000
14000
15000
16000
17000
19000
21000
23000
64000
68000
70000
71000
72000
73000
74000
75000
76000
77000
78000
79000
80000
81000
82000
83000
84000
86000
87000
89000
93000
97000
Subject
Condition
EM
Primary

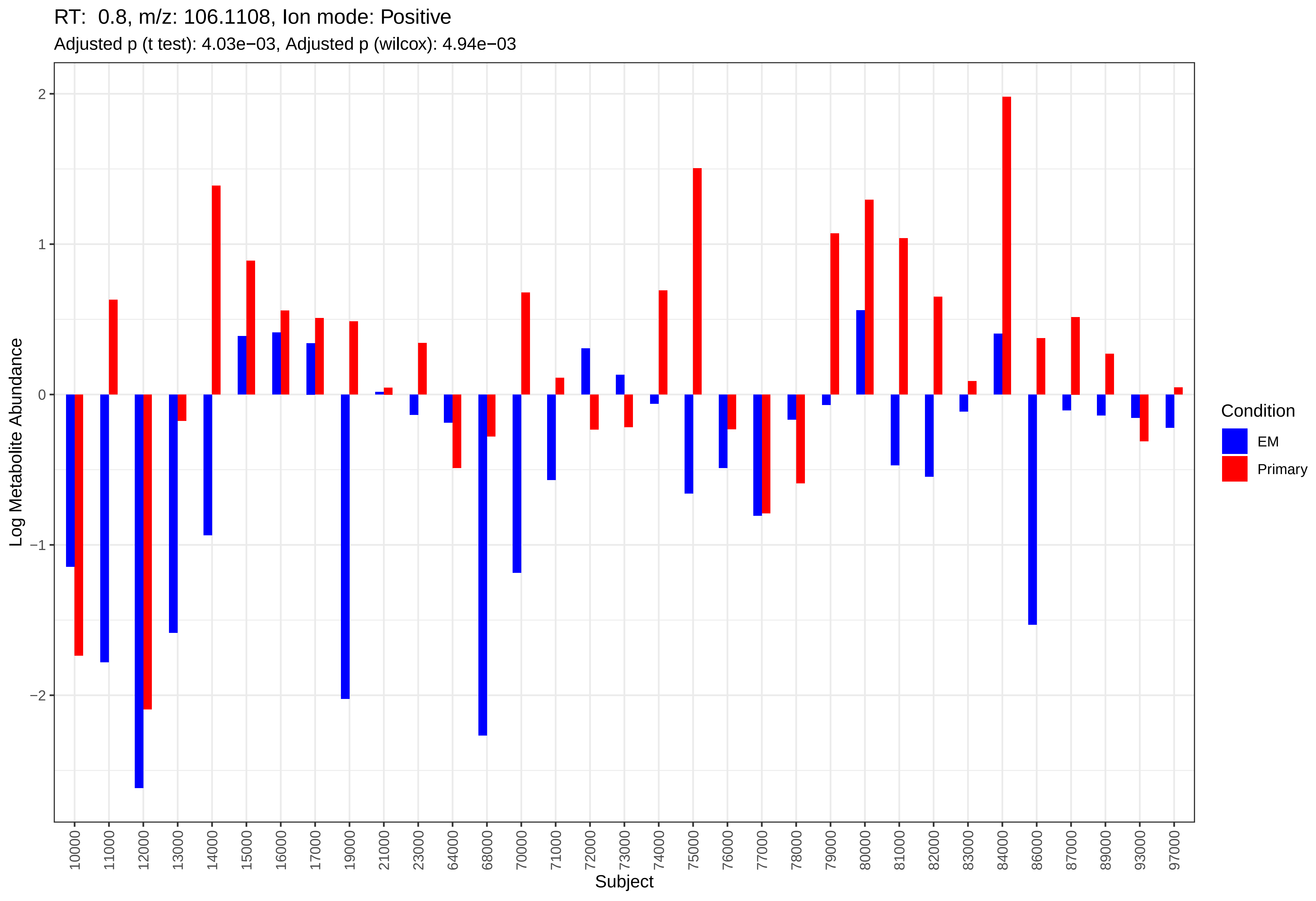

RT: 0.8, m/z: 106.1108, Ion mode: Positive
Adjusted p (t test): 4.03e−03, Adjusted p (wilcox): 4.94e−03
Log Metabolite Abundance
2
1
0
−1
−2
10000
11000
12000
13000
14000
15000
16000
17000
19000
21000
23000
64000
68000
70000
71000
72000
73000
74000
75000
76000
77000
78000
79000
80000
81000
82000
83000
84000
86000
87000
89000
93000
97000
Subject
Condition
EM
Primary

RT: 6.6, m/z: 155.1189, Ion mode: Positive

Adjusted p (t test): 4.13e−03, Adjusted p (wilcox): 4.27e−03

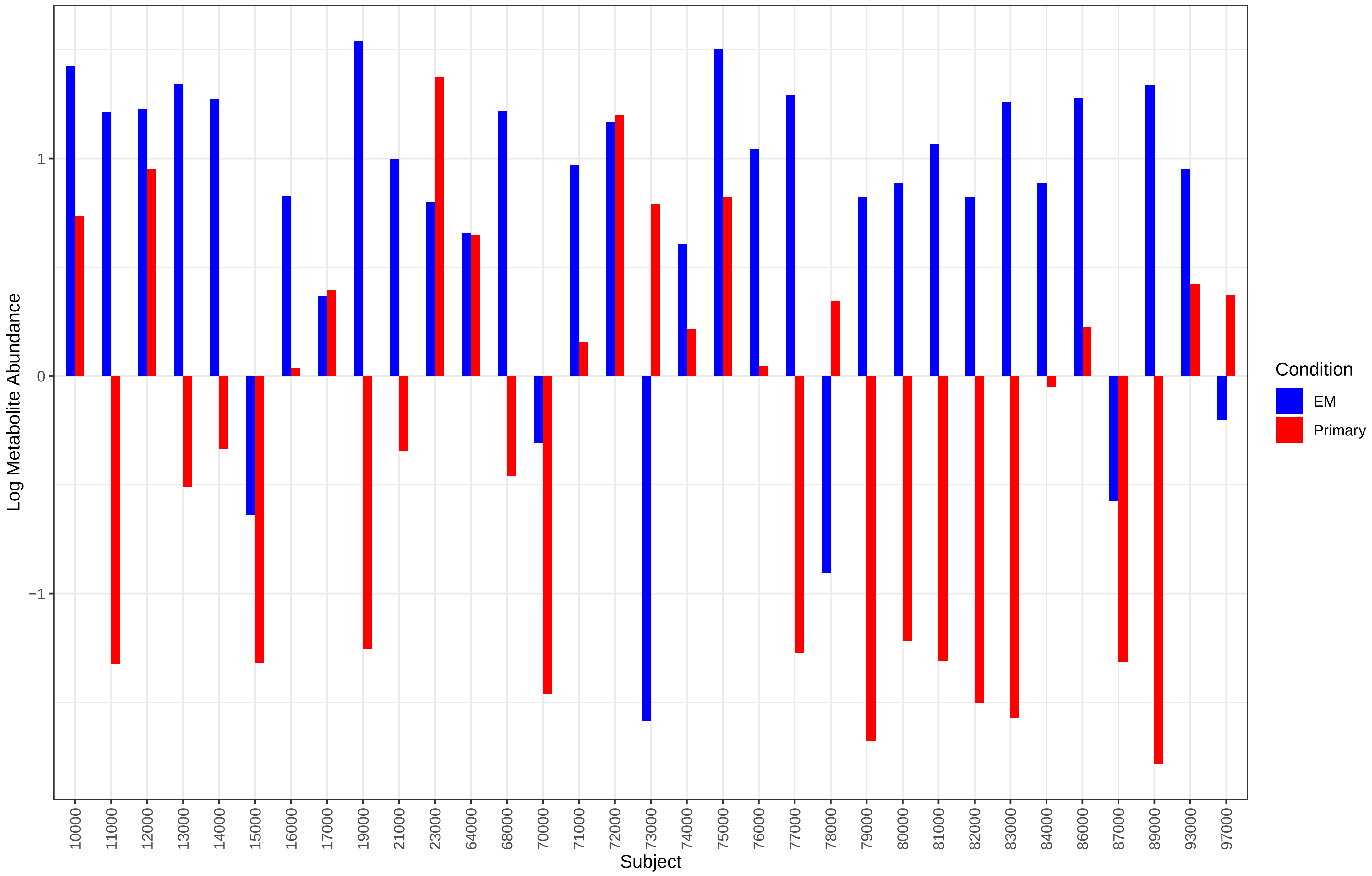

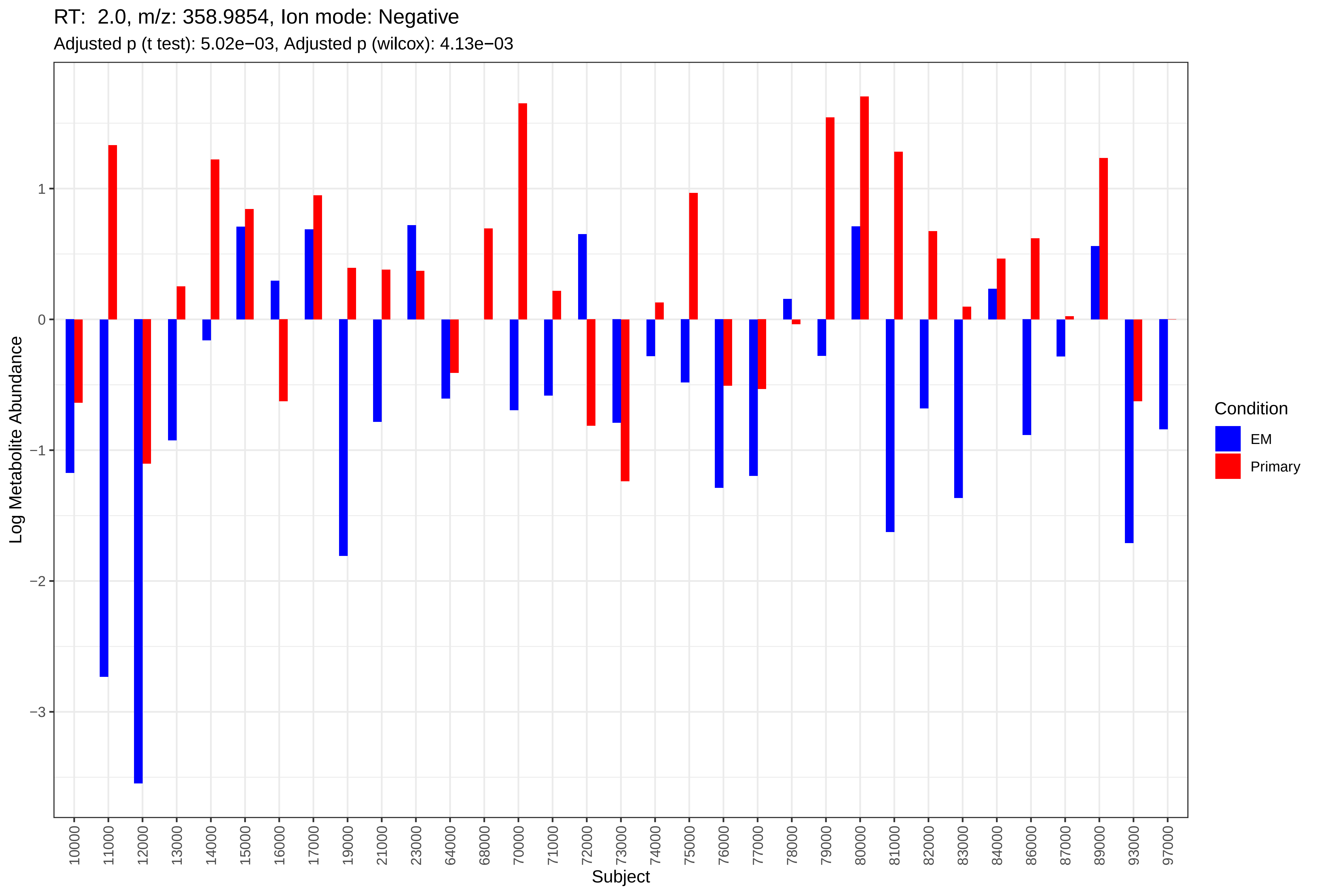
RT: 2.0, m/z: 358.9854, Ion mode: Negative
Adjusted p (t test): 5.02e−03, Adjusted p (wilcox): 4.13e−03
Log Metabolite Abundance
1
0
−1
−2
−3
Subject
10000
11000
12000
13000
14000
15000
16000
17000
19000
21000
23000
64000
68000
70000
71000
72000
73000
74000
75000
76000
77000
78000
79000
80000
81000
82000
83000
84000
86000
87000
89000
93000
97000
Condition
EM
Primary

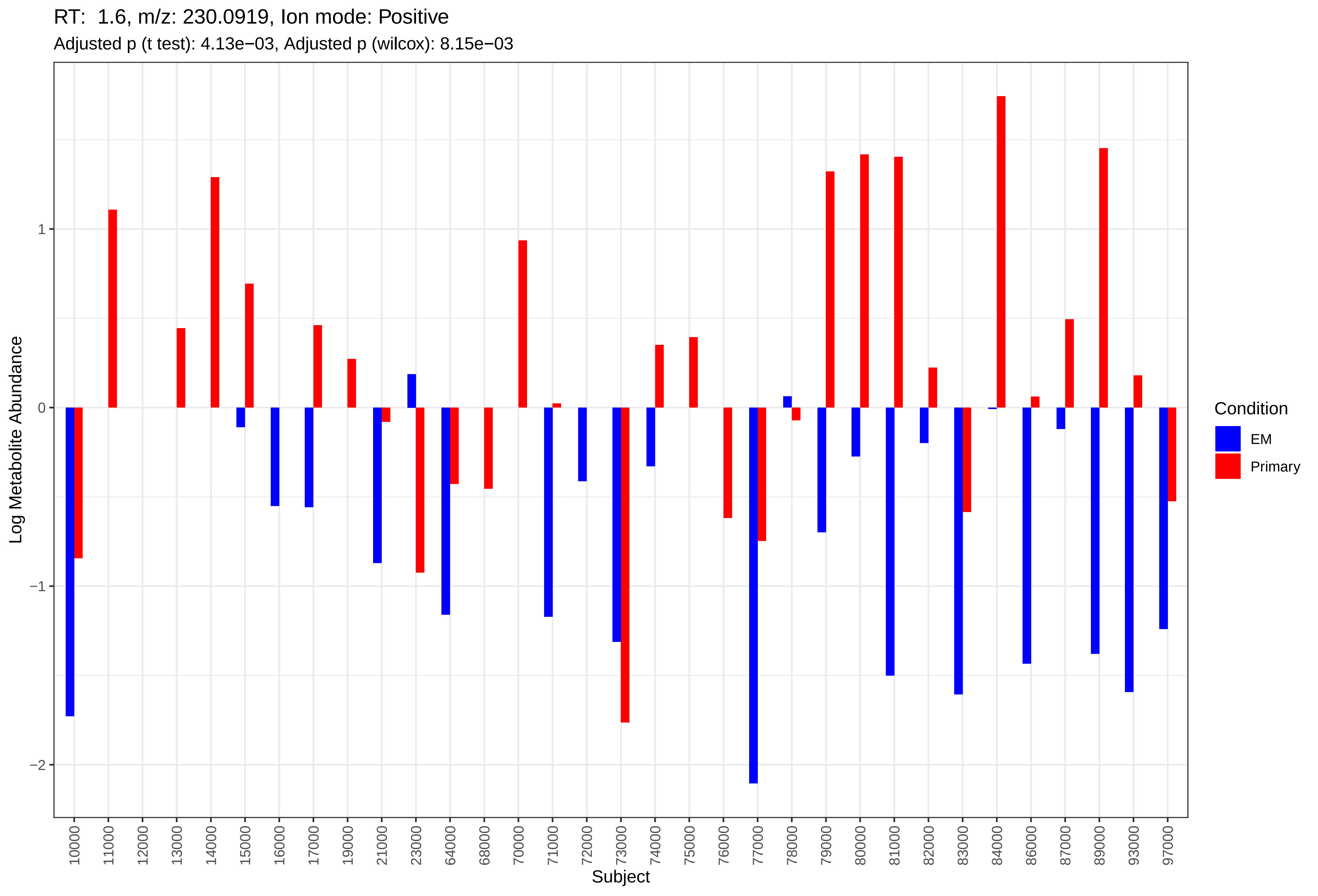

RT: 1.6, m/z: 230.0919, Ion mode: Positive
Adjusted p (t test): 4.13e−03, Adjusted p (wilcox): 8.15e−03
Log Metabolite Abundance
1
0
−1
−2
10000
11000
12000
13000
14000
15000
16000
17000
19000
21000
23000
64000
68000
70000
71000
72000
73000
74000
75000
76000
77000
78000
79000
80000
81000
82000
83000
84000
86000
87000
89000
93000
97000
Subject
Condition
EM
Primary

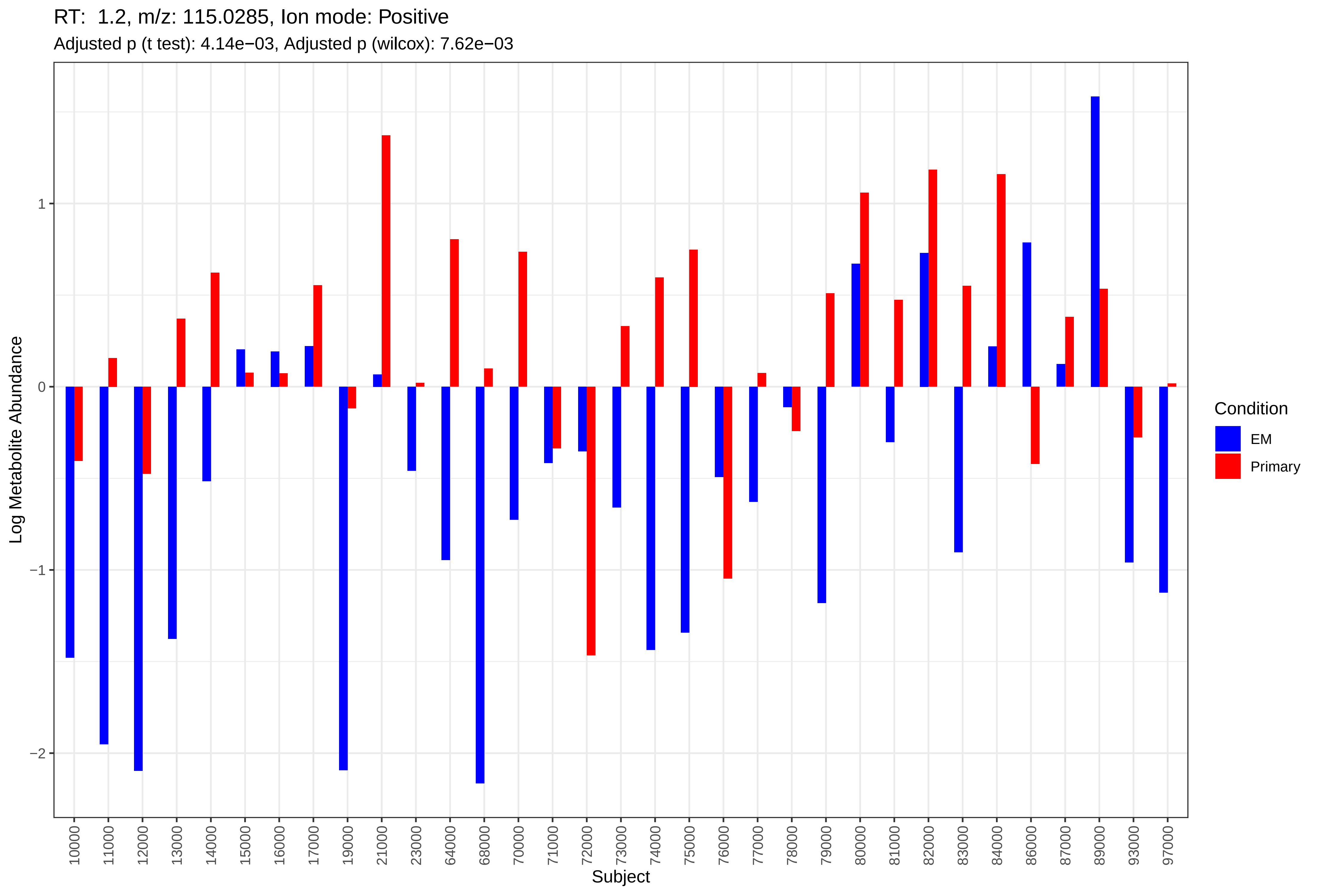
RT: 1.2, m/z: 115.0285, Ion mode: Positive
Adjusted p (t test): 4.14e−03, Adjusted p (wilcox): 7.62e−03
Log Metabolite Abundance
1
0
−1
−2
Subject
10000
11000
12000
13000
14000
15000
16000
17000
19000
21000
23000
64000
68000
70000
71000
72000
73000
74000
75000
76000
77000
78000
79000
80000
81000
82000
83000
84000
86000
87000
89000
93000
97000
Condition
EM
Primary

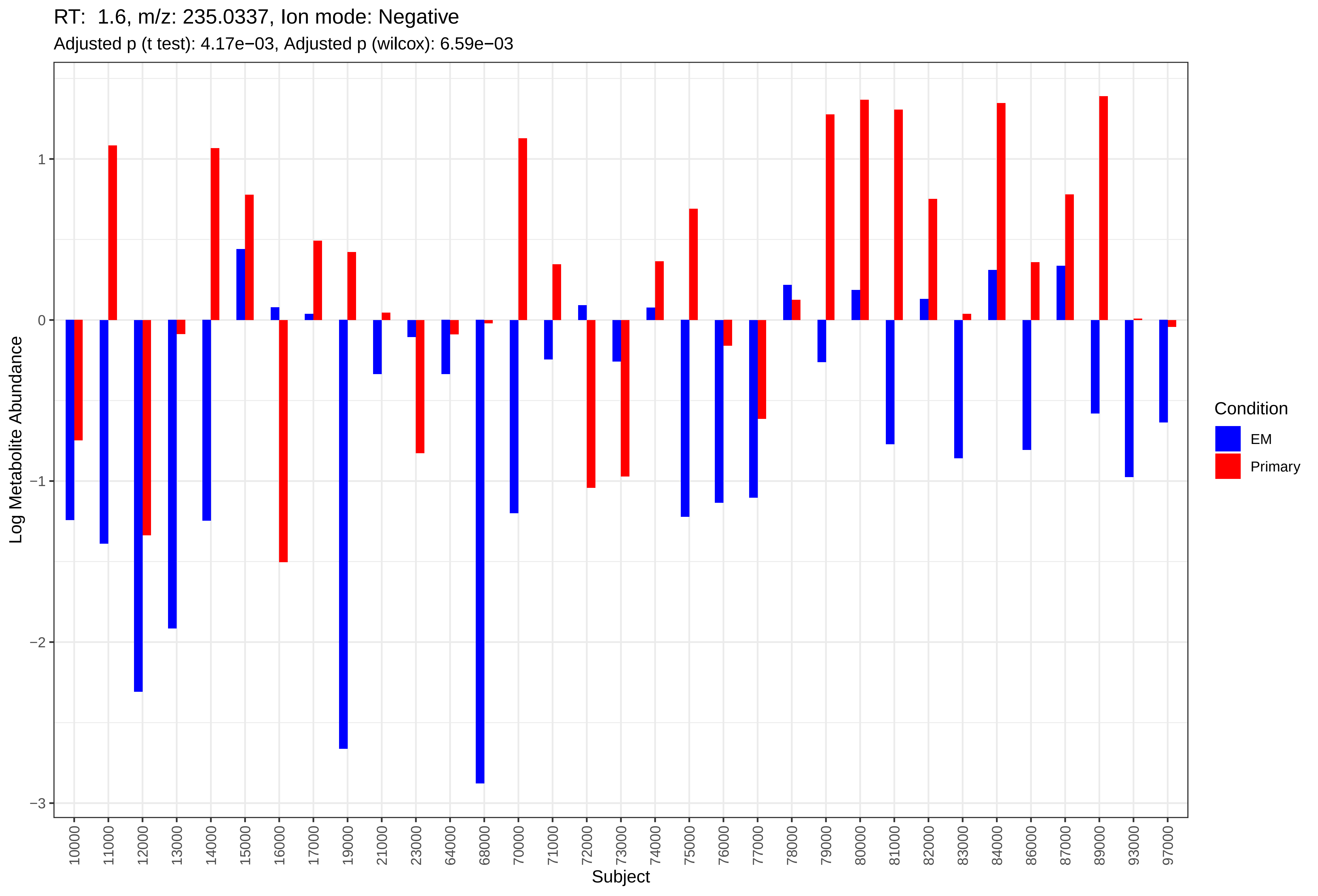
RT: 1.6, m/z: 235.0337, Ion mode: Negative
Adjusted p (t test): 4.17e−03, Adjusted p (wilcox): 6.59e−03
Log Metabolite Abundance
1
0
−1
−2
−3
10000
11000
12000
13000
14000
15000
16000
17000
19000
21000
23000
64000
68000
70000
71000
72000
73000
74000
75000
76000
77000
78000
79000
80000
81000
82000
83000
84000
86000
87000
89000
93000
97000
Subject
Condition
EM
Primary

RT: 8.2, m/z: 218.9997, Ion mode: Negative

Adjusted p (t test): 4.17e−03, Adjusted p (wilcox): 6.21e−03

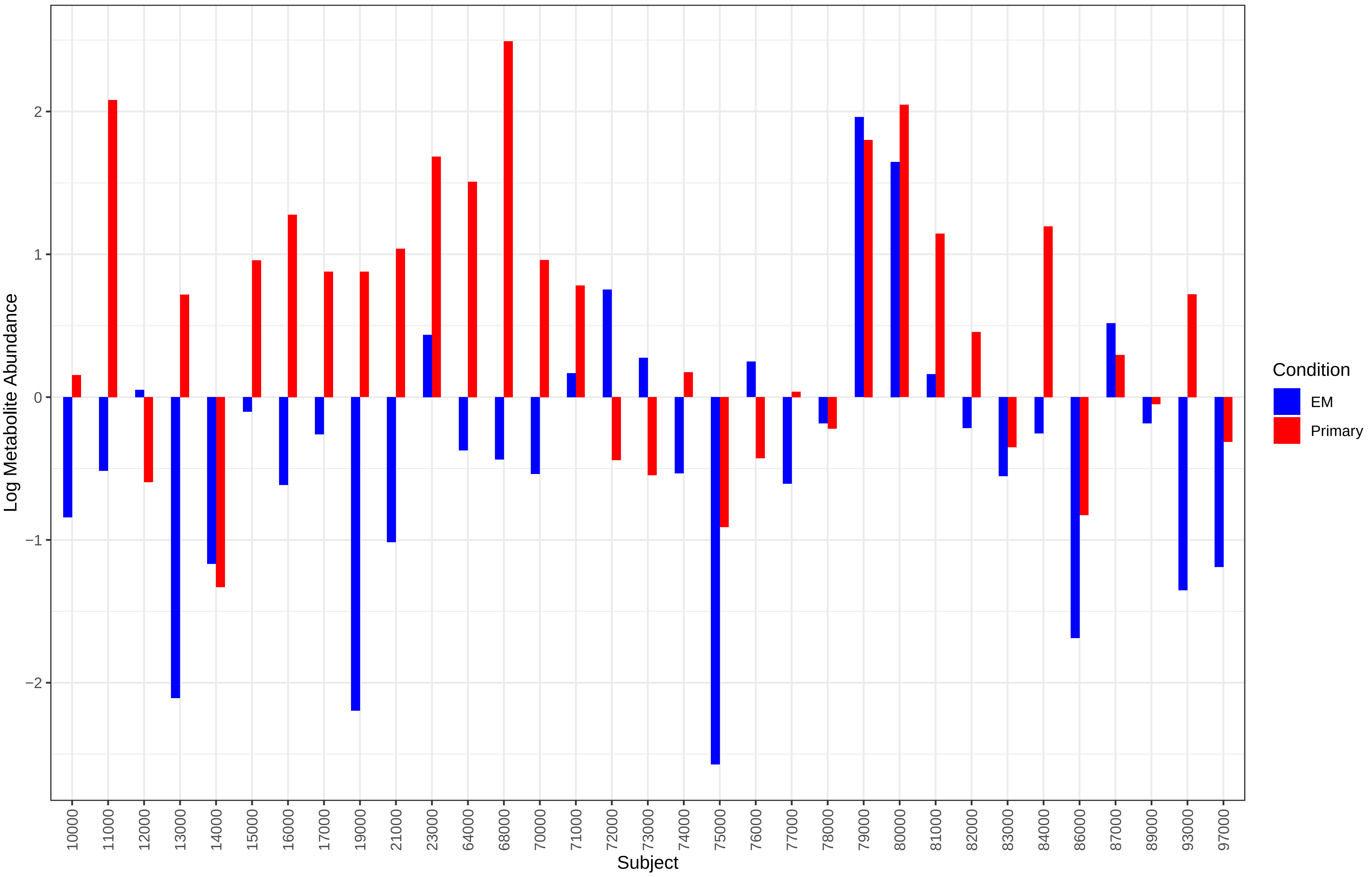

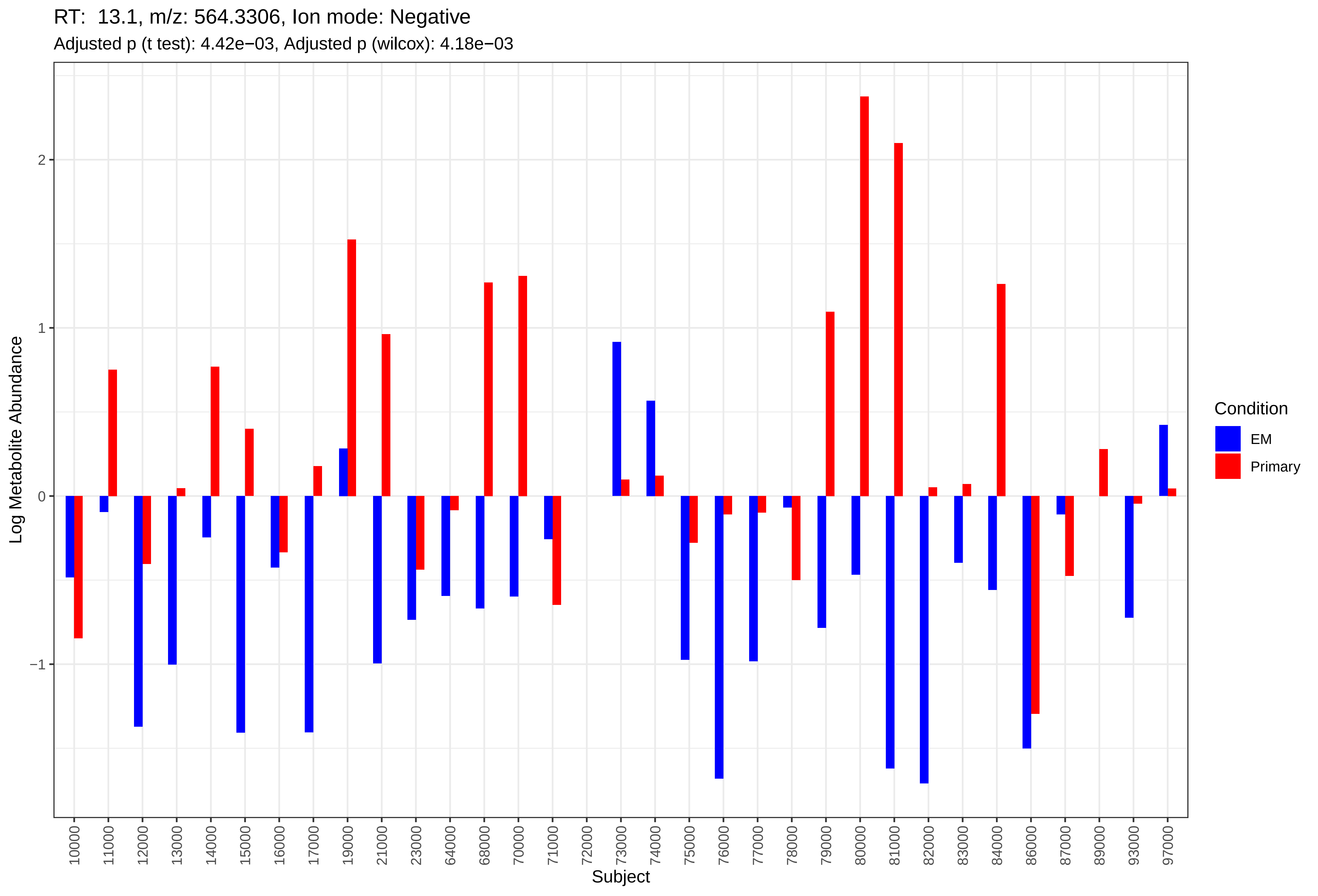
RT: 13.1, m/z: 564.3306, Ion mode: Negative
Adjusted p (t test): 4.42e−03, Adjusted p (wilcox): 4.18e−03
Log Metabolite Abundance
2
1
0
−1
Subject
10000
11000
12000
13000
14000
15000
16000
17000
19000
21000
23000
64000
68000
70000
71000
72000
73000
74000
75000
76000
77000
78000
79000
80000
81000
82000
83000
84000
86000
87000
89000
93000
97000
Condition
EM
Primary

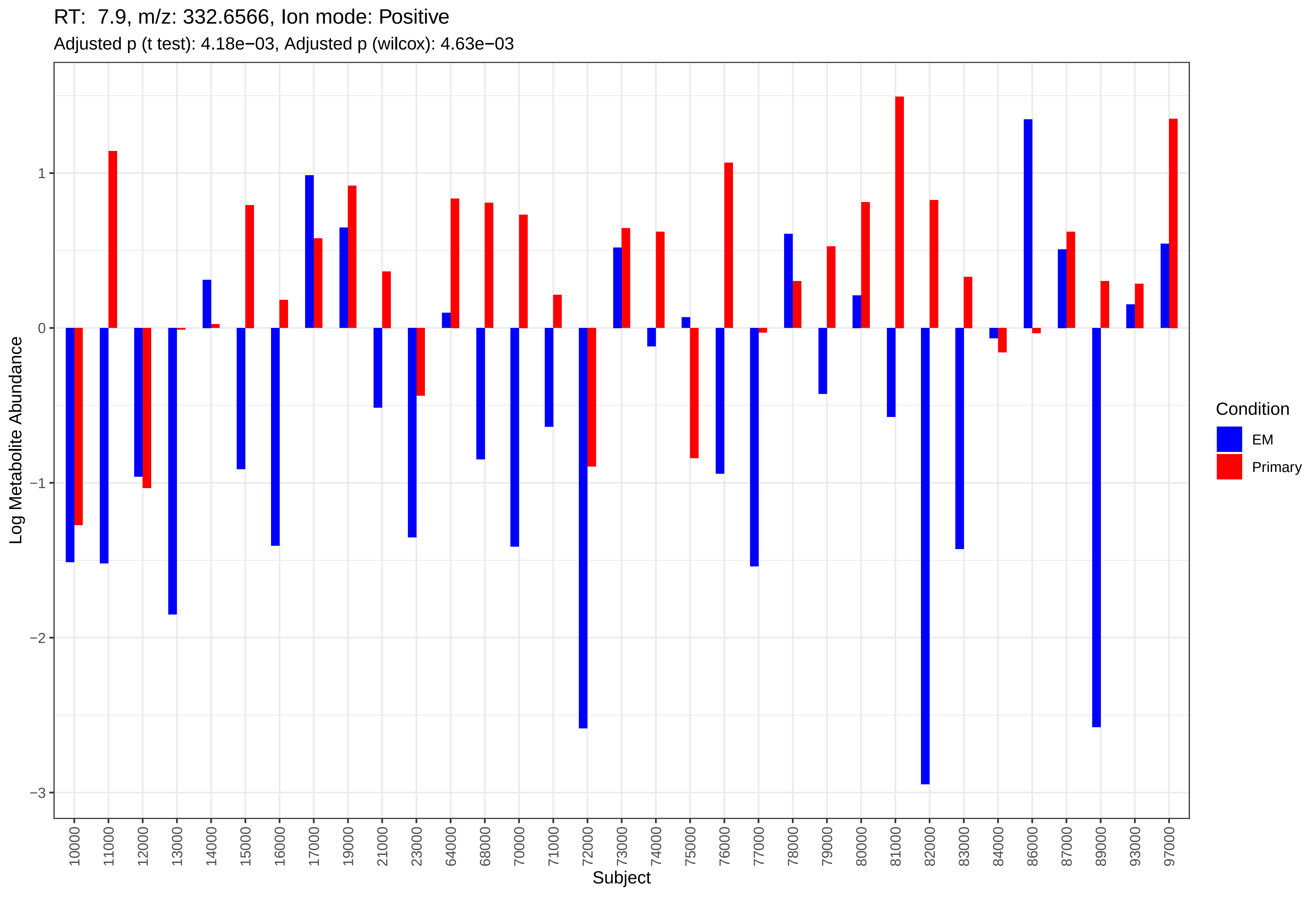
RT: 7.9, m/z: 332.6566, Ion mode: Positive
Adjusted p (t test): 4.18e−03, Adjusted p (wilcox): 4.63e−03
Log Metabolite Abundance
1
0
−1
−2
−3
10000
11000
12000
13000
14000
15000
16000
17000
19000
21000
23000
64000
68000
70000
71000
72000
73000
74000
75000
76000
77000
78000
79000
80000
81000
82000
83000
84000
86000
87000
89000
93000
97000
Subject
Condition
EM
Primary

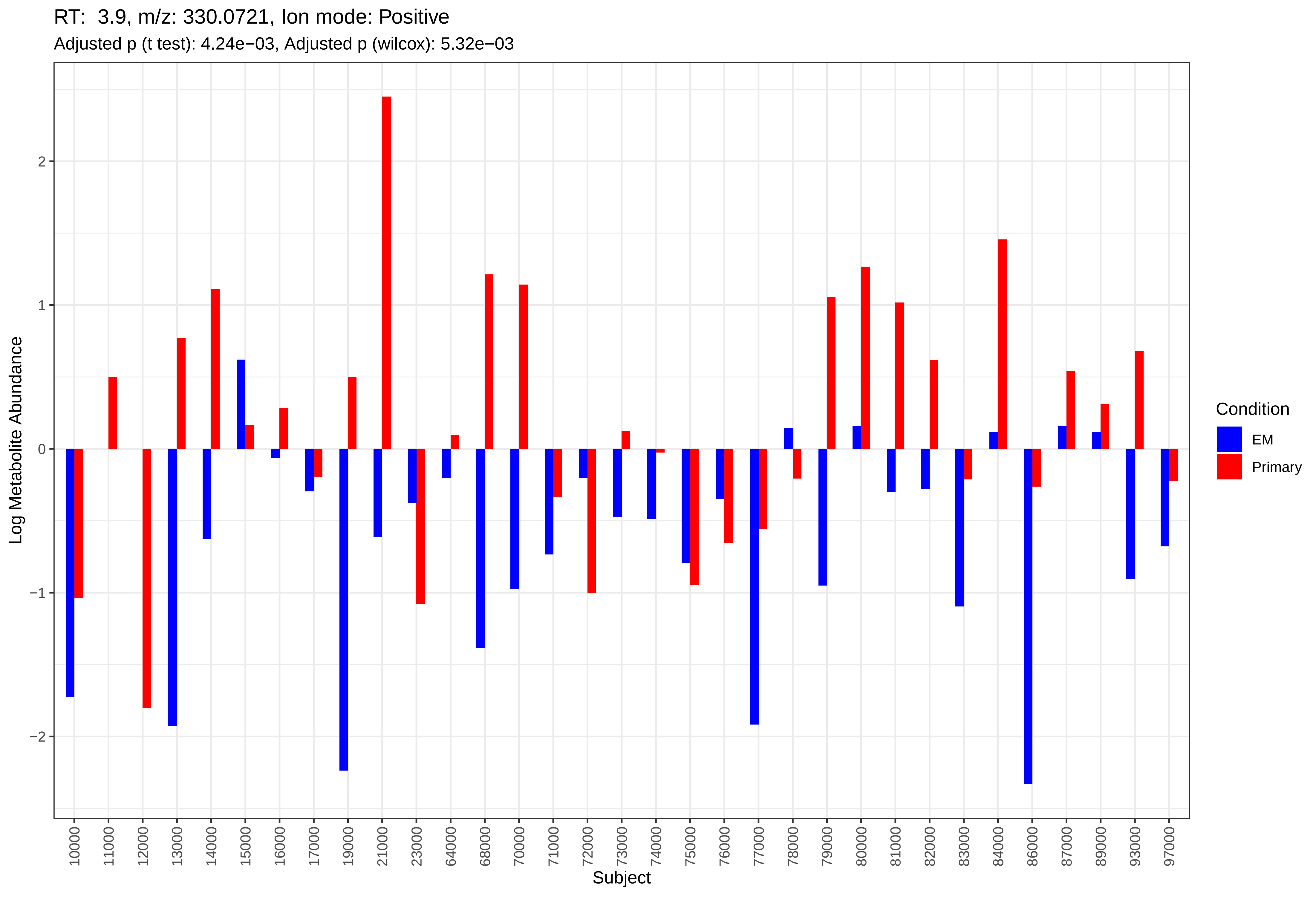

RT: 3.9, m/z: 330.0721, Ion mode: Positive
Adjusted p (t test): 4.24e−03, Adjusted p (wilcox): 5.32e−03
Log Metabolite Abundance
2
1
0
−1
−2
10000
11000
12000
13000
14000
15000
16000
17000
19000
21000
23000
64000
68000
70000
71000
72000
73000
74000
75000
76000
77000
78000
79000
80000
81000
82000
83000
84000
86000
87000
89000
93000
97000
Subject
Condition
EM
Primary

[M+Na−H] 21.9825 m/z adduct of 174.9913 m/z (RT: 1.2, m/z: 196.9732, Ion mode: Positive)

Adjusted p (t test): 4.24e−03, Adjusted p (wilcox): 8.15e−03

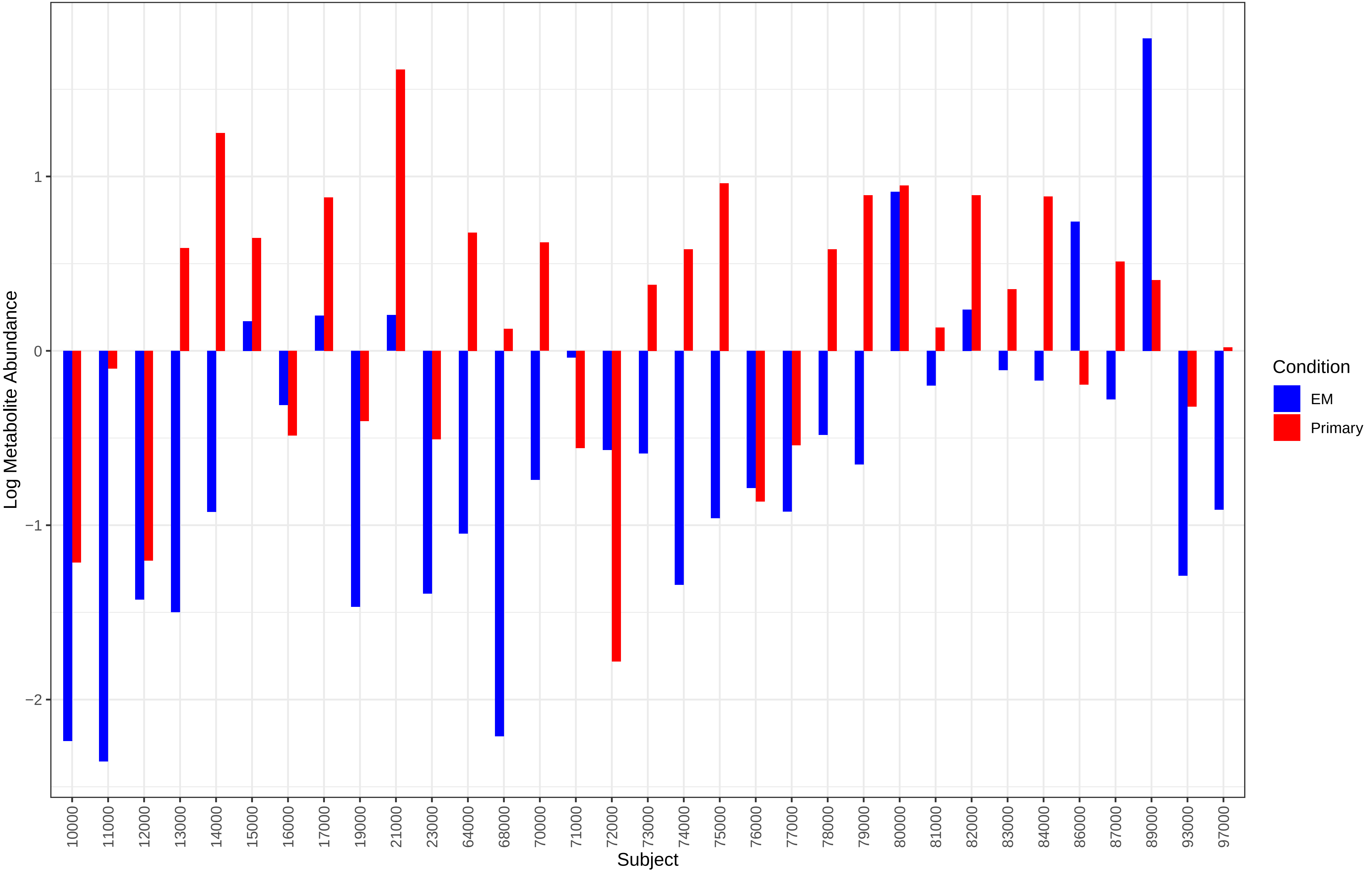

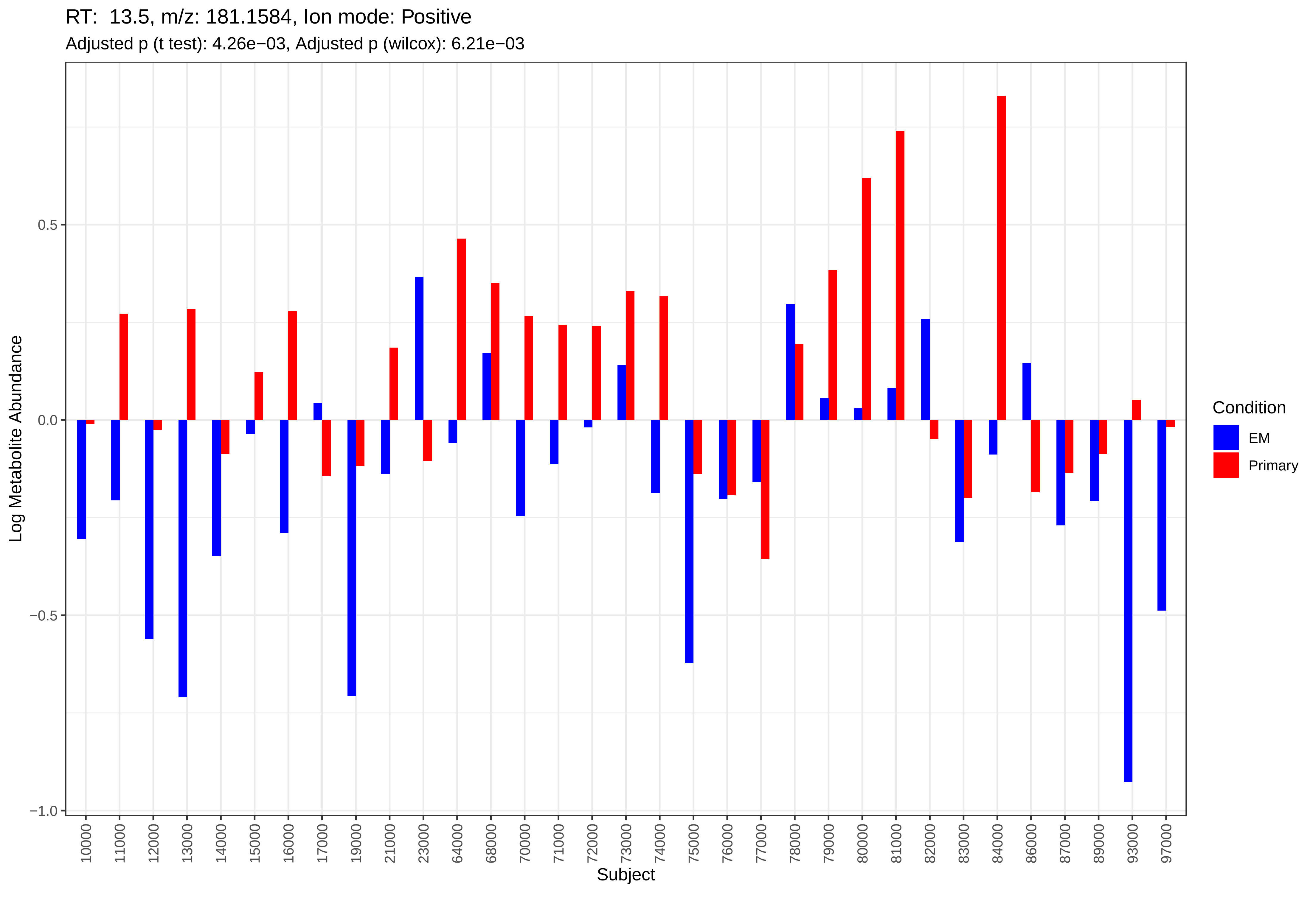
RT: 13.5, m/z: 181.1584, Ion mode: Positive
Adjusted p (t test): 4.26e−03, Adjusted p (wilcox): 6.21e−03
Log Metabolite Abundance
0.5
0.0
−0.5
−1.0
10000
11000
12000
13000
14000
15000
16000
17000
19000
21000
23000
64000
68000
70000
71000
72000
73000
74000
75000
76000
77000
78000
79000
80000
81000
82000
83000
84000
86000
87000
89000
93000
97000
Subject
Condition
EM
Primary

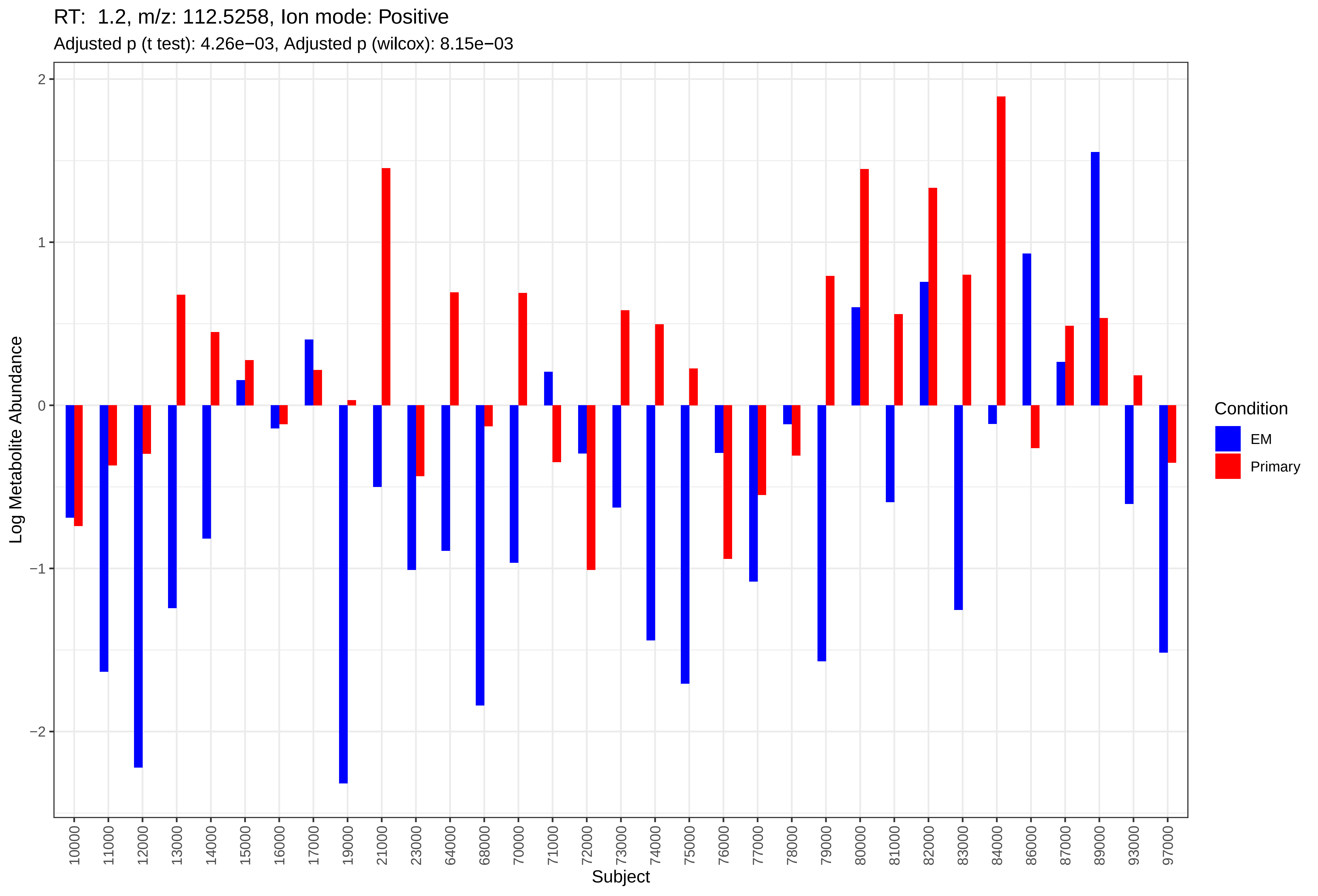

RT: 1.2, m/z: 112.5258, Ion mode: Positive
Adjusted p (t test): 4.26e−03, Adjusted p (wilcox): 8.15e−03
Log Metabolite Abundance
2
1
0
−1
−2
10000
11000
12000
13000
14000
15000
16000
17000
19000
21000
23000
64000
68000
70000
71000
72000
73000
74000
75000
76000
77000
78000
79000
80000
81000
82000
83000
84000
86000
87000
89000
93000
97000
Subject
Condition
EM
Primary

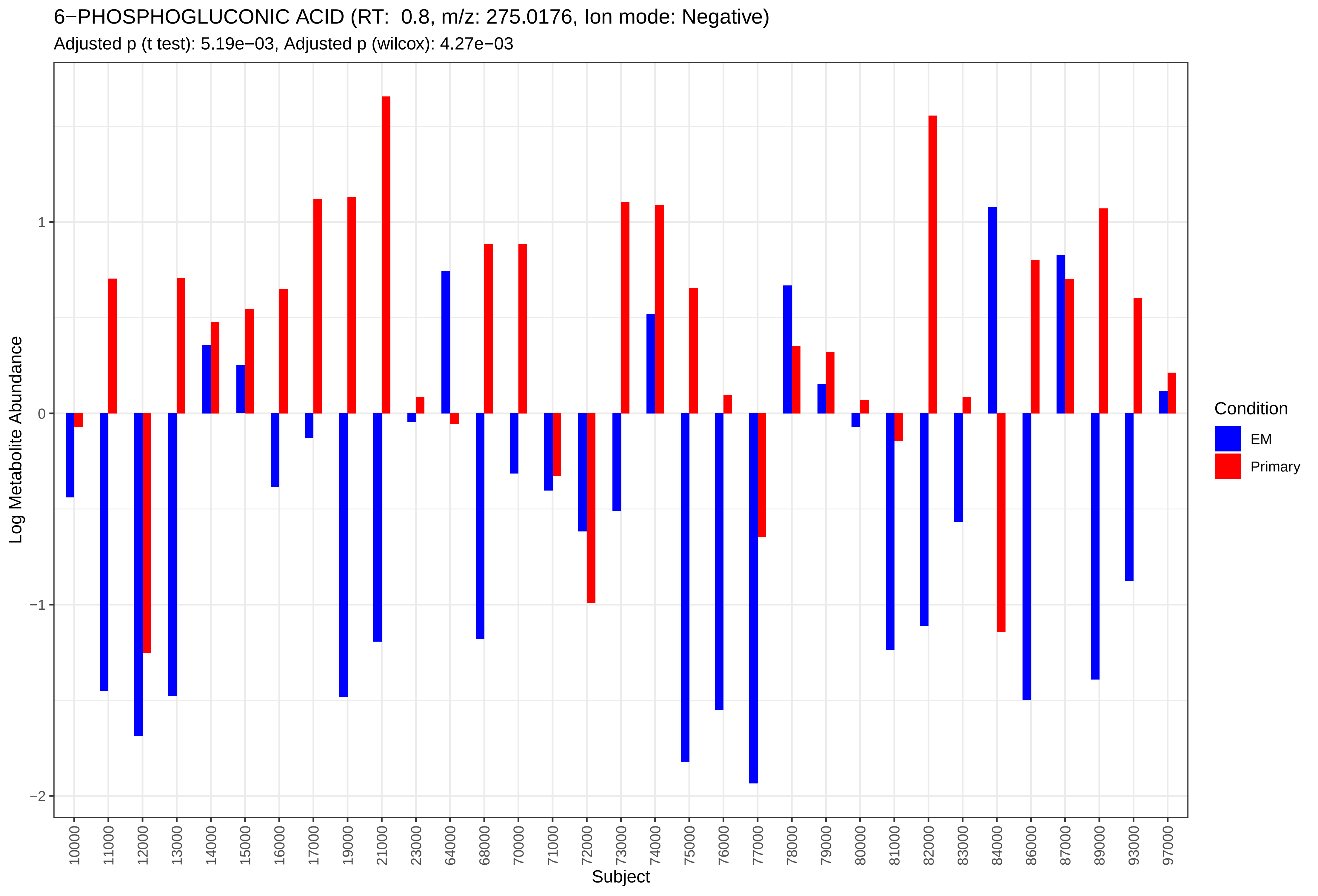

6−PHOSPHOGLUCONIC ACID (RT: 0.8, m/z: 275.0176, Ion mode: Negative)
Adjusted p (t test): 5.19e−03, Adjusted p (wilcox): 4.27e−03
Log Metabolite Abundance
1
0
−1
−2
10000
11000
12000
13000
14000
15000
16000
17000
19000
21000
23000
64000
68000
70000
71000
72000
73000
74000
75000
76000
77000
78000
79000
80000
81000
82000
83000
84000
86000
87000
89000
93000
97000
Subject
Condition
EM
Primary

RT: 2.0, m/z: 332.9768, Ion mode: Negative

Adjusted p (t test): 7.07e−03, Adjusted p (wilcox): 4.27e−03

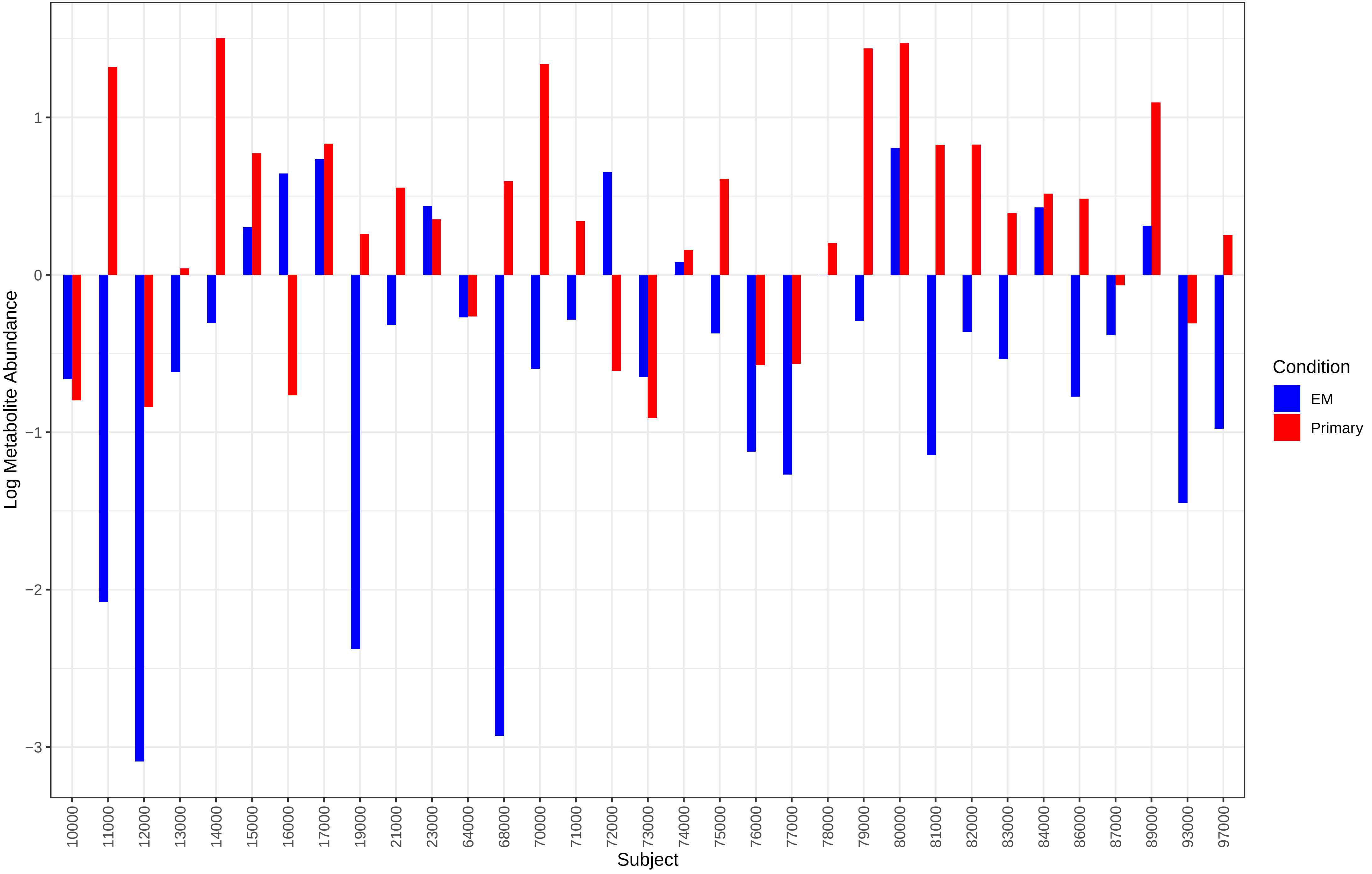

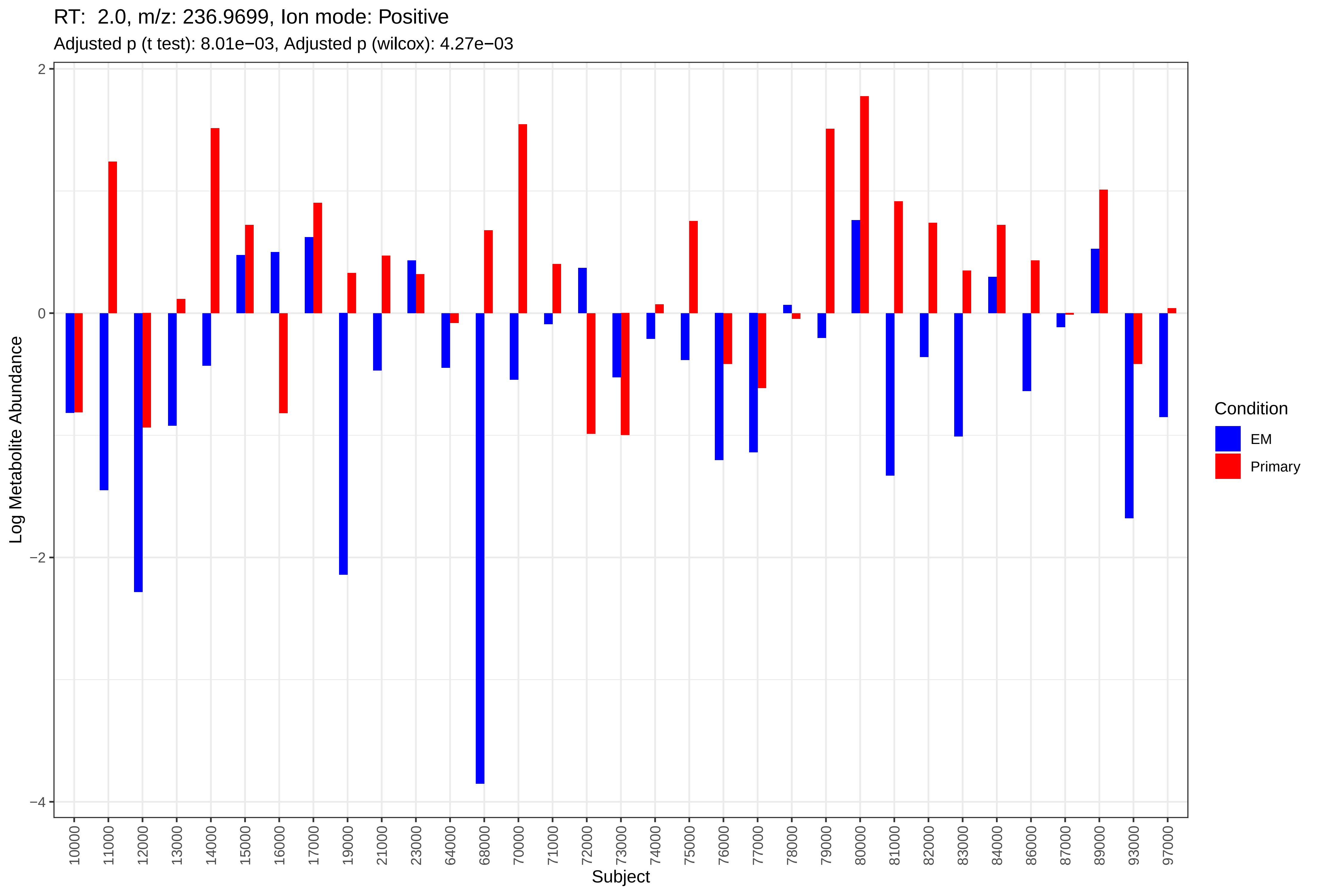

RT: 2.0, m/z: 236.9699, Ion mode: Positive
Adjusted p (t test): 8.01e−03, Adjusted p (wilcox): 4.27e−03
Log Metabolite Abundance
2
0
−2
−4
Subject
10000
11000
12000
13000
14000
15000
16000
17000
19000
21000
23000
64000
68000
70000
71000
72000
73000
74000
75000
76000
77000
78000
79000
80000
81000
82000
83000
84000
86000
87000
89000
93000
97000
Condition
EM
Primary

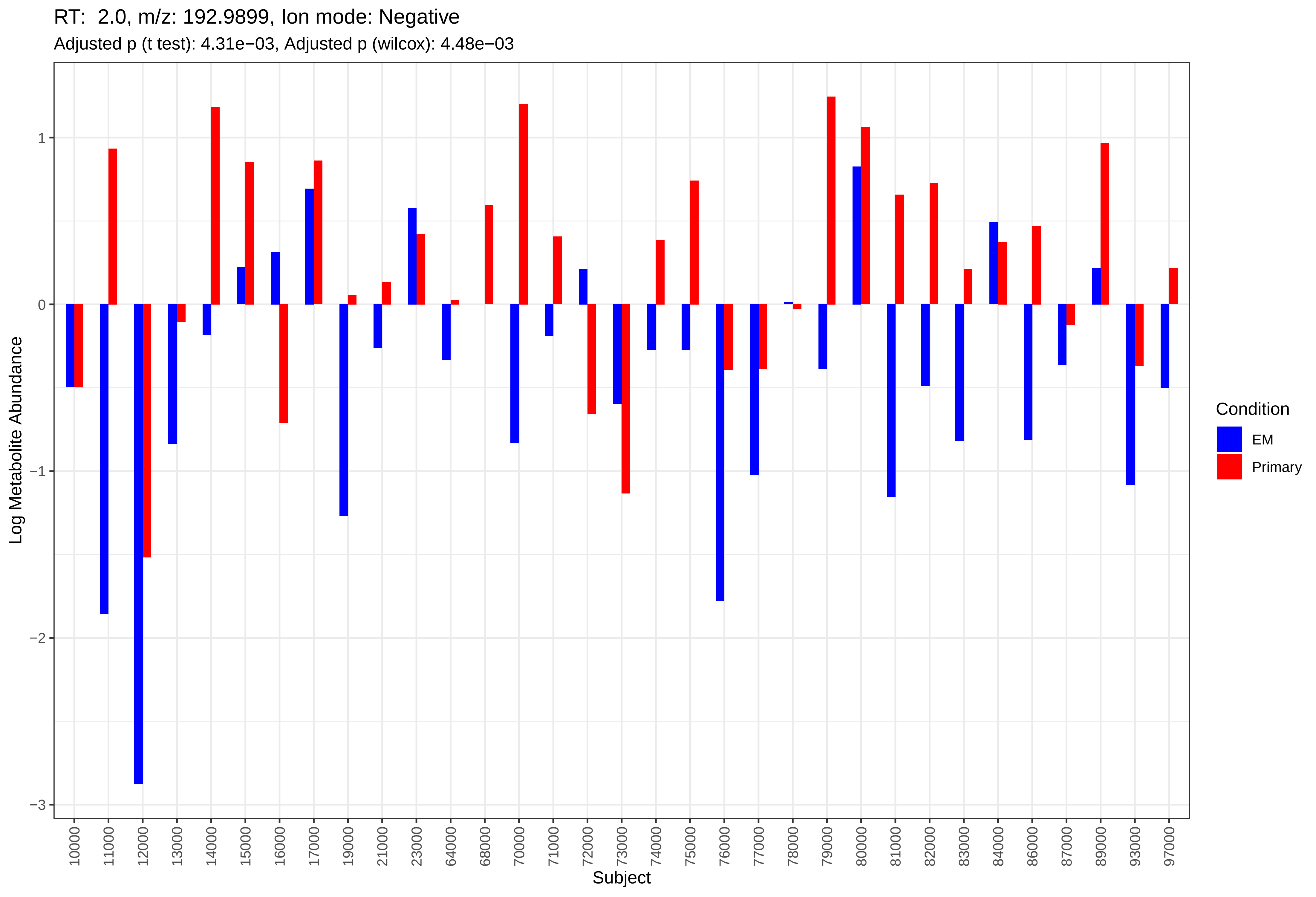

RT: 2.0, m/z: 192.9899, Ion mode: Negative
Adjusted p (t test): 4.31e−03, Adjusted p (wilcox): 4.48e−03
Log Metabolite Abundance
1
0
−1
−2
−3
10000
11000
12000
13000
14000
15000
16000
17000
19000
21000
23000
64000
68000
70000
71000
72000
73000
74000
75000
76000
77000
78000
79000
80000
81000
82000
83000
84000
86000
87000
89000
93000
97000
Subject
Condition
EM
Primary

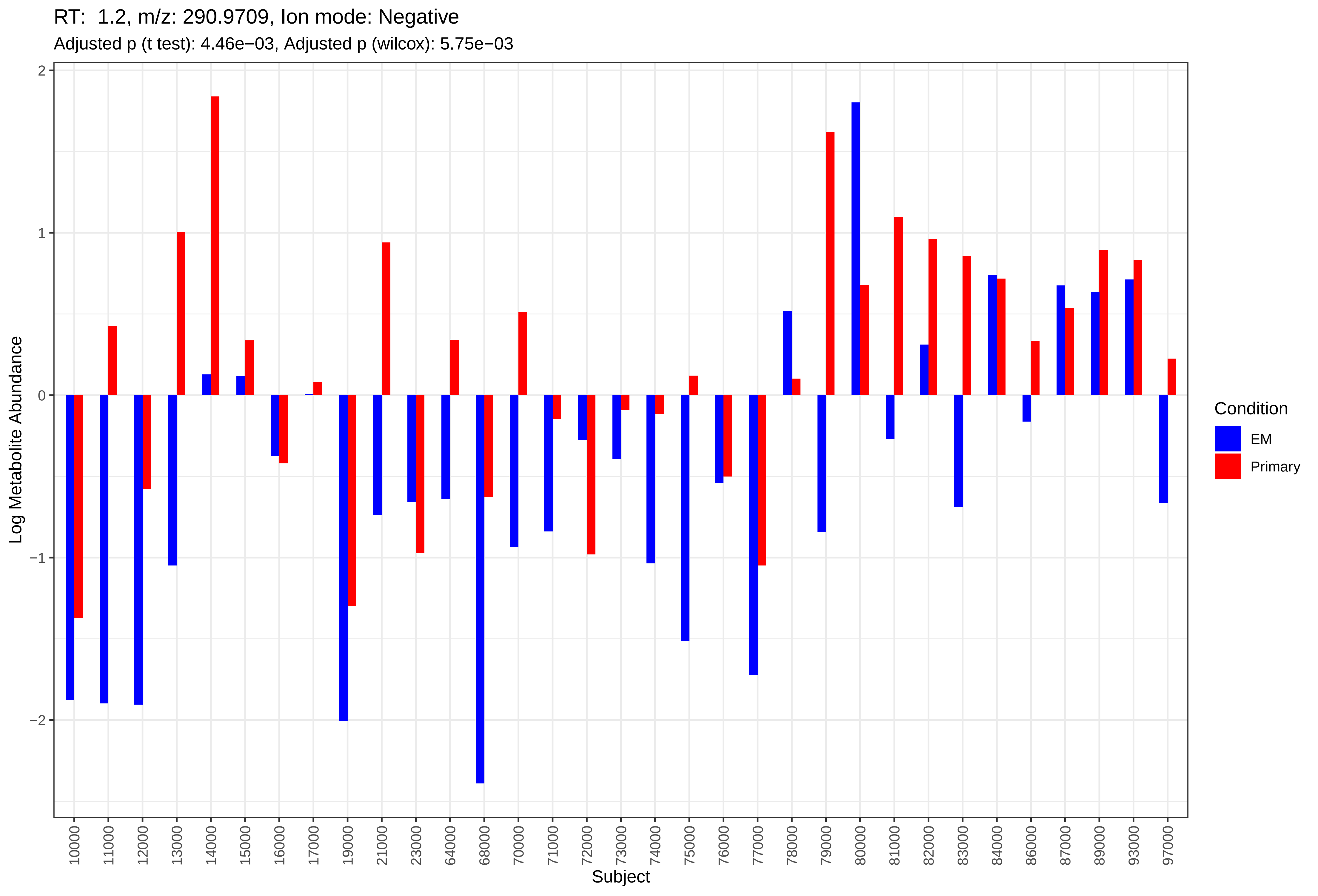
RT: 1.2, m/z: 290.9709, Ion mode: Negative
Adjusted p (t test): 4.46e−03, Adjusted p (wilcox): 5.75e−03
Log Metabolite Abundance
2
1
0
−1
−2
Subject
10000
11000
12000
13000
14000
15000
16000
17000
19000
21000
23000
64000
68000
70000
71000
72000
73000
74000
75000
76000
77000
78000
79000
80000
81000
82000
83000
84000
86000
87000
89000
93000
97000
Condition
EM
Primary

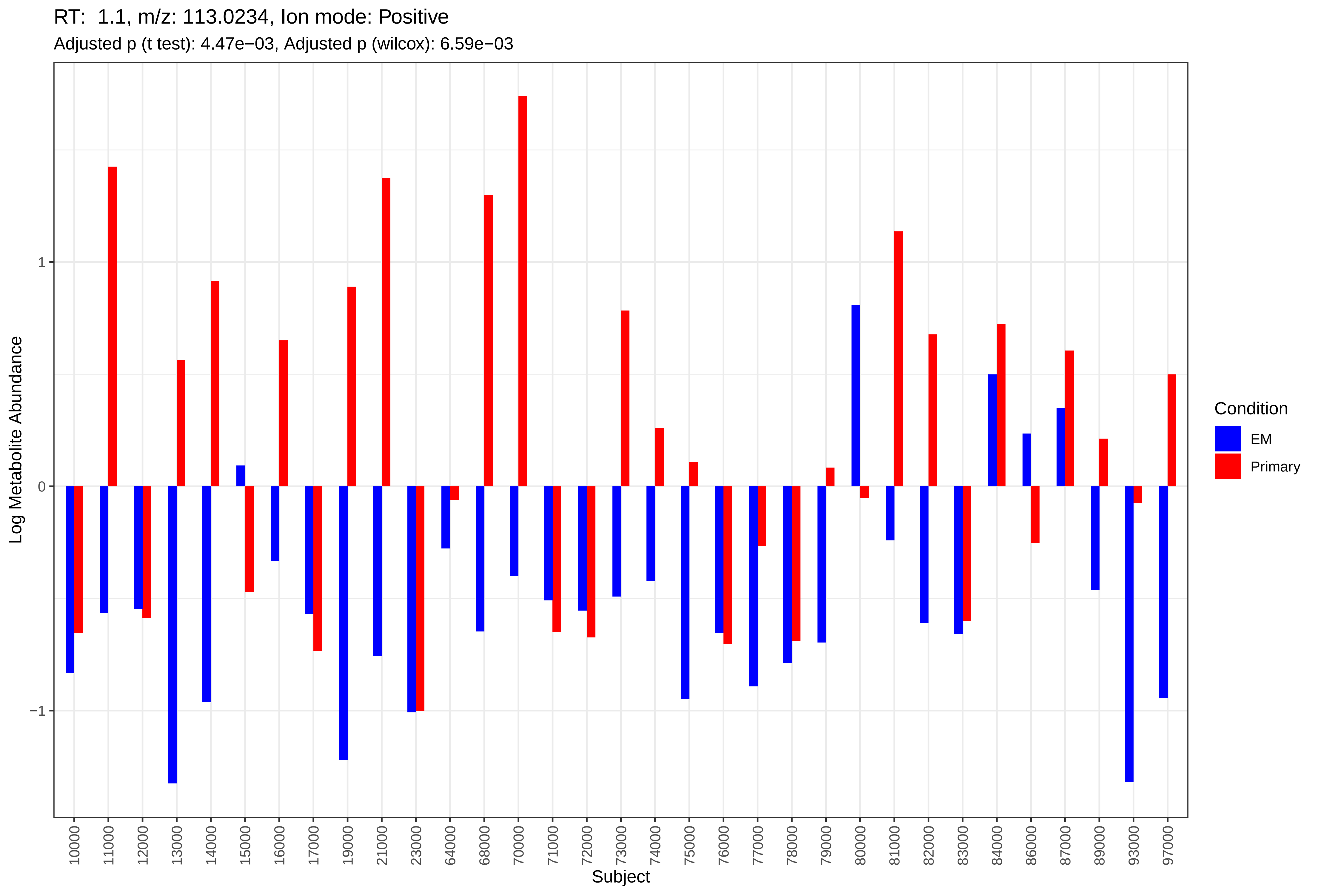
RT: 1.1, m/z: 113.0234, Ion mode: Positive
Adjusted p (t test): 4.47e−03, Adjusted p (wilcox): 6.59e−03
Log Metabolite Abundance
1
0
−1
Subject
10000
11000
12000
13000
14000
15000
16000
17000
19000
21000
23000
64000
68000
70000
71000
72000
73000
74000
75000
76000
77000
78000
79000
80000
81000
82000
83000
84000
86000
87000
89000
93000
97000
Condition
EM
Primary

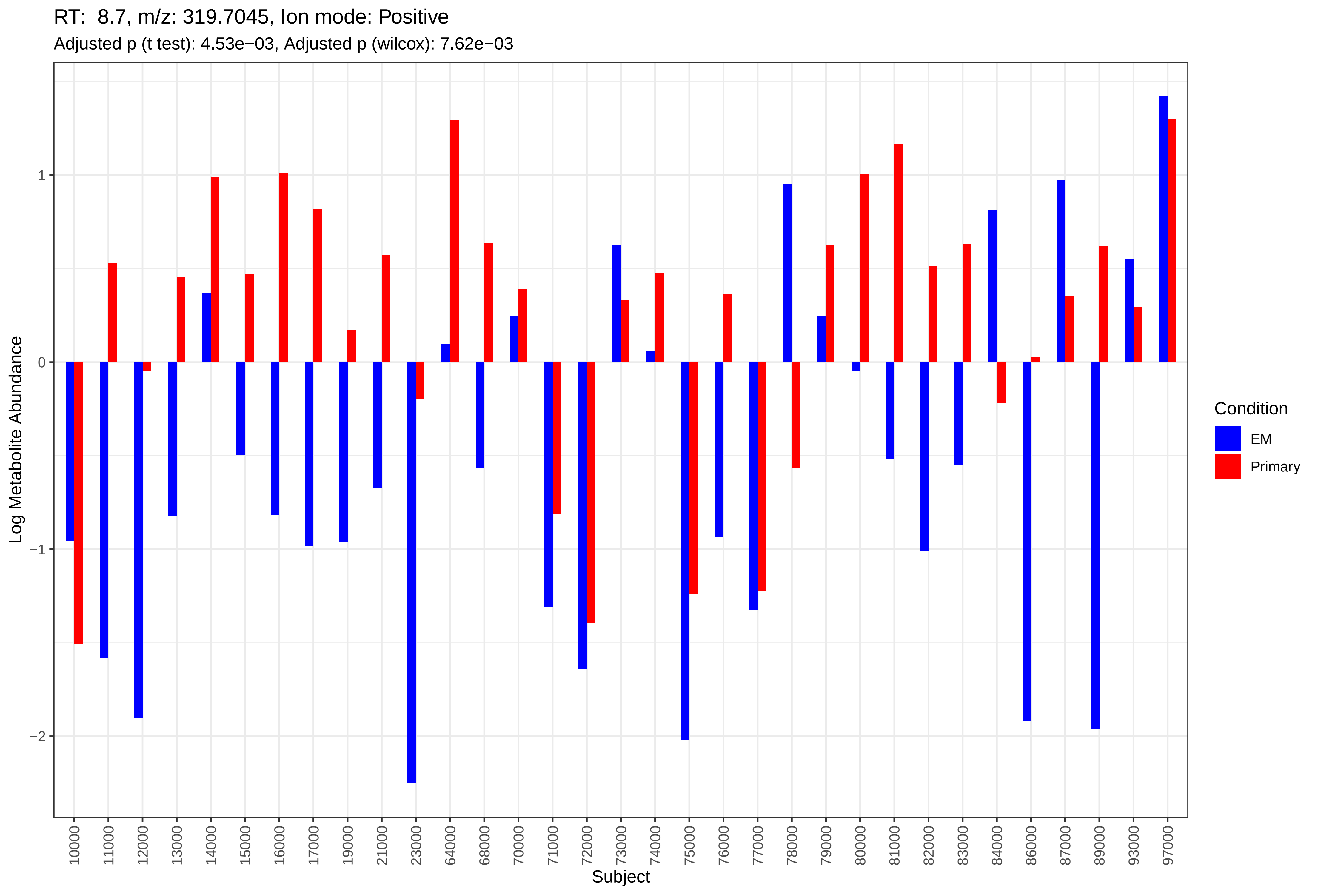
RT: 8.7, m/z: 319.7045, Ion mode: Positive
Adjusted p (t test): 4.53e−03, Adjusted p (wilcox): 7.62e−03
Log Metabolite Abundance
1
0
−1
−2
10000
11000
12000
13000
14000
15000
16000
17000
19000
21000
23000
64000
68000
70000
71000
72000
73000
74000
75000
76000
77000
78000
79000
80000
81000
82000
83000
84000
86000
87000
89000
93000
97000
Subject
Condition
EM
Primary

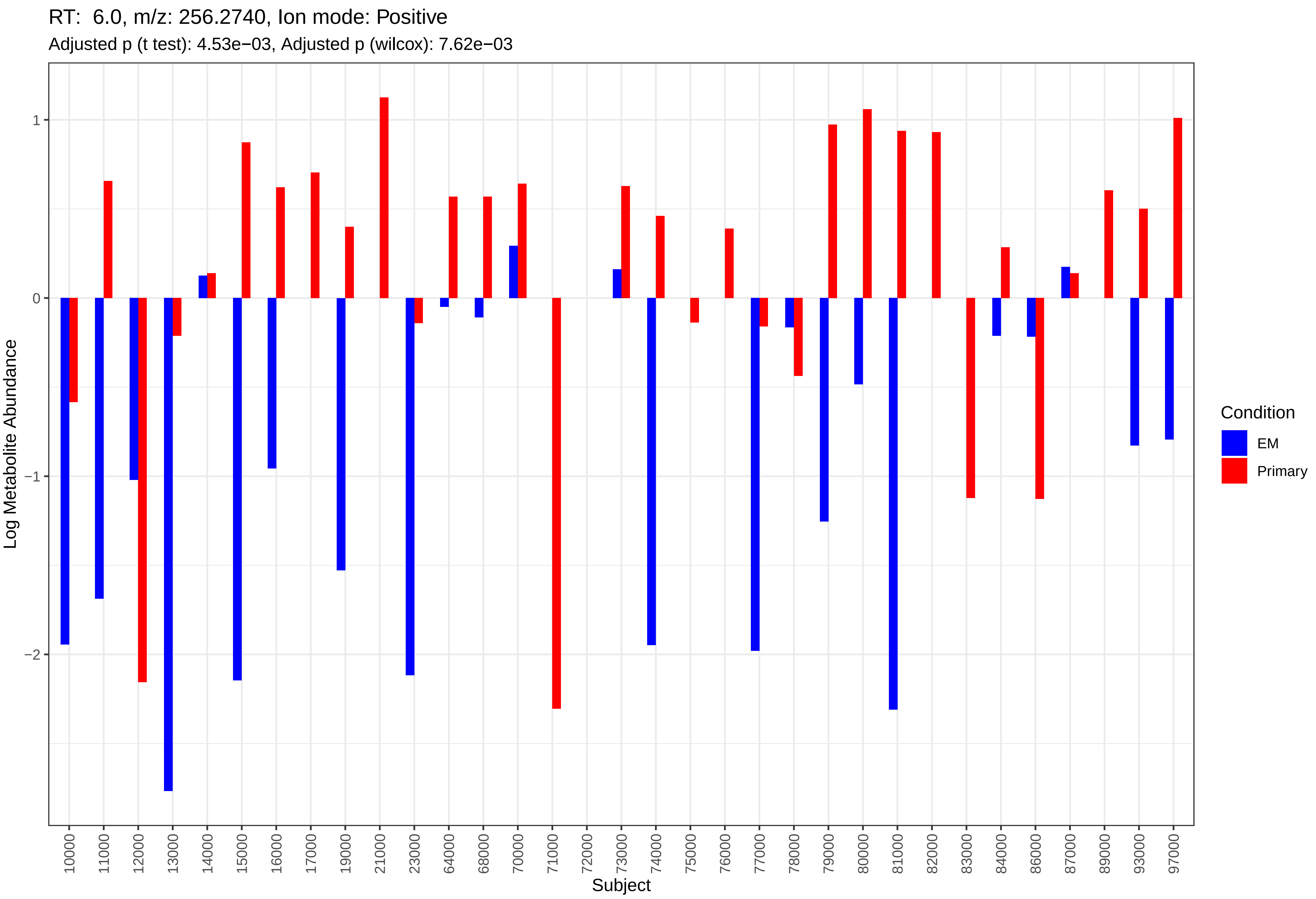
RT: 6.0, m/z: 256.2740, Ion mode: Positive
Adjusted p (t test): 4.53e−03, Adjusted p (wilcox): 7.62e−03
Log Metabolite Abundance
1
0
−1
−2
10000
11000
12000
13000
14000
15000
16000
17000
19000
21000
23000
64000
68000
70000
71000
72000
73000
74000
75000
76000
77000
78000
79000
80000
81000
82000
83000
84000
86000
87000
89000
93000
97000
Subject
Condition
EM
Primary

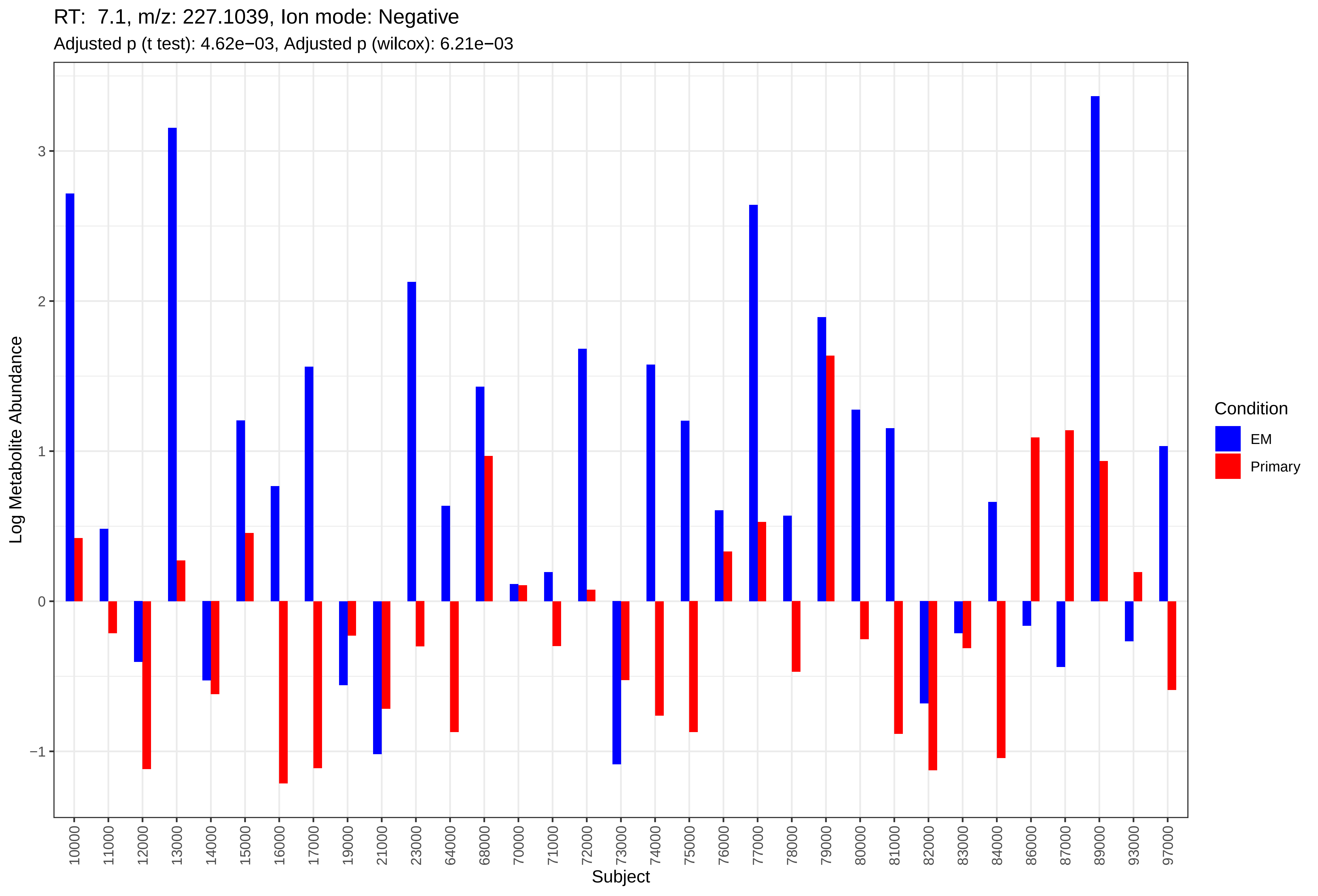
RT: 7.1, m/z: 227.1039, Ion mode: Negative
Adjusted p (t test): 4.62e−03, Adjusted p (wilcox): 6.21e−03
Log Metabolite Abundance
3
2
1
0
−1
10000
11000
12000
13000
14000
15000
16000
17000
19000
21000
23000
64000
68000
70000
71000
72000
73000
74000
75000
76000
77000
78000
79000
80000
81000
82000
83000
84000
86000
87000
89000
93000
97000
Subject
Condition
EM
Primary

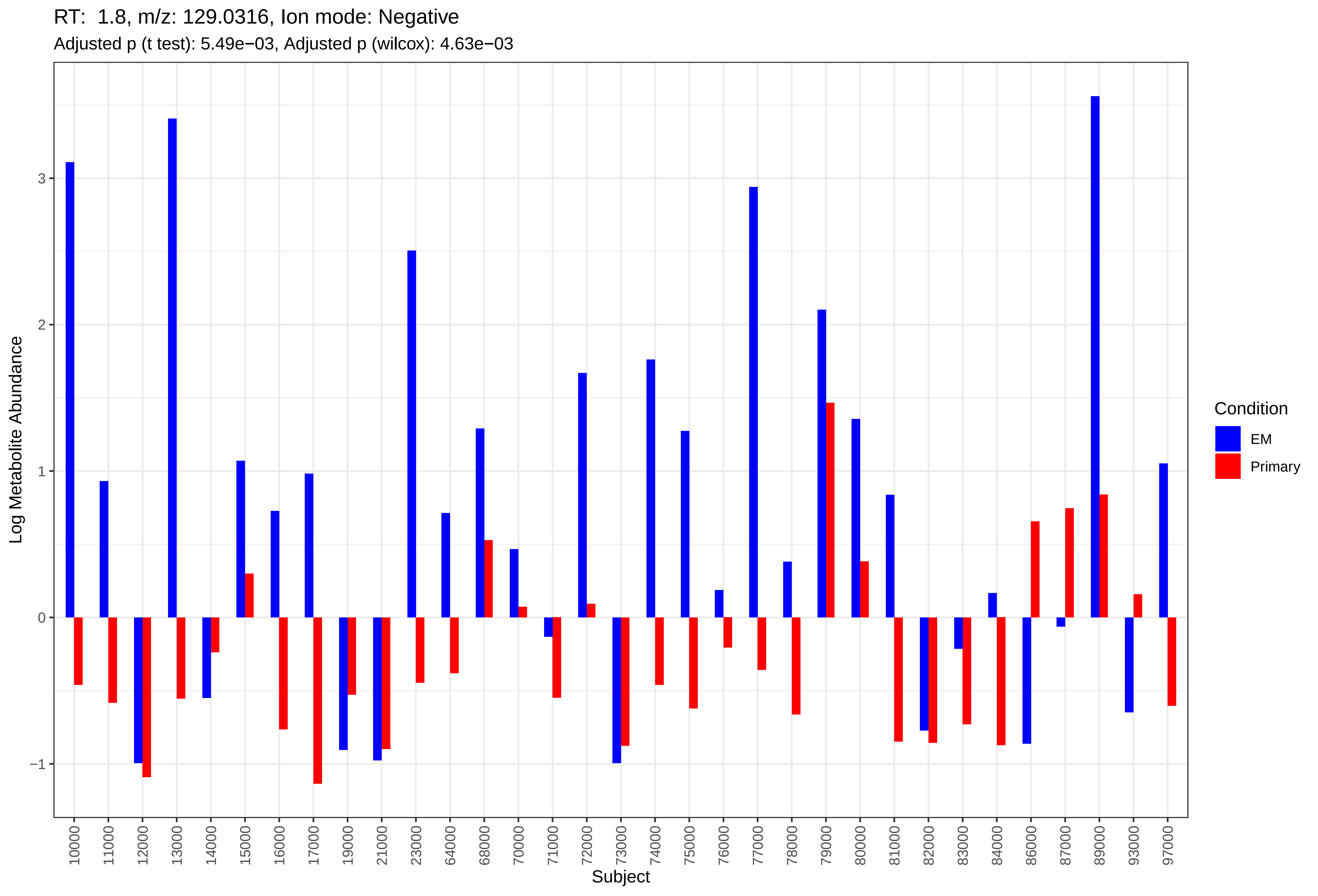

RT: 1.8, m/z: 129.0316, Ion mode: Negative
Adjusted p (t test): 5.49e−03, Adjusted p (wilcox): 4.63e−03
Log Metabolite Abundance
Subject
Condition
EM
Primary

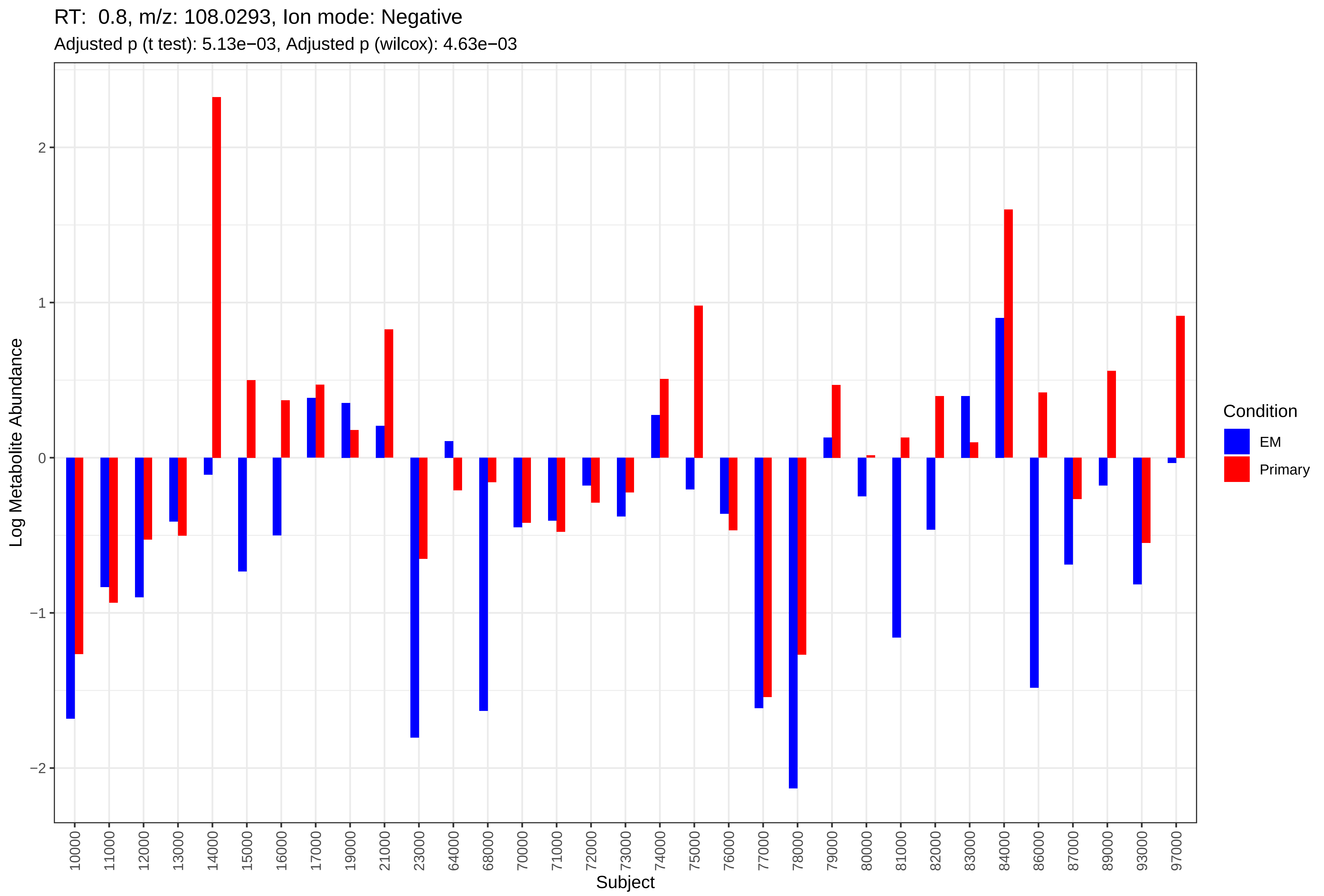
RT: 0.8, m/z: 108.0293, Ion mode: Negative
Adjusted p (t test): 5.13e−03, Adjusted p (wilcox): 4.63e−03
Log Metabolite Abundance
2
1
0
−1
−2
10000
11000
12000
13000
14000
15000
16000
17000
19000
21000
23000
64000
68000
70000
71000
72000
73000
74000
75000
76000
77000
78000
79000
80000
81000
82000
83000
84000
86000
87000
89000
93000
97000
Subject
Condition
EM
Primary

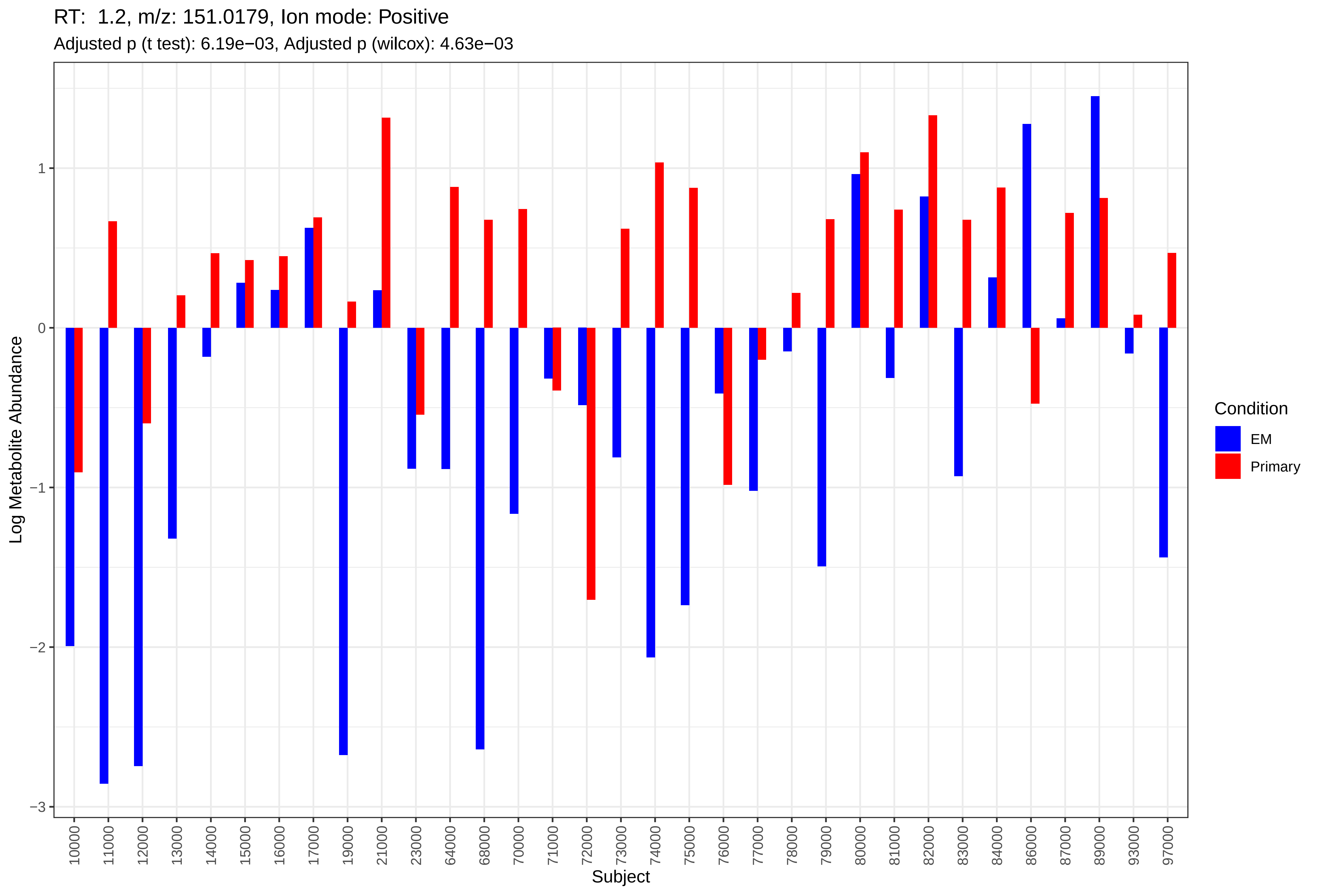
RT: 1.2, m/z: 151.0179, Ion mode: Positive
Adjusted p (t test): 6.19e−03, Adjusted p (wilcox): 4.63e−03
Log Metabolite Abundance
1
0
−1
−2
−3
10000
11000
12000
13000
14000
15000
16000
17000
19000
21000
23000
64000
68000
70000
71000
72000
73000
74000
75000
76000
77000
78000
79000
80000
81000
82000
83000
84000
86000
87000
89000
93000
97000
Subject
Condition
EM
Primary

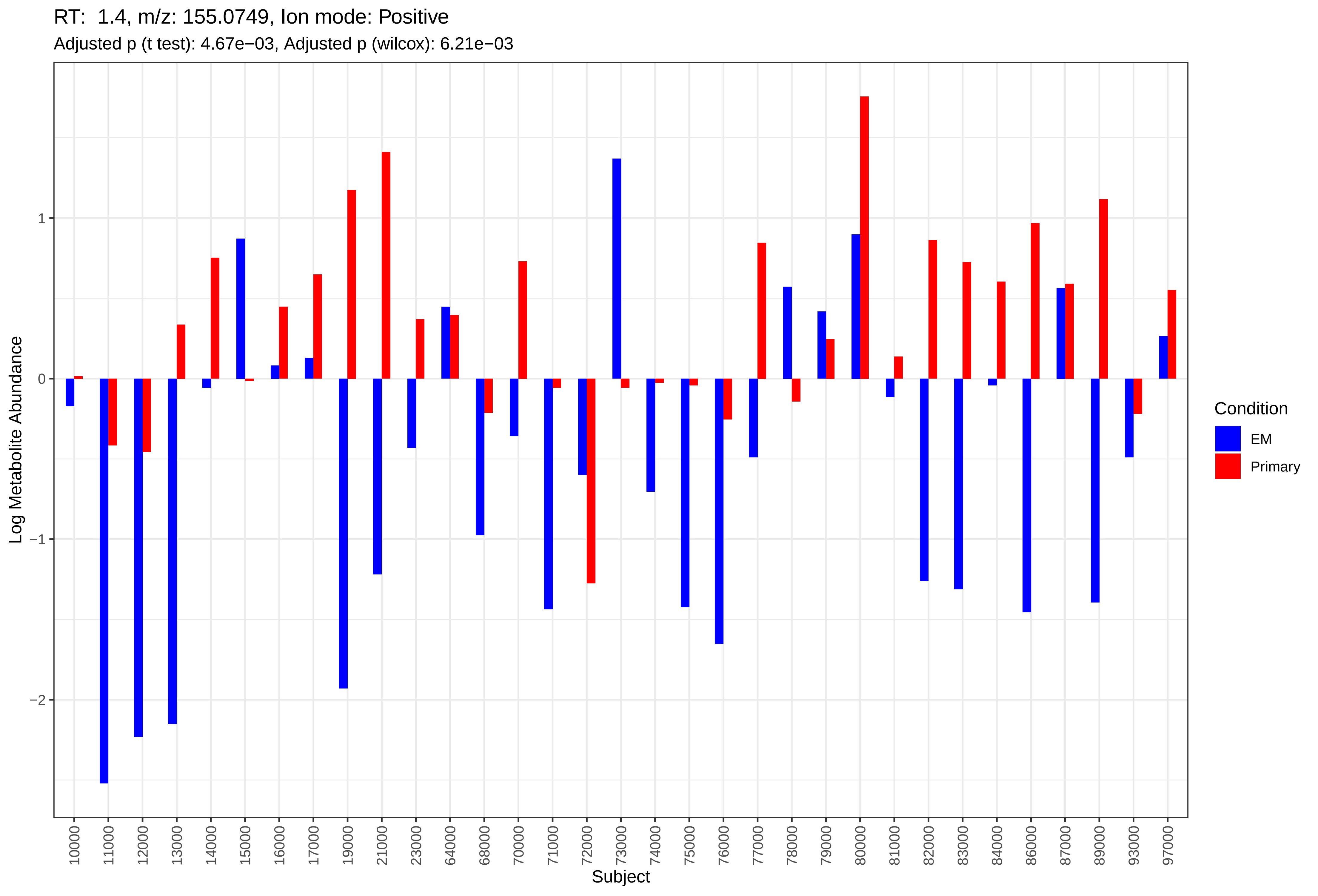
RT: 1.4, m/z: 155.0749, Ion mode: Positive
Adjusted p (t test): 4.67e−03, Adjusted p (wilcox): 6.21e−03
Log Metabolite Abundance
1
0
−1
−2
Subject
10000
11000
12000
13000
14000
15000
16000
17000
19000
21000
23000
64000
68000
70000
71000
72000
73000
74000
75000
76000
77000
78000
79000
80000
81000
82000
83000
84000
86000
87000
89000
93000
97000
Condition
EM
Primary

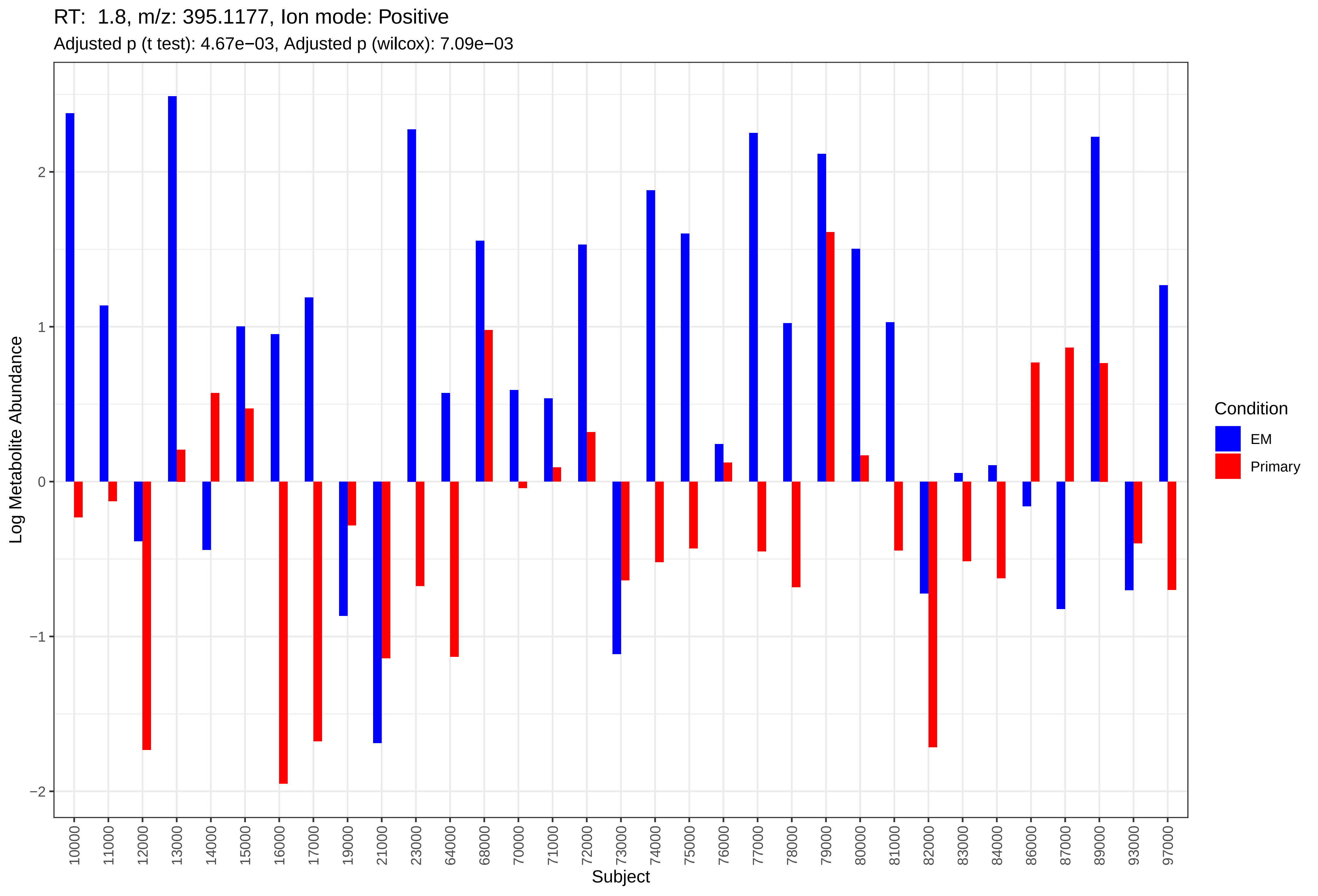
RT: 1.8, m/z: 395.1177, Ion mode: Positive
Adjusted p (t test): 4.67e−03, Adjusted p (wilcox): 7.09e−03
Log Metabolite Abundance
2
1
0
−1
−2
10000
11000
12000
13000
14000
15000
16000
17000
19000
21000
23000
64000
68000
70000
71000
72000
73000
74000
75000
76000
77000
78000
79000
80000
81000
82000
83000
84000
86000
87000
89000
93000
97000
Subject
Condition
EM
Primary

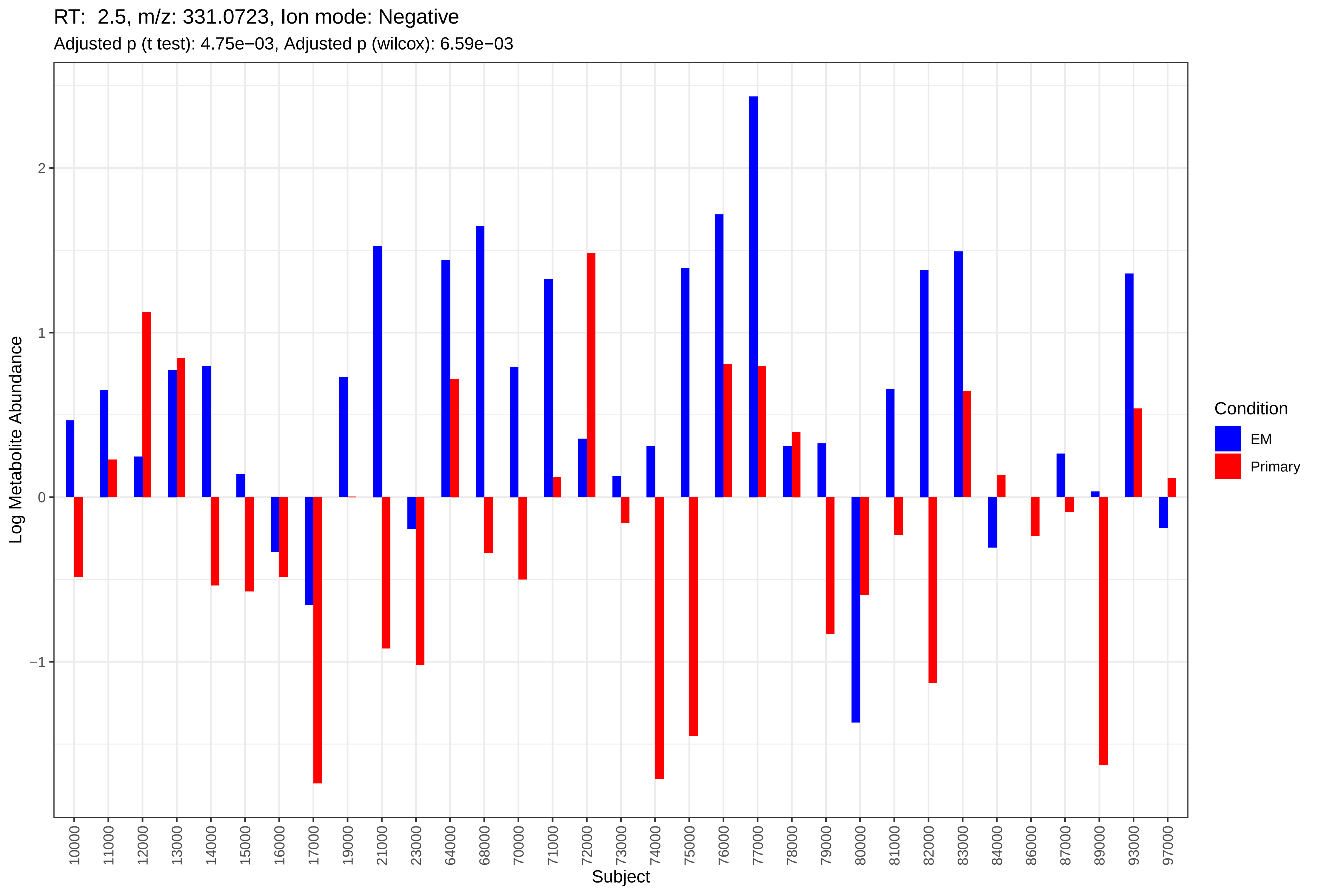

RT: 2.5, m/z: 331.0723, Ion mode: Negative
Adjusted p (t test): 4.75e−03, Adjusted p (wilcox): 6.59e−03
Log Metabolite Abundance
2
1
0
−1
10000
11000
12000
13000
14000
15000
16000
17000
19000
21000
23000
64000
68000
70000
71000
72000
73000
74000
75000
76000
77000
78000
79000
80000
81000
82000
83000
84000
86000
87000
89000
93000
97000
Subject
Condition
EM
Primary

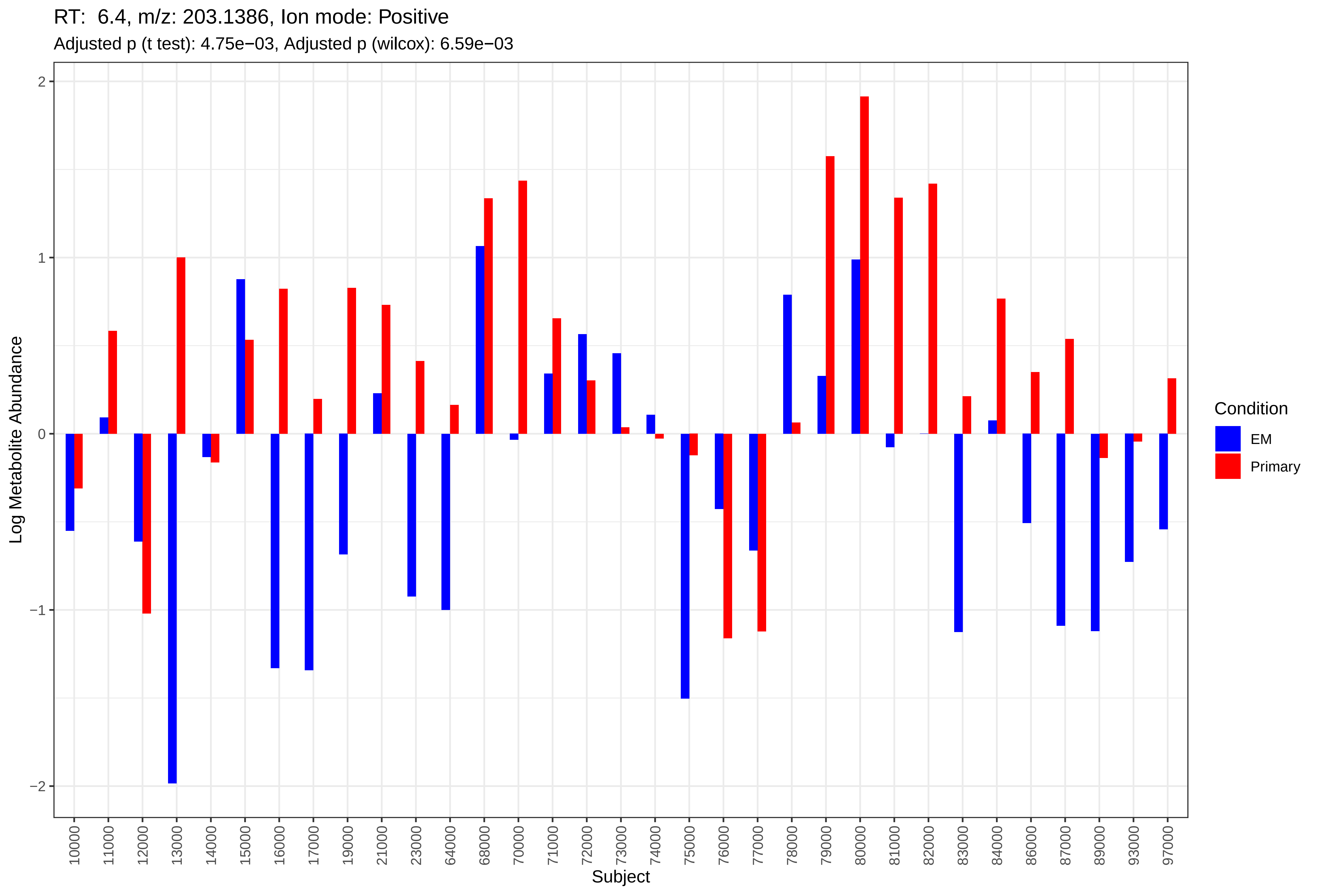
RT: 6.4, m/z: 203.1386, Ion mode: Positive
Adjusted p (t test): 4.75e−03, Adjusted p (wilcox): 6.59e−03
Log Metabolite Abundance
2
1
0
−1
−2
Subject
10000
11000
12000
13000
14000
15000
16000
17000
19000
21000
23000
64000
68000
70000
71000
72000
73000
74000
75000
76000
77000
78000
79000
80000
81000
82000
83000
84000
86000
87000
89000
93000
97000
Condition
EM
Primary

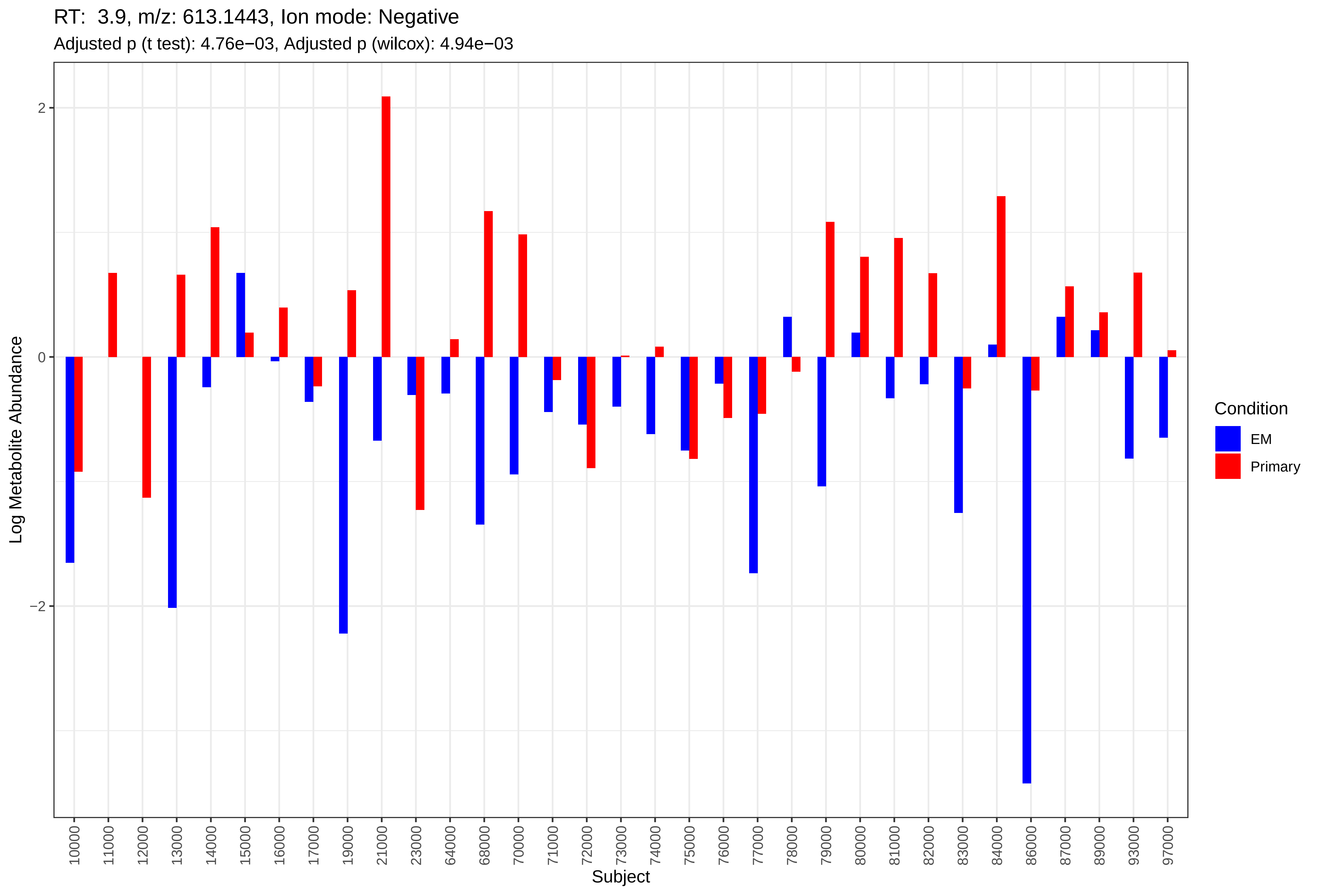

RT: 3.9, m/z: 613.1443, Ion mode: Negative
Adjusted p (t test): 4.76e−03, Adjusted p (wilcox): 4.94e−03
Log Metabolite Abundance
2
0
−2
Subject
10000
11000
12000
13000
14000
15000
16000
17000
19000
21000
23000
64000
68000
70000
71000
72000
73000
74000
75000
76000
77000
78000
79000
80000
81000
82000
83000
84000
86000
87000
89000
93000
97000
Condition
EM
Primary

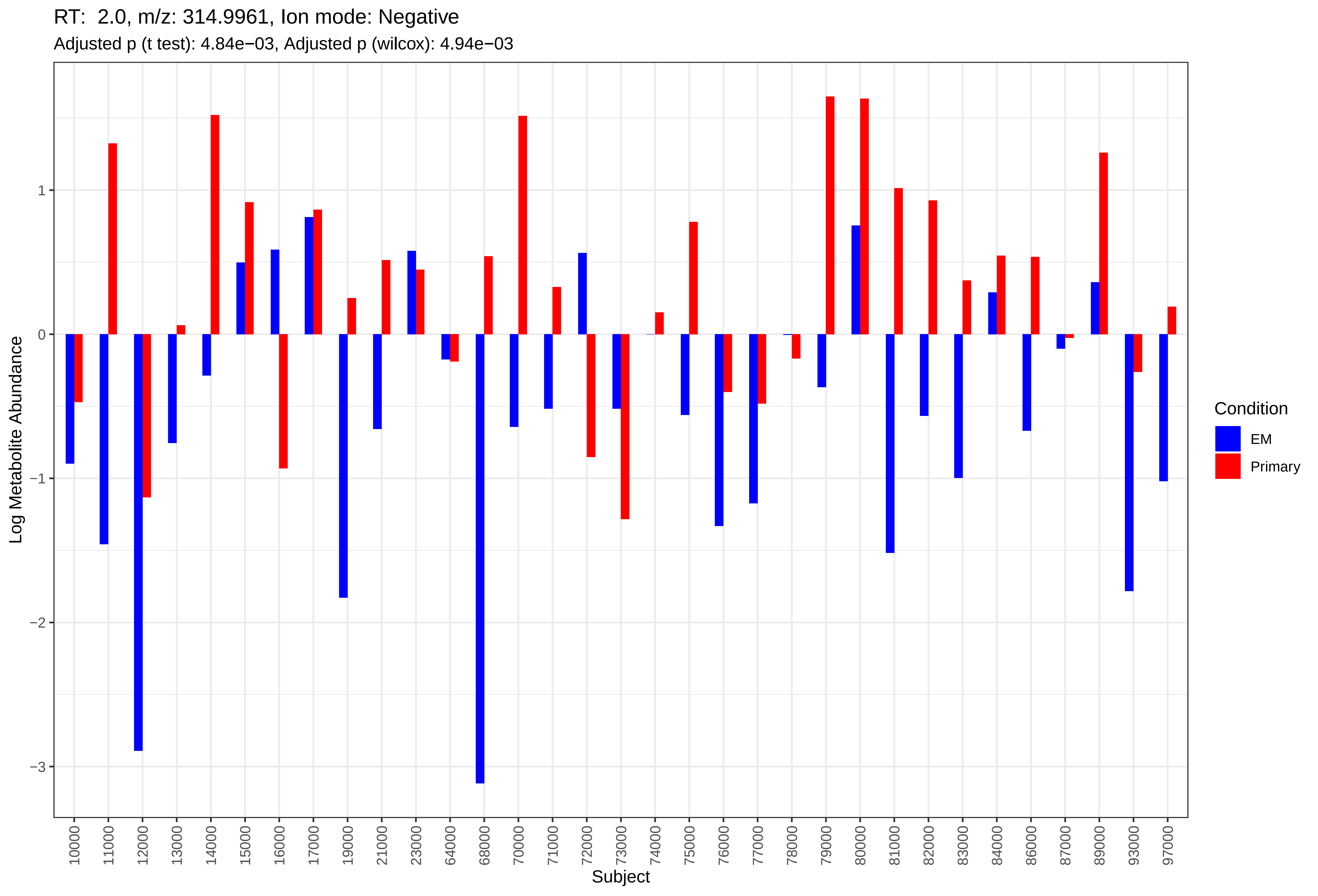

RT: 2.0, m/z: 314.9961, Ion mode: Negative
Adjusted p (t test): 4.84e−03, Adjusted p (wilcox): 4.94e−03
Log Metabolite Abundance
1
0
−1
−2
−3
10000
11000
12000
13000
14000
15000
16000
17000
19000
21000
23000
64000
68000
70000
71000
72000
73000
74000
75000
76000
77000
78000
79000
80000
81000
82000
83000
84000
86000
87000
89000
93000
97000
Subject
Condition
EM
Primary

LysoPE (18:1) (RT: 13.5, m/z: 478.2942, Ion mode: Negative)

Adjusted p (t test): 4.85e−03, Adjusted p (wilcox): 5.16e−03

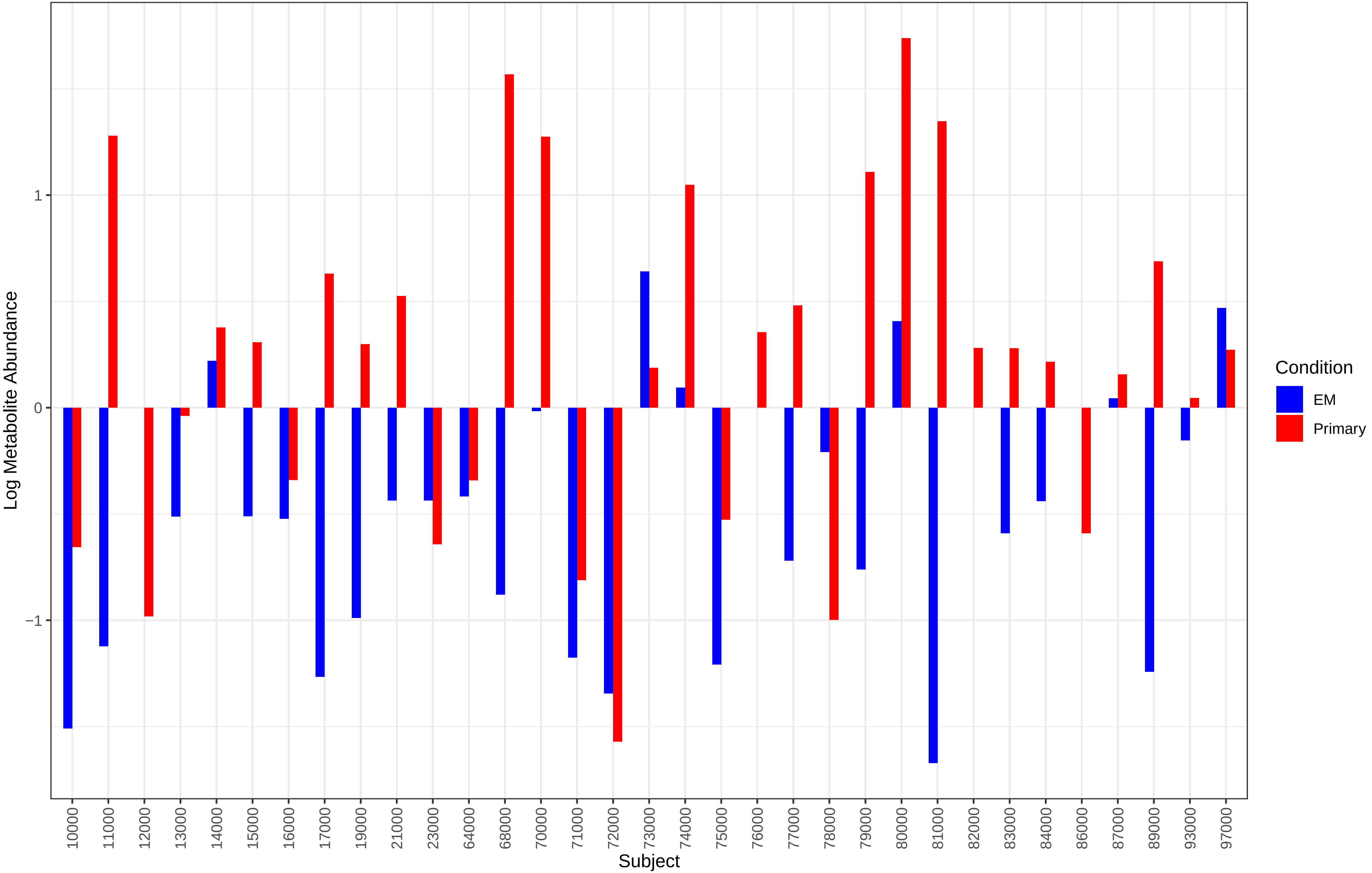

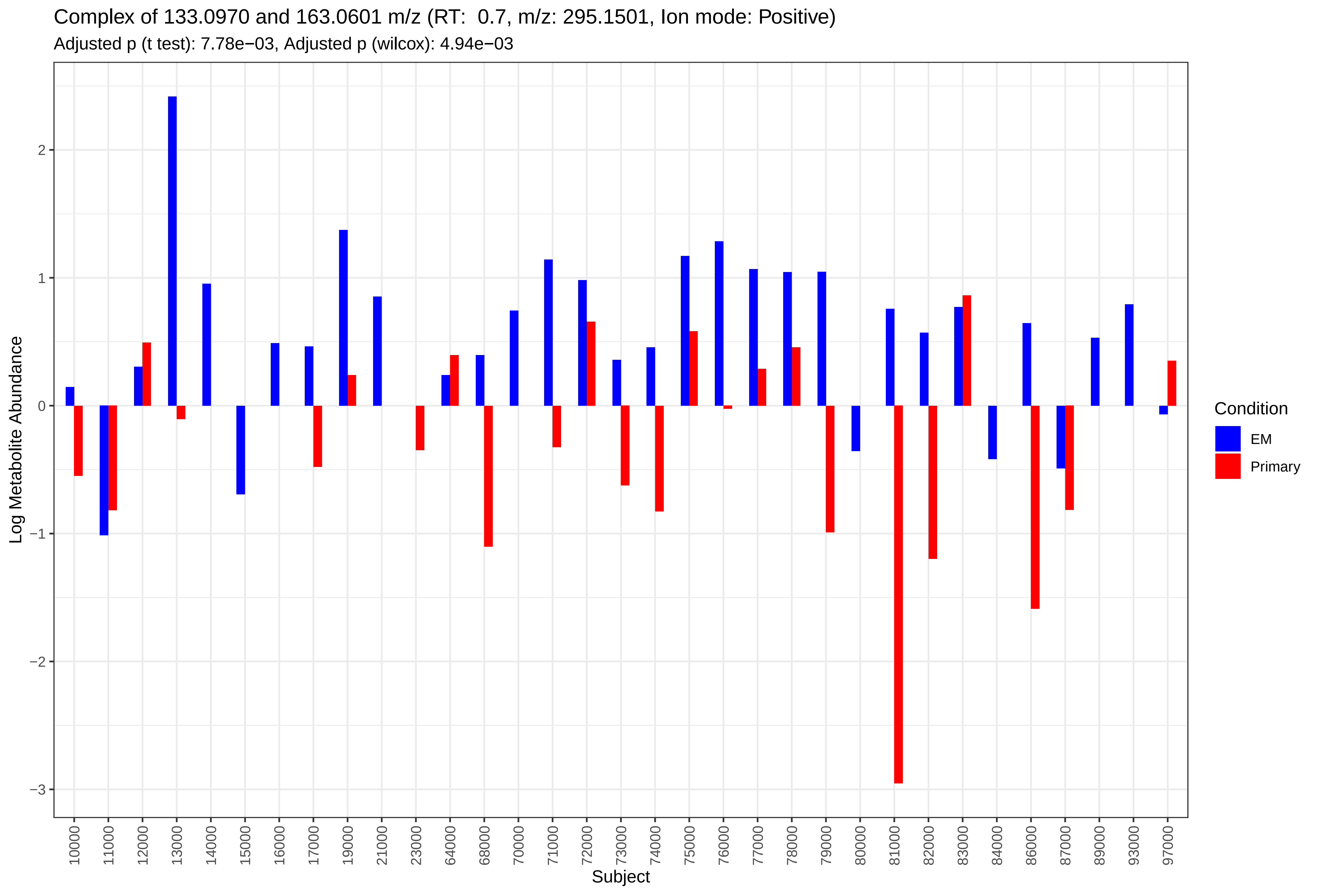
Complex of 133.0970 and 163.0601 m/z (RT: 0.7, m/z: 295.1501, Ion mode: Positive)
Adjusted p (t test): 7.78e−03, Adjusted p (wilcox): 4.94e−03
Log Metabolite Abundance
2
1
0
−1
−2
−3
10000
11000
12000
13000
14000
15000
16000
17000
19000
21000
23000
64000
68000
70000
71000
72000
73000
74000
75000
76000
77000
78000
79000
80000
81000
82000
83000
84000
86000
87000
89000
93000
97000
Subject
Condition
EM
Primary

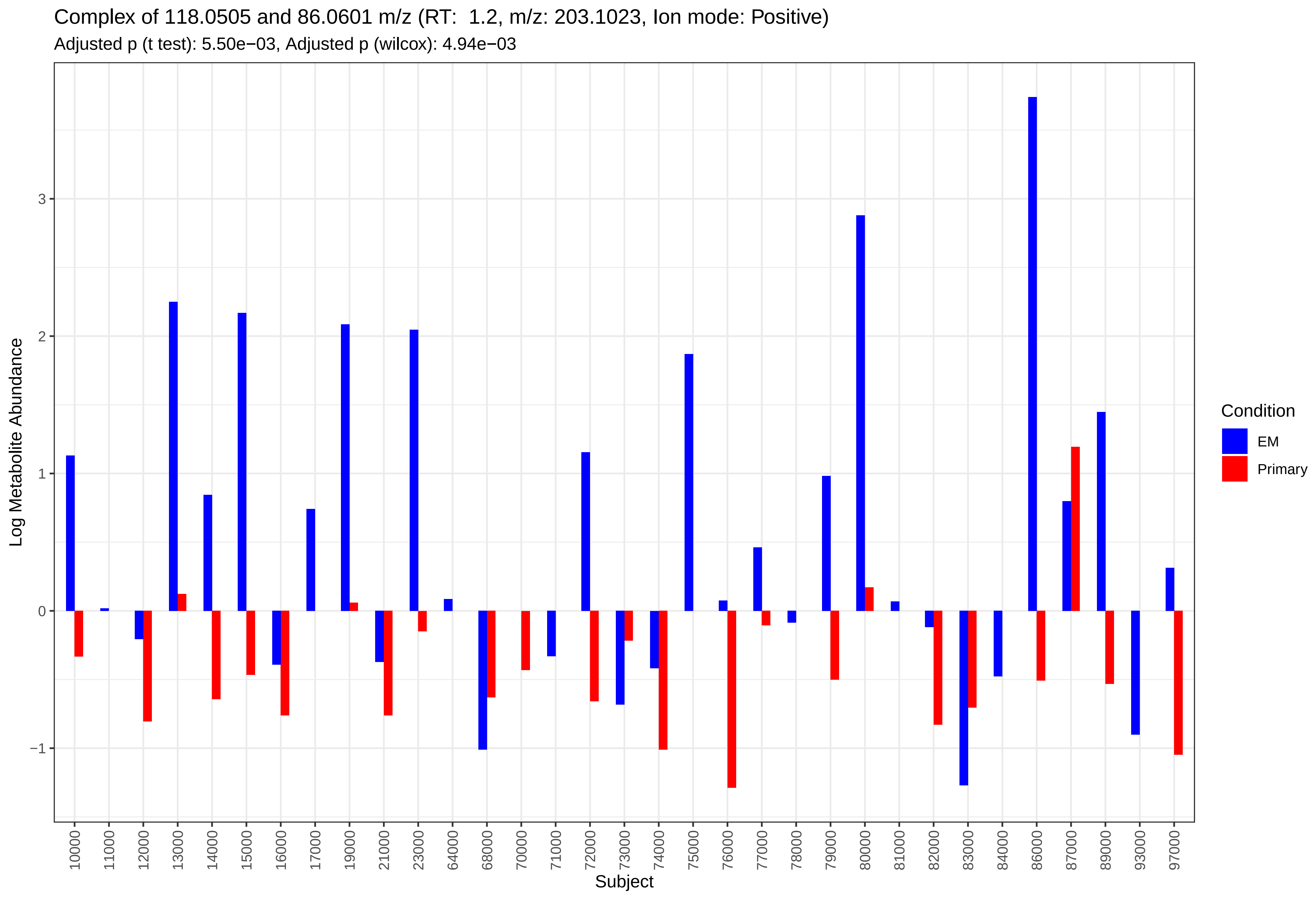

Complex of 118.0505 and 86.0601 m/z (RT: 1.2, m/z: 203.1023, Ion mode: Positive)
Adjusted p (t test): 5.50e−03, Adjusted p (wilcox): 4.94e−03
Log Metabolite Abundance
3
2
1
0
−1
Subject
10000
11000
12000
13000
14000
15000
16000
17000
19000
21000
23000
64000
68000
70000
71000
72000
73000
74000
75000
76000
77000
78000
79000
80000
81000
82000
83000
84000
86000
87000
89000
93000
97000
Condition
EM
Primary

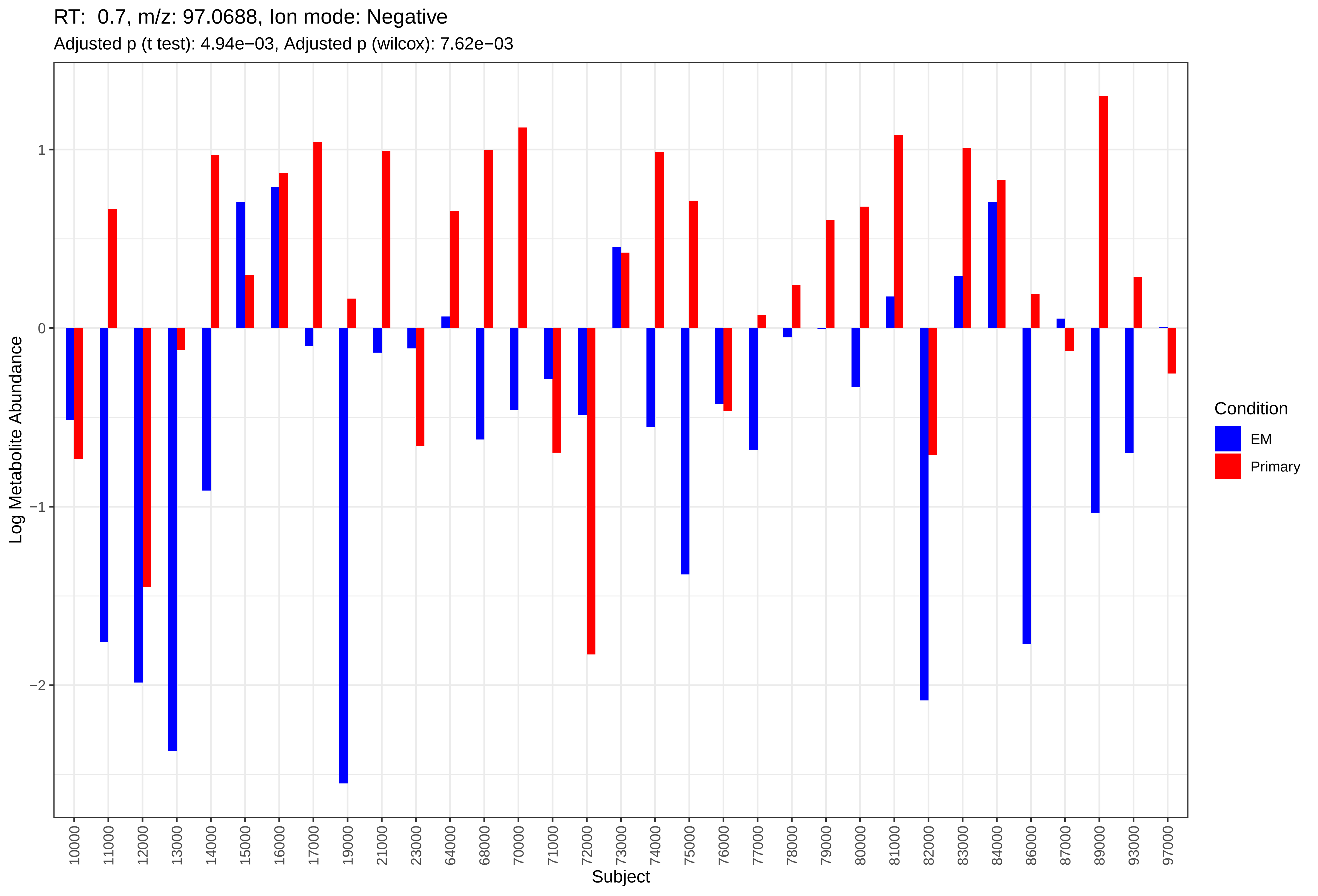
RT: 0.7, m/z: 97.0688, Ion mode: Negative
Adjusted p (t test): 4.94e−03, Adjusted p (wilcox): 7.62e−03
Log Metabolite Abundance
1
0
−1
−2
10000
11000
12000
13000
14000
15000
16000
17000
19000
21000
23000
64000
68000
70000
71000
72000
73000
74000
75000
76000
77000
78000
79000
80000
81000
82000
83000
84000
86000
87000
89000
93000
97000
Subject
Condition
EM
Primary

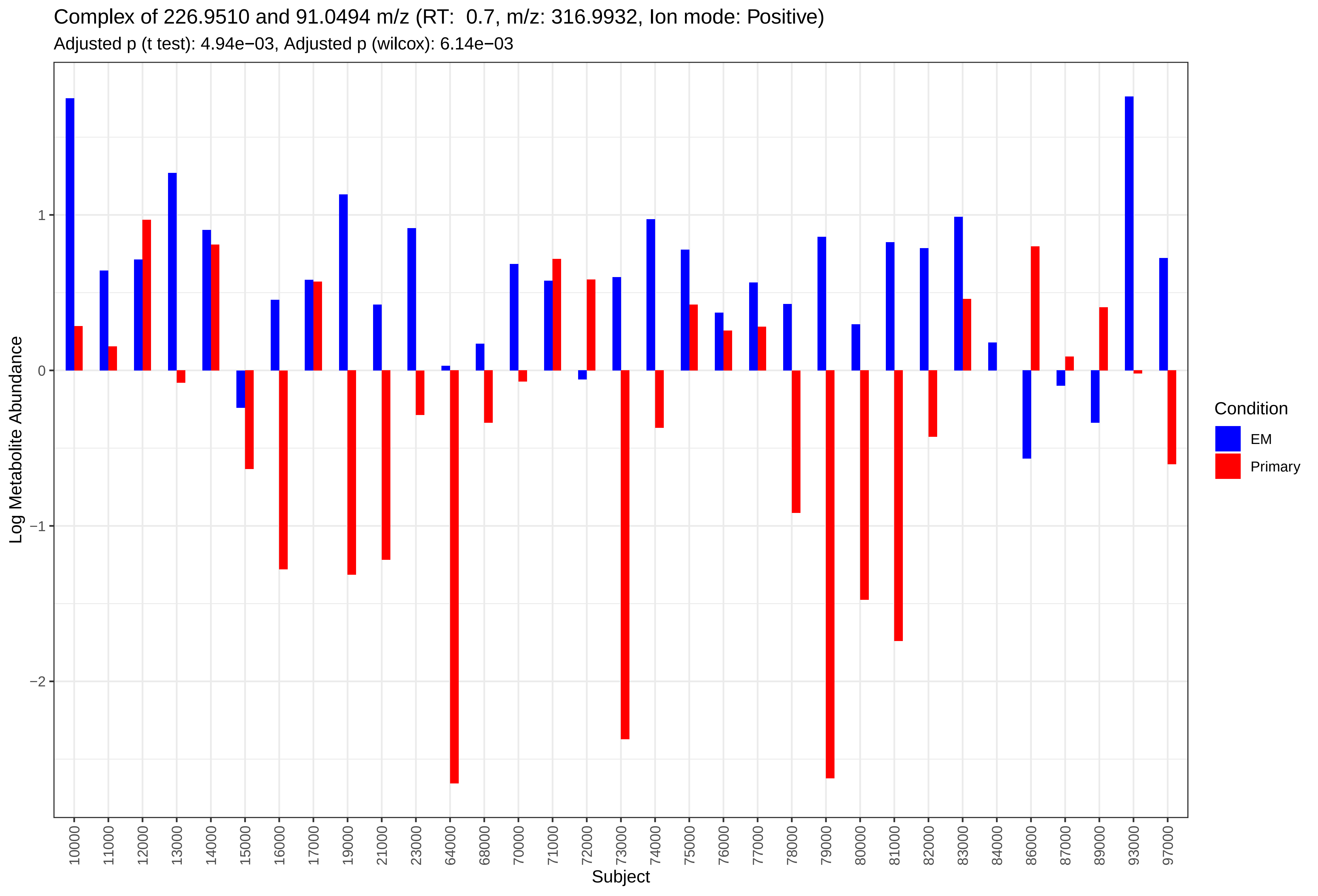

Complex of 226.9510 and 91.0494 m/z (RT: 0.7, m/z: 316.9932, Ion mode: Positive)
Adjusted p (t test): 4.94e−03, Adjusted p (wilcox): 6.14e−03
Log Metabolite Abundance
1
0
−1
−2
10000
11000
12000
13000
14000
15000
16000
17000
19000
21000
23000
64000
68000
70000
71000
72000
73000
74000
75000
76000
77000
78000
79000
80000
81000
82000
83000
84000
86000
87000
89000
93000
97000
Subject
Condition
EM
Primary

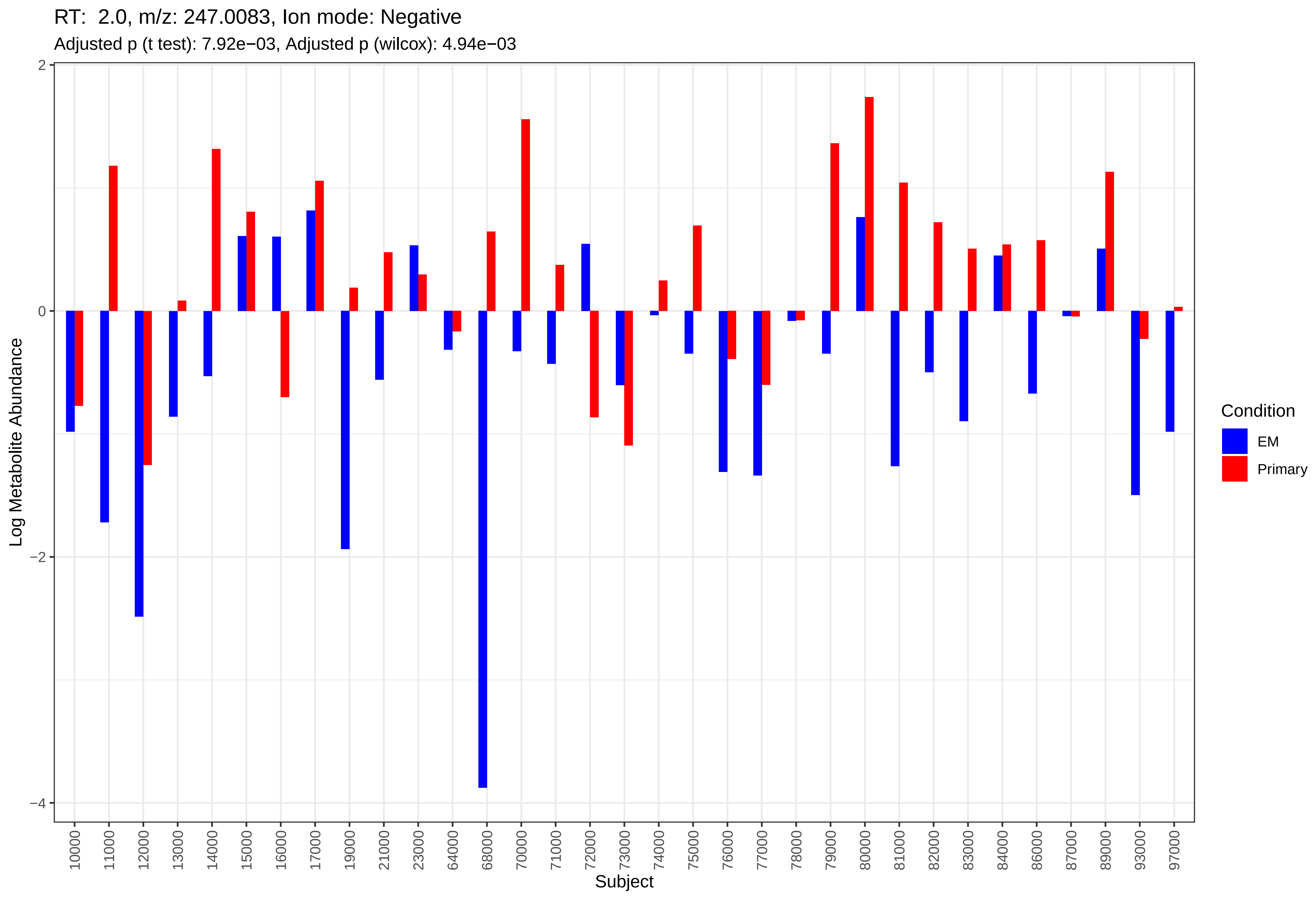
RT: 2.0, m/z: 247.0083, Ion mode: Negative
Adjusted p (t test): 7.92e−03, Adjusted p (wilcox): 4.94e−03
Log Metabolite Abundance
2
0
−2
−4
10000
11000
12000
13000
14000
15000
16000
17000
19000
21000
23000
64000
68000
70000
71000
72000
73000
74000
75000
76000
77000
78000
79000
80000
81000
82000
83000
84000
86000
87000
89000
93000
97000
Subject
Condition
EM
Primary

C6H12O6−HEXOSE/KETOSE/INOSITOL (RT: 0.7, m/z: 179.0563, Ion mode: Negative)

Adjusted p (t test): 7.64e−03, Adjusted p (wilcox): 4.94e−03

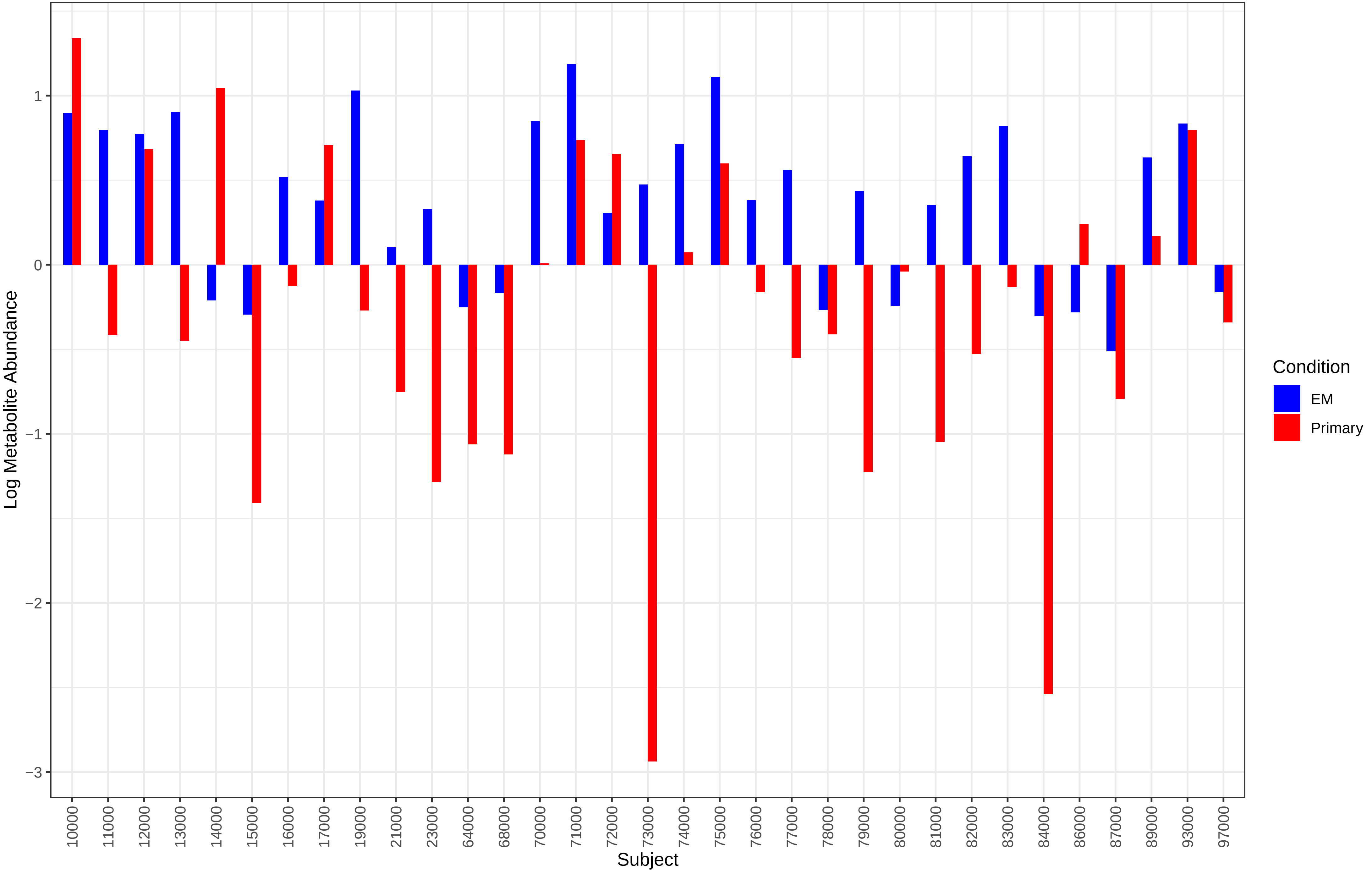

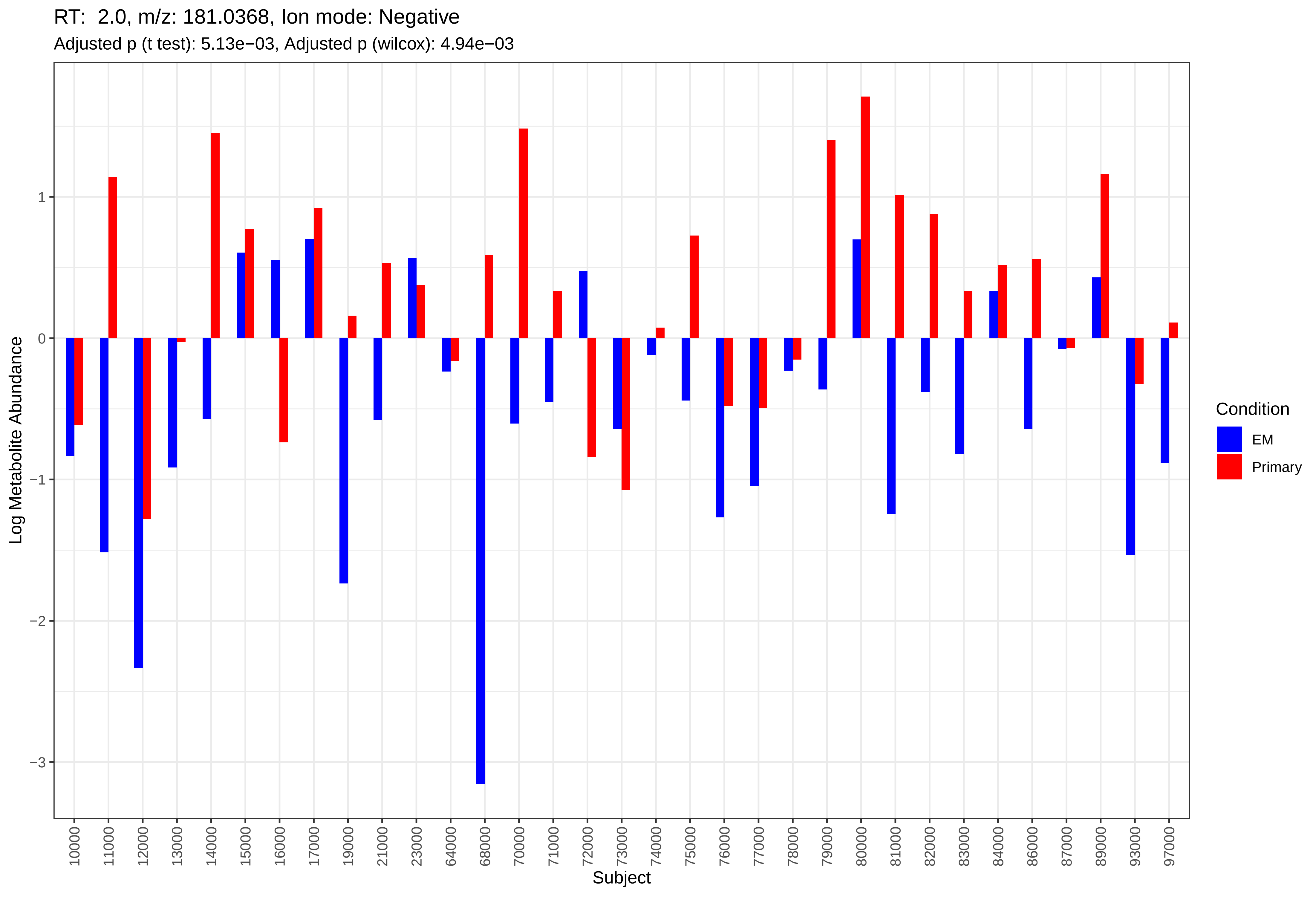
RT: 2.0, m/z: 181.0368, Ion mode: Negative
Adjusted p (t test): 5.13e−03, Adjusted p (wilcox): 4.94e−03
Log Metabolite Abundance
1
0
−1
−2
−3
Subject
10000
11000
12000
13000
14000
15000
16000
17000
19000
21000
23000
64000
68000
70000
71000
72000
73000
74000
75000
76000
77000
78000
79000
80000
81000
82000
83000
84000
86000
87000
89000
93000
97000
Condition
EM
Primary

RT: 10.2, m/z: 117.0697, Ion mode: Positive

Adjusted p (t test): 5.49e−03, Adjusted p (wilcox): 4.94e−03

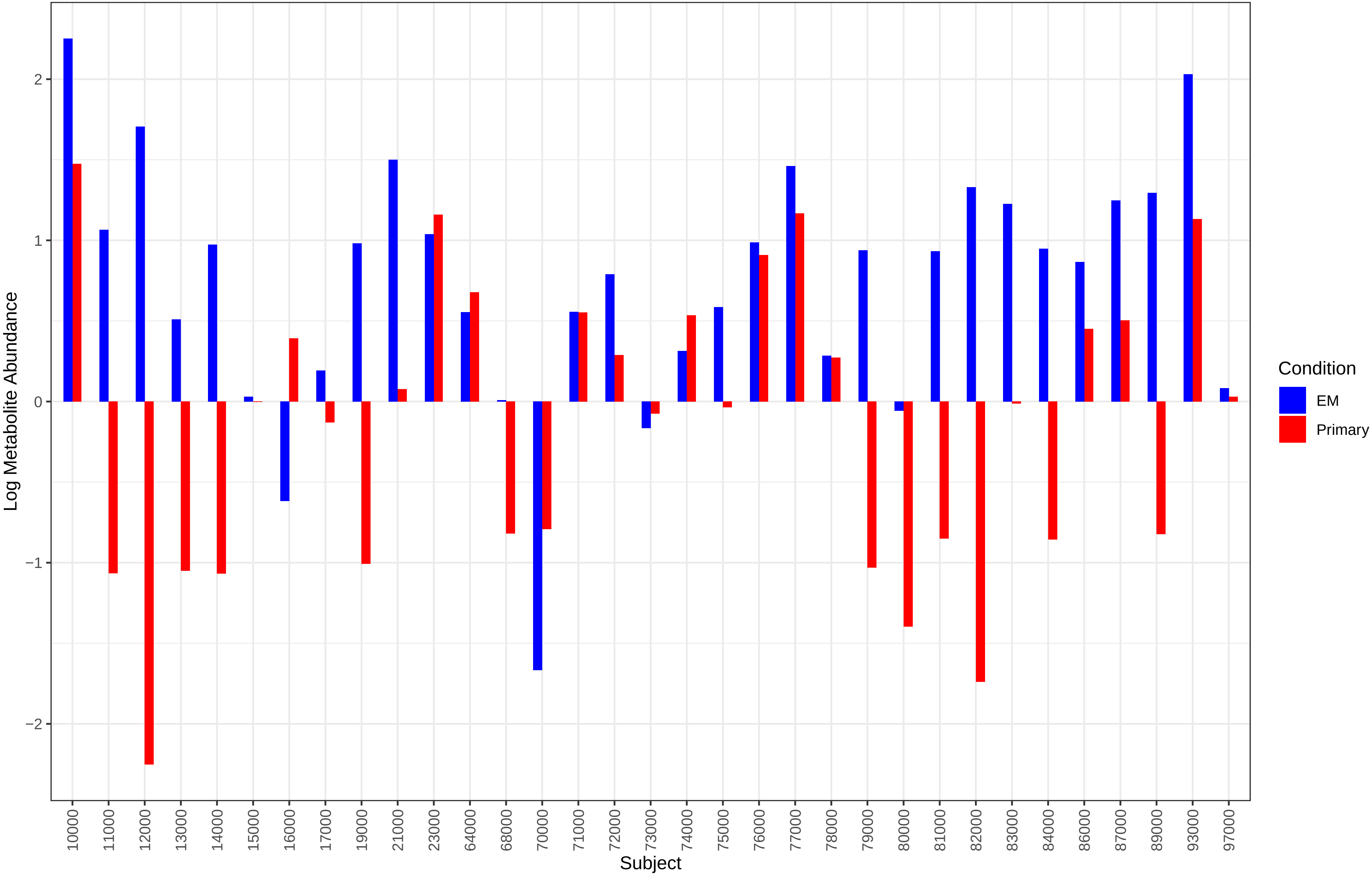

Isobutyrylcarnitine (RT: 7.4, m/z: 232.1538, Ion mode: Positive)
Adjusted p (t test): 8.88e−03, Adjusted p (wilcox): 4.94e−03

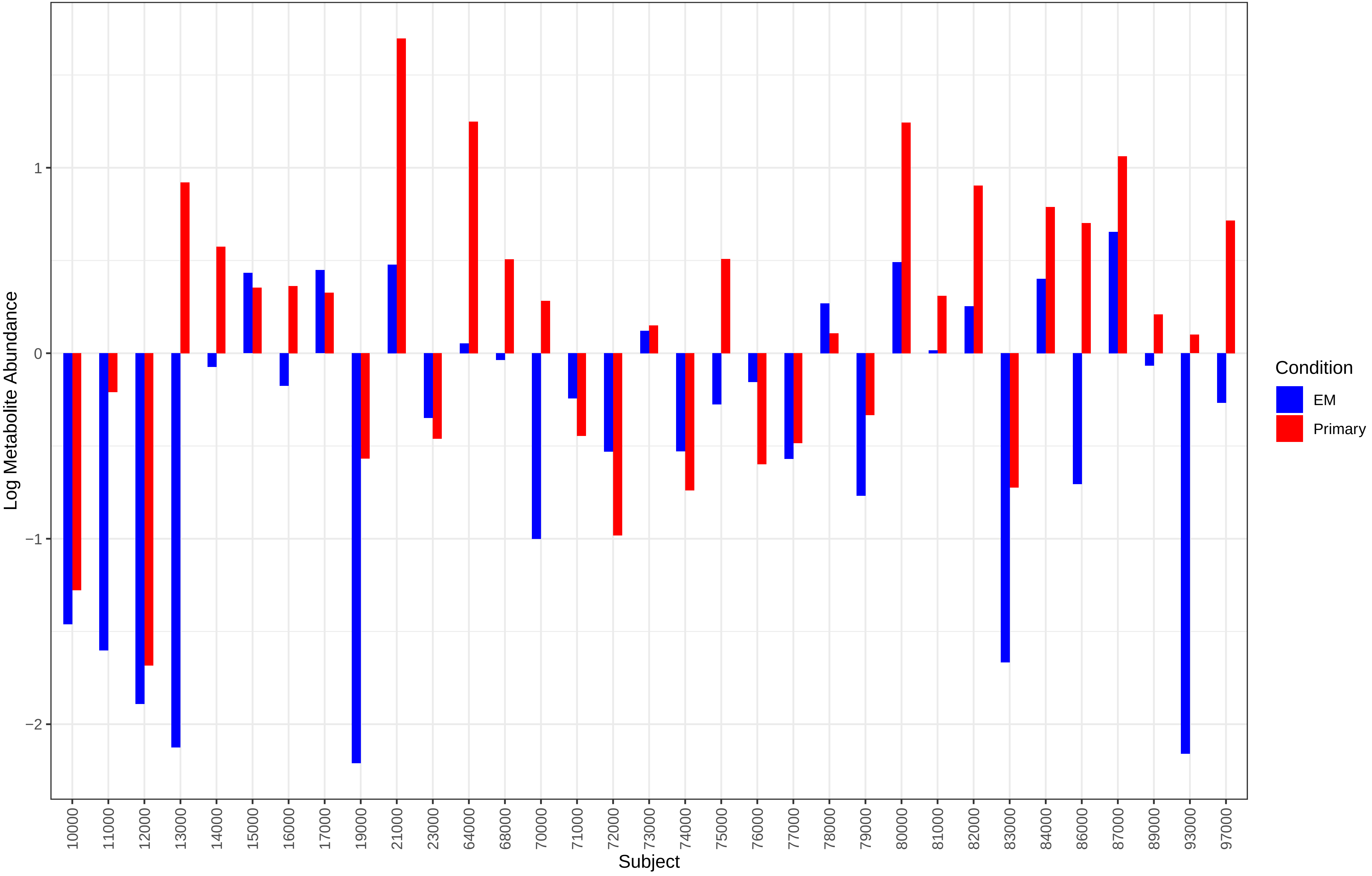

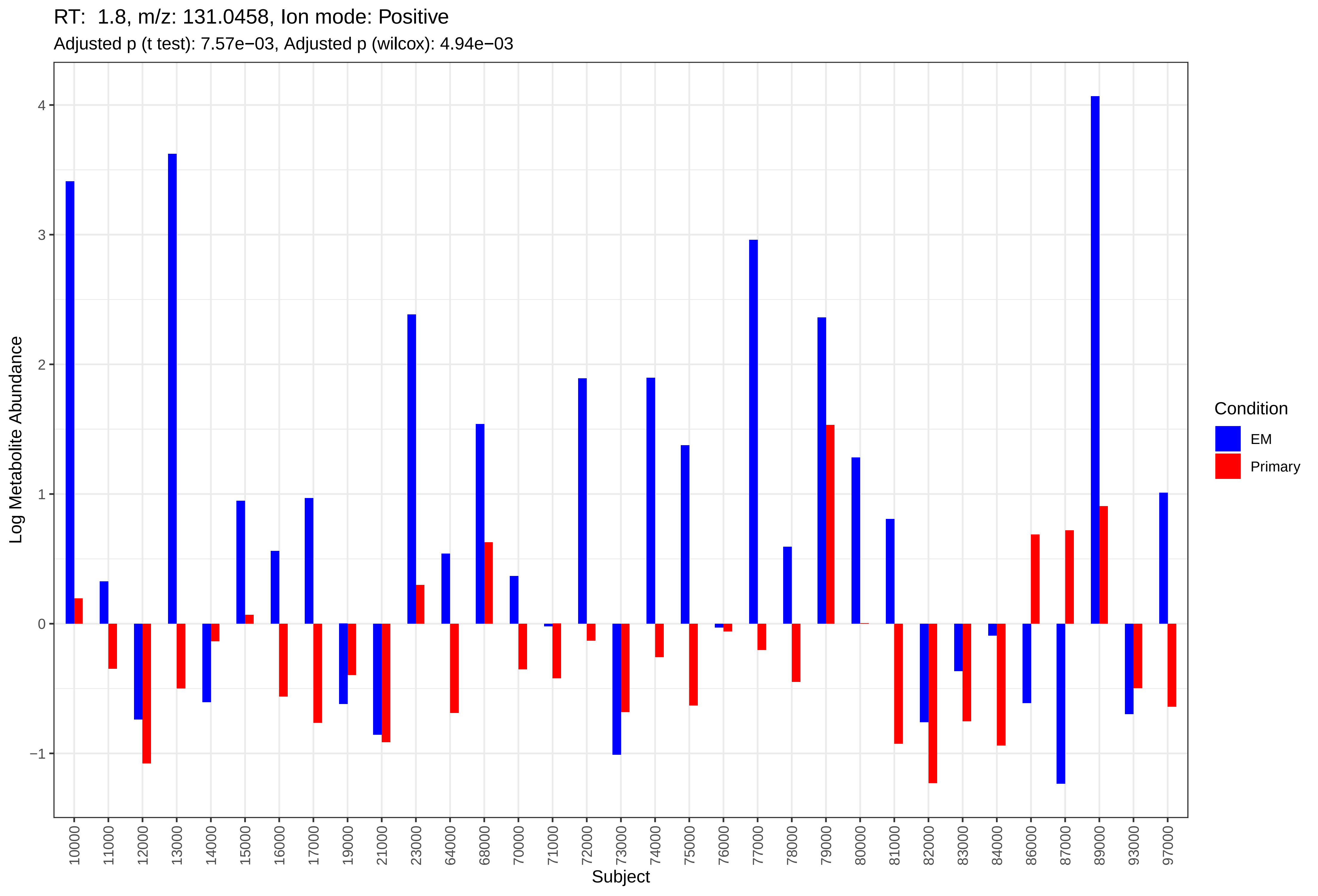
RT: 1.8, m/z: 131.0458, Ion mode: Positive
Adjusted p (t test): 7.57e−03, Adjusted p (wilcox): 4.94e−03
Log Metabolite Abundance
Subject
Condition
EM
Primary
4
3
2
1
0
−1
10000
11000
12000
13000
14000
15000
16000
17000
19000
21000
23000
64000
68000
70000
71000
72000
73000
74000
75000
76000
77000
78000
79000
80000
81000
82000
83000
84000
86000
87000
89000
93000
97000

RT: 0.7, m/z: 174.9530, Ion mode: Positive

Adjusted p (t test): 5.49e−03, Adjusted p (wilcox): 4.94e−03

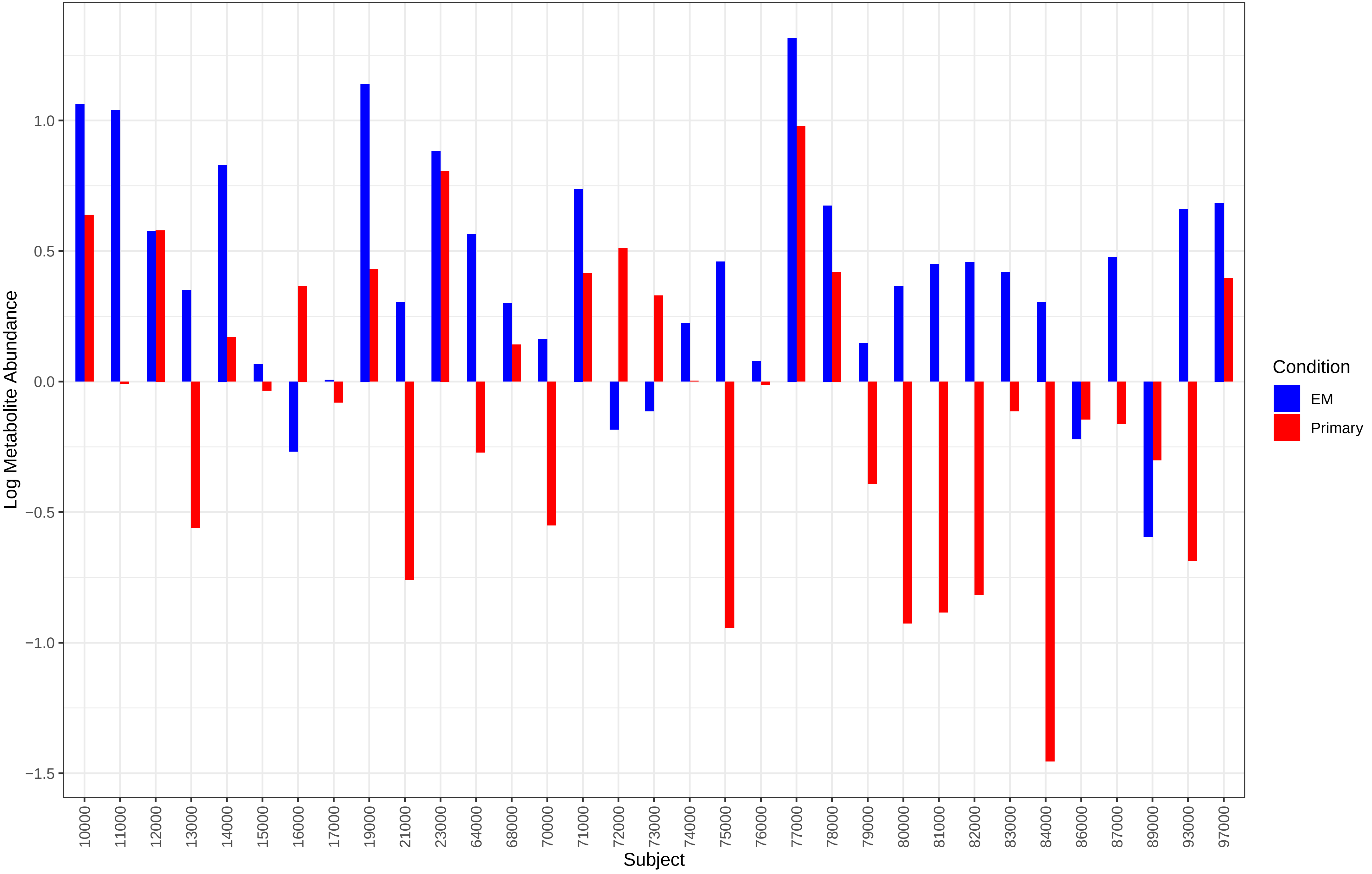

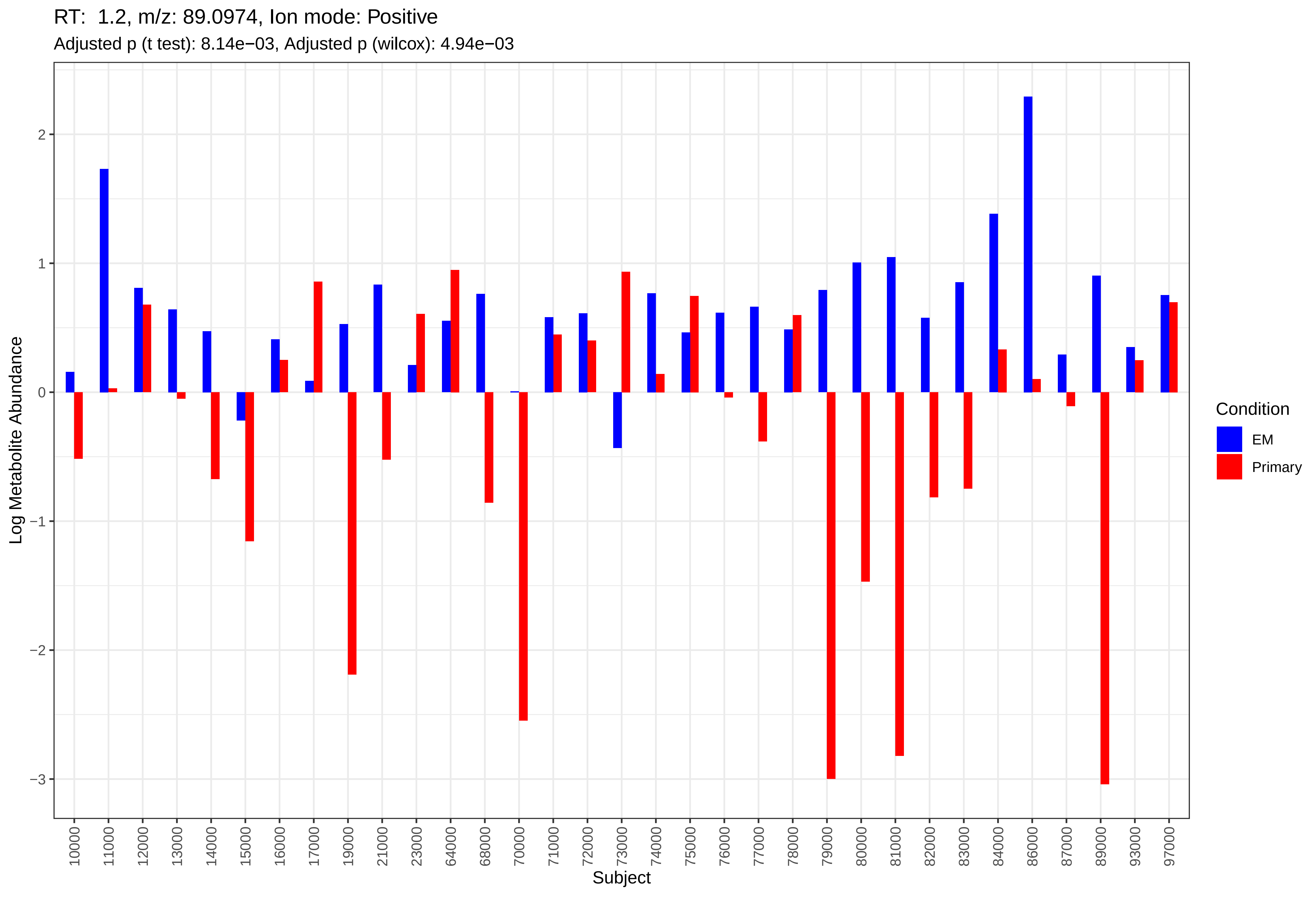

RT: 1.2, m/z: 89.0974, Ion mode: Positive
Adjusted p (t test): 8.14e−03, Adjusted p (wilcox): 4.94e−03
Log Metabolite Abundance
2
1
0
−1
−2
−3
10000
11000
12000
13000
14000
15000
16000
17000
19000
21000
23000
64000
68000
70000
71000
72000
73000
74000
75000
76000
77000
78000
79000
80000
81000
82000
83000
84000
86000
87000
89000
93000
97000
Subject
Condition
EM
Primary

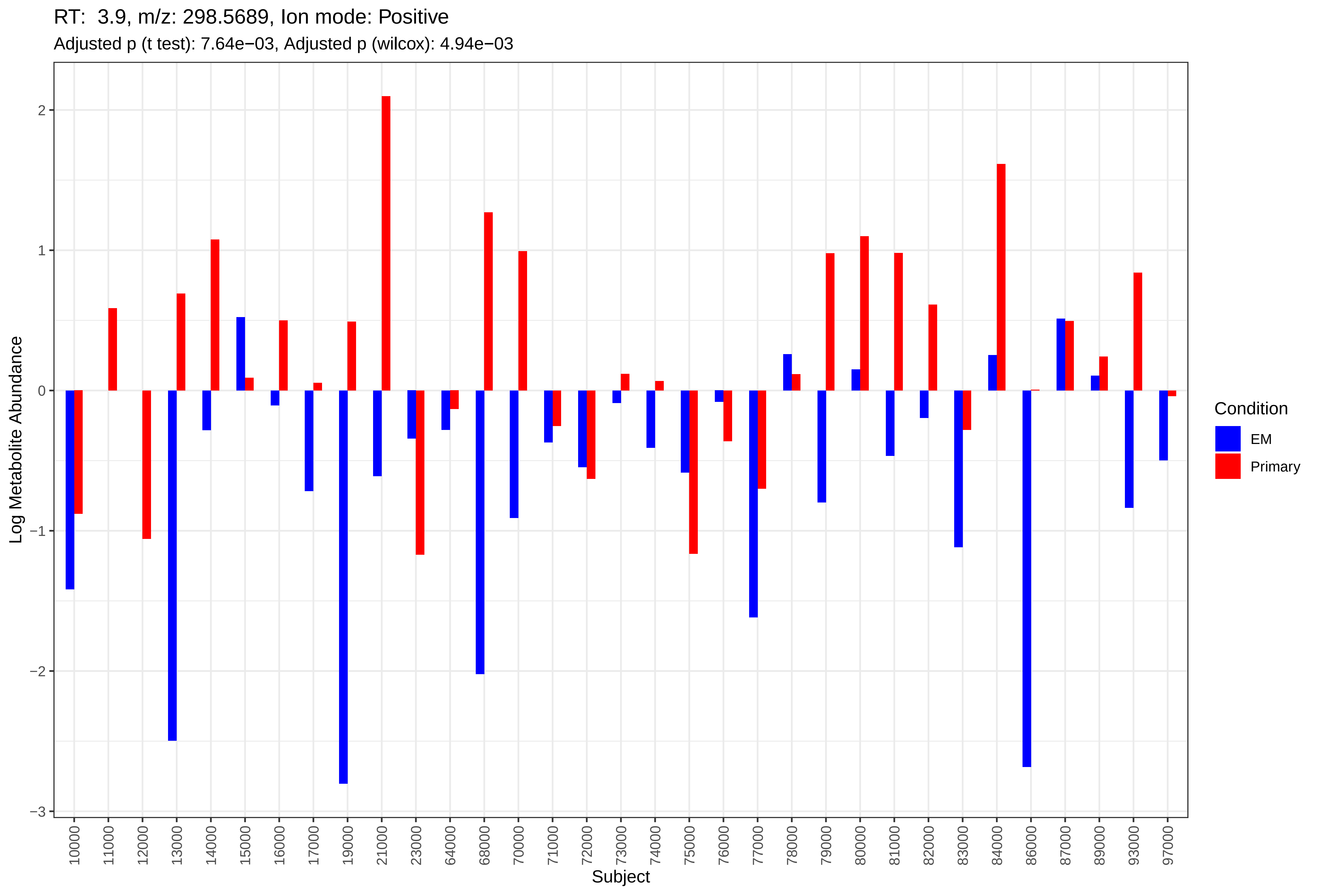

RT: 3.9, m/z: 298.5689, Ion mode: Positive
Adjusted p (t test): 7.64e−03, Adjusted p (wilcox): 4.94e−03
Log Metabolite Abundance
2
1
0
−1
−2
−3
10000
11000
12000
13000
14000
15000
16000
17000
19000
21000
23000
64000
68000
70000
71000
72000
73000
74000
75000
76000
77000
78000
79000
80000
81000
82000
83000
84000
86000
87000
89000
93000
97000
Subject
Condition
EM
Primary

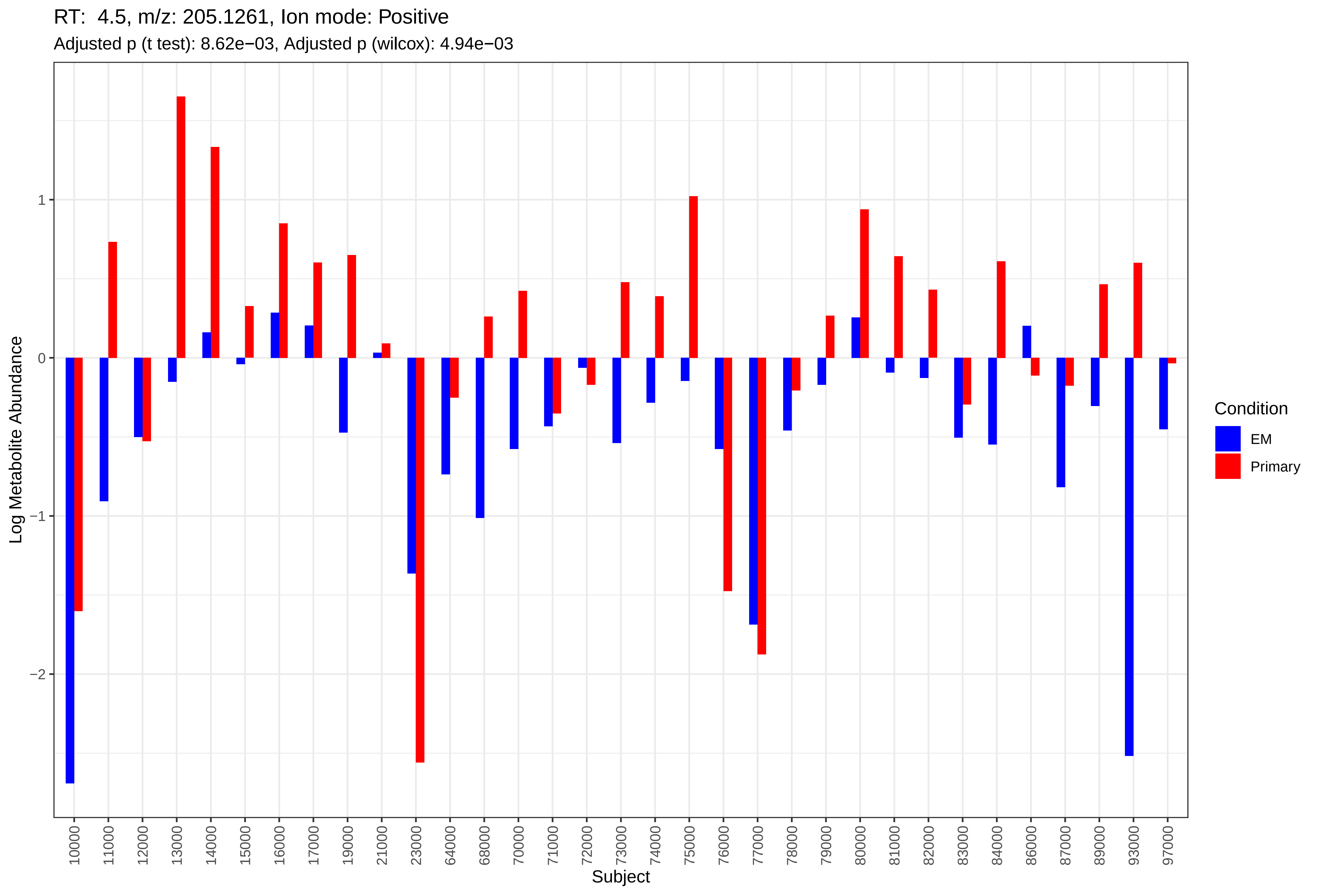

RT: 4.5, m/z: 205.1261, Ion mode: Positive
Adjusted p (t test): 8.62e−03, Adjusted p (wilcox): 4.94e−03
Log Metabolite Abundance
1
0
−1
−2
10000
11000
12000
13000
14000
15000
16000
17000
19000
21000
23000
64000
68000
70000
71000
72000
73000
74000
75000
76000
77000
78000
79000
80000
81000
82000
83000
84000
86000
87000
89000
93000
97000
Subject
Condition
EM
Primary

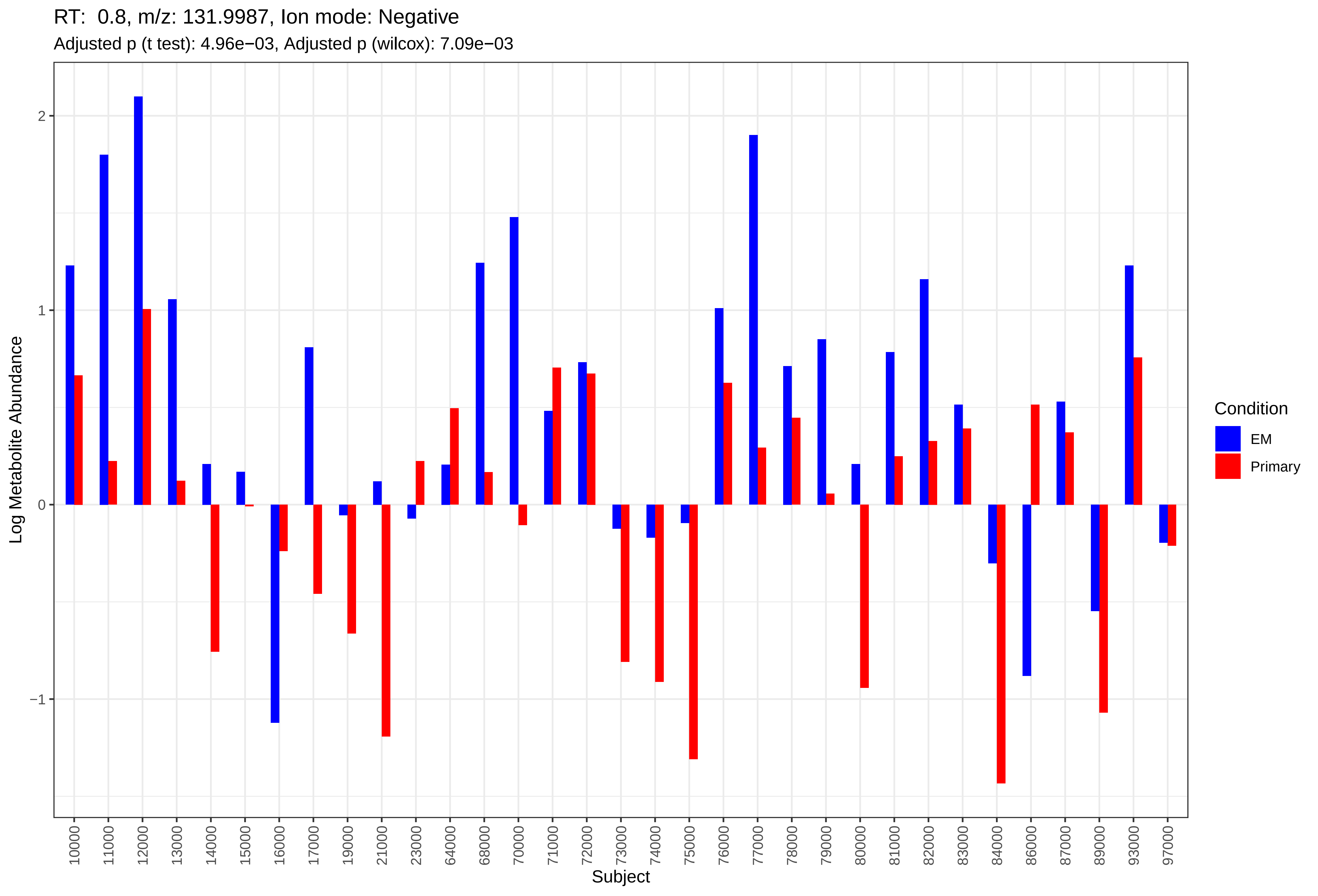
RT: 0.8, m/z: 131.9987, Ion mode: Negative
Adjusted p (t test): 4.96e−03, Adjusted p (wilcox): 7.09e−03
Log Metabolite Abundance
2
1
0
−1
Subject
10000
11000
12000
13000
14000
15000
16000
17000
19000
21000
23000
64000
68000
70000
71000
72000
73000
74000
75000
76000
77000
78000
79000
80000
81000
82000
83000
84000
86000
87000
89000
93000
97000
Condition
EM
Primary

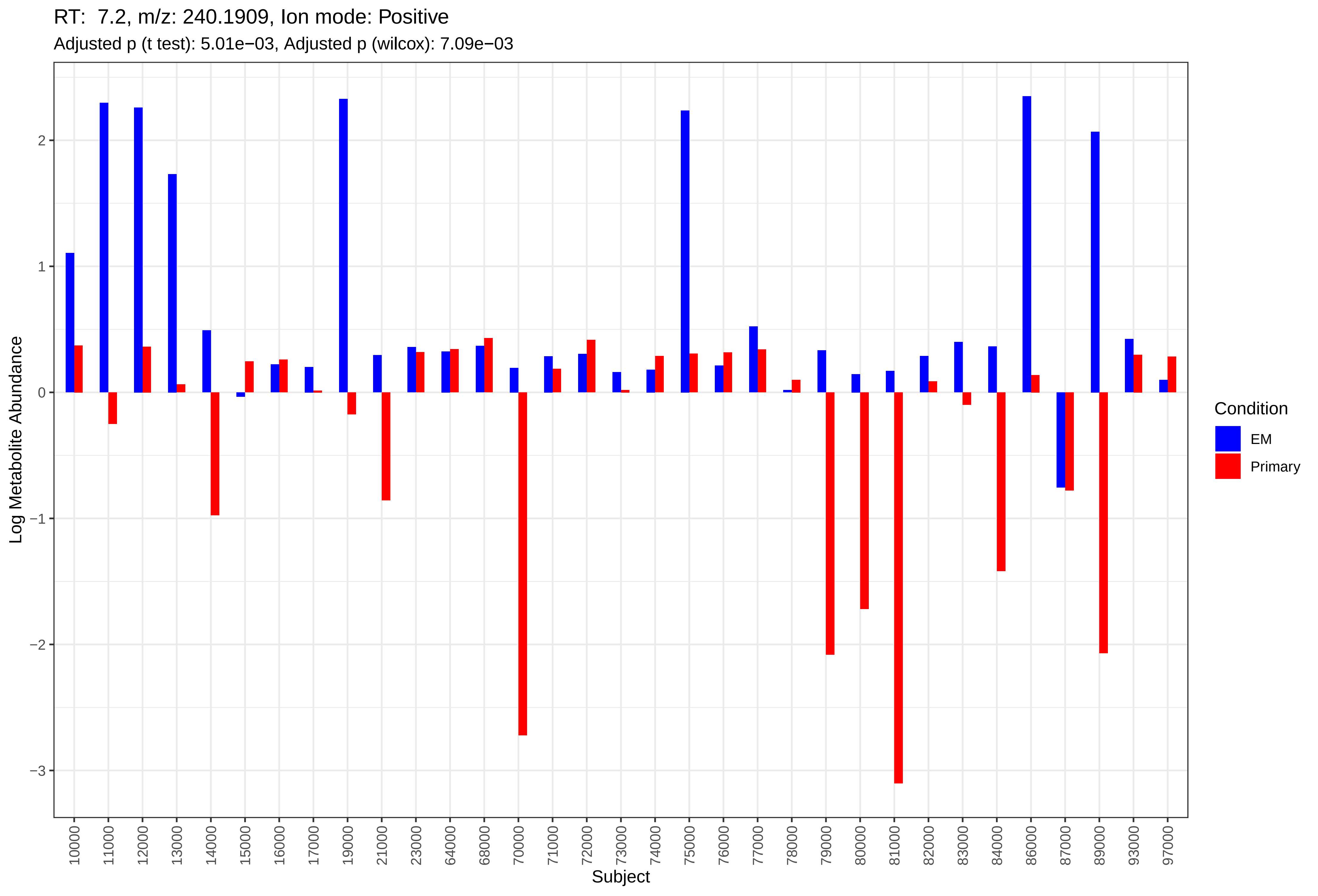

RT: 7.2, m/z: 240.1909, Ion mode: Positive
Adjusted p (t test): 5.01e−03, Adjusted p (wilcox): 7.09e−03
Log Metabolite Abundance
2
1
0
−1
−2
−3
10000
11000
12000
13000
14000
15000
16000
17000
19000
21000
23000
64000
68000
70000
71000
72000
73000
74000
75000
76000
77000
78000
79000
80000
81000
82000
83000
84000
86000
87000
89000
93000
97000
Subject
Condition
EM
Primary

RT: 2.9, m/z: 201.0868, Ion mode: Positive

Adjusted p (t test): 5.01e−03, Adjusted p (wilcox): 7.62e−03

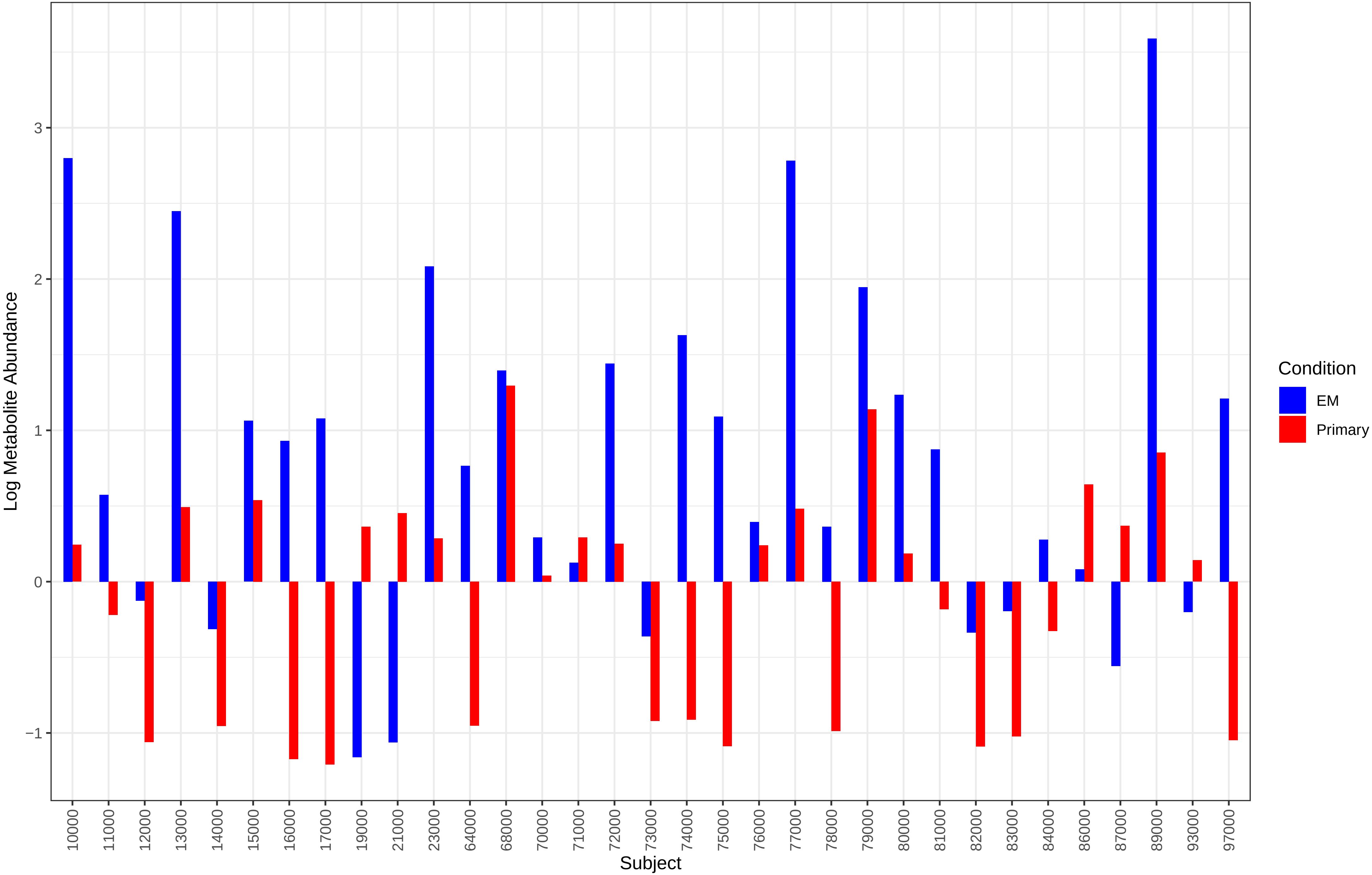

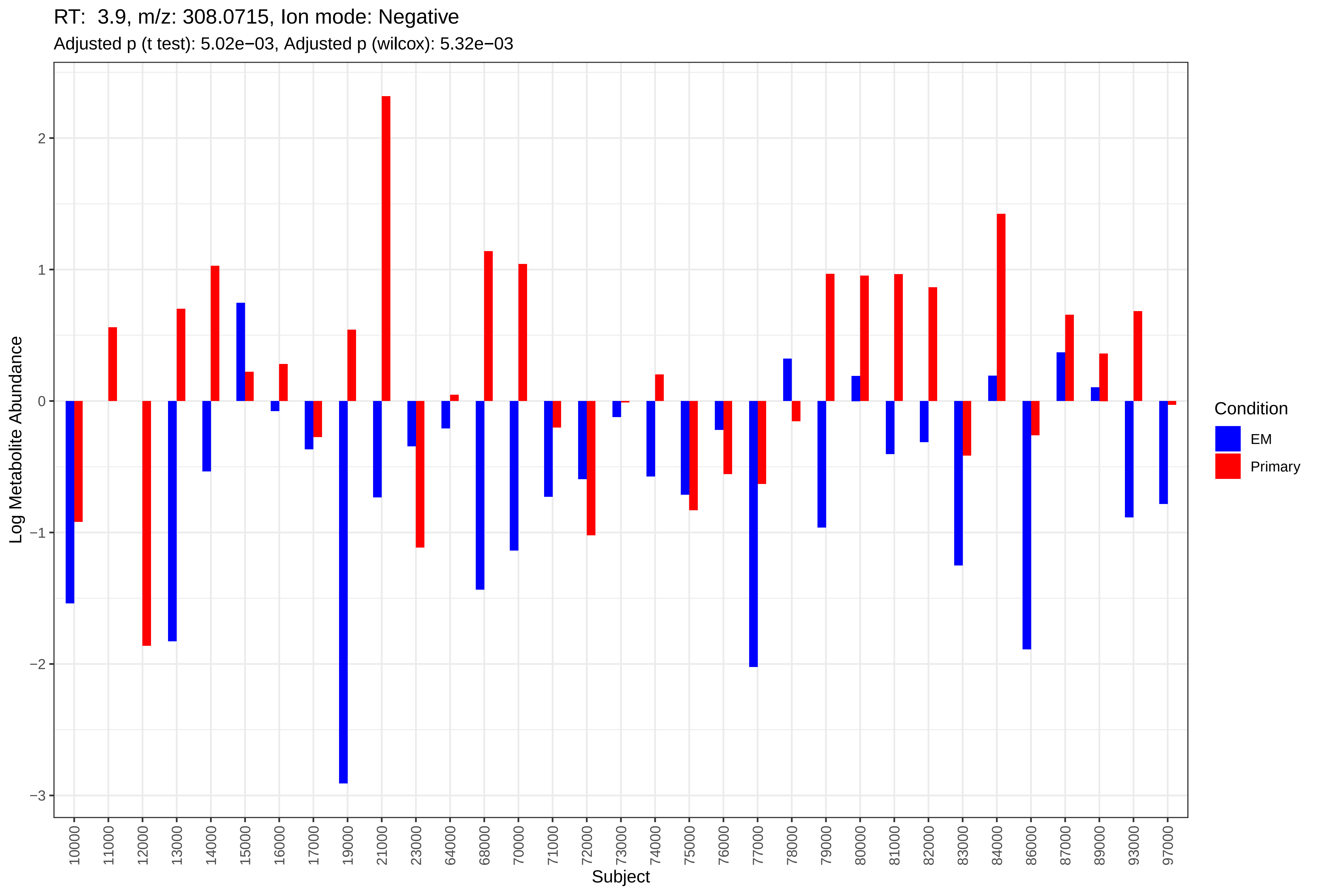
RT: 3.9, m/z: 308.0715, Ion mode: Negative
Adjusted p (t test): 5.02e−03, Adjusted p (wilcox): 5.32e−03
Log Metabolite Abundance
2
1
0
−1
−2
−3
10000
11000
12000
13000
14000
15000
16000
17000
19000
21000
23000
64000
68000
70000
71000
72000
73000
74000
75000
76000
77000
78000
79000
80000
81000
82000
83000
84000
86000
87000
89000
93000
97000
Subject
Condition
EM
Primary

RT: 12.2, m/z: 171.1376, Ion mode: Positive

Adjusted p (t test): 5.02e−03, Adjusted p (wilcox): 6.21e−03

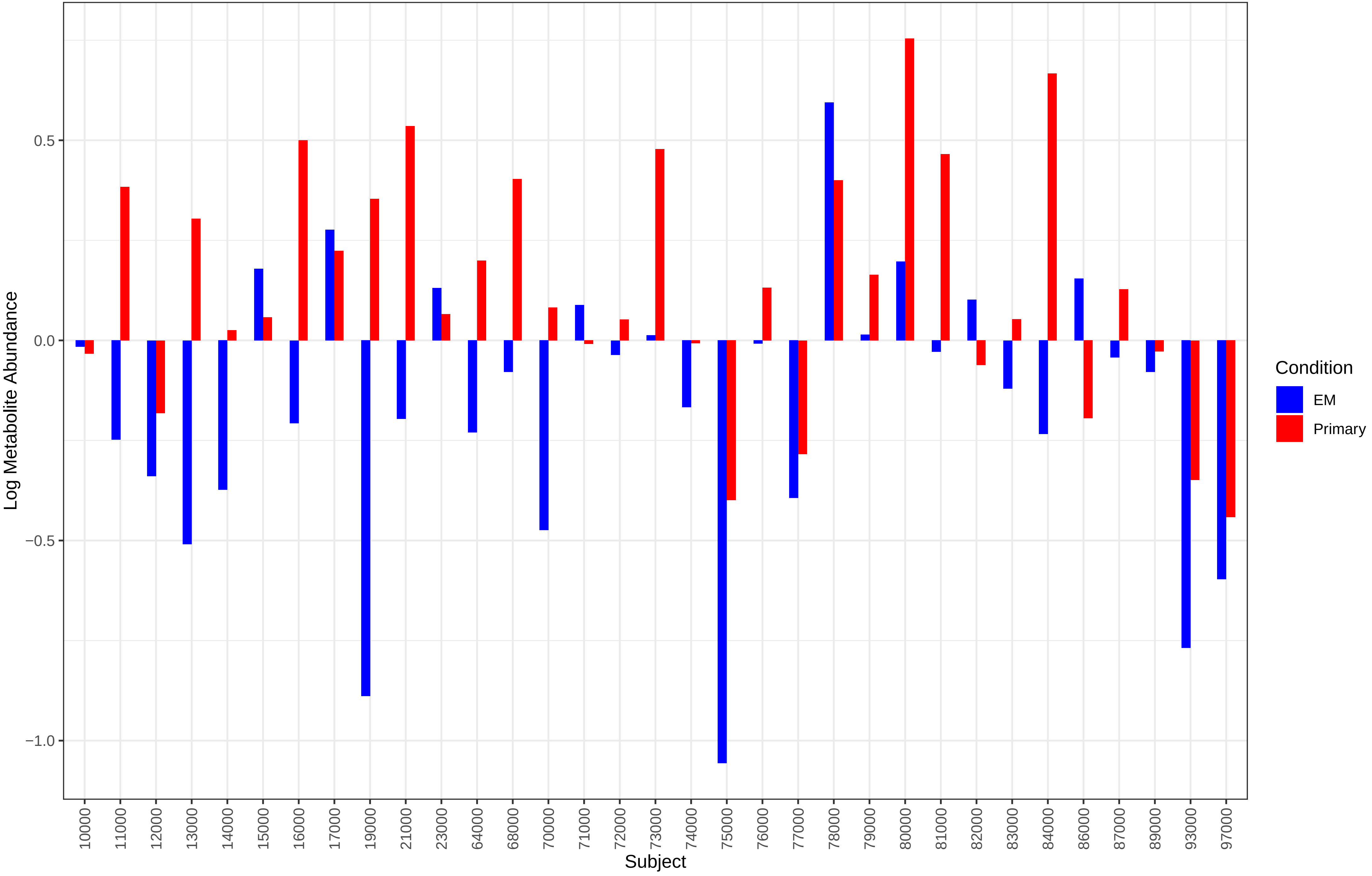

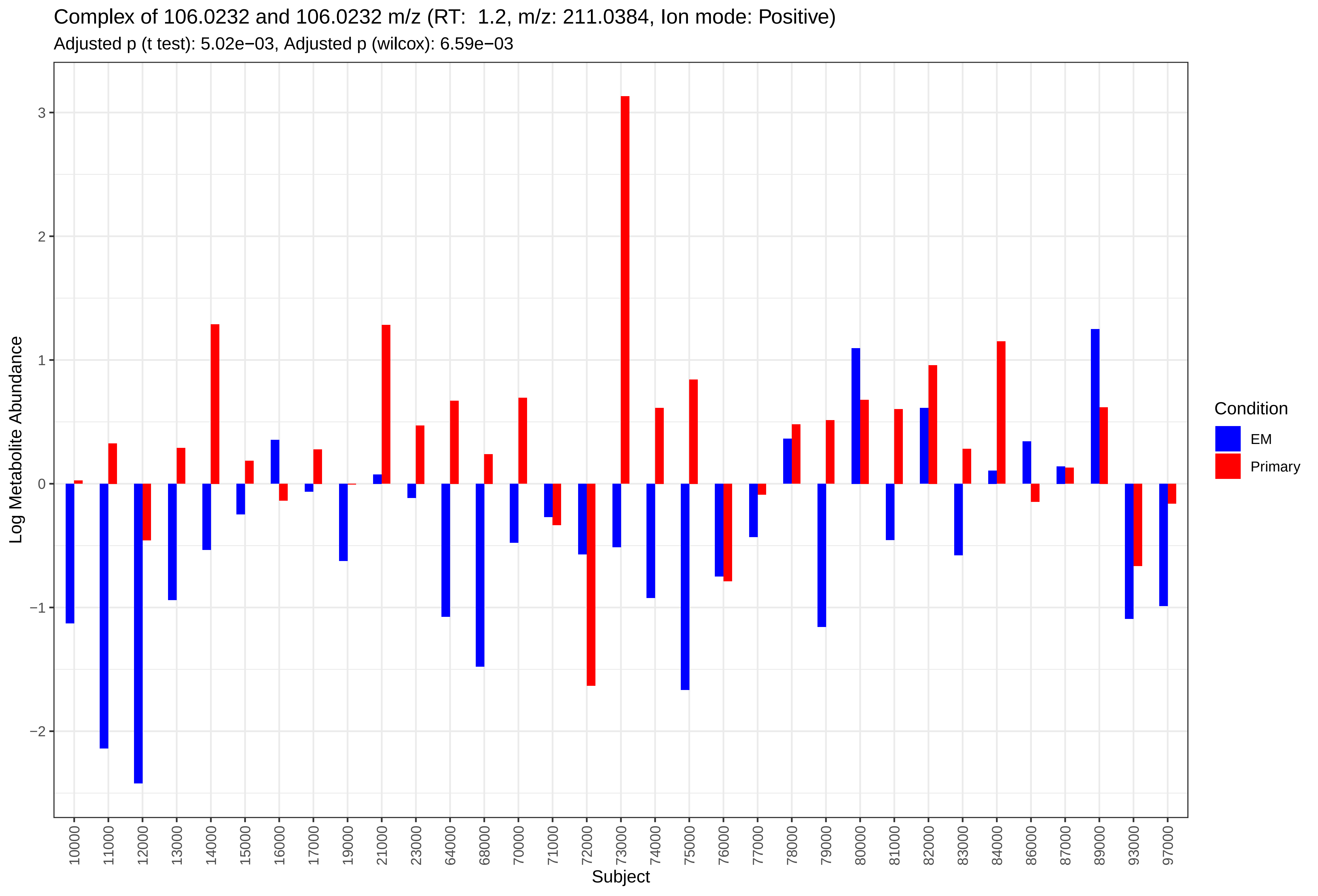

Complex of 106.0232 and 106.0232 m/z (RT: 1.2, m/z: 211.0384, Ion mode: Positive)
Adjusted p (t test): 5.02e−03, Adjusted p (wilcox): 6.59e−03
Log Metabolite Abundance
3
2
1
0
−1
−2
Subject
10000
11000
12000
13000
14000
15000
16000
17000
19000
21000
23000
64000
68000
70000
71000
72000
73000
74000
75000
76000
77000
78000
79000
80000
81000
82000
83000
84000
86000
87000
89000
93000
97000
Condition
EM
Primary

RT: 1.8, m/z: 271.9499, Ion mode: Negative

Adjusted p (t test): 5.04e−03, Adjusted p (wilcox): 7.62e−03

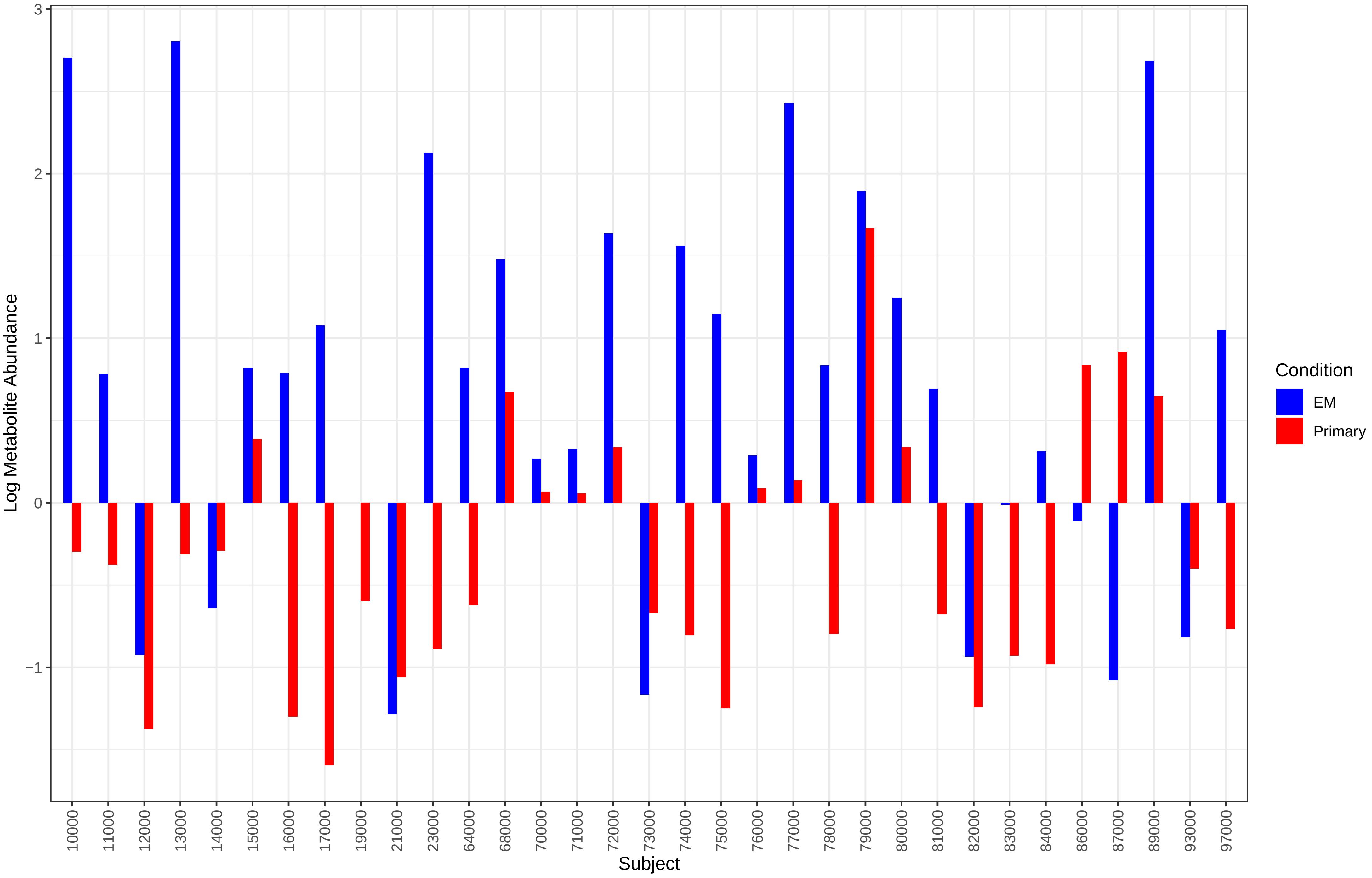

RT: 1.2, m/z: 217.0120, Ion mode: Negative

Adjusted p (t test): 5.13e−03, Adjusted p (wilcox): 6.36e−03

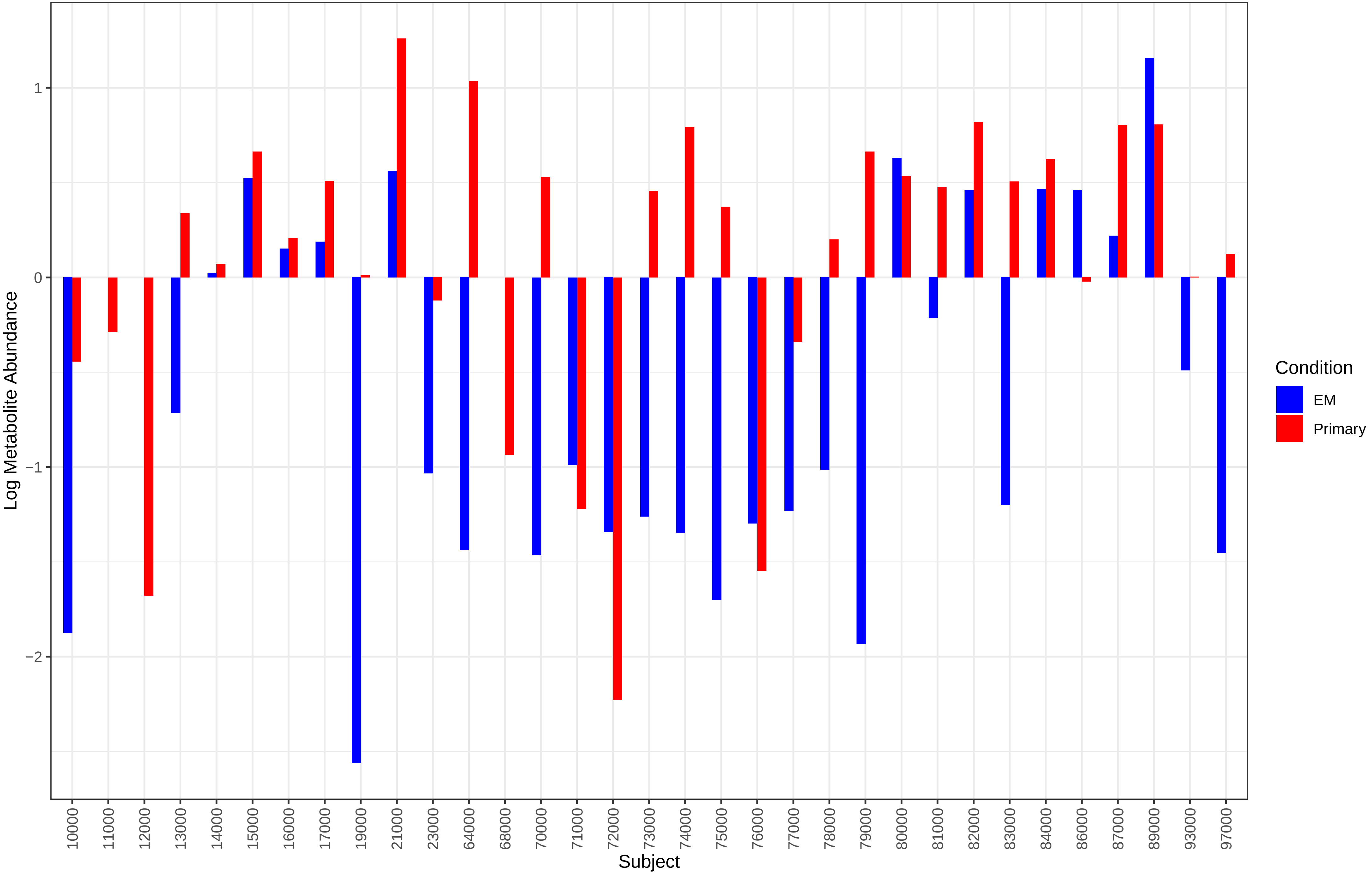

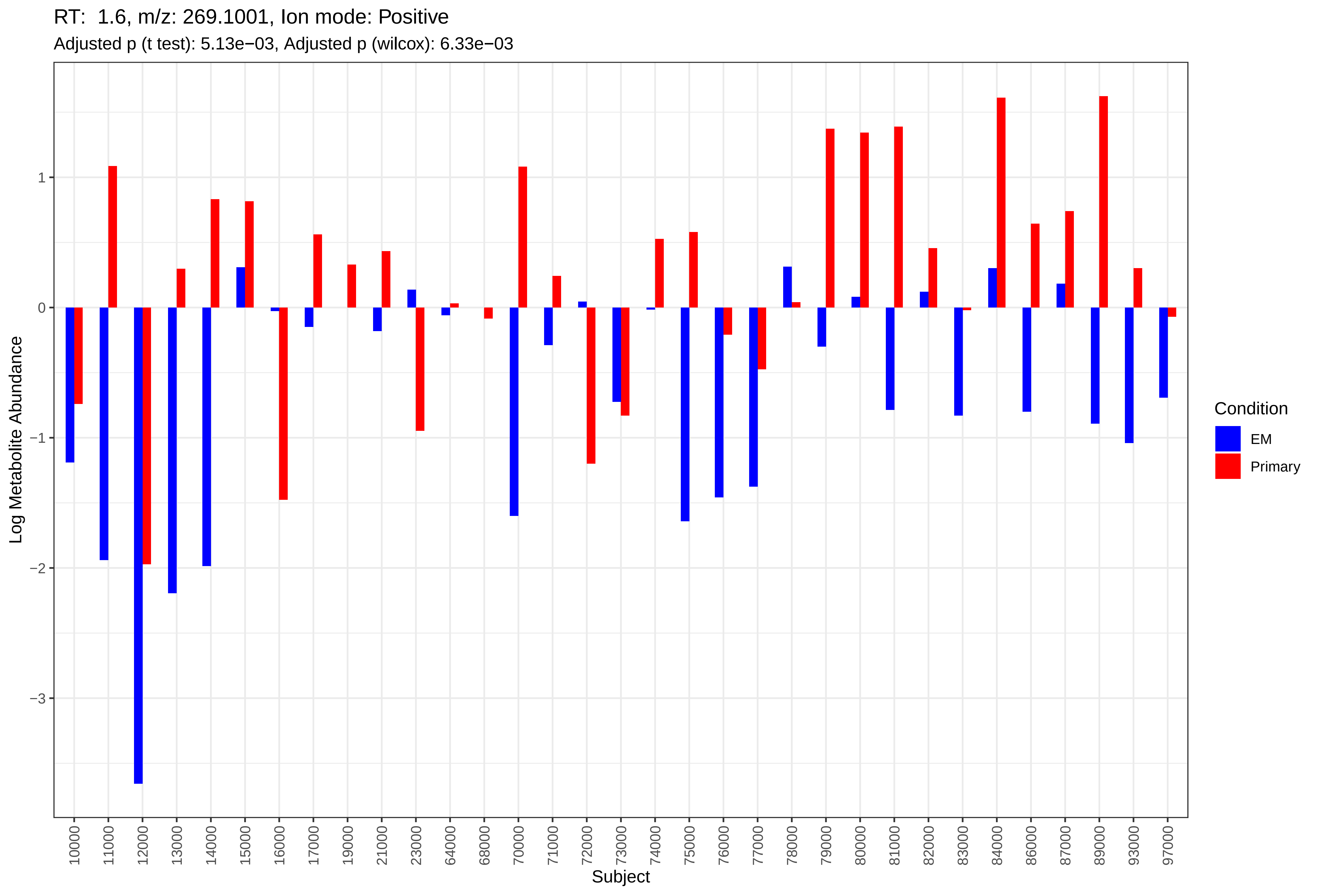
RT: 1.6, m/z: 269.1001, Ion mode: Positive
Adjusted p (t test): 5.13e−03, Adjusted p (wilcox): 6.33e−03
Log Metabolite Abundance
1
0
−1
−2
−3
10000
11000
12000
13000
14000
15000
16000
17000
19000
21000
23000
64000
68000
70000
71000
72000
73000
74000
75000
76000
77000
78000
79000
80000
81000
82000
83000
84000
86000
87000
89000
93000
97000
Subject
Condition
EM
Primary

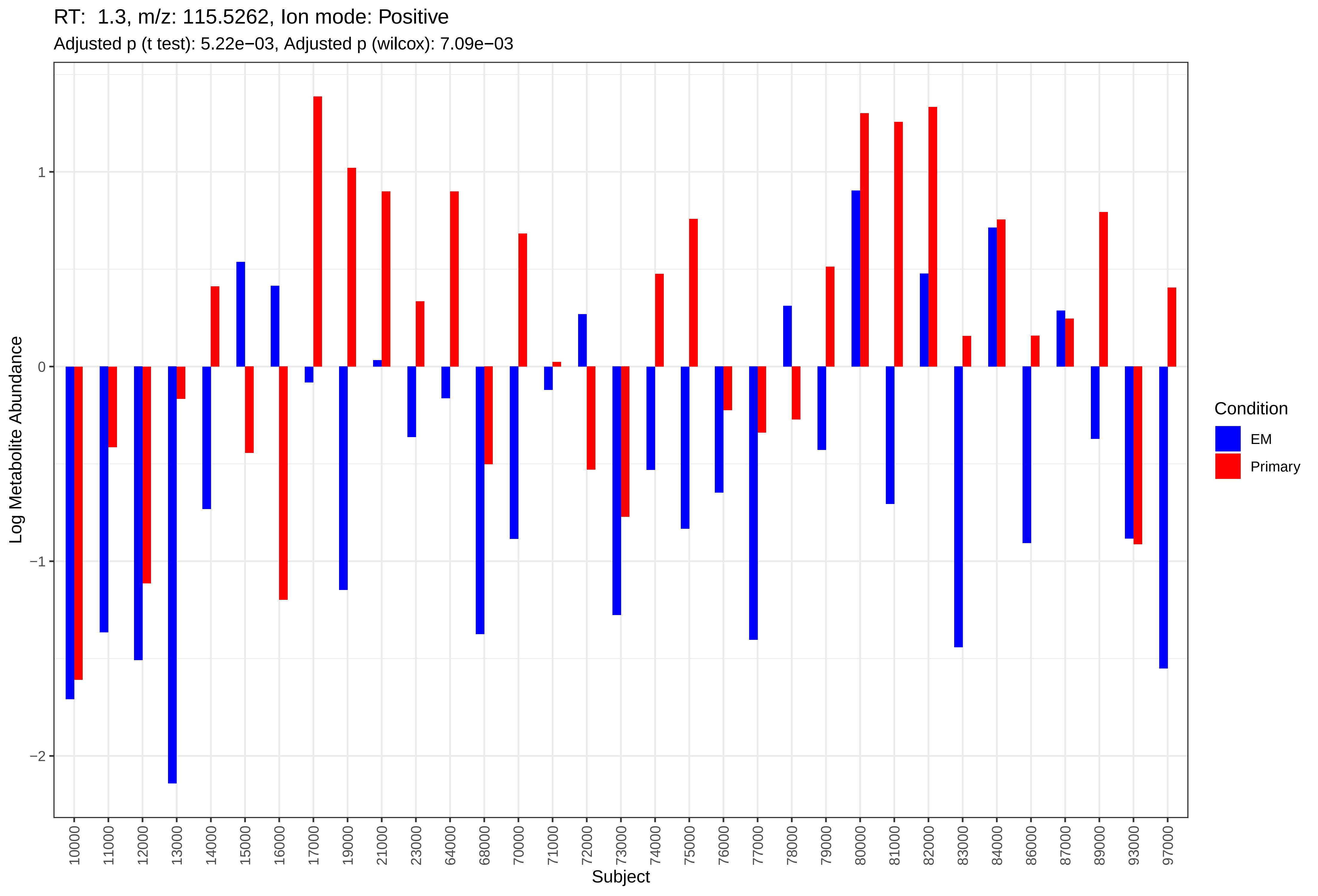
RT: 1.3, m/z: 115.5262, Ion mode: Positive
Adjusted p (t test): 5.22e−03, Adjusted p (wilcox): 7.09e−03
Log Metabolite Abundance
1
0
−1
−2
Subject
10000
11000
12000
13000
14000
15000
16000
17000
19000
21000
23000
64000
68000
70000
71000
72000
73000
74000
75000
76000
77000
78000
79000
80000
81000
82000
83000
84000
86000
87000
89000
93000
97000
Condition
EM
Primary

RT: 2.8, m/z: 183.0411, Ion mode: Negative

Adjusted p (t test): 5.24e−03, Adjusted p (wilcox): 6.36e−03

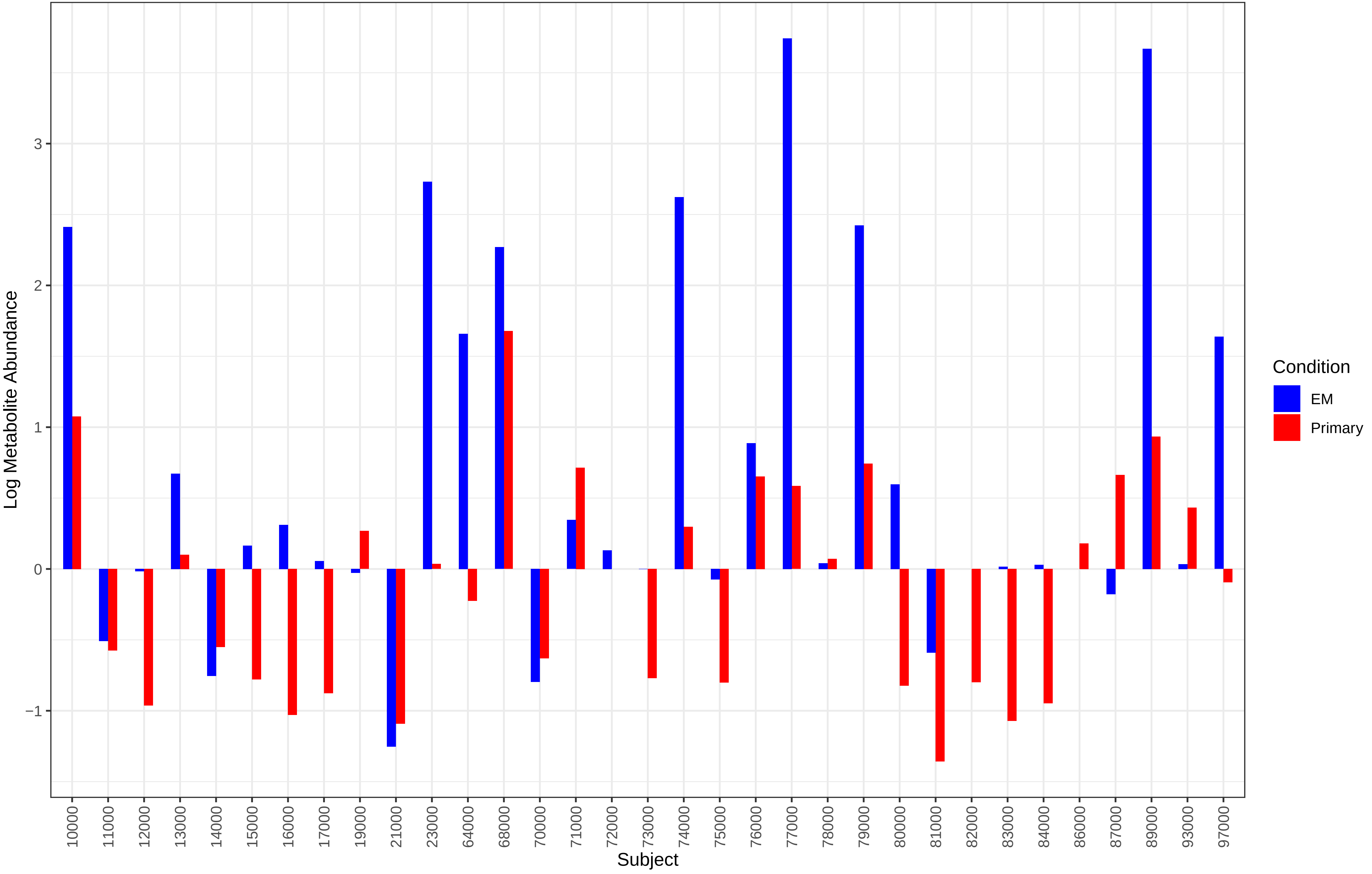

RT: 7.7, m/z: 709.7917, Ion mode: Positive

Adjusted p (t test): 8.14e−03, Adjusted p (wilcox): 5.29e−03

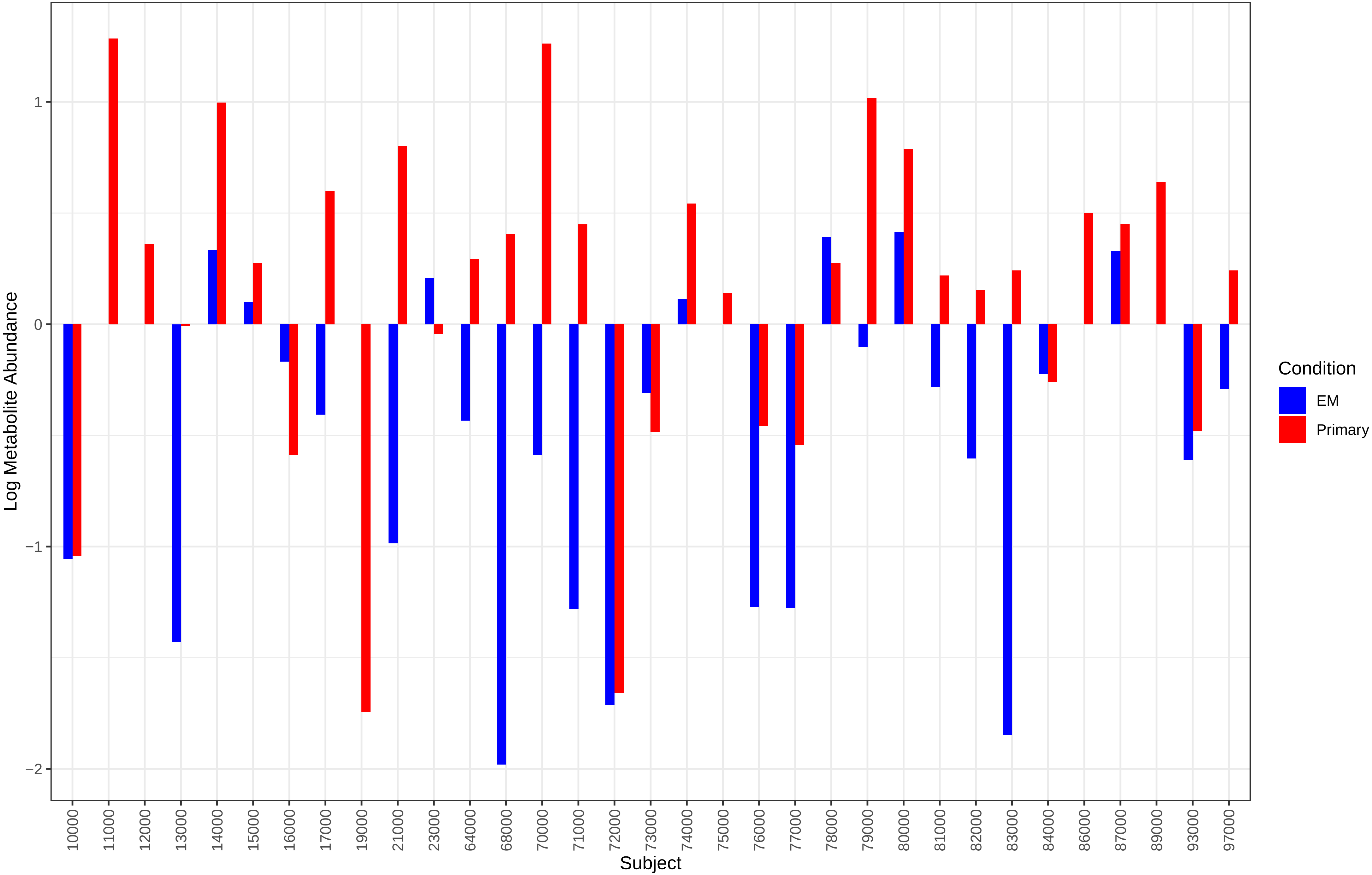

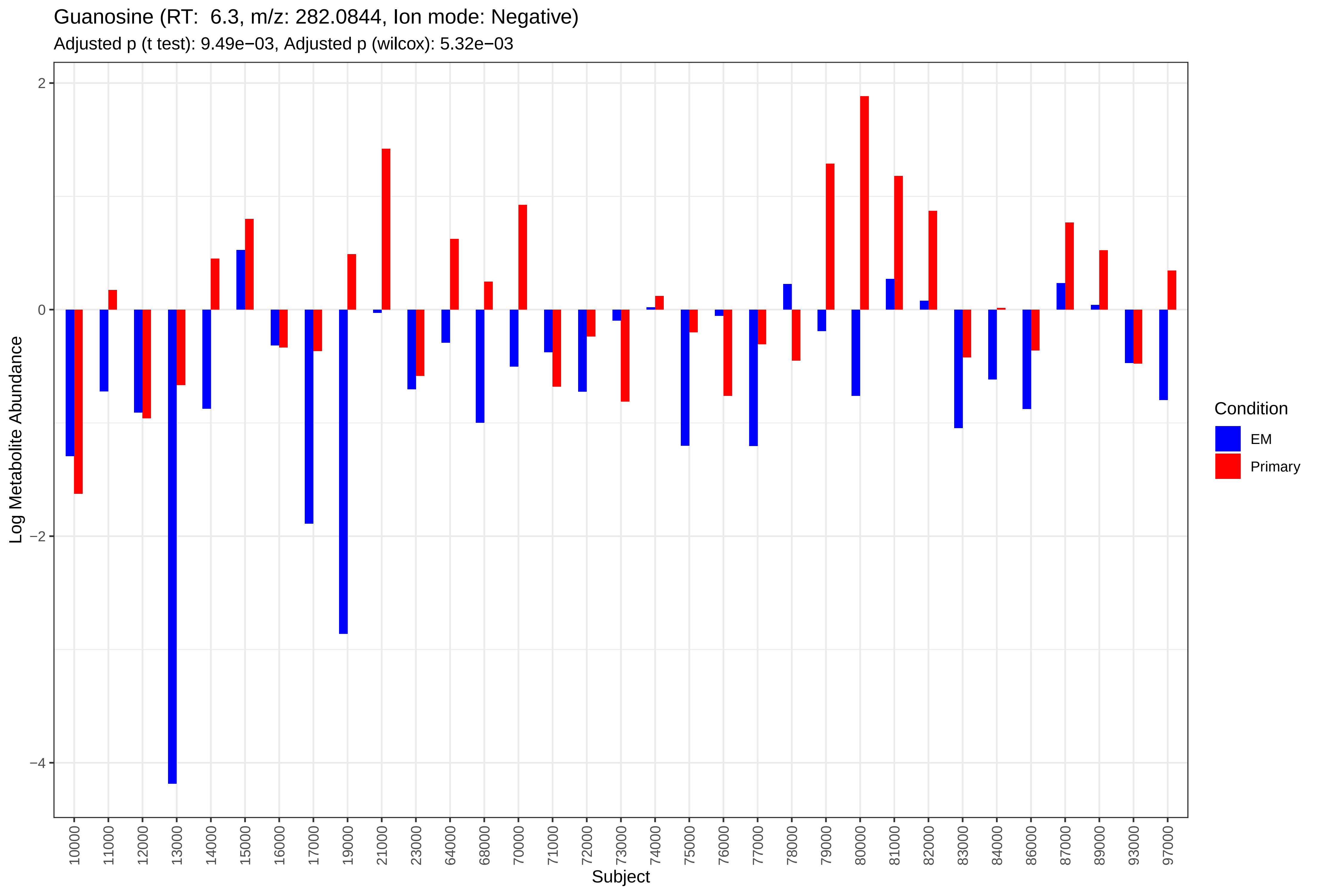
Guanosine (RT: 6.3, m/z: 282.0844, Ion mode: Negative)
Adjusted p (t test): 9.49e−03, Adjusted p (wilcox): 5.32e−03
Log Metabolite Abundance
2
0
−2
−4
Subject
10000
11000
12000
13000
14000
15000
16000
17000
19000
21000
23000
64000
68000
70000
71000
72000
73000
74000
75000
76000
77000
78000
79000
80000
81000
82000
83000
84000
86000
87000
89000
93000
97000
Condition
EM
Primary

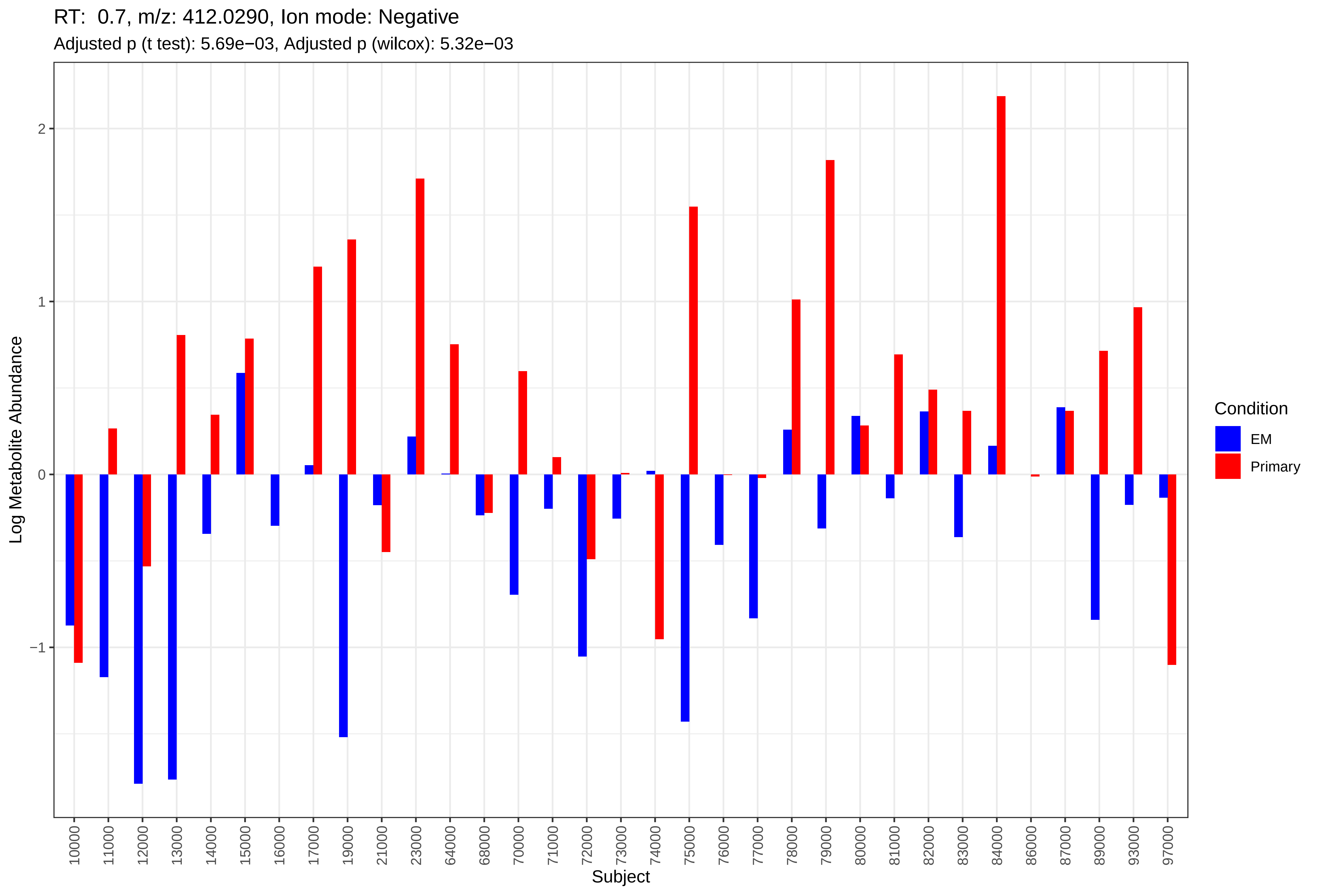

RT: 0.7, m/z: 412.0290, Ion mode: Negative
Adjusted p (t test): 5.69e−03, Adjusted p (wilcox): 5.32e−03
Log Metabolite Abundance
2
1
0
−1
Subject
10000
11000
12000
13000
14000
15000
16000
17000
19000
21000
23000
64000
68000
70000
71000
72000
73000
74000
75000
76000
77000
78000
79000
80000
81000
82000
83000
84000
86000
87000
89000
93000
97000
Condition
EM
Primary

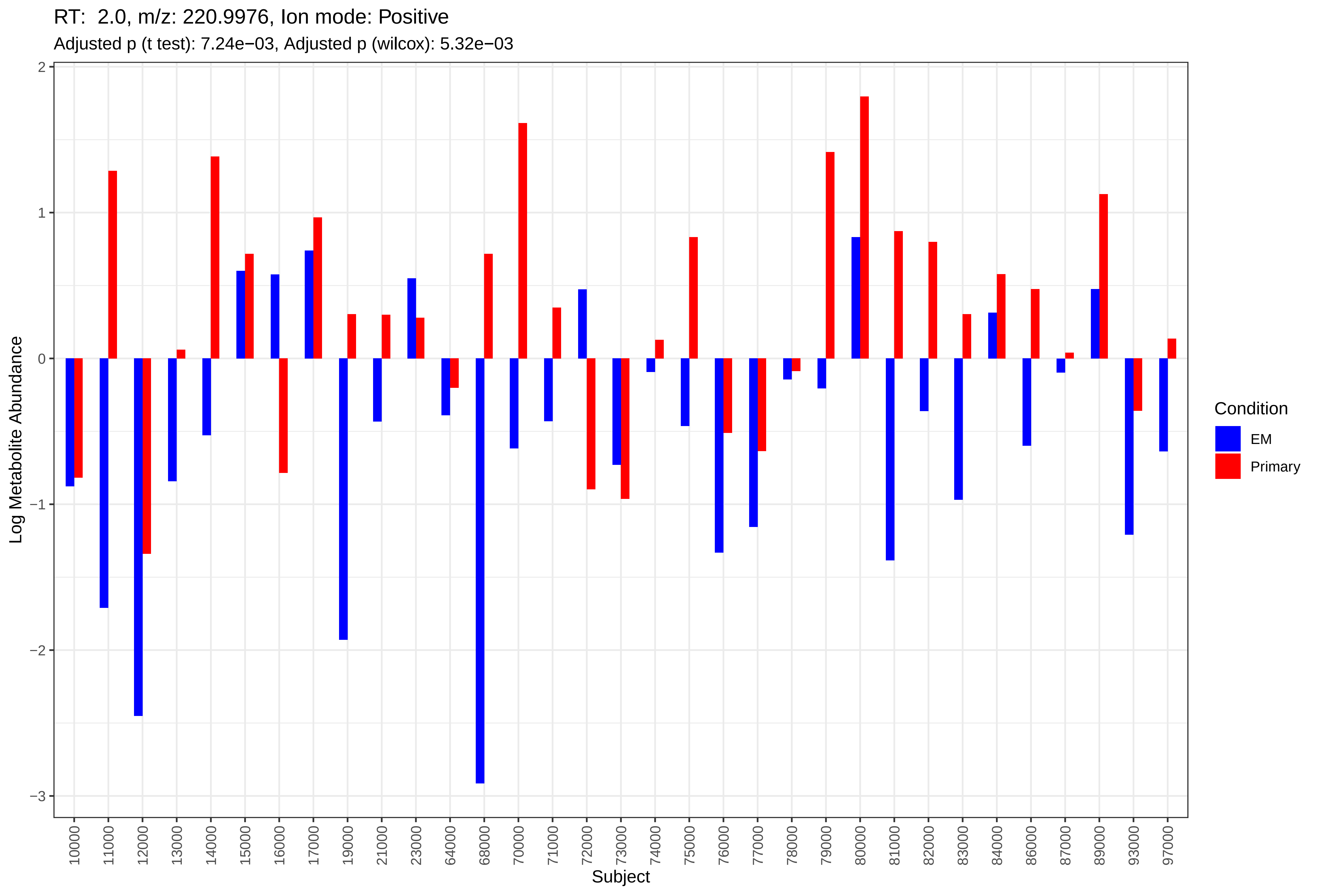

RT: 2.0, m/z: 220.9976, Ion mode: Positive
Adjusted p (t test): 7.24e−03, Adjusted p (wilcox): 5.32e−03
Log Metabolite Abundance
2
1
0
−1
−2
−3
10000
11000
12000
13000
14000
15000
16000
17000
19000
21000
23000
64000
68000
70000
71000
72000
73000
74000
75000
76000
77000
78000
79000
80000
81000
82000
83000
84000
86000
87000
89000
93000
97000
Subject
Condition
EM
Primary

RT: 2.4, m/z: 281.2005, Ion mode: Positive

Adjusted p (t test): 9.13e−03, Adjusted p (wilcox): 5.32e−03

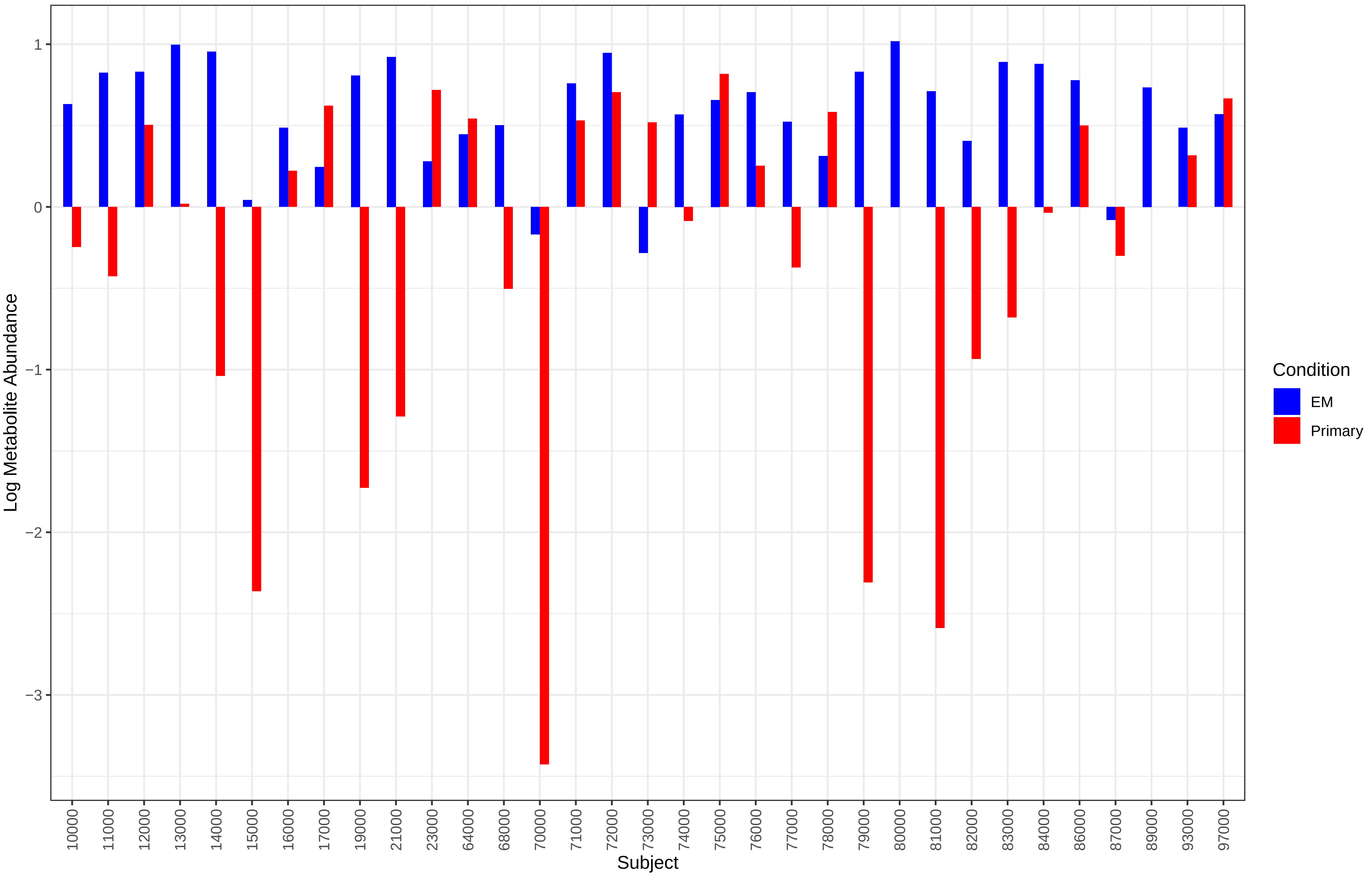

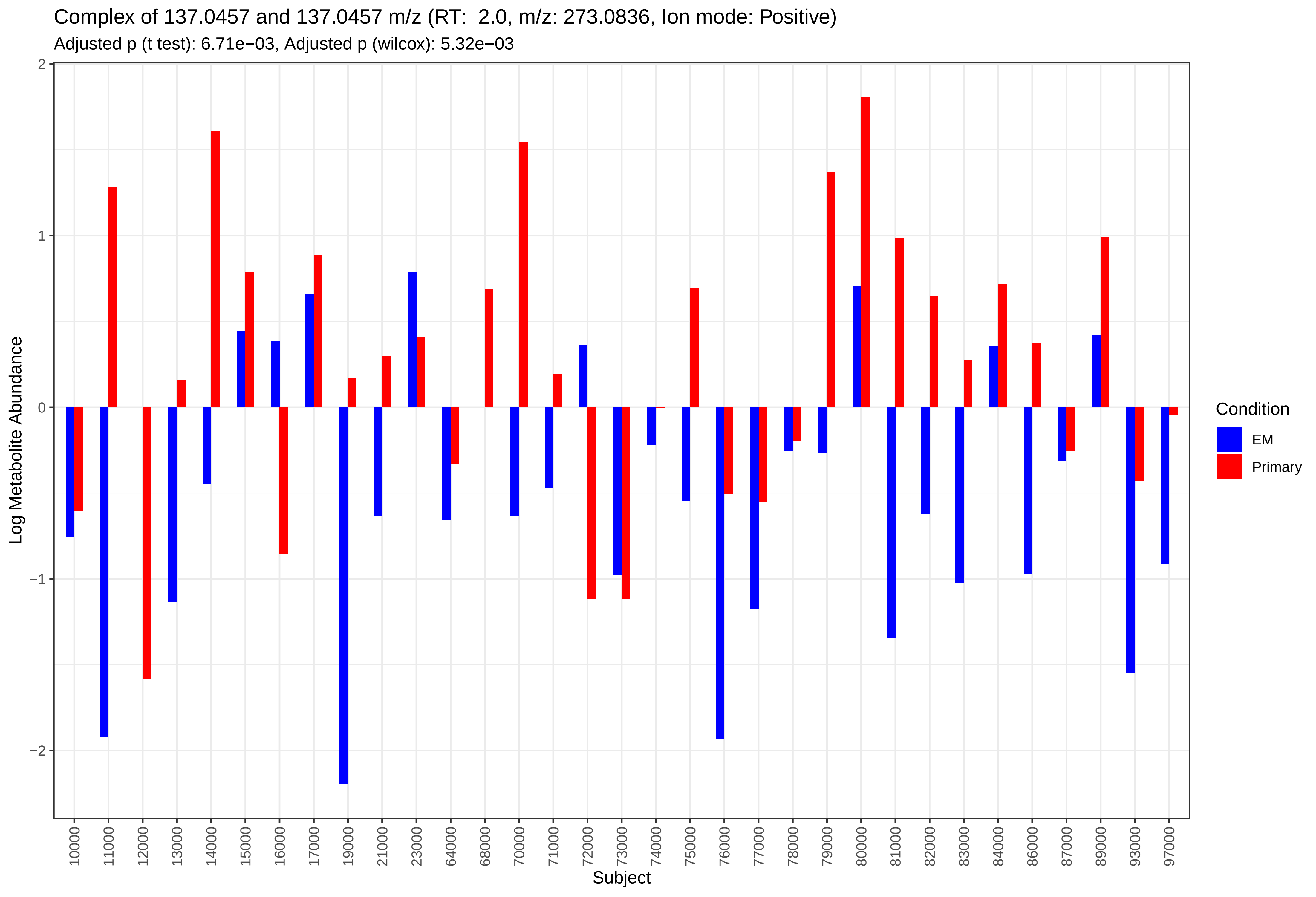

Complex of 137.0457 and 137.0457 m/z (RT: 2.0, m/z: 273.0836, Ion mode: Positive)
Adjusted p (t test): 6.71e−03, Adjusted p (wilcox): 5.32e−03
Log Metabolite Abundance
Subject
Condition
EM
Primary
2
1
0
−1
−2
10000
11000
12000
13000
14000
15000
16000
17000
19000
21000
23000
64000
68000
70000
71000
72000
73000
74000
75000
76000
77000
78000
79000
80000
81000
82000
83000
84000
86000
87000
89000
93000
97000

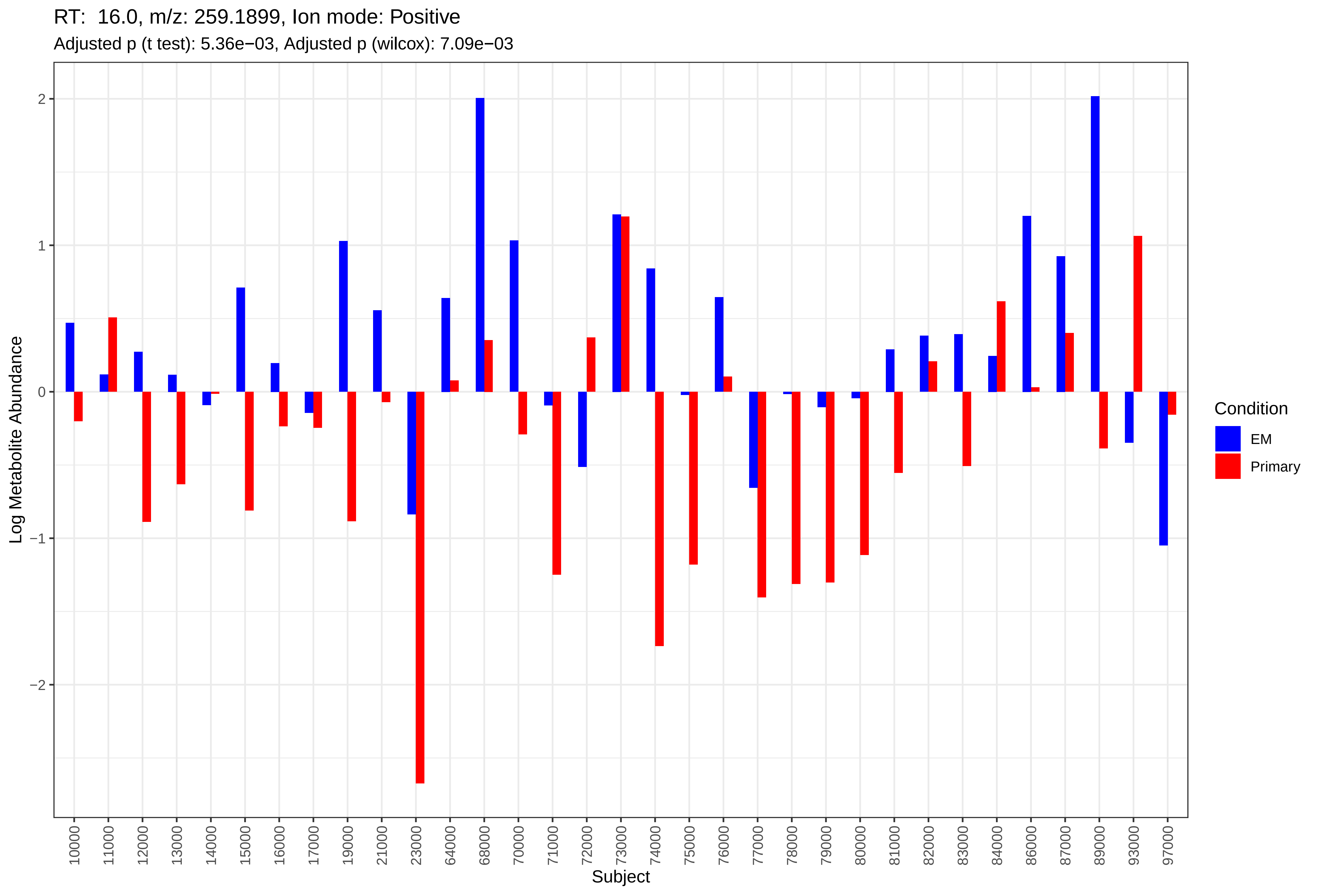
RT: 16.0, m/z: 259.1899, Ion mode: Positive
Adjusted p (t test): 5.36e−03, Adjusted p (wilcox): 7.09e−03
Log Metabolite Abundance
2
1
0
−1
−2
10000
11000
12000
13000
14000
15000
16000
17000
19000
21000
23000
64000
68000
70000
71000
72000
73000
74000
75000
76000
77000
78000
79000
80000
81000
82000
83000
84000
86000
87000
89000
93000
97000
Subject
Condition
EM
Primary

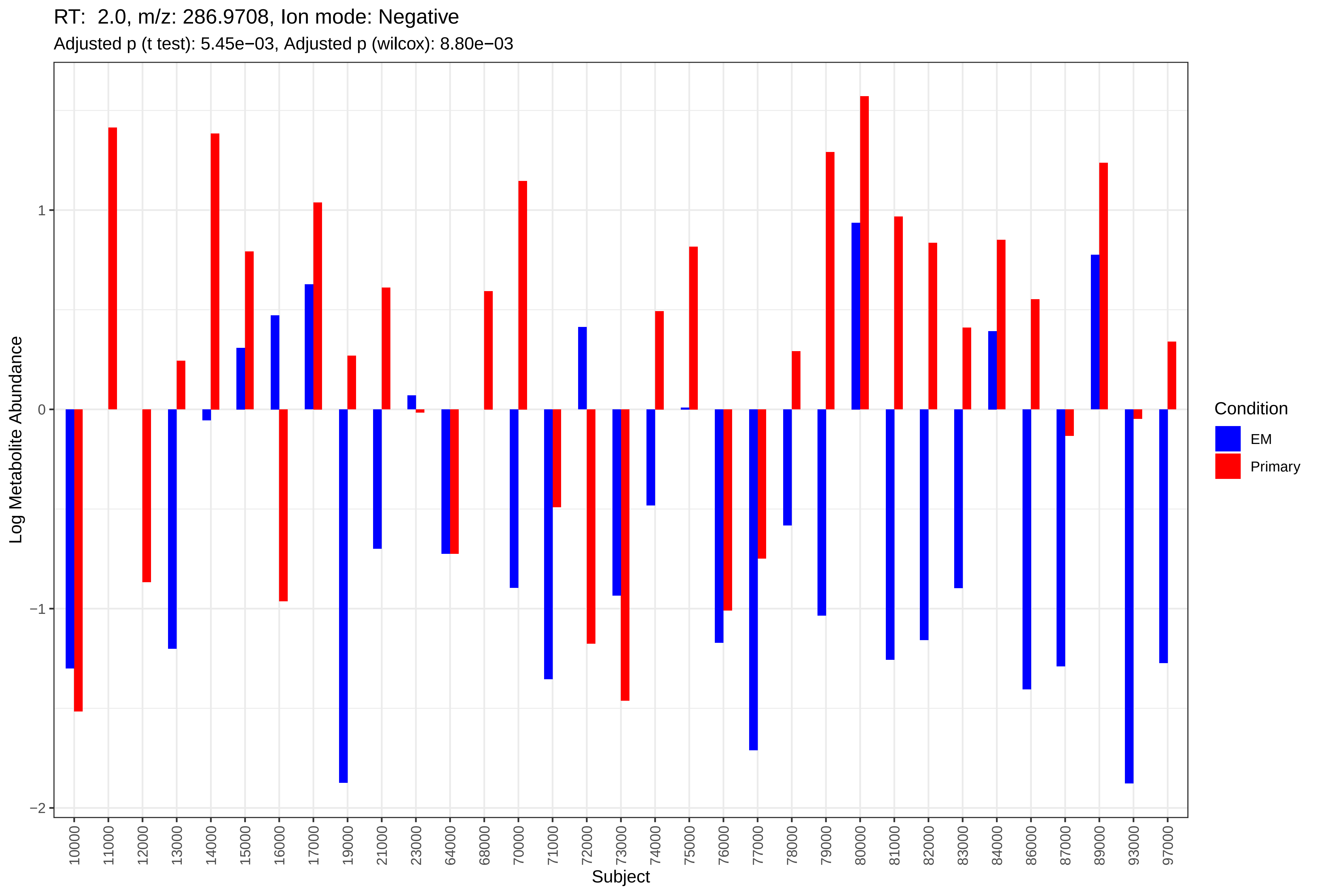
RT: 2.0, m/z: 286.9708, Ion mode: Negative
Adjusted p (t test): 5.45e−03, Adjusted p (wilcox): 8.80e−03
Log Metabolite Abundance
1
0
−1
−2
10000
11000
12000
13000
14000
15000
16000
17000
19000
21000
23000
64000
68000
70000
71000
72000
73000
74000
75000
76000
77000
78000
79000
80000
81000
82000
83000
84000
86000
87000
89000
93000
97000
Subject
Condition
EM
Primary

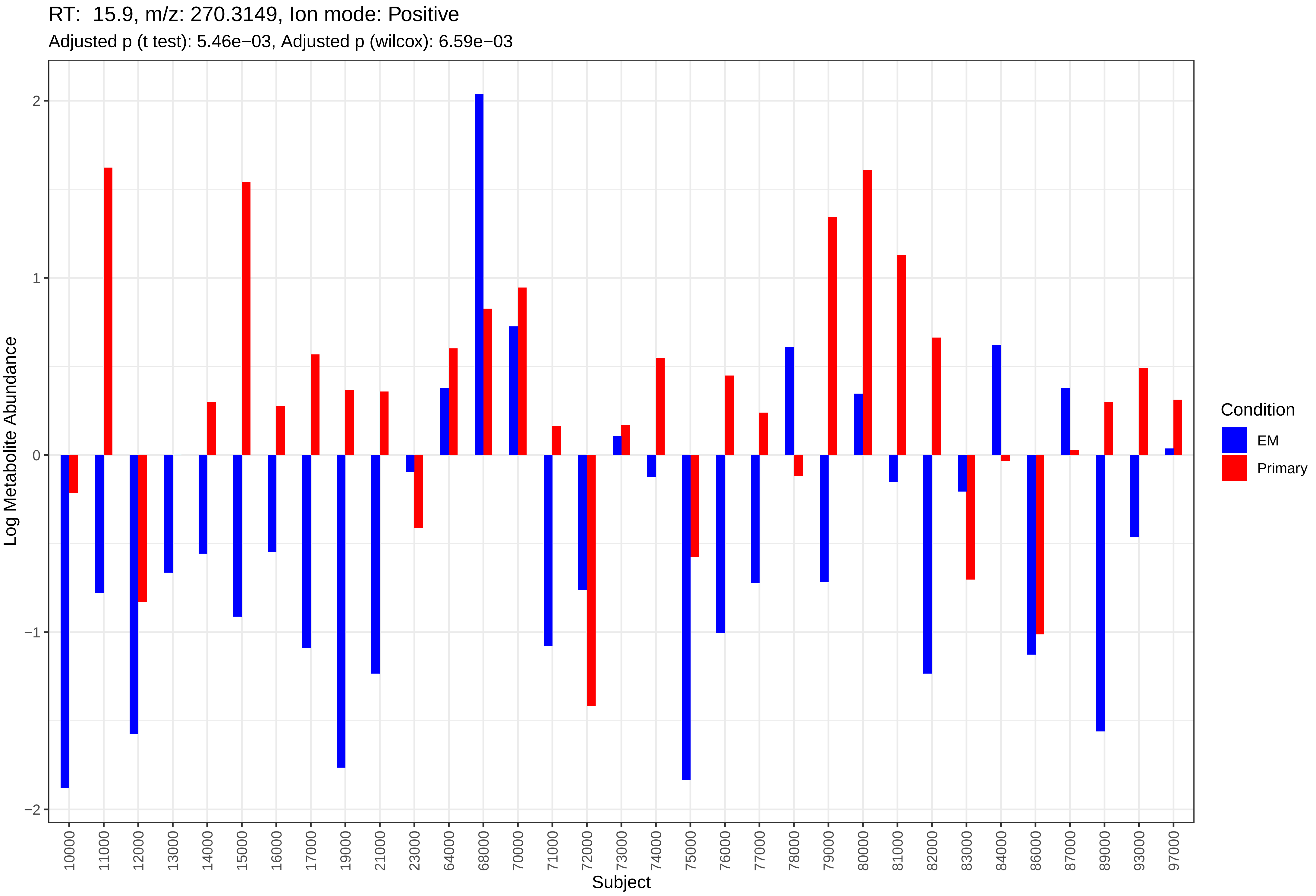
RT: 15.9, m/z: 270.3149, Ion mode: Positive
Adjusted p (t test): 5.46e−03, Adjusted p (wilcox): 6.59e−03
Log Metabolite Abundance
2
1
0
−1
−2
Subject
10000
11000
12000
13000
14000
15000
16000
17000
19000
21000
23000
64000
68000
70000
71000
72000
73000
74000
75000
76000
77000
78000
79000
80000
81000
82000
83000
84000
86000
87000
89000
93000
97000
Condition
EM
Primary

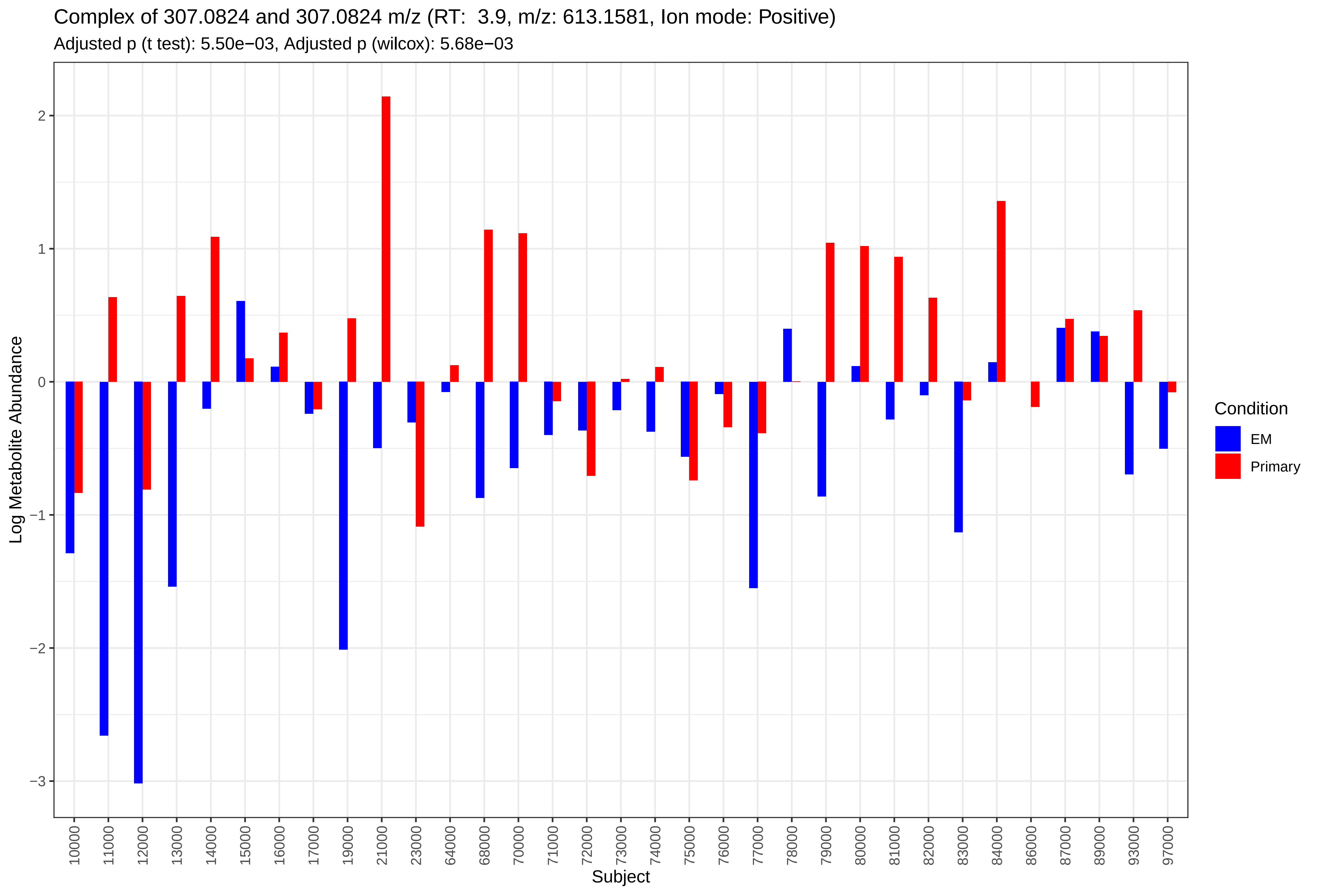

Complex of 307.0824 and 307.0824 m/z (RT: 3.9, m/z: 613.1581, Ion mode: Positive)
Adjusted p (t test): 5.50e−03, Adjusted p (wilcox): 5.68e−03
Log Metabolite Abundance
2
1
0
−1
−2
−3
Subject
10000
11000
12000
13000
14000
15000
16000
17000
19000
21000
23000
64000
68000
70000
71000
72000
73000
74000
75000
76000
77000
78000
79000
80000
81000
82000
83000
84000
86000
87000
89000
93000
97000
Condition
EM
Primary

RT: 1.8, m/z: 97.5205, Ion mode: Positive

Adjusted p (t test): 5.61e−03, Adjusted p (wilcox): 9.51e−03

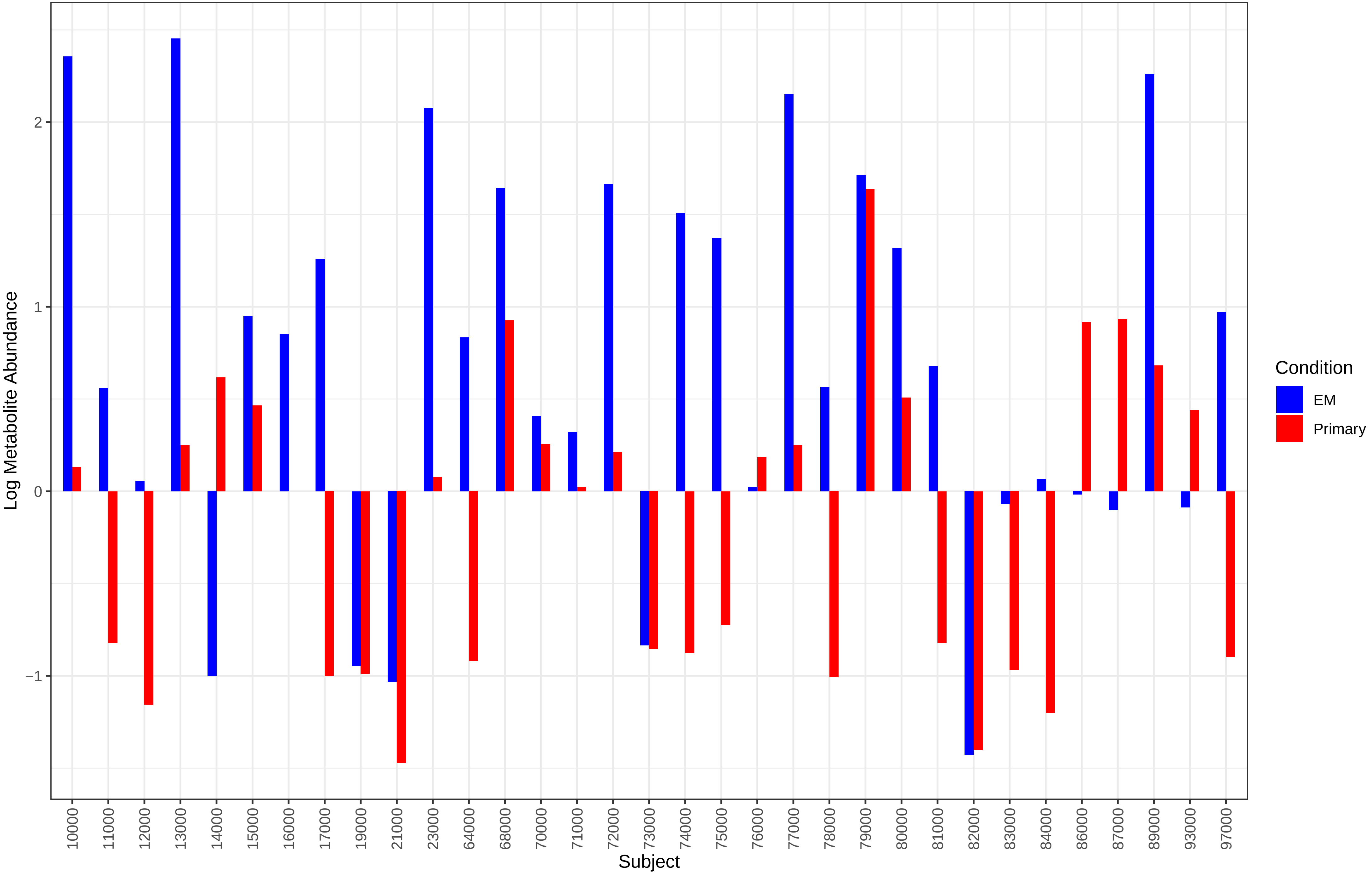

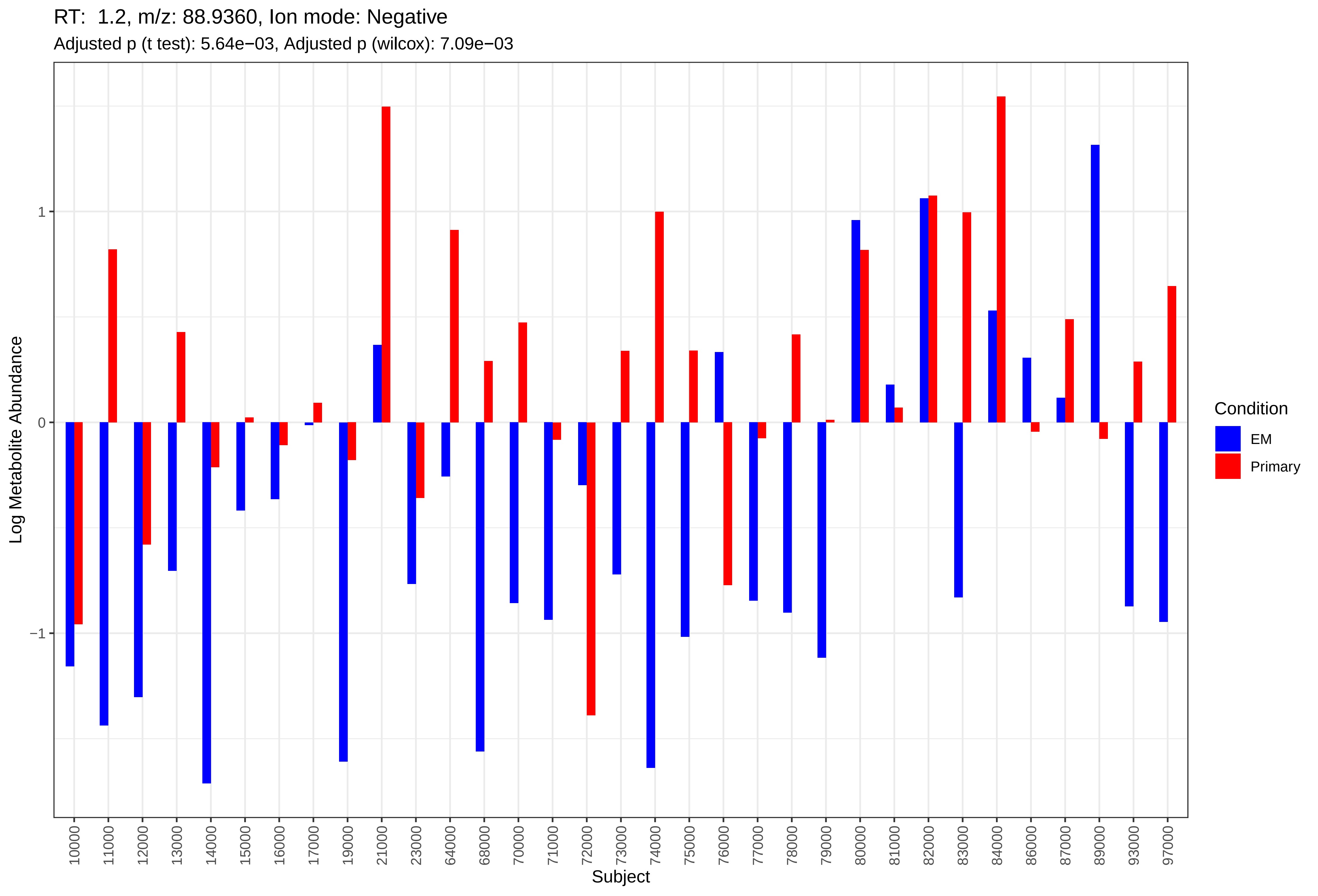

RT: 1.2, m/z: 88.9360, Ion mode: Negative
Adjusted p (t test): 5.64e−03, Adjusted p (wilcox): 7.09e−03
Log Metabolite Abundance
1
0
−1
Subject
10000
11000
12000
13000
14000
15000
16000
17000
19000
21000
23000
64000
68000
70000
71000
72000
73000
74000
75000
76000
77000
78000
79000
80000
81000
82000
83000
84000
86000
87000
89000
93000
97000
Condition
EM
Primary

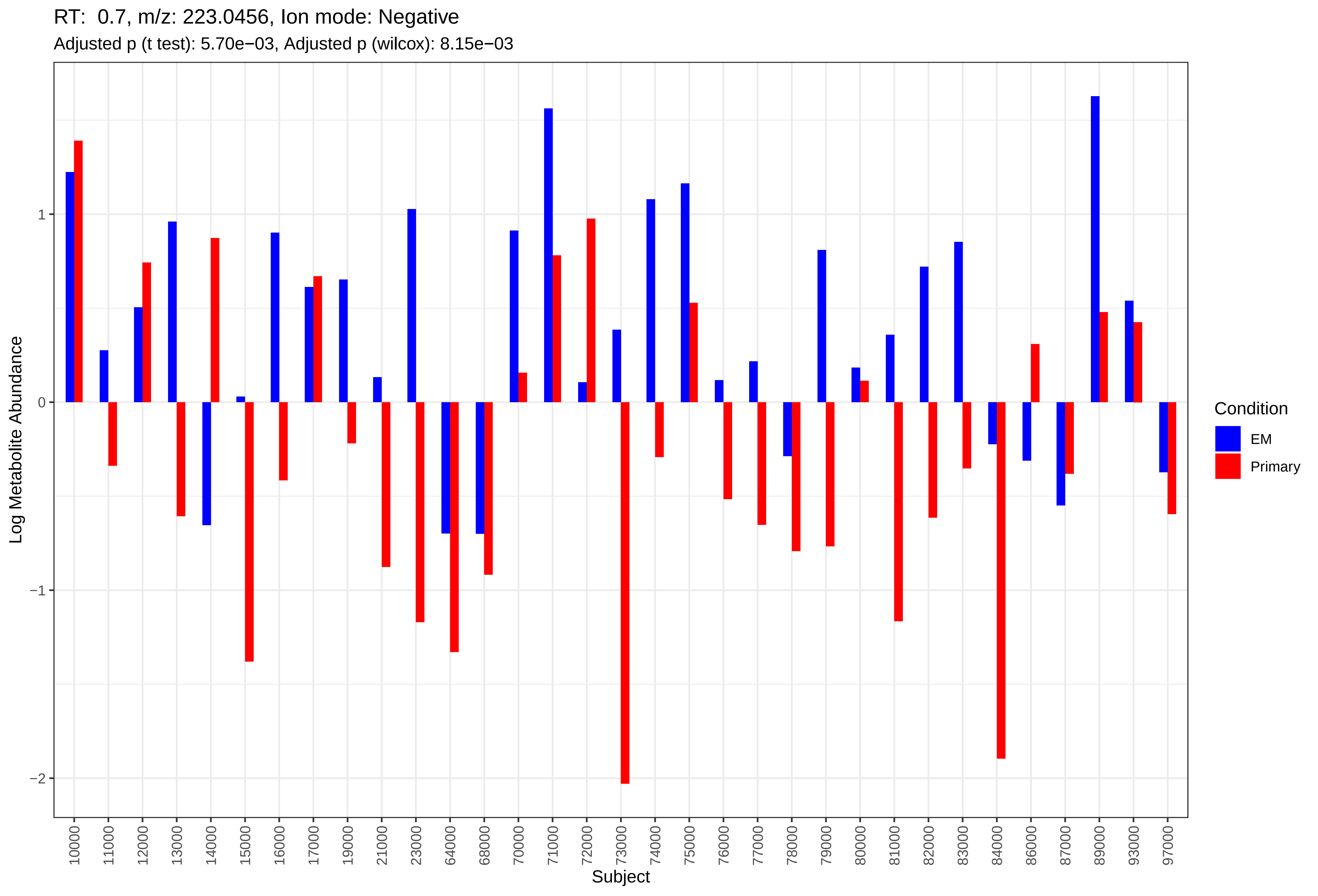
RT: 0.7, m/z: 223.0456, Ion mode: Negative
Adjusted p (t test): 5.70e−03, Adjusted p (wilcox): 8.15e−03
Log Metabolite Abundance
1
0
−1
−2
10000
11000
12000
13000
14000
15000
16000
17000
19000
21000
23000
64000
68000
70000
71000
72000
73000
74000
75000
76000
77000
78000
79000
80000
81000
82000
83000
84000
86000
87000
89000
93000
97000
Subject
Condition
EM
Primary

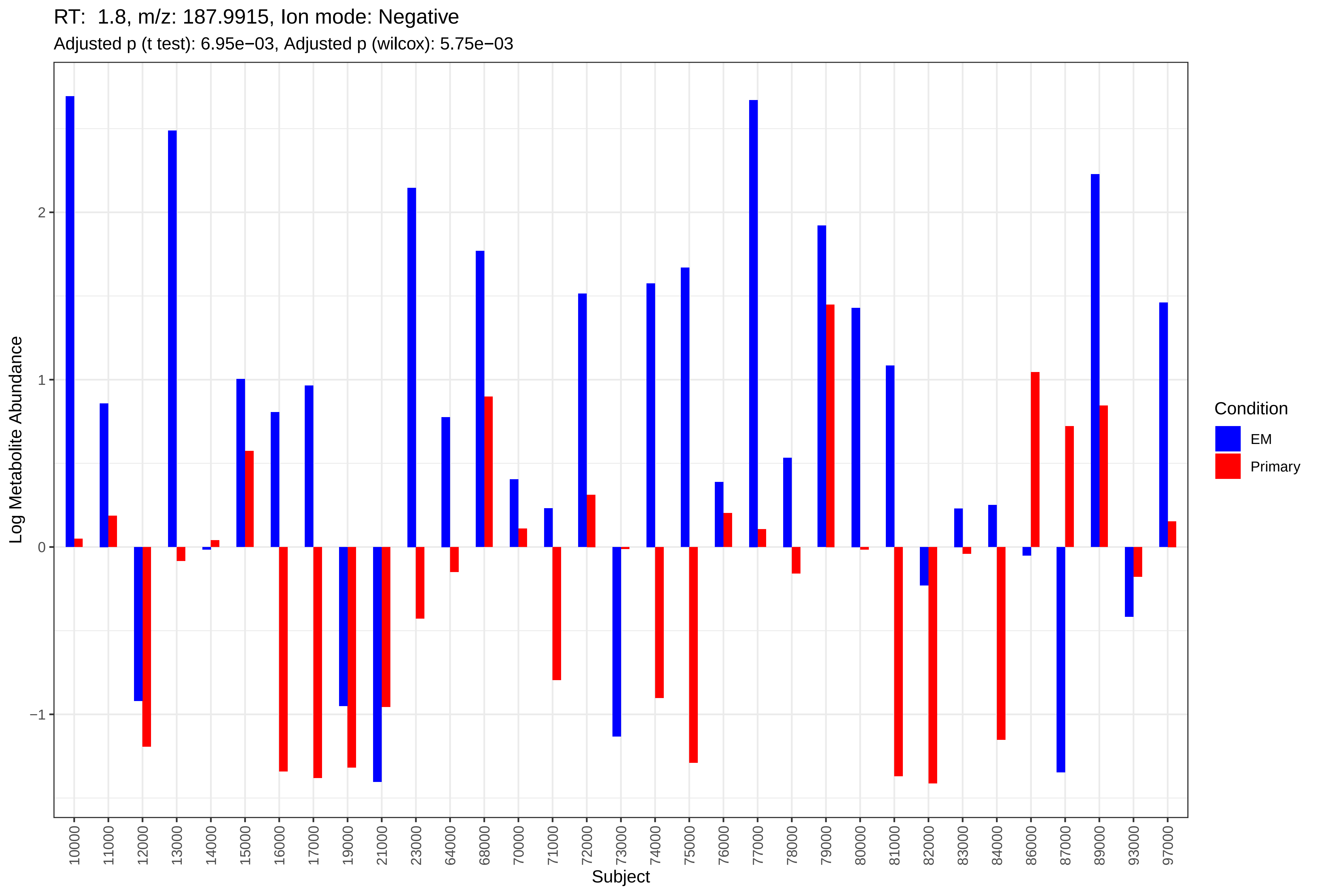

RT: 1.8, m/z: 187.9915, Ion mode: Negative
Adjusted p (t test): 6.95e−03, Adjusted p (wilcox): 5.75e−03
Log Metabolite Abundance
2
1
0
−1
10000
11000
12000
13000
14000
15000
16000
17000
19000
21000
23000
64000
68000
70000
71000
72000
73000
74000
75000
76000
77000
78000
79000
80000
81000
82000
83000
84000
86000
87000
89000
93000
97000
Subject
Condition
EM
Primary

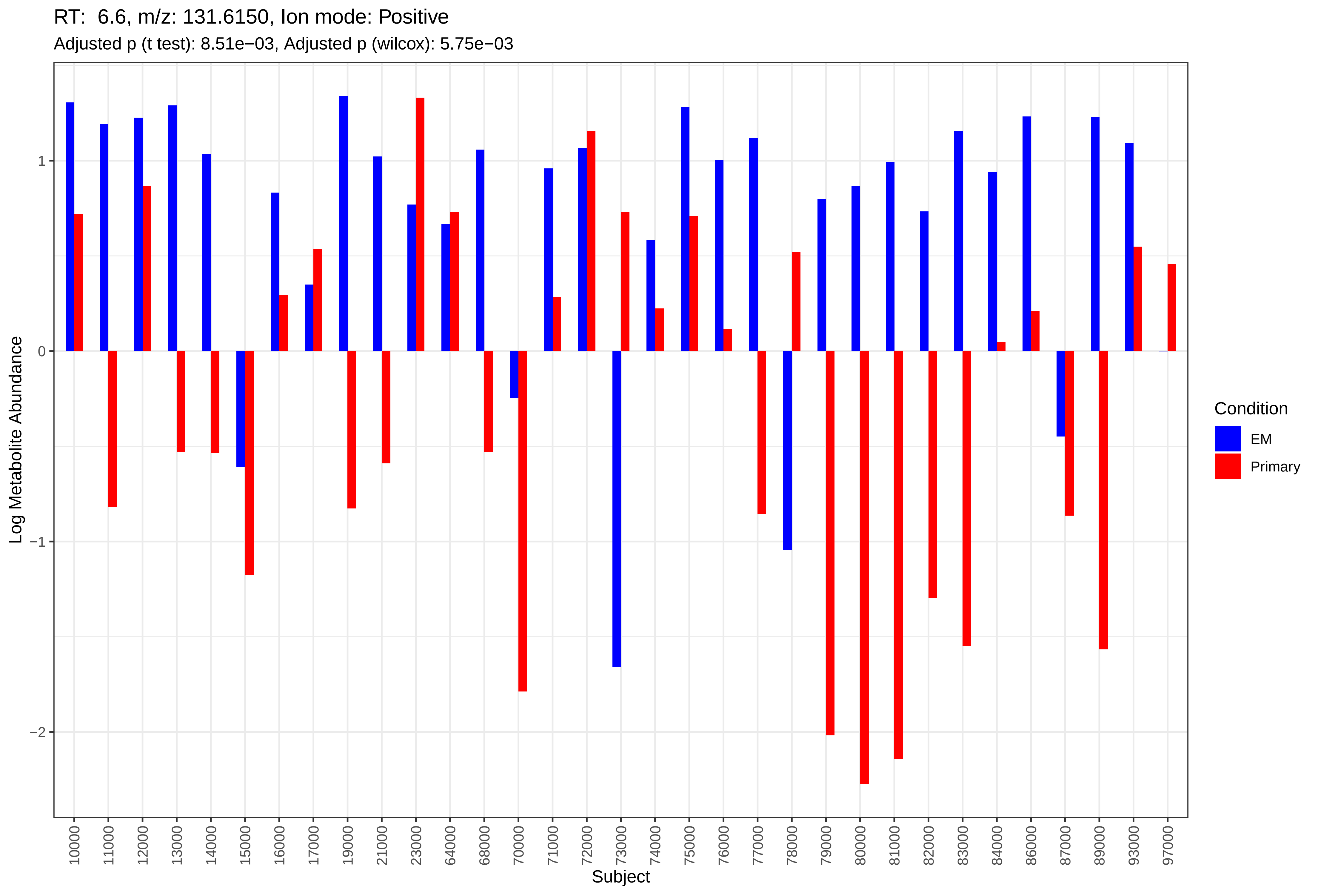

RT: 6.6, m/z: 131.6150, Ion mode: Positive
Adjusted p (t test): 8.51e−03, Adjusted p (wilcox): 5.75e−03
Log Metabolite Abundance
1
0
−1
−2
Subject
10000
11000
12000
13000
14000
15000
16000
17000
19000
21000
23000
64000
68000
70000
71000
72000
73000
74000
75000
76000
77000
78000
79000
80000
81000
82000
83000
84000
86000
87000
89000
93000
97000
Condition
EM
Primary

RT: 6.6, m/z: 309.2307, Ion mode: Positive

Adjusted p (t test): 5.79e−03, Adjusted p (wilcox): 6.59e−03

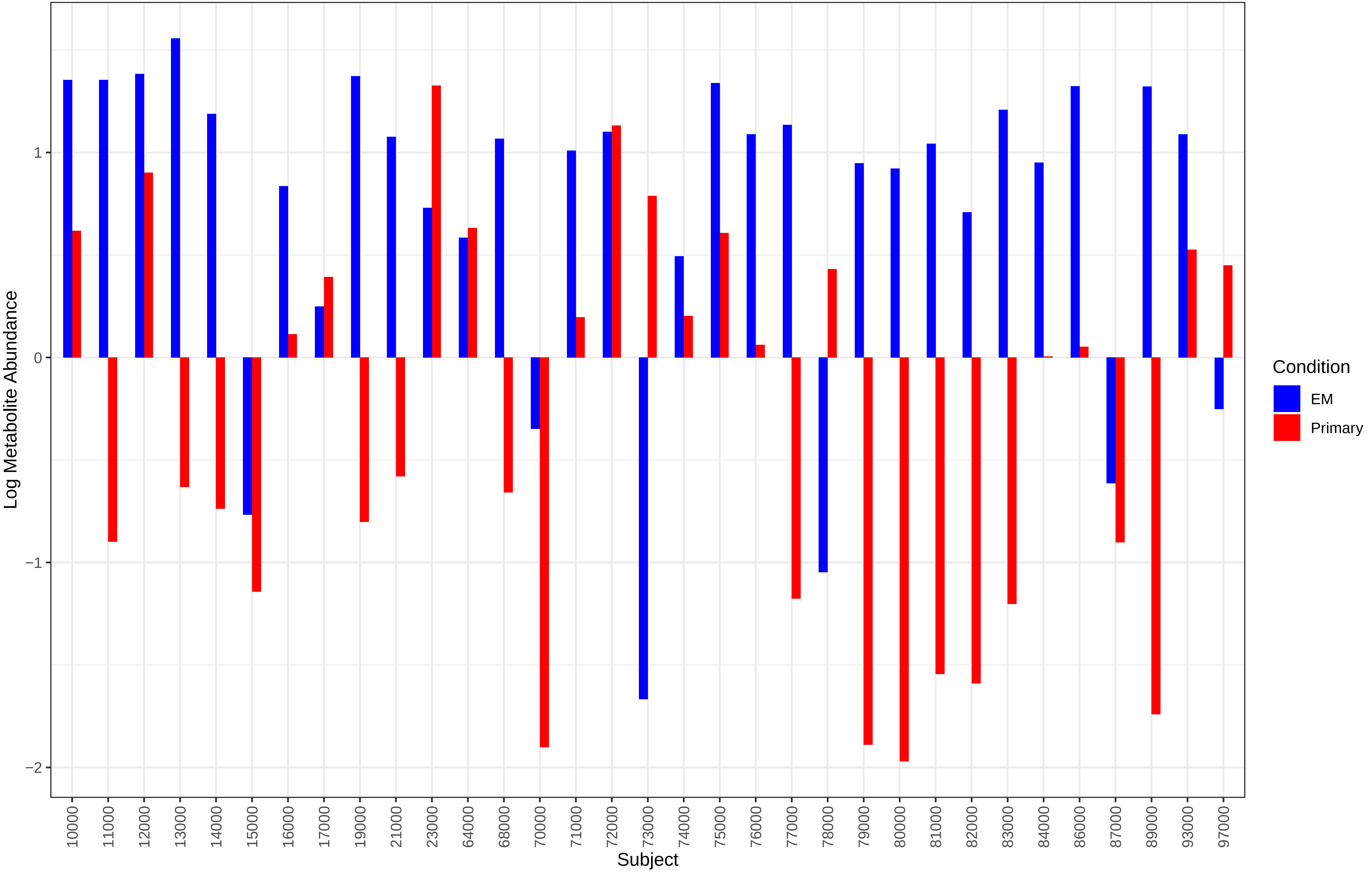

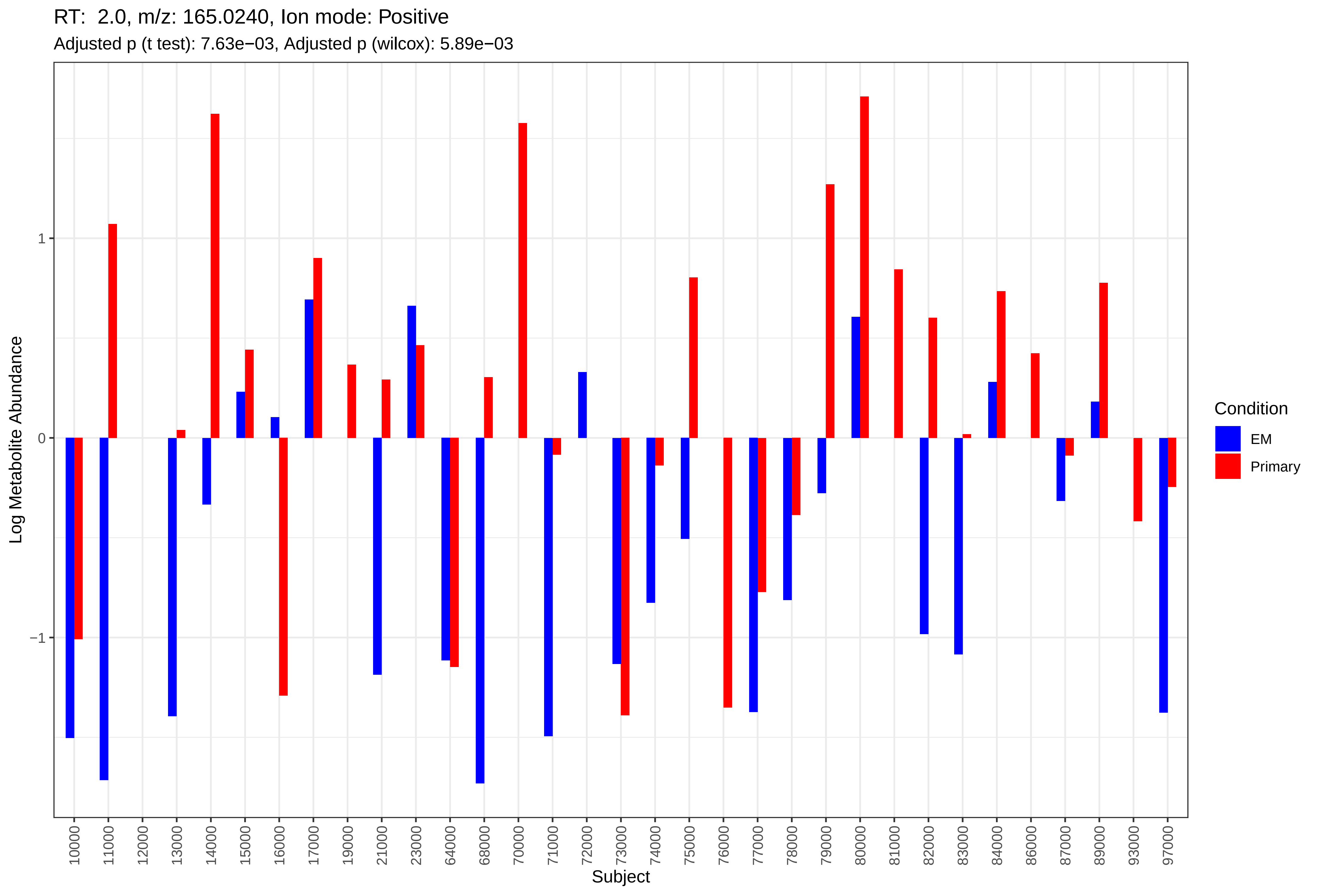
RT: 2.0, m/z: 165.0240, Ion mode: Positive
Adjusted p (t test): 7.63e−03, Adjusted p (wilcox): 5.89e−03
Log Metabolite Abundance
1
0
−1
10000
11000
12000
13000
14000
15000
16000
17000
19000
21000
23000
64000
68000
70000
71000
72000
73000
74000
75000
76000
77000
78000
79000
80000
81000
82000
83000
84000
86000
87000
89000
93000
97000
Subject
Condition
EM
Primary

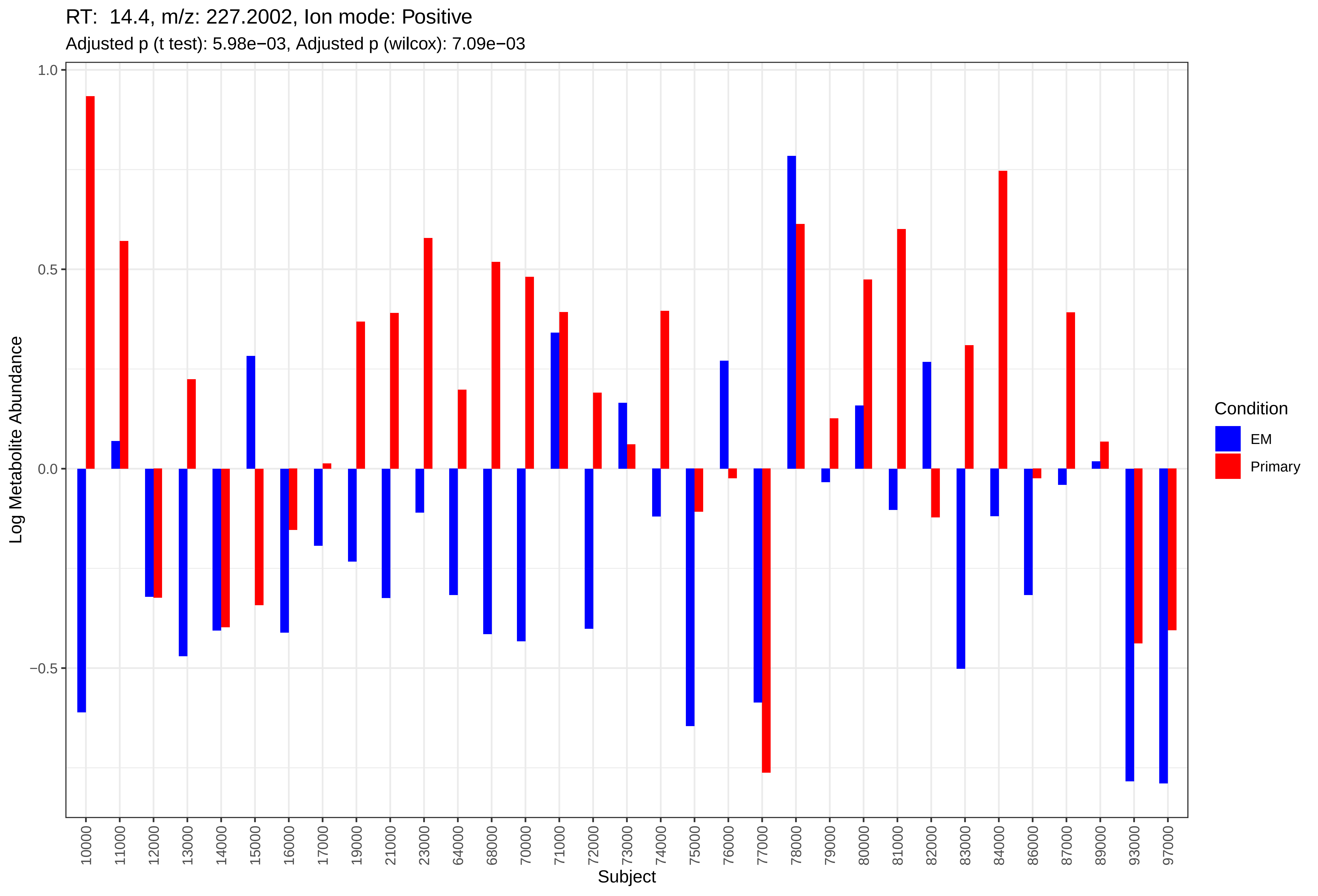

RT: 14.4, m/z: 227.2002, Ion mode: Positive
Adjusted p (t test): 5.98e−03, Adjusted p (wilcox): 7.09e−03
Log Metabolite Abundance
1.0
0.5
0.0
−0.5
Subject
10000
11000
12000
13000
14000
15000
16000
17000
19000
21000
23000
64000
68000
70000
71000
72000
73000
74000
75000
76000
77000
78000
79000
80000
81000
82000
83000
84000
86000
87000
89000
93000
97000
Condition
EM
Primary

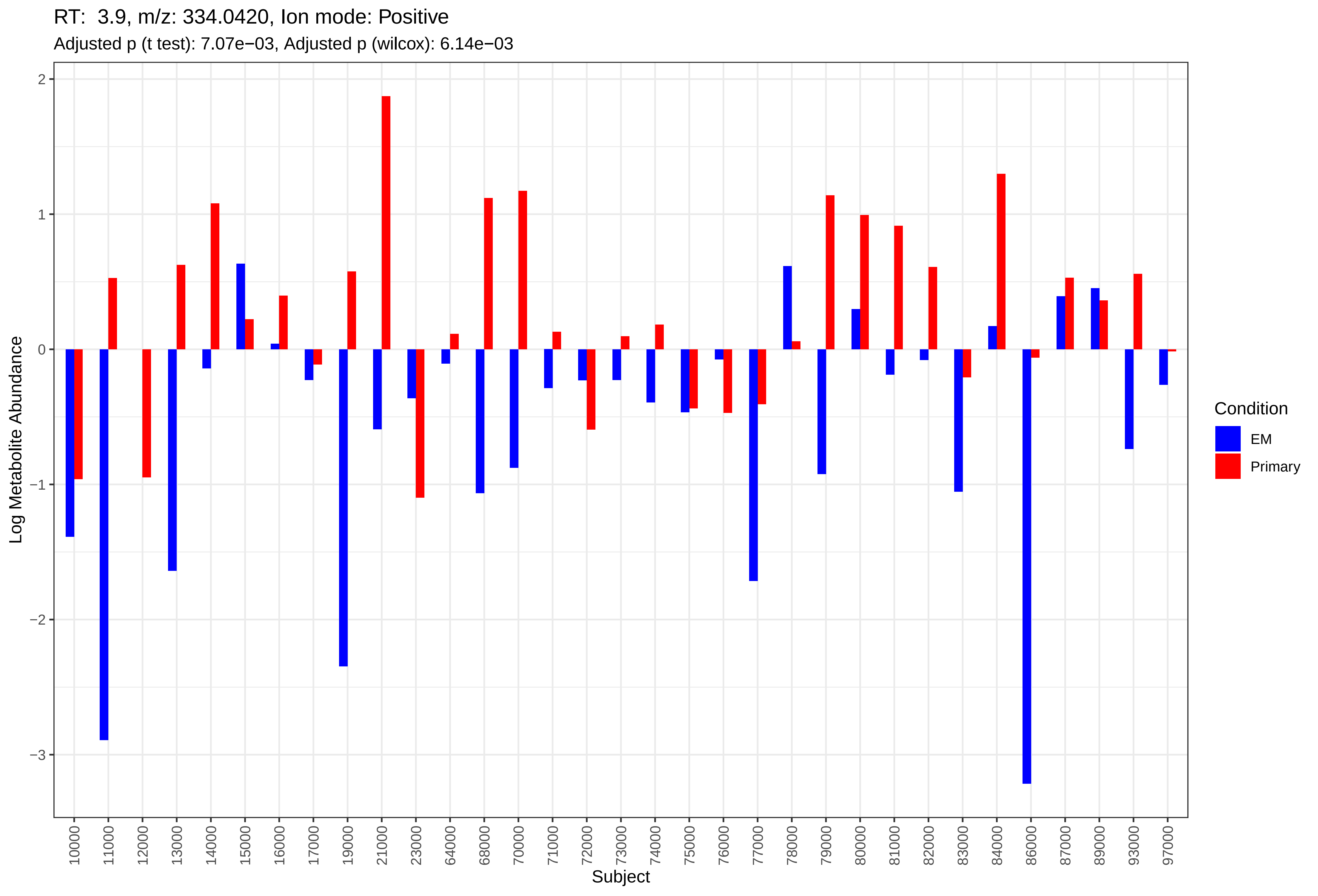

RT: 3.9, m/z: 334.0420, Ion mode: Positive
Adjusted p (t test): 7.07e−03, Adjusted p (wilcox): 6.14e−03
Log Metabolite Abundance
2
1
0
−1
−2
−3
Subject
10000
11000
12000
13000
14000
15000
16000
17000
19000
21000
23000
64000
68000
70000
71000
72000
73000
74000
75000
76000
77000
78000
79000
80000
81000
82000
83000
84000
86000
87000
89000
93000
97000
Condition
EM
Primary

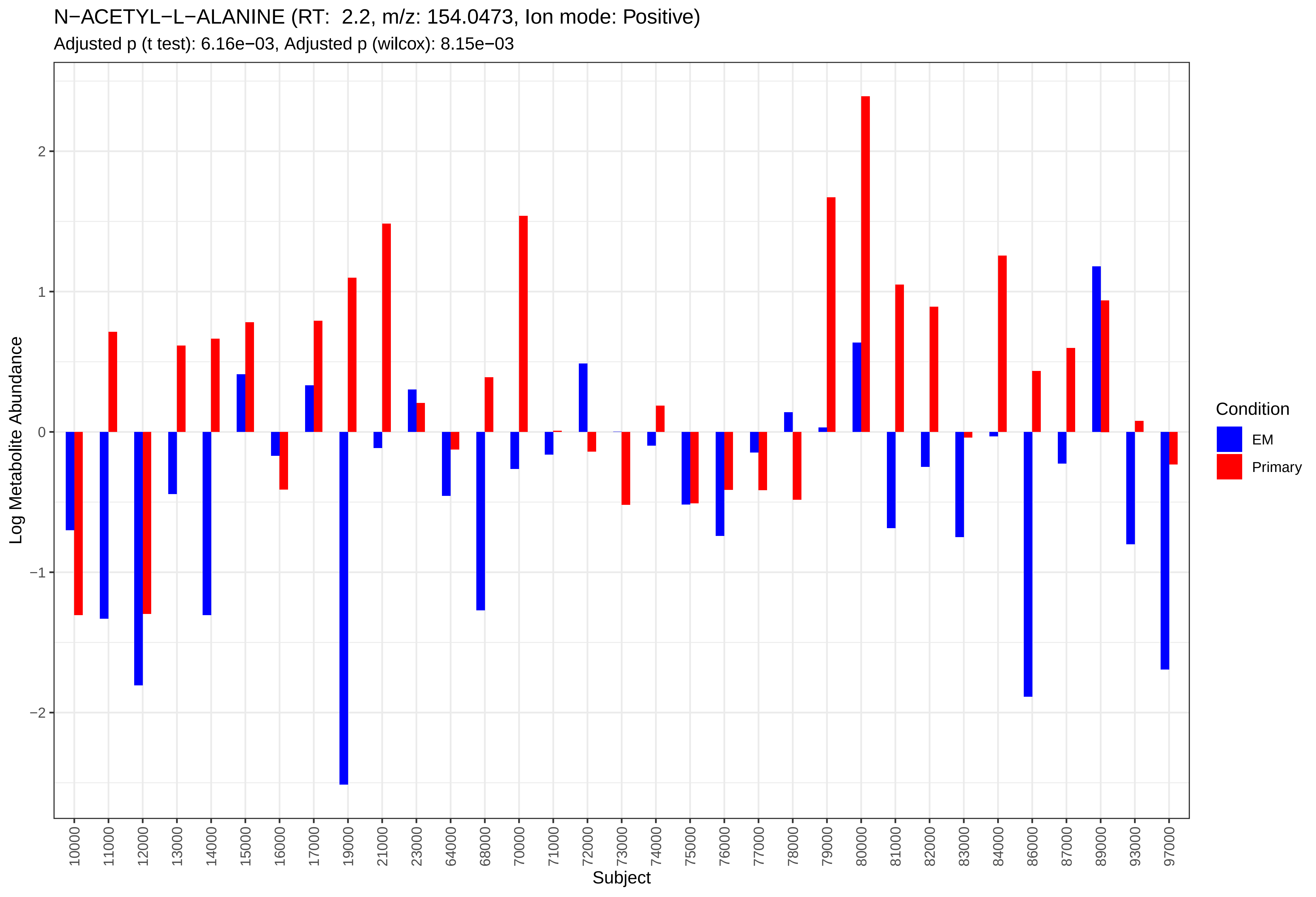
N−ACETYL−L−ALANINE (RT: 2.2, m/z: 154.0473, Ion mode: Positive)
Adjusted p (t test): 6.16e−03, Adjusted p (wilcox): 8.15e−03
Log Metabolite Abundance
2
1
0
−1
−2
10000
11000
12000
13000
14000
15000
16000
17000
19000
21000
23000
64000
68000
70000
71000
72000
73000
74000
75000
76000
77000
78000
79000
80000
81000
82000
83000
84000
86000
87000
89000
93000
97000
Subject
Condition
EM
Primary

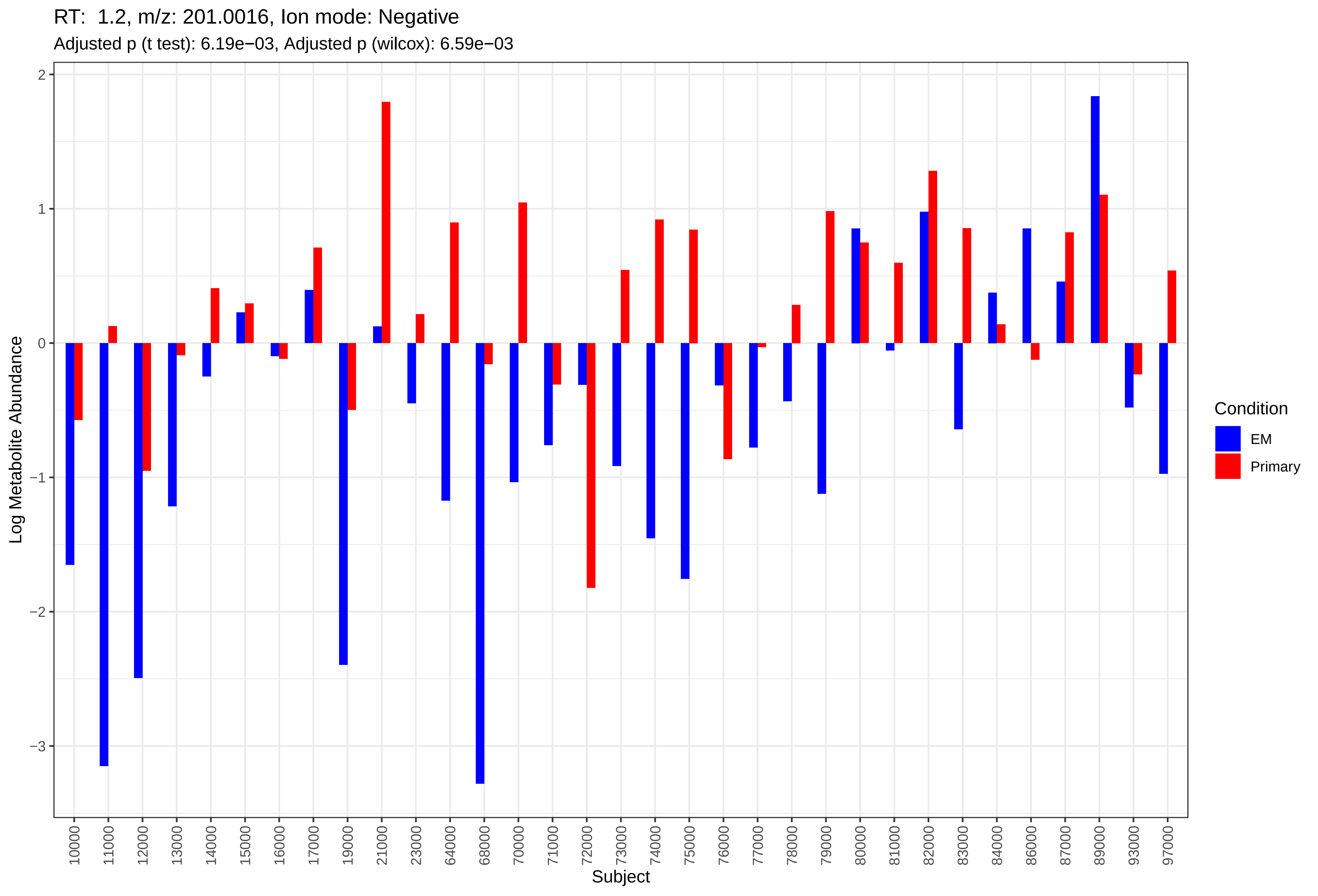
RT: 1.2, m/z: 201.0016, Ion mode: Negative
Adjusted p (t test): 6.19e−03, Adjusted p (wilcox): 6.59e−03
Log Metabolite Abundance
Subject
Condition
EM
Primary
2
1
0
−1
−2
−3
10000
11000
12000
13000
14000
15000
16000
17000
19000
21000
23000
64000
68000
70000
71000
72000
73000
74000
75000
76000
77000
78000
79000
80000
81000
82000
83000
84000
86000
87000
89000
93000
97000

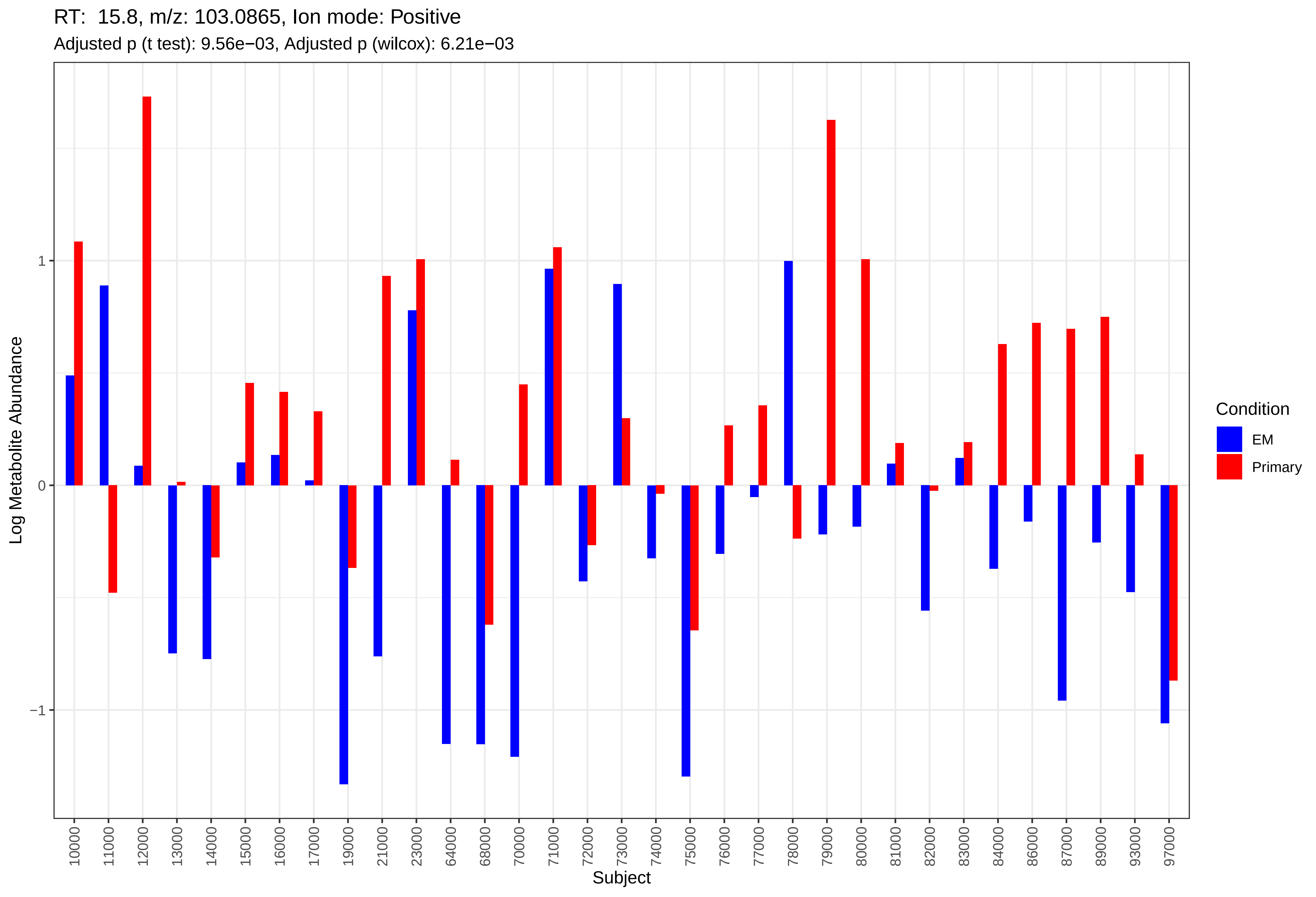

RT: 15.8, m/z: 103.0865, Ion mode: Positive
Adjusted p (t test): 9.56e−03, Adjusted p (wilcox): 6.21e−03
Log Metabolite Abundance
1
0
−1
Subject
10000
11000
12000
13000
14000
15000
16000
17000
19000
21000
23000
64000
68000
70000
71000
72000
73000
74000
75000
76000
77000
78000
79000
80000
81000
82000
83000
84000
86000
87000
89000
93000
97000
Condition
EM
Primary

RT: 0.9, m/z: 166.0270, Ion mode: Positive

Adjusted p (t test): 8.14e−03, Adjusted p (wilcox): 6.21e−03

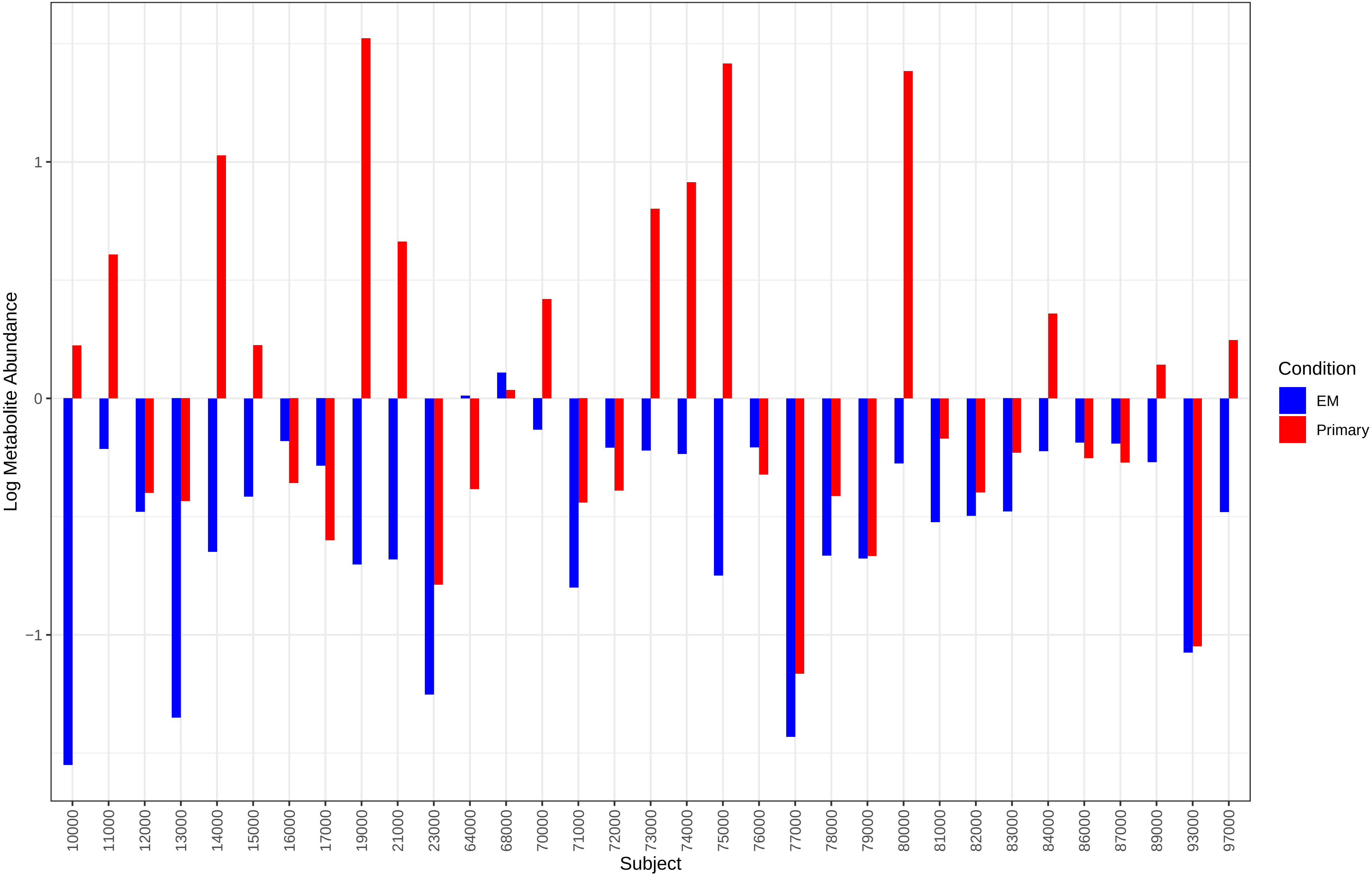

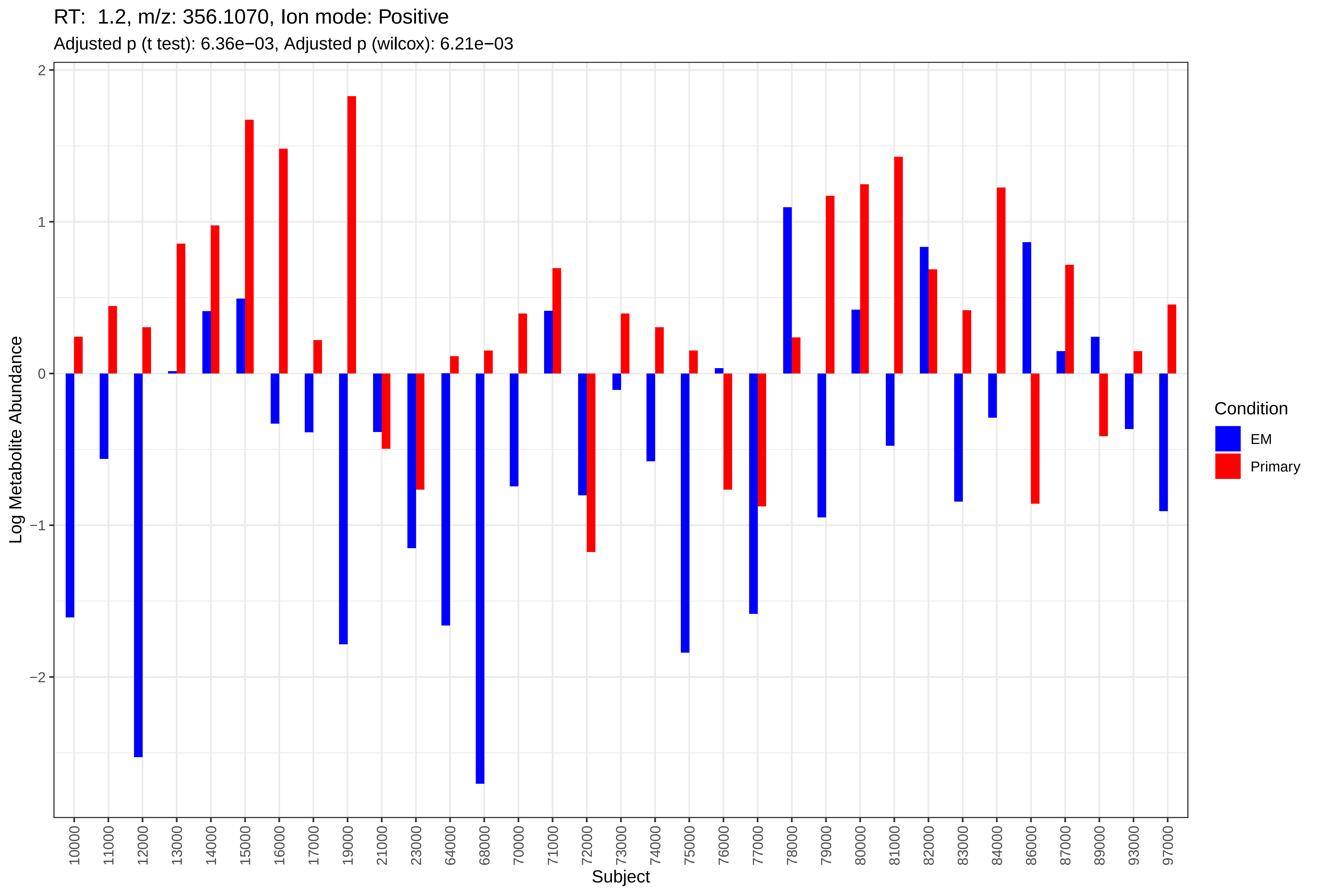

RT: 1.2, m/z: 356.1070, Ion mode: Positive
Adjusted p (t test): 6.36e−03, Adjusted p (wilcox): 6.21e−03
Log Metabolite Abundance
2
1
0
−1
−2
Subject
10000
11000
12000
13000
14000
15000
16000
17000
19000
21000
23000
64000
68000
70000
71000
72000
73000
74000
75000
76000
77000
78000
79000
80000
81000
82000
83000
84000
86000
87000
89000
93000
97000
Condition
EM
Primary

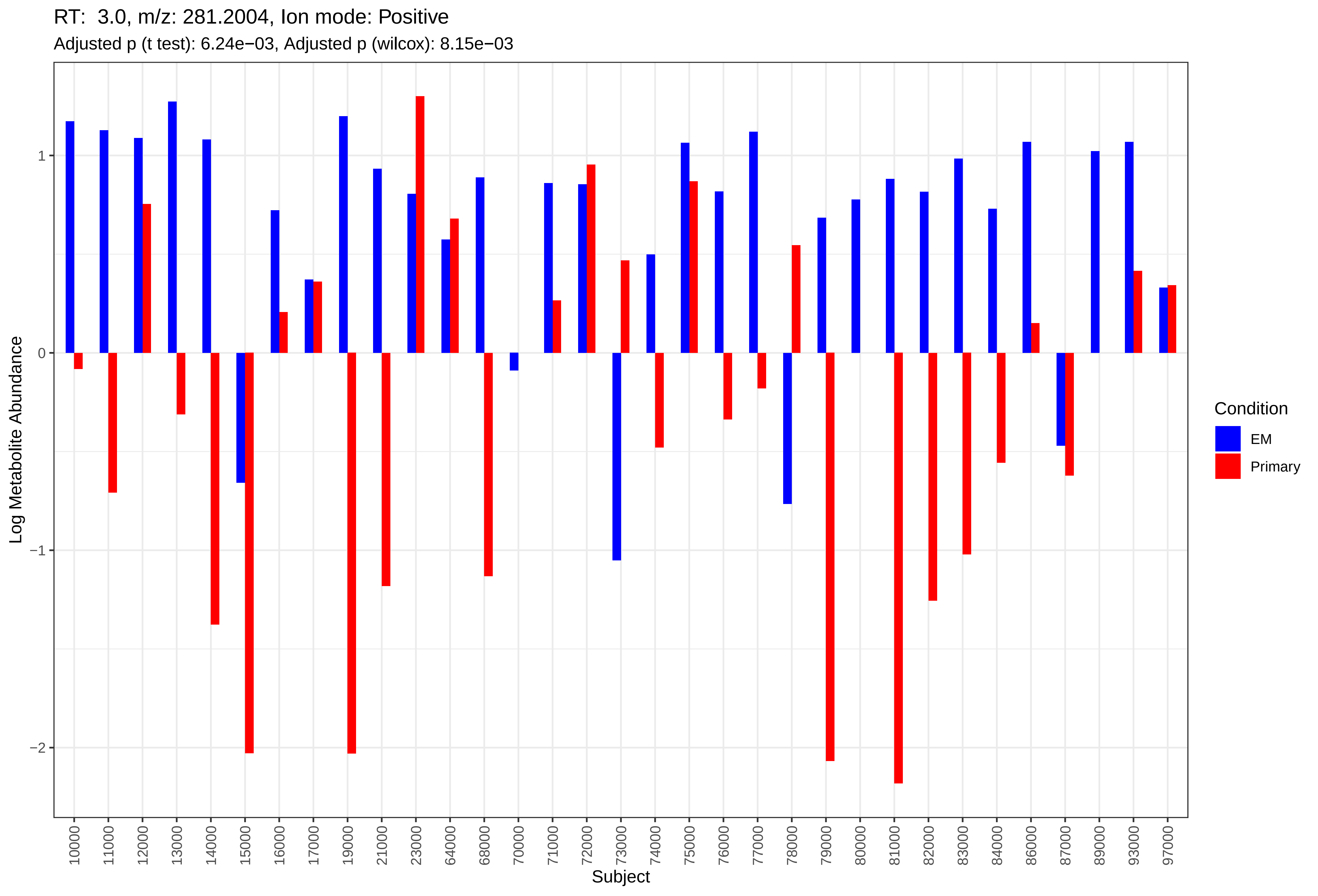
RT: 3.0, m/z: 281.2004, Ion mode: Positive
Adjusted p (t test): 6.24e−03, Adjusted p (wilcox): 8.15e−03
Log Metabolite Abundance
1
0
−1
−2
Subject
10000
11000
12000
13000
14000
15000
16000
17000
19000
21000
23000
64000
68000
70000
71000
72000
73000
74000
75000
76000
77000
78000
79000
80000
81000
82000
83000
84000
86000
87000
89000
93000
97000
Condition
EM
Primary

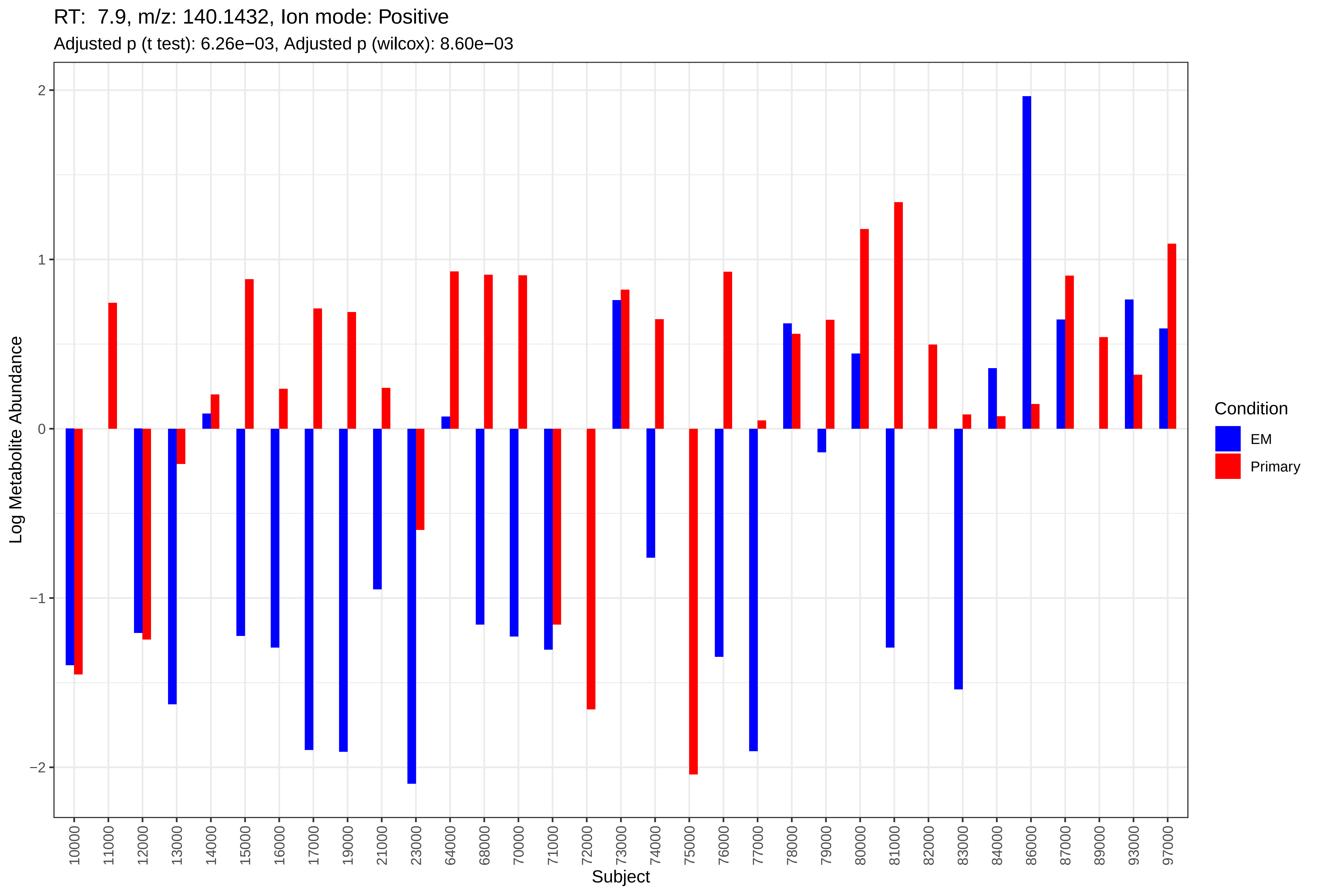

RT: 7.9, m/z: 140.1432, Ion mode: Positive
Adjusted p (t test): 6.26e−03, Adjusted p (wilcox): 8.60e−03
Log Metabolite Abundance
2
1
0
−1
−2
10000
11000
12000
13000
14000
15000
16000
17000
19000
21000
23000
64000
68000
70000
71000
72000
73000
74000
75000
76000
77000
78000
79000
80000
81000
82000
83000
84000
86000
87000
89000
93000
97000
Subject
Condition
EM
Primary

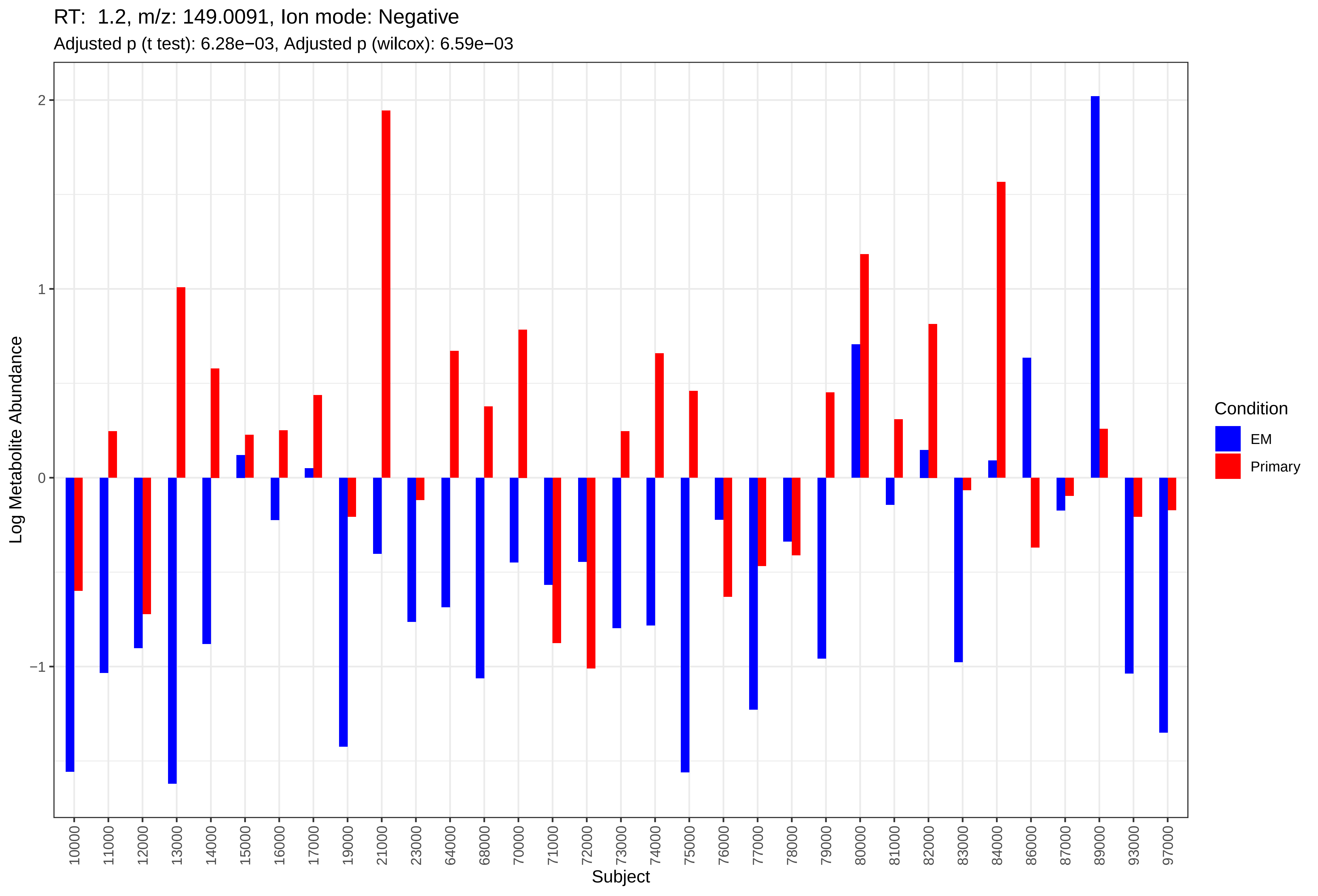

RT: 1.2, m/z: 149.0091, Ion mode: Negative
Adjusted p (t test): 6.28e−03, Adjusted p (wilcox): 6.59e−03
Log Metabolite Abundance
2
1
0
−1
10000
11000
12000
13000
14000
15000
16000
17000
19000
21000
23000
64000
68000
70000
71000
72000
73000
74000
75000
76000
77000
78000
79000
80000
81000
82000
83000
84000
86000
87000
89000
93000
97000
Subject
Condition
EM
Primary

RT: 1.2, m/z: 212.1026, Ion mode: Positive

Adjusted p (t test): 9.98e−03, Adjusted p (wilcox): 6.36e−03

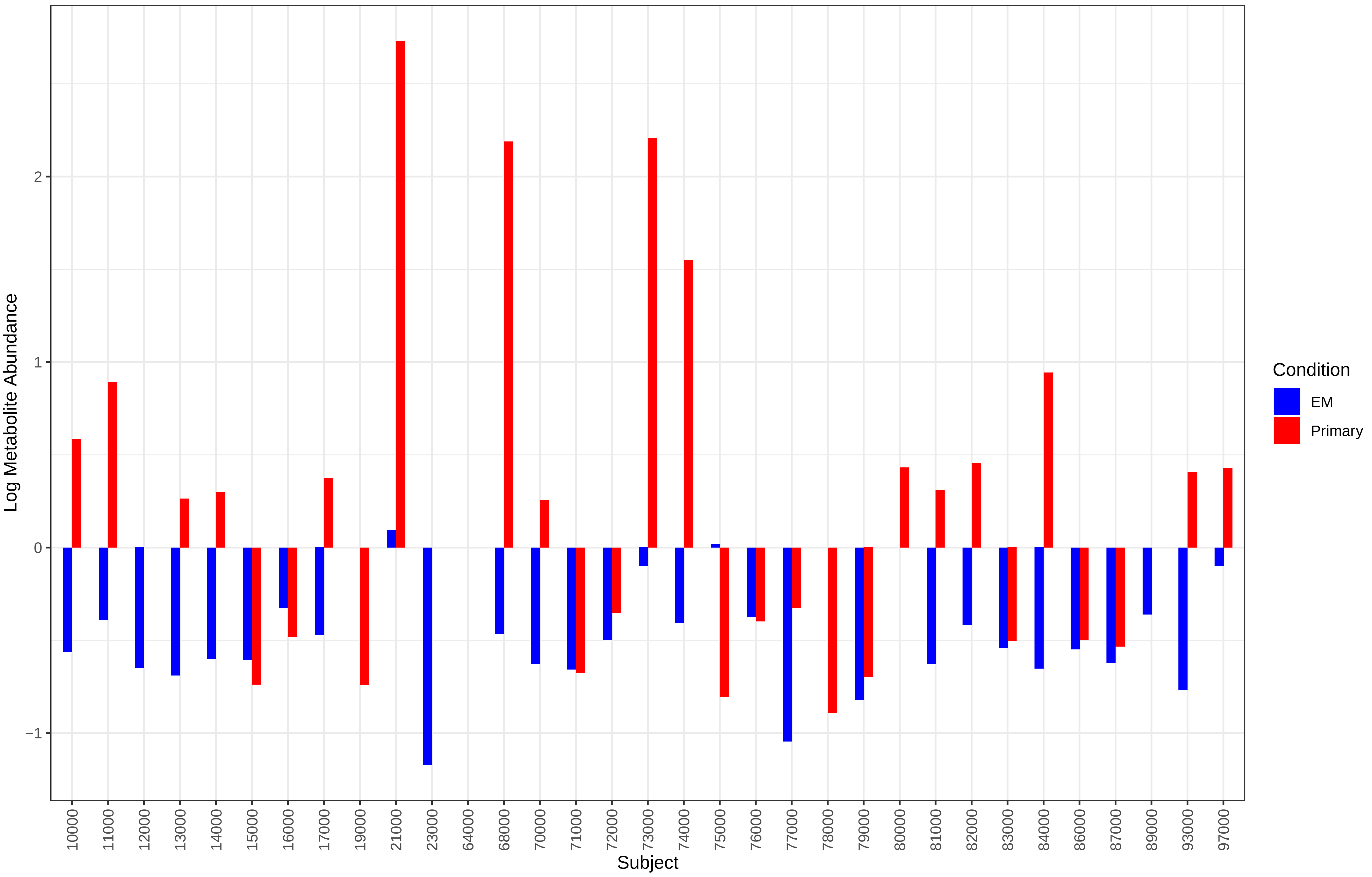

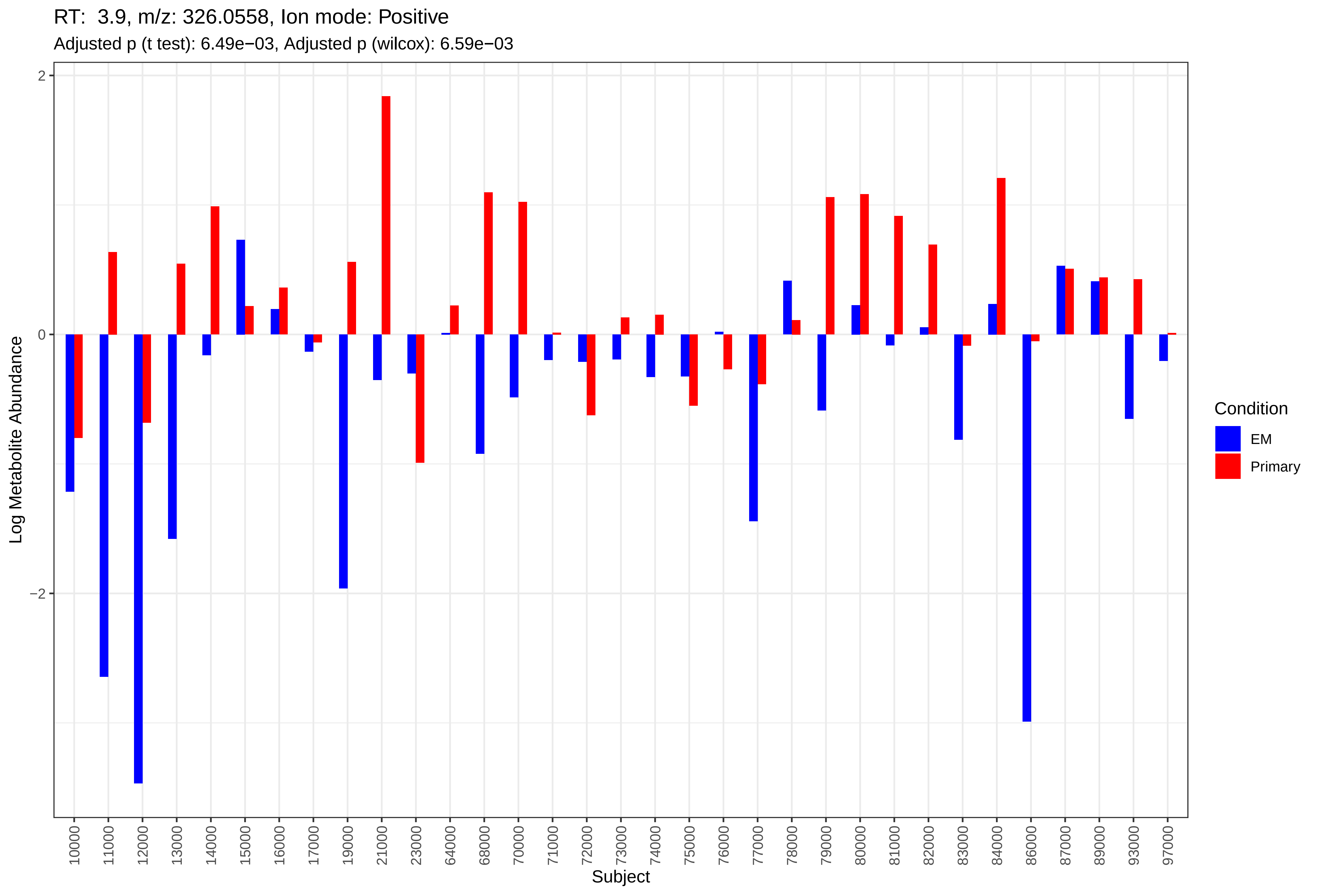

RT: 3.9, m/z: 326.0558, Ion mode: Positive
Adjusted p (t test): 6.49e−03, Adjusted p (wilcox): 6.59e−03
2
0
−2
Log Metabolite Abundance
10000
11000
12000
13000
14000
15000
16000
17000
19000
21000
23000
64000
68000
70000
71000
72000
73000
74000
75000
76000
77000
78000
79000
80000
81000
82000
83000
84000
86000
87000
89000
93000
97000
Subject
Condition
EM
Primary

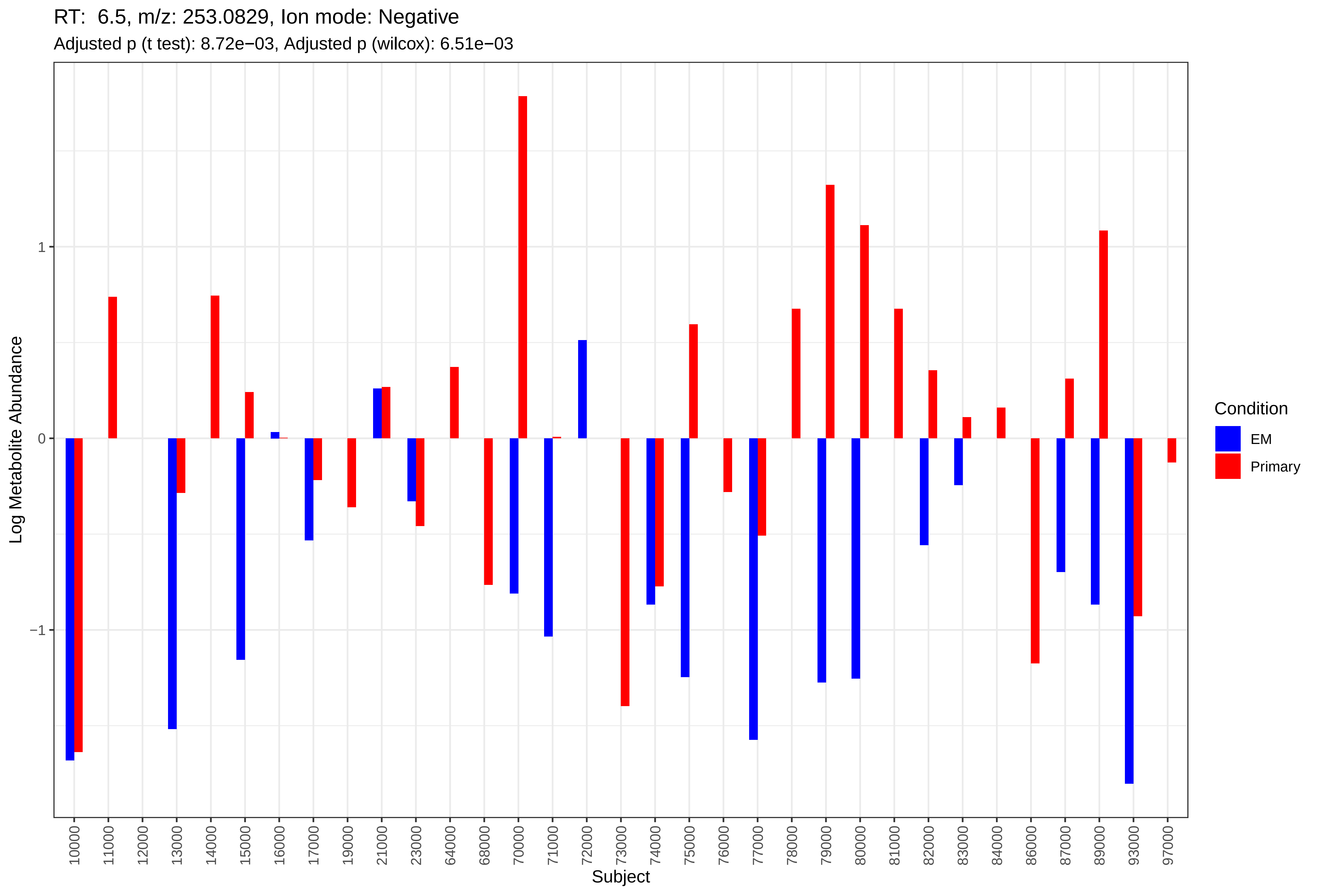
RT: 6.5, m/z: 253.0829, Ion mode: Negative
Adjusted p (t test): 8.72e−03, Adjusted p (wilcox): 6.51e−03
Log Metabolite Abundance
1
0
−1
Subject
10000
11000
12000
13000
14000
15000
16000
17000
19000
21000
23000
64000
68000
70000
71000
72000
73000
74000
75000
76000
77000
78000
79000
80000
81000
82000
83000
84000
86000
87000
89000
93000
97000
Condition
EM
Primary

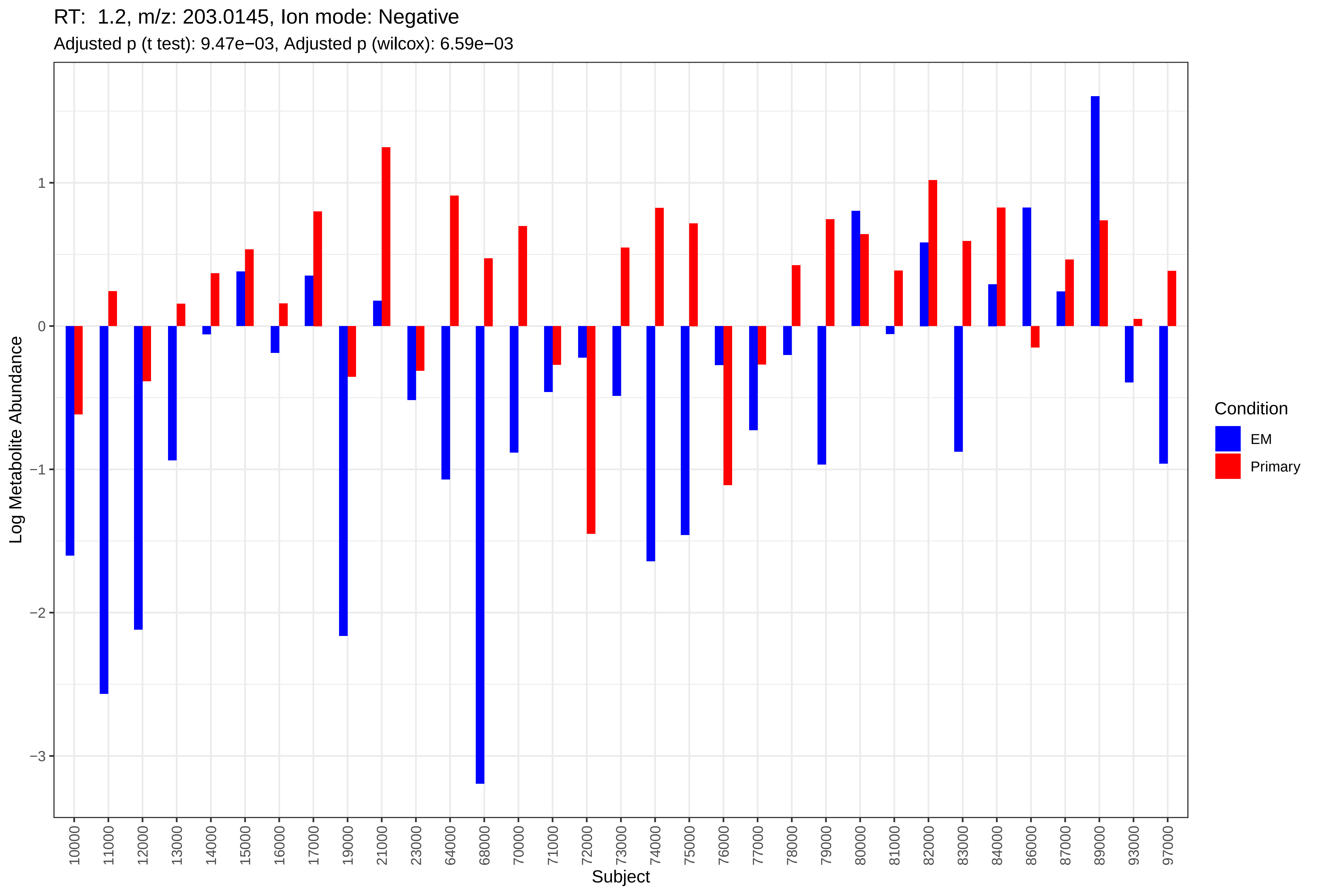
RT: 1.2, m/z: 203.0145, Ion mode: Negative
Adjusted p (t test): 9.47e−03, Adjusted p (wilcox): 6.59e−03
Log Metabolite Abundance
1
0
−1
−2
−3
10000
11000
12000
13000
14000
15000
16000
17000
19000
21000
23000
64000
68000
70000
71000
72000
73000
74000
75000
76000
77000
78000
79000
80000
81000
82000
83000
84000
86000
87000
89000
93000
97000
Subject
Condition
EM
Primary

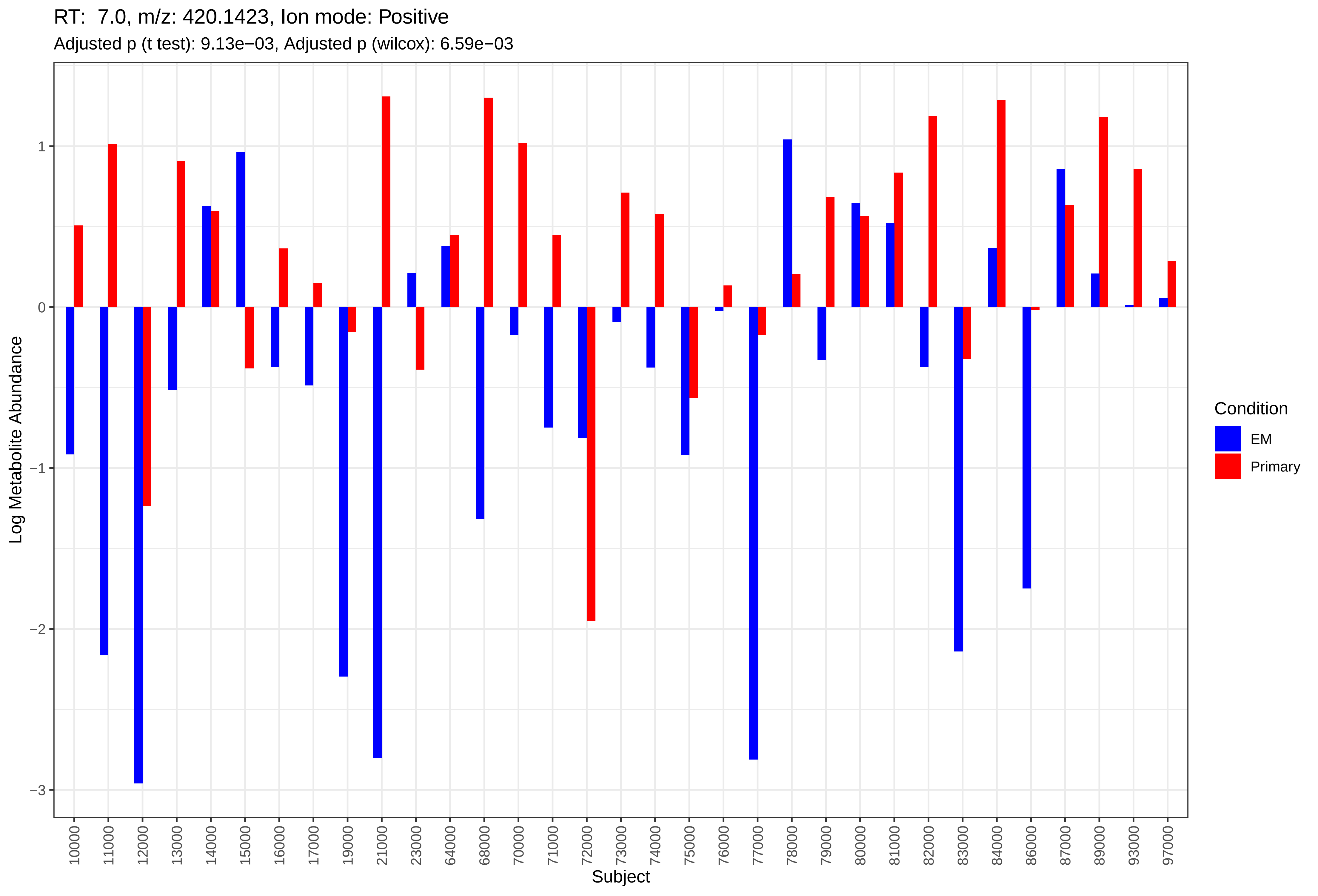

RT: 7.0, m/z: 420.1423, Ion mode: Positive
Adjusted p (t test): 9.13e−03, Adjusted p (wilcox): 6.59e−03
Log Metabolite Abundance
1
0
−1
−2
−3
10000
11000
12000
13000
14000
15000
16000
17000
19000
21000
23000
64000
68000
70000
71000
72000
73000
74000
75000
76000
77000
78000
79000
80000
81000
82000
83000
84000
86000
87000
89000
93000
97000
Subject
Condition
EM
Primary

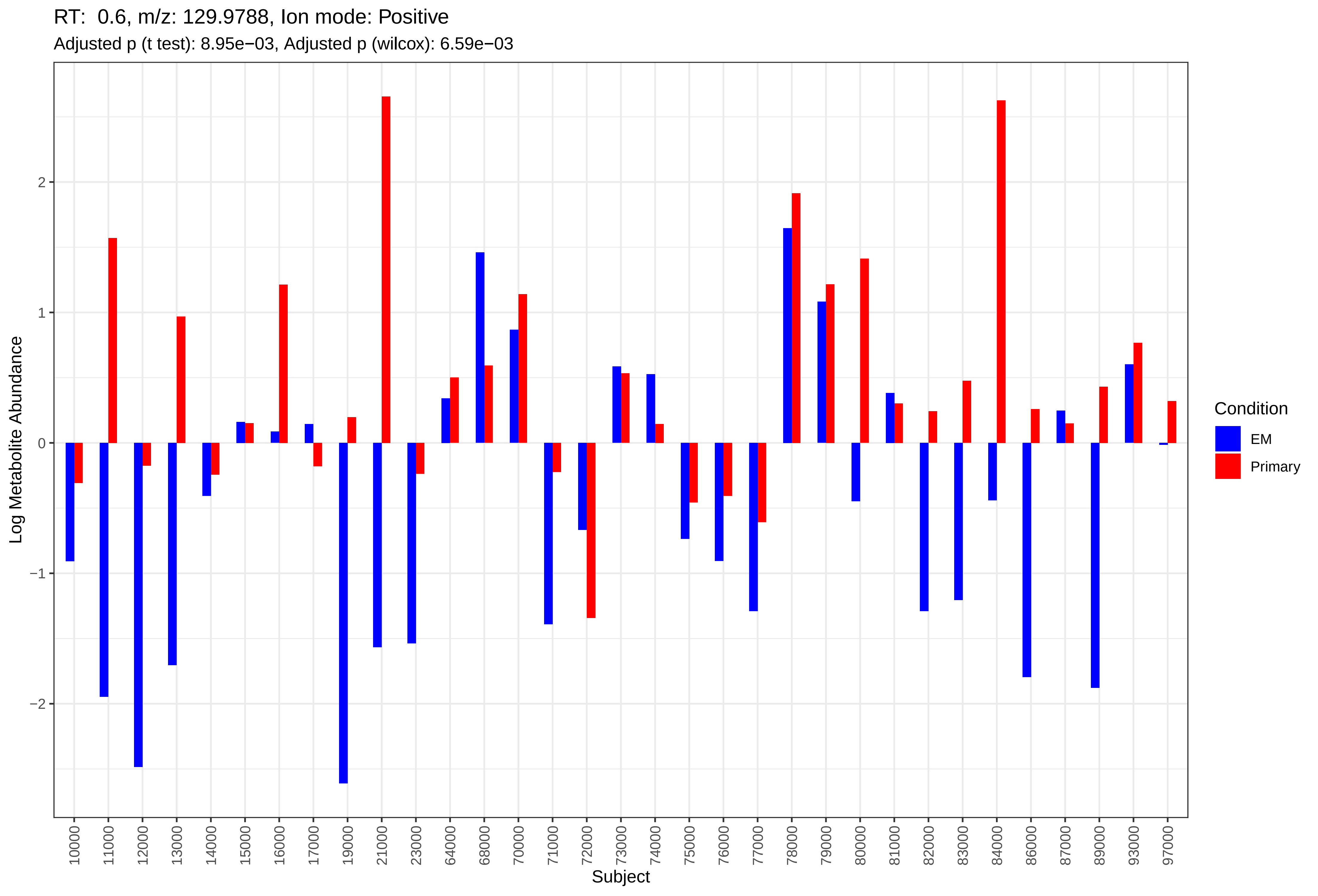

RT: 0.6, m/z: 129.9788, Ion mode: Positive
Adjusted p (t test): 8.95e−03, Adjusted p (wilcox): 6.59e−03
Log Metabolite Abundance
2
1
0
−1
−2
10000
11000
12000
13000
14000
15000
16000
17000
19000
21000
23000
64000
68000
70000
71000
72000
73000
74000
75000
76000
77000
78000
79000
80000
81000
82000
83000
84000
86000
87000
89000
93000
97000
Subject
Condition
EM
Primary

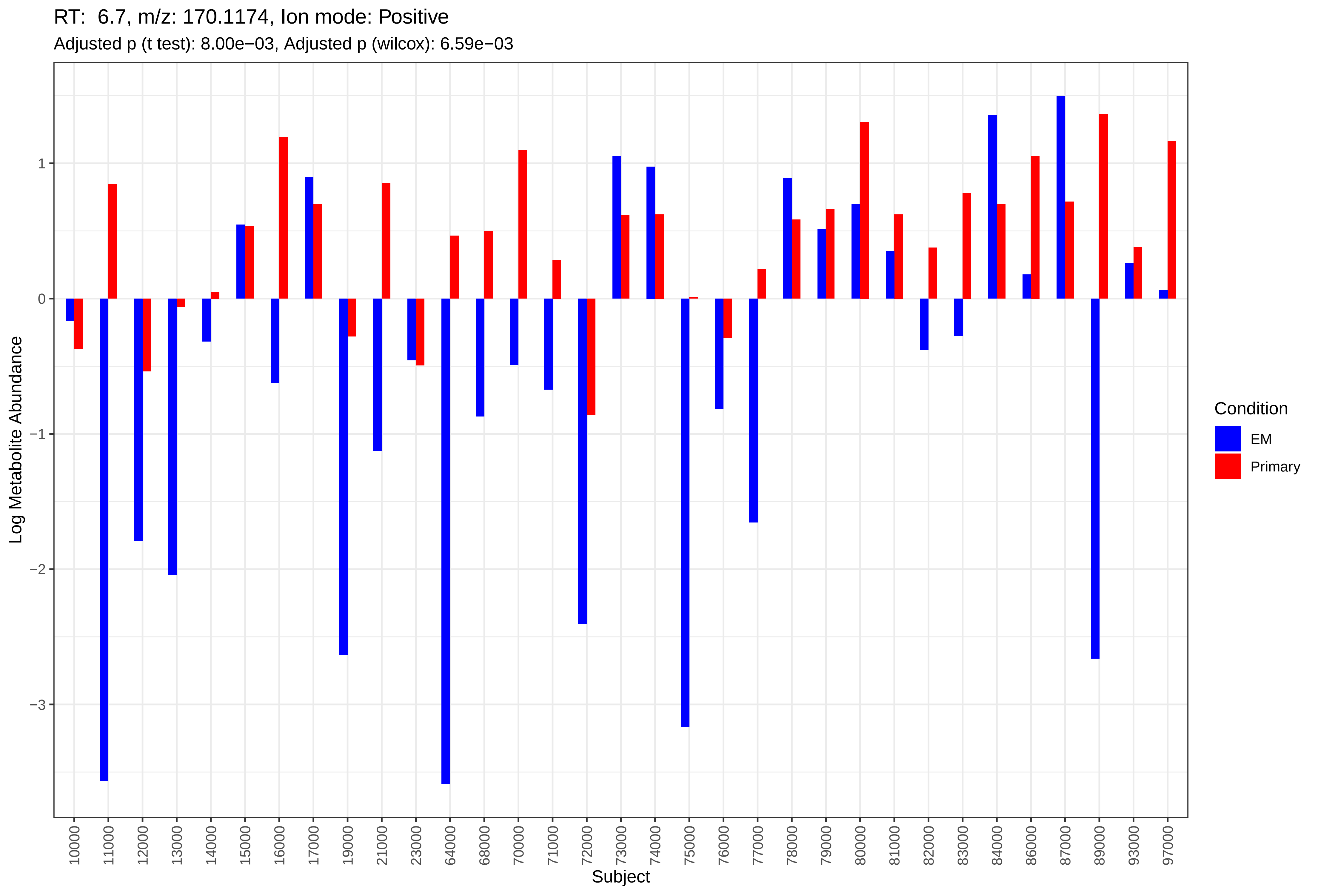

RT: 6.7, m/z: 170.1174, Ion mode: Positive
Adjusted p (t test): 8.00e−03, Adjusted p (wilcox): 6.59e−03
Log Metabolite Abundance
1
0
−1
−2
−3
10000
11000
12000
13000
14000
15000
16000
17000
19000
21000
23000
64000
68000
70000
71000
72000
73000
74000
75000
76000
77000
78000
79000
80000
81000
82000
83000
84000
86000
87000
89000
93000
97000
Subject
Condition
EM
Primary

Complex of 130.0498 and 156.0765 m/z (RT: 0.8, m/z: 285.1183, Ion mode: Positive)

Adjusted p (t test): 7.03e−03, Adjusted p (wilcox): 6.59e−03

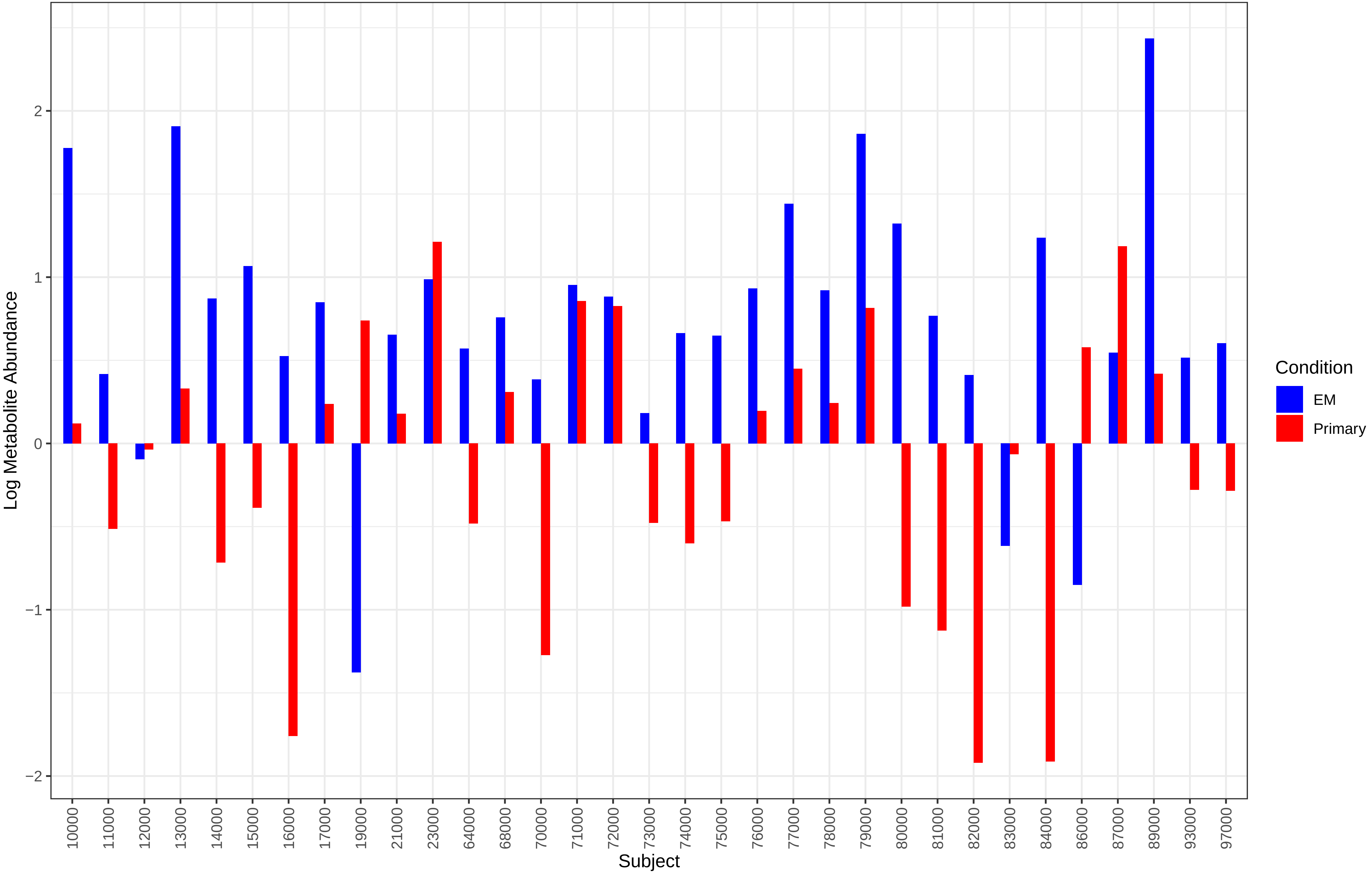

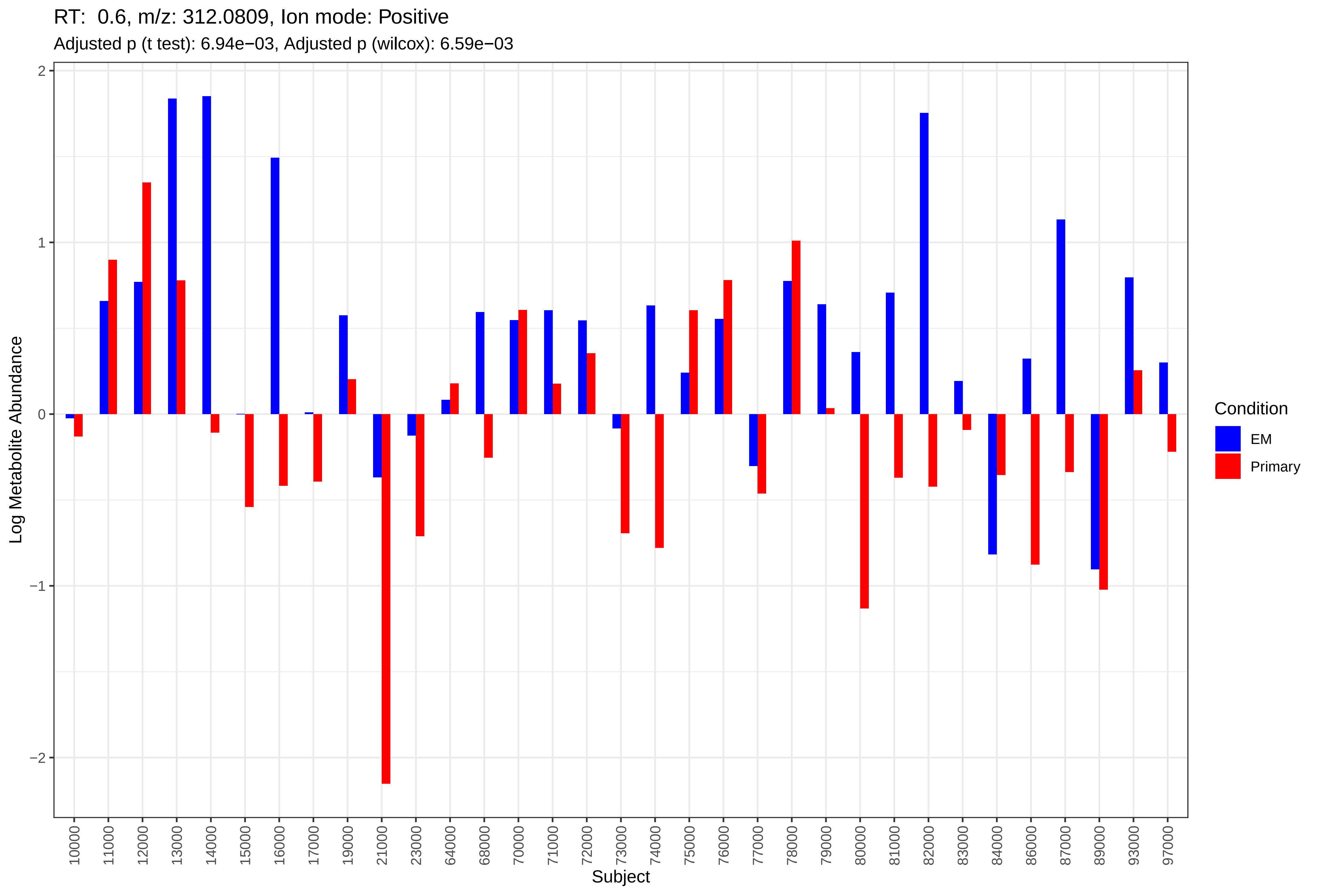
RT: 0.6, m/z: 312.0809, Ion mode: Positive
Adjusted p (t test): 6.94e−03, Adjusted p (wilcox): 6.59e−03
Log Metabolite Abundance
2
1
0
−1
−2
Subject
10000
11000
12000
13000
14000
15000
16000
17000
19000
21000
23000
64000
68000
70000
71000
72000
73000
74000
75000
76000
77000
78000
79000
80000
81000
82000
83000
84000
86000
87000
89000
93000
97000
Condition
EM
Primary

RT: 3.4, m/z: 172.1329, Ion mode: Positive

Adjusted p (t test): 8.95e−03, Adjusted p (wilcox): 6.59e−03

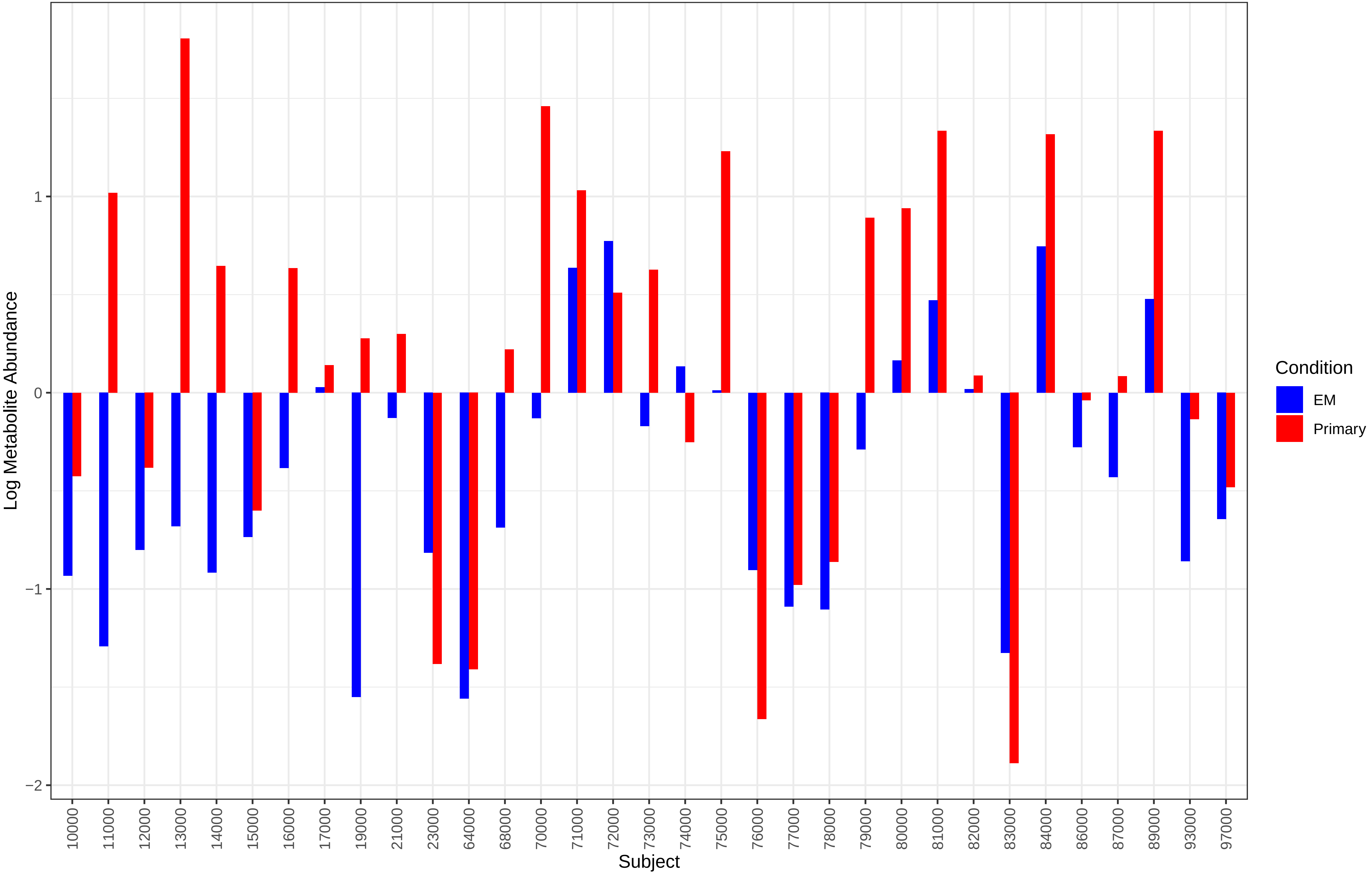

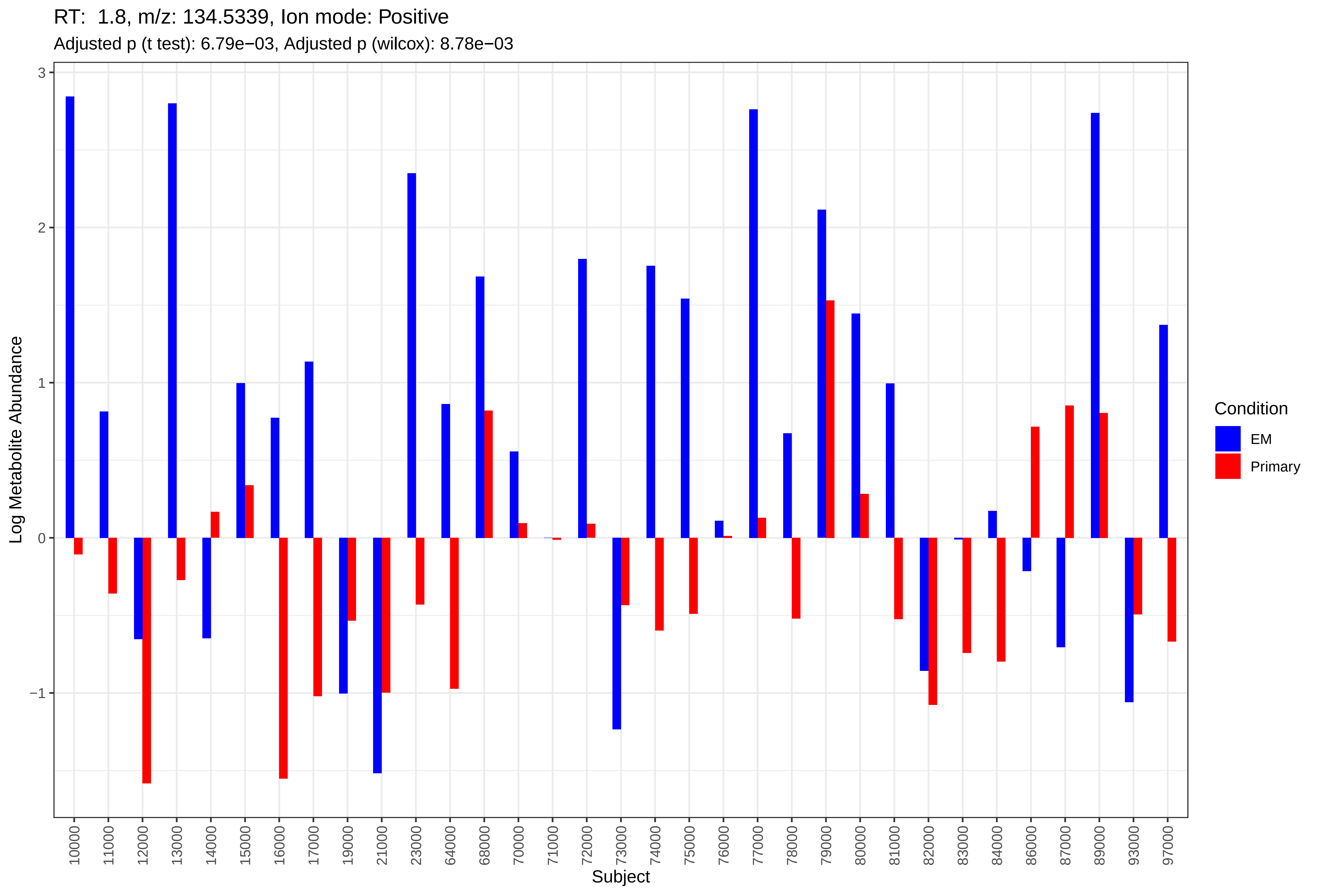
RT: 1.8, m/z: 134.5339, Ion mode: Positive
Adjusted p (t test): 6.79e−03, Adjusted p (wilcox): 8.78e−03
Log Metabolite Abundance
3
2
1
0
−1
Subject
10000
11000
12000
13000
14000
15000
16000
17000
19000
21000
23000
64000
68000
70000
71000
72000
73000
74000
75000
76000
77000
78000
79000
80000
81000
82000
83000
84000
86000
87000
89000
93000
97000
Condition
EM
Primary

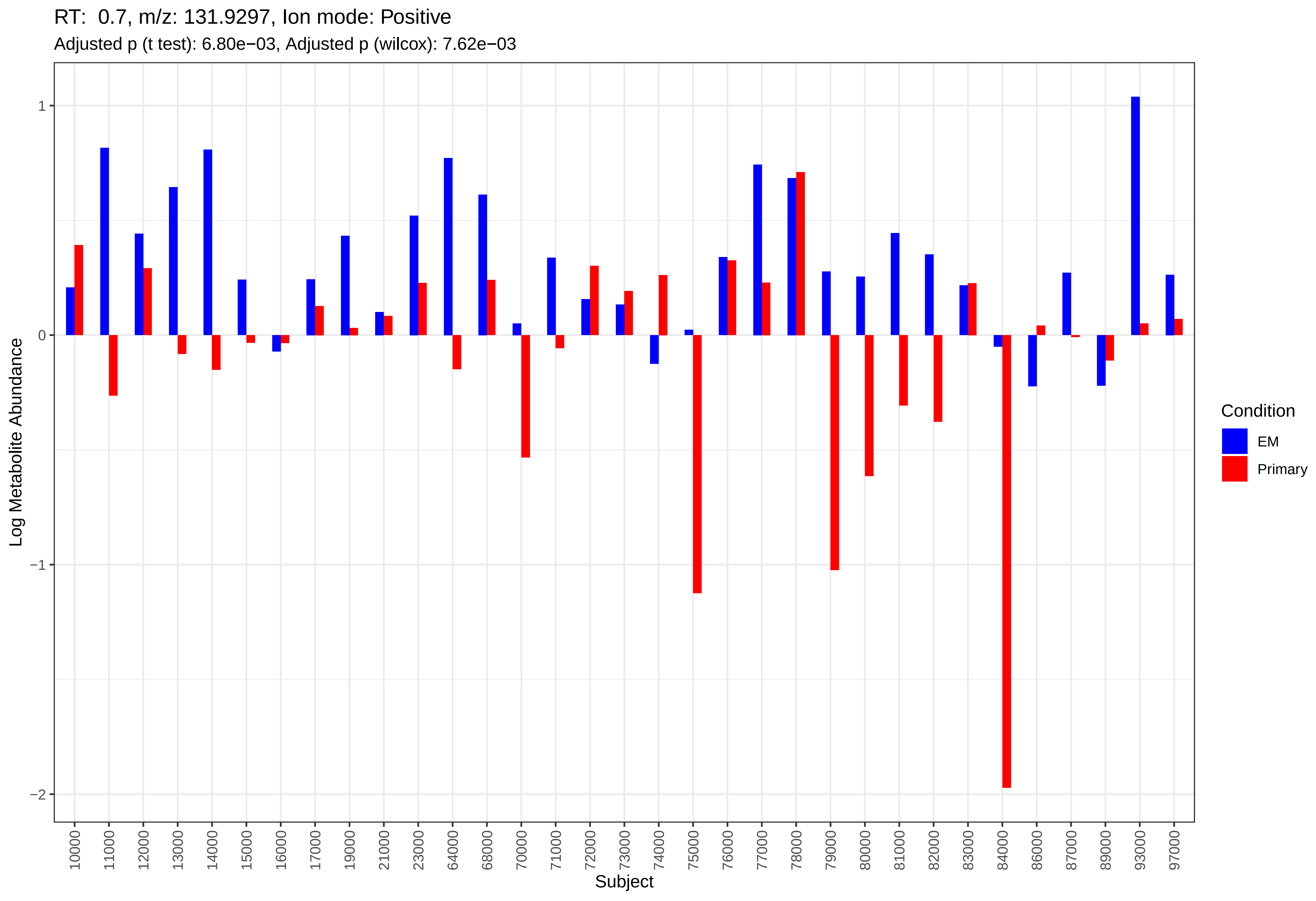
RT: 0.7, m/z: 131.9297, Ion mode: Positive
Adjusted p (t test): 6.80e−03, Adjusted p (wilcox): 7.62e−03
Log Metabolite Abundance
1
0
−1
−2
10000
11000
12000
13000
14000
15000
16000
17000
19000
21000
23000
64000
68000
70000
71000
72000
73000
74000
75000
76000
77000
78000
79000
80000
81000
82000
83000
84000
86000
87000
89000
93000
97000
Subject
Condition
EM
Primary

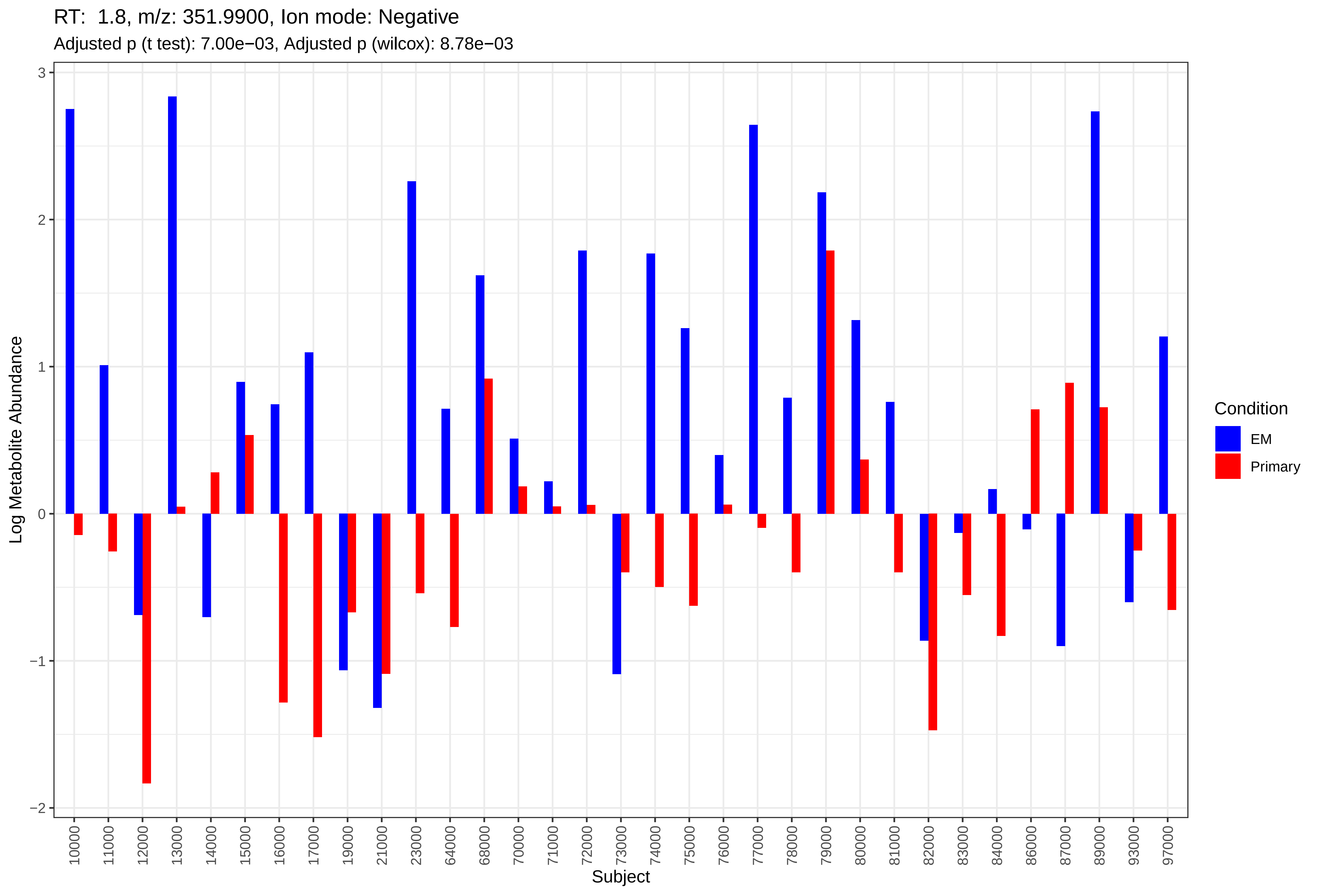

RT: 1.8, m/z: 351.9900, Ion mode: Negative
Adjusted p (t test): 7.00e−03, Adjusted p (wilcox): 8.78e−03
Log Metabolite Abundance
3
2
1
0
−1
−2
10000
11000
12000
13000
14000
15000
16000
17000
19000
21000
23000
64000
68000
70000
71000
72000
73000
74000
75000
76000
77000
78000
79000
80000
81000
82000
83000
84000
86000
87000
89000
93000
97000
Subject
Condition
EM
Primary

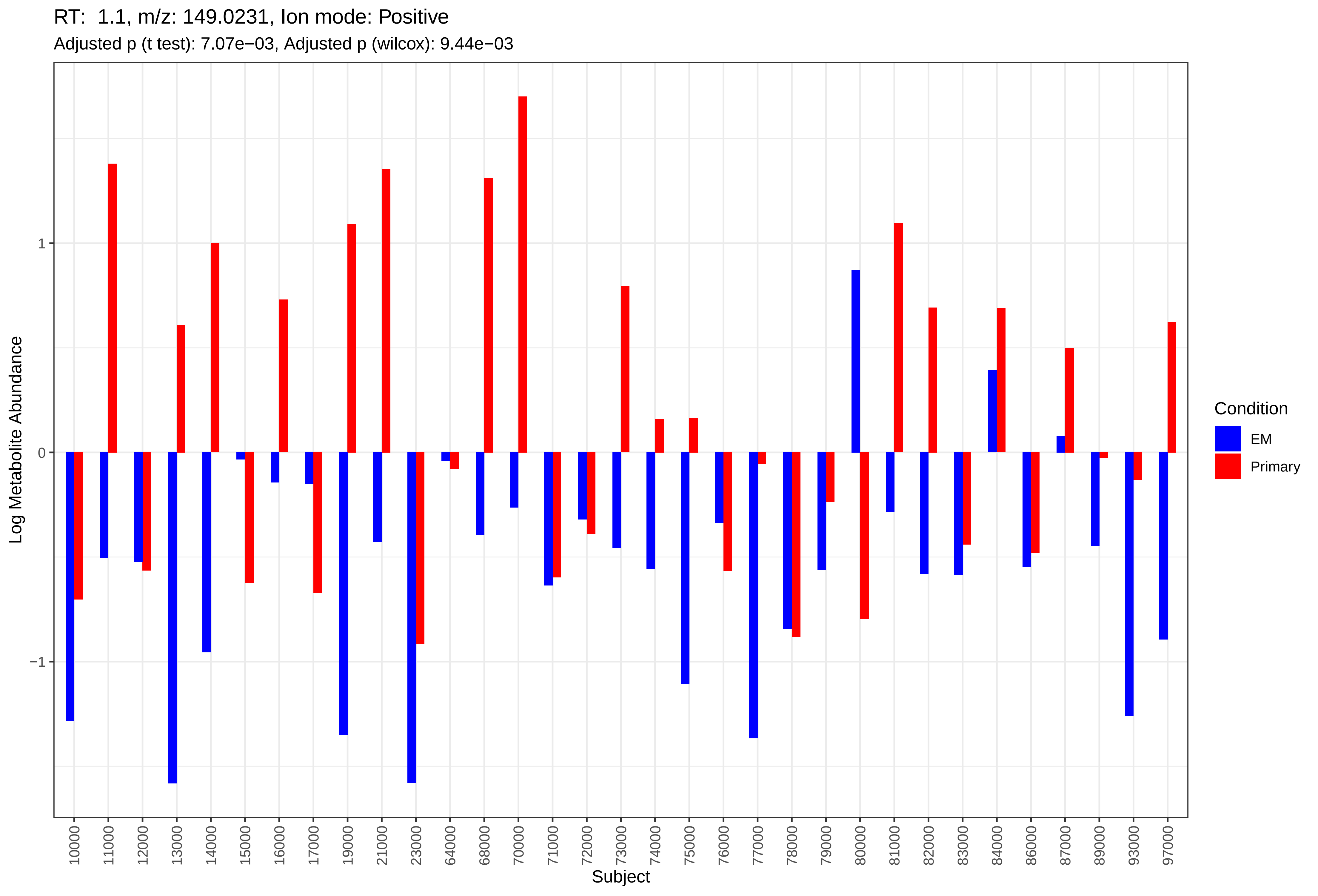

RT: 1.1, m/z: 149.0231, Ion mode: Positive
Adjusted p (t test): 7.07e−03, Adjusted p (wilcox): 9.44e−03
Log Metabolite Abundance
1
0
−1
10000
11000
12000
13000
14000
15000
16000
17000
19000
21000
23000
64000
68000
70000
71000
72000
73000
74000
75000
76000
77000
78000
79000
80000
81000
82000
83000
84000
86000
87000
89000
93000
97000
Subject
Condition
EM
Primary

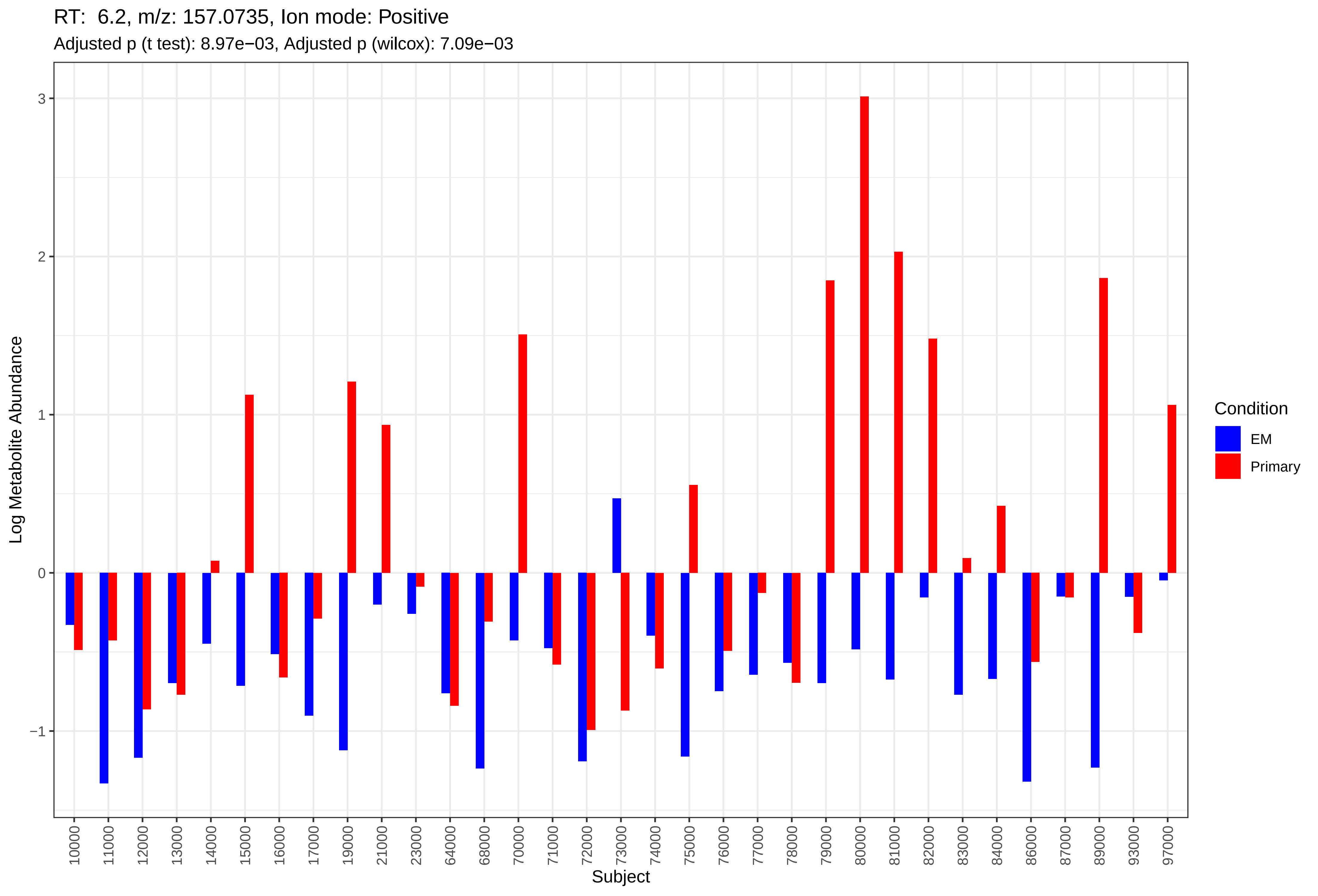
RT: 6.2, m/z: 157.0735, Ion mode: Positive
Adjusted p (t test): 8.97e−03, Adjusted p (wilcox): 7.09e−03
Log Metabolite Abundance
3
2
1
0
−1
10000
11000
12000
13000
14000
15000
16000
17000
19000
21000
23000
64000
68000
70000
71000
72000
73000
74000
75000
76000
77000
78000
79000
80000
81000
82000
83000
84000
86000
87000
89000
93000
97000
Subject
Condition
EM
Primary

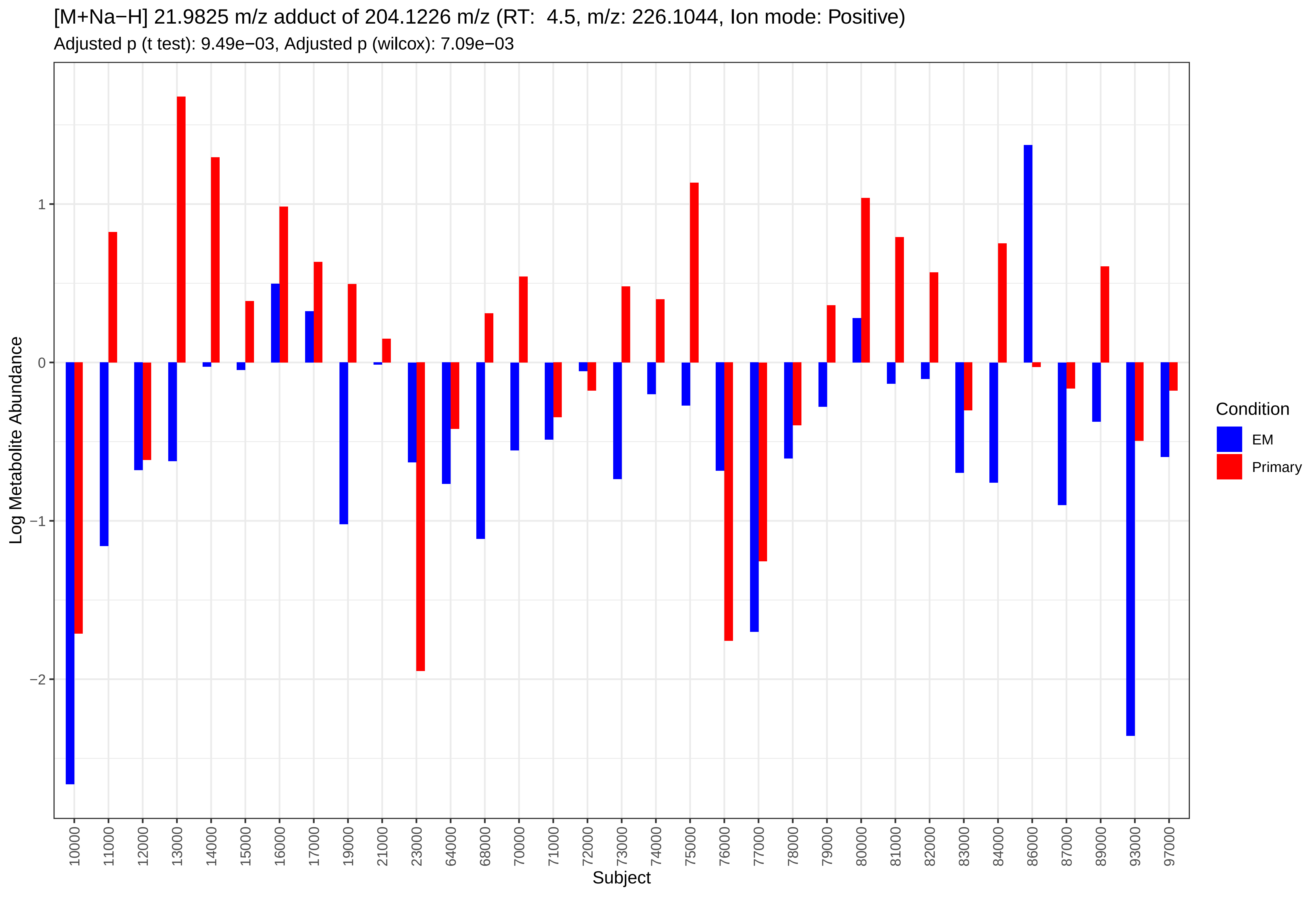

[M+Na−H] 21.9825 m/z adduct of 204.1226 m/z (RT: 4.5, m/z: 226.1044, Ion mode: Positive)
Adjusted p (t test): 9.49e−03, Adjusted p (wilcox): 7.09e−03
Log Metabolite Abundance
1
0
−1
−2
Subject
10000
11000
12000
13000
14000
15000
16000
17000
19000
21000
23000
64000
68000
70000
71000
72000
73000
74000
75000
76000
77000
78000
79000
80000
81000
82000
83000
84000
86000
87000
89000
93000
97000
Condition
EM
Primary

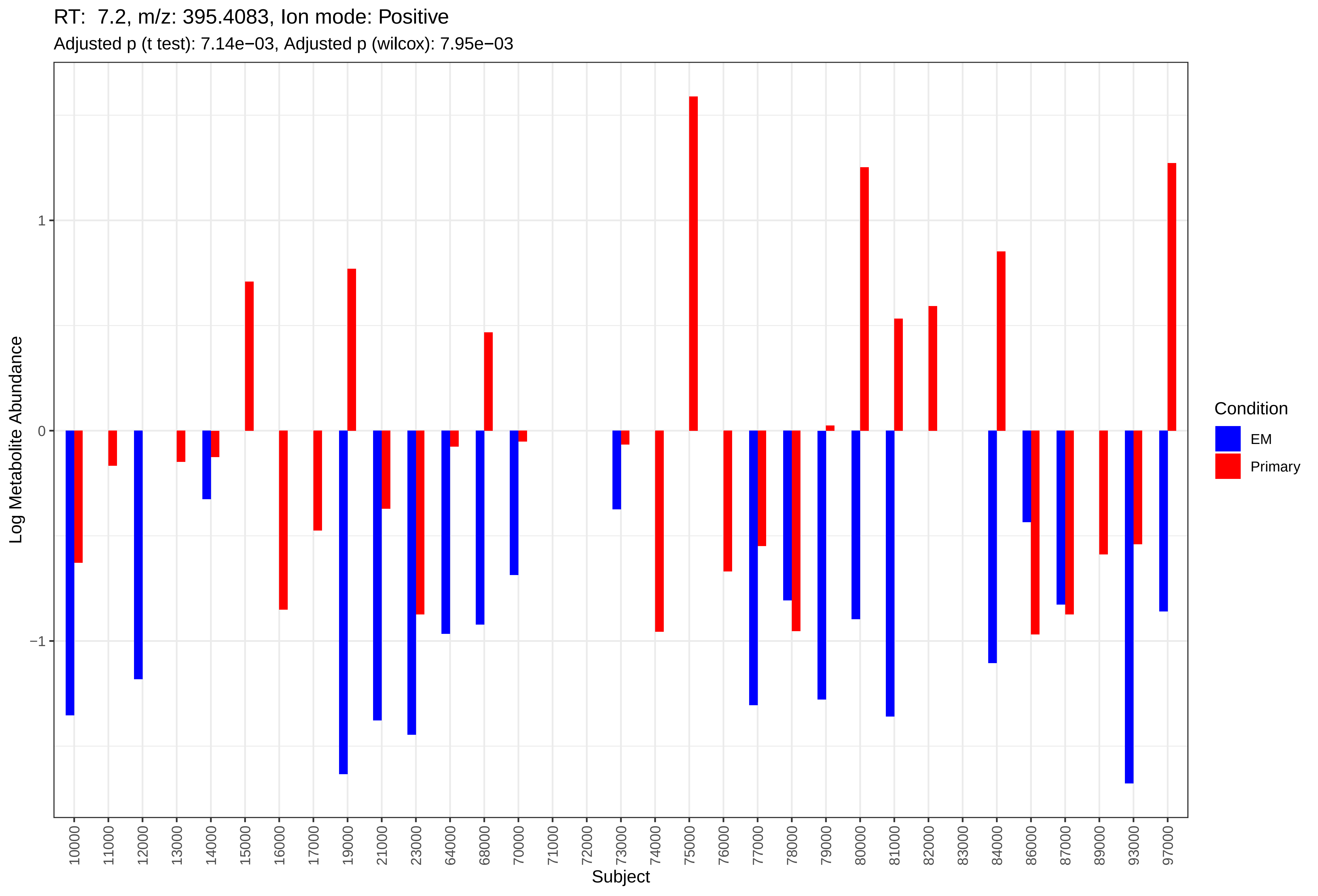
RT: 7.2, m/z: 395.4083, Ion mode: Positive
Adjusted p (t test): 7.14e−03, Adjusted p (wilcox): 7.95e−03
Log Metabolite Abundance
1
0
−1
Subject
10000
11000
12000
13000
14000
15000
16000
17000
19000
21000
23000
64000
68000
70000
71000
72000
73000
74000
75000
76000
77000
78000
79000
80000
81000
82000
83000
84000
86000
87000
89000
93000
97000
Condition
EM
Primary

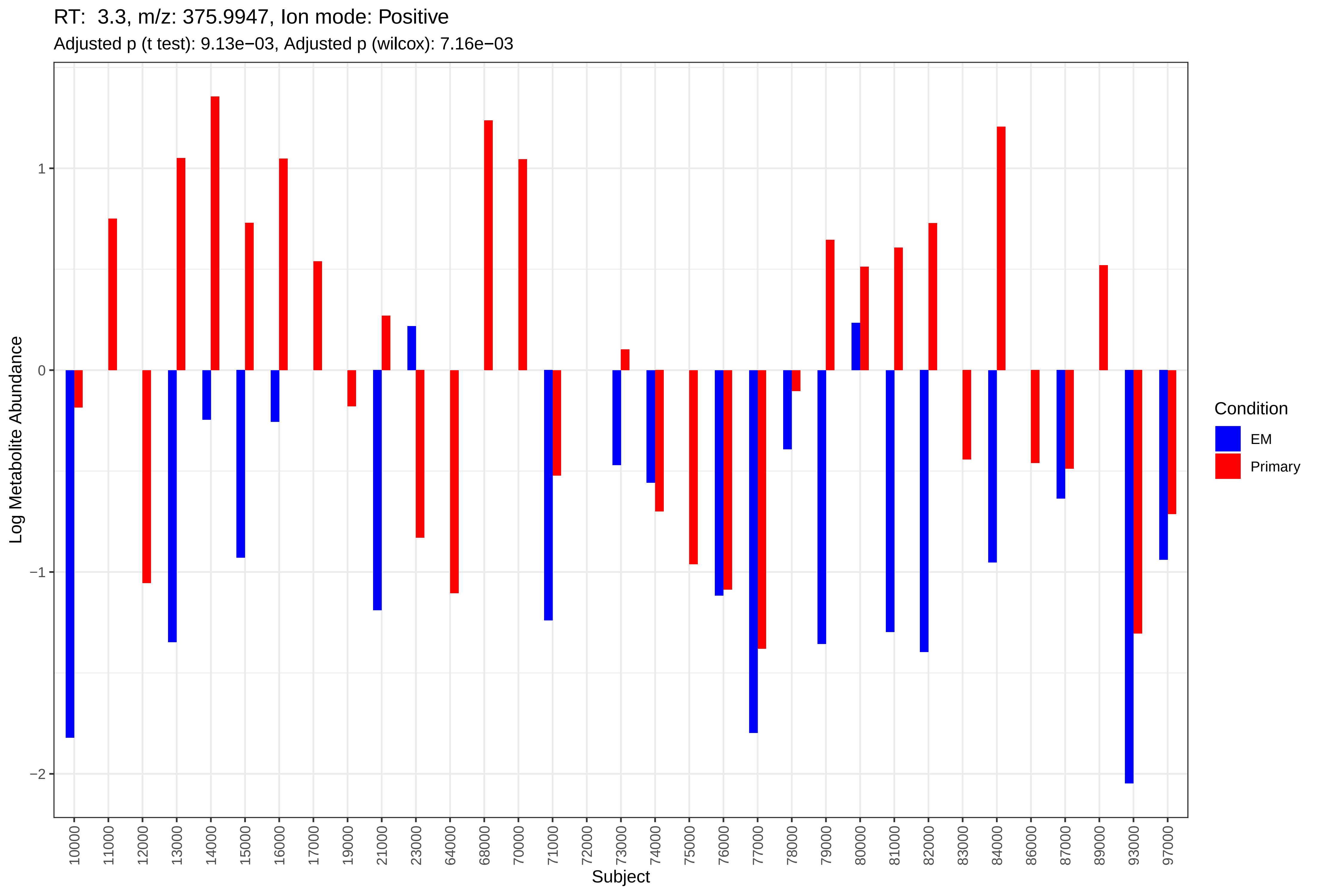

RT: 3.3, m/z: 375.9947, Ion mode: Positive
Adjusted p (t test): 9.13e−03, Adjusted p (wilcox): 7.16e−03
Log Metabolite Abundance
1
0
−1
−2
Subject
10000
11000
12000
13000
14000
15000
16000
17000
19000
21000
23000
64000
68000
70000
71000
72000
73000
74000
75000
76000
77000
78000
79000
80000
81000
82000
83000
84000
86000
87000
89000
93000
97000
Condition
EM
Primary

RT: 6.0, m/z: 108.1120, Ion mode: Positive

Adjusted p (t test): 9.19e−03, Adjusted p (wilcox): 7.16e−03

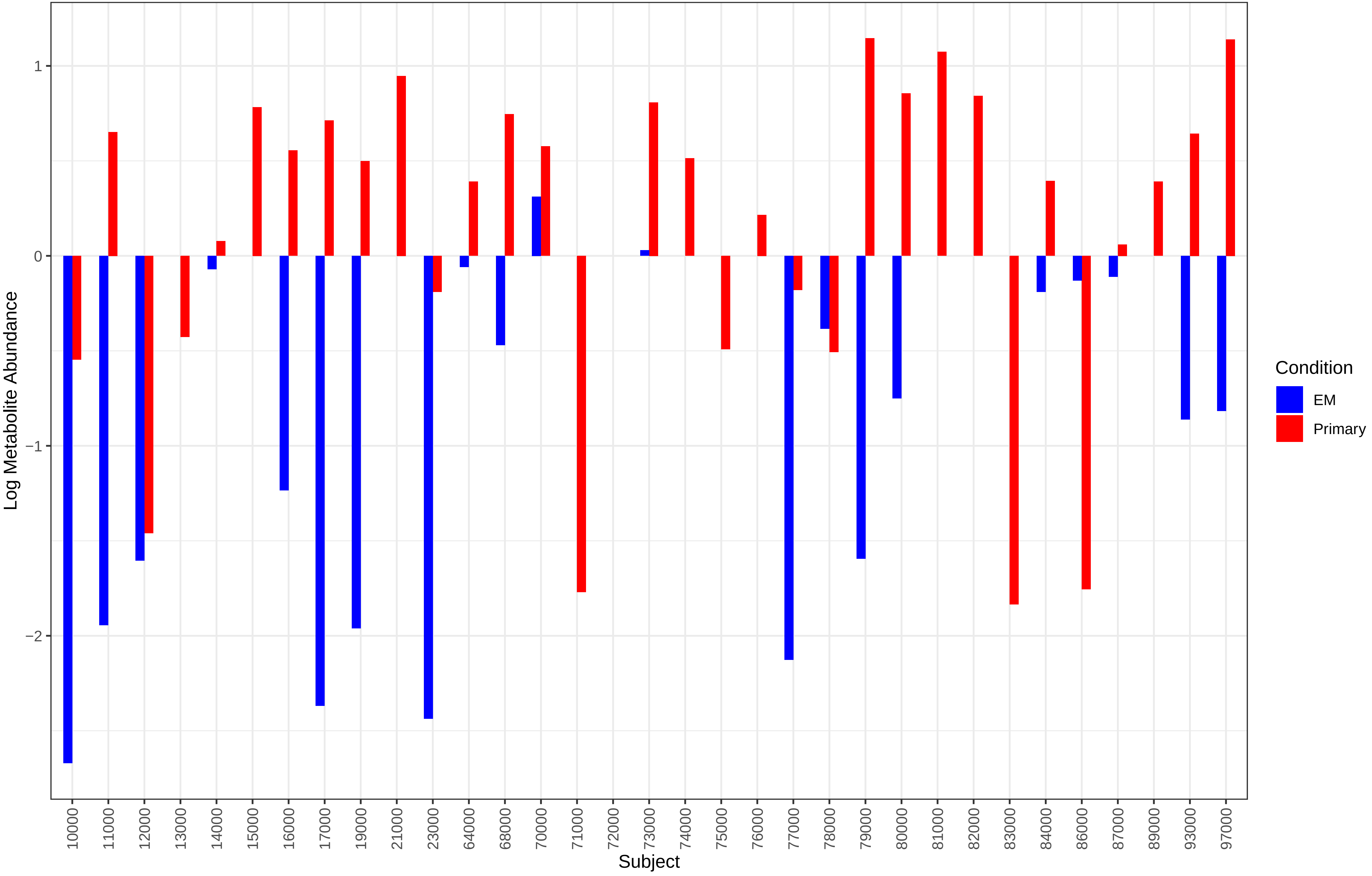

[M−HCOOH+H] −46.0055 m/z adduct of 139.0499 m/z (RT: 1.9, m/z: 93.0446, Ion mode: Positive)

Adjusted p (t test): 7.24e−03, Adjusted p (wilcox): 8.15e−03

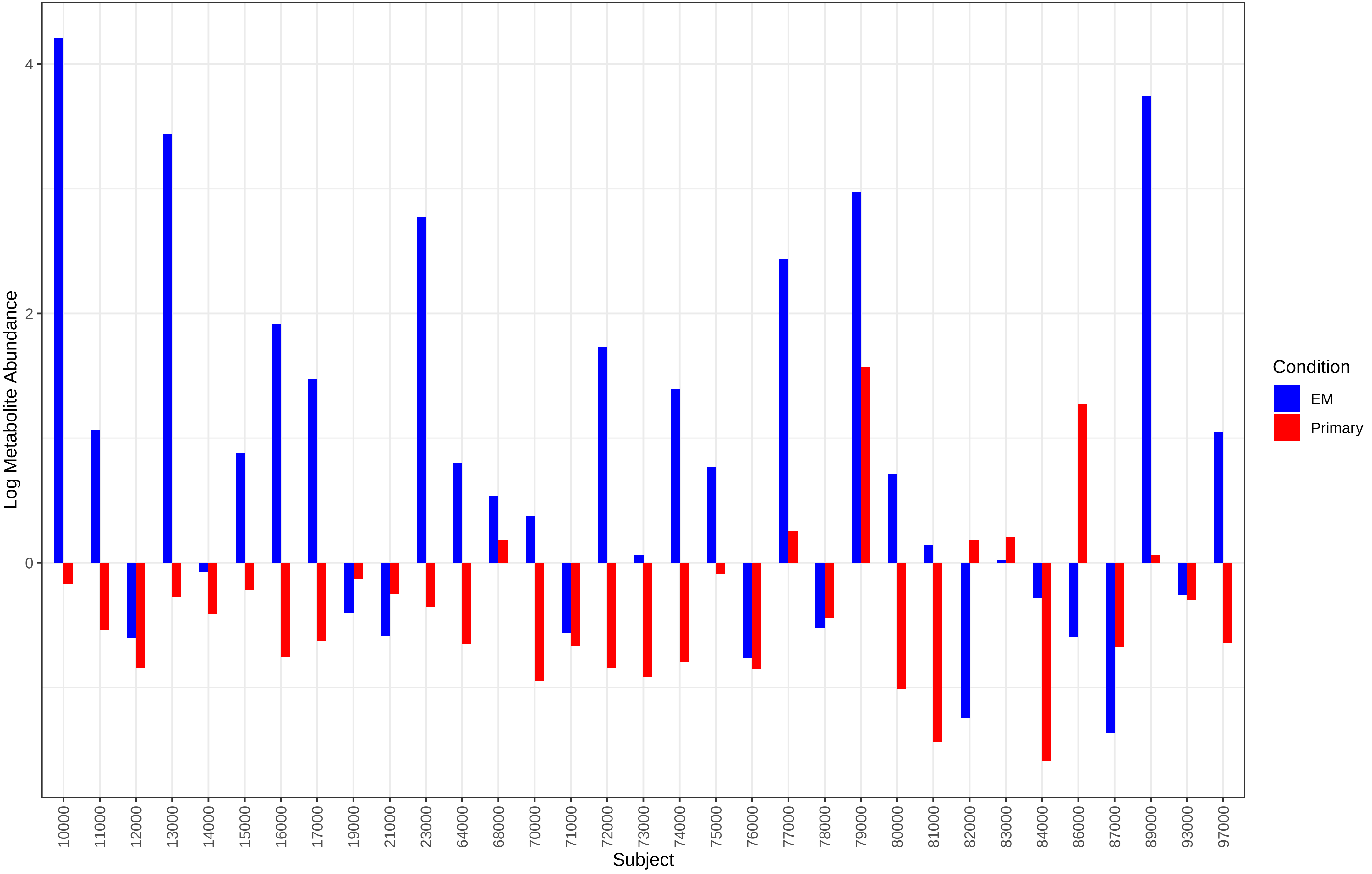

RT: 1.2, m/z: 263.0082, Ion mode: Negative

Adjusted p (t test): 7.31e−03, Adjusted p (wilcox): 8.15e−03

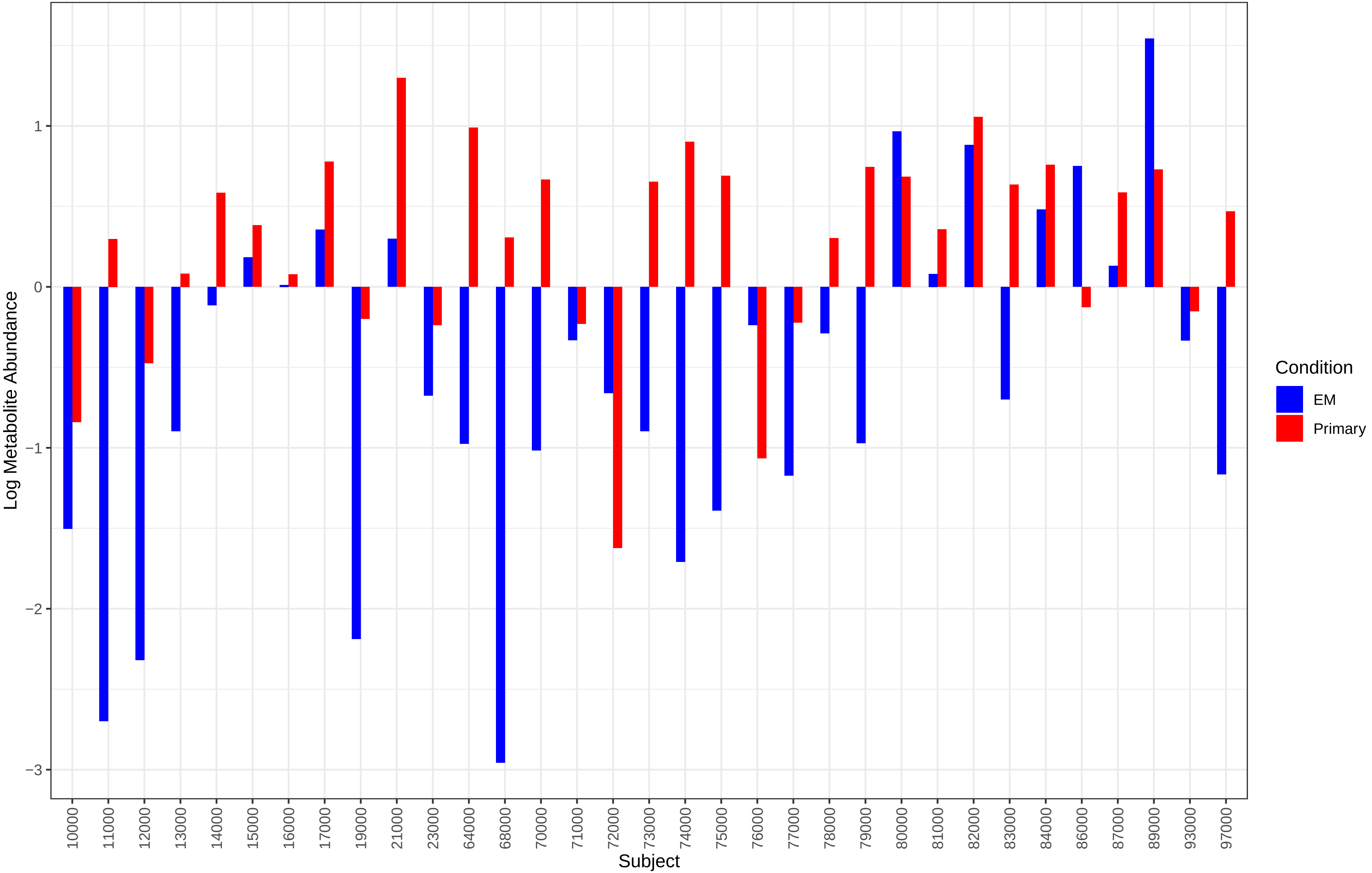

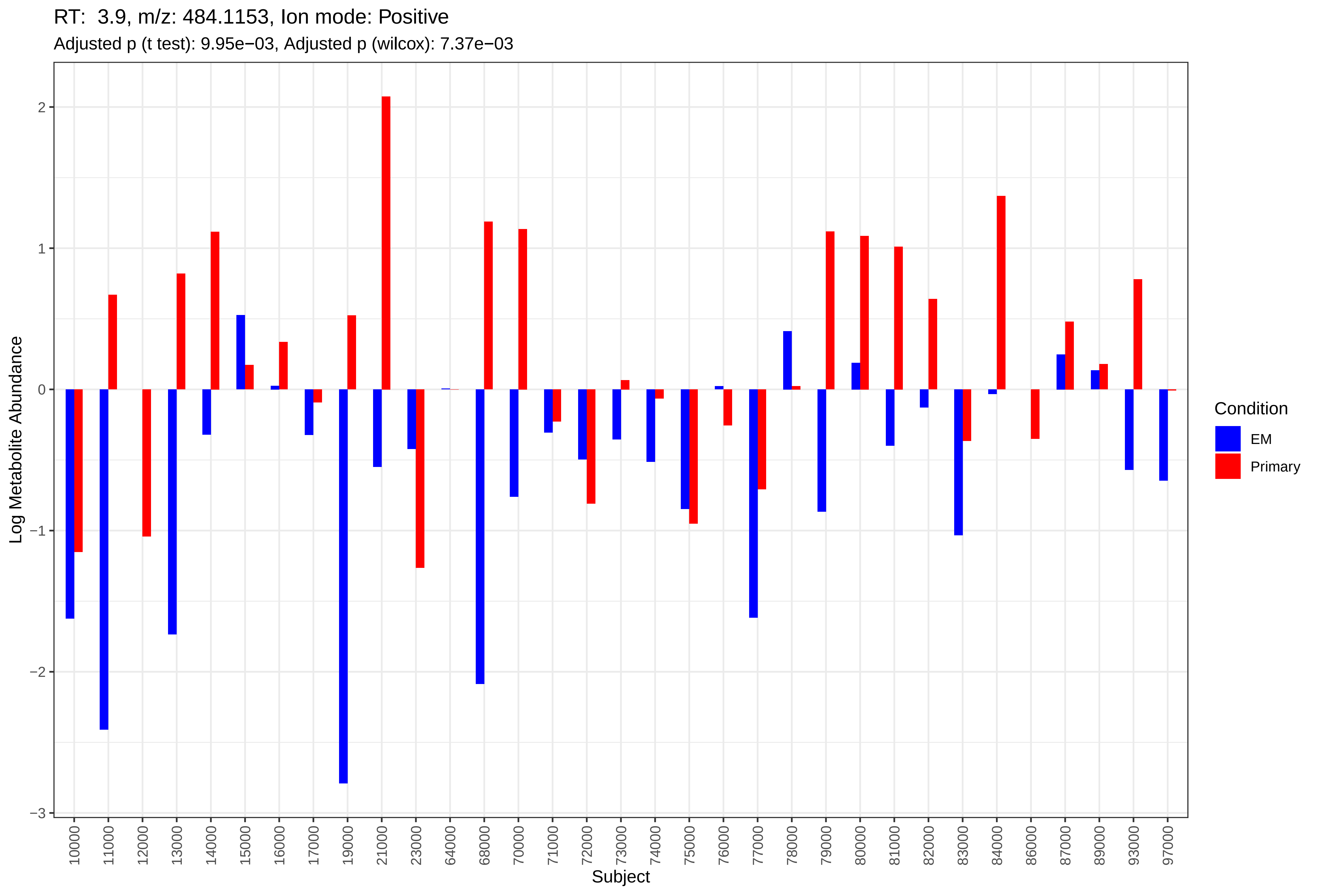
RT: 3.9, m/z: 484.1153, Ion mode: Positive
Adjusted p (t test): 9.95e−03, Adjusted p (wilcox): 7.37e−03
Log Metabolite Abundance
Subject
Condition
EM
Primary
2
1
0
−1
−2
−3
10000
11000
12000
13000
14000
15000
16000
17000
19000
21000
23000
64000
68000
70000
71000
72000
73000
74000
75000
76000
77000
78000
79000
80000
81000
82000
83000
84000
86000
87000
89000
93000
97000

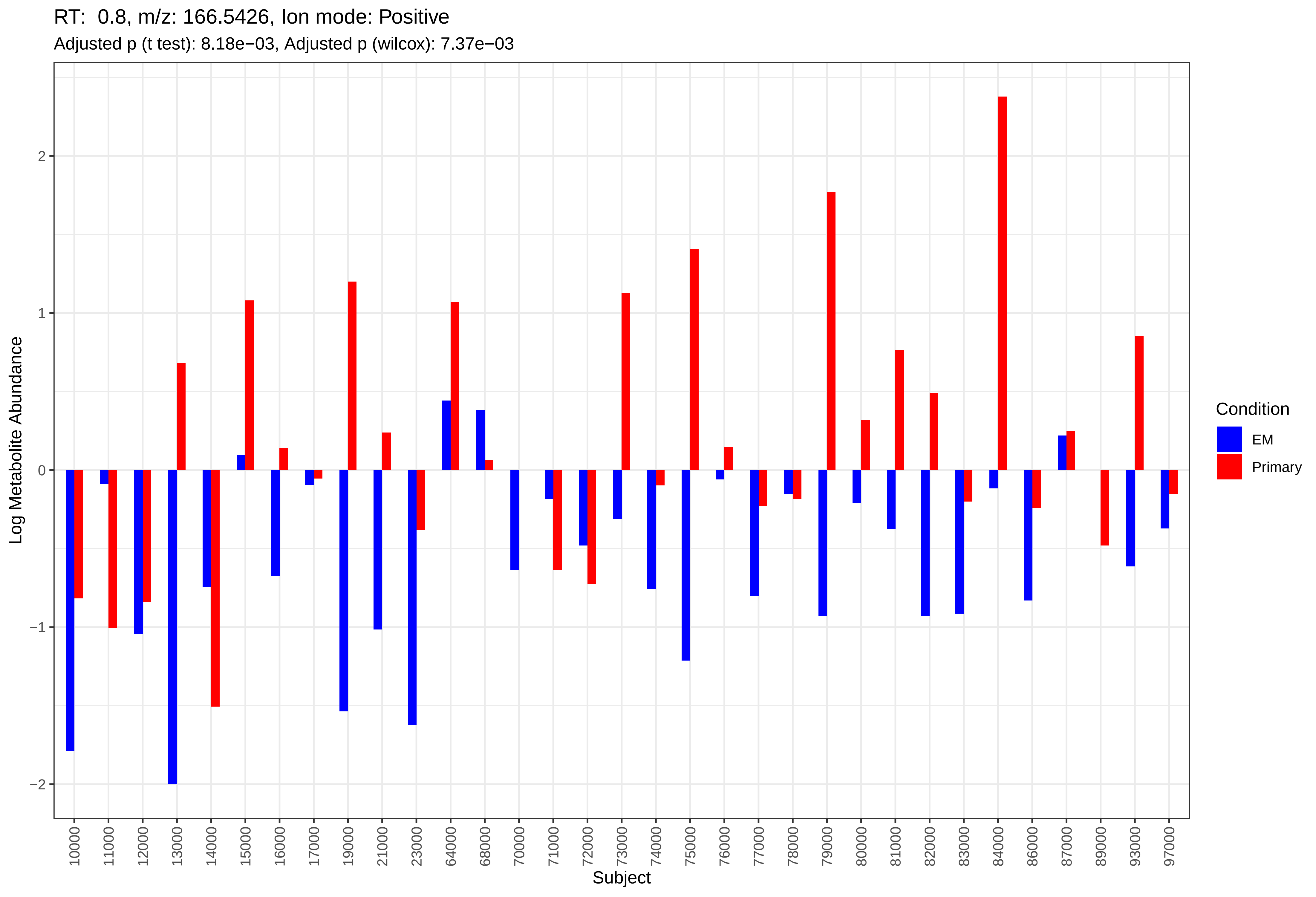

RT: 0.8, m/z: 166.5426, Ion mode: Positive
Adjusted p (t test): 8.18e−03, Adjusted p (wilcox): 7.37e−03
Log Metabolite Abundance
2
1
0
−1
−2
Subject
10000
11000
12000
13000
14000
15000
16000
17000
19000
21000
23000
64000
68000
70000
71000
72000
73000
74000
75000
76000
77000
78000
79000
80000
81000
82000
83000
84000
86000
87000
89000
93000
97000
Condition
EM
Primary

RT: 3.6, m/z: 157.0733, Ion mode: Positive

Adjusted p (t test): 7.45e−03, Adjusted p (wilcox): 7.62e−03

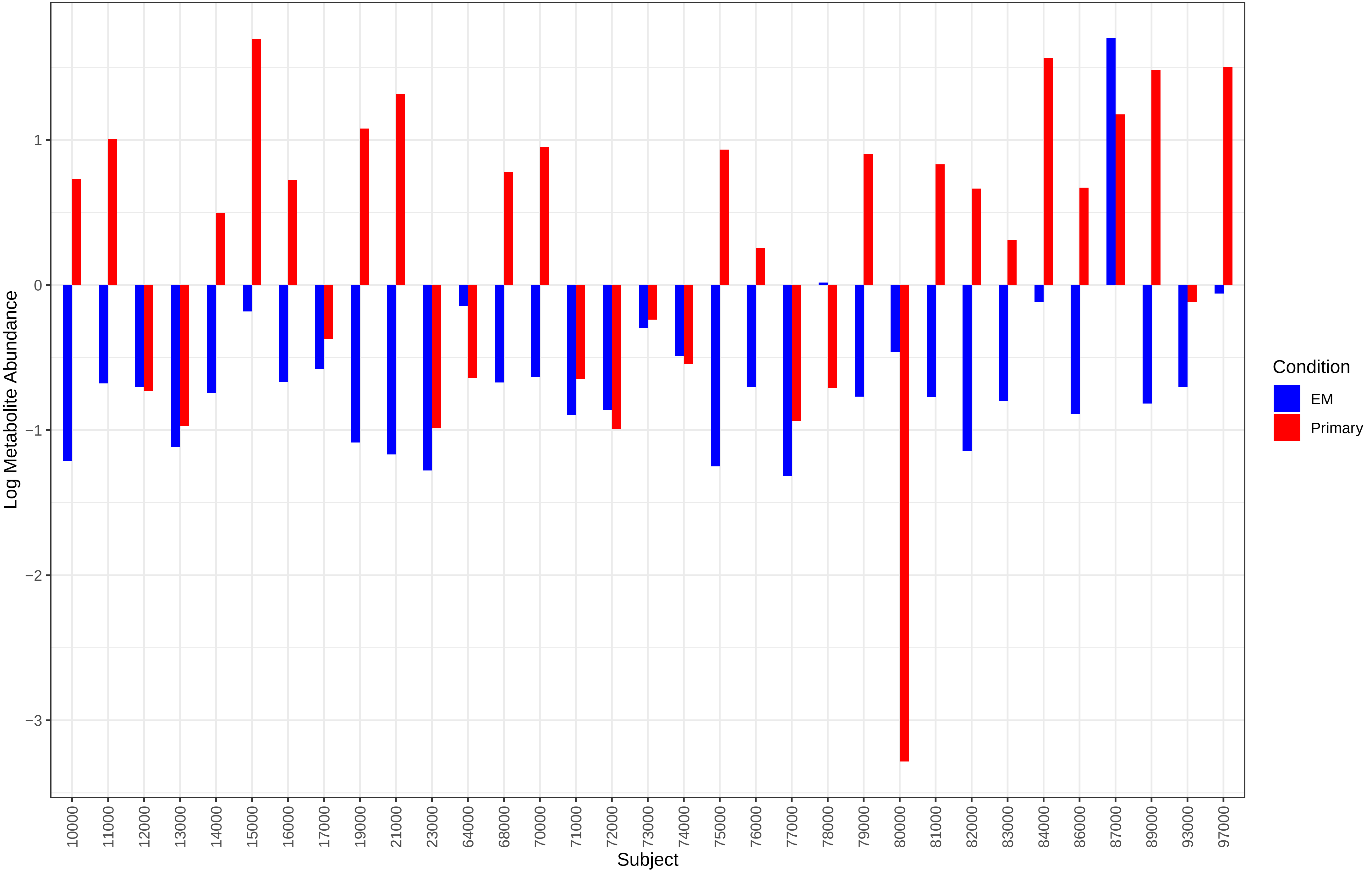

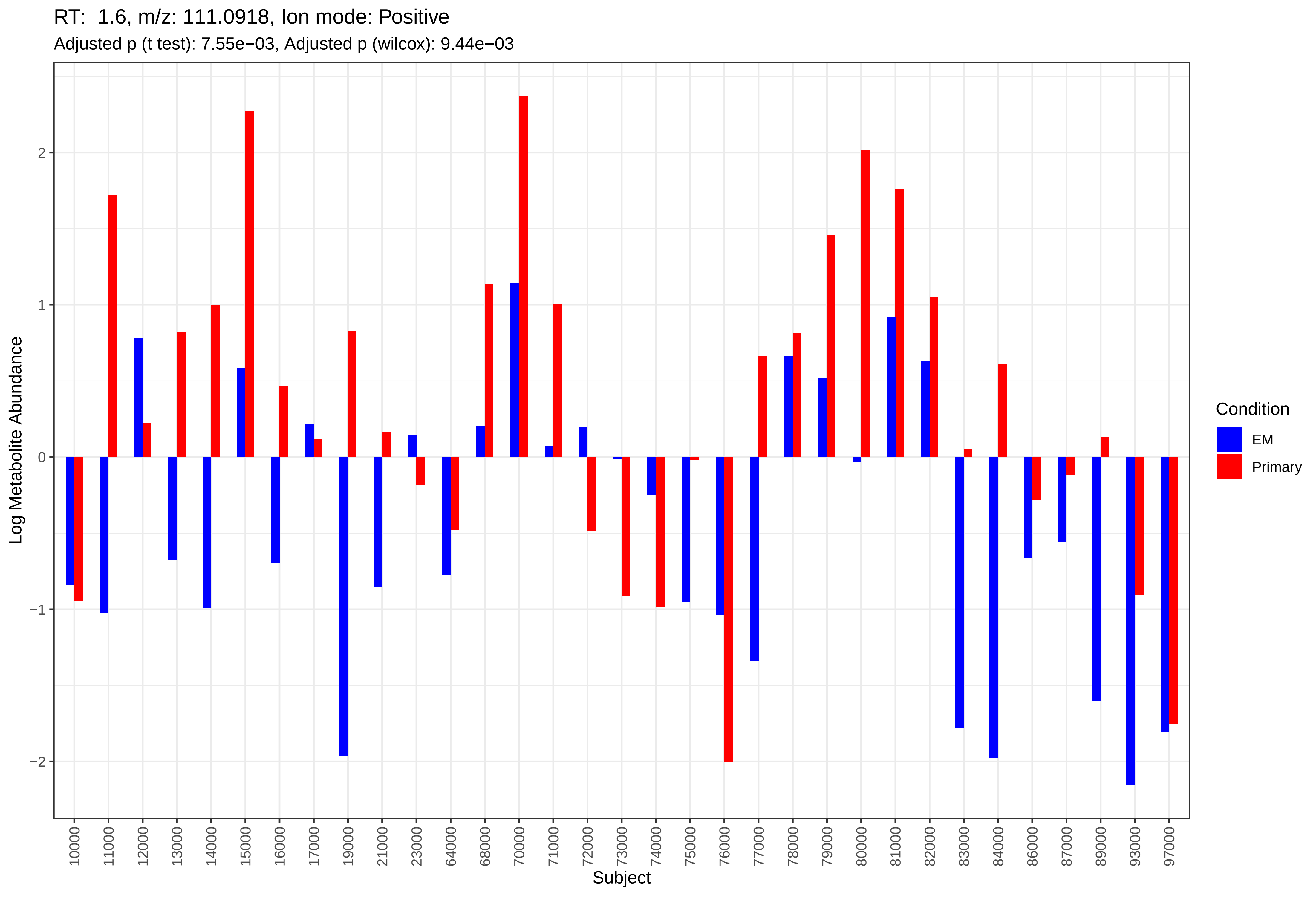

RT: 1.6, m/z: 111.0918, Ion mode: Positive
Adjusted p (t test): 7.55e−03, Adjusted p (wilcox): 9.44e−03
Log Metabolite Abundance
2
1
0
−1
−2
10000
11000
12000
13000
14000
15000
16000
17000
19000
21000
23000
64000
68000
70000
71000
72000
73000
74000
75000
76000
77000
78000
79000
80000
81000
82000
83000
84000
86000
87000
89000
93000
97000
Subject
Condition
EM
Primary

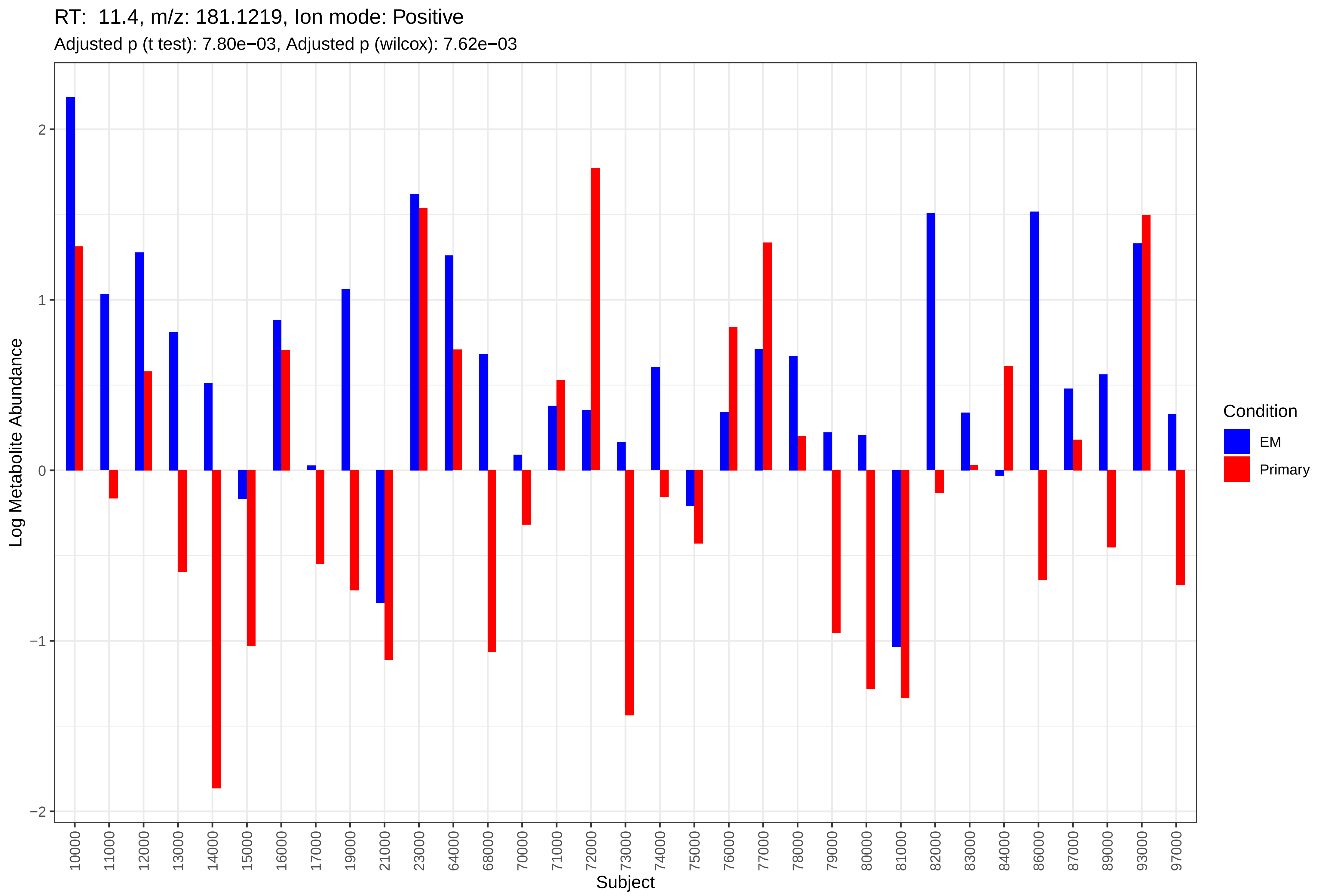
RT: 11.4, m/z: 181.1219, Ion mode: Positive
Adjusted p (t test): 7.80e−03, Adjusted p (wilcox): 7.62e−03
Log Metabolite Abundance
2
1
0
−1
−2
10000
11000
12000
13000
14000
15000
16000
17000
19000
21000
23000
64000
68000
70000
71000
72000
73000
74000
75000
76000
77000
78000
79000
80000
81000
82000
83000
84000
86000
87000
89000
93000
97000
Subject
Condition
EM
Primary

[M−NH3+H] −17.0265 m/z adduct of 155.0749 m/z (RT: 1.4, m/z: 138.0485, Ion mode: Positive)

Adjusted p (t test): 9.18e−03, Adjusted p (wilcox): 7.62e−03

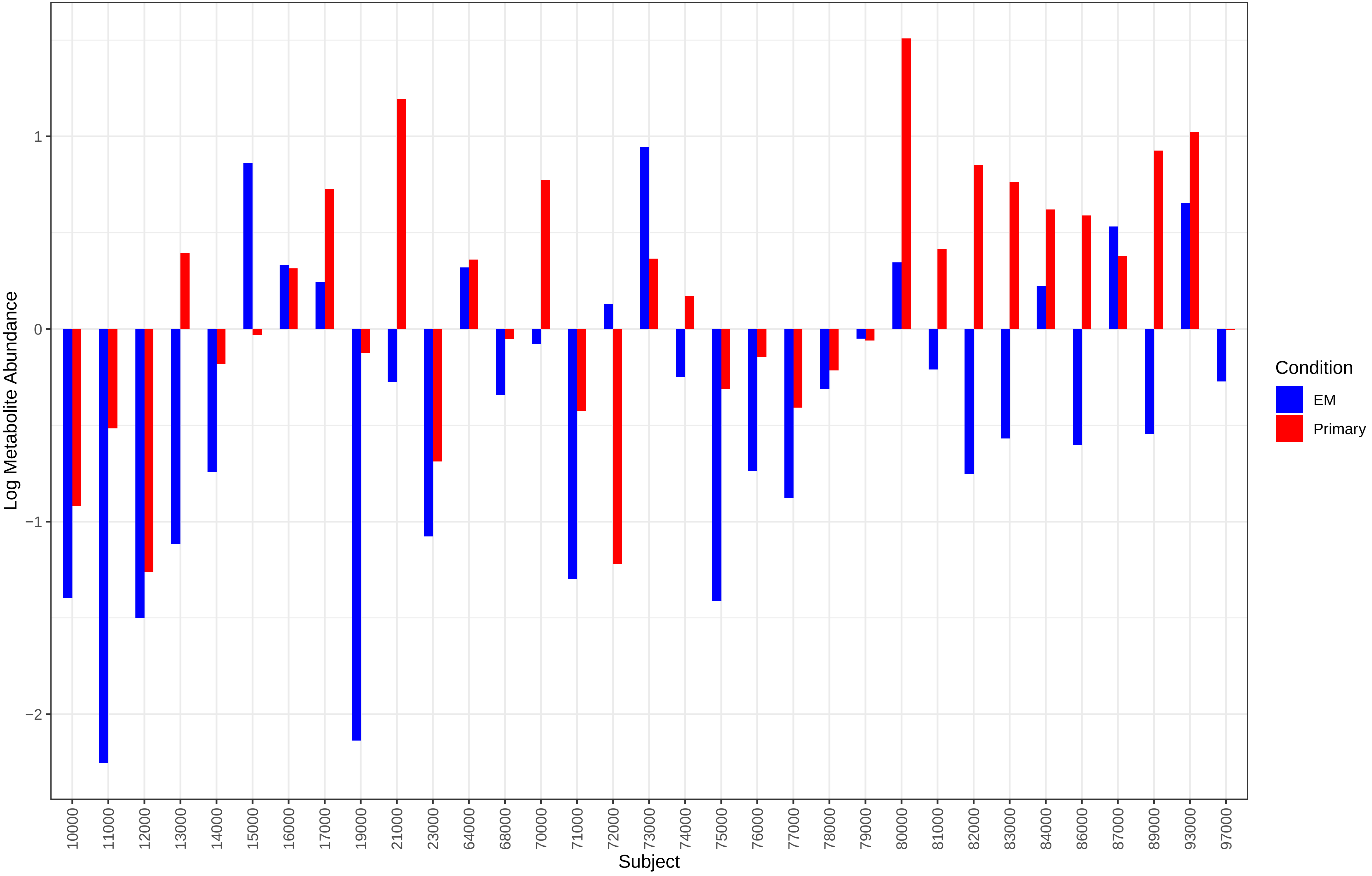

RT: 0.7, m/z: 227.1794, Ion mode: Positive

Adjusted p (t test): 8.33e−03, Adjusted p (wilcox): 7.62e−03

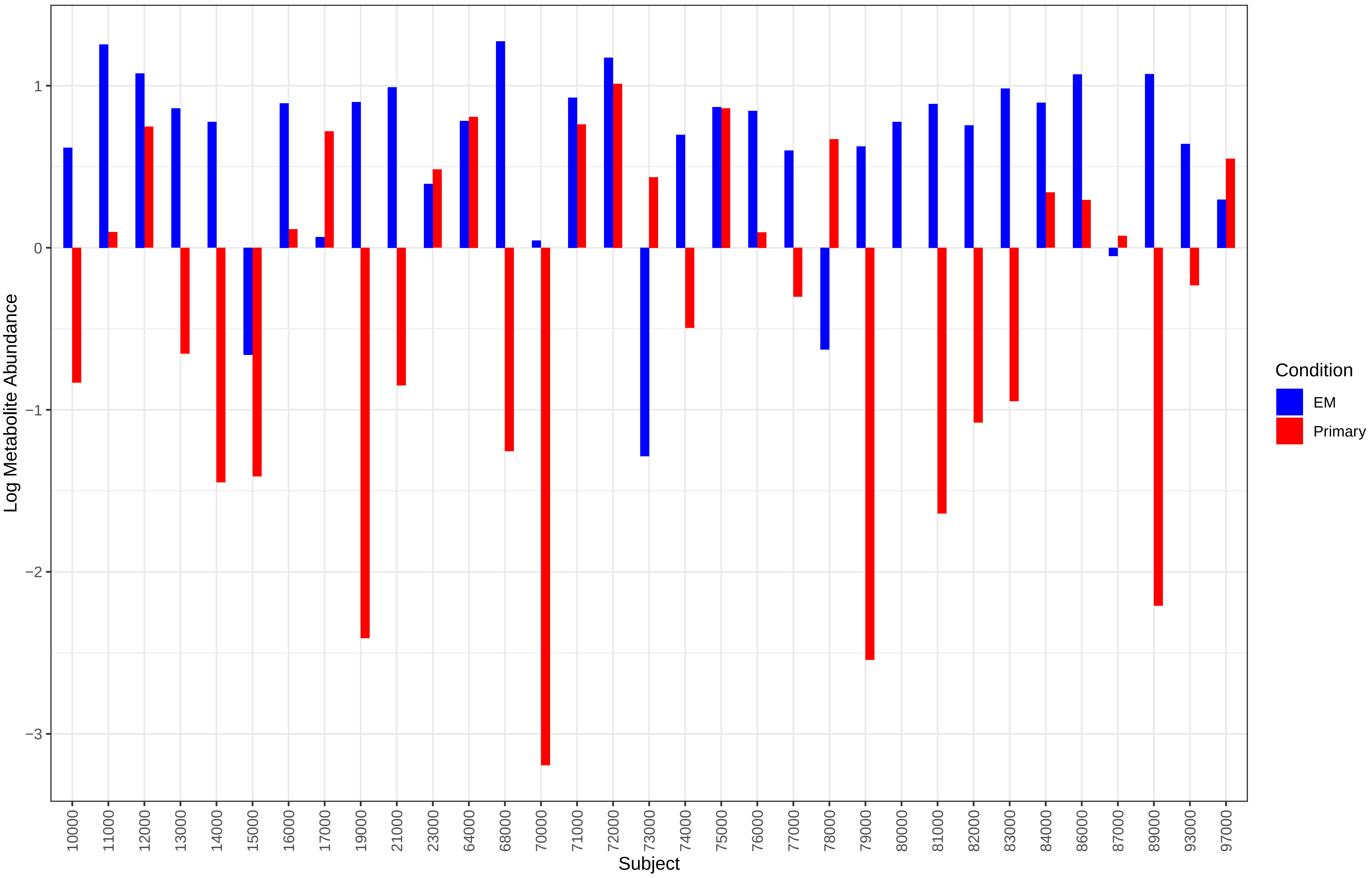

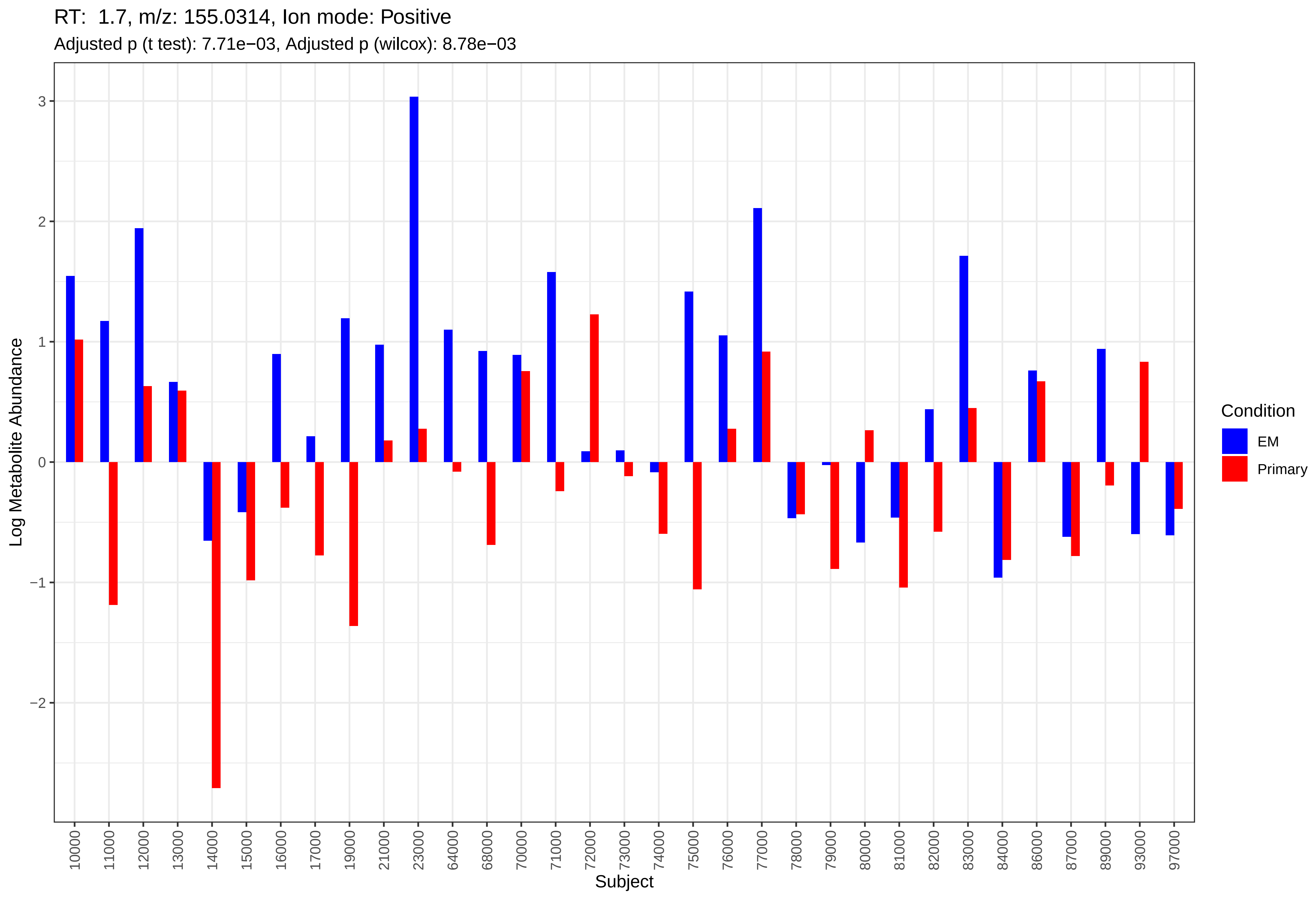
RT: 1.7, m/z: 155.0314, Ion mode: Positive
Adjusted p (t test): 7.71e−03, Adjusted p (wilcox): 8.78e−03
Log Metabolite Abundance
3
2
1
0
−1
−2
10000
11000
12000
13000
14000
15000
16000
17000
19000
21000
23000
64000
68000
70000
71000
72000
73000
74000
75000
76000
77000
78000
79000
80000
81000
82000
83000
84000
86000
87000
89000
93000
97000
Subject
Condition
EM
Primary

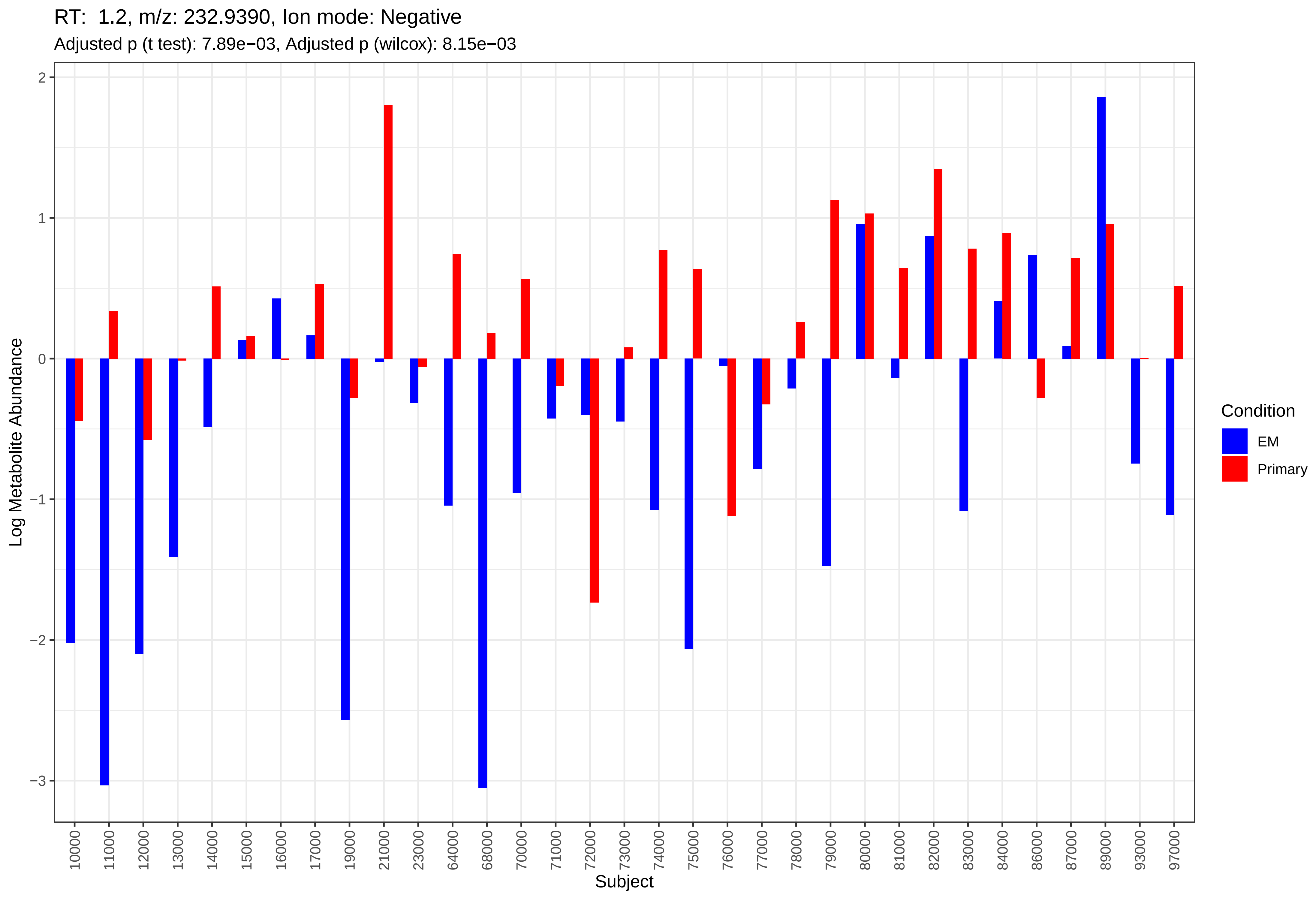

RT: 1.2, m/z: 232.9390, Ion mode: Negative
Adjusted p (t test): 7.89e−03, Adjusted p (wilcox): 8.15e−03
Log Metabolite Abundance
2
1
0
−1
−2
−3
Subject
10000
11000
12000
13000
14000
15000
16000
17000
19000
21000
23000
64000
68000
70000
71000
72000
73000
74000
75000
76000
77000
78000
79000
80000
81000
82000
83000
84000
86000
87000
89000
93000
97000
Condition
EM
Primary

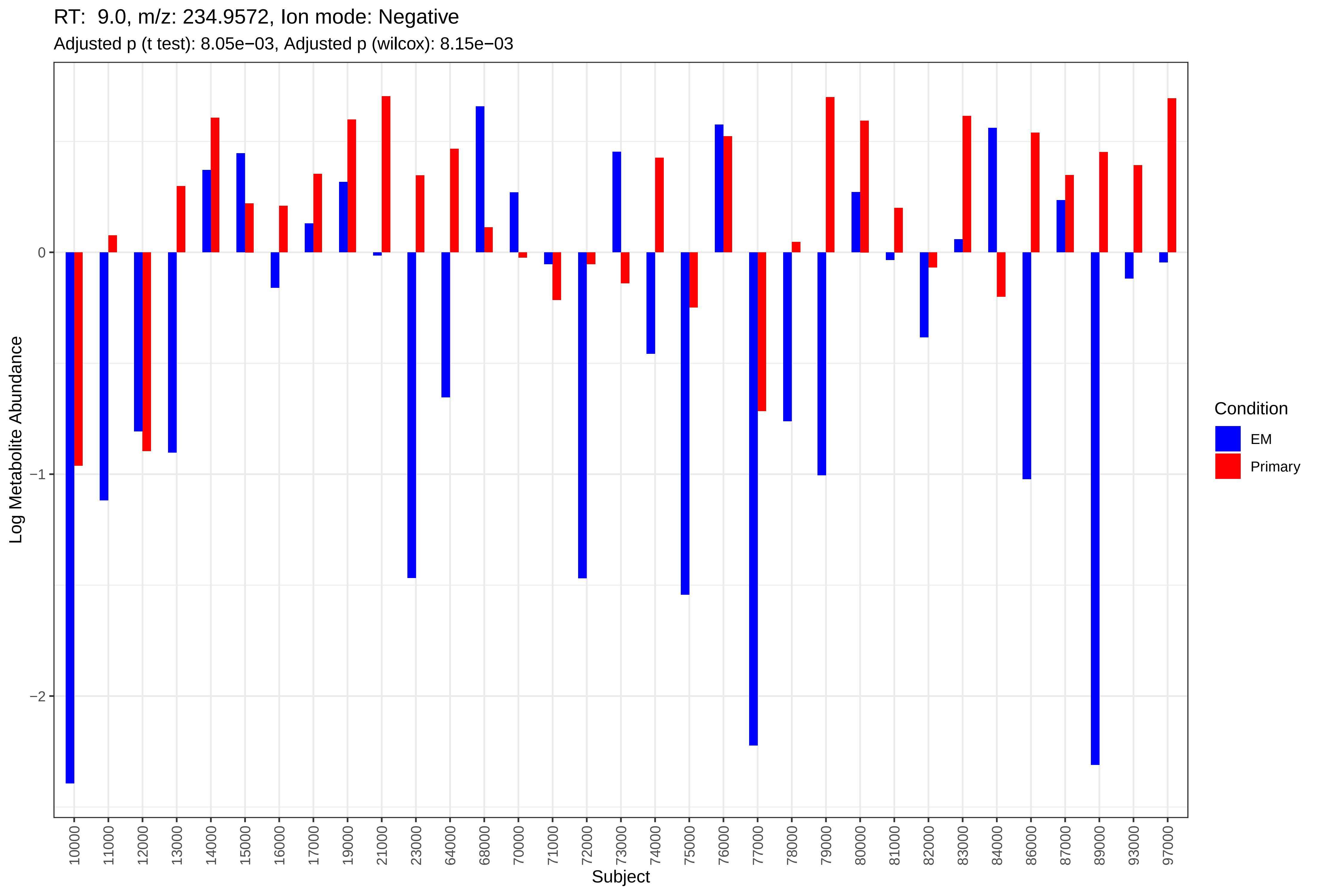
RT: 9.0, m/z: 234.9572, Ion mode: Negative
Adjusted p (t test): 8.05e−03, Adjusted p (wilcox): 8.15e−03
Log Metabolite Abundance
0
−1
−2
Subject
10000
11000
12000
13000
14000
15000
16000
17000
19000
21000
23000
64000
68000
70000
71000
72000
73000
74000
75000
76000
77000
78000
79000
80000
81000
82000
83000
84000
86000
87000
89000
93000
97000
Condition
EM
Primary

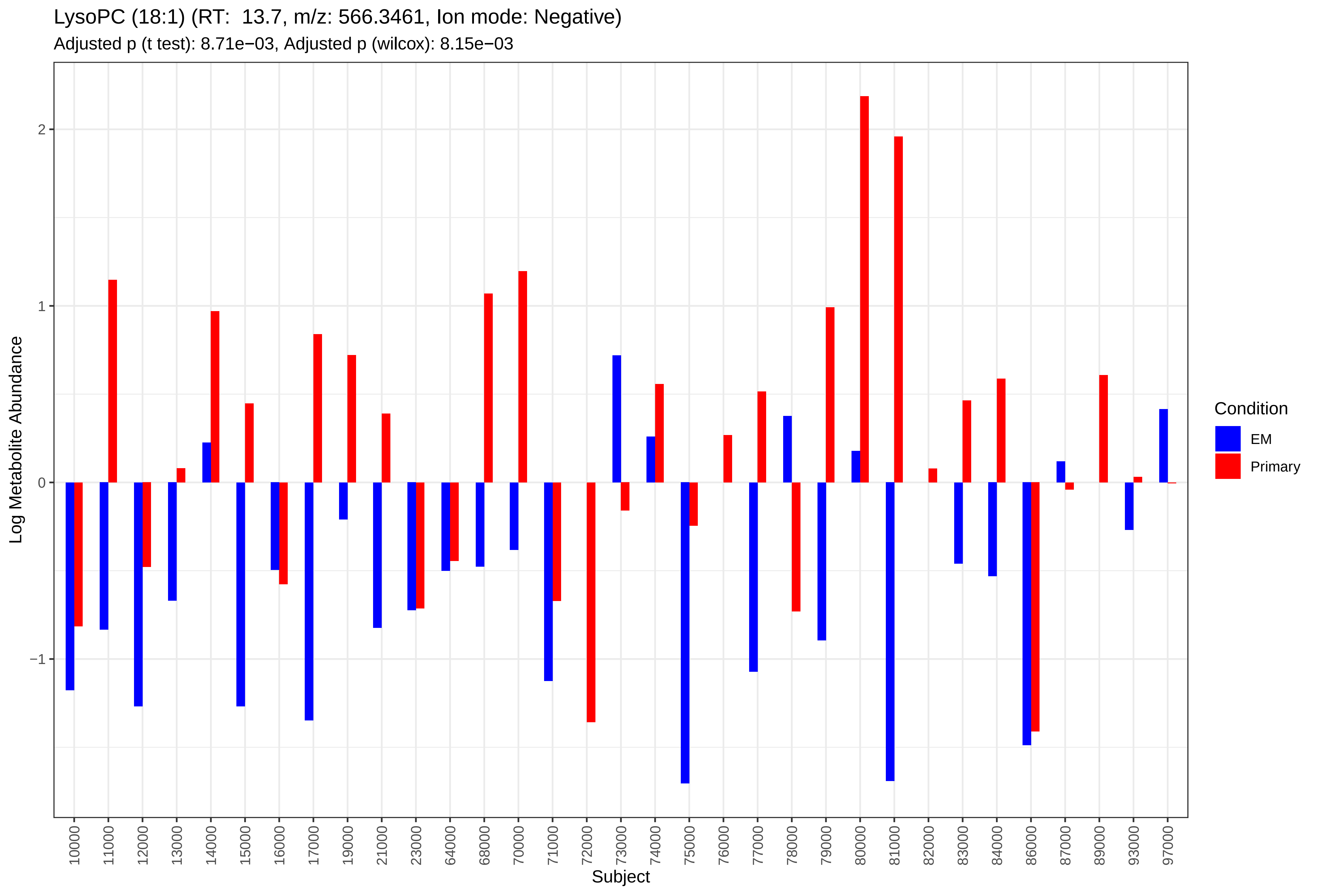
LysoPC (18:1) (RT: 13.7, m/z: 566.3461, Ion mode: Negative)
Adjusted p (t test): 8.71e−03, Adjusted p (wilcox): 8.15e−03
Log Metabolite Abundance
2
1
0
−1
Subject
10000
11000
12000
13000
14000
15000
16000
17000
19000
21000
23000
64000
68000
70000
71000
72000
73000
74000
75000
76000
77000
78000
79000
80000
81000
82000
83000
84000
86000
87000
89000
93000
97000
Condition
EM
Primary

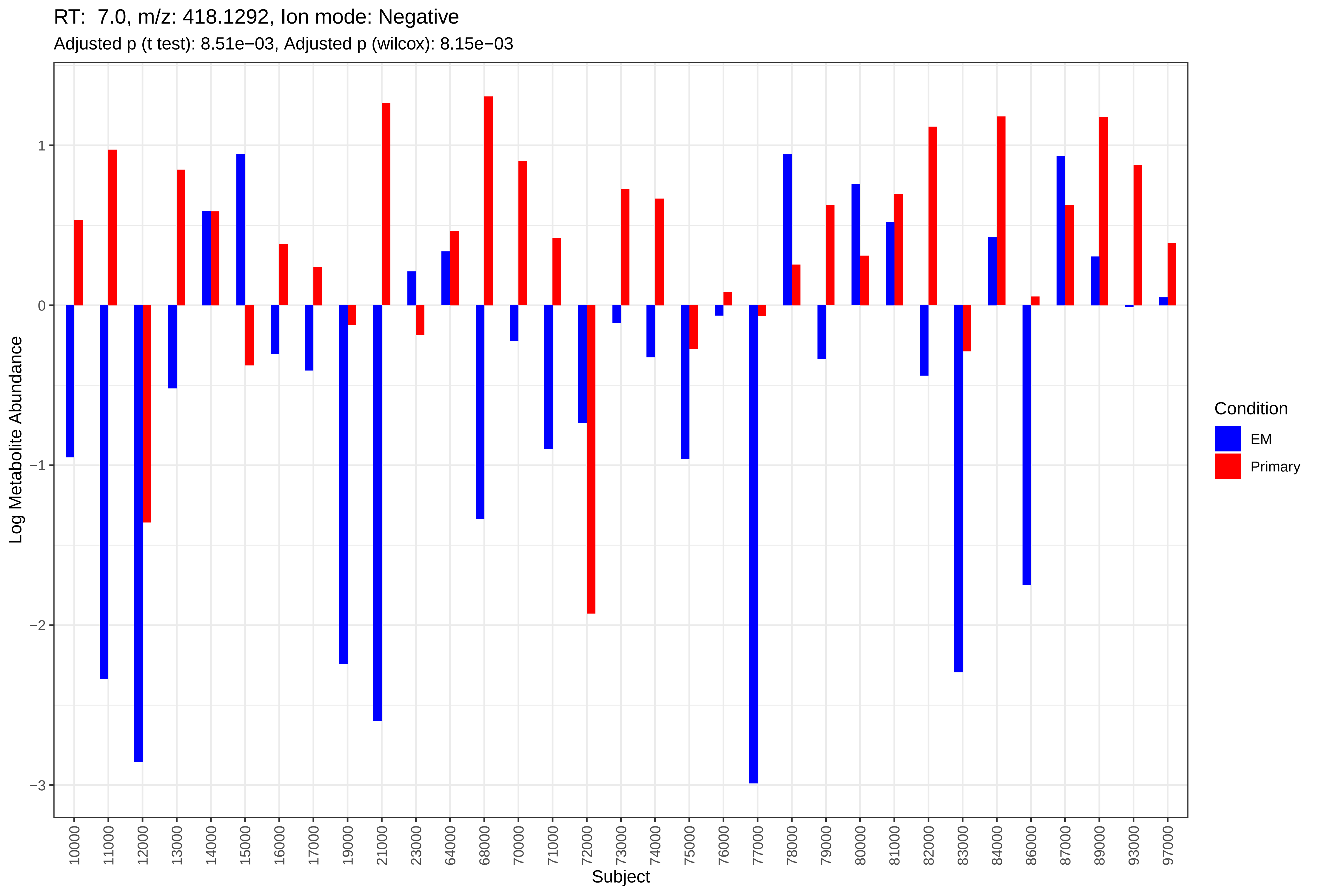

RT: 7.0, m/z: 418.1292, Ion mode: Negative
Adjusted p (t test): 8.51e−03, Adjusted p (wilcox): 8.15e−03
Log Metabolite Abundance
1
0
−1
−2
−3
Subject
10000
11000
12000
13000
14000
15000
16000
17000
19000
21000
23000
64000
68000
70000
71000
72000
73000
74000
75000
76000
77000
78000
79000
80000
81000
82000
83000
84000
86000
87000
89000
93000
97000
Condition
EM
Primary

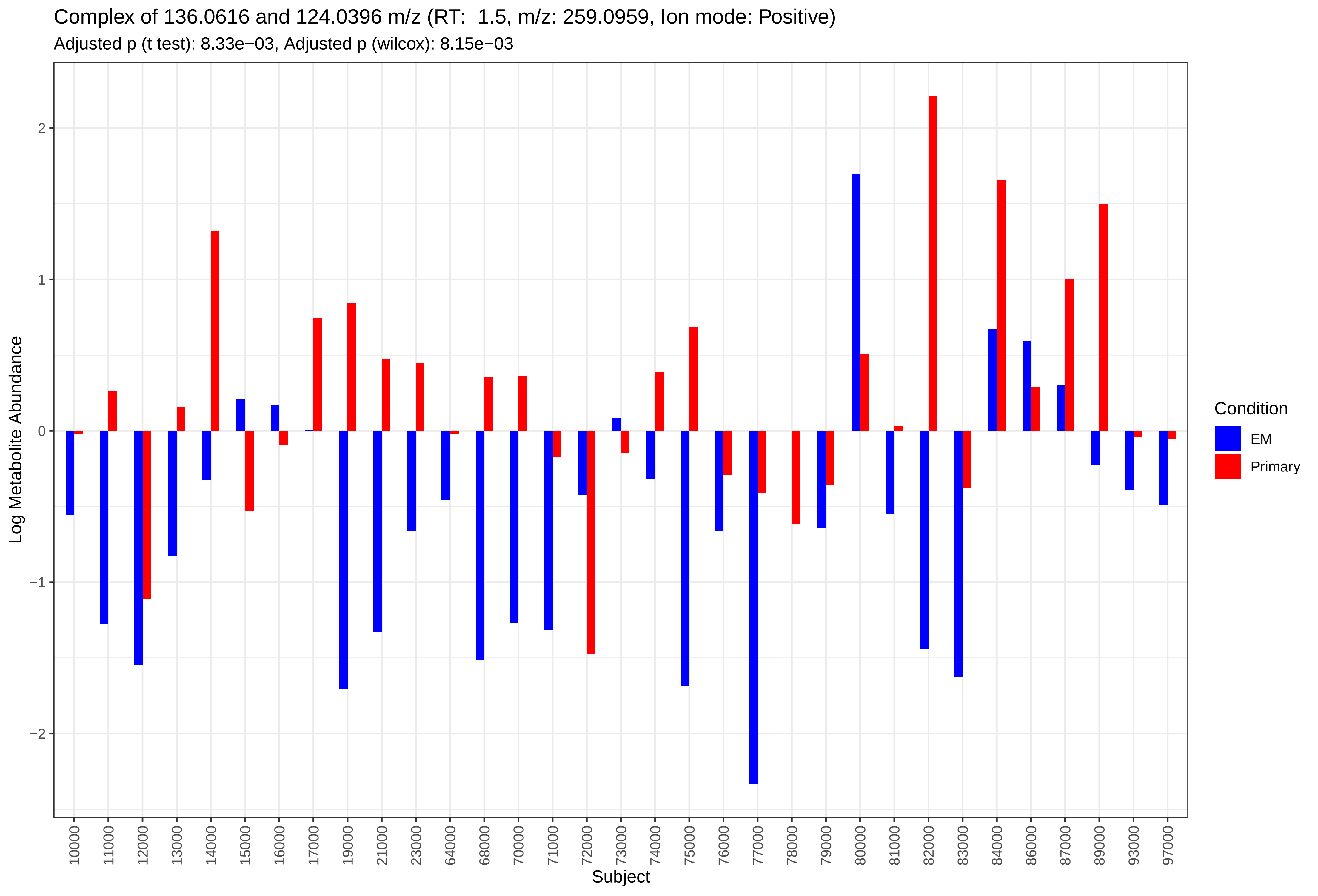
Complex of 136.0616 and 124.0396 m/z (RT: 1.5, m/z: 259.0959, Ion mode: Positive)
Adjusted p (t test): 8.33e−03, Adjusted p (wilcox): 8.15e−03
Log Metabolite Abundance
2
1
0
−1
−2
Subject
10000
11000
12000
13000
14000
15000
16000
17000
19000
21000
23000
64000
68000
70000
71000
72000
73000
74000
75000
76000
77000
78000
79000
80000
81000
82000
83000
84000
86000
87000
89000
93000
97000
Condition
EM
Primary

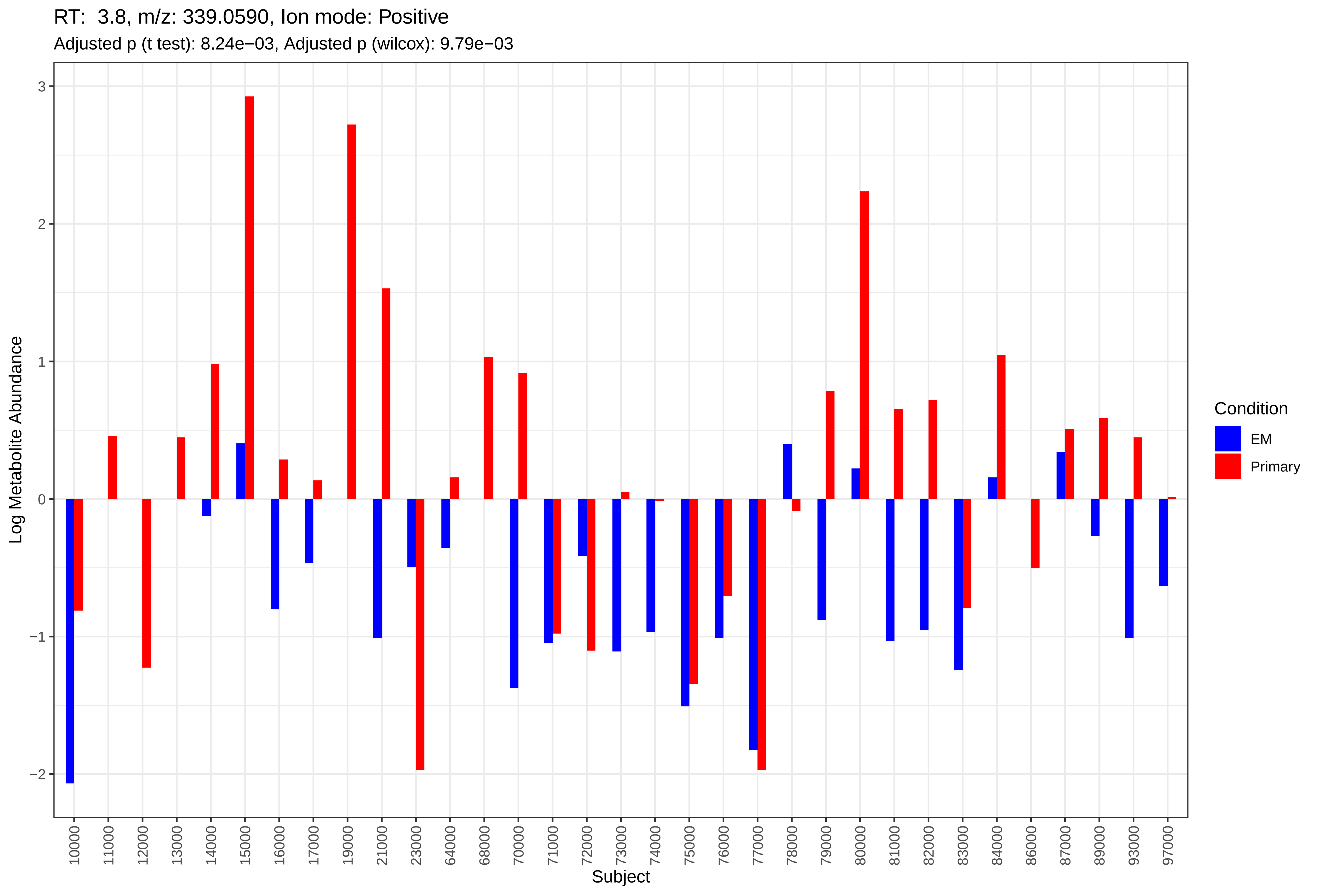
RT: 3.8, m/z: 339.0590, Ion mode: Positive
Adjusted p (t test): 8.24e−03, Adjusted p (wilcox): 9.79e−03
Log Metabolite Abundance
3
2
1
0
−1
−2
10000
11000
12000
13000
14000
15000
16000
17000
19000
21000
23000
64000
68000
70000
71000
72000
73000
74000
75000
76000
77000
78000
79000
80000
81000
82000
83000
84000
86000
87000
89000
93000
97000
Subject
Condition
EM
Primary

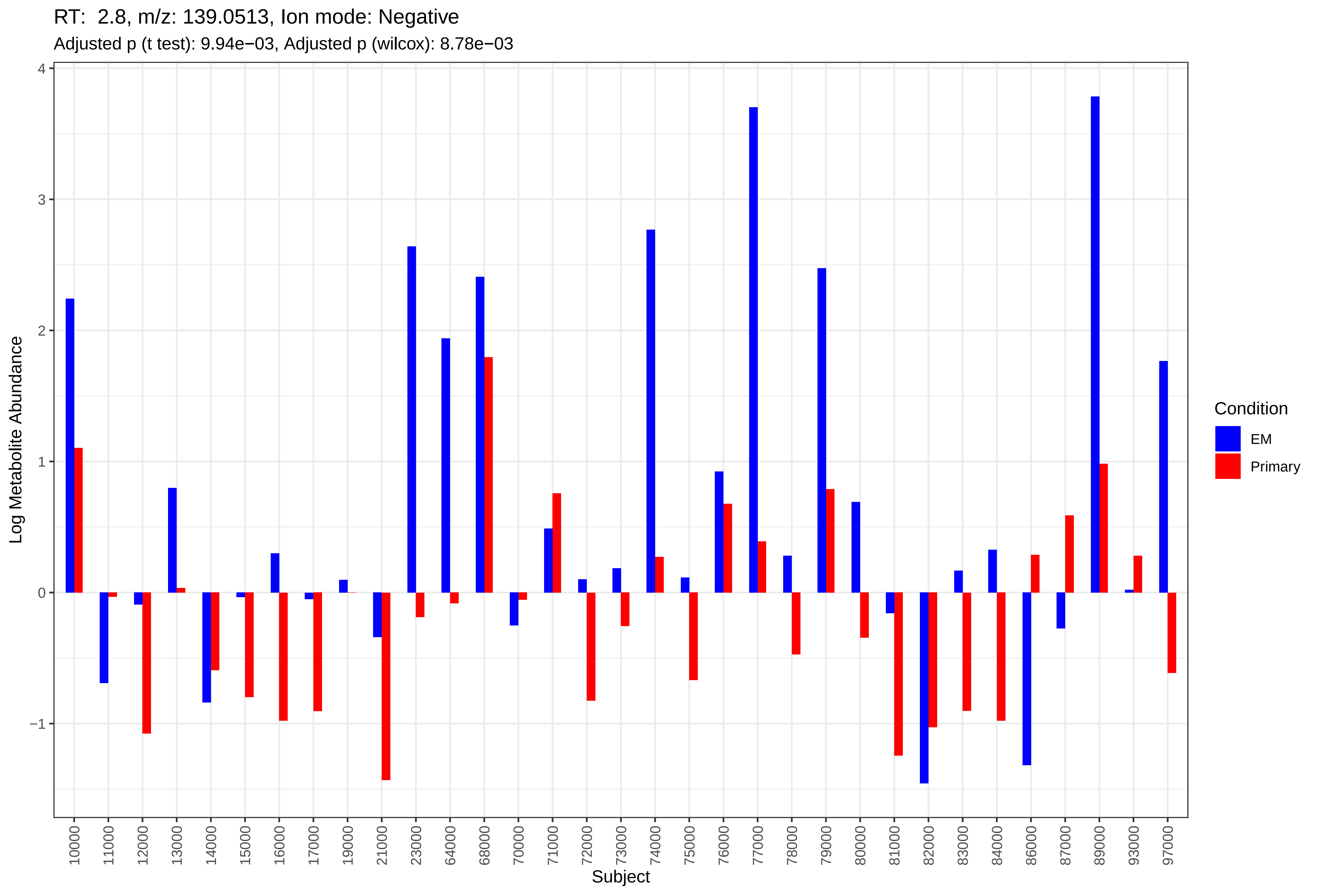

RT: 2.8, m/z: 139.0513, Ion mode: Negative
Adjusted p (t test): 9.94e−03, Adjusted p (wilcox): 8.78e−03
Log Metabolite Abundance
4
3
2
1
0
−1
10000
11000
12000
13000
14000
15000
16000
17000
19000
21000
23000
64000
68000
70000
71000
72000
73000
74000
75000
76000
77000
78000
79000
80000
81000
82000
83000
84000
86000
87000
89000
93000
97000
Subject
Condition
EM
Primary

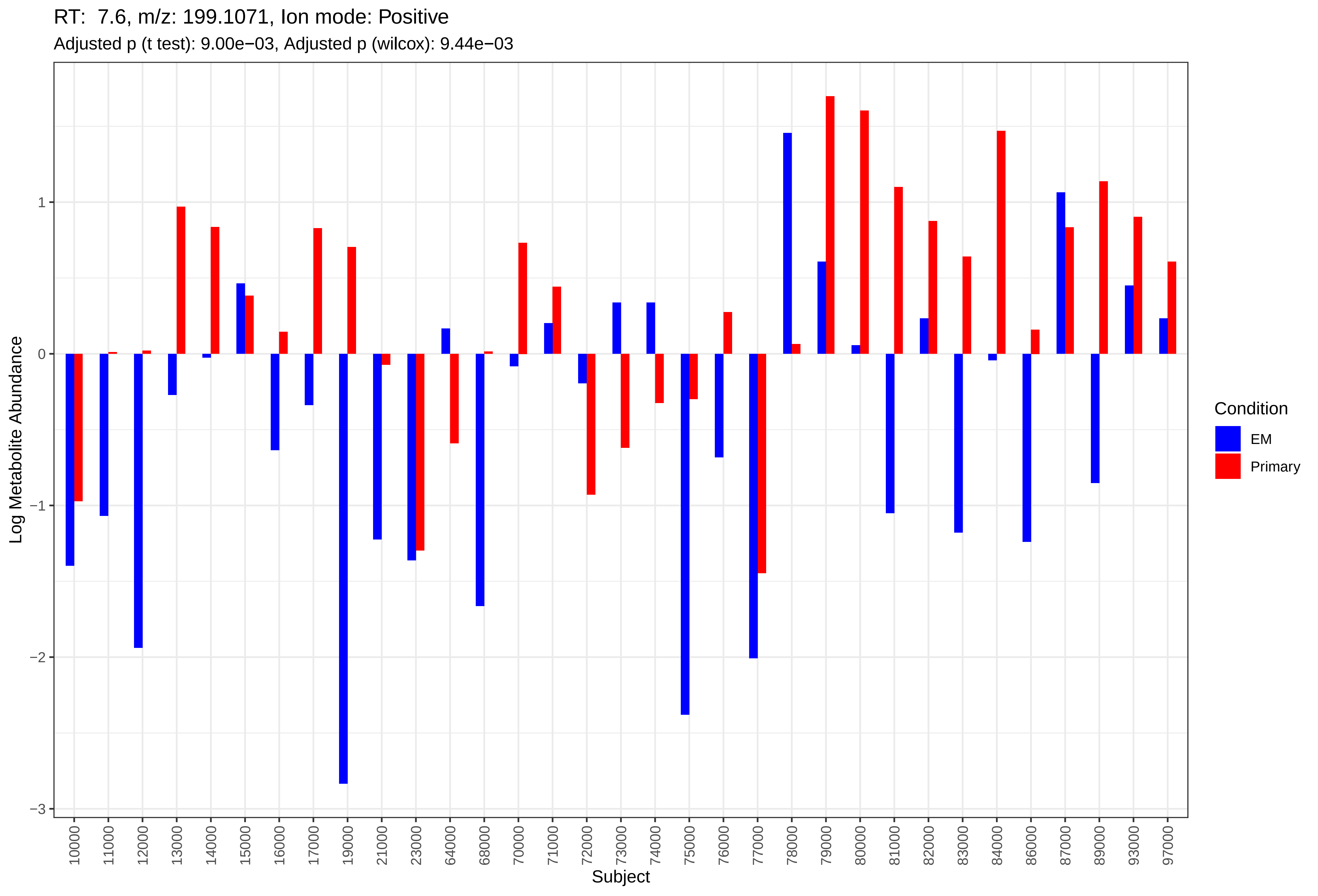
RT: 7.6, m/z: 199.1071, Ion mode: Positive
Adjusted p (t test): 9.00e−03, Adjusted p (wilcox): 9.44e−03
Log Metabolite Abundance
1
0
−1
−2
−3
10000
11000
12000
13000
14000
15000
16000
17000
19000
21000
23000
64000
68000
70000
71000
72000
73000
74000
75000
76000
77000
78000
79000
80000
81000
82000
83000
84000
86000
87000
89000
93000
97000
Subject
Condition
EM
Primary

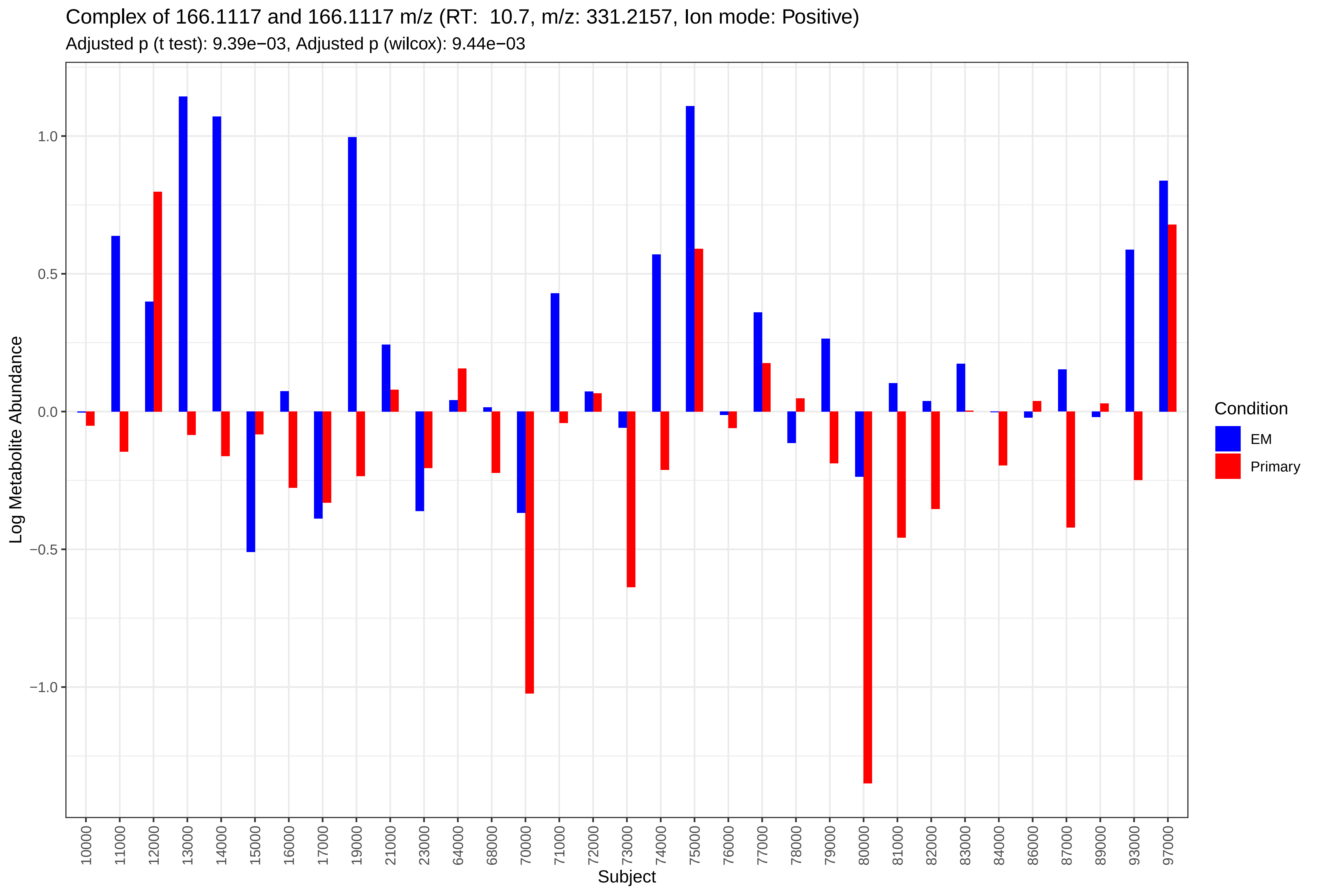

Complex of 166.1117 and 166.1117 m/z (RT: 10.7, m/z: 331.2157, Ion mode: Positive)
Adjusted p (t test): 9.39e−03, Adjusted p (wilcox): 9.44e−03
Log Metabolite Abundance
1.0
0.5
0.0
−0.5
−1.0
Subject
10000
11000
12000
13000
14000
15000
16000
17000
19000
21000
23000
64000
68000
70000
71000
72000
73000
74000
75000
76000
77000
78000
79000
80000
81000
82000
83000
84000
86000
87000
89000
93000
97000
Condition
EM
Primary

RT: 0.6, m/z: 150.9829, Ion mode: Negative

Adjusted p (t test): 9.56e−03, Adjusted p (wilcox): 9.44e−03

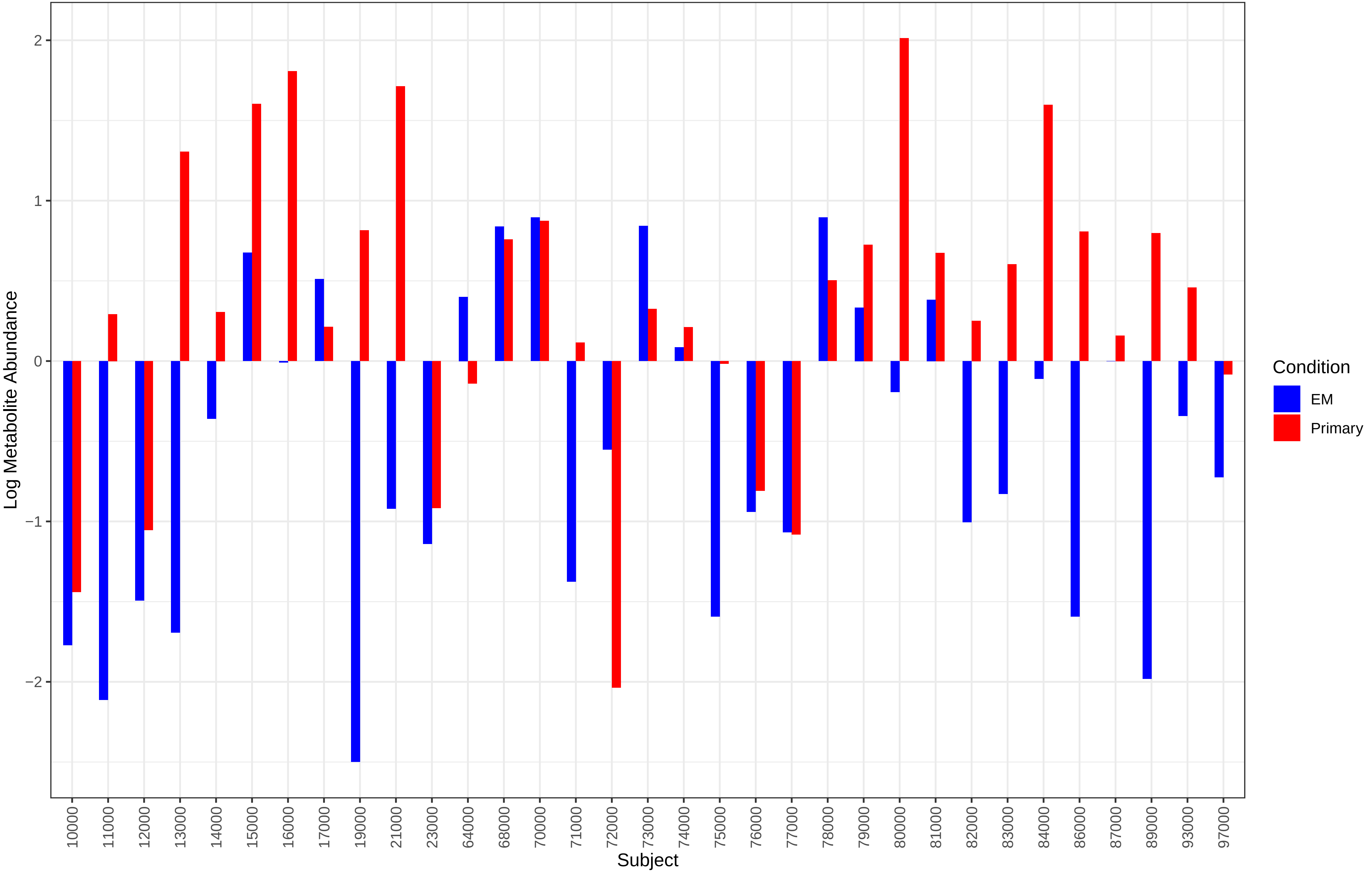

RT: 6.4, m/z: 290.0852, Ion mode: Positive

Adjusted p (t test): 9.54e−03, Adjusted p (wilcox): 9.82e−03

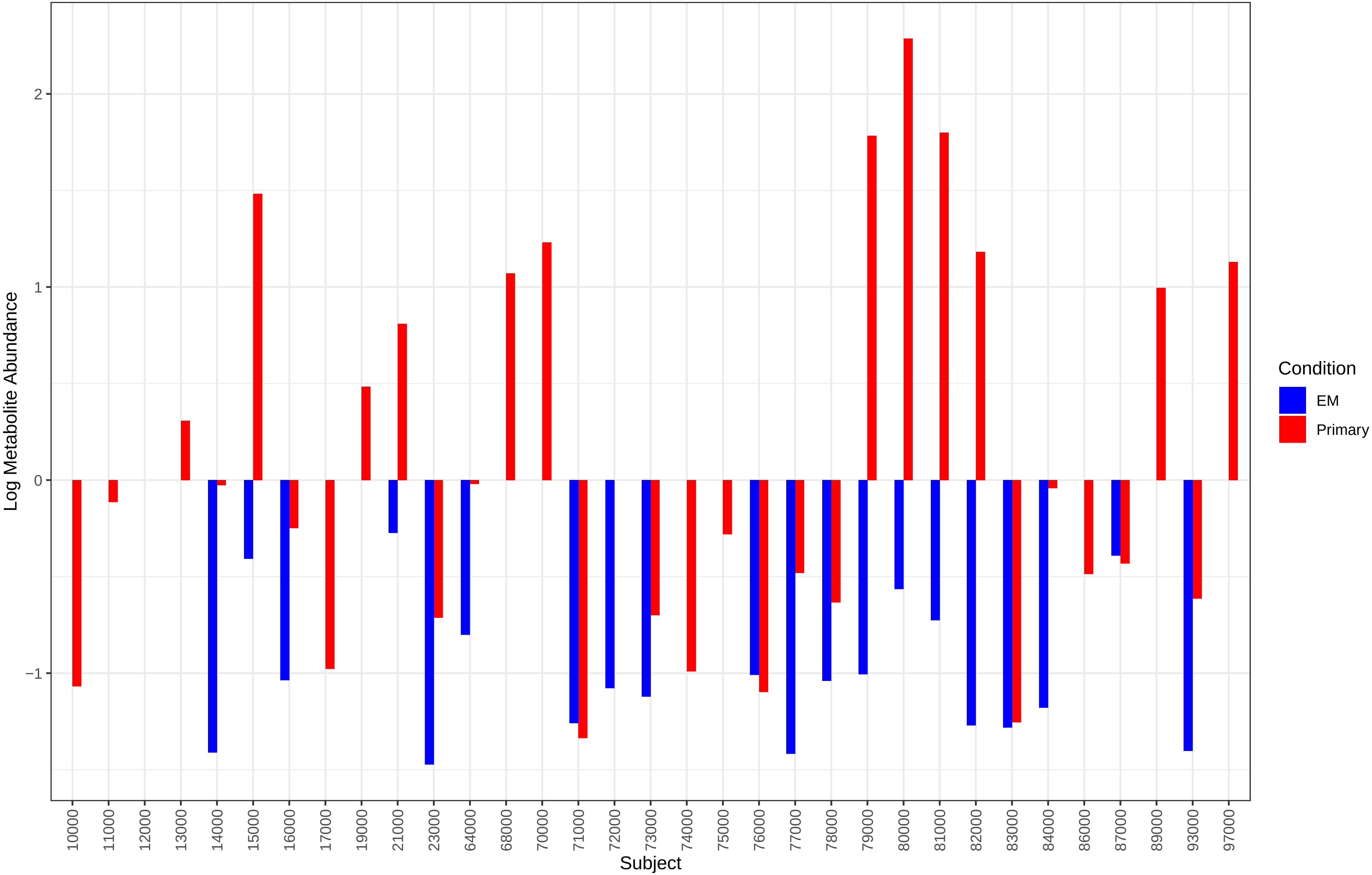

Supplement: S4 Fig — Bar charts reporting all differentially abundant metabolites between primary melanoma and matched EM. (PDF) [file pone.0240849.s004.pdf]
