# Supplementary material for: Metabolomics of primary cutaneous melanoma and matched adjacent extratumoral microenvironment
Source: PLoS One. 2020 Oct 27;15(10):e0240849. doi: 10.1371/journal.pone.0240849 (PMC7591037; doi:10.1371/journal.pone.0240849)
Supplement: S6 Fig — Bar charts reporting all differentially abundant metabolites between metastatic melanoma and EM. (PDF) [file pone.0240849.s006.pdf]

S6 Fig
All differentially abundant metabolites
Metastasis vs. EM

Supplement to

"Metabolomics of primary cutaneous melanoma and matched adjacent extratumoral microenvironment"

Nicholas J. Taylor, Irina Gaynanova, Eric A. Welsh, Timothy J. Garrett, Chris Beecher,
Ritin Sharma, John Koomen, Keiran S.M. Smalley, Steven A. Eschrich, Jane L. Messina, Peter A. Kanetsky

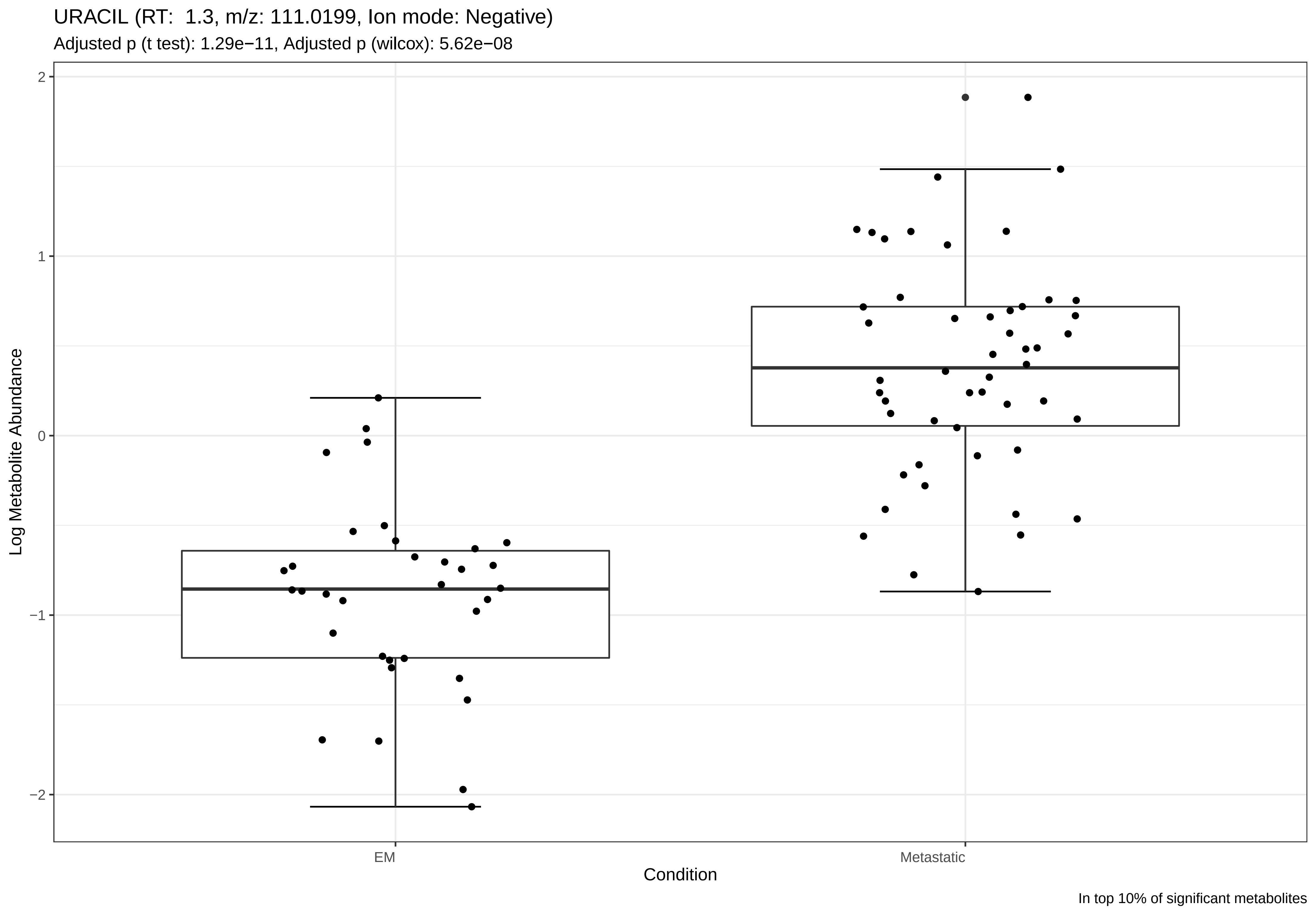
URACIL (RT: 1.3, m/z: 111.0199, Ion mode: Negative)
Adjusted p (t test): 1.29e−11, Adjusted p (wilcox): 5.62e−08
Log Metabolite Abundance
2
1
0
−1
−2
EM
Metastatic
Condition
In top 10% of significant metabolites

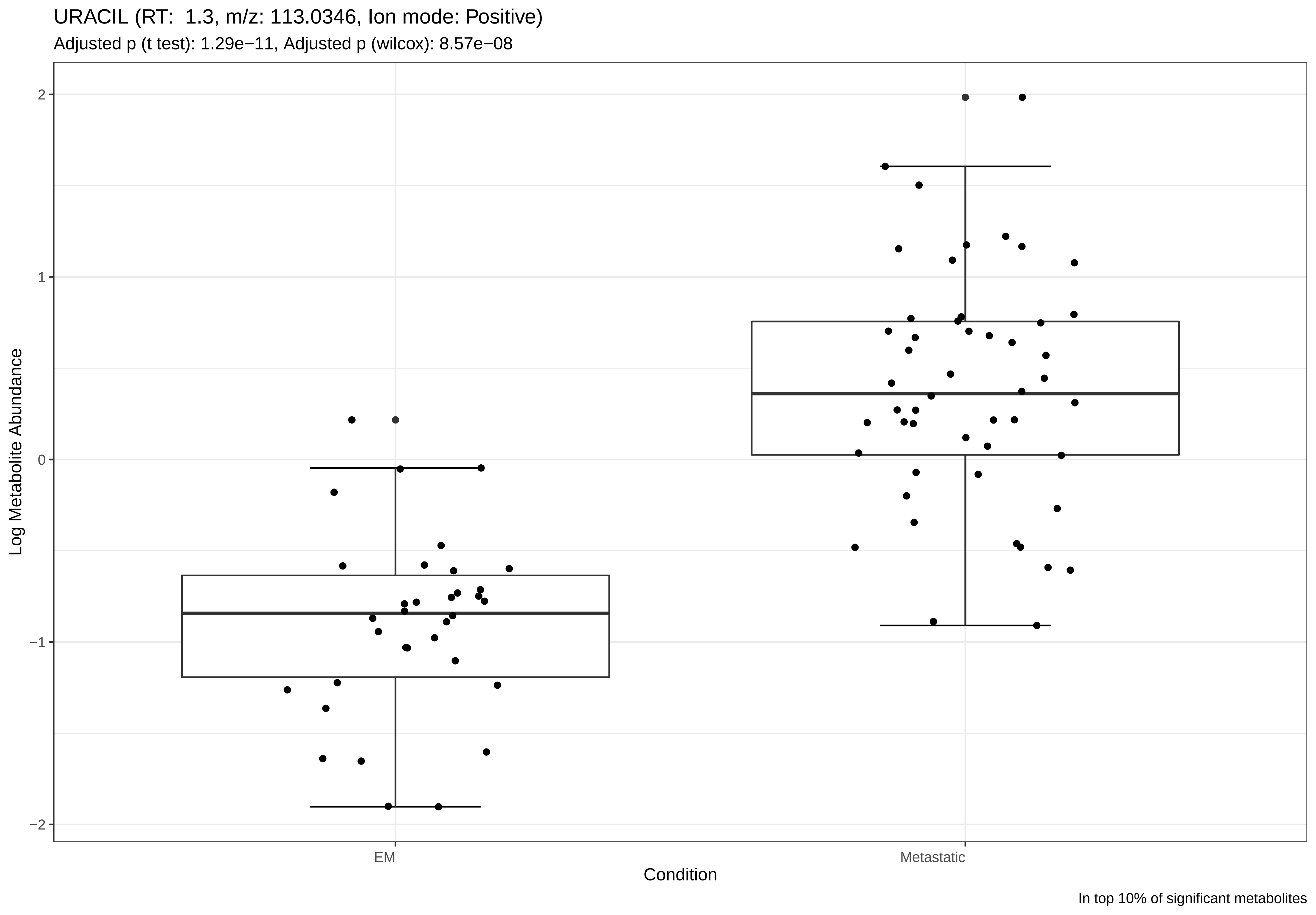
URACIL (RT: 1.3, m/z: 113.0346, Ion mode: Positive)
Adjusted p (t test): 1.29e−11, Adjusted p (wilcox): 8.57e−08
Log Metabolite Abundance
2
1
0
−1
−2
EM
Metastatic
Condition
In top 10% of significant metabolites

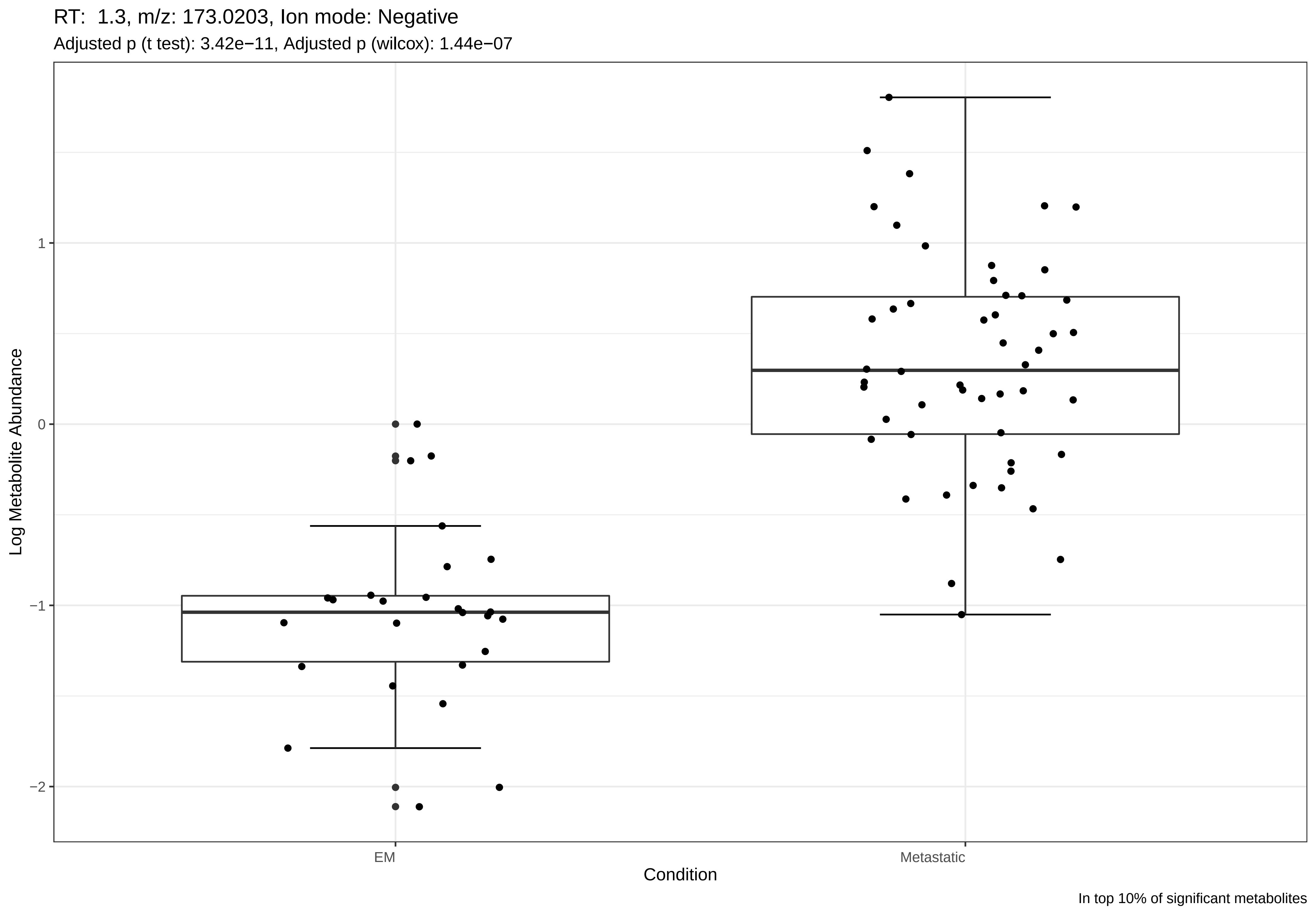

RT: 1.3, m/z: 173.0203, Ion mode: Negative
Adjusted p (t test): 3.42e−11, Adjusted p (wilcox): 1.44e−07
Log Metabolite Abundance
1
0
−1
−2
EM
Metastatic
Condition
In top 10% of significant metabolites

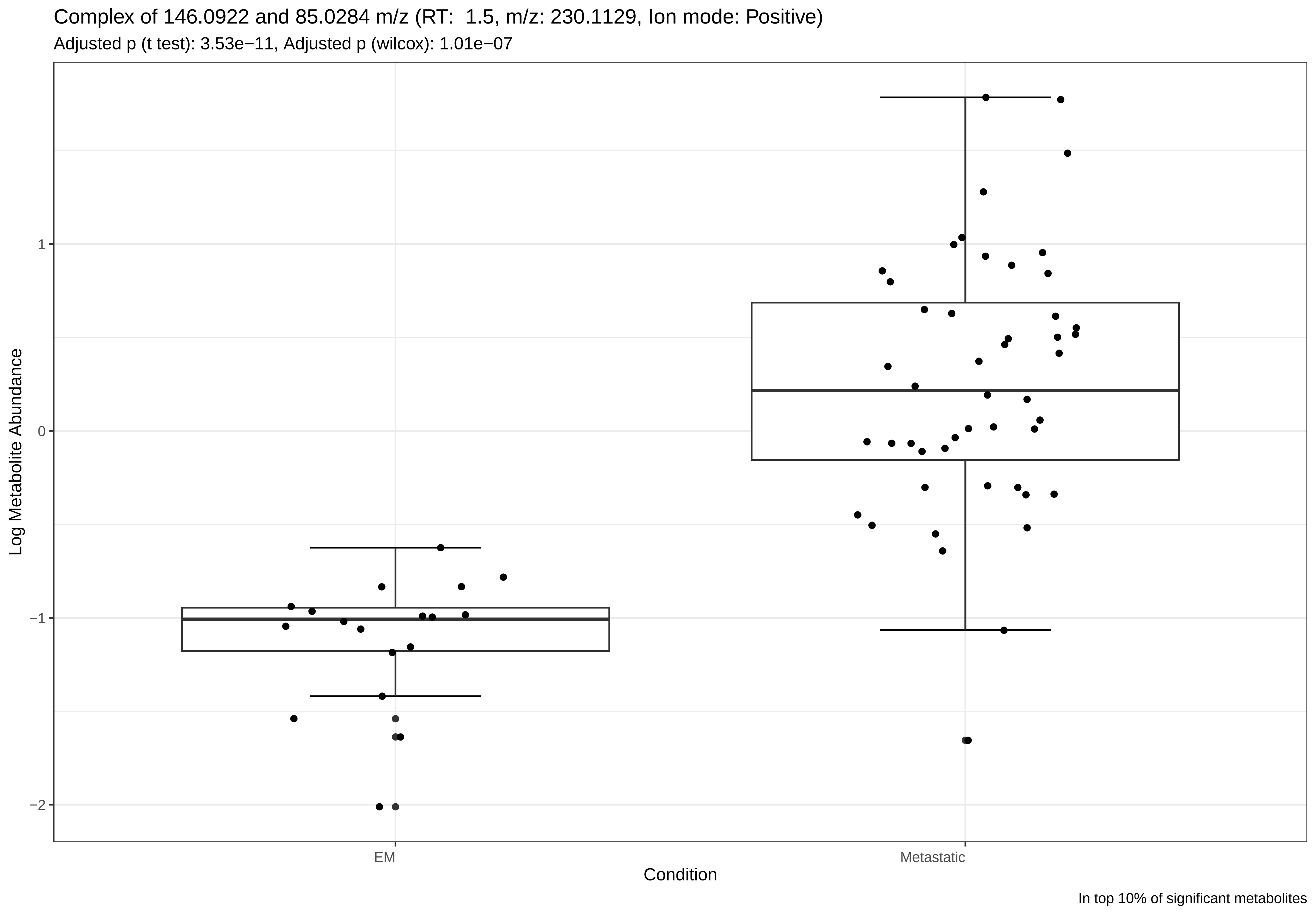

Complex of 146.0922 and 85.0284 m/z (RT: 1.5, m/z: 230.1129, Ion mode: Positive)
Adjusted p (t test): 3.53e−11, Adjusted p (wilcox): 1.01e−07
Log Metabolite Abundance
1
0
−1
−2
EM
Metastatic
Condition
In top 10% of significant metabolites

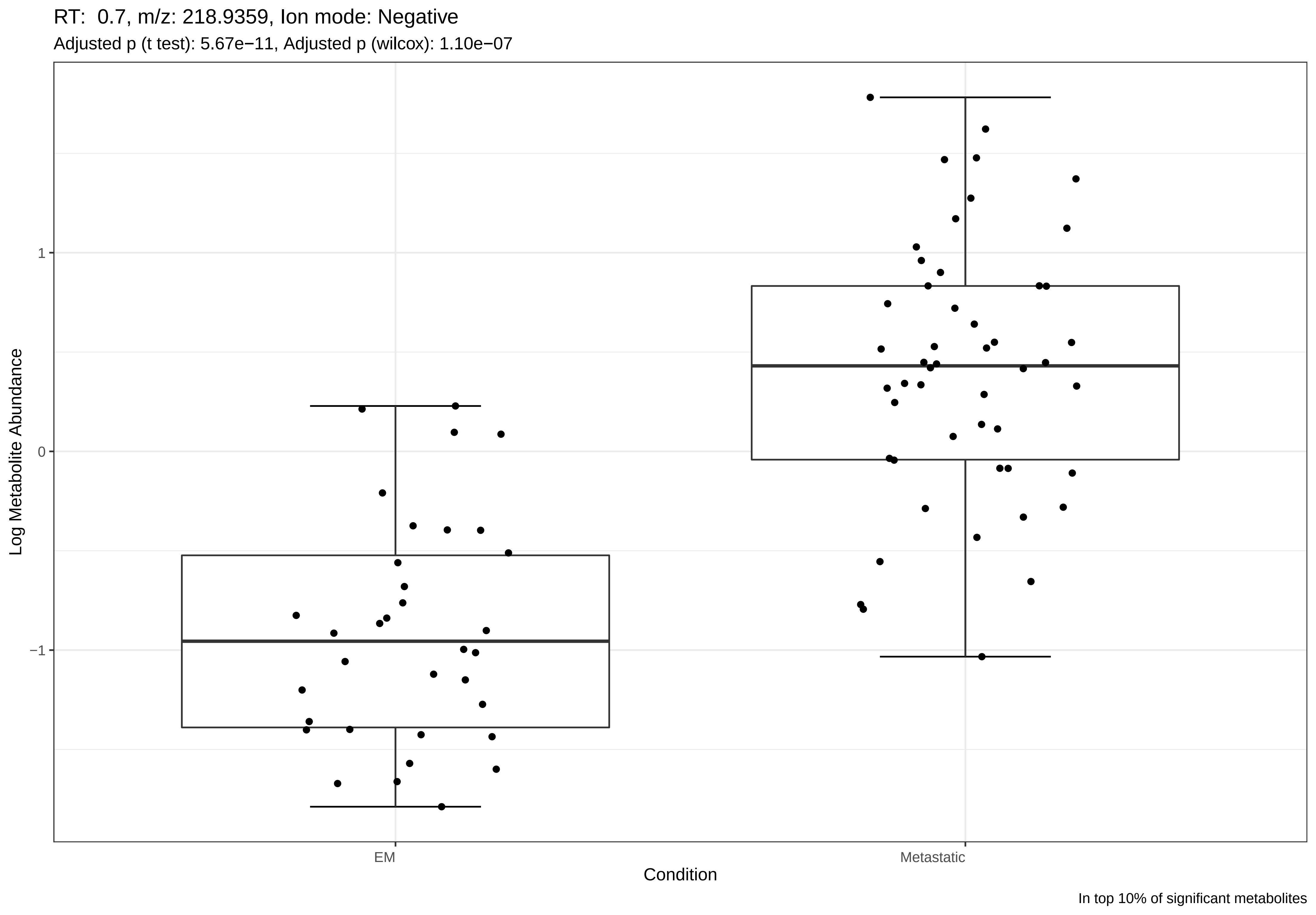
RT: 0.7, m/z: 218.9359, Ion mode: Negative
Adjusted p (t test): 5.67e−11, Adjusted p (wilcox): 1.10e−07
Log Metabolite Abundance
1
0
−1
EM
Metastatic
Condition
In top 10% of significant metabolites

RT: 0.7, m/z: 296.8818, Ion mode: Negative

Adjusted p (t test): 5.67e−11, Adjusted p (wilcox): 1.10e−07

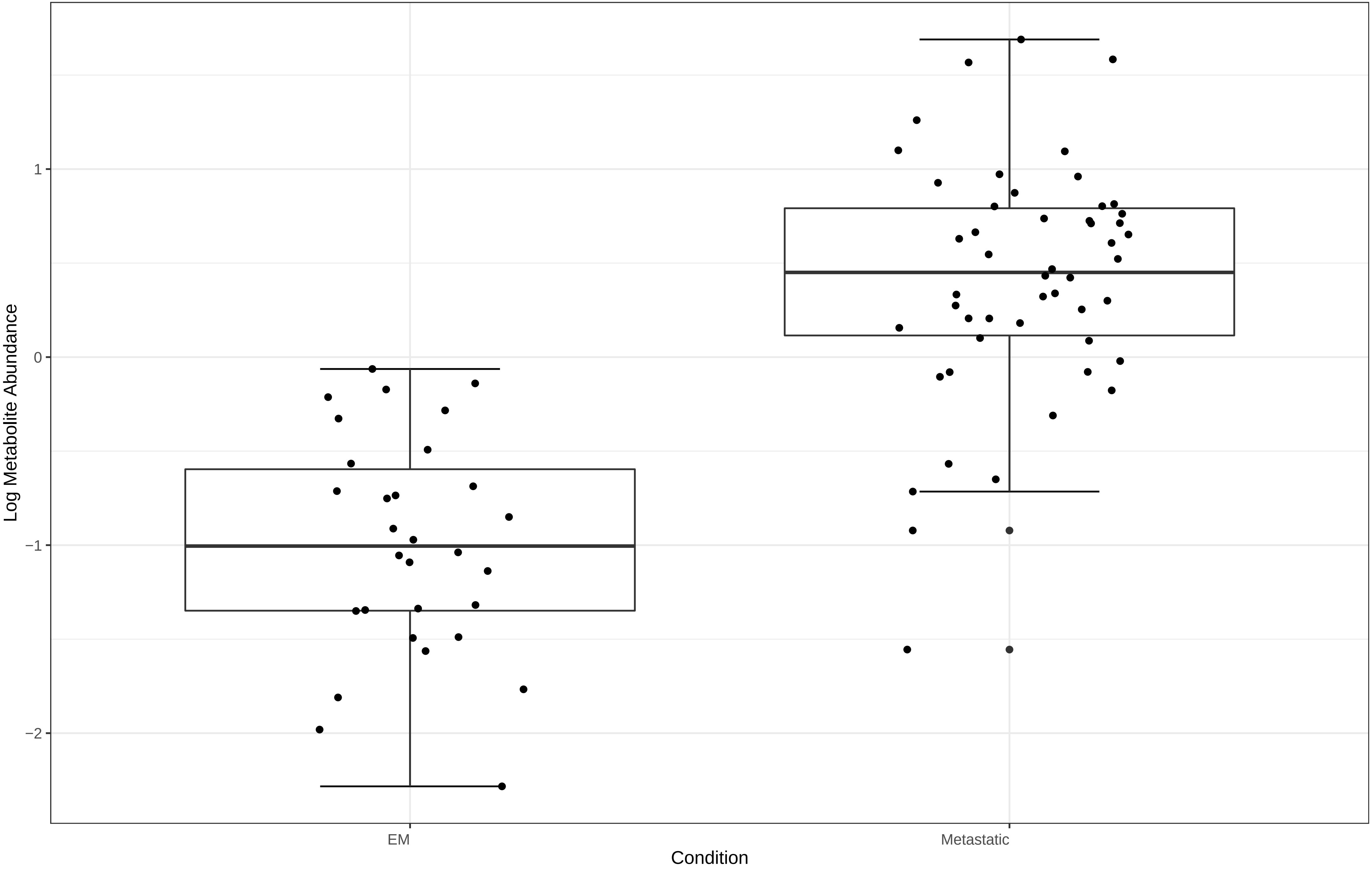

In top 10% of significant metabolites

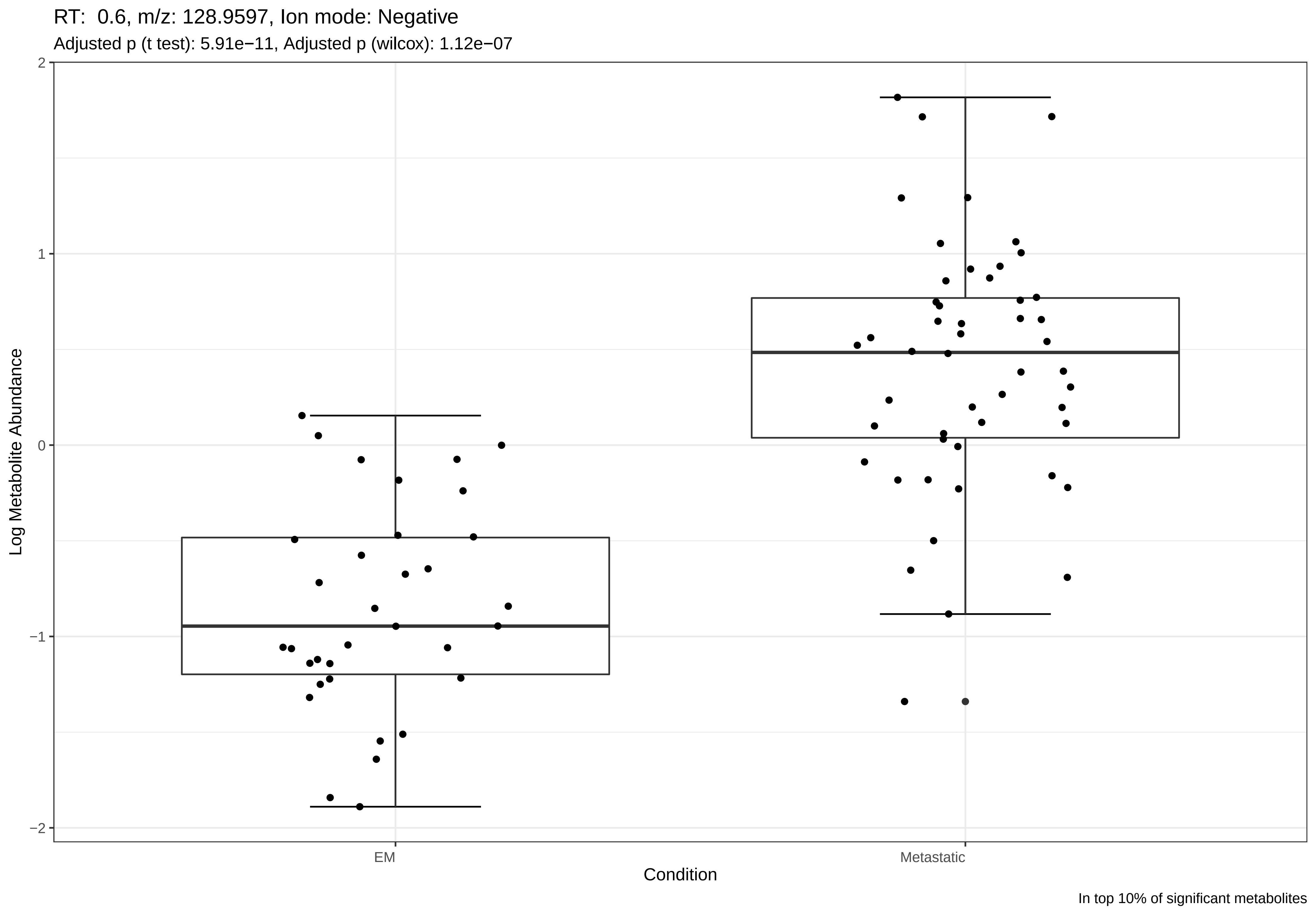
RT: 0.6, m/z: 128.9597, Ion mode: Negative
Adjusted p (t test): 5.91e−11, Adjusted p (wilcox): 1.12e−07
Log Metabolite Abundance
2
1
0
−1
−2
EM
Metastatic
Condition
In top 10% of significant metabolites

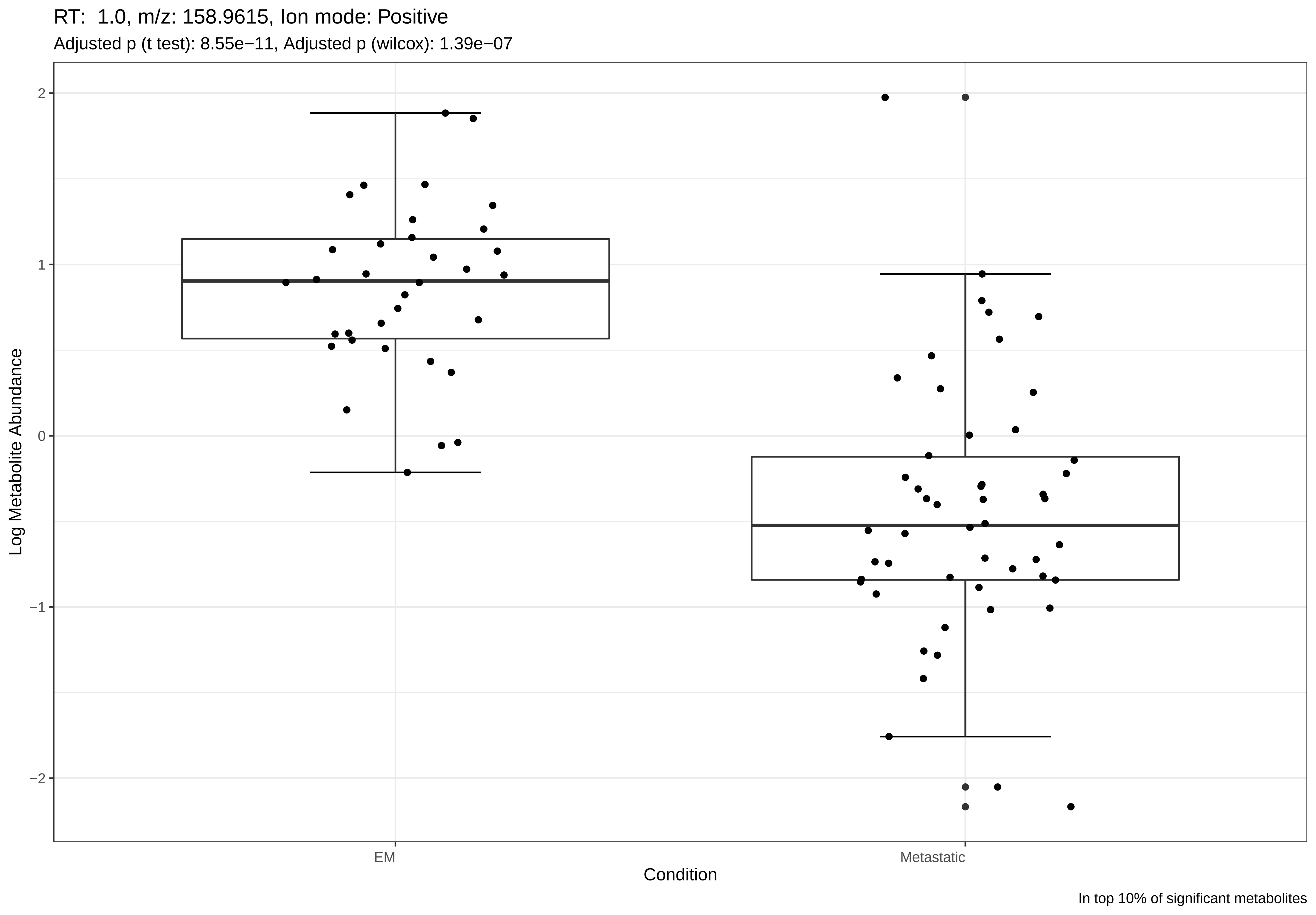

RT: 1.0, m/z: 158.9615, Ion mode: Positive
Adjusted p (t test): 8.55e−11, Adjusted p (wilcox): 1.39e−07
Log Metabolite Abundance
2
1
0
−1
−2
EM
Metastatic
Condition
In top 10% of significant metabolites

RT: 1.3, m/z: 212.9581, Ion mode: Positive

Adjusted p (t test): 8.55e−11, Adjusted p (wilcox): 2.13e−07

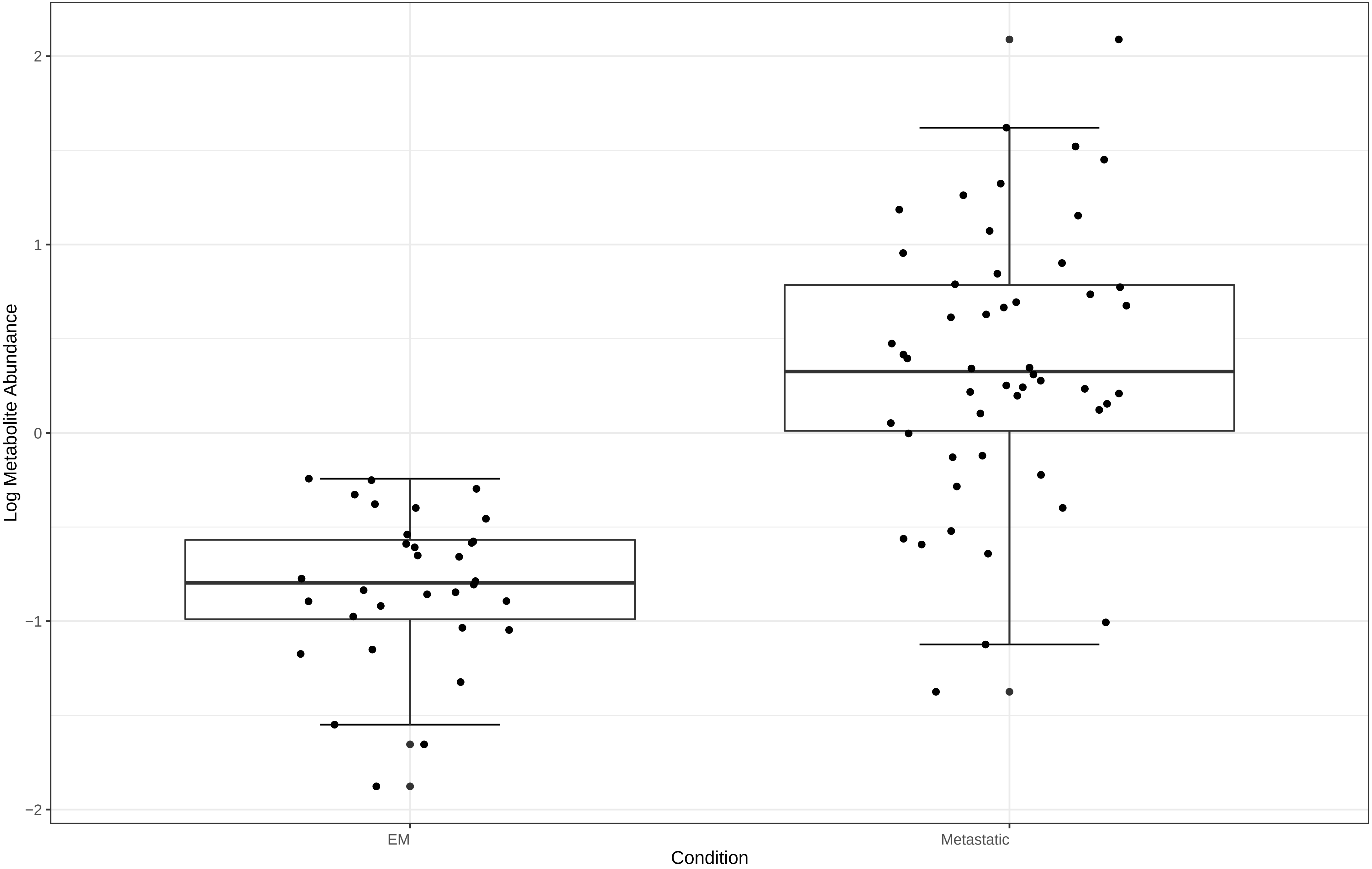

In top 10% of significant metabolites

Complex of 102.9702 and 113.0346 m/z (RT: 1.3, m/z: 214.9969, Ion mode: Positive)

Adjusted p (t test): 8.55e−11, Adjusted p (wilcox): 5.95e−07

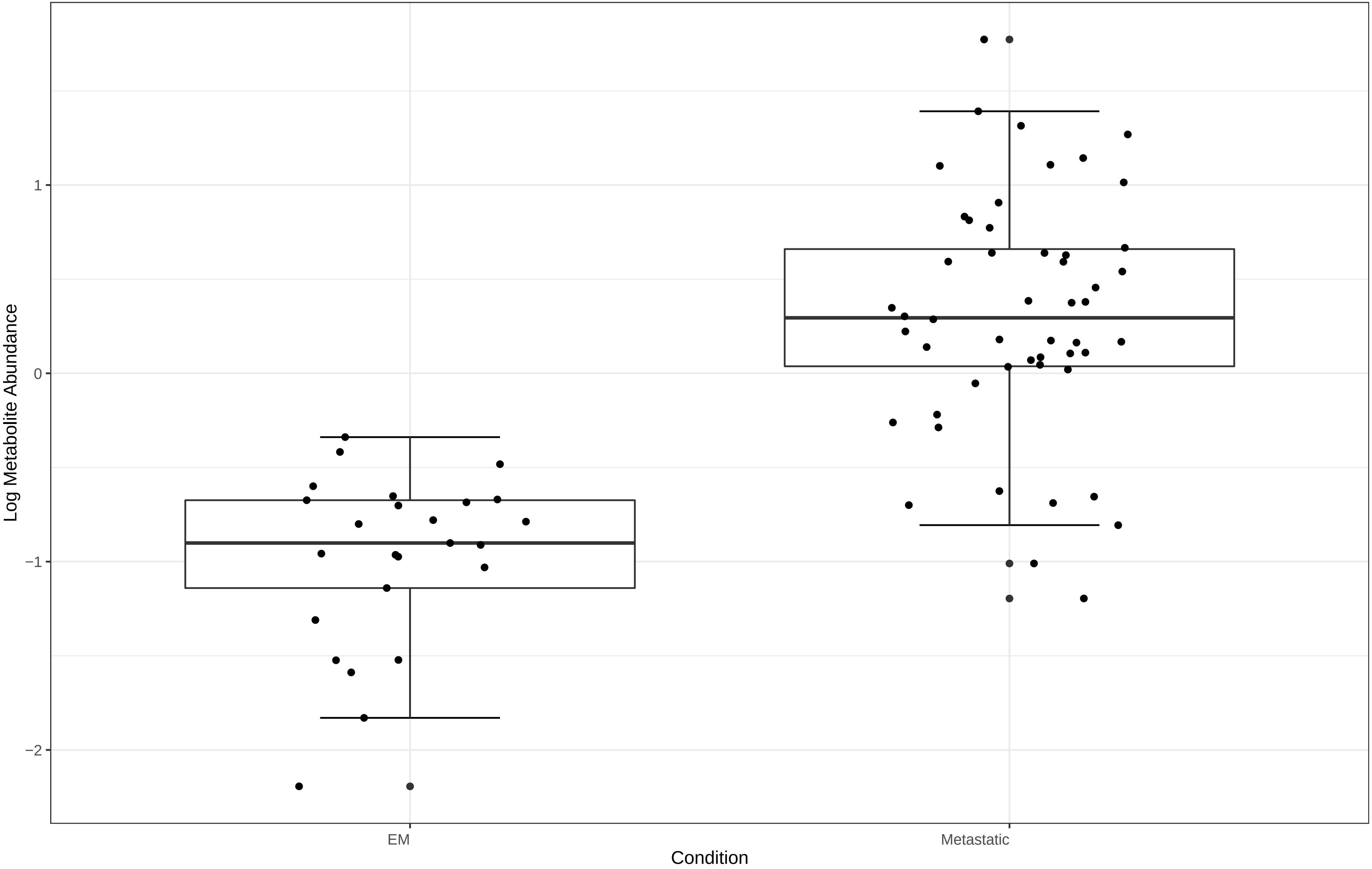

In top 10% of significant metabolites

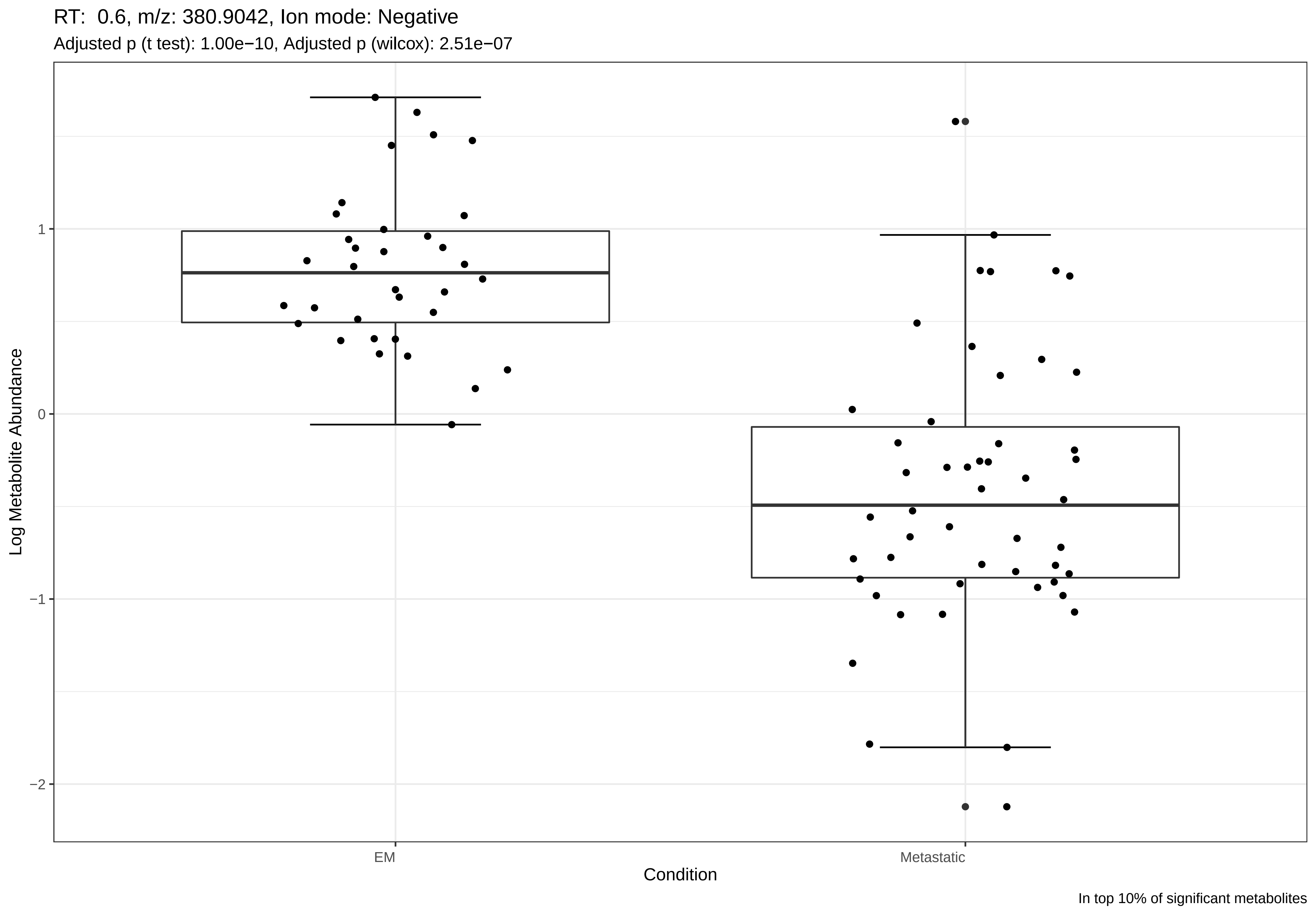
RT: 0.6, m/z: 380.9042, Ion mode: Negative
Adjusted p (t test): 1.00e−10, Adjusted p (wilcox): 2.51e−07
Log Metabolite Abundance
1
0
−1
−2
EM
Metastatic
Condition
In top 10% of significant metabolites

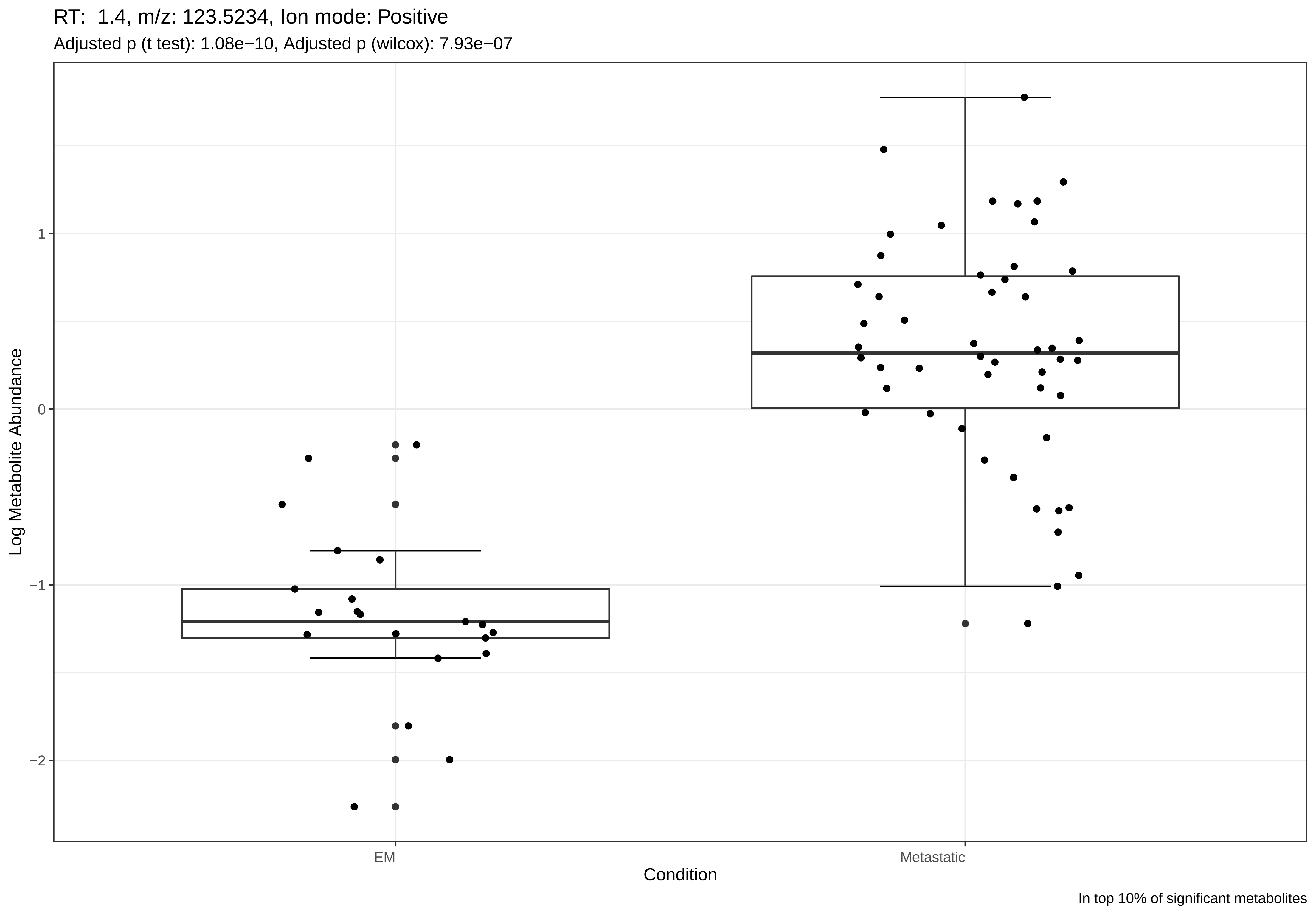

RT: 1.4, m/z: 123.5234, Ion mode: Positive
Adjusted p (t test): 1.08e−10, Adjusted p (wilcox): 7.93e−07
Log Metabolite Abundance
1
0
−1
−2
EM
Metastatic
Condition
In top 10% of significant metabolites

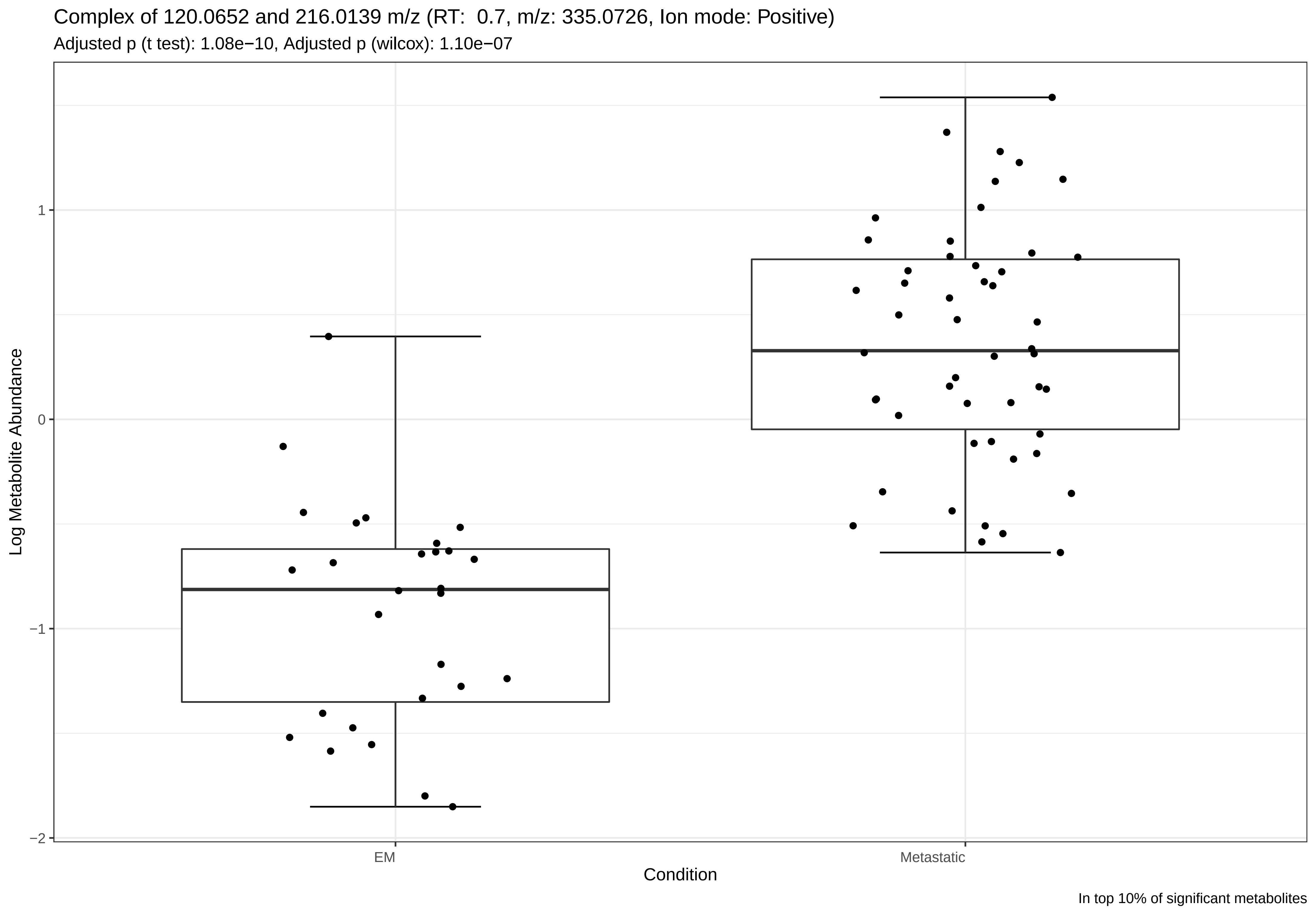

Complex of 120.0652 and 216.0139 m/z (RT: 0.7, m/z: 335.0726, Ion mode: Positive)
Adjusted p (t test): 1.08e−10, Adjusted p (wilcox): 1.10e−07
Log Metabolite Abundance
1
0
−1
−2
EM
Metastatic
Condition
In top 10% of significant metabolites

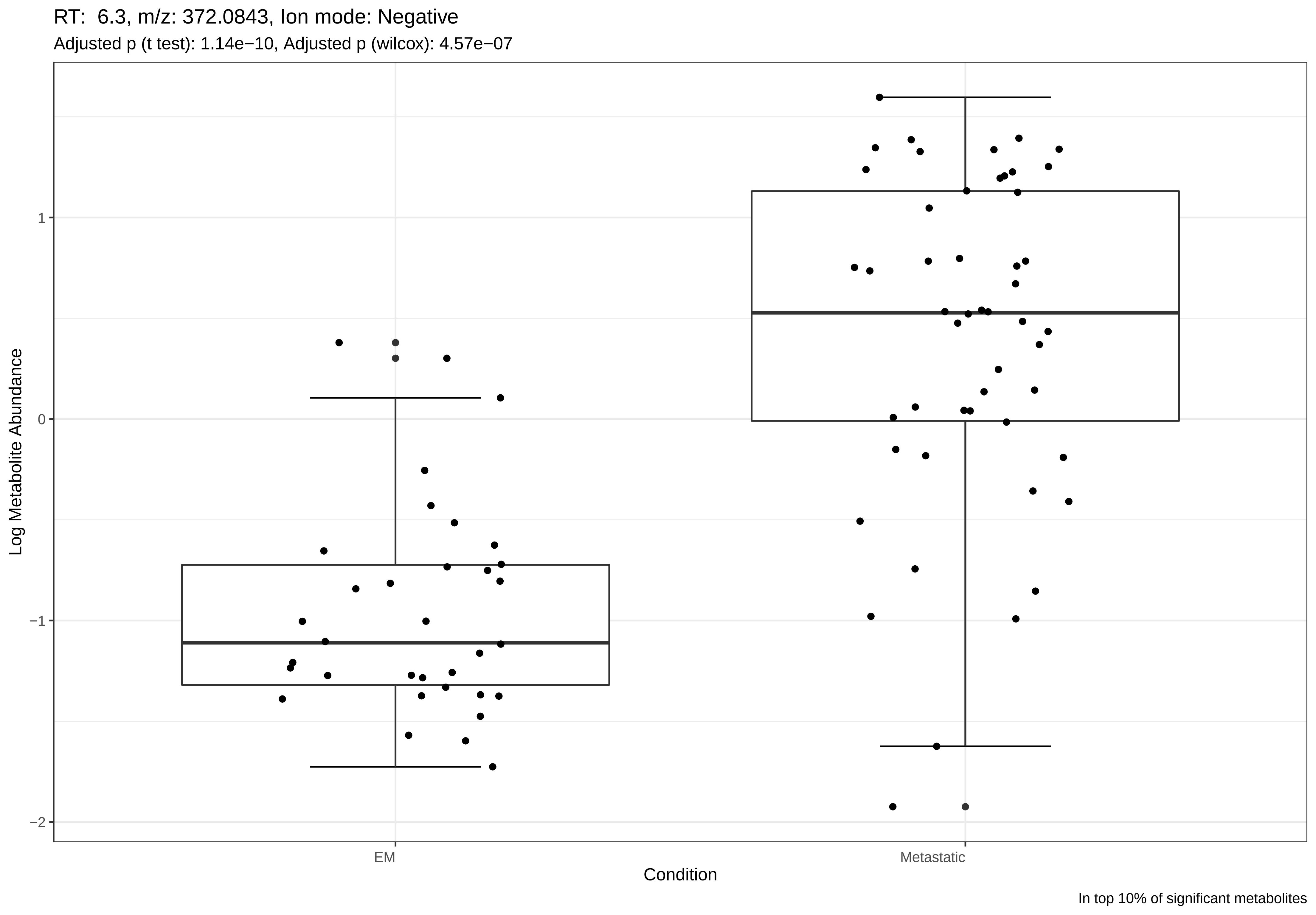

RT: 6.3, m/z: 372.0843, Ion mode: Negative
Adjusted p (t test): 1.14e−10, Adjusted p (wilcox): 4.57e−07
Log Metabolite Abundance
1
0
−1
−2
EM
Metastatic
Condition
In top 10% of significant metabolites

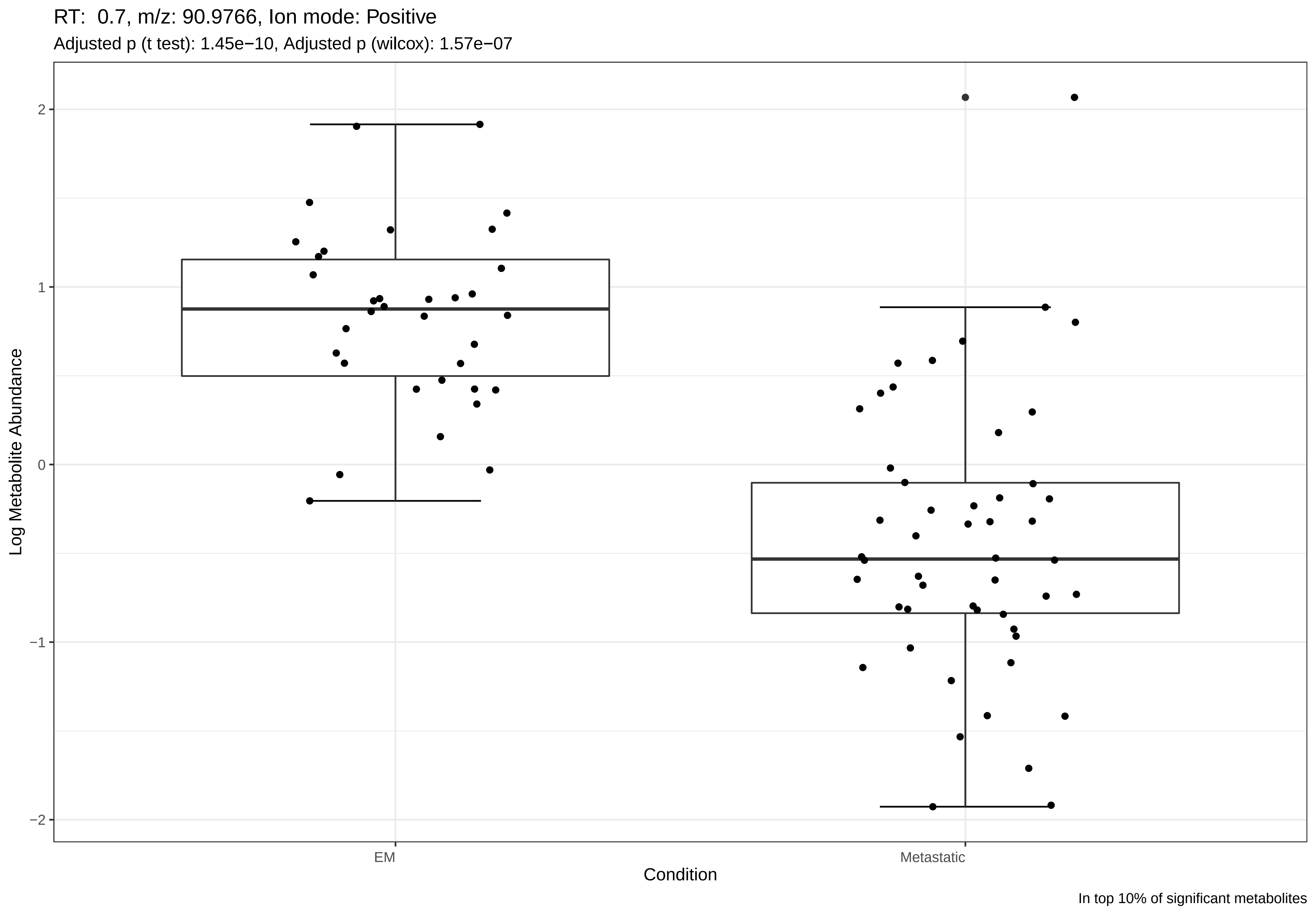
RT: 0.7, m/z: 90.9766, Ion mode: Positive
Adjusted p (t test): 1.45e−10, Adjusted p (wilcox): 1.57e−07
Log Metabolite Abundance
2
1
0
−1
−2
EM
Metastatic
Condition
In top 10% of significant metabolites

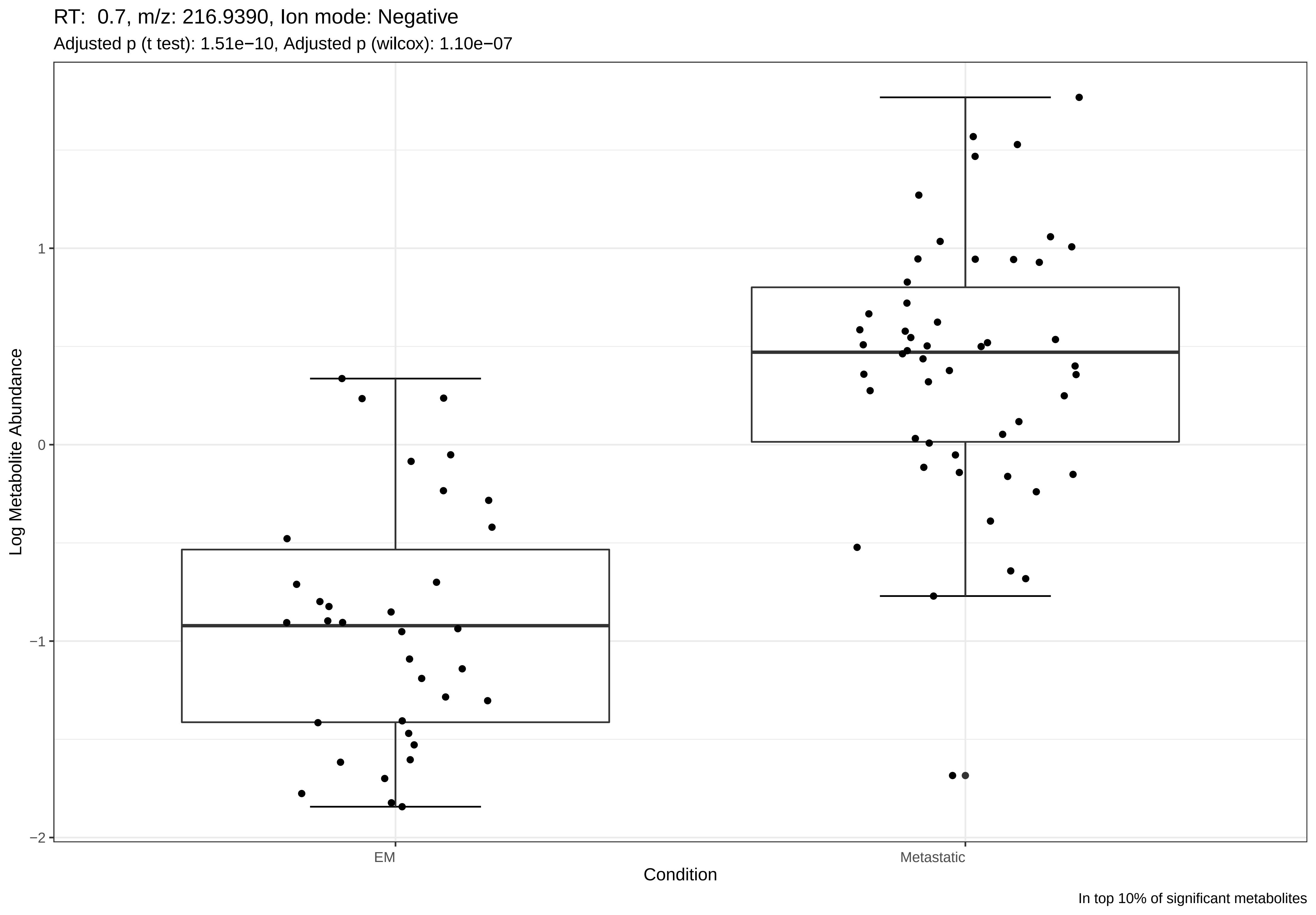

RT: 0.7, m/z: 216.9390, Ion mode: Negative
Adjusted p (t test): 1.51e−10, Adjusted p (wilcox): 1.10e−07
Log Metabolite Abundance
1
0
−1
−2
EM
Metastatic
Condition
In top 10% of significant metabolites

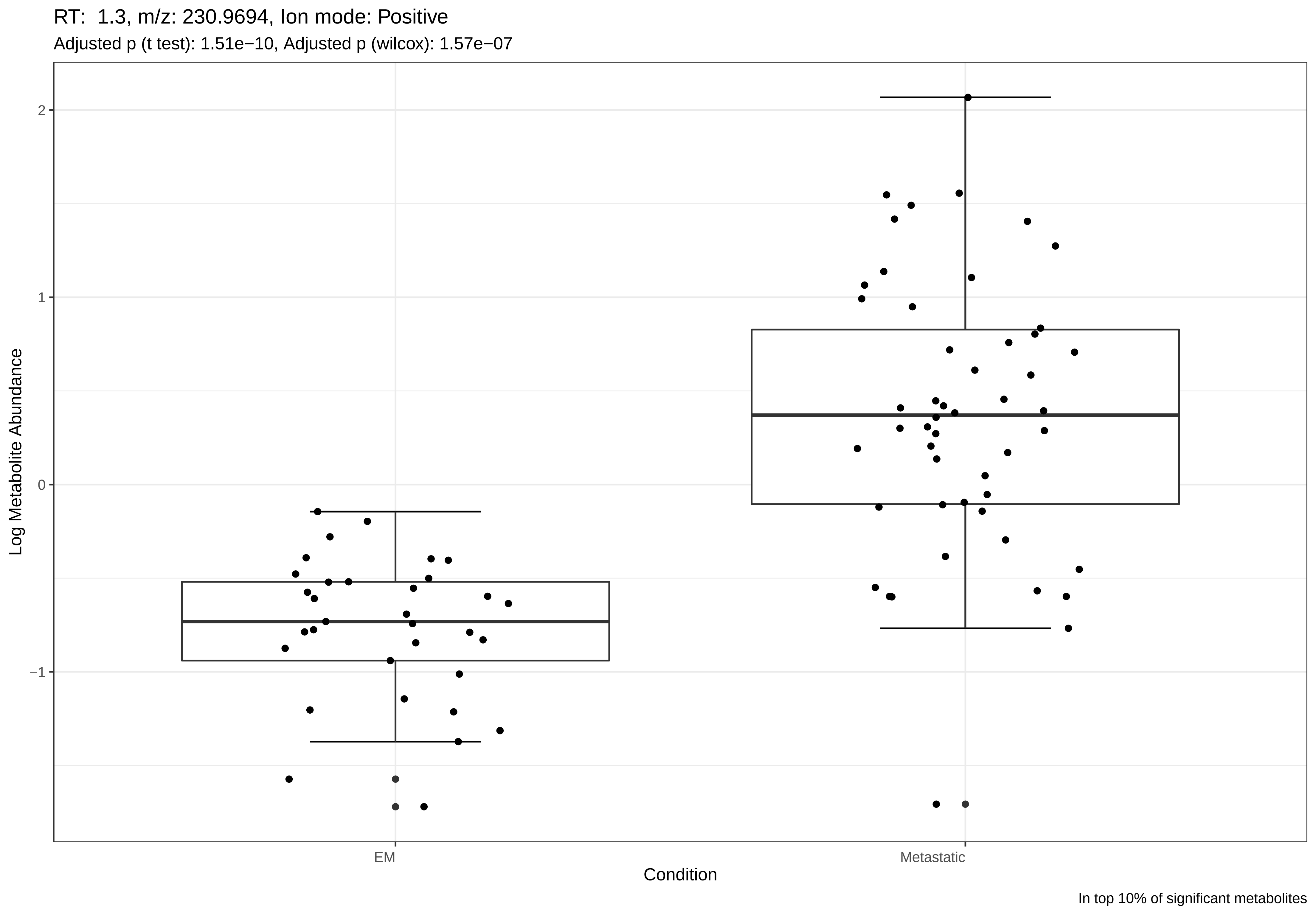

RT: 1.3, m/z: 230.9694, Ion mode: Positive
Adjusted p (t test): 1.51e−10, Adjusted p (wilcox): 1.57e−07
Log Metabolite Abundance
2
1
0
−1
EM
Metastatic
Condition
In top 10% of significant metabolites

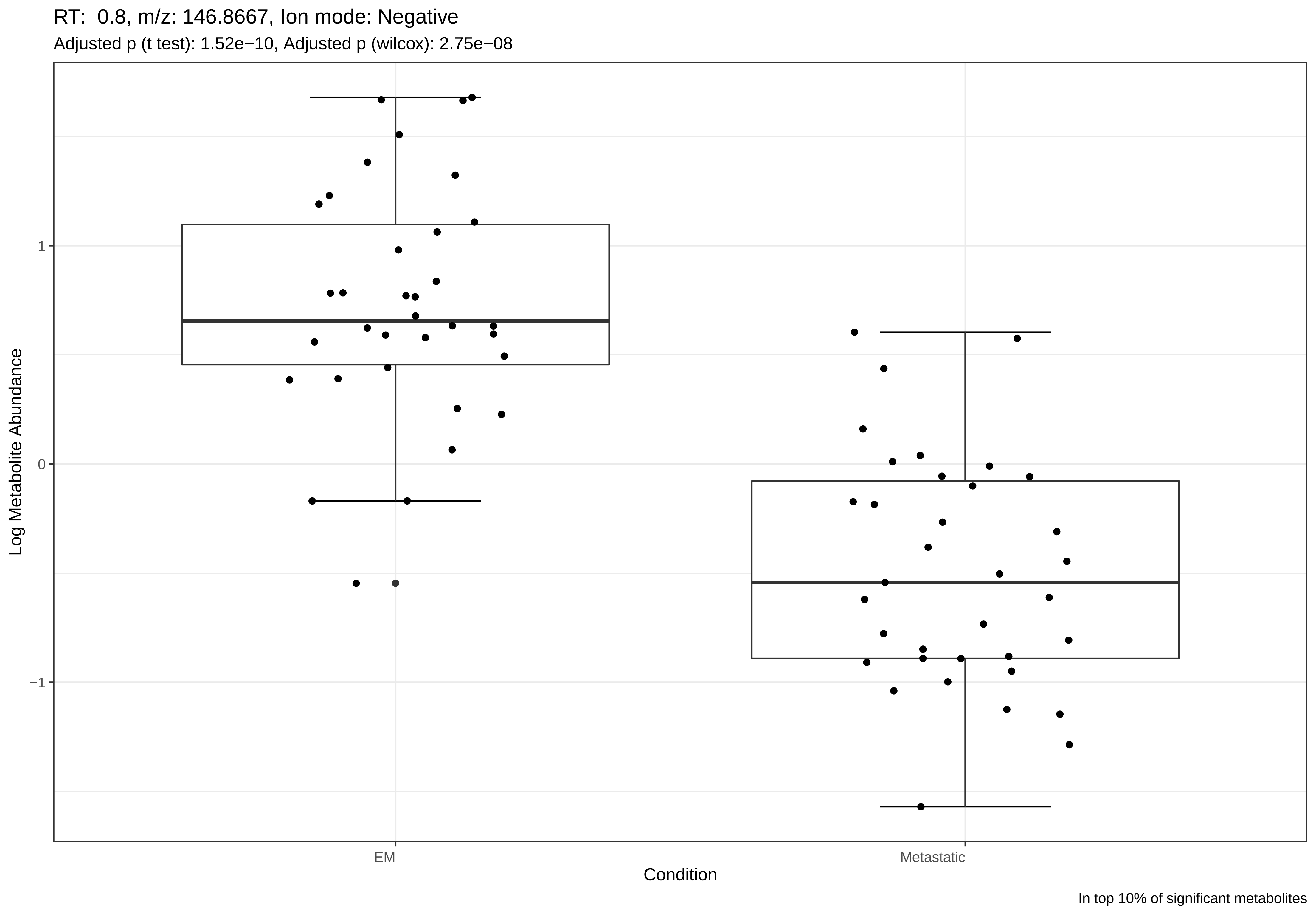

RT: 0.8, m/z: 146.8667, Ion mode: Negative
Adjusted p (t test): 1.52e−10, Adjusted p (wilcox): 2.75e−08
Log Metabolite Abundance
1
0
−1
EM
Metastatic
Condition
In top 10% of significant metabolites

CYTOSINE (RT: 0.8, m/z: 112.0505, Ion mode: Positive)

Adjusted p (t test): 1.52e−10, Adjusted p (wilcox): 1.10e−07

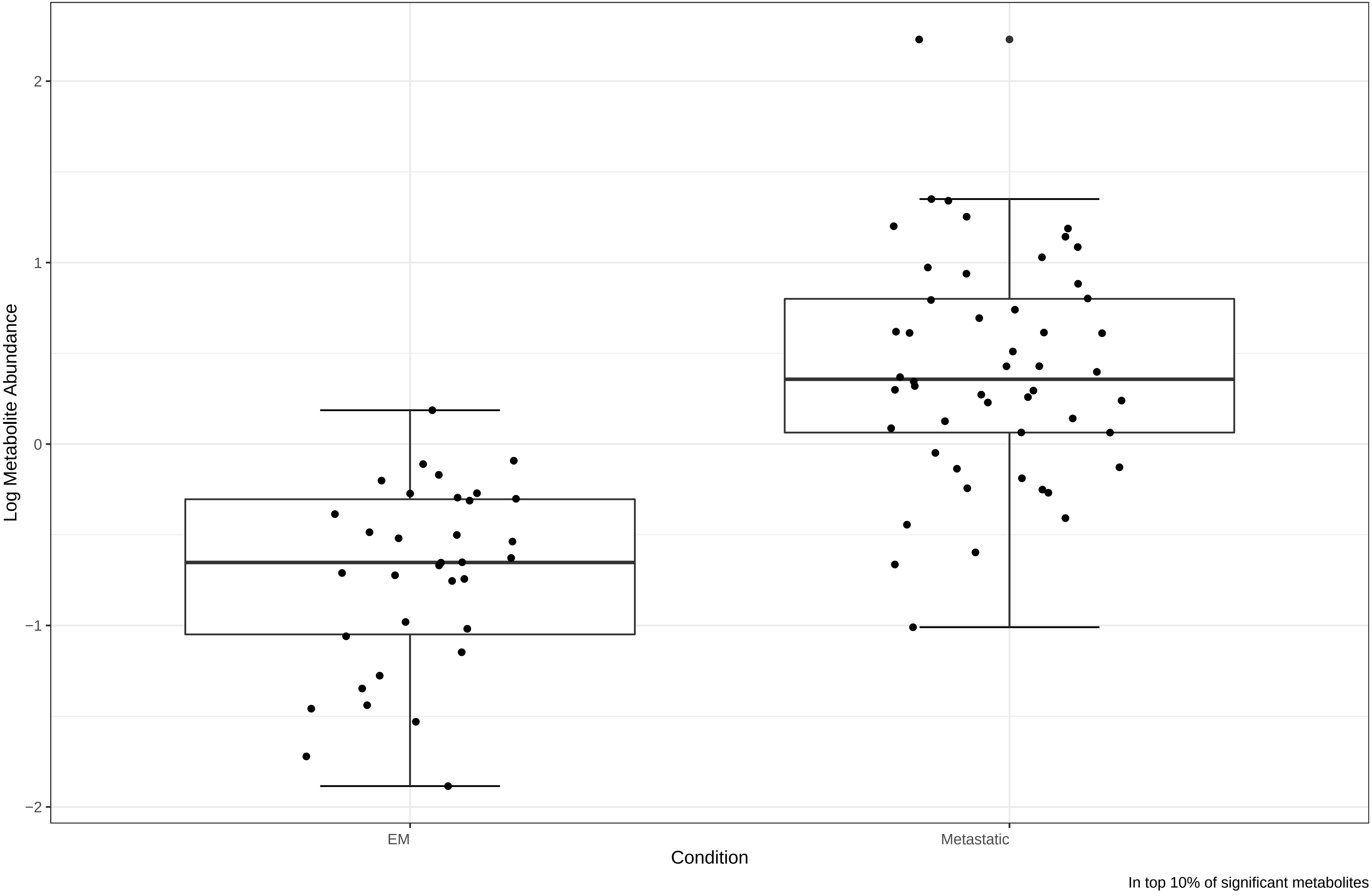

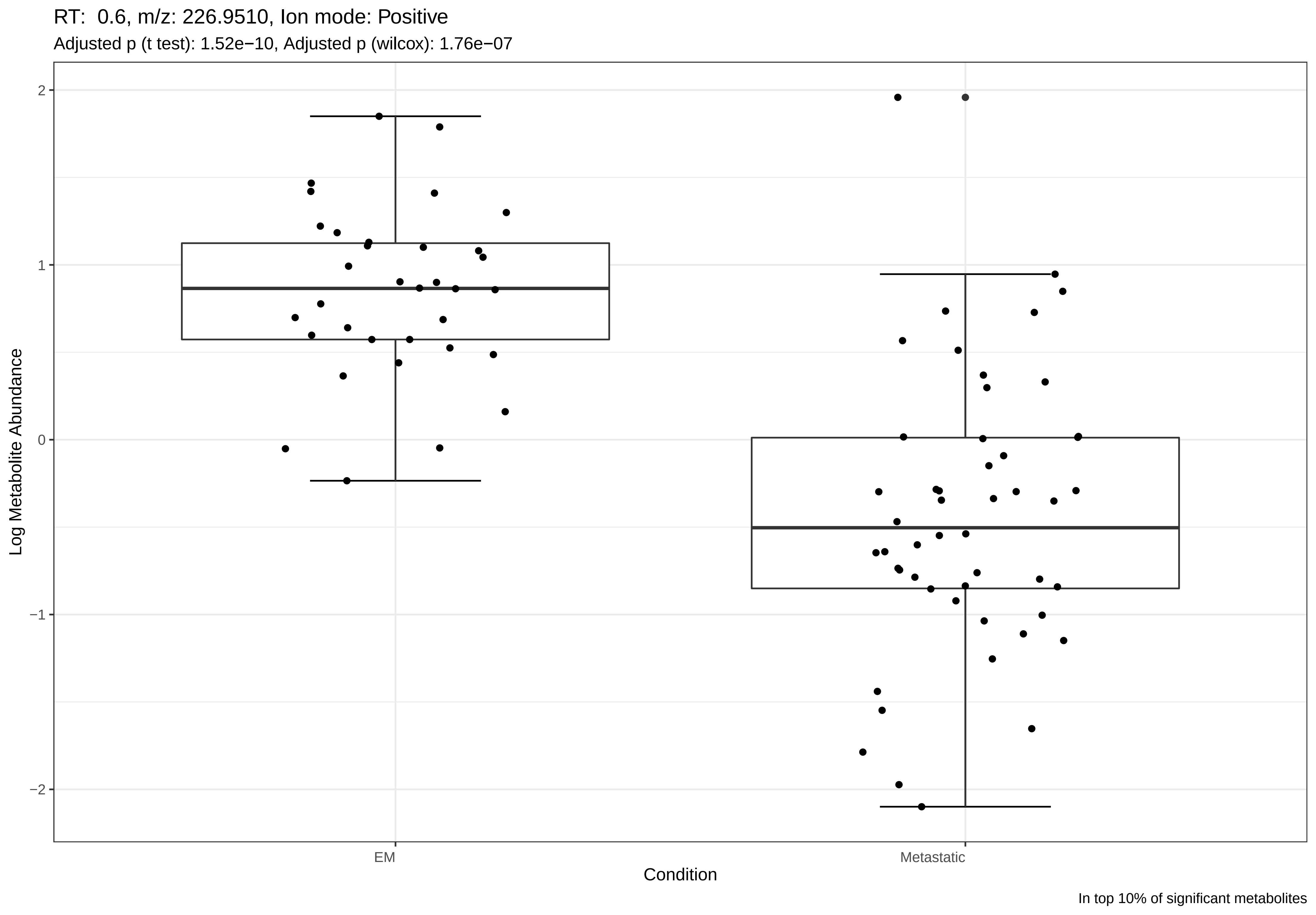
RT: 0.6, m/z: 226.9510, Ion mode: Positive
Adjusted p (t test): 1.52e−10, Adjusted p (wilcox): 1.76e−07
Log Metabolite Abundance
2
1
0
−1
−2
EM
Metastatic
Condition
In top 10% of significant metabolites

RT: 0.8, m/z: 247.0570, Ion mode: Positive

Adjusted p (t test): 1.54e−10, Adjusted p (wilcox): 1.10e−07

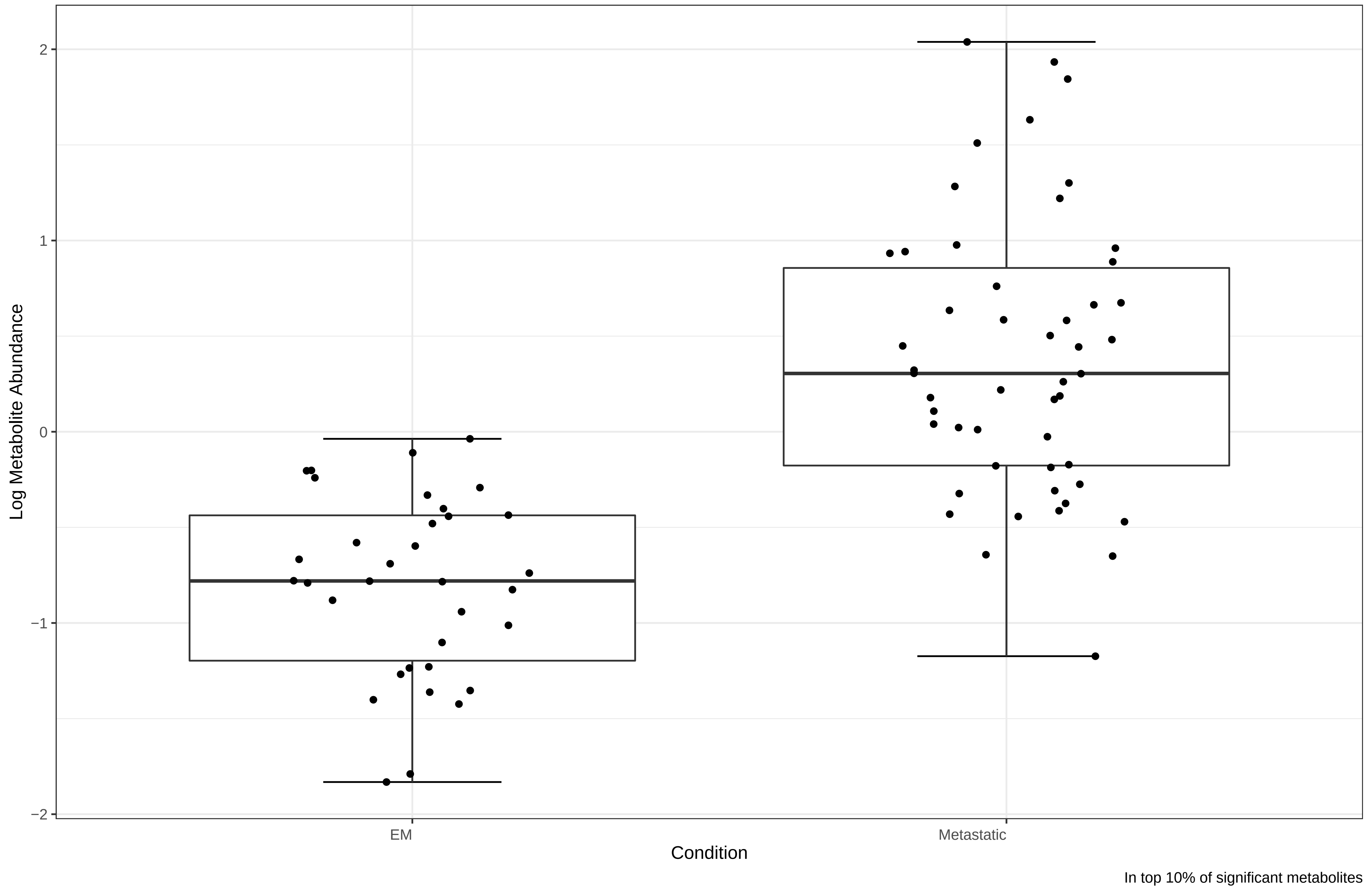

RT: 6.3, m/z: 374.0977, Ion mode: Positive

Adjusted p (t test): 1.89e−10, Adjusted p (wilcox): 7.06e−07

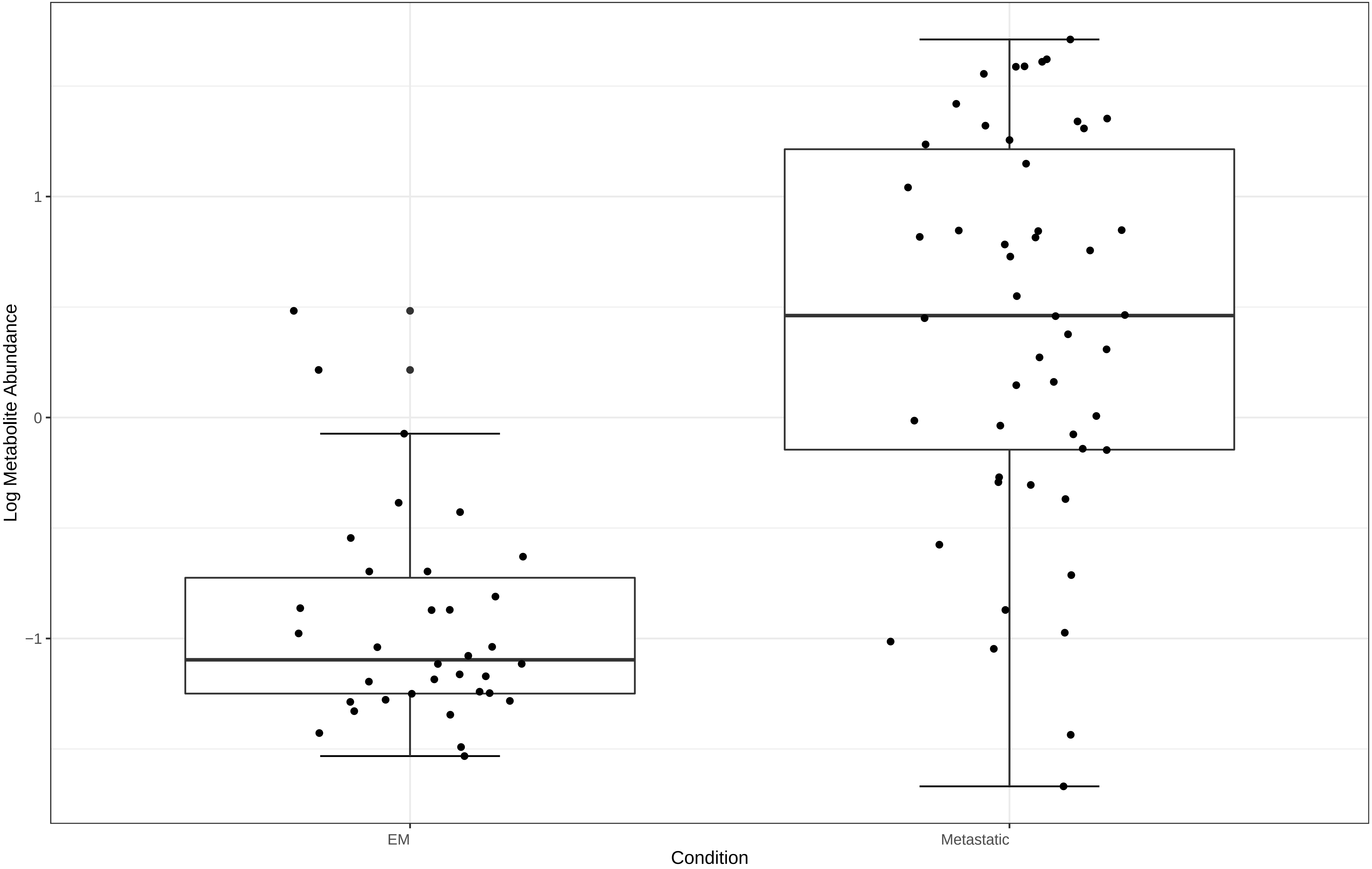

In top 10% of significant metabolites

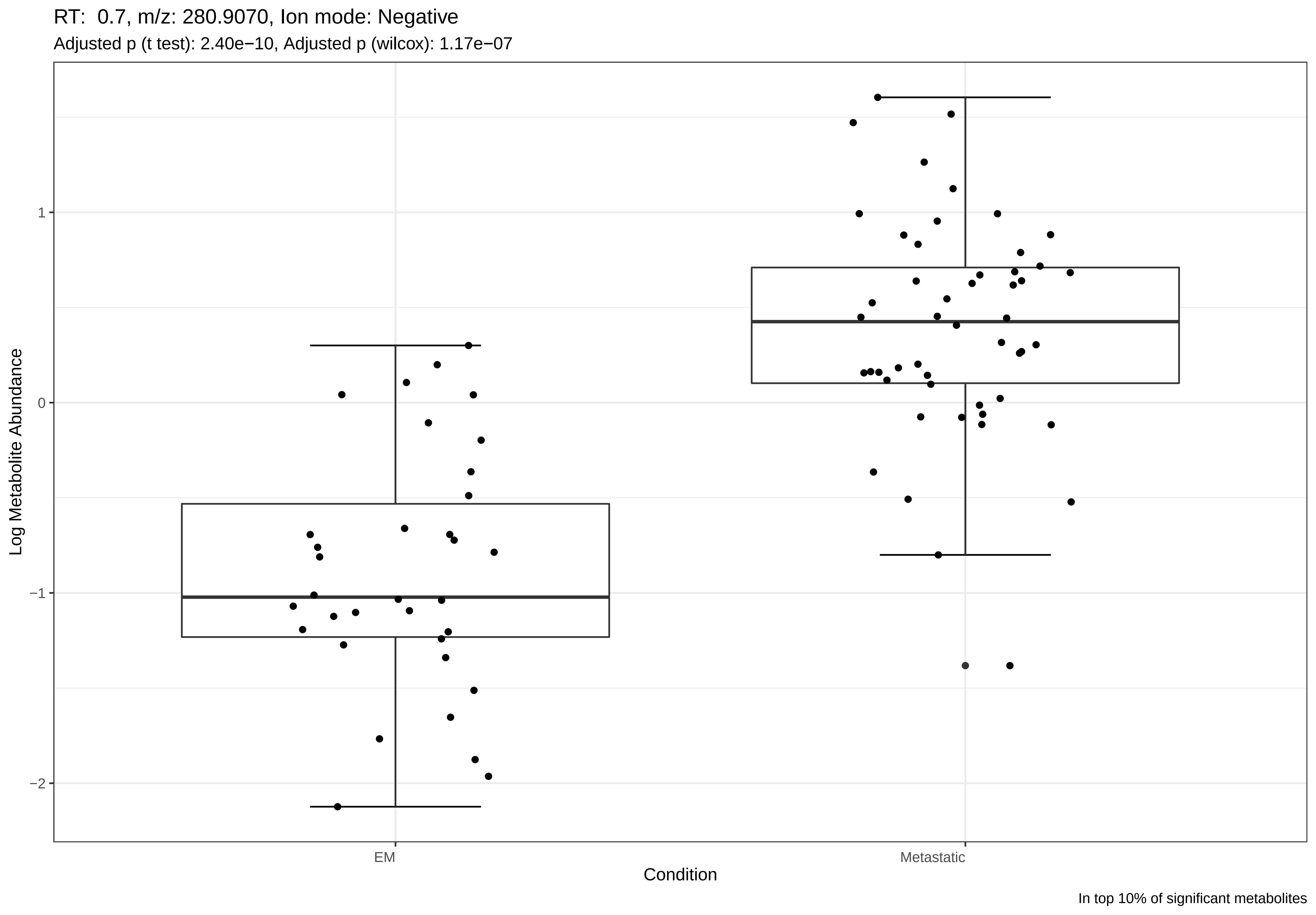
RT: 0.7, m/z: 280.9070, Ion mode: Negative
Adjusted p (t test): 2.40e−10, Adjusted p (wilcox): 1.17e−07
Log Metabolite Abundance
1
0
−1
−2
EM
Metastatic
Condition
In top 10% of significant metabolites

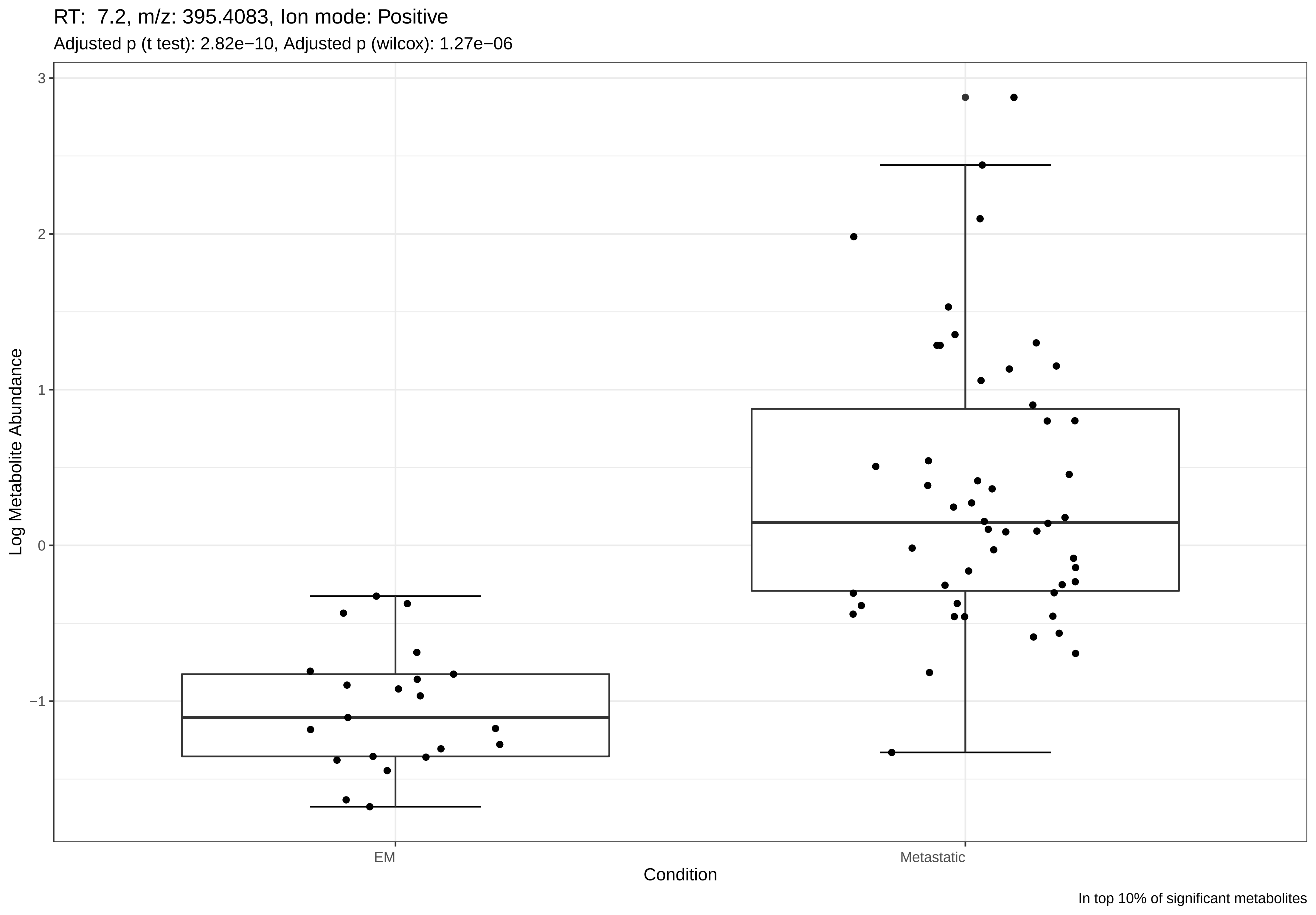
RT: 7.2, m/z: 395.4083, Ion mode: Positive
Adjusted p (t test): 2.82e−10, Adjusted p (wilcox): 1.27e−06
Log Metabolite Abundance
3
2
1
0
−1
EM
Metastatic
Condition
In top 10% of significant metabolites

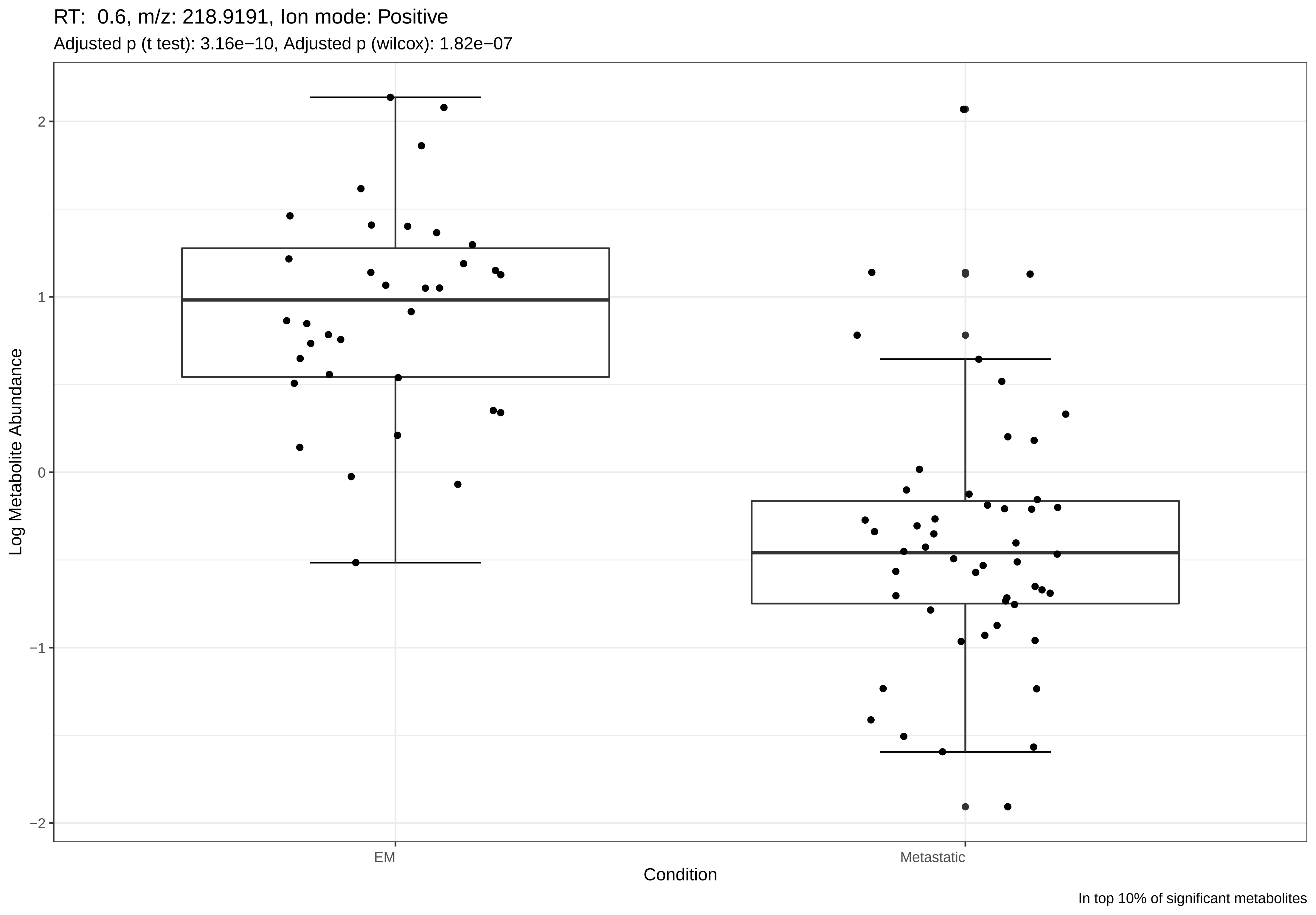

RT: 0.6, m/z: 218.9191, Ion mode: Positive
Adjusted p (t test): 3.16e−10, Adjusted p (wilcox): 1.82e−07
Log Metabolite Abundance
2
1
0
−1
−2
EM
Metastatic
Condition
In top 10% of significant metabolites

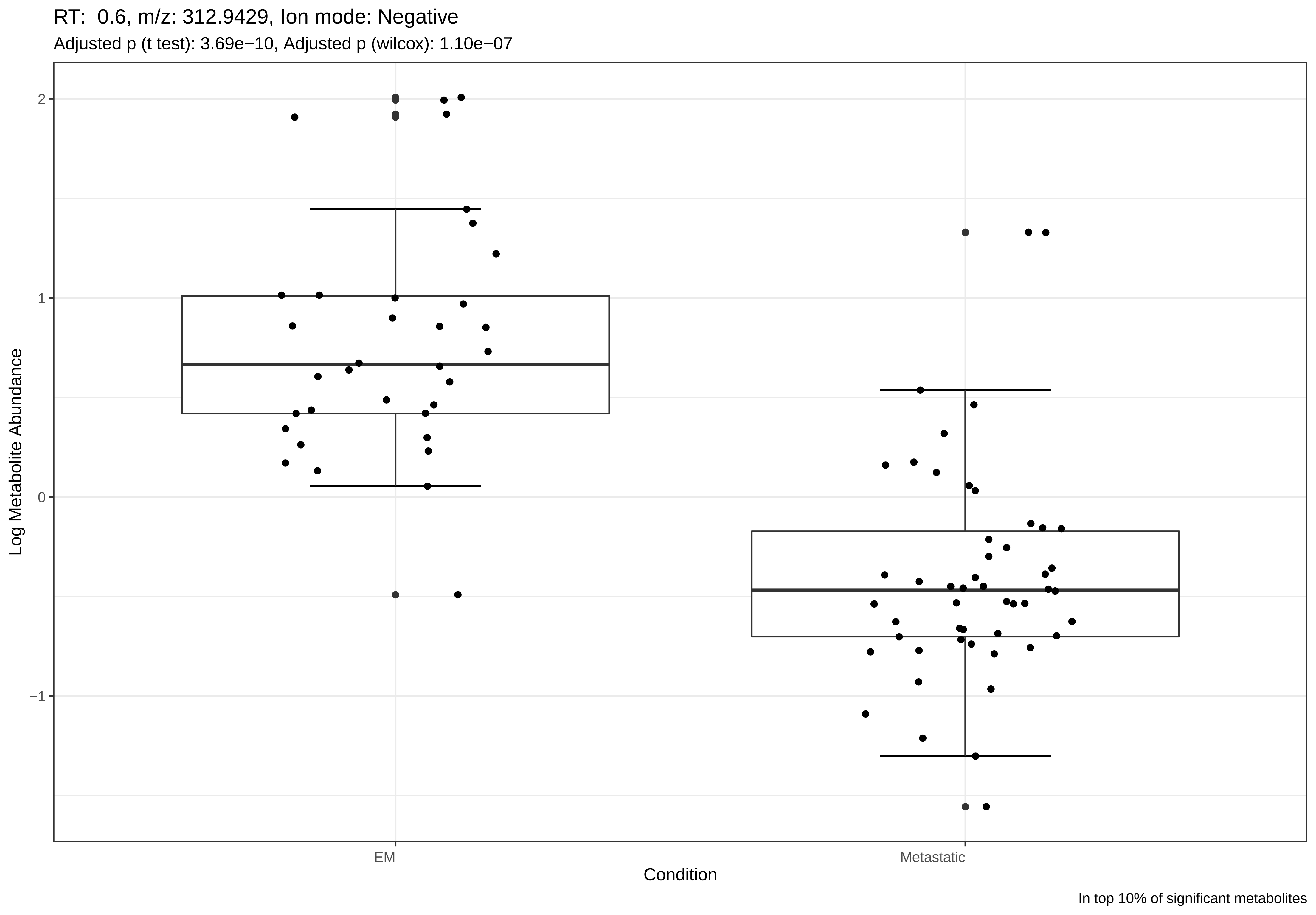
RT: 0.6, m/z: 312.9429, Ion mode: Negative
Adjusted p (t test): 3.69e−10, Adjusted p (wilcox): 1.10e−07
2
1
0
−1
Log Metabolite Abundance
EM
Metastatic
Condition
In top 10% of significant metabolites

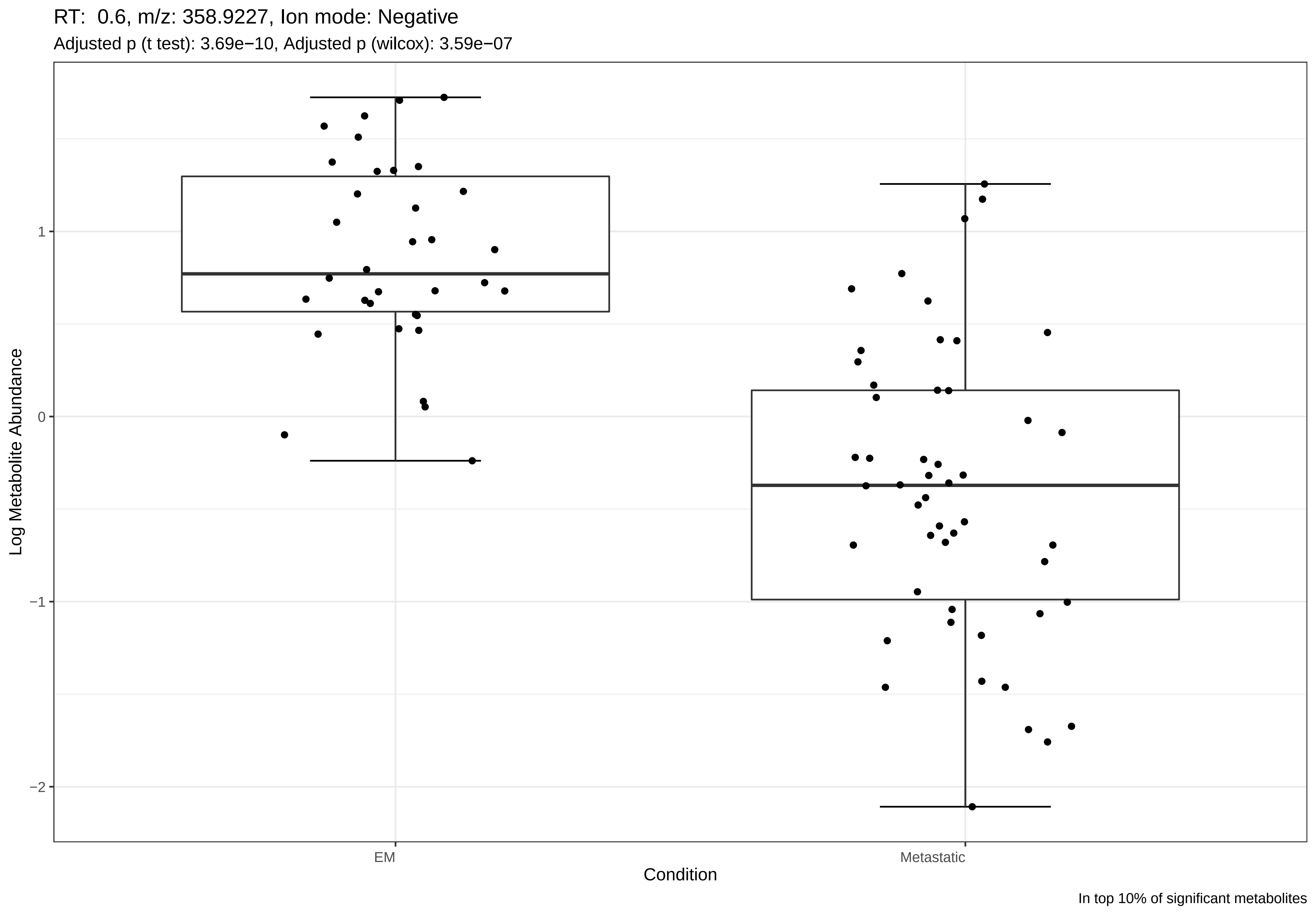

RT: 0.6, m/z: 358.9227, Ion mode: Negative
Adjusted p (t test): 3.69e−10, Adjusted p (wilcox): 3.59e−07
Log Metabolite Abundance
1
0
−1
−2
EM
Metastatic
Condition
In top 10% of significant metabolites

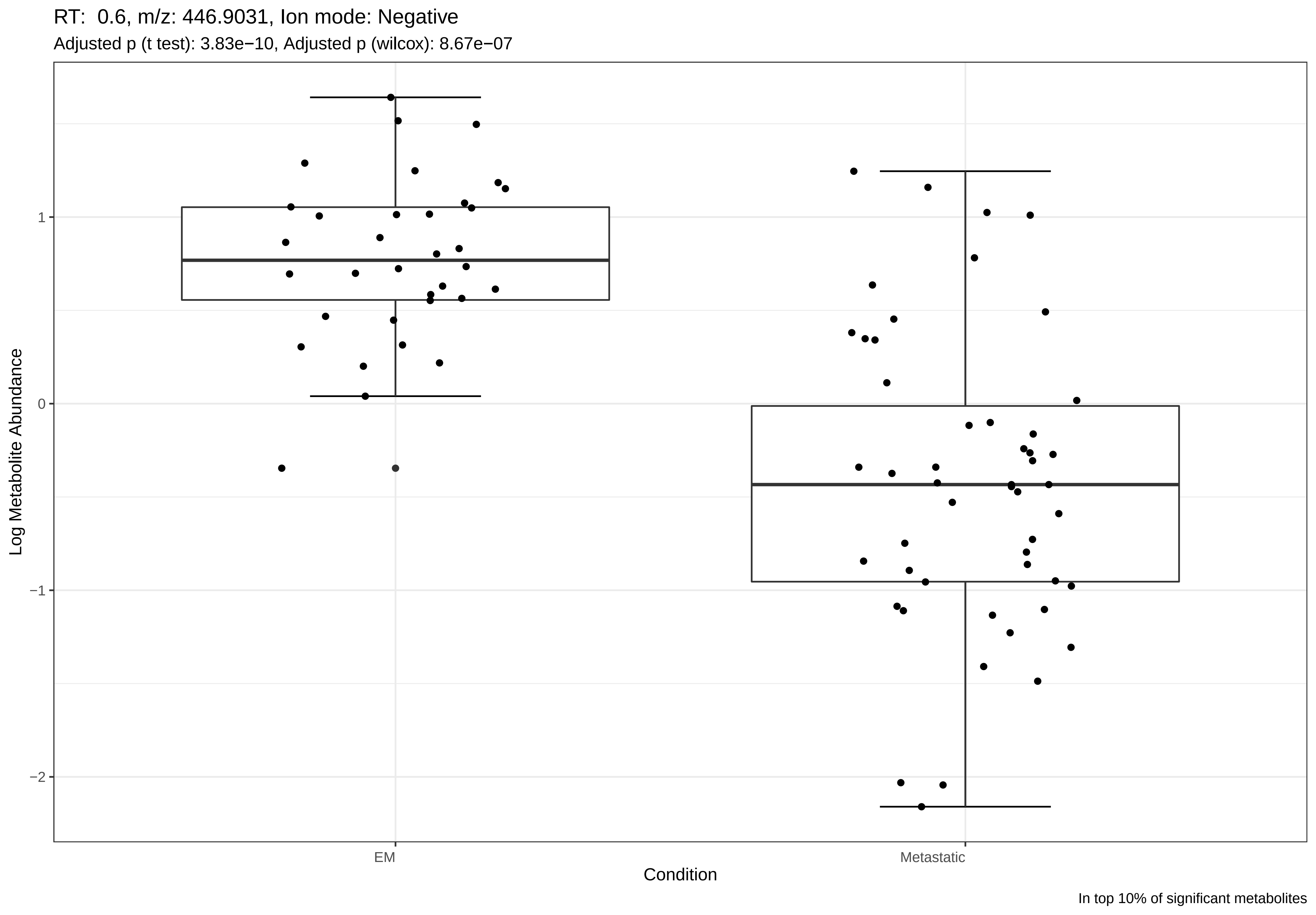
RT: 0.6, m/z: 446.9031, Ion mode: Negative
Adjusted p (t test): 3.83e−10, Adjusted p (wilcox): 8.67e−07
Log Metabolite Abundance
1
0
−1
−2
EM
Metastatic
Condition
In top 10% of significant metabolites

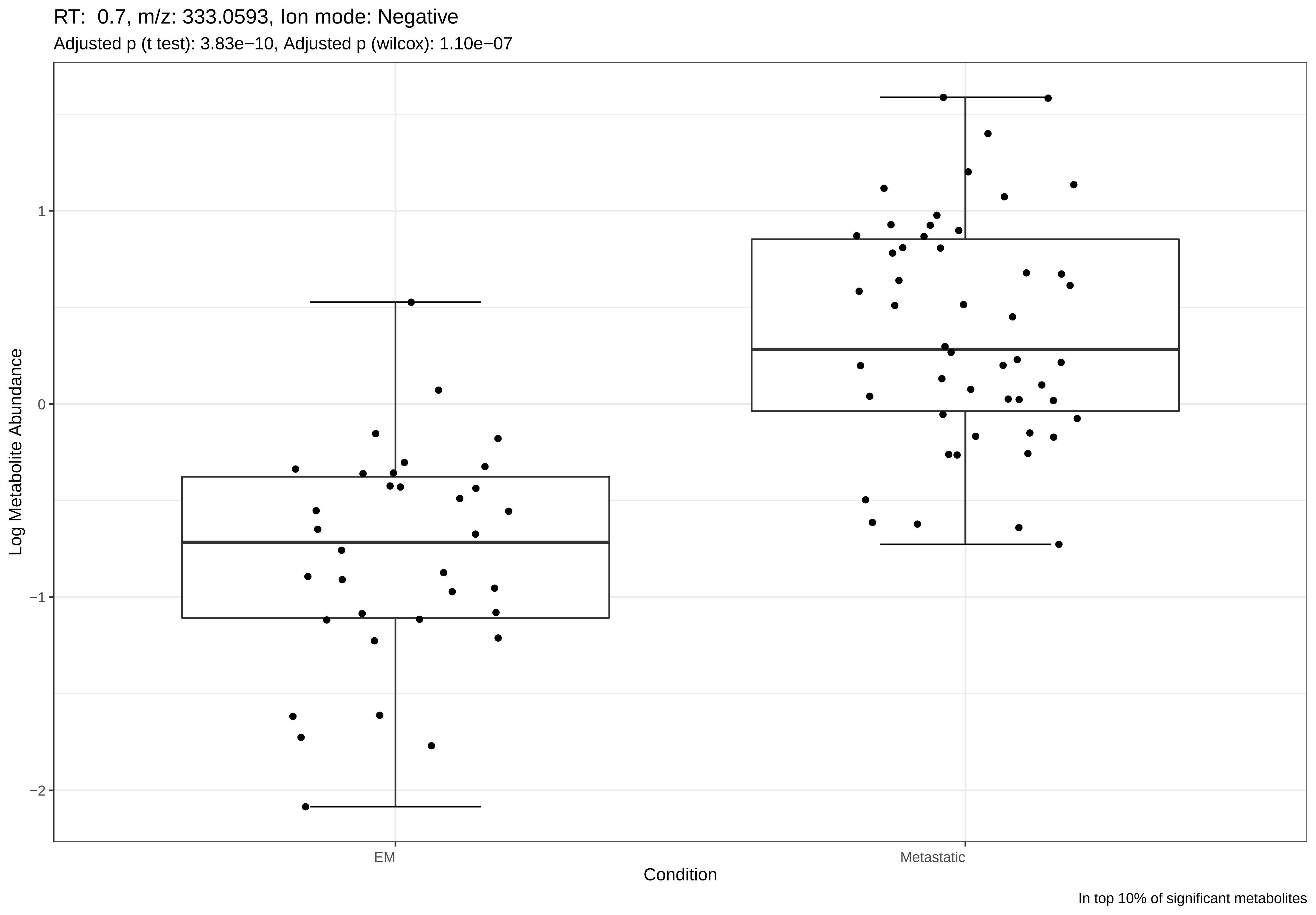
RT: 0.7, m/z: 333.0593, Ion mode: Negative
Adjusted p (t test): 3.83e−10, Adjusted p (wilcox): 1.10e−07
Log Metabolite Abundance
1
0
−1
−2
EM
Metastatic
Condition
In top 10% of significant metabolites

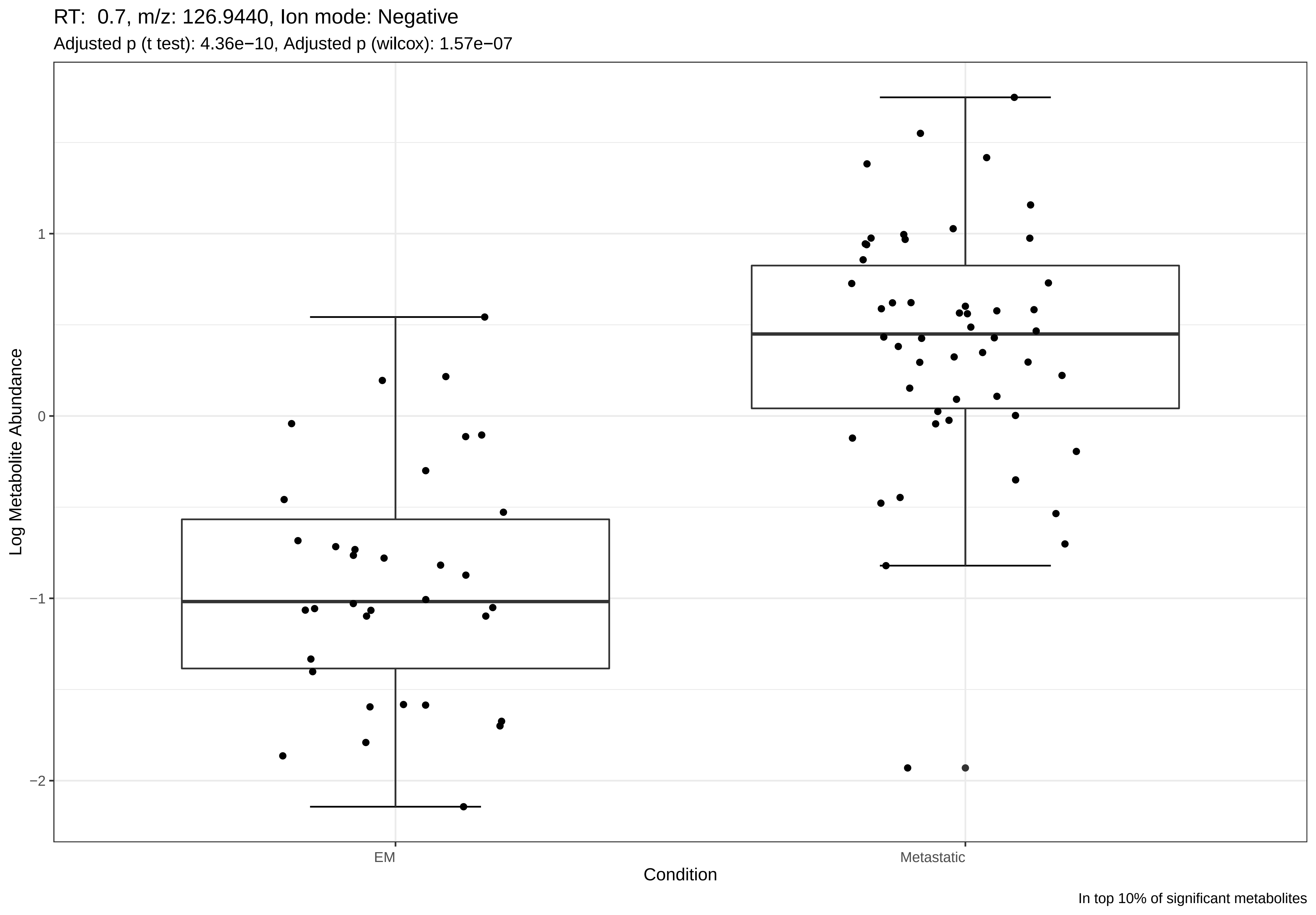
RT: 0.7, m/z: 126.9440, Ion mode: Negative
Adjusted p (t test): 4.36e−10, Adjusted p (wilcox): 1.57e−07
Log Metabolite Abundance
1
0
−1
−2
EM
Metastatic
Condition
In top 10% of significant metabolites

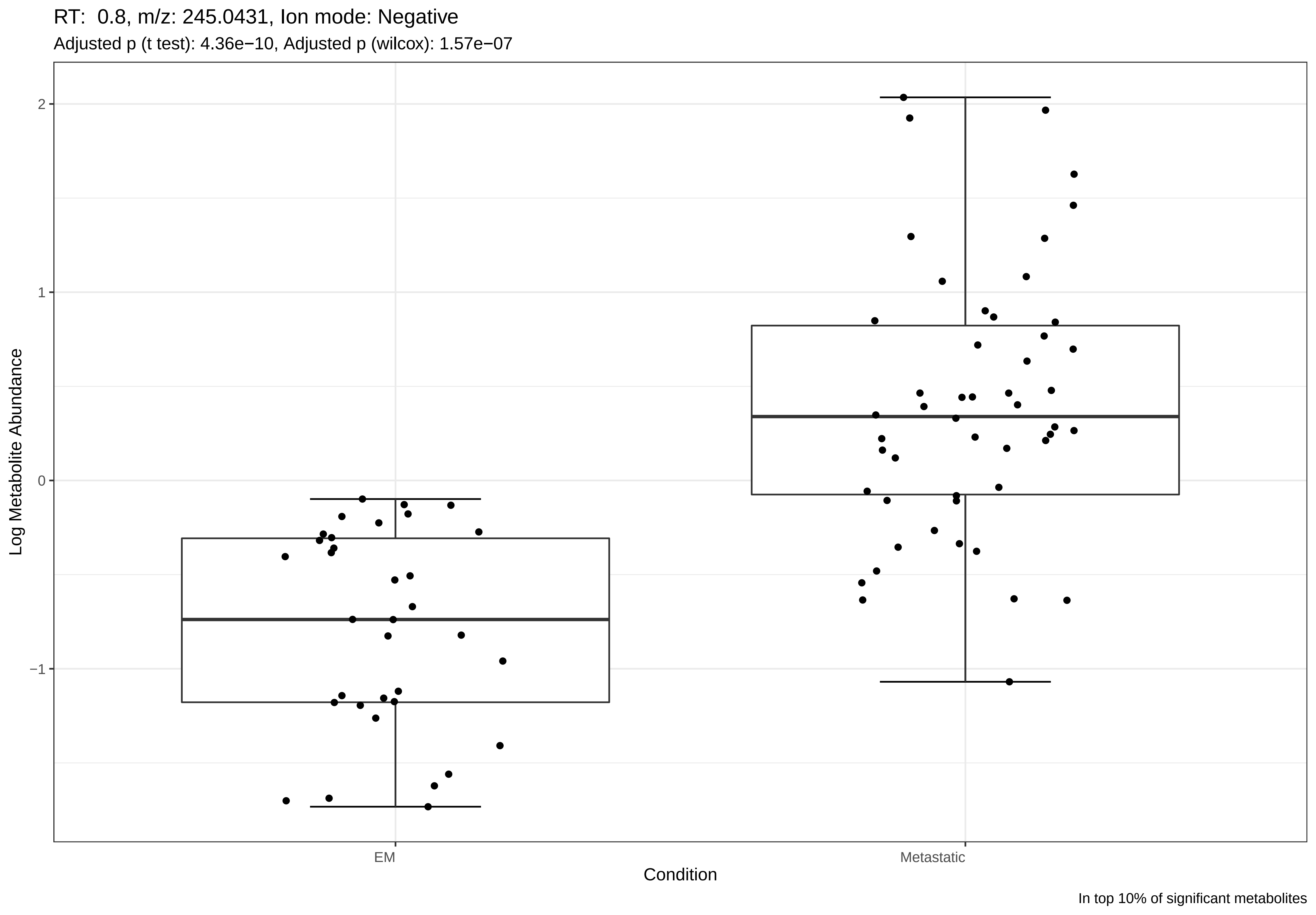

RT: 0.8, m/z: 245.0431, Ion mode: Negative
Adjusted p (t test): 4.36e−10, Adjusted p (wilcox): 1.57e−07
Log Metabolite Abundance
2
1
0
−1
EM
Metastatic
Condition
In top 10% of significant metabolites

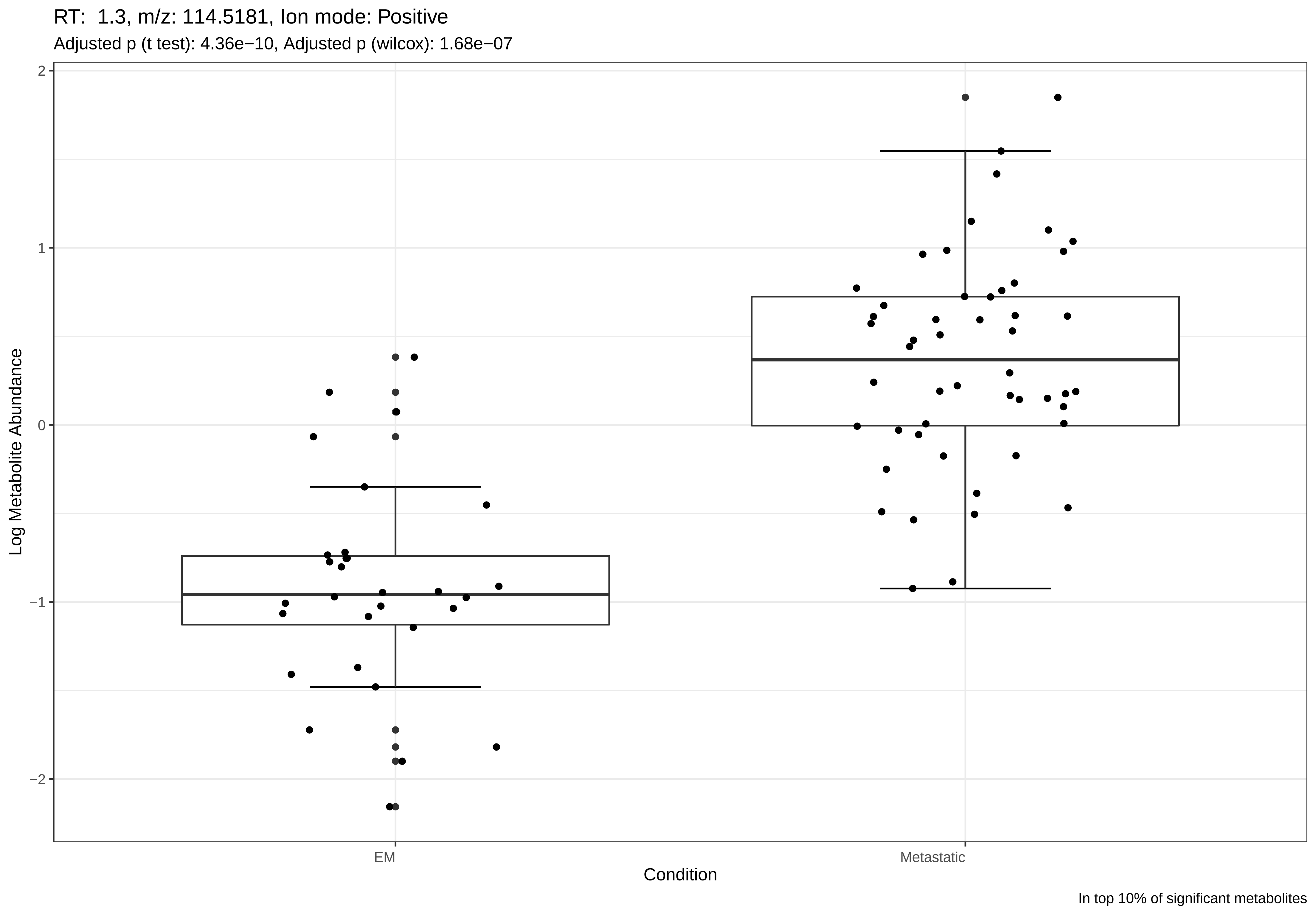

RT: 1.3, m/z: 114.5181, Ion mode: Positive
Adjusted p (t test): 4.36e−10, Adjusted p (wilcox): 1.68e−07
Log Metabolite Abundance
2
1
0
−1
−2
EM
Metastatic
Condition
In top 10% of significant metabolites

RT: 0.6, m/z: 316.9480, Ion mode: Negative

Adjusted p (t test): 4.62e−10, Adjusted p (wilcox): 4.06e−07

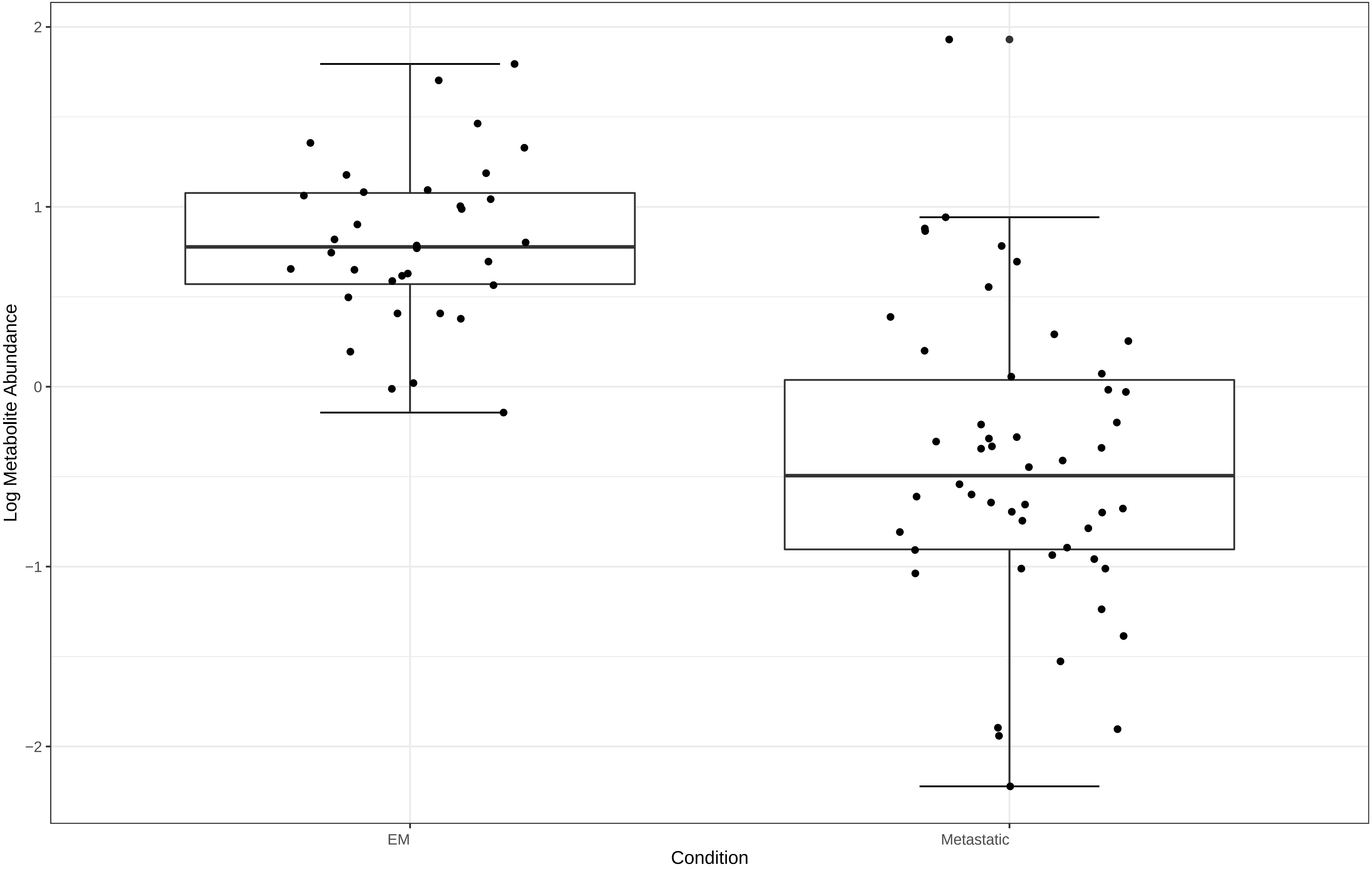

In top 10% of significant metabolites

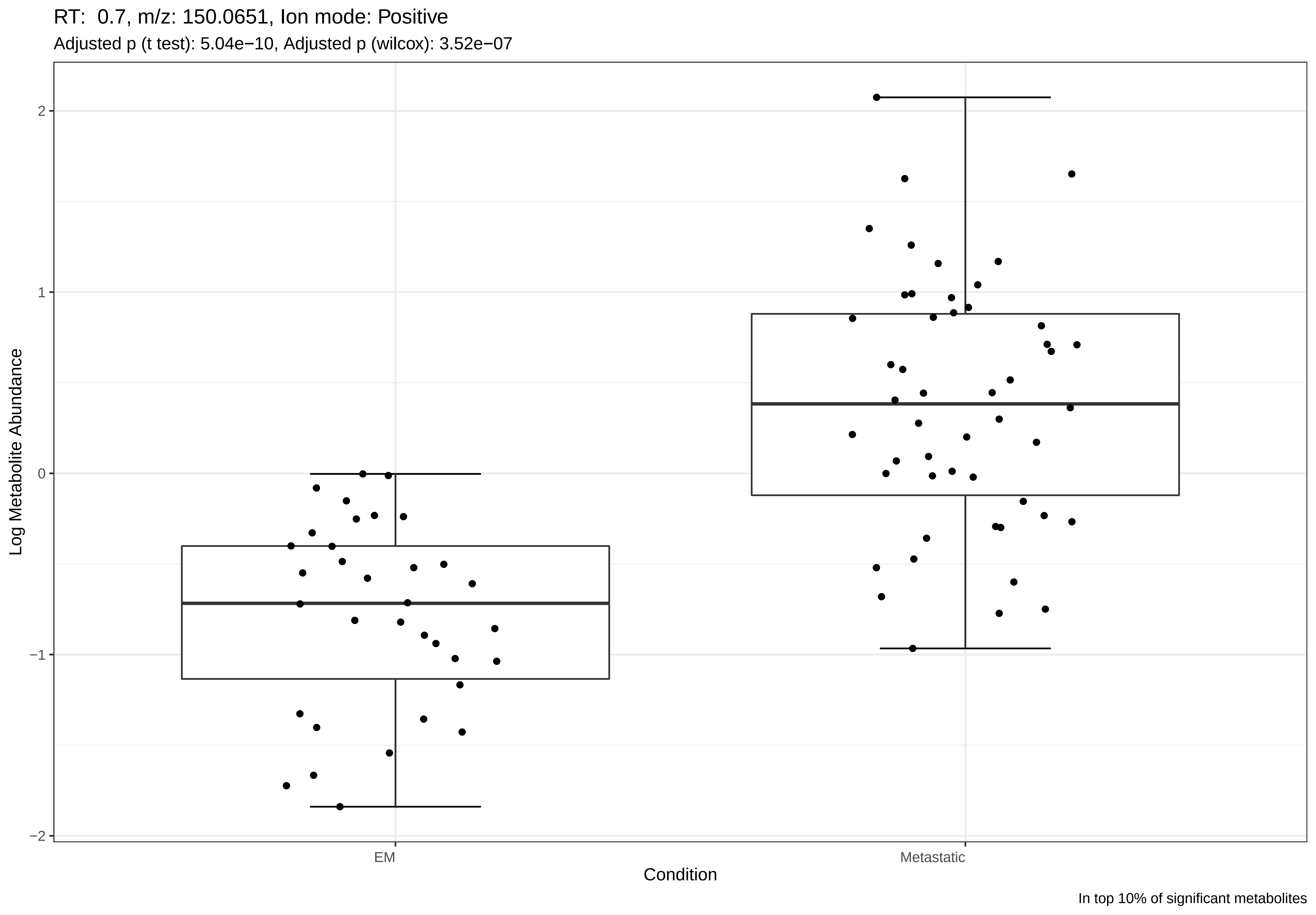
RT: 0.7, m/z: 150.0651, Ion mode: Positive
Adjusted p (t test): 5.04e−10, Adjusted p (wilcox): 3.52e−07
Log Metabolite Abundance
2
1
0
−1
−2
EM
Metastatic
Condition
In top 10% of significant metabolites

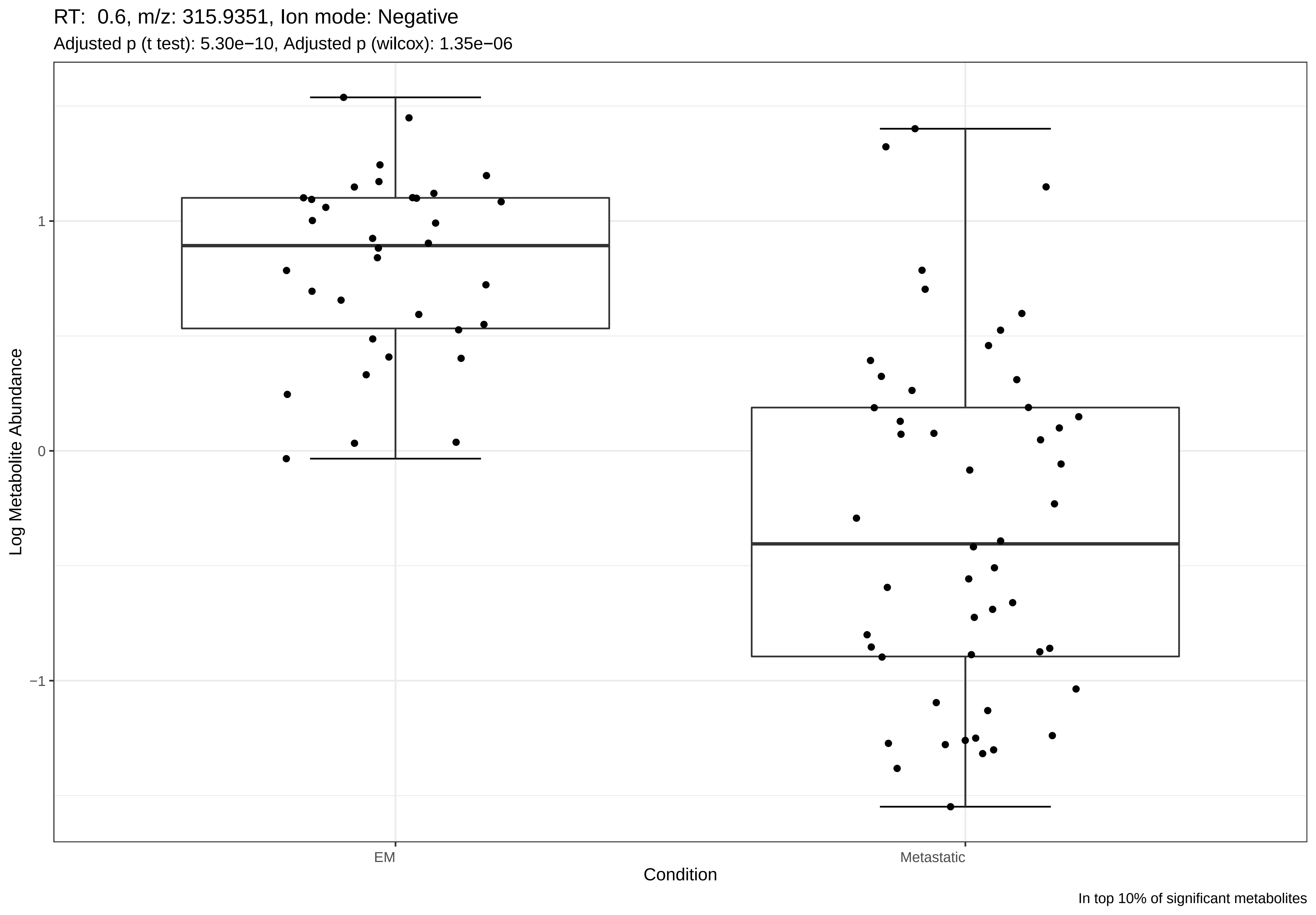
RT: 0.6, m/z: 315.9351, Ion mode: Negative
Adjusted p (t test): 5.30e−10, Adjusted p (wilcox): 1.35e−06
Log Metabolite Abundance
1
0
−1
EM
Metastatic
Condition
In top 10% of significant metabolites

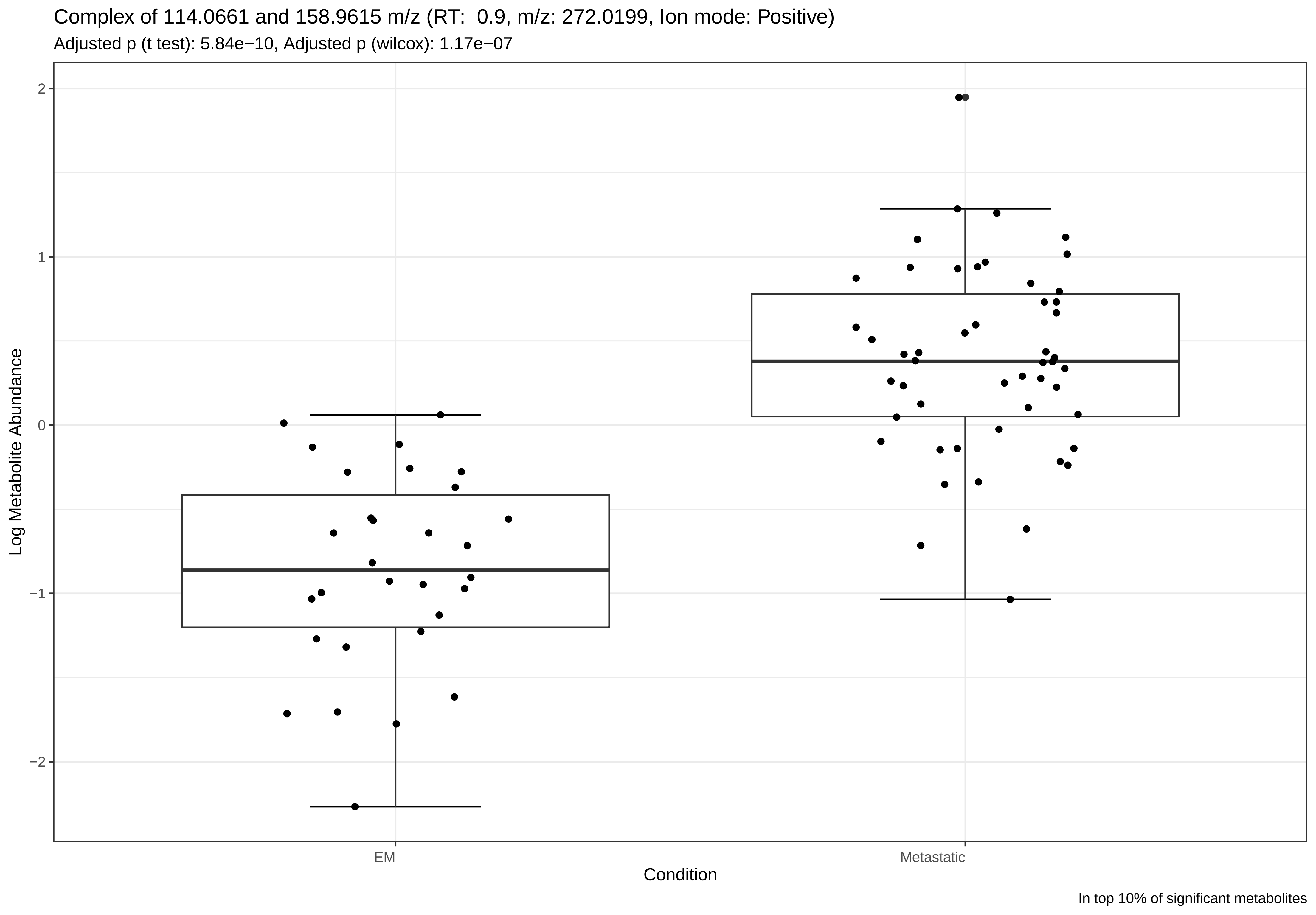

Complex of 114.0661 and 158.9615 m/z (RT: 0.9, m/z: 272.0199, Ion mode: Positive)
Adjusted p (t test): 5.84e−10, Adjusted p (wilcox): 1.17e−07
Log Metabolite Abundance
2
1
0
−1
−2
EM
Metastatic
Condition
In top 10% of significant metabolites

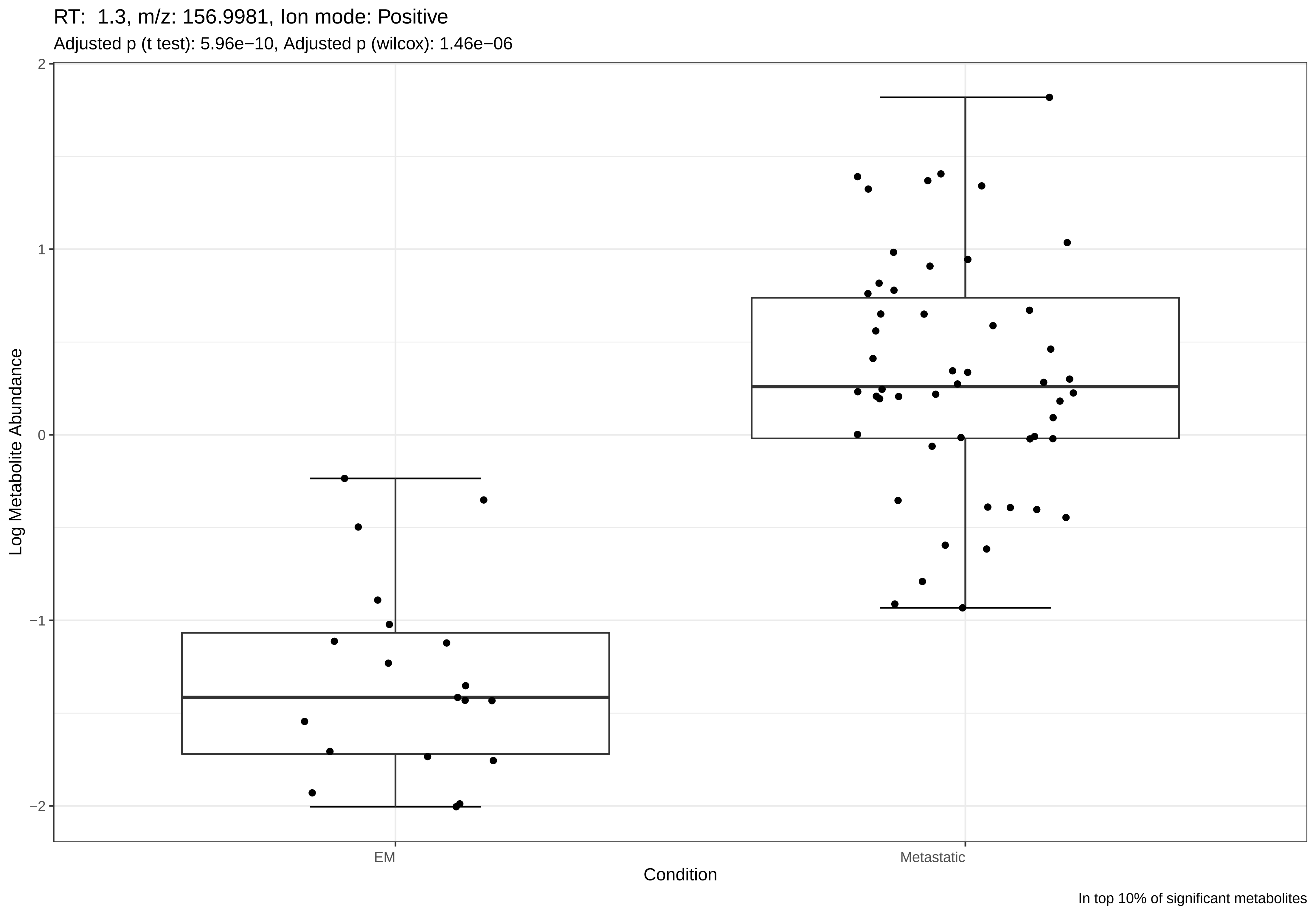
RT: 1.3, m/z: 156.9981, Ion mode: Positive
Adjusted p (t test): 5.96e−10, Adjusted p (wilcox): 1.46e−06
Log Metabolite Abundance
2
1
0
−1
−2
EM
Metastatic
Condition
In top 10% of significant metabolites

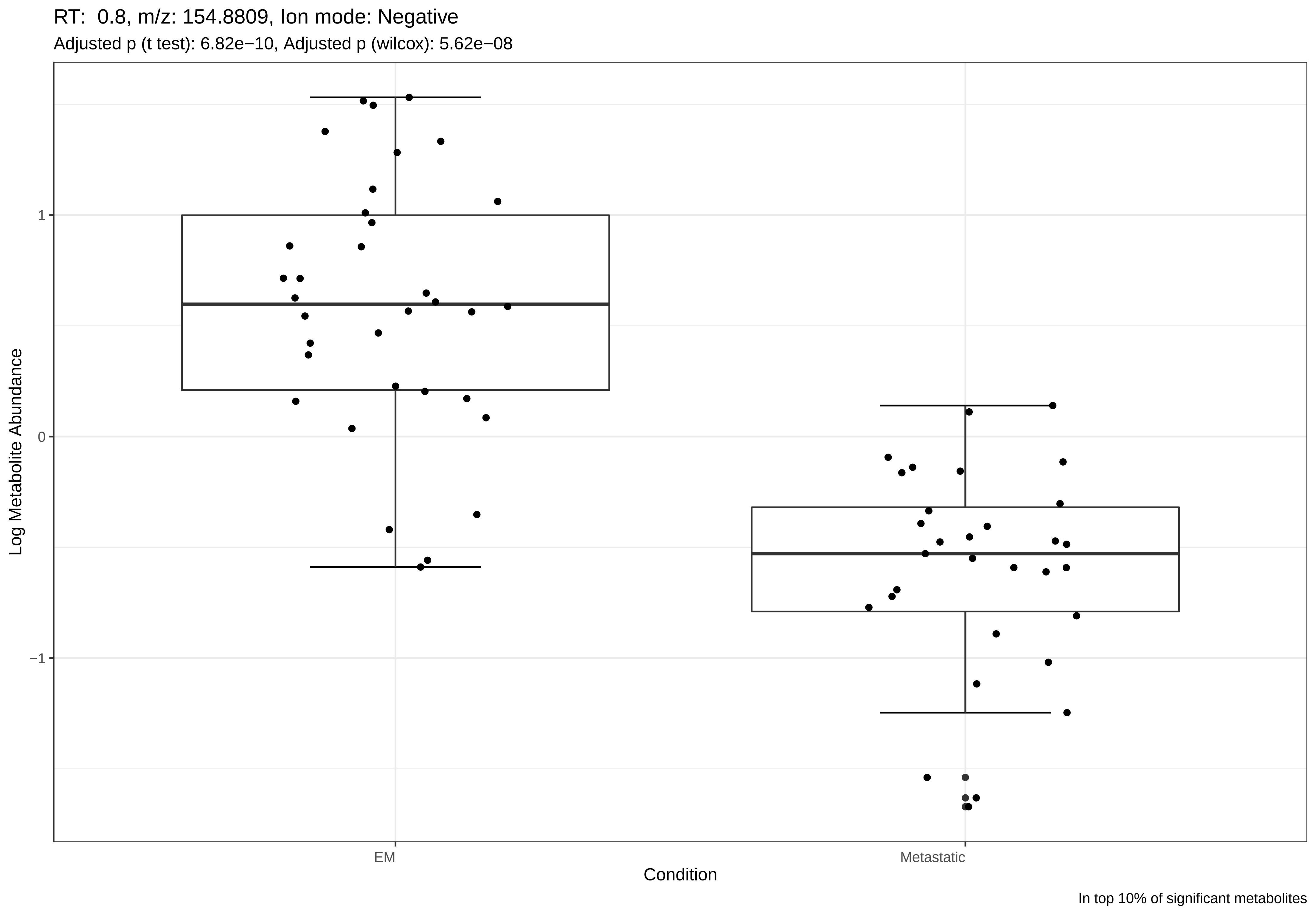

RT: 0.8, m/z: 154.8809, Ion mode: Negative
Adjusted p (t test): 6.82e−10, Adjusted p (wilcox): 5.62e−08
Log Metabolite Abundance
1
0
−1
EM
Metastatic
Condition
In top 10% of significant metabolites

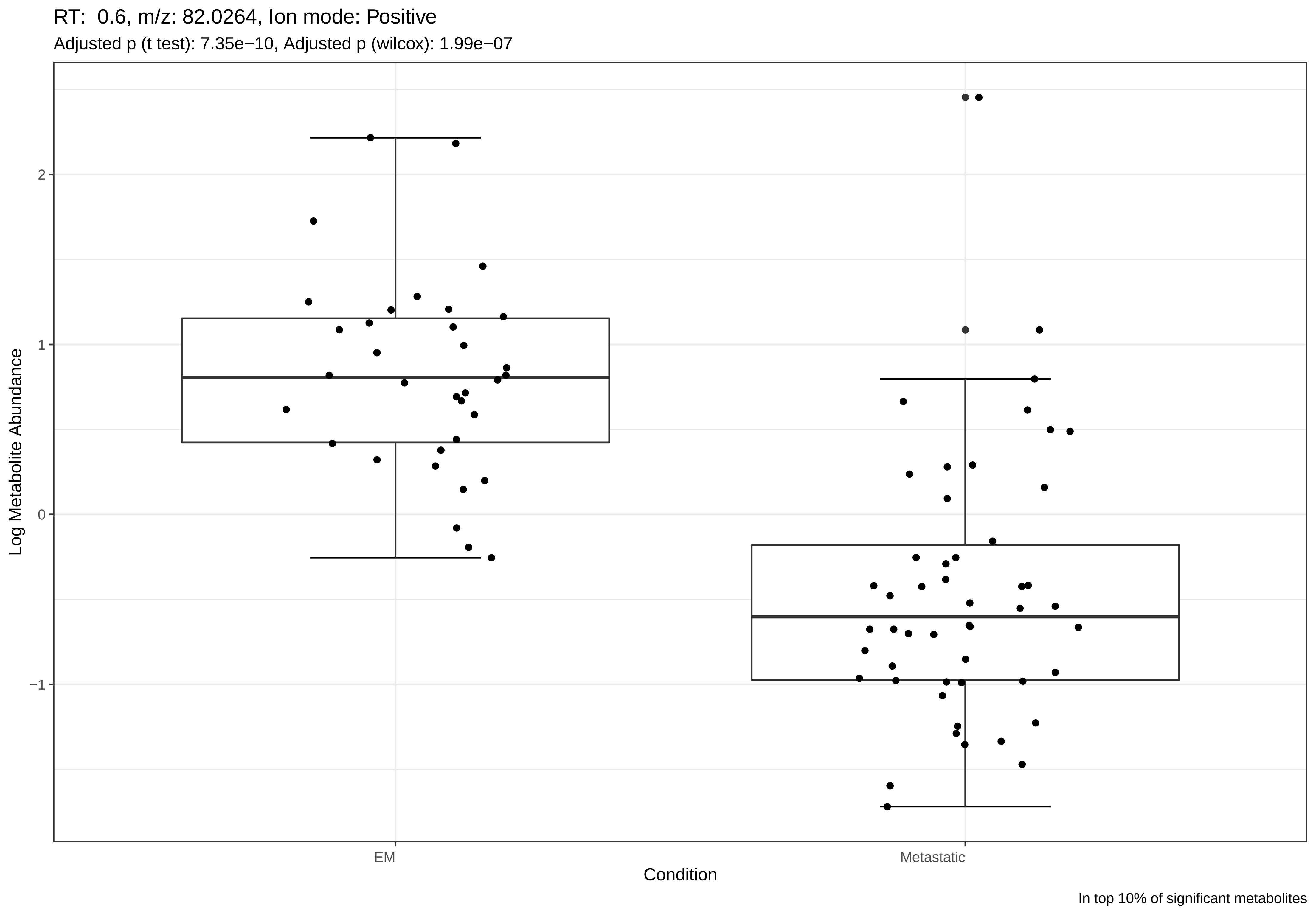

RT: 0.6, m/z: 82.0264, Ion mode: Positive
Adjusted p (t test): 7.35e−10, Adjusted p (wilcox): 1.99e−07
Log Metabolite Abundance
2
1
0
−1
EM
Metastatic
Condition
In top 10% of significant metabolites

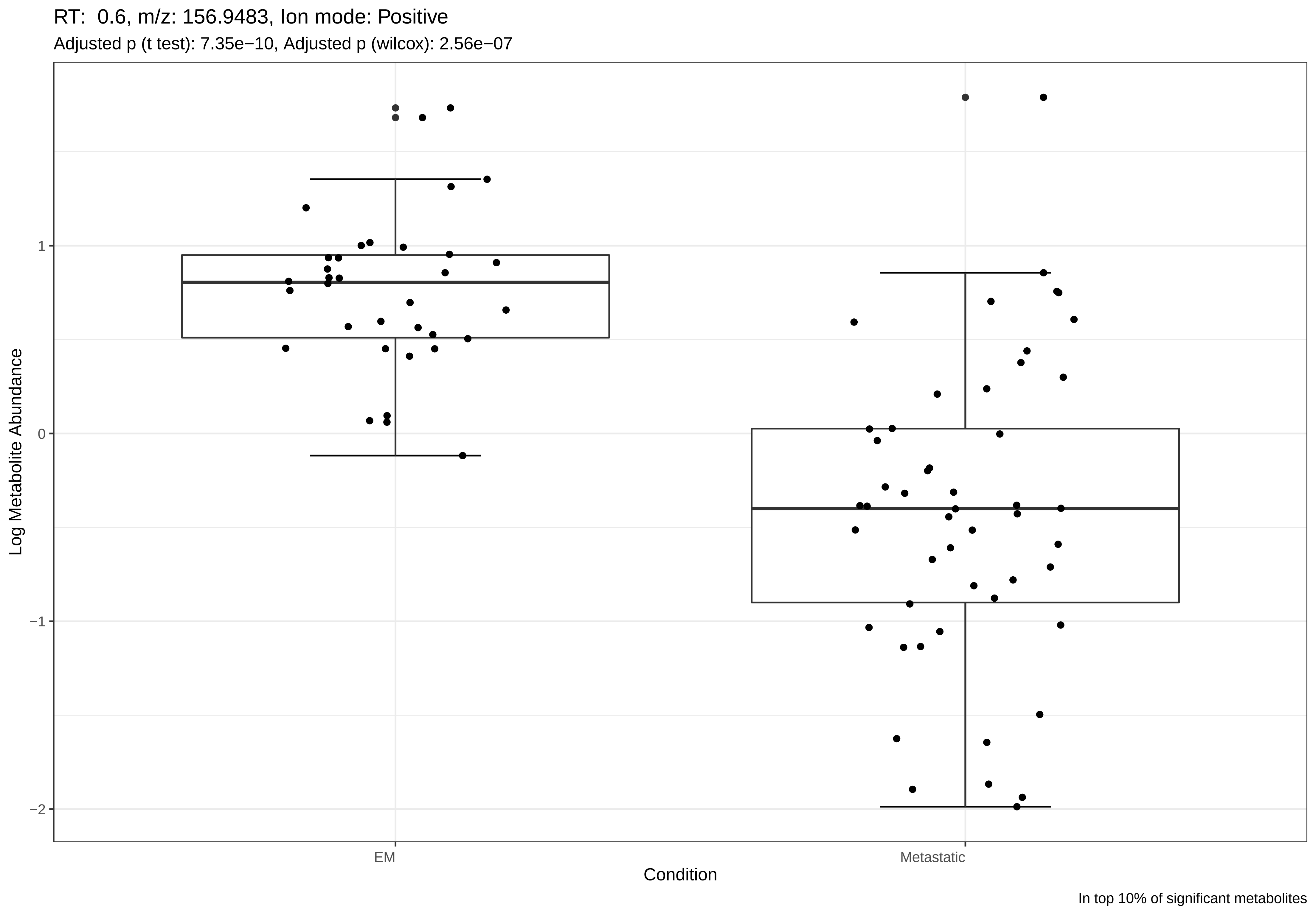
RT: 0.6, m/z: 156.9483, Ion mode: Positive
Adjusted p (t test): 7.35e−10, Adjusted p (wilcox): 2.56e−07
Log Metabolite Abundance
1
0
−1
−2
EM
Metastatic
Condition
In top 10% of significant metabolites

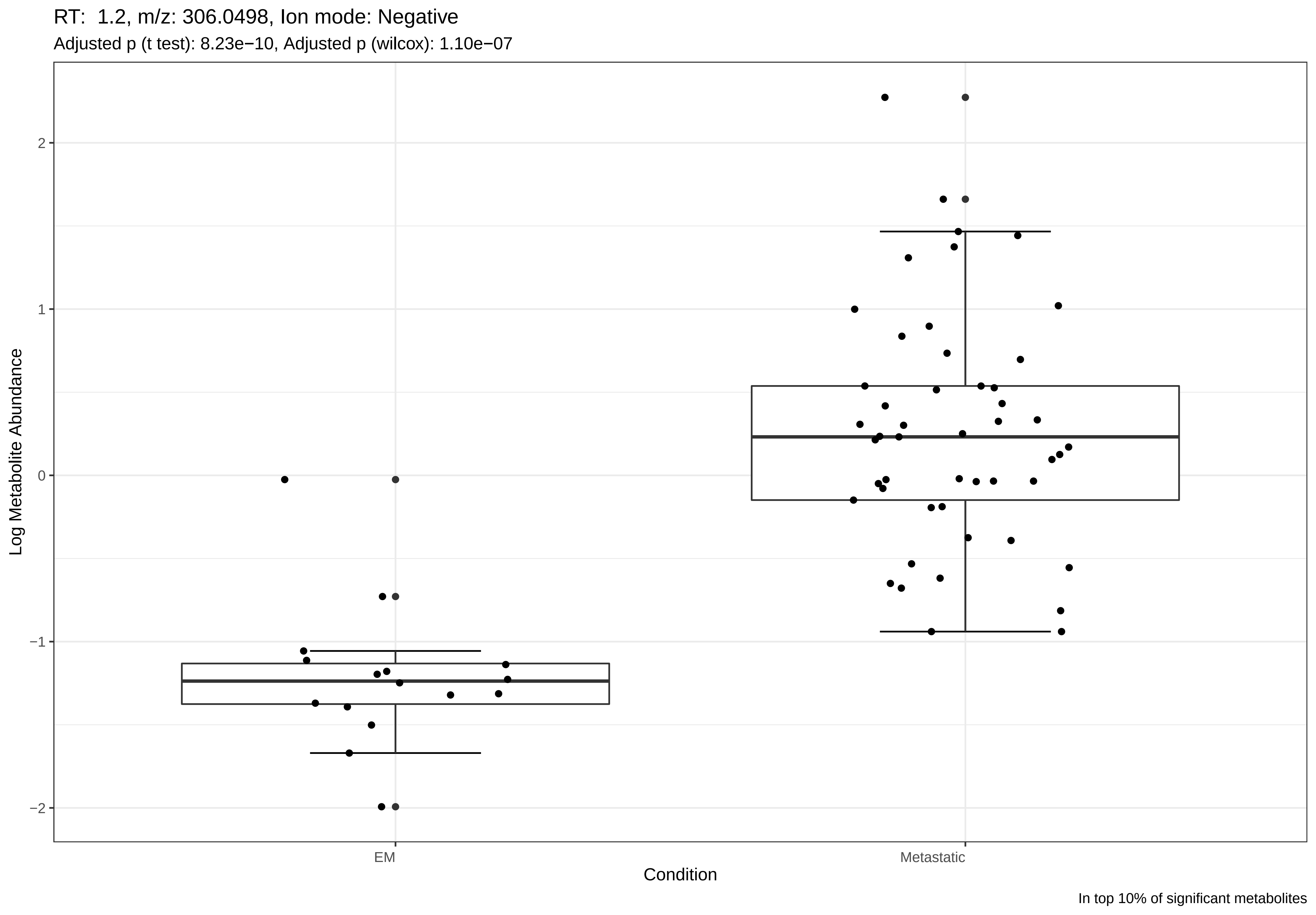
RT: 1.2, m/z: 306.0498, Ion mode: Negative
Adjusted p (t test): 8.23e−10, Adjusted p (wilcox): 1.10e−07
Log Metabolite Abundance
2
1
0
−1
−2
EM
Metastatic
Condition
In top 10% of significant metabolites

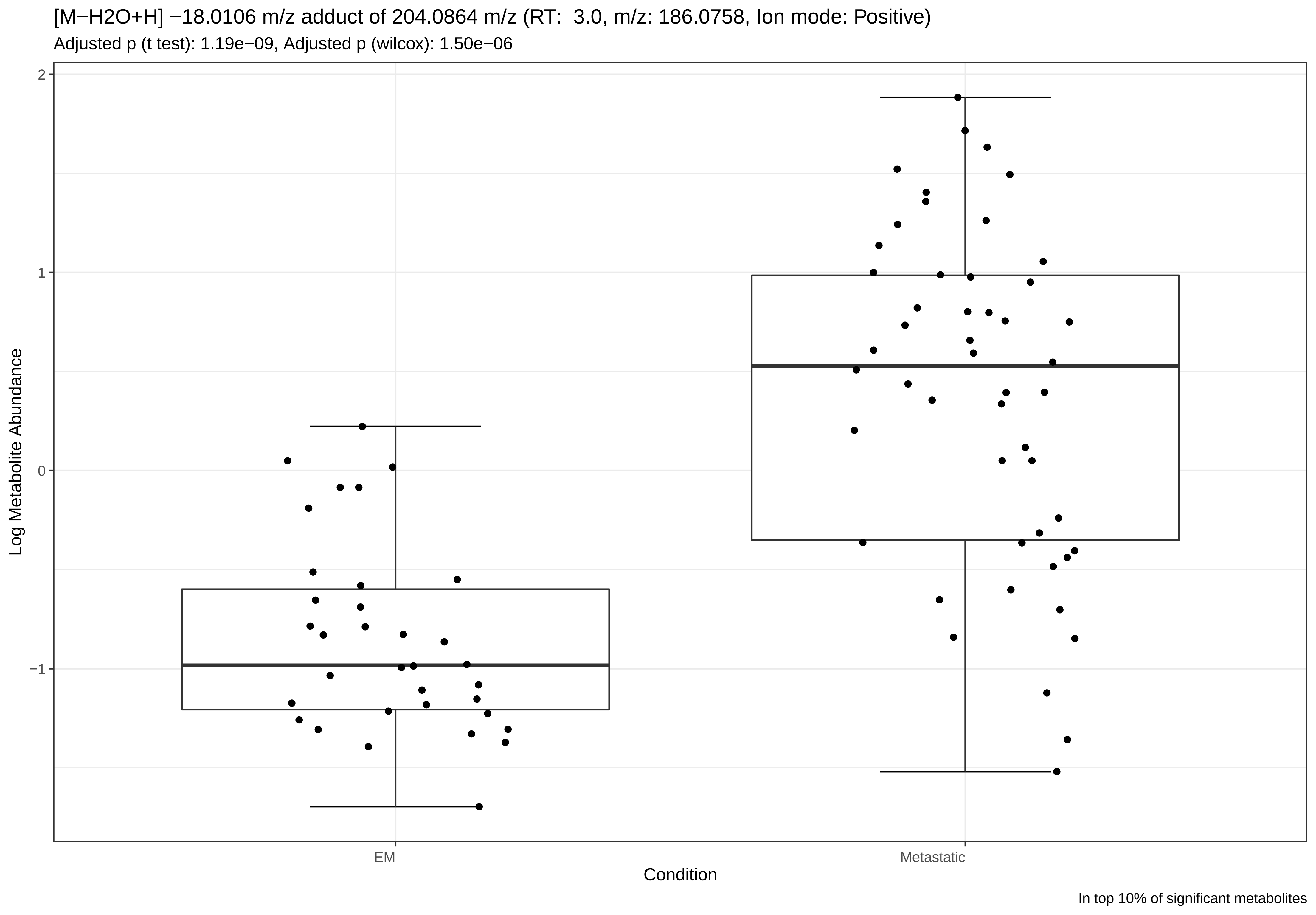
[M−H2O+H] −18.0106 m/z adduct of 204.0864 m/z (RT: 3.0, m/z: 186.0758, Ion mode: Positive)
Adjusted p (t test): 1.19e−09, Adjusted p (wilcox): 1.50e−06
Log Metabolite Abundance
2
1
0
−1
EM
Metastatic
Condition
In top 10% of significant metabolites

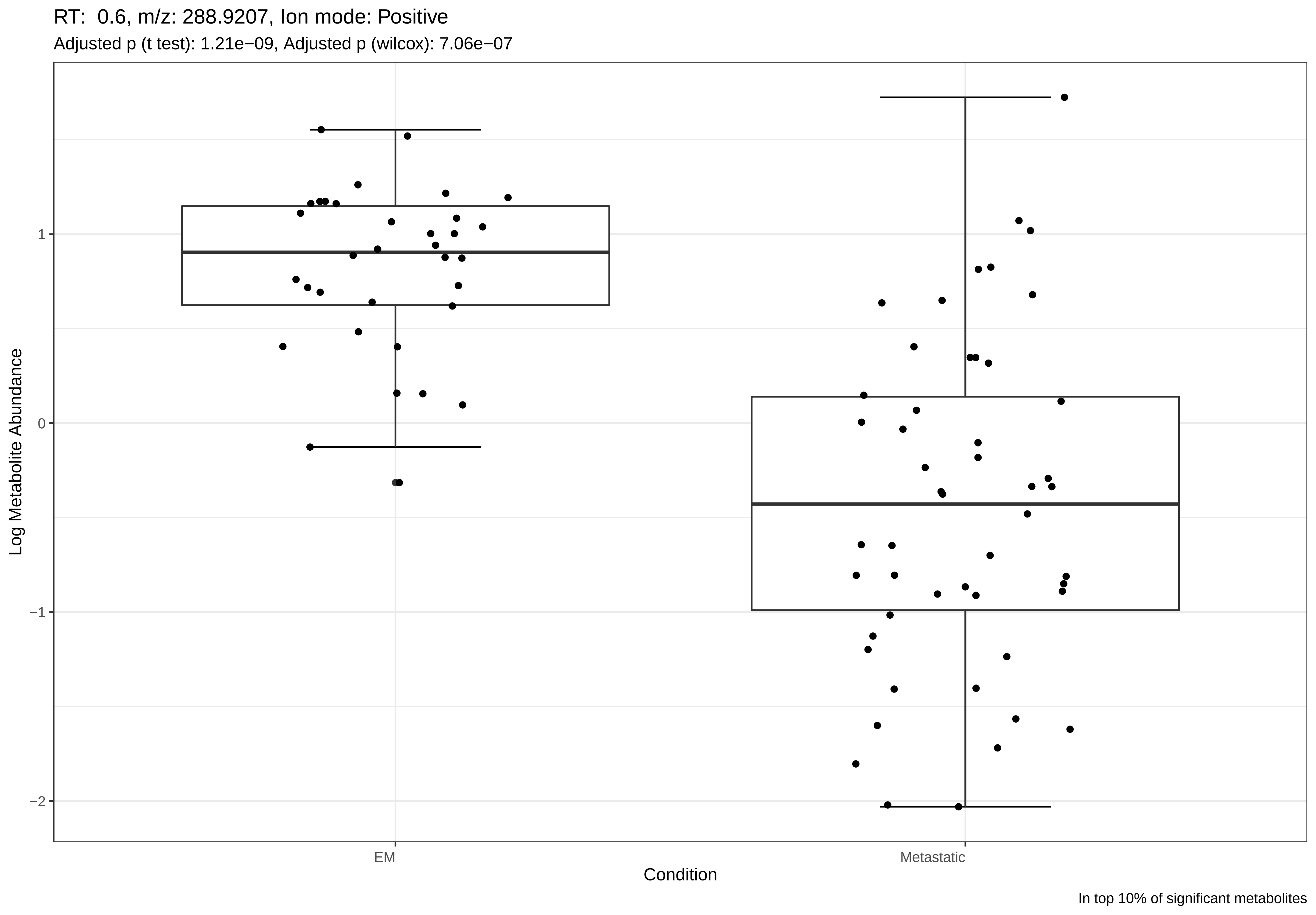

RT: 0.6, m/z: 288.9207, Ion mode: Positive
Adjusted p (t test): 1.21e−09, Adjusted p (wilcox): 7.06e−07
Log Metabolite Abundance
1
0
−1
−2
EM
Metastatic
Condition
In top 10% of significant metabolites

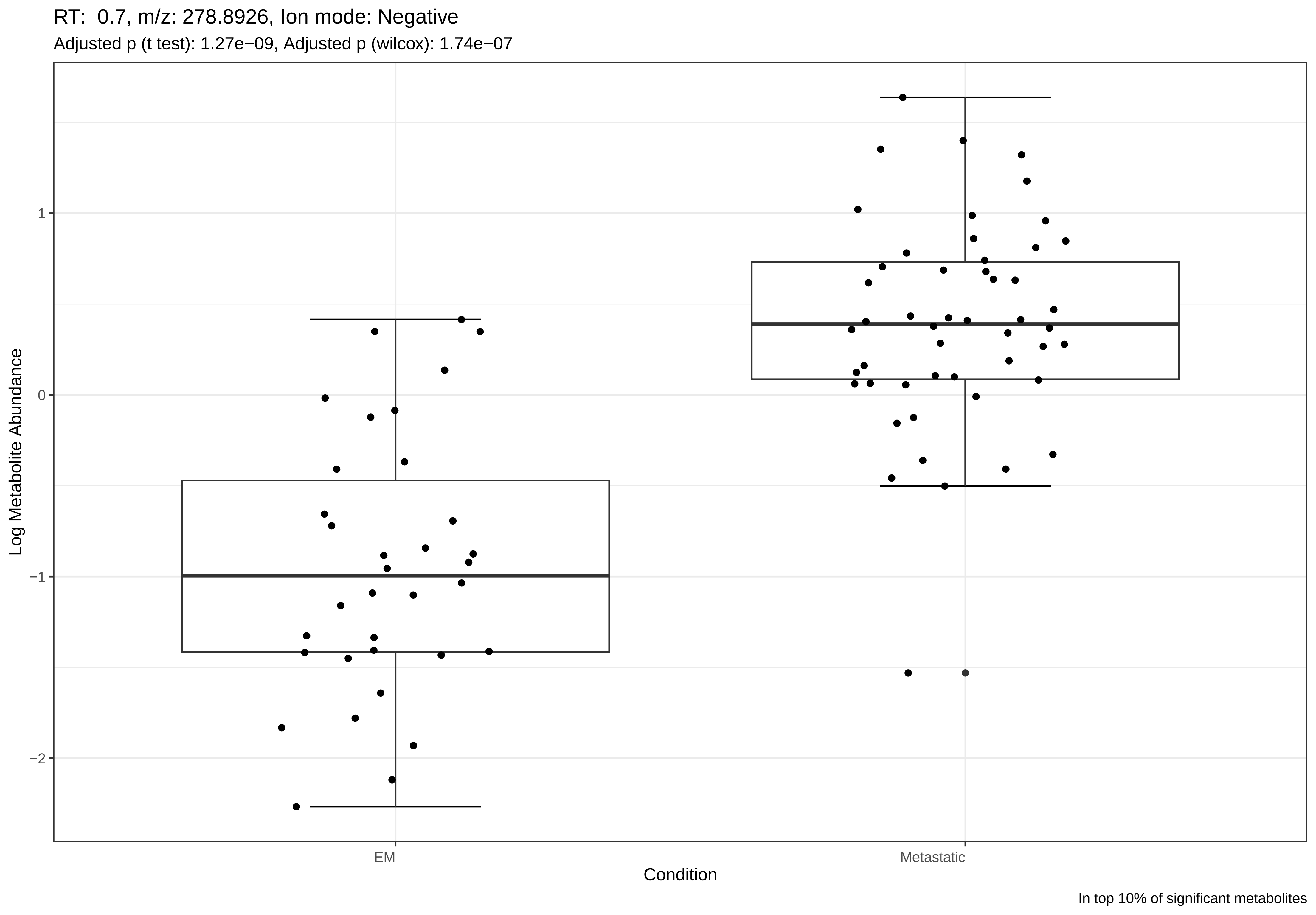

RT: 0.7, m/z: 278.8926, Ion mode: Negative
Adjusted p (t test): 1.27e−09, Adjusted p (wilcox): 1.74e−07
Log Metabolite Abundance
1
0
−1
−2
EM
Metastatic
Condition
In top 10% of significant metabolites

RT: 0.6, m/z: 432.8575, Ion mode: Negative

Adjusted p (t test): 1.36e−09, Adjusted p (wilcox): 1.39e−07

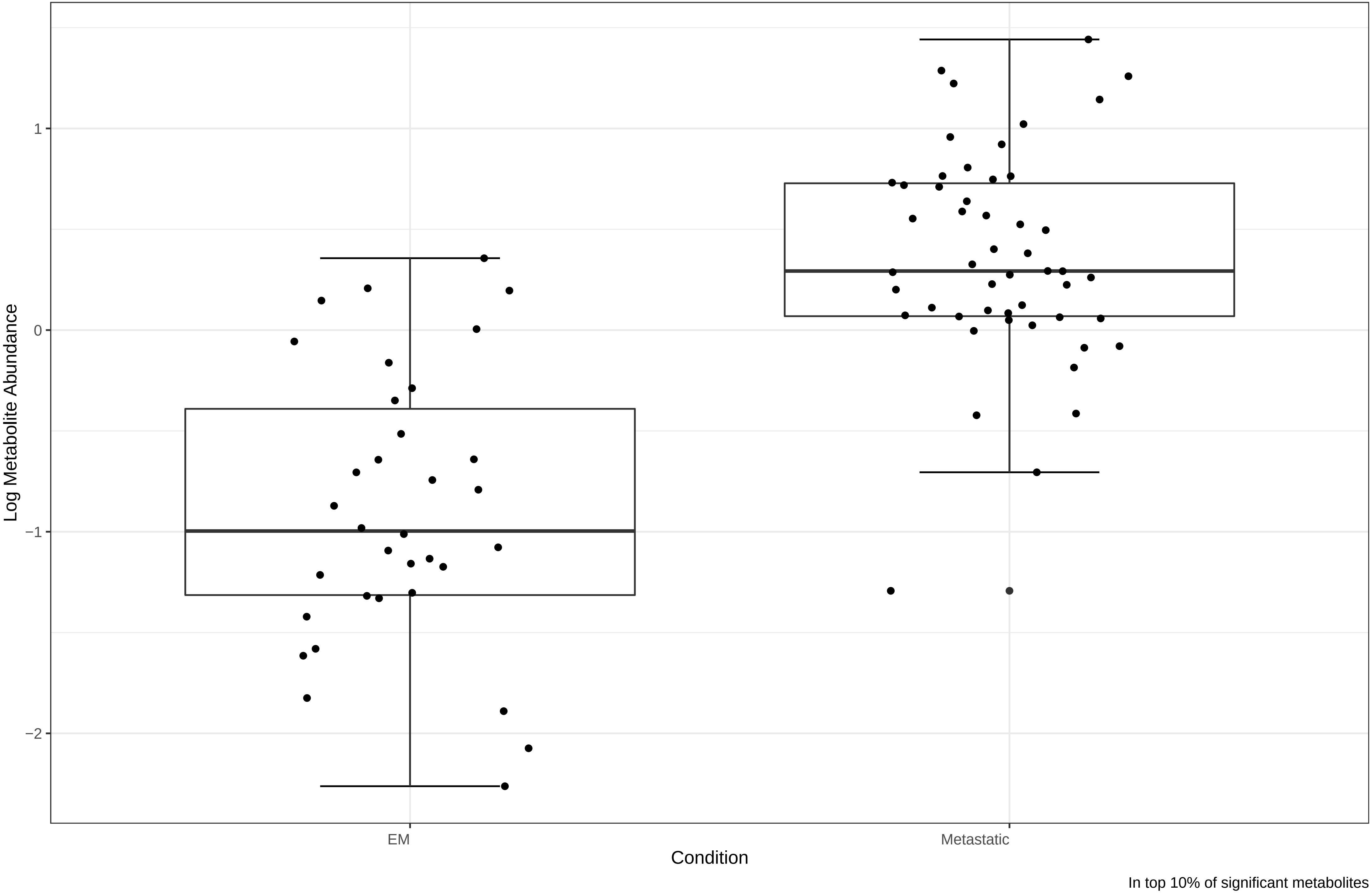

RT: 3.3, m/z: 204.0863, Ion mode: Positive

Adjusted p (t test): 1.41e−09, Adjusted p (wilcox): 1.07e−06

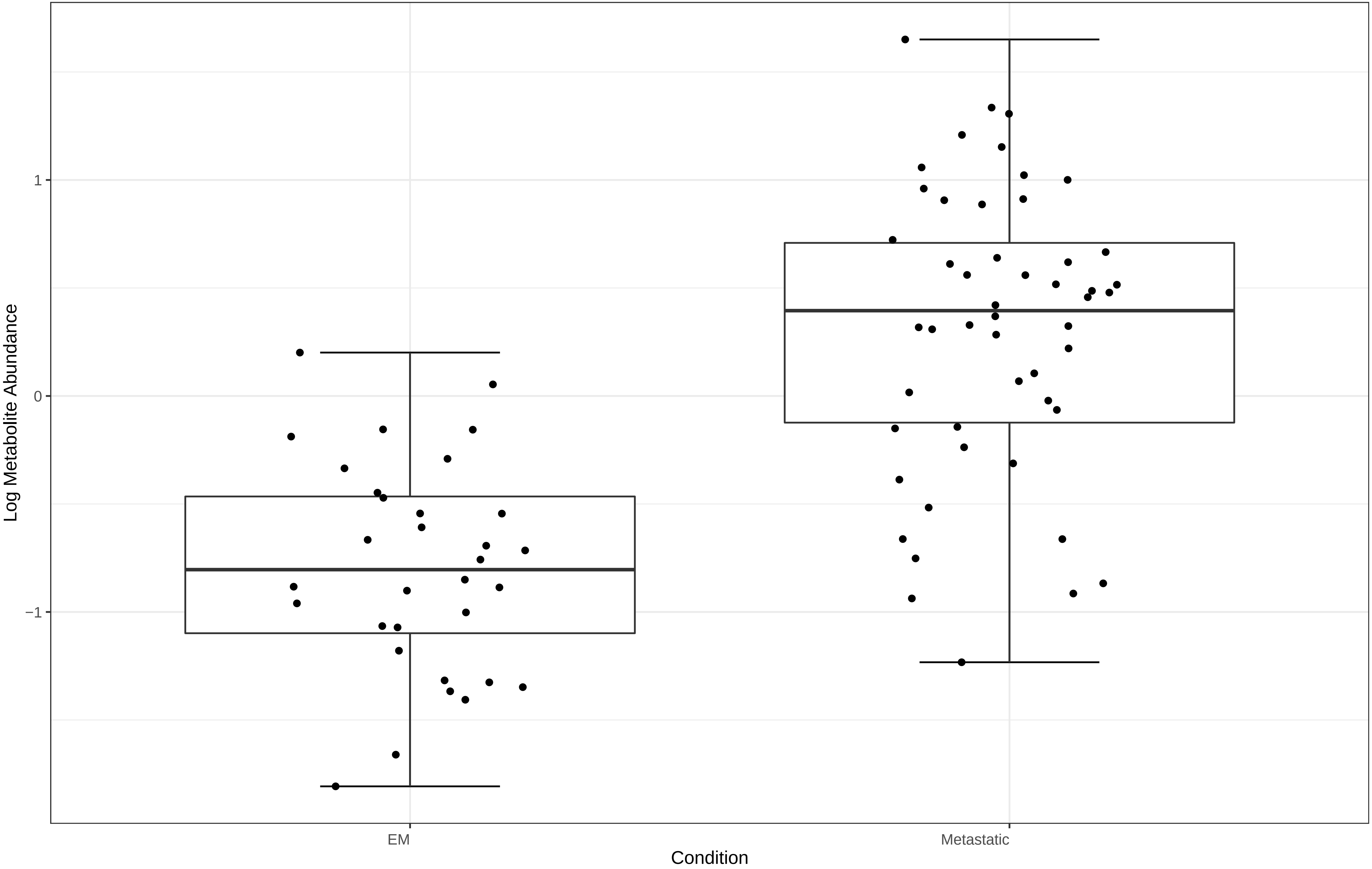

In top 10% of significant metabolites

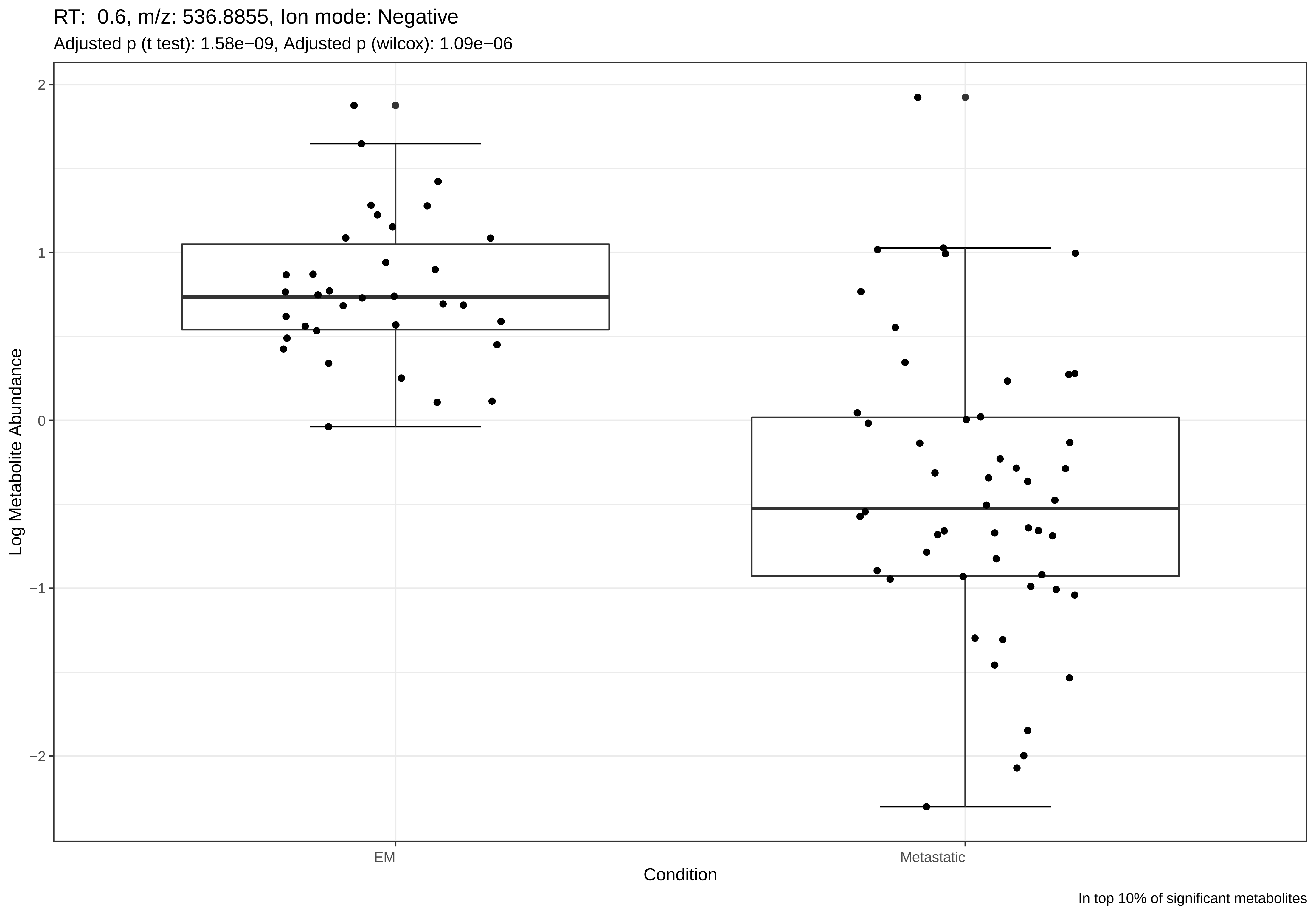
RT: 0.6, m/z: 536.8855, Ion mode: Negative
Adjusted p (t test): 1.58e−09, Adjusted p (wilcox): 1.09e−06
Log Metabolite Abundance
2
1
0
−1
−2
EM
Metastatic
Condition
In top 10% of significant metabolites

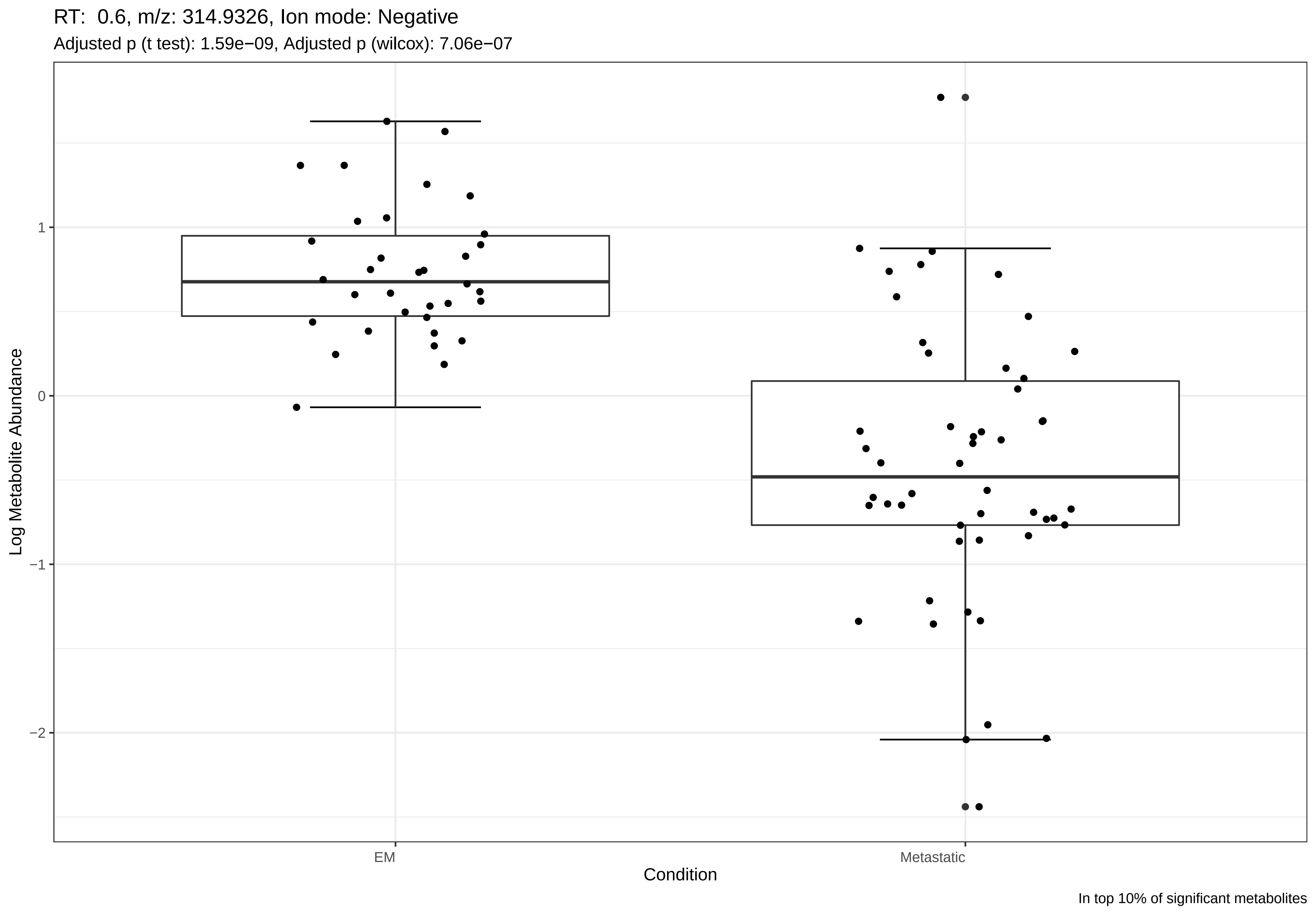
RT: 0.6, m/z: 314.9326, Ion mode: Negative
Adjusted p (t test): 1.59e−09, Adjusted p (wilcox): 7.06e−07
Log Metabolite Abundance
1
0
−1
−2
EM
Metastatic
Condition
In top 10% of significant metabolites

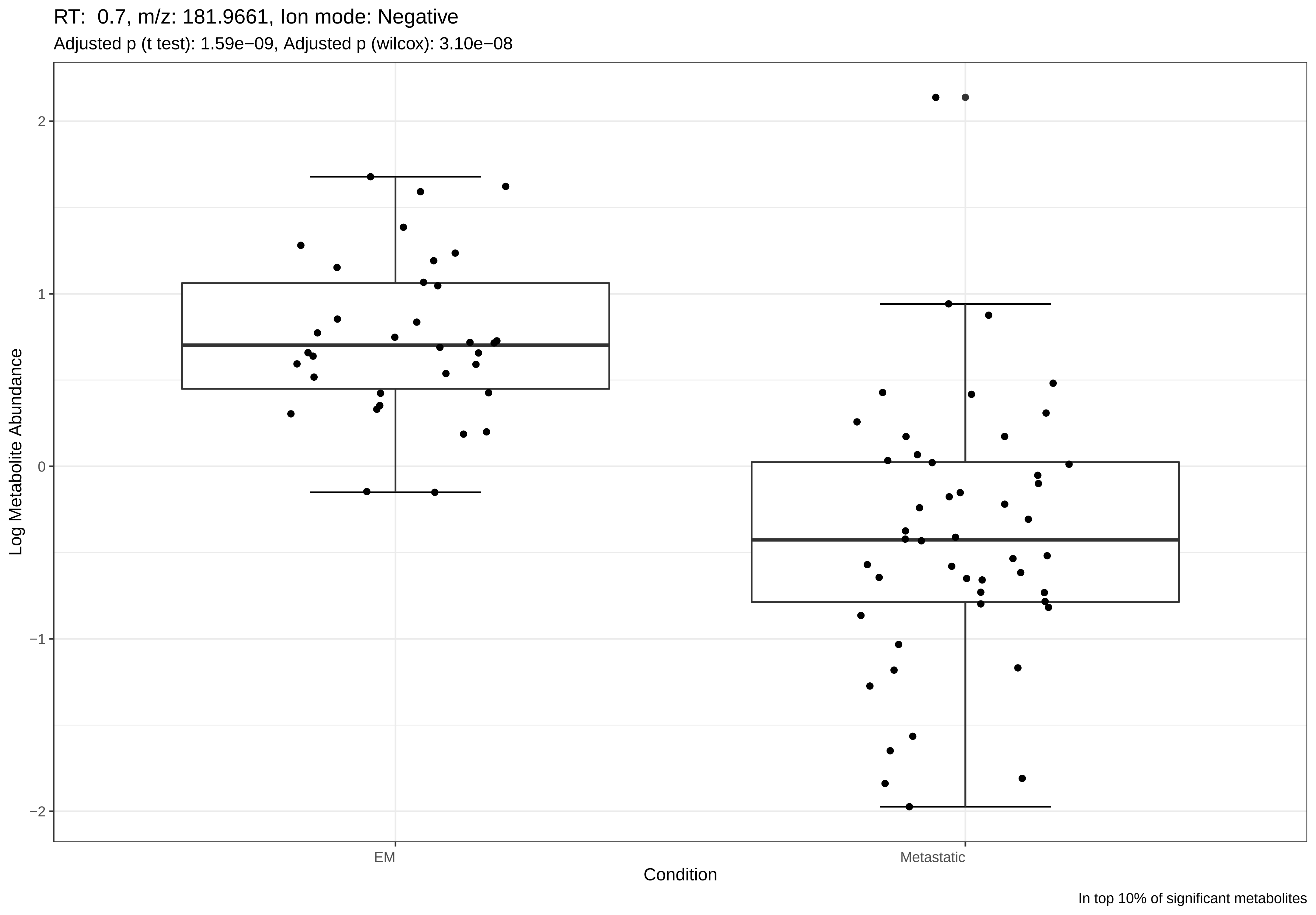
RT: 0.7, m/z: 181.9661, Ion mode: Negative
Adjusted p (t test): 1.59e−09, Adjusted p (wilcox): 3.10e−08
Log Metabolite Abundance
2
1
0
−1
−2
EM
Metastatic
Condition
In top 10% of significant metabolites

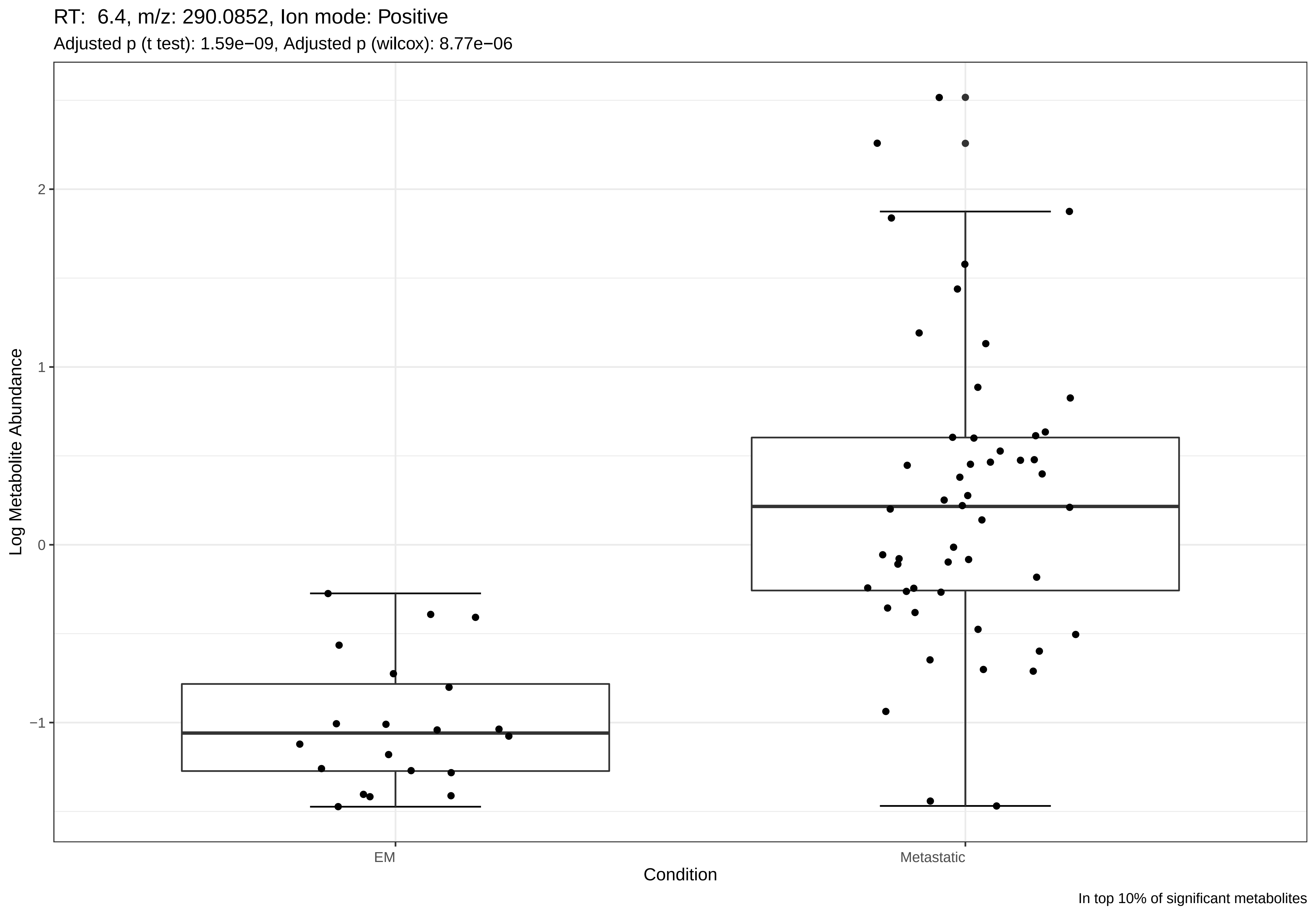
RT: 6.4, m/z: 290.0852, Ion mode: Positive
Adjusted p (t test): 1.59e−09, Adjusted p (wilcox): 8.77e−06
Log Metabolite Abundance
2
1
0
−1
EM
Metastatic
Condition
In top 10% of significant metabolites

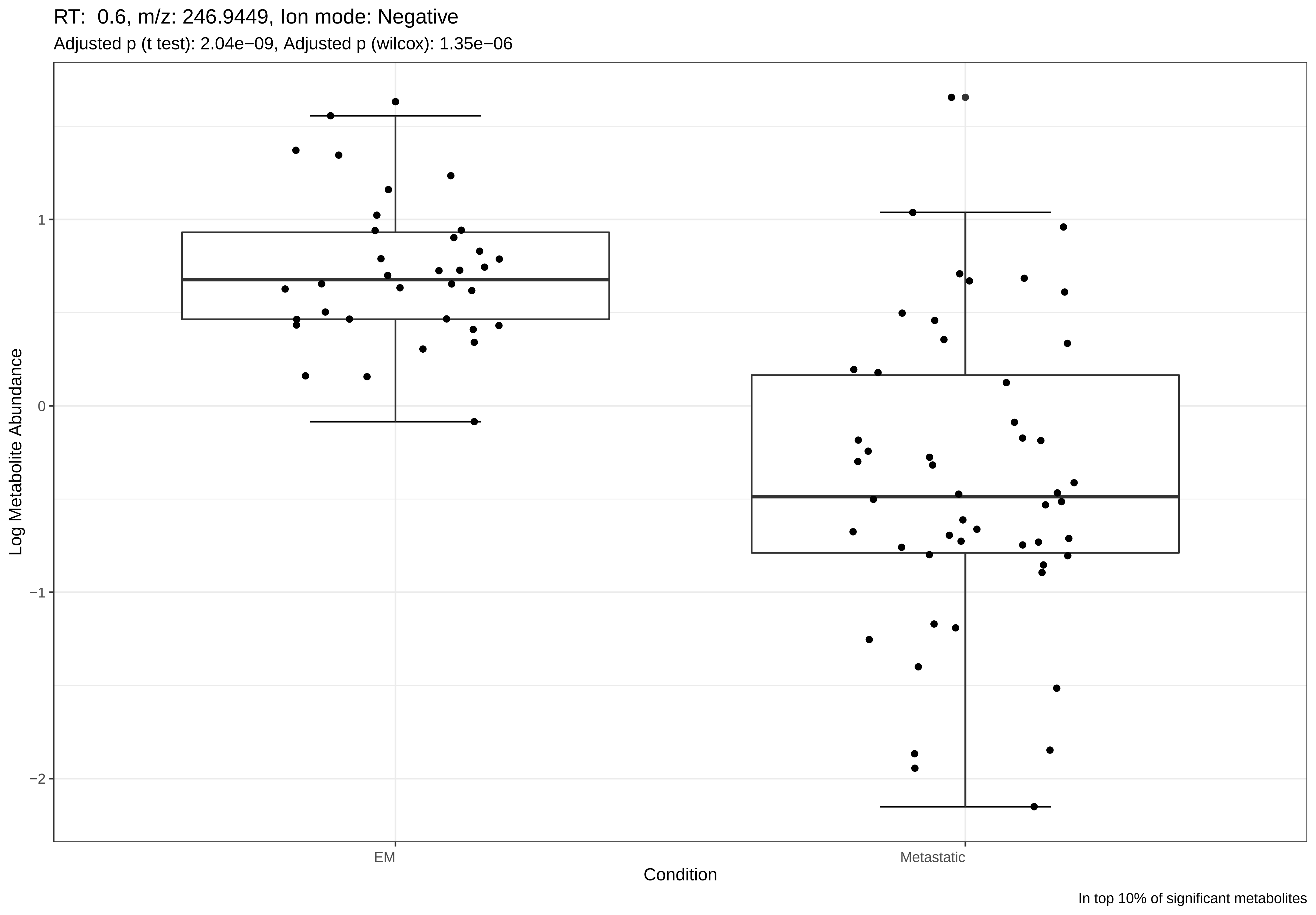
RT: 0.6, m/z: 246.9449, Ion mode: Negative
Adjusted p (t test): 2.04e−09, Adjusted p (wilcox): 1.35e−06
Log Metabolite Abundance
1
0
−1
−2
EM
Metastatic
Condition
In top 10% of significant metabolites

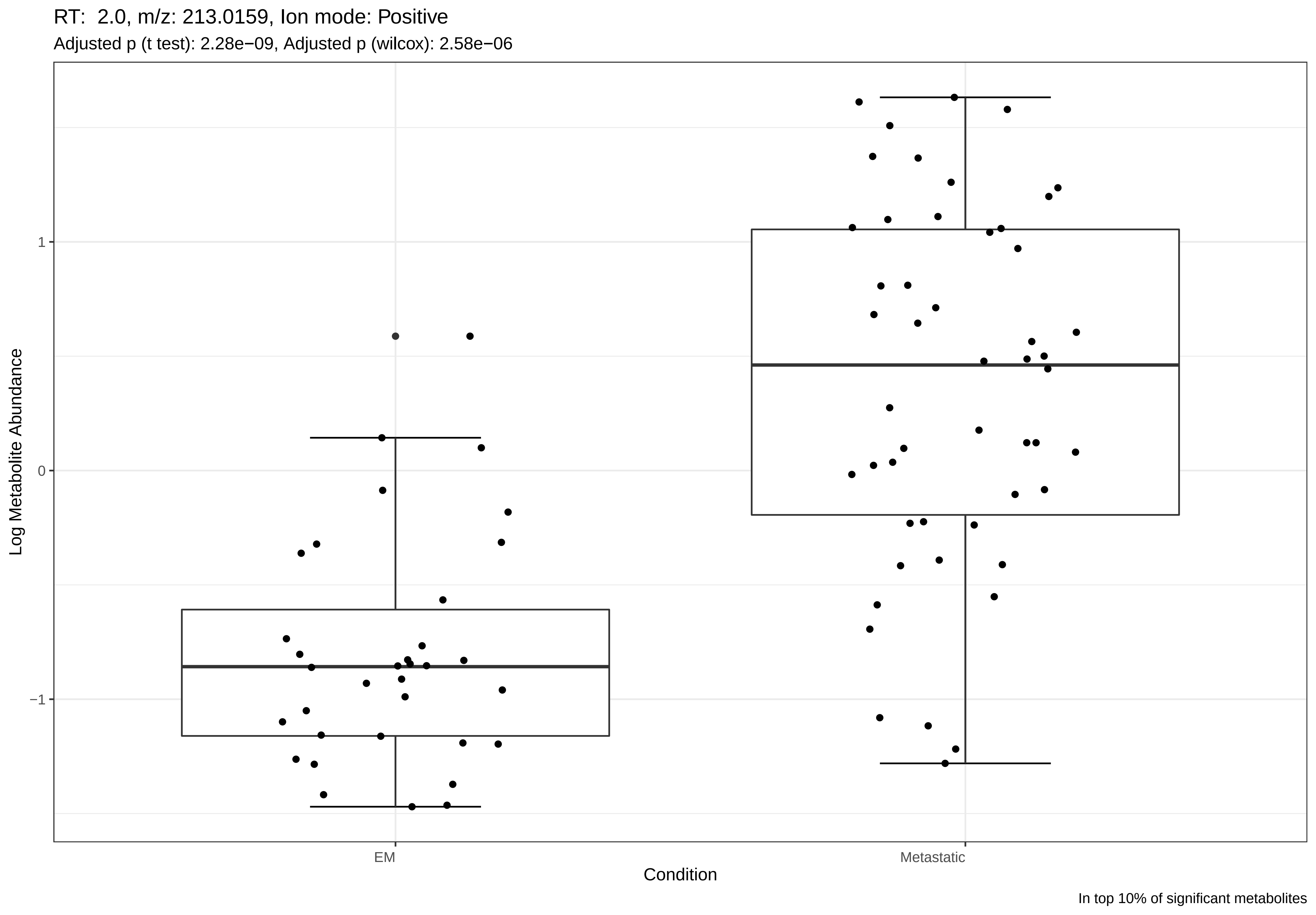

RT: 2.0, m/z: 213.0159, Ion mode: Positive
Adjusted p (t test): 2.28e−09, Adjusted p (wilcox): 2.58e−06
Log Metabolite Abundance
1
0
−1
EM
Metastatic
Condition
In top 10% of significant metabolites

RT: 6.3, m/z: 256.1775, Ion mode: Positive

Adjusted p (t test): 2.63e−09, Adjusted p (wilcox): 5.13e−06

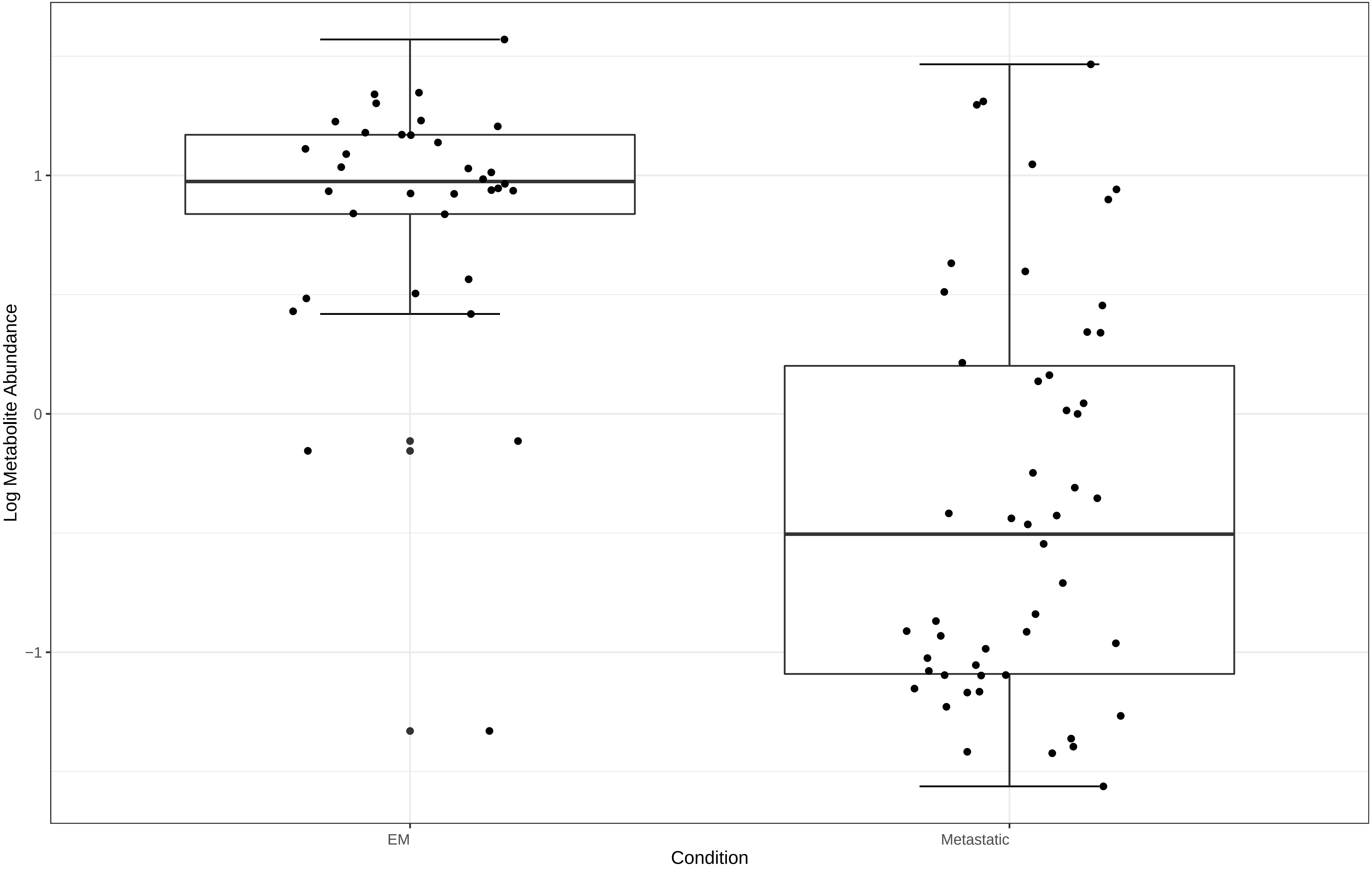

In top 10% of significant metabolites

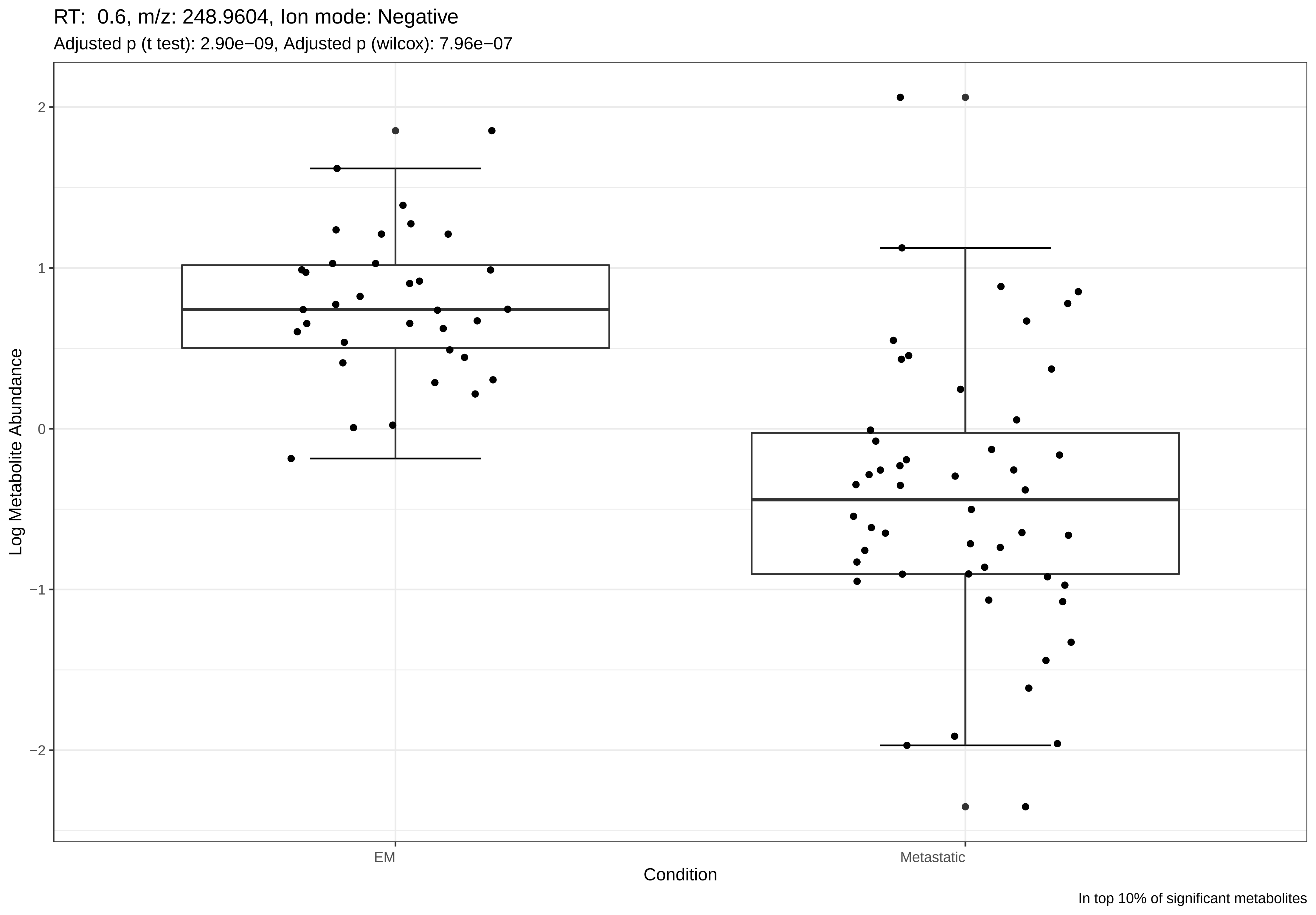
RT: 0.6, m/z: 248.9604, Ion mode: Negative
Adjusted p (t test): 2.90e−09, Adjusted p (wilcox): 7.96e−07
Log Metabolite Abundance
2
1
0
−1
−2
EM
Metastatic
Condition
In top 10% of significant metabolites

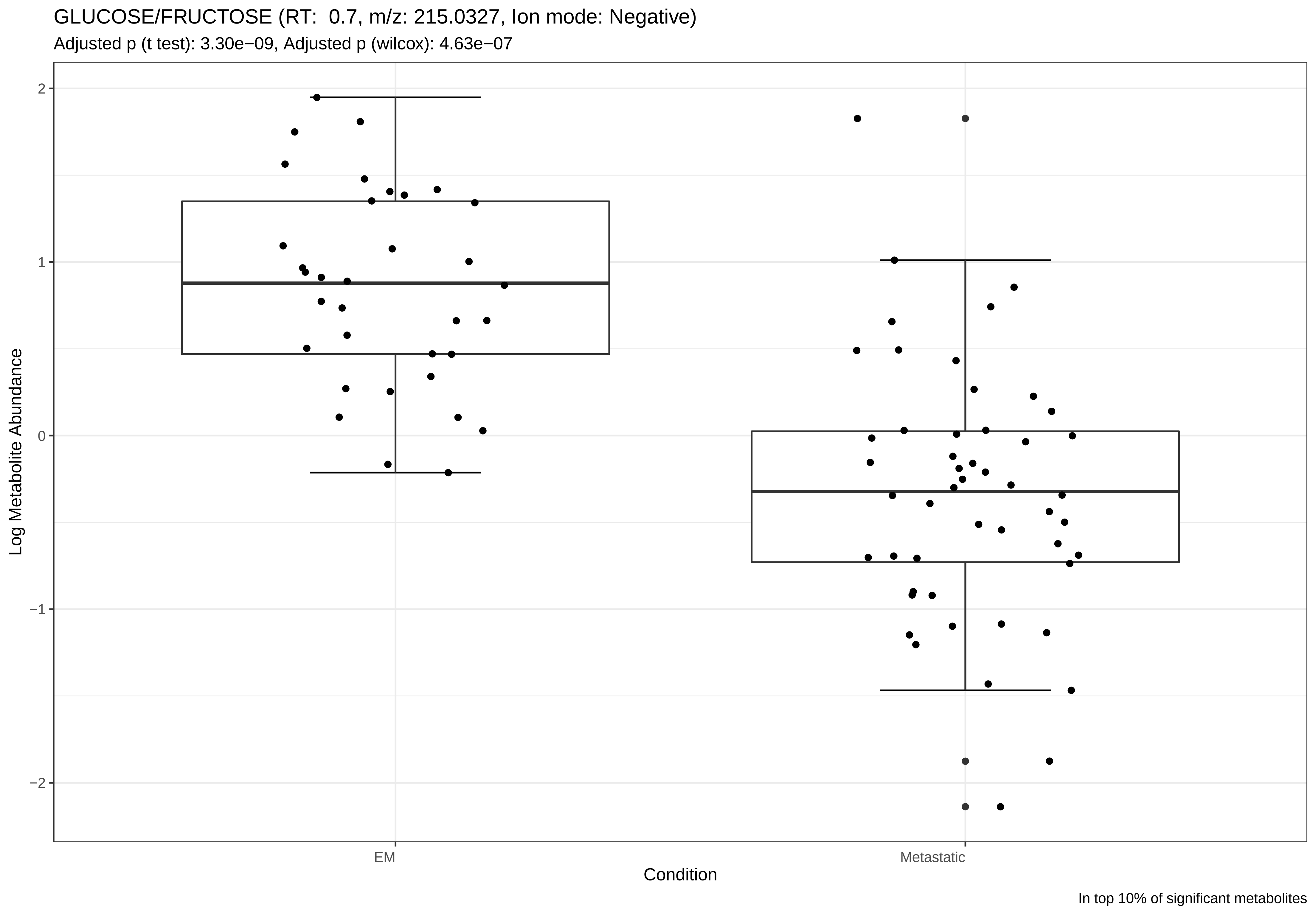
GLUCOSE/FRUCTOSE (RT: 0.7, m/z: 215.0327, Ion mode: Negative)
Adjusted p (t test): 3.30e−09, Adjusted p (wilcox): 4.63e−07
Log Metabolite Abundance
2
1
0
−1
−2
EM
Metastatic
Condition
In top 10% of significant metabolites

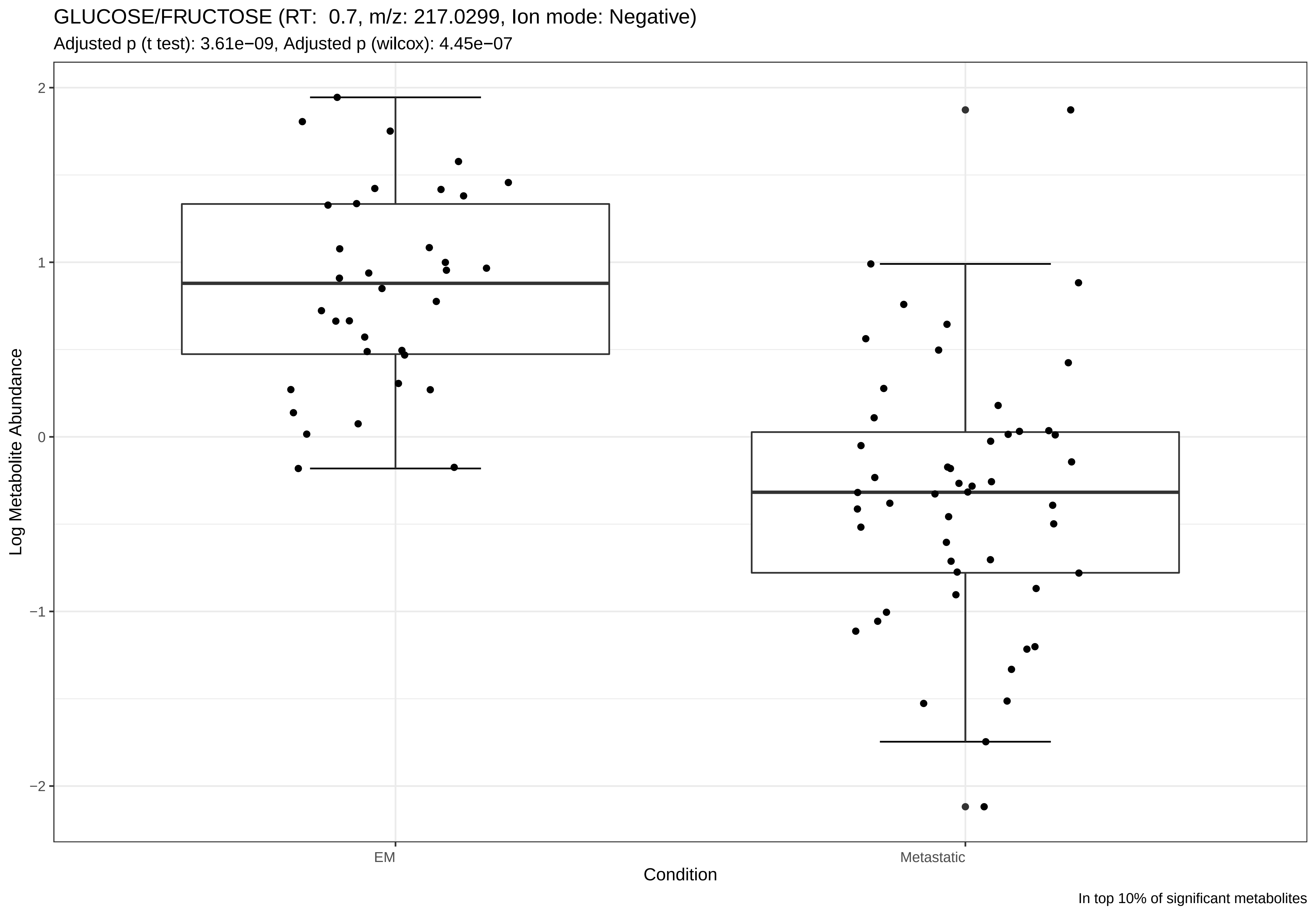

GLUCOSE/FRUCTOSE (RT: 0.7, m/z: 217.0299, Ion mode: Negative)
Adjusted p (t test): 3.61e−09, Adjusted p (wilcox): 4.45e−07
Log Metabolite Abundance
2
1
0
−1
−2
EM
Metastatic
Condition
In top 10% of significant metabolites

RT: 0.9, m/z: 577.0958, Ion mode: Negative

Adjusted p (t test): 3.61e−09, Adjusted p (wilcox): 2.09e−07

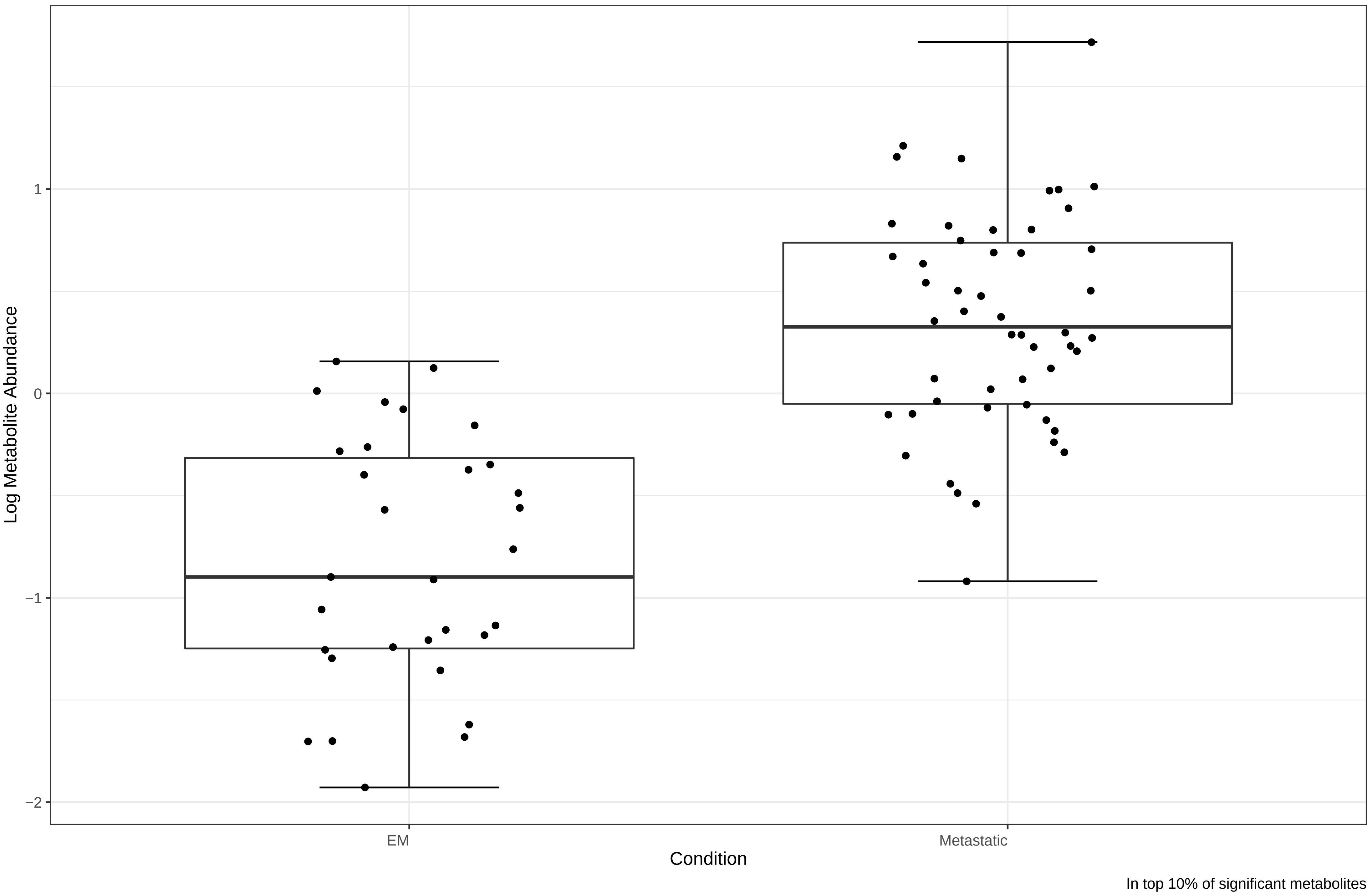

RT: 0.7, m/z: 92.9278, Ion mode: Negative

Adjusted p (t test): 4.91e−09, Adjusted p (wilcox): 3.90e−07

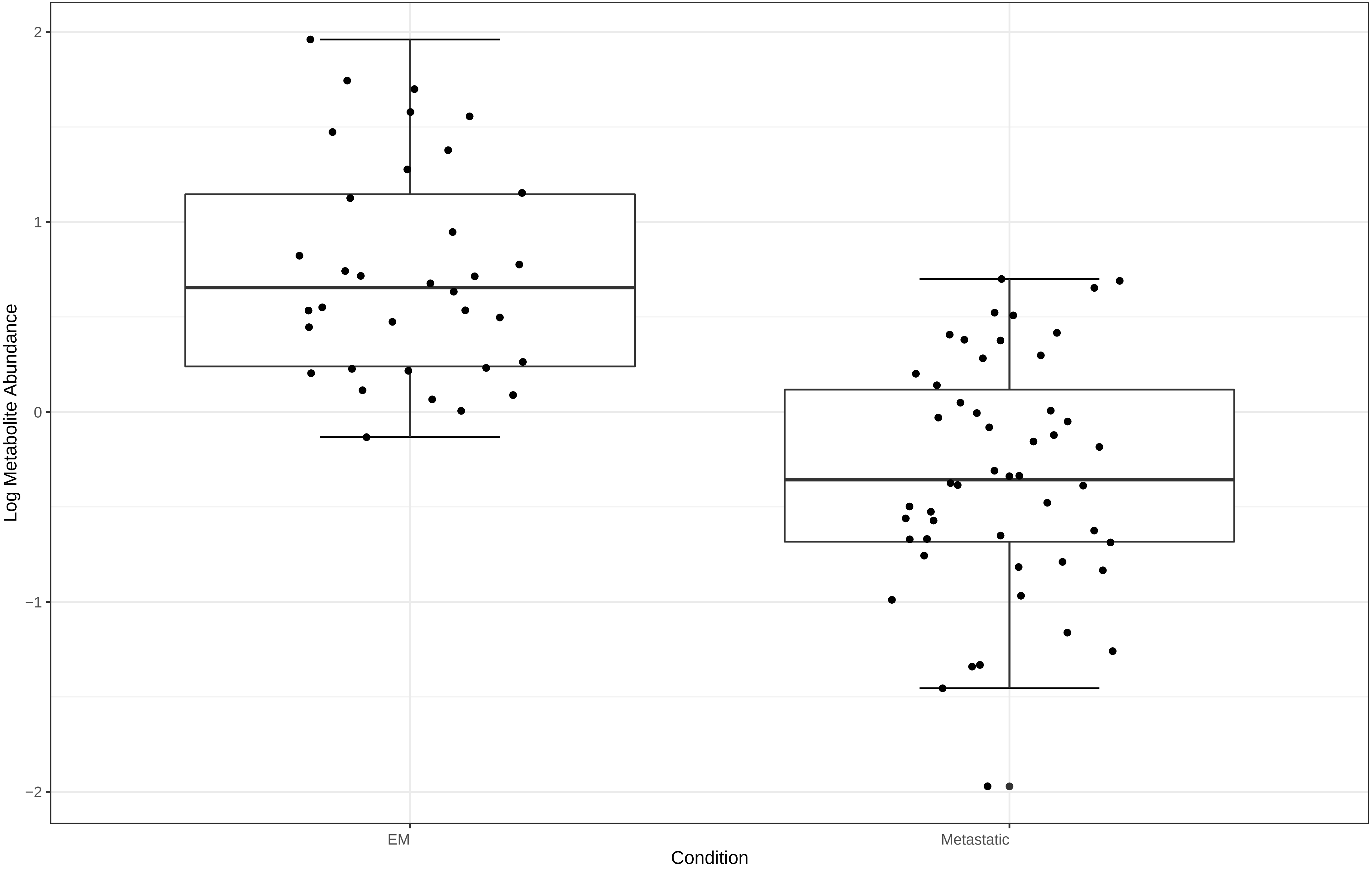

In top 10% of significant metabolites

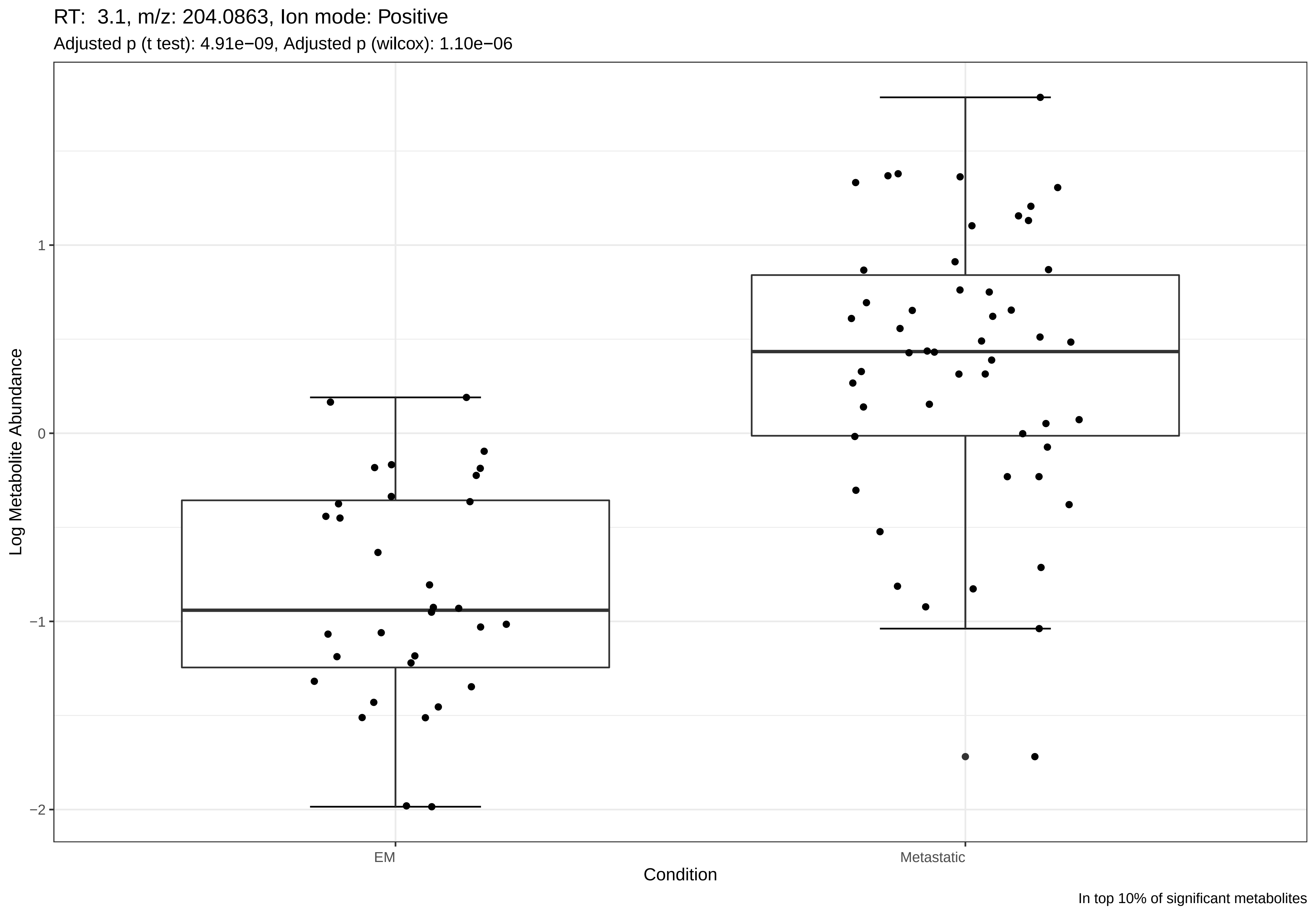

RT: 3.1, m/z: 204.0863, Ion mode: Positive
Adjusted p (t test): 4.91e−09, Adjusted p (wilcox): 1.10e−06
Log Metabolite Abundance
1
0
−1
−2
EM
Metastatic
Condition
In top 10% of significant metabolites

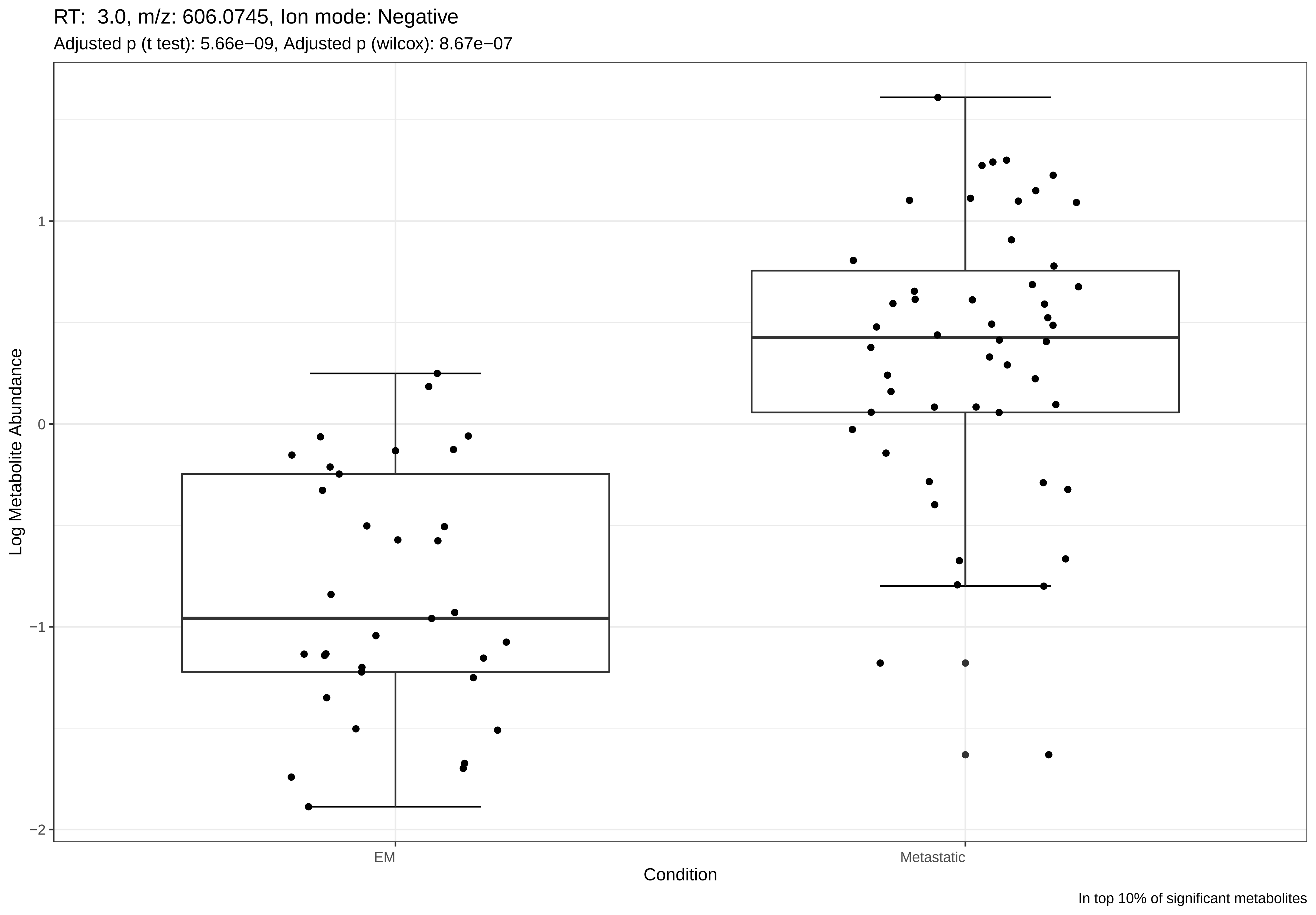
RT: 3.0, m/z: 606.0745, Ion mode: Negative
Adjusted p (t test): 5.66e−09, Adjusted p (wilcox): 8.67e−07
Log Metabolite Abundance
1
0
−1
−2
EM
Metastatic
Condition
In top 10% of significant metabolites

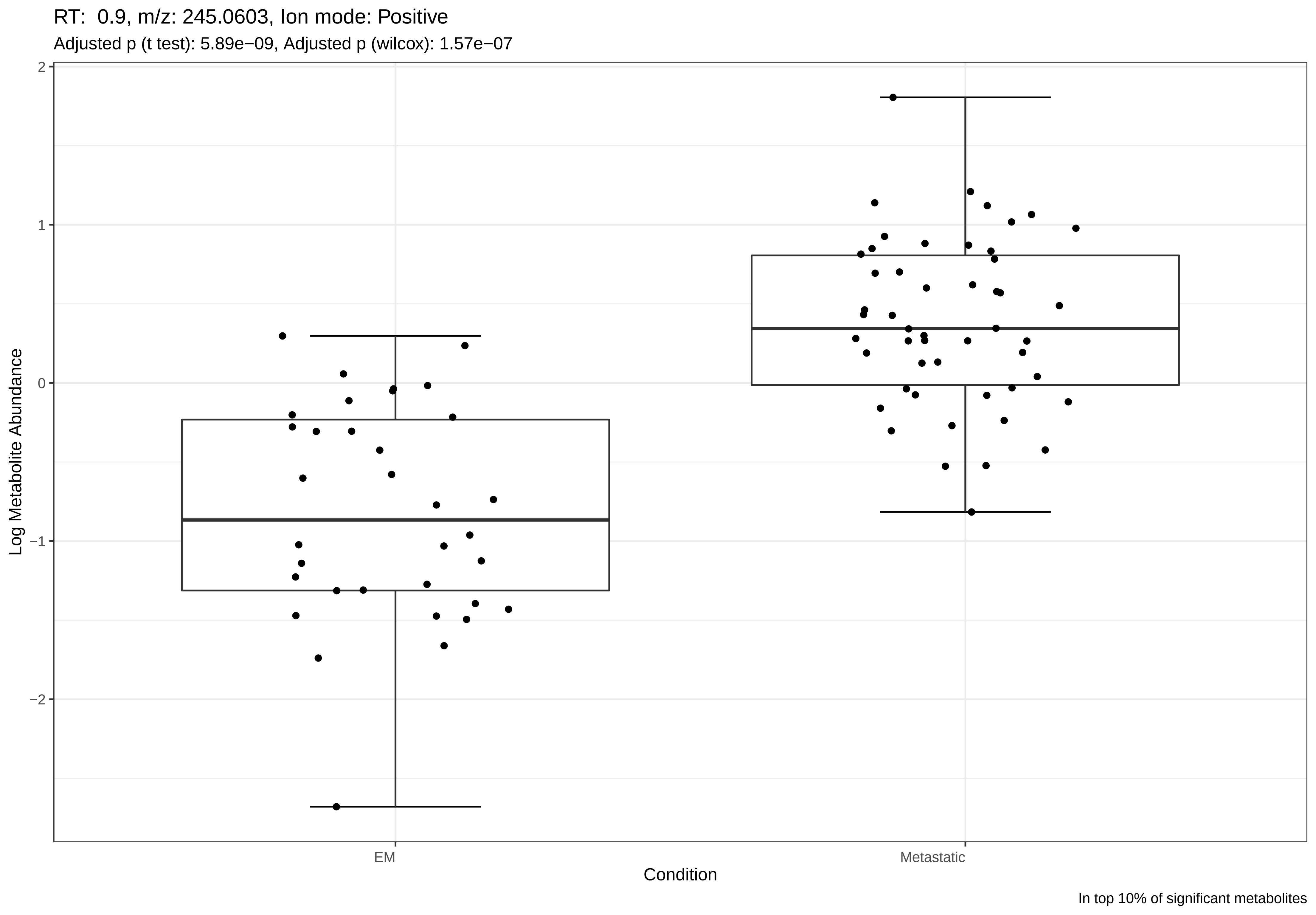

RT: 0.9, m/z: 245.0603, Ion mode: Positive
Adjusted p (t test): 5.89e−09, Adjusted p (wilcox): 1.57e−07
Log Metabolite Abundance
2
1
0
−1
−2
EM
Metastatic
Condition
In top 10% of significant metabolites

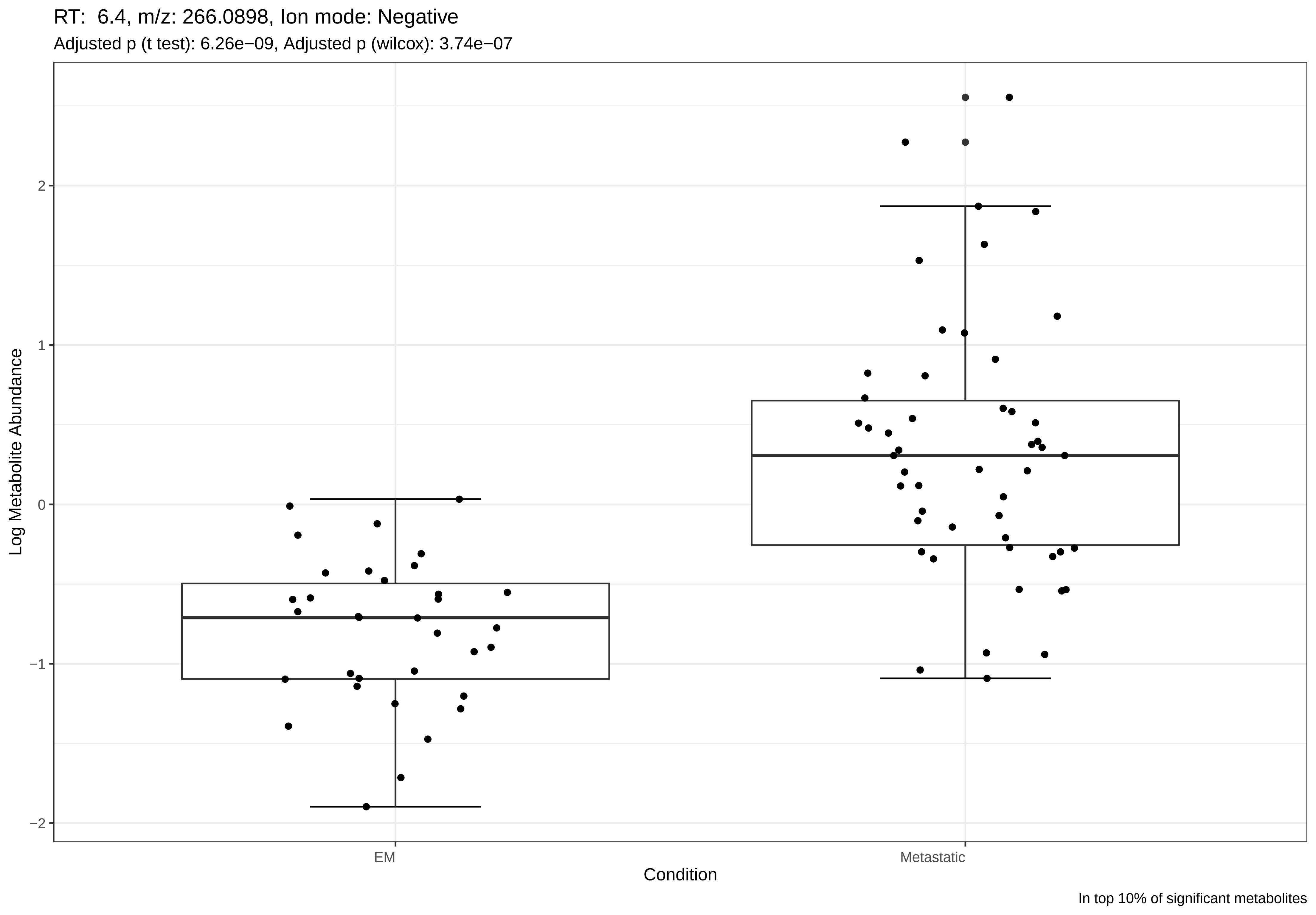

RT: 6.4, m/z: 266.0898, Ion mode: Negative
Adjusted p (t test): 6.26e−09, Adjusted p (wilcox): 3.74e−07
Log Metabolite Abundance
2
1
0
−1
−2
EM
Metastatic
Condition
In top 10% of significant metabolites

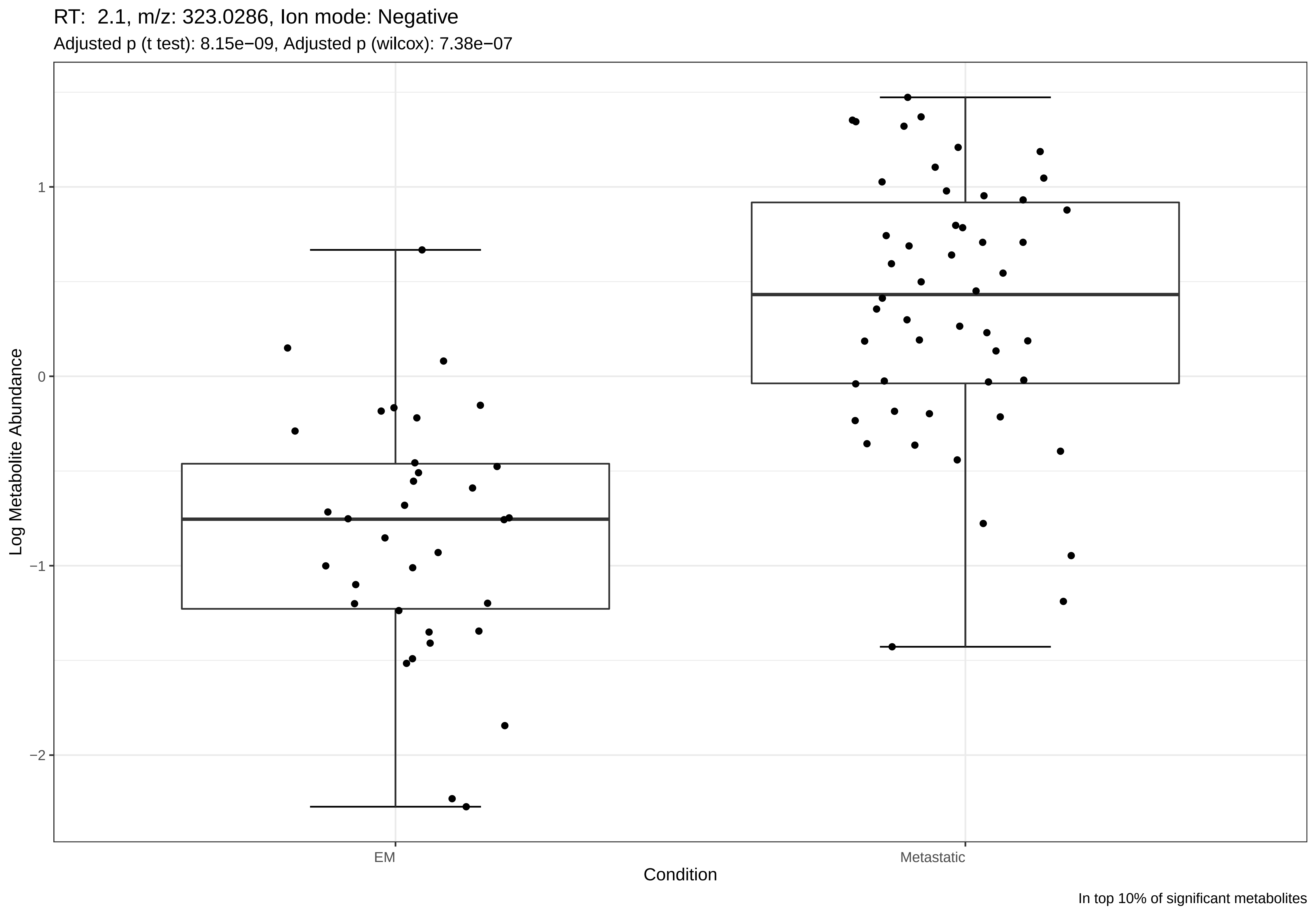
RT: 2.1, m/z: 323.0286, Ion mode: Negative
Adjusted p (t test): 8.15e−09, Adjusted p (wilcox): 7.38e−07
Log Metabolite Abundance
1
0
−1
−2
EM
Metastatic
Condition
In top 10% of significant metabolites

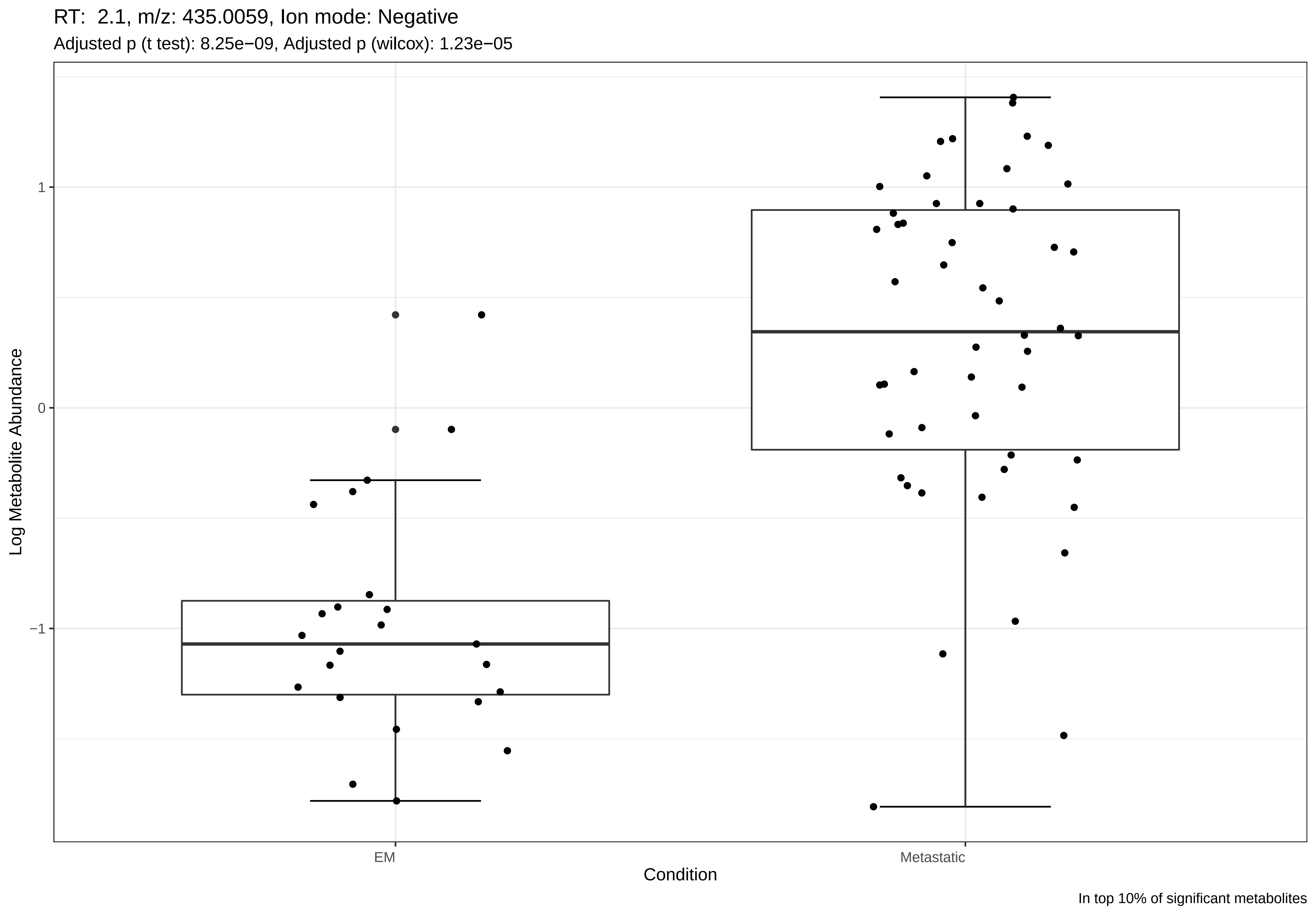

RT: 2.1, m/z: 435.0059, Ion mode: Negative
Adjusted p (t test): 8.25e−09, Adjusted p (wilcox): 1.23e−05
Log Metabolite Abundance
1
0
−1
EM
Metastatic
Condition
In top 10% of significant metabolites

RT: 2.0, m/z: 347.0240, Ion mode: Positive

Adjusted p (t test): 8.25e−09, Adjusted p (wilcox): 8.91e−06

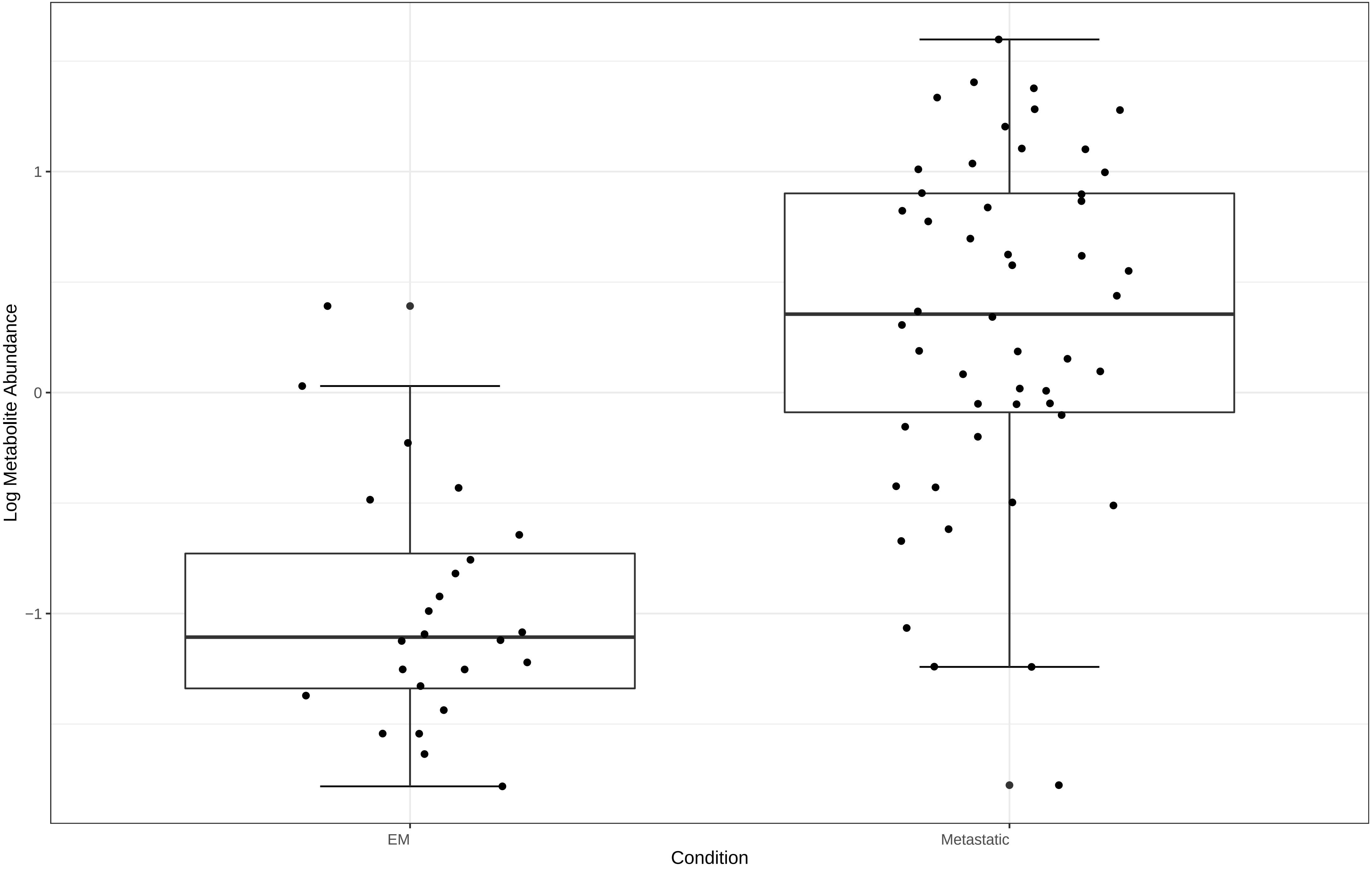

In top 10% of significant metabolites

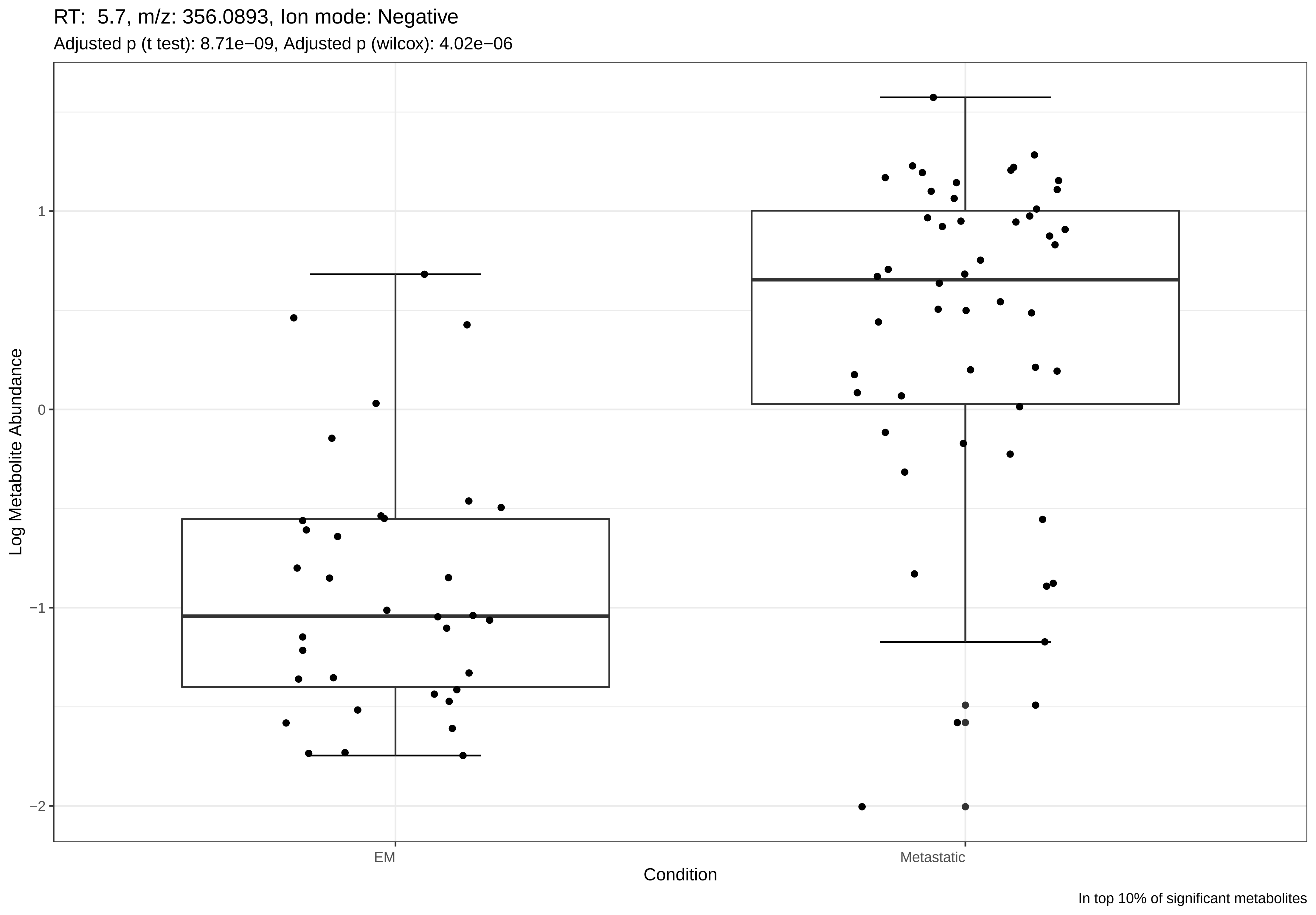

RT: 5.7, m/z: 356.0893, Ion mode: Negative
Adjusted p (t test): 8.71e−09, Adjusted p (wilcox): 4.02e−06
Log Metabolite Abundance
1
0
−1
−2
EM
Metastatic
Condition
In top 10% of significant metabolites

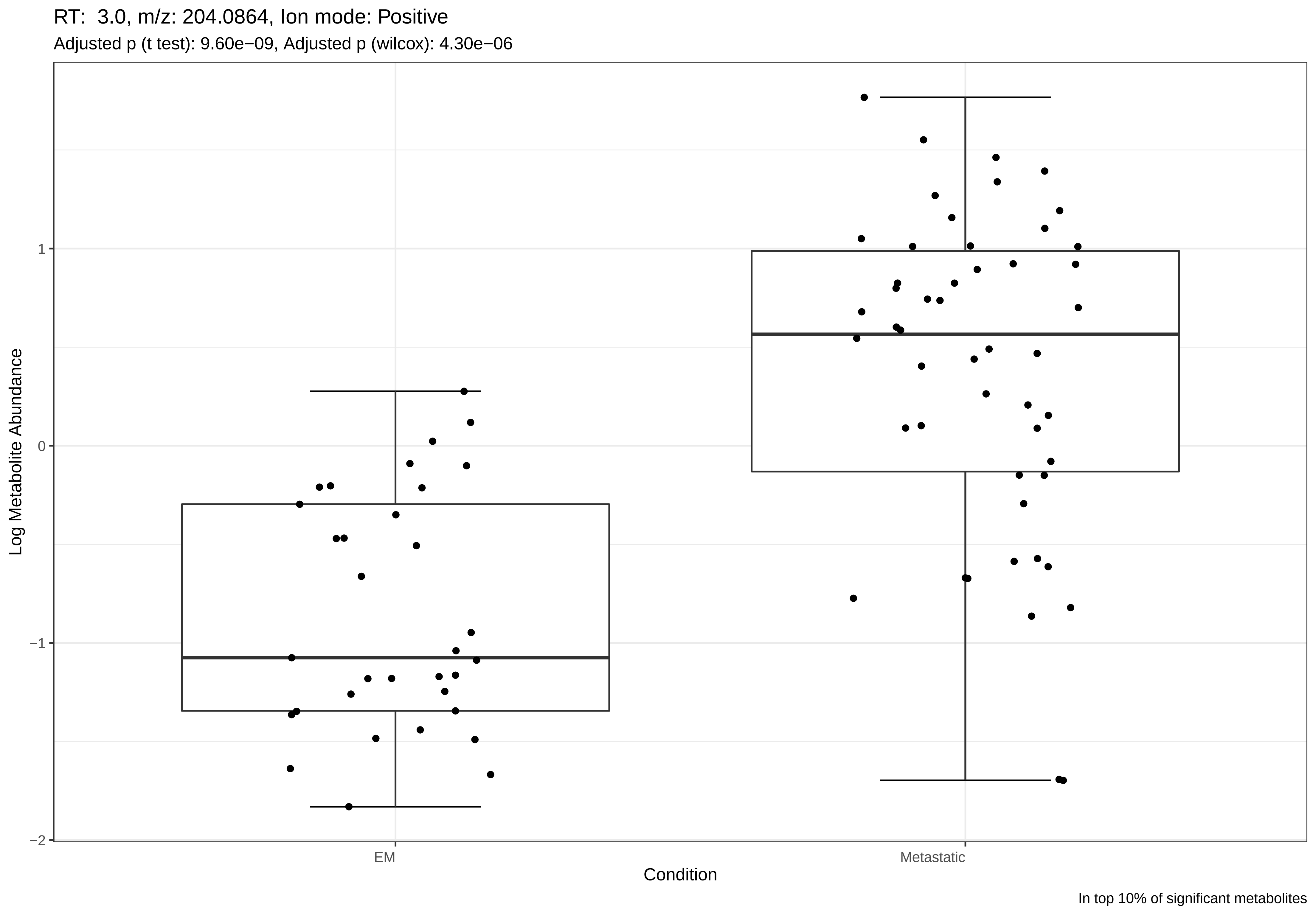
RT: 3.0, m/z: 204.0864, Ion mode: Positive
Adjusted p (t test): 9.60e−09, Adjusted p (wilcox): 4.30e−06
Log Metabolite Abundance
1
0
−1
−2
EM
Metastatic
Condition
In top 10% of significant metabolites

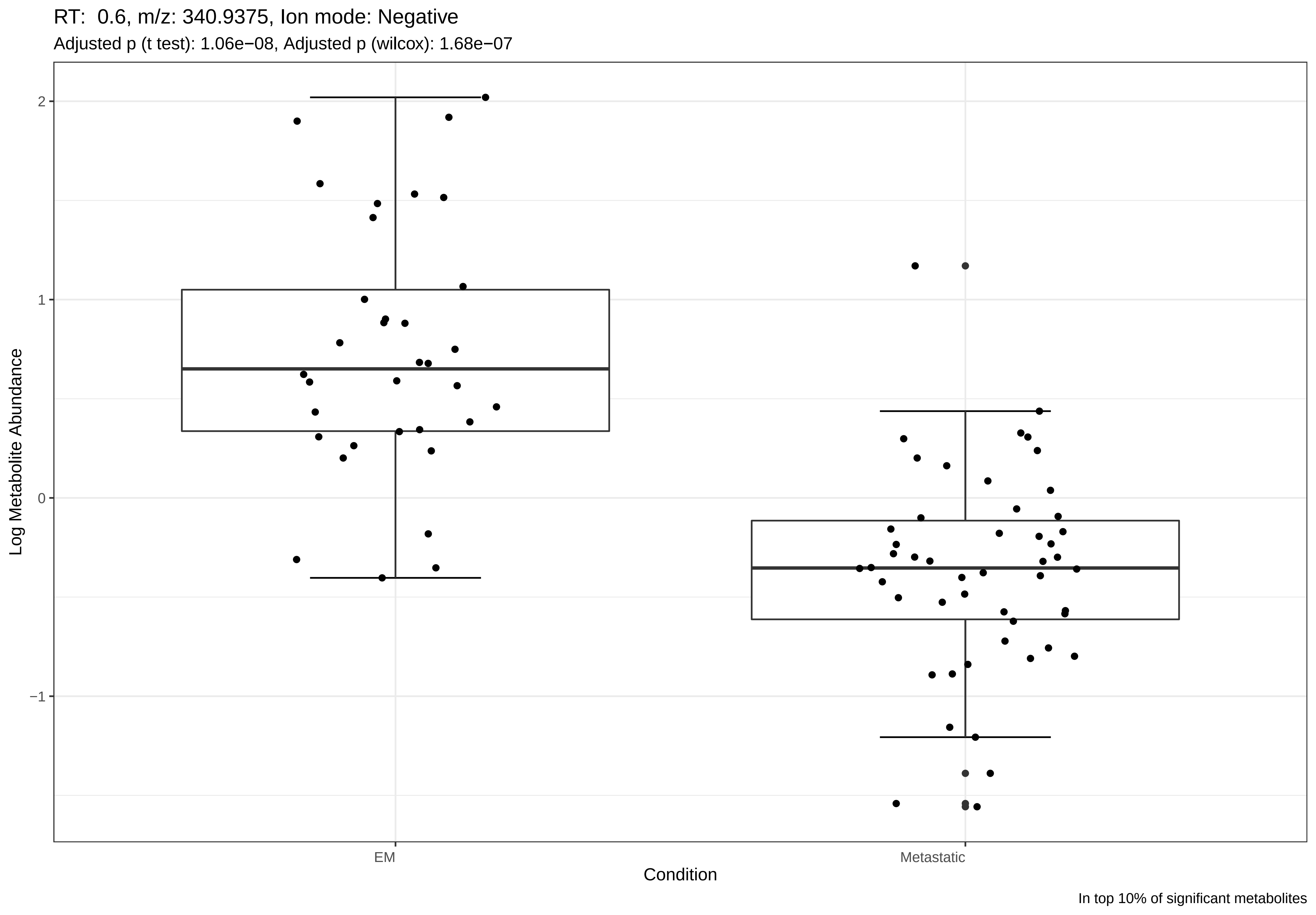
RT: 0.6, m/z: 340.9375, Ion mode: Negative
Adjusted p (t test): 1.06e−08, Adjusted p (wilcox): 1.68e−07
Log Metabolite Abundance
2
1
0
−1
EM
Metastatic
Condition
In top 10% of significant metabolites

RT: 2.0, m/z: 332.0589, Ion mode: Negative

Adjusted p (t test): 1.06e−08, Adjusted p (wilcox): 7.39e−06

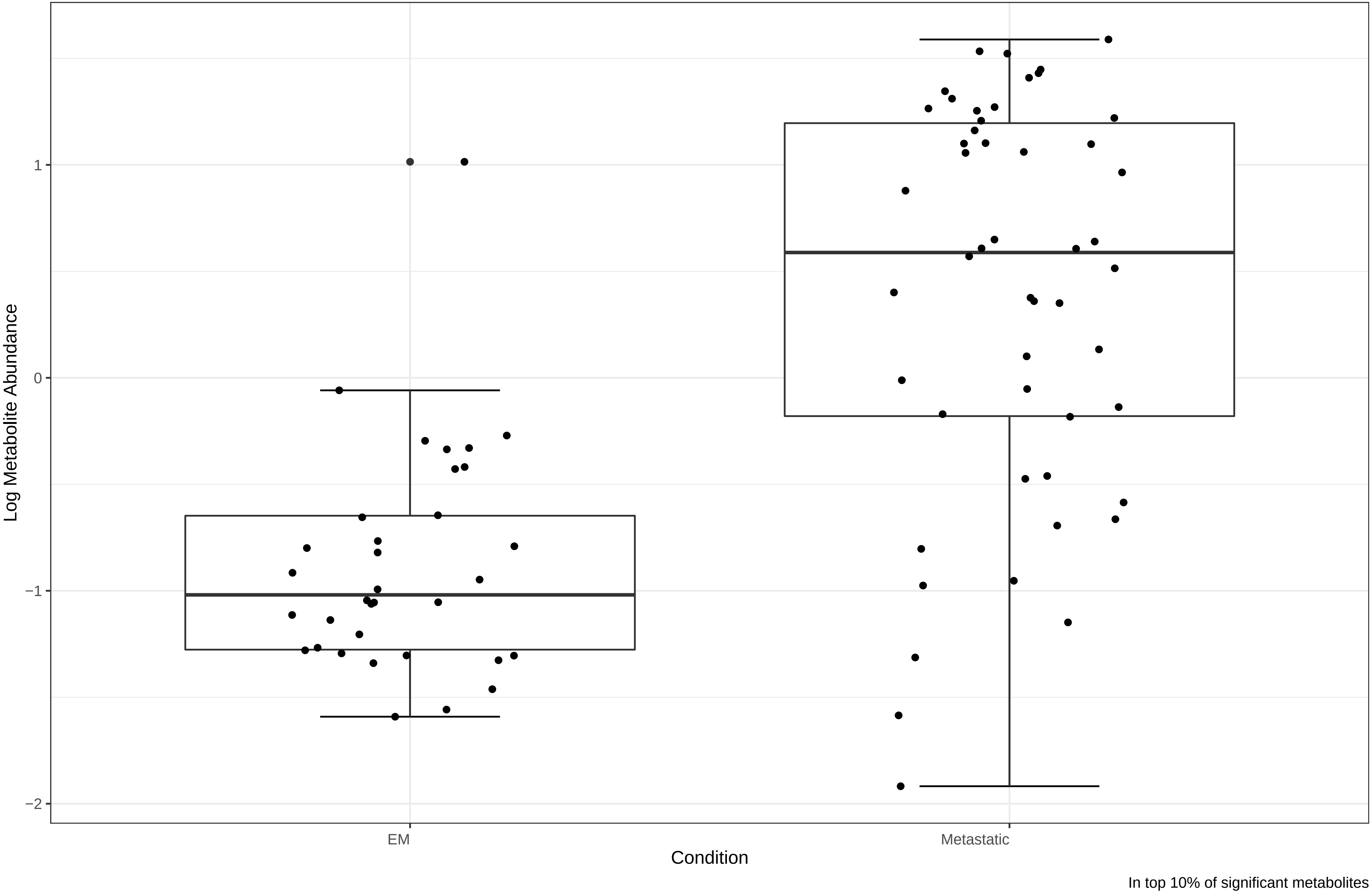

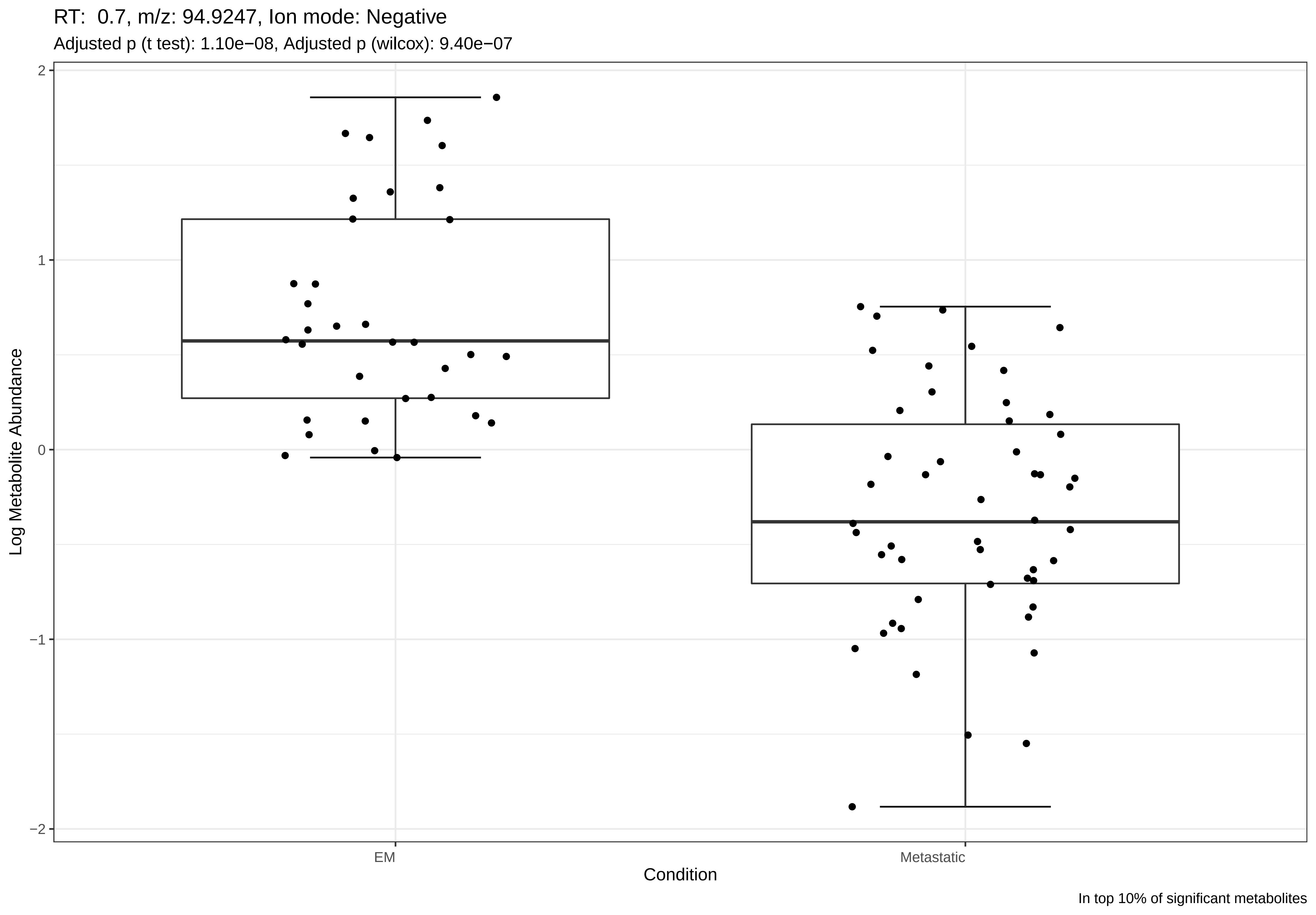
RT: 0.7, m/z: 94.9247, Ion mode: Negative
Adjusted p (t test): 1.10e−08, Adjusted p (wilcox): 9.40e−07
Log Metabolite Abundance
2
1
0
−1
−2
EM
Metastatic
Condition
In top 10% of significant metabolites

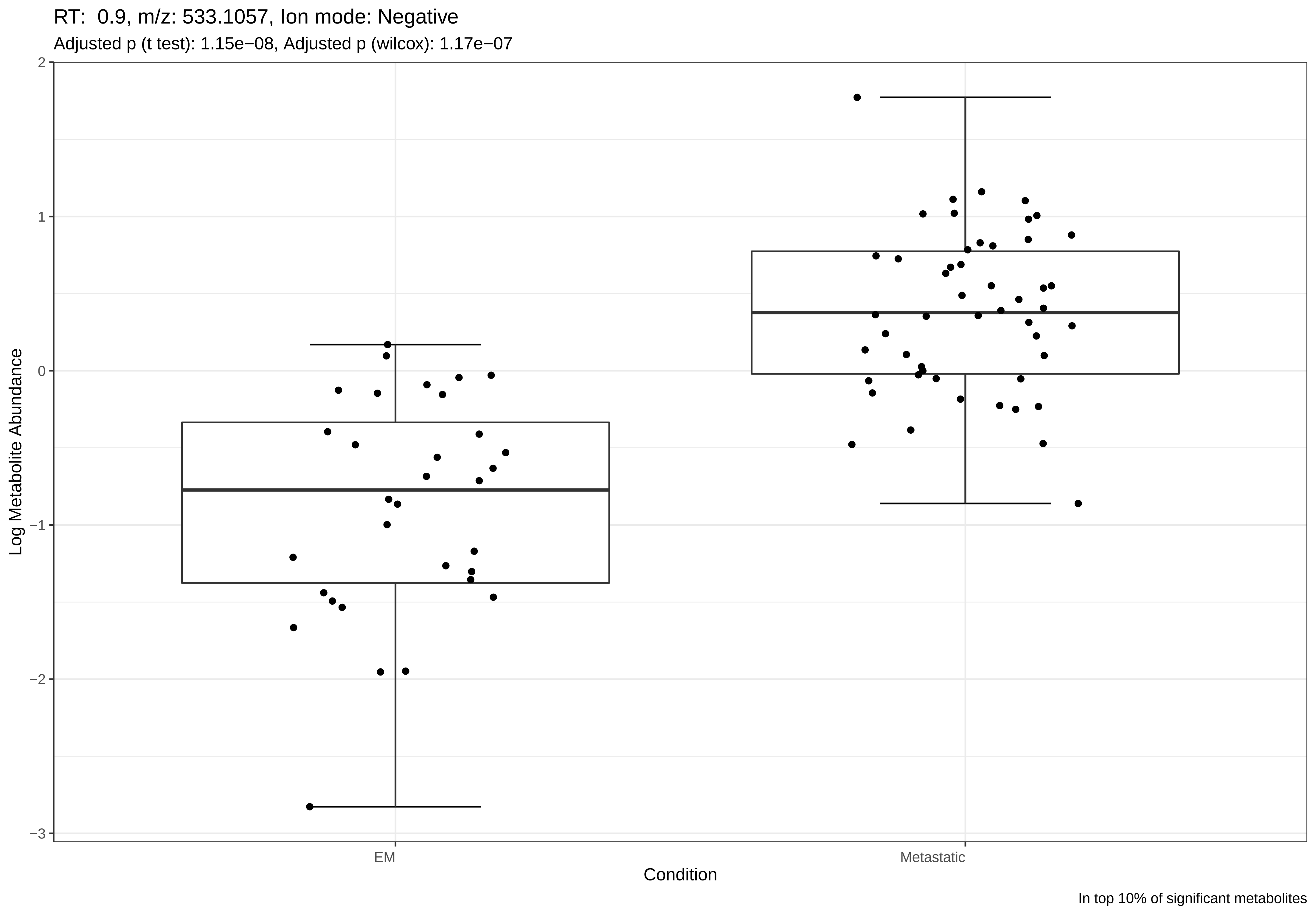

RT: 0.9, m/z: 533.1057, Ion mode: Negative
Adjusted p (t test): 1.15e−08, Adjusted p (wilcox): 1.17e−07
2
1
0
−1
−2
−3
Log Metabolite Abundance
EM
Metastatic
Condition
In top 10% of significant metabolites

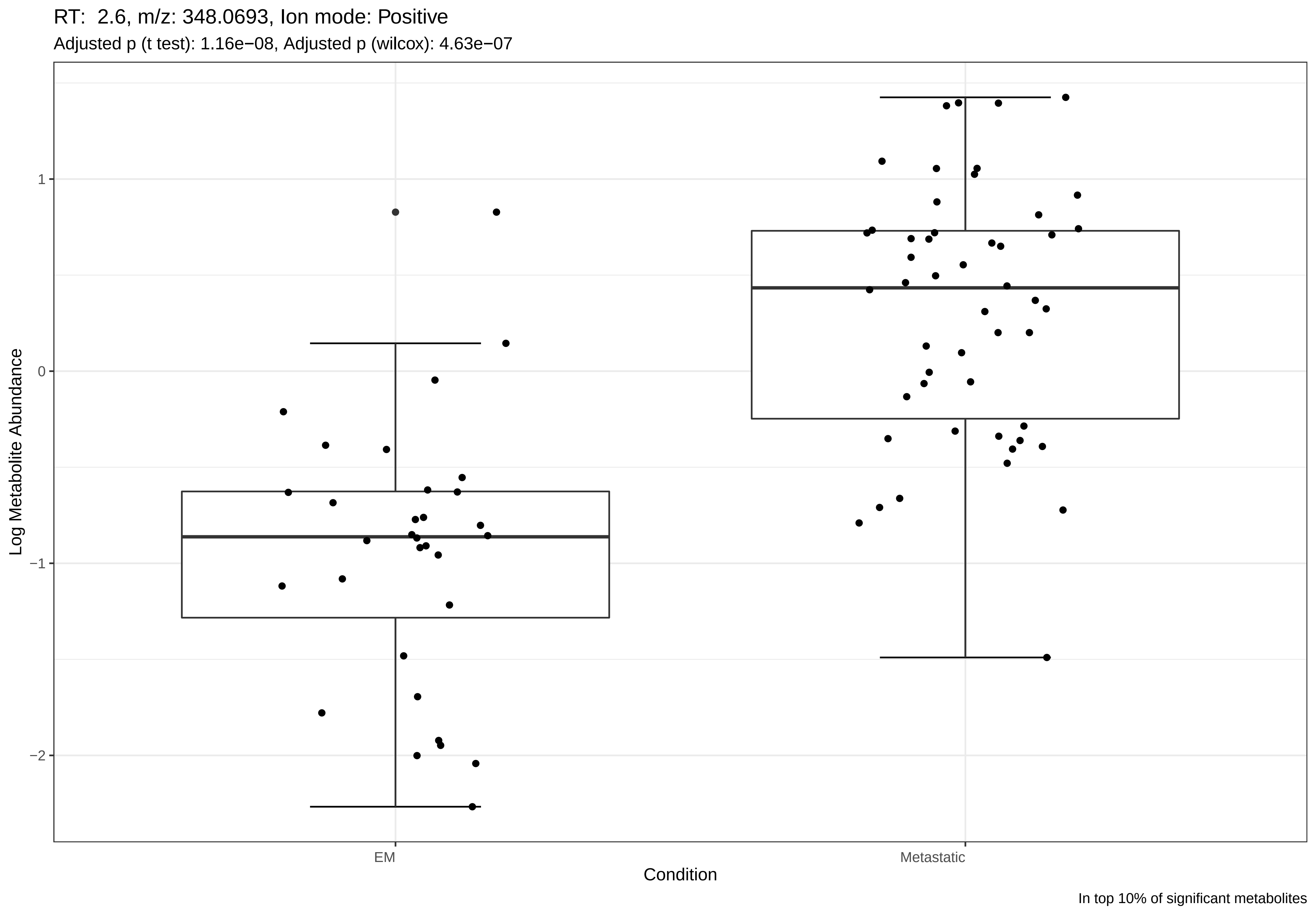
RT: 2.6, m/z: 348.0693, Ion mode: Positive
Adjusted p (t test): 1.16e−08, Adjusted p (wilcox): 4.63e−07
Log Metabolite Abundance
1
0
−1
−2
EM
Metastatic
Condition
In top 10% of significant metabolites

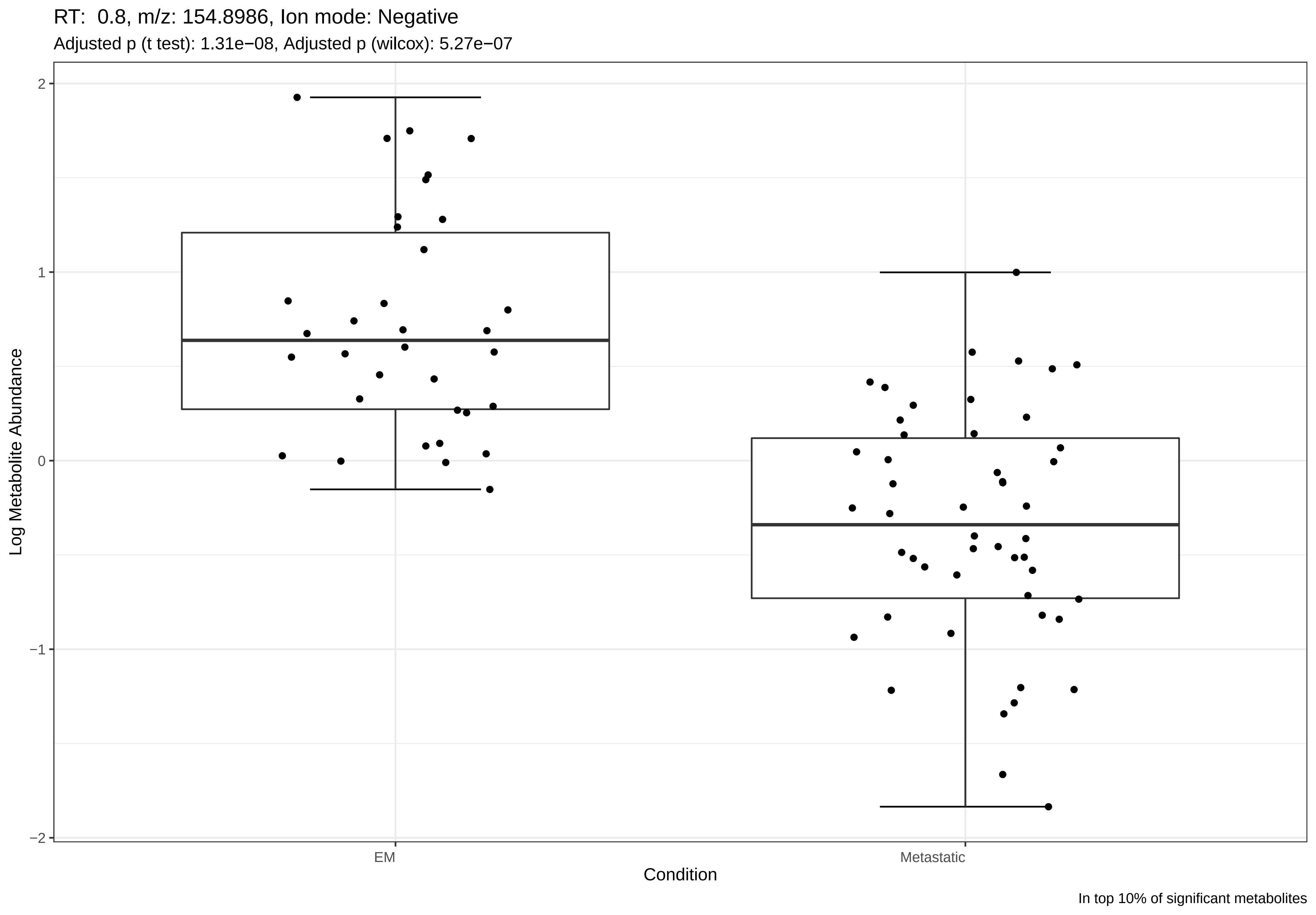
RT: 0.8, m/z: 154.8986, Ion mode: Negative
Adjusted p (t test): 1.31e−08, Adjusted p (wilcox): 5.27e−07
Log Metabolite Abundance
2
1
0
−1
−2
EM
Metastatic
Condition
In top 10% of significant metabolites

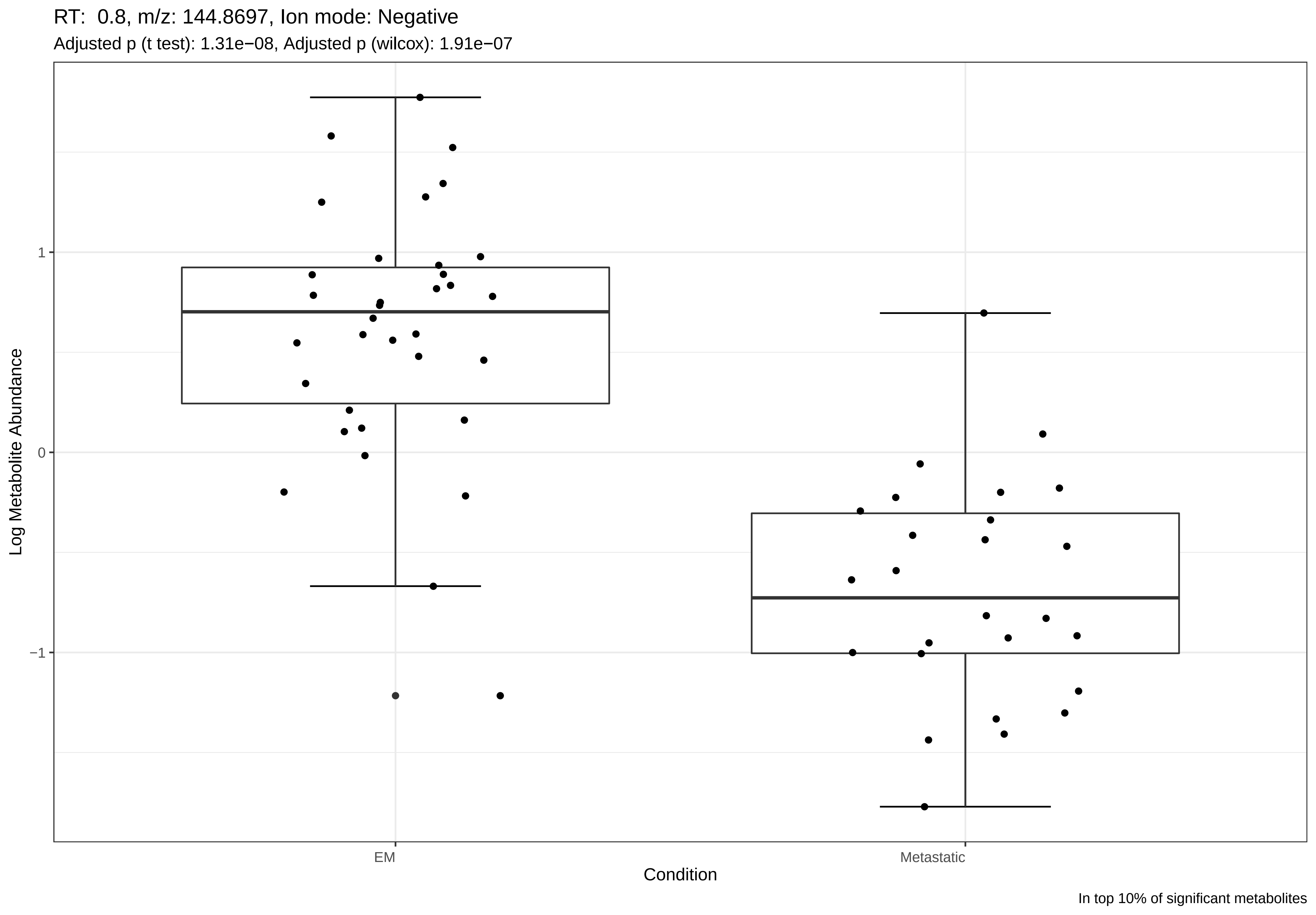
RT: 0.8, m/z: 144.8697, Ion mode: Negative
Adjusted p (t test): 1.31e−08, Adjusted p (wilcox): 1.91e−07
Log Metabolite Abundance
1
0
−1
EM
Metastatic
Condition
In top 10% of significant metabolites

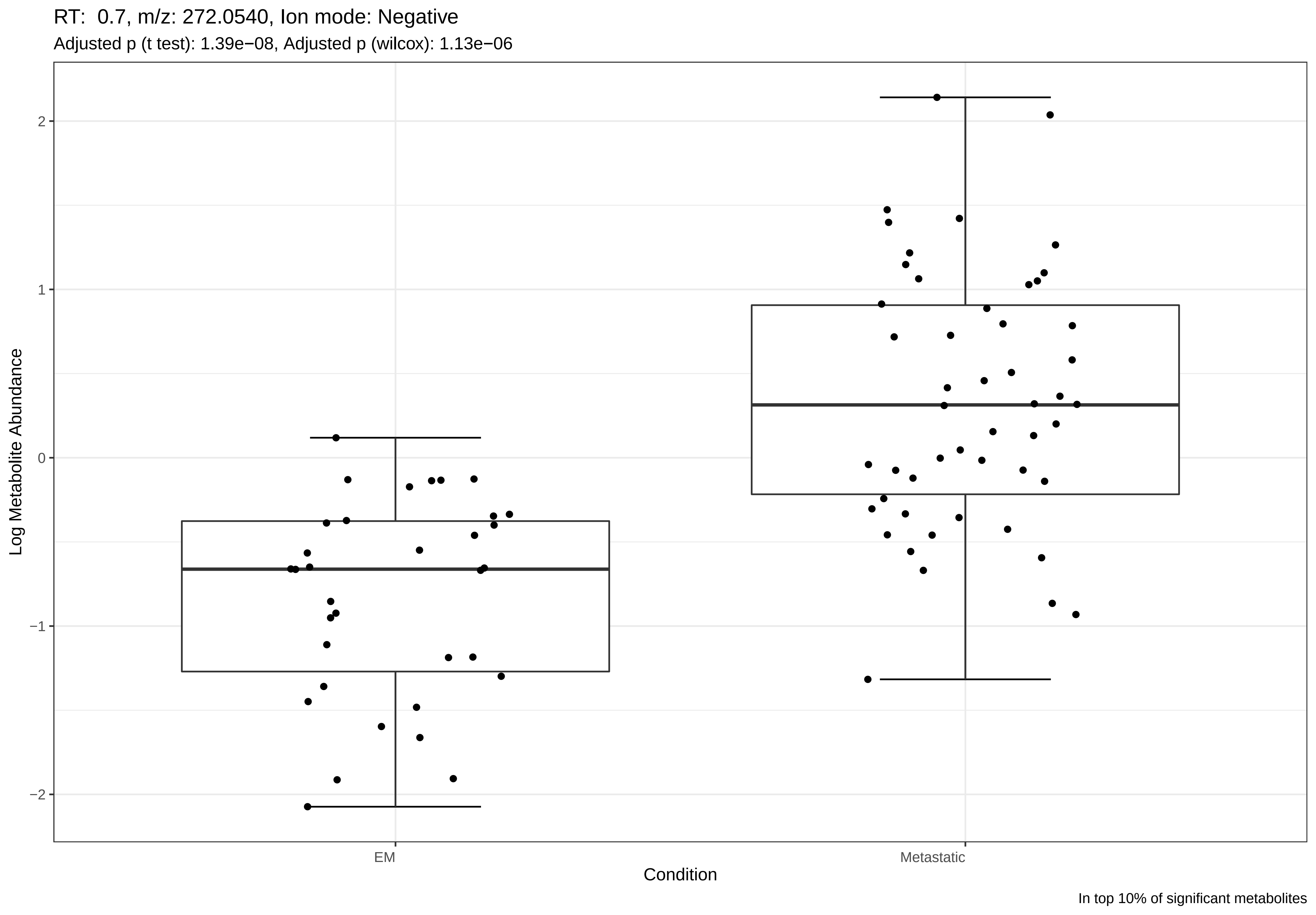
RT: 0.7, m/z: 272.0540, Ion mode: Negative
Adjusted p (t test): 1.39e−08, Adjusted p (wilcox): 1.13e−06
Log Metabolite Abundance
2
1
0
−1
−2
EM
Metastatic
Condition
In top 10% of significant metabolites

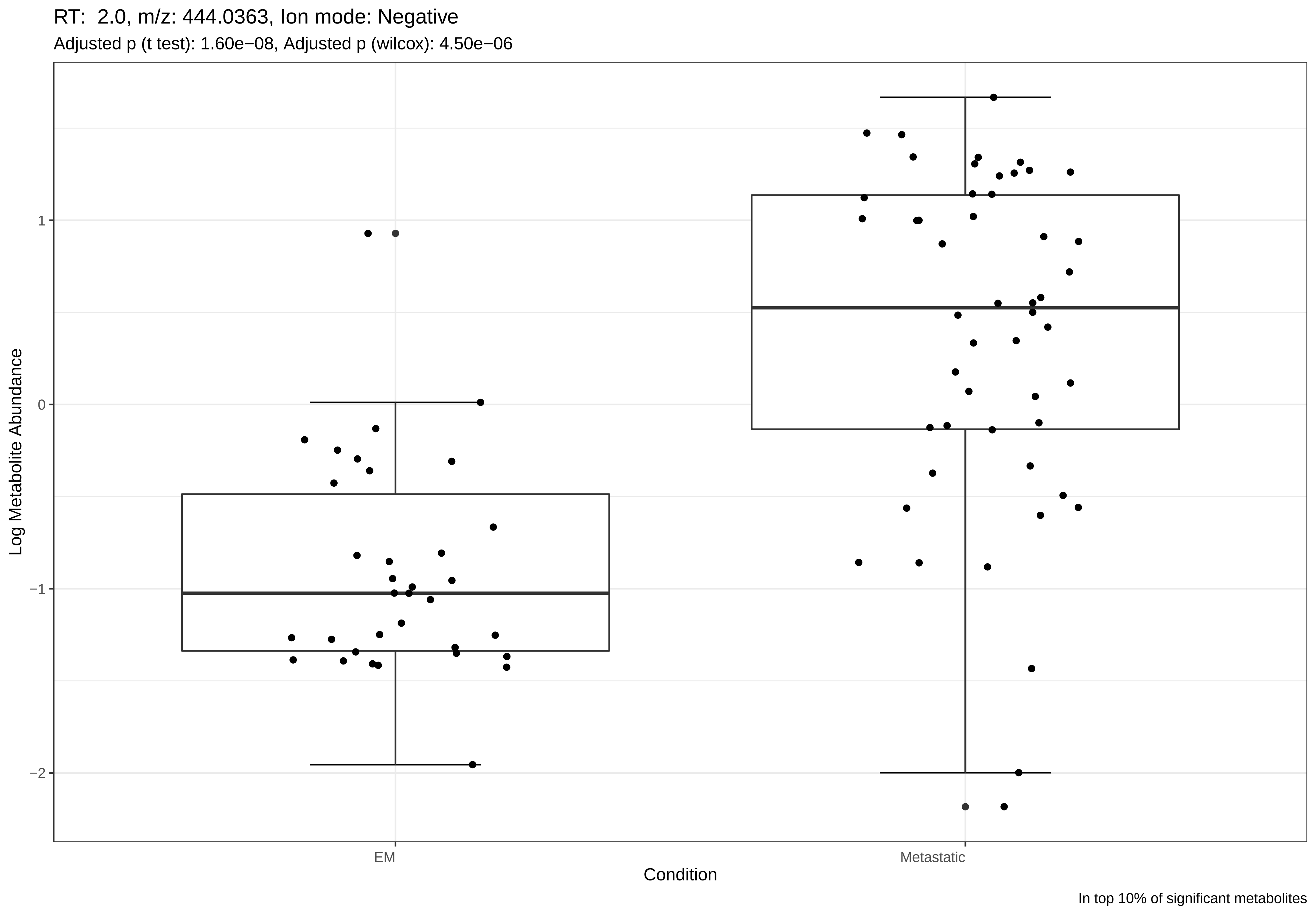

RT: 2.0, m/z: 444.0363, Ion mode: Negative
Adjusted p (t test): 1.60e−08, Adjusted p (wilcox): 4.50e−06
Log Metabolite Abundance
1
0
−1
−2
EM
Metastatic
Condition
In top 10% of significant metabolites

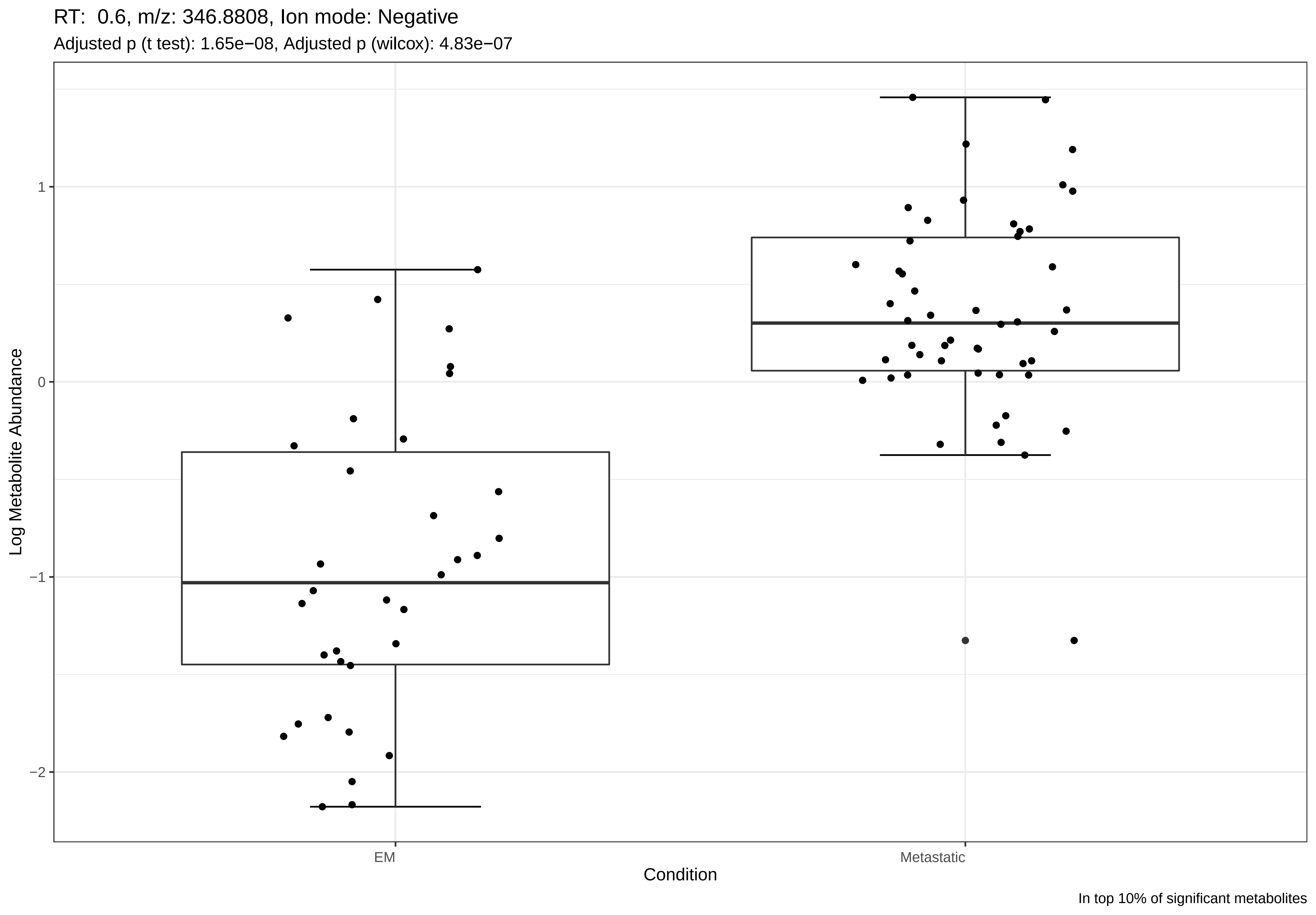

RT: 0.6, m/z: 346.8808, Ion mode: Negative
Adjusted p (t test): 1.65e−08, Adjusted p (wilcox): 4.83e−07
Log Metabolite Abundance
1
0
−1
−2
EM
Metastatic
Condition
In top 10% of significant metabolites

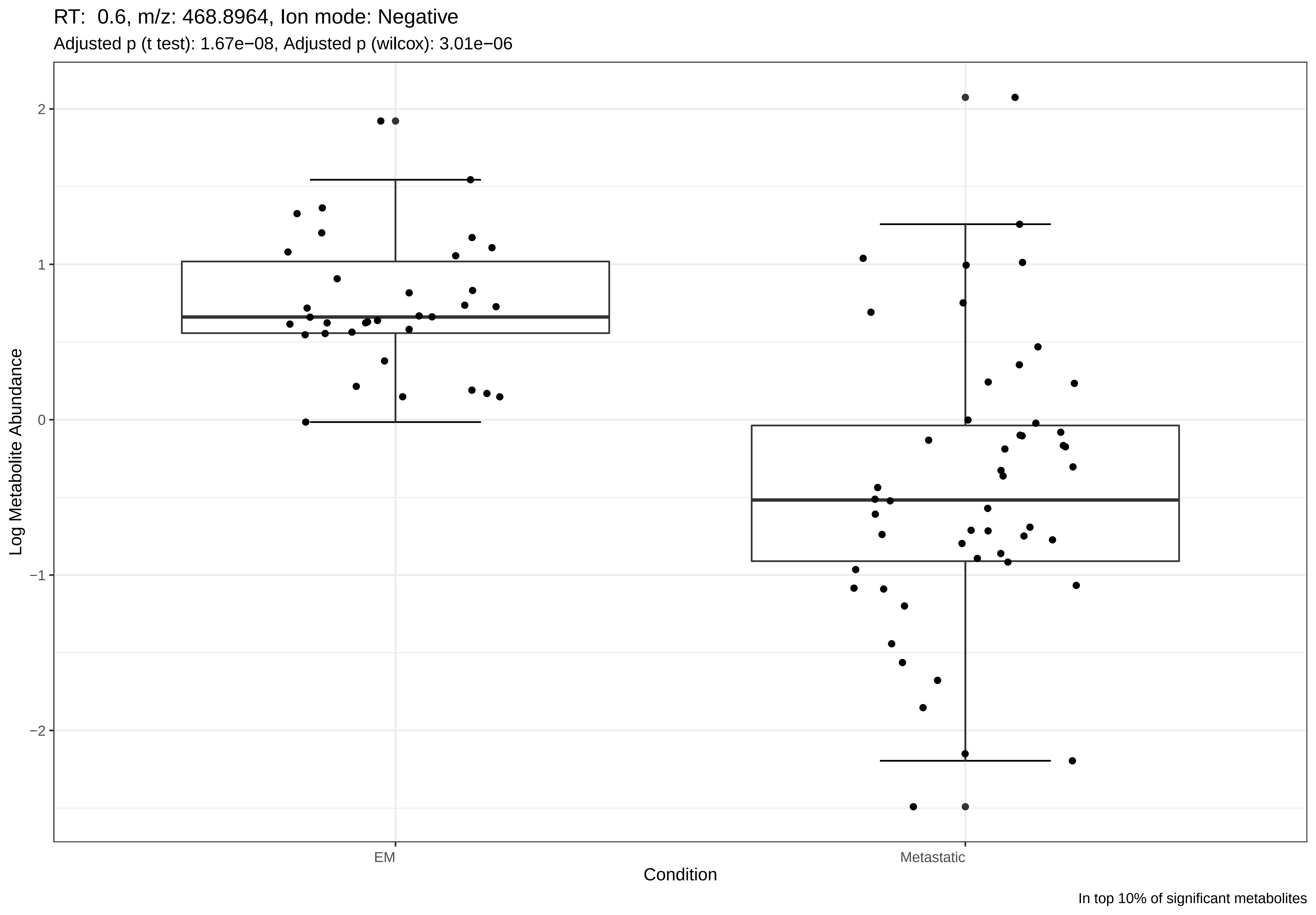
RT: 0.6, m/z: 468.8964, Ion mode: Negative
Adjusted p (t test): 1.67e−08, Adjusted p (wilcox): 3.01e−06
Log Metabolite Abundance
2
1
0
−1
−2
EM
Metastatic
Condition
In top 10% of significant metabolites

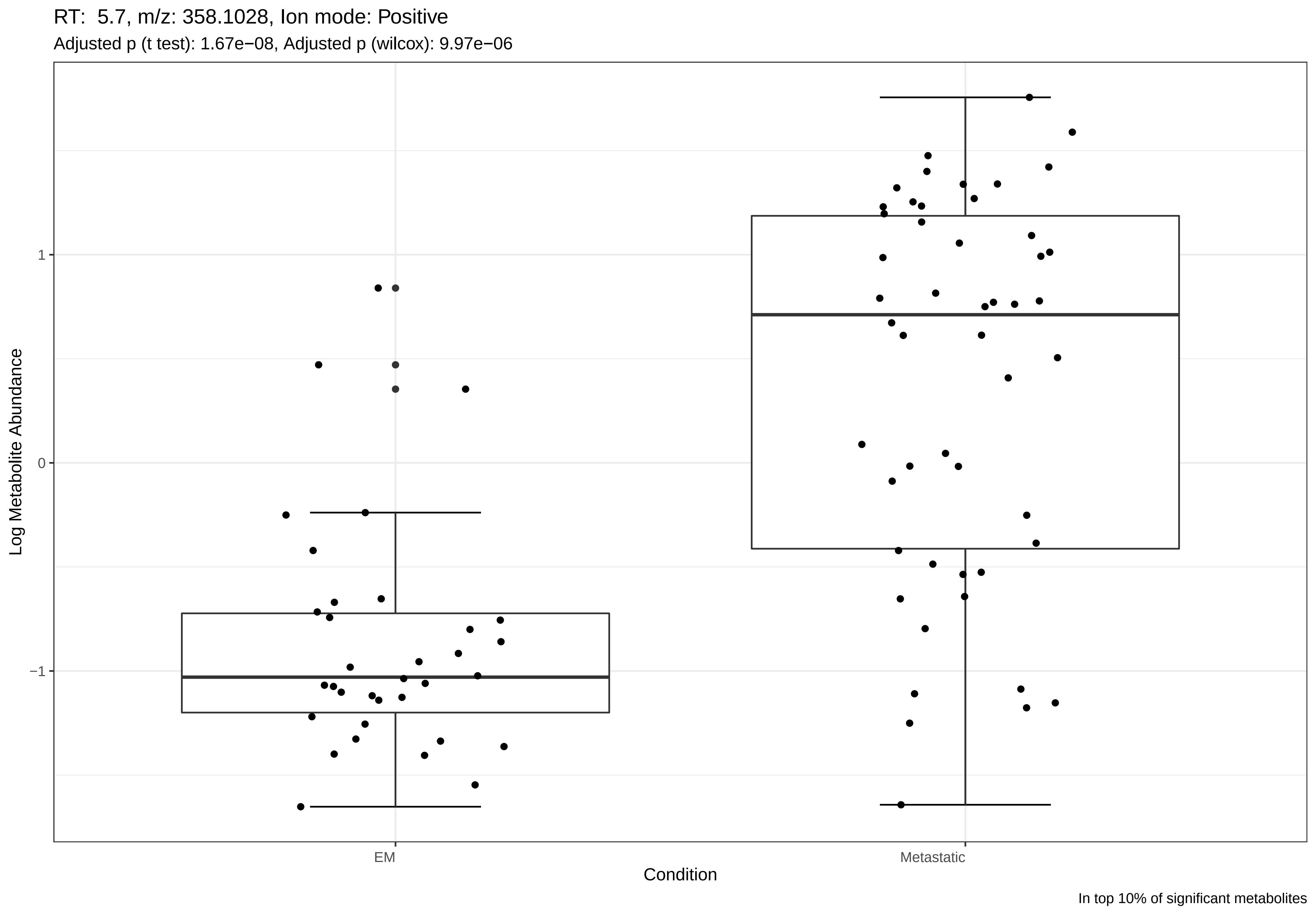

RT: 5.7, m/z: 358.1028, Ion mode: Positive
Adjusted p (t test): 1.67e−08, Adjusted p (wilcox): 9.97e−06
Log Metabolite Abundance
1
0
−1
EM
Metastatic
Condition
In top 10% of significant metabolites

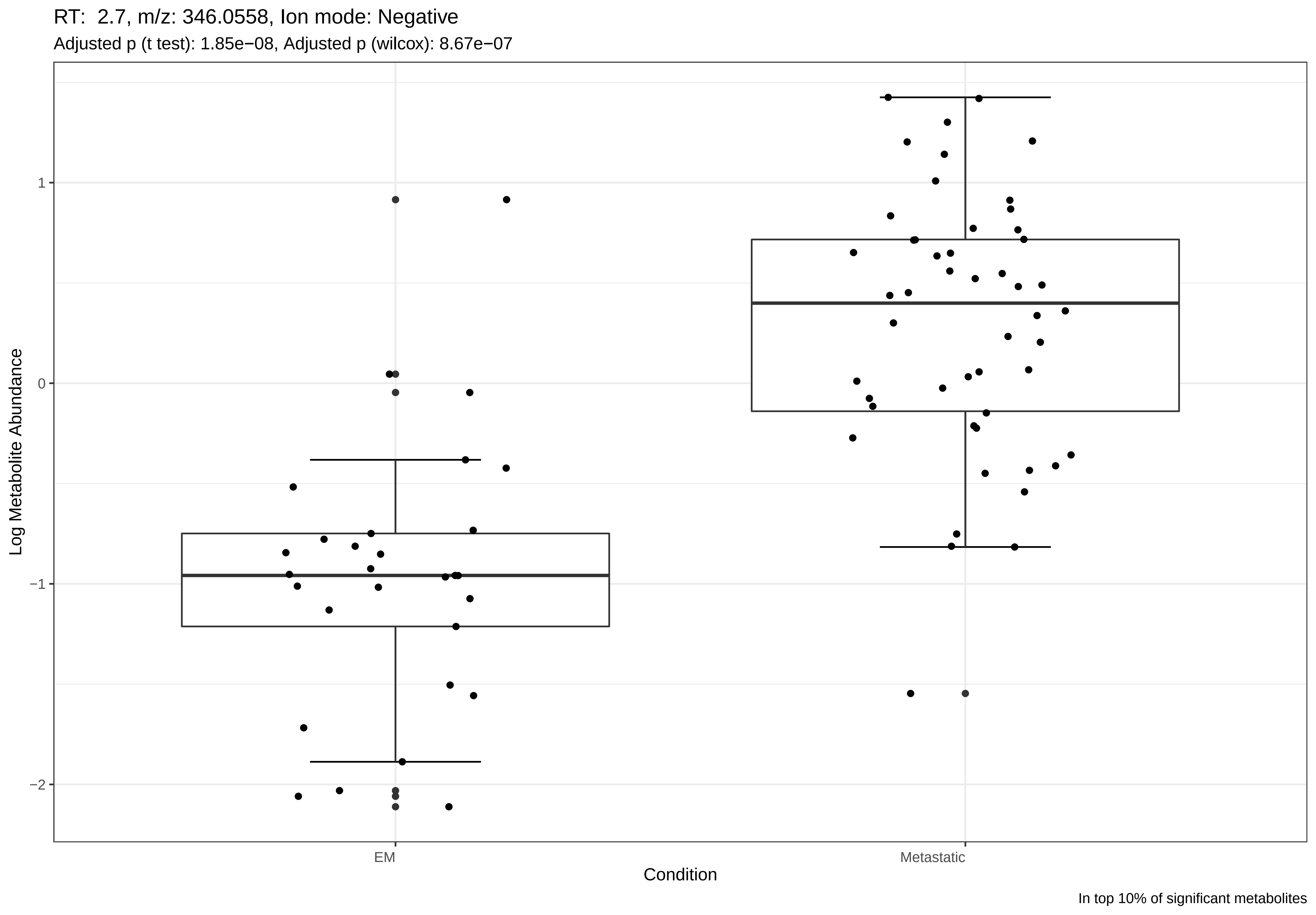
RT: 2.7, m/z: 346.0558, Ion mode: Negative
Adjusted p (t test): 1.85e−08, Adjusted p (wilcox): 8.67e−07
Log Metabolite Abundance
1
0
−1
−2
EM
Metastatic
Condition
In top 10% of significant metabolites

RT: 0.7, m/z: 228.0639, Ion mode: Negative

Adjusted p (t test): 1.98e−08, Adjusted p (wilcox): 9.40e−07

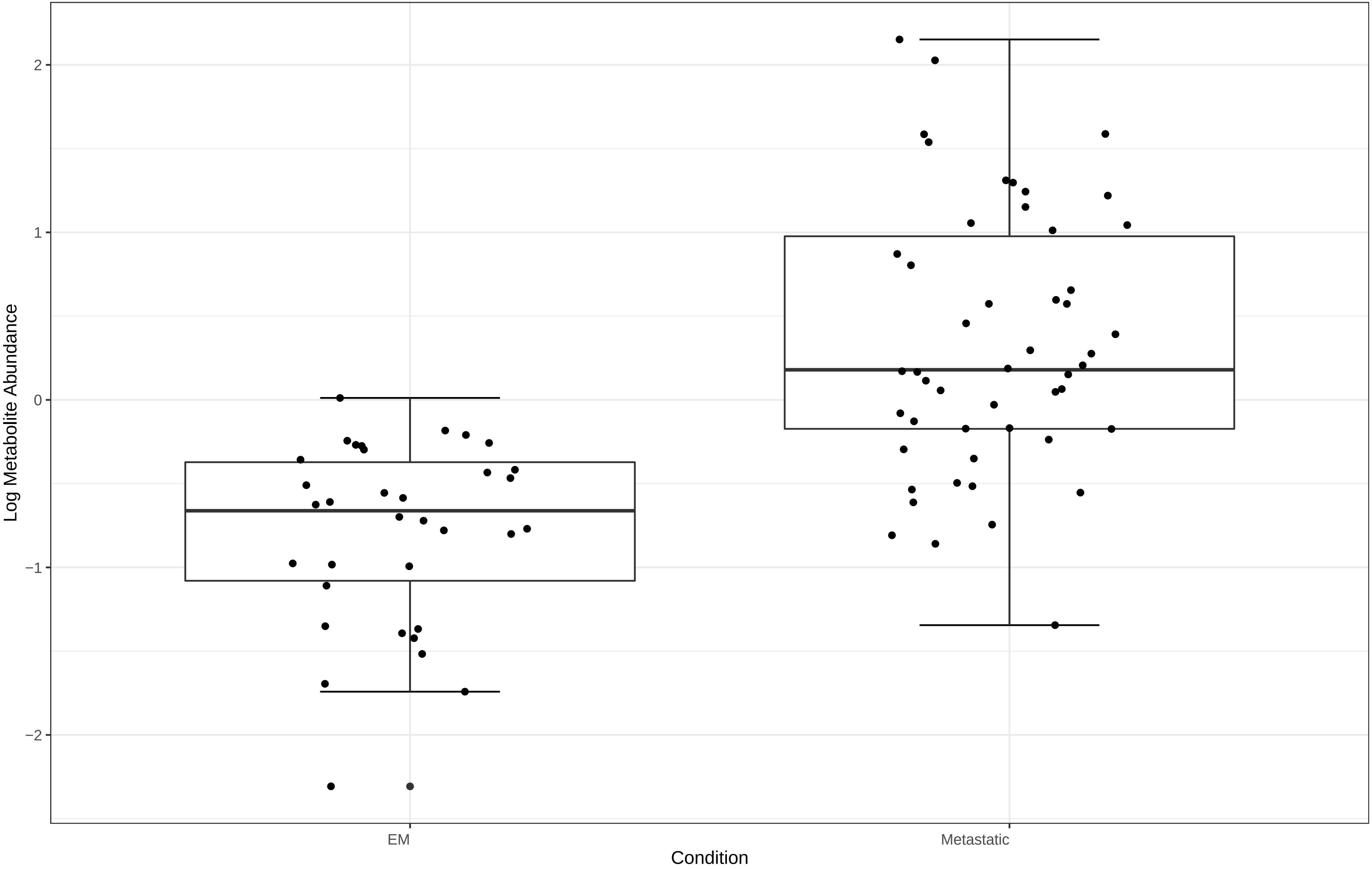

In top 10% of significant metabolites

RT: 6.4, m/z: 268.1033, Ion mode: Positive

Adjusted p (t test): 1.98e−08, Adjusted p (wilcox): 3.01e−06

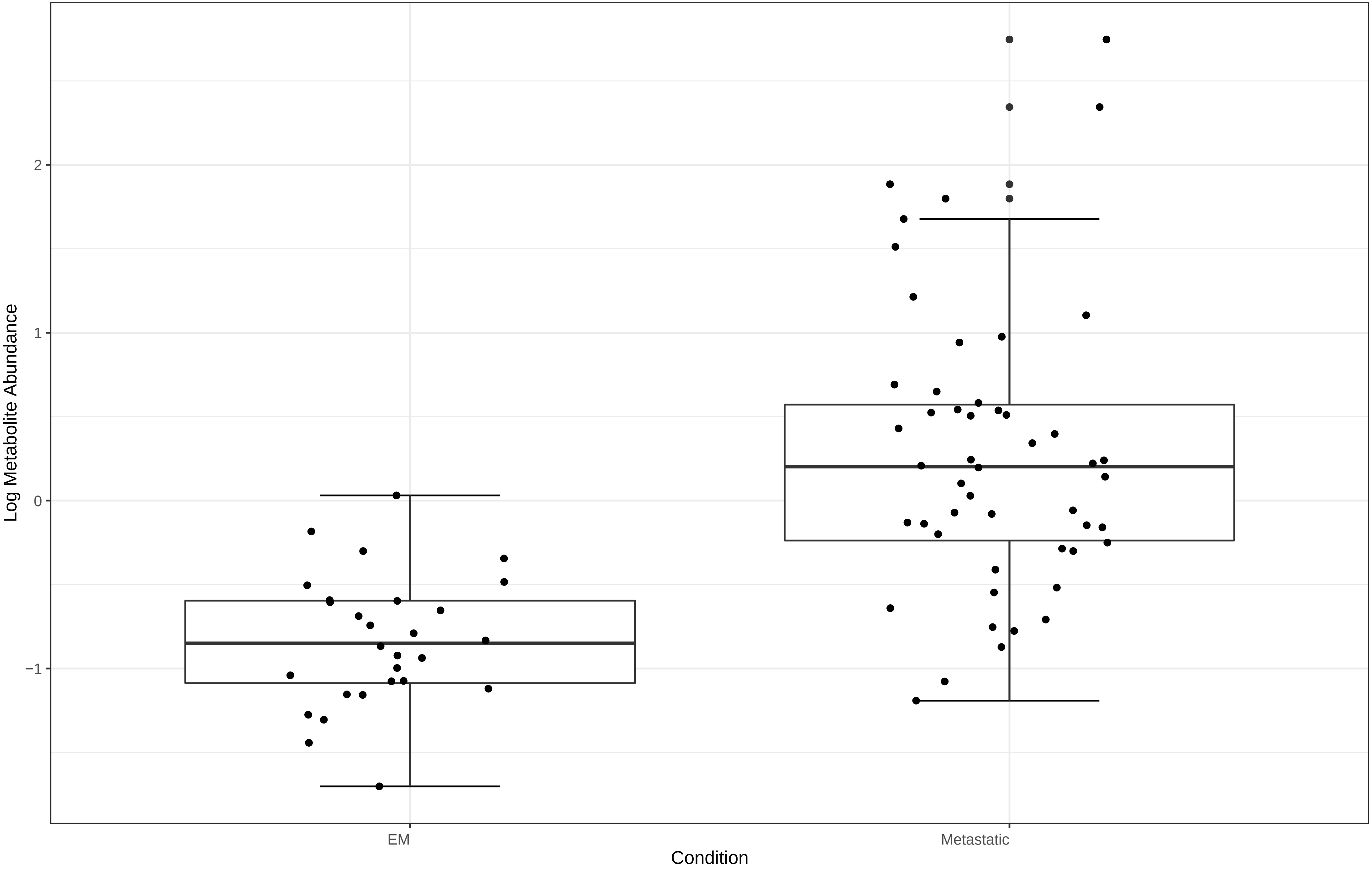

In top 10% of significant metabolites

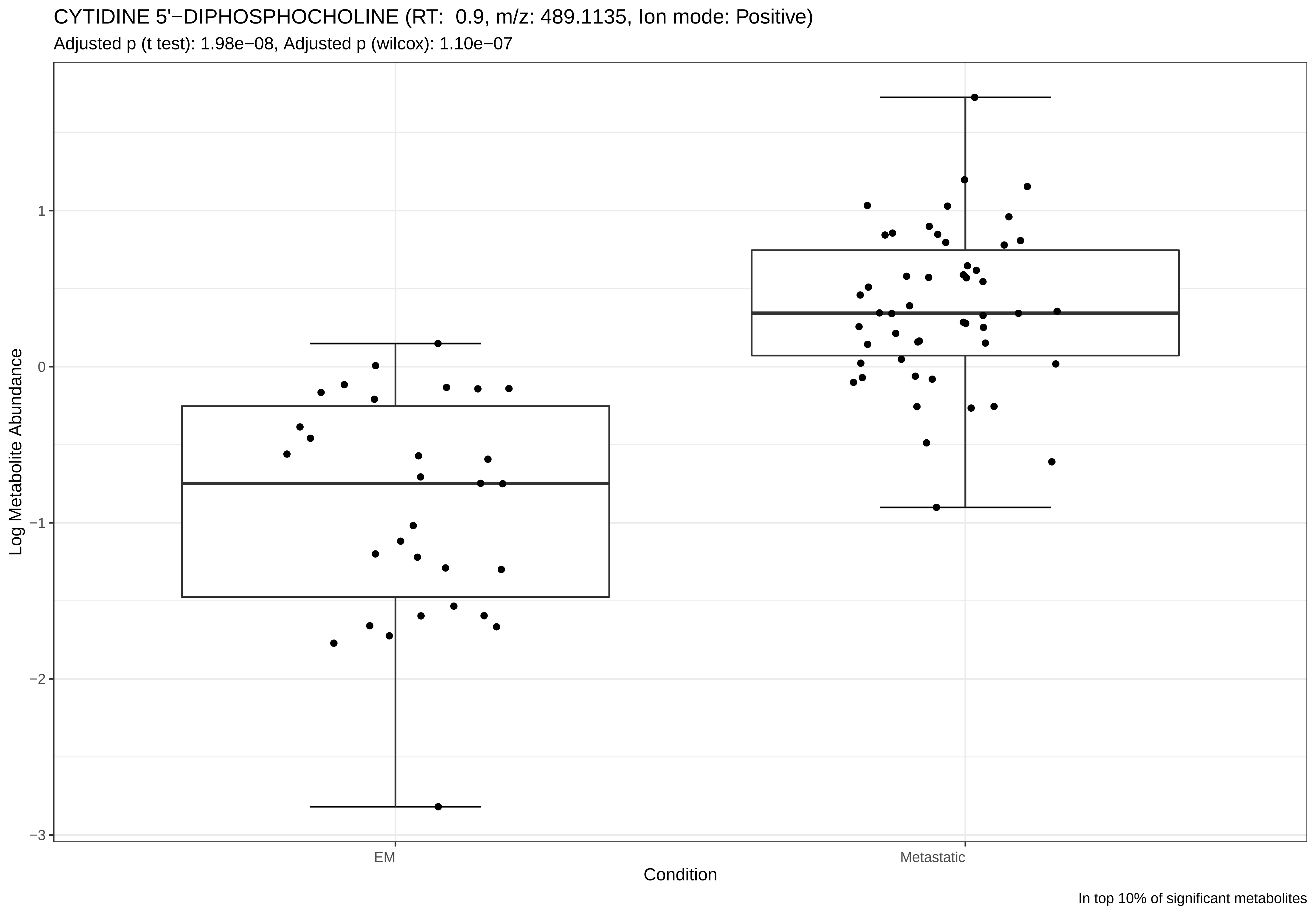

CYTIDINE 5'−DIPHOSPHOCHOLINE (RT: 0.9, m/z: 489.1135, Ion mode: Positive)
Adjusted p (t test): 1.98e−08, Adjusted p (wilcox): 1.10e−07
Log Metabolite Abundance
1
0
−1
−2
−3
EM
Metastatic
Condition
In top 10% of significant metabolites

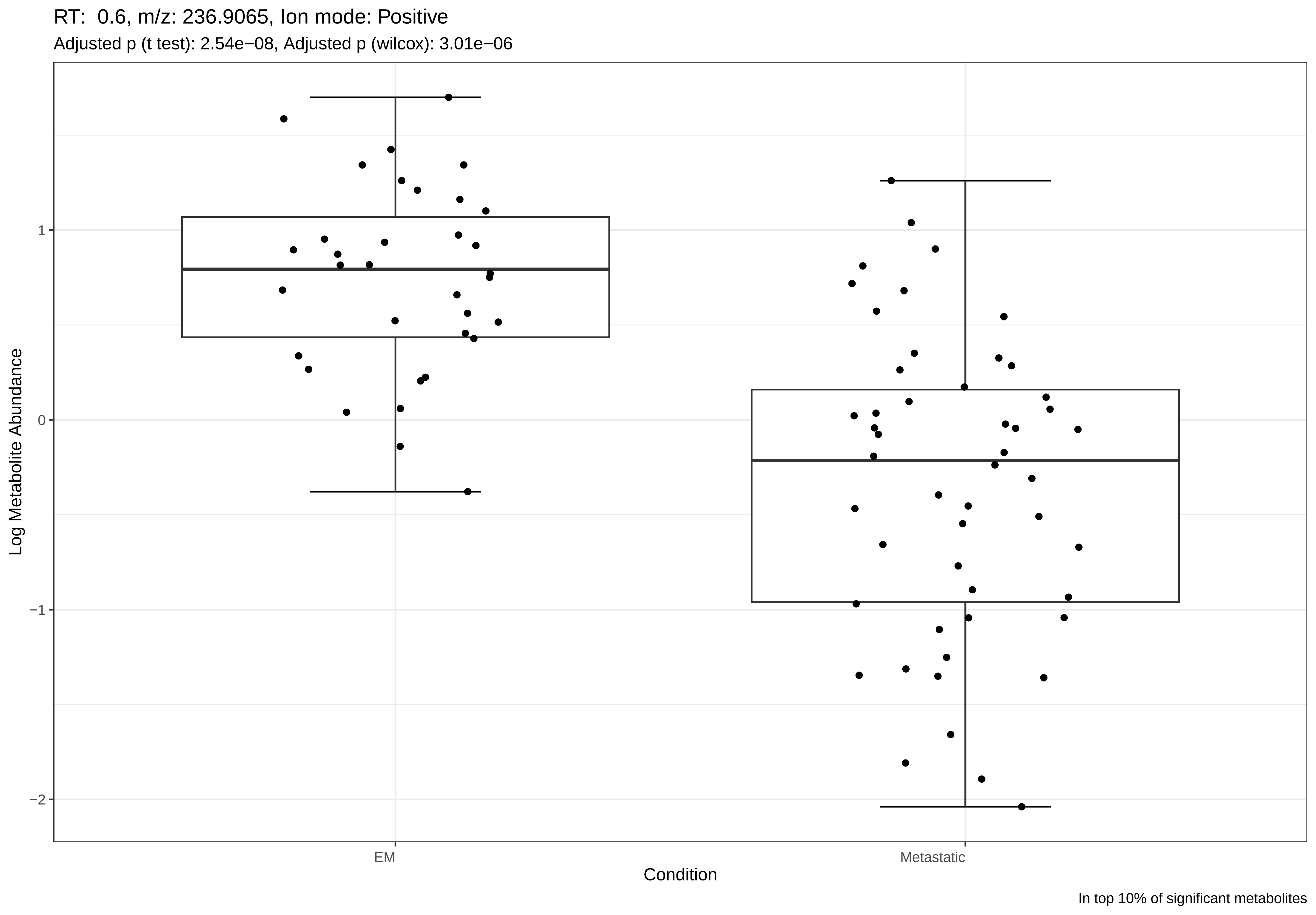
RT: 0.6, m/z: 236.9065, Ion mode: Positive
Adjusted p (t test): 2.54e−08, Adjusted p (wilcox): 3.01e−06
Log Metabolite Abundance
1
0
−1
−2
EM
Metastatic
Condition
In top 10% of significant metabolites

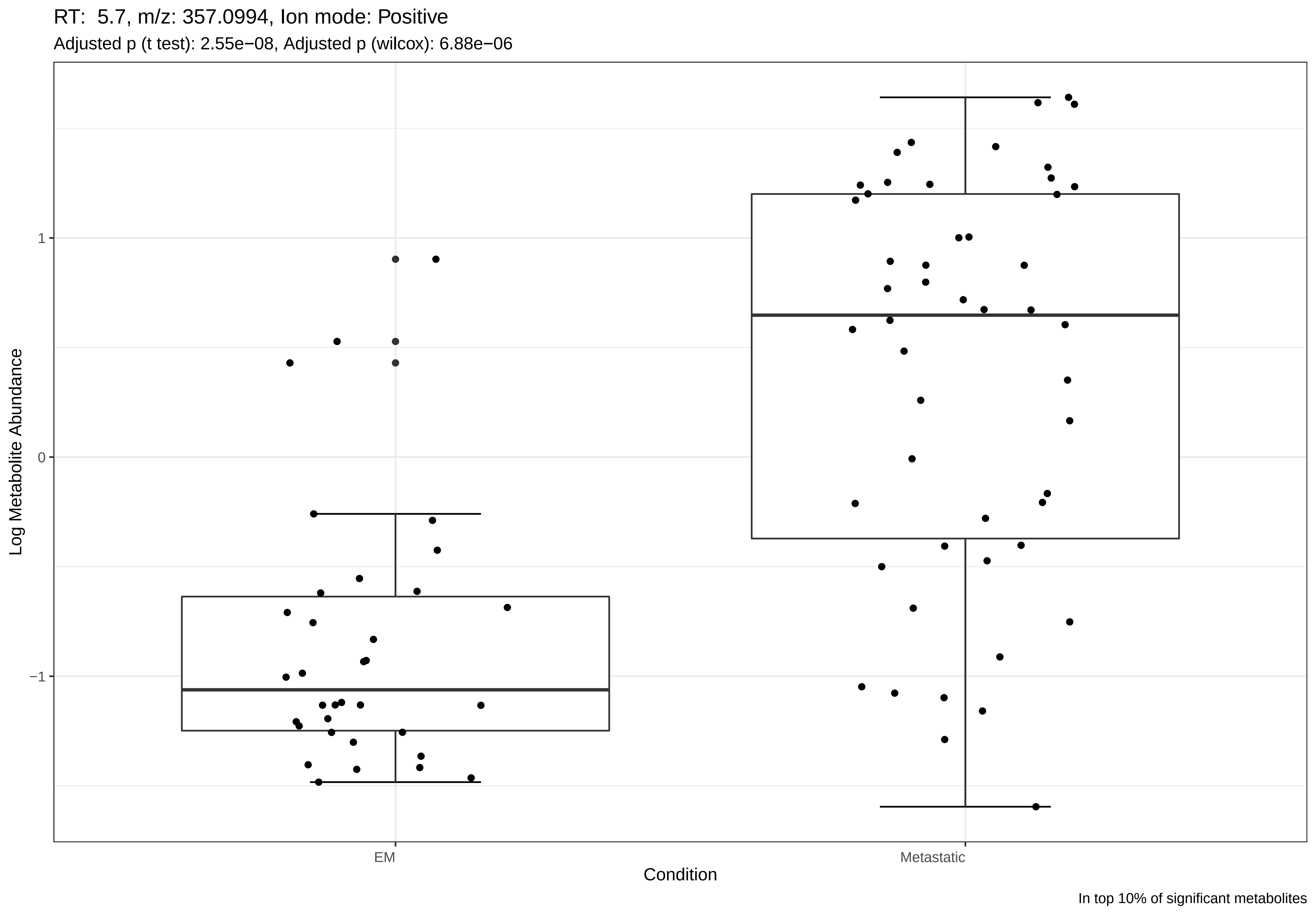

RT: 5.7, m/z: 357.0994, Ion mode: Positive
Adjusted p (t test): 2.55e−08, Adjusted p (wilcox): 6.88e−06
Log Metabolite Abundance
1
0
−1
EM
Metastatic
Condition
In top 10% of significant metabolites

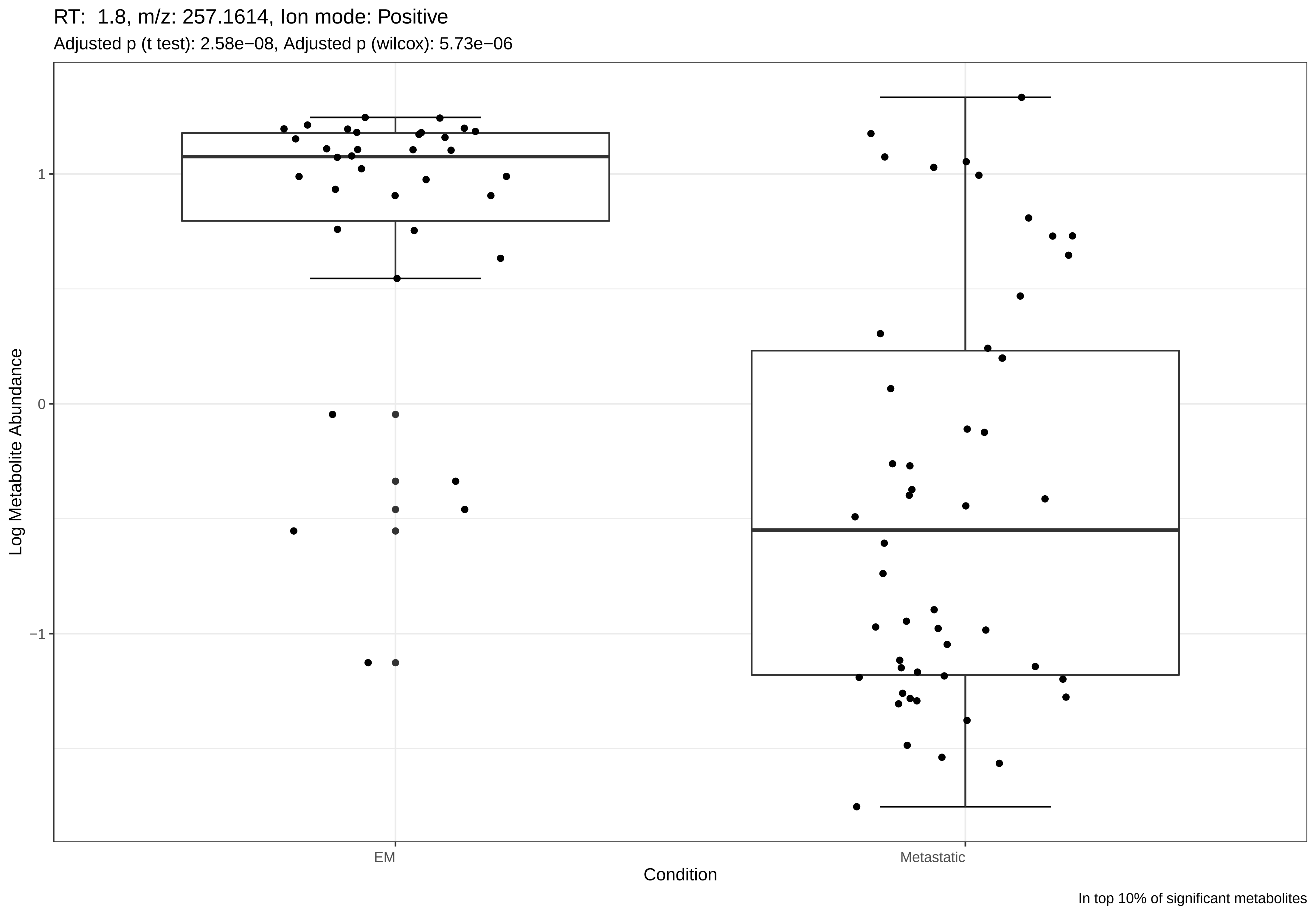
RT: 1.8, m/z: 257.1614, Ion mode: Positive
Adjusted p (t test): 2.58e−08, Adjusted p (wilcox): 5.73e−06
Log Metabolite Abundance
1
0
−1
EM
Metastatic
Condition
In top 10% of significant metabolites

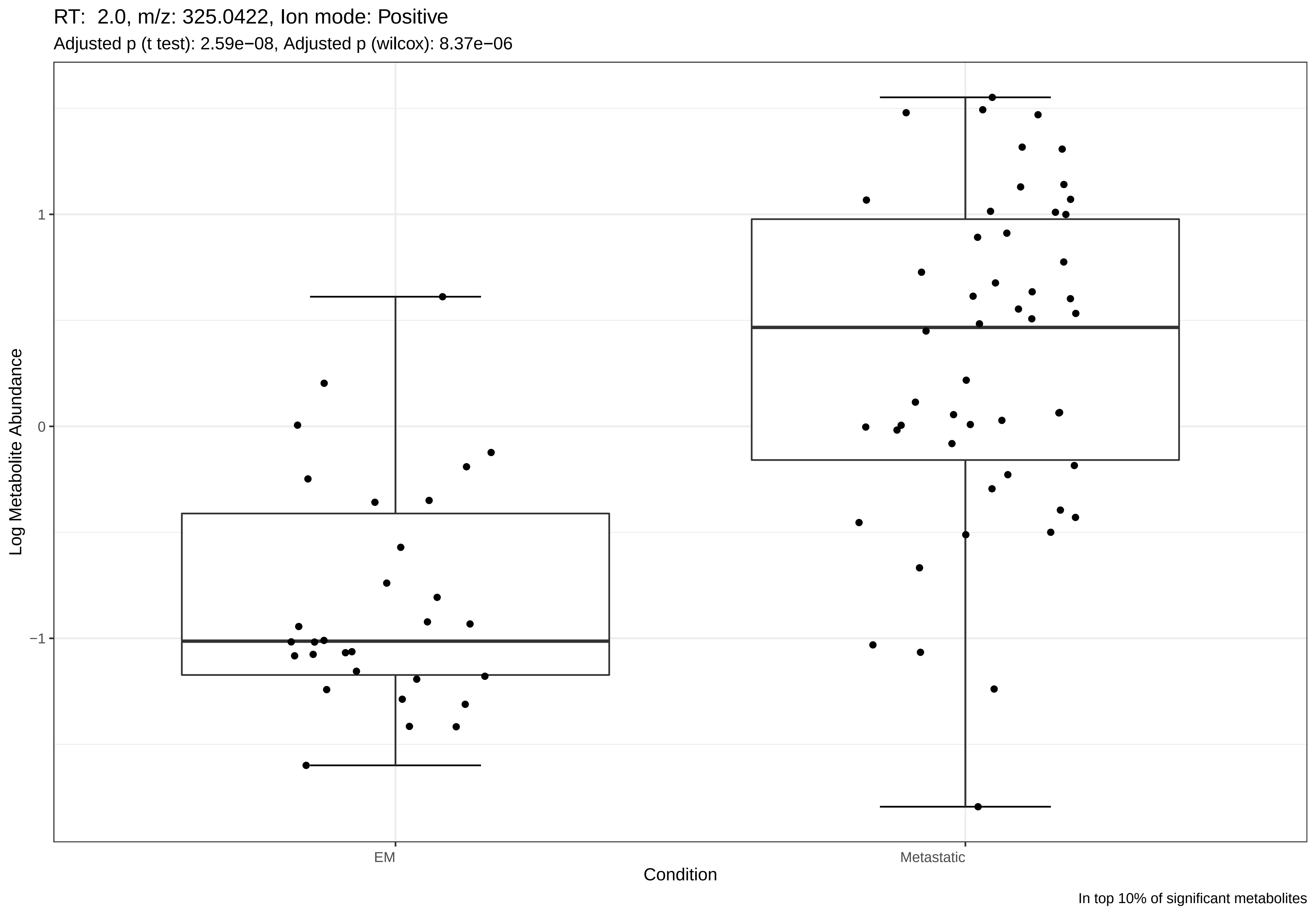

RT: 2.0, m/z: 325.0422, Ion mode: Positive
Adjusted p (t test): 2.59e−08, Adjusted p (wilcox): 8.37e−06
Log Metabolite Abundance
1
0
−1
EM
Metastatic
Condition
In top 10% of significant metabolites

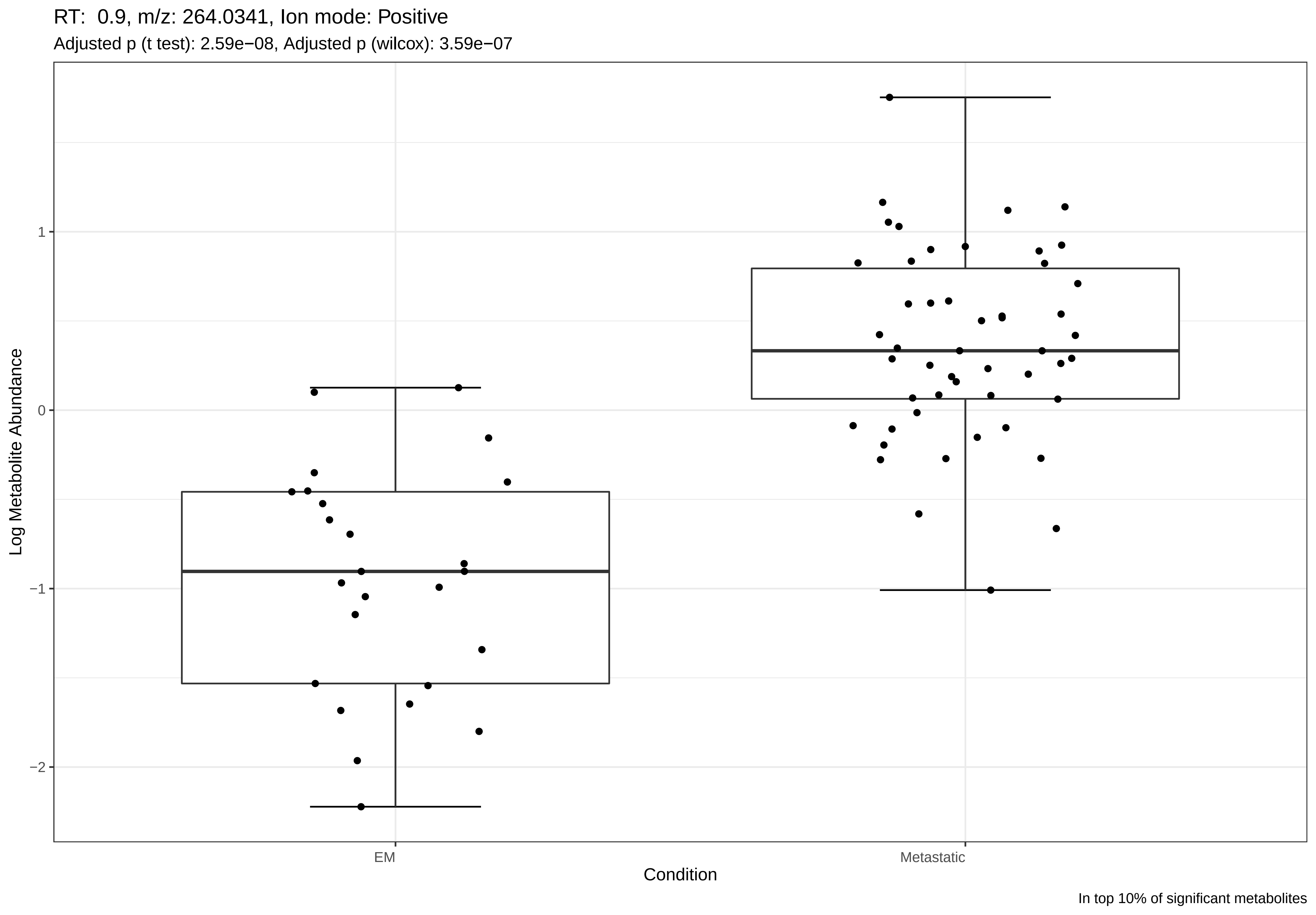
RT: 0.9, m/z: 264.0341, Ion mode: Positive
Adjusted p (t test): 2.59e−08, Adjusted p (wilcox): 3.59e−07
Log Metabolite Abundance
1
0
−1
−2
EM
Metastatic
Condition
In top 10% of significant metabolites

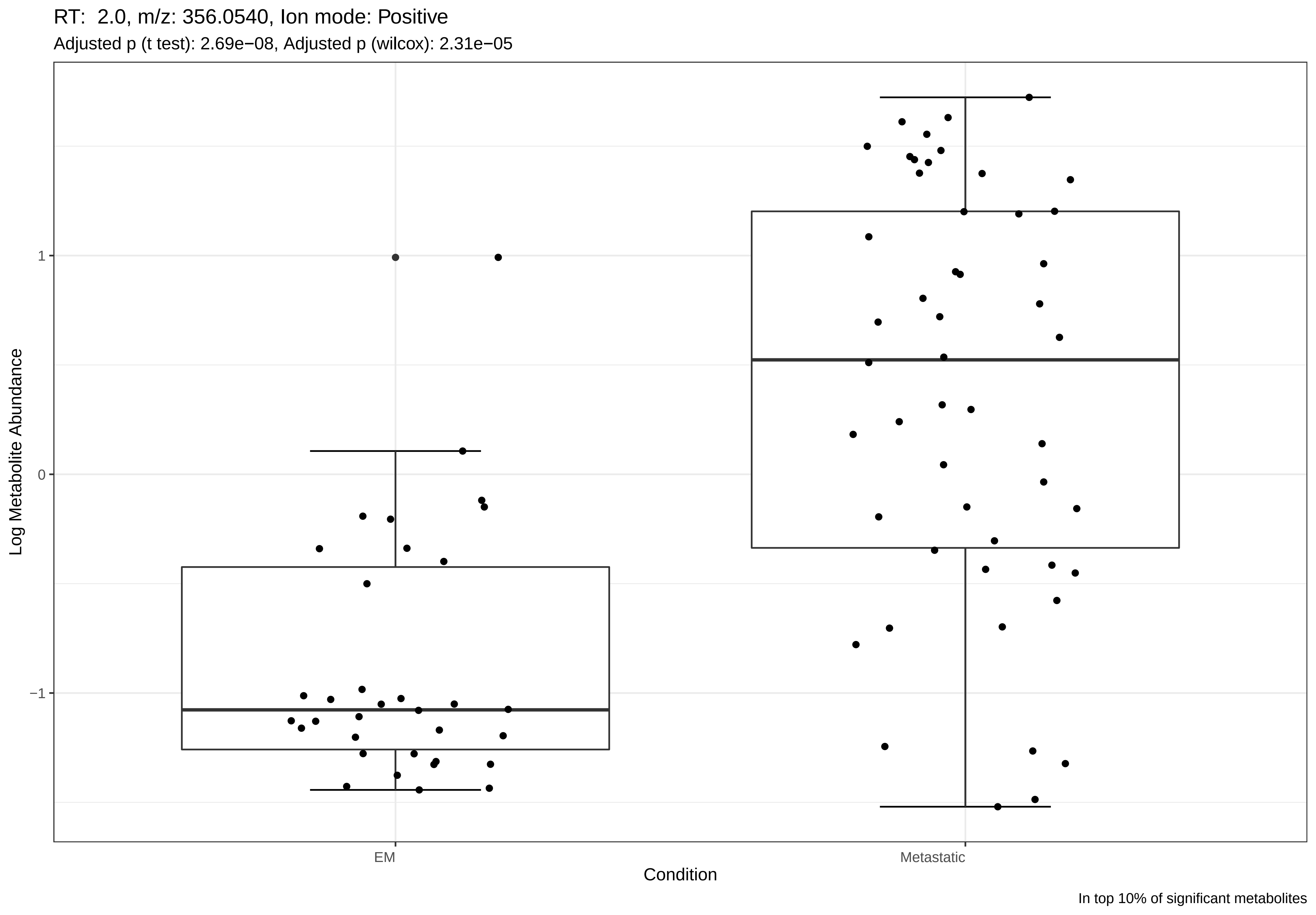

RT: 2.0, m/z: 356.0540, Ion mode: Positive
Adjusted p (t test): 2.69e−08, Adjusted p (wilcox): 2.31e−05
Log Metabolite Abundance
1
0
−1
EM
Metastatic
Condition
In top 10% of significant metabolites

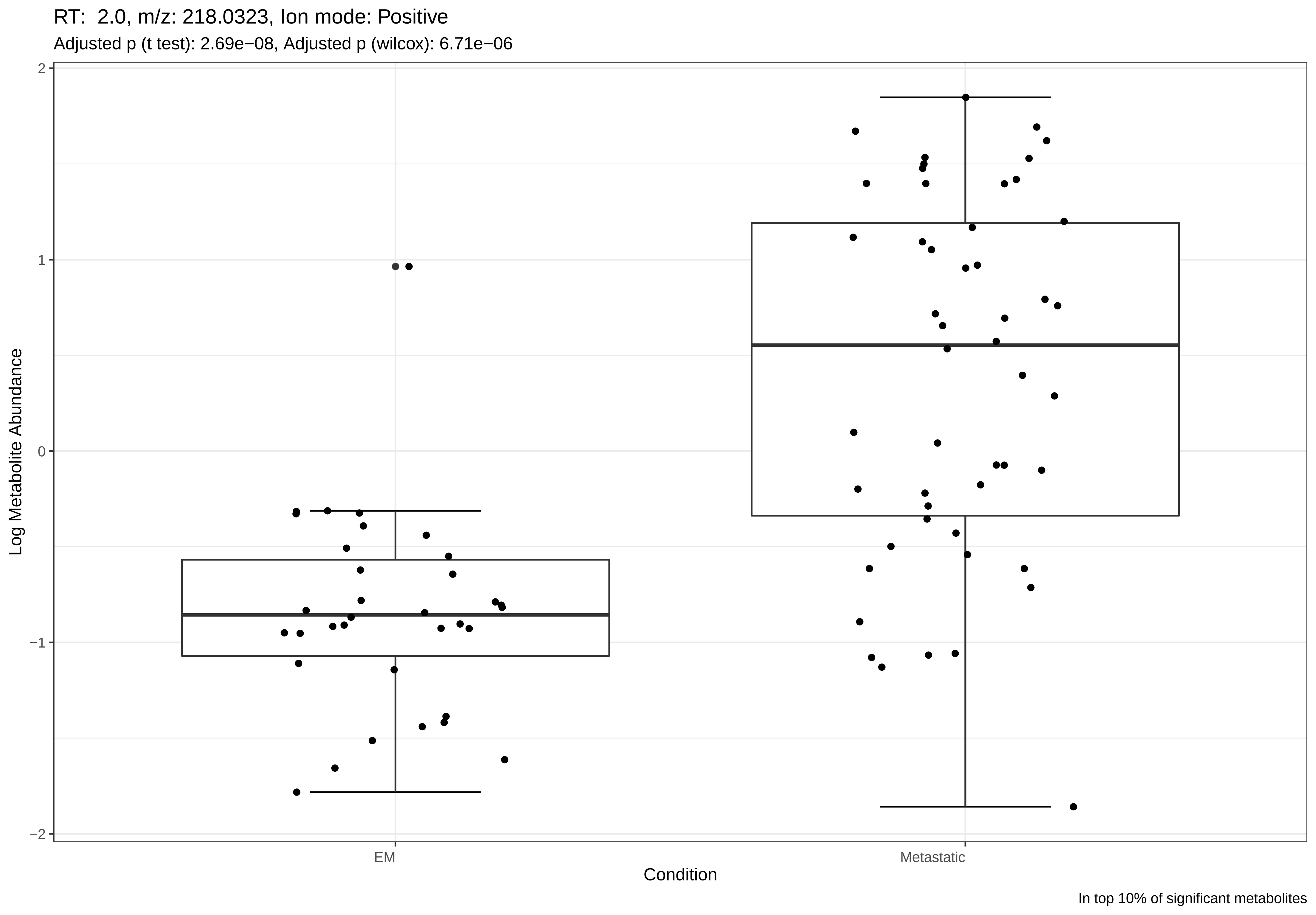

RT: 2.0, m/z: 218.0323, Ion mode: Positive
Adjusted p (t test): 2.69e−08, Adjusted p (wilcox): 6.71e−06
Log Metabolite Abundance
2
1
0
−1
−2
EM
Metastatic
Condition
In top 10% of significant metabolites

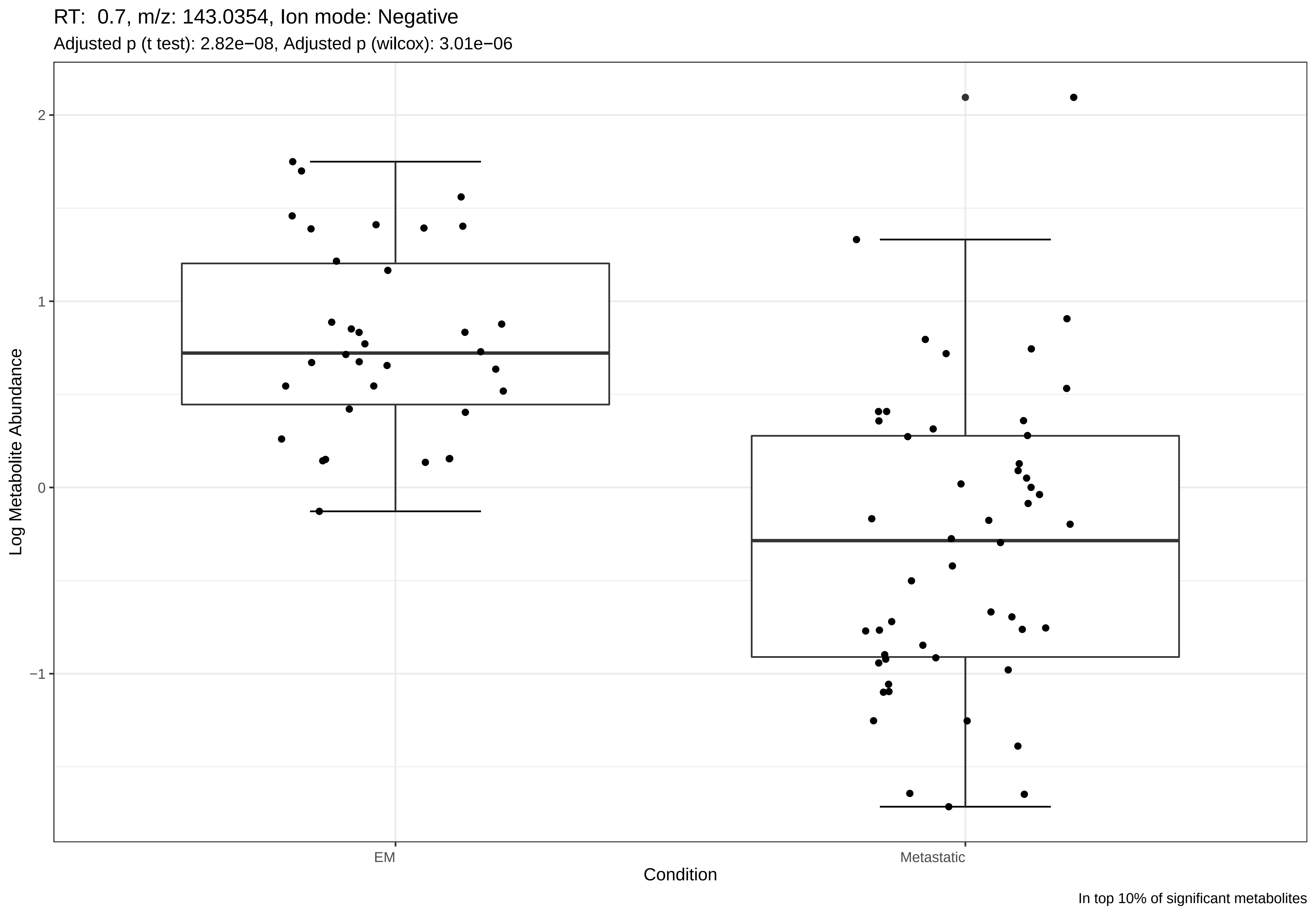
RT: 0.7, m/z: 143.0354, Ion mode: Negative
Adjusted p (t test): 2.82e−08, Adjusted p (wilcox): 3.01e−06
Log Metabolite Abundance
2
1
0
−1
EM
Metastatic
Condition
In top 10% of significant metabolites

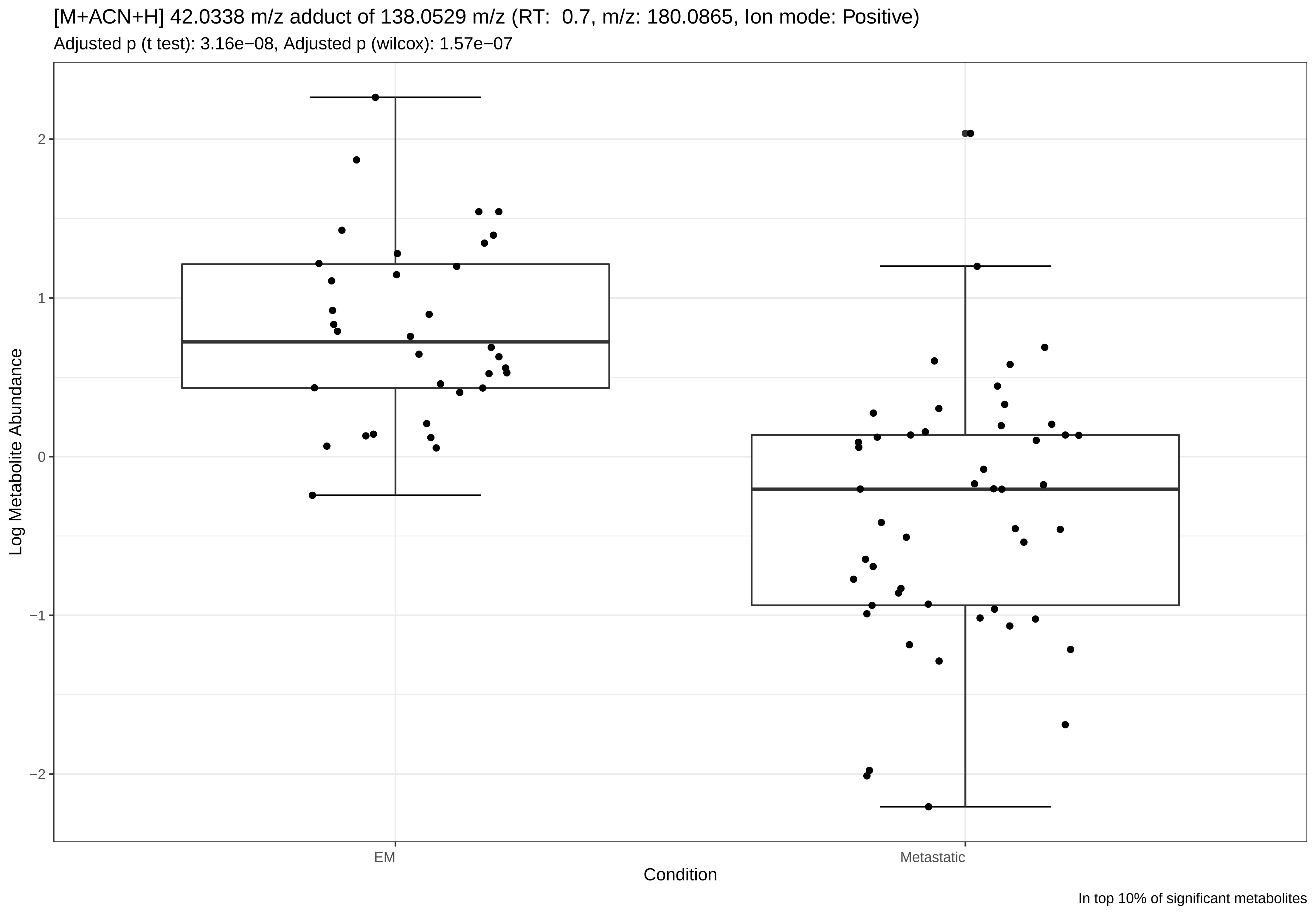

[M+ACN+H] 42.0338 m/z adduct of 138.0529 m/z (RT: 0.7, m/z: 180.0865, Ion mode: Positive)
Adjusted p (t test): 3.16e−08, Adjusted p (wilcox): 1.57e−07
Log Metabolite Abundance
2
1
0
−1
−2
EM
Metastatic
Condition
In top 10% of significant metabolites

RT: 1.4, m/z: 126.0268, Ion mode: Positive

Adjusted p (t test): 3.17e−08, Adjusted p (wilcox): 6.11e−06

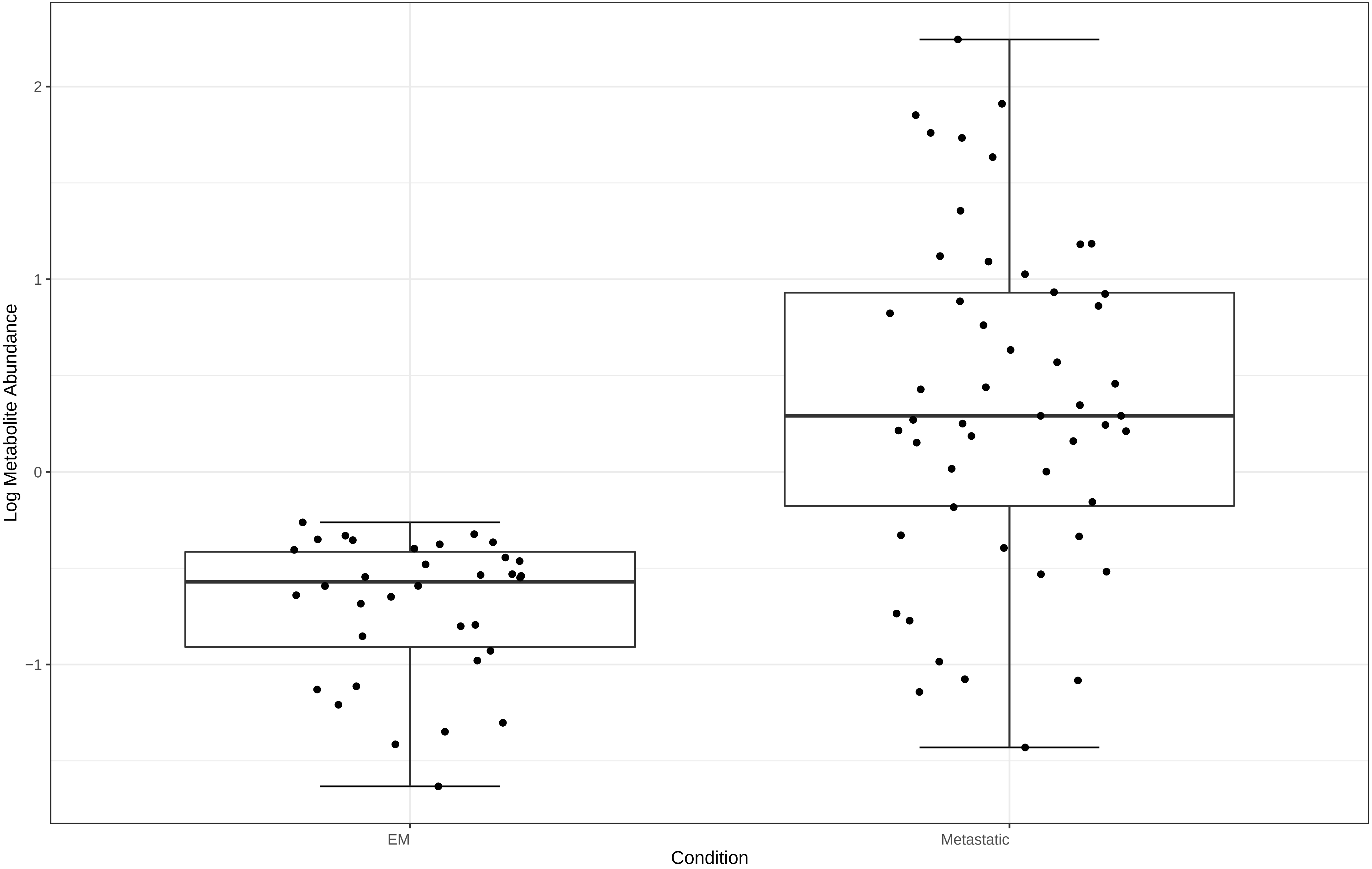

In top 10% of significant metabolites

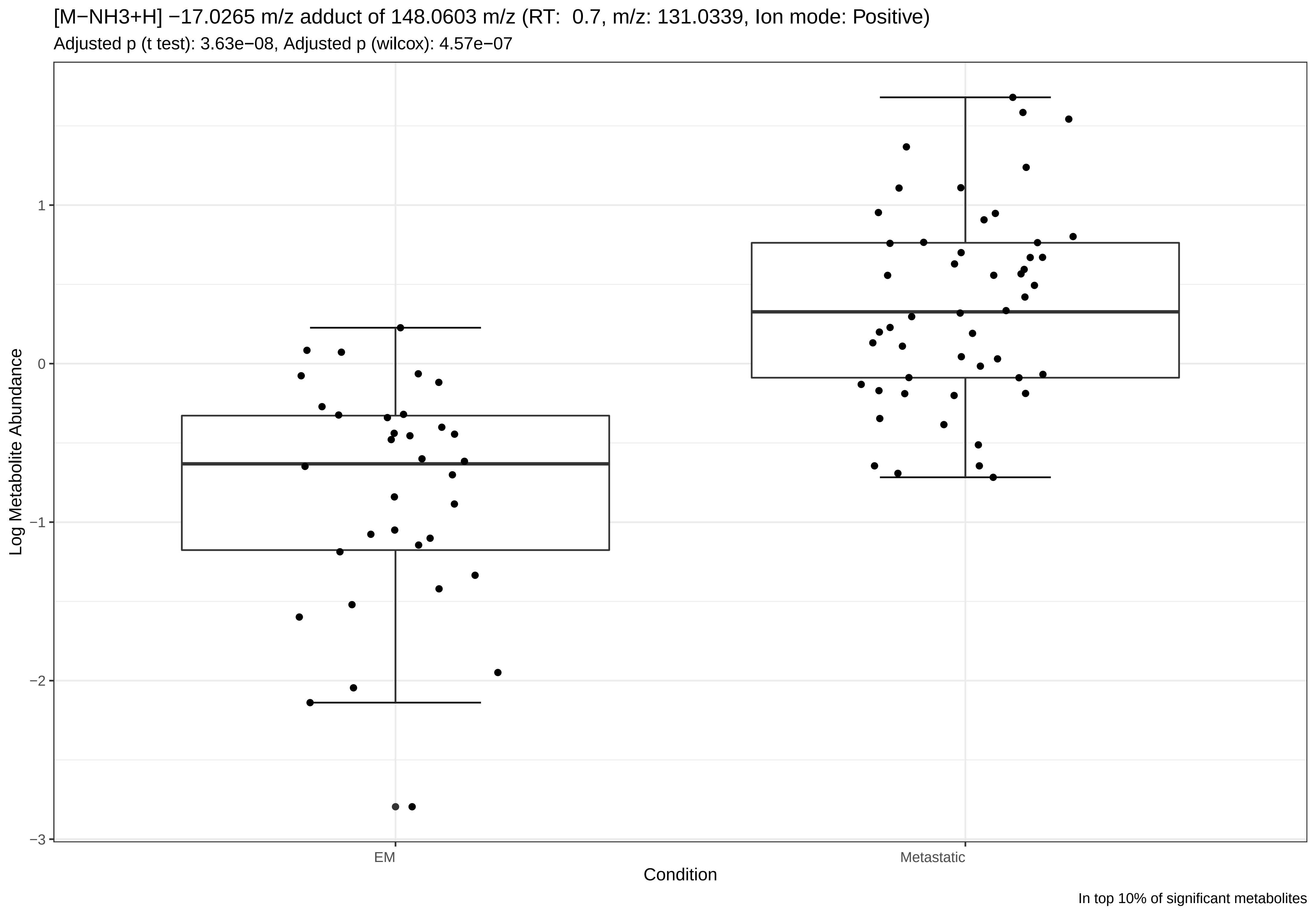

[M−NH3+H] −17.0265 m/z adduct of 148.0603 m/z (RT: 0.7, m/z: 131.0339, Ion mode: Positive)
Adjusted p (t test): 3.63e−08, Adjusted p (wilcox): 4.57e−07
Log Metabolite Abundance
1
0
−1
−2
−3
EM
Metastatic
Condition
In top 10% of significant metabolites

RT: 0.7, m/z: 113.0243, Ion mode: Negative

Adjusted p (t test): 4.28e−08, Adjusted p (wilcox): 3.10e−06

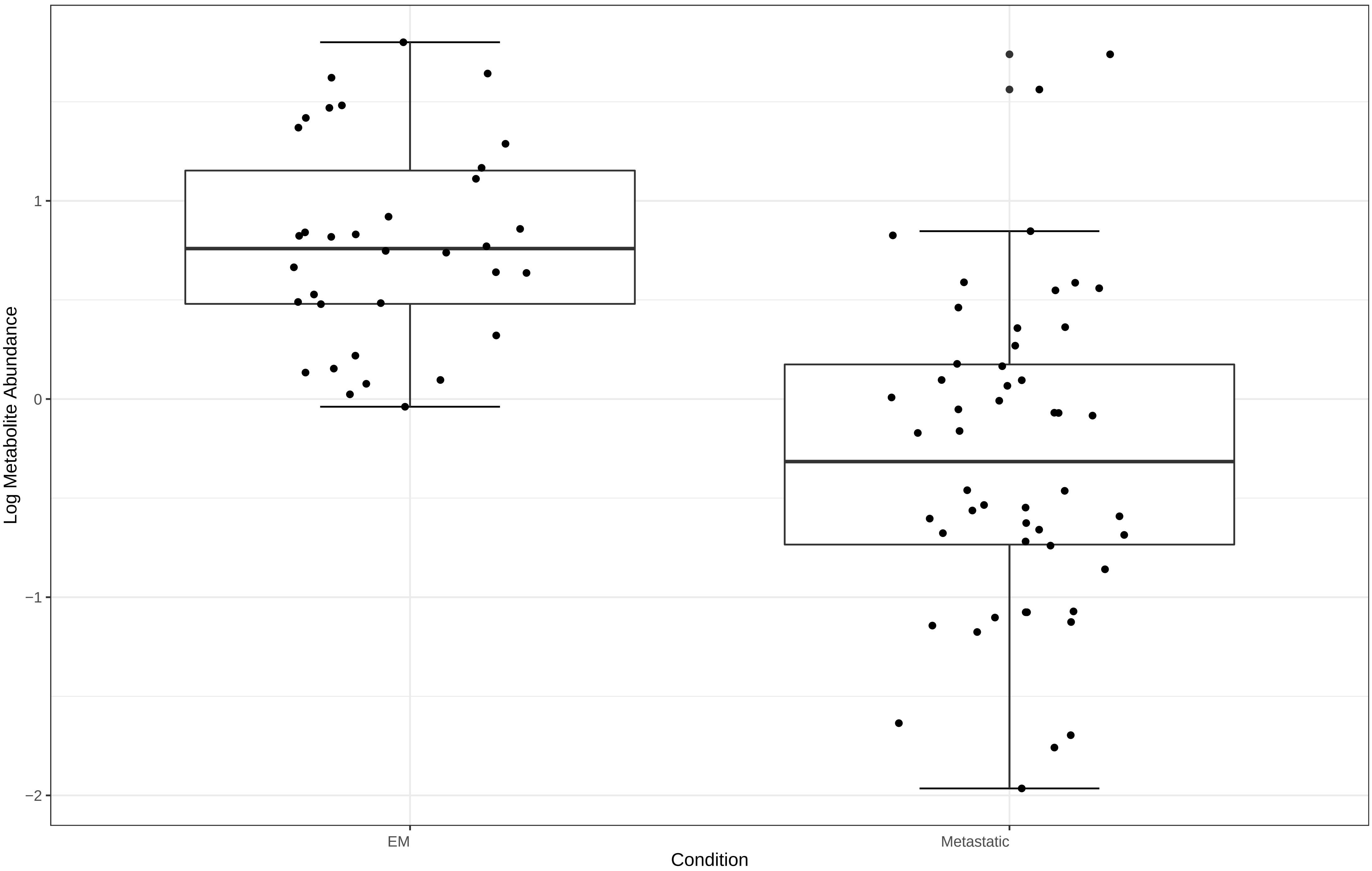

In top 10% of significant metabolites

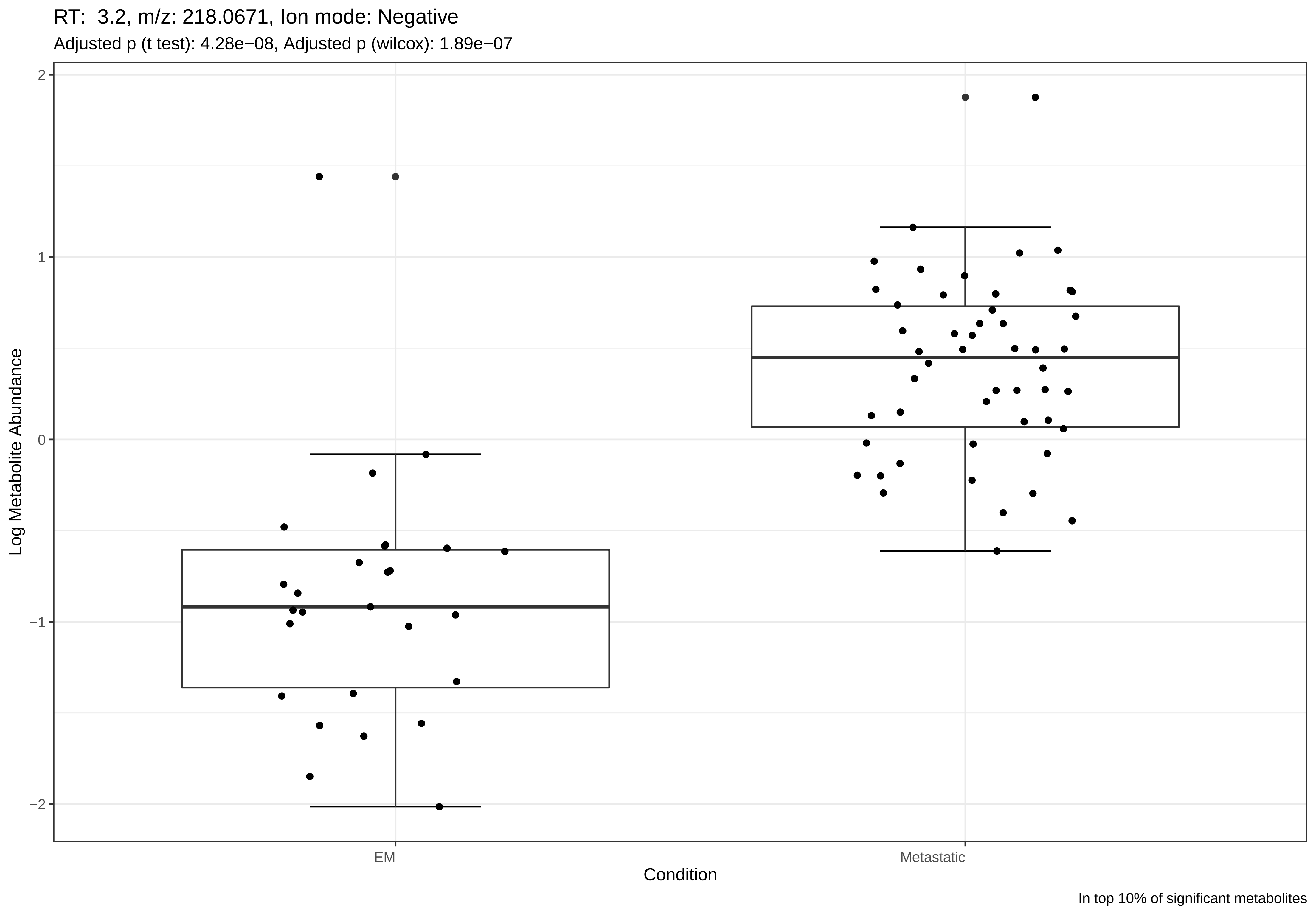
RT: 3.2, m/z: 218.0671, Ion mode: Negative
Adjusted p (t test): 4.28e−08, Adjusted p (wilcox): 1.89e−07
2
1
0
−1
−2
Log Metabolite Abundance
EM
Metastatic
Condition
In top 10% of significant metabolites

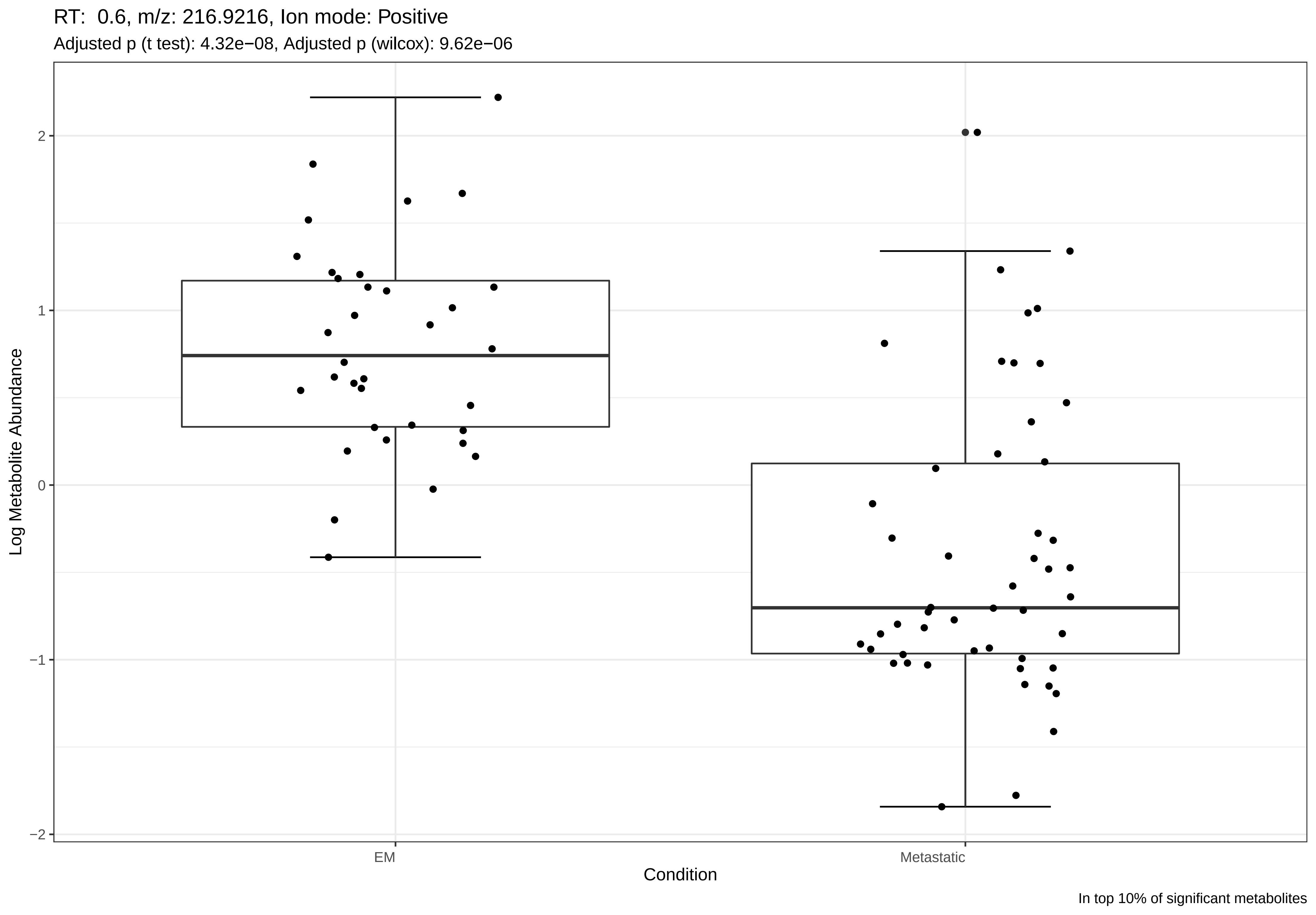
RT: 0.6, m/z: 216.9216, Ion mode: Positive
Adjusted p (t test): 4.32e−08, Adjusted p (wilcox): 9.62e−06
Log Metabolite Abundance
2
1
0
−1
−2
EM
Metastatic
Condition
In top 10% of significant metabolites

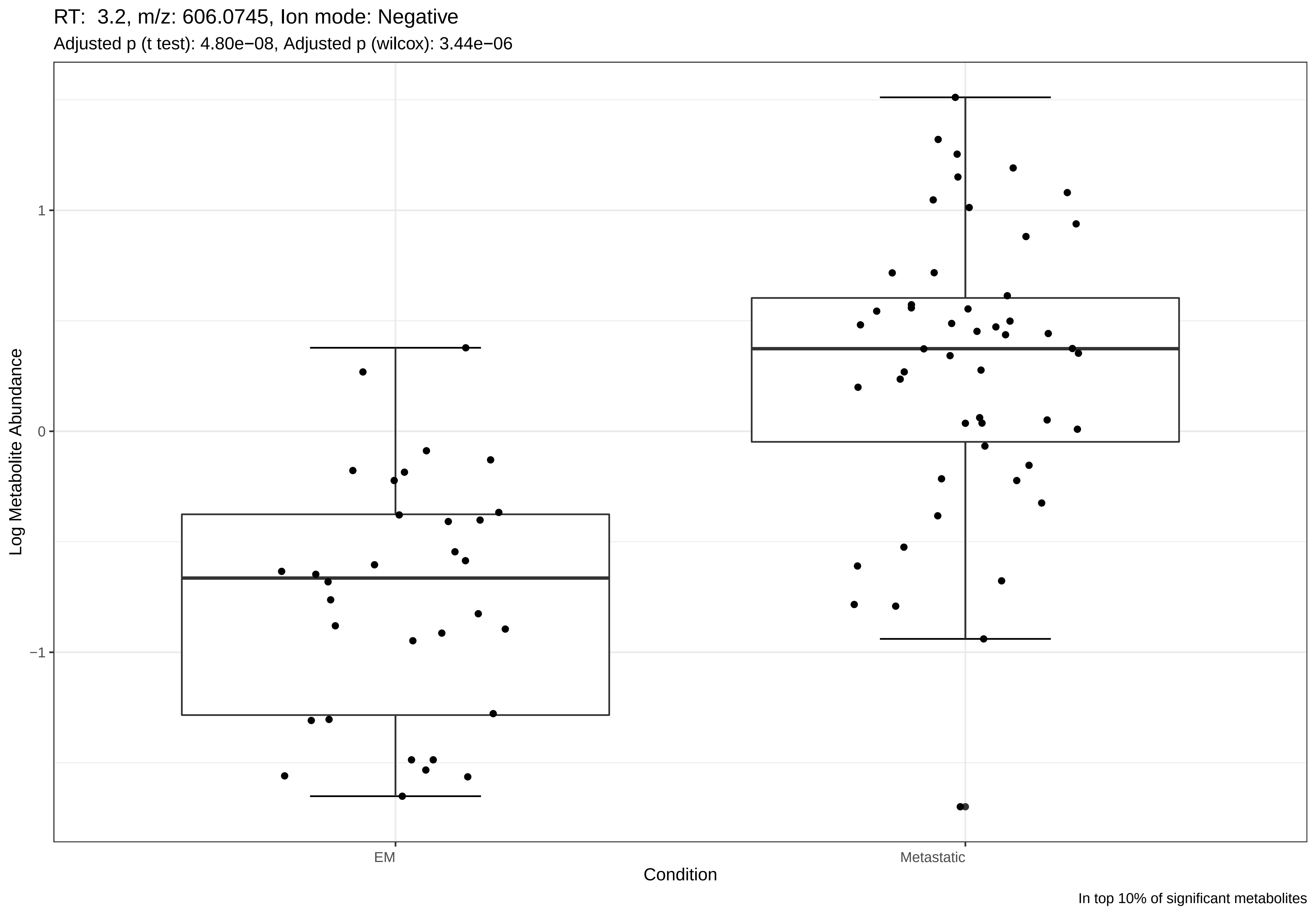
RT: 3.2, m/z: 606.0745, Ion mode: Negative
Adjusted p (t test): 4.80e−08, Adjusted p (wilcox): 3.44e−06
Log Metabolite Abundance
1
0
−1
EM
Metastatic
Condition
In top 10% of significant metabolites

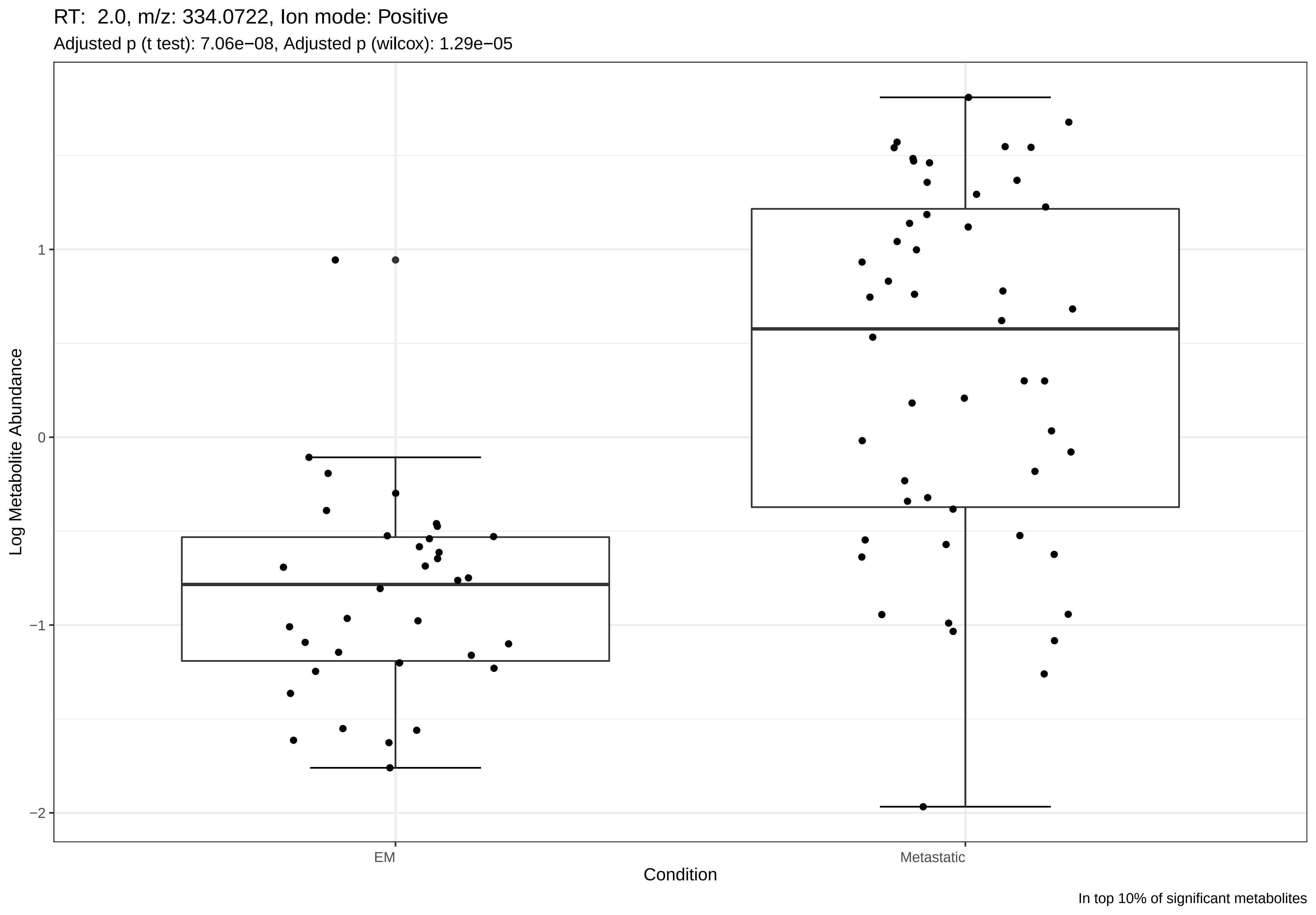
RT: 2.0, m/z: 334.0722, Ion mode: Positive
Adjusted p (t test): 7.06e−08, Adjusted p (wilcox): 1.29e−05
Log Metabolite Abundance
1
0
−1
−2
EM
Metastatic
Condition
In top 10% of significant metabolites

RT: 0.7, m/z: 96.9218, Ion mode: Negative

Adjusted p (t test): 7.12e−08, Adjusted p (wilcox): 2.98e−06

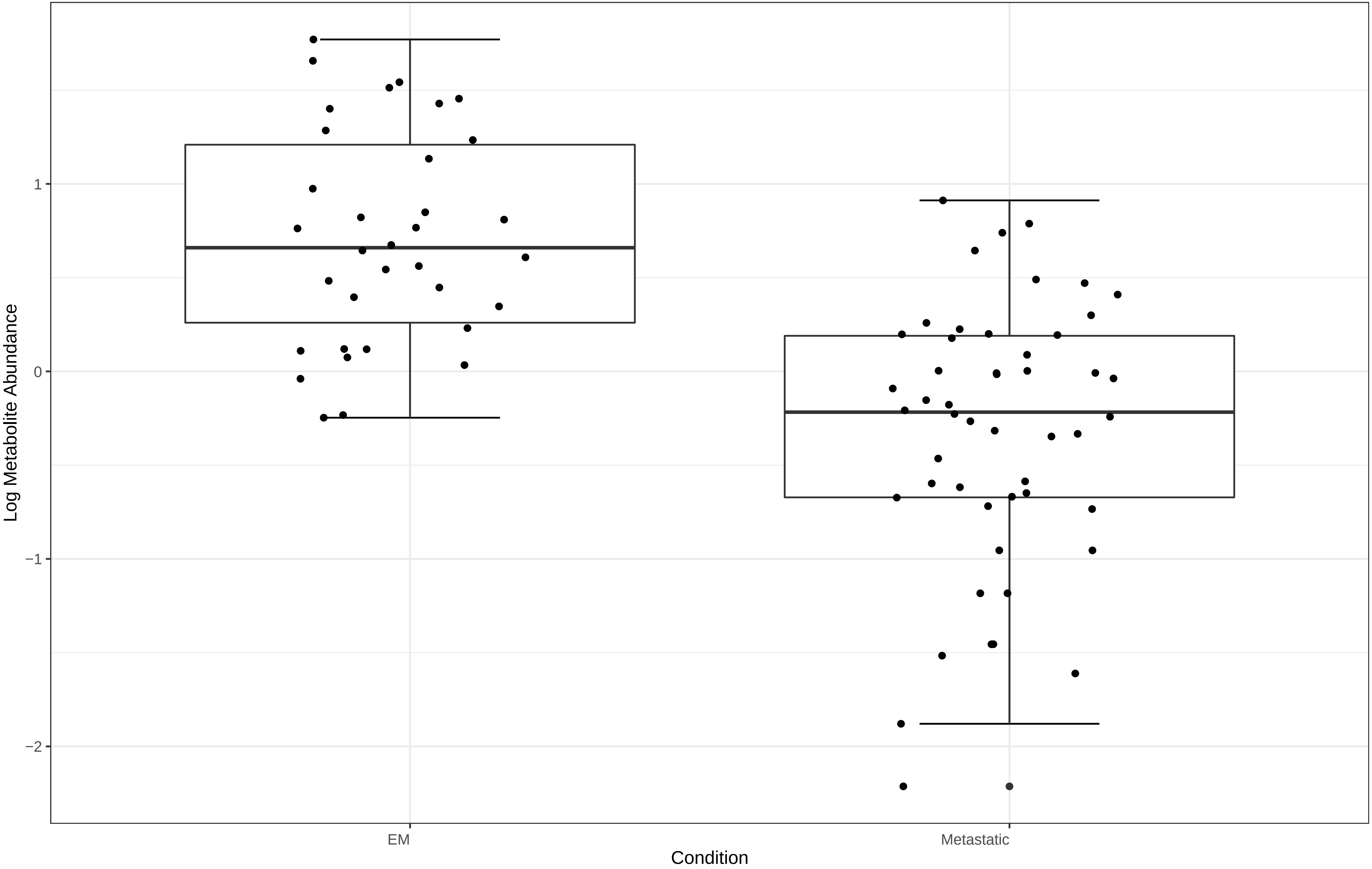

In top 10% of significant metabolites

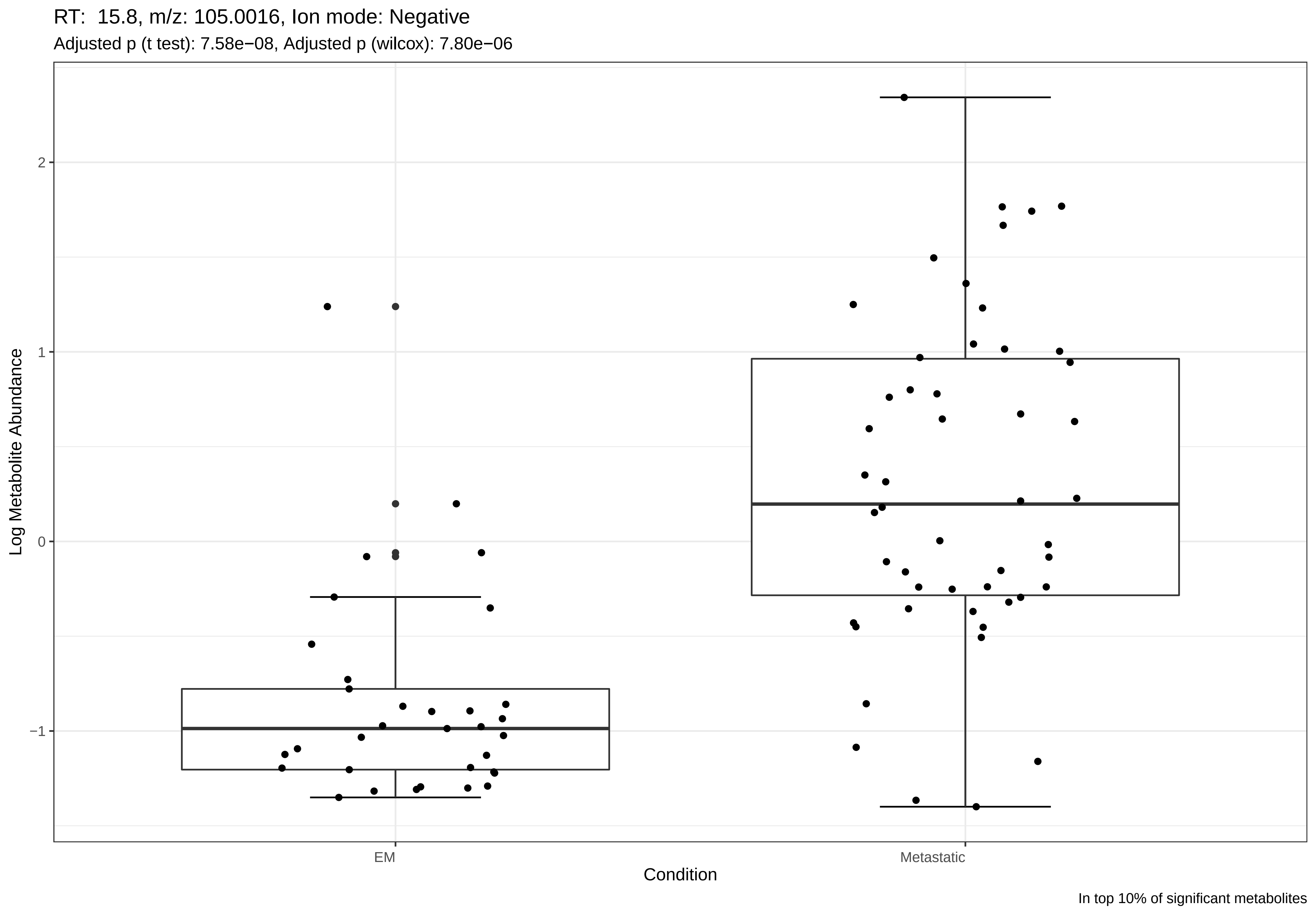
RT: 15.8, m/z: 105.0016, Ion mode: Negative
Adjusted p (t test): 7.58e−08, Adjusted p (wilcox): 7.80e−06
Log Metabolite Abundance
2
1
0
−1
EM
Metastatic
Condition
In top 10% of significant metabolites

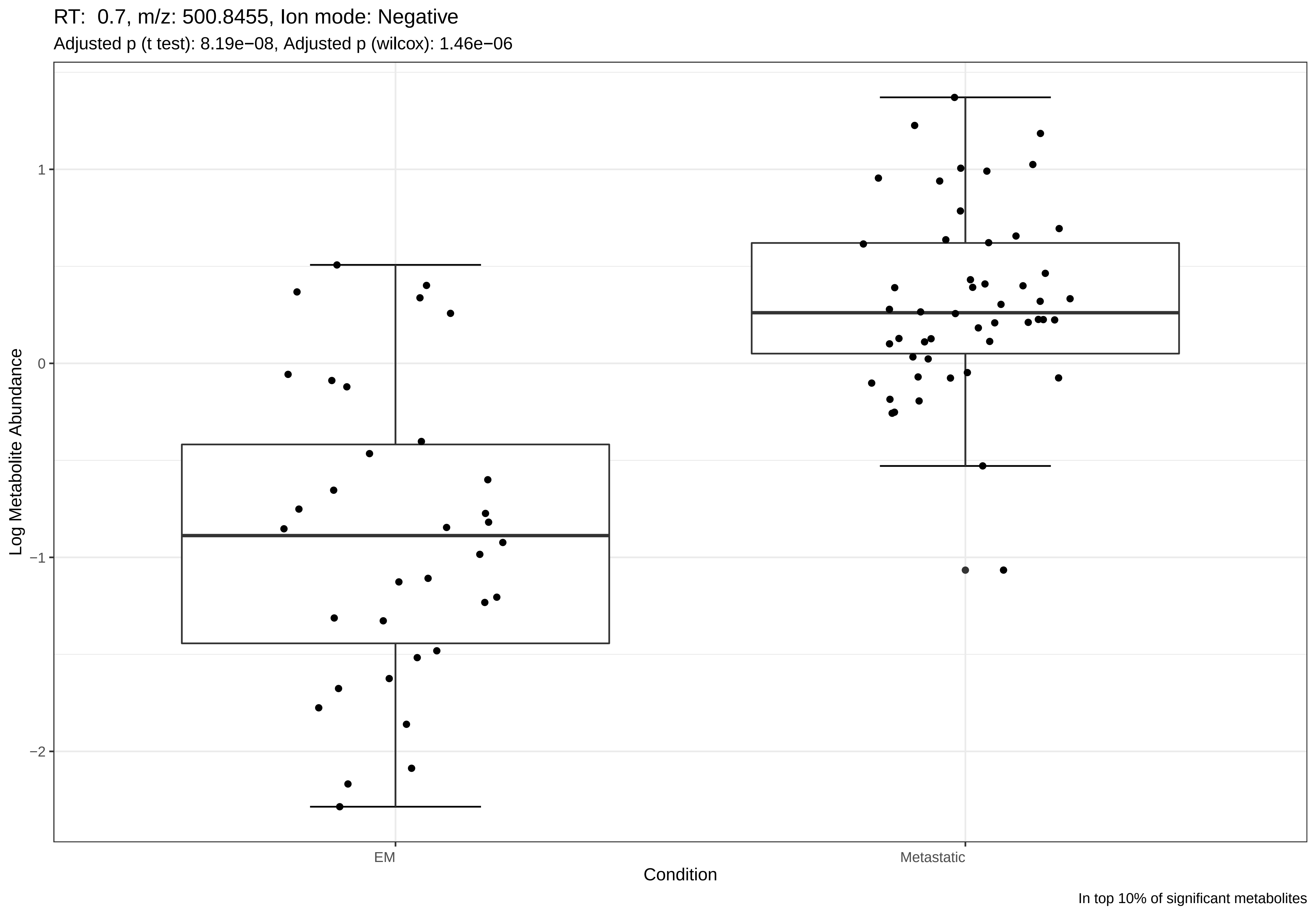

RT: 0.7, m/z: 500.8455, Ion mode: Negative
Adjusted p (t test): 8.19e−08, Adjusted p (wilcox): 1.46e−06
Log Metabolite Abundance
1
0
−1
−2
EM
Metastatic
Condition
In top 10% of significant metabolites

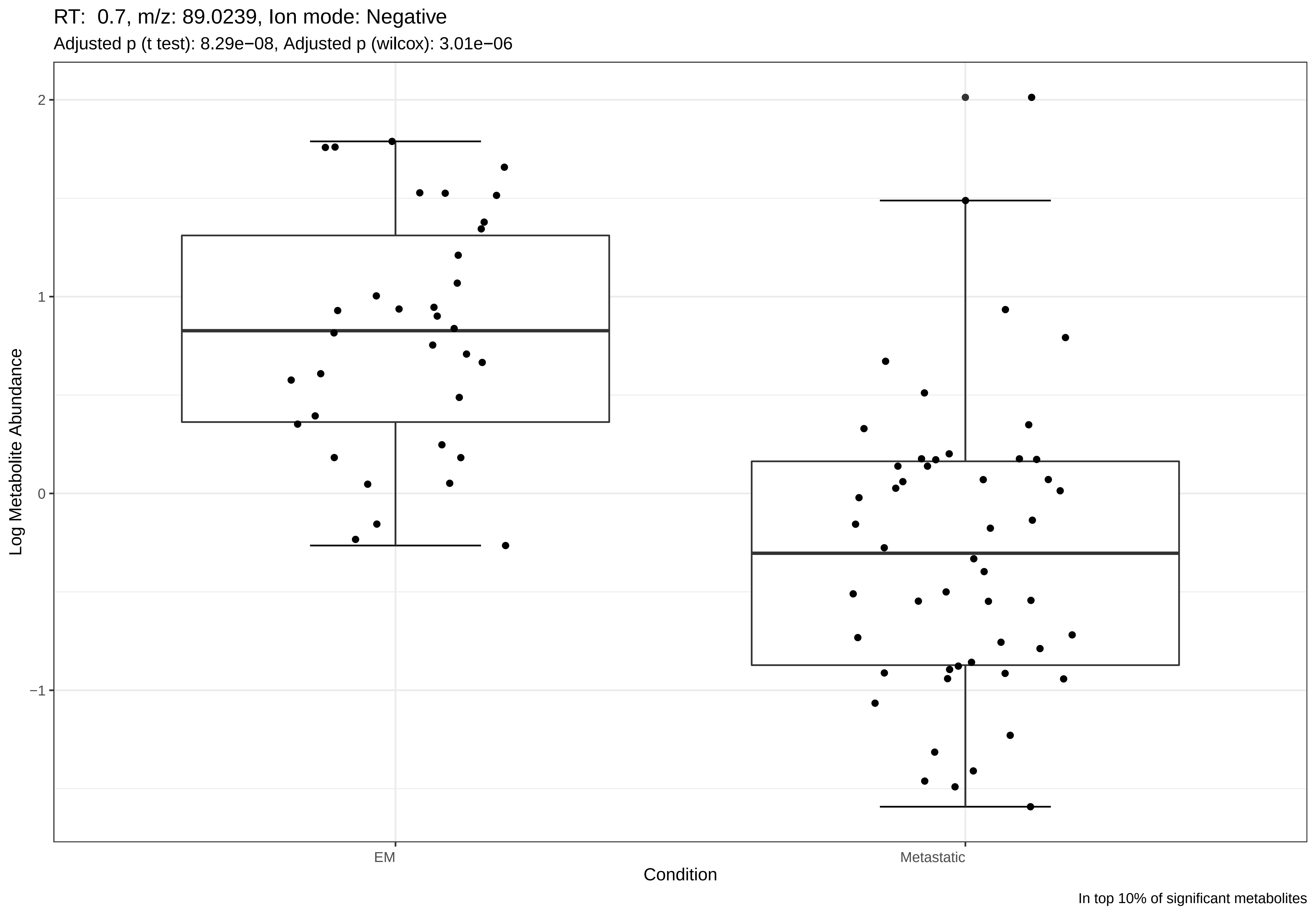

RT: 0.7, m/z: 89.0239, Ion mode: Negative
Adjusted p (t test): 8.29e−08, Adjusted p (wilcox): 3.01e−06
Log Metabolite Abundance
2
1
0
−1
EM
Metastatic
Condition
In top 10% of significant metabolites

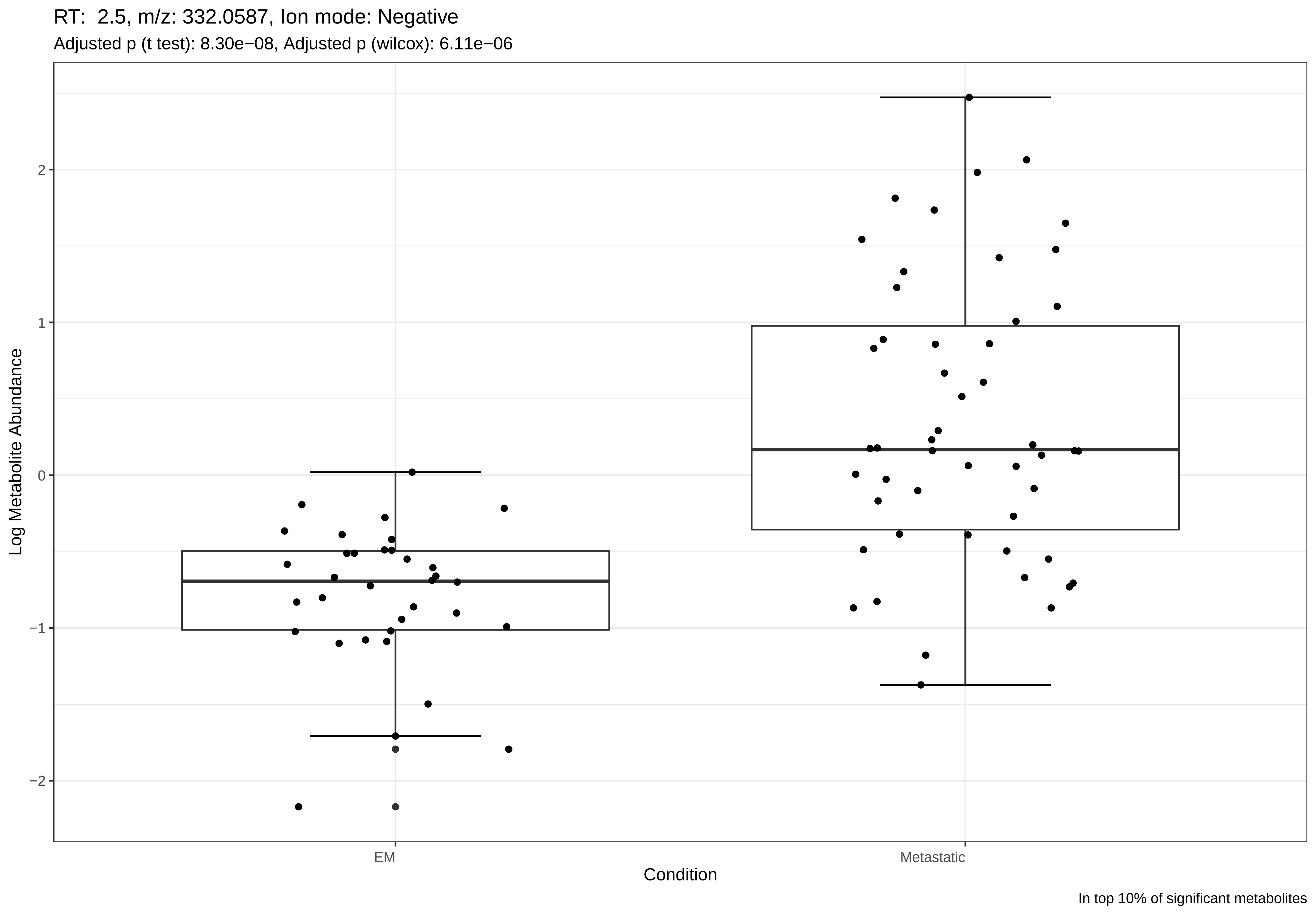

RT: 2.5, m/z: 332.0587, Ion mode: Negative
Adjusted p (t test): 8.30e−08, Adjusted p (wilcox): 6.11e−06
Log Metabolite Abundance
2
1
0
−1
−2
EM
Metastatic
Condition
In top 10% of significant metabolites

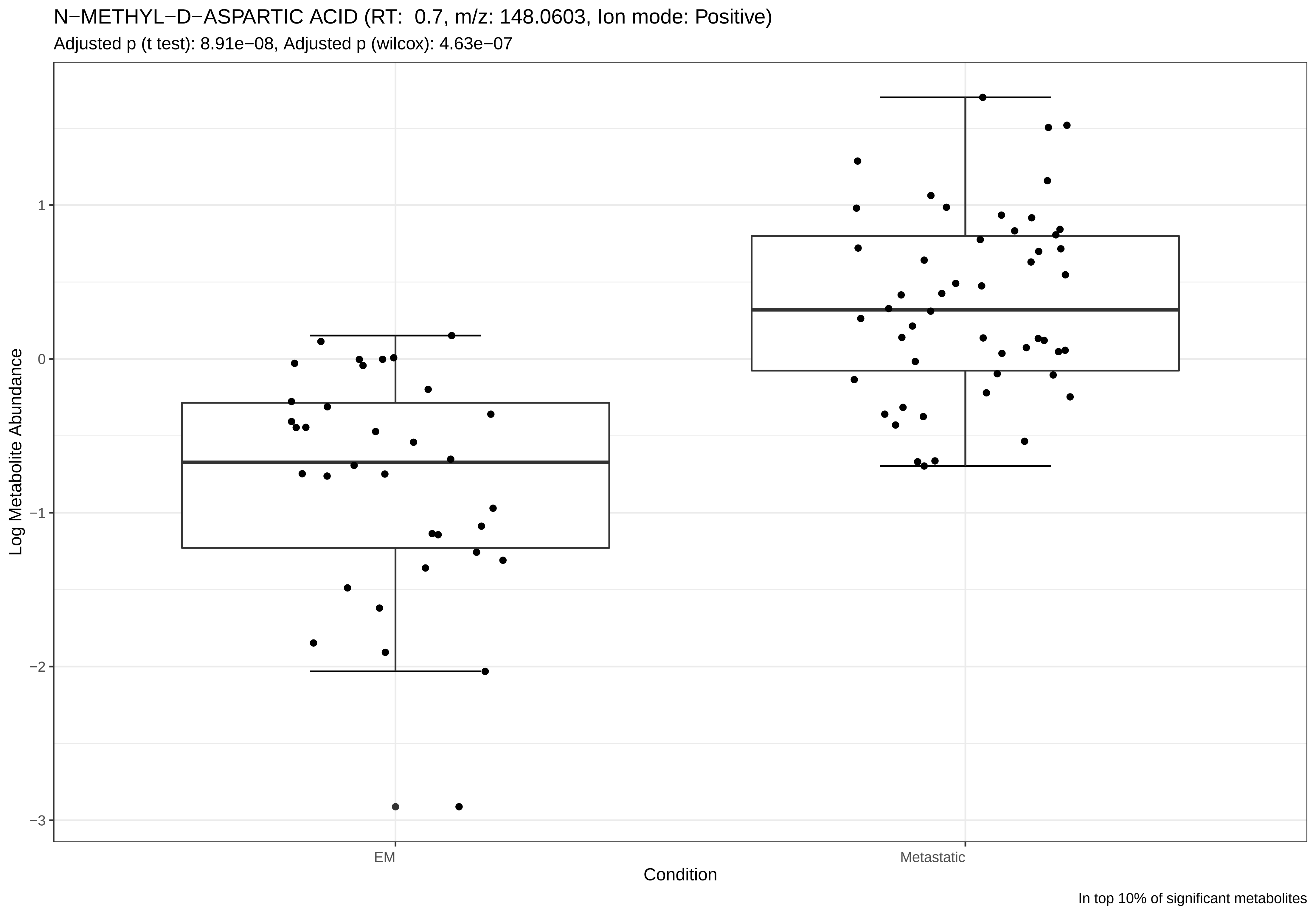

N−METHYL−D−ASPARTIC ACID (RT: 0.7, m/z: 148.0603, Ion mode: Positive)
Adjusted p (t test): 8.91e−08, Adjusted p (wilcox): 4.63e−07
Log Metabolite Abundance
1
0
−1
−2
−3
EM
Metastatic
Condition
In top 10% of significant metabolites

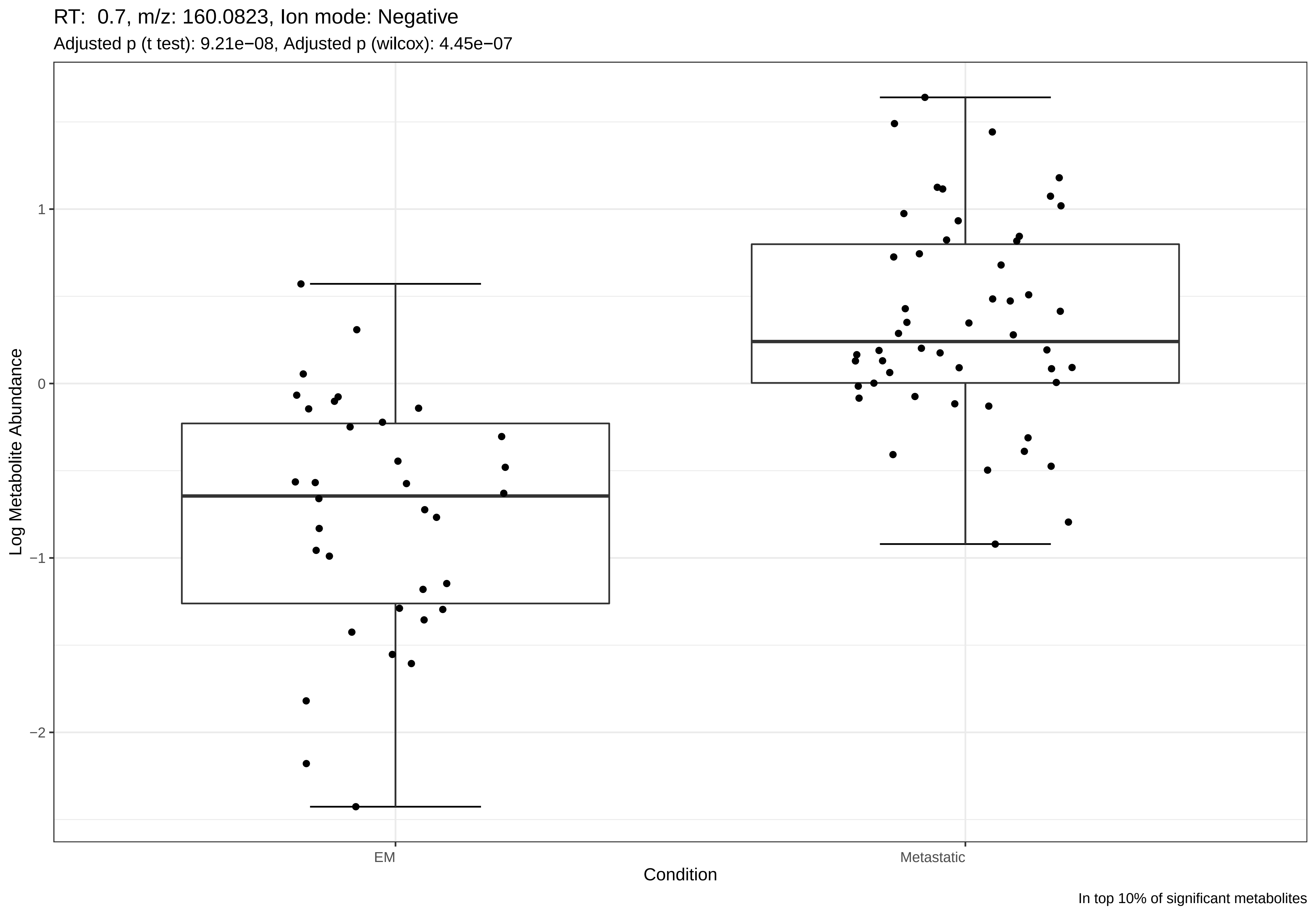
RT: 0.7, m/z: 160.0823, Ion mode: Negative
Adjusted p (t test): 9.21e−08, Adjusted p (wilcox): 4.45e−07
Log Metabolite Abundance
1
0
−1
−2
EM
Metastatic
Condition
In top 10% of significant metabolites

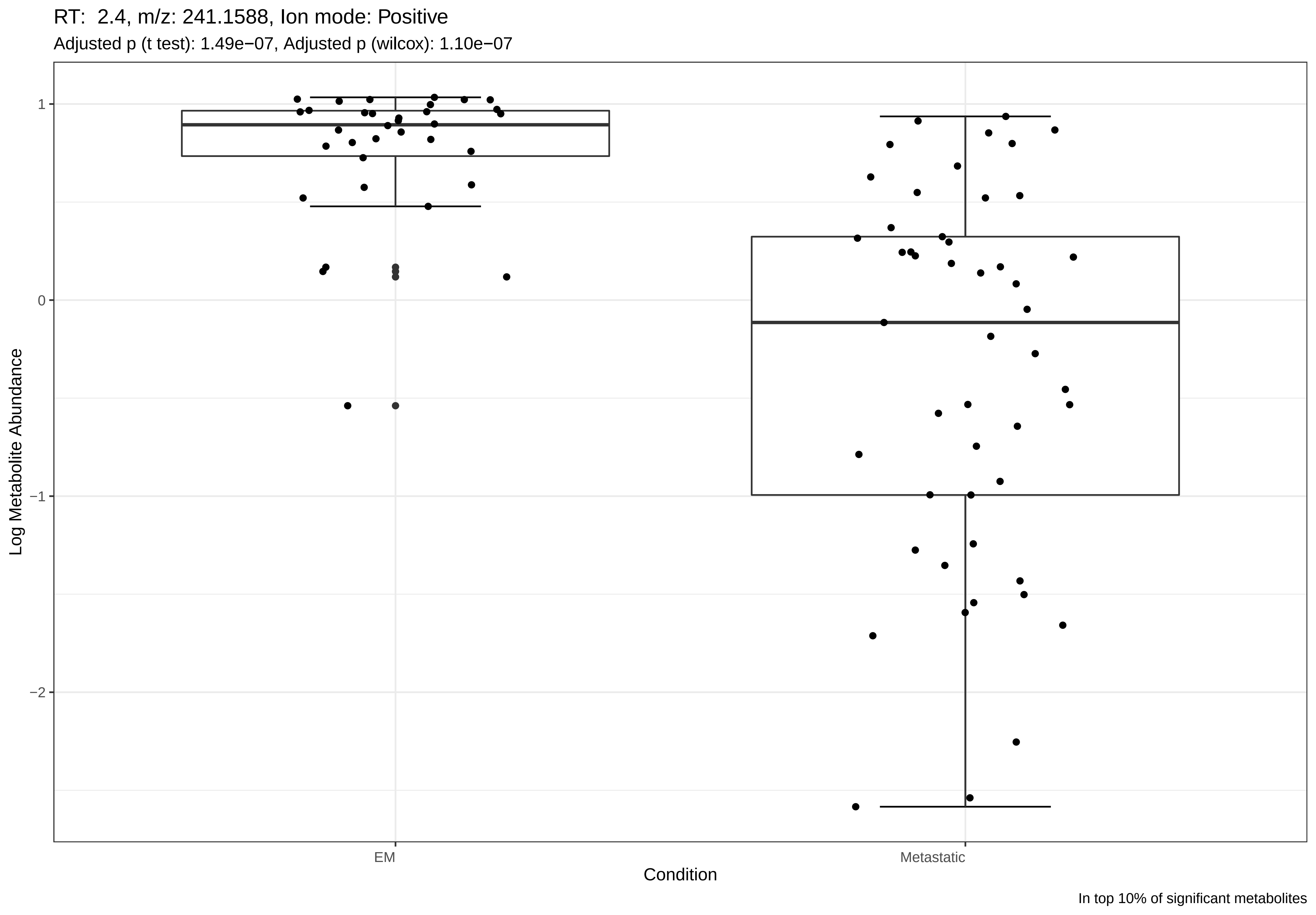

RT: 2.4, m/z: 241.1588, Ion mode: Positive
Adjusted p (t test): 1.49e−07, Adjusted p (wilcox): 1.10e−07
Log Metabolite Abundance
1
0
−1
−2
EM
Metastatic
Condition
In top 10% of significant metabolites

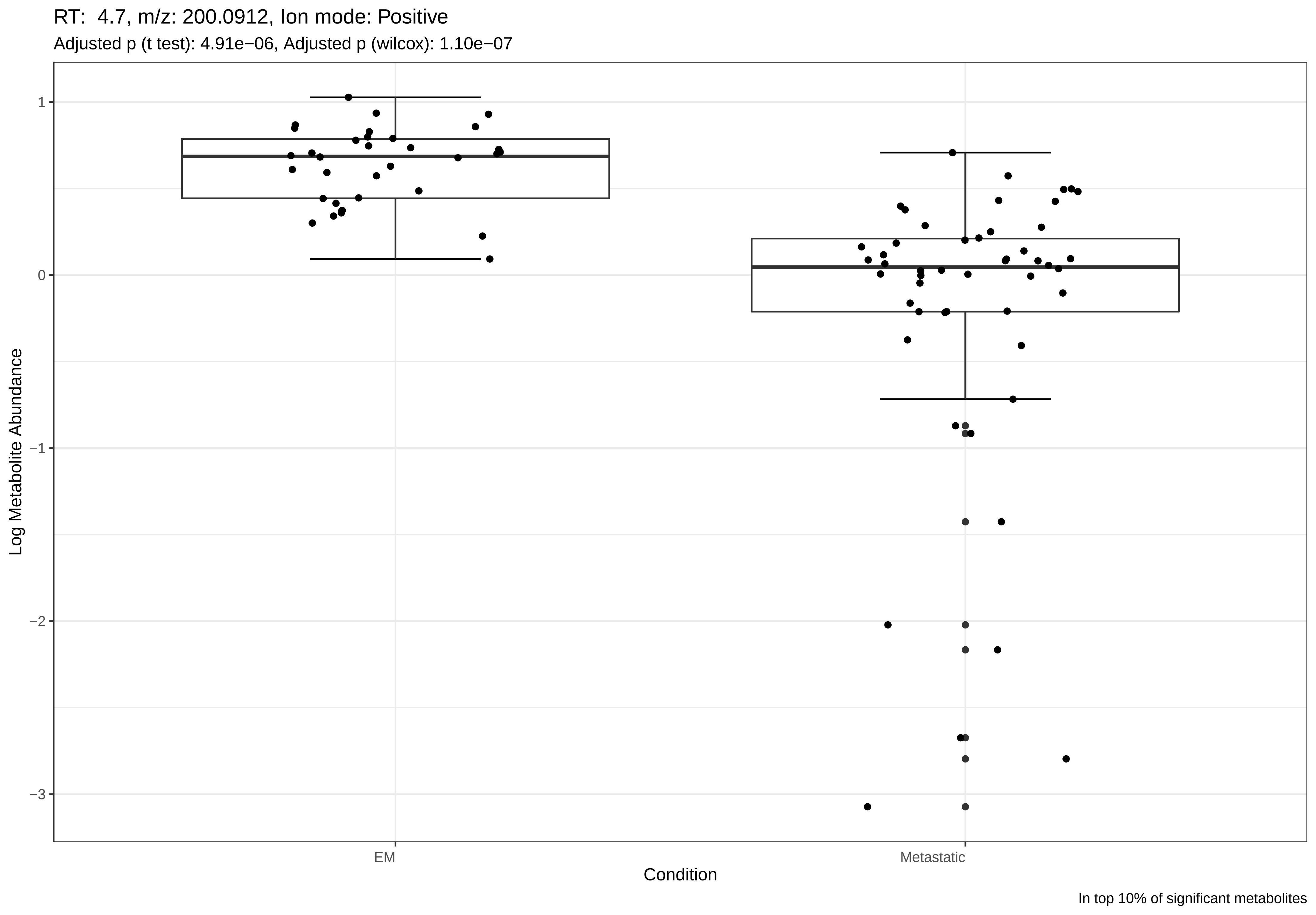
RT: 4.7, m/z: 200.0912, Ion mode: Positive
Adjusted p (t test): 4.91e−06, Adjusted p (wilcox): 1.10e−07
Log Metabolite Abundance
1
0
−1
−2
−3
EM
Metastatic
Condition
In top 10% of significant metabolites

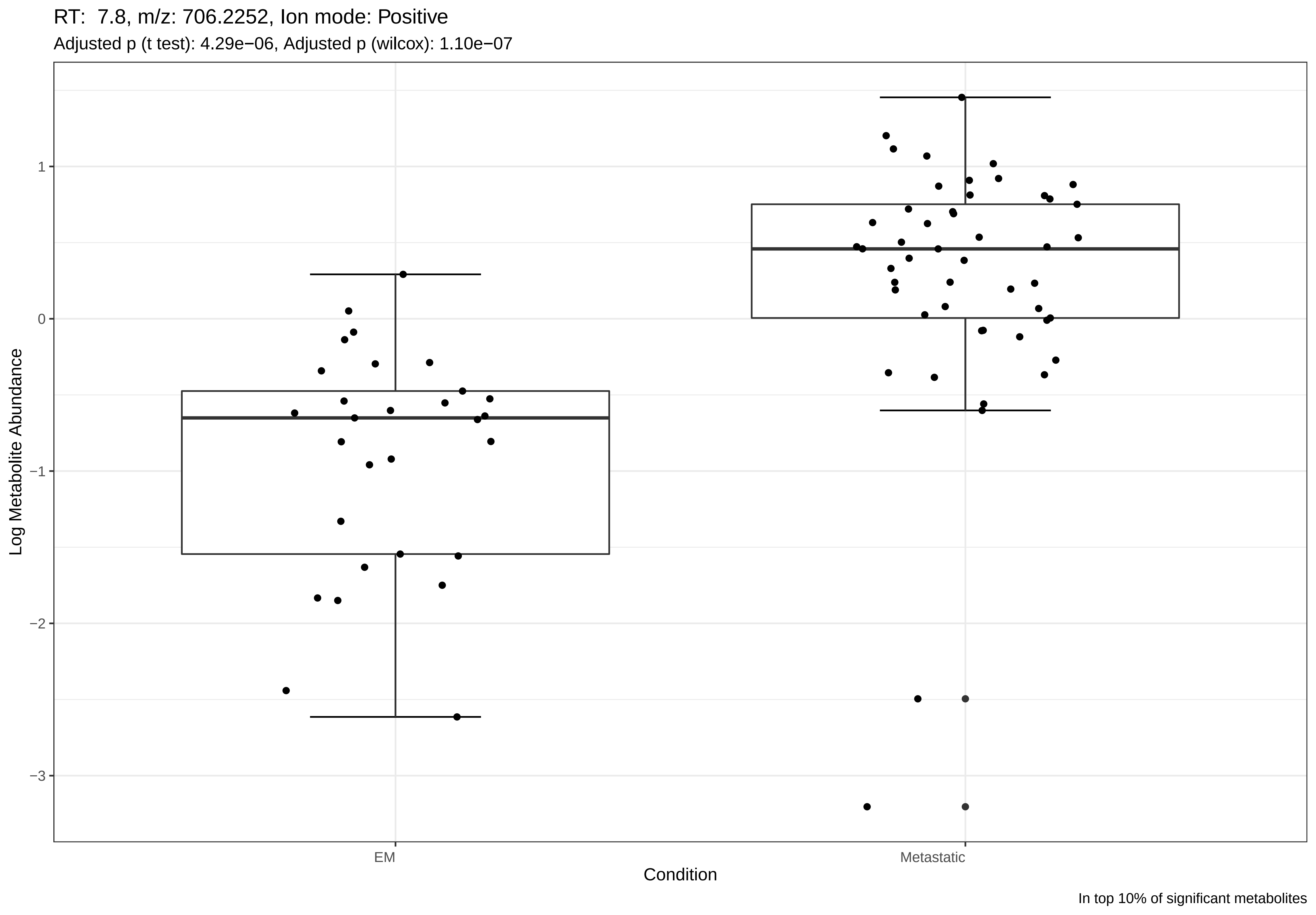
RT: 7.8, m/z: 706.2252, Ion mode: Positive
Adjusted p (t test): 4.29e−06, Adjusted p (wilcox): 1.10e−07
1
0
−1
−2
−3
Log Metabolite Abundance
EM
Metastatic
Condition
In top 10% of significant metabolites

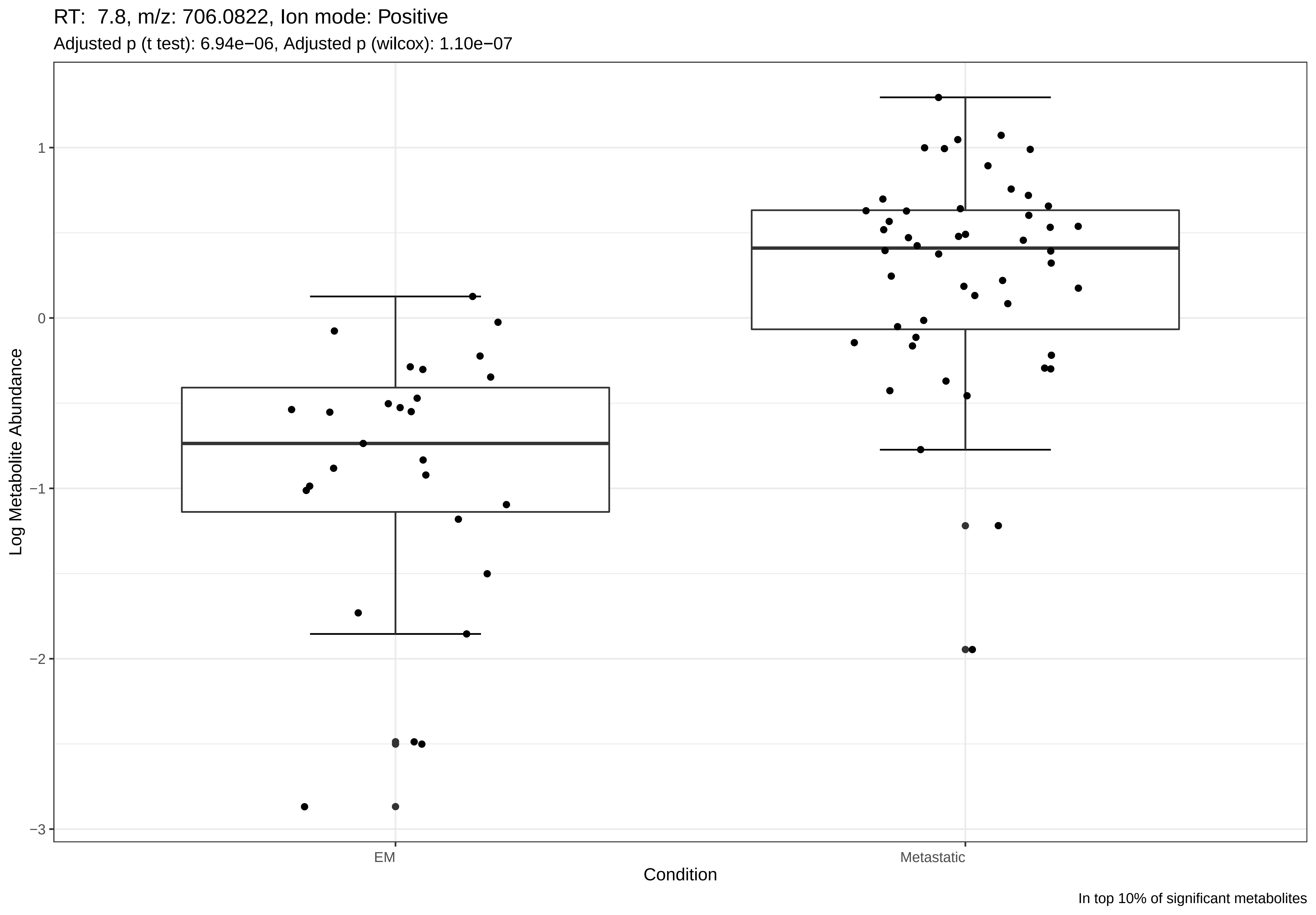

RT: 7.8, m/z: 706.0822, Ion mode: Positive
Adjusted p (t test): 6.94e−06, Adjusted p (wilcox): 1.10e−07
Log Metabolite Abundance
1
0
−1
−2
−3
EM
Metastatic
Condition
In top 10% of significant metabolites

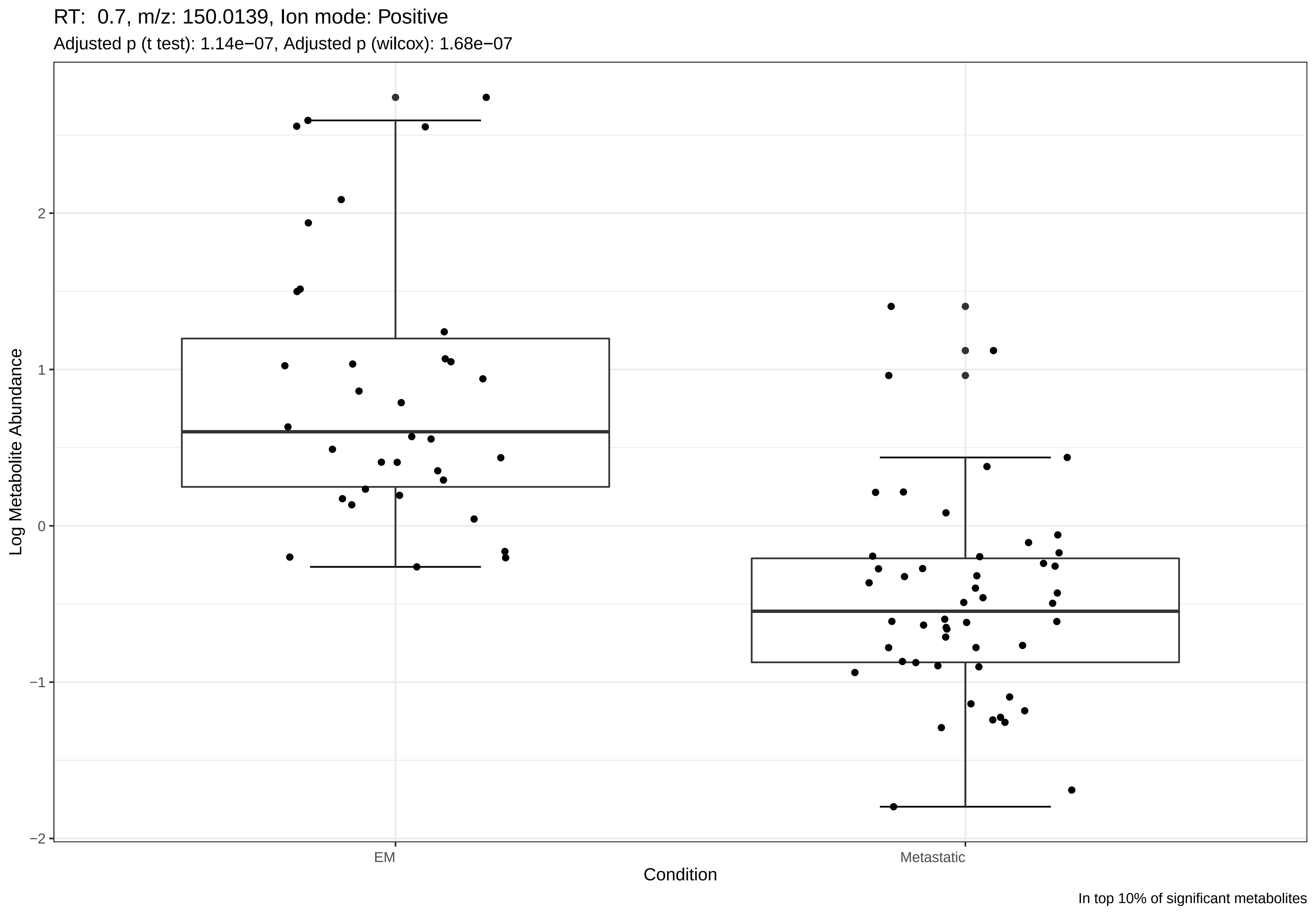
RT: 0.7, m/z: 150.0139, Ion mode: Positive
Adjusted p (t test): 1.14e−07, Adjusted p (wilcox): 1.68e−07
Log Metabolite Abundance
2
1
0
−1
−2
EM
Metastatic
Condition
In top 10% of significant metabolites

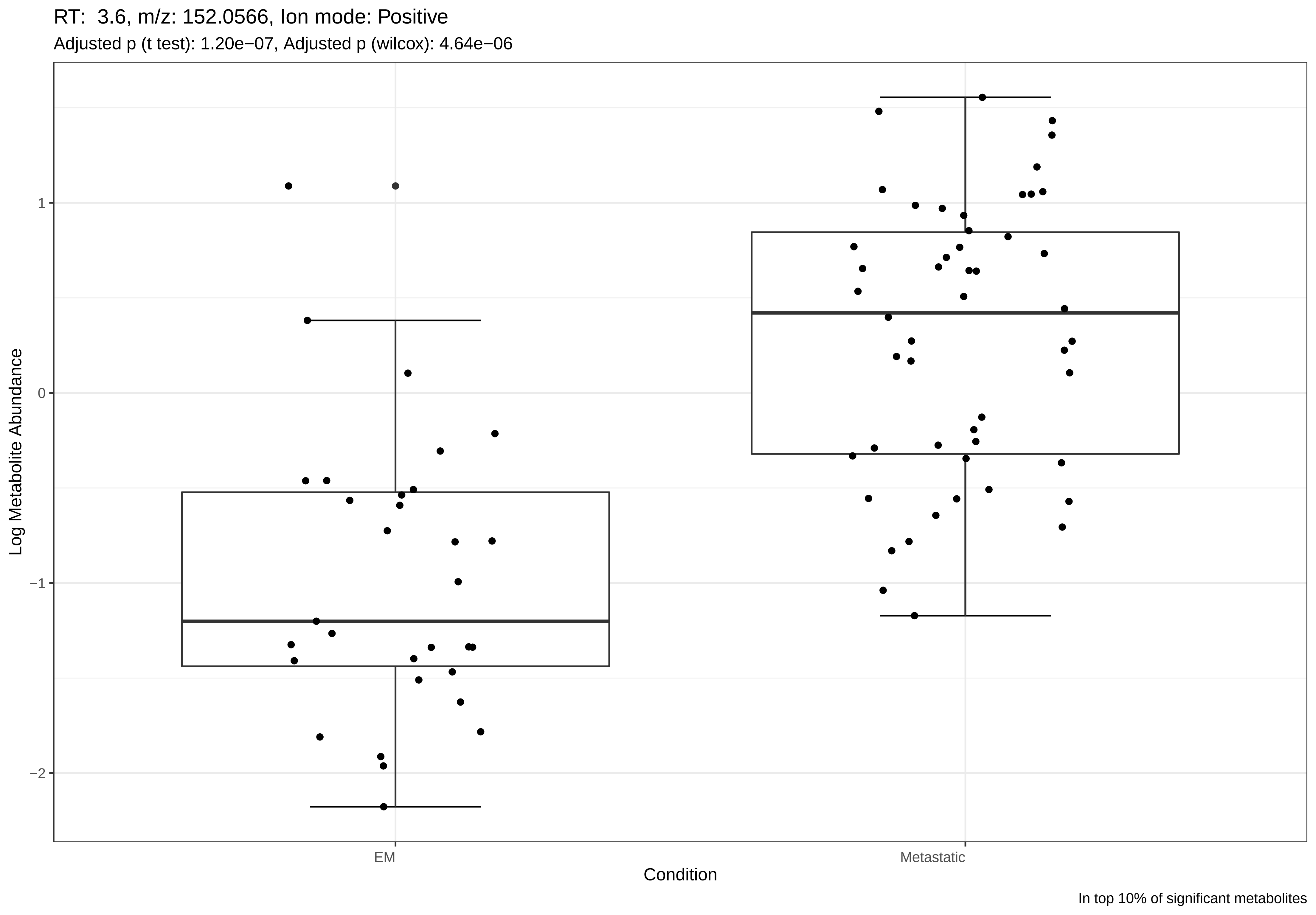

RT: 3.6, m/z: 152.0566, Ion mode: Positive
Adjusted p (t test): 1.20e−07, Adjusted p (wilcox): 4.64e−06
Log Metabolite Abundance
1
0
−1
−2
EM
Metastatic
Condition
In top 10% of significant metabolites

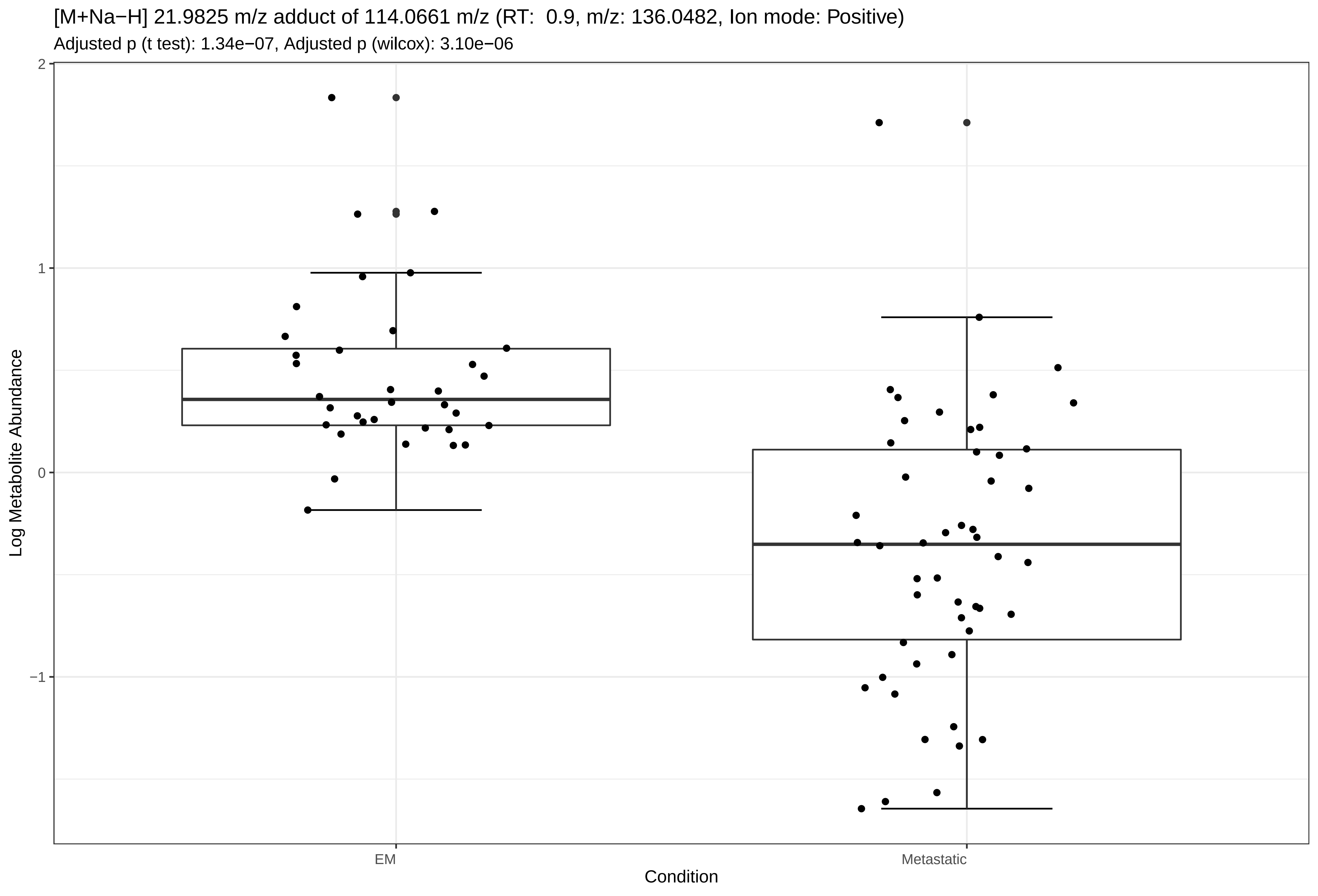
[M+Na−H] 21.9825 m/z adduct of 114.0661 m/z (RT: 0.9, m/z: 136.0482, Ion mode: Positive)
Adjusted p (t test): 1.34e−07, Adjusted p (wilcox): 3.10e−06
Log Metabolite Abundance
2
1
0
−1
EM
Metastatic
Condition

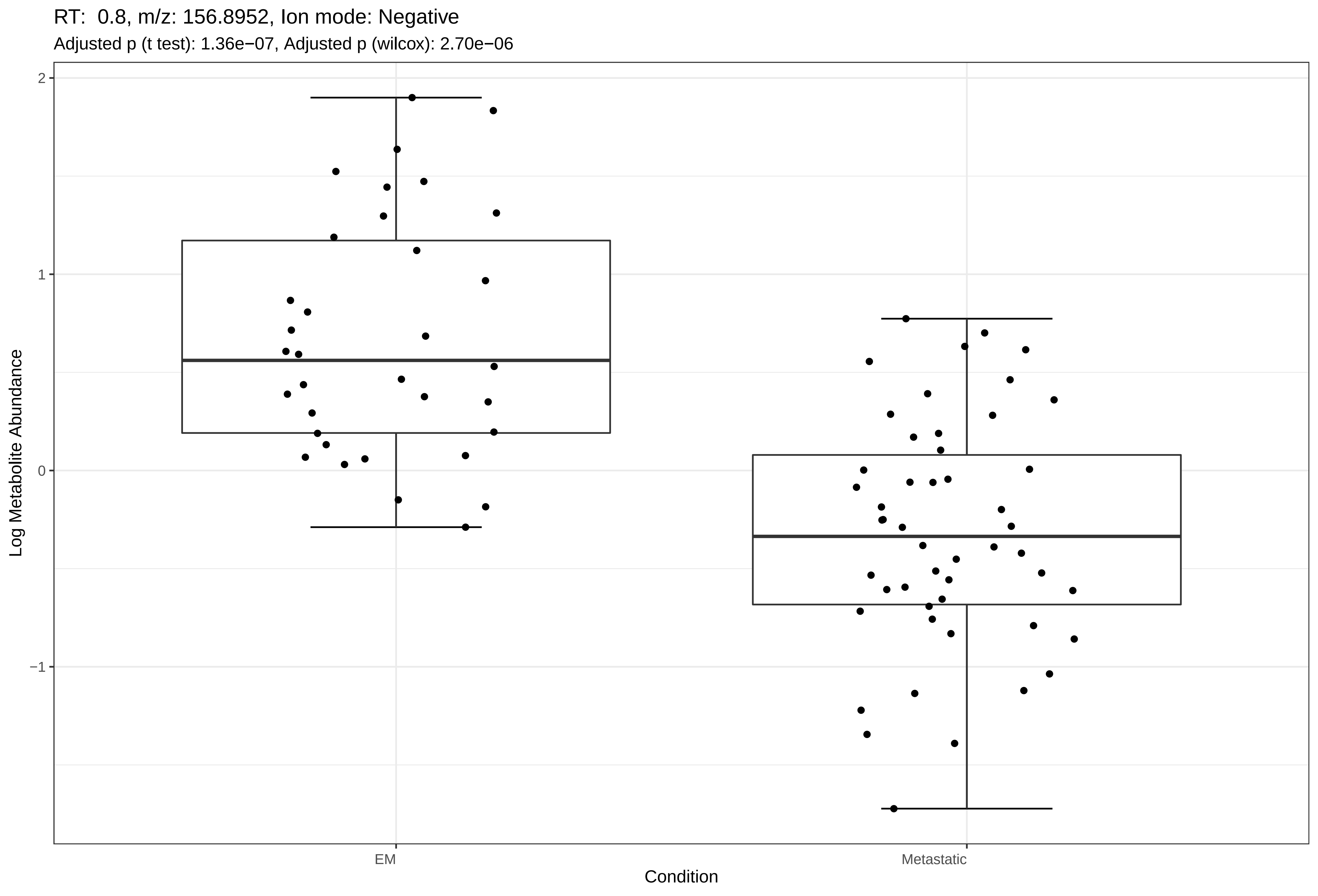

RT: 0.8, m/z: 156.8952, Ion mode: Negative
Adjusted p (t test): 1.36e−07, Adjusted p (wilcox): 2.70e−06
Log Metabolite Abundance
2
1
0
−1
EM
Metastatic
Condition

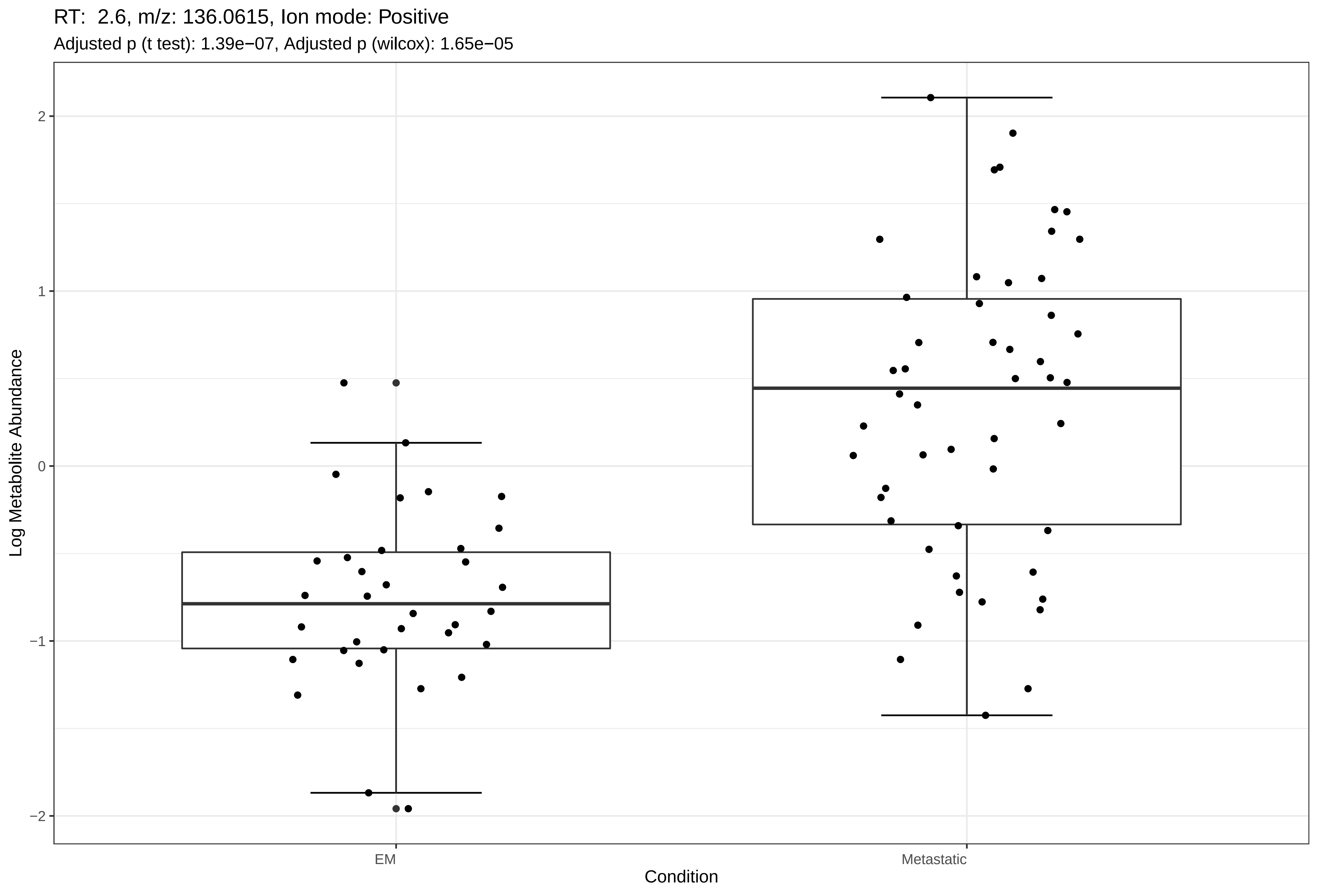

RT: 2.6, m/z: 136.0615, Ion mode: Positive
Adjusted p (t test): 1.39e−07, Adjusted p (wilcox): 1.65e−05
Log Metabolite Abundance
2
1
0
−1
−2
EM
Metastatic
Condition

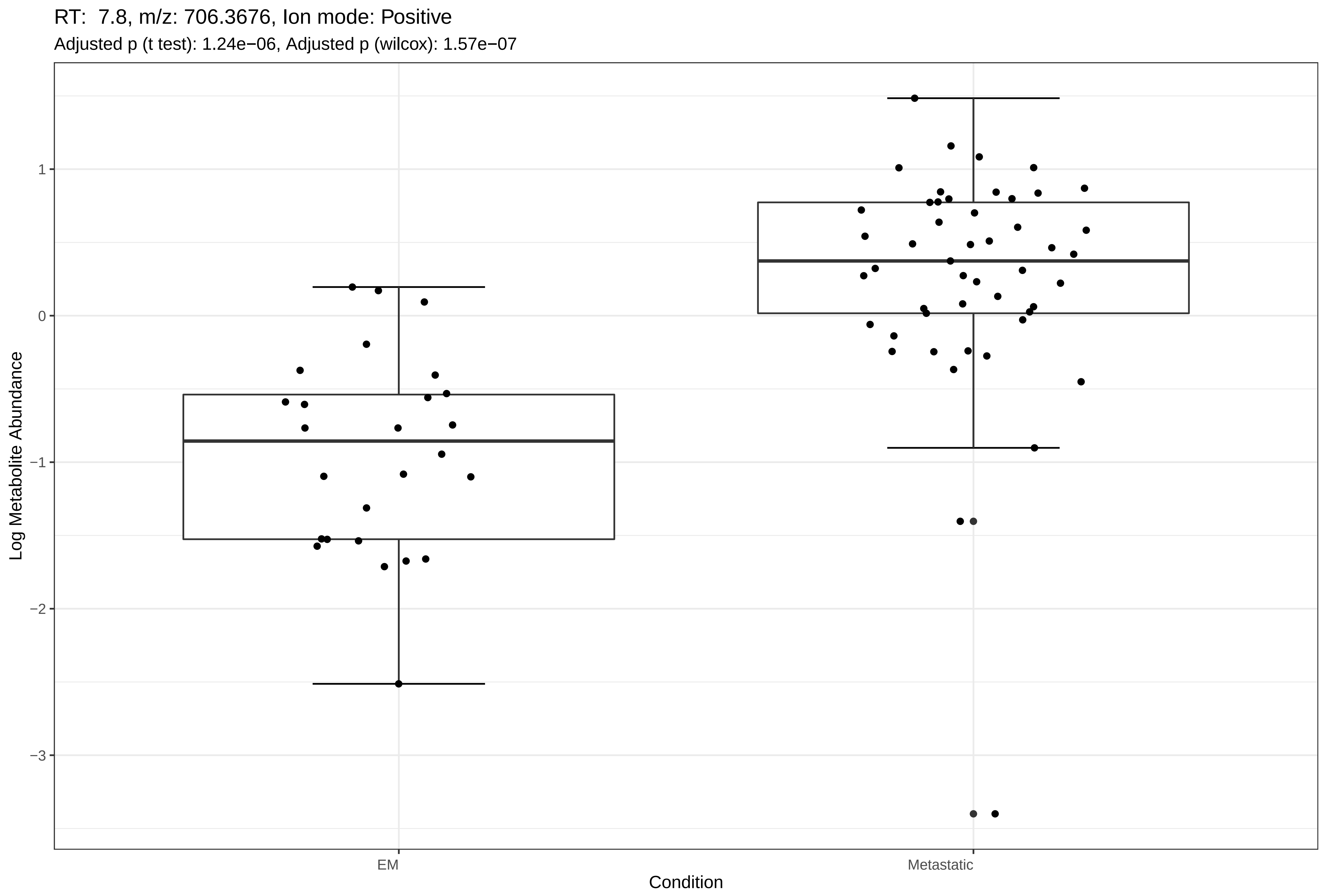
RT: 7.8, m/z: 706.3676, Ion mode: Positive
Adjusted p (t test): 1.24e−06, Adjusted p (wilcox): 1.57e−07
Log Metabolite Abundance
1
0
−1
−2
−3
EM
Metastatic
Condition

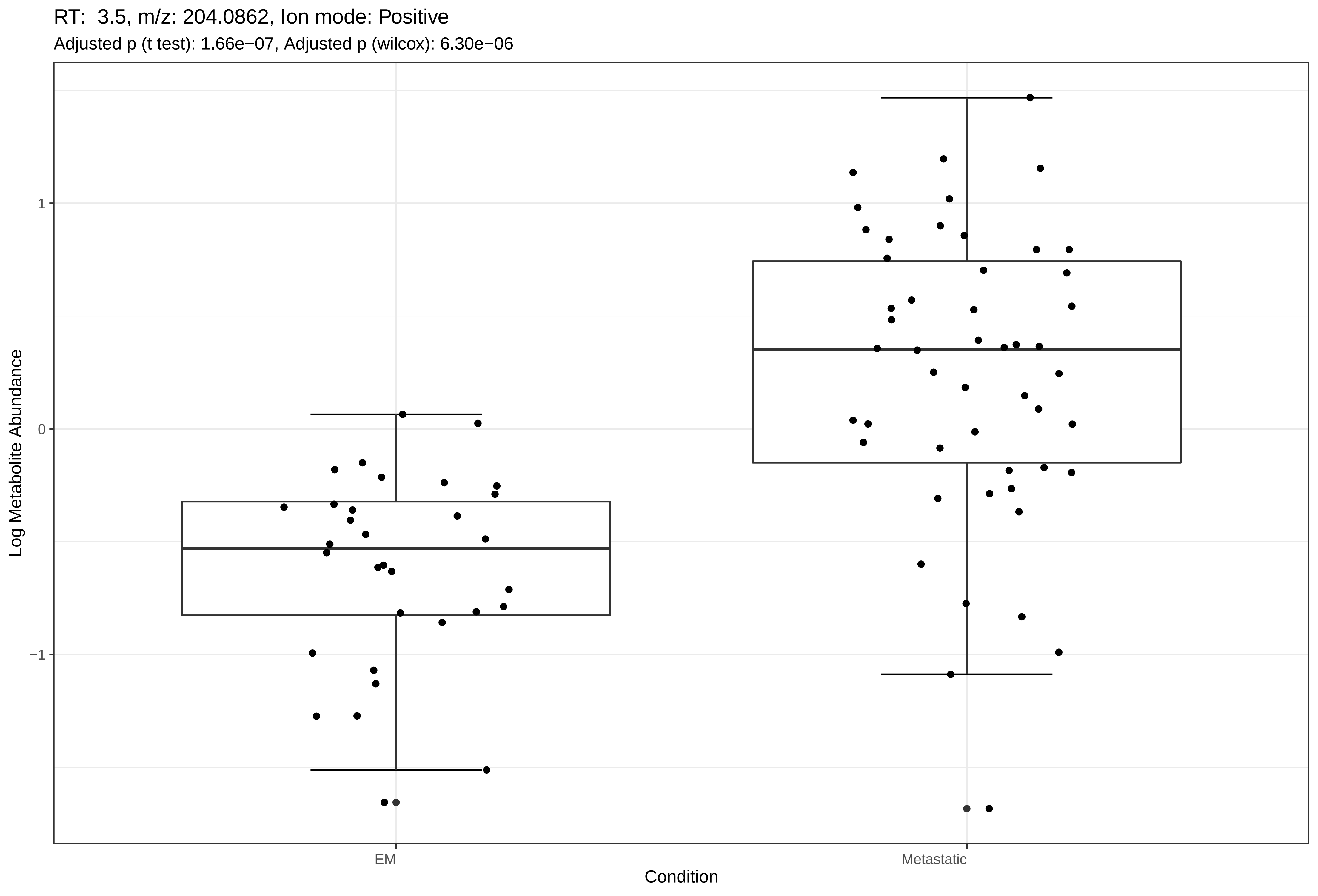

RT: 3.5, m/z: 204.0862, Ion mode: Positive
Adjusted p (t test): 1.66e−07, Adjusted p (wilcox): 6.30e−06
Log Metabolite Abundance
1
0
−1
EM
Metastatic
Condition

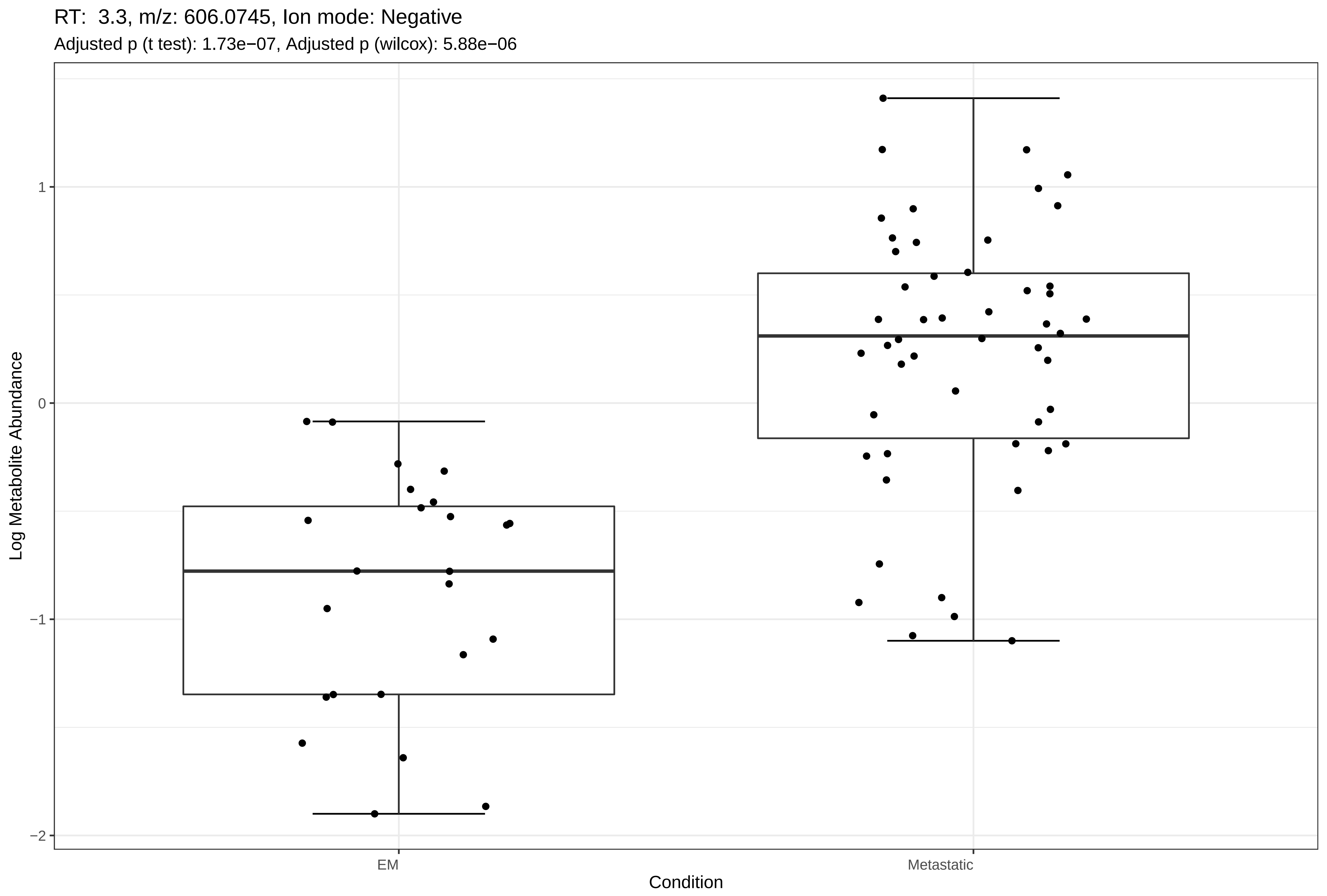
RT: 3.3, m/z: 606.0745, Ion mode: Negative
Adjusted p (t test): 1.73e−07, Adjusted p (wilcox): 5.88e−06
Log Metabolite Abundance
1
0
−1
−2
EM
Metastatic
Condition

RT: 6.5, m/z: 197.1498, Ion mode: Positive

Adjusted p (t test): 2.89e−05, Adjusted p (wilcox): 1.76e−07

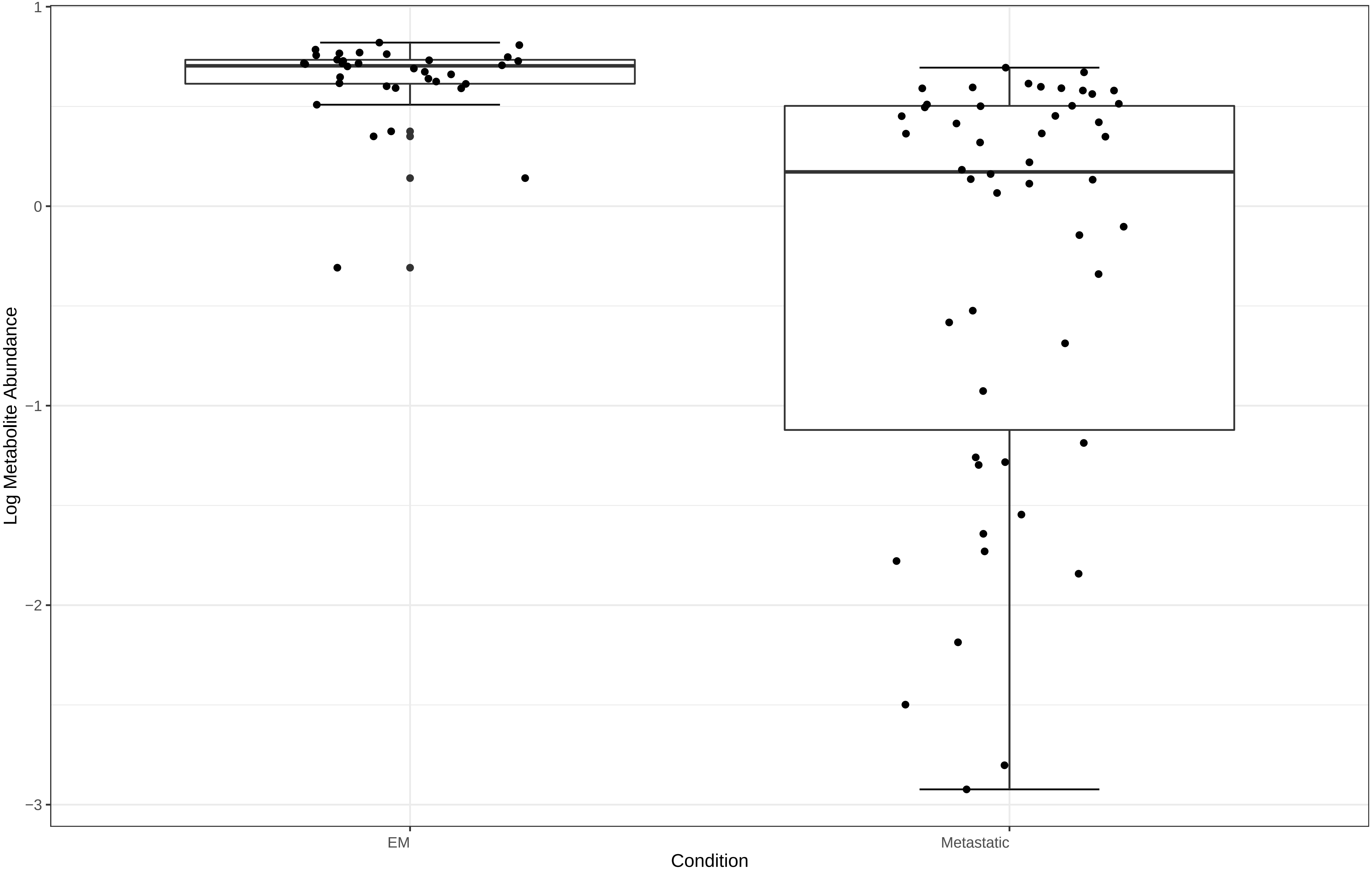

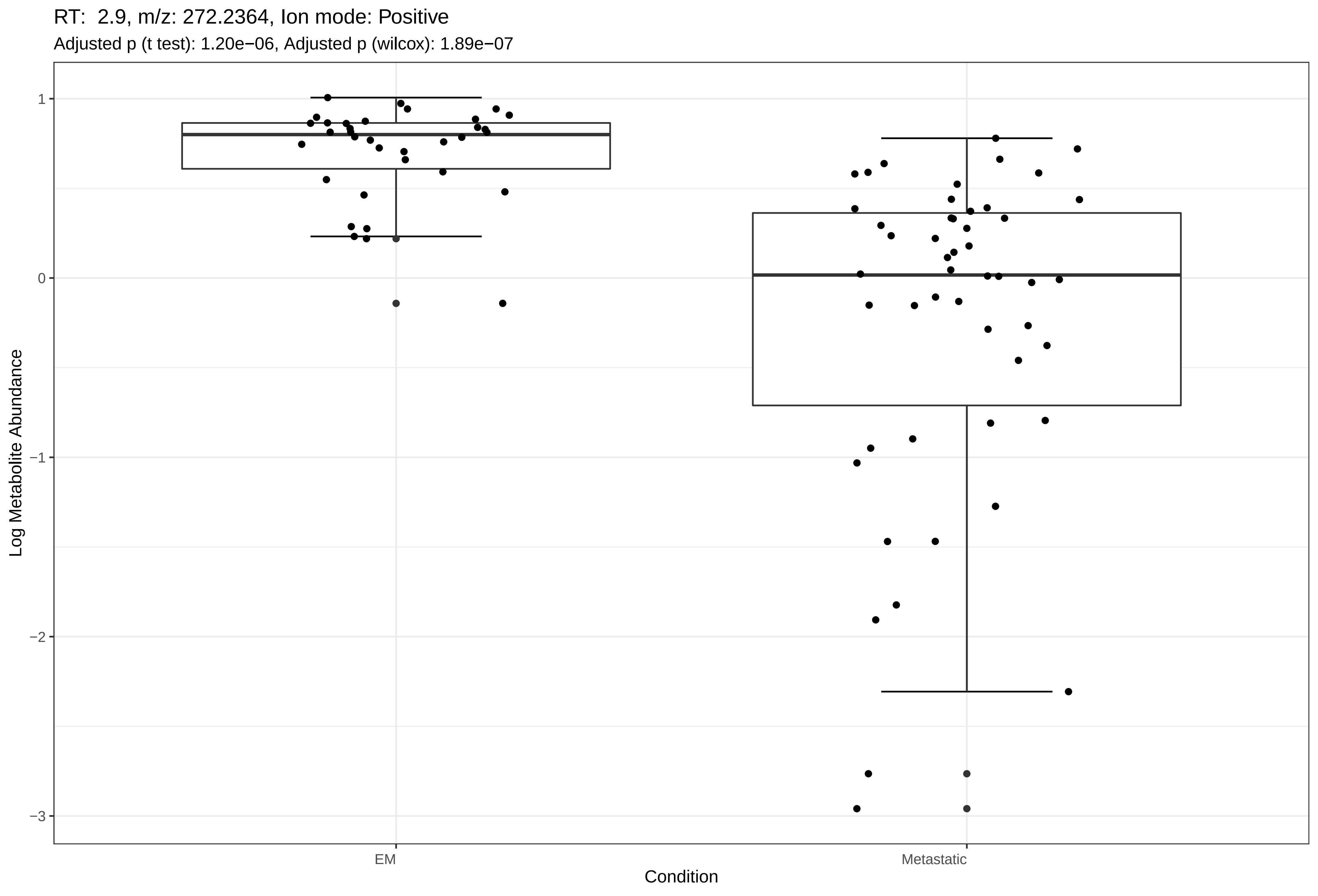
RT: 2.9, m/z: 272.2364, Ion mode: Positive
Adjusted p (t test): 1.20e−06, Adjusted p (wilcox): 1.89e−07
Log Metabolite Abundance
1
0
−1
−2
−3
EM
Metastatic
Condition

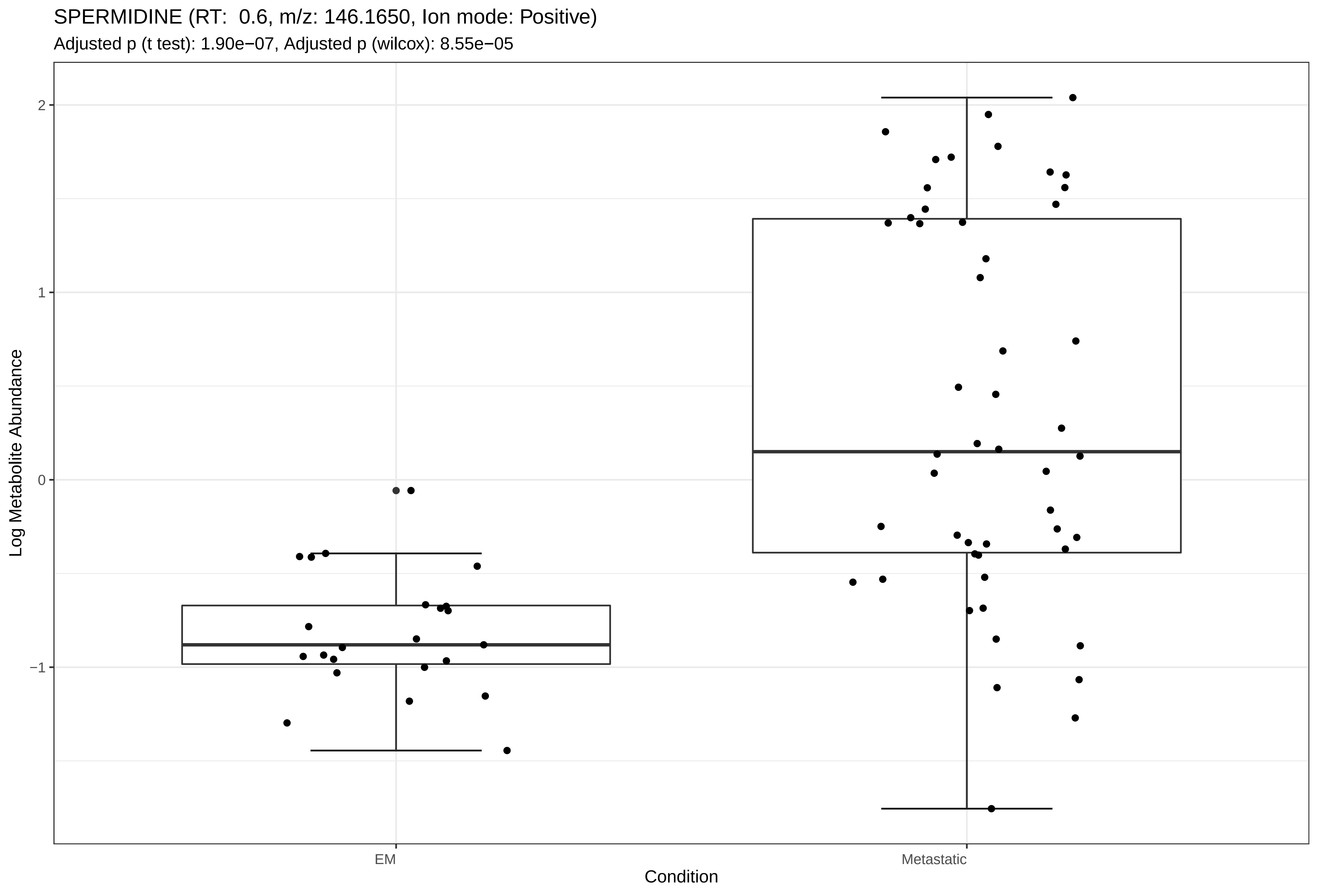

SPERMIDINE (RT: 0.6, m/z: 146.1650, Ion mode: Positive)
Adjusted p (t test): 1.90e−07, Adjusted p (wilcox): 8.55e−05
Log Metabolite Abundance
2
1
0
−1
EM
Metastatic
Condition

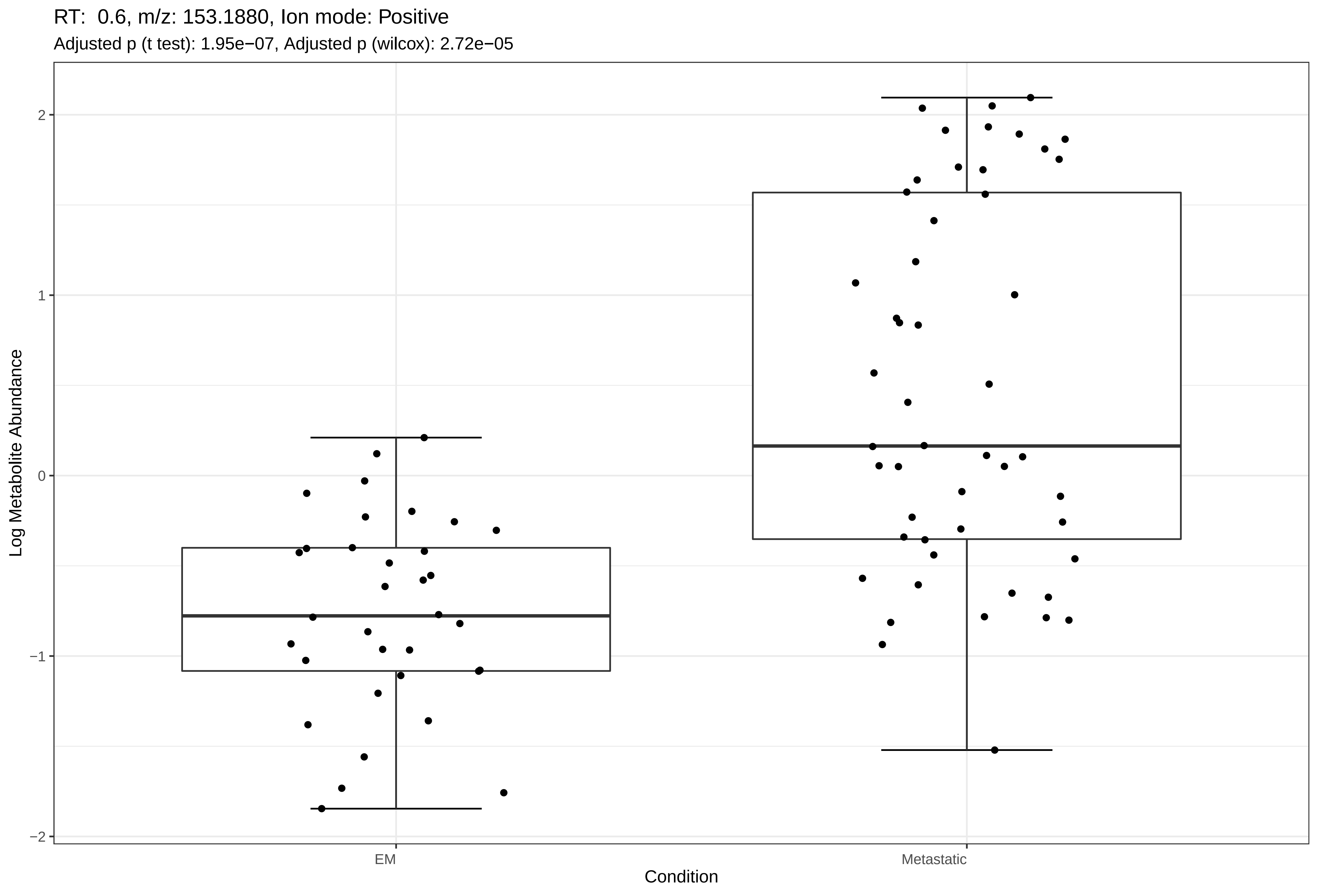
RT: 0.6, m/z: 153.1880, Ion mode: Positive
Adjusted p (t test): 1.95e−07, Adjusted p (wilcox): 2.72e−05
Log Metabolite Abundance
2
1
0
−1
−2
EM
Metastatic
Condition

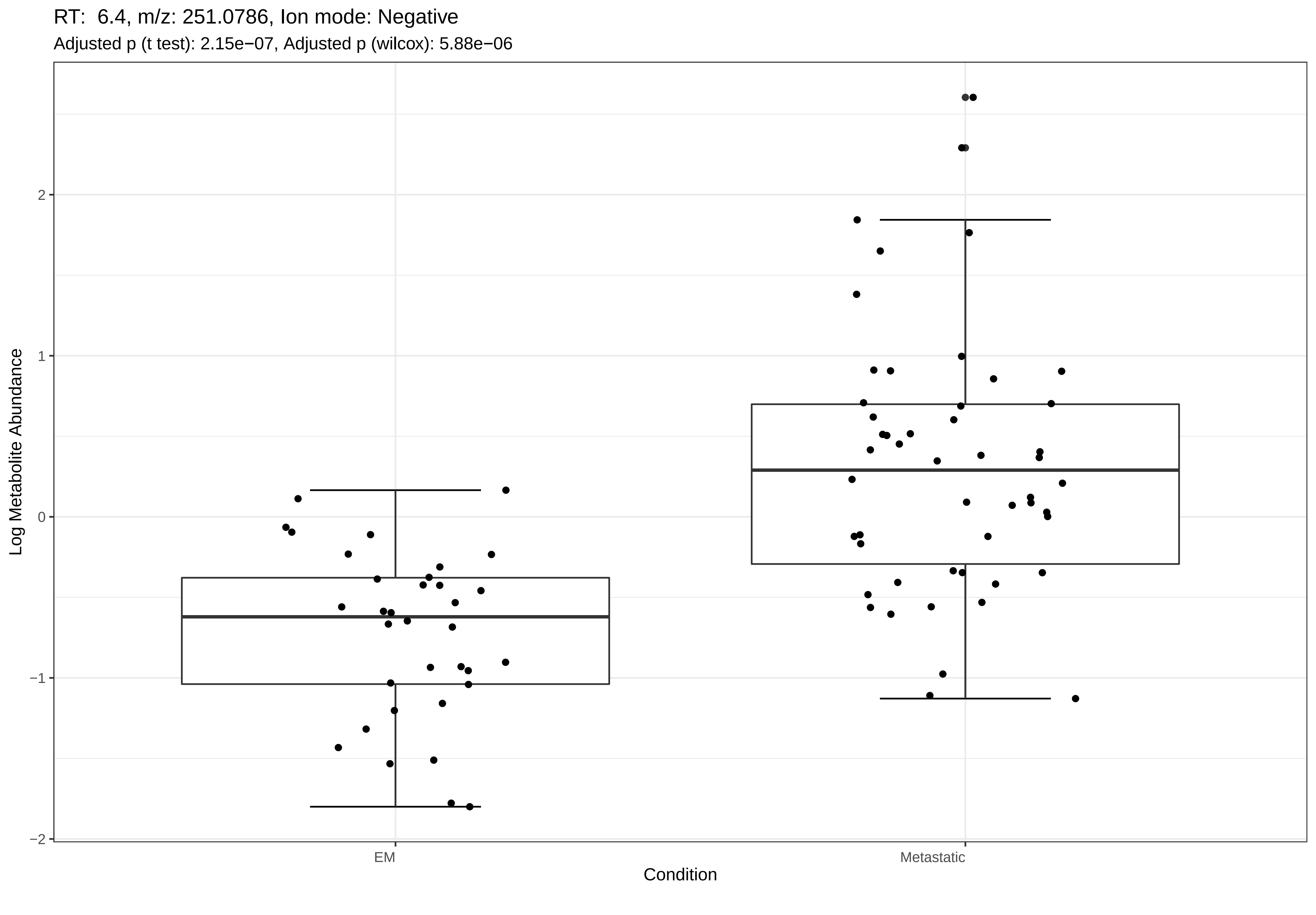

RT: 6.4, m/z: 251.0786, Ion mode: Negative
Adjusted p (t test): 2.15e−07, Adjusted p (wilcox): 5.88e−06
Log Metabolite Abundance
2
1
0
−1
−2
EM
Metastatic
Condition

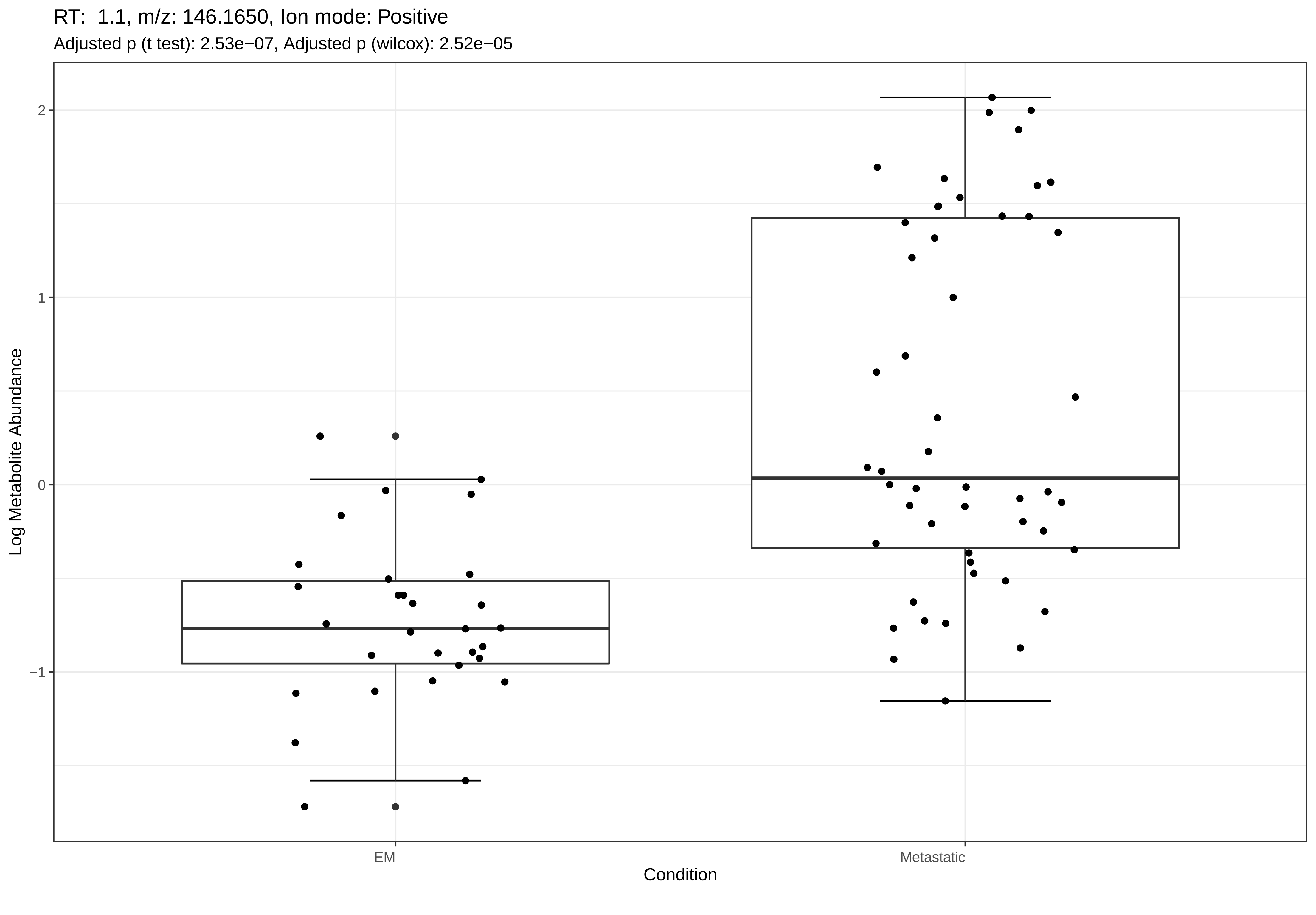
RT: 1.1, m/z: 146.1650, Ion mode: Positive
Adjusted p (t test): 2.53e−07, Adjusted p (wilcox): 2.52e−05
Log Metabolite Abundance
2
1
0
−1
EM
Metastatic
Condition

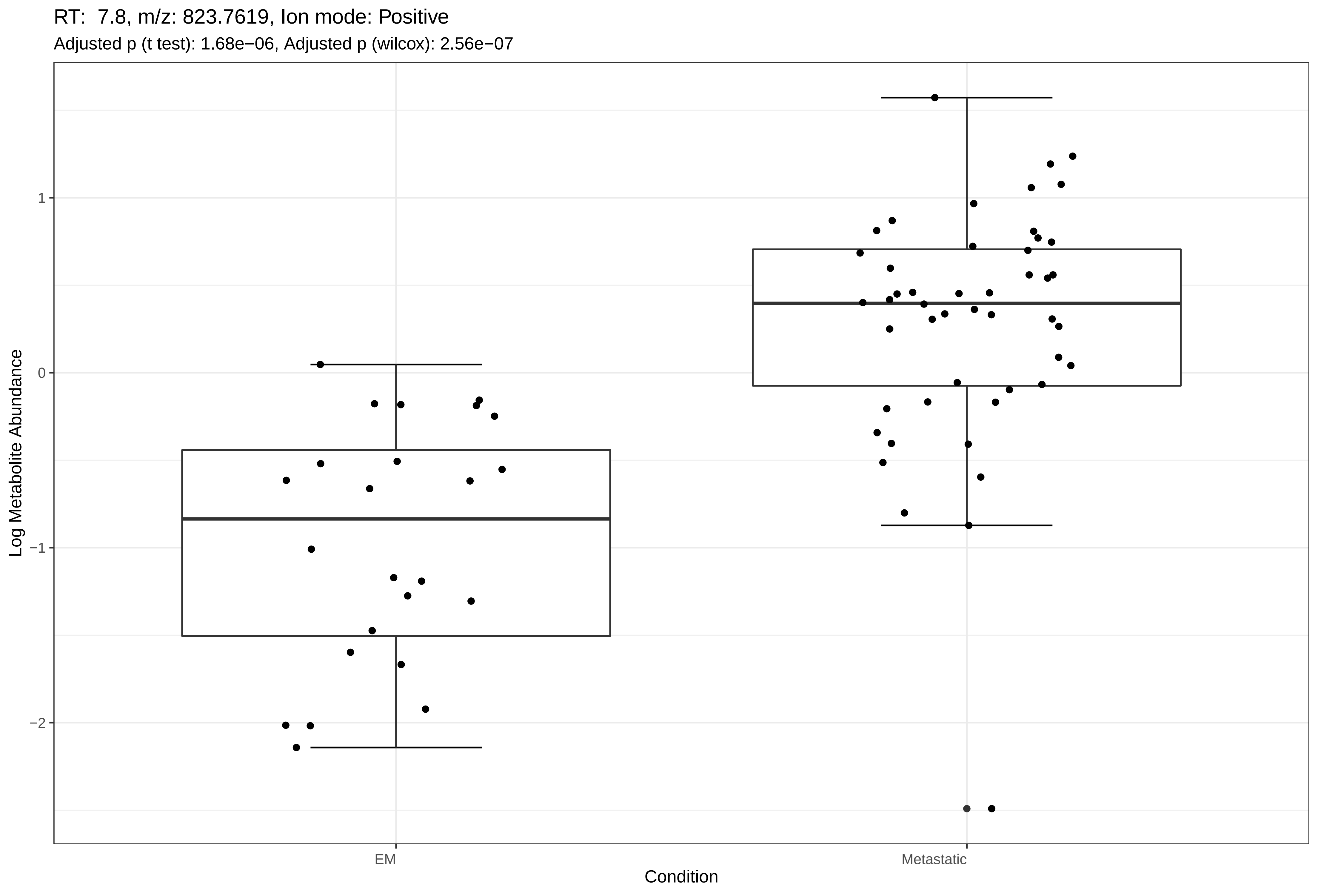

RT: 7.8, m/z: 823.7619, Ion mode: Positive
Adjusted p (t test): 1.68e−06, Adjusted p (wilcox): 2.56e−07
Log Metabolite Abundance
1
0
−1
−2
EM
Metastatic
Condition

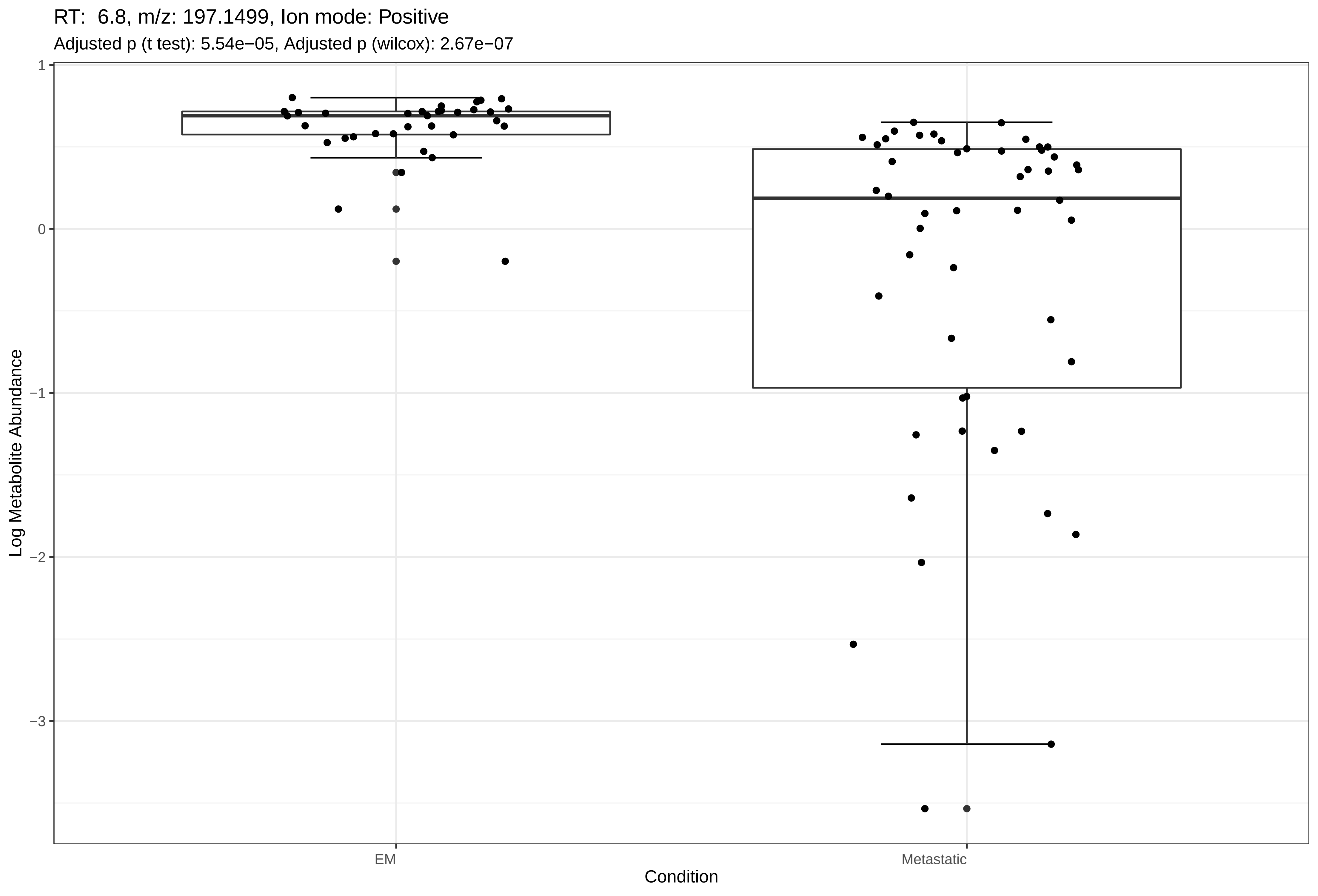
RT: 6.8, m/z: 197.1499, Ion mode: Positive
Adjusted p (t test): 5.54e−05, Adjusted p (wilcox): 2.67e−07
Log Metabolite Abundance
1
0
−1
−2
−3
EM
Metastatic
Condition

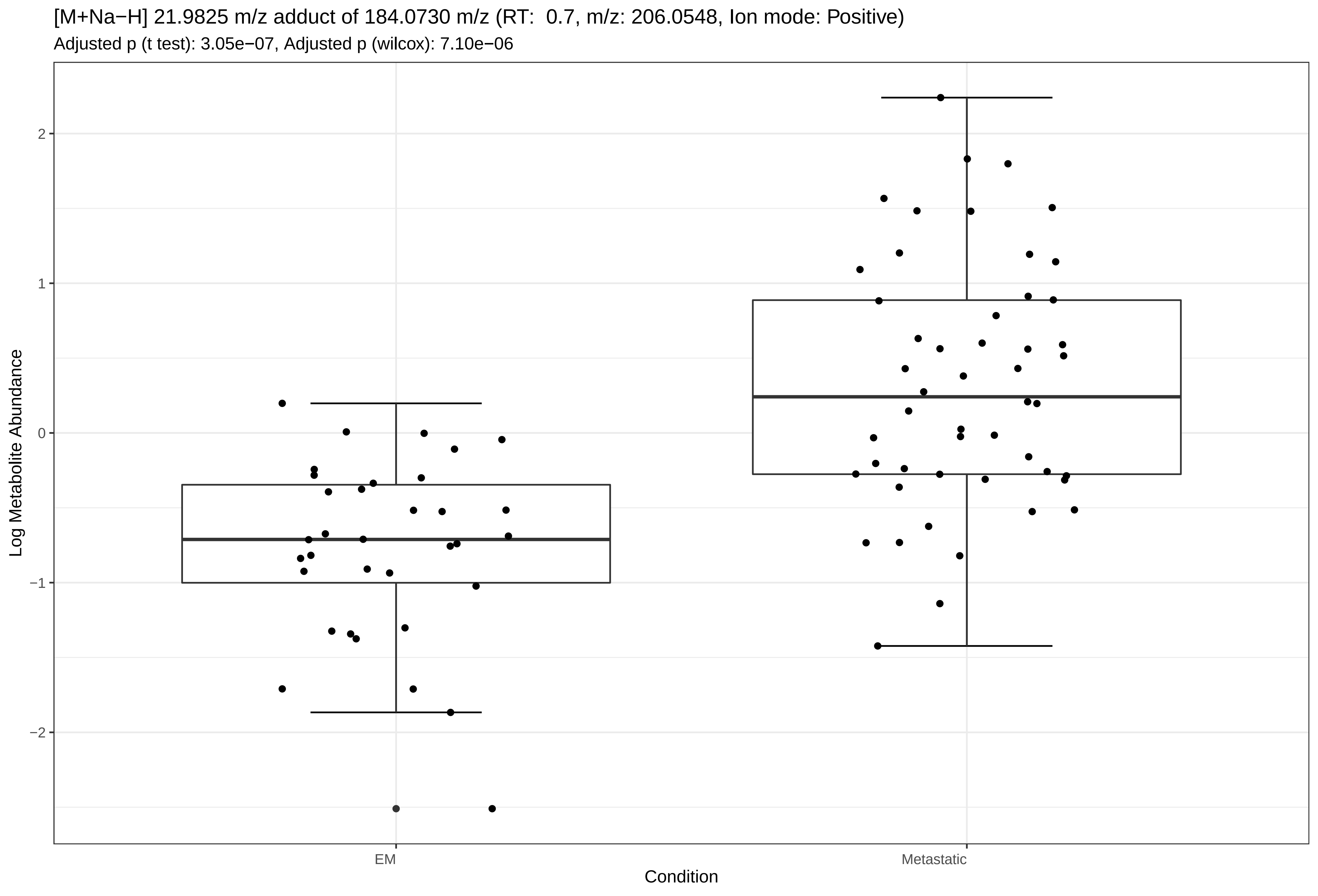

[M+Na−H] 21.9825 m/z adduct of 184.0730 m/z (RT: 0.7, m/z: 206.0548, Ion mode: Positive)
Adjusted p (t test): 3.05e−07, Adjusted p (wilcox): 7.10e−06
Log Metabolite Abundance
2
1
0
−1
−2
EM
Metastatic
Condition

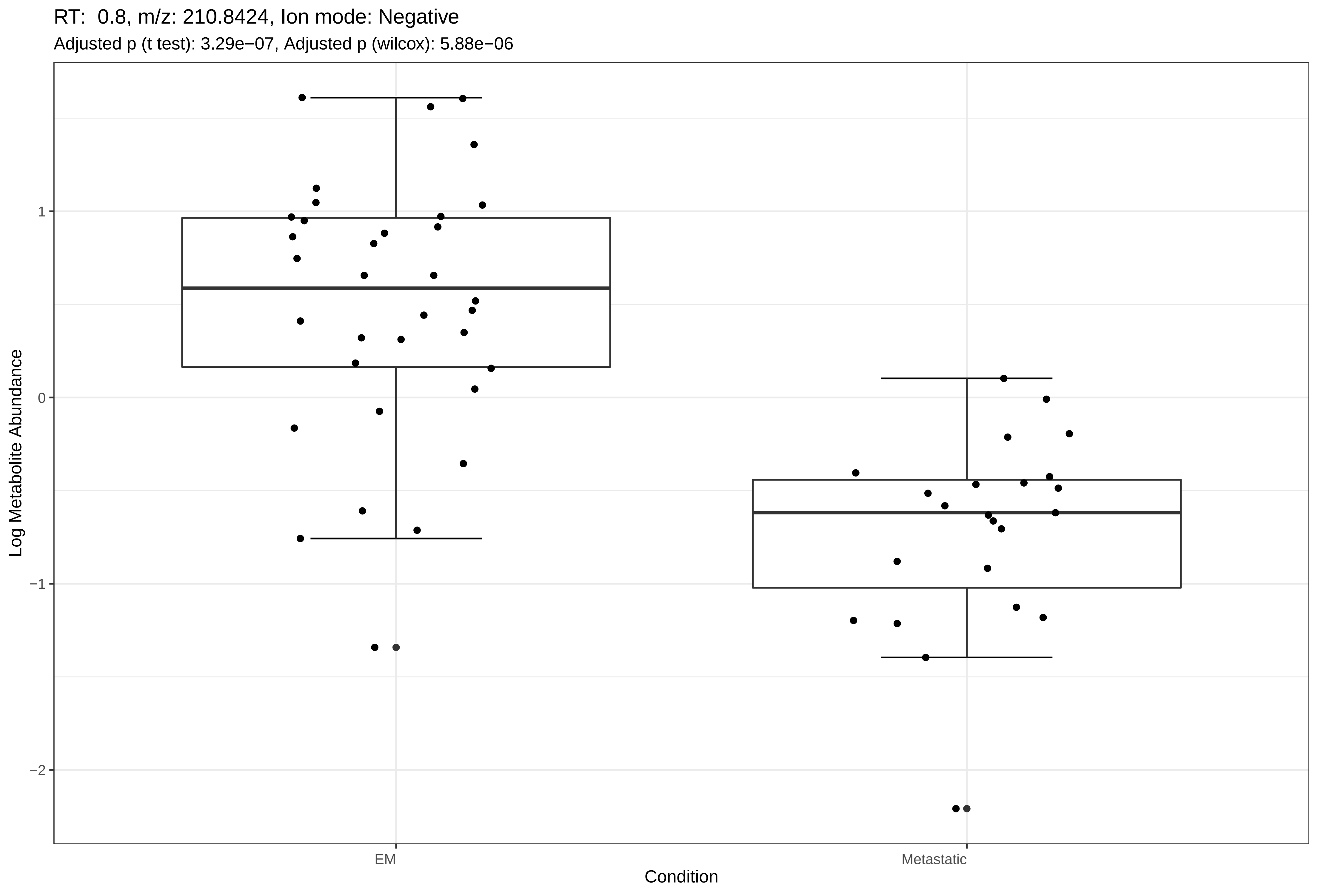
RT: 0.8, m/z: 210.8424, Ion mode: Negative
Adjusted p (t test): 3.29e−07, Adjusted p (wilcox): 5.88e−06
Log Metabolite Abundance
1
0
−1
−2
EM
Metastatic
Condition

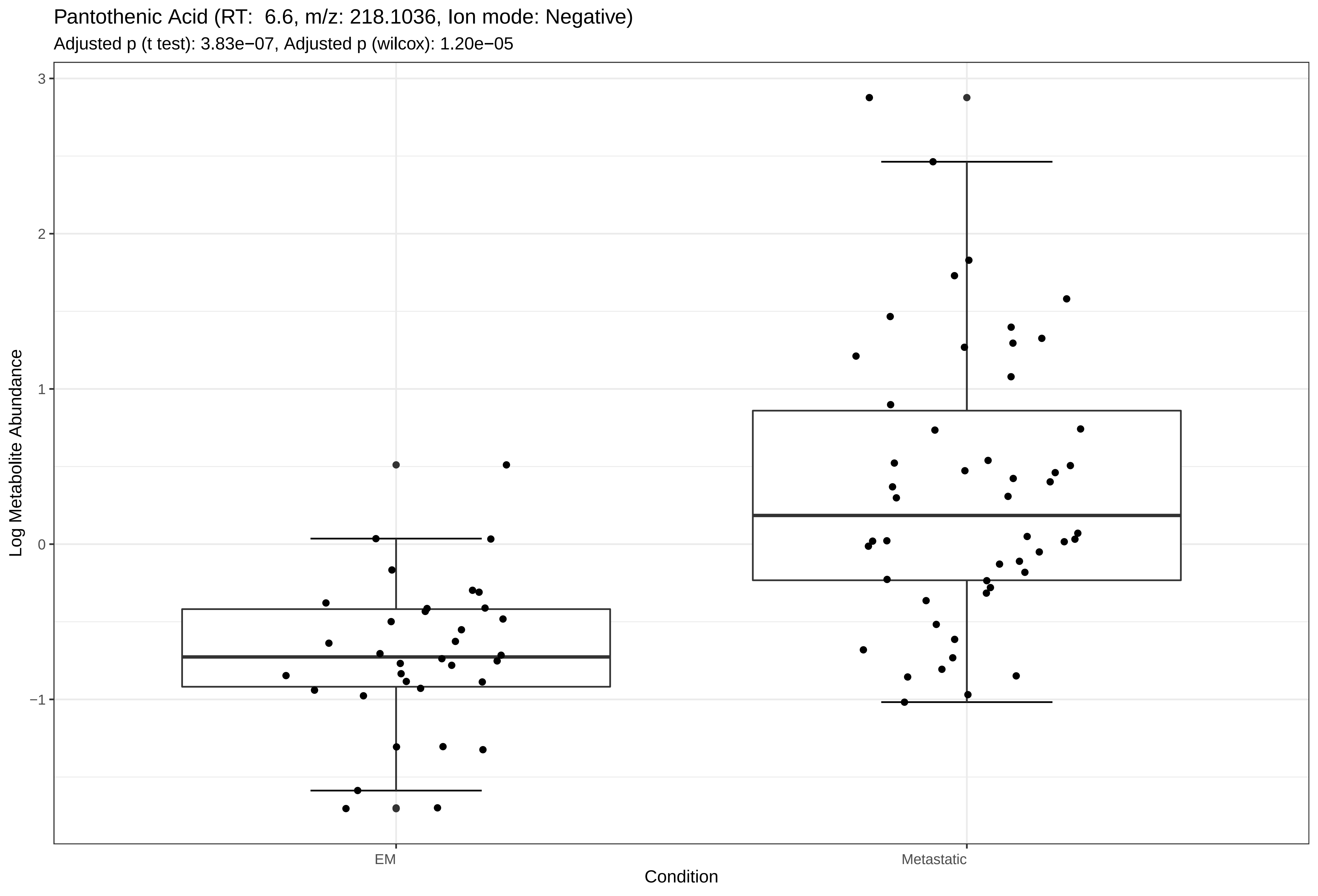

Pantothenic Acid (RT: 6.6, m/z: 218.1036, Ion mode: Negative)
Adjusted p (t test): 3.83e−07, Adjusted p (wilcox): 1.20e−05
Log Metabolite Abundance
3
2
1
0
−1
EM
Metastatic
Condition

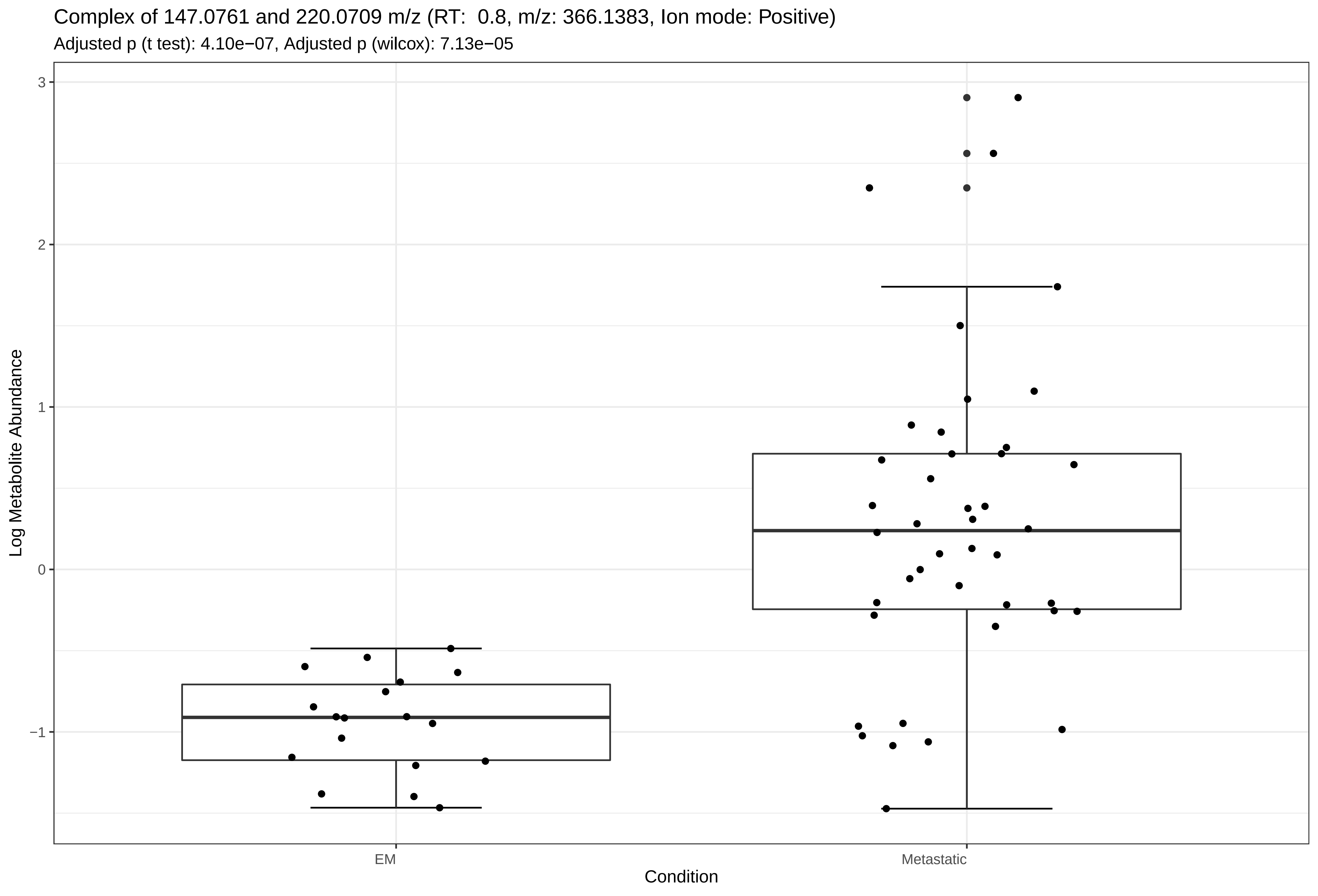

Complex of 147.0761 and 220.0709 m/z (RT: 0.8, m/z: 366.1383, Ion mode: Positive)
Adjusted p (t test): 4.10e−07, Adjusted p (wilcox): 7.13e−05
Log Metabolite Abundance
3
2
1
0
−1
EM
Metastatic
Condition

RT: 2.6, m/z: 182.1310, Ion mode: Positive

Adjusted p (t test): 1.98e−04, Adjusted p (wilcox): 4.23e−07

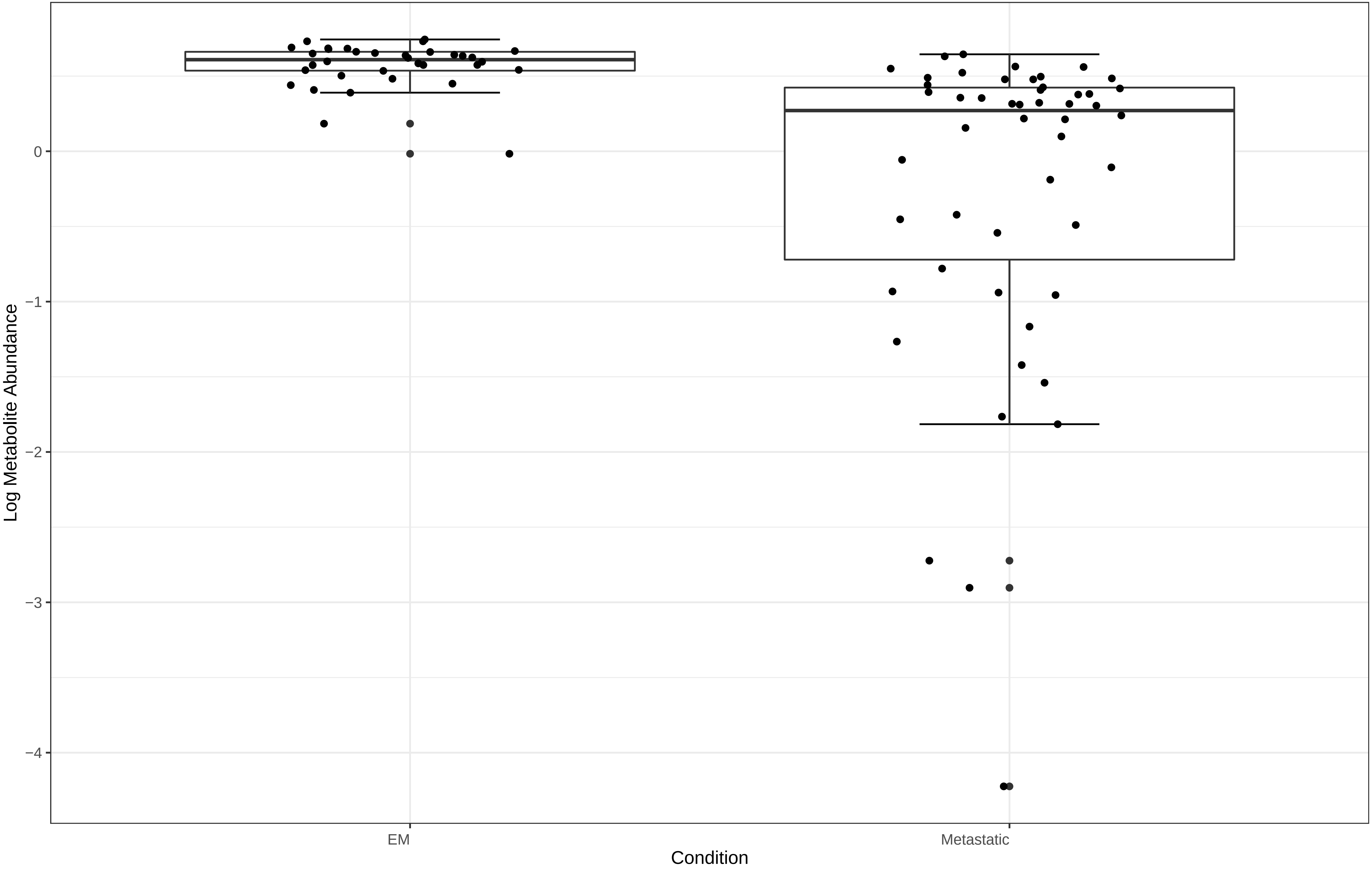

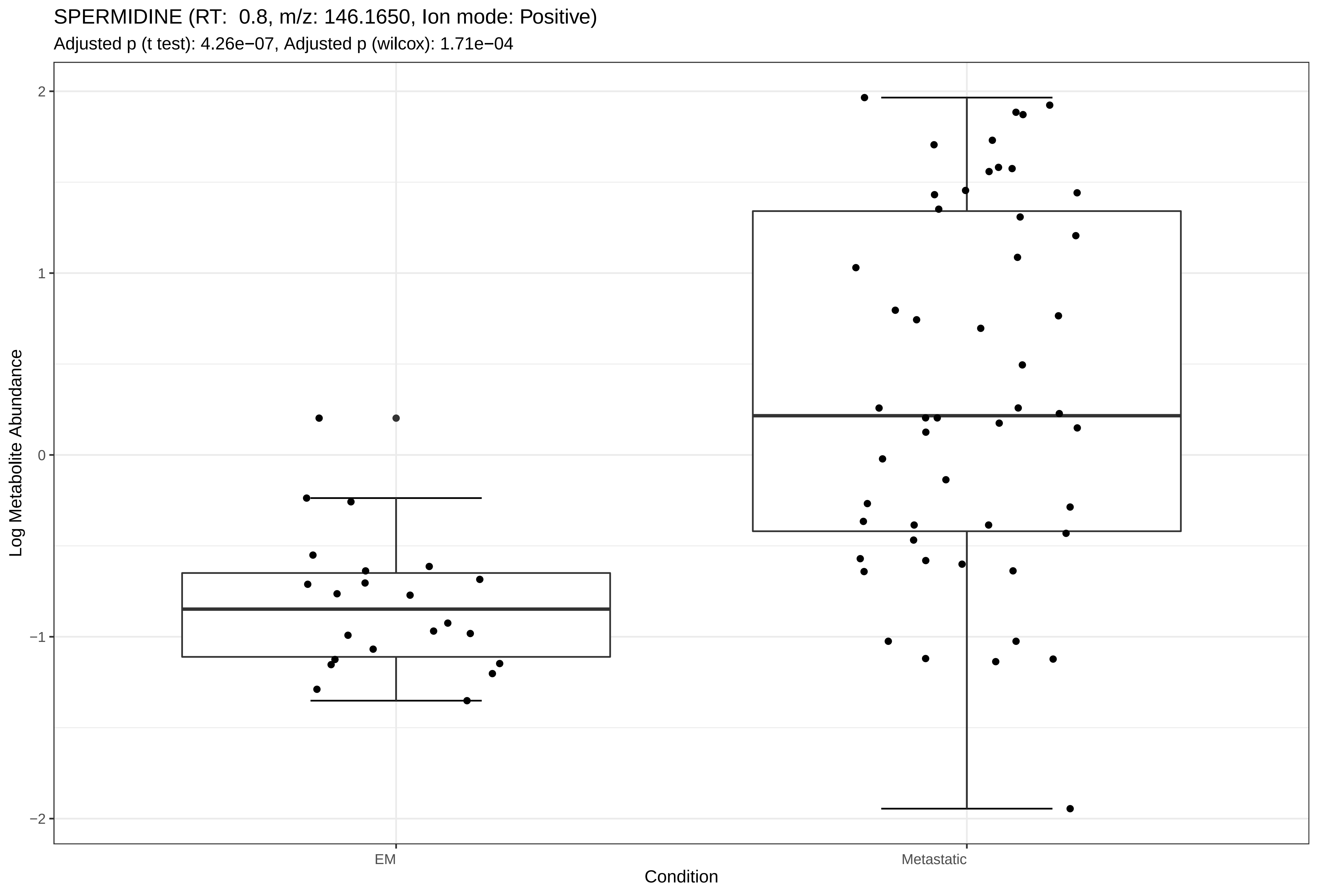
SPERMIDINE (RT: 0.8, m/z: 146.1650, Ion mode: Positive)
Adjusted p (t test): 4.26e−07, Adjusted p (wilcox): 1.71e−04
Log Metabolite Abundance
2
1
0
−1
−2
EM
Metastatic
Condition

RT: 0.7, m/z: 96.9693, Ion mode: Negative

Adjusted p (t test): 1.35e−06, Adjusted p (wilcox): 4.45e−07

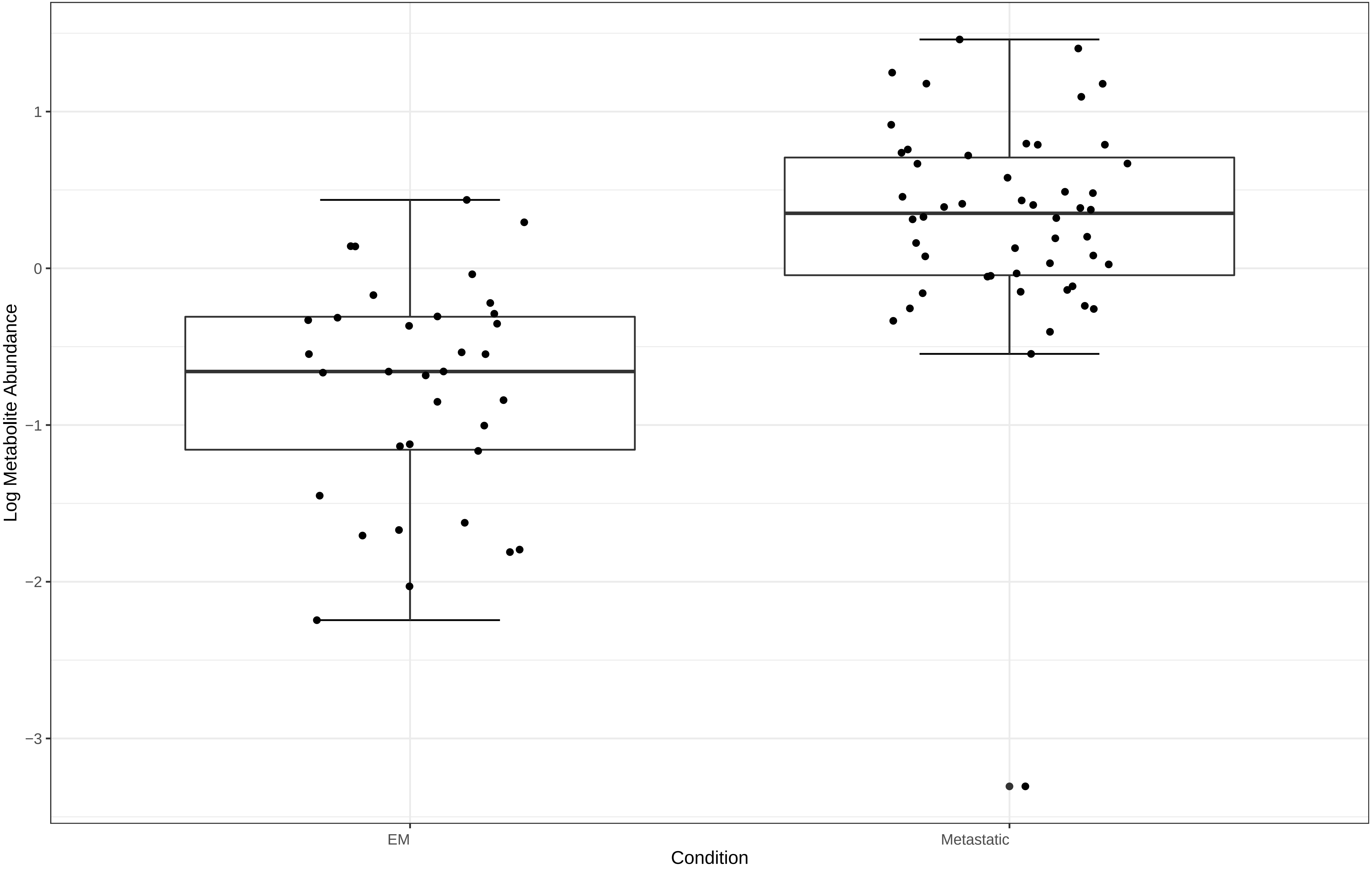

RT: 4.5, m/z: 204.1226, Ion mode: Positive

Adjusted p (t test): 4.75e−04, Adjusted p (wilcox): 4.45e−07

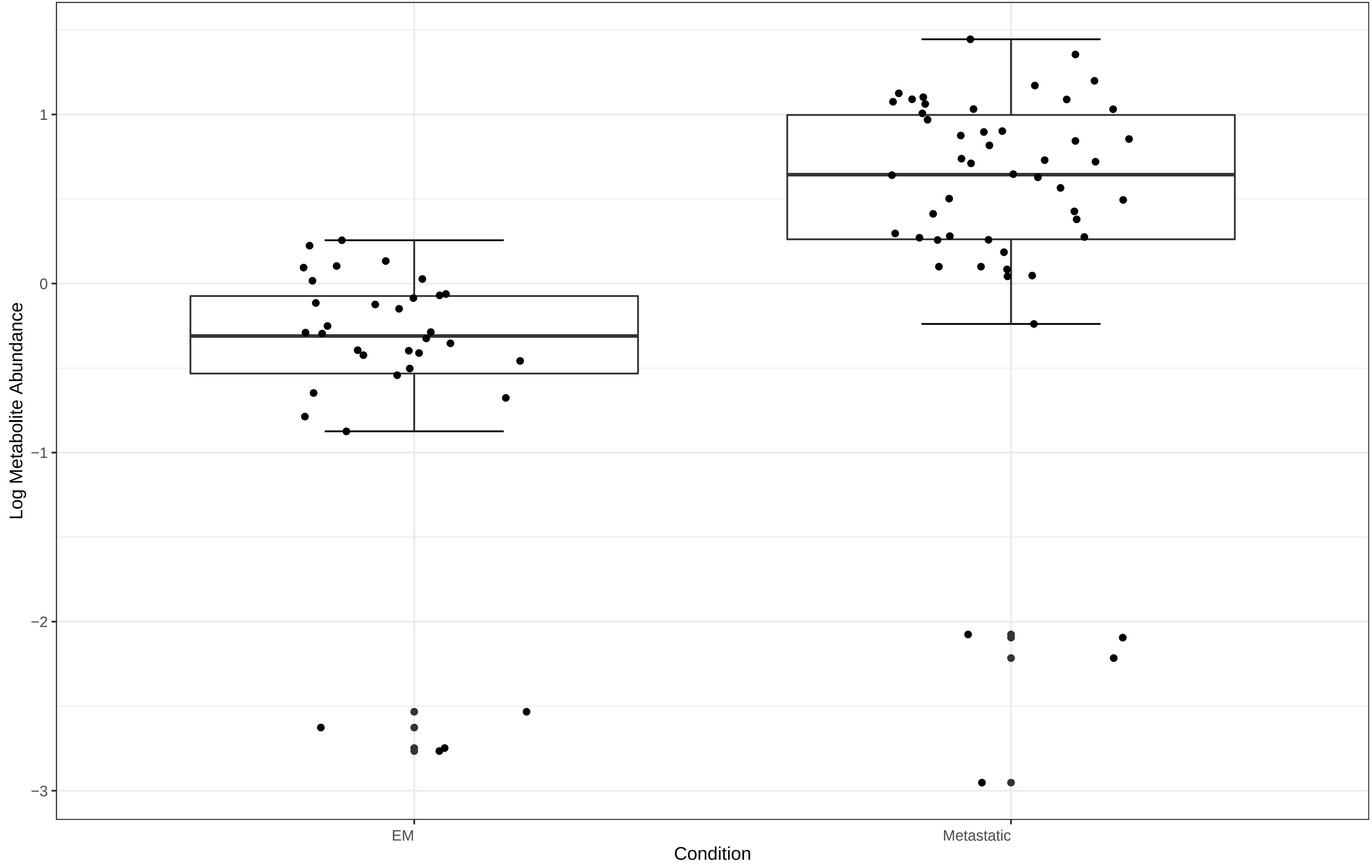

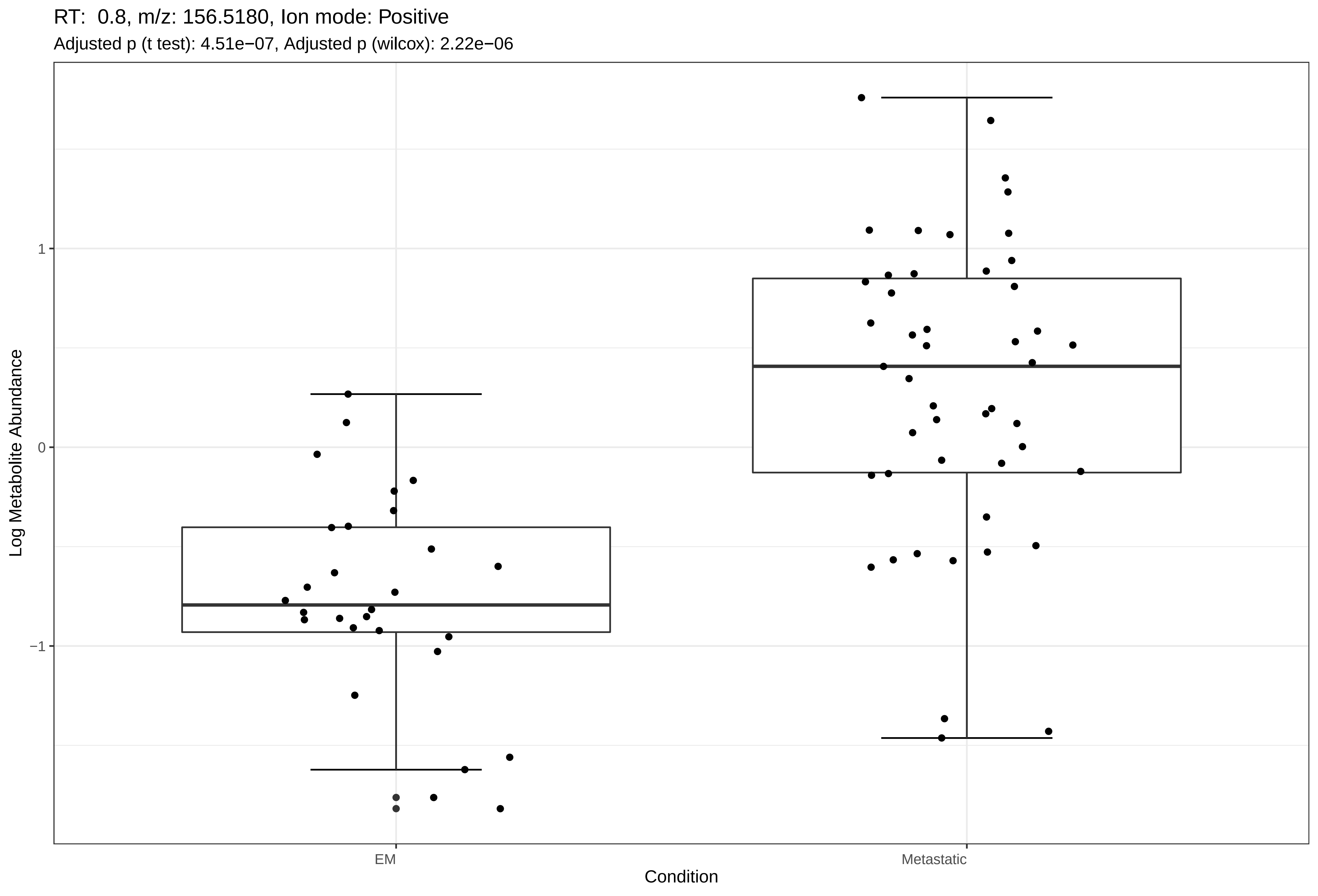
RT: 0.8, m/z: 156.5180, Ion mode: Positive
Adjusted p (t test): 4.51e−07, Adjusted p (wilcox): 2.22e−06
Log Metabolite Abundance
1
0
−1
EM
Metastatic
Condition

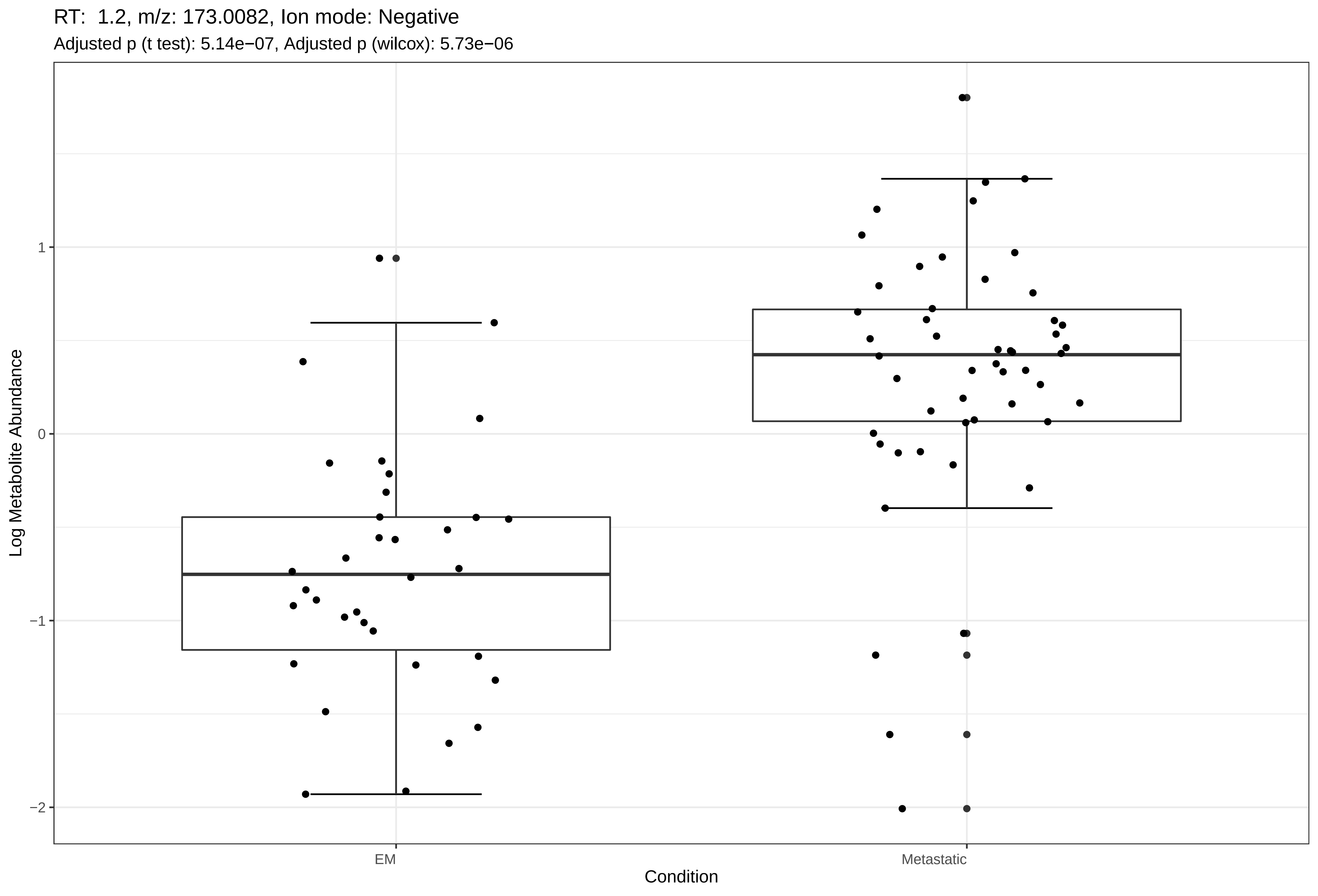

RT: 1.2, m/z: 173.0082, Ion mode: Negative
Adjusted p (t test): 5.14e−07, Adjusted p (wilcox): 5.73e−06
Log Metabolite Abundance
1
0
−1
−2
EM
Metastatic
Condition

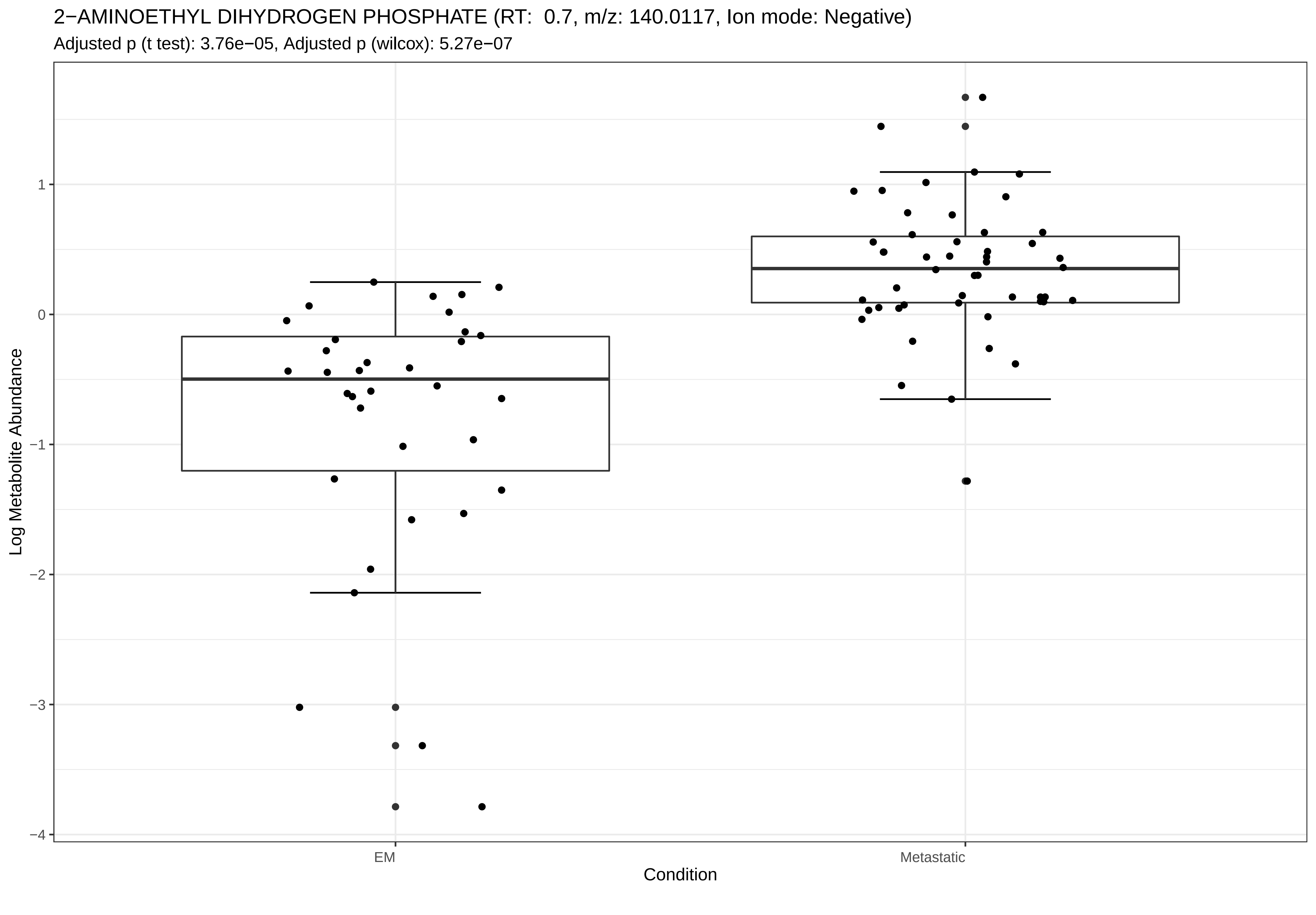
2−AMINOETHYL DIHYDROGEN PHOSPHATE (RT: 0.7, m/z: 140.0117, Ion mode: Negative)
Adjusted p (t test): 3.76e−05, Adjusted p (wilcox): 5.27e−07
Log Metabolite Abundance
1
0
−1
−2
−3
−4
EM
Metastatic
Condition

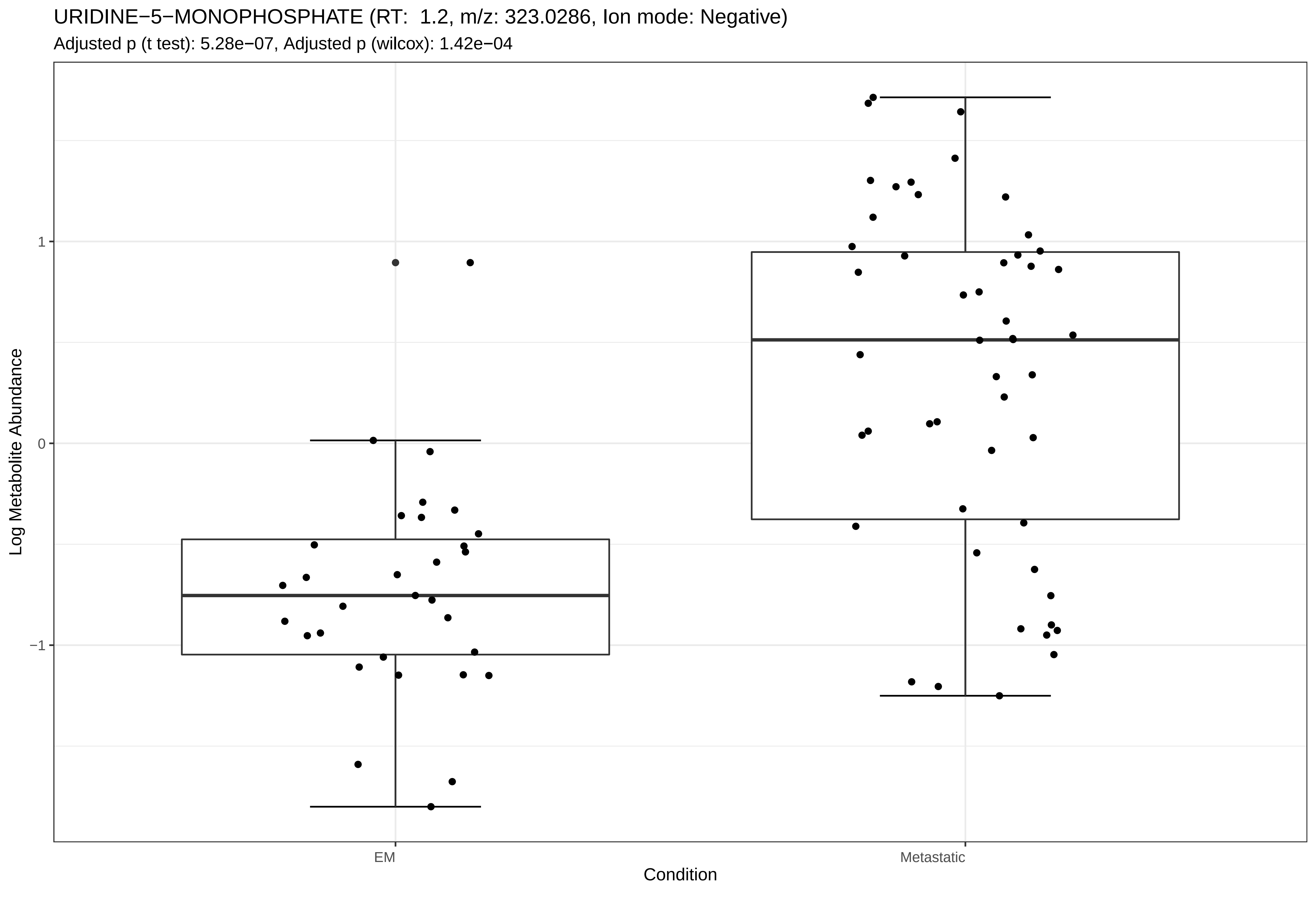
URIDINE−5−MONOPHOSPHATE (RT: 1.2, m/z: 323.0286, Ion mode: Negative)
Adjusted p (t test): 5.28e−07, Adjusted p (wilcox): 1.42e−04
Log Metabolite Abundance
1
0
−1
EM
Metastatic
Condition

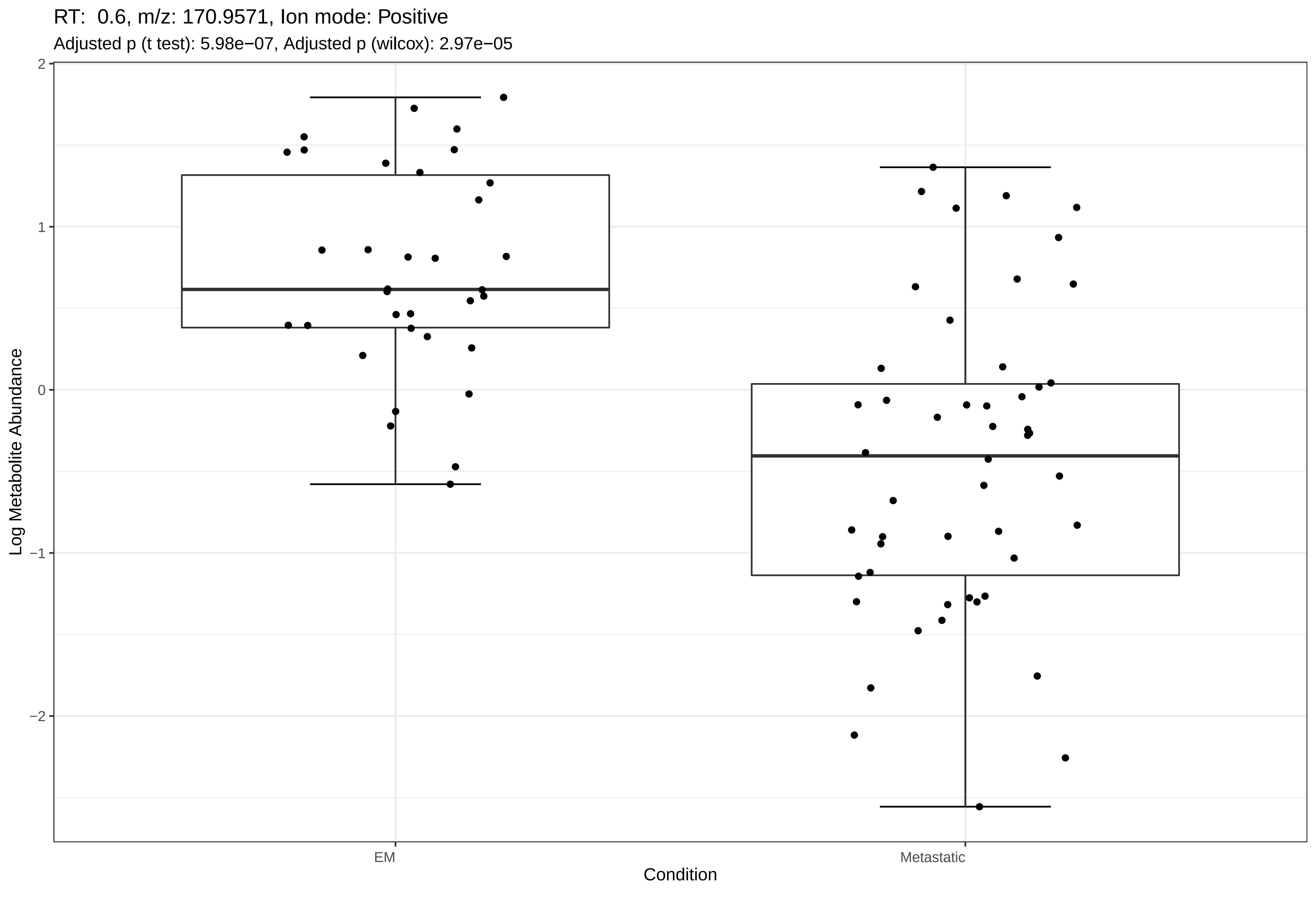
RT: 0.6, m/z: 170.9571, Ion mode: Positive
Adjusted p (t test): 5.98e−07, Adjusted p (wilcox): 2.97e−05
Log Metabolite Abundance
2
1
0
−1
−2
EM
Metastatic
Condition

RT: 1.8, m/z: 256.1582, Ion mode: Positive

Adjusted p (t test): 5.98e−07, Adjusted p (wilcox): 6.88e−06

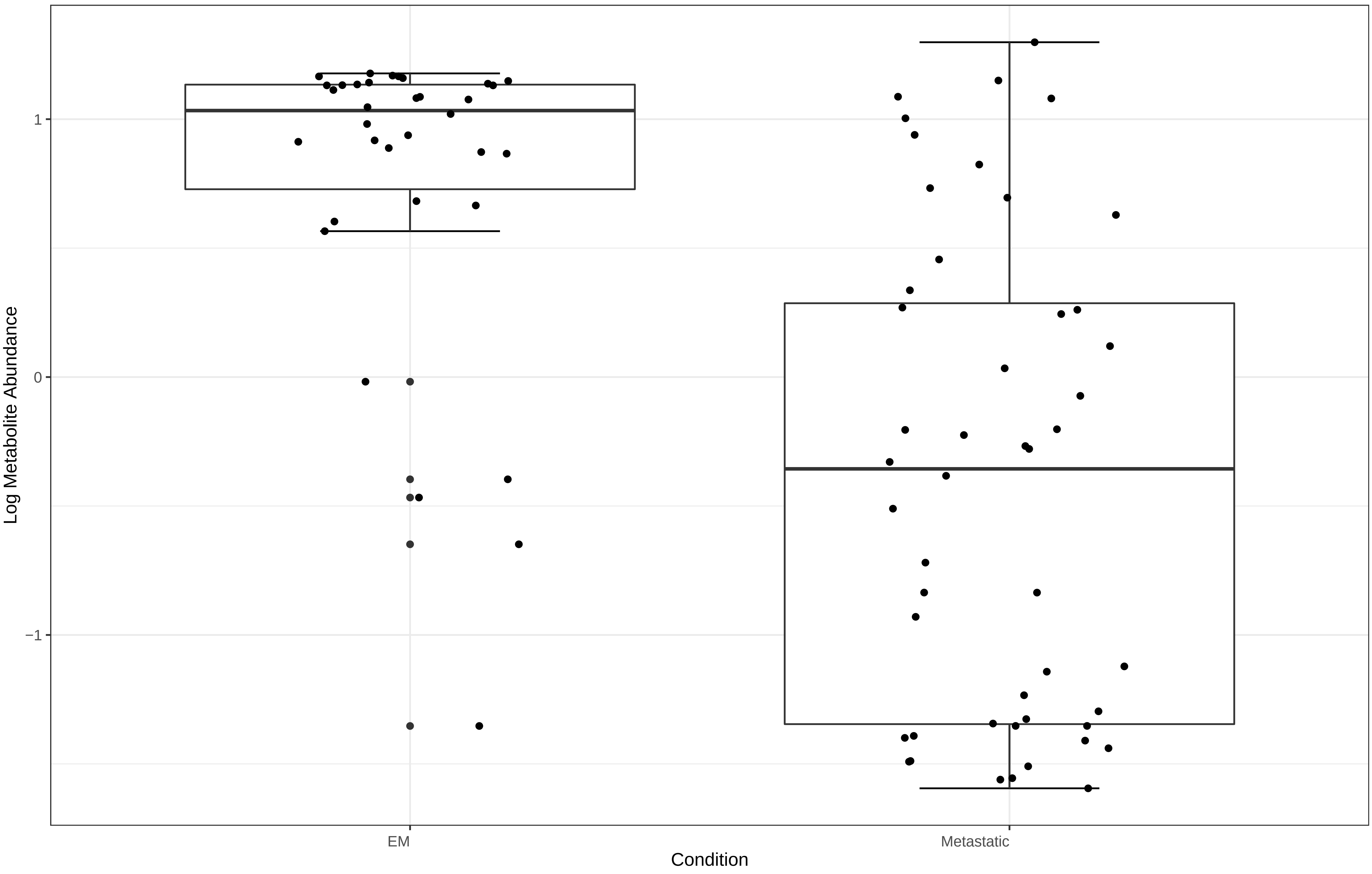

RT: 9.5, m/z: 204.0861, Ion mode: Positive

Adjusted p (t test): 6.22e−07, Adjusted p (wilcox): 6.29e−06

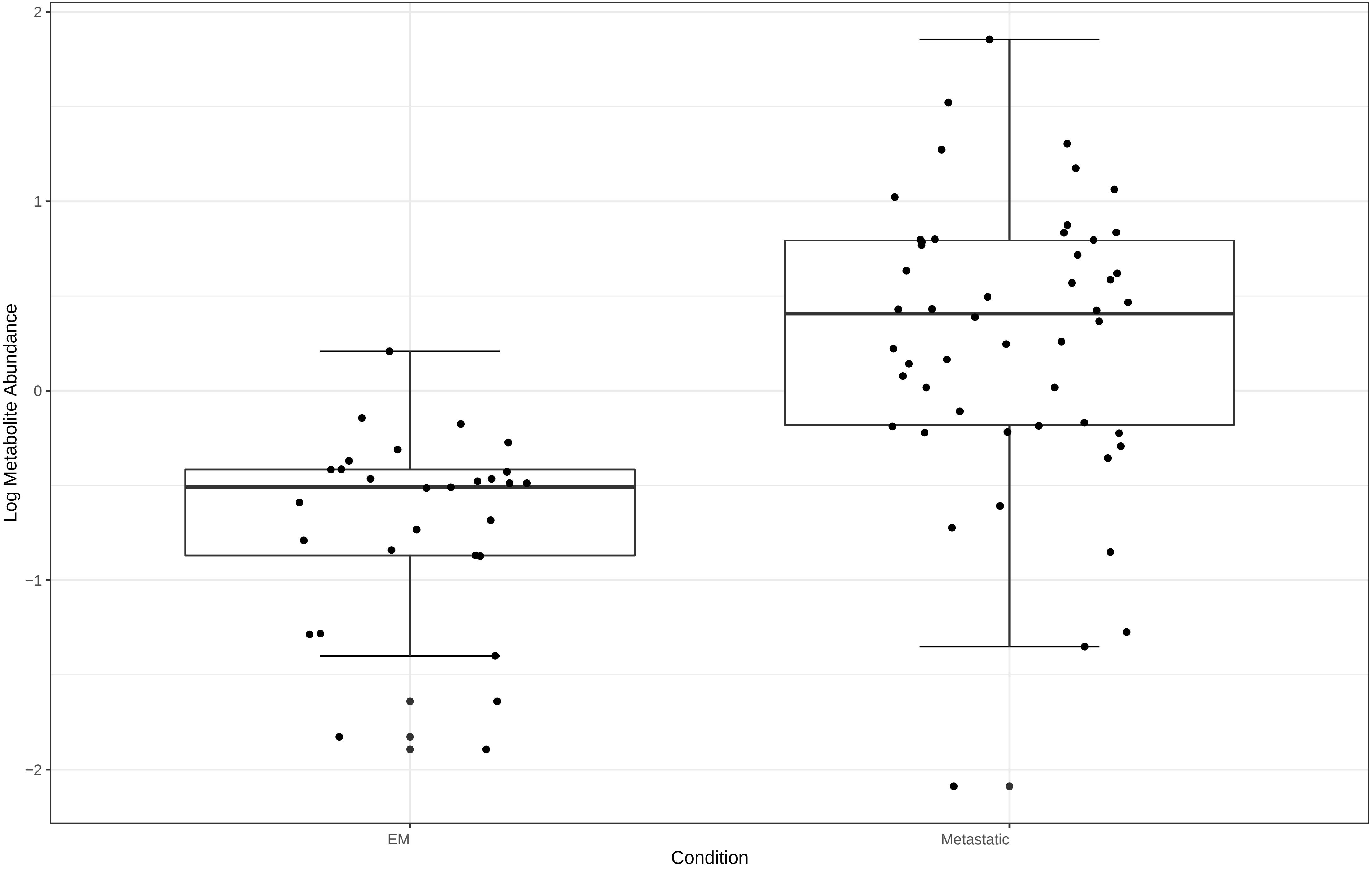

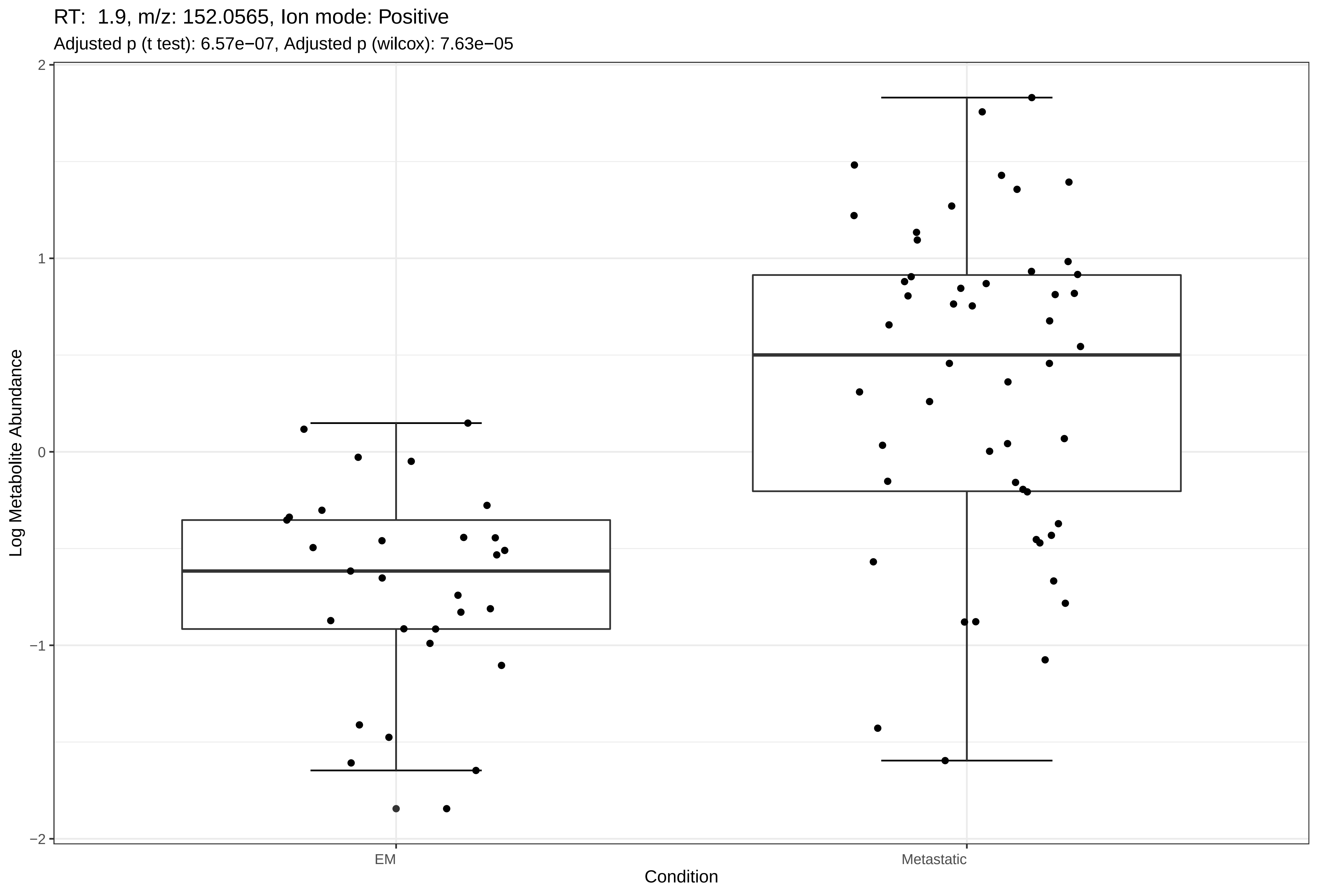
RT: 1.9, m/z: 152.0565, Ion mode: Positive
Adjusted p (t test): 6.57e−07, Adjusted p (wilcox): 7.63e−05
Log Metabolite Abundance
2
1
0
−1
−2
EM
Metastatic
Condition

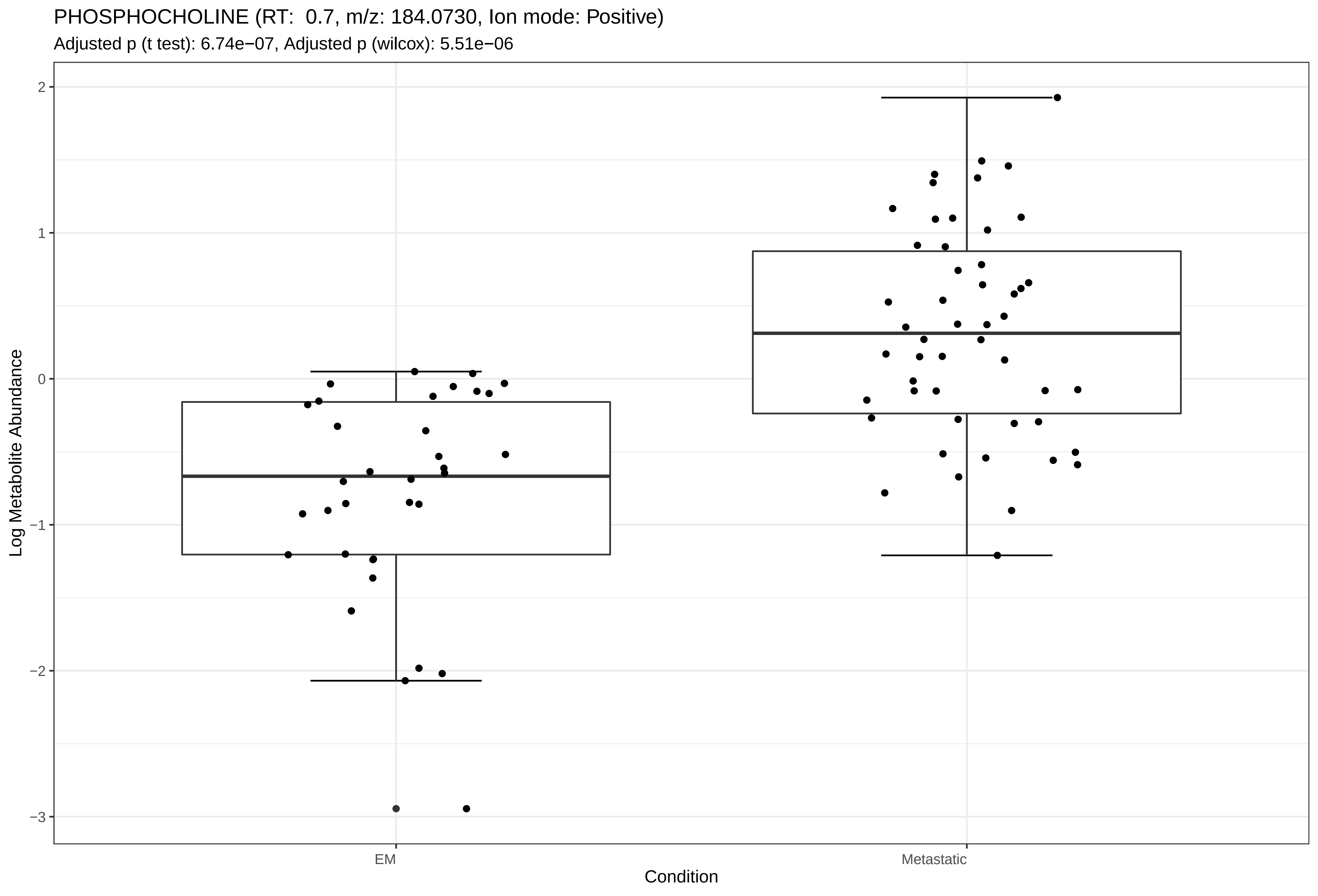

PHOSPHOCHOLINE (RT: 0.7, m/z: 184.0730, Ion mode: Positive)
Adjusted p (t test): 6.74e−07, Adjusted p (wilcox): 5.51e−06
Log Metabolite Abundance
2
1
0
−1
−2
−3
EM
Metastatic
Condition

RT: 0.7, m/z: 186.0761, Ion mode: Positive

Adjusted p (t test): 6.95e−07, Adjusted p (wilcox): 1.15e−05

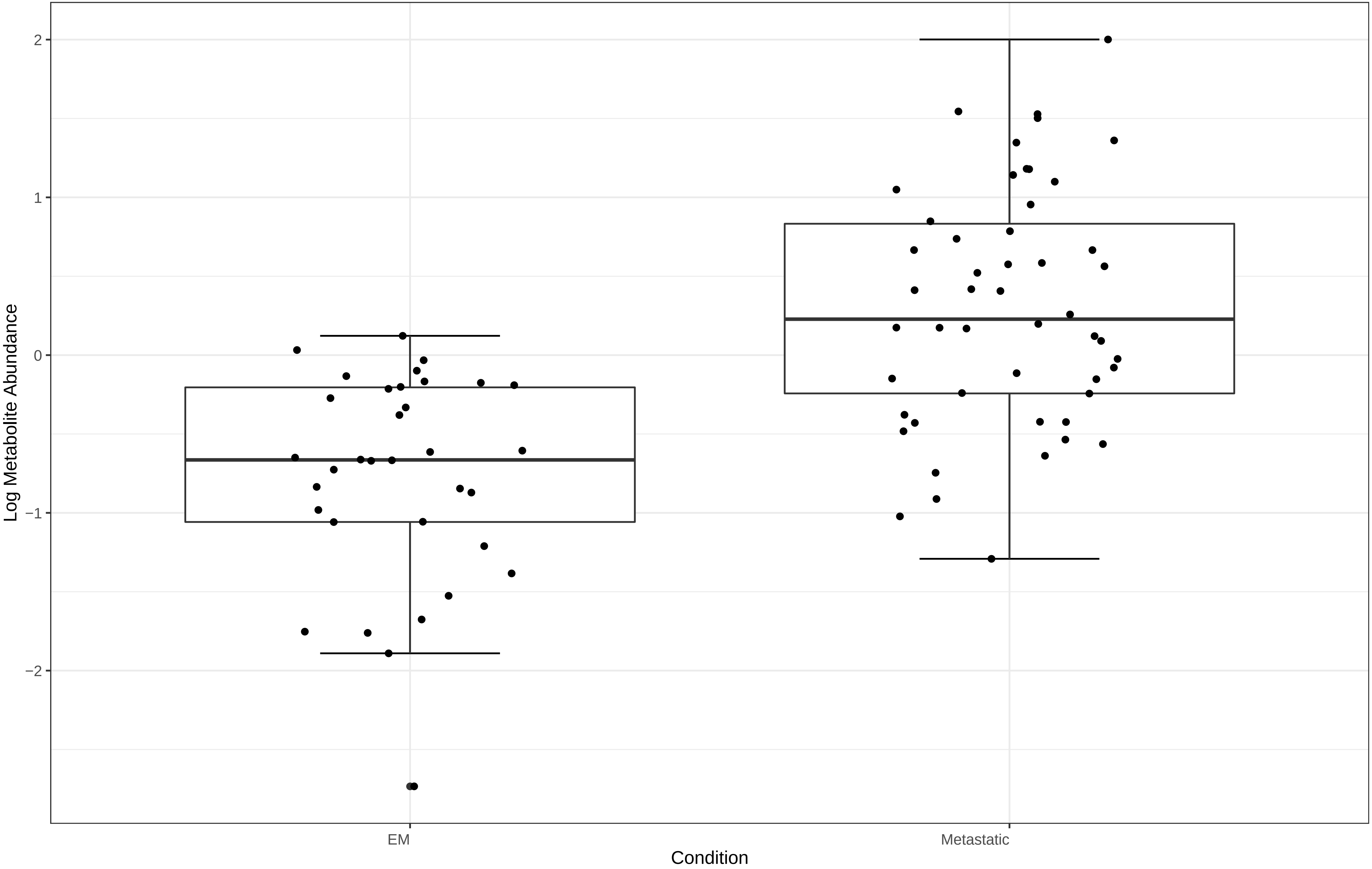

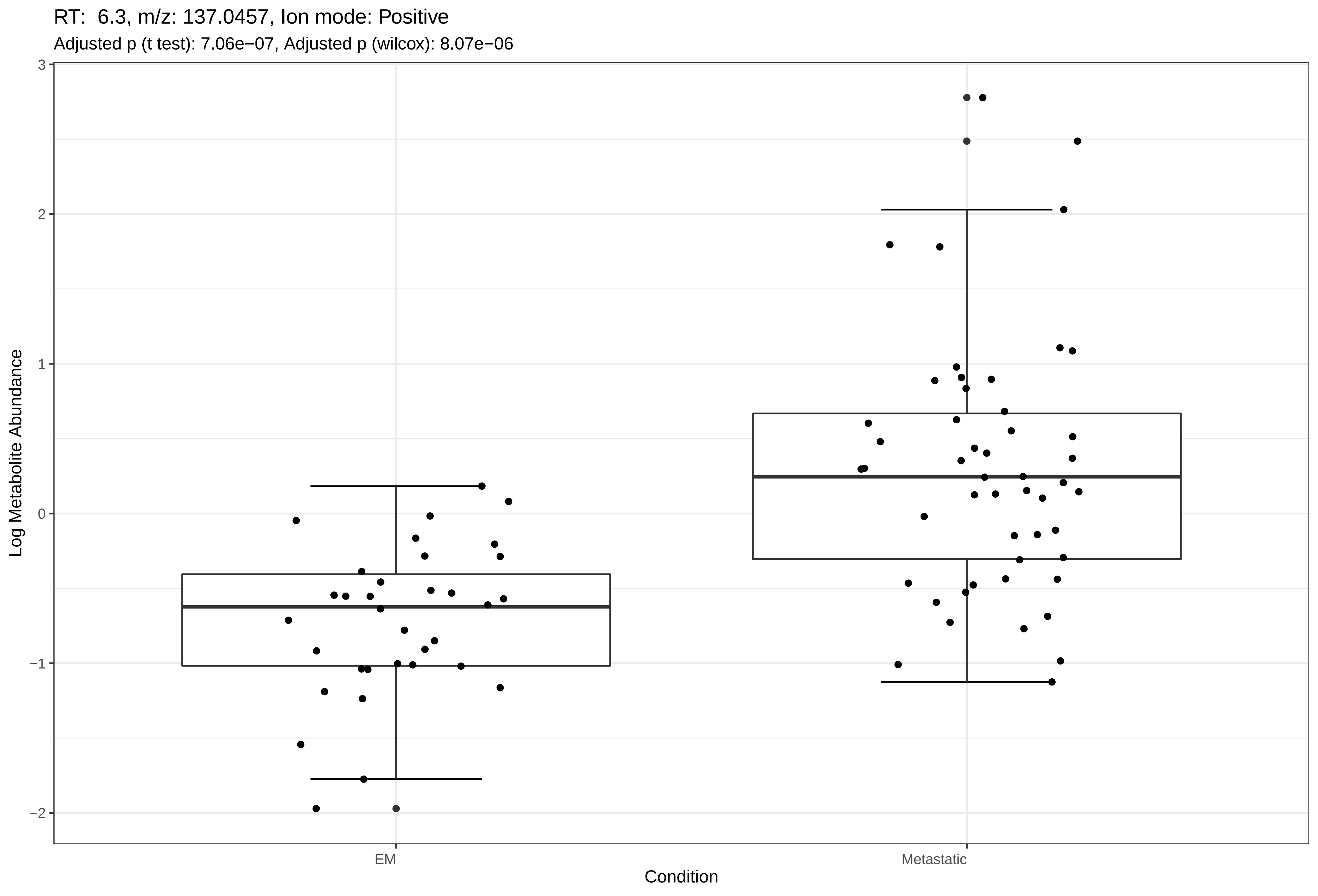

RT: 6.3, m/z: 137.0457, Ion mode: Positive
Adjusted p (t test): 7.06e−07, Adjusted p (wilcox): 8.07e−06
Log Metabolite Abundance
3
2
1
0
−1
−2
EM
Metastatic
Condition

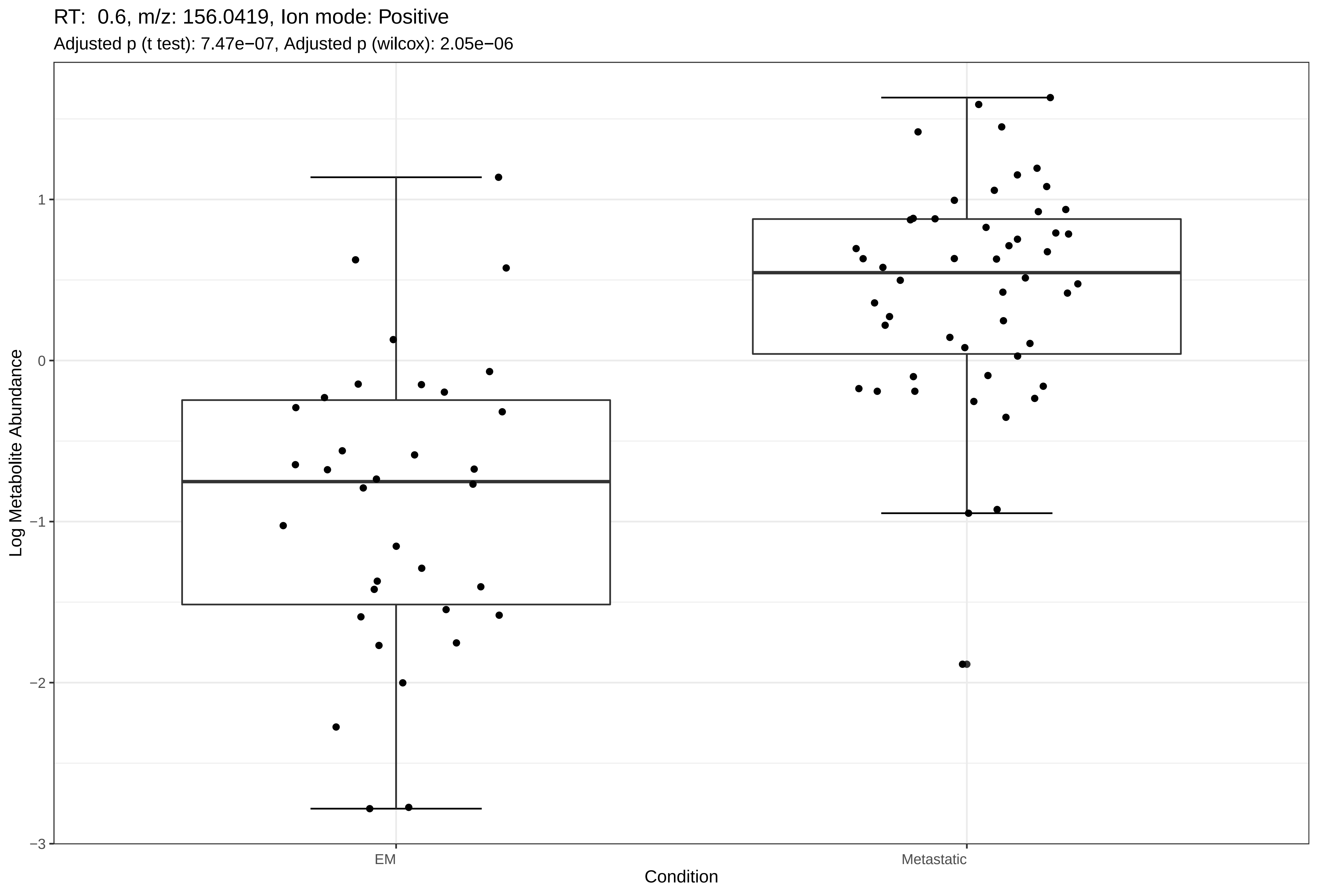

RT: 0.6, m/z: 156.0419, Ion mode: Positive
Adjusted p (t test): 7.47e−07, Adjusted p (wilcox): 2.05e−06
Log Metabolite Abundance
1
0
−1
−2
−3
EM
Metastatic
Condition

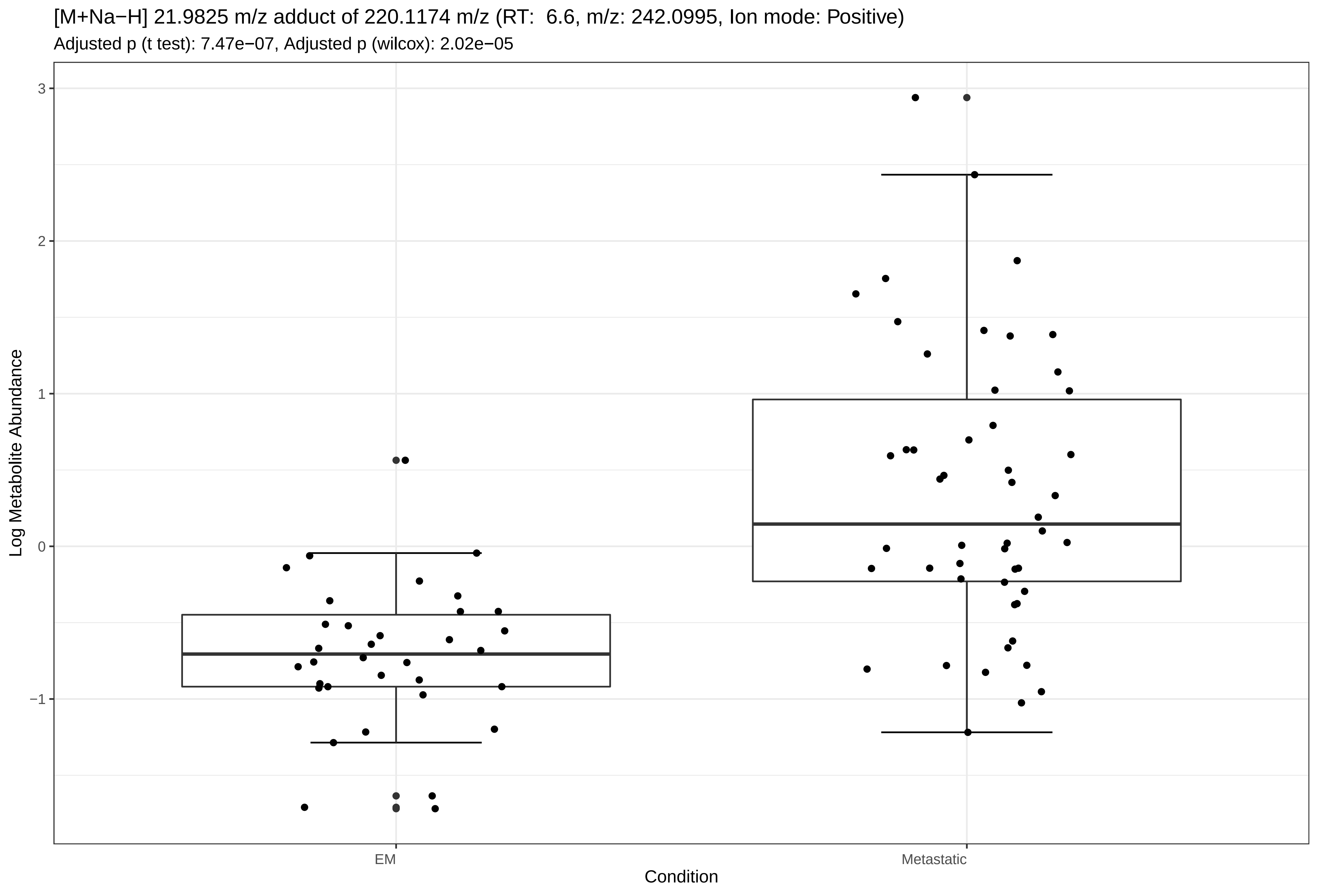
[M+Na−H] 21.9825 m/z adduct of 220.1174 m/z (RT: 6.6, m/z: 242.0995, Ion mode: Positive)
Adjusted p (t test): 7.47e−07, Adjusted p (wilcox): 2.02e−05
Log Metabolite Abundance
3
2
1
0
−1
EM
Metastatic
Condition

RT: 0.9, m/z: 123.5941, Ion mode: Positive

Adjusted p (t test): 7.65e−07, Adjusted p (wilcox): 7.10e−06

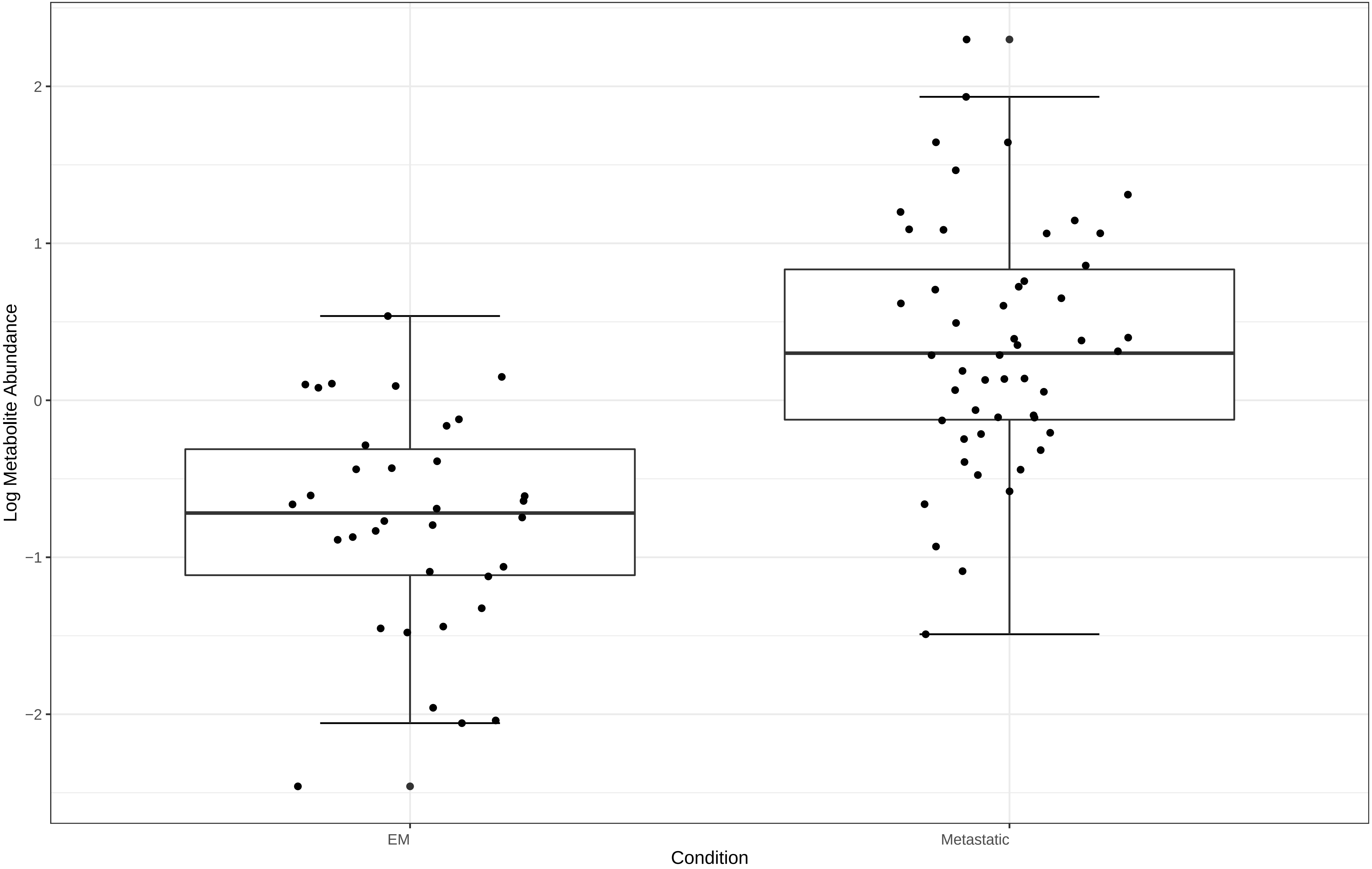

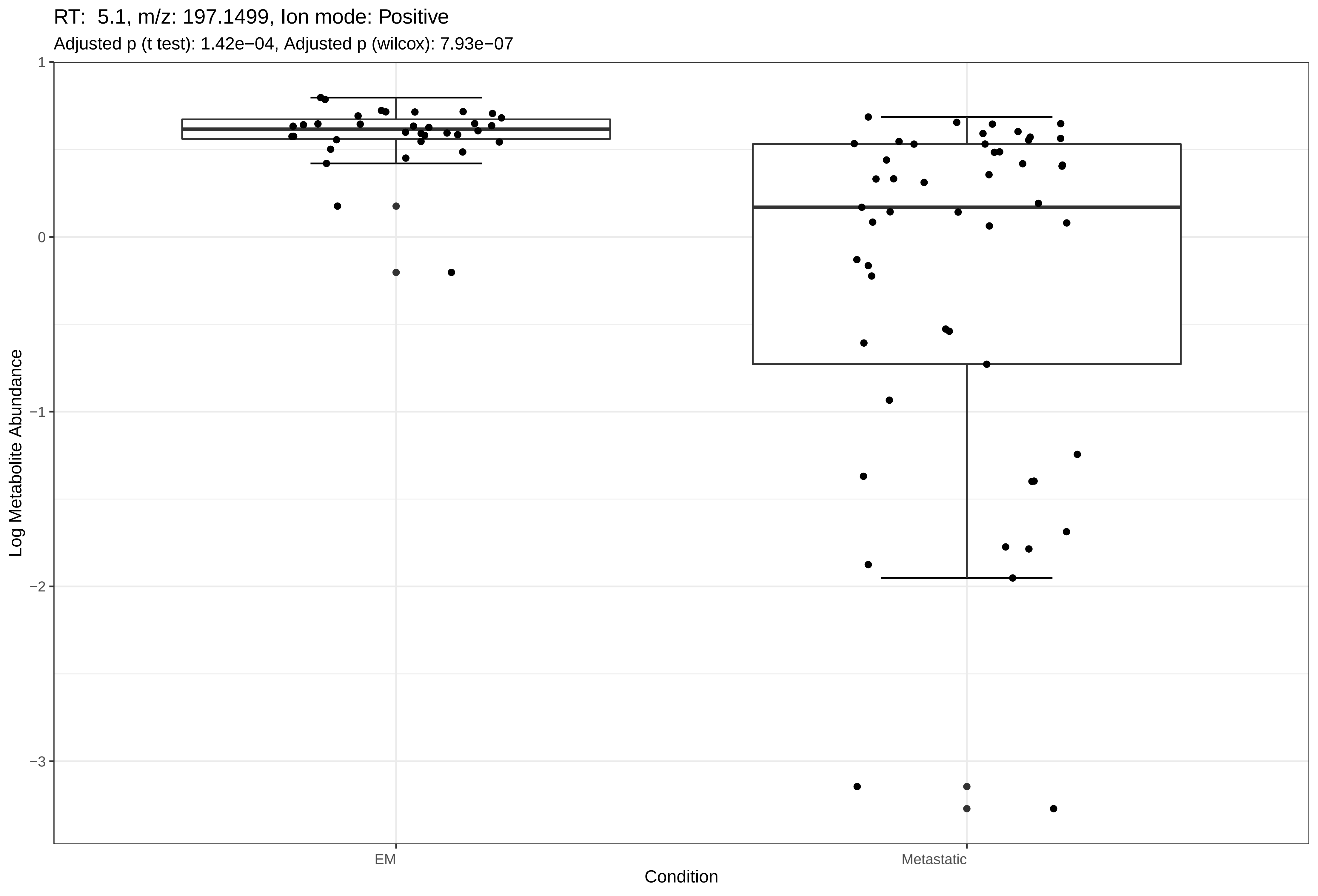
RT: 5.1, m/z: 197.1499, Ion mode: Positive
Adjusted p (t test): 1.42e−04, Adjusted p (wilcox): 7.93e−07
1
0
−1
−2
−3
Log Metabolite Abundance
EM
Metastatic
Condition

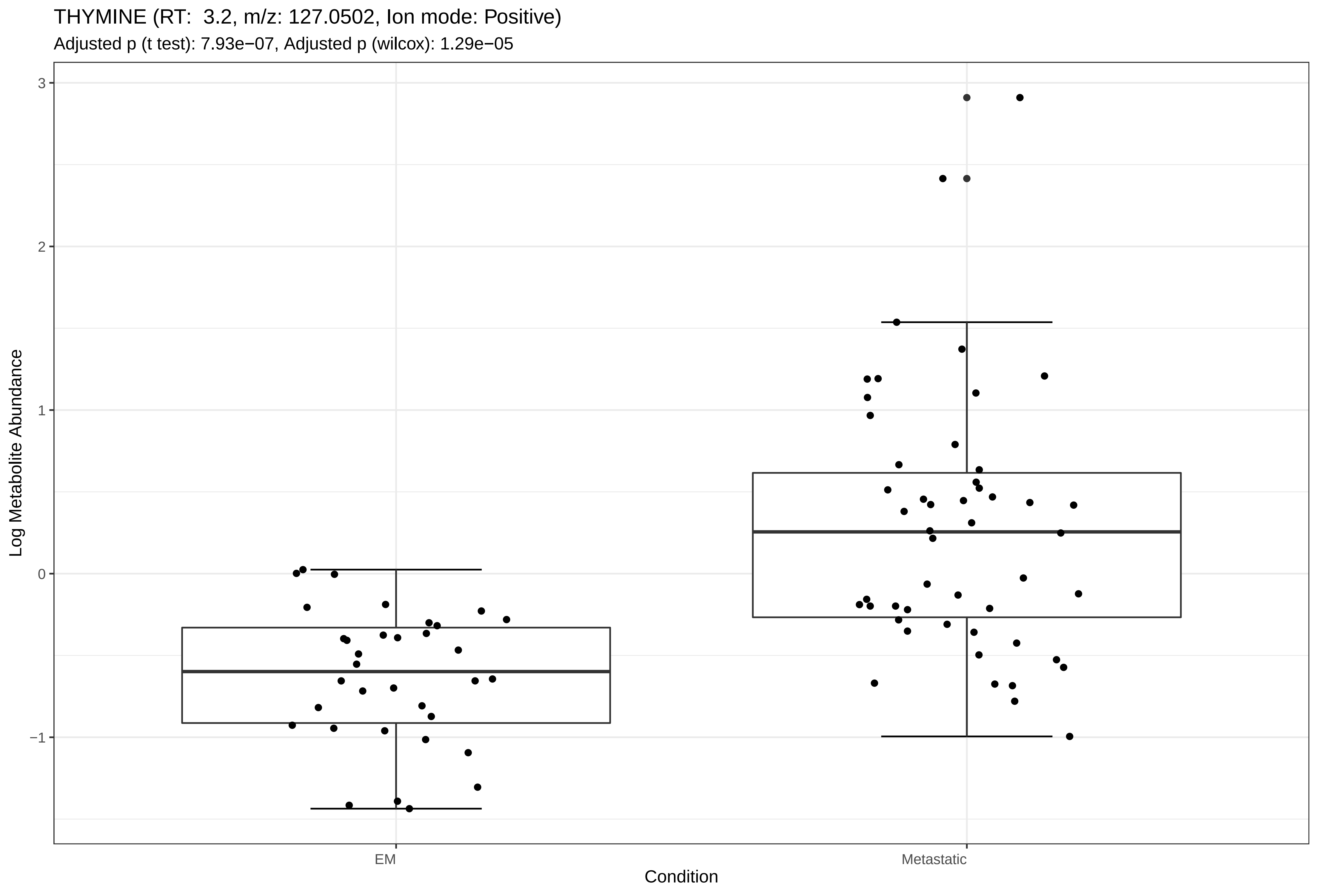

THYMINE (RT: 3.2, m/z: 127.0502, Ion mode: Positive)
Adjusted p (t test): 7.93e−07, Adjusted p (wilcox): 1.29e−05
Log Metabolite Abundance
3
2
1
0
−1
EM
Metastatic
Condition

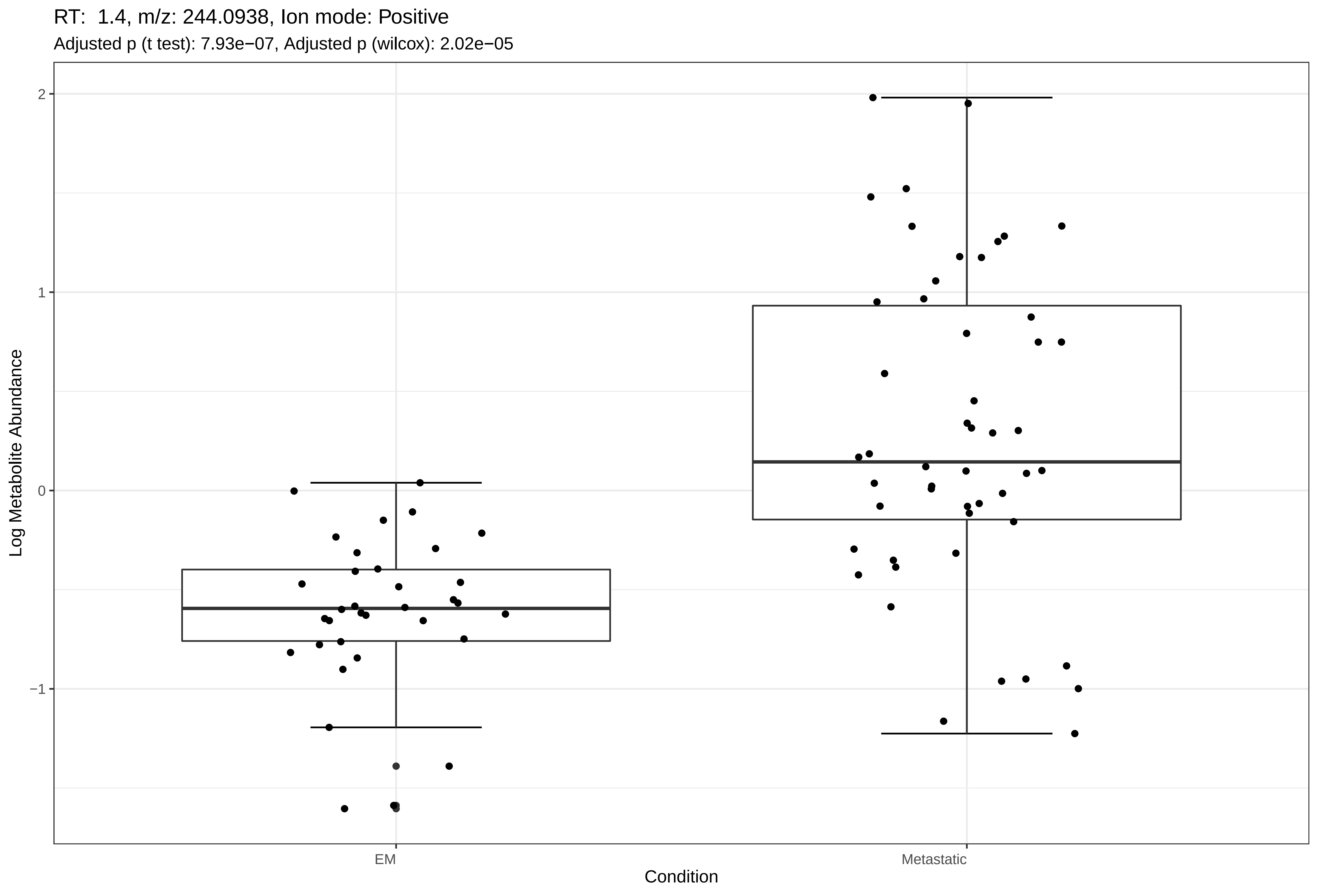
RT: 1.4, m/z: 244.0938, Ion mode: Positive
Adjusted p (t test): 7.93e−07, Adjusted p (wilcox): 2.02e−05
Log Metabolite Abundance
2
1
0
−1
EM
Metastatic
Condition

RT: 0.8, m/z: 74.9613, Ion mode: Negative

Adjusted p (t test): 8.48e−07, Adjusted p (wilcox): 3.29e−05

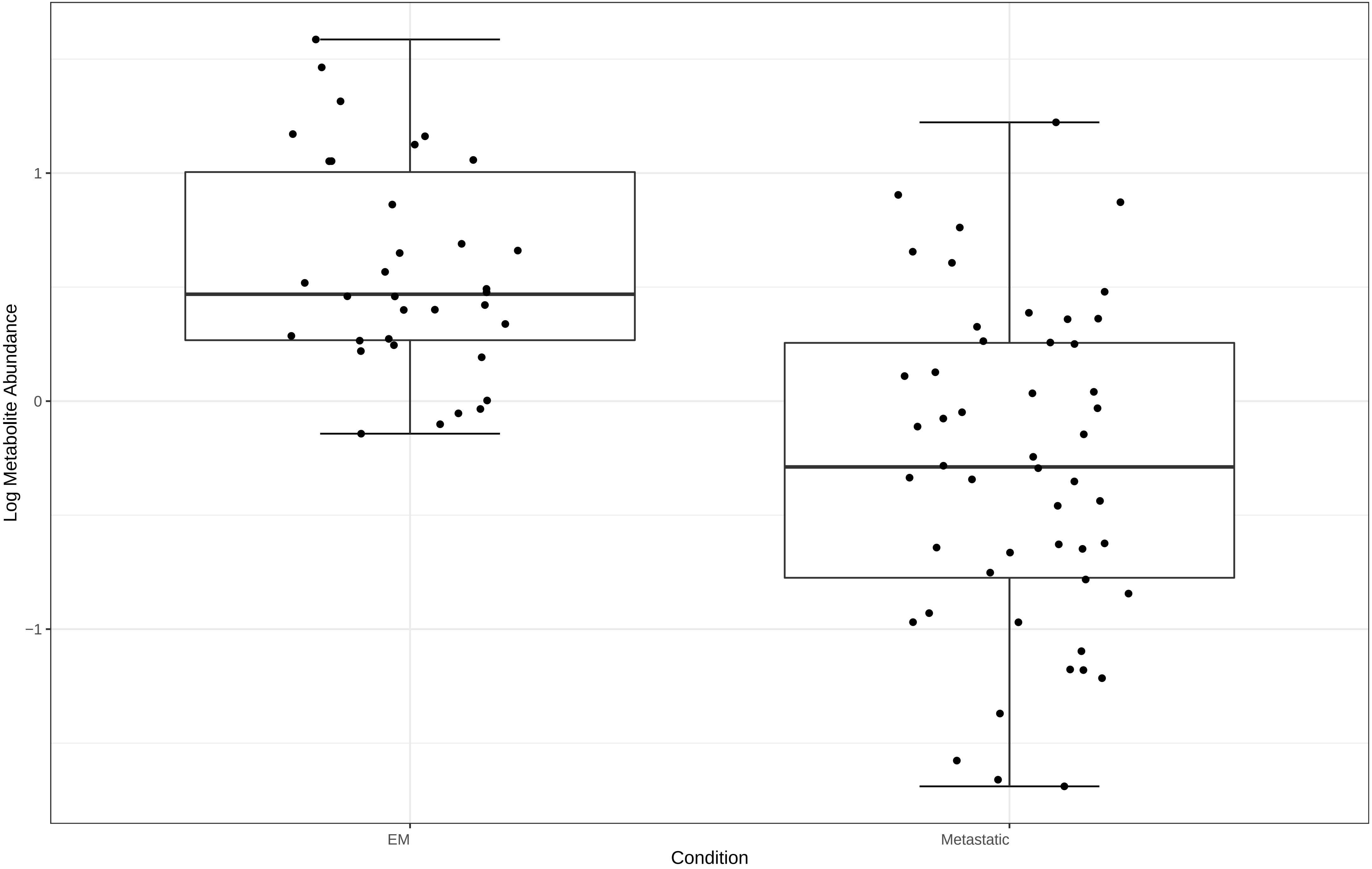

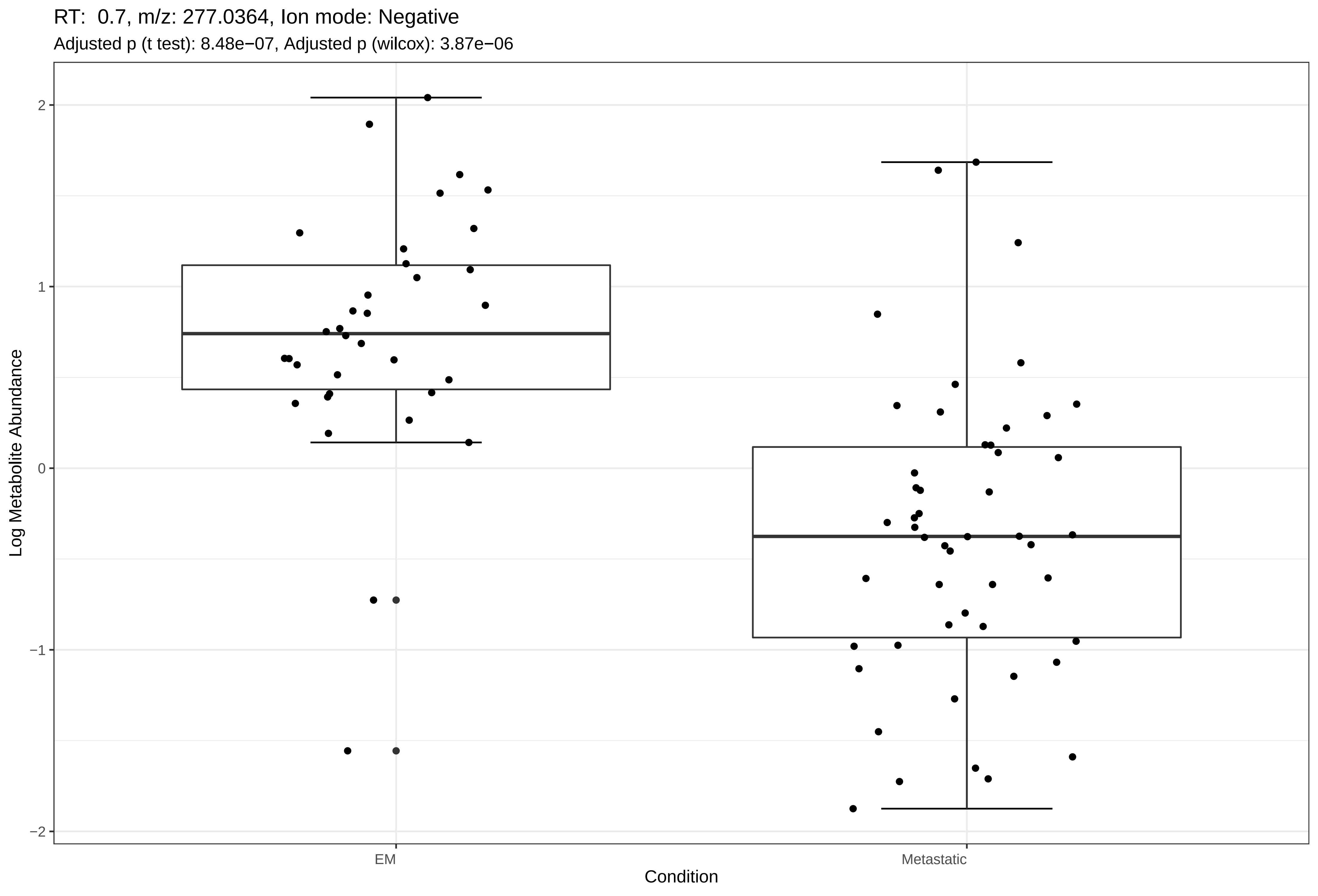
RT: 0.7, m/z: 277.0364, Ion mode: Negative
Adjusted p (t test): 8.48e−07, Adjusted p (wilcox): 3.87e−06
Log Metabolite Abundance
2
1
0
−1
−2
EM
Metastatic
Condition

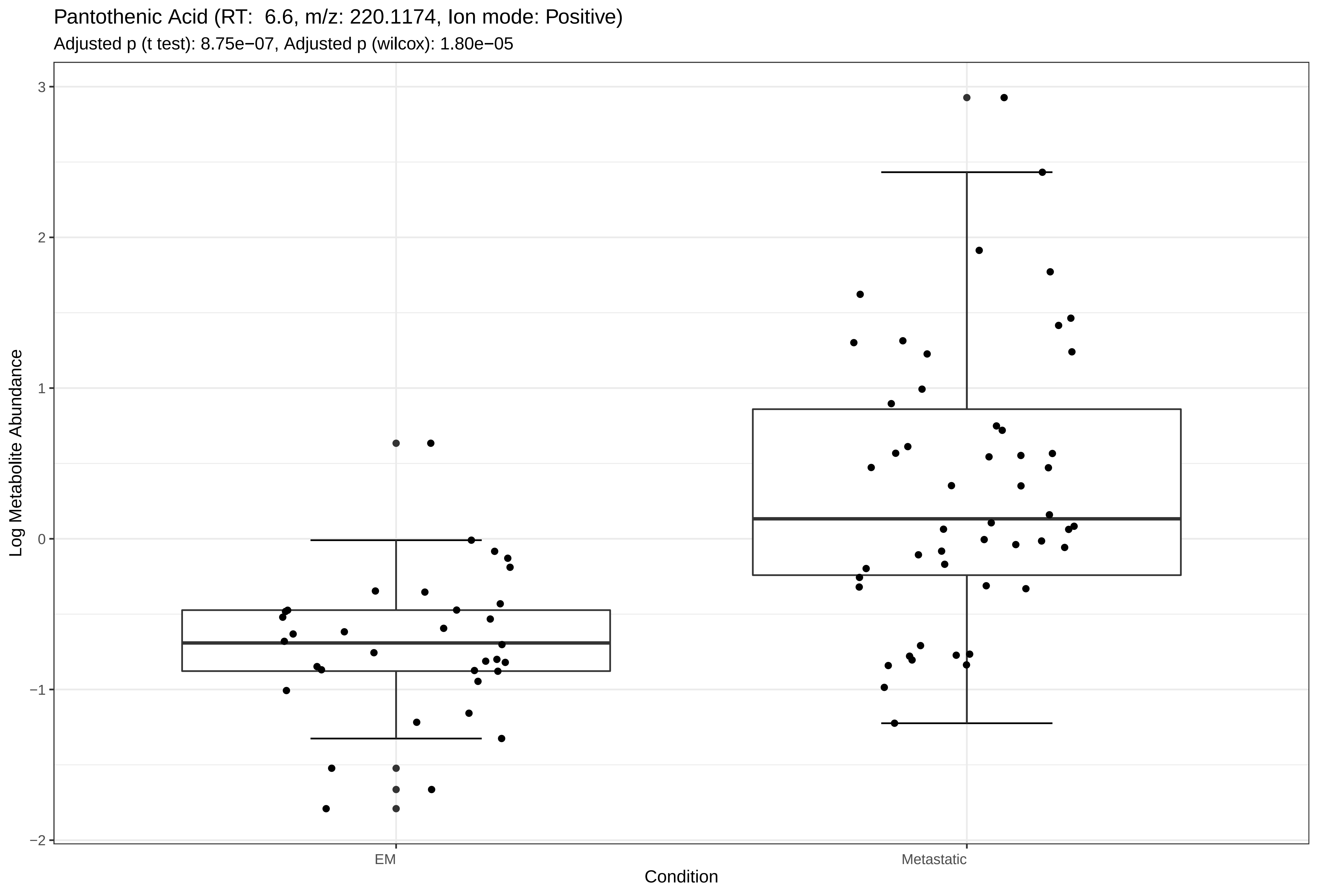
Pantothenic Acid (RT: 6.6, m/z: 220.1174, Ion mode: Positive)
Adjusted p (t test): 8.75e−07, Adjusted p (wilcox): 1.80e−05
Log Metabolite Abundance
3
2
1
0
−1
−2
EM
Metastatic
Condition

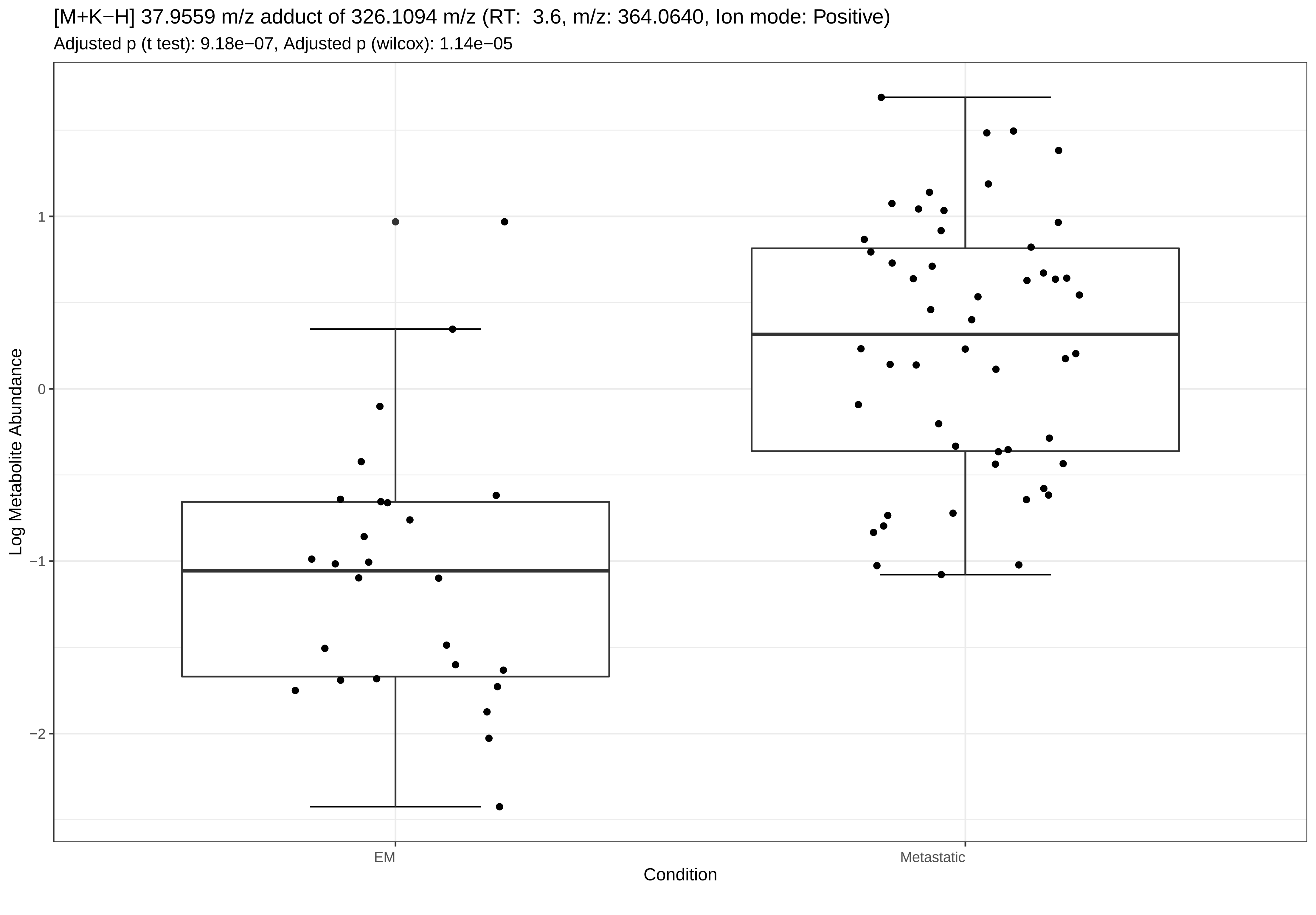
[M+K−H] 37.9559 m/z adduct of 326.1094 m/z (RT: 3.6, m/z: 364.0640, Ion mode: Positive)
Adjusted p (t test): 9.18e−07, Adjusted p (wilcox): 1.14e−05
Log Metabolite Abundance
1
0
−1
−2
EM
Metastatic
Condition

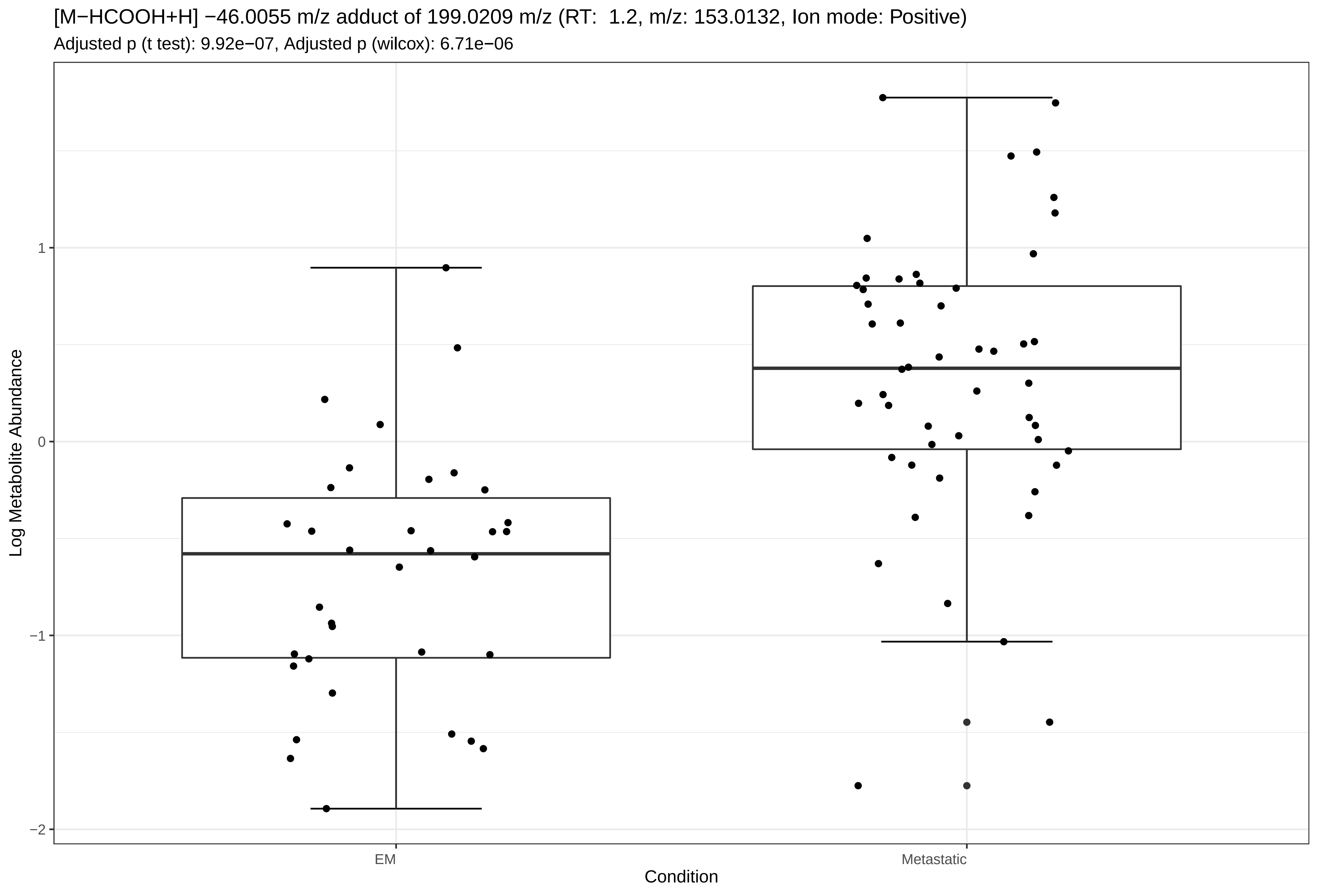

[M−HCOOH+H] −46.0055 m/z adduct of 199.0209 m/z (RT: 1.2, m/z: 153.0132, Ion mode: Positive)
Adjusted p (t test): 9.92e−07, Adjusted p (wilcox): 6.71e−06
Log Metabolite Abundance
1
0
−1
−2
EM
Metastatic
Condition

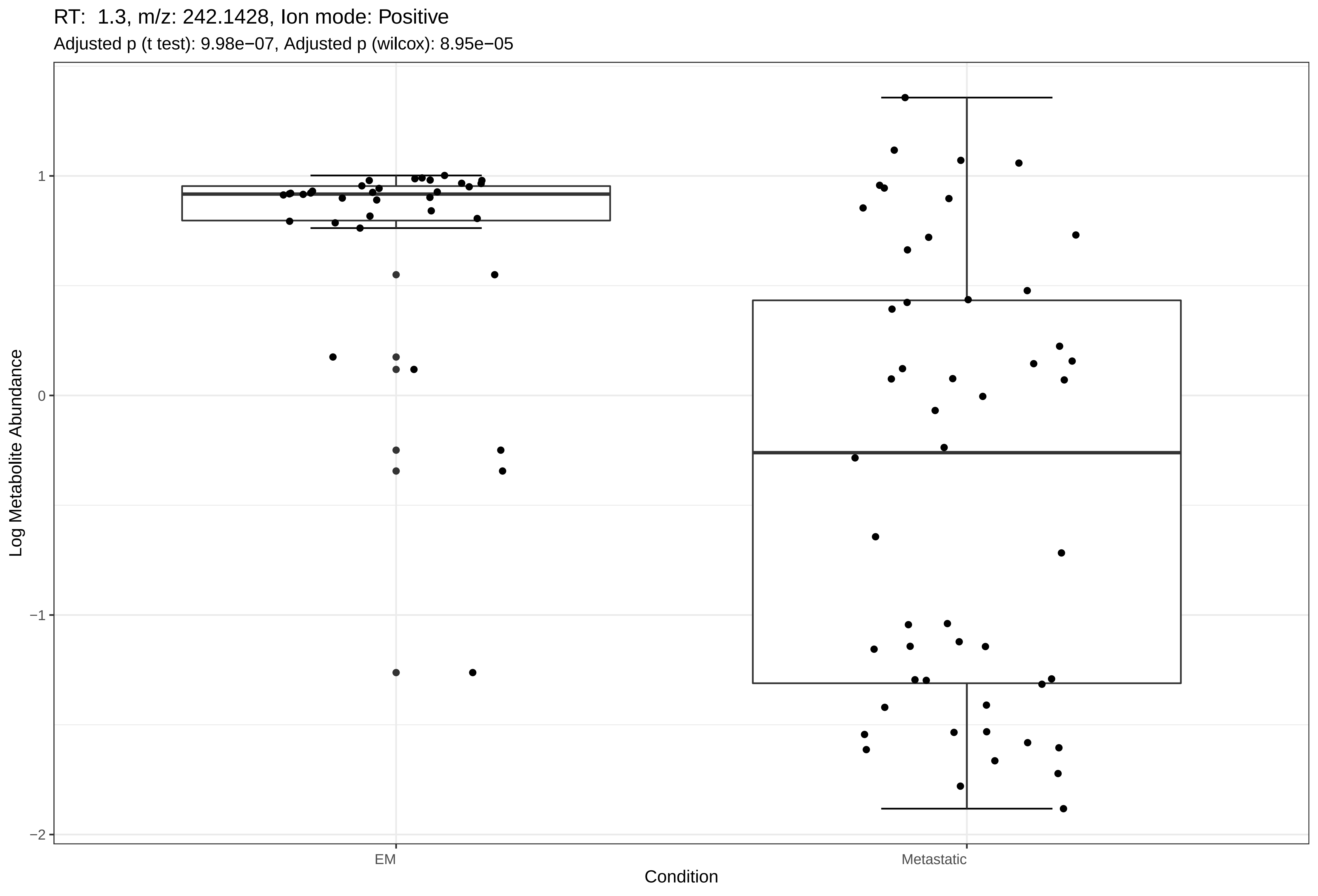

RT: 1.3, m/z: 242.1428, Ion mode: Positive
Adjusted p (t test): 9.98e−07, Adjusted p (wilcox): 8.95e−05
Log Metabolite Abundance
1
0
−1
−2
EM
Metastatic
Condition

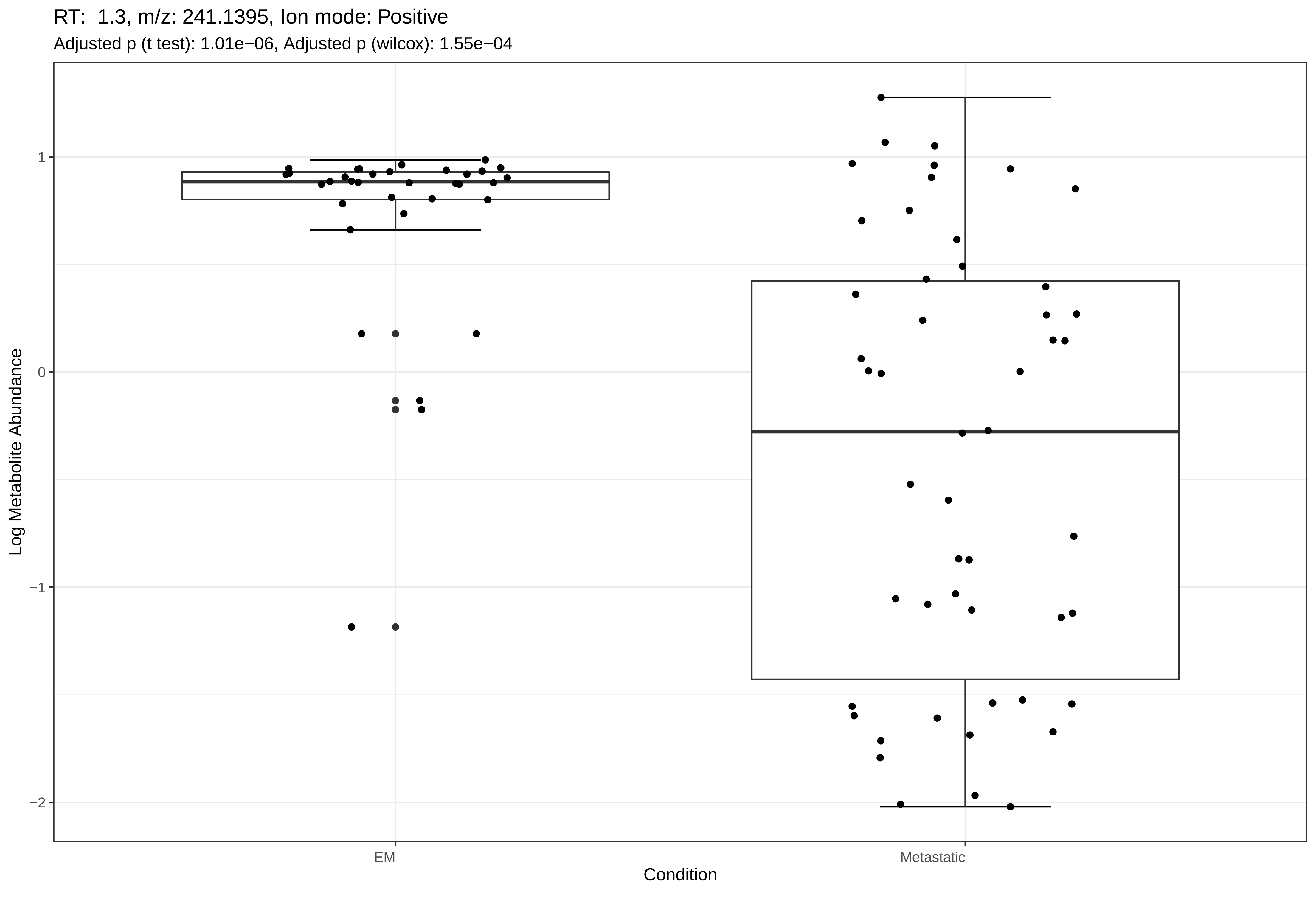

RT: 1.3, m/z: 241.1395, Ion mode: Positive
Adjusted p (t test): 1.01e−06, Adjusted p (wilcox): 1.55e−04
Log Metabolite Abundance
1
0
−1
−2
EM
Metastatic
Condition

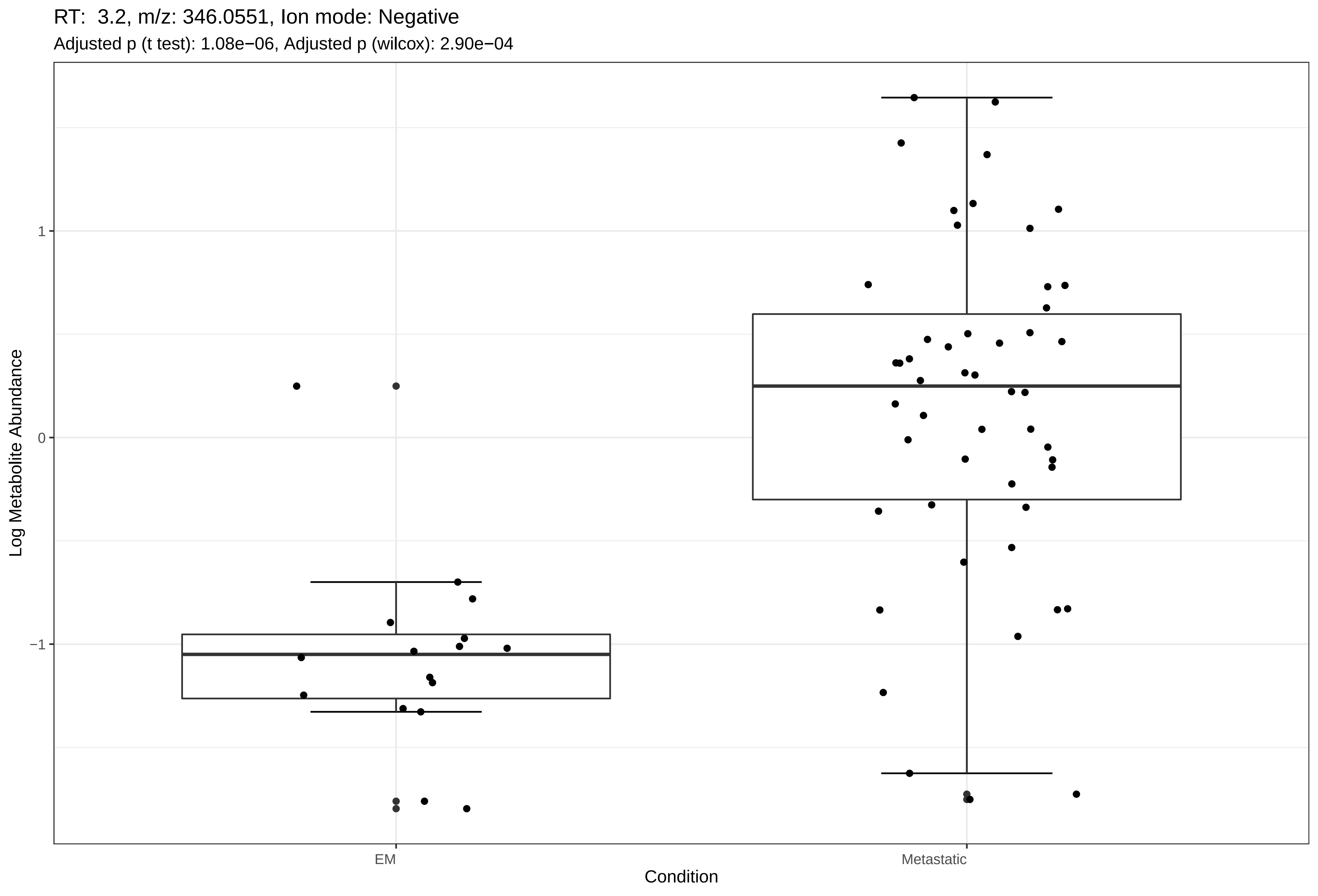
RT: 3.2, m/z: 346.0551, Ion mode: Negative
Adjusted p (t test): 1.08e−06, Adjusted p (wilcox): 2.90e−04
Log Metabolite Abundance
1
0
−1
EM
Metastatic
Condition

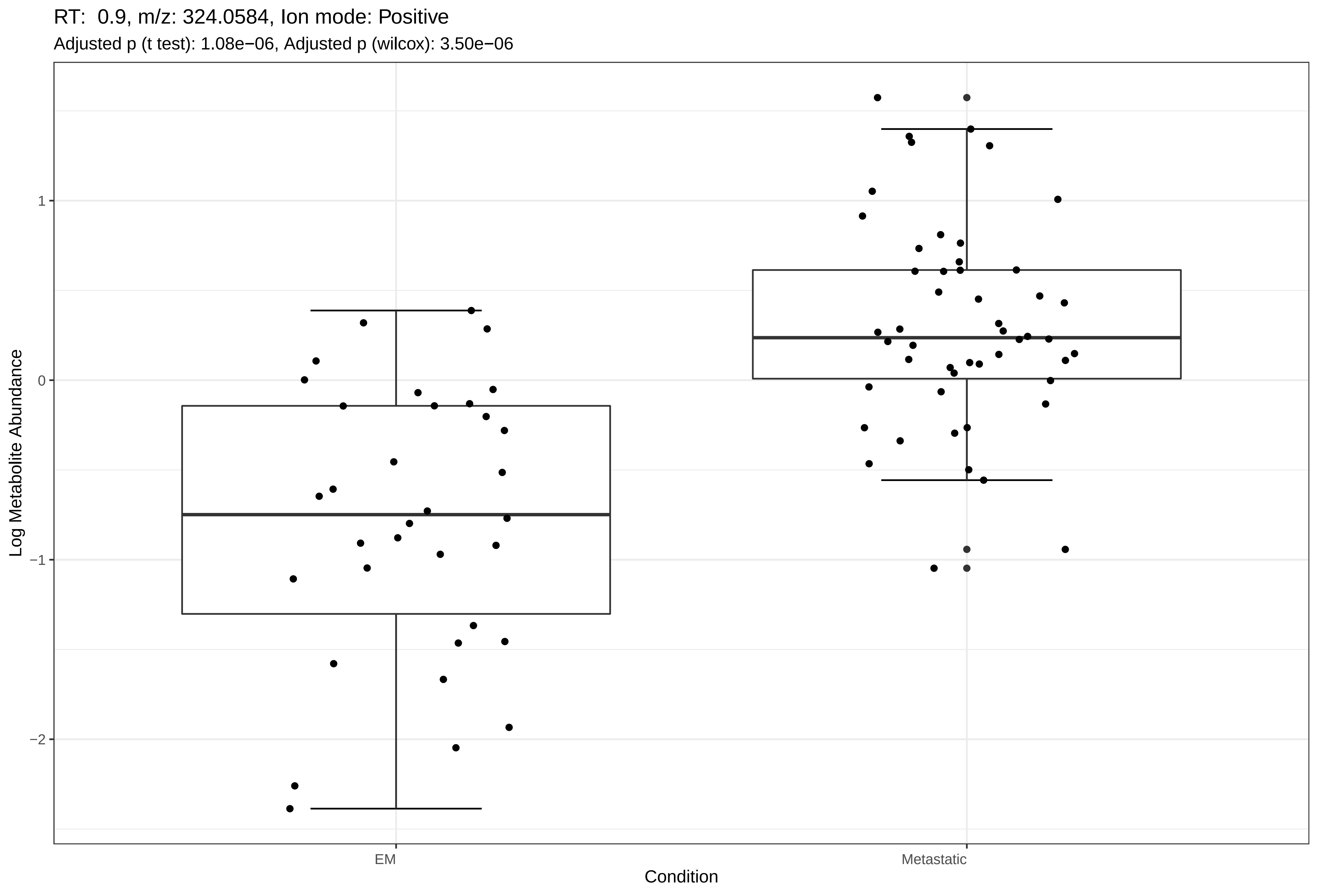
RT: 0.9, m/z: 324.0584, Ion mode: Positive
Adjusted p (t test): 1.08e−06, Adjusted p (wilcox): 3.50e−06
Log Metabolite Abundance
1
0
−1
−2
EM
Metastatic
Condition

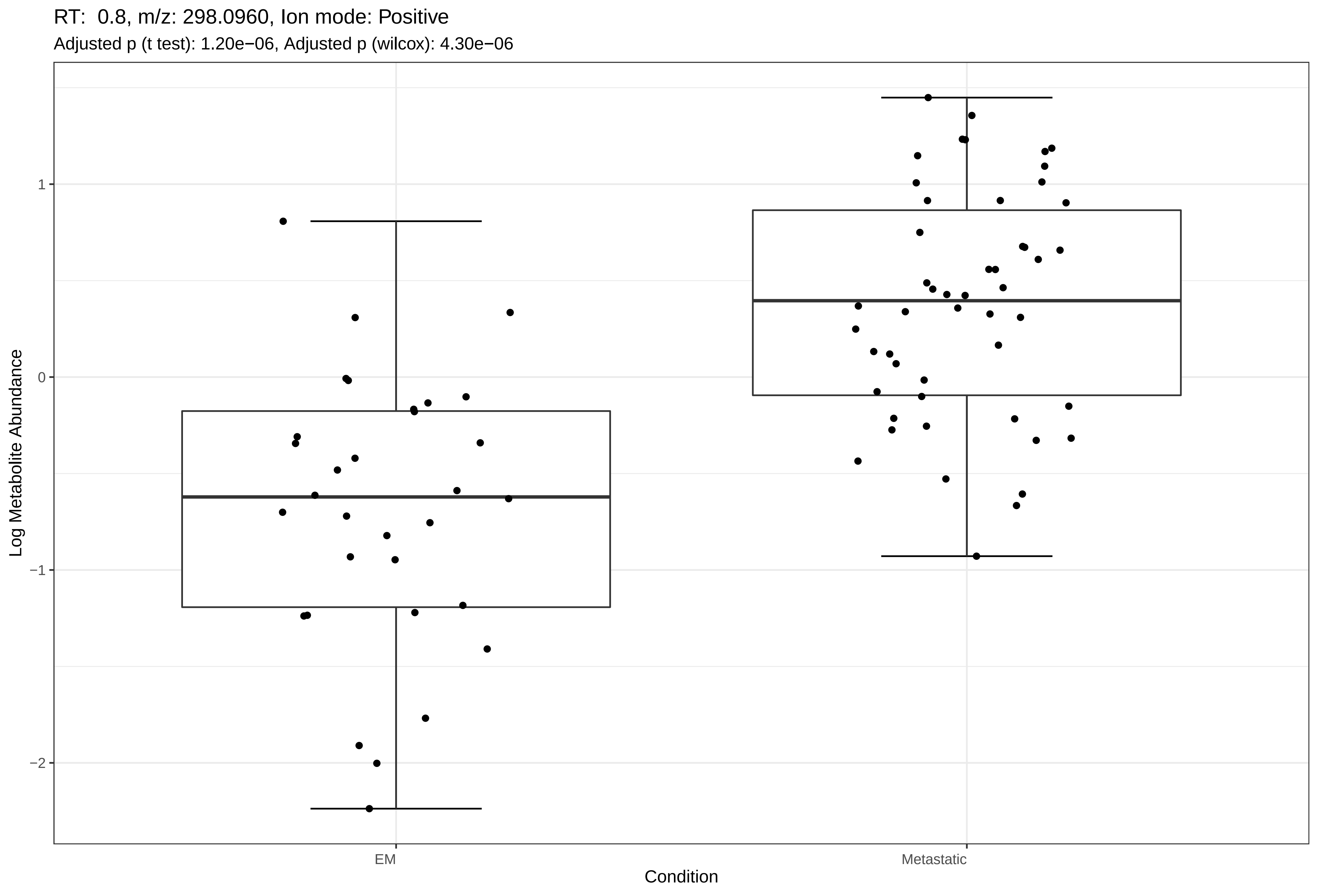
RT: 0.8, m/z: 298.0960, Ion mode: Positive
Adjusted p (t test): 1.20e−06, Adjusted p (wilcox): 4.30e−06
Log Metabolite Abundance
1
0
−1
−2
EM
Metastatic
Condition

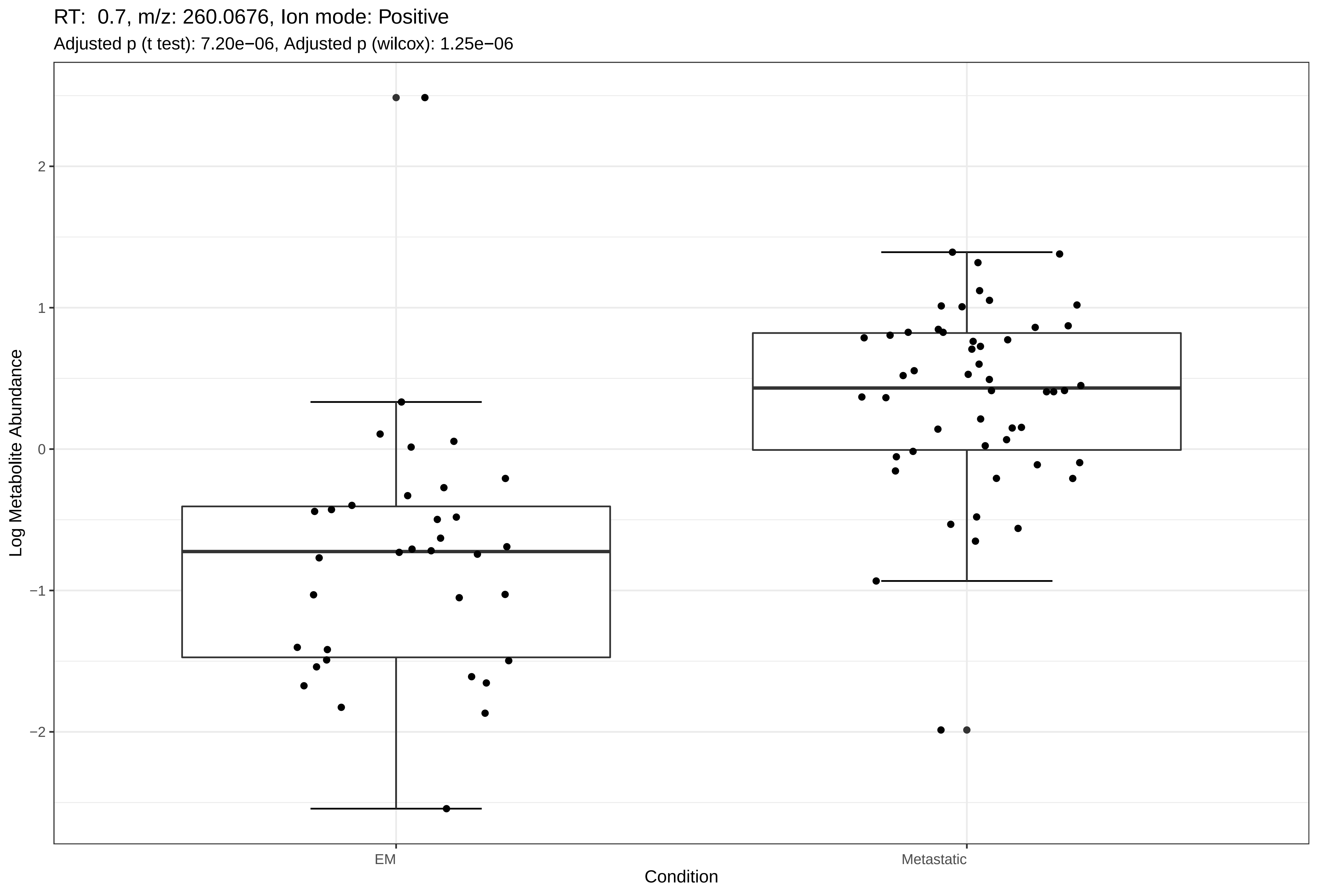
RT: 0.7, m/z: 260.0676, Ion mode: Positive
Adjusted p (t test): 7.20e−06, Adjusted p (wilcox): 1.25e−06
Log Metabolite Abundance
2
1
0
−1
−2
EM
Metastatic
Condition

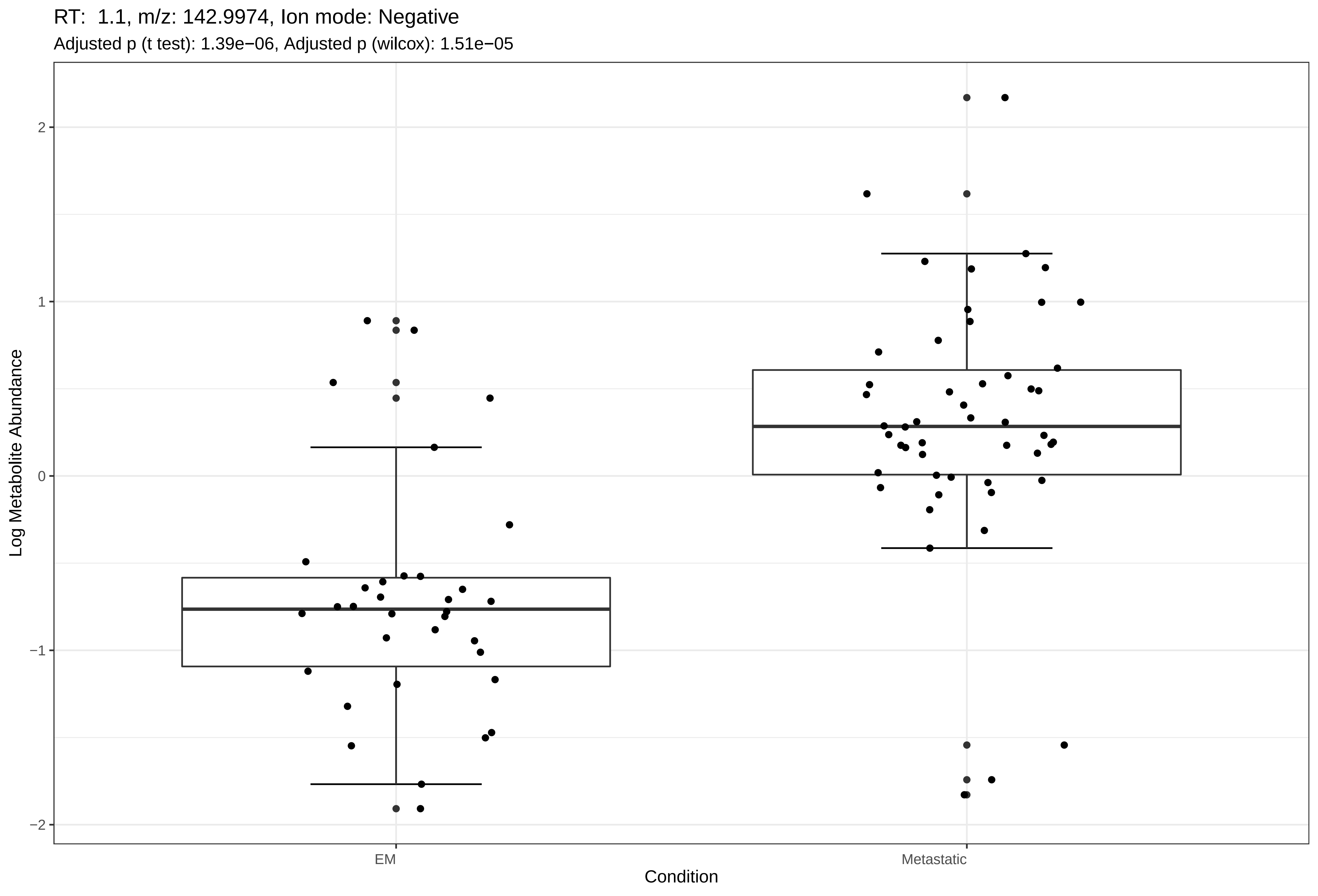
RT: 1.1, m/z: 142.9974, Ion mode: Negative
Adjusted p (t test): 1.39e−06, Adjusted p (wilcox): 1.51e−05
Log Metabolite Abundance
2
1
0
−1
−2
EM
Metastatic
Condition

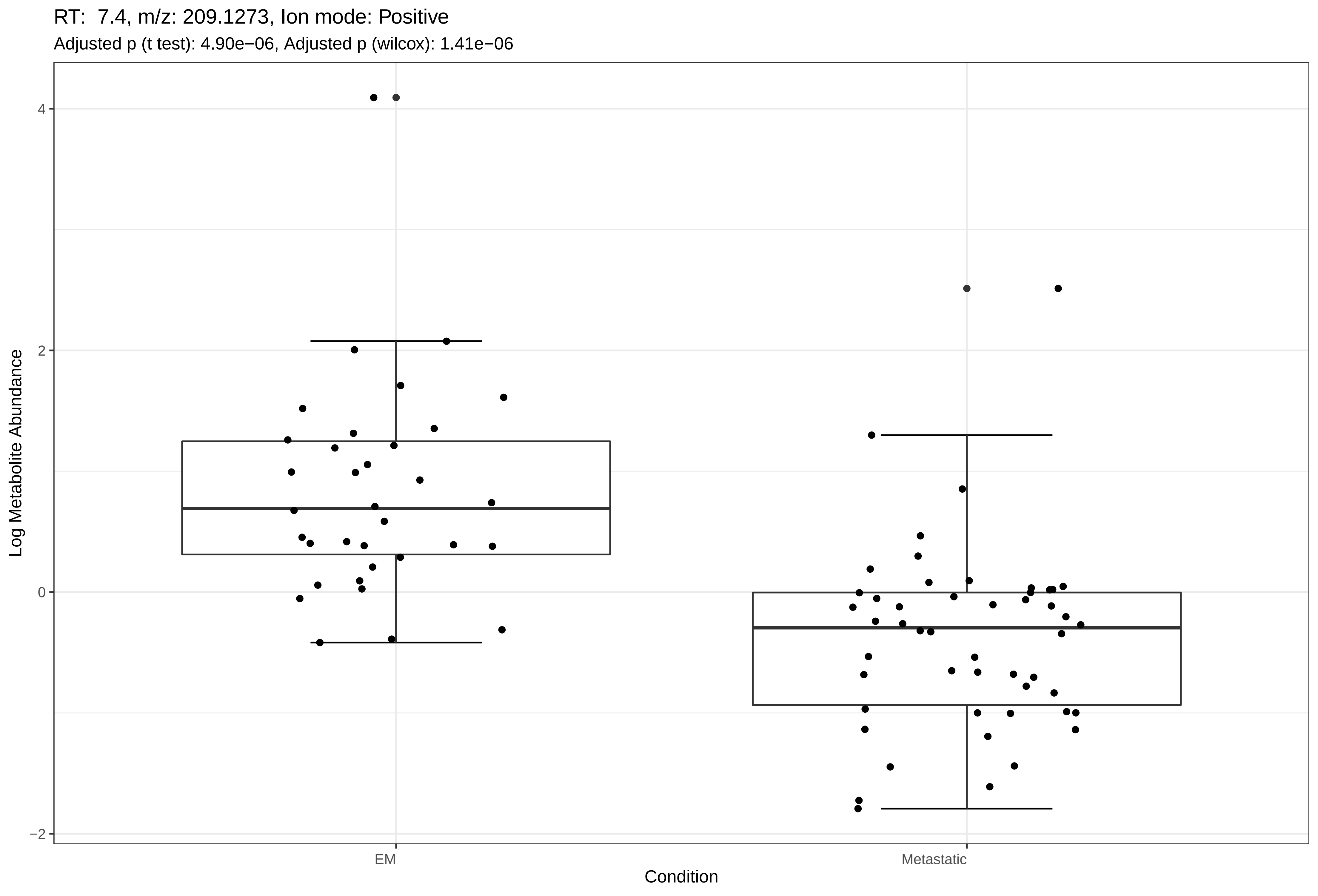
RT: 7.4, m/z: 209.1273, Ion mode: Positive
Adjusted p (t test): 4.90e−06, Adjusted p (wilcox): 1.41e−06
Log Metabolite Abundance
4
2
0
−2
EM
Metastatic
Condition

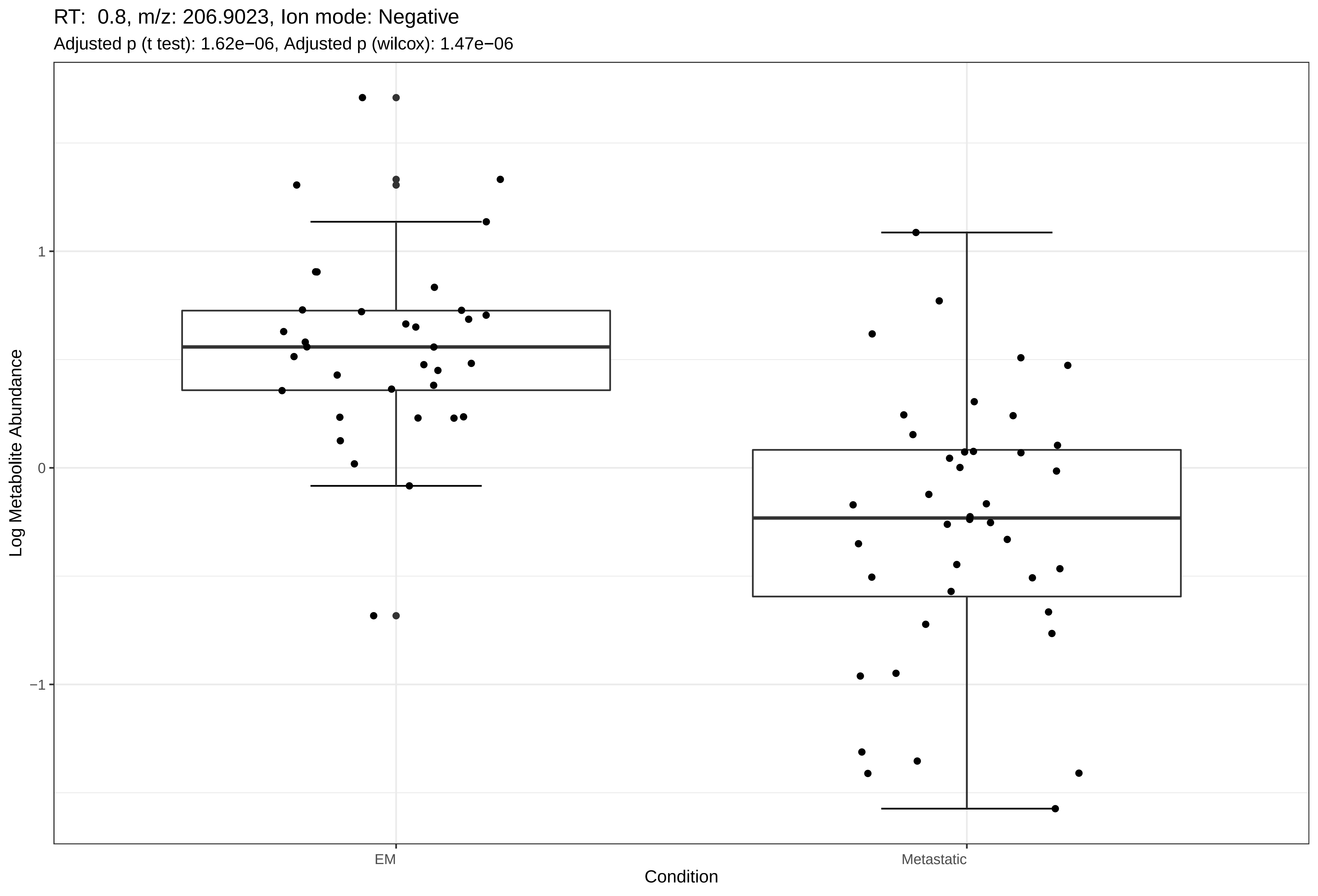

RT: 0.8, m/z: 206.9023, Ion mode: Negative
Adjusted p (t test): 1.62e−06, Adjusted p (wilcox): 1.47e−06
Log Metabolite Abundance
1
0
−1
EM
Metastatic
Condition

[M+K−H] 37.9559 m/z adduct of 139.0500 m/z (RT: 2.9, m/z: 177.0069, Ion mode: Positive)

Adjusted p (t test): 6.89e−03, Adjusted p (wilcox): 1.50e−06

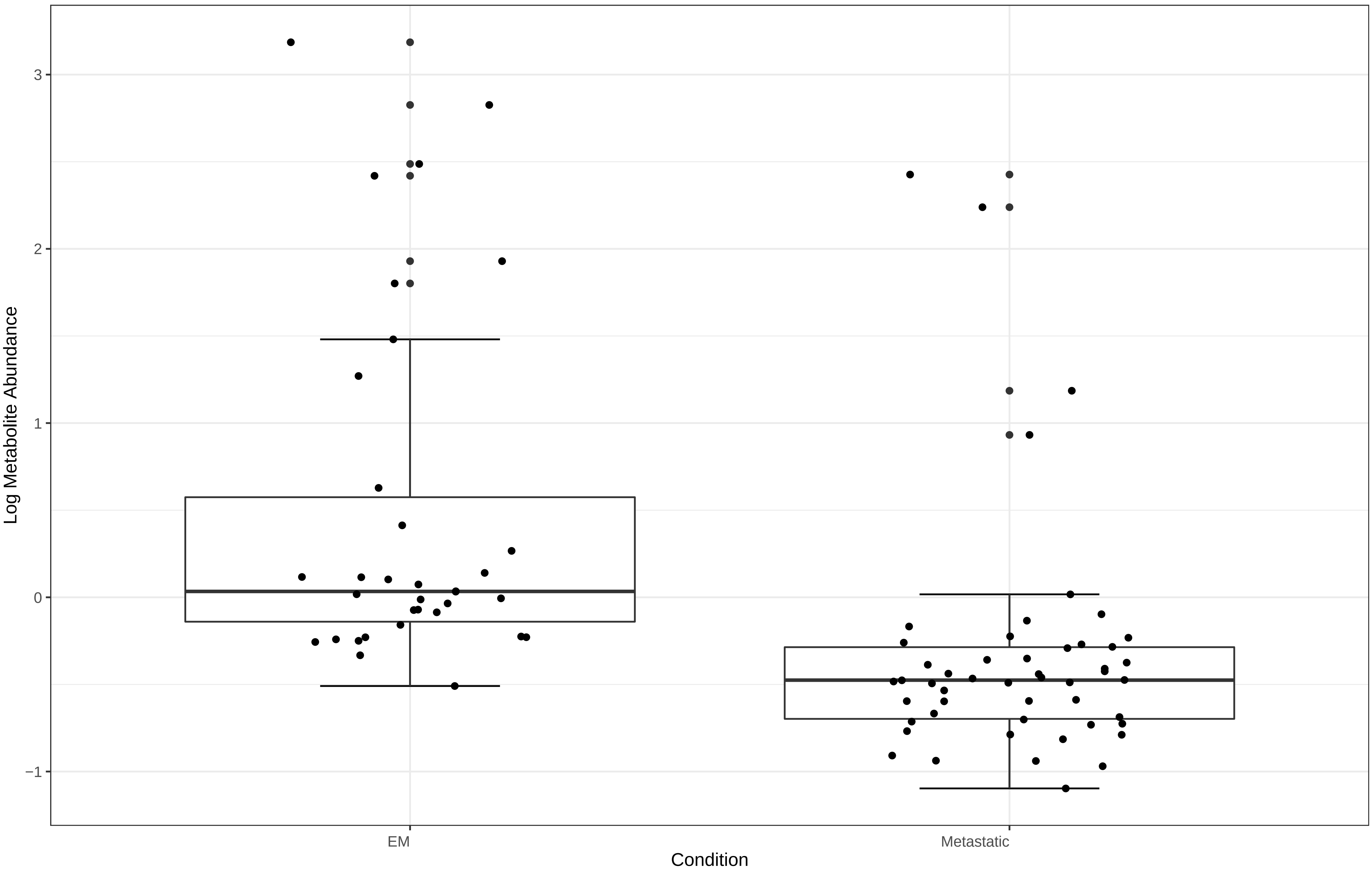

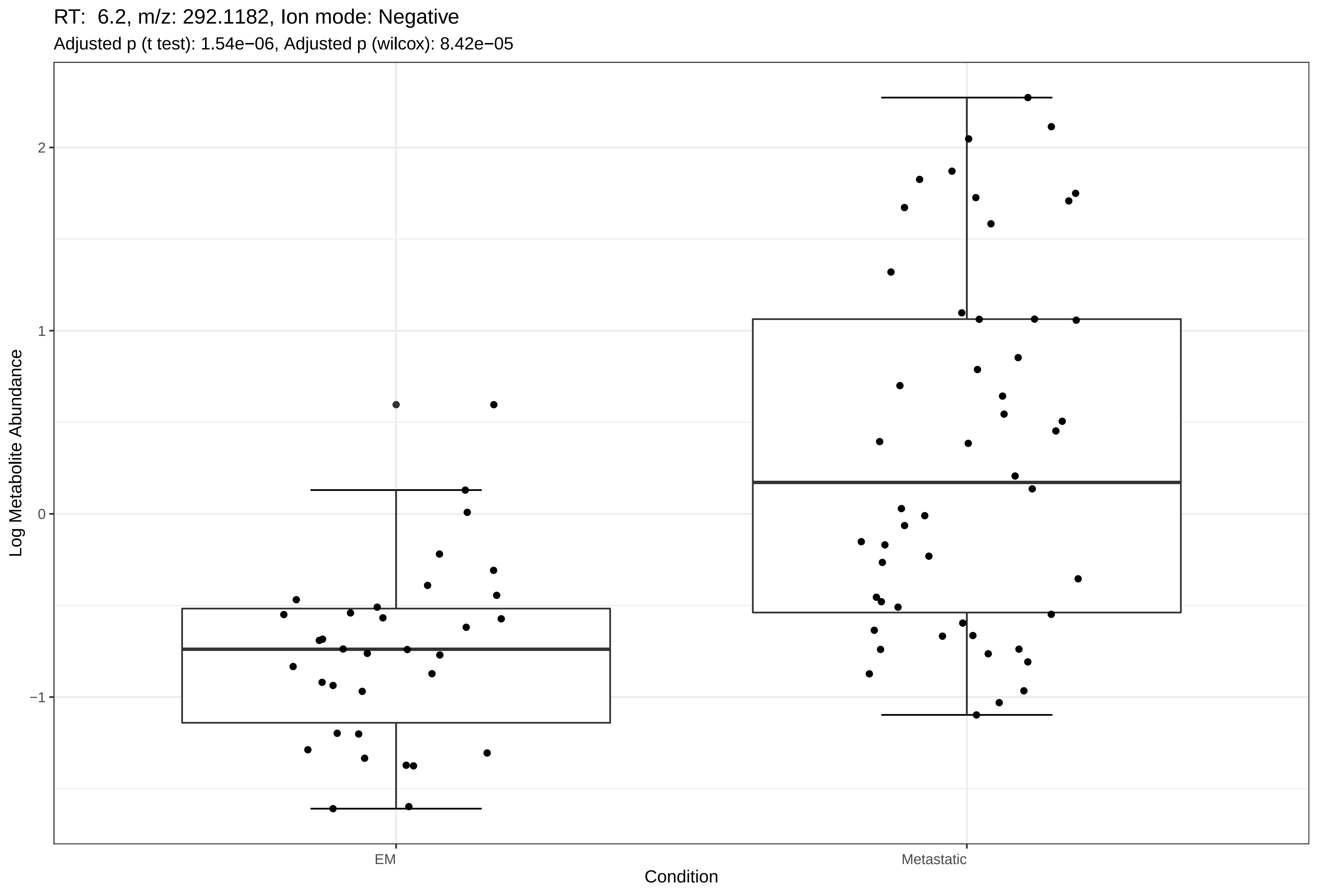

RT: 6.2, m/z: 292.1182, Ion mode: Negative
Adjusted p (t test): 1.54e−06, Adjusted p (wilcox): 8.42e−05
Log Metabolite Abundance
2
1
0
−1
EM
Metastatic
Condition

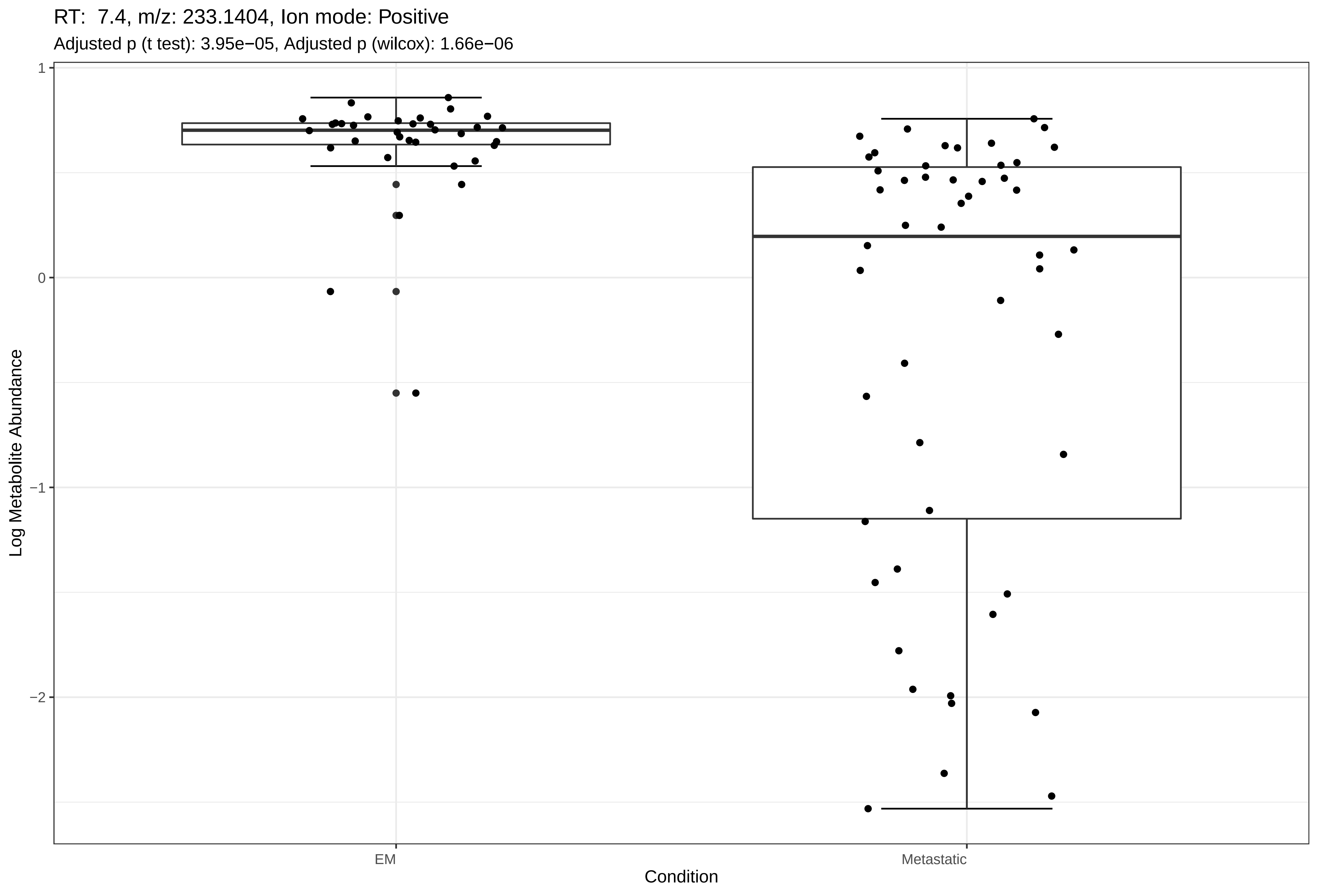

RT: 7.4, m/z: 233.1404, Ion mode: Positive
Adjusted p (t test): 3.95e−05, Adjusted p (wilcox): 1.66e−06
1
0
−1
−2
Log Metabolite Abundance
EM
Metastatic
Condition

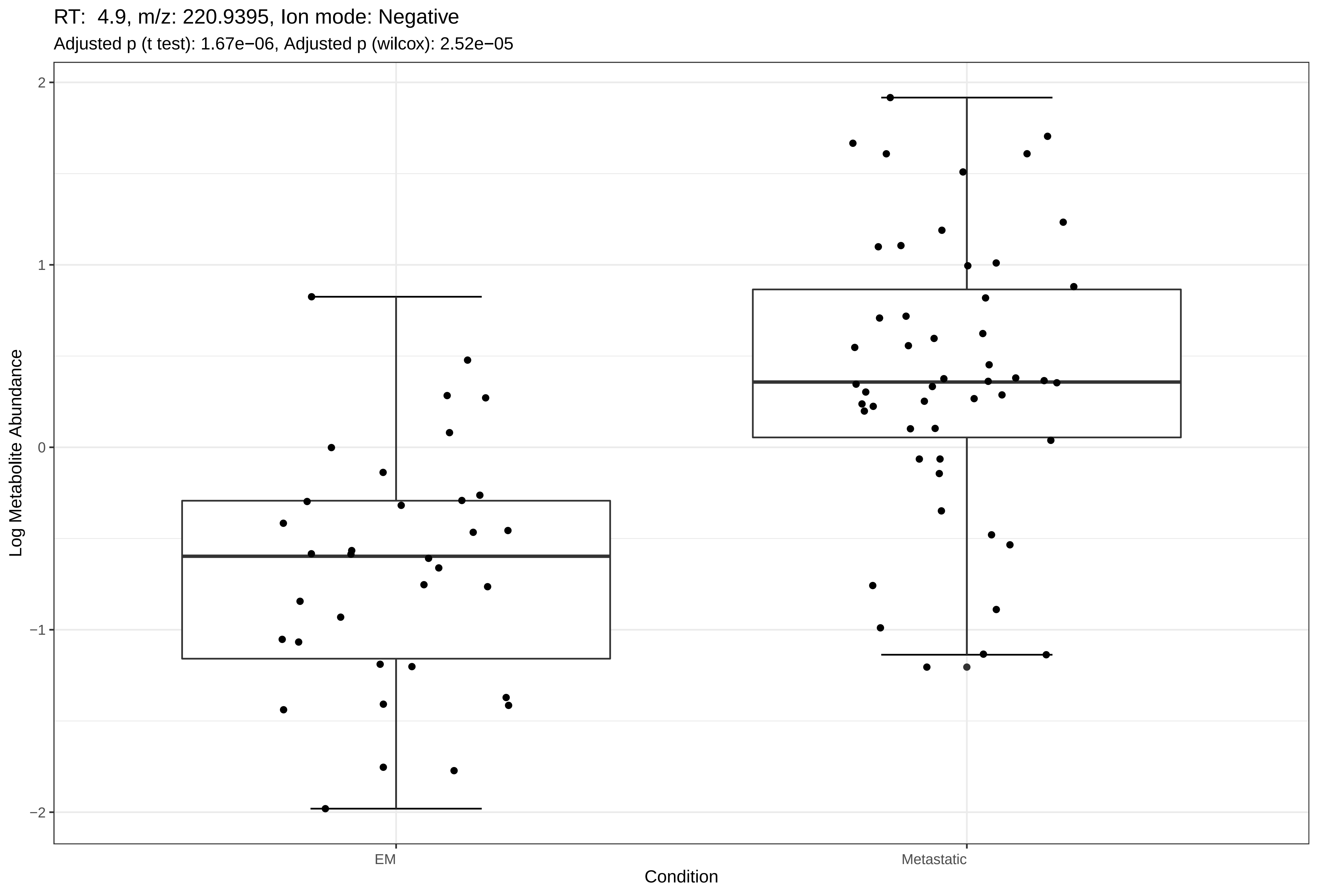

RT: 4.9, m/z: 220.9395, Ion mode: Negative
Adjusted p (t test): 1.67e−06, Adjusted p (wilcox): 2.52e−05
Log Metabolite Abundance
2
1
0
−1
−2
EM
Metastatic
Condition

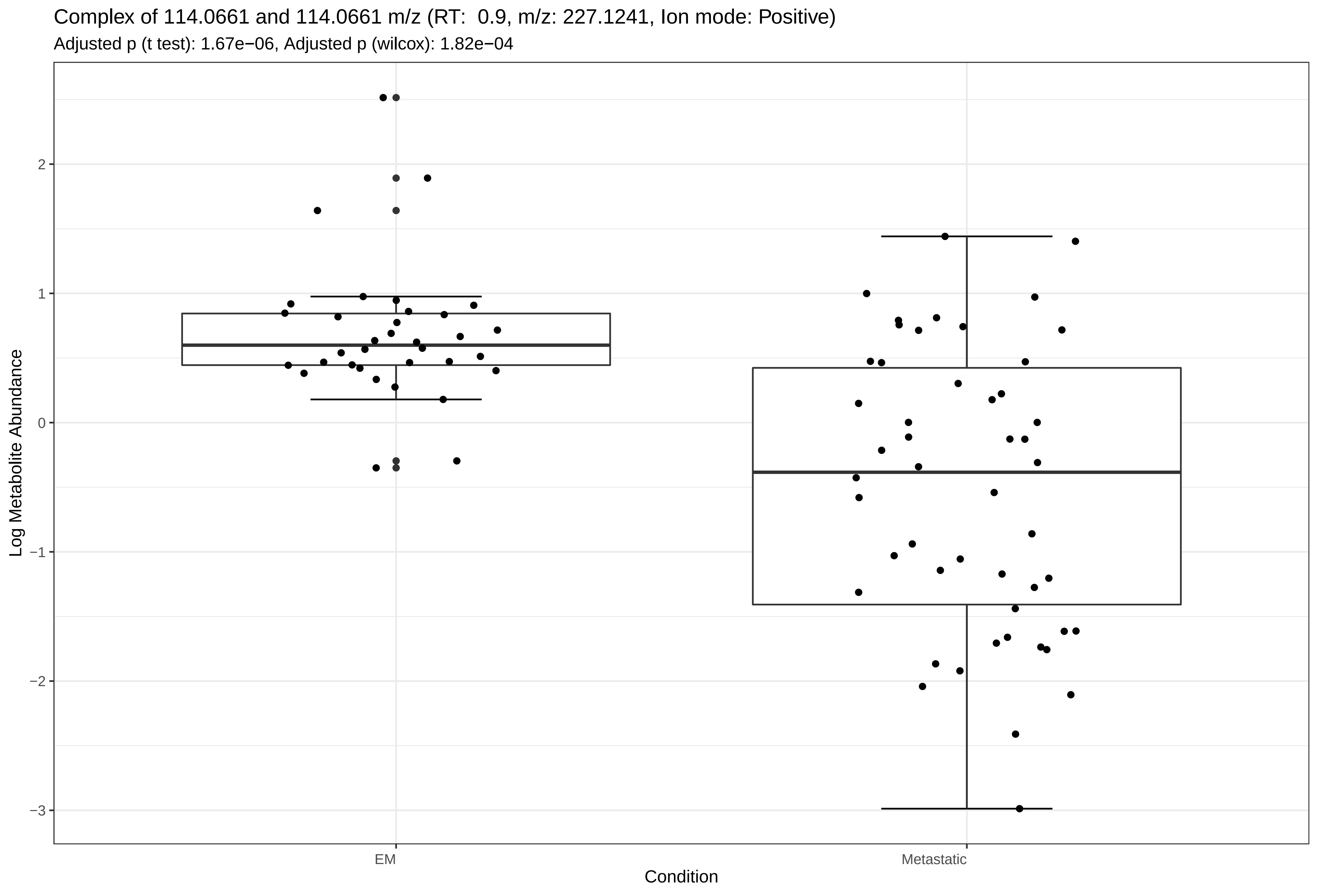

Complex of 114.0661 and 114.0661 m/z (RT: 0.9, m/z: 227.1241, Ion mode: Positive)
Adjusted p (t test): 1.67e−06, Adjusted p (wilcox): 1.82e−04
Log Metabolite Abundance
2
1
0
−1
−2
−3
EM
Metastatic
Condition

RT: 3.7, m/z: 362.0508, Ion mode: Negative

Adjusted p (t test): 1.68e−06, Adjusted p (wilcox): 6.43e−06

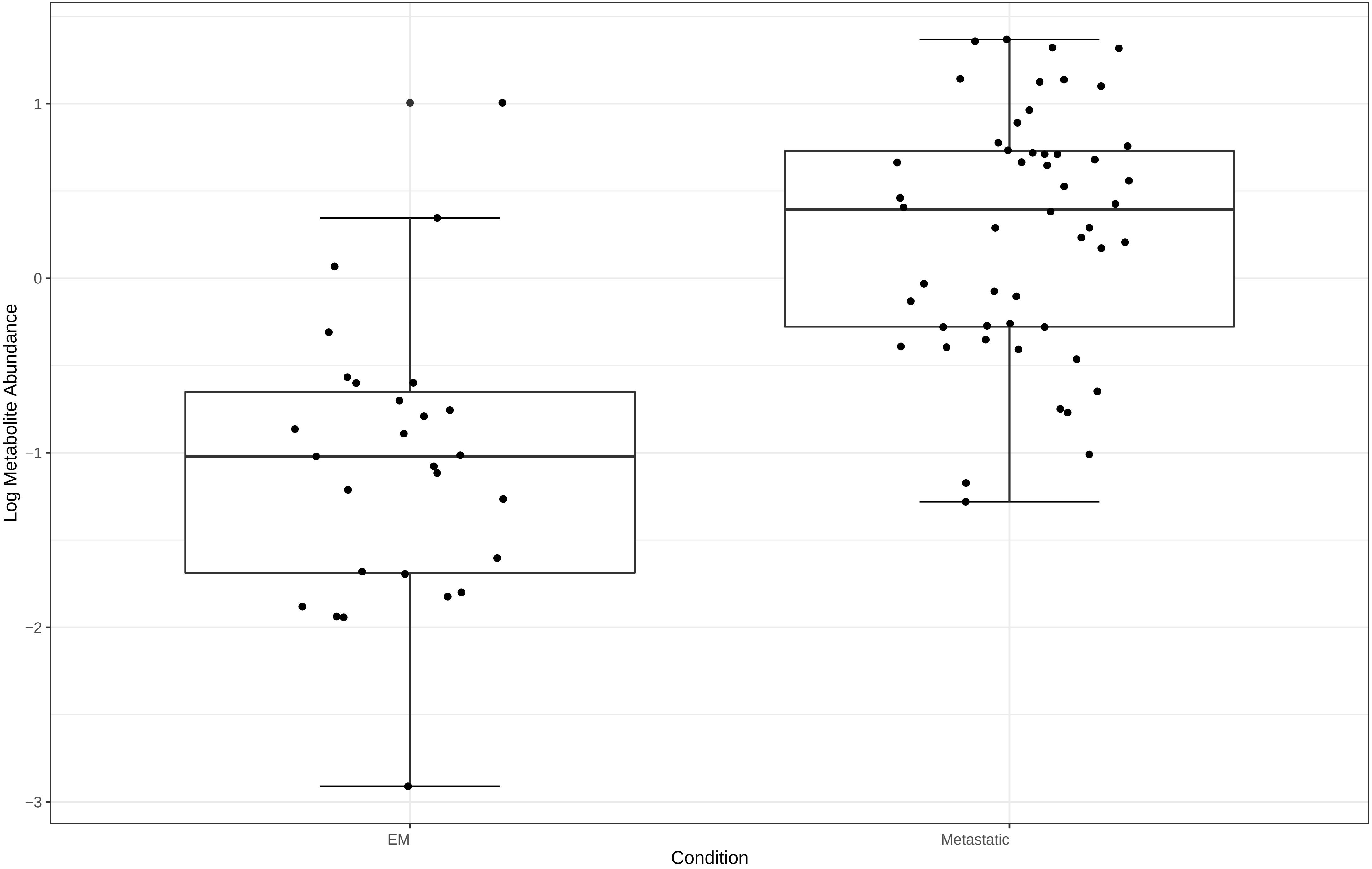

RT: 0.8, m/z: 150.8867, Ion mode: Negative

Adjusted p (t test): 1.74e−06, Adjusted p (wilcox): 1.32e−05

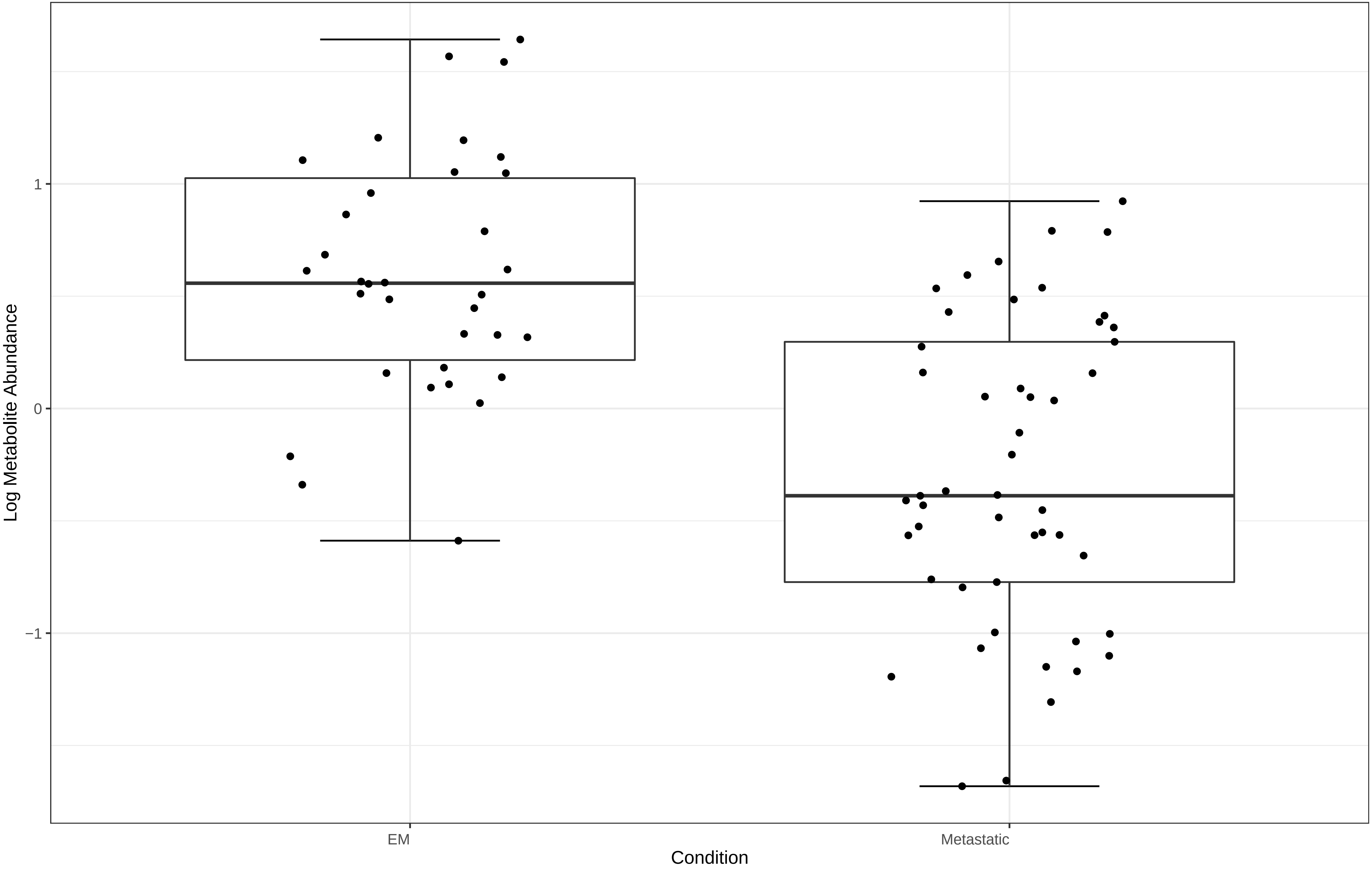

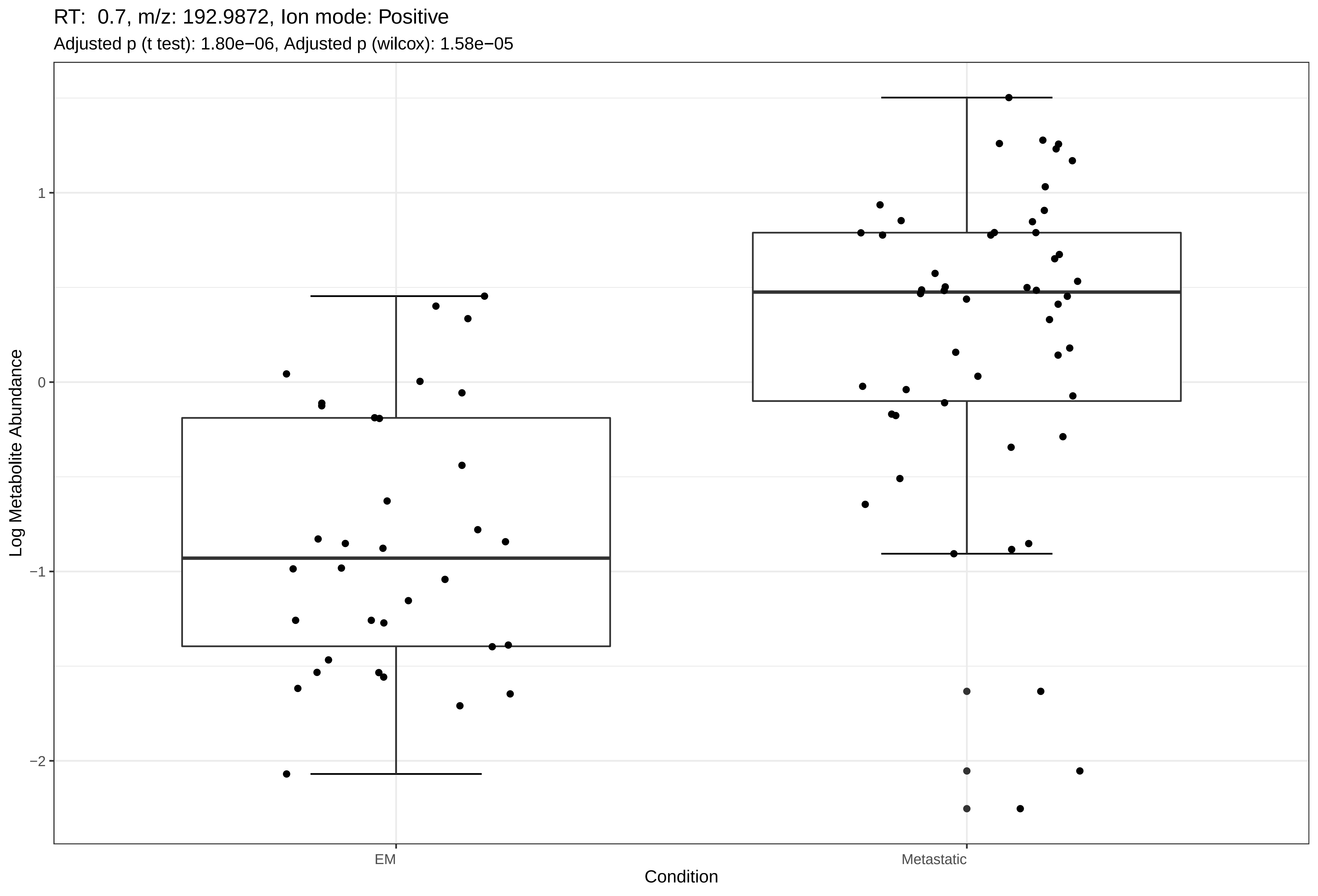

RT: 0.7, m/z: 192.9872, Ion mode: Positive
Adjusted p (t test): 1.80e−06, Adjusted p (wilcox): 1.58e−05
Log Metabolite Abundance
1
0
−1
−2
EM
Metastatic
Condition

RT: 0.8, m/z: 200.0753, Ion mode: Positive

Adjusted p (t test): 2.11e−06, Adjusted p (wilcox): 2.58e−06

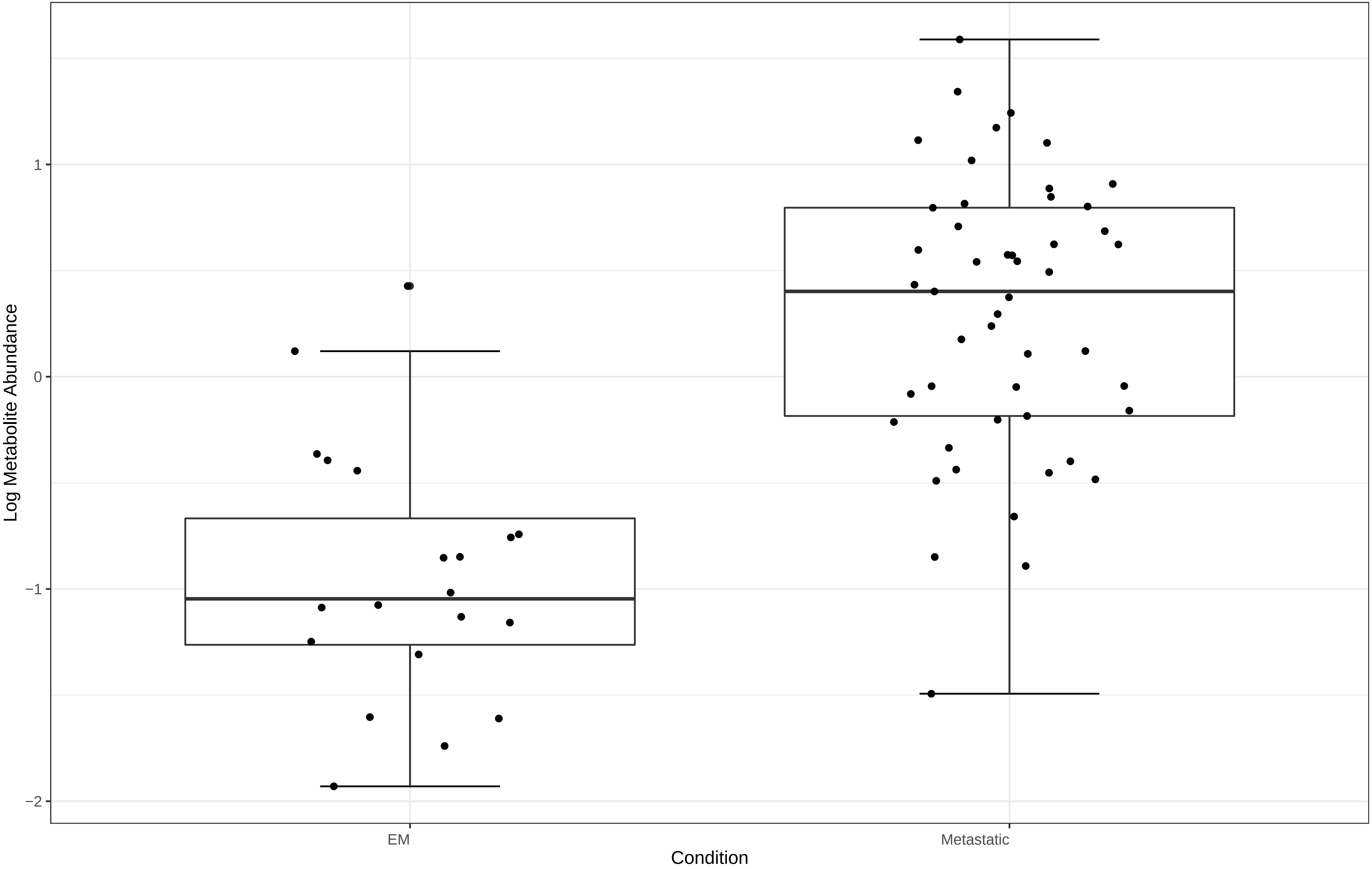

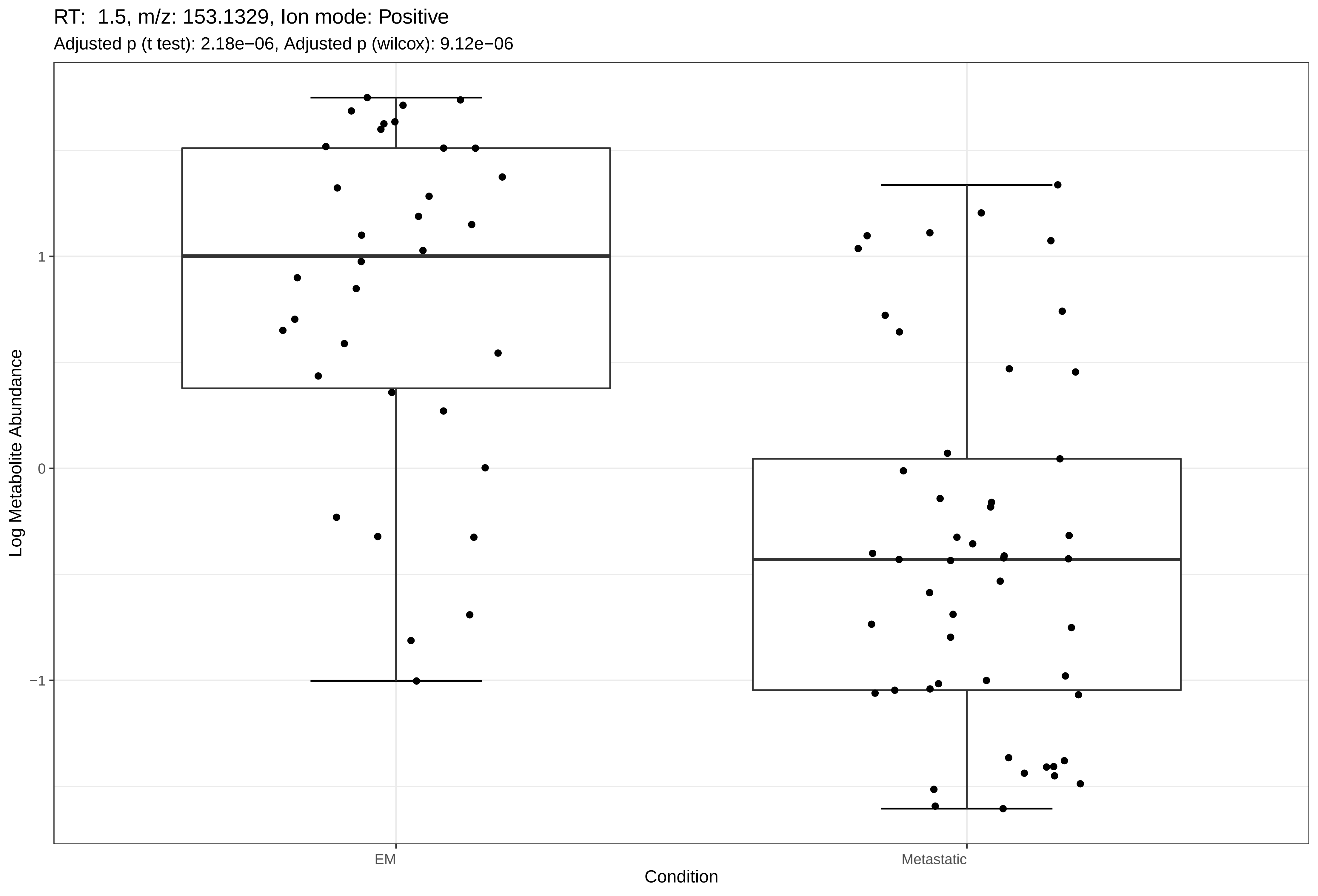

RT: 1.5, m/z: 153.1329, Ion mode: Positive
Adjusted p (t test): 2.18e−06, Adjusted p (wilcox): 9.12e−06
Log Metabolite Abundance
1
0
−1
EM
Metastatic
Condition

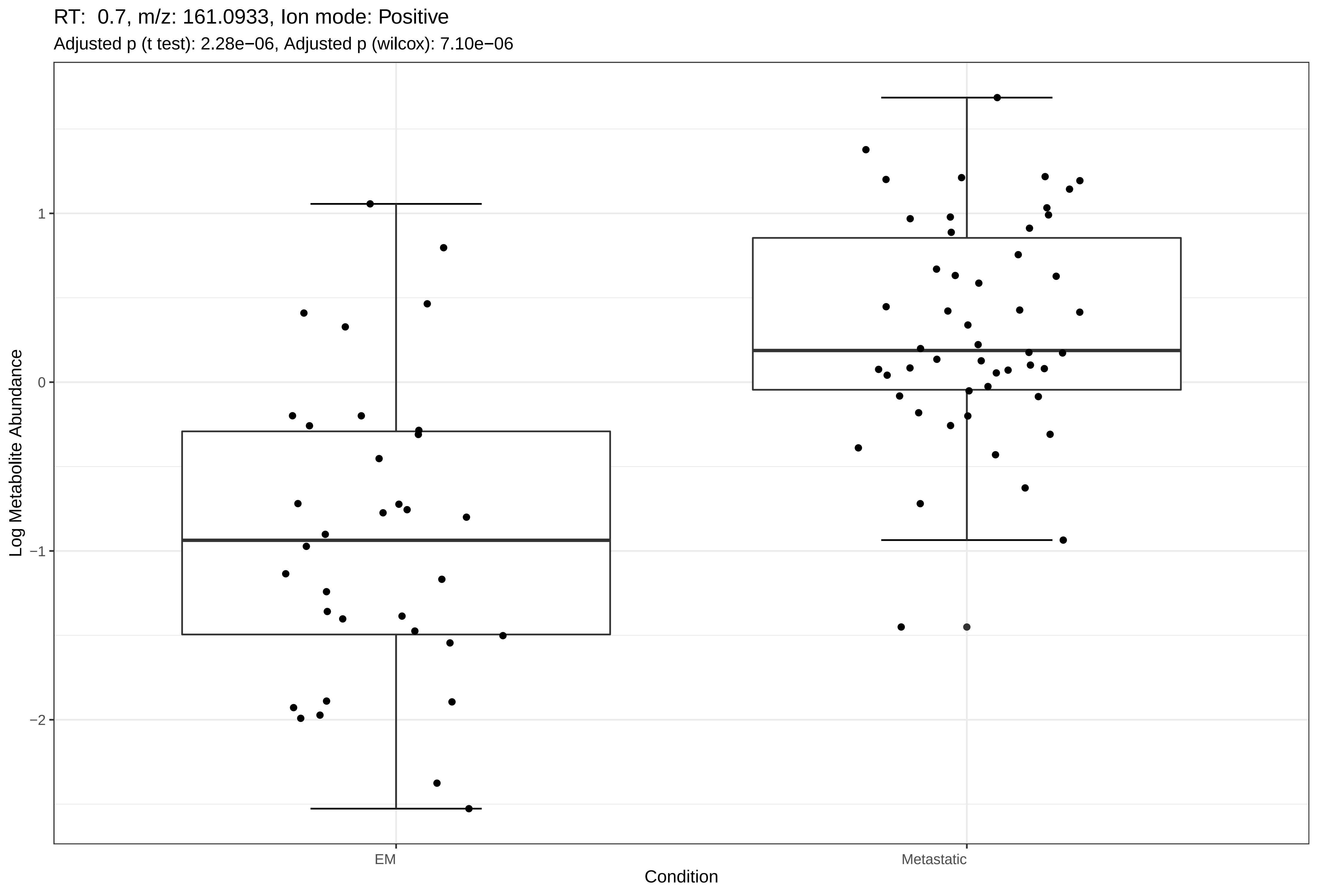

RT: 0.7, m/z: 161.0933, Ion mode: Positive
Adjusted p (t test): 2.28e−06, Adjusted p (wilcox): 7.10e−06
Log Metabolite Abundance
1
0
−1
−2
EM
Metastatic
Condition

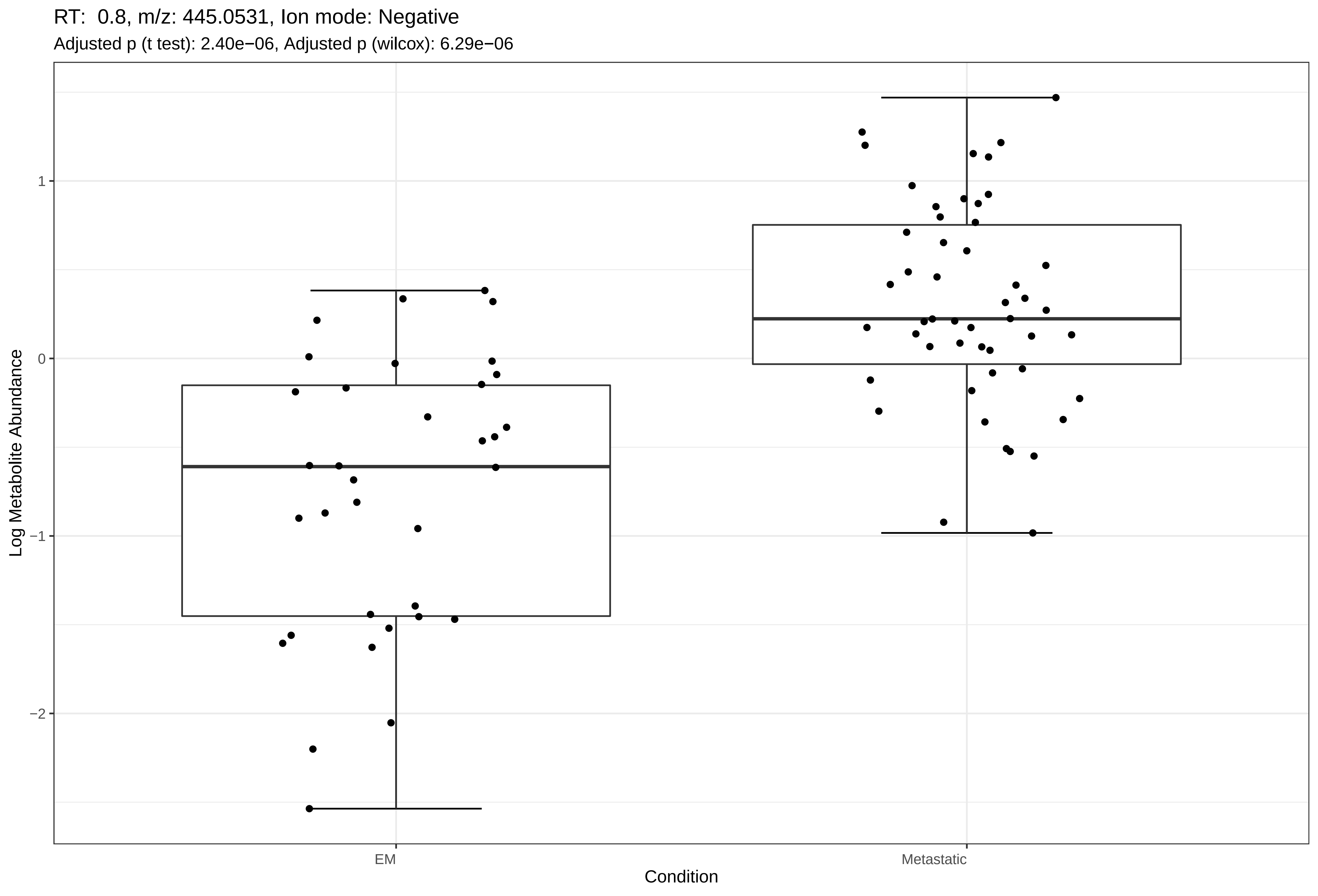

RT: 0.8, m/z: 445.0531, Ion mode: Negative
Adjusted p (t test): 2.40e−06, Adjusted p (wilcox): 6.29e−06
Log Metabolite Abundance
1
0
−1
−2
EM
Metastatic
Condition

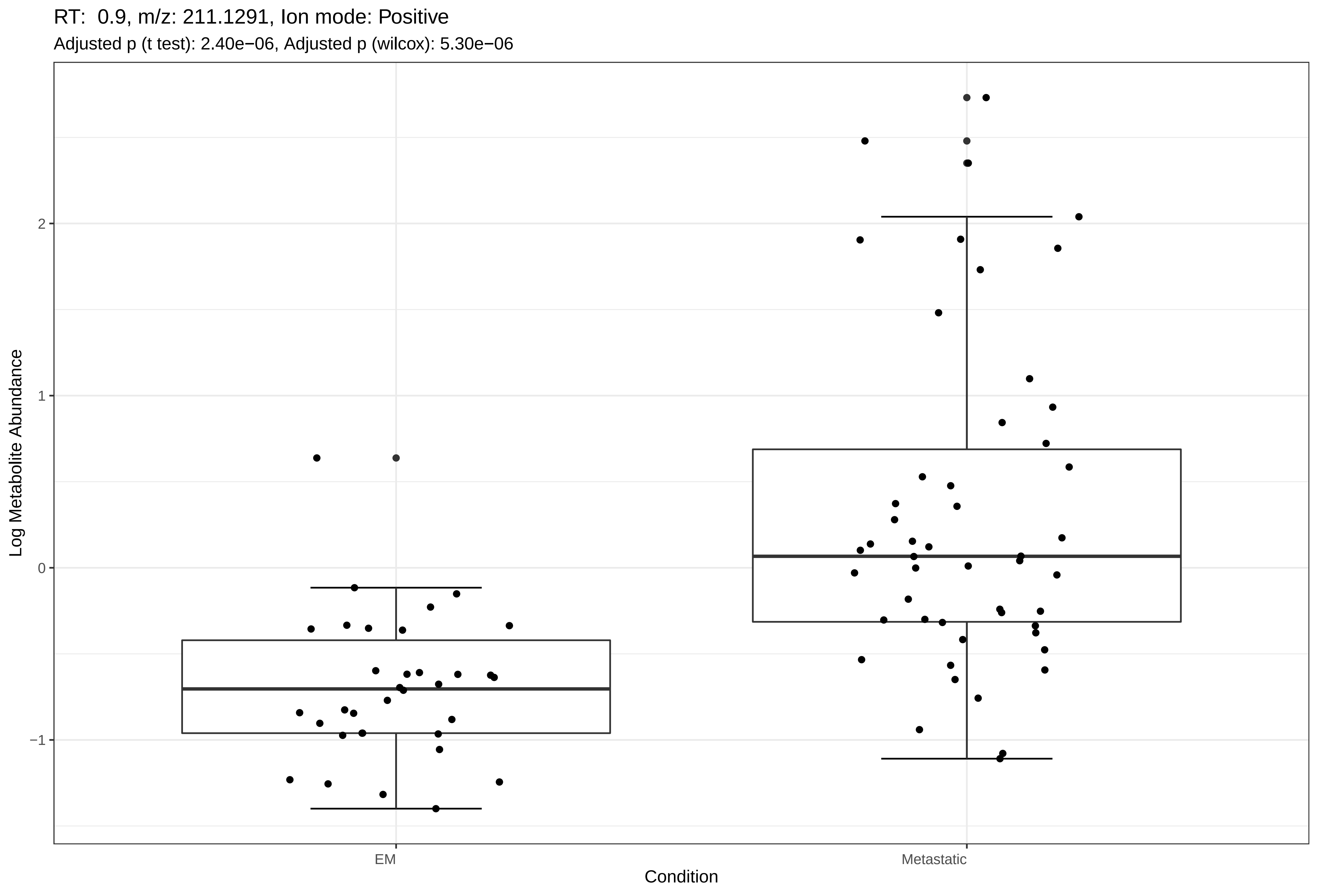
RT: 0.9, m/z: 211.1291, Ion mode: Positive
Adjusted p (t test): 2.40e−06, Adjusted p (wilcox): 5.30e−06
Log Metabolite Abundance
2
1
0
−1
EM
Metastatic
Condition

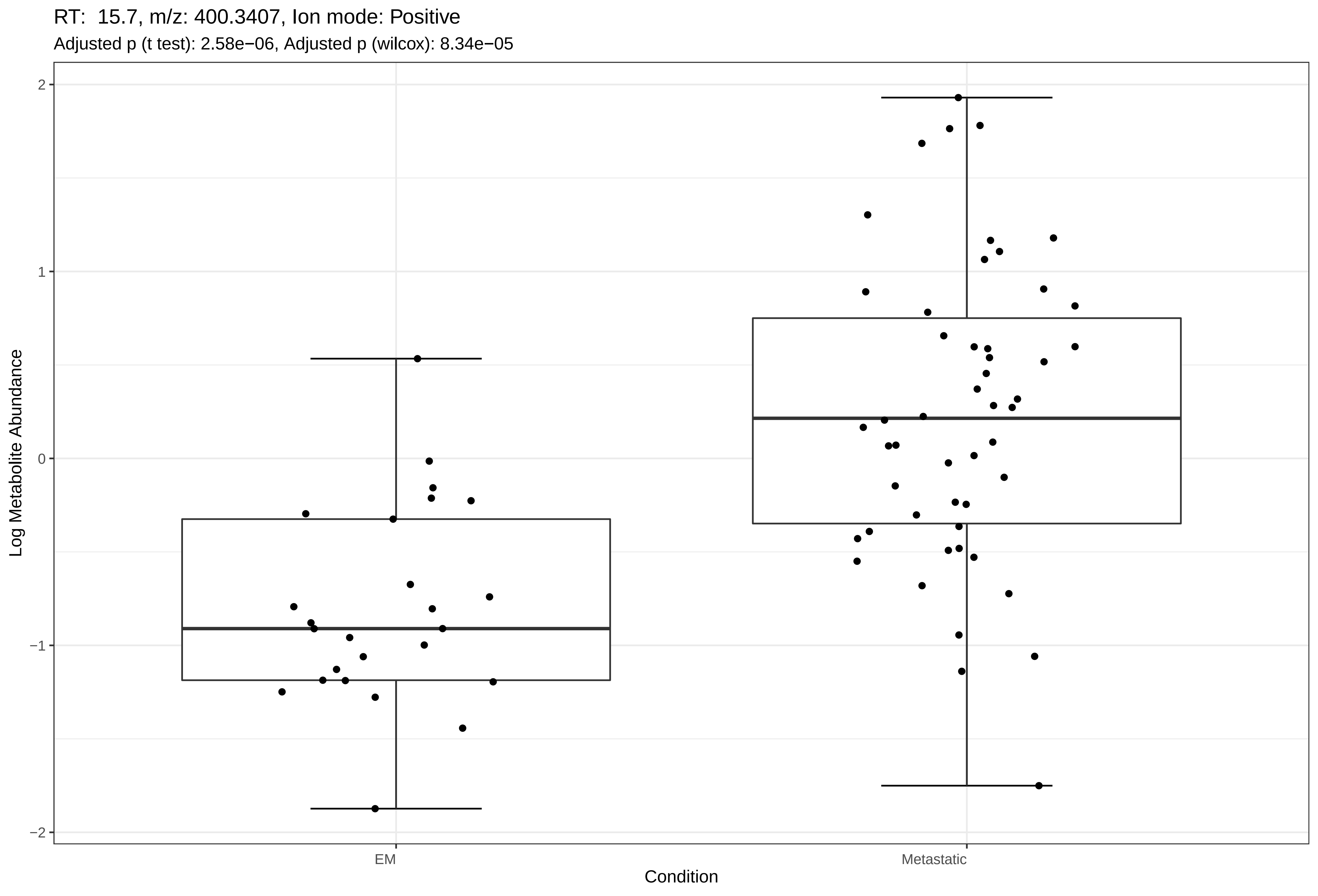

RT: 15.7, m/z: 400.3407, Ion mode: Positive
Adjusted p (t test): 2.58e−06, Adjusted p (wilcox): 8.34e−05
Log Metabolite Abundance
2
1
0
−1
−2
EM
Metastatic
Condition

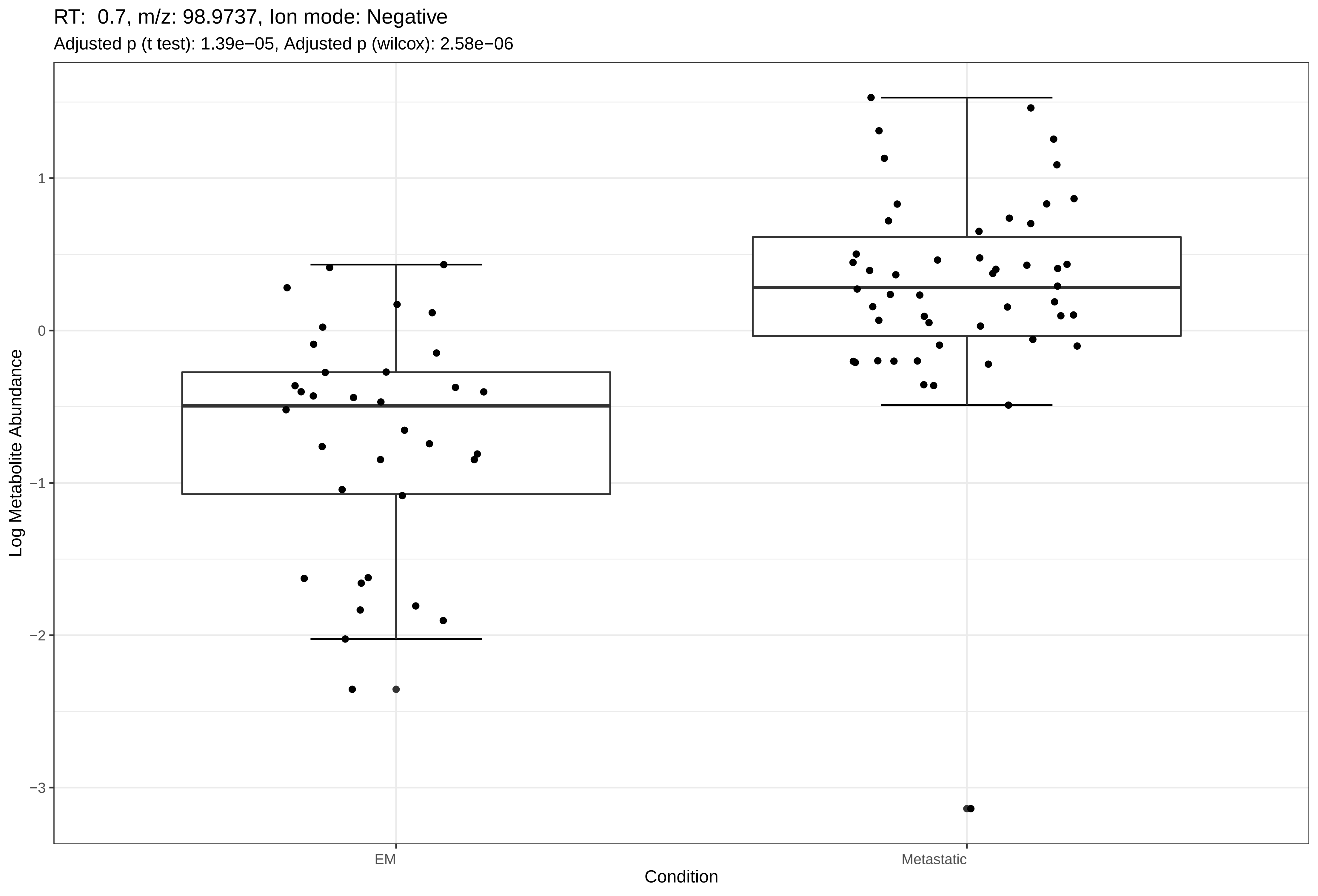
RT: 0.7, m/z: 98.9737, Ion mode: Negative
Adjusted p (t test): 1.39e−05, Adjusted p (wilcox): 2.58e−06
Log Metabolite Abundance
1
0
−1
−2
−3
EM
Metastatic
Condition

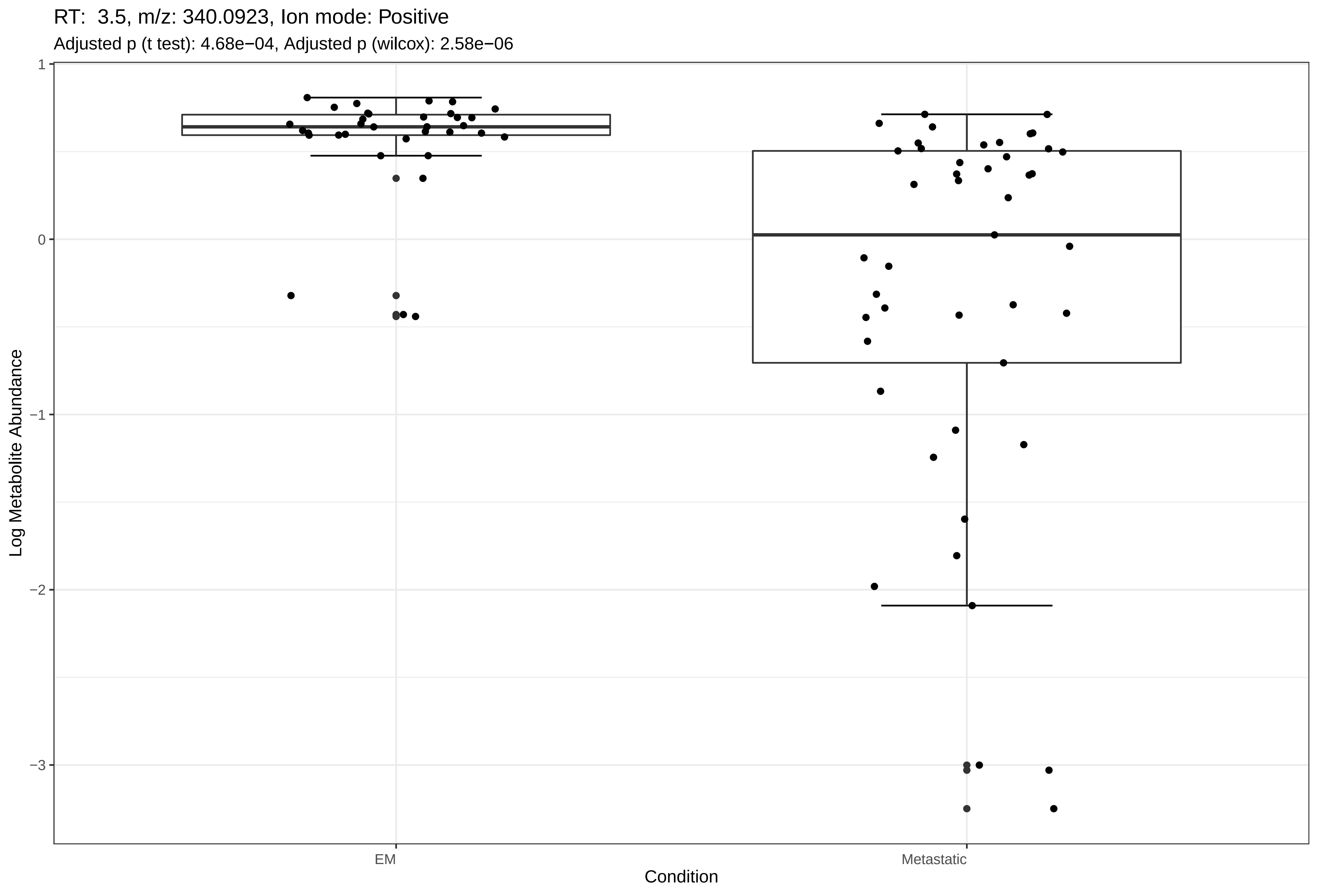

RT: 3.5, m/z: 340.0923, Ion mode: Positive
Adjusted p (t test): 4.68e−04, Adjusted p (wilcox): 2.58e−06
Log Metabolite Abundance
1
0
−1
−2
−3
EM
Metastatic
Condition

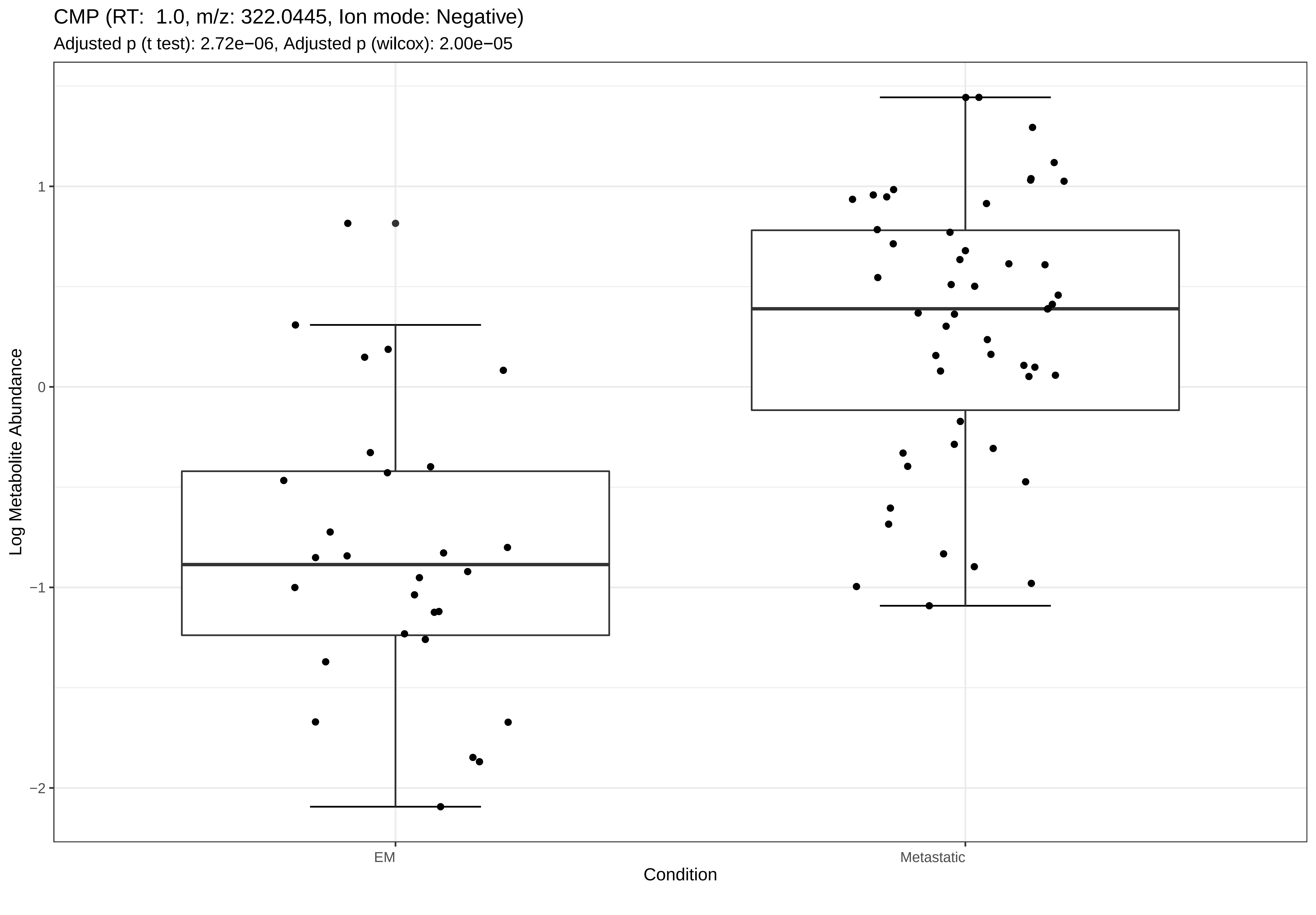

CMP (RT: 1.0, m/z: 322.0445, Ion mode: Negative)
Adjusted p (t test): 2.72e−06, Adjusted p (wilcox): 2.00e−05
Log Metabolite Abundance
1
0
−1
−2
EM
Metastatic
Condition

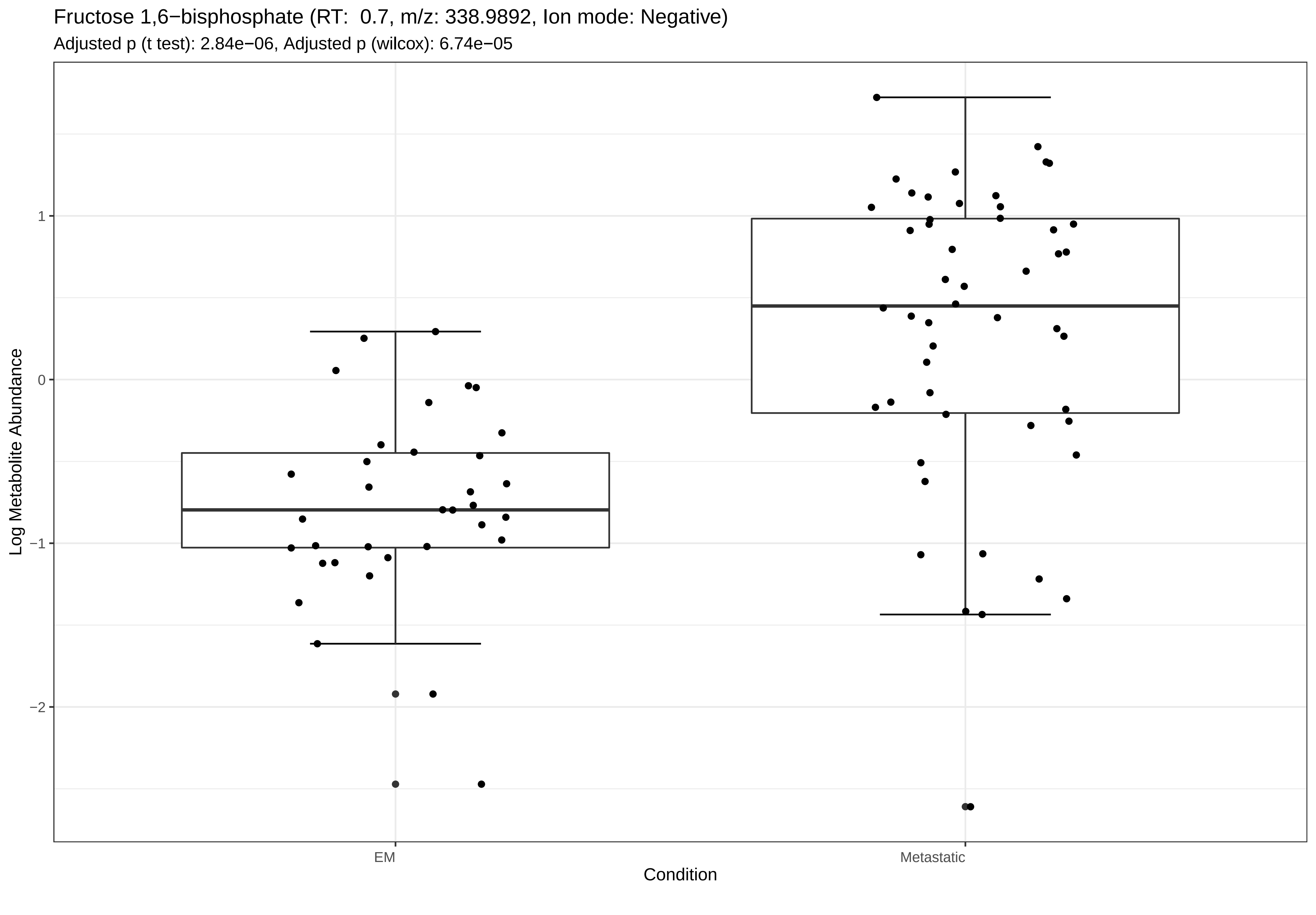
Fructose 1,6−bisphosphate (RT: 0.7, m/z: 338.9892, Ion mode: Negative)
Adjusted p (t test): 2.84e−06, Adjusted p (wilcox): 6.74e−05
Log Metabolite Abundance
1
0
−1
−2
EM
Metastatic
Condition

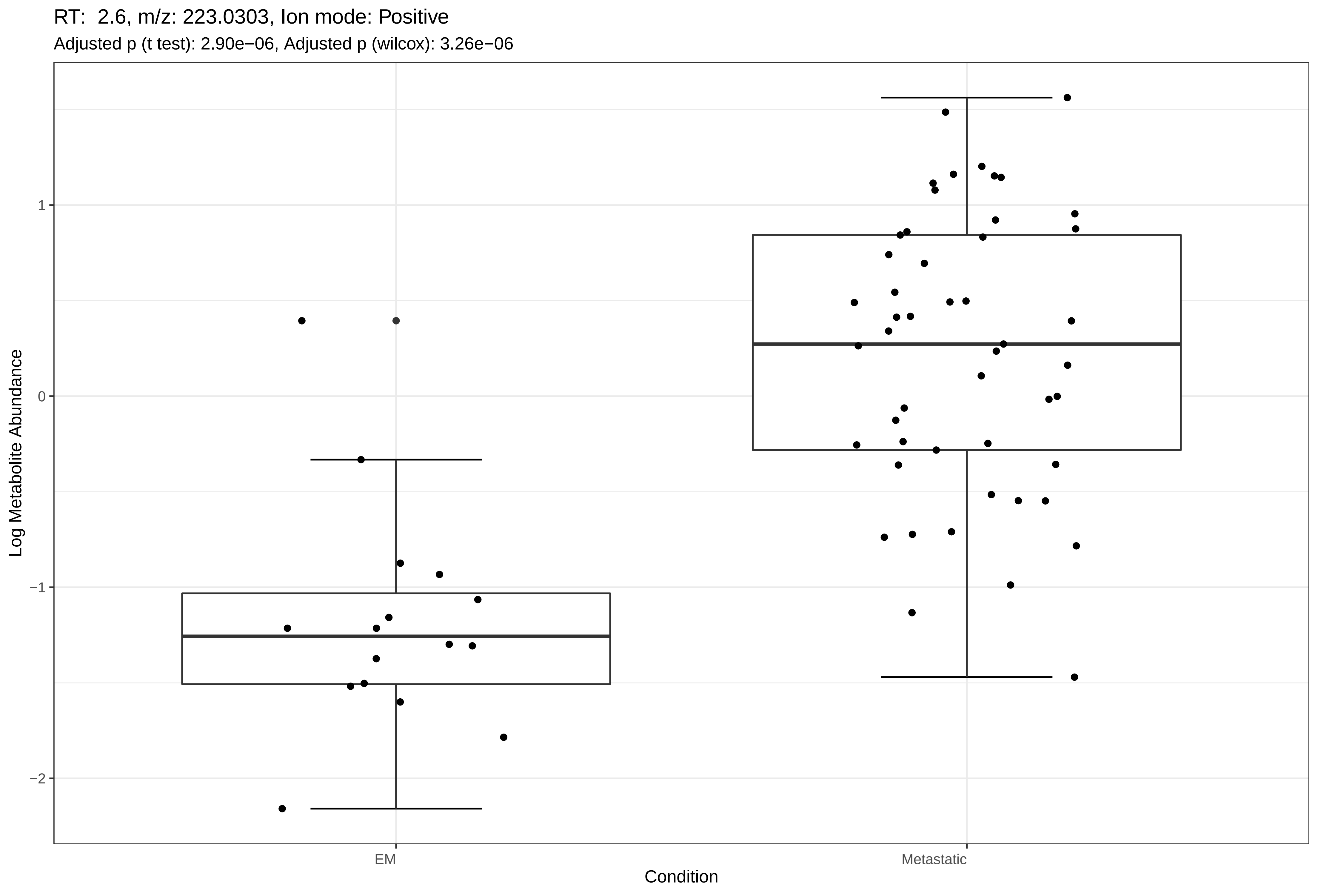

RT: 2.6, m/z: 223.0303, Ion mode: Positive
Adjusted p (t test): 2.90e−06, Adjusted p (wilcox): 3.26e−06
Log Metabolite Abundance
1
0
−1
−2
EM
Metastatic
Condition

RT: 6.2, m/z: 313.5563, Ion mode: Negative

Adjusted p (t test): 3.70e−04, Adjusted p (wilcox): 3.10e−06

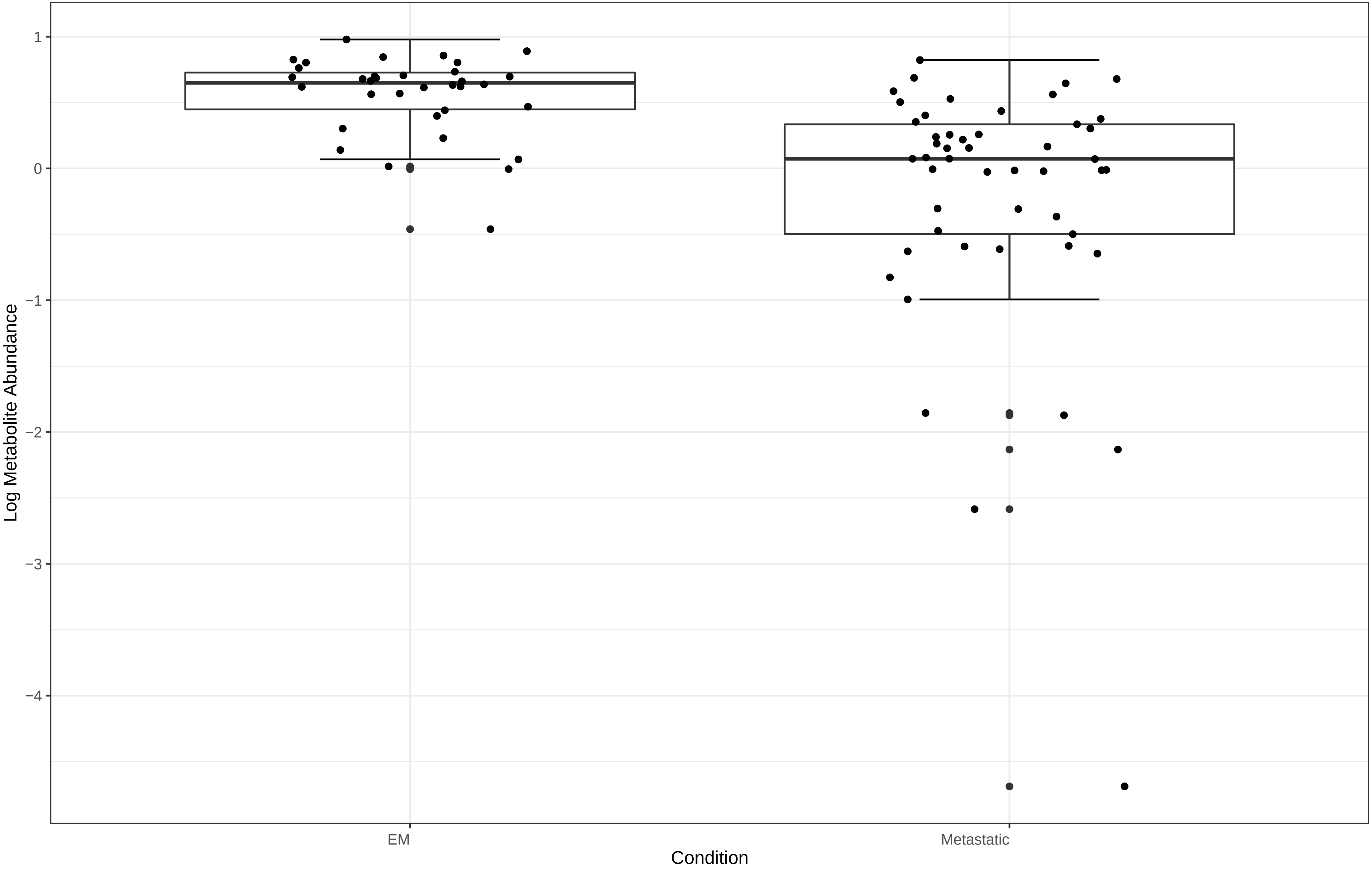

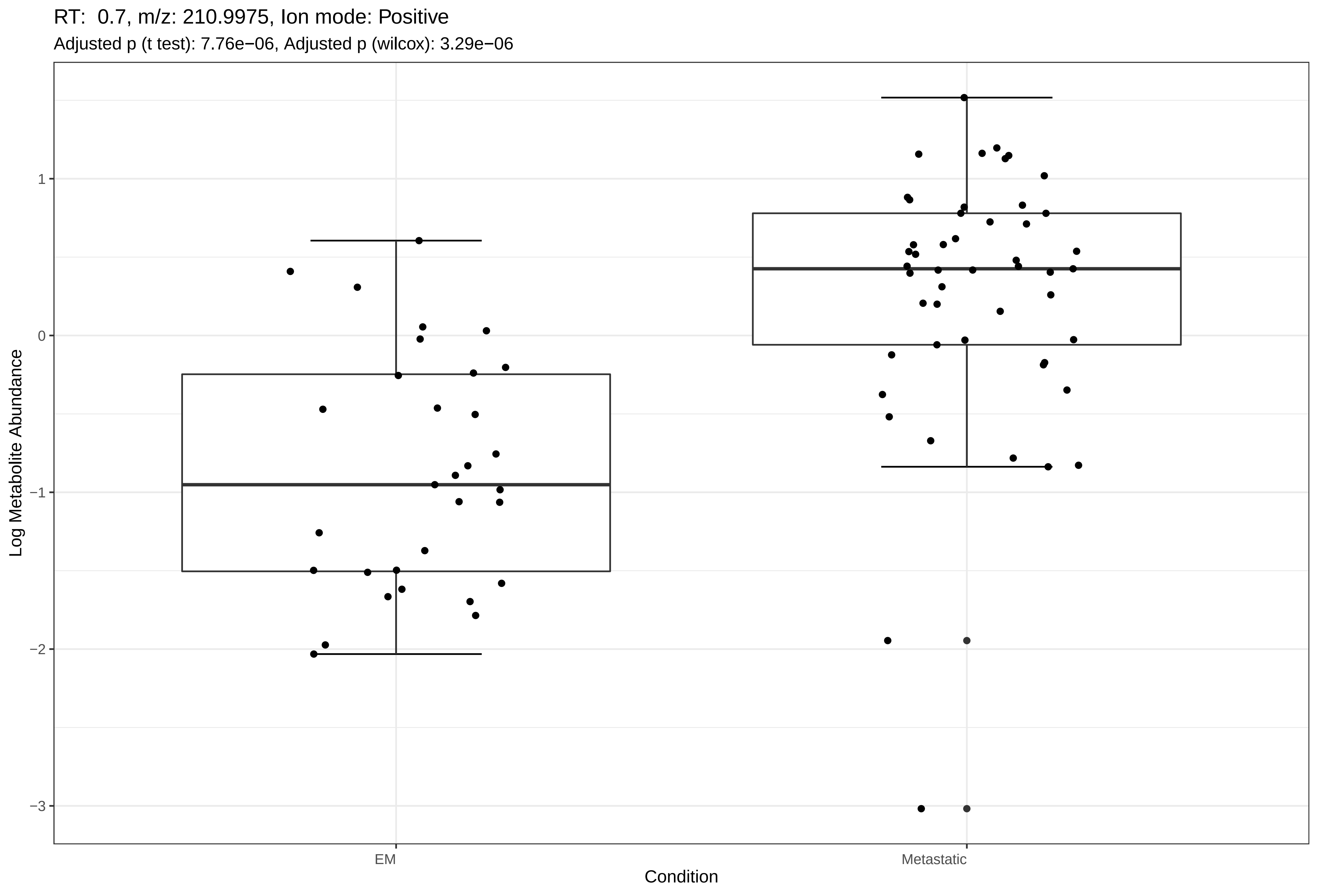

RT: 0.7, m/z: 210.9975, Ion mode: Positive
Adjusted p (t test): 7.76e−06, Adjusted p (wilcox): 3.29e−06
Log Metabolite Abundance
1
0
−1
−2
−3
EM
Metastatic
Condition

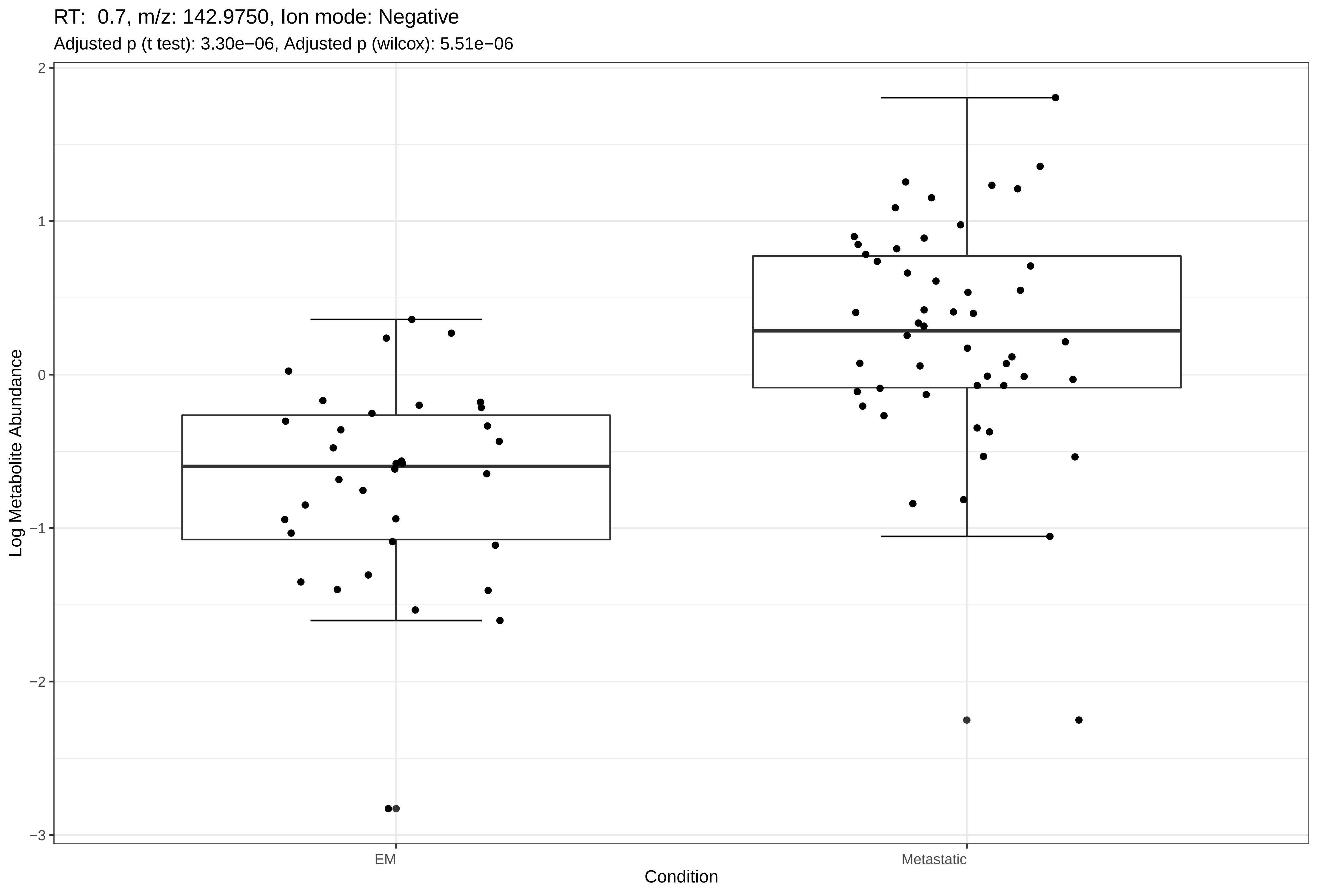

RT: 0.7, m/z: 142.9750, Ion mode: Negative
Adjusted p (t test): 3.30e−06, Adjusted p (wilcox): 5.51e−06
Log Metabolite Abundance
2
1
0
−1
−2
−3
EM
Metastatic
Condition

RT: 0.7, m/z: 80.9479, Ion mode: Positive

Adjusted p (t test): 3.31e−06, Adjusted p (wilcox): 1.20e−04

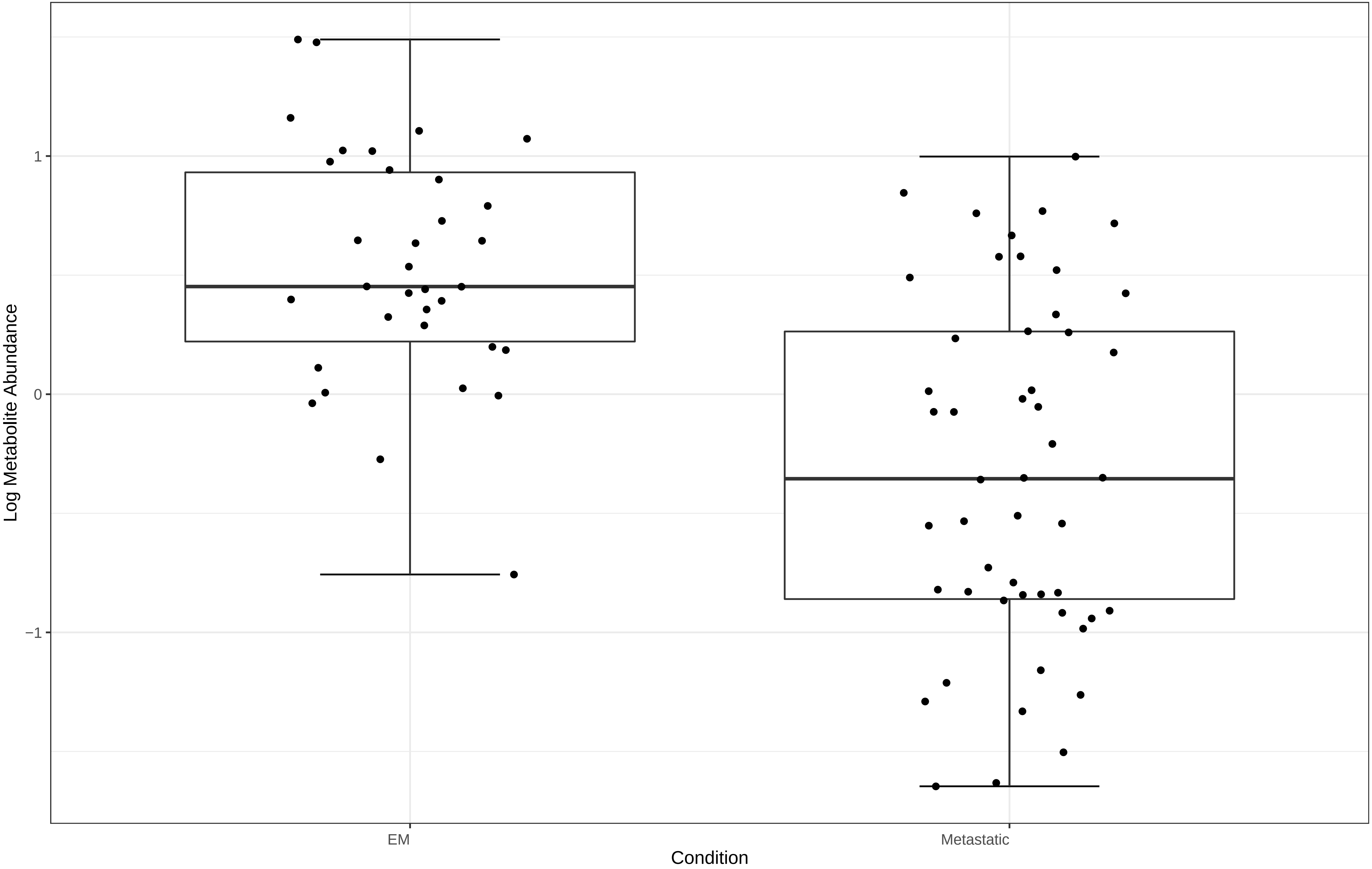

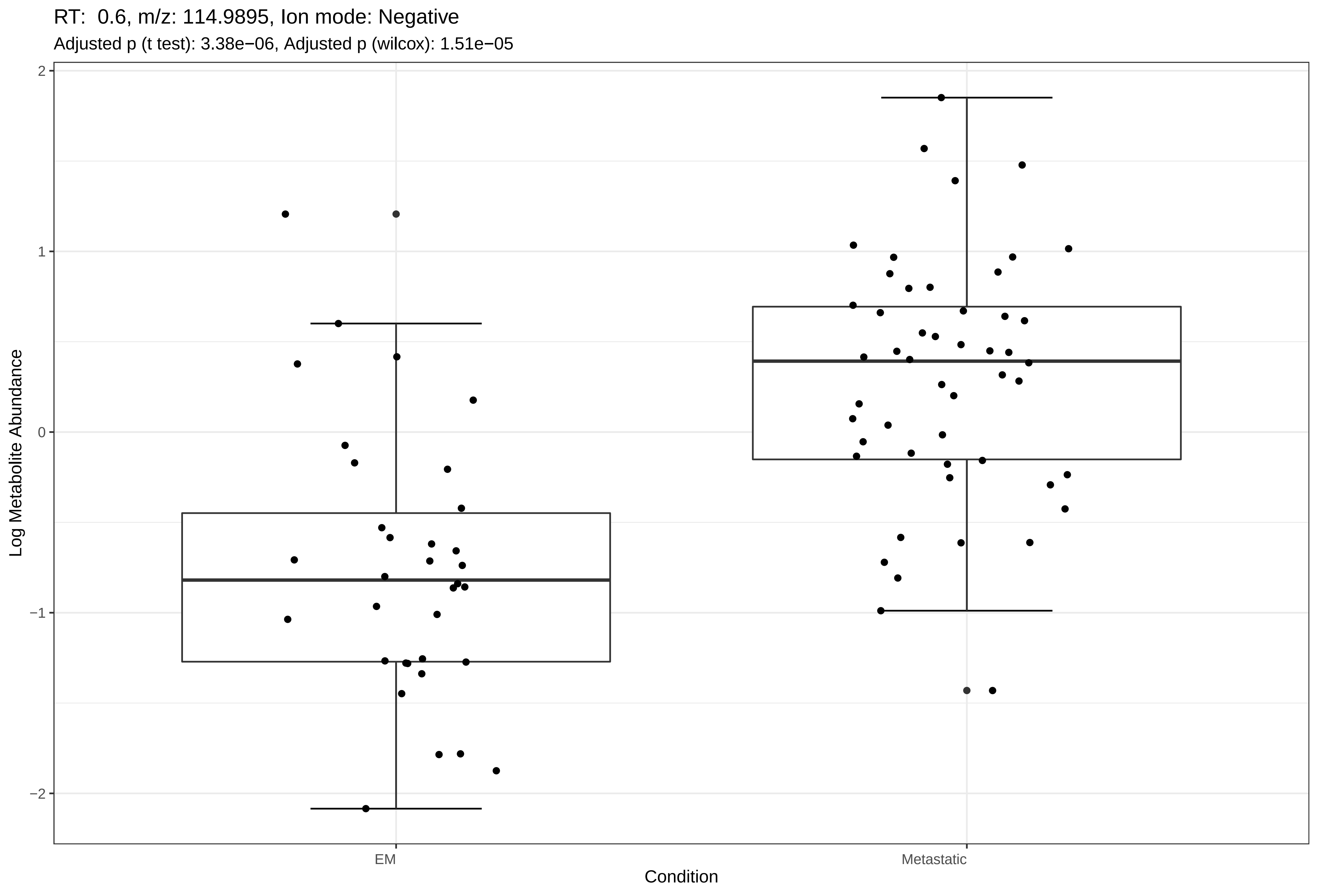
RT: 0.6, m/z: 114.9895, Ion mode: Negative
Adjusted p (t test): 3.38e−06, Adjusted p (wilcox): 1.51e−05
Log Metabolite Abundance
2
1
0
−1
−2
EM
Metastatic
Condition

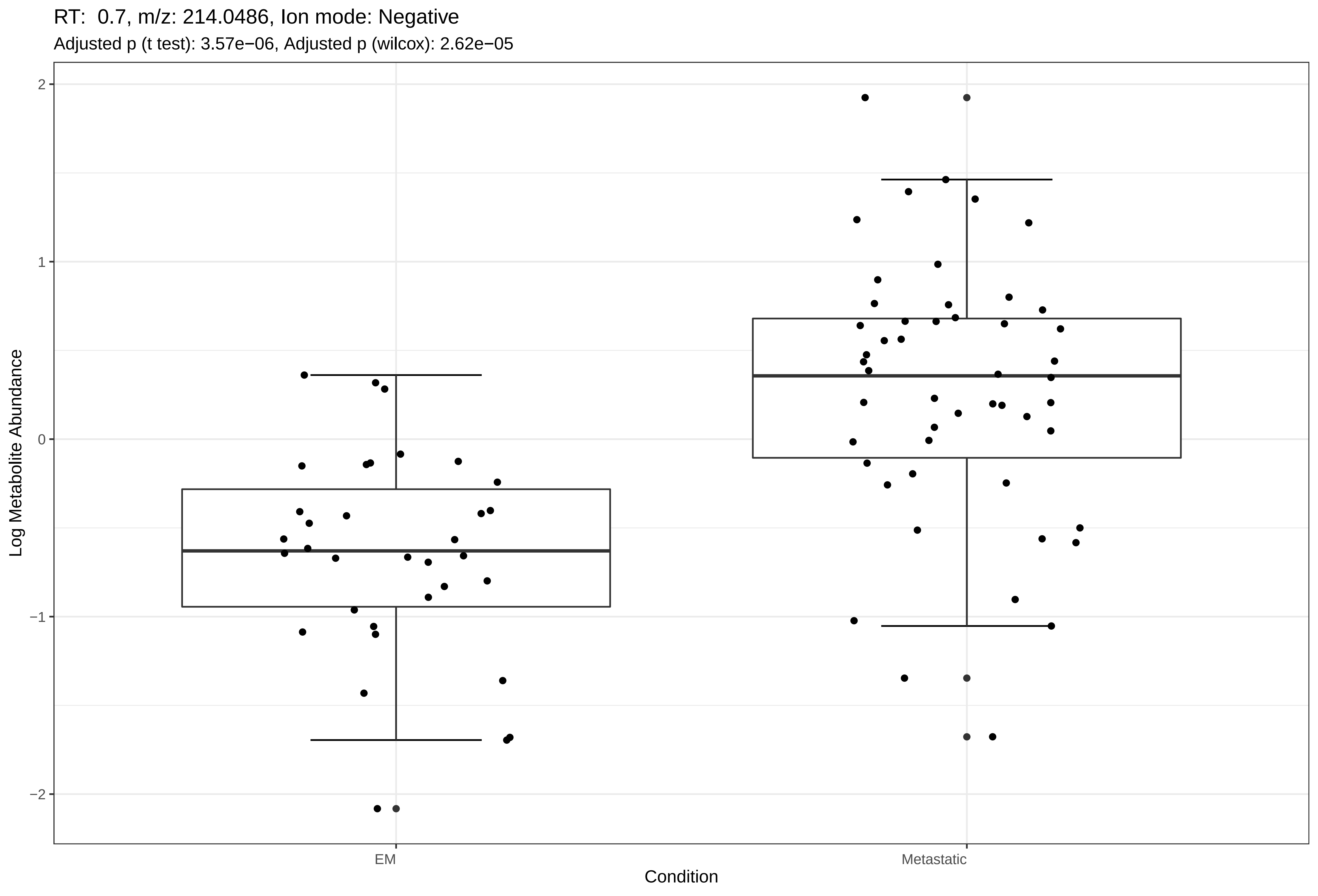
RT: 0.7, m/z: 214.0486, Ion mode: Negative
Adjusted p (t test): 3.57e−06, Adjusted p (wilcox): 2.62e−05
Log Metabolite Abundance
2
1
0
−1
−2
EM
Metastatic
Condition

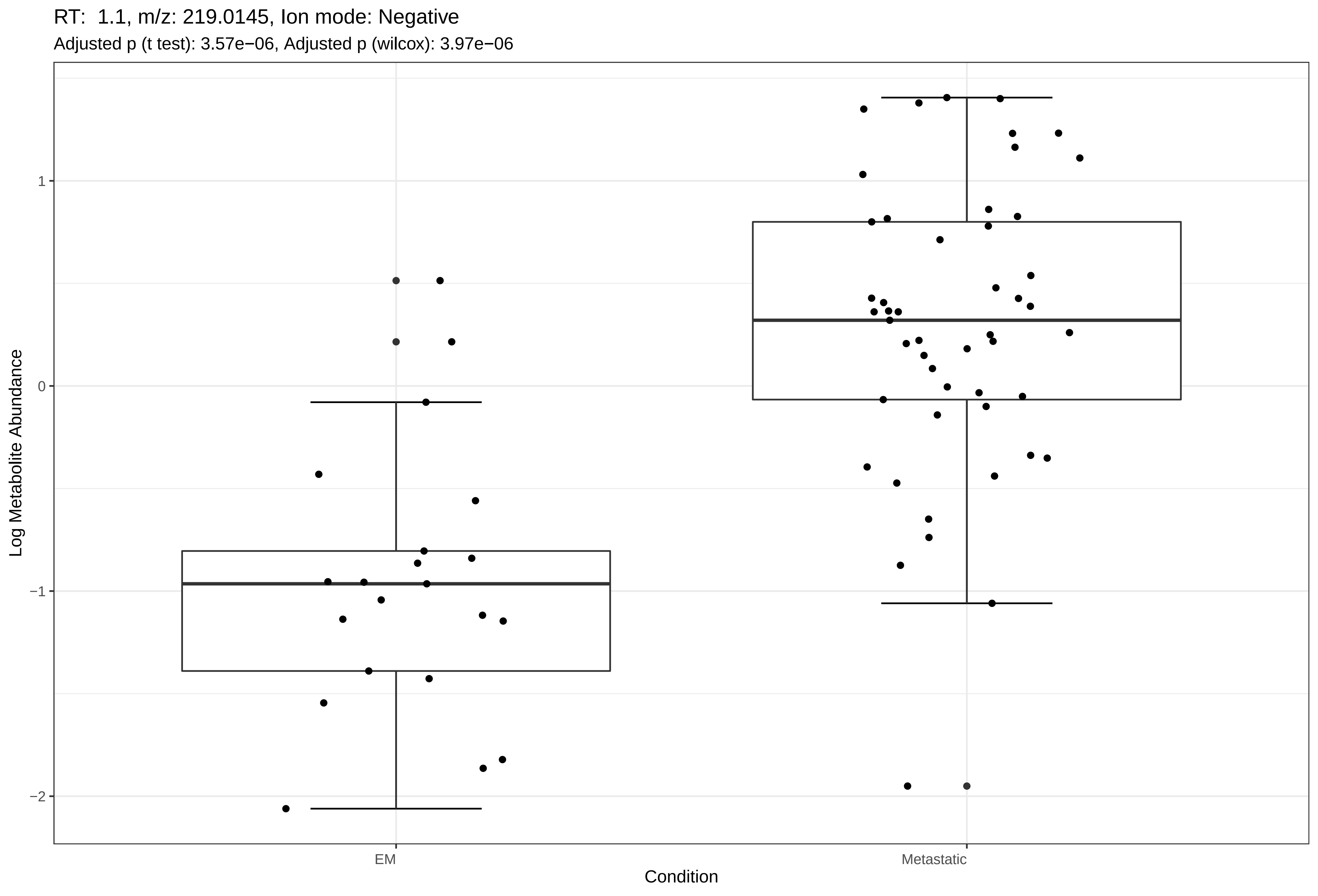
RT: 1.1, m/z: 219.0145, Ion mode: Negative
Adjusted p (t test): 3.57e−06, Adjusted p (wilcox): 3.97e−06
Log Metabolite Abundance
1
0
−1
−2
EM
Metastatic
Condition

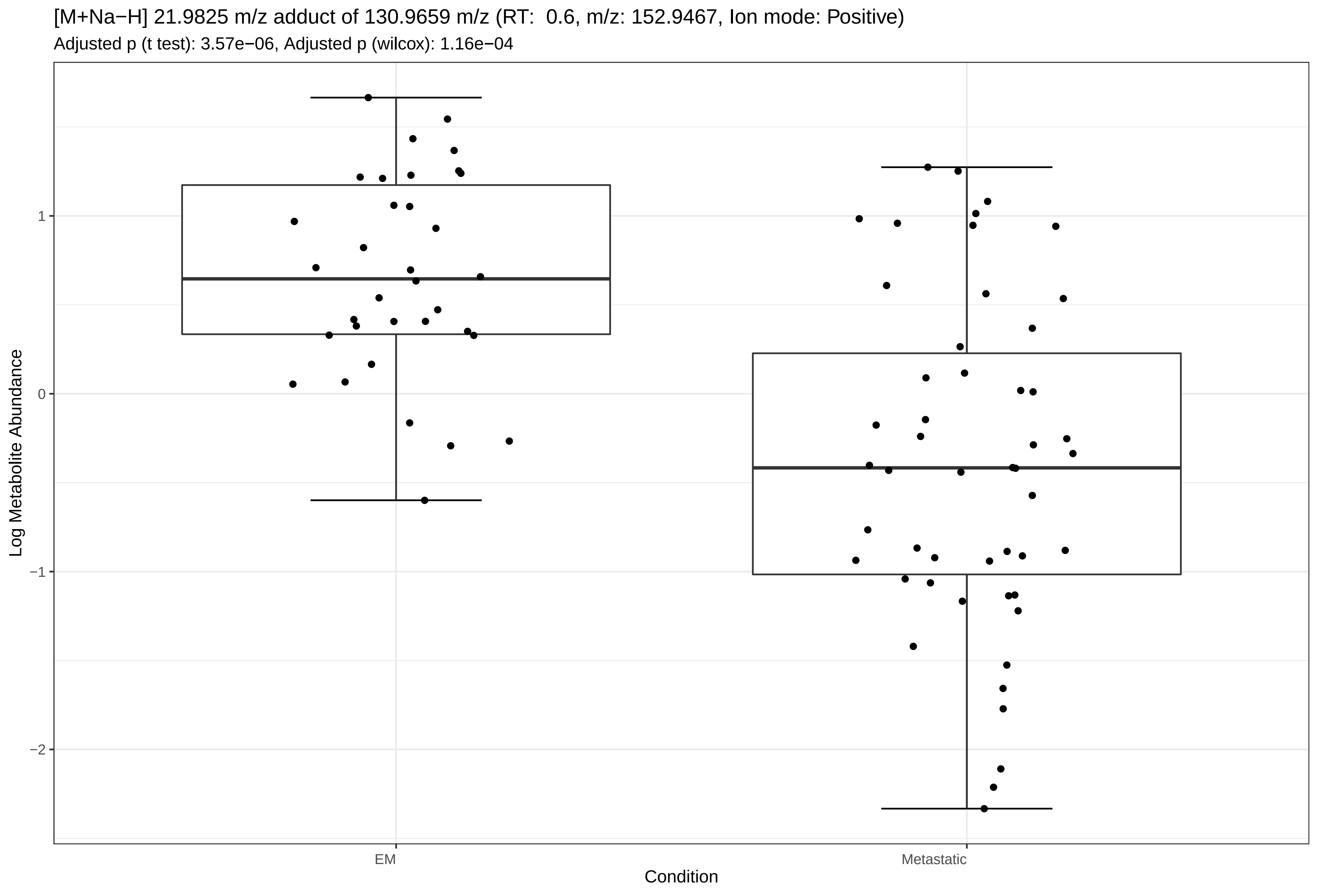

[M+Na−H] 21.9825 m/z adduct of 130.9659 m/z (RT: 0.6, m/z: 152.9467, Ion mode: Positive)
Adjusted p (t test): 3.57e−06, Adjusted p (wilcox): 1.16e−04
Log Metabolite Abundance
1
0
−1
−2
EM
Metastatic
Condition

Complex of 135.0027 and 79.0205 m/z (RT: 1.2, m/z: 213.0163, Ion mode: Positive)

Adjusted p (t test): 3.88e−06, Adjusted p (wilcox): 1.66e−04

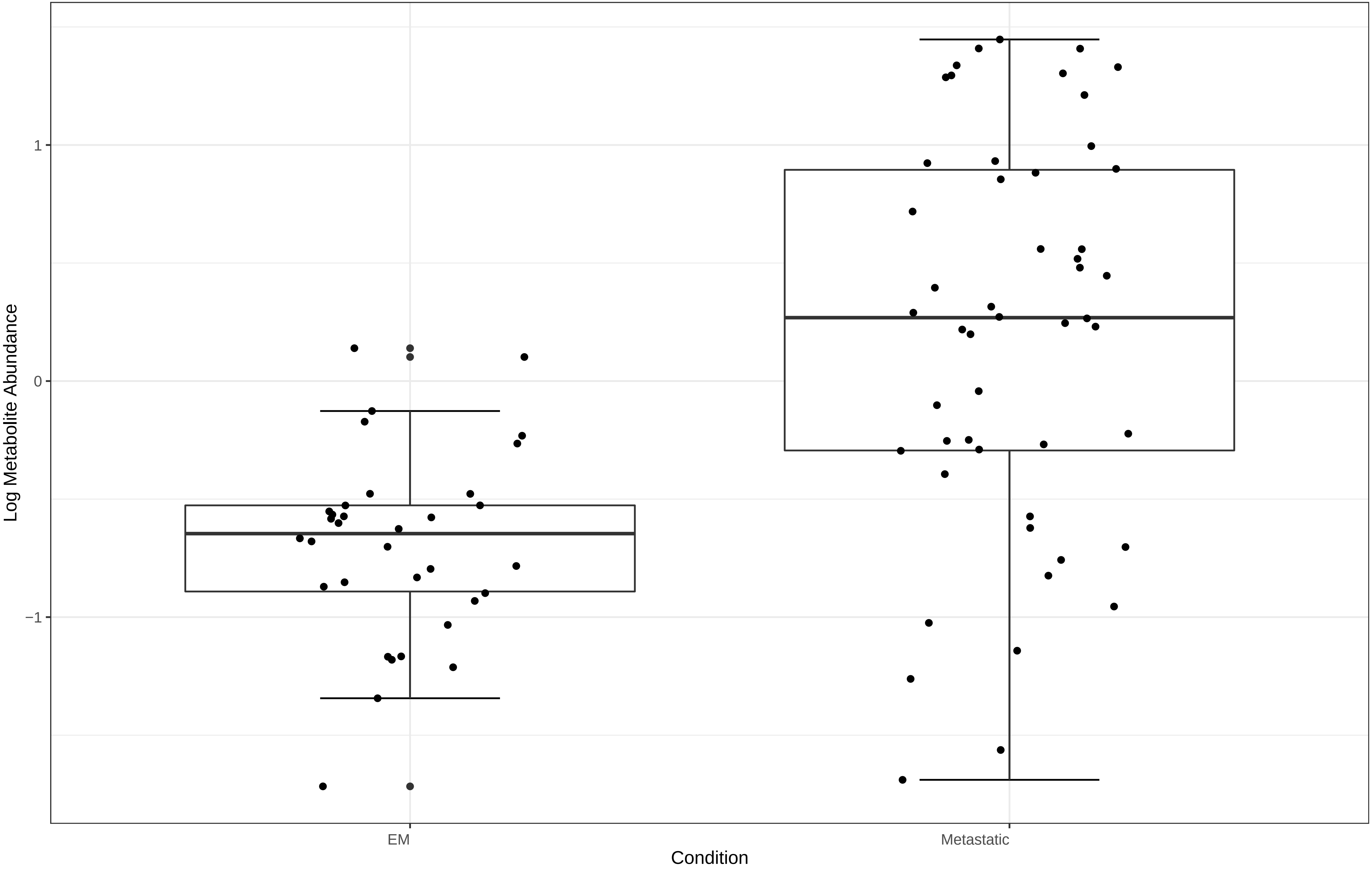

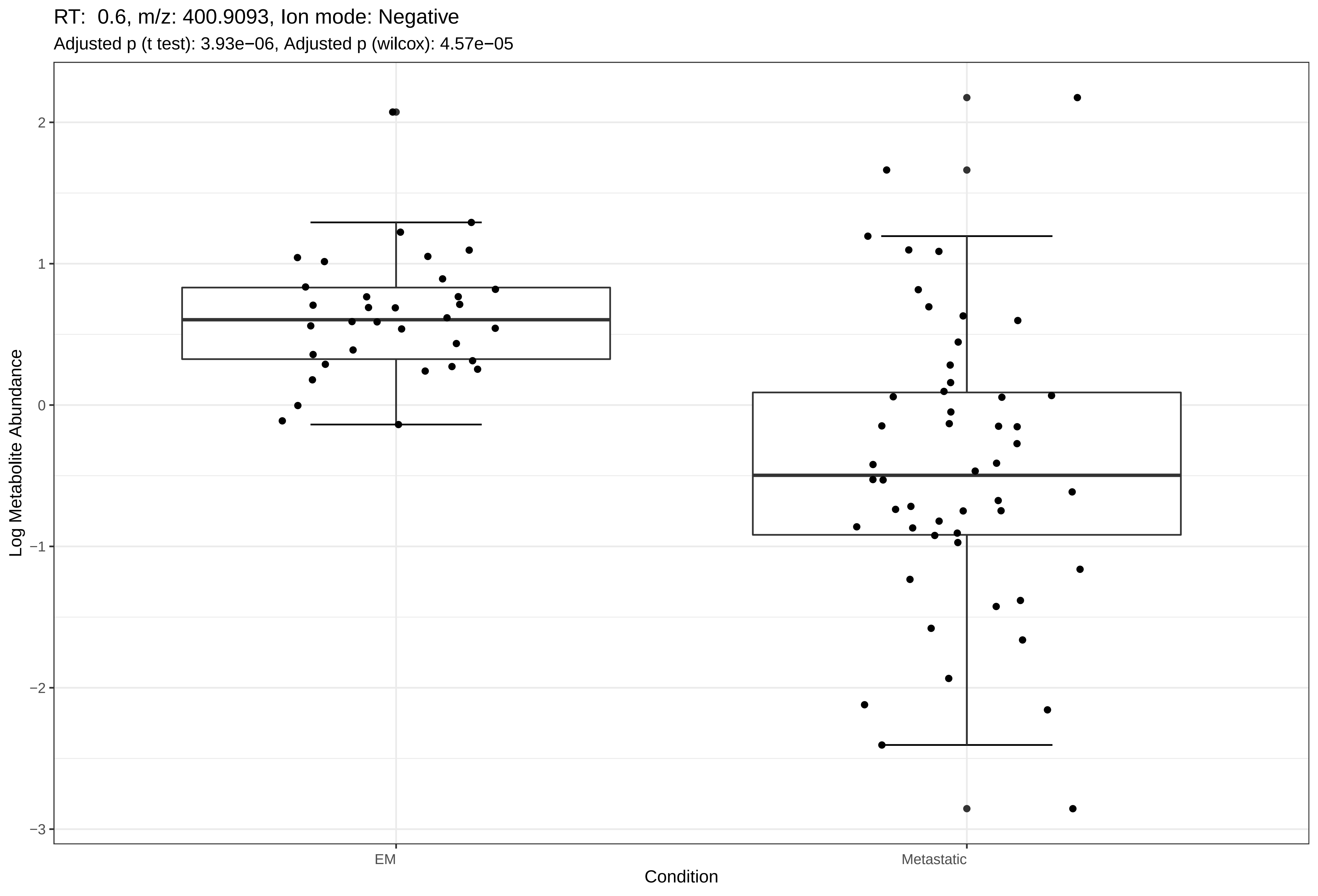

RT: 0.6, m/z: 400.9093, Ion mode: Negative
Adjusted p (t test): 3.93e−06, Adjusted p (wilcox): 4.57e−05
Log Metabolite Abundance
2
1
0
−1
−2
−3
EM
Metastatic
Condition

RT: 6.4, m/z: 275.0738, Ion mode: Positive

Adjusted p (t test): 3.98e−06, Adjusted p (wilcox): 2.84e−05

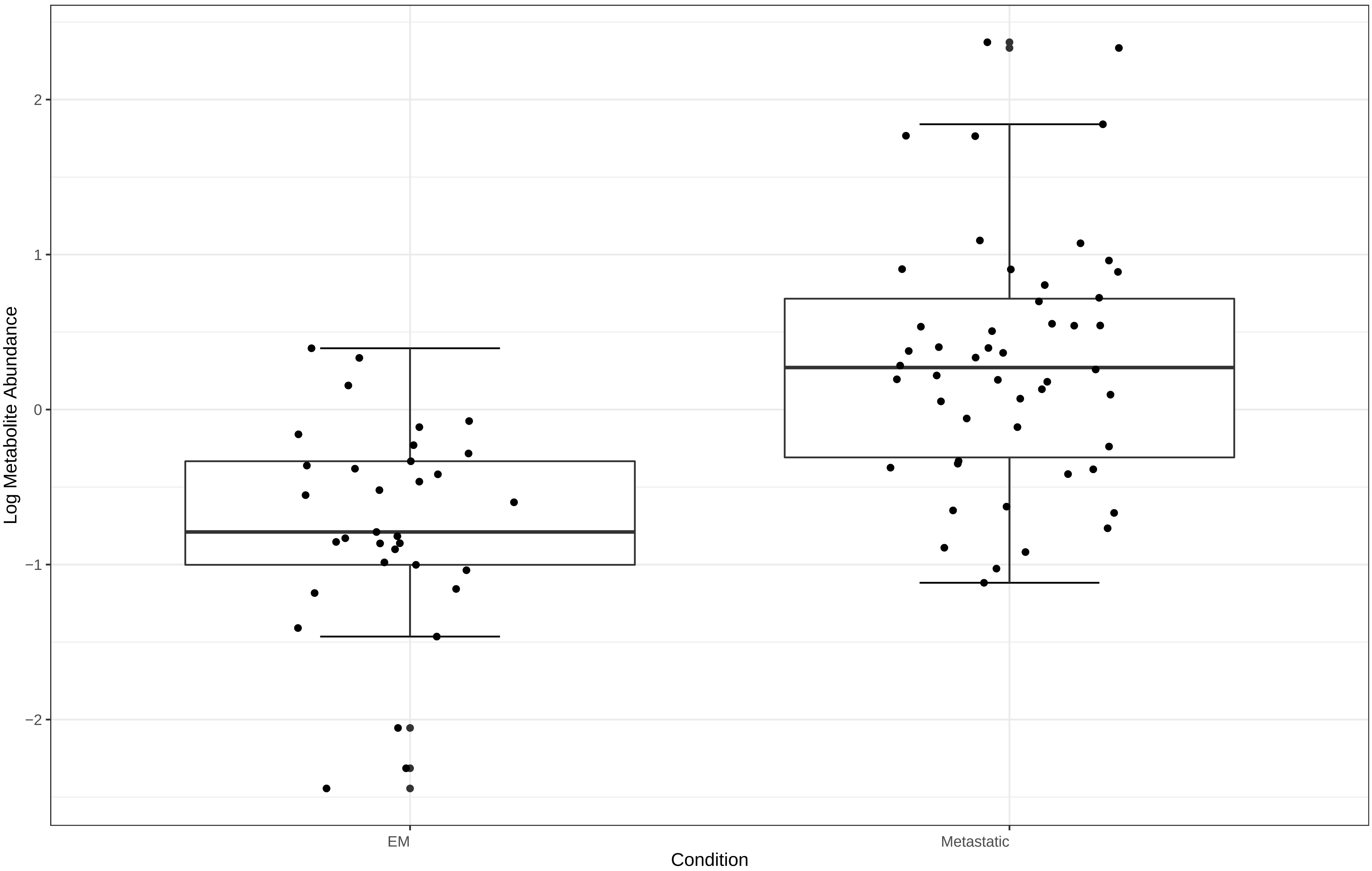

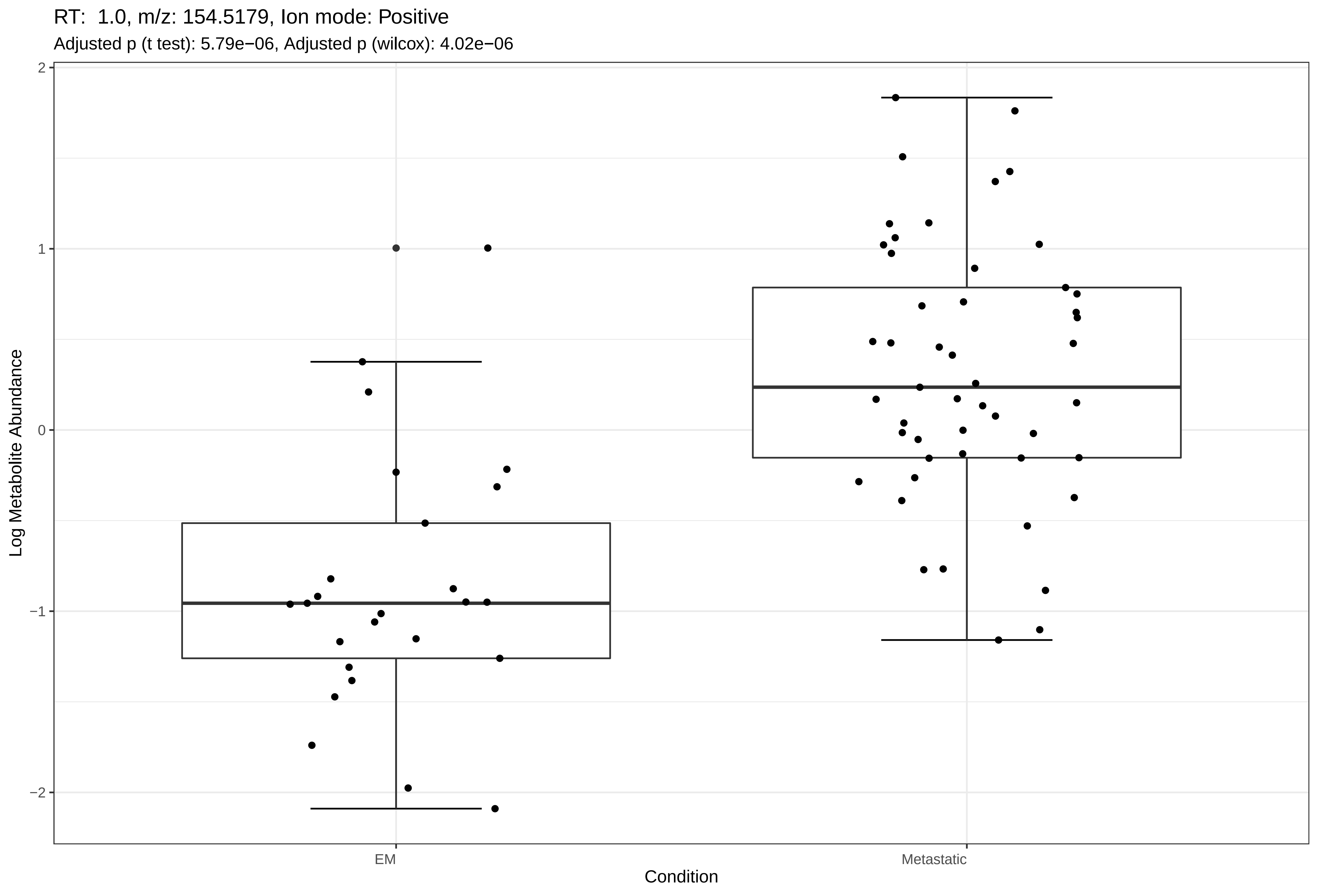

RT: 1.0, m/z: 154.5179, Ion mode: Positive
Adjusted p (t test): 5.79e−06, Adjusted p (wilcox): 4.02e−06
Log Metabolite Abundance
2
1
0
−1
−2
EM
Metastatic
Condition

RT: 1.9, m/z: 182.1309, Ion mode: Positive

Adjusted p (t test): 1.28e−05, Adjusted p (wilcox): 4.04e−06

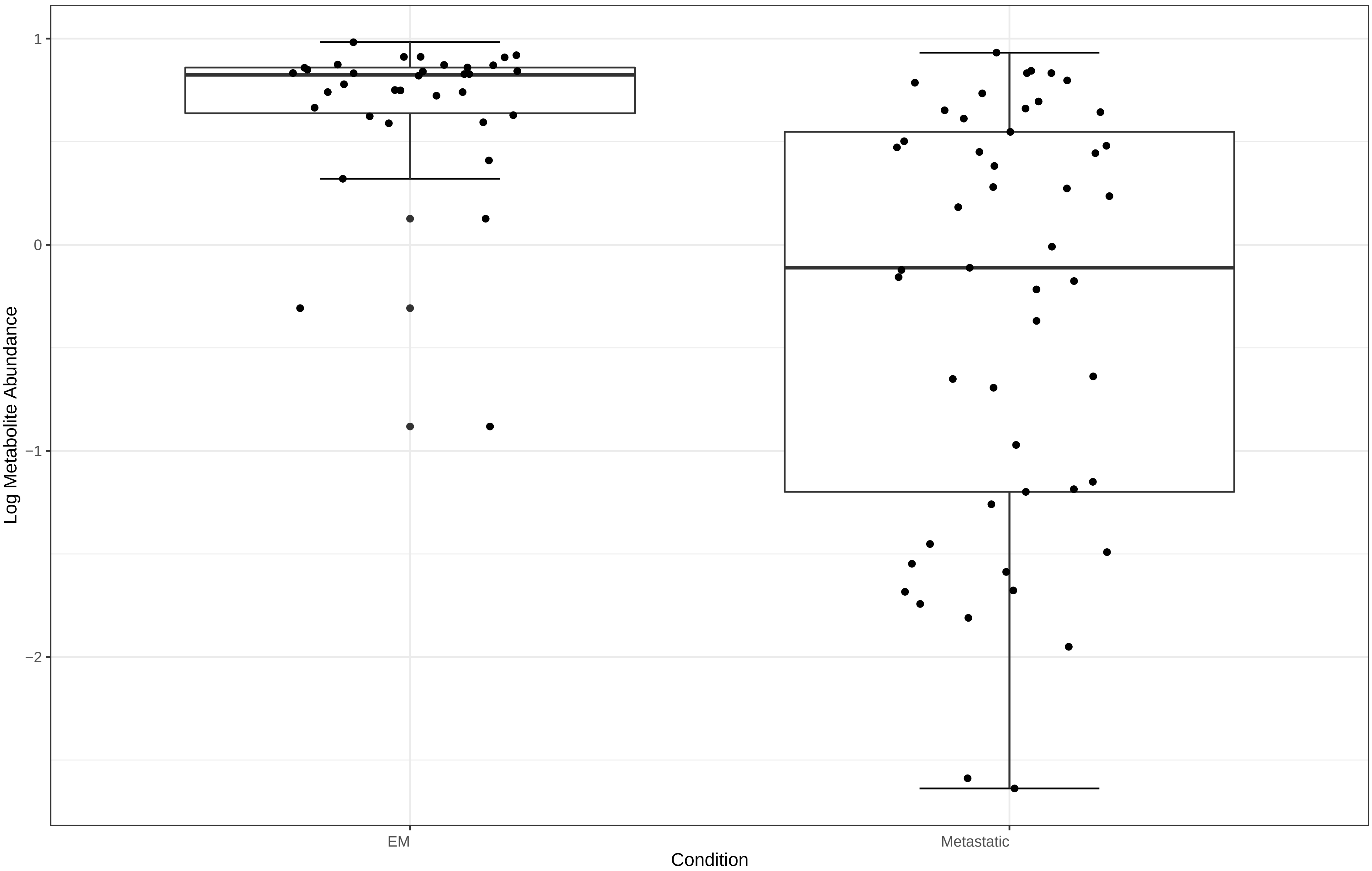

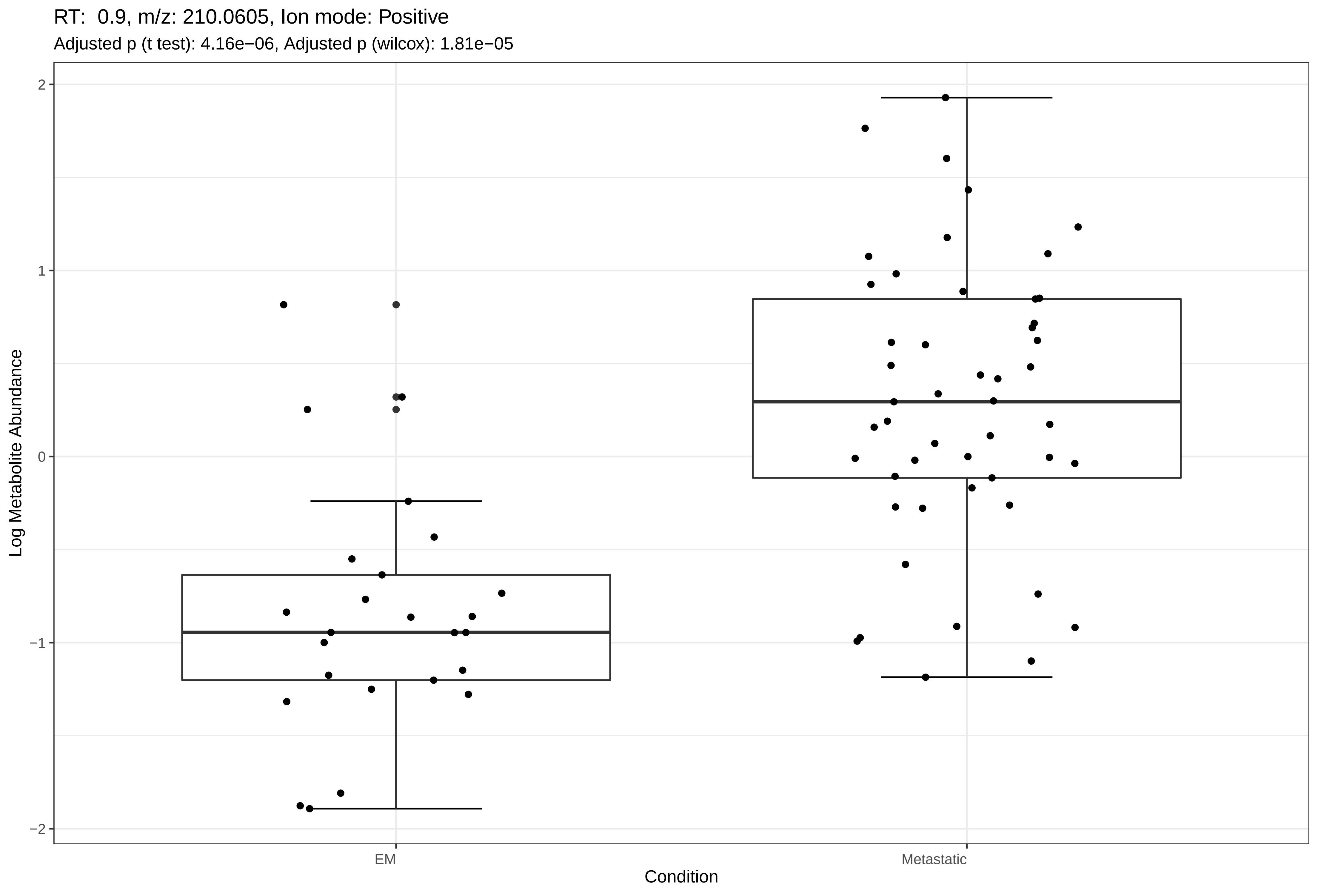

RT: 0.9, m/z: 210.0605, Ion mode: Positive
Adjusted p (t test): 4.16e−06, Adjusted p (wilcox): 1.81e−05
Log Metabolite Abundance
2
1
0
−1
−2
EM
Metastatic
Condition

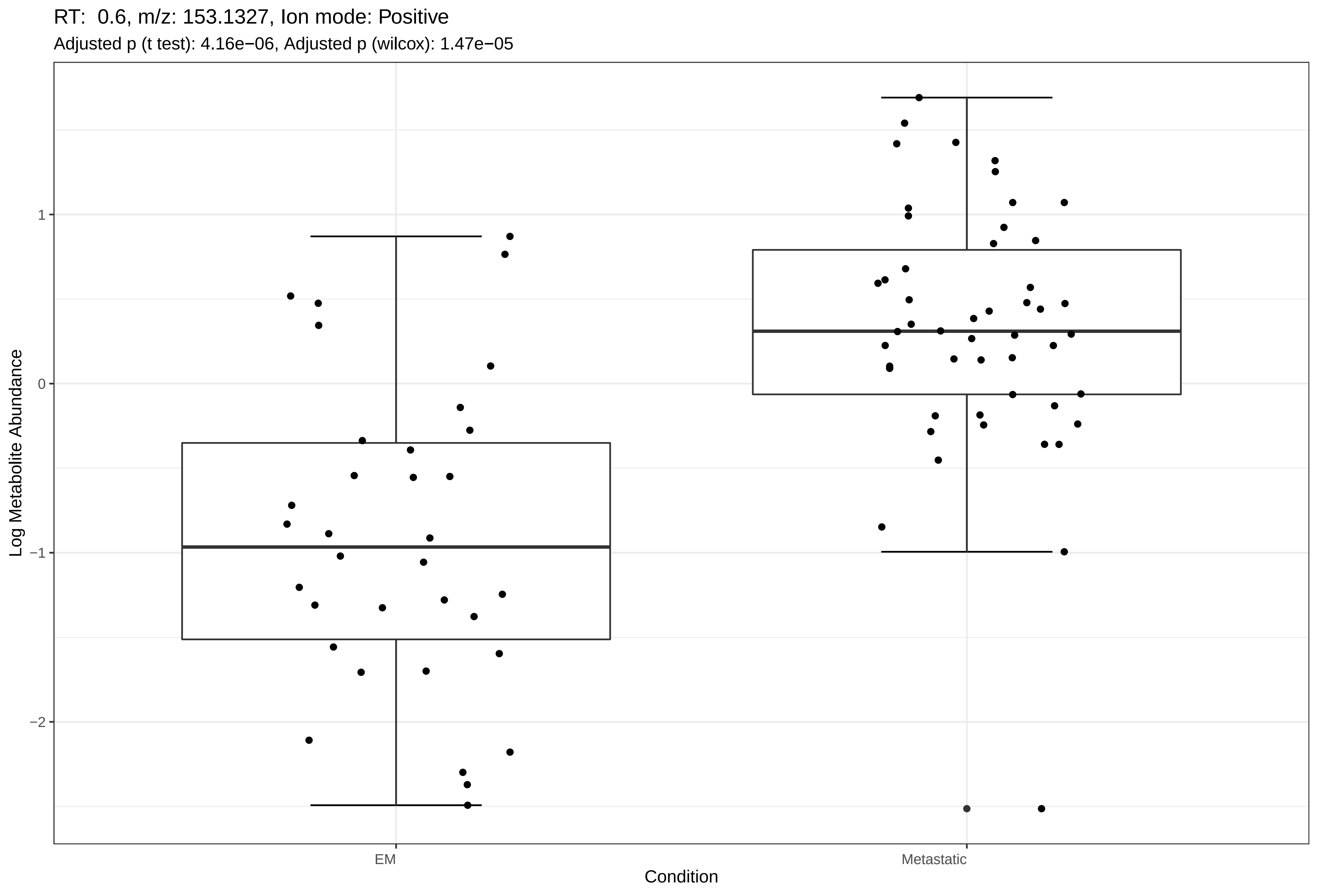
RT: 0.6, m/z: 153.1327, Ion mode: Positive
Adjusted p (t test): 4.16e−06, Adjusted p (wilcox): 1.47e−05
Log Metabolite Abundance
1
0
−1
−2
EM
Metastatic
Condition

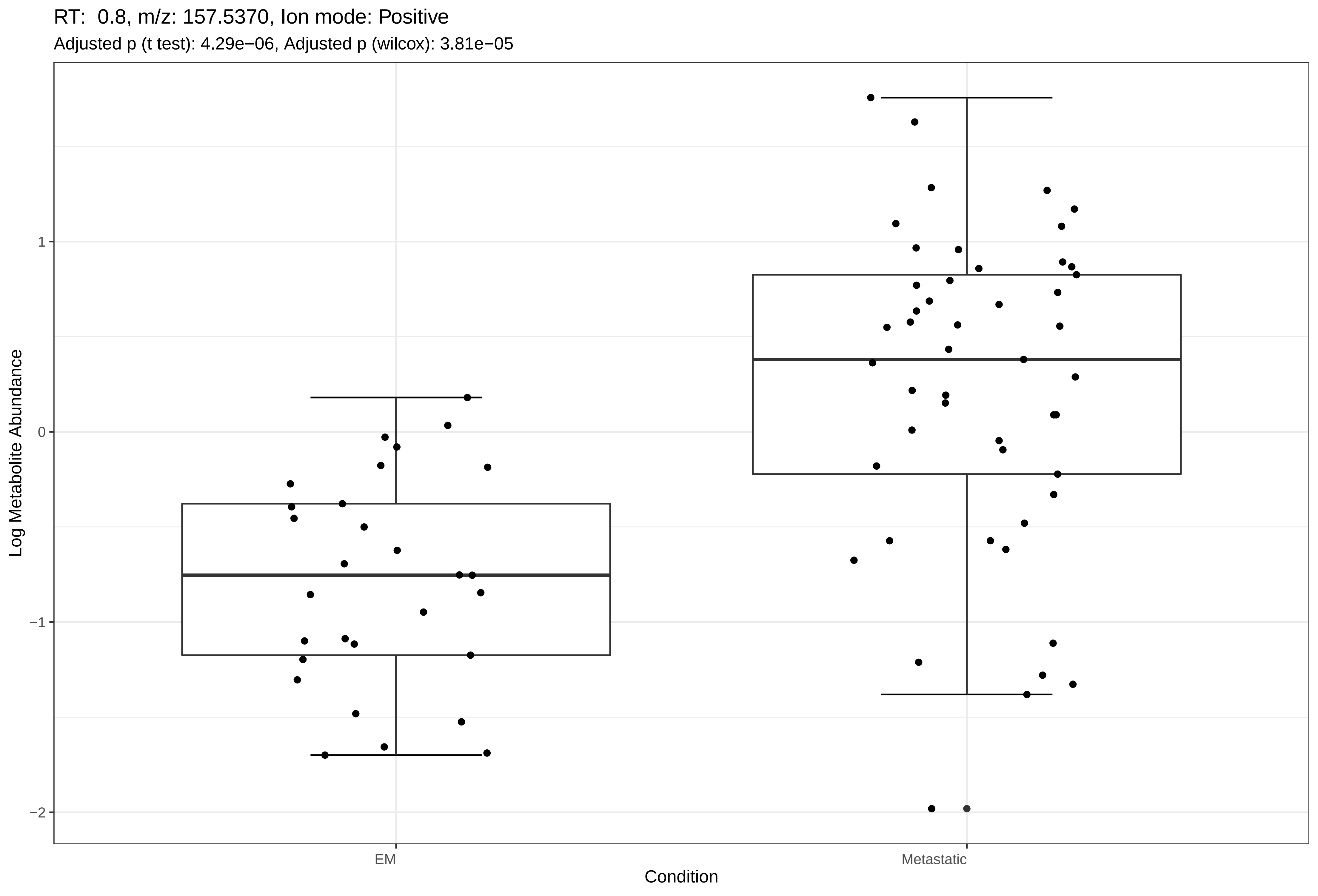
RT: 0.8, m/z: 157.5370, Ion mode: Positive
Adjusted p (t test): 4.29e−06, Adjusted p (wilcox): 3.81e−05
Log Metabolite Abundance
1
0
−1
−2
EM
Metastatic
Condition

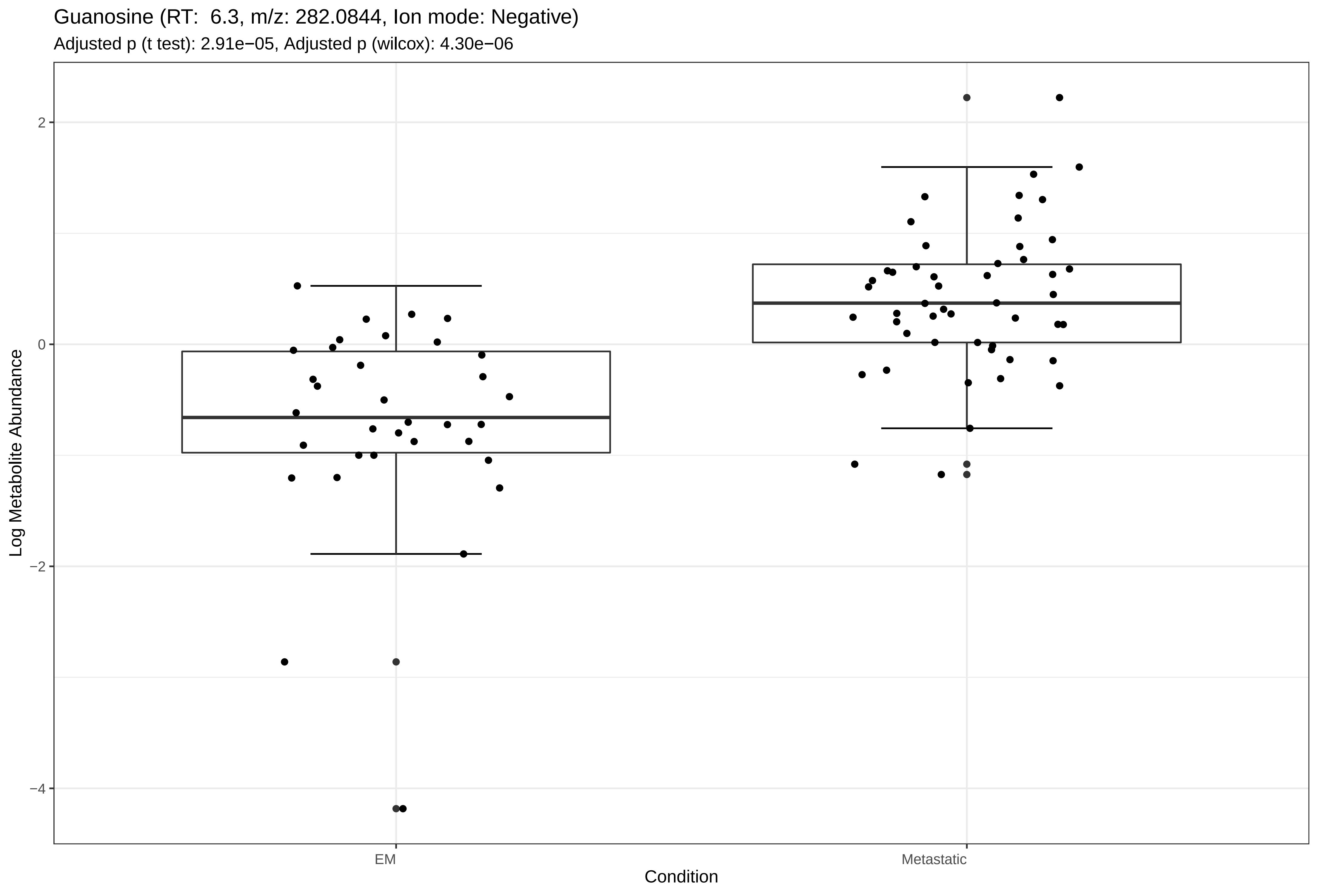
Guanosine (RT: 6.3, m/z: 282.0844, Ion mode: Negative)
Adjusted p (t test): 2.91e−05, Adjusted p (wilcox): 4.30e−06
Log Metabolite Abundance
2
0
−2
−4
EM
Metastatic
Condition

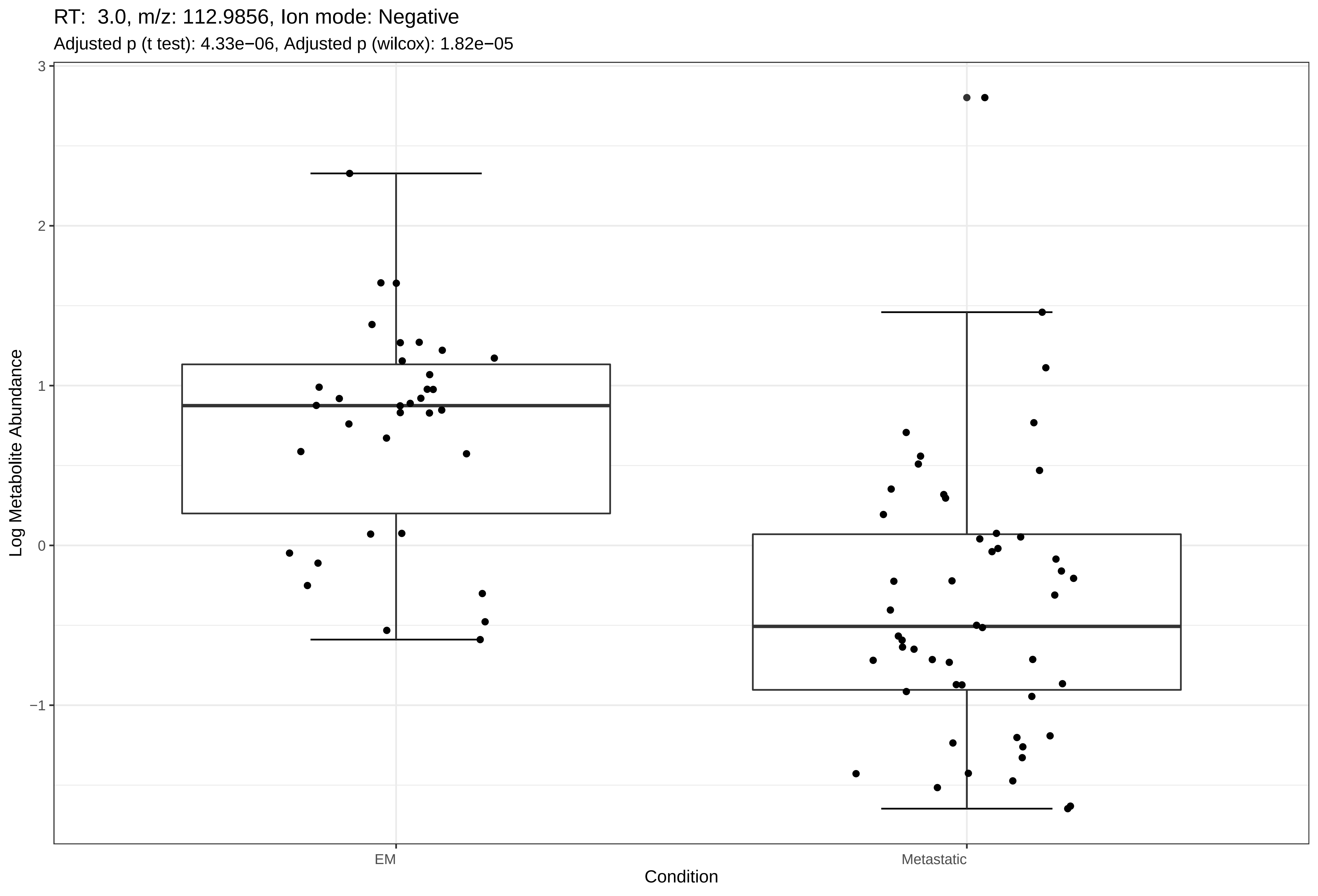
RT: 3.0, m/z: 112.9856, Ion mode: Negative
Adjusted p (t test): 4.33e−06, Adjusted p (wilcox): 1.82e−05
3
2
1
0
−1
Log Metabolite Abundance
EM
Metastatic
Condition

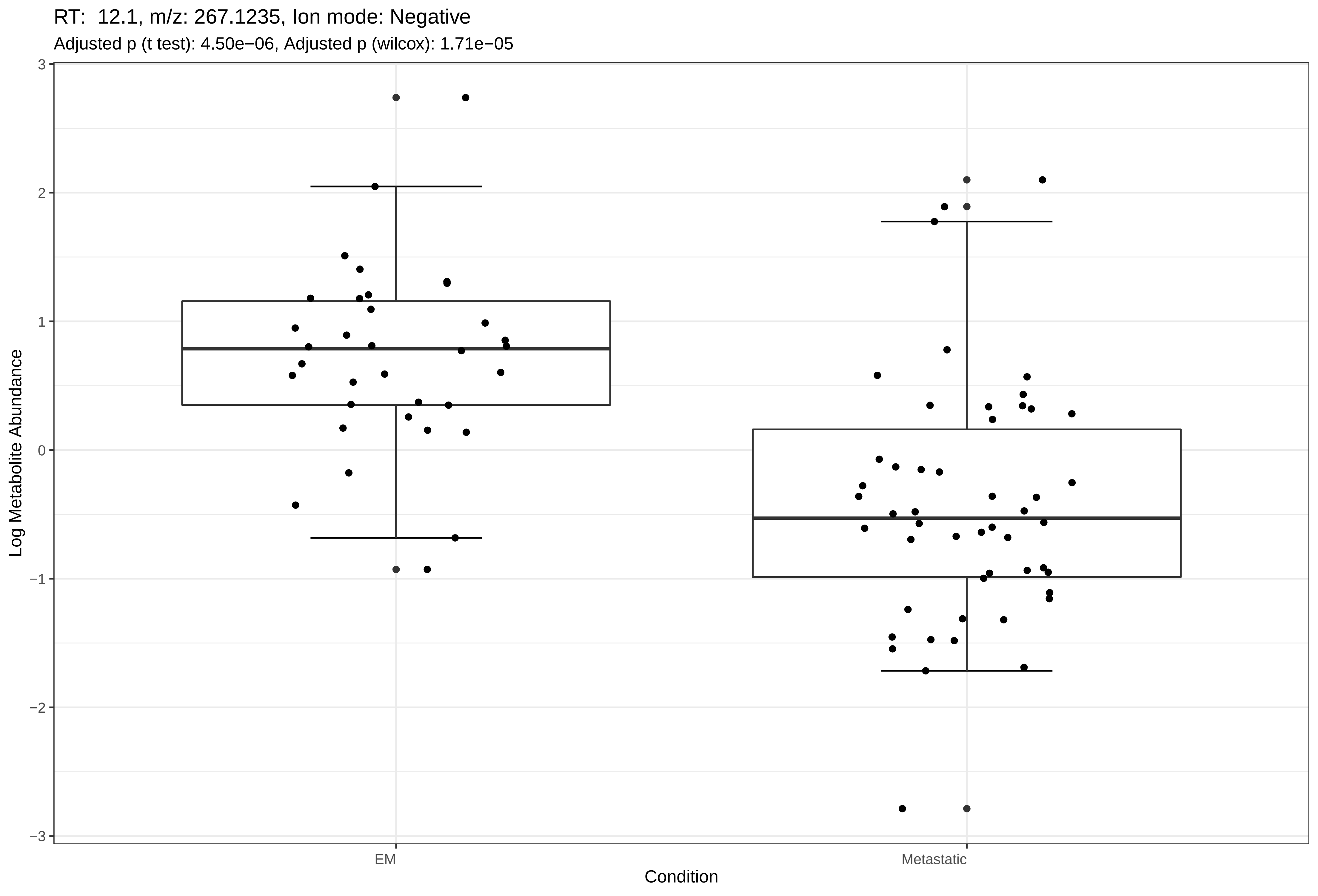

RT: 12.1, m/z: 267.1235, Ion mode: Negative
Adjusted p (t test): 4.50e−06, Adjusted p (wilcox): 1.71e−05
Log Metabolite Abundance
3
2
1
0
−1
−2
−3
EM
Metastatic
Condition

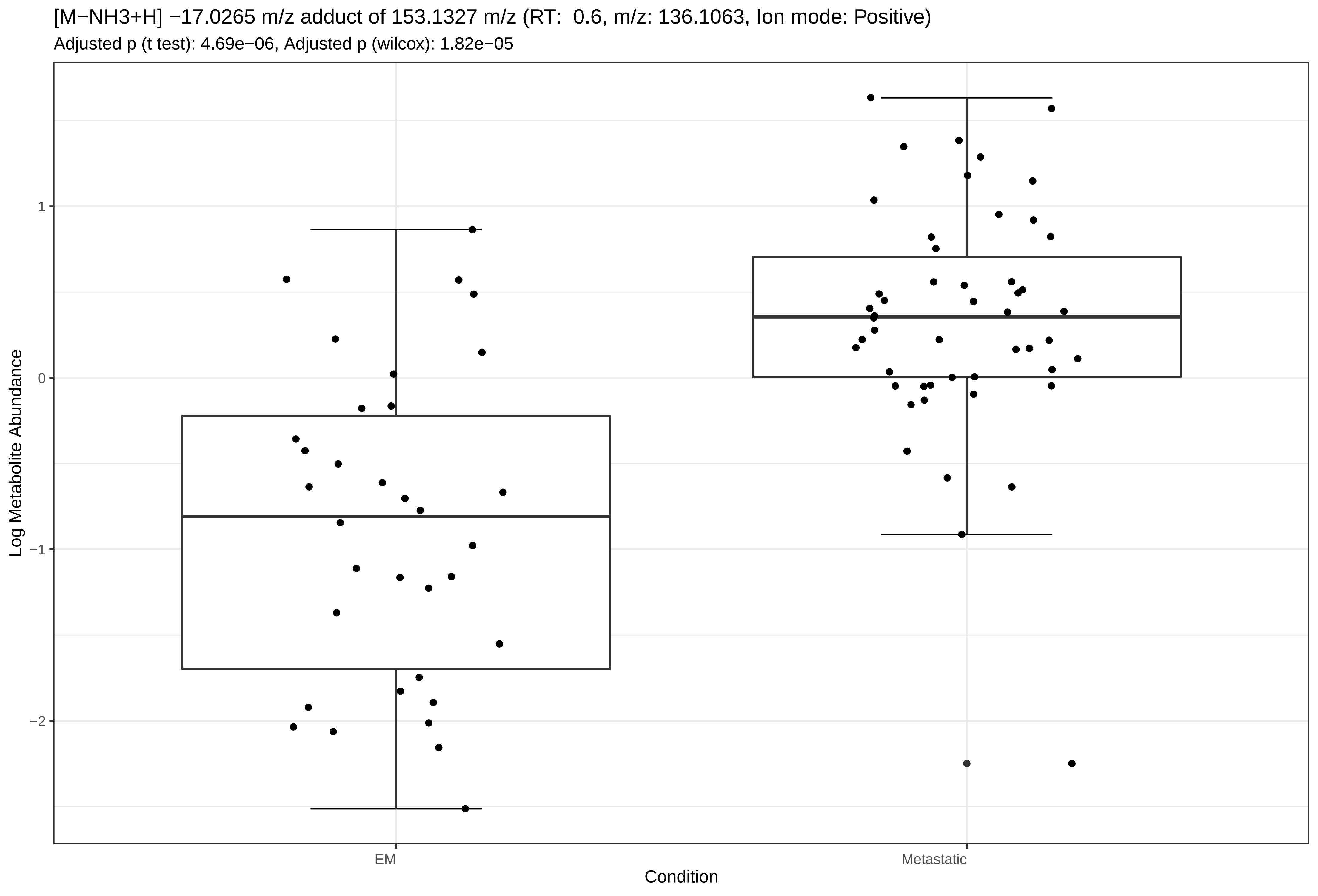
[M−NH3+H] −17.0265 m/z adduct of 153.1327 m/z (RT: 0.6, m/z: 136.1063, Ion mode: Positive)
Adjusted p (t test): 4.69e−06, Adjusted p (wilcox): 1.82e−05
Log Metabolite Abundance
1
0
−1
−2
EM
Metastatic
Condition

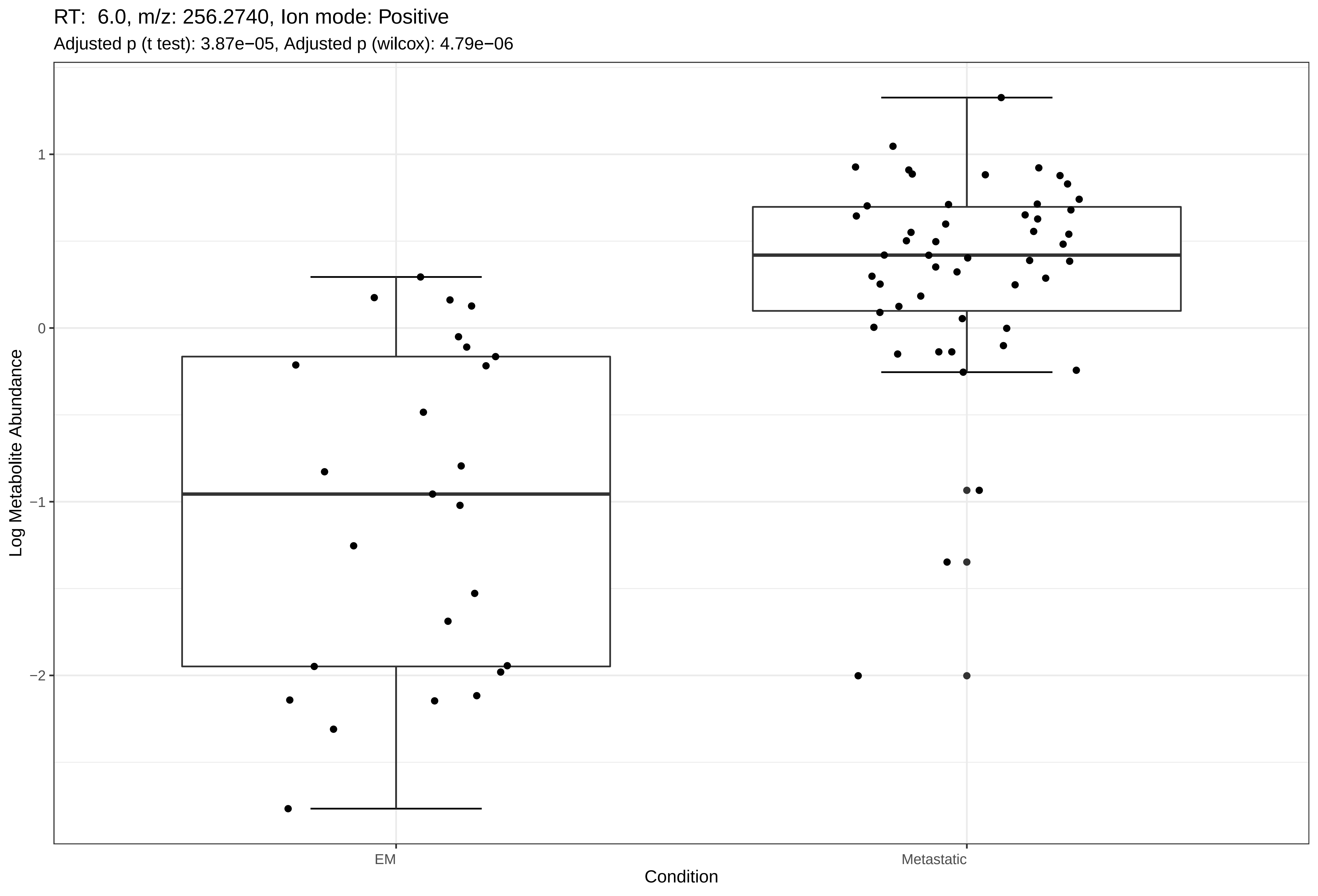
RT: 6.0, m/z: 256.2740, Ion mode: Positive
Adjusted p (t test): 3.87e−05, Adjusted p (wilcox): 4.79e−06
Log Metabolite Abundance
1
0
−1
−2
EM
Metastatic
Condition

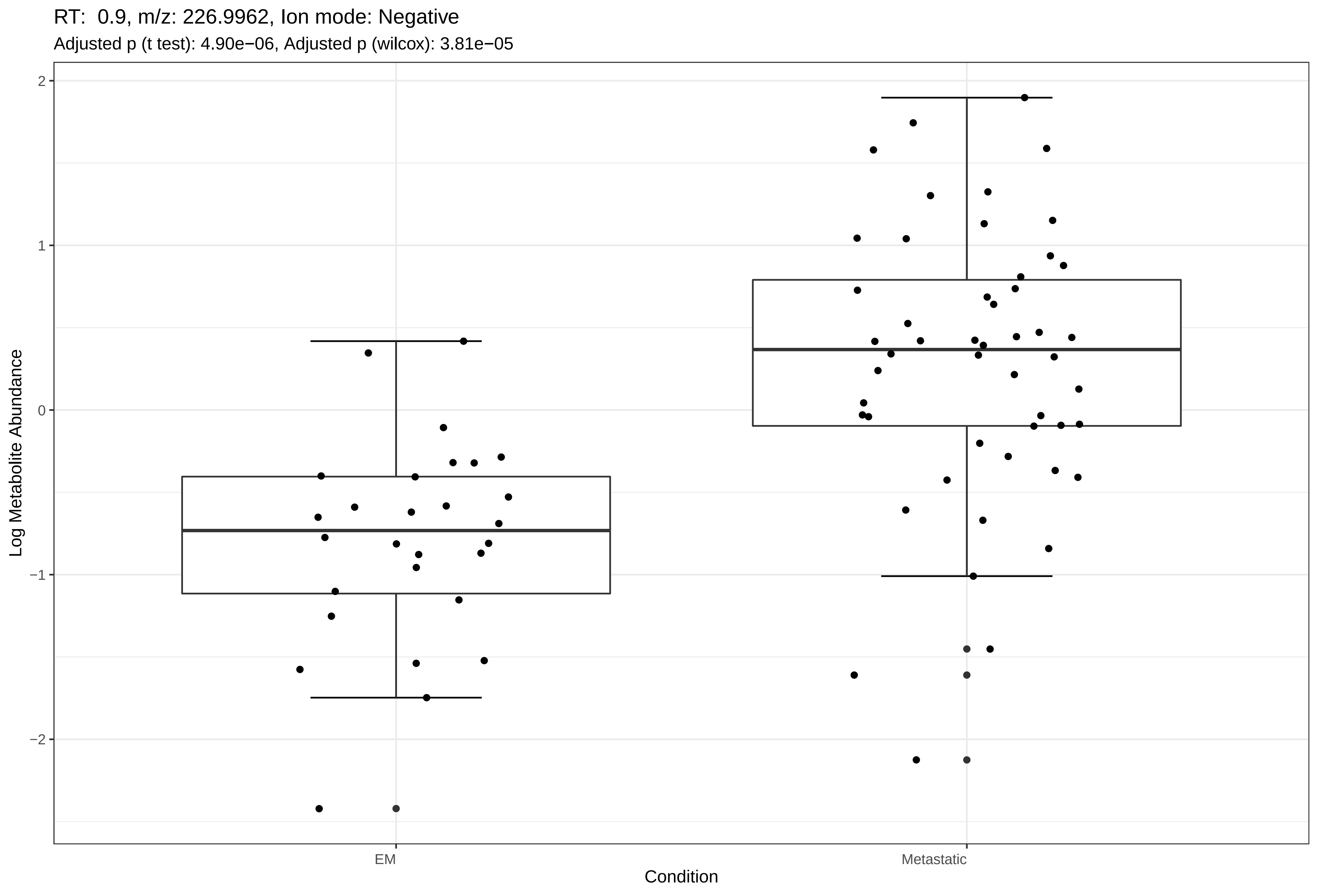
RT: 0.9, m/z: 226.9962, Ion mode: Negative
Adjusted p (t test): 4.90e−06, Adjusted p (wilcox): 3.81e−05
Log Metabolite Abundance
2
1
0
−1
−2
EM
Metastatic
Condition

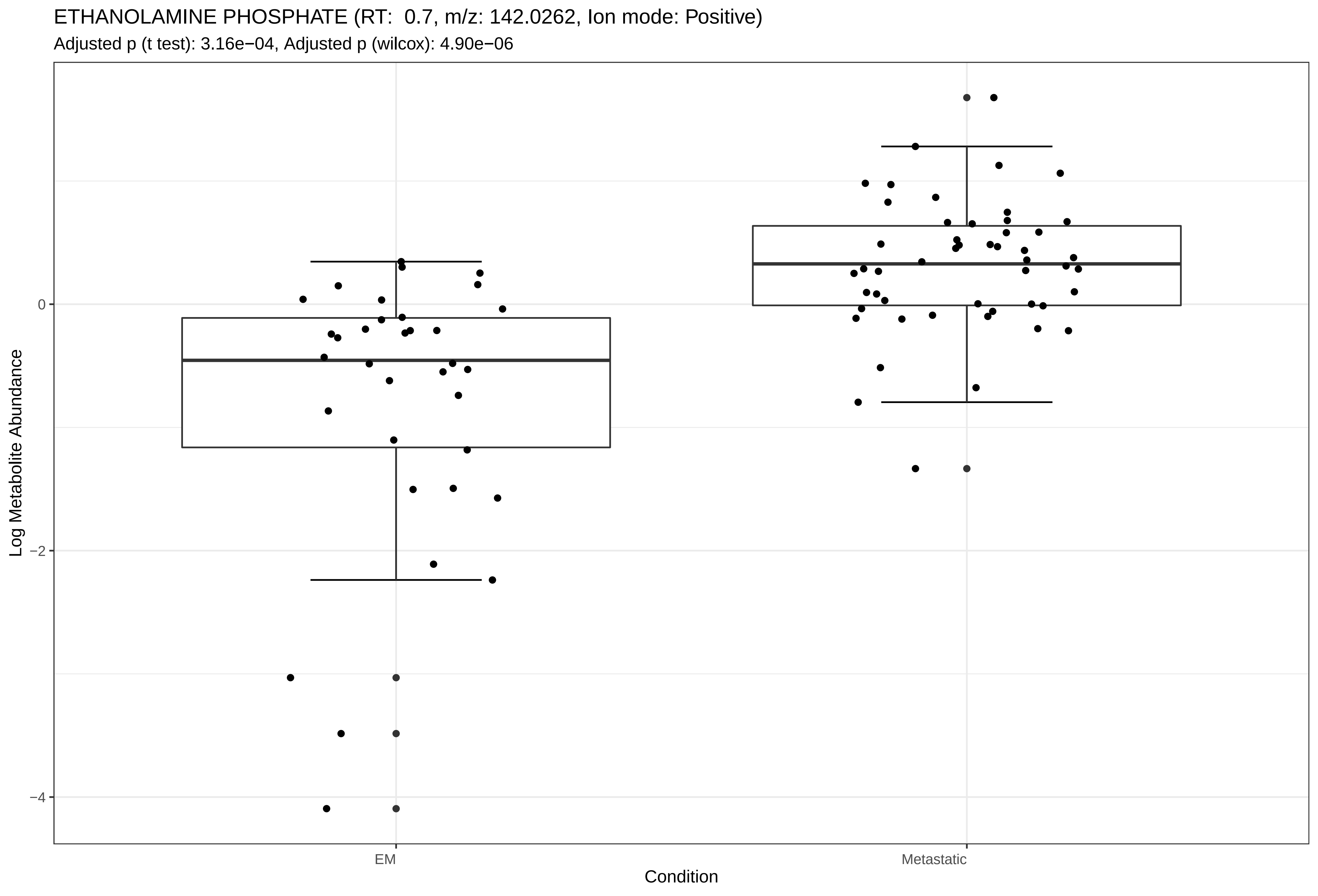

ETHANOLAMINE PHOSPHATE (RT: 0.7, m/z: 142.0262, Ion mode: Positive)
Adjusted p (t test): 3.16e−04, Adjusted p (wilcox): 4.90e−06
Log Metabolite Abundance
0
−2
−4
EM
Metastatic
Condition

RT: 0.5, m/z: 321.2607, Ion mode: Positive

Adjusted p (t test): 4.91e−06, Adjusted p (wilcox): 1.29e−05

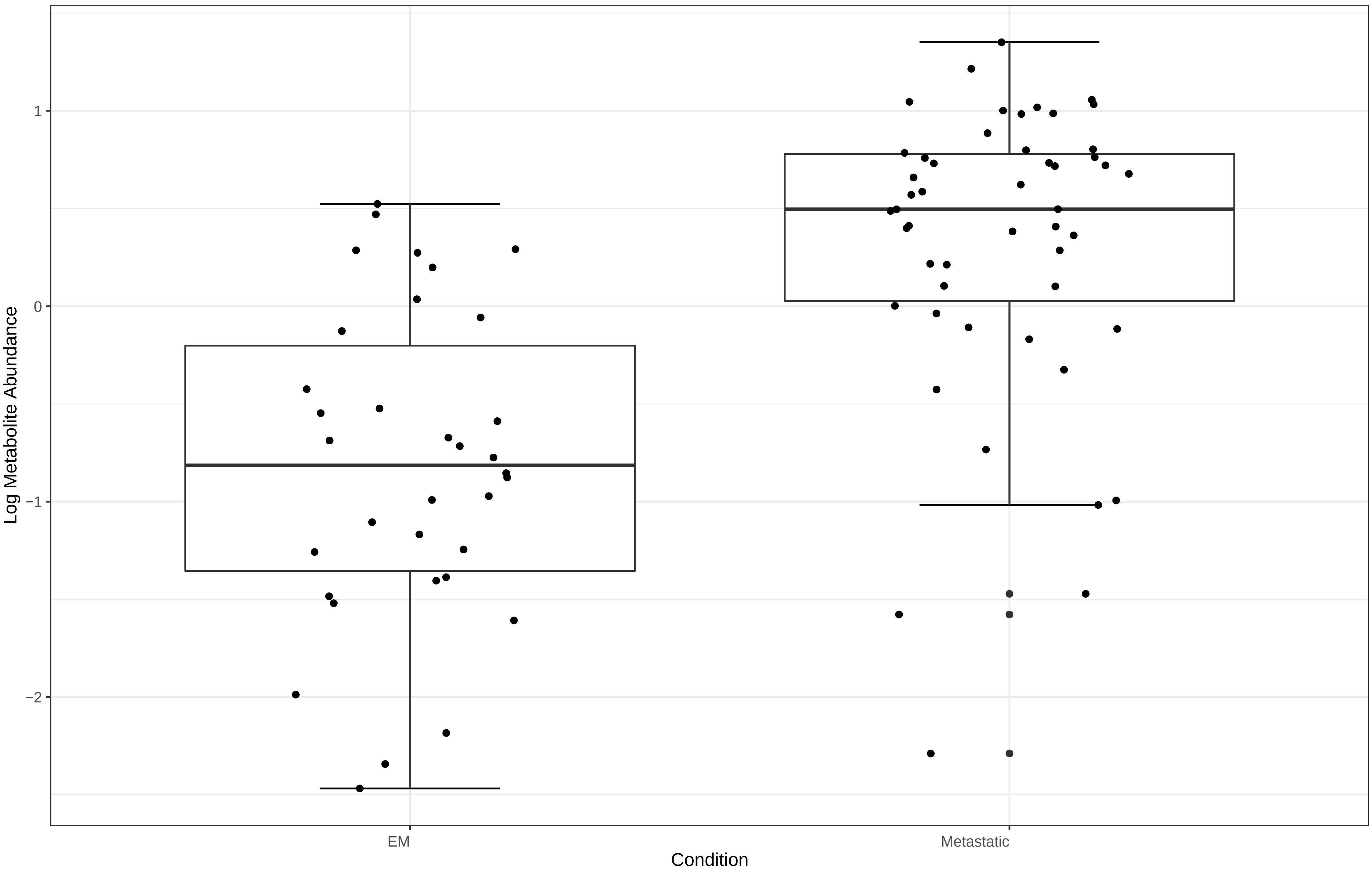

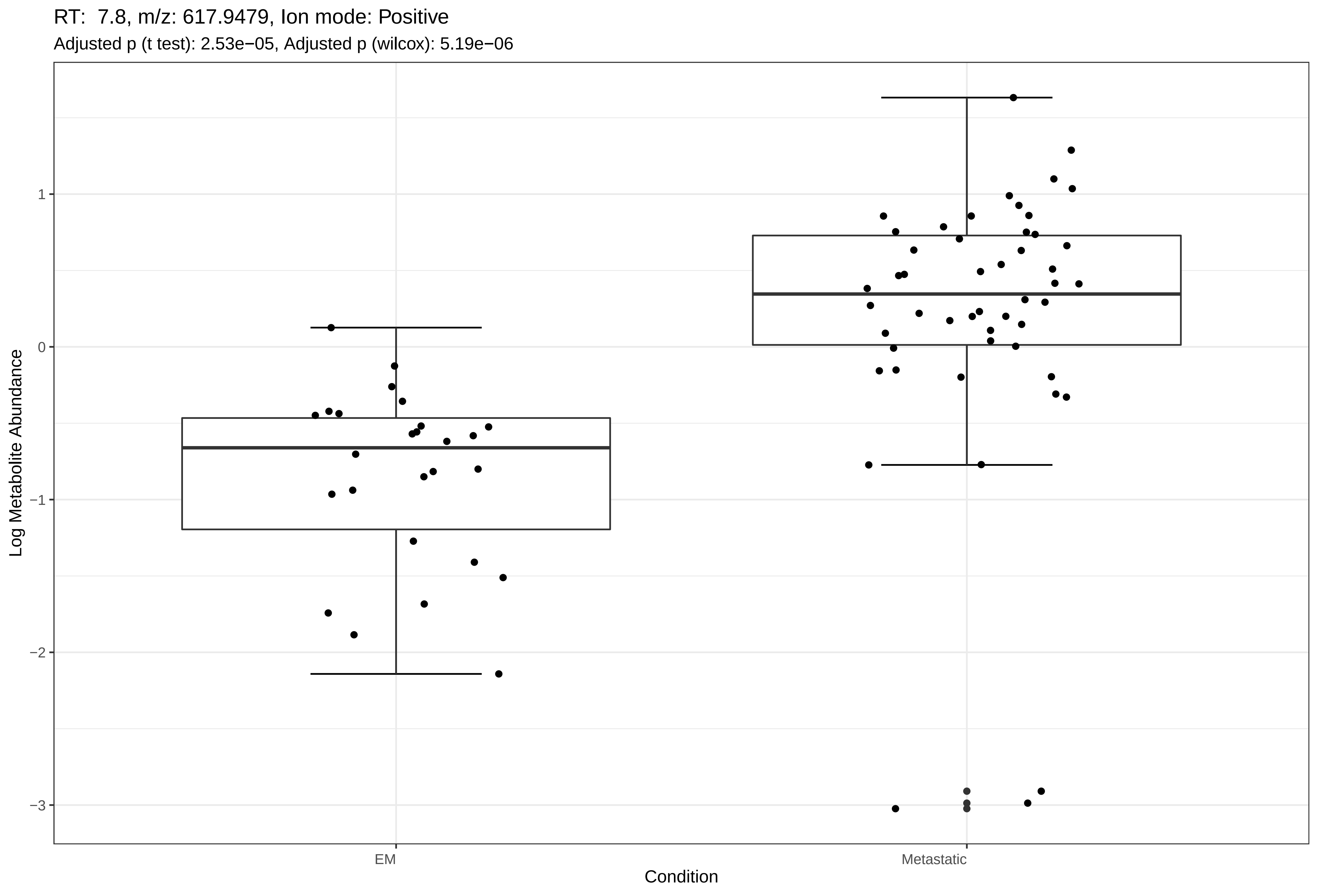
RT: 7.8, m/z: 617.9479, Ion mode: Positive
Adjusted p (t test): 2.53e−05, Adjusted p (wilcox): 5.19e−06
Log Metabolite Abundance
1
0
−1
−2
−3
EM
Metastatic
Condition

RT: 0.8, m/z: 149.9402, Ion mode: Positive

Adjusted p (t test): 5.26e−06, Adjusted p (wilcox): 5.08e−05

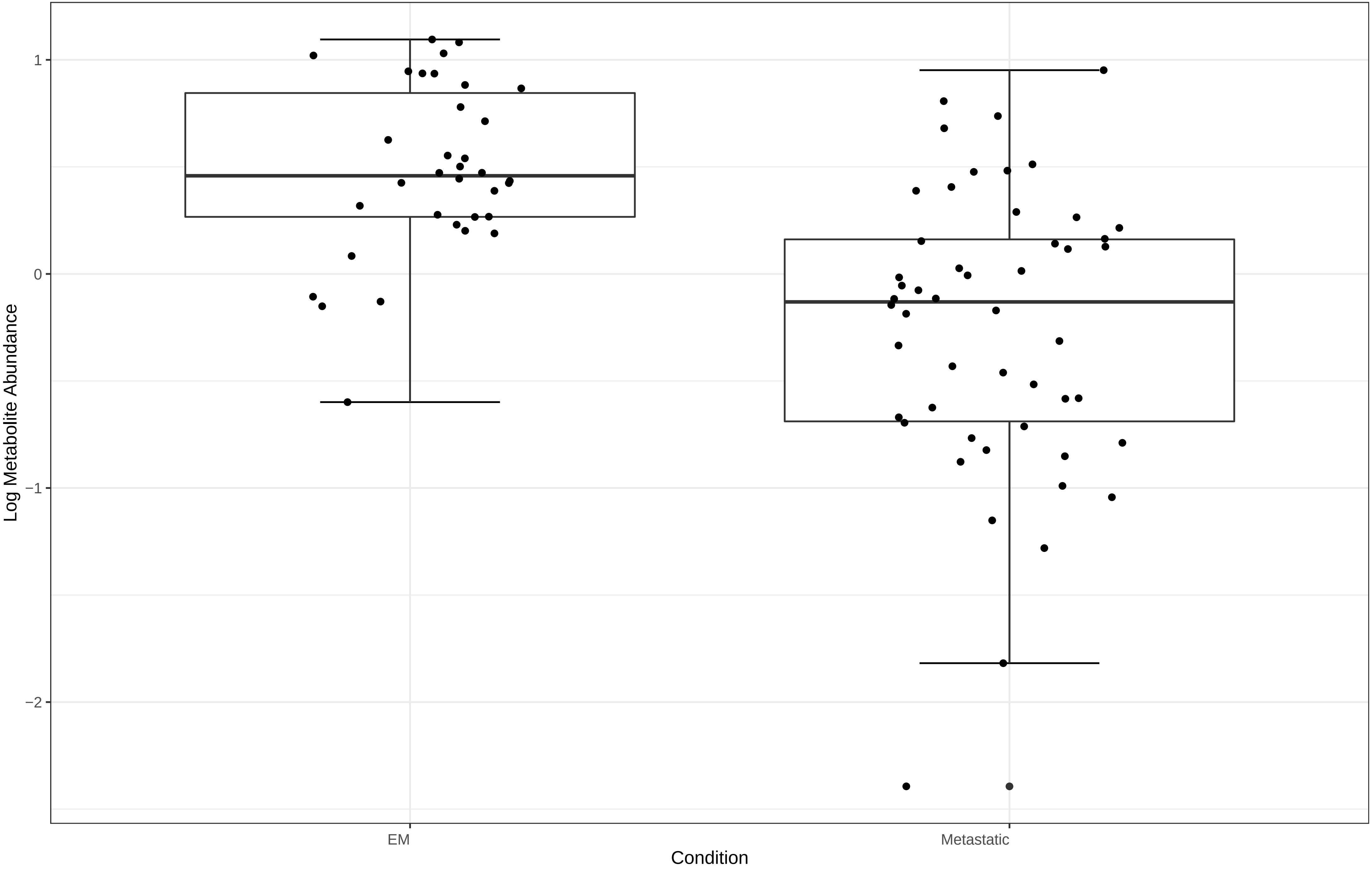

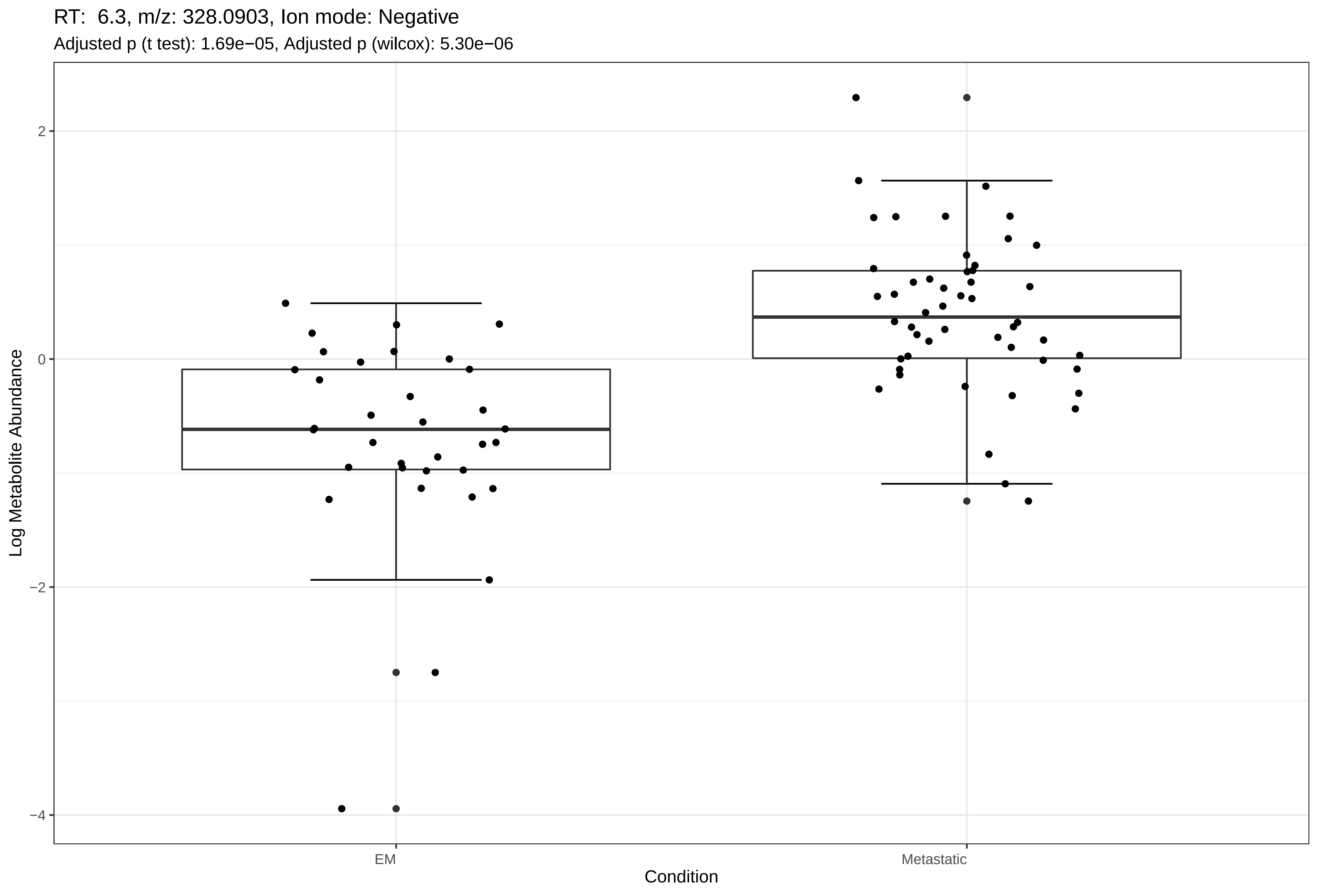
RT: 6.3, m/z: 328.0903, Ion mode: Negative
Adjusted p (t test): 1.69e−05, Adjusted p (wilcox): 5.30e−06
Log Metabolite Abundance
2
0
−2
−4
EM
Metastatic
Condition

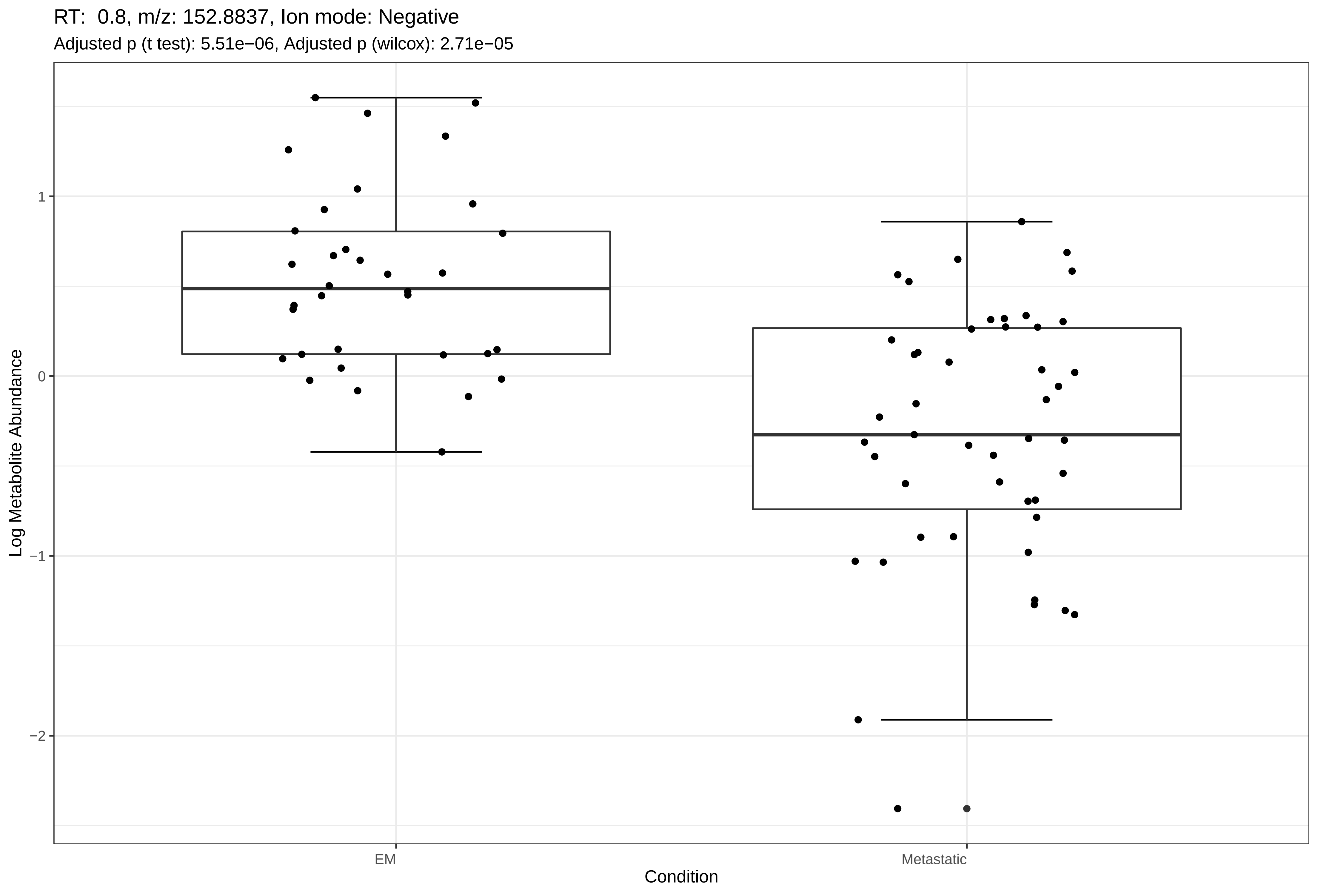
RT: 0.8, m/z: 152.8837, Ion mode: Negative
Adjusted p (t test): 5.51e−06, Adjusted p (wilcox): 2.71e−05
Log Metabolite Abundance
1
0
−1
−2
EM
Metastatic
Condition

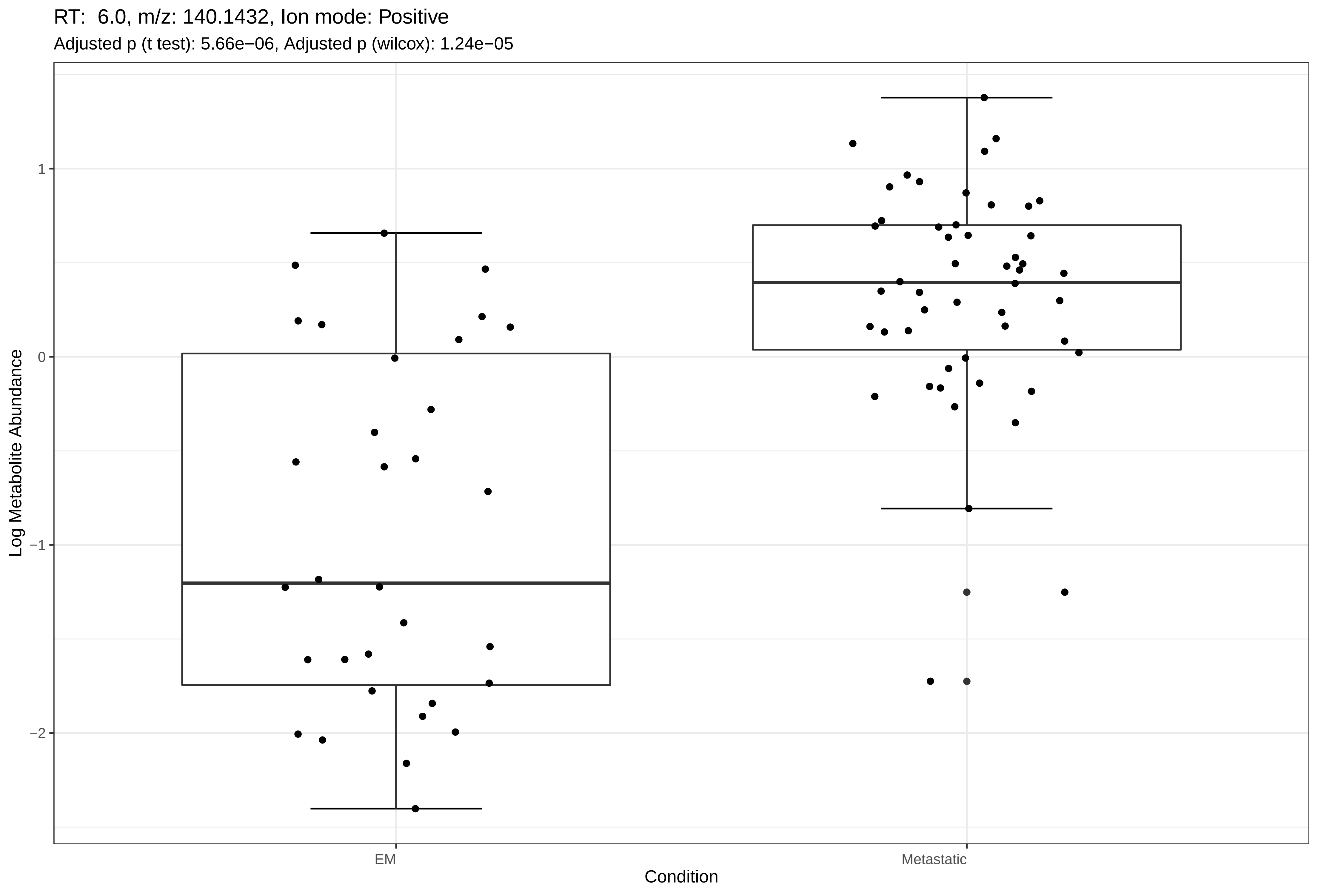
RT: 6.0, m/z: 140.1432, Ion mode: Positive
Adjusted p (t test): 5.66e−06, Adjusted p (wilcox): 1.24e−05
Log Metabolite Abundance
1
0
−1
−2
EM
Metastatic
Condition

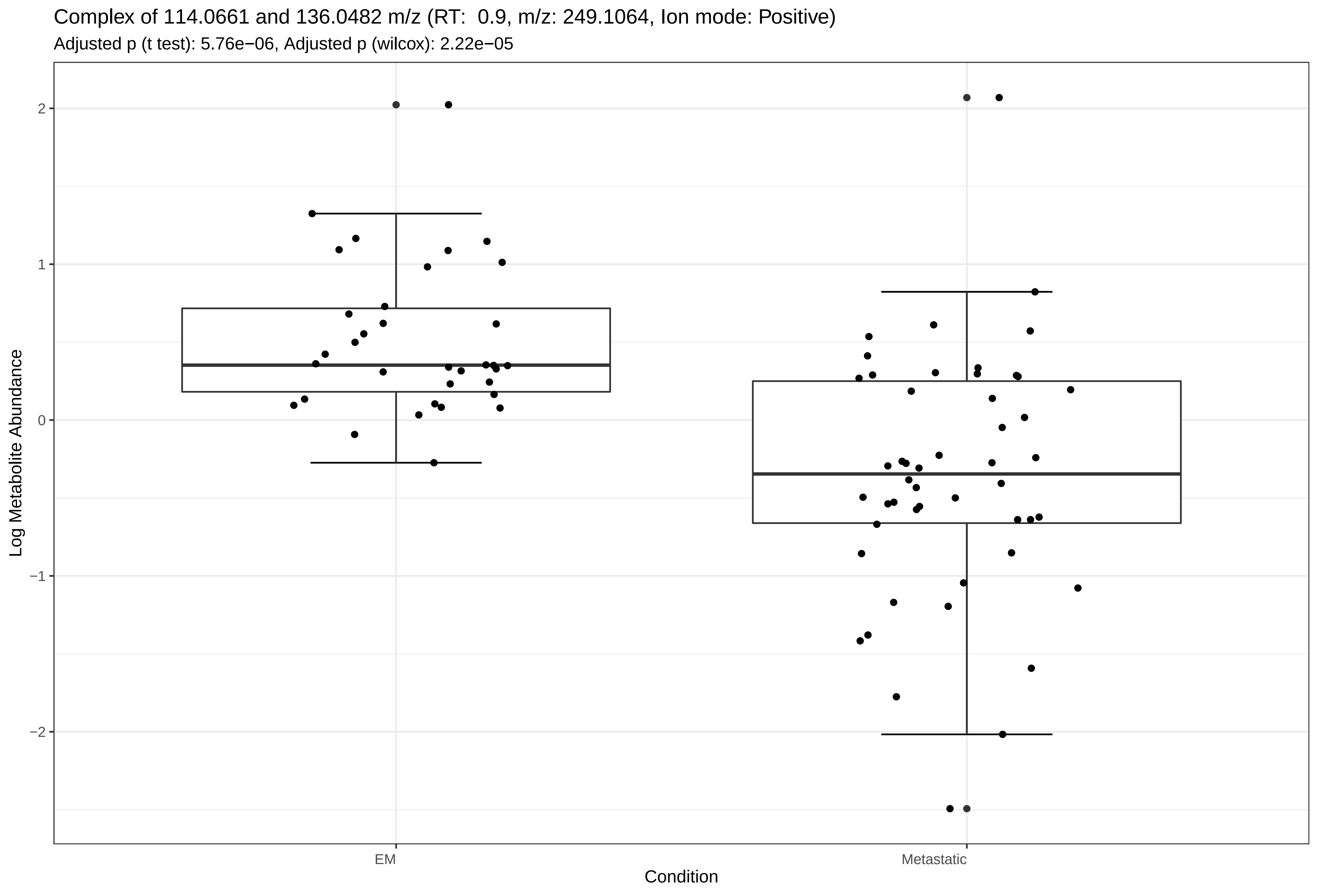

Complex of 114.0661 and 136.0482 m/z (RT: 0.9, m/z: 249.1064, Ion mode: Positive)
Adjusted p (t test): 5.76e−06, Adjusted p (wilcox): 2.22e−05
Log Metabolite Abundance
2
1
0
−1
−2
EM
Metastatic
Condition

RT: 6.2, m/z: 394.0619, Ion mode: Negative

Adjusted p (t test): 5.11e−05, Adjusted p (wilcox): 5.88e−06

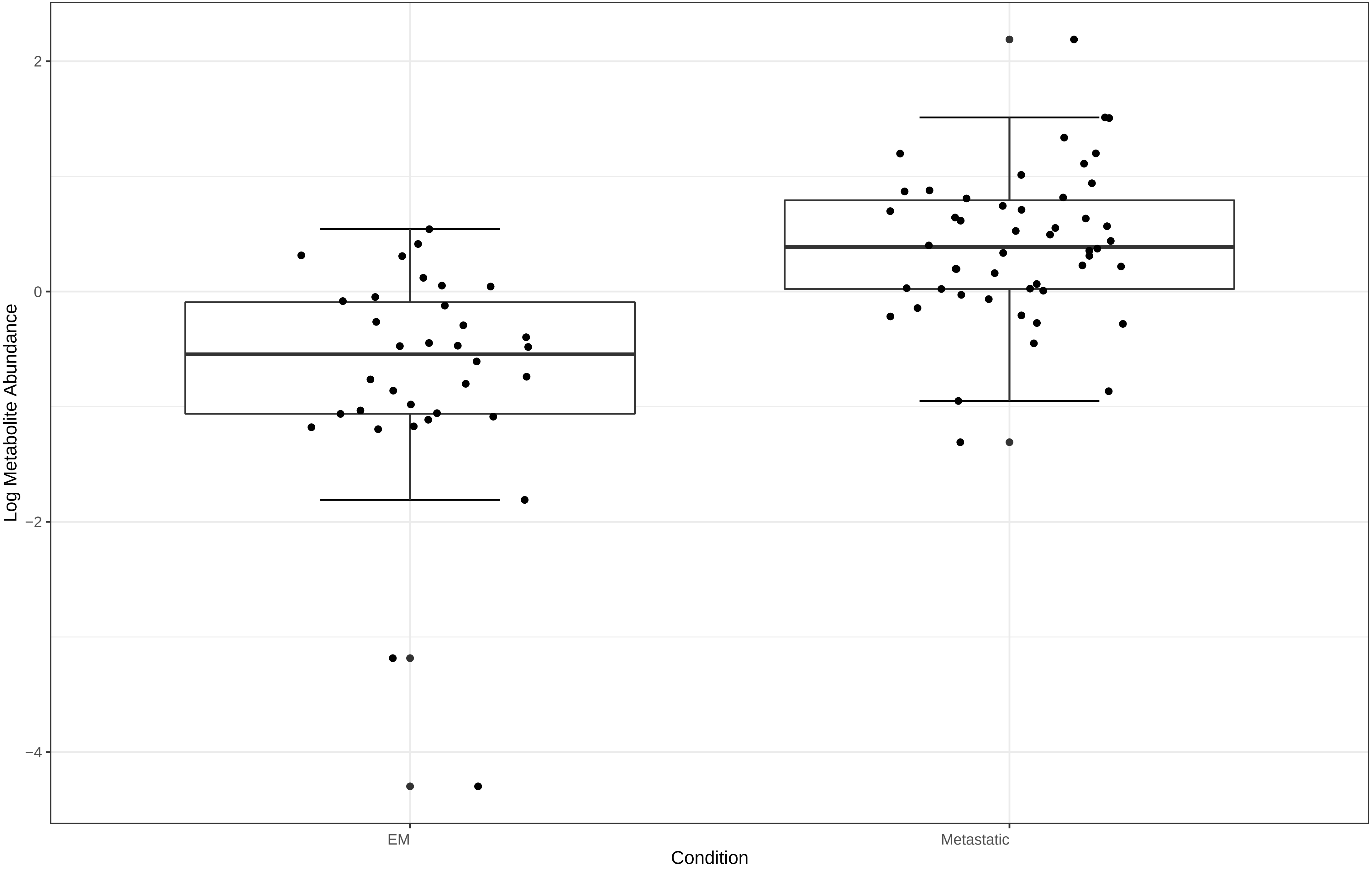

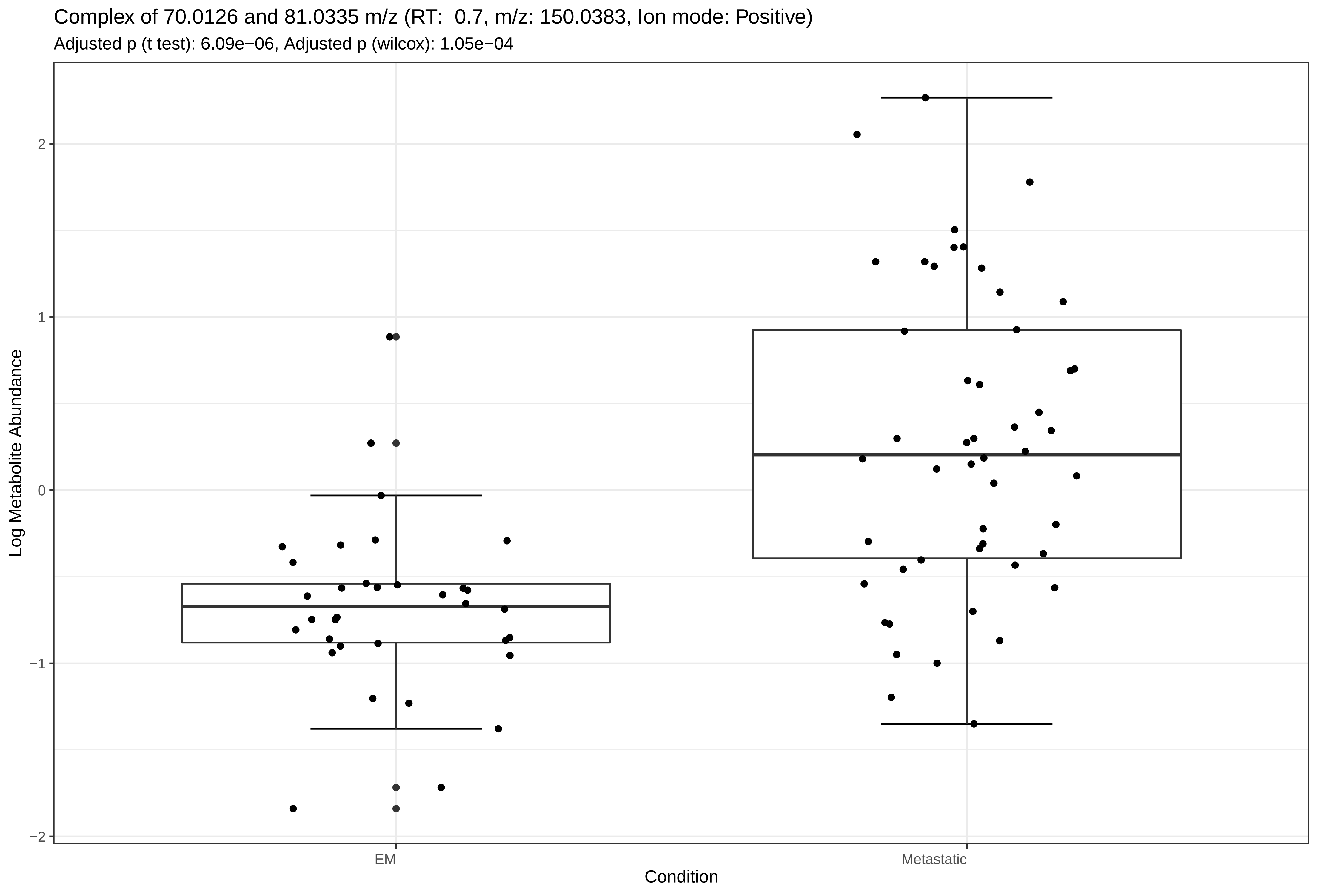
Complex of 70.0126 and 81.0335 m/z (RT: 0.7, m/z: 150.0383, Ion mode: Positive)
Adjusted p (t test): 6.09e−06, Adjusted p (wilcox): 1.05e−04
Log Metabolite Abundance
2
1
0
−1
−2
EM
Metastatic
Condition

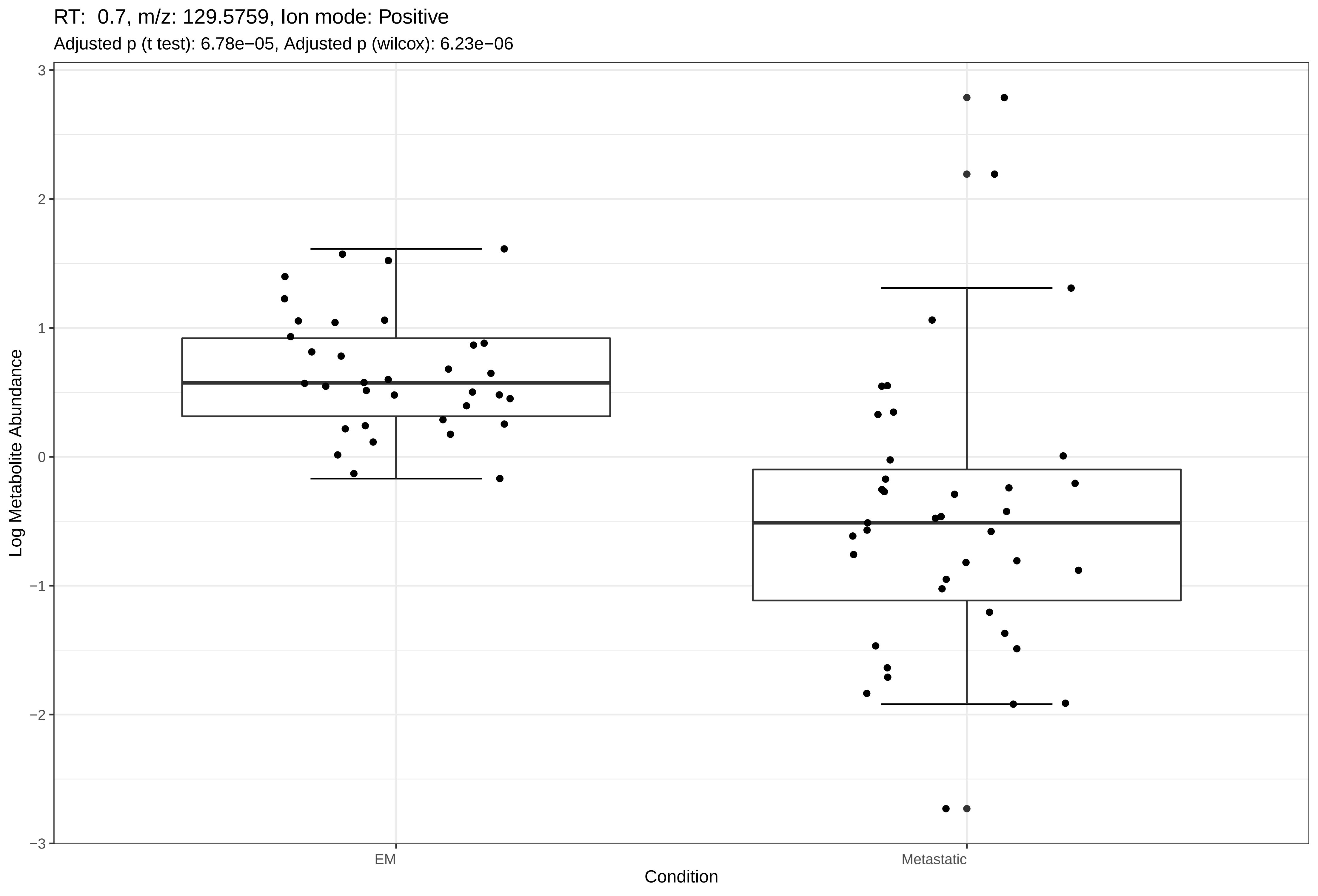

RT: 0.7, m/z: 129.5759, Ion mode: Positive
Adjusted p (t test): 6.78e−05, Adjusted p (wilcox): 6.23e−06
Log Metabolite Abundance
3
2
1
0
−1
−2
−3
EM
Metastatic
Condition

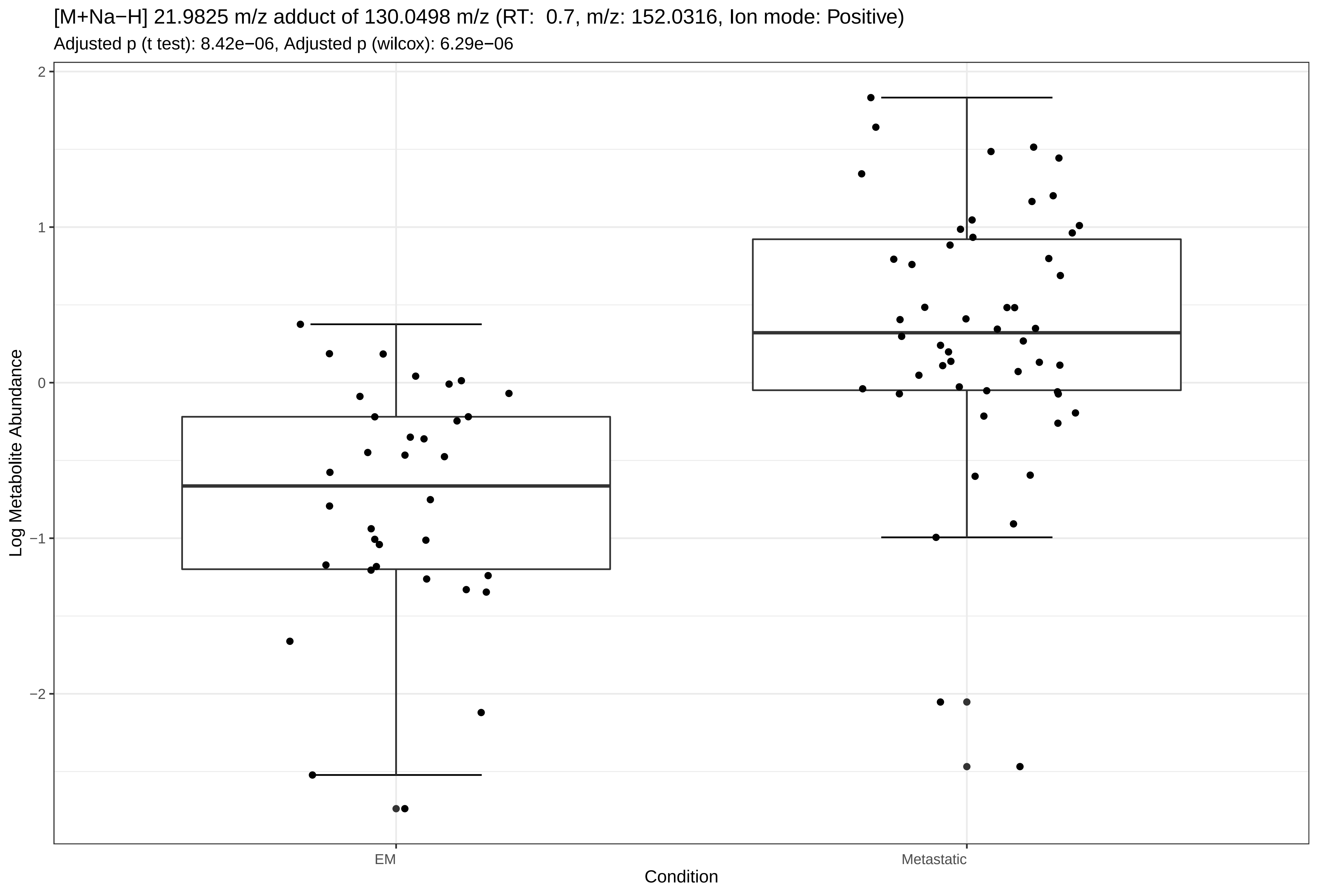

[M+Na−H] 21.9825 m/z adduct of 130.0498 m/z (RT: 0.7, m/z: 152.0316, Ion mode: Positive)
Adjusted p (t test): 8.42e−06, Adjusted p (wilcox): 6.29e−06
Log Metabolite Abundance
2
1
0
−1
−2
EM
Metastatic
Condition

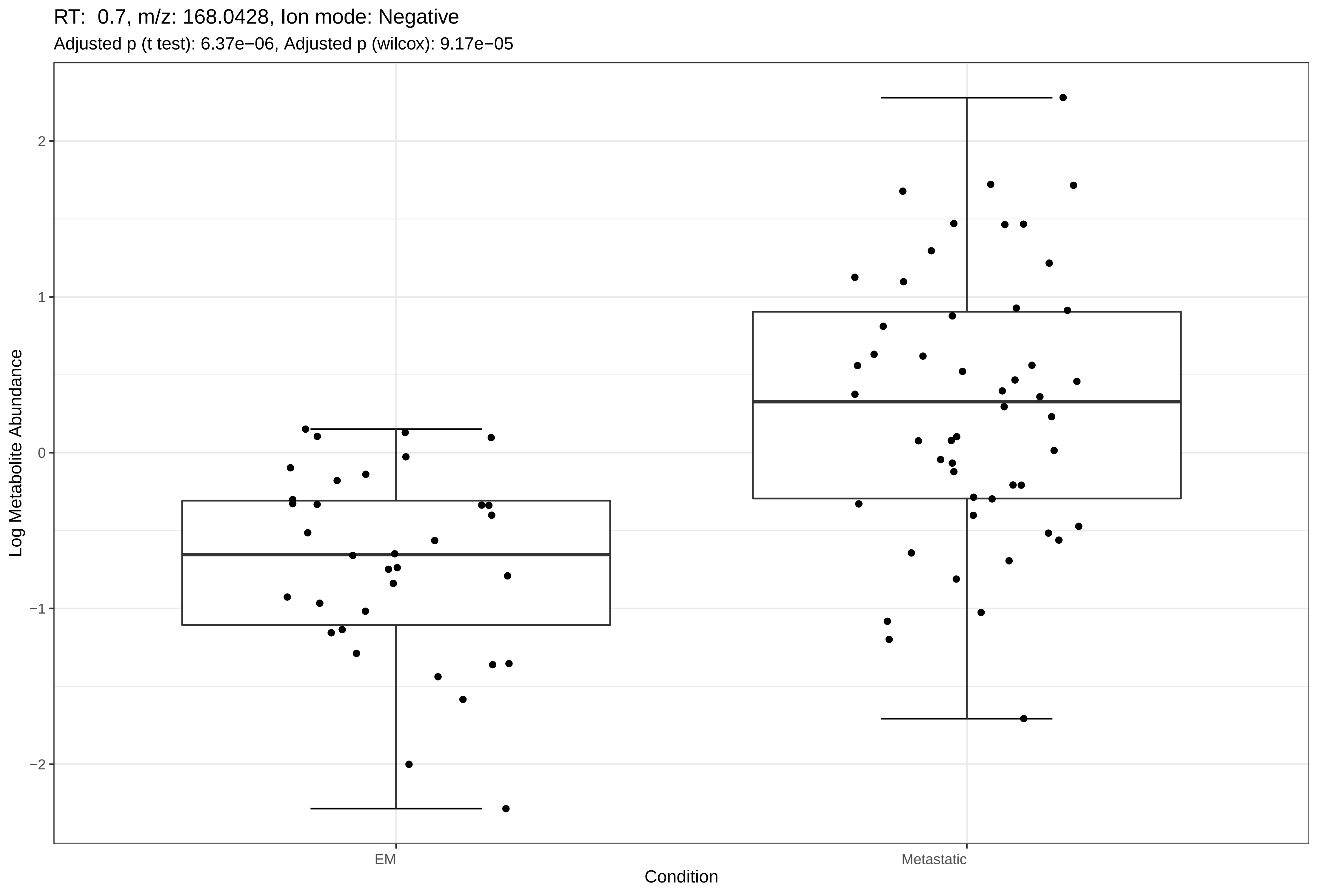

RT: 0.7, m/z: 168.0428, Ion mode: Negative
Adjusted p (t test): 6.37e−06, Adjusted p (wilcox): 9.17e−05
Log Metabolite Abundance
2
1
0
−1
−2
EM
Metastatic
Condition

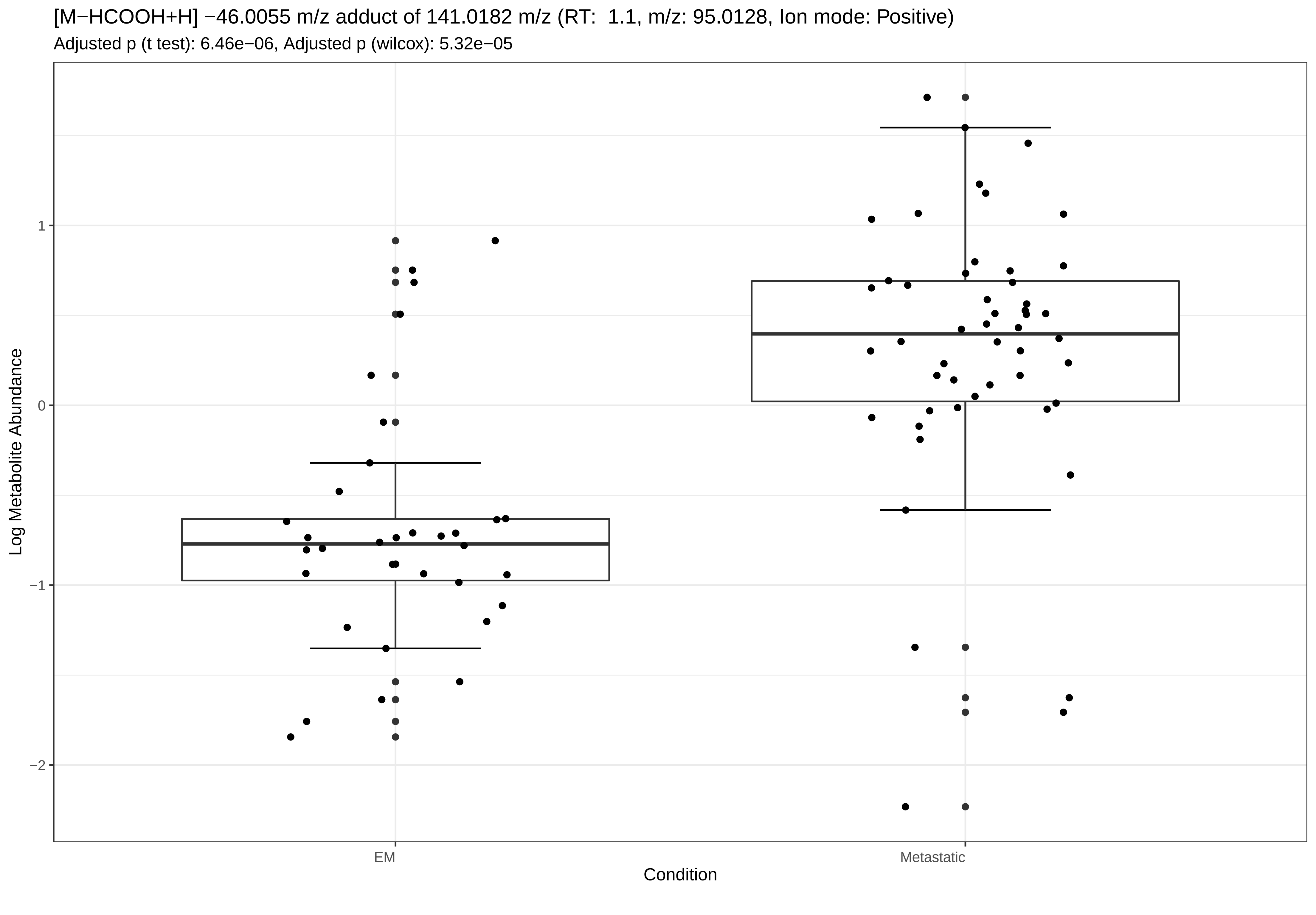

[M−HCOOH+H] −46.0055 m/z adduct of 141.0182 m/z (RT: 1.1, m/z: 95.0128, Ion mode: Positive)
Adjusted p (t test): 6.46e−06, Adjusted p (wilcox): 5.32e−05
Log Metabolite Abundance
1
0
−1
−2
EM
Metastatic
Condition

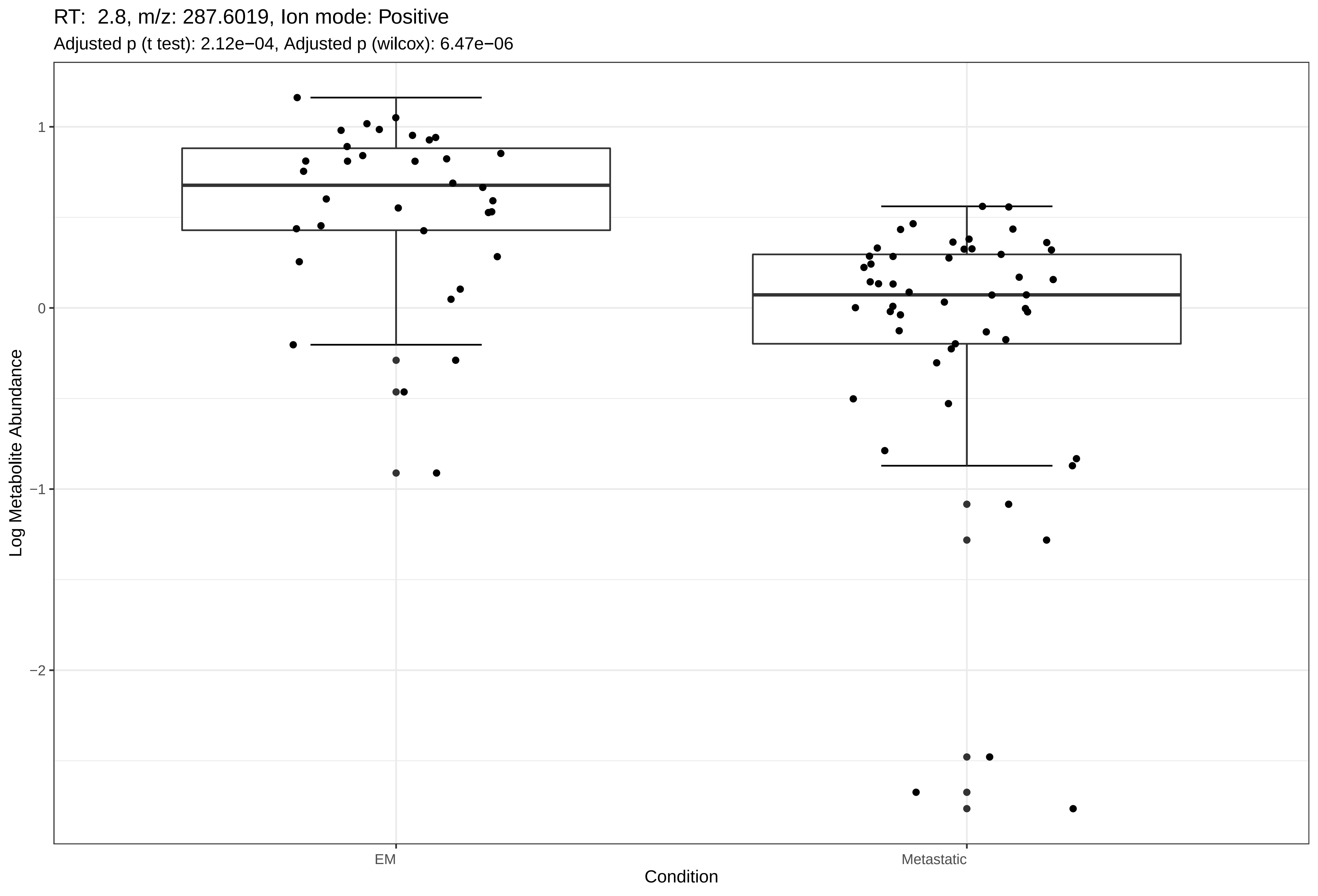
RT: 2.8, m/z: 287.6019, Ion mode: Positive
Adjusted p (t test): 2.12e−04, Adjusted p (wilcox): 6.47e−06
Log Metabolite Abundance
1
0
−1
−2
EM
Metastatic
Condition

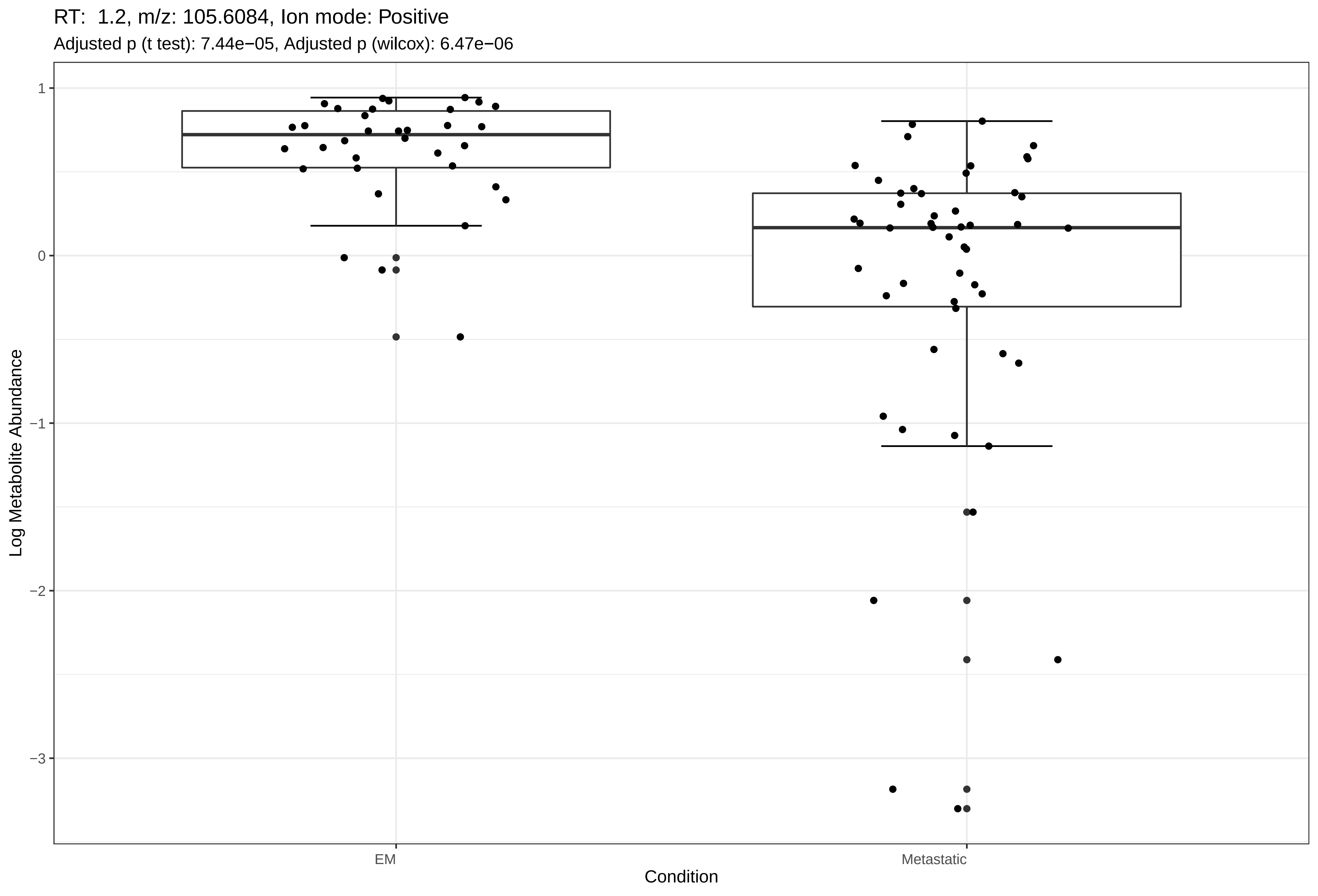
RT: 1.2, m/z: 105.6084, Ion mode: Positive
Adjusted p (t test): 7.44e−05, Adjusted p (wilcox): 6.47e−06
Log Metabolite Abundance
1
0
−1
−2
−3
EM
Metastatic
Condition

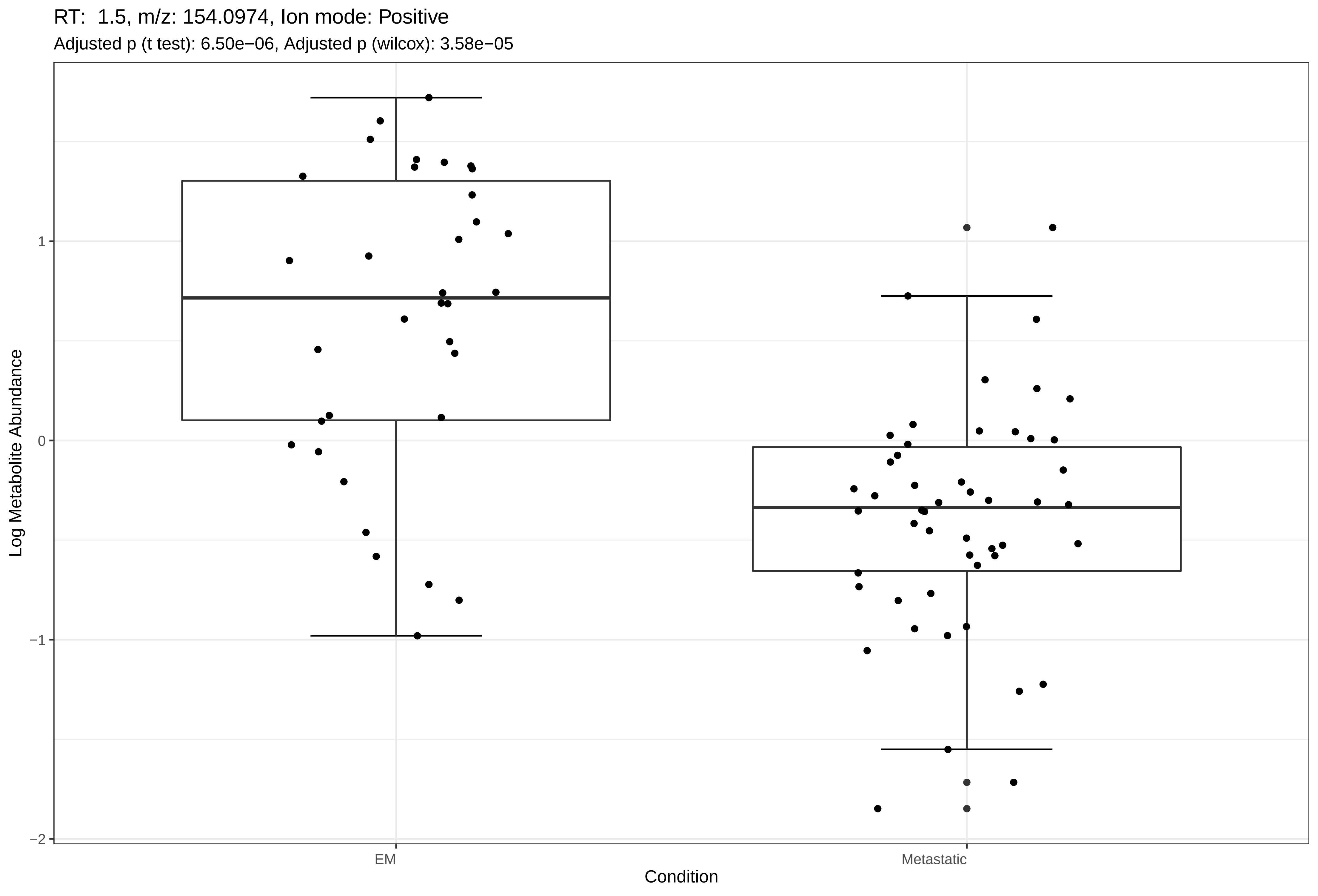
RT: 1.5, m/z: 154.0974, Ion mode: Positive
Adjusted p (t test): 6.50e−06, Adjusted p (wilcox): 3.58e−05
Log Metabolite Abundance
1
0
−1
−2
EM
Metastatic
Condition

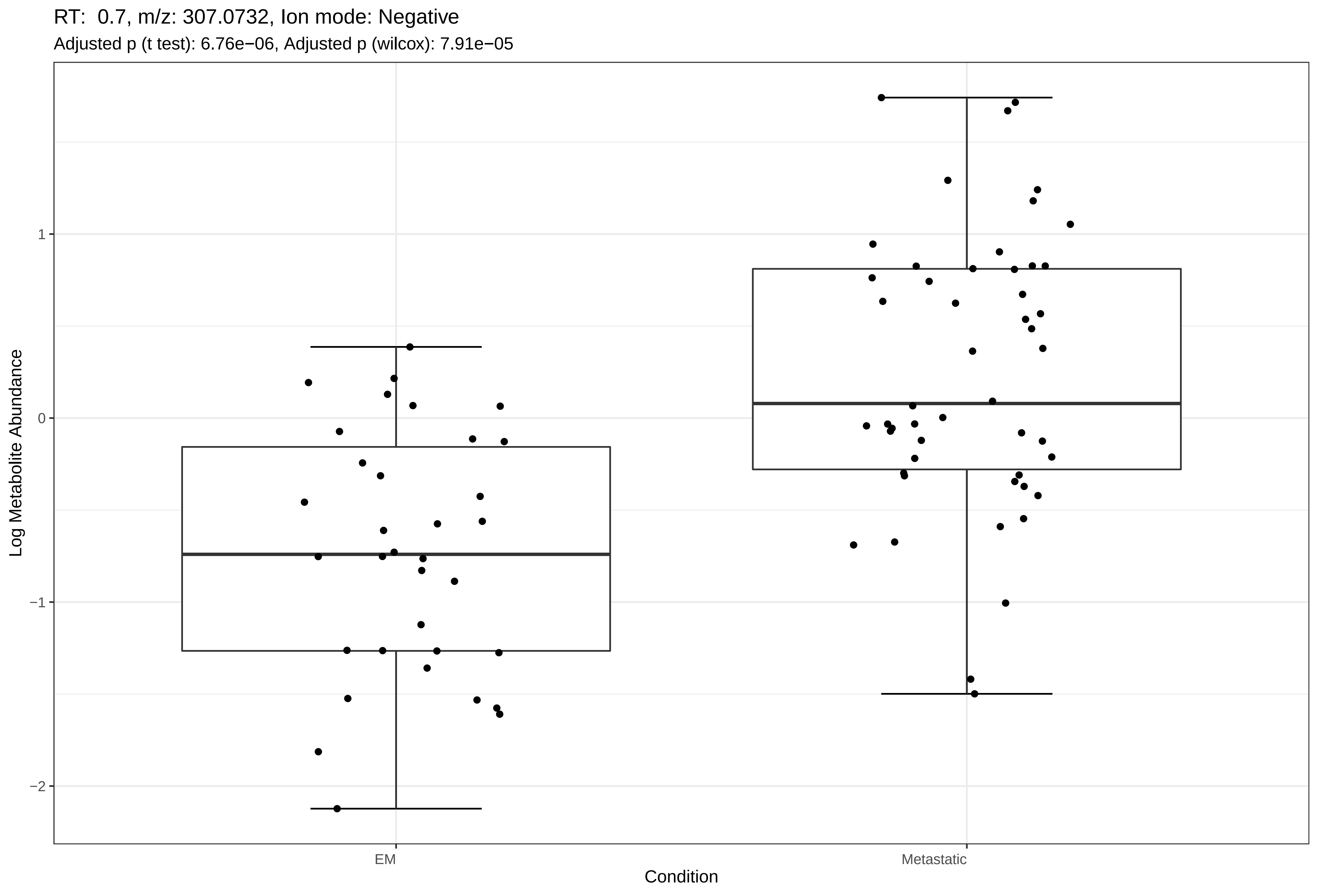

RT: 0.7, m/z: 307.0732, Ion mode: Negative
Adjusted p (t test): 6.76e−06, Adjusted p (wilcox): 7.91e−05
Log Metabolite Abundance
1
0
−1
−2
EM
Metastatic
Condition

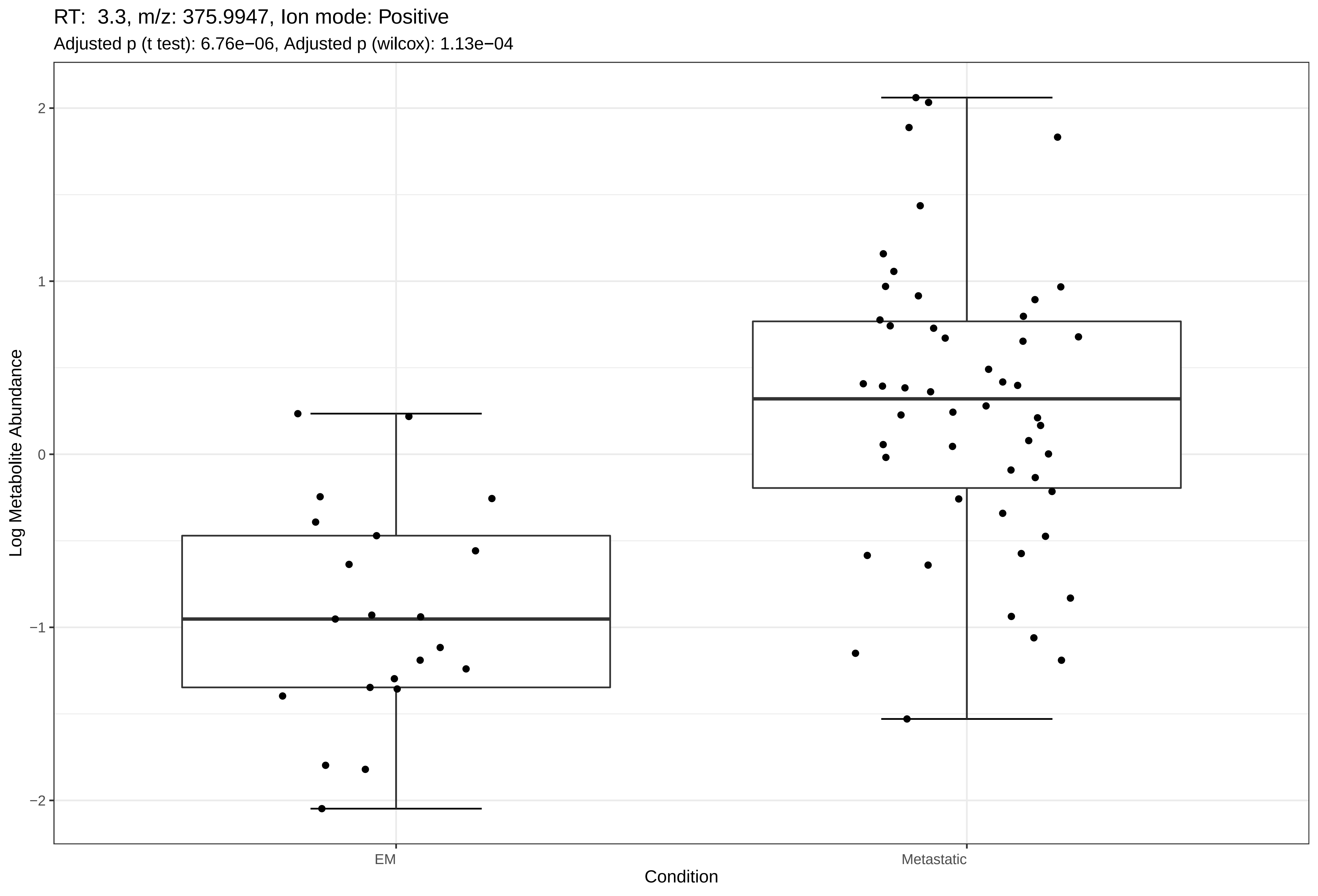
RT: 3.3, m/z: 375.9947, Ion mode: Positive
Adjusted p (t test): 6.76e−06, Adjusted p (wilcox): 1.13e−04
Log Metabolite Abundance
2
1
0
−1
−2
EM
Metastatic
Condition

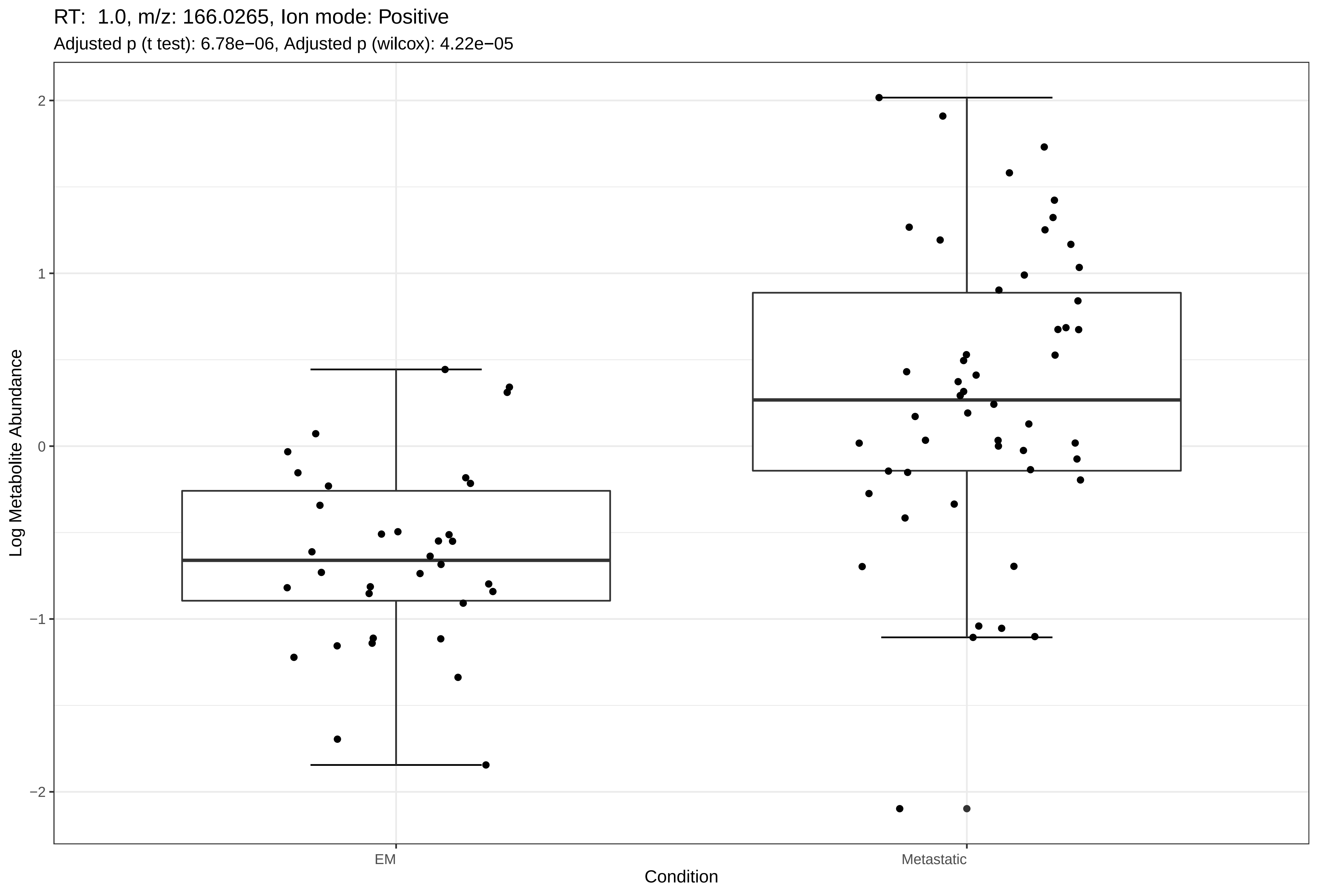
RT: 1.0, m/z: 166.0265, Ion mode: Positive
Adjusted p (t test): 6.78e−06, Adjusted p (wilcox): 4.22e−05
Log Metabolite Abundance
2
1
0
−1
−2
EM
Metastatic
Condition

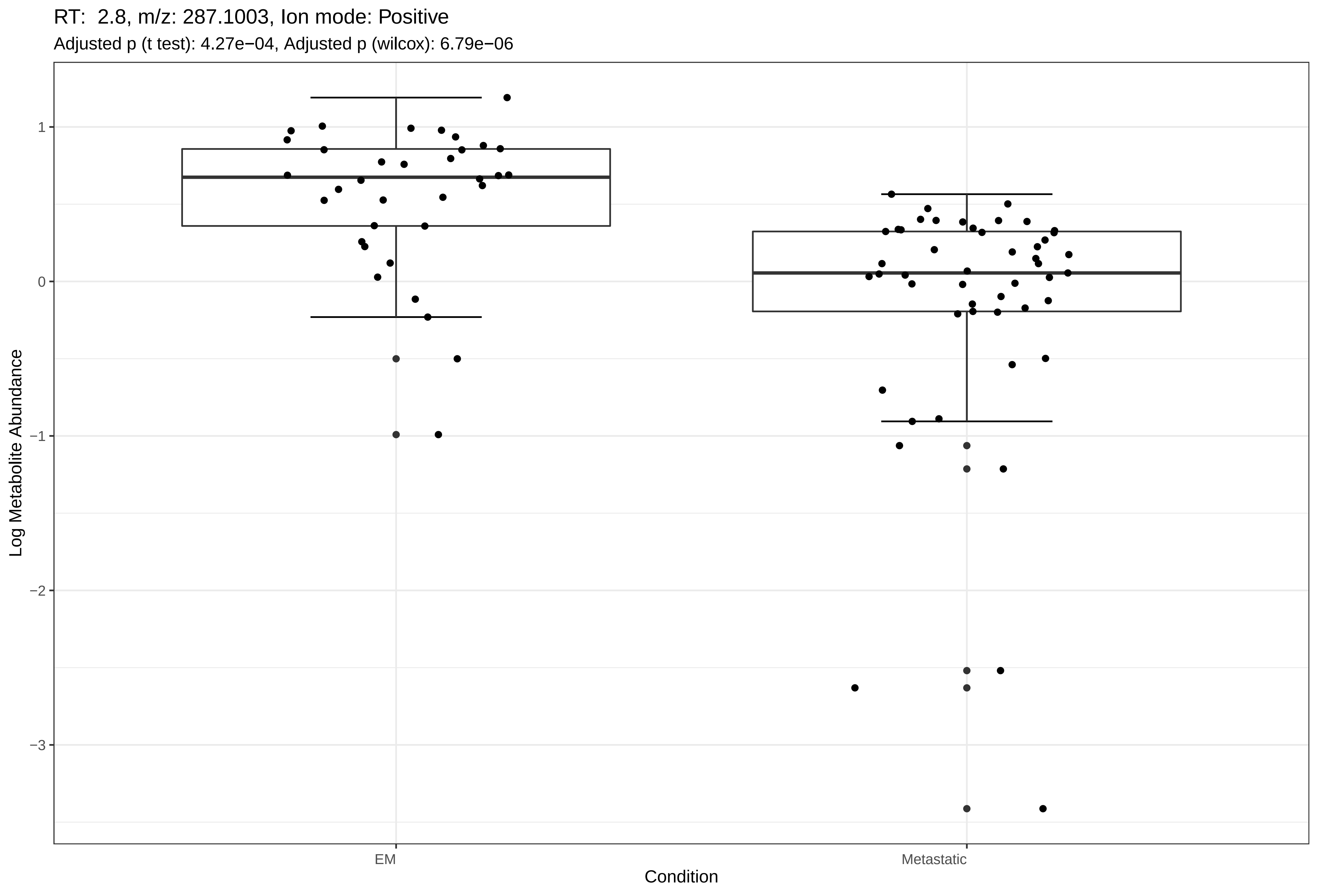
RT: 2.8, m/z: 287.1003, Ion mode: Positive
Adjusted p (t test): 4.27e−04, Adjusted p (wilcox): 6.79e−06
Log Metabolite Abundance
1
0
−1
−2
−3
EM
Metastatic
Condition

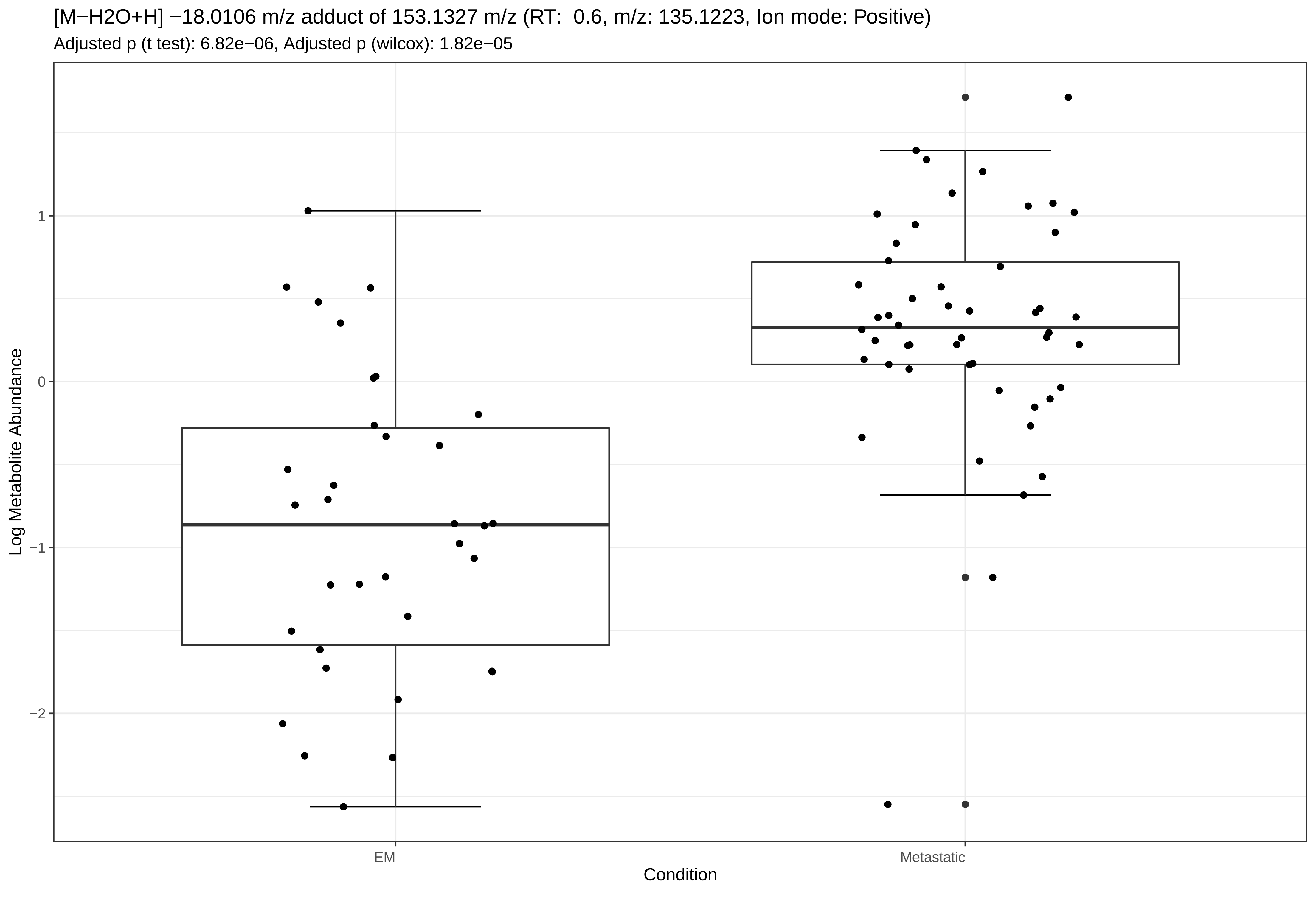

[M−H2O+H] −18.0106 m/z adduct of 153.1327 m/z (RT: 0.6, m/z: 135.1223, Ion mode: Positive)
Adjusted p (t test): 6.82e−06, Adjusted p (wilcox): 1.82e−05
Log Metabolite Abundance
1
0
−1
−2
EM
Metastatic
Condition

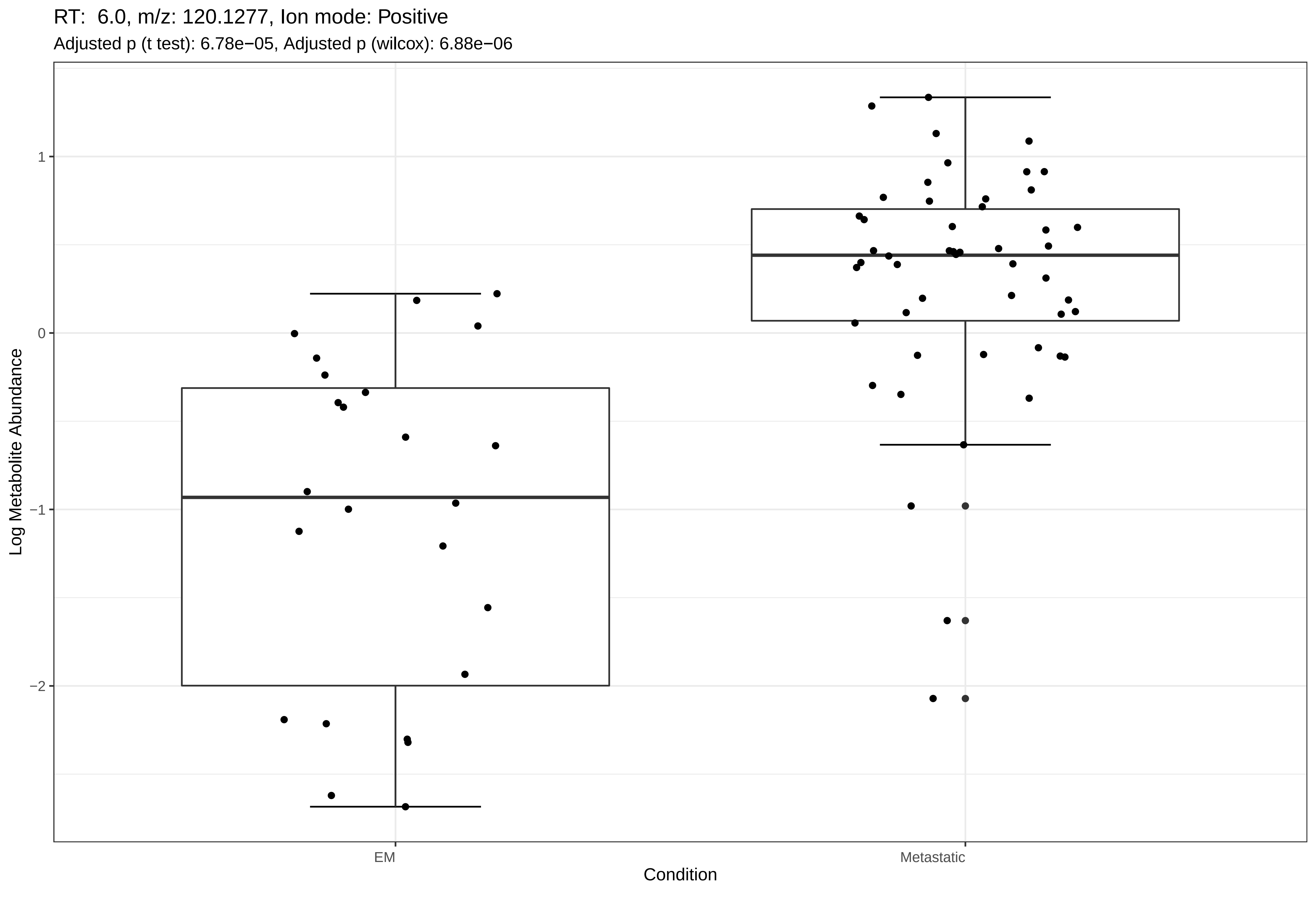

RT: 6.0, m/z: 120.1277, Ion mode: Positive
Adjusted p (t test): 6.78e−05, Adjusted p (wilcox): 6.88e−06
Log Metabolite Abundance
1
0
−1
−2
EM
Metastatic
Condition

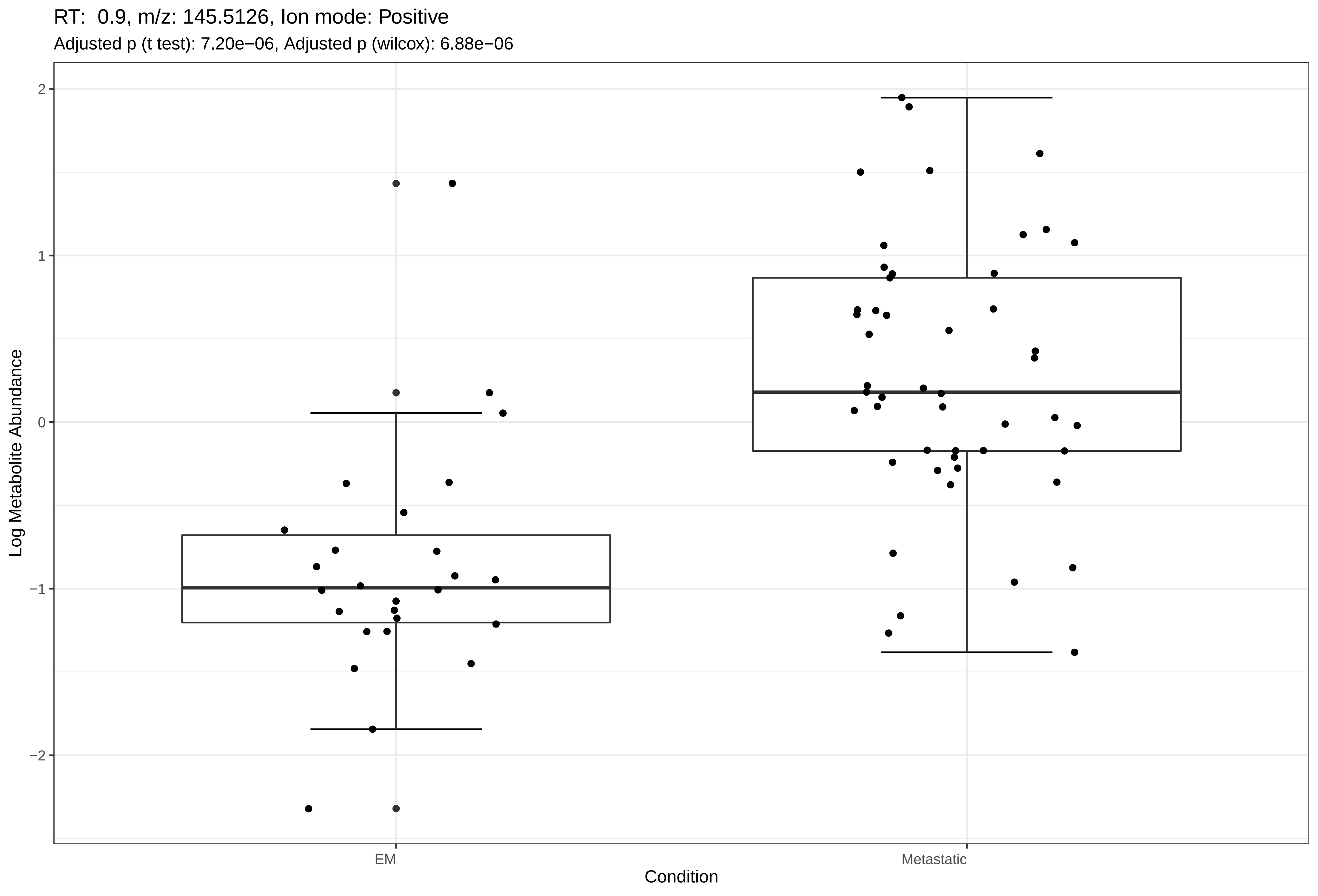
RT: 0.9, m/z: 145.5126, Ion mode: Positive
Adjusted p (t test): 7.20e−06, Adjusted p (wilcox): 6.88e−06
Log Metabolite Abundance
2
1
0
−1
−2
EM
Metastatic
Condition

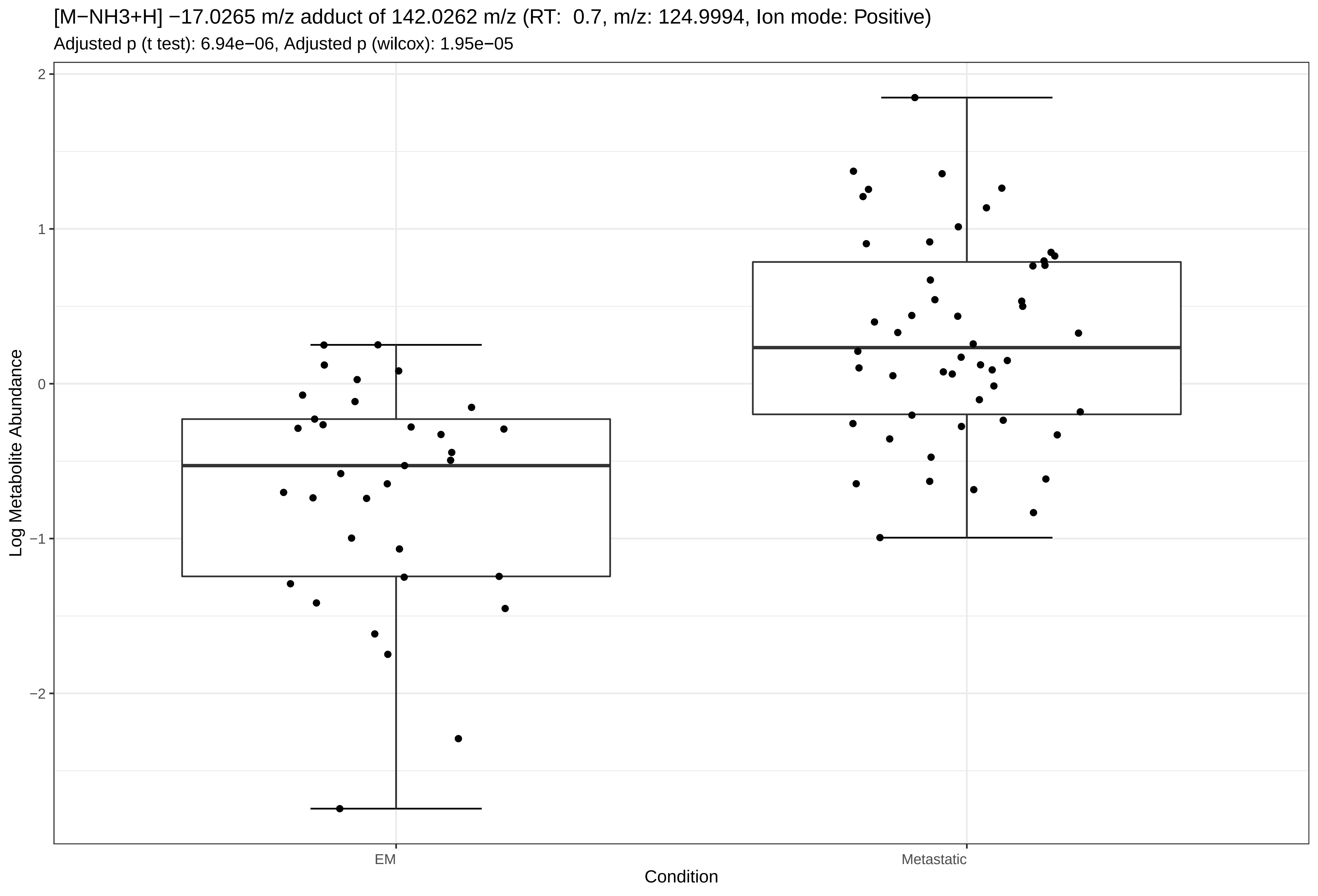

[M−NH3+H] −17.0265 m/z adduct of 142.0262 m/z (RT: 0.7, m/z: 124.9994, Ion mode: Positive)
Adjusted p (t test): 6.94e−06, Adjusted p (wilcox): 1.95e−05
2
1
0
−1
−2
Log Metabolite Abundance
EM
Metastatic
Condition

RT: 1.8, m/z: 177.5466, Ion mode: Positive

Adjusted p (t test): 6.98e−06, Adjusted p (wilcox): 3.52e−05

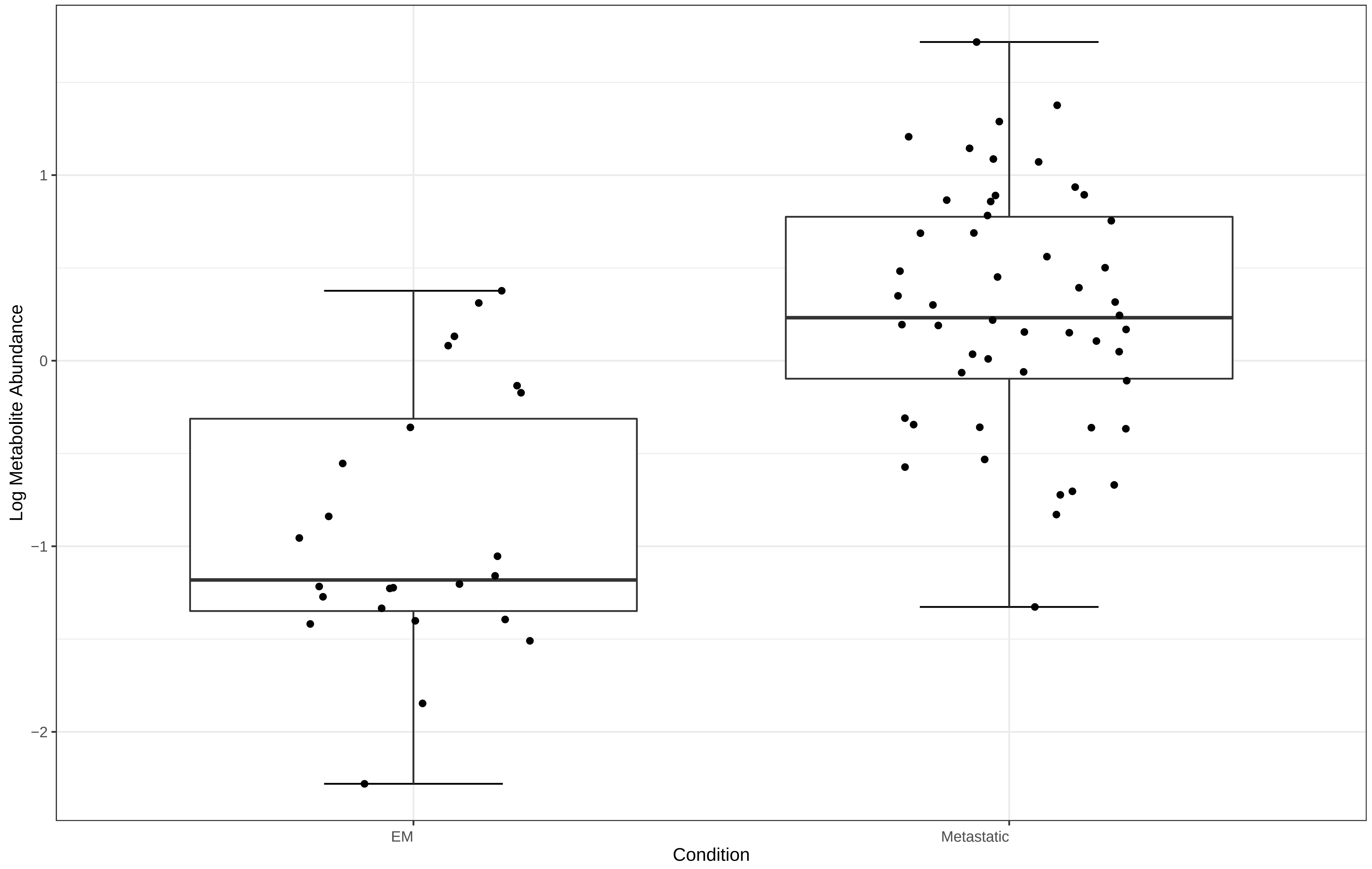

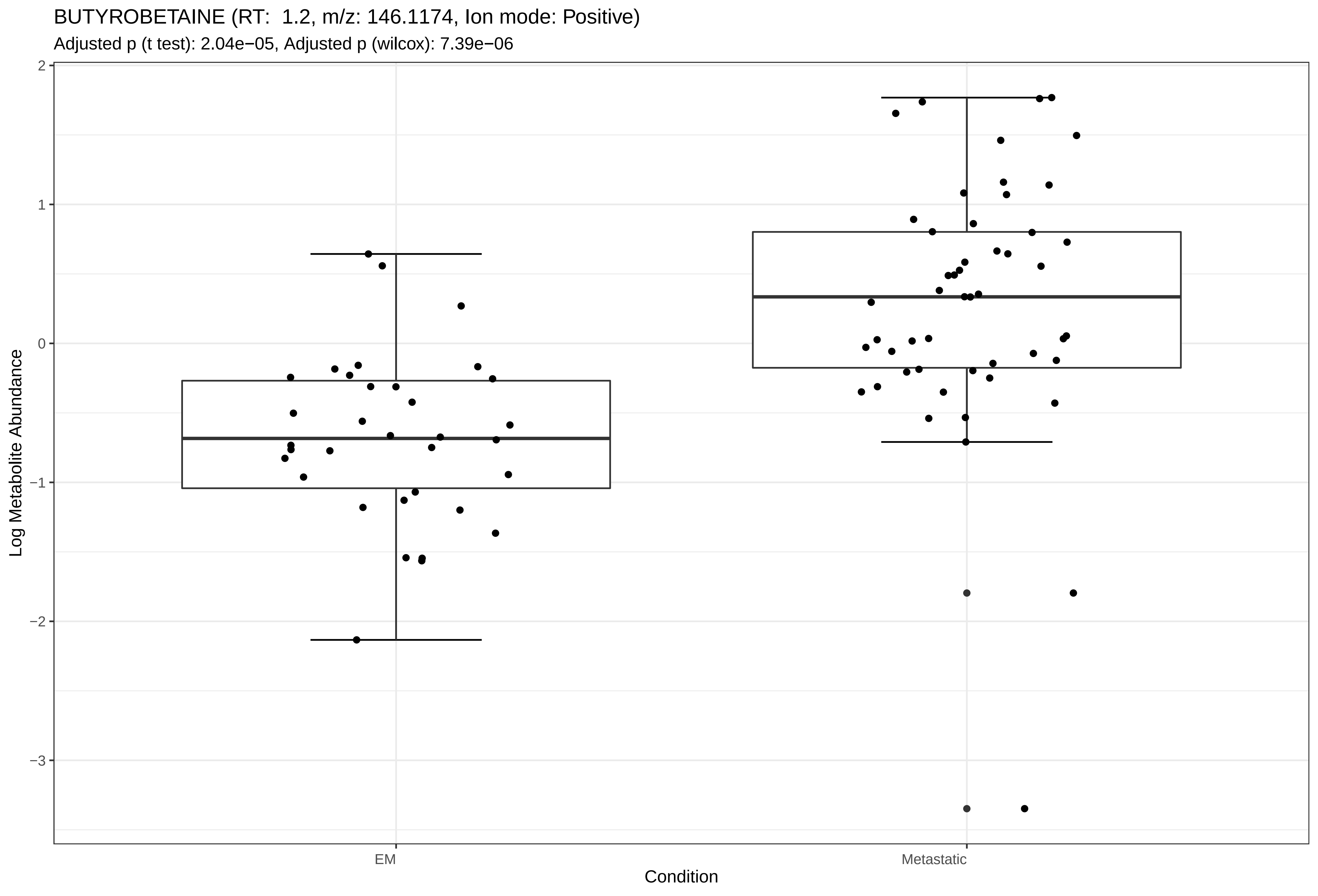

BUTYROBETAINE (RT: 1.2, m/z: 146.1174, Ion mode: Positive)
Adjusted p (t test): 2.04e−05, Adjusted p (wilcox): 7.39e−06
2
1
0
−1
−2
−3
Log Metabolite Abundance
EM
Metastatic
Condition

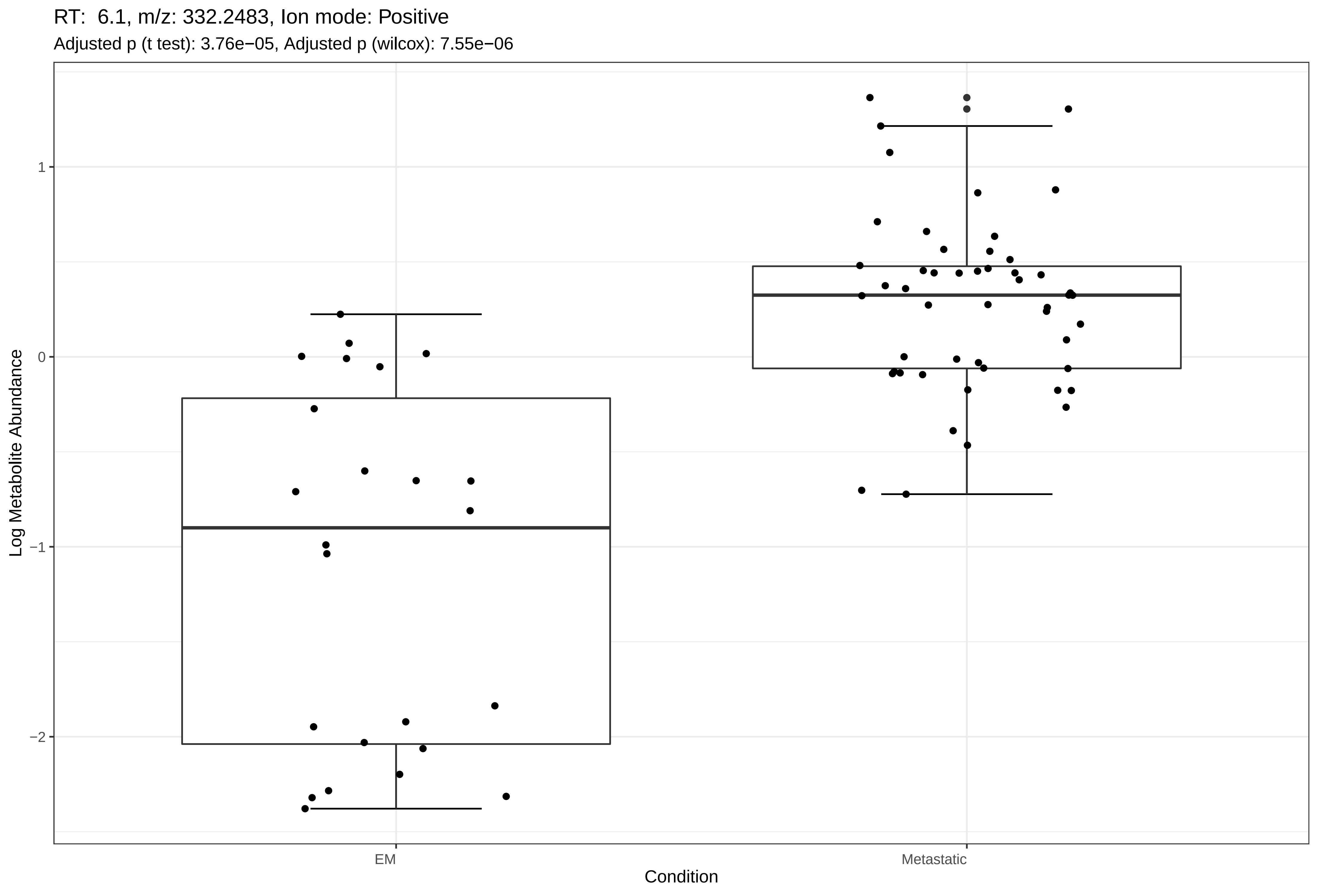
RT: 6.1, m/z: 332.2483, Ion mode: Positive
Adjusted p (t test): 3.76e−05, Adjusted p (wilcox): 7.55e−06
Log Metabolite Abundance
1
0
−1
−2
EM
Metastatic
Condition

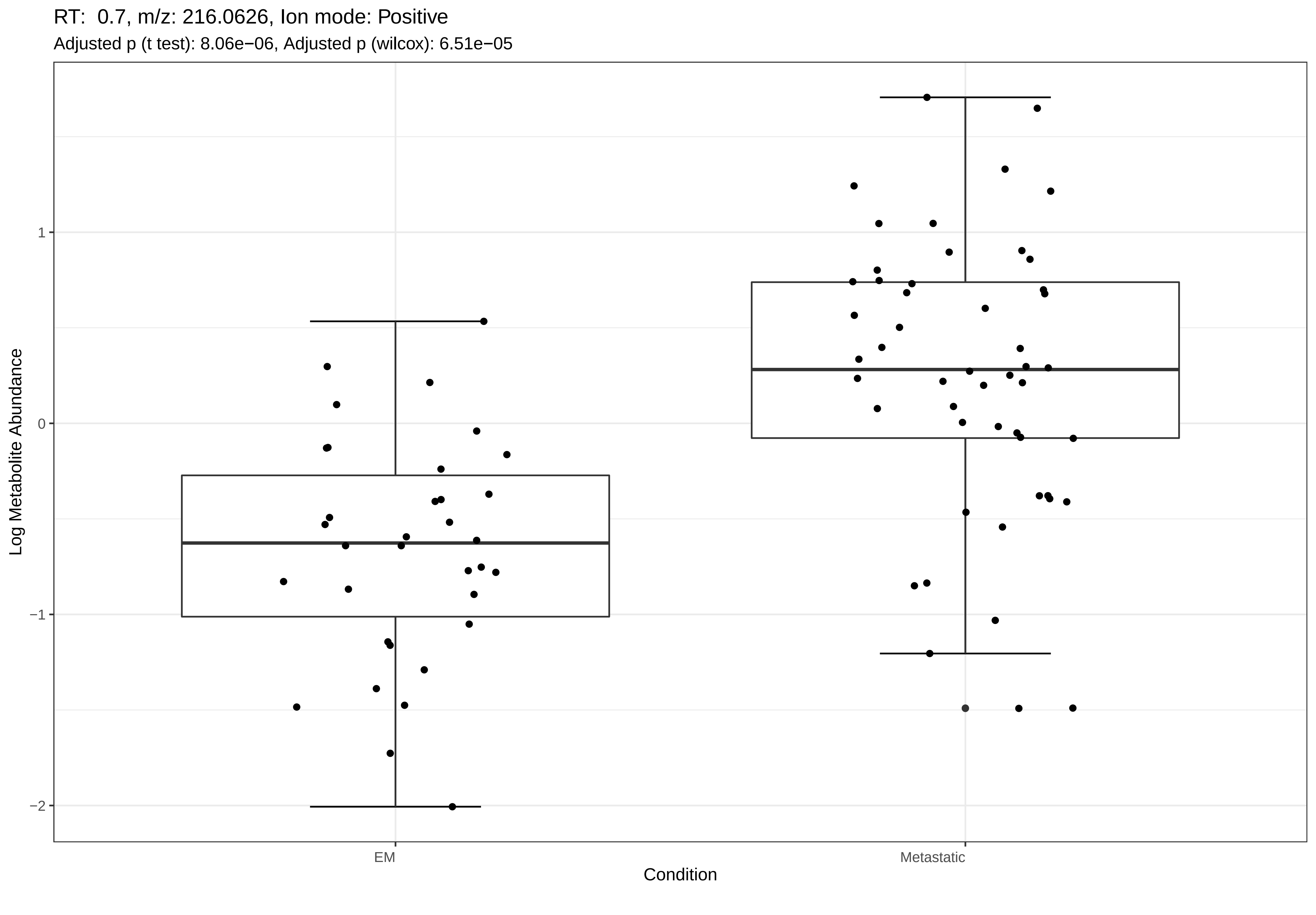

RT: 0.7, m/z: 216.0626, Ion mode: Positive
Adjusted p (t test): 8.06e−06, Adjusted p (wilcox): 6.51e−05
Log Metabolite Abundance
1
0
−1
−2
EM
Metastatic
Condition

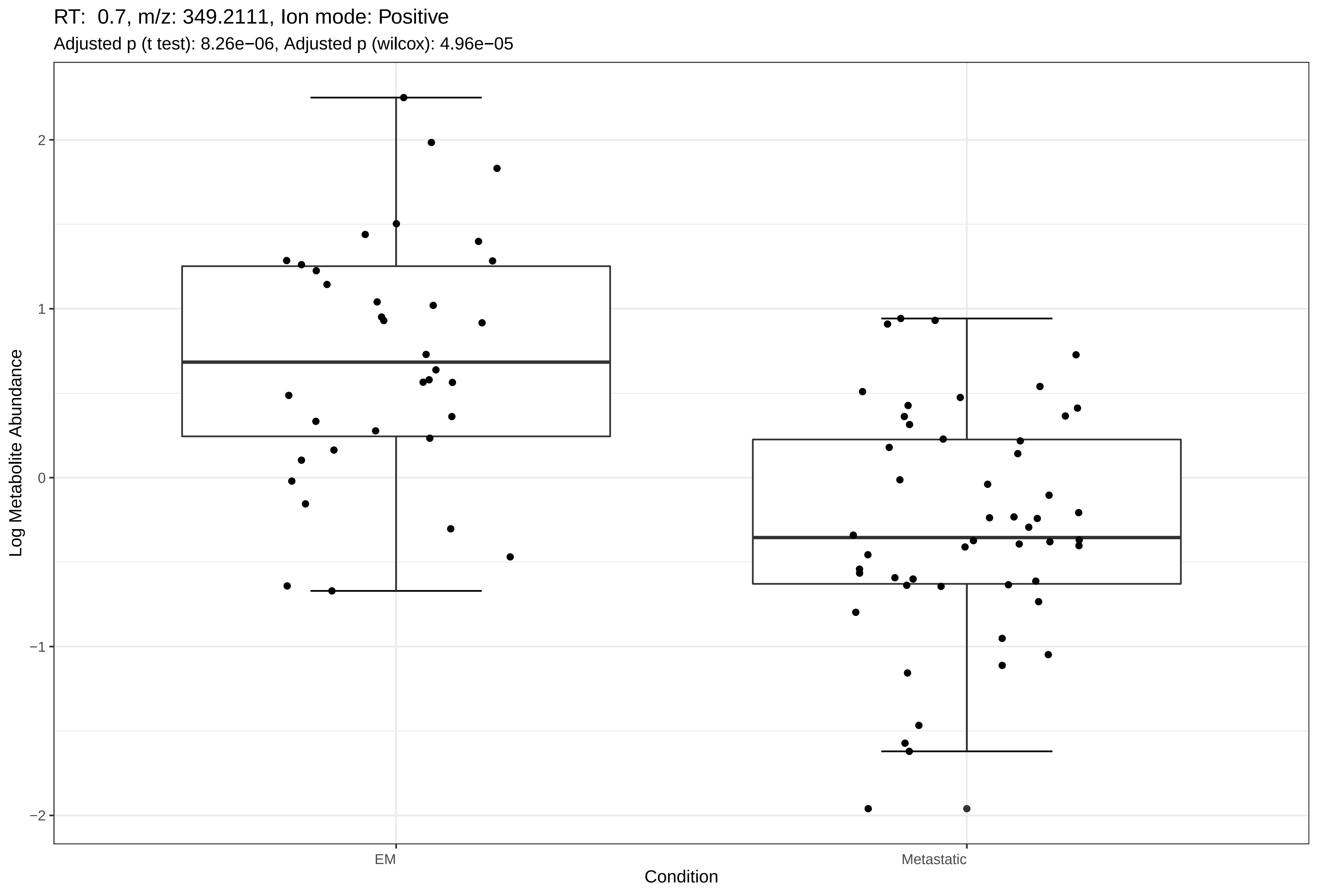
RT: 0.7, m/z: 349.2111, Ion mode: Positive
Adjusted p (t test): 8.26e−06, Adjusted p (wilcox): 4.96e−05
Log Metabolite Abundance
2
1
0
−1
−2
EM
Metastatic
Condition

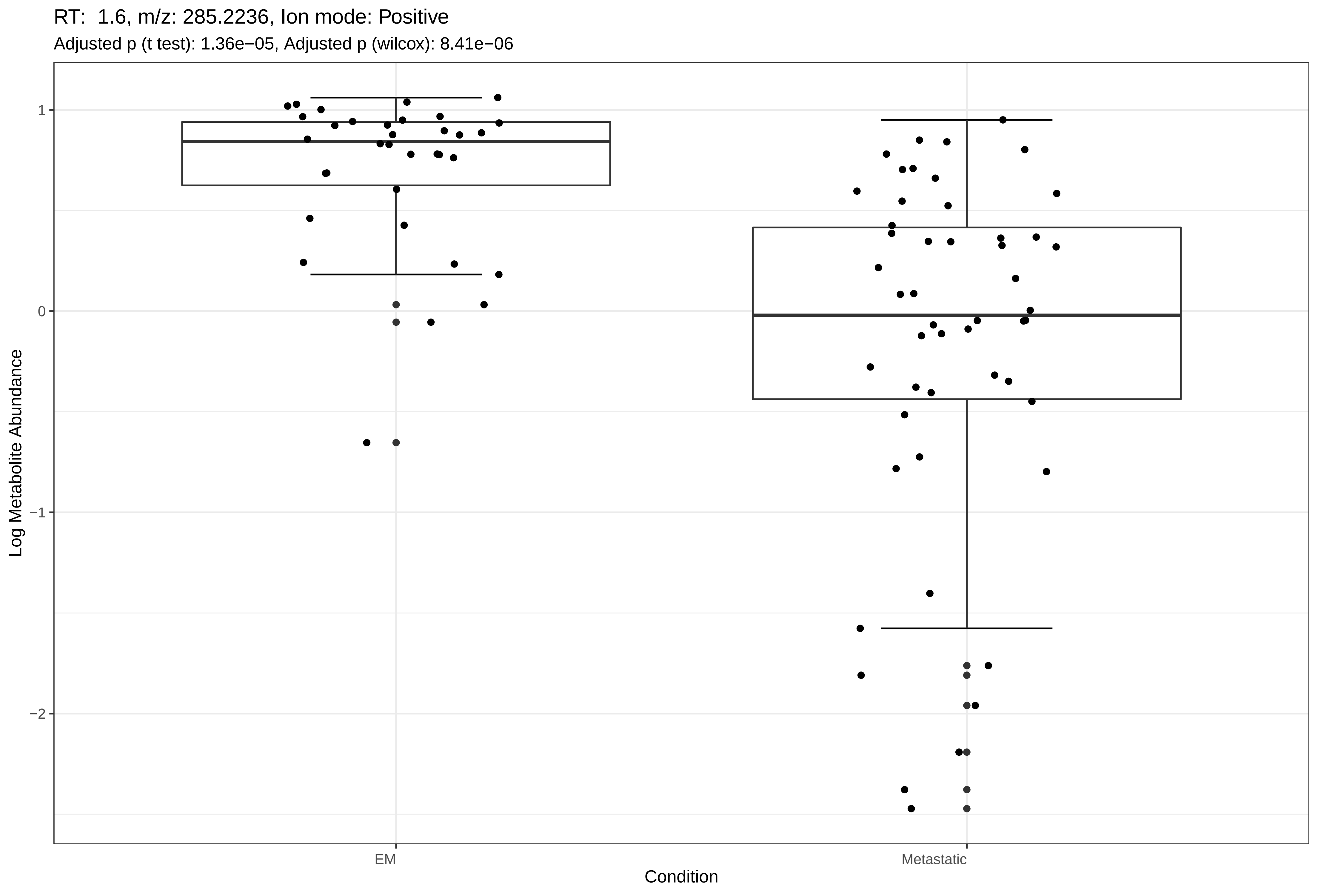

RT: 1.6, m/z: 285.2236, Ion mode: Positive
Adjusted p (t test): 1.36e−05, Adjusted p (wilcox): 8.41e−06
1
0
−1
−2
Log Metabolite Abundance
EM
Metastatic
Condition

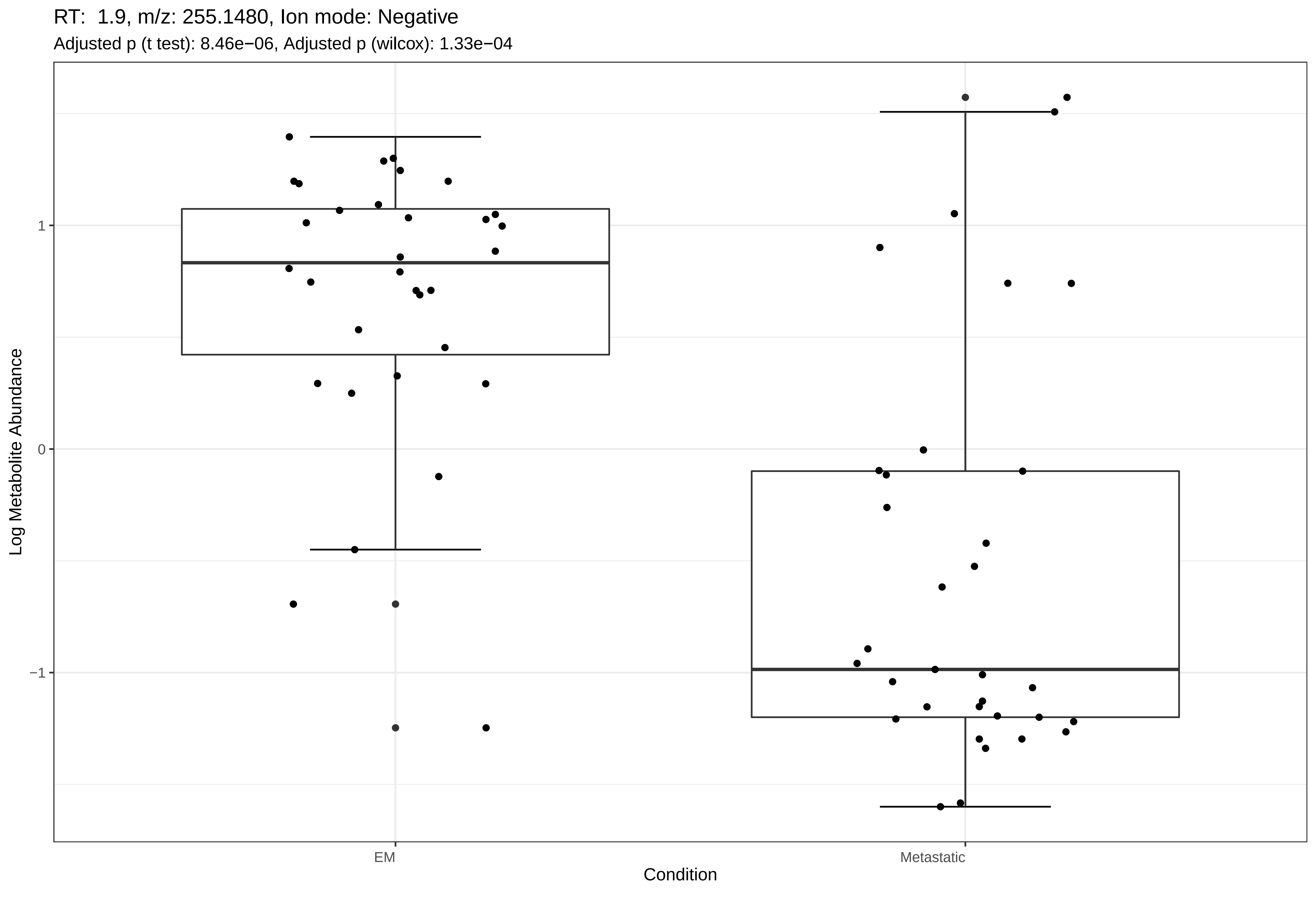
RT: 1.9, m/z: 255.1480, Ion mode: Negative
Adjusted p (t test): 8.46e−06, Adjusted p (wilcox): 1.33e−04
Log Metabolite Abundance
1
0
−1
EM
Metastatic
Condition

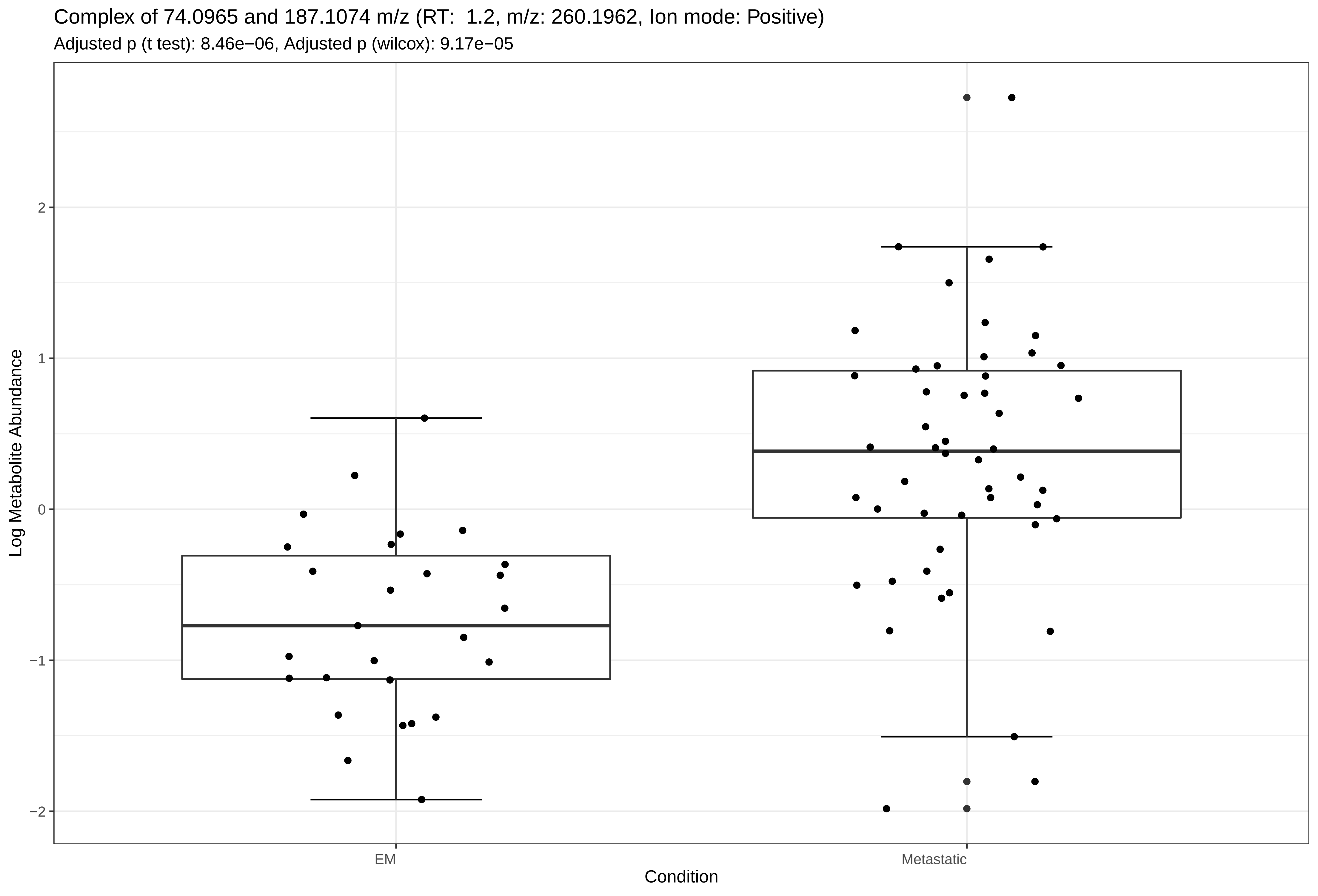

Complex of 74.0965 and 187.1074 m/z (RT: 1.2, m/z: 260.1962, Ion mode: Positive)
Adjusted p (t test): 8.46e−06, Adjusted p (wilcox): 9.17e−05
Log Metabolite Abundance
2
1
0
−1
−2
EM
Metastatic
Condition

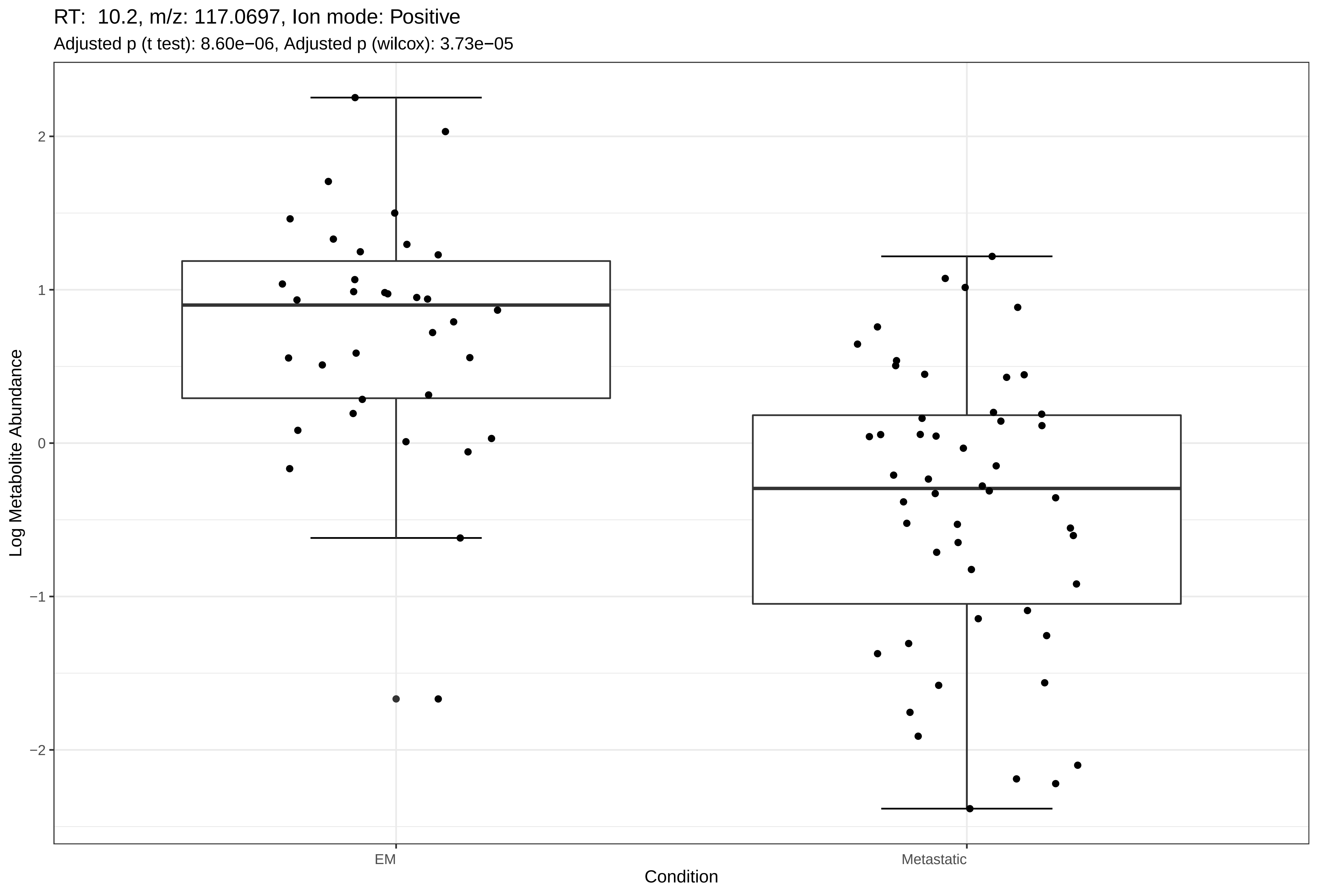
RT: 10.2, m/z: 117.0697, Ion mode: Positive
Adjusted p (t test): 8.60e−06, Adjusted p (wilcox): 3.73e−05
Log Metabolite Abundance
2
1
0
−1
−2
EM
Metastatic
Condition

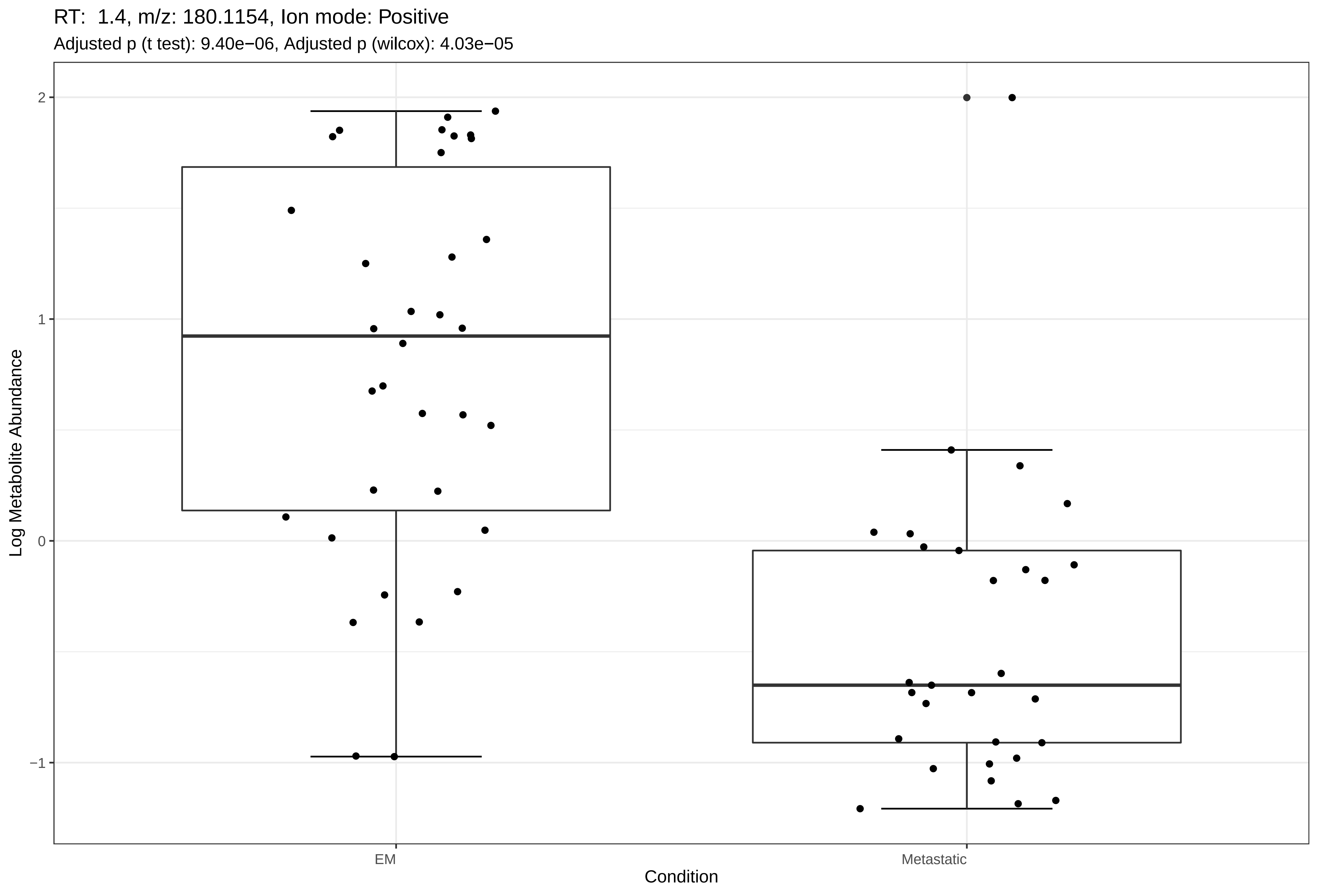

RT: 1.4, m/z: 180.1154, Ion mode: Positive
Adjusted p (t test): 9.40e−06, Adjusted p (wilcox): 4.03e−05
Log Metabolite Abundance
2
1
0
−1
EM
Metastatic
Condition

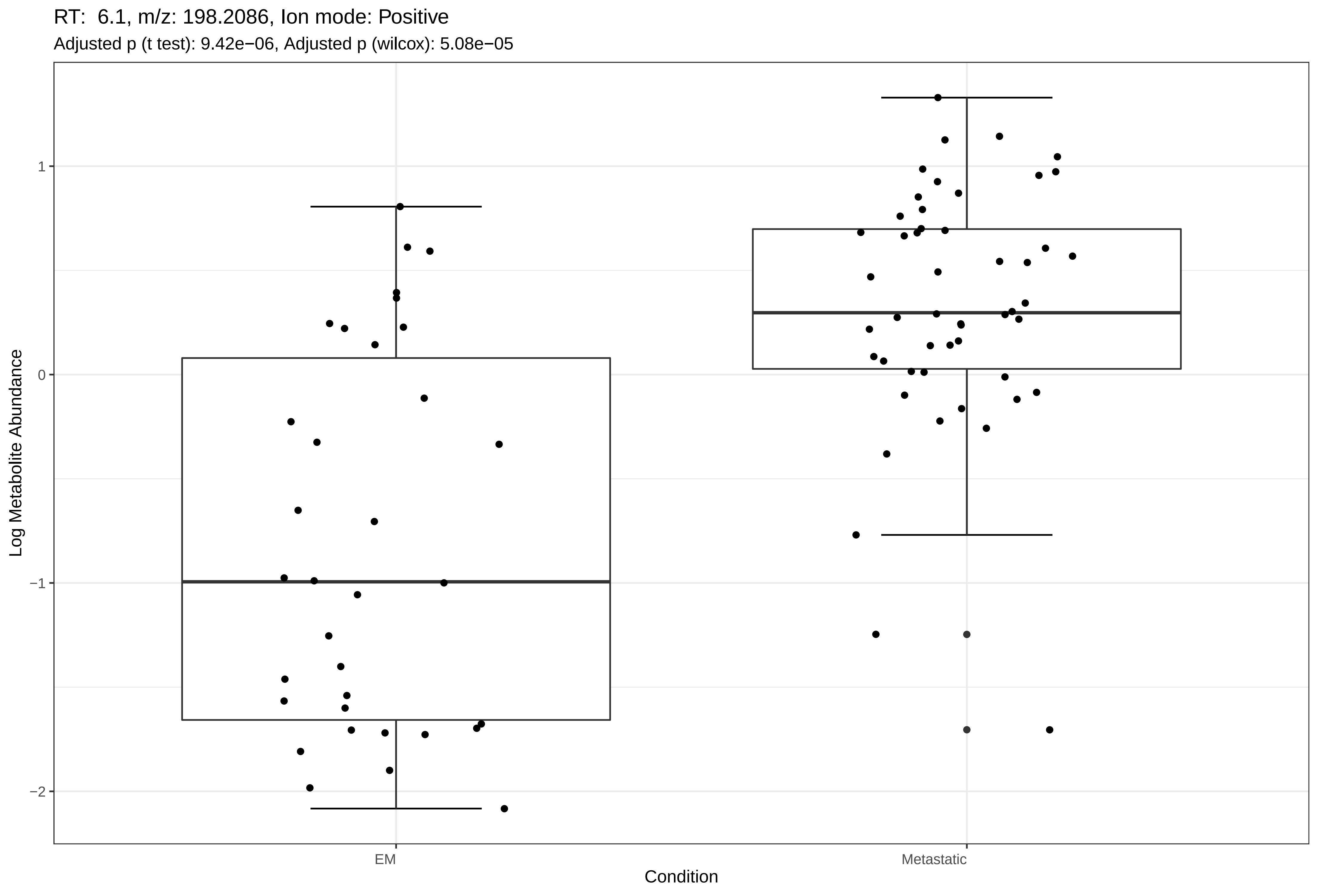
RT: 6.1, m/z: 198.2086, Ion mode: Positive
Adjusted p (t test): 9.42e−06, Adjusted p (wilcox): 5.08e−05
Log Metabolite Abundance
1
0
−1
−2
EM
Metastatic
Condition

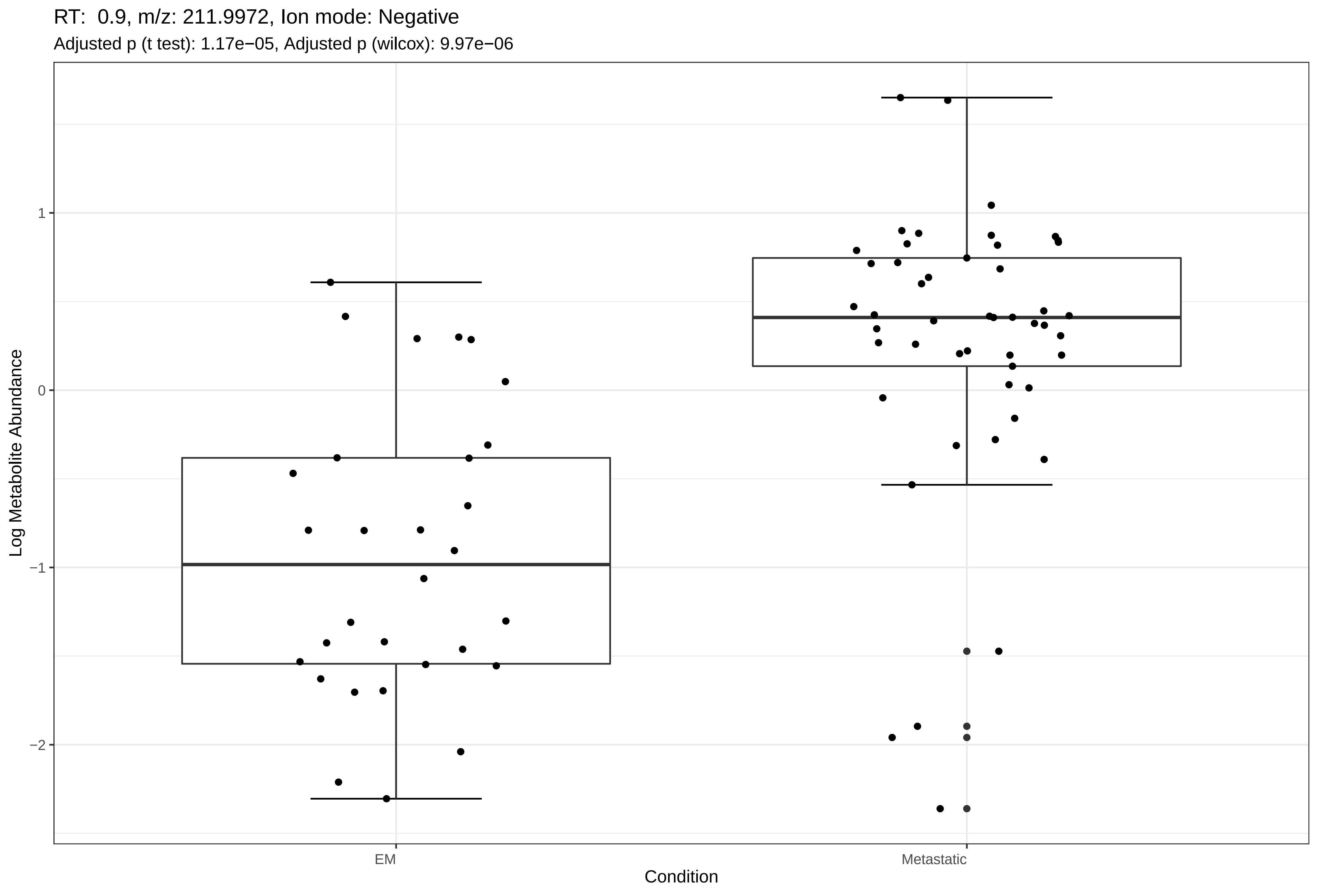
RT: 0.9, m/z: 211.9972, Ion mode: Negative
Adjusted p (t test): 1.17e−05, Adjusted p (wilcox): 9.97e−06
Log Metabolite Abundance
1
0
−1
−2
EM
Metastatic
Condition

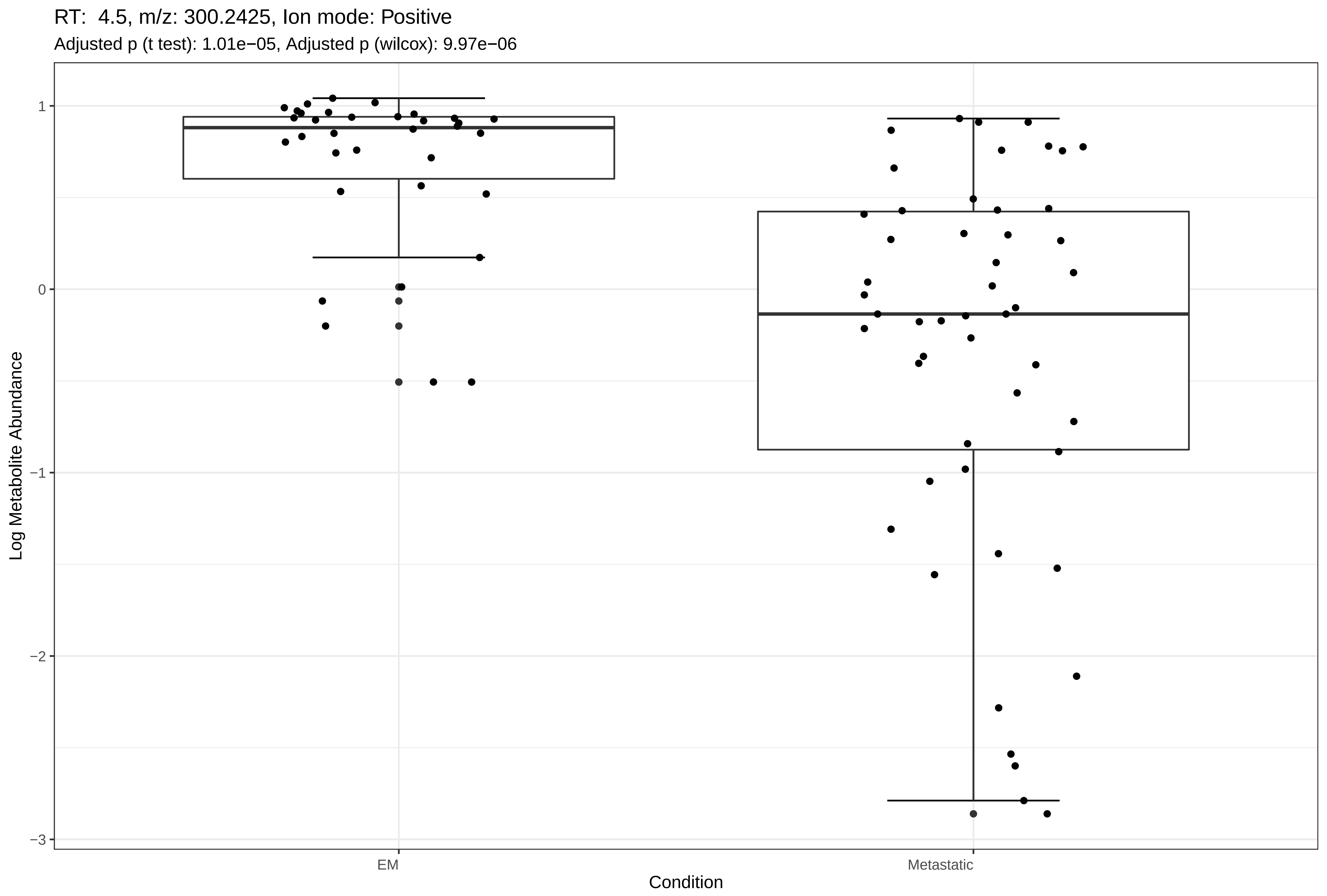

RT: 4.5, m/z: 300.2425, Ion mode: Positive
Adjusted p (t test): 1.01e−05, Adjusted p (wilcox): 9.97e−06
Log Metabolite Abundance
1
0
−1
−2
−3
EM
Metastatic
Condition

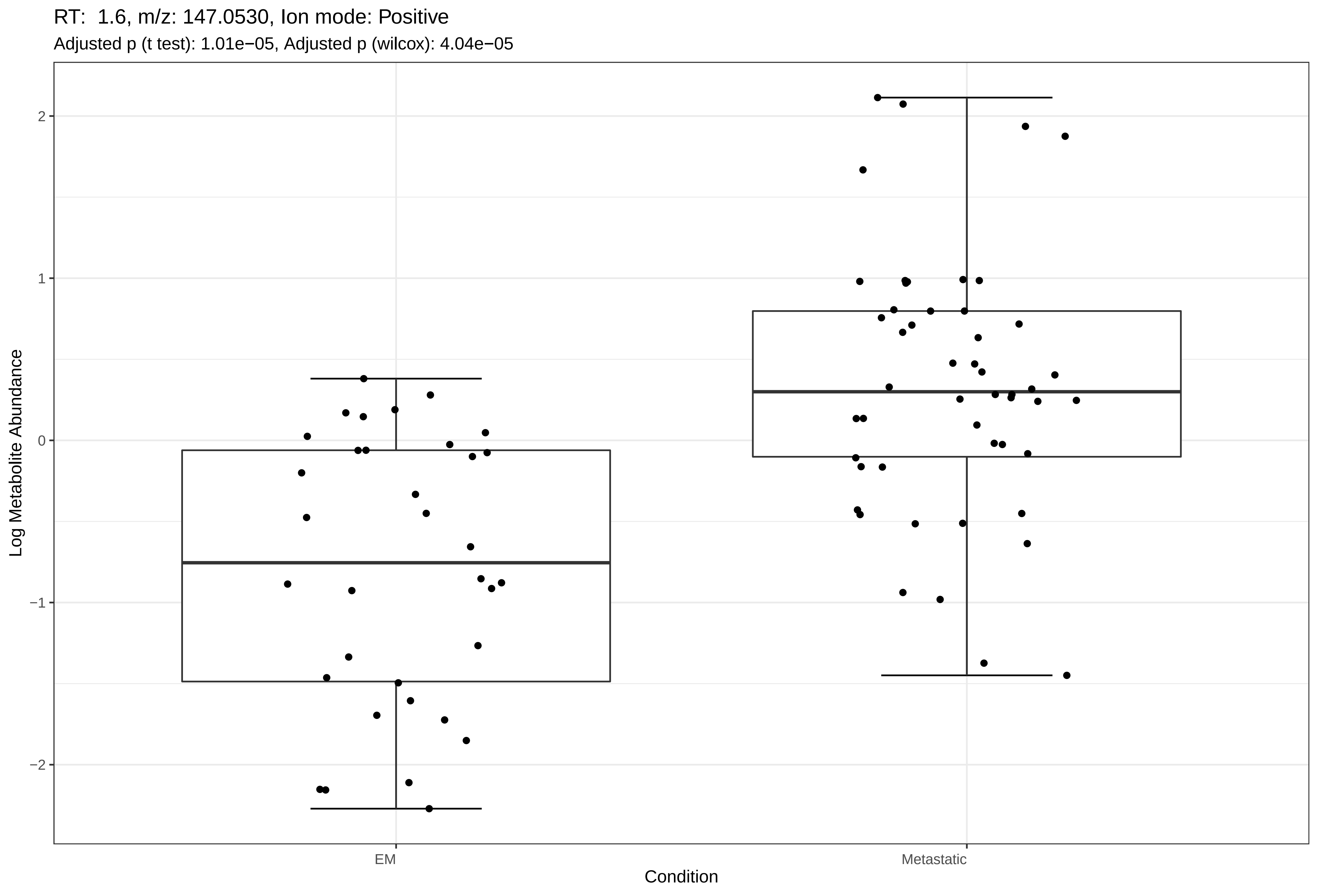
RT: 1.6, m/z: 147.0530, Ion mode: Positive
Adjusted p (t test): 1.01e−05, Adjusted p (wilcox): 4.04e−05
Log Metabolite Abundance
2
1
0
−1
−2
EM
Metastatic
Condition

RT: 2.1, m/z: 212.0523, Ion mode: Positive

Adjusted p (t test): 1.05e−05, Adjusted p (wilcox): 1.82e−05

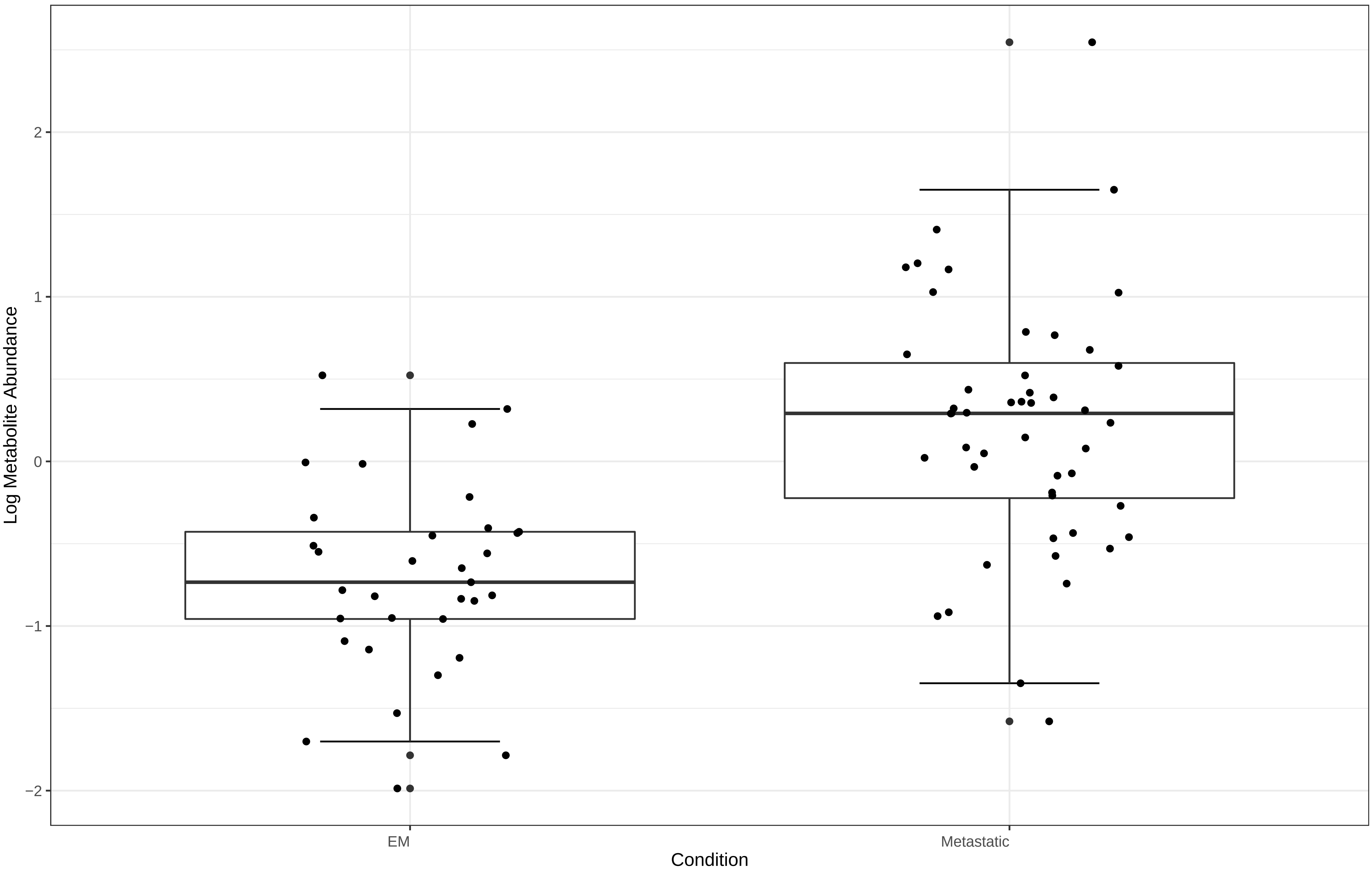

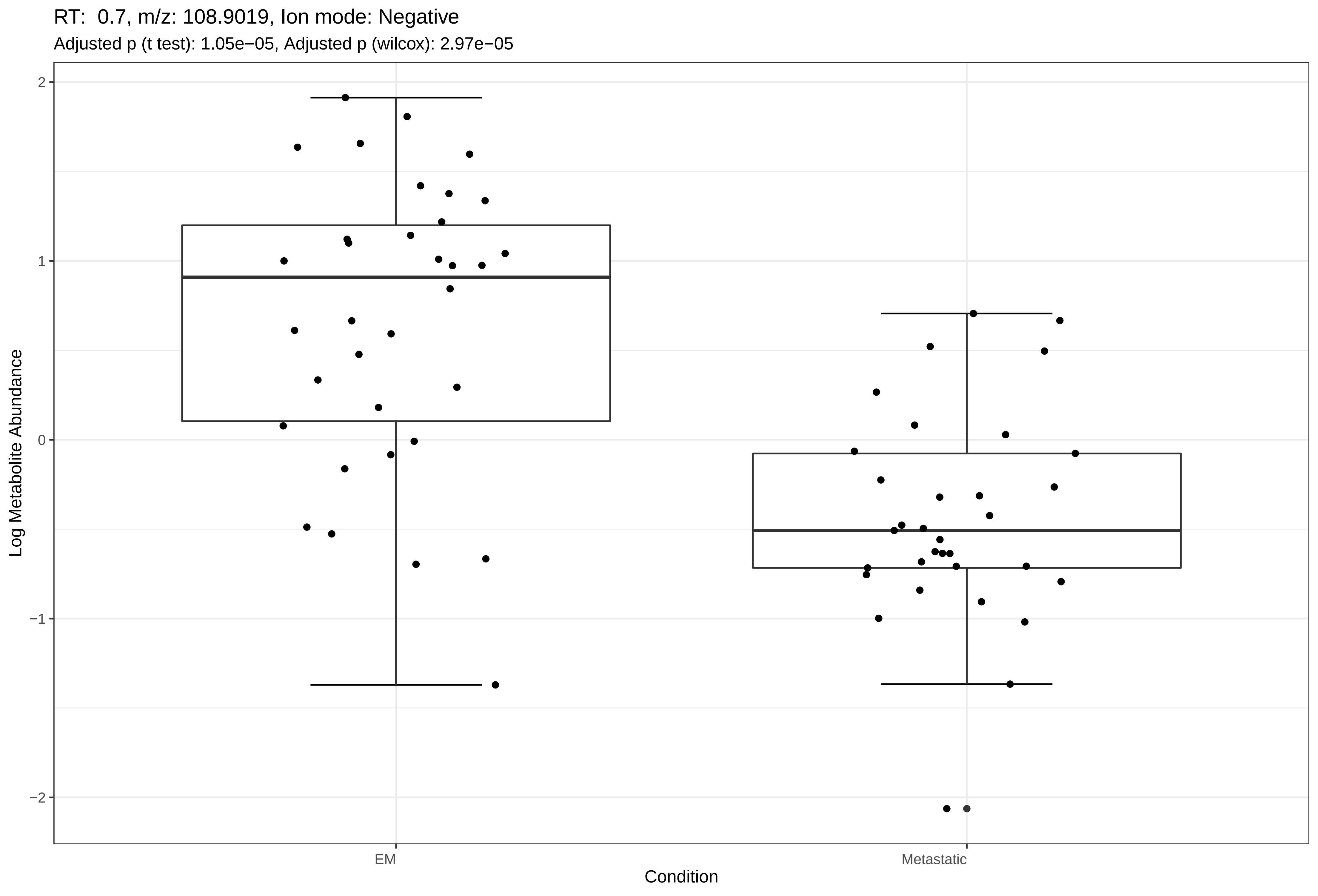

RT: 0.7, m/z: 108.9019, Ion mode: Negative
Adjusted p (t test): 1.05e−05, Adjusted p (wilcox): 2.97e−05
Log Metabolite Abundance
2
1
0
−1
−2
EM
Metastatic
Condition

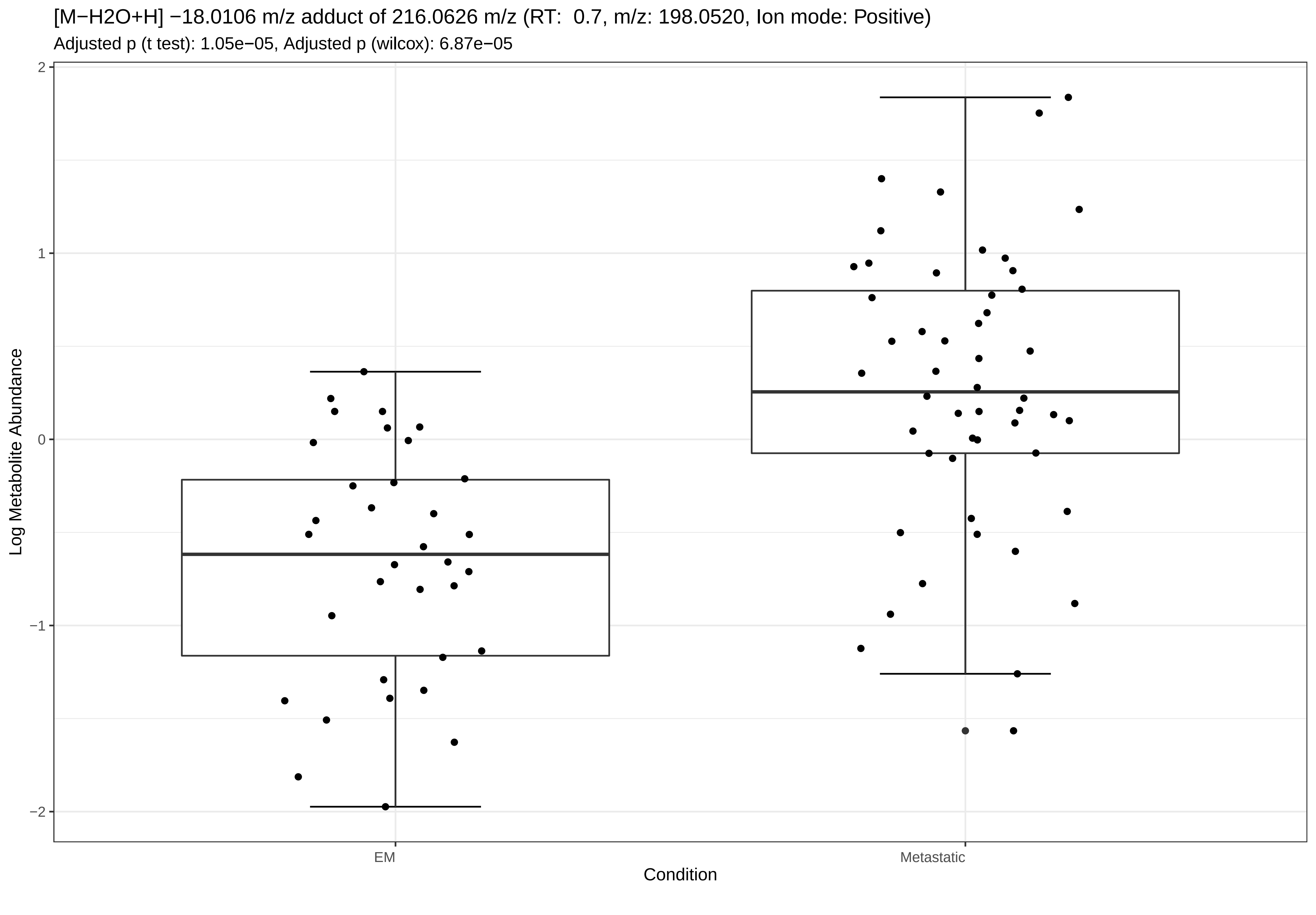

[M−H2O+H] −18.0106 m/z adduct of 216.0626 m/z (RT: 0.7, m/z: 198.0520, Ion mode: Positive)
Adjusted p (t test): 1.05e−05, Adjusted p (wilcox): 6.87e−05
Log Metabolite Abundance
2
1
0
−1
−2
EM
Metastatic
Condition

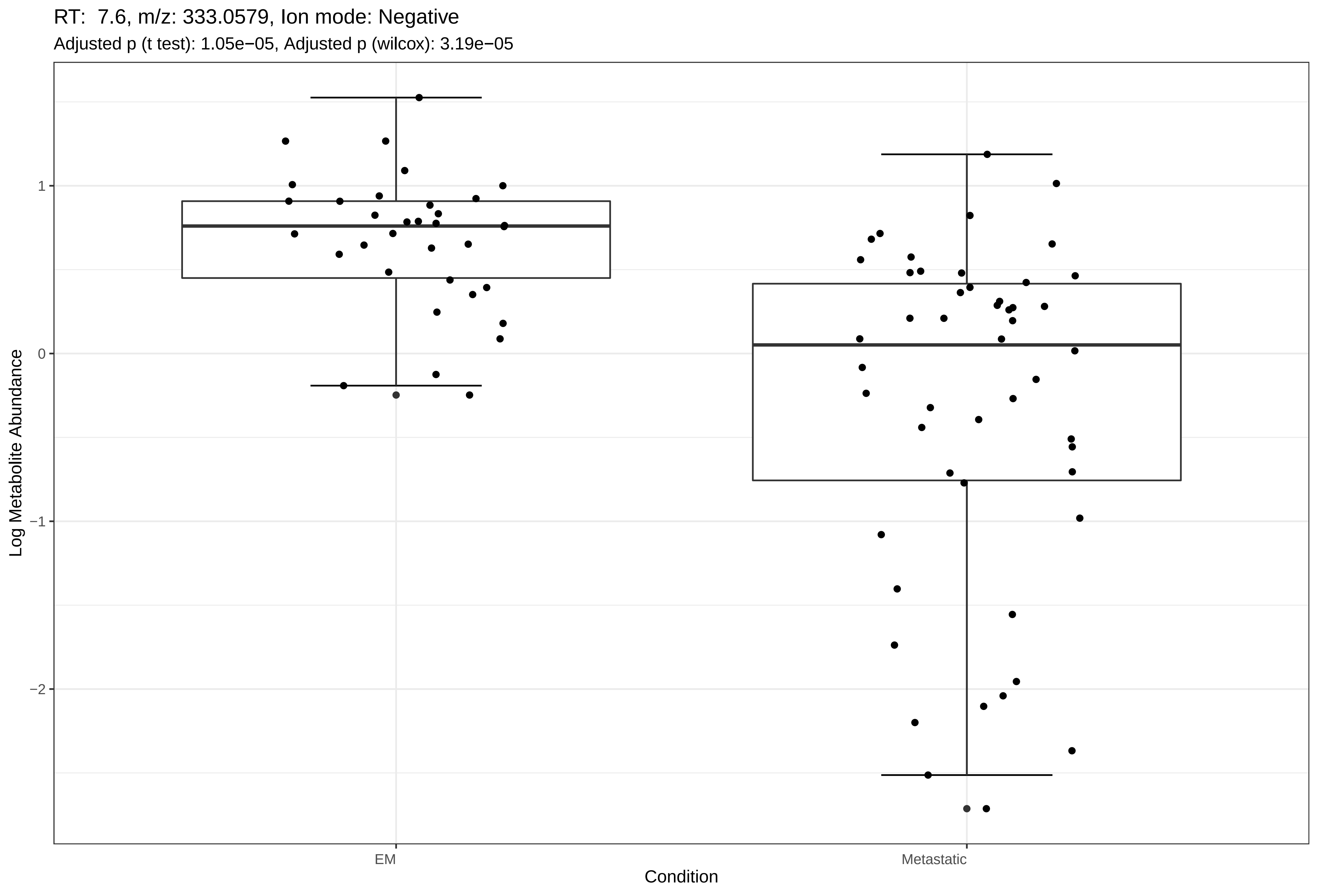

RT: 7.6, m/z: 333.0579, Ion mode: Negative
Adjusted p (t test): 1.05e−05, Adjusted p (wilcox): 3.19e−05
Log Metabolite Abundance
1
0
−1
−2
EM
Metastatic
Condition

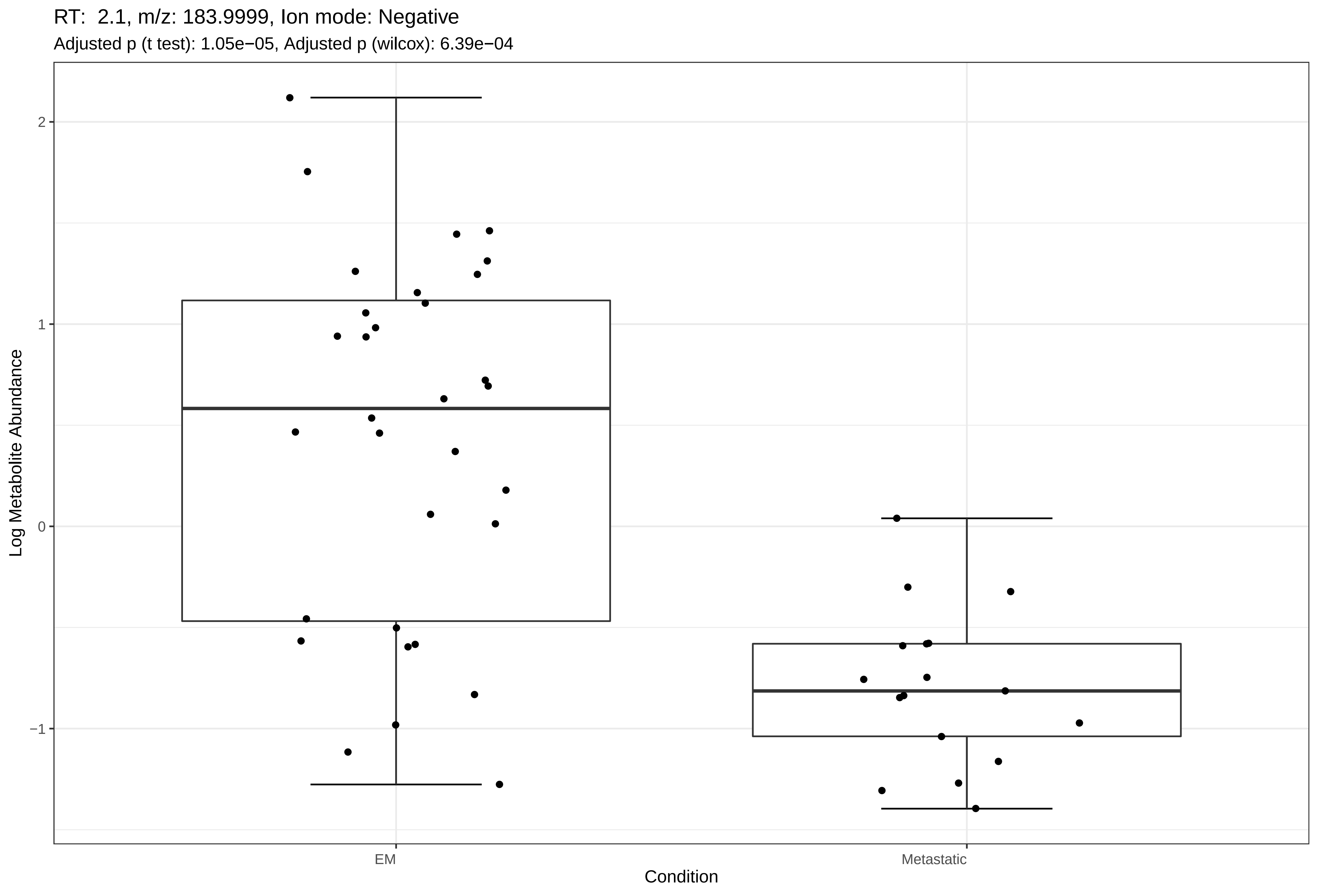

RT: 2.1, m/z: 183.9999, Ion mode: Negative
Adjusted p (t test): 1.05e−05, Adjusted p (wilcox): 6.39e−04
Log Metabolite Abundance
2
1
0
−1
EM
Metastatic
Condition

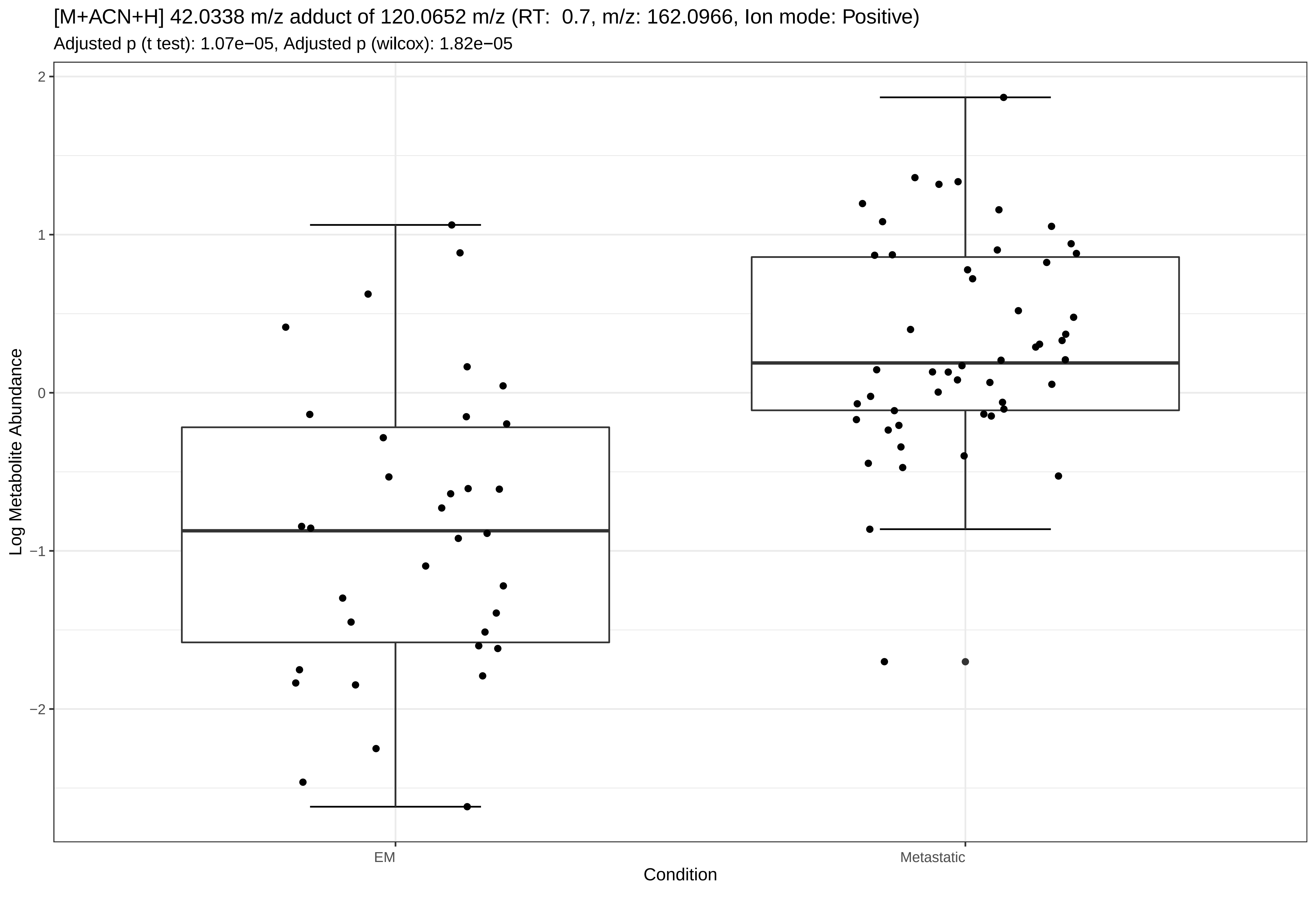
[M+ACN+H] 42.0338 m/z adduct of 120.0652 m/z (RT: 0.7, m/z: 162.0966, Ion mode: Positive)
Adjusted p (t test): 1.07e−05, Adjusted p (wilcox): 1.82e−05
Log Metabolite Abundance
2
1
0
−1
−2
EM
Metastatic
Condition

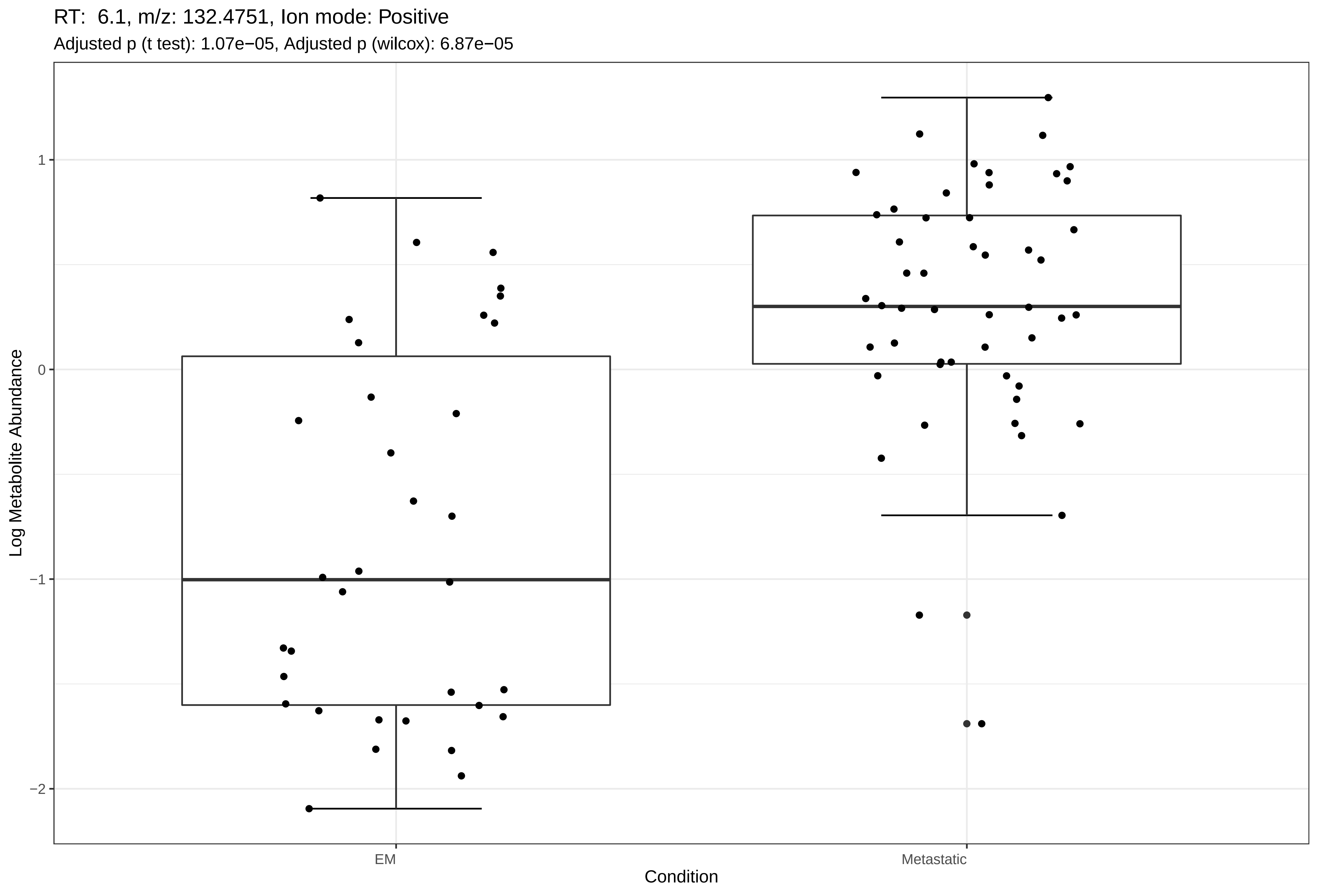
RT: 6.1, m/z: 132.4751, Ion mode: Positive
Adjusted p (t test): 1.07e−05, Adjusted p (wilcox): 6.87e−05
Log Metabolite Abundance
1
0
−1
−2
EM
Metastatic
Condition

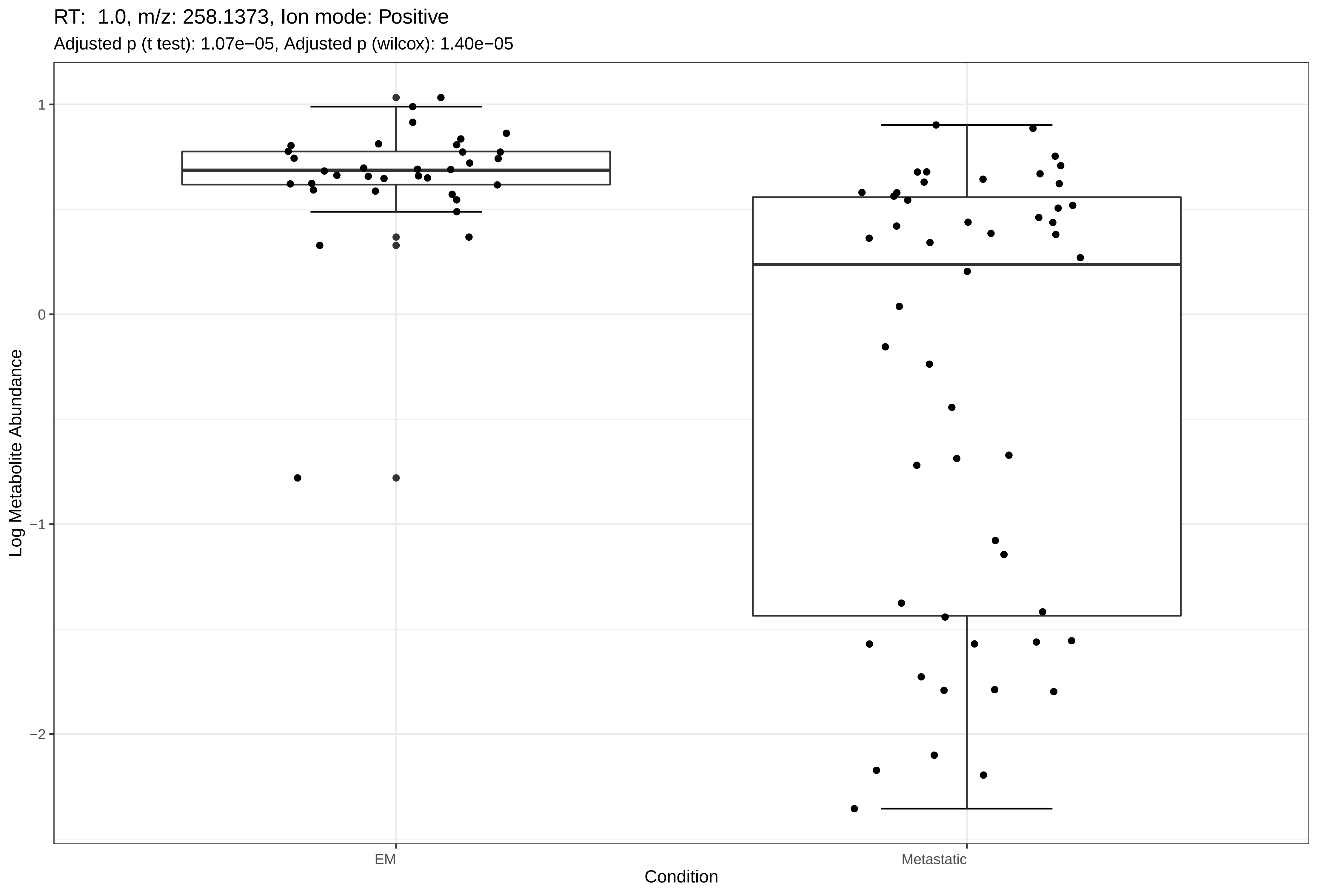
RT: 1.0, m/z: 258.1373, Ion mode: Positive
Adjusted p (t test): 1.07e−05, Adjusted p (wilcox): 1.40e−05
Log Metabolite Abundance
1
0
−1
−2
EM
Metastatic
Condition

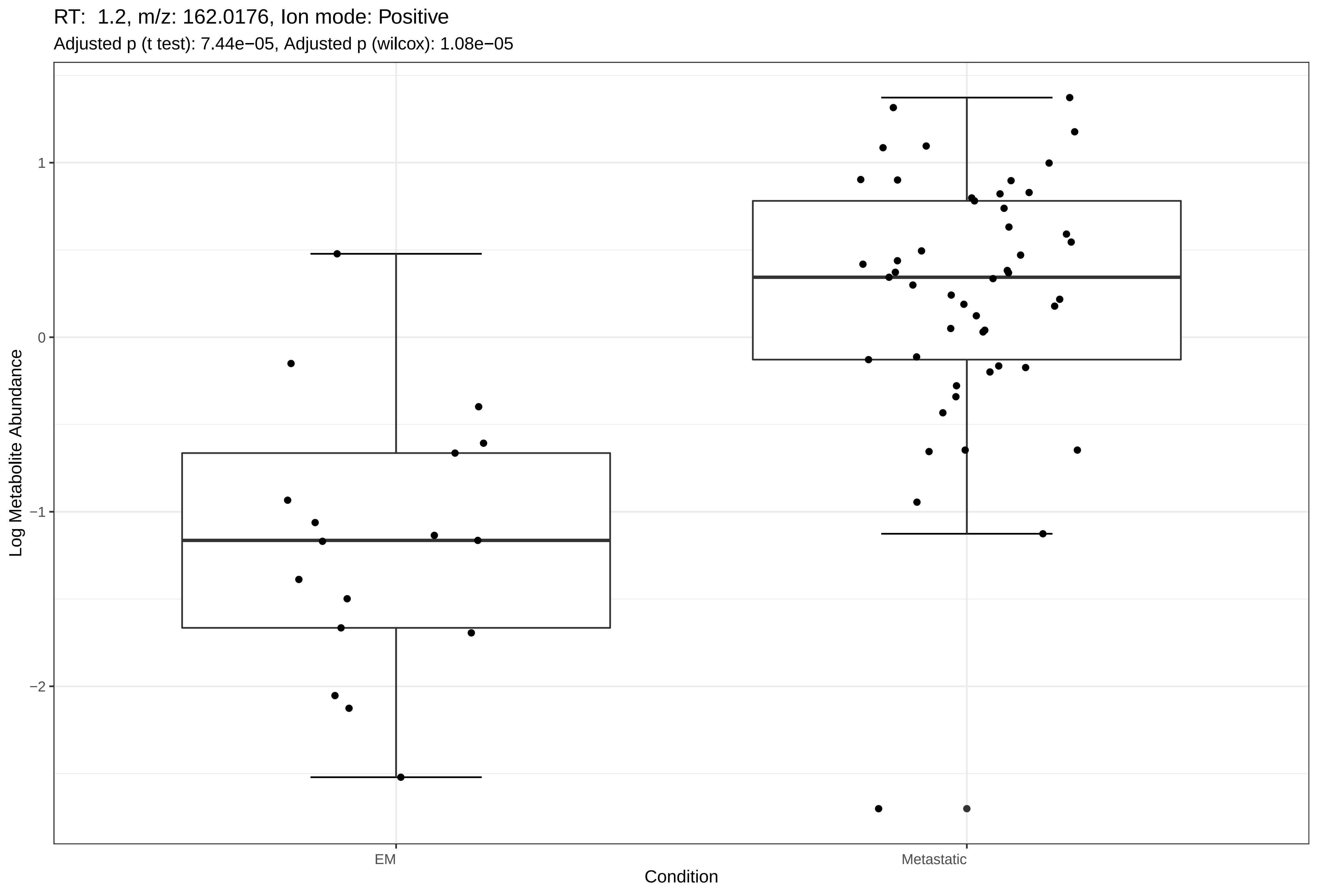
RT: 1.2, m/z: 162.0176, Ion mode: Positive
Adjusted p (t test): 7.44e−05, Adjusted p (wilcox): 1.08e−05
Log Metabolite Abundance
1
0
−1
−2
EM
Metastatic
Condition

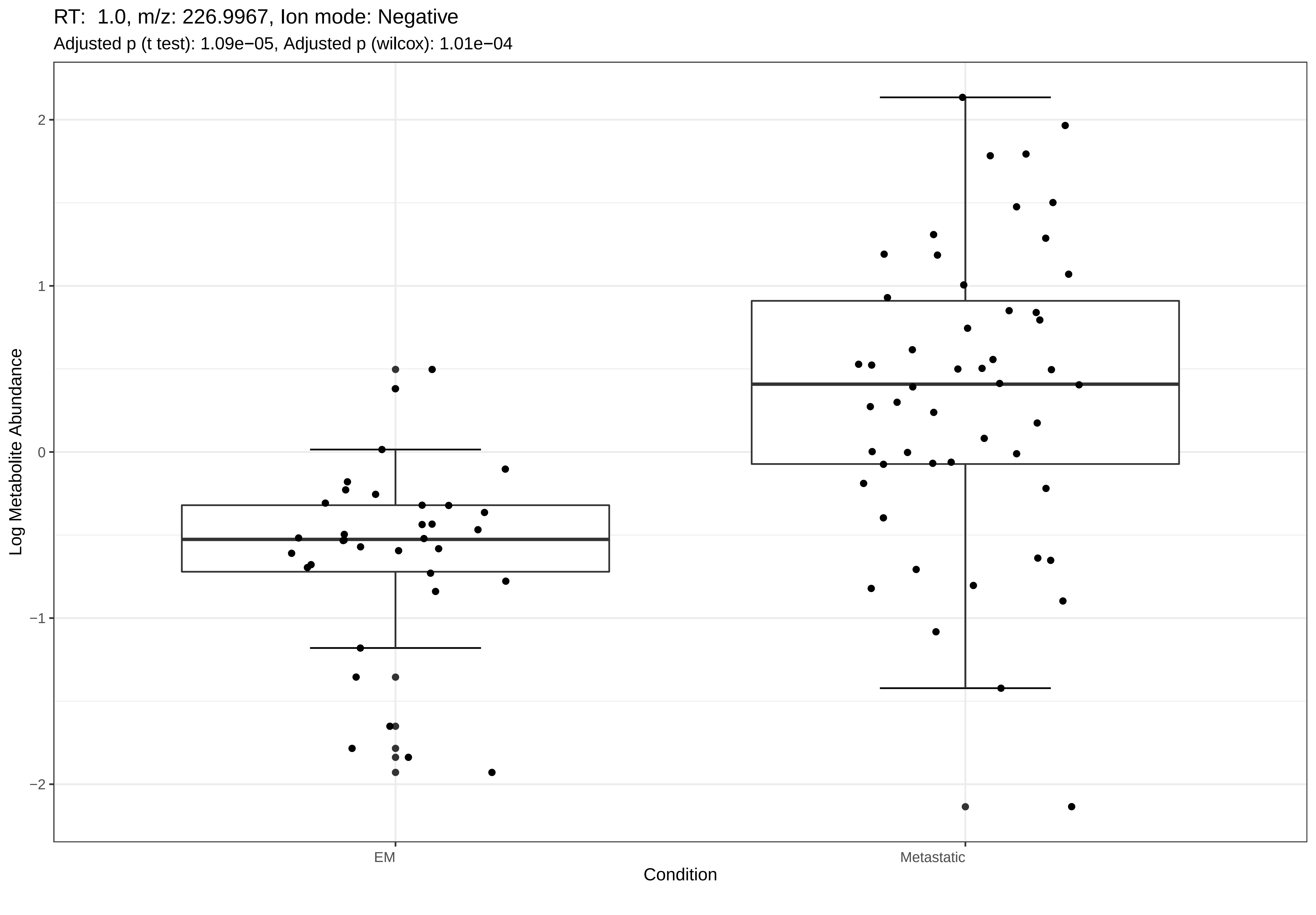
RT: 1.0, m/z: 226.9967, Ion mode: Negative
Adjusted p (t test): 1.09e−05, Adjusted p (wilcox): 1.01e−04
Log Metabolite Abundance
2
1
0
−1
−2
EM
Metastatic
Condition

RT: 4.8, m/z: 220.9395, Ion mode: Negative

Adjusted p (t test): 1.09e−05, Adjusted p (wilcox): 6.74e−05

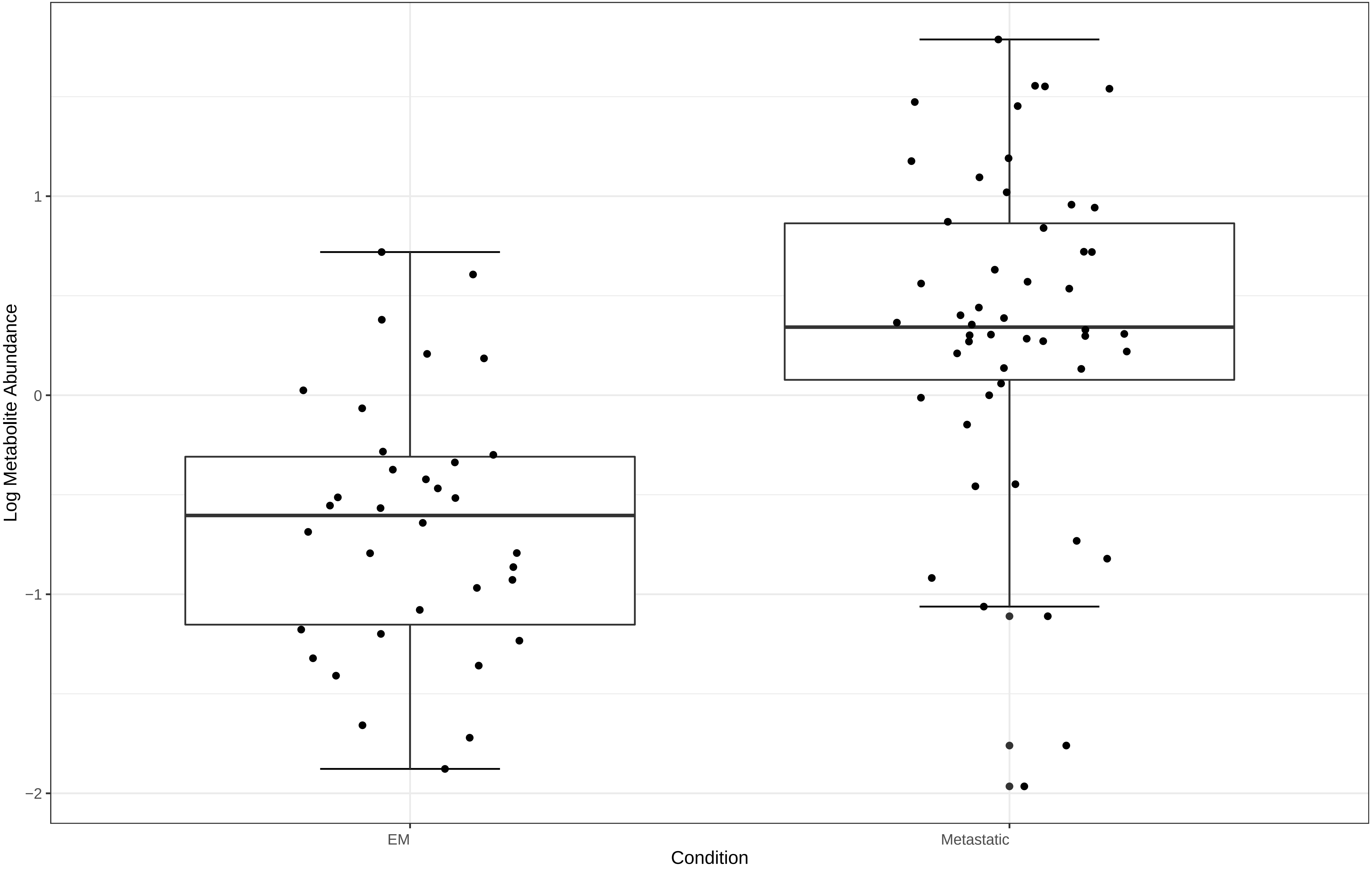

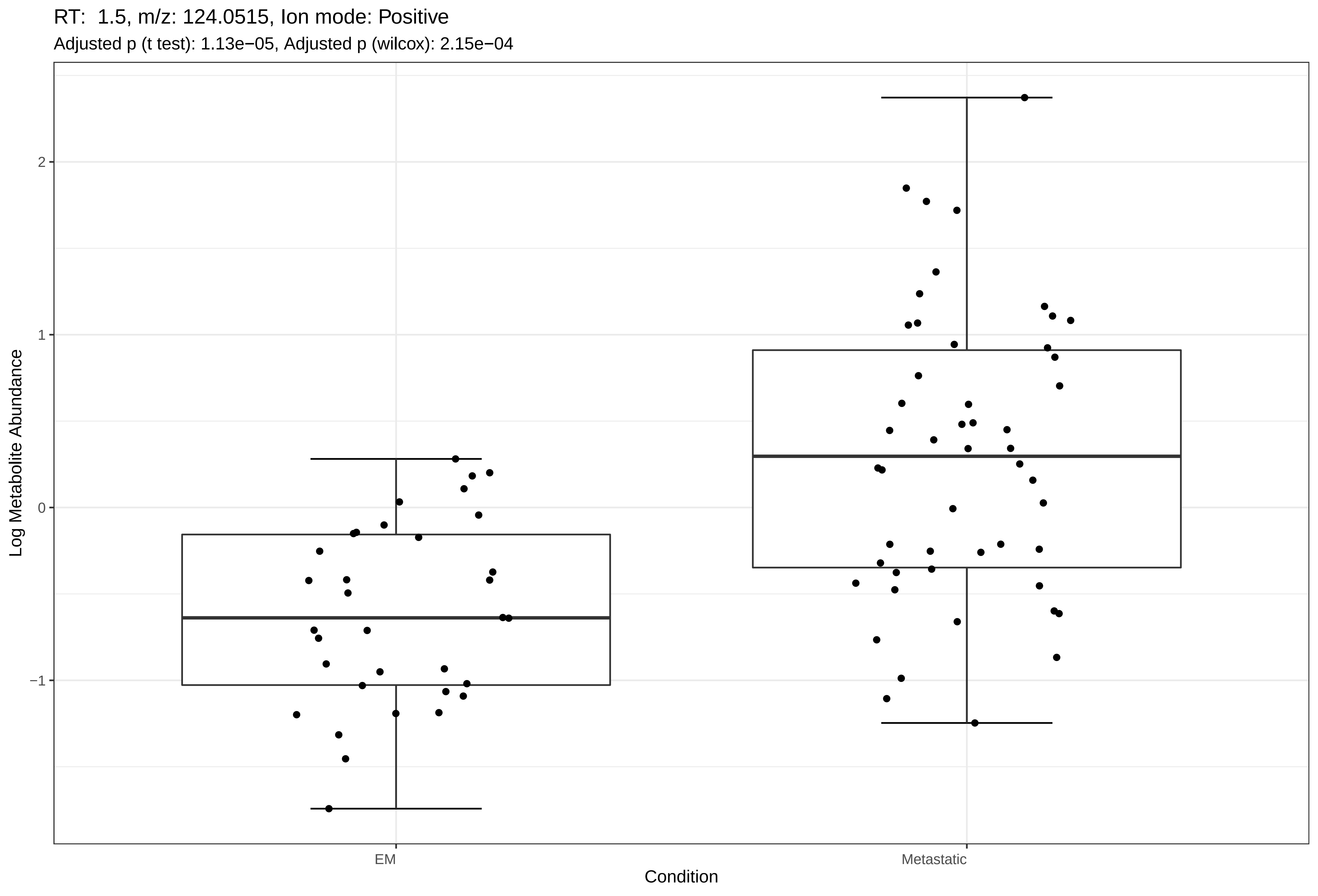

RT: 1.5, m/z: 124.0515, Ion mode: Positive
Adjusted p (t test): 1.13e−05, Adjusted p (wilcox): 2.15e−04
Log Metabolite Abundance
2
1
0
−1
EM
Metastatic
Condition

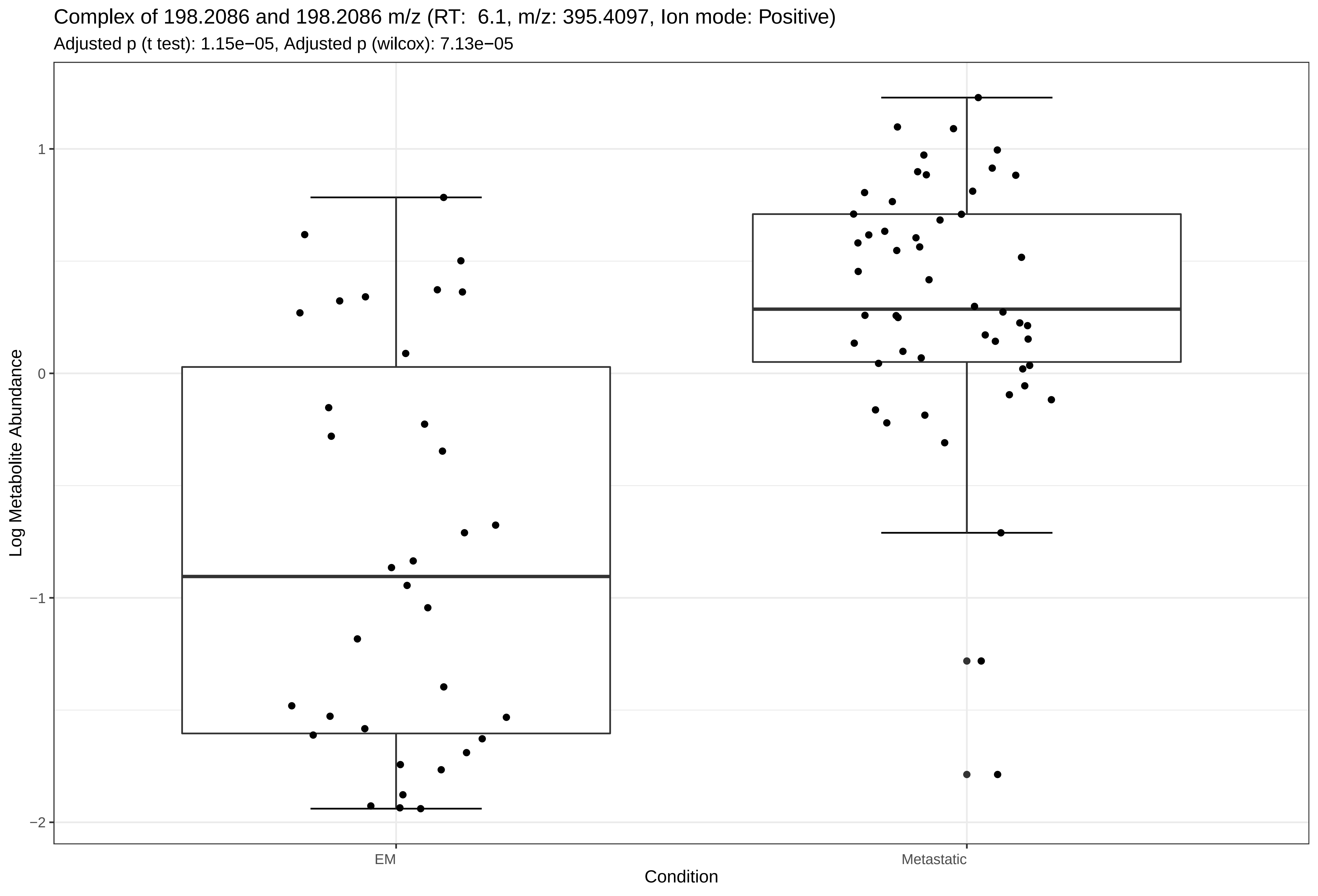

Complex of 198.2086 and 198.2086 m/z (RT: 6.1, m/z: 395.4097, Ion mode: Positive)
Adjusted p (t test): 1.15e−05, Adjusted p (wilcox): 7.13e−05
Log Metabolite Abundance
1
0
−1
−2
EM
Metastatic
Condition

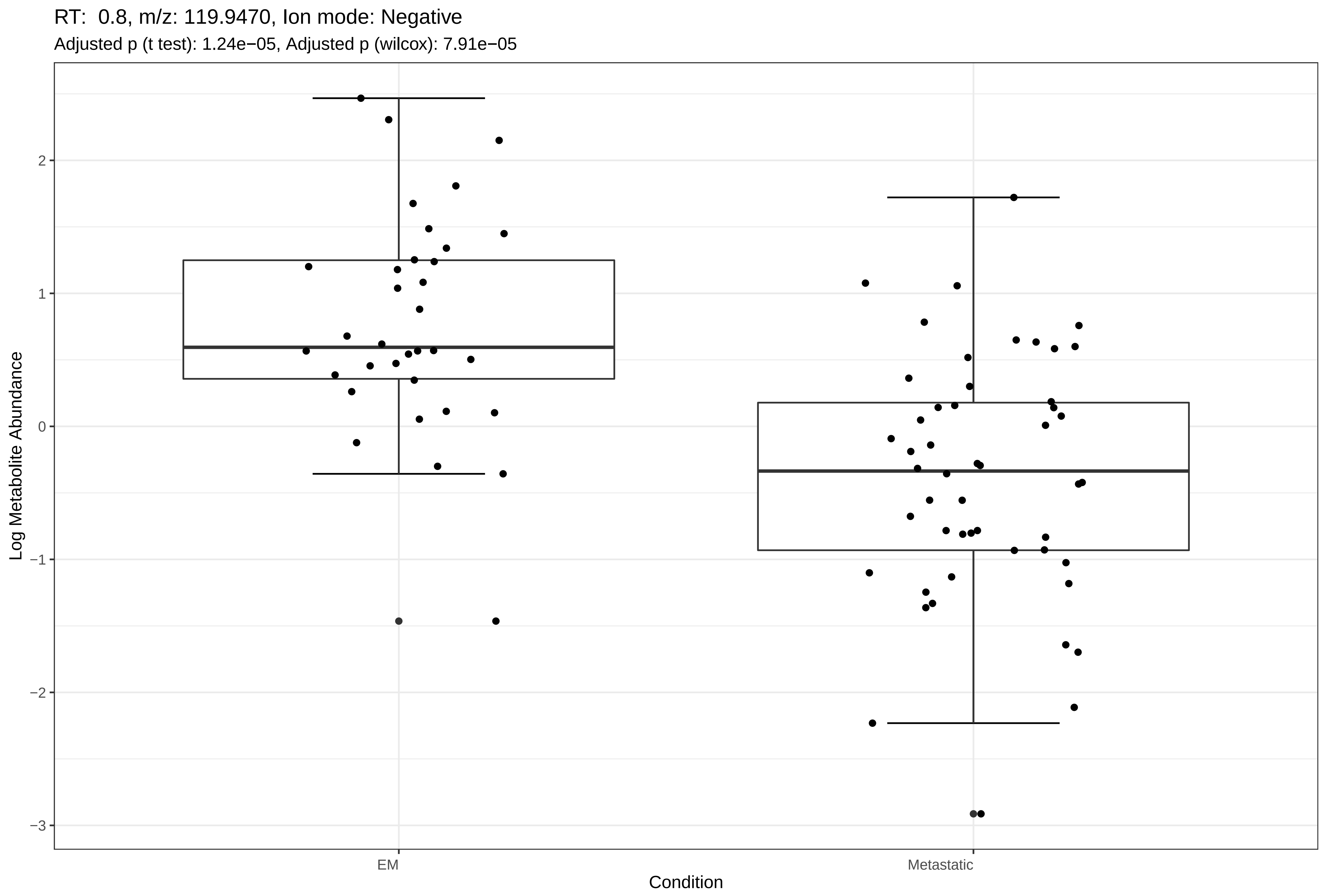
RT: 0.8, m/z: 119.9470, Ion mode: Negative
Adjusted p (t test): 1.24e−05, Adjusted p (wilcox): 7.91e−05
Log Metabolite Abundance
2
1
0
−1
−2
−3
EM
Metastatic
Condition

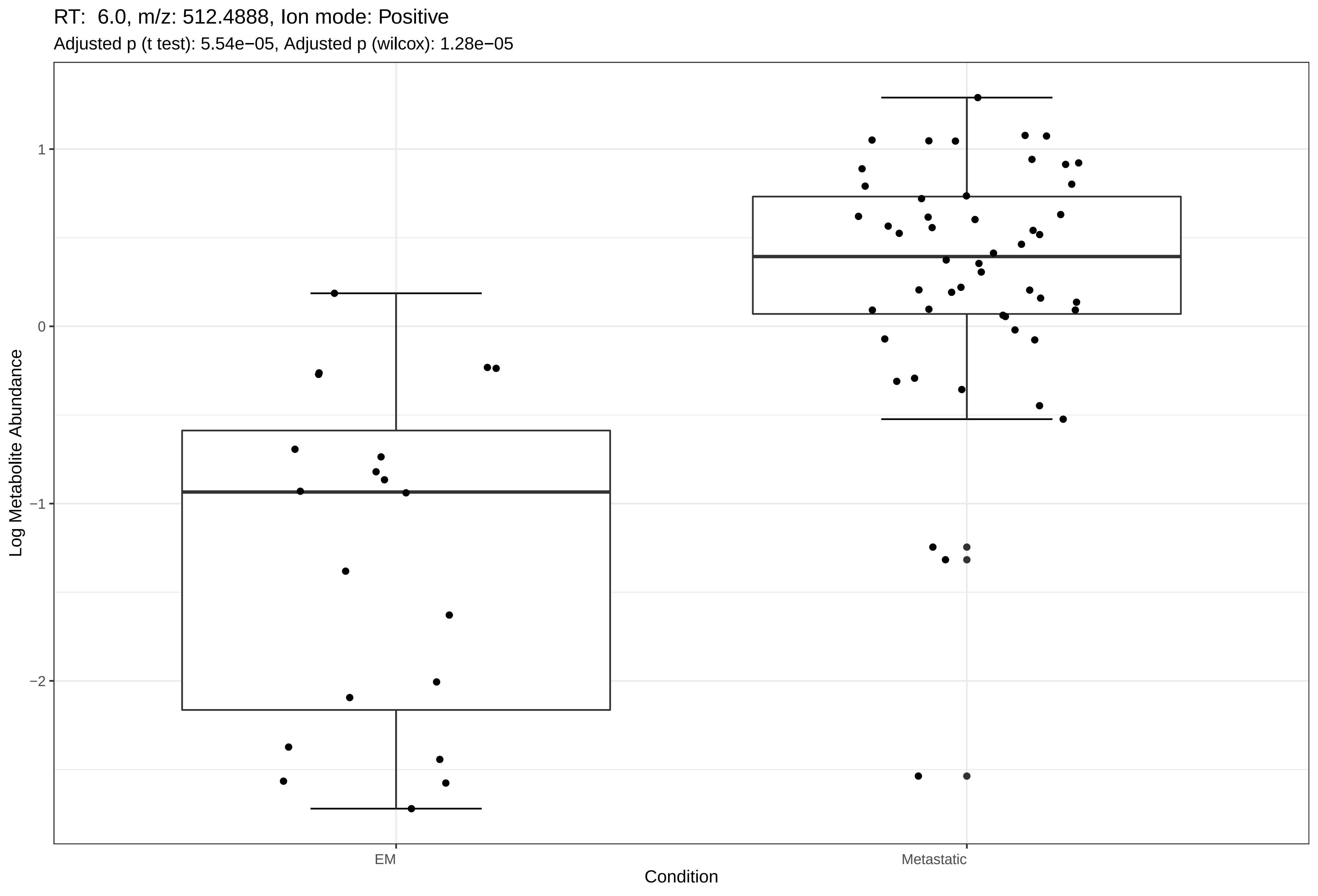
RT: 6.0, m/z: 512.4888, Ion mode: Positive
Adjusted p (t test): 5.54e−05, Adjusted p (wilcox): 1.28e−05
Log Metabolite Abundance
1
0
−1
−2
EM
Metastatic
Condition

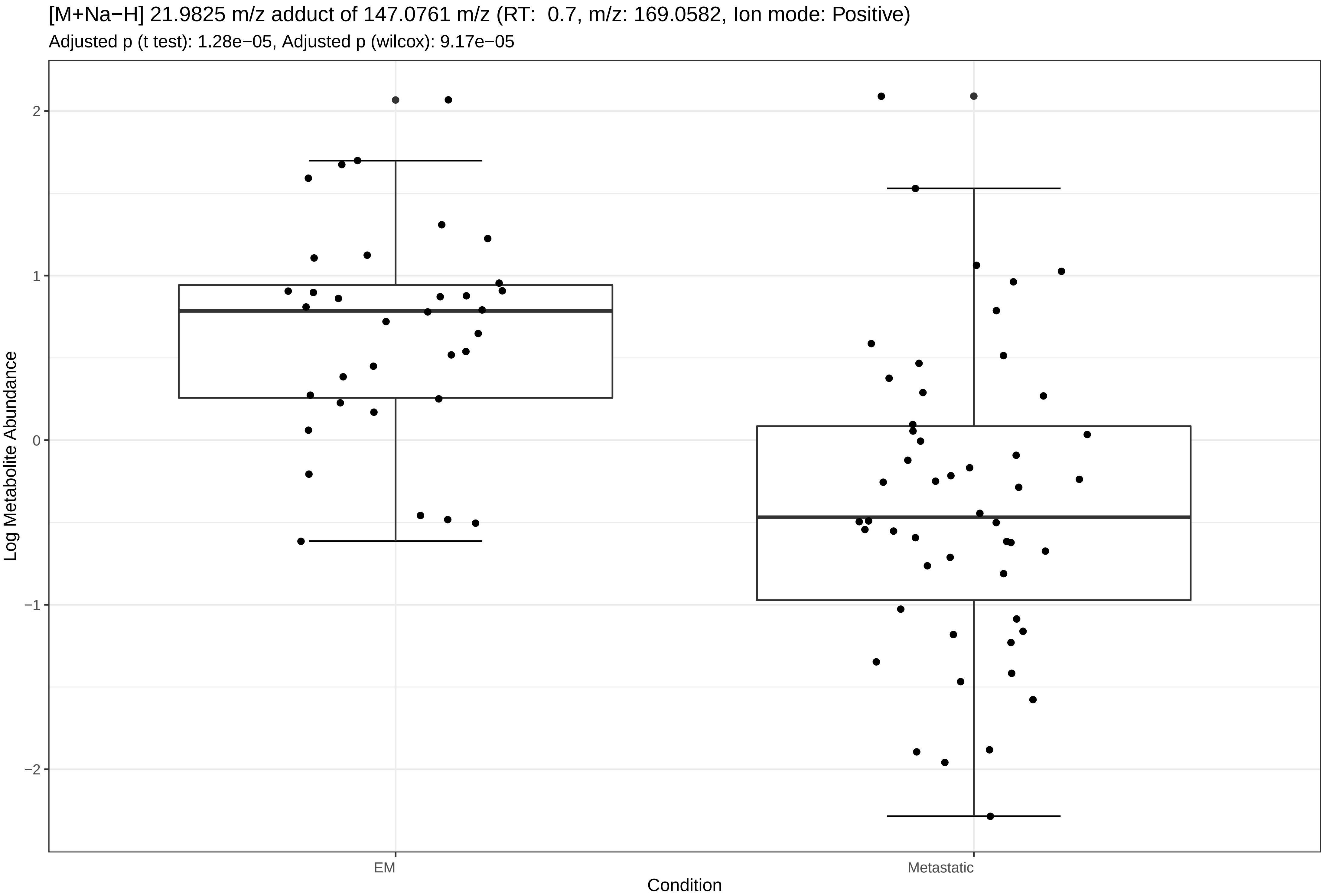
[M+Na−H] 21.9825 m/z adduct of 147.0761 m/z (RT: 0.7, m/z: 169.0582, Ion mode: Positive)
Adjusted p (t test): 1.28e−05, Adjusted p (wilcox): 9.17e−05
Log Metabolite Abundance
2
1
0
−1
−2
EM
Metastatic
Condition

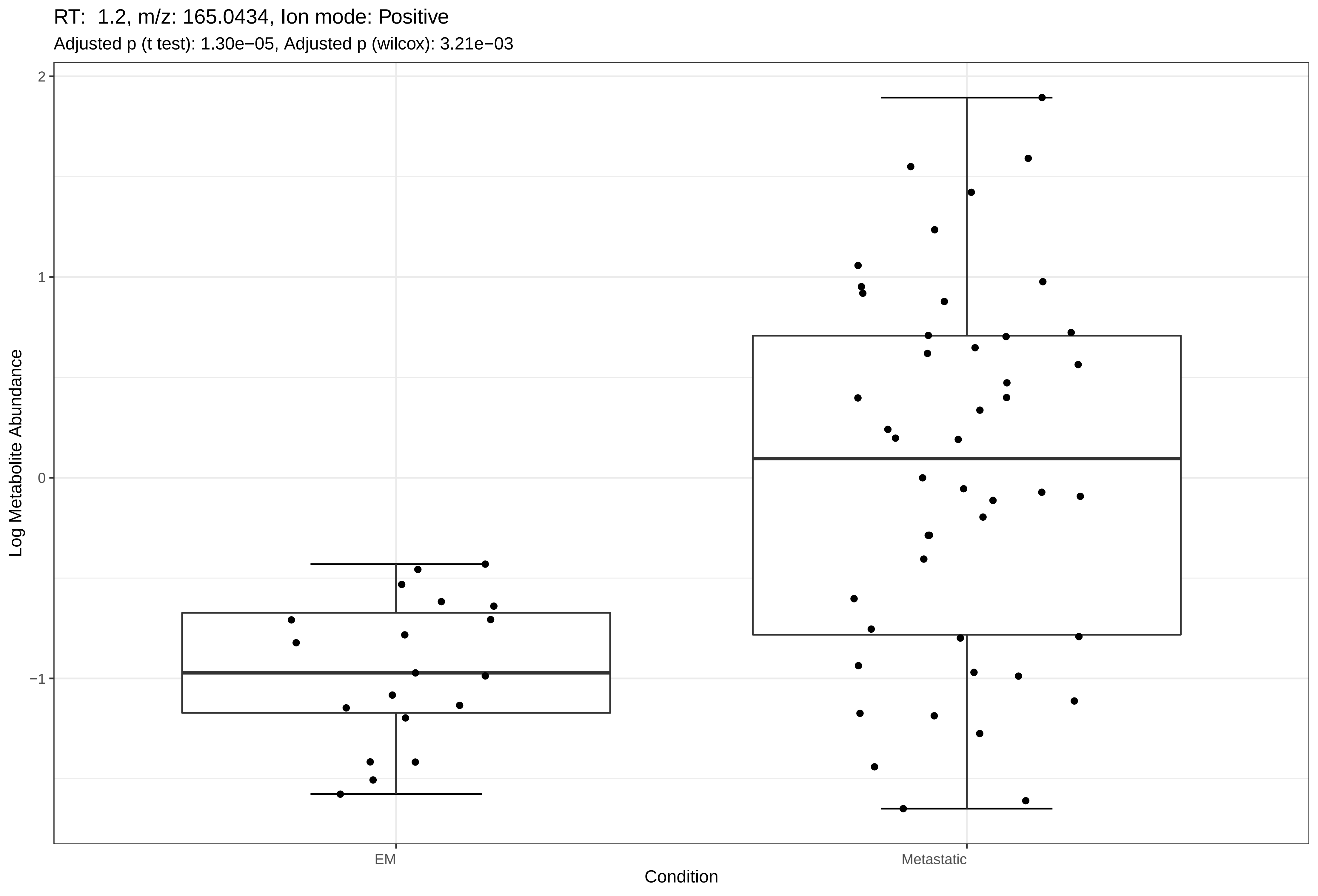
RT: 1.2, m/z: 165.0434, Ion mode: Positive
Adjusted p (t test): 1.30e−05, Adjusted p (wilcox): 3.21e−03
Log Metabolite Abundance
2
1
0
−1
EM
Metastatic
Condition

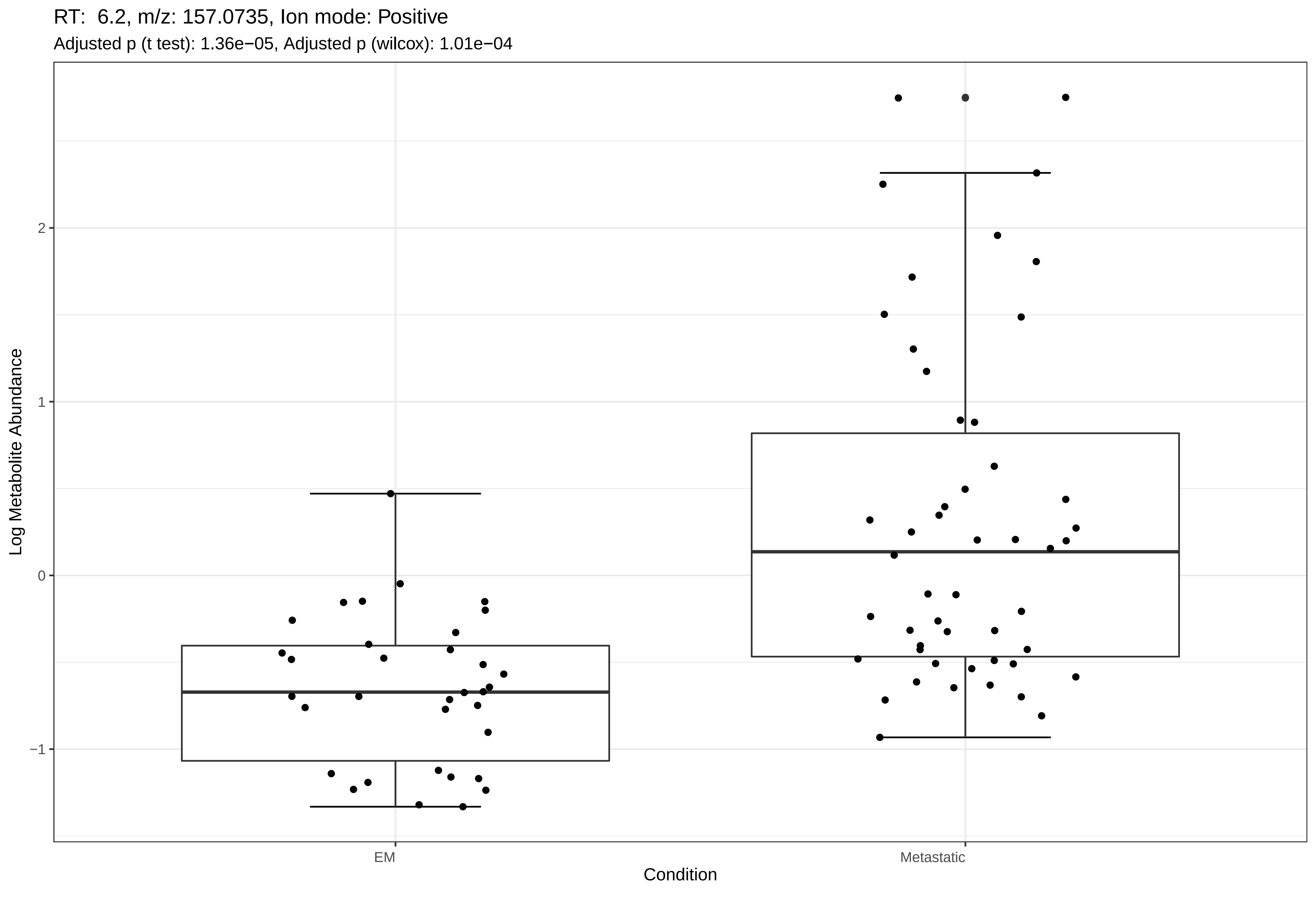

RT: 6.2, m/z: 157.0735, Ion mode: Positive
Adjusted p (t test): 1.36e−05, Adjusted p (wilcox): 1.01e−04
Log Metabolite Abundance
2
1
0
−1
EM
Metastatic
Condition

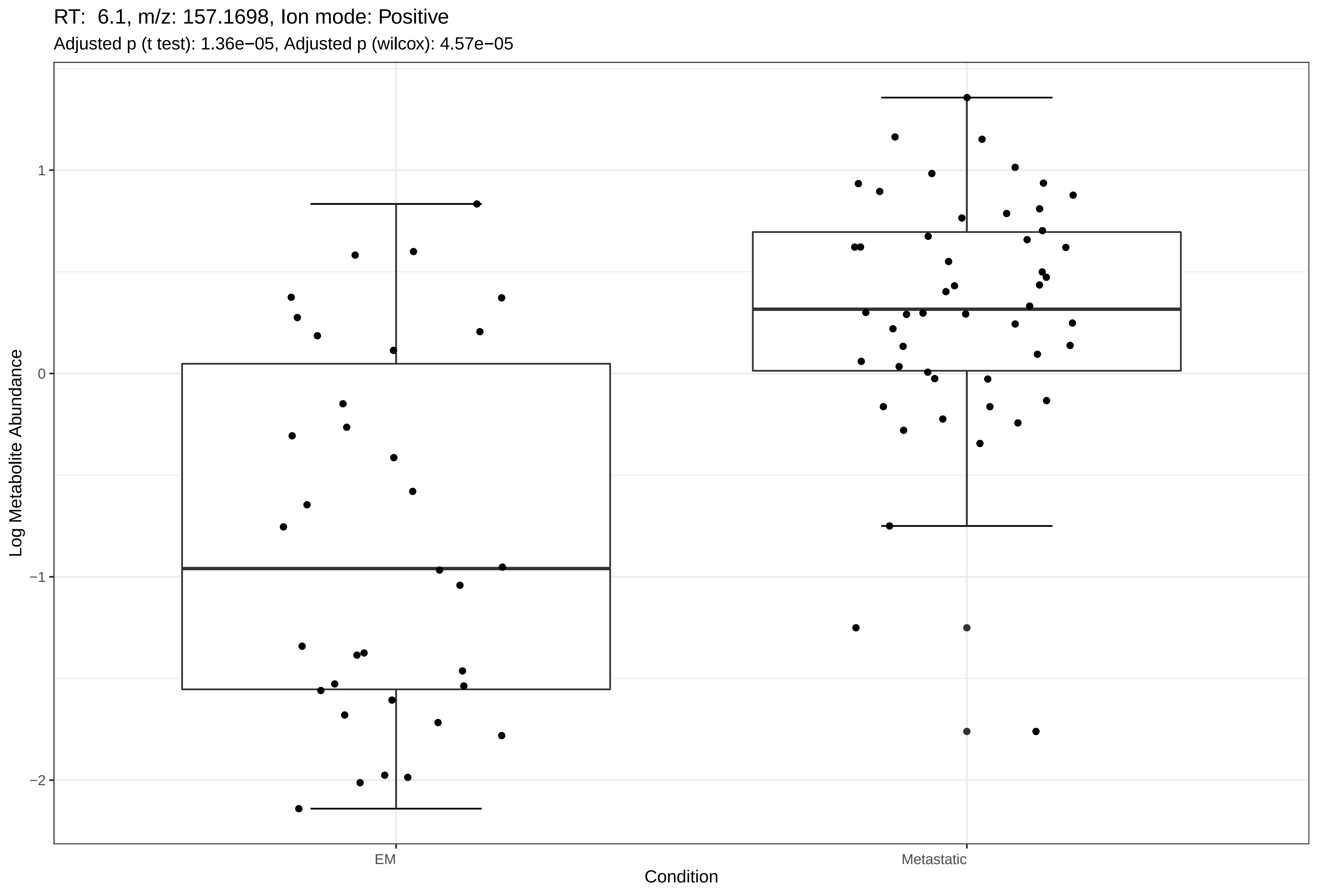

RT: 6.1, m/z: 157.1698, Ion mode: Positive
Adjusted p (t test): 1.36e−05, Adjusted p (wilcox): 4.57e−05
Log Metabolite Abundance
1
0
−1
−2
EM
Metastatic
Condition

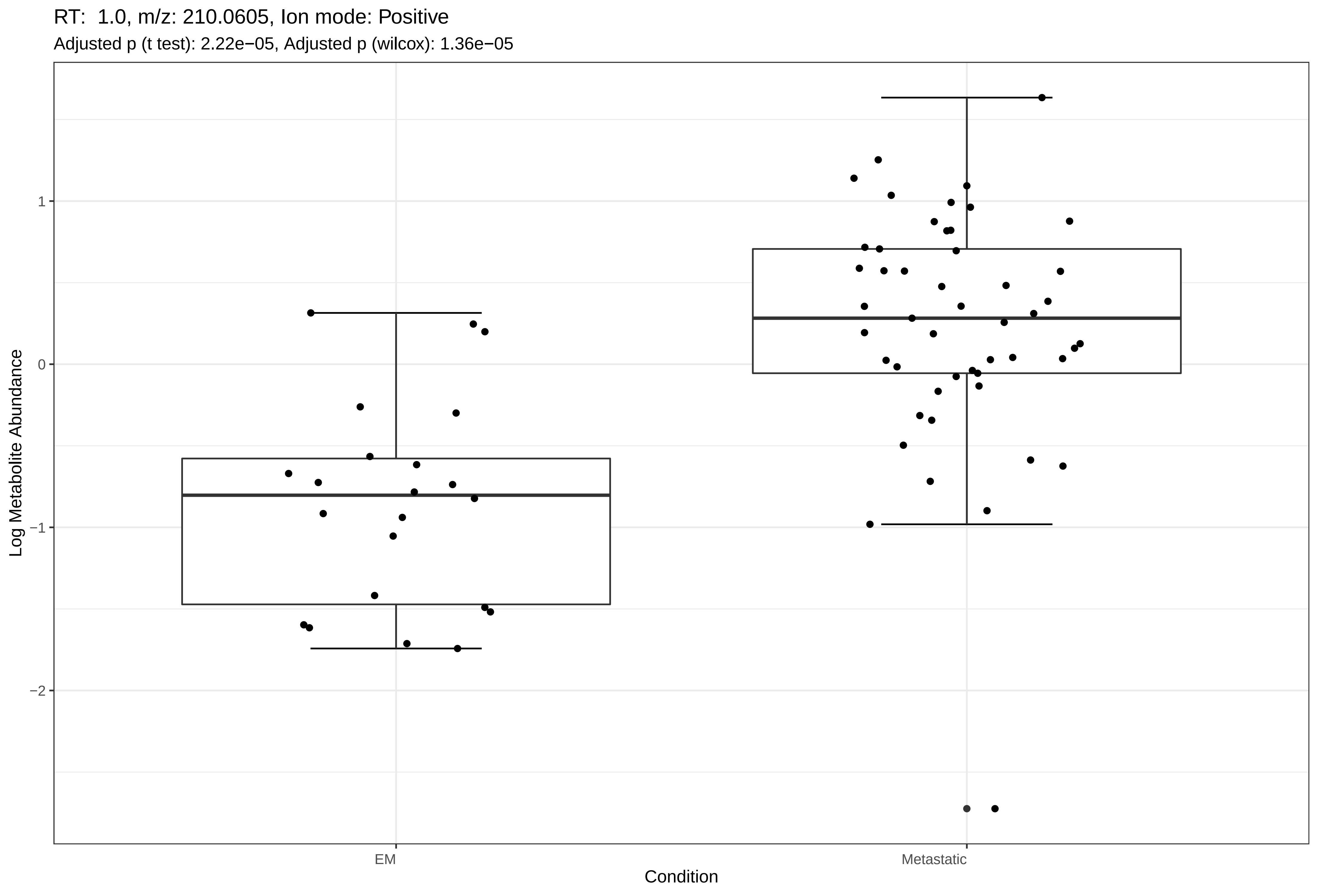
RT: 1.0, m/z: 210.0605, Ion mode: Positive
Adjusted p (t test): 2.22e−05, Adjusted p (wilcox): 1.36e−05
Log Metabolite Abundance
1
0
−1
−2
EM
Metastatic
Condition

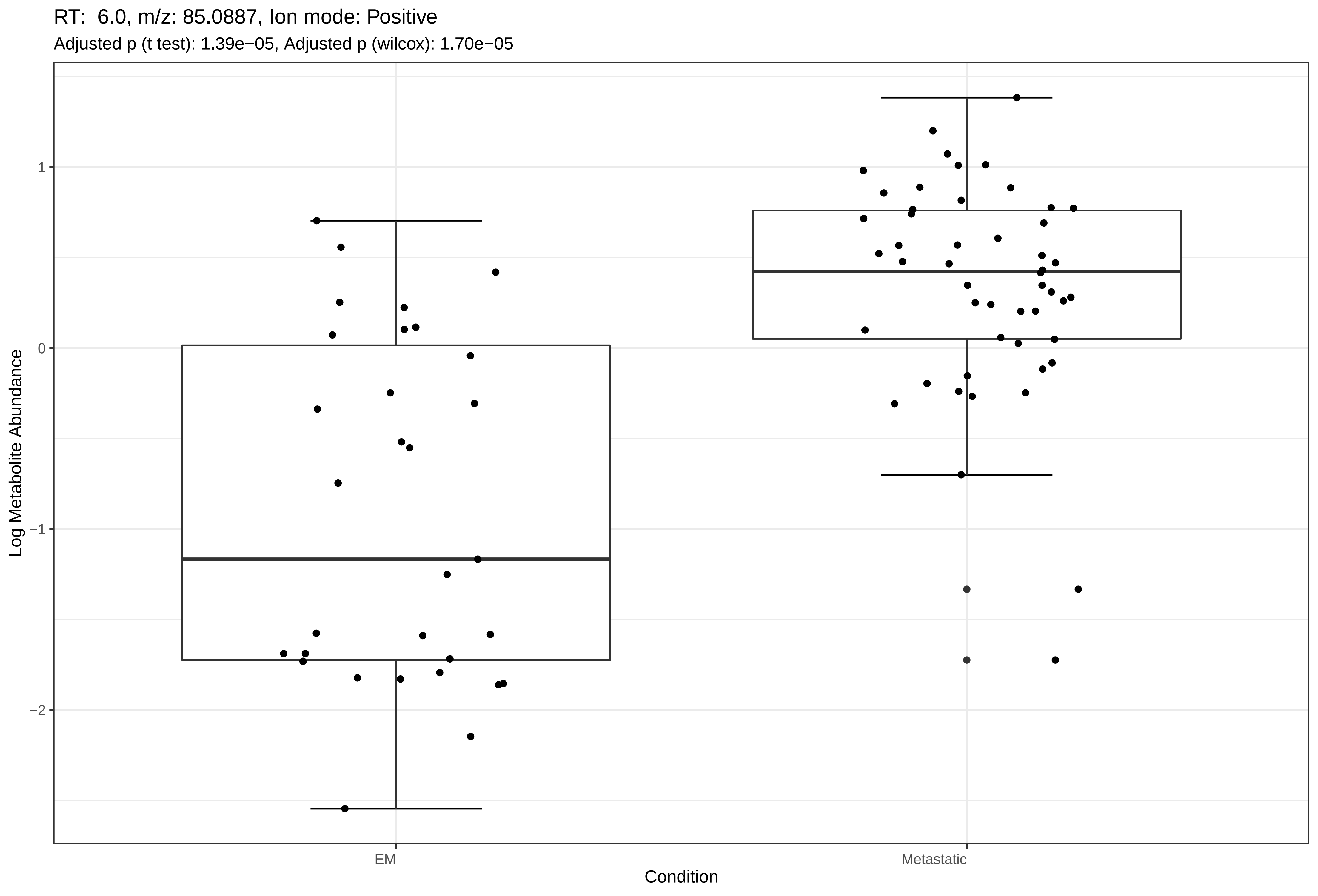
RT: 6.0, m/z: 85.0887, Ion mode: Positive
Adjusted p (t test): 1.39e−05, Adjusted p (wilcox): 1.70e−05
Log Metabolite Abundance
1
0
−1
−2
EM
Metastatic
Condition

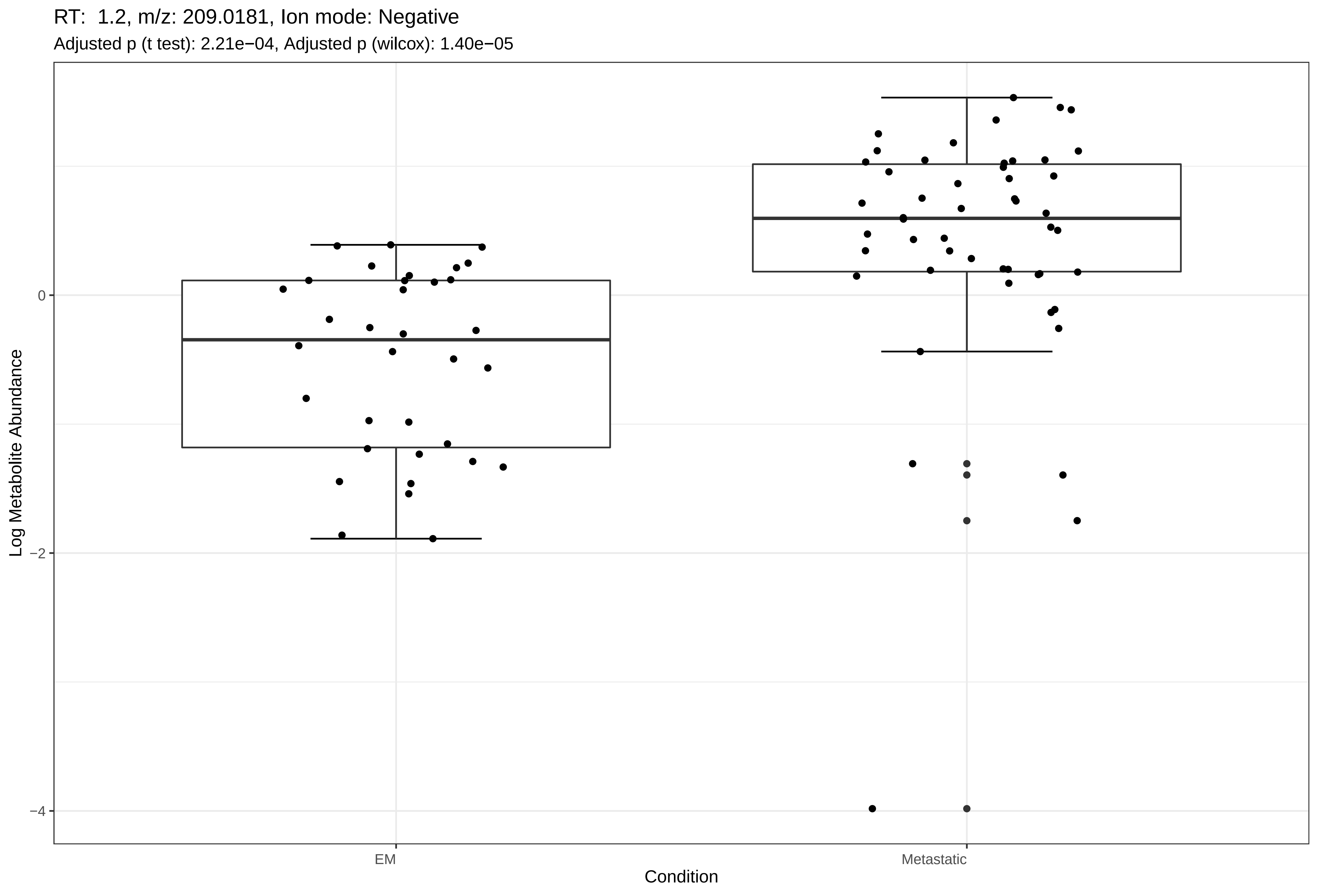

RT: 1.2, m/z: 209.0181, Ion mode: Negative
Adjusted p (t test): 2.21e−04, Adjusted p (wilcox): 1.40e−05
Log Metabolite Abundance
0
−2
−4
EM
Metastatic
Condition

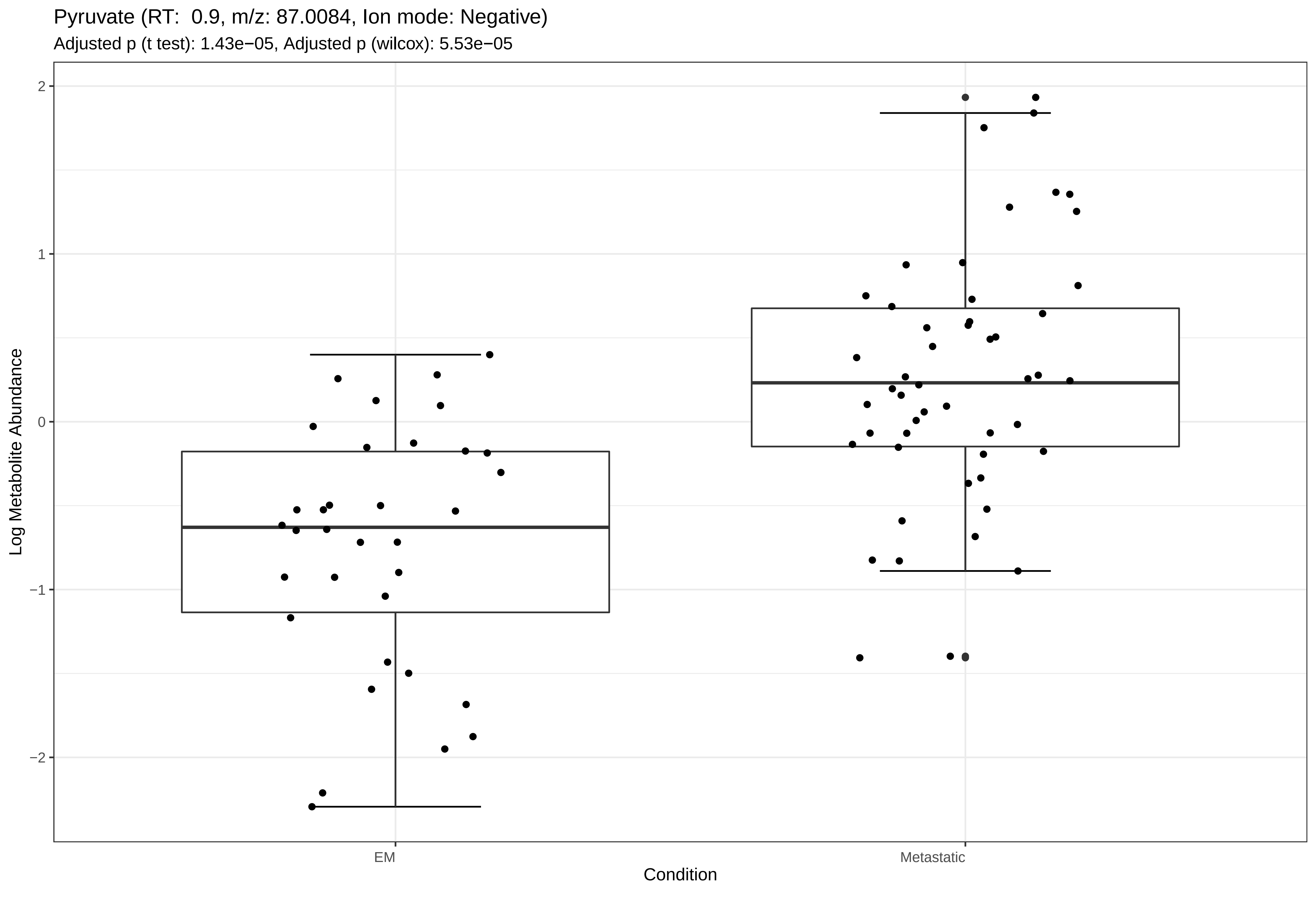
Pyruvate (RT: 0.9, m/z: 87.0084, Ion mode: Negative)
Adjusted p (t test): 1.43e−05, Adjusted p (wilcox): 5.53e−05
Log Metabolite Abundance
2
1
0
−1
−2
EM
Metastatic
Condition

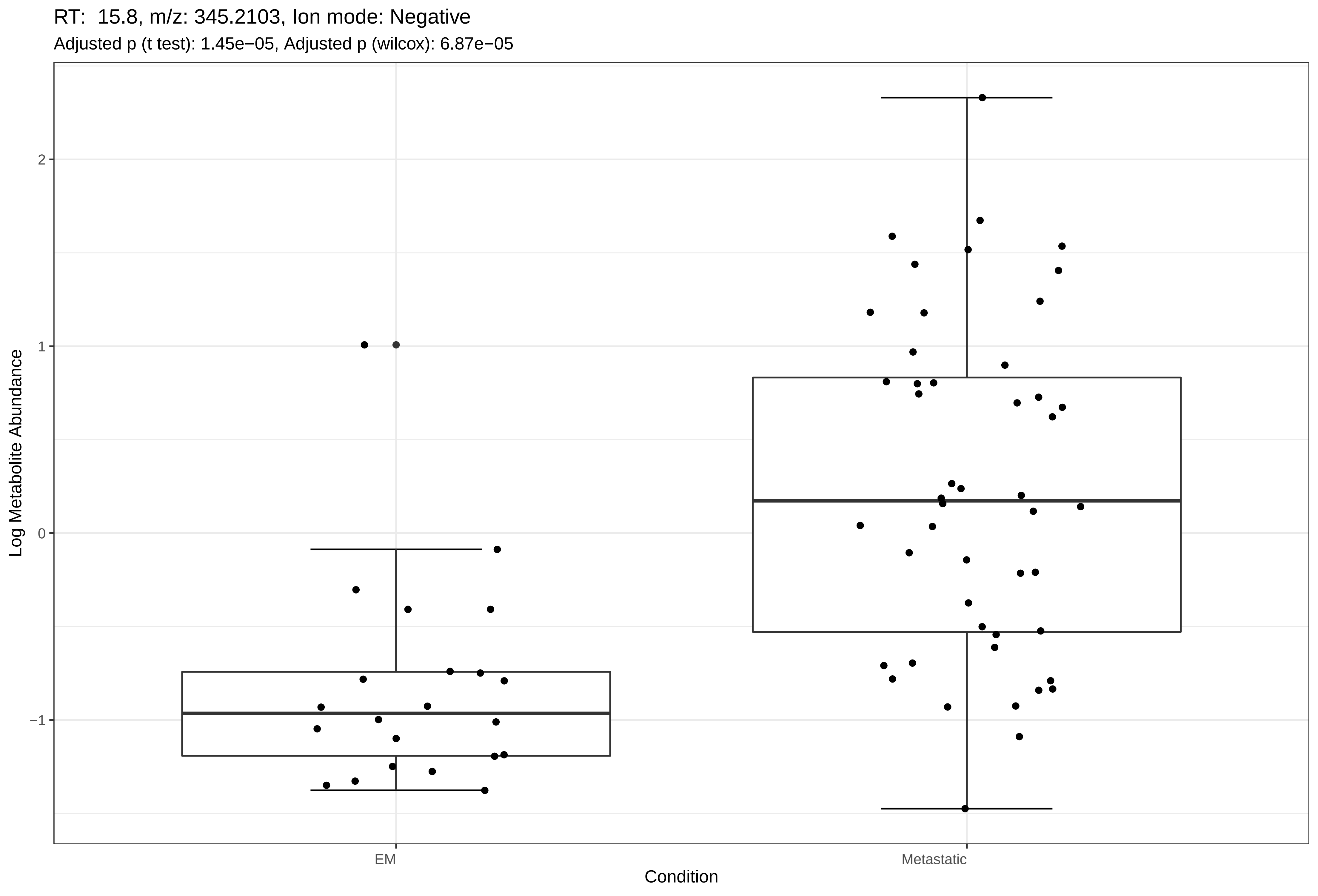

RT: 15.8, m/z: 345.2103, Ion mode: Negative
Adjusted p (t test): 1.45e−05, Adjusted p (wilcox): 6.87e−05
Log Metabolite Abundance
2
1
0
−1
EM
Metastatic
Condition

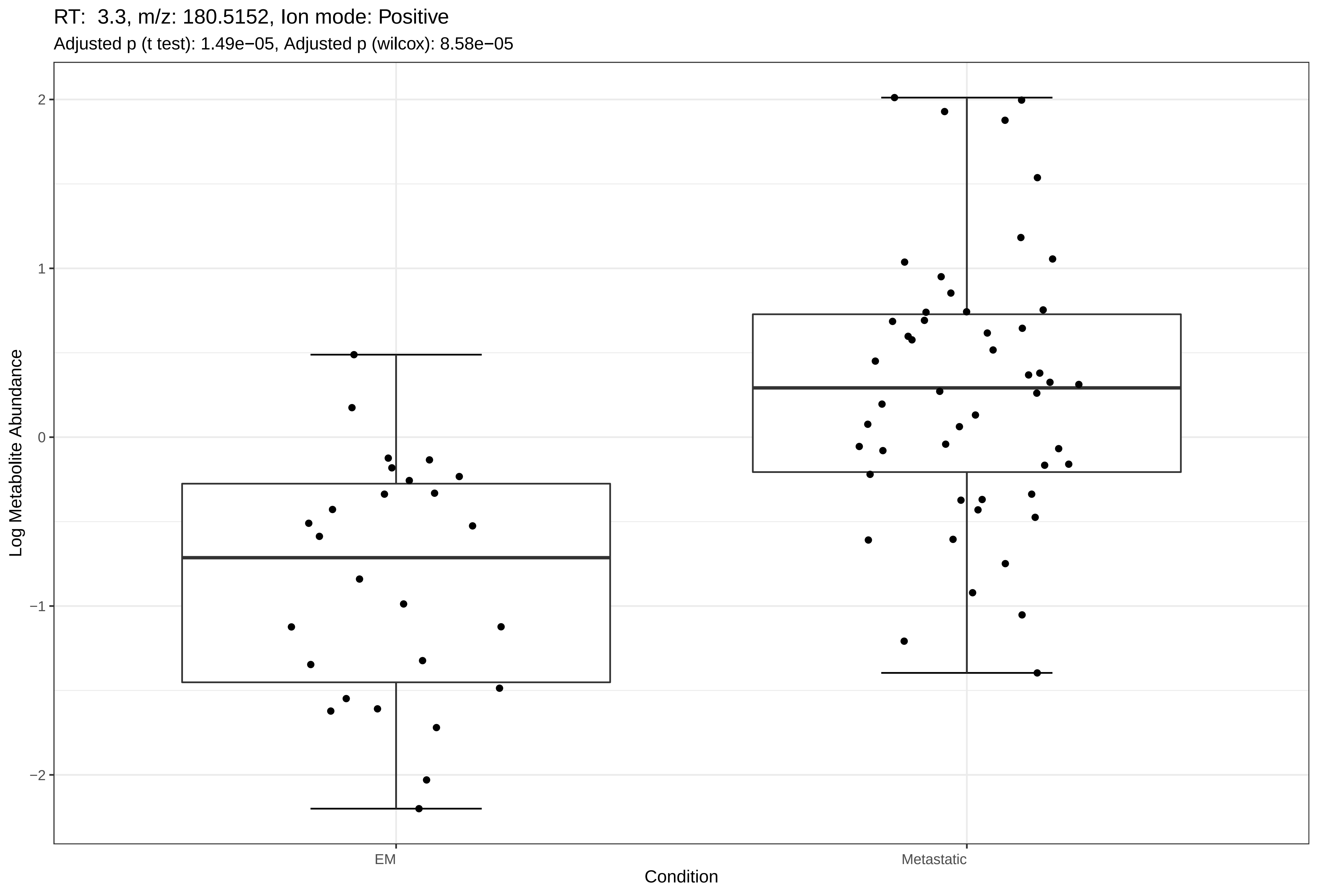

RT: 3.3, m/z: 180.5152, Ion mode: Positive
Adjusted p (t test): 1.49e−05, Adjusted p (wilcox): 8.58e−05
Log Metabolite Abundance
2
1
0
−1
−2
EM
Metastatic
Condition

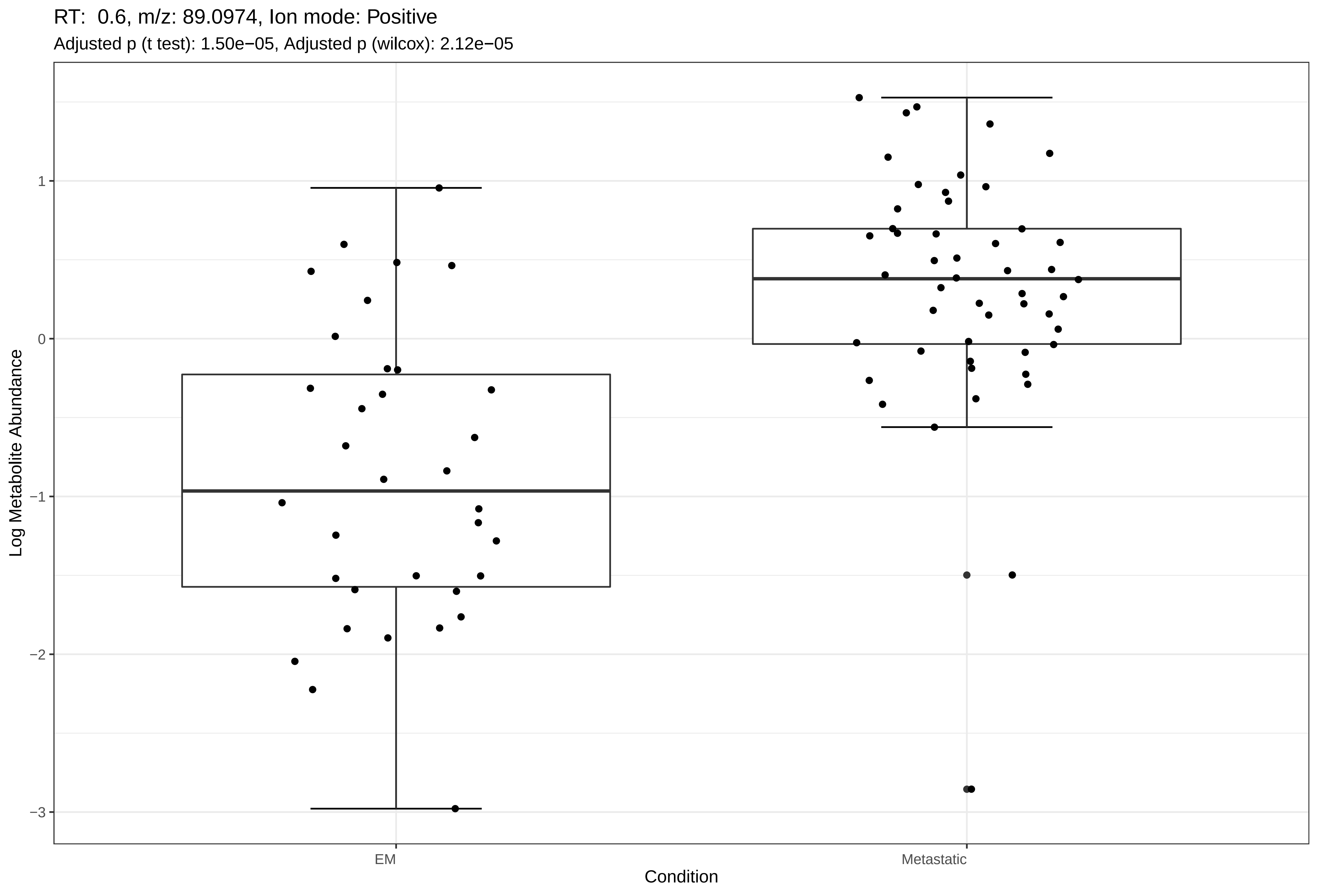
RT: 0.6, m/z: 89.0974, Ion mode: Positive
Adjusted p (t test): 1.50e−05, Adjusted p (wilcox): 2.12e−05
Log Metabolite Abundance
1
0
−1
−2
−3
EM
Metastatic
Condition

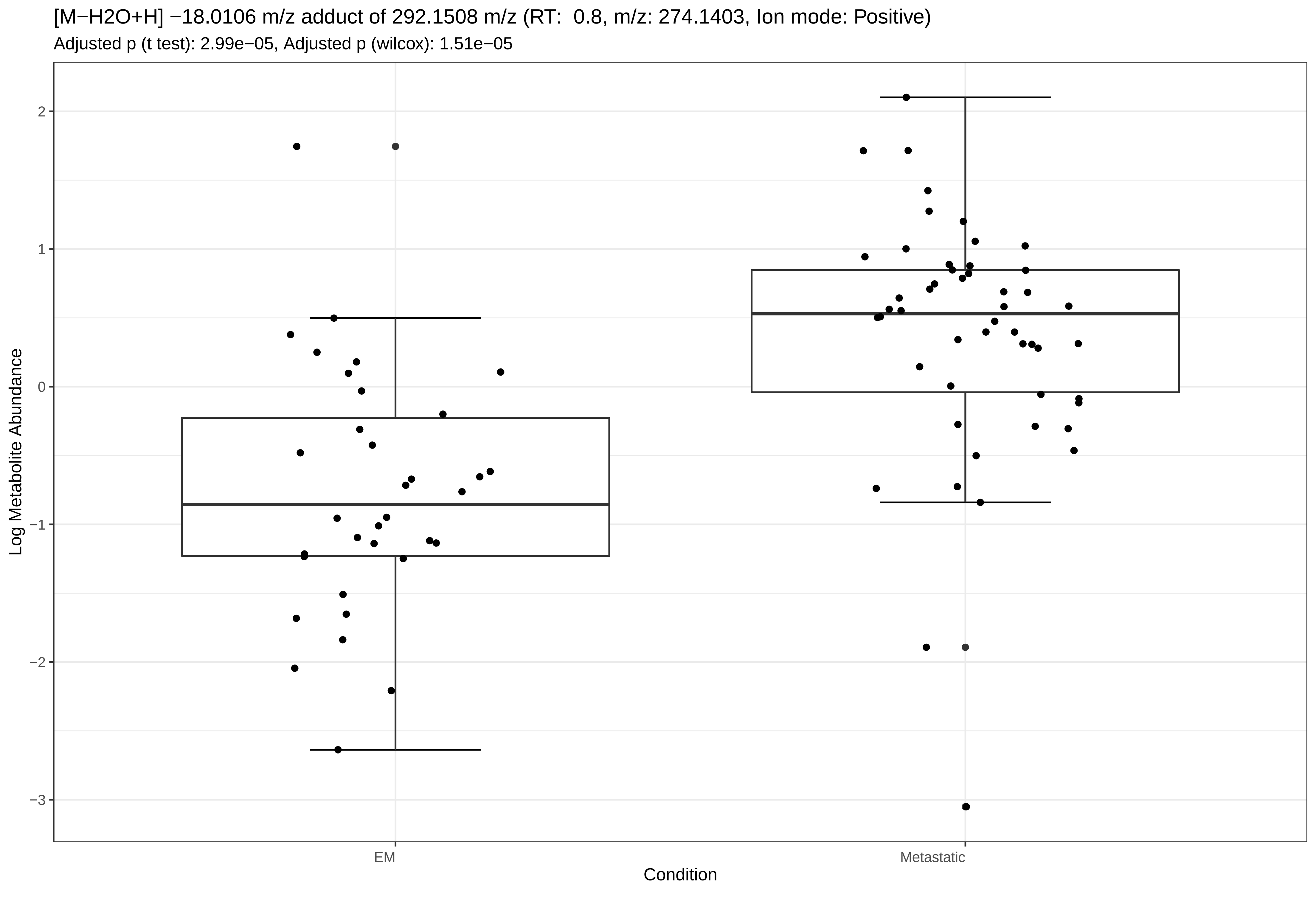
[M−H2O+H] −18.0106 m/z adduct of 292.1508 m/z (RT: 0.8, m/z: 274.1403, Ion mode: Positive)
Adjusted p (t test): 2.99e−05, Adjusted p (wilcox): 1.51e−05
Log Metabolite Abundance
2
1
0
−1
−2
−3
EM
Metastatic
Condition

[M+Na−H] 21.9825 m/z adduct of 148.0603 m/z (RT: 0.7, m/z: 170.0421, Ion mode: Positive)

Adjusted p (t test): 2.99e−05, Adjusted p (wilcox): 1.51e−05

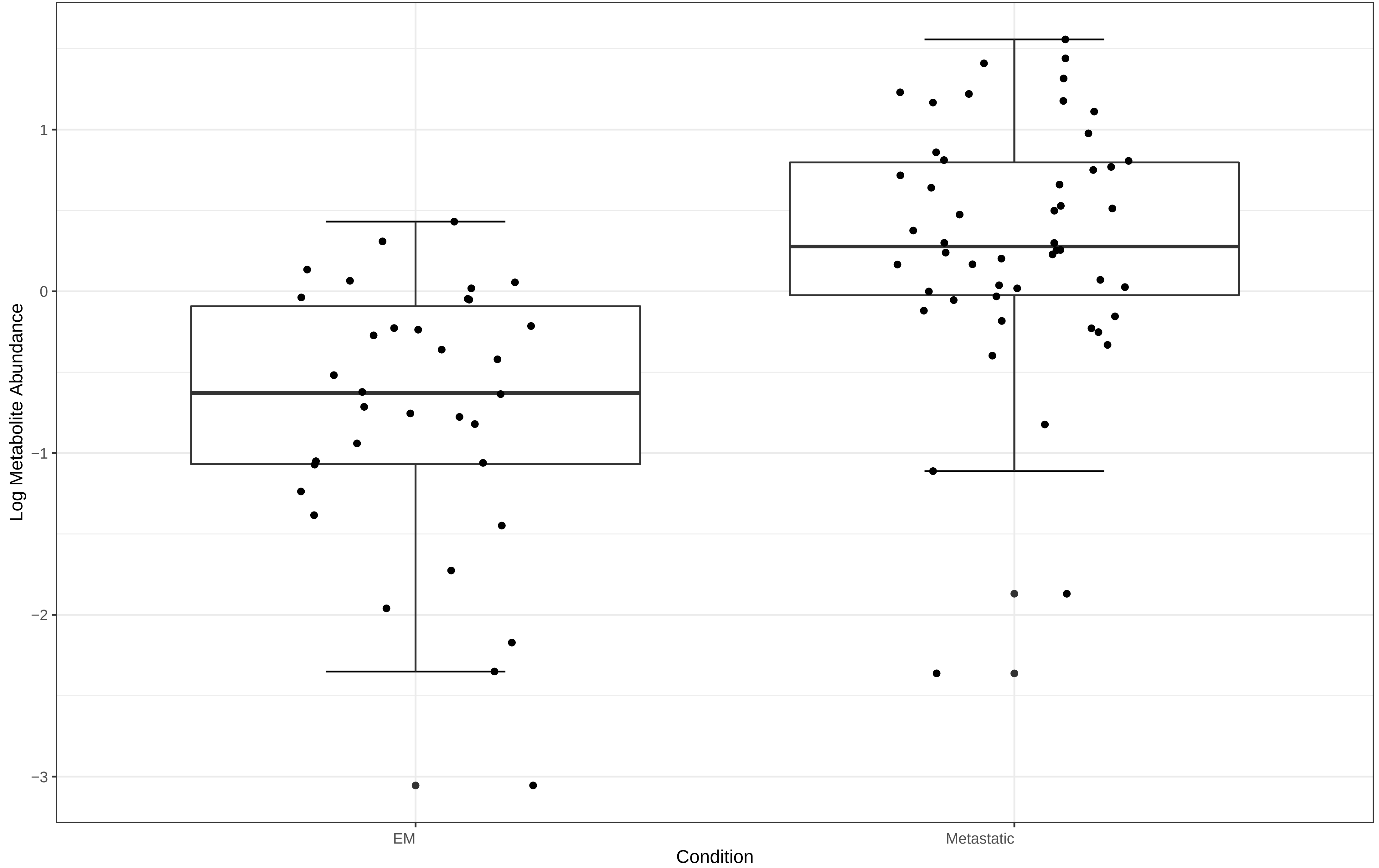

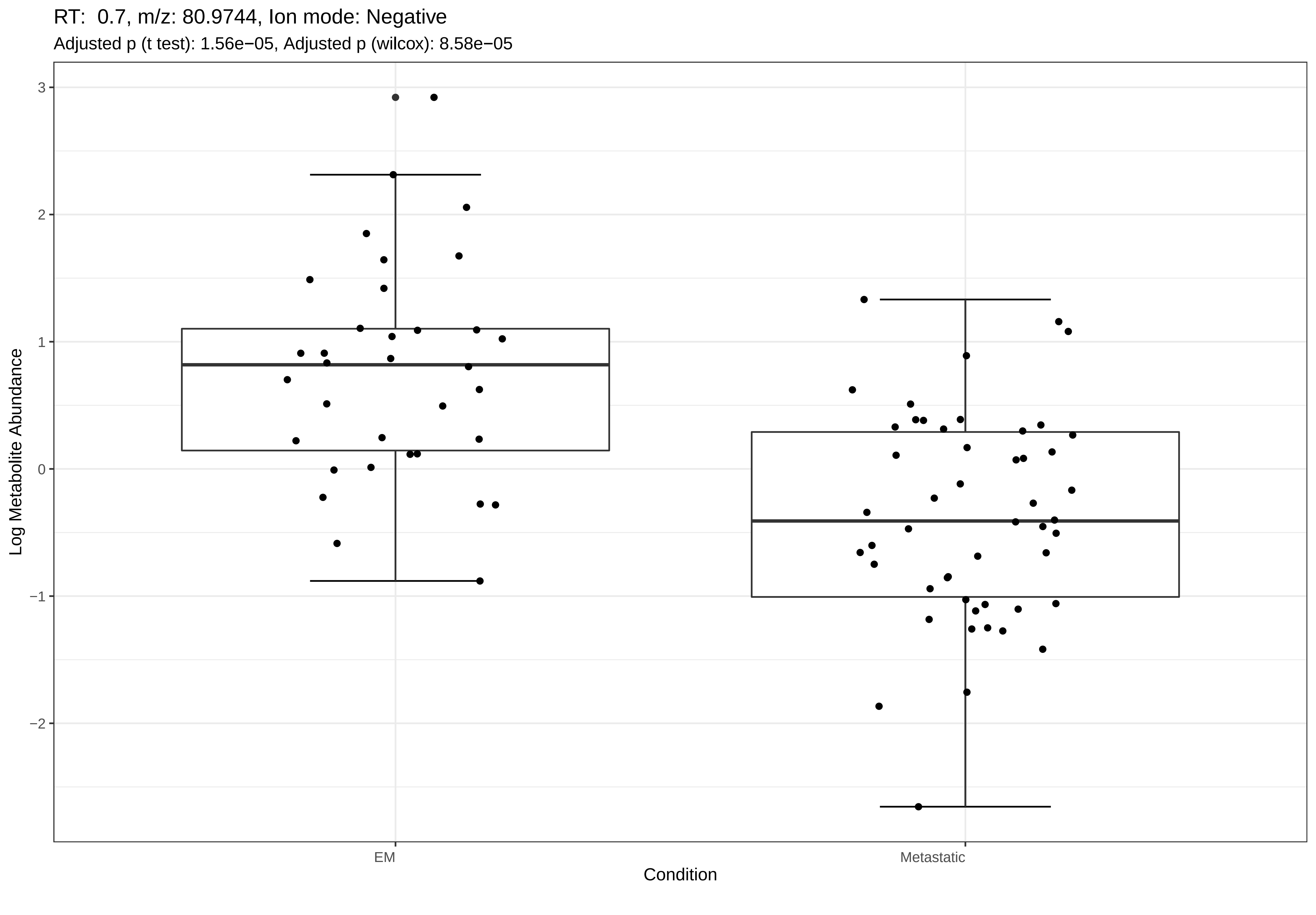

RT: 0.7, m/z: 80.9744, Ion mode: Negative
Adjusted p (t test): 1.56e−05, Adjusted p (wilcox): 8.58e−05
Log Metabolite Abundance
3
2
1
0
−1
−2
EM
Metastatic
Condition

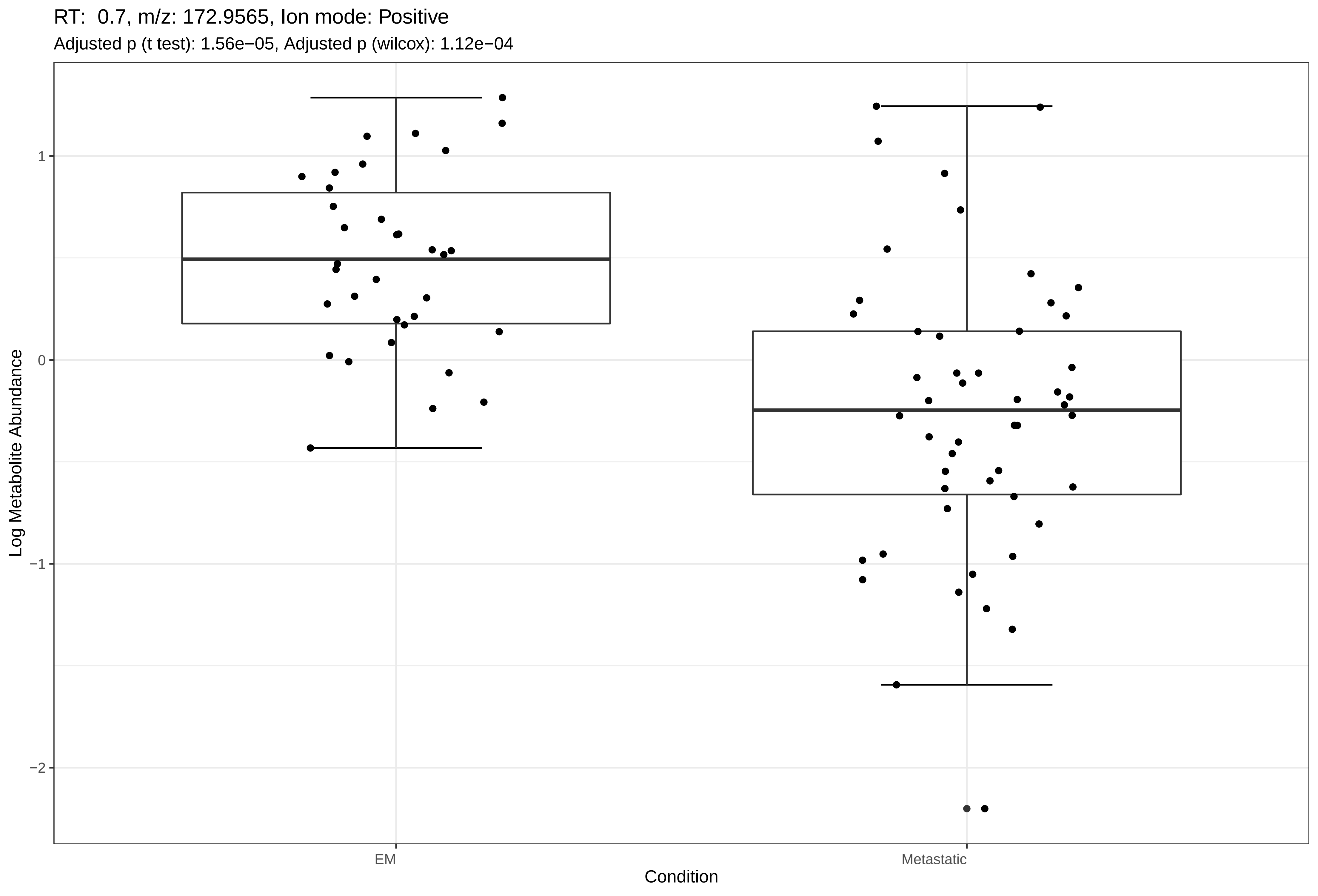

RT: 0.7, m/z: 172.9565, Ion mode: Positive
Adjusted p (t test): 1.56e−05, Adjusted p (wilcox): 1.12e−04
Log Metabolite Abundance
1
0
−1
−2
EM
Metastatic
Condition

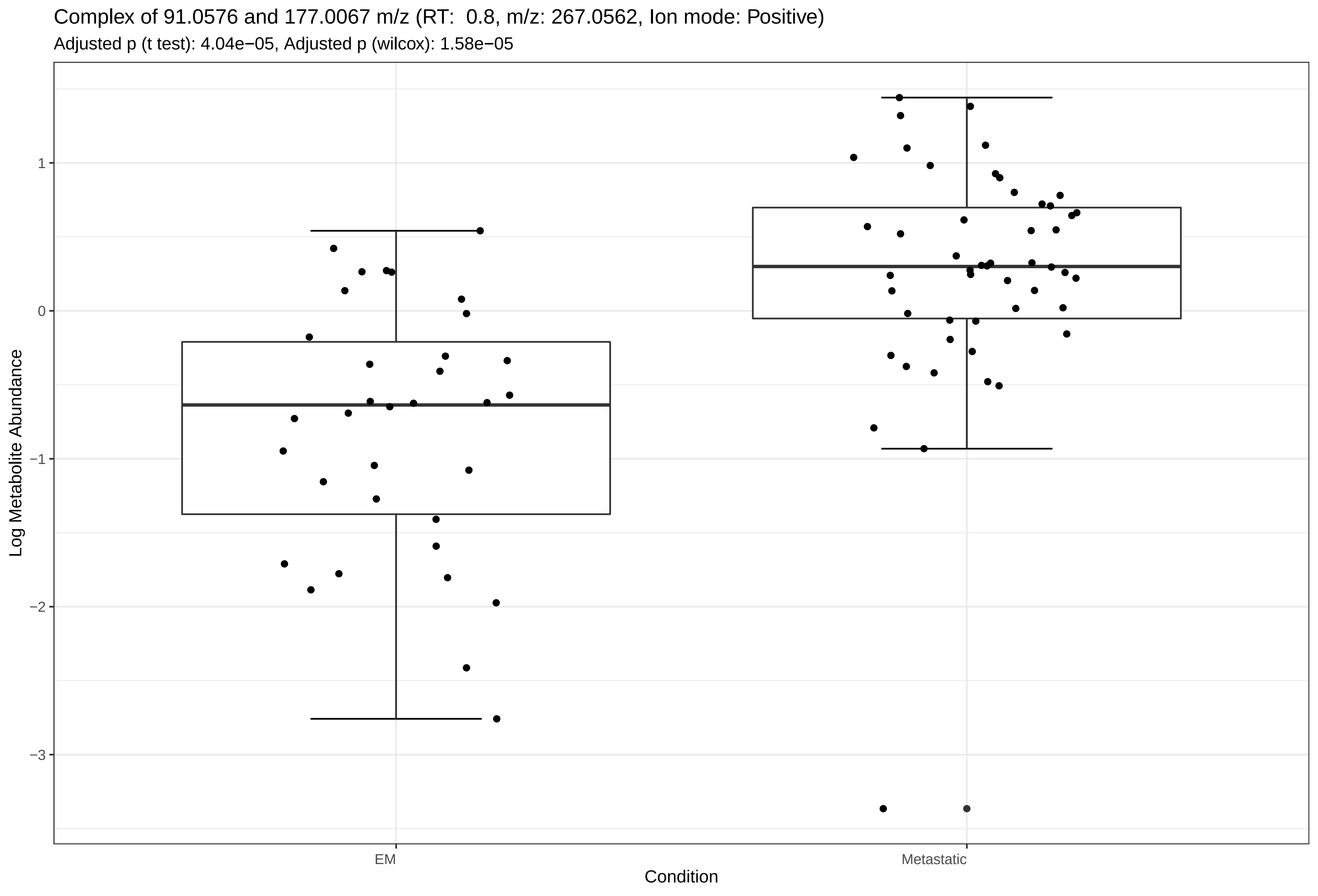
Complex of 91.0576 and 177.0067 m/z (RT: 0.8, m/z: 267.0562, Ion mode: Positive)
Adjusted p (t test): 4.04e−05, Adjusted p (wilcox): 1.58e−05
Log Metabolite Abundance
1
0
−1
−2
−3
EM
Metastatic
Condition

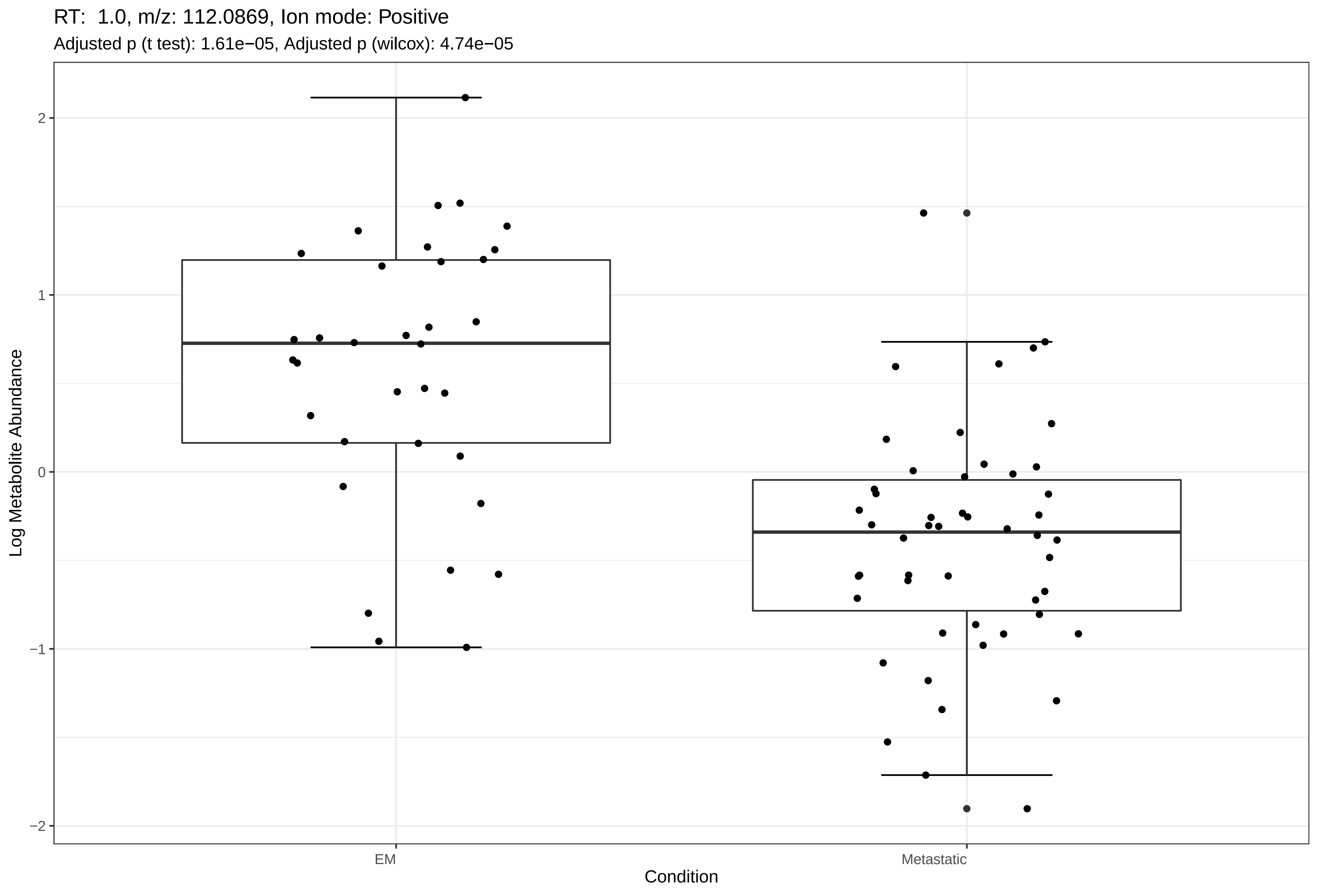
RT: 1.0, m/z: 112.0869, Ion mode: Positive
Adjusted p (t test): 1.61e−05, Adjusted p (wilcox): 4.74e−05
Log Metabolite Abundance
2
1
0
−1
−2
EM
Metastatic
Condition

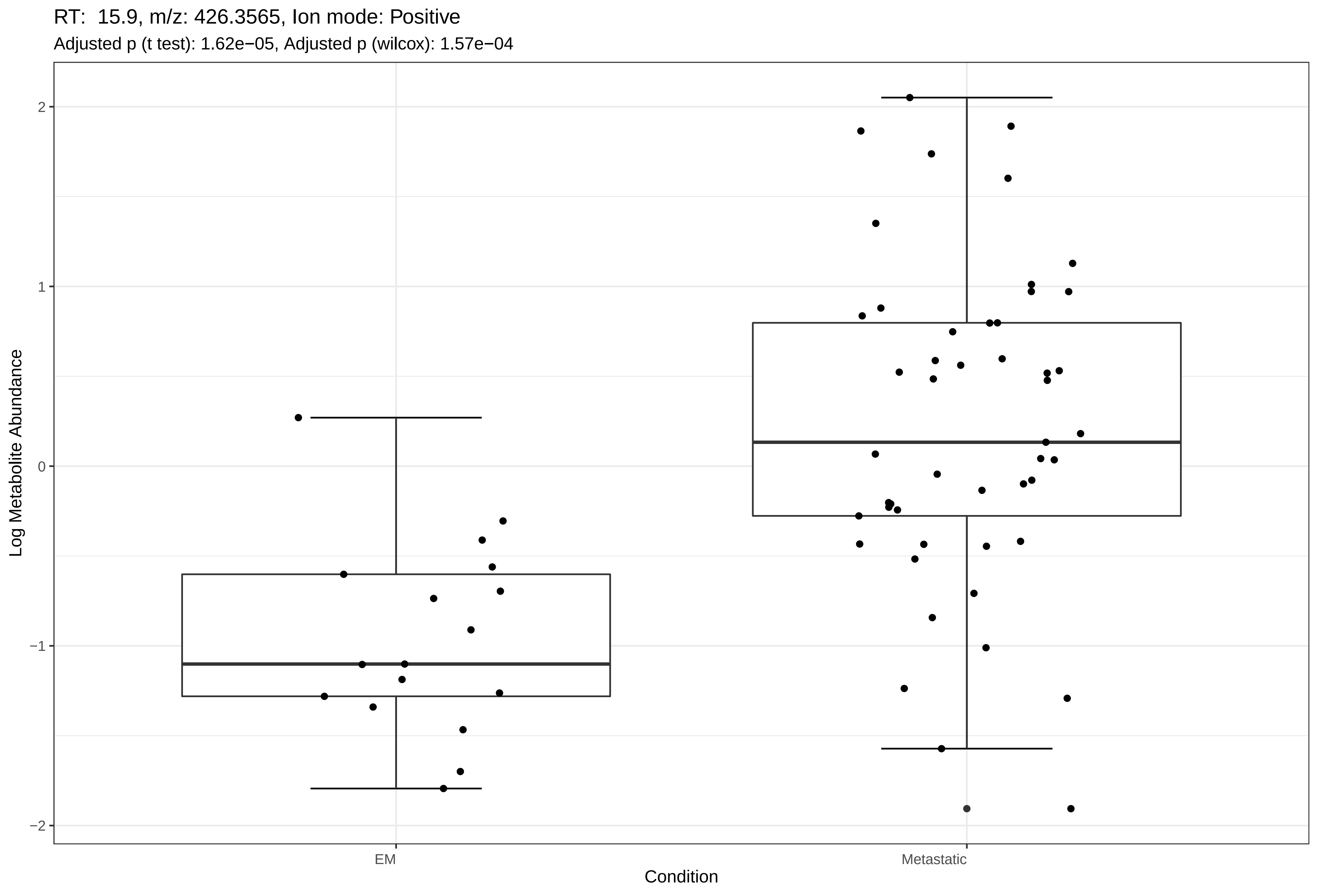

RT: 15.9, m/z: 426.3565, Ion mode: Positive
Adjusted p (t test): 1.62e−05, Adjusted p (wilcox): 1.57e−04
Log Metabolite Abundance
2
1
0
−1
−2
EM
Metastatic
Condition

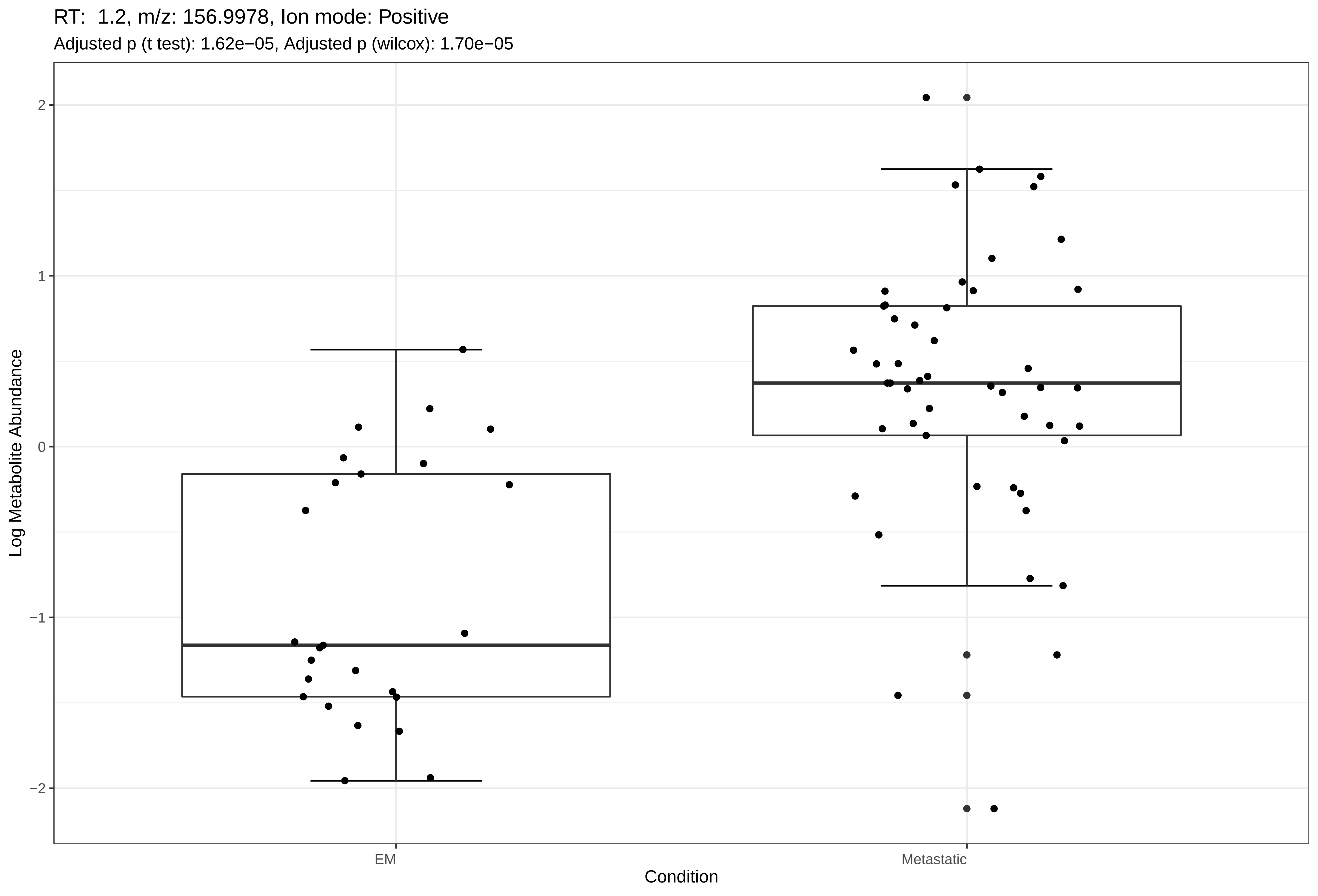
RT: 1.2, m/z: 156.9978, Ion mode: Positive
Adjusted p (t test): 1.62e−05, Adjusted p (wilcox): 1.70e−05
Log Metabolite Abundance
2
1
0
−1
−2
EM
Metastatic
Condition

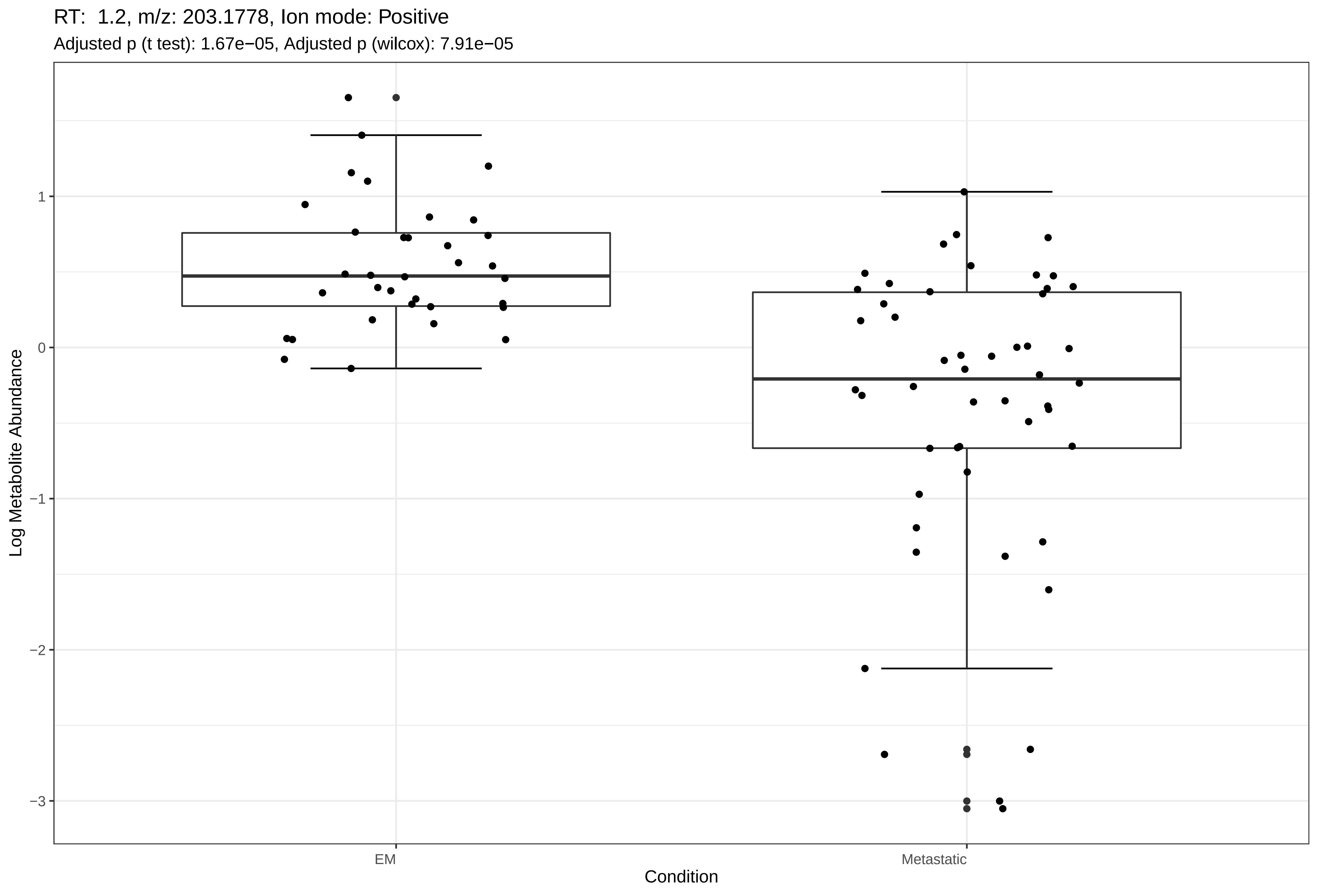

RT: 1.2, m/z: 203.1778, Ion mode: Positive
Adjusted p (t test): 1.67e−05, Adjusted p (wilcox): 7.91e−05
Log Metabolite Abundance
1
0
−1
−2
−3
EM
Metastatic
Condition

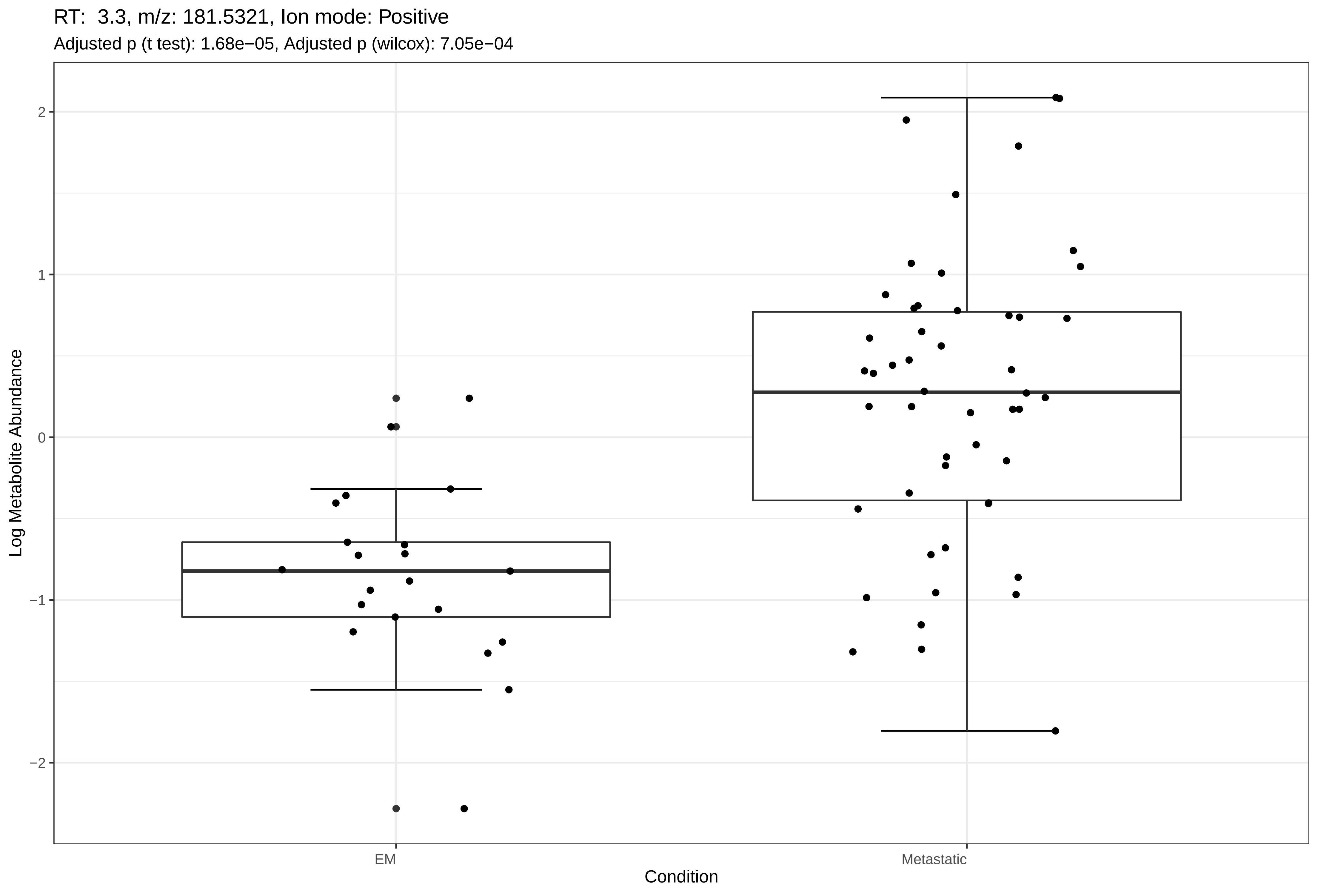

RT: 3.3, m/z: 181.5321, Ion mode: Positive
Adjusted p (t test): 1.68e−05, Adjusted p (wilcox): 7.05e−04
Log Metabolite Abundance
2
1
0
−1
−2
EM
Metastatic
Condition

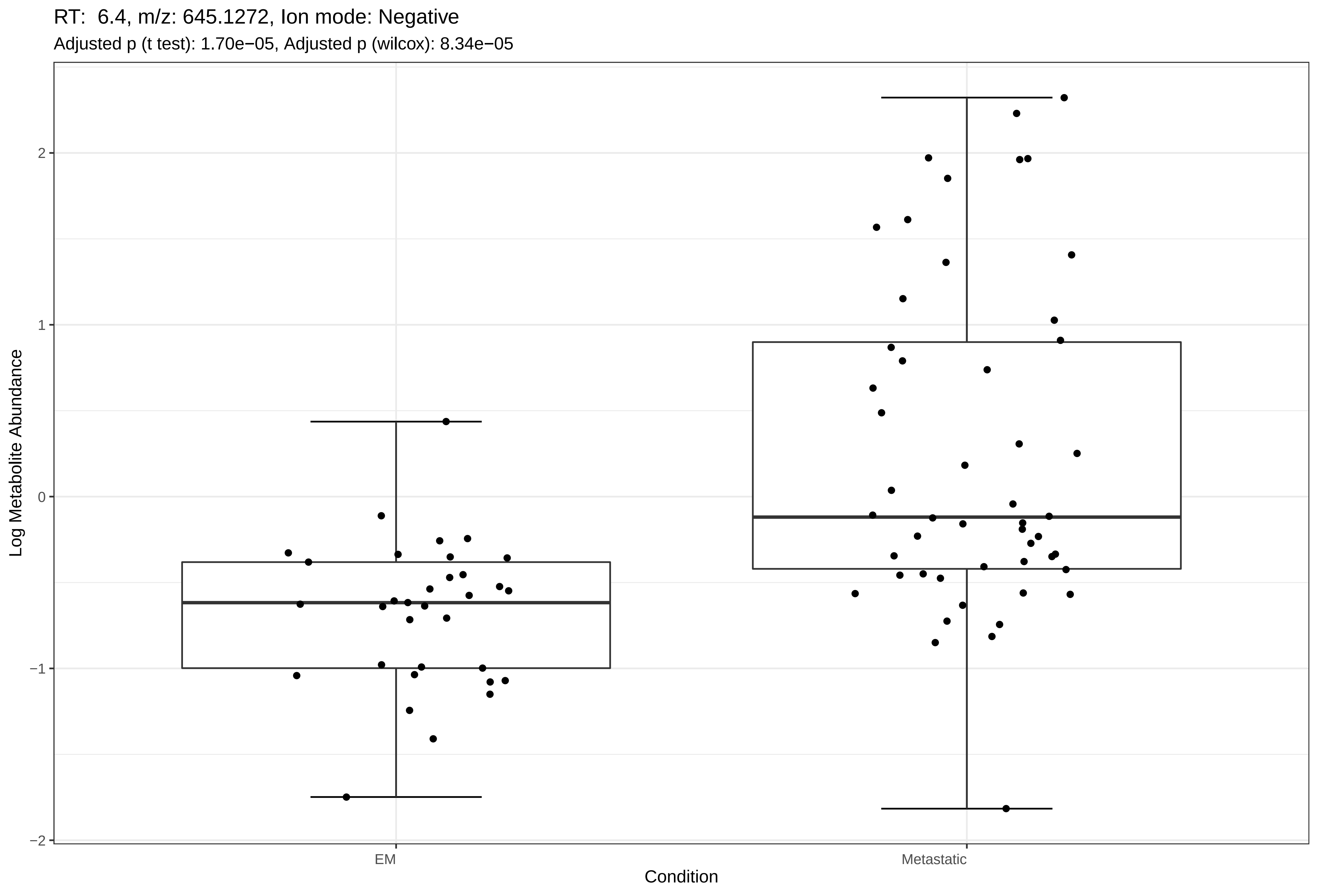
RT: 6.4, m/z: 645.1272, Ion mode: Negative
Adjusted p (t test): 1.70e−05, Adjusted p (wilcox): 8.34e−05
Log Metabolite Abundance
2
1
0
−1
−2
EM
Metastatic
Condition

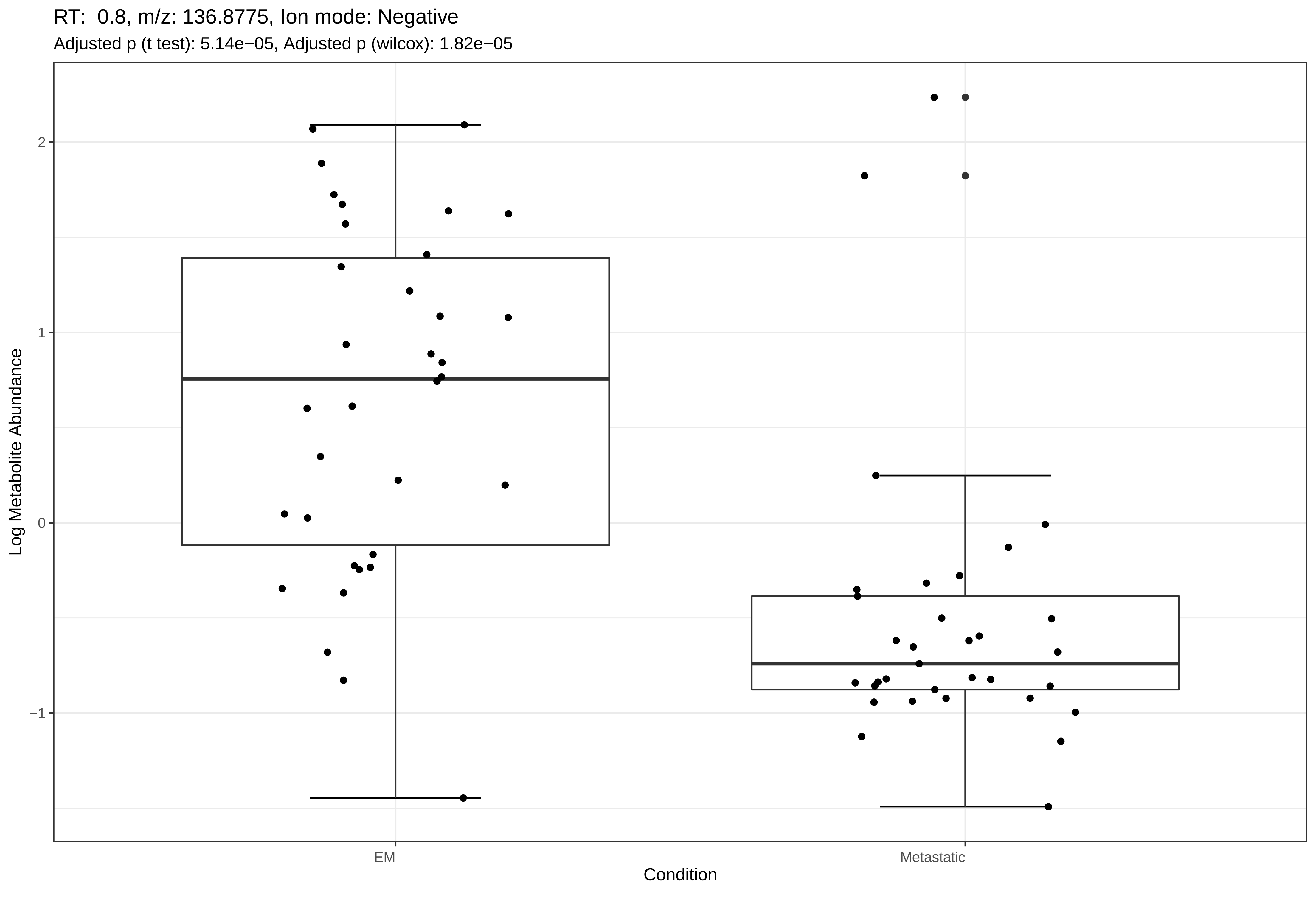

RT: 0.8, m/z: 136.8775, Ion mode: Negative
Adjusted p (t test): 5.14e−05, Adjusted p (wilcox): 1.82e−05
Log Metabolite Abundance
2
1
0
−1
EM
Metastatic
Condition

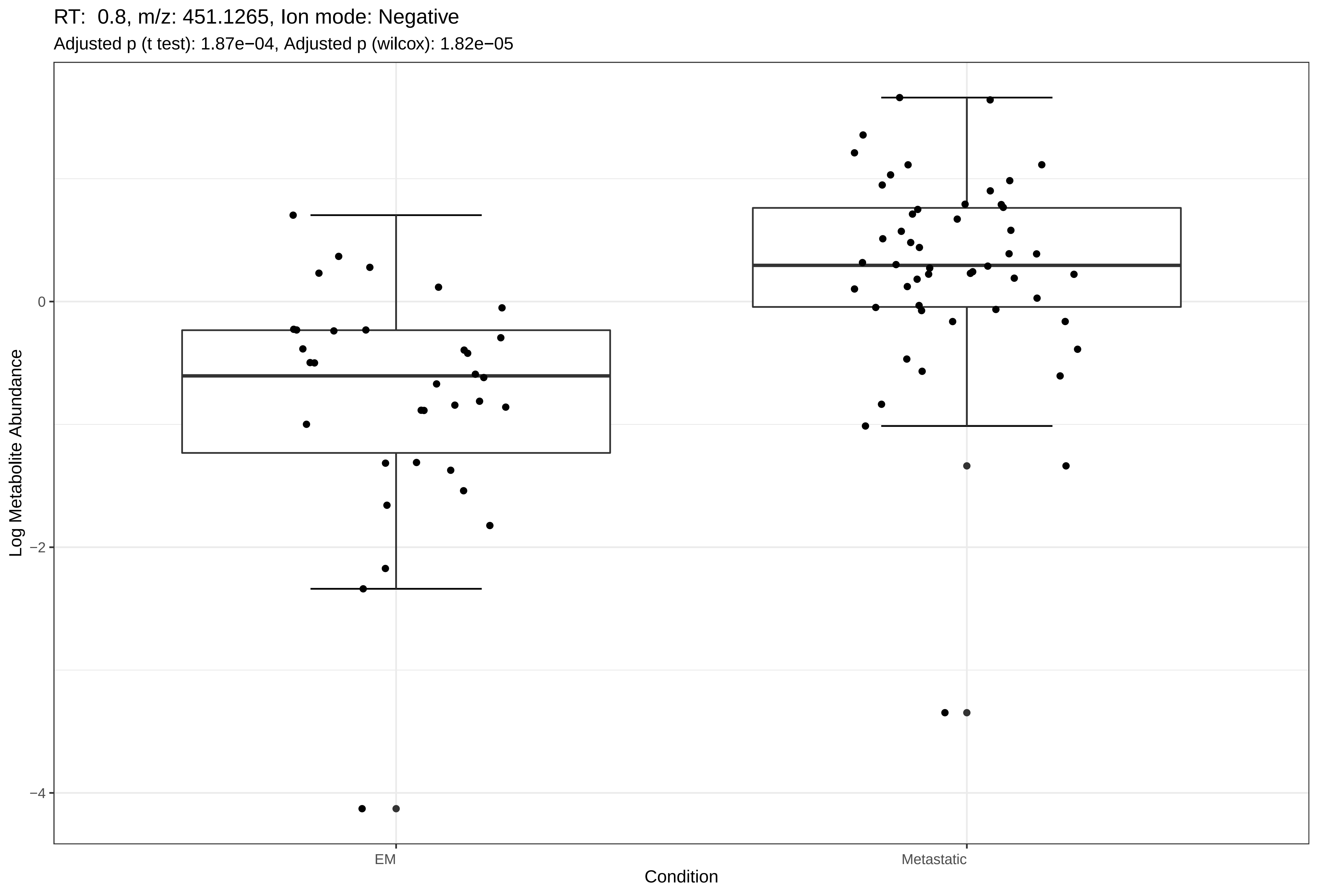

RT: 0.8, m/z: 451.1265, Ion mode: Negative
Adjusted p (t test): 1.87e−04, Adjusted p (wilcox): 1.82e−05
Log Metabolite Abundance
0
−2
−4
EM
Metastatic
Condition

RT: 3.9, m/z: 316.6141, Ion mode: Positive

Adjusted p (t test): 7.29e−03, Adjusted p (wilcox): 1.90e−05

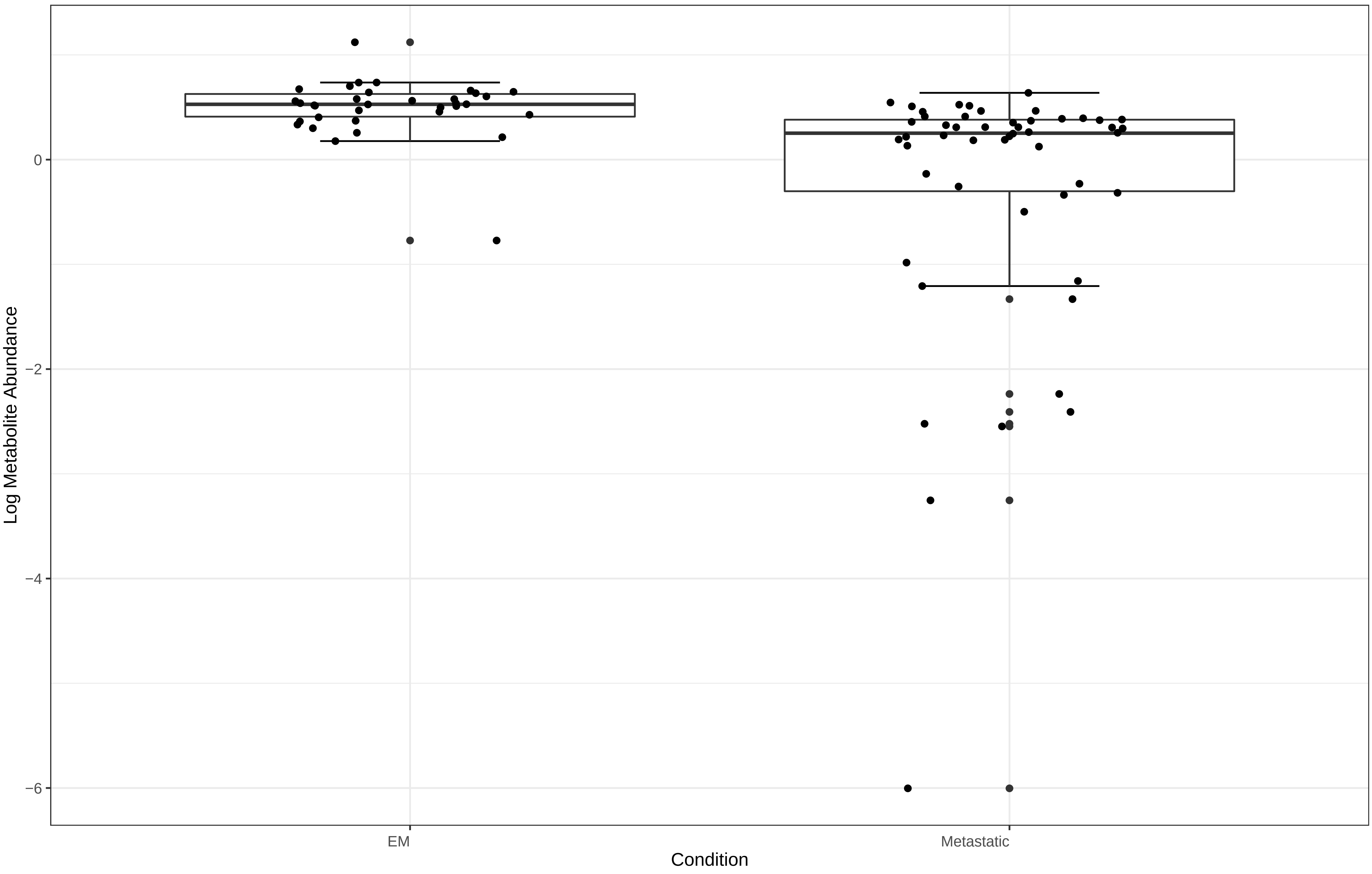

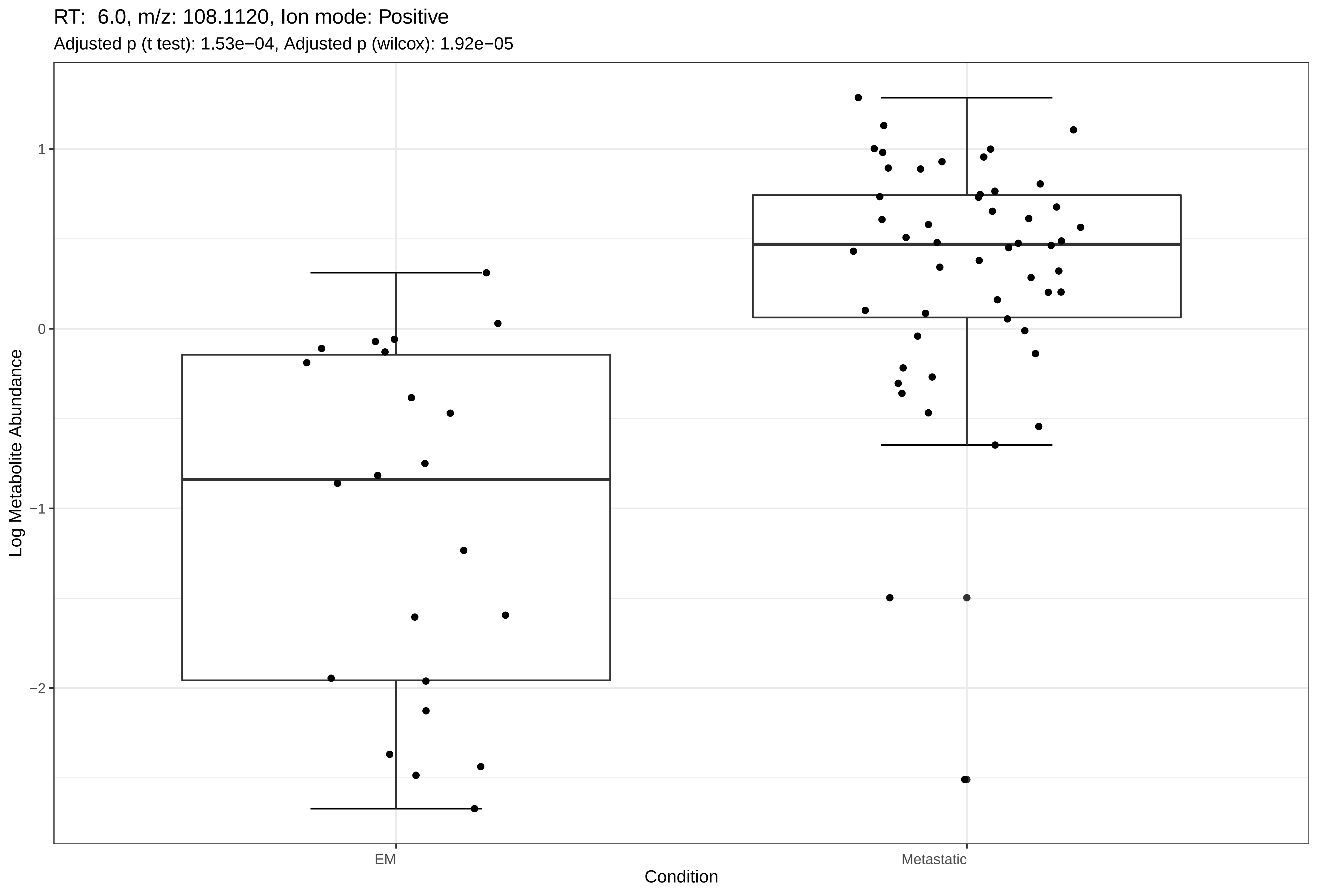

RT: 6.0, m/z: 108.1120, Ion mode: Positive
Adjusted p (t test): 1.53e−04, Adjusted p (wilcox): 1.92e−05
Log Metabolite Abundance
1
0
−1
−2
EM
Metastatic
Condition

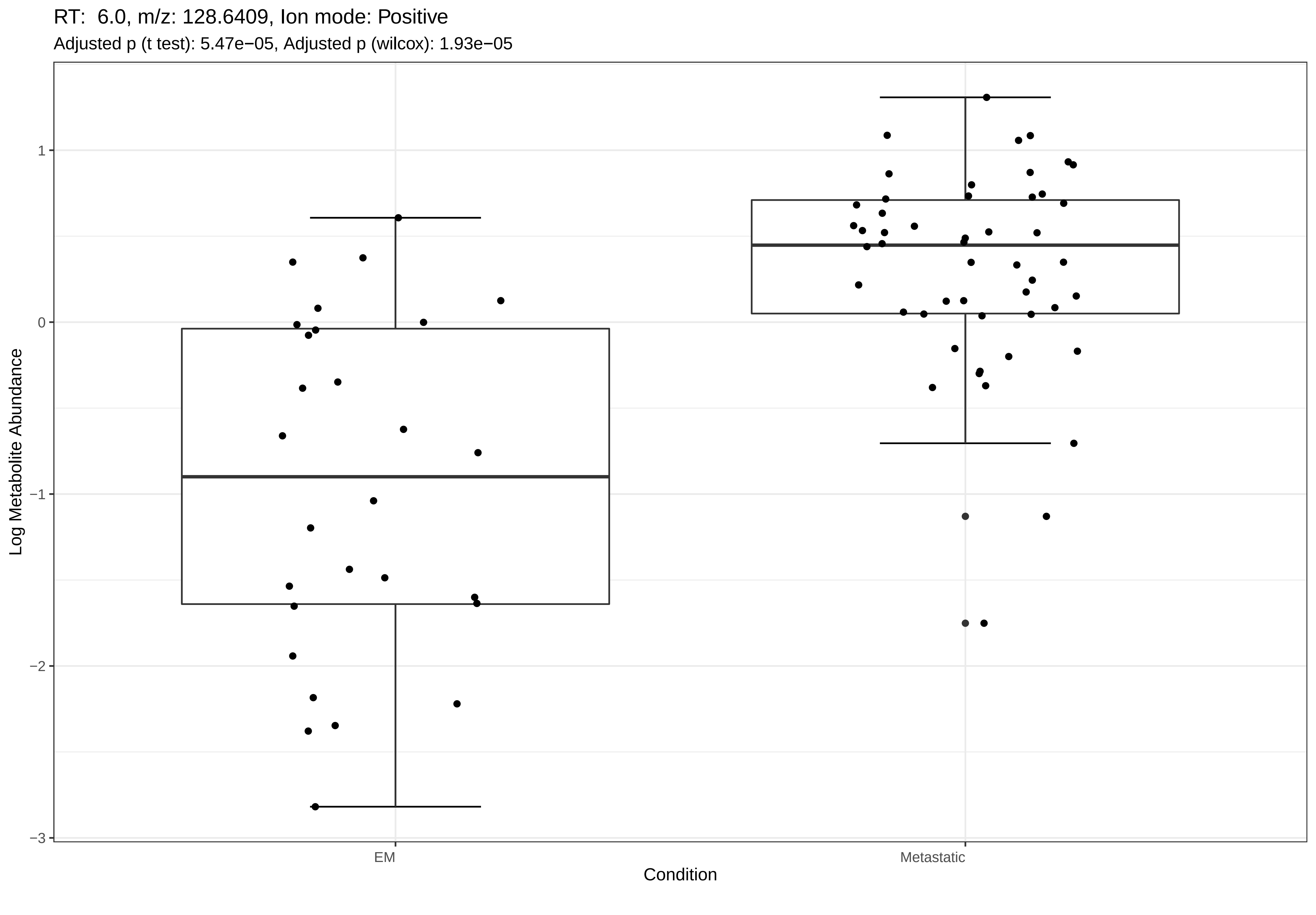
RT: 6.0, m/z: 128.6409, Ion mode: Positive
Adjusted p (t test): 5.47e−05, Adjusted p (wilcox): 1.93e−05
Log Metabolite Abundance
1
0
−1
−2
−3
EM
Metastatic
Condition

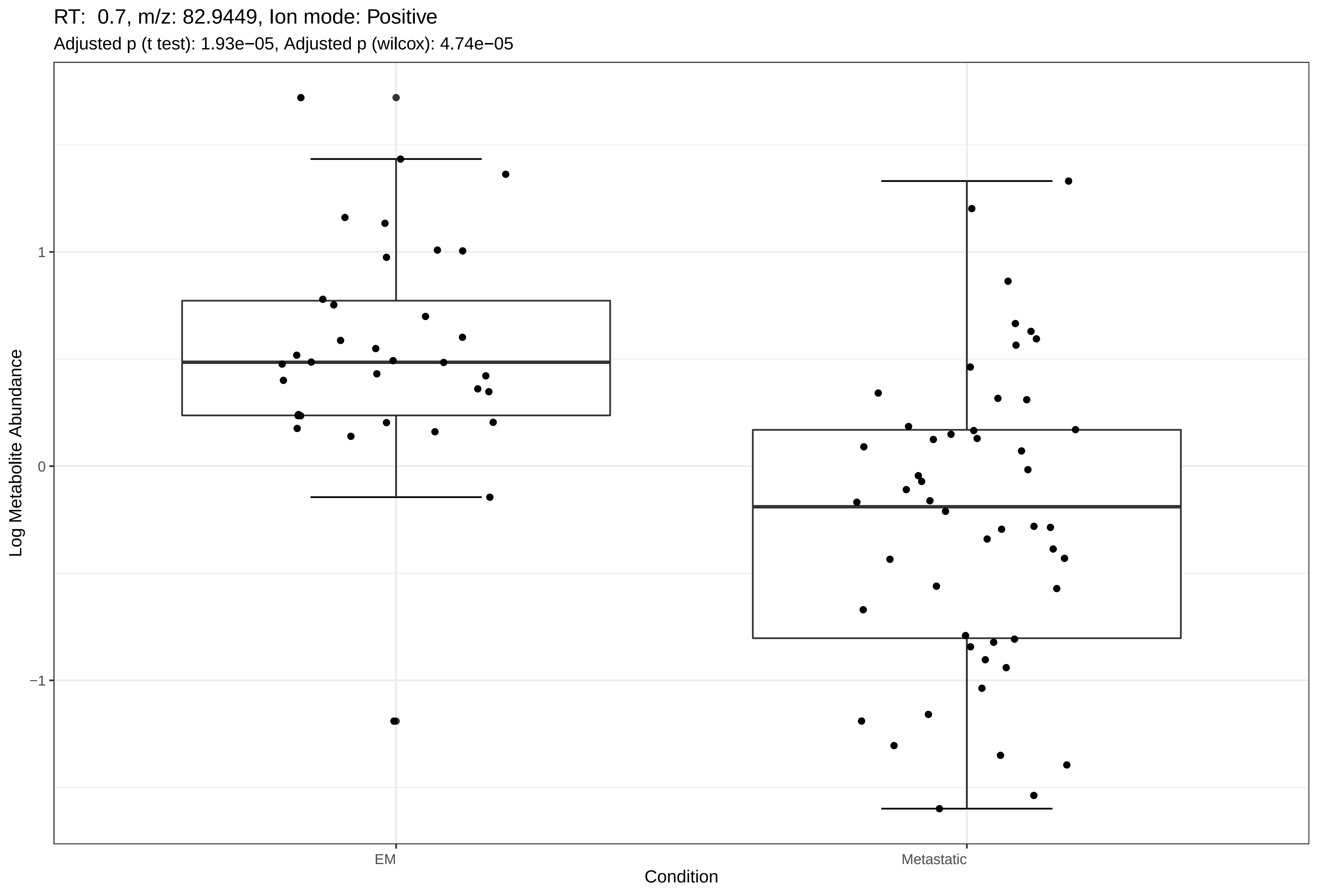

RT: 0.7, m/z: 82.9449, Ion mode: Positive
Adjusted p (t test): 1.93e−05, Adjusted p (wilcox): 4.74e−05
Log Metabolite Abundance
1
0
−1
EM
Metastatic
Condition

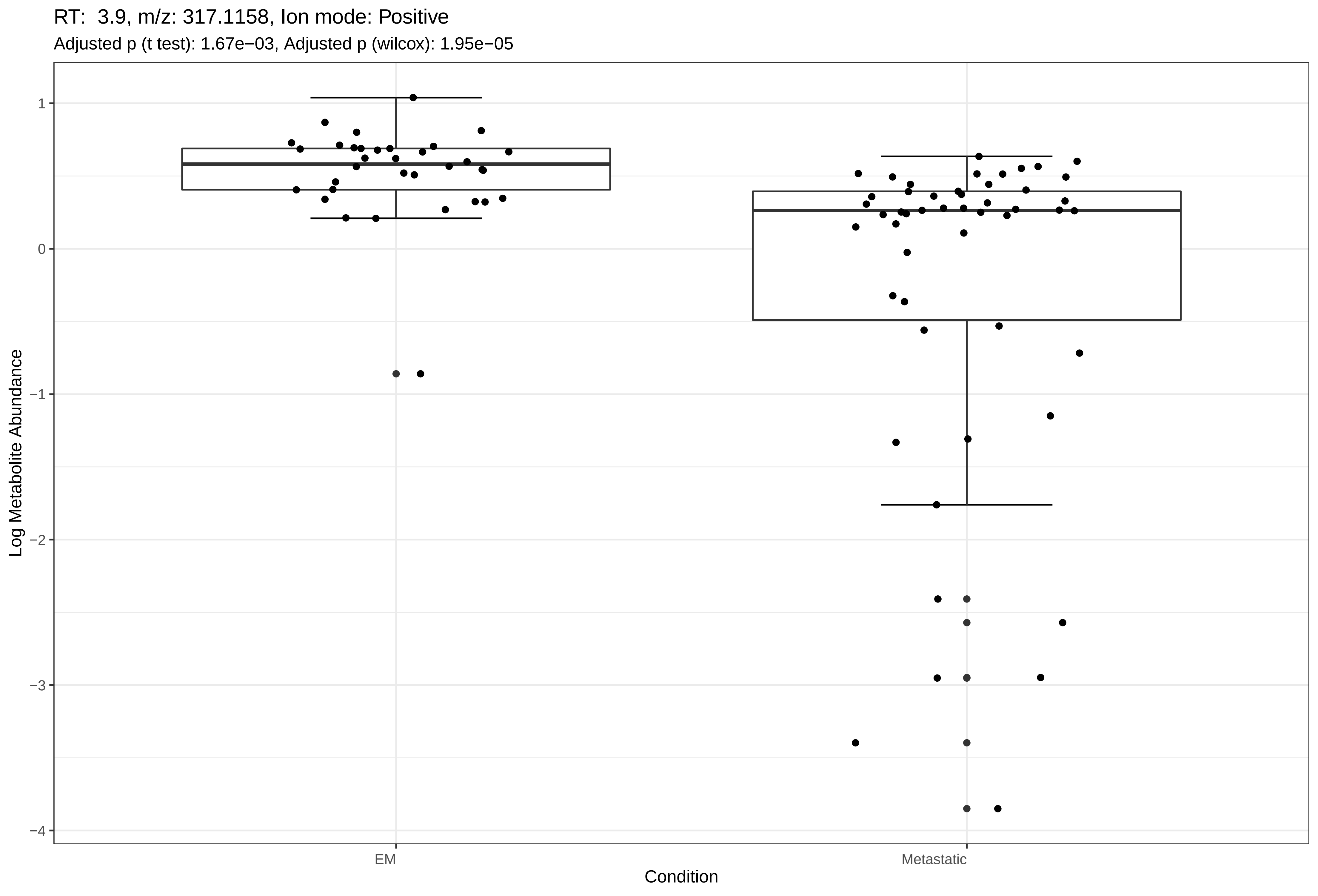
RT: 3.9, m/z: 317.1158, Ion mode: Positive
Adjusted p (t test): 1.67e−03, Adjusted p (wilcox): 1.95e−05
Log Metabolite Abundance
1
0
−1
−2
−3
−4
EM
Metastatic
Condition

RT: 3.9, m/z: 257.1003, Ion mode: Positive

Adjusted p (t test): 5.22e−05, Adjusted p (wilcox): 1.95e−05

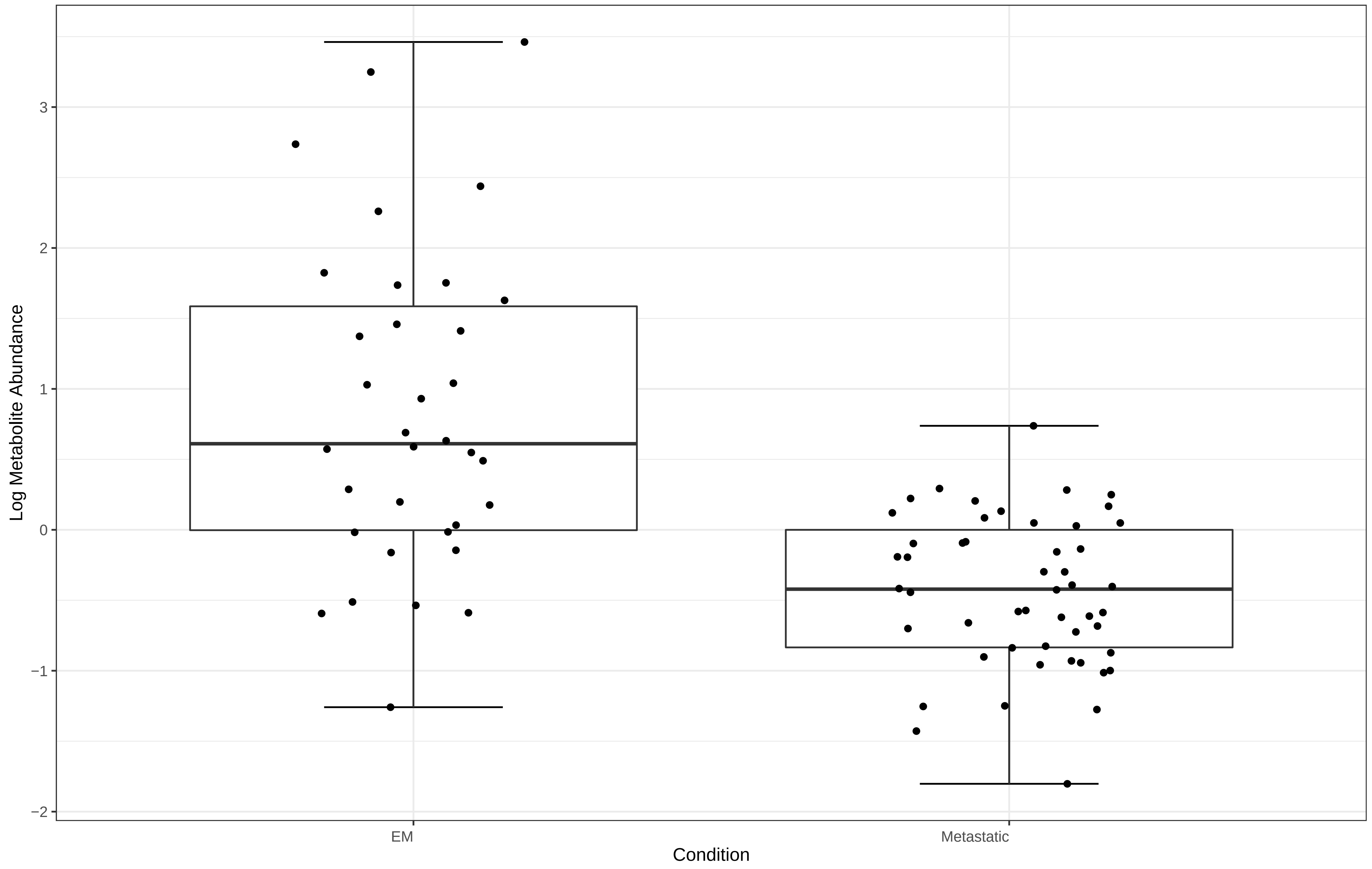

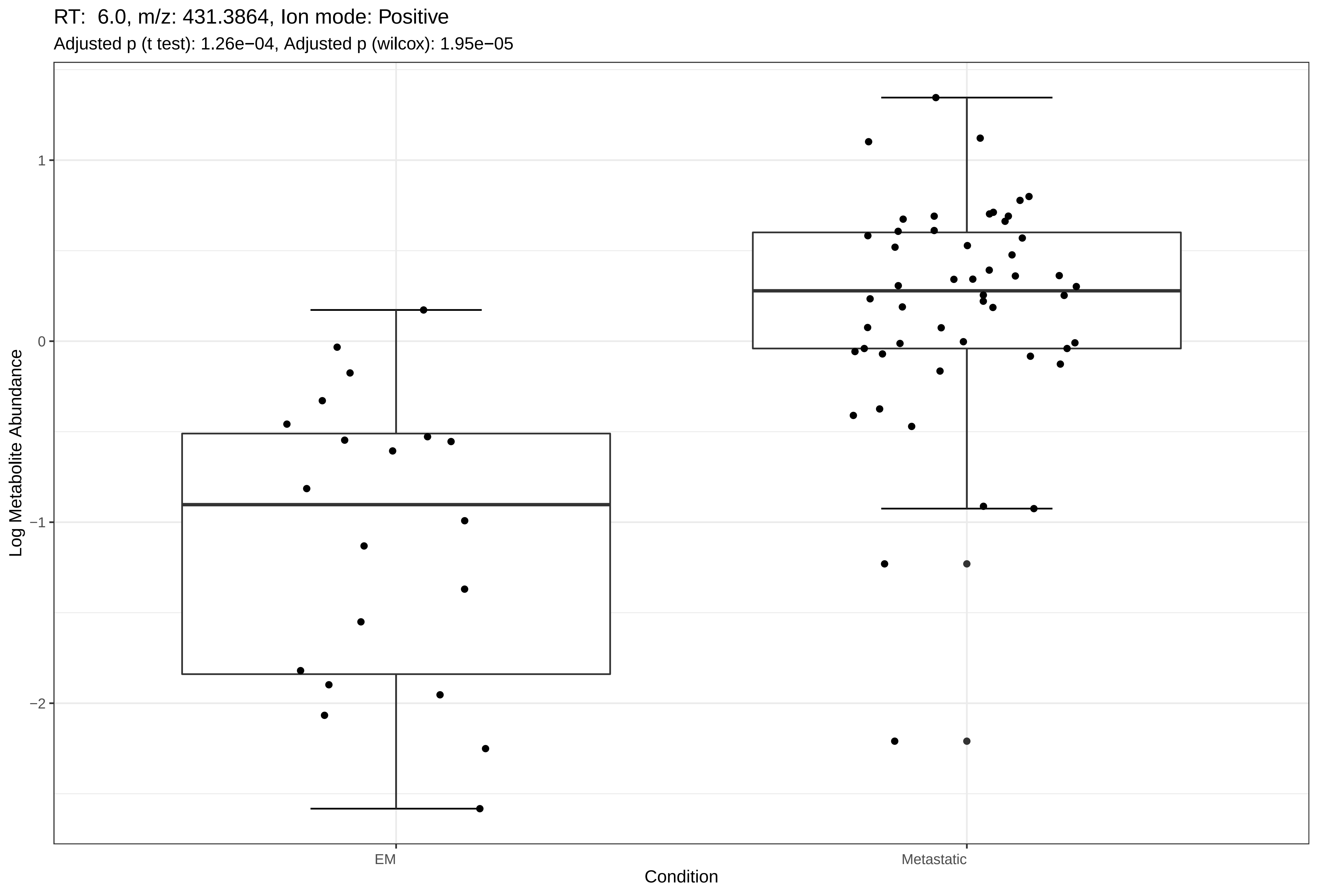

RT: 6.0, m/z: 431.3864, Ion mode: Positive
Adjusted p (t test): 1.26e−04, Adjusted p (wilcox): 1.95e−05
Log Metabolite Abundance
1
0
−1
−2
EM
Metastatic
Condition

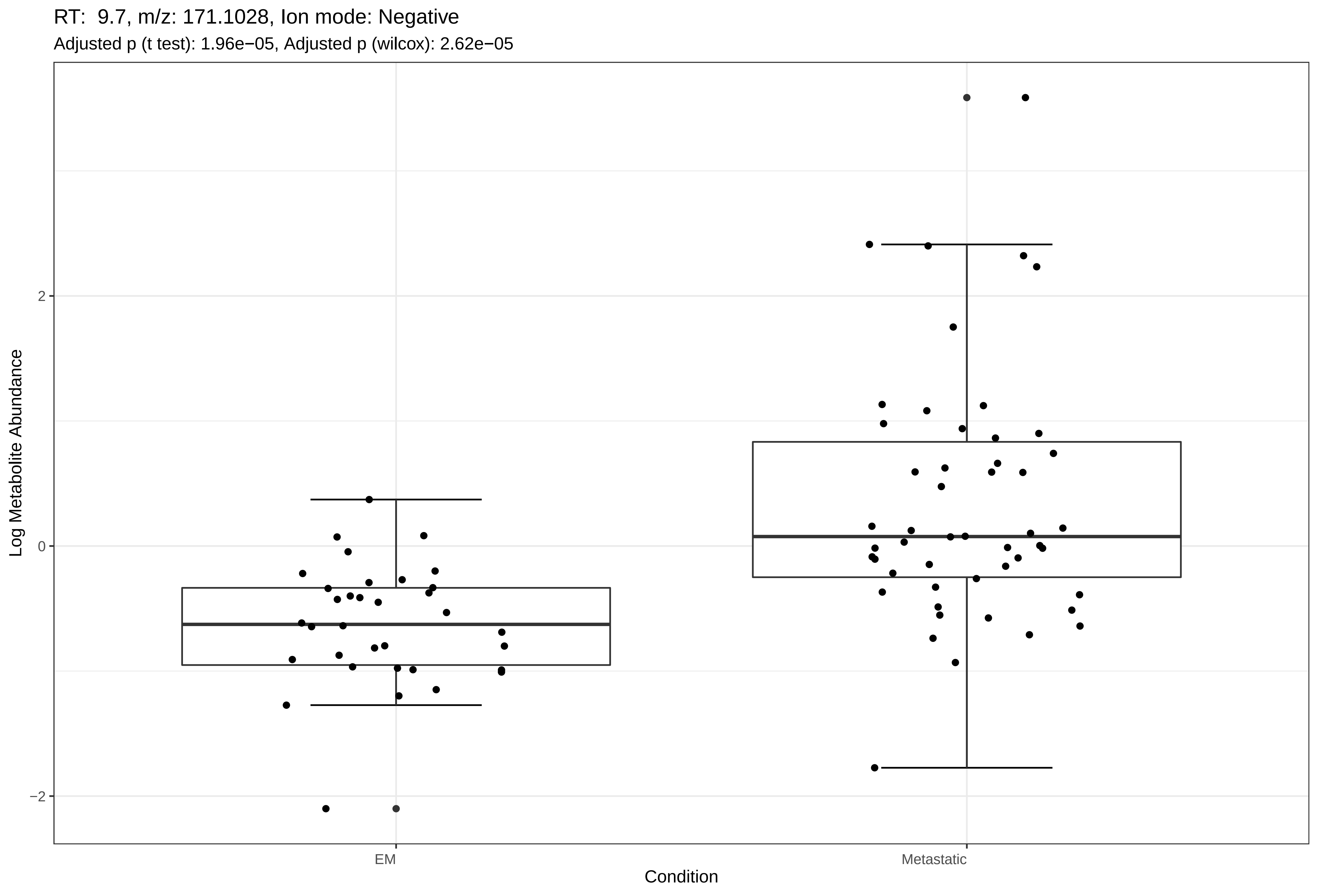
RT: 9.7, m/z: 171.1028, Ion mode: Negative
Adjusted p (t test): 1.96e−05, Adjusted p (wilcox): 2.62e−05
Log Metabolite Abundance
2
0
−2
EM
Metastatic
Condition

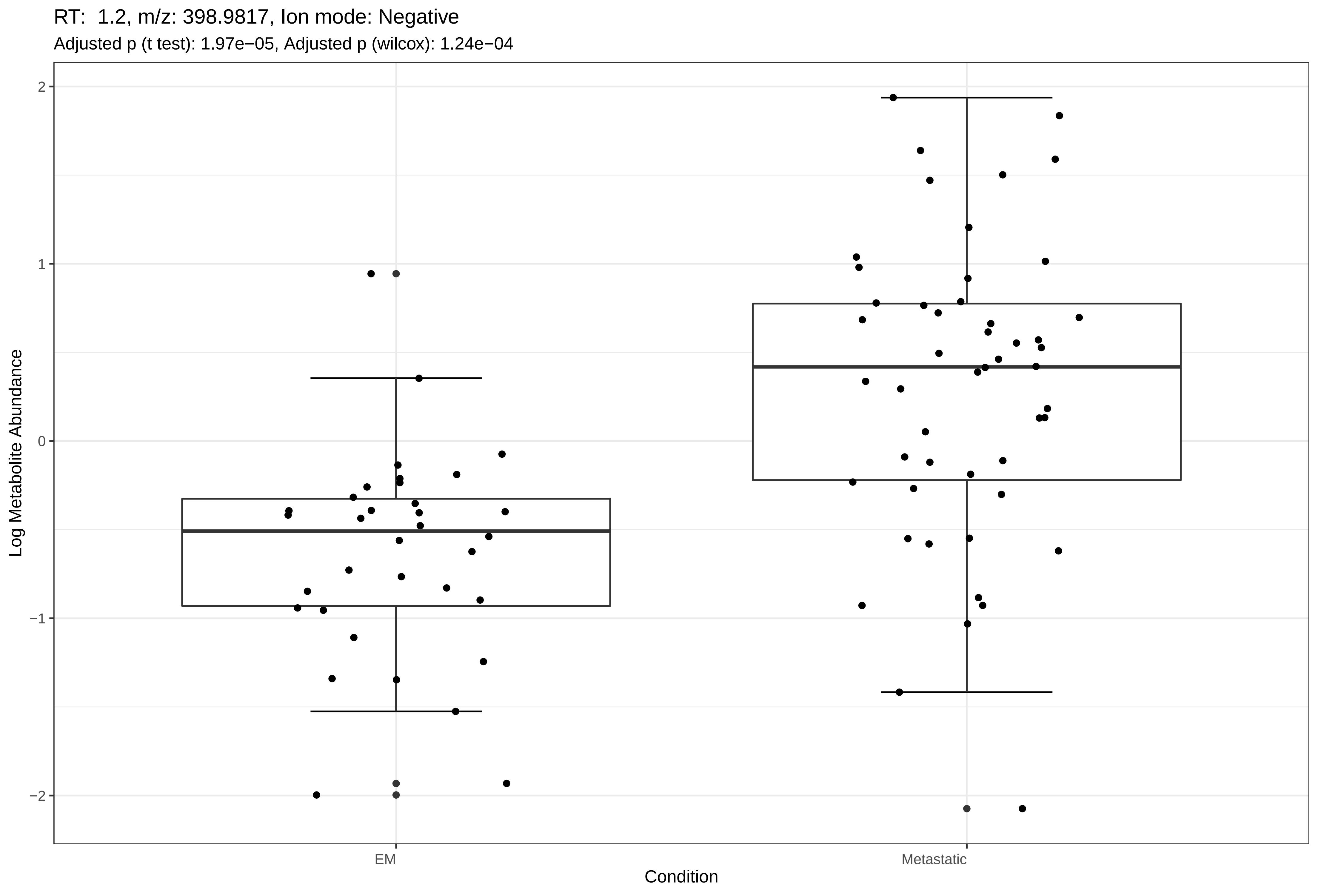
RT: 1.2, m/z: 398.9817, Ion mode: Negative
Adjusted p (t test): 1.97e−05, Adjusted p (wilcox): 1.24e−04
Log Metabolite Abundance
2
1
0
−1
−2
EM
Metastatic
Condition

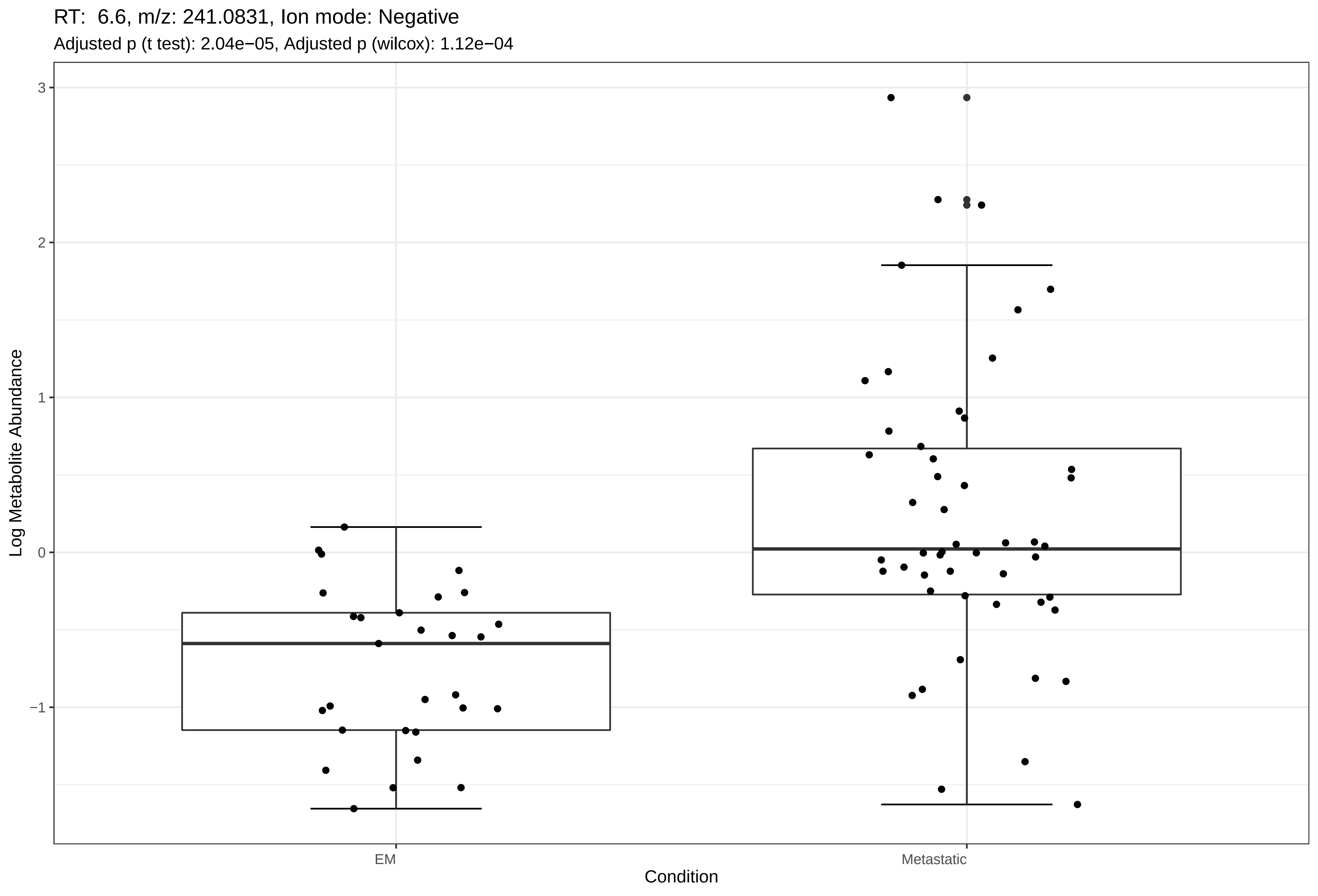
RT: 6.6, m/z: 241.0831, Ion mode: Negative
Adjusted p (t test): 2.04e−05, Adjusted p (wilcox): 1.12e−04
Log Metabolite Abundance
3
2
1
0
−1
EM
Metastatic
Condition

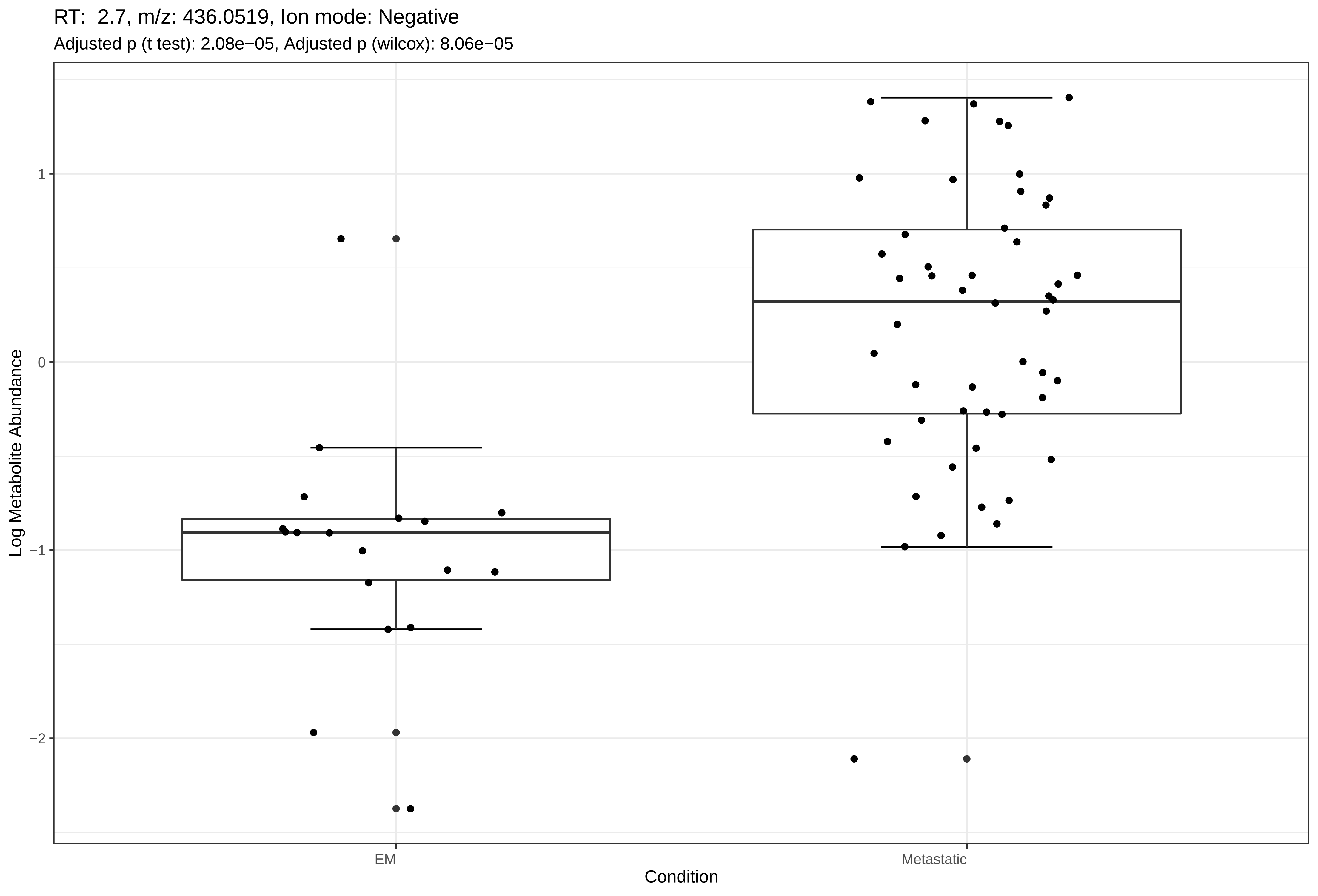
RT: 2.7, m/z: 436.0519, Ion mode: Negative
Adjusted p (t test): 2.08e−05, Adjusted p (wilcox): 8.06e−05
Log Metabolite Abundance
1
0
−1
−2
EM
Metastatic
Condition

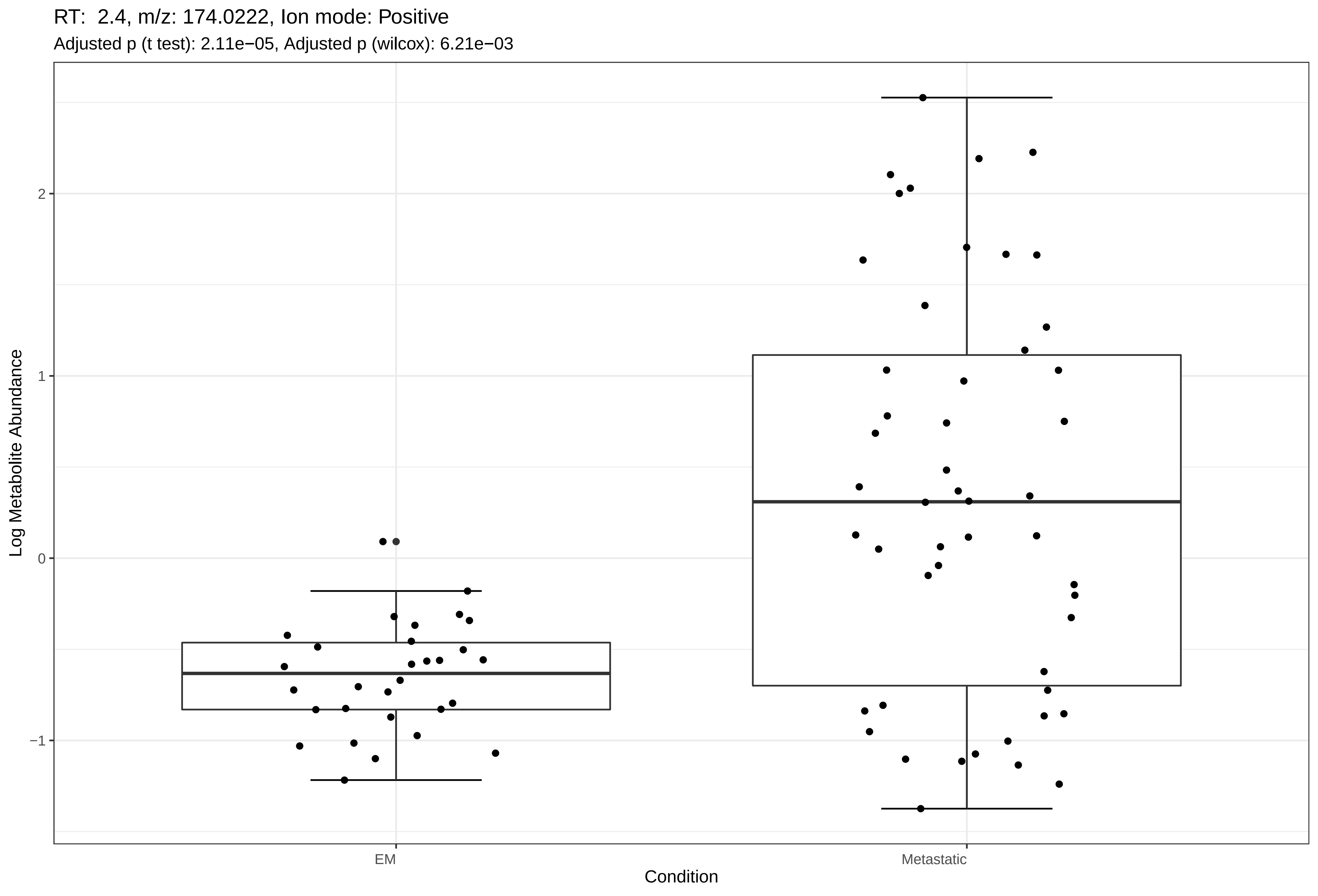

RT: 2.4, m/z: 174.0222, Ion mode: Positive
Adjusted p (t test): 2.11e−05, Adjusted p (wilcox): 6.21e−03
Log Metabolite Abundance
2
1
0
−1
EM
Metastatic
Condition

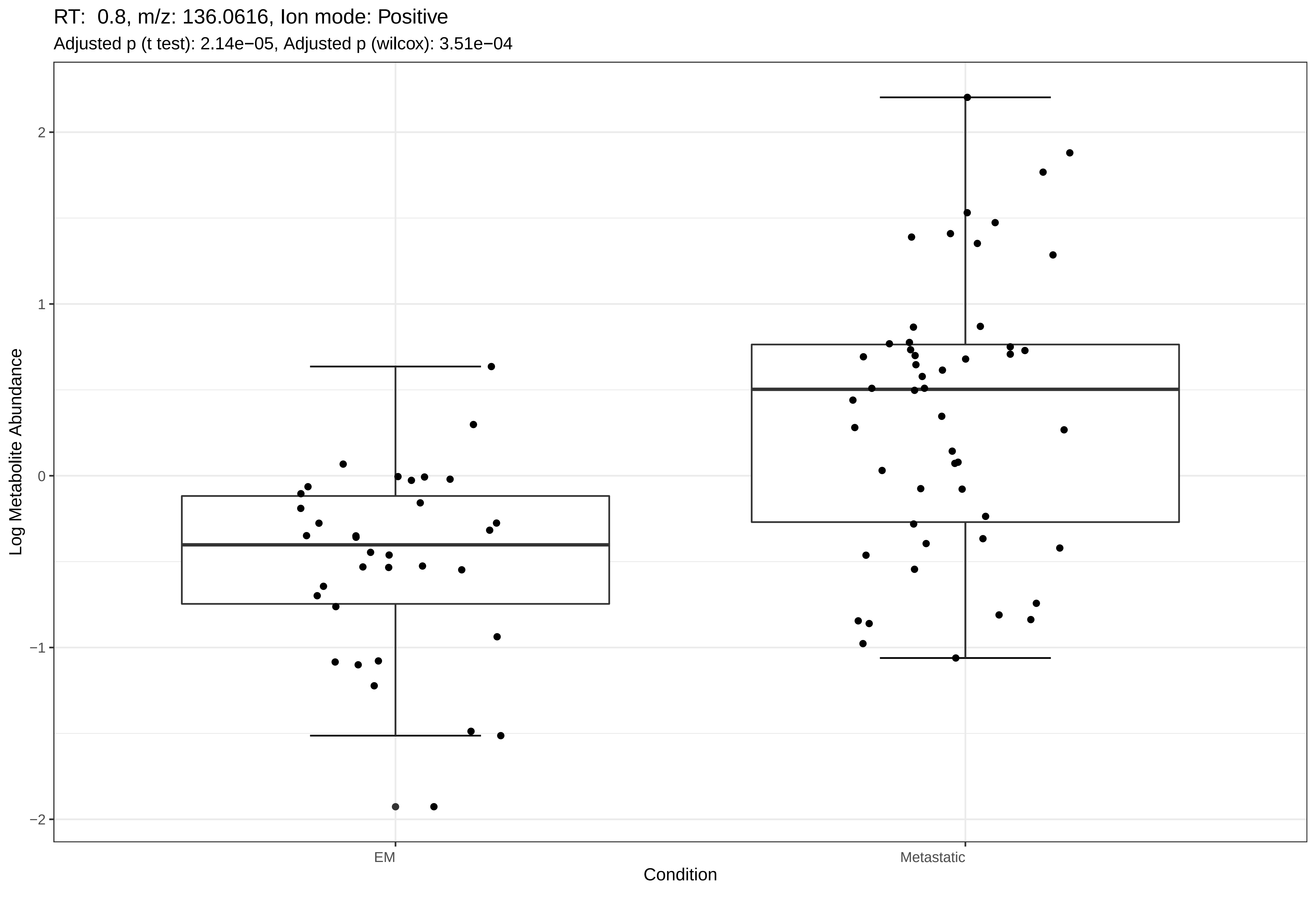

RT: 0.8, m/z: 136.0616, Ion mode: Positive
Adjusted p (t test): 2.14e−05, Adjusted p (wilcox): 3.51e−04
Log Metabolite Abundance
2
1
0
−1
−2
EM
Metastatic
Condition

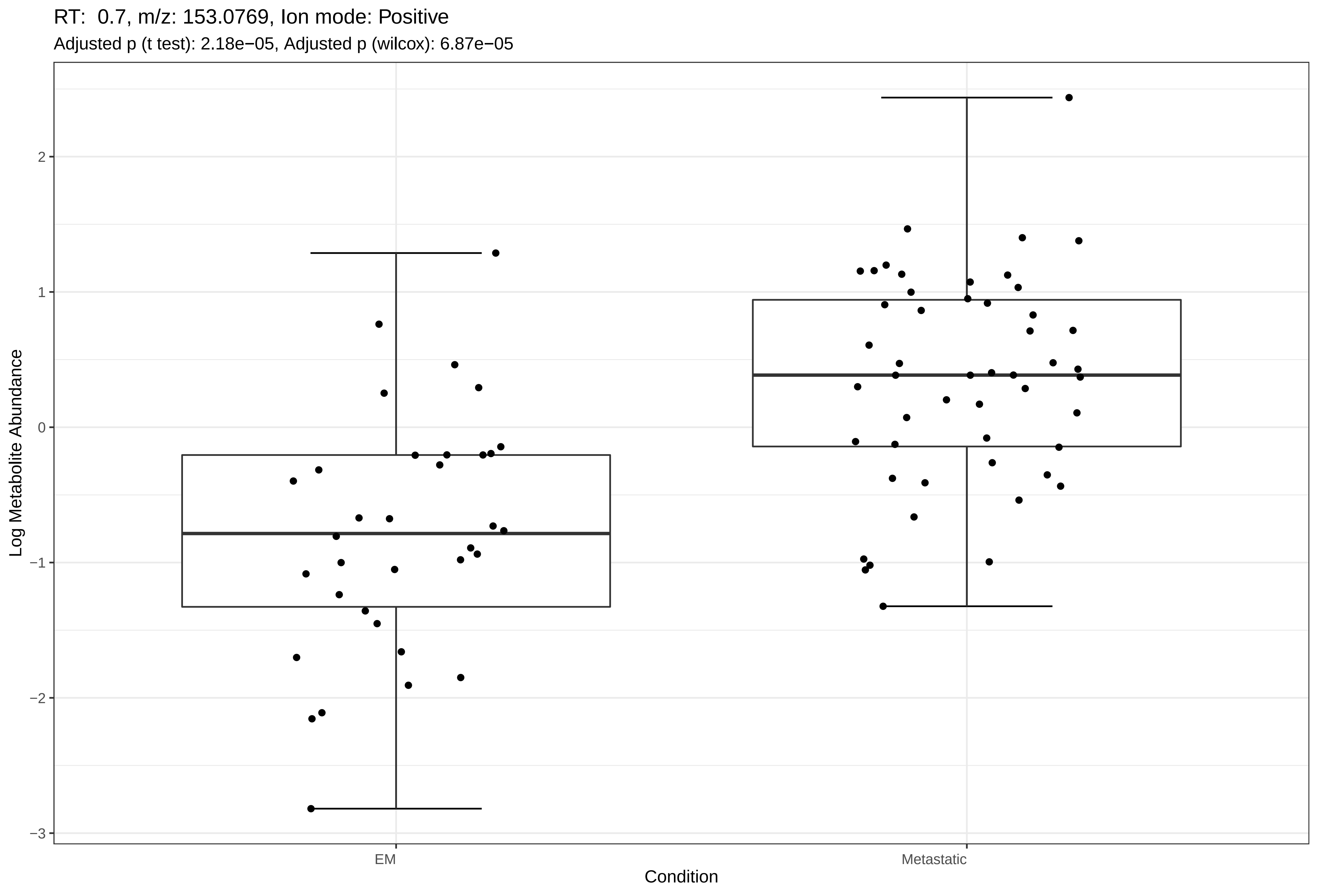

RT: 0.7, m/z: 153.0769, Ion mode: Positive
Adjusted p (t test): 2.18e−05, Adjusted p (wilcox): 6.87e−05
Log Metabolite Abundance
2
1
0
−1
−2
−3
EM
Metastatic
Condition

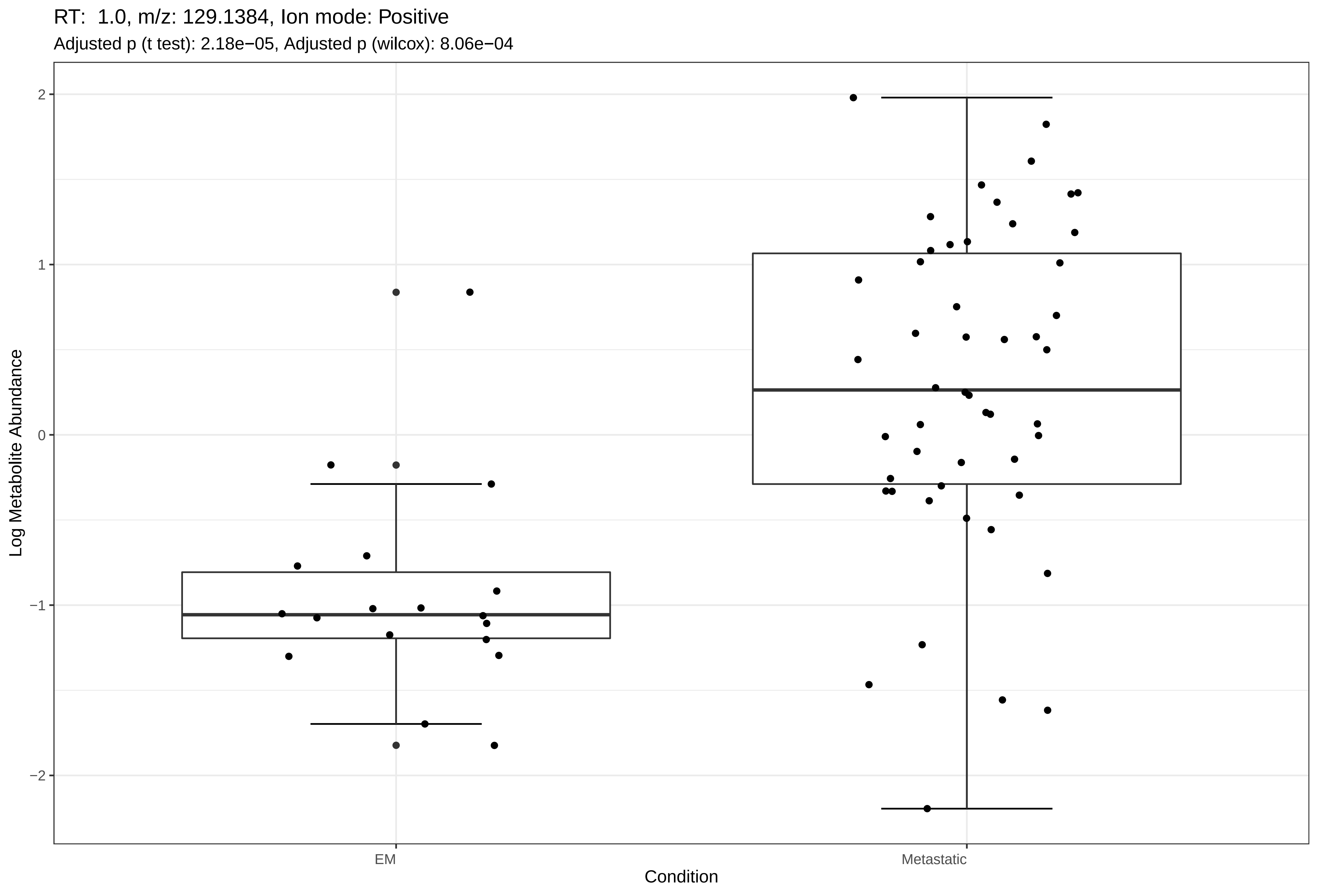

RT: 1.0, m/z: 129.1384, Ion mode: Positive
Adjusted p (t test): 2.18e−05, Adjusted p (wilcox): 8.06e−04
Log Metabolite Abundance
2
1
0
−1
−2
EM
Metastatic
Condition

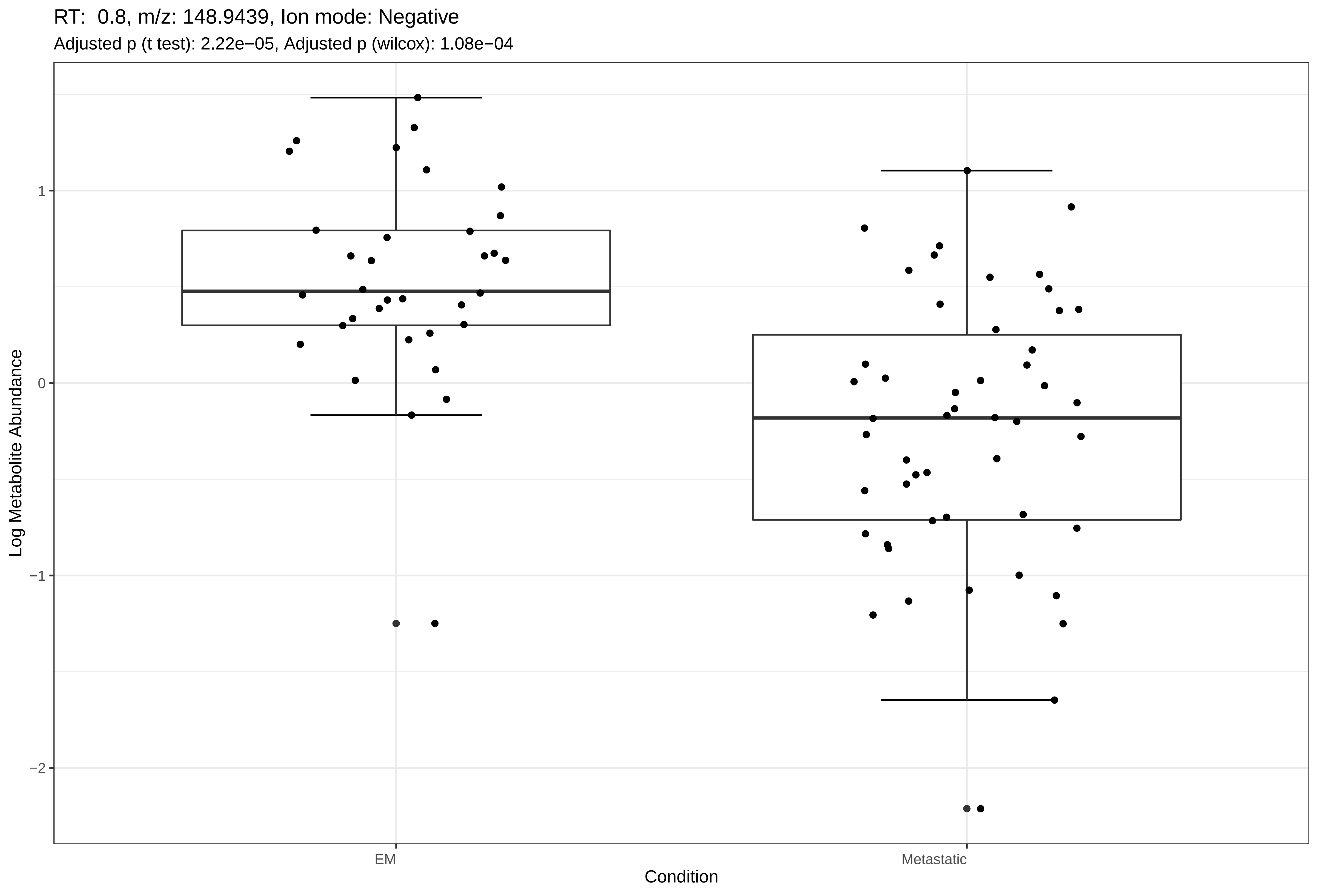

RT: 0.8, m/z: 148.9439, Ion mode: Negative
Adjusted p (t test): 2.22e−05, Adjusted p (wilcox): 1.08e−04
Log Metabolite Abundance
1
0
−1
−2
EM
Metastatic
Condition

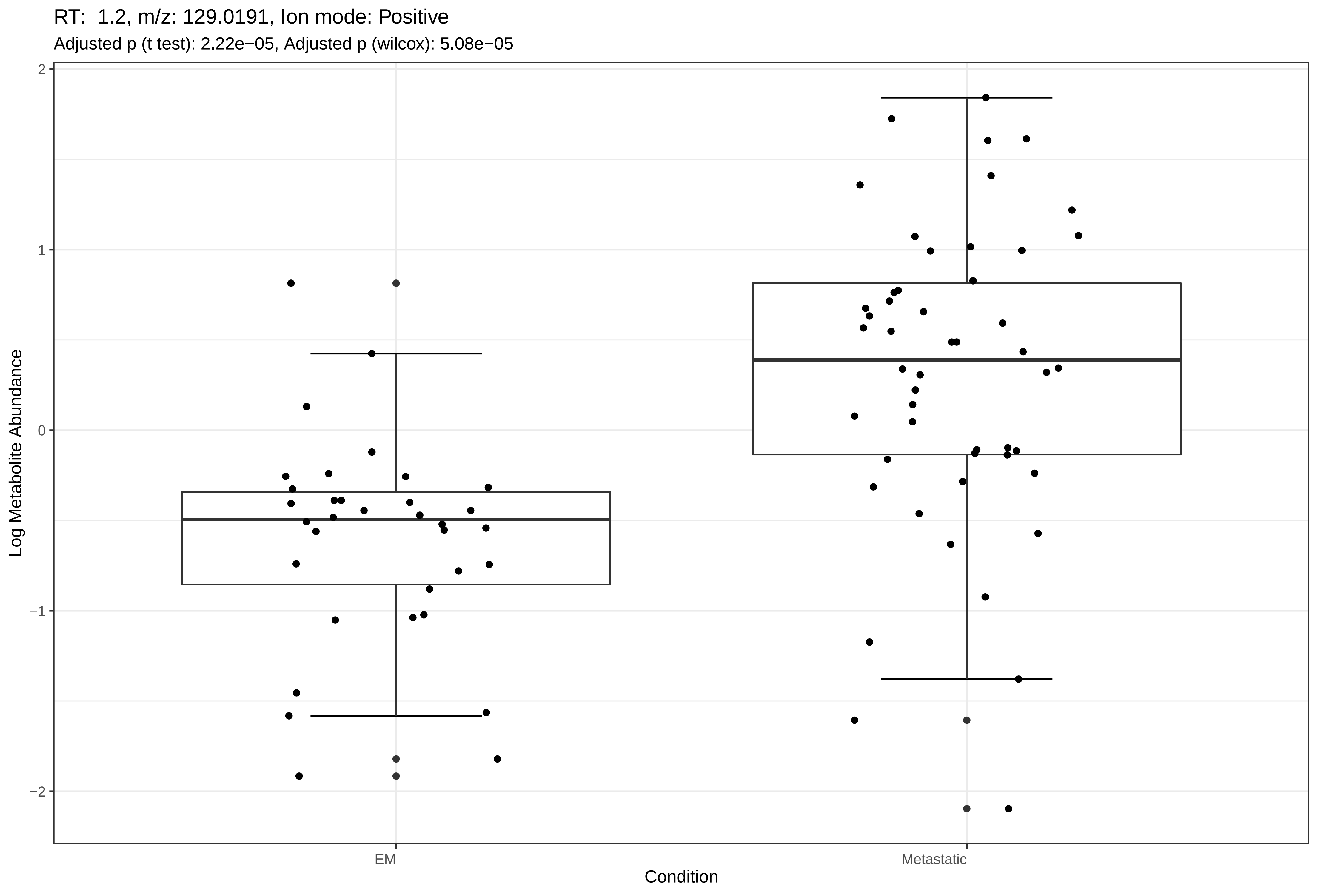
RT: 1.2, m/z: 129.0191, Ion mode: Positive
Adjusted p (t test): 2.22e−05, Adjusted p (wilcox): 5.08e−05
Log Metabolite Abundance
2
1
0
−1
−2
EM
Metastatic
Condition

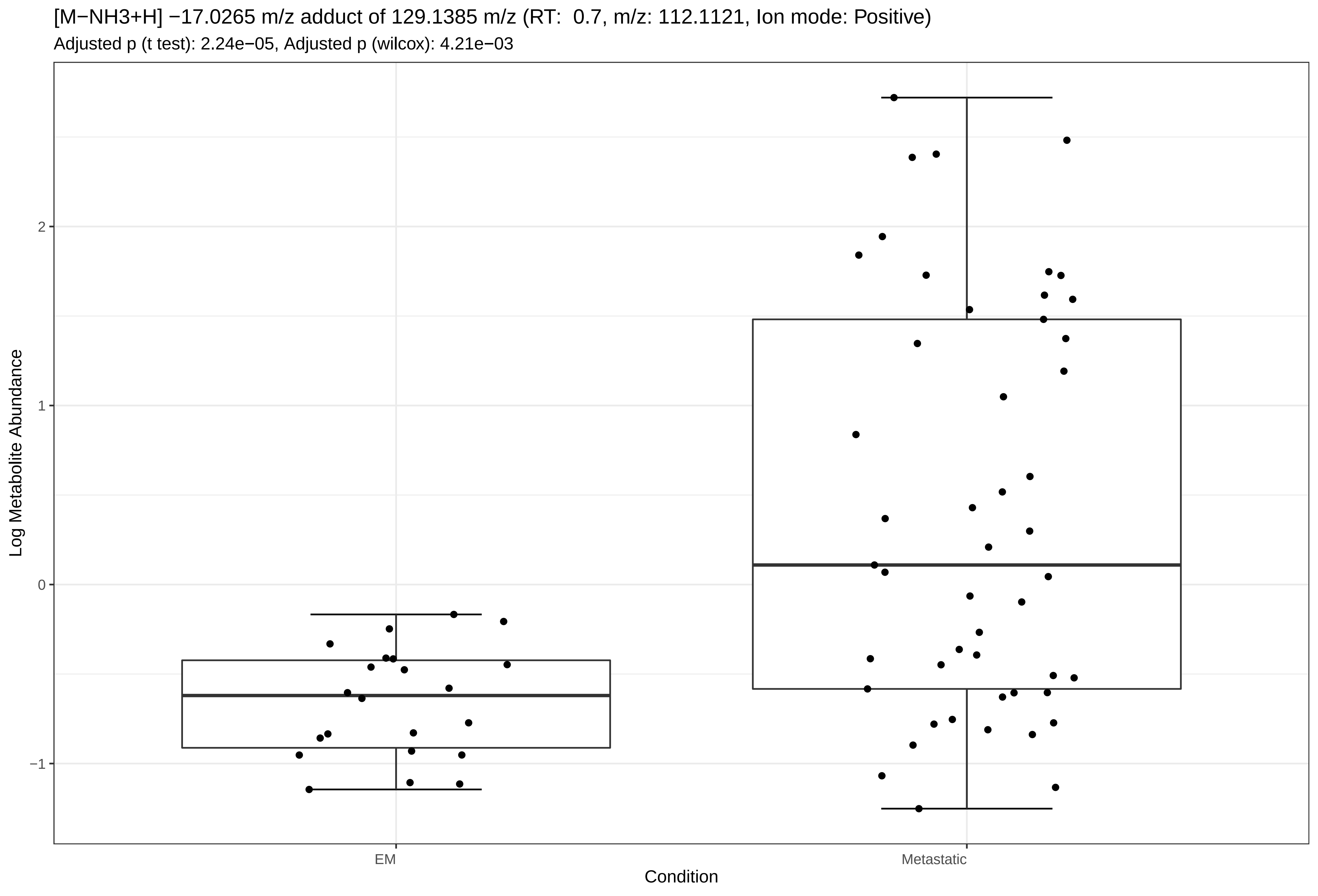

[M−NH3+H] −17.0265 m/z adduct of 129.1385 m/z (RT: 0.7, m/z: 112.1121, Ion mode: Positive)
Adjusted p (t test): 2.24e−05, Adjusted p (wilcox): 4.21e−03
Log Metabolite Abundance
2
1
0
−1
EM
Metastatic
Condition

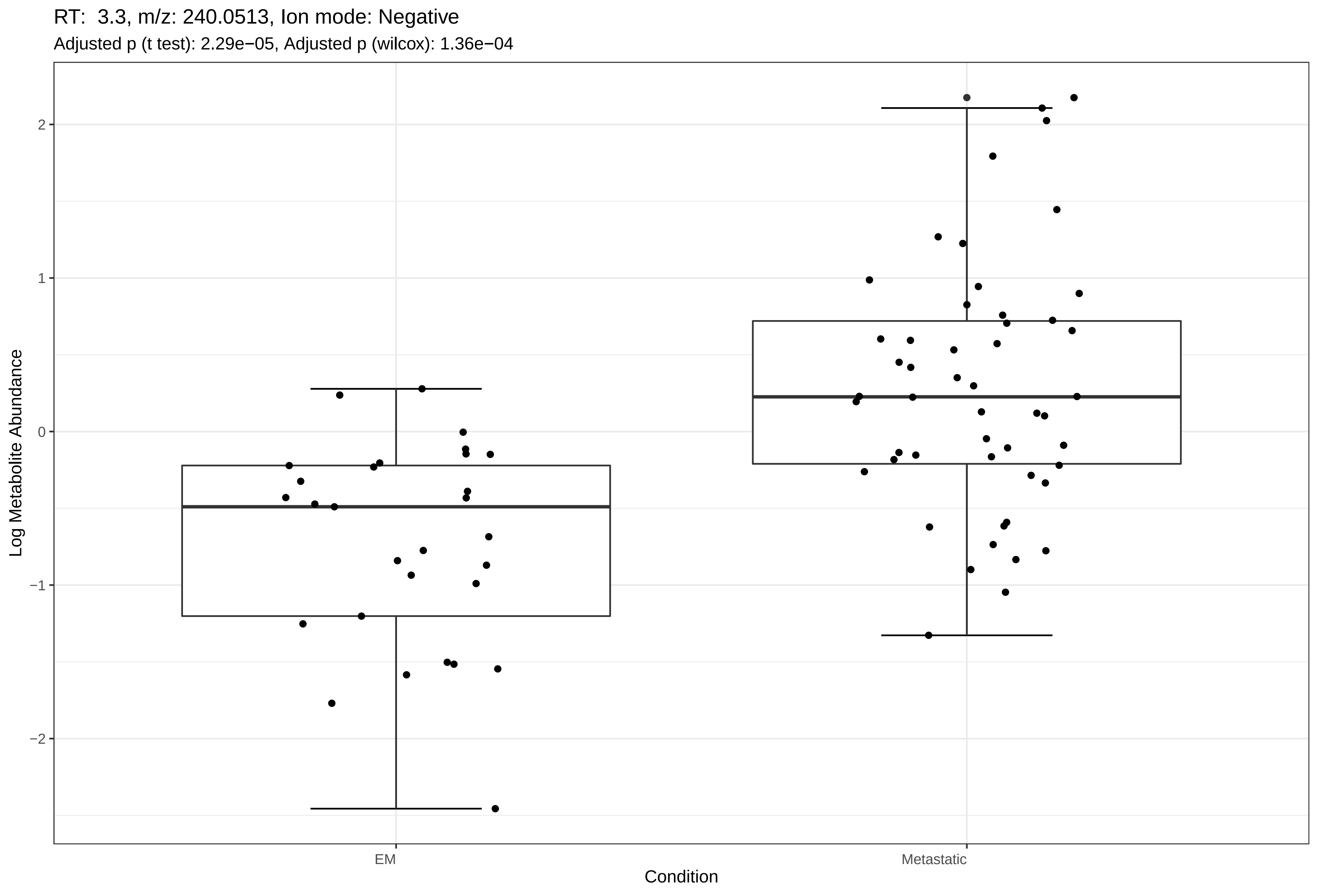
RT: 3.3, m/z: 240.0513, Ion mode: Negative
Adjusted p (t test): 2.29e−05, Adjusted p (wilcox): 1.36e−04
Log Metabolite Abundance
2
1
0
−1
−2
EM
Metastatic
Condition

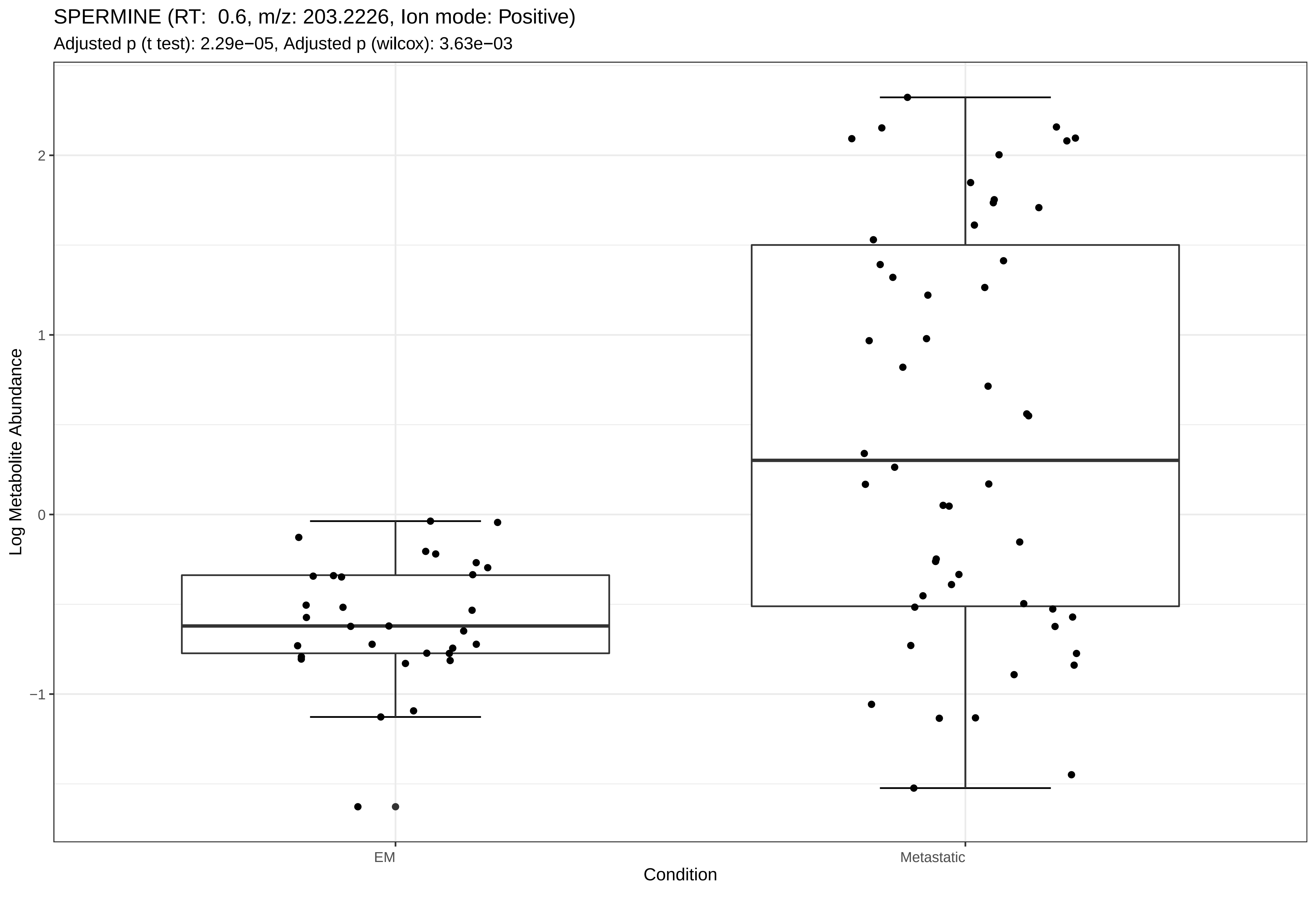
SPERMINE (RT: 0.6, m/z: 203.2226, Ion mode: Positive)
Adjusted p (t test): 2.29e−05, Adjusted p (wilcox): 3.63e−03
Log Metabolite Abundance
2
1
0
−1
EM
Metastatic
Condition

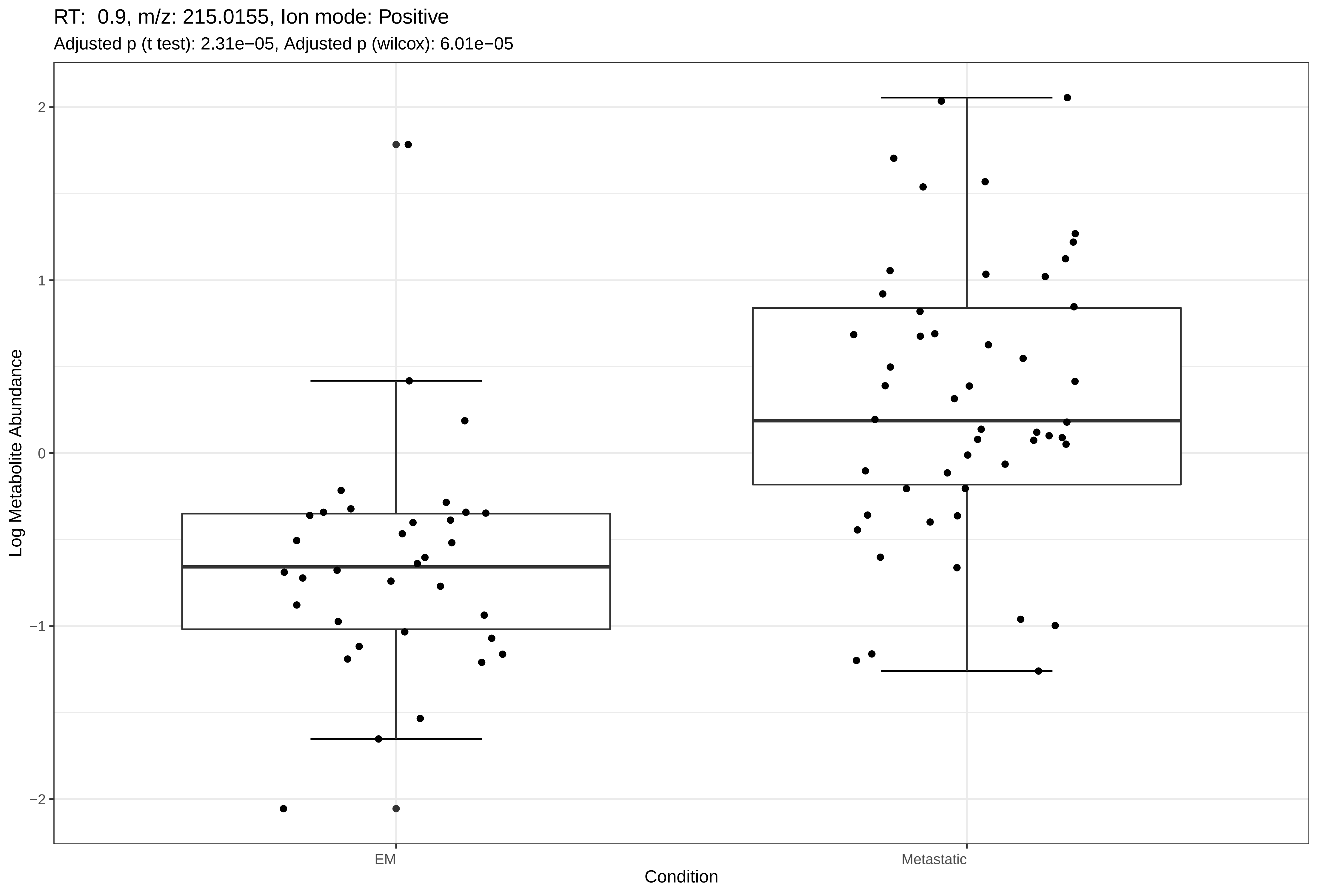

RT: 0.9, m/z: 215.0155, Ion mode: Positive
Adjusted p (t test): 2.31e−05, Adjusted p (wilcox): 6.01e−05
Log Metabolite Abundance
2
1
0
−1
−2
EM
Metastatic
Condition

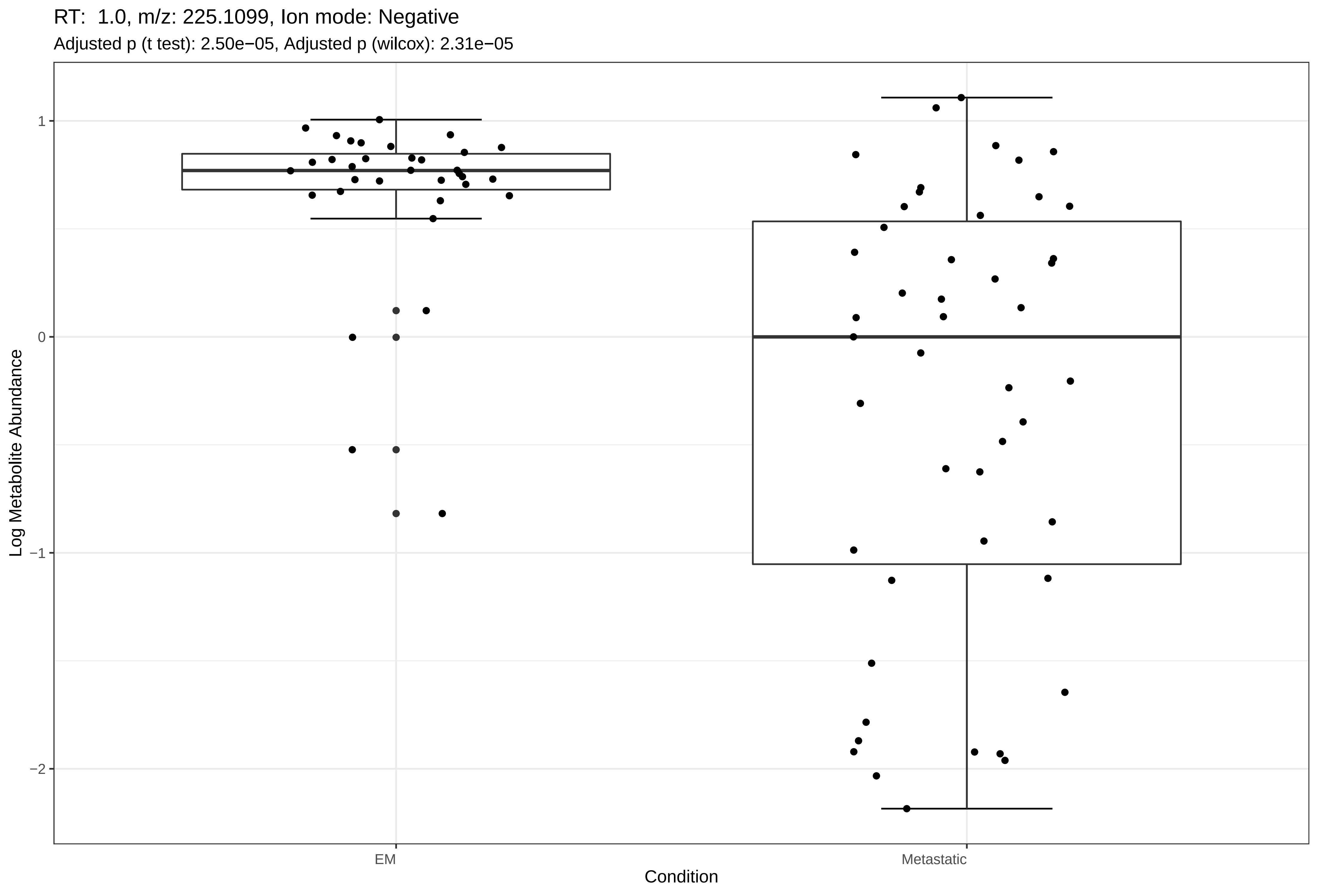
RT: 1.0, m/z: 225.1099, Ion mode: Negative
Adjusted p (t test): 2.50e−05, Adjusted p (wilcox): 2.31e−05
Log Metabolite Abundance
1
0
−1
−2
EM
Metastatic
Condition

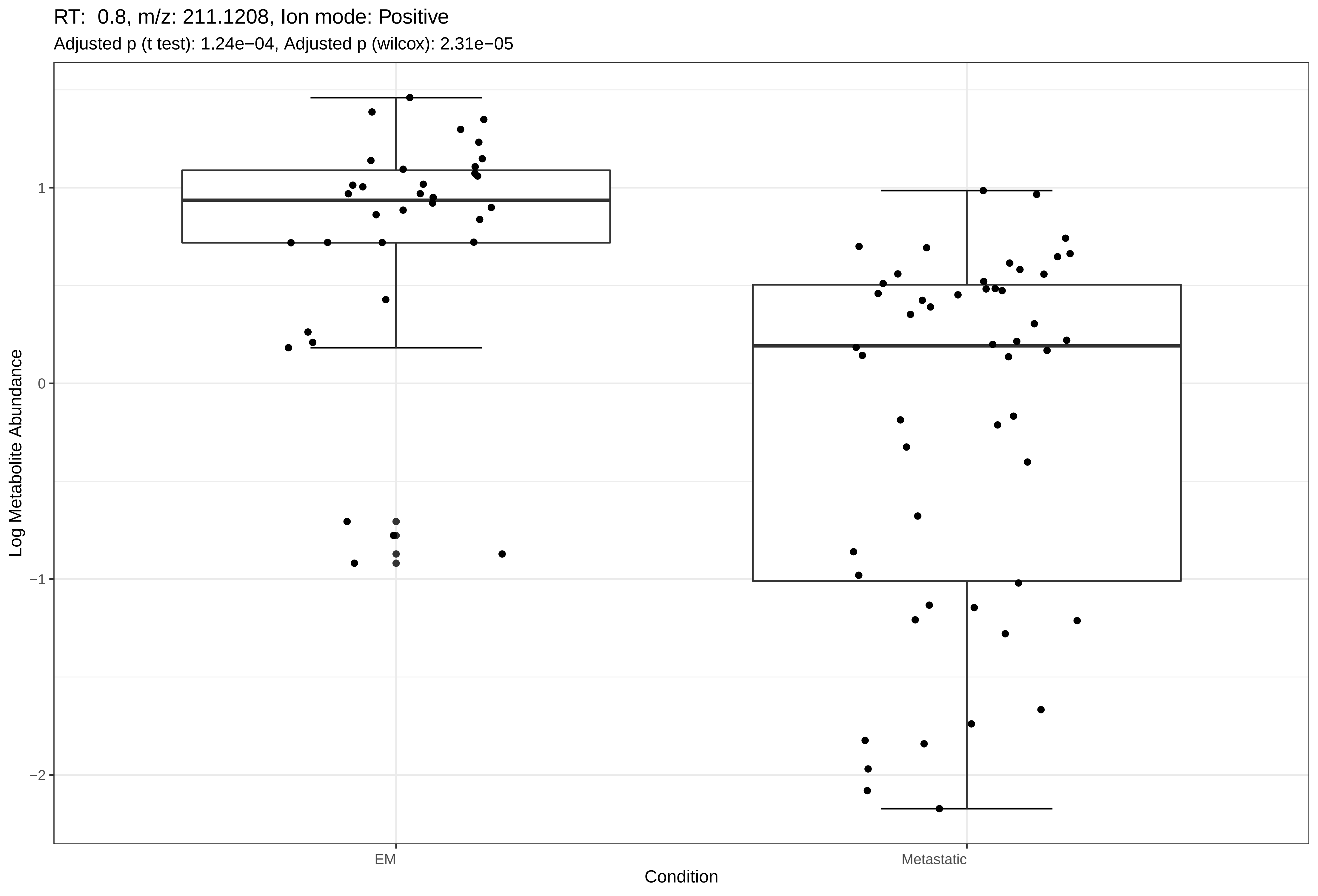

RT: 0.8, m/z: 211.1208, Ion mode: Positive
Adjusted p (t test): 1.24e−04, Adjusted p (wilcox): 2.31e−05
Log Metabolite Abundance
1
0
−1
−2
EM
Metastatic
Condition

Complex of 130.0498 and 156.0765 m/z (RT: 0.8, m/z: 285.1183, Ion mode: Positive)
Adjusted p (t test): 2.33e−05, Adjusted p (wilcox): 3.47e−05

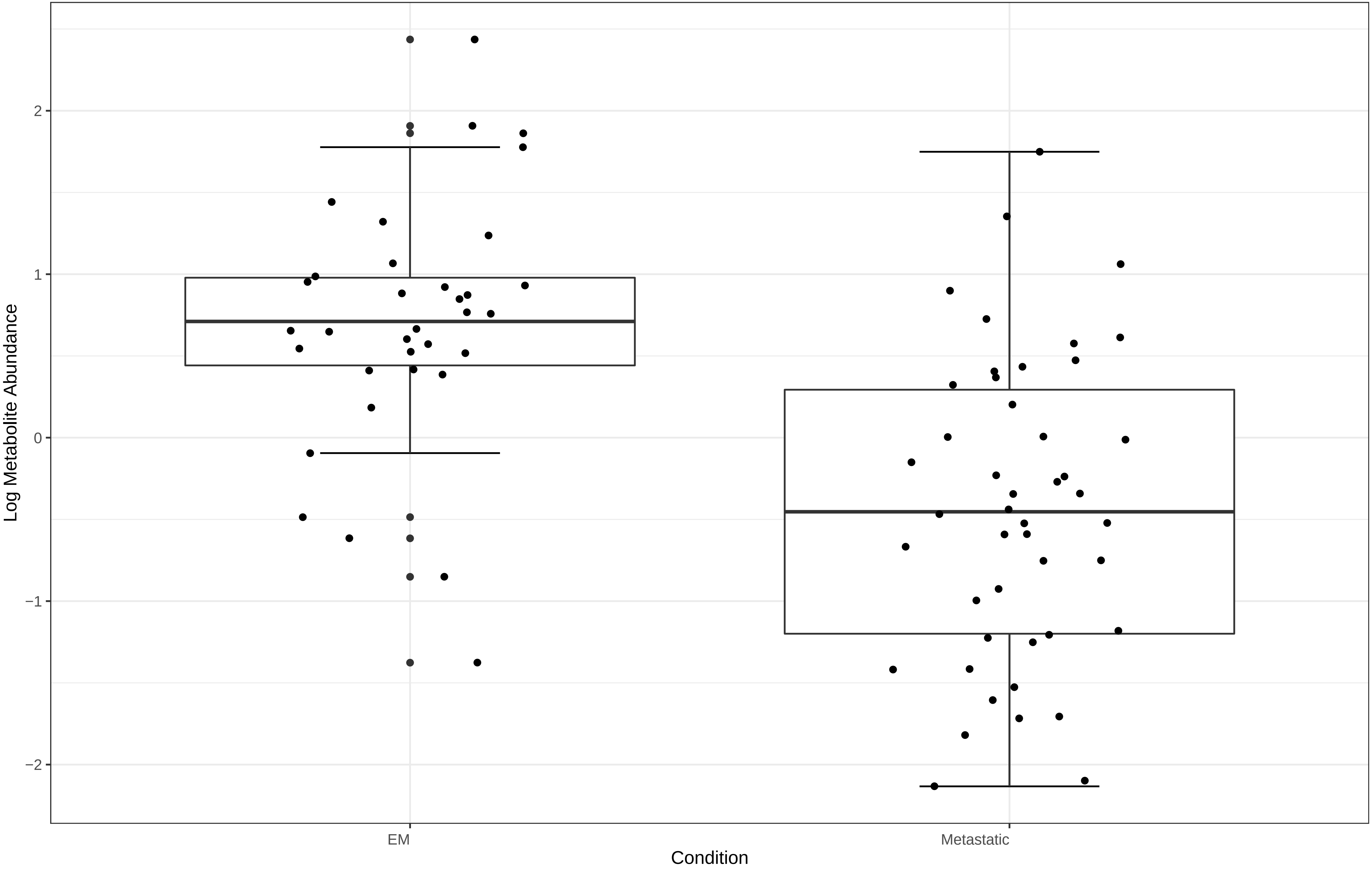

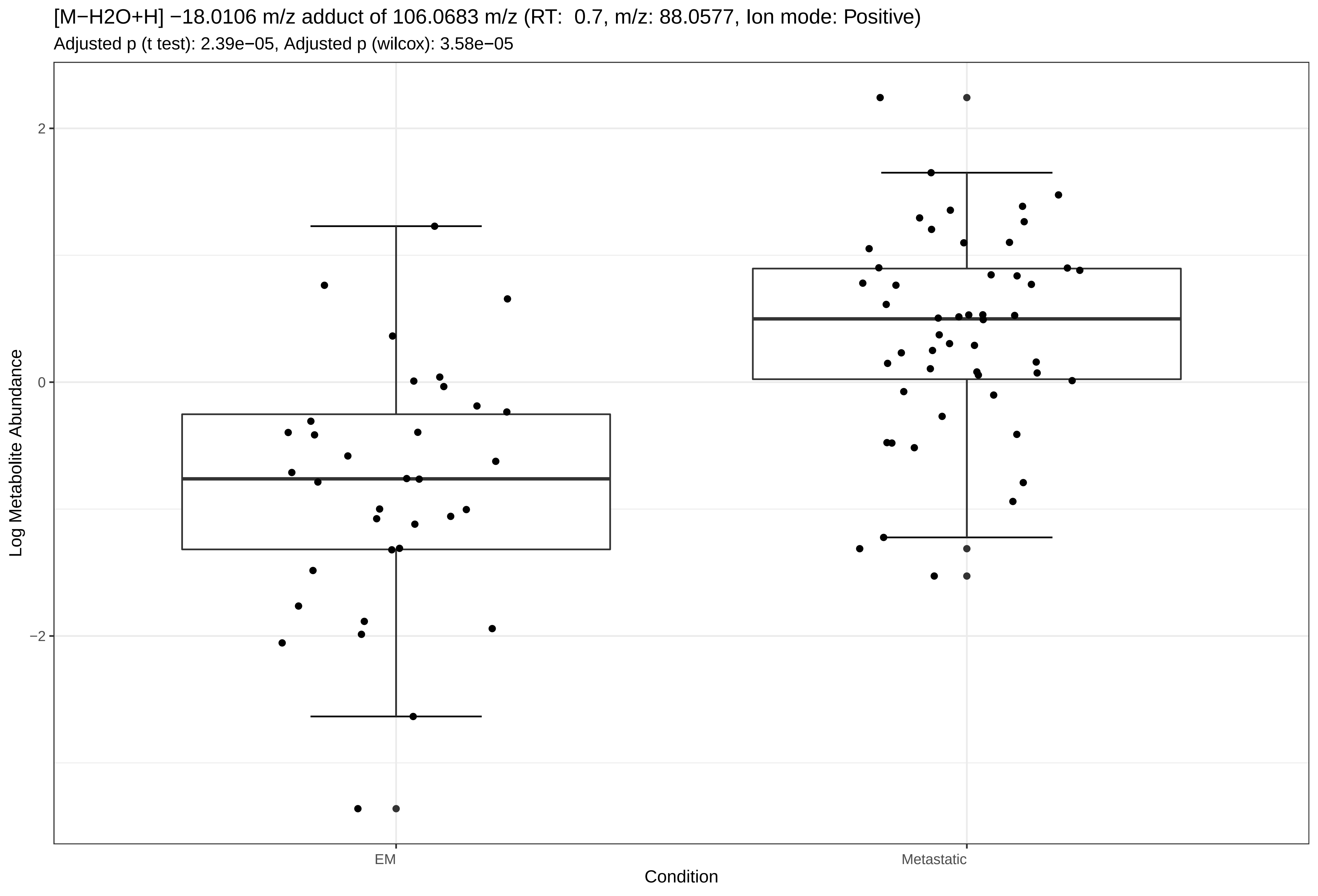

[M−H2O+H] −18.0106 m/z adduct of 106.0683 m/z (RT: 0.7, m/z: 88.0577, Ion mode: Positive)
Adjusted p (t test): 2.39e−05, Adjusted p (wilcox): 3.58e−05
Log Metabolite Abundance
2
0
−2
EM
Metastatic
Condition

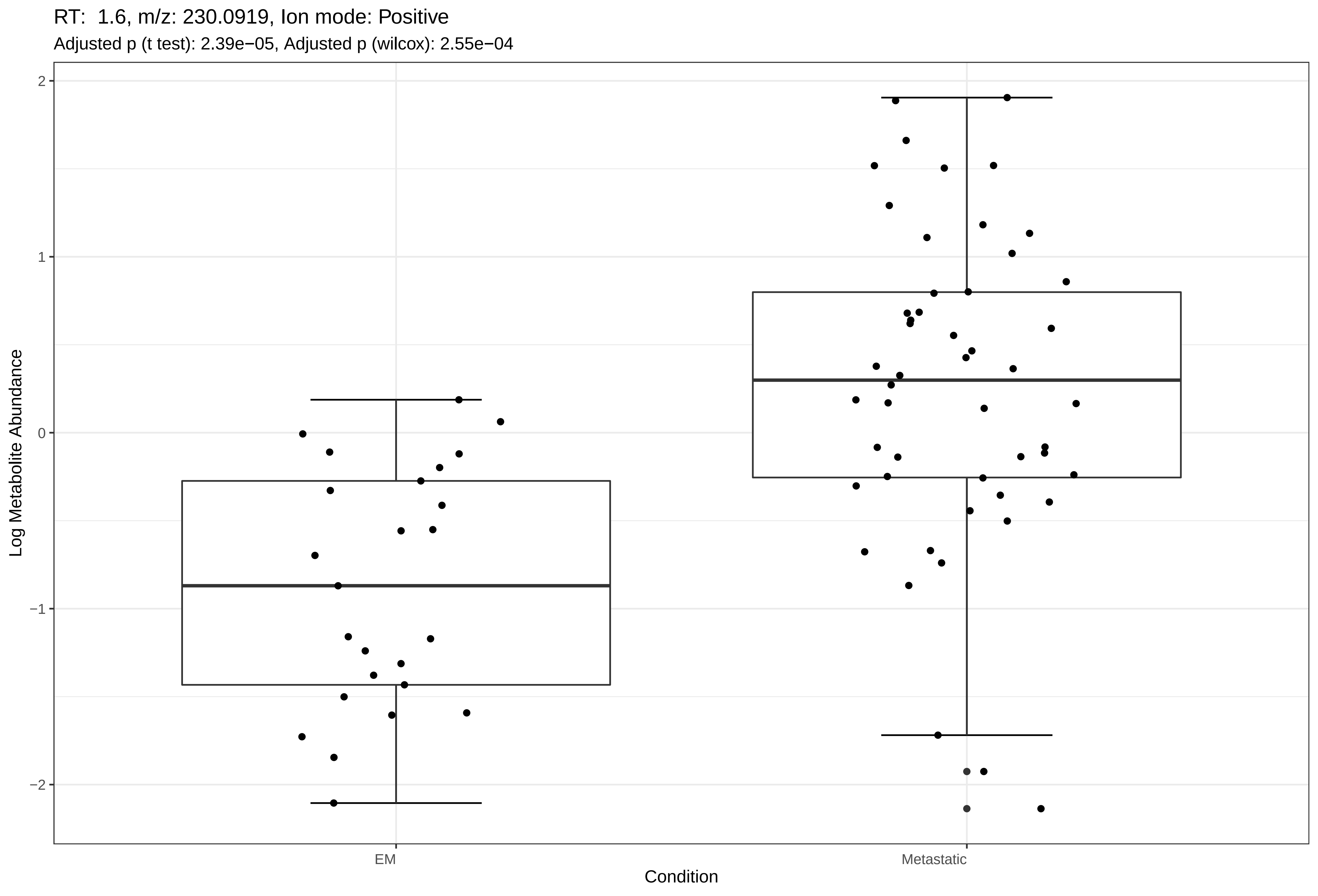
RT: 1.6, m/z: 230.0919, Ion mode: Positive
Adjusted p (t test): 2.39e−05, Adjusted p (wilcox): 2.55e−04
Log Metabolite Abundance
2
1
0
−1
−2
EM
Metastatic
Condition

RT: 7.0, m/z: 295.1967, Ion mode: Positive

Adjusted p (t test): 2.39e−05, Adjusted p (wilcox): 5.08e−05

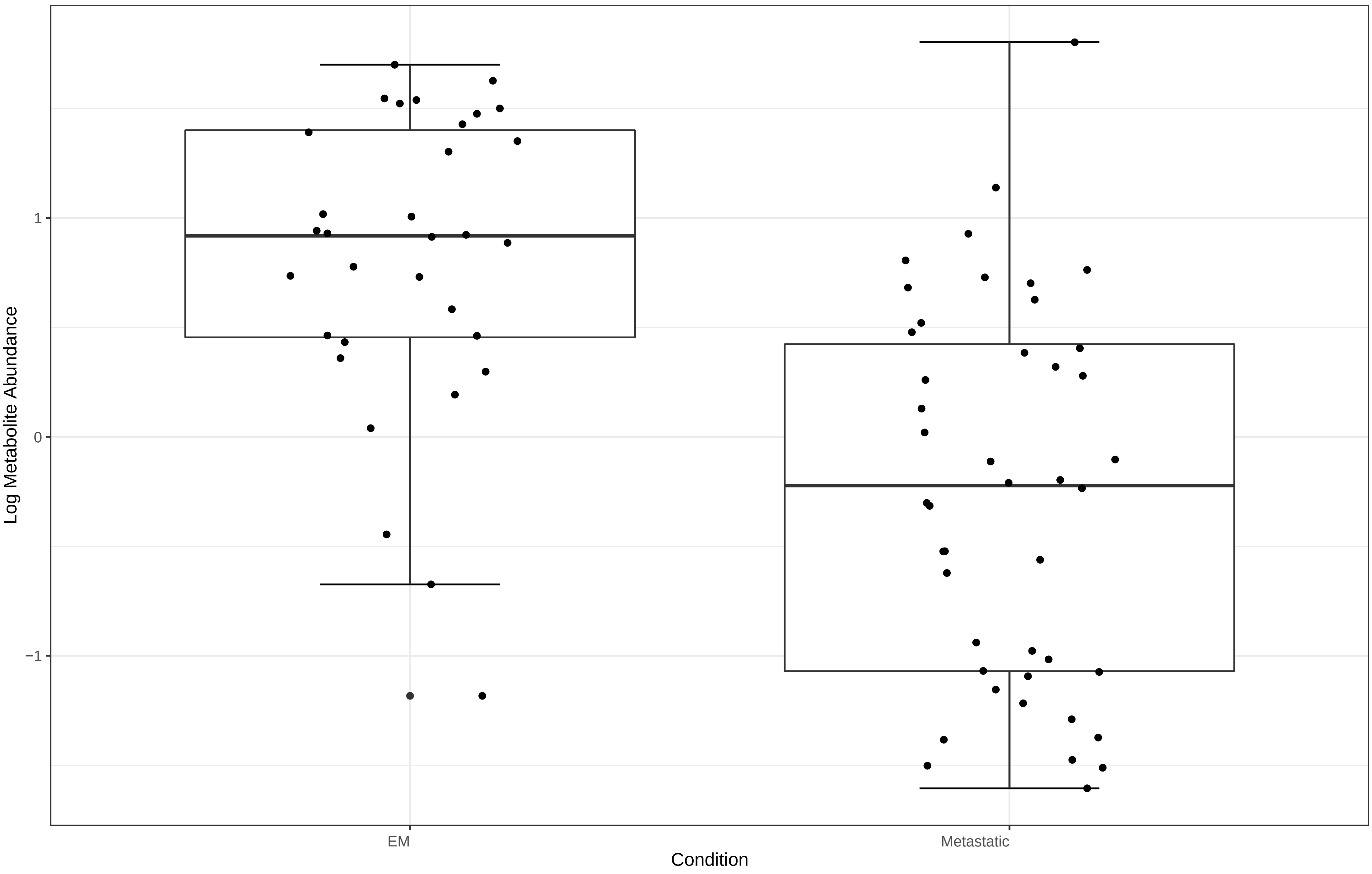

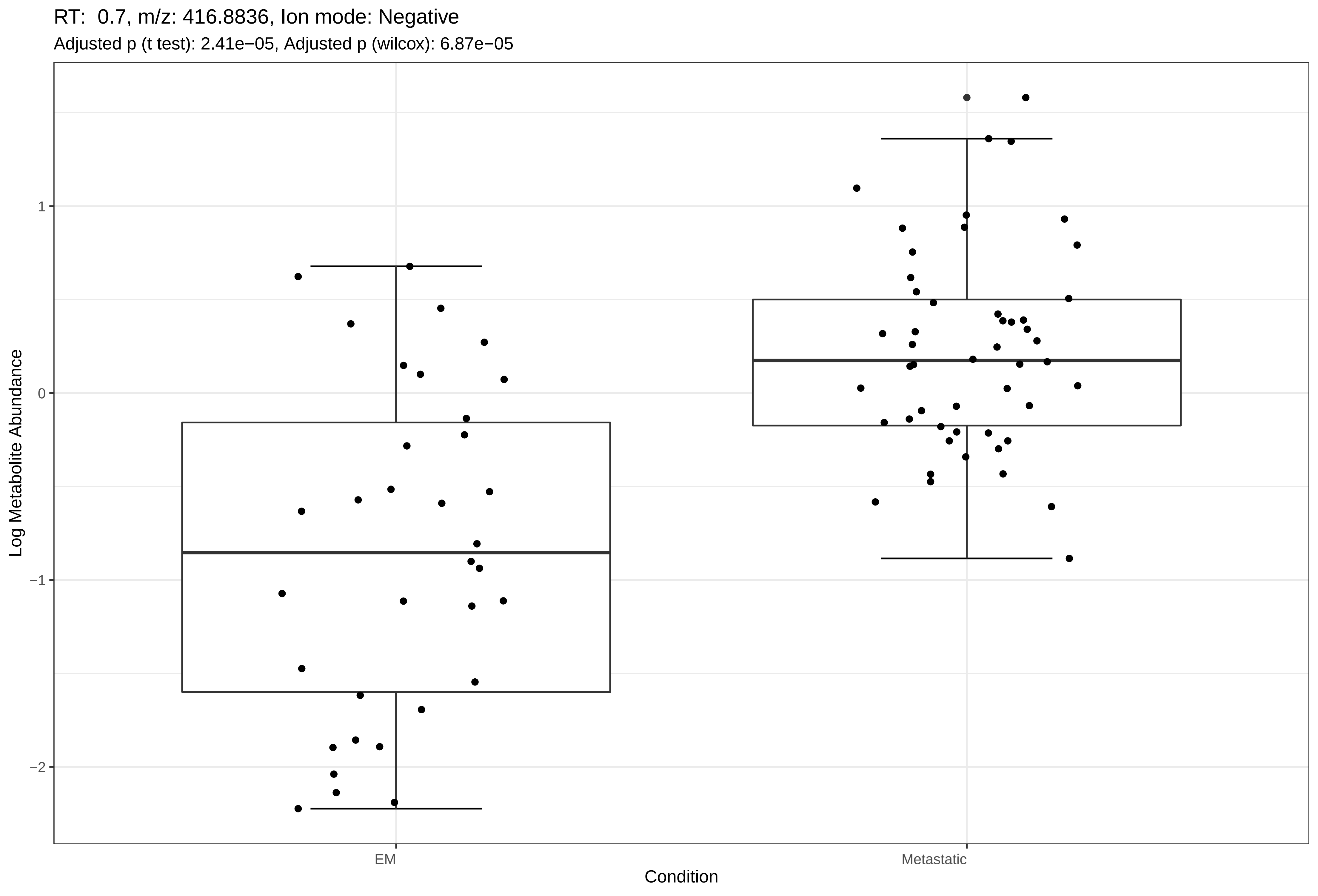
RT: 0.7, m/z: 416.8836, Ion mode: Negative
Adjusted p (t test): 2.41e−05, Adjusted p (wilcox): 6.87e−05
Log Metabolite Abundance
1
0
−1
−2
EM
Metastatic
Condition

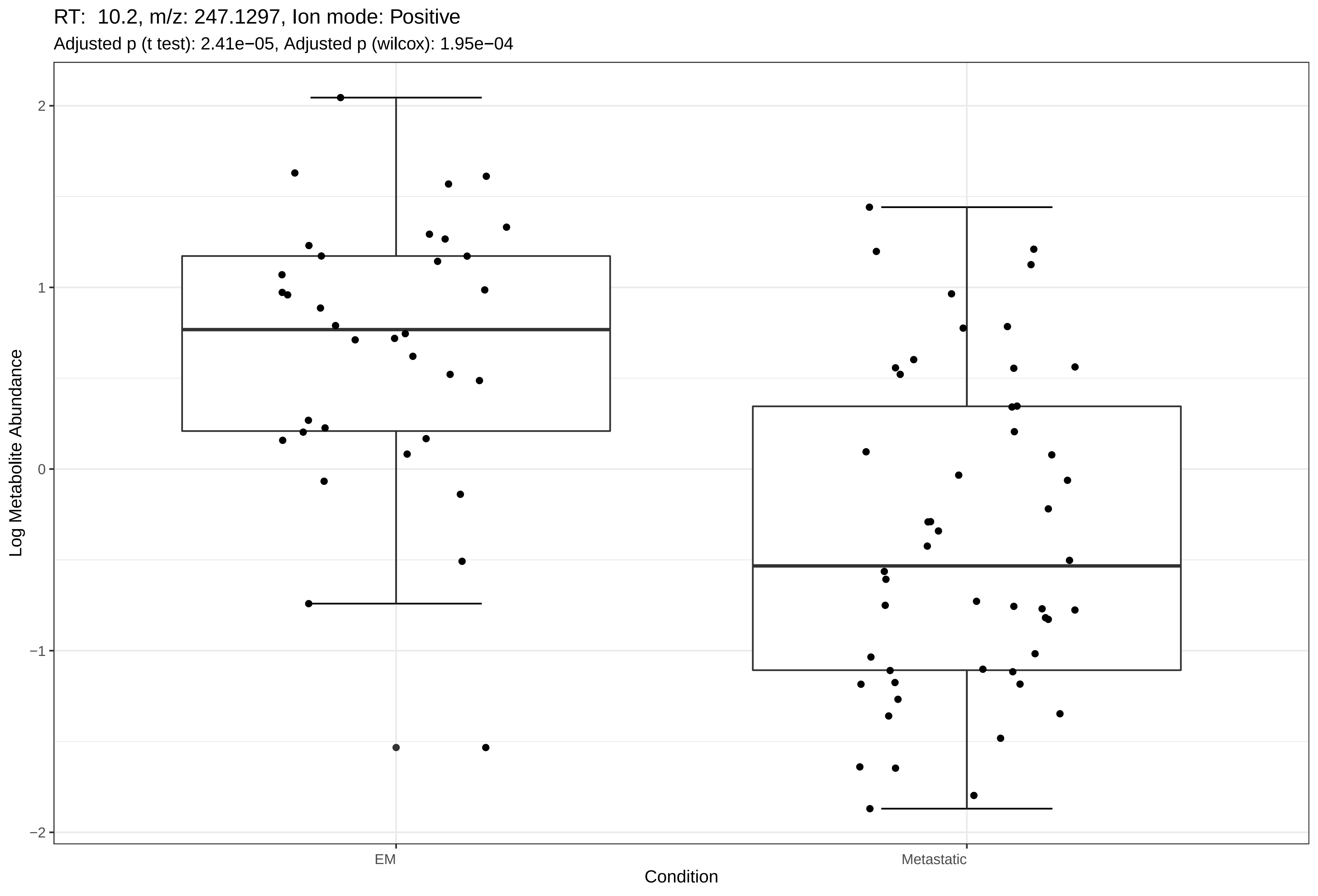

RT: 10.2, m/z: 247.1297, Ion mode: Positive
Adjusted p (t test): 2.41e−05, Adjusted p (wilcox): 1.95e−04
Log Metabolite Abundance
2
1
0
−1
−2
EM
Metastatic
Condition

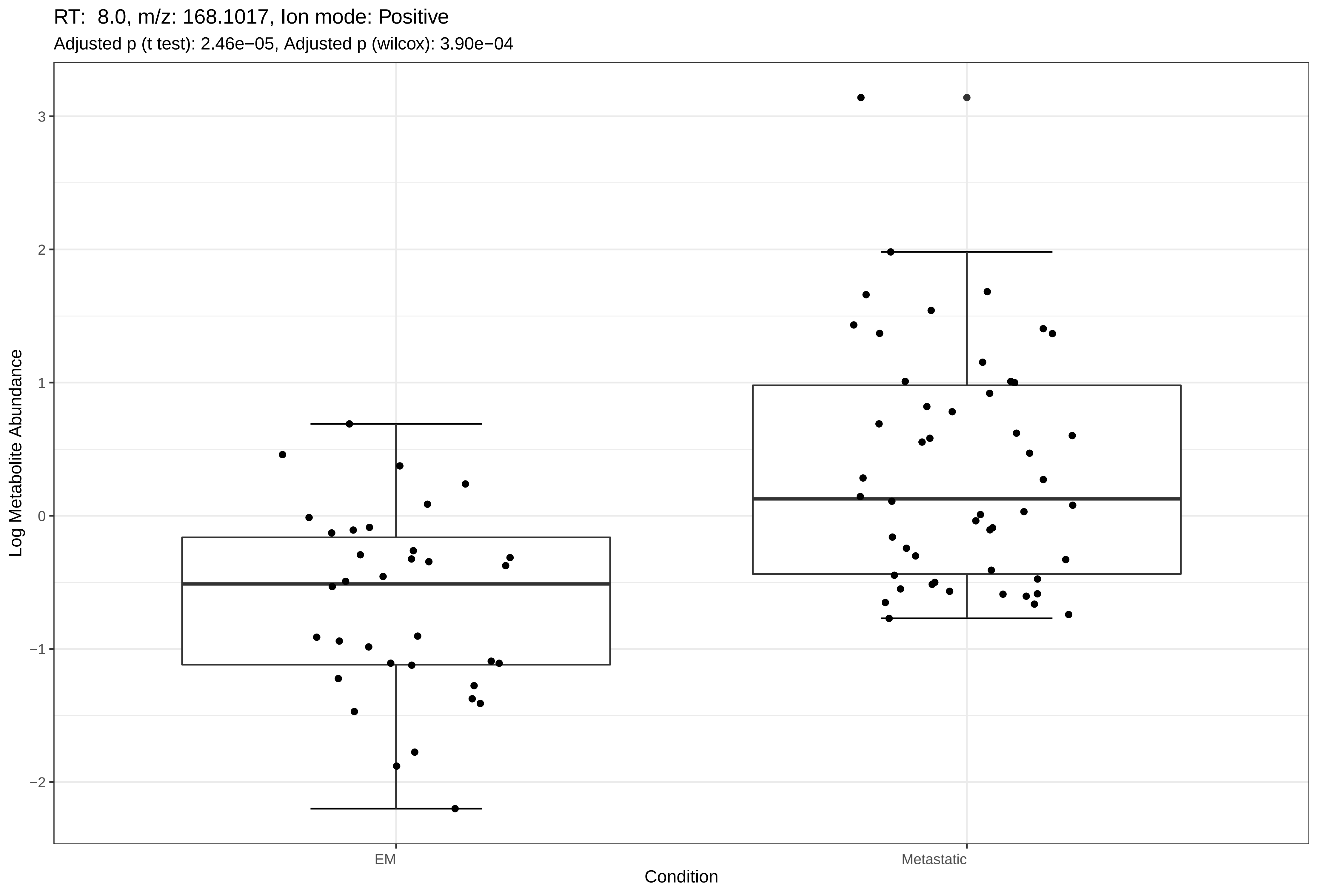
RT: 8.0, m/z: 168.1017, Ion mode: Positive
Adjusted p (t test): 2.46e−05, Adjusted p (wilcox): 3.90e−04
Log Metabolite Abundance
3
2
1
0
−1
−2
EM
Metastatic
Condition

RT: 1.0, m/z: 245.0778, Ion mode: Negative

Adjusted p (t test): 2.47e−05, Adjusted p (wilcox): 1.73e−03

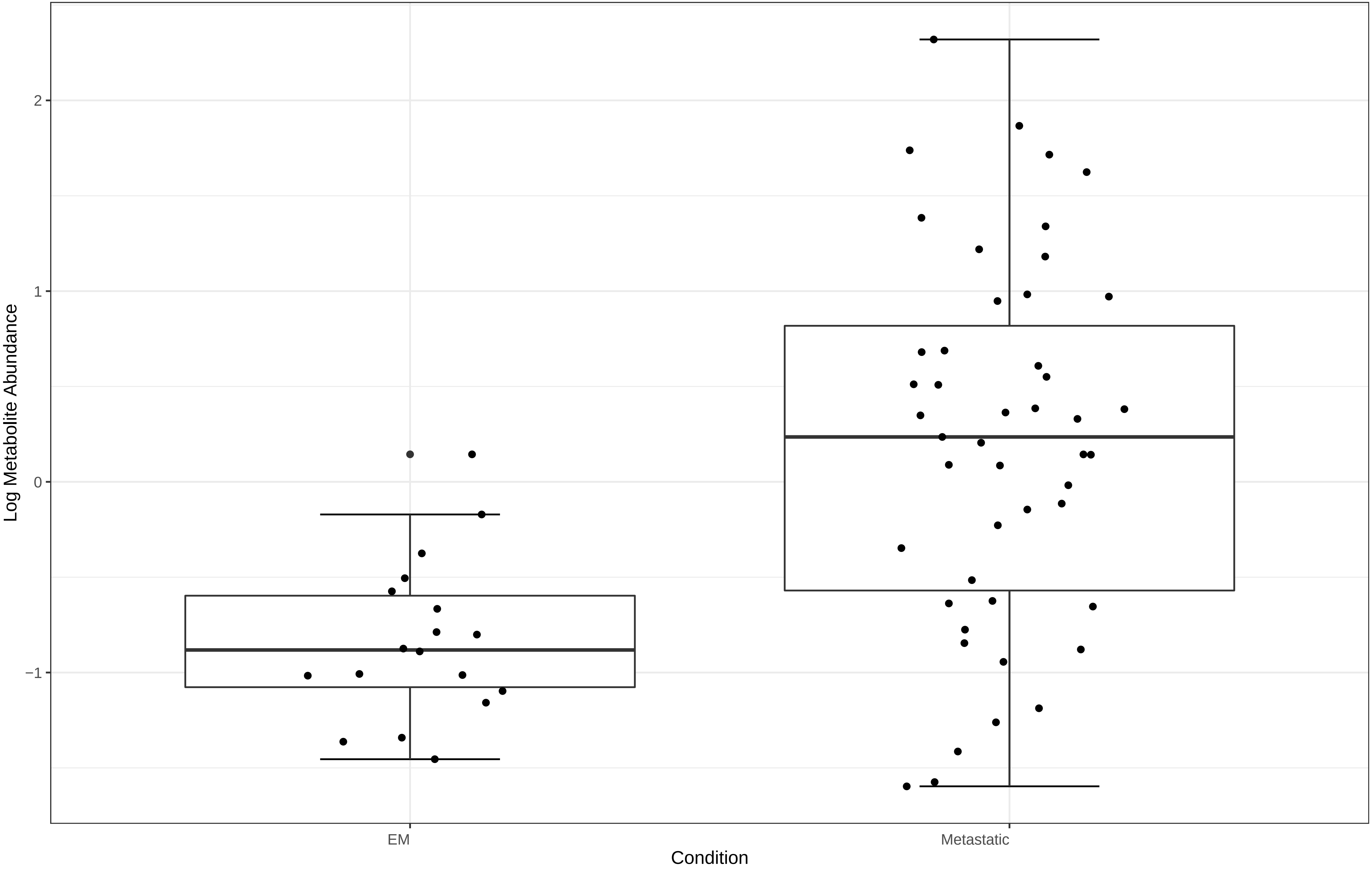

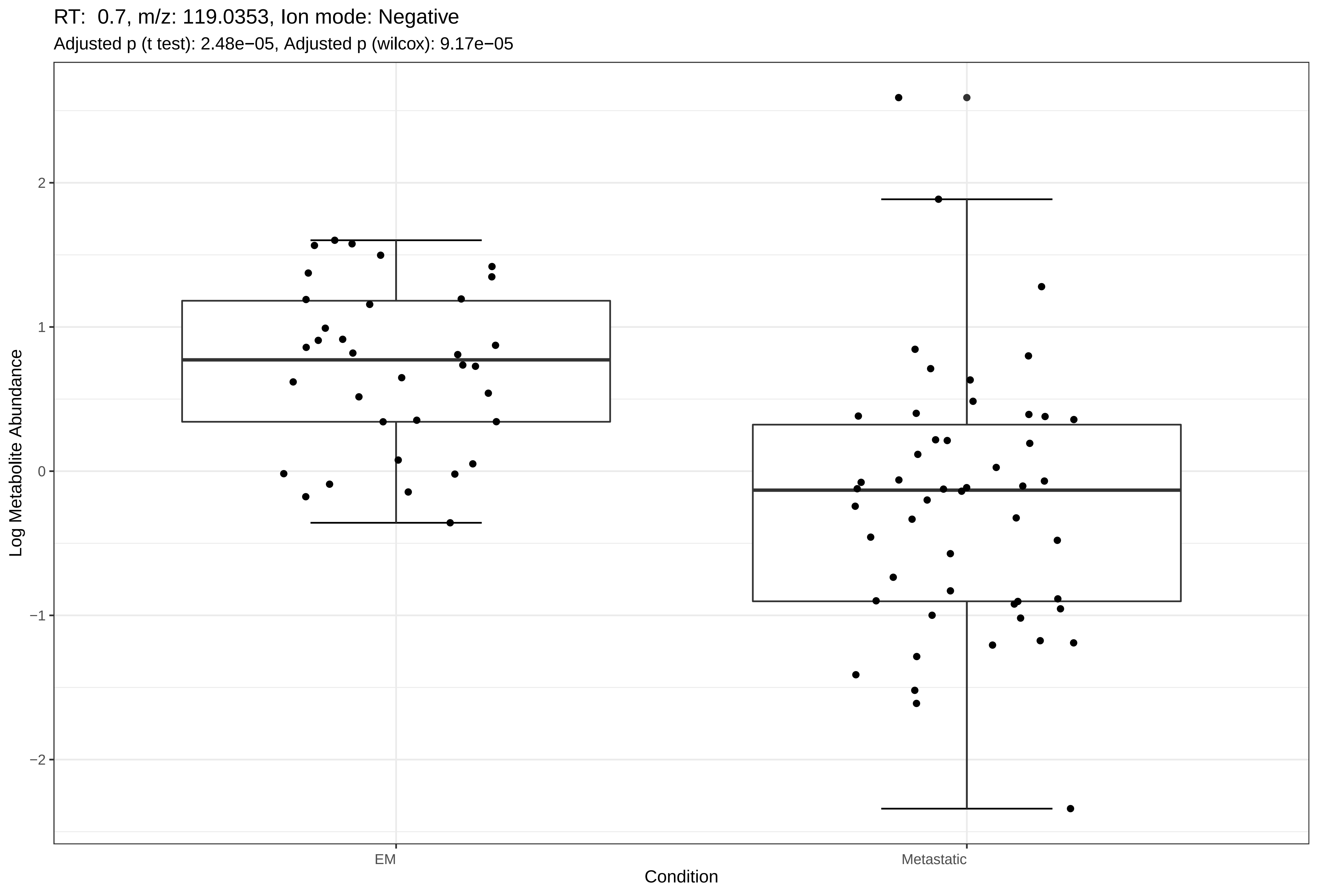
RT: 0.7, m/z: 119.0353, Ion mode: Negative
Adjusted p (t test): 2.48e−05, Adjusted p (wilcox): 9.17e−05
Log Metabolite Abundance
2
1
0
−1
−2
EM
Metastatic
Condition

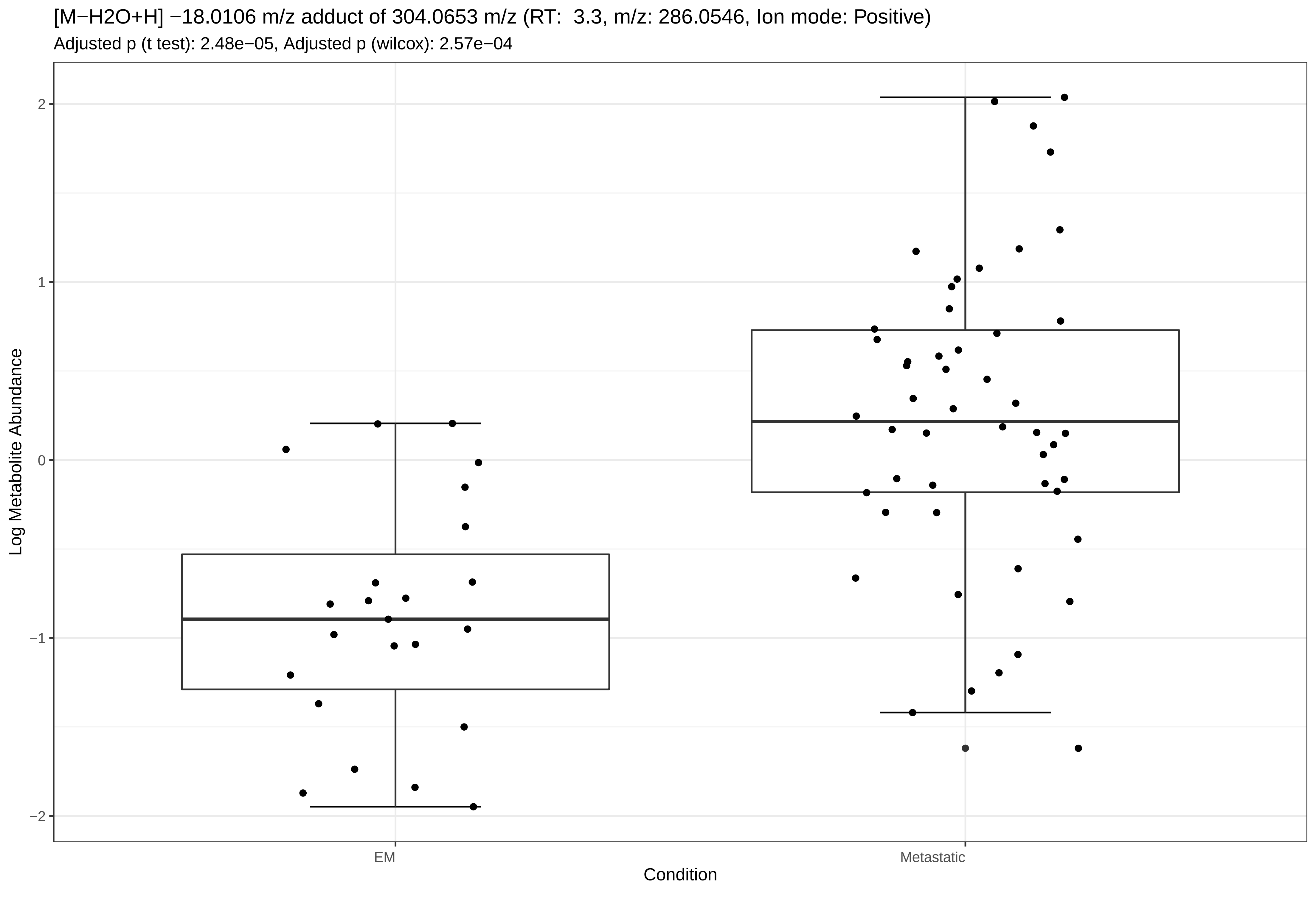

[M−H2O+H] −18.0106 m/z adduct of 304.0653 m/z (RT: 3.3, m/z: 286.0546, Ion mode: Positive)
Adjusted p (t test): 2.48e−05, Adjusted p (wilcox): 2.57e−04
Log Metabolite Abundance
2
1
0
−1
−2
EM
Metastatic
Condition

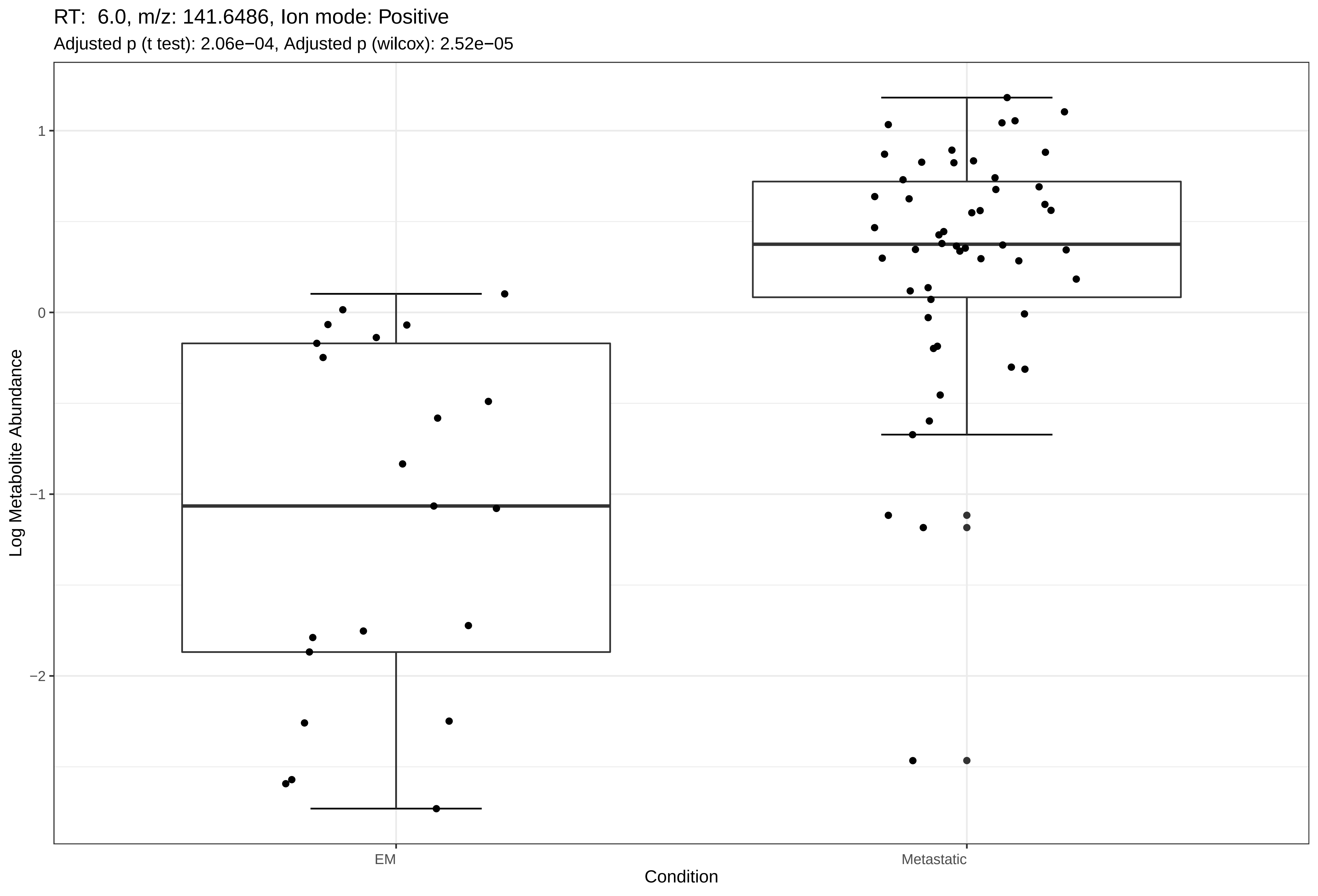

RT: 6.0, m/z: 141.6486, Ion mode: Positive
Adjusted p (t test): 2.06e−04, Adjusted p (wilcox): 2.52e−05
Log Metabolite Abundance
1
0
−1
−2
EM
Metastatic
Condition

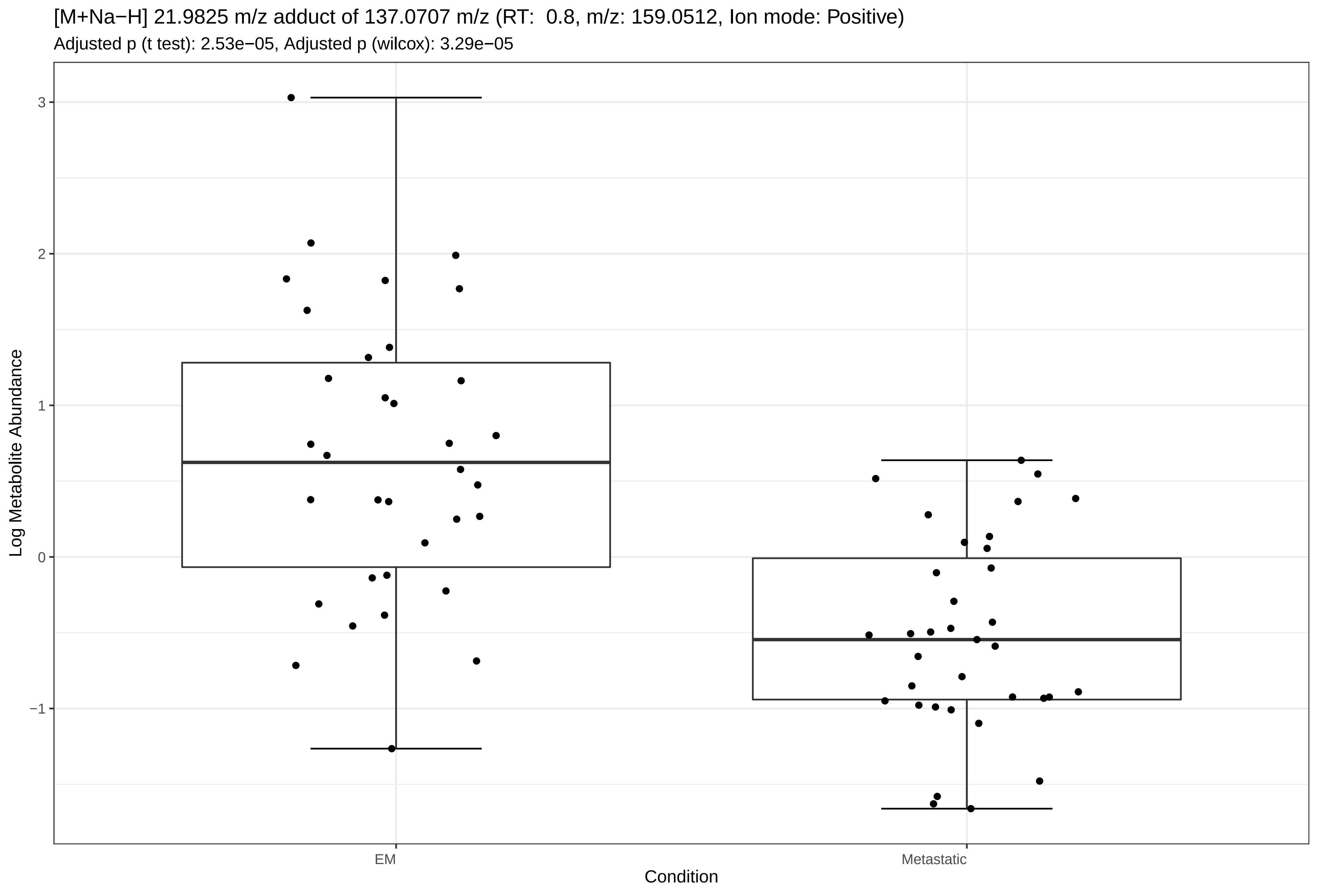
[M+Na−H] 21.9825 m/z adduct of 137.0707 m/z (RT: 0.8, m/z: 159.0512, Ion mode: Positive)
Adjusted p (t test): 2.53e−05, Adjusted p (wilcox): 3.29e−05
Log Metabolite Abundance
3
2
1
0
−1
EM
Metastatic
Condition

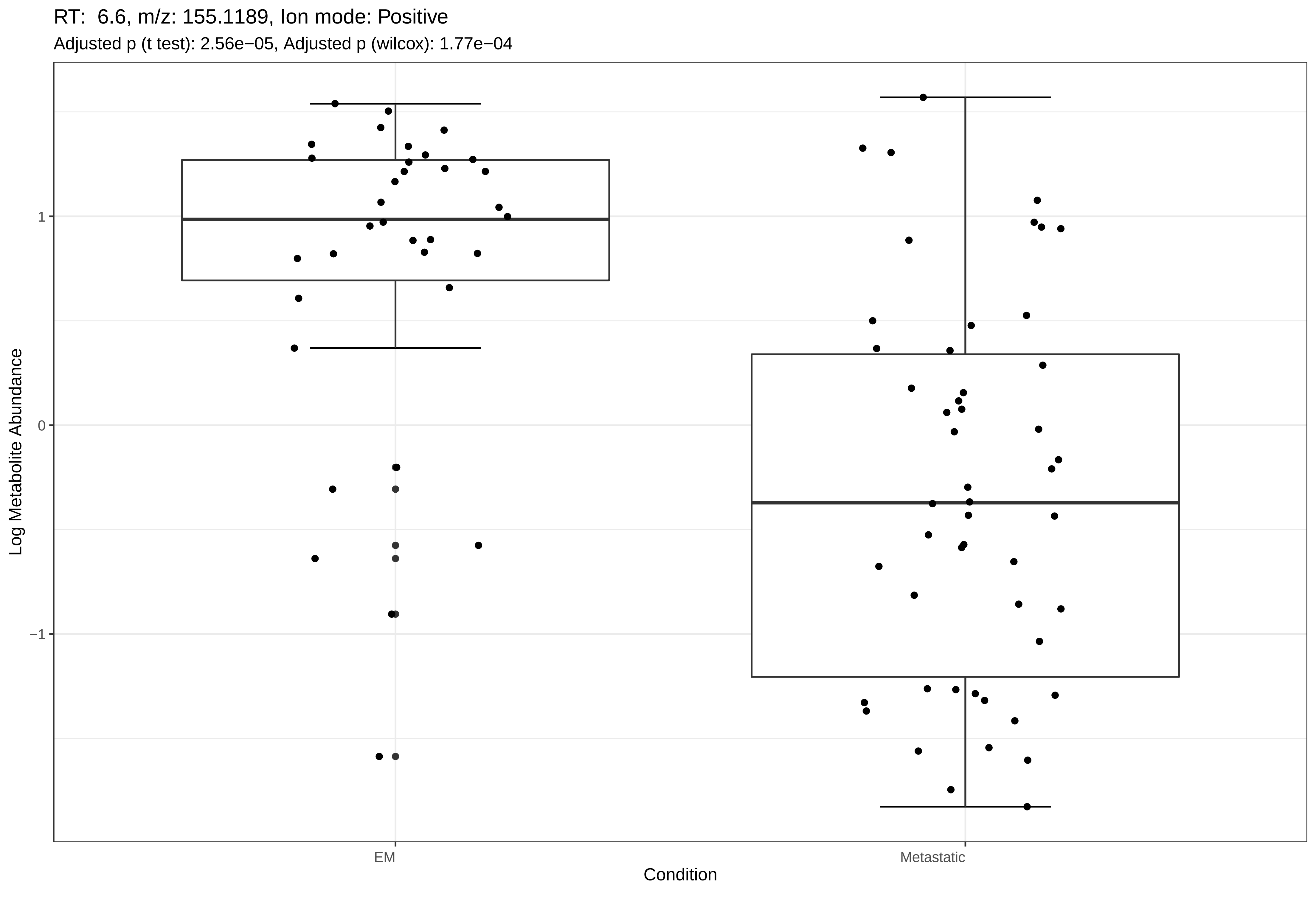
RT: 6.6, m/z: 155.1189, Ion mode: Positive
Adjusted p (t test): 2.56e−05, Adjusted p (wilcox): 1.77e−04
Log Metabolite Abundance
1
0
−1
EM
Metastatic
Condition

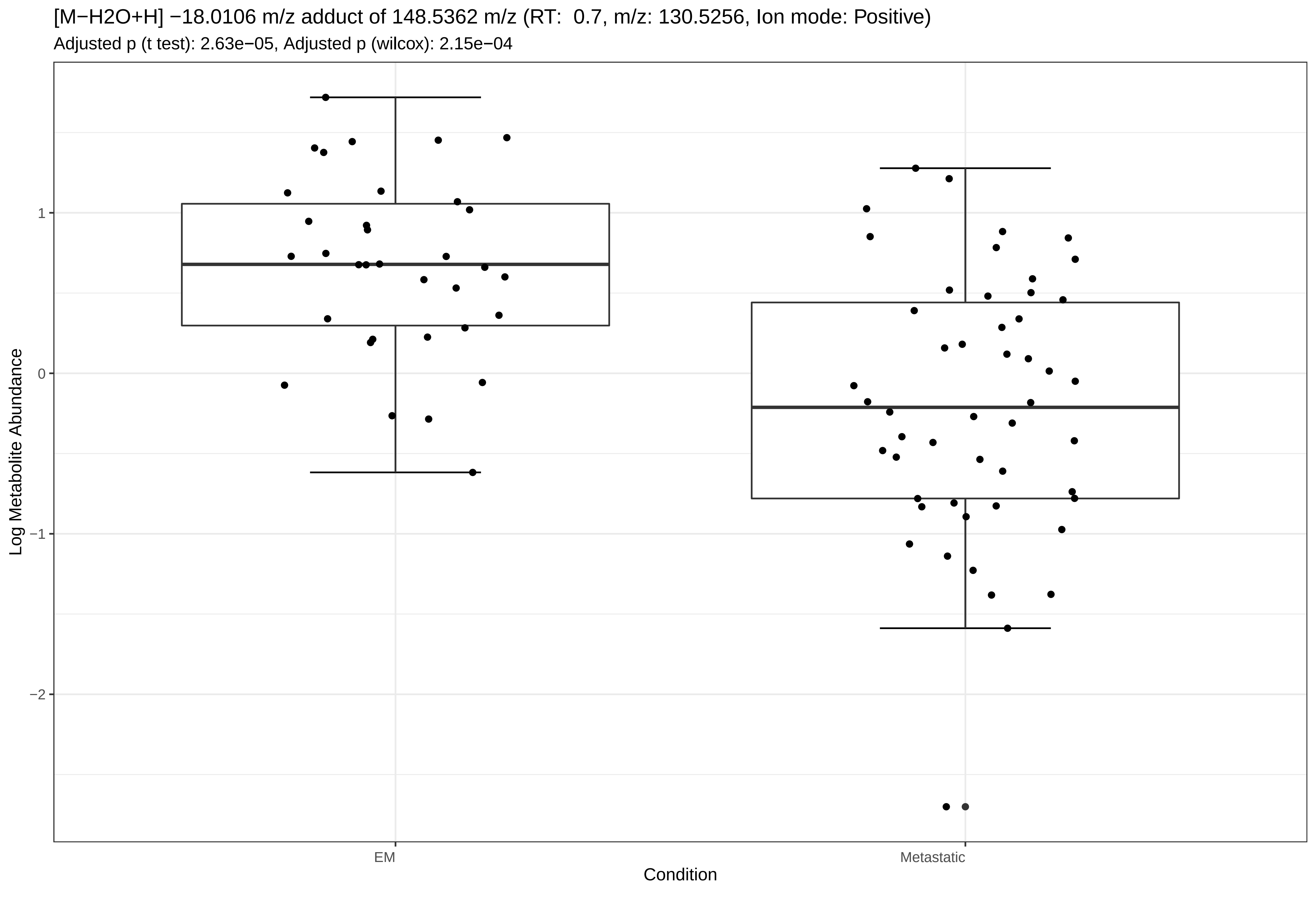
[M−H2O+H] −18.0106 m/z adduct of 148.5362 m/z (RT: 0.7, m/z: 130.5256, Ion mode: Positive)
Adjusted p (t test): 2.63e−05, Adjusted p (wilcox): 2.15e−04
Log Metabolite Abundance
1
0
−1
−2
EM
Metastatic
Condition

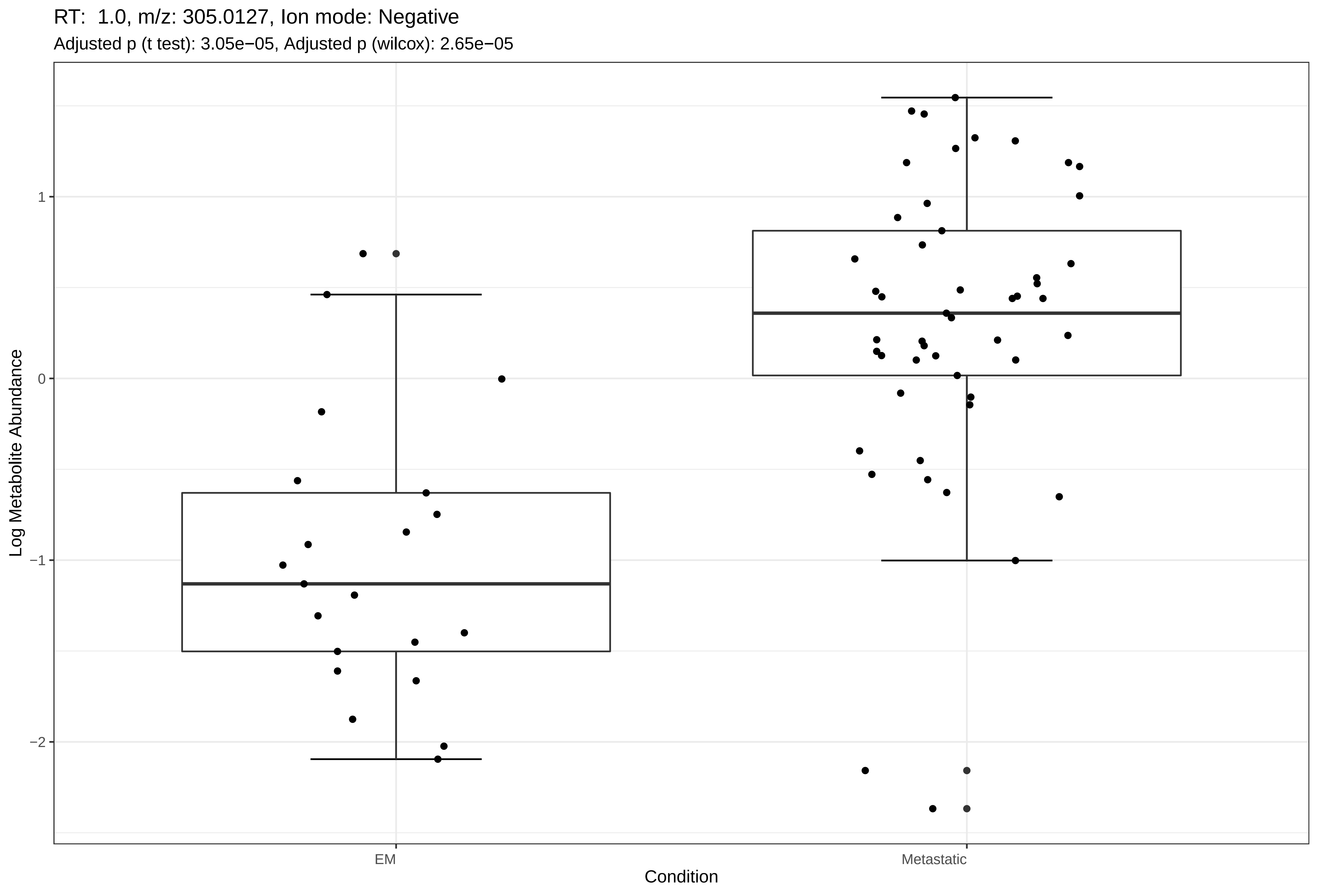

RT: 1.0, m/z: 305.0127, Ion mode: Negative
Adjusted p (t test): 3.05e−05, Adjusted p (wilcox): 2.65e−05
Log Metabolite Abundance
1
0
−1
−2
EM
Metastatic
Condition

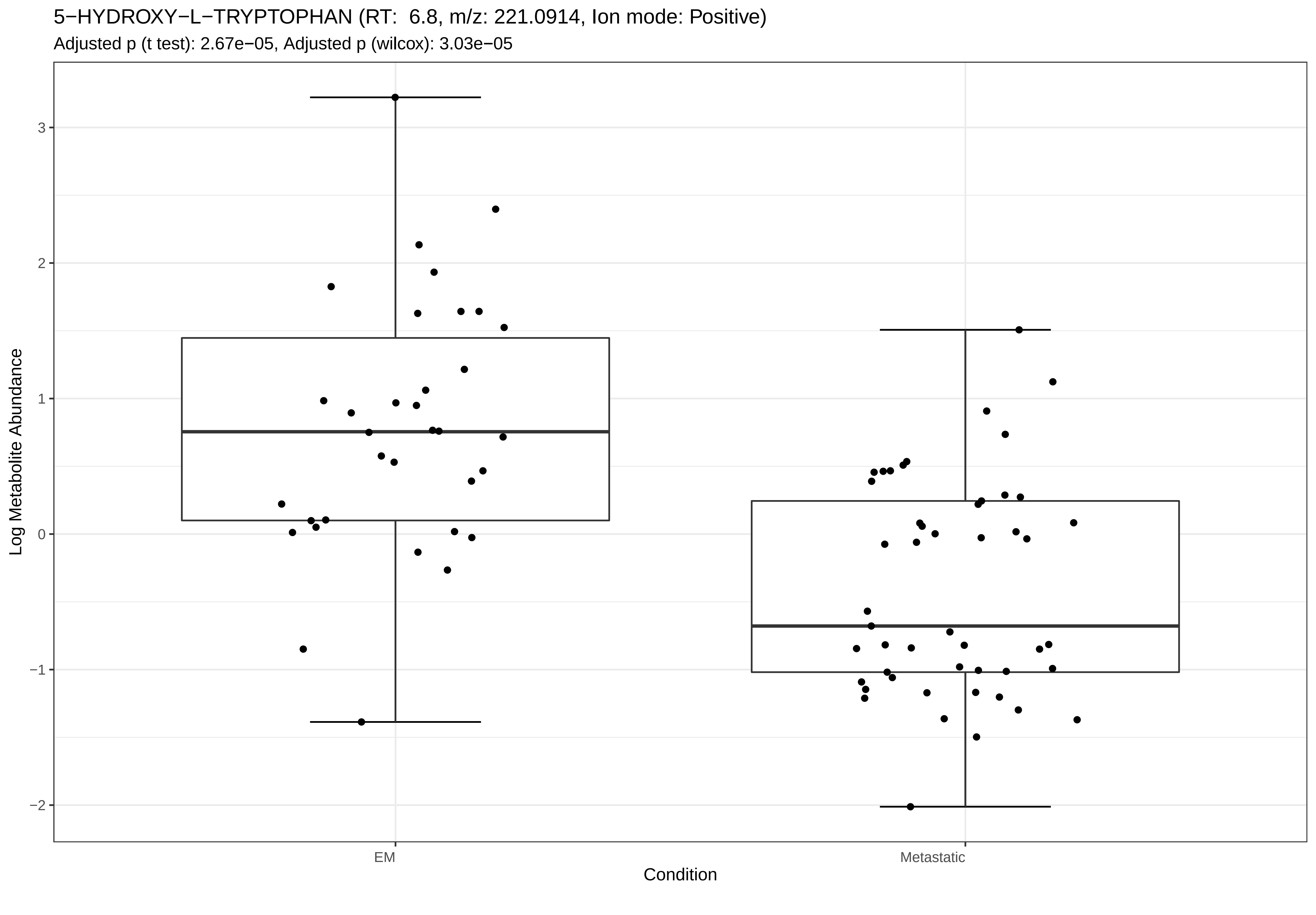

5−HYDROXY−L−TRYPTOPHAN (RT: 6.8, m/z: 221.0914, Ion mode: Positive)
Adjusted p (t test): 2.67e−05, Adjusted p (wilcox): 3.03e−05
Log Metabolite Abundance
3
2
1
0
−1
−2
EM
Metastatic
Condition

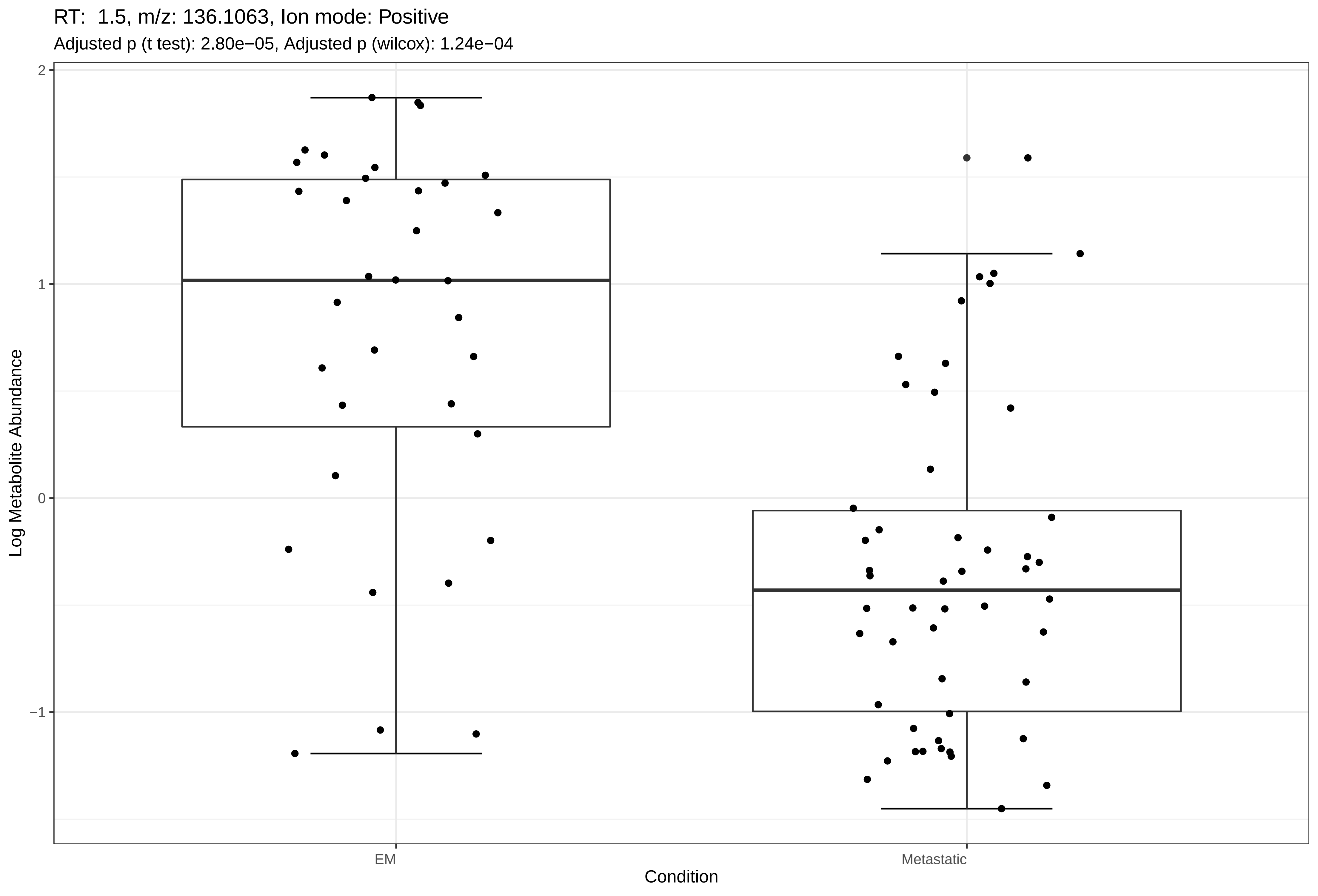

RT: 1.5, m/z: 136.1063, Ion mode: Positive
Adjusted p (t test): 2.80e−05, Adjusted p (wilcox): 1.24e−04
Log Metabolite Abundance
2
1
0
−1
EM
Metastatic
Condition

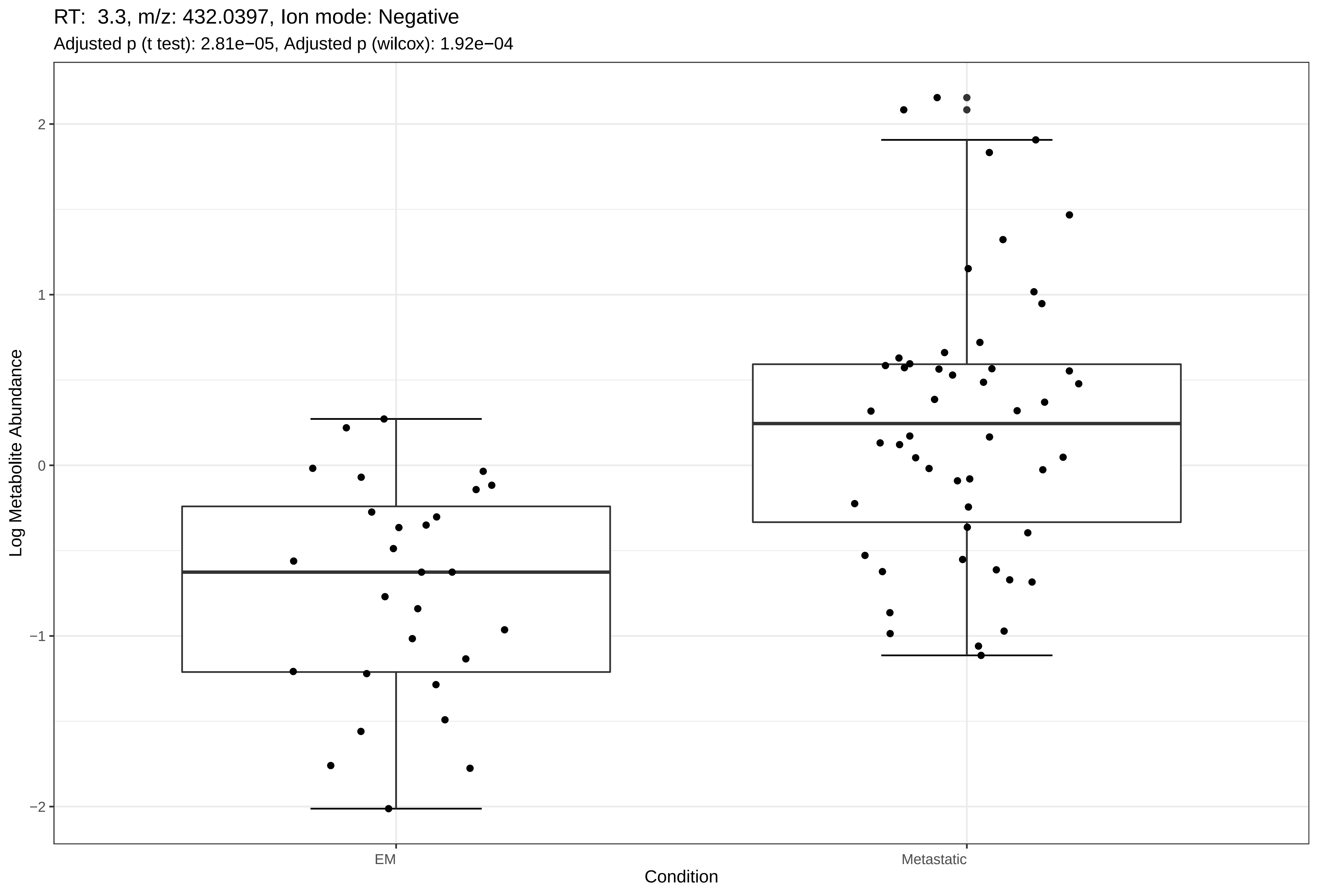

RT: 3.3, m/z: 432.0397, Ion mode: Negative
Adjusted p (t test): 2.81e−05, Adjusted p (wilcox): 1.92e−04
Log Metabolite Abundance
2
1
0
−1
−2
EM
Metastatic
Condition

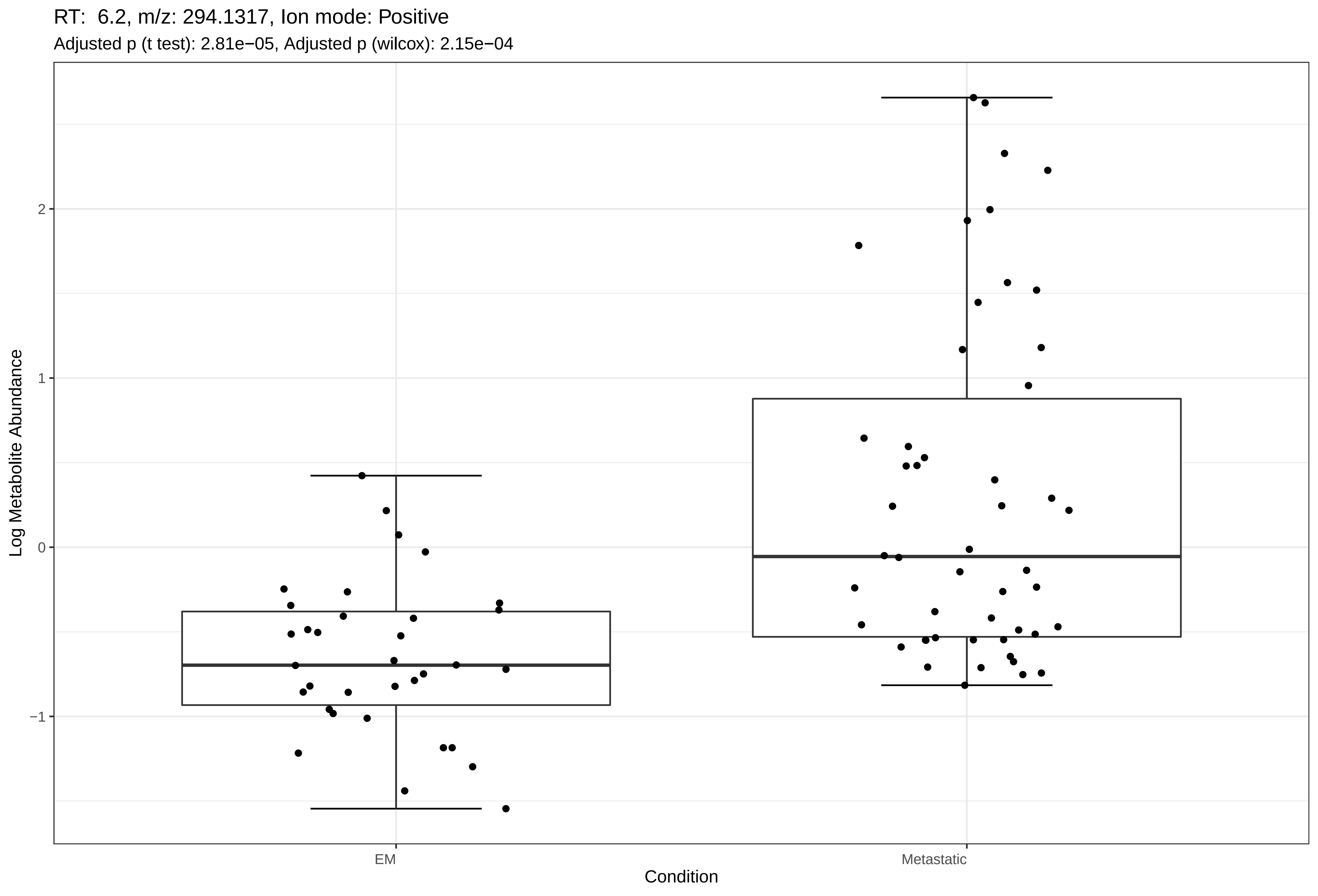

RT: 6.2, m/z: 294.1317, Ion mode: Positive
Adjusted p (t test): 2.81e−05, Adjusted p (wilcox): 2.15e−04
Log Metabolite Abundance
2
1
0
−1
EM
Metastatic
Condition

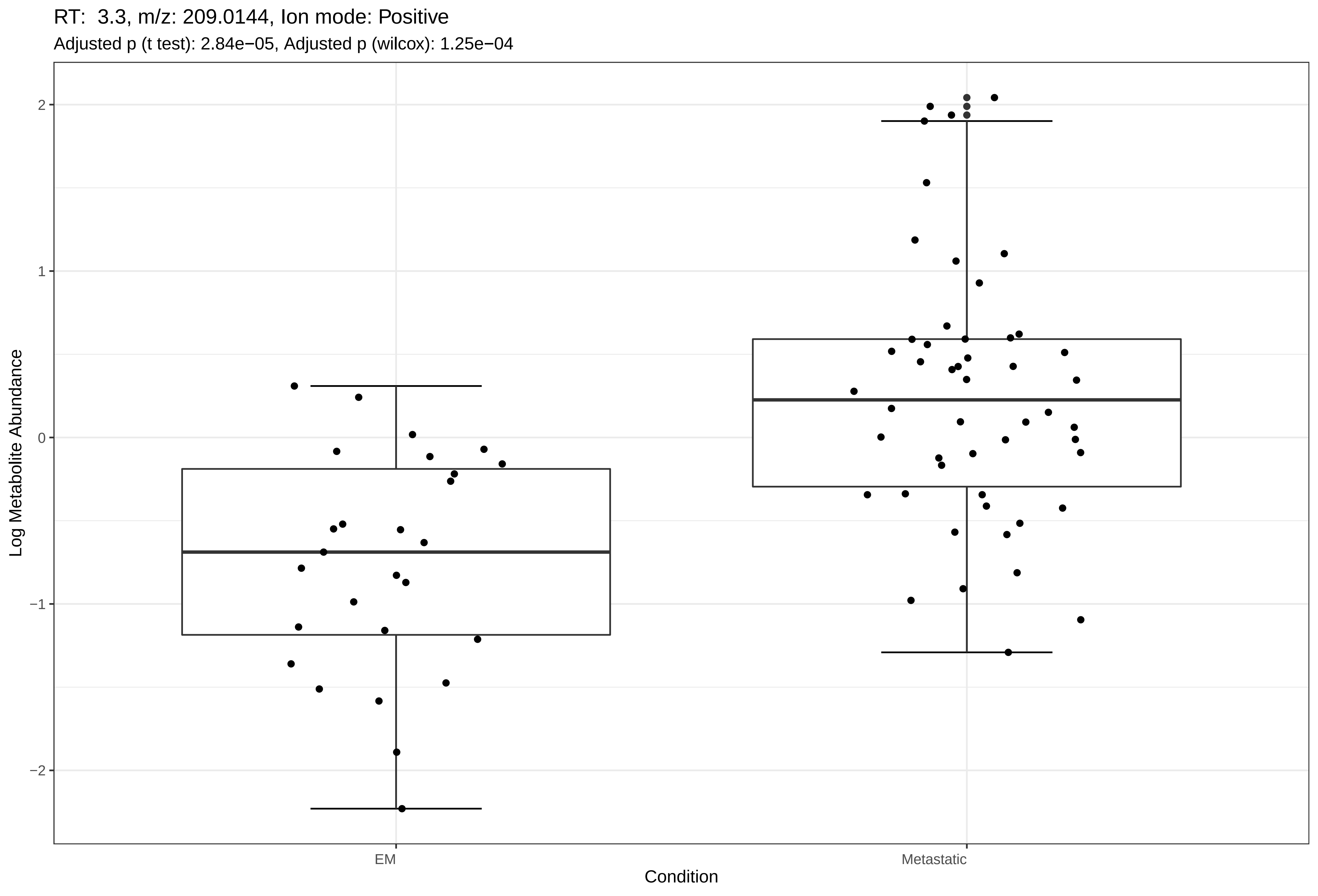
RT: 3.3, m/z: 209.0144, Ion mode: Positive
Adjusted p (t test): 2.84e−05, Adjusted p (wilcox): 1.25e−04
Log Metabolite Abundance
2
1
0
−1
−2
EM
Metastatic
Condition

RT: 6.1, m/z: 397.0312, Ion mode: Negative

Adjusted p (t test): 1.83e−04, Adjusted p (wilcox): 2.84e−05

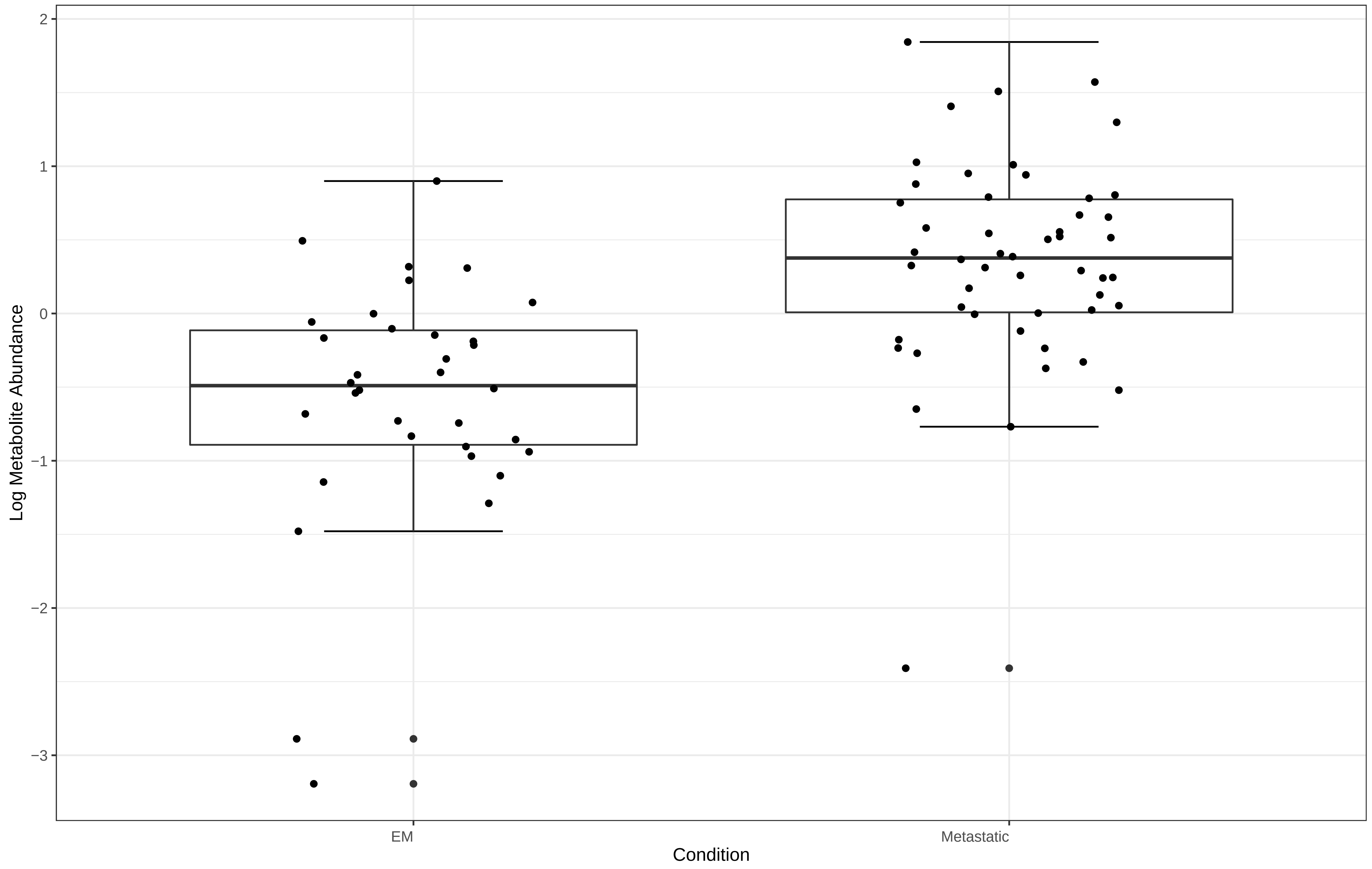

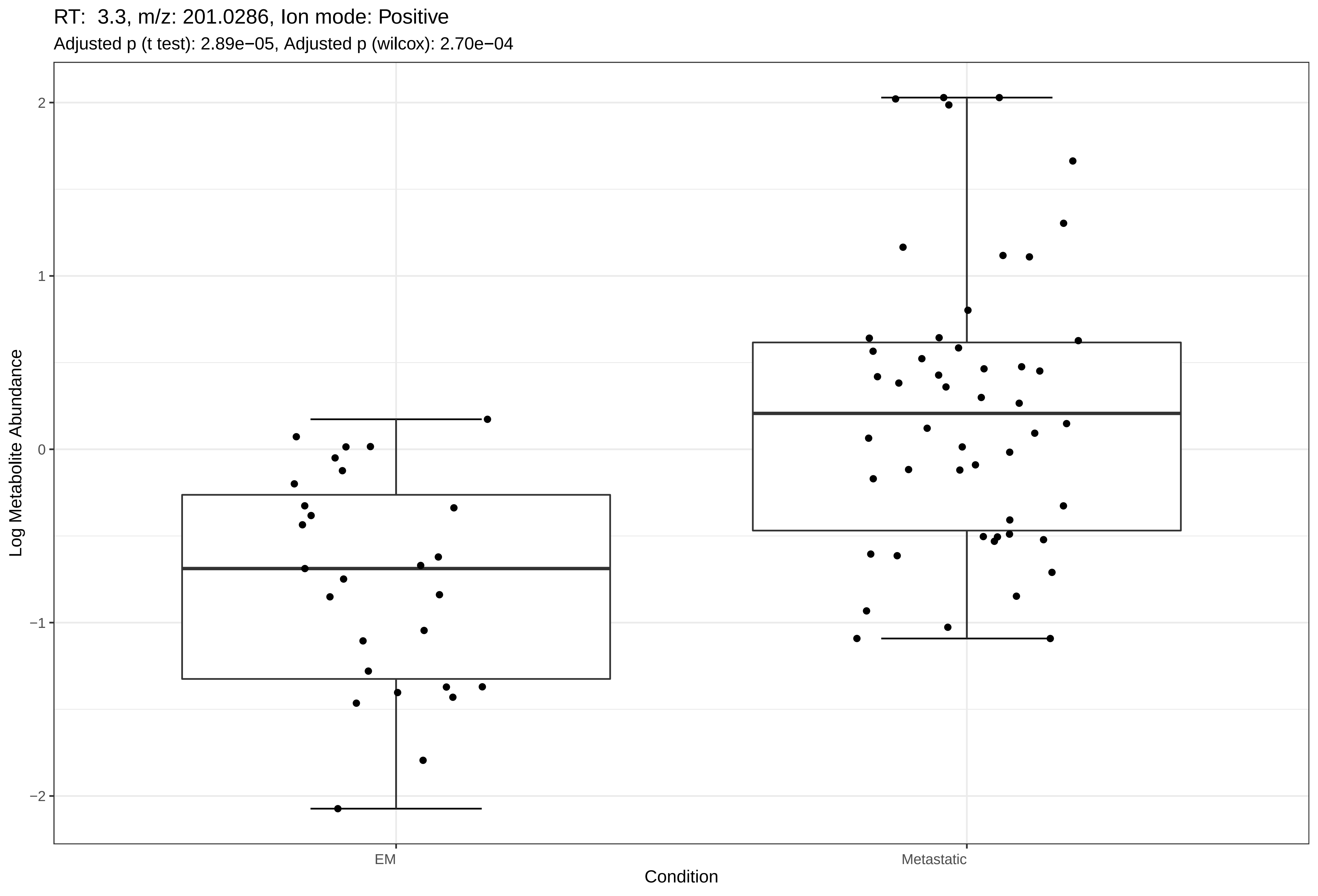
RT: 3.3, m/z: 201.0286, Ion mode: Positive
Adjusted p (t test): 2.89e−05, Adjusted p (wilcox): 2.70e−04
Log Metabolite Abundance
2
1
0
−1
−2
EM
Metastatic
Condition

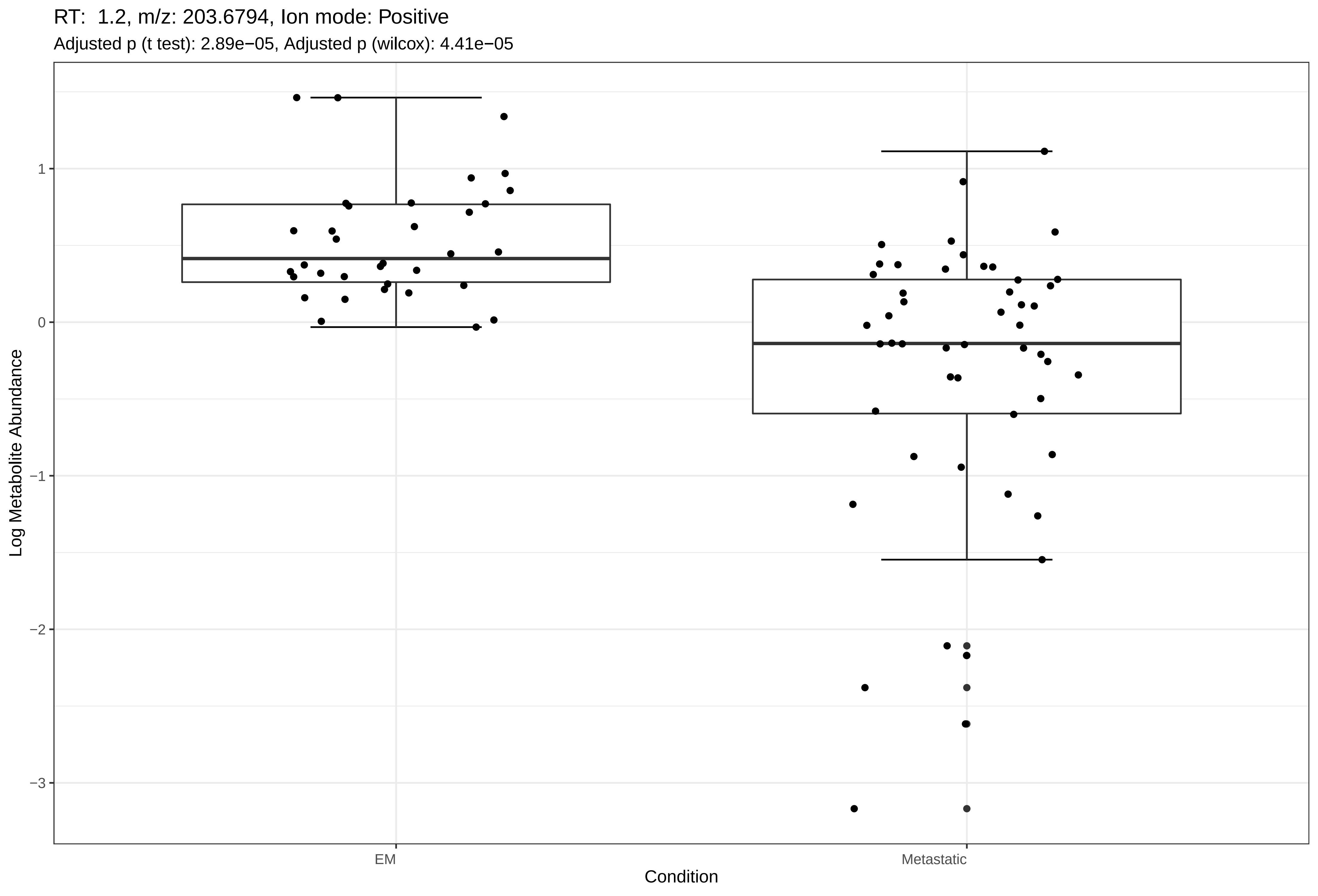

RT: 1.2, m/z: 203.6794, Ion mode: Positive
Adjusted p (t test): 2.89e−05, Adjusted p (wilcox): 4.41e−05
Log Metabolite Abundance
1
0
−1
−2
−3
EM
Metastatic
Condition

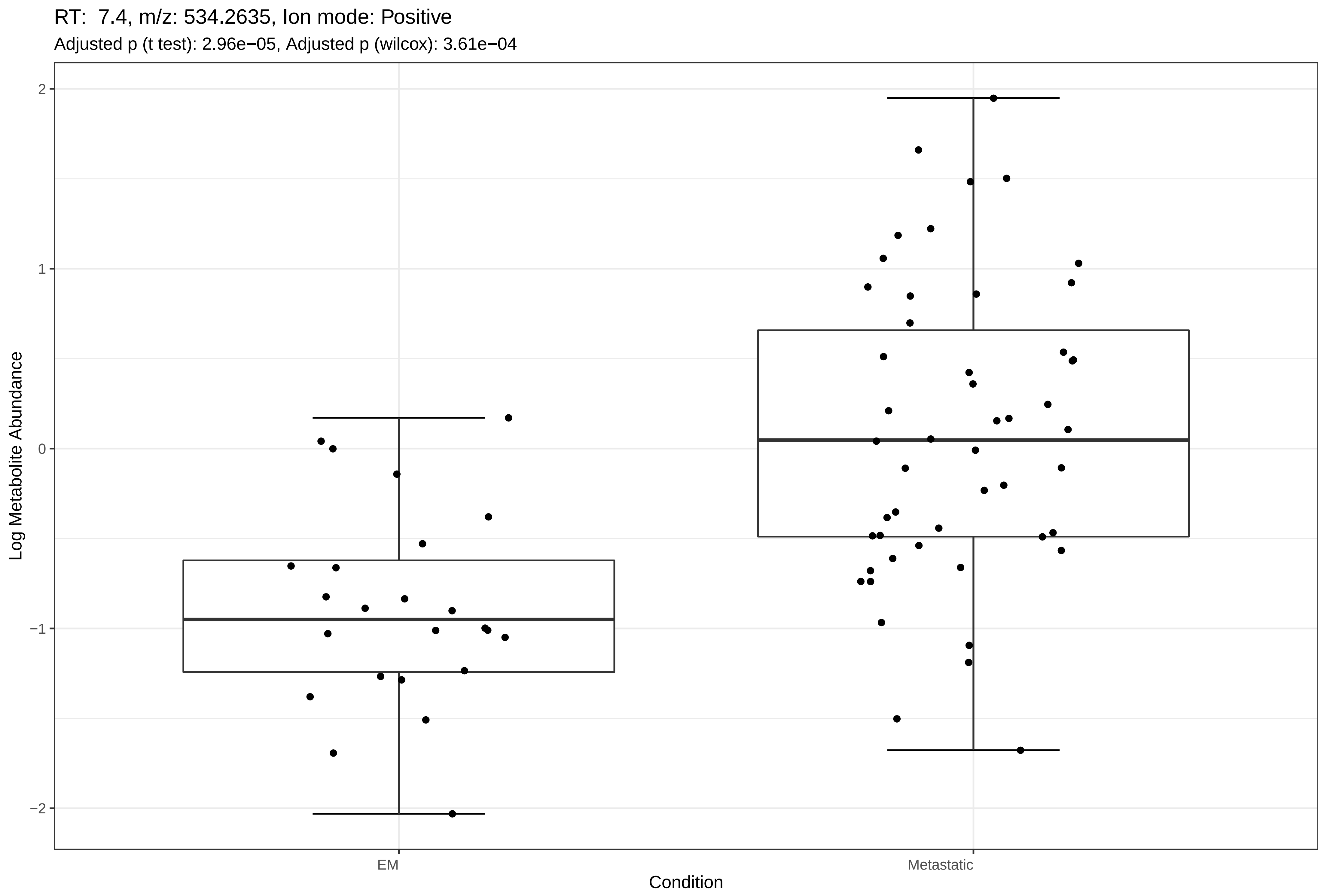
RT: 7.4, m/z: 534.2635, Ion mode: Positive
Adjusted p (t test): 2.96e−05, Adjusted p (wilcox): 3.61e−04
Log Metabolite Abundance
2
1
0
−1
−2
EM
Metastatic
Condition

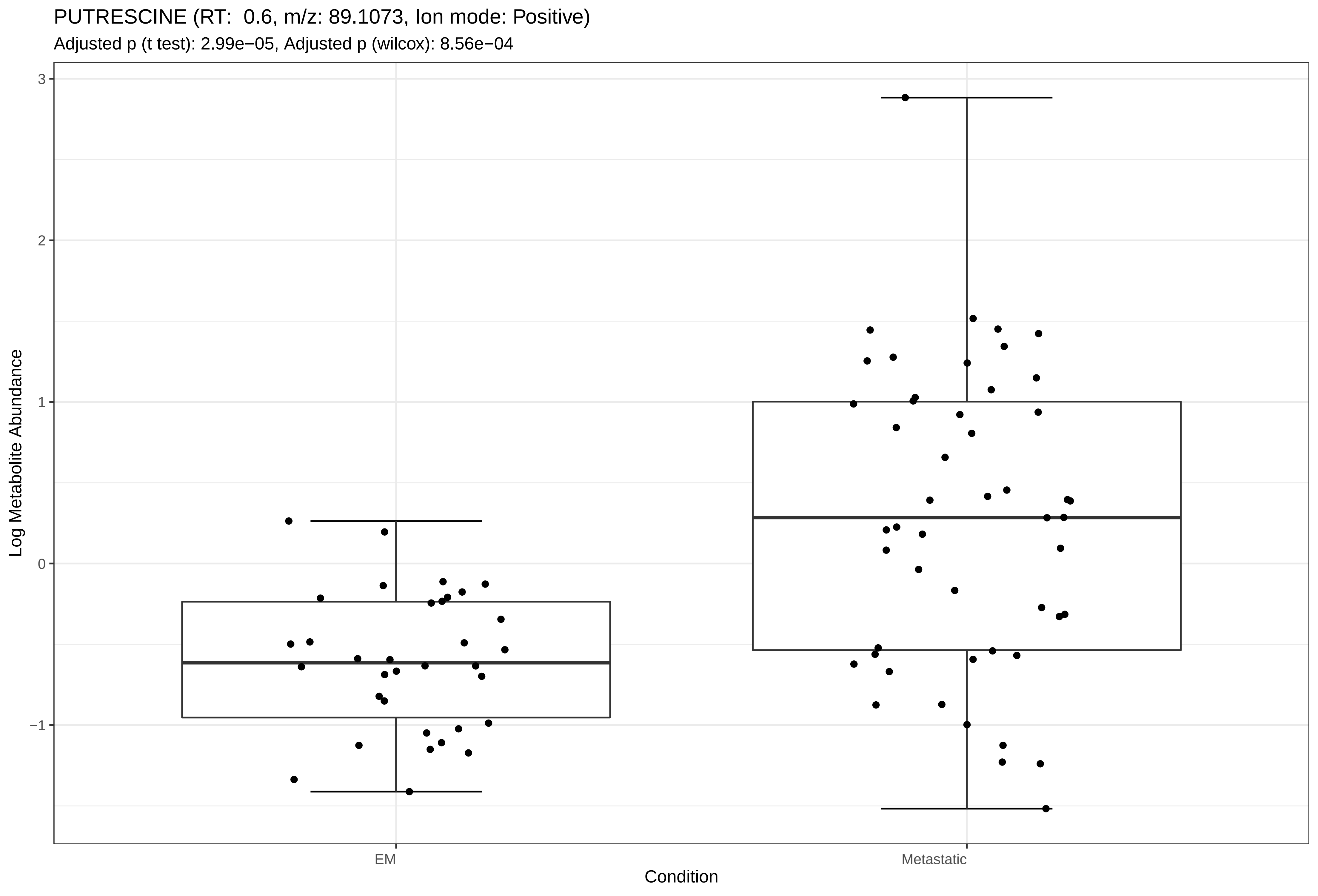
PUTRESCINE (RT: 0.6, m/z: 89.1073, Ion mode: Positive)
Adjusted p (t test): 2.99e−05, Adjusted p (wilcox): 8.56e−04
Log Metabolite Abundance
3
2
1
0
−1
EM
Metastatic
Condition

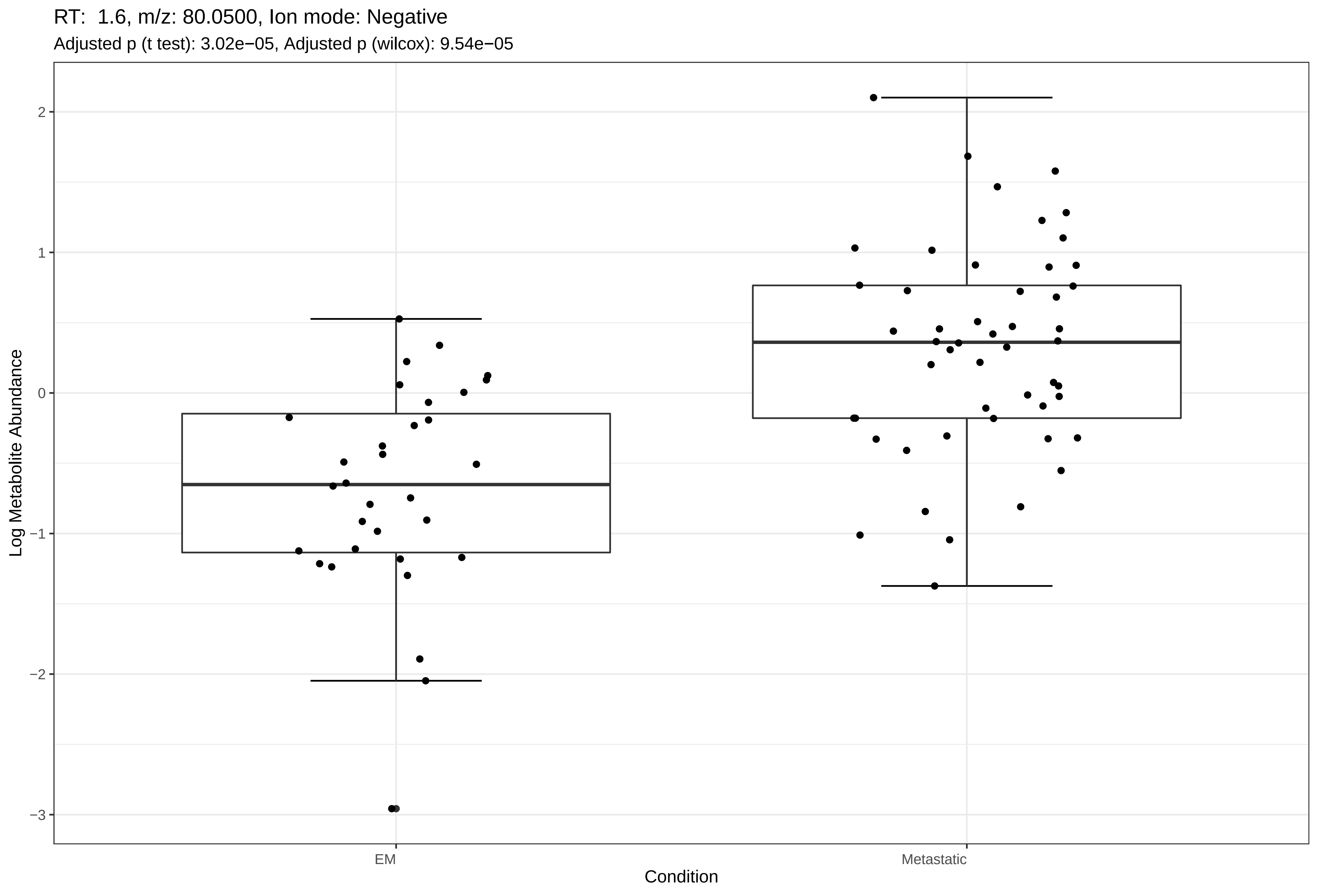
RT: 1.6, m/z: 80.0500, Ion mode: Negative
Adjusted p (t test): 3.02e−05, Adjusted p (wilcox): 9.54e−05
Log Metabolite Abundance
2
1
0
−1
−2
−3
EM
Metastatic
Condition

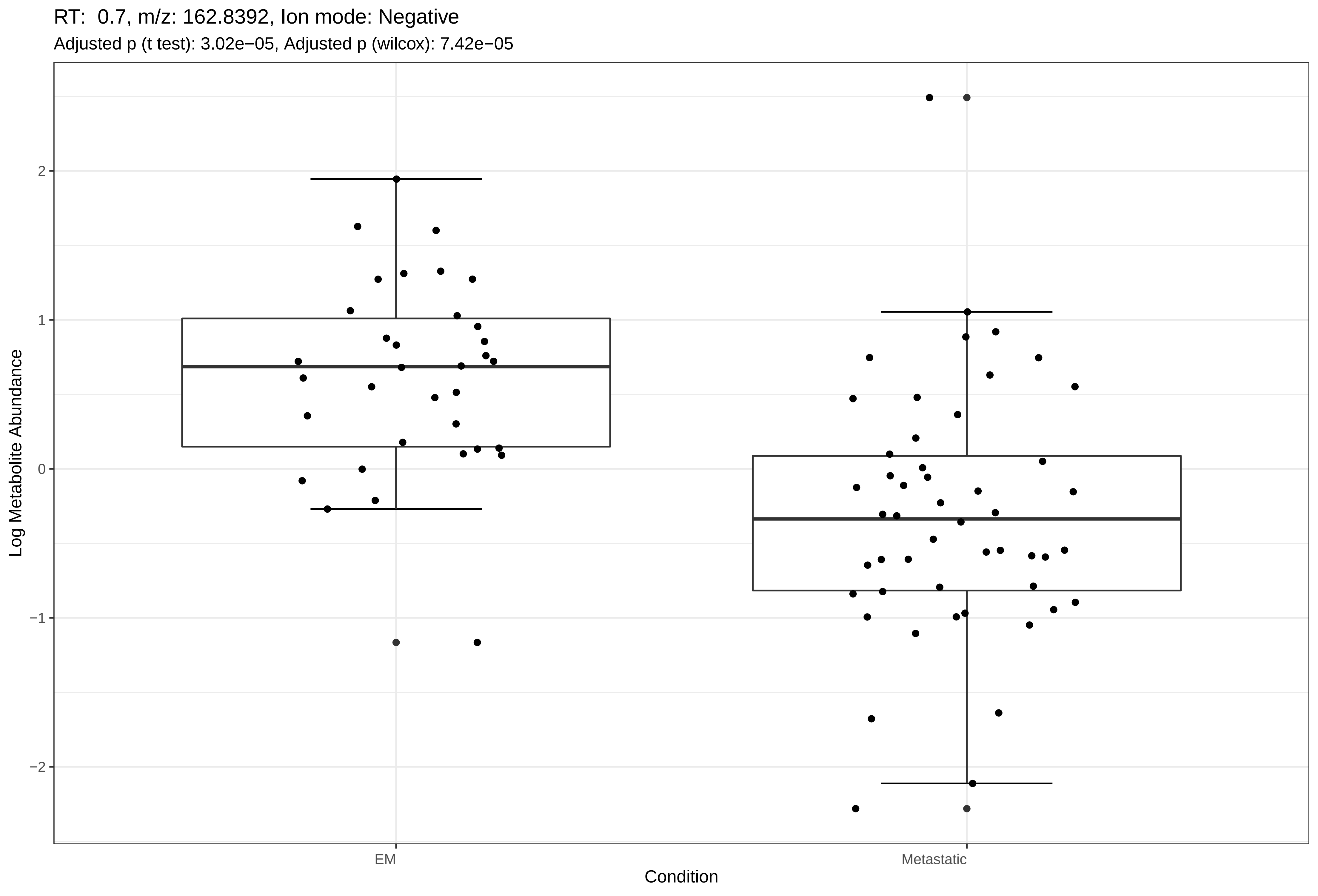

RT: 0.7, m/z: 162.8392, Ion mode: Negative
Adjusted p (t test): 3.02e−05, Adjusted p (wilcox): 7.42e−05
Log Metabolite Abundance
2
1
0
−1
−2
EM
Metastatic
Condition

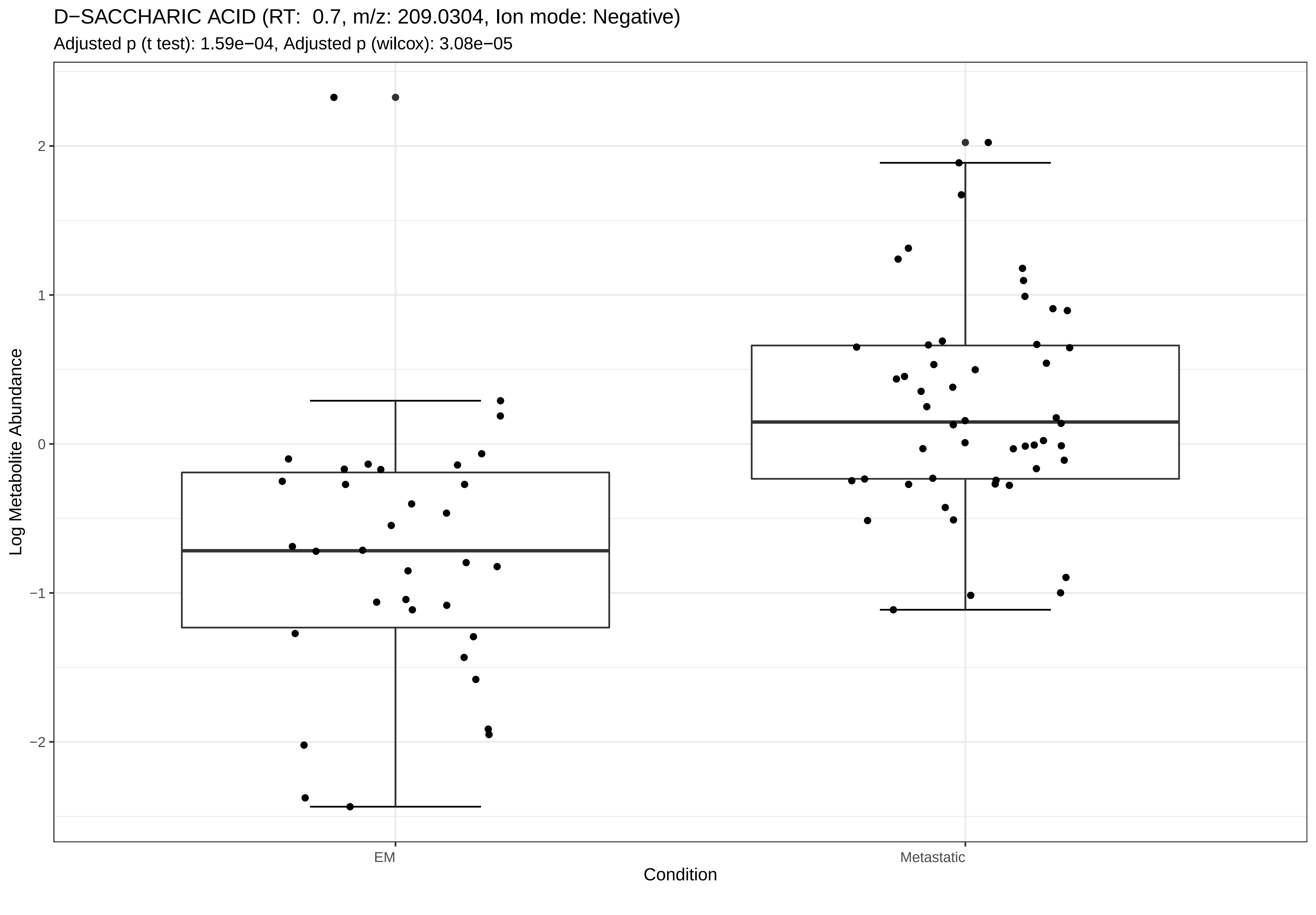

D−SACCHARIC ACID (RT: 0.7, m/z: 209.0304, Ion mode: Negative)
Adjusted p (t test): 1.59e−04, Adjusted p (wilcox): 3.08e−05
Log Metabolite Abundance
2
1
0
−1
−2
EM
Metastatic
Condition

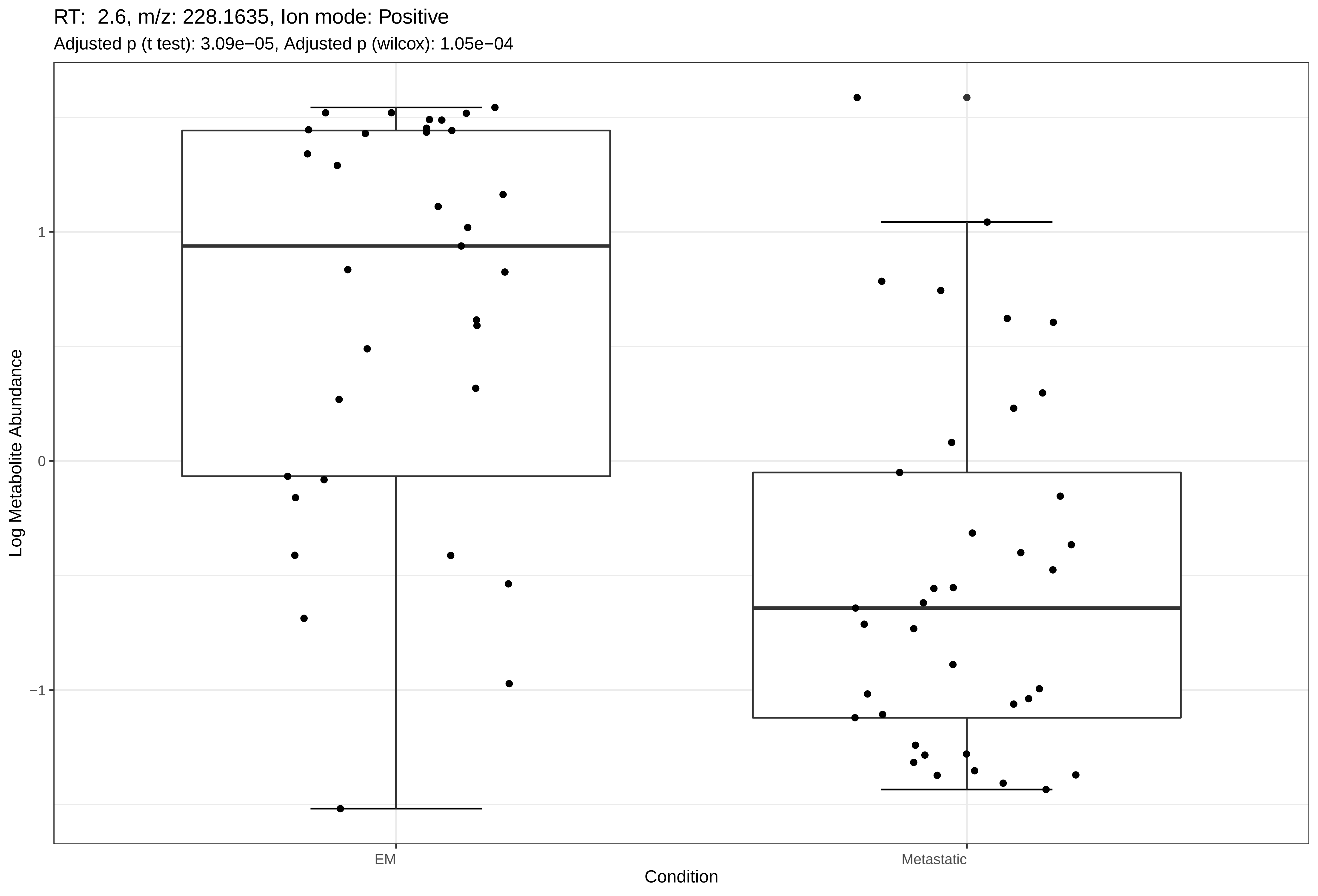
RT: 2.6, m/z: 228.1635, Ion mode: Positive
Adjusted p (t test): 3.09e−05, Adjusted p (wilcox): 1.05e−04
Log Metabolite Abundance
1
0
−1
EM
Metastatic
Condition

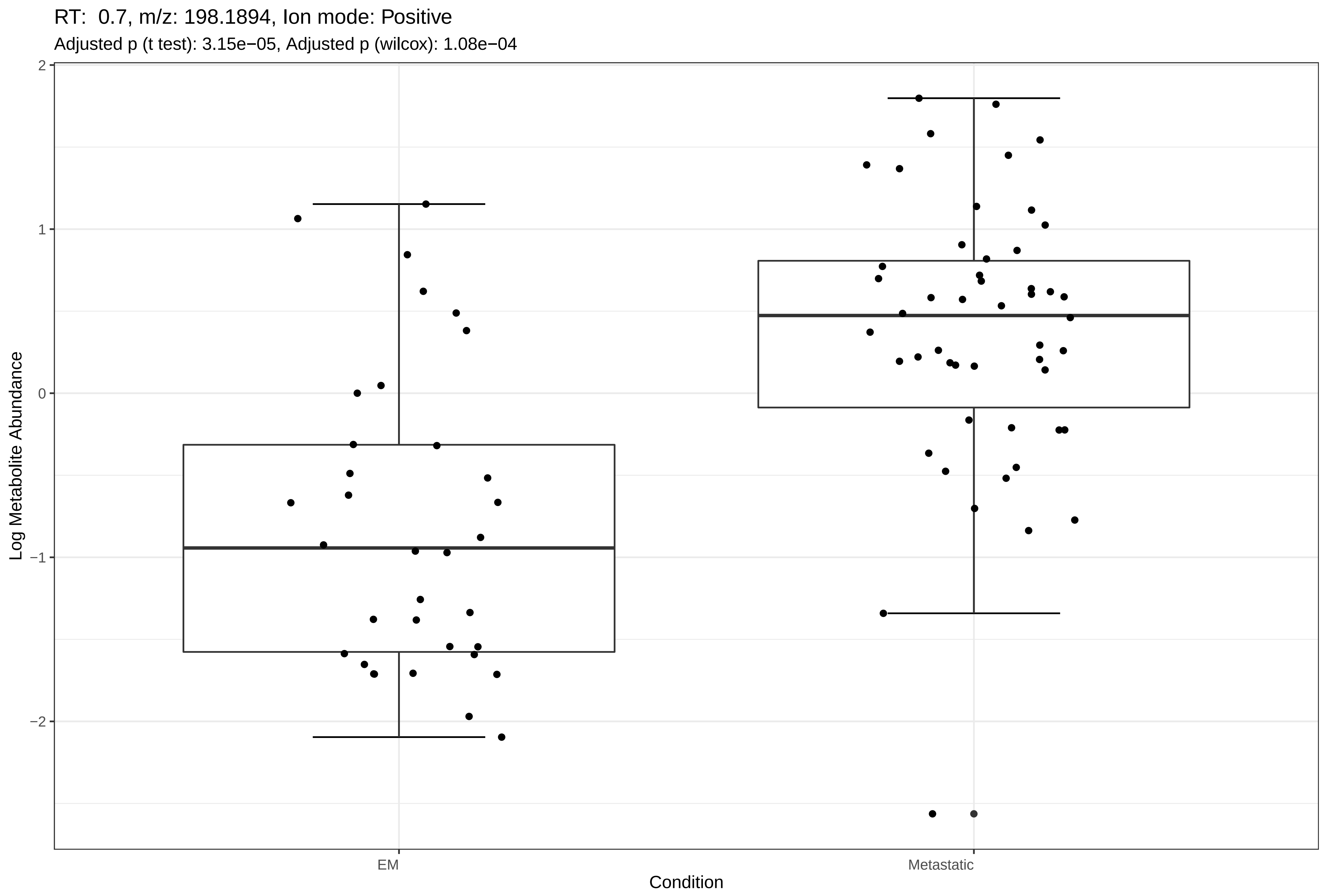

RT: 0.7, m/z: 198.1894, Ion mode: Positive
Adjusted p (t test): 3.15e−05, Adjusted p (wilcox): 1.08e−04
Log Metabolite Abundance
2
1
0
−1
−2
EM
Metastatic
Condition

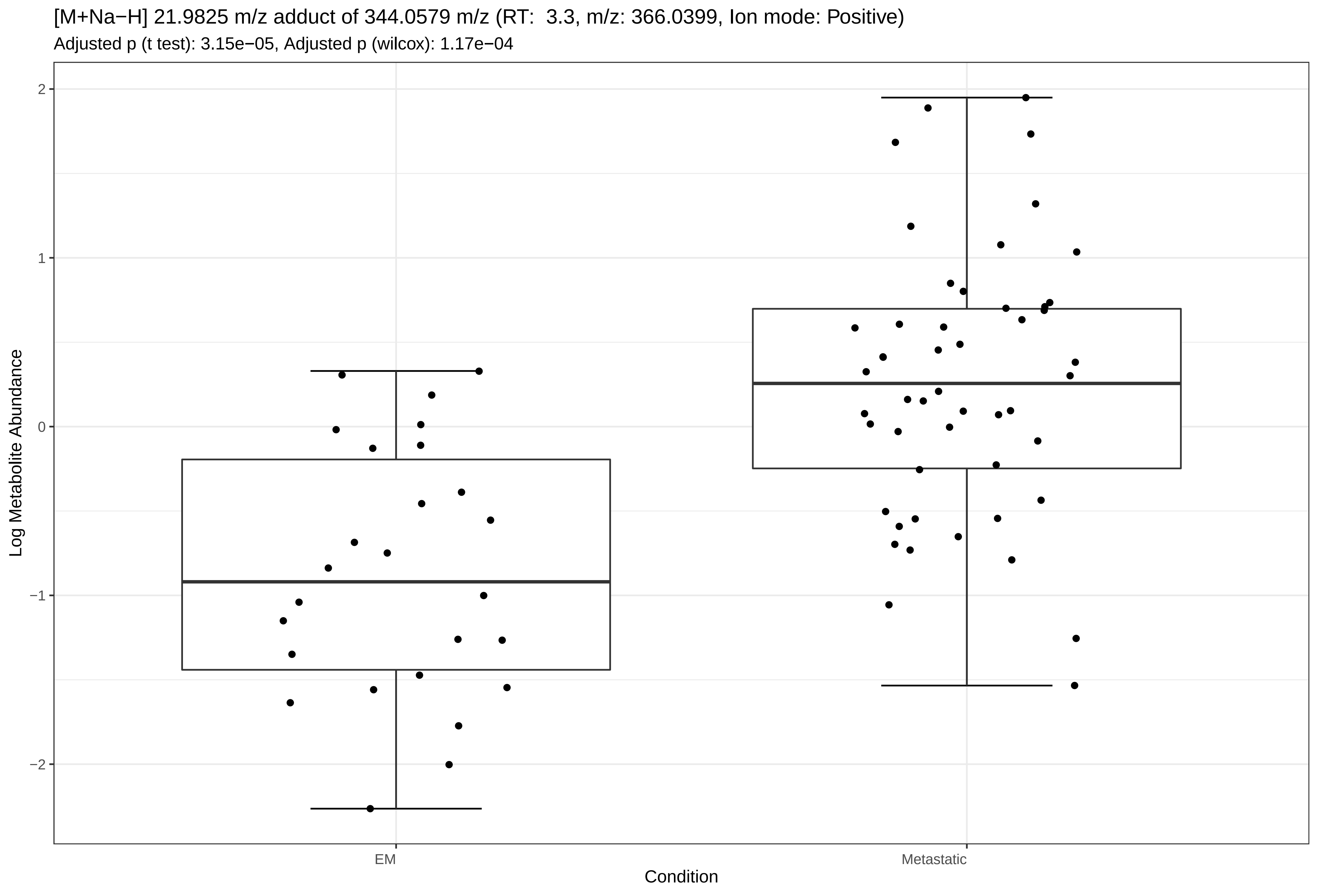
[M+Na−H] 21.9825 m/z adduct of 344.0579 m/z (RT: 3.3, m/z: 366.0399, Ion mode: Positive)
Adjusted p (t test): 3.15e−05, Adjusted p (wilcox): 1.17e−04
Log Metabolite Abundance
2
1
0
−1
−2
EM
Metastatic
Condition

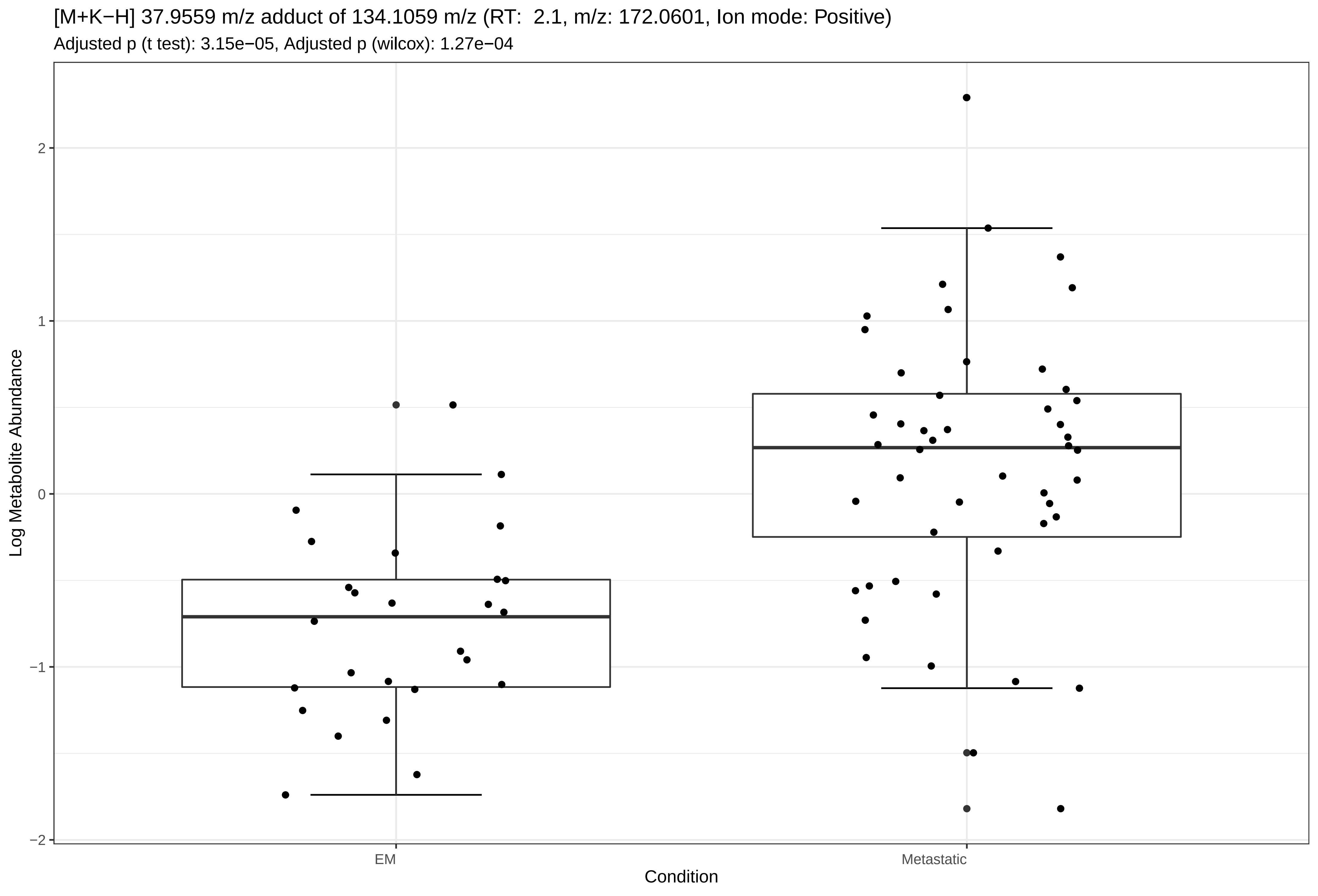

[M+K−H] 37.9559 m/z adduct of 134.1059 m/z (RT: 2.1, m/z: 172.0601, Ion mode: Positive)
Adjusted p (t test): 3.15e−05, Adjusted p (wilcox): 1.27e−04
Log Metabolite Abundance
2
1
0
−1
−2
EM
Metastatic
Condition

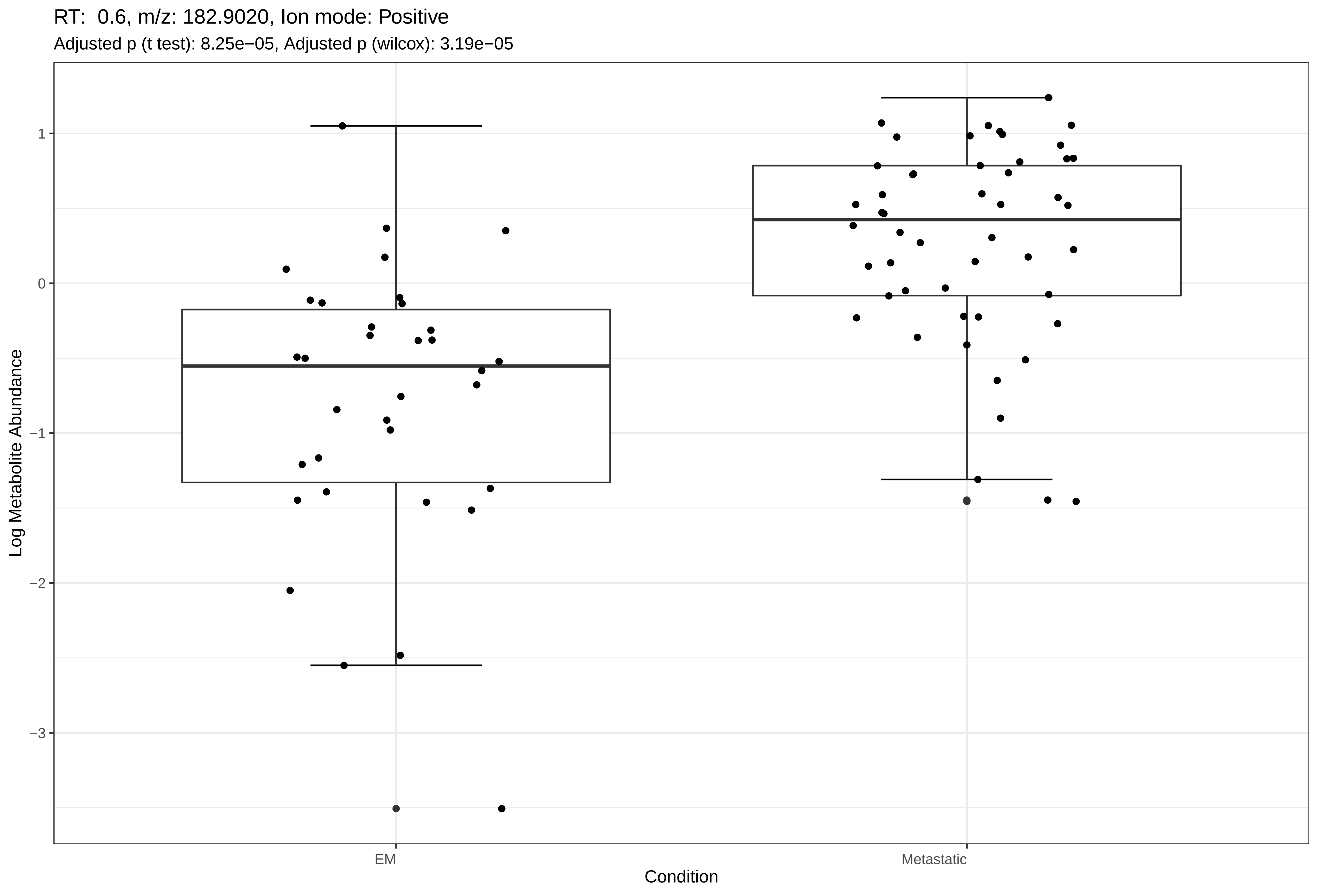

RT: 0.6, m/z: 182.9020, Ion mode: Positive
Adjusted p (t test): 8.25e−05, Adjusted p (wilcox): 3.19e−05
Log Metabolite Abundance
1
0
−1
−2
−3
EM
Metastatic
Condition

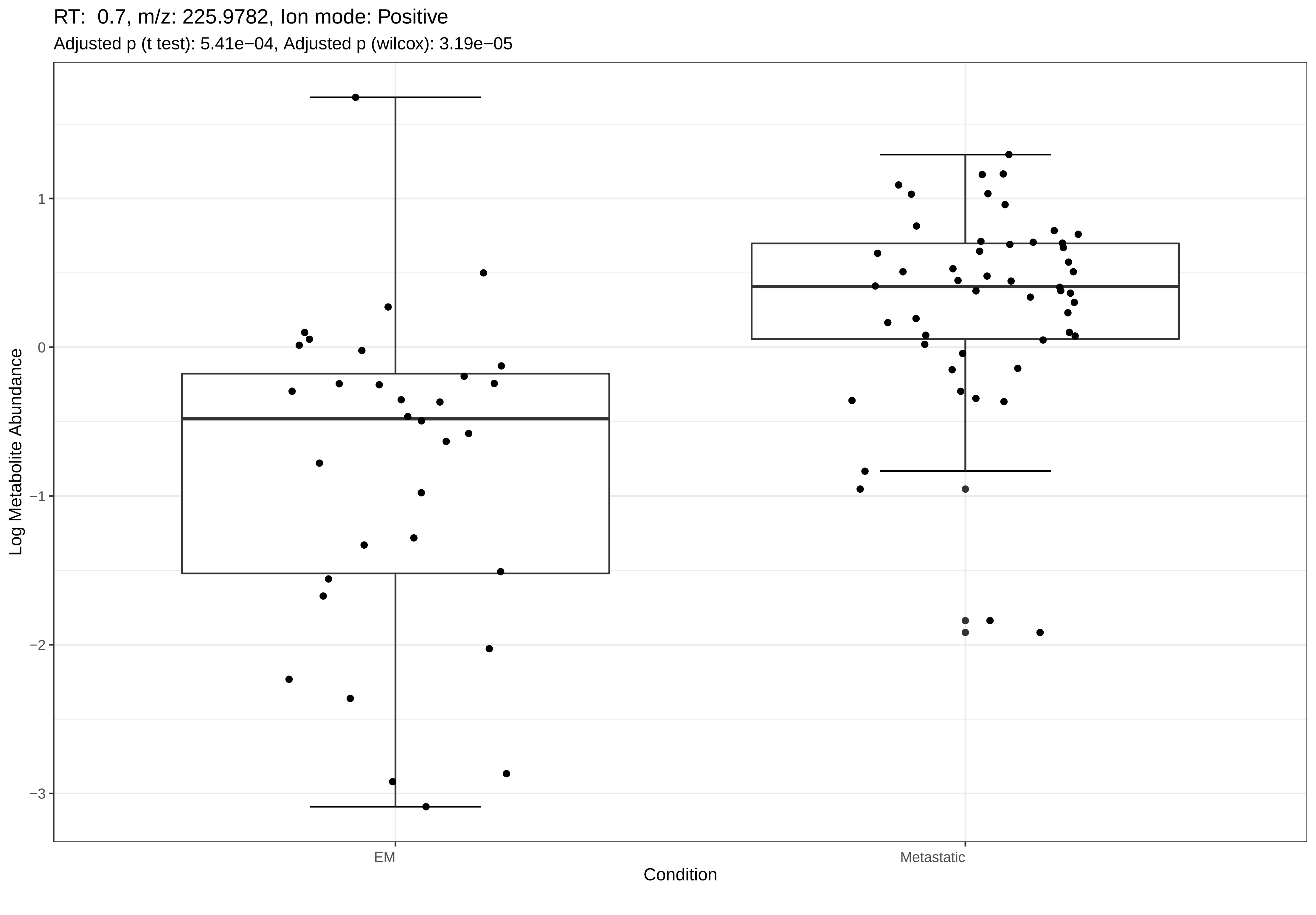

RT: 0.7, m/z: 225.9782, Ion mode: Positive
Adjusted p (t test): 5.41e−04, Adjusted p (wilcox): 3.19e−05
Log Metabolite Abundance
1
0
−1
−2
−3
EM
Metastatic
Condition

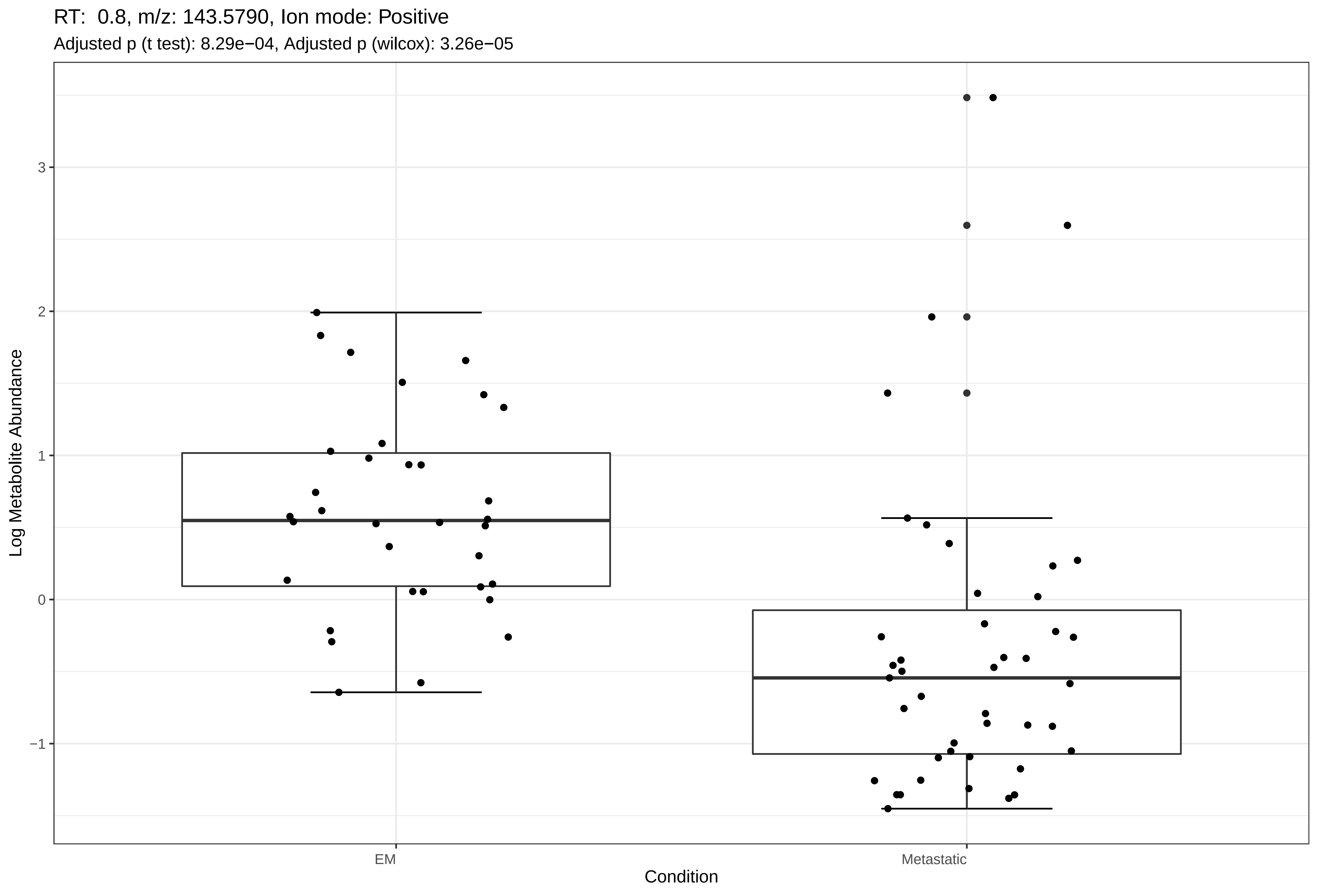

RT: 0.8, m/z: 143.5790, Ion mode: Positive
Adjusted p (t test): 8.29e−04, Adjusted p (wilcox): 3.26e−05
Log Metabolite Abundance
3
2
1
0
−1
EM
Metastatic
Condition

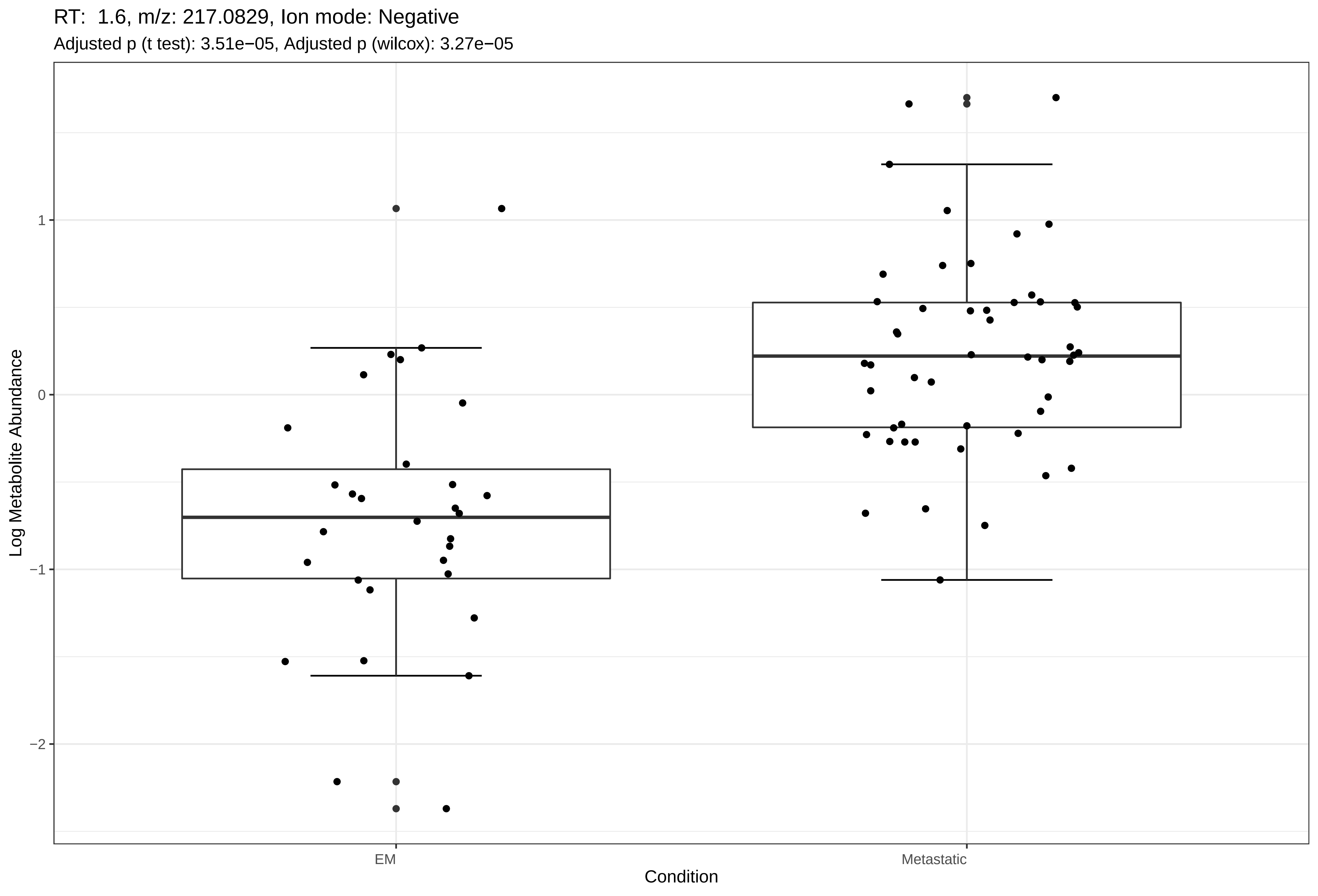
RT: 1.6, m/z: 217.0829, Ion mode: Negative
Adjusted p (t test): 3.51e−05, Adjusted p (wilcox): 3.27e−05
Log Metabolite Abundance
1
0
−1
−2
EM
Metastatic
Condition

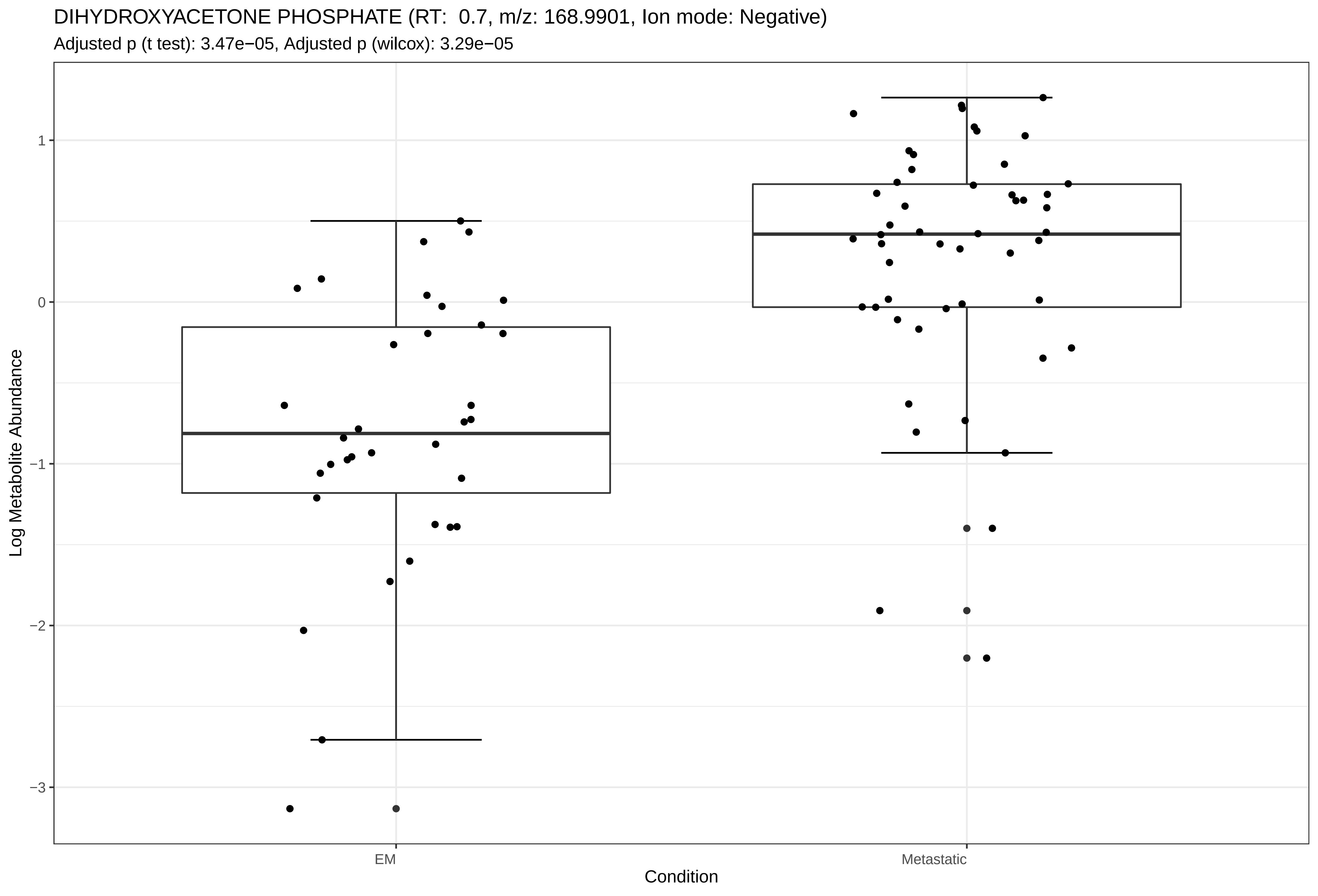
DIHYDROXYACETONE PHOSPHATE (RT: 0.7, m/z: 168.9901, Ion mode: Negative)
Adjusted p (t test): 3.47e−05, Adjusted p (wilcox): 3.29e−05
Log Metabolite Abundance
1
0
−1
−2
−3
EM
Metastatic
Condition

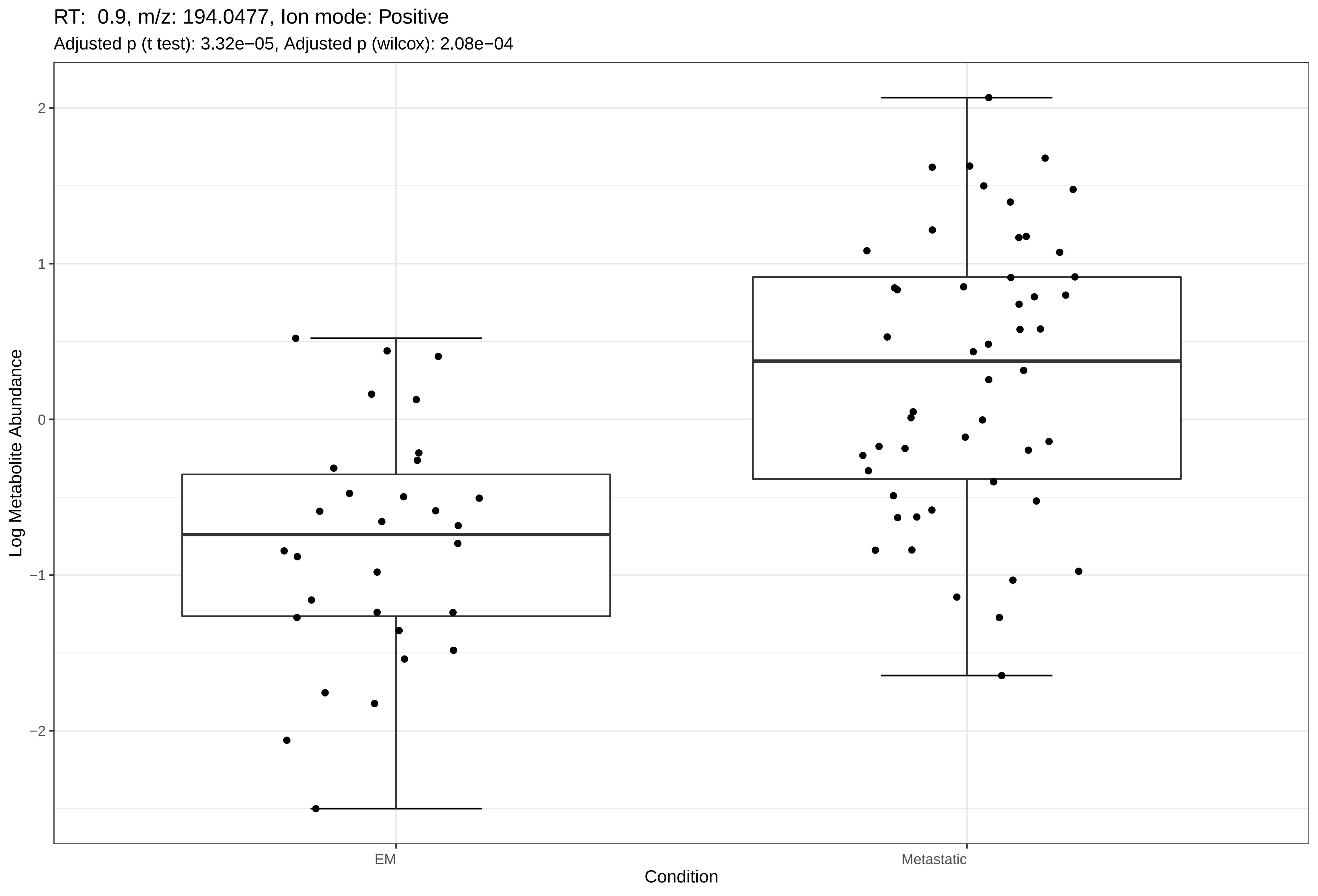

RT: 0.9, m/z: 194.0477, Ion mode: Positive
Adjusted p (t test): 3.32e−05, Adjusted p (wilcox): 2.08e−04
Log Metabolite Abundance
2
1
0
−1
−2
EM
Metastatic
Condition

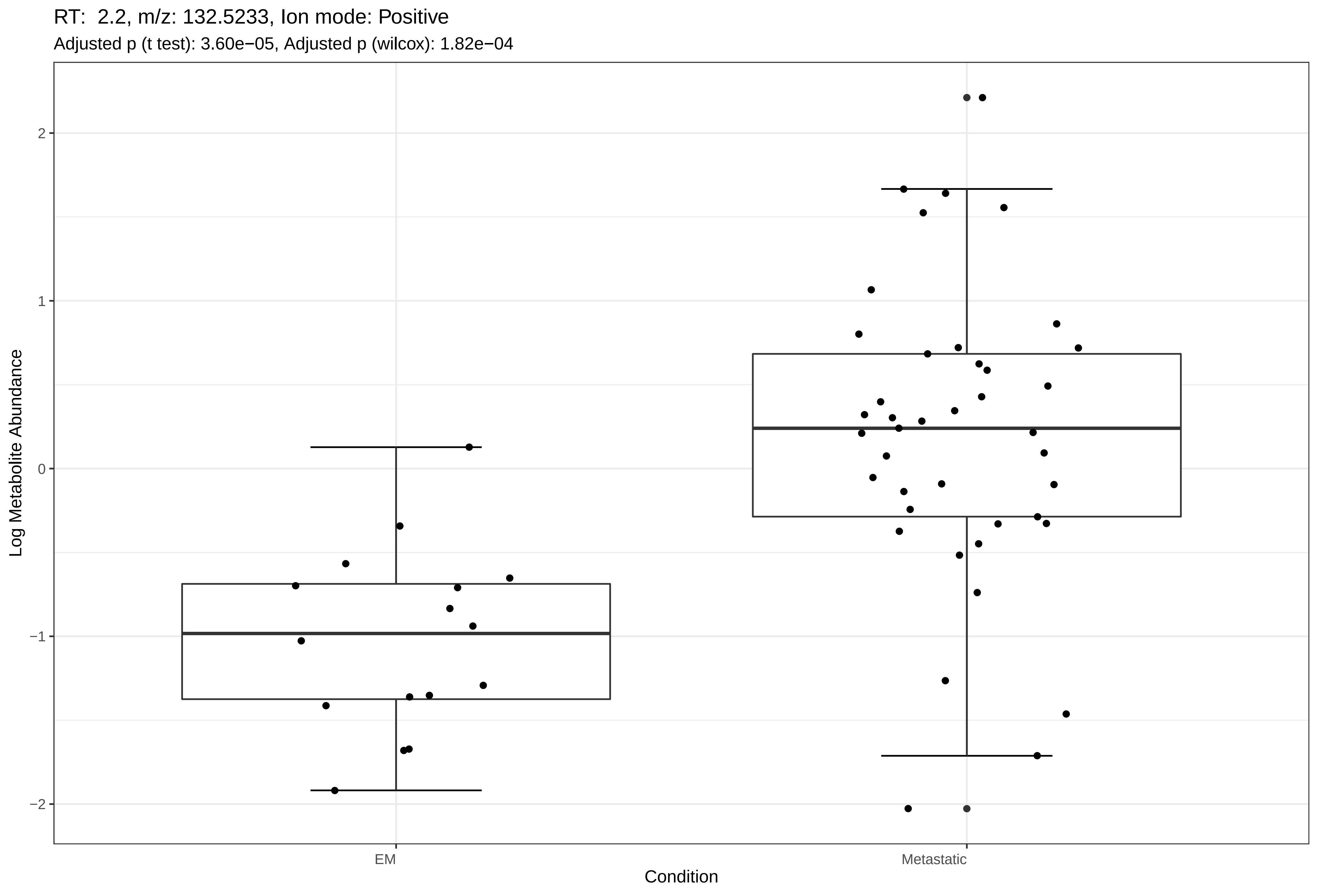

RT: 2.2, m/z: 132.5233, Ion mode: Positive
Adjusted p (t test): 3.60e−05, Adjusted p (wilcox): 1.82e−04
Log Metabolite Abundance
2
1
0
−1
−2
EM
Metastatic
Condition

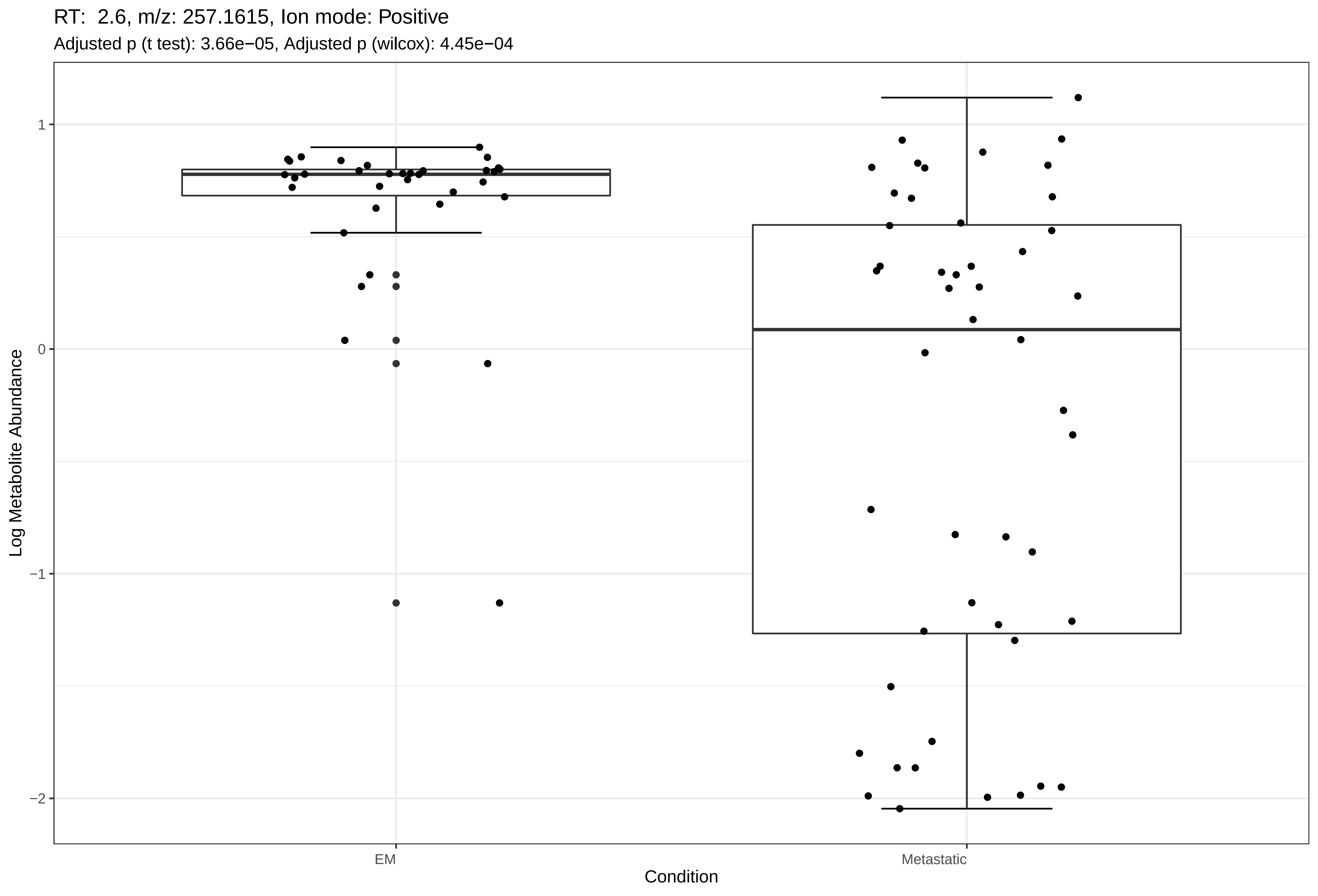
RT: 2.6, m/z: 257.1615, Ion mode: Positive
Adjusted p (t test): 3.66e−05, Adjusted p (wilcox): 4.45e−04
Log Metabolite Abundance
1
0
−1
−2
EM
Metastatic
Condition

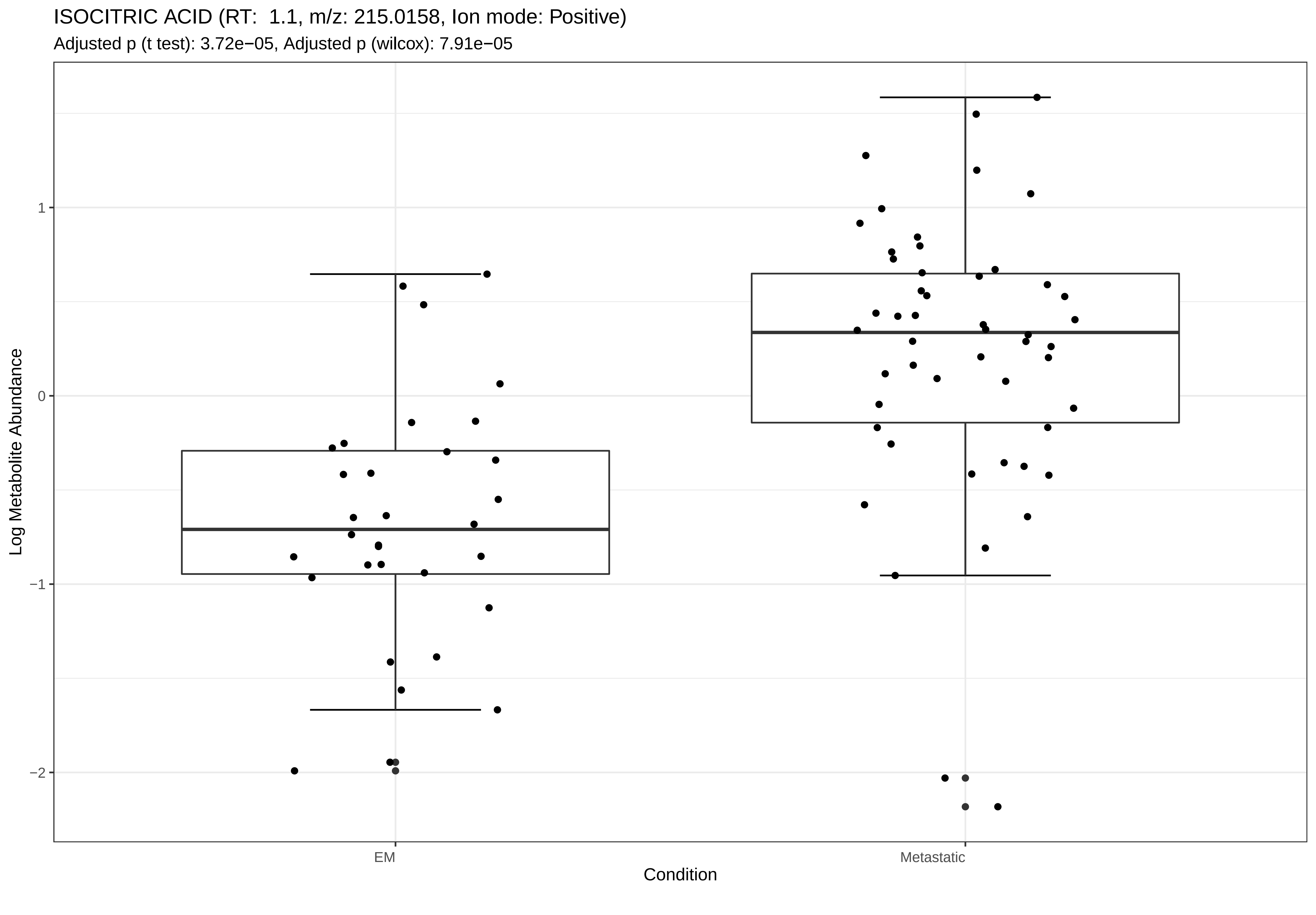

ISOCITRIC ACID (RT: 1.1, m/z: 215.0158, Ion mode: Positive)
Adjusted p (t test): 3.72e−05, Adjusted p (wilcox): 7.91e−05
Log Metabolite Abundance
1
0
−1
−2
EM
Metastatic
Condition

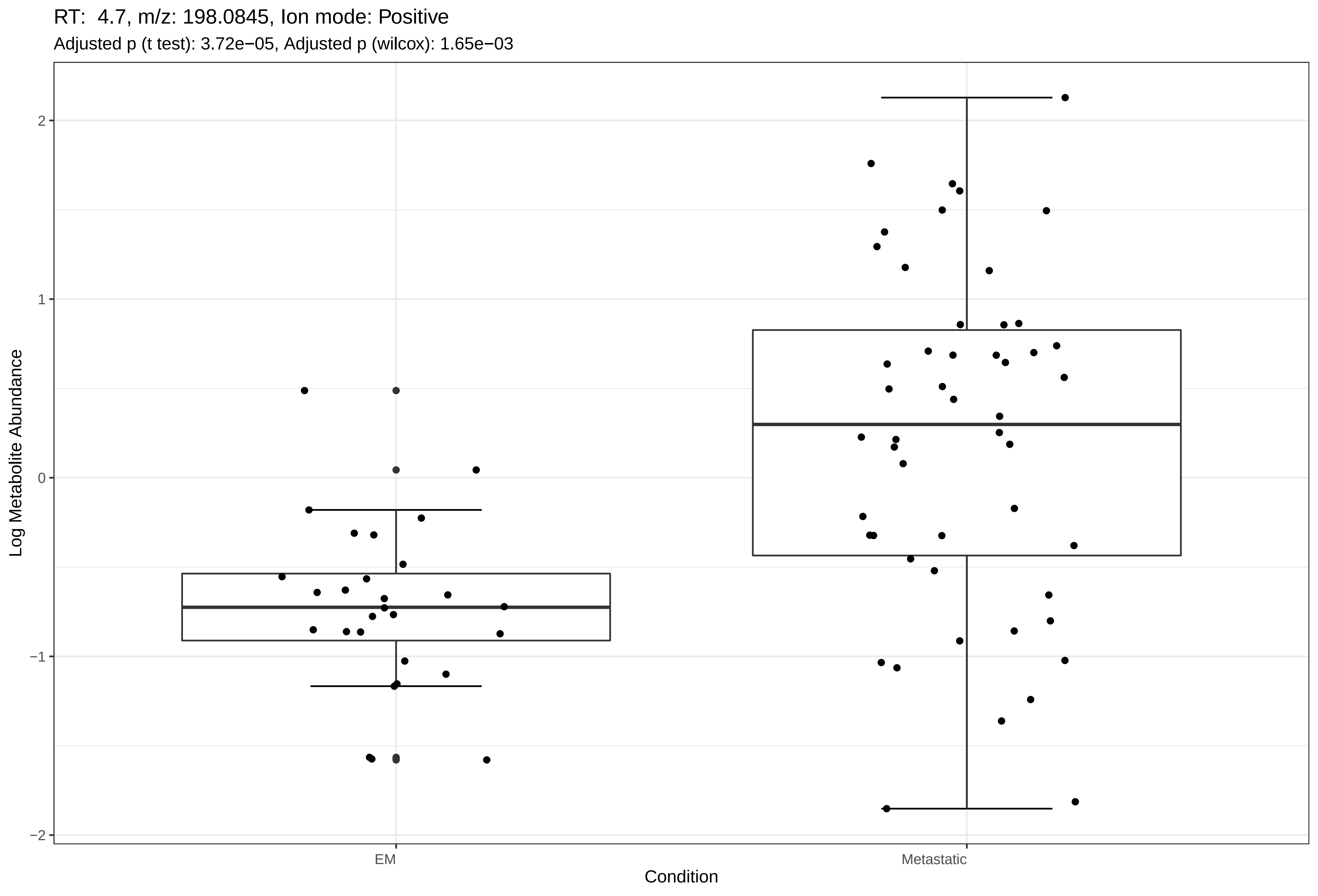

RT: 4.7, m/z: 198.0845, Ion mode: Positive
Adjusted p (t test): 3.72e−05, Adjusted p (wilcox): 1.65e−03
Log Metabolite Abundance
2
1
0
−1
−2
EM
Metastatic
Condition

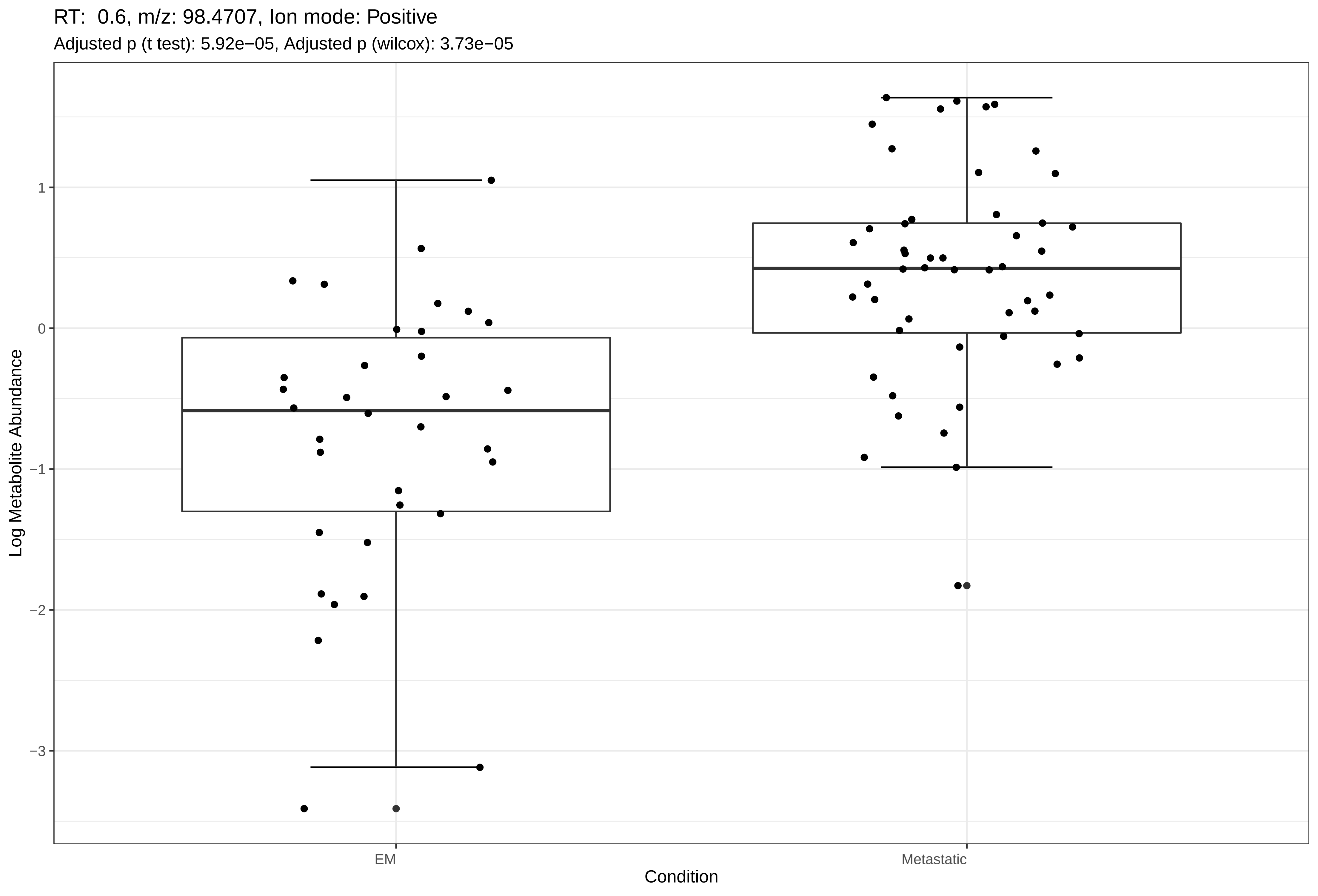
RT: 0.6, m/z: 98.4707, Ion mode: Positive
Adjusted p (t test): 5.92e−05, Adjusted p (wilcox): 3.73e−05
Log Metabolite Abundance
1
0
−1
−2
−3
EM
Metastatic
Condition

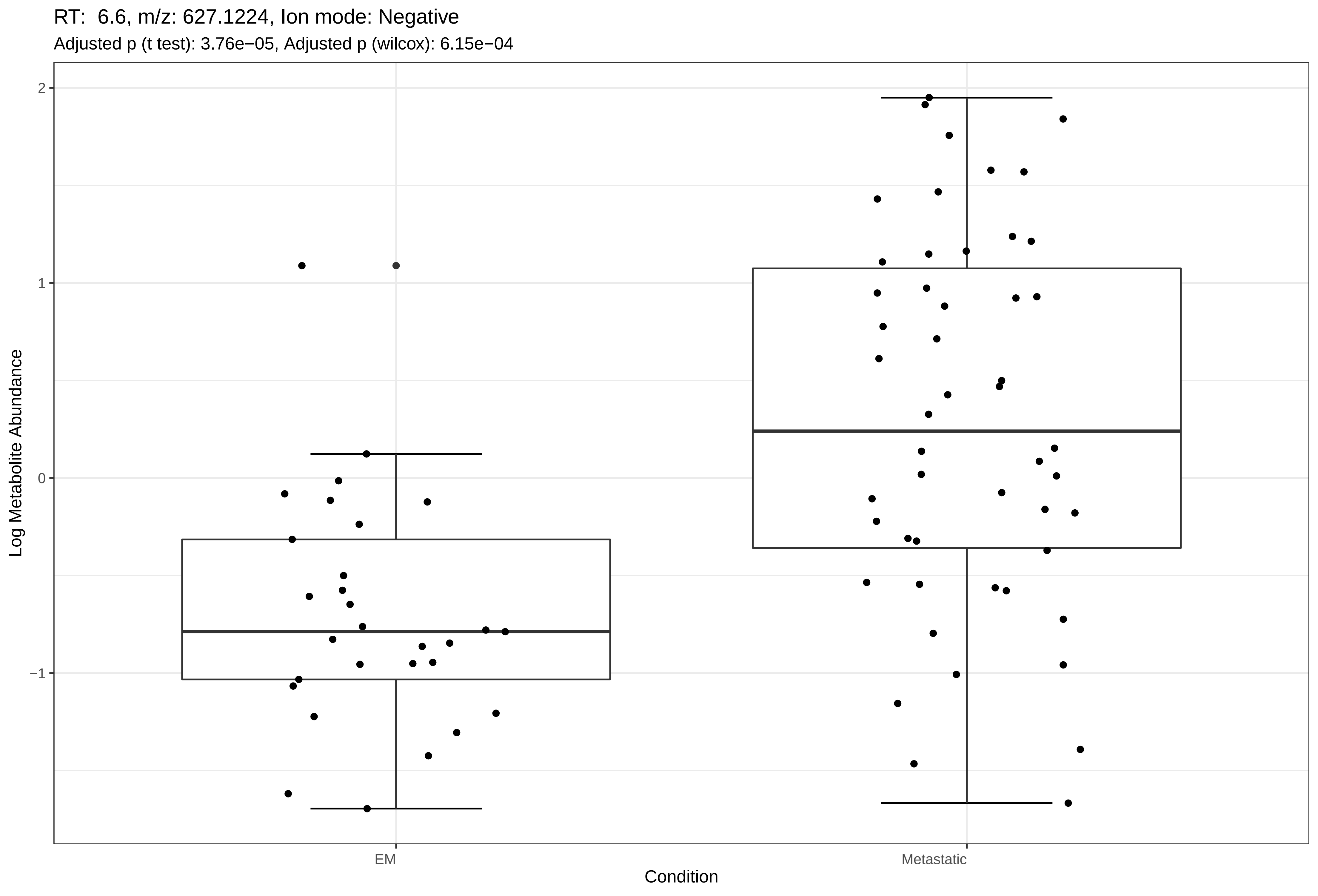

RT: 6.6, m/z: 627.1224, Ion mode: Negative
Adjusted p (t test): 3.76e−05, Adjusted p (wilcox): 6.15e−04
Log Metabolite Abundance
2
1
0
−1
EM
Metastatic
Condition

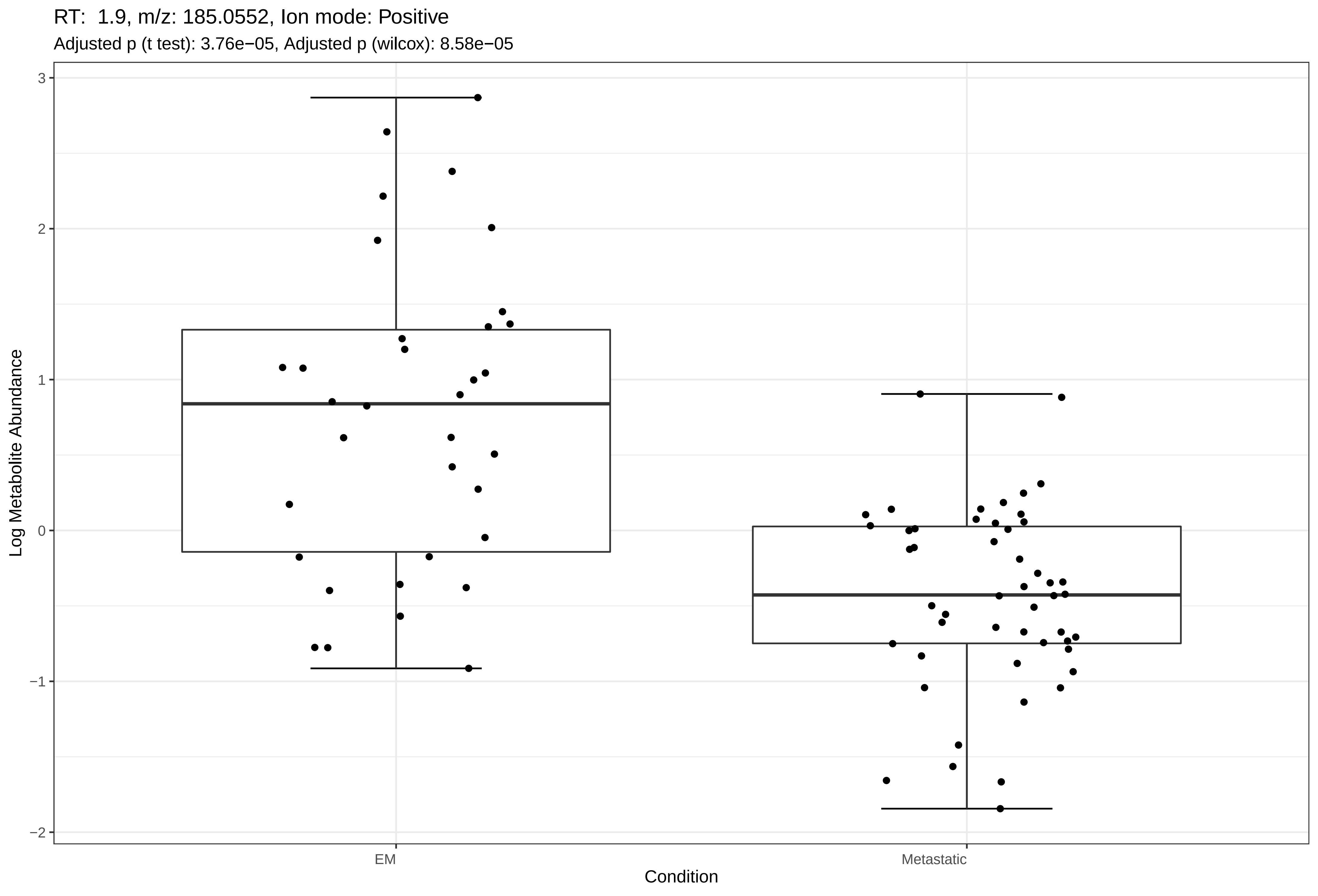

RT: 1.9, m/z: 185.0552, Ion mode: Positive
Adjusted p (t test): 3.76e−05, Adjusted p (wilcox): 8.58e−05
Log Metabolite Abundance
3
2
1
0
−1
−2
EM
Metastatic
Condition

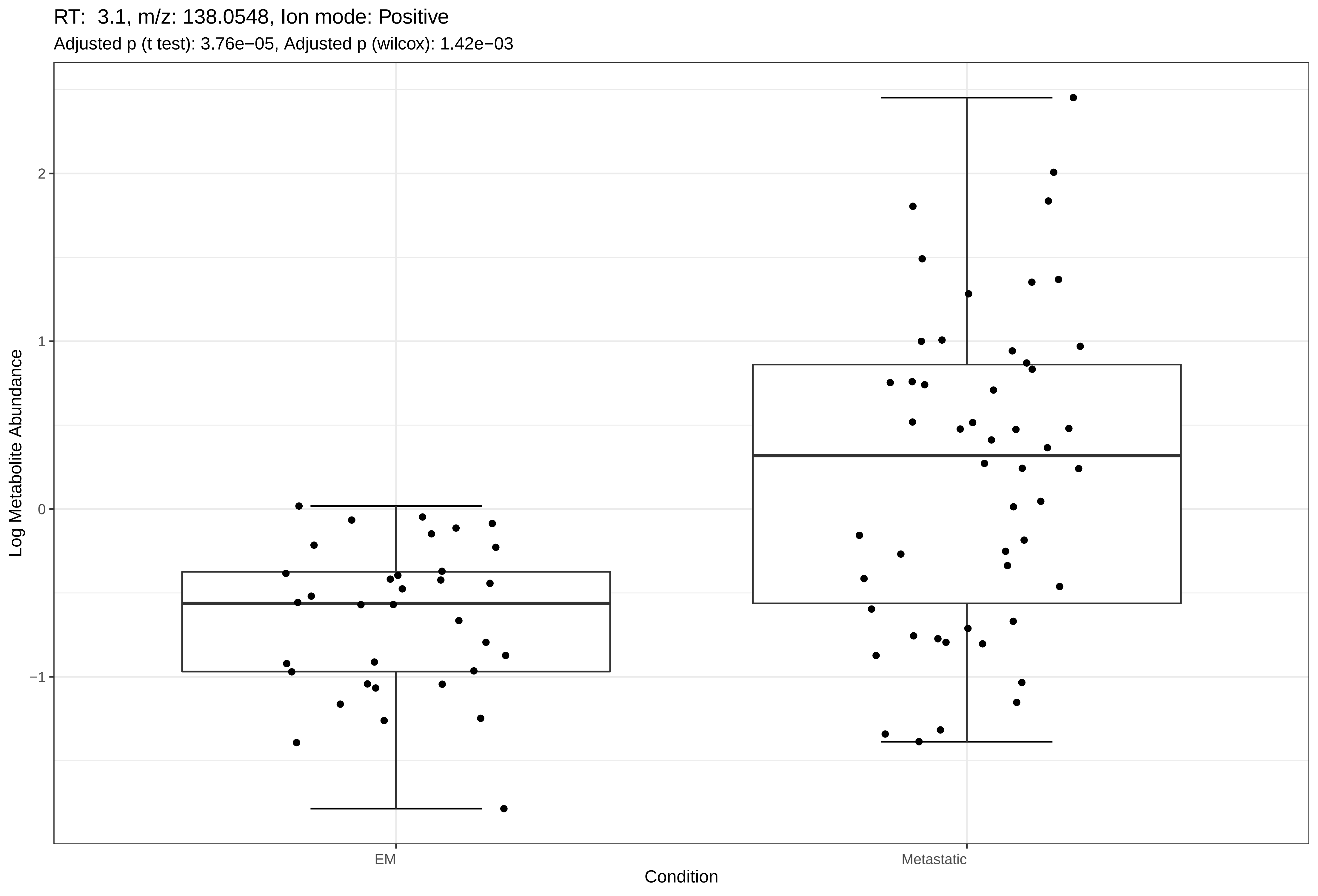

RT: 3.1, m/z: 138.0548, Ion mode: Positive
Adjusted p (t test): 3.76e−05, Adjusted p (wilcox): 1.42e−03
Log Metabolite Abundance
2
1
0
−1
EM
Metastatic
Condition

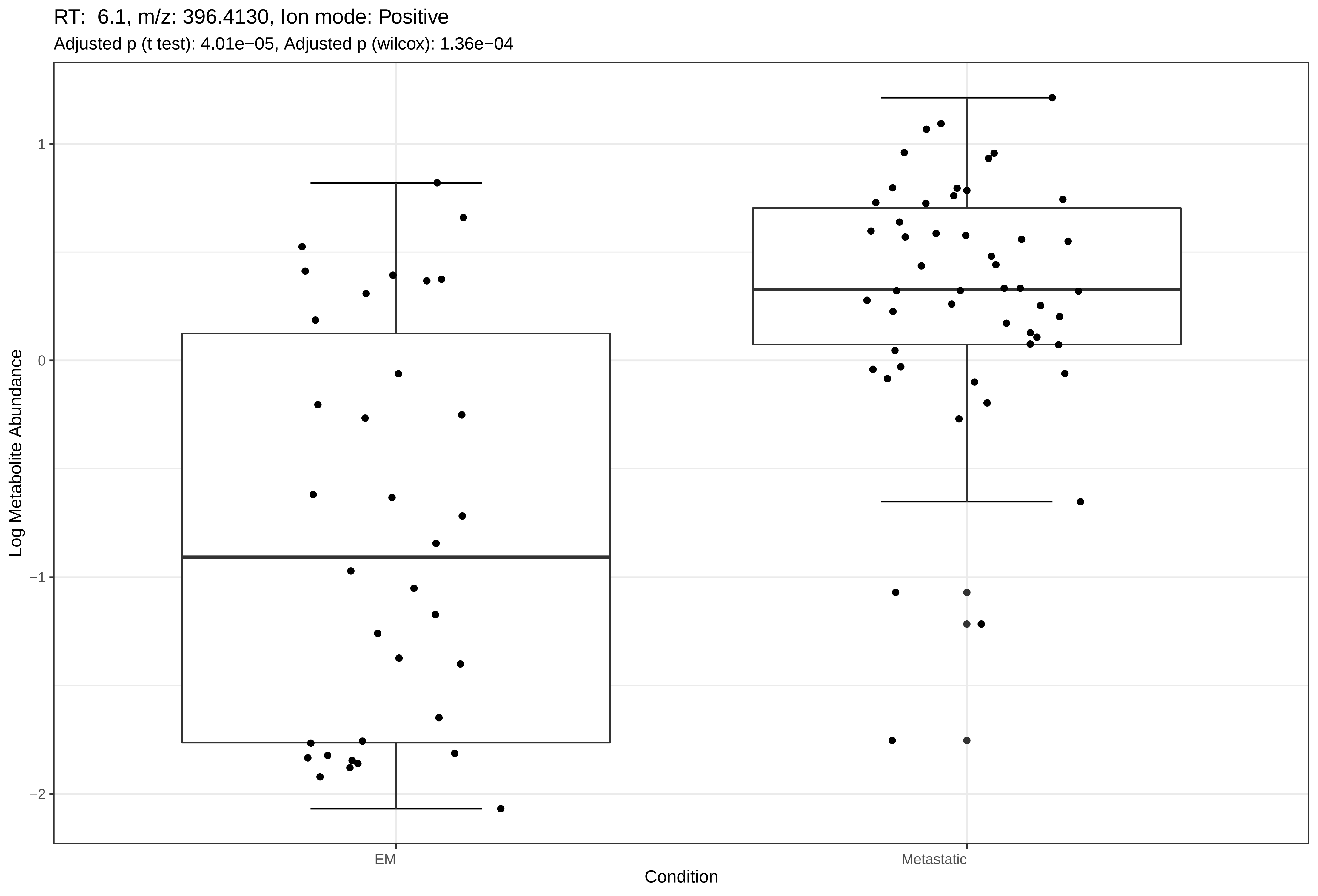
RT: 6.1, m/z: 396.4130, Ion mode: Positive
Adjusted p (t test): 4.01e−05, Adjusted p (wilcox): 1.36e−04
Log Metabolite Abundance
1
0
−1
−2
EM
Metastatic
Condition

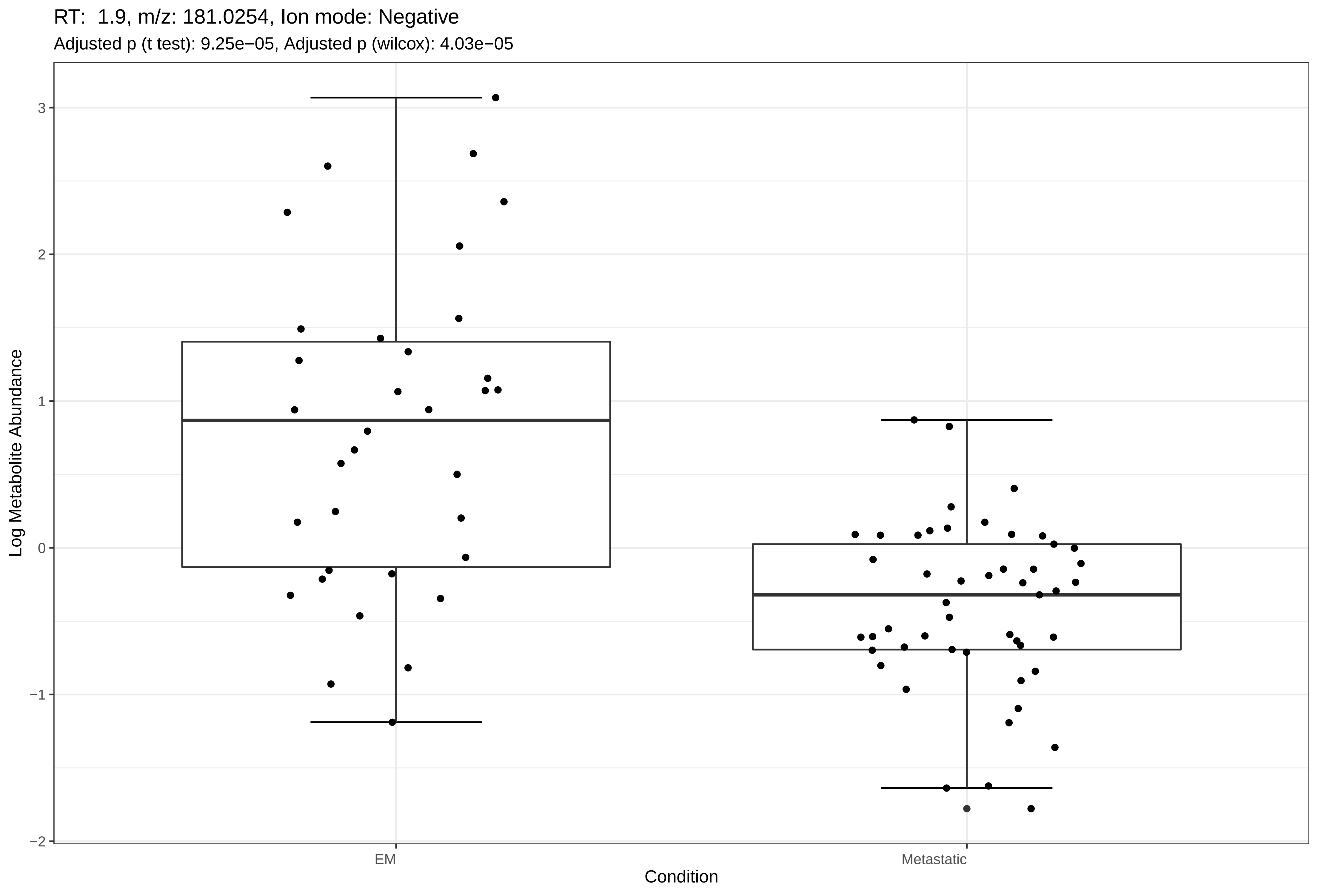
RT: 1.9, m/z: 181.0254, Ion mode: Negative
Adjusted p (t test): 9.25e−05, Adjusted p (wilcox): 4.03e−05
Log Metabolite Abundance
3
2
1
0
−1
−2
EM
Metastatic
Condition

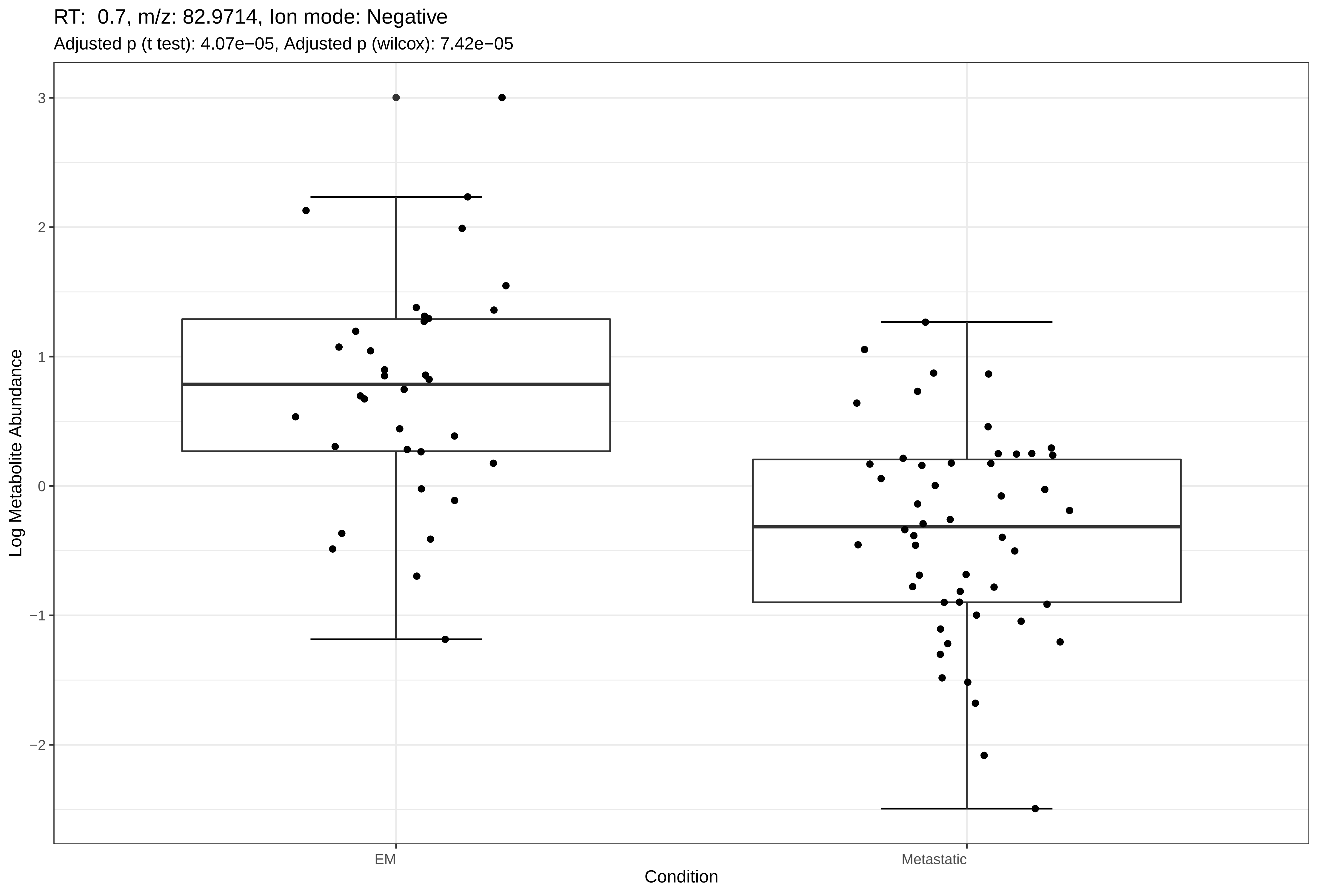
RT: 0.7, m/z: 82.9714, Ion mode: Negative
Adjusted p (t test): 4.07e−05, Adjusted p (wilcox): 7.42e−05
Log Metabolite Abundance
3
2
1
0
−1
−2
EM
Metastatic
Condition

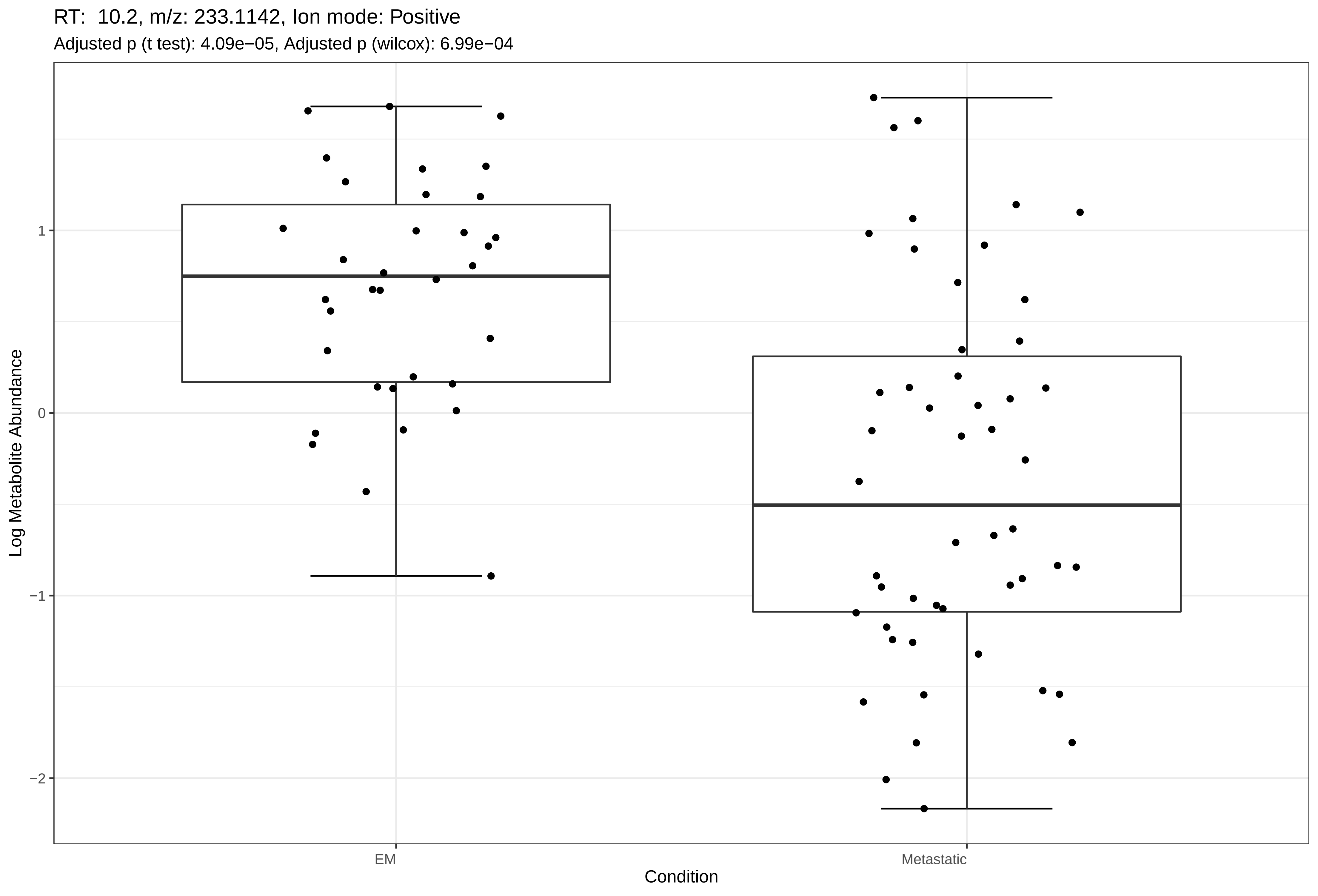

RT: 10.2, m/z: 233.1142, Ion mode: Positive
Adjusted p (t test): 4.09e−05, Adjusted p (wilcox): 6.99e−04
Log Metabolite Abundance
1
0
−1
−2
EM
Metastatic
Condition

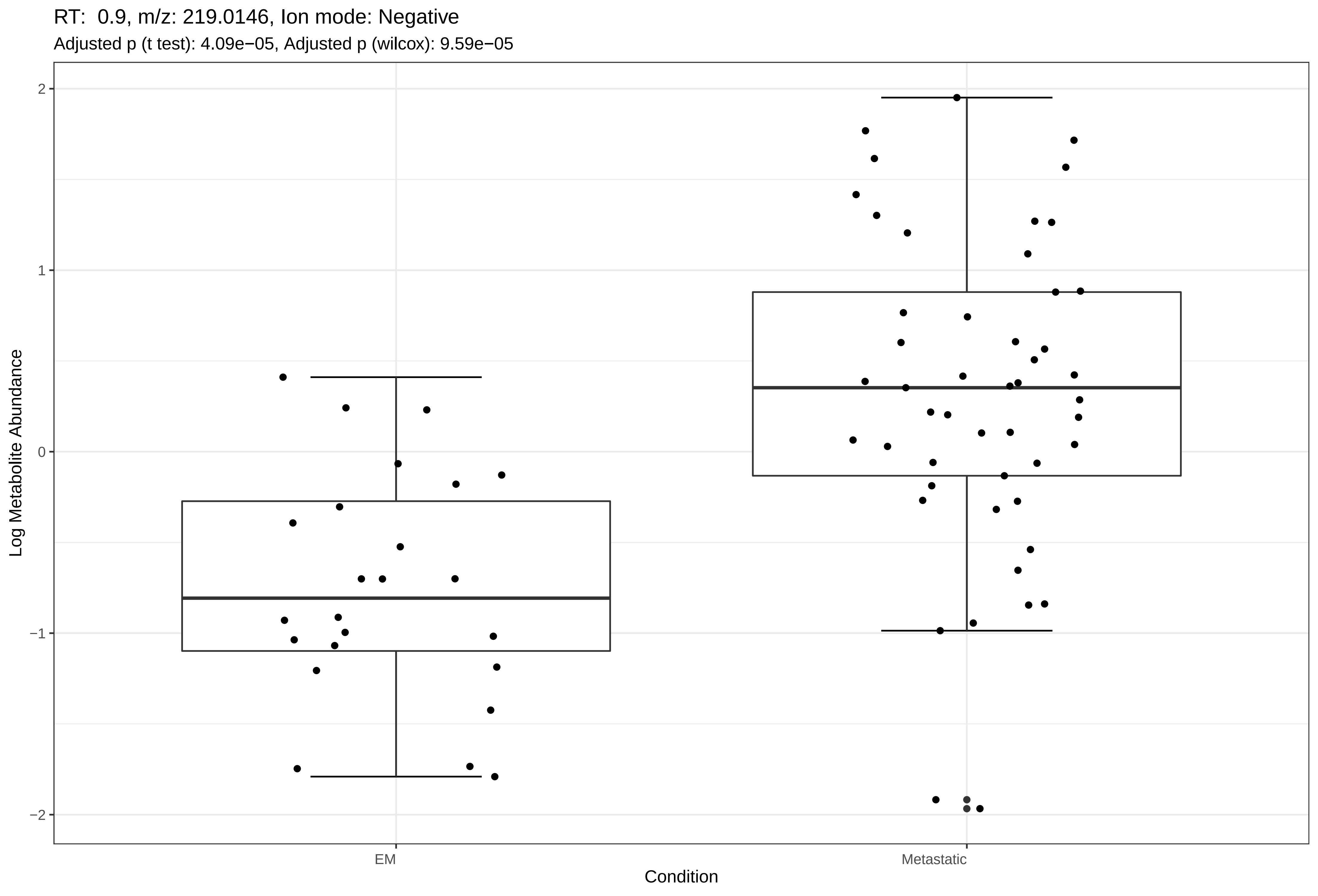
RT: 0.9, m/z: 219.0146, Ion mode: Negative
Adjusted p (t test): 4.09e−05, Adjusted p (wilcox): 9.59e−05
Log Metabolite Abundance
2
1
0
−1
−2
EM
Metastatic
Condition

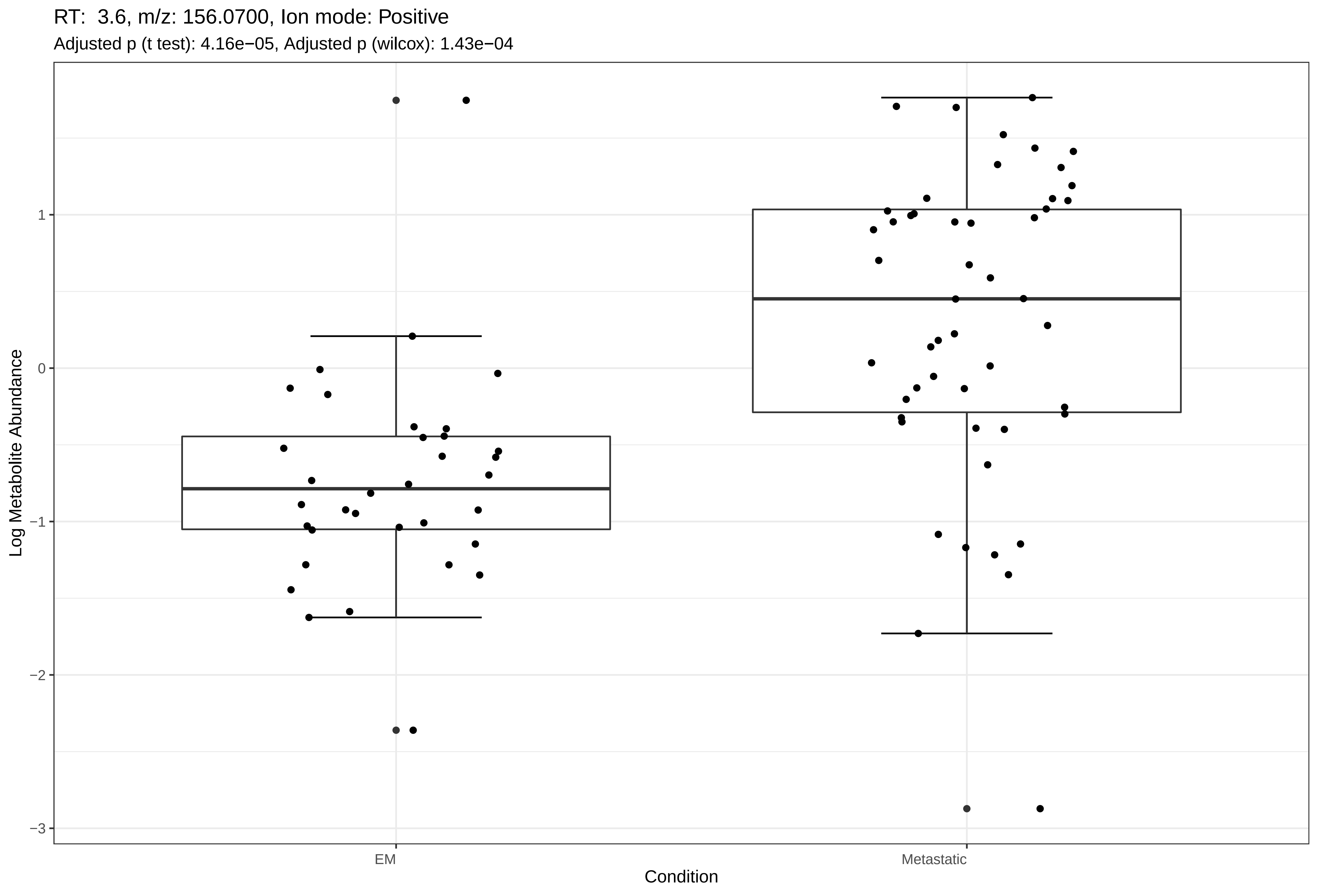

RT: 3.6, m/z: 156.0700, Ion mode: Positive
Adjusted p (t test): 4.16e−05, Adjusted p (wilcox): 1.43e−04
Log Metabolite Abundance
1
0
−1
−2
−3
EM
Metastatic
Condition

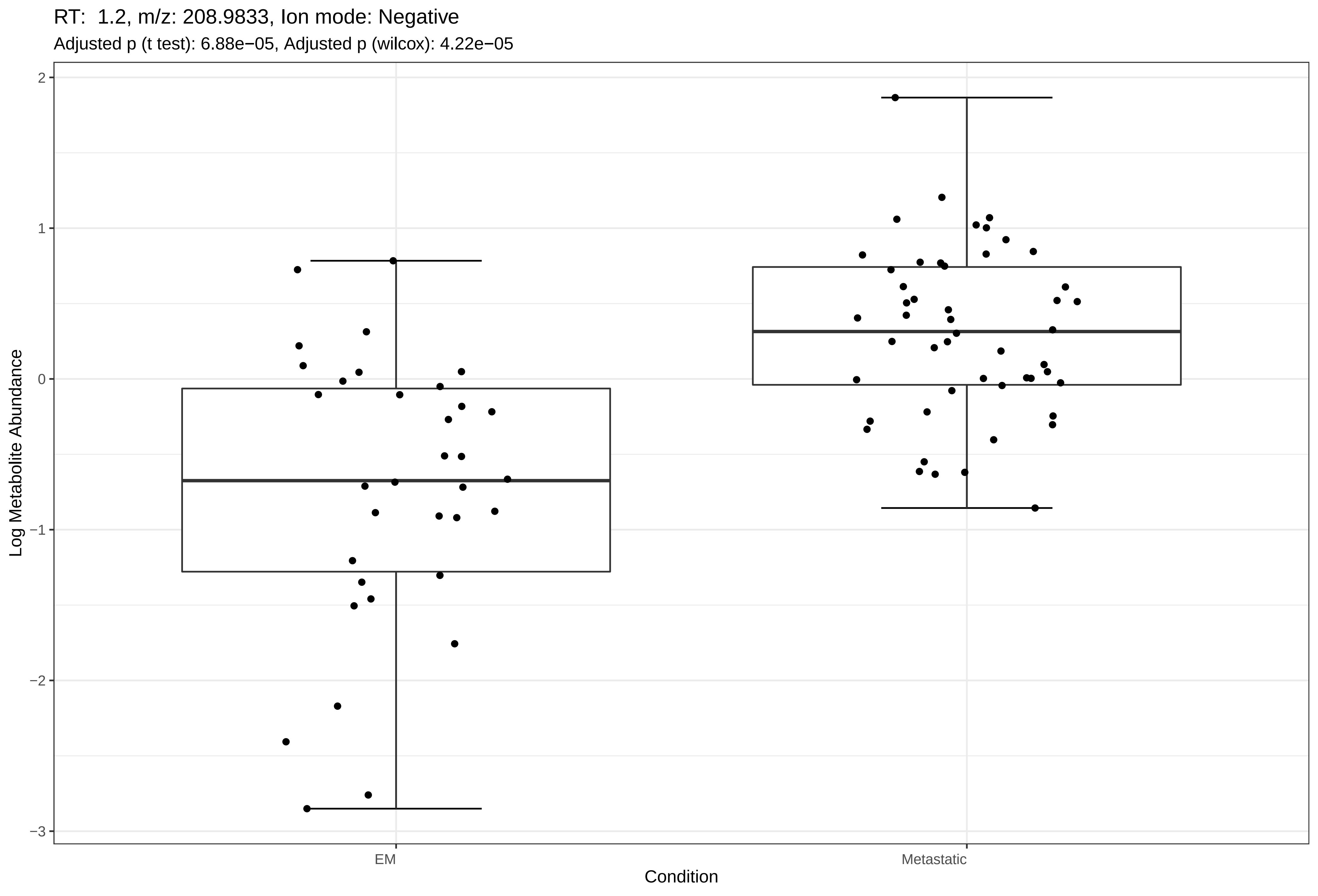
RT: 1.2, m/z: 208.9833, Ion mode: Negative
Adjusted p (t test): 6.88e−05, Adjusted p (wilcox): 4.22e−05
Log Metabolite Abundance
2
1
0
−1
−2
−3
EM
Metastatic
Condition

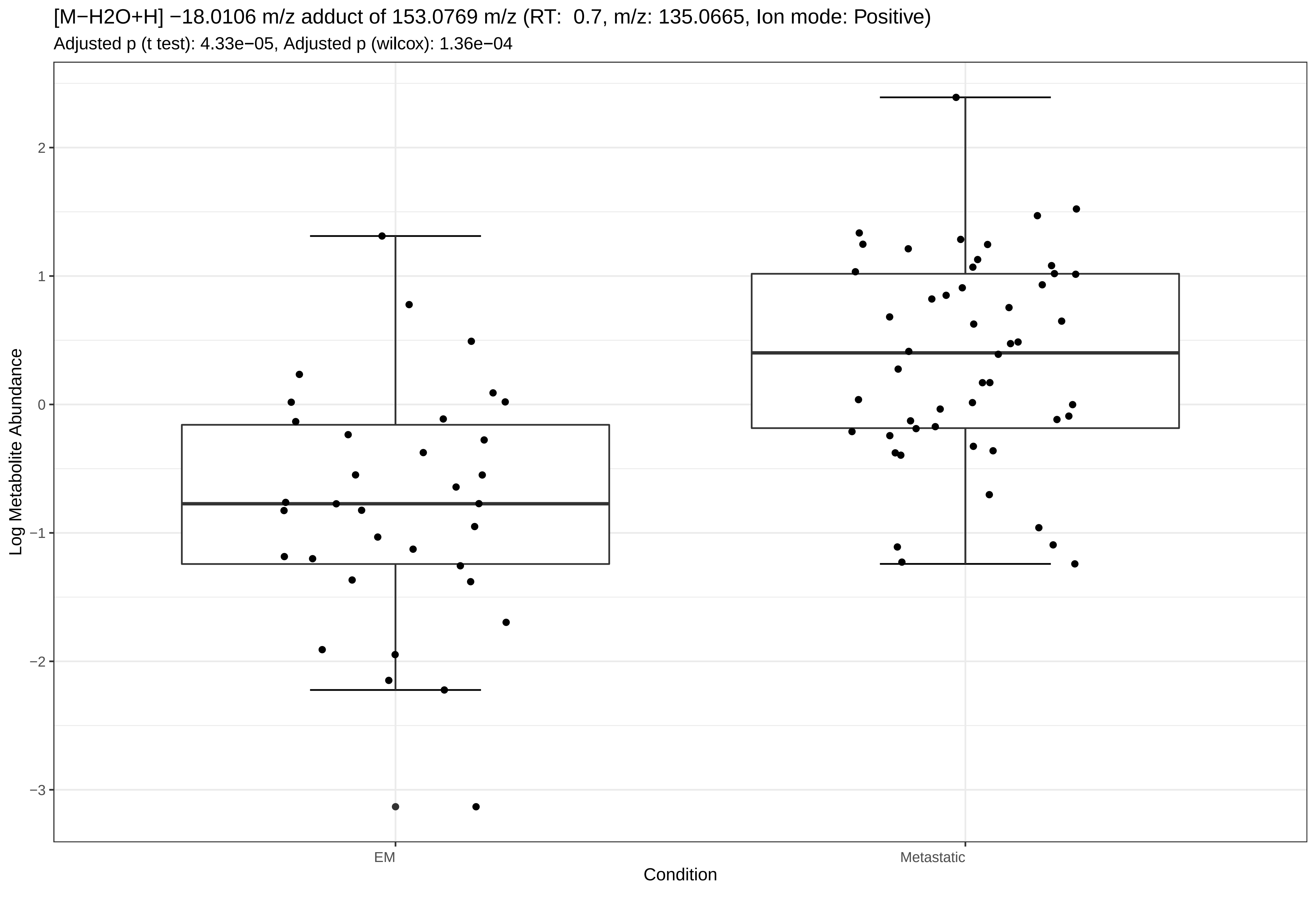
[M−H2O+H] −18.0106 m/z adduct of 153.0769 m/z (RT: 0.7, m/z: 135.0665, Ion mode: Positive)
Adjusted p (t test): 4.33e−05, Adjusted p (wilcox): 1.36e−04
Log Metabolite Abundance
2
1
0
−1
−2
−3
EM
Metastatic
Condition

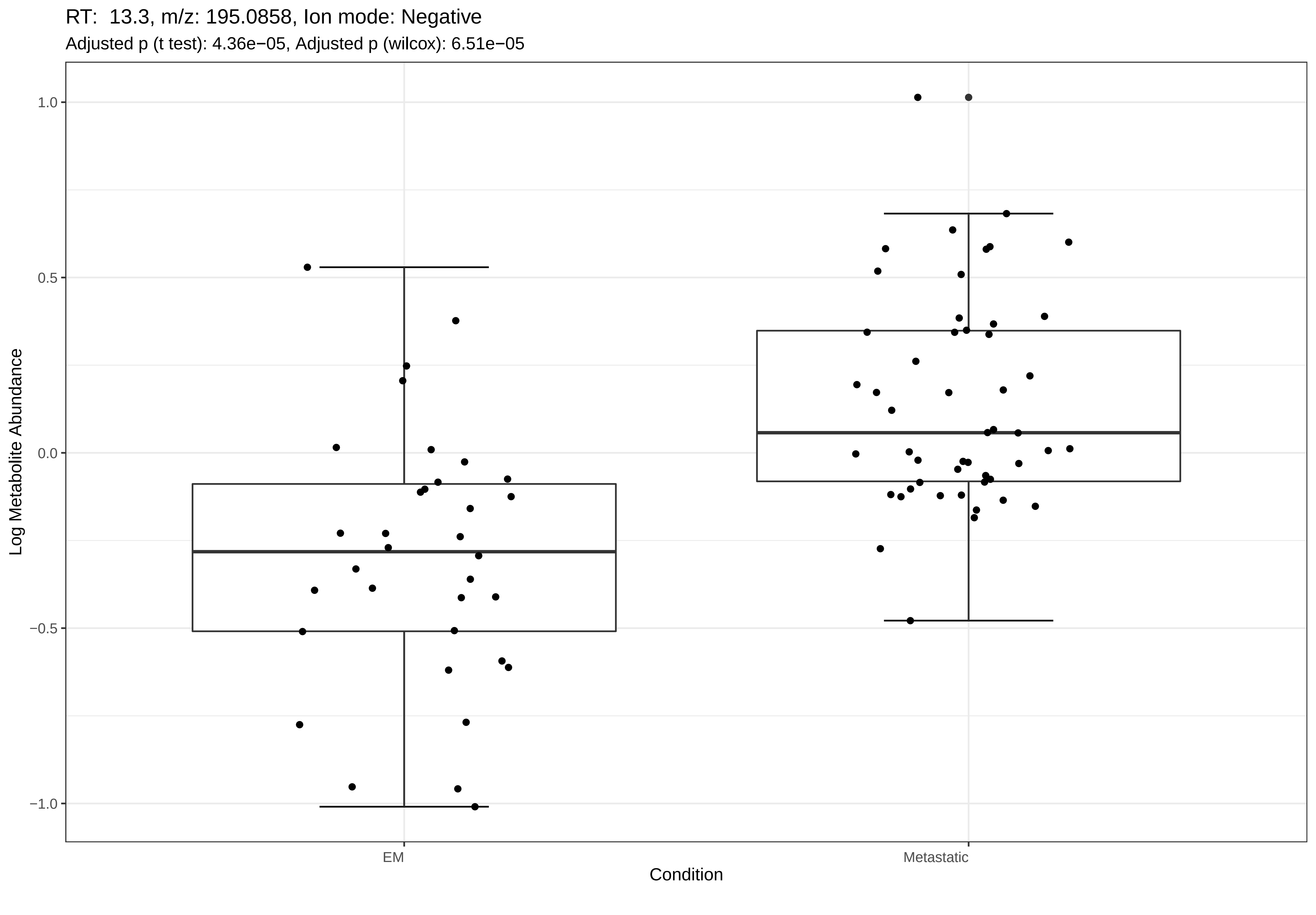

RT: 13.3, m/z: 195.0858, Ion mode: Negative
Adjusted p (t test): 4.36e−05, Adjusted p (wilcox): 6.51e−05
Log Metabolite Abundance
1.0
0.5
0.0
−0.5
−1.0
EM
Metastatic
Condition

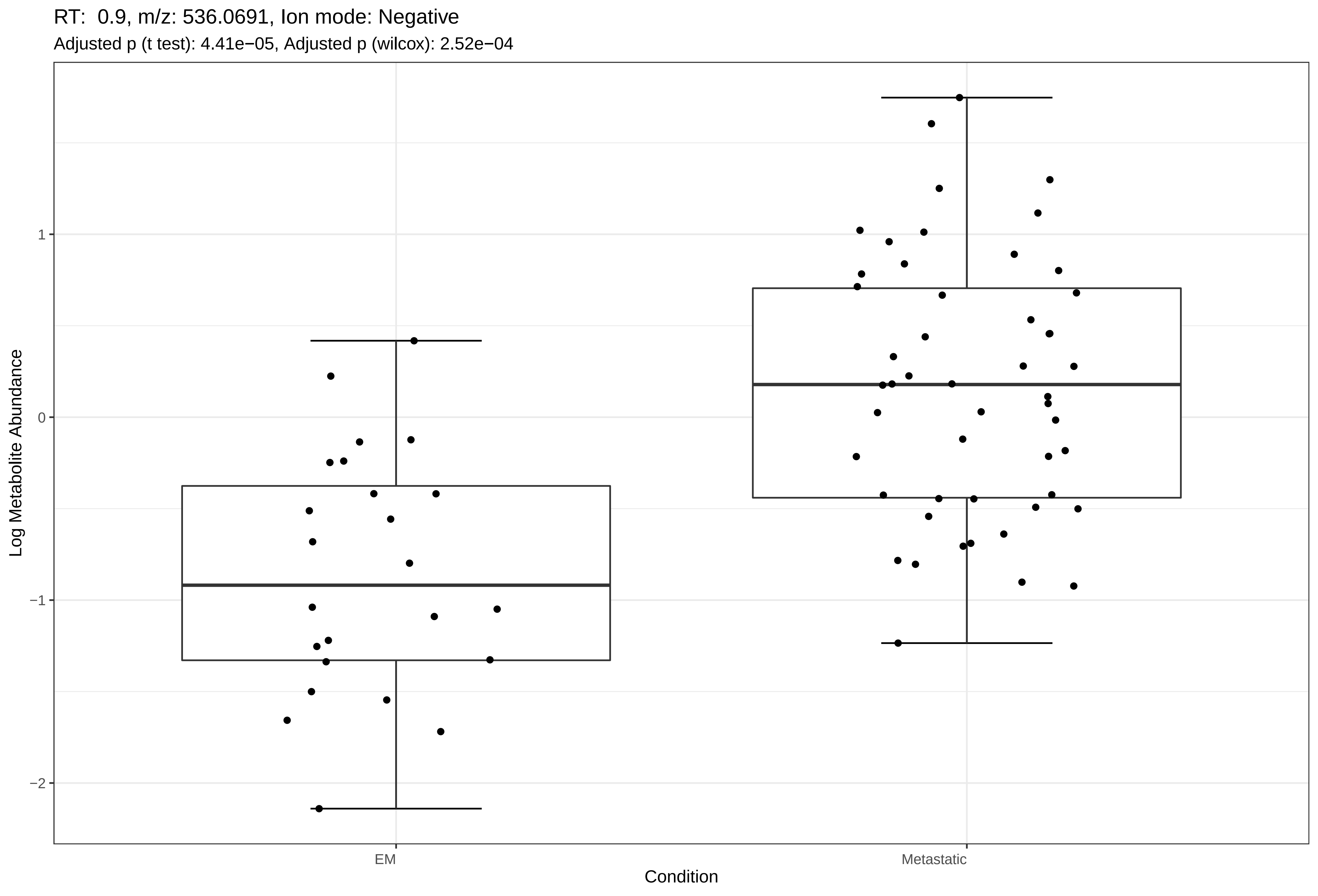
RT: 0.9, m/z: 536.0691, Ion mode: Negative
Adjusted p (t test): 4.41e−05, Adjusted p (wilcox): 2.52e−04
Log Metabolite Abundance
1
0
−1
−2
EM
Metastatic
Condition

RT: 1.6, m/z: 125.0709, Ion mode: Positive

Adjusted p (t test): 4.41e−05, Adjusted p (wilcox): 6.56e−04

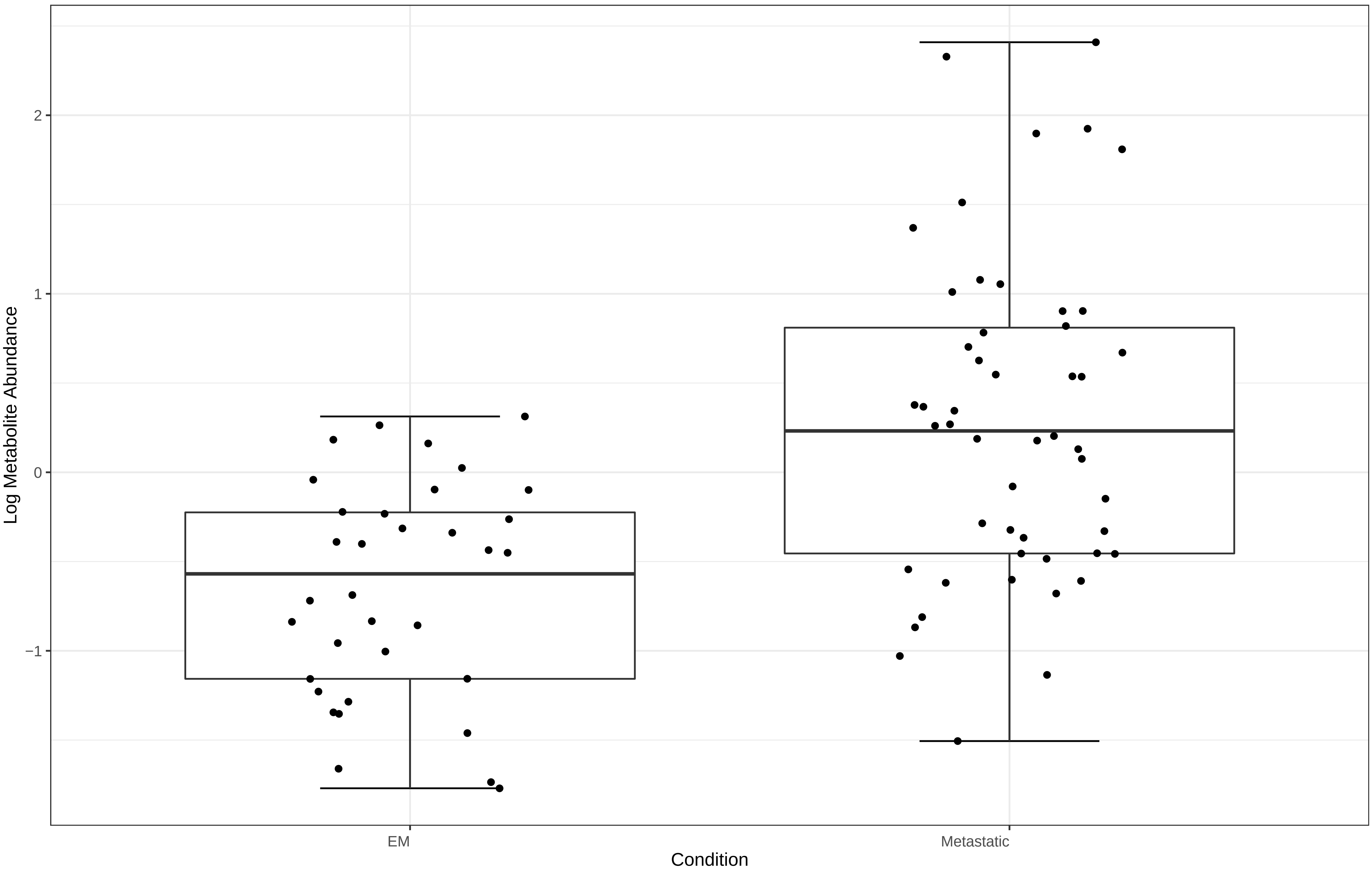

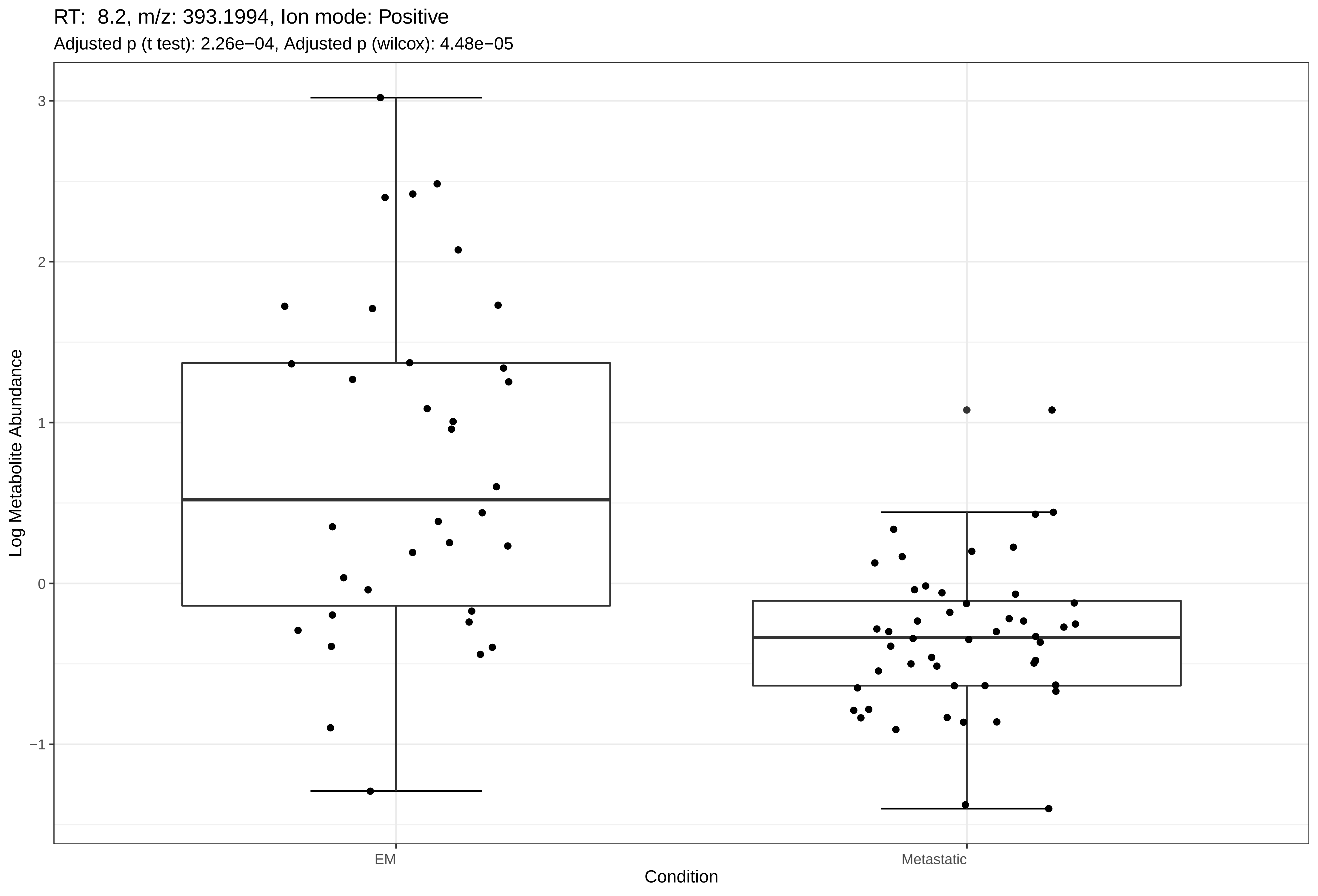

RT: 8.2, m/z: 393.1994, Ion mode: Positive
Adjusted p (t test): 2.26e−04, Adjusted p (wilcox): 4.48e−05
Log Metabolite Abundance
3
2
1
0
−1
EM
Metastatic
Condition

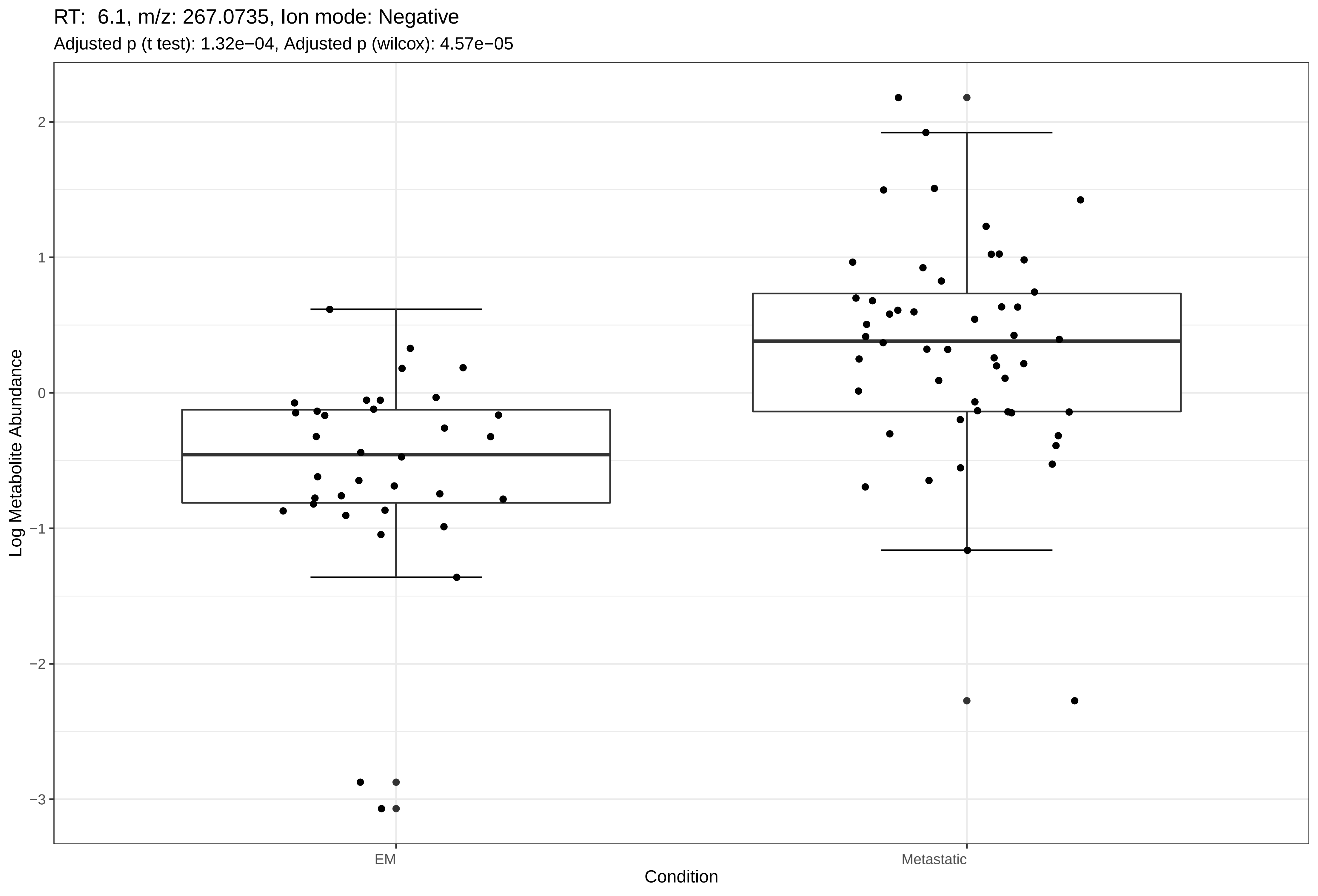
RT: 6.1, m/z: 267.0735, Ion mode: Negative
Adjusted p (t test): 1.32e−04, Adjusted p (wilcox): 4.57e−05
Log Metabolite Abundance
2
1
0
−1
−2
−3
EM
Metastatic
Condition

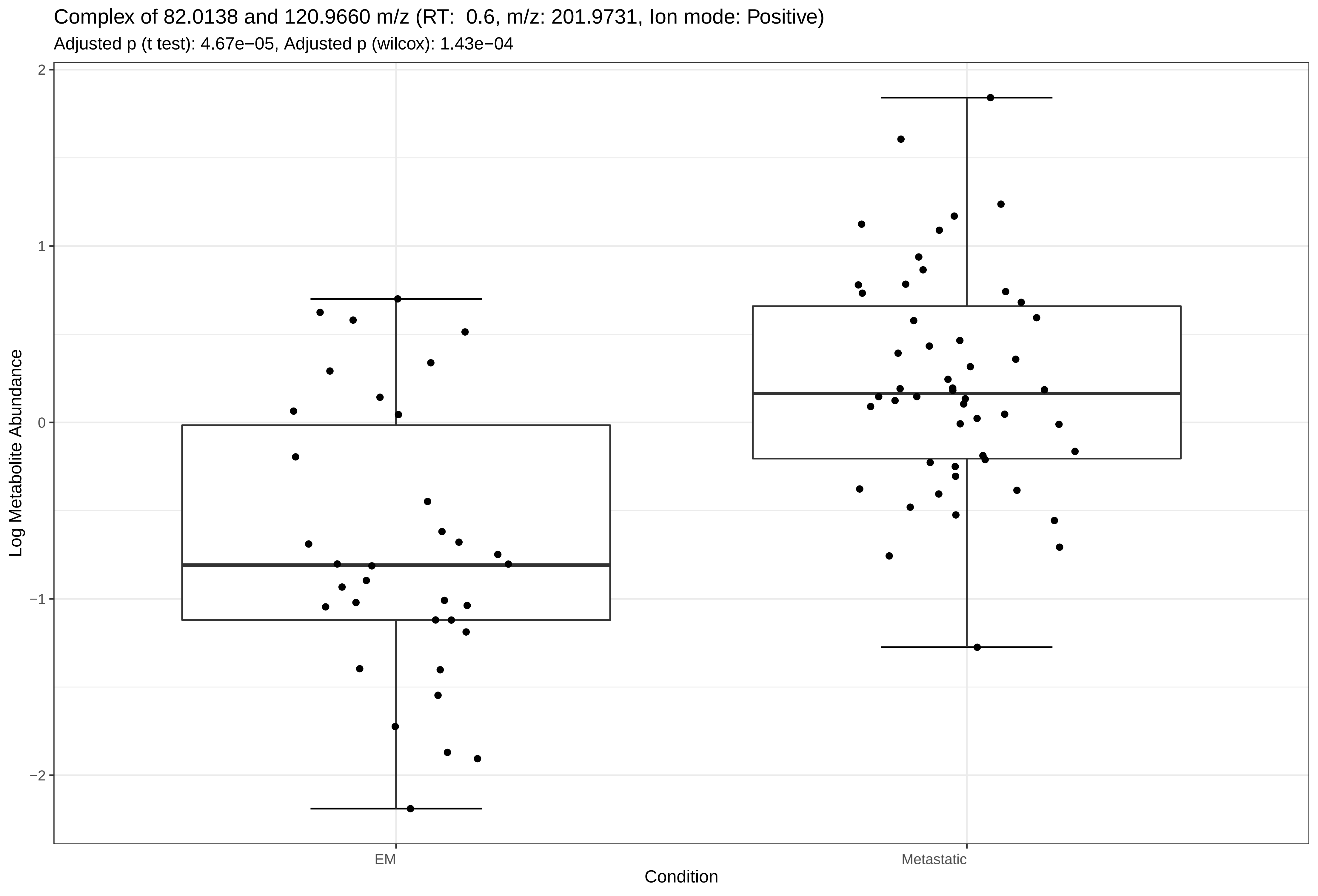
Complex of 82.0138 and 120.9660 m/z (RT: 0.6, m/z: 201.9731, Ion mode: Positive)
Adjusted p (t test): 4.67e−05, Adjusted p (wilcox): 1.43e−04
2
1
0
−1
−2
Log Metabolite Abundance
EM
Metastatic
Condition

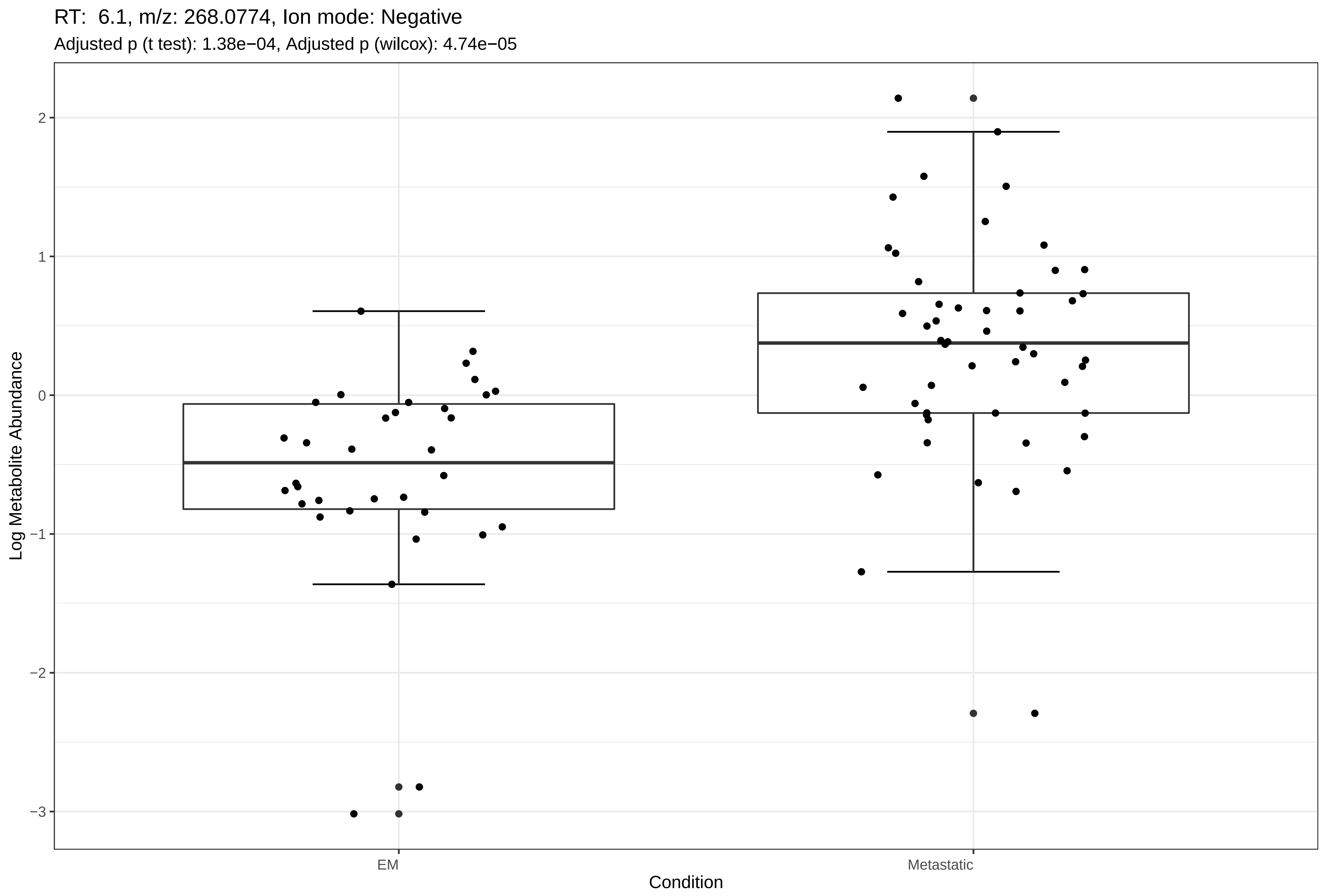

RT: 6.1, m/z: 268.0774, Ion mode: Negative
Adjusted p (t test): 1.38e−04, Adjusted p (wilcox): 4.74e−05
Log Metabolite Abundance
2
1
0
−1
−2
−3
EM
Metastatic
Condition

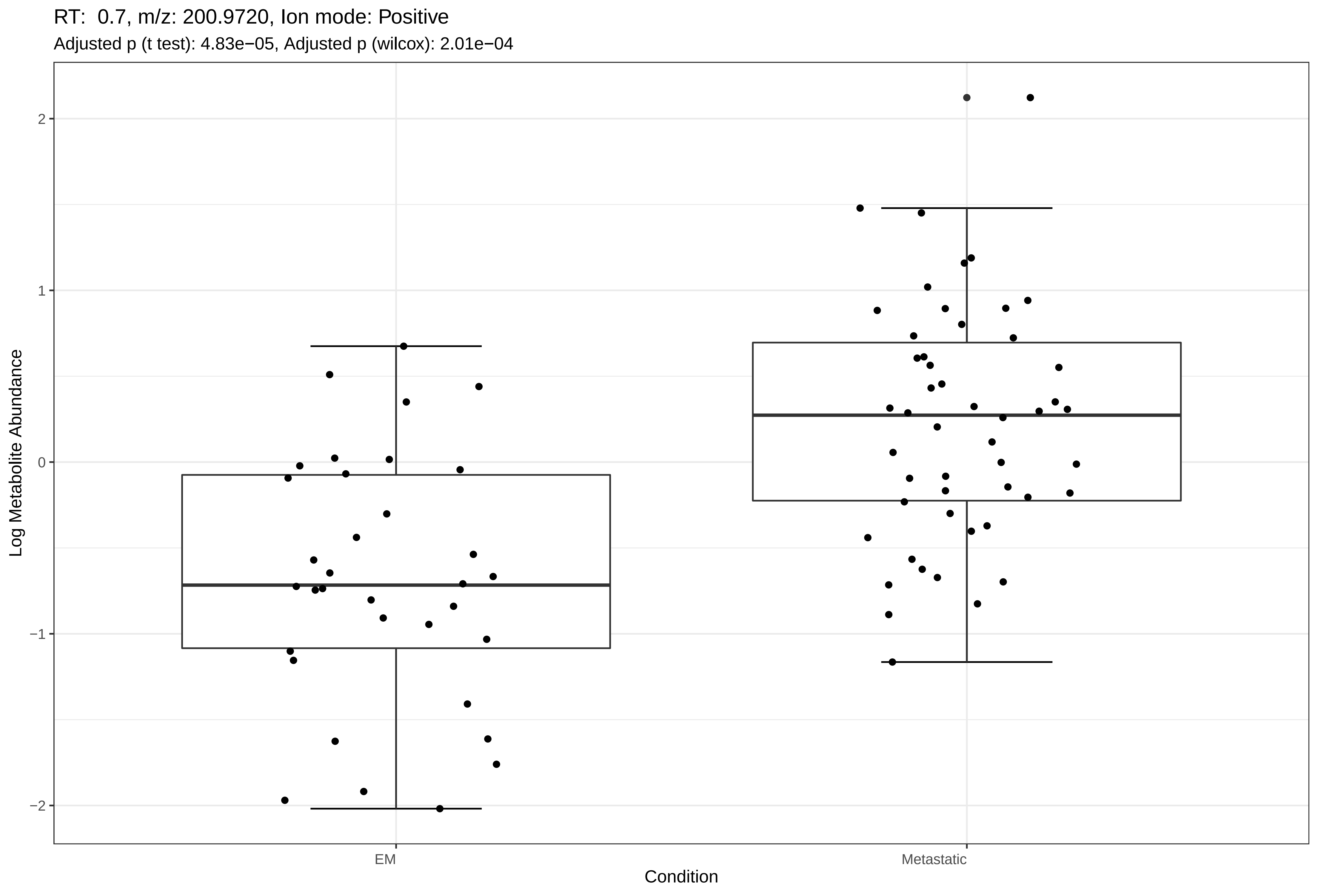
RT: 0.7, m/z: 200.9720, Ion mode: Positive
Adjusted p (t test): 4.83e−05, Adjusted p (wilcox): 2.01e−04
Log Metabolite Abundance
2
1
0
−1
−2
EM
Metastatic
Condition

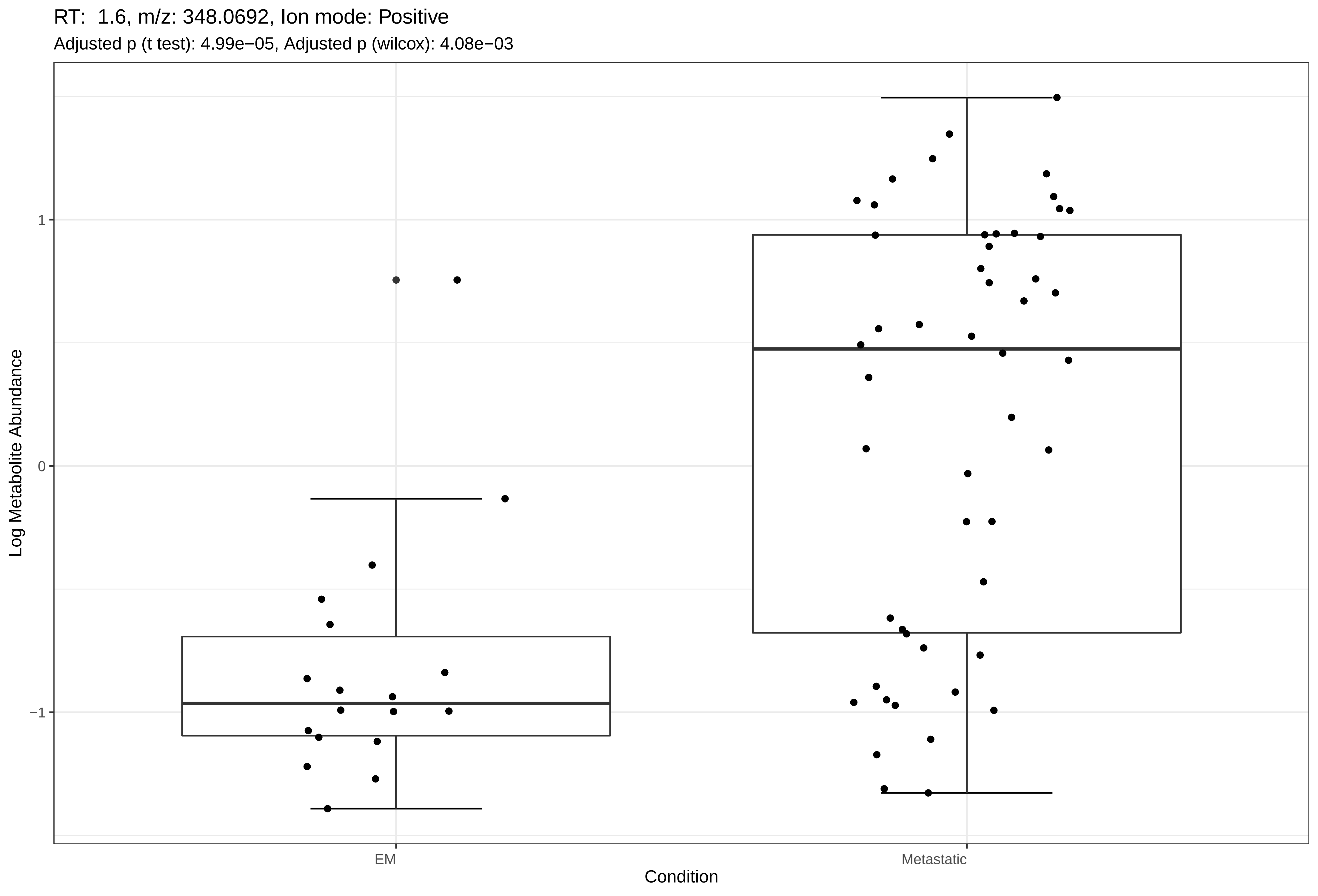
RT: 1.6, m/z: 348.0692, Ion mode: Positive
Adjusted p (t test): 4.99e−05, Adjusted p (wilcox): 4.08e−03
Log Metabolite Abundance
1
0
−1
EM
Metastatic
Condition

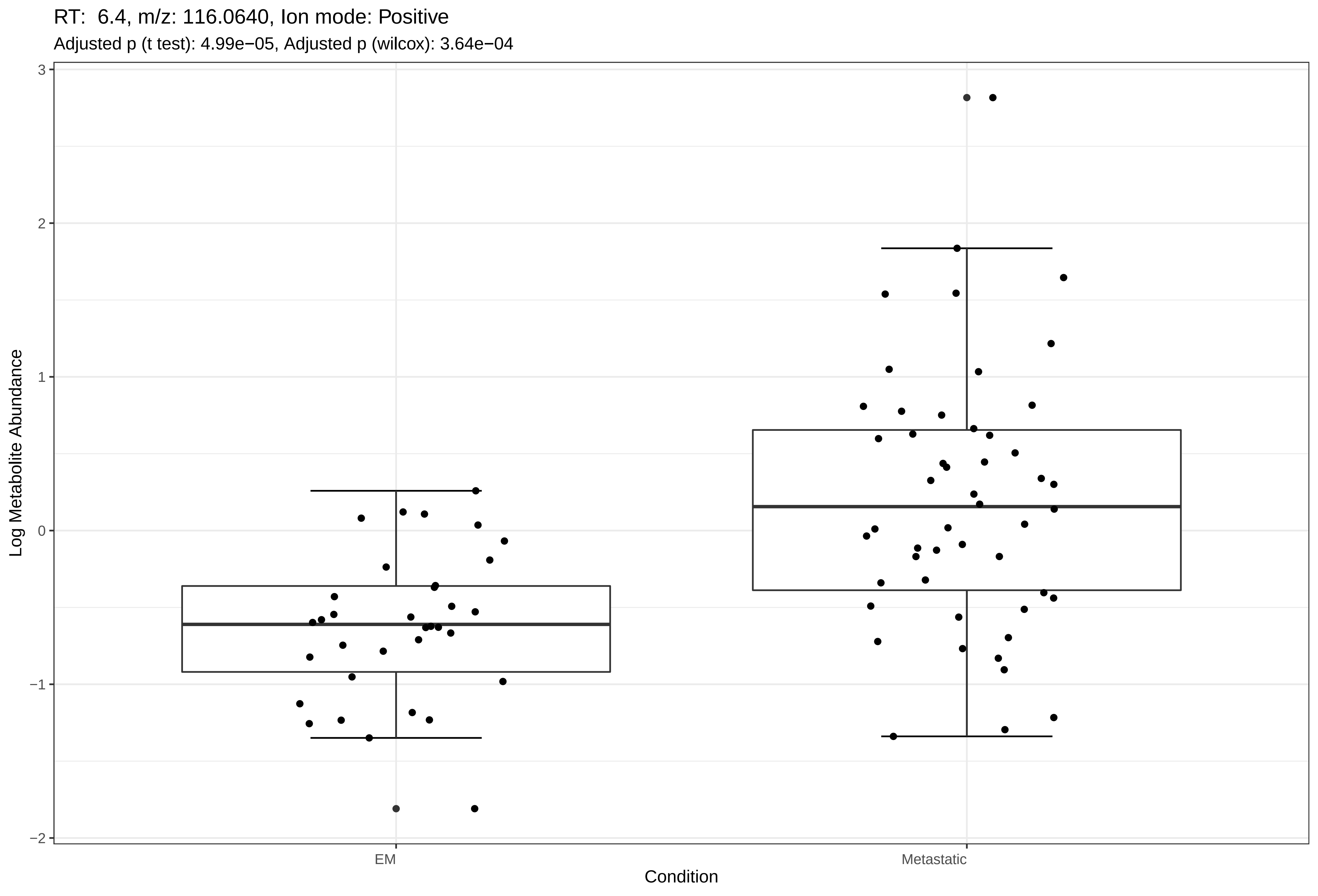

RT: 6.4, m/z: 116.0640, Ion mode: Positive
Adjusted p (t test): 4.99e−05, Adjusted p (wilcox): 3.64e−04
Log Metabolite Abundance
3
2
1
0
−1
−2
EM
Metastatic
Condition

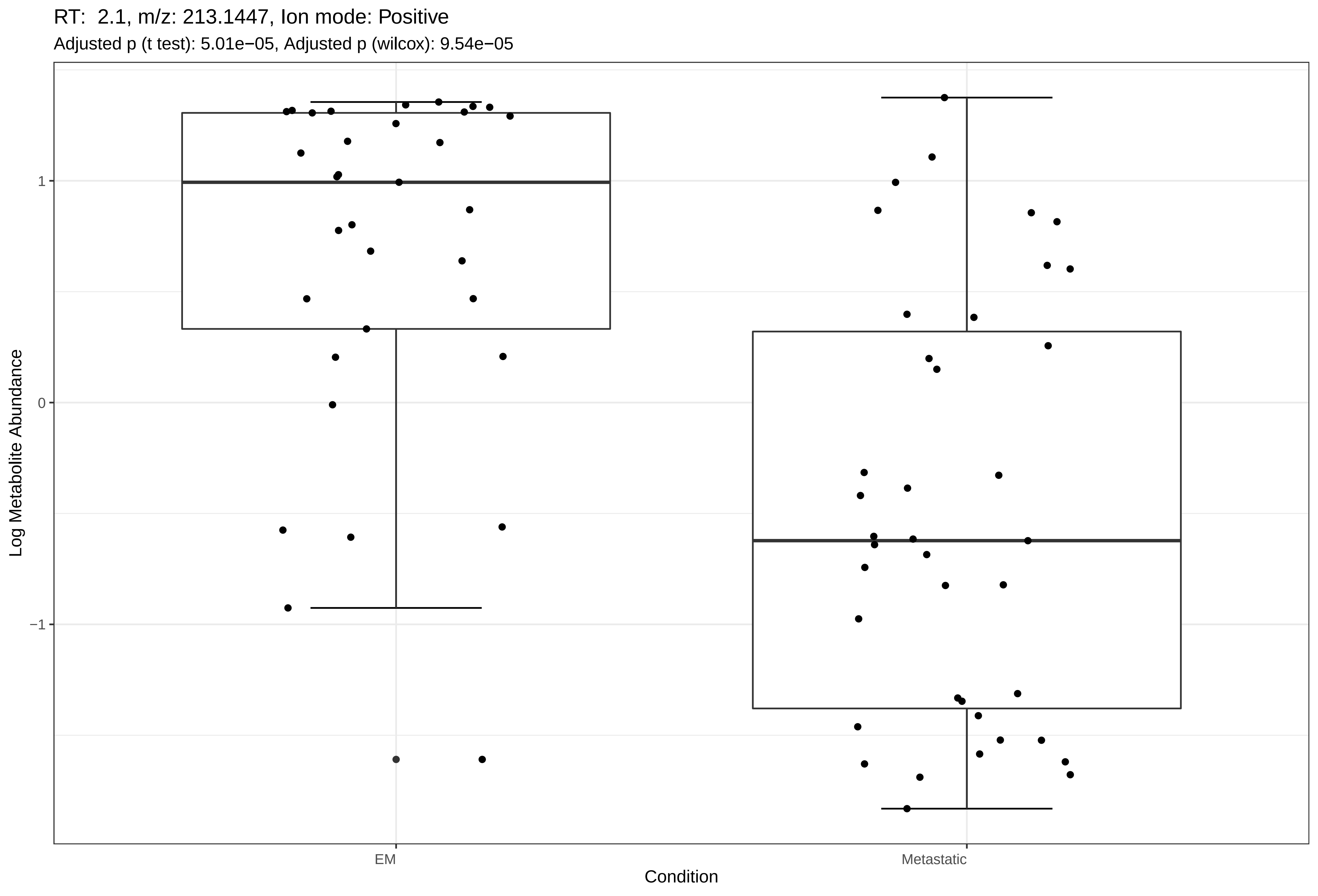

RT: 2.1, m/z: 213.1447, Ion mode: Positive
Adjusted p (t test): 5.01e−05, Adjusted p (wilcox): 9.54e−05
Log Metabolite Abundance
1
0
−1
EM
Metastatic
Condition

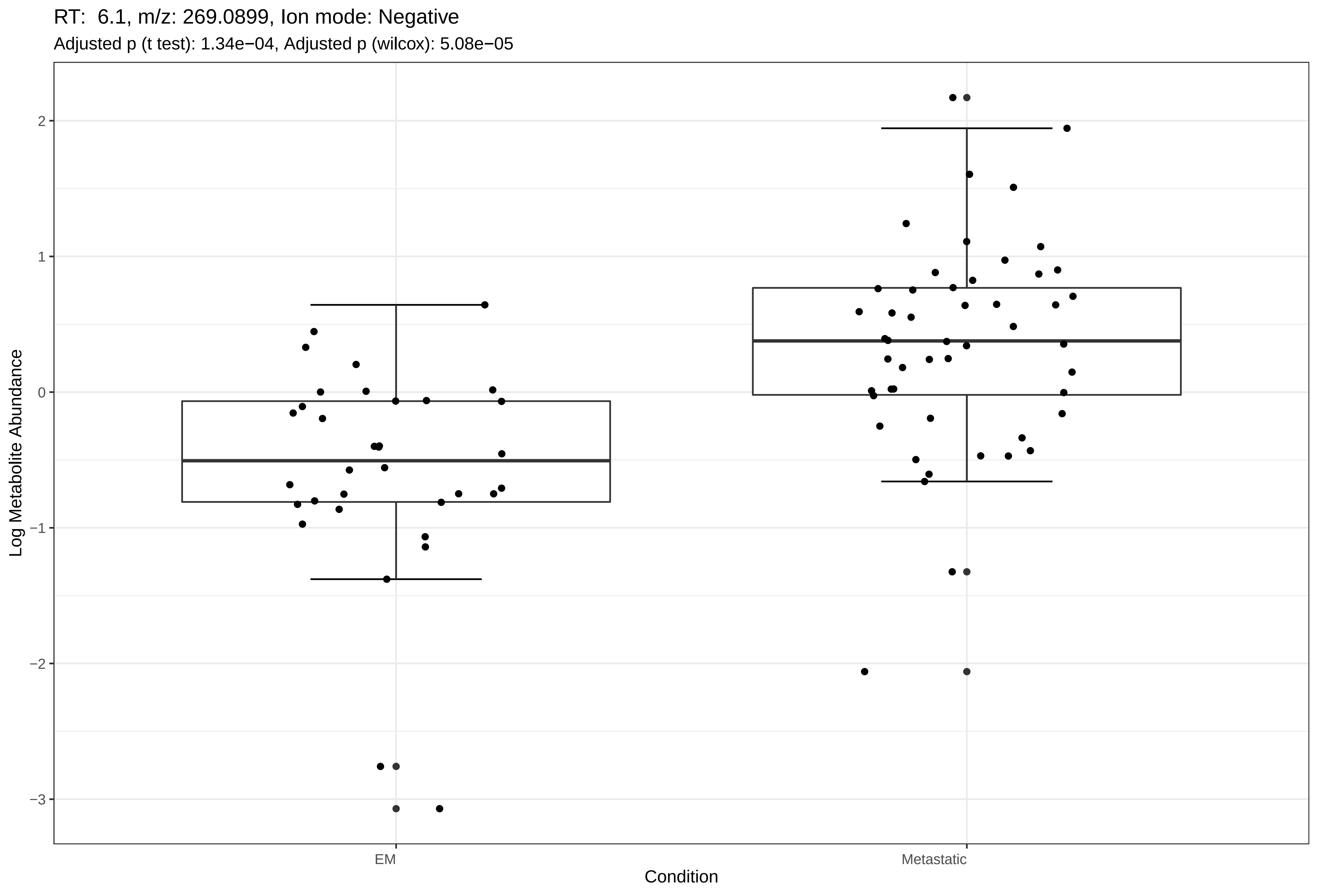

RT: 6.1, m/z: 269.0899, Ion mode: Negative
Adjusted p (t test): 1.34e−04, Adjusted p (wilcox): 5.08e−05
Log Metabolite Abundance
2
1
0
−1
−2
−3
EM
Metastatic
Condition

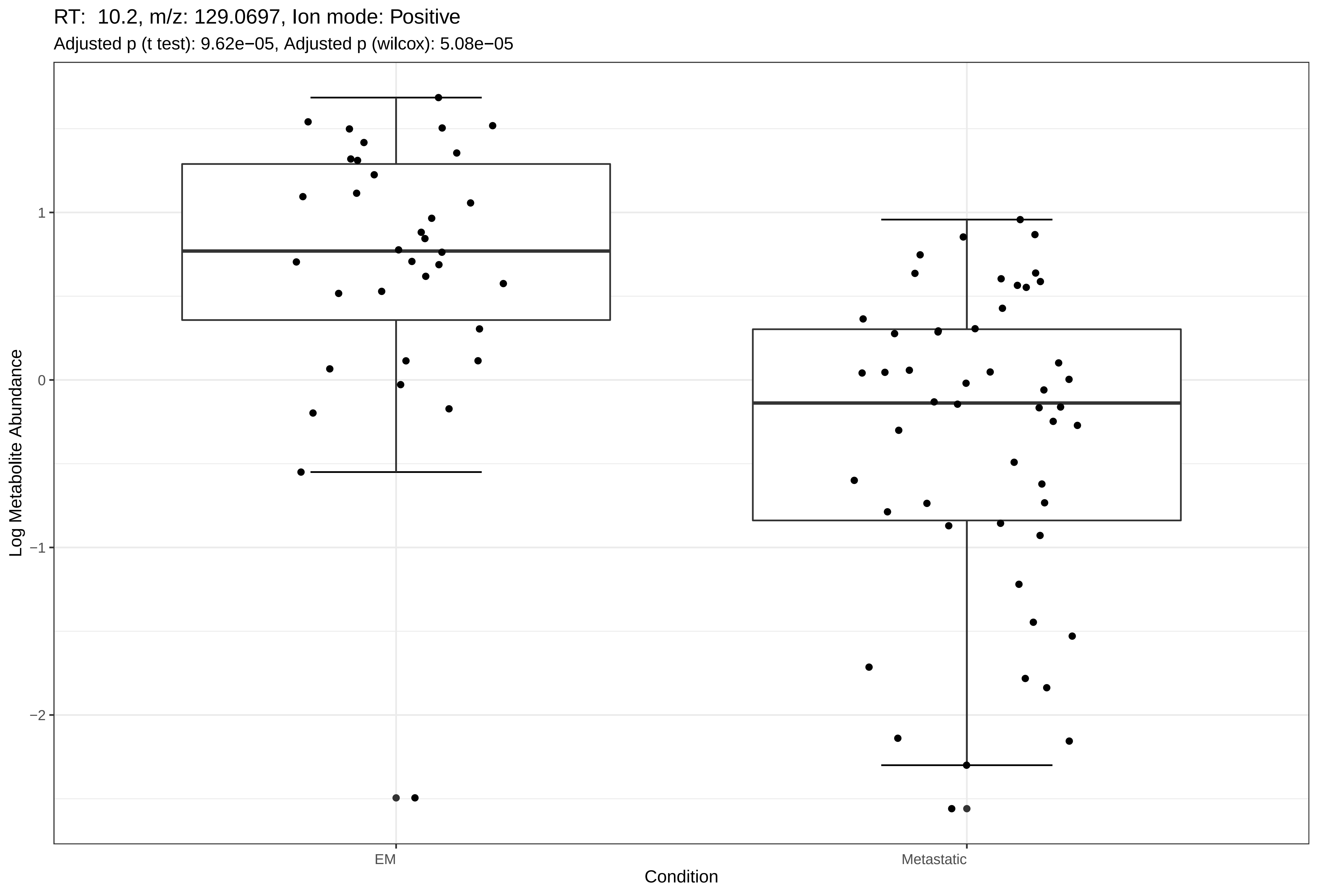
RT: 10.2, m/z: 129.0697, Ion mode: Positive
Adjusted p (t test): 9.62e−05, Adjusted p (wilcox): 5.08e−05
Log Metabolite Abundance
1
0
−1
−2
EM
Metastatic
Condition

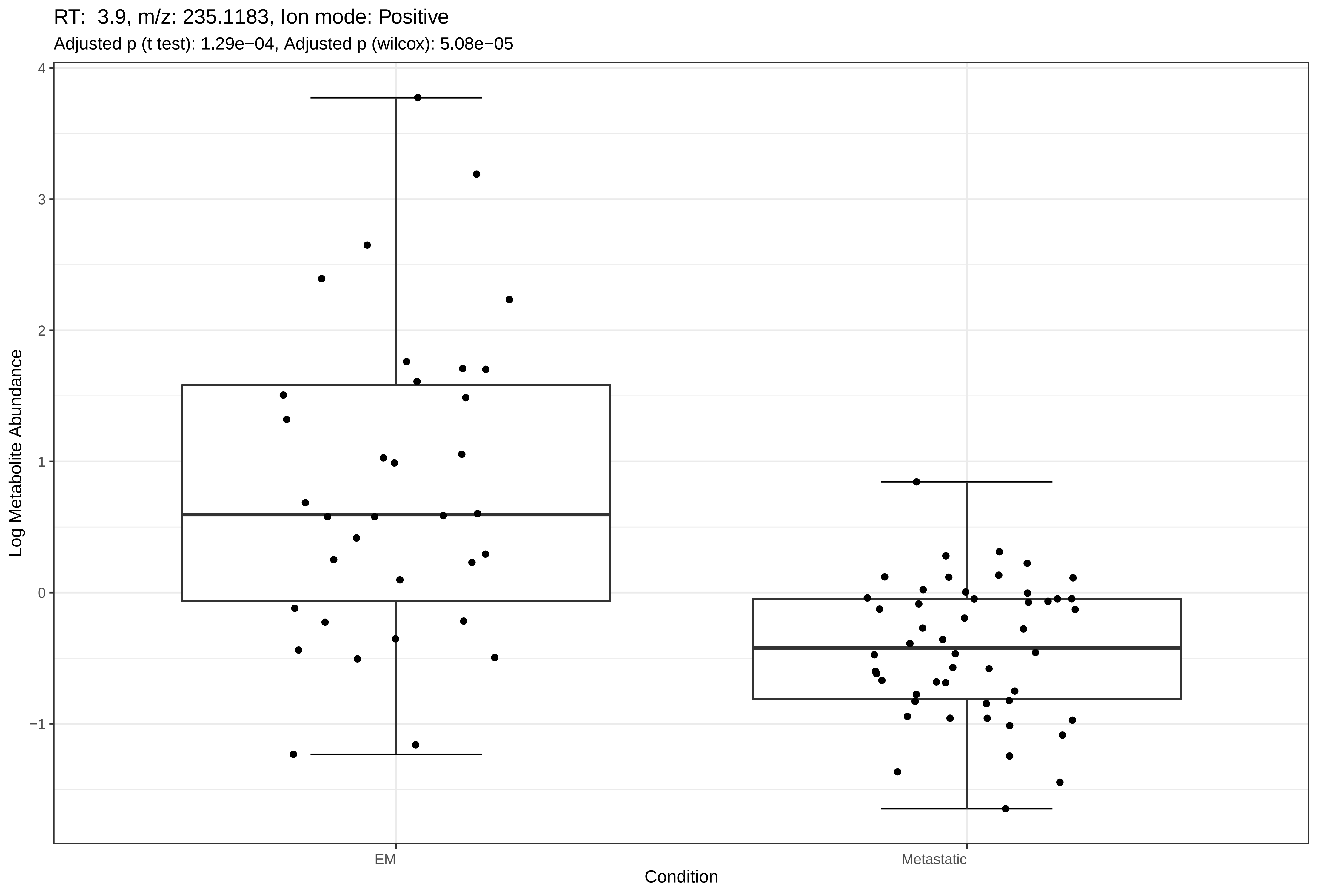
RT: 3.9, m/z: 235.1183, Ion mode: Positive
Adjusted p (t test): 1.29e−04, Adjusted p (wilcox): 5.08e−05
Log Metabolite Abundance
4
3
2
1
0
−1
EM
Metastatic
Condition

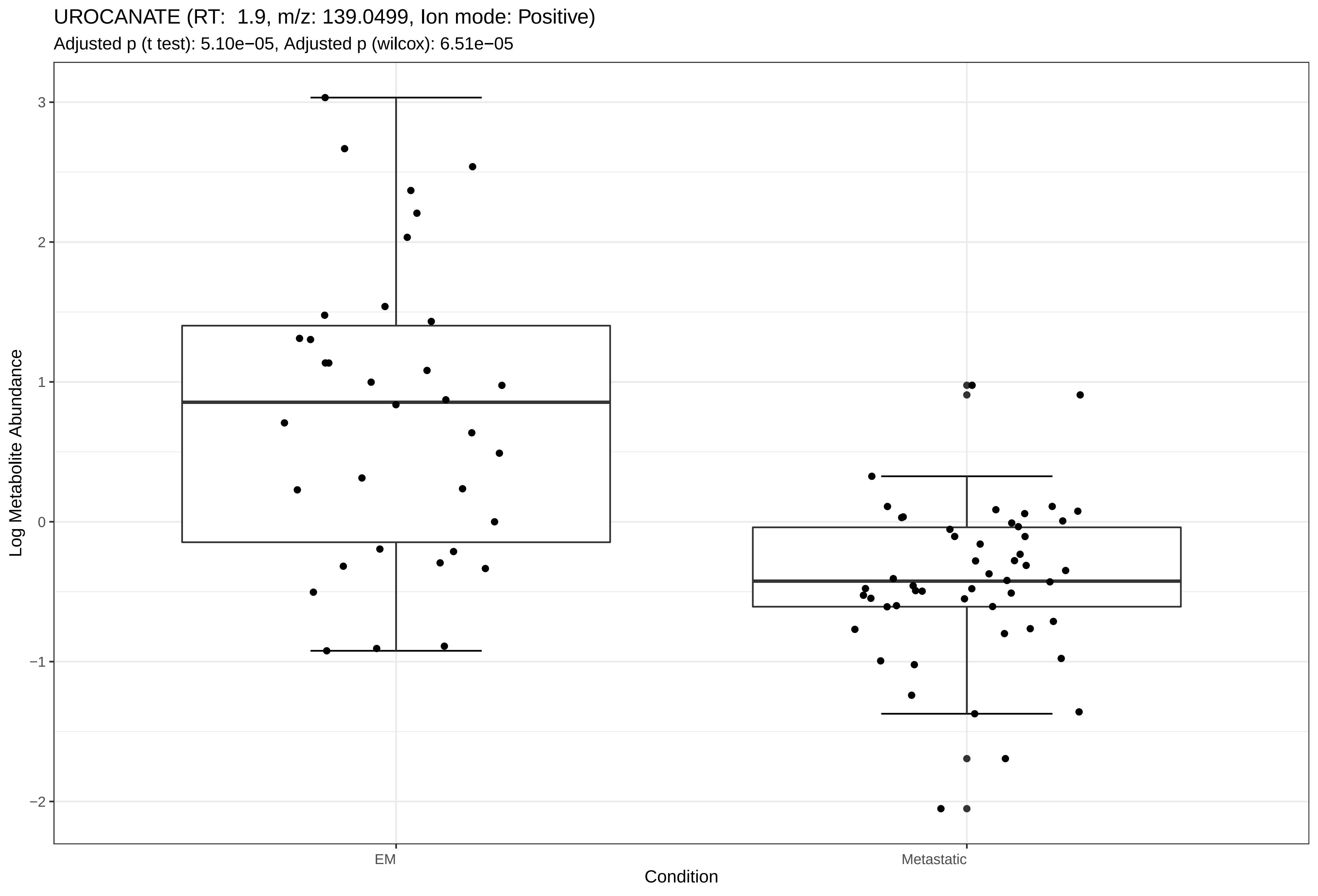

UROCANATE (RT: 1.9, m/z: 139.0499, Ion mode: Positive)
Adjusted p (t test): 5.10e−05, Adjusted p (wilcox): 6.51e−05
Log Metabolite Abundance
3
2
1
0
−1
−2
EM
Metastatic
Condition

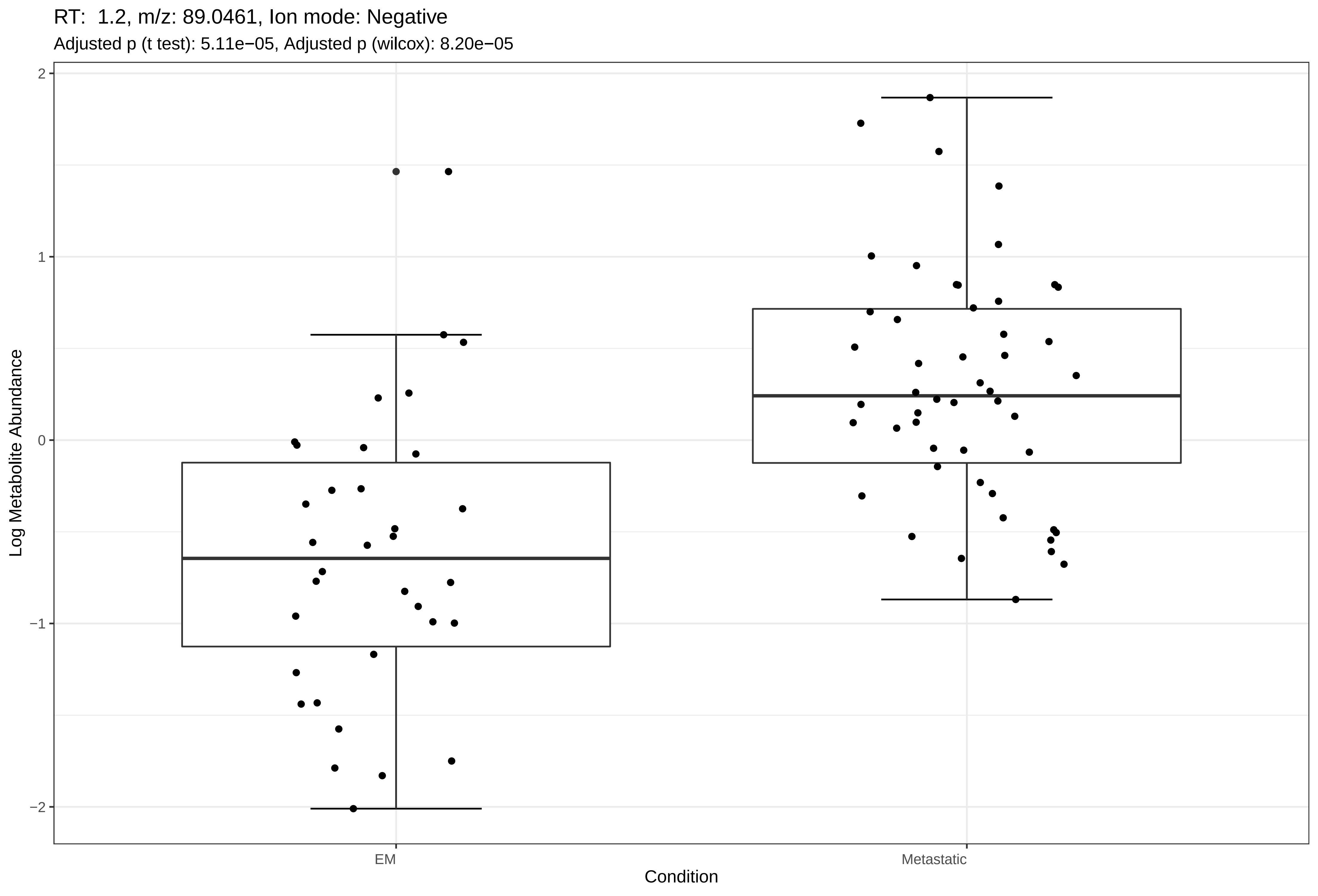

RT: 1.2, m/z: 89.0461, Ion mode: Negative
Adjusted p (t test): 5.11e−05, Adjusted p (wilcox): 8.20e−05
Log Metabolite Abundance
2
1
0
−1
−2
EM
Metastatic
Condition

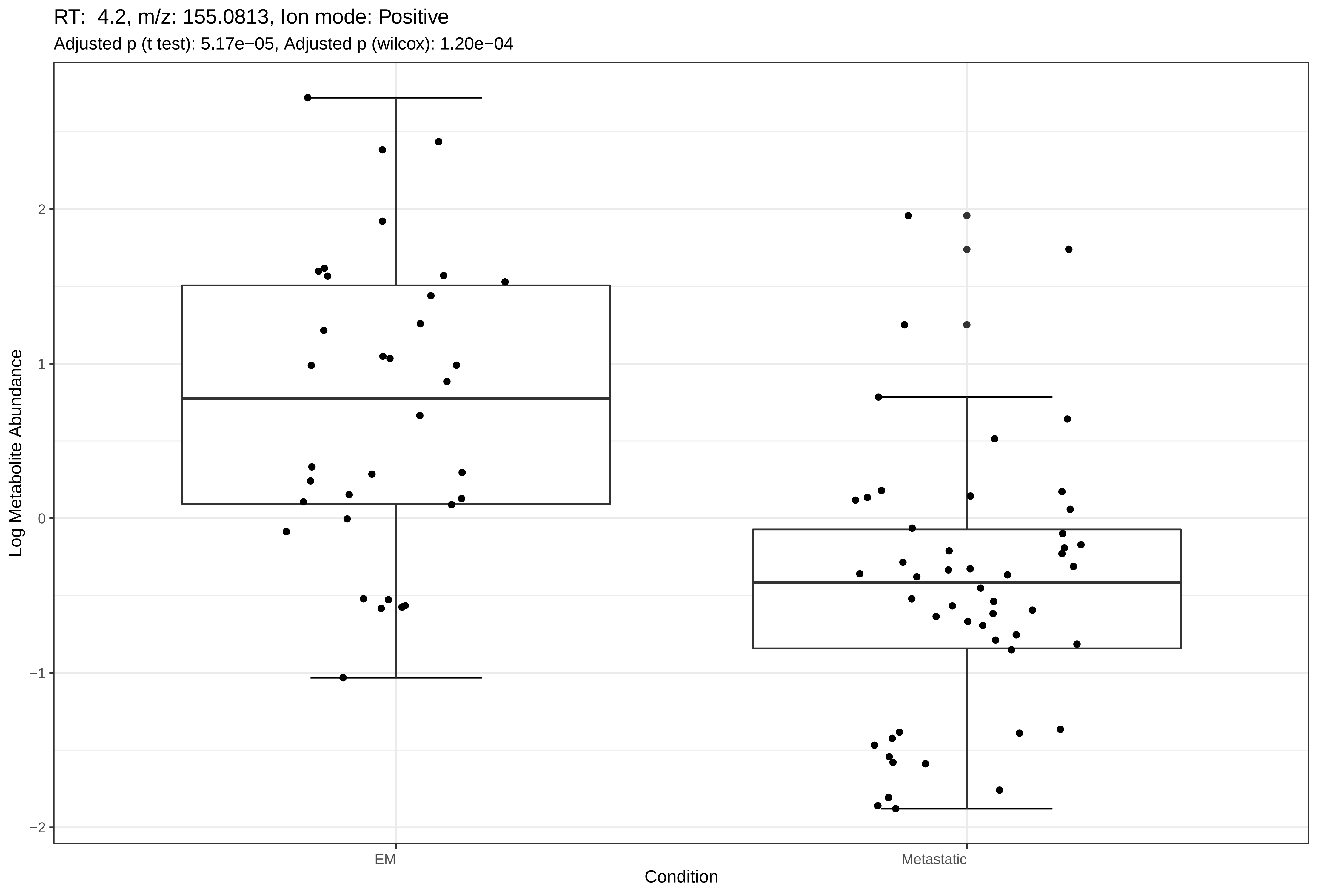
RT: 4.2, m/z: 155.0813, Ion mode: Positive
Adjusted p (t test): 5.17e−05, Adjusted p (wilcox): 1.20e−04
Log Metabolite Abundance
2
1
0
−1
−2
EM
Metastatic
Condition

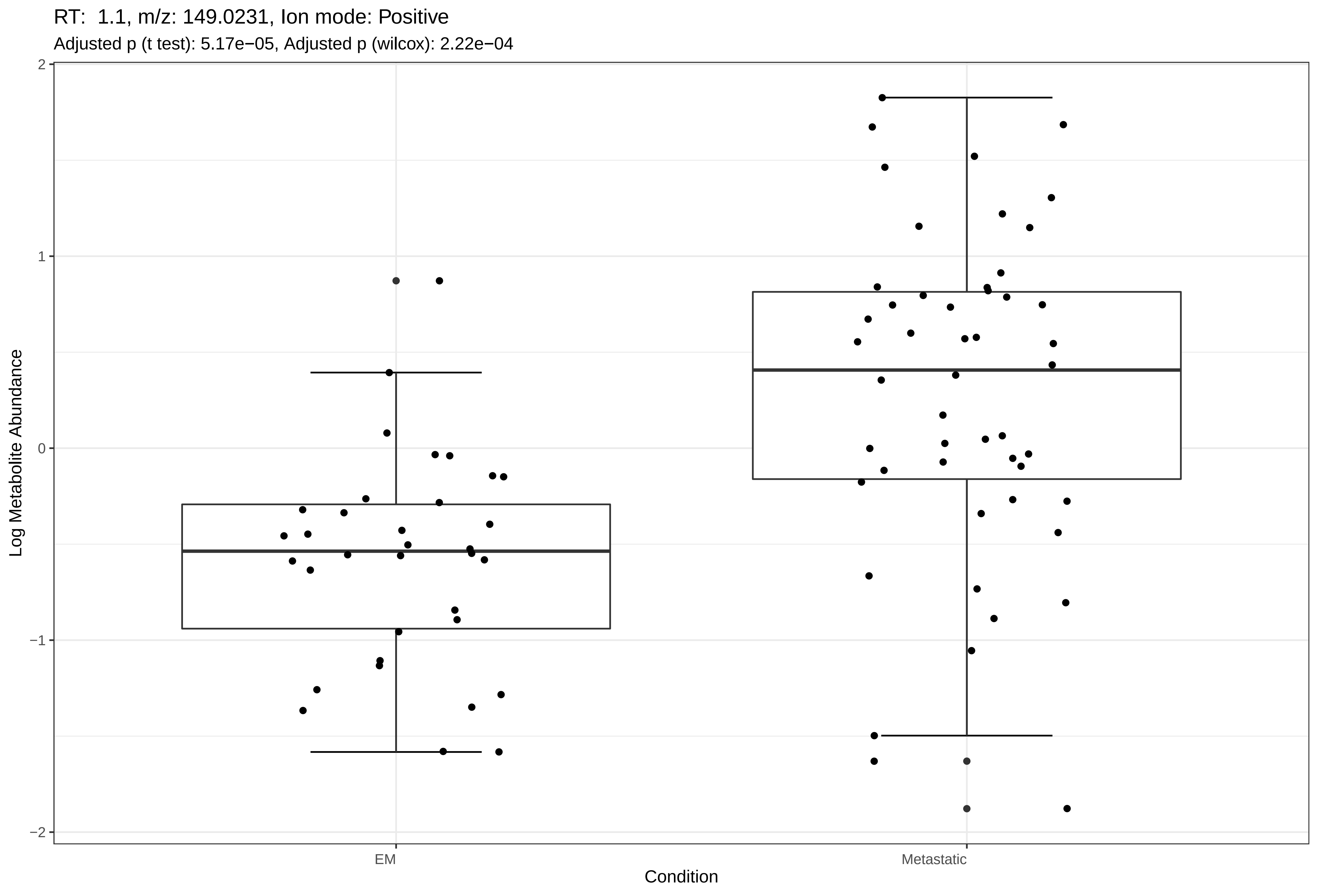

RT: 1.1, m/z: 149.0231, Ion mode: Positive
Adjusted p (t test): 5.17e−05, Adjusted p (wilcox): 2.22e−04
Log Metabolite Abundance
2
1
0
−1
−2
EM
Metastatic
Condition

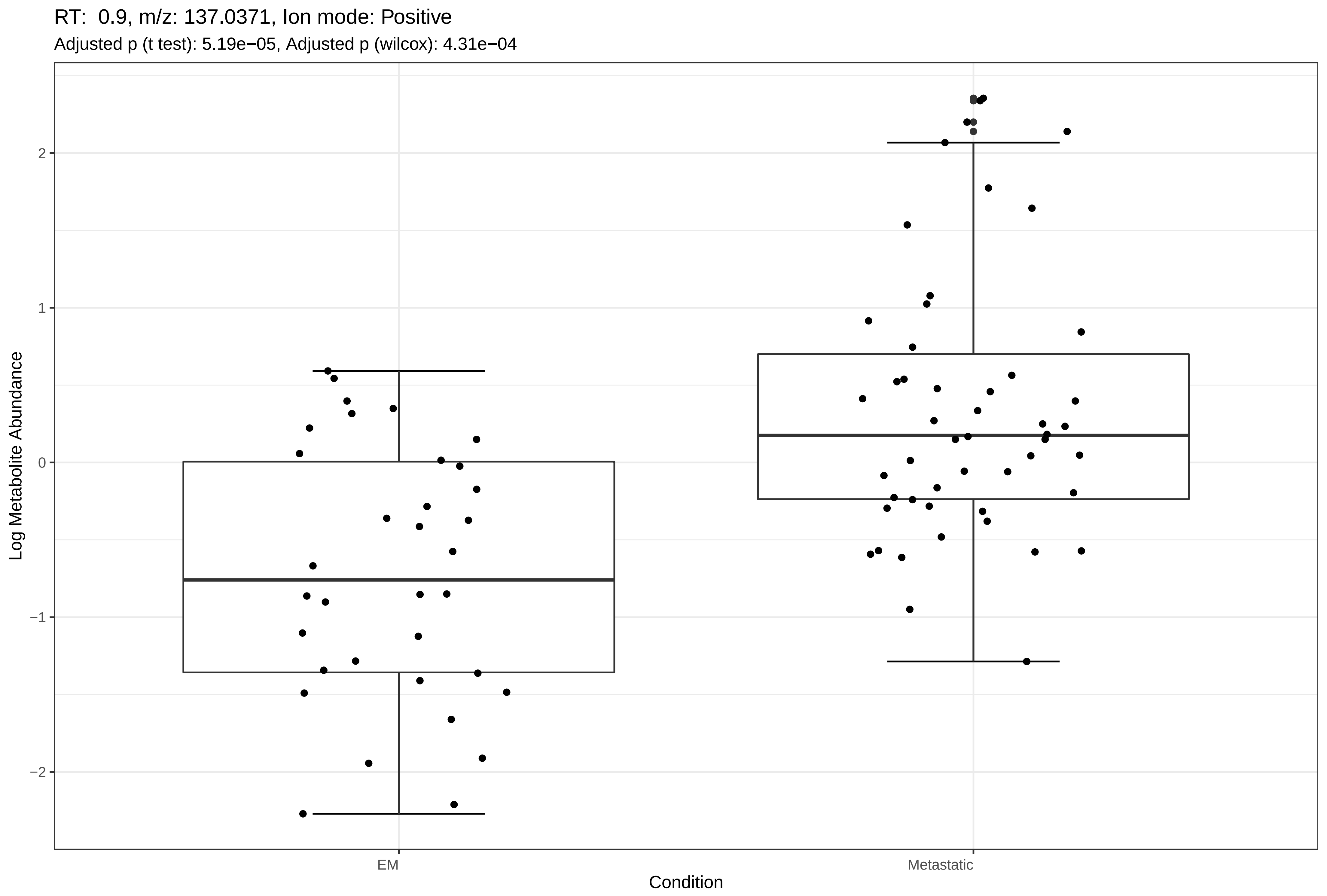
RT: 0.9, m/z: 137.0371, Ion mode: Positive
Adjusted p (t test): 5.19e−05, Adjusted p (wilcox): 4.31e−04
Log Metabolite Abundance
2
1
0
−1
−2
EM
Metastatic
Condition

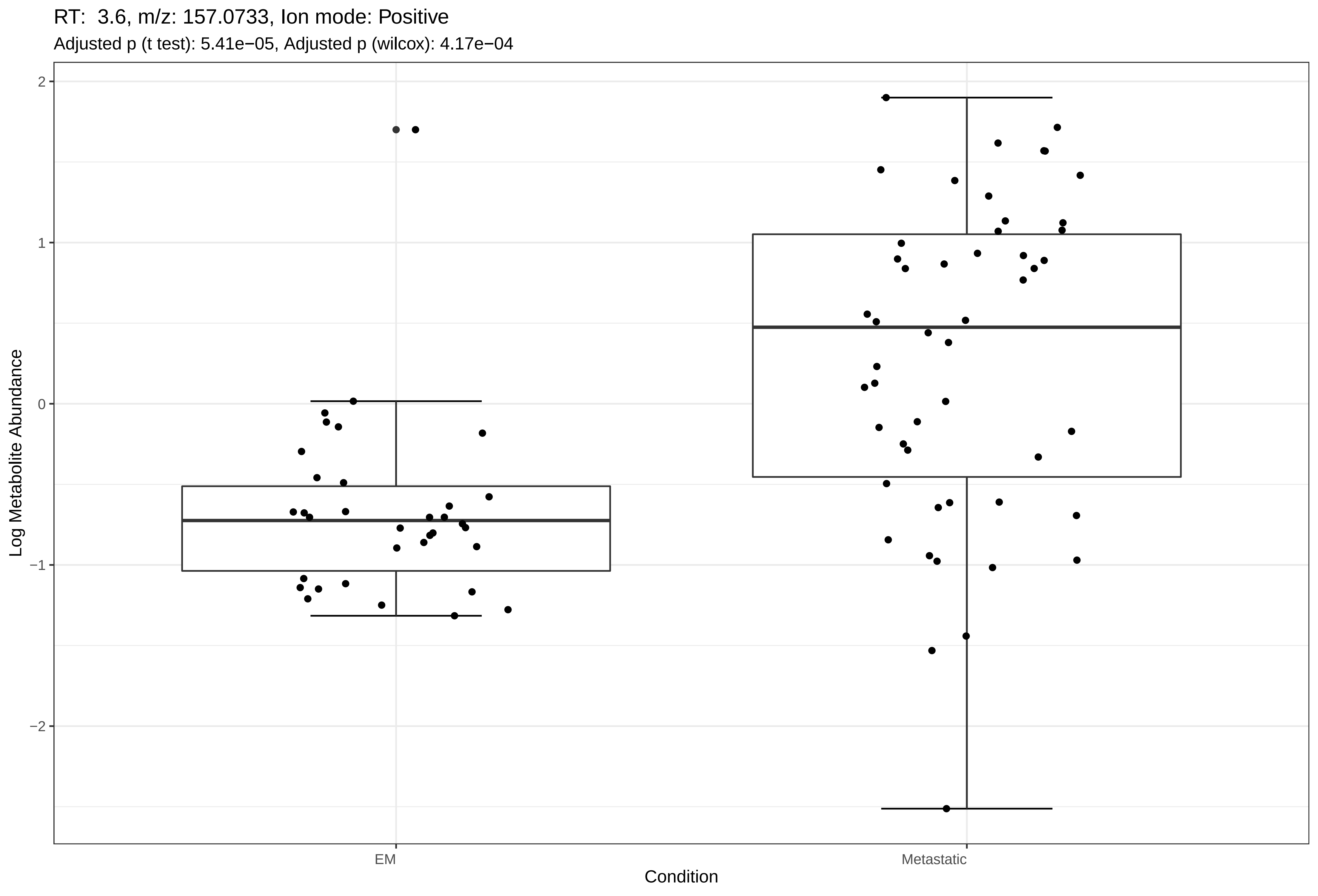
RT: 3.6, m/z: 157.0733, Ion mode: Positive
Adjusted p (t test): 5.41e−05, Adjusted p (wilcox): 4.17e−04
Log Metabolite Abundance
2
1
0
−1
−2
EM
Metastatic
Condition

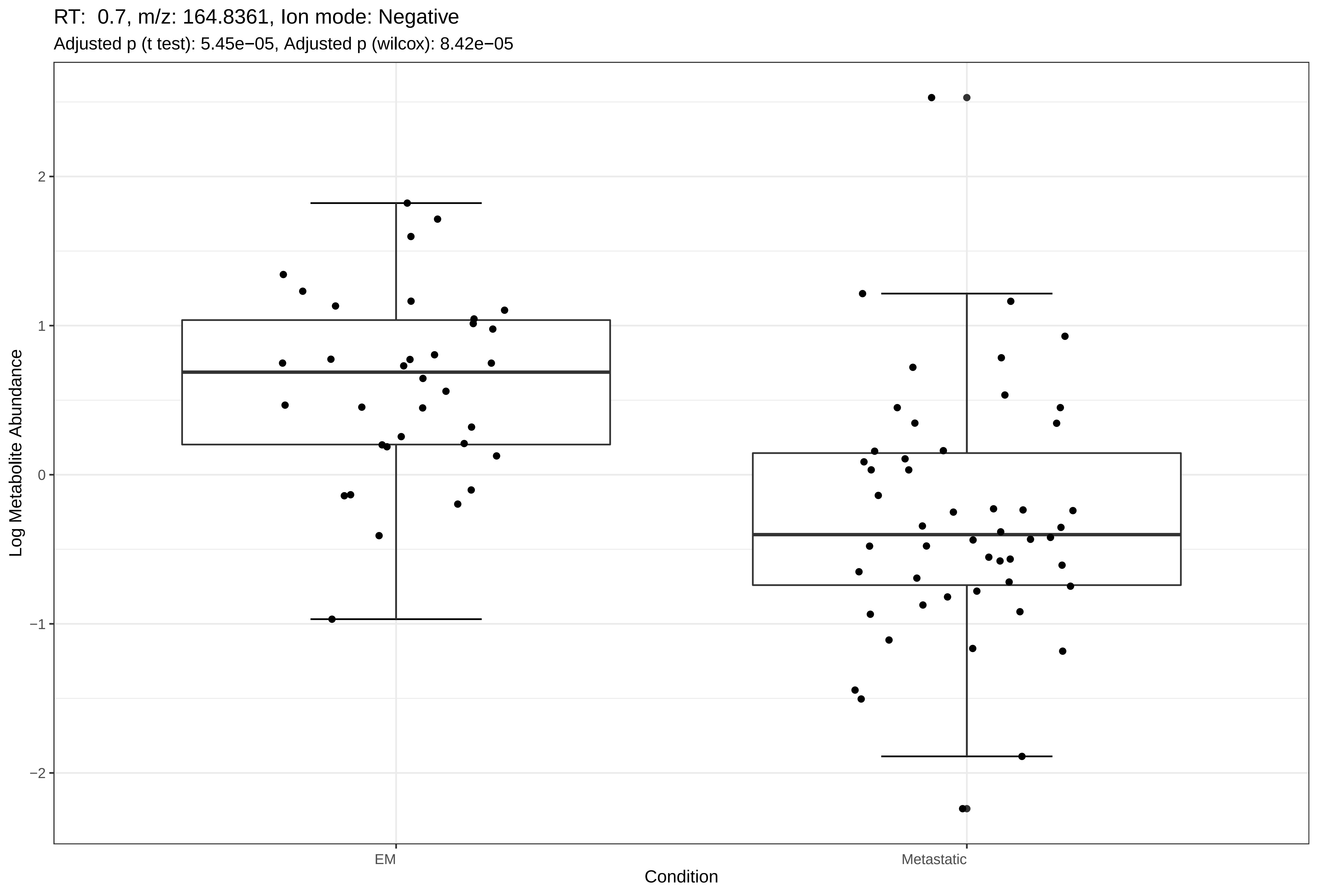
RT: 0.7, m/z: 164.8361, Ion mode: Negative
Adjusted p (t test): 5.45e−05, Adjusted p (wilcox): 8.42e−05
Log Metabolite Abundance
2
1
0
−1
−2
EM
Metastatic
Condition

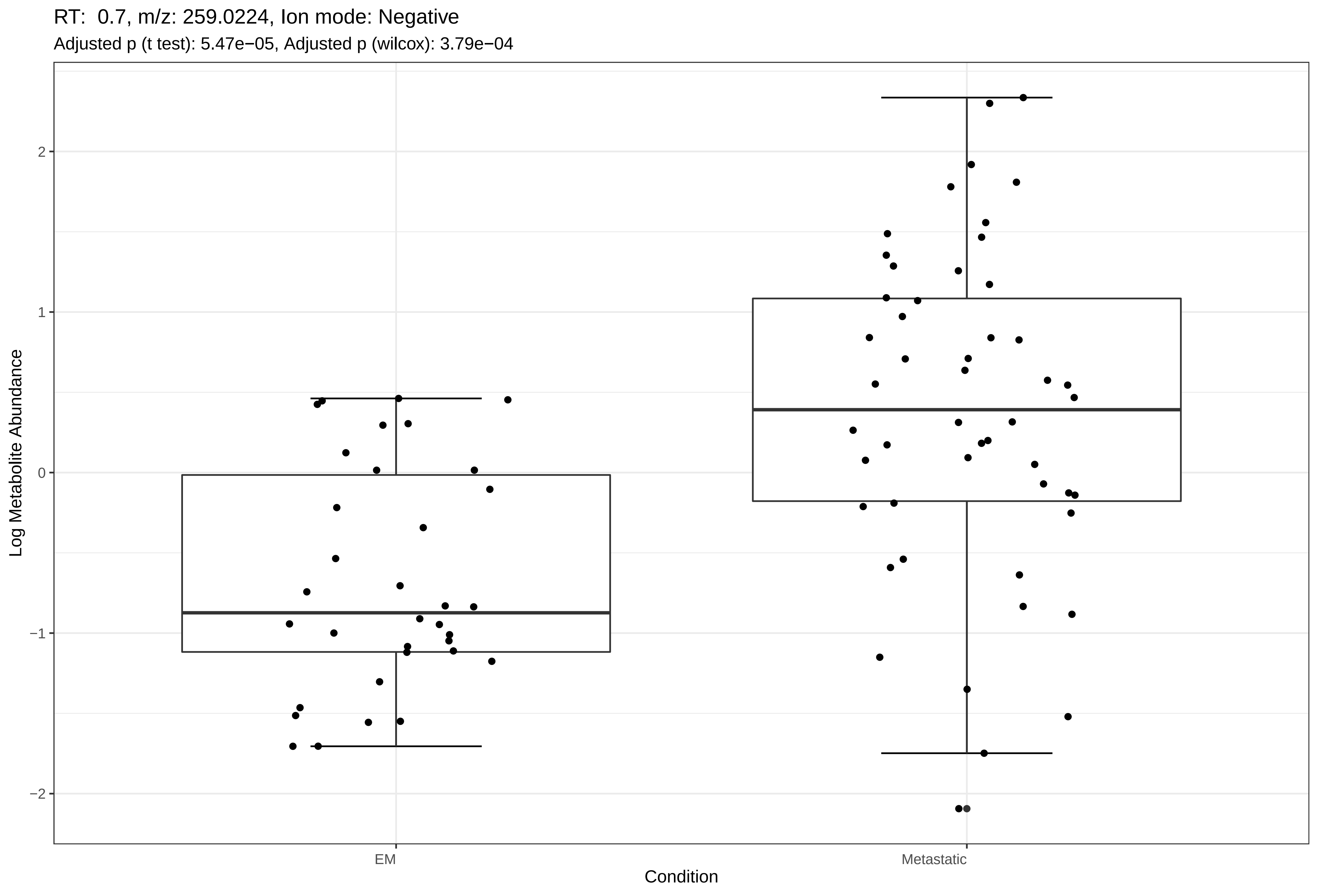
RT: 0.7, m/z: 259.0224, Ion mode: Negative
Adjusted p (t test): 5.47e−05, Adjusted p (wilcox): 3.79e−04
Log Metabolite Abundance
2
1
0
−1
−2
EM
Metastatic
Condition

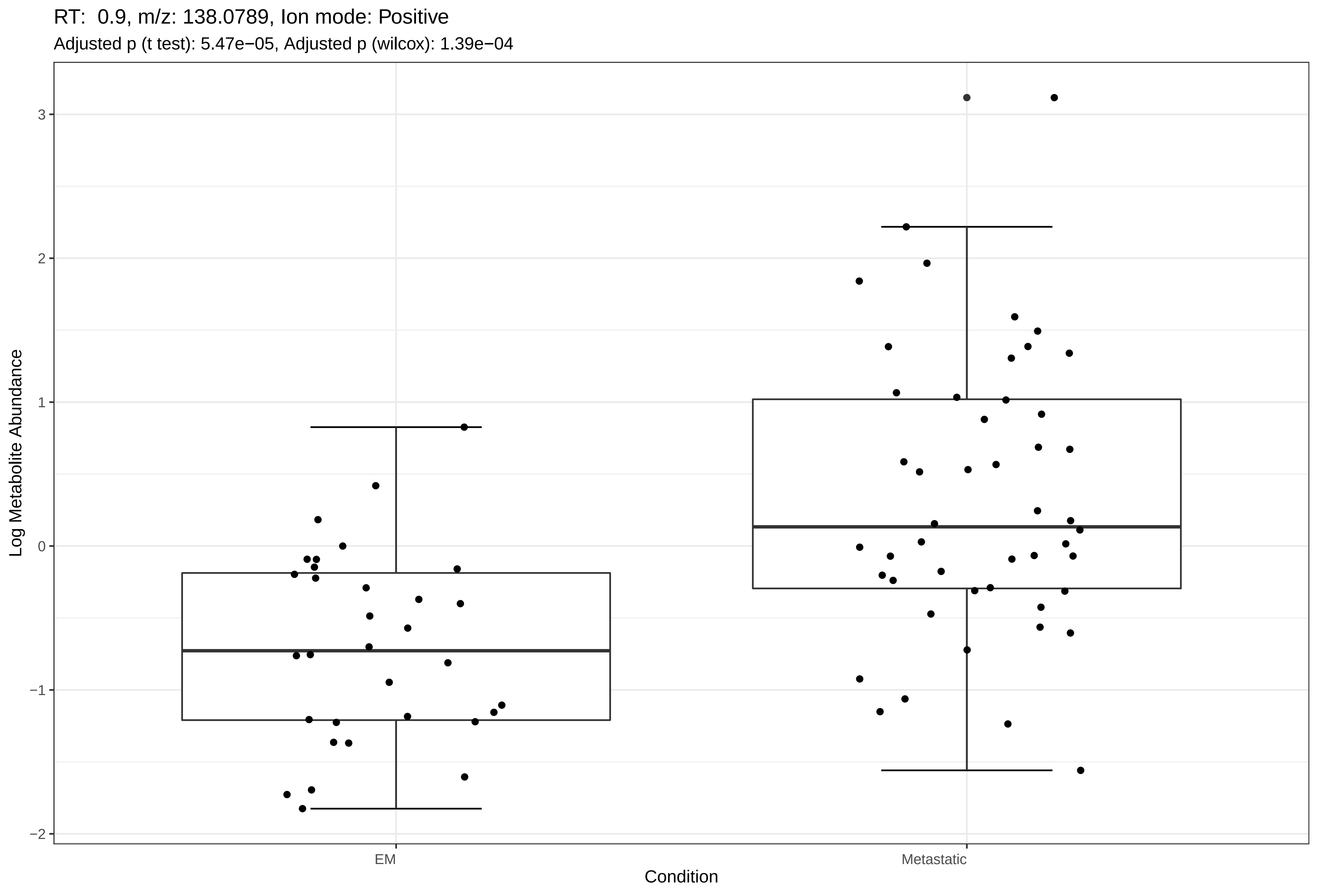
RT: 0.9, m/z: 138.0789, Ion mode: Positive
Adjusted p (t test): 5.47e−05, Adjusted p (wilcox): 1.39e−04
Log Metabolite Abundance
3
2
1
0
−1
−2
EM
Metastatic
Condition

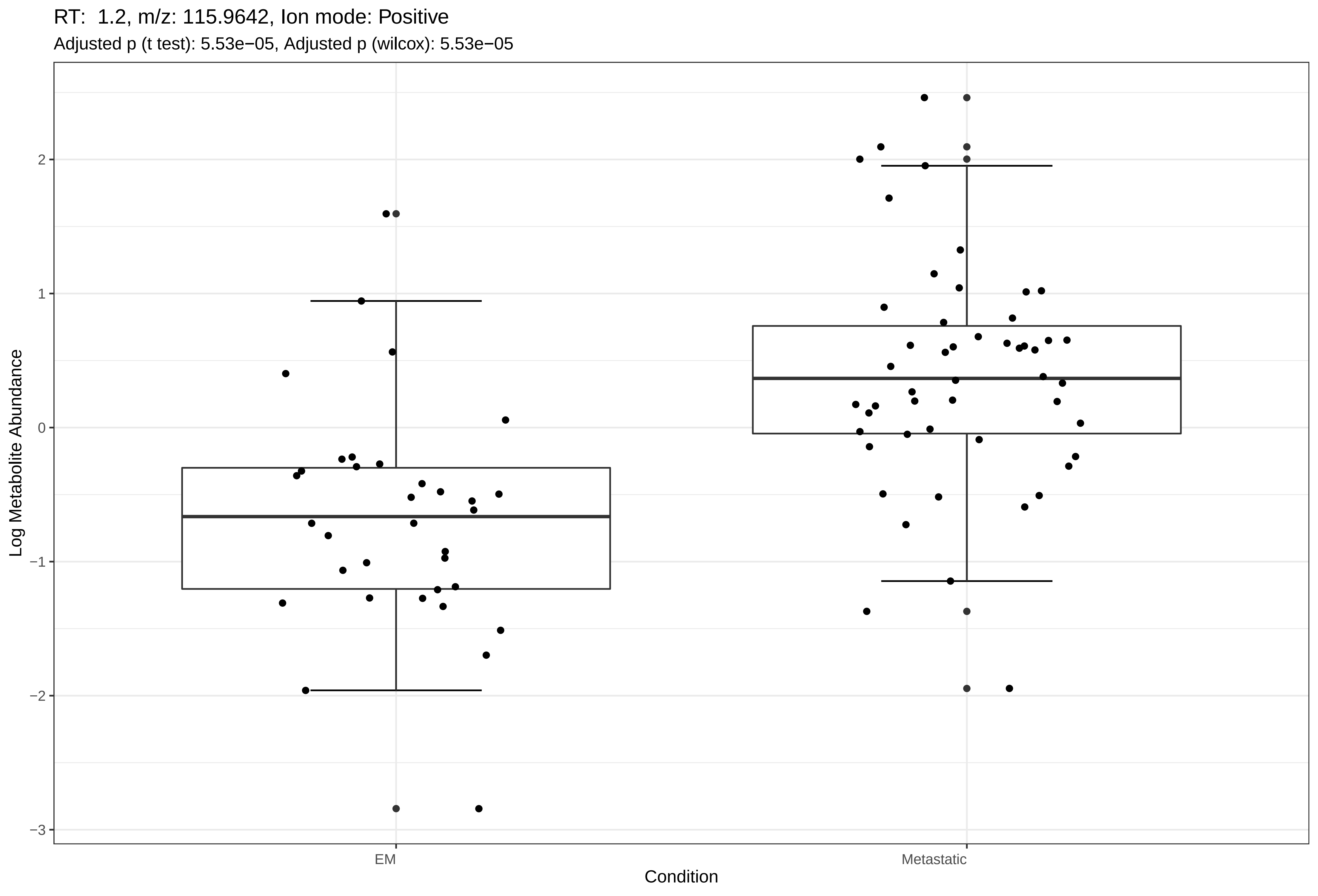

RT: 1.2, m/z: 115.9642, Ion mode: Positive
Adjusted p (t test): 5.53e−05, Adjusted p (wilcox): 5.53e−05
Log Metabolite Abundance
2
1
0
−1
−2
−3
EM
Metastatic
Condition

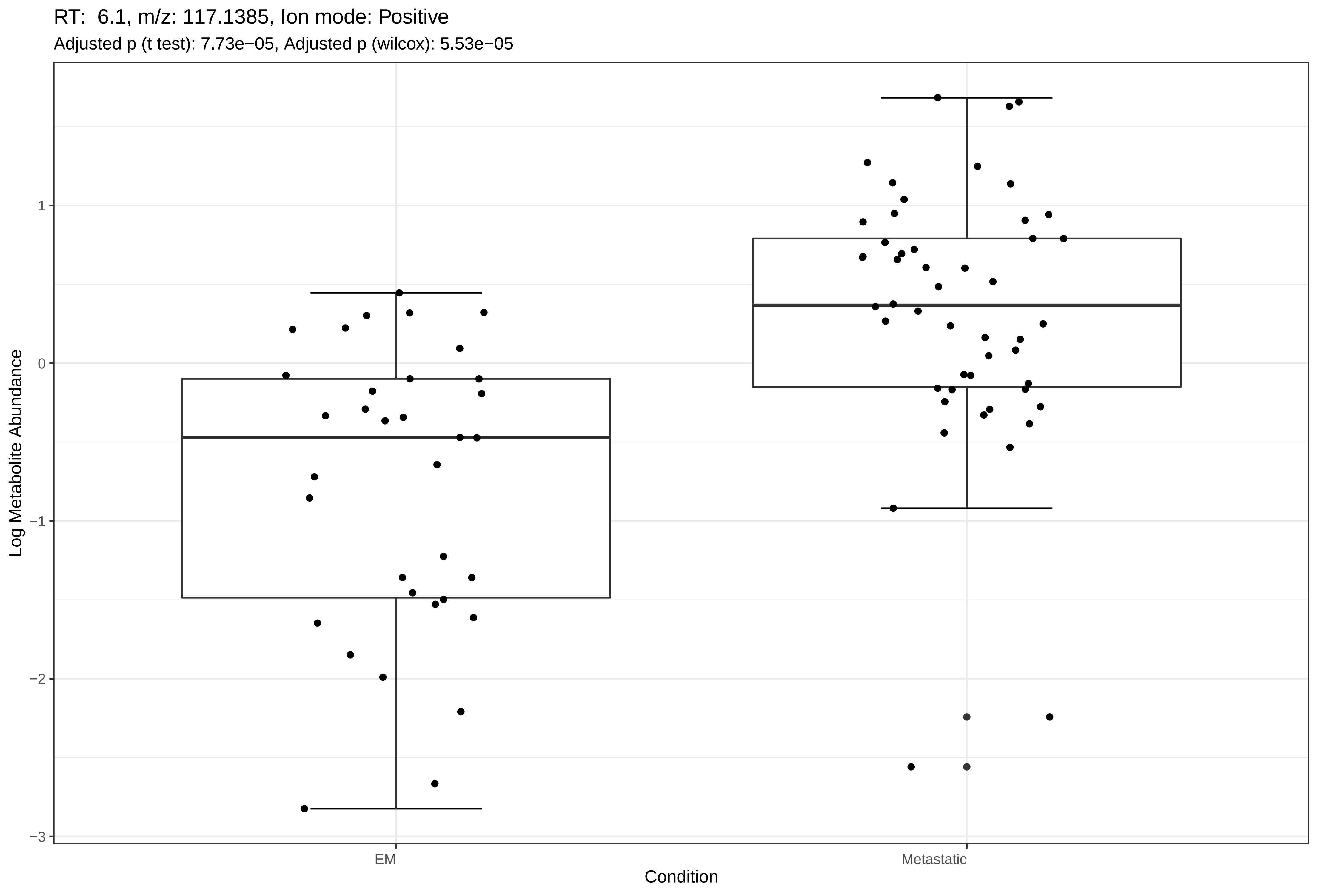

RT: 6.1, m/z: 117.1385, Ion mode: Positive
Adjusted p (t test): 7.73e−05, Adjusted p (wilcox): 5.53e−05
Log Metabolite Abundance
1
0
−1
−2
−3
EM
Metastatic
Condition

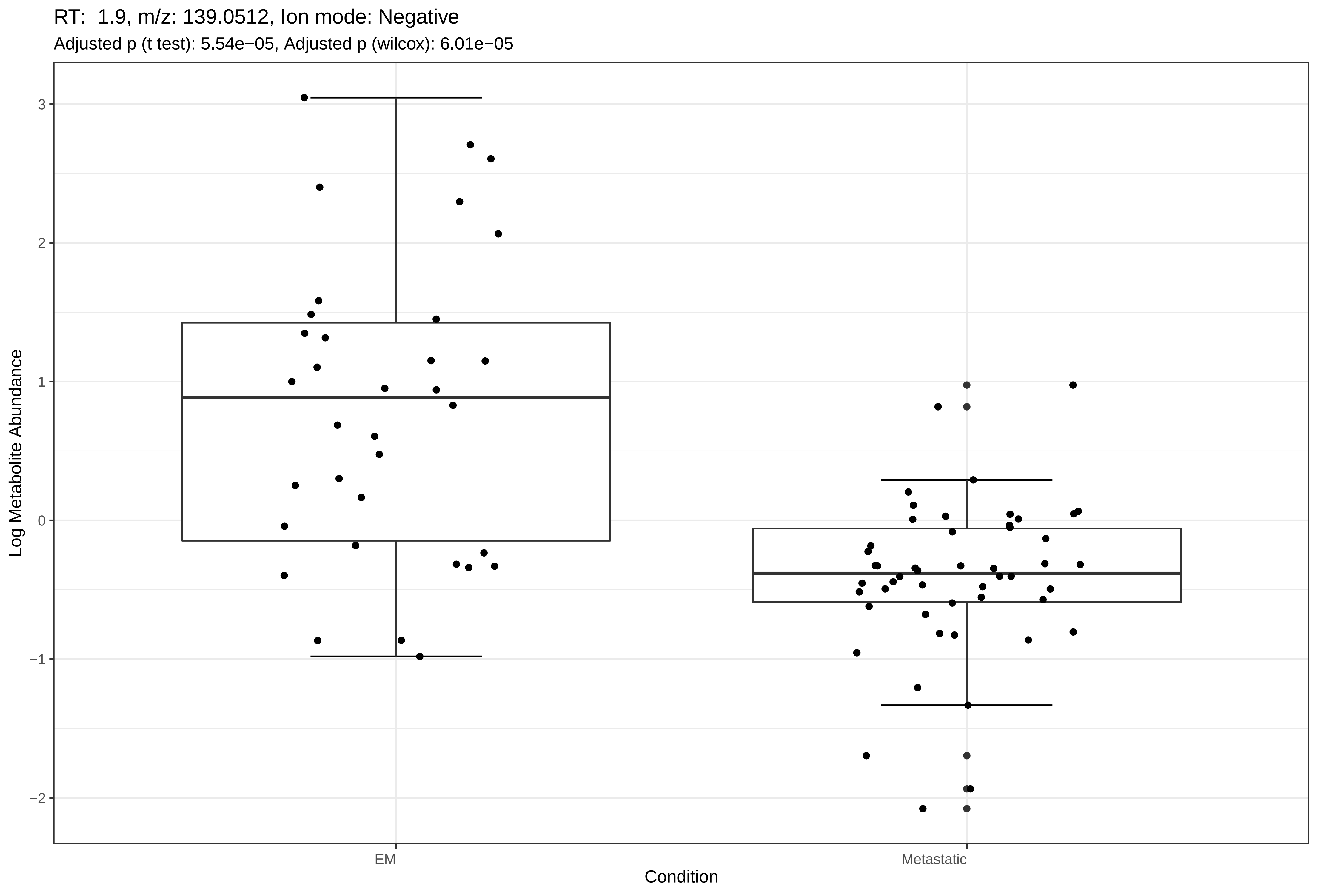
RT: 1.9, m/z: 139.0512, Ion mode: Negative
Adjusted p (t test): 5.54e−05, Adjusted p (wilcox): 6.01e−05
Log Metabolite Abundance
3
2
1
0
−1
−2
EM
Metastatic
Condition

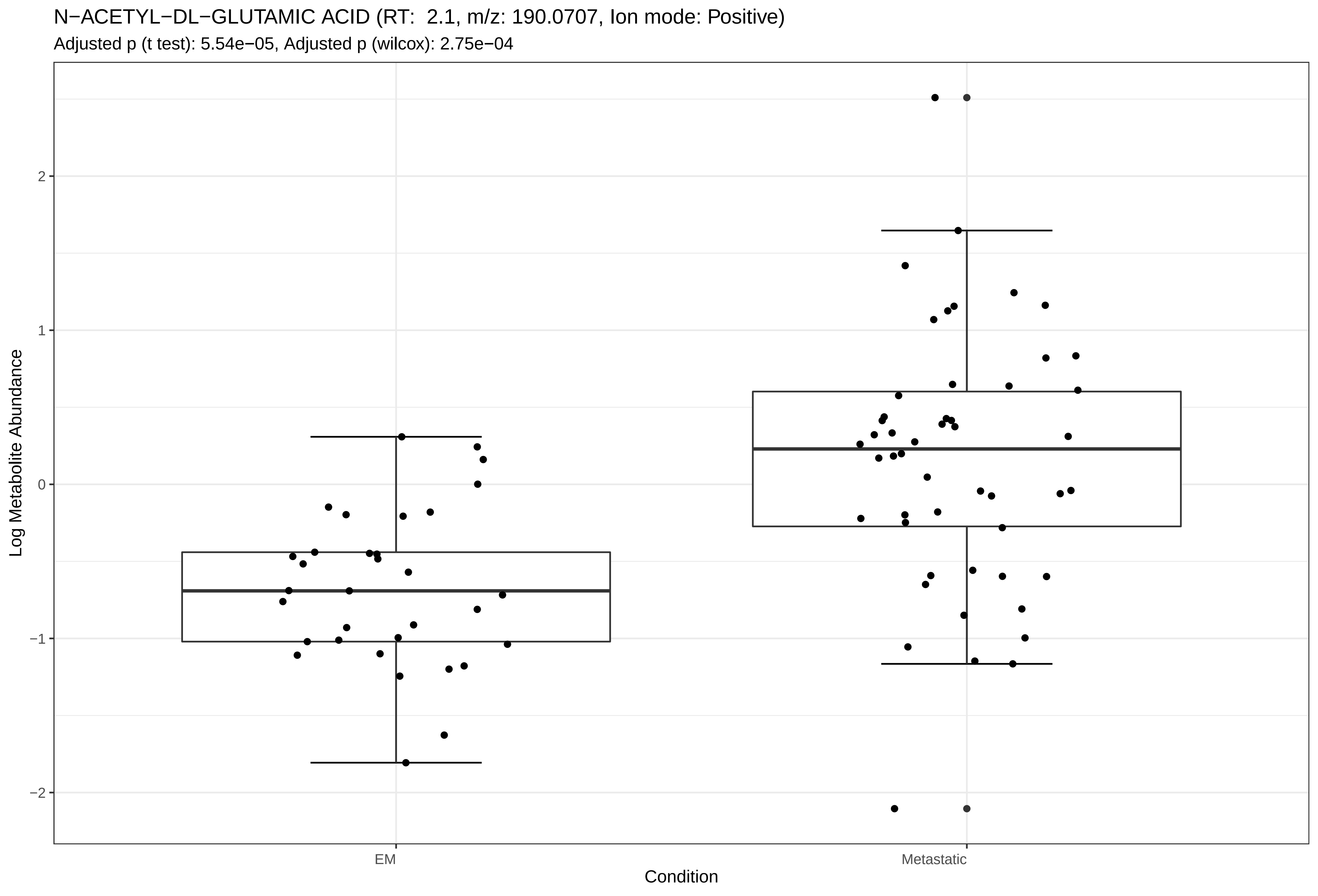
N−ACETYL−DL−GLUTAMIC ACID (RT: 2.1, m/z: 190.0707, Ion mode: Positive)
Adjusted p (t test): 5.54e−05, Adjusted p (wilcox): 2.75e−04
Log Metabolite Abundance
2
1
0
−1
−2
EM
Metastatic
Condition

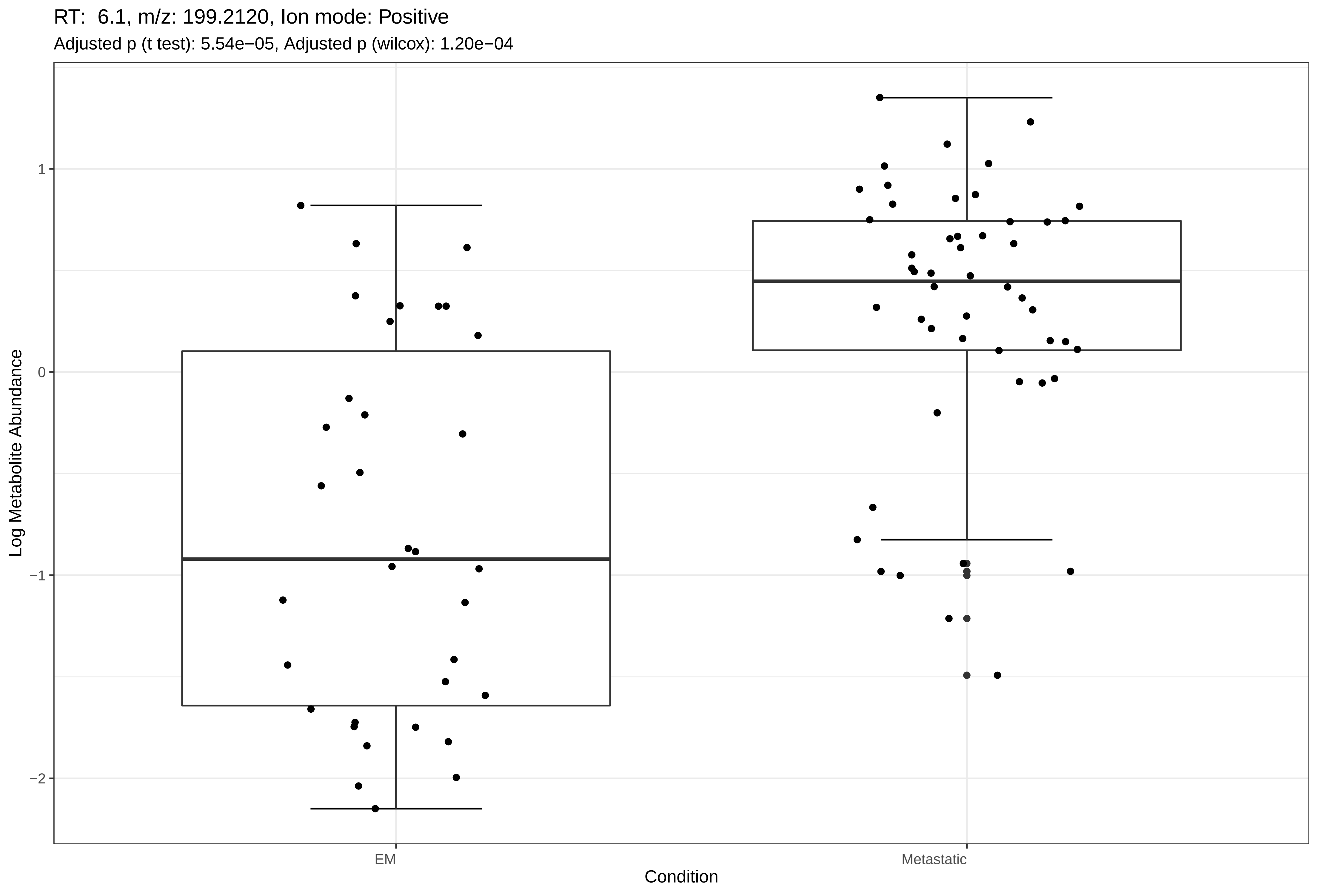

RT: 6.1, m/z: 199.2120, Ion mode: Positive
Adjusted p (t test): 5.54e−05, Adjusted p (wilcox): 1.20e−04
Log Metabolite Abundance
1
0
−1
−2
EM
Metastatic
Condition

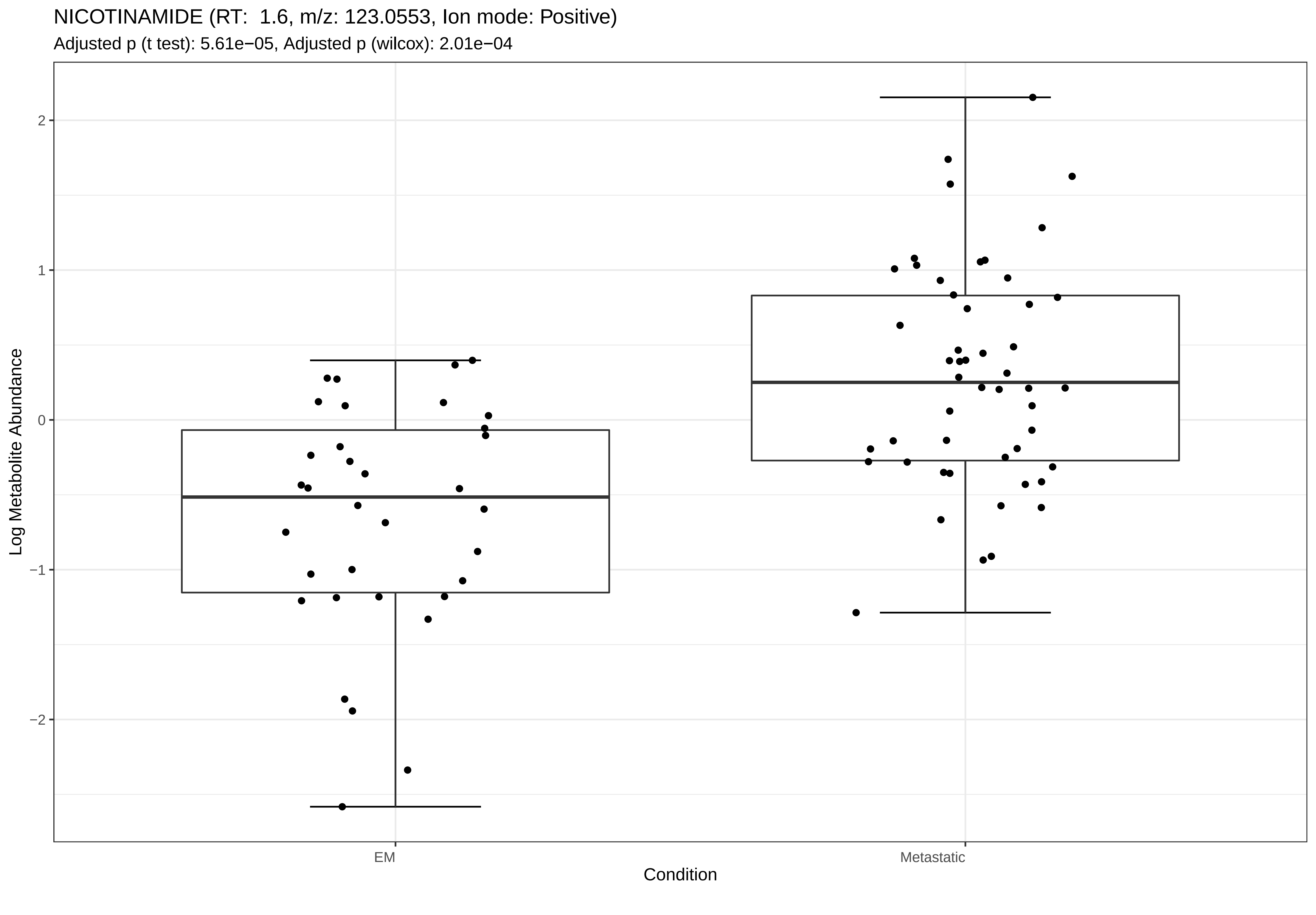

NICOTINAMIDE (RT: 1.6, m/z: 123.0553, Ion mode: Positive)
Adjusted p (t test): 5.61e−05, Adjusted p (wilcox): 2.01e−04
Log Metabolite Abundance
2
1
0
−1
−2
EM
Metastatic
Condition

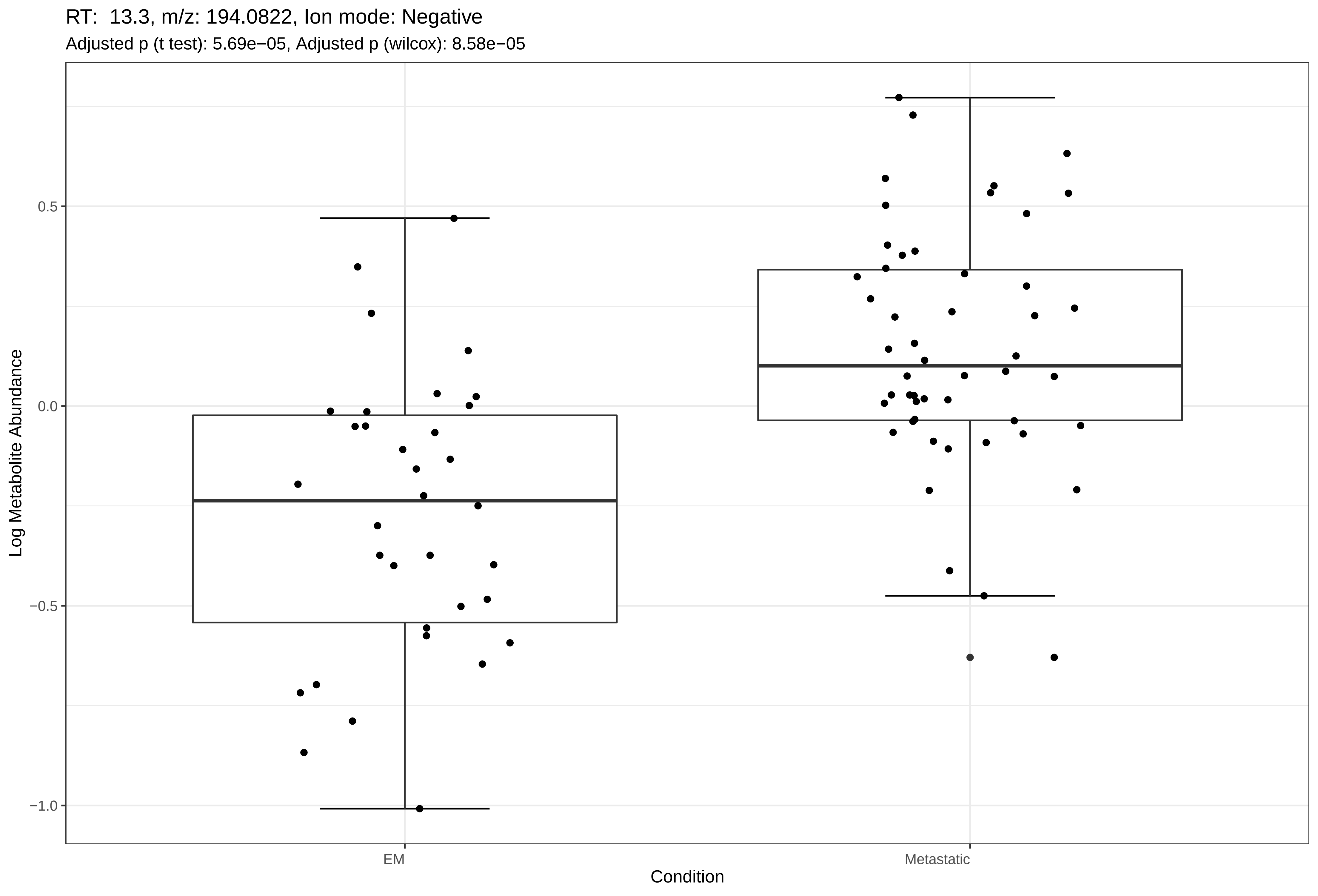

RT: 13.3, m/z: 194.0822, Ion mode: Negative
Adjusted p (t test): 5.69e−05, Adjusted p (wilcox): 8.58e−05
Log Metabolite Abundance
0.5
0.0
−0.5
−1.0
EM
Metastatic
Condition

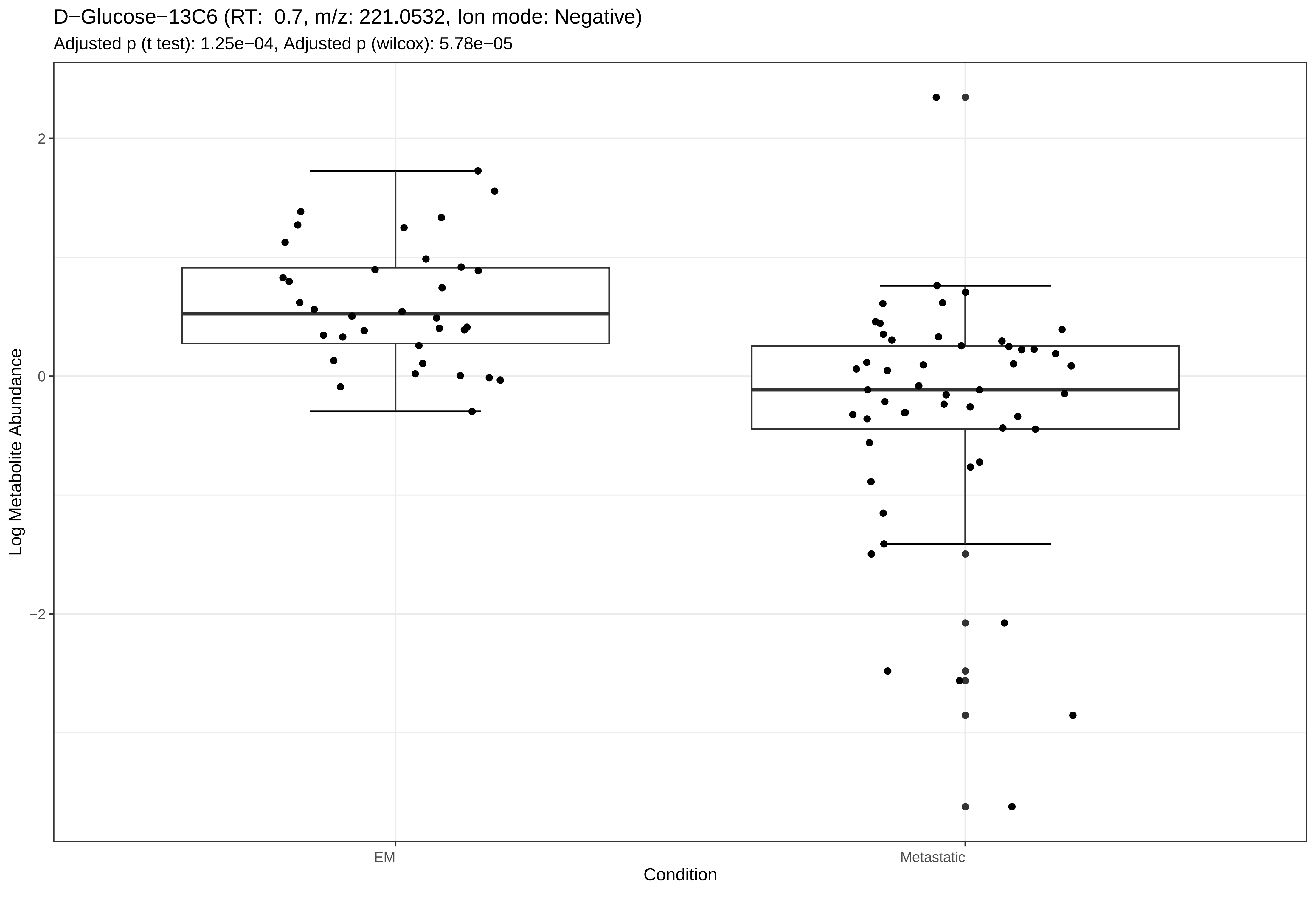
D−Glucose−13C6 (RT: 0.7, m/z: 221.0532, Ion mode: Negative)
Adjusted p (t test): 1.25e−04, Adjusted p (wilcox): 5.78e−05
Log Metabolite Abundance
2
0
−2
EM
Metastatic
Condition

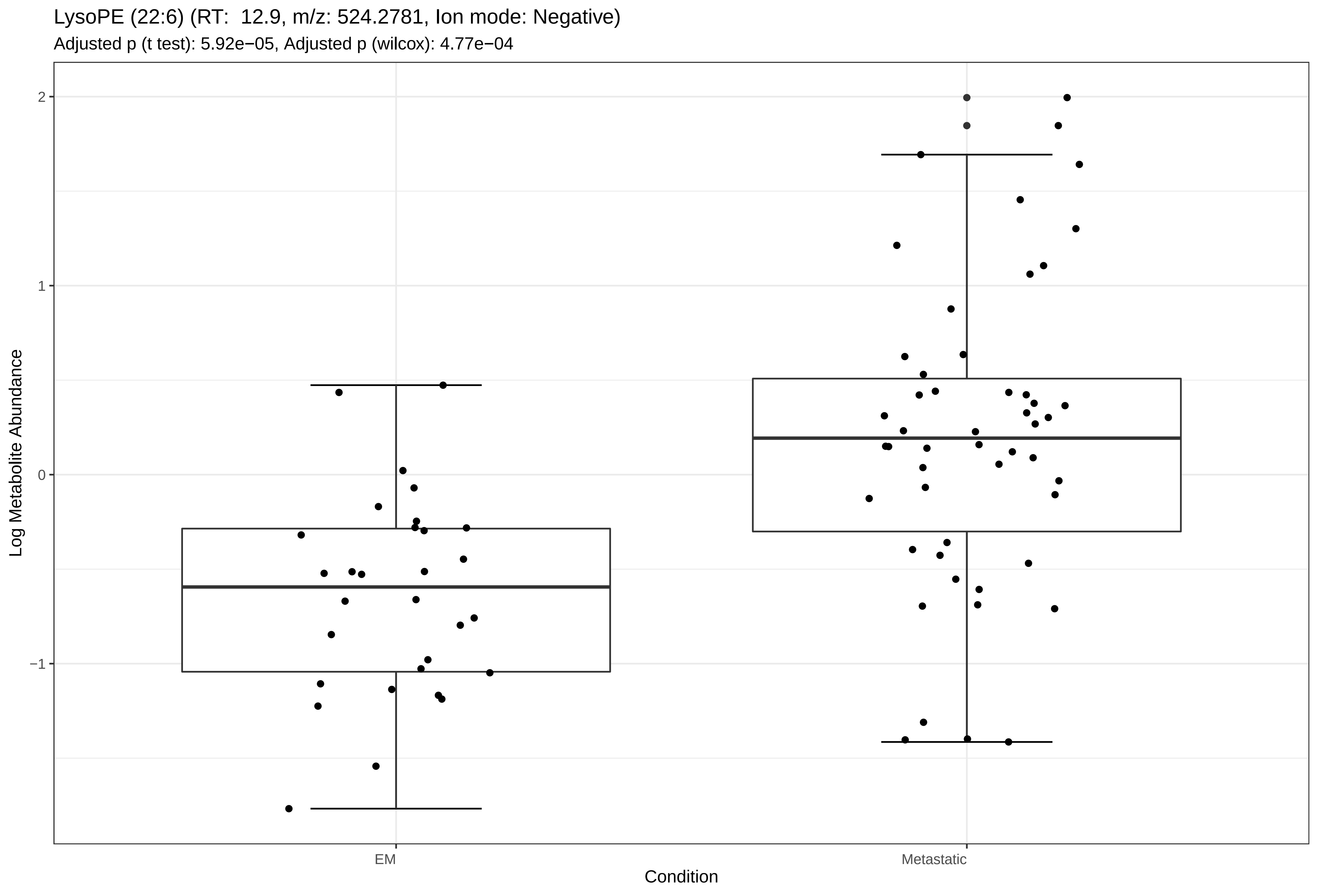

LysoPE (22:6) (RT: 12.9, m/z: 524.2781, Ion mode: Negative)
Adjusted p (t test): 5.92e−05, Adjusted p (wilcox): 4.77e−04
Log Metabolite Abundance
2
1
0
−1
EM
Metastatic
Condition

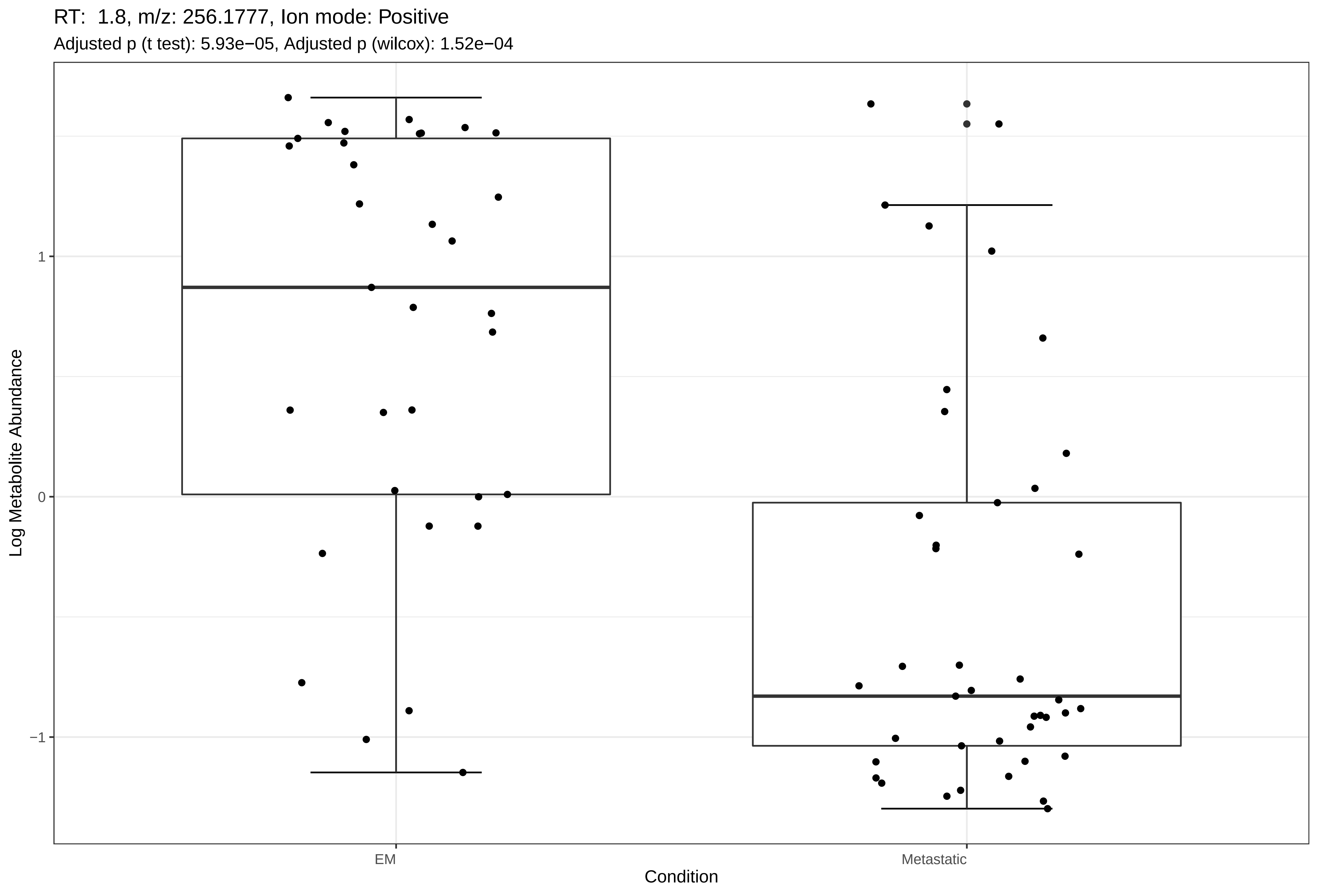
RT: 1.8, m/z: 256.1777, Ion mode: Positive
Adjusted p (t test): 5.93e−05, Adjusted p (wilcox): 1.52e−04
Log Metabolite Abundance
1
0
−1
EM
Metastatic
Condition

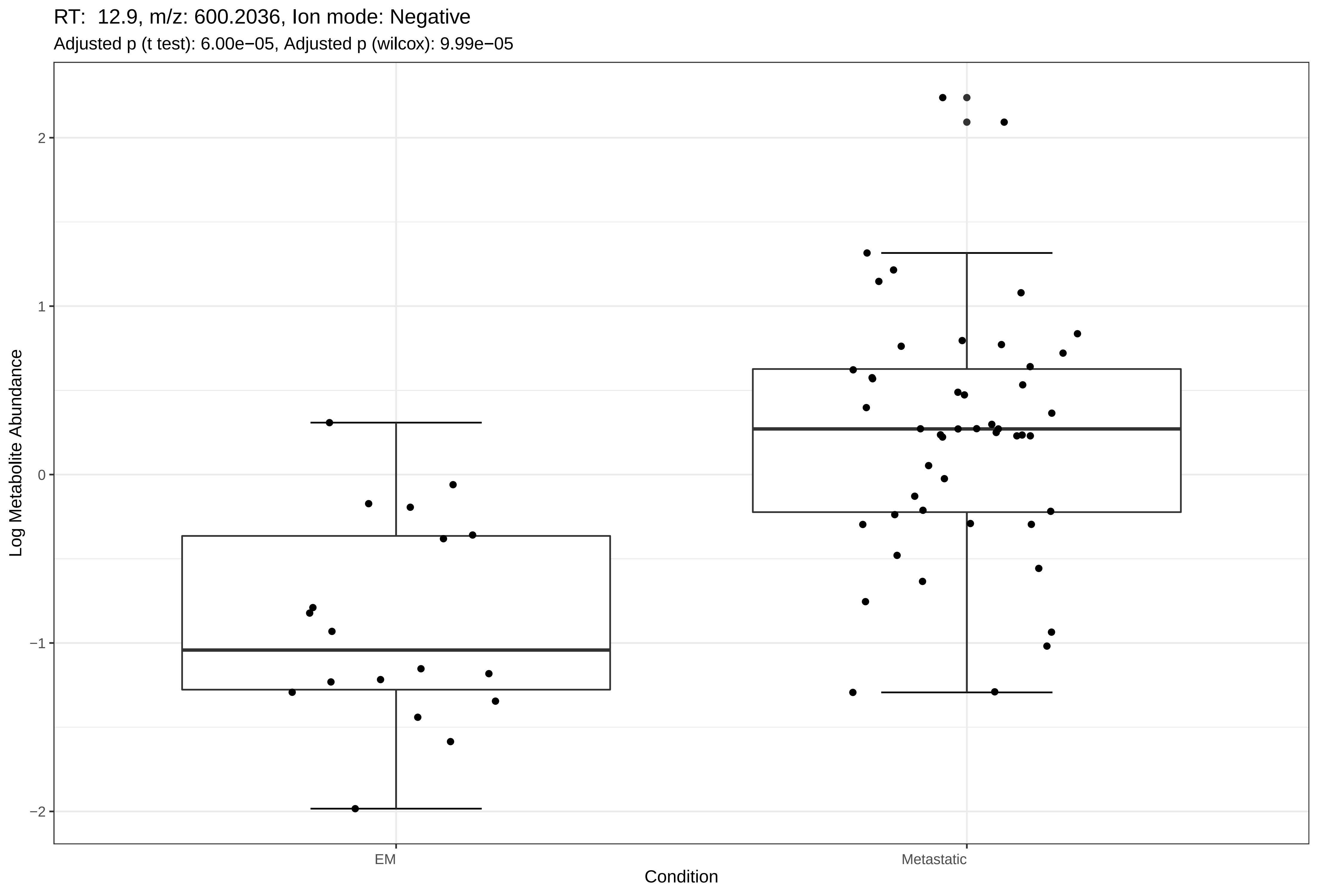
RT: 12.9, m/z: 600.2036, Ion mode: Negative
Adjusted p (t test): 6.00e−05, Adjusted p (wilcox): 9.99e−05
Log Metabolite Abundance
2
1
0
−1
−2
EM
Metastatic
Condition

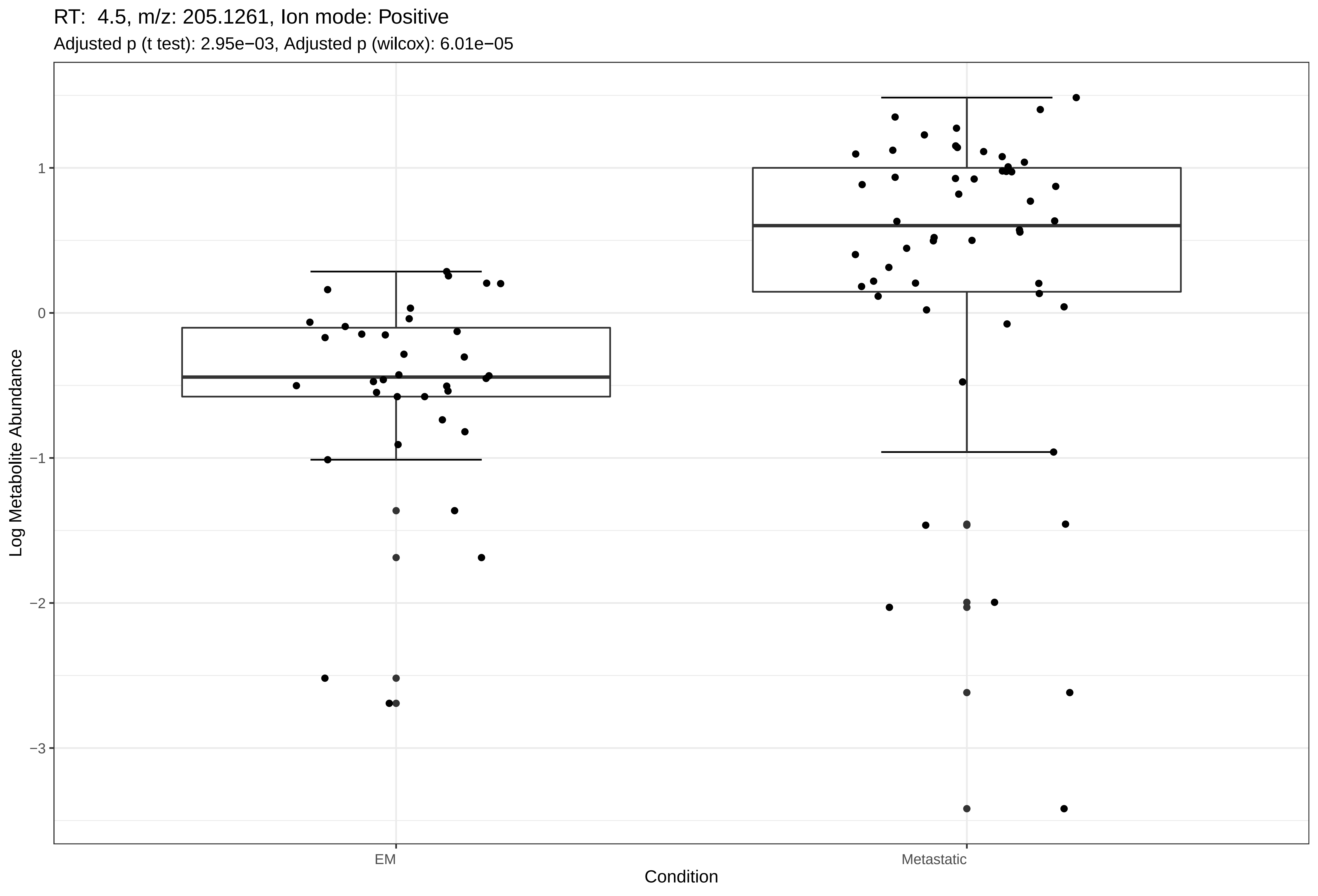
RT: 4.5, m/z: 205.1261, Ion mode: Positive
Adjusted p (t test): 2.95e−03, Adjusted p (wilcox): 6.01e−05
Log Metabolite Abundance
1
0
−1
−2
−3
EM
Metastatic
Condition

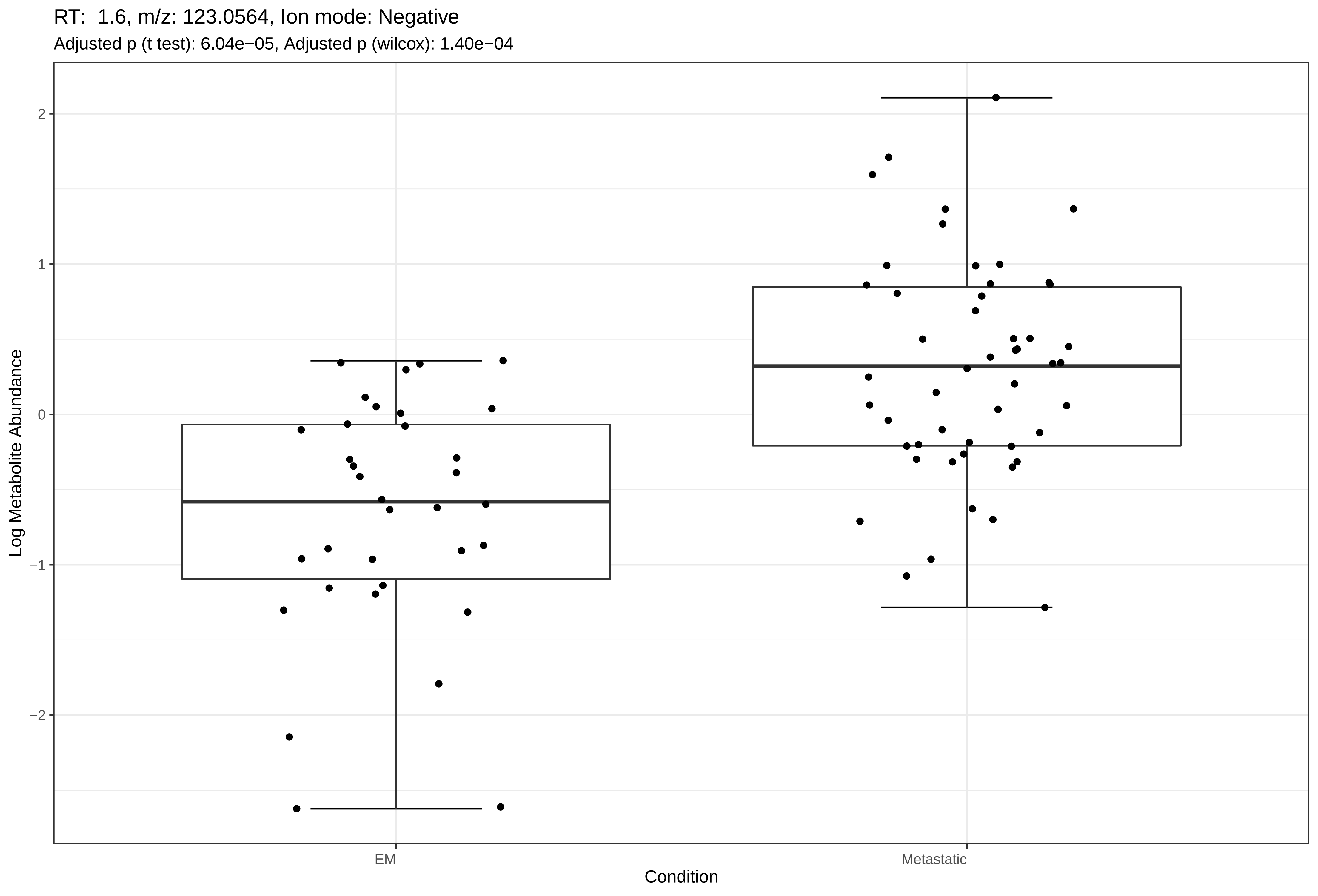

RT: 1.6, m/z: 123.0564, Ion mode: Negative
Adjusted p (t test): 6.04e−05, Adjusted p (wilcox): 1.40e−04
Log Metabolite Abundance
2
1
0
−1
−2
EM
Metastatic
Condition

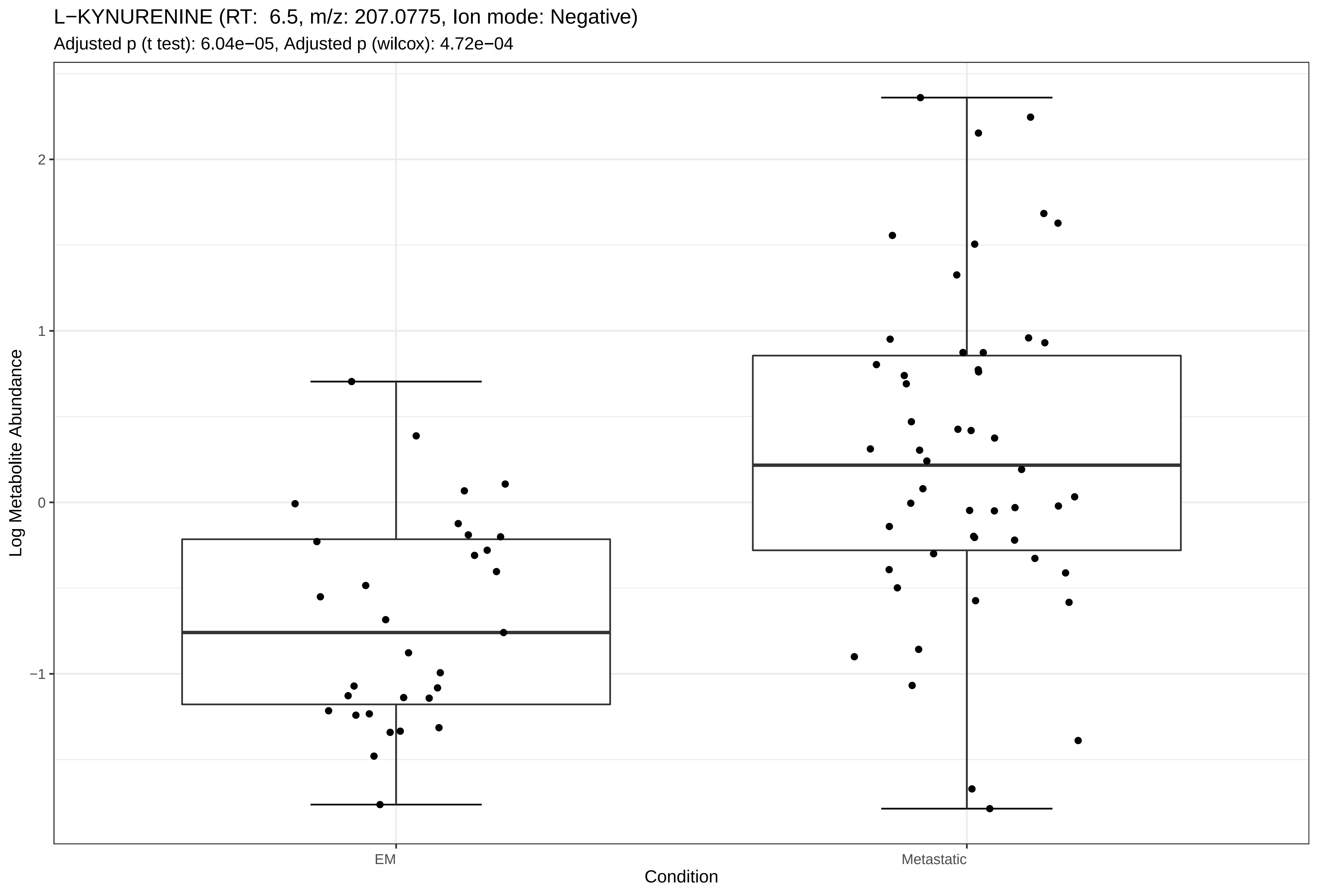

L−KYNURENINE (RT: 6.5, m/z: 207.0775, Ion mode: Negative)
Adjusted p (t test): 6.04e−05, Adjusted p (wilcox): 4.72e−04
2
1
0
−1
Log Metabolite Abundance
EM
Metastatic
Condition

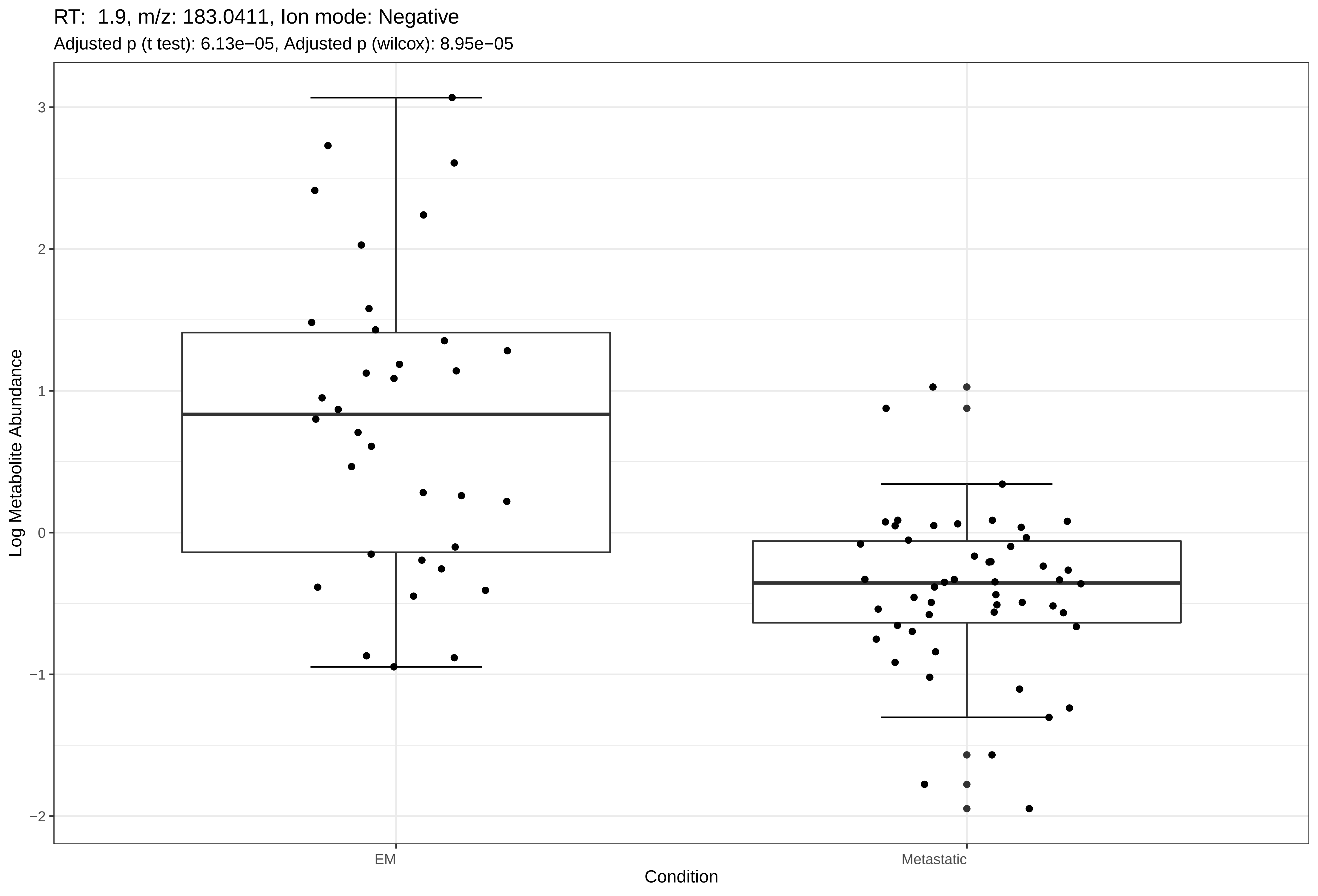
RT: 1.9, m/z: 183.0411, Ion mode: Negative
Adjusted p (t test): 6.13e−05, Adjusted p (wilcox): 8.95e−05
Log Metabolite Abundance
3
2
1
0
−1
−2
EM
Metastatic
Condition

RT: 0.9, m/z: 241.1048, Ion mode: Negative

Adjusted p (t test): 6.15e−05, Adjusted p (wilcox): 2.76e−03

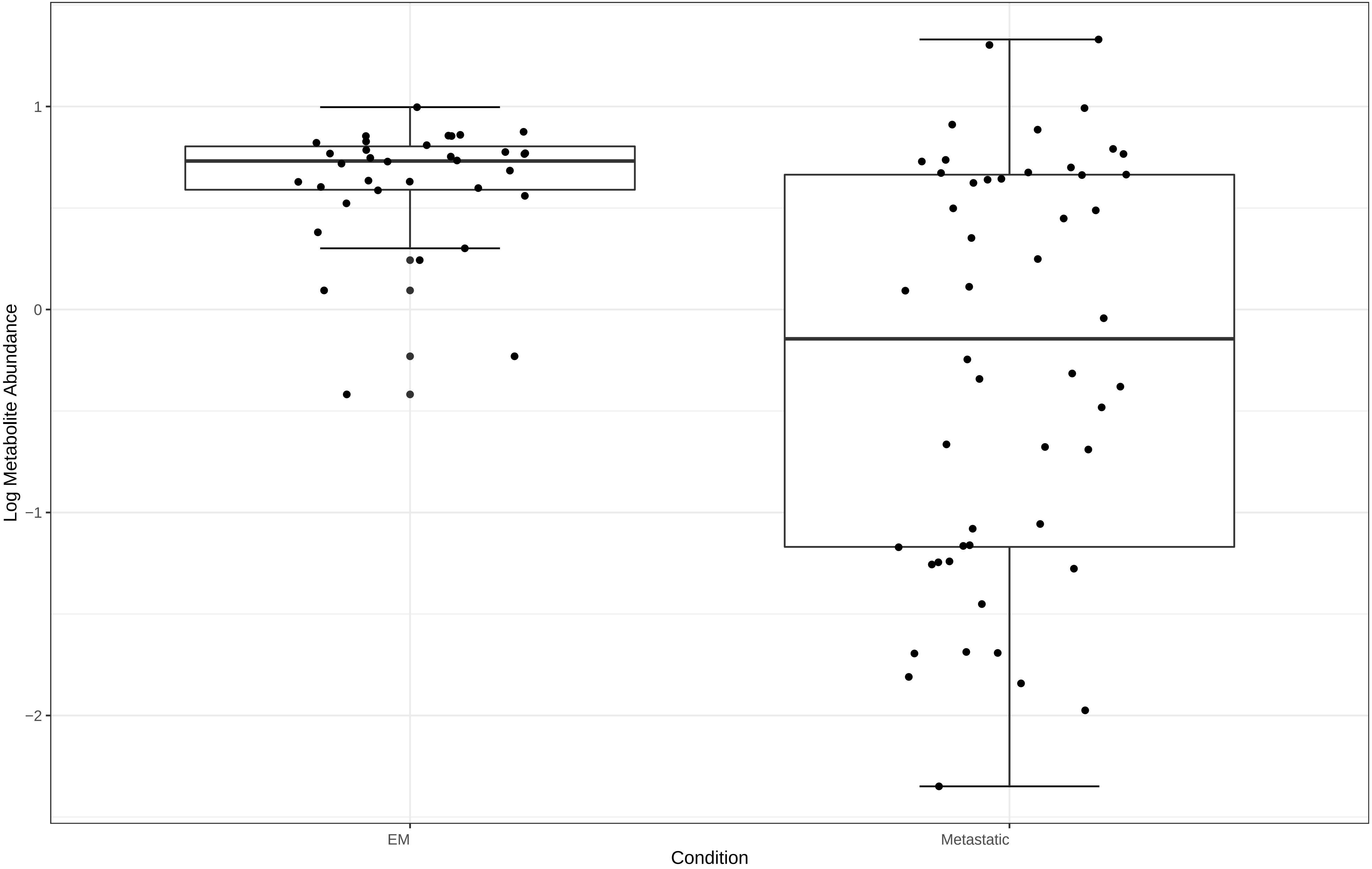

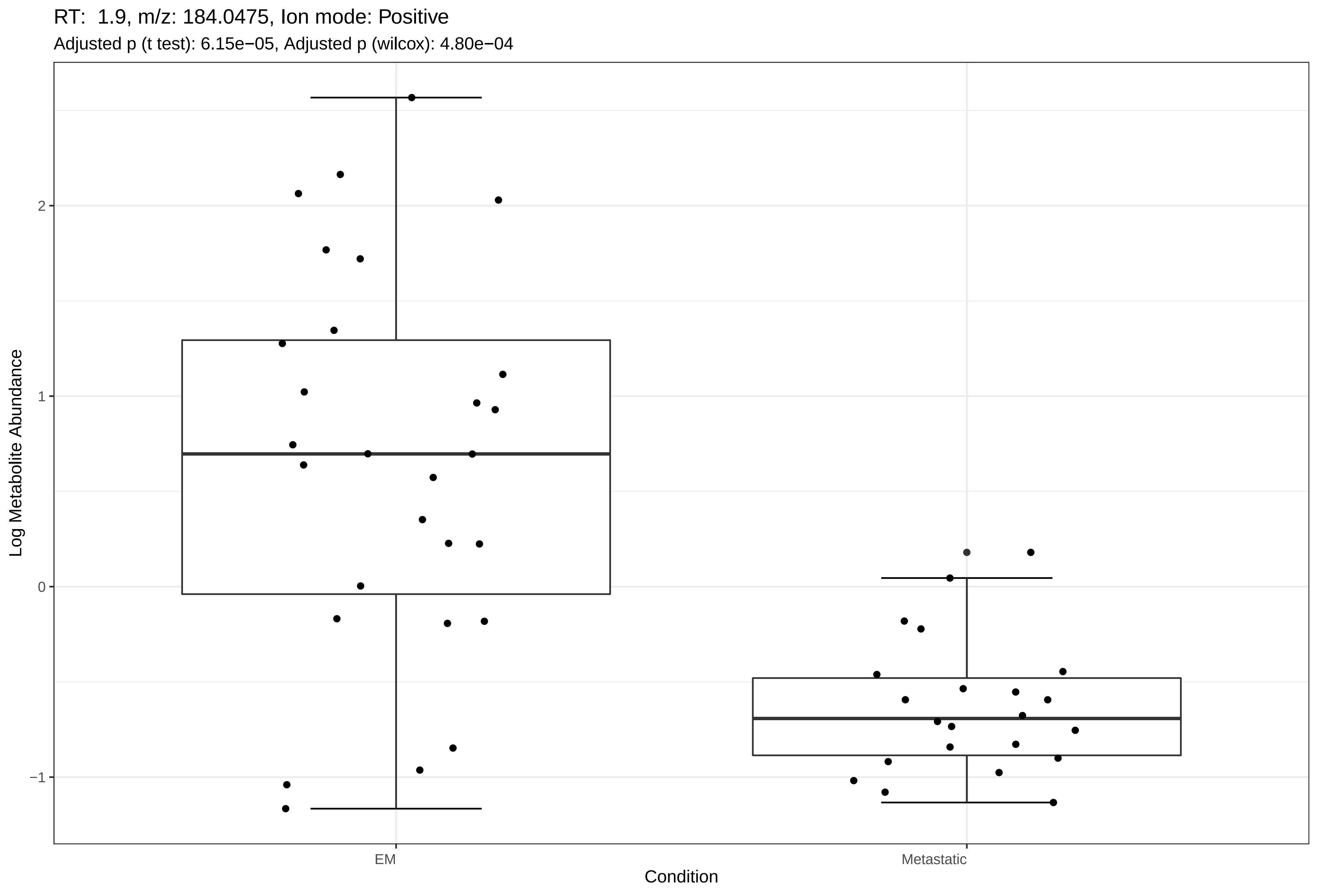
RT: 1.9, m/z: 184.0475, Ion mode: Positive
Adjusted p (t test): 6.15e−05, Adjusted p (wilcox): 4.80e−04
Log Metabolite Abundance
2
1
0
−1
EM
Metastatic
Condition

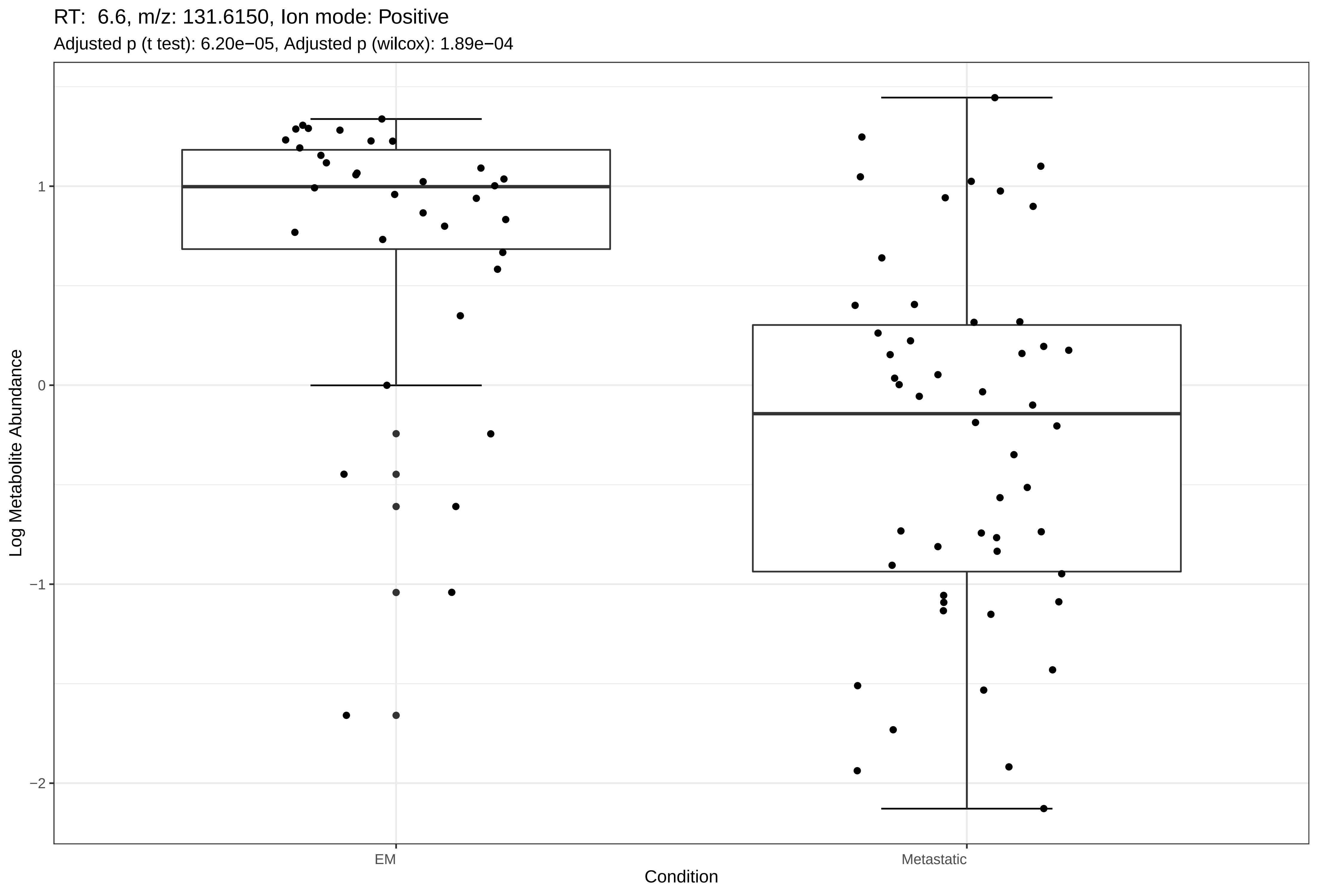
RT: 6.6, m/z: 131.6150, Ion mode: Positive
Adjusted p (t test): 6.20e−05, Adjusted p (wilcox): 1.89e−04
Log Metabolite Abundance
1
0
−1
−2
EM
Metastatic
Condition

[M−H2O+H] −18.0106 m/z adduct of 322.0759 m/z (RT: 3.3, m/z: 304.0653, Ion mode: Positive)

Adjusted p (t test): 6.36e−05, Adjusted p (wilcox): 3.93e−04

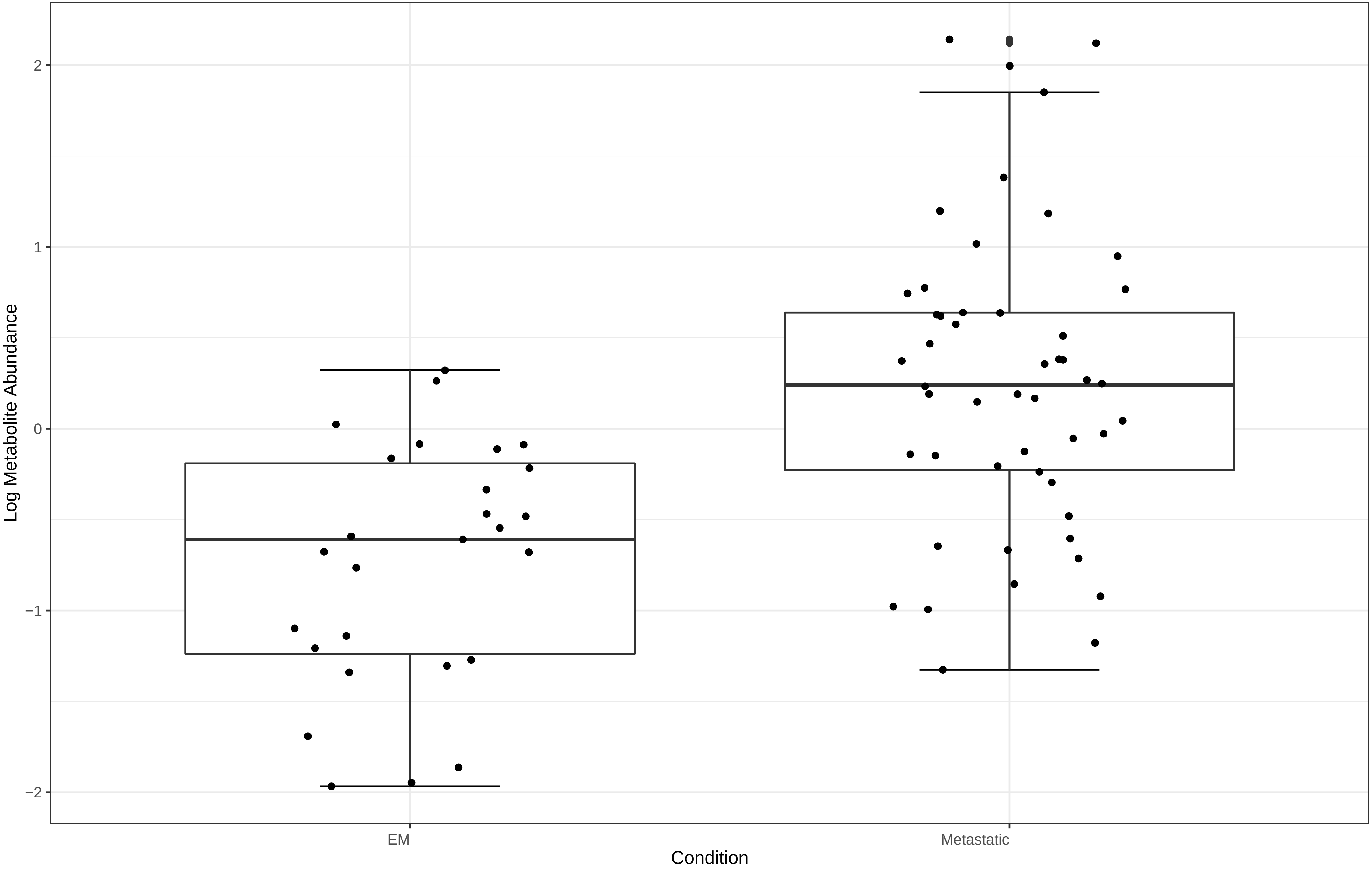

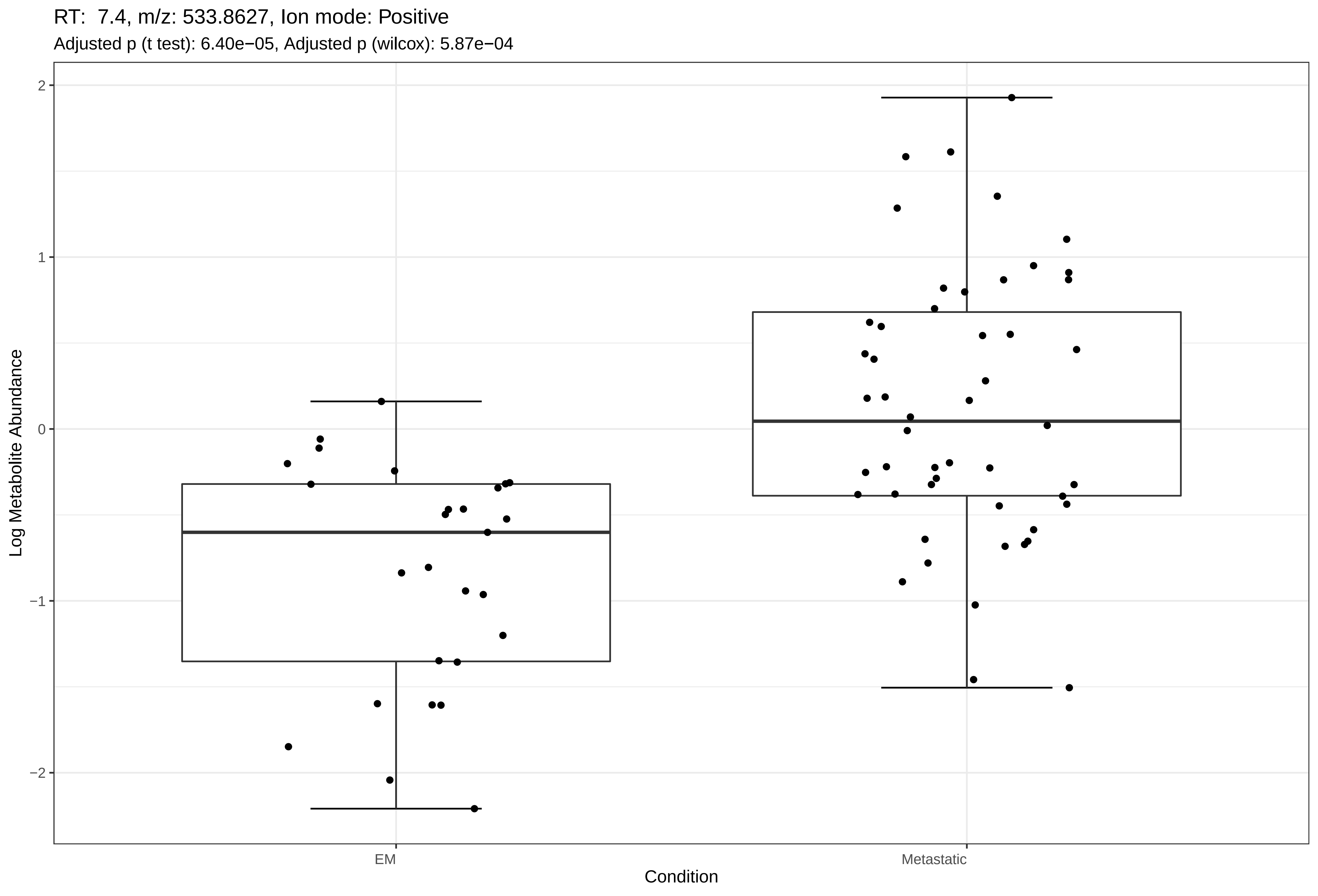

RT: 7.4, m/z: 533.8627, Ion mode: Positive
Adjusted p (t test): 6.40e−05, Adjusted p (wilcox): 5.87e−04
2
1
0
−1
−2
Log Metabolite Abundance
EM
Metastatic
Condition

RT: 1.7, m/z: 130.1227, Ion mode: Positive

Adjusted p (t test): 6.42e−05, Adjusted p (wilcox): 9.79e−05

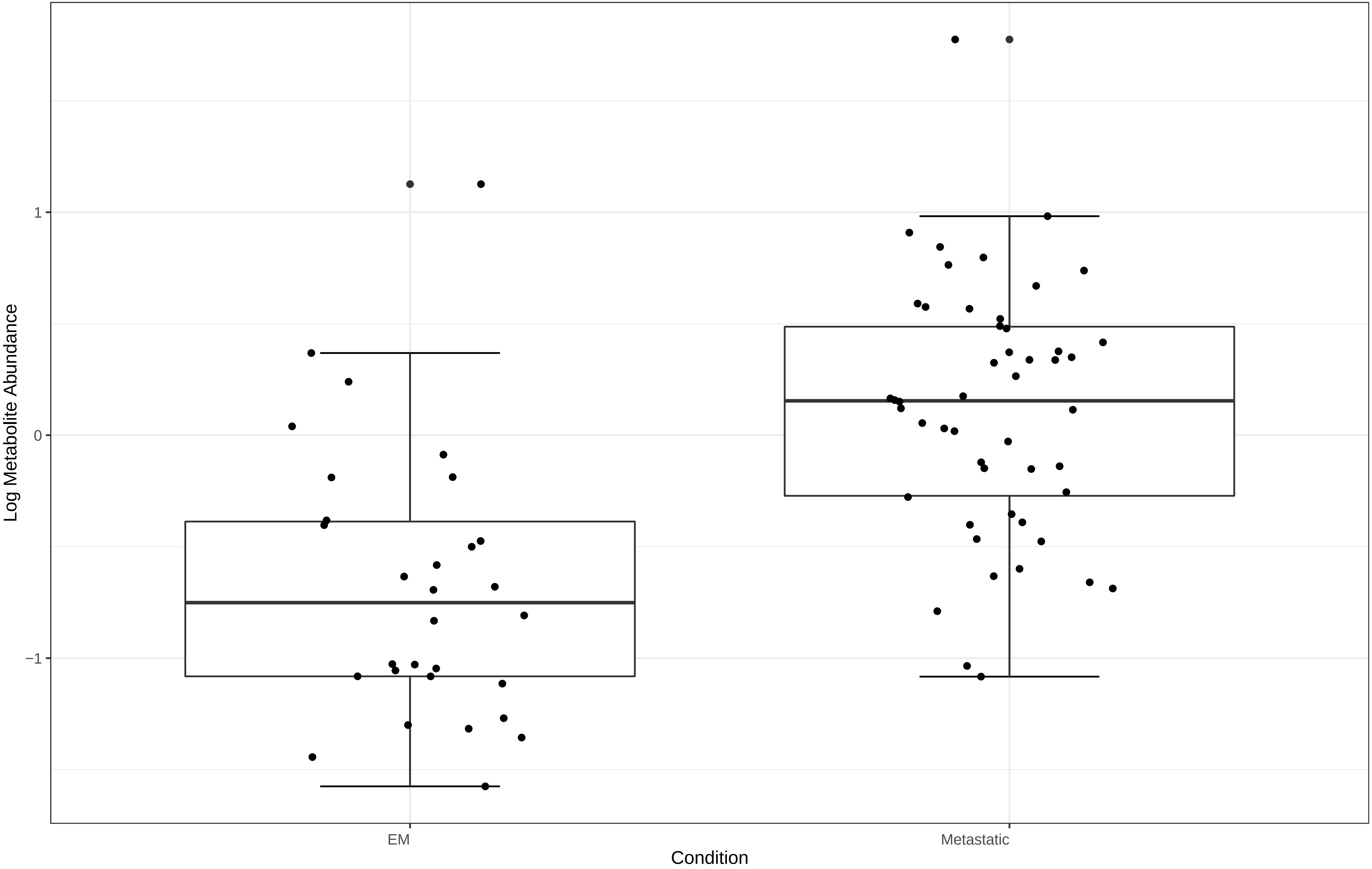

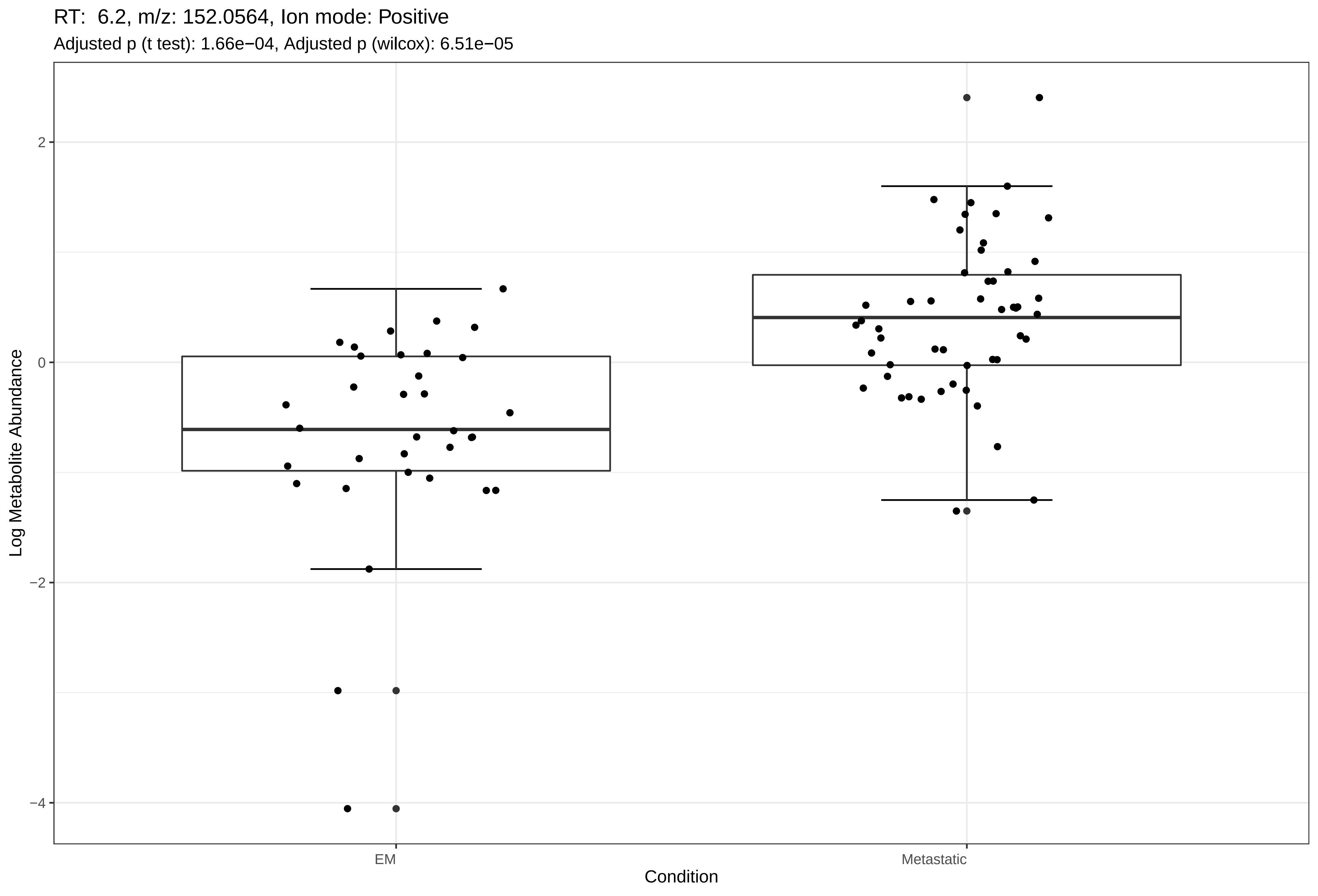

RT: 6.2, m/z: 152.0564, Ion mode: Positive
Adjusted p (t test): 1.66e−04, Adjusted p (wilcox): 6.51e−05
Log Metabolite Abundance
2
0
−2
−4
EM
Metastatic
Condition

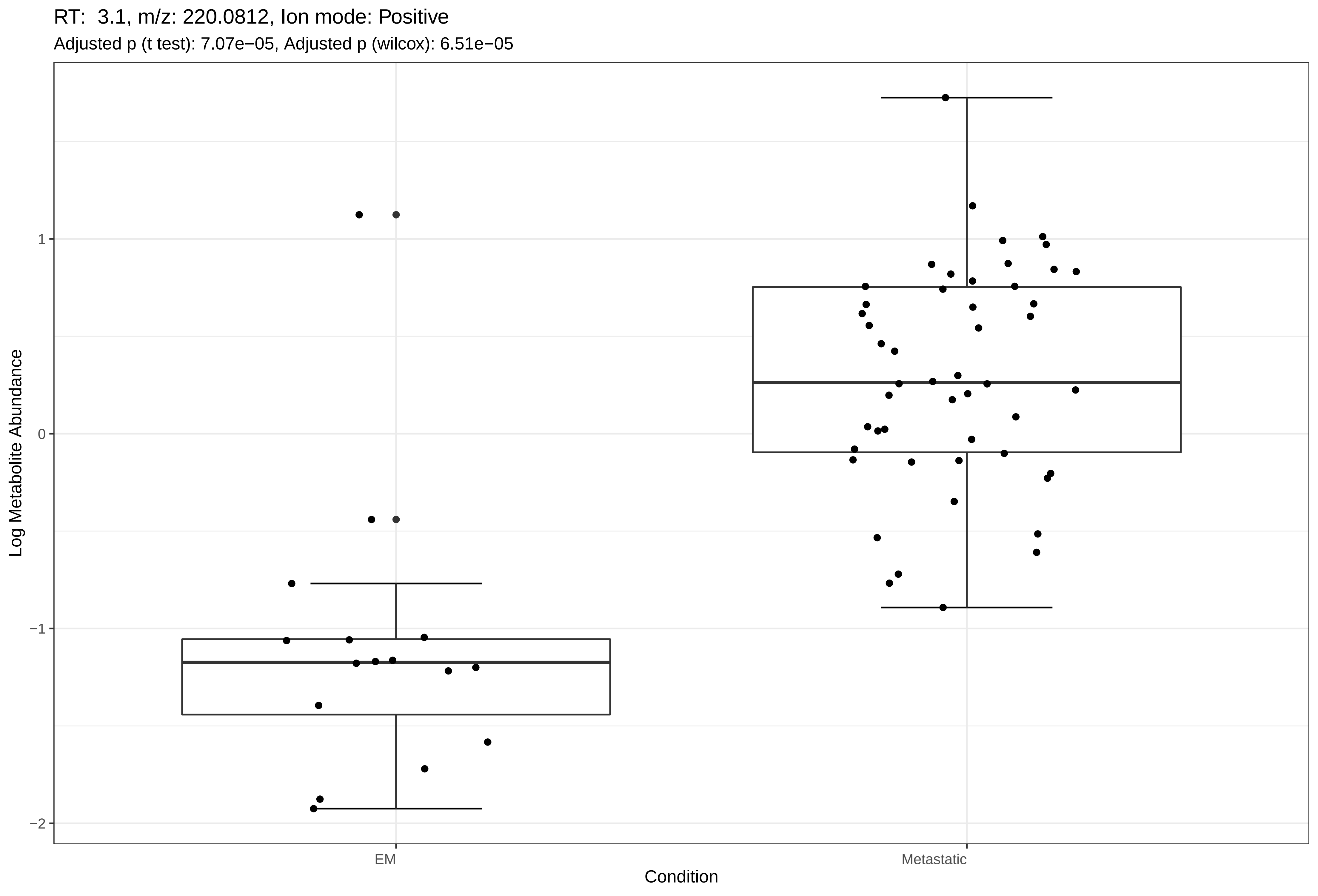

RT: 3.1, m/z: 220.0812, Ion mode: Positive
Adjusted p (t test): 7.07e−05, Adjusted p (wilcox): 6.51e−05
Log Metabolite Abundance
1
0
−1
−2
EM
Metastatic
Condition

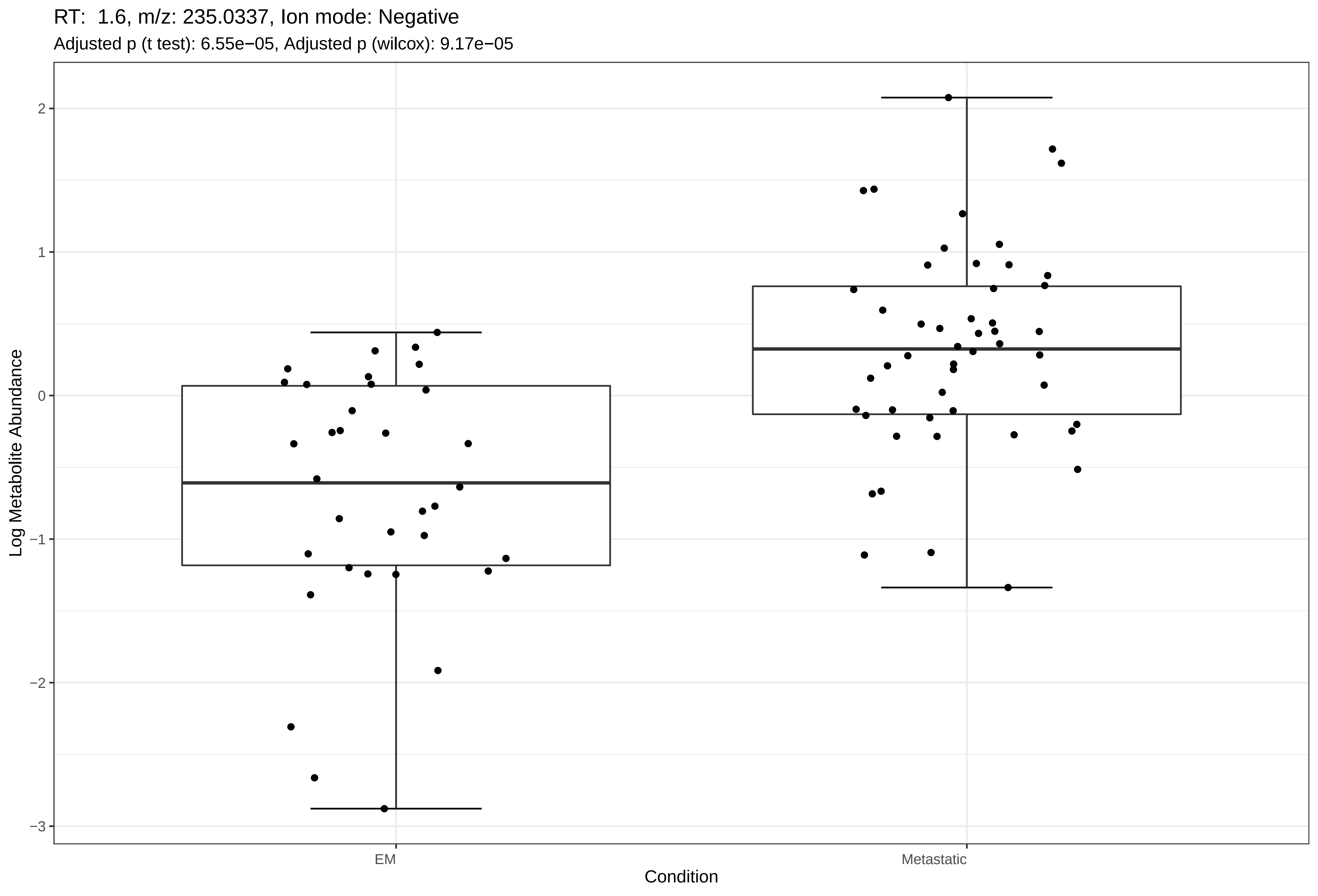

RT: 1.6, m/z: 235.0337, Ion mode: Negative
Adjusted p (t test): 6.55e−05, Adjusted p (wilcox): 9.17e−05
Log Metabolite Abundance
2
1
0
−1
−2
−3
EM
Metastatic
Condition

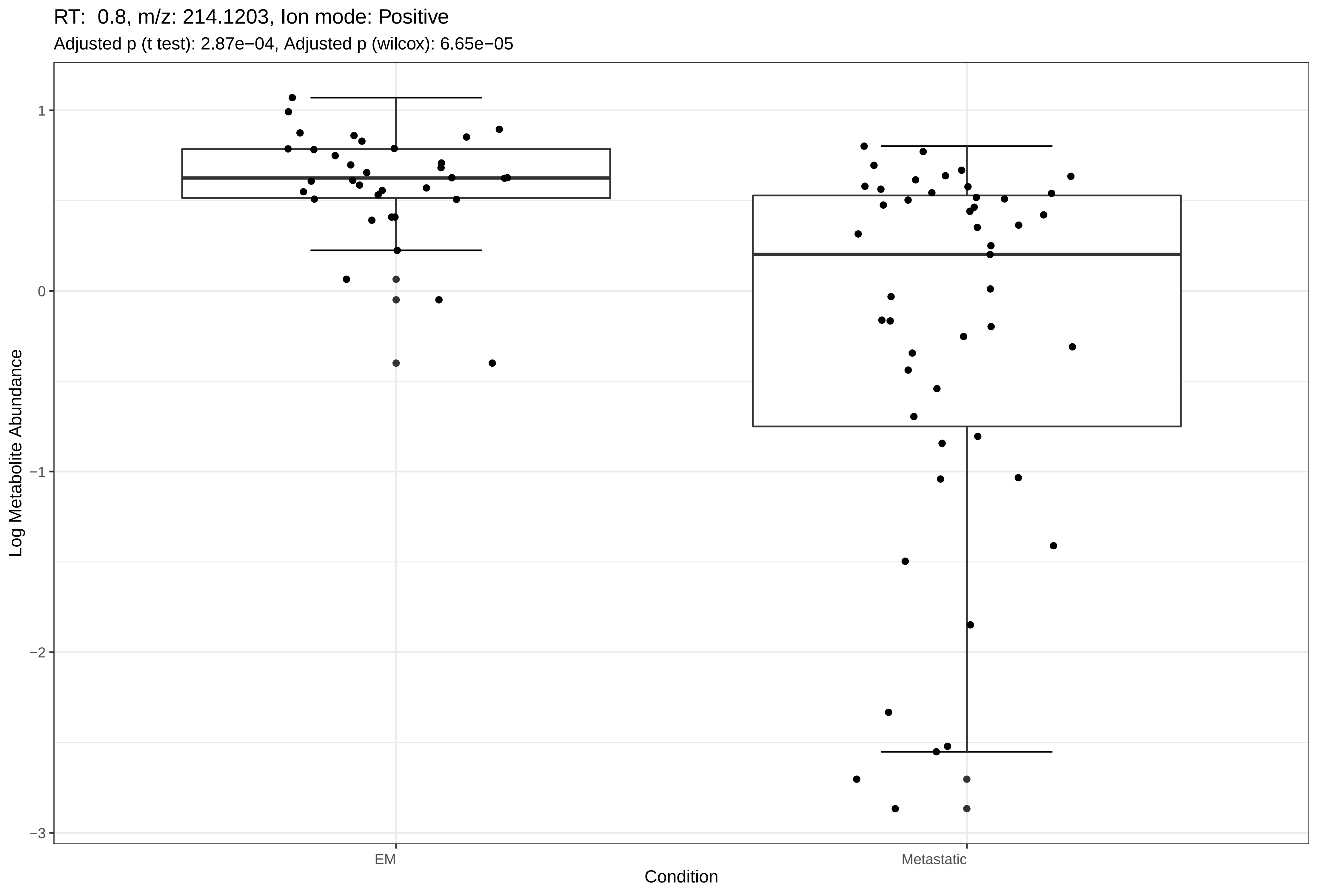

RT: 0.8, m/z: 214.1203, Ion mode: Positive
Adjusted p (t test): 2.87e−04, Adjusted p (wilcox): 6.65e−05
Log Metabolite Abundance
1
0
−1
−2
−3
EM
Metastatic
Condition

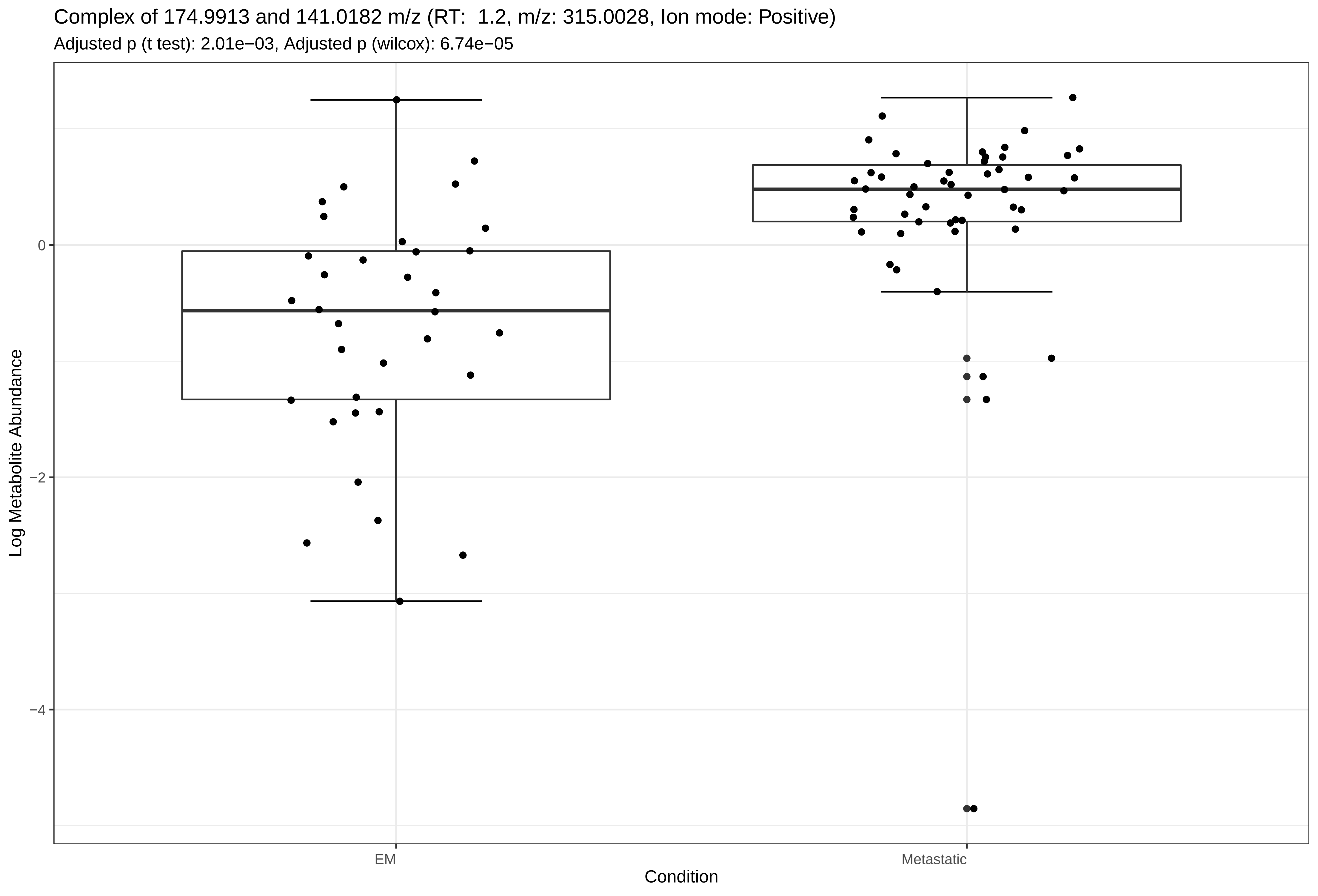

Complex of 174.9913 and 141.0182 m/z (RT: 1.2, m/z: 315.0028, Ion mode: Positive)
Adjusted p (t test): 2.01e−03, Adjusted p (wilcox): 6.74e−05
Log Metabolite Abundance
0
−2
−4
EM
Metastatic
Condition

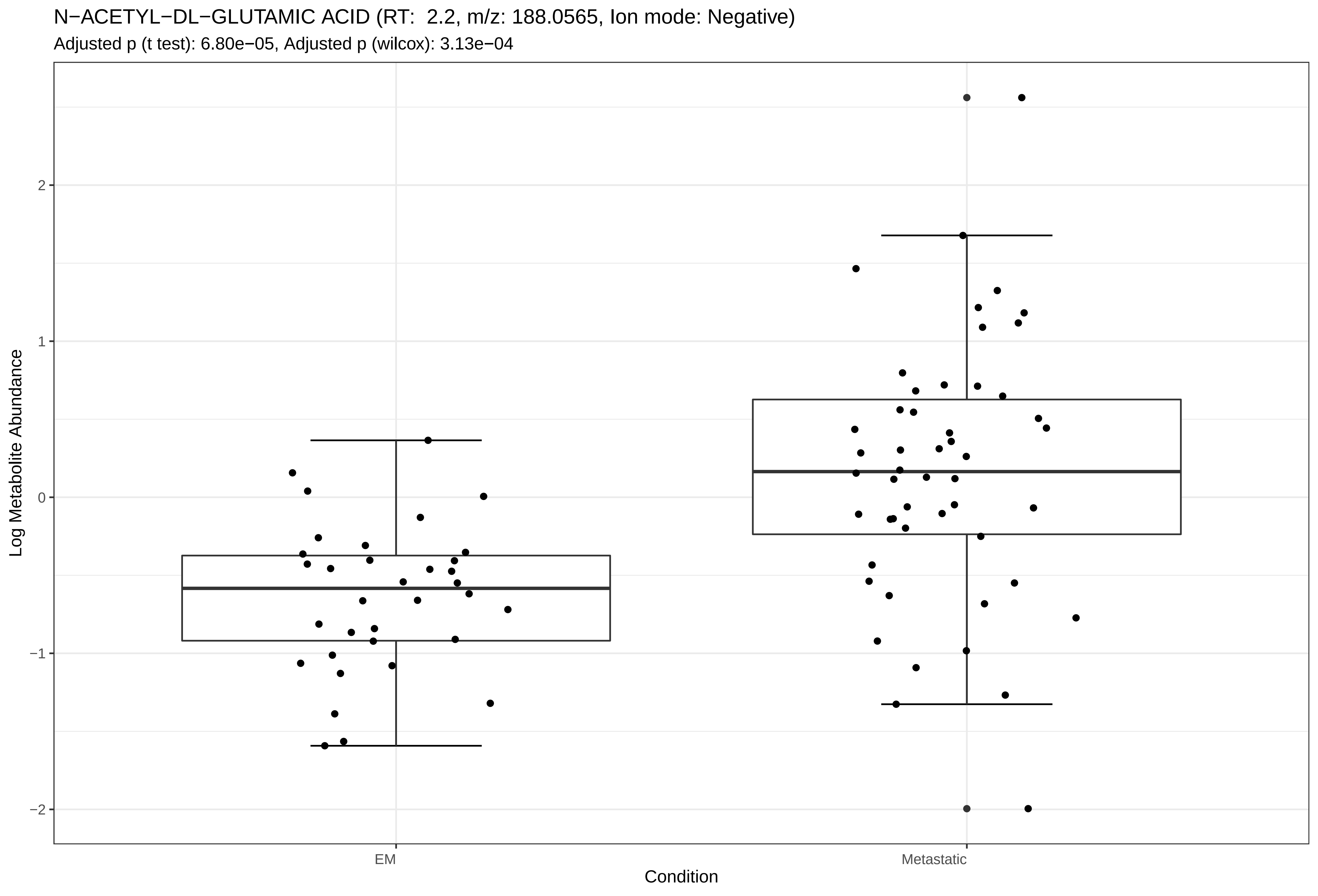

N−ACETYL−DL−GLUTAMIC ACID (RT: 2.2, m/z: 188.0565, Ion mode: Negative)
Adjusted p (t test): 6.80e−05, Adjusted p (wilcox): 3.13e−04
Log Metabolite Abundance
2
1
0
−1
−2
EM
Metastatic
Condition

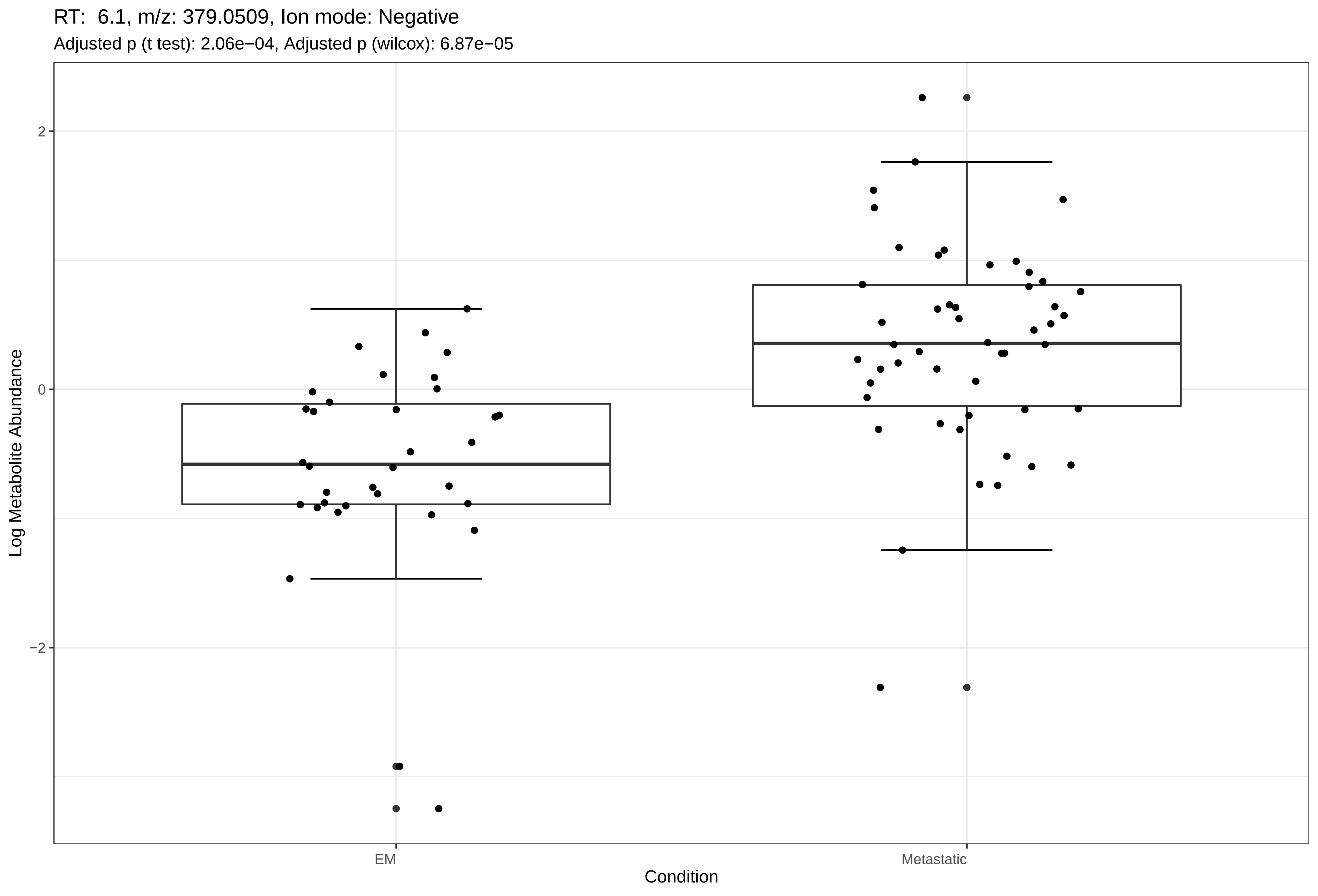
RT: 6.1, m/z: 379.0509, Ion mode: Negative
Adjusted p (t test): 2.06e−04, Adjusted p (wilcox): 6.87e−05
2
0
−2
Log Metabolite Abundance
EM
Metastatic
Condition

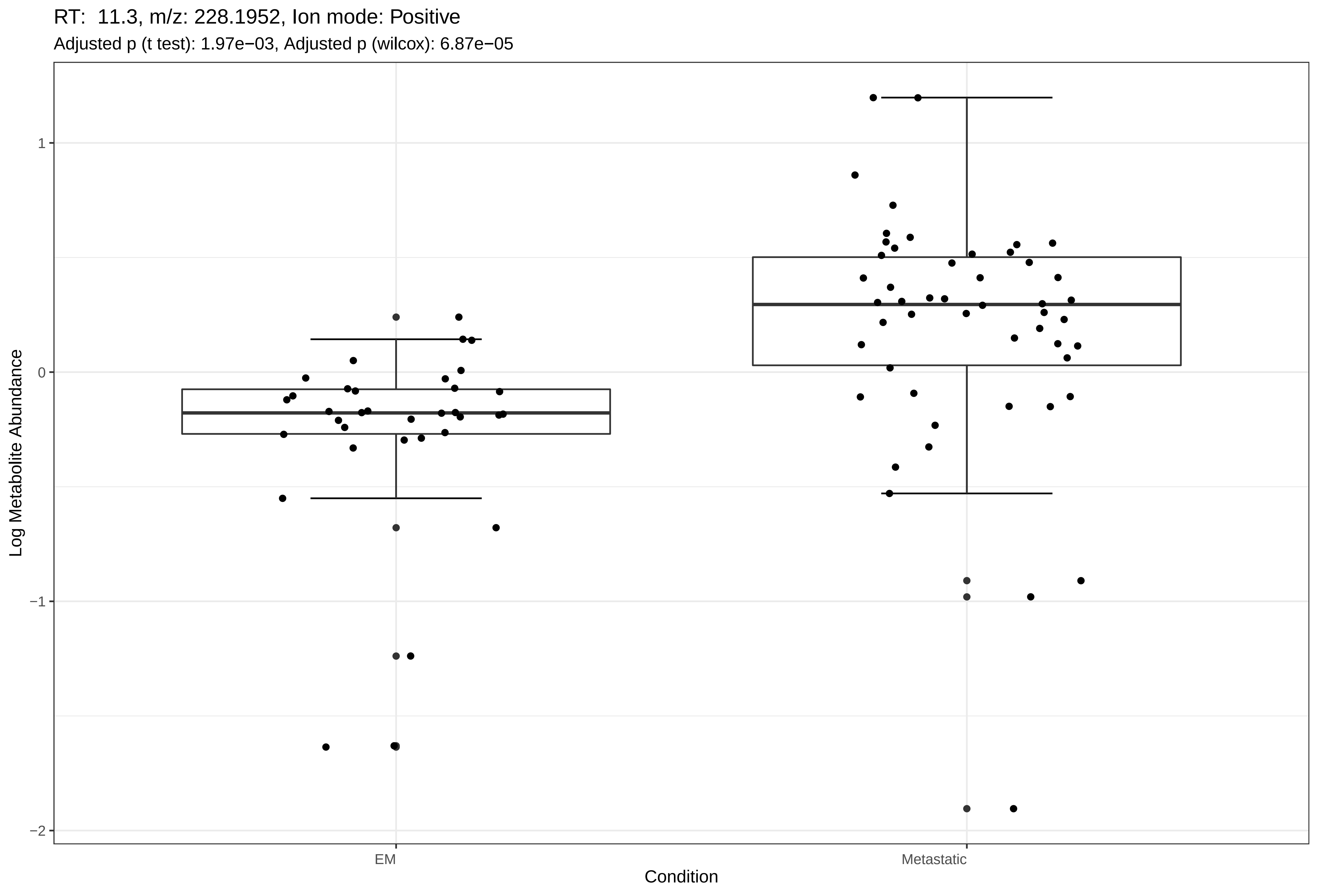
RT: 11.3, m/z: 228.1952, Ion mode: Positive
Adjusted p (t test): 1.97e−03, Adjusted p (wilcox): 6.87e−05
Log Metabolite Abundance
1
0
−1
−2
EM
Metastatic
Condition

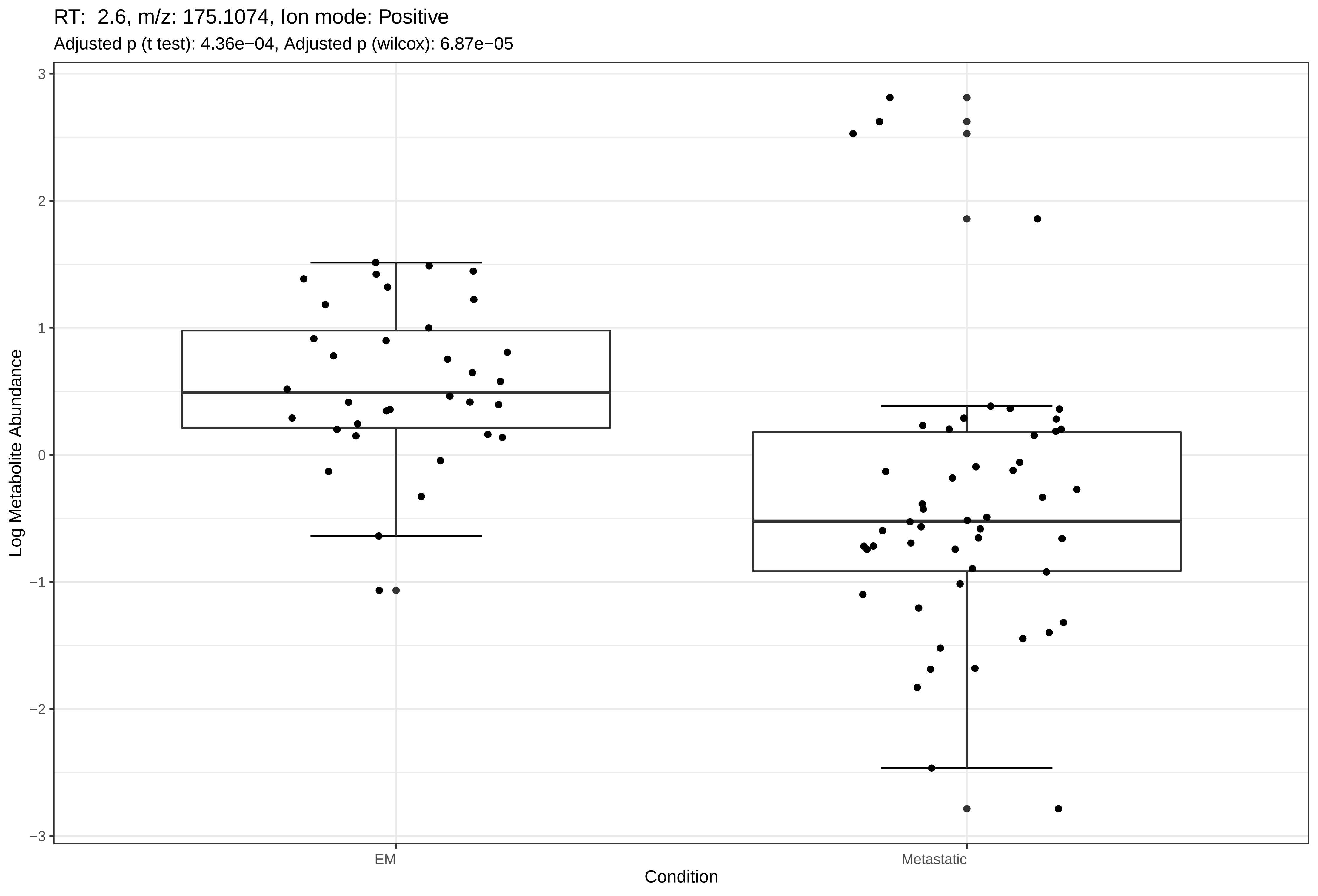

RT: 2.6, m/z: 175.1074, Ion mode: Positive
Adjusted p (t test): 4.36e−04, Adjusted p (wilcox): 6.87e−05
Log Metabolite Abundance
3
2
1
0
−1
−2
−3
EM
Metastatic
Condition

[M+Na−H] 21.9825 m/z adduct of 204.1226 m/z (RT: 4.5, m/z: 226.1044, Ion mode: Positive)

Adjusted p (t test): 1.46e−03, Adjusted p (wilcox): 6.87e−05

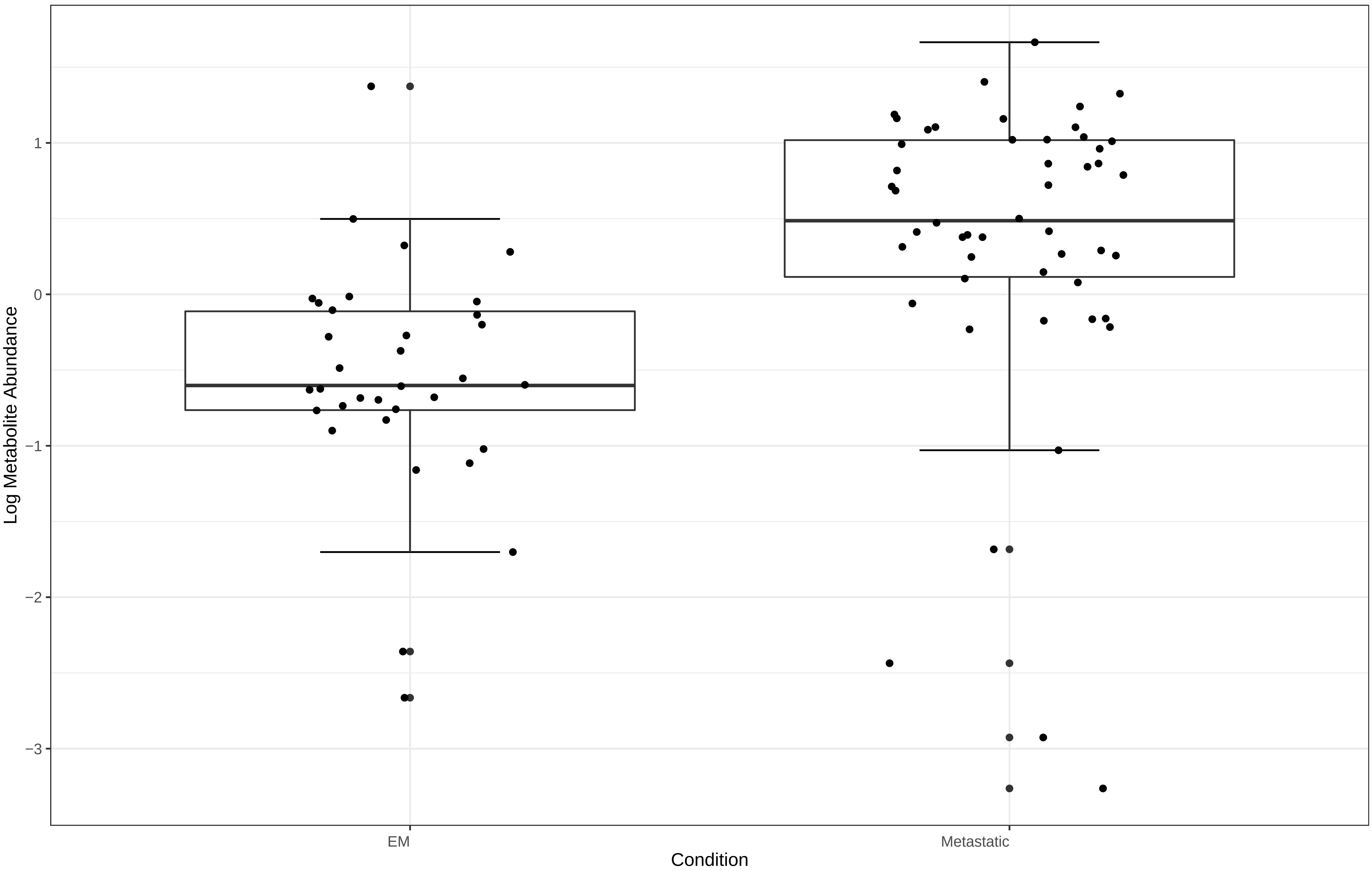

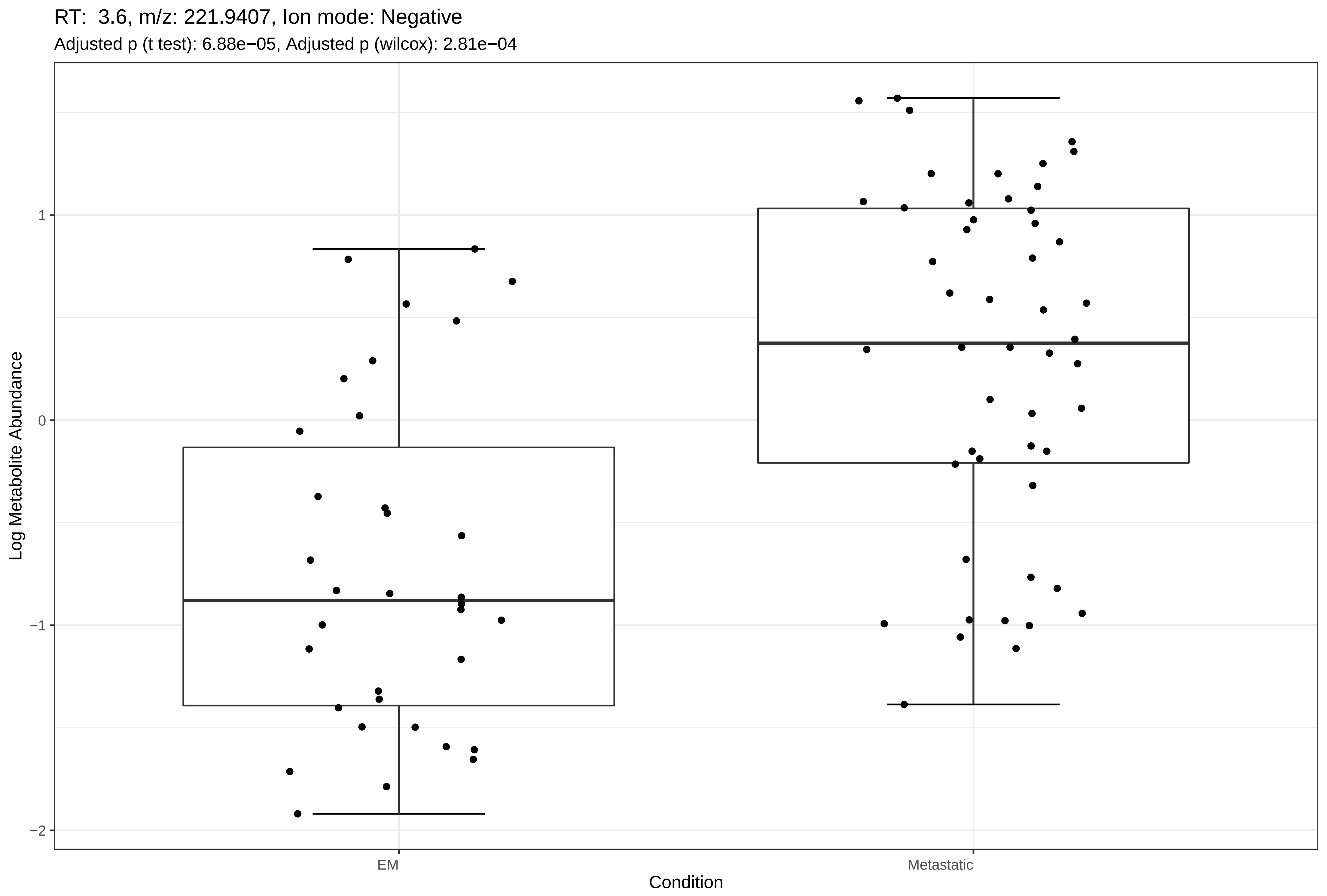

RT: 3.6, m/z: 221.9407, Ion mode: Negative
Adjusted p (t test): 6.88e−05, Adjusted p (wilcox): 2.81e−04
Log Metabolite Abundance
1
0
−1
−2
EM
Metastatic
Condition

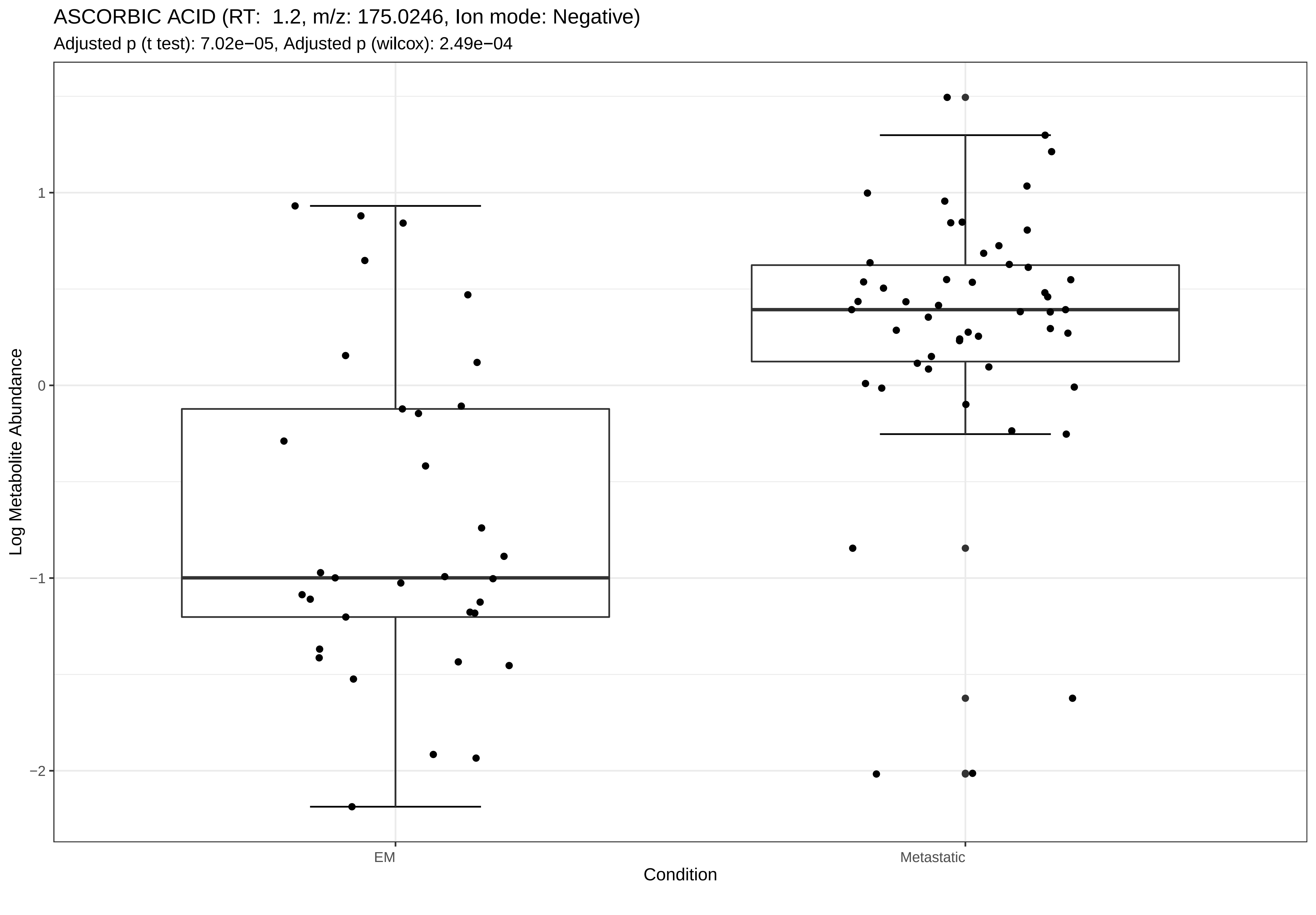
ASCORBIC ACID (RT: 1.2, m/z: 175.0246, Ion mode: Negative)
Adjusted p (t test): 7.02e−05, Adjusted p (wilcox): 2.49e−04
Log Metabolite Abundance
1
0
−1
−2
EM
Metastatic
Condition

RT: 10.5, m/z: 449.2761, Ion mode: Negative

Adjusted p (t test): 7.08e−05, Adjusted p (wilcox): 7.22e−04

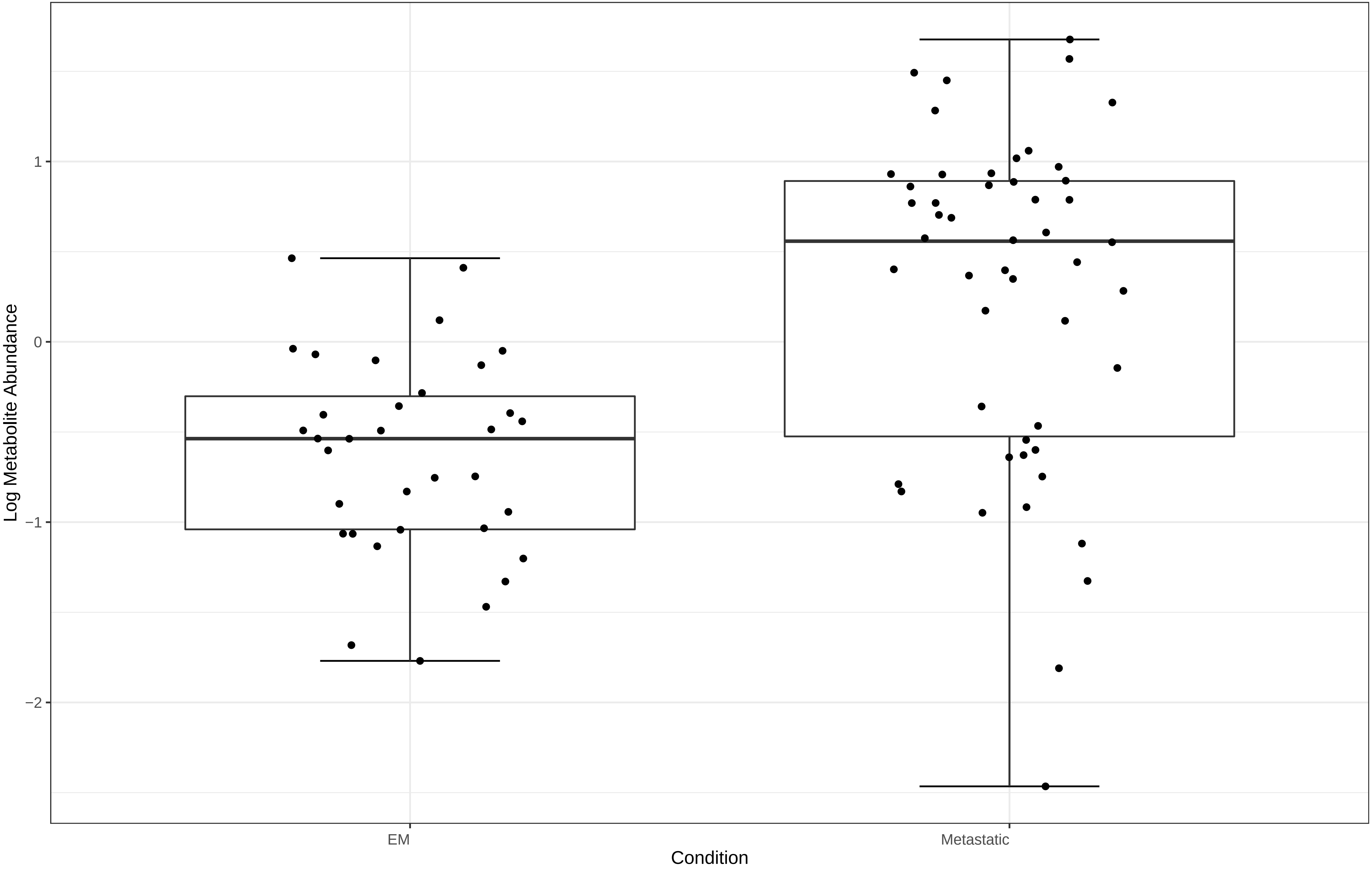

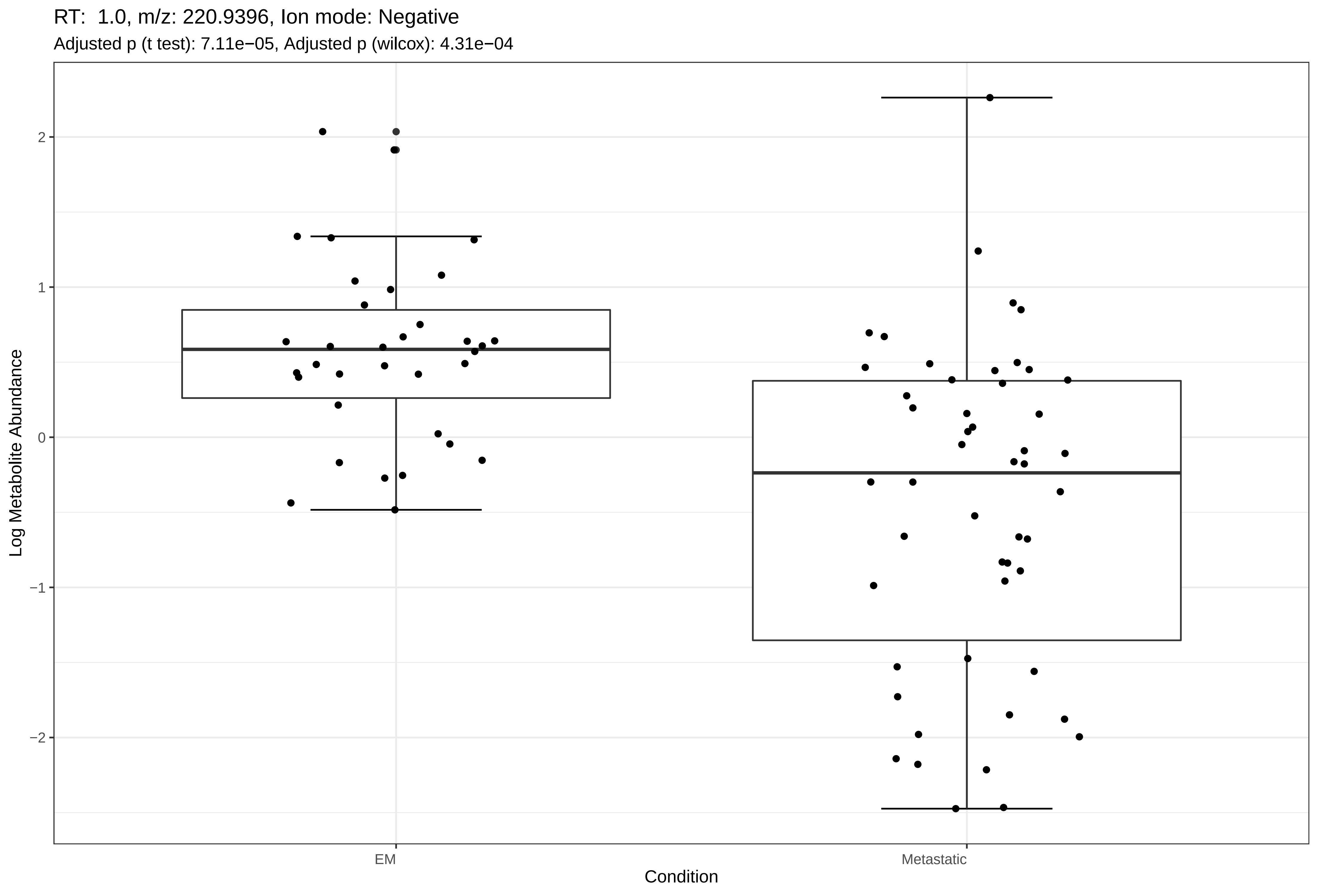

RT: 1.0, m/z: 220.9396, Ion mode: Negative
Adjusted p (t test): 7.11e−05, Adjusted p (wilcox): 4.31e−04
Log Metabolite Abundance
2
1
0
−1
−2
EM
Metastatic
Condition

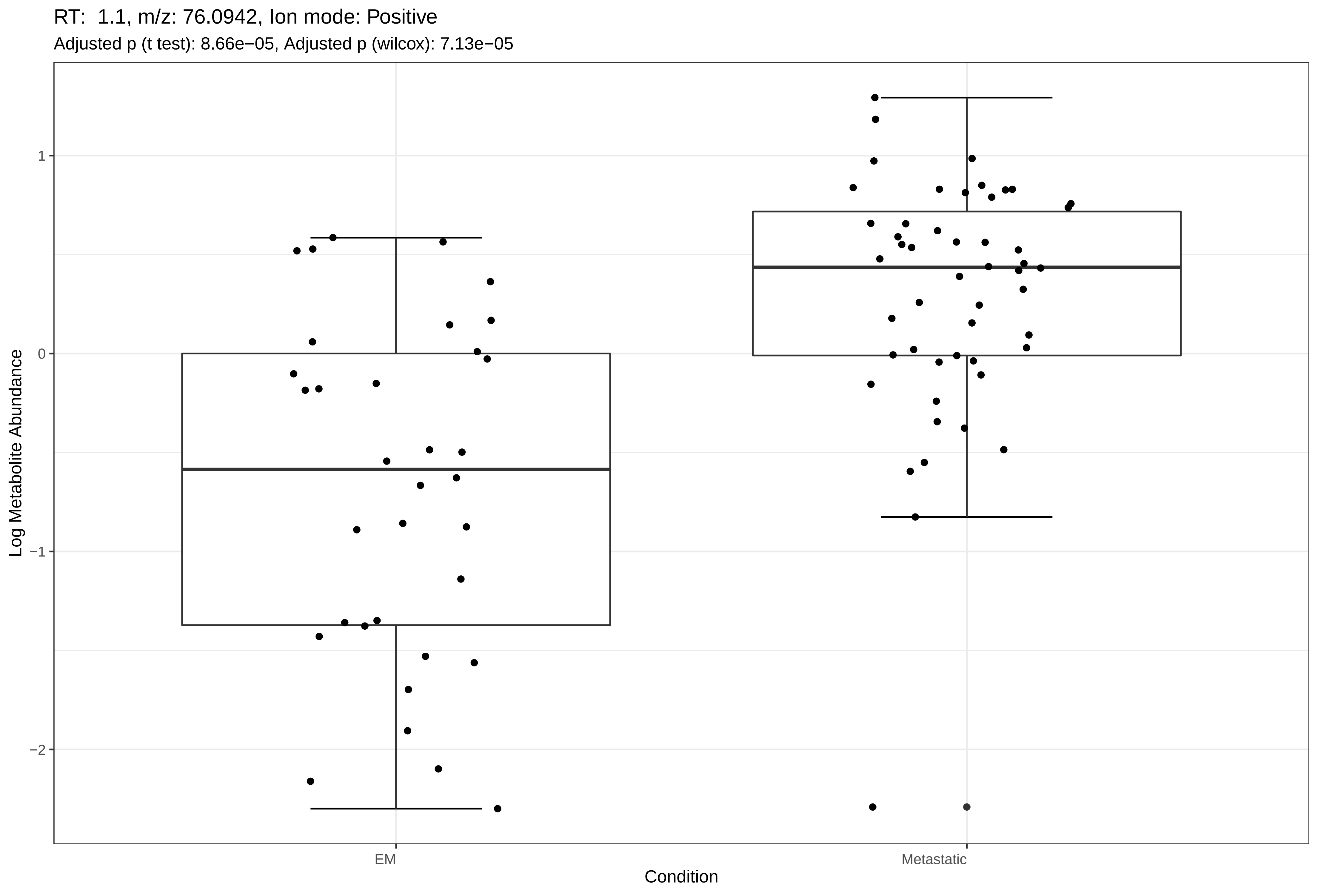
RT: 1.1, m/z: 76.0942, Ion mode: Positive
Adjusted p (t test): 8.66e−05, Adjusted p (wilcox): 7.13e−05
Log Metabolite Abundance
1
0
−1
−2
EM
Metastatic
Condition

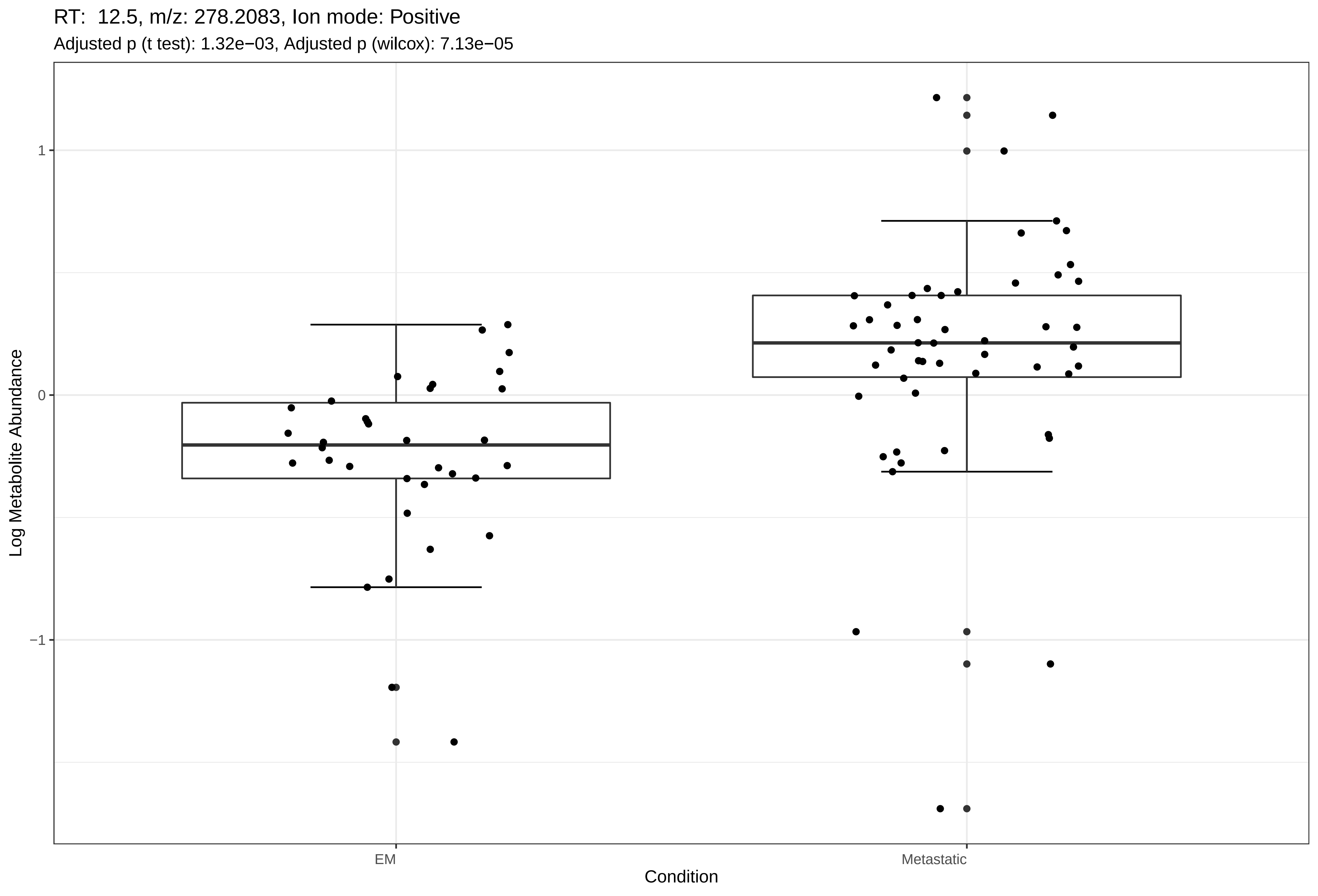

RT: 12.5, m/z: 278.2083, Ion mode: Positive
Adjusted p (t test): 1.32e−03, Adjusted p (wilcox): 7.13e−05
Log Metabolite Abundance
1
0
−1
EM
Metastatic
Condition

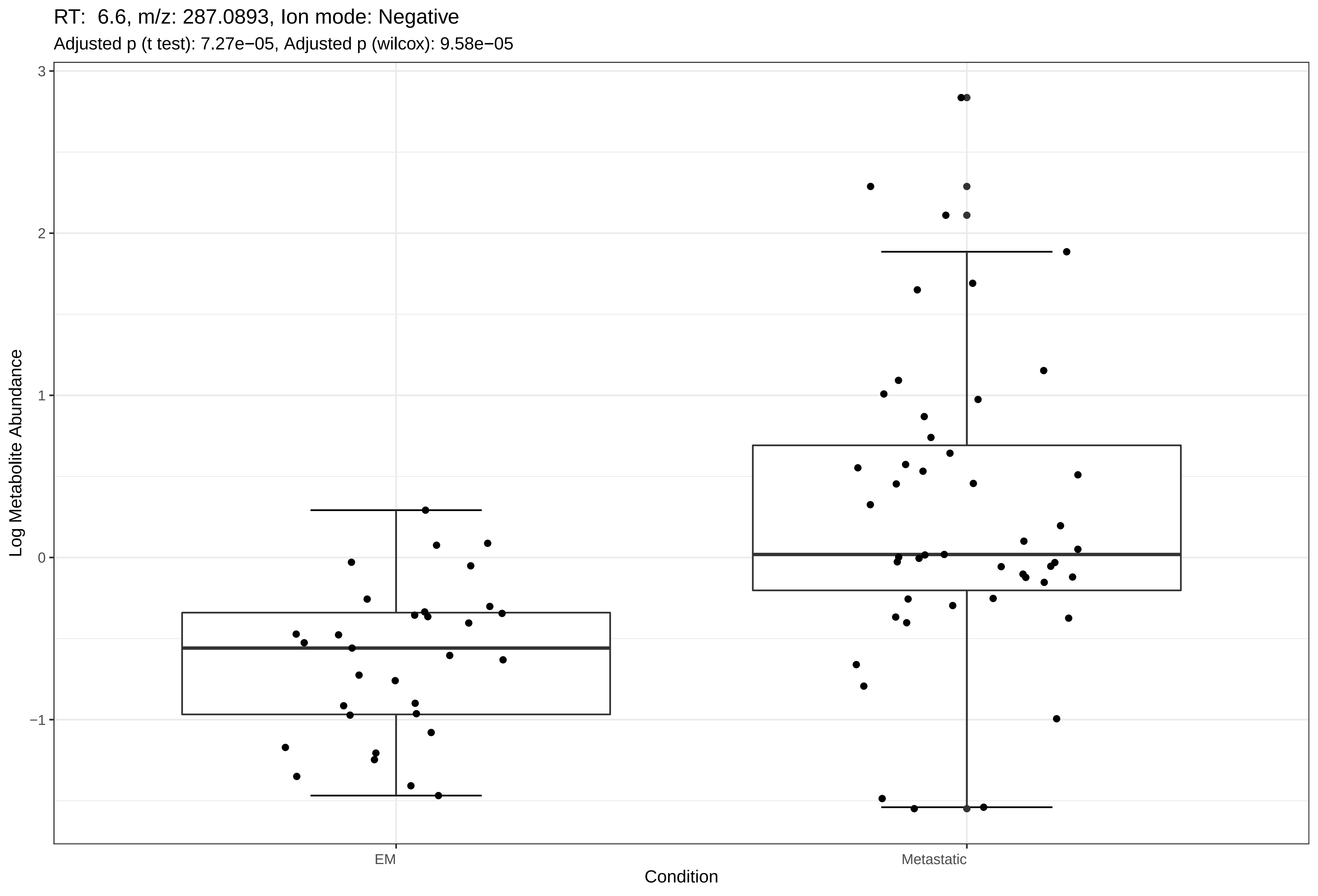
RT: 6.6, m/z: 287.0893, Ion mode: Negative
Adjusted p (t test): 7.27e−05, Adjusted p (wilcox): 9.58e−05
Log Metabolite Abundance
3
2
1
0
−1
EM
Metastatic
Condition

RT: 10.1, m/z: 143.1428, Ion mode: Positive

Adjusted p (t test): 7.30e−05, Adjusted p (wilcox): 4.77e−04

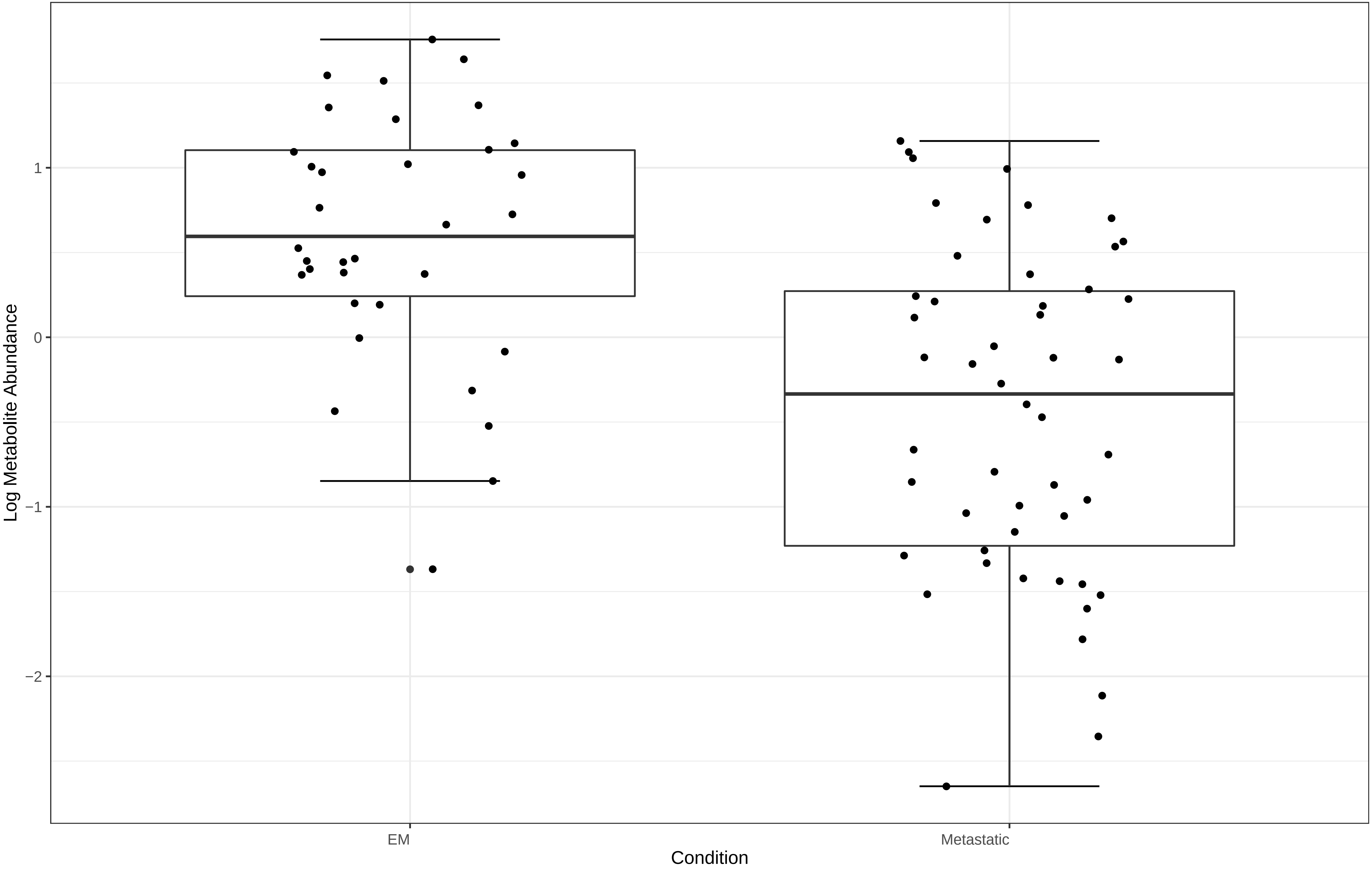

L−KYNURENINE (RT: 6.4, m/z: 209.0916, Ion mode: Positive)

Adjusted p (t test): 7.38e−05, Adjusted p (wilcox): 5.87e−04

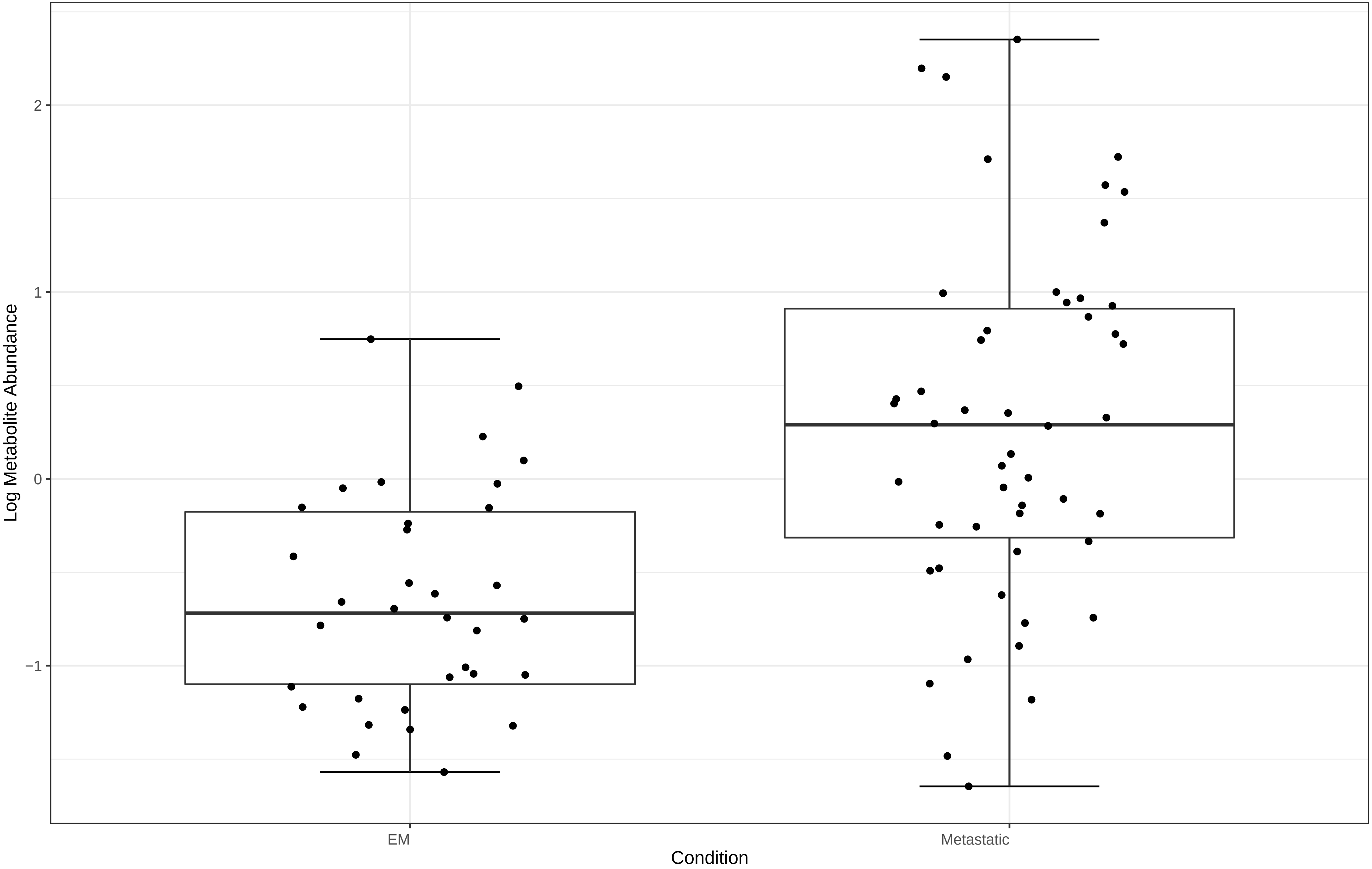

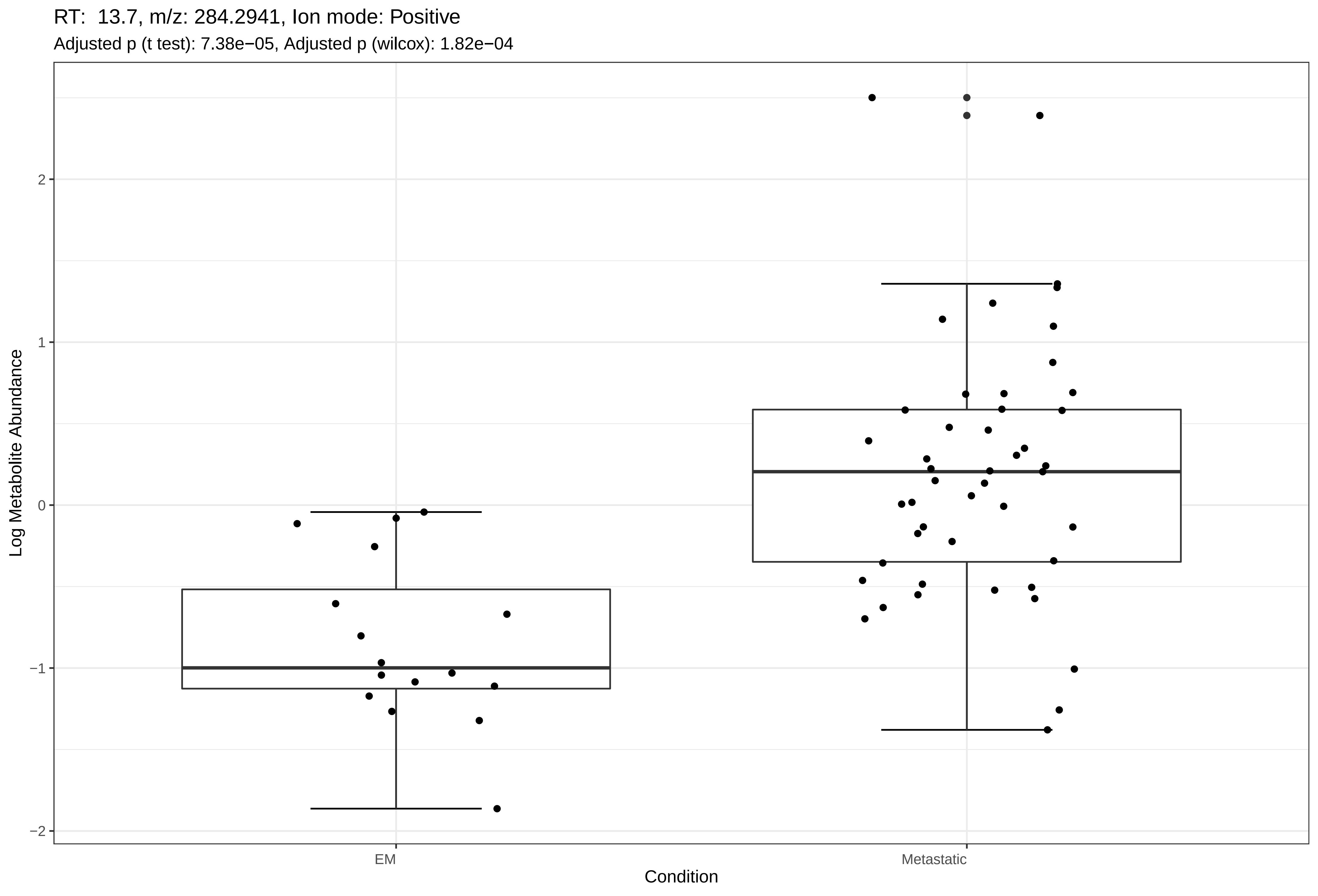

RT: 13.7, m/z: 284.2941, Ion mode: Positive
Adjusted p (t test): 7.38e−05, Adjusted p (wilcox): 1.82e−04
Log Metabolite Abundance
2
1
0
−1
−2
EM
Metastatic
Condition

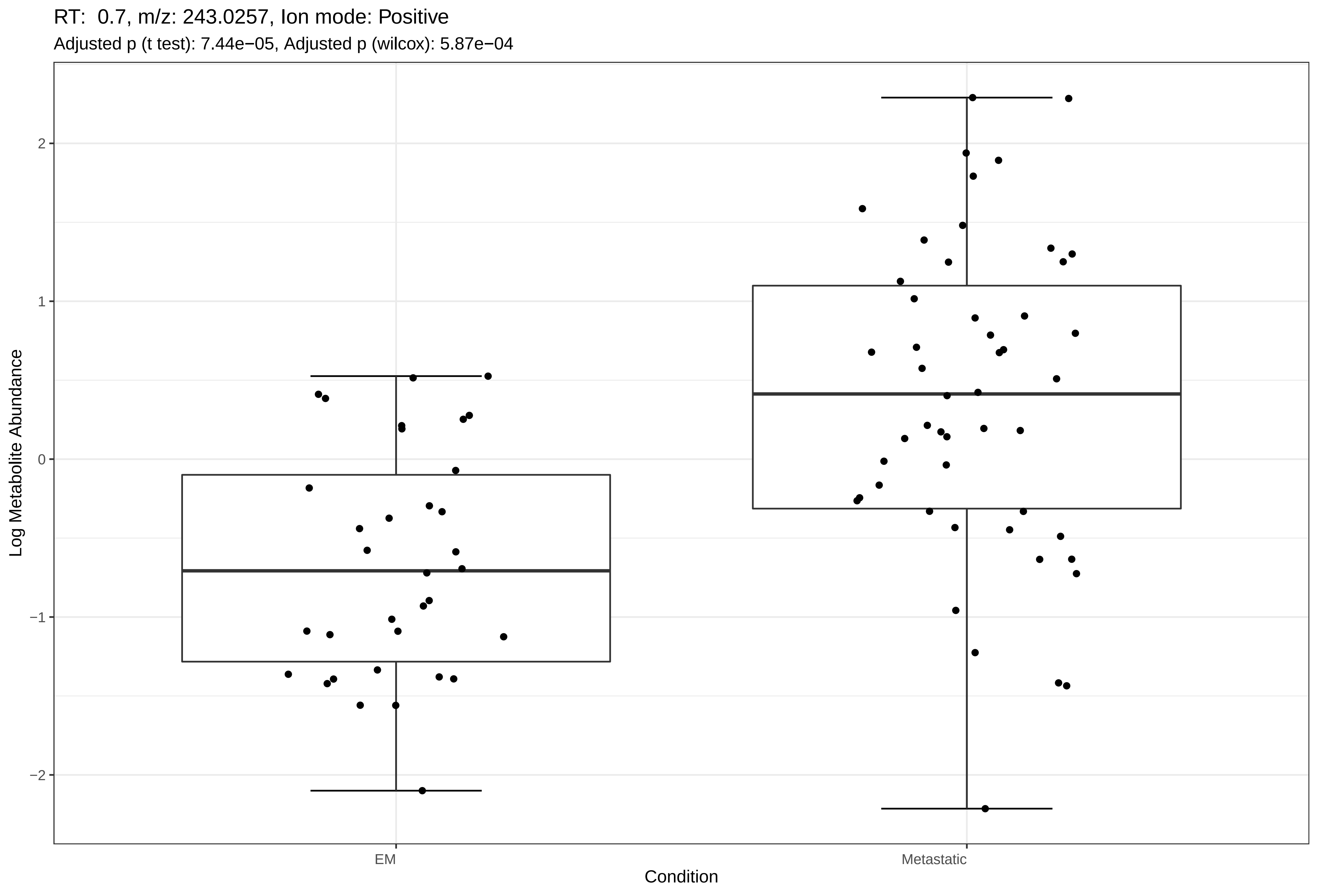
RT: 0.7, m/z: 243.0257, Ion mode: Positive
Adjusted p (t test): 7.44e−05, Adjusted p (wilcox): 5.87e−04
Log Metabolite Abundance
2
1
0
−1
−2
EM
Metastatic
Condition

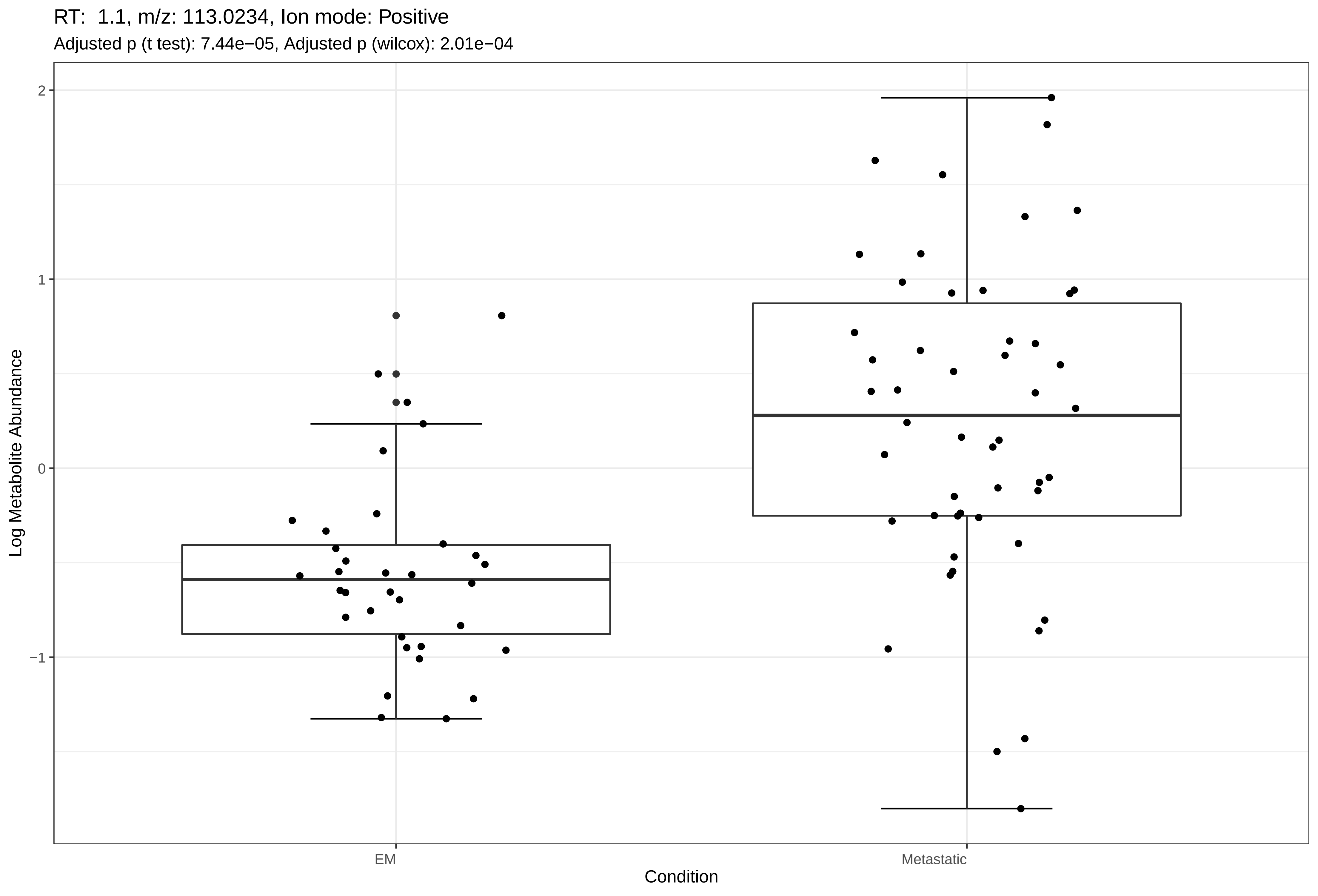
RT: 1.1, m/z: 113.0234, Ion mode: Positive
Adjusted p (t test): 7.44e−05, Adjusted p (wilcox): 2.01e−04
Log Metabolite Abundance
2
1
0
−1
EM
Metastatic
Condition

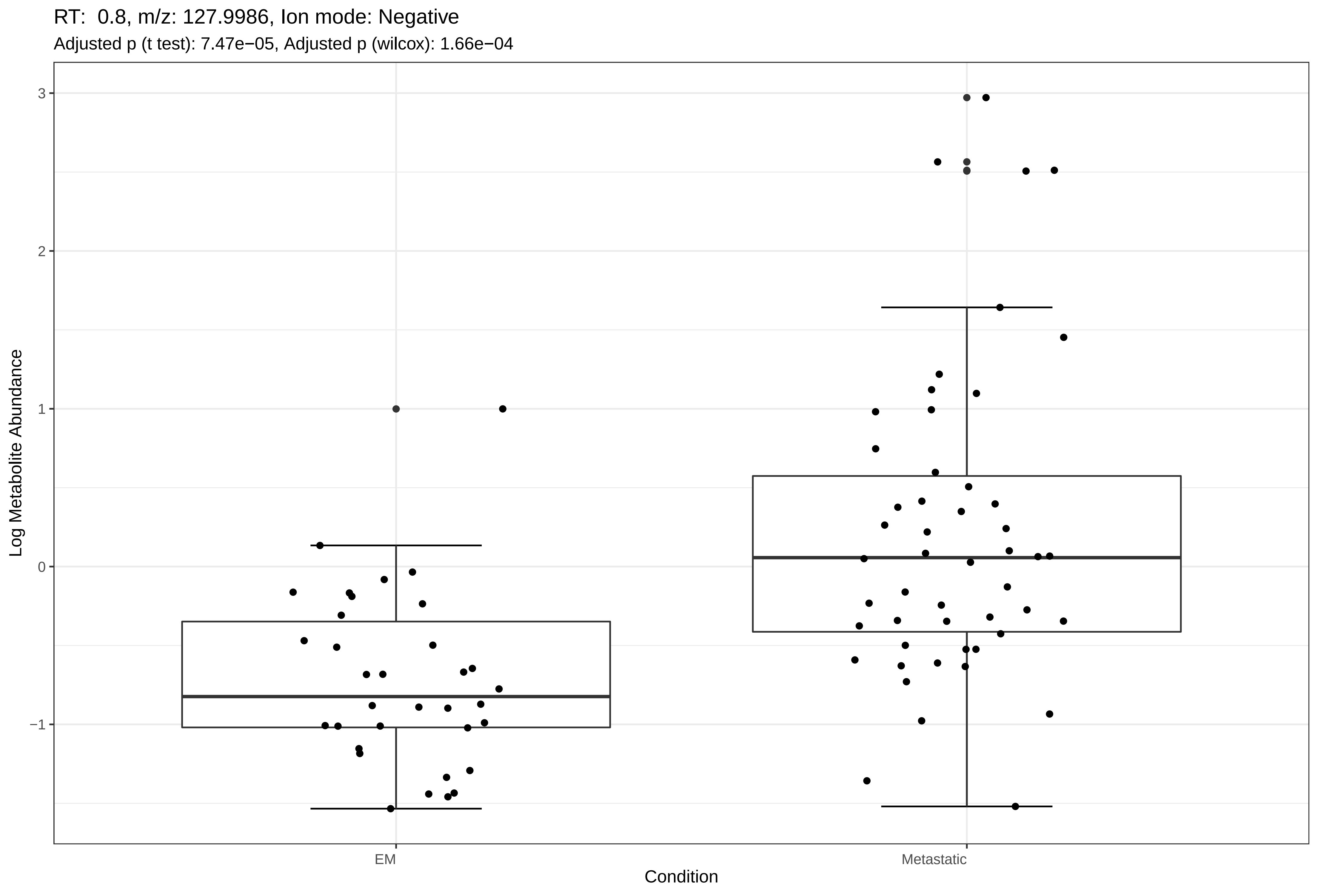

RT: 0.8, m/z: 127.9986, Ion mode: Negative
Adjusted p (t test): 7.47e−05, Adjusted p (wilcox): 1.66e−04
Log Metabolite Abundance
3
2
1
0
−1
EM
Metastatic
Condition

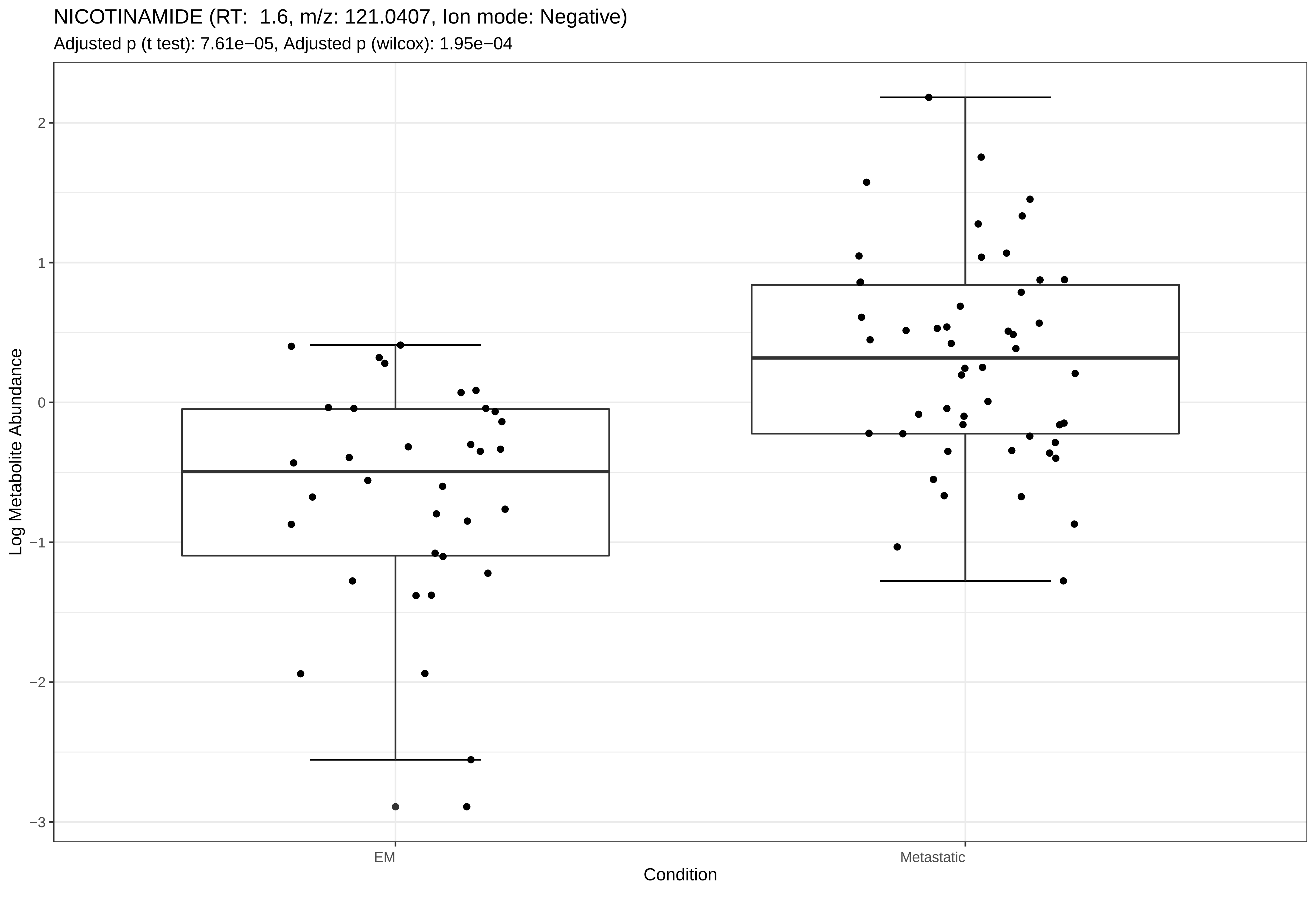
NICOTINAMIDE (RT: 1.6, m/z: 121.0407, Ion mode: Negative)
Adjusted p (t test): 7.61e−05, Adjusted p (wilcox): 1.95e−04
Log Metabolite Abundance
2
1
0
−1
−2
−3
EM
Metastatic
Condition

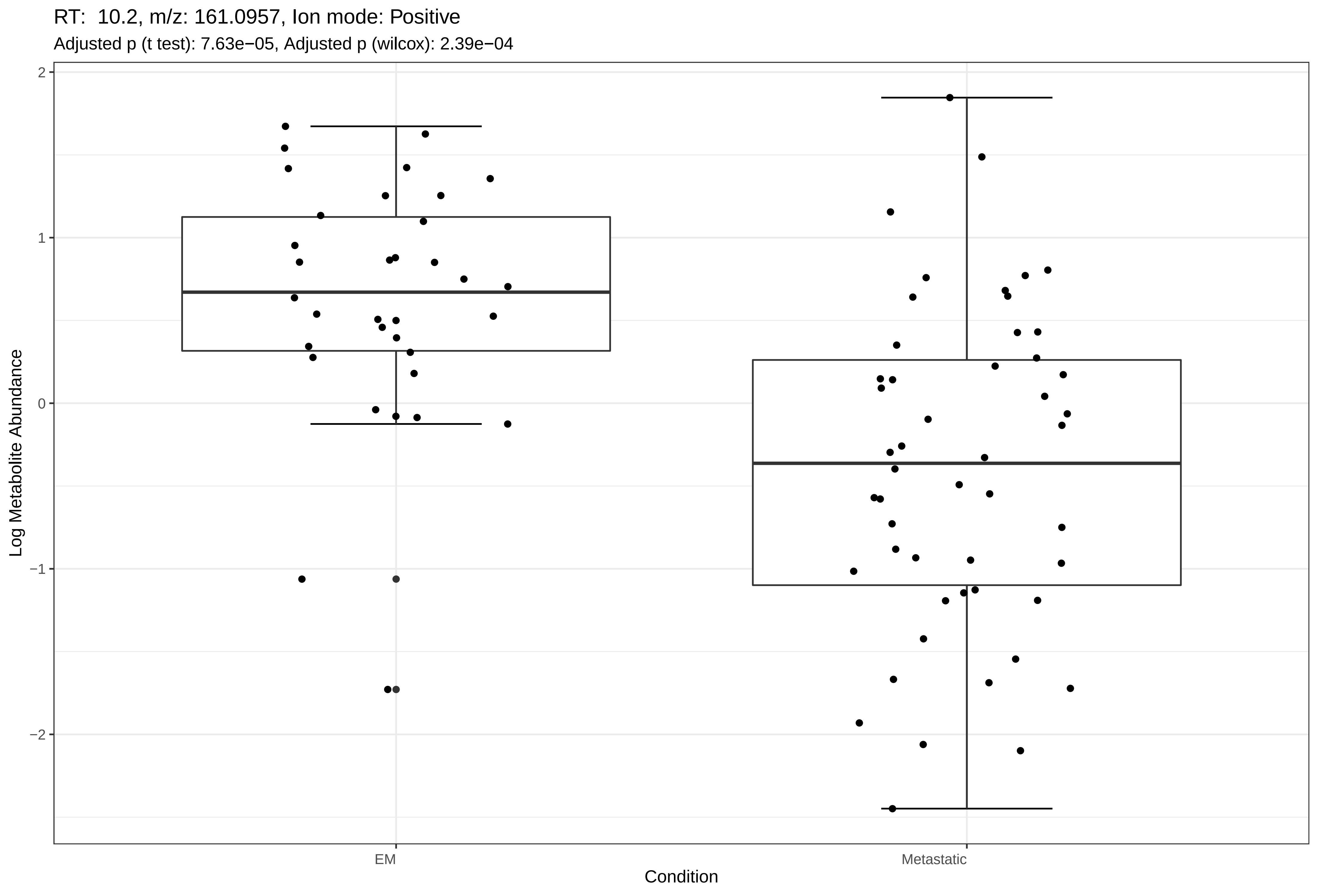
RT: 10.2, m/z: 161.0957, Ion mode: Positive
Adjusted p (t test): 7.63e−05, Adjusted p (wilcox): 2.39e−04
Log Metabolite Abundance
2
1
0
−1
−2
EM
Metastatic
Condition

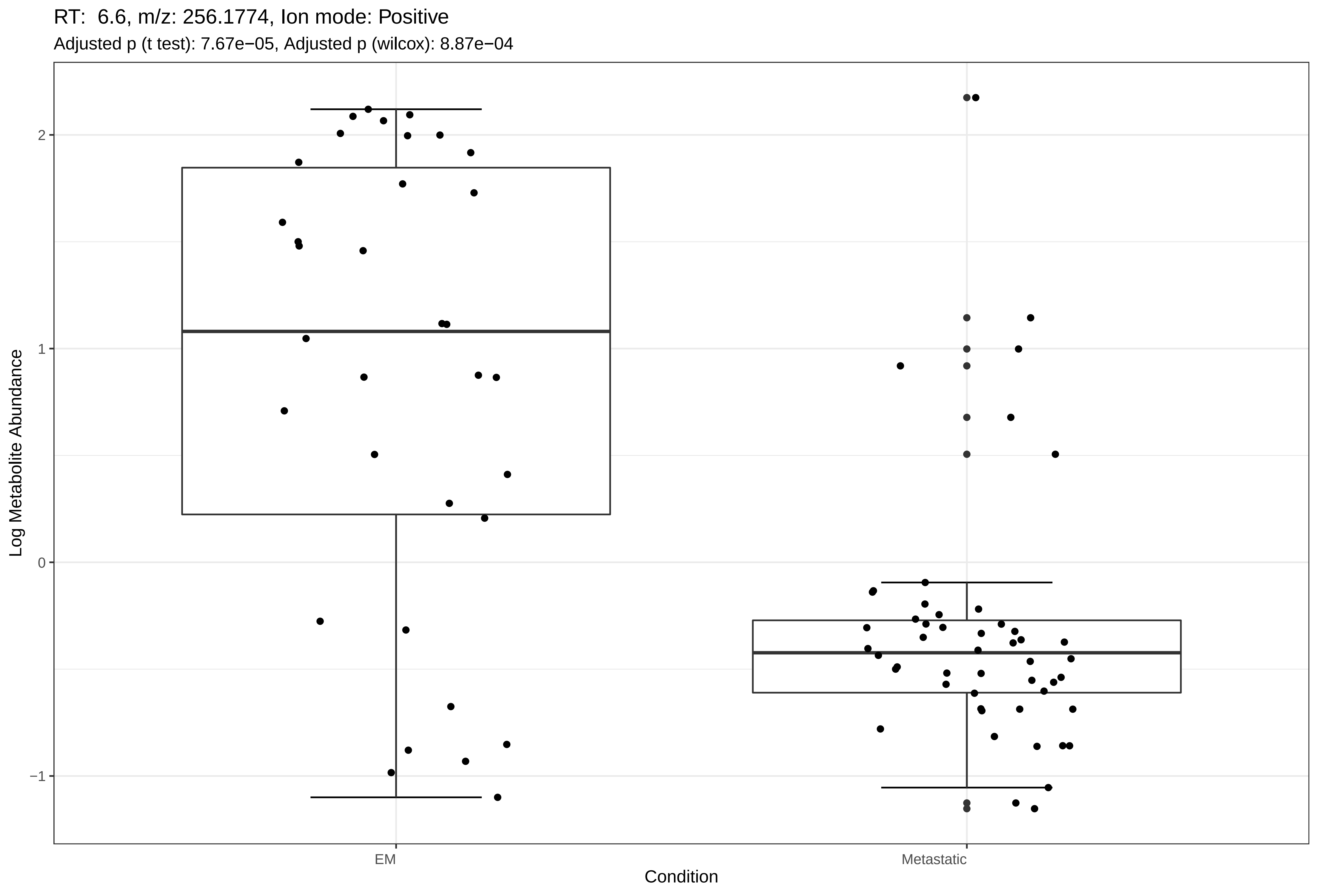

RT: 6.6, m/z: 256.1774, Ion mode: Positive
Adjusted p (t test): 7.67e−05, Adjusted p (wilcox): 8.87e−04
Log Metabolite Abundance
2
1
0
−1
EM
Metastatic
Condition

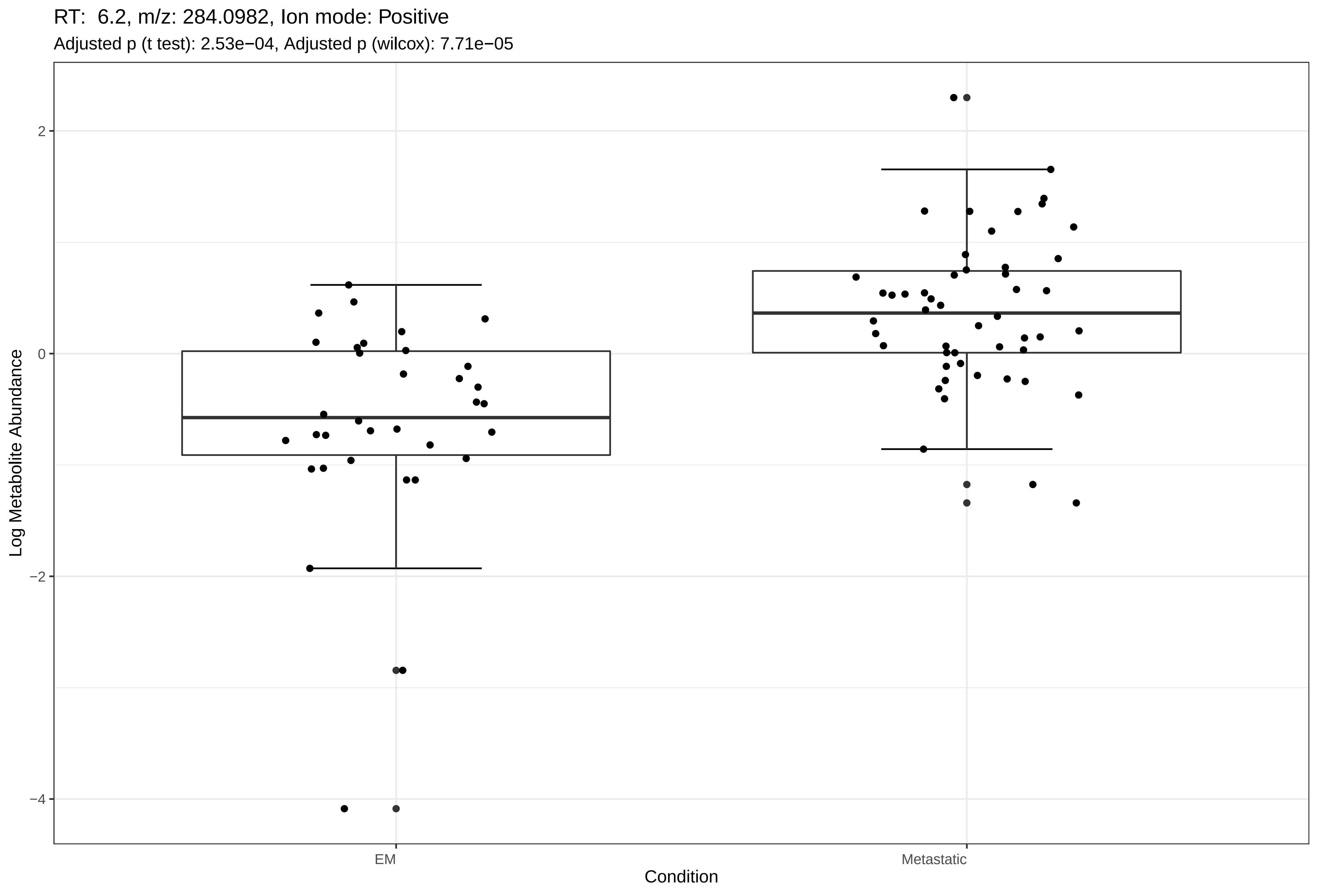

RT: 6.2, m/z: 284.0982, Ion mode: Positive
Adjusted p (t test): 2.53e−04, Adjusted p (wilcox): 7.71e−05
Log Metabolite Abundance
2
0
−2
−4
EM
Metastatic
Condition

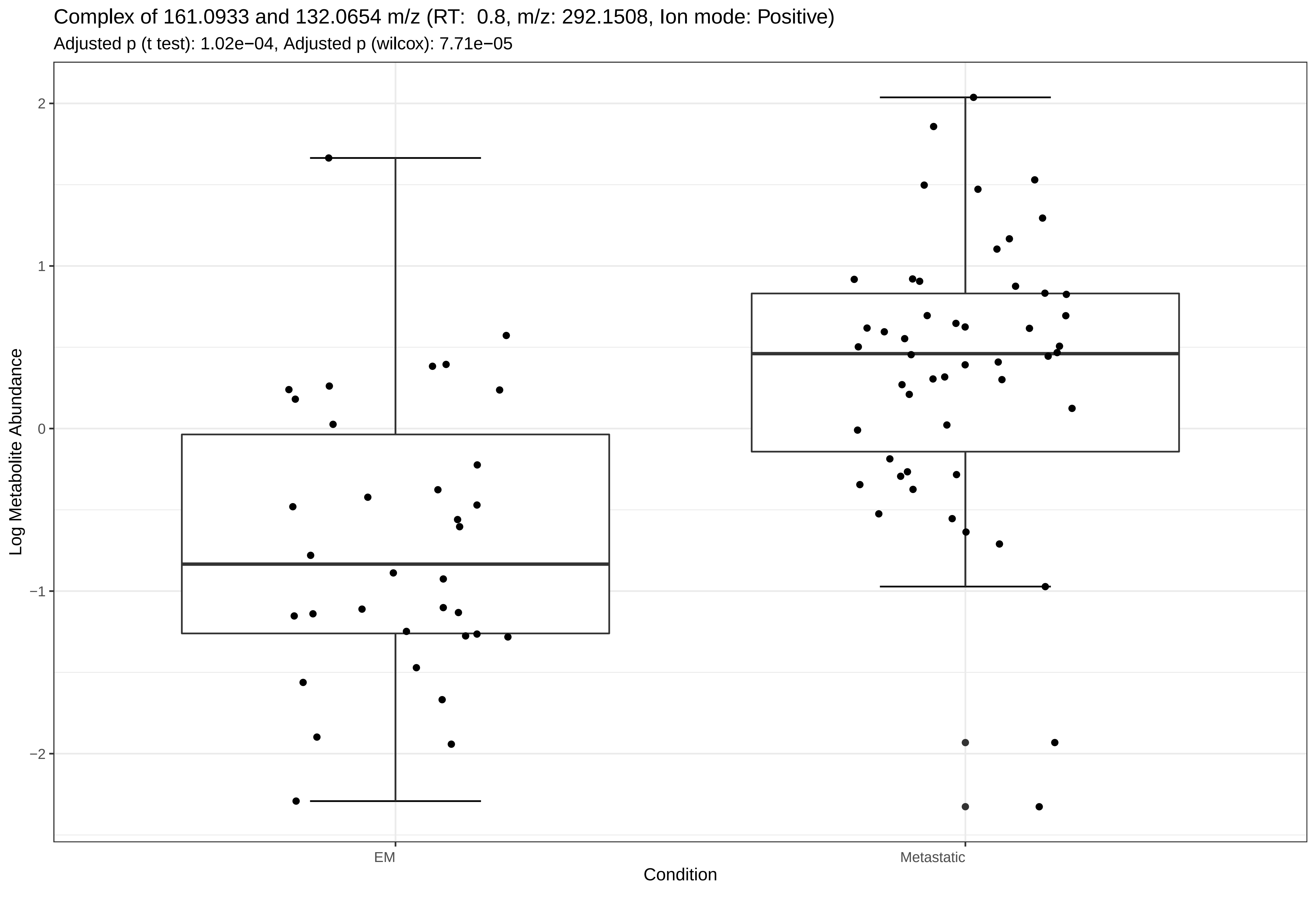

Complex of 161.0933 and 132.0654 m/z (RT: 0.8, m/z: 292.1508, Ion mode: Positive)
Adjusted p (t test): 1.02e−04, Adjusted p (wilcox): 7.71e−05
Log Metabolite Abundance
2
1
0
−1
−2
EM
Metastatic
Condition

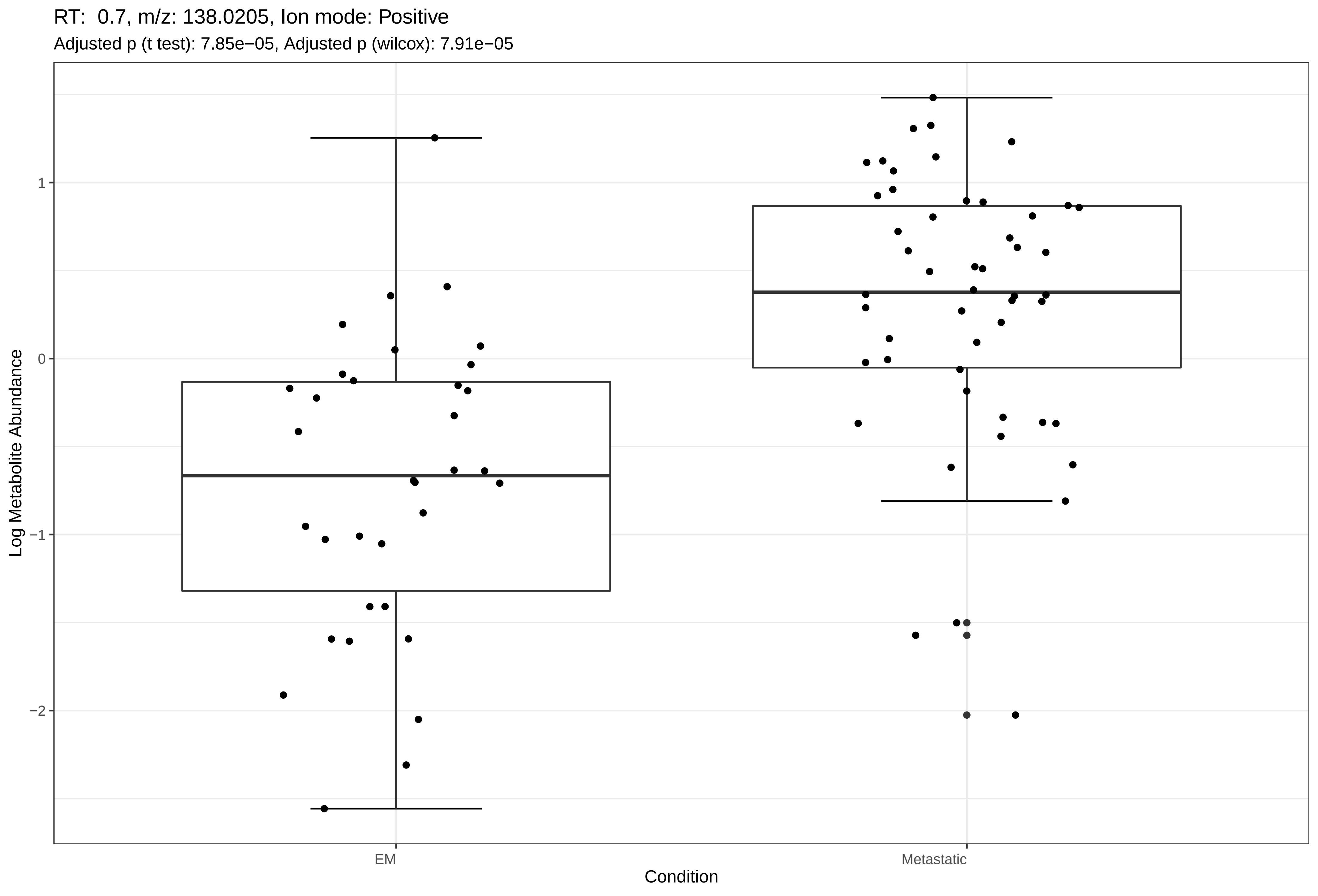

RT: 0.7, m/z: 138.0205, Ion mode: Positive
Adjusted p (t test): 7.85e−05, Adjusted p (wilcox): 7.91e−05
Log Metabolite Abundance
1
0
−1
−2
EM
Metastatic
Condition

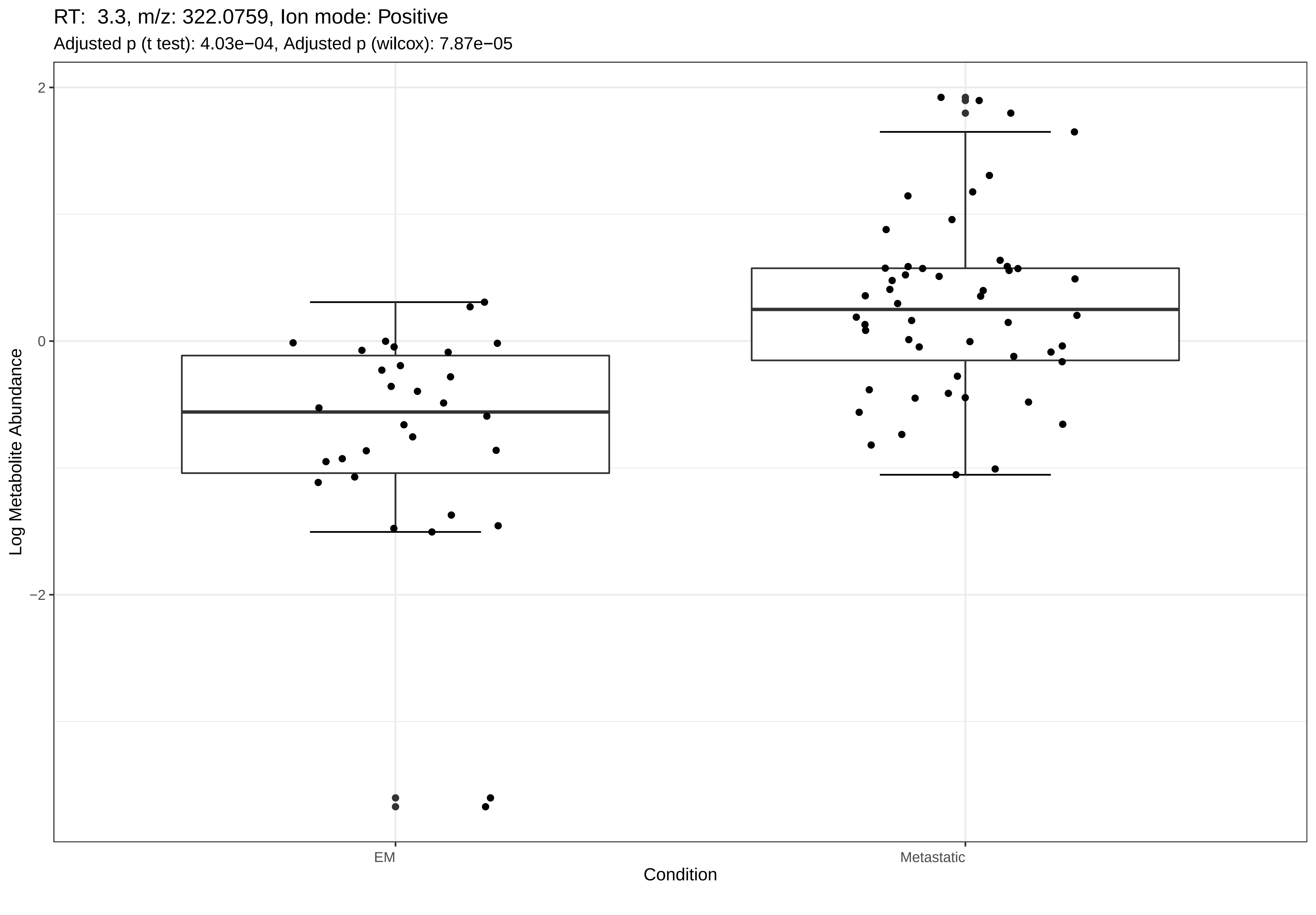
RT: 3.3, m/z: 322.0759, Ion mode: Positive
Adjusted p (t test): 4.03e−04, Adjusted p (wilcox): 7.87e−05
Log Metabolite Abundance
2
0
−2
EM
Metastatic
Condition

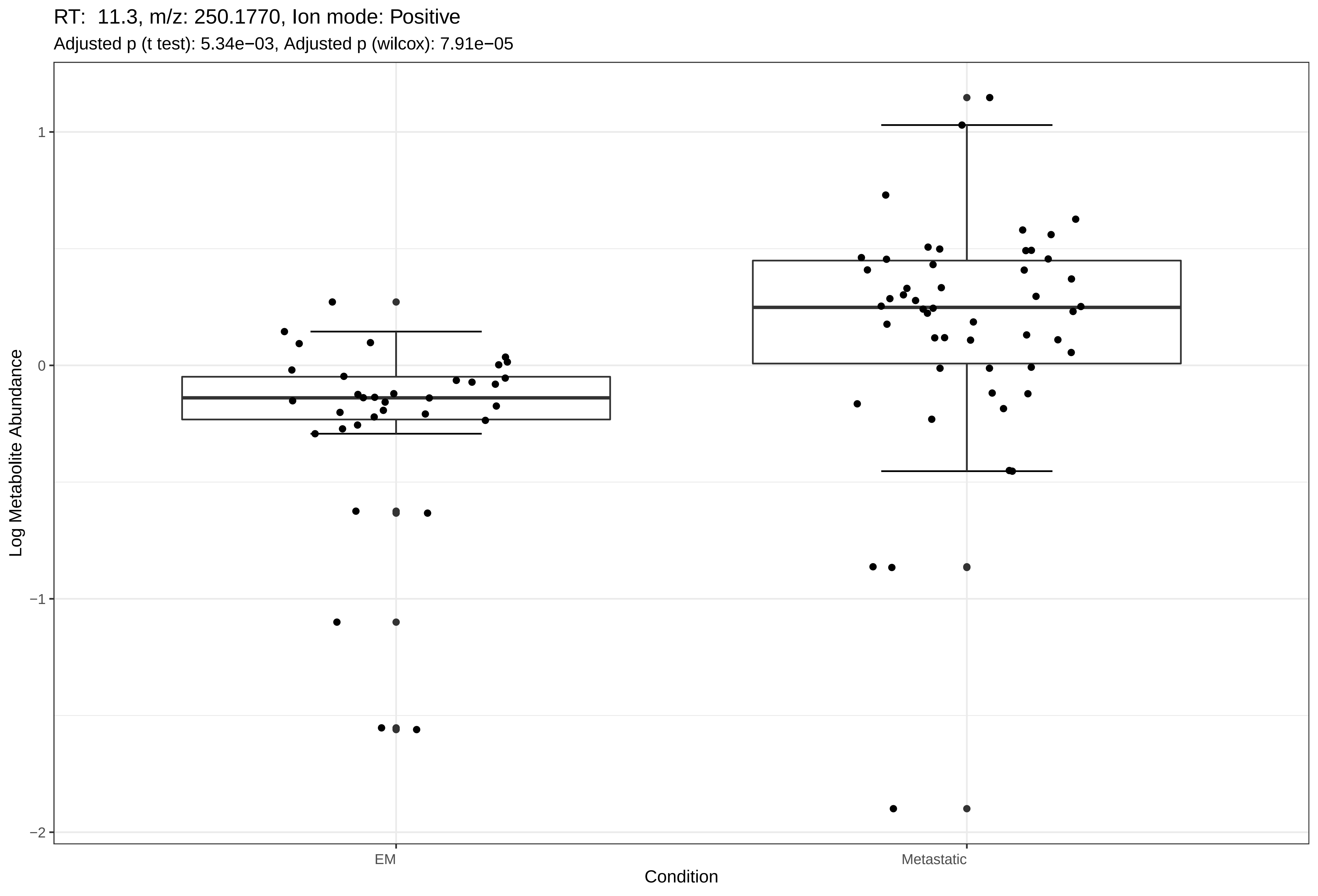

RT: 11.3, m/z: 250.1770, Ion mode: Positive
Adjusted p (t test): 5.34e−03, Adjusted p (wilcox): 7.91e−05
Log Metabolite Abundance
1
0
−1
−2
EM
Metastatic
Condition

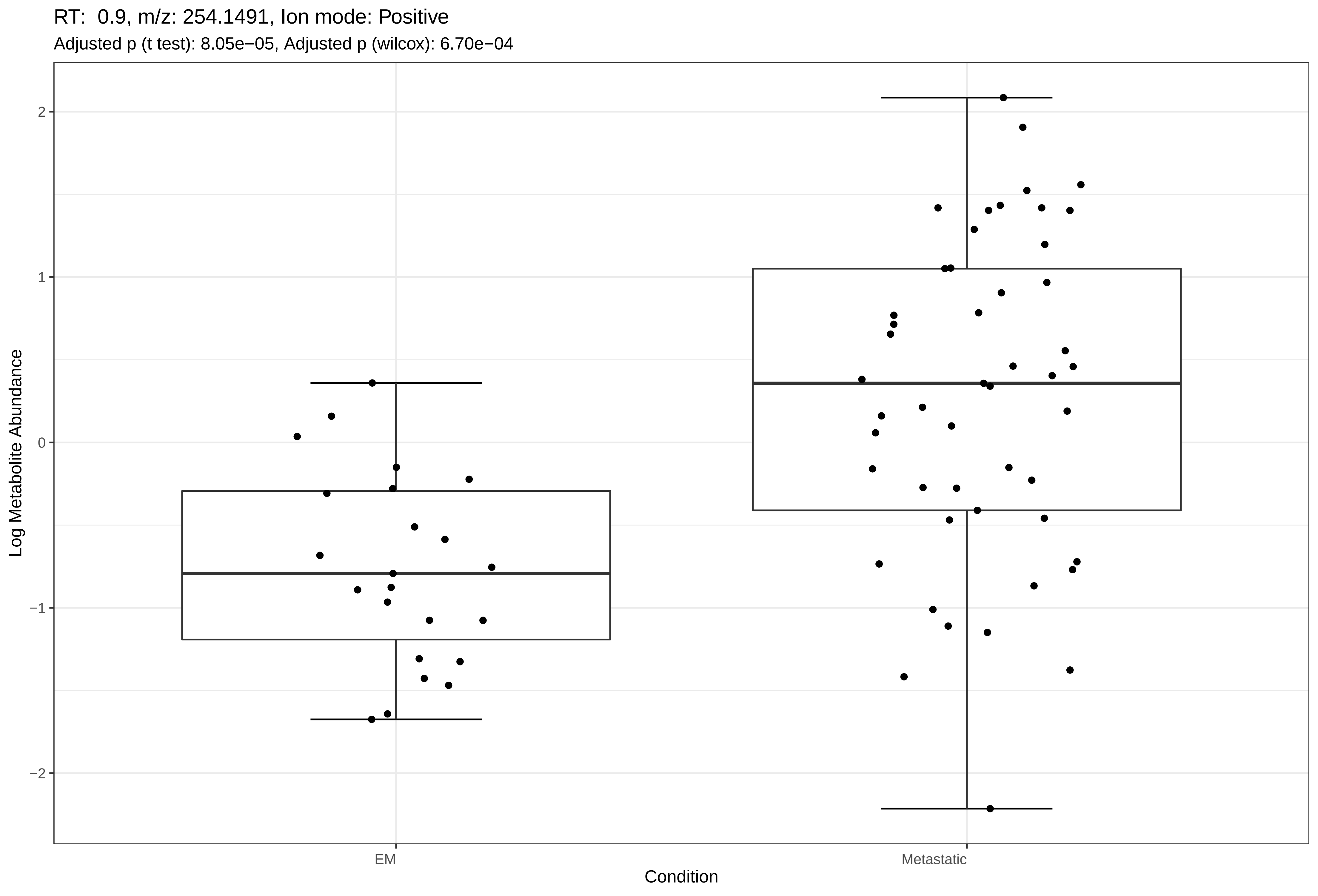

RT: 0.9, m/z: 254.1491, Ion mode: Positive
Adjusted p (t test): 8.05e−05, Adjusted p (wilcox): 6.70e−04
Log Metabolite Abundance
2
1
0
−1
−2
EM
Metastatic
Condition

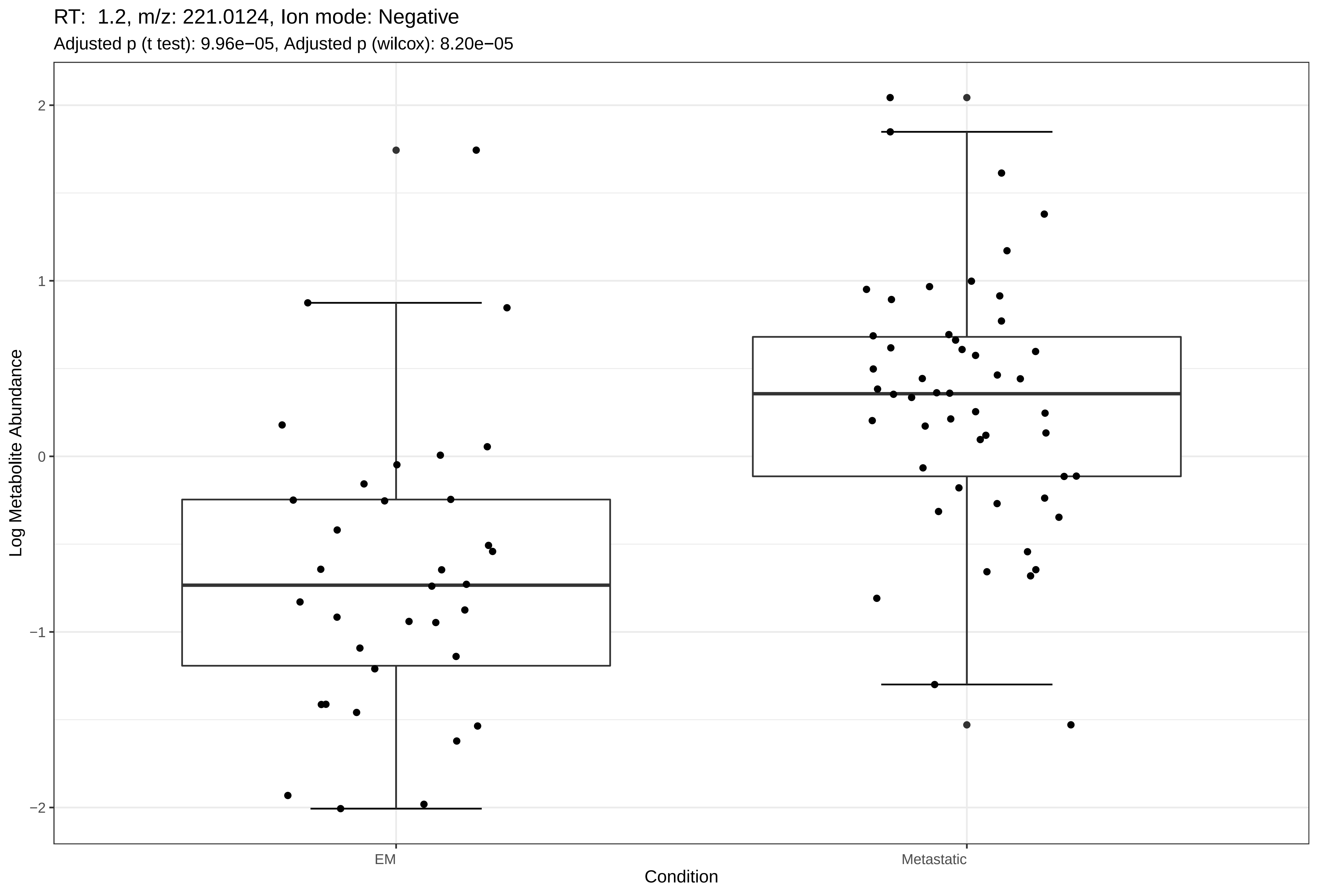

RT: 1.2, m/z: 221.0124, Ion mode: Negative
Adjusted p (t test): 9.96e−05, Adjusted p (wilcox): 8.20e−05
Log Metabolite Abundance
2
1
0
−1
−2
EM
Metastatic
Condition

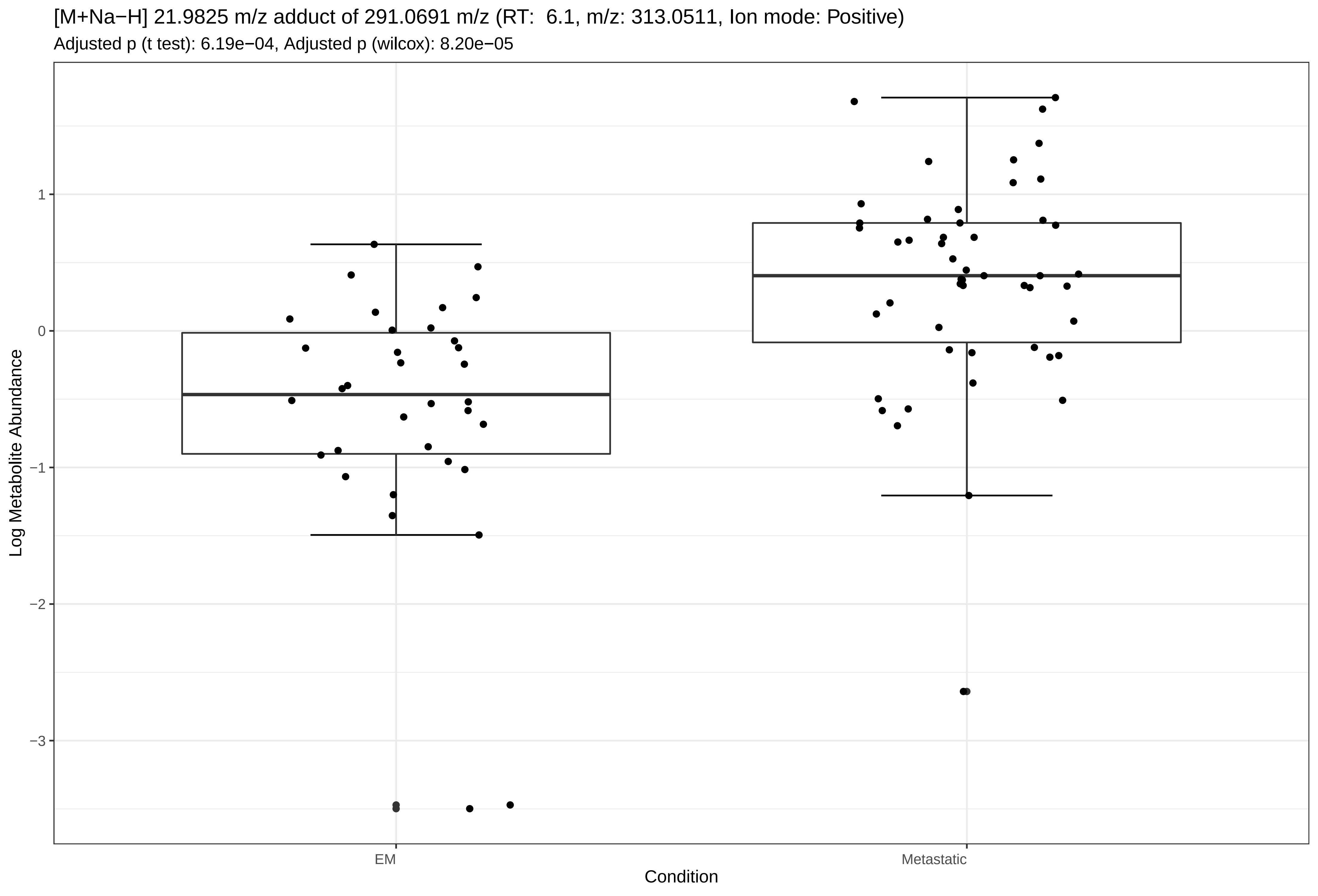

[M+Na−H] 21.9825 m/z adduct of 291.0691 m/z (RT: 6.1, m/z: 313.0511, Ion mode: Positive)
Adjusted p (t test): 6.19e−04, Adjusted p (wilcox): 8.20e−05
Log Metabolite Abundance
1
0
−1
−2
−3
EM
Metastatic
Condition

Complex of 118.0862 and 269.1001 m/z (RT: 1.6, m/z: 386.1788, Ion mode: Positive)

Adjusted p (t test): 8.25e−05, Adjusted p (wilcox): 1.36e−04

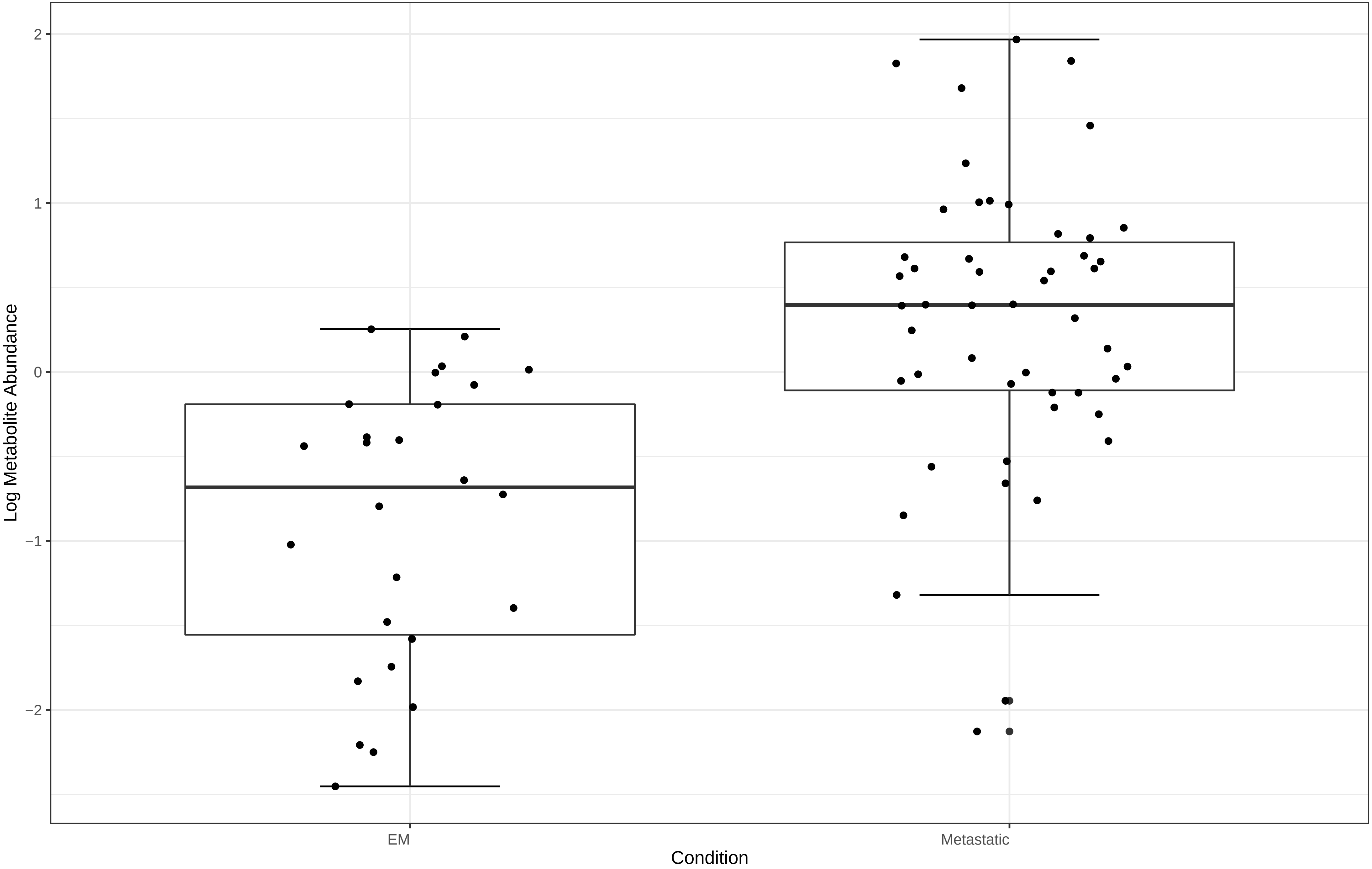

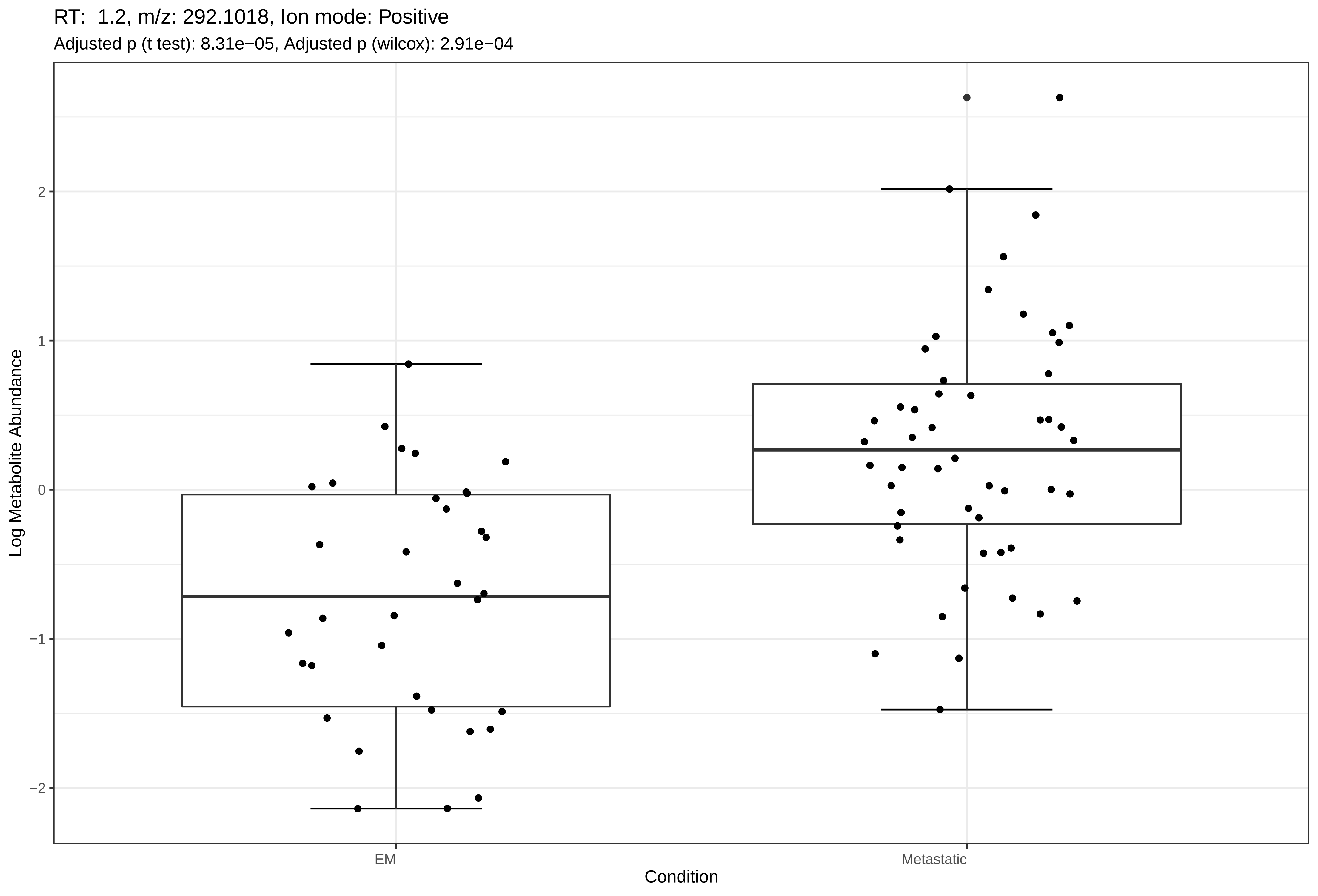

RT: 1.2, m/z: 292.1018, Ion mode: Positive
Adjusted p (t test): 8.31e−05, Adjusted p (wilcox): 2.91e−04
Log Metabolite Abundance
2
1
0
−1
−2
EM
Metastatic
Condition

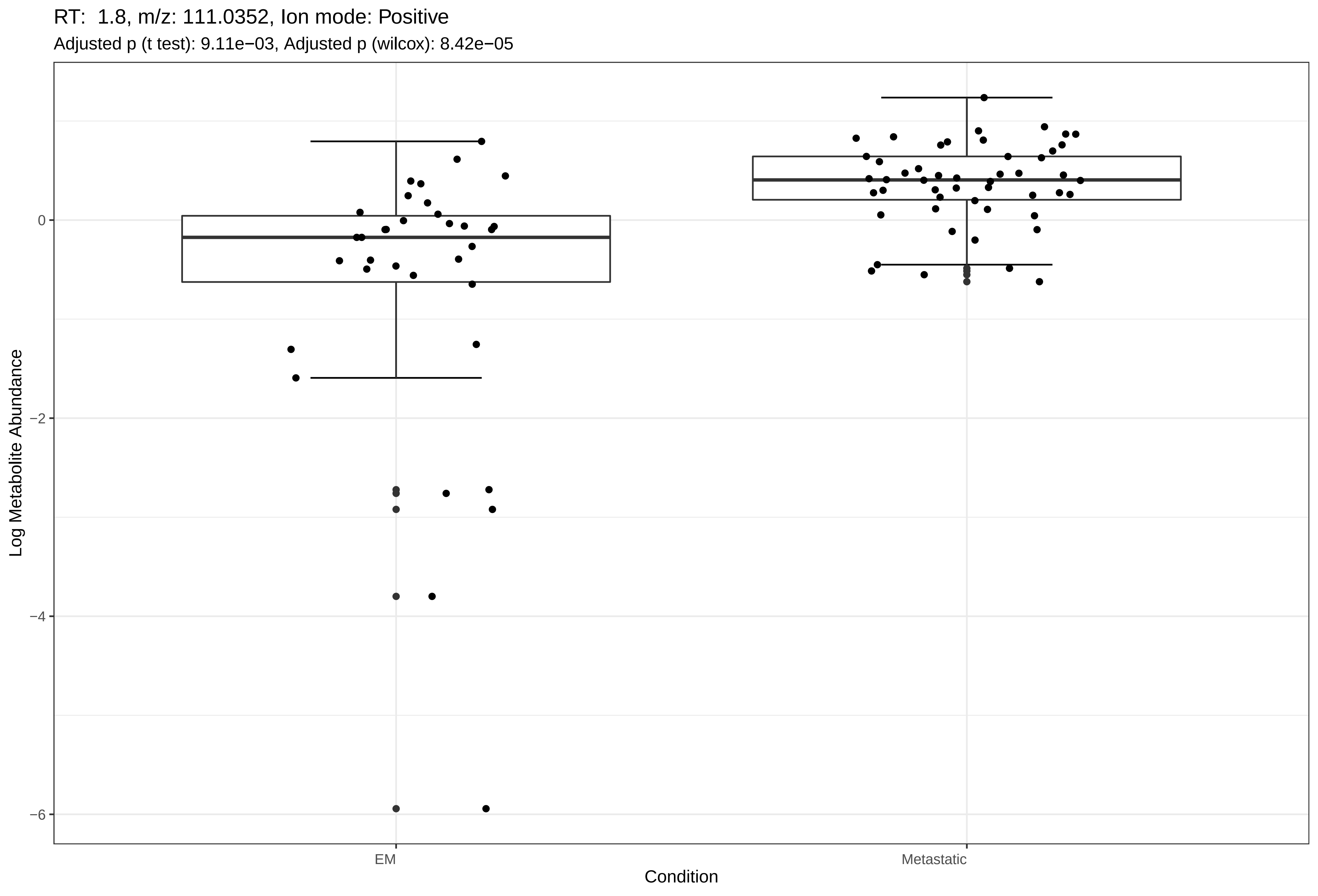

RT: 1.8, m/z: 111.0352, Ion mode: Positive
Adjusted p (t test): 9.11e−03, Adjusted p (wilcox): 8.42e−05
Log Metabolite Abundance
0
−2
−4
−6
EM
Metastatic
Condition

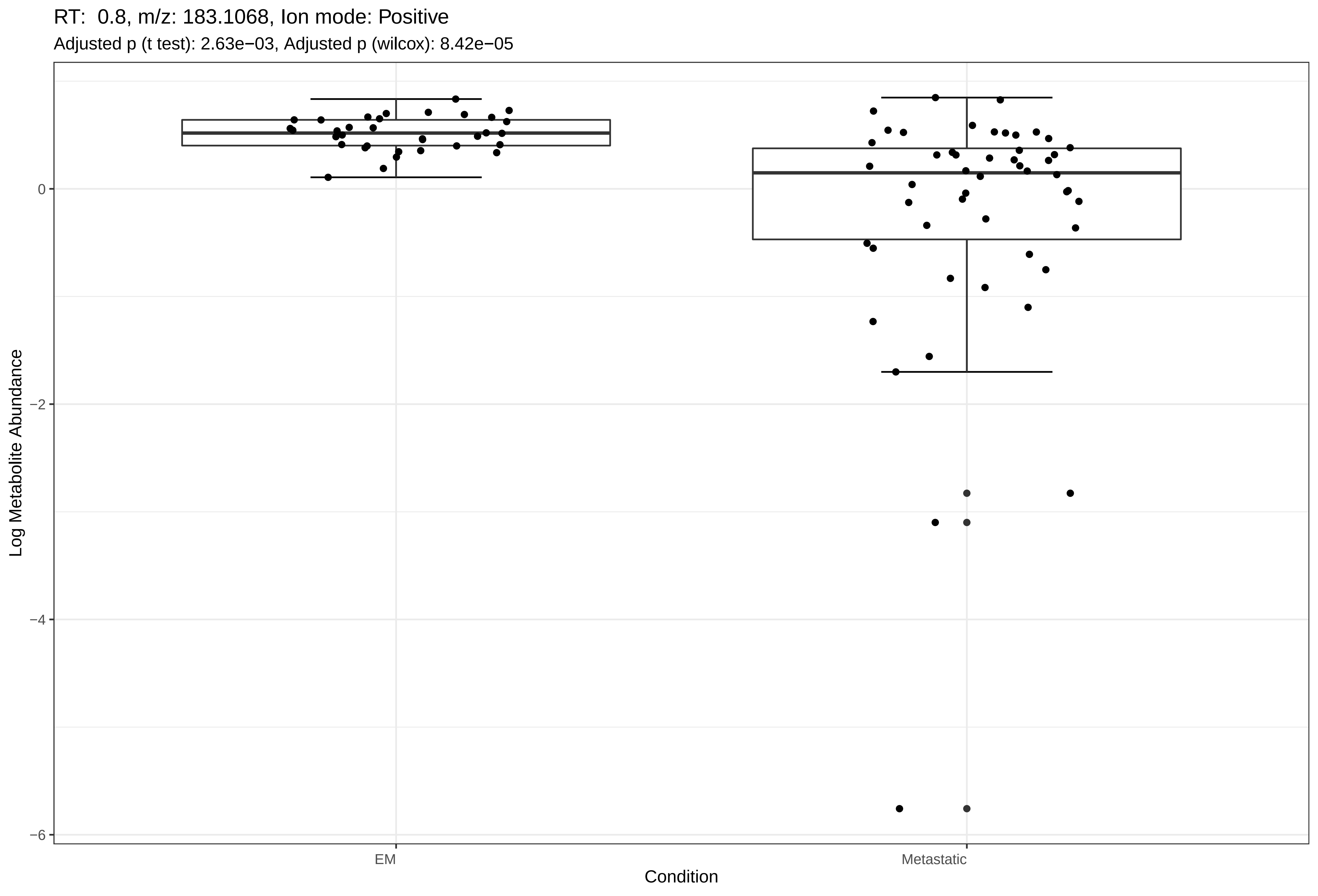

RT: 0.8, m/z: 183.1068, Ion mode: Positive
Adjusted p (t test): 2.63e−03, Adjusted p (wilcox): 8.42e−05
Log Metabolite Abundance
0
−2
−4
−6
EM
Metastatic
Condition

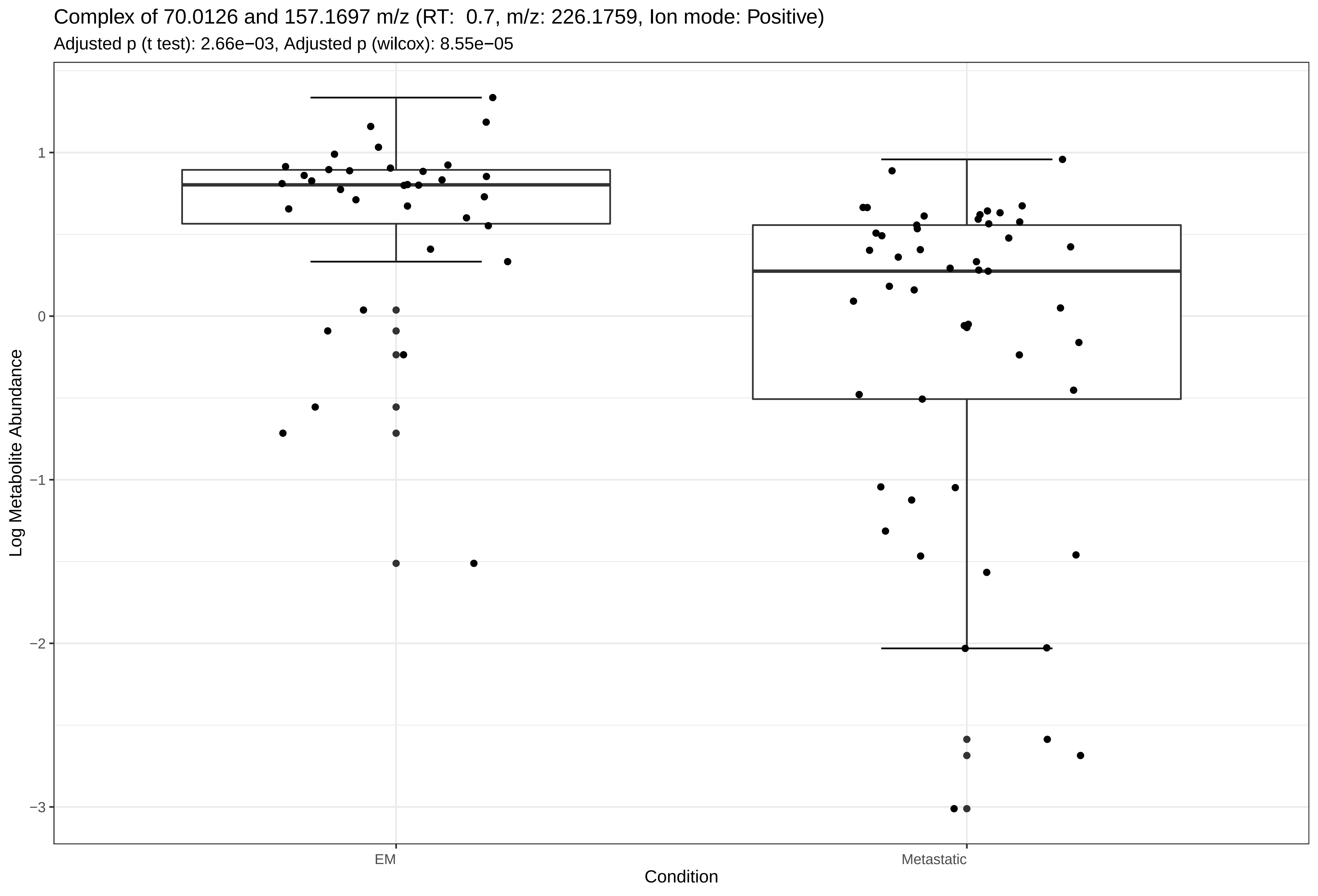

Complex of 70.0126 and 157.1697 m/z (RT: 0.7, m/z: 226.1759, Ion mode: Positive)
Adjusted p (t test): 2.66e−03, Adjusted p (wilcox): 8.55e−05
Log Metabolite Abundance
1
0
−1
−2
−3
EM
Metastatic
Condition

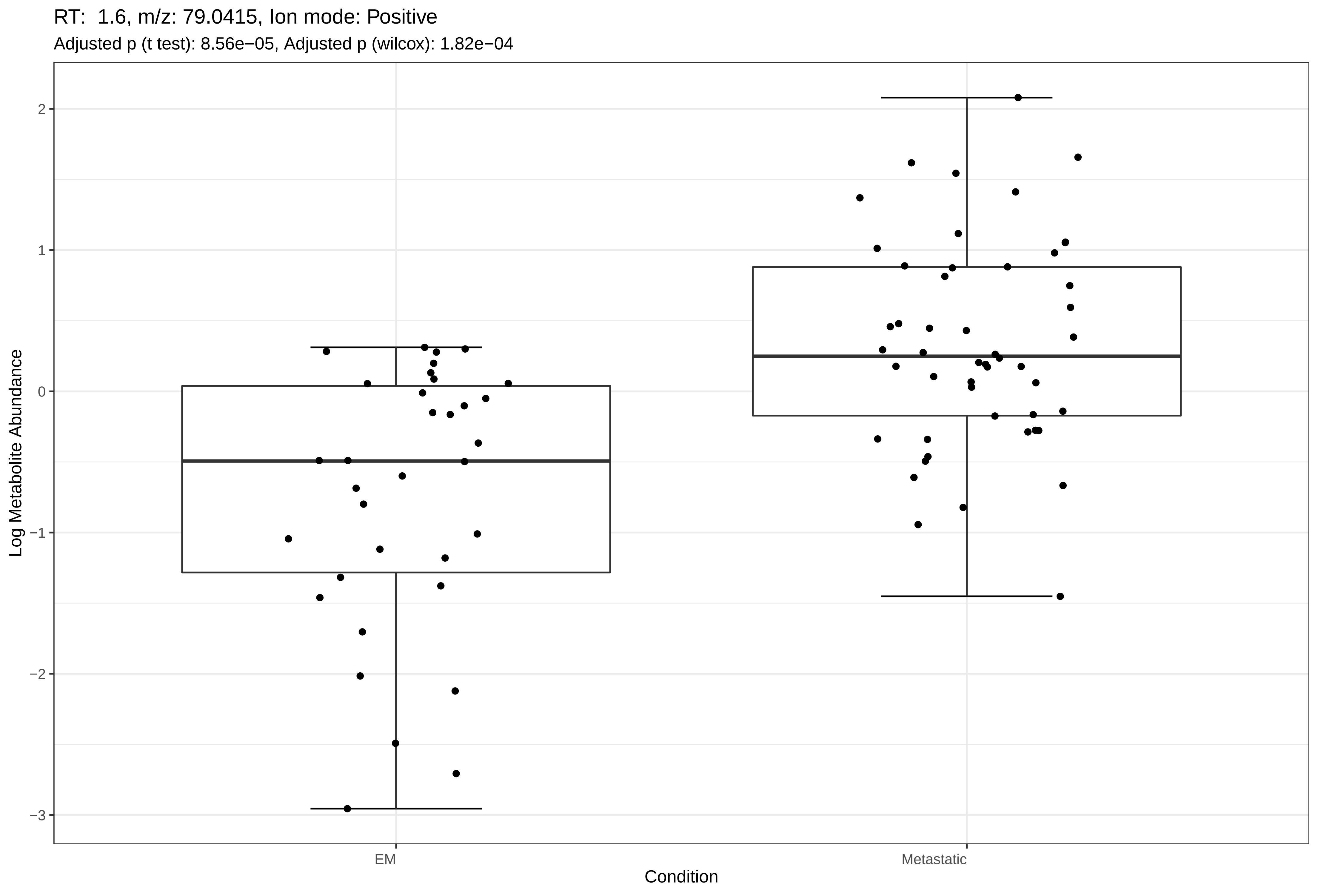

RT: 1.6, m/z: 79.0415, Ion mode: Positive
Adjusted p (t test): 8.56e−05, Adjusted p (wilcox): 1.82e−04
Log Metabolite Abundance
2
1
0
−1
−2
−3
EM
Metastatic
Condition

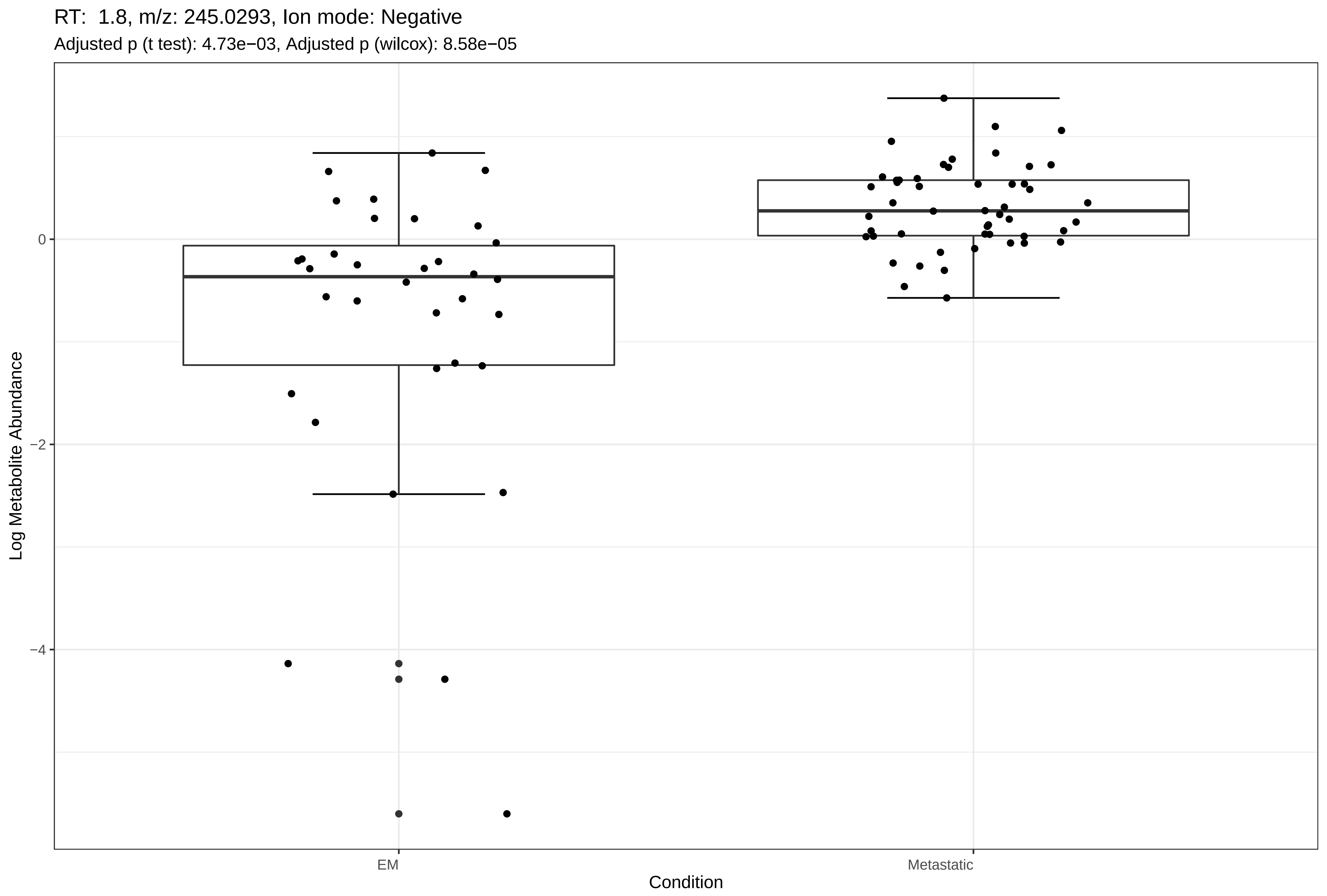
RT: 1.8, m/z: 245.0293, Ion mode: Negative
Adjusted p (t test): 4.73e−03, Adjusted p (wilcox): 8.58e−05
Log Metabolite Abundance
0
−2
−4
EM
Metastatic
Condition

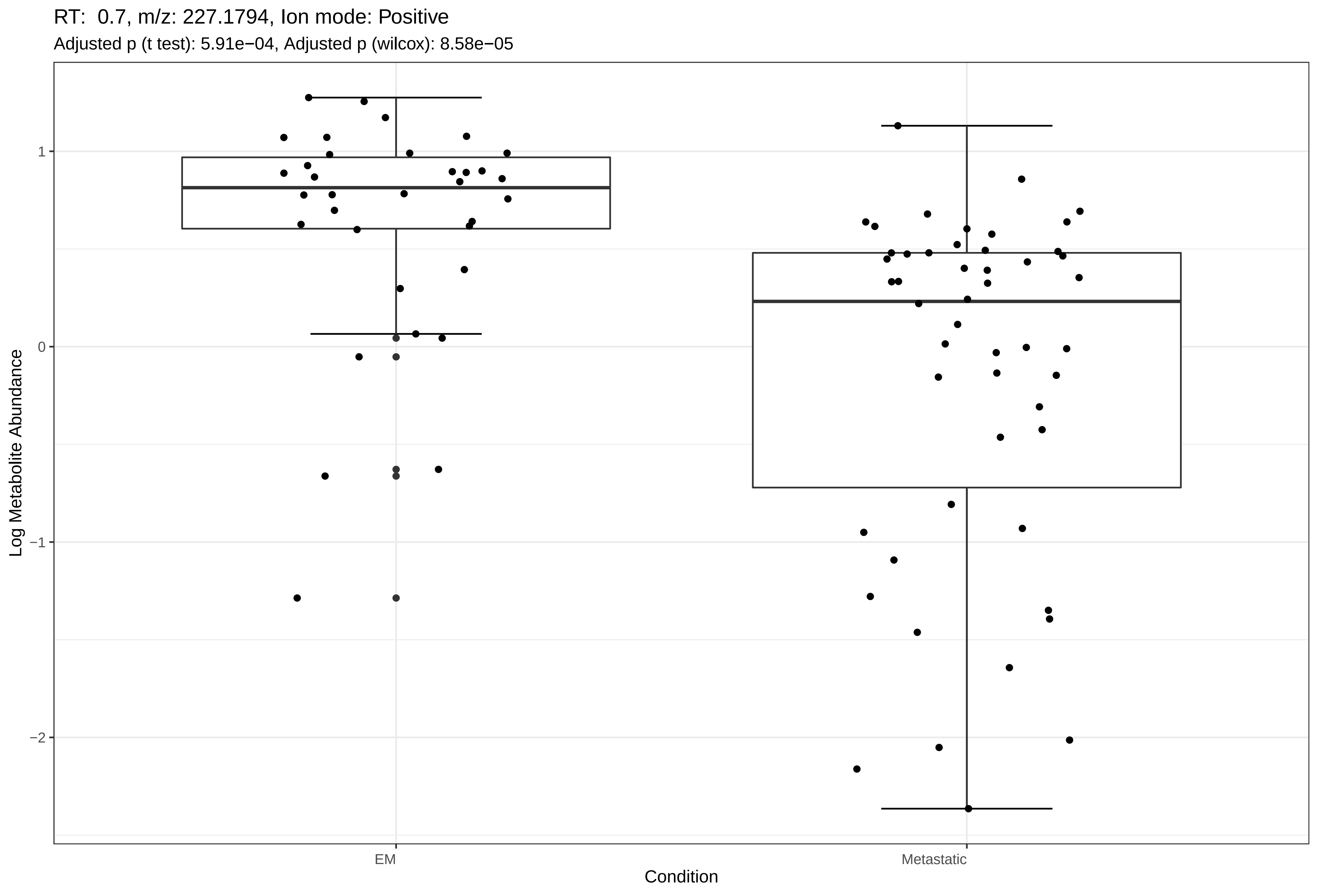
RT: 0.7, m/z: 227.1794, Ion mode: Positive
Adjusted p (t test): 5.91e−04, Adjusted p (wilcox): 8.58e−05
Log Metabolite Abundance
1
0
−1
−2
EM
Metastatic
Condition

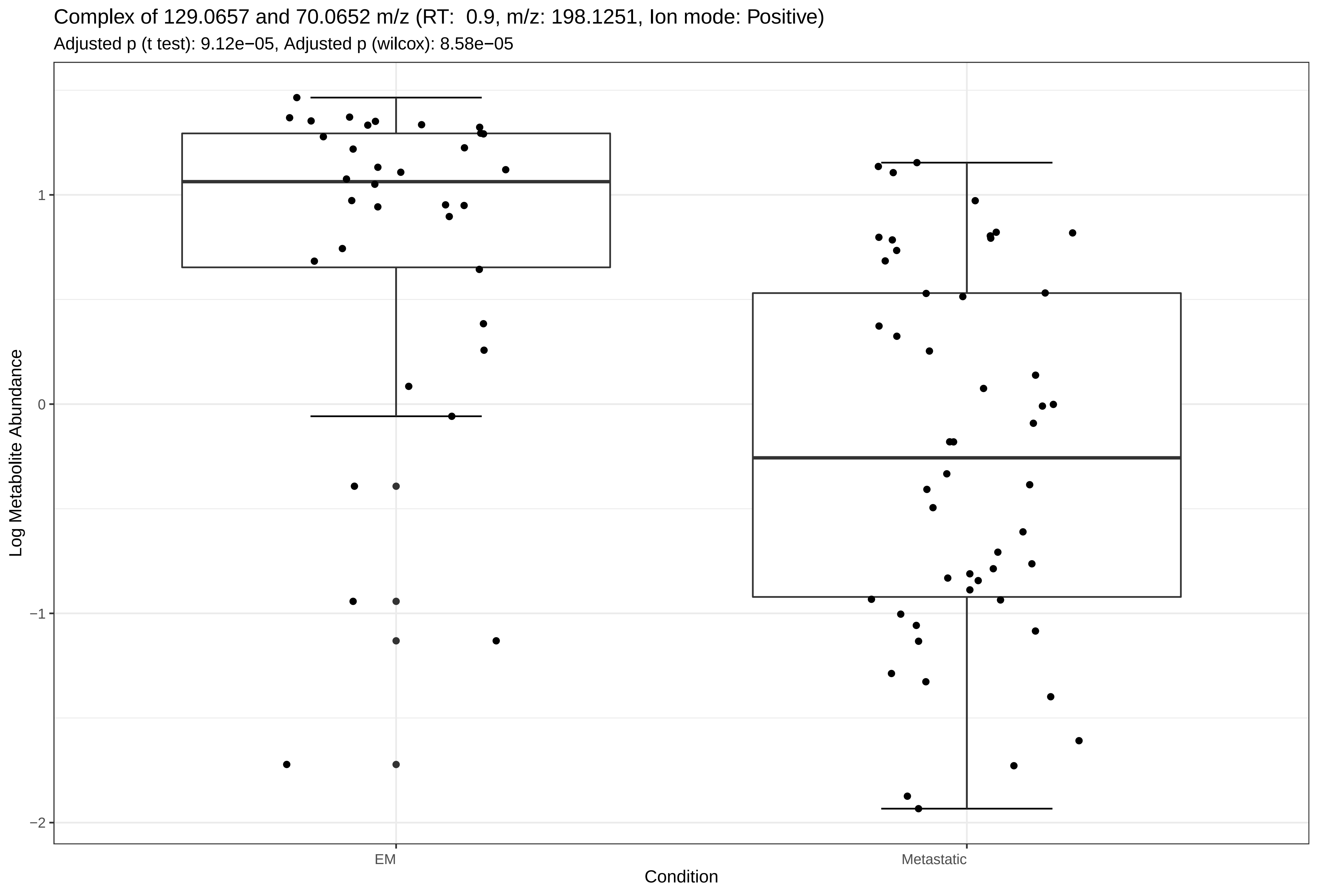
Complex of 129.0657 and 70.0652 m/z (RT: 0.9, m/z: 198.1251, Ion mode: Positive)
Adjusted p (t test): 9.12e−05, Adjusted p (wilcox): 8.58e−05
Log Metabolite Abundance
1
0
−1
−2
EM
Metastatic
Condition

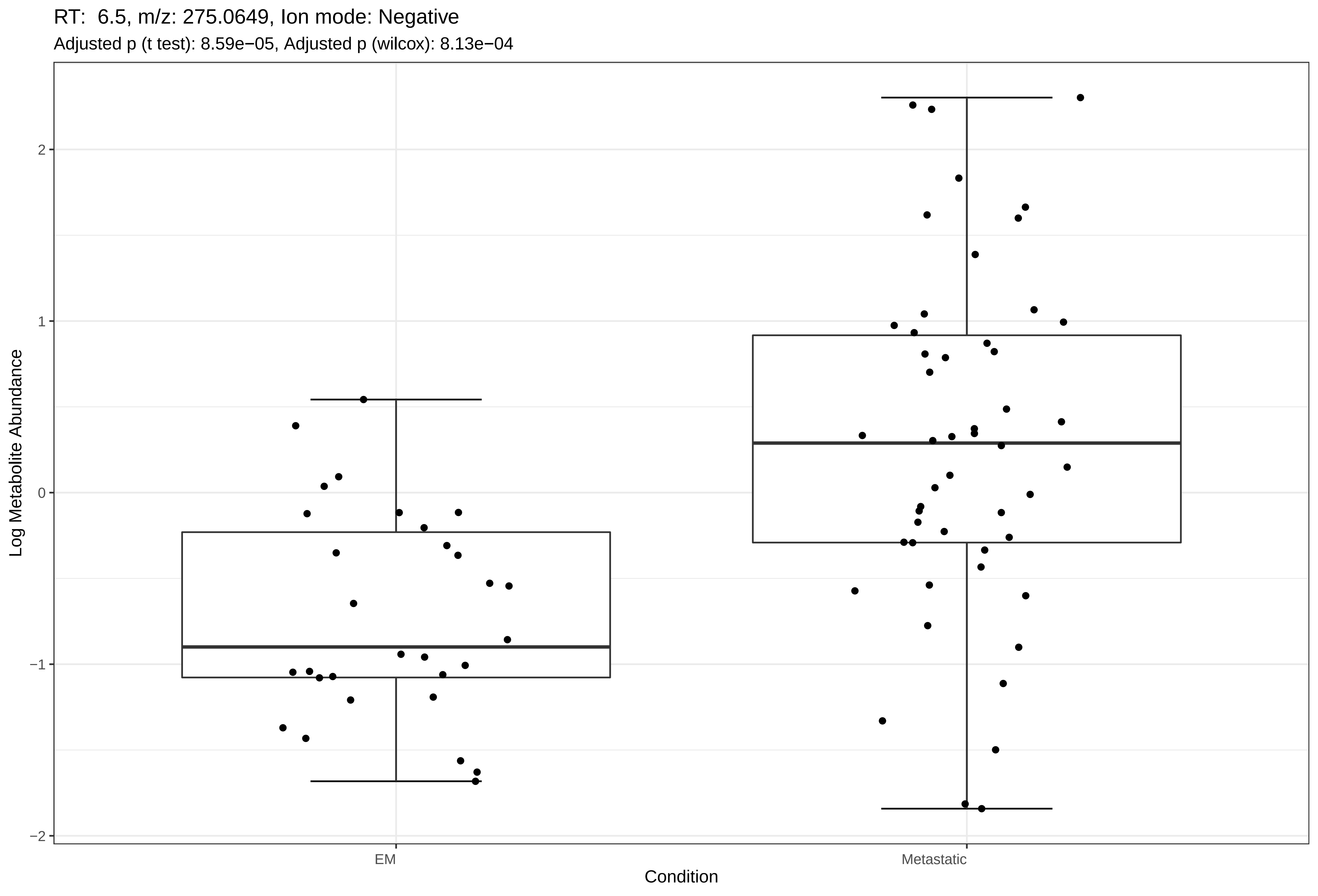
RT: 6.5, m/z: 275.0649, Ion mode: Negative
Adjusted p (t test): 8.59e−05, Adjusted p (wilcox): 8.13e−04
Log Metabolite Abundance
2
1
0
−1
−2
EM
Metastatic
Condition

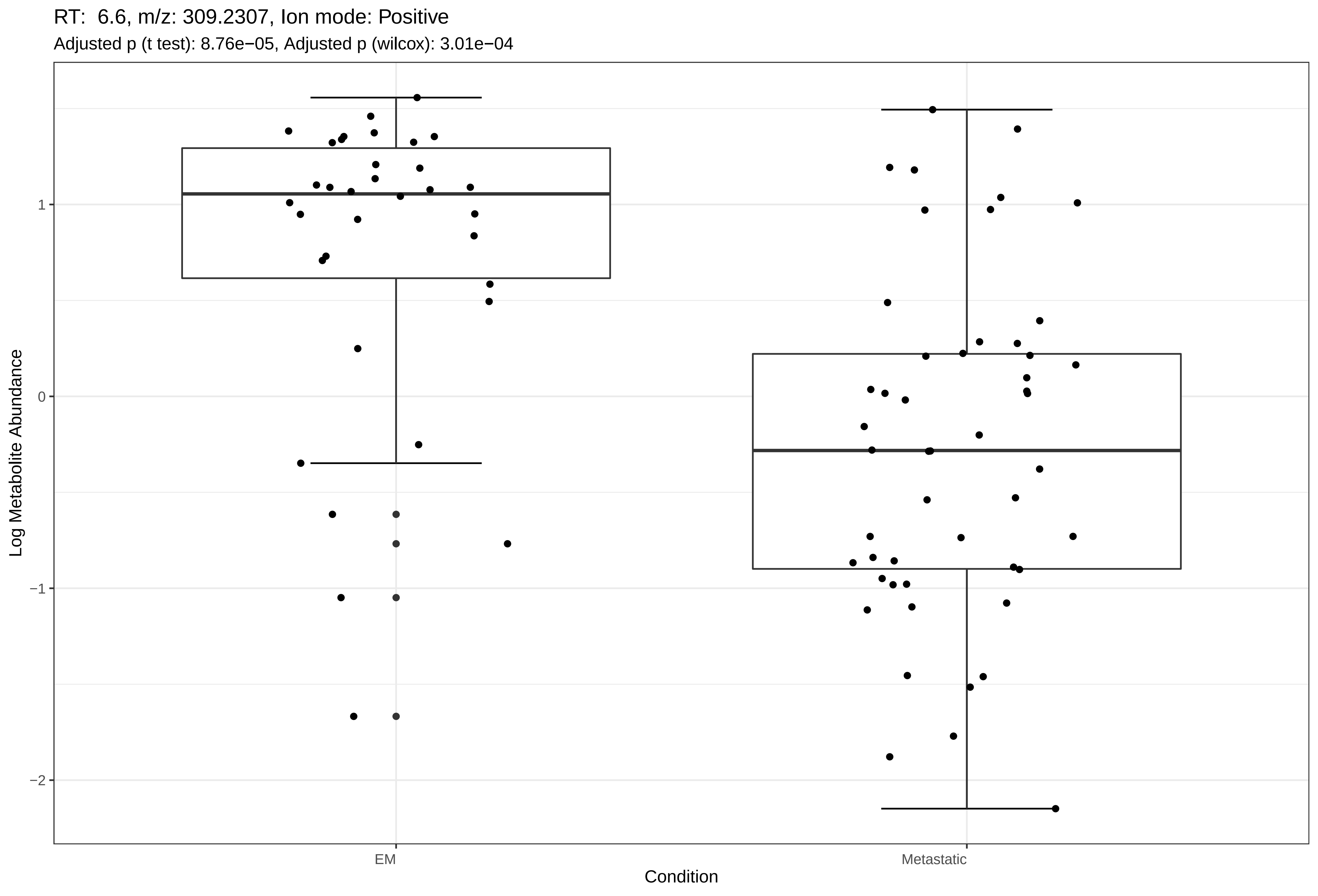

RT: 6.6, m/z: 309.2307, Ion mode: Positive
Adjusted p (t test): 8.76e−05, Adjusted p (wilcox): 3.01e−04
Log Metabolite Abundance
1
0
−1
−2
EM
Metastatic
Condition

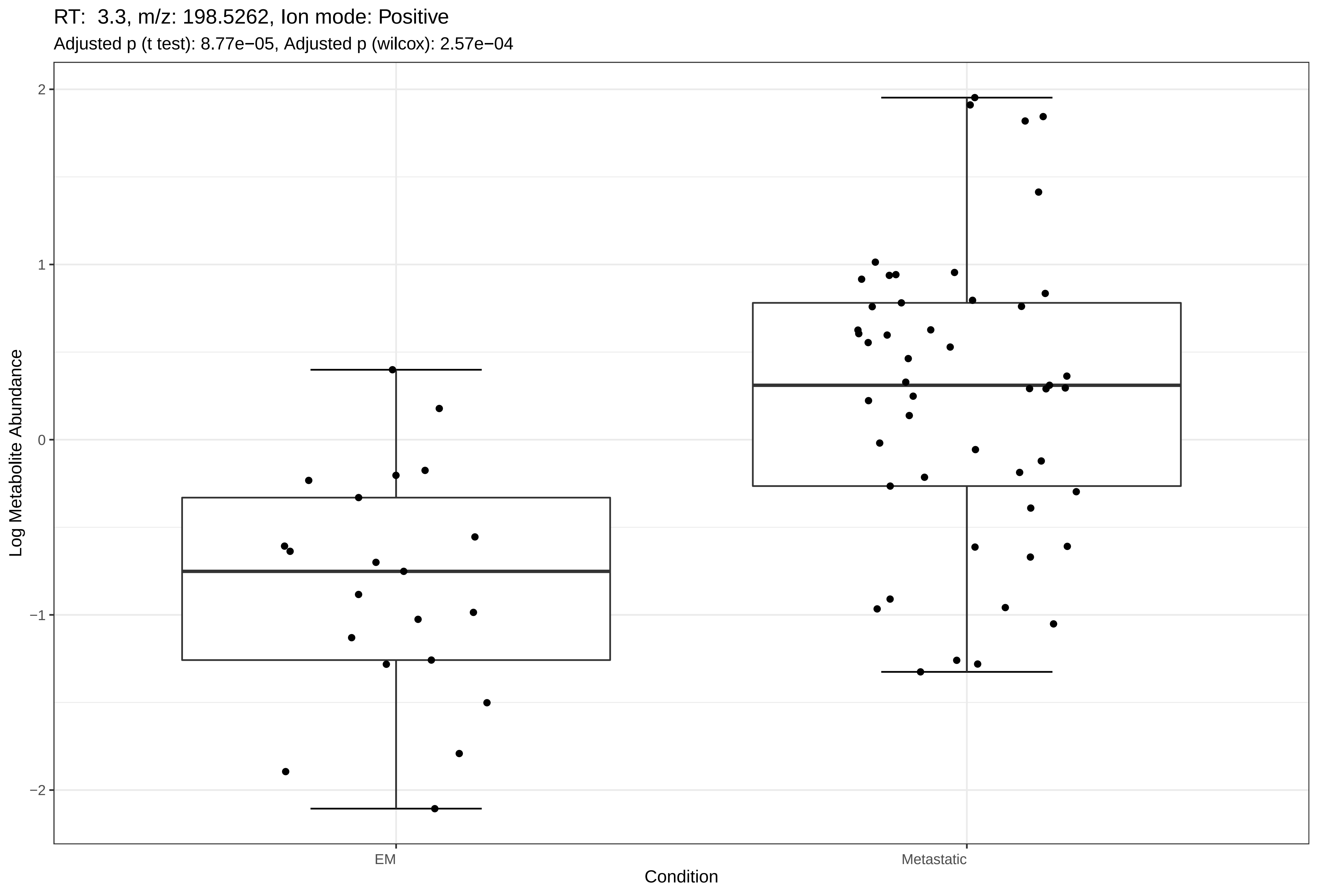

RT: 3.3, m/z: 198.5262, Ion mode: Positive
Adjusted p (t test): 8.77e−05, Adjusted p (wilcox): 2.57e−04
Log Metabolite Abundance
2
1
0
−1
−2
EM
Metastatic
Condition

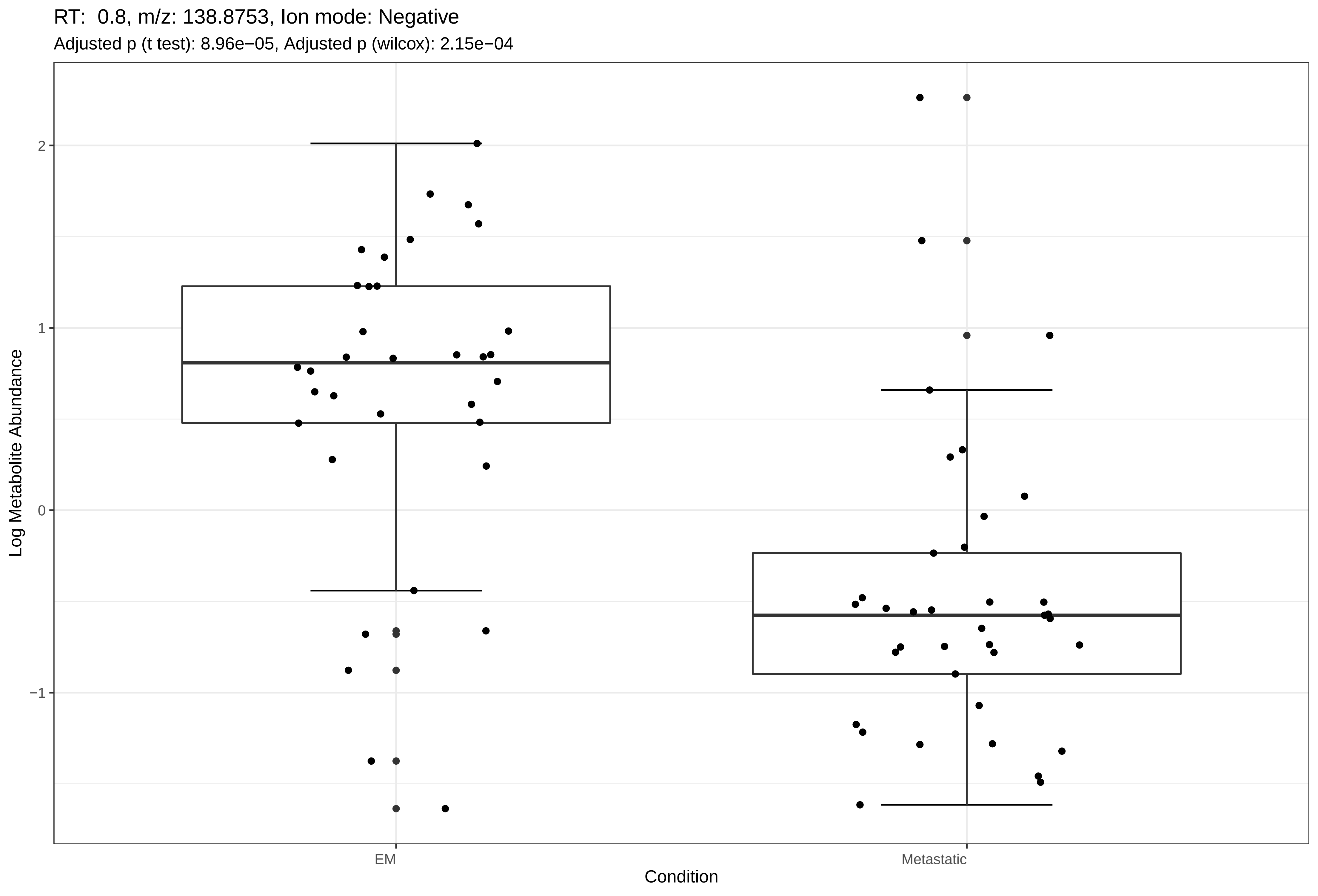

RT: 0.8, m/z: 138.8753, Ion mode: Negative
Adjusted p (t test): 8.96e−05, Adjusted p (wilcox): 2.15e−04
Log Metabolite Abundance
2
1
0
−1
EM
Metastatic
Condition

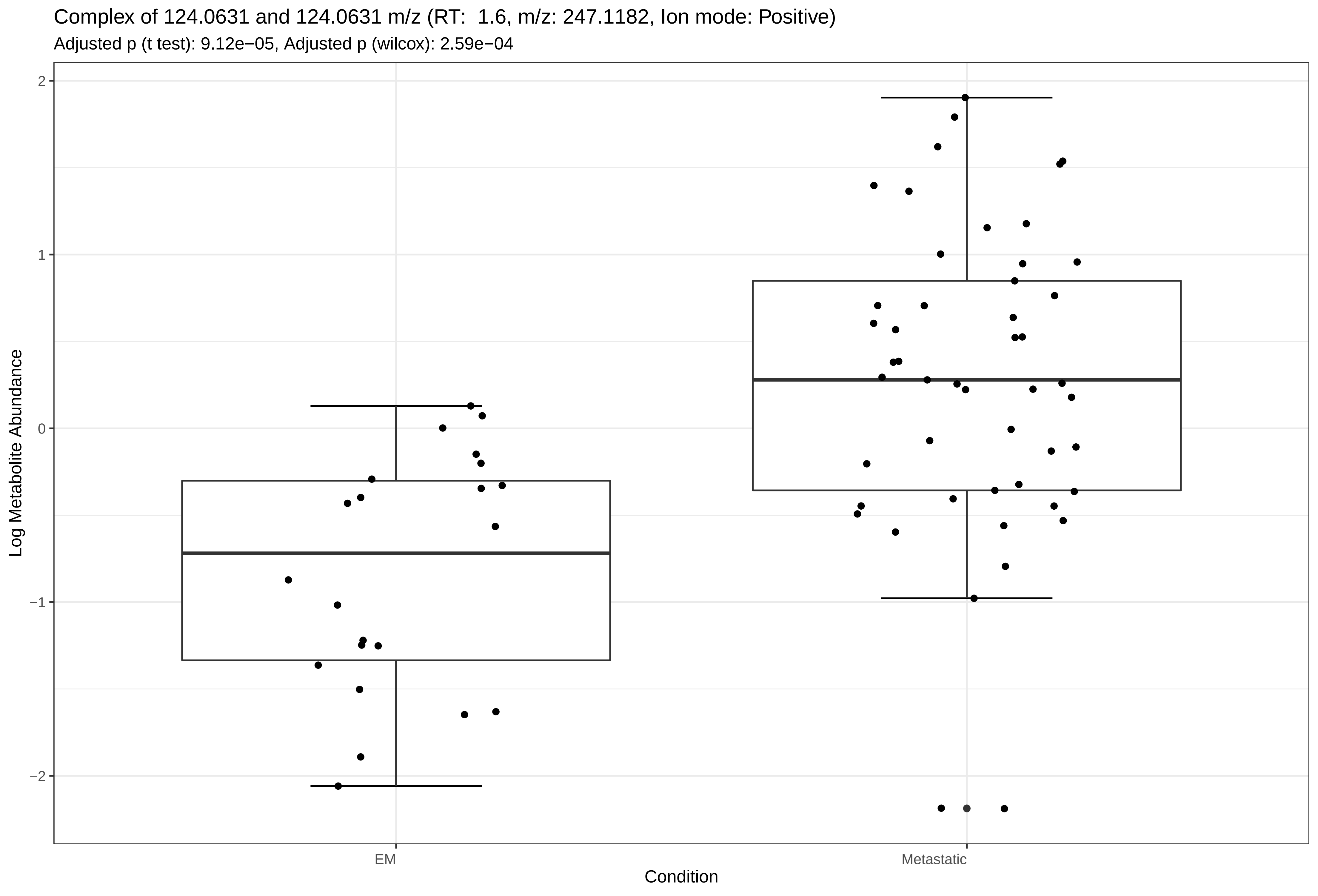
Complex of 124.0631 and 124.0631 m/z (RT: 1.6, m/z: 247.1182, Ion mode: Positive)
Adjusted p (t test): 9.12e−05, Adjusted p (wilcox): 2.59e−04
Log Metabolite Abundance
2
1
0
−1
−2
EM
Metastatic
Condition

DIHYDROXYACETONE PHOSPHATE (RT: 0.7, m/z: 171.0051, Ion mode: Positive)

Adjusted p (t test): 9.17e−05, Adjusted p (wilcox): 1.28e−04

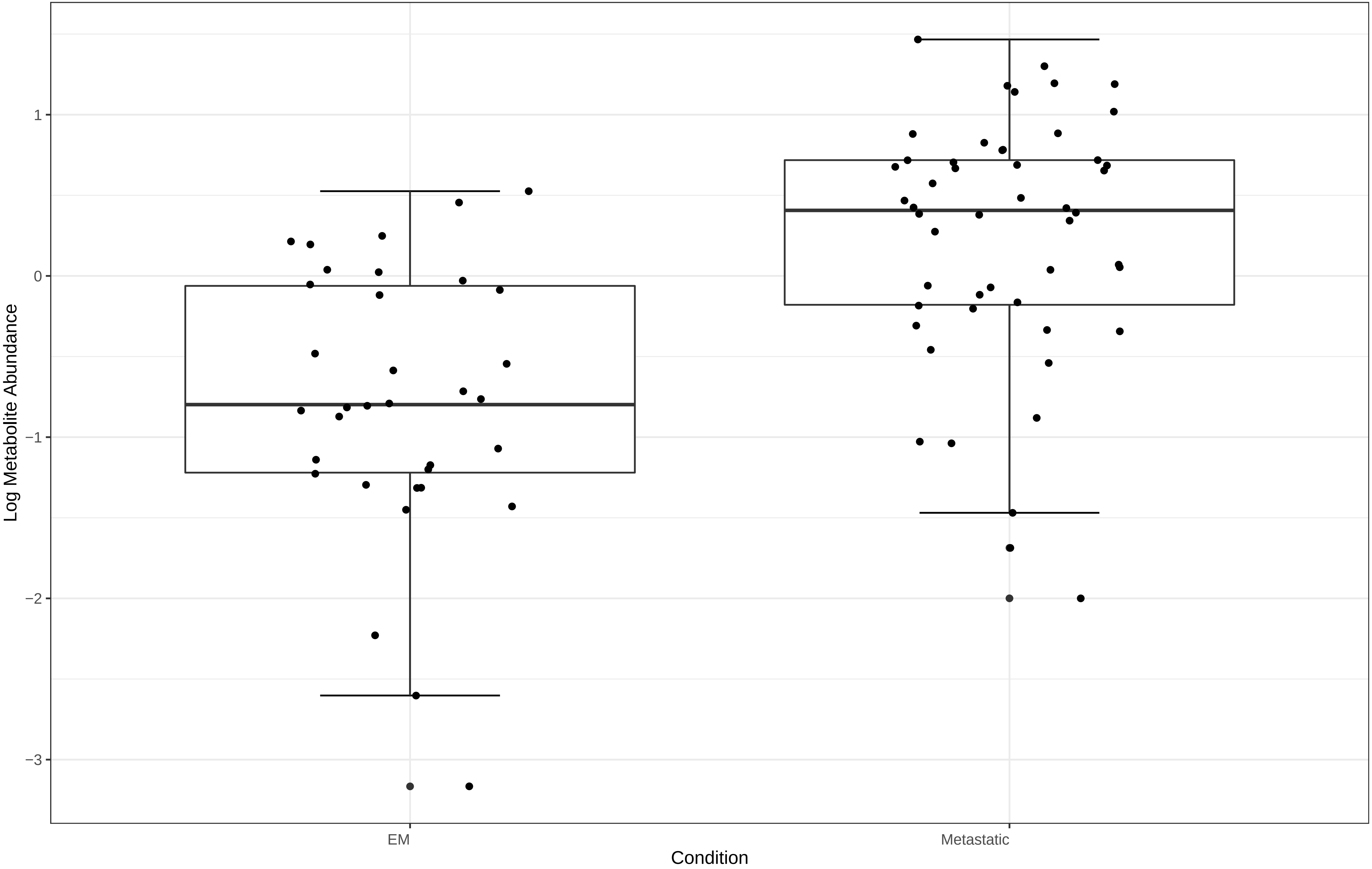

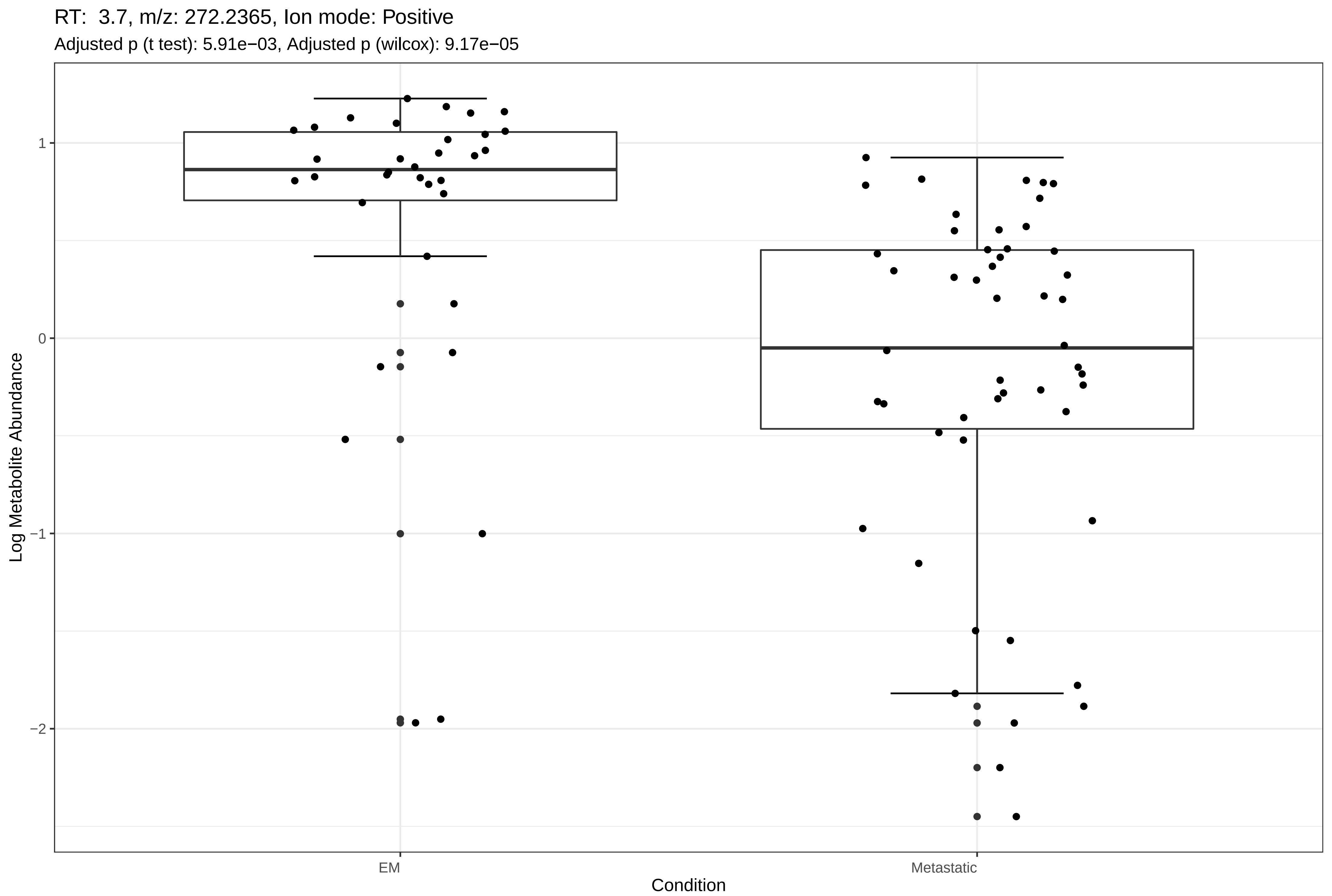
RT: 3.7, m/z: 272.2365, Ion mode: Positive
Adjusted p (t test): 5.91e−03, Adjusted p (wilcox): 9.17e−05
Log Metabolite Abundance
1
0
−1
−2
EM
Metastatic
Condition

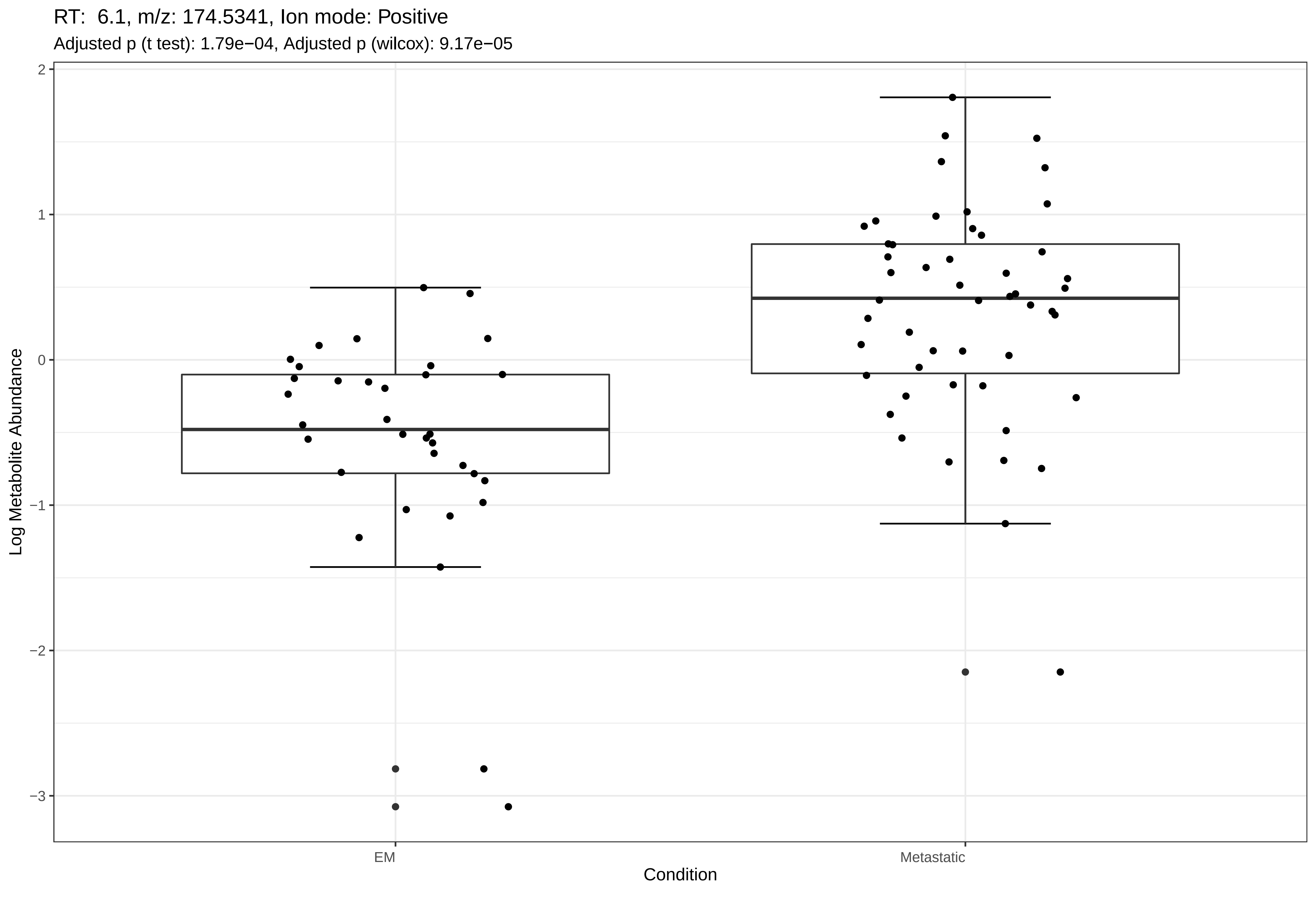
RT: 6.1, m/z: 174.5341, Ion mode: Positive
Adjusted p (t test): 1.79e−04, Adjusted p (wilcox): 9.17e−05
Log Metabolite Abundance
2
1
0
−1
−2
−3
EM
Metastatic
Condition

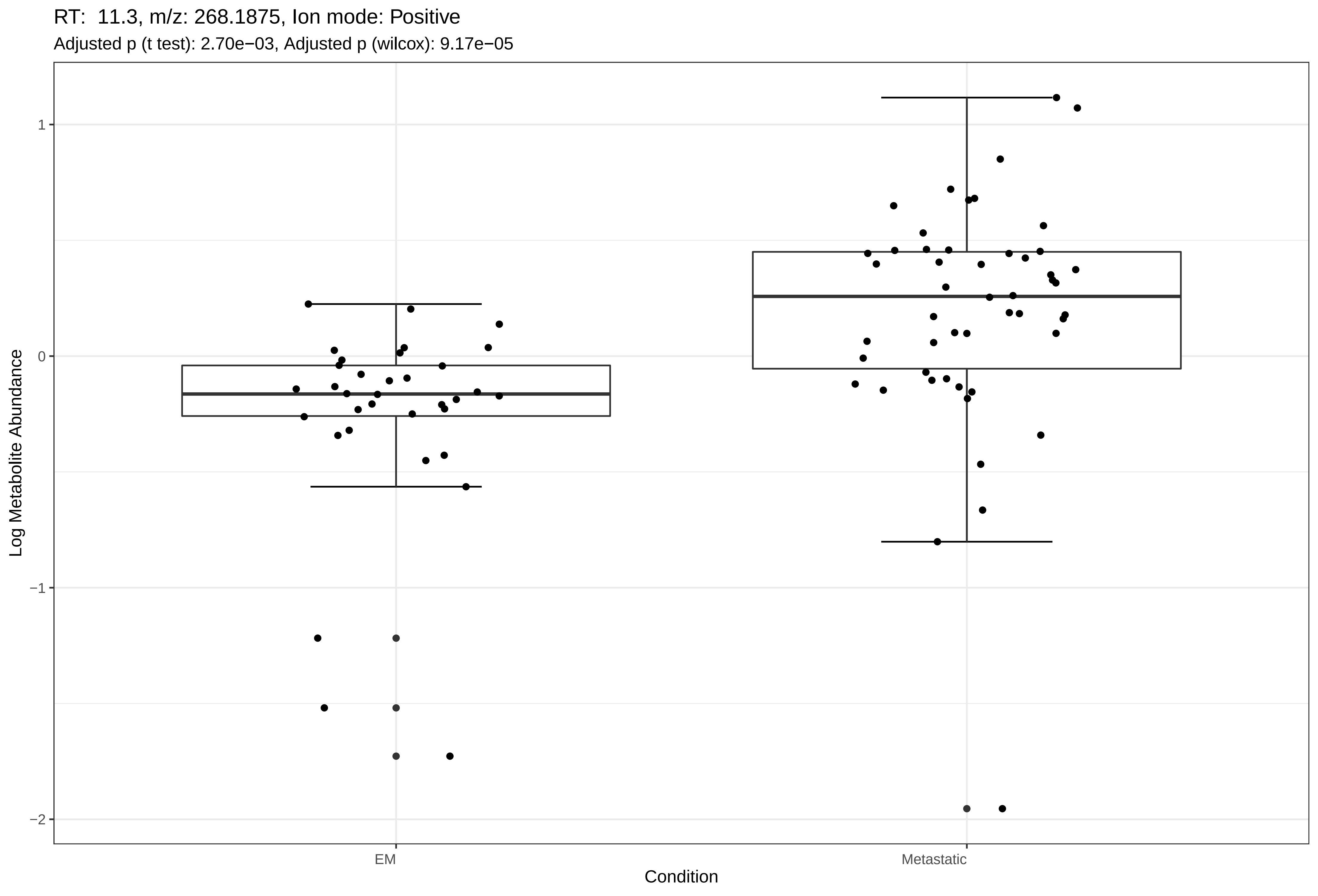
RT: 11.3, m/z: 268.1875, Ion mode: Positive
Adjusted p (t test): 2.70e−03, Adjusted p (wilcox): 9.17e−05
Log Metabolite Abundance
1
0
−1
−2
EM
Metastatic
Condition

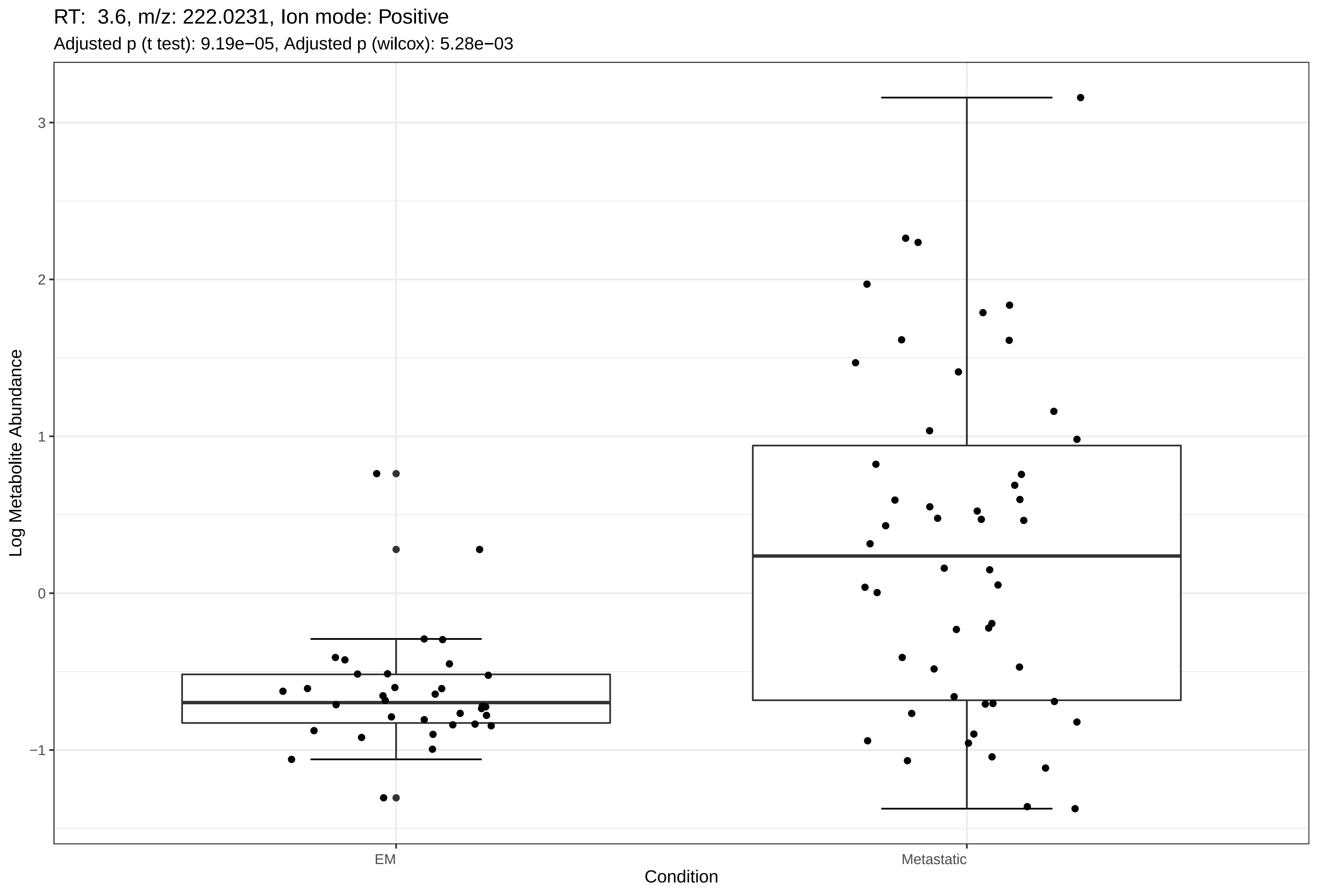
RT: 3.6, m/z: 222.0231, Ion mode: Positive
Adjusted p (t test): 9.19e−05, Adjusted p (wilcox): 5.28e−03
Log Metabolite Abundance
3
2
1
0
−1
EM
Metastatic
Condition

RT: 6.1, m/z: 135.0307, Ion mode: Negative

Adjusted p (t test): 9.19e−05, Adjusted p (wilcox): 1.20e−04

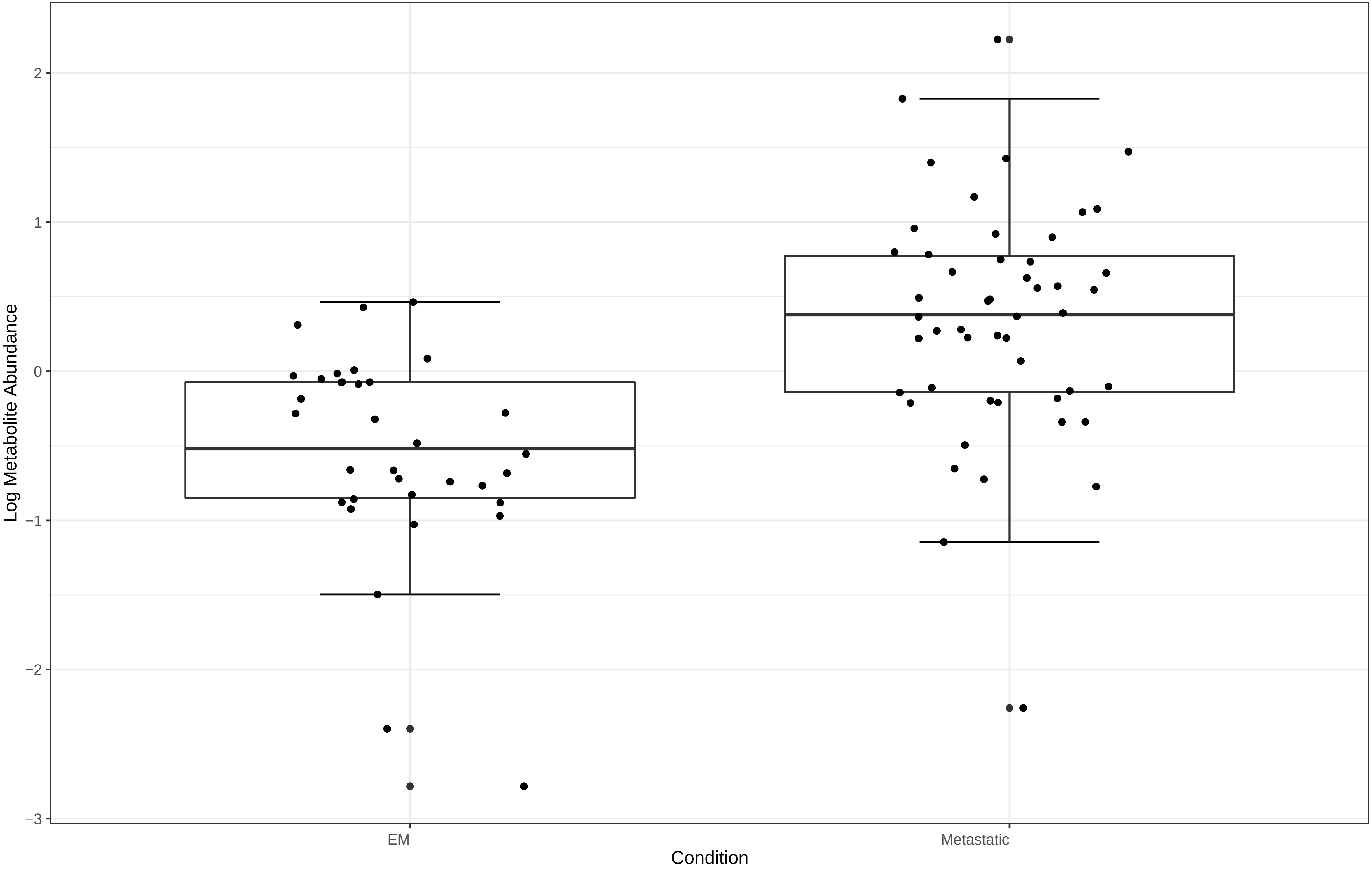

[M−HCOOH+H] −46.0055 m/z adduct of 322.0759 m/z (RT: 3.3, m/z: 276.0704, Ion mode: Positive)

Adjusted p (t test): 9.36e−05, Adjusted p (wilcox): 1.93e−03

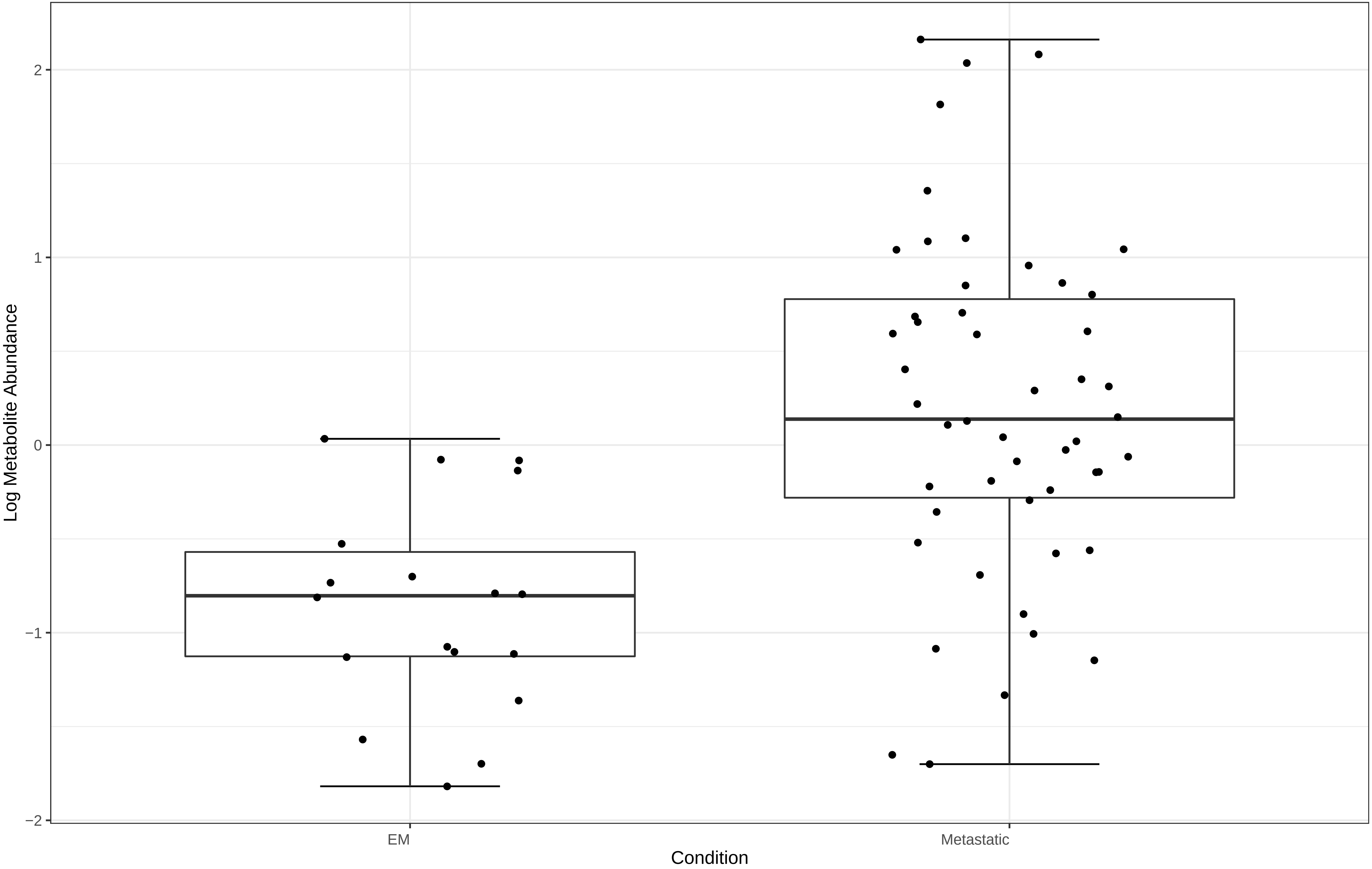

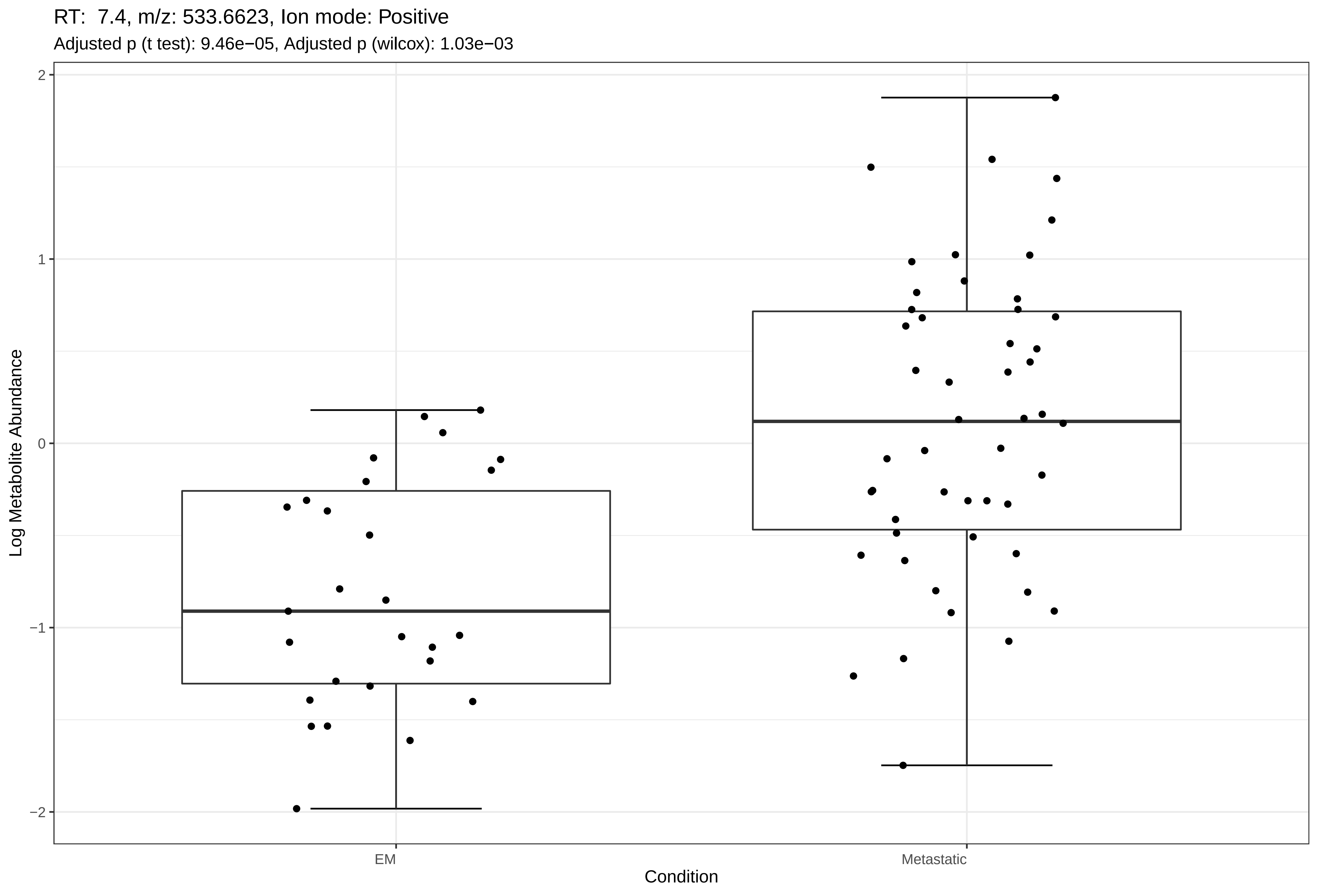
RT: 7.4, m/z: 533.6623, Ion mode: Positive
Adjusted p (t test): 9.46e−05, Adjusted p (wilcox): 1.03e−03
Log Metabolite Abundance
2
1
0
−1
−2
EM
Metastatic
Condition

RT: 1.2, m/z: 87.0084, Ion mode: Negative

Adjusted p (t test): 1.42e−04, Adjusted p (wilcox): 9.54e−05

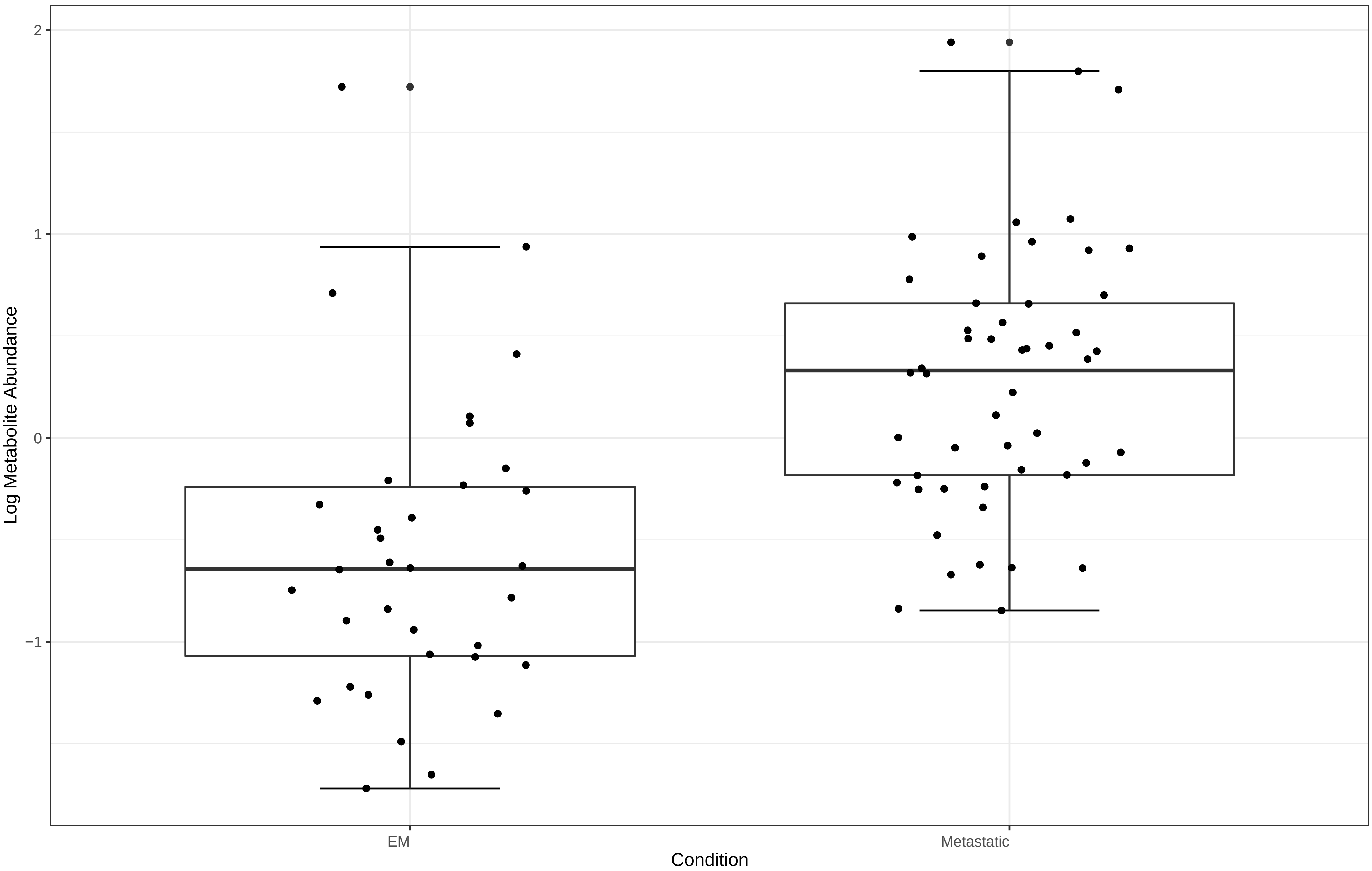

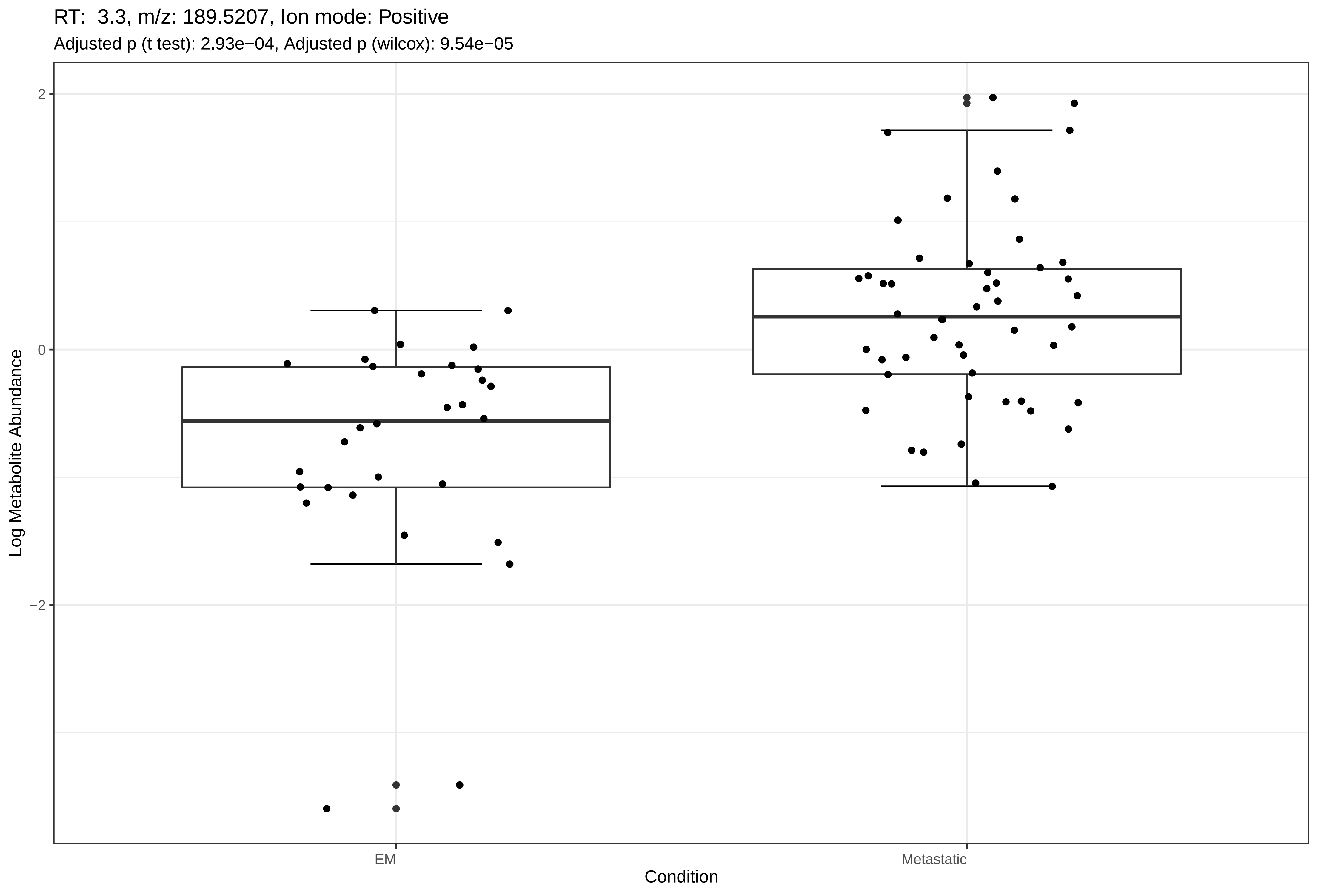
RT: 3.3, m/z: 189.5207, Ion mode: Positive
Adjusted p (t test): 2.93e−04, Adjusted p (wilcox): 9.54e−05
2
0
−2
Log Metabolite Abundance
EM
Metastatic
Condition

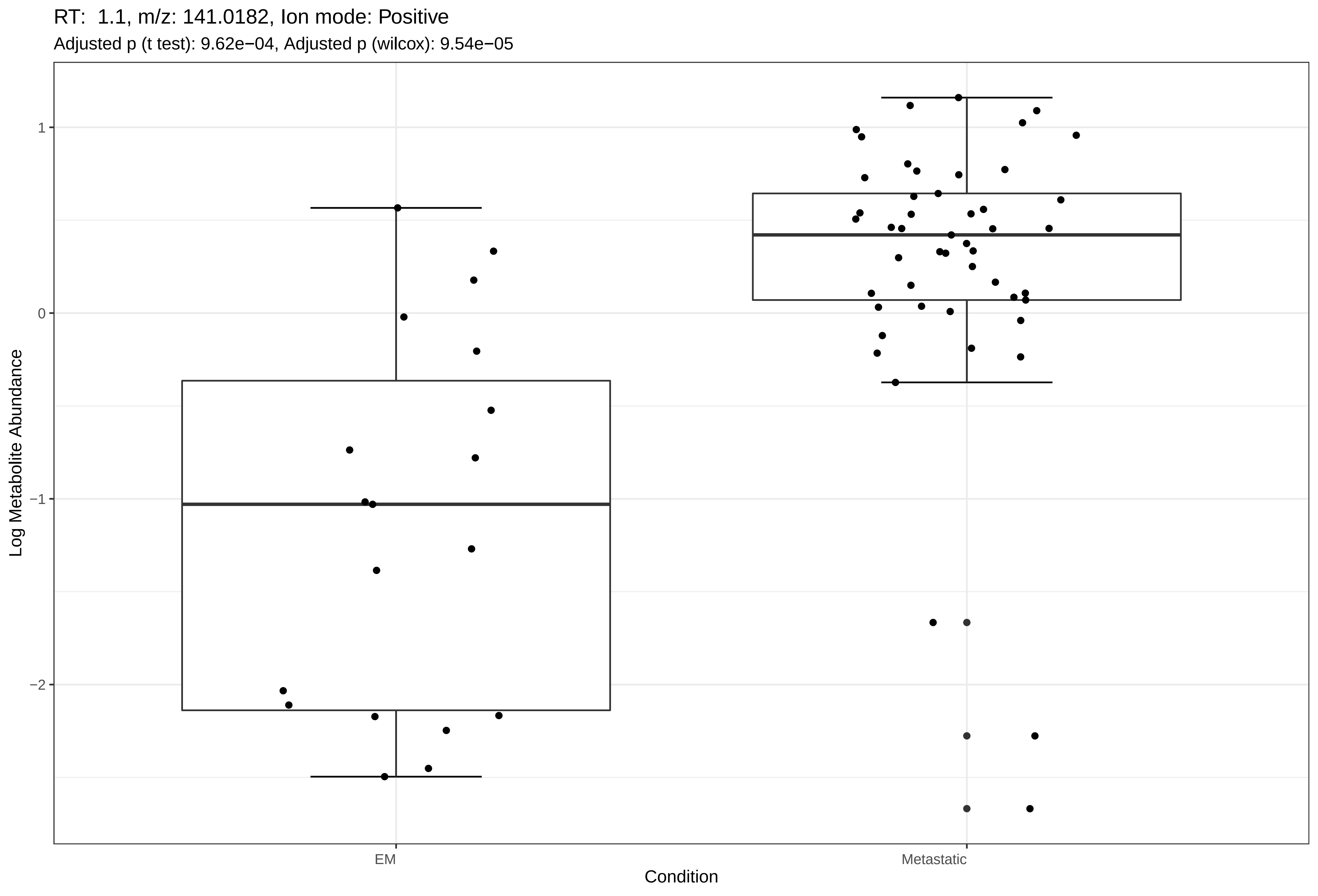

RT: 1.1, m/z: 141.0182, Ion mode: Positive
Adjusted p (t test): 9.62e−04, Adjusted p (wilcox): 9.54e−05
Log Metabolite Abundance
1
0
−1
−2
EM
Metastatic
Condition

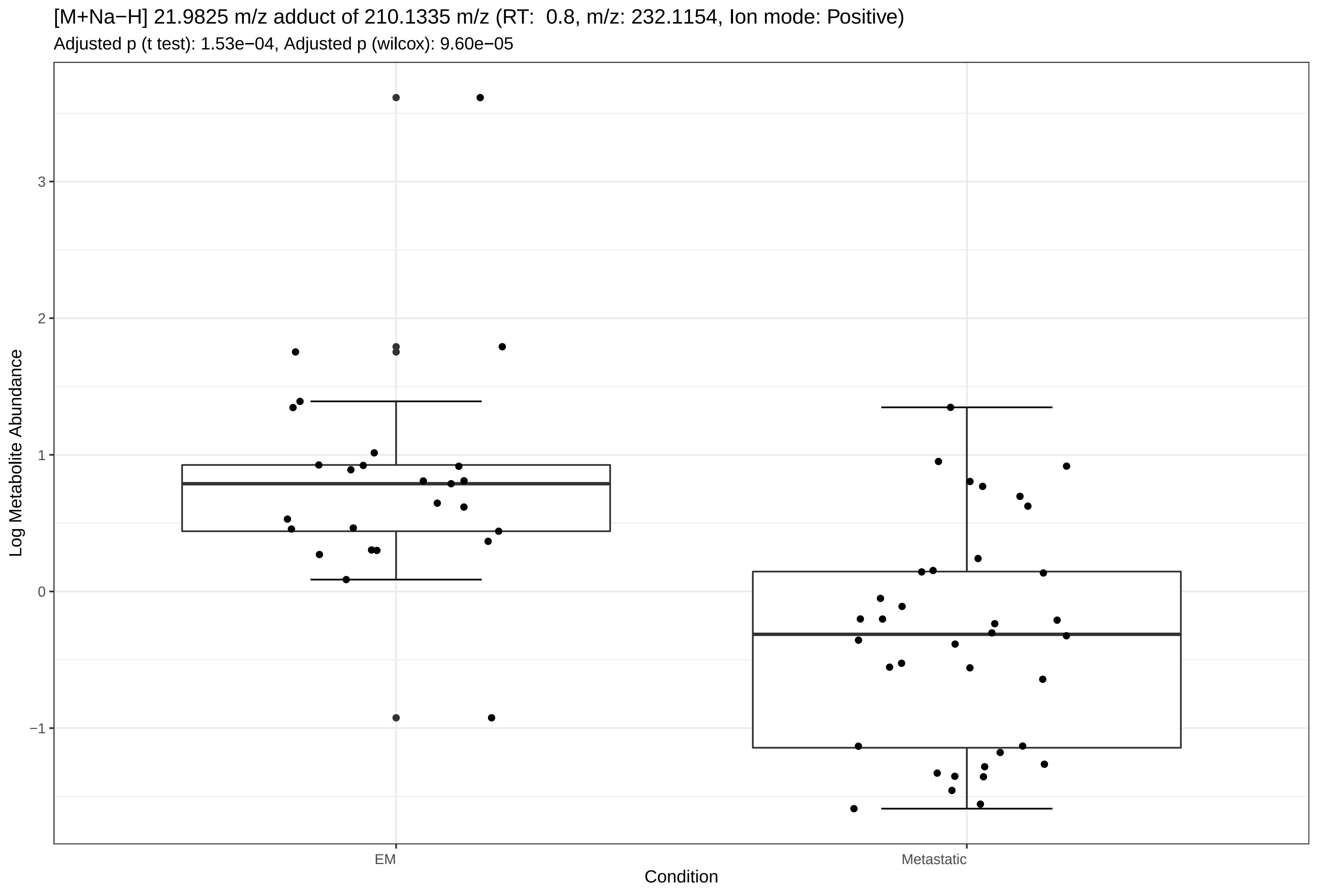
[M+Na−H] 21.9825 m/z adduct of 210.1335 m/z (RT: 0.8, m/z: 232.1154, Ion mode: Positive)
Adjusted p (t test): 1.53e−04, Adjusted p (wilcox): 9.60e−05
Log Metabolite Abundance
3
2
1
0
−1
EM
Metastatic
Condition

[M−NH3+H] −17.0265 m/z adduct of 209.0916 m/z (RT: 6.5, m/z: 192.0652, Ion mode: Positive)
Adjusted p (t test): 9.71e−05, Adjusted p (wilcox): 8.05e−04

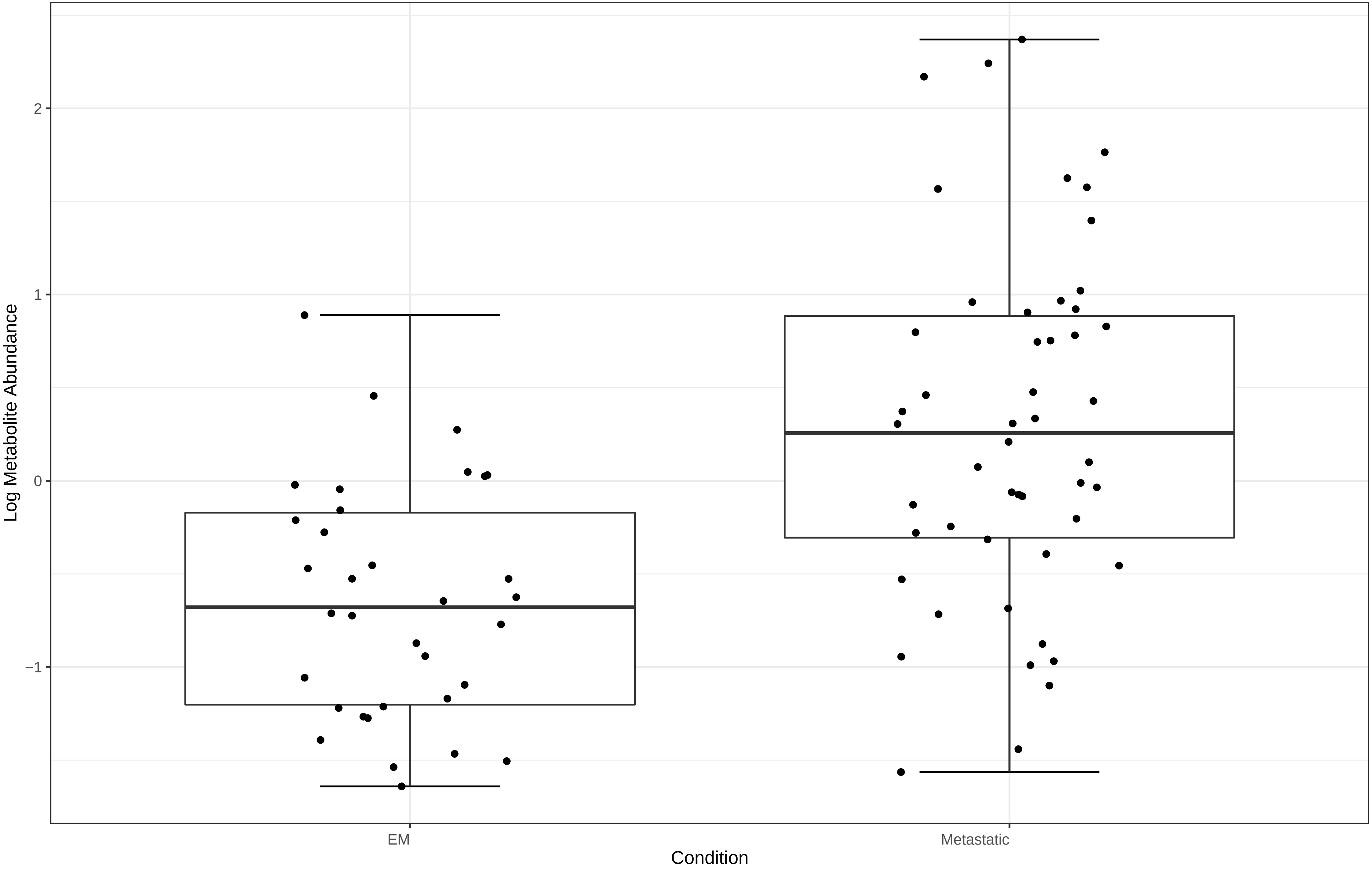

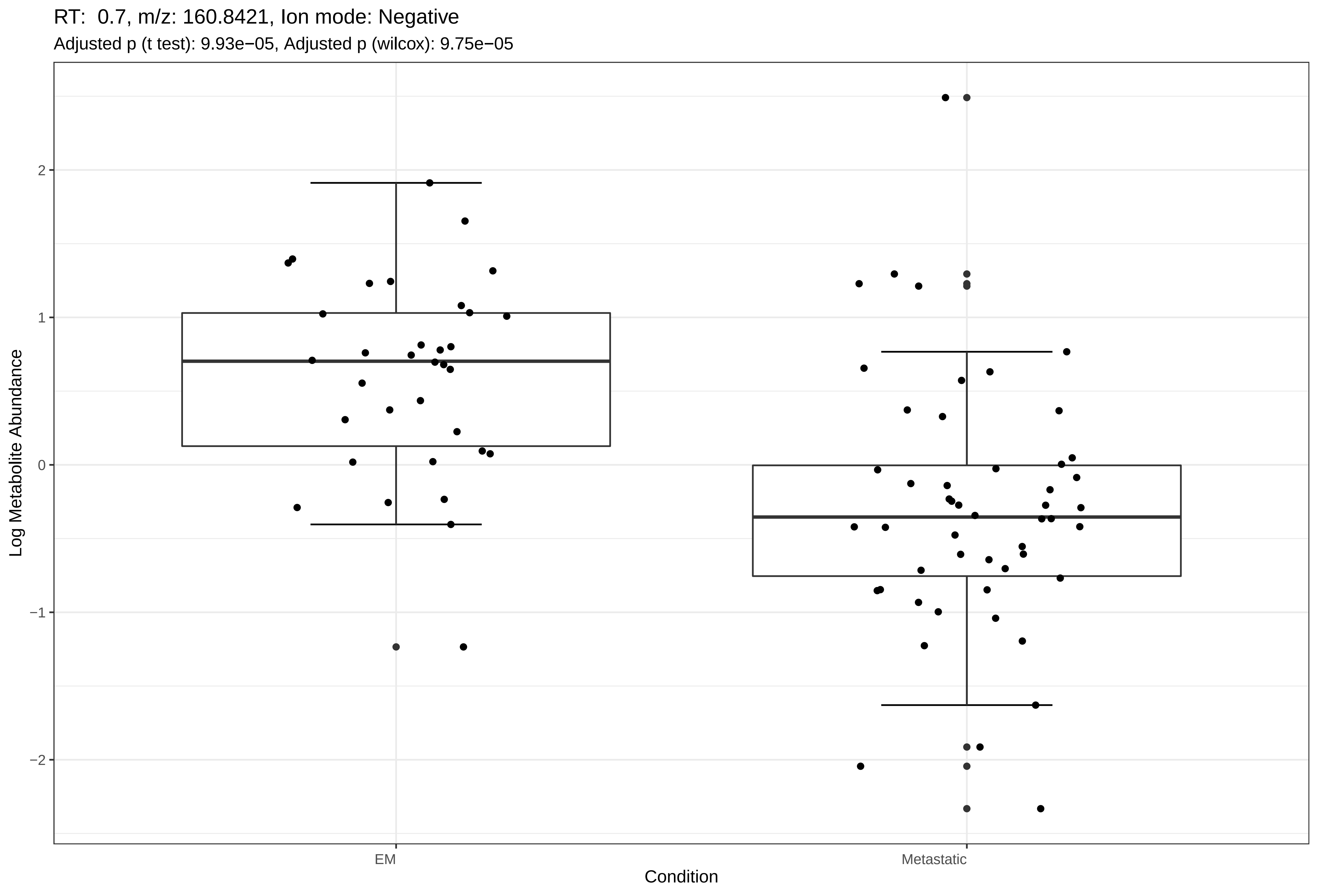
RT: 0.7, m/z: 160.8421, Ion mode: Negative
Adjusted p (t test): 9.93e−05, Adjusted p (wilcox): 9.75e−05
Log Metabolite Abundance
2
1
0
−1
−2
EM
Metastatic
Condition

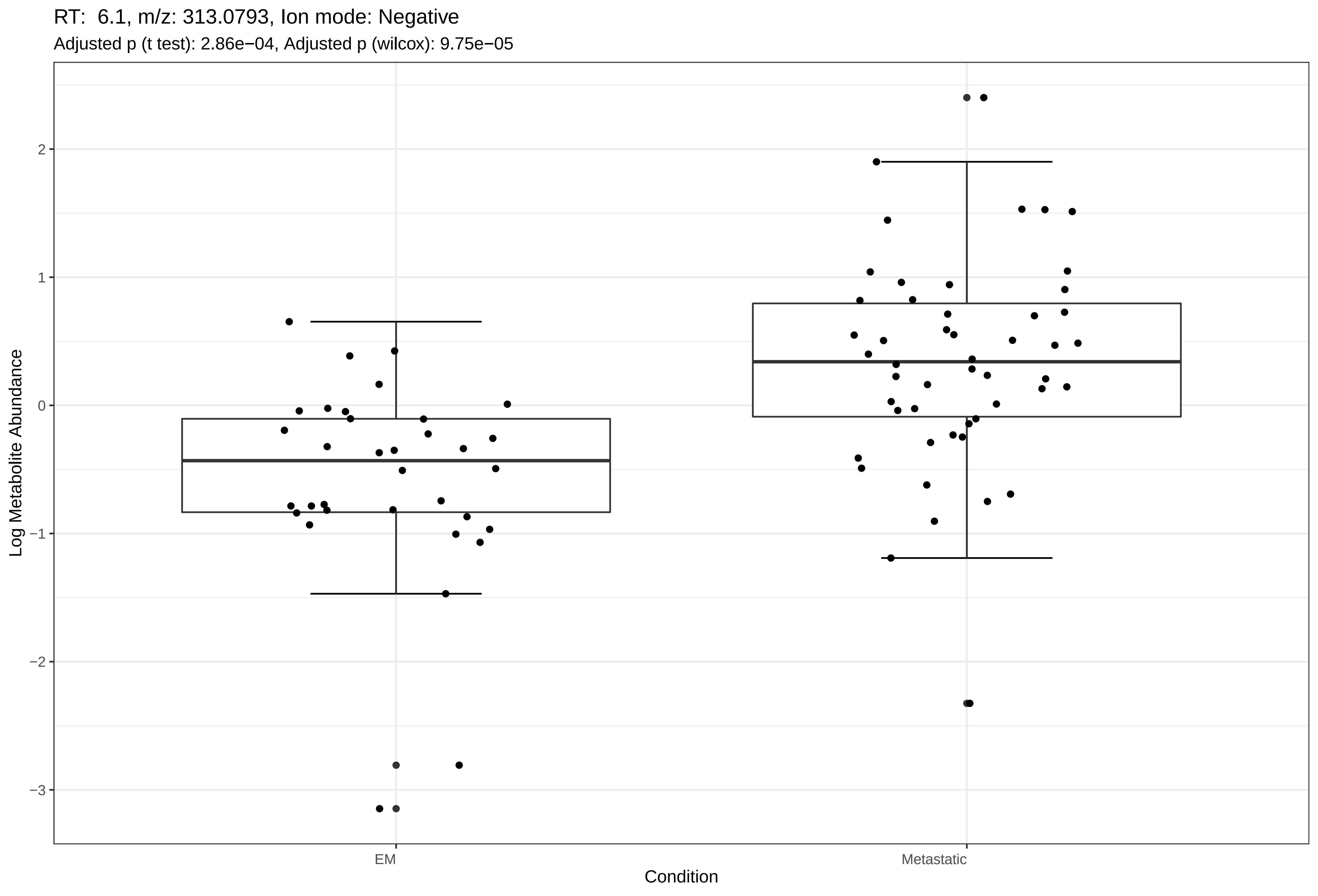

RT: 6.1, m/z: 313.0793, Ion mode: Negative
Adjusted p (t test): 2.86e−04, Adjusted p (wilcox): 9.75e−05
Log Metabolite Abundance
2
1
0
−1
−2
−3
EM
Metastatic
Condition

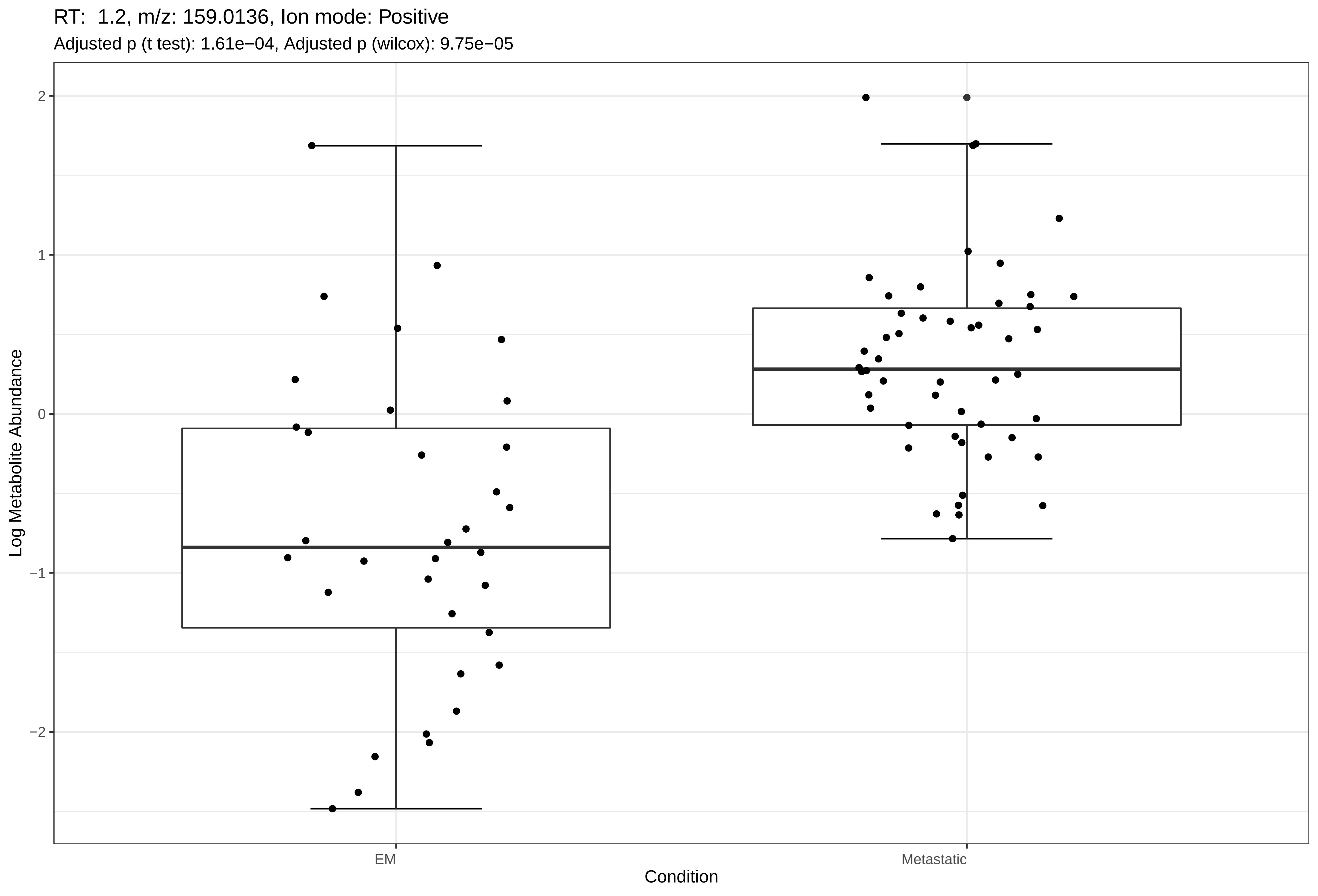
RT: 1.2, m/z: 159.0136, Ion mode: Positive
Adjusted p (t test): 1.61e−04, Adjusted p (wilcox): 9.75e−05
Log Metabolite Abundance
2
1
0
−1
−2
EM
Metastatic
Condition

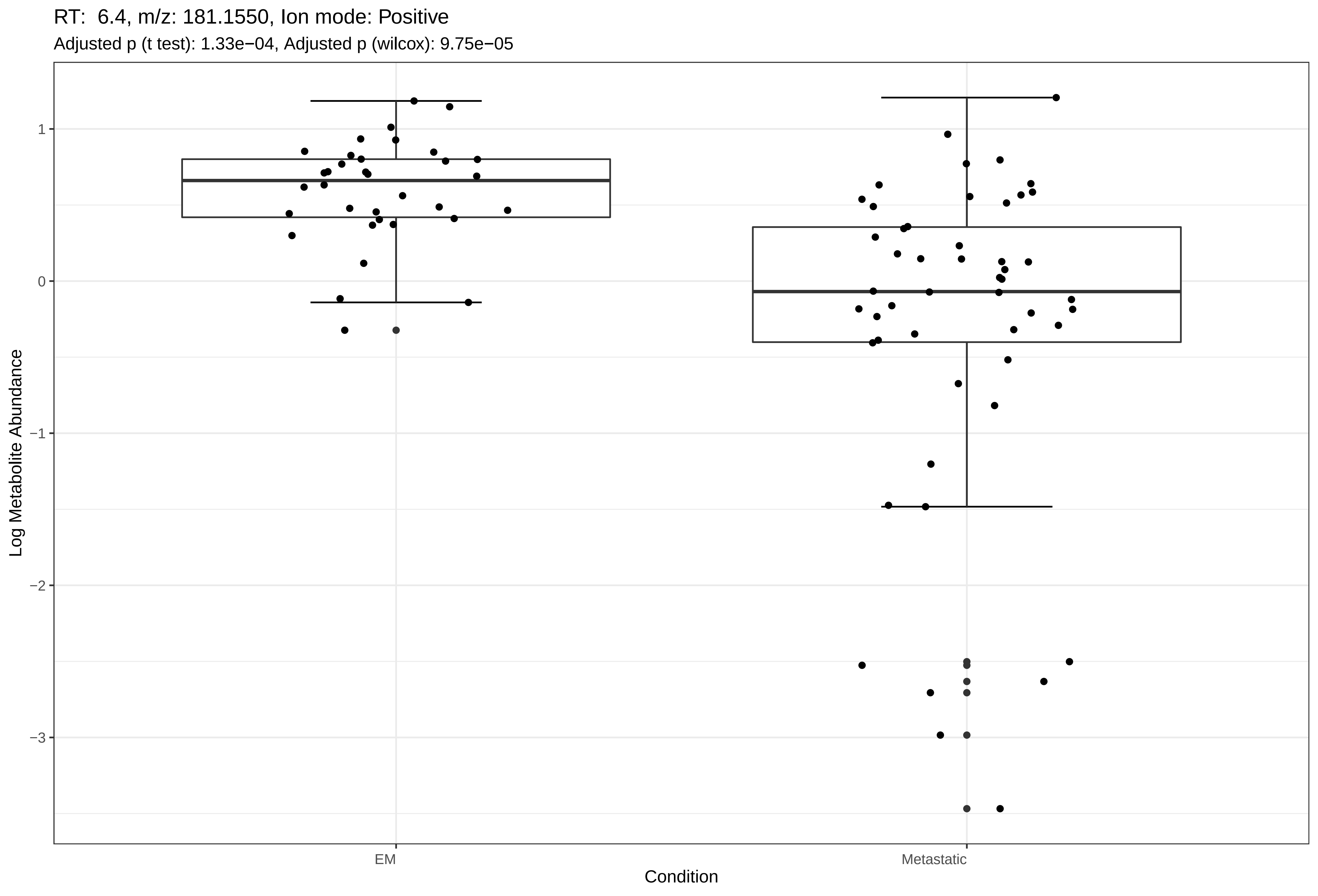
RT: 6.4, m/z: 181.1550, Ion mode: Positive
Adjusted p (t test): 1.33e−04, Adjusted p (wilcox): 9.75e−05
Log Metabolite Abundance
1
0
−1
−2
−3
EM
Metastatic
Condition

RT: 0.7, m/z: 120.9660, Ion mode: Positive

Adjusted p (t test): 9.76e−05, Adjusted p (wilcox): 1.60e−04

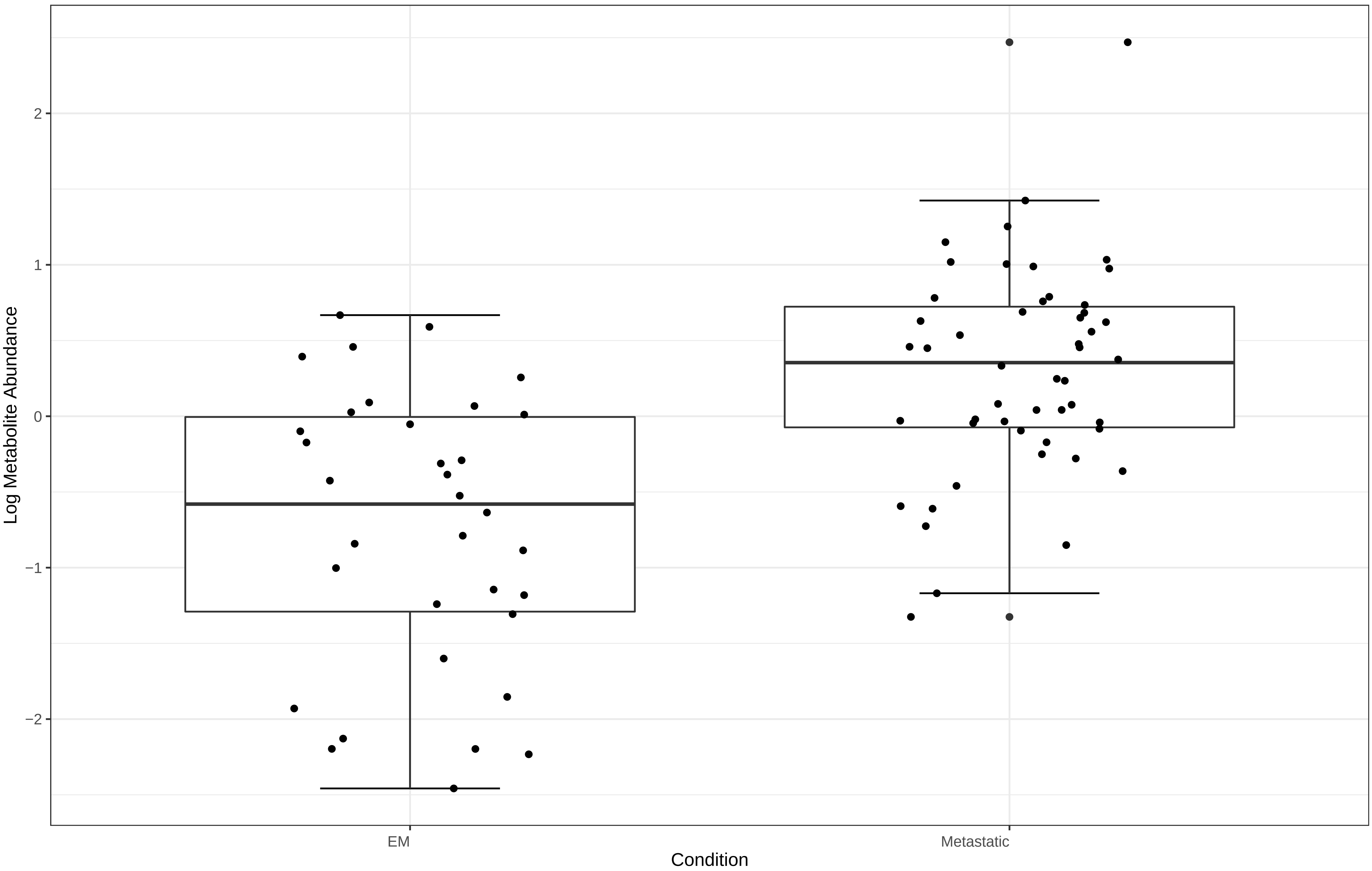

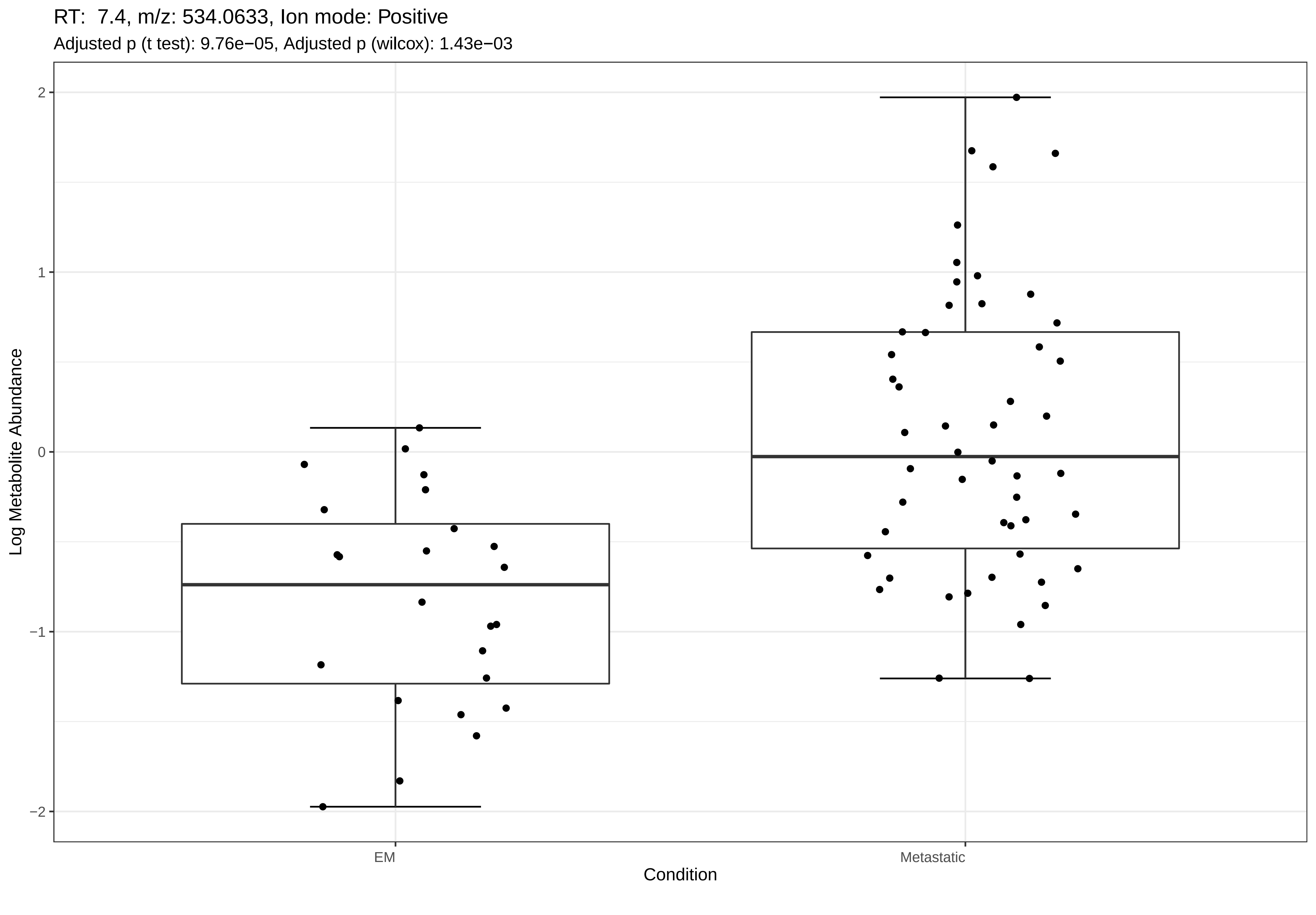

RT: 7.4, m/z: 534.0633, Ion mode: Positive
Adjusted p (t test): 9.76e−05, Adjusted p (wilcox): 1.43e−03
Log Metabolite Abundance
2
1
0
−1
−2
EM
Metastatic
Condition

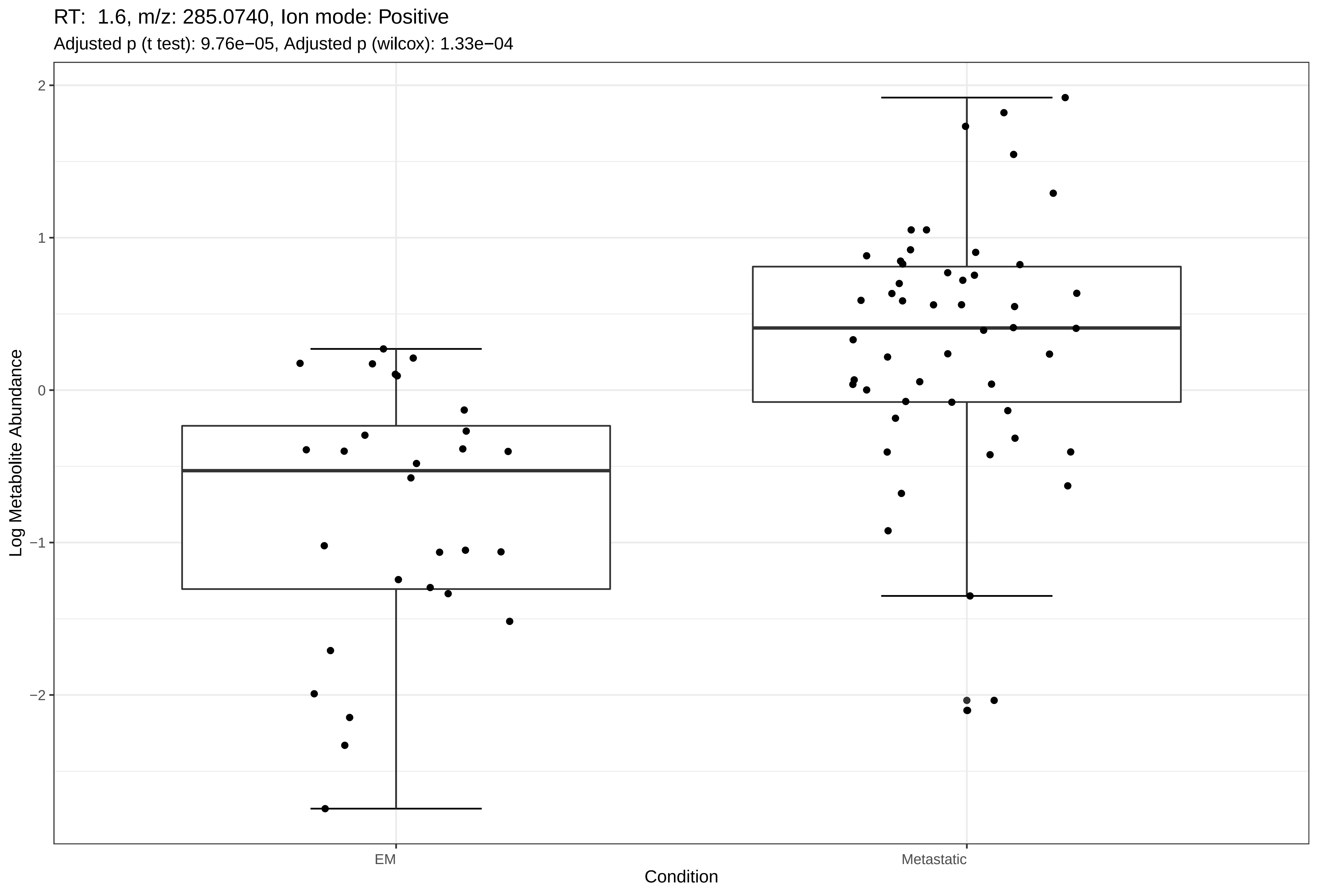
RT: 1.6, m/z: 285.0740, Ion mode: Positive
Adjusted p (t test): 9.76e−05, Adjusted p (wilcox): 1.33e−04
2
1
0
−1
−2
Log Metabolite Abundance
EM
Metastatic
Condition

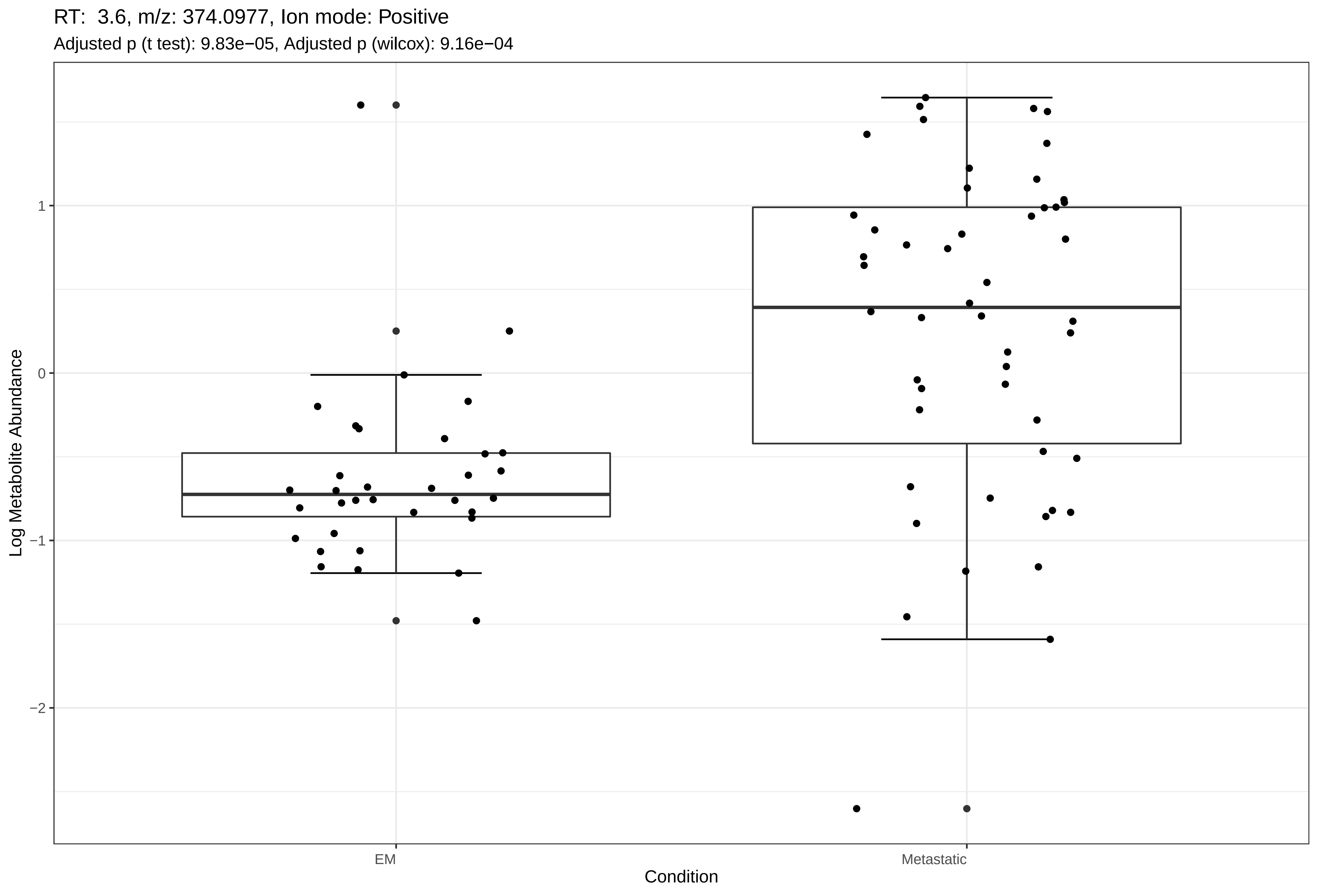
RT: 3.6, m/z: 374.0977, Ion mode: Positive
Adjusted p (t test): 9.83e−05, Adjusted p (wilcox): 9.16e−04
Log Metabolite Abundance
1
0
−1
−2
EM
Metastatic
Condition

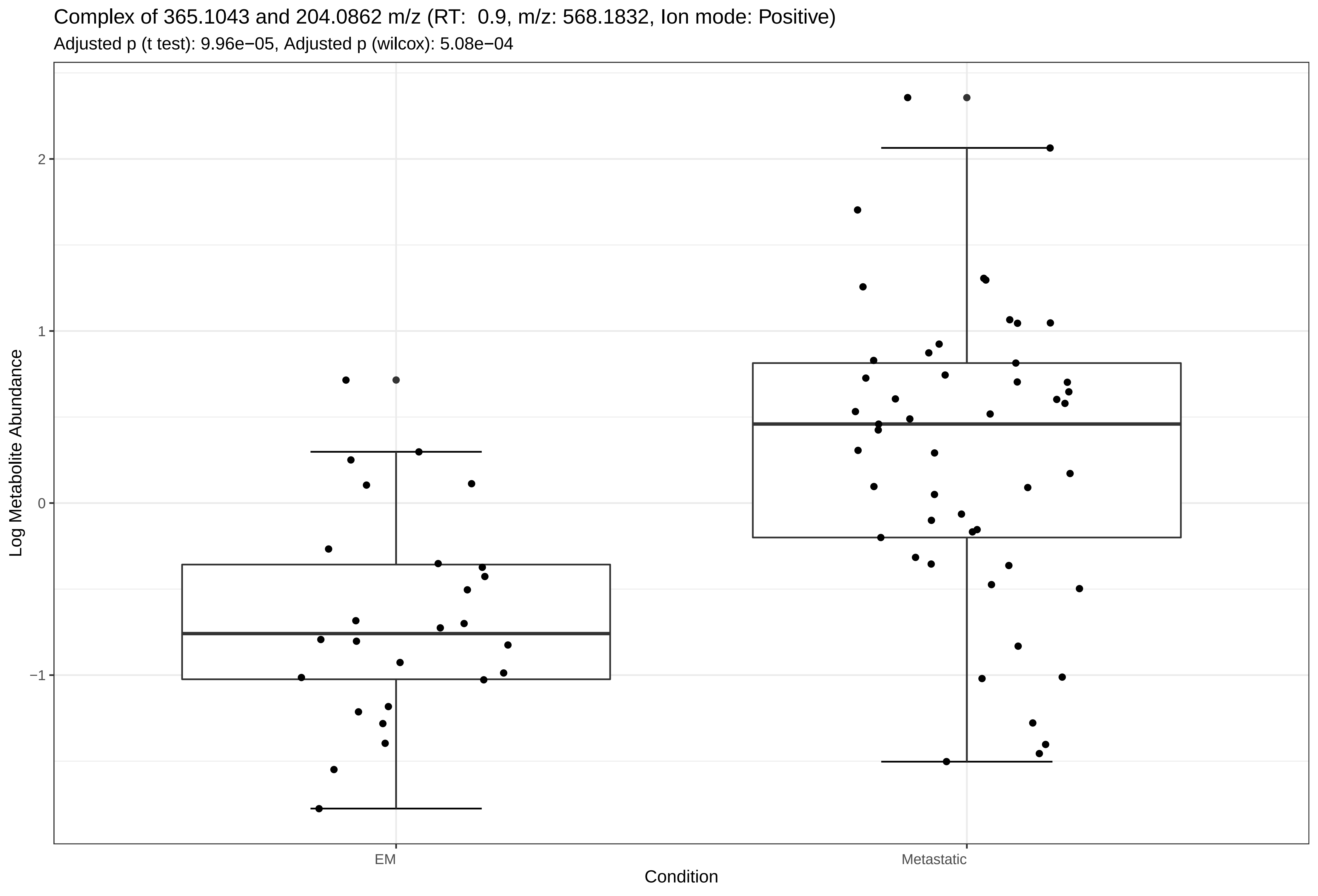

Complex of 365.1043 and 204.0862 m/z (RT: 0.9, m/z: 568.1832, Ion mode: Positive)
Adjusted p (t test): 9.96e−05, Adjusted p (wilcox): 5.08e−04
Log Metabolite Abundance
2
1
0
−1
EM
Metastatic
Condition

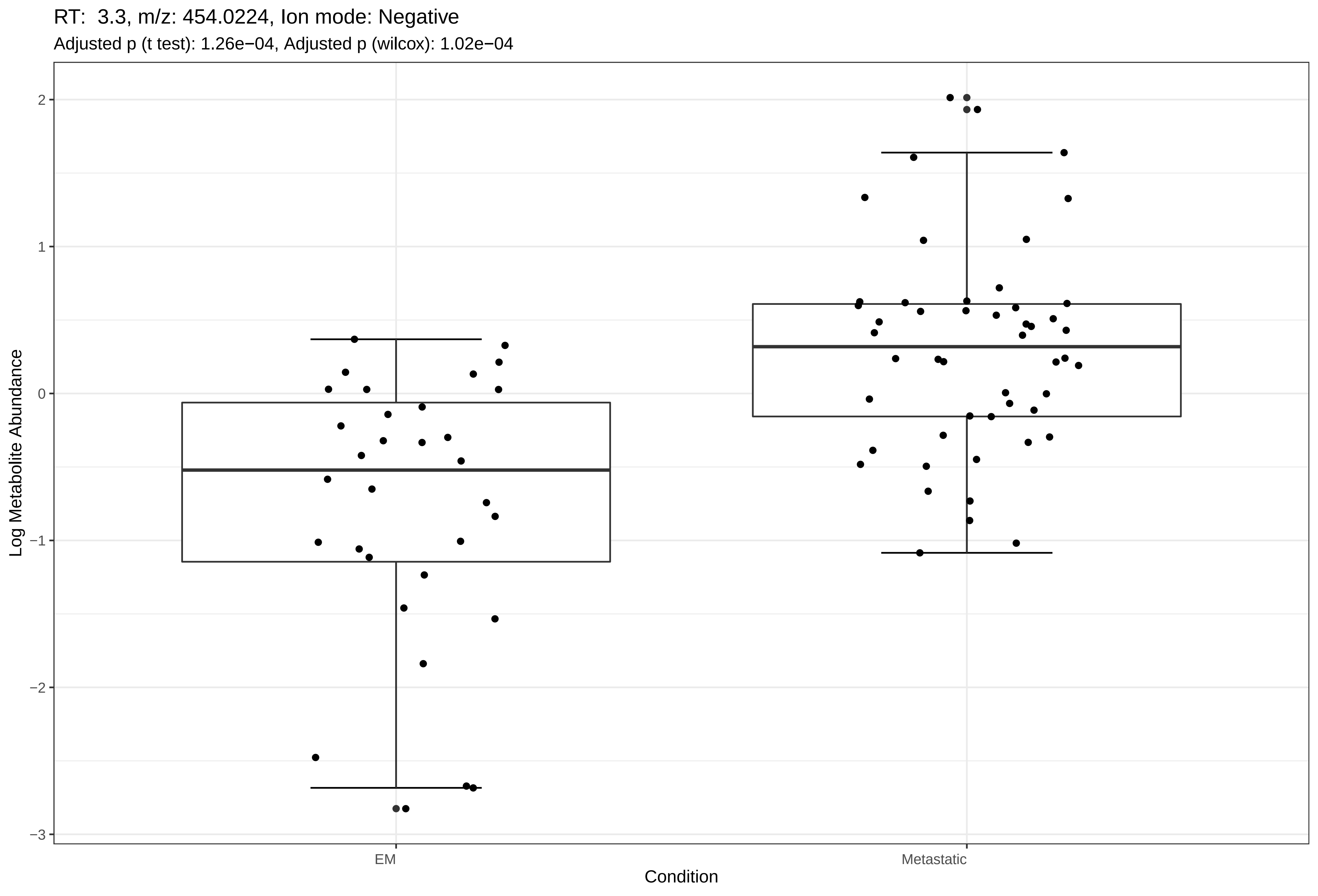
RT: 3.3, m/z: 454.0224, Ion mode: Negative
Adjusted p (t test): 1.26e−04, Adjusted p (wilcox): 1.02e−04
Log Metabolite Abundance
2
1
0
−1
−2
−3
EM
Metastatic
Condition

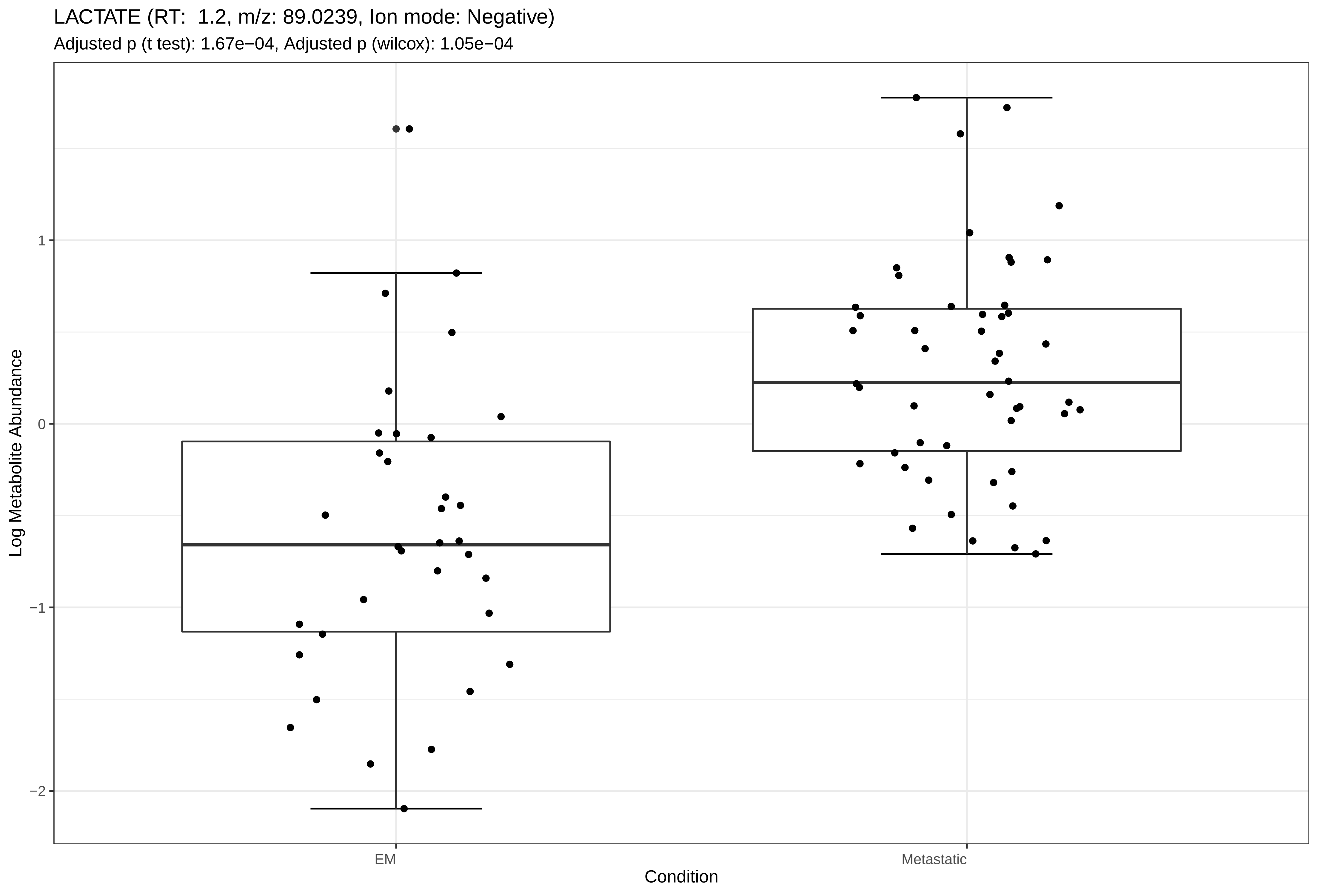

LACTATE (RT: 1.2, m/z: 89.0239, Ion mode: Negative)
Adjusted p (t test): 1.67e−04, Adjusted p (wilcox): 1.05e−04
Log Metabolite Abundance
1
0
−1
−2
EM
Metastatic
Condition

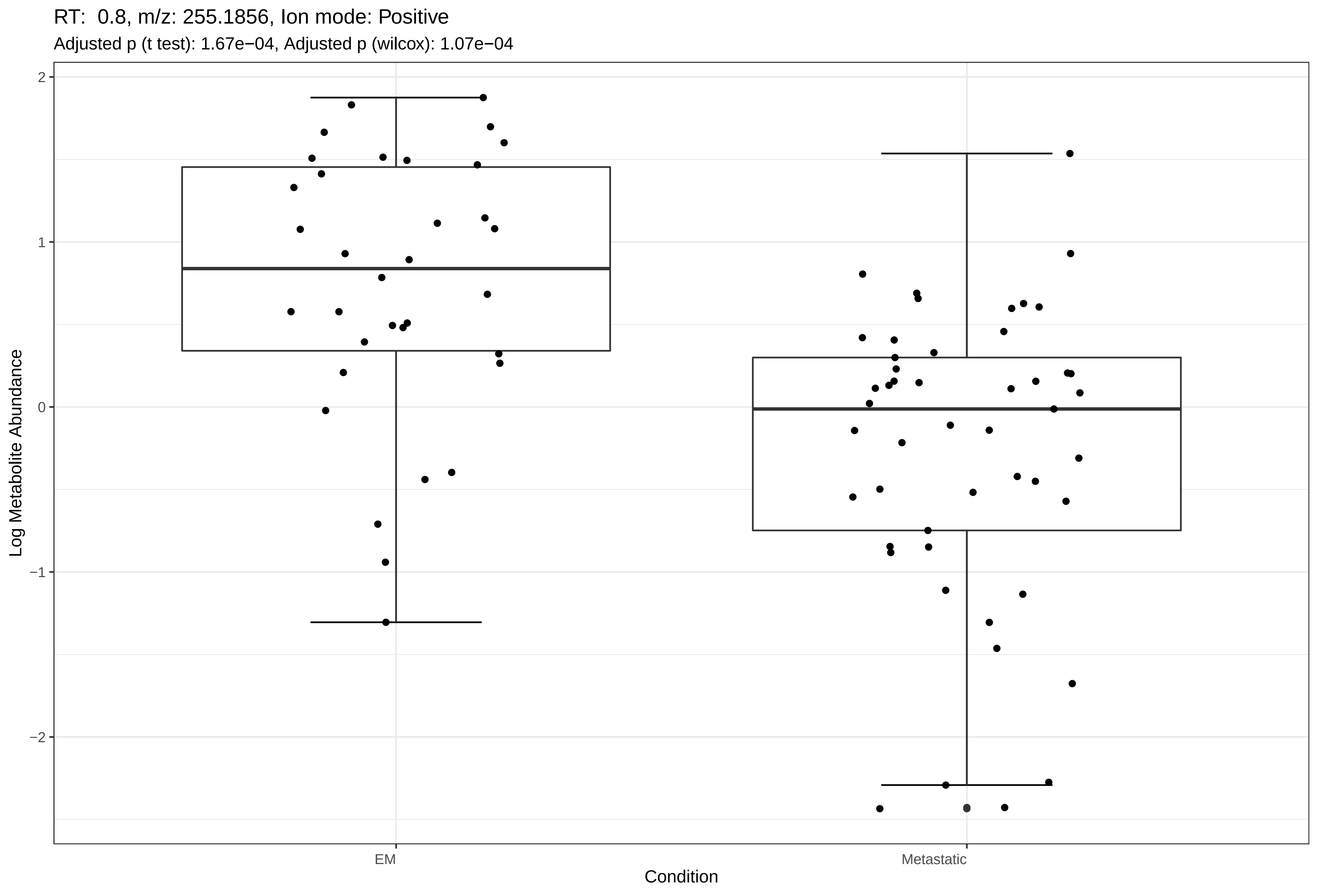
RT: 0.8, m/z: 255.1856, Ion mode: Positive
Adjusted p (t test): 1.67e−04, Adjusted p (wilcox): 1.07e−04
Log Metabolite Abundance
2
1
0
−1
−2
EM
Metastatic
Condition

RT: 0.7, m/z: 193.9296, Ion mode: Positive

Adjusted p (t test): 1.07e−04, Adjusted p (wilcox): 1.72e−04

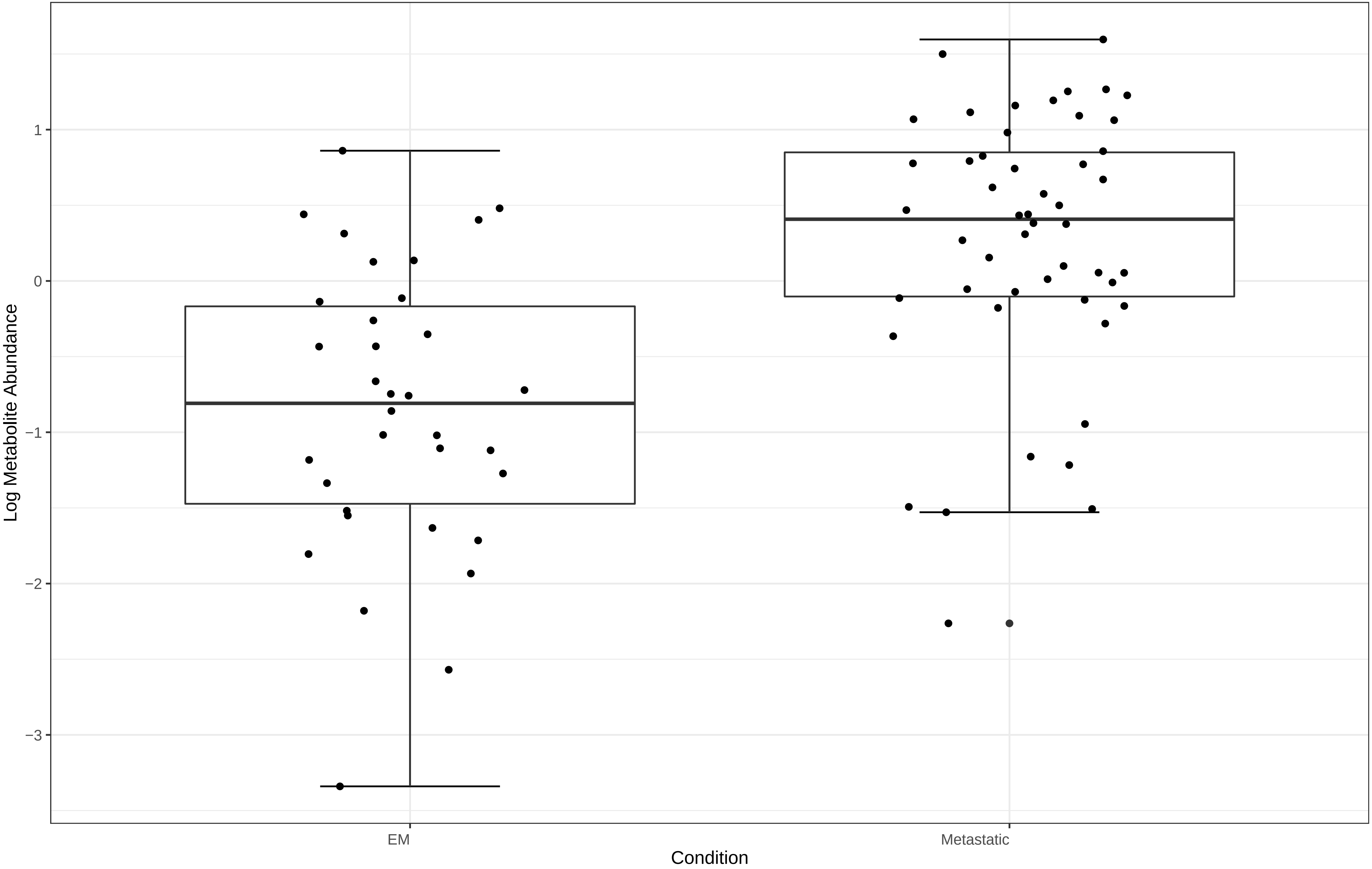

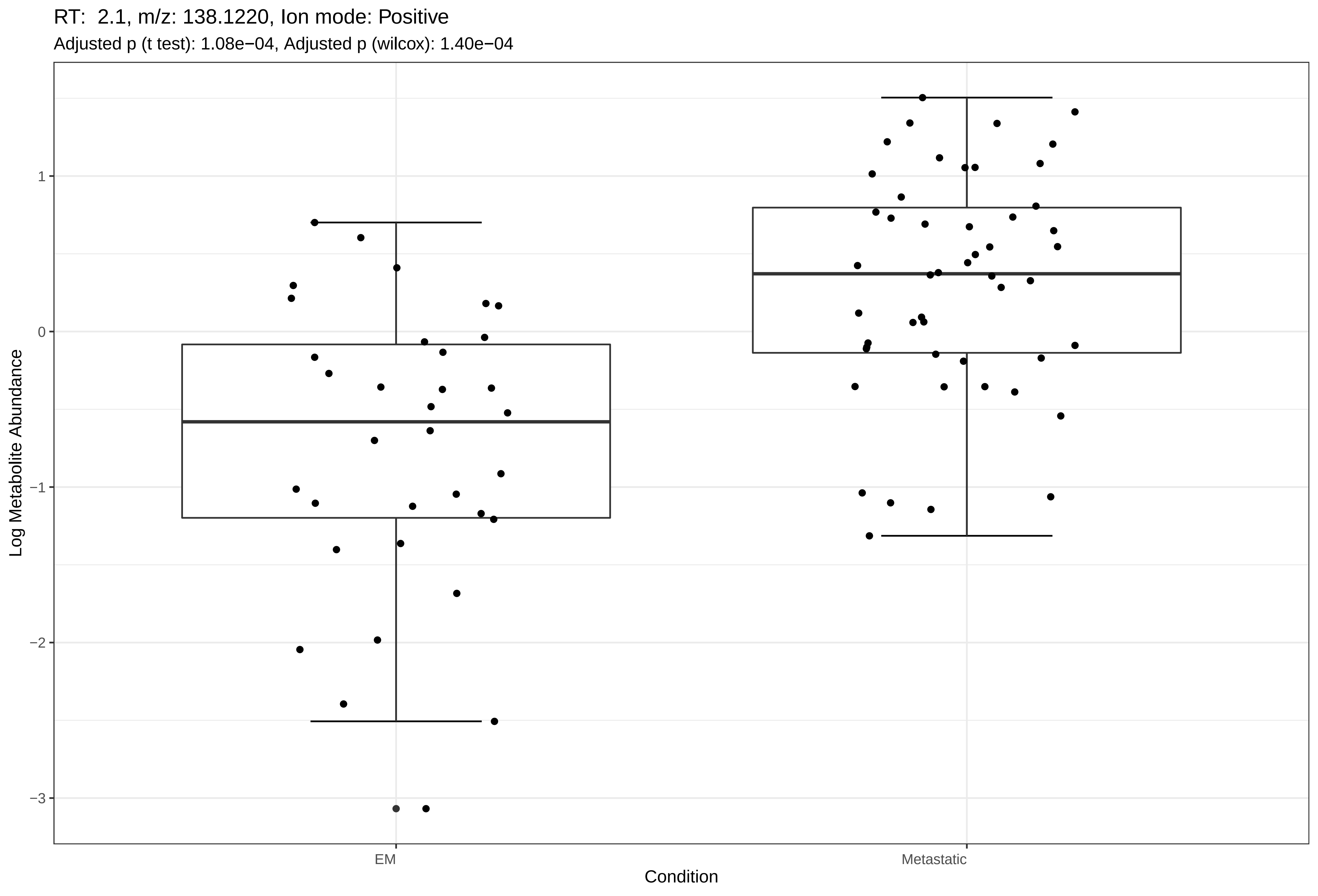
RT: 2.1, m/z: 138.1220, Ion mode: Positive
Adjusted p (t test): 1.08e−04, Adjusted p (wilcox): 1.40e−04
Log Metabolite Abundance
1
0
−1
−2
−3
EM
Metastatic
Condition

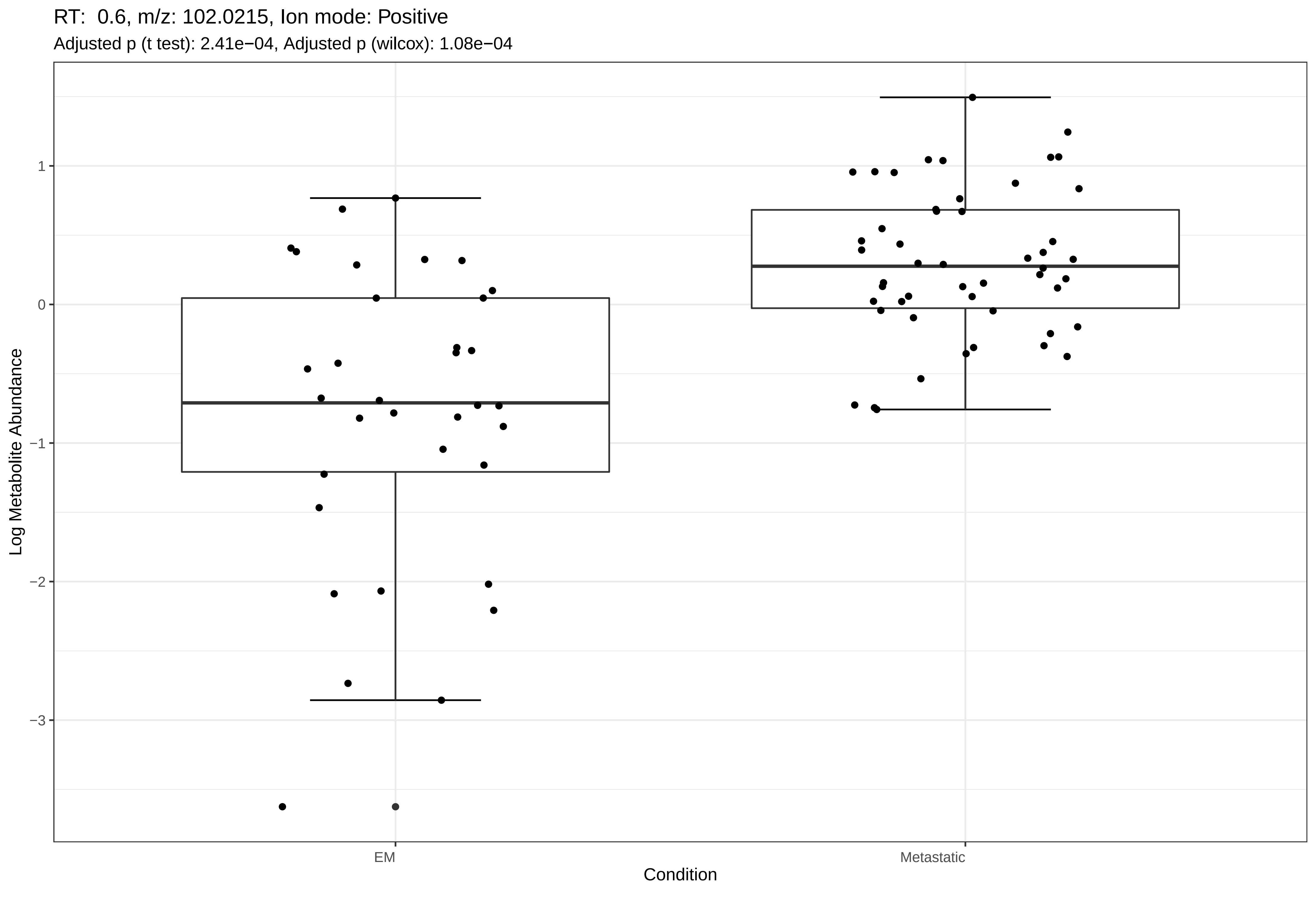
RT: 0.6, m/z: 102.0215, Ion mode: Positive
Adjusted p (t test): 2.41e−04, Adjusted p (wilcox): 1.08e−04
Log Metabolite Abundance
1
0
−1
−2
−3
EM
Metastatic
Condition

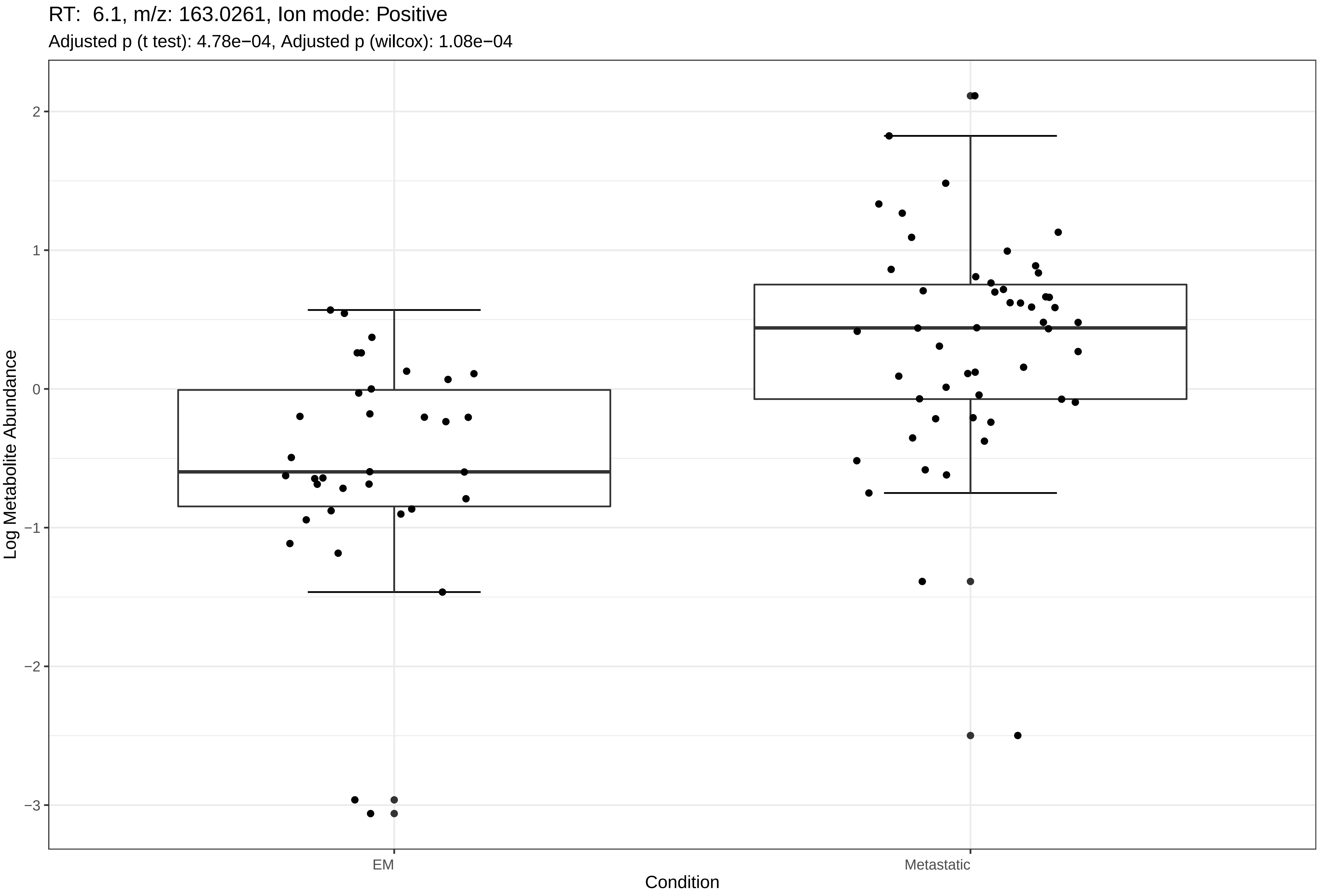

RT: 6.1, m/z: 163.0261, Ion mode: Positive
Adjusted p (t test): 4.78e−04, Adjusted p (wilcox): 1.08e−04
Log Metabolite Abundance
2
1
0
−1
−2
−3
EM
Metastatic
Condition

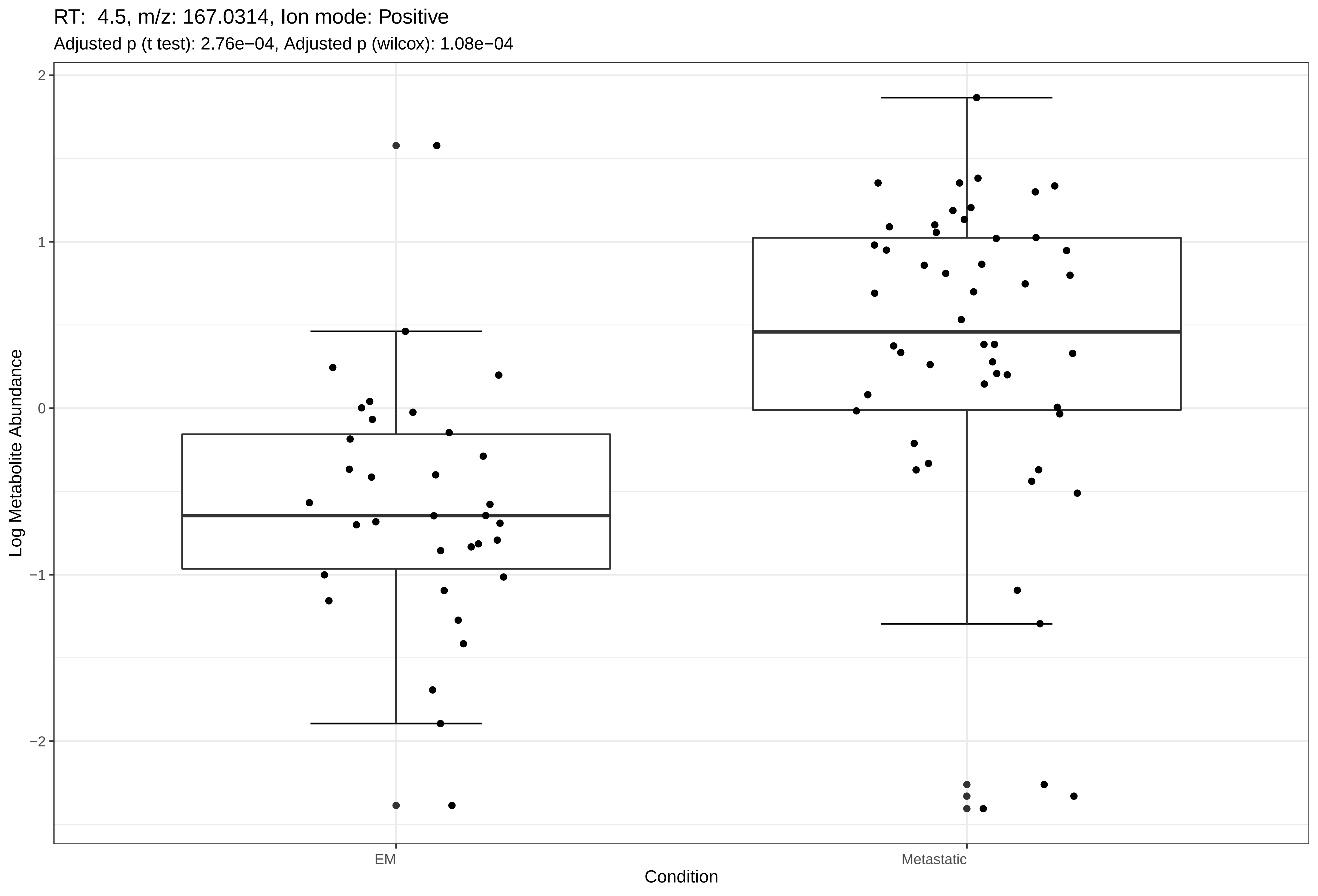

RT: 4.5, m/z: 167.0314, Ion mode: Positive
Adjusted p (t test): 2.76e−04, Adjusted p (wilcox): 1.08e−04
Log Metabolite Abundance
2
1
0
−1
−2
EM
Metastatic
Condition

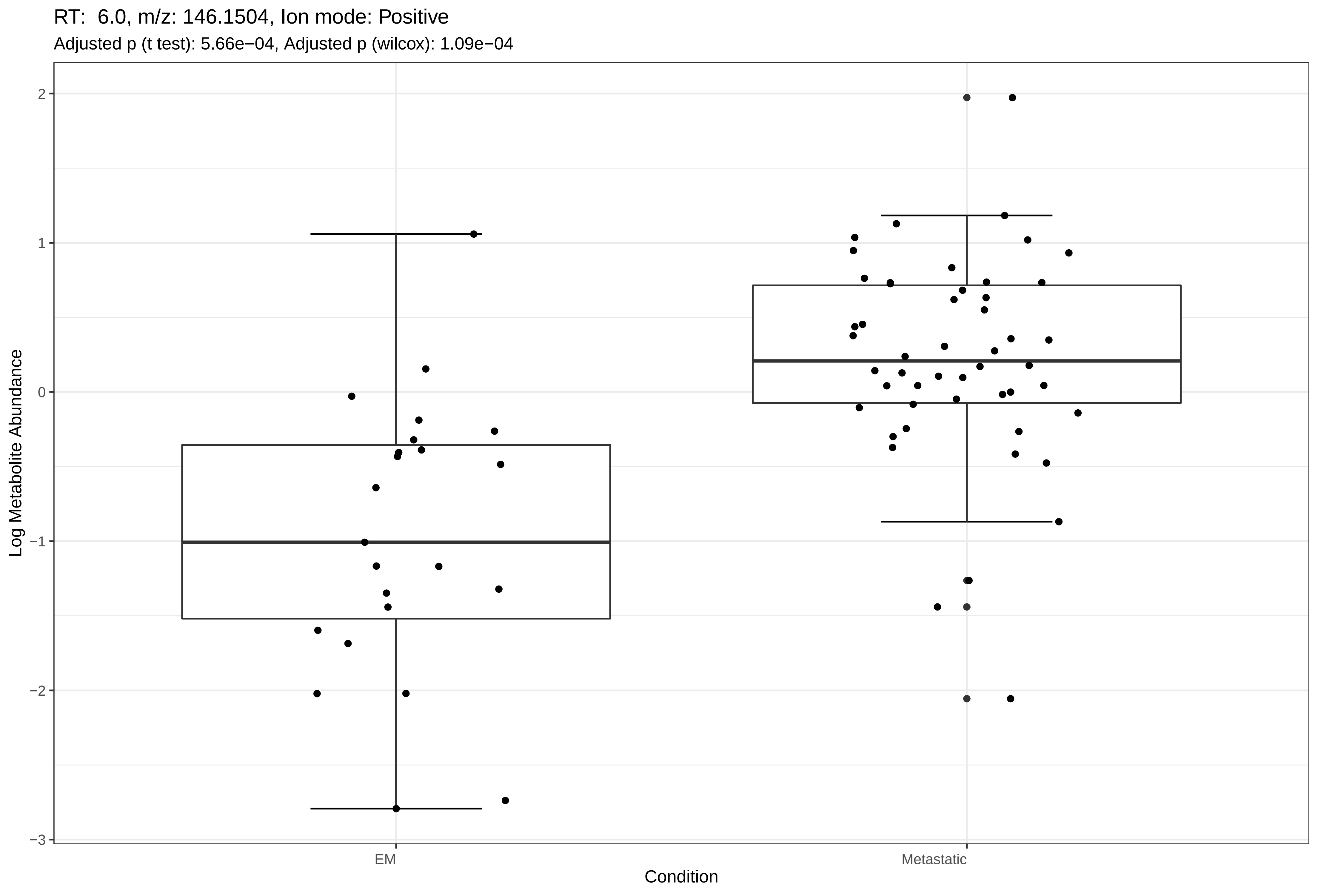
RT: 6.0, m/z: 146.1504, Ion mode: Positive
Adjusted p (t test): 5.66e−04, Adjusted p (wilcox): 1.09e−04
Log Metabolite Abundance
2
1
0
−1
−2
−3
EM
Metastatic
Condition

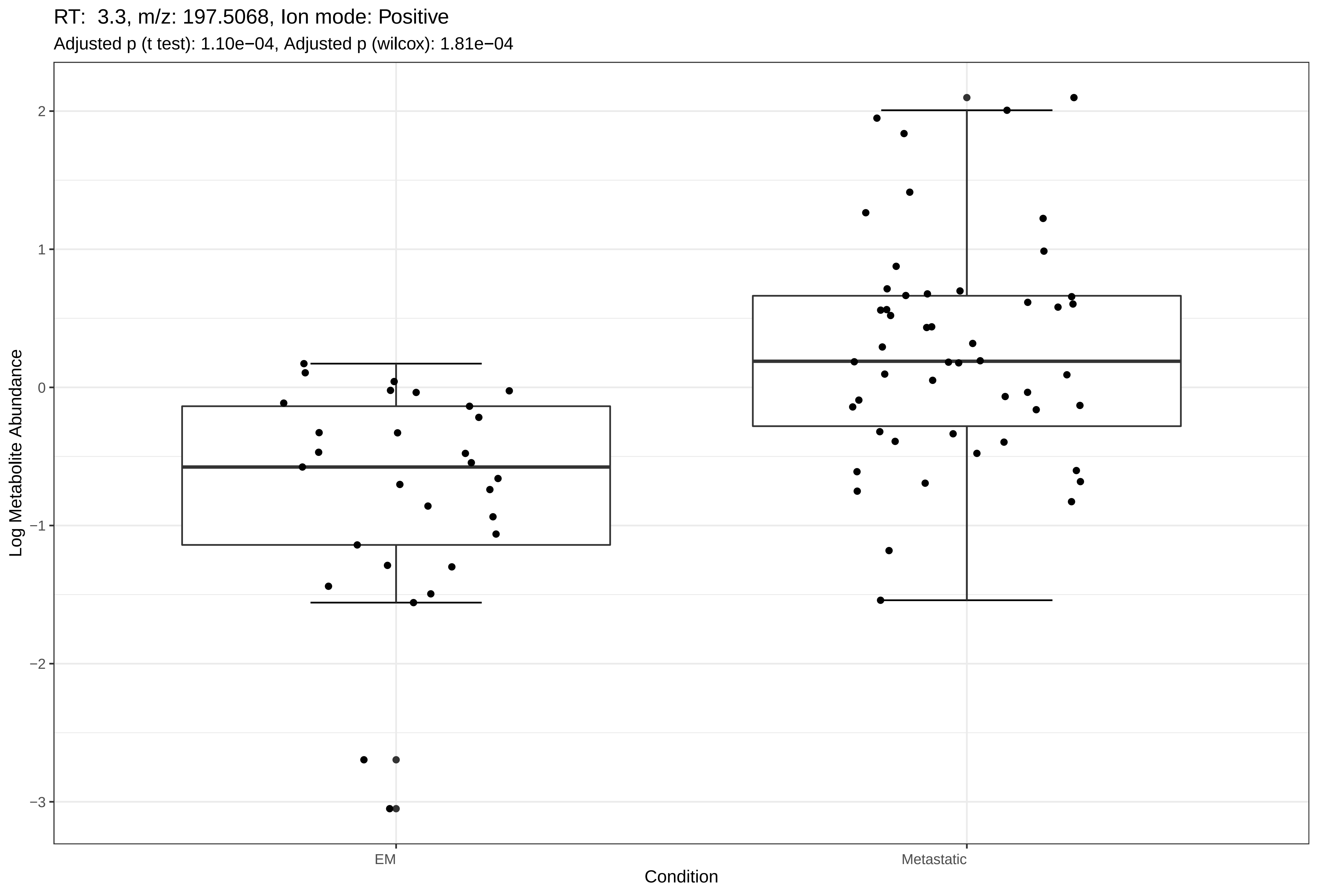
RT: 3.3, m/z: 197.5068, Ion mode: Positive
Adjusted p (t test): 1.10e−04, Adjusted p (wilcox): 1.81e−04
Log Metabolite Abundance
2
1
0
−1
−2
−3
EM
Metastatic
Condition

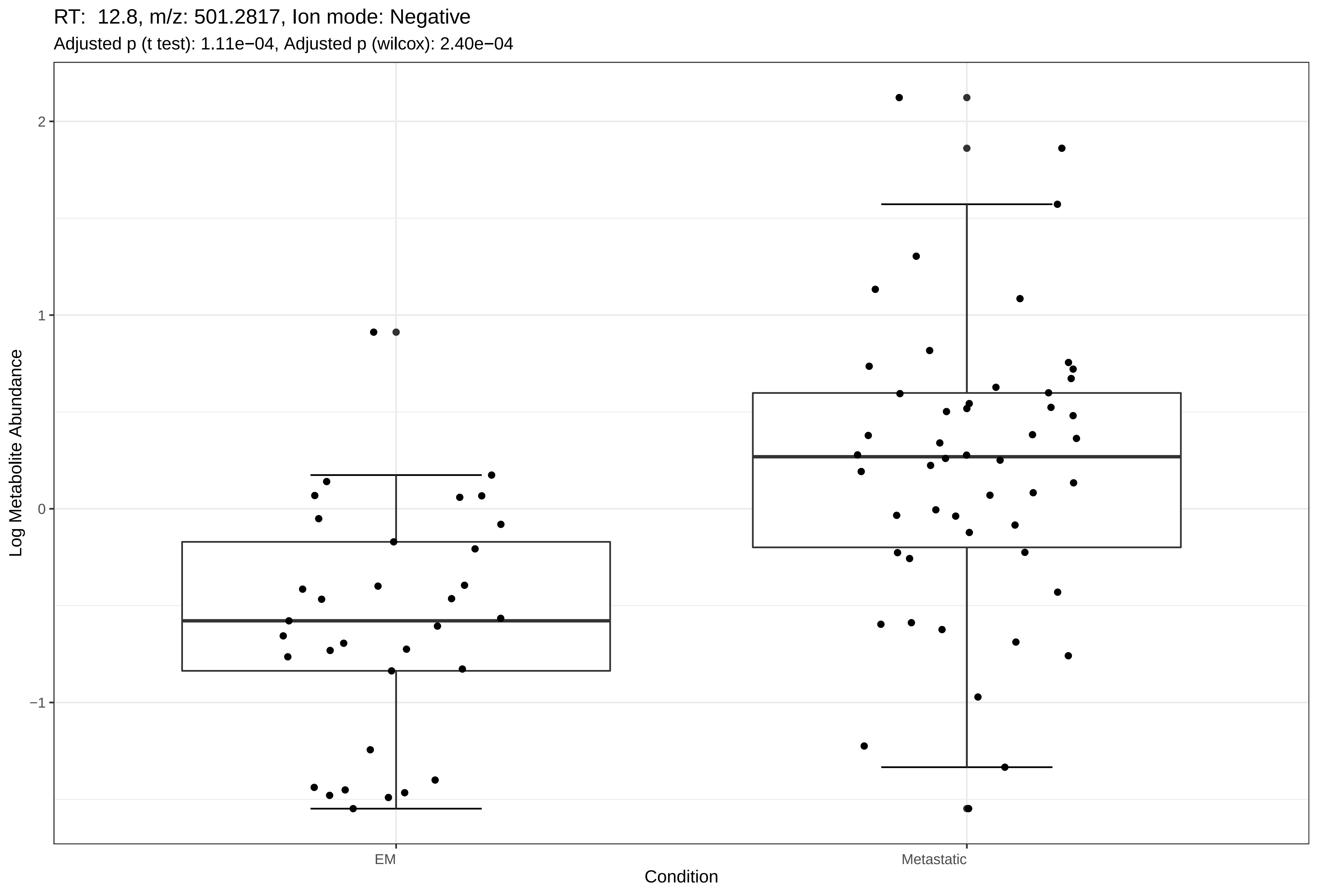
RT: 12.8, m/z: 501.2817, Ion mode: Negative
Adjusted p (t test): 1.11e−04, Adjusted p (wilcox): 2.40e−04
Log Metabolite Abundance
2
1
0
−1
EM
Metastatic
Condition

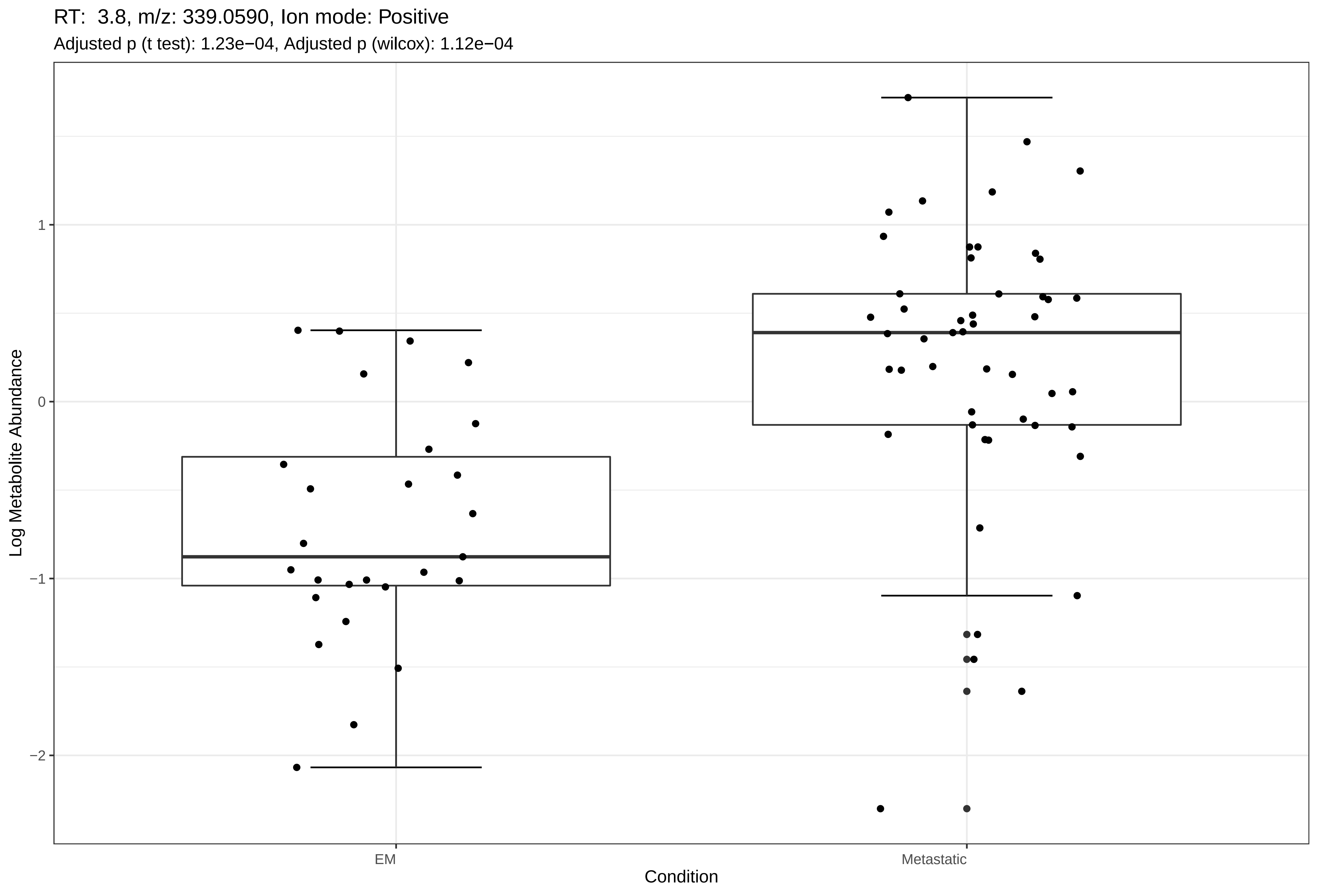

RT: 3.8, m/z: 339.0590, Ion mode: Positive
Adjusted p (t test): 1.23e−04, Adjusted p (wilcox): 1.12e−04
Log Metabolite Abundance
1
0
−1
−2
EM
Metastatic
Condition

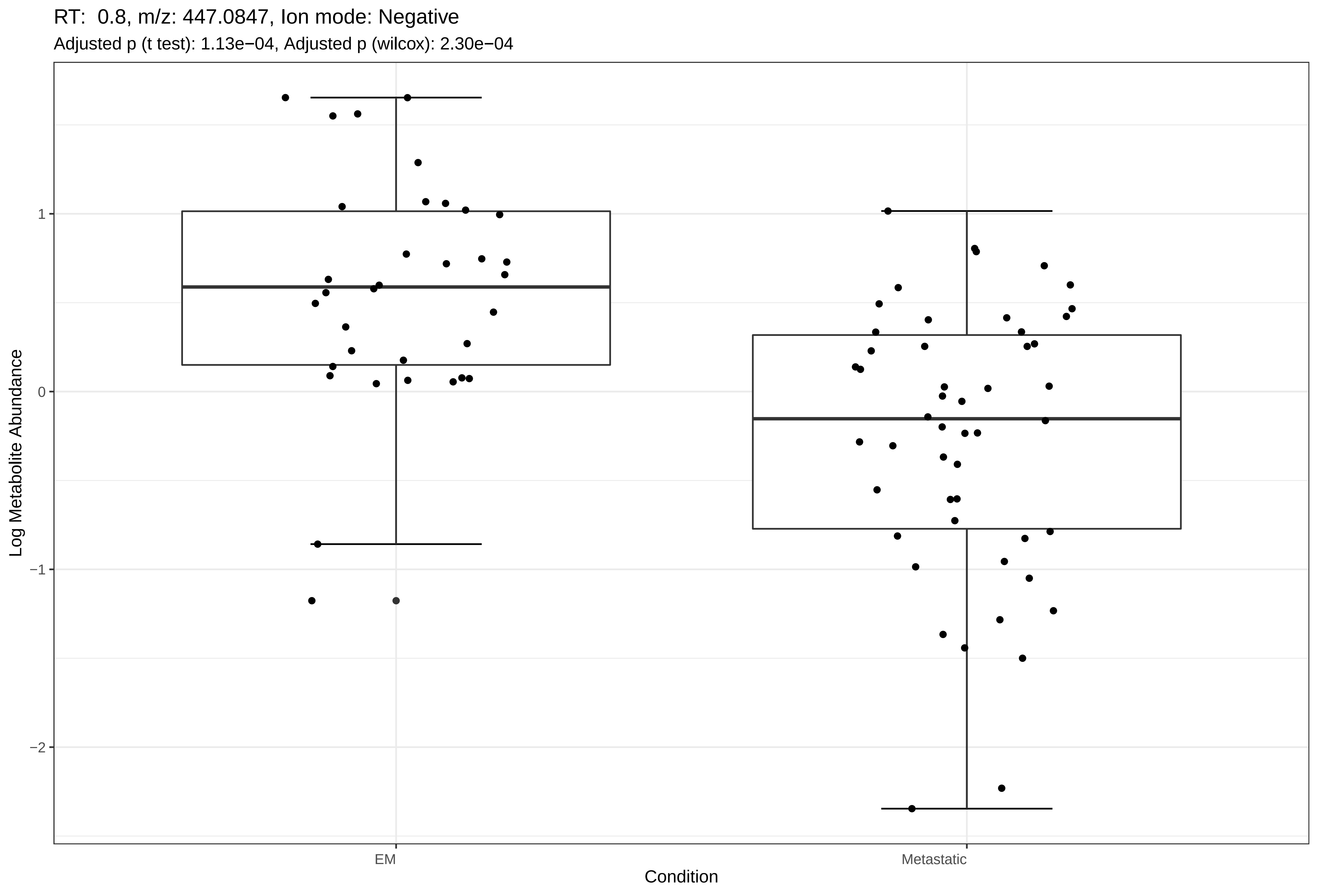

RT: 0.8, m/z: 447.0847, Ion mode: Negative
Adjusted p (t test): 1.13e−04, Adjusted p (wilcox): 2.30e−04
Log Metabolite Abundance
1
0
−1
−2
EM
Metastatic
Condition

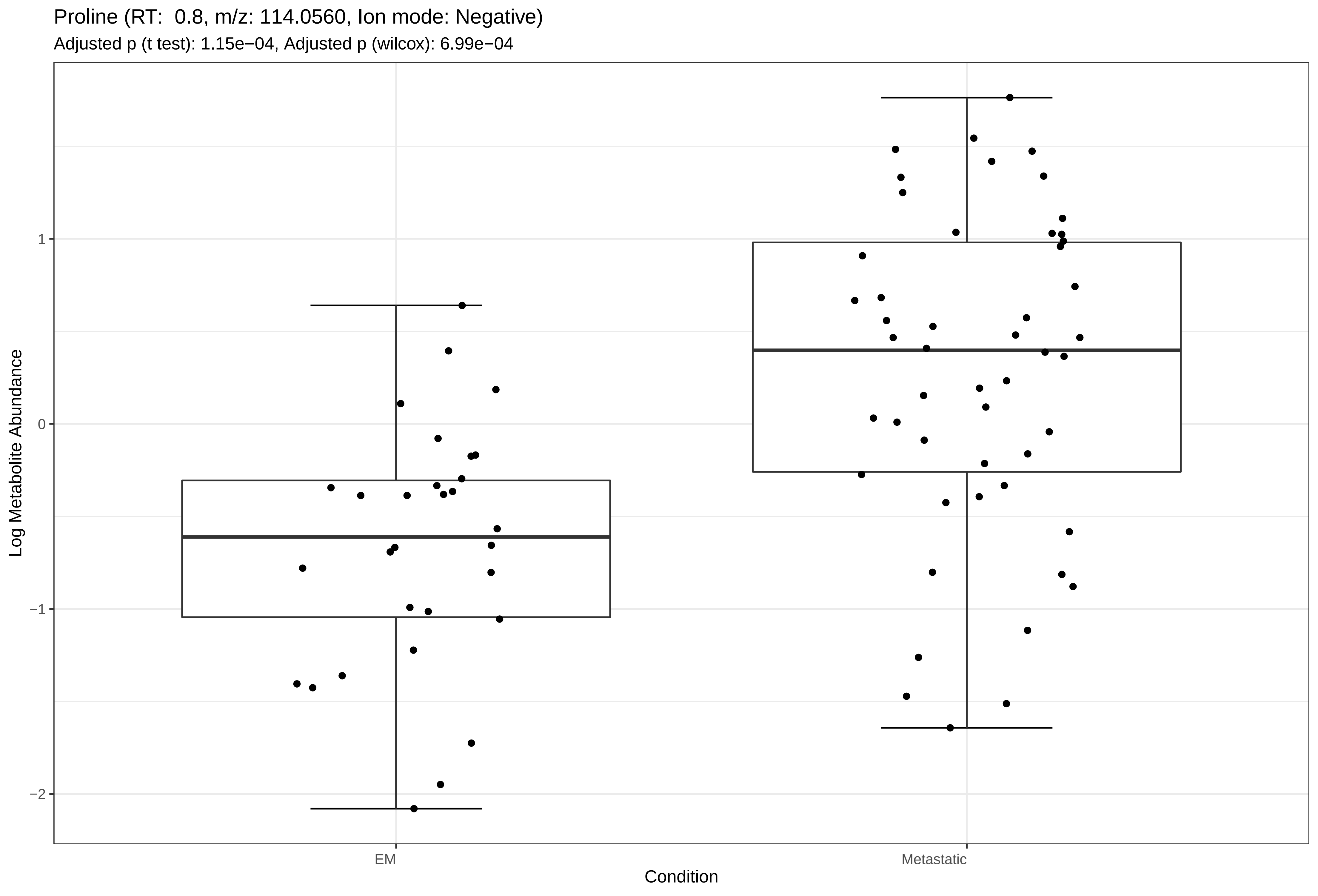
Proline (RT: 0.8, m/z: 114.0560, Ion mode: Negative)
Adjusted p (t test): 1.15e−04, Adjusted p (wilcox): 6.99e−04
Log Metabolite Abundance
1
0
−1
−2
EM
Metastatic
Condition

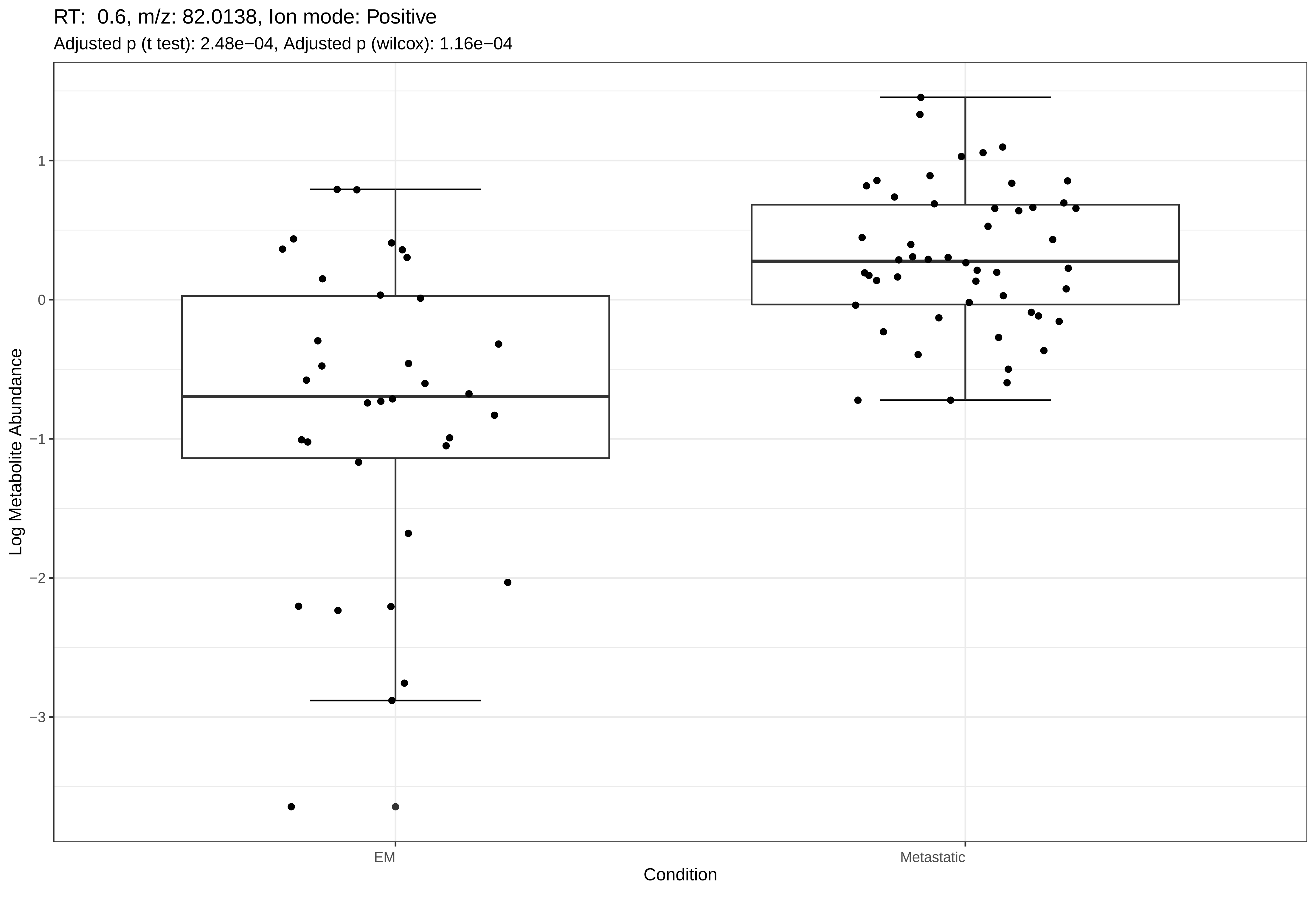

RT: 0.6, m/z: 82.0138, Ion mode: Positive
Adjusted p (t test): 2.48e−04, Adjusted p (wilcox): 1.16e−04
Log Metabolite Abundance
1
0
−1
−2
−3
EM
Metastatic
Condition

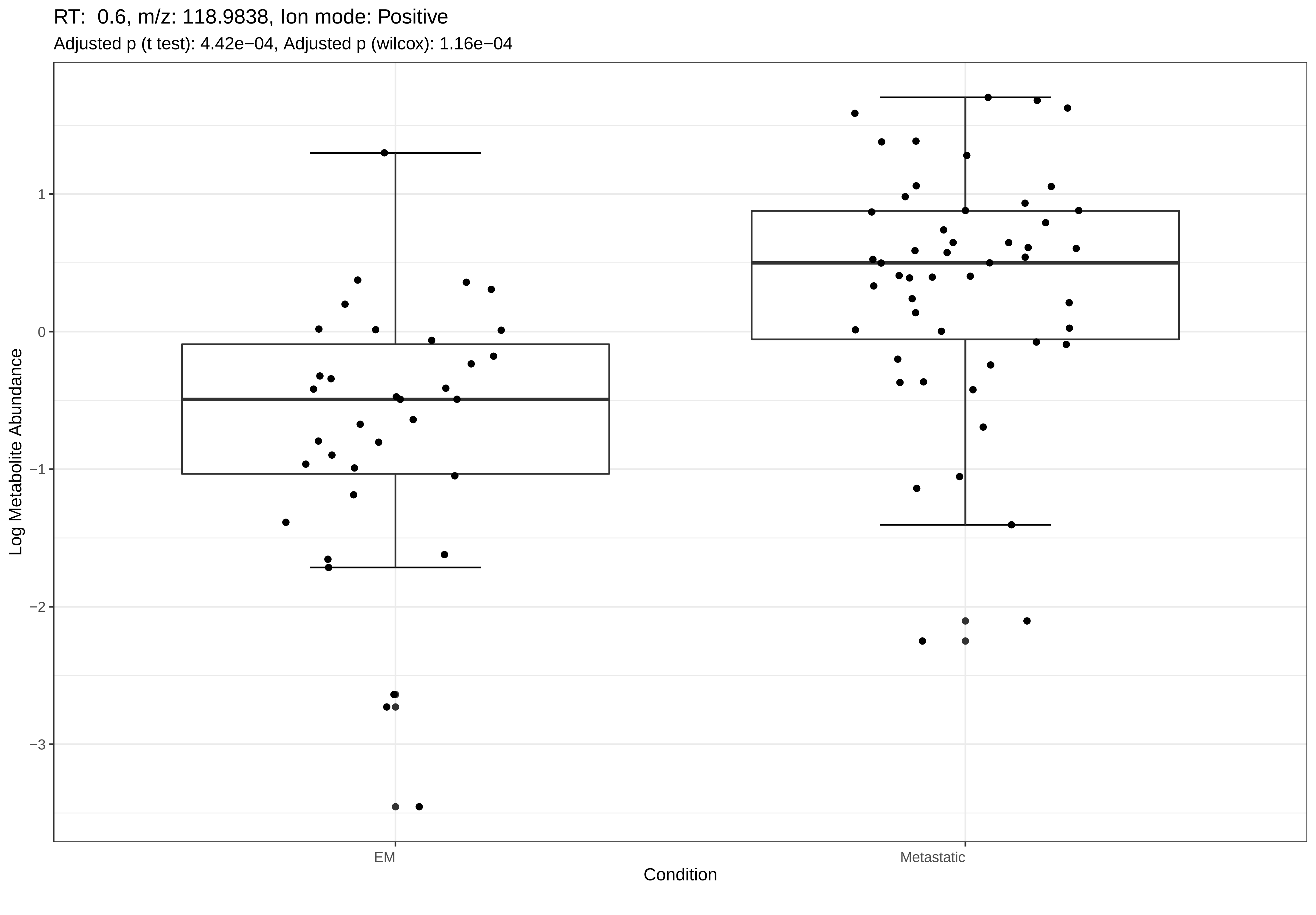
RT: 0.6, m/z: 118.9838, Ion mode: Positive
Adjusted p (t test): 4.42e−04, Adjusted p (wilcox): 1.16e−04
Log Metabolite Abundance
1
0
−1
−2
−3
EM
Metastatic
Condition

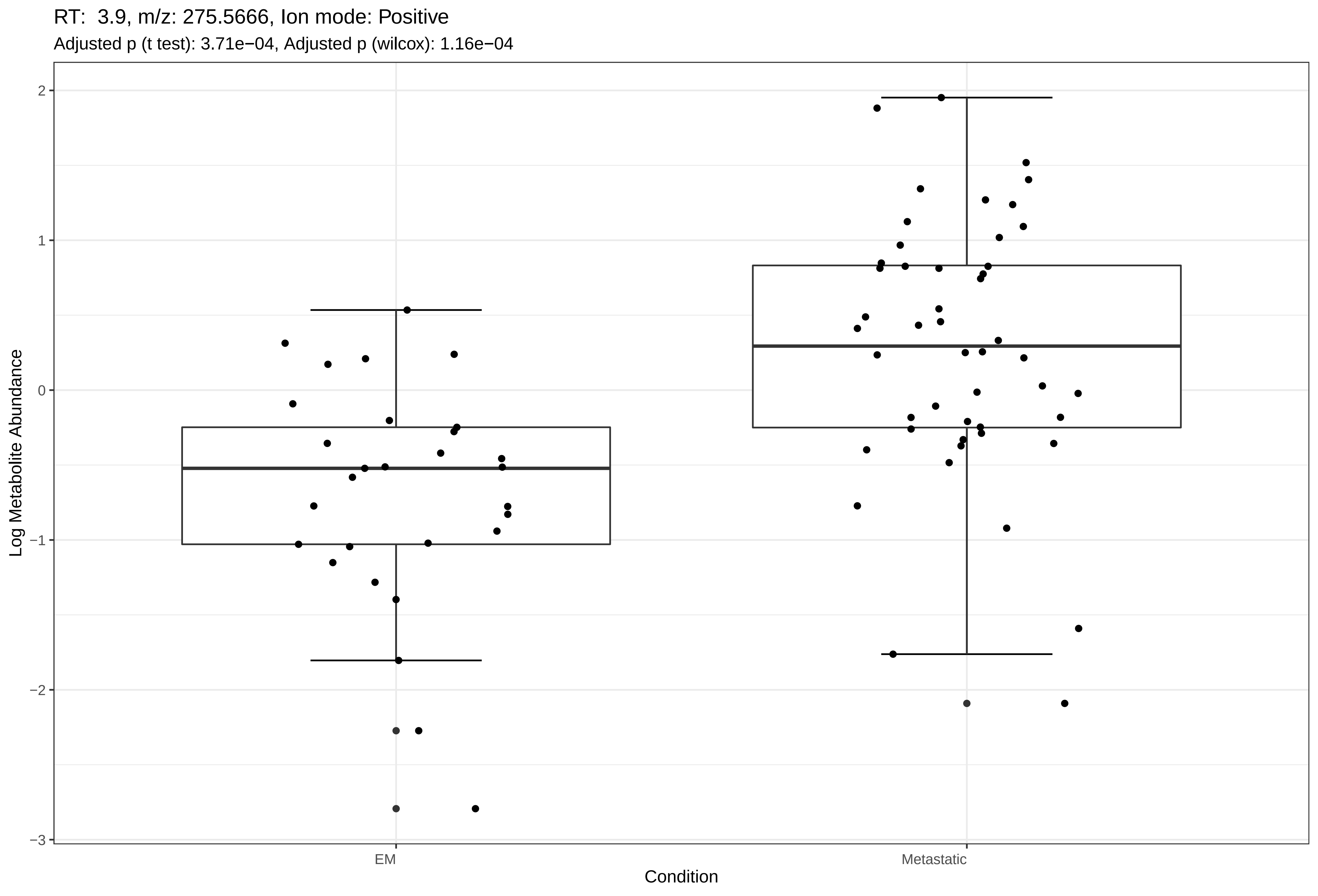
RT: 3.9, m/z: 275.5666, Ion mode: Positive
Adjusted p (t test): 3.71e−04, Adjusted p (wilcox): 1.16e−04
Log Metabolite Abundance
2
1
0
−1
−2
−3
EM
Metastatic
Condition

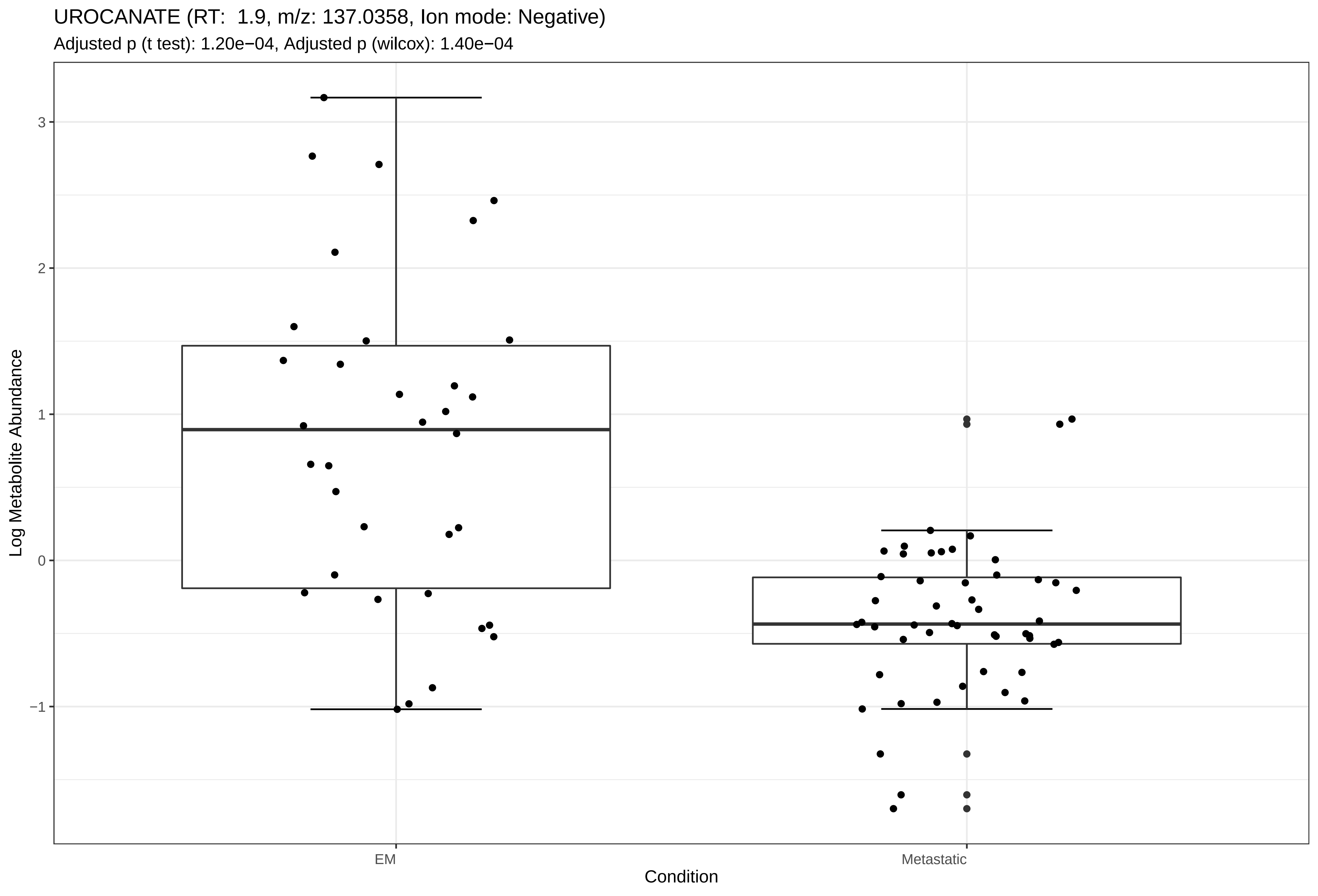
UROCANATE (RT: 1.9, m/z: 137.0358, Ion mode: Negative)
Adjusted p (t test): 1.20e−04, Adjusted p (wilcox): 1.40e−04
Log Metabolite Abundance
3
2
1
0
−1
EM
Metastatic
Condition

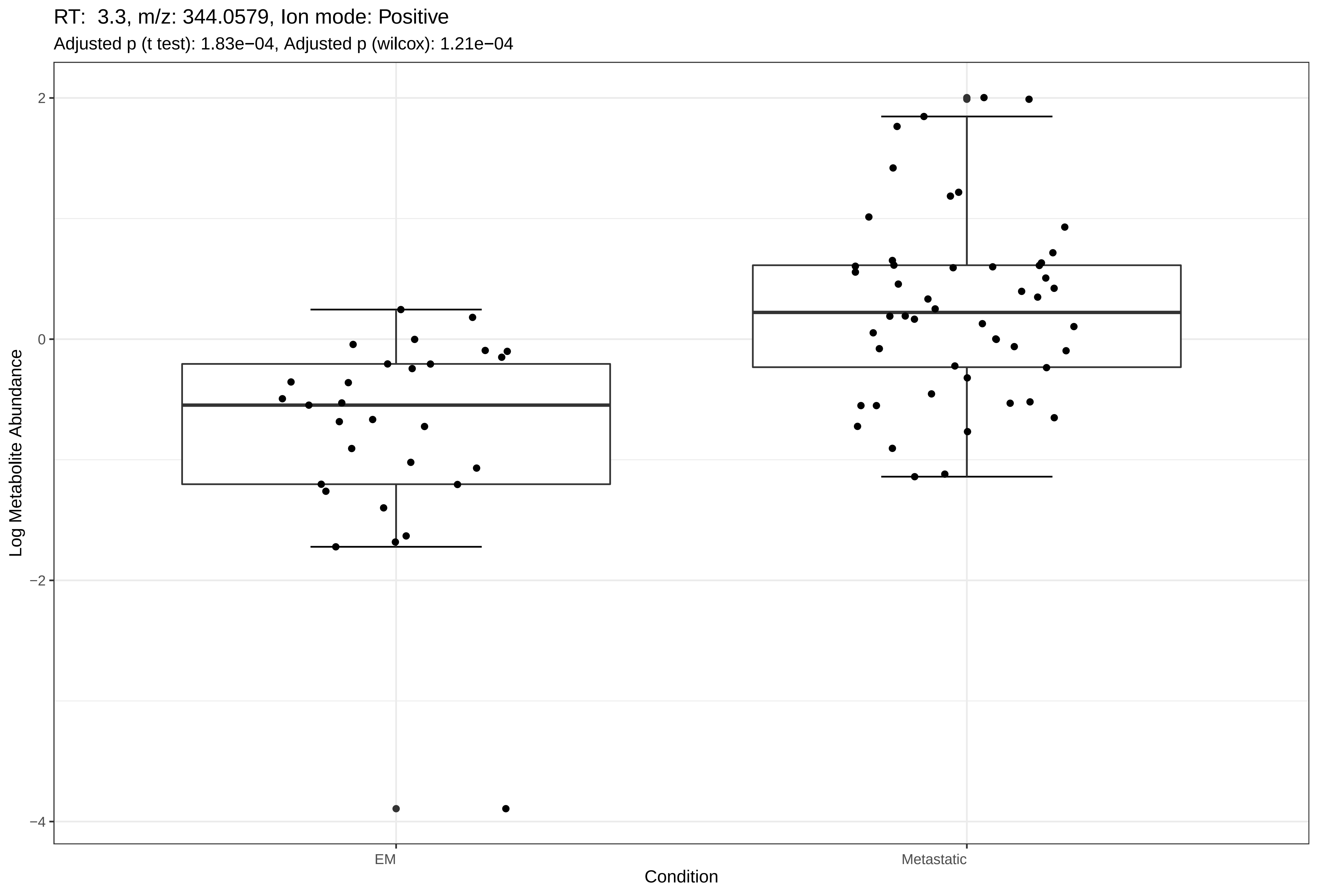

RT: 3.3, m/z: 344.0579, Ion mode: Positive
Adjusted p (t test): 1.83e−04, Adjusted p (wilcox): 1.21e−04
2
0
−2
−4
Log Metabolite Abundance
EM
Metastatic
Condition

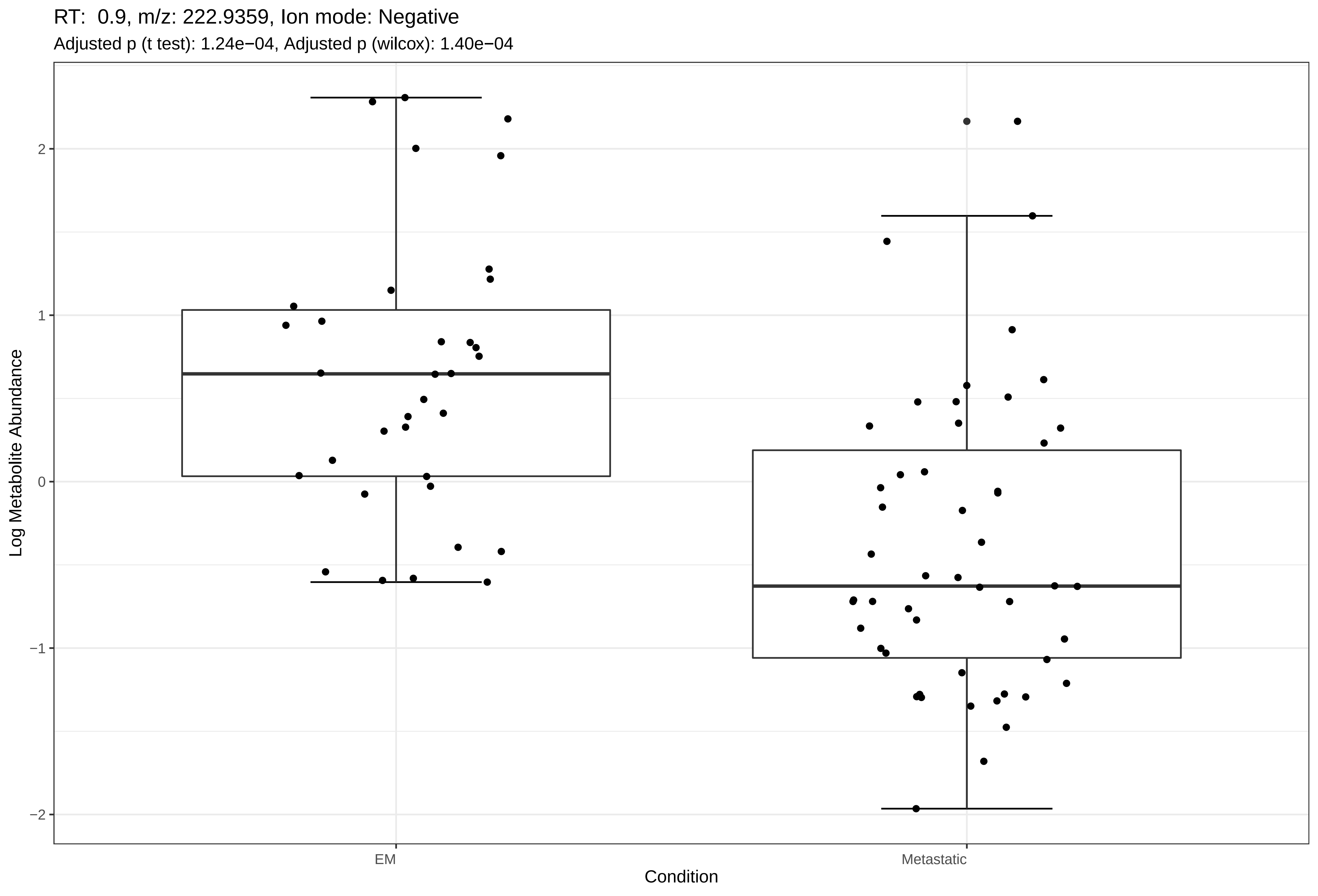
RT: 0.9, m/z: 222.9359, Ion mode: Negative
Adjusted p (t test): 1.24e−04, Adjusted p (wilcox): 1.40e−04
Log Metabolite Abundance
2
1
0
−1
−2
EM
Metastatic
Condition

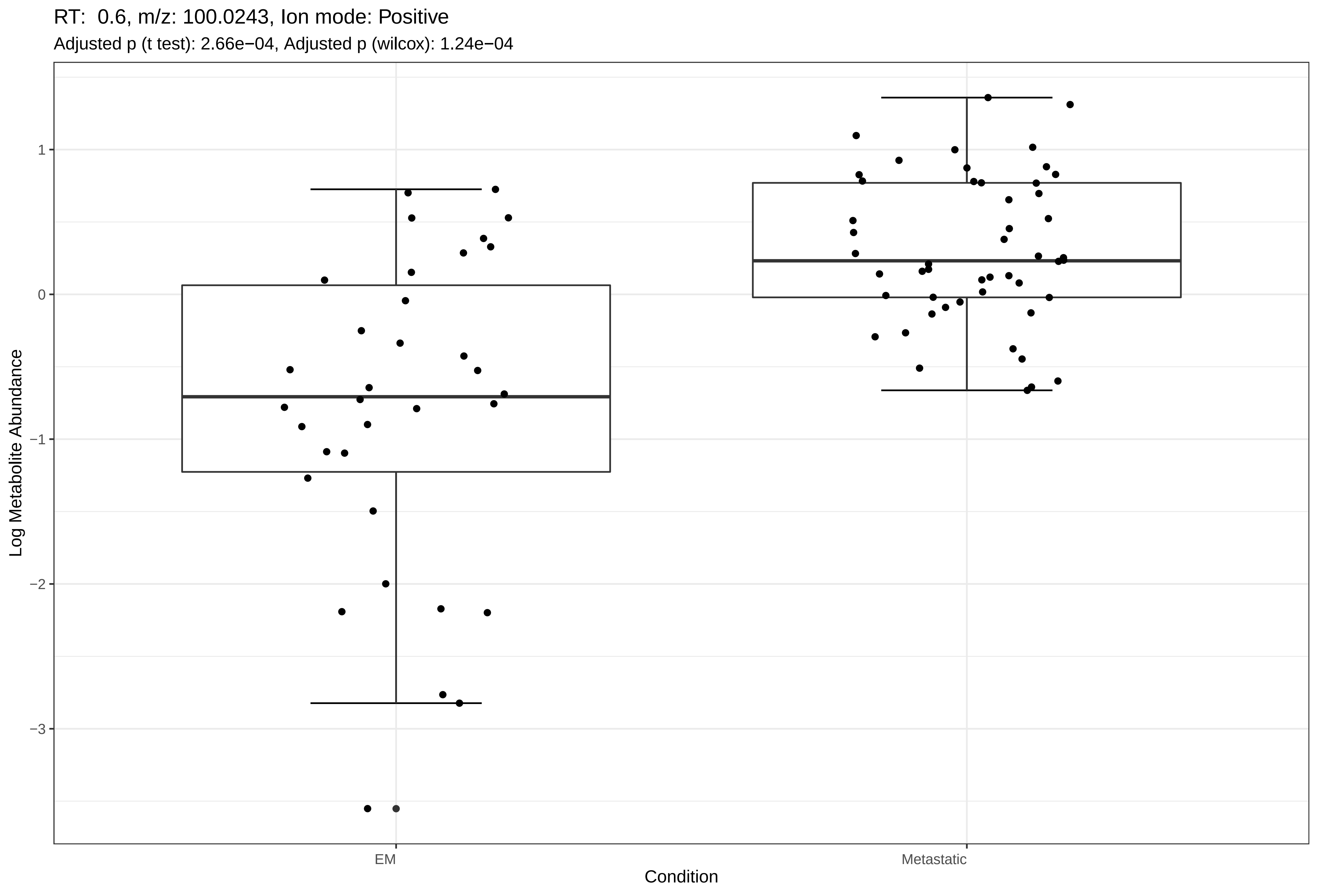
RT: 0.6, m/z: 100.0243, Ion mode: Positive
Adjusted p (t test): 2.66e−04, Adjusted p (wilcox): 1.24e−04
Log Metabolite Abundance
1
0
−1
−2
−3
EM
Metastatic
Condition

[M+K−H] 37.9559 m/z adduct of 174.9913 m/z (RT: 1.2, m/z: 212.9453, Ion mode: Positive)

Adjusted p (t test): 1.97e−04, Adjusted p (wilcox): 1.24e−04

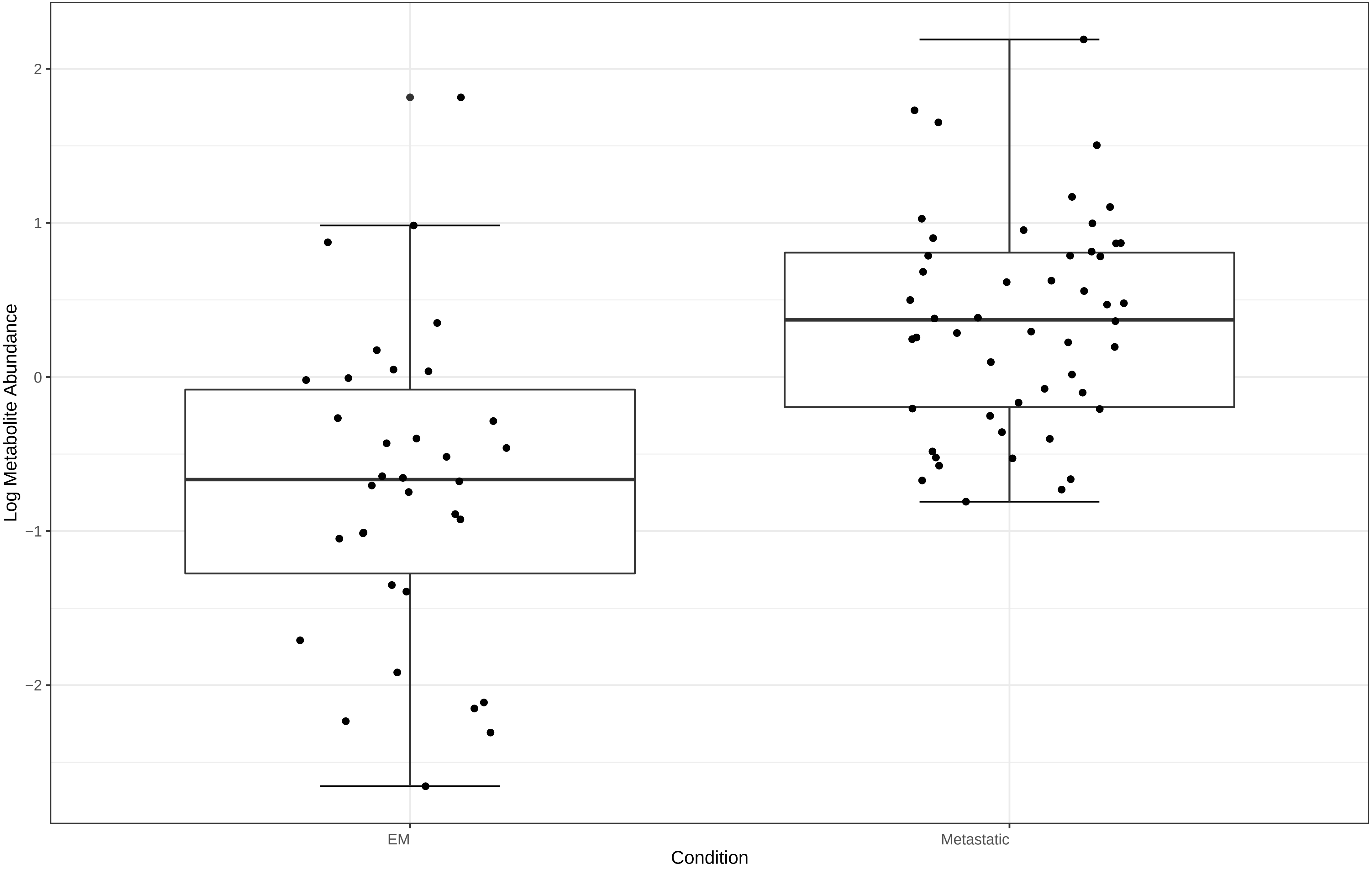

[M+Na−H] 21.9825 m/z adduct of 284.0982 m/z (RT: 6.2, m/z: 306.0799, Ion mode: Positive)

Adjusted p (t test): 5.71e−04, Adjusted p (wilcox): 1.24e−04

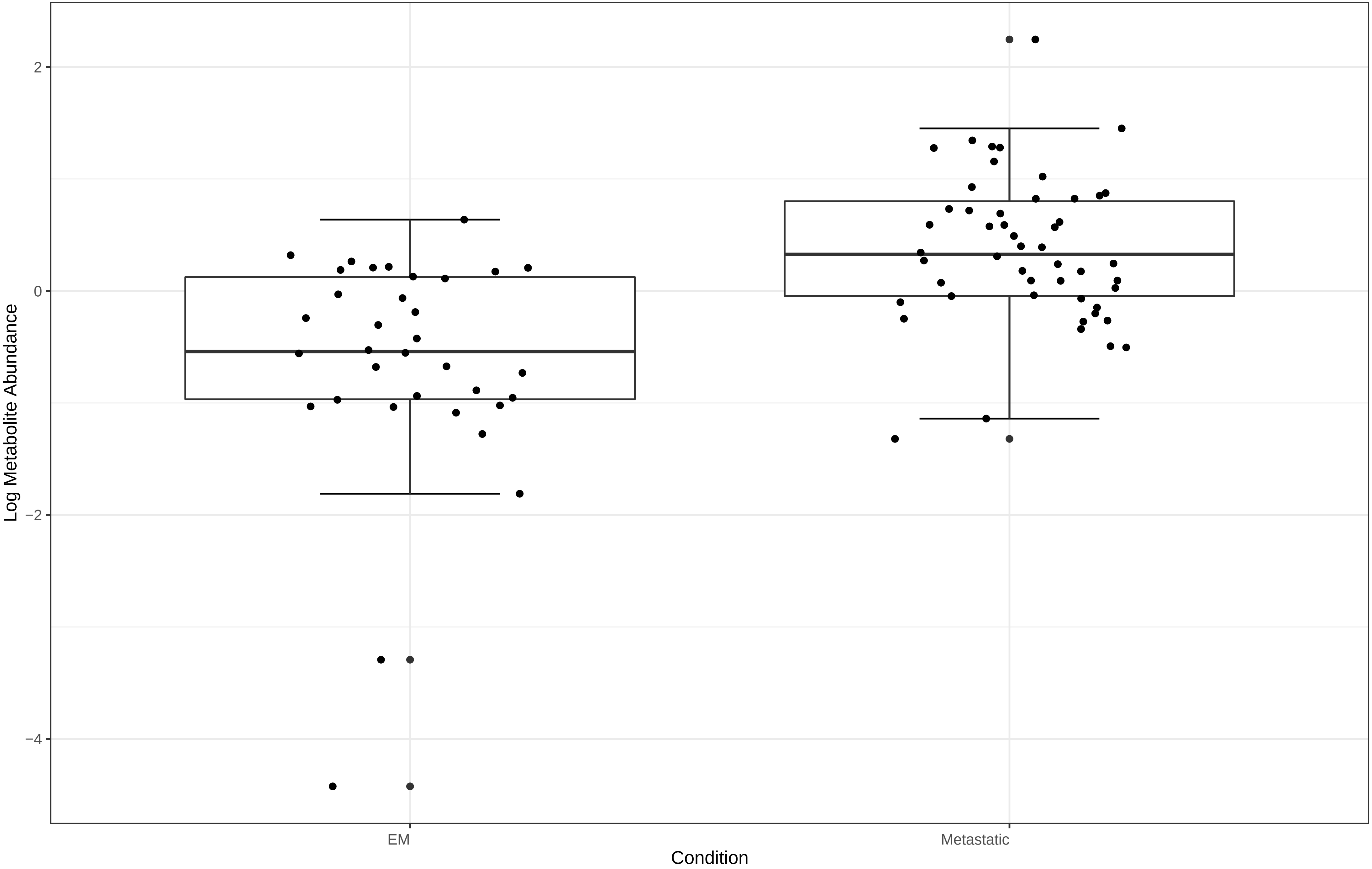

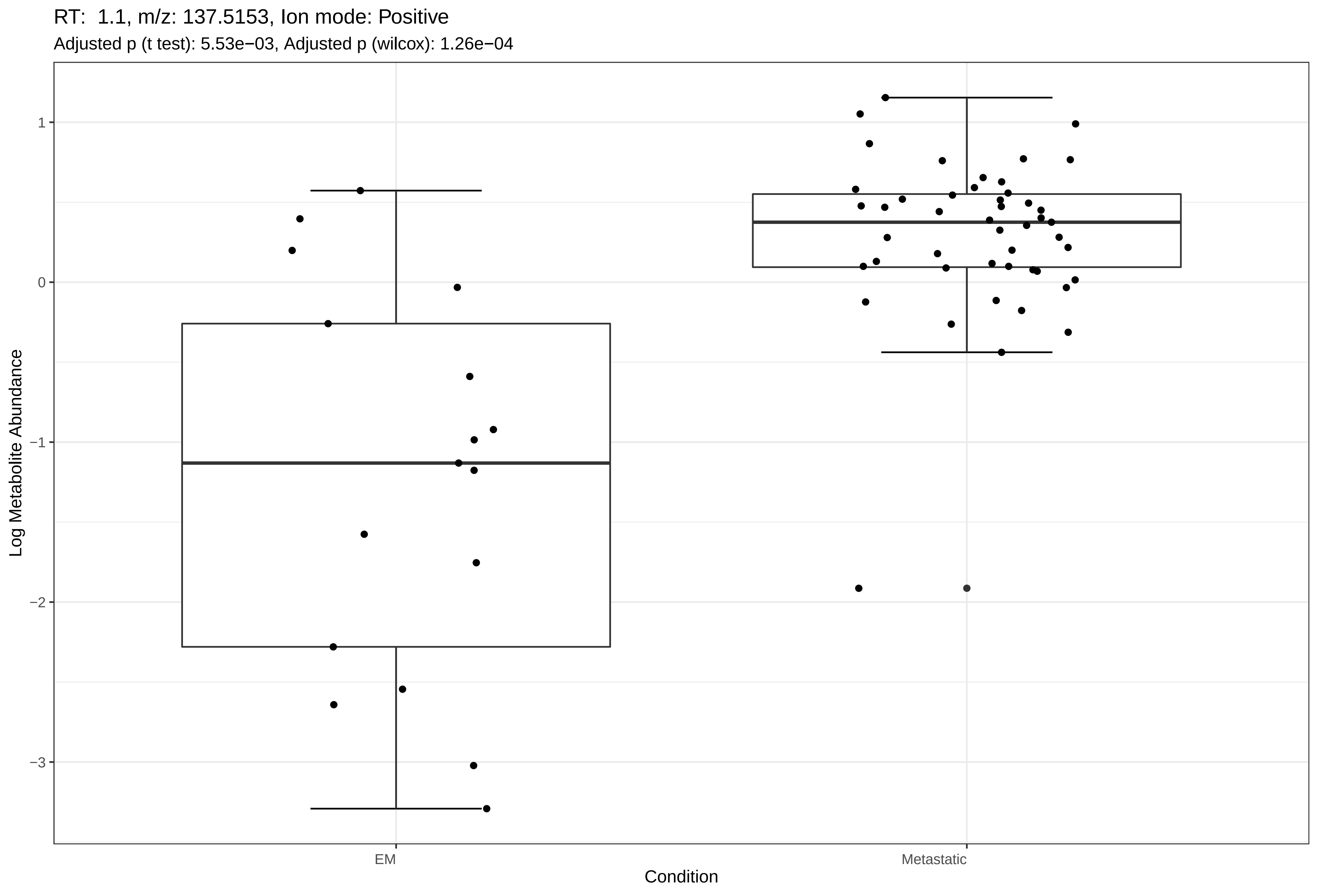
RT: 1.1, m/z: 137.5153, Ion mode: Positive
Adjusted p (t test): 5.53e−03, Adjusted p (wilcox): 1.26e−04
Log Metabolite Abundance
1
0
−1
−2
−3
EM
Metastatic
Condition

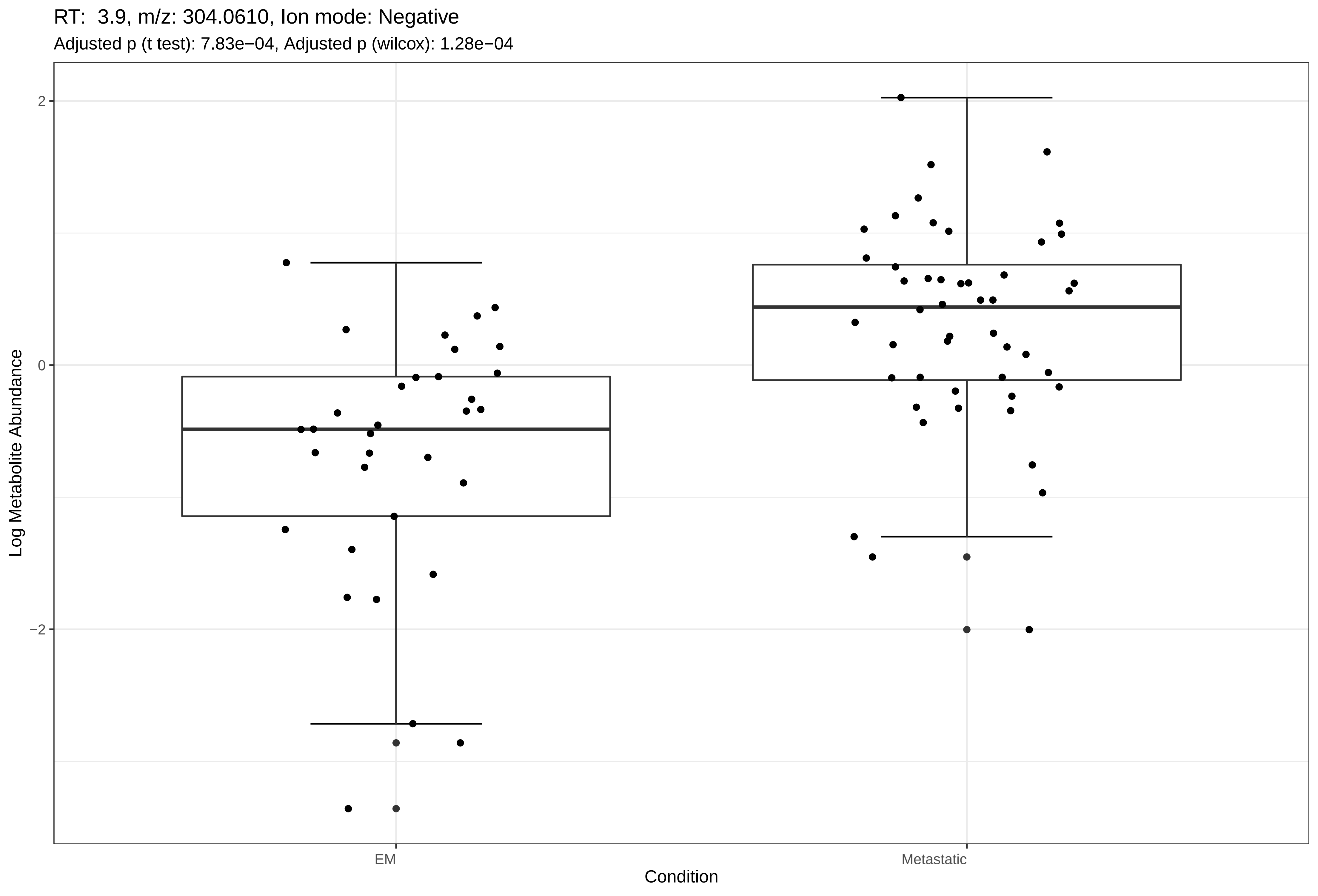
RT: 3.9, m/z: 304.0610, Ion mode: Negative
Adjusted p (t test): 7.83e−04, Adjusted p (wilcox): 1.28e−04
Log Metabolite Abundance
2
0
−2
EM
Metastatic
Condition

RT: 3.9, m/z: 319.0609, Ion mode: Positive

Adjusted p (t test): 6.05e−04, Adjusted p (wilcox): 1.28e−04

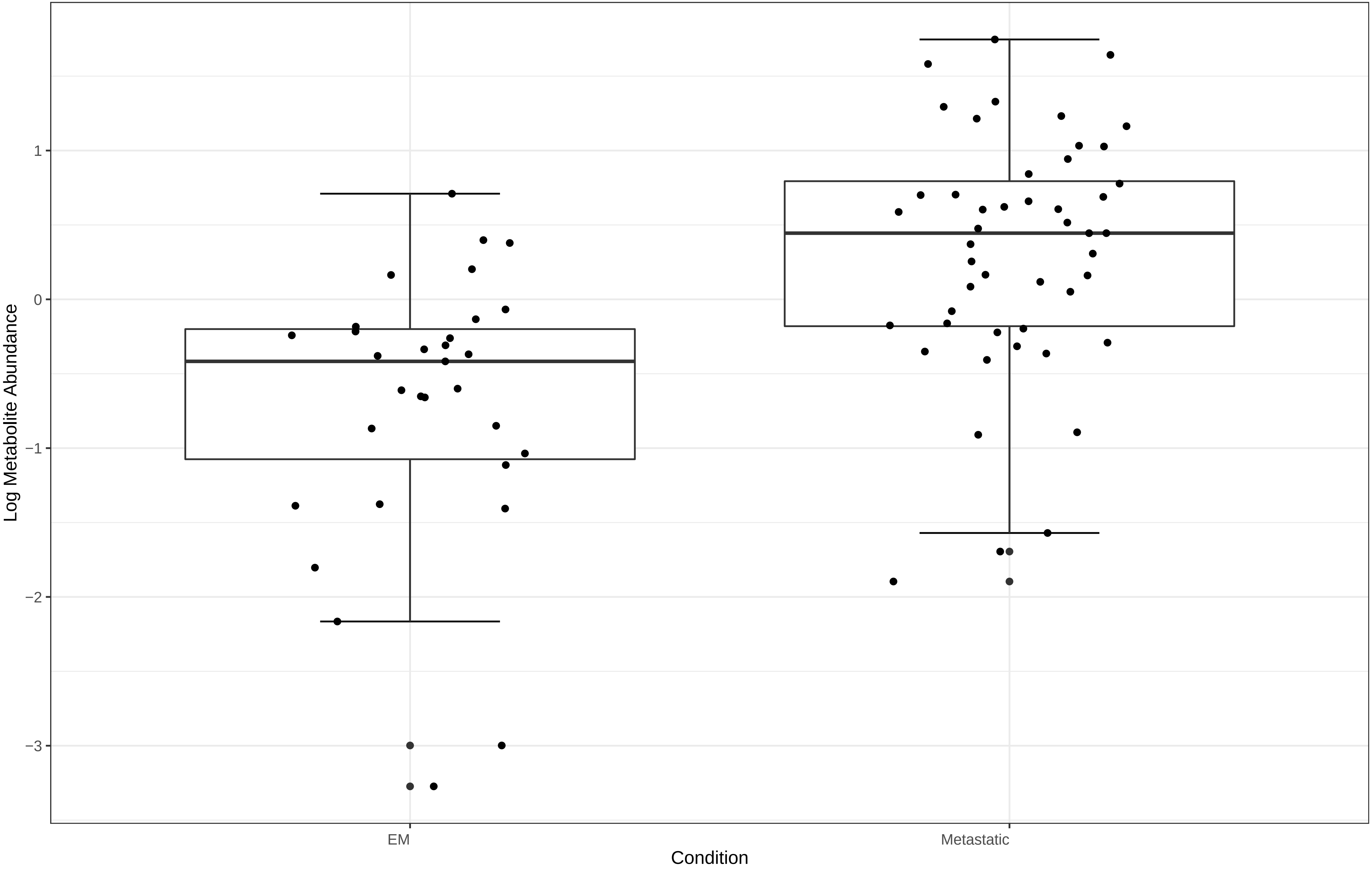

RT: 1.2, m/z: 163.5888, Ion mode: Positive

Adjusted p (t test): 1.30e−04, Adjusted p (wilcox): 1.41e−04

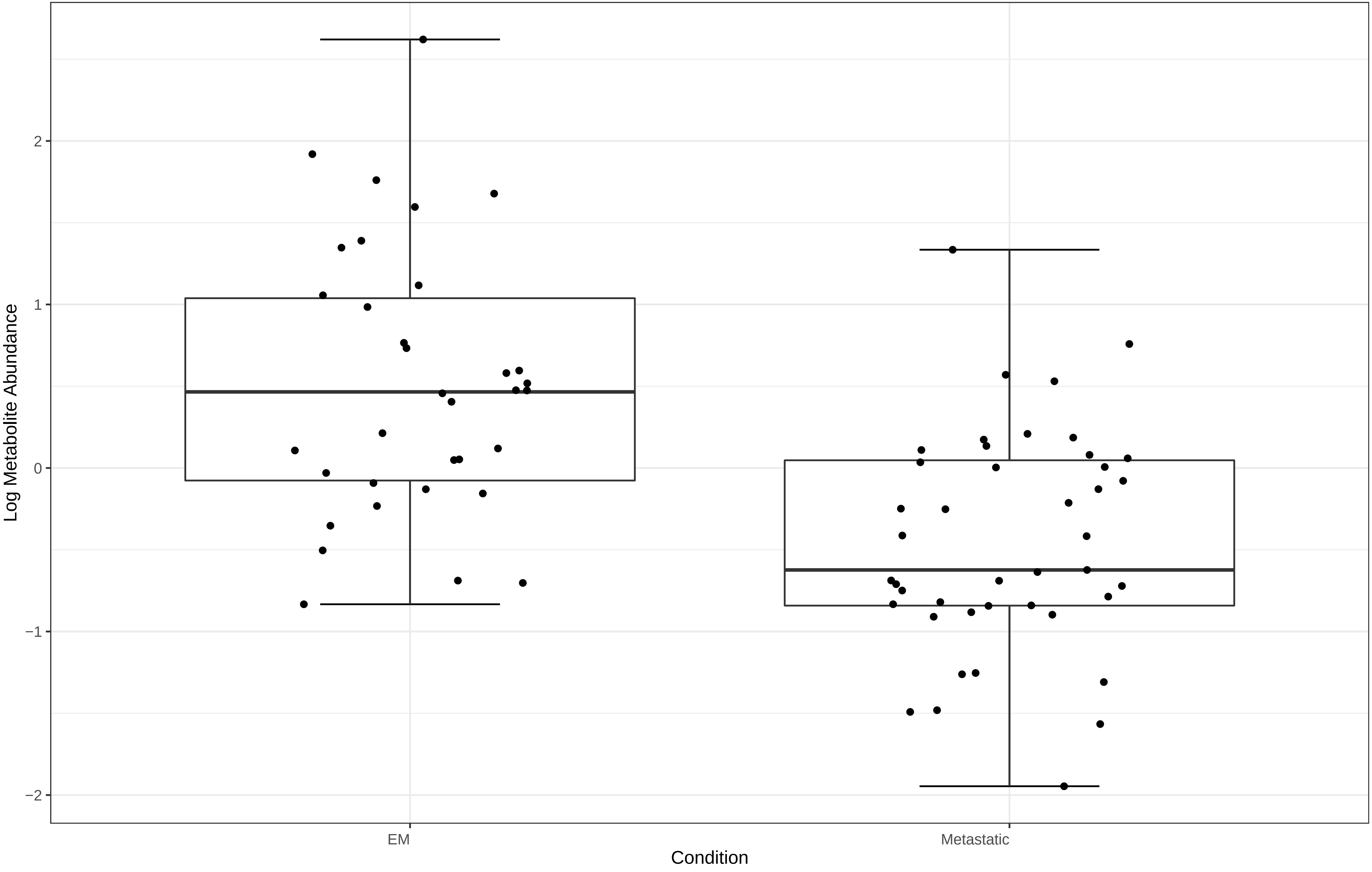

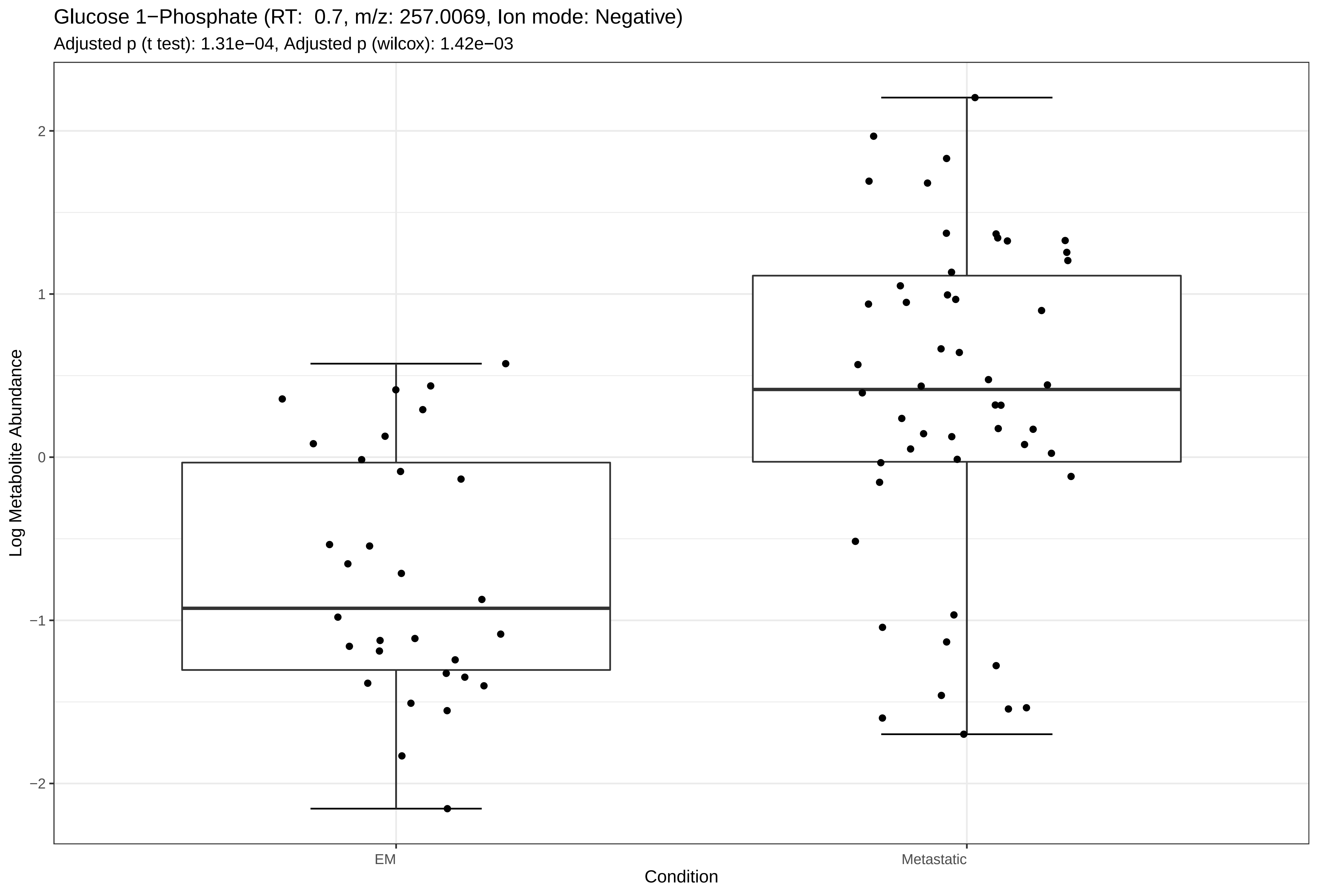

Glucose 1−Phosphate (RT: 0.7, m/z: 257.0069, Ion mode: Negative)
Adjusted p (t test): 1.31e−04, Adjusted p (wilcox): 1.42e−03
Log Metabolite Abundance
2
1
0
−1
−2
EM
Metastatic
Condition

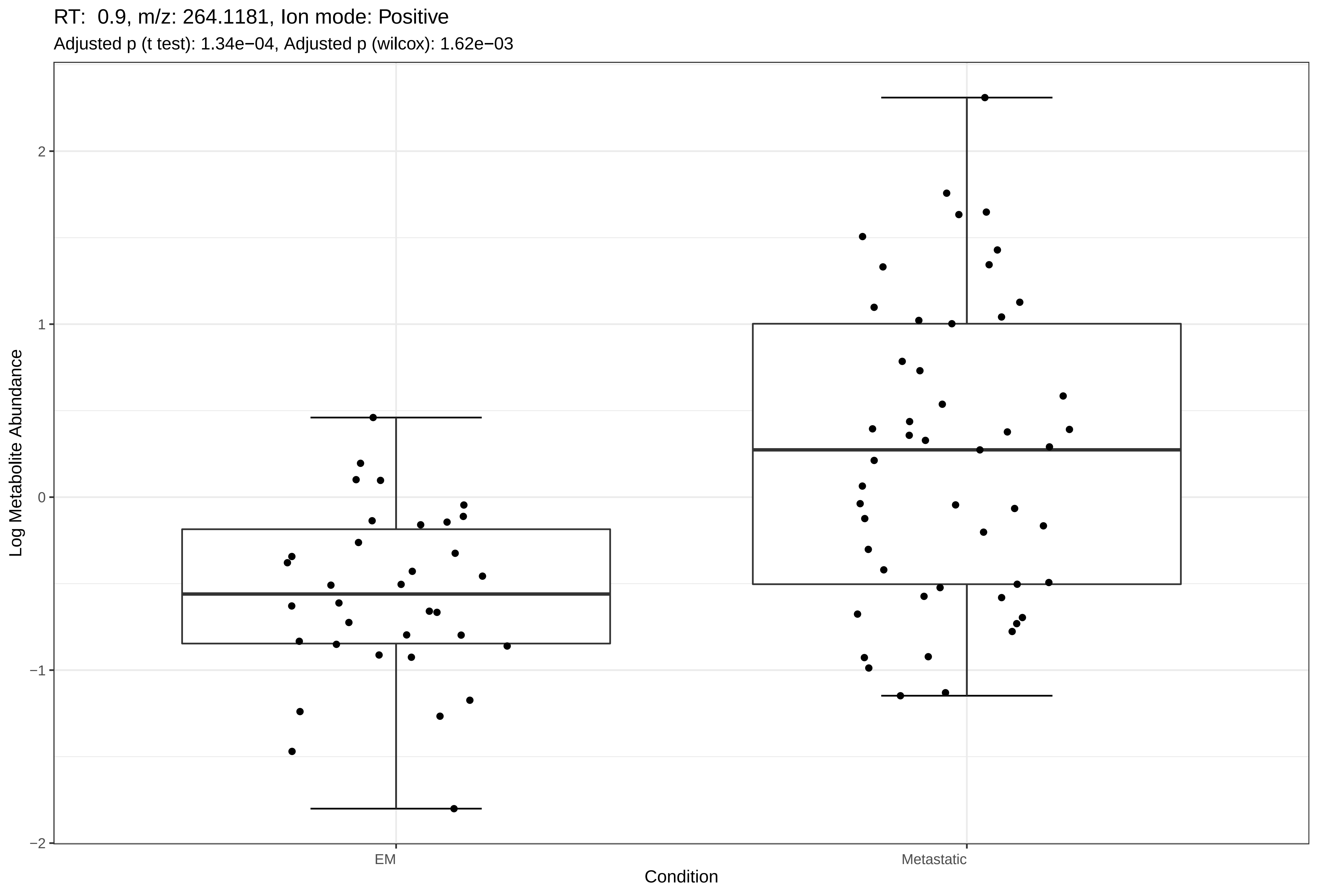

RT: 0.9, m/z: 264.1181, Ion mode: Positive
Adjusted p (t test): 1.34e−04, Adjusted p (wilcox): 1.62e−03
Log Metabolite Abundance
2
1
0
−1
−2
EM
Metastatic
Condition

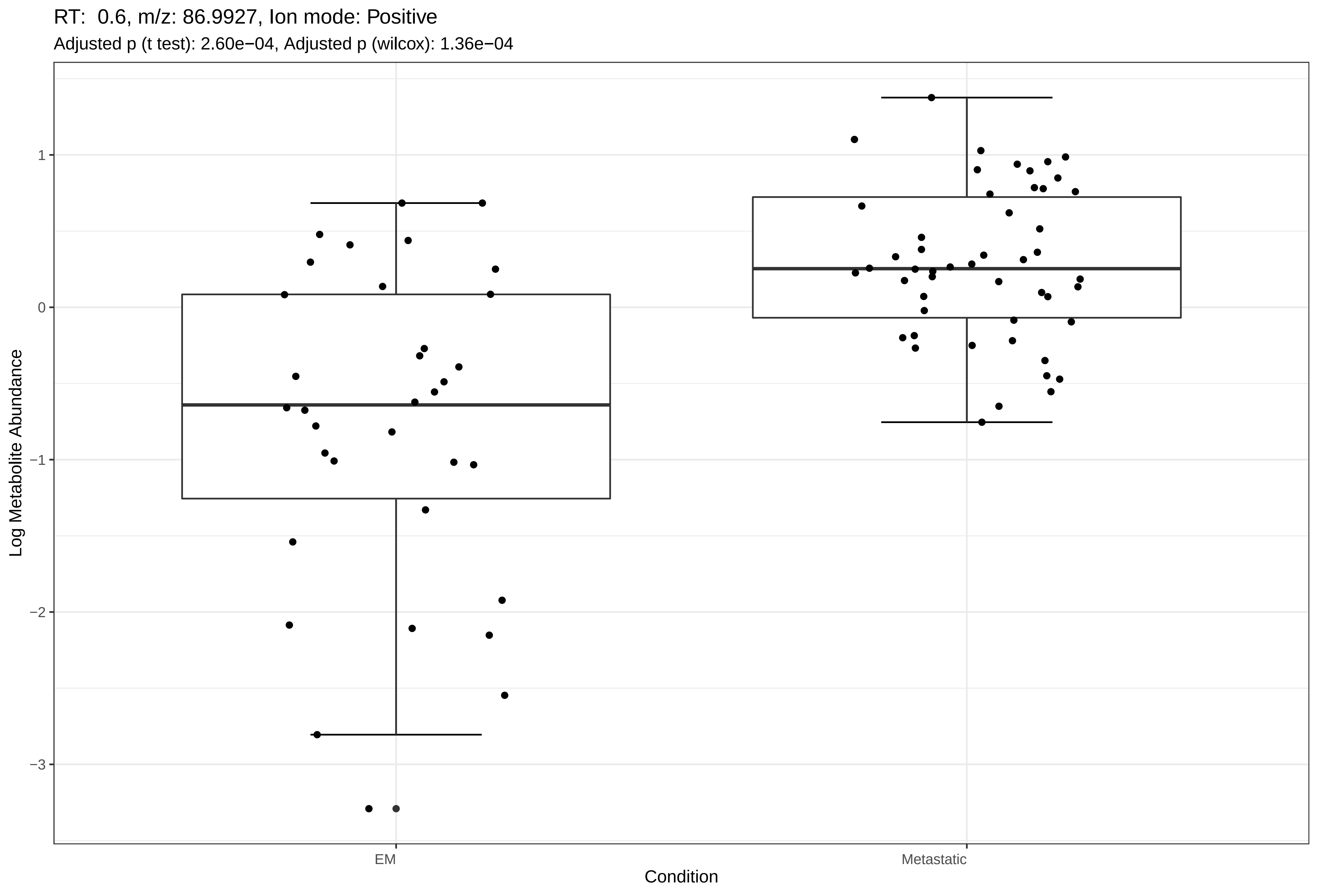

RT: 0.6, m/z: 86.9927, Ion mode: Positive
Adjusted p (t test): 2.60e−04, Adjusted p (wilcox): 1.36e−04
Log Metabolite Abundance
1
0
−1
−2
−3
EM
Metastatic
Condition

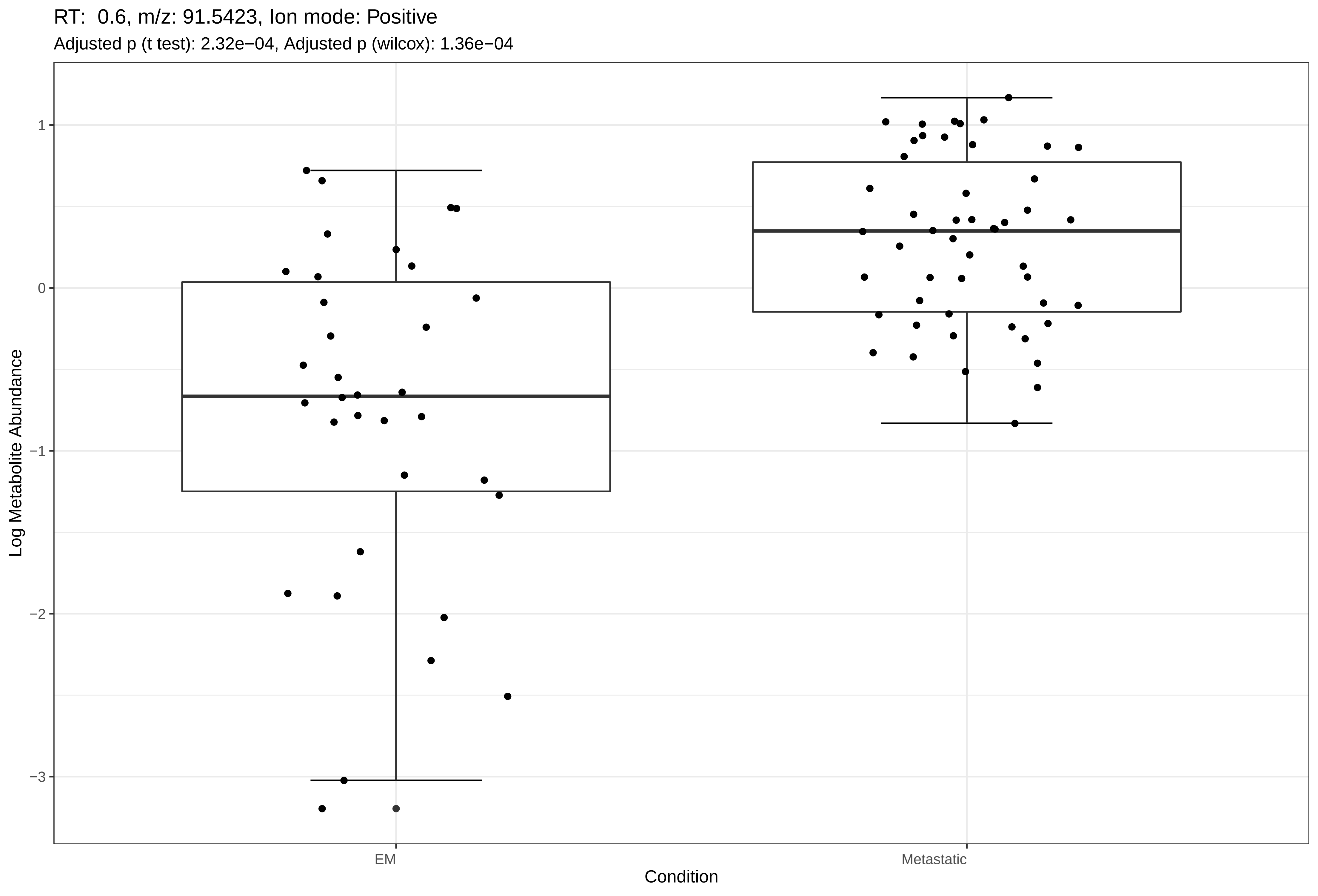

RT: 0.6, m/z: 91.5423, Ion mode: Positive
Adjusted p (t test): 2.32e−04, Adjusted p (wilcox): 1.36e−04
Log Metabolite Abundance
1
0
−1
−2
−3
EM
Metastatic
Condition

[M−H2O+H] −18.0106 m/z adduct of 139.0499 m/z (RT: 1.9, m/z: 121.0390, Ion mode: Positive)

Adjusted p (t test): 2.80e−04, Adjusted p (wilcox): 1.36e−04

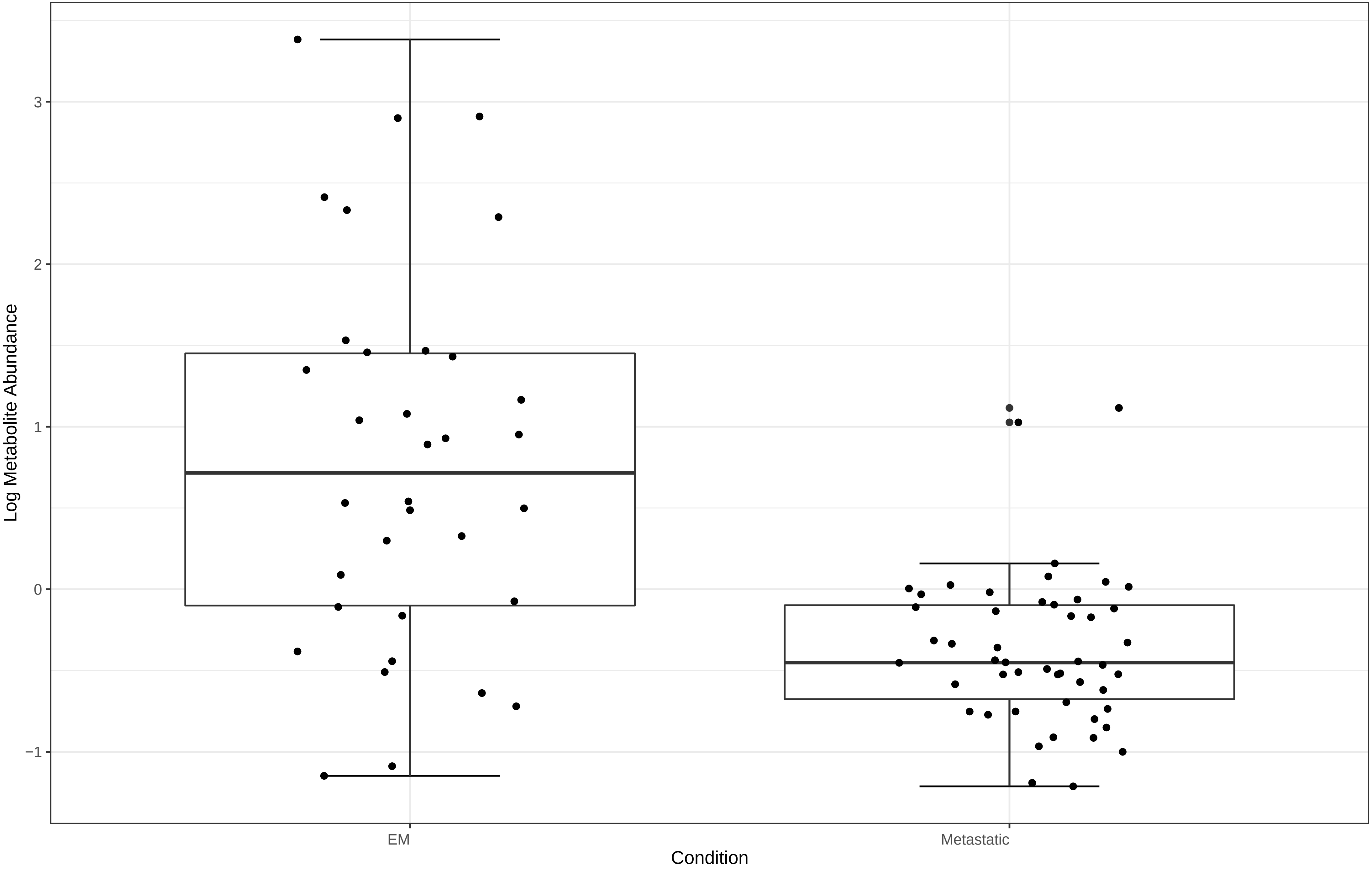

[M−HCOOH+H] −46.0055 m/z adduct of 129.0196 m/z (RT: 0.6, m/z: 83.0143, Ion mode: Positive)

Adjusted p (t test): 2.67e−04, Adjusted p (wilcox): 1.36e−04

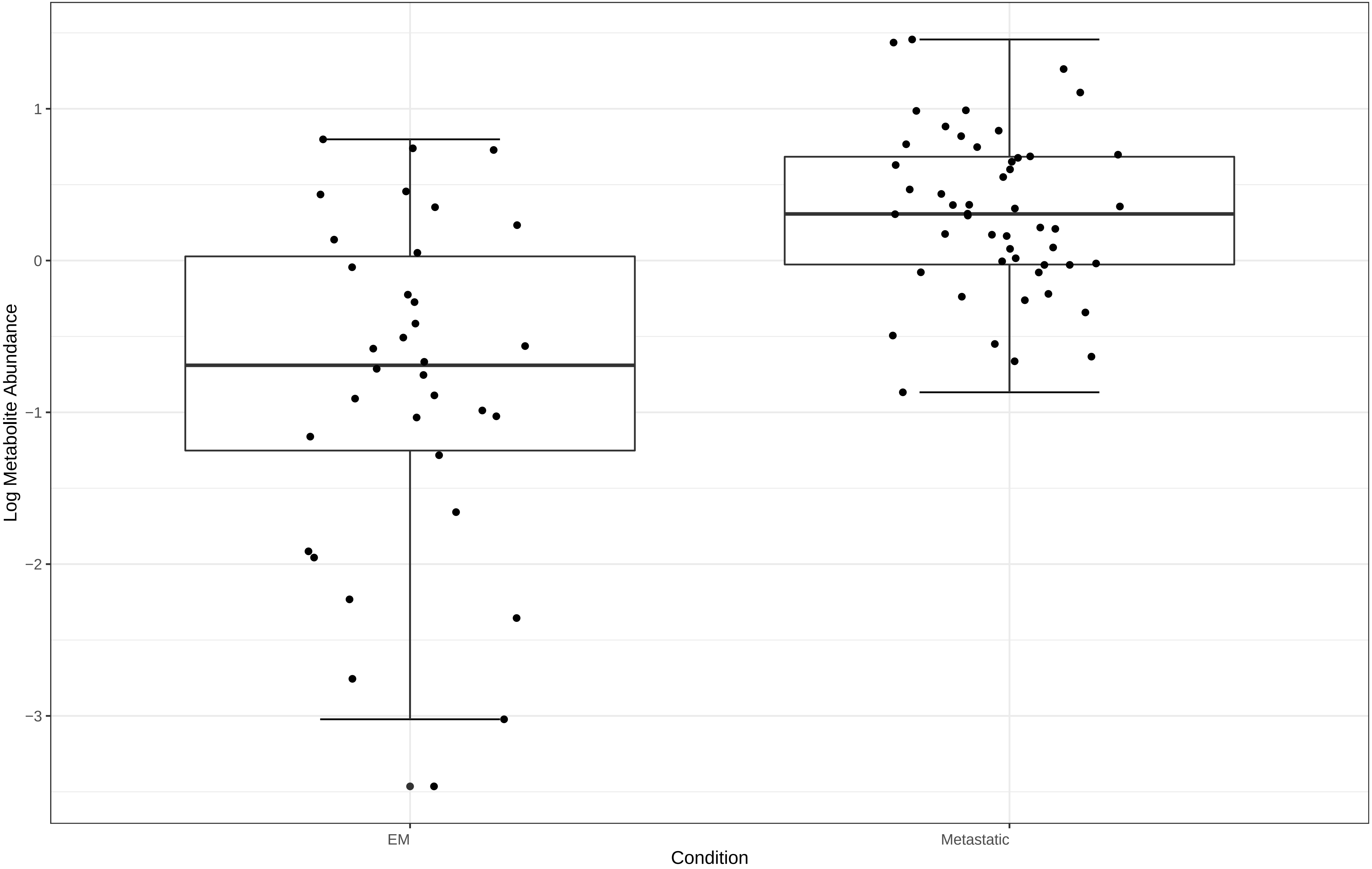

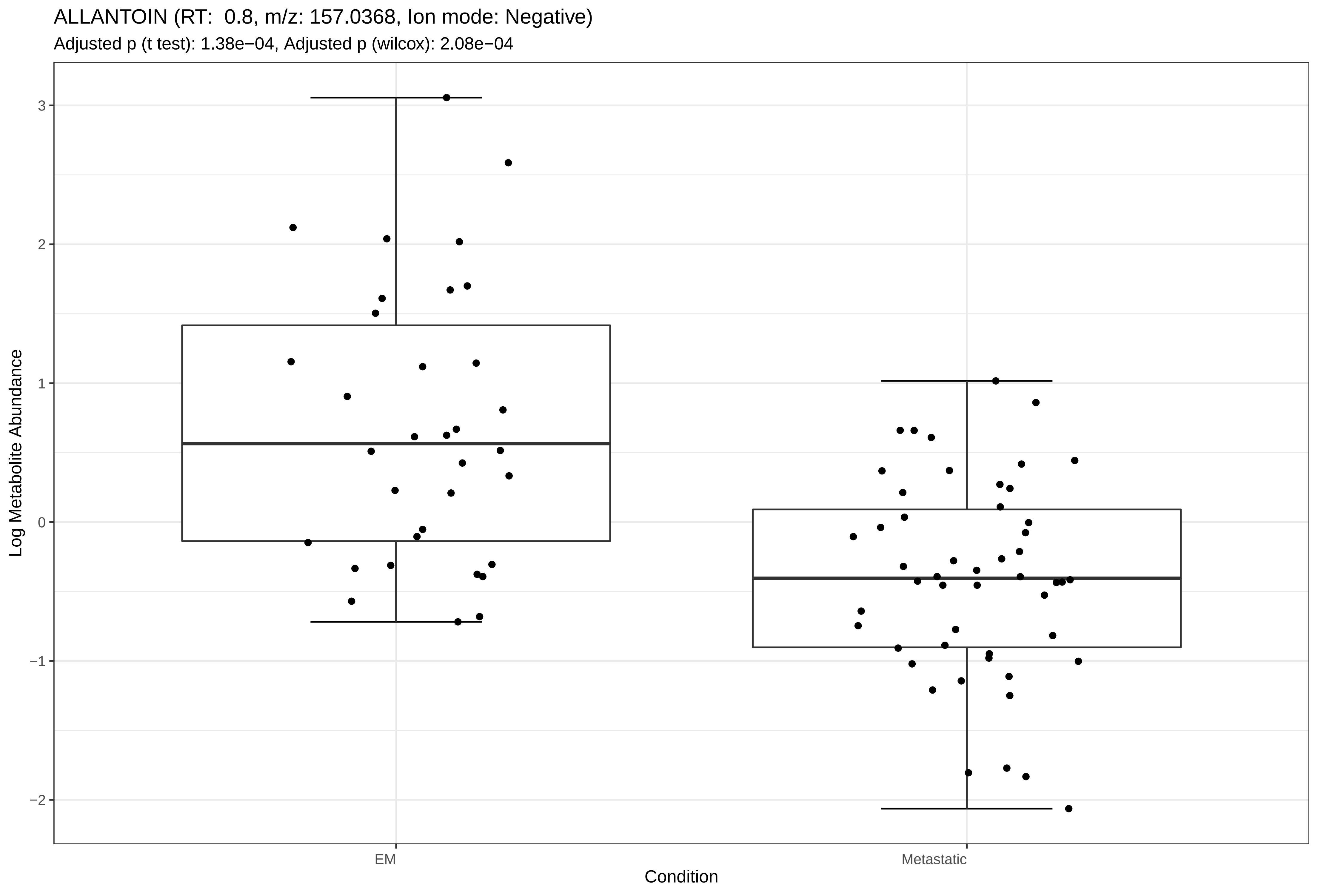
ALLANTOIN (RT: 0.8, m/z: 157.0368, Ion mode: Negative)
Adjusted p (t test): 1.38e−04, Adjusted p (wilcox): 2.08e−04
Log Metabolite Abundance
3
2
1
0
−1
−2
EM
Metastatic
Condition

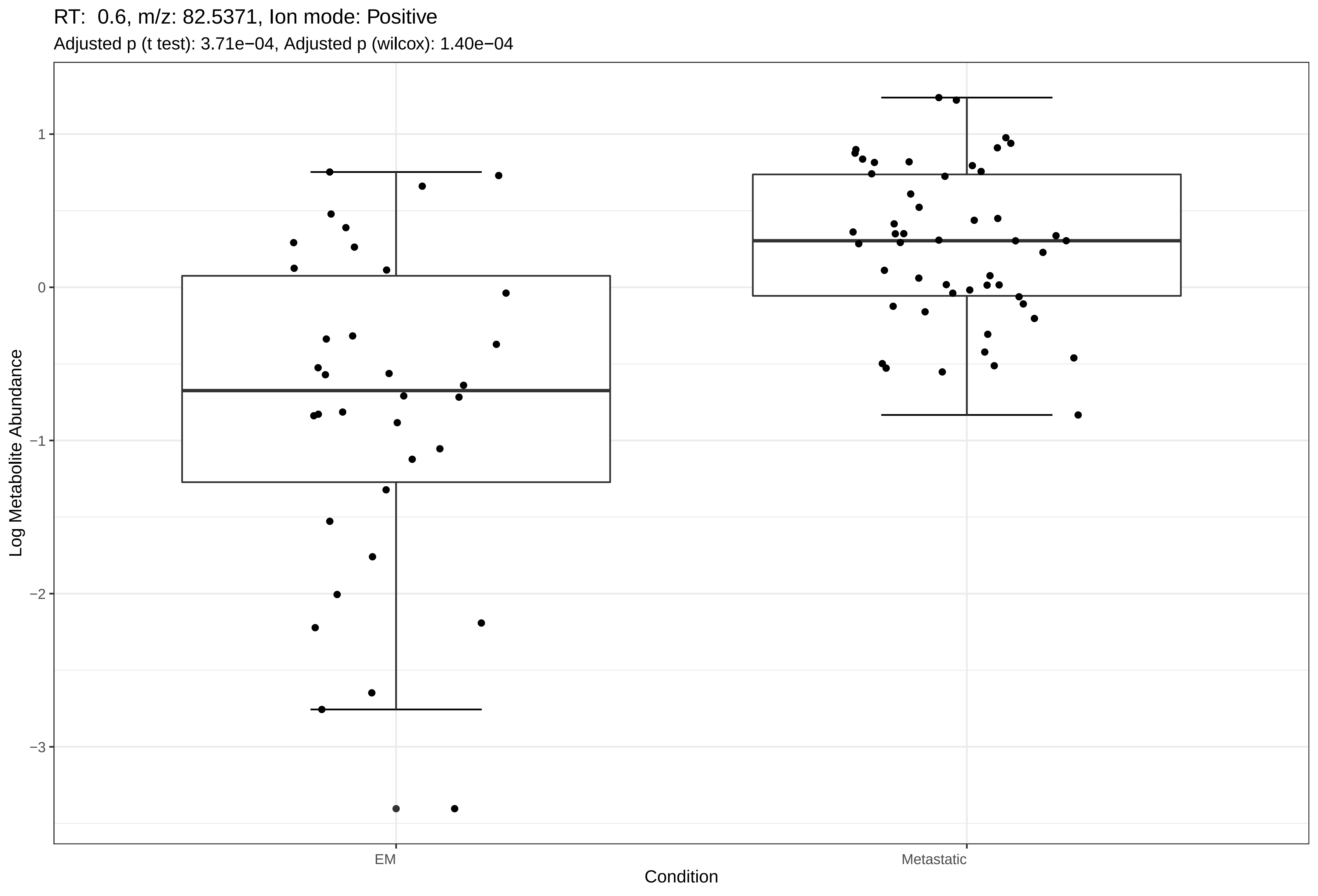

RT: 0.6, m/z: 82.5371, Ion mode: Positive
Adjusted p (t test): 3.71e−04, Adjusted p (wilcox): 1.40e−04
Log Metabolite Abundance
1
0
−1
−2
−3
EM
Metastatic
Condition

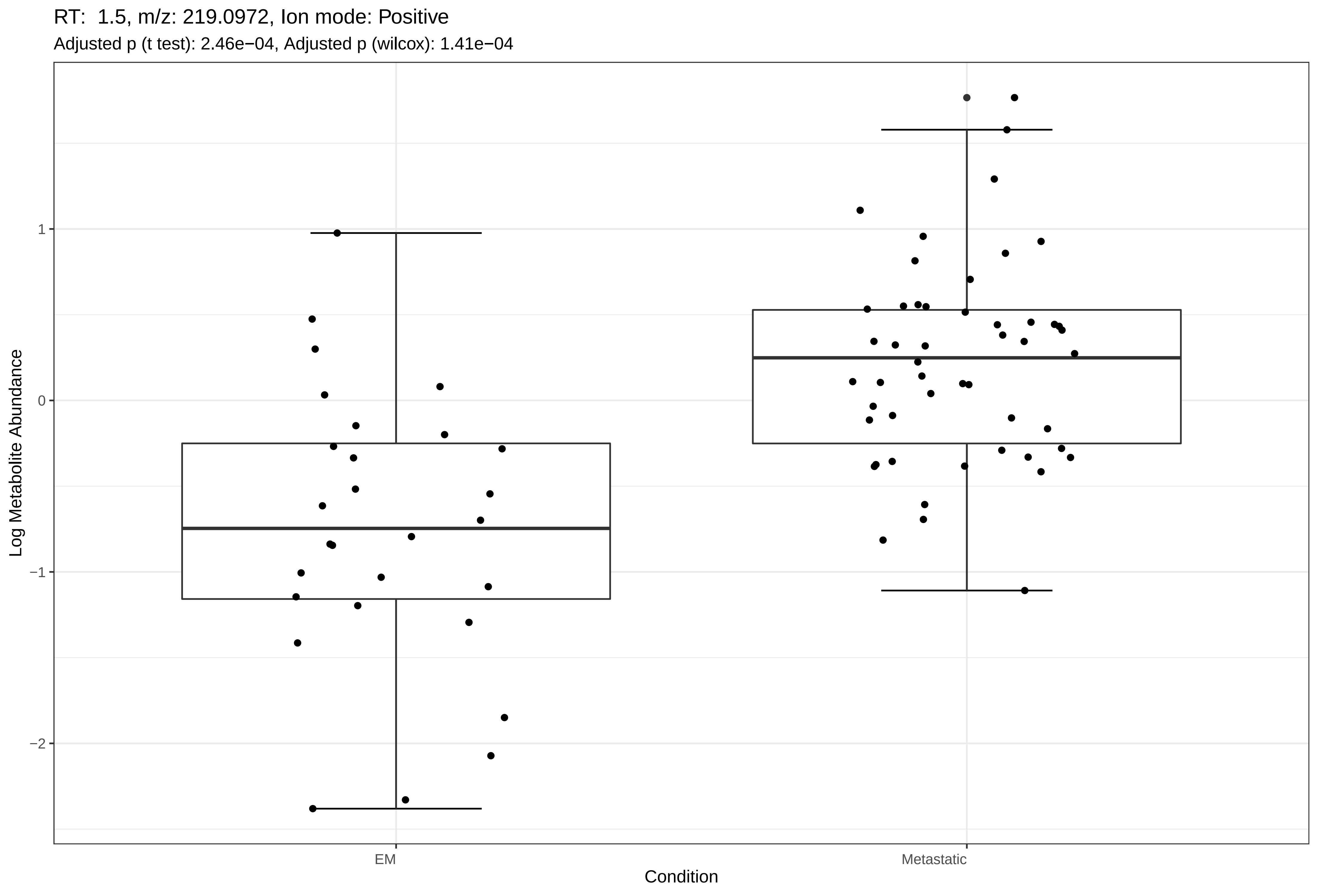
RT: 1.5, m/z: 219.0972, Ion mode: Positive
Adjusted p (t test): 2.46e−04, Adjusted p (wilcox): 1.41e−04
Log Metabolite Abundance
1
0
−1
−2
EM
Metastatic
Condition

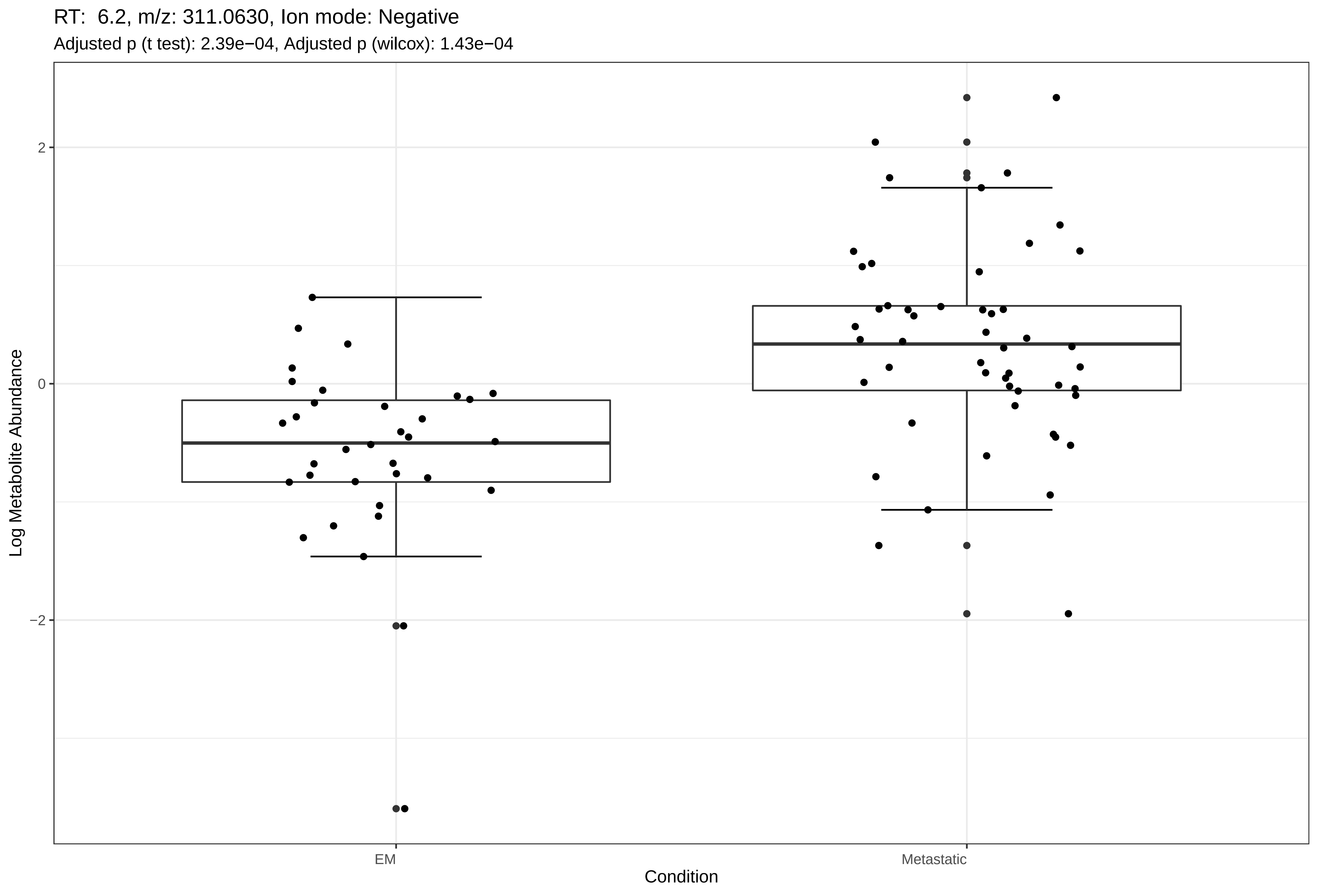
RT: 6.2, m/z: 311.0630, Ion mode: Negative
Adjusted p (t test): 2.39e−04, Adjusted p (wilcox): 1.43e−04
Log Metabolite Abundance
2
0
−2
EM
Metastatic
Condition

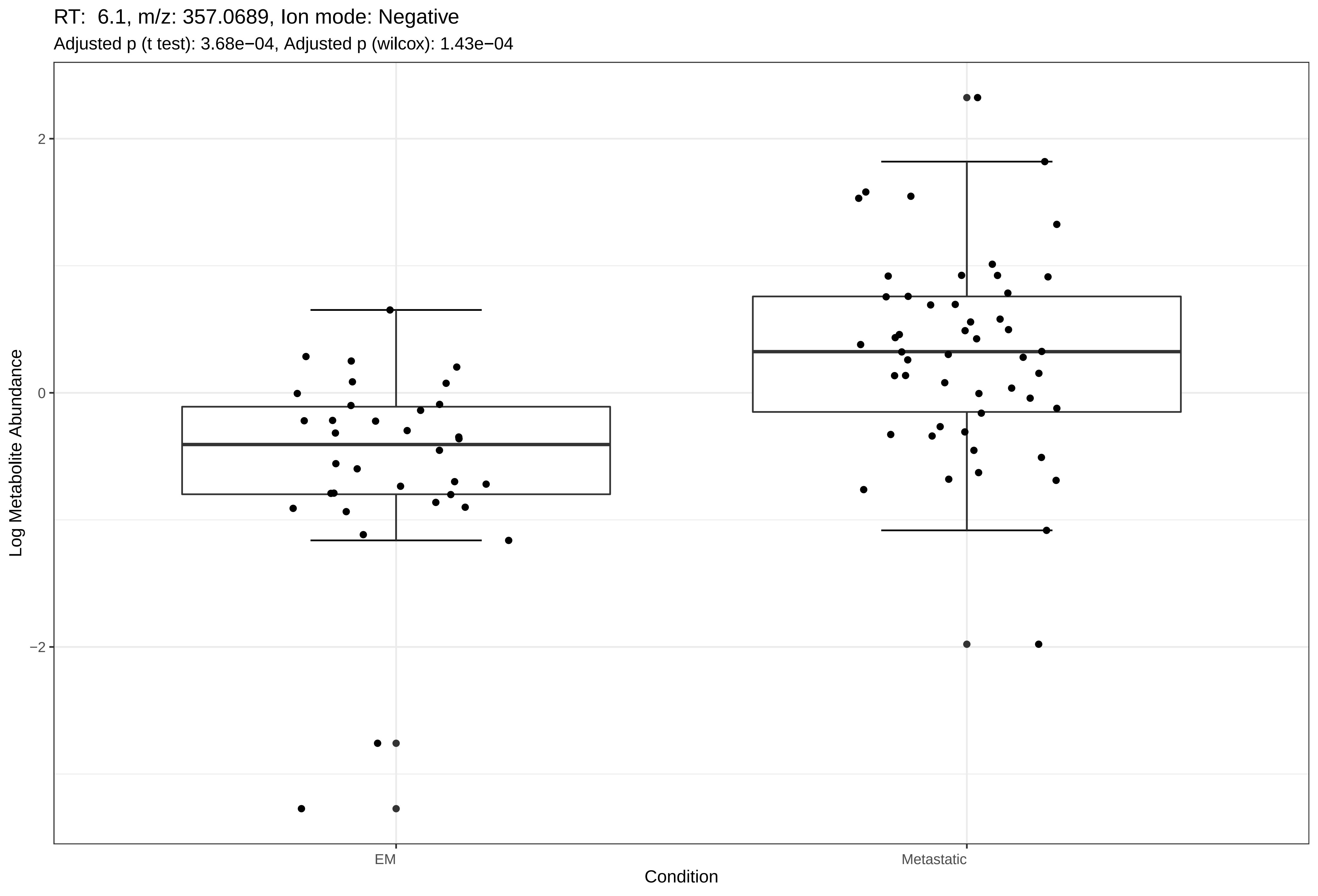
RT: 6.1, m/z: 357.0689, Ion mode: Negative
Adjusted p (t test): 3.68e−04, Adjusted p (wilcox): 1.43e−04
Log Metabolite Abundance
2
0
−2
EM
Metastatic
Condition

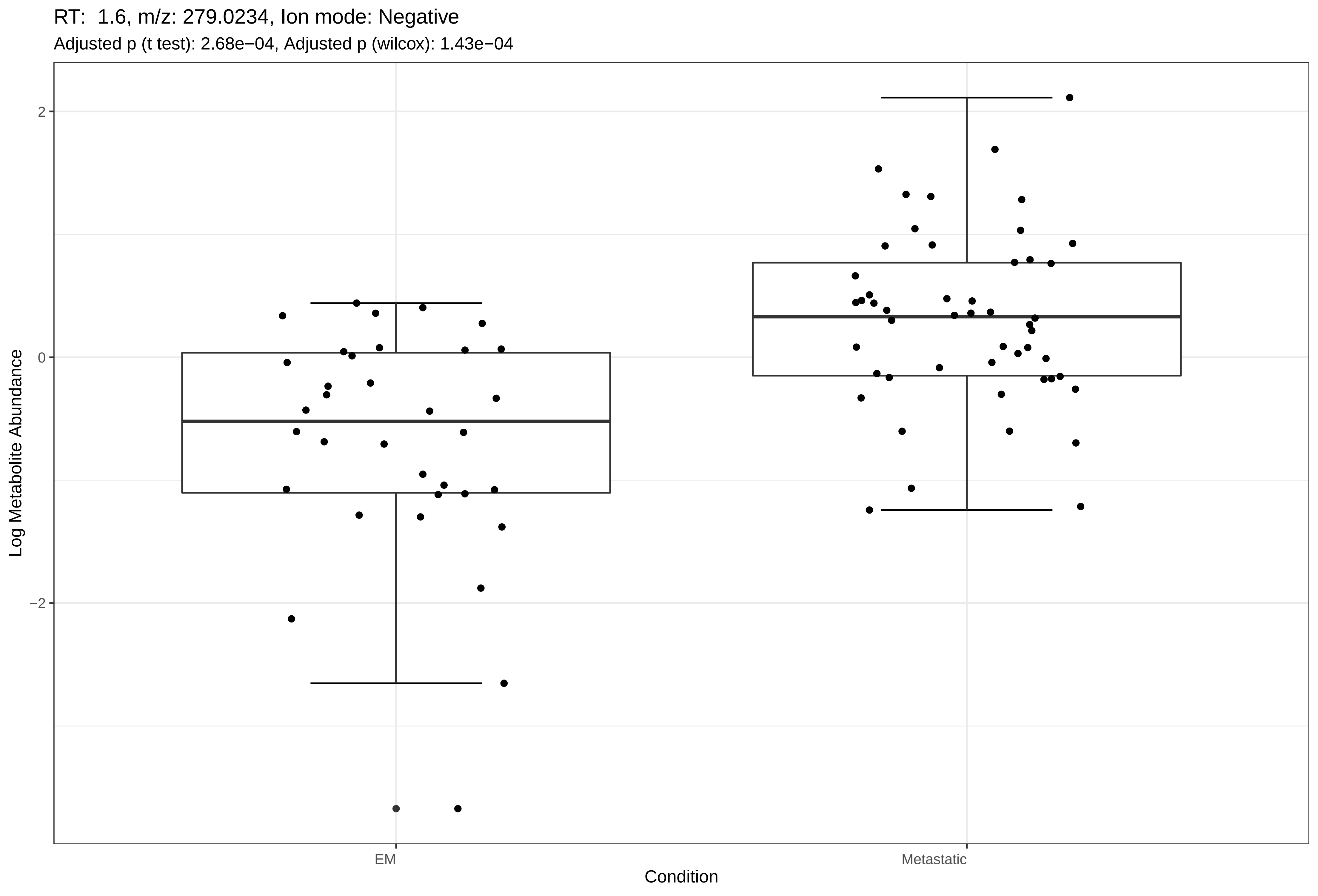
RT: 1.6, m/z: 279.0234, Ion mode: Negative
Adjusted p (t test): 2.68e−04, Adjusted p (wilcox): 1.43e−04
2
0
−2
Log Metabolite Abundance
EM
Metastatic
Condition

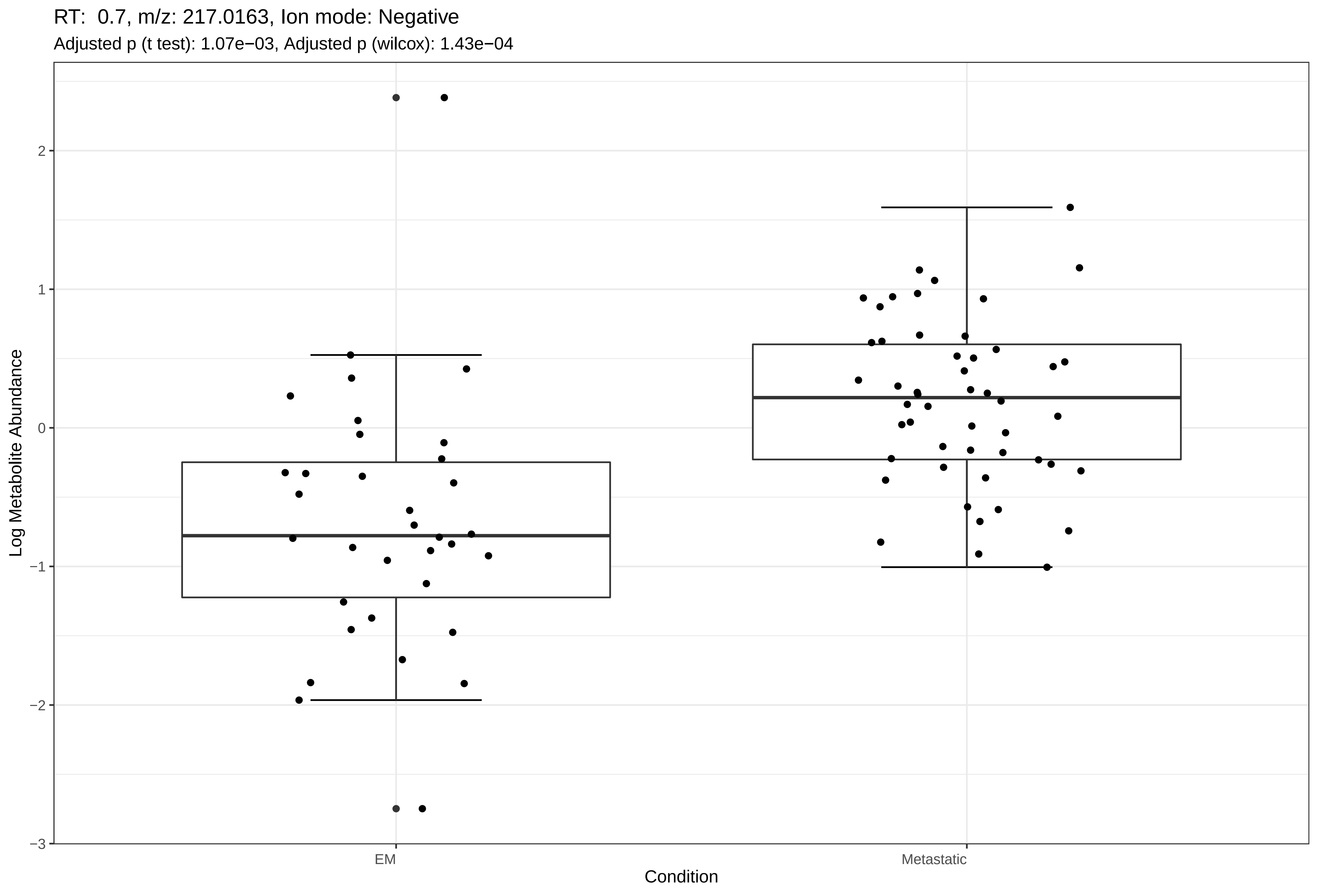
RT: 0.7, m/z: 217.0163, Ion mode: Negative
Adjusted p (t test): 1.07e−03, Adjusted p (wilcox): 1.43e−04
Log Metabolite Abundance
2
1
0
−1
−2
−3
EM
Metastatic
Condition

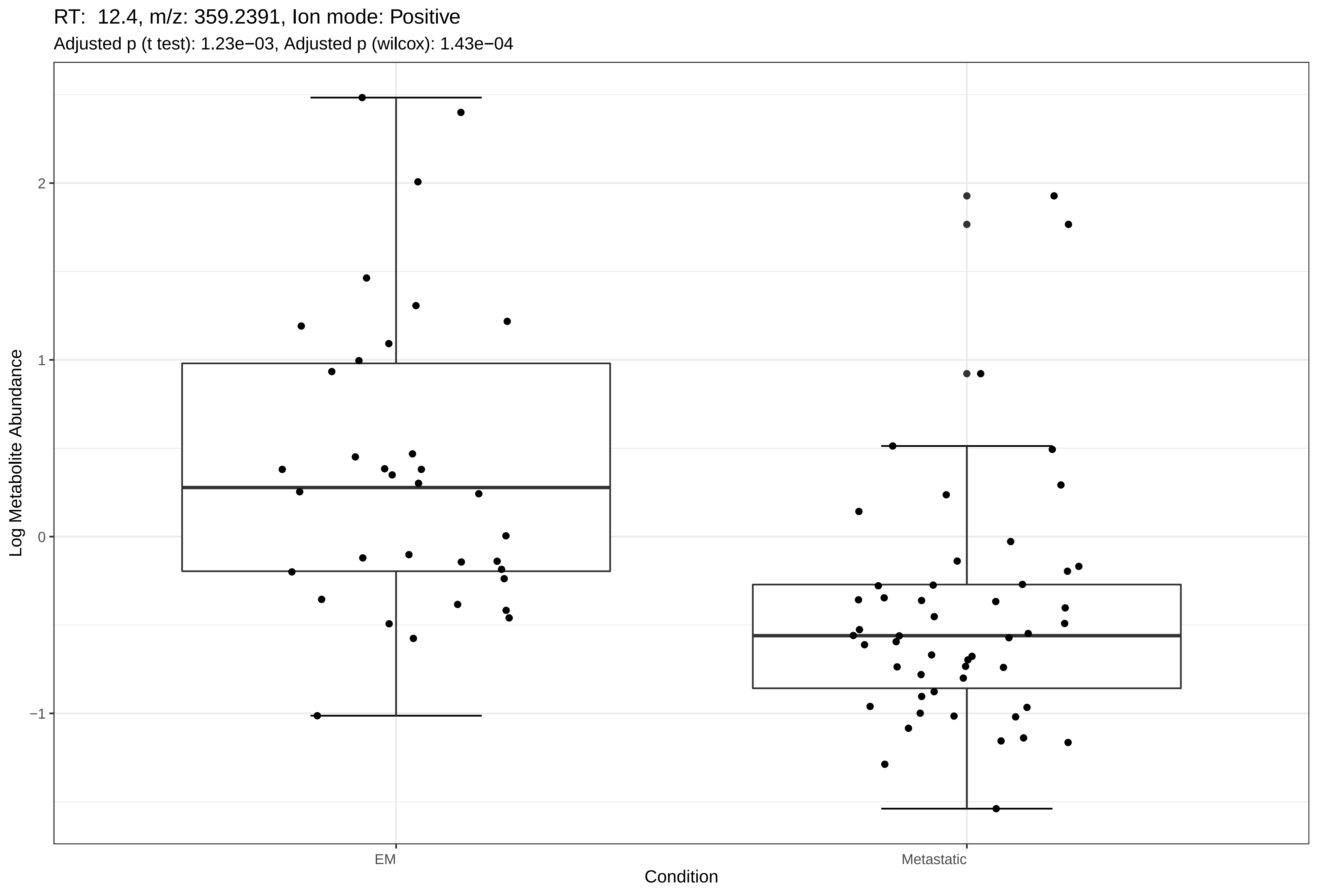

RT: 12.4, m/z: 359.2391, Ion mode: Positive
Adjusted p (t test): 1.23e−03, Adjusted p (wilcox): 1.43e−04
Log Metabolite Abundance
2
1
0
−1
EM
Metastatic
Condition

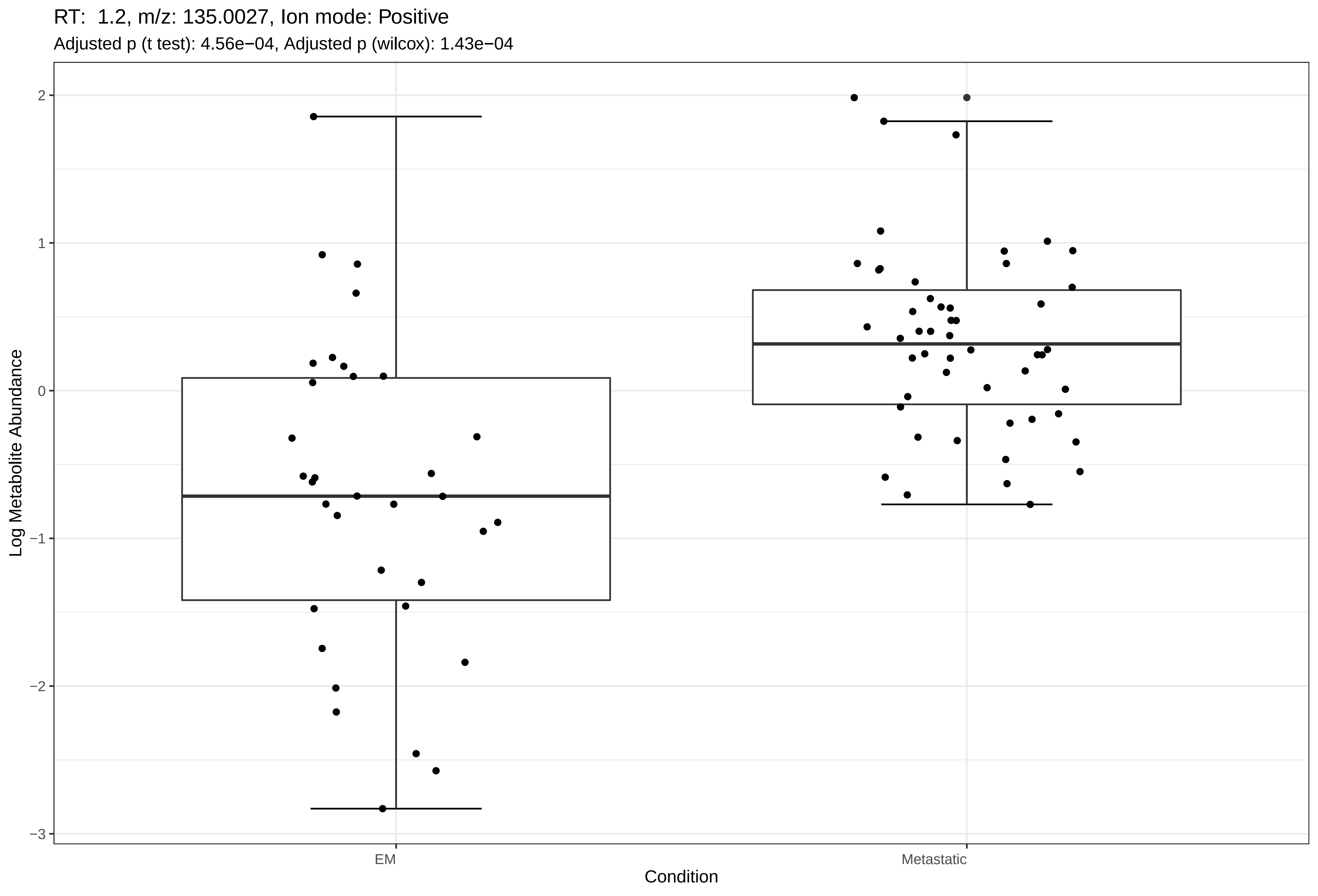
RT: 1.2, m/z: 135.0027, Ion mode: Positive
Adjusted p (t test): 4.56e−04, Adjusted p (wilcox): 1.43e−04
Log Metabolite Abundance
2
1
0
−1
−2
−3
EM
Metastatic
Condition

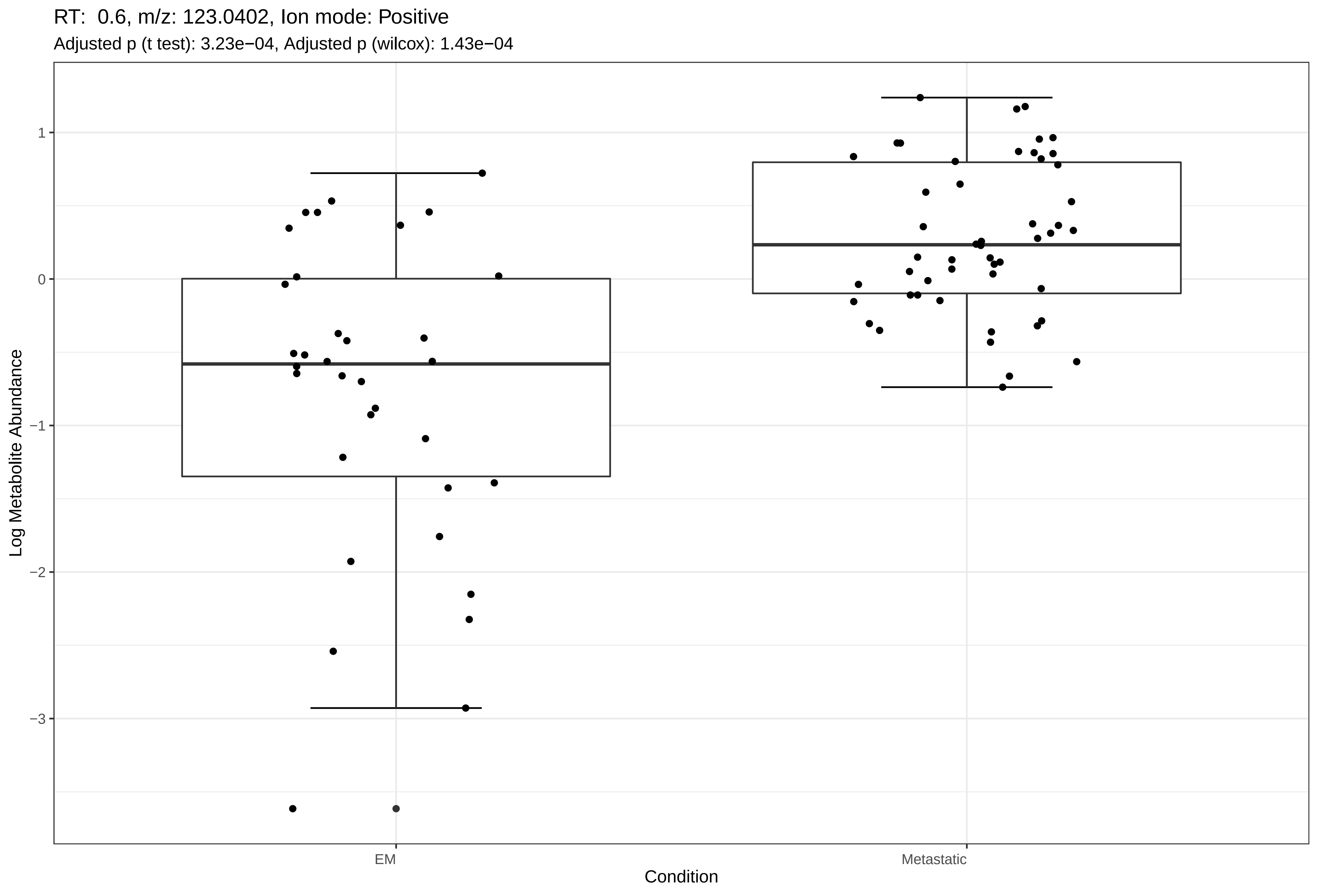
RT: 0.6, m/z: 123.0402, Ion mode: Positive
Adjusted p (t test): 3.23e−04, Adjusted p (wilcox): 1.43e−04
Log Metabolite Abundance
1
0
−1
−2
−3
EM
Metastatic
Condition

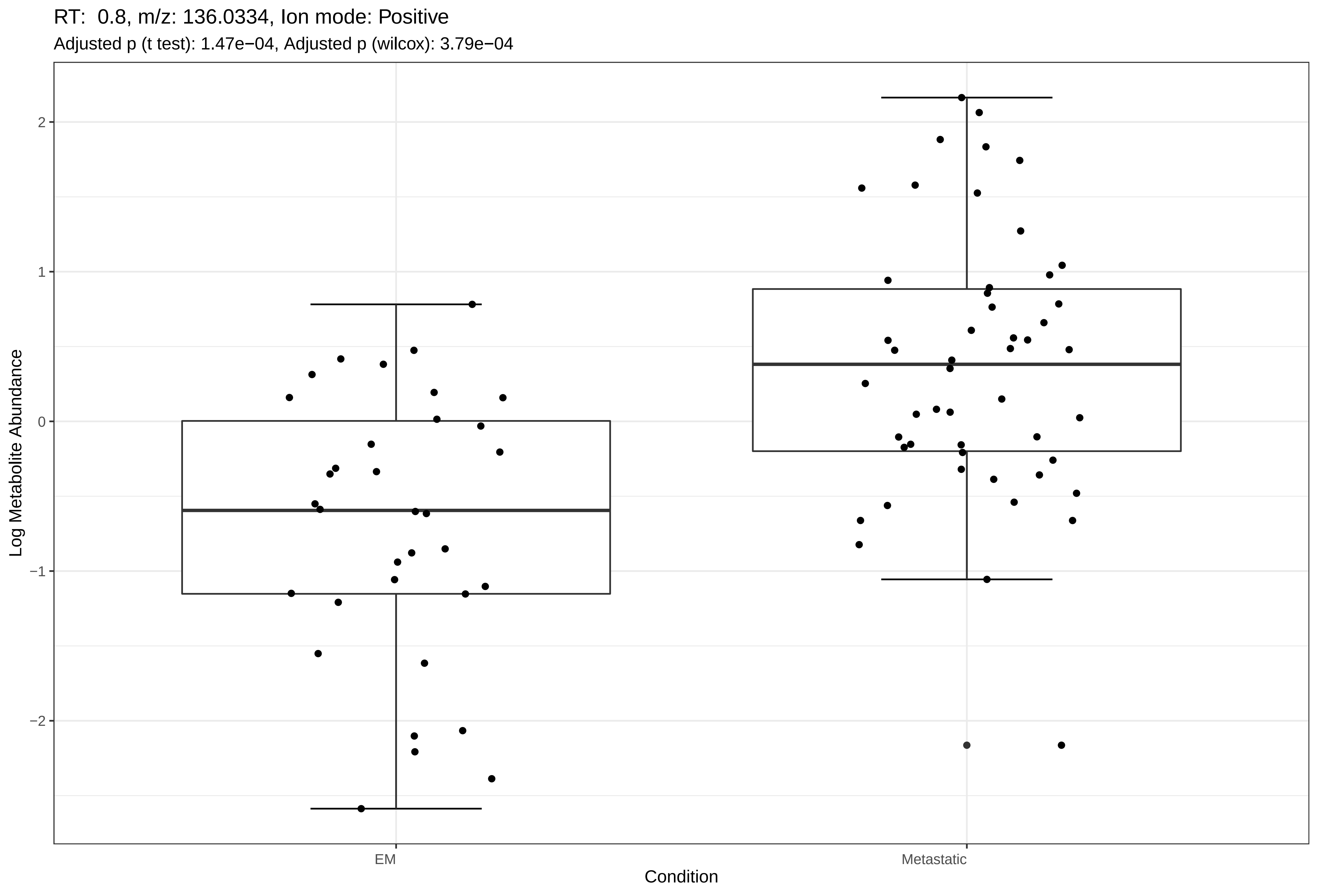
RT: 0.8, m/z: 136.0334, Ion mode: Positive
Adjusted p (t test): 1.47e−04, Adjusted p (wilcox): 3.79e−04
Log Metabolite Abundance
2
1
0
−1
−2
EM
Metastatic
Condition

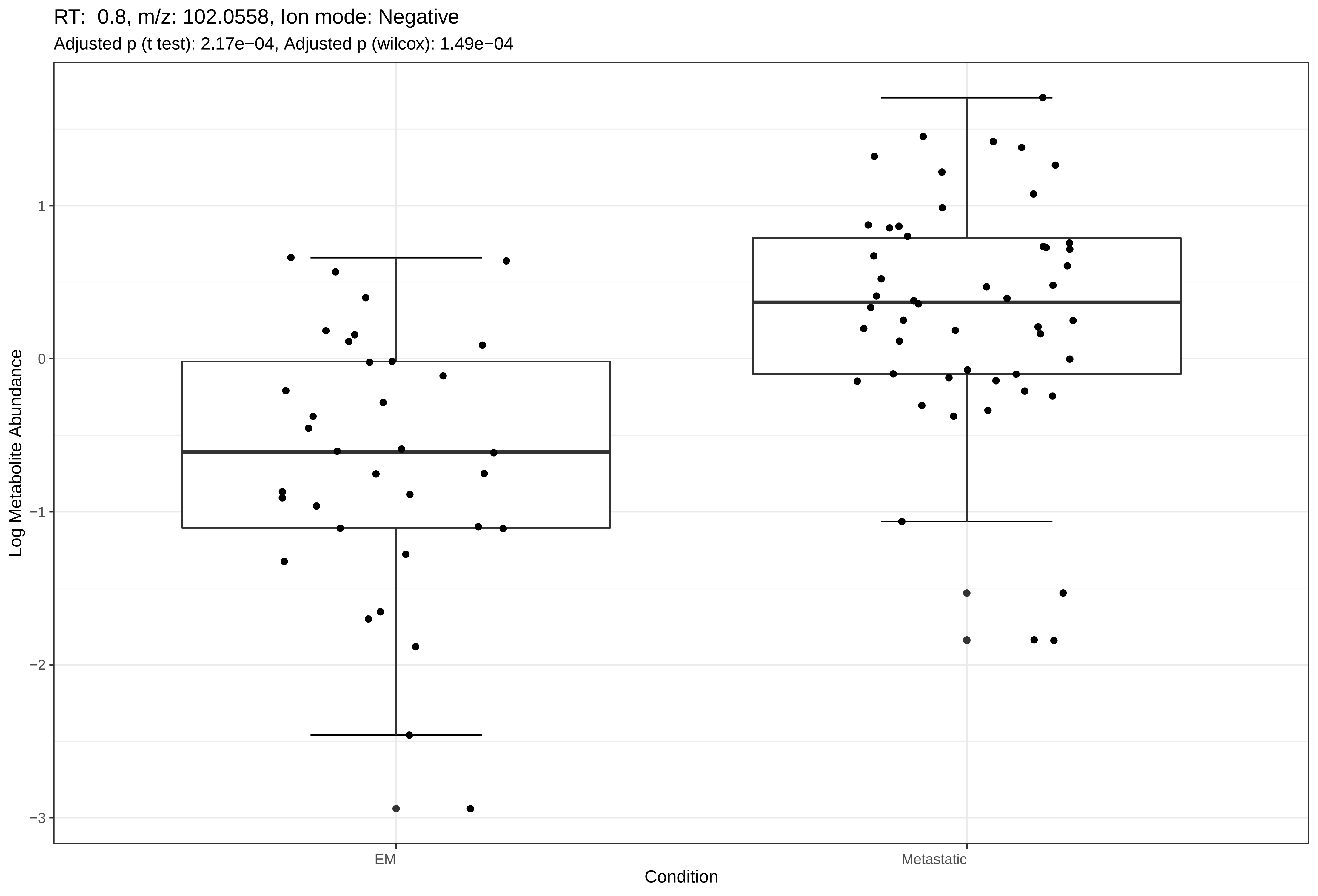

RT: 0.8, m/z: 102.0558, Ion mode: Negative
Adjusted p (t test): 2.17e−04, Adjusted p (wilcox): 1.49e−04
Log Metabolite Abundance
1
0
−1
−2
−3
EM
Metastatic
Condition

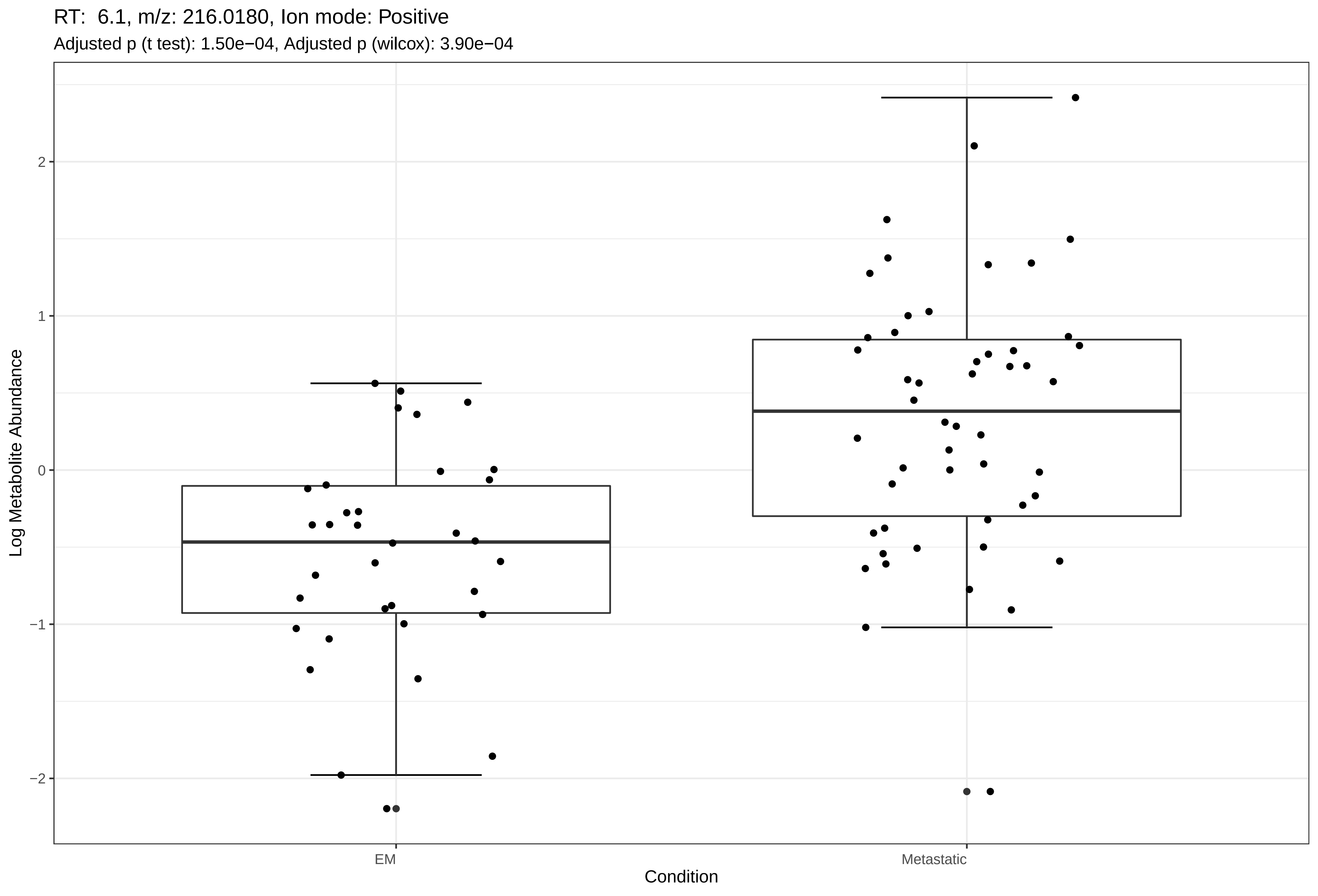
RT: 6.1, m/z: 216.0180, Ion mode: Positive
Adjusted p (t test): 1.50e−04, Adjusted p (wilcox): 3.90e−04
Log Metabolite Abundance
2
1
0
−1
−2
EM
Metastatic
Condition

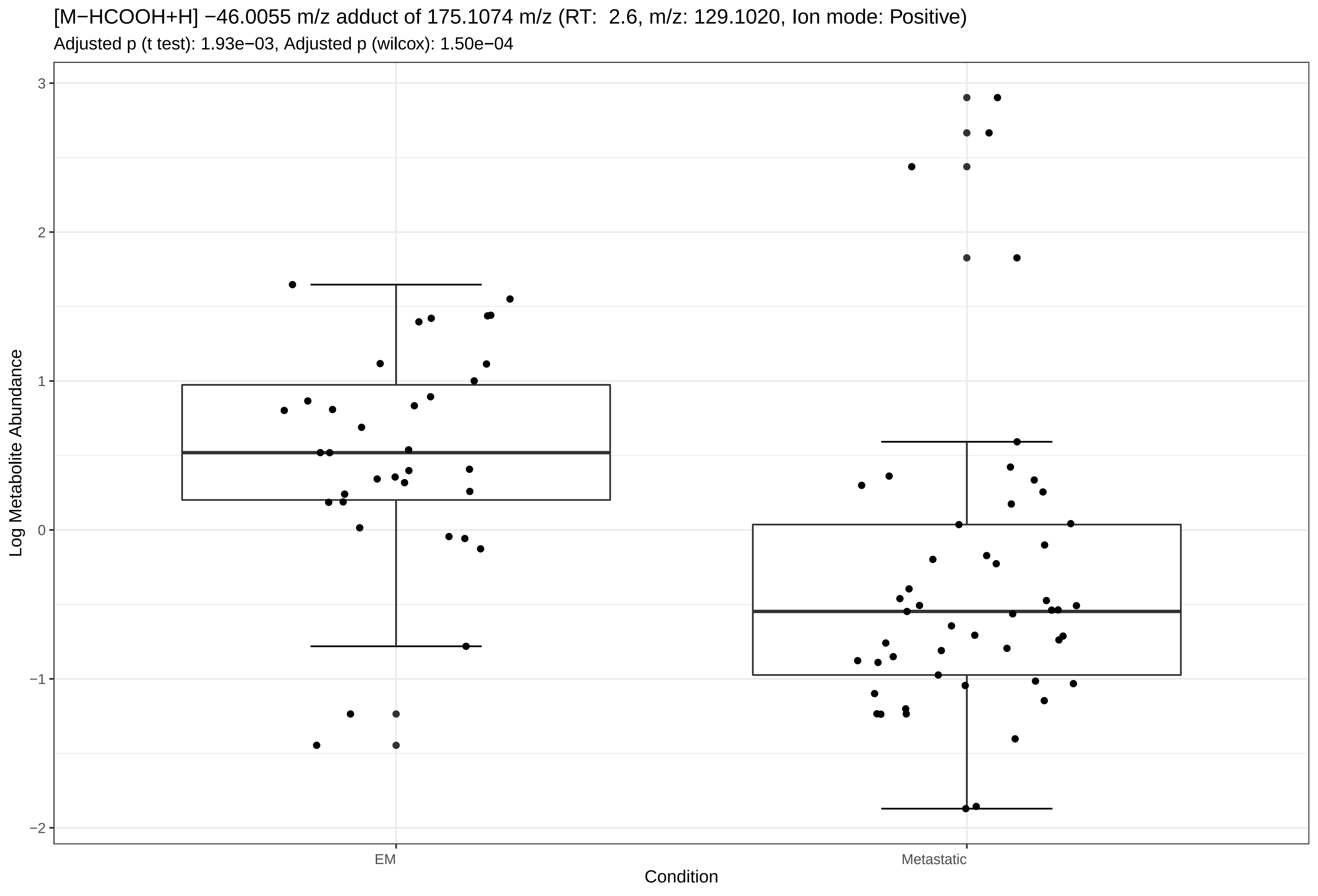

[M−HCOOH+H] −46.0055 m/z adduct of 175.1074 m/z (RT: 2.6, m/z: 129.1020, Ion mode: Positive)
Adjusted p (t test): 1.93e−03, Adjusted p (wilcox): 1.50e−04
Log Metabolite Abundance
3
2
1
0
−1
−2
EM
Metastatic
Condition

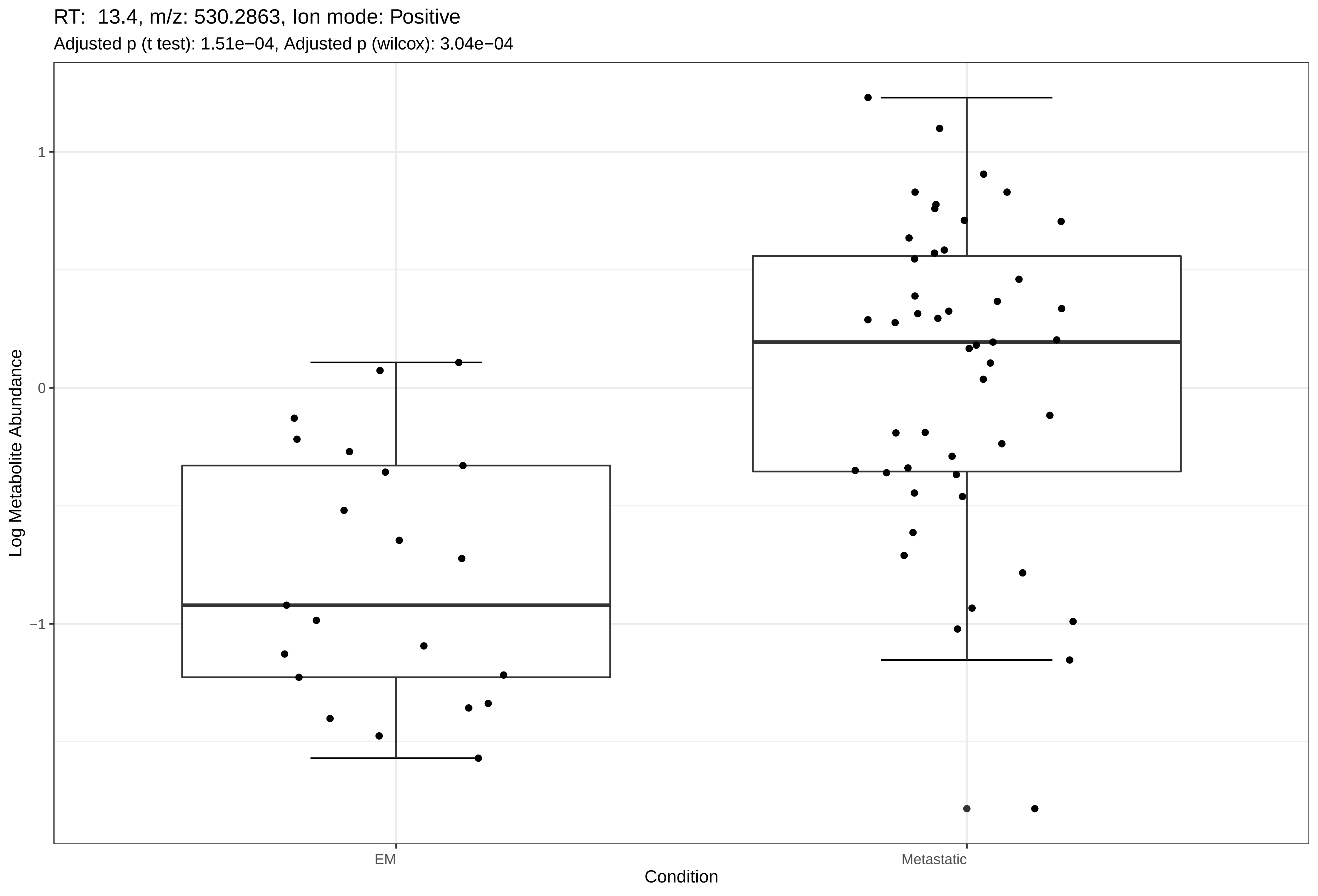
RT: 13.4, m/z: 530.2863, Ion mode: Positive
Adjusted p (t test): 1.51e−04, Adjusted p (wilcox): 3.04e−04
Log Metabolite Abundance
1
0
−1
EM
Metastatic
Condition

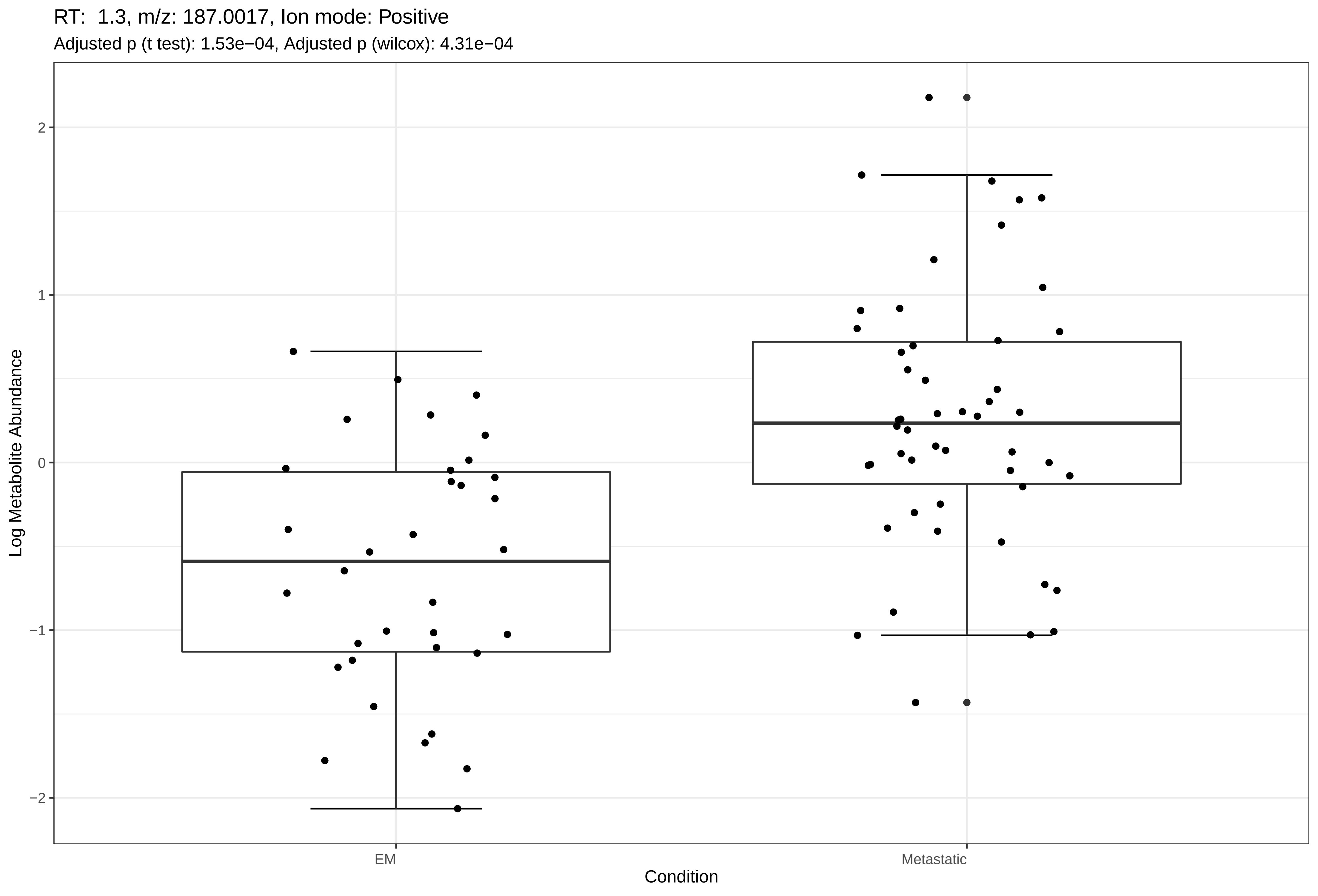
RT: 1.3, m/z: 187.0017, Ion mode: Positive
Adjusted p (t test): 1.53e−04, Adjusted p (wilcox): 4.31e−04
Log Metabolite Abundance
2
1
0
−1
−2
EM
Metastatic
Condition

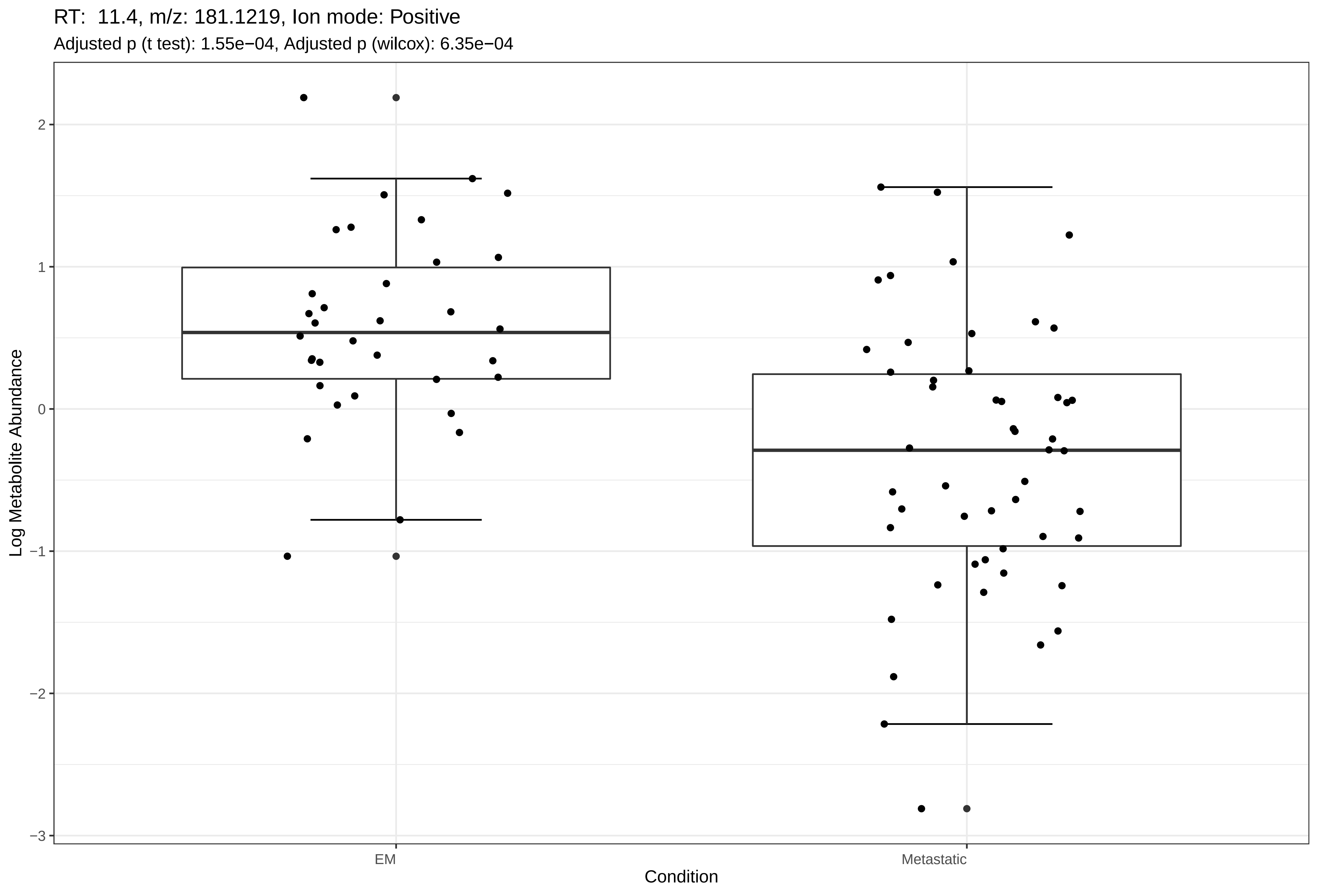
RT: 11.4, m/z: 181.1219, Ion mode: Positive
Adjusted p (t test): 1.55e−04, Adjusted p (wilcox): 6.35e−04
Log Metabolite Abundance
2
1
0
−1
−2
−3
EM
Metastatic
Condition

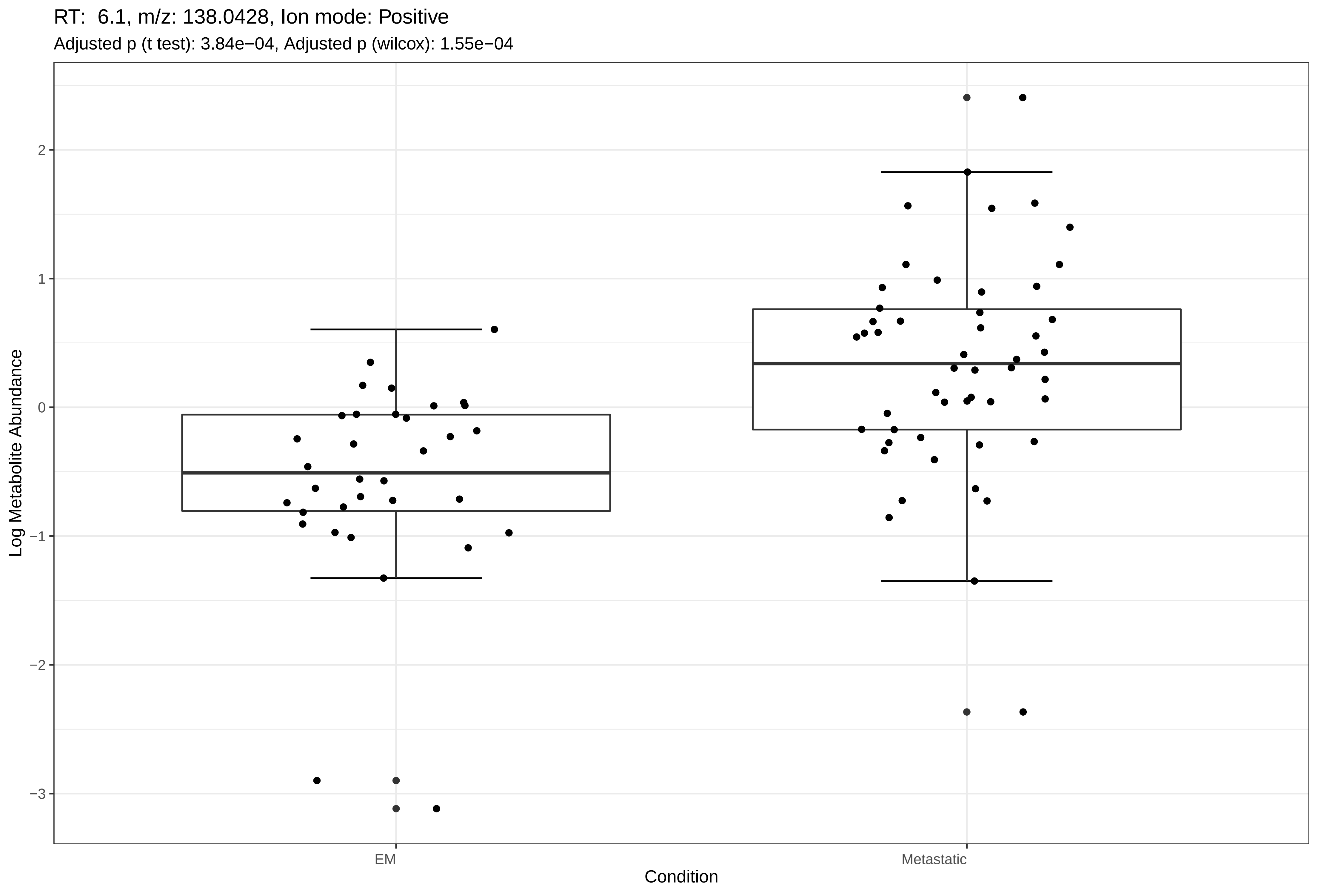
RT: 6.1, m/z: 138.0428, Ion mode: Positive
Adjusted p (t test): 3.84e−04, Adjusted p (wilcox): 1.55e−04
Log Metabolite Abundance
2
1
0
−1
−2
−3
EM
Metastatic
Condition

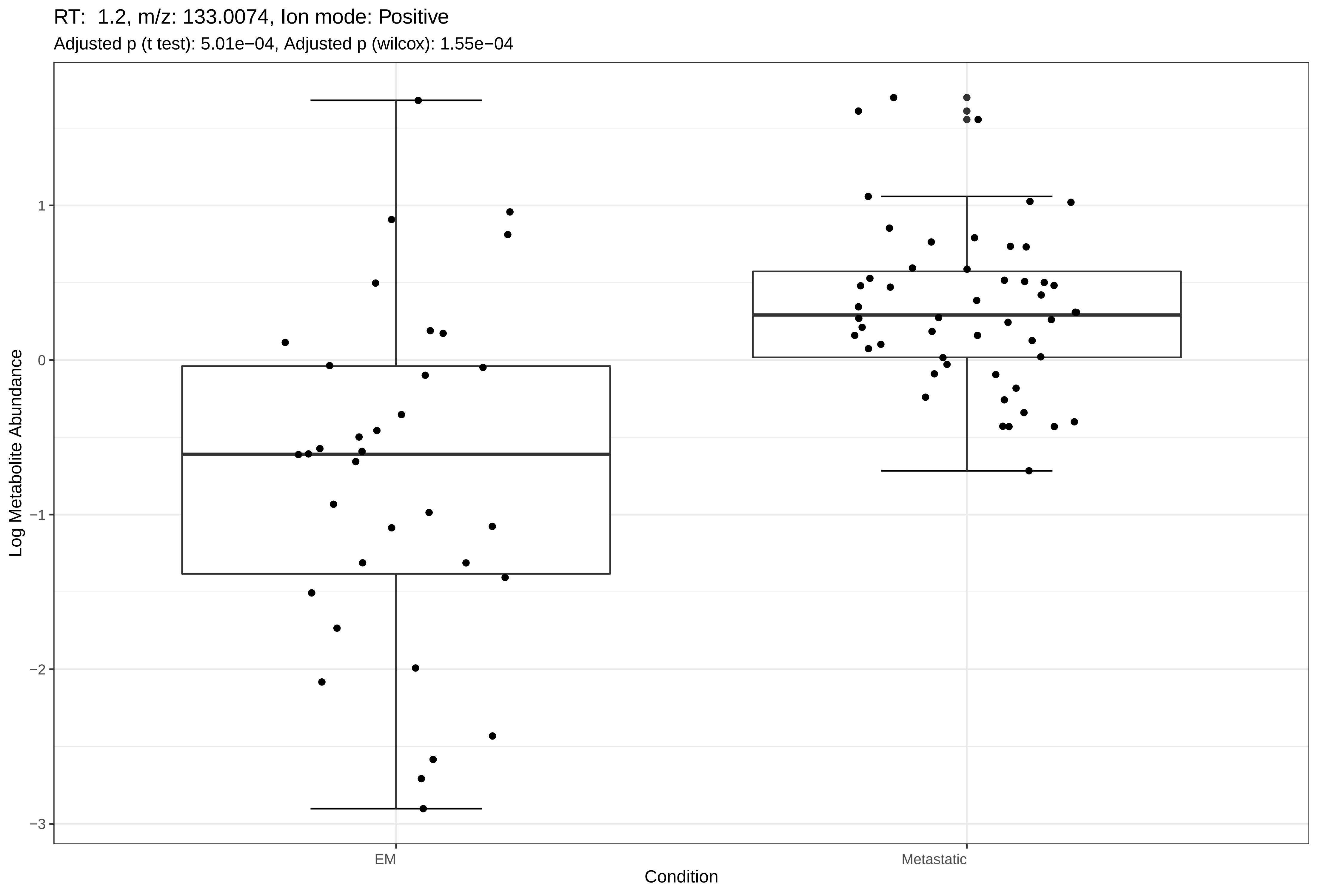
RT: 1.2, m/z: 133.0074, Ion mode: Positive
Adjusted p (t test): 5.01e−04, Adjusted p (wilcox): 1.55e−04
Log Metabolite Abundance
1
0
−1
−2
−3
EM
Metastatic
Condition

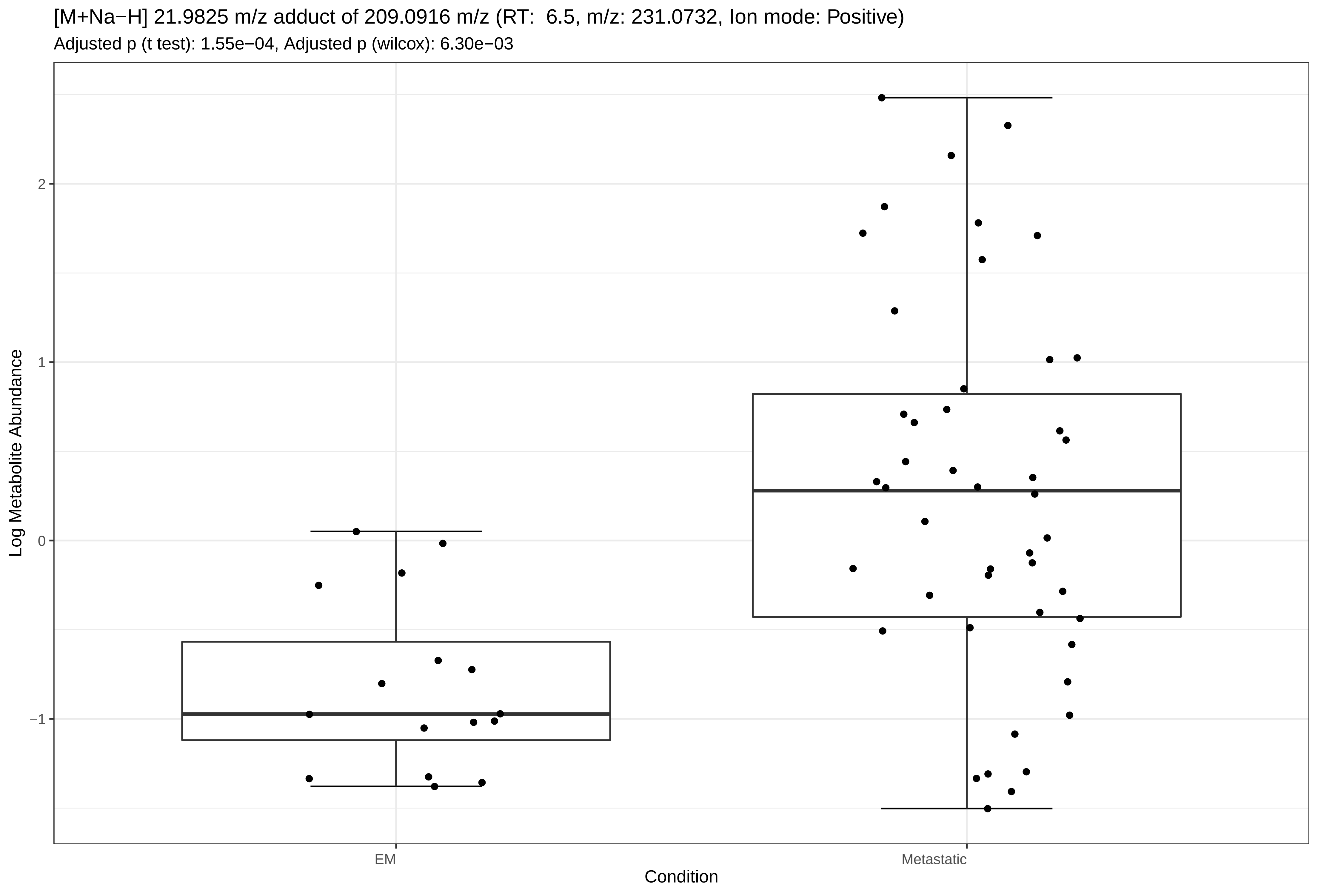
[M+Na−H] 21.9825 m/z adduct of 209.0916 m/z (RT: 6.5, m/z: 231.0732, Ion mode: Positive)
Adjusted p (t test): 1.55e−04, Adjusted p (wilcox): 6.30e−03
Log Metabolite Abundance
2
1
0
−1
EM
Metastatic
Condition

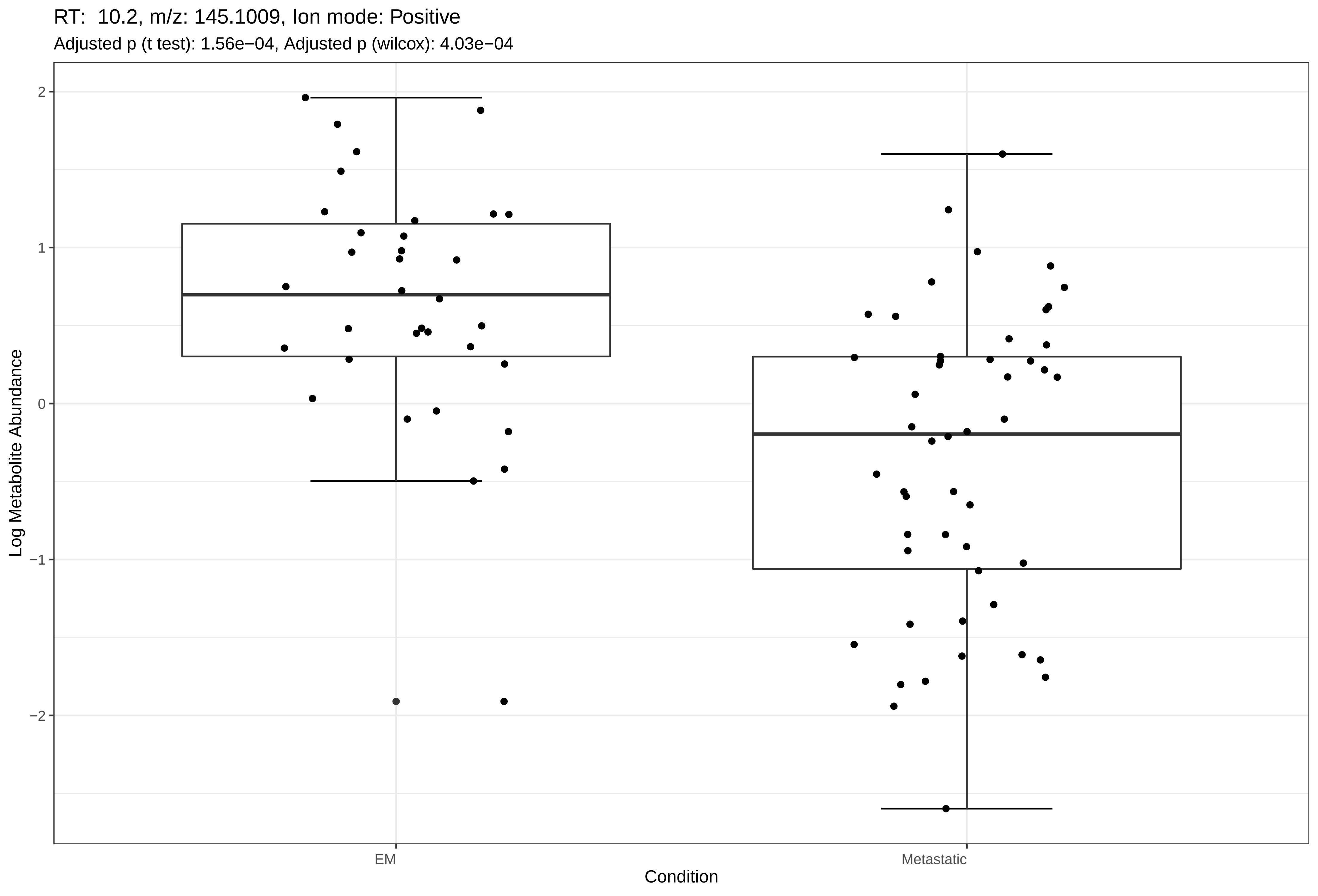
RT: 10.2, m/z: 145.1009, Ion mode: Positive
Adjusted p (t test): 1.56e−04, Adjusted p (wilcox): 4.03e−04
Log Metabolite Abundance
2
1
0
−1
−2
EM
Metastatic
Condition

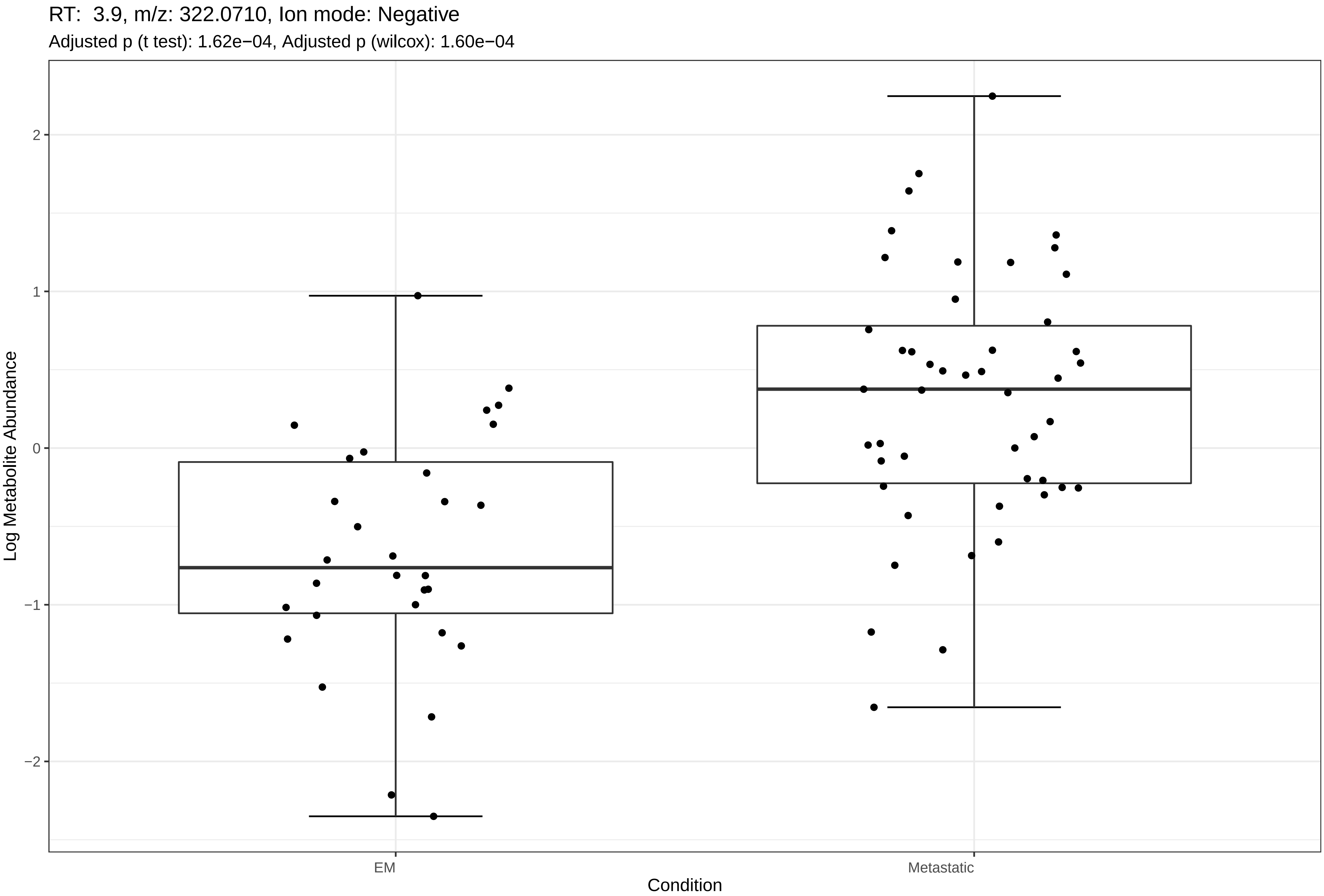
RT: 3.9, m/z: 322.0710, Ion mode: Negative
Adjusted p (t test): 1.62e−04, Adjusted p (wilcox): 1.60e−04
Log Metabolite Abundance
2
1
0
−1
−2
EM
Metastatic
Condition

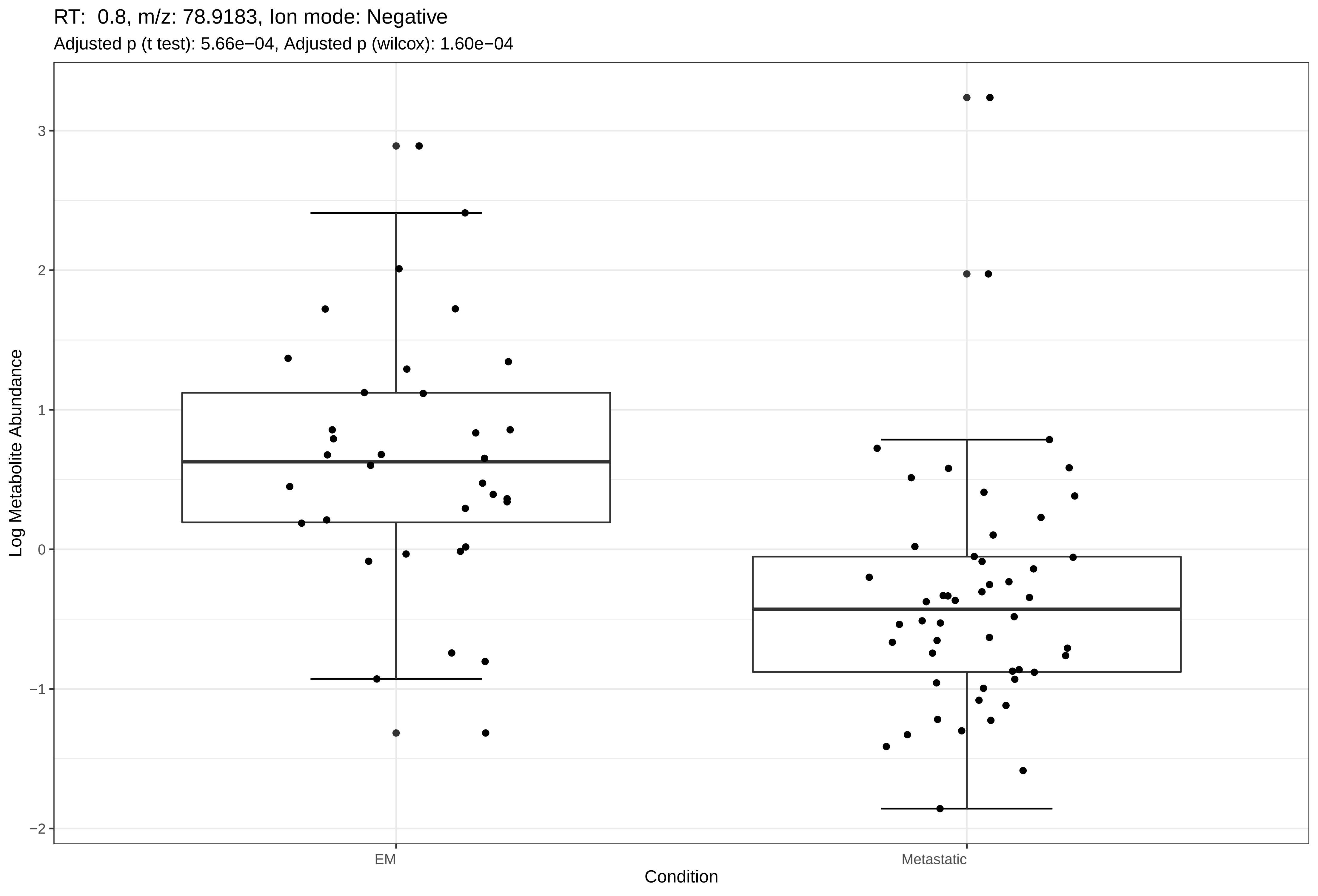
RT: 0.8, m/z: 78.9183, Ion mode: Negative
Adjusted p (t test): 5.66e−04, Adjusted p (wilcox): 1.60e−04
Log Metabolite Abundance
3
2
1
0
−1
−2
EM
Metastatic
Condition

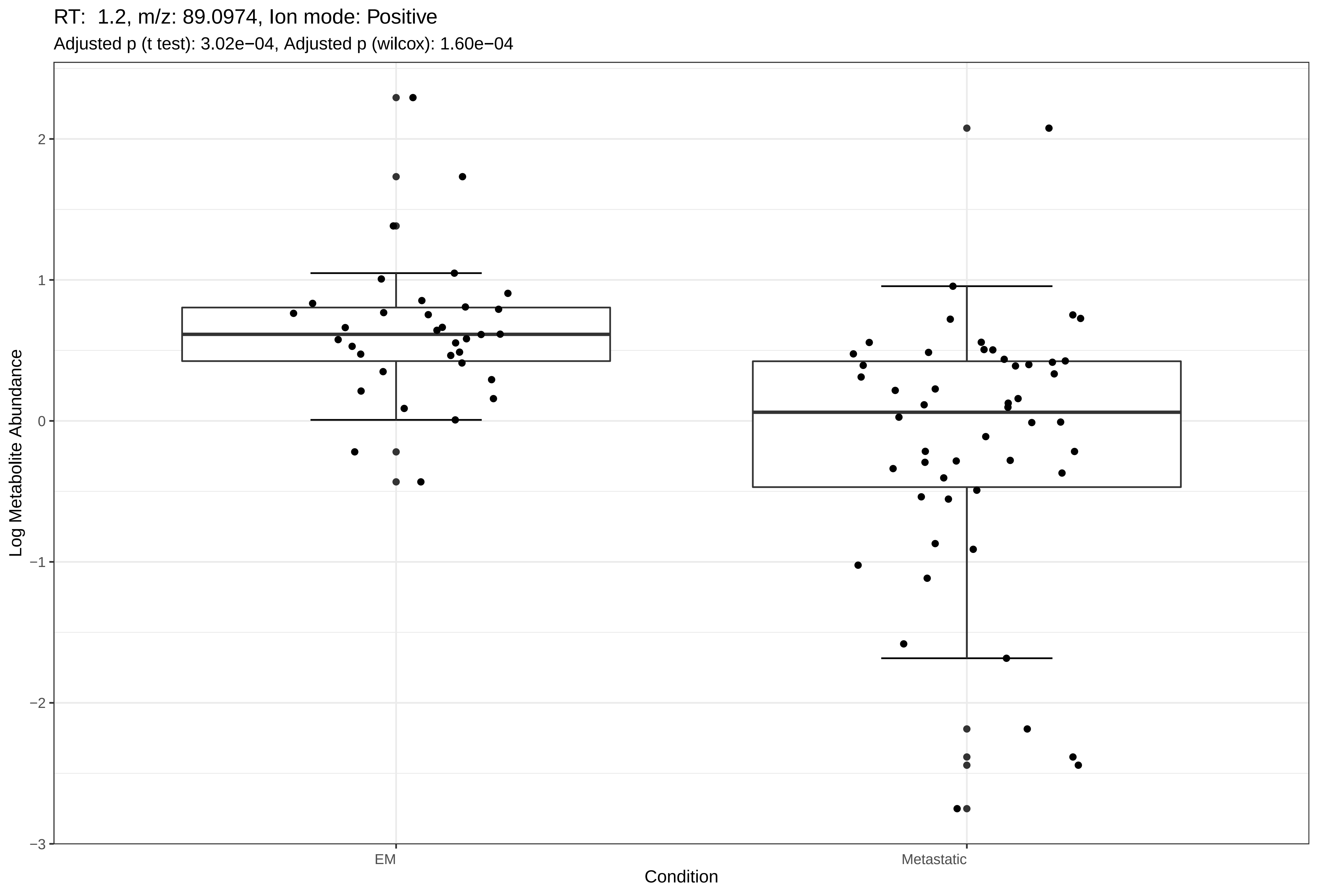

RT: 1.2, m/z: 89.0974, Ion mode: Positive
Adjusted p (t test): 3.02e−04, Adjusted p (wilcox): 1.60e−04
Log Metabolite Abundance
2
1
0
−1
−2
−3
EM
Metastatic
Condition

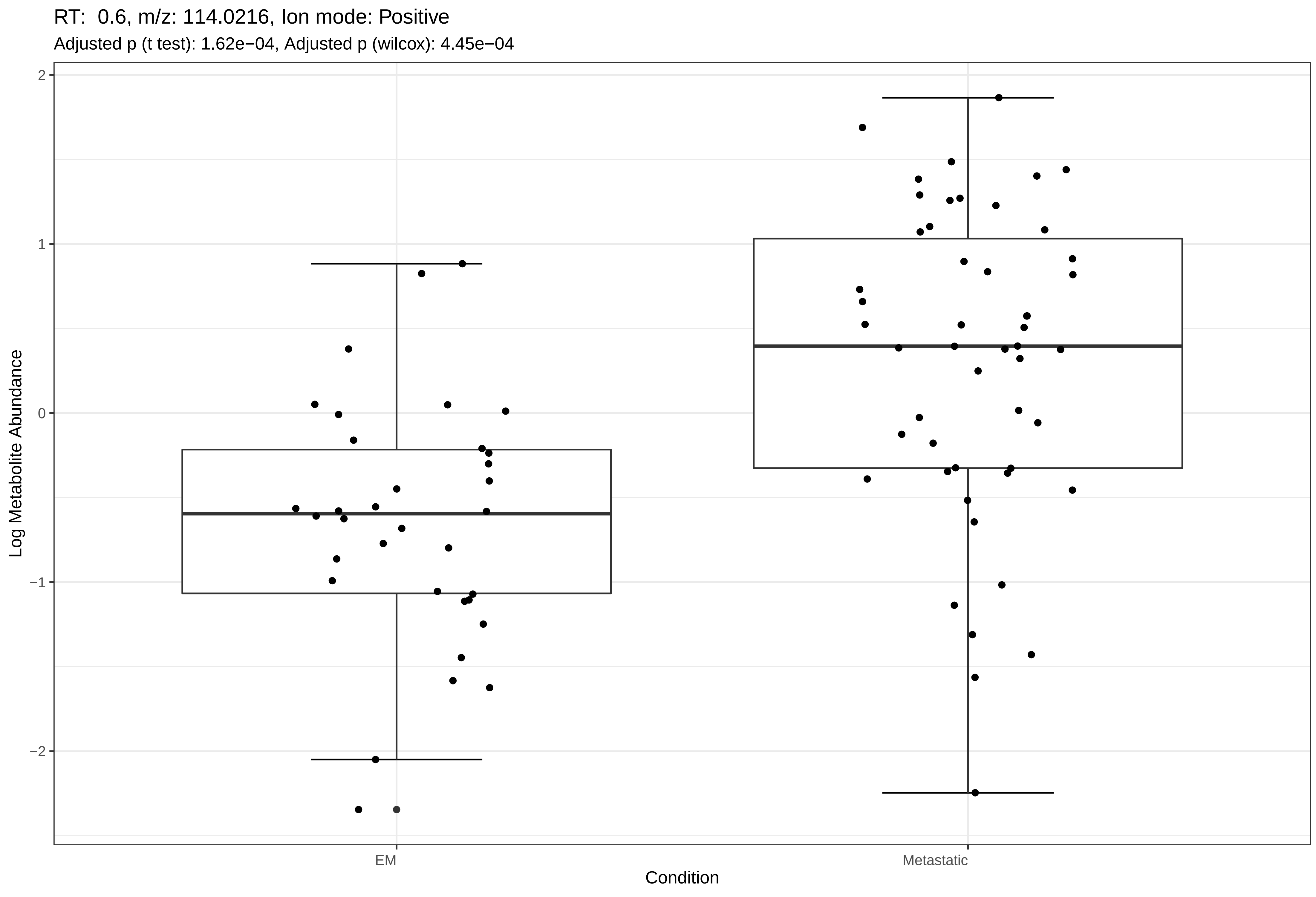
RT: 0.6, m/z: 114.0216, Ion mode: Positive
Adjusted p (t test): 1.62e−04, Adjusted p (wilcox): 4.45e−04
Log Metabolite Abundance
2
1
0
−1
−2
EM
Metastatic
Condition

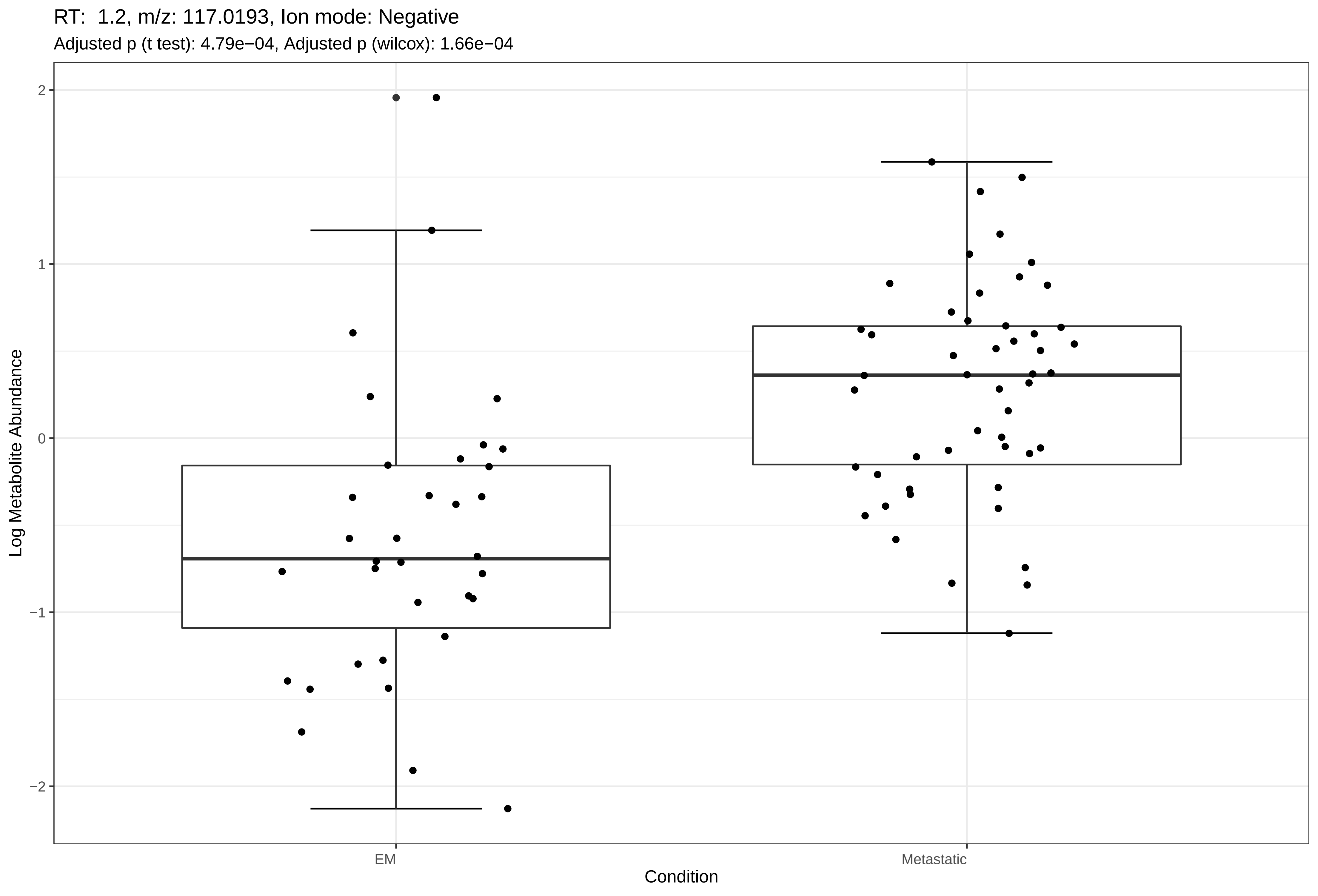

RT: 1.2, m/z: 117.0193, Ion mode: Negative
Adjusted p (t test): 4.79e−04, Adjusted p (wilcox): 1.66e−04
Log Metabolite Abundance
2
1
0
−1
−2
EM
Metastatic
Condition

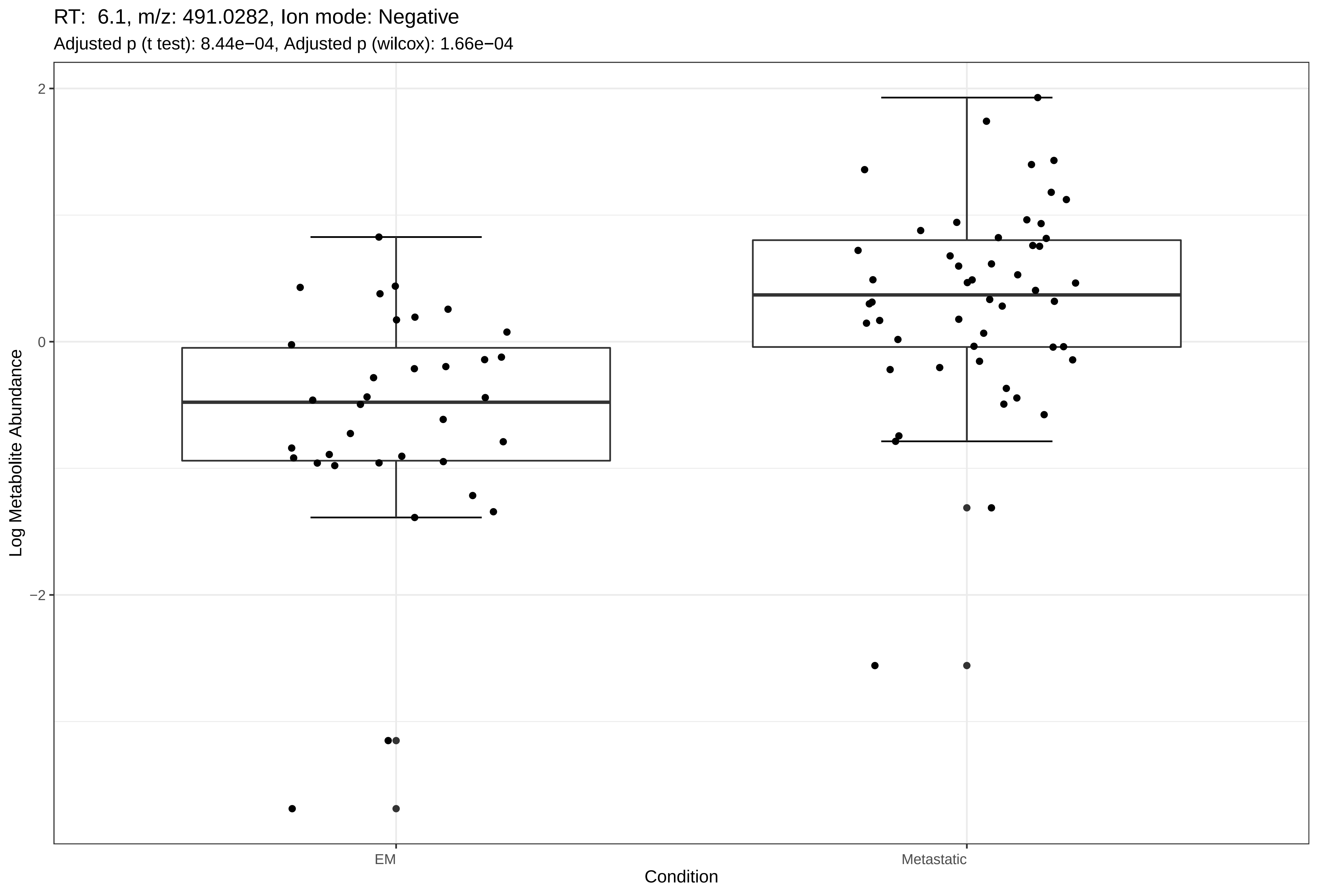
RT: 6.1, m/z: 491.0282, Ion mode: Negative
Adjusted p (t test): 8.44e−04, Adjusted p (wilcox): 1.66e−04
Log Metabolite Abundance
2
0
−2
EM
Metastatic
Condition

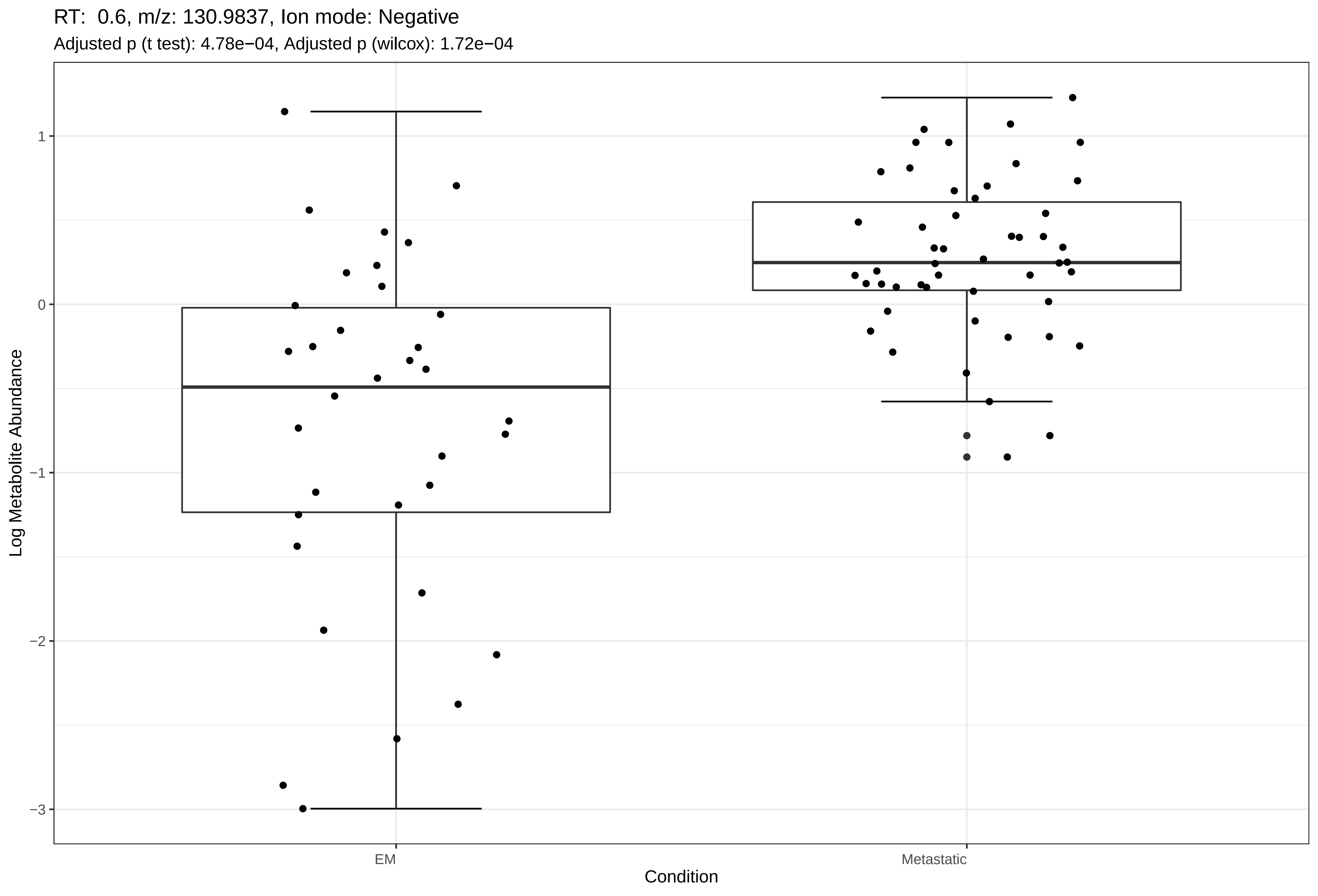
RT: 0.6, m/z: 130.9837, Ion mode: Negative
Adjusted p (t test): 4.78e−04, Adjusted p (wilcox): 1.72e−04
Log Metabolite Abundance
1
0
−1
−2
−3
EM
Metastatic
Condition

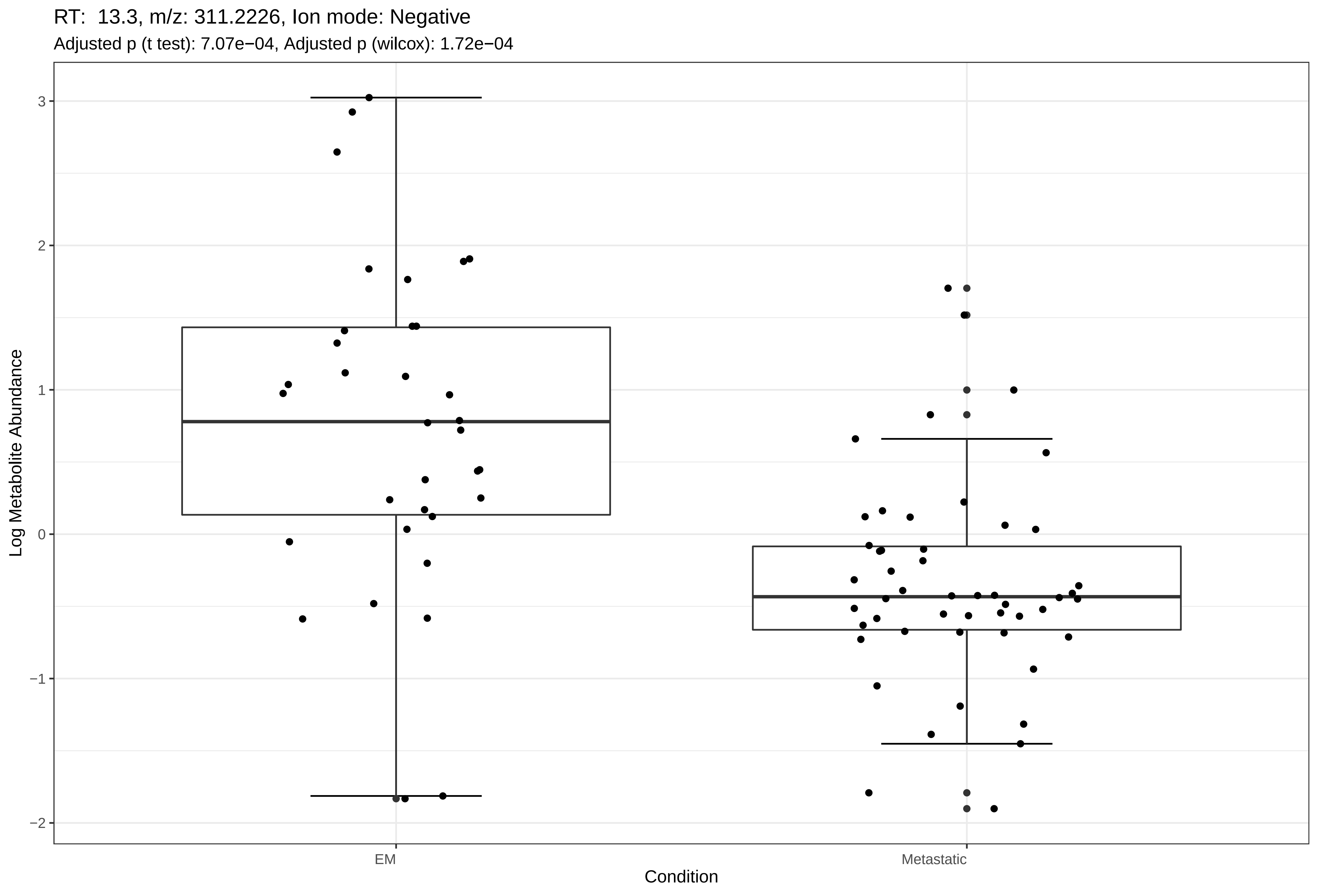
RT: 13.3, m/z: 311.2226, Ion mode: Negative
Adjusted p (t test): 7.07e−04, Adjusted p (wilcox): 1.72e−04
Log Metabolite Abundance
3
2
1
0
−1
−2
EM
Metastatic
Condition

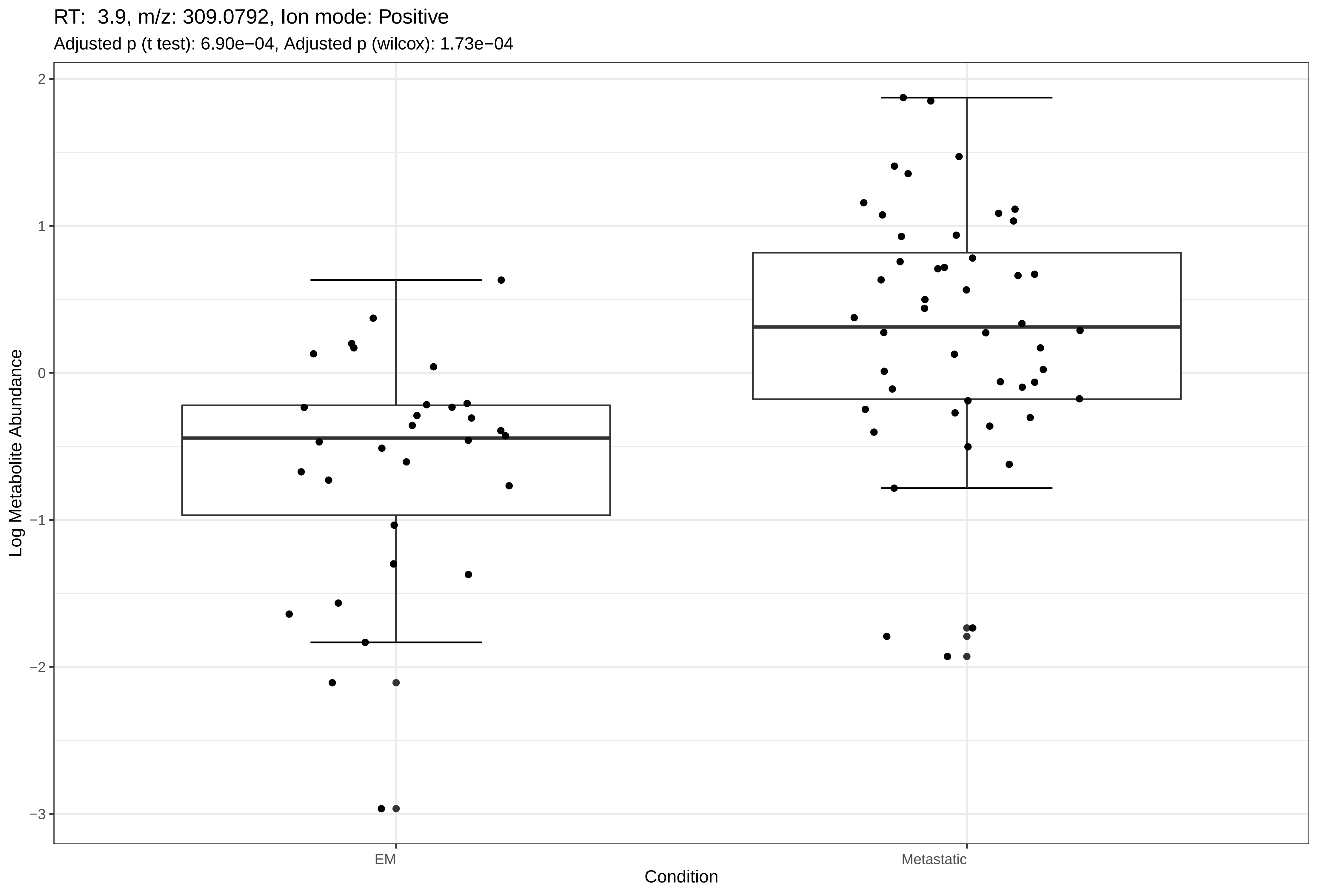
RT: 3.9, m/z: 309.0792, Ion mode: Positive
Adjusted p (t test): 6.90e−04, Adjusted p (wilcox): 1.73e−04
Log Metabolite Abundance
2
1
0
−1
−2
−3
EM
Metastatic
Condition

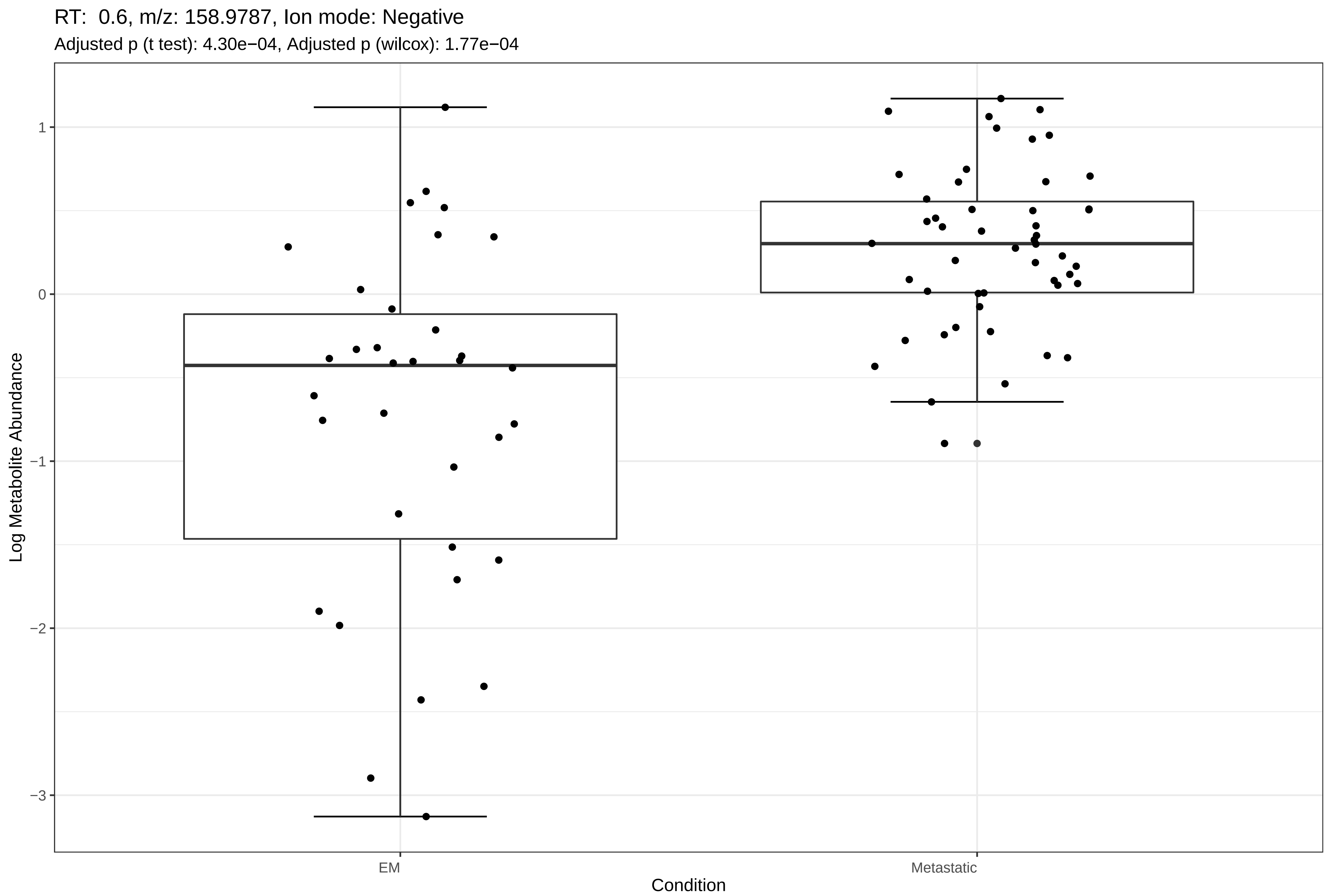
RT: 0.6, m/z: 158.9787, Ion mode: Negative
Adjusted p (t test): 4.30e−04, Adjusted p (wilcox): 1.77e−04
Log Metabolite Abundance
1
0
−1
−2
−3
EM
Metastatic
Condition

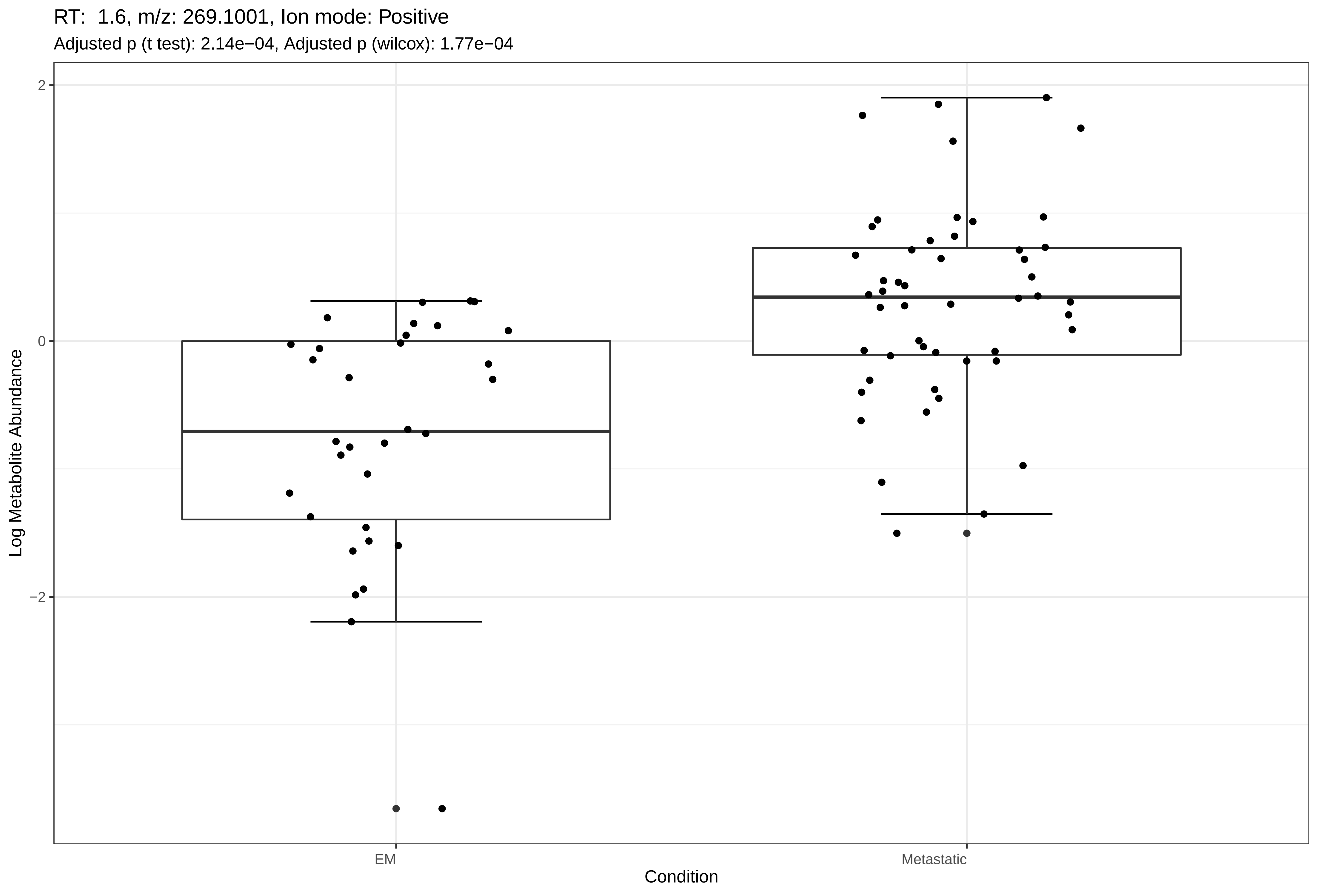
RT: 1.6, m/z: 269.1001, Ion mode: Positive
Adjusted p (t test): 2.14e−04, Adjusted p (wilcox): 1.77e−04
Log Metabolite Abundance
2
0
−2
EM
Metastatic
Condition

RT: 1.4, m/z: 173.0918, Ion mode: Positive

Adjusted p (t test): 2.26e−04, Adjusted p (wilcox): 1.77e−04

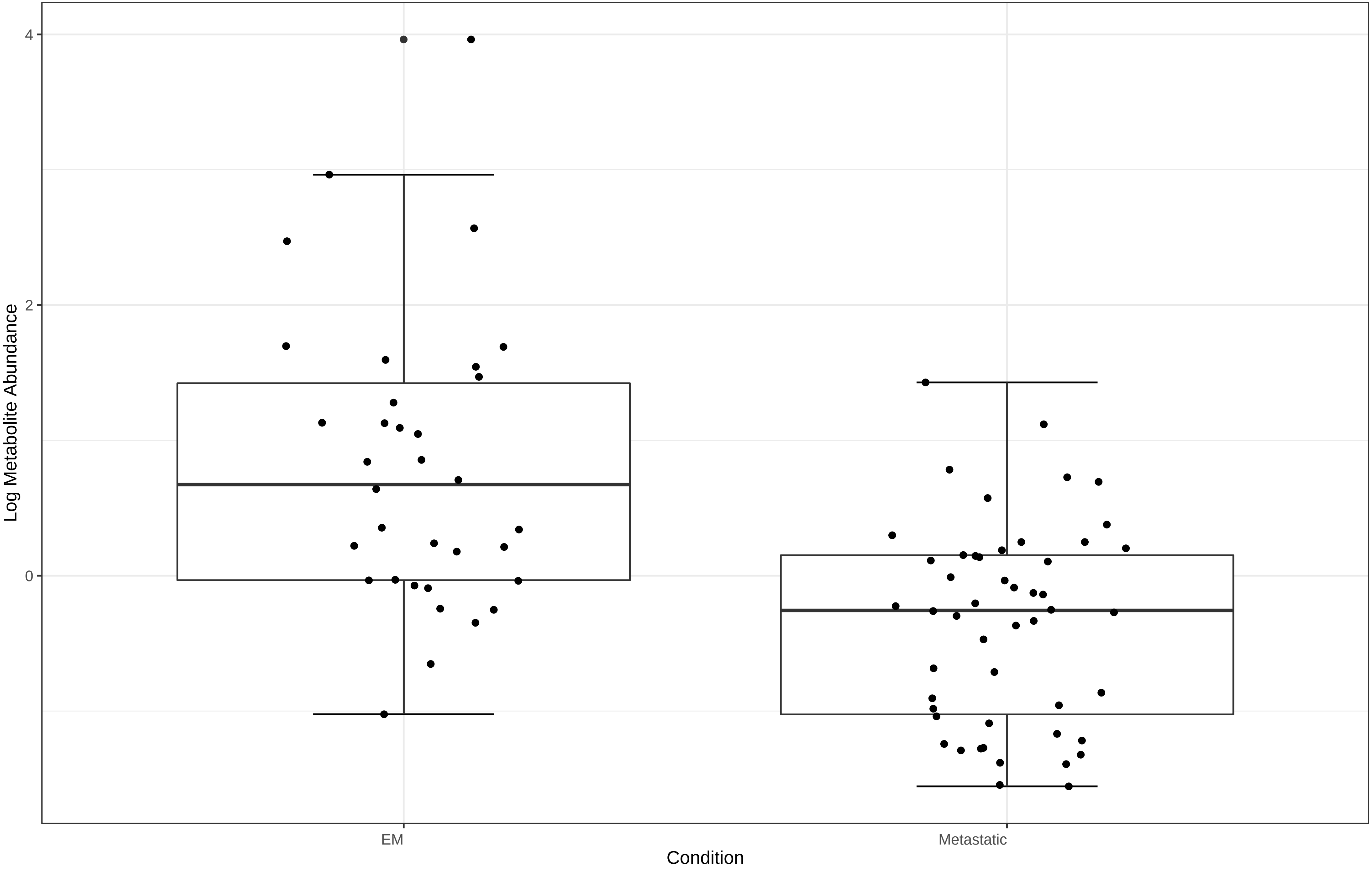

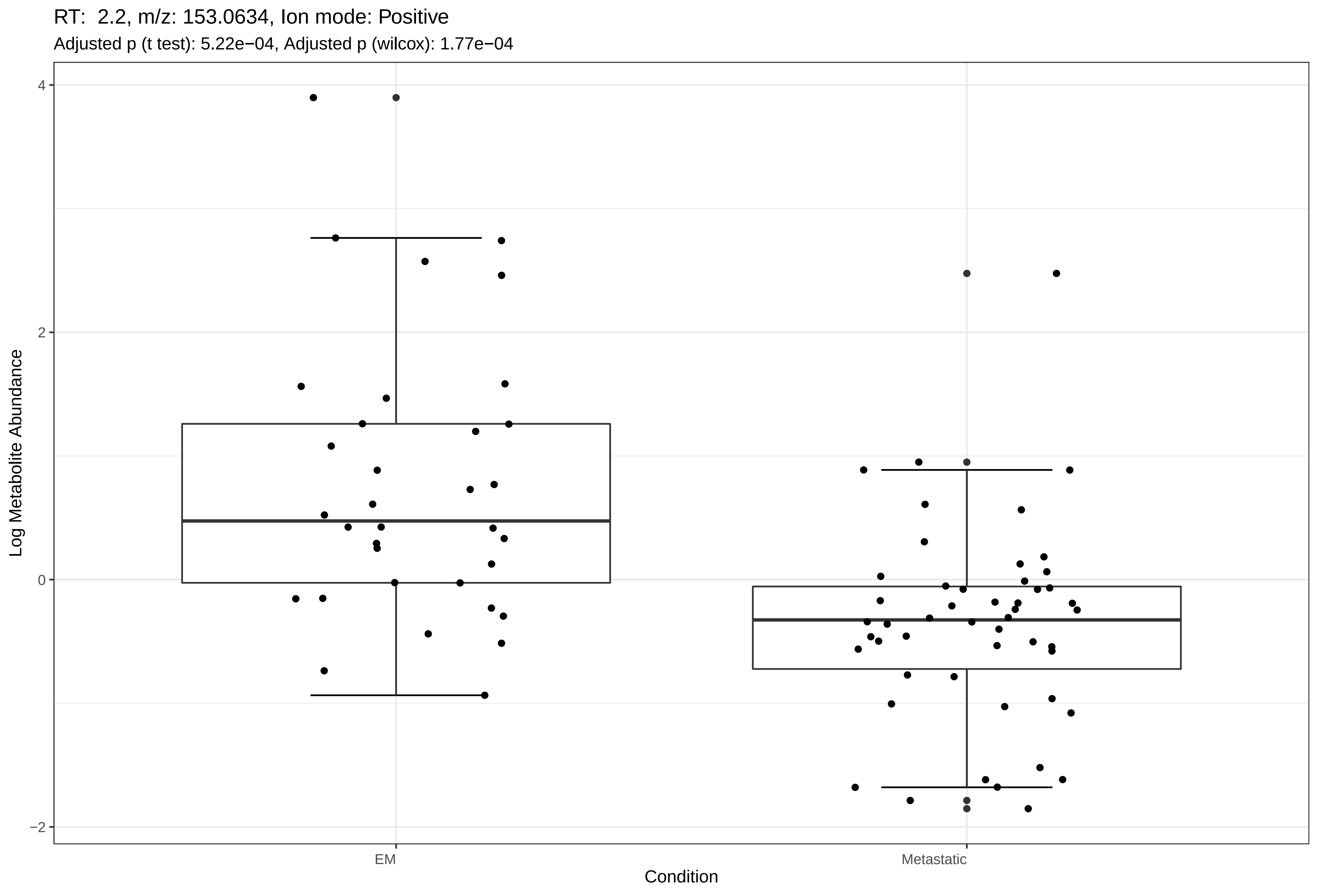

RT: 2.2, m/z: 153.0634, Ion mode: Positive
Adjusted p (t test): 5.22e−04, Adjusted p (wilcox): 1.77e−04
Log Metabolite Abundance
4
2
0
−2
EM
Metastatic
Condition

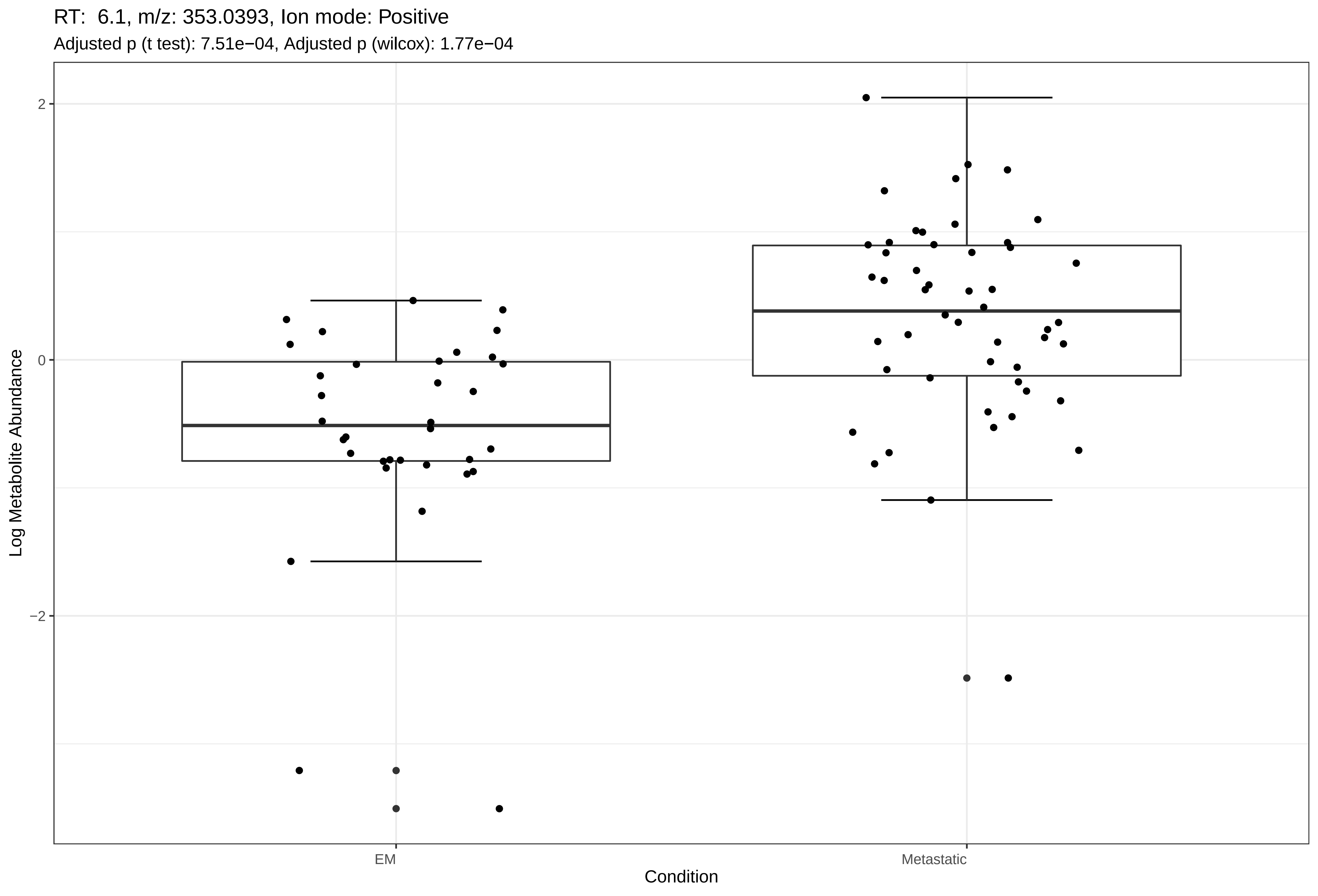
RT: 6.1, m/z: 353.0393, Ion mode: Positive
Adjusted p (t test): 7.51e−04, Adjusted p (wilcox): 1.77e−04
Log Metabolite Abundance
2
0
−2
EM
Metastatic
Condition

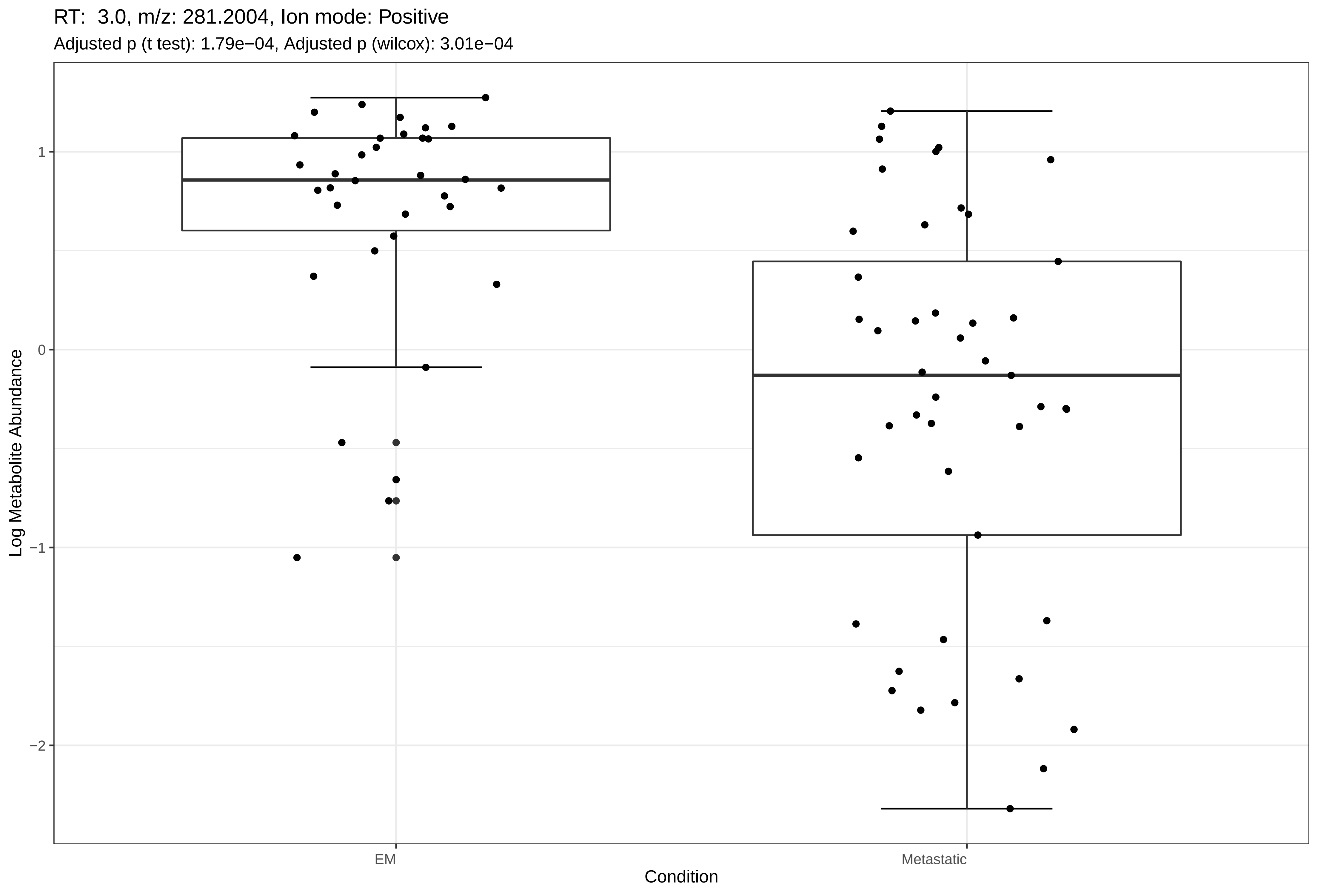

RT: 3.0, m/z: 281.2004, Ion mode: Positive
Adjusted p (t test): 1.79e−04, Adjusted p (wilcox): 3.01e−04
Log Metabolite Abundance
1
0
−1
−2
EM
Metastatic
Condition

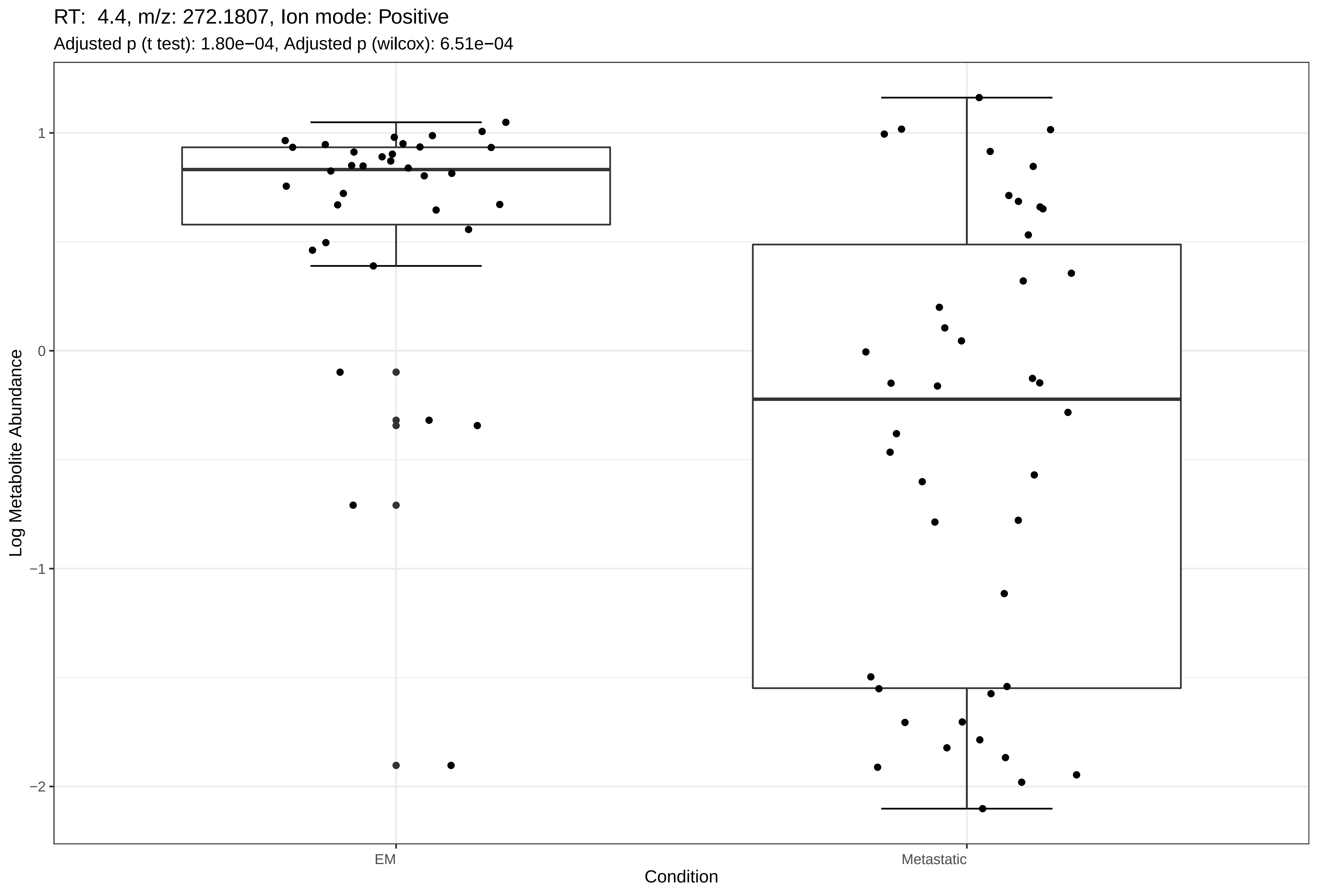
RT: 4.4, m/z: 272.1807, Ion mode: Positive
Adjusted p (t test): 1.80e−04, Adjusted p (wilcox): 6.51e−04
Log Metabolite Abundance
1
0
−1
−2
EM
Metastatic
Condition

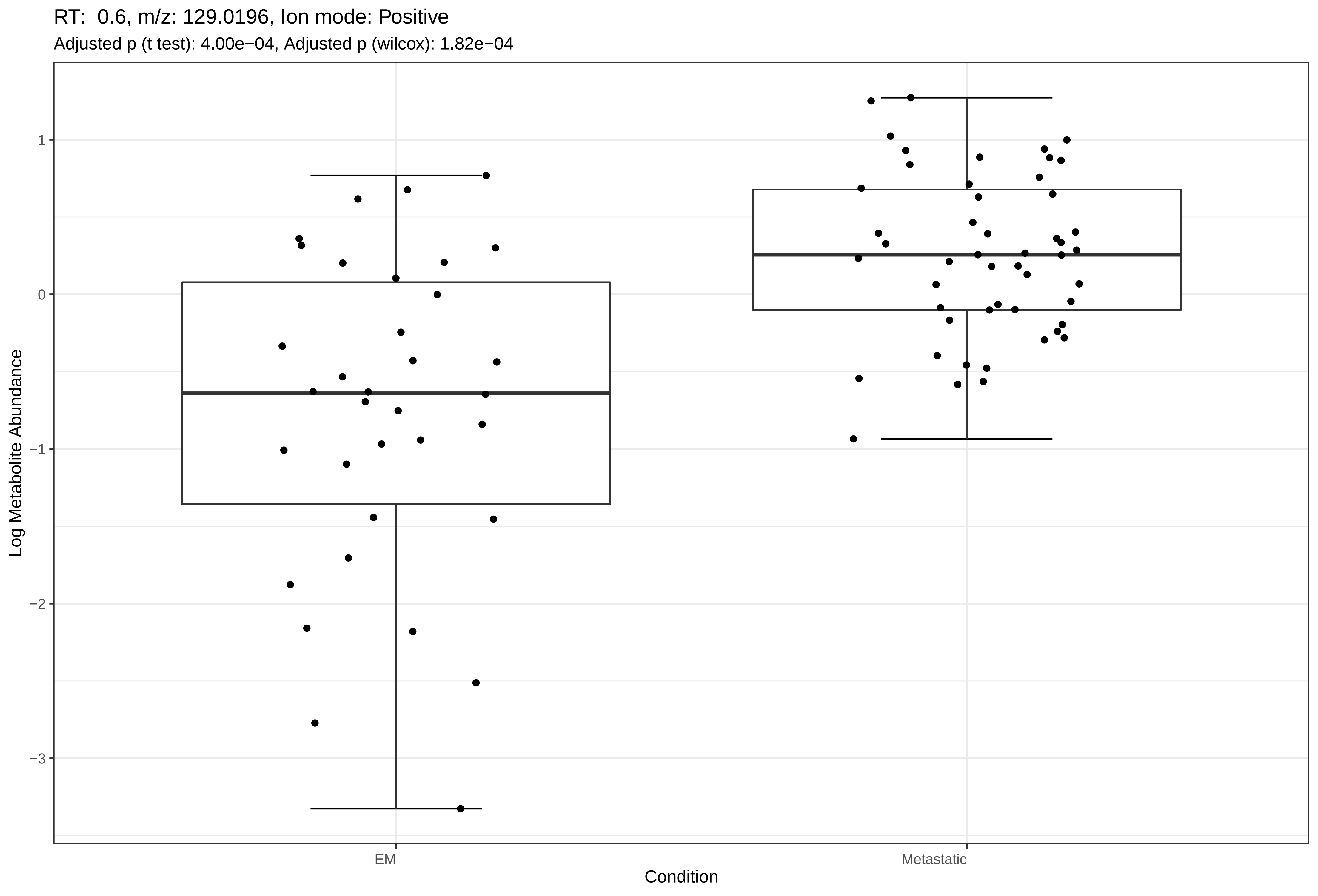
RT: 0.6, m/z: 129.0196, Ion mode: Positive
Adjusted p (t test): 4.00e−04, Adjusted p (wilcox): 1.82e−04
Log Metabolite Abundance
1
0
−1
−2
−3
EM
Metastatic
Condition

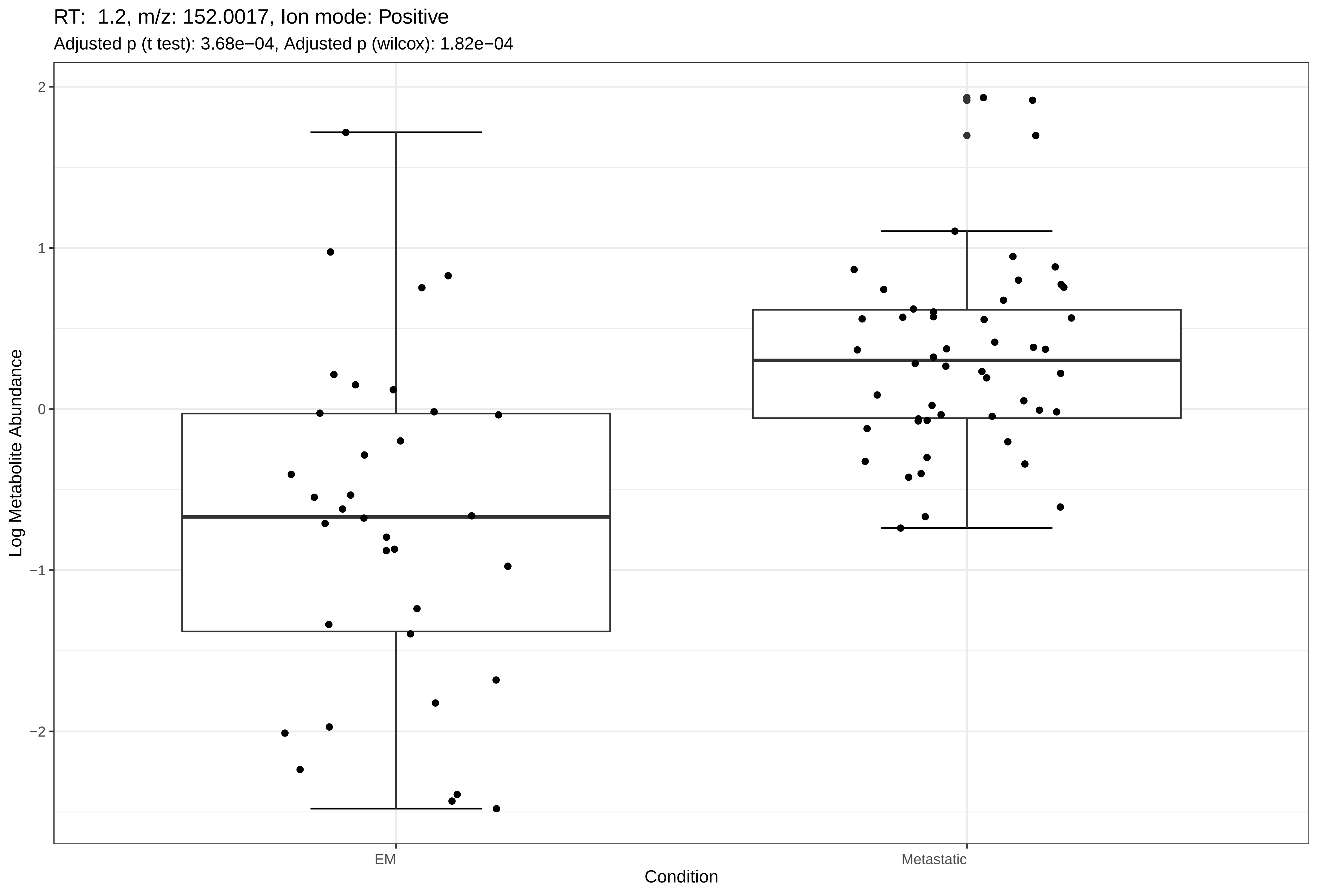

RT: 1.2, m/z: 152.0017, Ion mode: Positive
Adjusted p (t test): 3.68e−04, Adjusted p (wilcox): 1.82e−04
Log Metabolite Abundance
2
1
0
−1
−2
EM
Metastatic
Condition

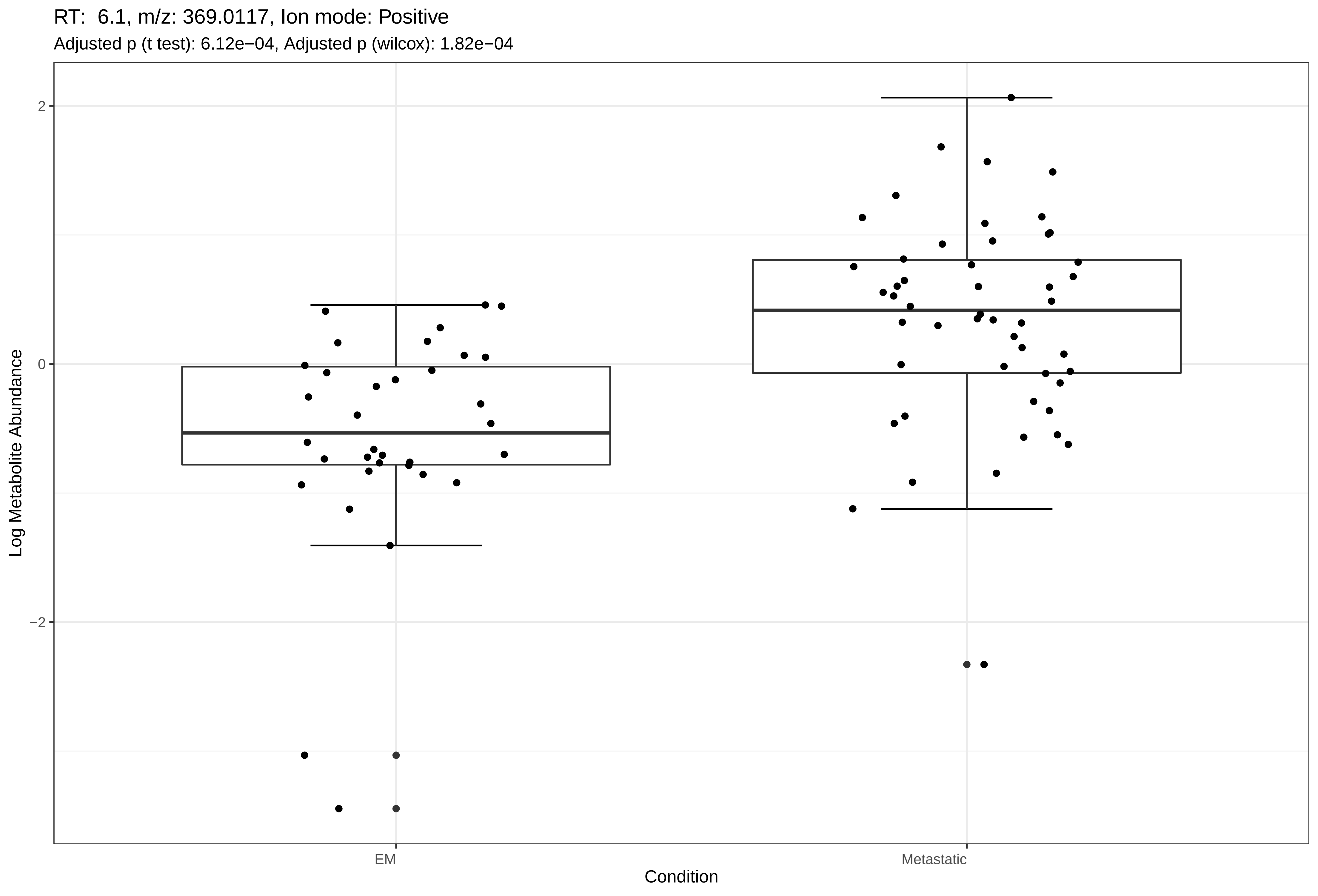

RT: 6.1, m/z: 369.0117, Ion mode: Positive
Adjusted p (t test): 6.12e−04, Adjusted p (wilcox): 1.82e−04
2
0
−2
Log Metabolite Abundance
EM
Metastatic
Condition

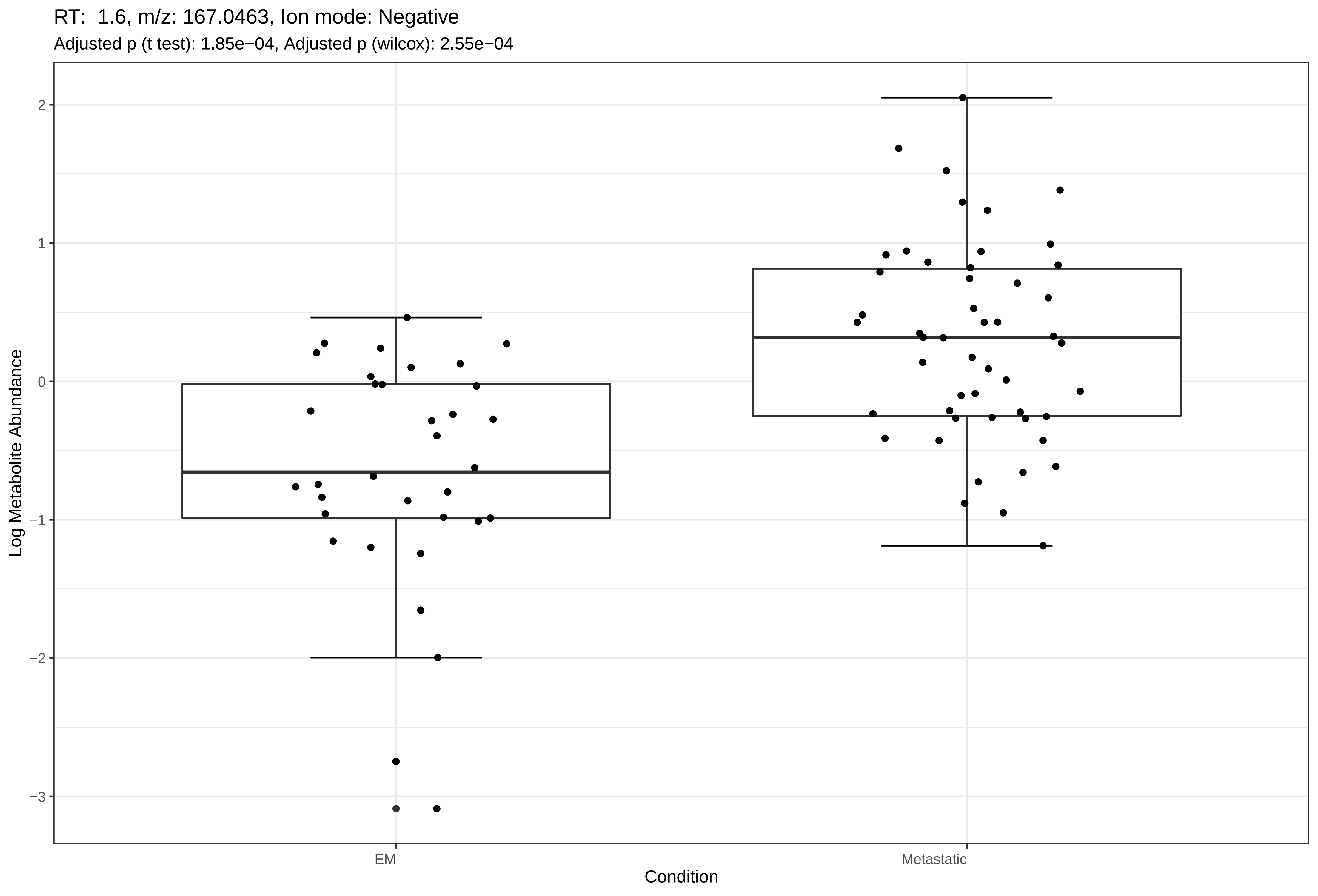
RT: 1.6, m/z: 167.0463, Ion mode: Negative
Adjusted p (t test): 1.85e−04, Adjusted p (wilcox): 2.55e−04
Log Metabolite Abundance
2
1
0
−1
−2
−3
EM
Metastatic
Condition

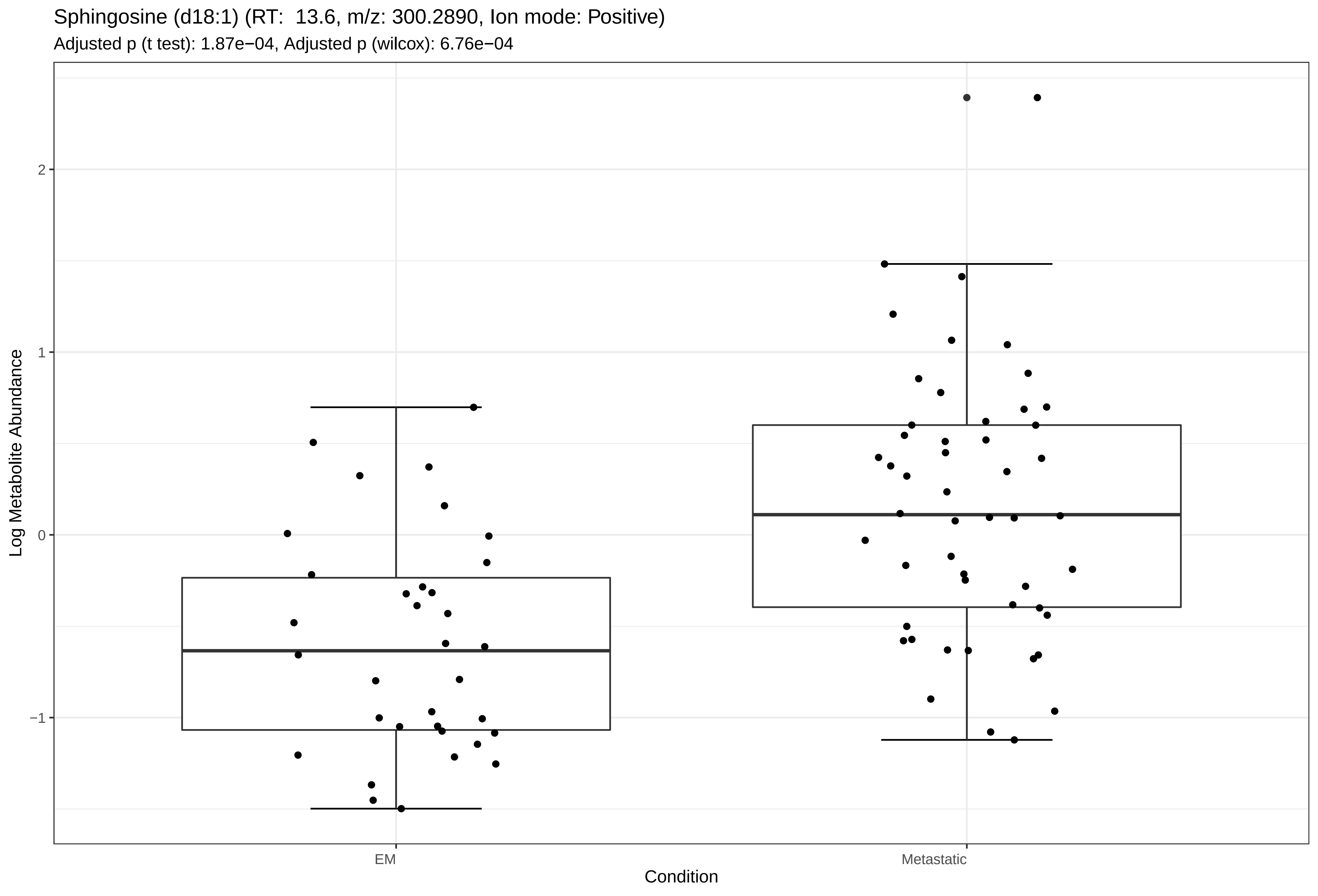

Sphingosine (d18:1) (RT: 13.6, m/z: 300.2890, Ion mode: Positive)
Adjusted p (t test): 1.87e−04, Adjusted p (wilcox): 6.76e−04
Log Metabolite Abundance
2
1
0
−1
EM
Metastatic
Condition

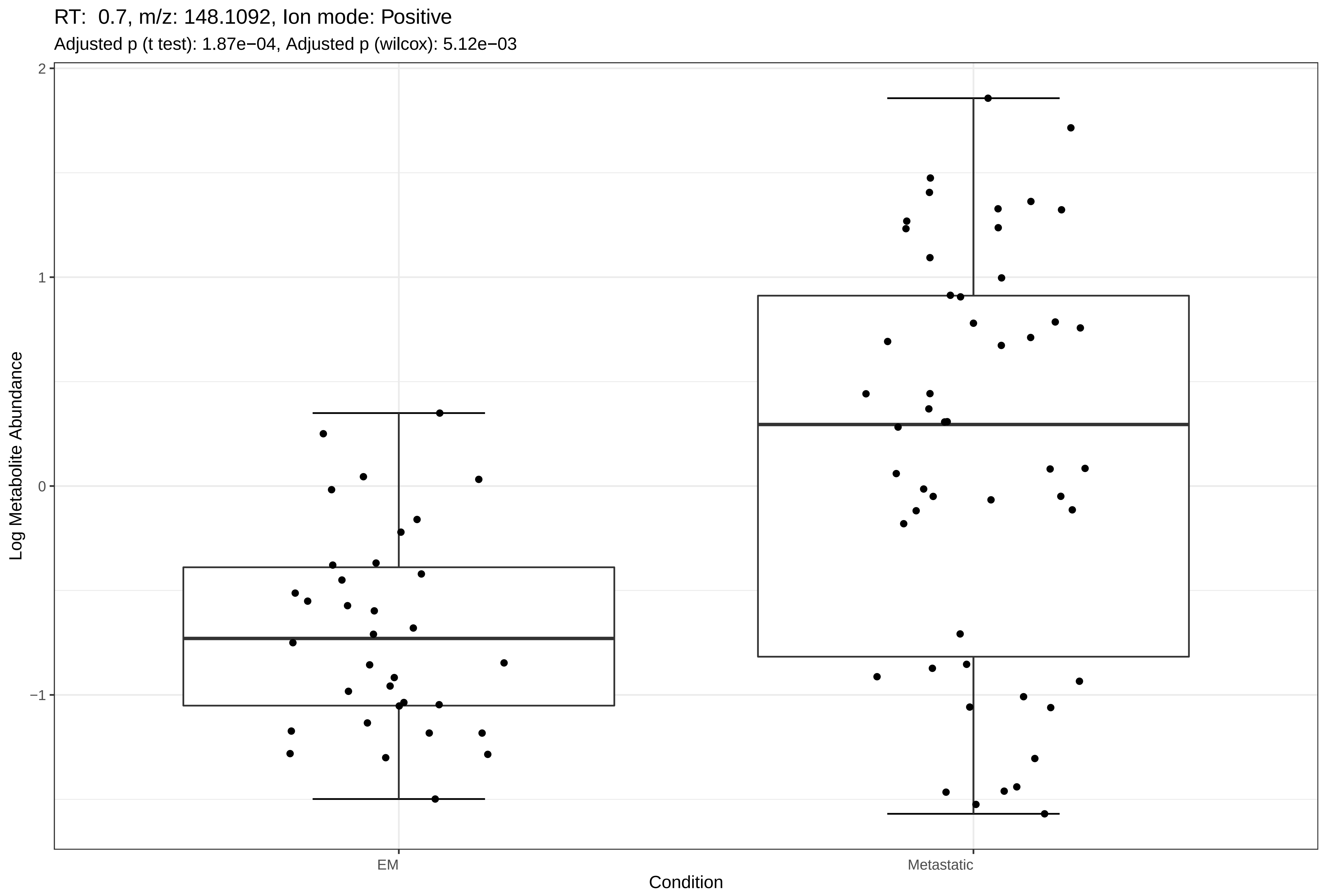

RT: 0.7, m/z: 148.1092, Ion mode: Positive
Adjusted p (t test): 1.87e−04, Adjusted p (wilcox): 5.12e−03
Log Metabolite Abundance
2
1
0
−1
EM
Metastatic
Condition

RT: 3.9, m/z: 182.5277, Ion mode: Positive

Adjusted p (t test): 9.38e−04, Adjusted p (wilcox): 1.89e−04

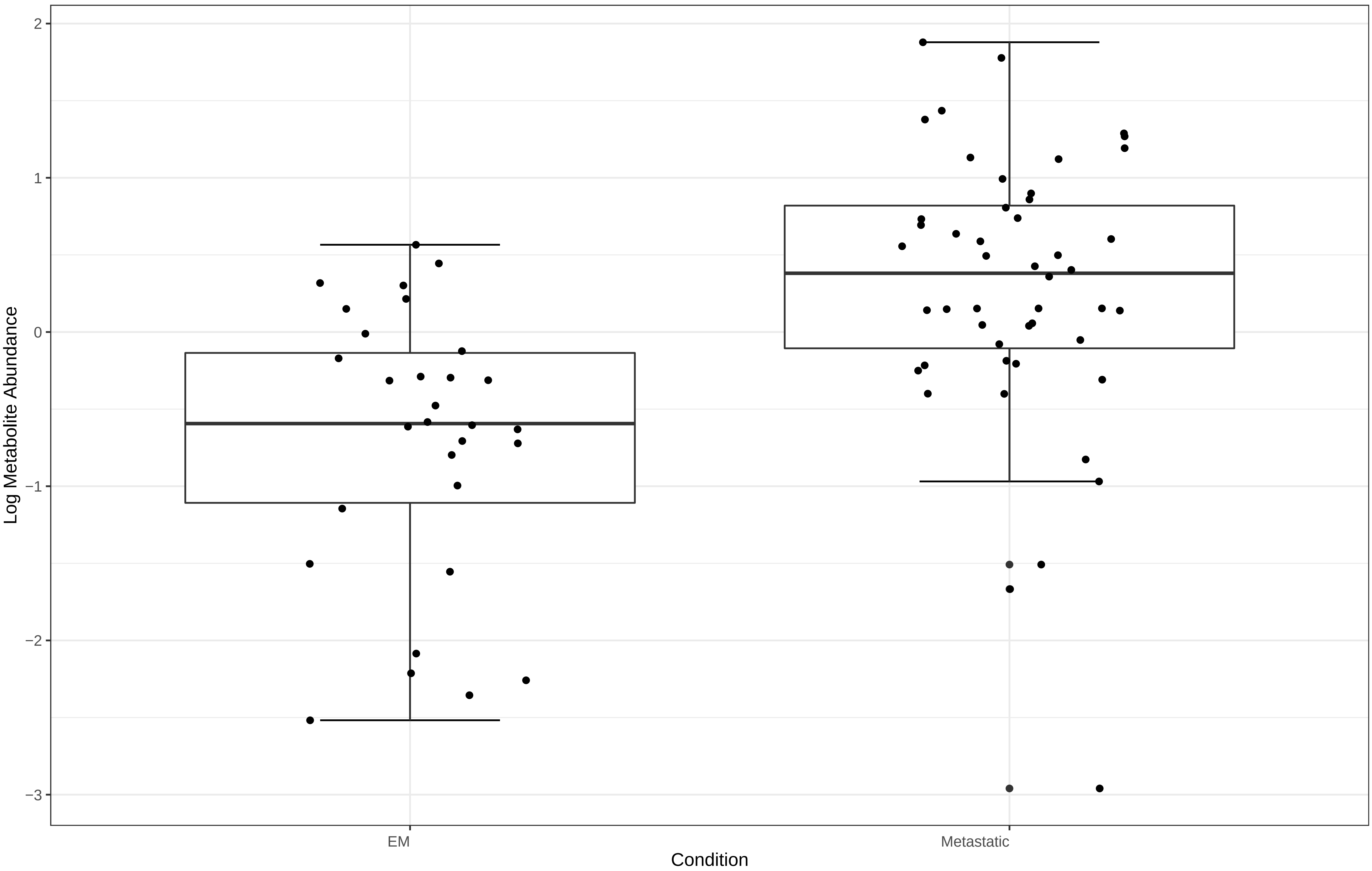

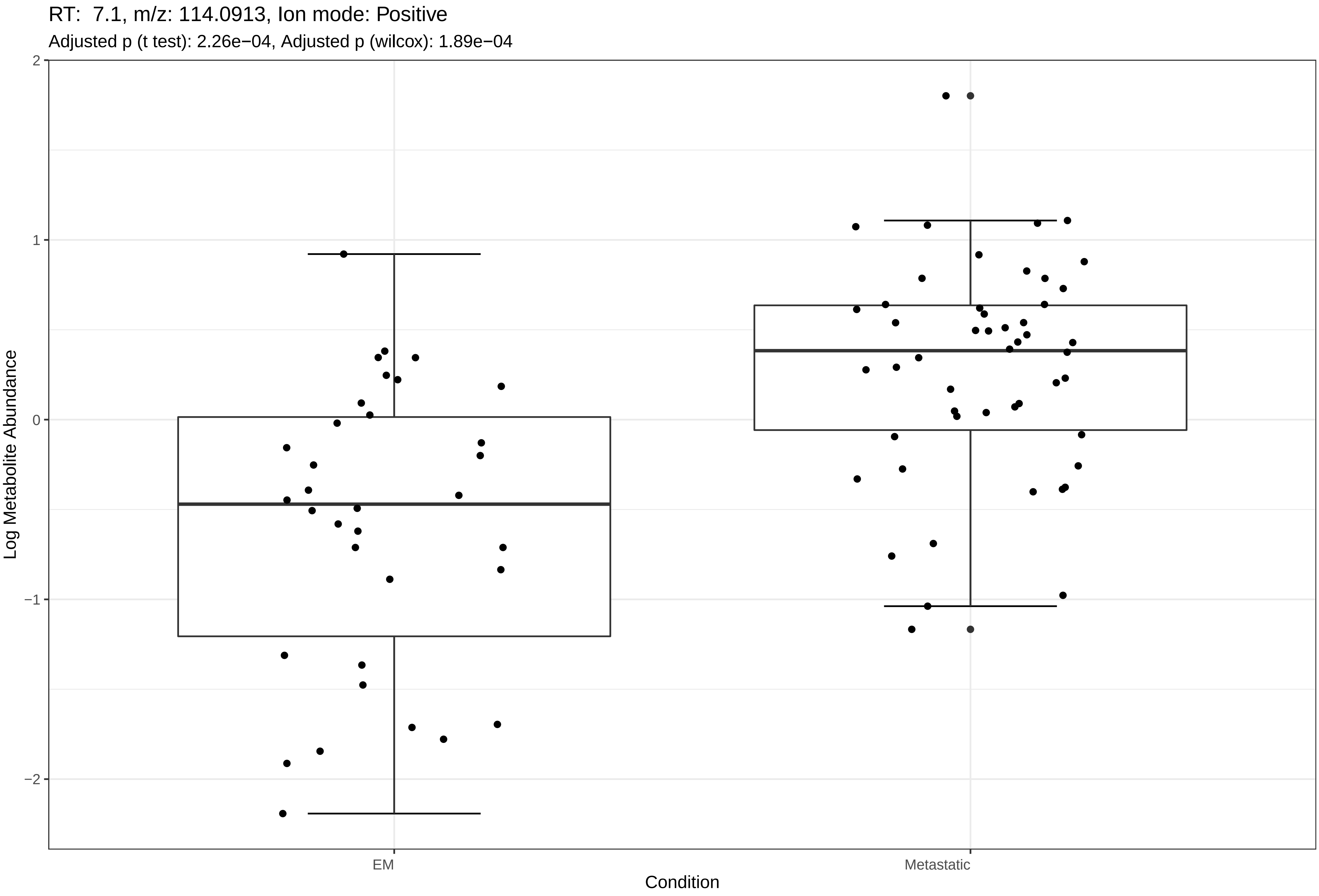
RT: 7.1, m/z: 114.0913, Ion mode: Positive
Adjusted p (t test): 2.26e−04, Adjusted p (wilcox): 1.89e−04
Log Metabolite Abundance
2
1
0
−1
−2
EM
Metastatic
Condition

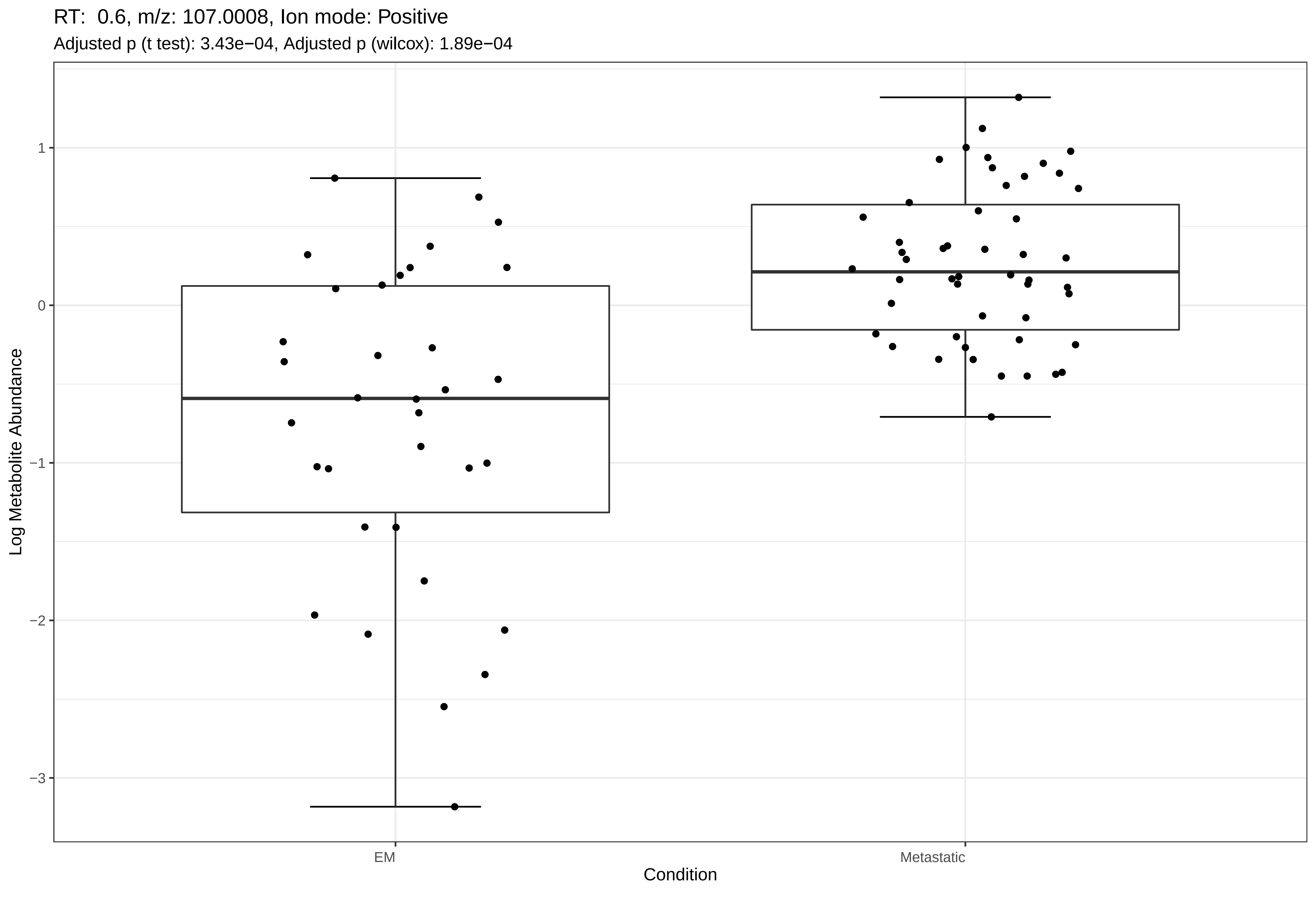

RT: 0.6, m/z: 107.0008, Ion mode: Positive
Adjusted p (t test): 3.43e−04, Adjusted p (wilcox): 1.89e−04
Log Metabolite Abundance
1
0
−1
−2
−3
EM
Metastatic
Condition

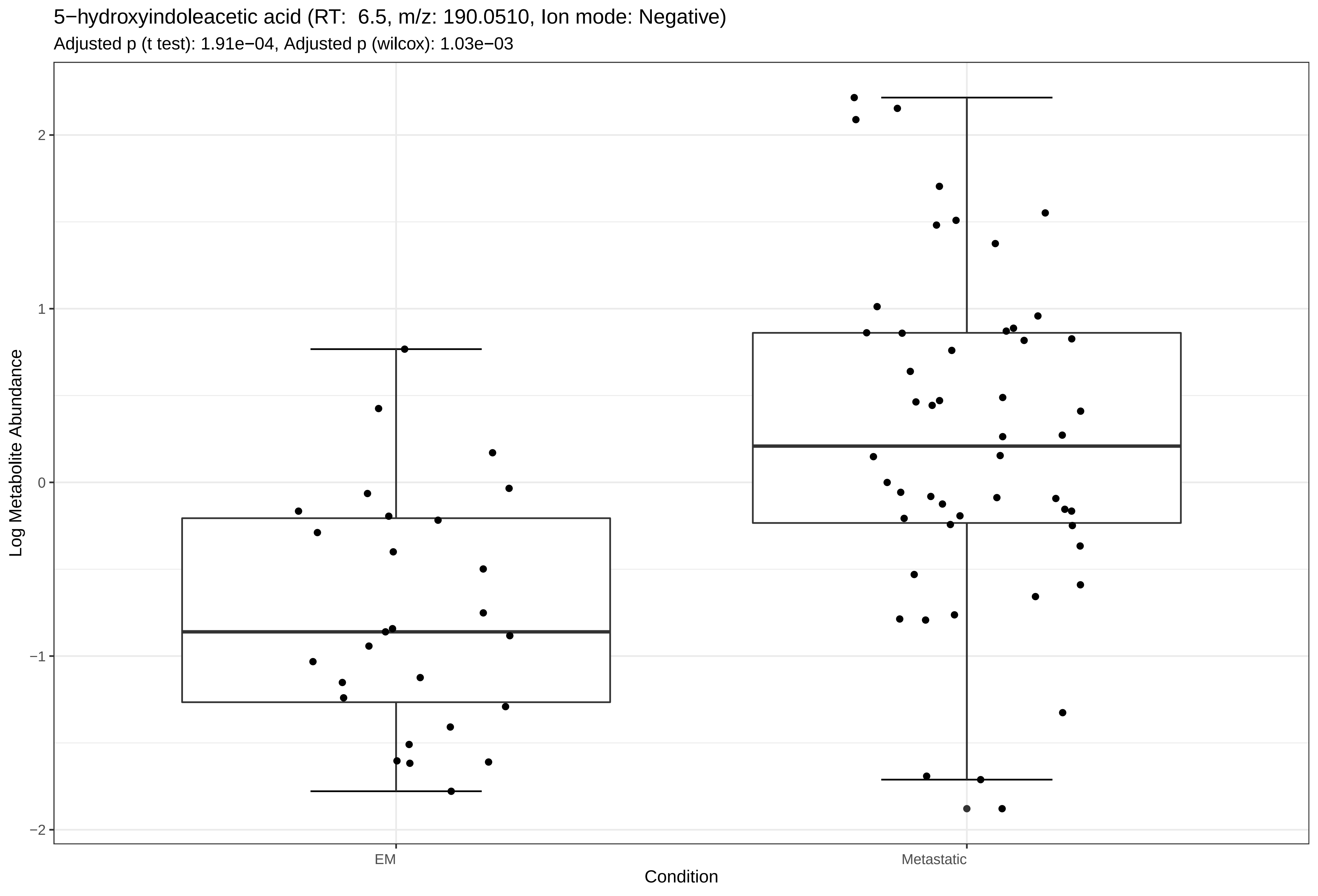

5−hydroxyindoleacetic acid (RT: 6.5, m/z: 190.0510, Ion mode: Negative)
Adjusted p (t test): 1.91e−04, Adjusted p (wilcox): 1.03e−03
Log Metabolite Abundance
2
1
0
−1
−2
EM
Metastatic
Condition

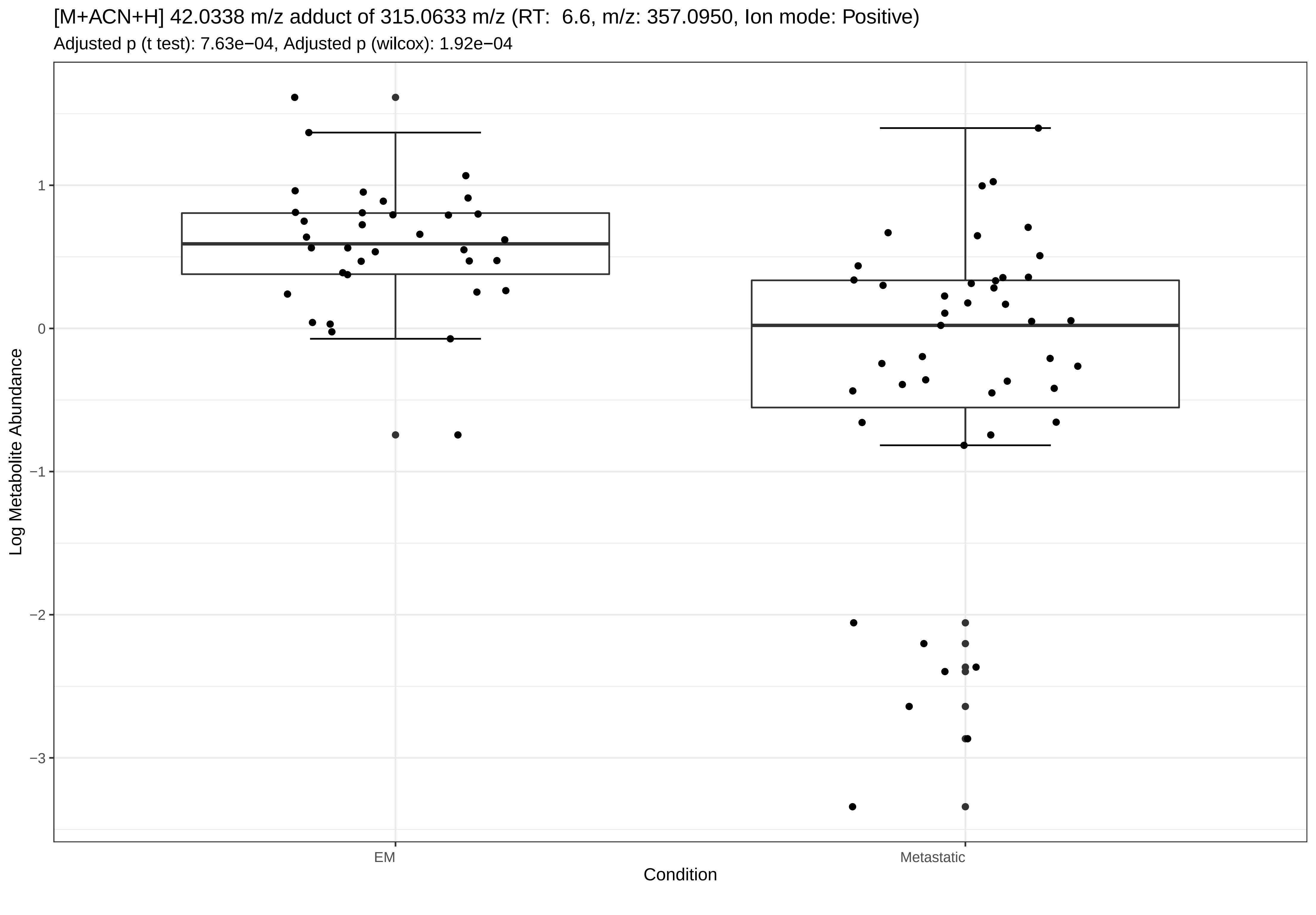

[M+ACN+H] 42.0338 m/z adduct of 315.0633 m/z (RT: 6.6, m/z: 357.0950, Ion mode: Positive)
Adjusted p (t test): 7.63e−04, Adjusted p (wilcox): 1.92e−04
Log Metabolite Abundance
1
0
−1
−2
−3
EM
Metastatic
Condition

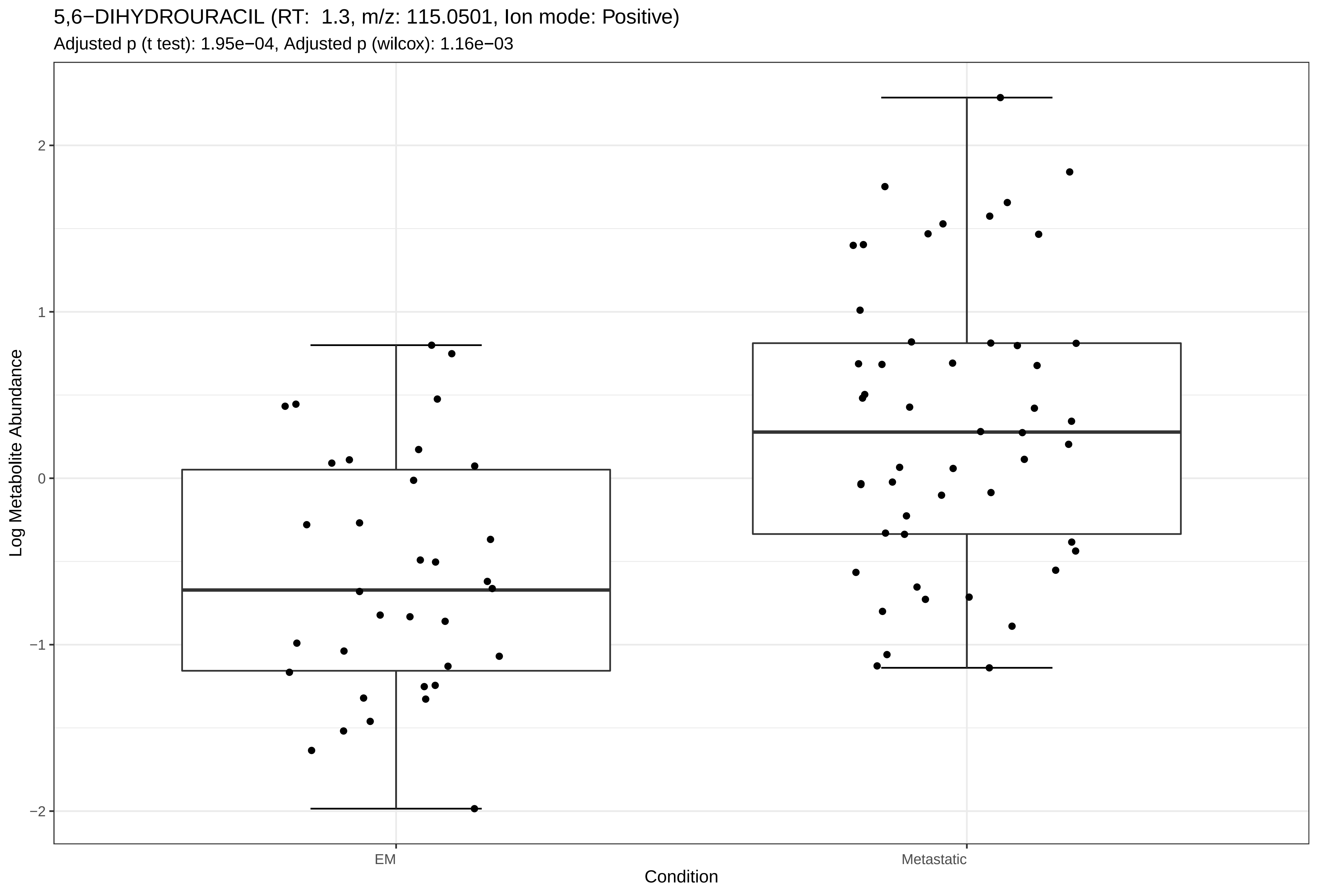
5,6−DIHYDROURACIL (RT: 1.3, m/z: 115.0501, Ion mode: Positive)
Adjusted p (t test): 1.95e−04, Adjusted p (wilcox): 1.16e−03
Log Metabolite Abundance
2
1
0
−1
−2
EM
Metastatic
Condition

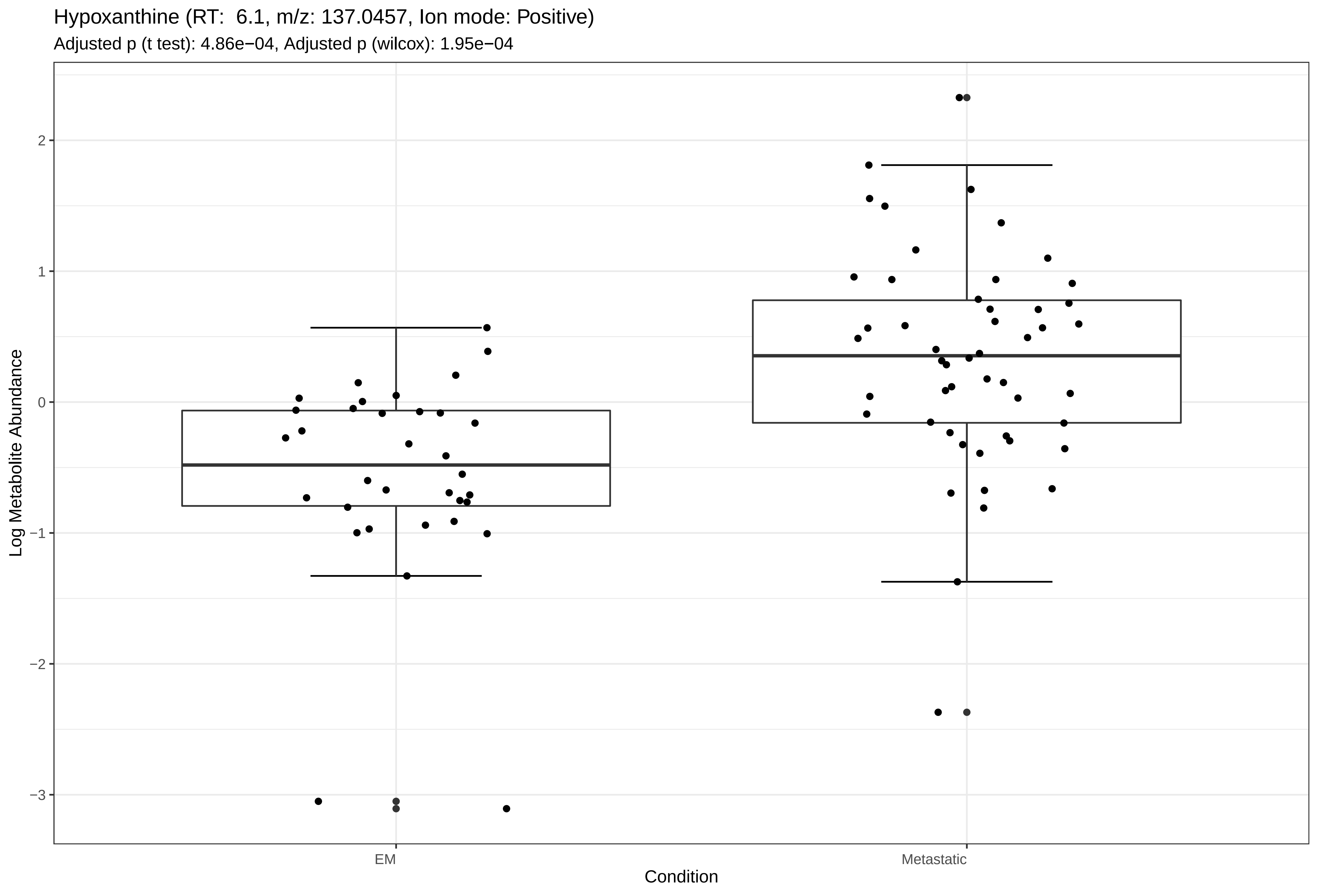

Hypoxanthine (RT: 6.1, m/z: 137.0457, Ion mode: Positive)
Adjusted p (t test): 4.86e−04, Adjusted p (wilcox): 1.95e−04
Log Metabolite Abundance
2
1
0
−1
−2
−3
EM
Metastatic
Condition

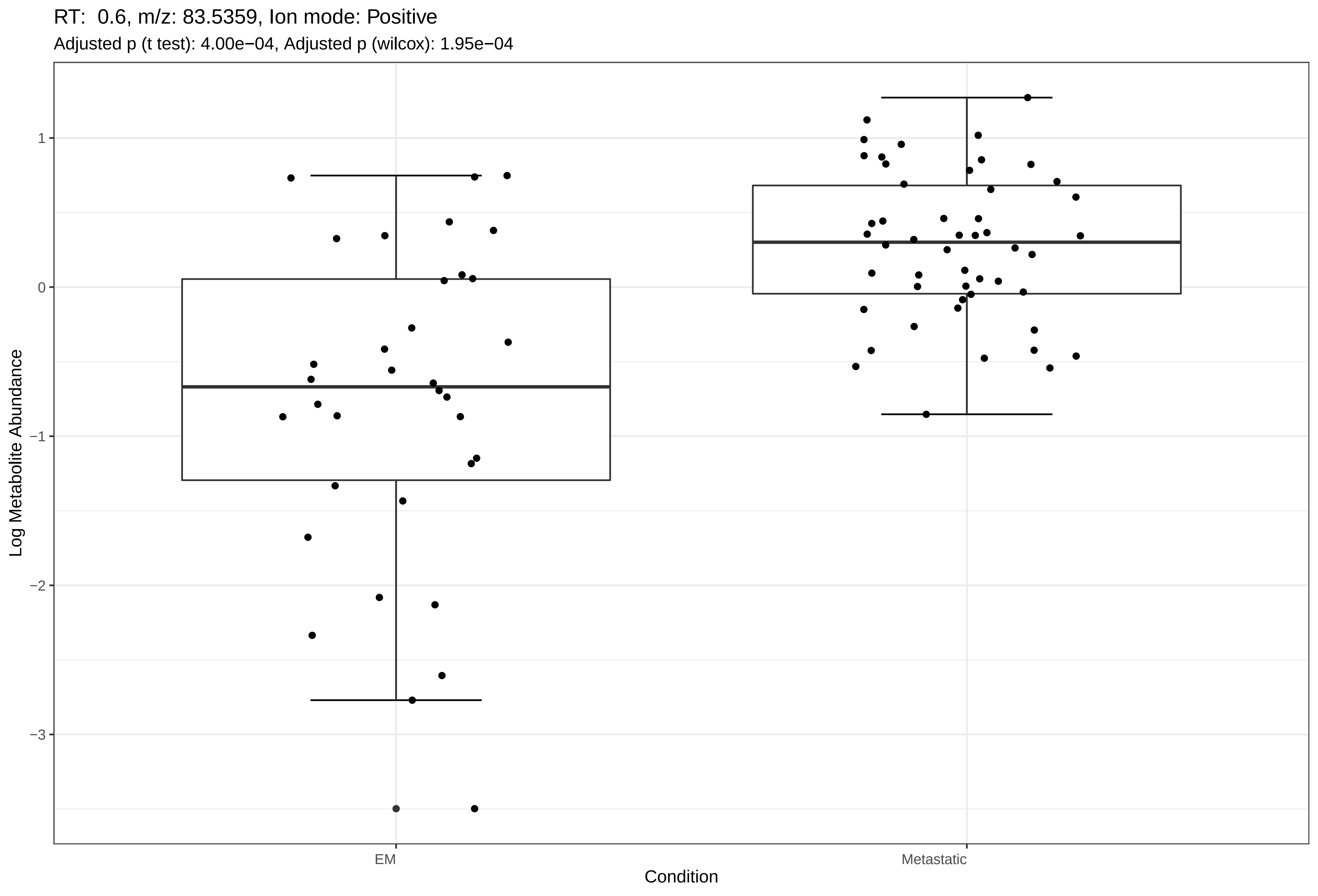
RT: 0.6, m/z: 83.5359, Ion mode: Positive
Adjusted p (t test): 4.00e−04, Adjusted p (wilcox): 1.95e−04
Log Metabolite Abundance
1
0
−1
−2
−3
EM
Metastatic
Condition

RT: 0.6, m/z: 71.0292, Ion mode: Positive

Adjusted p (t test): 3.27e−04, Adjusted p (wilcox): 1.95e−04

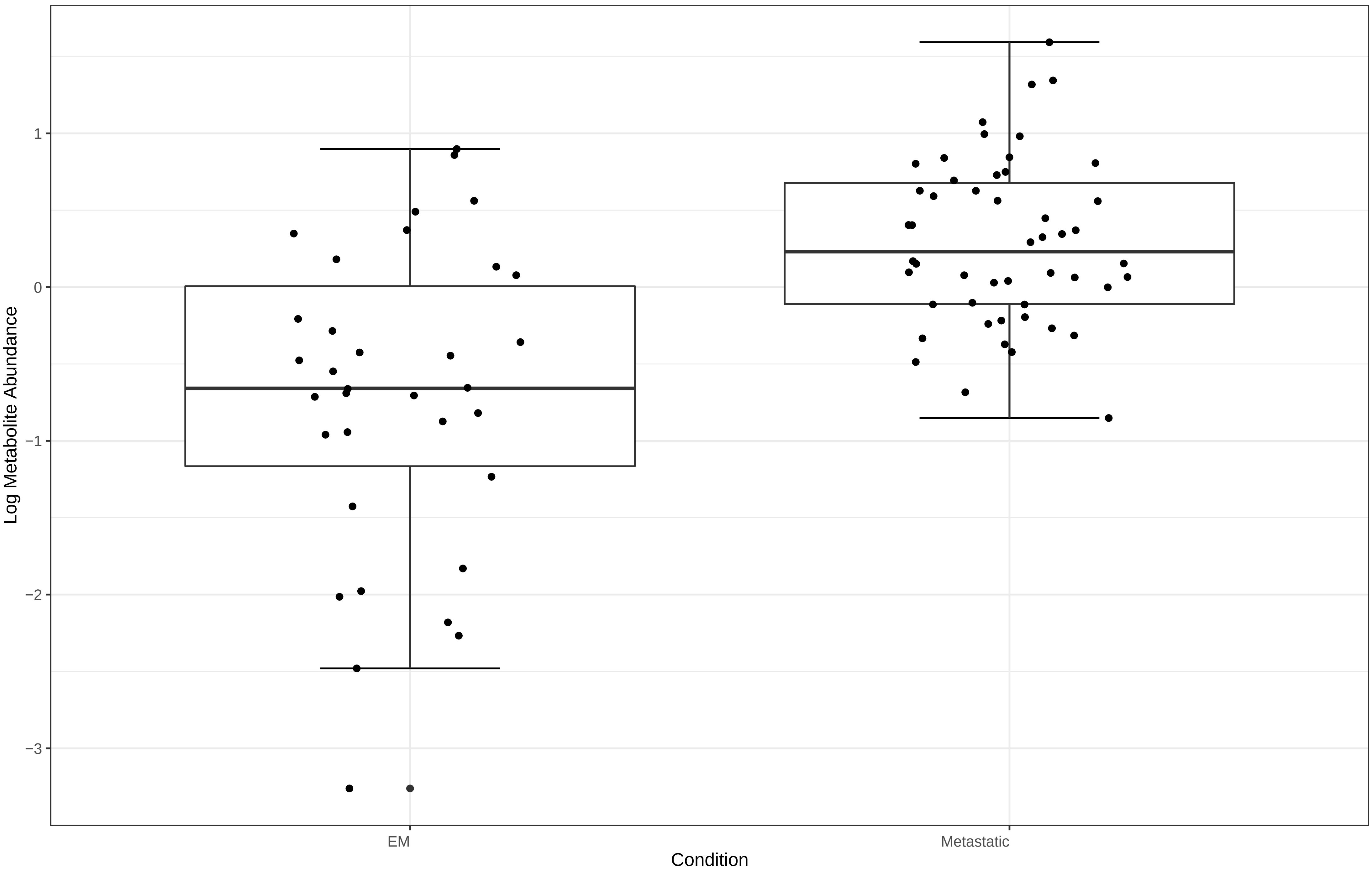

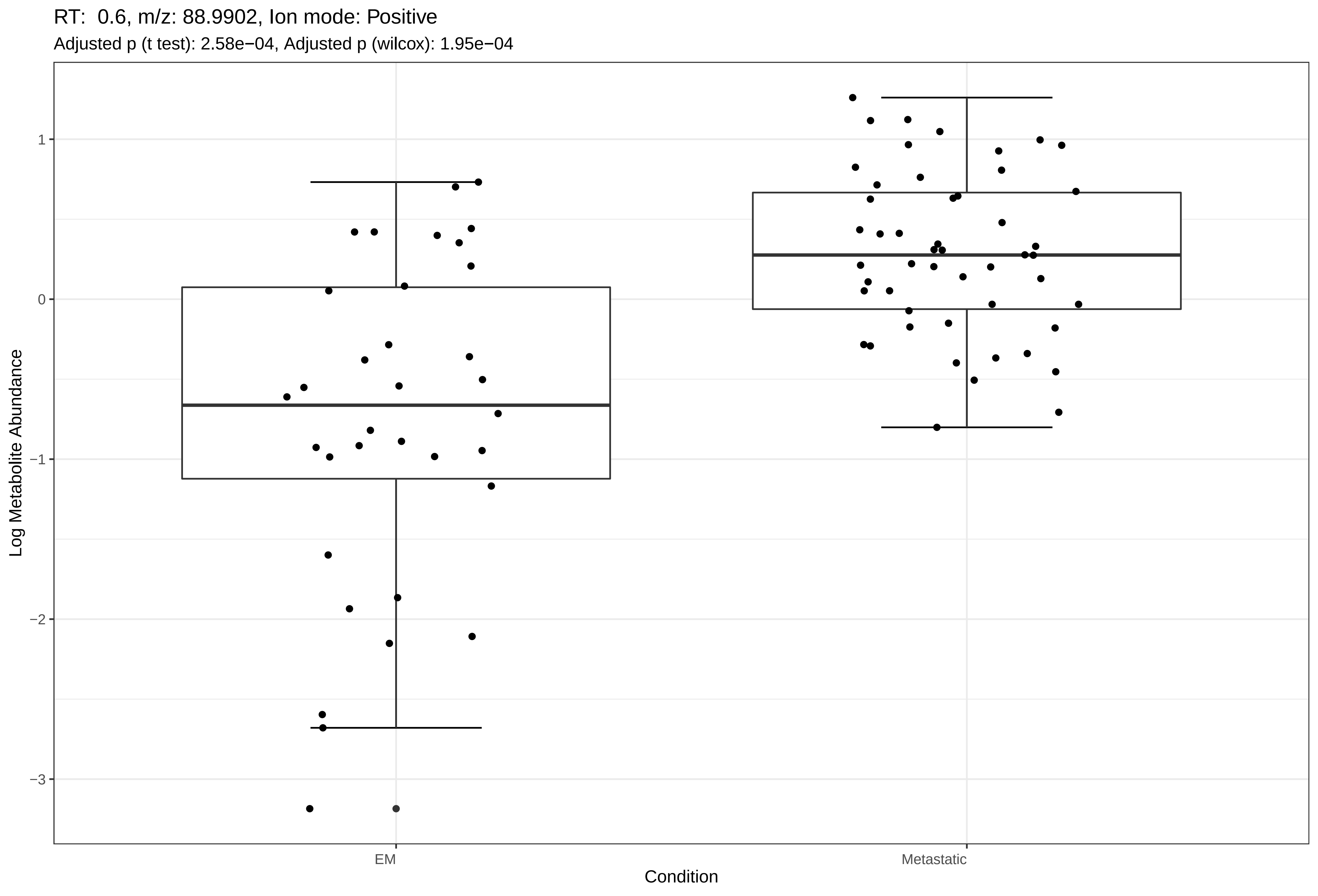

RT: 0.6, m/z: 88.9902, Ion mode: Positive
Adjusted p (t test): 2.58e−04, Adjusted p (wilcox): 1.95e−04
Log Metabolite Abundance
1
0
−1
−2
−3
EM
Metastatic
Condition

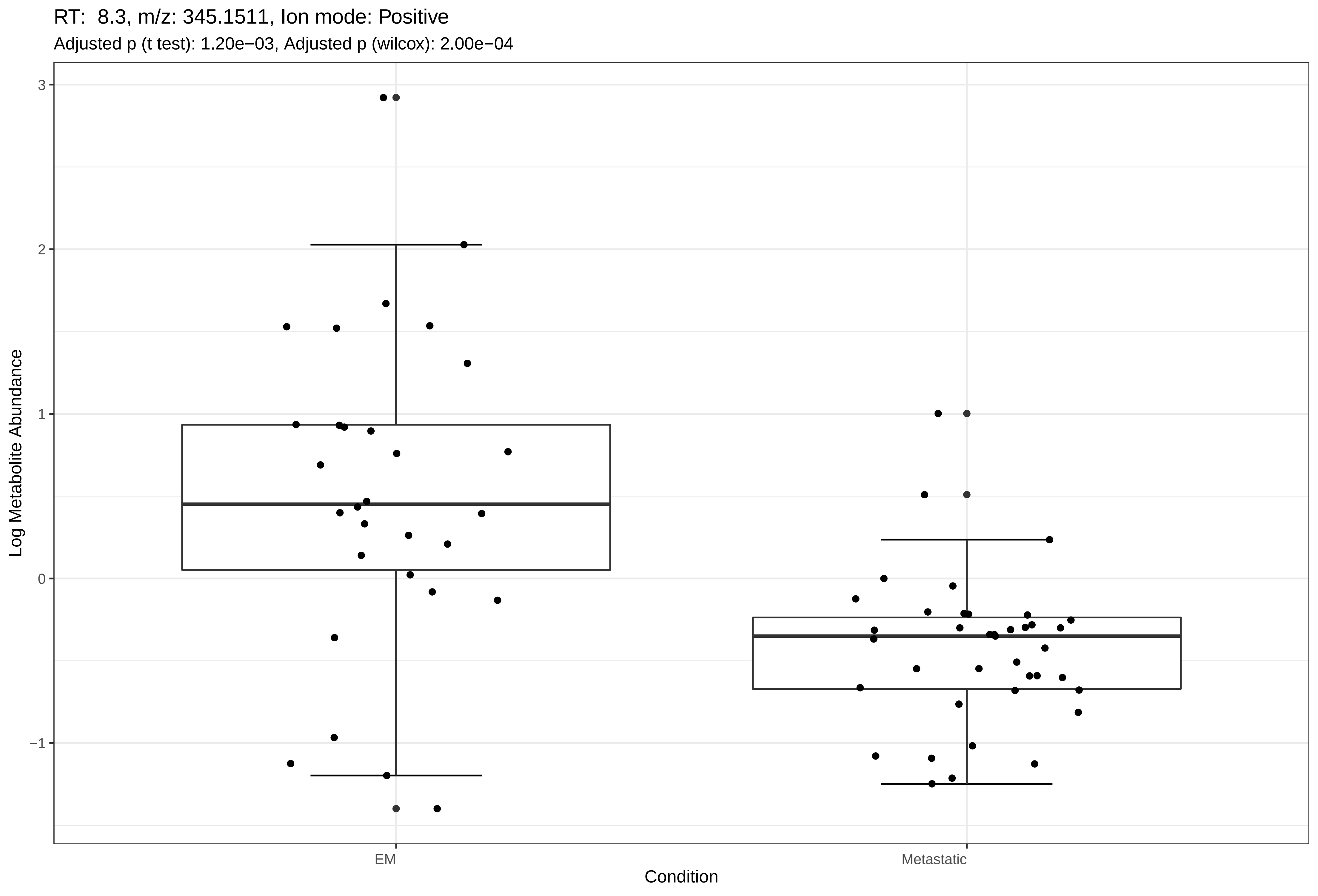
RT: 8.3, m/z: 345.1511, Ion mode: Positive
Adjusted p (t test): 1.20e−03, Adjusted p (wilcox): 2.00e−04
Log Metabolite Abundance
3
2
1
0
−1
EM
Metastatic
Condition

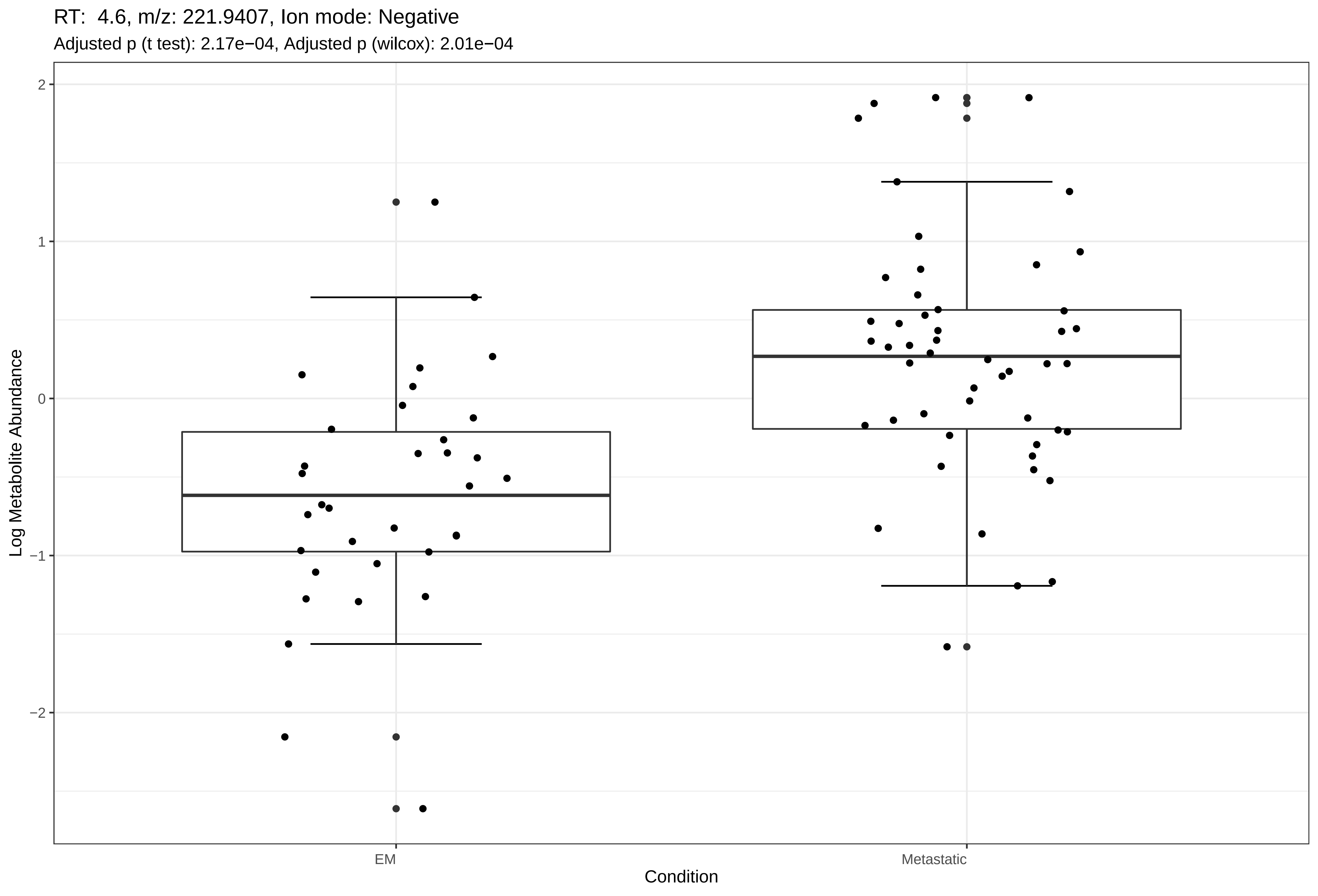

RT: 4.6, m/z: 221.9407, Ion mode: Negative
Adjusted p (t test): 2.17e−04, Adjusted p (wilcox): 2.01e−04
Log Metabolite Abundance
2
1
0
−1
−2
EM
Metastatic
Condition

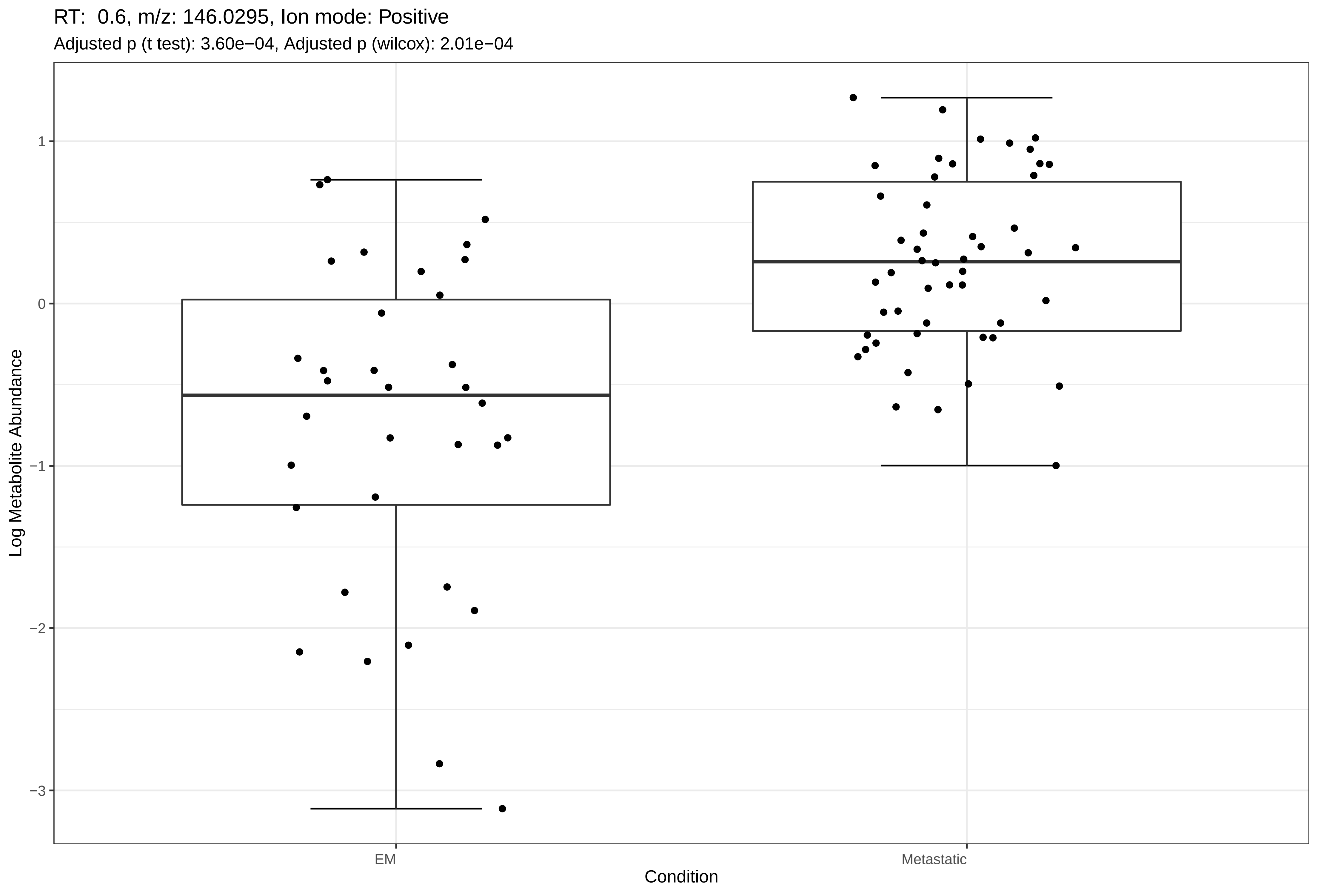

RT: 0.6, m/z: 146.0295, Ion mode: Positive
Adjusted p (t test): 3.60e−04, Adjusted p (wilcox): 2.01e−04
Log Metabolite Abundance
1
0
−1
−2
−3
EM
Metastatic
Condition

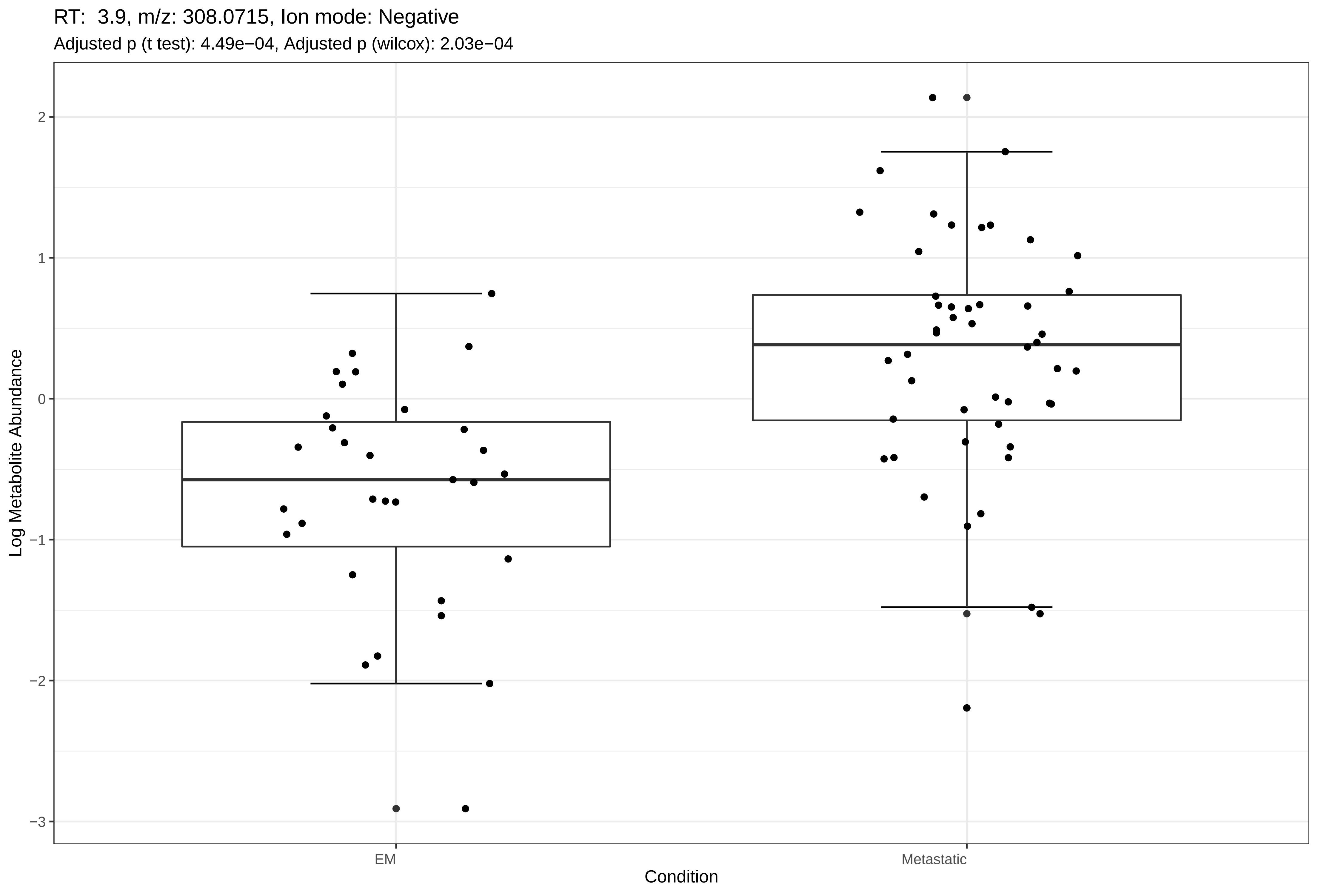
RT: 3.9, m/z: 308.0715, Ion mode: Negative
Adjusted p (t test): 4.49e−04, Adjusted p (wilcox): 2.03e−04
Log Metabolite Abundance
2
1
0
−1
−2
−3
EM
Metastatic
Condition

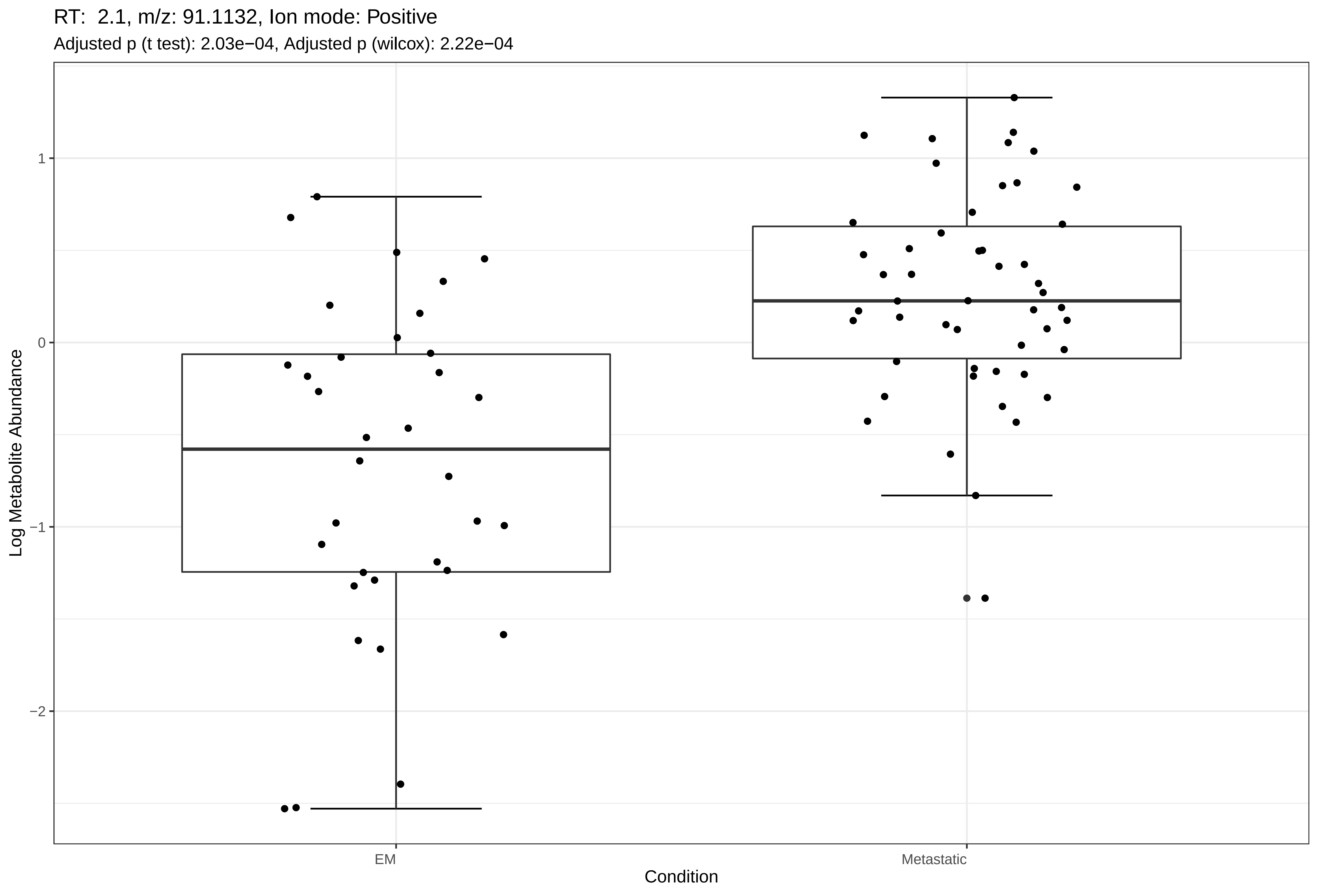

RT: 2.1, m/z: 91.1132, Ion mode: Positive
Adjusted p (t test): 2.03e−04, Adjusted p (wilcox): 2.22e−04
Log Metabolite Abundance
1
0
−1
−2
EM
Metastatic
Condition

[M+Na−H] 21.9825 m/z adduct of 160.1036 m/z (RT: 2.1, m/z: 182.0857, Ion mode: Positive)

Adjusted p (t test): 2.03e−04, Adjusted p (wilcox): 2.30e−04

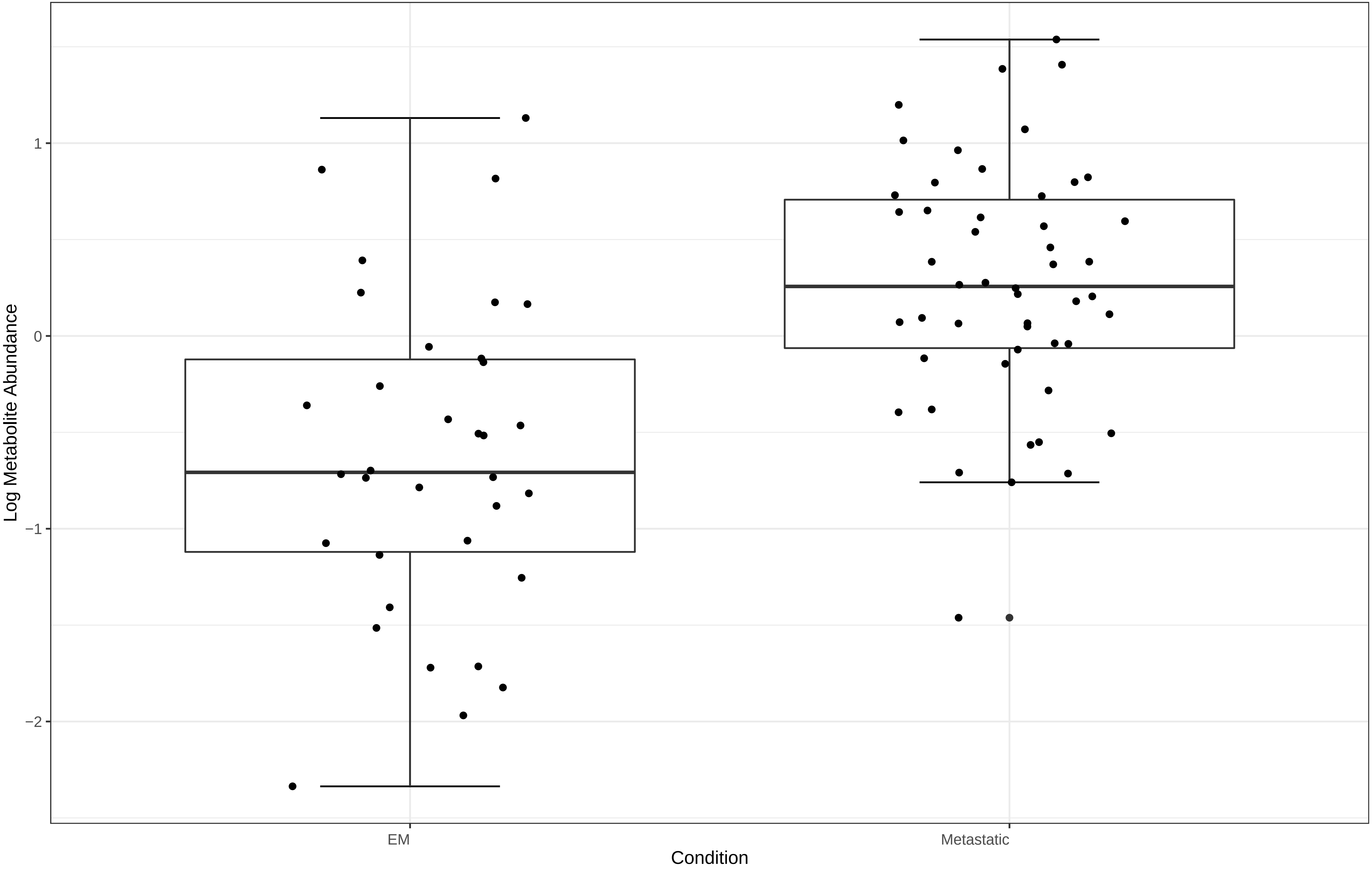

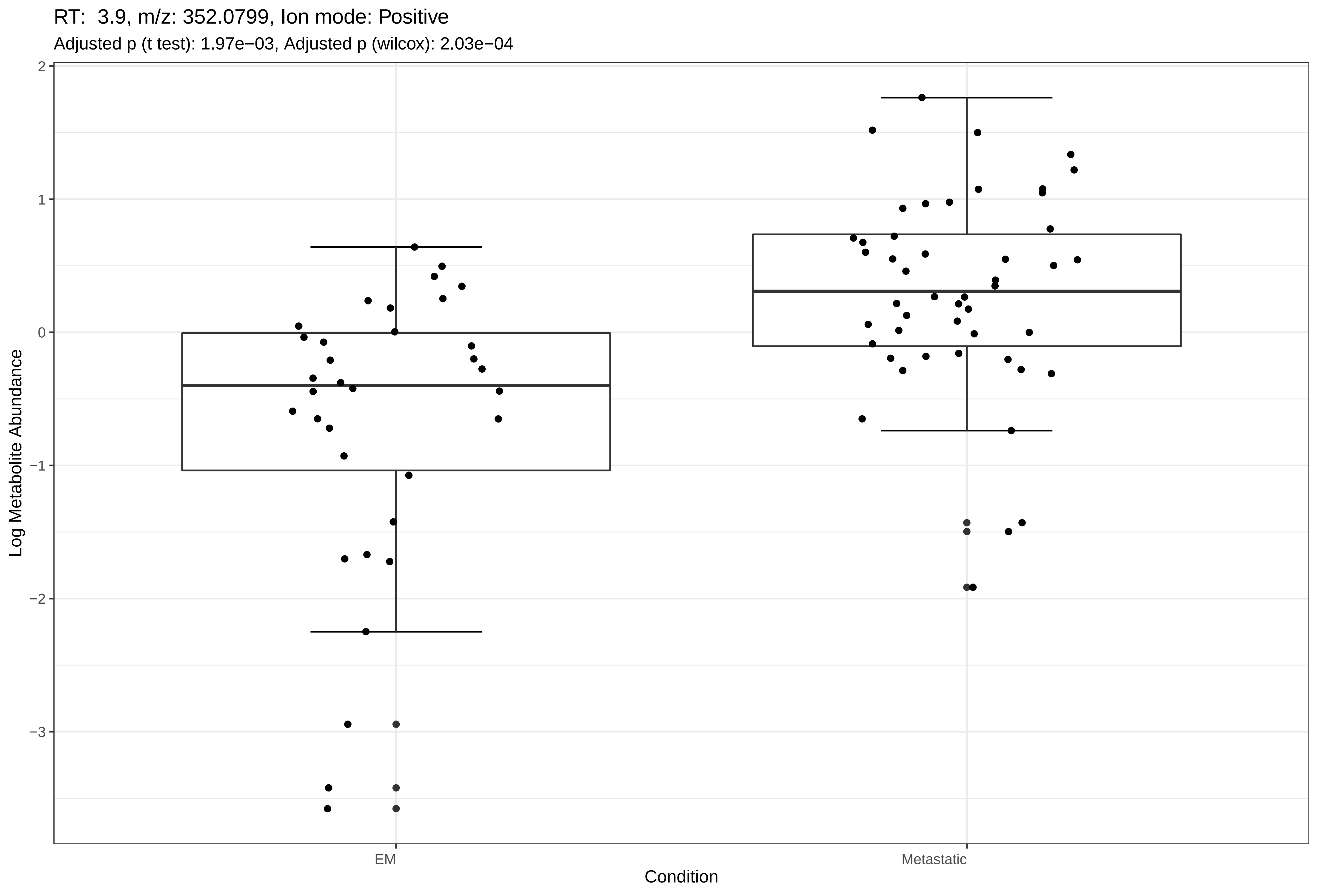
RT: 3.9, m/z: 352.0799, Ion mode: Positive
Adjusted p (t test): 1.97e−03, Adjusted p (wilcox): 2.03e−04
Log Metabolite Abundance
2
1
0
−1
−2
−3
EM
Metastatic
Condition

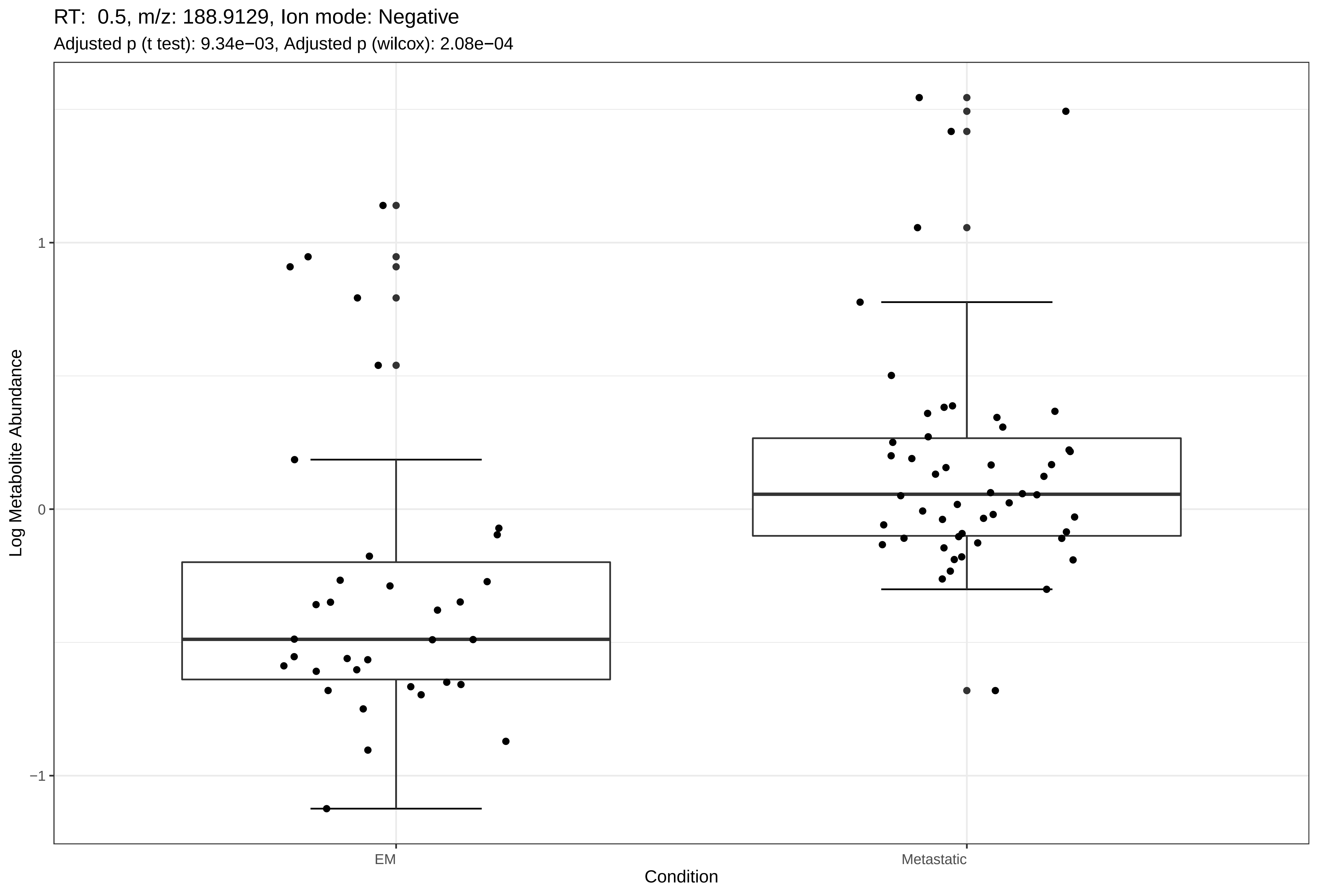

RT: 0.5, m/z: 188.9129, Ion mode: Negative
Adjusted p (t test): 9.34e−03, Adjusted p (wilcox): 2.08e−04
Log Metabolite Abundance
1
0
−1
EM
Metastatic
Condition

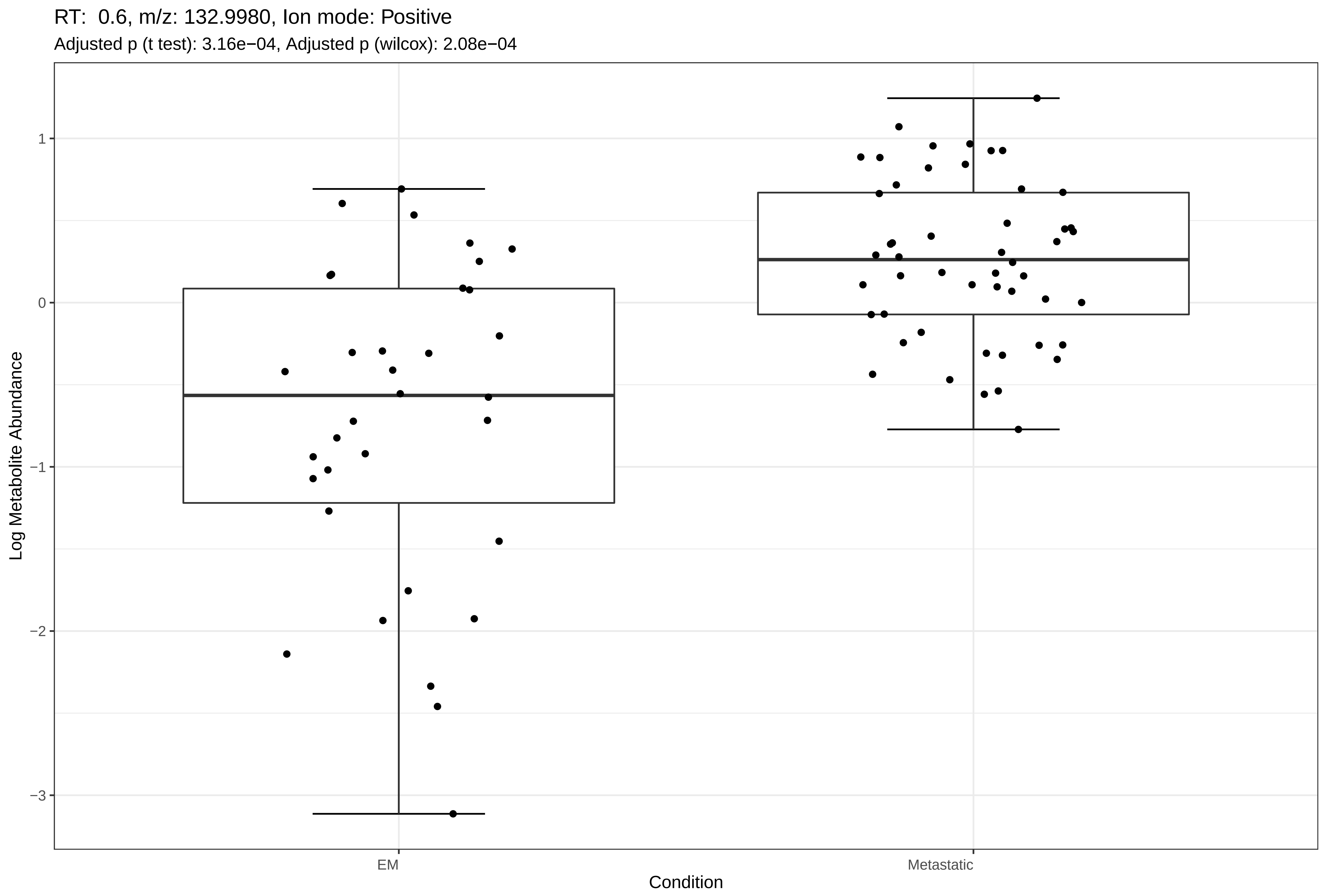

RT: 0.6, m/z: 132.9980, Ion mode: Positive
Adjusted p (t test): 3.16e−04, Adjusted p (wilcox): 2.08e−04
Log Metabolite Abundance
1
0
−1
−2
−3
EM
Metastatic
Condition

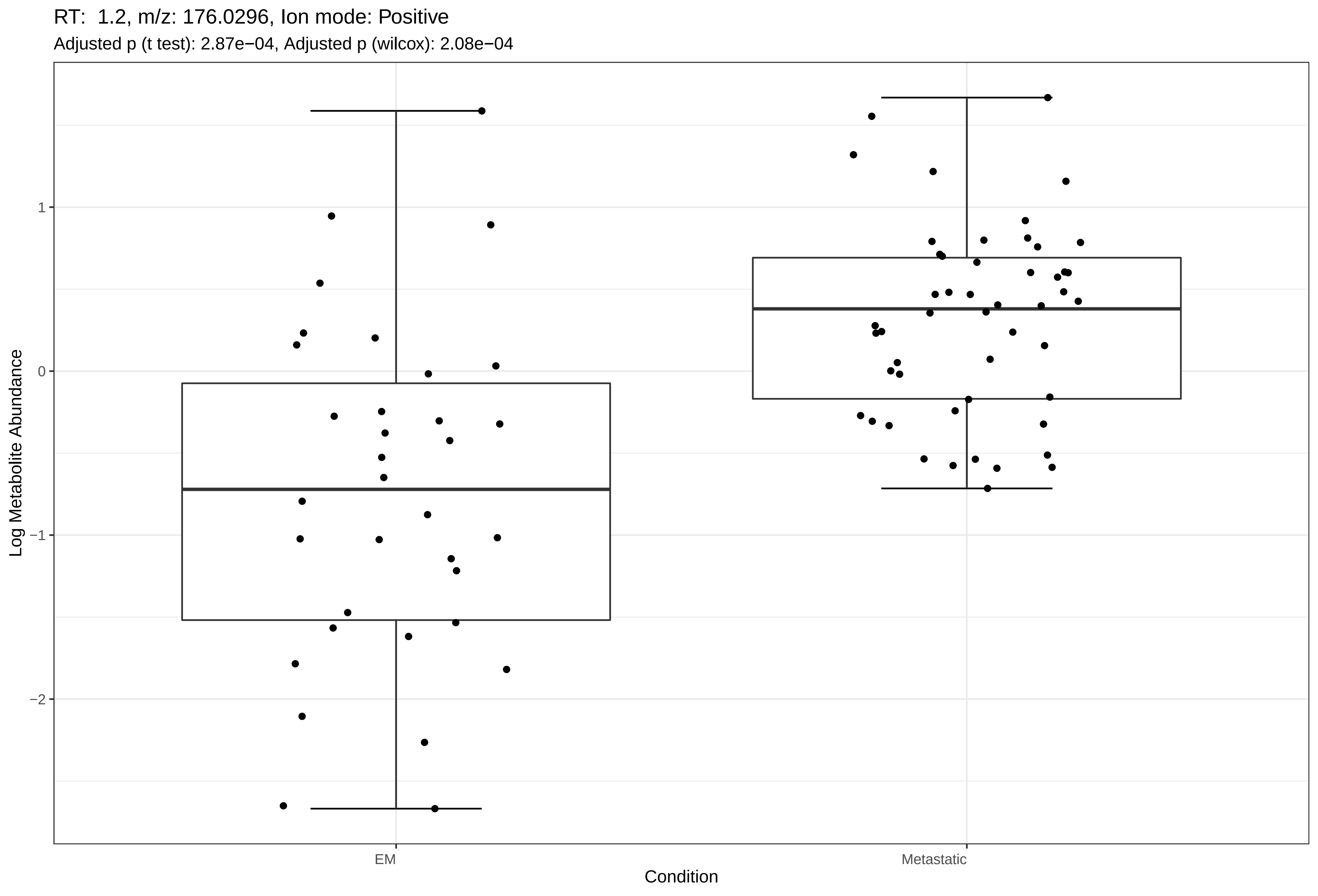

RT: 1.2, m/z: 176.0296, Ion mode: Positive
Adjusted p (t test): 2.87e−04, Adjusted p (wilcox): 2.08e−04
Log Metabolite Abundance
1
0
−1
−2
EM
Metastatic
Condition

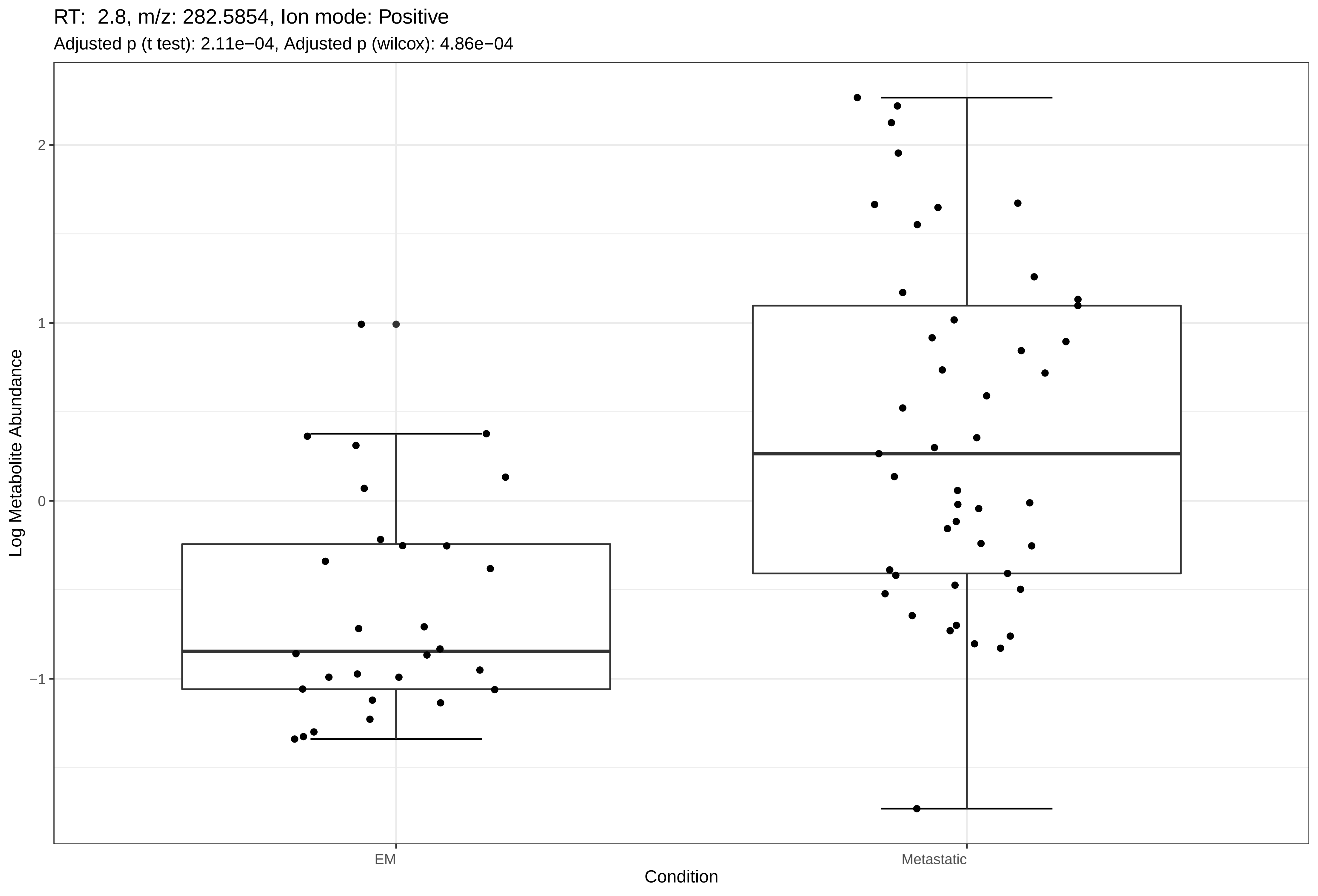

RT: 2.8, m/z: 282.5854, Ion mode: Positive
Adjusted p (t test): 2.11e−04, Adjusted p (wilcox): 4.86e−04
Log Metabolite Abundance
2
1
0
−1
EM
Metastatic
Condition

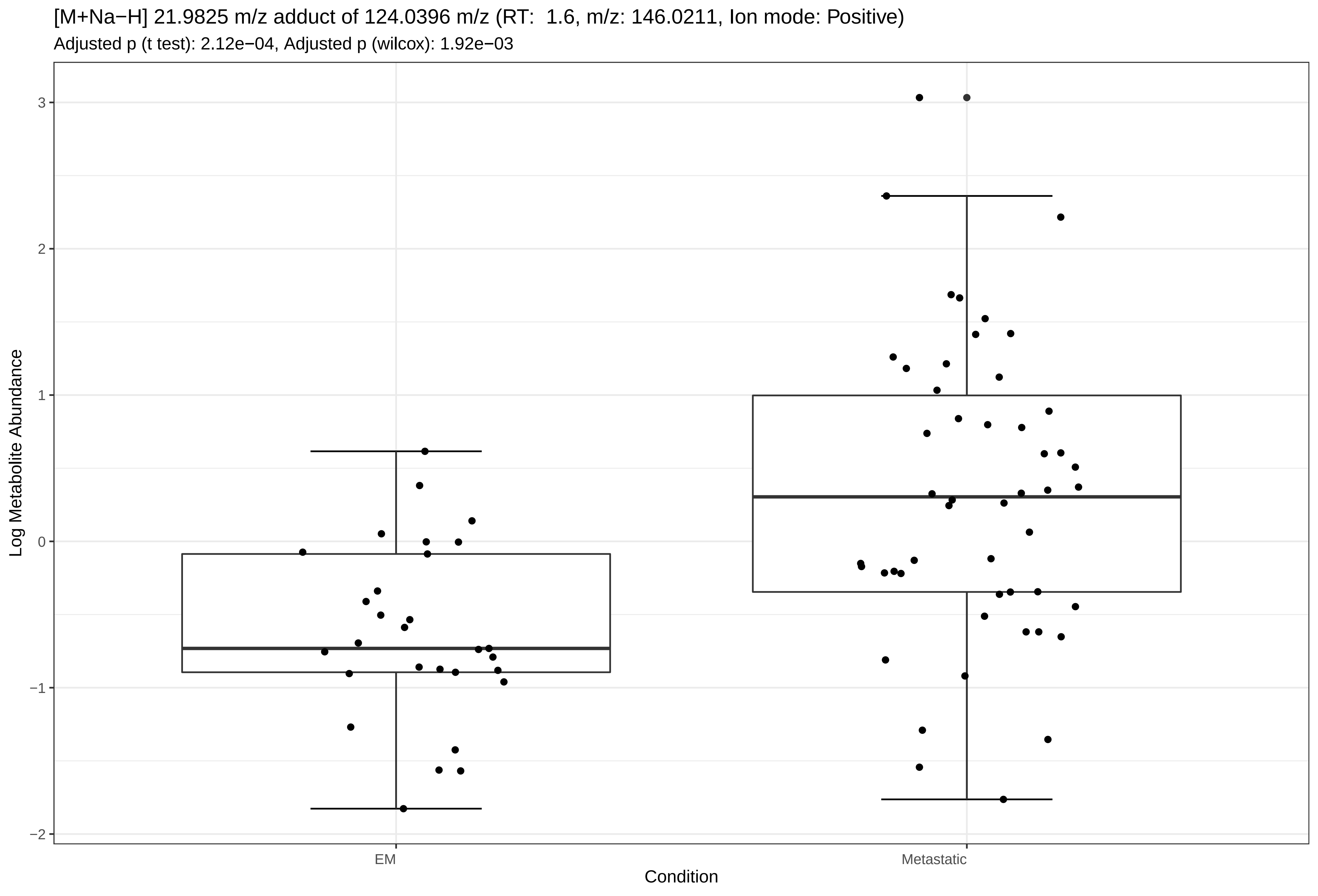

[M+Na−H] 21.9825 m/z adduct of 124.0396 m/z (RT: 1.6, m/z: 146.0211, Ion mode: Positive)
Adjusted p (t test): 2.12e−04, Adjusted p (wilcox): 1.92e−03
Log Metabolite Abundance
3
2
1
0
−1
−2
EM
Metastatic
Condition

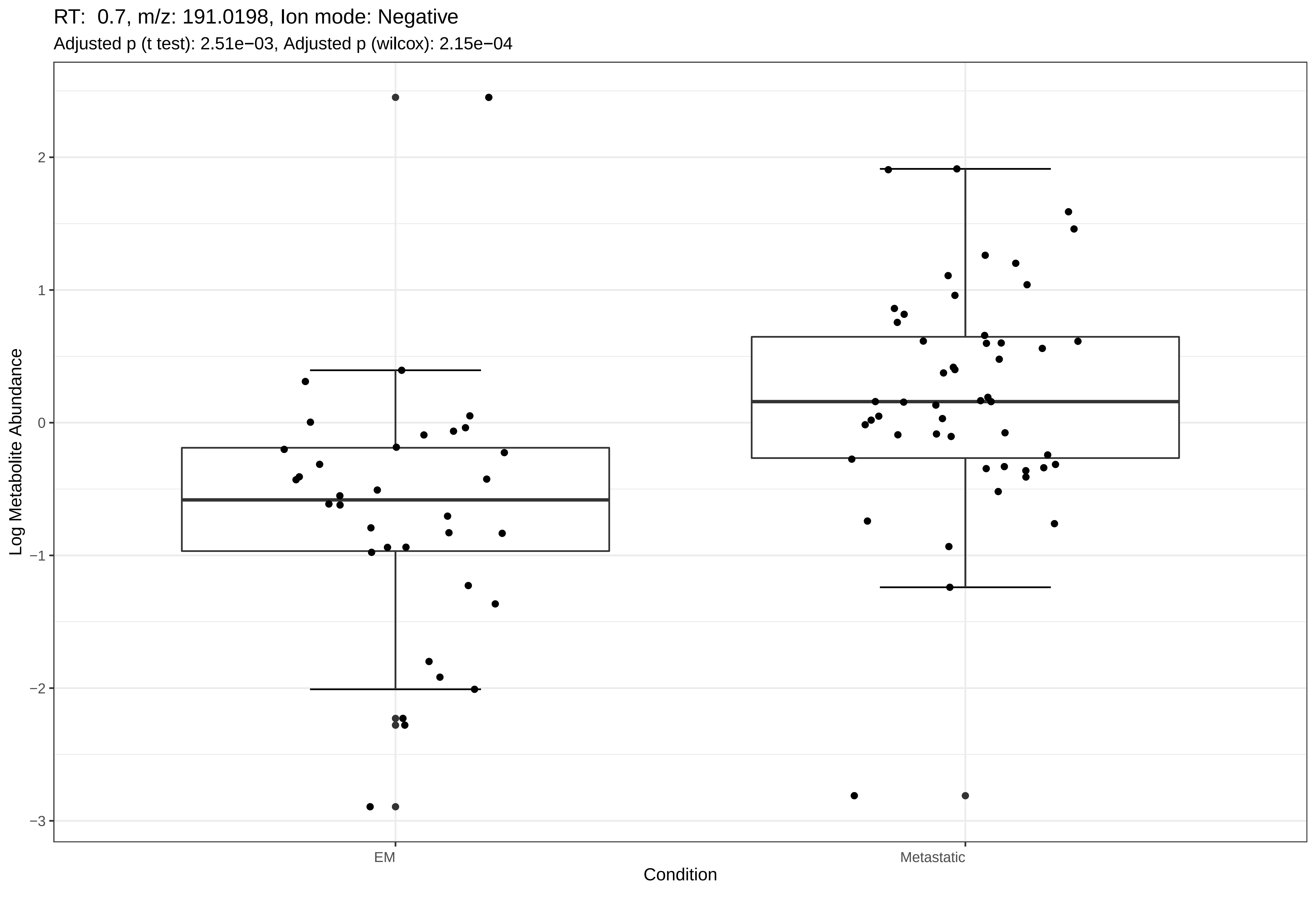
RT: 0.7, m/z: 191.0198, Ion mode: Negative
Adjusted p (t test): 2.51e−03, Adjusted p (wilcox): 2.15e−04
Log Metabolite Abundance
2
1
0
−1
−2
−3
EM
Metastatic
Condition

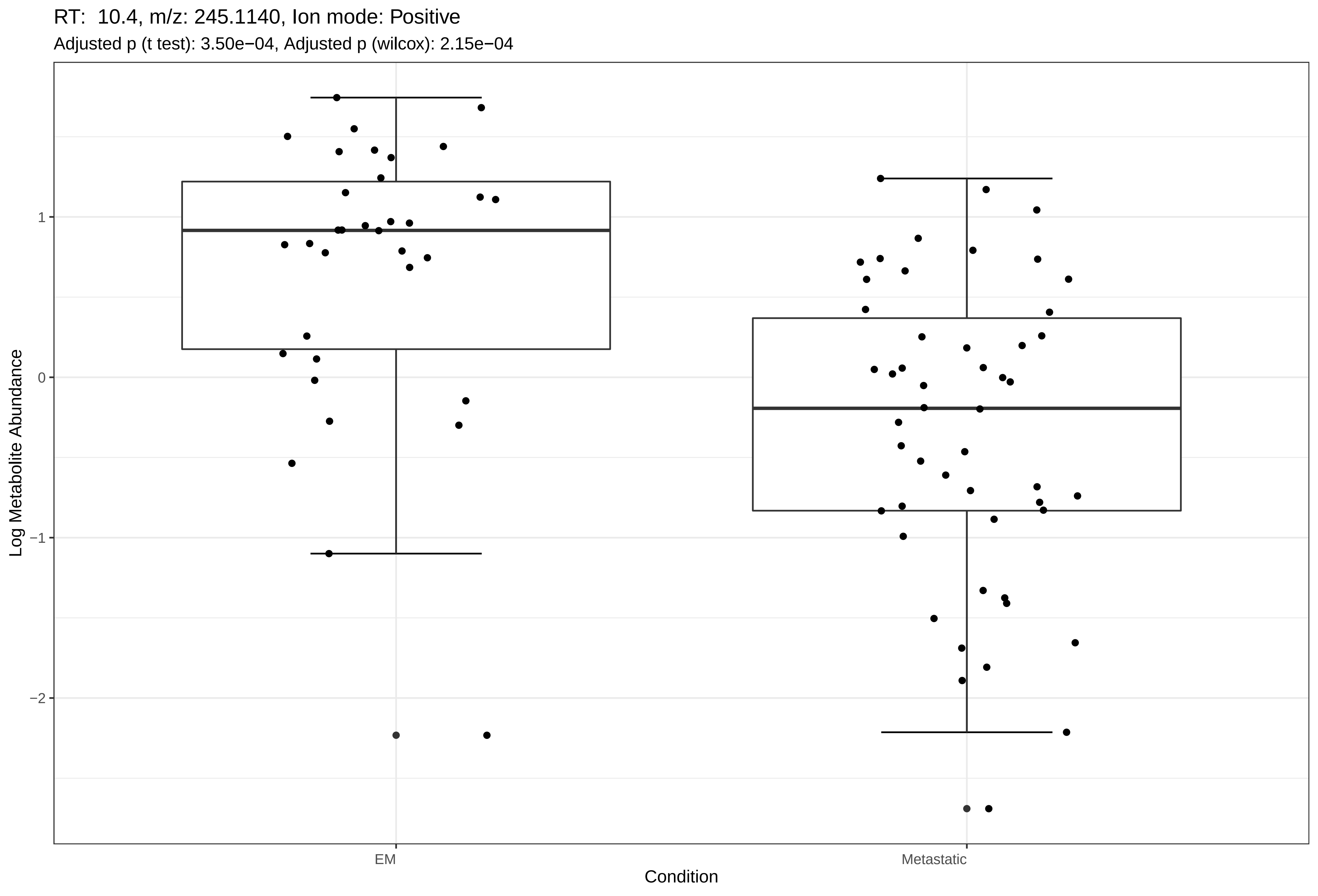

RT: 10.4, m/z: 245.1140, Ion mode: Positive
Adjusted p (t test): 3.50e−04, Adjusted p (wilcox): 2.15e−04
Log Metabolite Abundance
1
0
−1
−2
EM
Metastatic
Condition

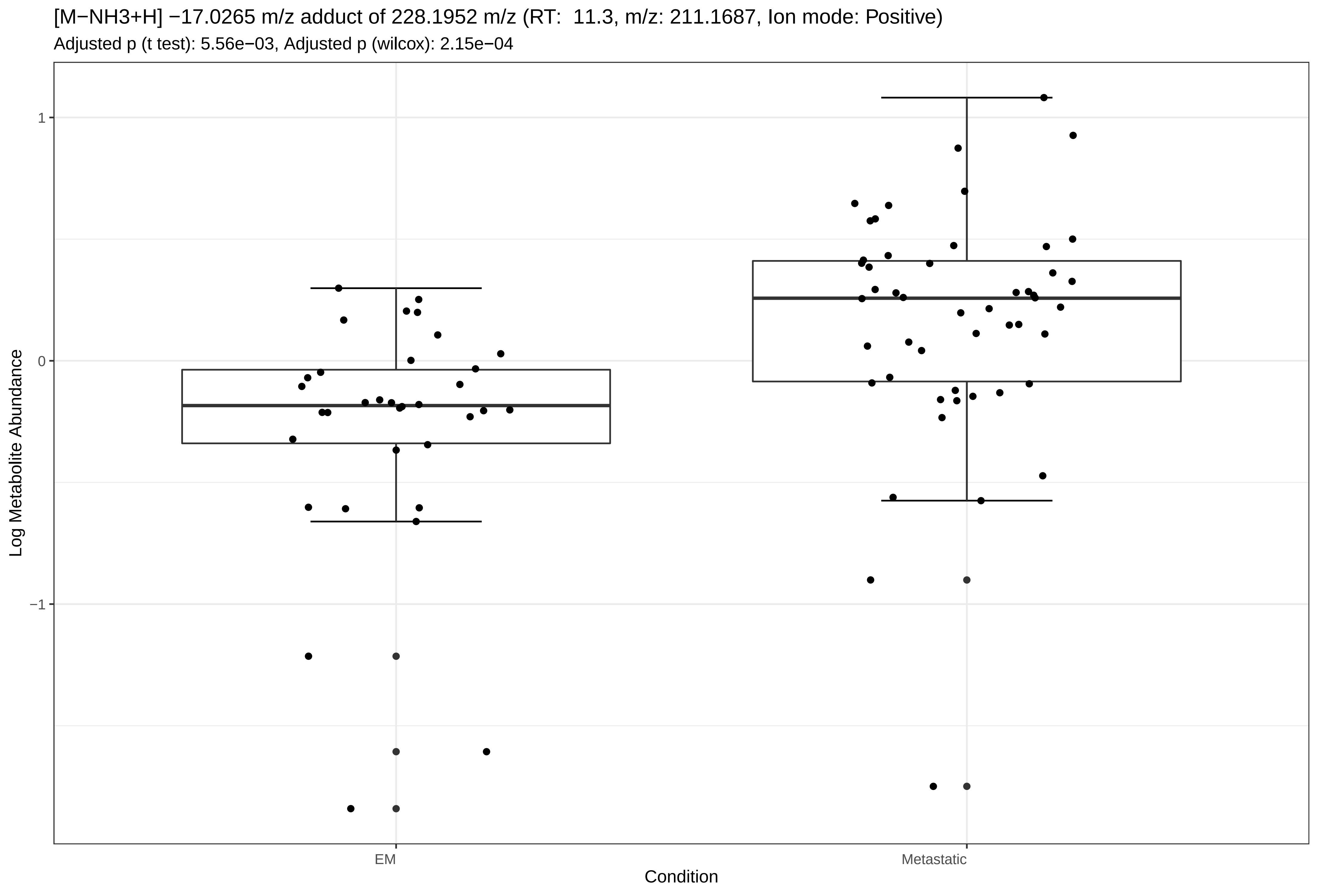

[M−NH3+H] −17.0265 m/z adduct of 228.1952 m/z (RT: 11.3, m/z: 211.1687, Ion mode: Positive)
Adjusted p (t test): 5.56e−03, Adjusted p (wilcox): 2.15e−04
Log Metabolite Abundance
1
0
−1
EM
Metastatic
Condition

LysoPE (20:4) (RT: 12.8, m/z: 500.2787, Ion mode: Negative)

Adjusted p (t test): 2.16e−04, Adjusted p (wilcox): 3.64e−04

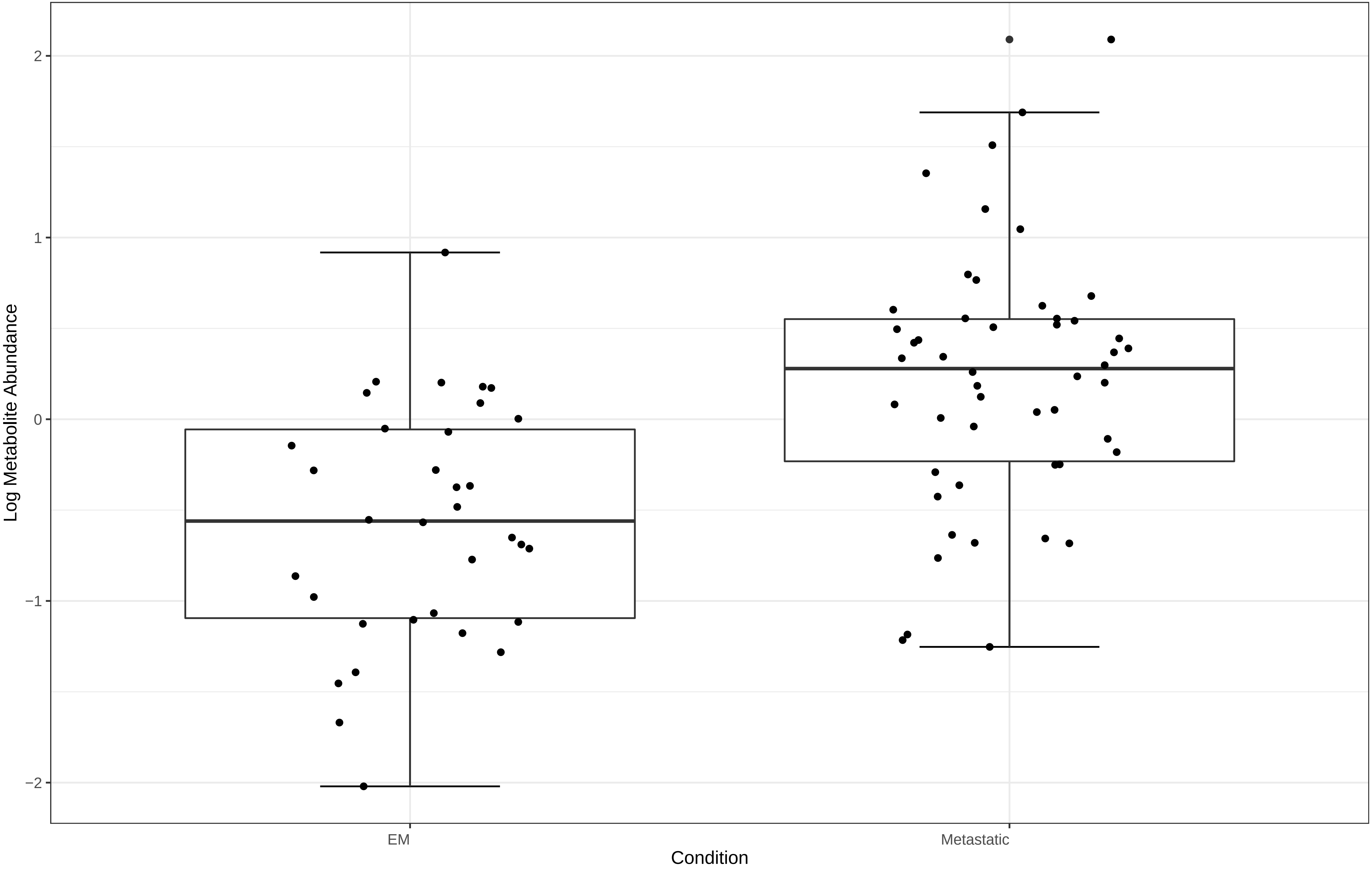

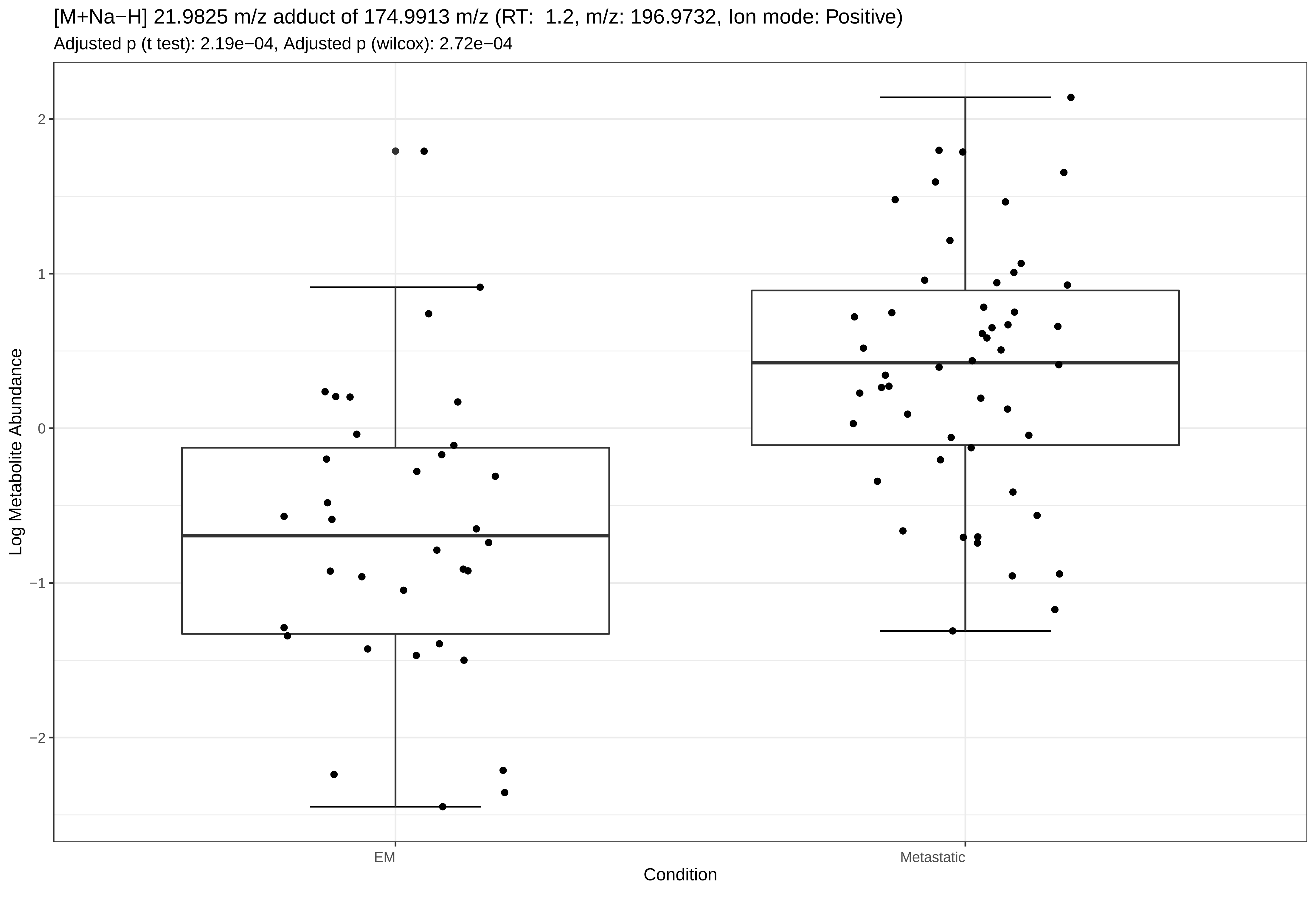

[M+Na−H] 21.9825 m/z adduct of 174.9913 m/z (RT: 1.2, m/z: 196.9732, Ion mode: Positive)
Adjusted p (t test): 2.19e−04, Adjusted p (wilcox): 2.72e−04
Log Metabolite Abundance
2
1
0
−1
−2
EM
Metastatic
Condition

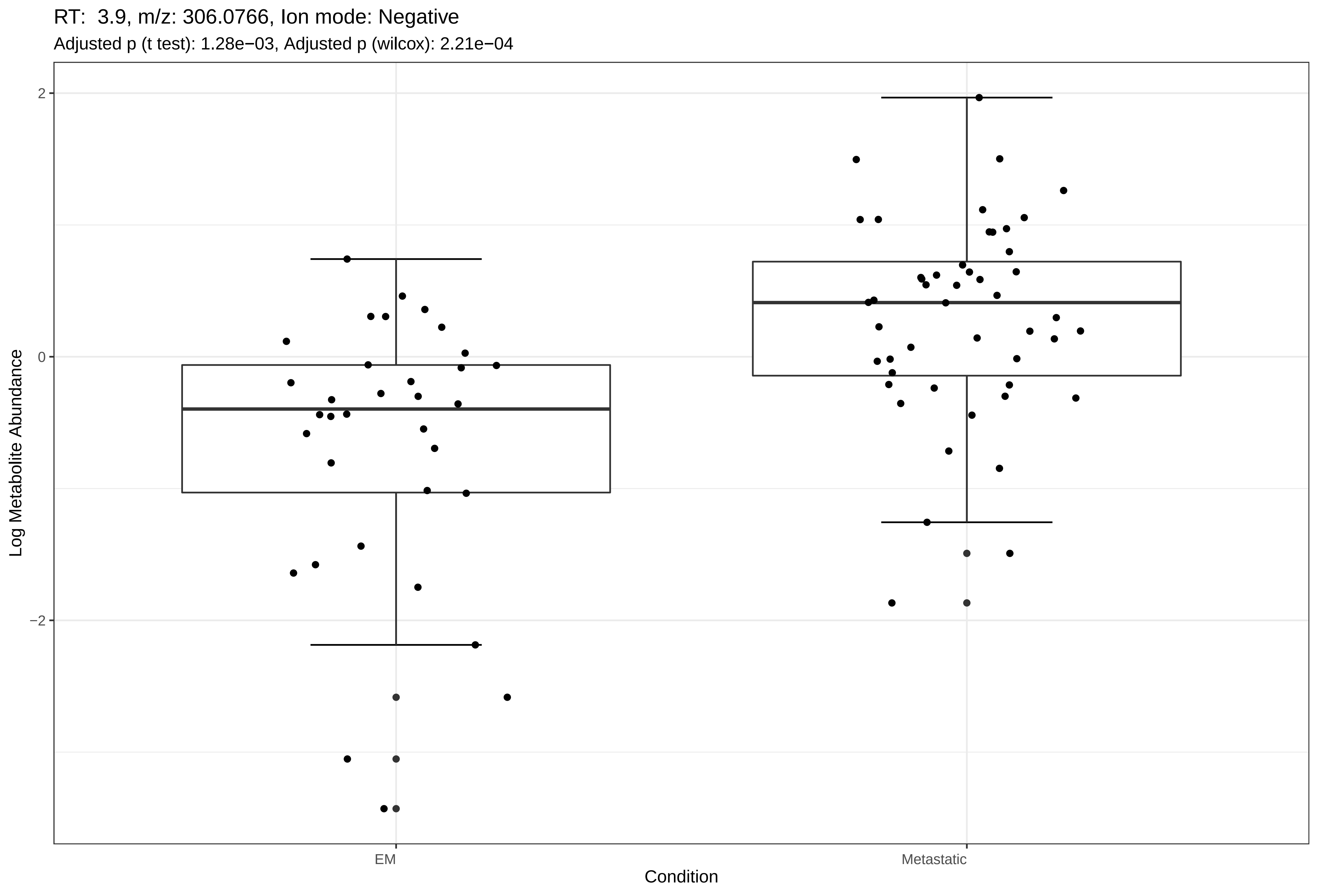
RT: 3.9, m/z: 306.0766, Ion mode: Negative
Adjusted p (t test): 1.28e−03, Adjusted p (wilcox): 2.21e−04
Log Metabolite Abundance
2
0
−2
EM
Metastatic
Condition

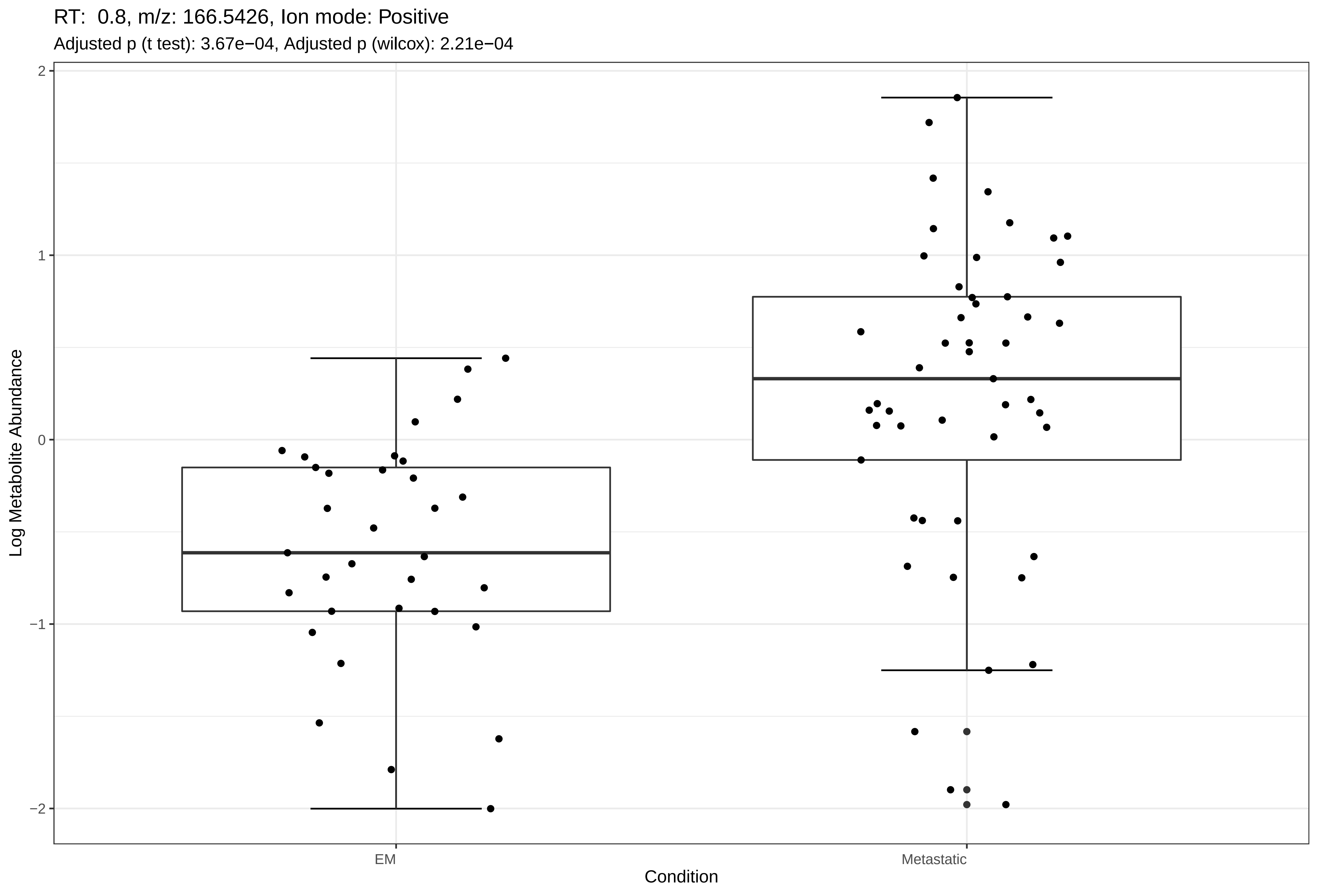

RT: 0.8, m/z: 166.5426, Ion mode: Positive
Adjusted p (t test): 3.67e−04, Adjusted p (wilcox): 2.21e−04
Log Metabolite Abundance
2
1
0
−1
−2
EM
Metastatic
Condition

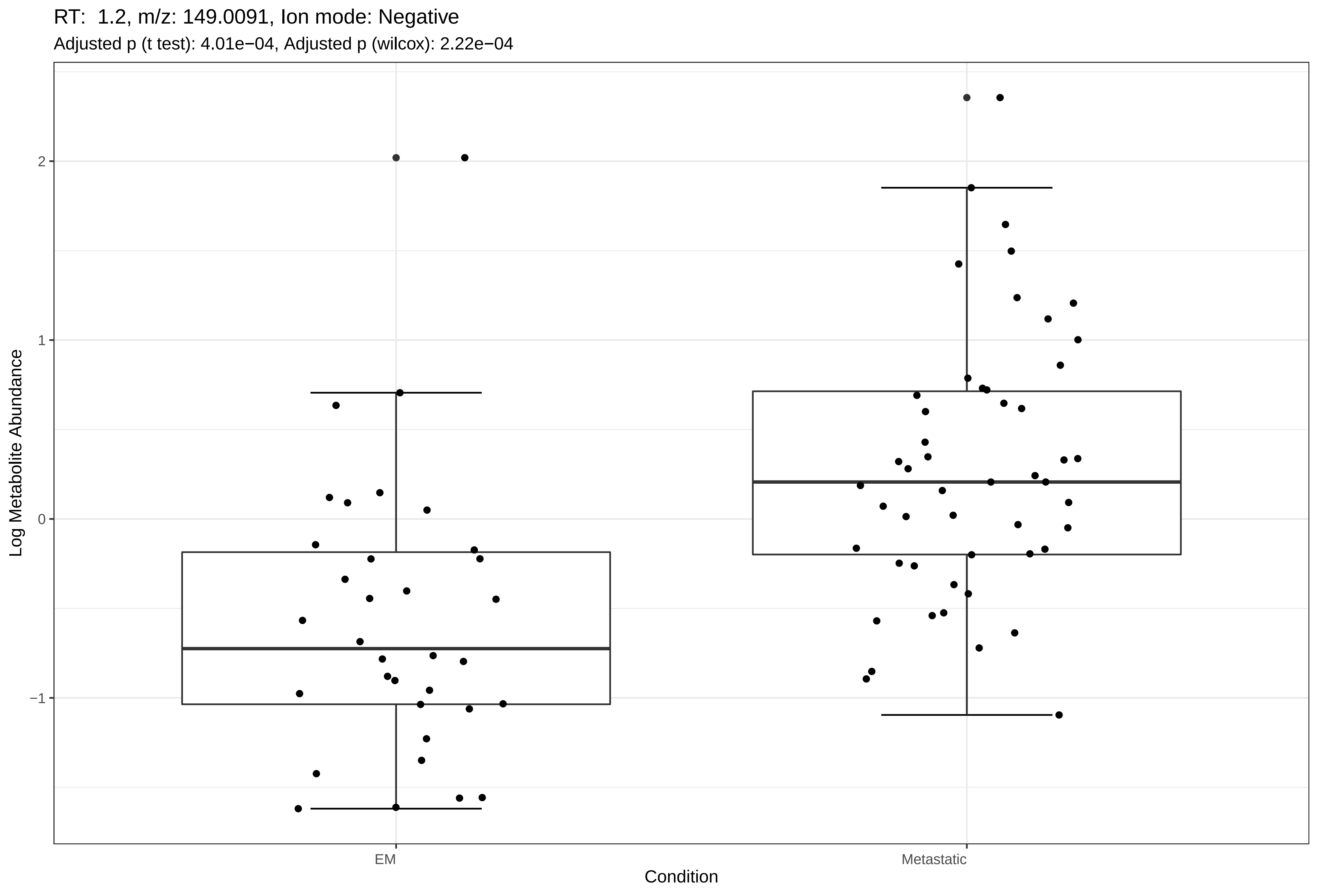
RT: 1.2, m/z: 149.0091, Ion mode: Negative
Adjusted p (t test): 4.01e−04, Adjusted p (wilcox): 2.22e−04
Log Metabolite Abundance
2
1
0
−1
EM
Metastatic
Condition

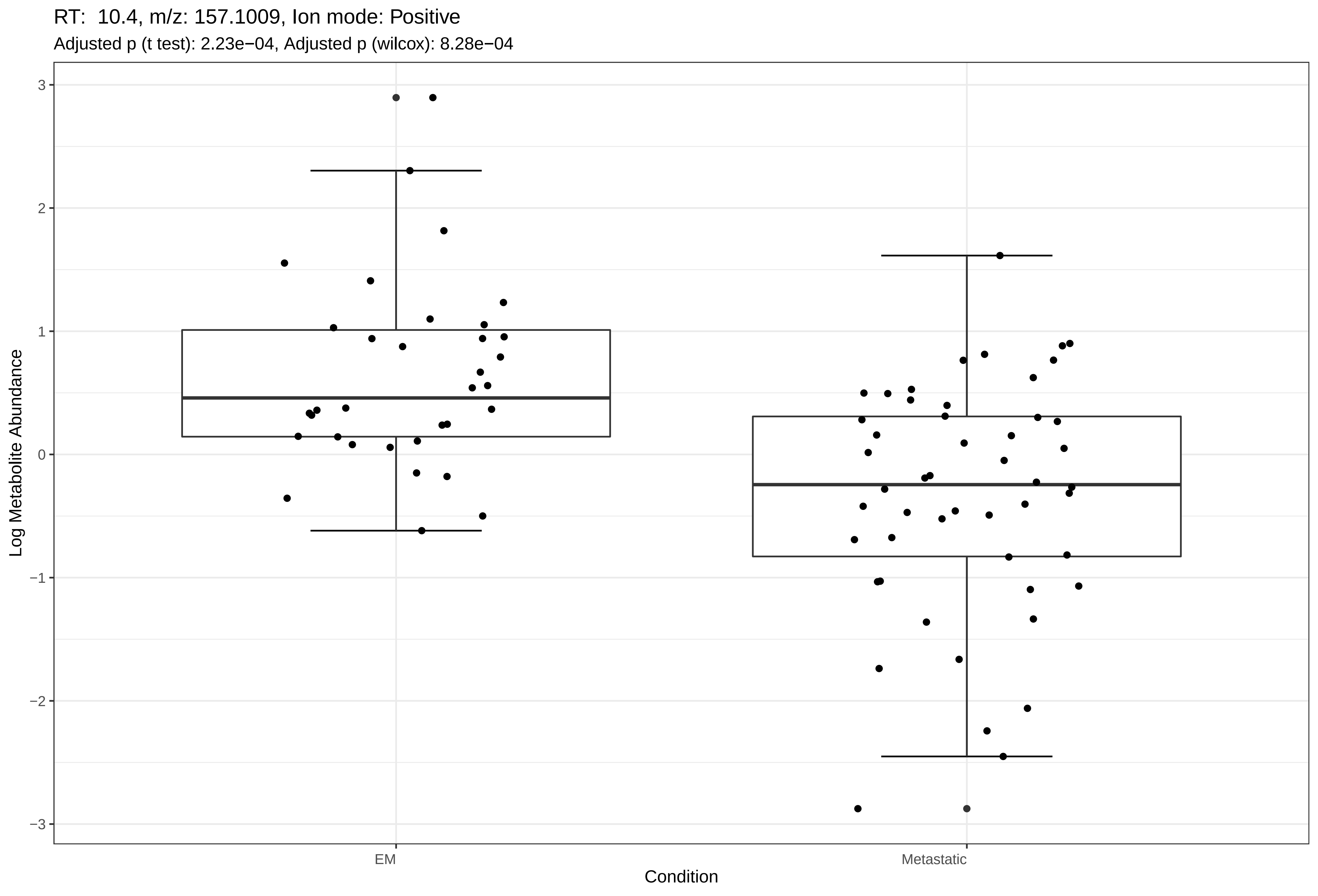
RT: 10.4, m/z: 157.1009, Ion mode: Positive
Adjusted p (t test): 2.23e−04, Adjusted p (wilcox): 8.28e−04
Log Metabolite Abundance
3
2
1
0
−1
−2
−3
EM
Metastatic
Condition

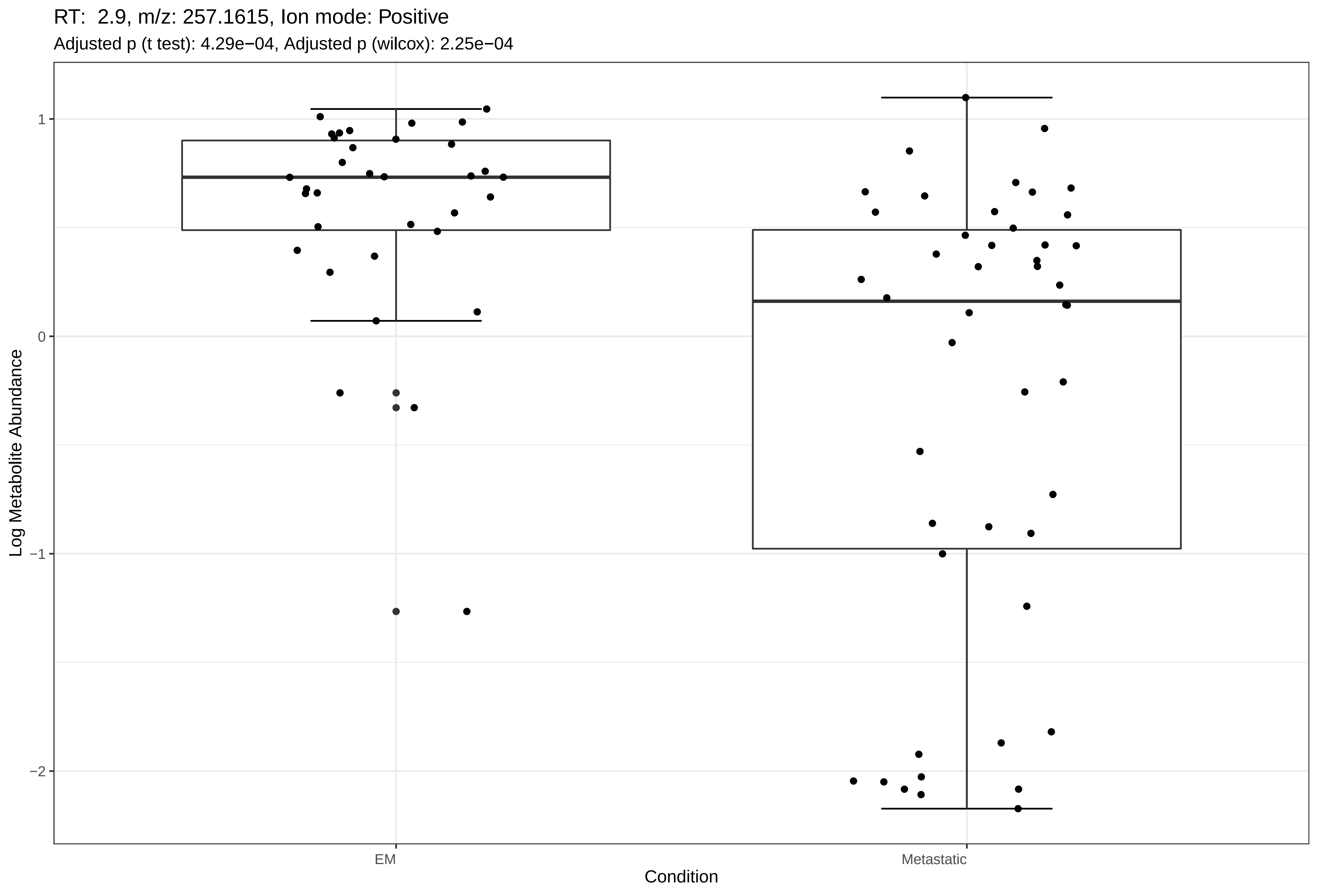

RT: 2.9, m/z: 257.1615, Ion mode: Positive
Adjusted p (t test): 4.29e−04, Adjusted p (wilcox): 2.25e−04
Log Metabolite Abundance
1
0
−1
−2
EM
Metastatic
Condition

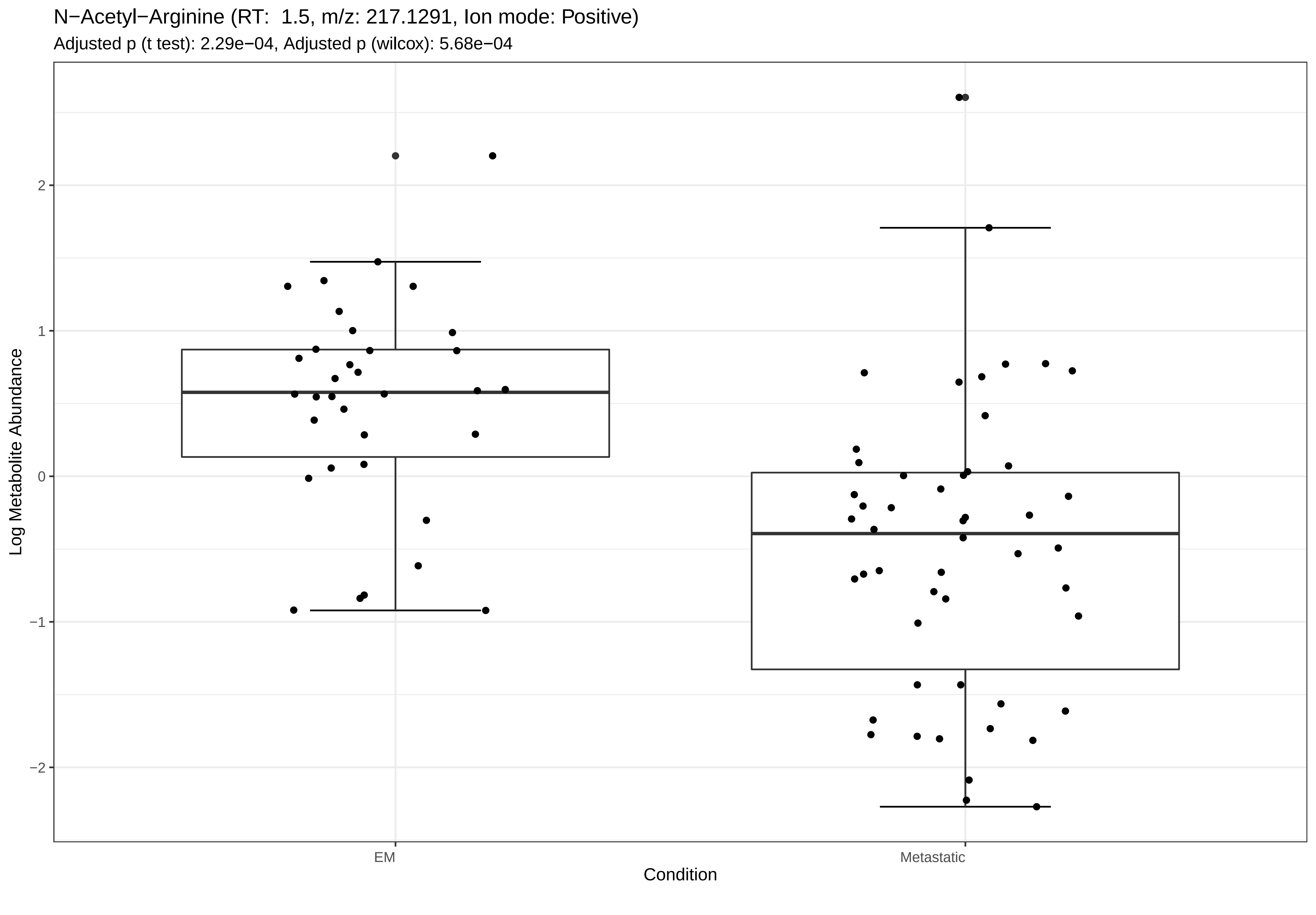

N−Acetyl−Arginine (RT: 1.5, m/z: 217.1291, Ion mode: Positive)
Adjusted p (t test): 2.29e−04, Adjusted p (wilcox): 5.68e−04
Log Metabolite Abundance
2
1
0
−1
−2
EM
Metastatic
Condition

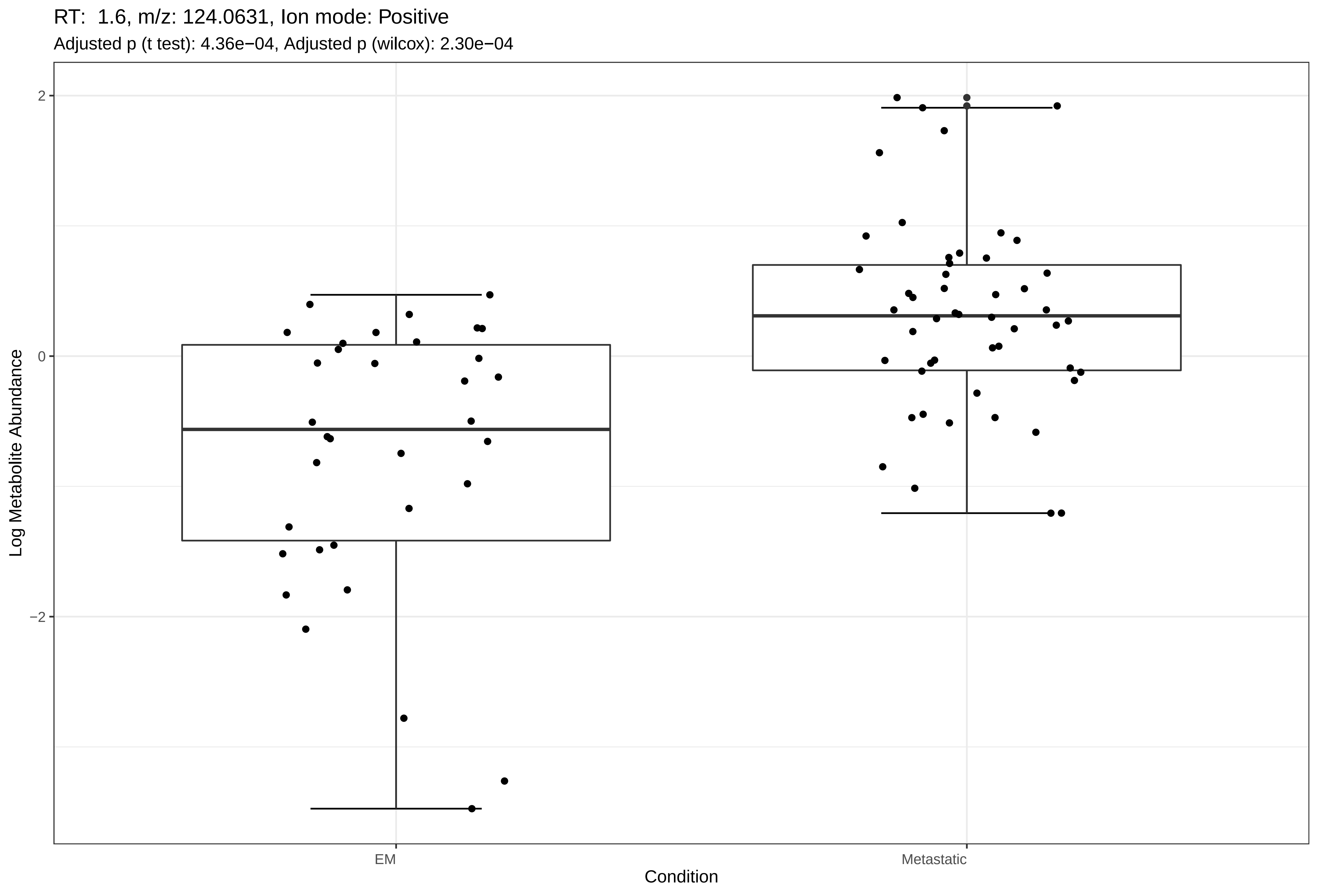

RT: 1.6, m/z: 124.0631, Ion mode: Positive
Adjusted p (t test): 4.36e−04, Adjusted p (wilcox): 2.30e−04
Log Metabolite Abundance
2
0
−2
EM
Metastatic
Condition

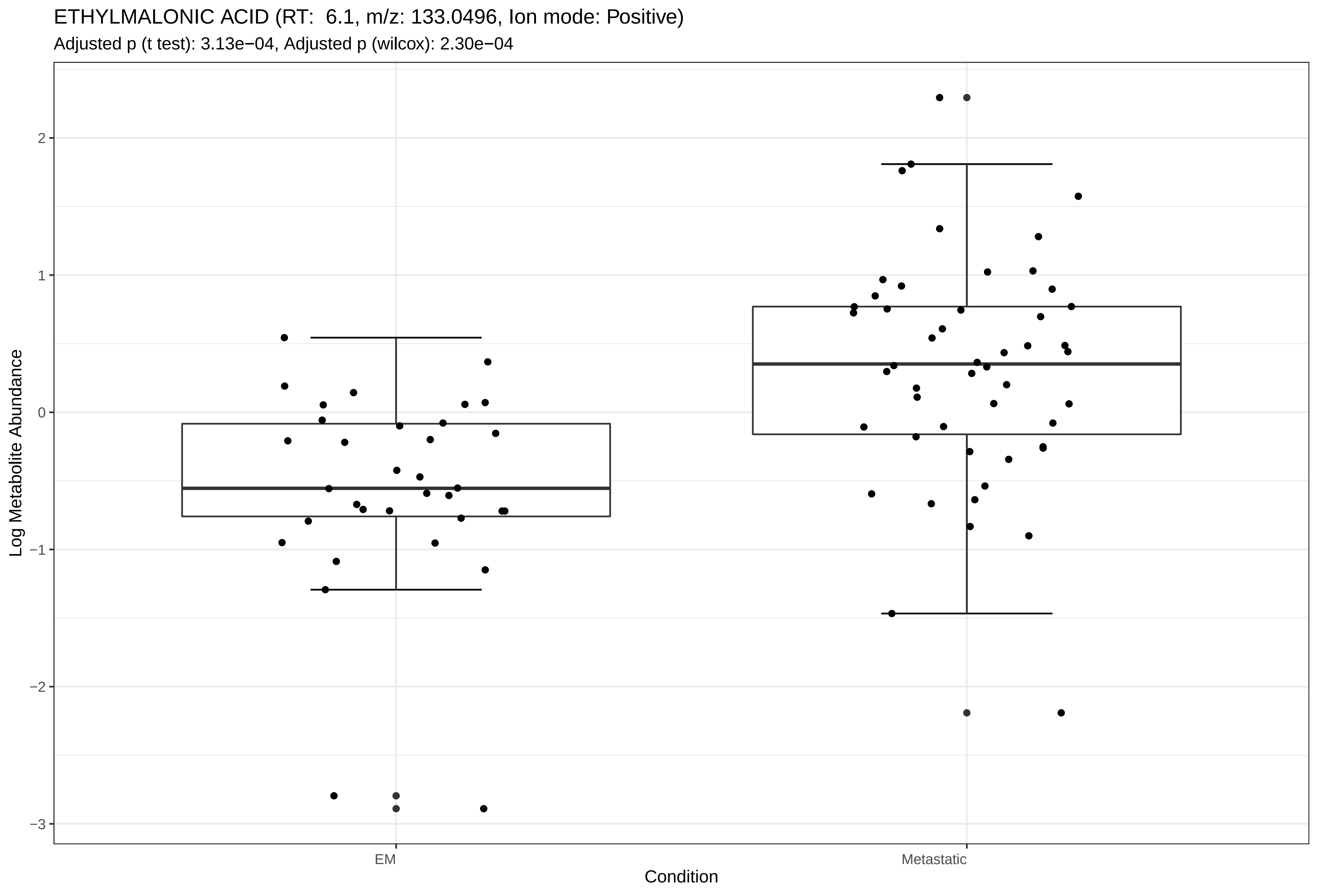

ETHYLMALONIC ACID (RT: 6.1, m/z: 133.0496, Ion mode: Positive)
Adjusted p (t test): 3.13e−04, Adjusted p (wilcox): 2.30e−04
Log Metabolite Abundance
2
1
0
−1
−2
−3
EM
Metastatic
Condition

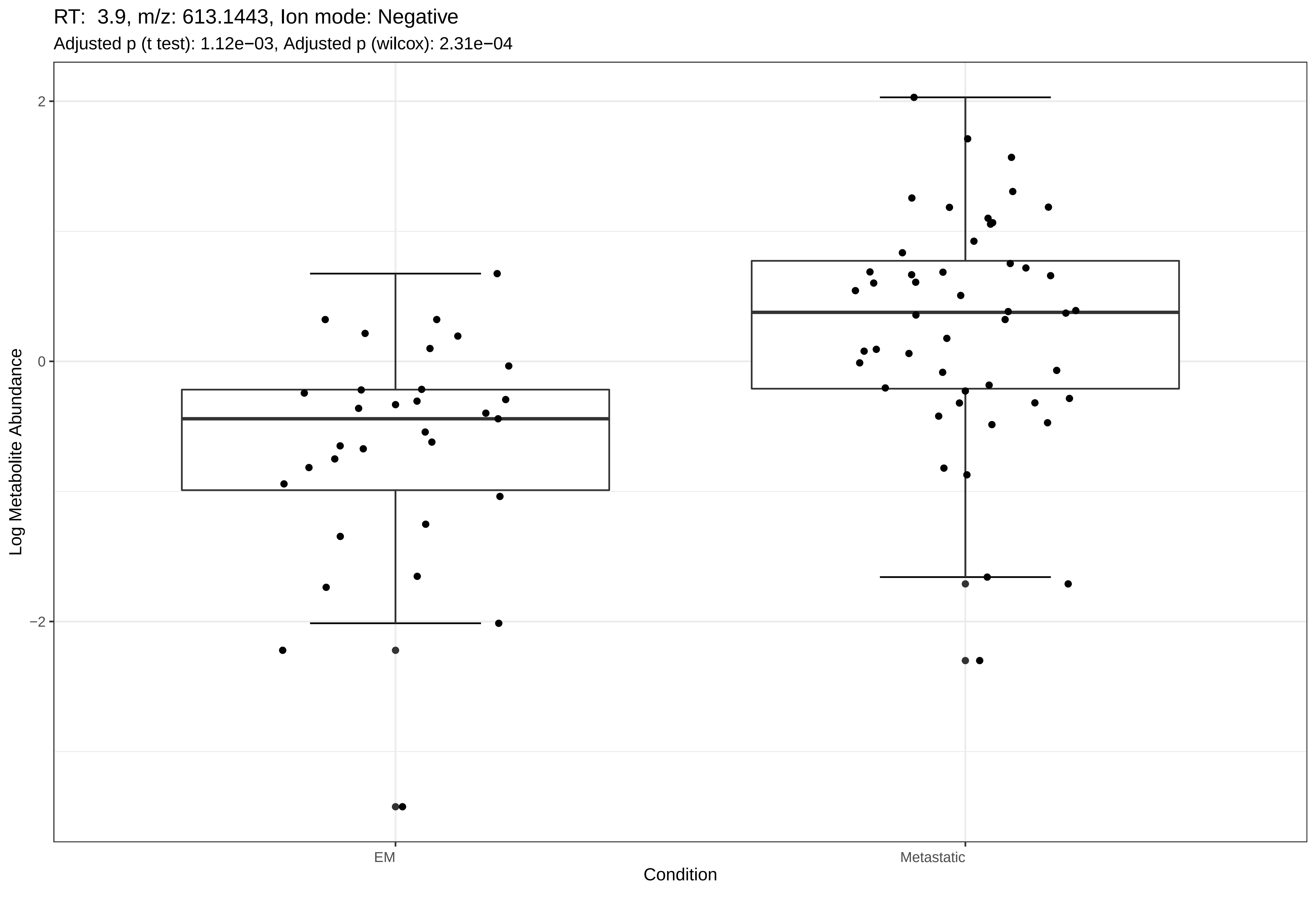
RT: 3.9, m/z: 613.1443, Ion mode: Negative
Adjusted p (t test): 1.12e−03, Adjusted p (wilcox): 2.31e−04
2
0
−2
Log Metabolite Abundance
EM
Metastatic
Condition

RT: 6.1, m/z: 325.0322, Ion mode: Negative

Adjusted p (t test): 2.37e−03, Adjusted p (wilcox): 2.39e−04

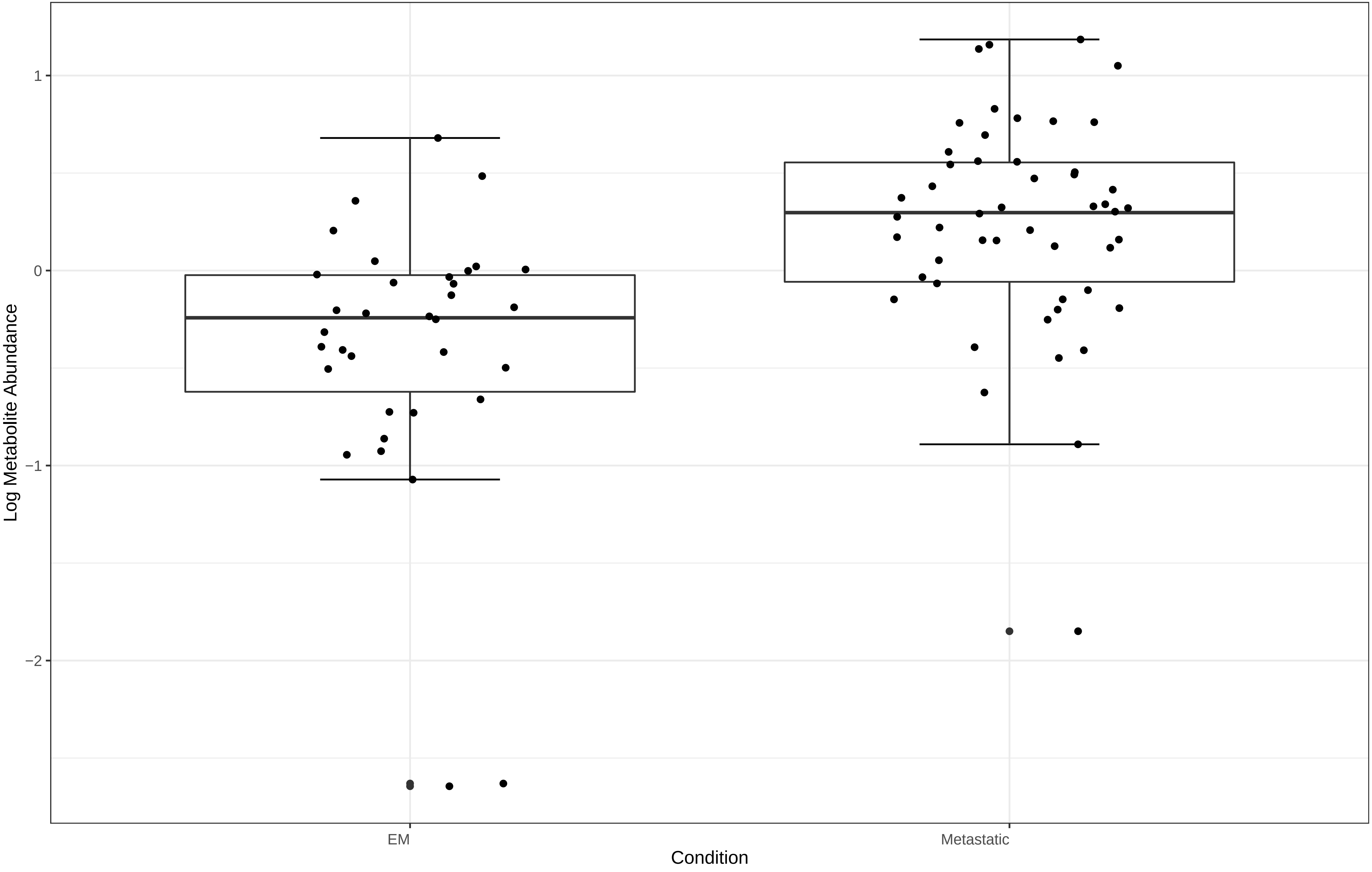

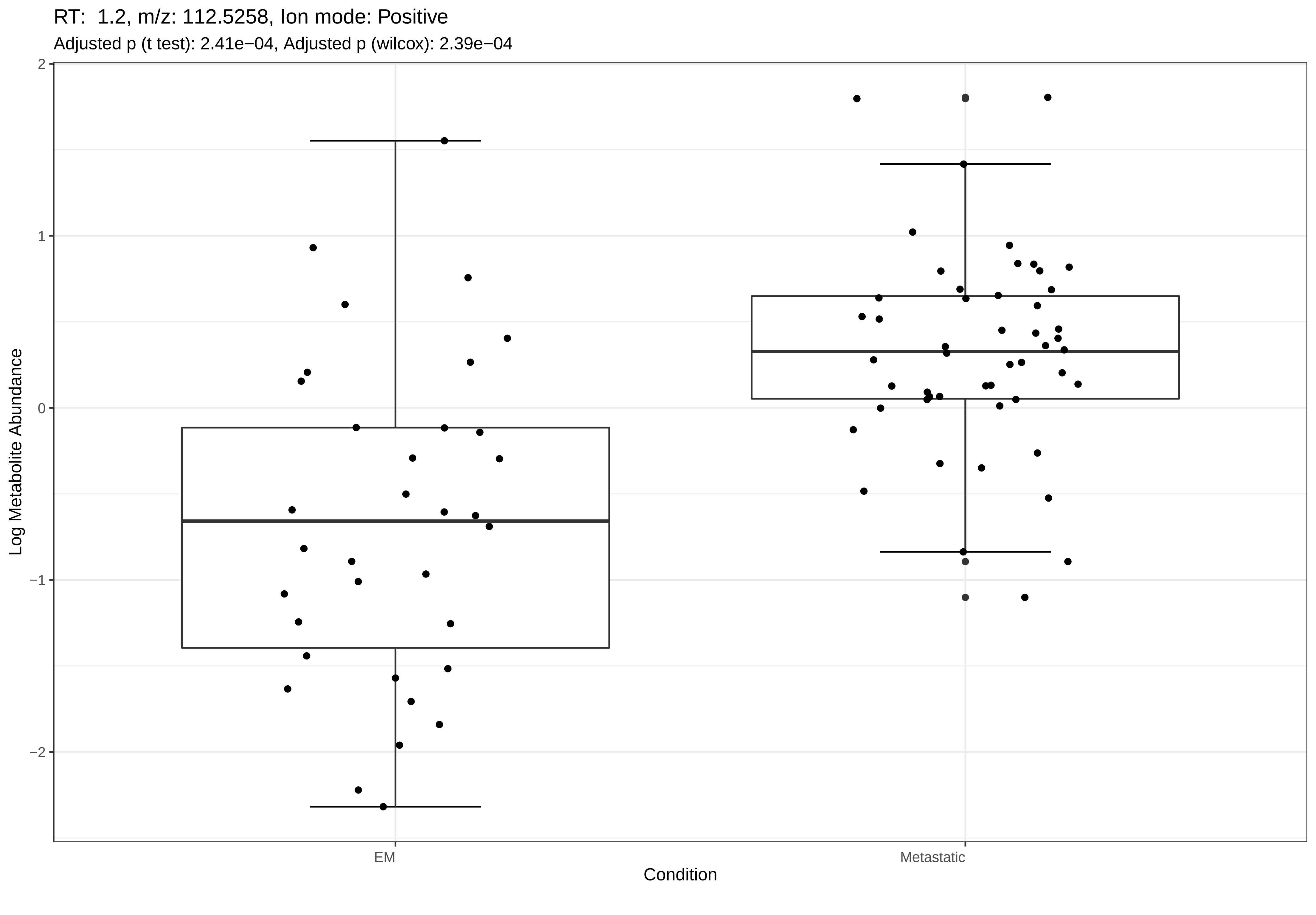

RT: 1.2, m/z: 112.5258, Ion mode: Positive
Adjusted p (t test): 2.41e−04, Adjusted p (wilcox): 2.39e−04
Log Metabolite Abundance
2
1
0
−1
−2
EM
Metastatic
Condition

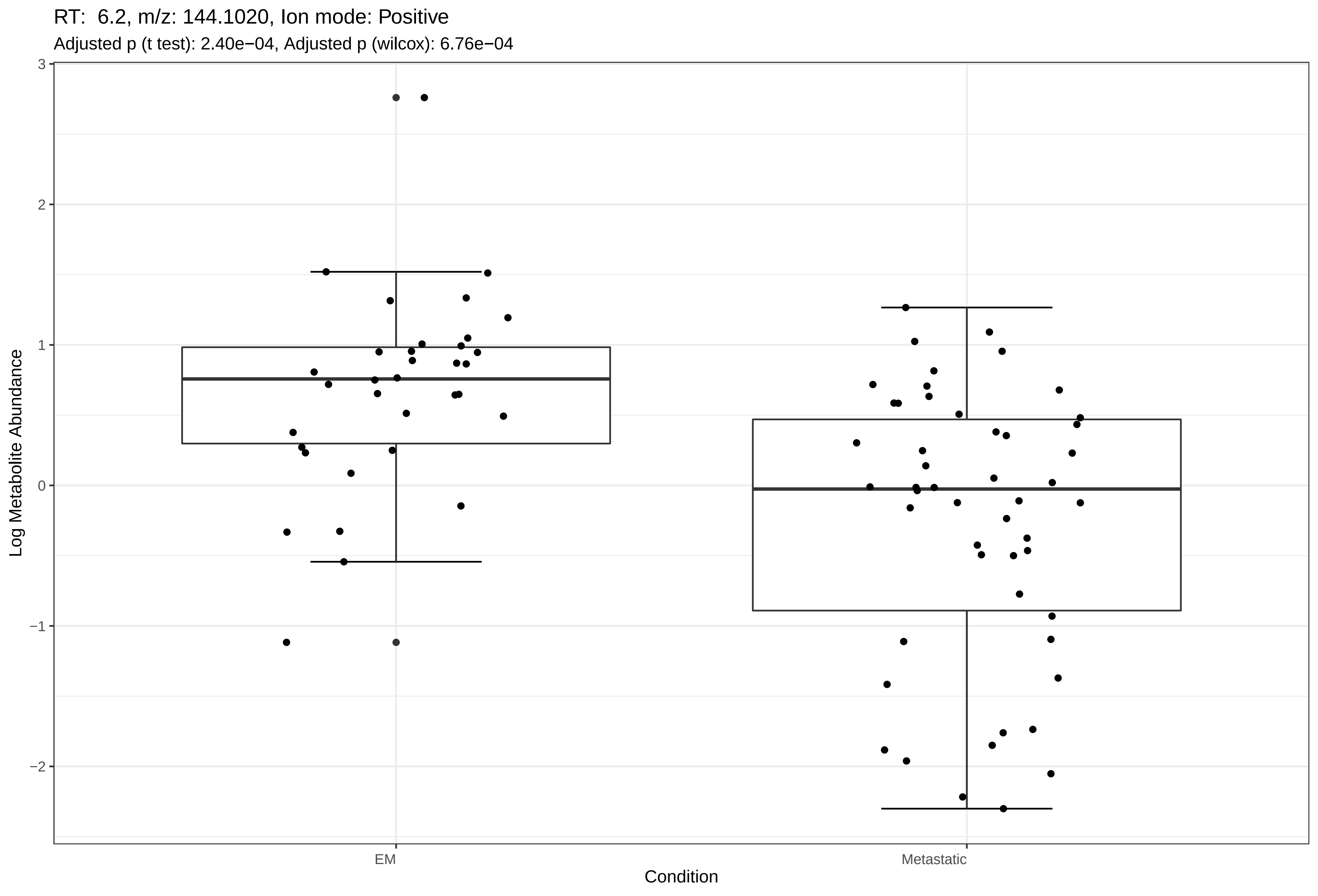
RT: 6.2, m/z: 144.1020, Ion mode: Positive
Adjusted p (t test): 2.40e−04, Adjusted p (wilcox): 6.76e−04
Log Metabolite Abundance
3
2
1
0
−1
−2
EM
Metastatic
Condition

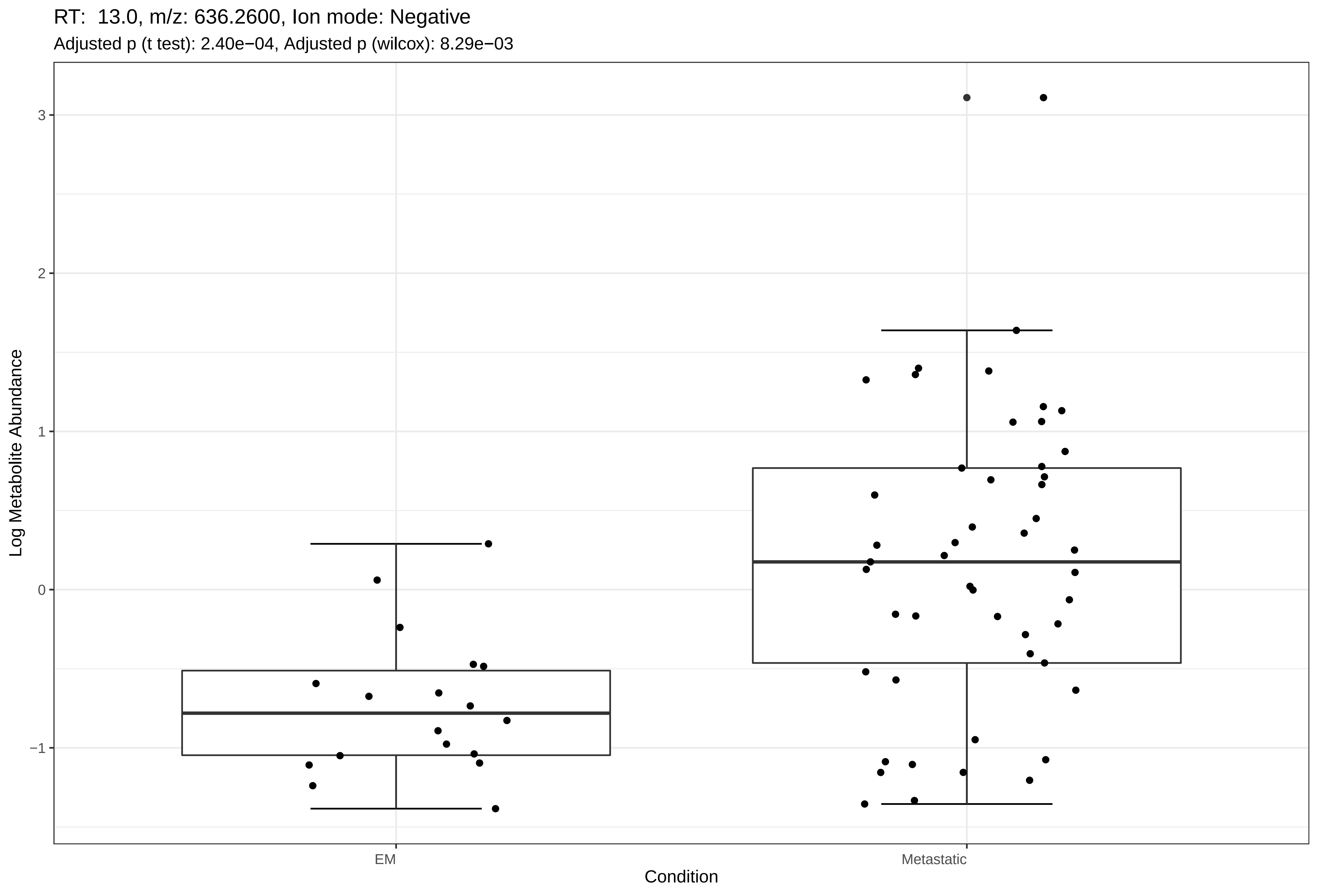

RT: 13.0, m/z: 636.2600, Ion mode: Negative
Adjusted p (t test): 2.40e−04, Adjusted p (wilcox): 8.29e−03
Log Metabolite Abundance
3
2
1
0
−1
EM
Metastatic
Condition

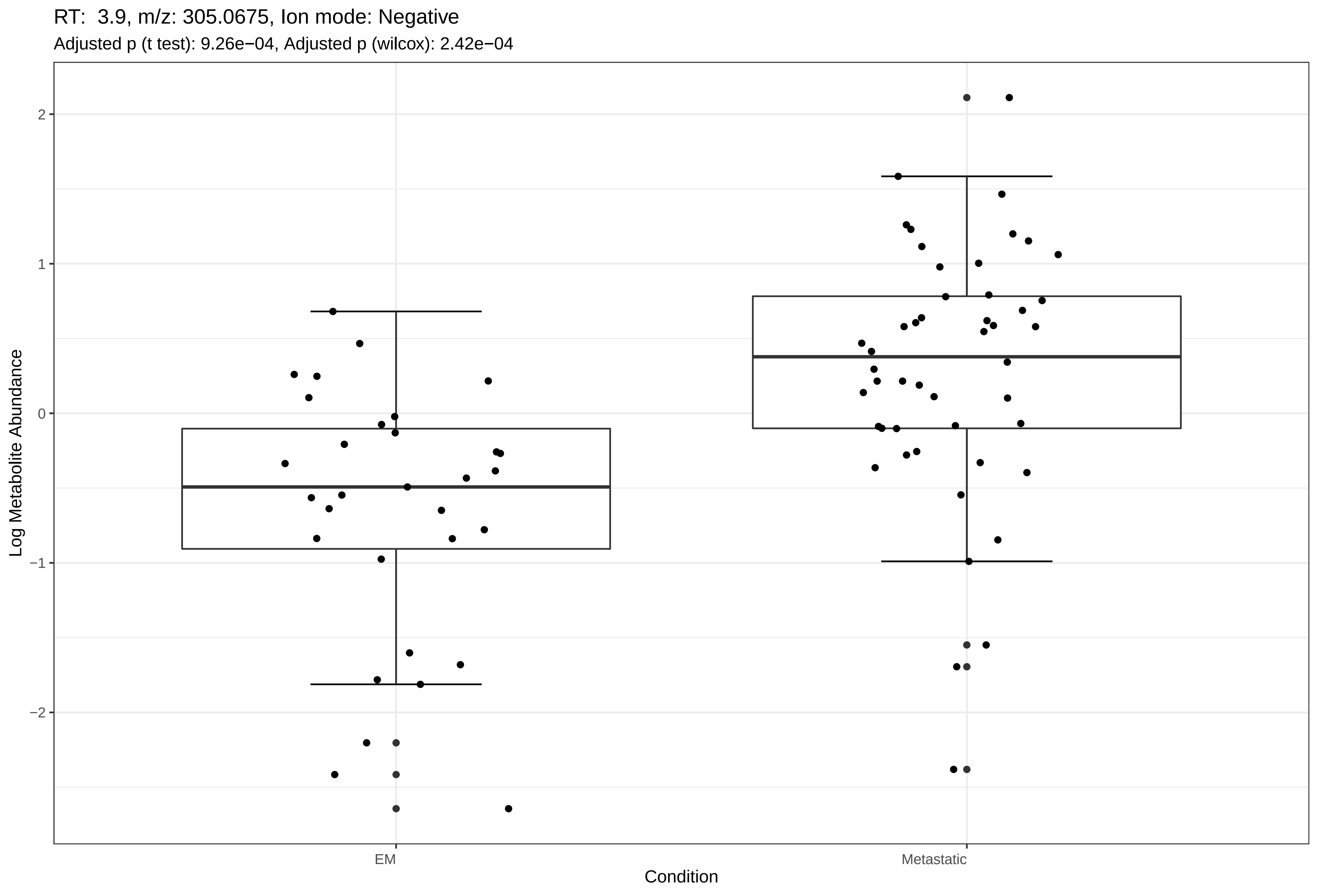

RT: 3.9, m/z: 305.0675, Ion mode: Negative
Adjusted p (t test): 9.26e−04, Adjusted p (wilcox): 2.42e−04
Log Metabolite Abundance
2
1
0
−1
−2
EM
Metastatic
Condition

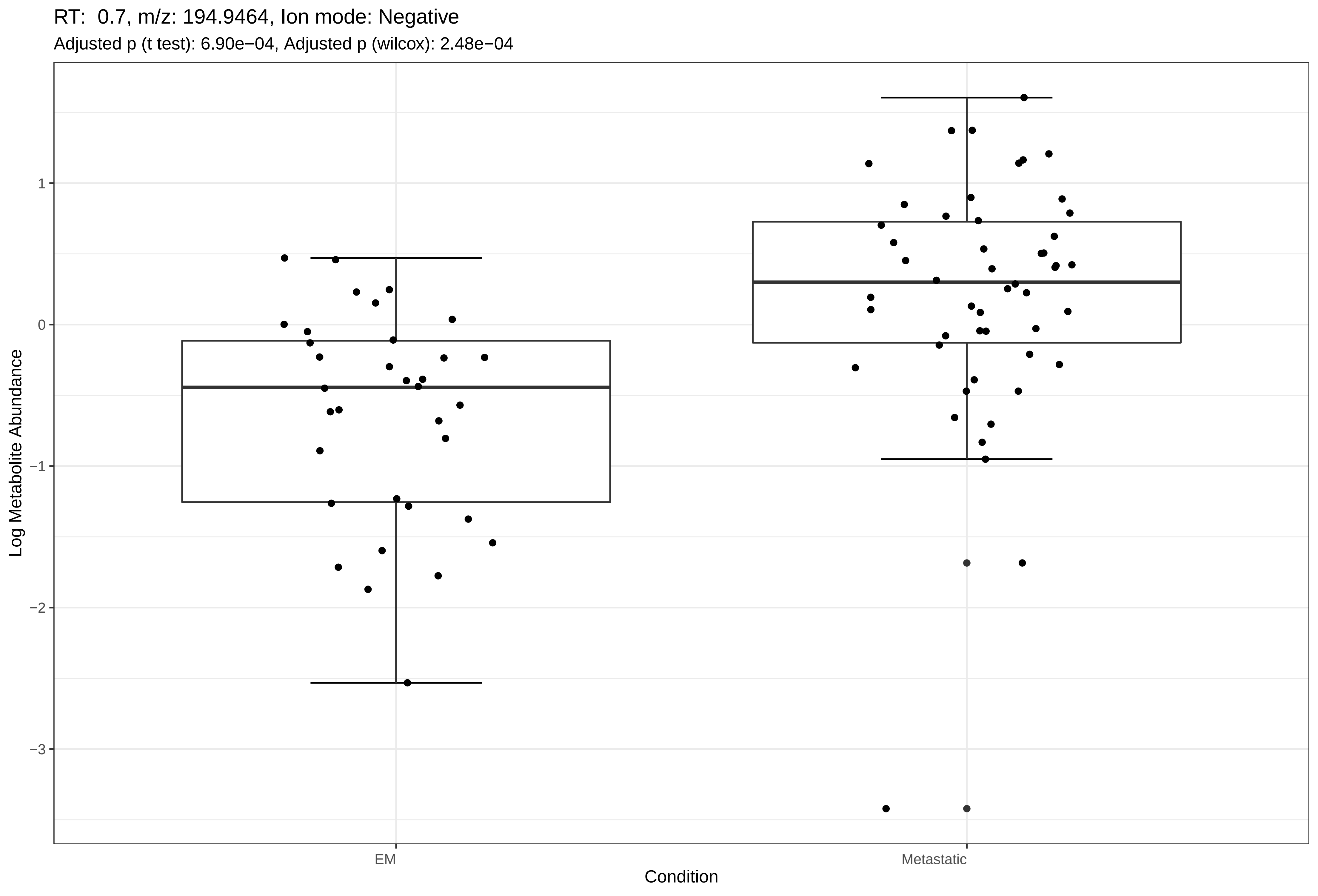
RT: 0.7, m/z: 194.9464, Ion mode: Negative
Adjusted p (t test): 6.90e−04, Adjusted p (wilcox): 2.48e−04
Log Metabolite Abundance
1
0
−1
−2
−3
EM
Metastatic
Condition

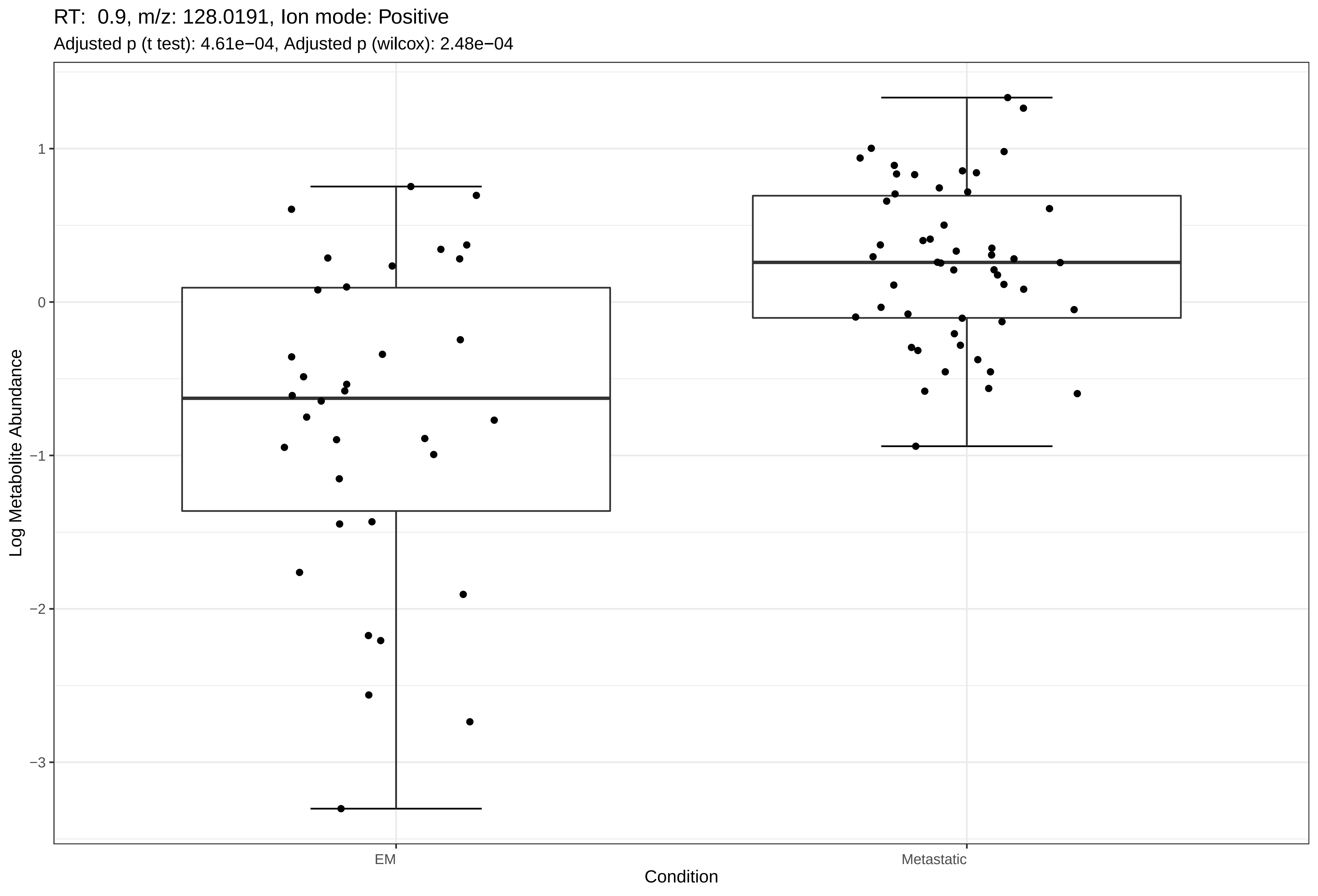
RT: 0.9, m/z: 128.0191, Ion mode: Positive
Adjusted p (t test): 4.61e−04, Adjusted p (wilcox): 2.48e−04
Log Metabolite Abundance
1
0
−1
−2
−3
EM
Metastatic
Condition

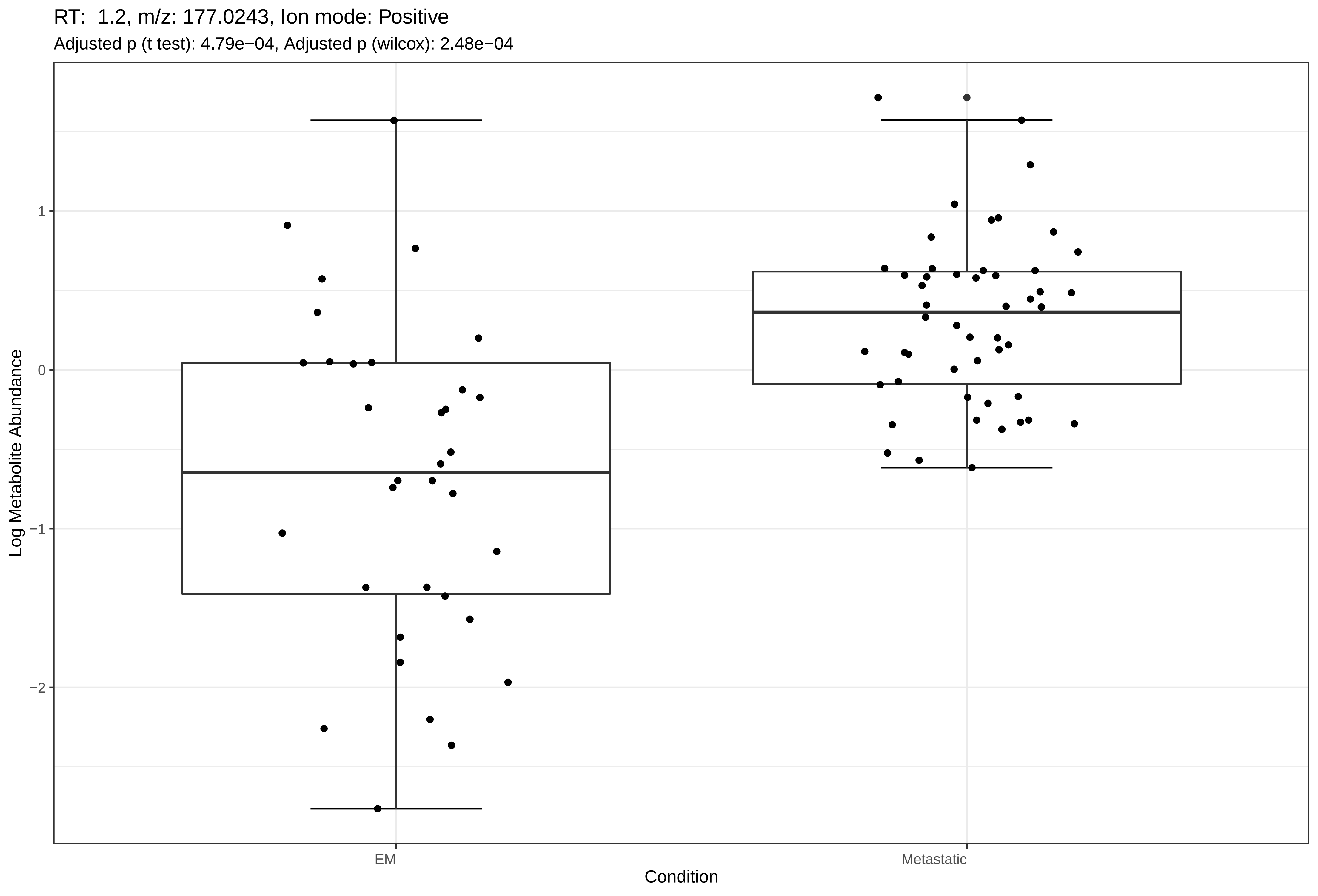
RT: 1.2, m/z: 177.0243, Ion mode: Positive
Adjusted p (t test): 4.79e−04, Adjusted p (wilcox): 2.48e−04
Log Metabolite Abundance
1
0
−1
−2
EM
Metastatic
Condition

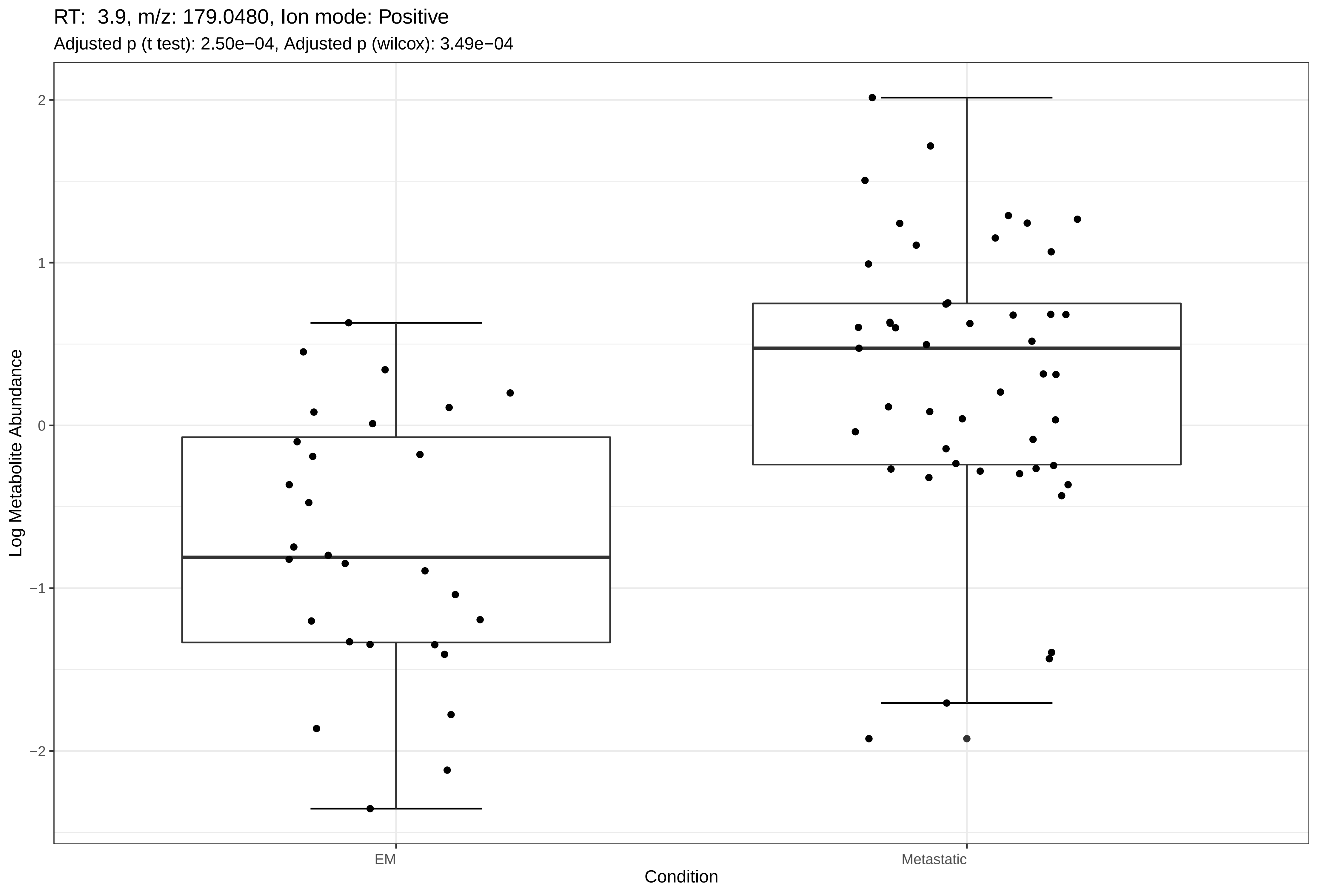
RT: 3.9, m/z: 179.0480, Ion mode: Positive
Adjusted p (t test): 2.50e−04, Adjusted p (wilcox): 3.49e−04
Log Metabolite Abundance
2
1
0
−1
−2
EM
Metastatic
Condition

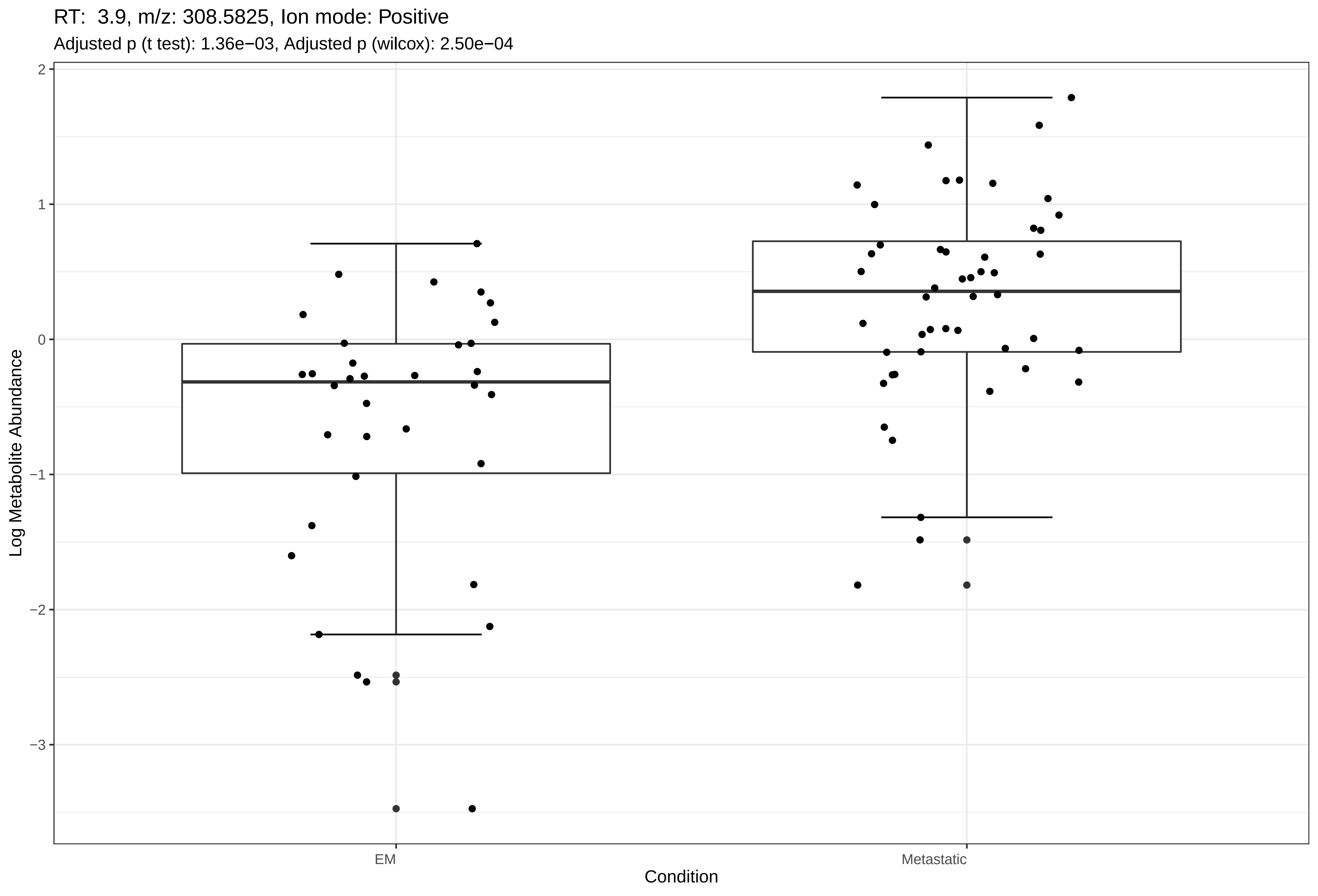

RT: 3.9, m/z: 308.5825, Ion mode: Positive
Adjusted p (t test): 1.36e−03, Adjusted p (wilcox): 2.50e−04
Log Metabolite Abundance
2
1
0
−1
−2
−3
EM
Metastatic
Condition

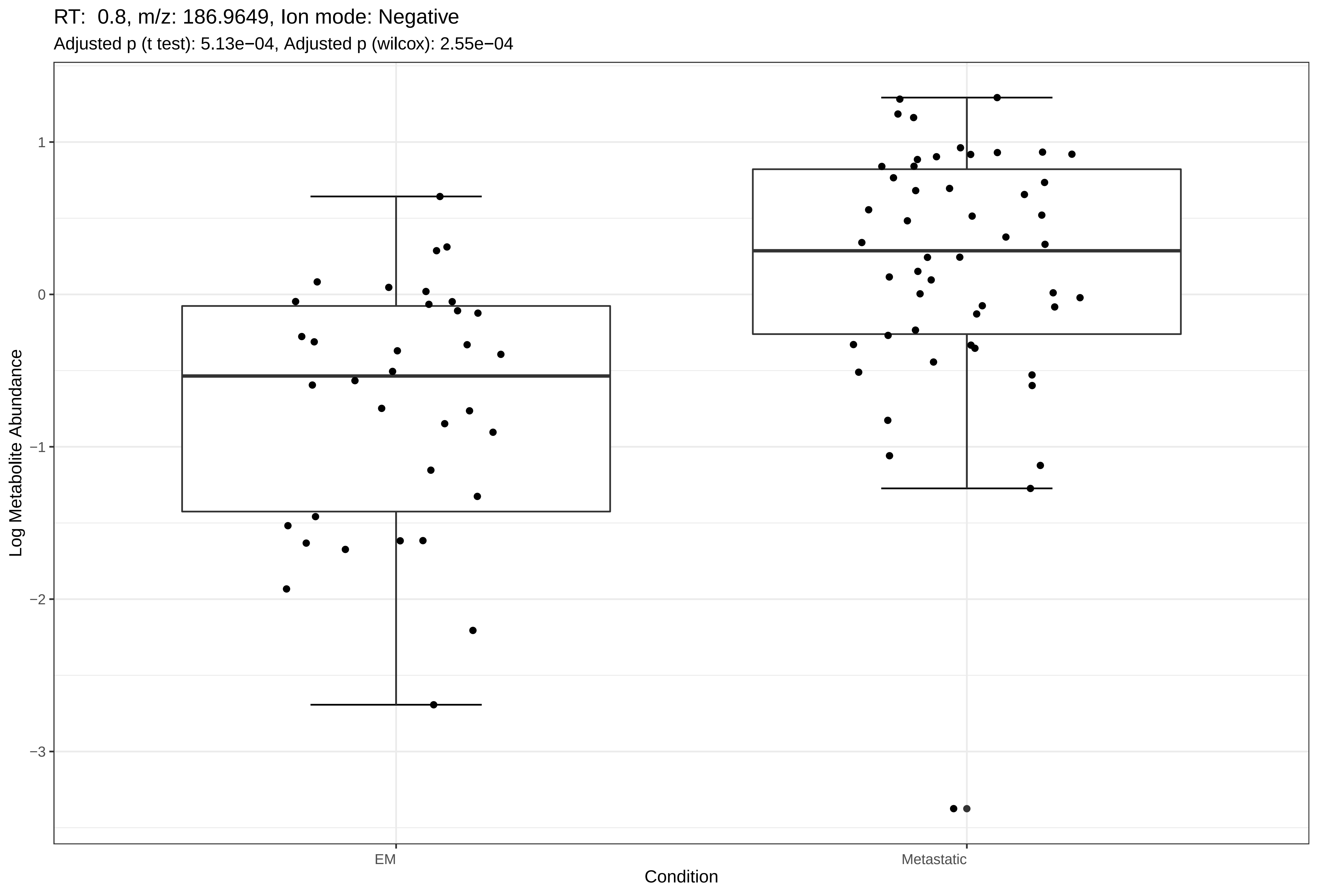
RT: 0.8, m/z: 186.9649, Ion mode: Negative
Adjusted p (t test): 5.13e−04, Adjusted p (wilcox): 2.55e−04
Log Metabolite Abundance
1
0
−1
−2
−3
EM
Metastatic
Condition

RT: 1.1, m/z: 123.1029, Ion mode: Positive

Adjusted p (t test): 3.39e−04, Adjusted p (wilcox): 2.55e−04

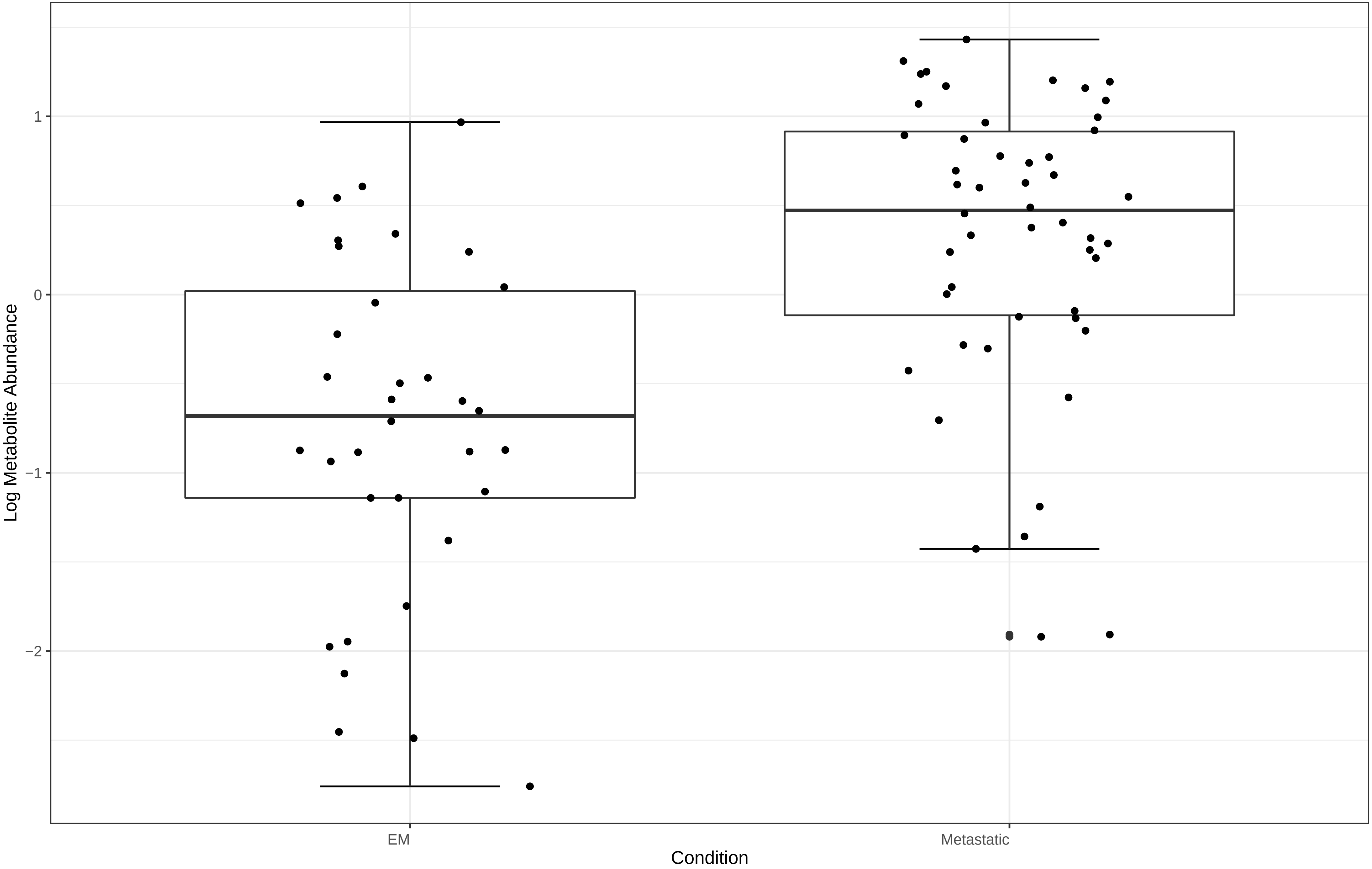

RT: 1.2, m/z: 193.0017, Ion mode: Positive

Adjusted p (t test): 3.91e−04, Adjusted p (wilcox): 2.55e−04

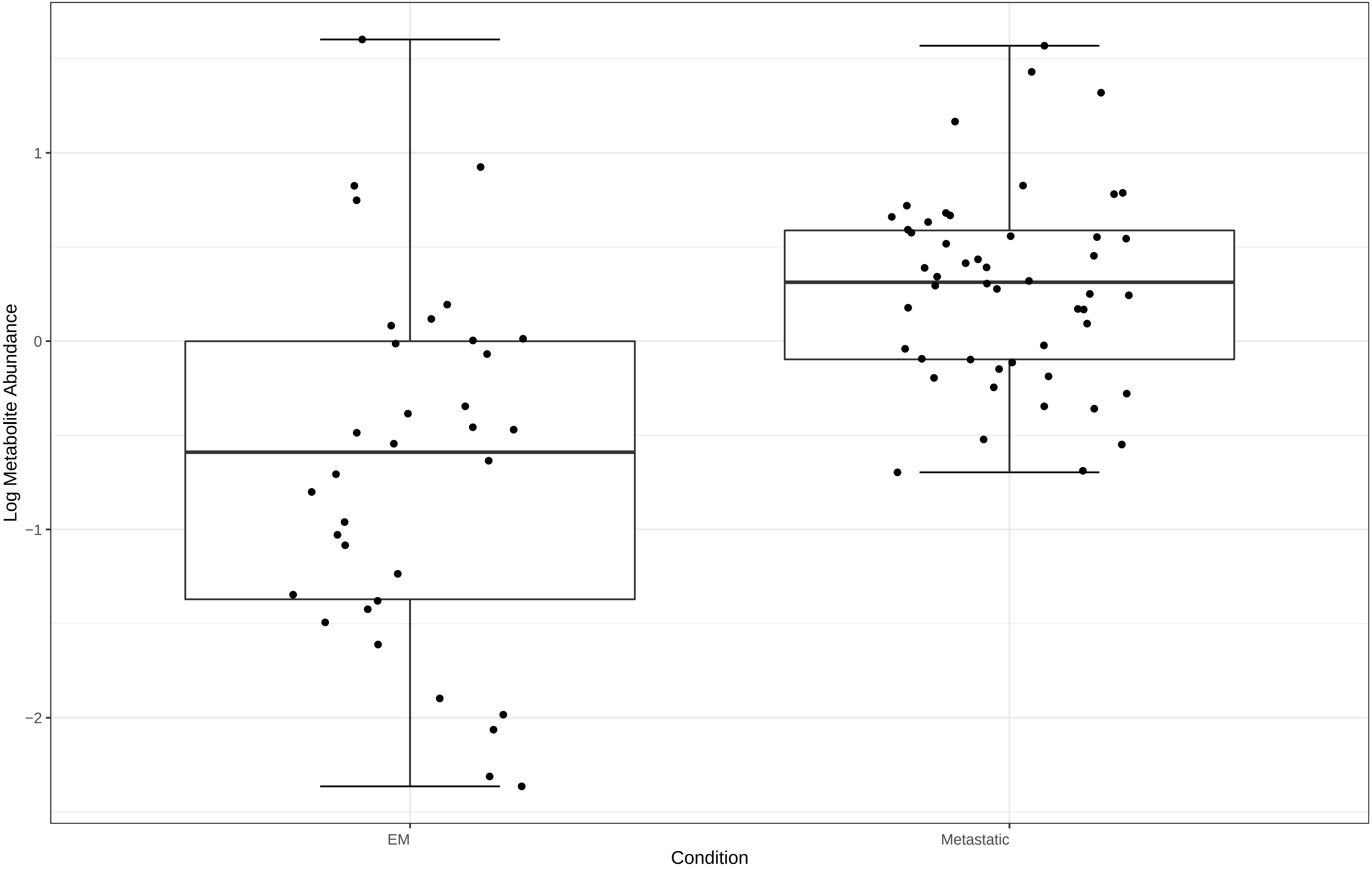

RT: 0.6, m/z: 106.0041, Ion mode: Positive

Adjusted p (t test): 3.84e−04, Adjusted p (wilcox): 2.55e−04

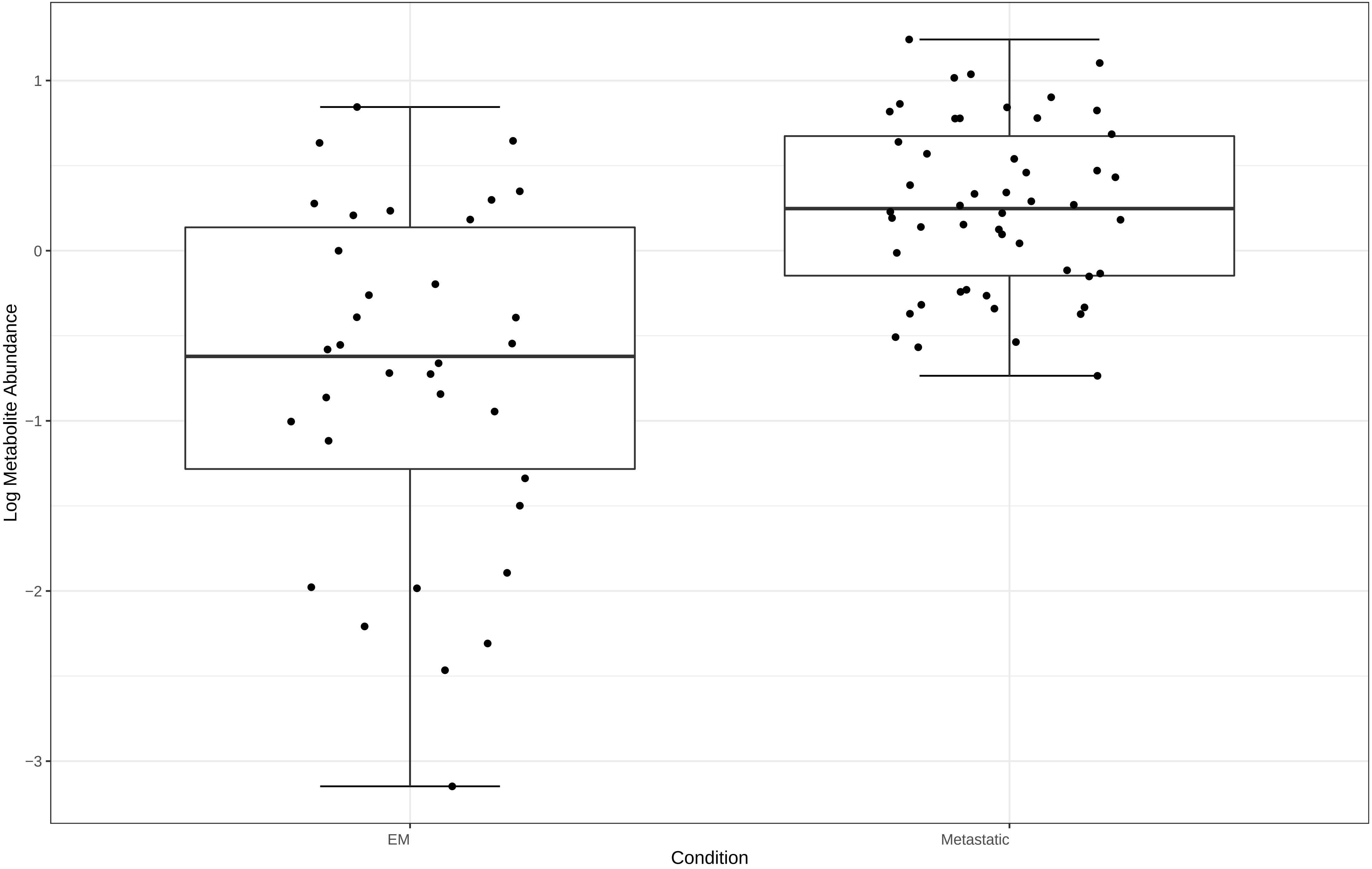

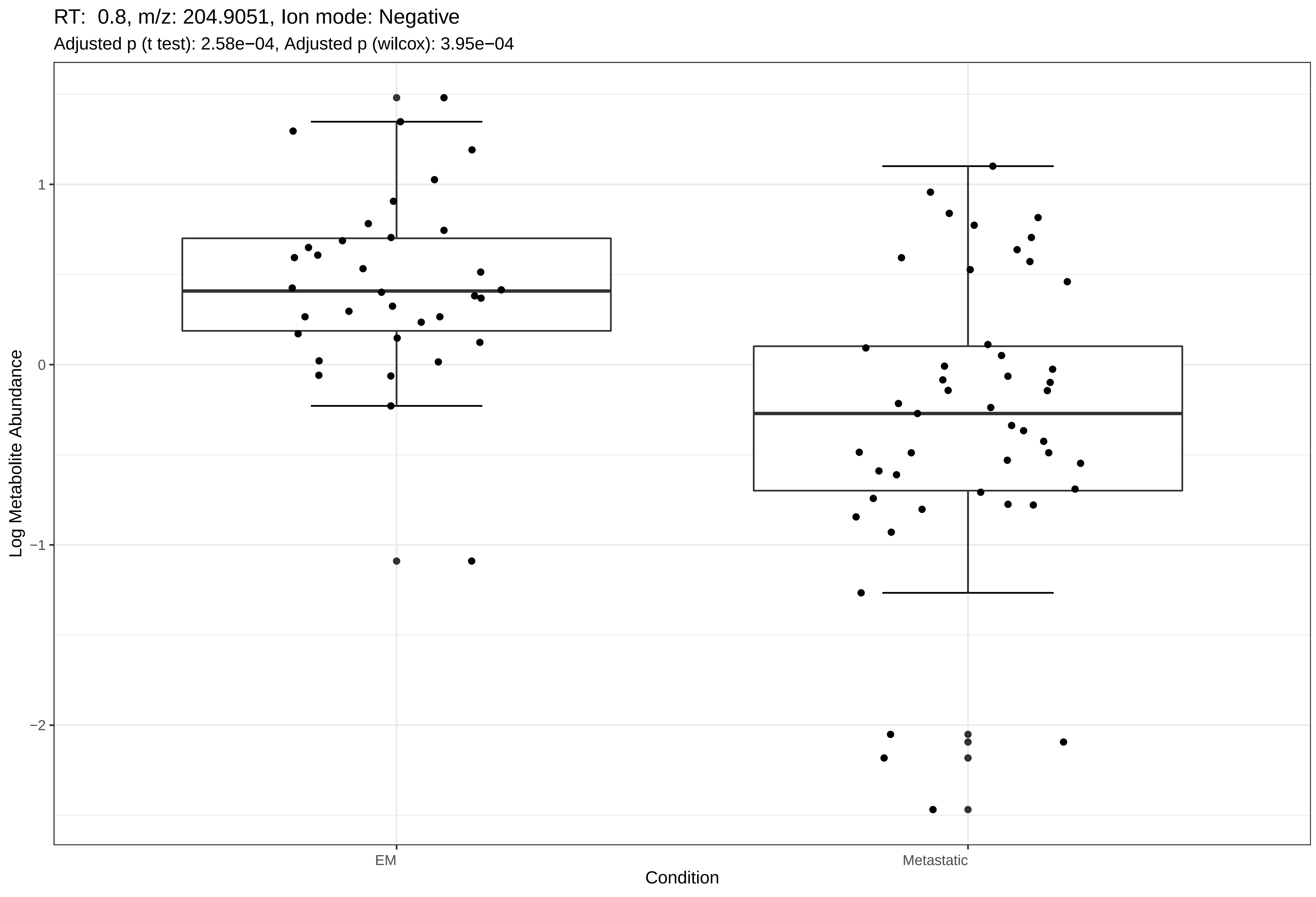

RT: 0.8, m/z: 204.9051, Ion mode: Negative
Adjusted p (t test): 2.58e−04, Adjusted p (wilcox): 3.95e−04
Log Metabolite Abundance
1
0
−1
−2
EM
Metastatic
Condition

RT: 4.4, m/z: 220.9395, Ion mode: Negative

Adjusted p (t test): 2.60e−04, Adjusted p (wilcox): 4.77e−04

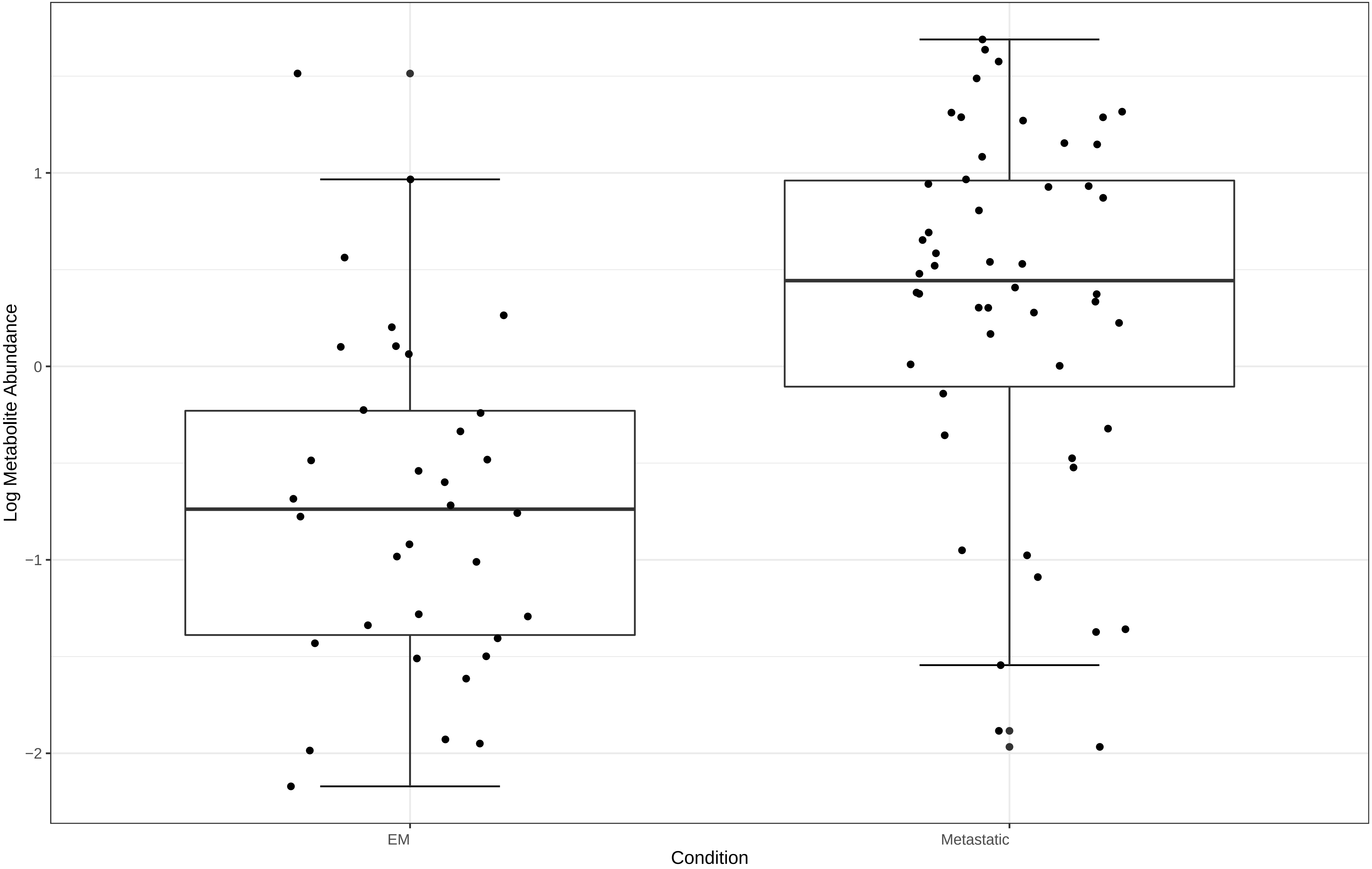

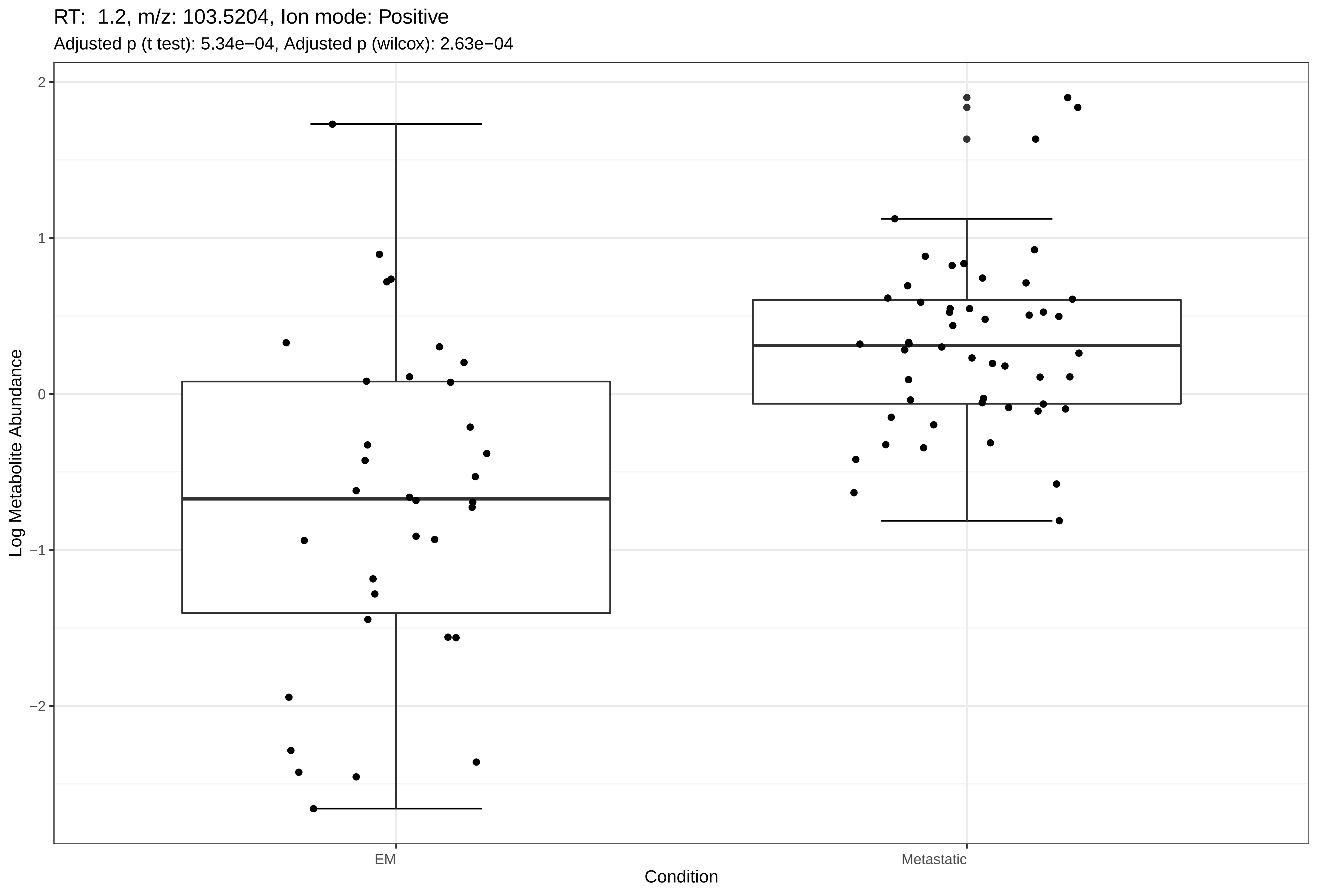
RT: 1.2, m/z: 103.5204, Ion mode: Positive
Adjusted p (t test): 5.34e−04, Adjusted p (wilcox): 2.63e−04
Log Metabolite Abundance
2
1
0
−1
−2
EM
Metastatic
Condition

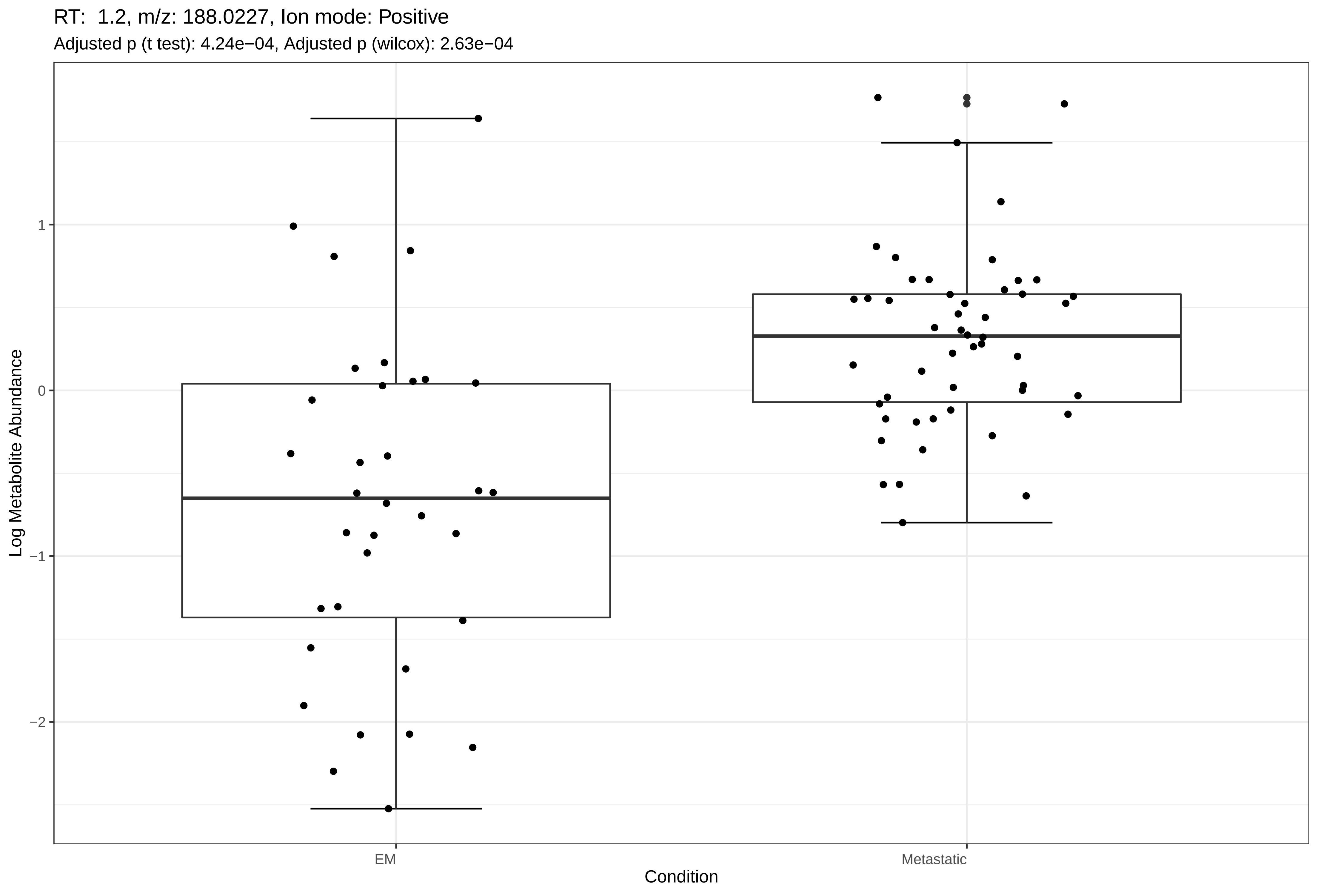

RT: 1.2, m/z: 188.0227, Ion mode: Positive
Adjusted p (t test): 4.24e−04, Adjusted p (wilcox): 2.63e−04
Log Metabolite Abundance
1
0
−1
−2
EM
Metastatic
Condition

RT: 1.2, m/z: 165.0069, Ion mode: Positive

Adjusted p (t test): 5.09e−04, Adjusted p (wilcox): 2.63e−04

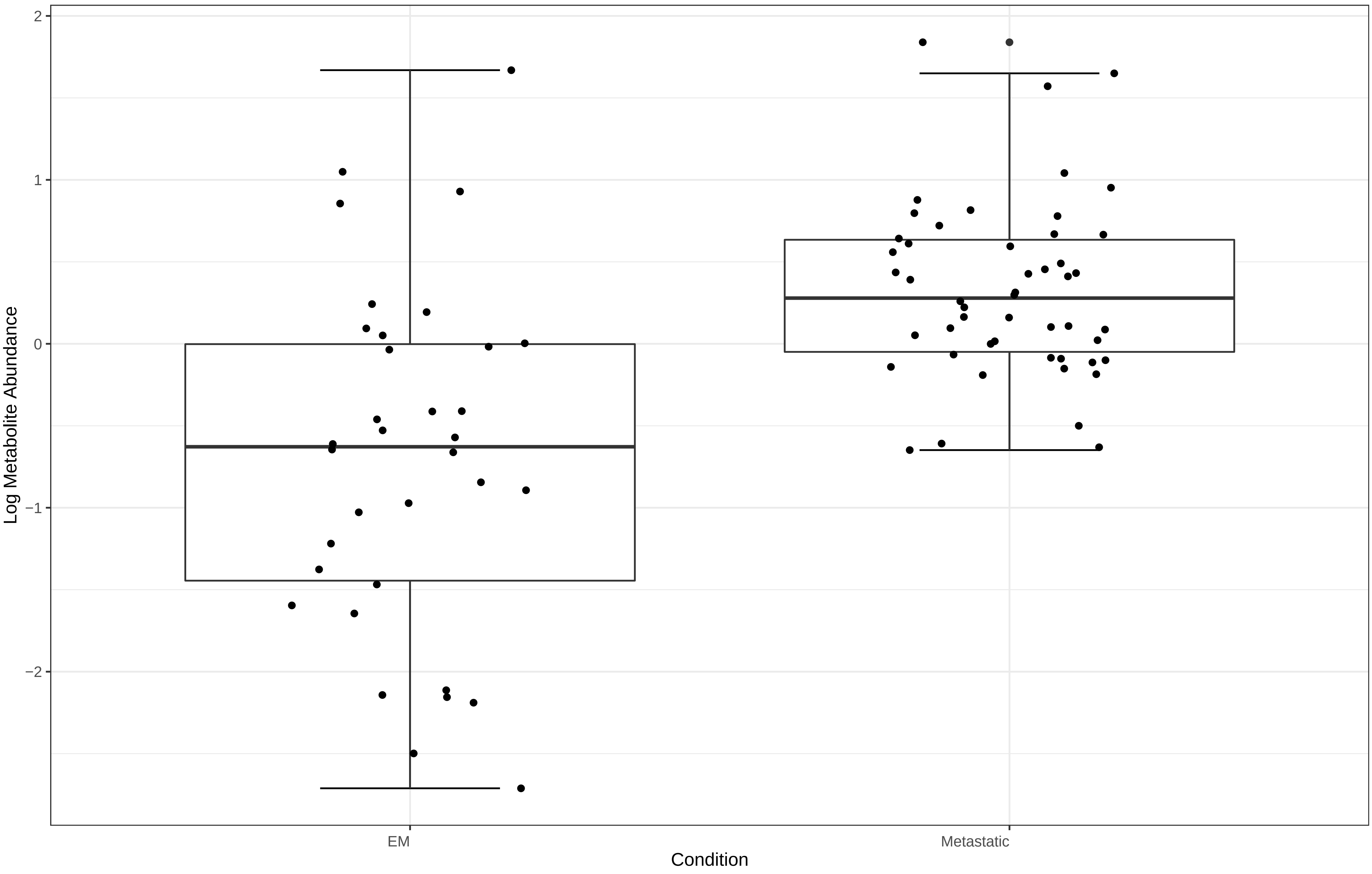

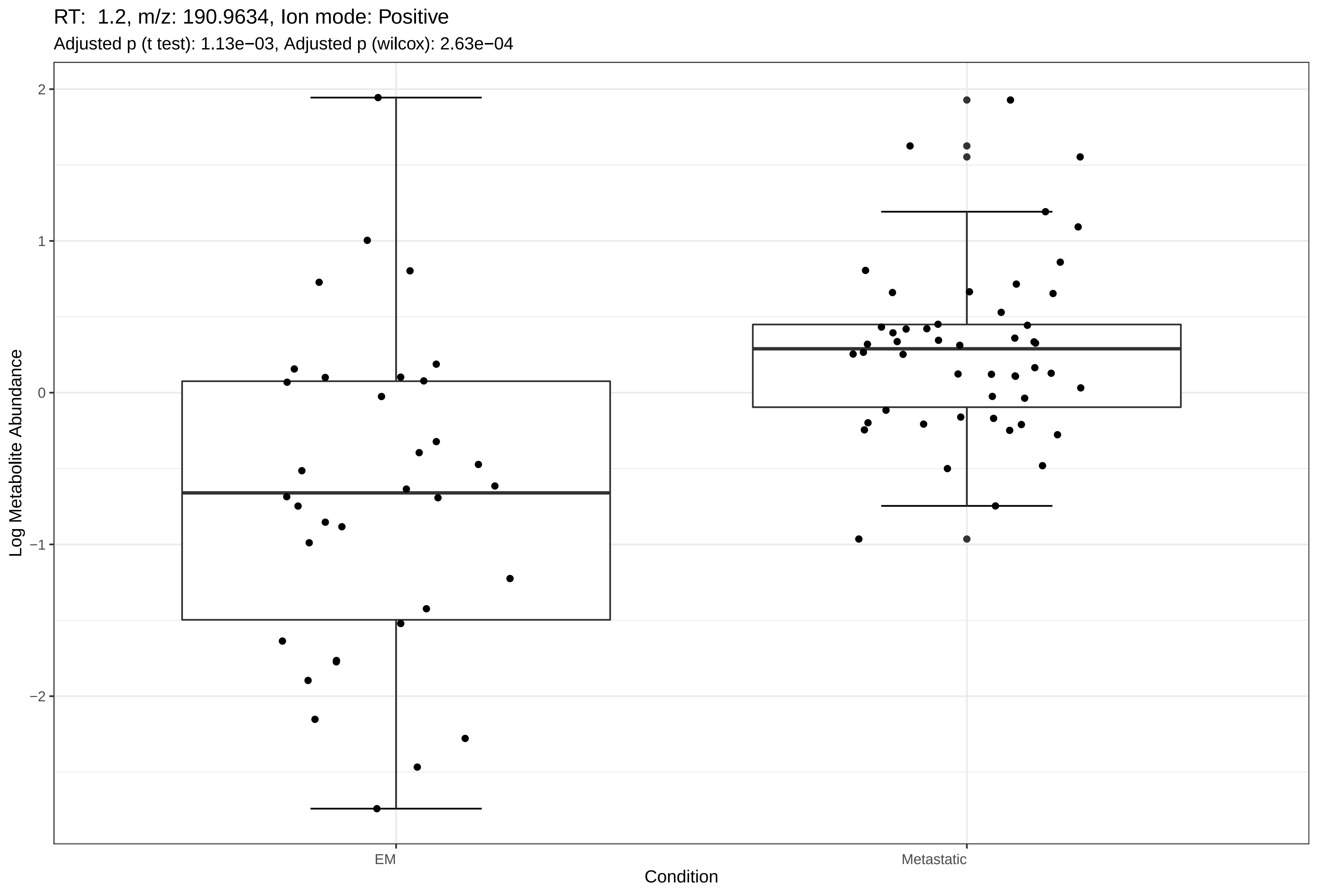

RT: 1.2, m/z: 190.9634, Ion mode: Positive
Adjusted p (t test): 1.13e−03, Adjusted p (wilcox): 2.63e−04
2
1
0
−1
−2
Log Metabolite Abundance
EM
Metastatic
Condition

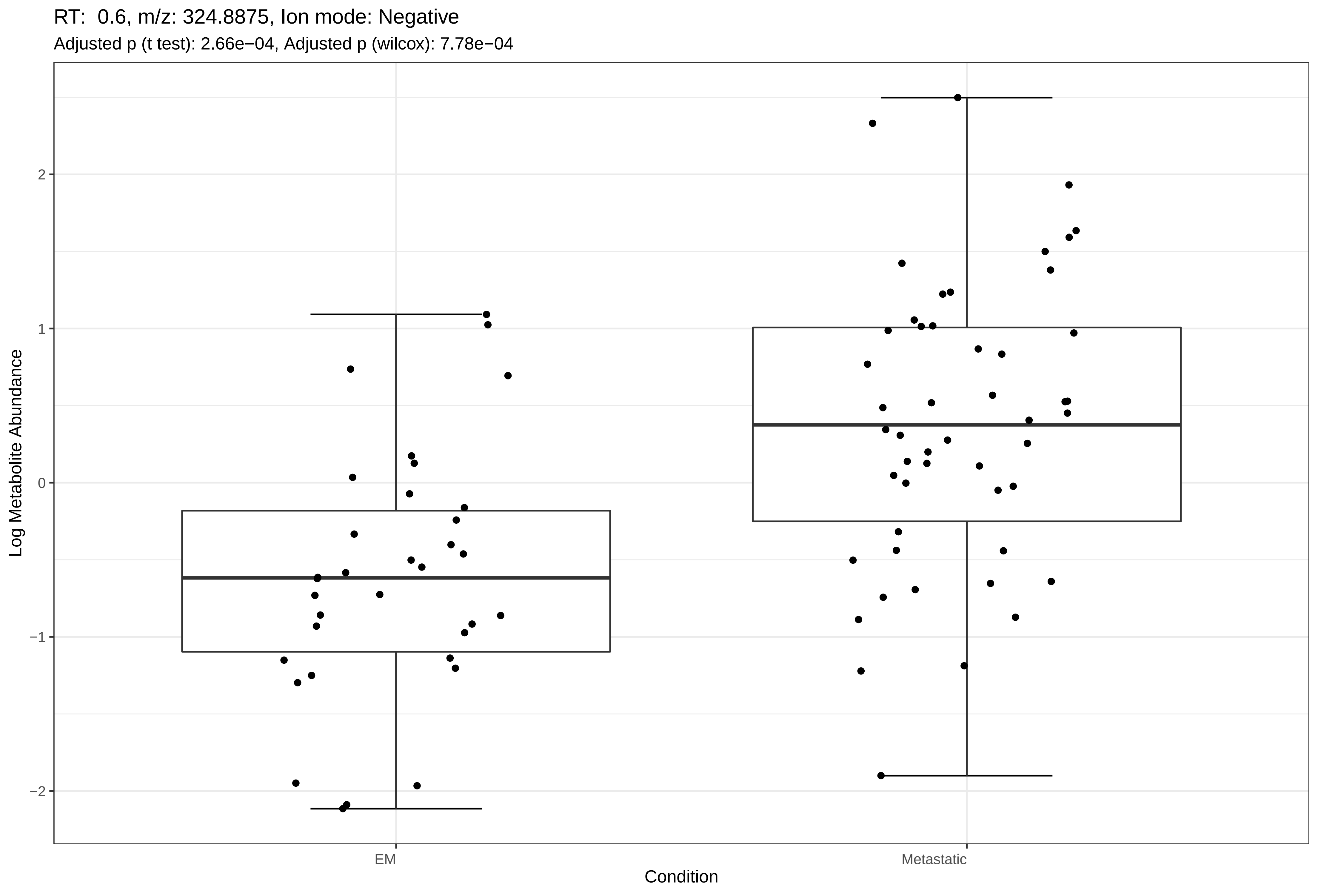
RT: 0.6, m/z: 324.8875, Ion mode: Negative
Adjusted p (t test): 2.66e−04, Adjusted p (wilcox): 7.78e−04
Log Metabolite Abundance
2
1
0
−1
−2
EM
Metastatic
Condition

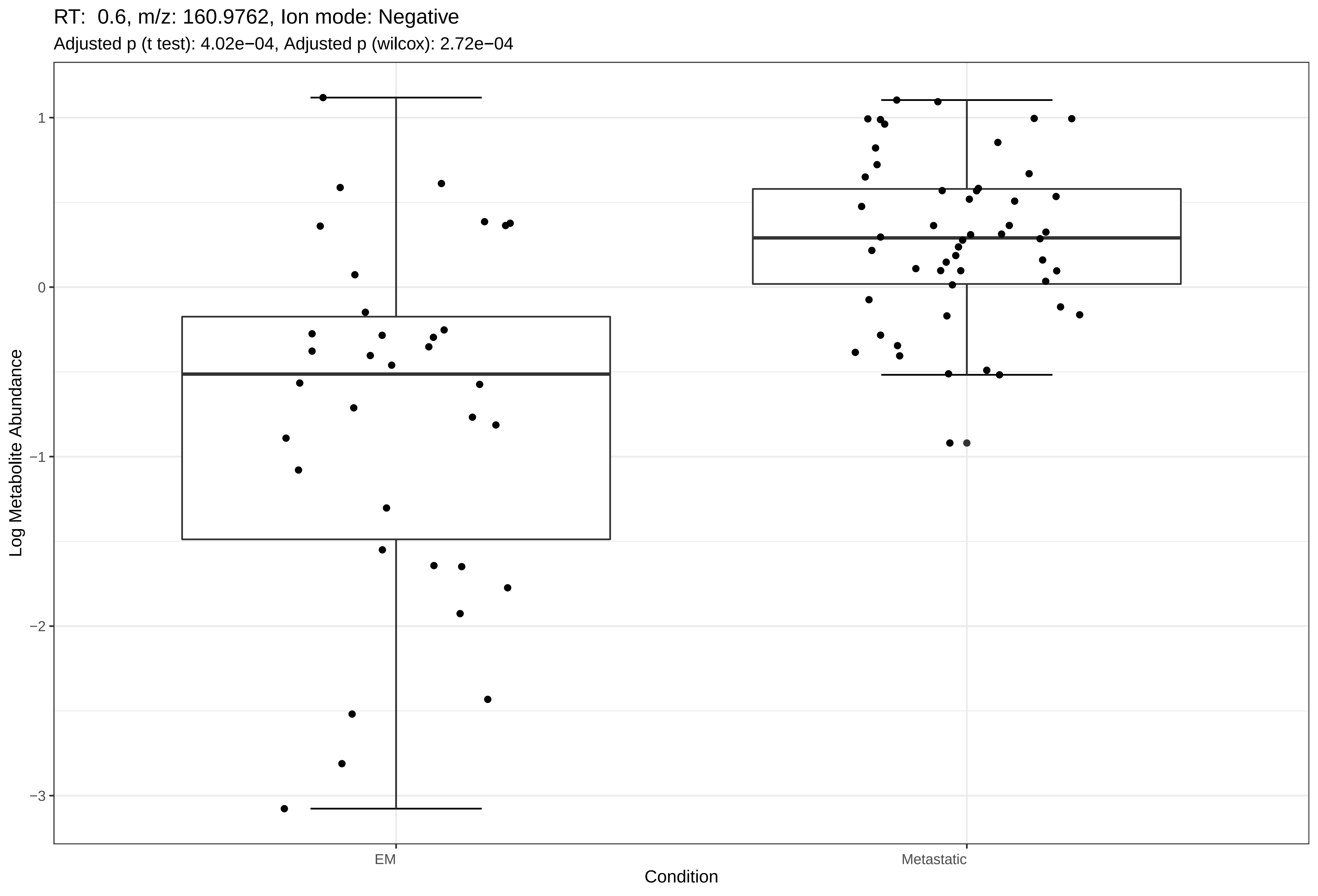

RT: 0.6, m/z: 160.9762, Ion mode: Negative
Adjusted p (t test): 4.02e−04, Adjusted p (wilcox): 2.72e−04
Log Metabolite Abundance
1
0
−1
−2
−3
EM
Metastatic
Condition

RT: 0.6, m/z: 105.0033, Ion mode: Positive

Adjusted p (t test): 3.68e−04, Adjusted p (wilcox): 2.72e−04

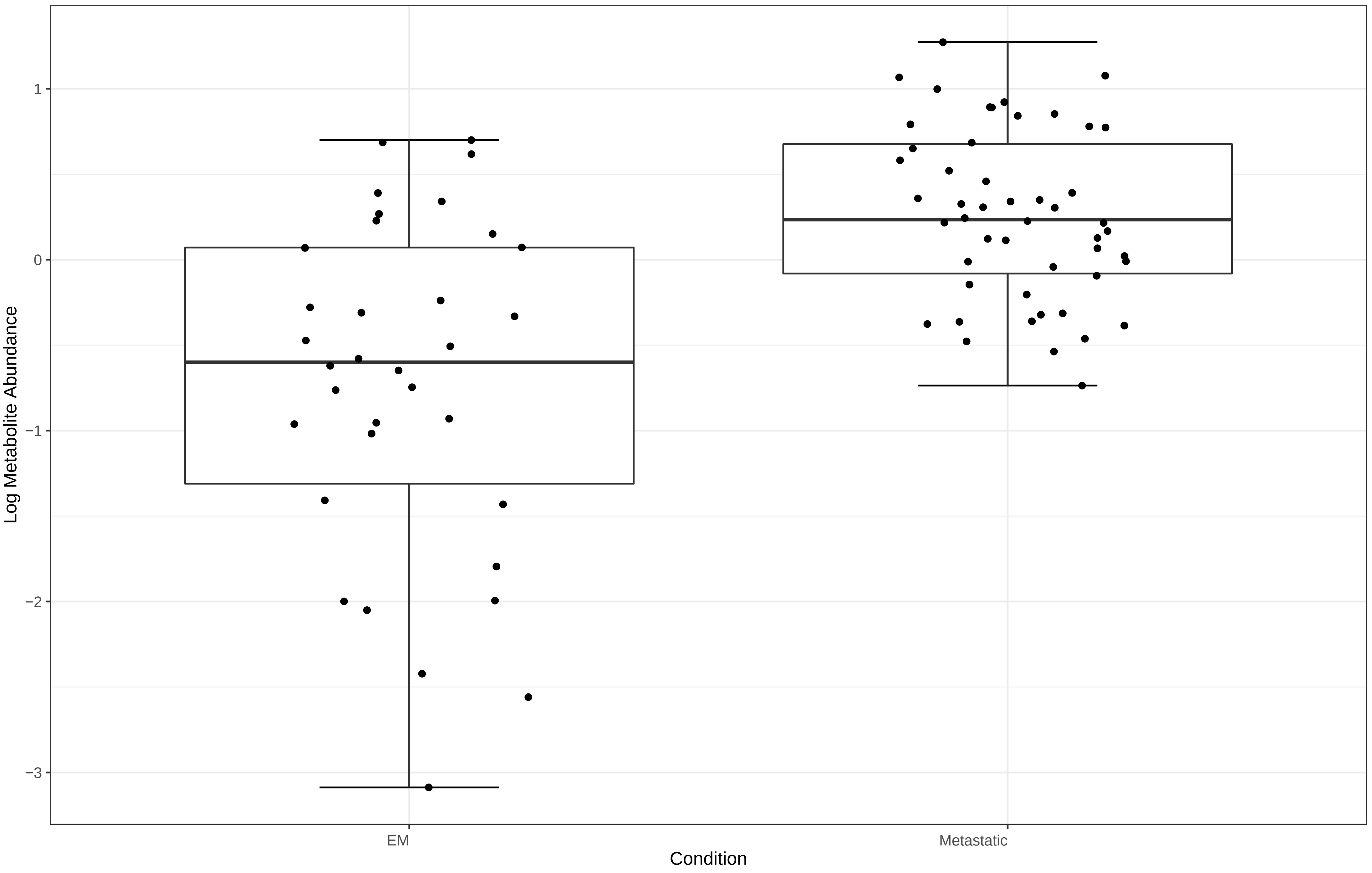

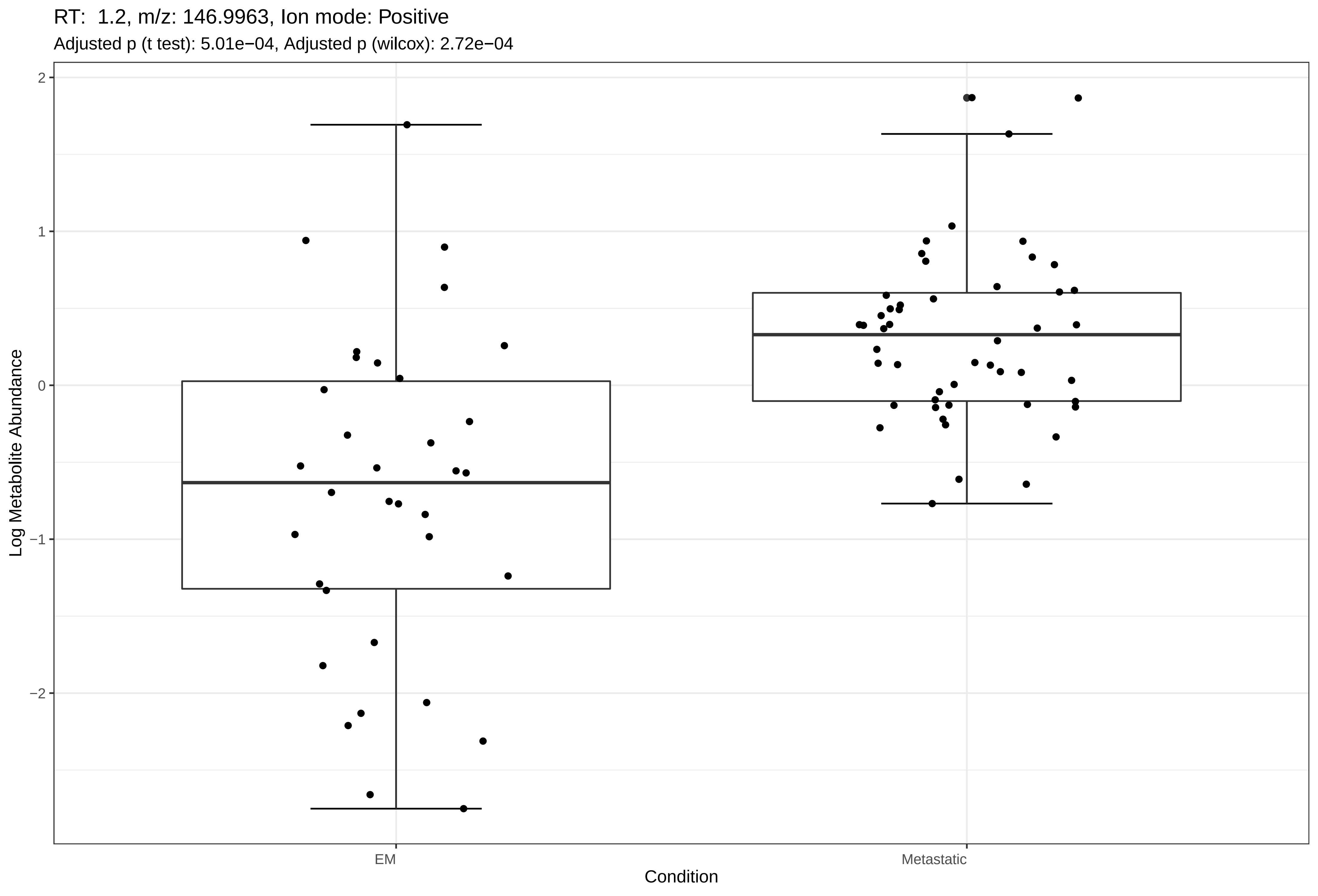
RT: 1.2, m/z: 146.9963, Ion mode: Positive
Adjusted p (t test): 5.01e−04, Adjusted p (wilcox): 2.72e−04
Log Metabolite Abundance
2
1
0
−1
−2
EM
Metastatic
Condition

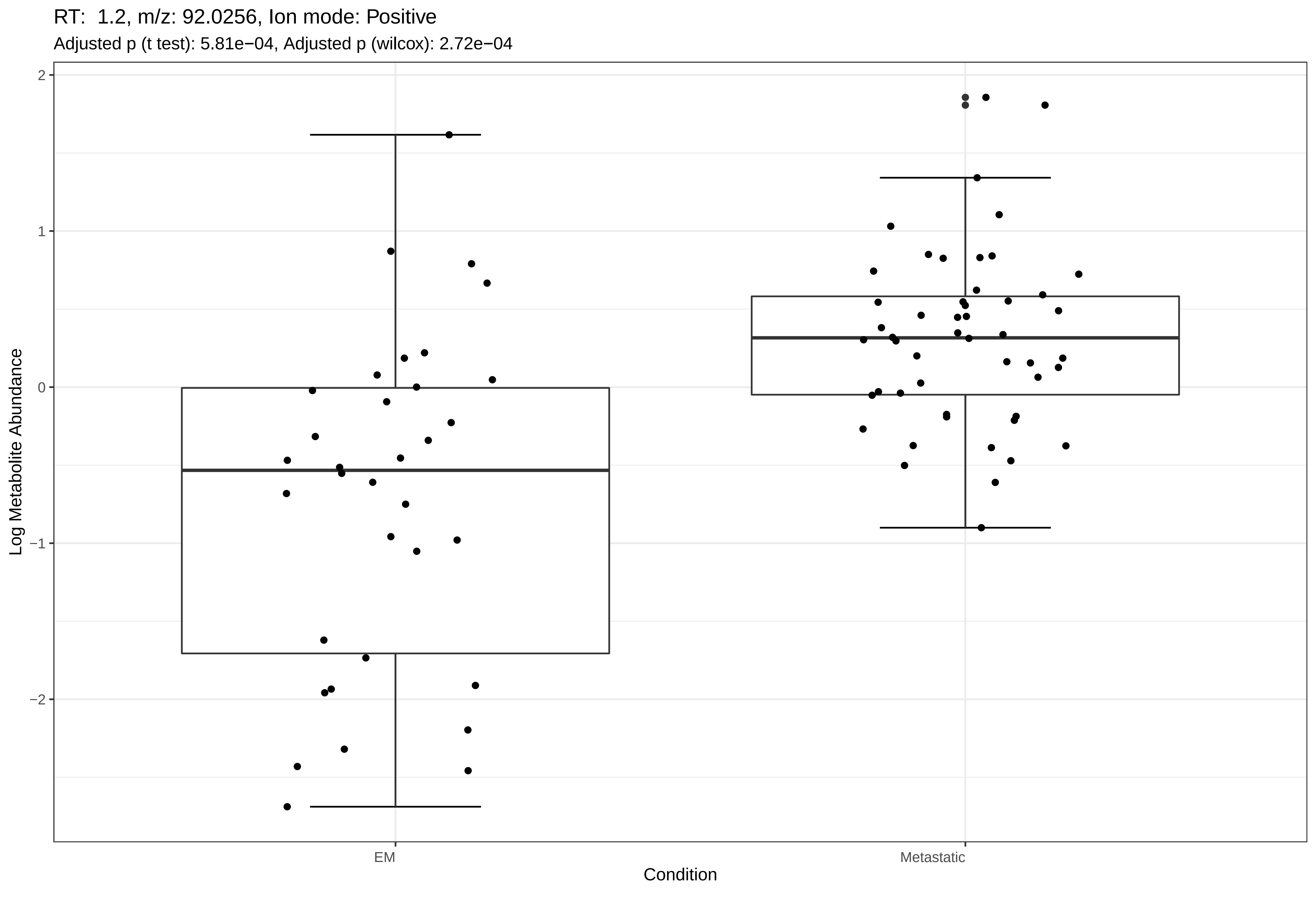

RT: 1.2, m/z: 92.0256, Ion mode: Positive
Adjusted p (t test): 5.81e−04, Adjusted p (wilcox): 2.72e−04
Log Metabolite Abundance
2
1
0
−1
−2
EM
Metastatic
Condition

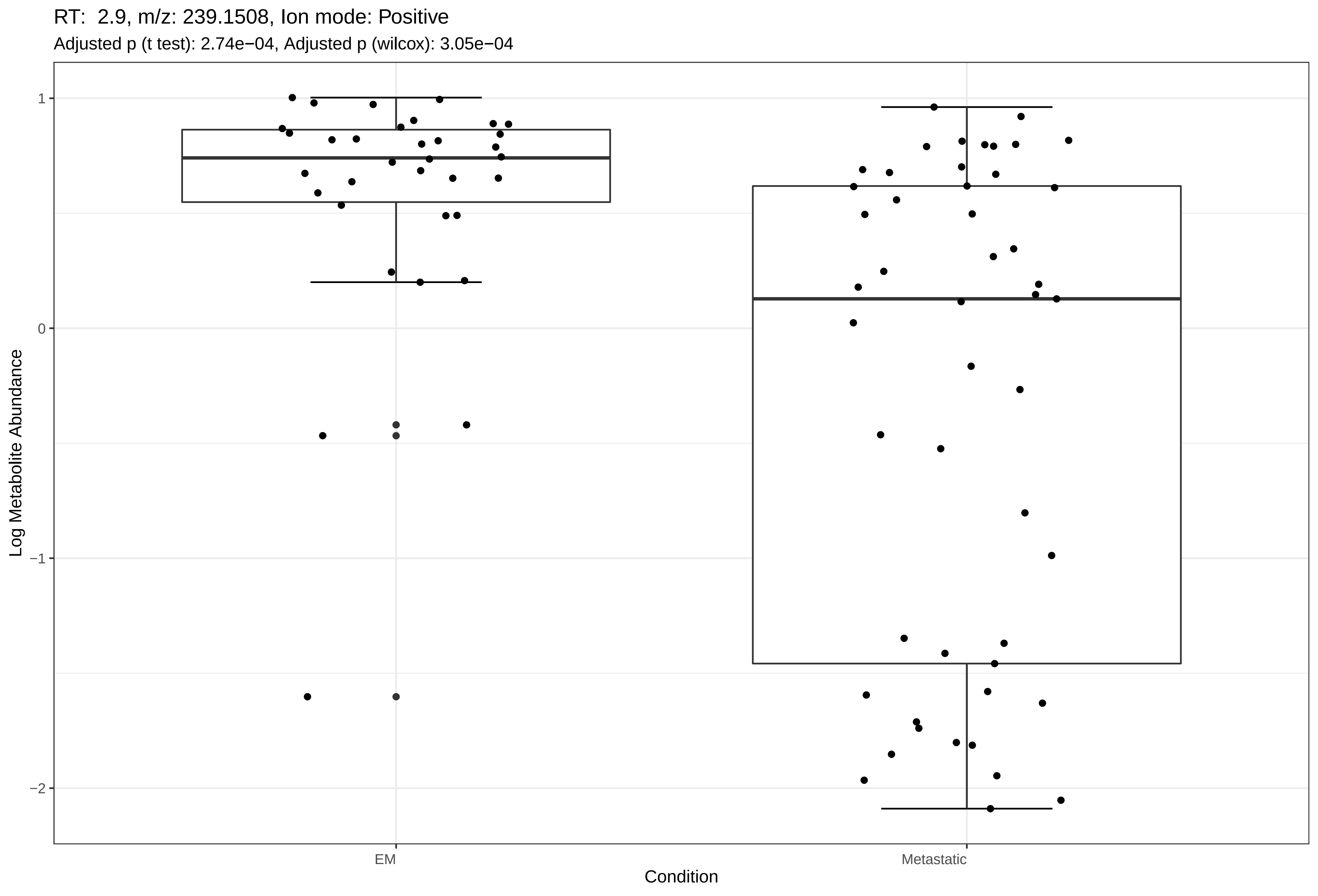
RT: 2.9, m/z: 239.1508, Ion mode: Positive
Adjusted p (t test): 2.74e−04, Adjusted p (wilcox): 3.05e−04
Log Metabolite Abundance
1
0
−1
−2
EM
Metastatic
Condition

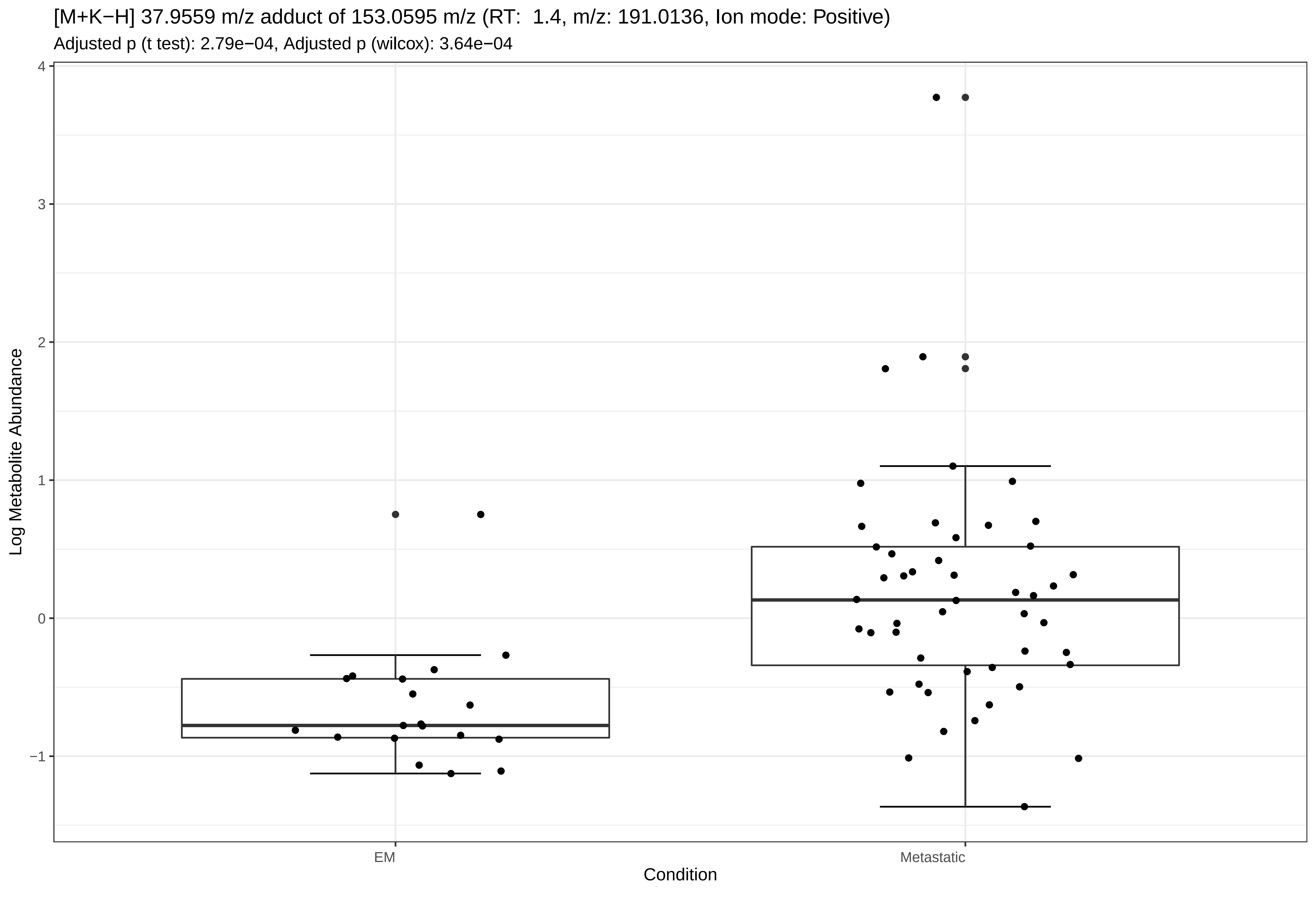

[M+K−H] 37.9559 m/z adduct of 153.0595 m/z (RT: 1.4, m/z: 191.0136, Ion mode: Positive)
Adjusted p (t test): 2.79e−04, Adjusted p (wilcox): 3.64e−04
Log Metabolite Abundance
4
3
2
1
0
−1
EM
Metastatic
Condition

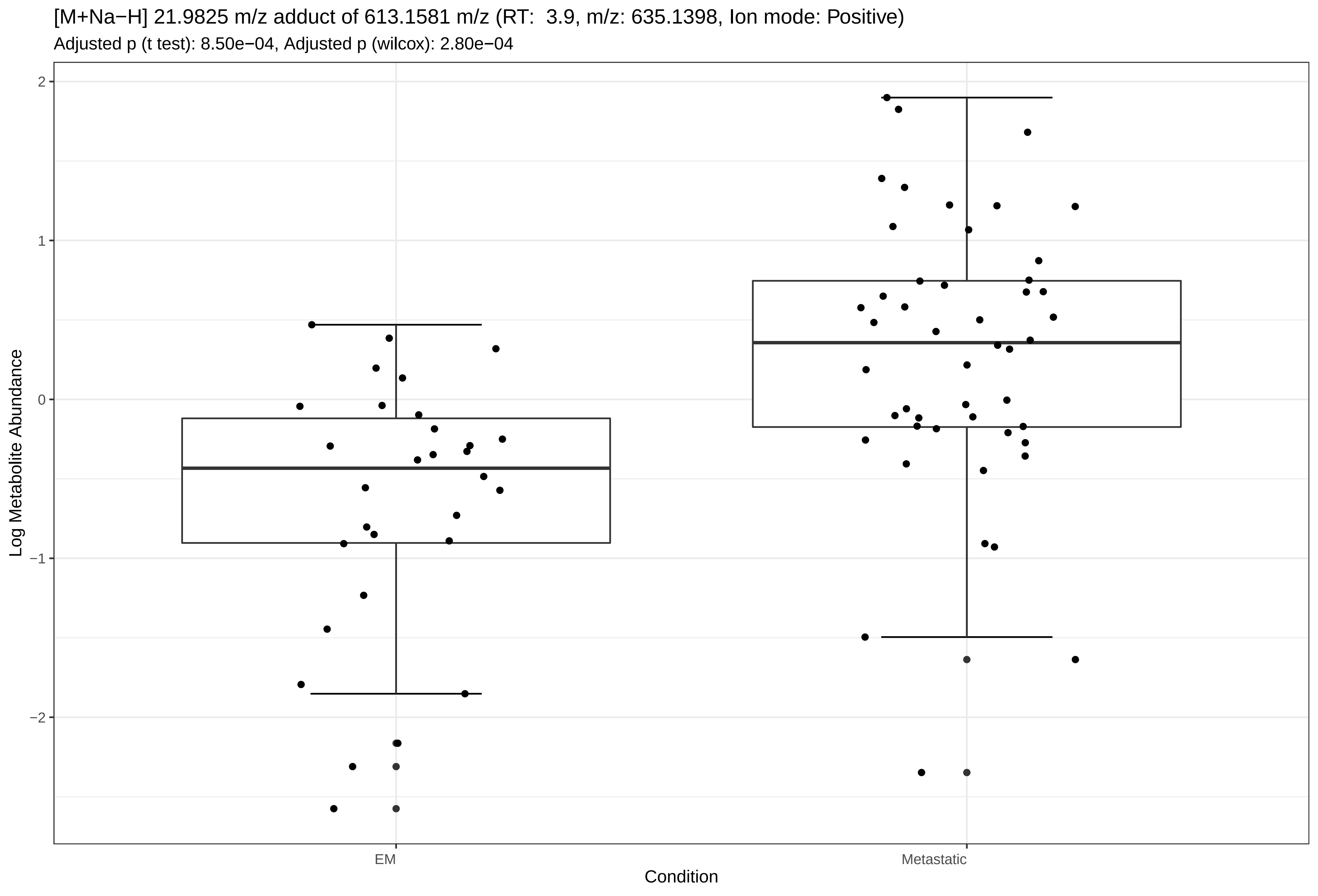
[M+Na−H] 21.9825 m/z adduct of 613.1581 m/z (RT: 3.9, m/z: 635.1398, Ion mode: Positive)
Adjusted p (t test): 8.50e−04, Adjusted p (wilcox): 2.80e−04
Log Metabolite Abundance
2
1
0
−1
−2
EM
Metastatic
Condition

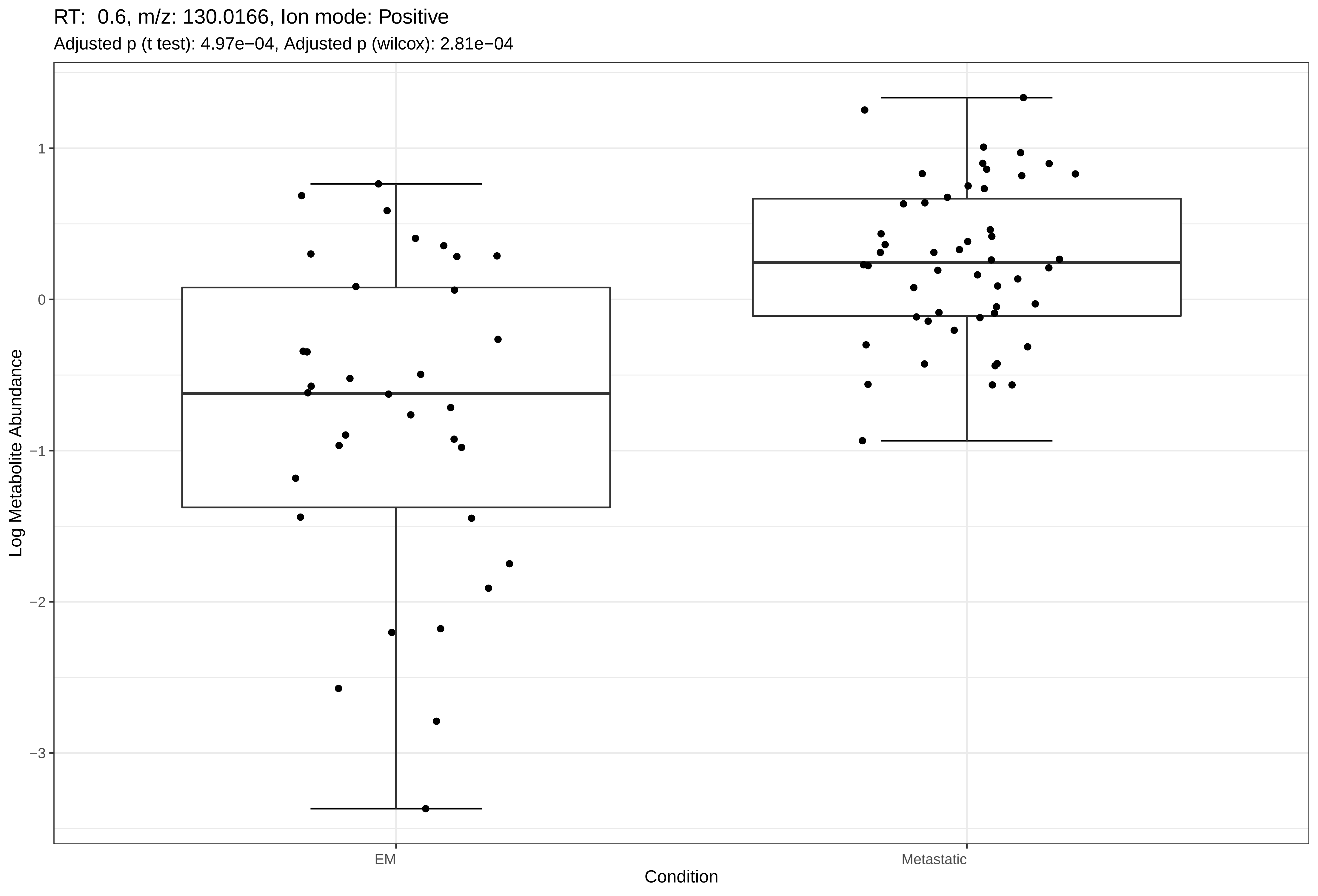
RT: 0.6, m/z: 130.0166, Ion mode: Positive
Adjusted p (t test): 4.97e−04, Adjusted p (wilcox): 2.81e−04
Log Metabolite Abundance
1
0
−1
−2
−3
EM
Metastatic
Condition

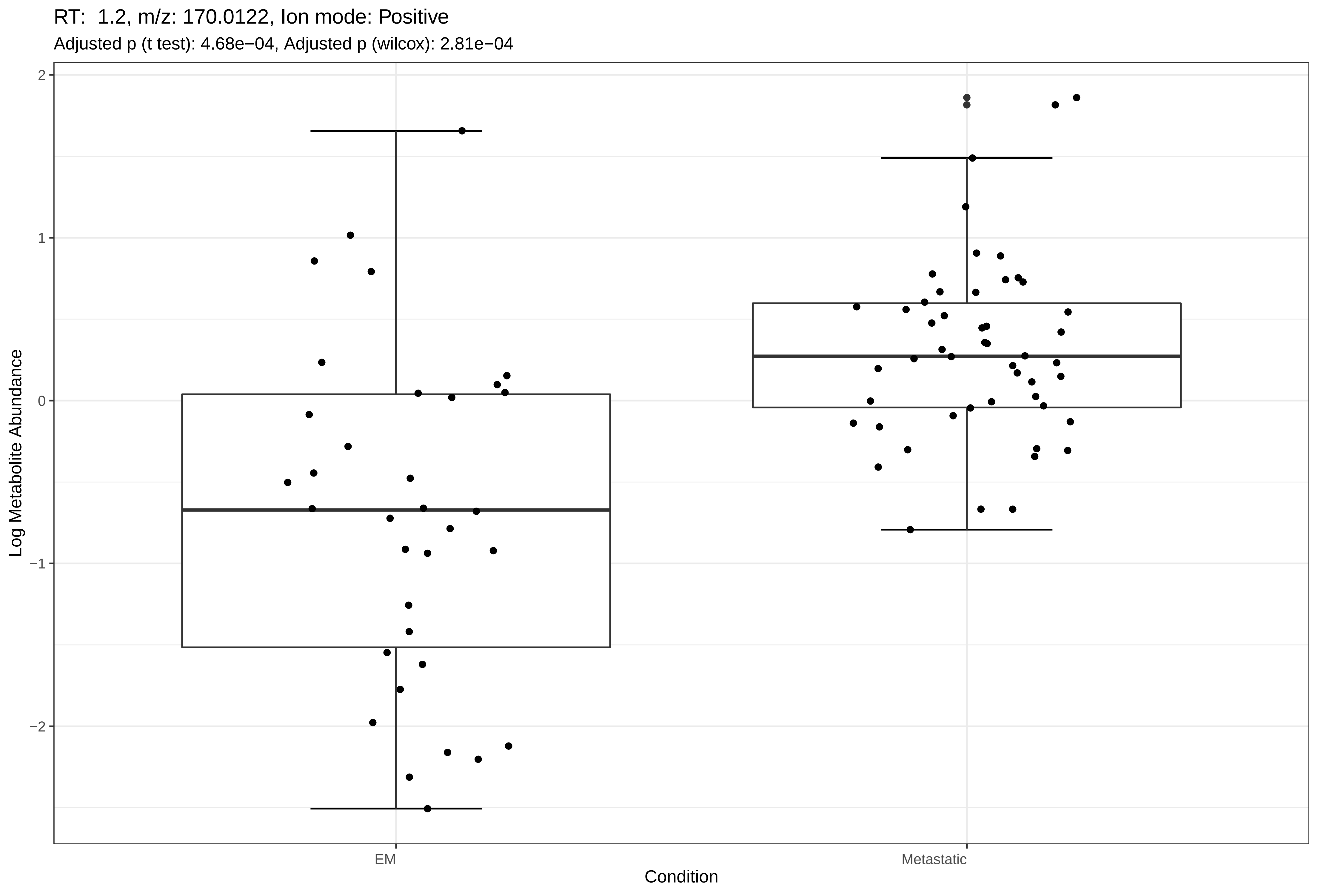

RT: 1.2, m/z: 170.0122, Ion mode: Positive
Adjusted p (t test): 4.68e−04, Adjusted p (wilcox): 2.81e−04
Log Metabolite Abundance
2
1
0
−1
−2
EM
Metastatic
Condition

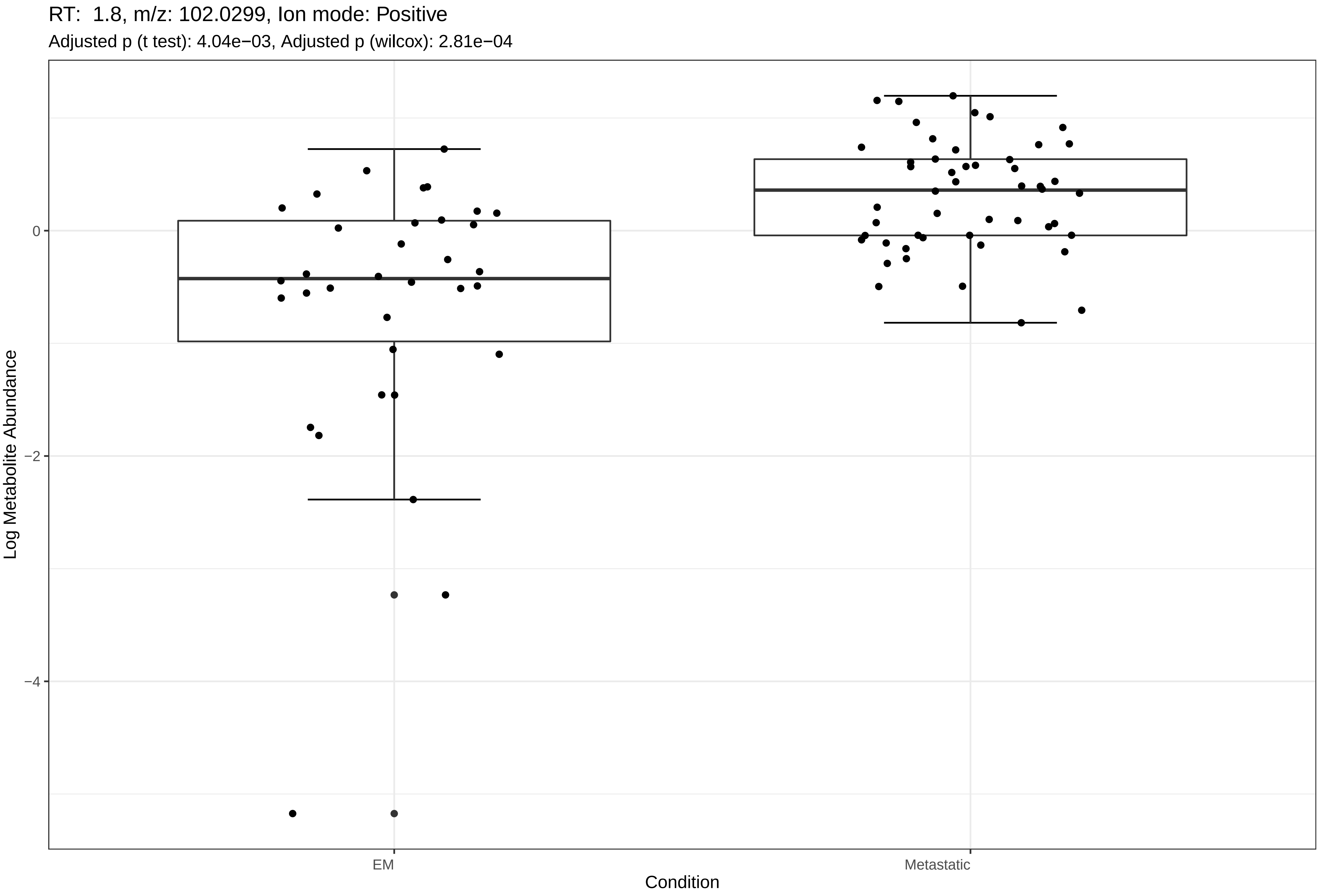
RT: 1.8, m/z: 102.0299, Ion mode: Positive
Adjusted p (t test): 4.04e−03, Adjusted p (wilcox): 2.81e−04
Log Metabolite Abundance
0
−2
−4
EM
Metastatic
Condition

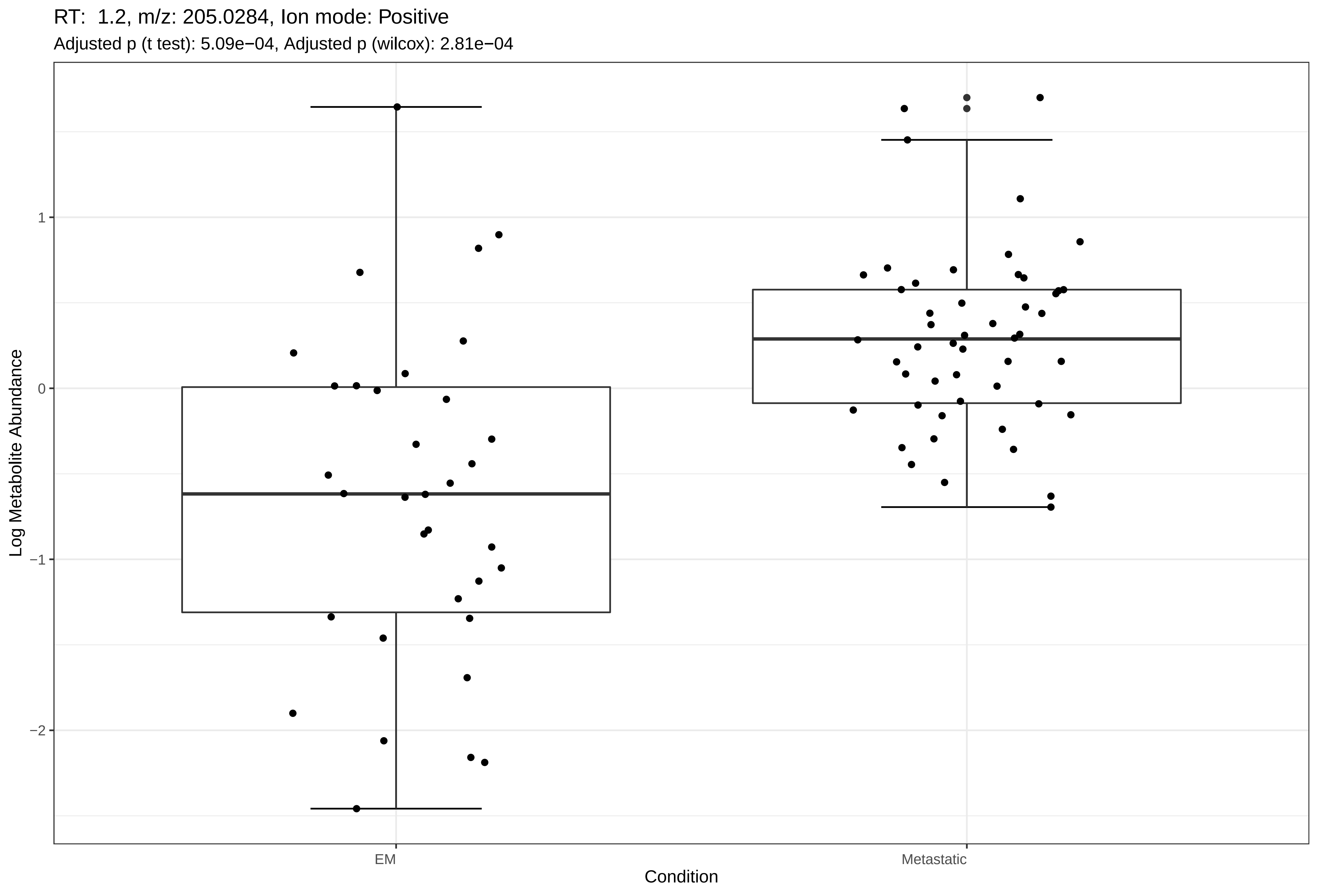
RT: 1.2, m/z: 205.0284, Ion mode: Positive
Adjusted p (t test): 5.09e−04, Adjusted p (wilcox): 2.81e−04
Log Metabolite Abundance
1
0
−1
−2
EM
Metastatic
Condition

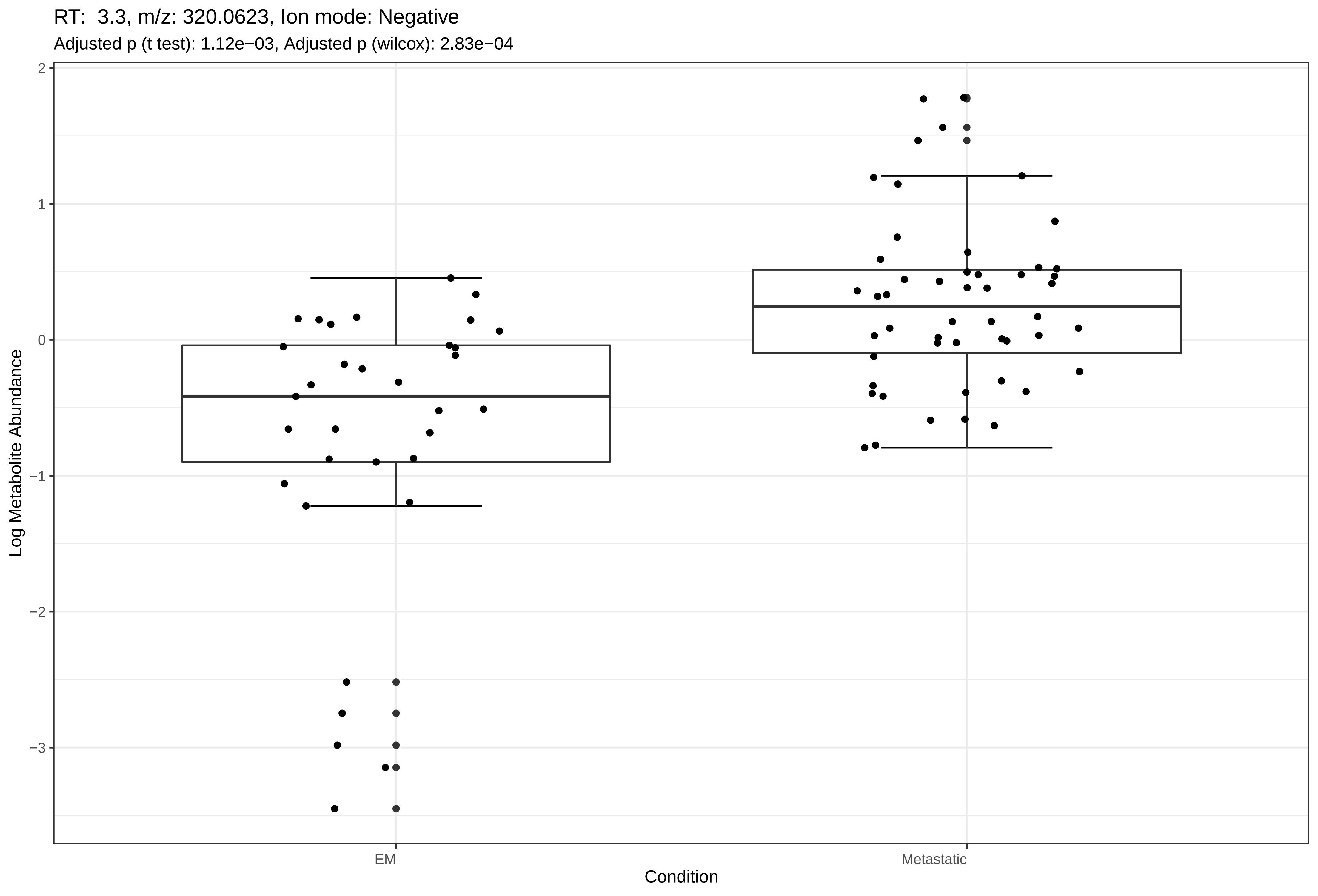

RT: 3.3, m/z: 320.0623, Ion mode: Negative
Adjusted p (t test): 1.12e−03, Adjusted p (wilcox): 2.83e−04
2
1
0
−1
−2
−3
Log Metabolite Abundance
EM
Metastatic
Condition

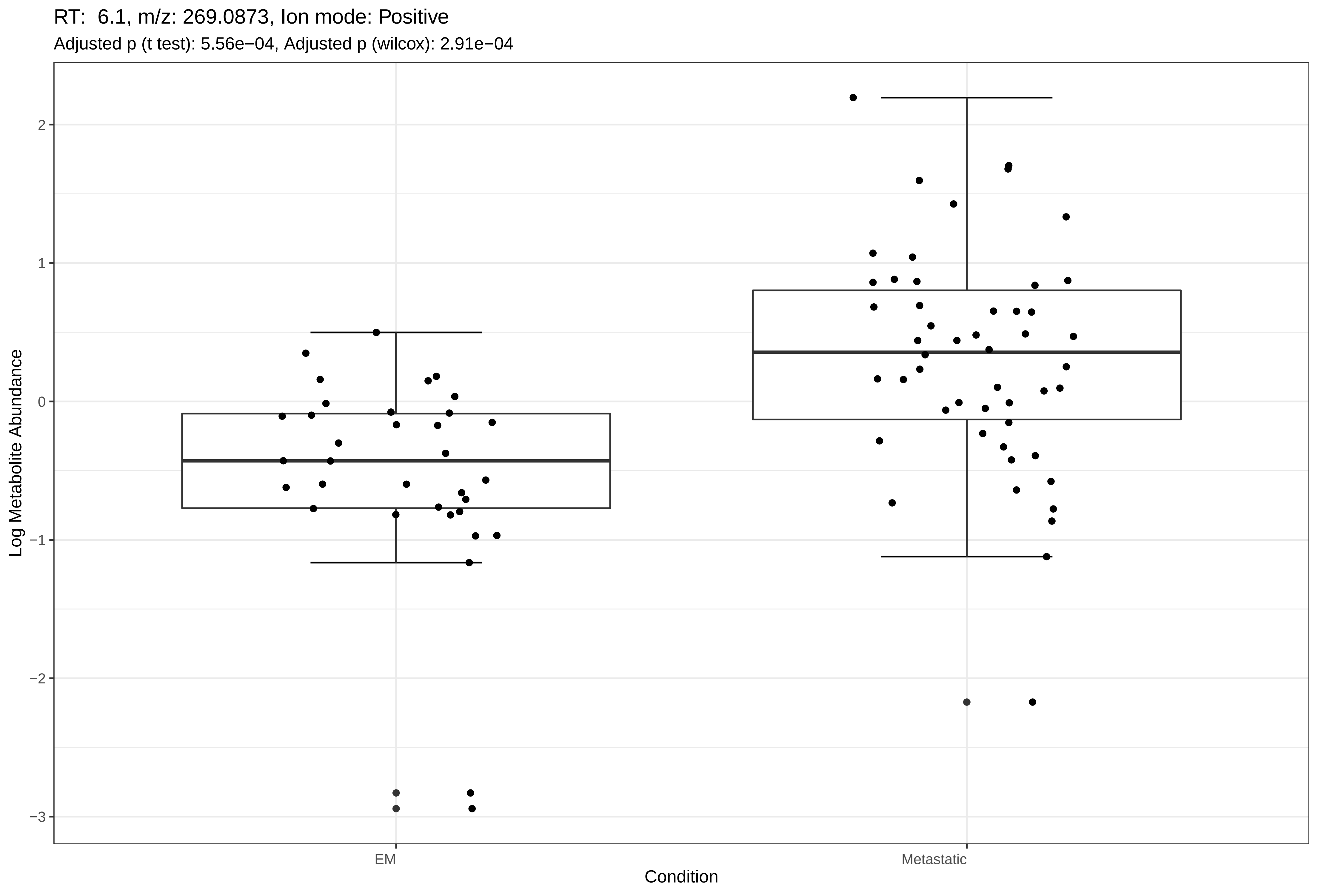
RT: 6.1, m/z: 269.0873, Ion mode: Positive
Adjusted p (t test): 5.56e−04, Adjusted p (wilcox): 2.91e−04
Log Metabolite Abundance
2
1
0
−1
−2
−3
EM
Metastatic
Condition

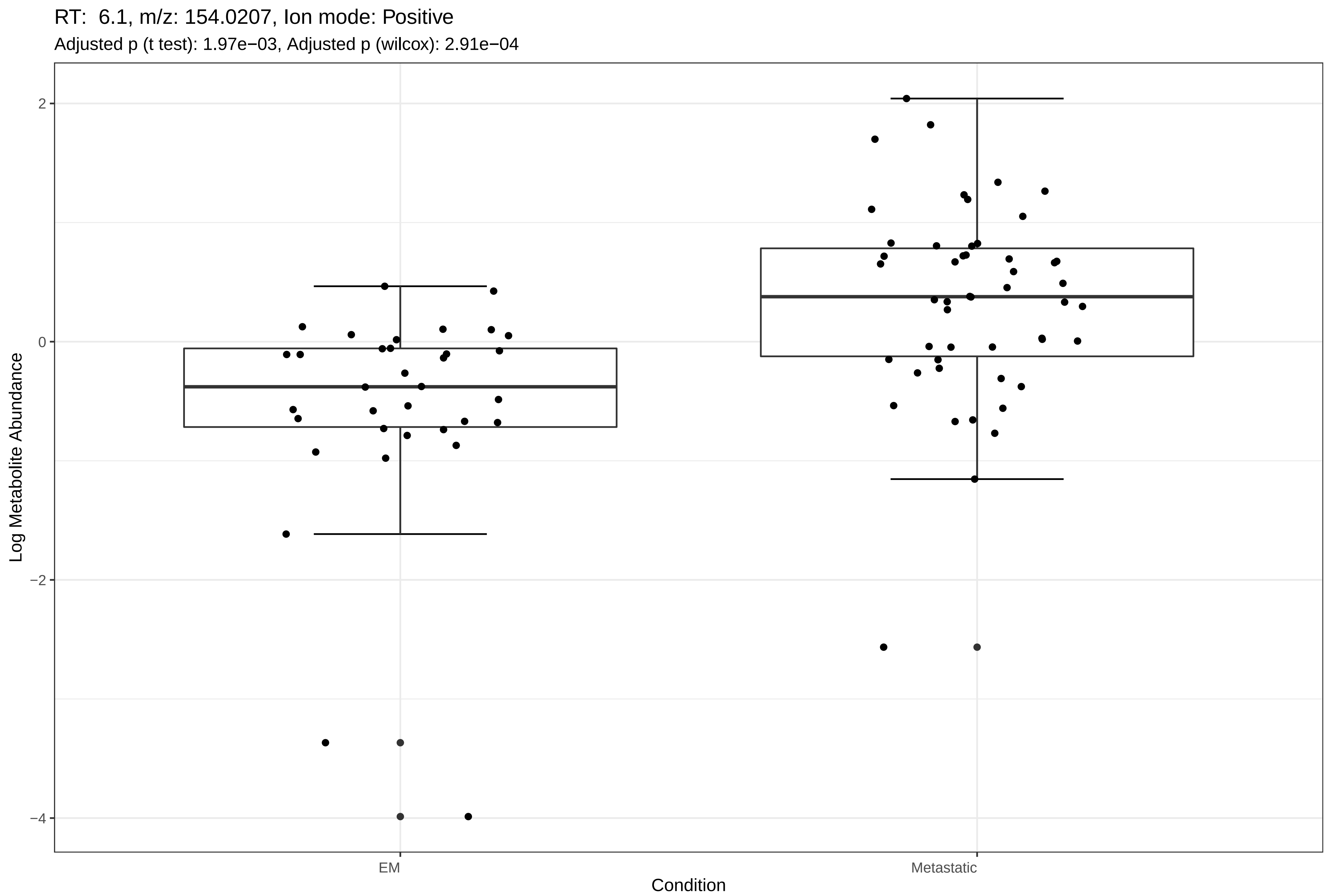
RT: 6.1, m/z: 154.0207, Ion mode: Positive
Adjusted p (t test): 1.97e−03, Adjusted p (wilcox): 2.91e−04
Log Metabolite Abundance
2
0
−2
−4
EM
Metastatic
Condition

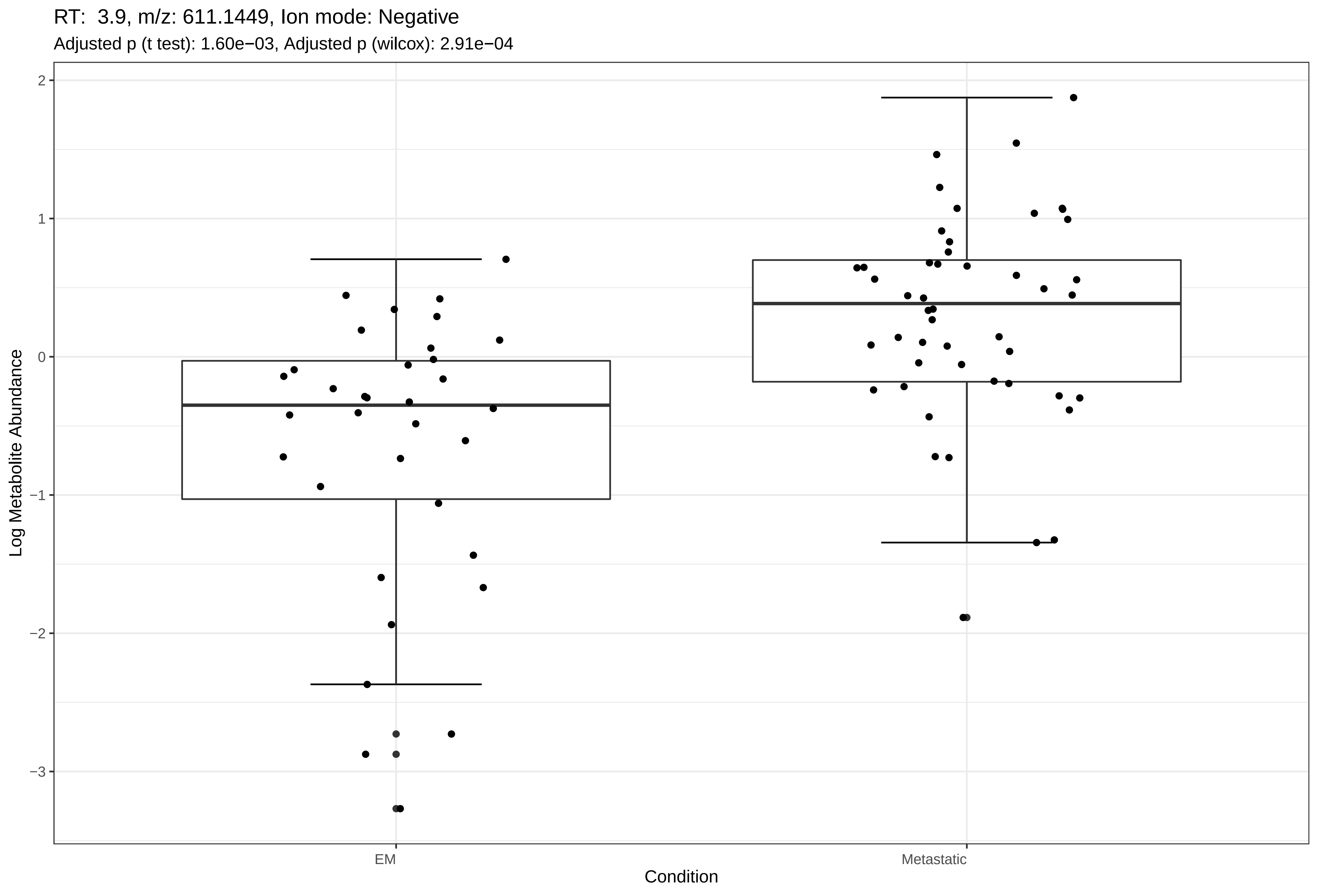

RT: 3.9, m/z: 611.1449, Ion mode: Negative
Adjusted p (t test): 1.60e−03, Adjusted p (wilcox): 2.91e−04
Log Metabolite Abundance
2
1
0
−1
−2
−3
EM
Metastatic
Condition

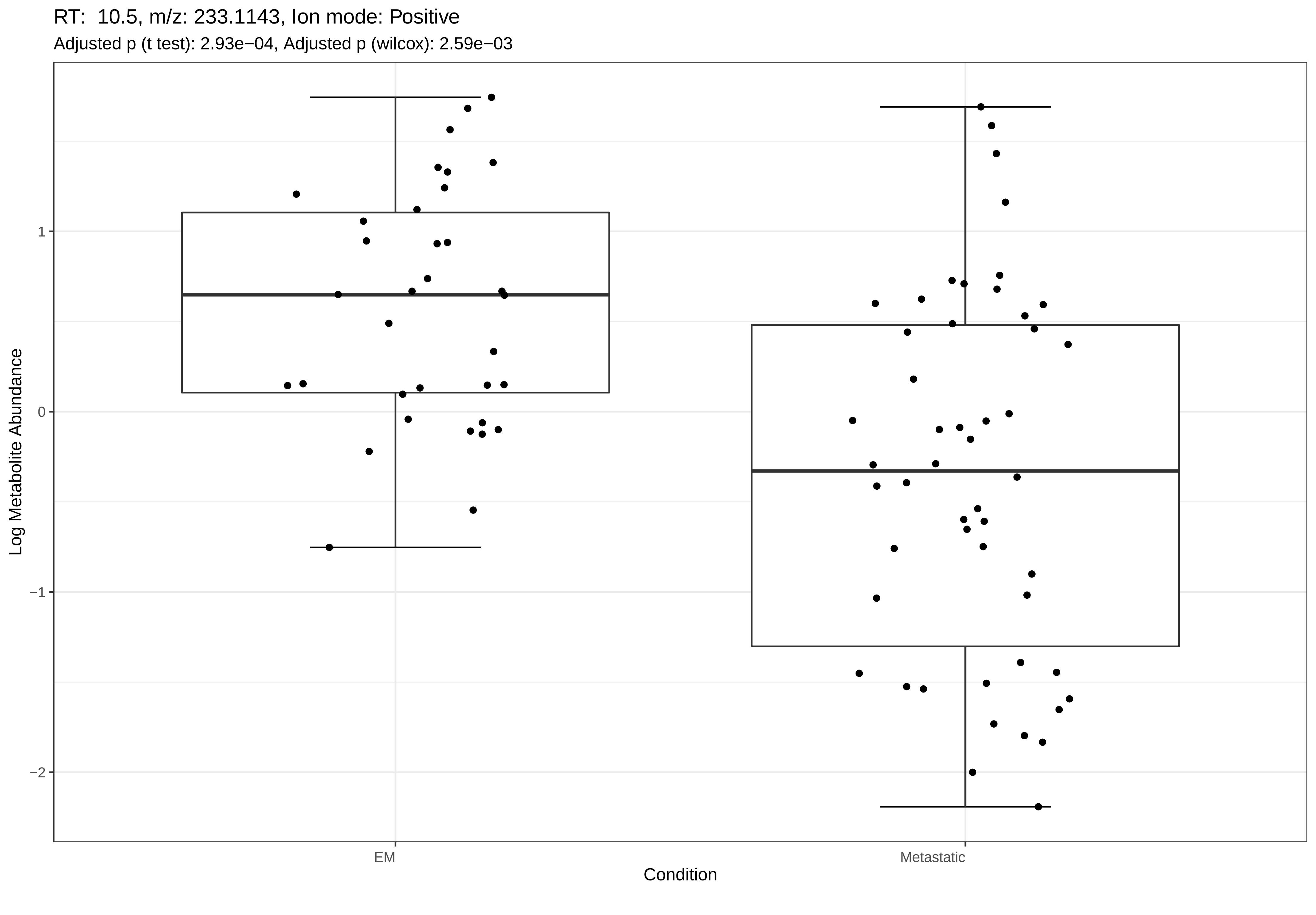
RT: 10.5, m/z: 233.1143, Ion mode: Positive
Adjusted p (t test): 2.93e−04, Adjusted p (wilcox): 2.59e−03
Log Metabolite Abundance
1
0
−1
−2
EM
Metastatic
Condition

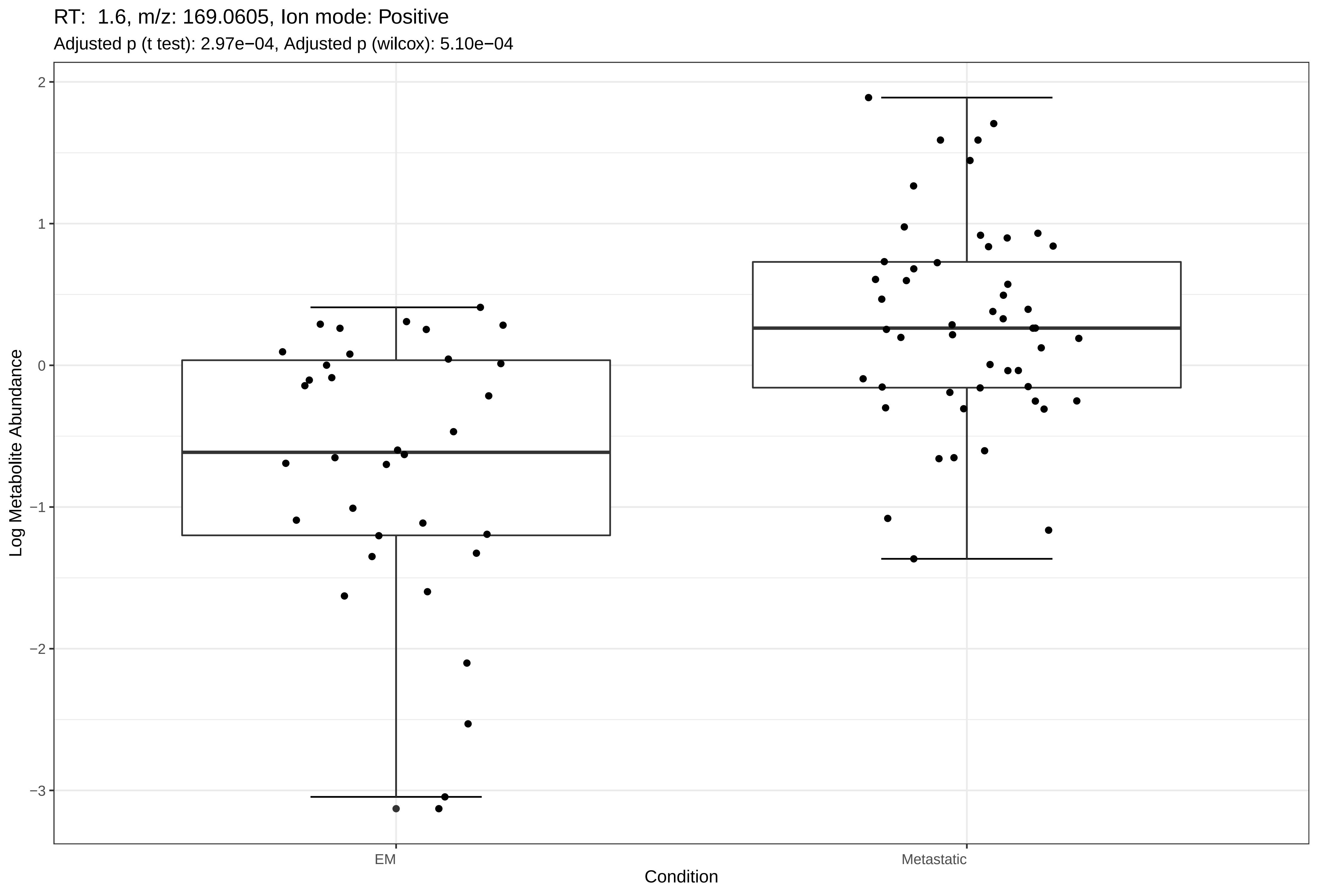

RT: 1.6, m/z: 169.0605, Ion mode: Positive
Adjusted p (t test): 2.97e−04, Adjusted p (wilcox): 5.10e−04
Log Metabolite Abundance
2
1
0
−1
−2
−3
EM
Metastatic
Condition

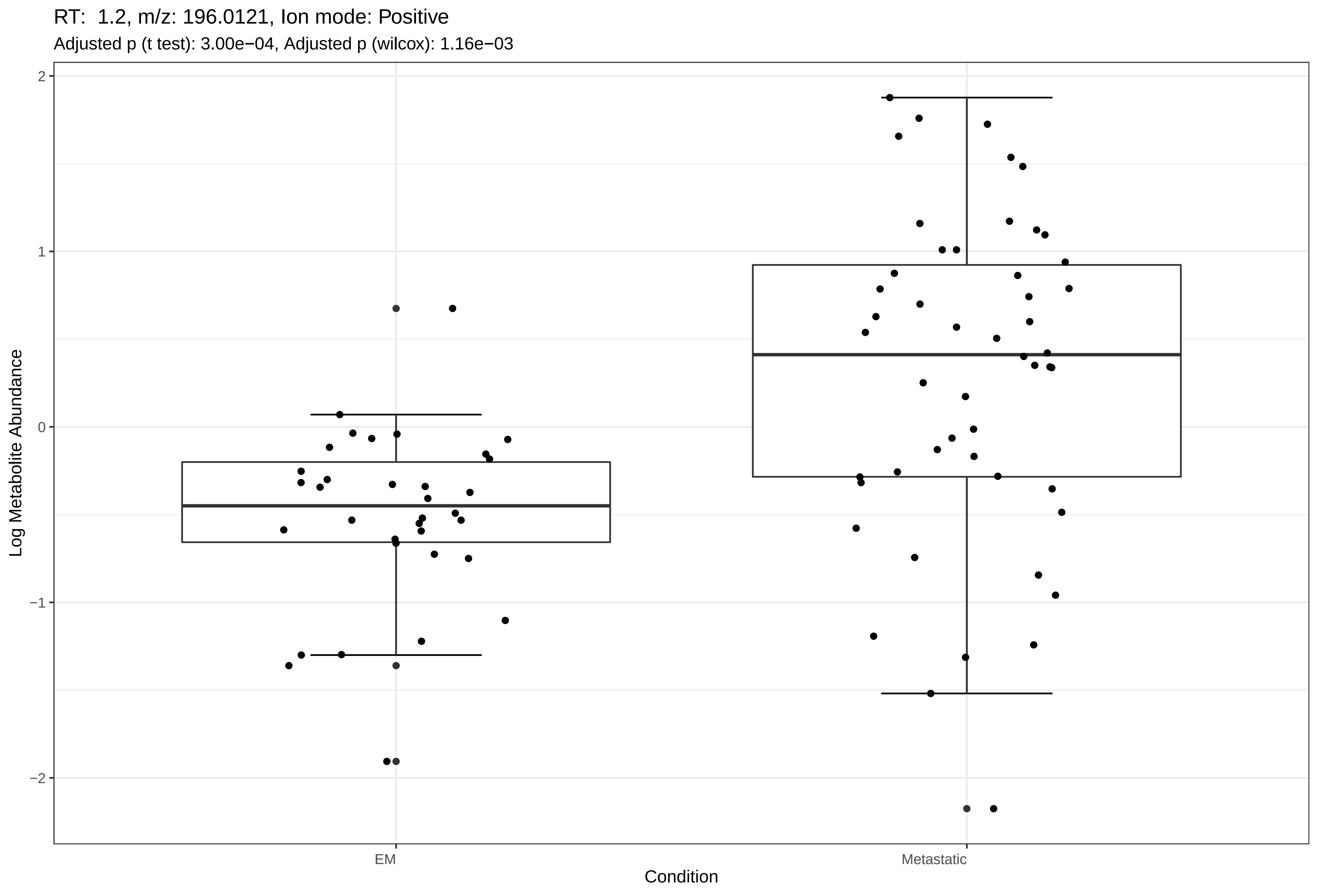

RT: 1.2, m/z: 196.0121, Ion mode: Positive
Adjusted p (t test): 3.00e−04, Adjusted p (wilcox): 1.16e−03
Log Metabolite Abundance
2
1
0
−1
−2
EM
Metastatic
Condition

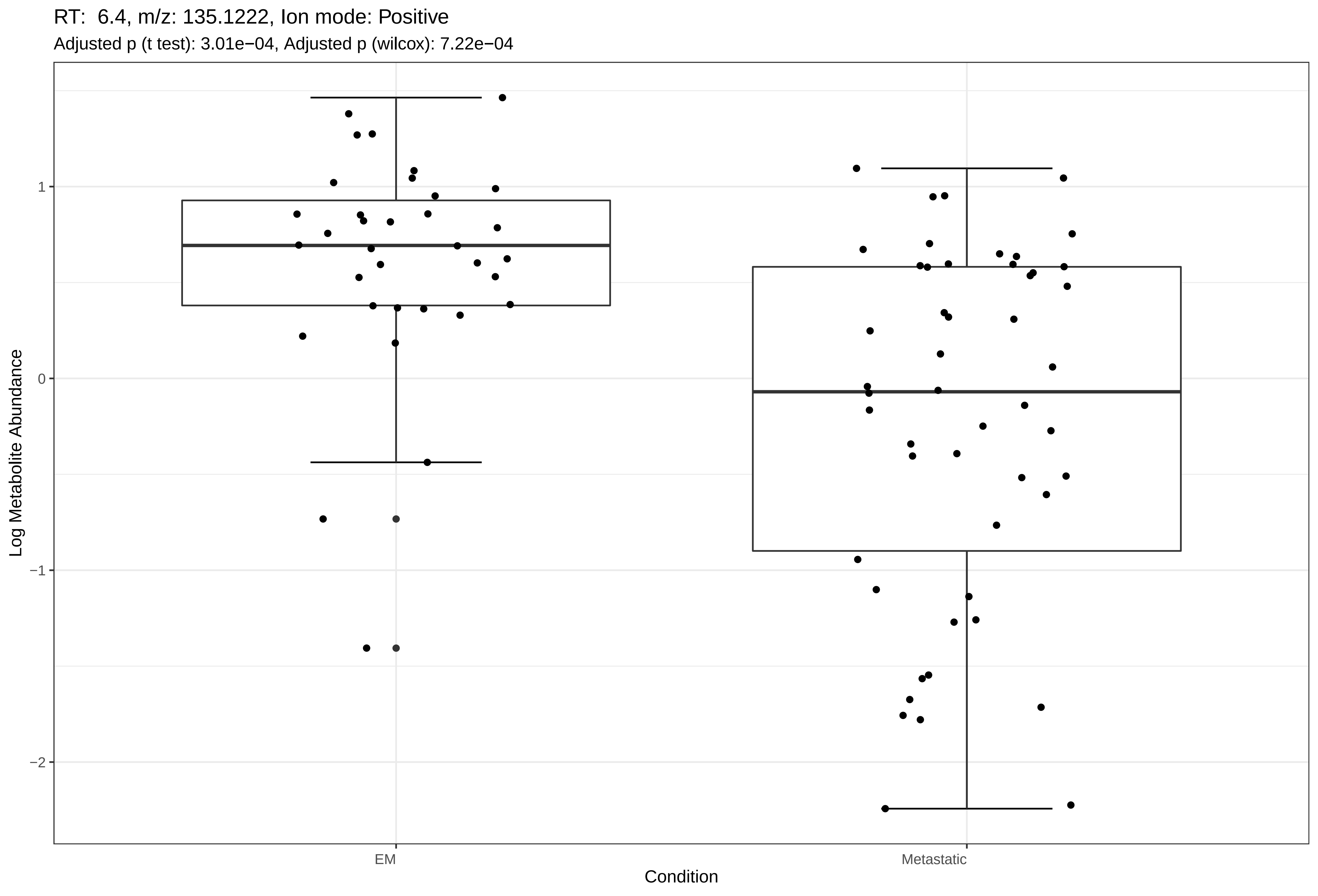

RT: 6.4, m/z: 135.1222, Ion mode: Positive
Adjusted p (t test): 3.01e−04, Adjusted p (wilcox): 7.22e−04
Log Metabolite Abundance
1
0
−1
−2
EM
Metastatic
Condition

LACTATE−dimer (RT: 1.2, m/z: 201.0384, Ion mode: Negative)

Adjusted p (t test): 6.23e−04, Adjusted p (wilcox): 3.01e−04

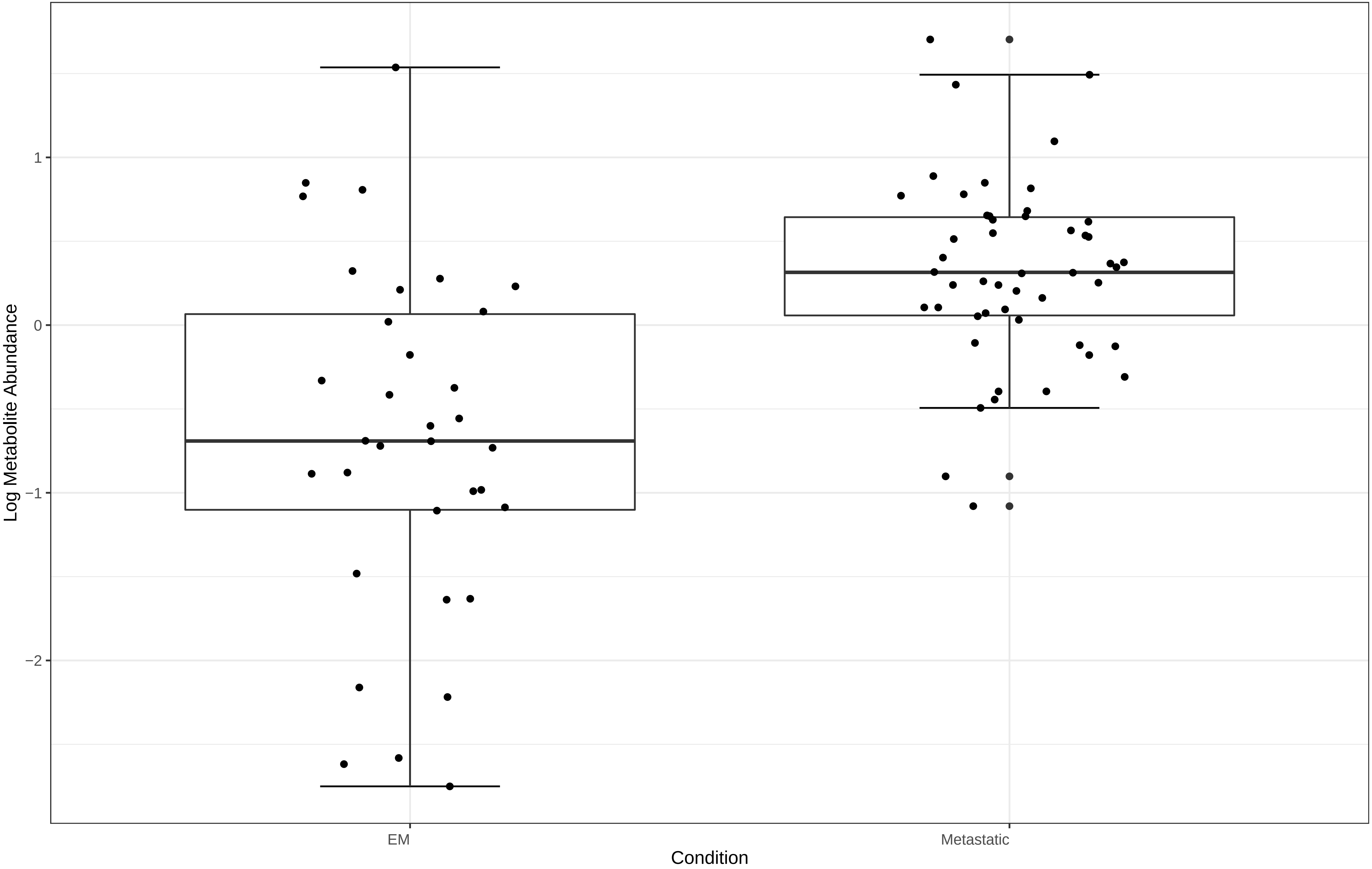

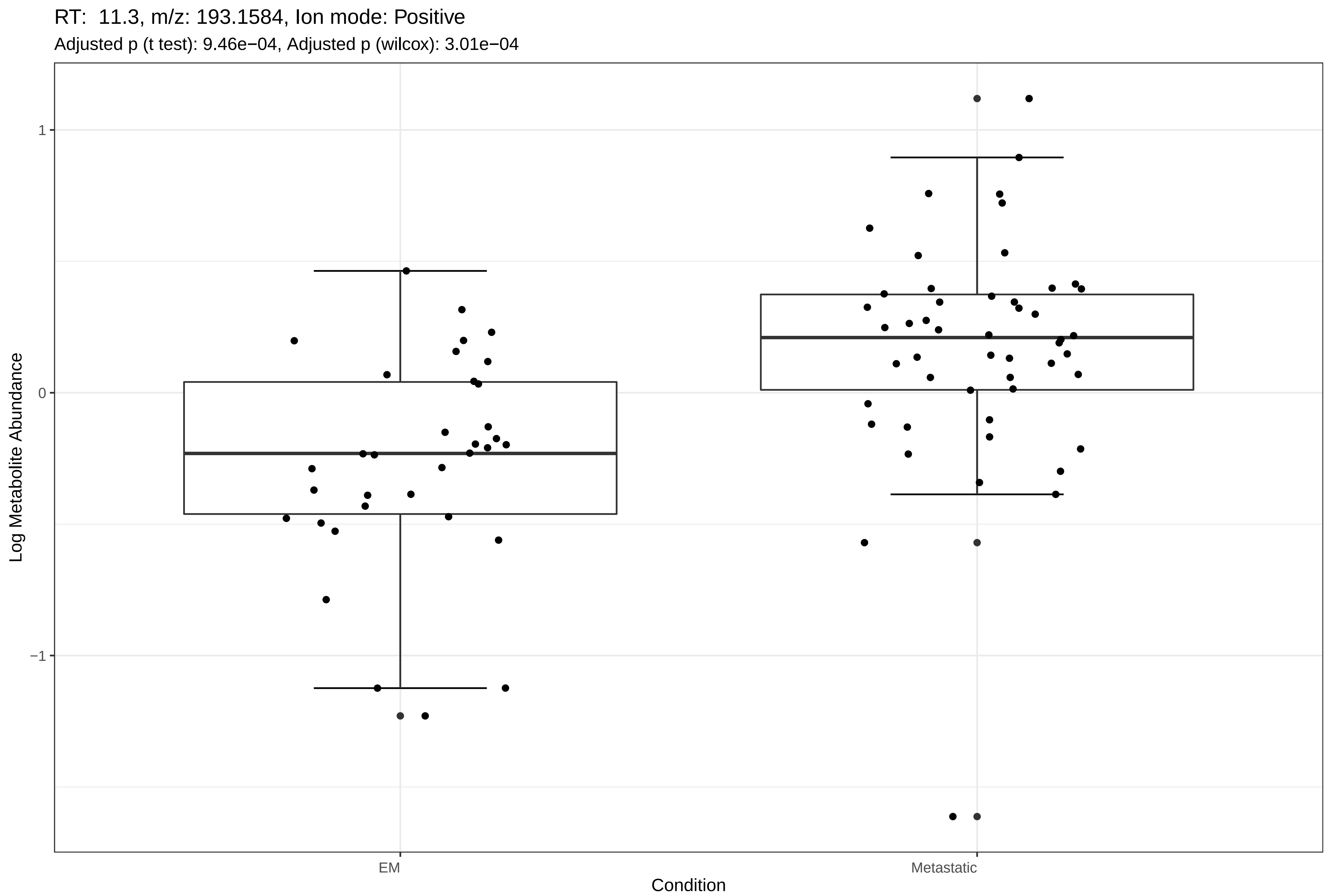
RT: 11.3, m/z: 193.1584, Ion mode: Positive
Adjusted p (t test): 9.46e−04, Adjusted p (wilcox): 3.01e−04
Log Metabolite Abundance
1
0
−1
EM
Metastatic
Condition

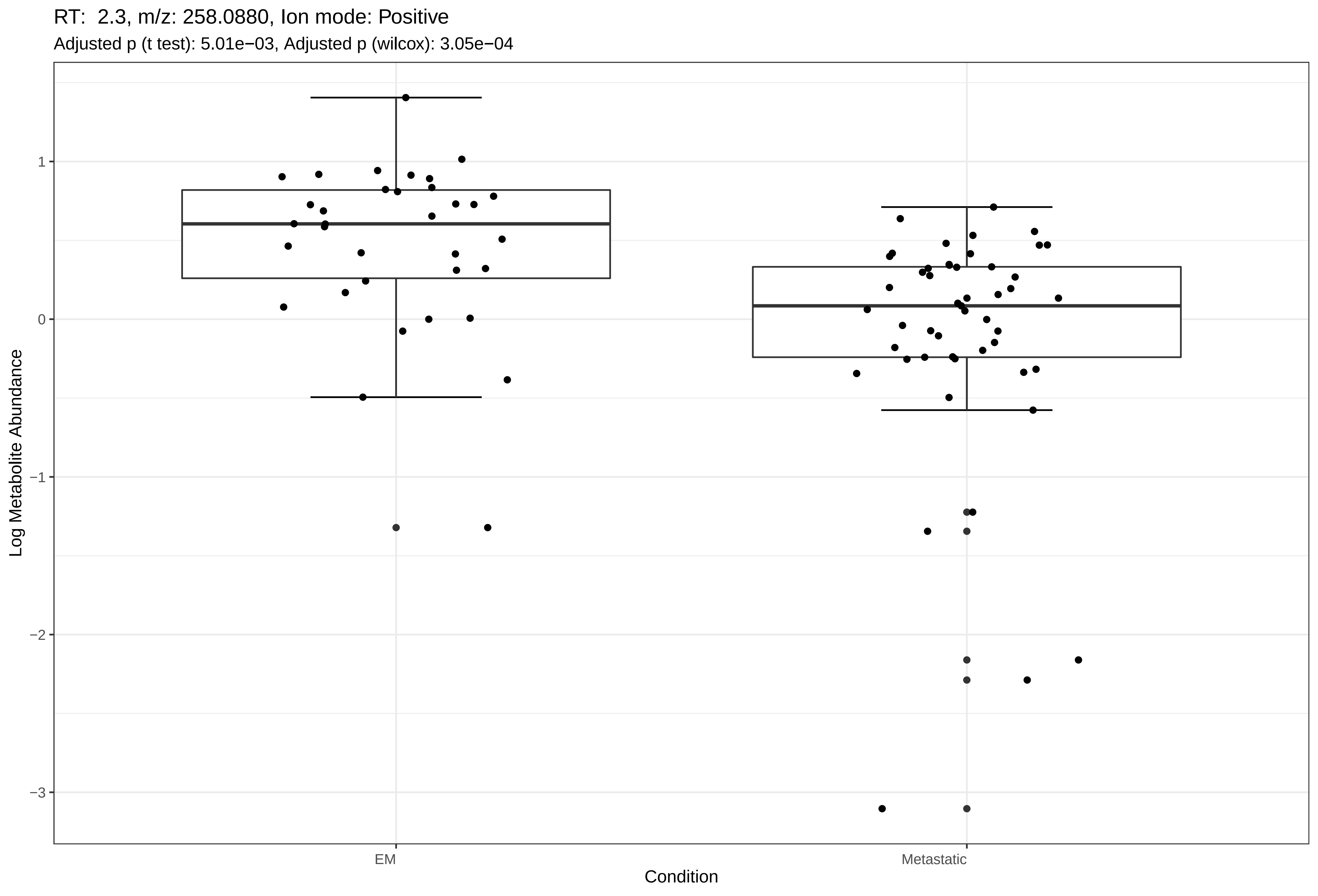

RT: 2.3, m/z: 258.0880, Ion mode: Positive
Adjusted p (t test): 5.01e−03, Adjusted p (wilcox): 3.05e−04
Log Metabolite Abundance
1
0
−1
−2
−3
EM
Metastatic
Condition

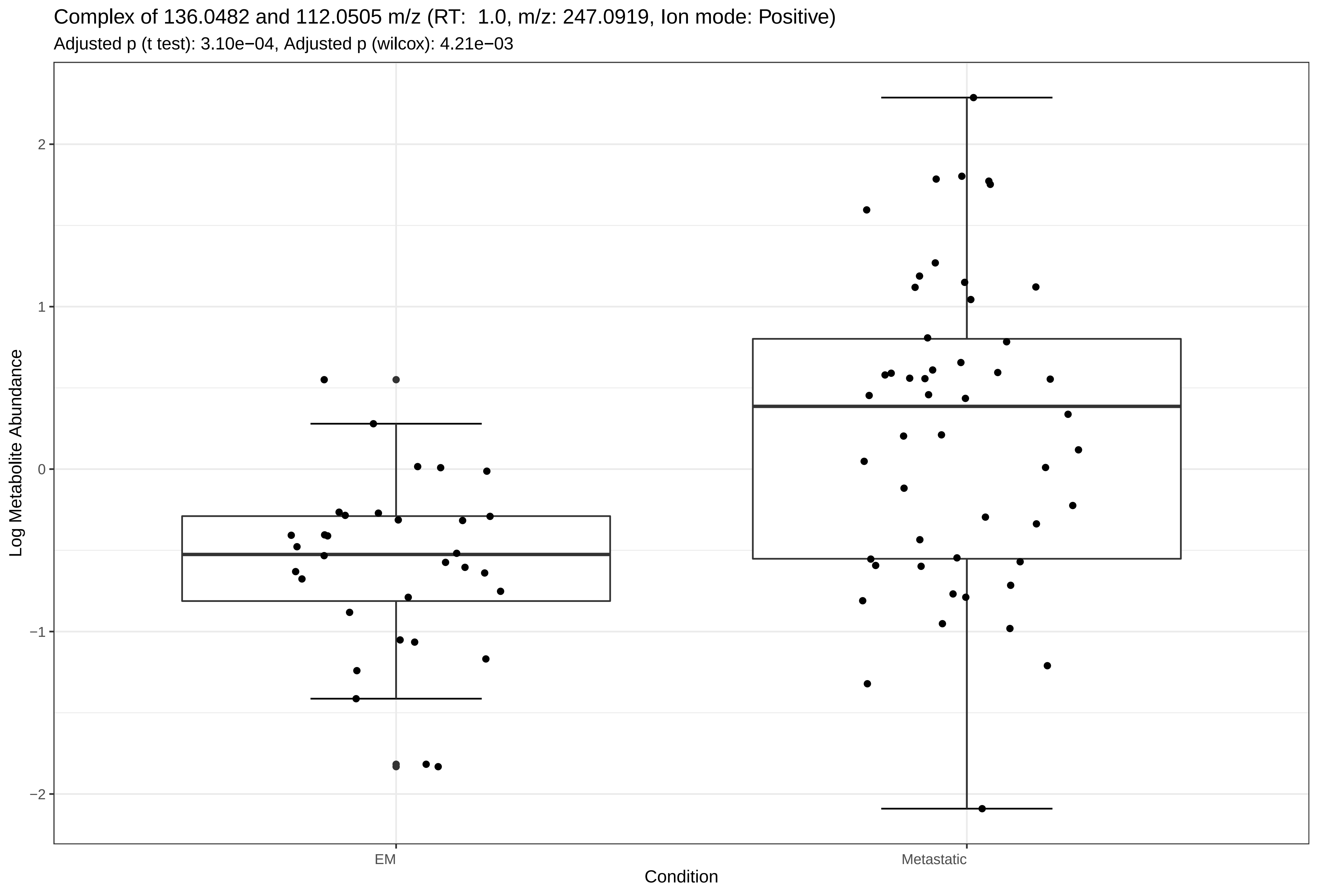

Complex of 136.0482 and 112.0505 m/z (RT: 1.0, m/z: 247.0919, Ion mode: Positive)
Adjusted p (t test): 3.10e−04, Adjusted p (wilcox): 4.21e−03
Log Metabolite Abundance
2
1
0
−1
−2
EM
Metastatic
Condition

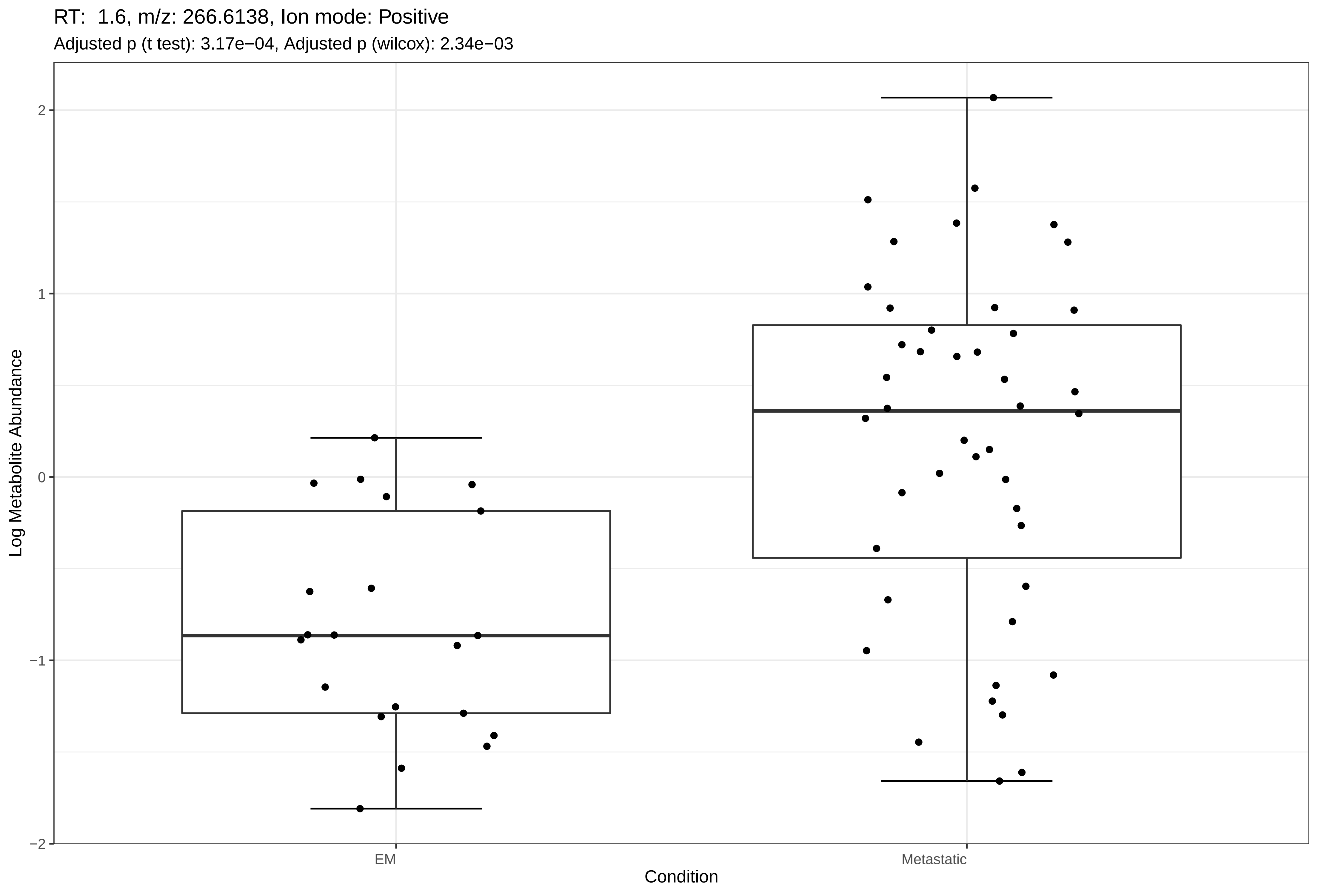
RT: 1.6, m/z: 266.6138, Ion mode: Positive
Adjusted p (t test): 3.17e−04, Adjusted p (wilcox): 2.34e−03
Log Metabolite Abundance
2
1
0
−1
−2
EM
Metastatic
Condition

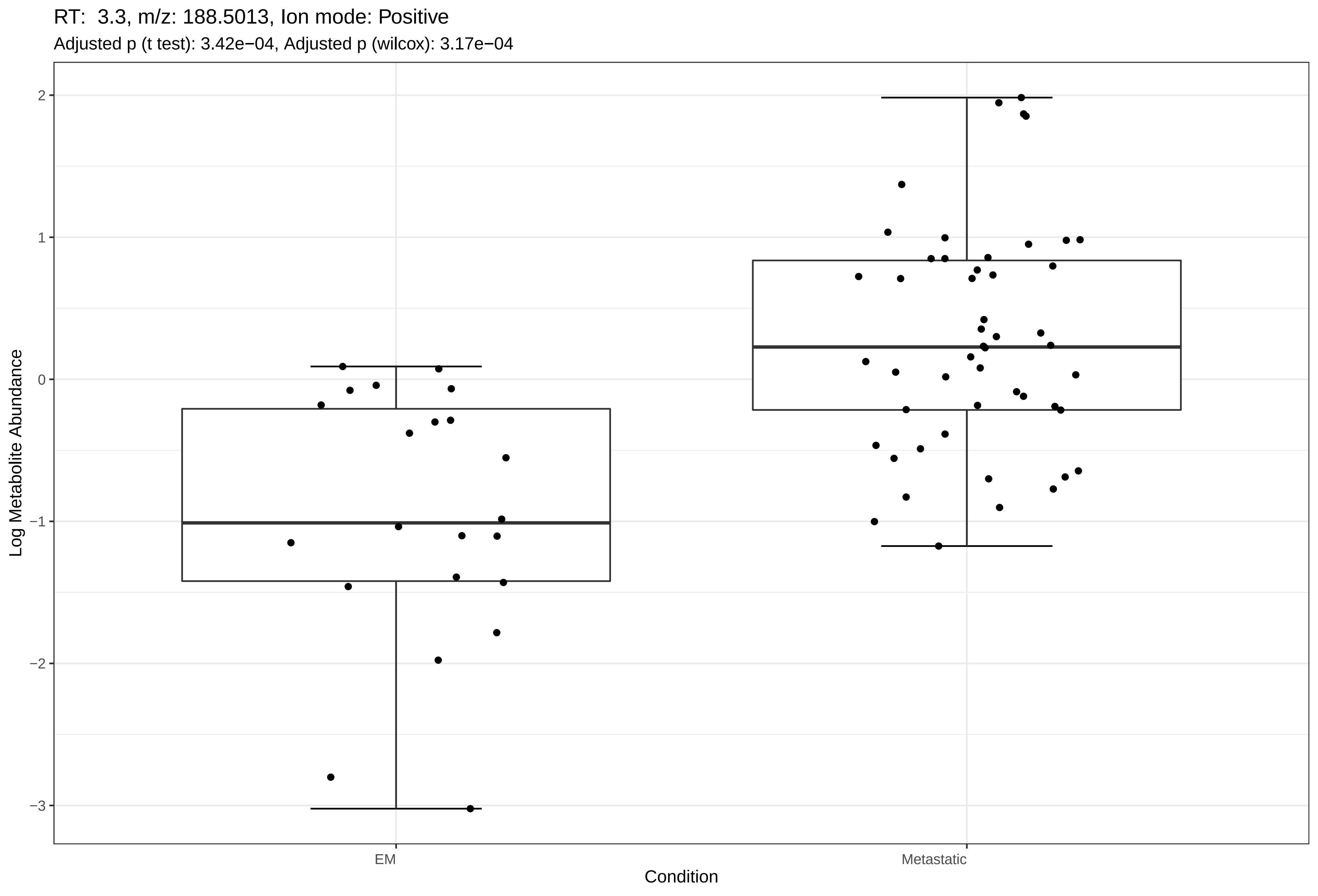

RT: 3.3, m/z: 188.5013, Ion mode: Positive
Adjusted p (t test): 3.42e−04, Adjusted p (wilcox): 3.17e−04
Log Metabolite Abundance
2
1
0
−1
−2
−3
EM
Metastatic
Condition

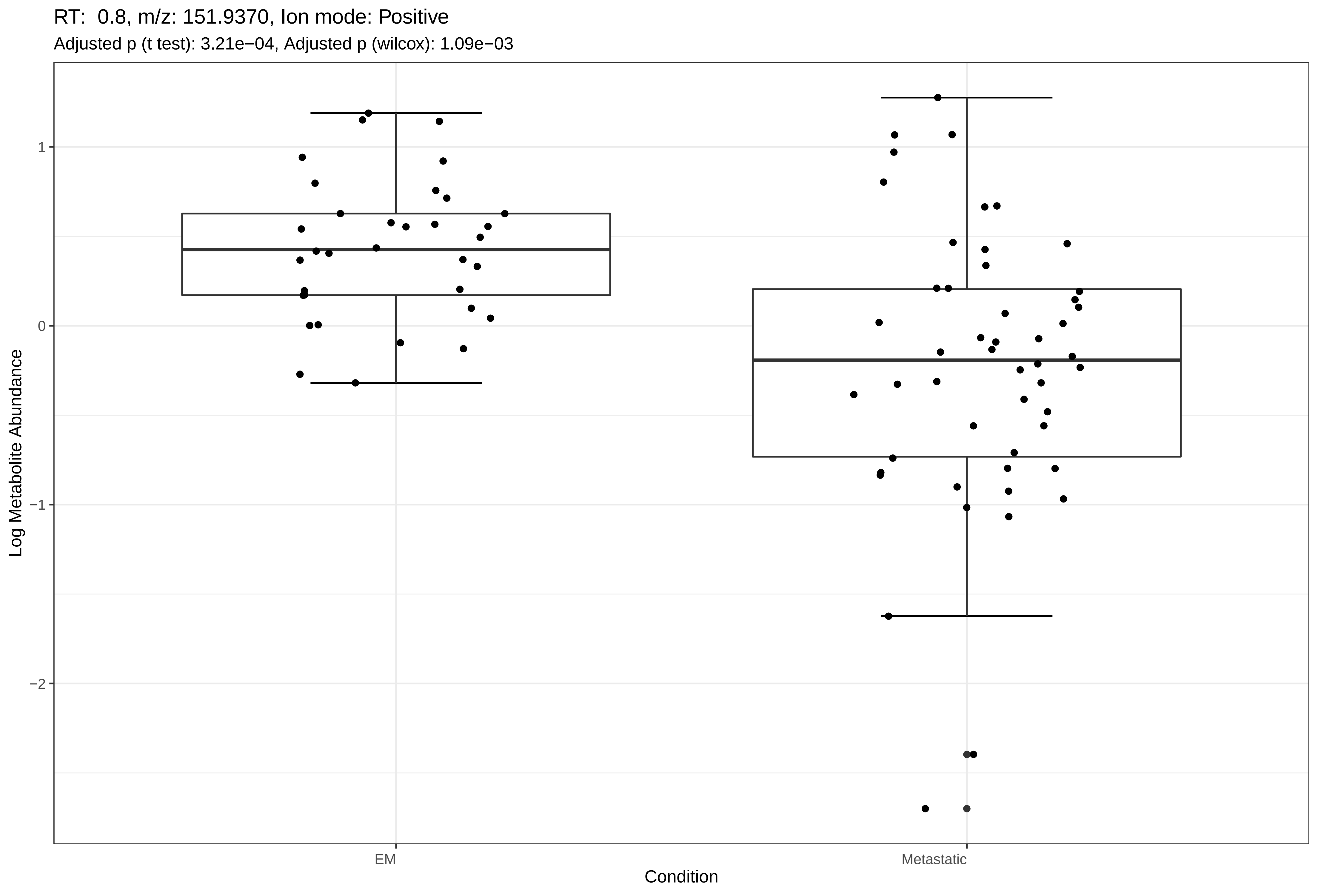
RT: 0.8, m/z: 151.9370, Ion mode: Positive
Adjusted p (t test): 3.21e−04, Adjusted p (wilcox): 1.09e−03
Log Metabolite Abundance
1
0
−1
−2
EM
Metastatic
Condition

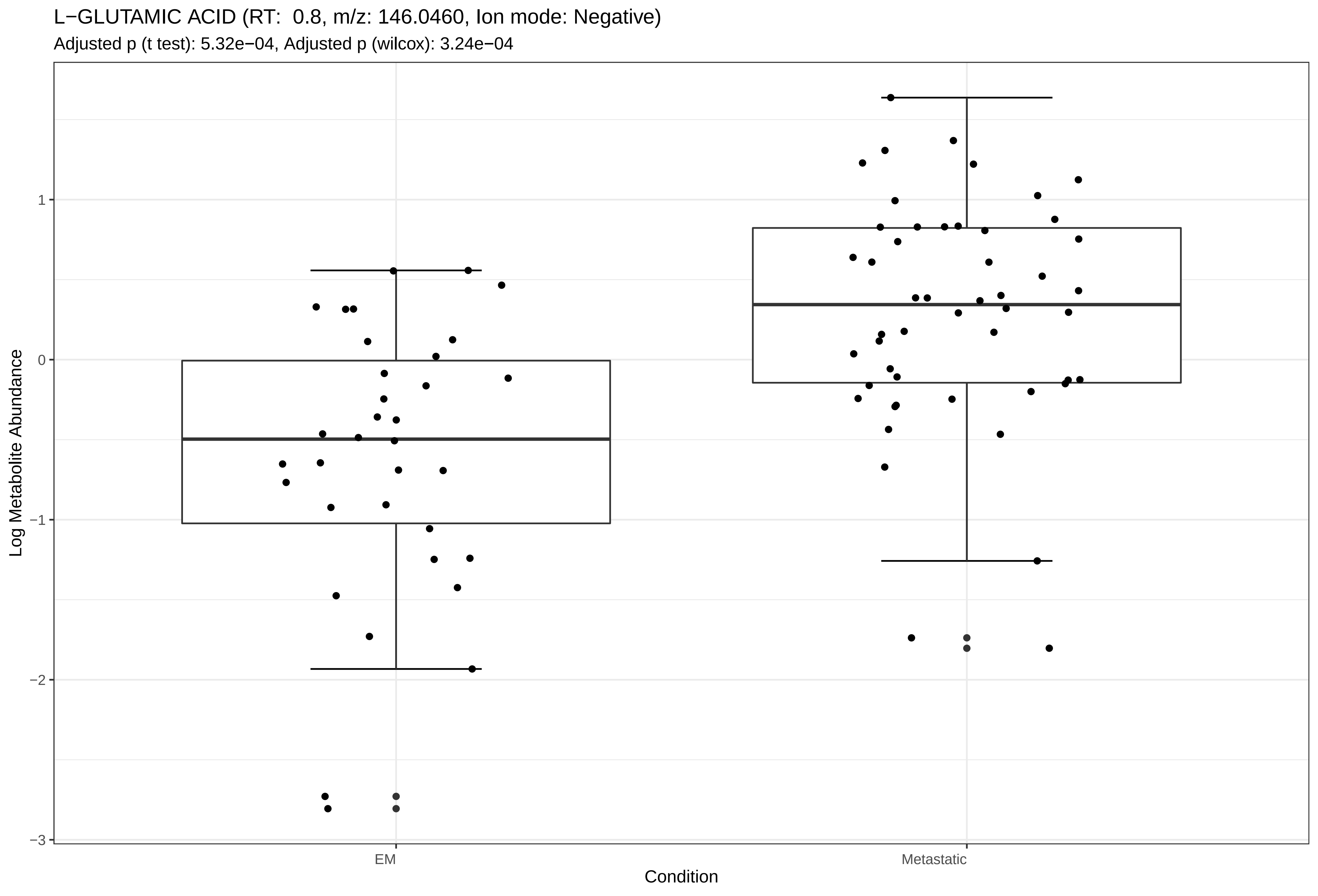

L−GLUTAMIC ACID (RT: 0.8, m/z: 146.0460, Ion mode: Negative)
Adjusted p (t test): 5.32e−04, Adjusted p (wilcox): 3.24e−04
Log Metabolite Abundance
1
0
−1
−2
−3
EM
Metastatic
Condition

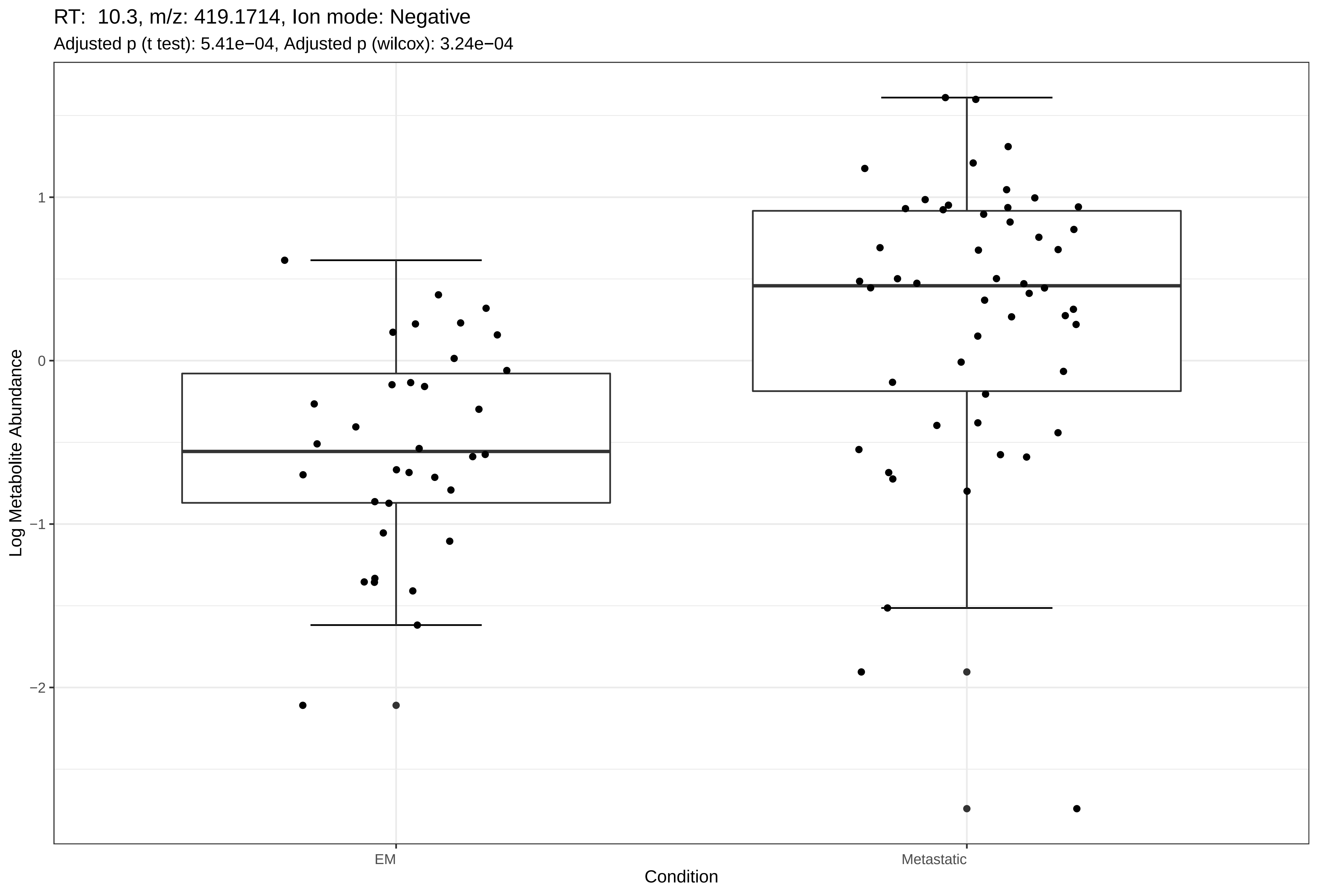
RT: 10.3, m/z: 419.1714, Ion mode: Negative
Adjusted p (t test): 5.41e−04, Adjusted p (wilcox): 3.24e−04
Log Metabolite Abundance
1
0
−1
−2
EM
Metastatic
Condition

RT: 0.6, m/z: 101.0248, Ion mode: Positive

Adjusted p (t test): 4.79e−04, Adjusted p (wilcox): 3.24e−04

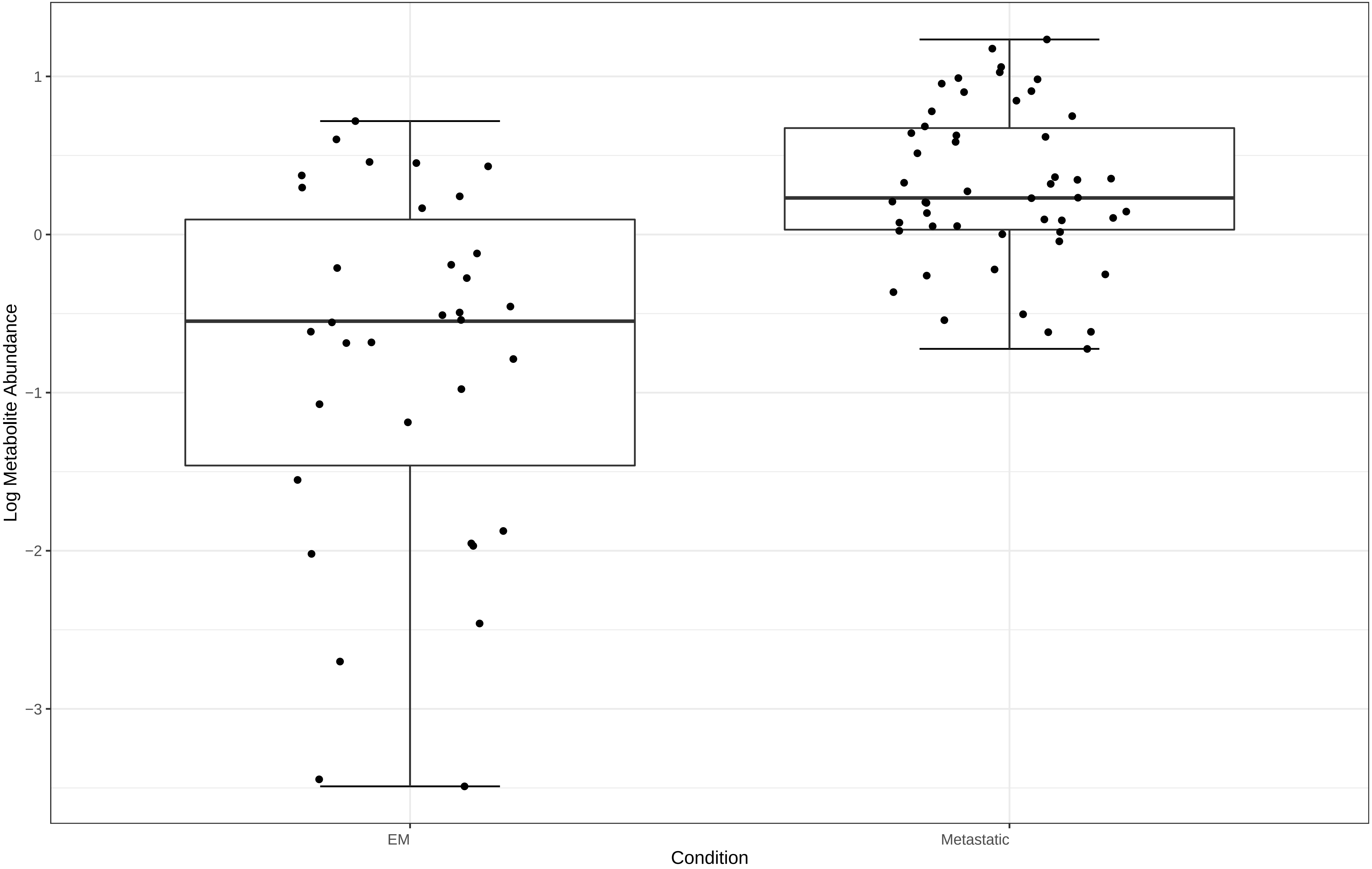

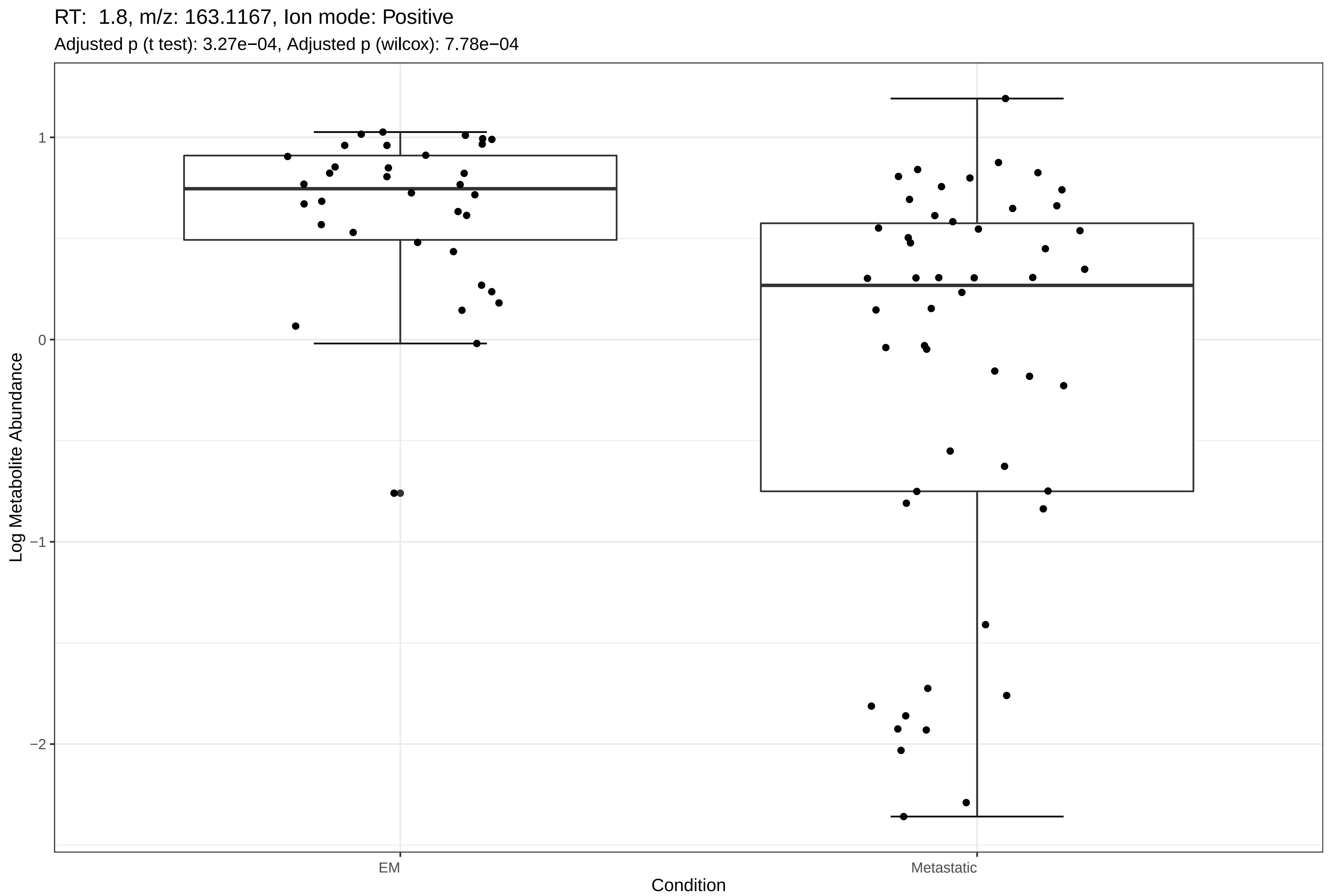

RT: 1.8, m/z: 163.1167, Ion mode: Positive
Adjusted p (t test): 3.27e−04, Adjusted p (wilcox): 7.78e−04
Log Metabolite Abundance
1
0
−1
−2
EM
Metastatic
Condition

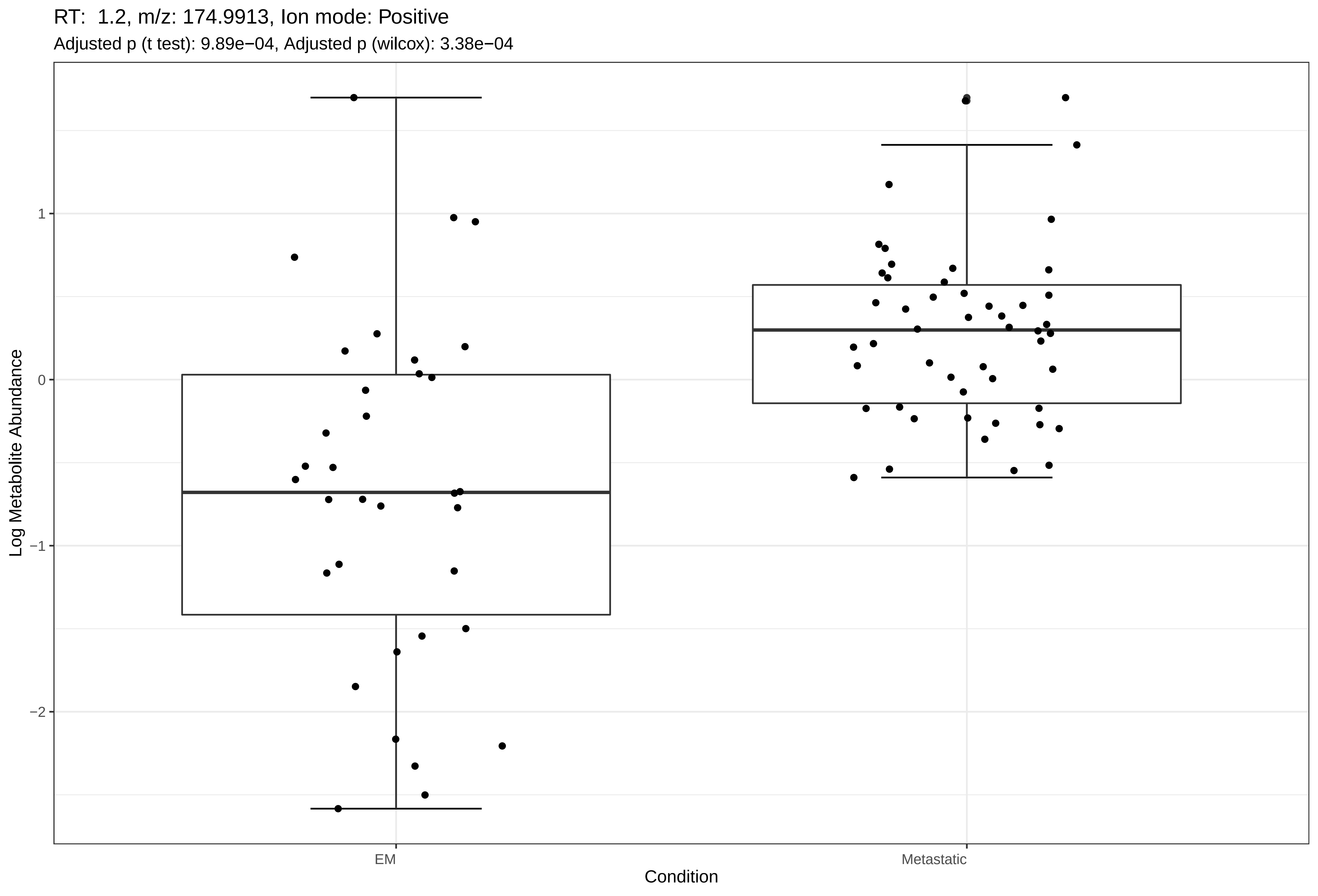
RT: 1.2, m/z: 174.9913, Ion mode: Positive
Adjusted p (t test): 9.89e−04, Adjusted p (wilcox): 3.38e−04
Log Metabolite Abundance
1
0
−1
−2
EM
Metastatic
Condition

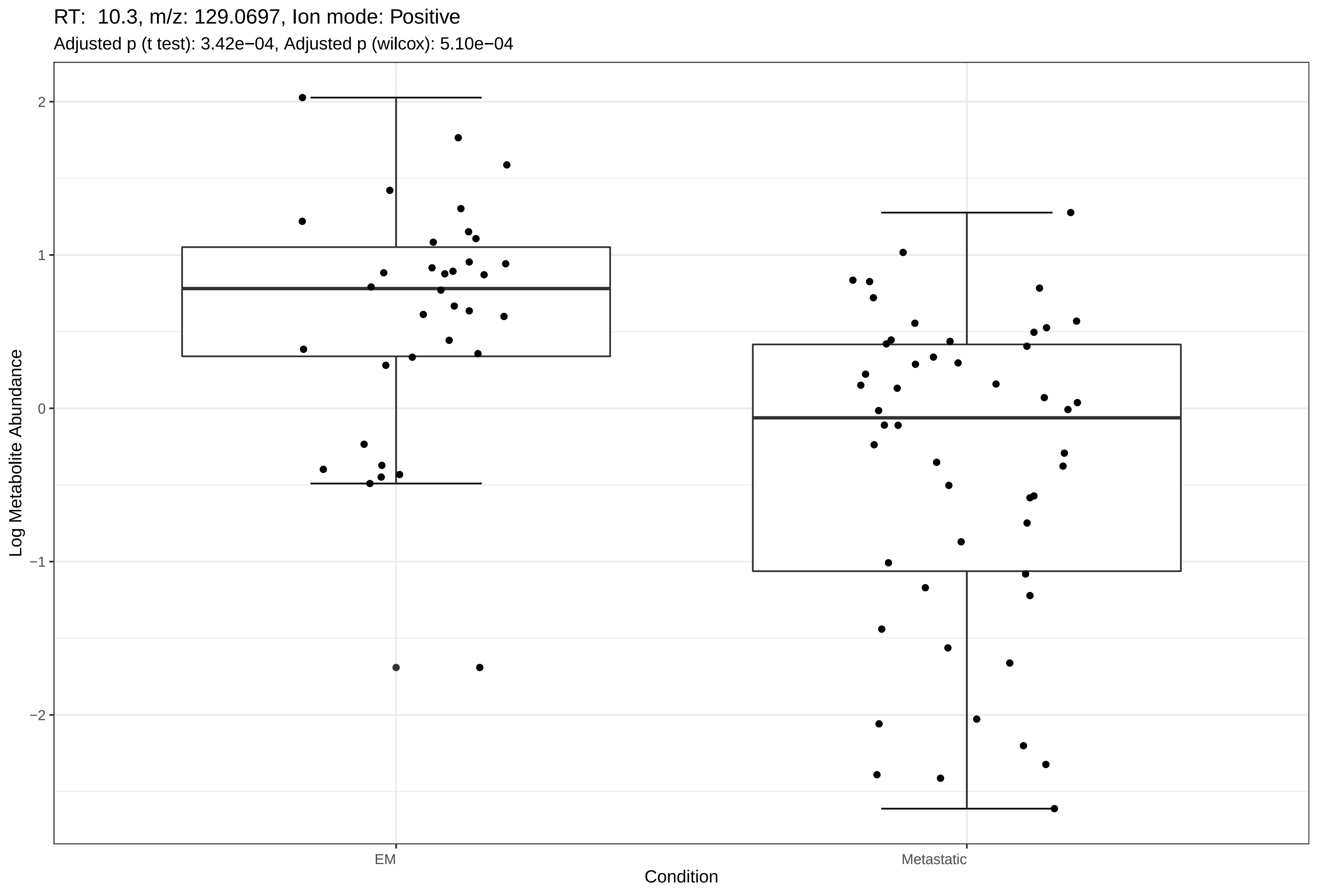
RT: 10.3, m/z: 129.0697, Ion mode: Positive
Adjusted p (t test): 3.42e−04, Adjusted p (wilcox): 5.10e−04
Log Metabolite Abundance
2
1
0
−1
−2
EM
Metastatic
Condition

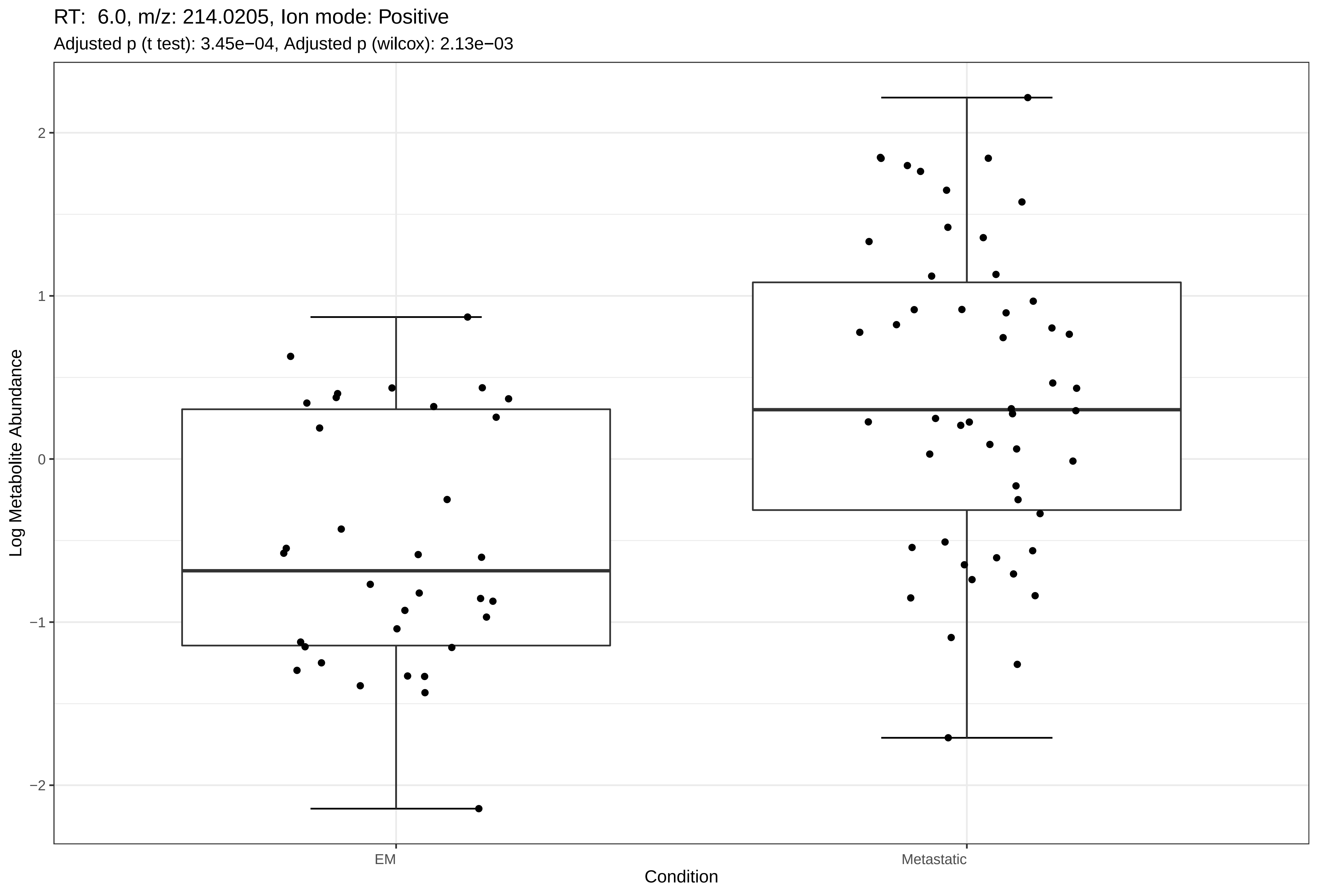

RT: 6.0, m/z: 214.0205, Ion mode: Positive
Adjusted p (t test): 3.45e−04, Adjusted p (wilcox): 2.13e−03
Log Metabolite Abundance
2
1
0
−1
−2
EM
Metastatic
Condition

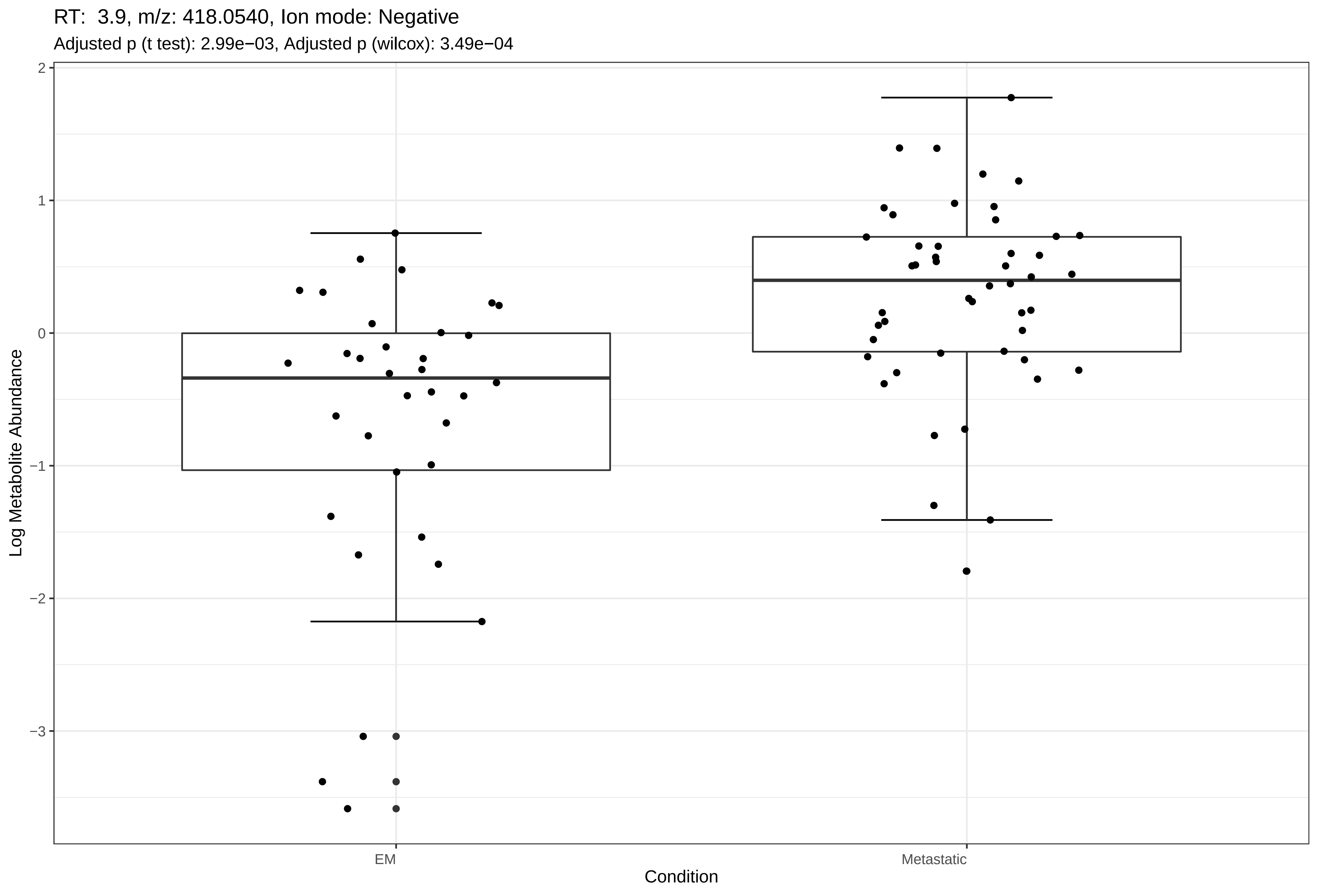

RT: 3.9, m/z: 418.0540, Ion mode: Negative
Adjusted p (t test): 2.99e−03, Adjusted p (wilcox): 3.49e−04
Log Metabolite Abundance
2
1
0
−1
−2
−3
EM
Metastatic
Condition

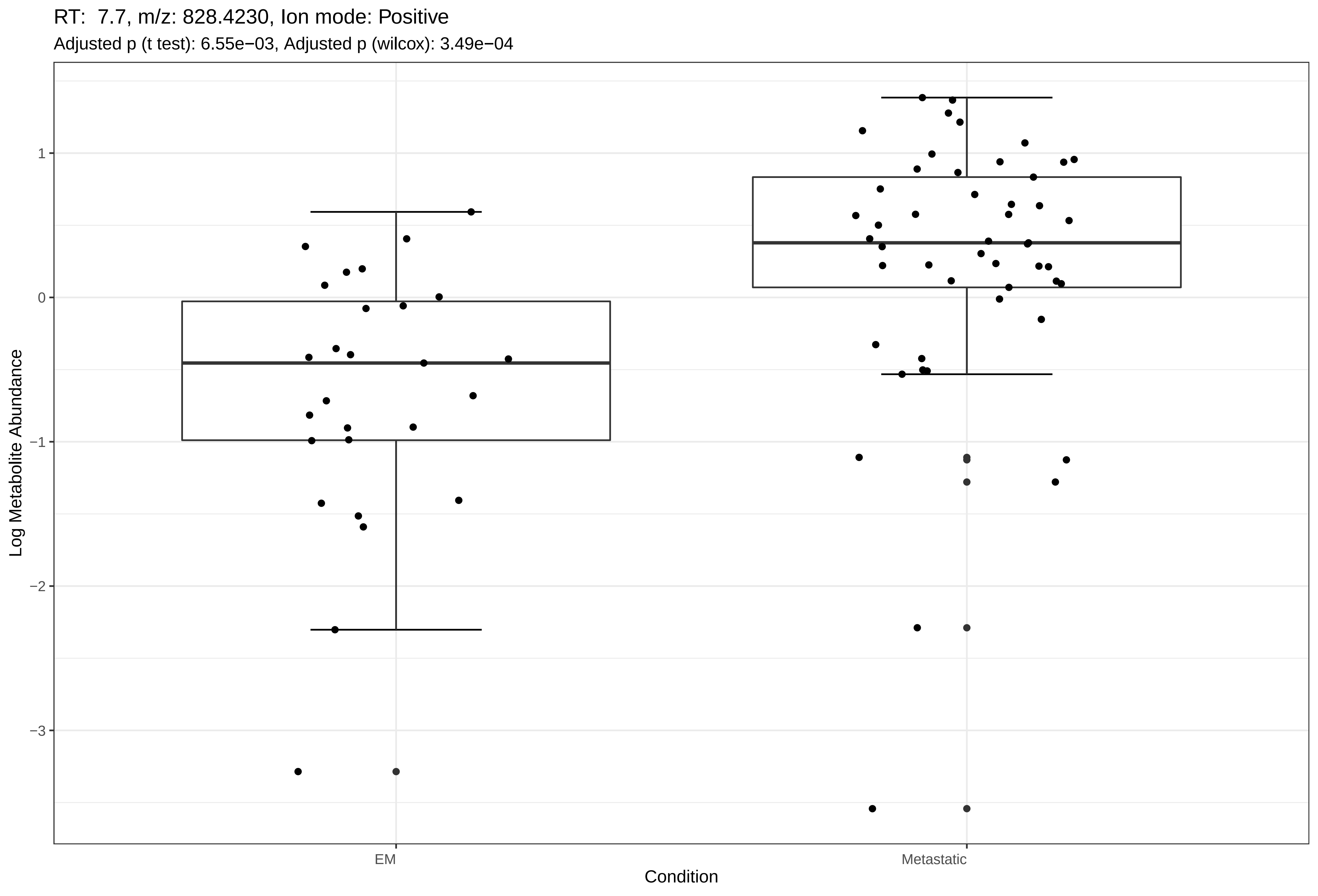

RT: 7.7, m/z: 828.4230, Ion mode: Positive
Adjusted p (t test): 6.55e−03, Adjusted p (wilcox): 3.49e−04
Log Metabolite Abundance
1
0
−1
−2
−3
EM
Metastatic
Condition

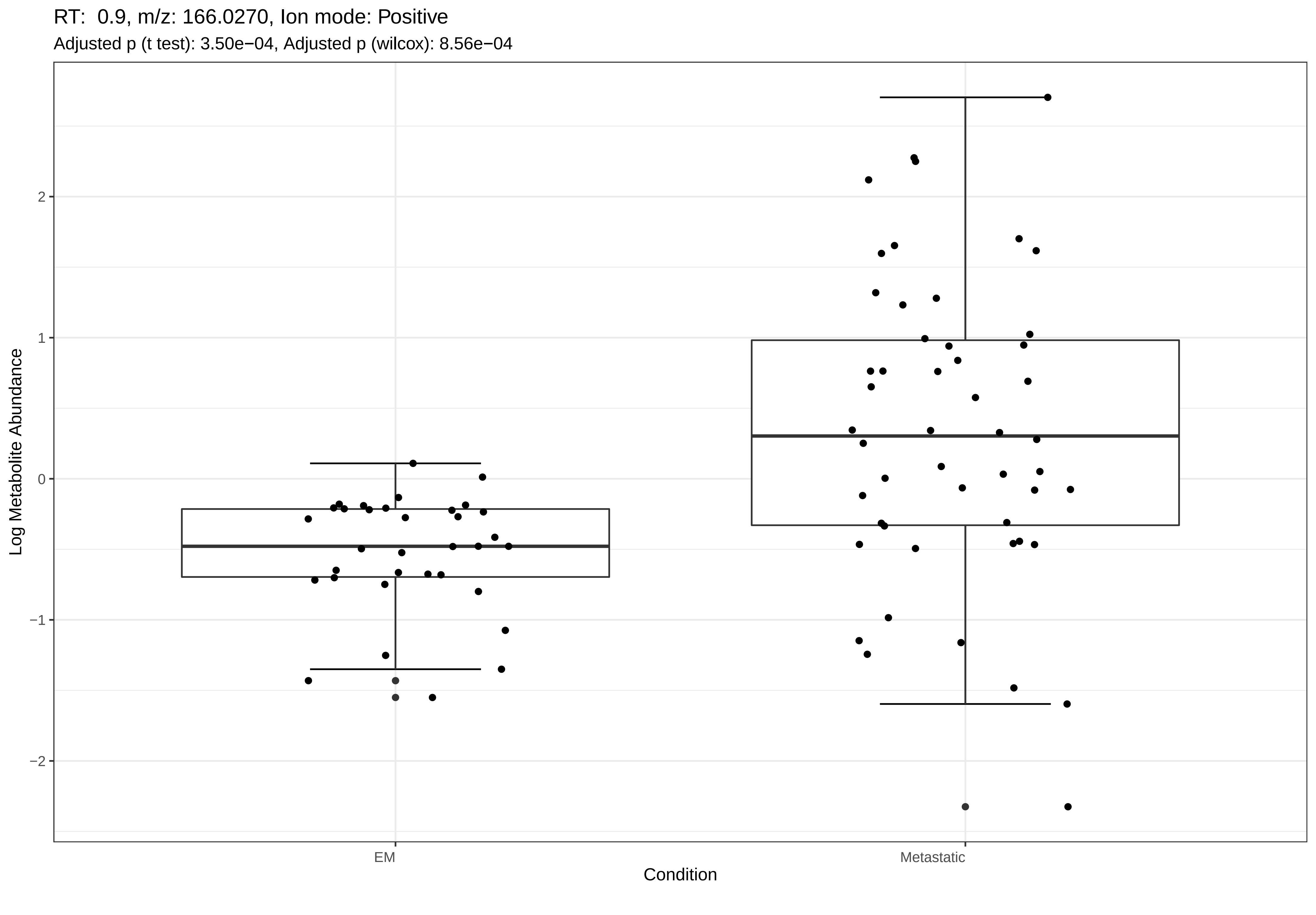
RT: 0.9, m/z: 166.0270, Ion mode: Positive
Adjusted p (t test): 3.50e−04, Adjusted p (wilcox): 8.56e−04
Log Metabolite Abundance
2
1
0
−1
−2
EM
Metastatic
Condition

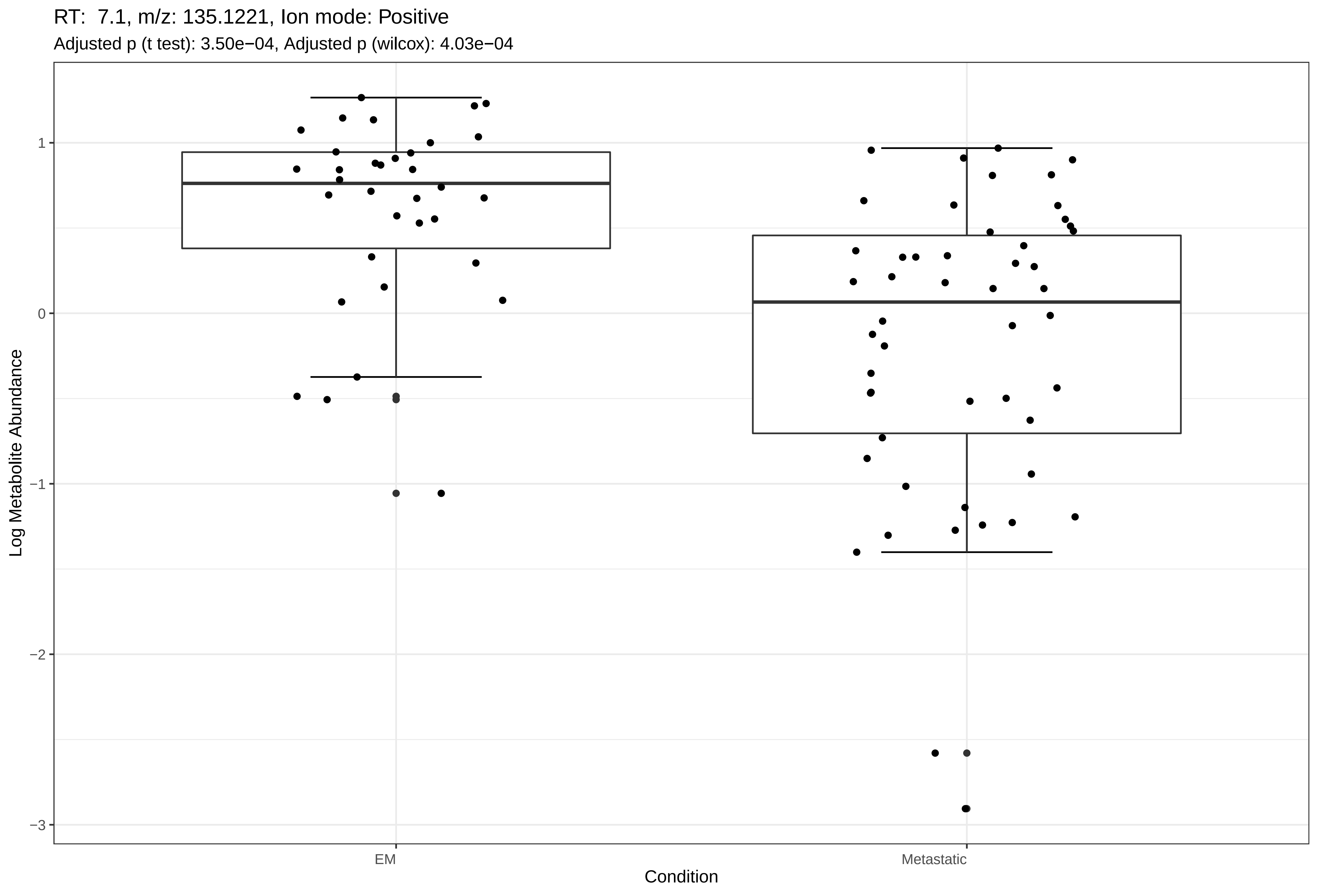

RT: 7.1, m/z: 135.1221, Ion mode: Positive
Adjusted p (t test): 3.50e−04, Adjusted p (wilcox): 4.03e−04
Log Metabolite Abundance
1
0
−1
−2
−3
EM
Metastatic
Condition

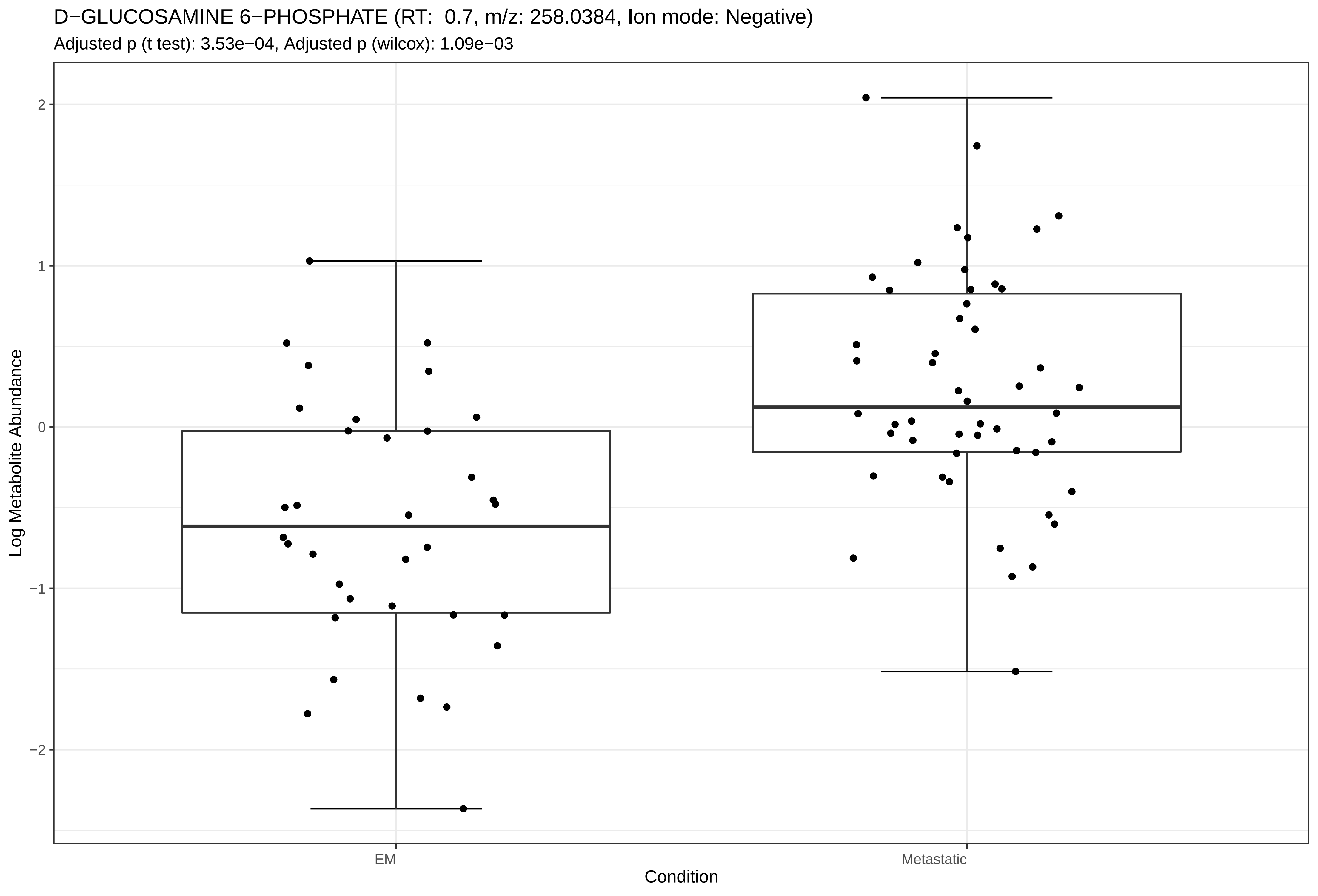

D−GLUCOSAMINE 6−PHOSPHATE (RT: 0.7, m/z: 258.0384, Ion mode: Negative)
Adjusted p (t test): 3.53e−04, Adjusted p (wilcox): 1.09e−03
Log Metabolite Abundance
2
1
0
−1
−2
EM
Metastatic
Condition

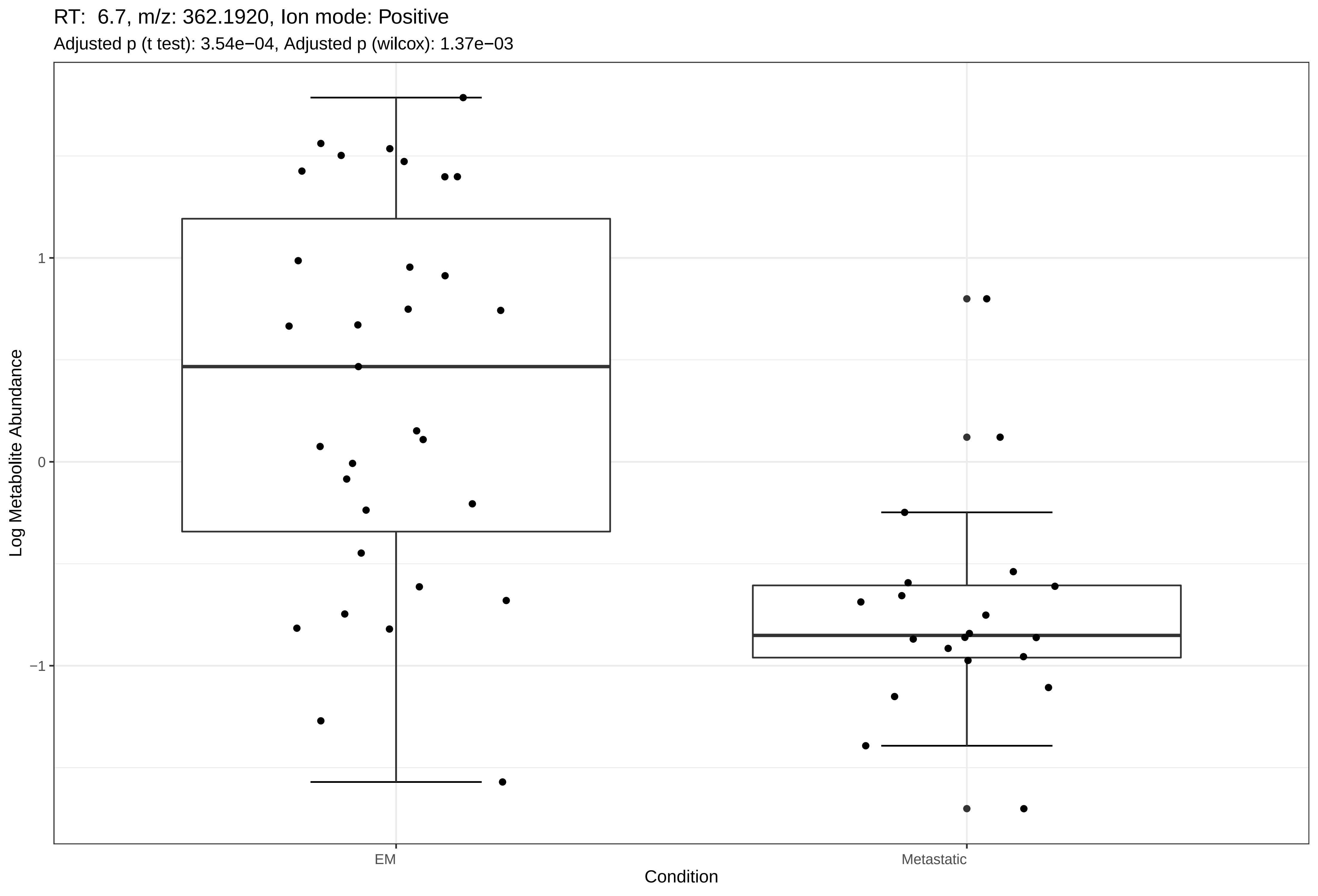

RT: 6.7, m/z: 362.1920, Ion mode: Positive
Adjusted p (t test): 3.54e−04, Adjusted p (wilcox): 1.37e−03
Log Metabolite Abundance
1
0
−1
EM
Metastatic
Condition

RT: 7.2, m/z: 240.1909, Ion mode: Positive

Adjusted p (t test): 3.56e−04, Adjusted p (wilcox): 4.77e−04

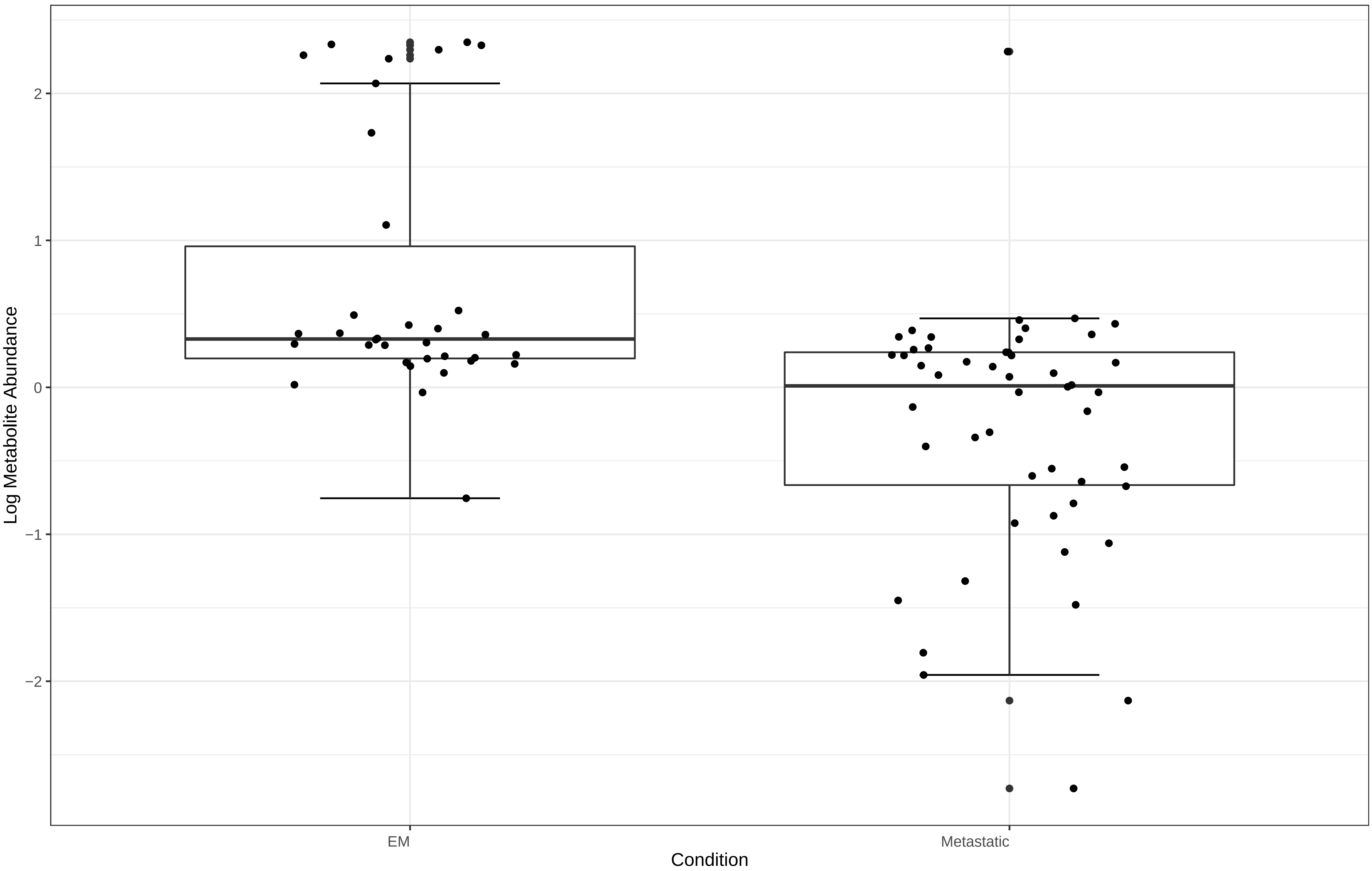

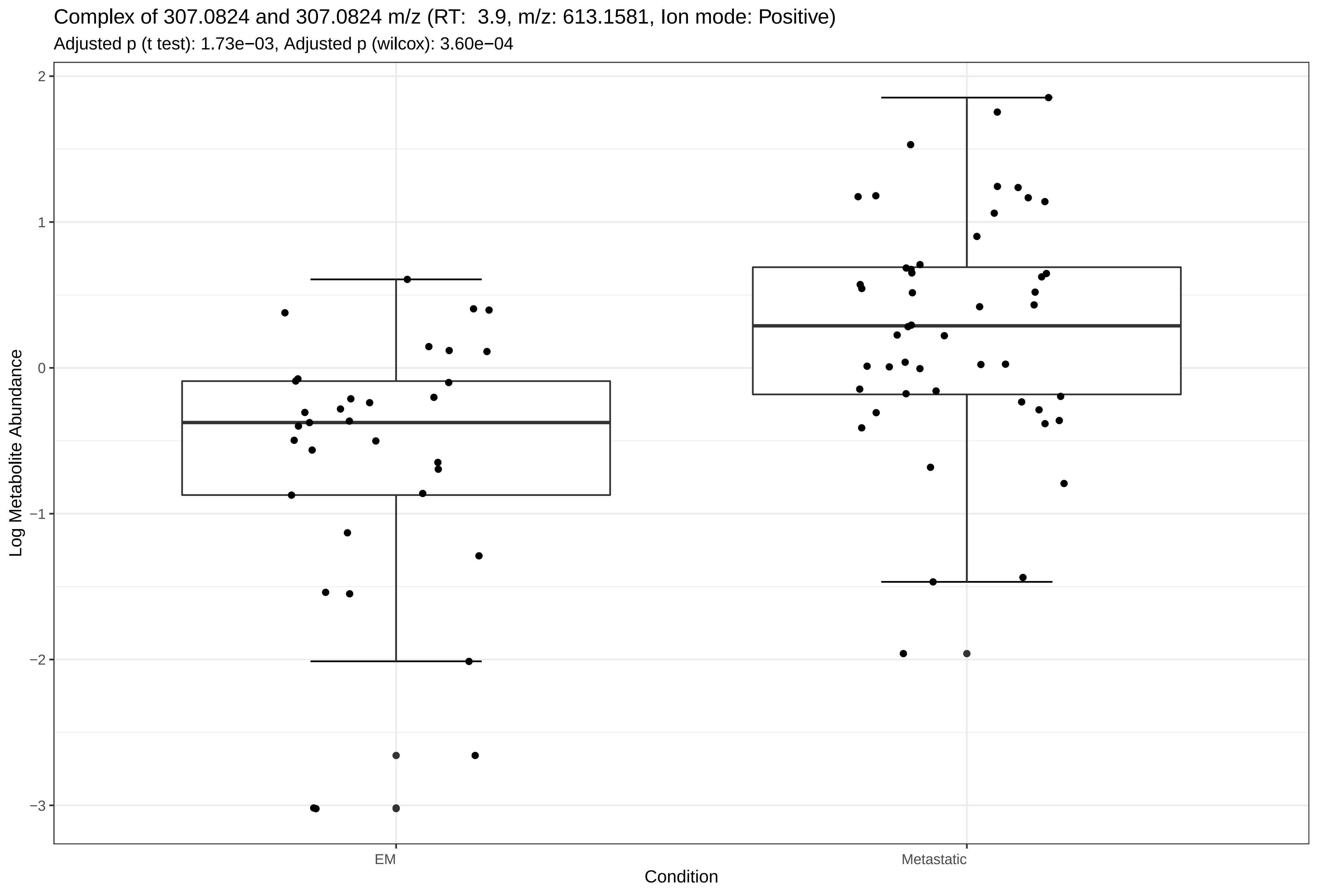
Complex of 307.0824 and 307.0824 m/z (RT: 3.9, m/z: 613.1581, Ion mode: Positive)
Adjusted p (t test): 1.73e−03, Adjusted p (wilcox): 3.60e−04
Log Metabolite Abundance
2
1
0
−1
−2
−3
EM
Metastatic
Condition

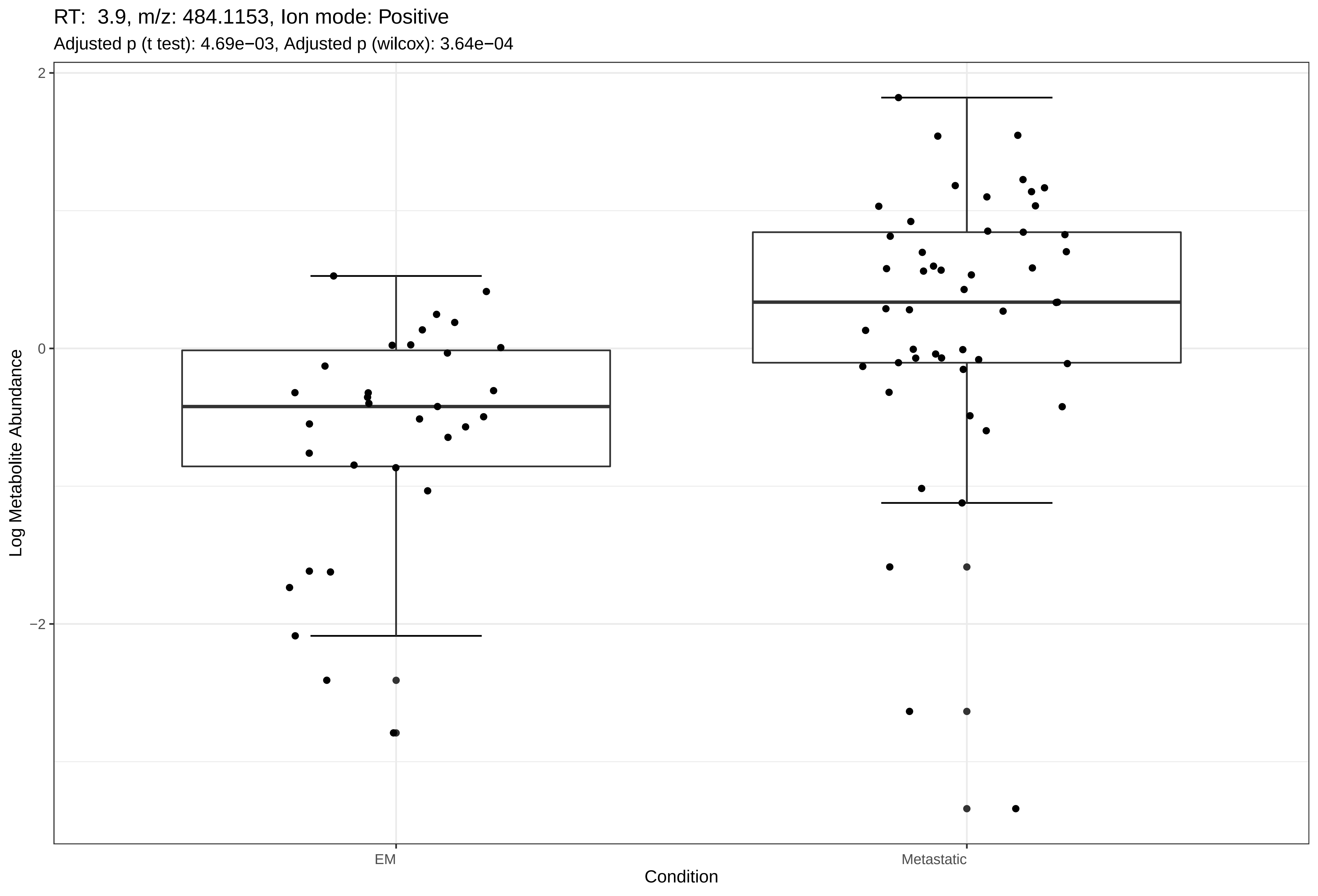
RT: 3.9, m/z: 484.1153, Ion mode: Positive
Adjusted p (t test): 4.69e−03, Adjusted p (wilcox): 3.64e−04
Log Metabolite Abundance
2
0
−2
EM
Metastatic
Condition

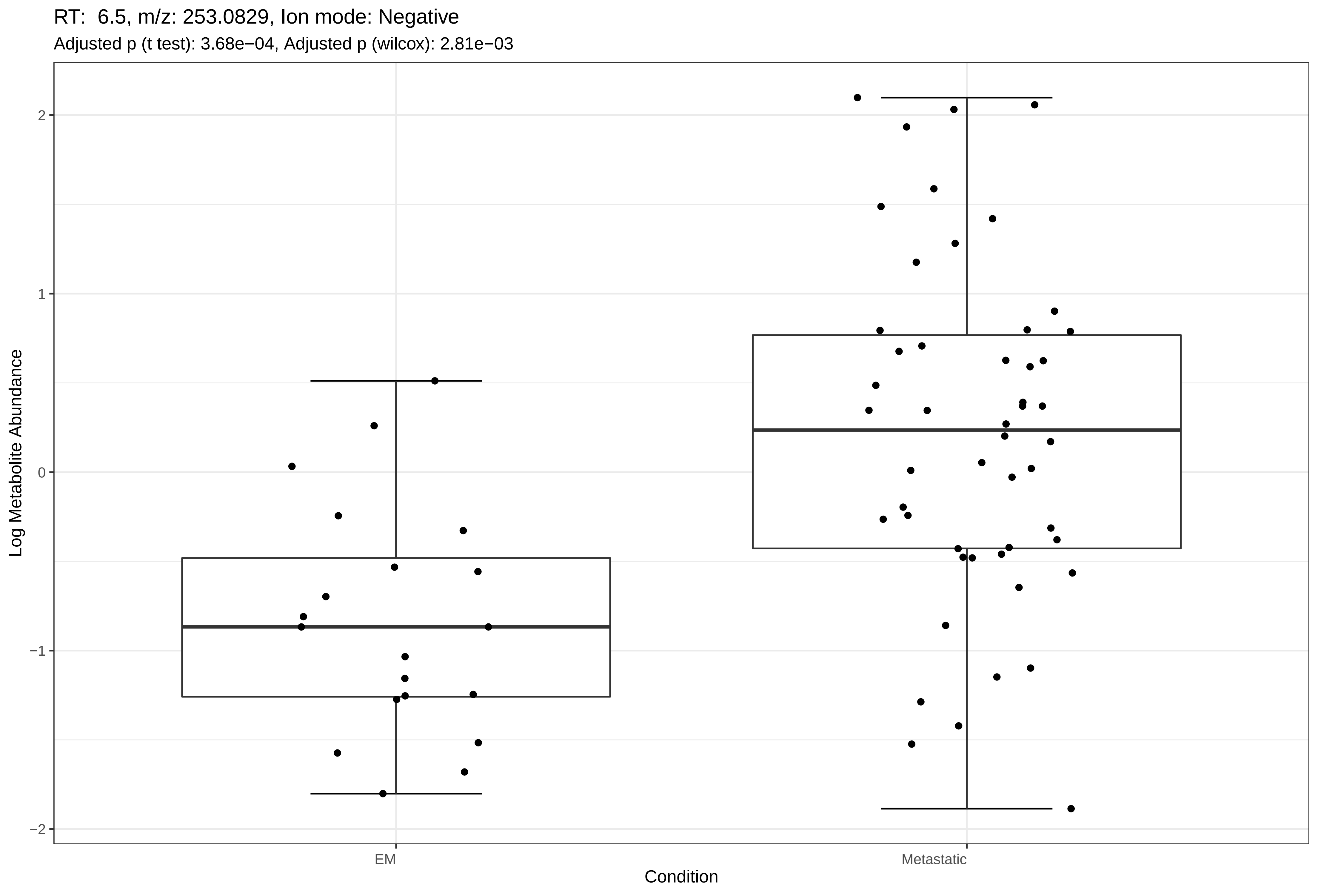
RT: 6.5, m/z: 253.0829, Ion mode: Negative
Adjusted p (t test): 3.68e−04, Adjusted p (wilcox): 2.81e−03
Log Metabolite Abundance
2
1
0
−1
−2
EM
Metastatic
Condition

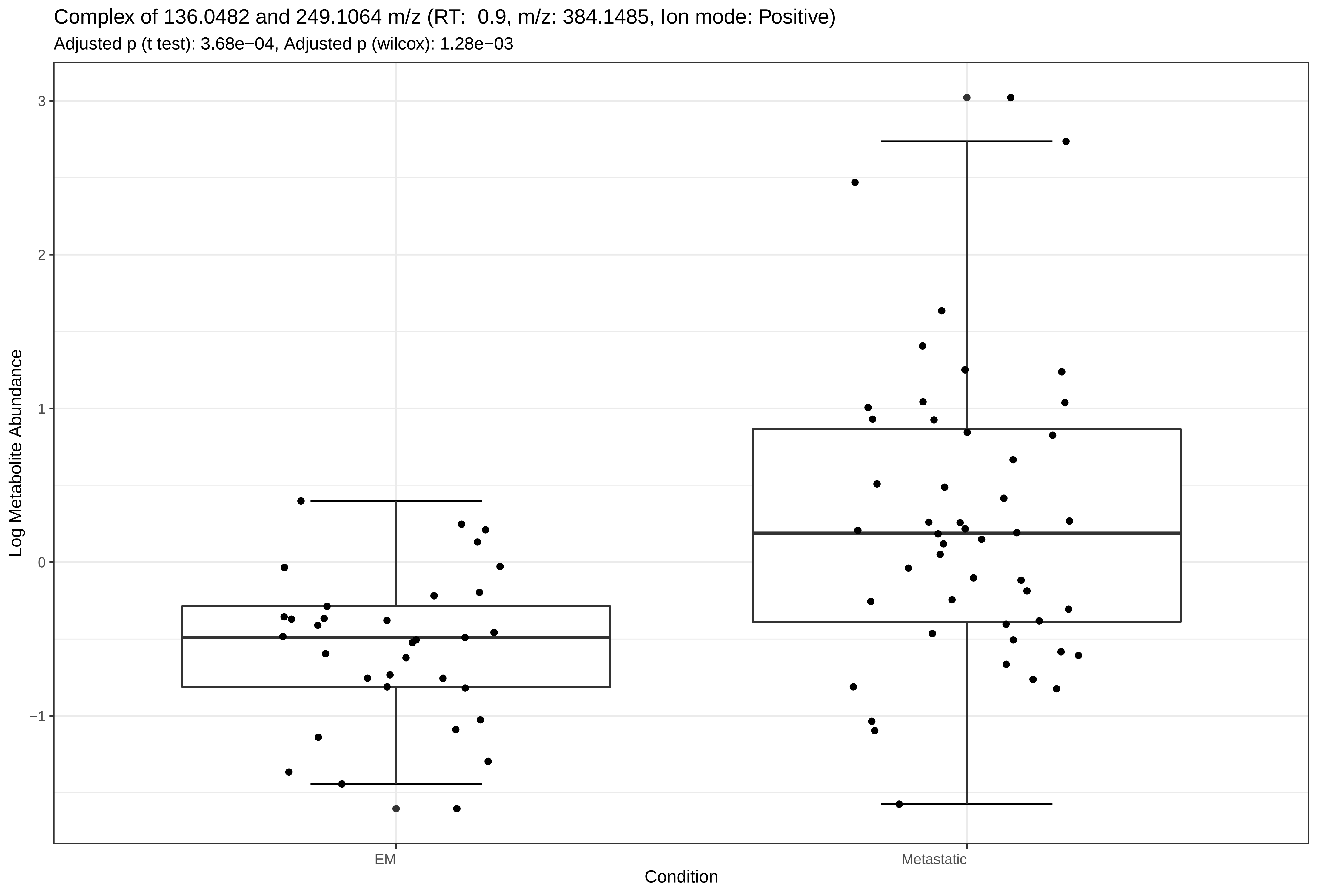
Complex of 136.0482 and 249.1064 m/z (RT: 0.9, m/z: 384.1485, Ion mode: Positive)
Adjusted p (t test): 3.68e−04, Adjusted p (wilcox): 1.28e−03
Log Metabolite Abundance
3
2
1
0
−1
EM
Metastatic
Condition

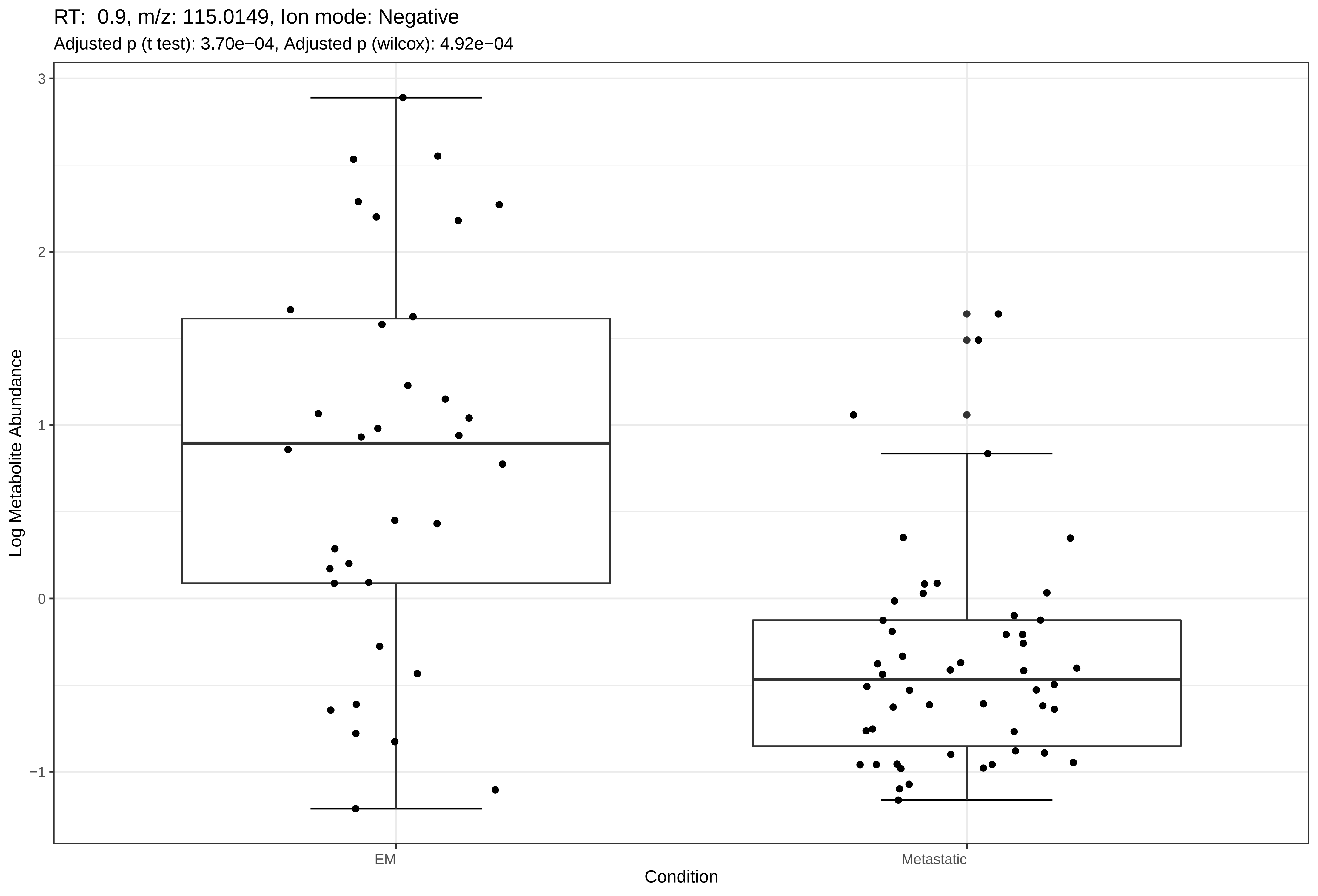
RT: 0.9, m/z: 115.0149, Ion mode: Negative
Adjusted p (t test): 3.70e−04, Adjusted p (wilcox): 4.92e−04
Log Metabolite Abundance
3
2
1
0
−1
EM
Metastatic
Condition

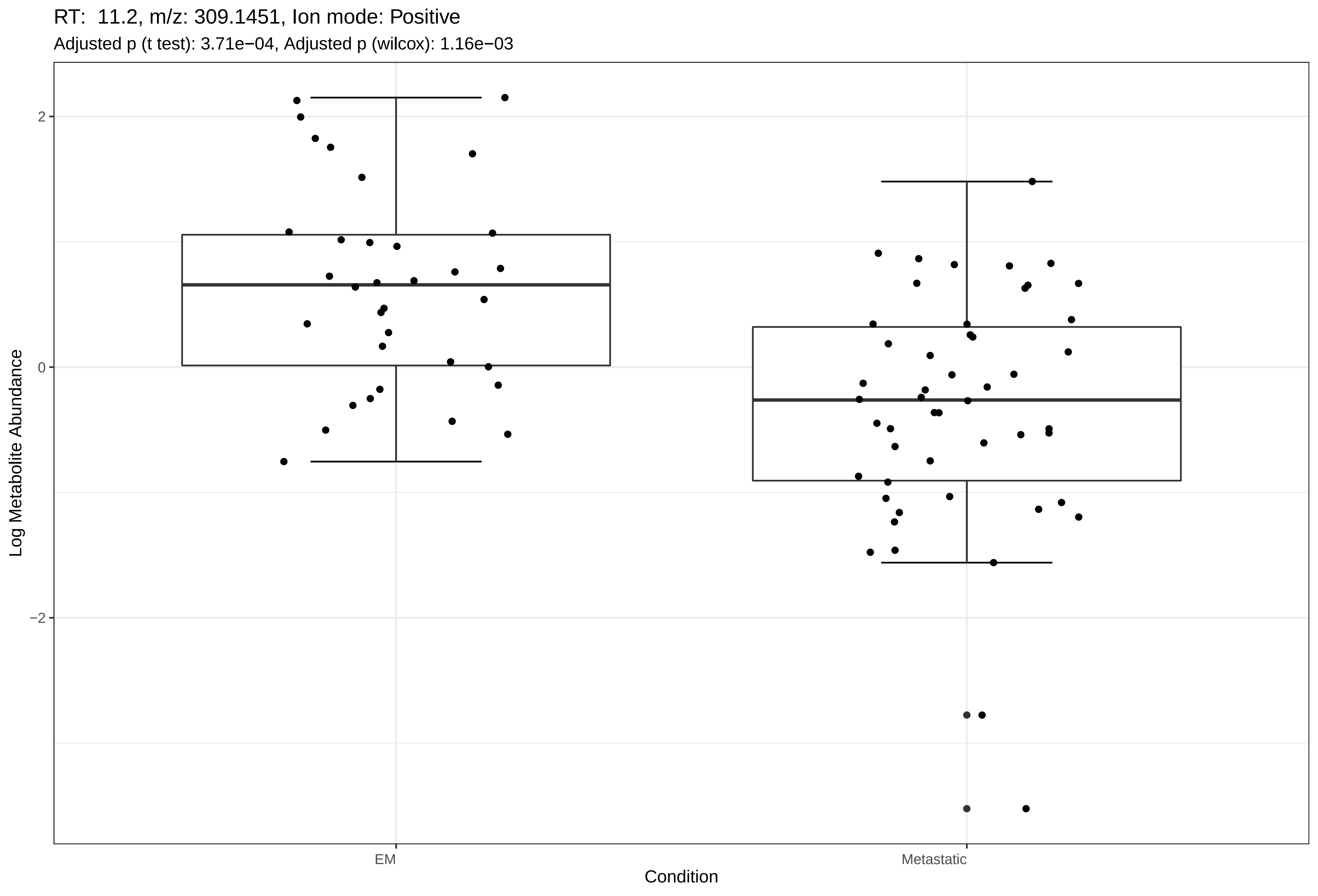

RT: 11.2, m/z: 309.1451, Ion mode: Positive
Adjusted p (t test): 3.71e−04, Adjusted p (wilcox): 1.16e−03
Log Metabolite Abundance
2
0
−2
EM
Metastatic
Condition

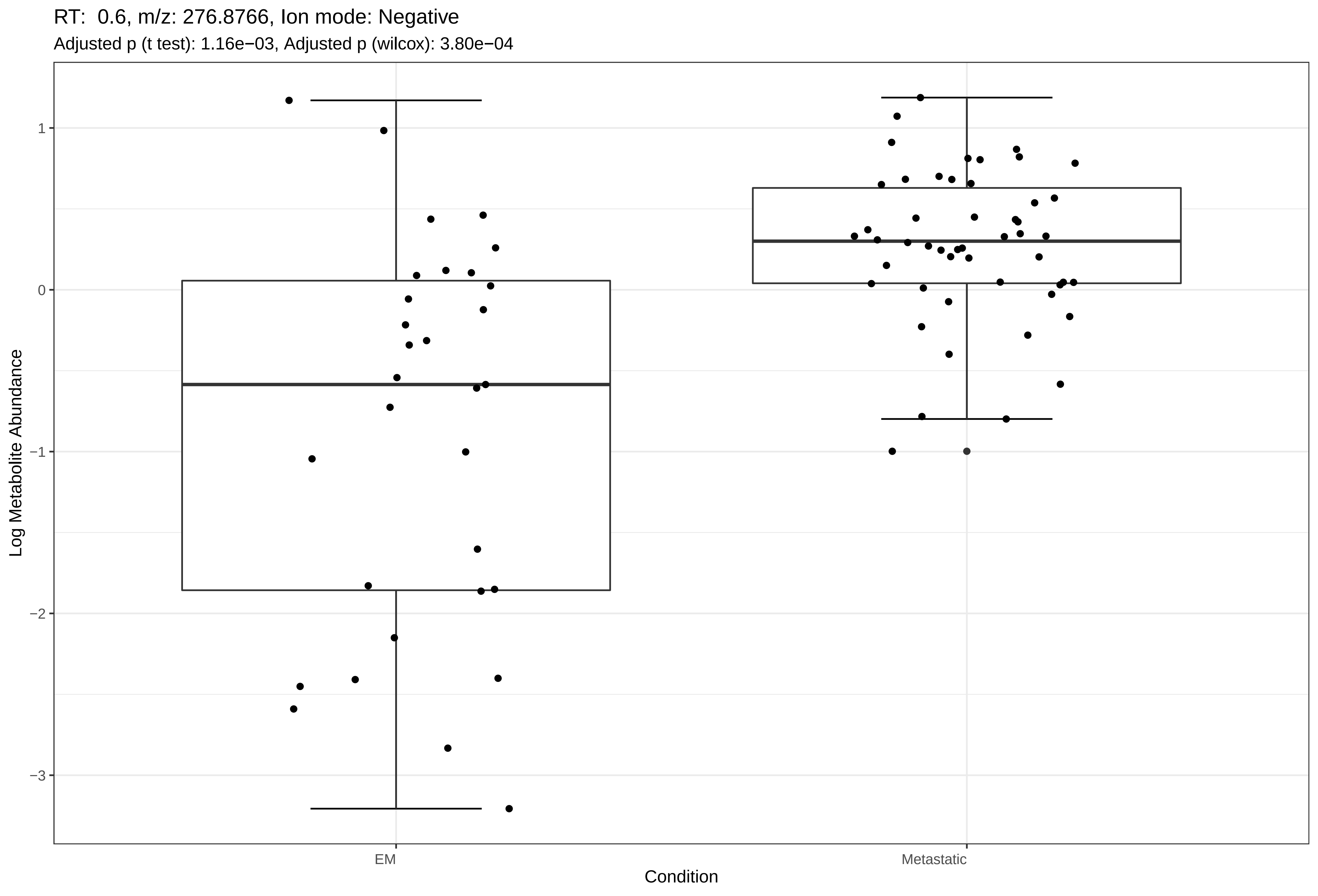

RT: 0.6, m/z: 276.8766, Ion mode: Negative
Adjusted p (t test): 1.16e−03, Adjusted p (wilcox): 3.80e−04
Log Metabolite Abundance
1
0
−1
−2
−3
EM
Metastatic
Condition

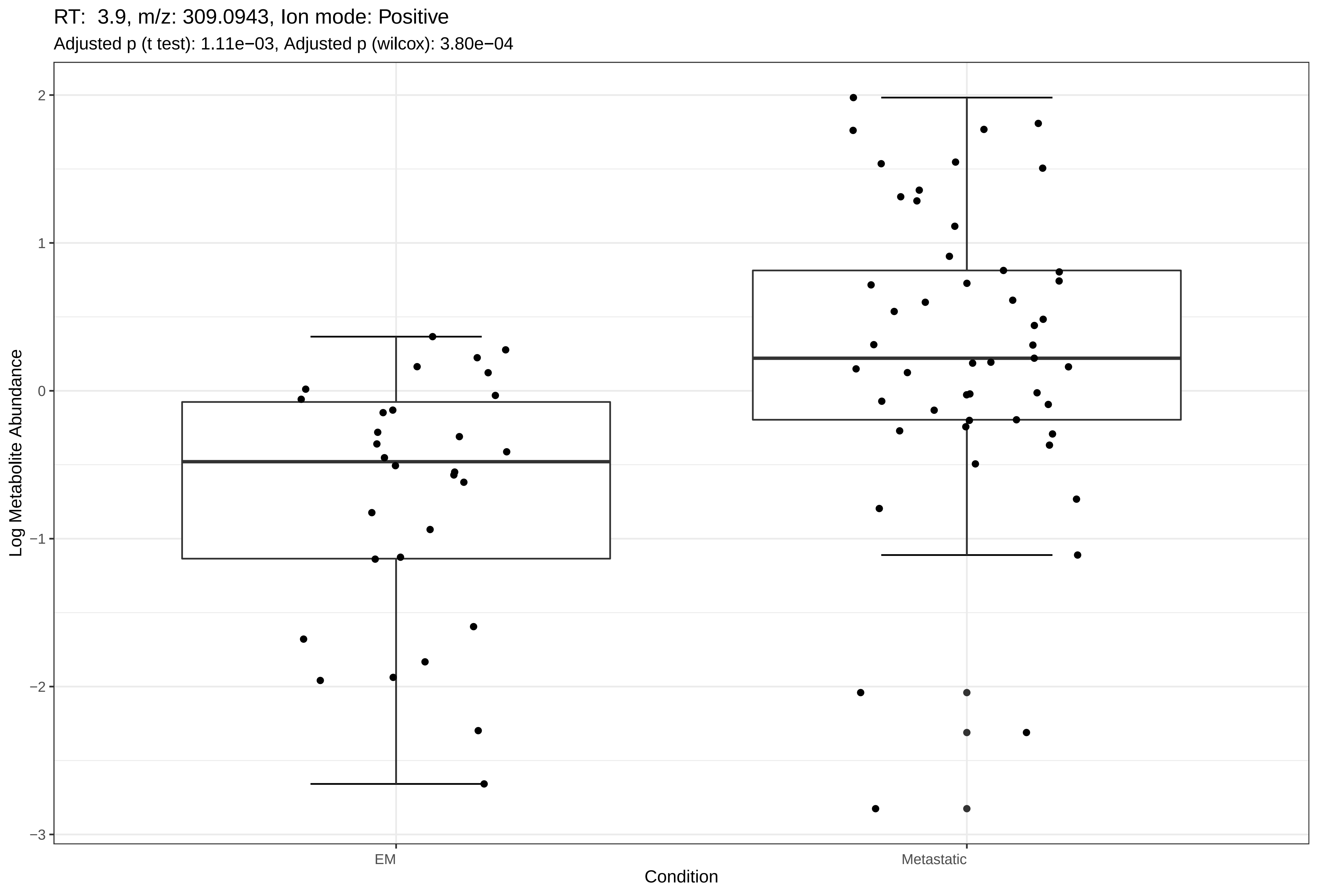
RT: 3.9, m/z: 309.0943, Ion mode: Positive
Adjusted p (t test): 1.11e−03, Adjusted p (wilcox): 3.80e−04
Log Metabolite Abundance
2
1
0
−1
−2
−3
EM
Metastatic
Condition

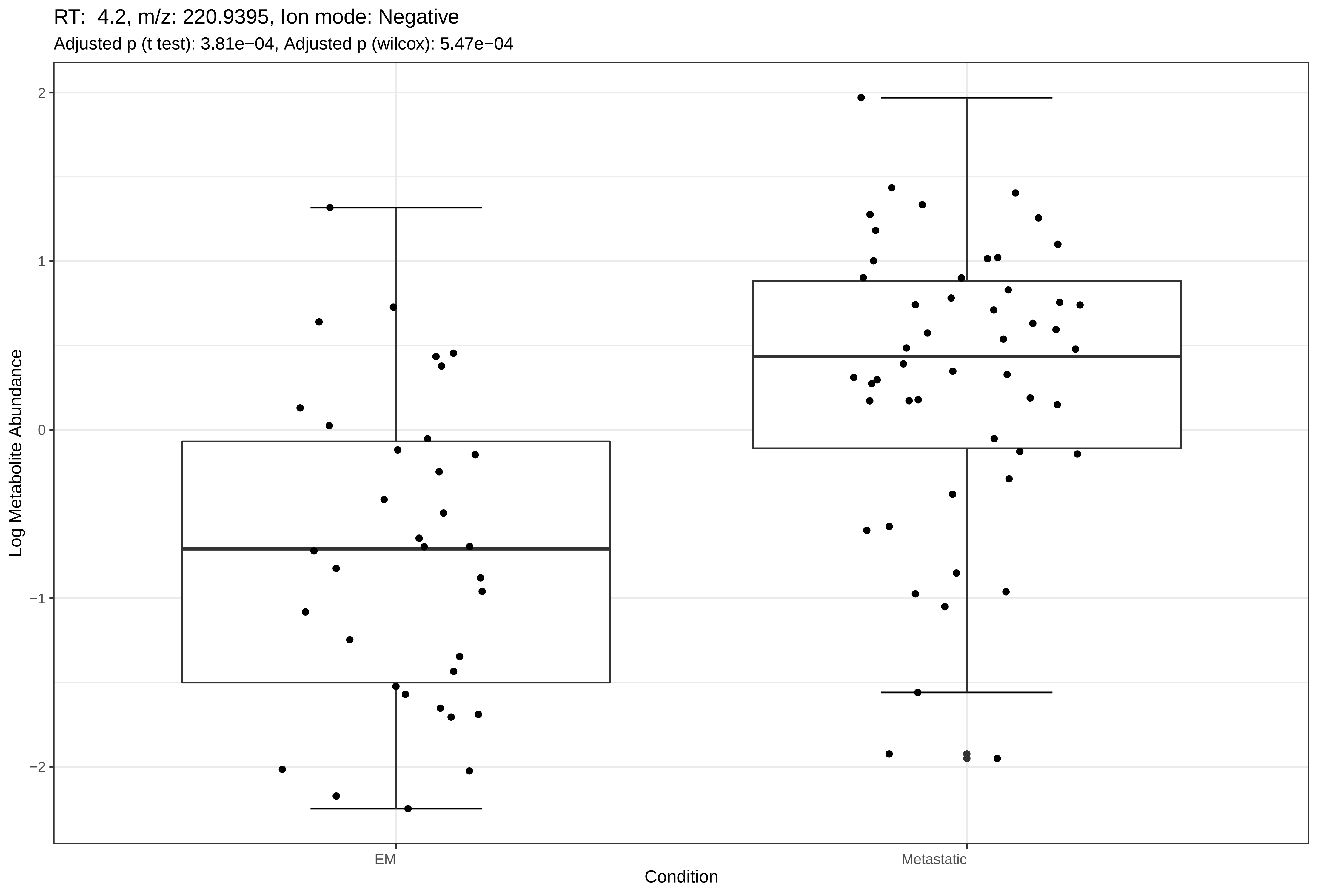

RT: 4.2, m/z: 220.9395, Ion mode: Negative
Adjusted p (t test): 3.81e−04, Adjusted p (wilcox): 5.47e−04
2
1
0
−1
−2
Log Metabolite Abundance
EM
Metastatic
Condition

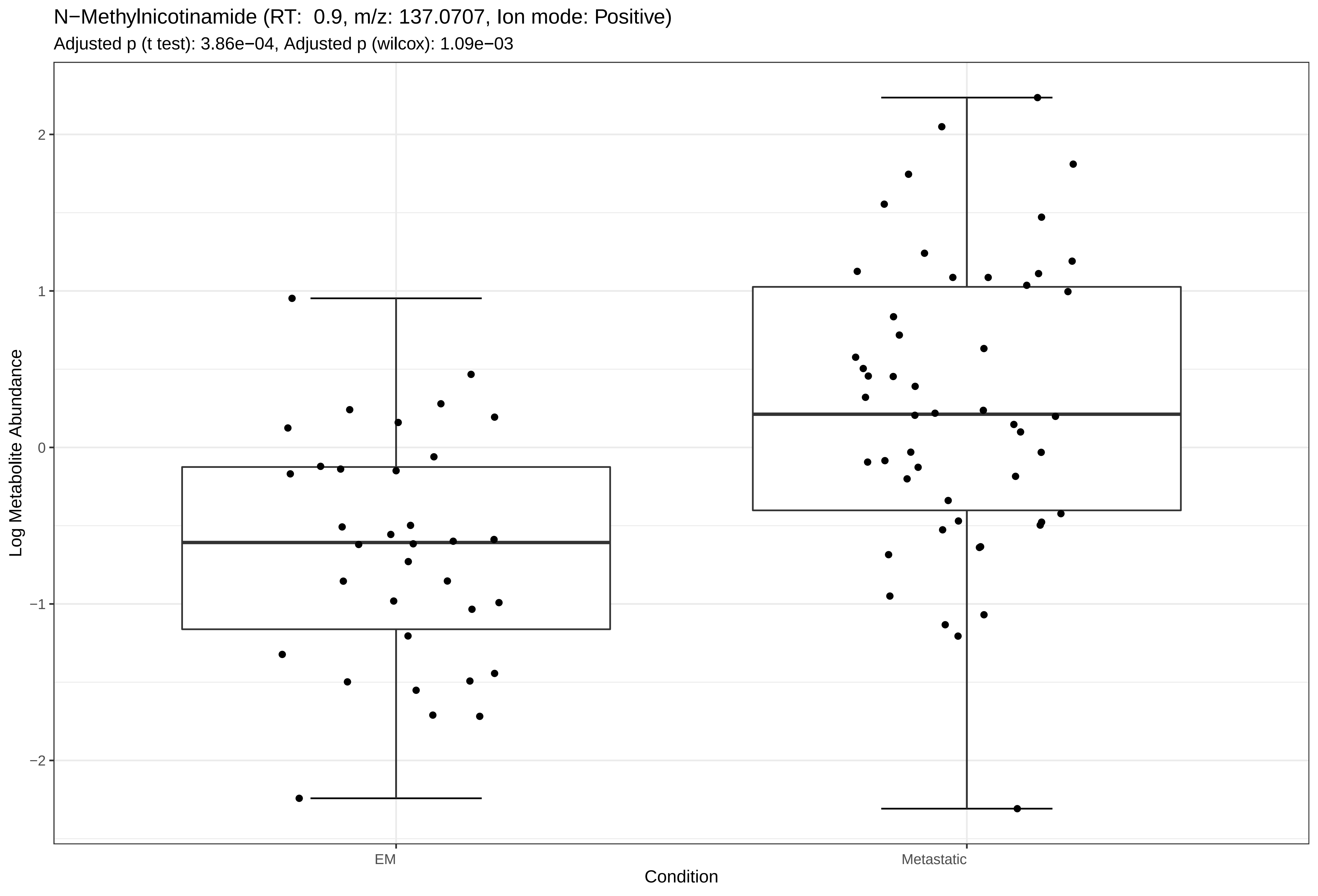
N−Methylnicotinamide (RT: 0.9, m/z: 137.0707, Ion mode: Positive)
Adjusted p (t test): 3.86e−04, Adjusted p (wilcox): 1.09e−03
Log Metabolite Abundance
2
1
0
−1
−2
EM
Metastatic
Condition

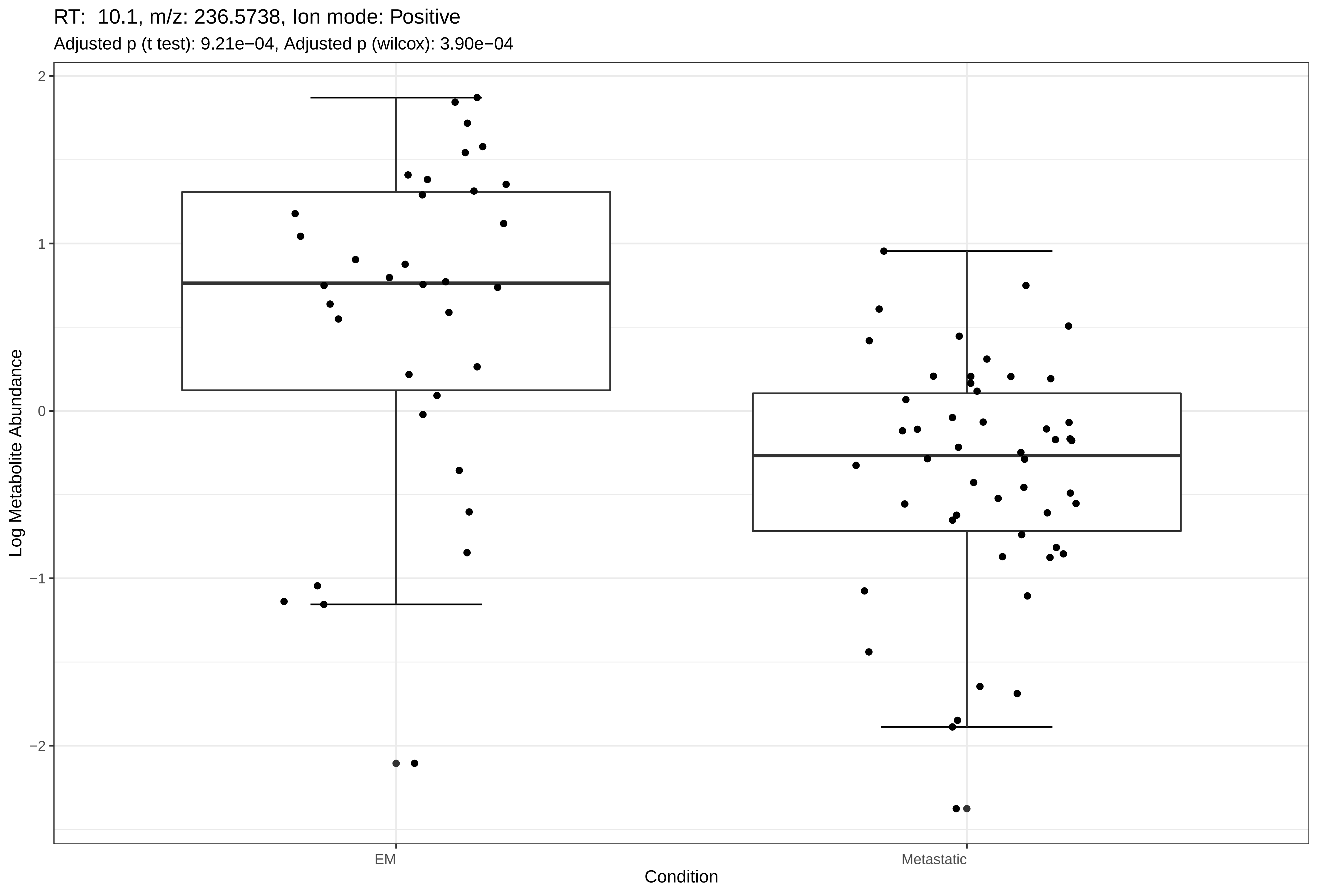
RT: 10.1, m/z: 236.5738, Ion mode: Positive
Adjusted p (t test): 9.21e−04, Adjusted p (wilcox): 3.90e−04
Log Metabolite Abundance
2
1
0
−1
−2
EM
Metastatic
Condition

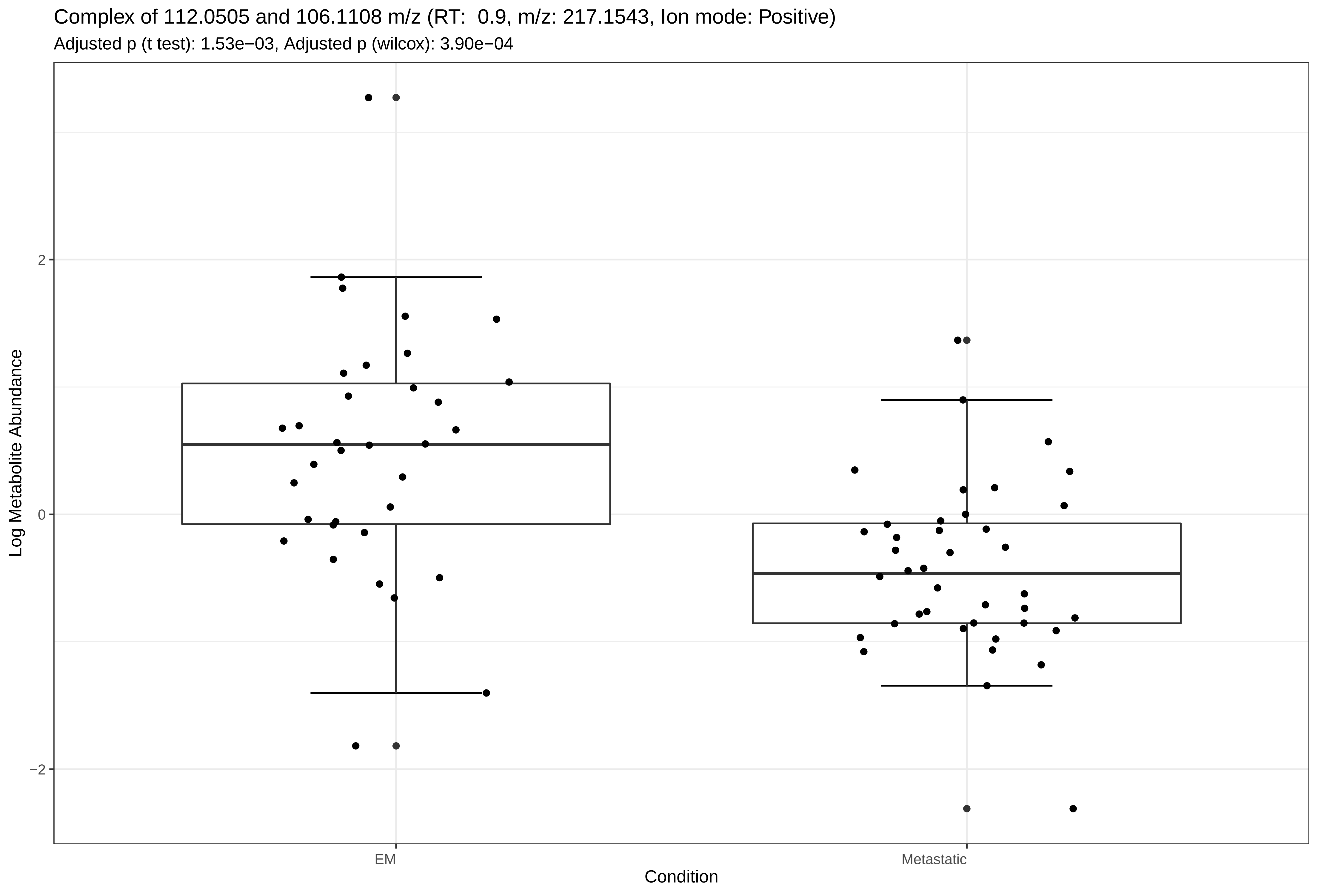

Complex of 112.0505 and 106.1108 m/z (RT: 0.9, m/z: 217.1543, Ion mode: Positive)
Adjusted p (t test): 1.53e−03, Adjusted p (wilcox): 3.90e−04
Log Metabolite Abundance
2
0
−2
EM
Metastatic
Condition

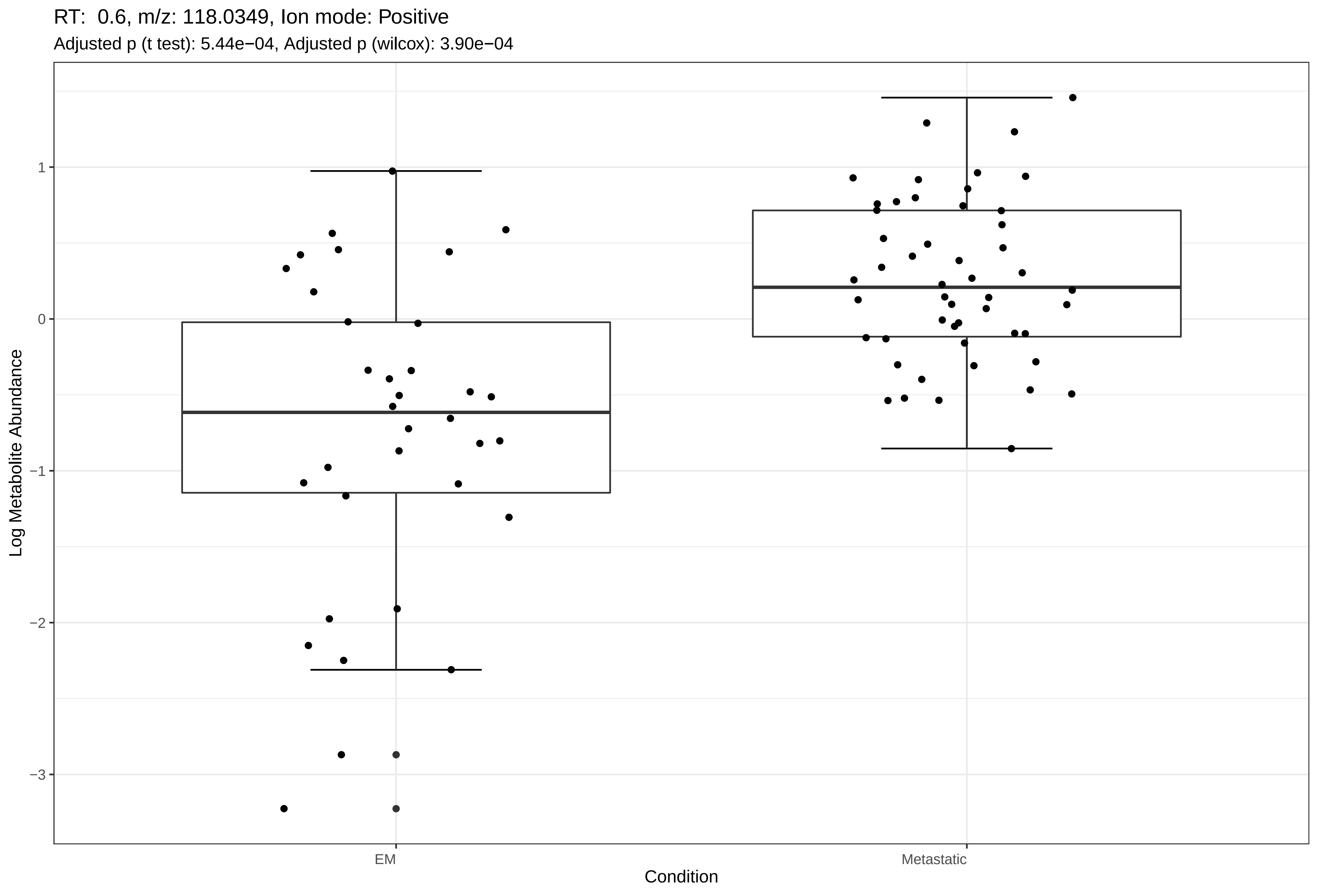
RT: 0.6, m/z: 118.0349, Ion mode: Positive
Adjusted p (t test): 5.44e−04, Adjusted p (wilcox): 3.90e−04
Log Metabolite Abundance
1
0
−1
−2
−3
EM
Metastatic
Condition

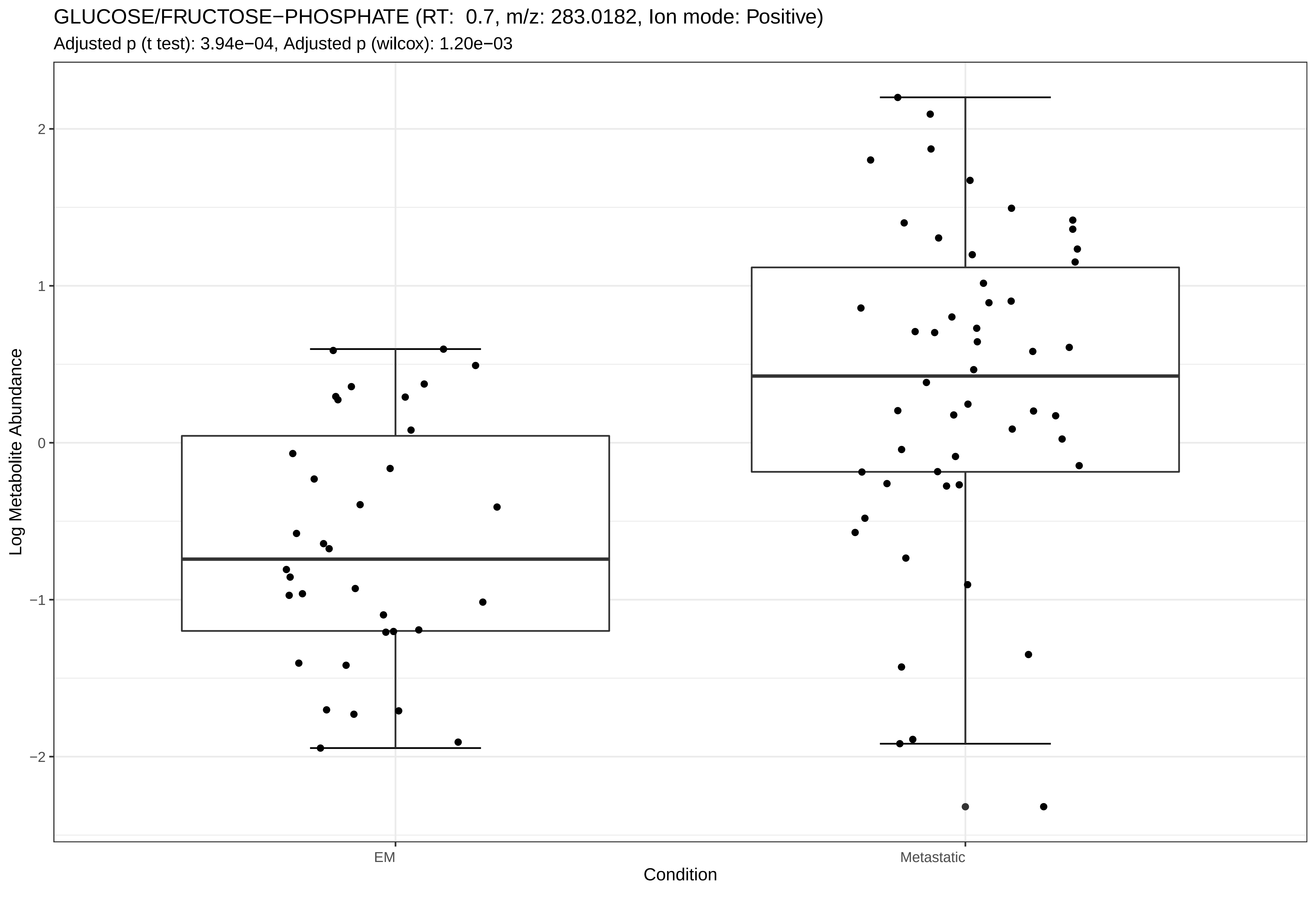
GLUCOSE/FRUCTOSE−PHOSPHATE (RT: 0.7, m/z: 283.0182, Ion mode: Positive)
Adjusted p (t test): 3.94e−04, Adjusted p (wilcox): 1.20e−03
Log Metabolite Abundance
2
1
0
−1
−2
EM
Metastatic
Condition

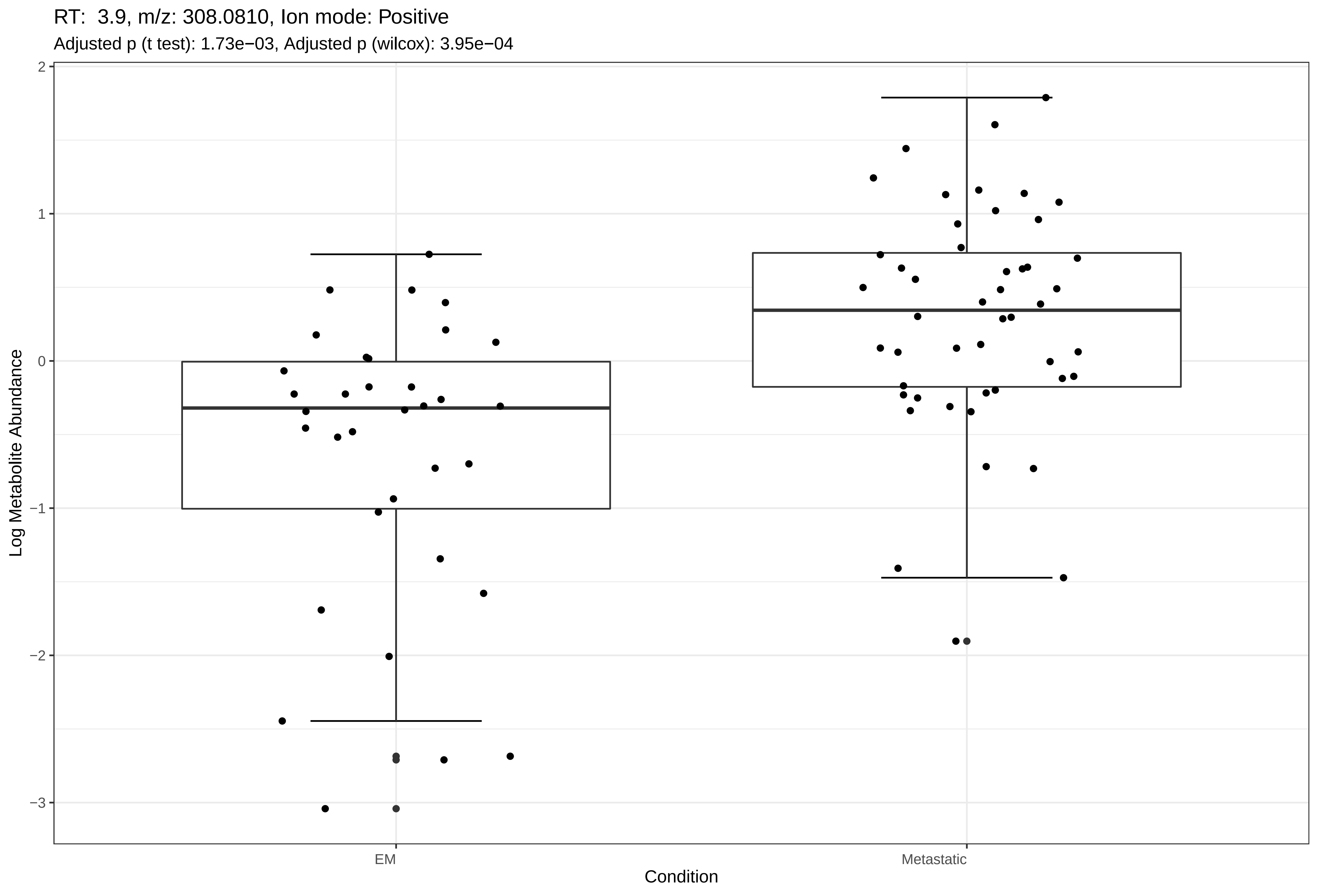

RT: 3.9, m/z: 308.0810, Ion mode: Positive
Adjusted p (t test): 1.73e−03, Adjusted p (wilcox): 3.95e−04
Log Metabolite Abundance
2
1
0
−1
−2
−3
EM
Metastatic
Condition

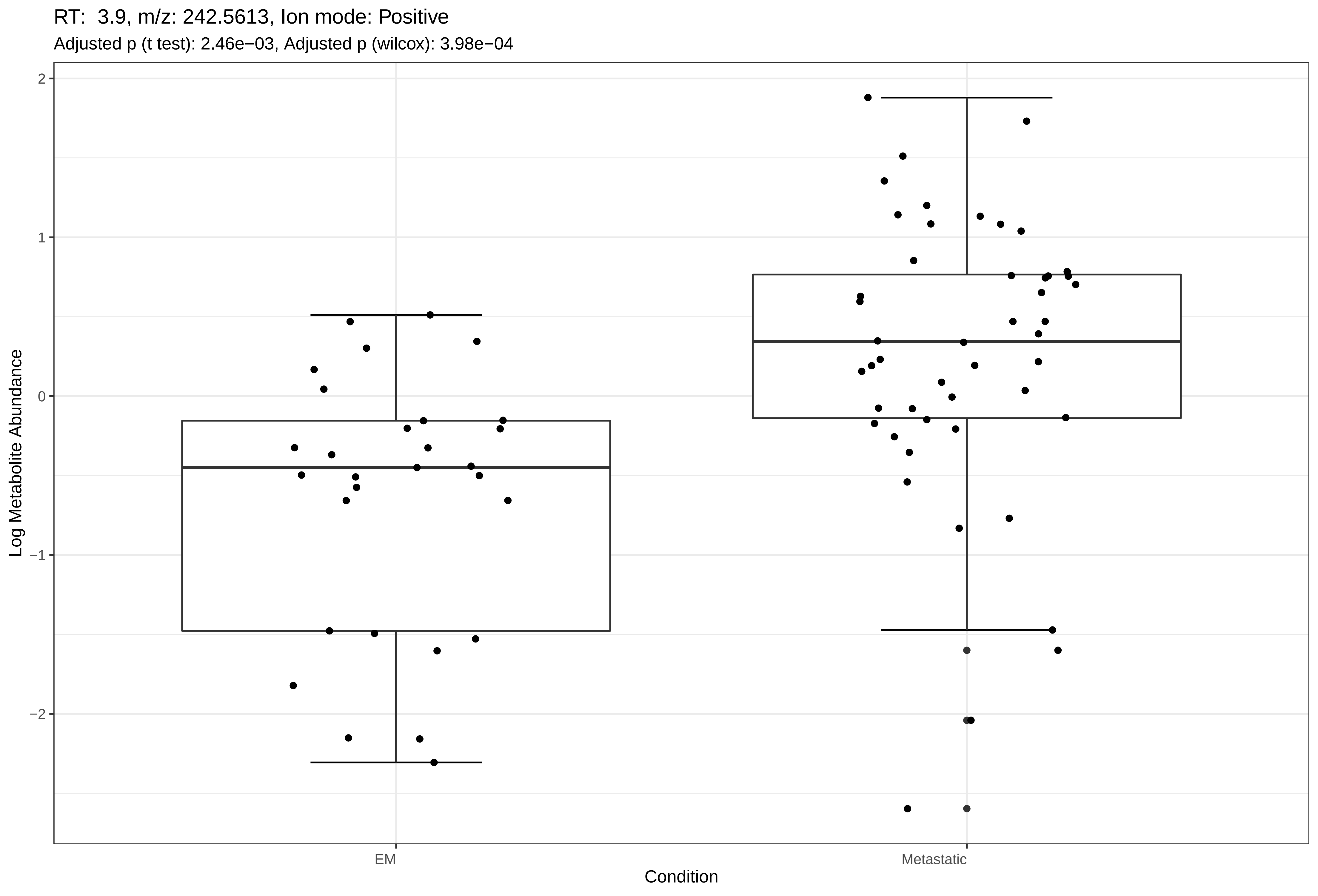
RT: 3.9, m/z: 242.5613, Ion mode: Positive
Adjusted p (t test): 2.46e−03, Adjusted p (wilcox): 3.98e−04
Log Metabolite Abundance
2
1
0
−1
−2
EM
Metastatic
Condition

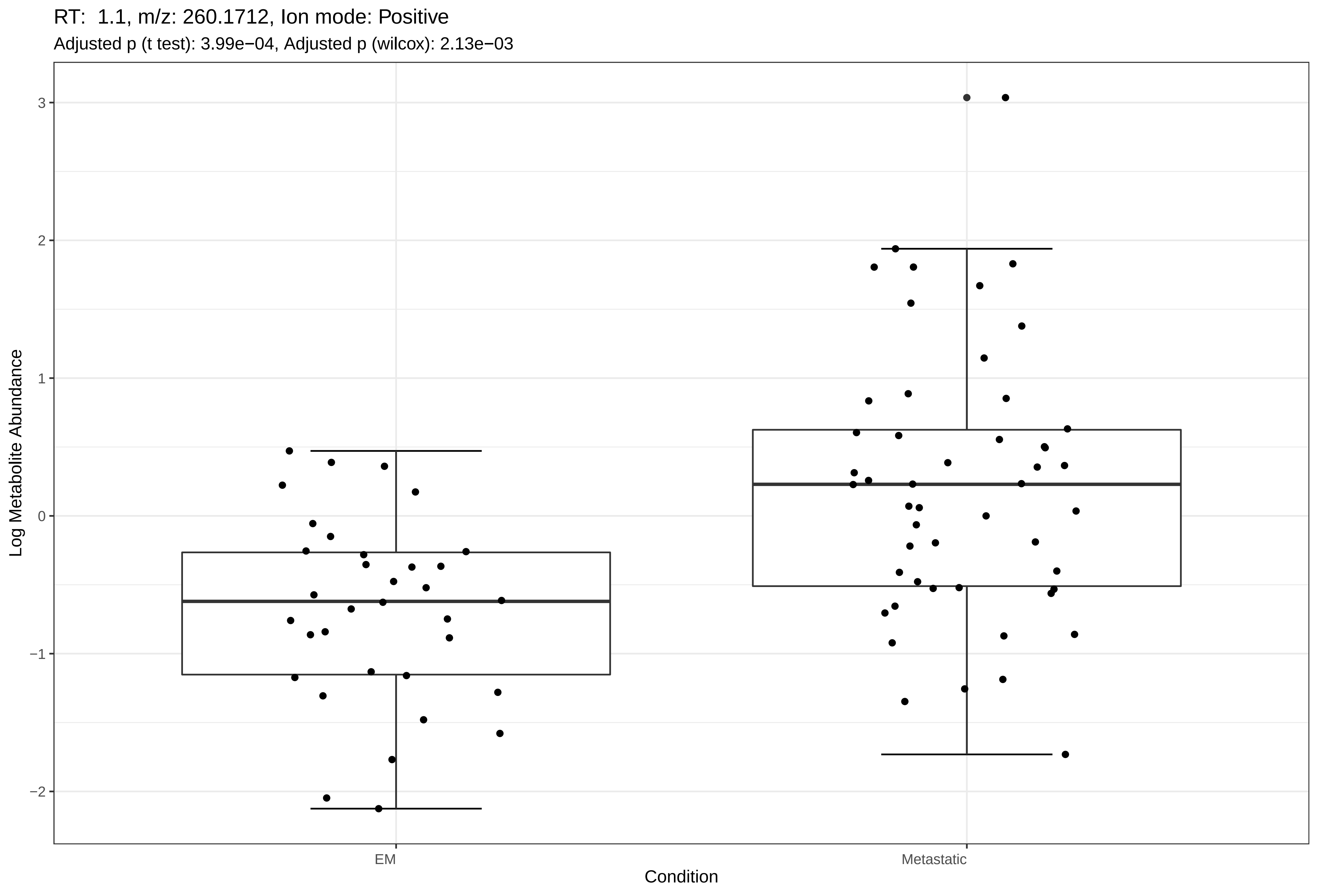
RT: 1.1, m/z: 260.1712, Ion mode: Positive
Adjusted p (t test): 3.99e−04, Adjusted p (wilcox): 2.13e−03
Log Metabolite Abundance
3
2
1
0
−1
−2
EM
Metastatic
Condition

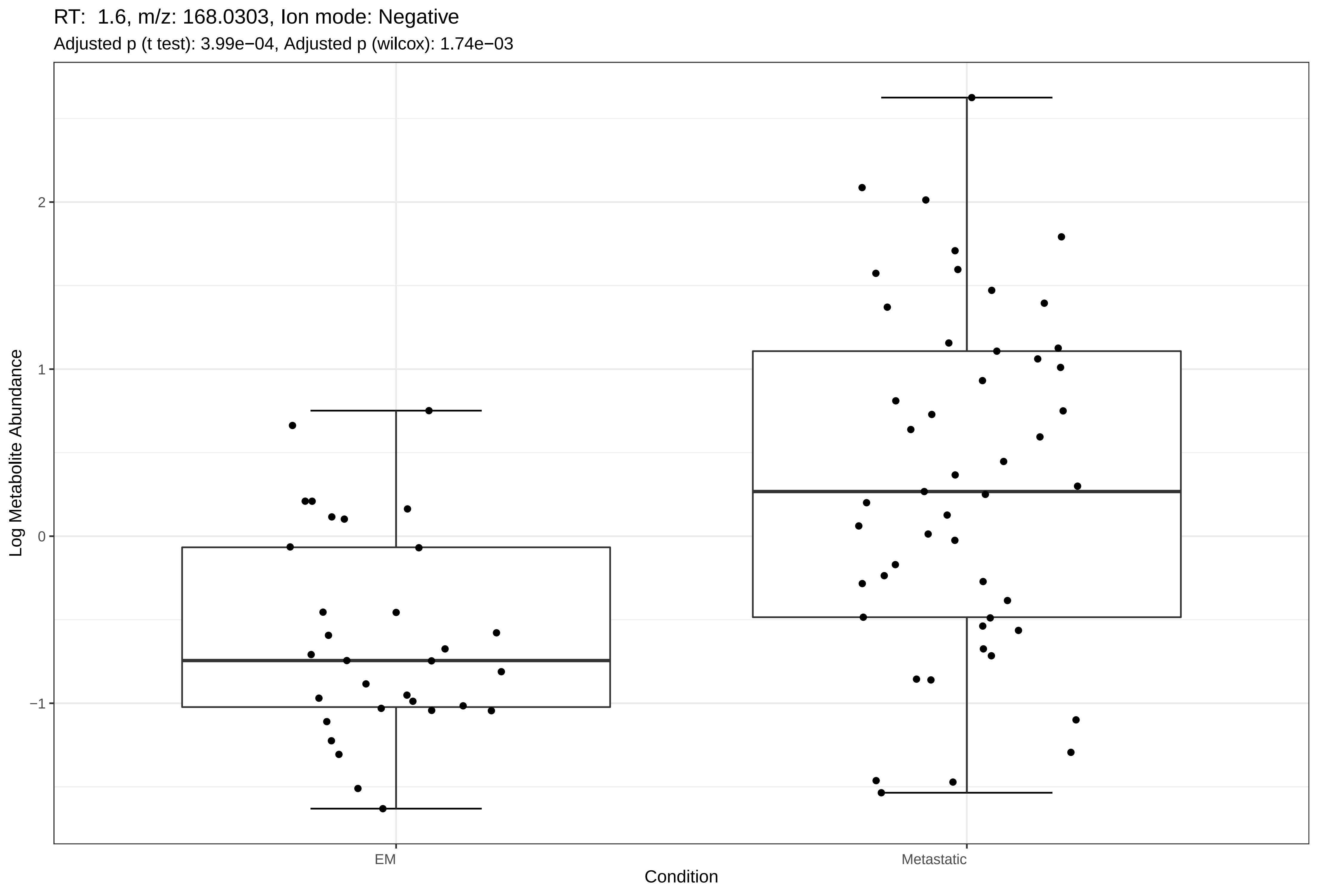
RT: 1.6, m/z: 168.0303, Ion mode: Negative
Adjusted p (t test): 3.99e−04, Adjusted p (wilcox): 1.74e−03
Log Metabolite Abundance
2
1
0
−1
EM
Metastatic
Condition

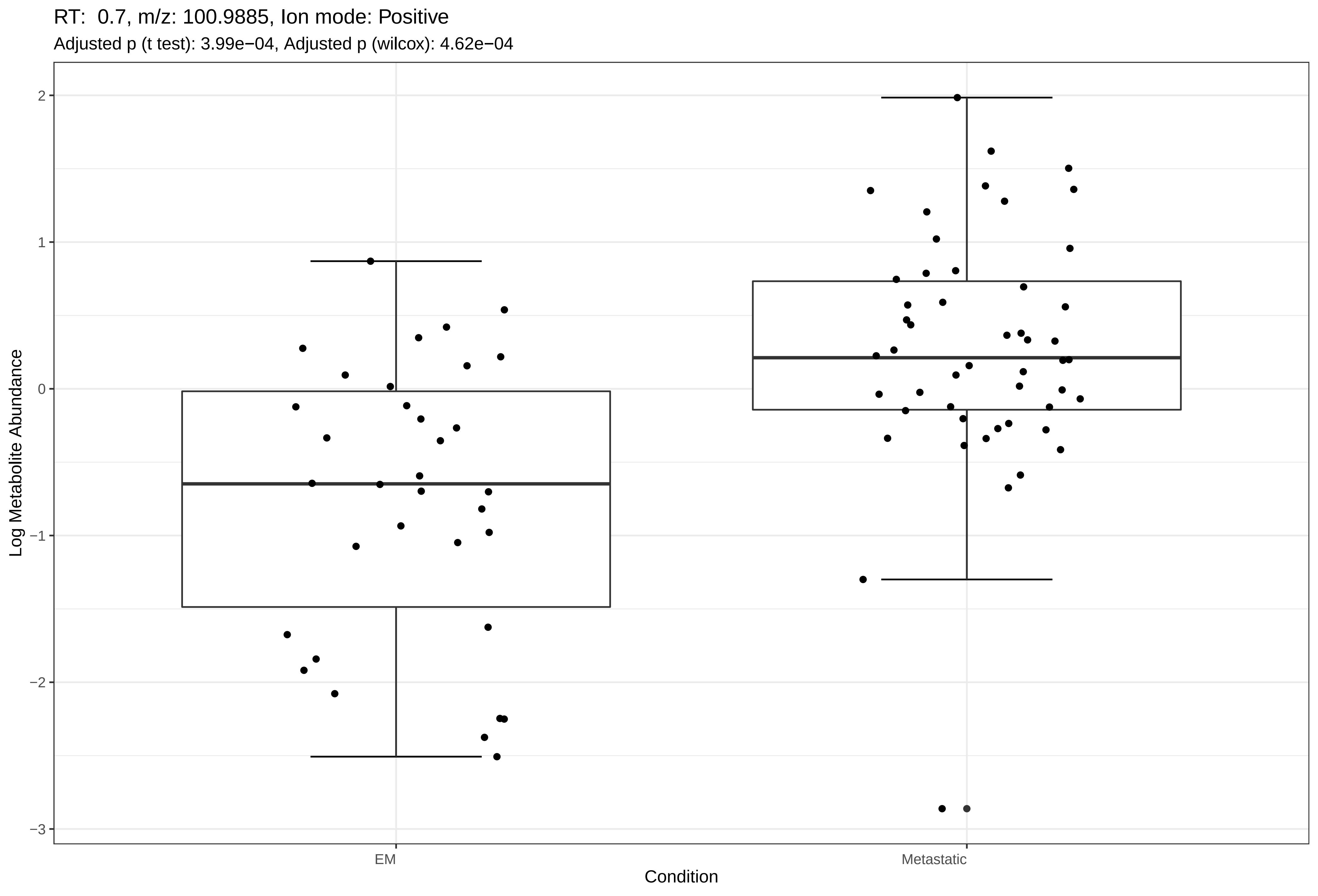

RT: 0.7, m/z: 100.9885, Ion mode: Positive
Adjusted p (t test): 3.99e−04, Adjusted p (wilcox): 4.62e−04
Log Metabolite Abundance
2
1
0
−1
−2
−3
EM
Metastatic
Condition

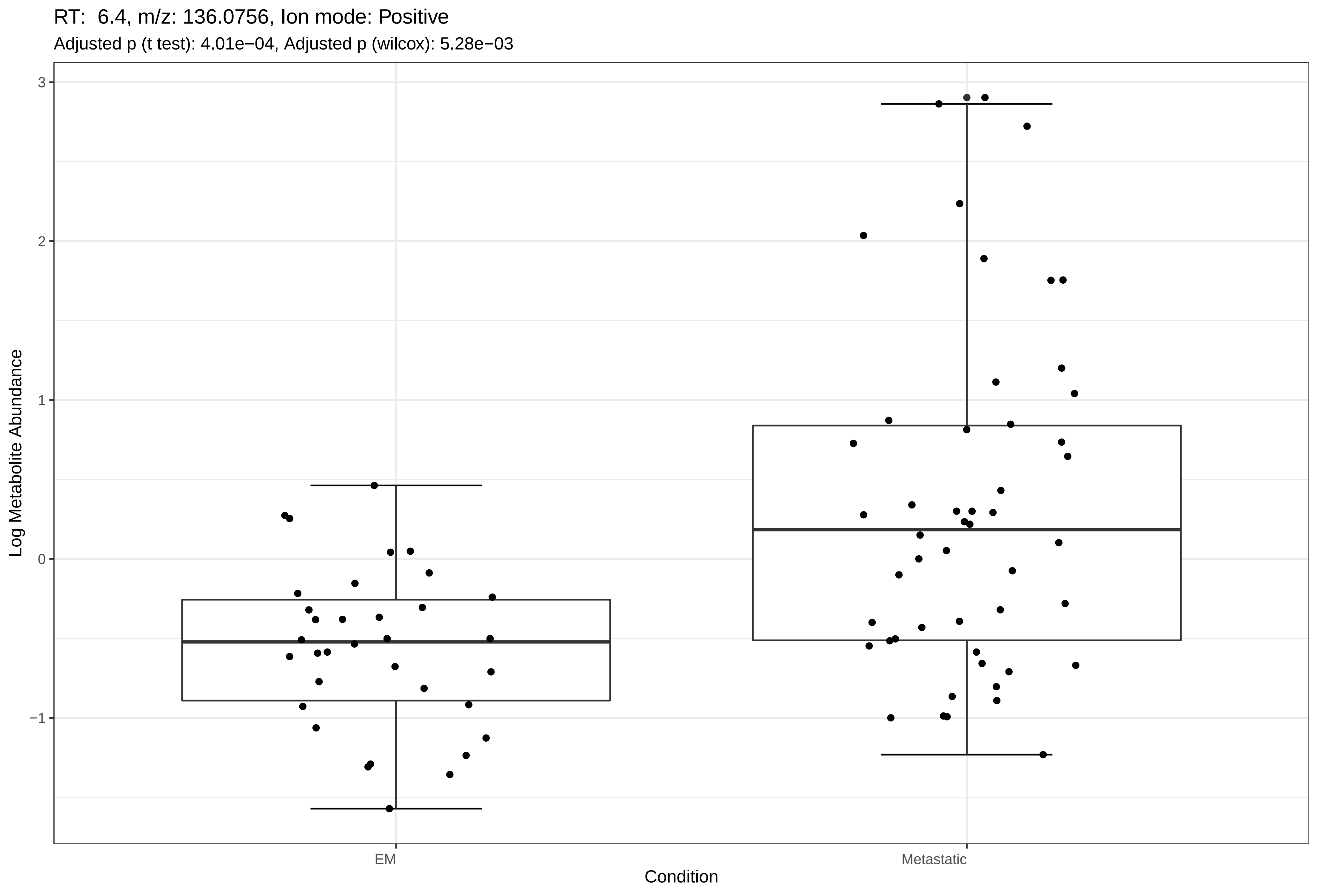
RT: 6.4, m/z: 136.0756, Ion mode: Positive
Adjusted p (t test): 4.01e−04, Adjusted p (wilcox): 5.28e−03
3
2
1
0
−1
Log Metabolite Abundance
EM
Metastatic
Condition

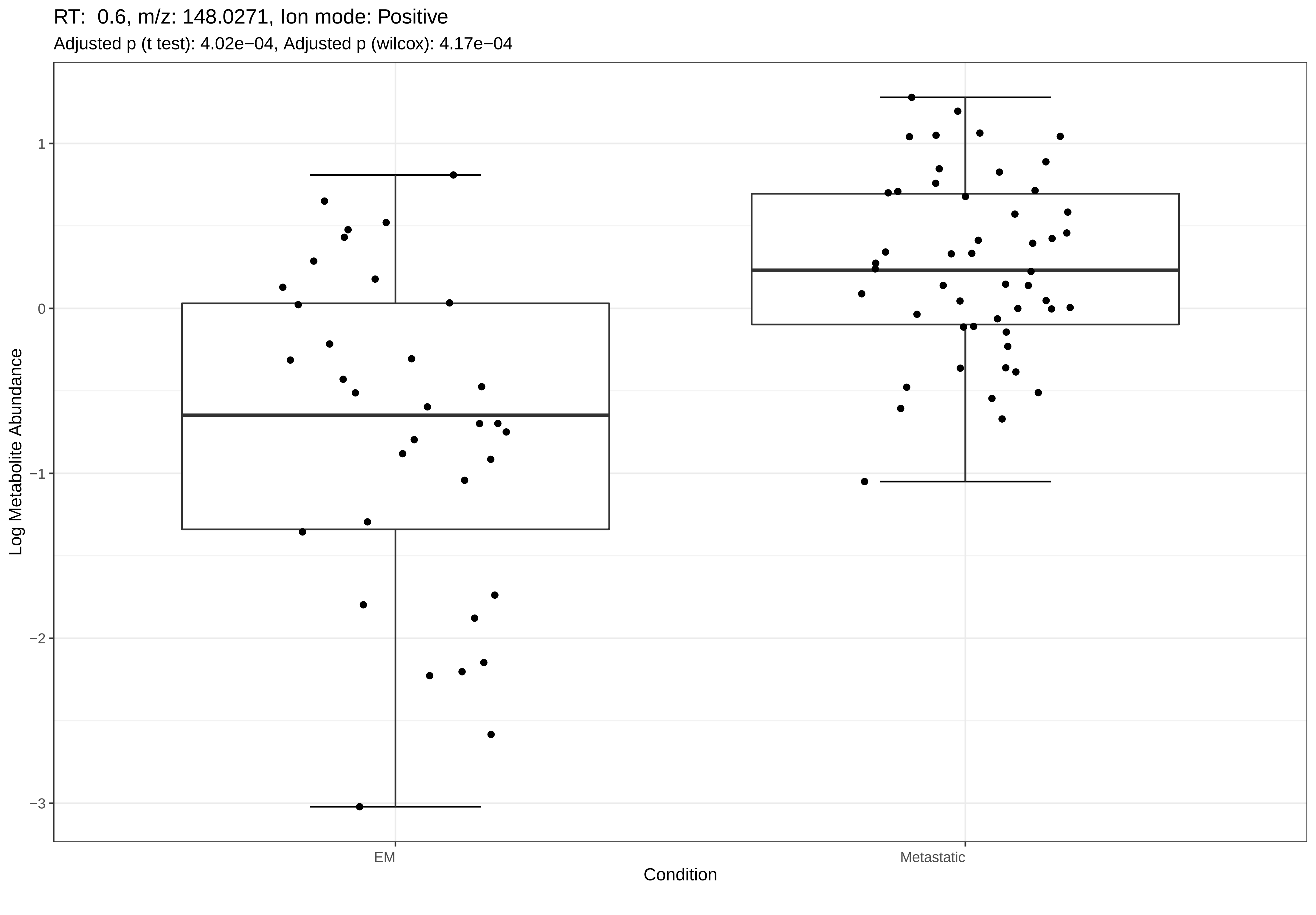
RT: 0.6, m/z: 148.0271, Ion mode: Positive
Adjusted p (t test): 4.02e−04, Adjusted p (wilcox): 4.17e−04
Log Metabolite Abundance
1
0
−1
−2
−3
EM
Metastatic
Condition

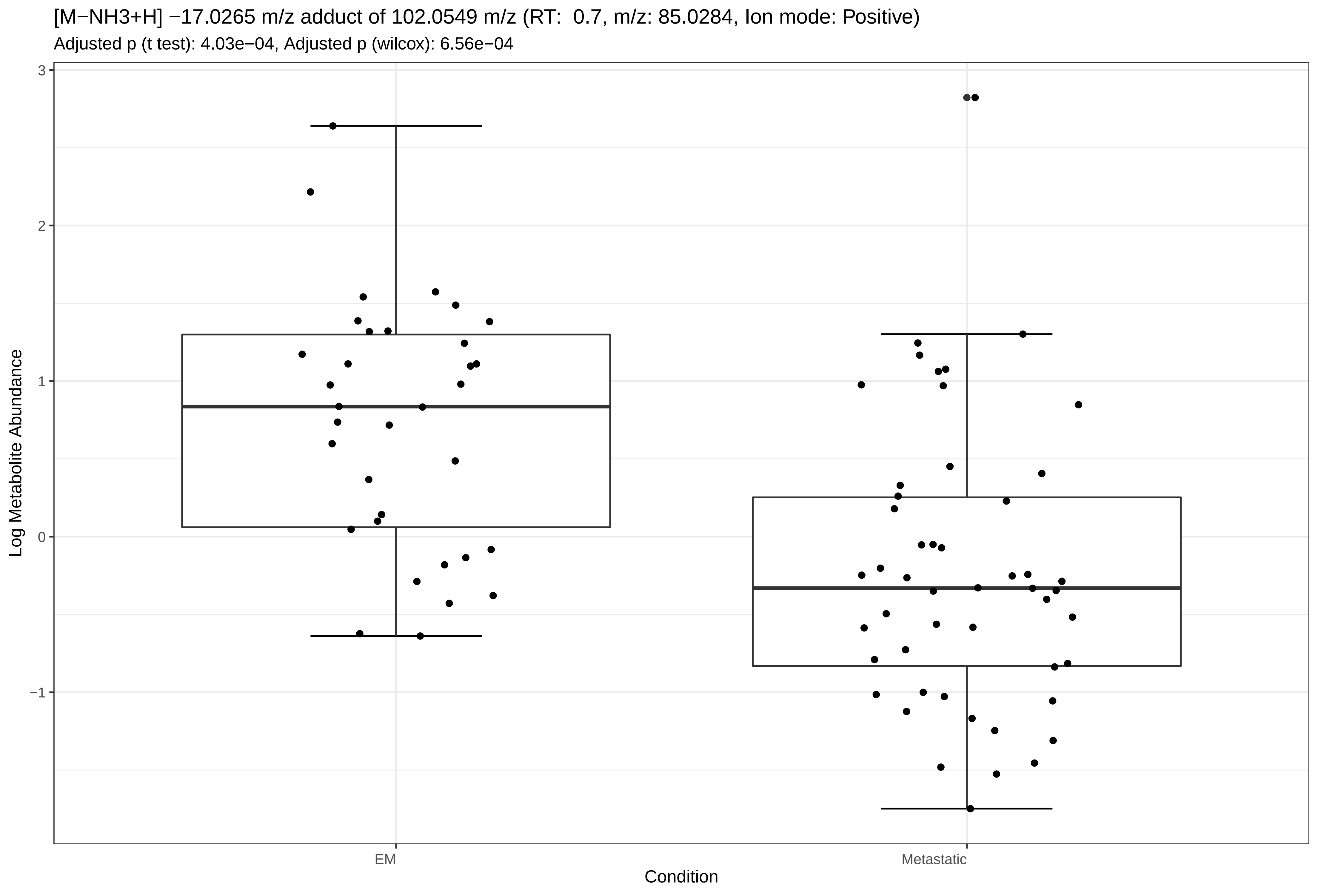

[M−NH3+H] −17.0265 m/z adduct of 102.0549 m/z (RT: 0.7, m/z: 85.0284, Ion mode: Positive)
Adjusted p (t test): 4.03e−04, Adjusted p (wilcox): 6.56e−04
Log Metabolite Abundance
3
2
1
0
−1
EM
Metastatic
Condition

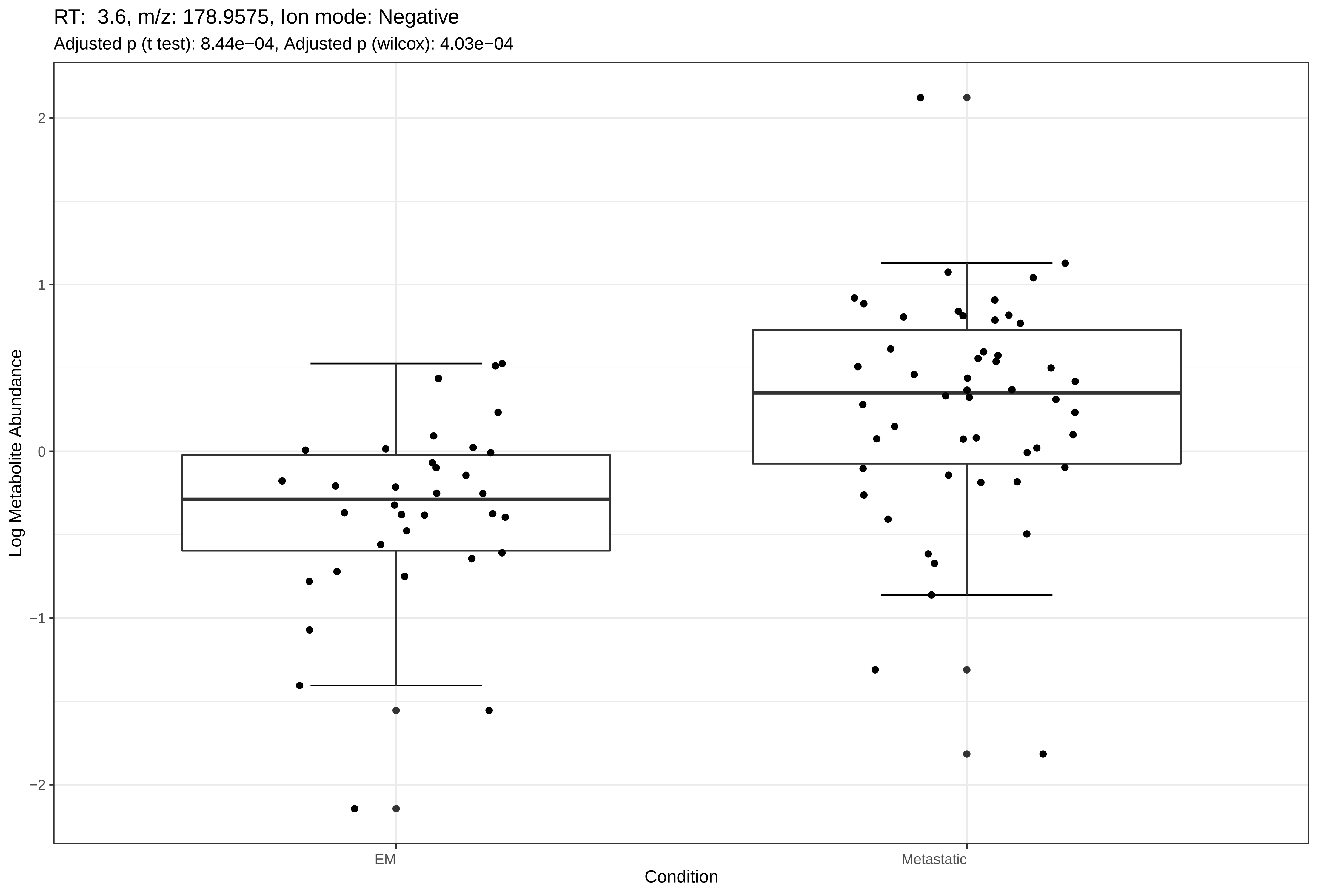

RT: 3.6, m/z: 178.9575, Ion mode: Negative
Adjusted p (t test): 8.44e−04, Adjusted p (wilcox): 4.03e−04
Log Metabolite Abundance
2
1
0
−1
−2
EM
Metastatic
Condition

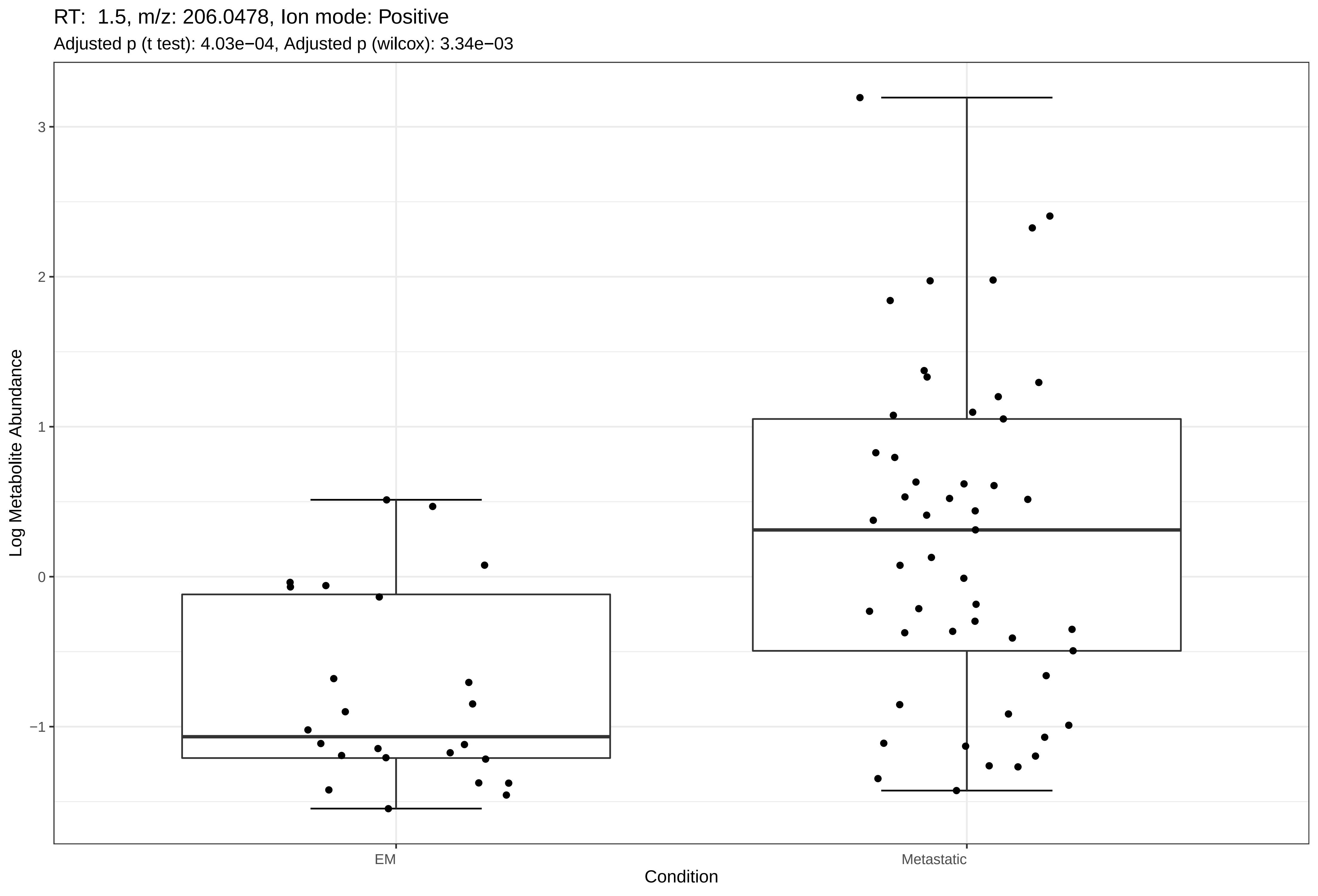
RT: 1.5, m/z: 206.0478, Ion mode: Positive
Adjusted p (t test): 4.03e−04, Adjusted p (wilcox): 3.34e−03
Log Metabolite Abundance
3
2
1
0
−1
EM
Metastatic
Condition

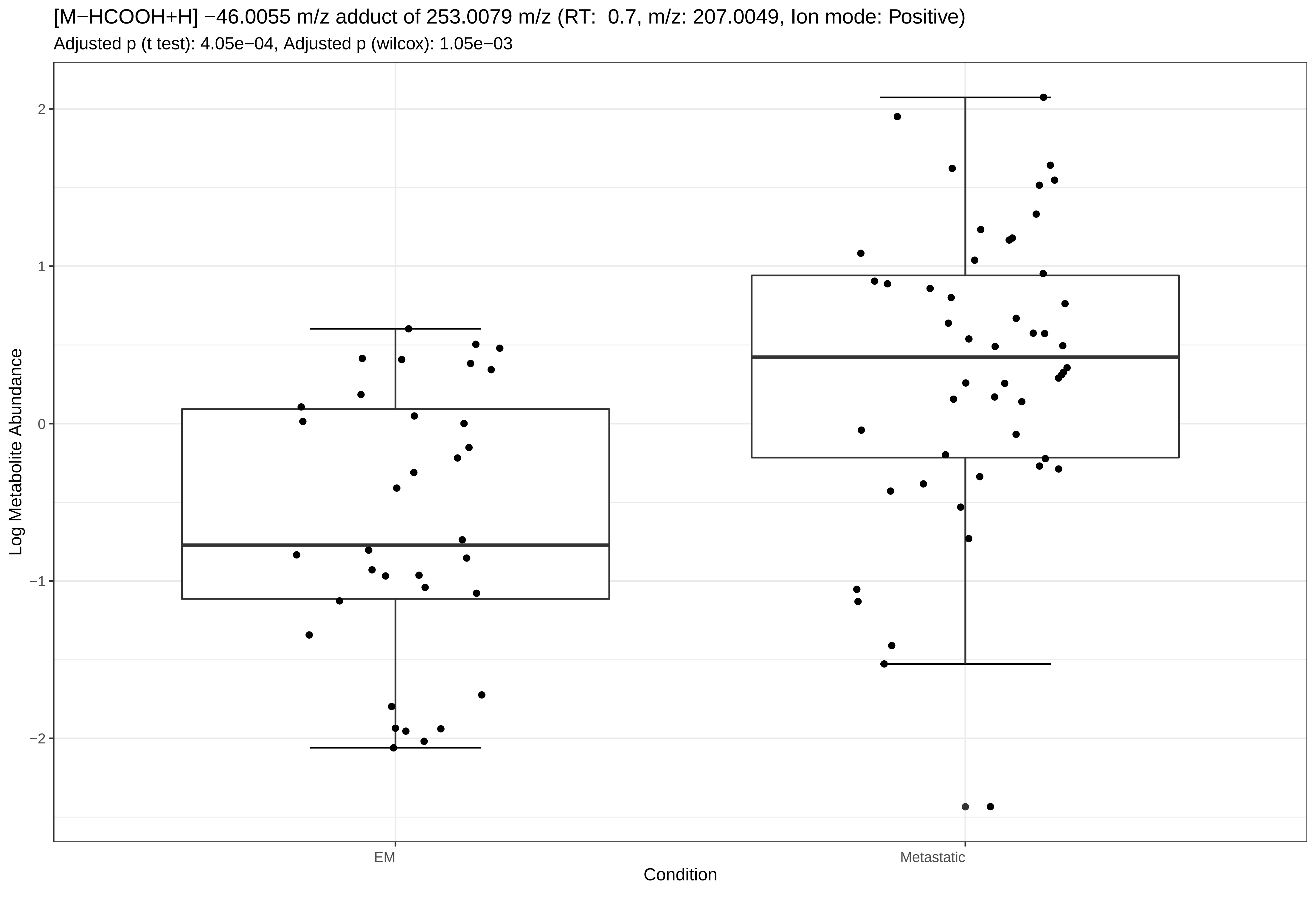
[M−HCOOH+H] −46.0055 m/z adduct of 253.0079 m/z (RT: 0.7, m/z: 207.0049, Ion mode: Positive)
Adjusted p (t test): 4.05e−04, Adjusted p (wilcox): 1.05e−03
Log Metabolite Abundance
2
1
0
−1
−2
EM
Metastatic
Condition

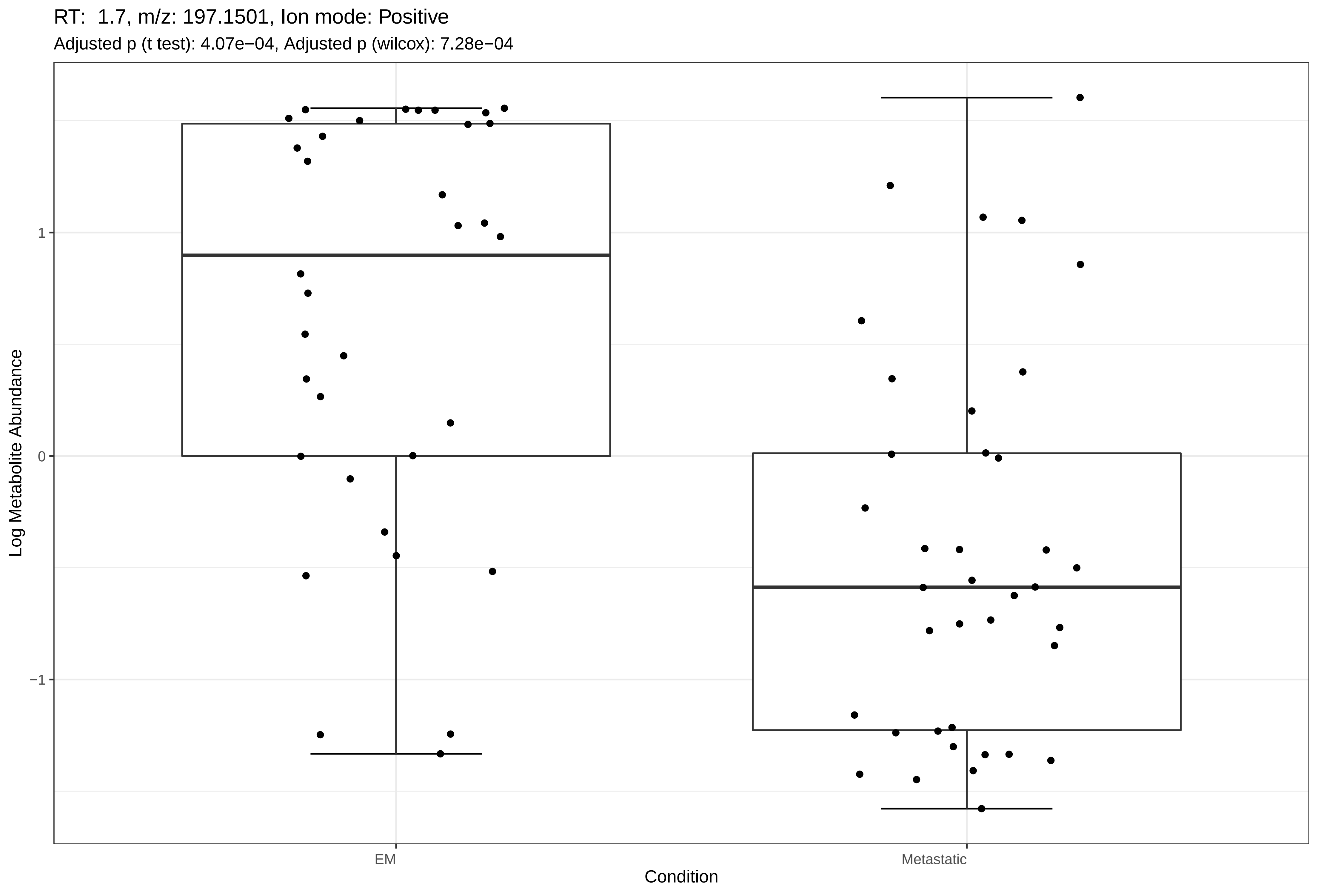

RT: 1.7, m/z: 197.1501, Ion mode: Positive
Adjusted p (t test): 4.07e−04, Adjusted p (wilcox): 7.28e−04
Log Metabolite Abundance
1
0
−1
EM
Metastatic
Condition

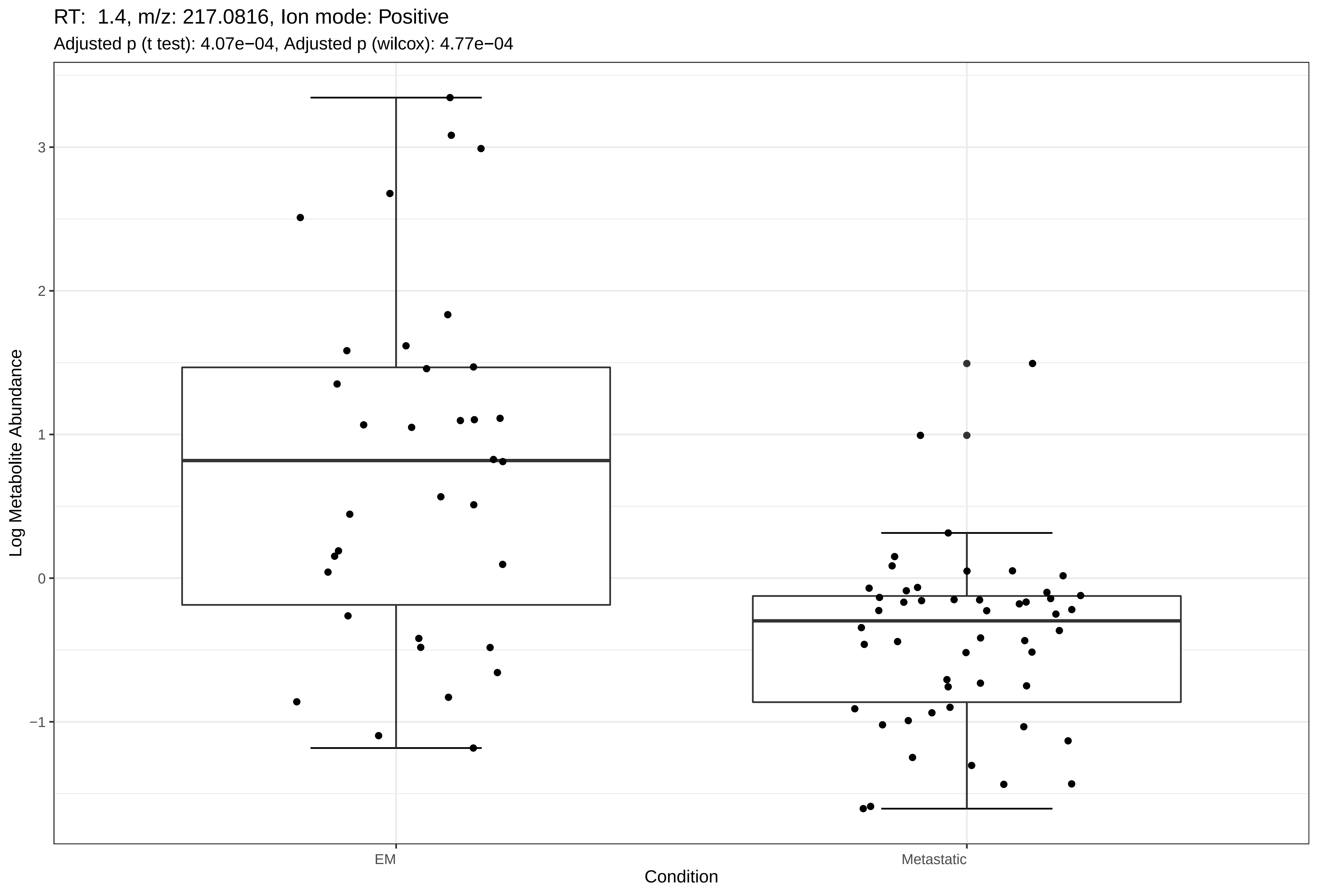

RT: 1.4, m/z: 217.0816, Ion mode: Positive
Adjusted p (t test): 4.07e−04, Adjusted p (wilcox): 4.77e−04
Log Metabolite Abundance
3
2
1
0
−1
EM
Metastatic
Condition

RT: 5.9, m/z: 197.1499, Ion mode: Positive

Adjusted p (t test): 4.14e−04, Adjusted p (wilcox): 6.70e−04

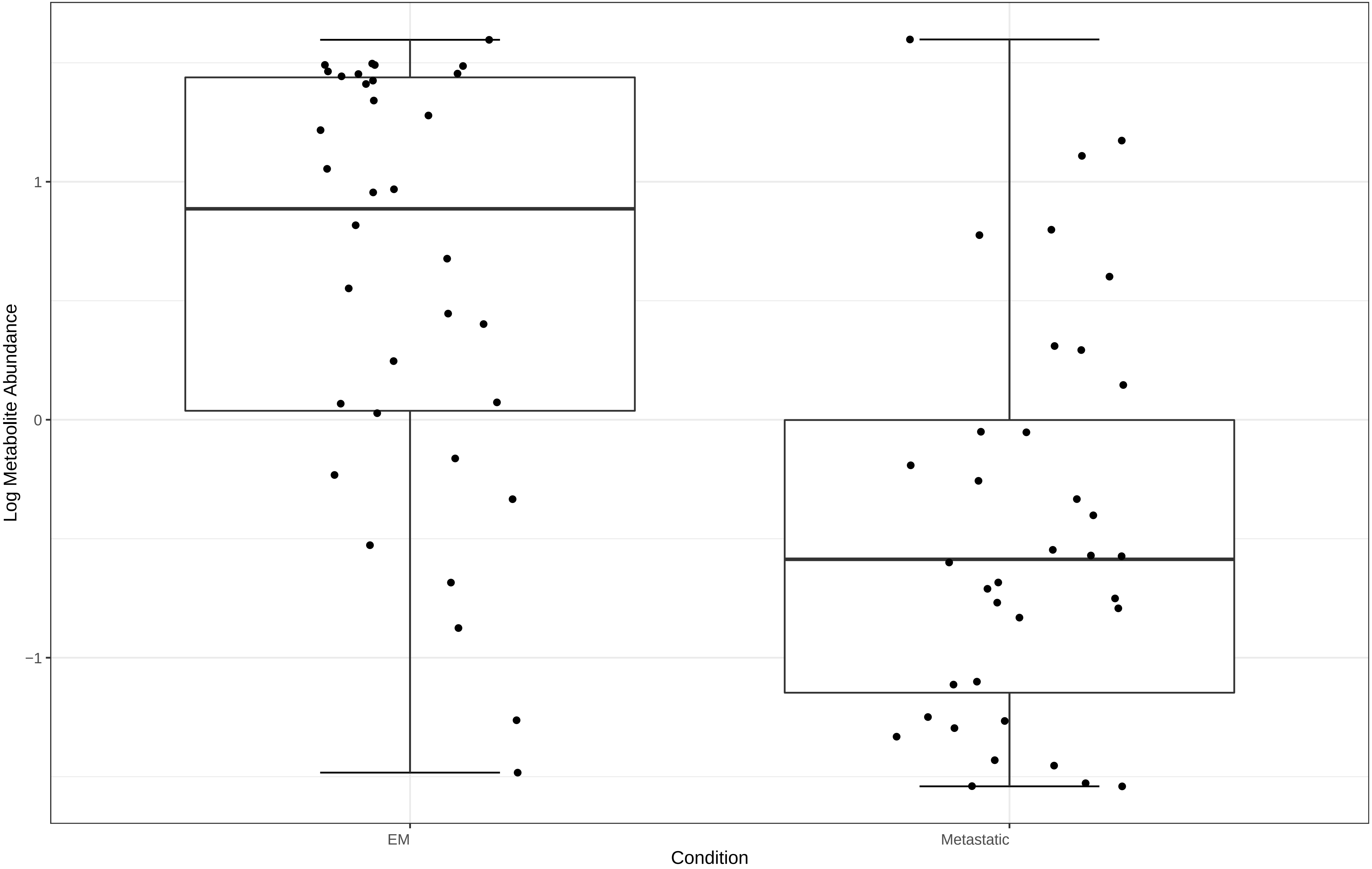

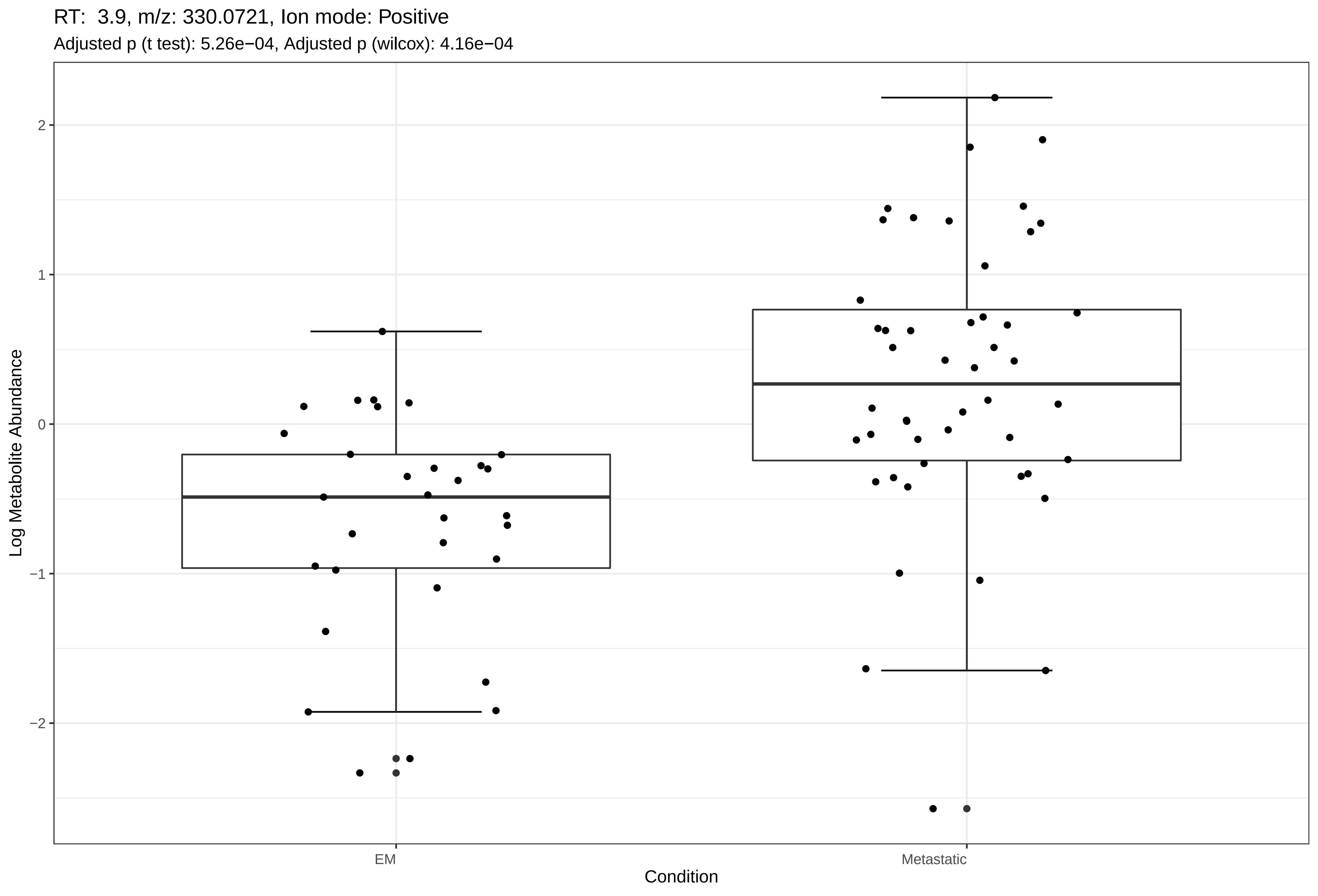
RT: 3.9, m/z: 330.0721, Ion mode: Positive
Adjusted p (t test): 5.26e−04, Adjusted p (wilcox): 4.16e−04
Log Metabolite Abundance
2
1
0
−1
−2
EM
Metastatic
Condition

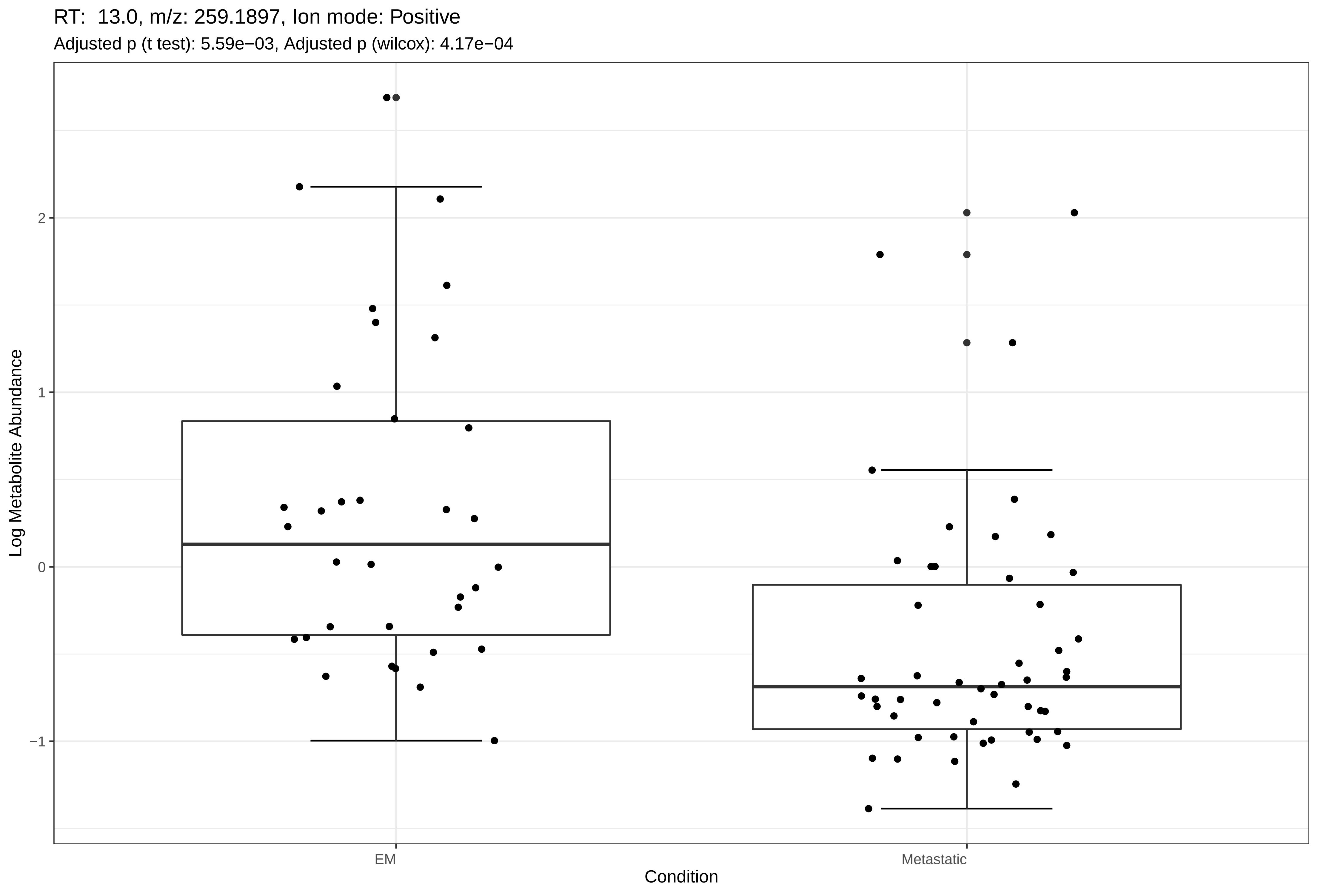

RT: 13.0, m/z: 259.1897, Ion mode: Positive
Adjusted p (t test): 5.59e−03, Adjusted p (wilcox): 4.17e−04
Log Metabolite Abundance
2
1
0
−1
EM
Metastatic
Condition

RT: 0.6, m/z: 112.0062, Ion mode: Positive

Adjusted p (t test): 5.61e−04, Adjusted p (wilcox): 4.17e−04

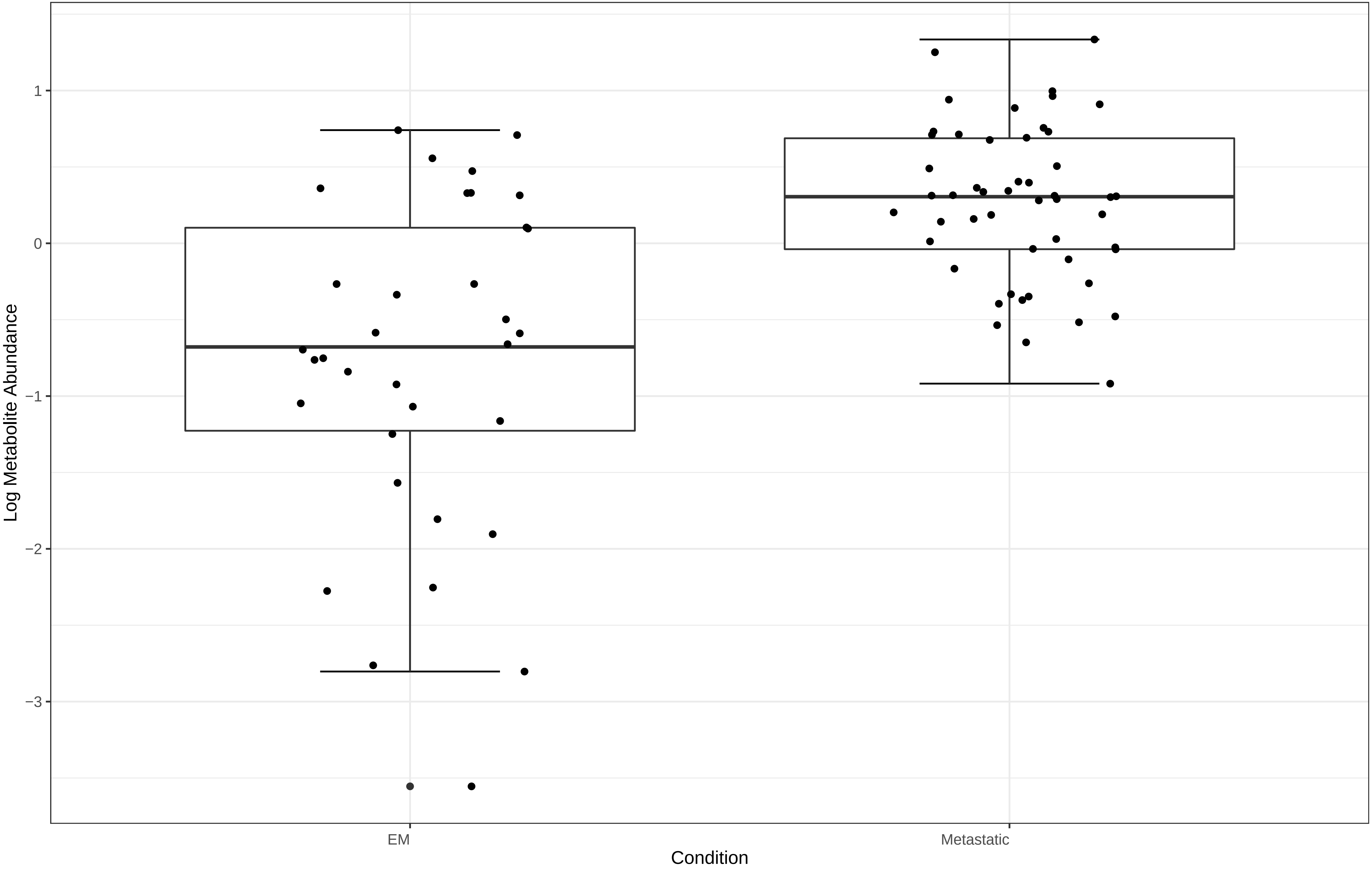

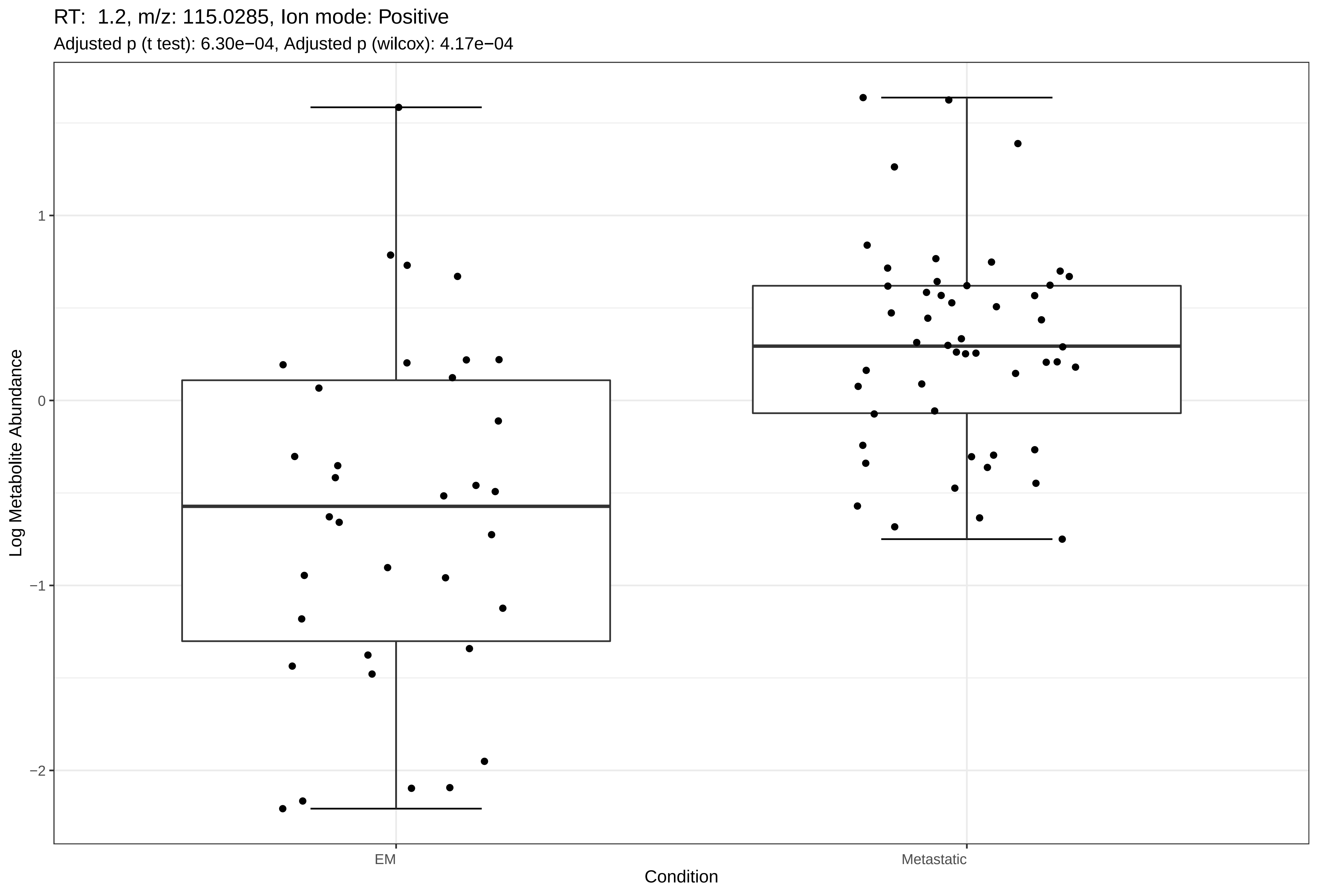

RT: 1.2, m/z: 115.0285, Ion mode: Positive
Adjusted p (t test): 6.30e−04, Adjusted p (wilcox): 4.17e−04
Log Metabolite Abundance
1
0
−1
−2
EM
Metastatic
Condition

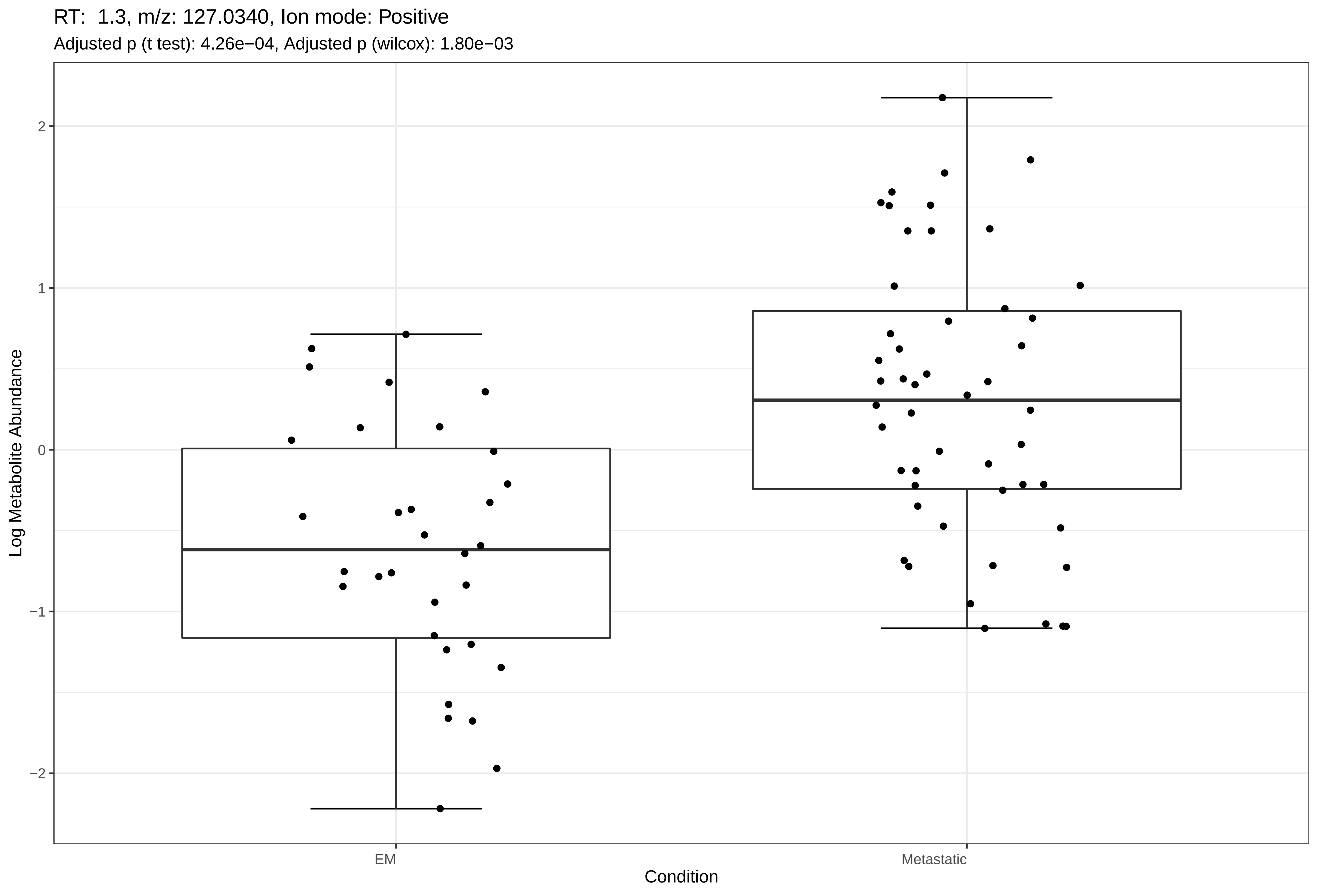
RT: 1.3, m/z: 127.0340, Ion mode: Positive
Adjusted p (t test): 4.26e−04, Adjusted p (wilcox): 1.80e−03
Log Metabolite Abundance
2
1
0
−1
−2
EM
Metastatic
Condition

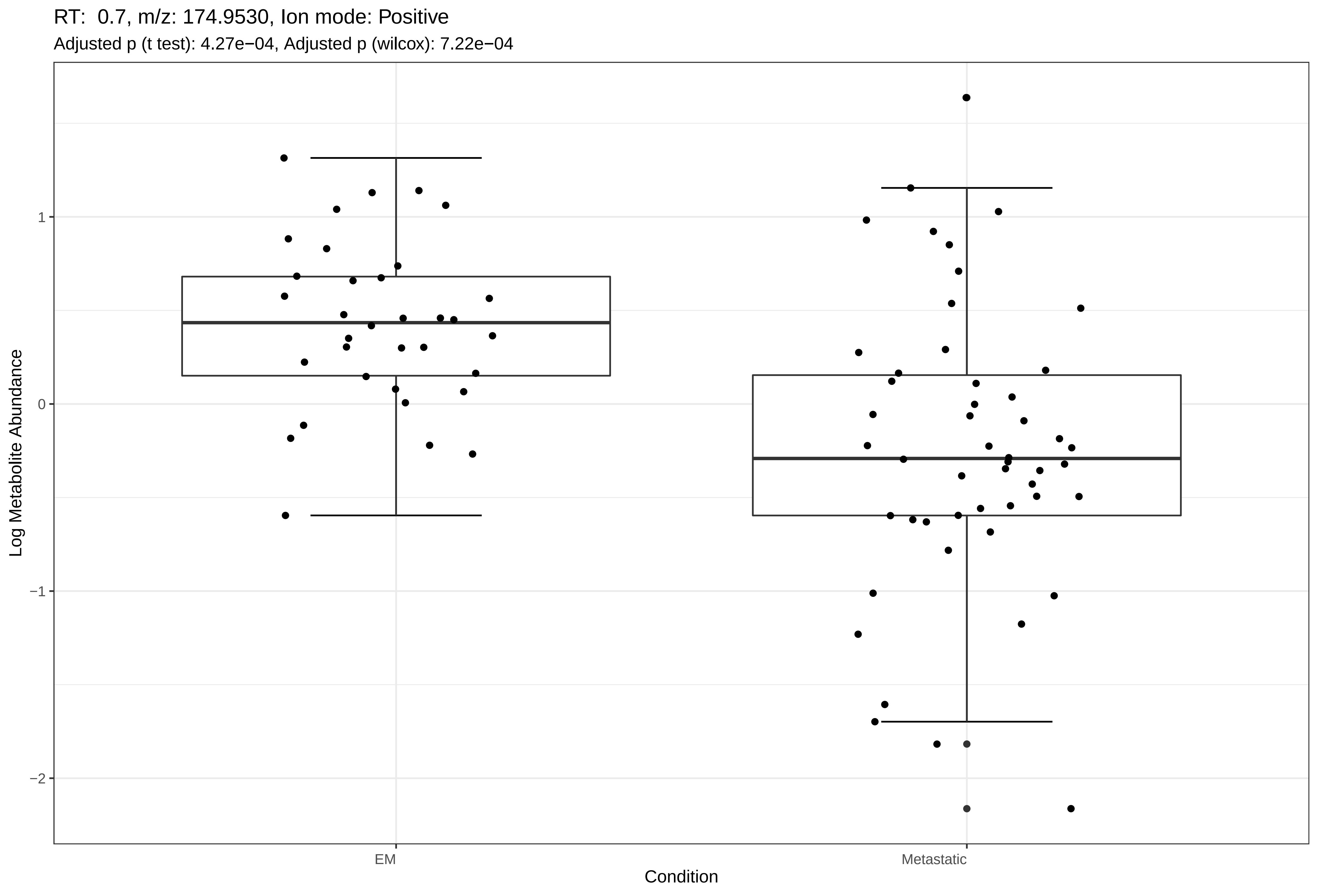
RT: 0.7, m/z: 174.9530, Ion mode: Positive
Adjusted p (t test): 4.27e−04, Adjusted p (wilcox): 7.22e−04
Log Metabolite Abundance
1
0
−1
−2
EM
Metastatic
Condition

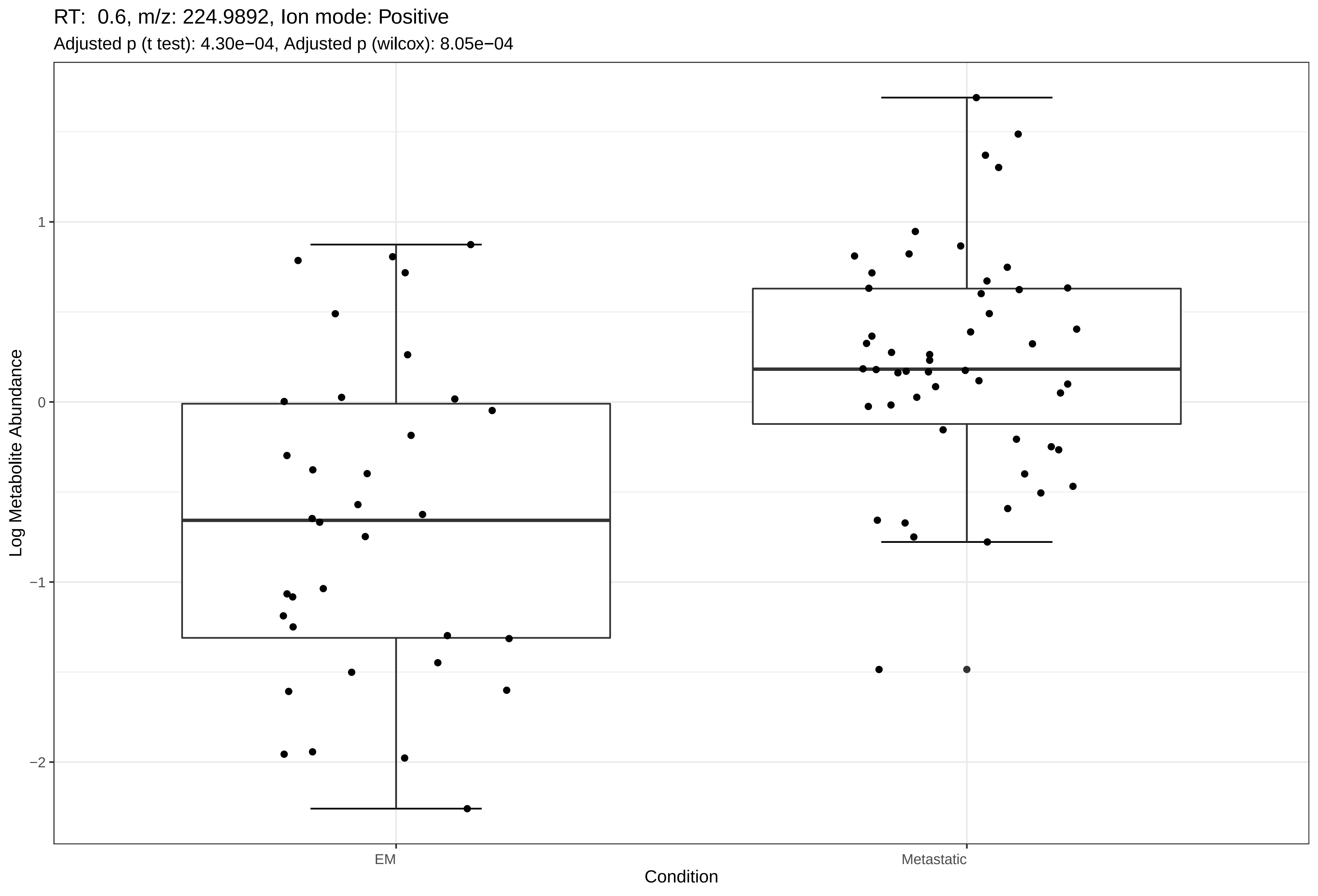
RT: 0.6, m/z: 224.9892, Ion mode: Positive
Adjusted p (t test): 4.30e−04, Adjusted p (wilcox): 8.05e−04
Log Metabolite Abundance
1
0
−1
−2
EM
Metastatic
Condition

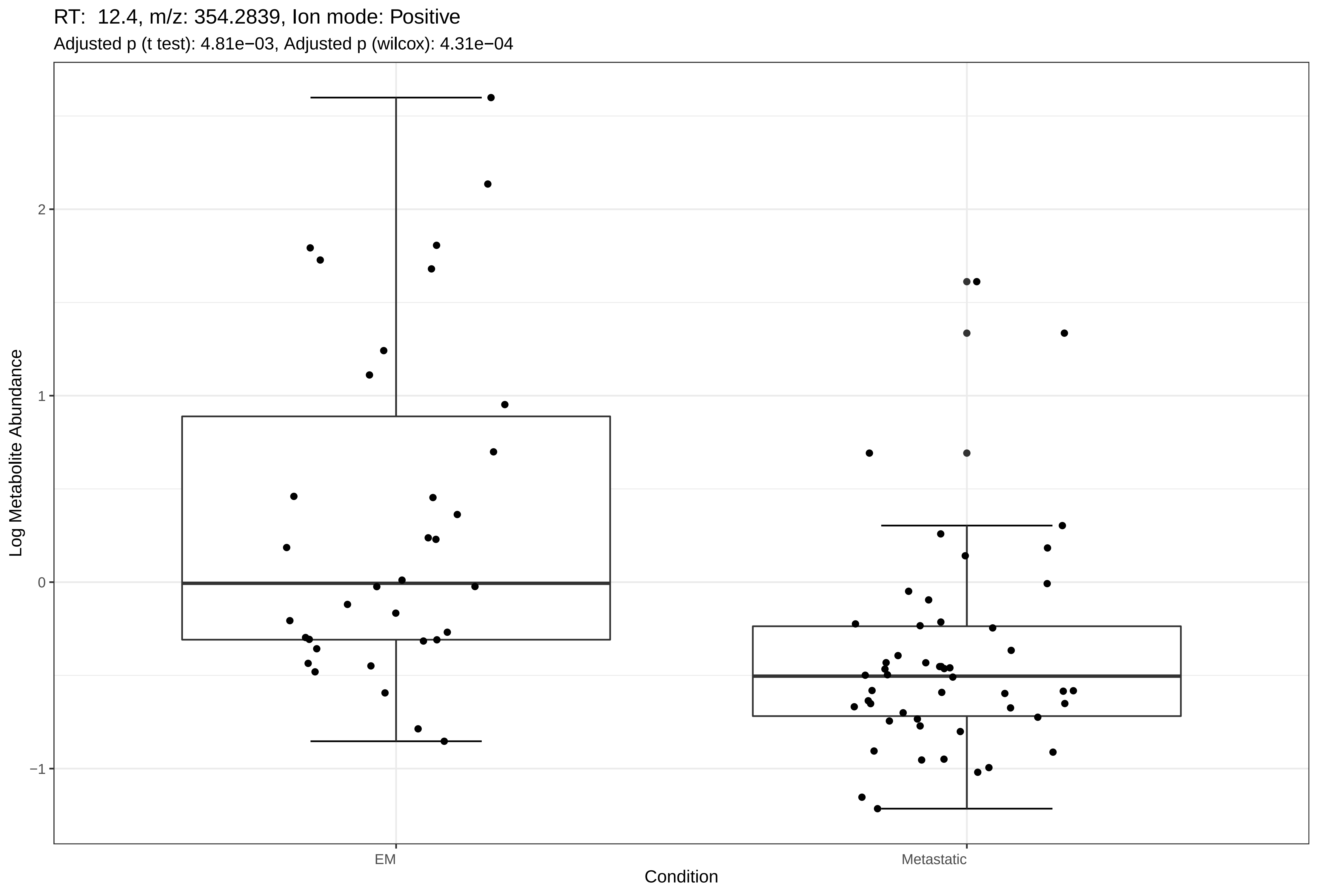

RT: 12.4, m/z: 354.2839, Ion mode: Positive
Adjusted p (t test): 4.81e−03, Adjusted p (wilcox): 4.31e−04
Log Metabolite Abundance
2
1
0
−1
EM
Metastatic
Condition

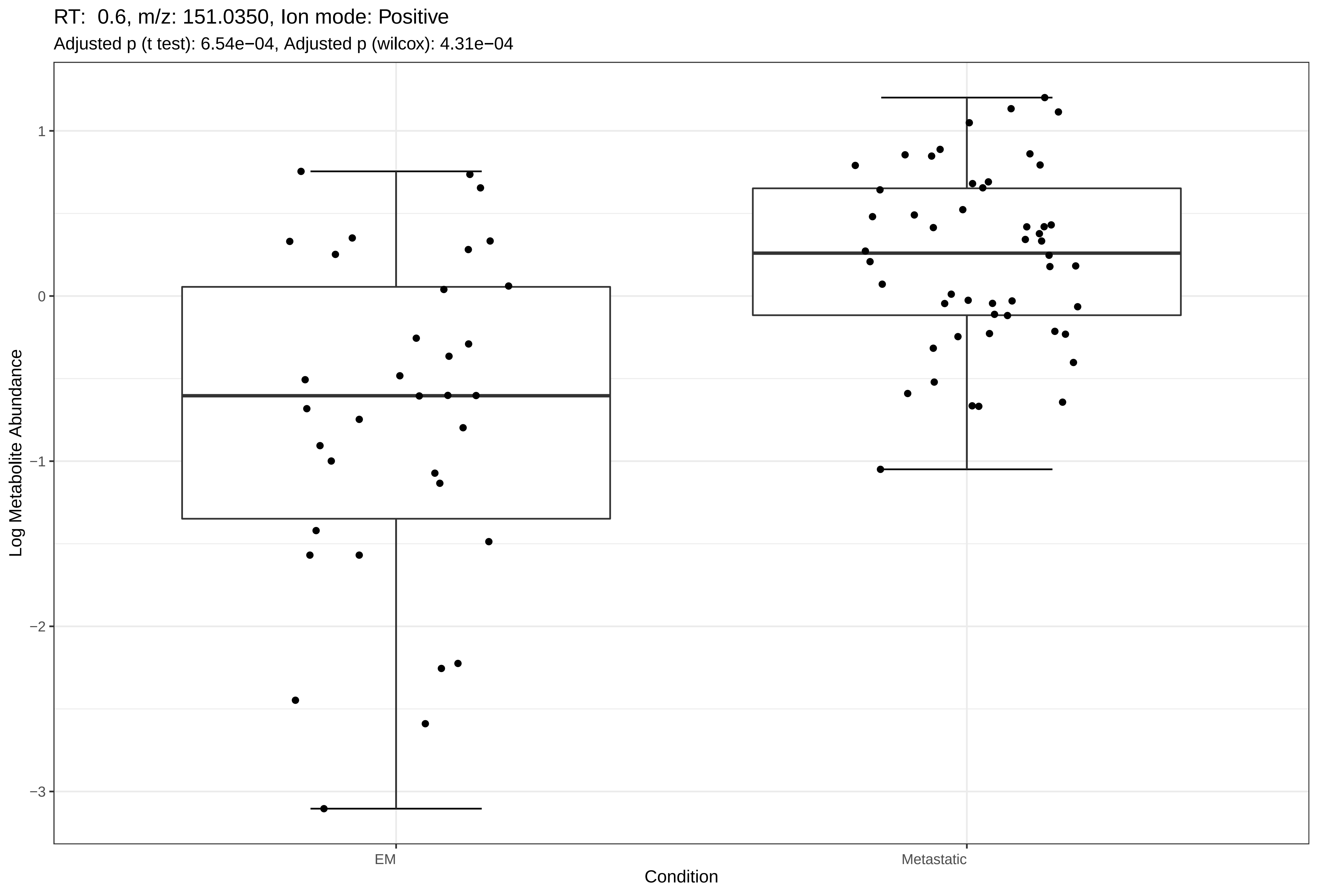

RT: 0.6, m/z: 151.0350, Ion mode: Positive
Adjusted p (t test): 6.54e−04, Adjusted p (wilcox): 4.31e−04
Log Metabolite Abundance
1
0
−1
−2
−3
EM
Metastatic
Condition

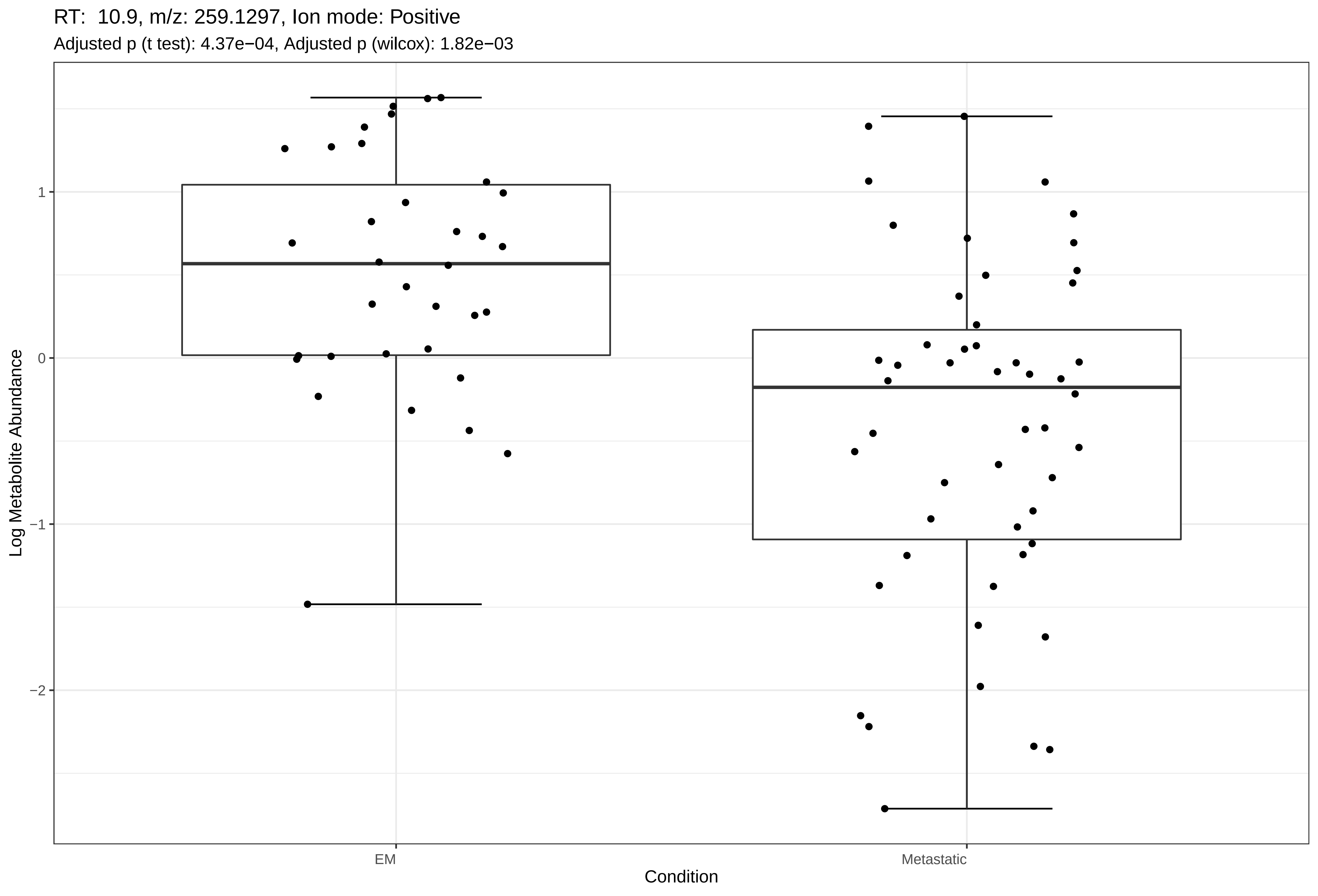

RT: 10.9, m/z: 259.1297, Ion mode: Positive
Adjusted p (t test): 4.37e−04, Adjusted p (wilcox): 1.82e−03
Log Metabolite Abundance
1
0
−1
−2
EM
Metastatic
Condition

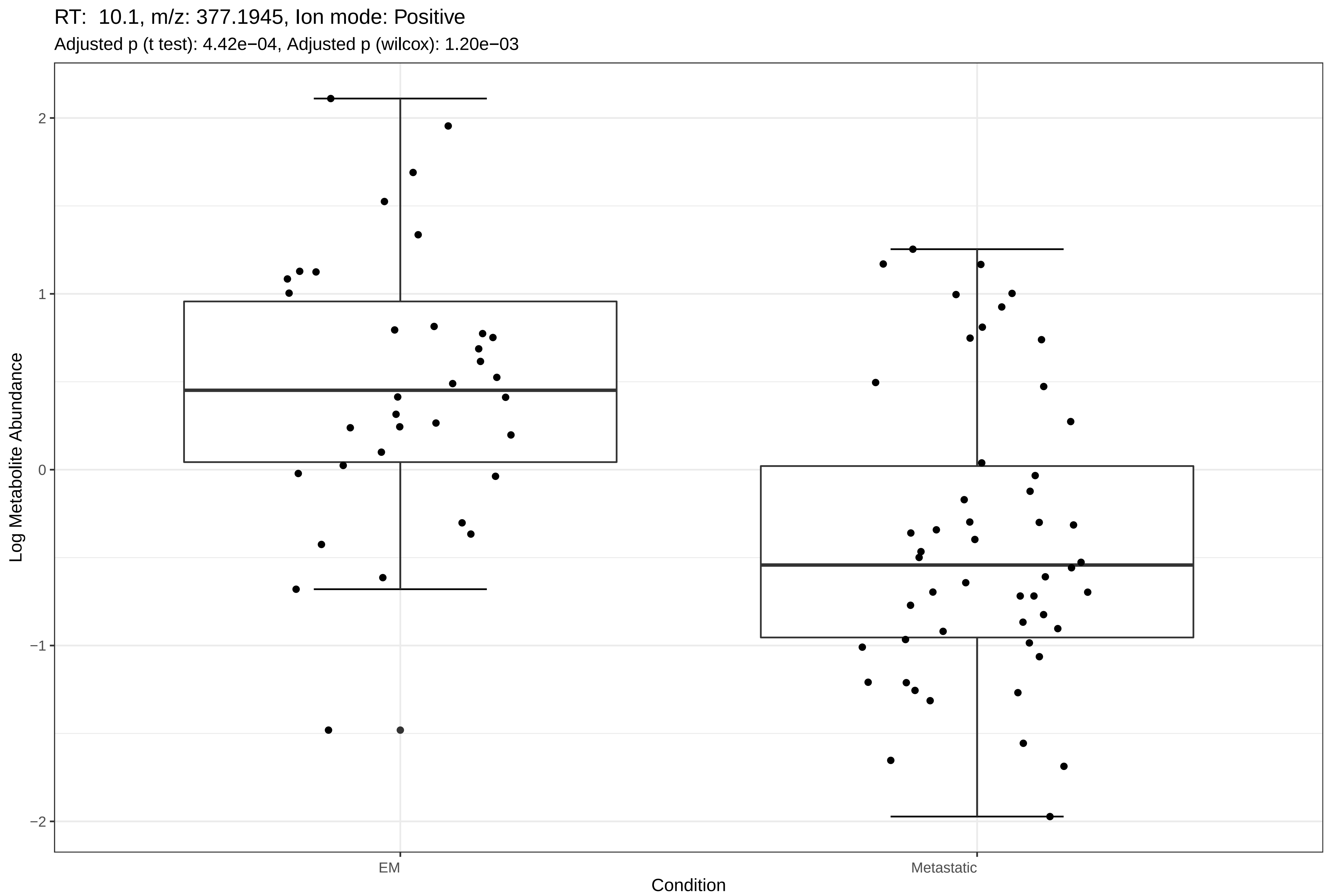

RT: 10.1, m/z: 377.1945, Ion mode: Positive
Adjusted p (t test): 4.42e−04, Adjusted p (wilcox): 1.20e−03
Log Metabolite Abundance
2
1
0
−1
−2
EM
Metastatic
Condition

RT: 0.8, m/z: 209.0213, Ion mode: Negative

Adjusted p (t test): 8.40e−04, Adjusted p (wilcox): 4.45e−04

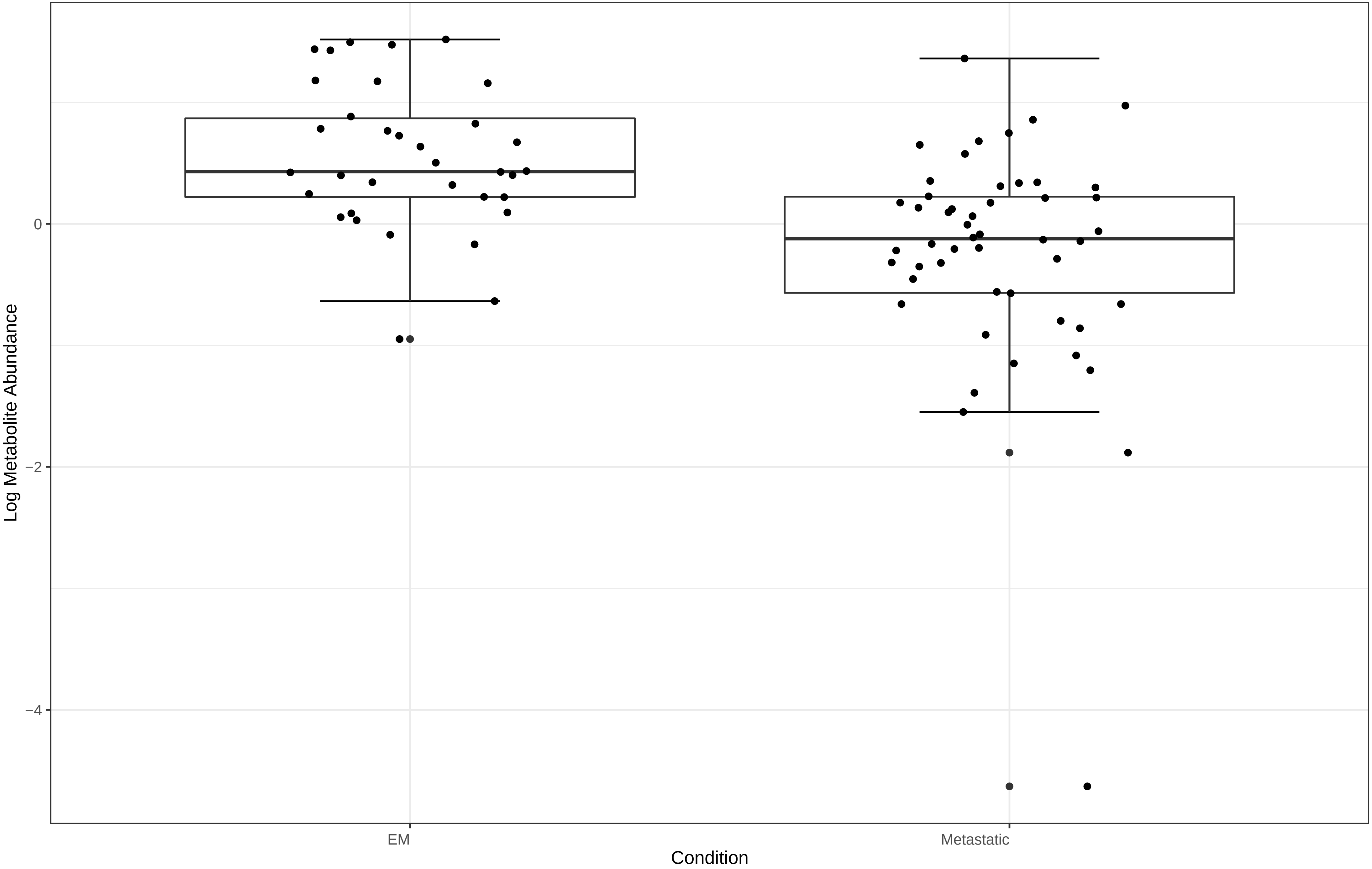

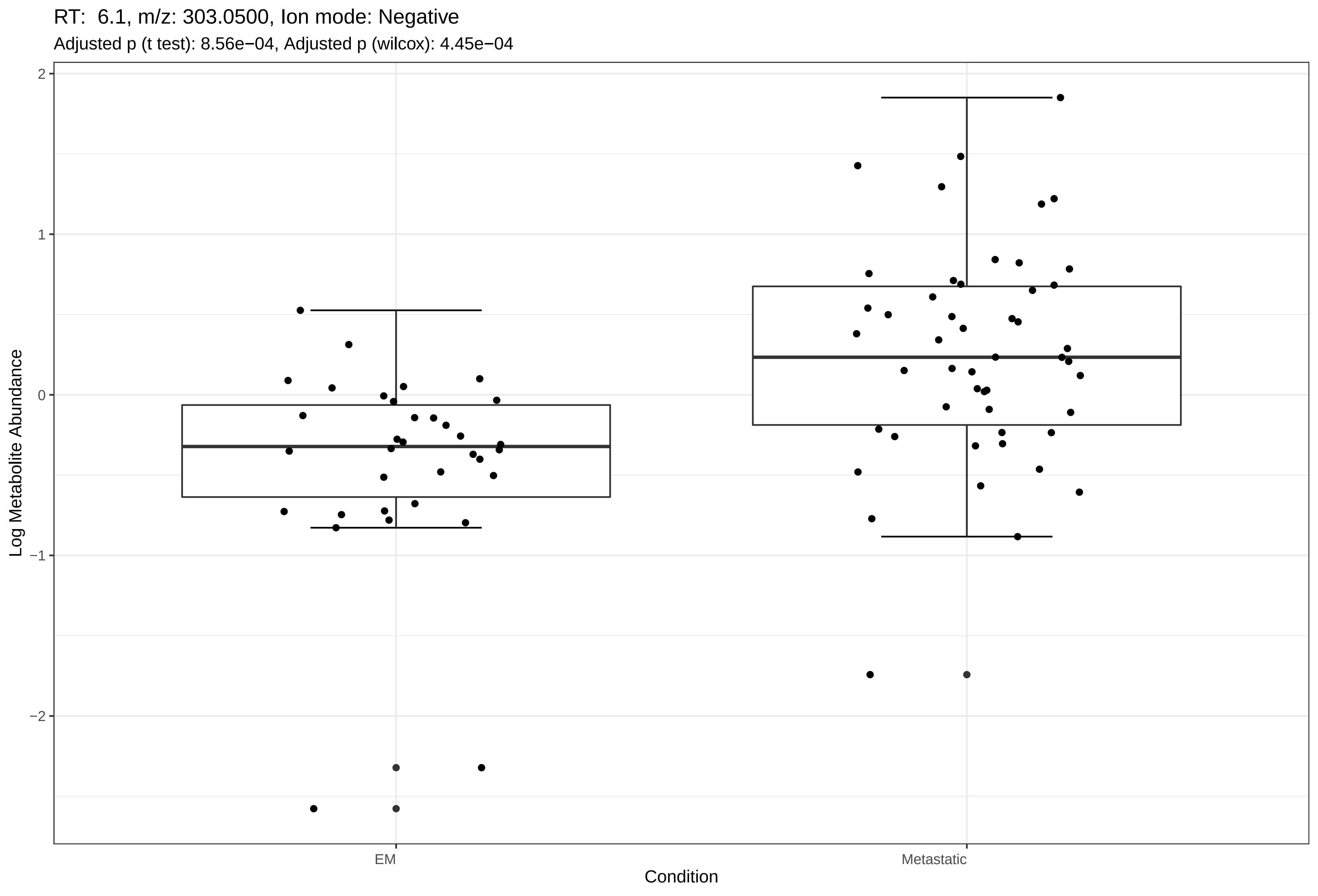
RT: 6.1, m/z: 303.0500, Ion mode: Negative
Adjusted p (t test): 8.56e−04, Adjusted p (wilcox): 4.45e−04
Log Metabolite Abundance
2
1
0
−1
−2
EM
Metastatic
Condition

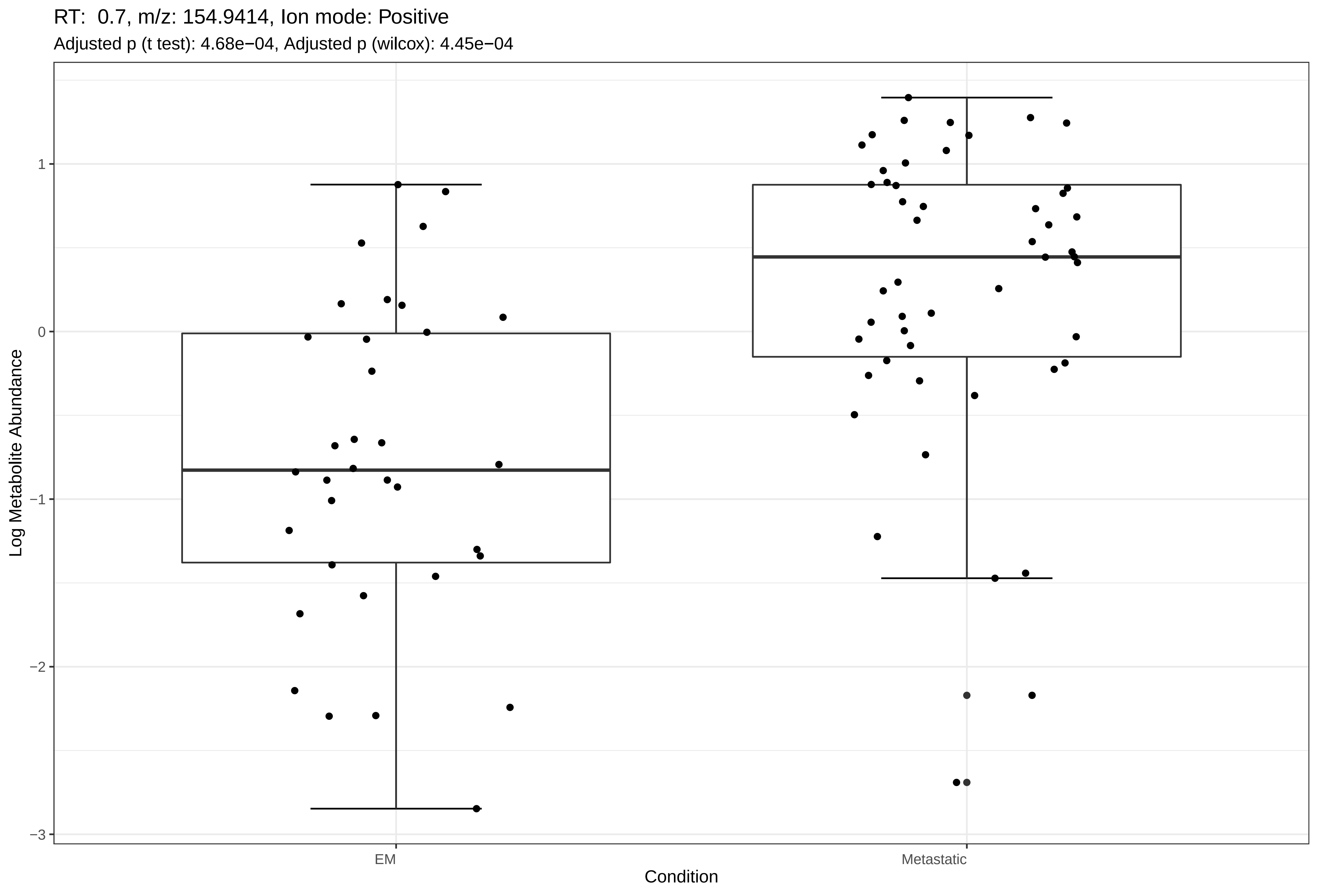

RT: 0.7, m/z: 154.9414, Ion mode: Positive
Adjusted p (t test): 4.68e−04, Adjusted p (wilcox): 4.45e−04
Log Metabolite Abundance
1
0
−1
−2
−3
EM
Metastatic
Condition

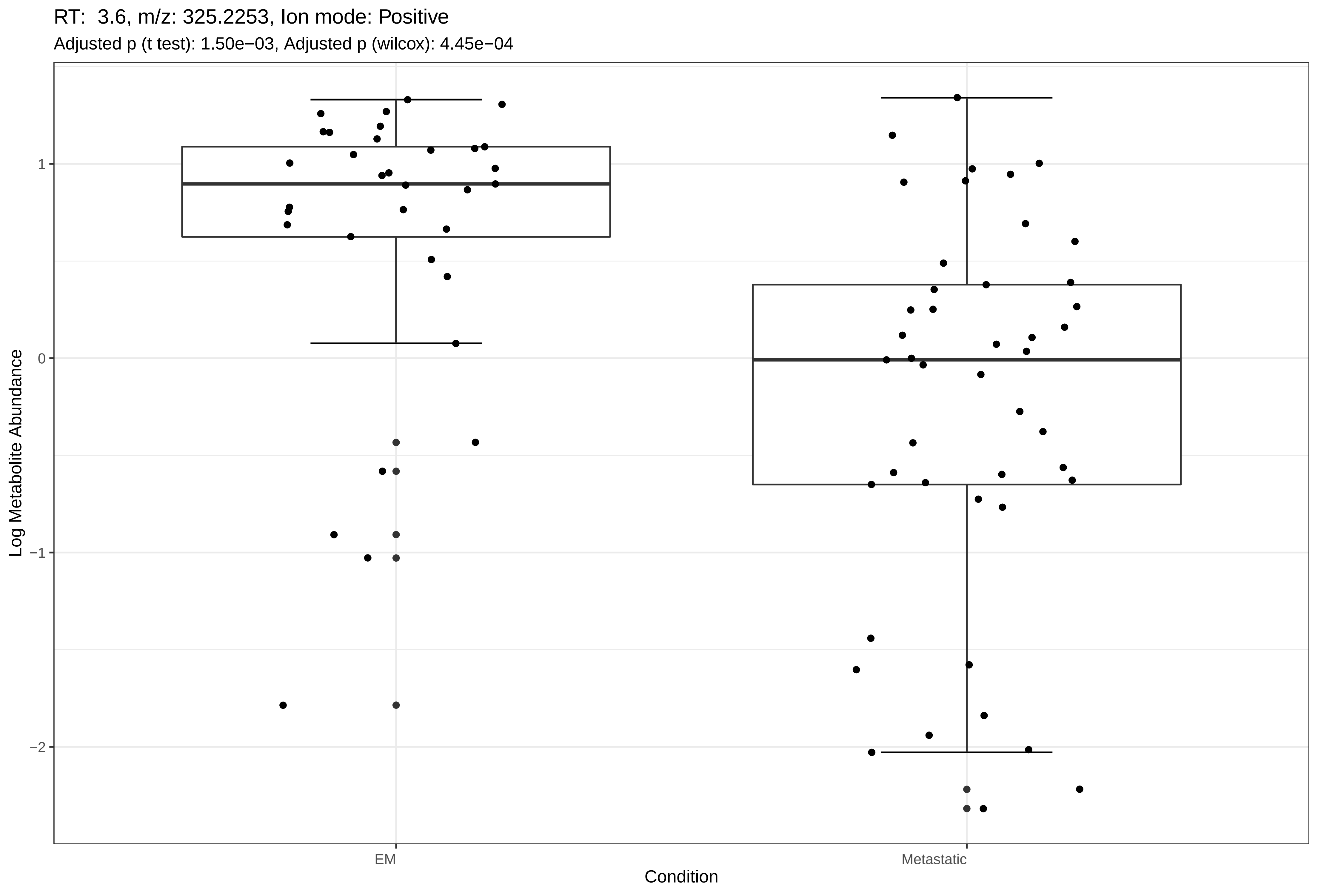

RT: 3.6, m/z: 325.2253, Ion mode: Positive
Adjusted p (t test): 1.50e−03, Adjusted p (wilcox): 4.45e−04
Log Metabolite Abundance
1
0
−1
−2
EM
Metastatic
Condition

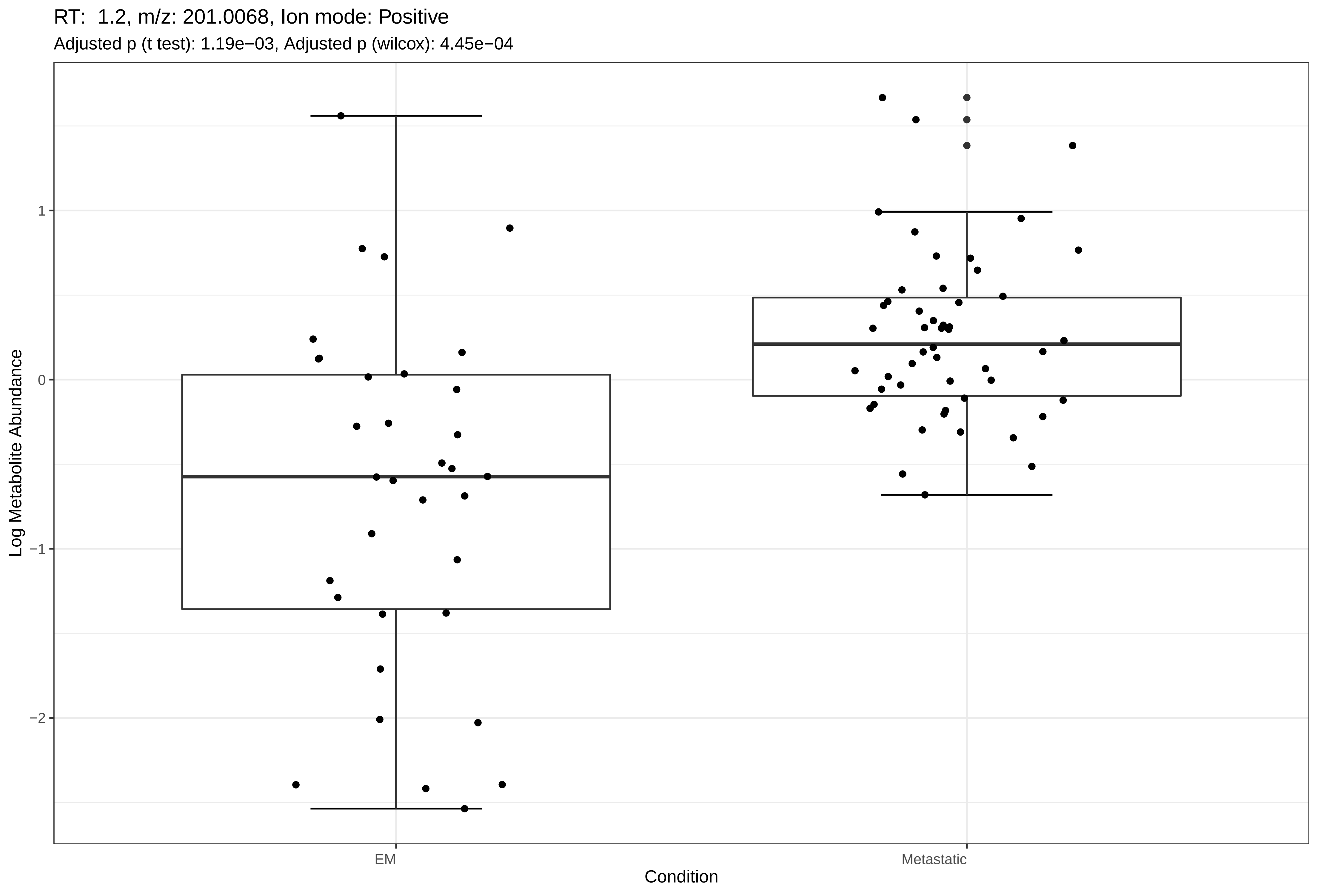
RT: 1.2, m/z: 201.0068, Ion mode: Positive
Adjusted p (t test): 1.19e−03, Adjusted p (wilcox): 4.45e−04
Log Metabolite Abundance
1
0
−1
−2
EM
Metastatic
Condition

RT: 0.7, m/z: 200.9468, Ion mode: Positive

Adjusted p (t test): 2.13e−03, Adjusted p (wilcox): 4.45e−04

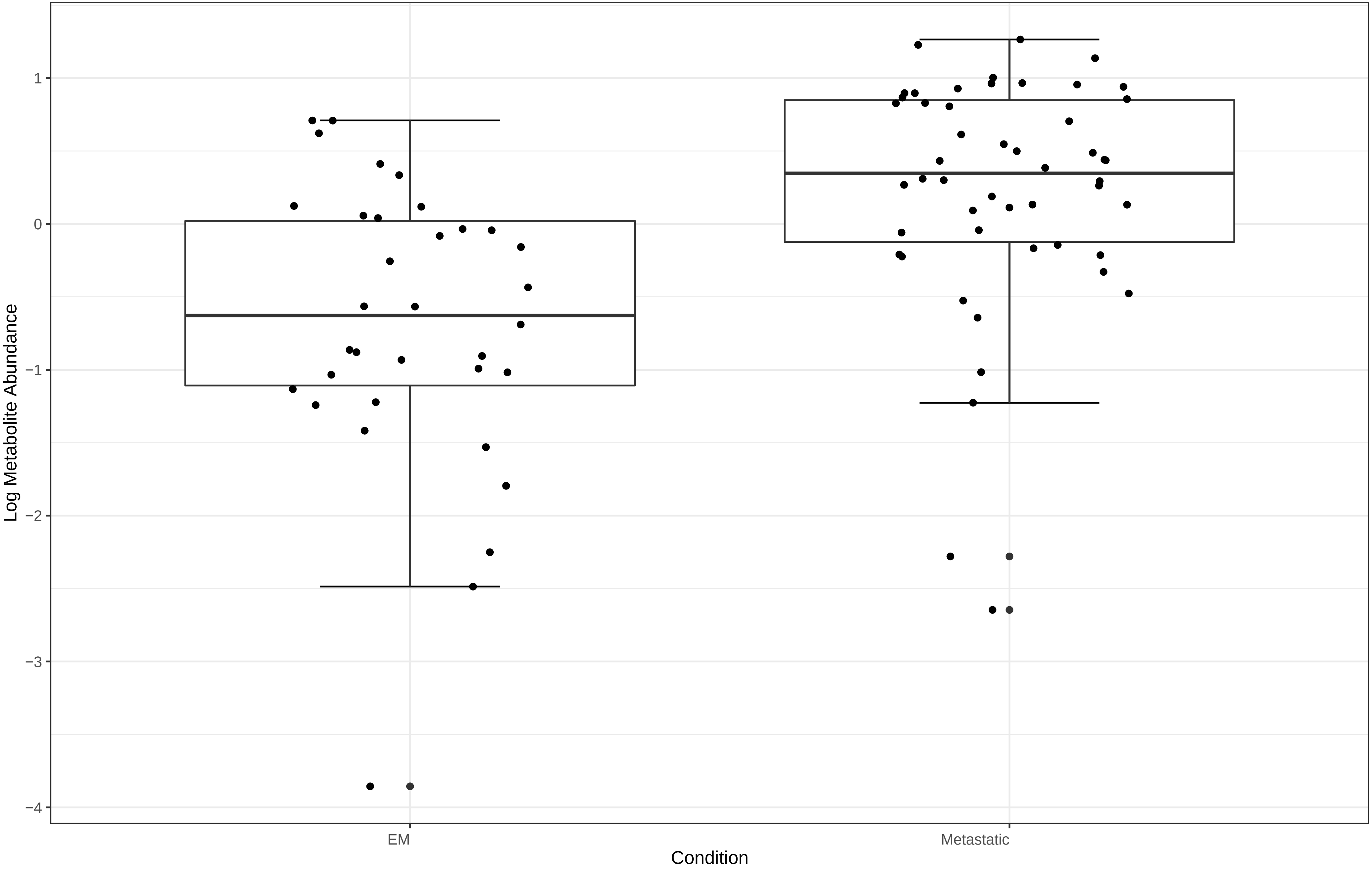

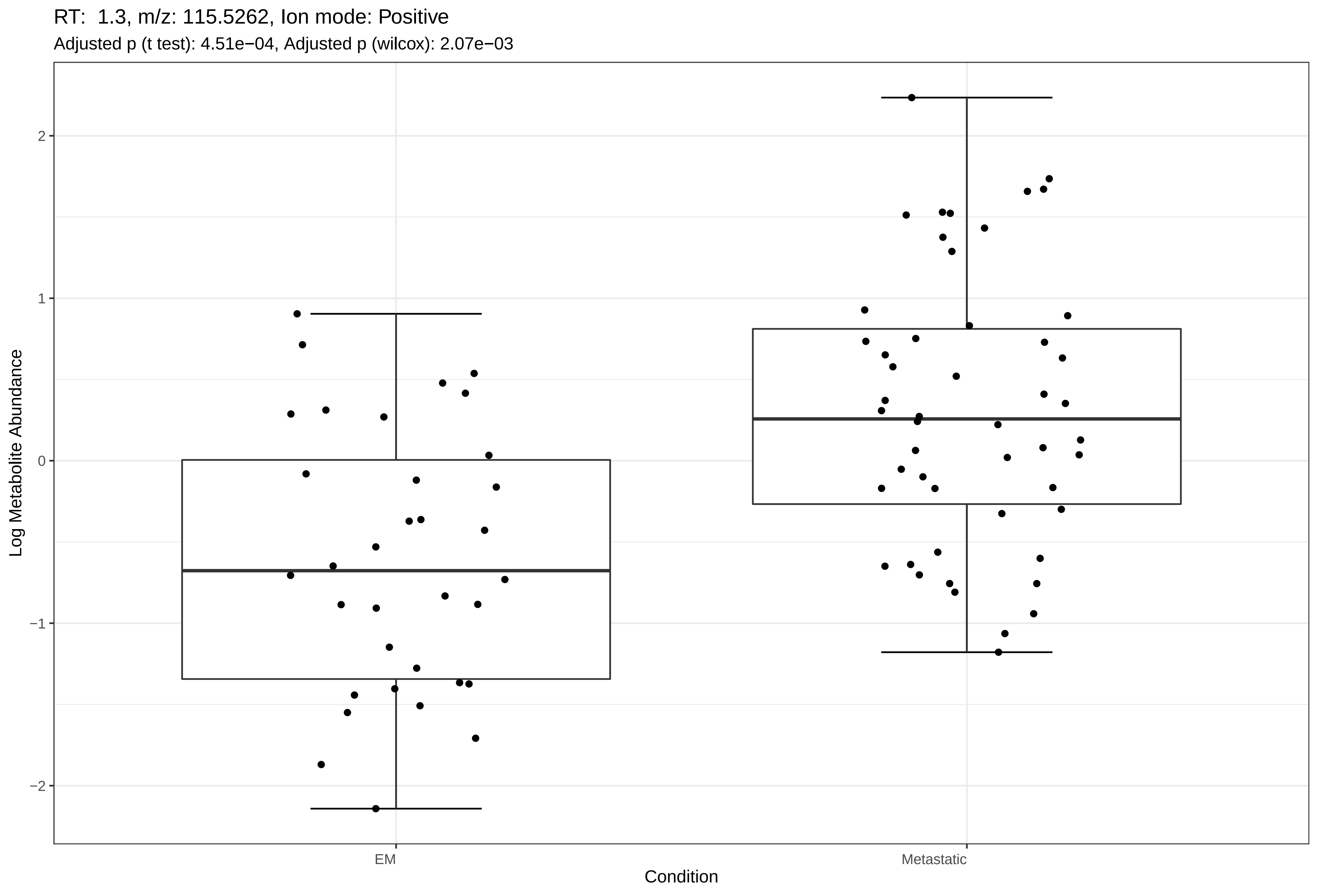

RT: 1.3, m/z: 115.5262, Ion mode: Positive
Adjusted p (t test): 4.51e−04, Adjusted p (wilcox): 2.07e−03
Log Metabolite Abundance
2
1
0
−1
−2
EM
Metastatic
Condition

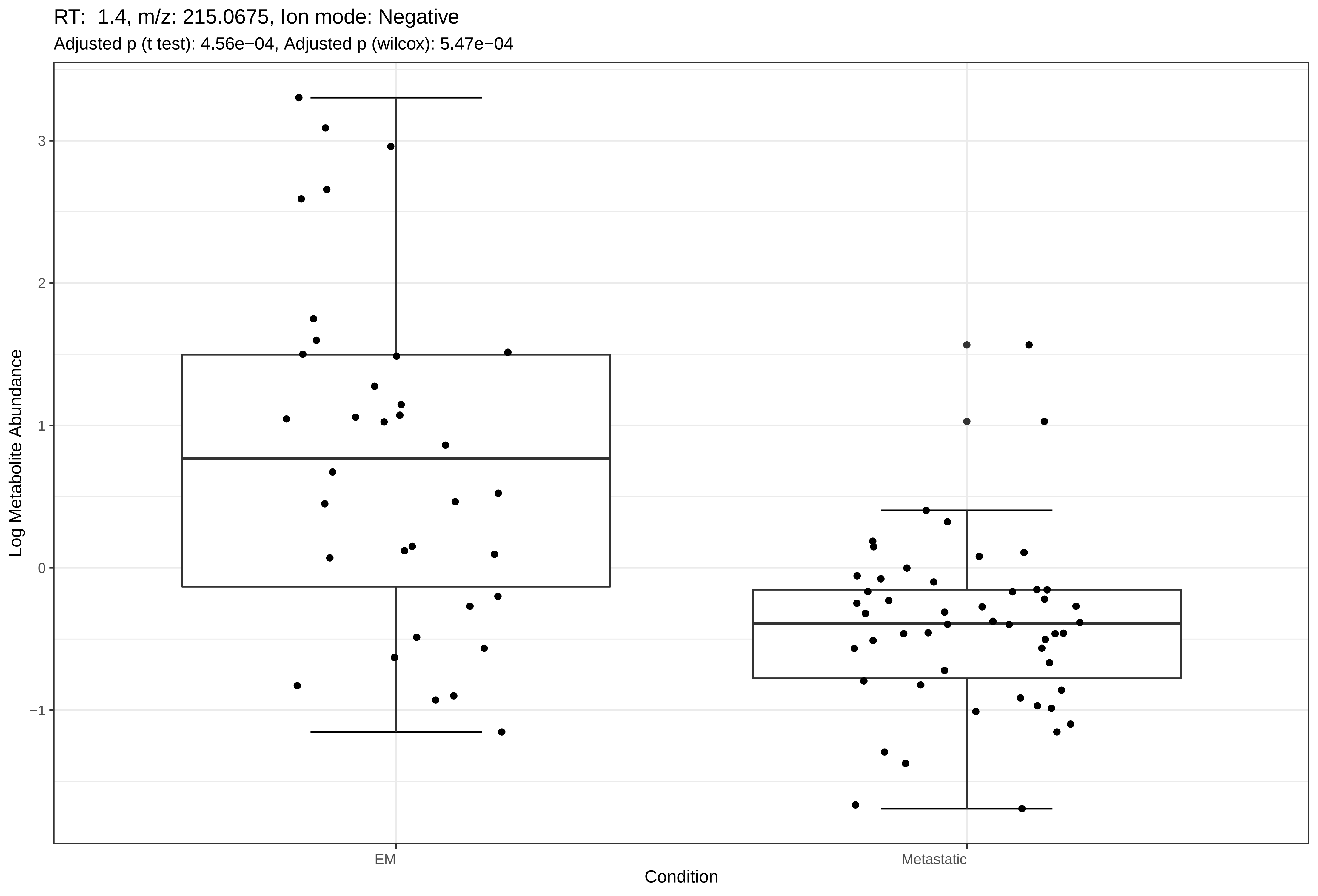

RT: 1.4, m/z: 215.0675, Ion mode: Negative
Adjusted p (t test): 4.56e−04, Adjusted p (wilcox): 5.47e−04
Log Metabolite Abundance
3
2
1
0
−1
EM
Metastatic
Condition

[M+Na−H] 21.9825 m/z adduct of 169.0605 m/z (RT: 1.6, m/z: 191.0424, Ion mode: Positive)

Adjusted p (t test): 4.60e−04, Adjusted p (wilcox): 5.47e−04

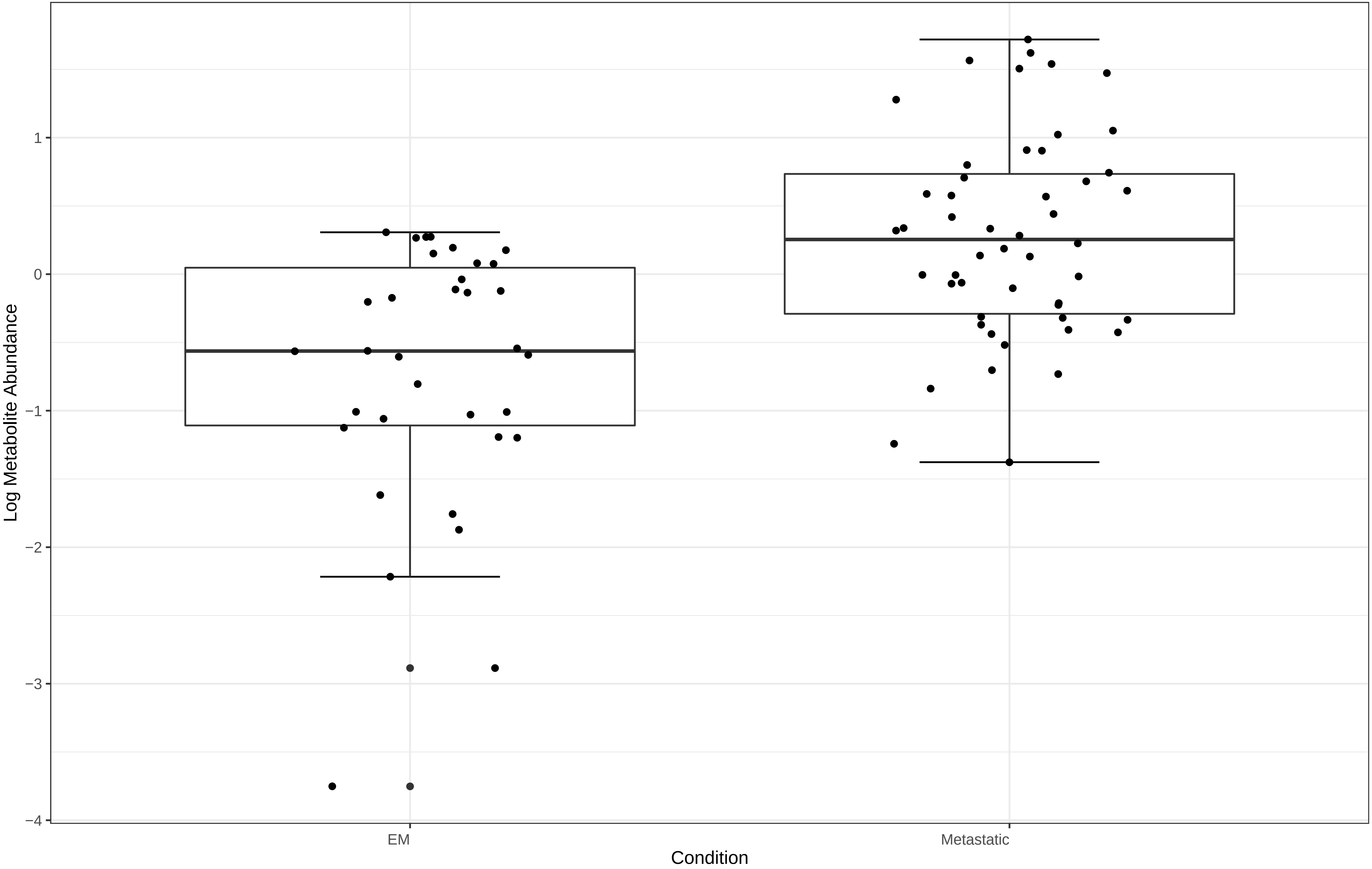

RT: 3.9, m/z: 334.0420, Ion mode: Positive

Adjusted p (t test): 2.62e−03, Adjusted p (wilcox): 4.62e−04

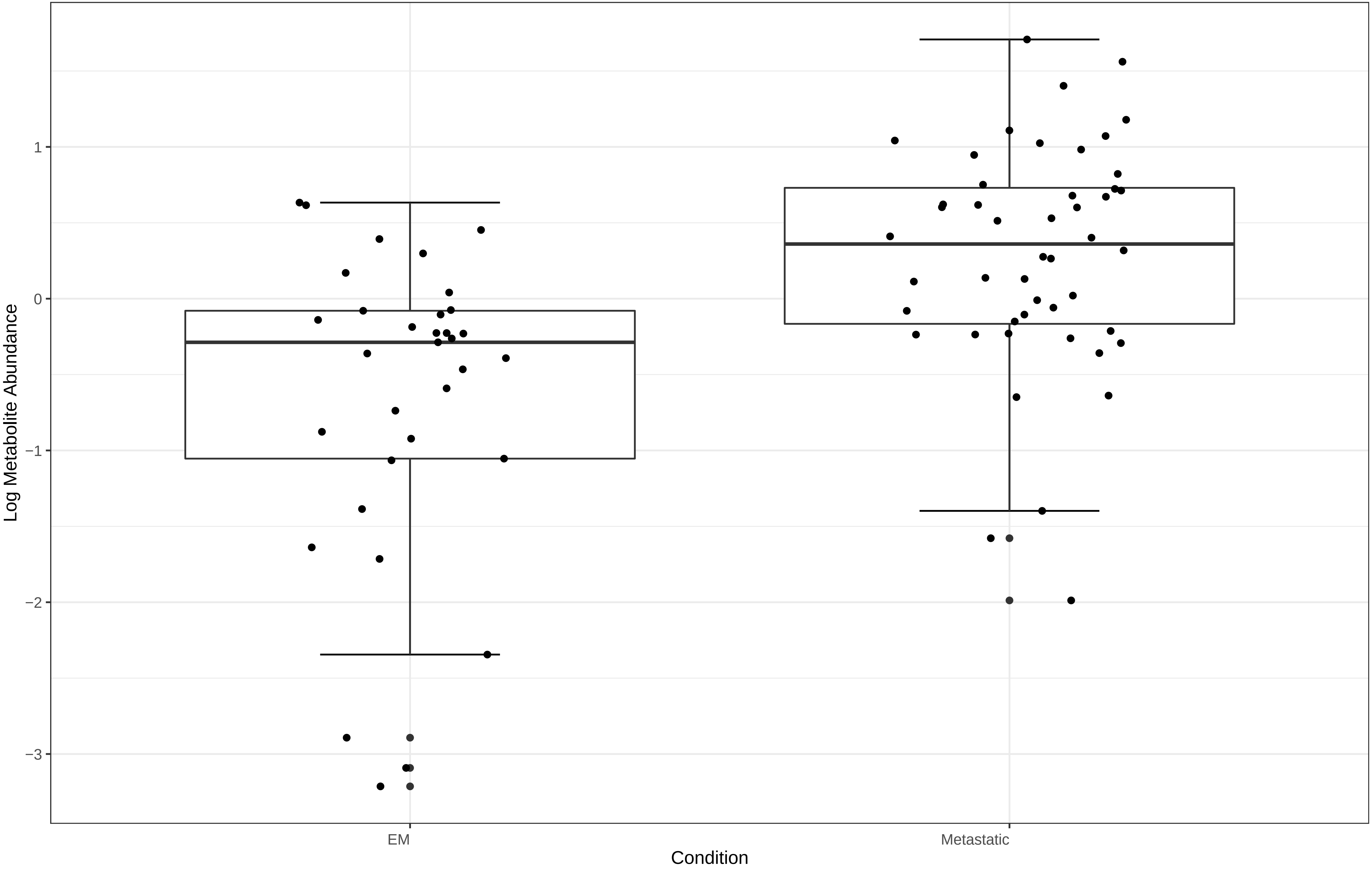

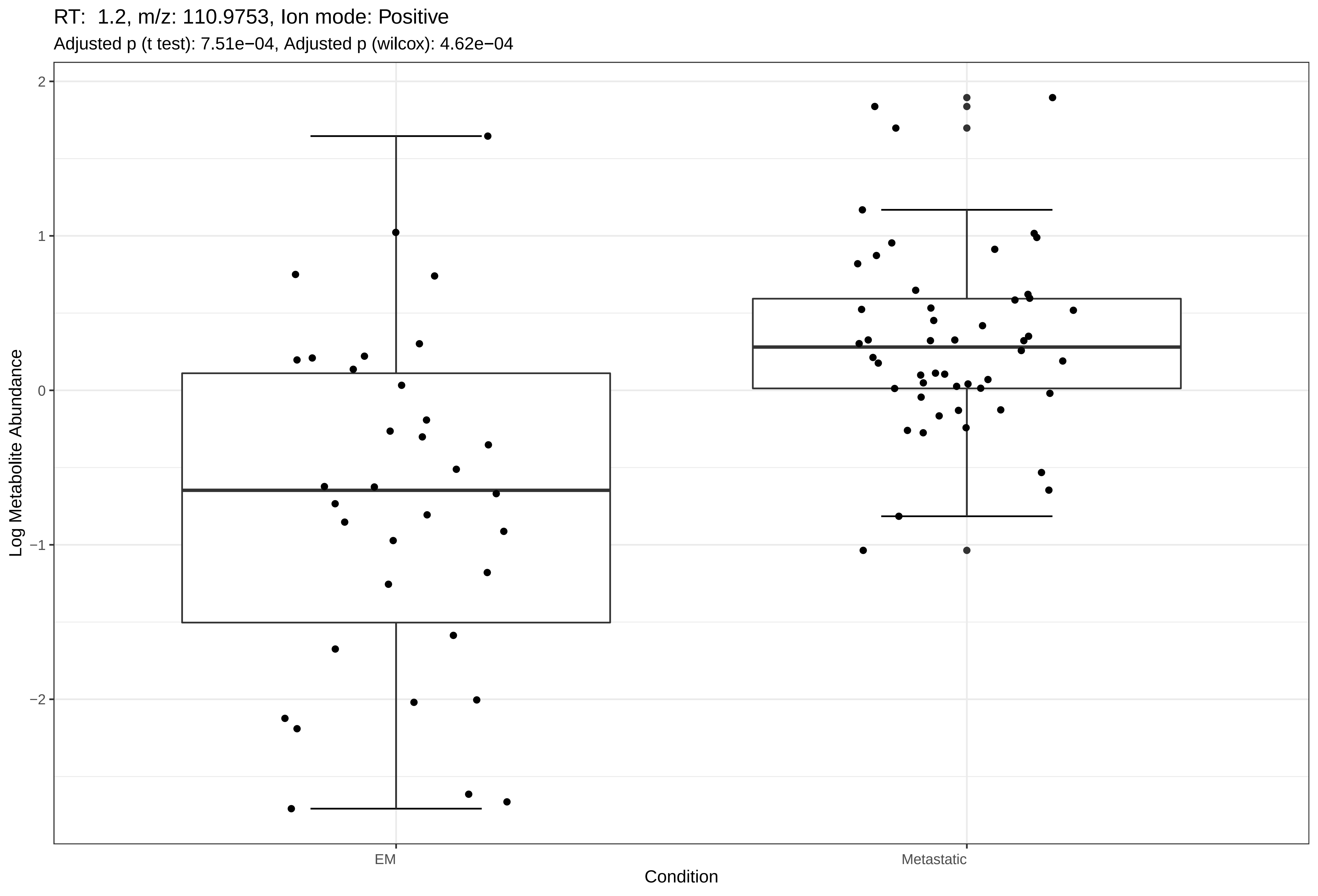
RT: 1.2, m/z: 110.9753, Ion mode: Positive
Adjusted p (t test): 7.51e−04, Adjusted p (wilcox): 4.62e−04
2
1
0
−1
−2
Log Metabolite Abundance
EM
Metastatic
Condition

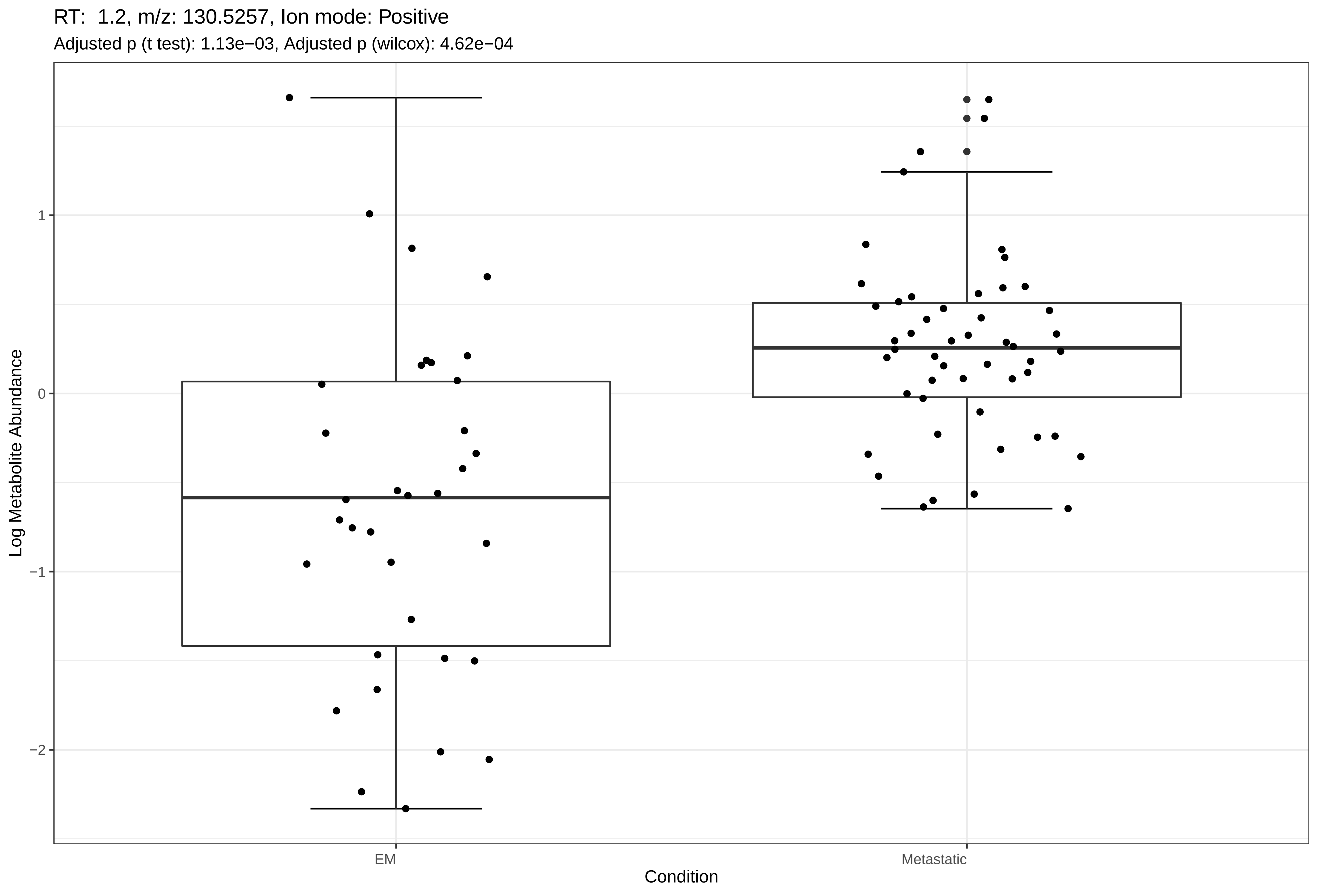
RT: 1.2, m/z: 130.5257, Ion mode: Positive
Adjusted p (t test): 1.13e−03, Adjusted p (wilcox): 4.62e−04
Log Metabolite Abundance
1
0
−1
−2
EM
Metastatic
Condition

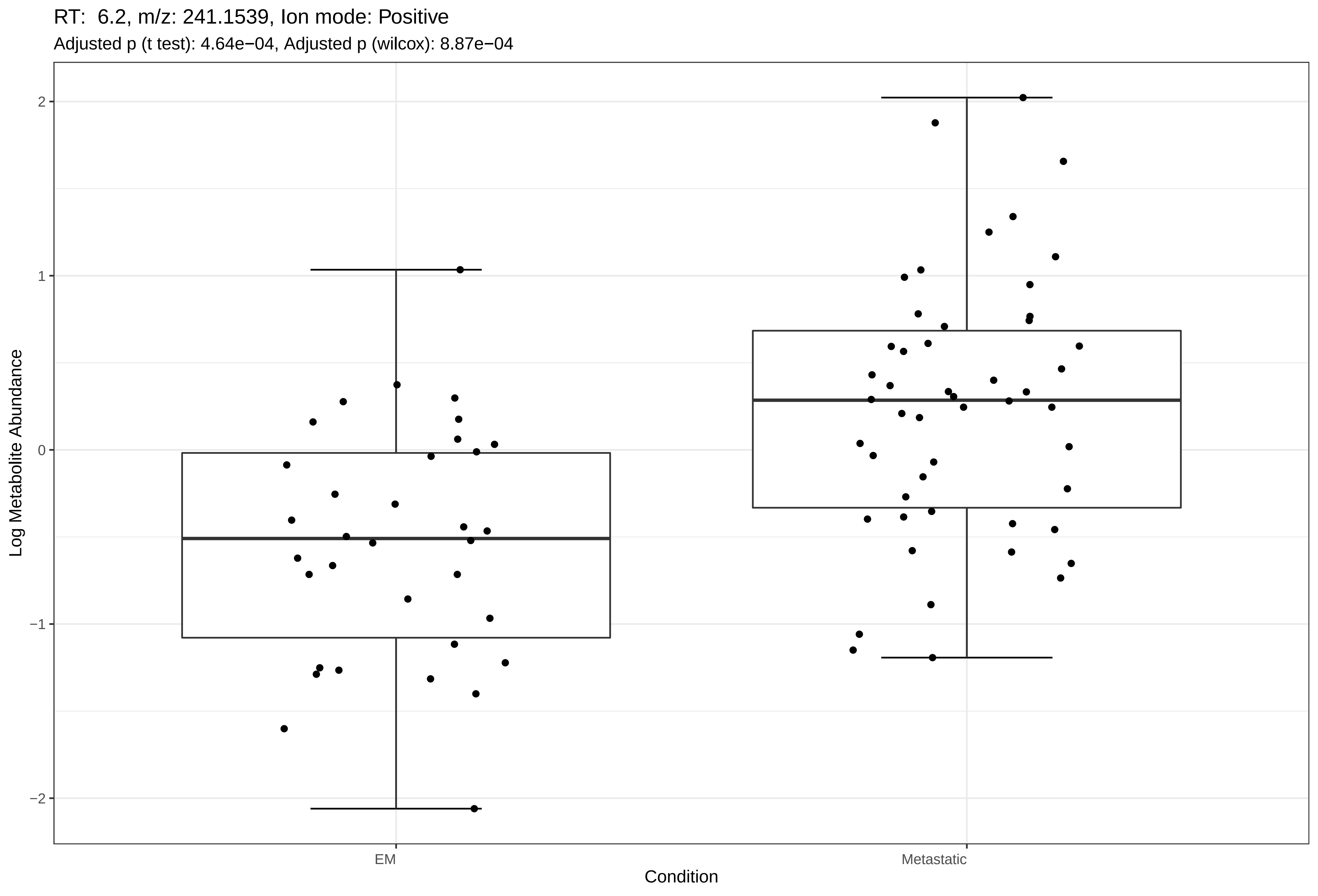
RT: 6.2, m/z: 241.1539, Ion mode: Positive
Adjusted p (t test): 4.64e−04, Adjusted p (wilcox): 8.87e−04
Log Metabolite Abundance
2
1
0
−1
−2
EM
Metastatic
Condition

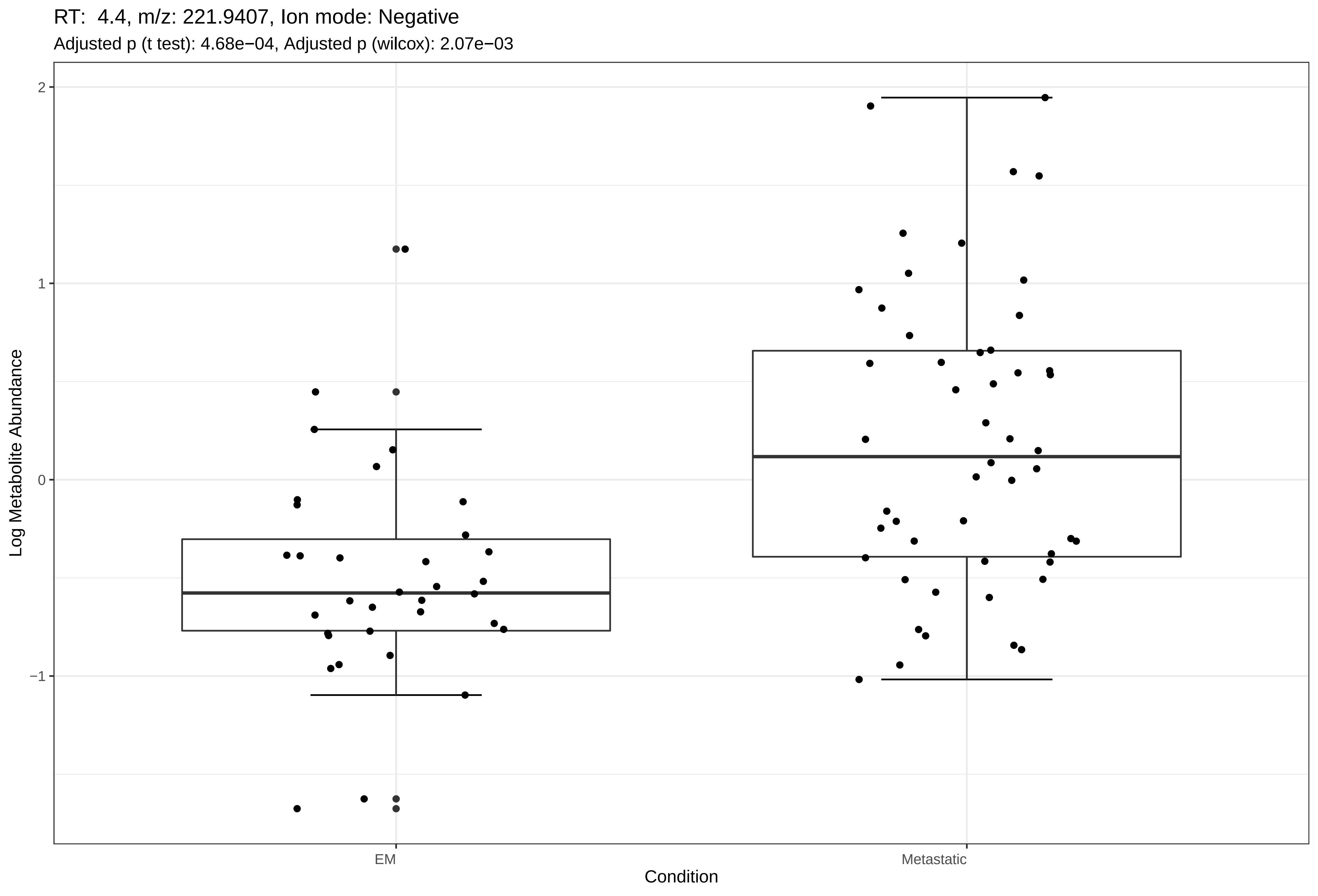
RT: 4.4, m/z: 221.9407, Ion mode: Negative
Adjusted p (t test): 4.68e−04, Adjusted p (wilcox): 2.07e−03
Log Metabolite Abundance
2
1
0
−1
EM
Metastatic
Condition

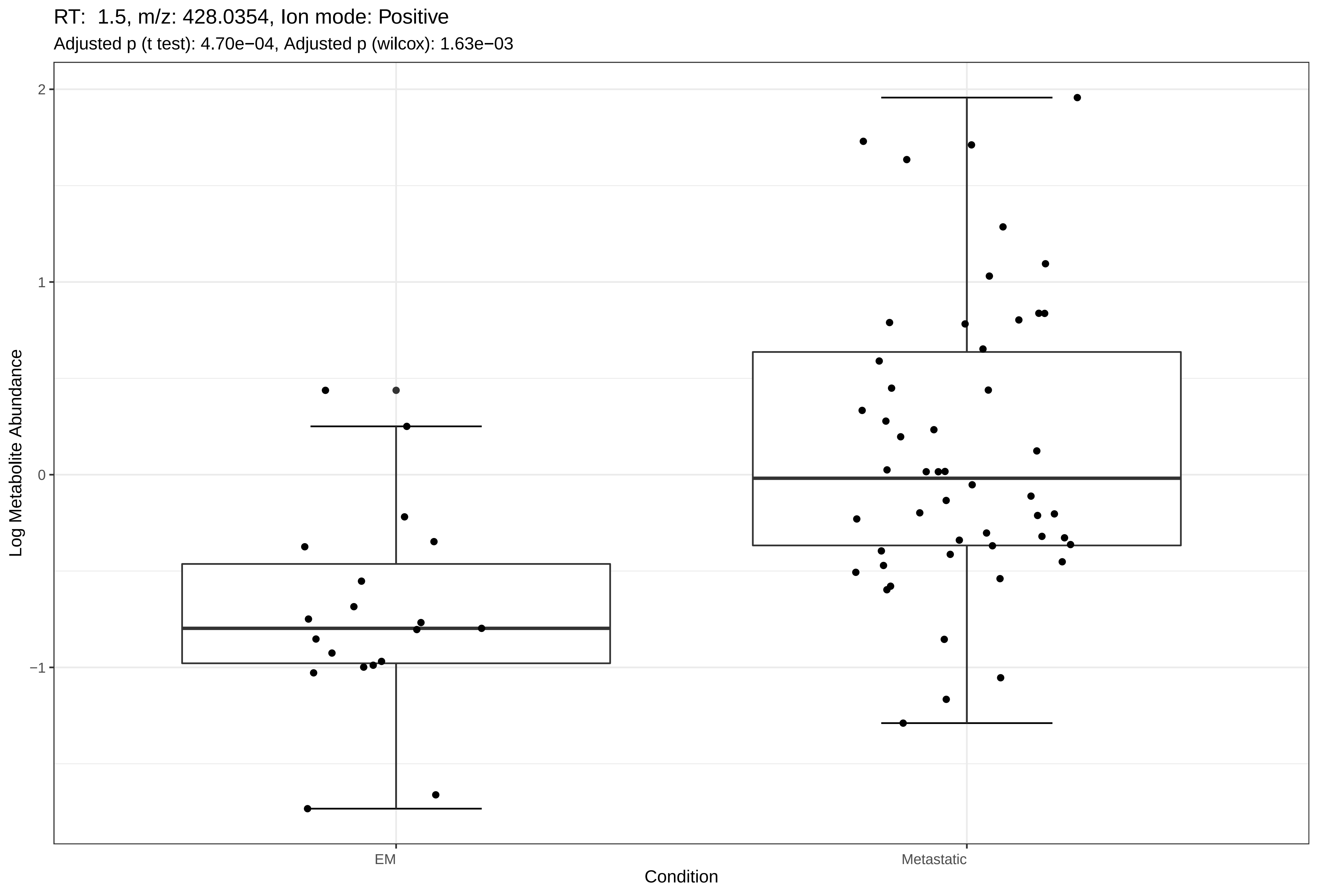
RT: 1.5, m/z: 428.0354, Ion mode: Positive
Adjusted p (t test): 4.70e−04, Adjusted p (wilcox): 1.63e−03
Log Metabolite Abundance
2
1
0
−1
EM
Metastatic
Condition

Orthophosphate (RT: 0.8, m/z: 98.9842, Ion mode: Positive)

Adjusted p (t test): 5.68e−04, Adjusted p (wilcox): 4.77e−04

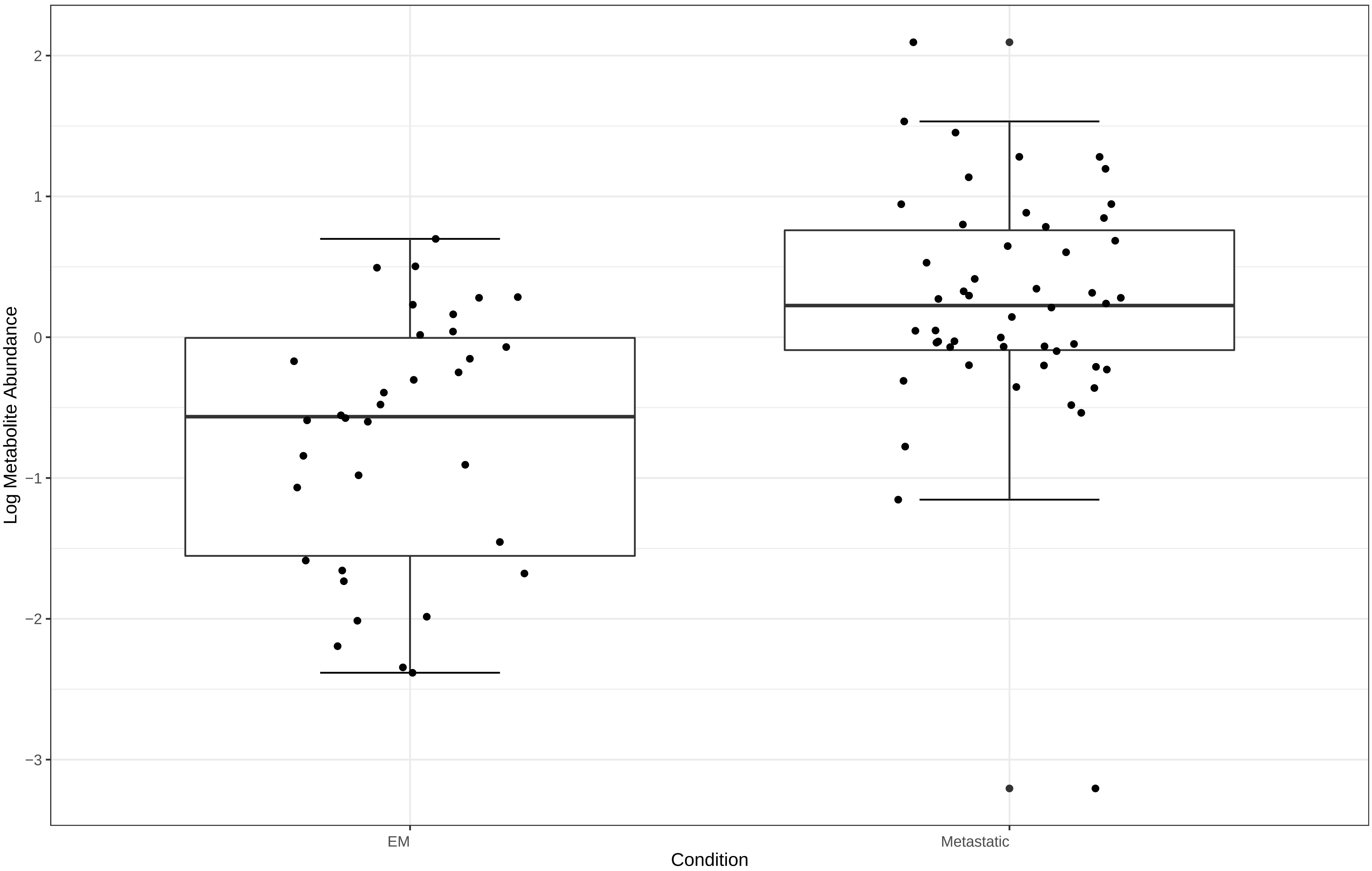

RT: 7.6, m/z: 282.1072, Ion mode: Negative

Adjusted p (t test): 4.85e−04, Adjusted p (wilcox): 6.99e−04

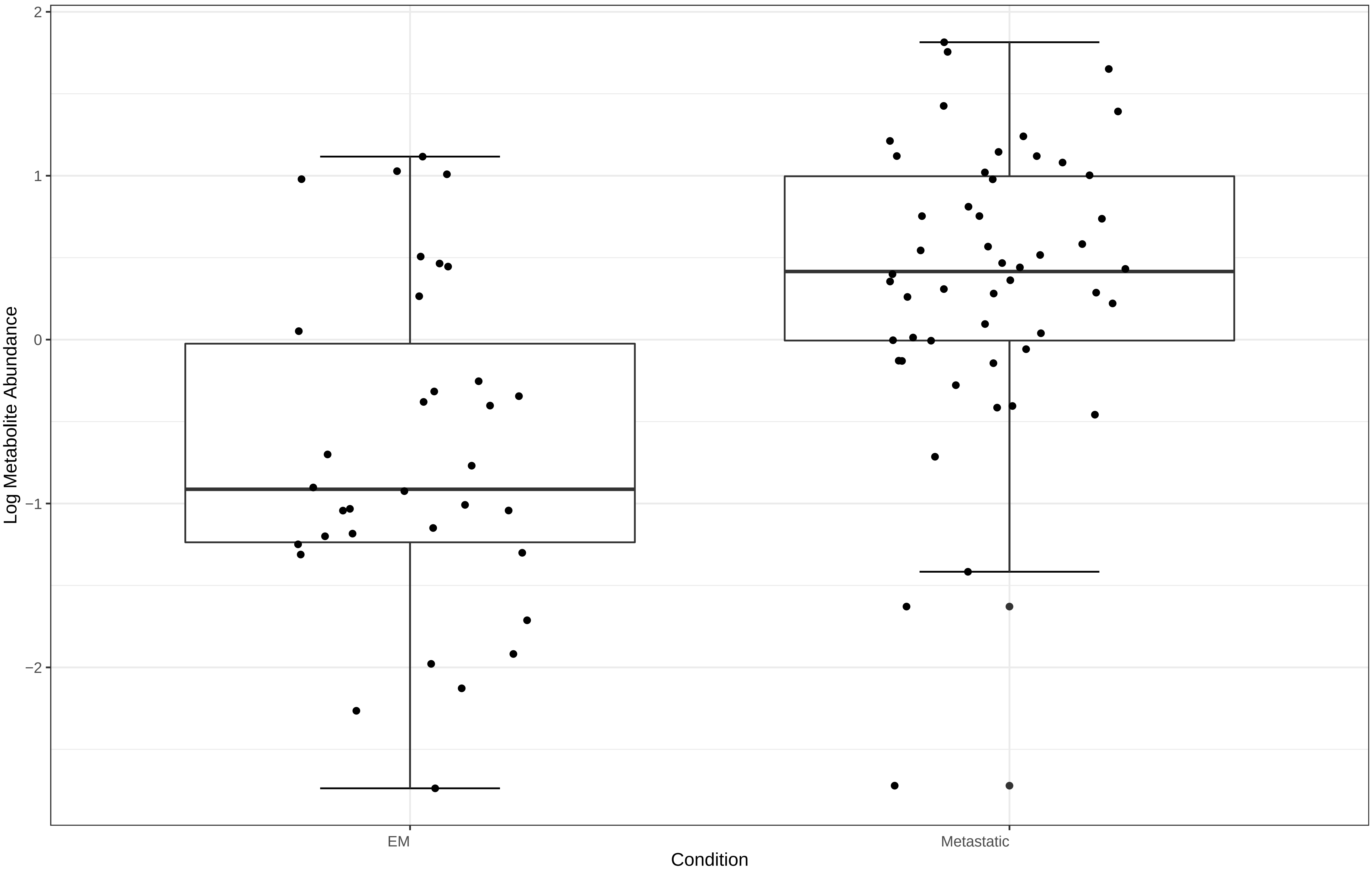

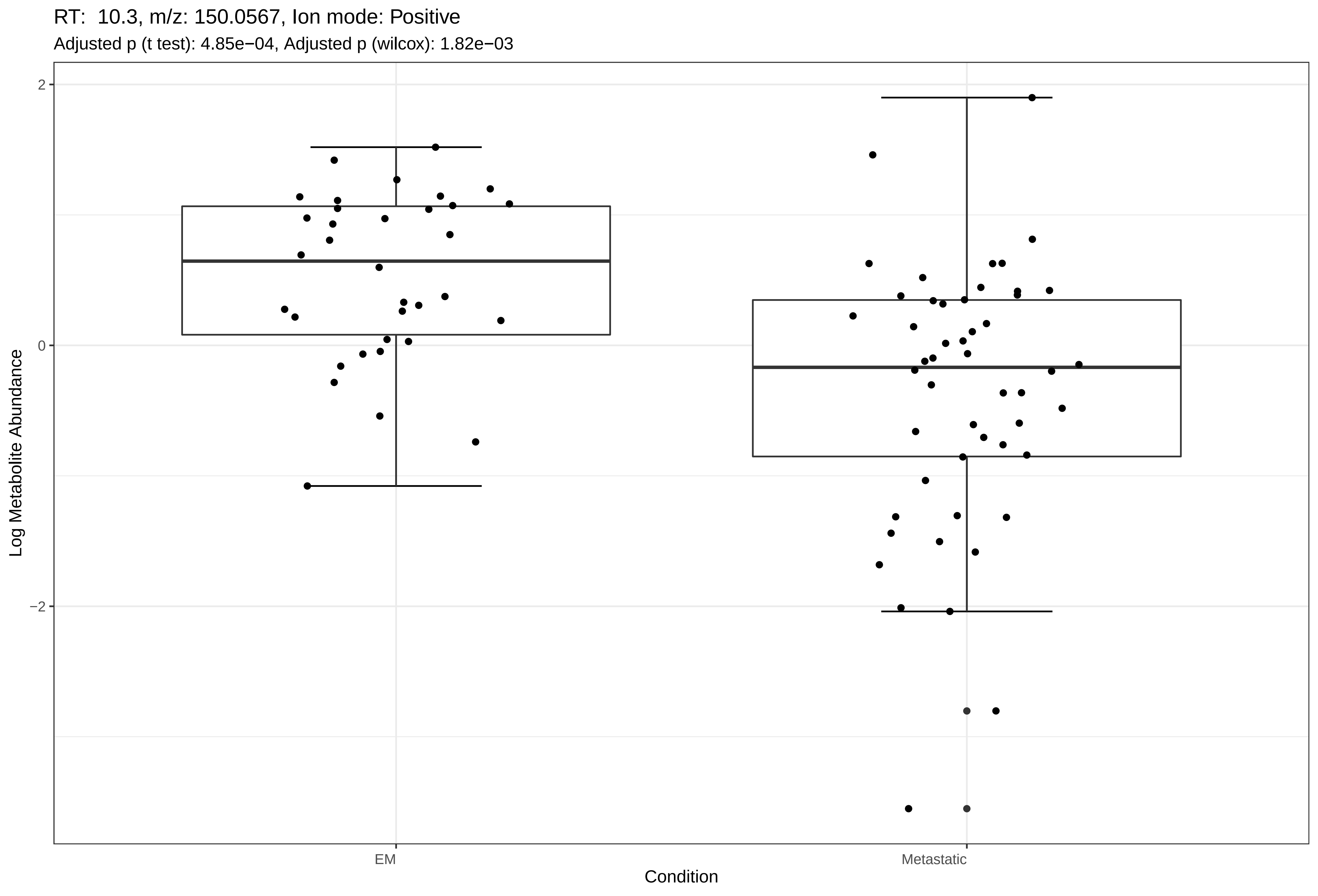
RT: 10.3, m/z: 150.0567, Ion mode: Positive
Adjusted p (t test): 4.85e−04, Adjusted p (wilcox): 1.82e−03
Log Metabolite Abundance
2
0
−2
EM
Metastatic
Condition

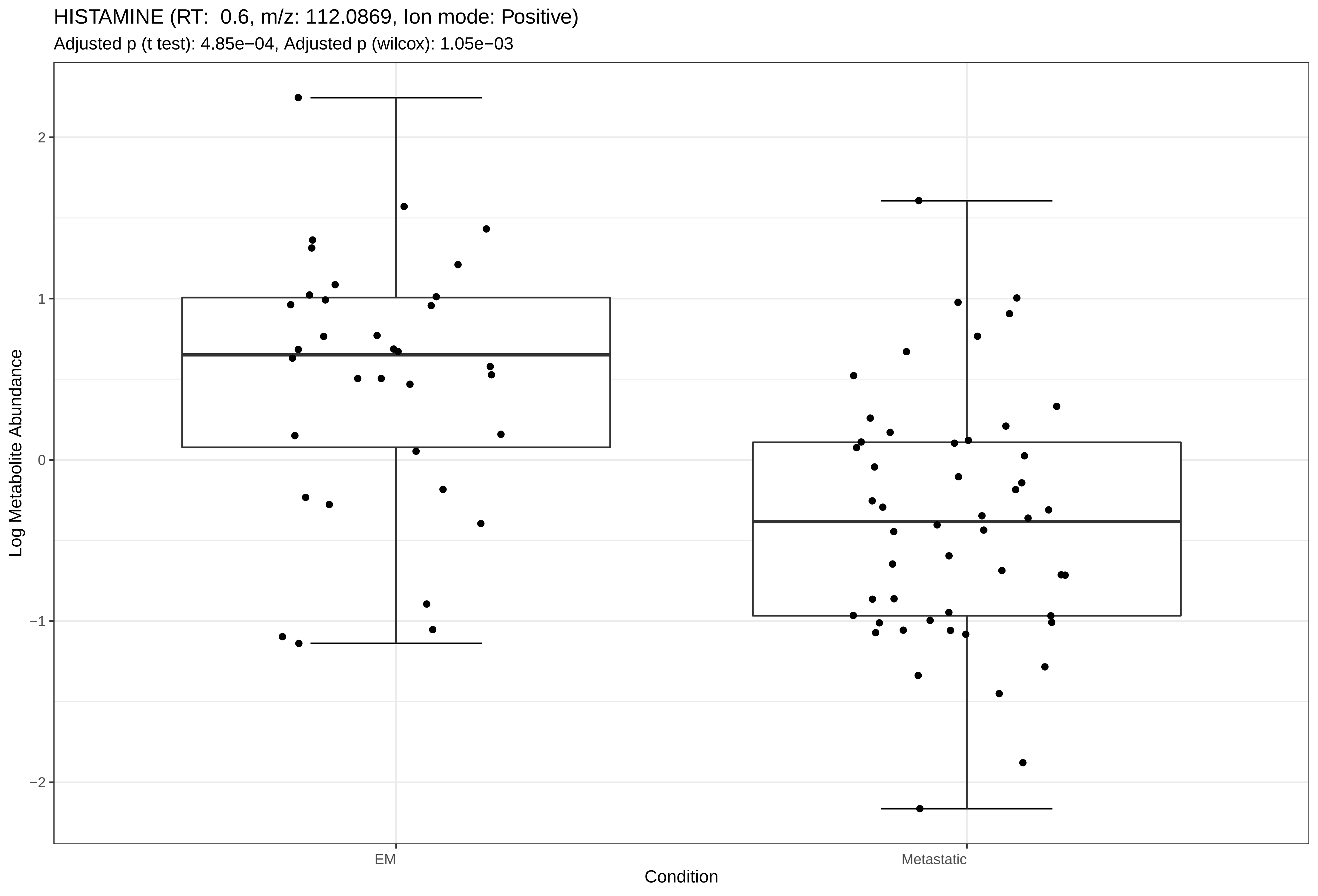

HISTAMINE (RT: 0.6, m/z: 112.0869, Ion mode: Positive)
Adjusted p (t test): 4.85e−04, Adjusted p (wilcox): 1.05e−03
Log Metabolite Abundance
2
1
0
−1
−2
EM
Metastatic
Condition

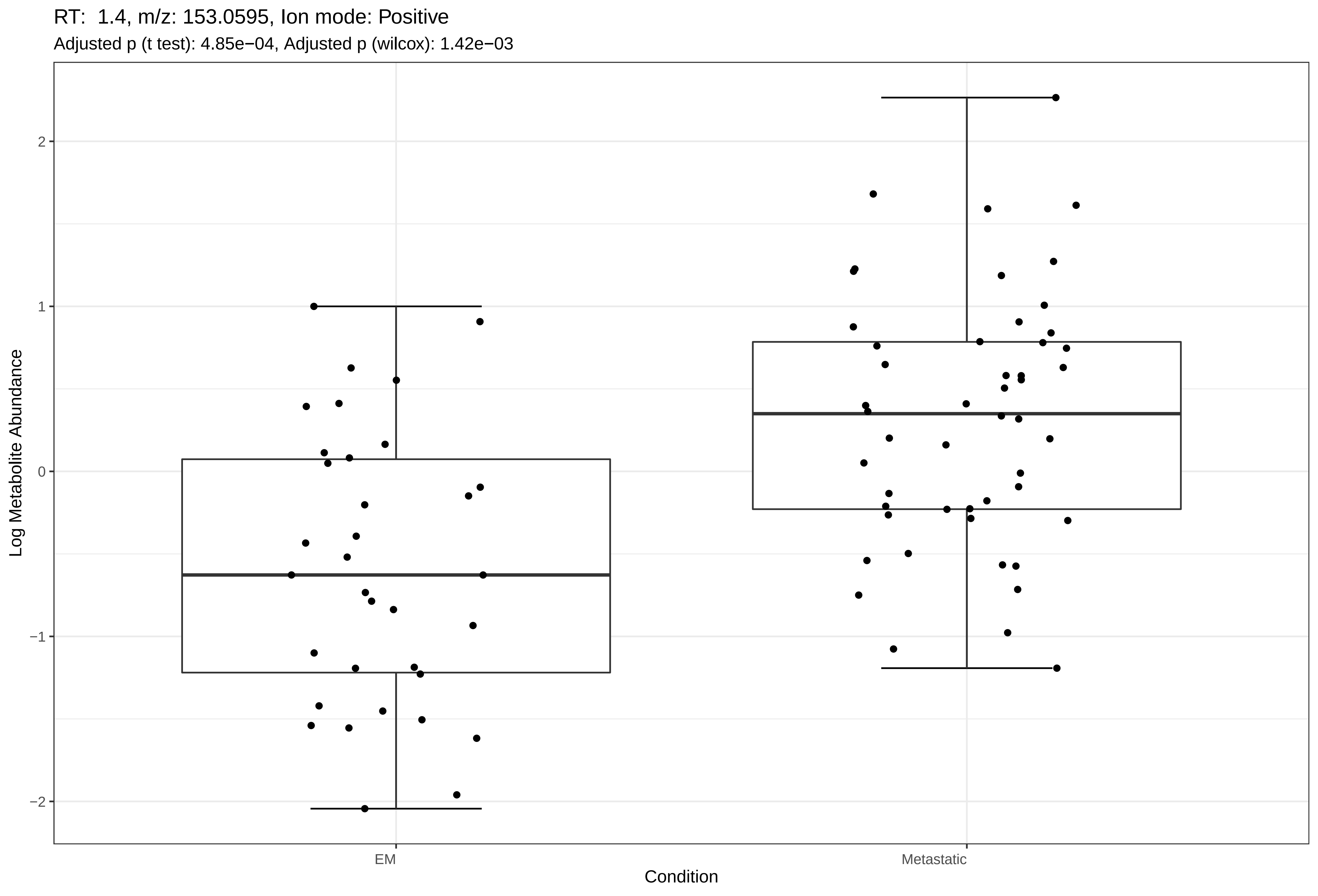

RT: 1.4, m/z: 153.0595, Ion mode: Positive
Adjusted p (t test): 4.85e−04, Adjusted p (wilcox): 1.42e−03
Log Metabolite Abundance
2
1
0
−1
−2
EM
Metastatic
Condition

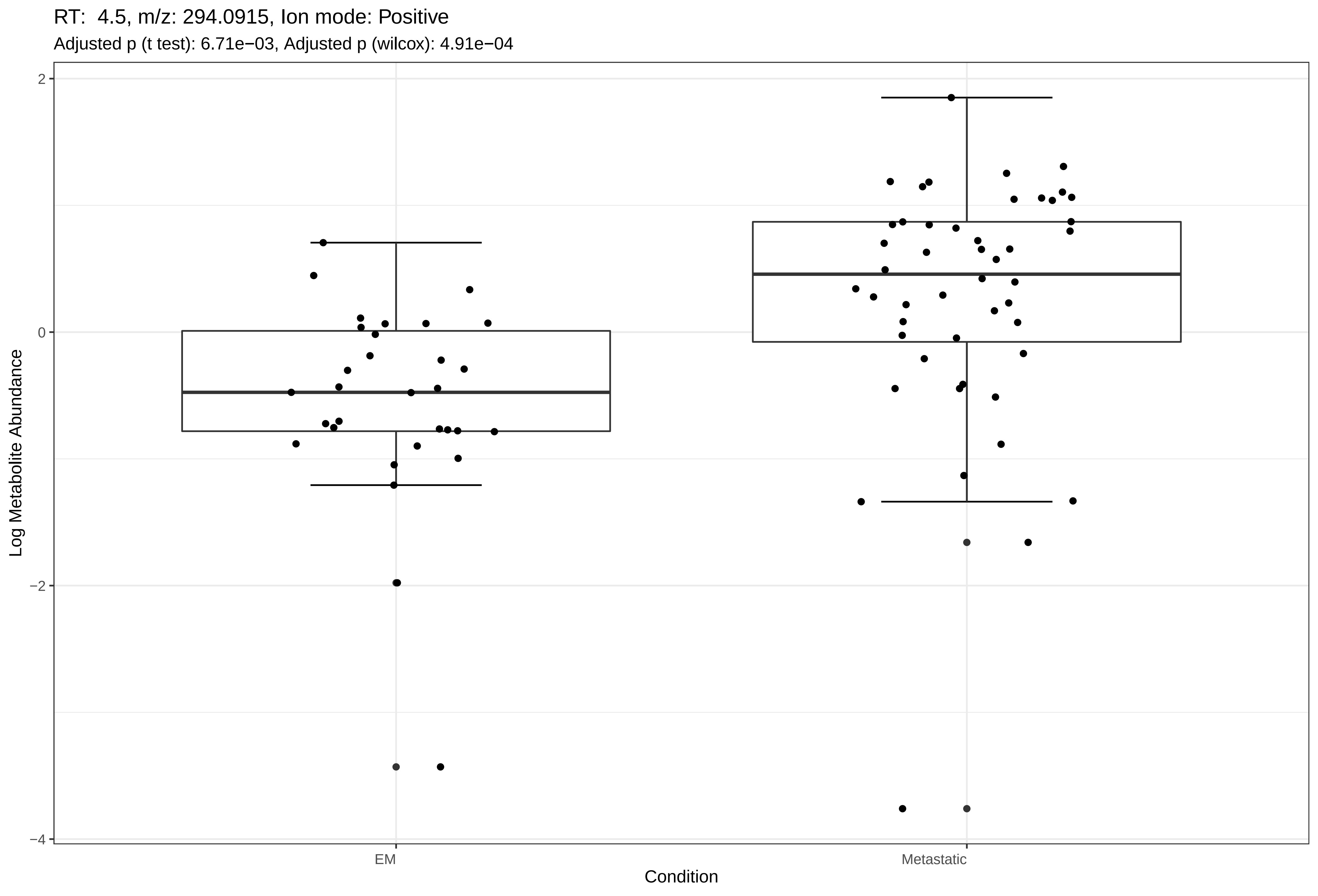
RT: 4.5, m/z: 294.0915, Ion mode: Positive
Adjusted p (t test): 6.71e−03, Adjusted p (wilcox): 4.91e−04
2
0
−2
−4
Log Metabolite Abundance
EM
Metastatic
Condition

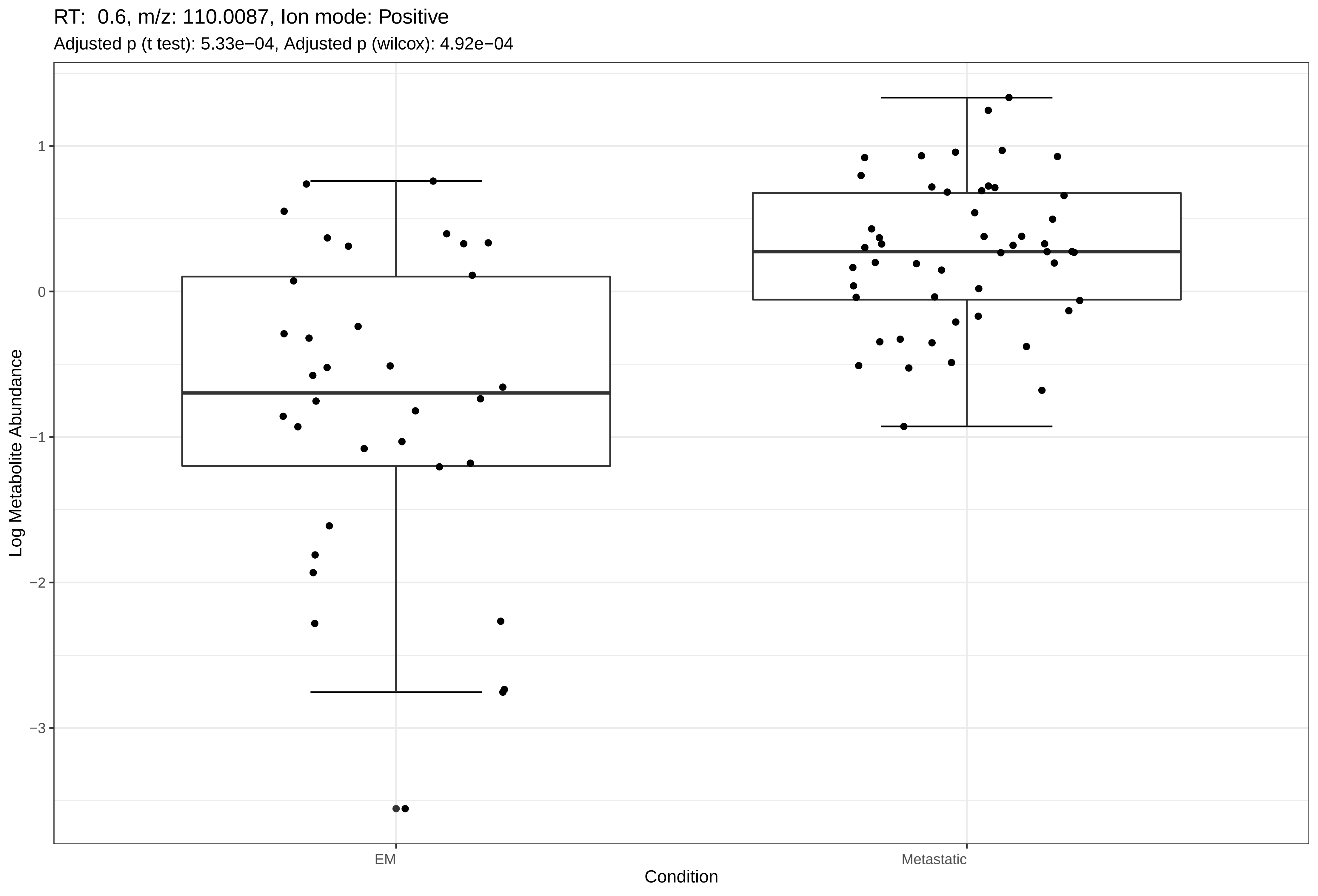
RT: 0.6, m/z: 110.0087, Ion mode: Positive
Adjusted p (t test): 5.33e−04, Adjusted p (wilcox): 4.92e−04
Log Metabolite Abundance
1
0
−1
−2
−3
EM
Metastatic
Condition

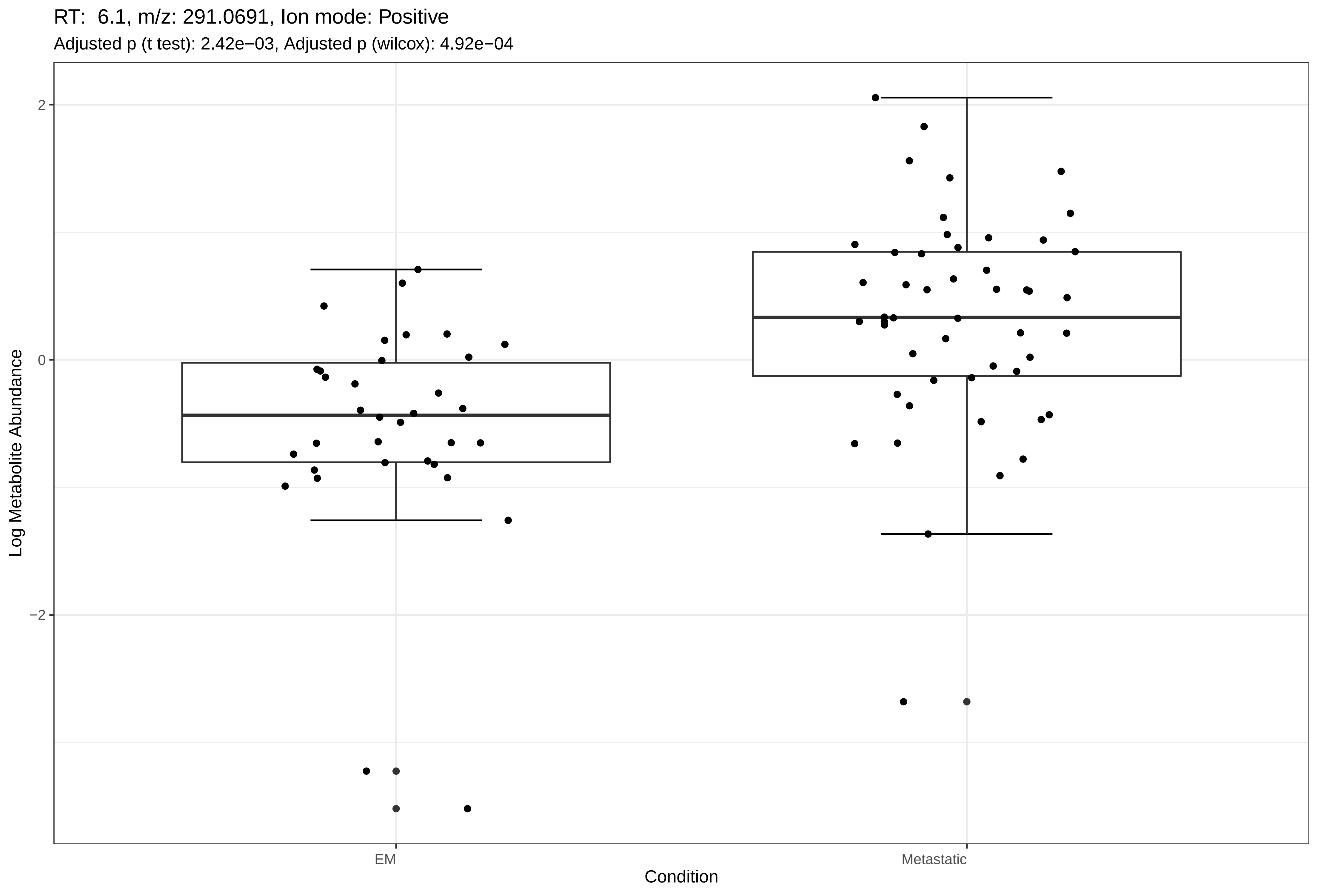
RT: 6.1, m/z: 291.0691, Ion mode: Positive
Adjusted p (t test): 2.42e−03, Adjusted p (wilcox): 4.92e−04
Log Metabolite Abundance
2
0
−2
EM
Metastatic
Condition

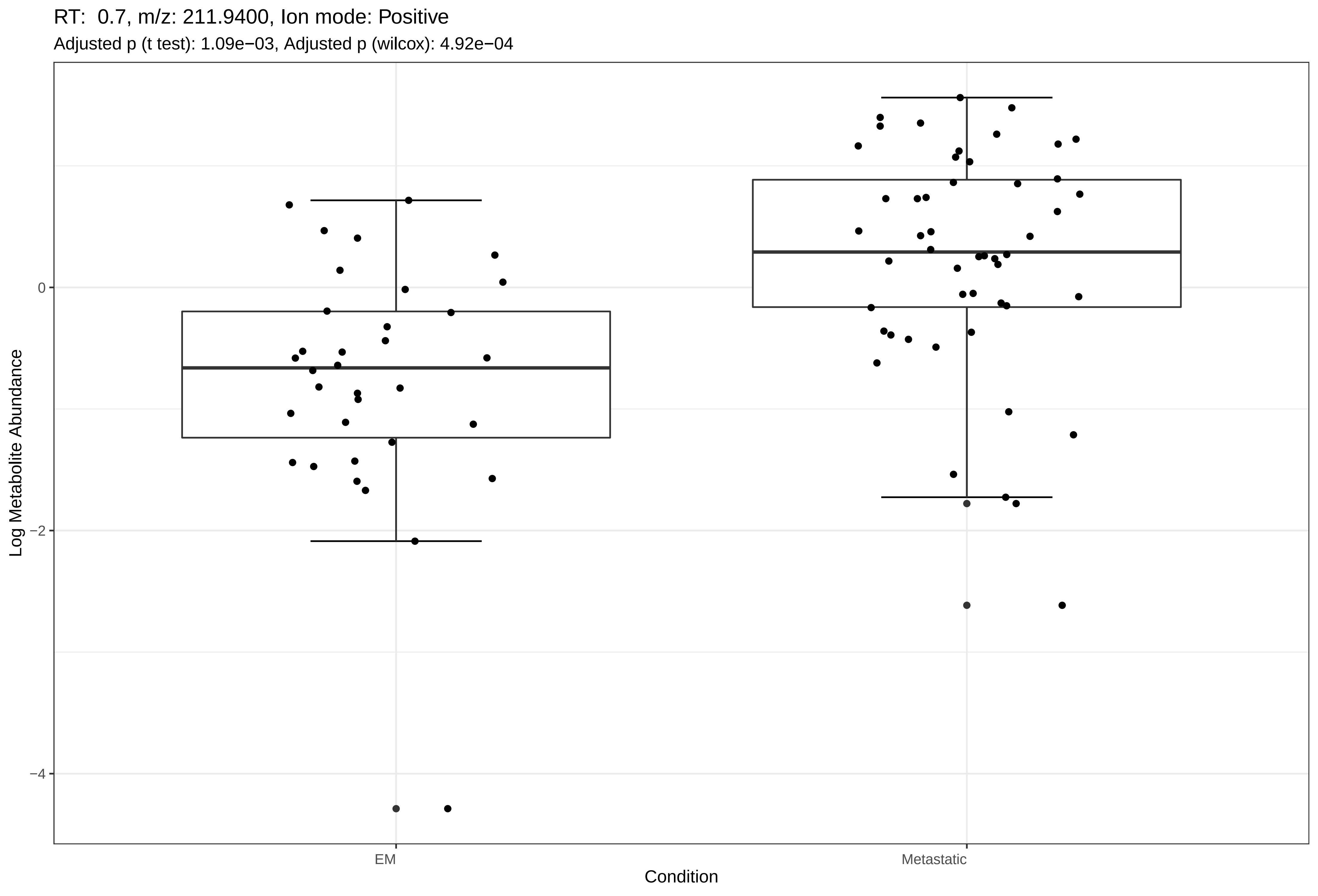

RT: 0.7, m/z: 211.9400, Ion mode: Positive
Adjusted p (t test): 1.09e−03, Adjusted p (wilcox): 4.92e−04
Log Metabolite Abundance
0
−2
−4
EM
Metastatic
Condition

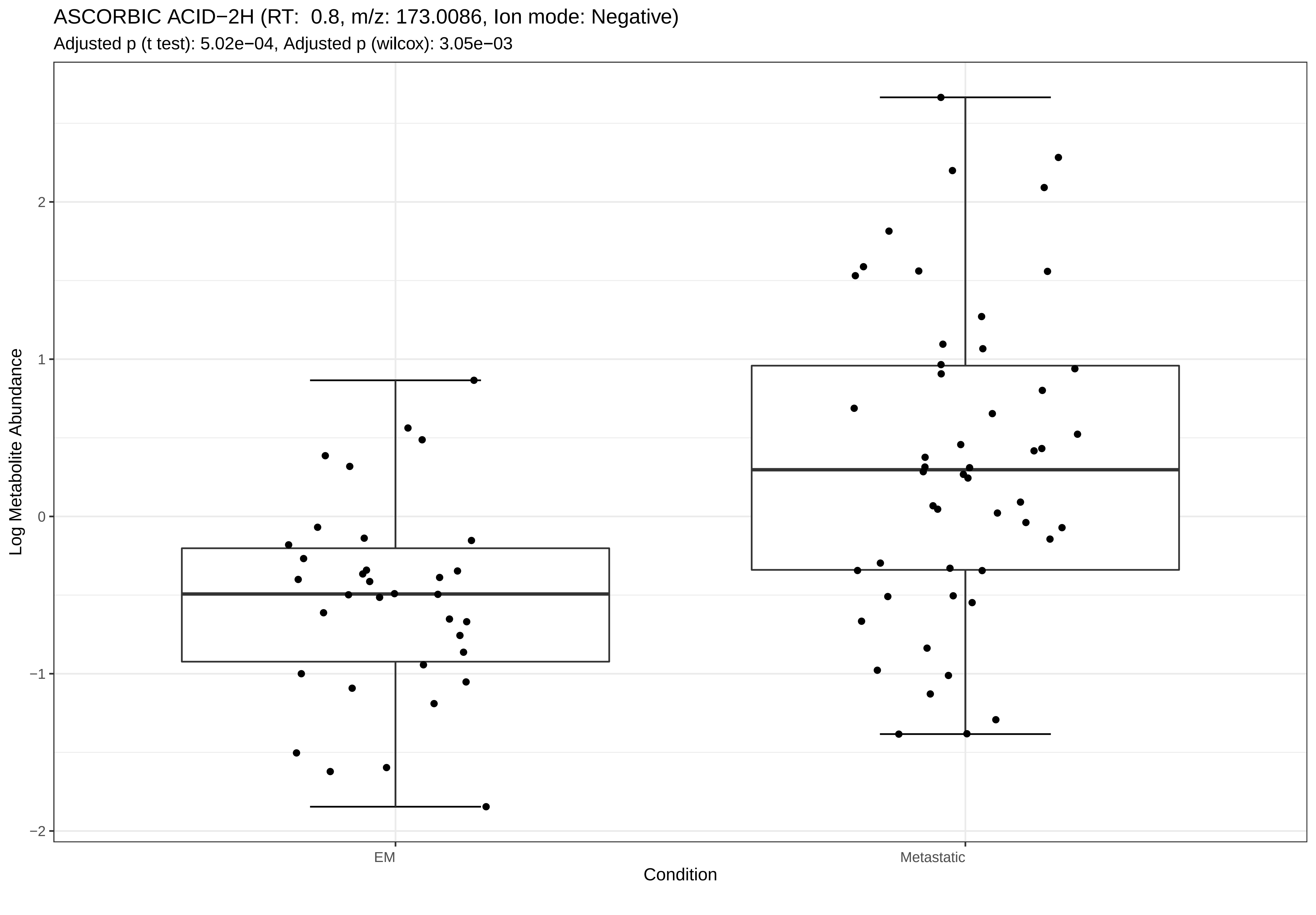

ASCORBIC ACID−2H (RT: 0.8, m/z: 173.0086, Ion mode: Negative)
Adjusted p (t test): 5.02e−04, Adjusted p (wilcox): 3.05e−03
Log Metabolite Abundance
2
1
0
−1
−2
EM
Metastatic
Condition

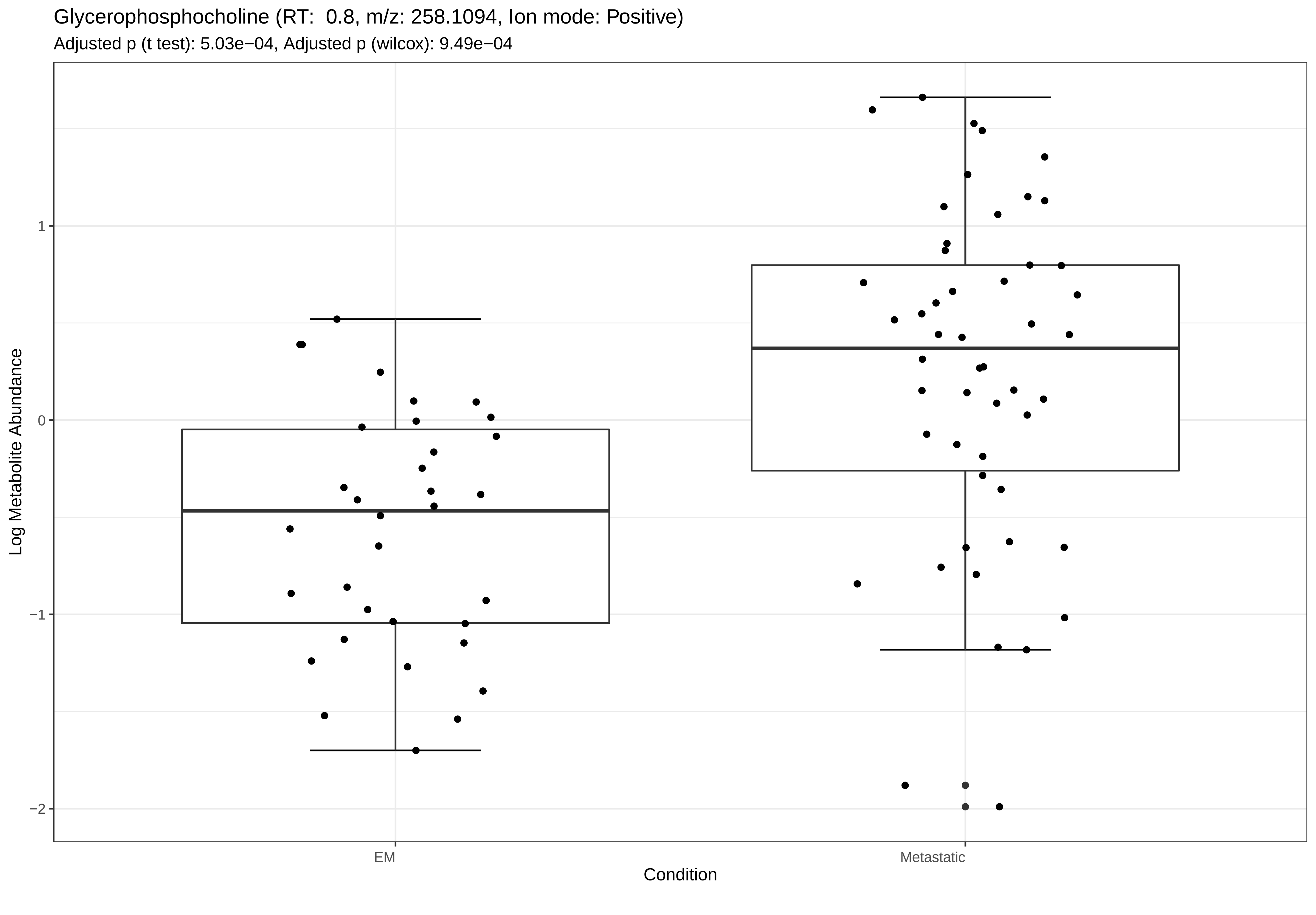
Glycerophosphocholine (RT: 0.8, m/z: 258.1094, Ion mode: Positive)
Adjusted p (t test): 5.03e−04, Adjusted p (wilcox): 9.49e−04
Log Metabolite Abundance
1
0
−1
−2
EM
Metastatic
Condition

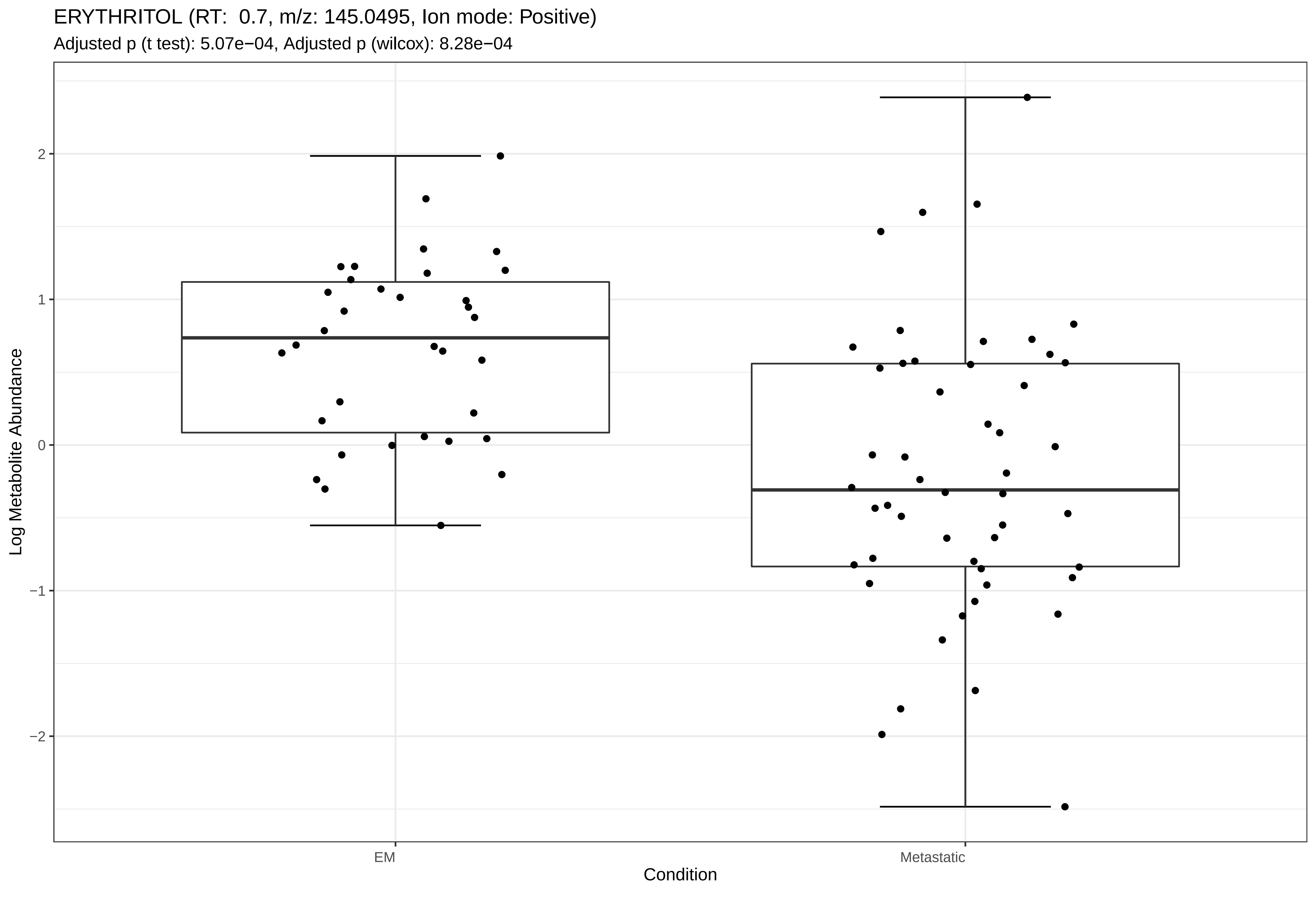
ERYTHRITOL (RT: 0.7, m/z: 145.0495, Ion mode: Positive)
Adjusted p (t test): 5.07e−04, Adjusted p (wilcox): 8.28e−04
Log Metabolite Abundance
2
1
0
−1
−2
EM
Metastatic
Condition

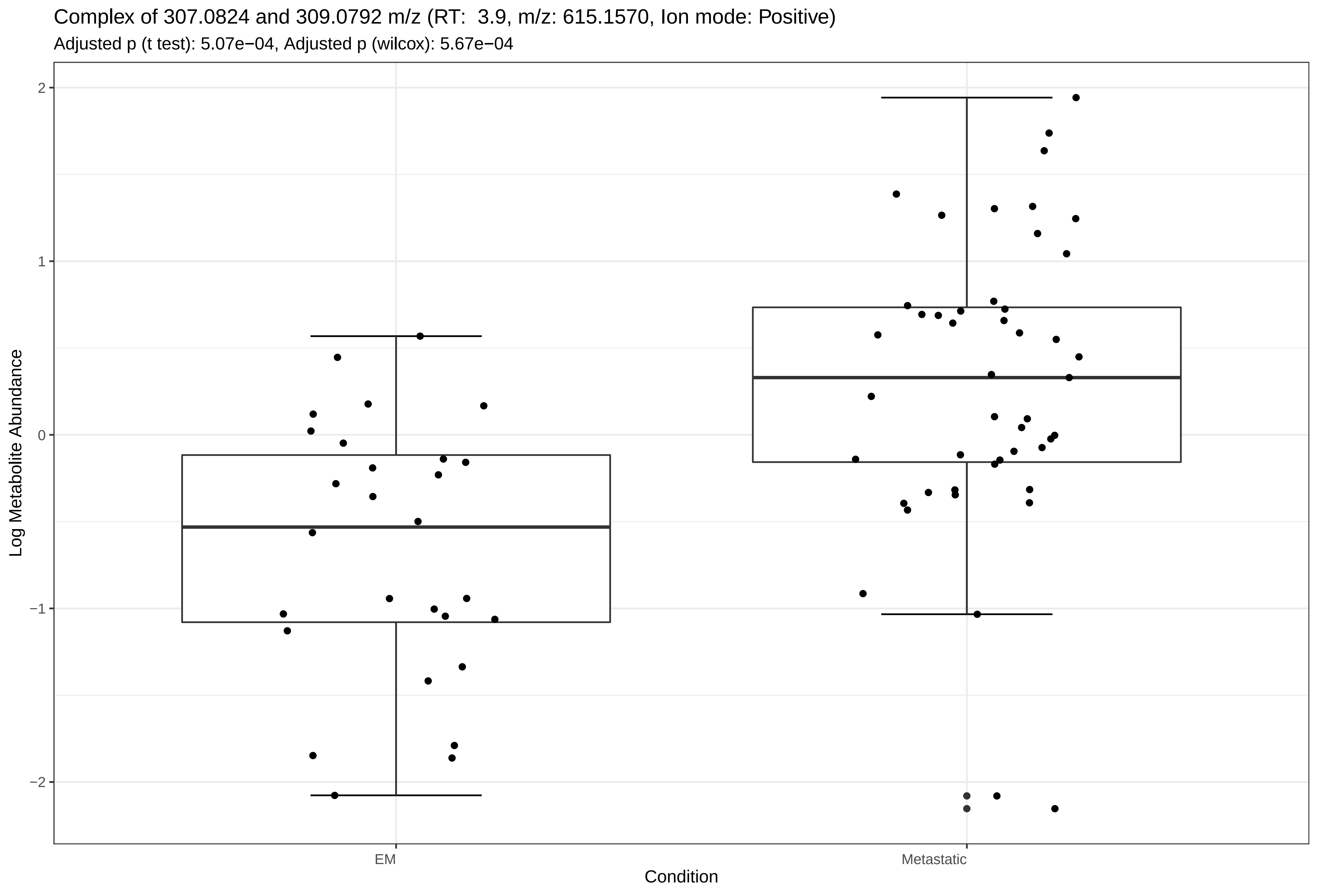

Complex of 307.0824 and 309.0792 m/z (RT: 3.9, m/z: 615.1570, Ion mode: Positive)
Adjusted p (t test): 5.07e−04, Adjusted p (wilcox): 5.67e−04
Log Metabolite Abundance
2
1
0
−1
−2
EM
Metastatic
Condition

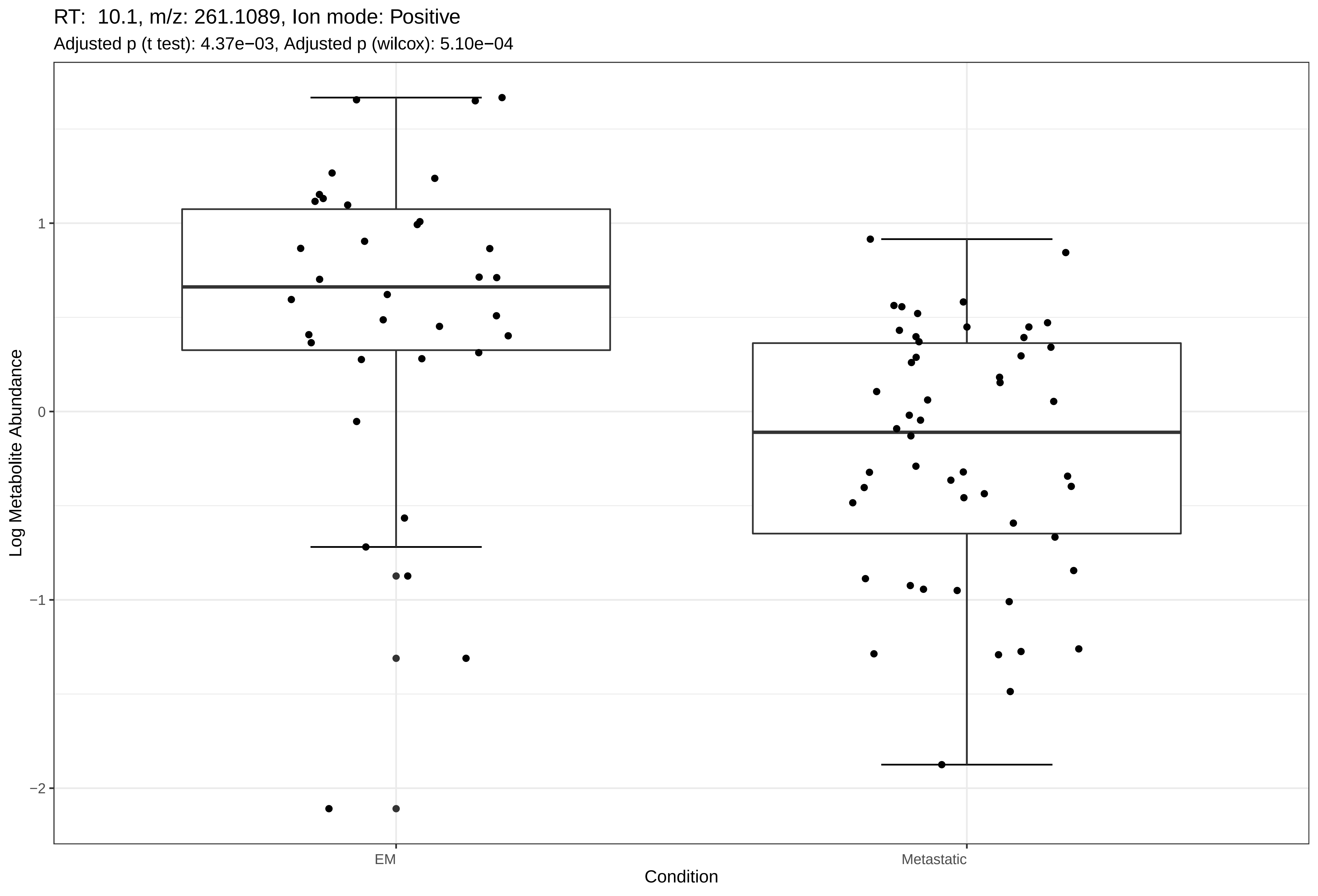

RT: 10.1, m/z: 261.1089, Ion mode: Positive
Adjusted p (t test): 4.37e−03, Adjusted p (wilcox): 5.10e−04
Log Metabolite Abundance
1
0
−1
−2
EM
Metastatic
Condition

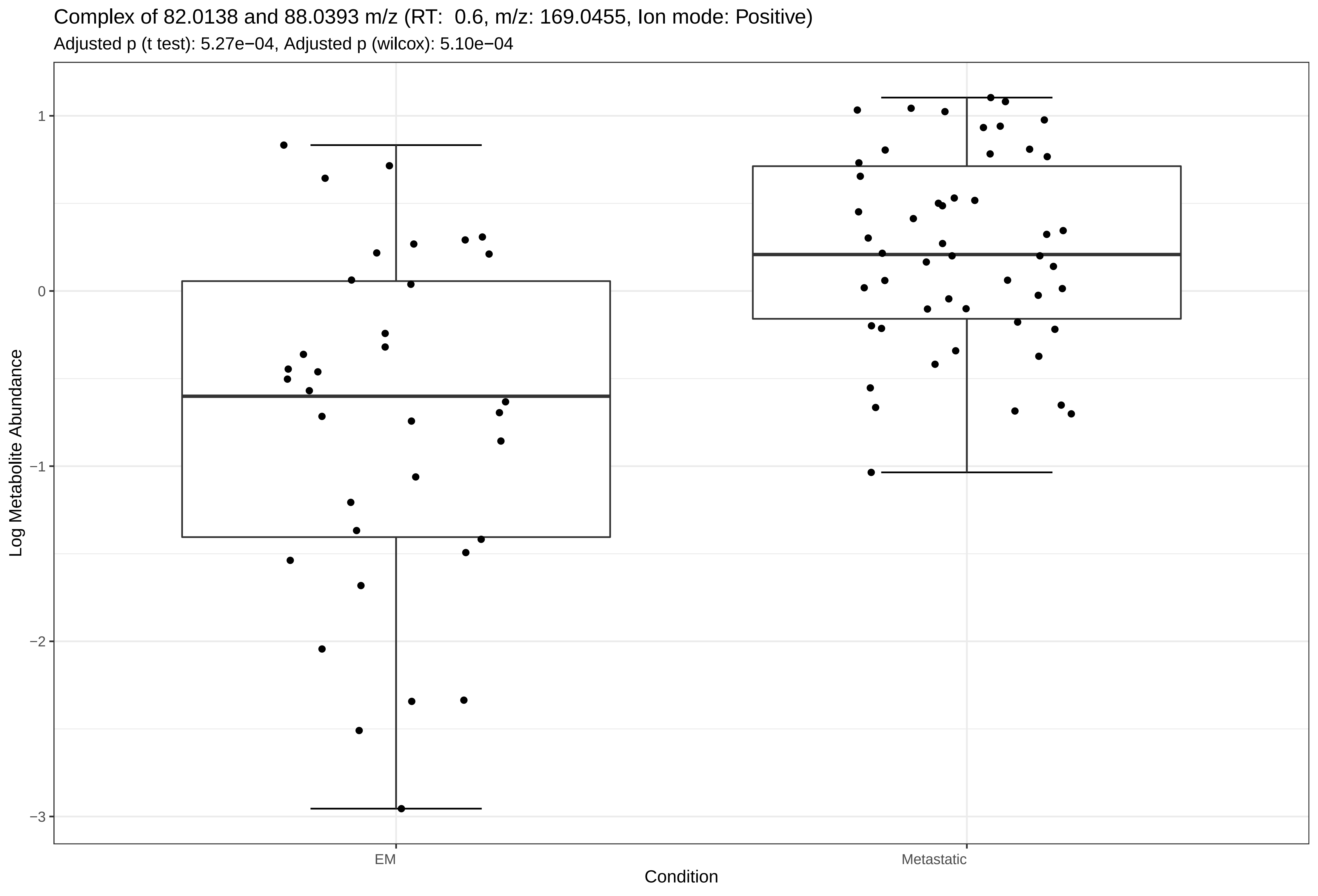

Complex of 82.0138 and 88.0393 m/z (RT: 0.6, m/z: 169.0455, Ion mode: Positive)
Adjusted p (t test): 5.27e−04, Adjusted p (wilcox): 5.10e−04
Log Metabolite Abundance
1
0
−1
−2
−3
EM
Metastatic
Condition

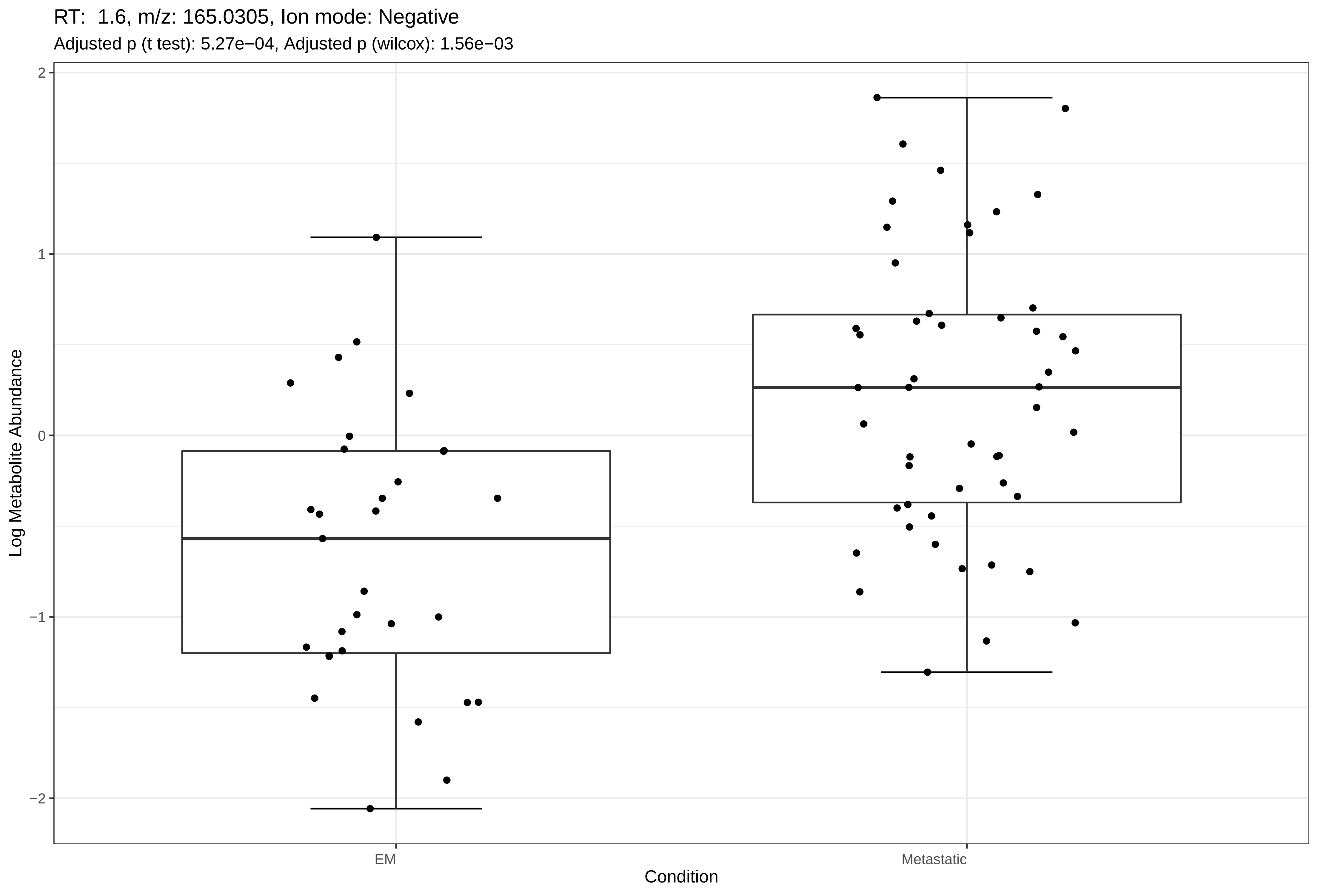
RT: 1.6, m/z: 165.0305, Ion mode: Negative
Adjusted p (t test): 5.27e−04, Adjusted p (wilcox): 1.56e−03
Log Metabolite Abundance
2
1
0
−1
−2
EM
Metastatic
Condition

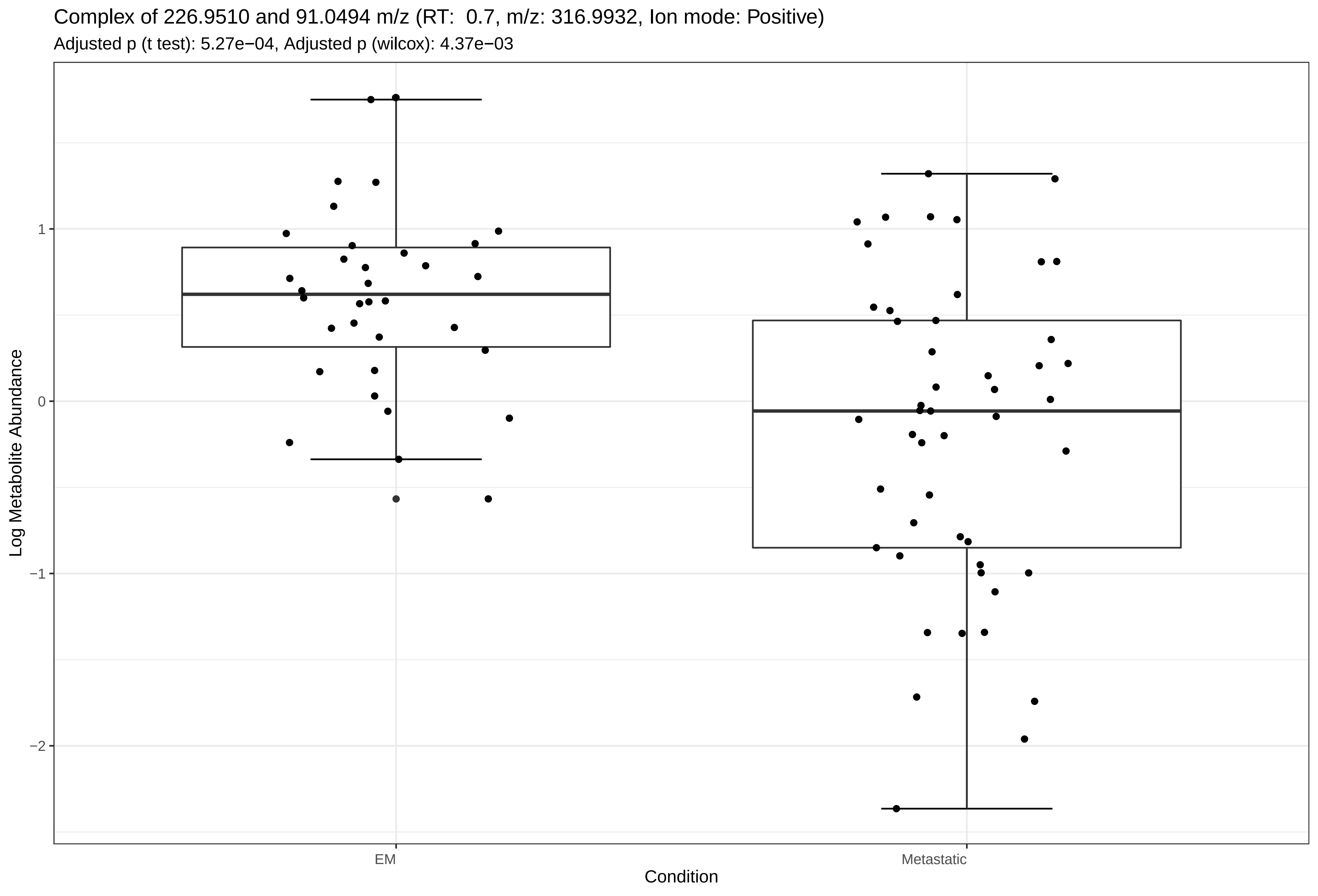

Complex of 226.9510 and 91.0494 m/z (RT: 0.7, m/z: 316.9932, Ion mode: Positive)
Adjusted p (t test): 5.27e−04, Adjusted p (wilcox): 4.37e−03
Log Metabolite Abundance
1
0
−1
−2
EM
Metastatic
Condition

RT: 9.8, m/z: 163.1114, Ion mode: Positive

Adjusted p (t test): 1.99e−03, Adjusted p (wilcox): 5.30e−04

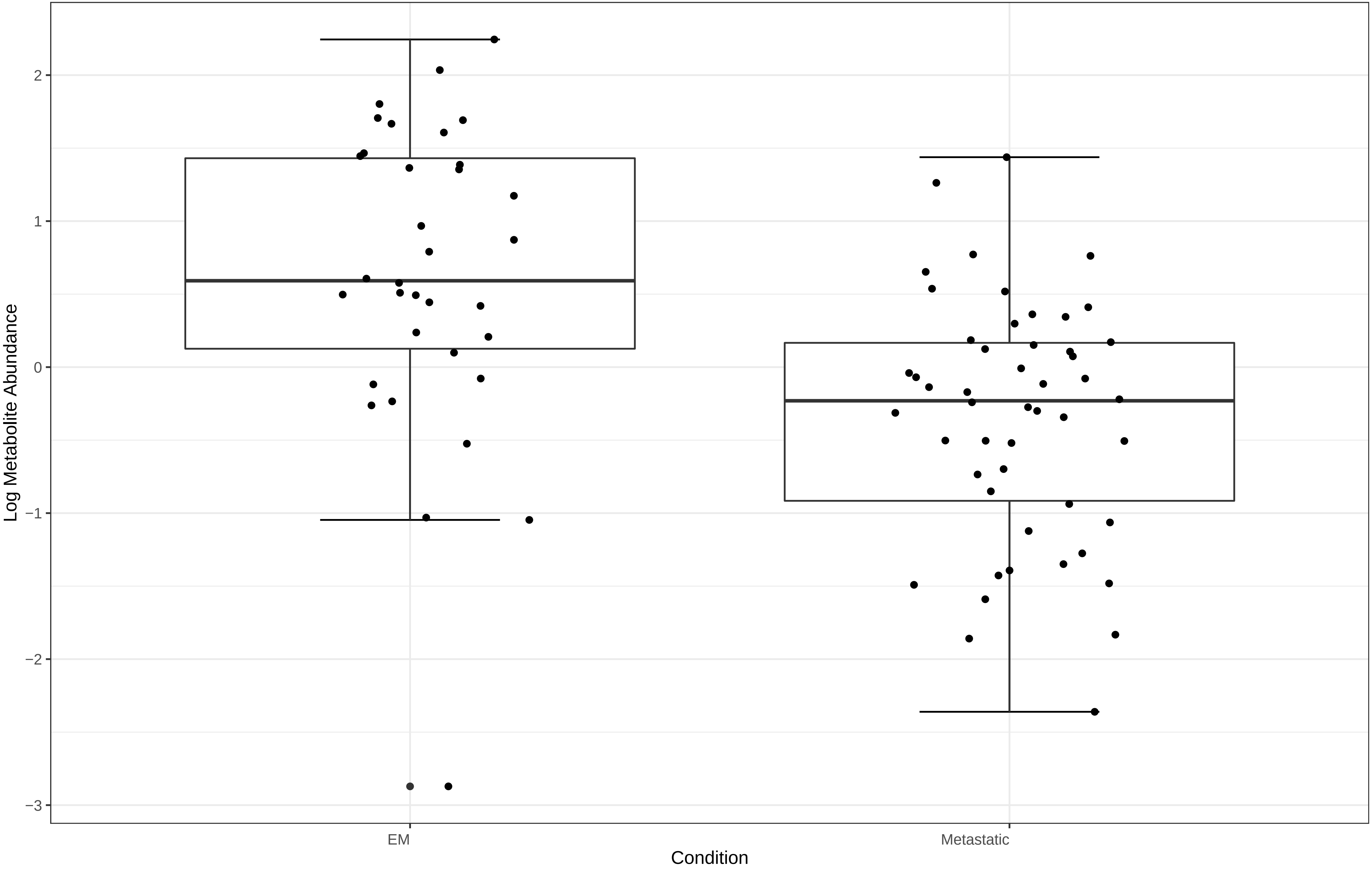

RT: 6.1, m/z: 142.0622, Ion mode: Positive

Adjusted p (t test): 4.36e−03, Adjusted p (wilcox): 5.30e−04

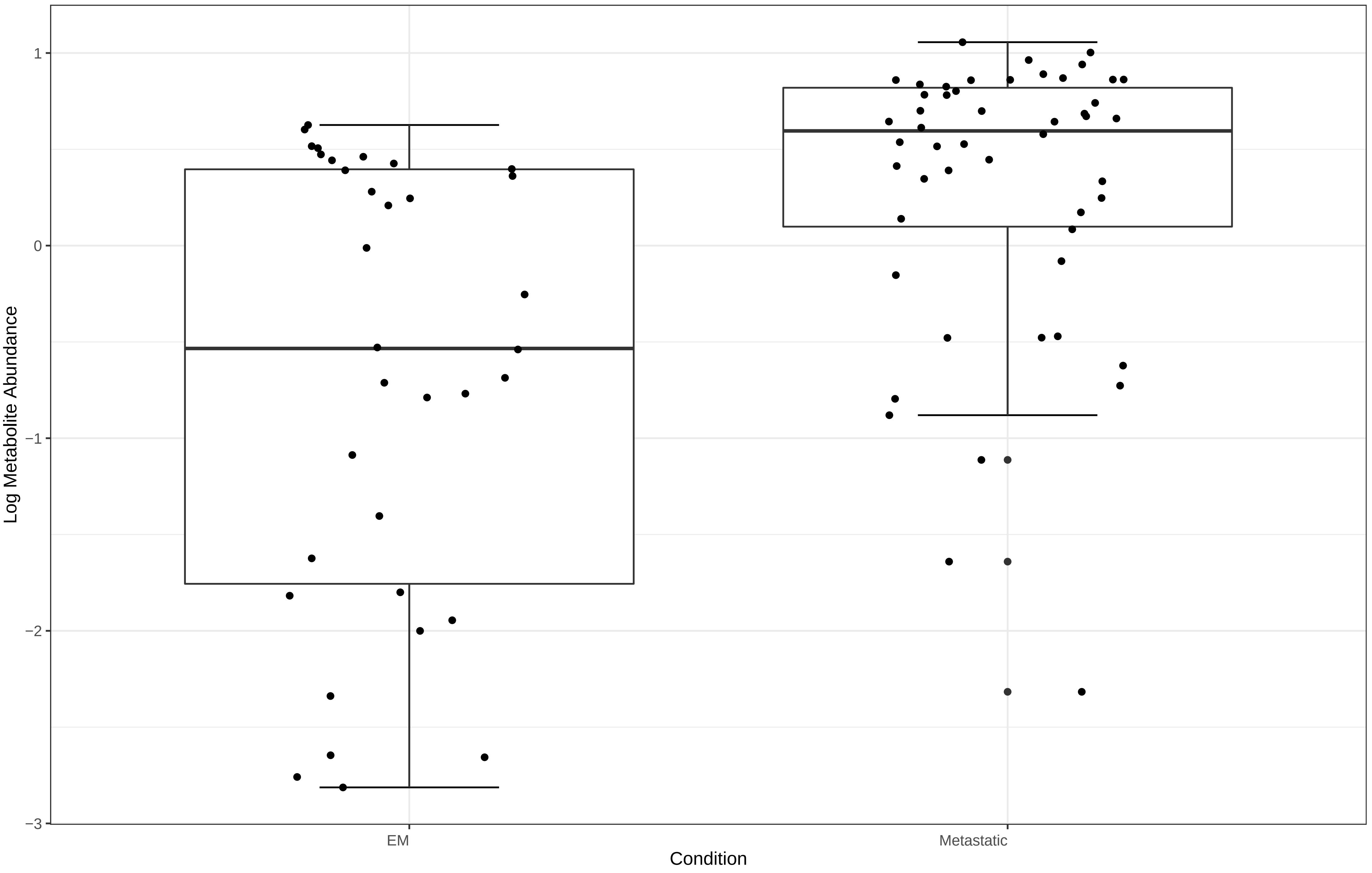

RT: 0.6, m/z: 123.0138, Ion mode: Positive

Adjusted p (t test): 5.71e−04, Adjusted p (wilcox): 5.30e−04

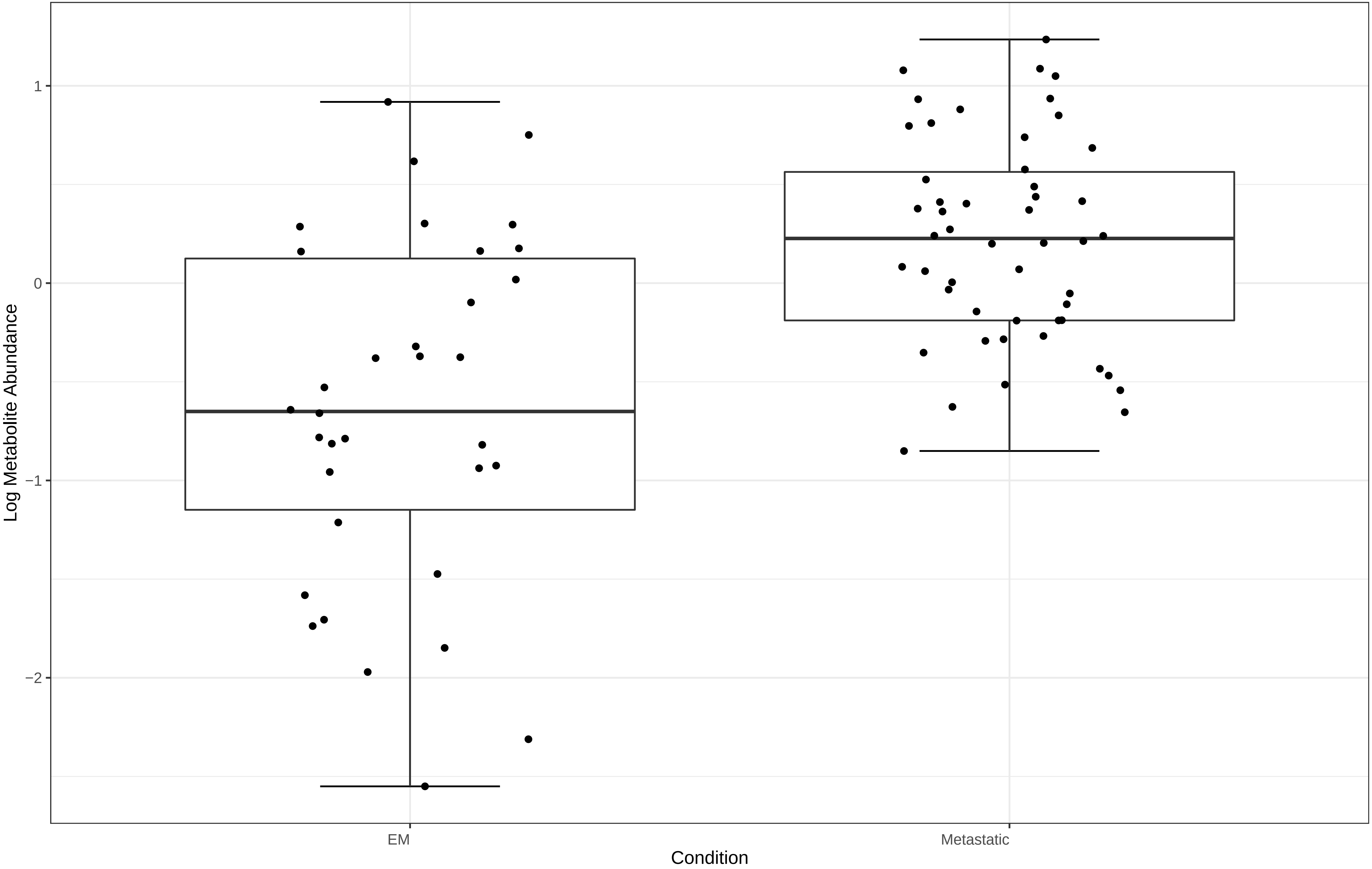

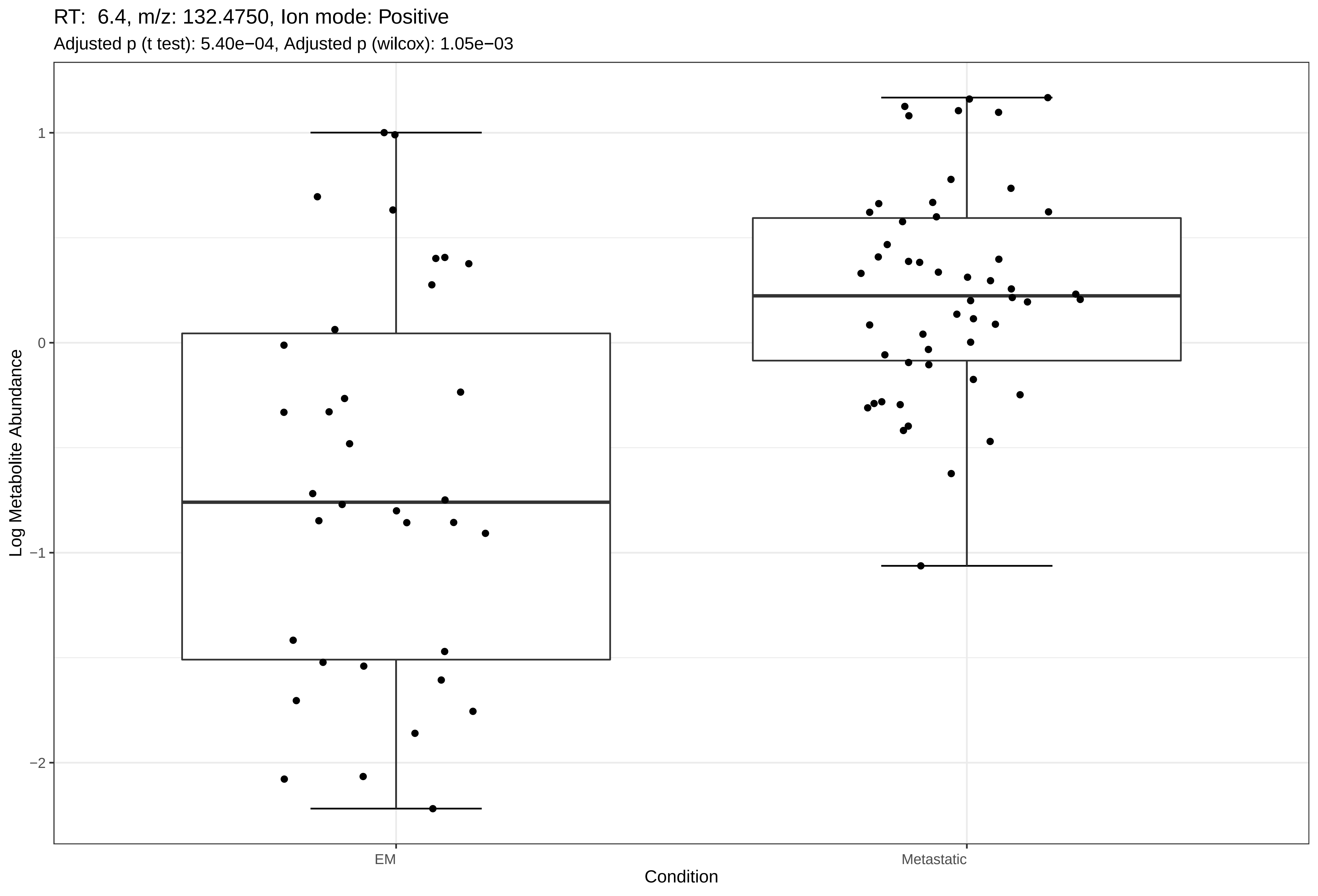

RT: 6.4, m/z: 132.4750, Ion mode: Positive
Adjusted p (t test): 5.40e−04, Adjusted p (wilcox): 1.05e−03
Log Metabolite Abundance
1
0
−1
−2
EM
Metastatic
Condition

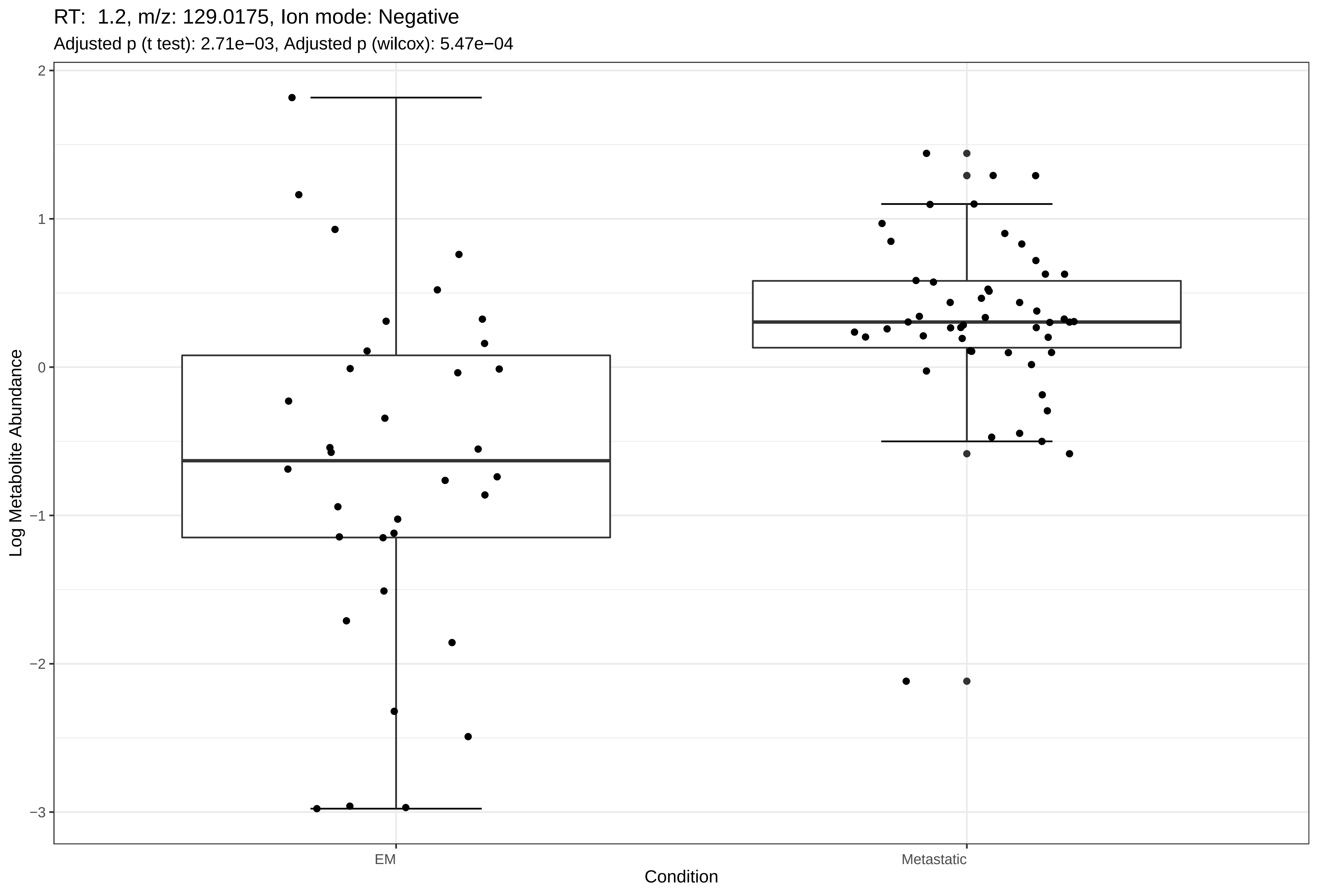
RT: 1.2, m/z: 129.0175, Ion mode: Negative
Adjusted p (t test): 2.71e−03, Adjusted p (wilcox): 5.47e−04
Log Metabolite Abundance
2
1
0
−1
−2
−3
EM
Metastatic
Condition

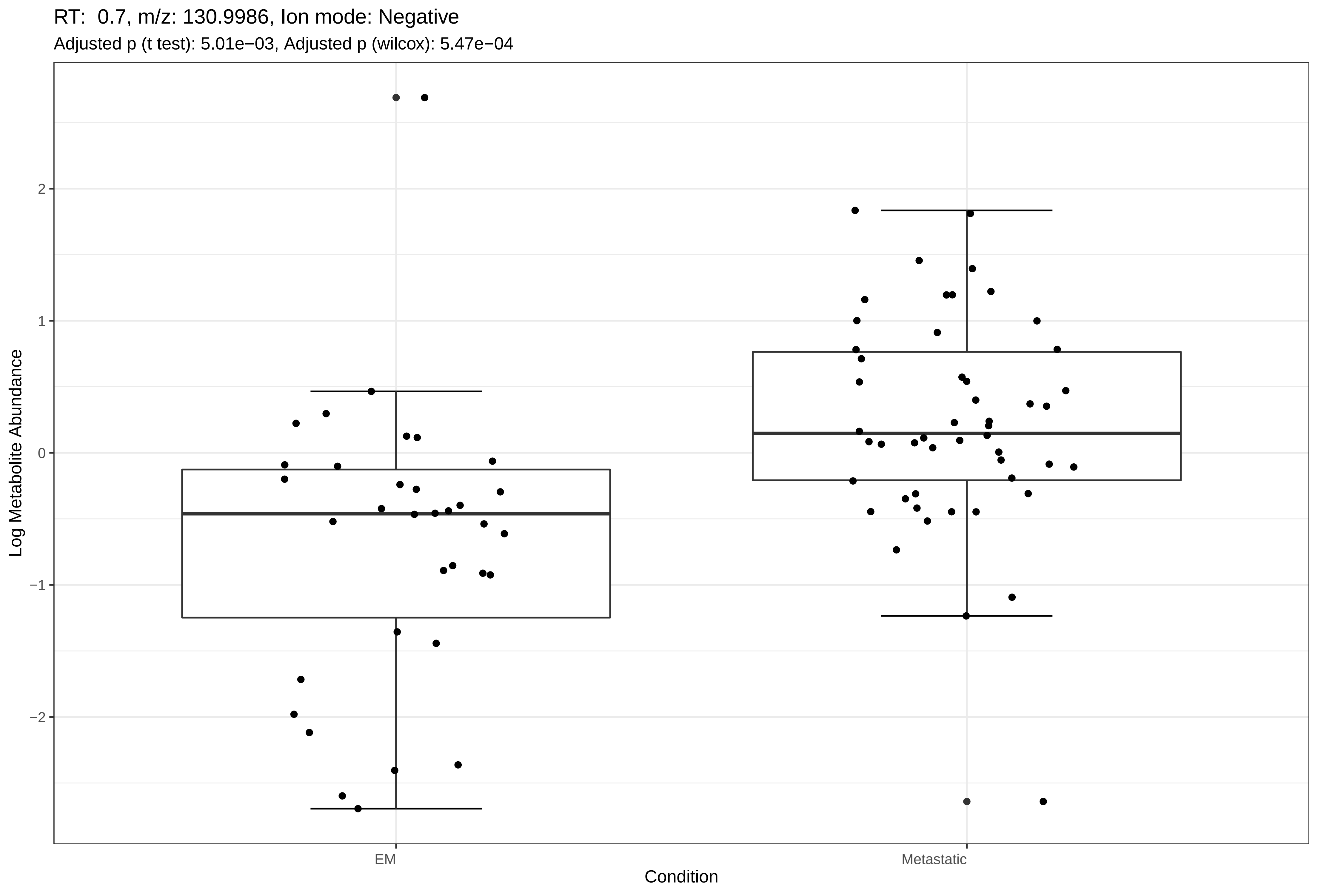
RT: 0.7, m/z: 130.9986, Ion mode: Negative
Adjusted p (t test): 5.01e−03, Adjusted p (wilcox): 5.47e−04
Log Metabolite Abundance
2
1
0
−1
−2
EM
Metastatic
Condition

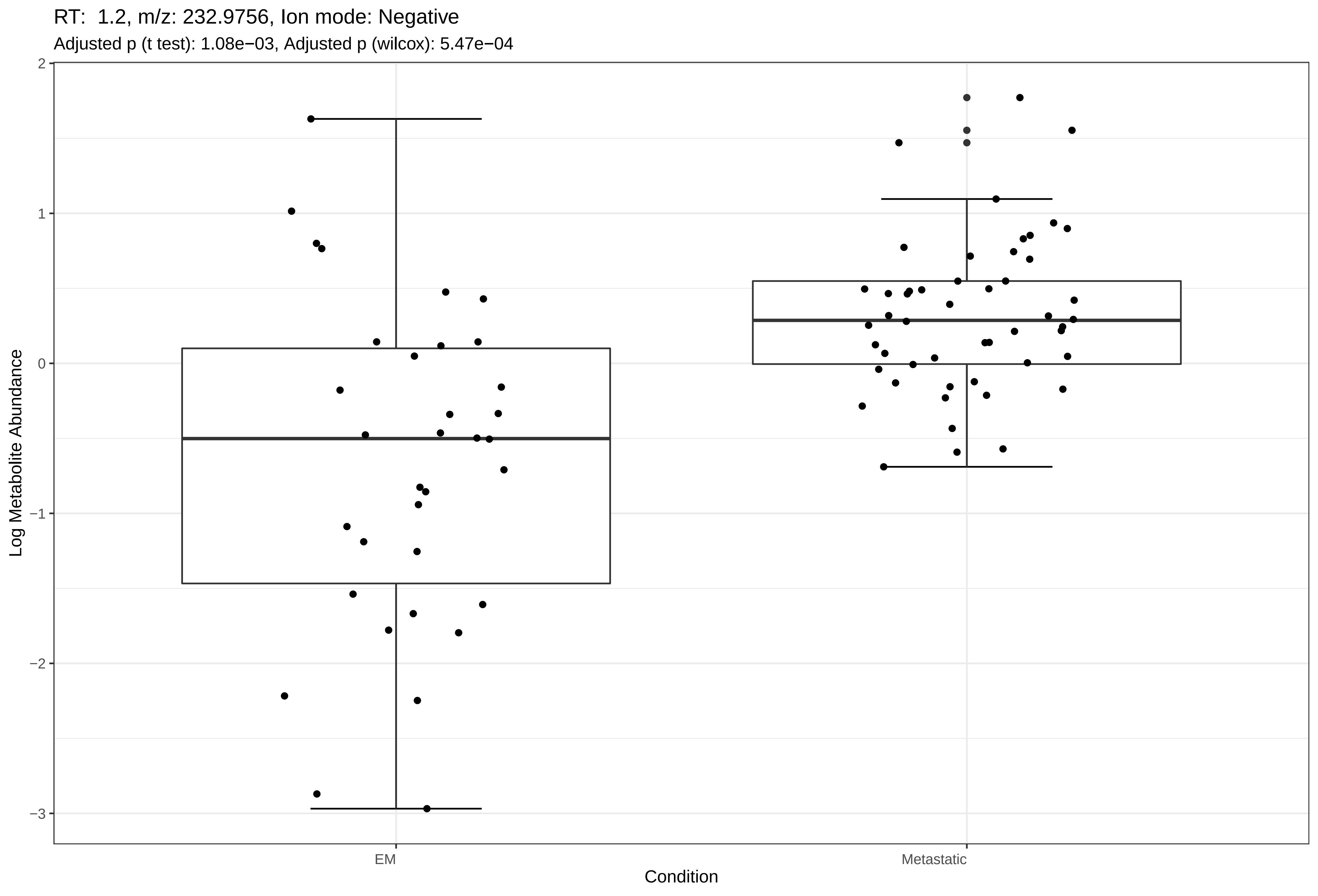
RT: 1.2, m/z: 232.9756, Ion mode: Negative
Adjusted p (t test): 1.08e−03, Adjusted p (wilcox): 5.47e−04
Log Metabolite Abundance
2
1
0
−1
−2
−3
EM
Metastatic
Condition

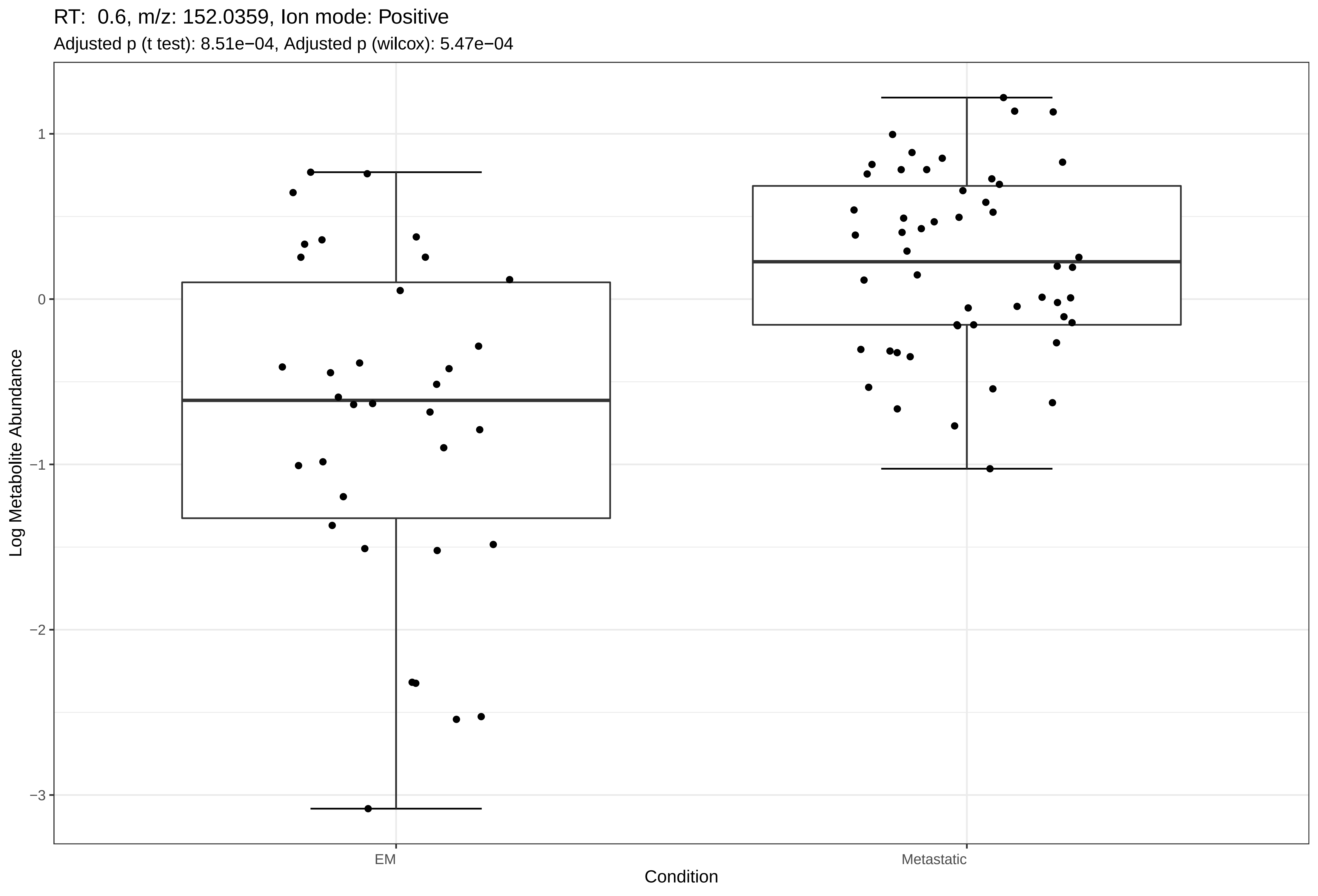
RT: 0.6, m/z: 152.0359, Ion mode: Positive
Adjusted p (t test): 8.51e−04, Adjusted p (wilcox): 5.47e−04
Log Metabolite Abundance
1
0
−1
−2
−3
EM
Metastatic
Condition

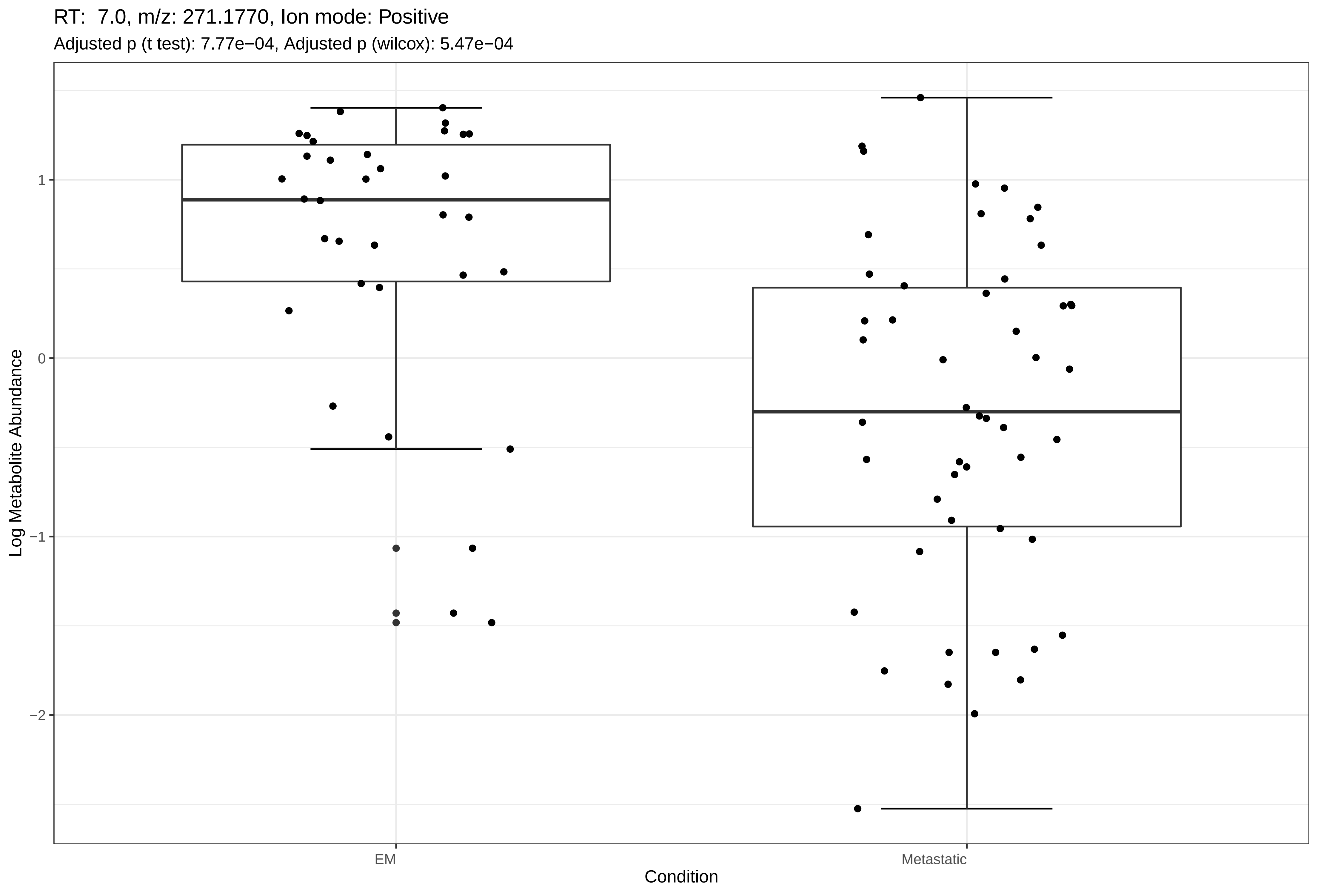

RT: 7.0, m/z: 271.1770, Ion mode: Positive
Adjusted p (t test): 7.77e−04, Adjusted p (wilcox): 5.47e−04
Log Metabolite Abundance
1
0
−1
−2
EM
Metastatic
Condition

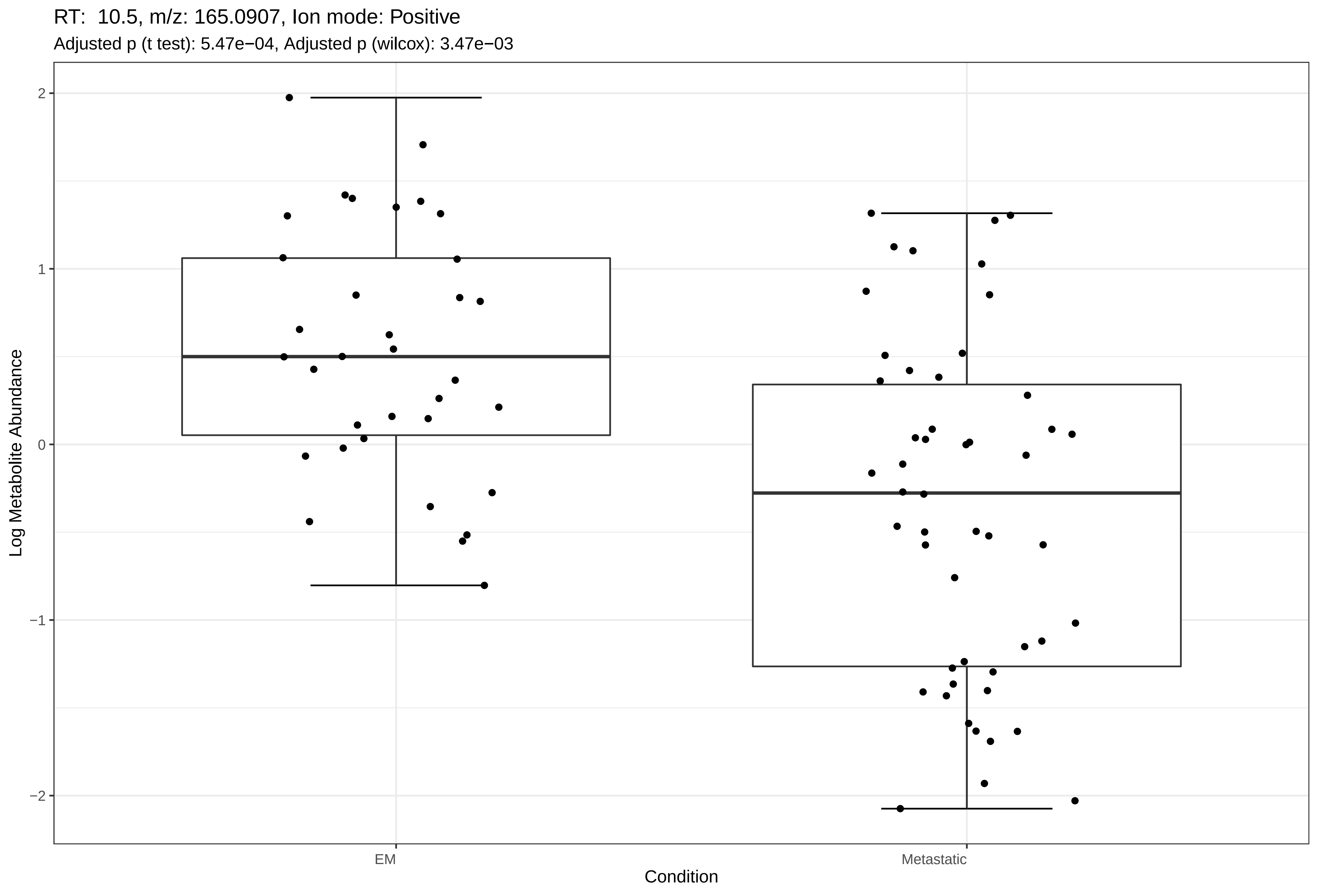

RT: 10.5, m/z: 165.0907, Ion mode: Positive
Adjusted p (t test): 5.47e−04, Adjusted p (wilcox): 3.47e−03
Log Metabolite Abundance
2
1
0
−1
−2
EM
Metastatic
Condition

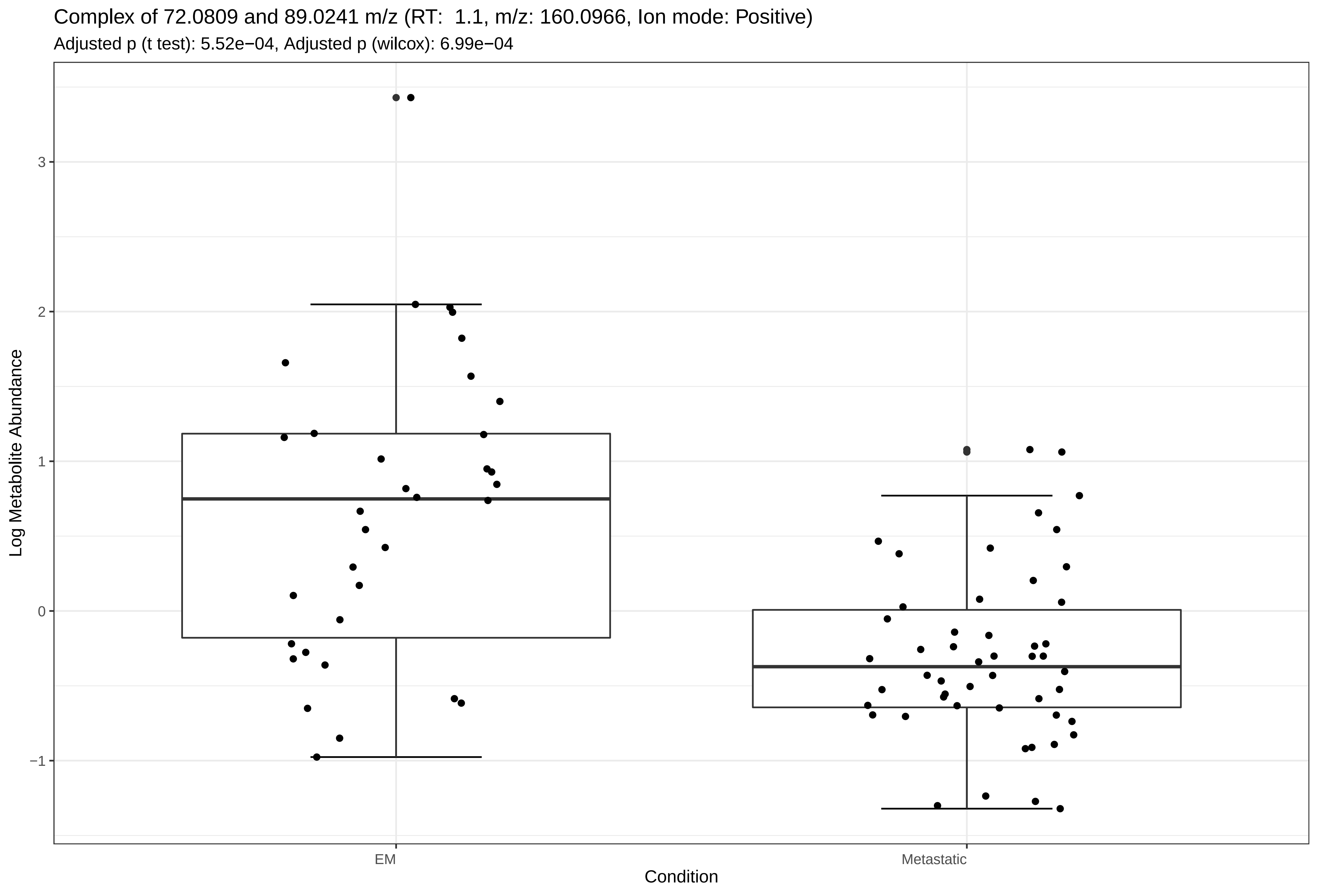
Complex of 72.0809 and 89.0241 m/z (RT: 1.1, m/z: 160.0966, Ion mode: Positive)
Adjusted p (t test): 5.52e−04, Adjusted p (wilcox): 6.99e−04
Log Metabolite Abundance
3
2
1
0
−1
EM
Metastatic
Condition

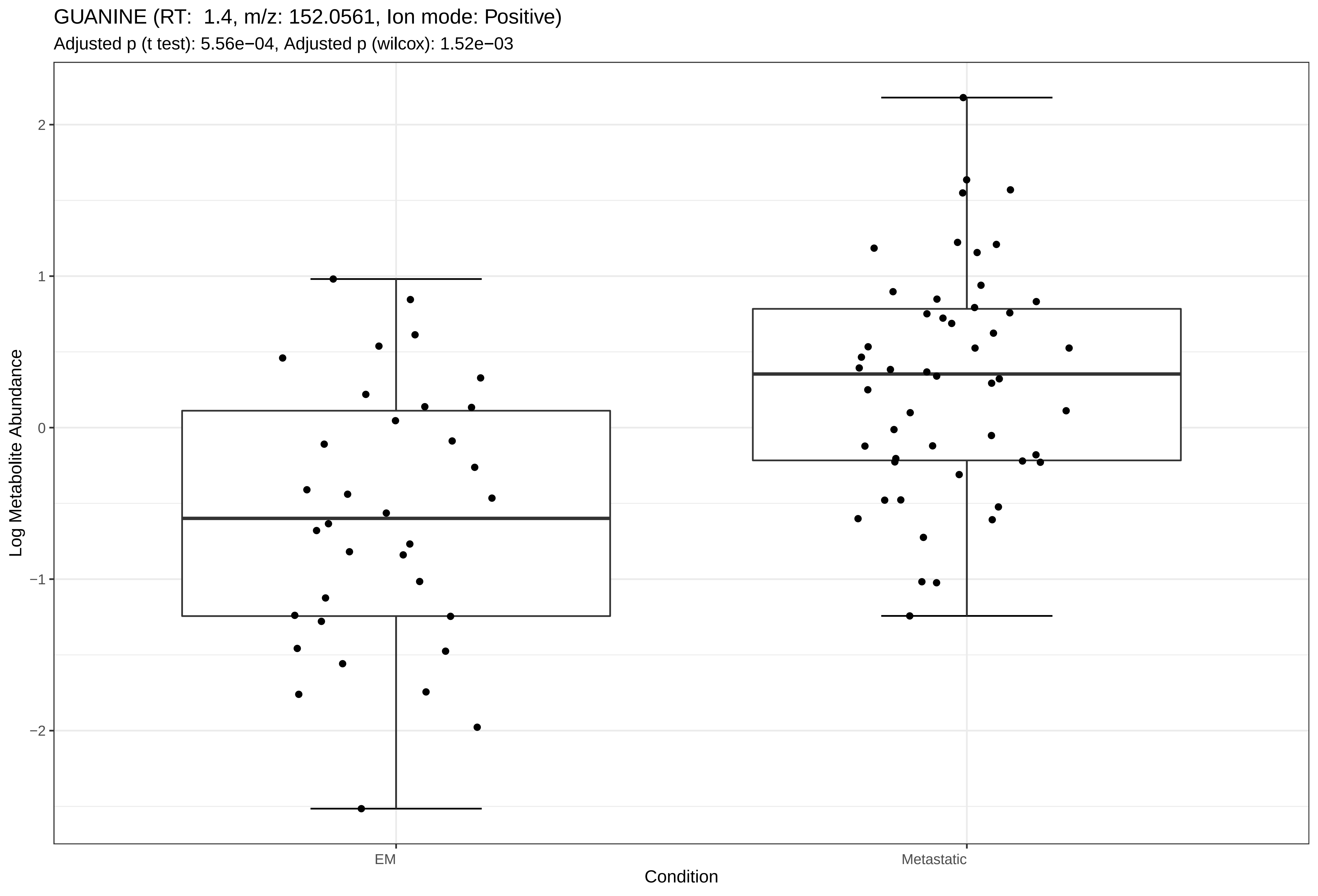
GUANINE (RT: 1.4, m/z: 152.0561, Ion mode: Positive)
Adjusted p (t test): 5.56e−04, Adjusted p (wilcox): 1.52e−03
Log Metabolite Abundance
2
1
0
−1
−2
EM
Metastatic
Condition

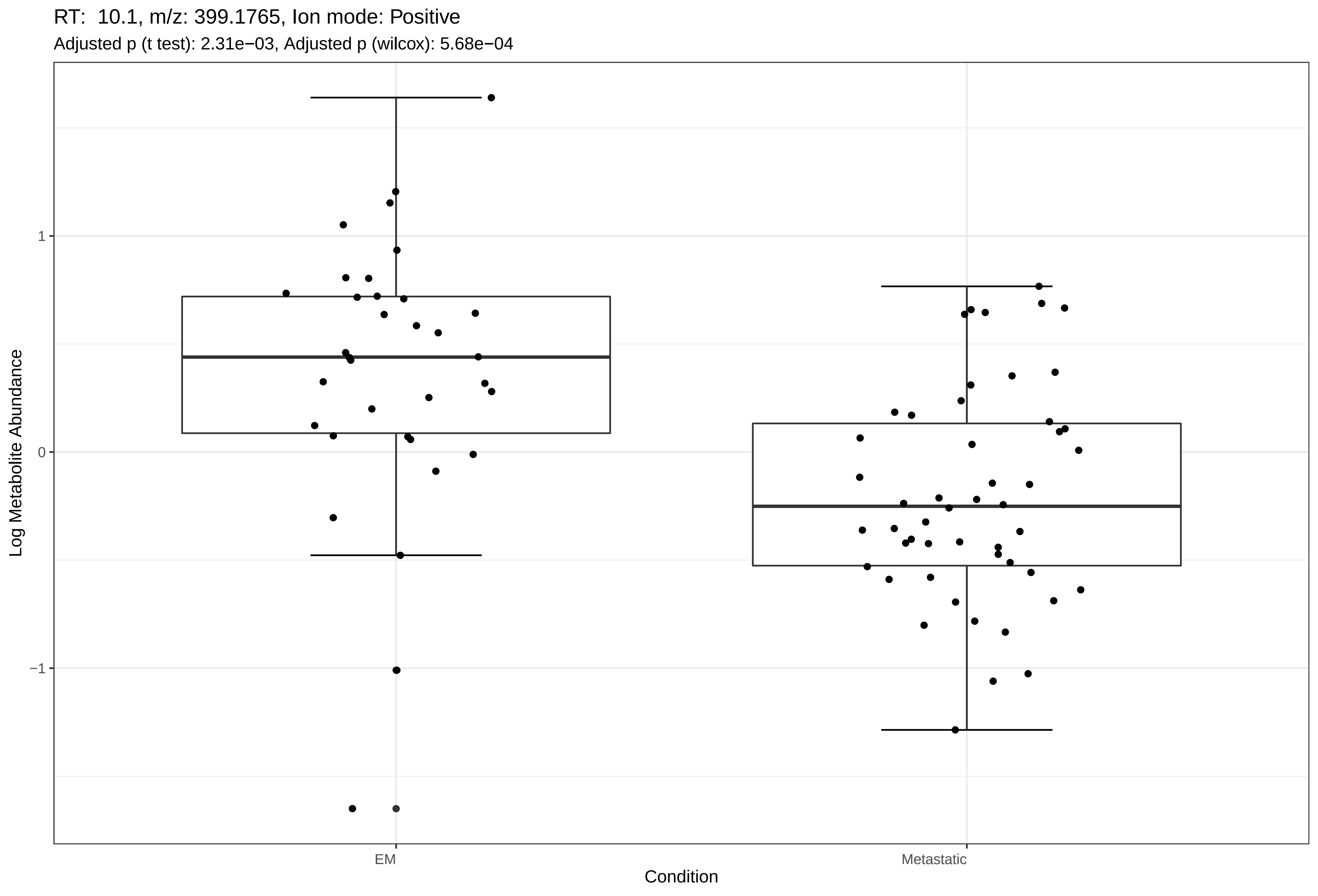
RT: 10.1, m/z: 399.1765, Ion mode: Positive
Adjusted p (t test): 2.31e−03, Adjusted p (wilcox): 5.68e−04
Log Metabolite Abundance
1
0
−1
EM
Metastatic
Condition

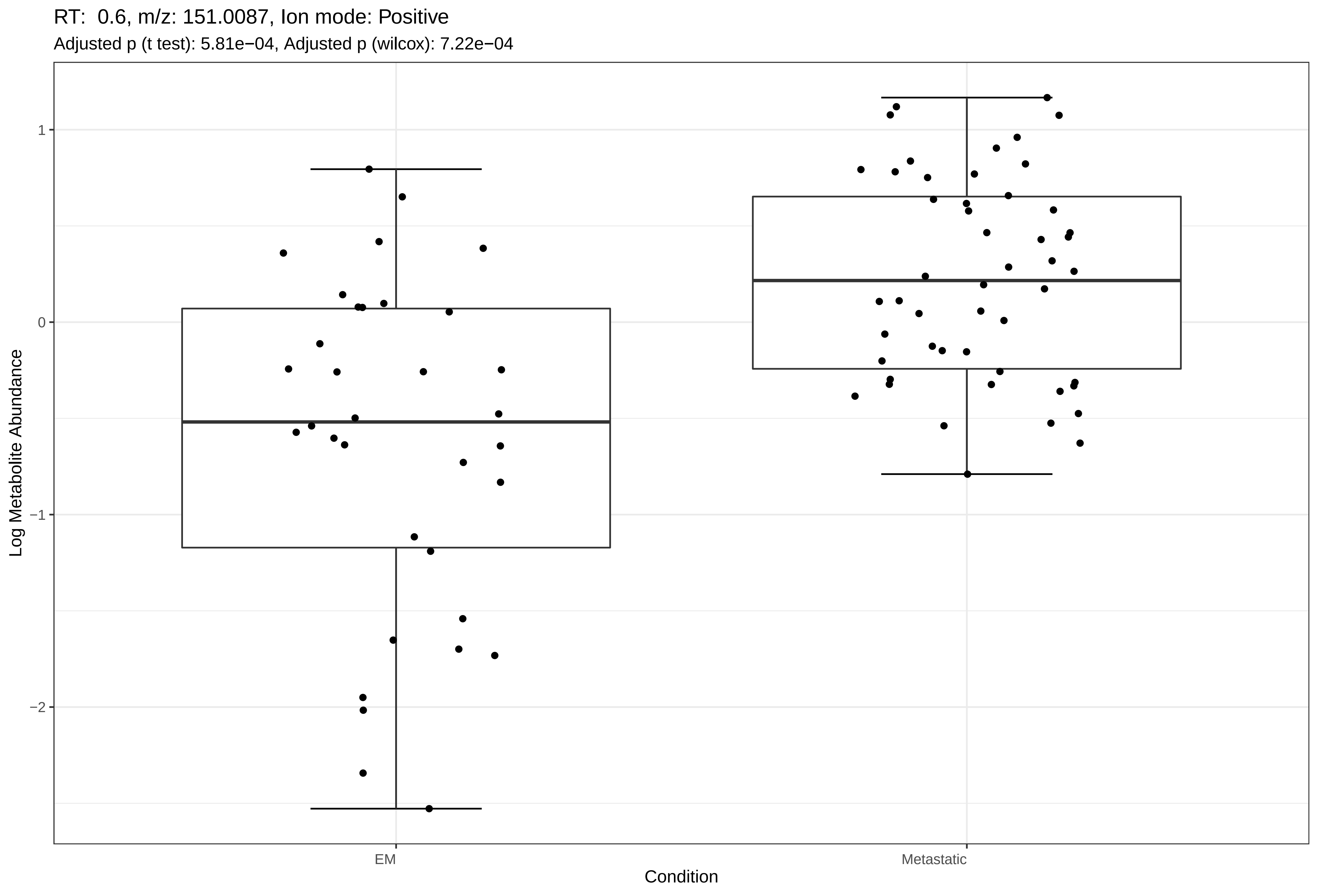
RT: 0.6, m/z: 151.0087, Ion mode: Positive
Adjusted p (t test): 5.81e−04, Adjusted p (wilcox): 7.22e−04
Log Metabolite Abundance
1
0
−1
−2
EM
Metastatic
Condition

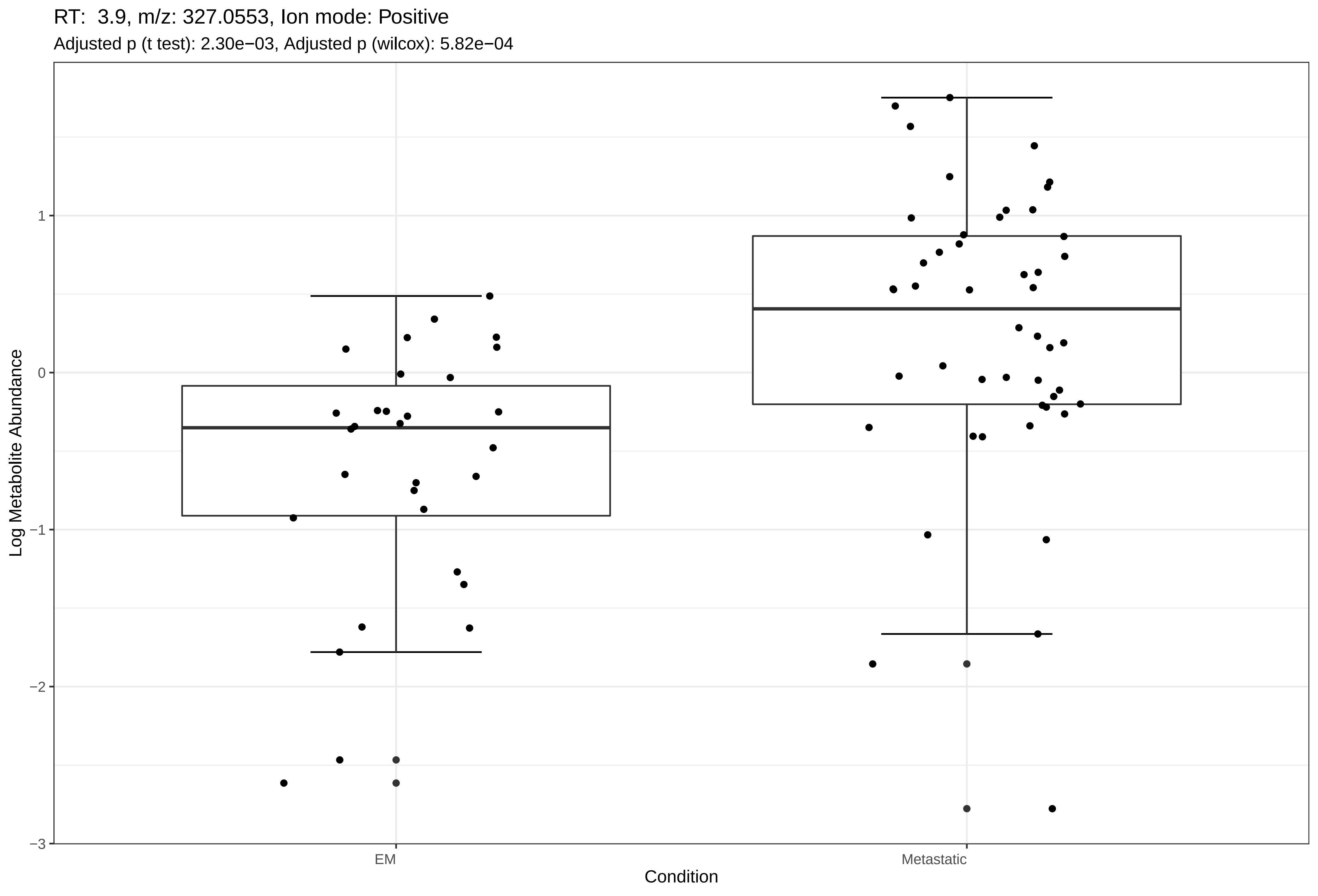
RT: 3.9, m/z: 327.0553, Ion mode: Positive
Adjusted p (t test): 2.30e−03, Adjusted p (wilcox): 5.82e−04
Log Metabolite Abundance
1
0
−1
−2
−3
EM
Metastatic
Condition

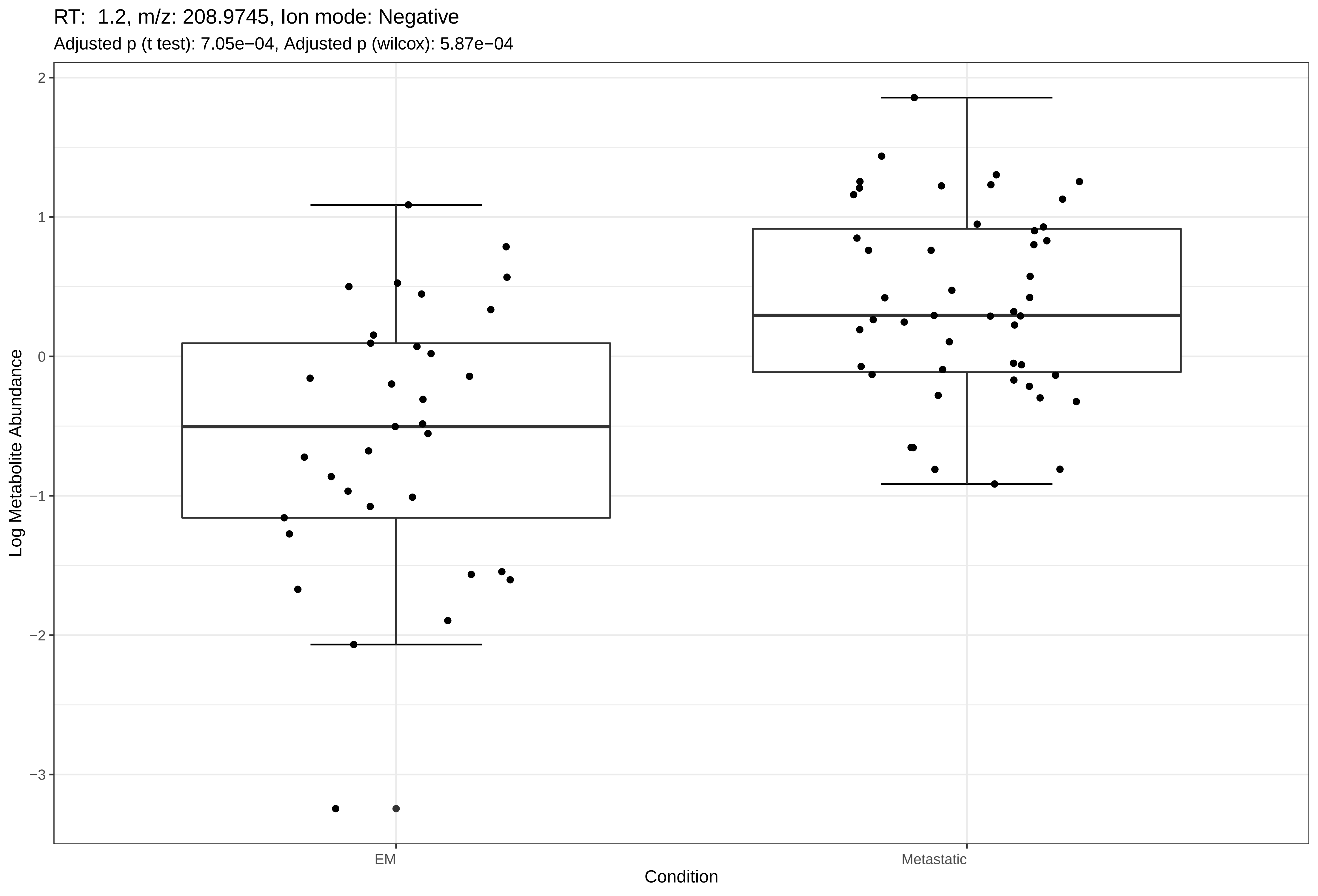
RT: 1.2, m/z: 208.9745, Ion mode: Negative
Adjusted p (t test): 7.05e−04, Adjusted p (wilcox): 5.87e−04
Log Metabolite Abundance
2
1
0
−1
−2
−3
EM
Metastatic
Condition

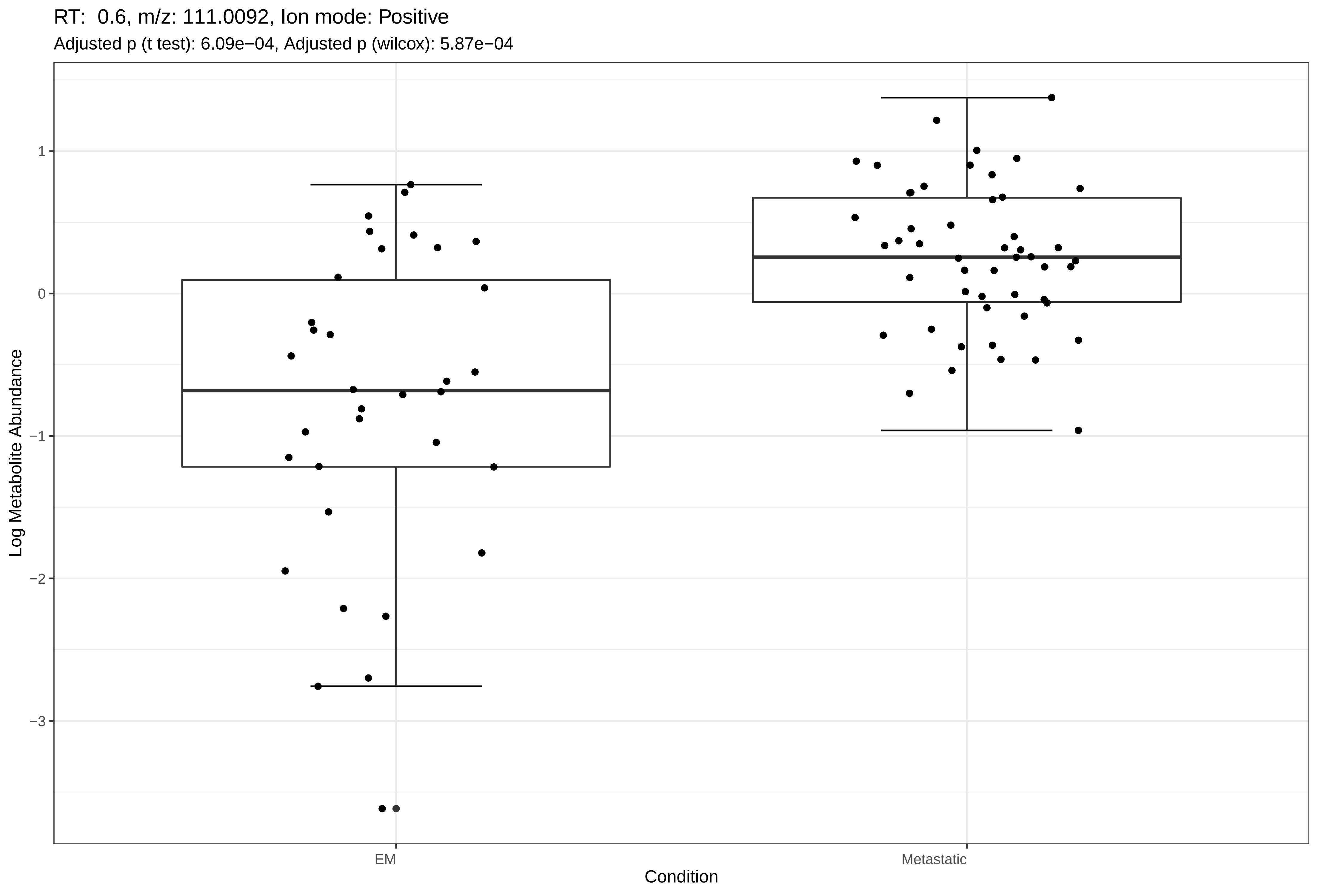

RT: 0.6, m/z: 111.0092, Ion mode: Positive
Adjusted p (t test): 6.09e−04, Adjusted p (wilcox): 5.87e−04
Log Metabolite Abundance
1
0
−1
−2
−3
EM
Metastatic
Condition

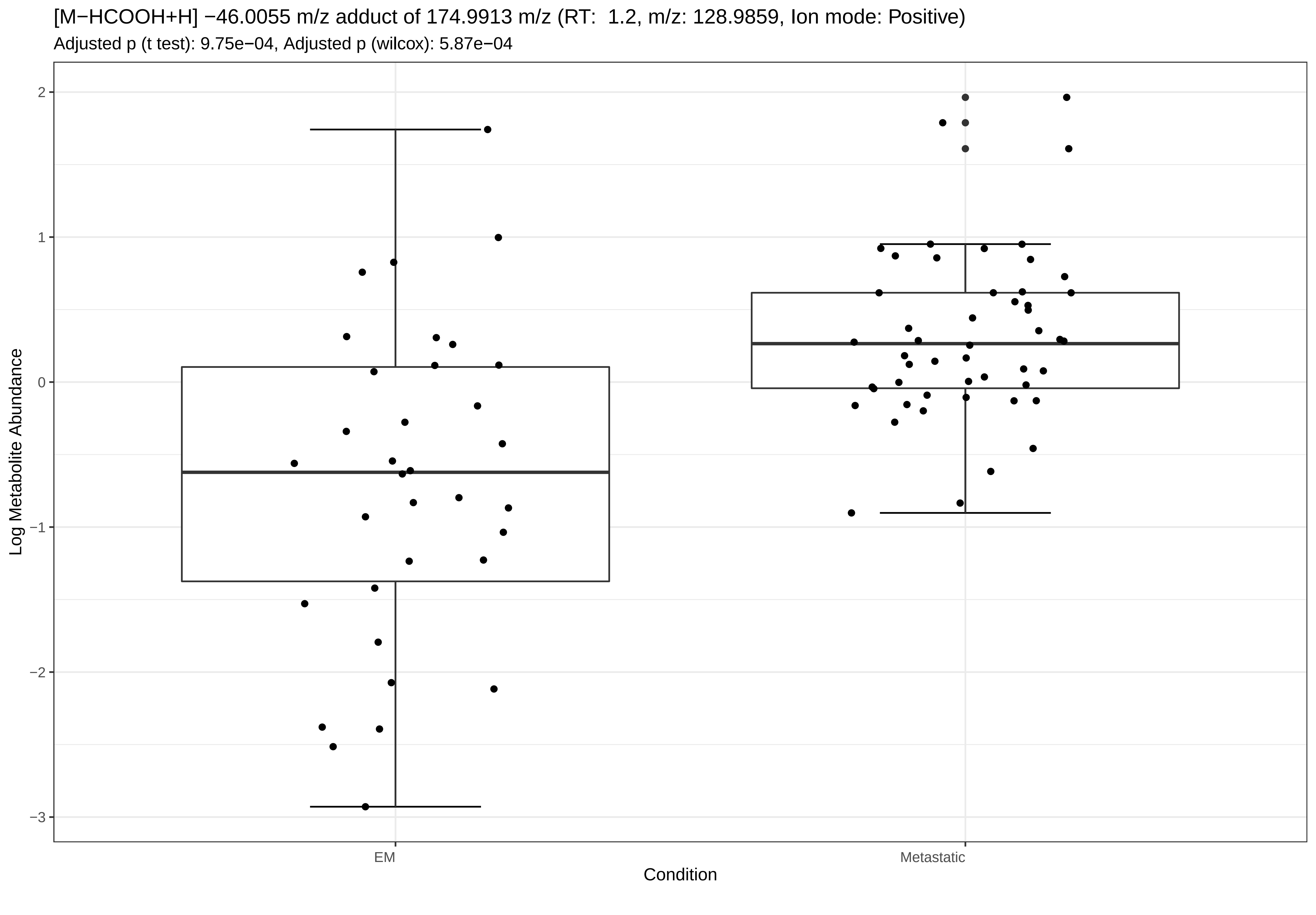

[M−HCOOH+H] −46.0055 m/z adduct of 174.9913 m/z (RT: 1.2, m/z: 128.9859, Ion mode: Positive)
Adjusted p (t test): 9.75e−04, Adjusted p (wilcox): 5.87e−04
Log Metabolite Abundance
2
1
0
−1
−2
−3
EM
Metastatic
Condition

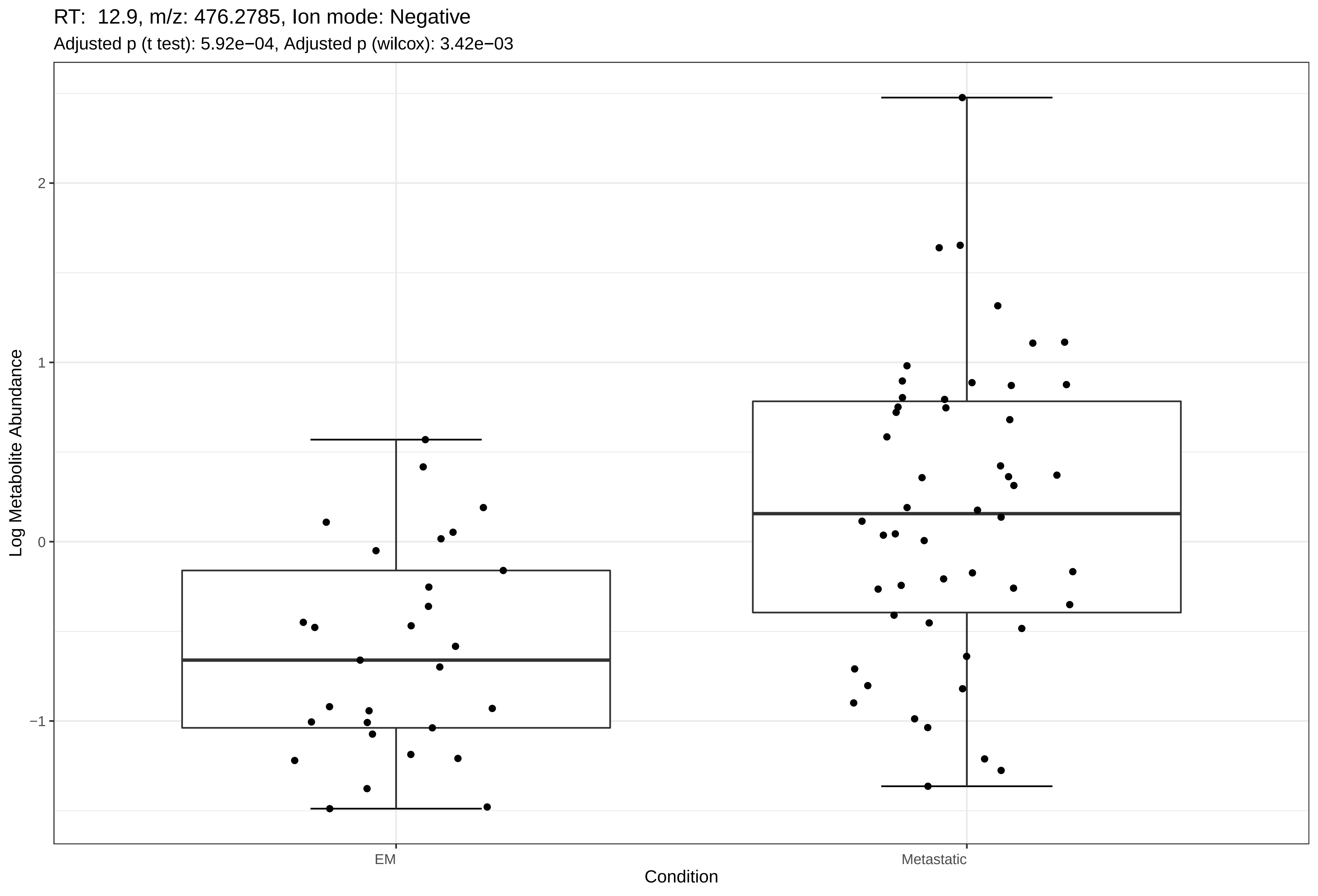

RT: 12.9, m/z: 476.2785, Ion mode: Negative
Adjusted p (t test): 5.92e−04, Adjusted p (wilcox): 3.42e−03
Log Metabolite Abundance
2
1
0
−1
EM
Metastatic
Condition

RT: 1.0, m/z: 211.1767, Ion mode: Positive

Adjusted p (t test): 5.97e−04, Adjusted p (wilcox): 7.78e−04

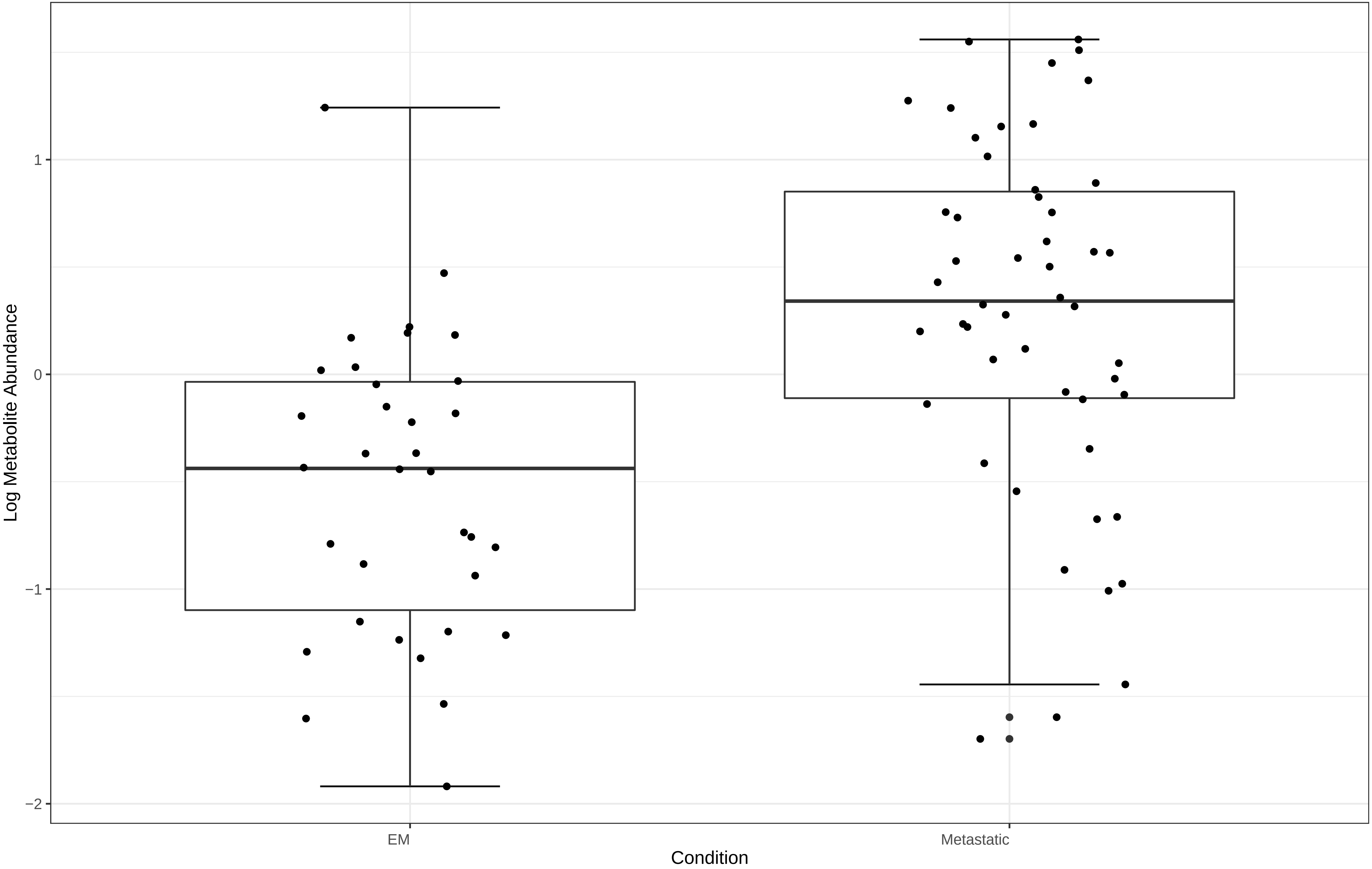

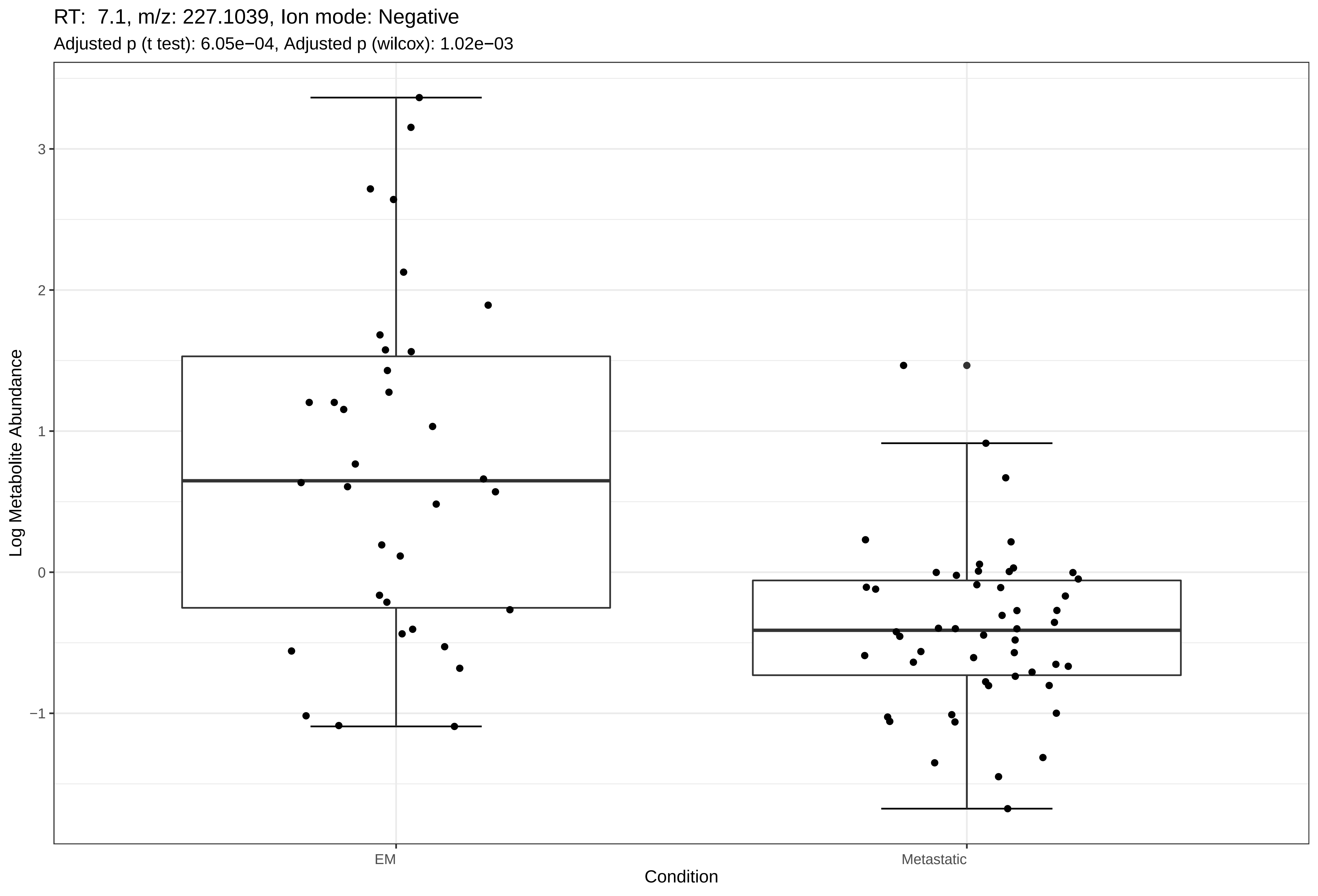
RT: 7.1, m/z: 227.1039, Ion mode: Negative
Adjusted p (t test): 6.05e−04, Adjusted p (wilcox): 1.02e−03
Log Metabolite Abundance
3
2
1
0
−1
EM
Metastatic
Condition

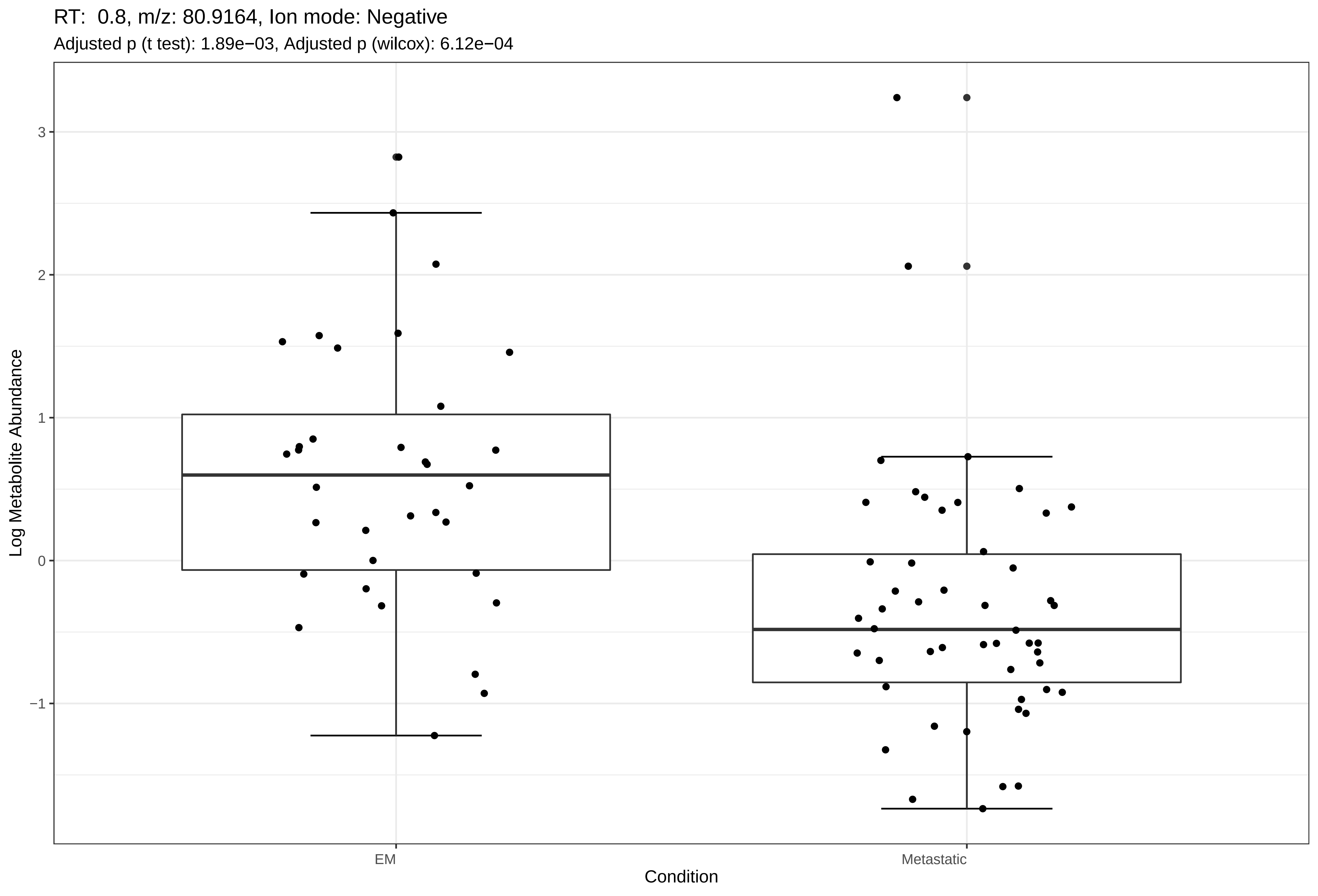

RT: 0.8, m/z: 80.9164, Ion mode: Negative
Adjusted p (t test): 1.89e−03, Adjusted p (wilcox): 6.12e−04
Log Metabolite Abundance
3
2
1
0
−1
EM
Metastatic
Condition

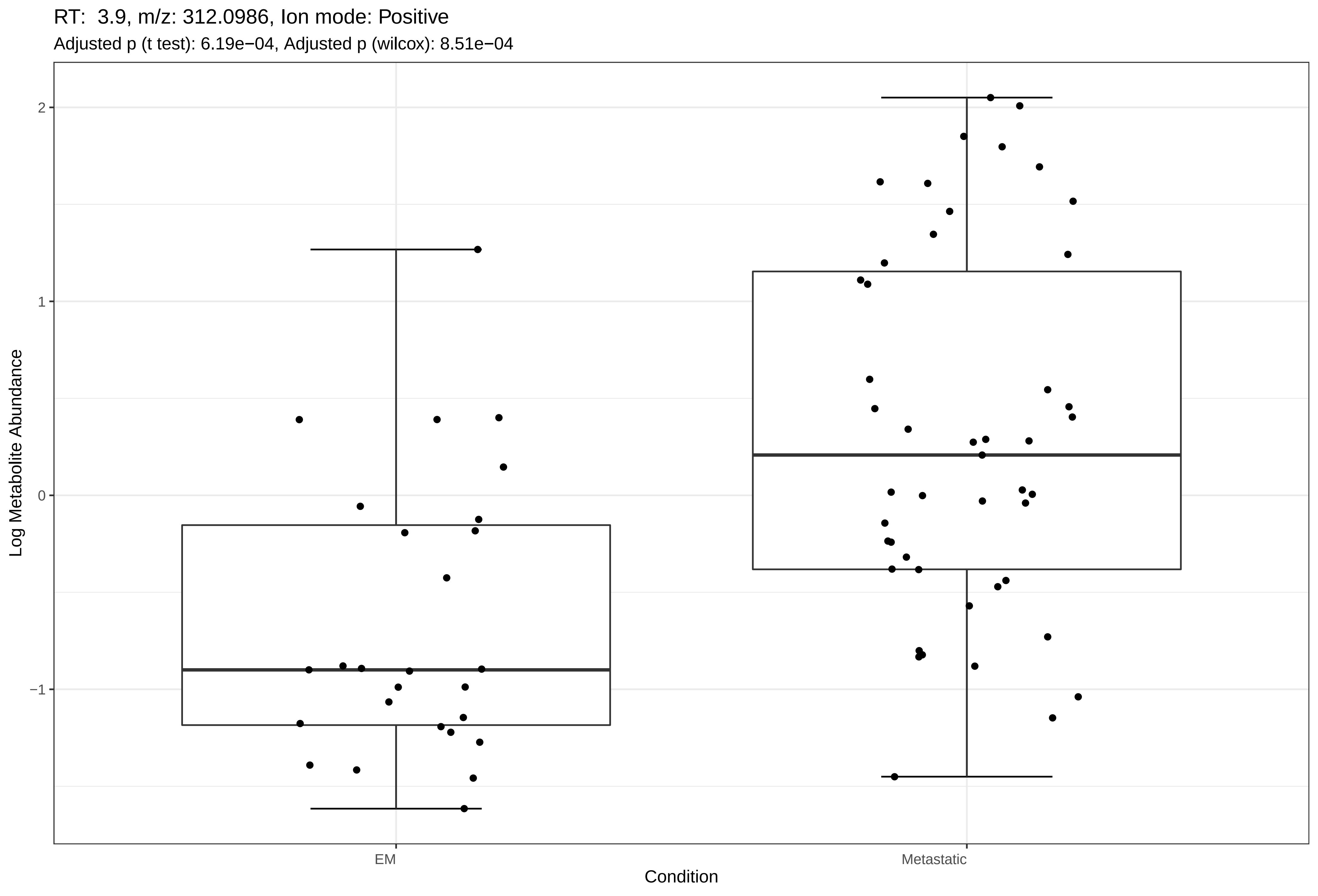
RT: 3.9, m/z: 312.0986, Ion mode: Positive
Adjusted p (t test): 6.19e−04, Adjusted p (wilcox): 8.51e−04
Log Metabolite Abundance
2
1
0
−1
EM
Metastatic
Condition

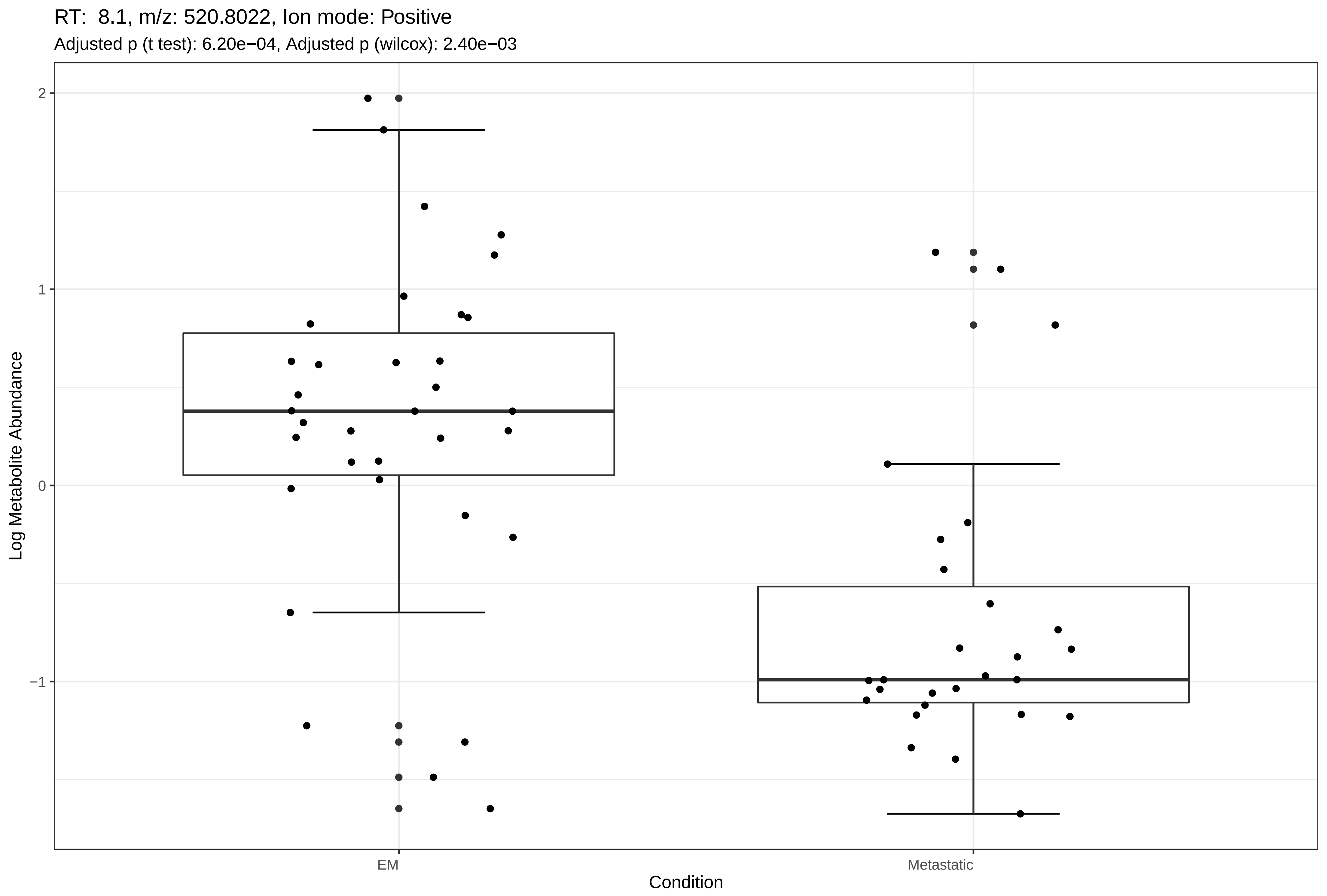
RT: 8.1, m/z: 520.8022, Ion mode: Positive
Adjusted p (t test): 6.20e−04, Adjusted p (wilcox): 2.40e−03
Log Metabolite Abundance
2
1
0
−1
EM
Metastatic
Condition

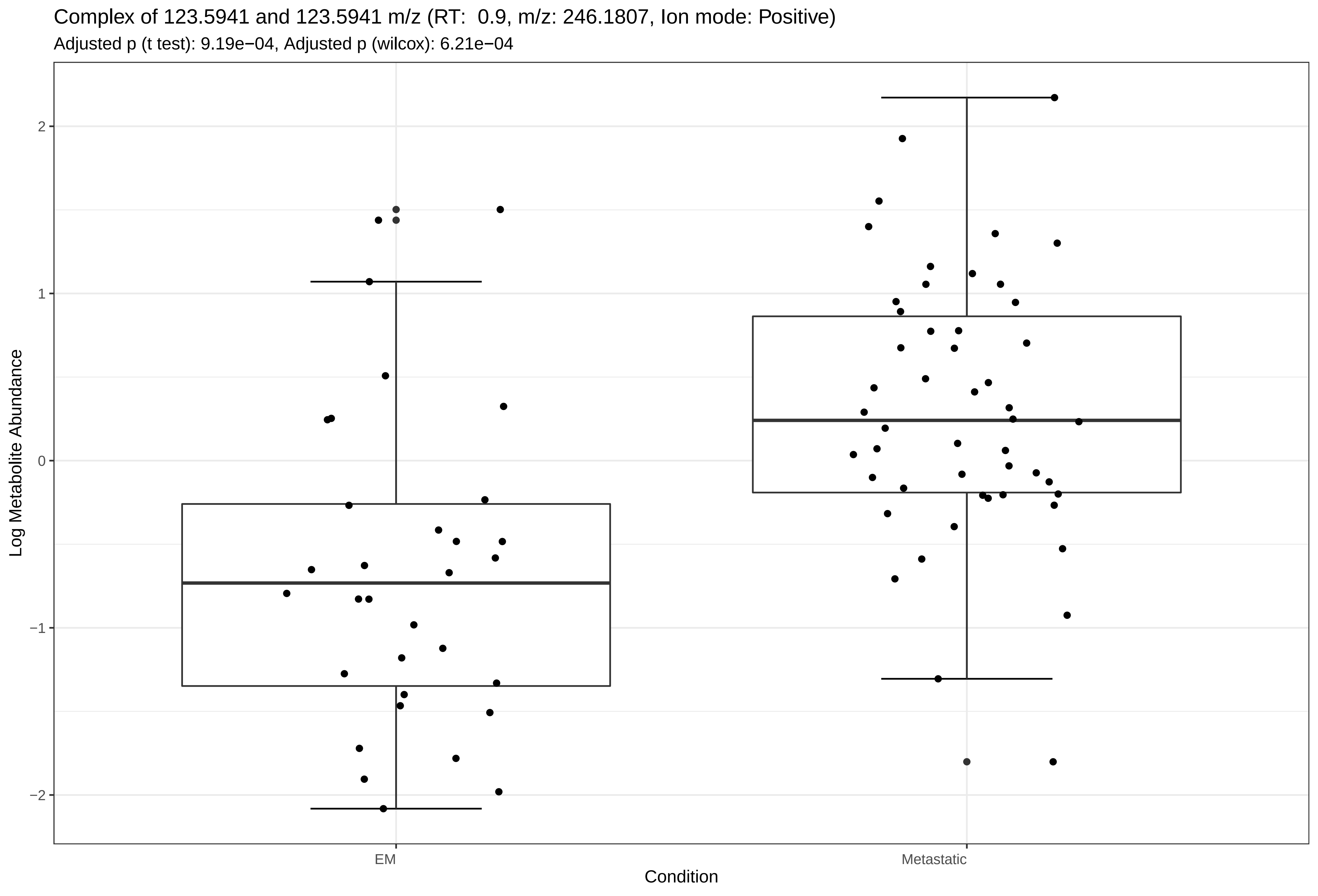

Complex of 123.5941 and 123.5941 m/z (RT: 0.9, m/z: 246.1807, Ion mode: Positive)
Adjusted p (t test): 9.19e−04, Adjusted p (wilcox): 6.21e−04
Log Metabolite Abundance
2
1
0
−1
−2
EM
Metastatic
Condition

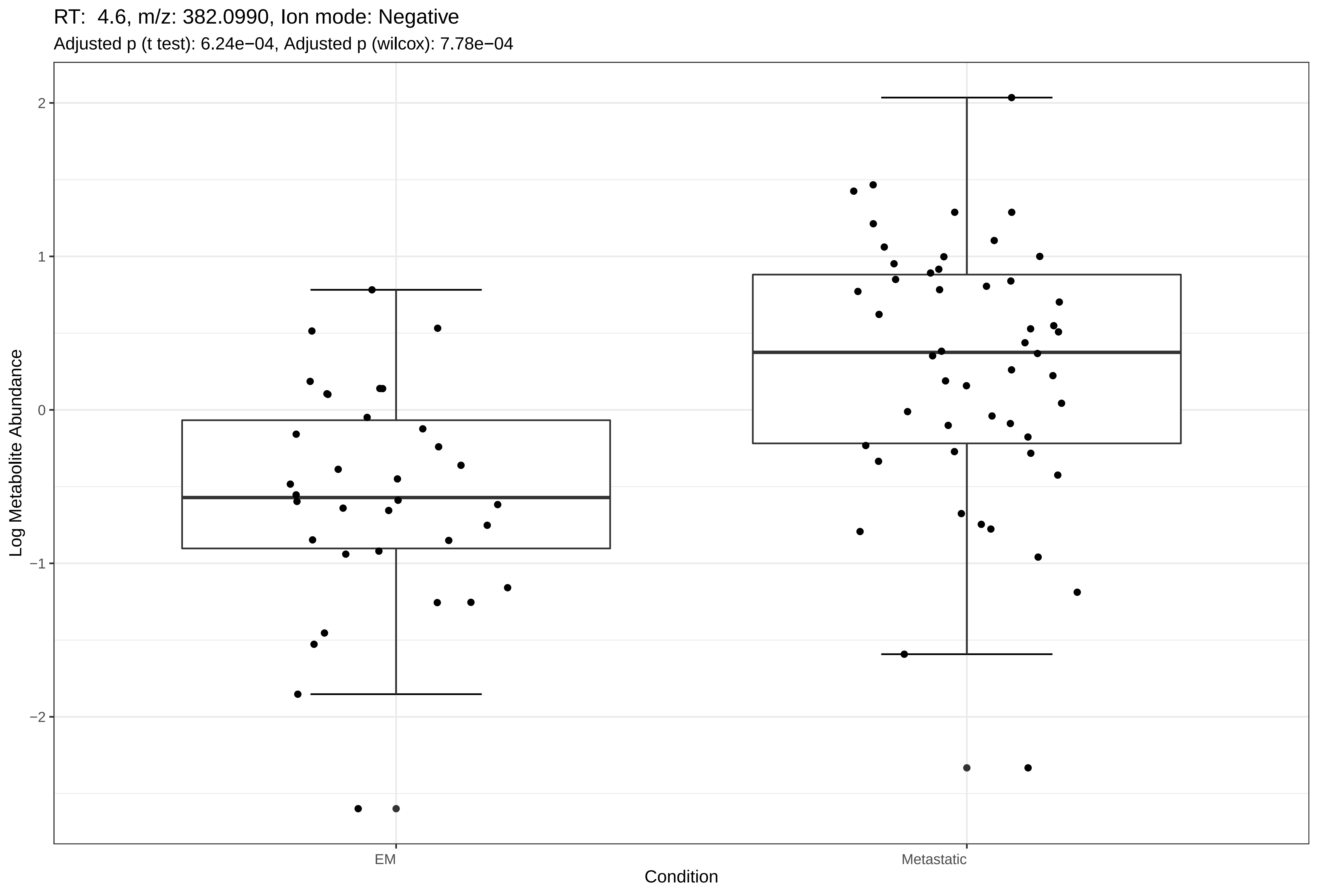

RT: 4.6, m/z: 382.0990, Ion mode: Negative
Adjusted p (t test): 6.24e−04, Adjusted p (wilcox): 7.78e−04
Log Metabolite Abundance
2
1
0
−1
−2
EM
Metastatic
Condition

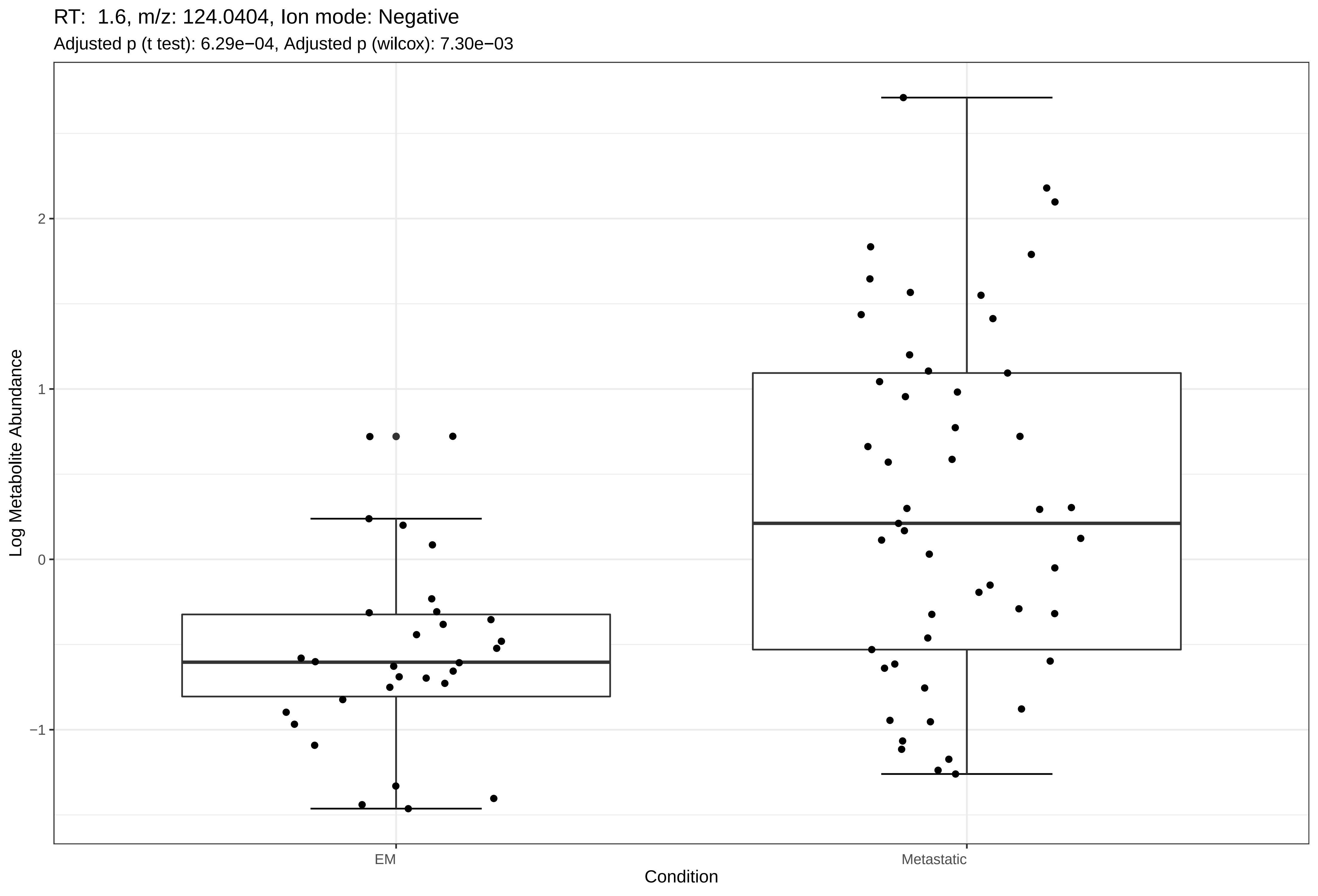
RT: 1.6, m/z: 124.0404, Ion mode: Negative
Adjusted p (t test): 6.29e−04, Adjusted p (wilcox): 7.30e−03
Log Metabolite Abundance
2
1
0
−1
EM
Metastatic
Condition

RT: 1.4, m/z: 141.0783, Ion mode: Positive

Adjusted p (t test): 6.34e−04, Adjusted p (wilcox): 1.93e−03

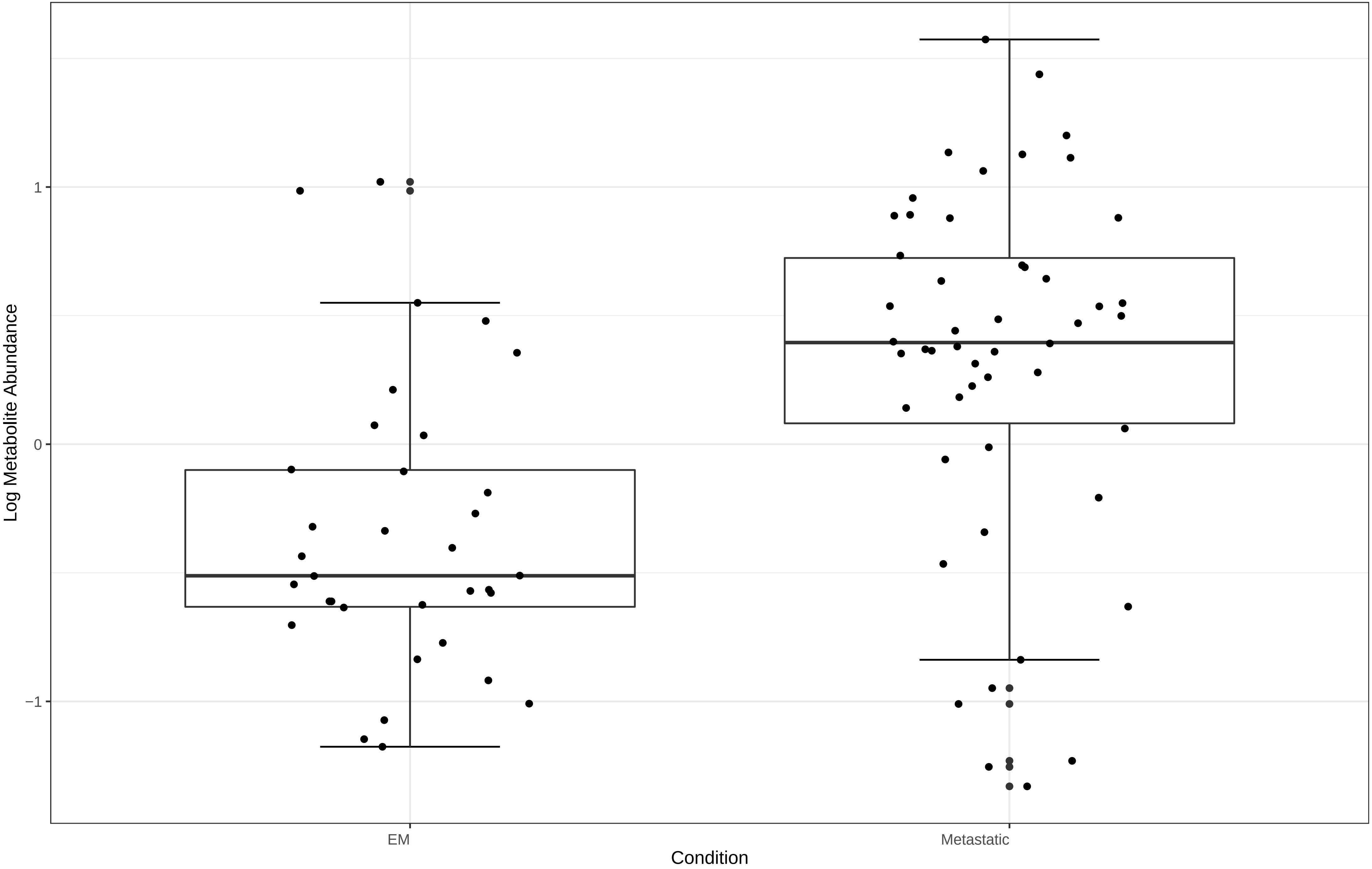

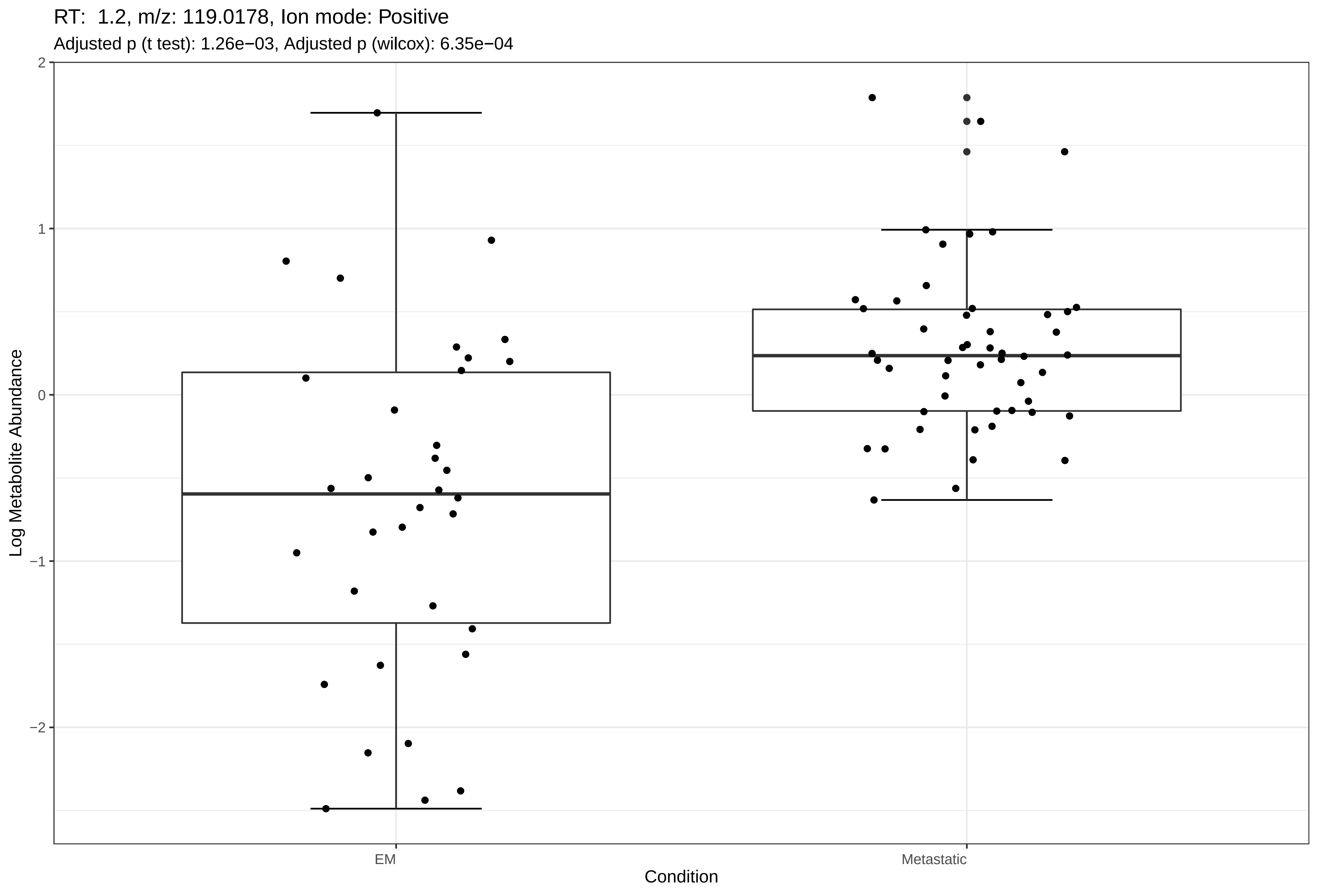

RT: 1.2, m/z: 119.0178, Ion mode: Positive
Adjusted p (t test): 1.26e−03, Adjusted p (wilcox): 6.35e−04
Log Metabolite Abundance
2
1
0
−1
−2
EM
Metastatic
Condition

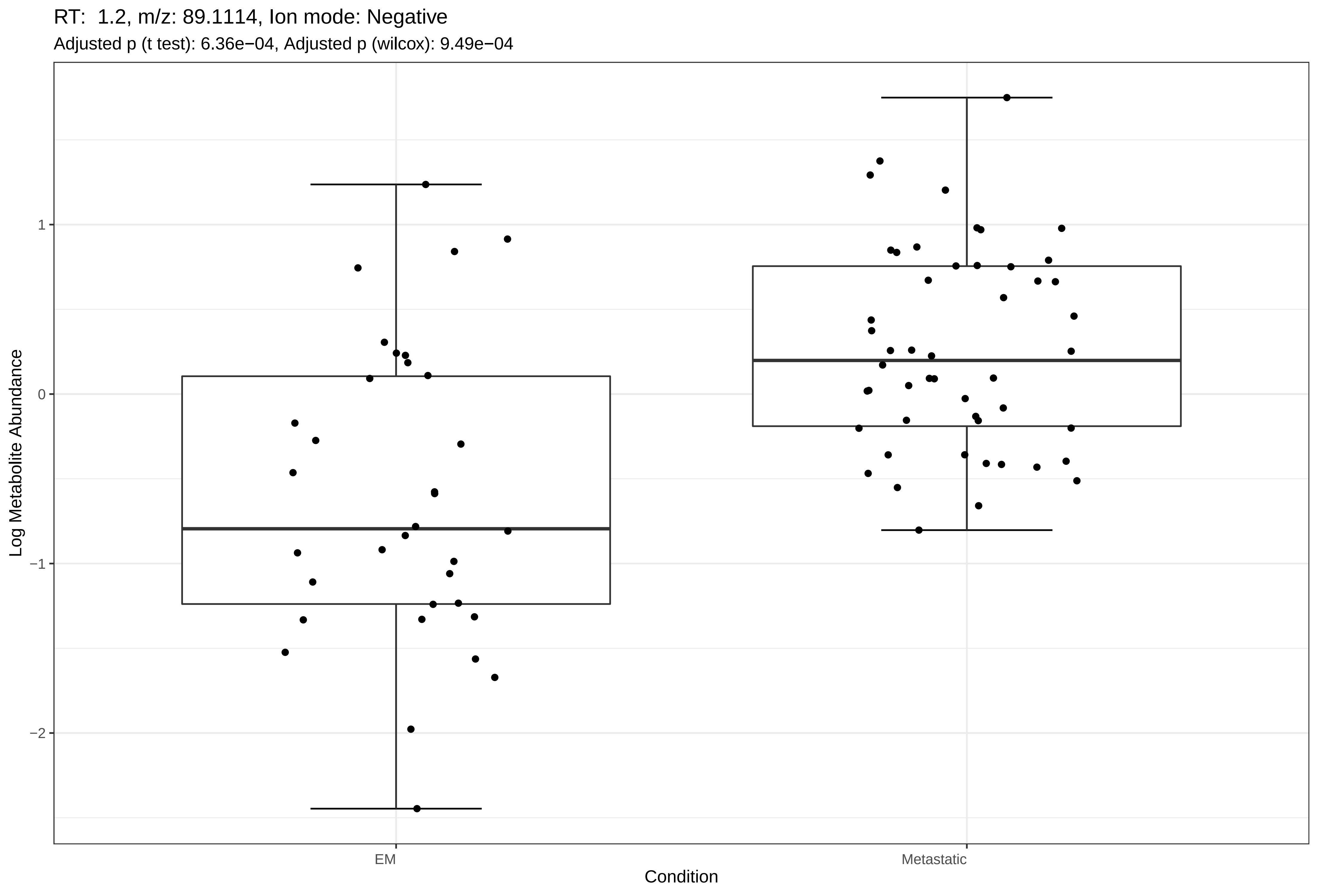

RT: 1.2, m/z: 89.1114, Ion mode: Negative
Adjusted p (t test): 6.36e−04, Adjusted p (wilcox): 9.49e−04
Log Metabolite Abundance
1
0
−1
−2
EM
Metastatic
Condition

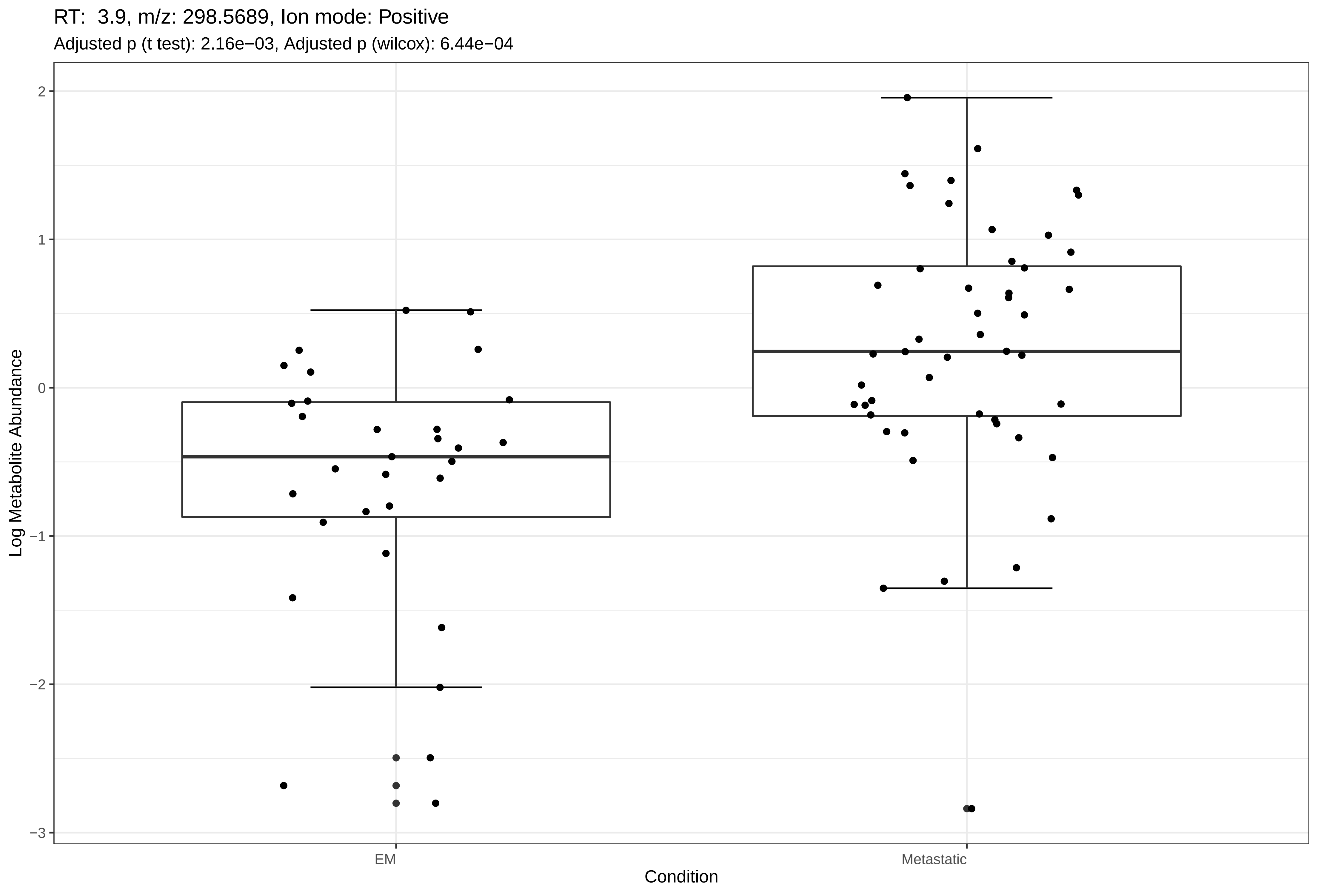
RT: 3.9, m/z: 298.5689, Ion mode: Positive
Adjusted p (t test): 2.16e−03, Adjusted p (wilcox): 6.44e−04
Log Metabolite Abundance
2
1
0
−1
−2
−3
EM
Metastatic
Condition

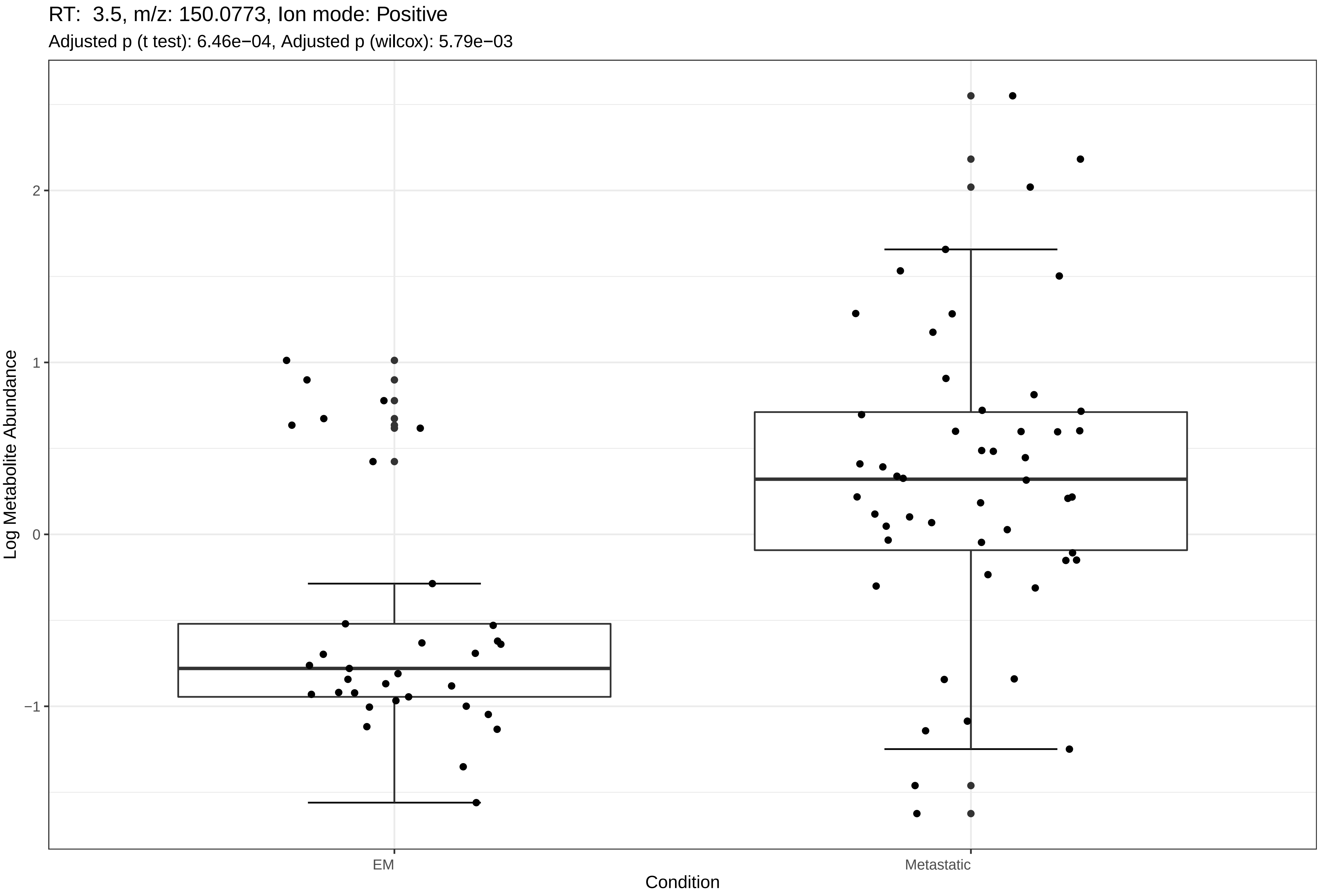

RT: 3.5, m/z: 150.0773, Ion mode: Positive
Adjusted p (t test): 6.46e−04, Adjusted p (wilcox): 5.79e−03
Log Metabolite Abundance
2
1
0
−1
EM
Metastatic
Condition

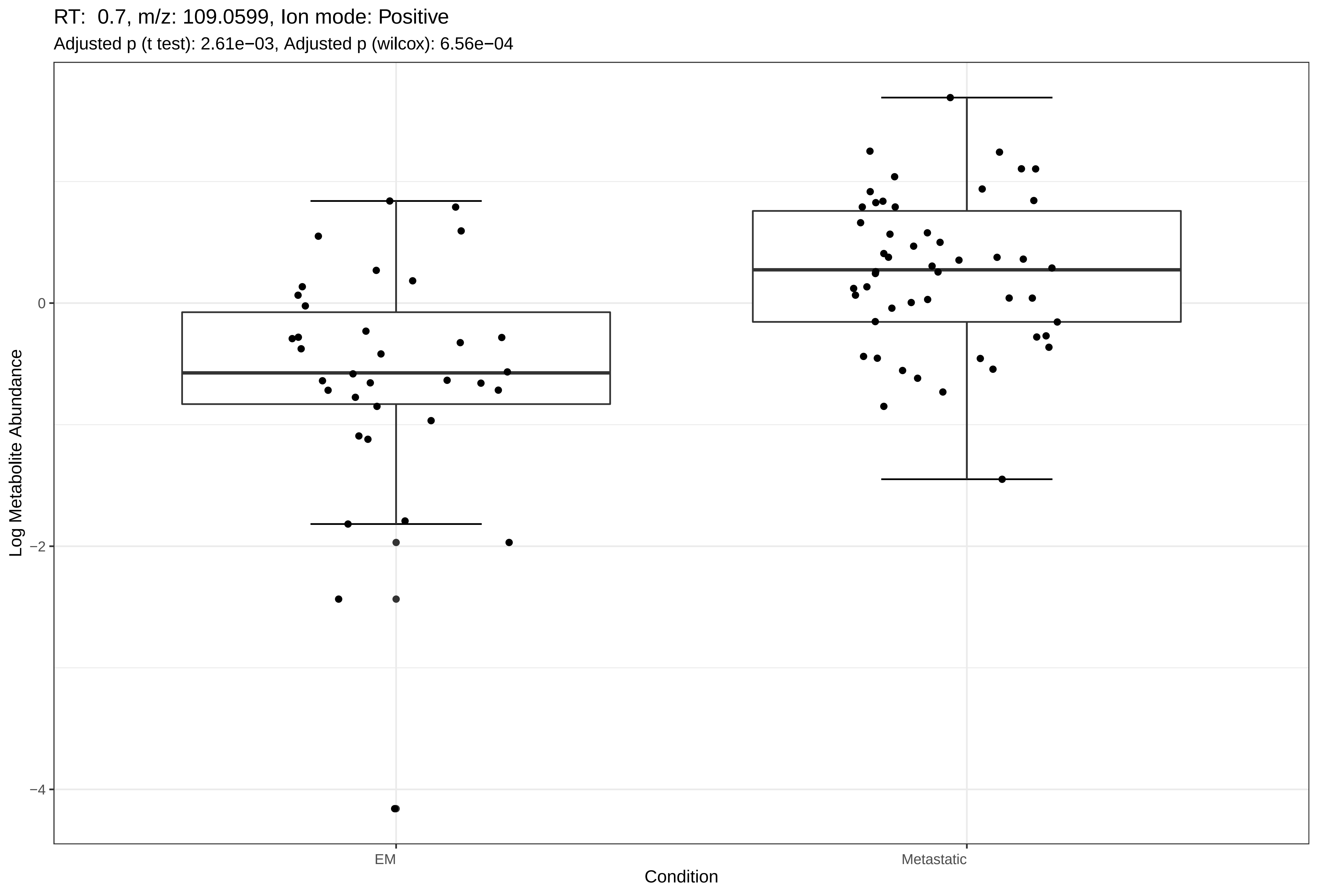

RT: 0.7, m/z: 109.0599, Ion mode: Positive
Adjusted p (t test): 2.61e−03, Adjusted p (wilcox): 6.56e−04
Log Metabolite Abundance
0
−2
−4
EM
Metastatic
Condition

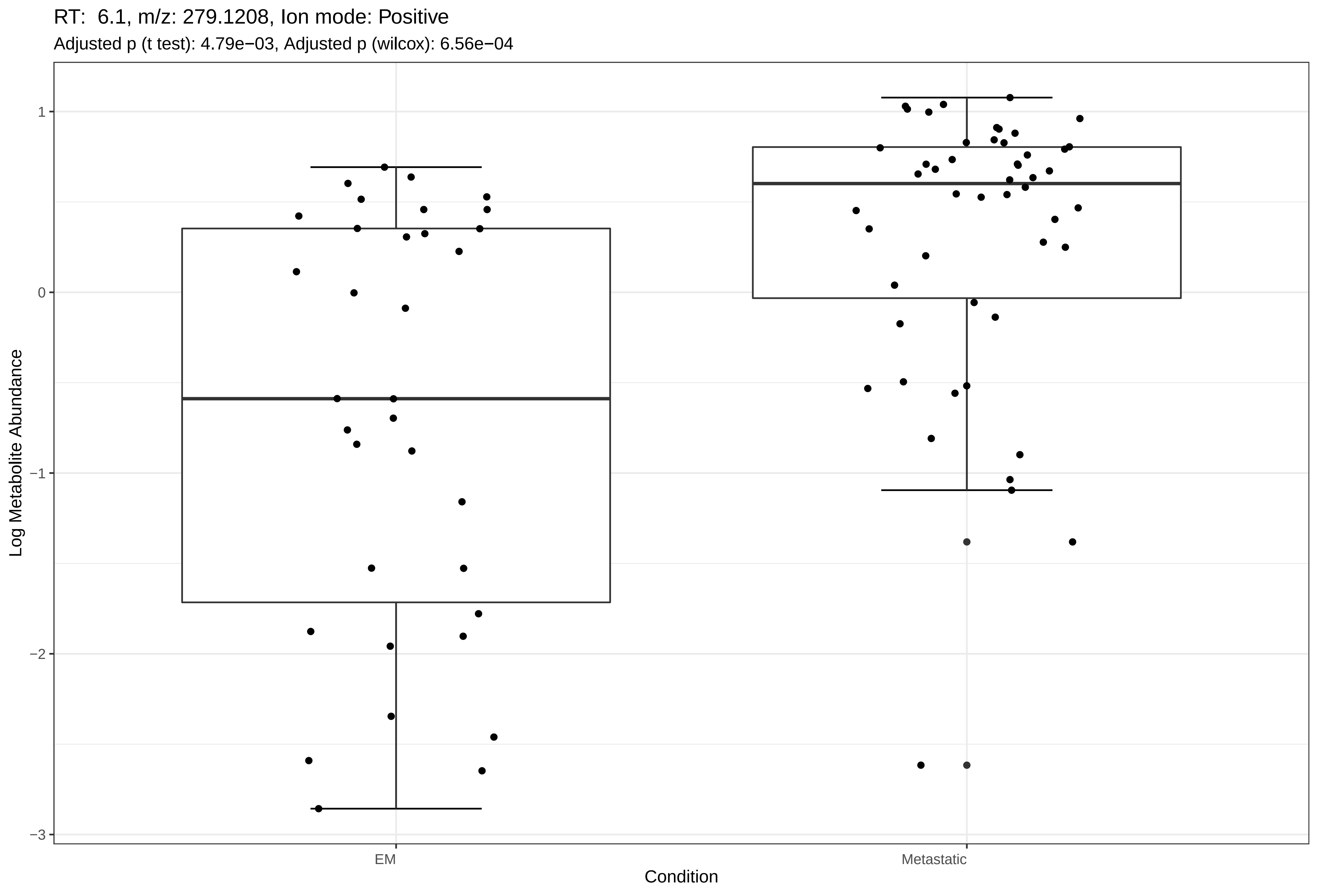

RT: 6.1, m/z: 279.1208, Ion mode: Positive
Adjusted p (t test): 4.79e−03, Adjusted p (wilcox): 6.56e−04
Log Metabolite Abundance
1
0
−1
−2
−3
EM
Metastatic
Condition

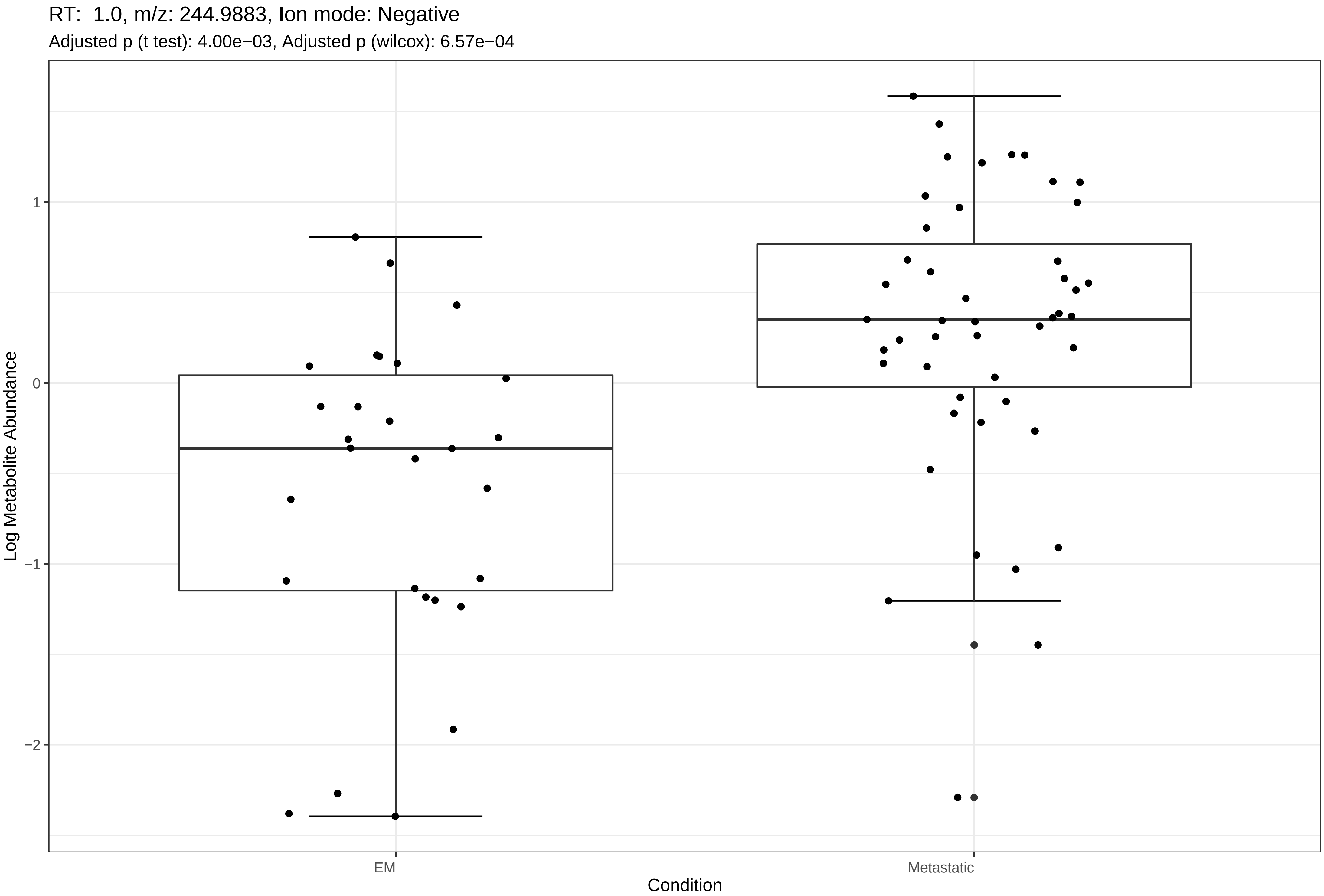
RT: 1.0, m/z: 244.9883, Ion mode: Negative
Adjusted p (t test): 4.00e−03, Adjusted p (wilcox): 6.57e−04
Log Metabolite Abundance
1
0
−1
−2
EM
Metastatic
Condition

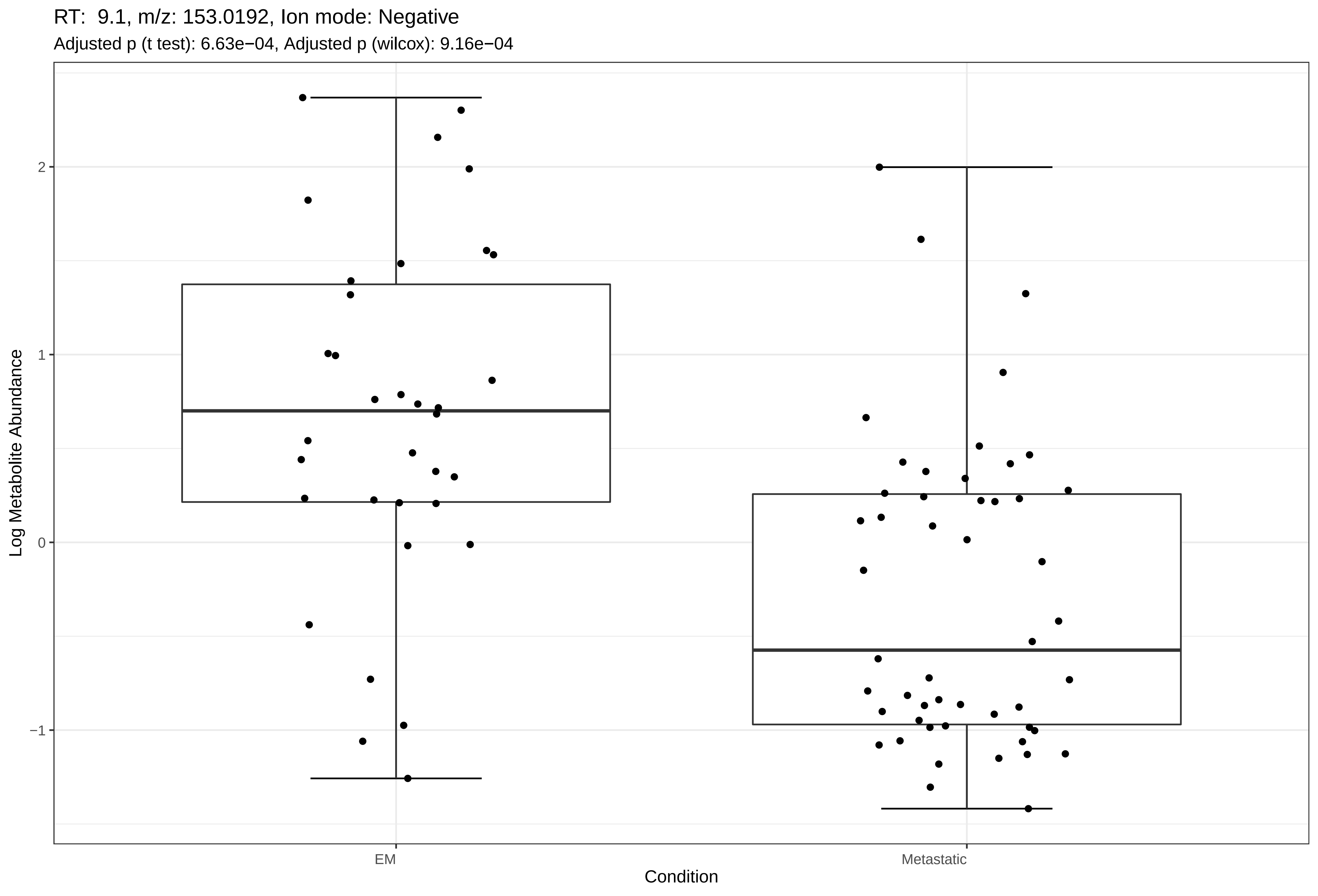

RT: 9.1, m/z: 153.0192, Ion mode: Negative
Adjusted p (t test): 6.63e−04, Adjusted p (wilcox): 9.16e−04
Log Metabolite Abundance
2
1
0
−1
EM
Metastatic
Condition

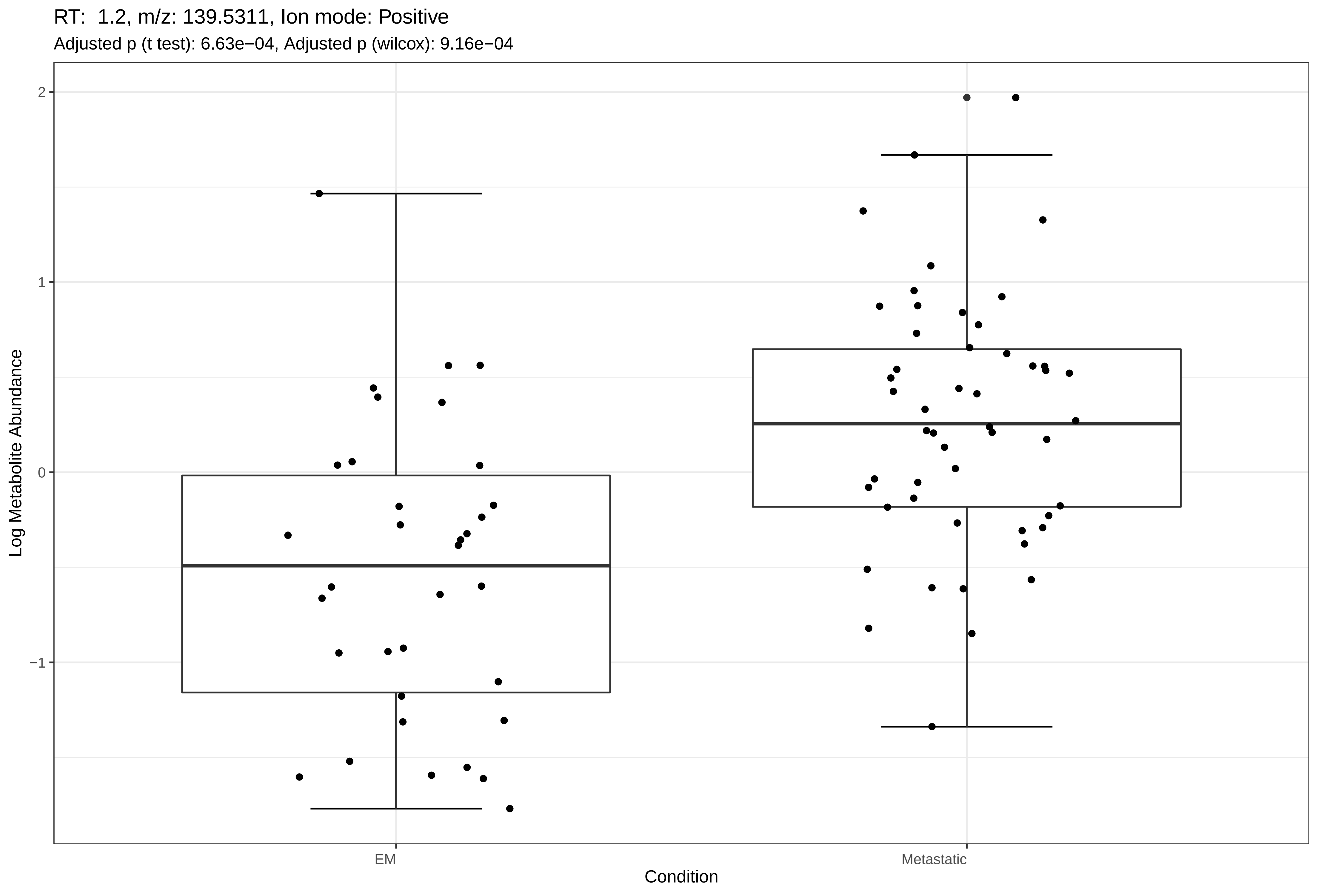
RT: 1.2, m/z: 139.5311, Ion mode: Positive
Adjusted p (t test): 6.63e−04, Adjusted p (wilcox): 9.16e−04
Log Metabolite Abundance
2
1
0
−1
EM
Metastatic
Condition

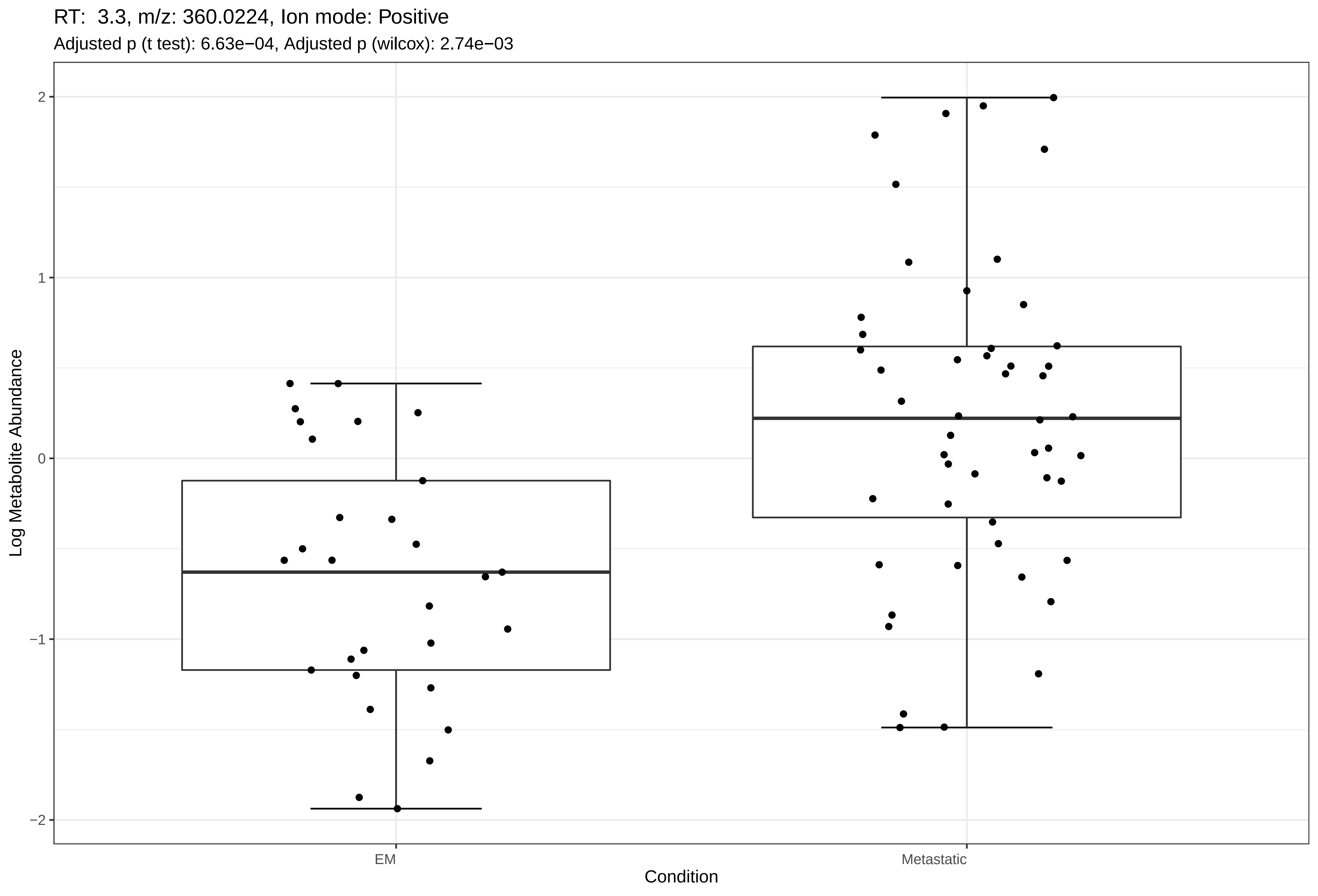
RT: 3.3, m/z: 360.0224, Ion mode: Positive
Adjusted p (t test): 6.63e−04, Adjusted p (wilcox): 2.74e−03
Log Metabolite Abundance
2
1
0
−1
−2
EM
Metastatic
Condition

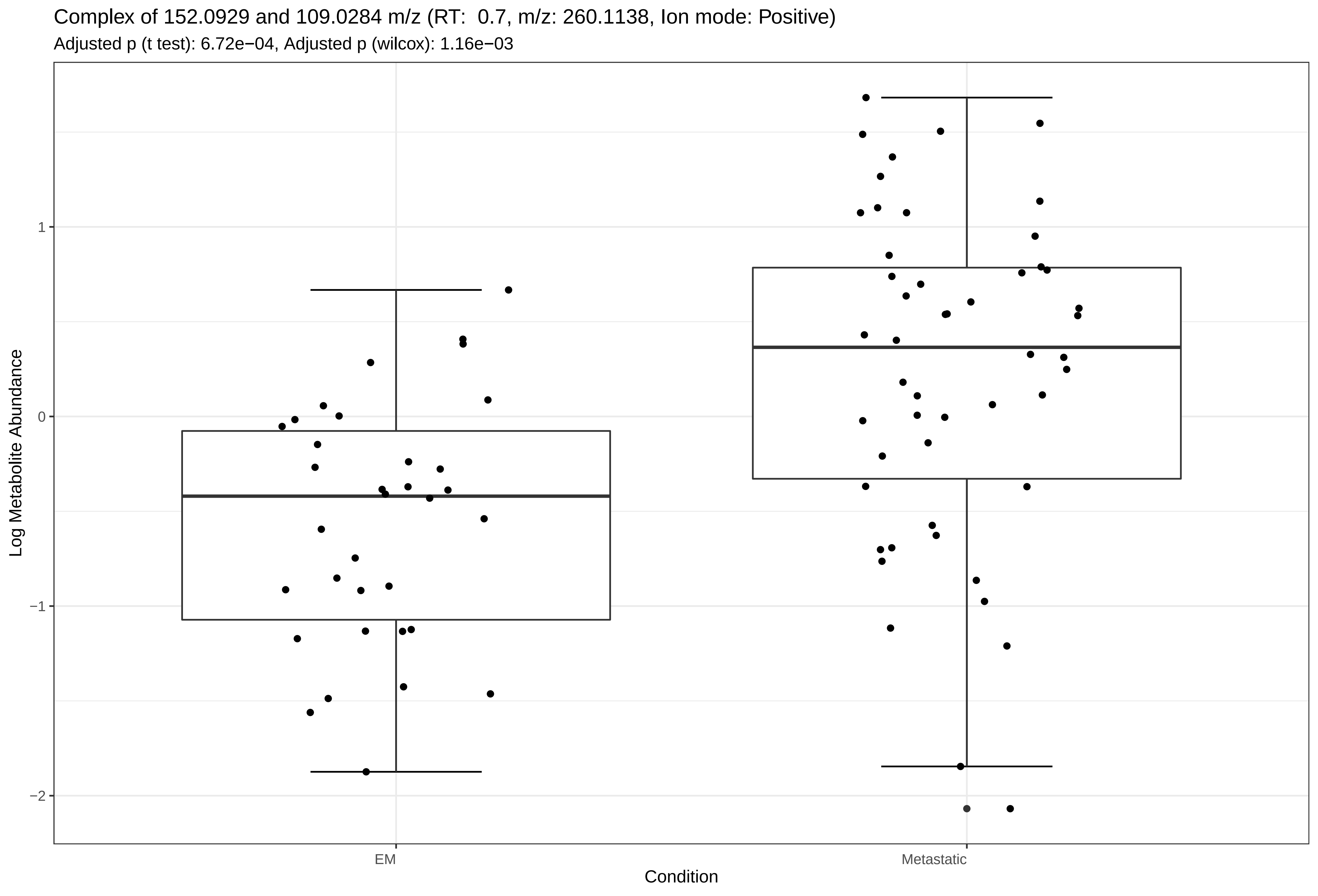
Complex of 152.0929 and 109.0284 m/z (RT: 0.7, m/z: 260.1138, Ion mode: Positive)
Adjusted p (t test): 6.72e−04, Adjusted p (wilcox): 1.16e−03
Log Metabolite Abundance
1
0
−1
−2
EM
Metastatic
Condition

RT: 2.9, m/z: 201.0868, Ion mode: Positive

Adjusted p (t test): 6.72e−04, Adjusted p (wilcox): 8.87e−04

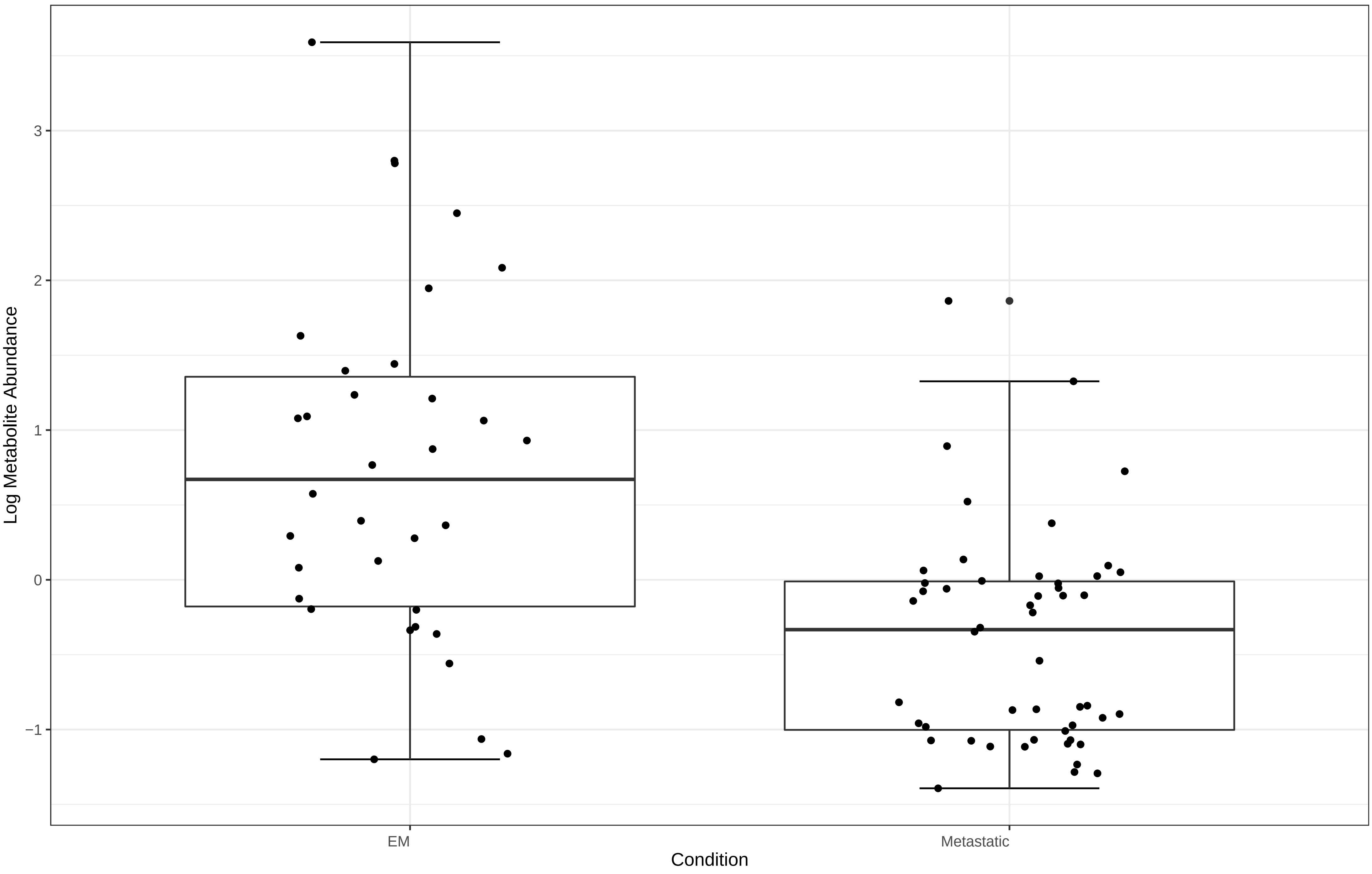

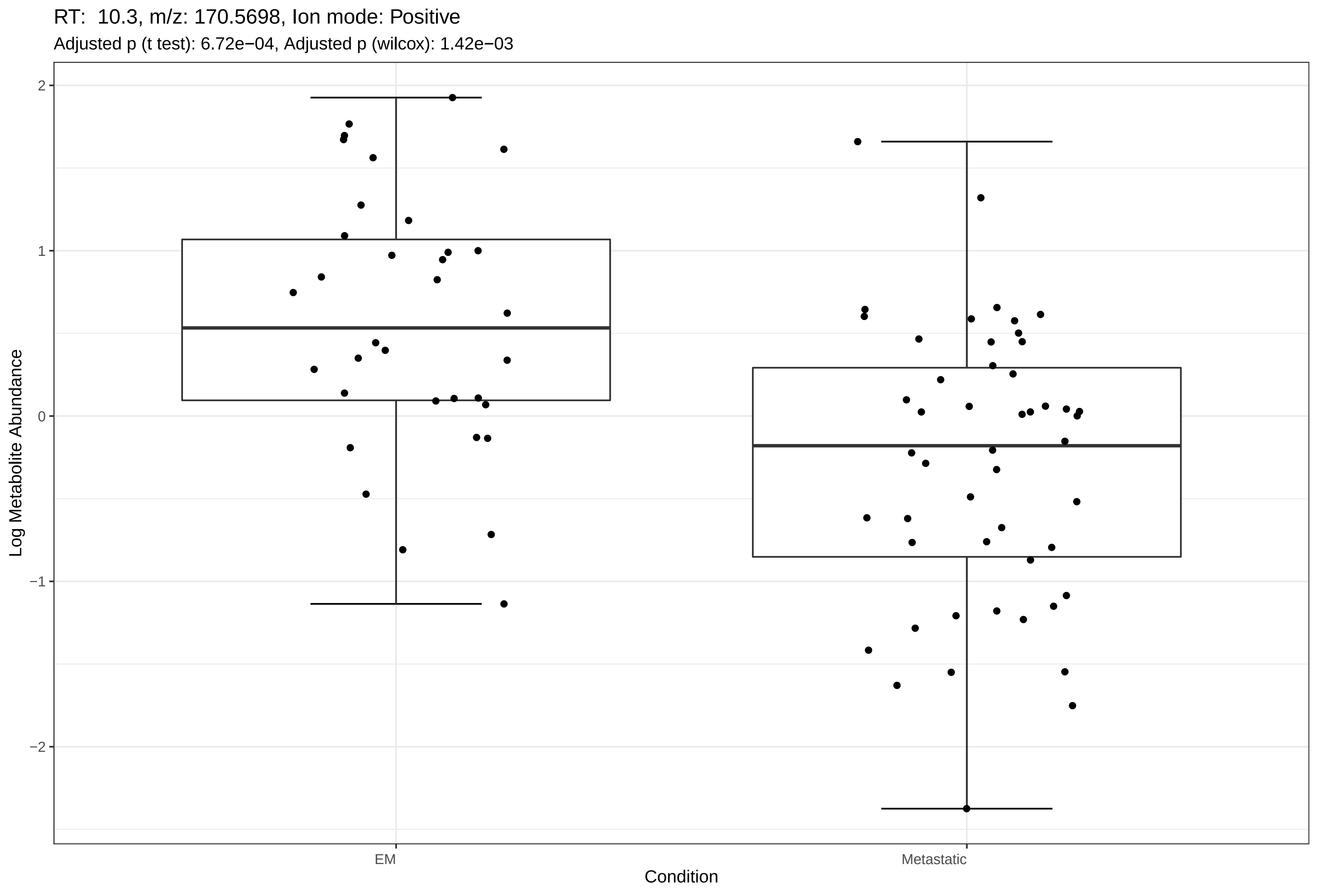
RT: 10.3, m/z: 170.5698, Ion mode: Positive
Adjusted p (t test): 6.72e−04, Adjusted p (wilcox): 1.42e−03
Log Metabolite Abundance
2
1
0
−1
−2
EM
Metastatic
Condition

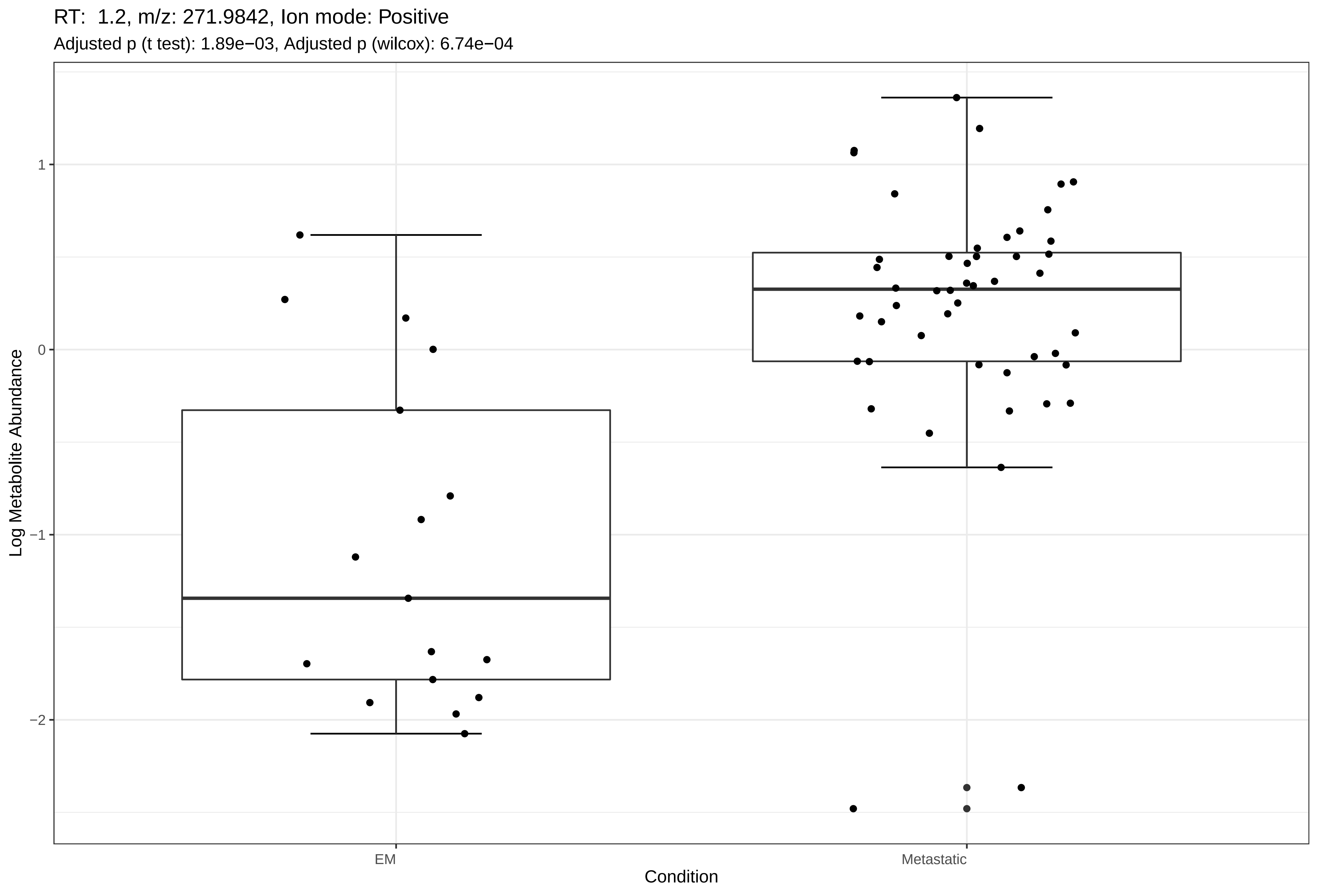
RT: 1.2, m/z: 271.9842, Ion mode: Positive
Adjusted p (t test): 1.89e−03, Adjusted p (wilcox): 6.74e−04
Log Metabolite Abundance
1
0
−1
−2
EM
Metastatic
Condition

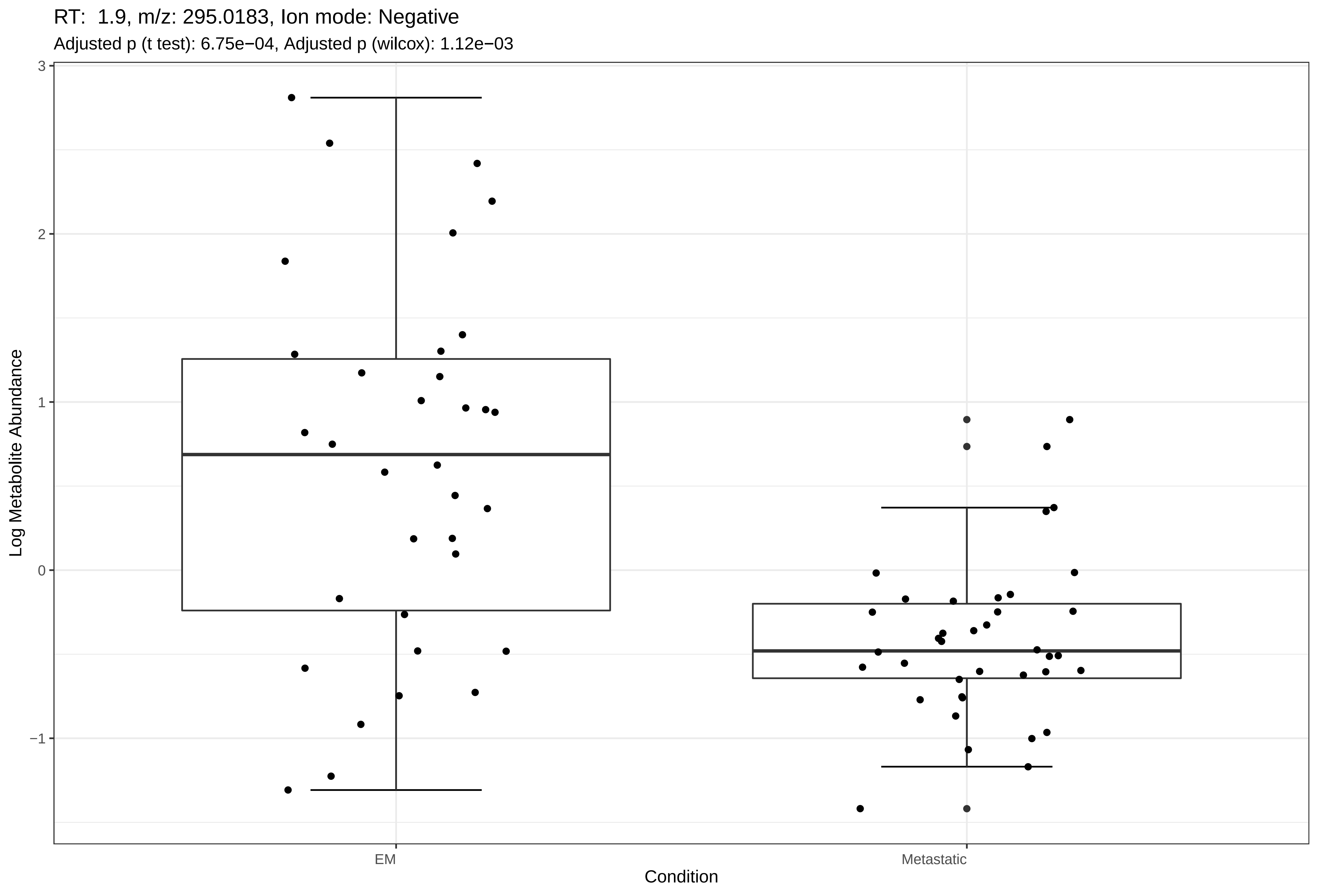
RT: 1.9, m/z: 295.0183, Ion mode: Negative
Adjusted p (t test): 6.75e−04, Adjusted p (wilcox): 1.12e−03
Log Metabolite Abundance
3
2
1
0
−1
EM
Metastatic
Condition

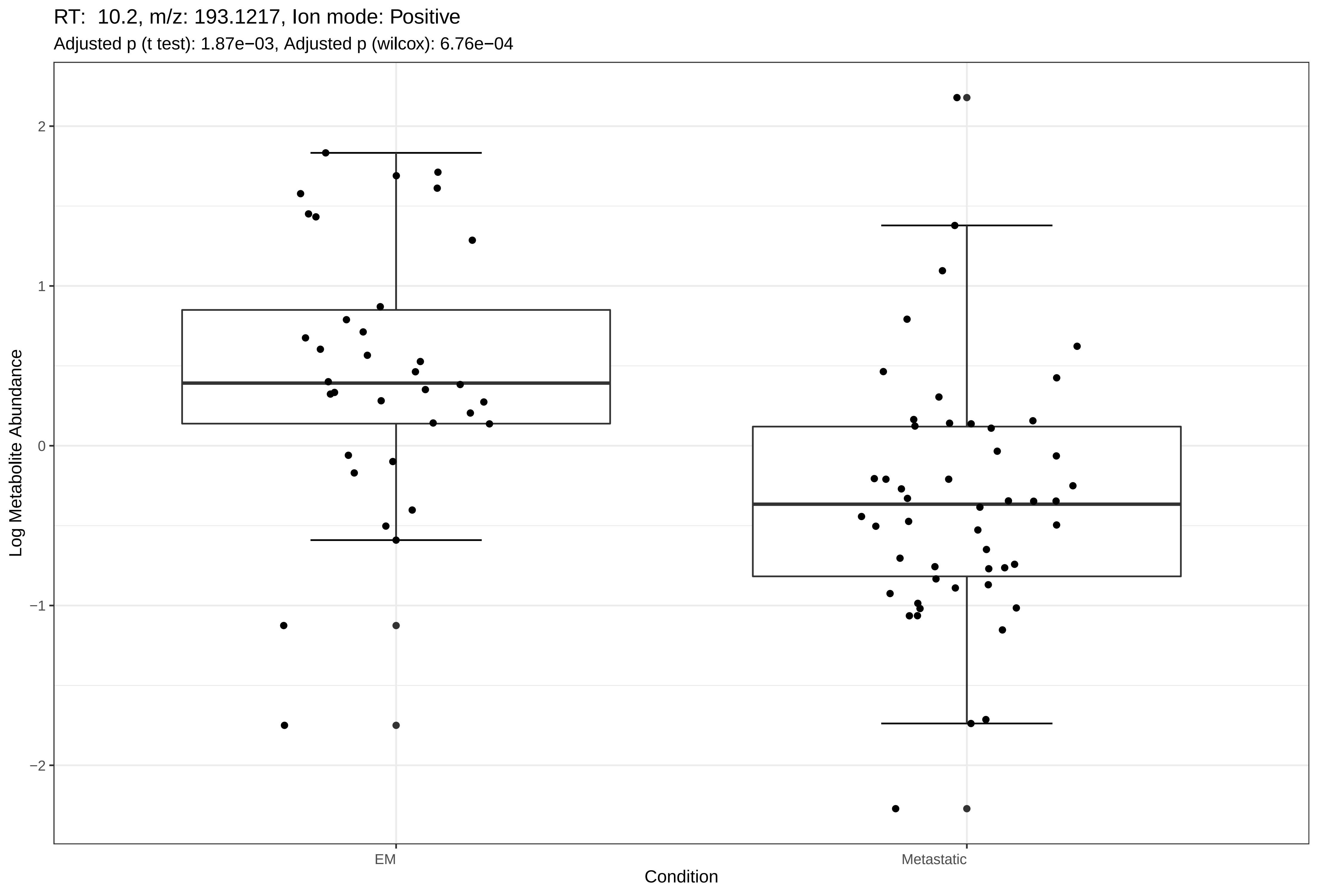
RT: 10.2, m/z: 193.1217, Ion mode: Positive
Adjusted p (t test): 1.87e−03, Adjusted p (wilcox): 6.76e−04
Log Metabolite Abundance
2
1
0
−1
−2
EM
Metastatic
Condition

[M+K−H] 37.9559 m/z adduct of 377.1945 m/z (RT: 10.1, m/z: 415.1504, Ion mode: Positive)

Adjusted p (t test): 8.00e−03, Adjusted p (wilcox): 6.76e−04

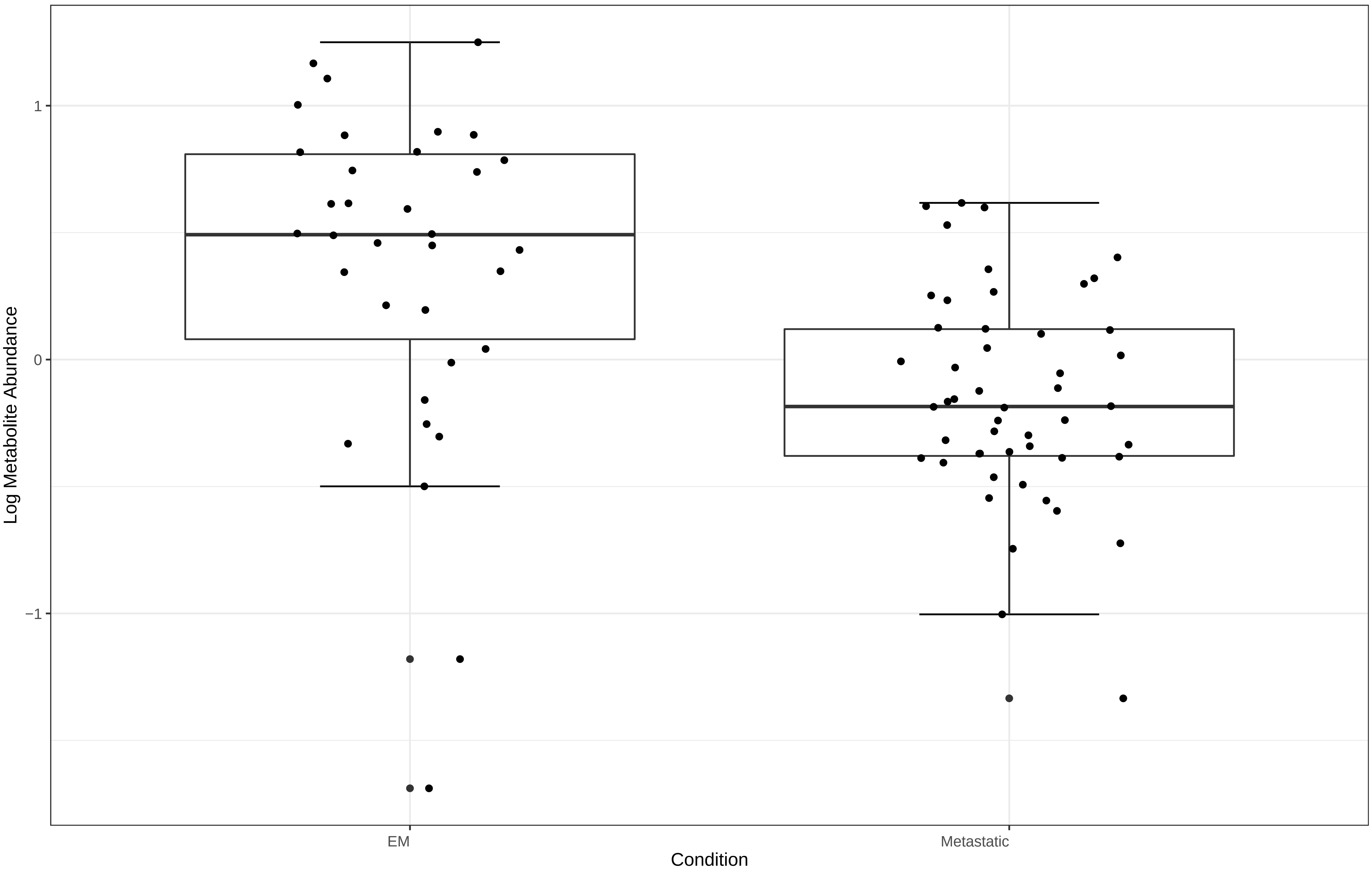

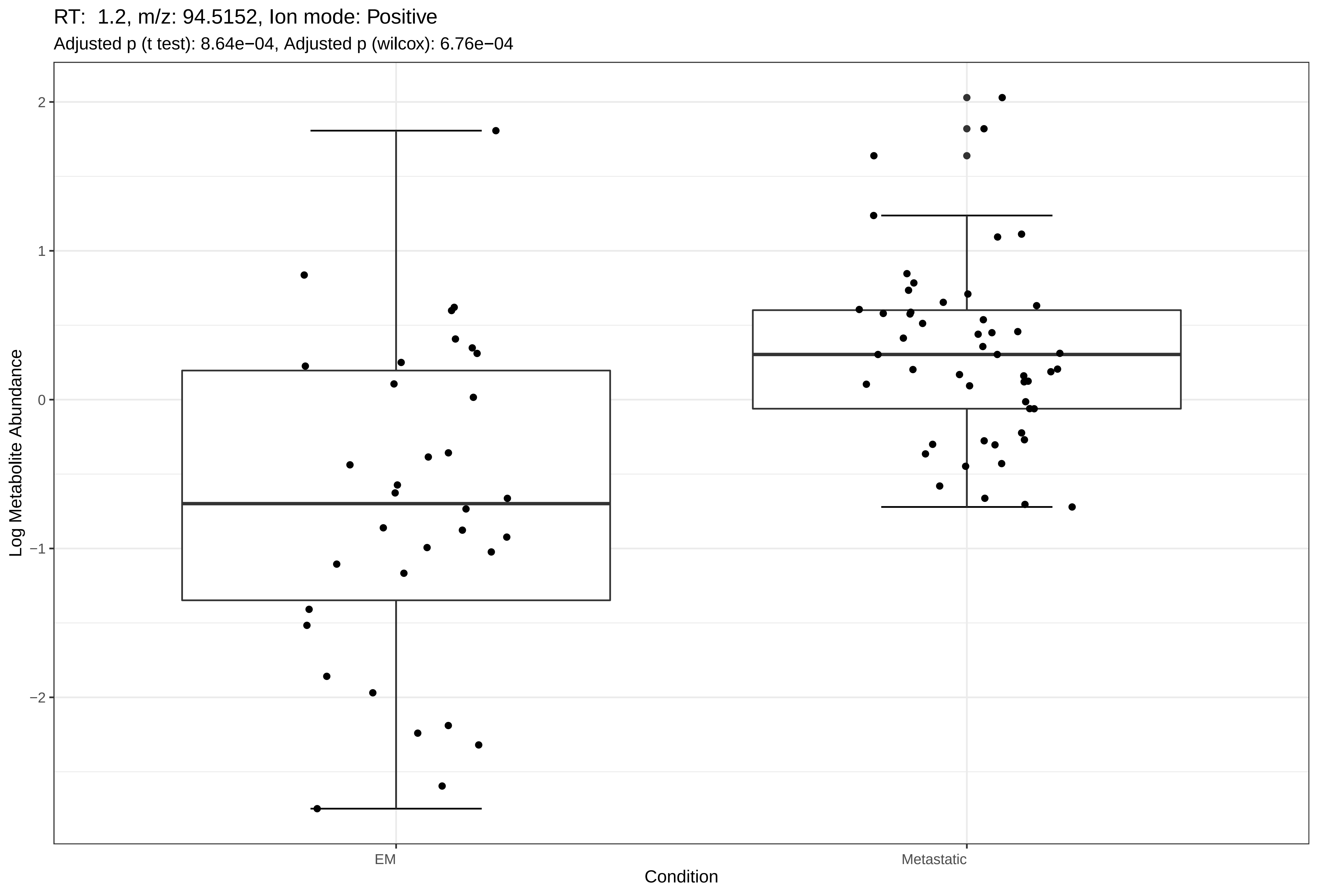

RT: 1.2, m/z: 94.5152, Ion mode: Positive
Adjusted p (t test): 8.64e−04, Adjusted p (wilcox): 6.76e−04
Log Metabolite Abundance
2
1
0
−1
−2
EM
Metastatic
Condition

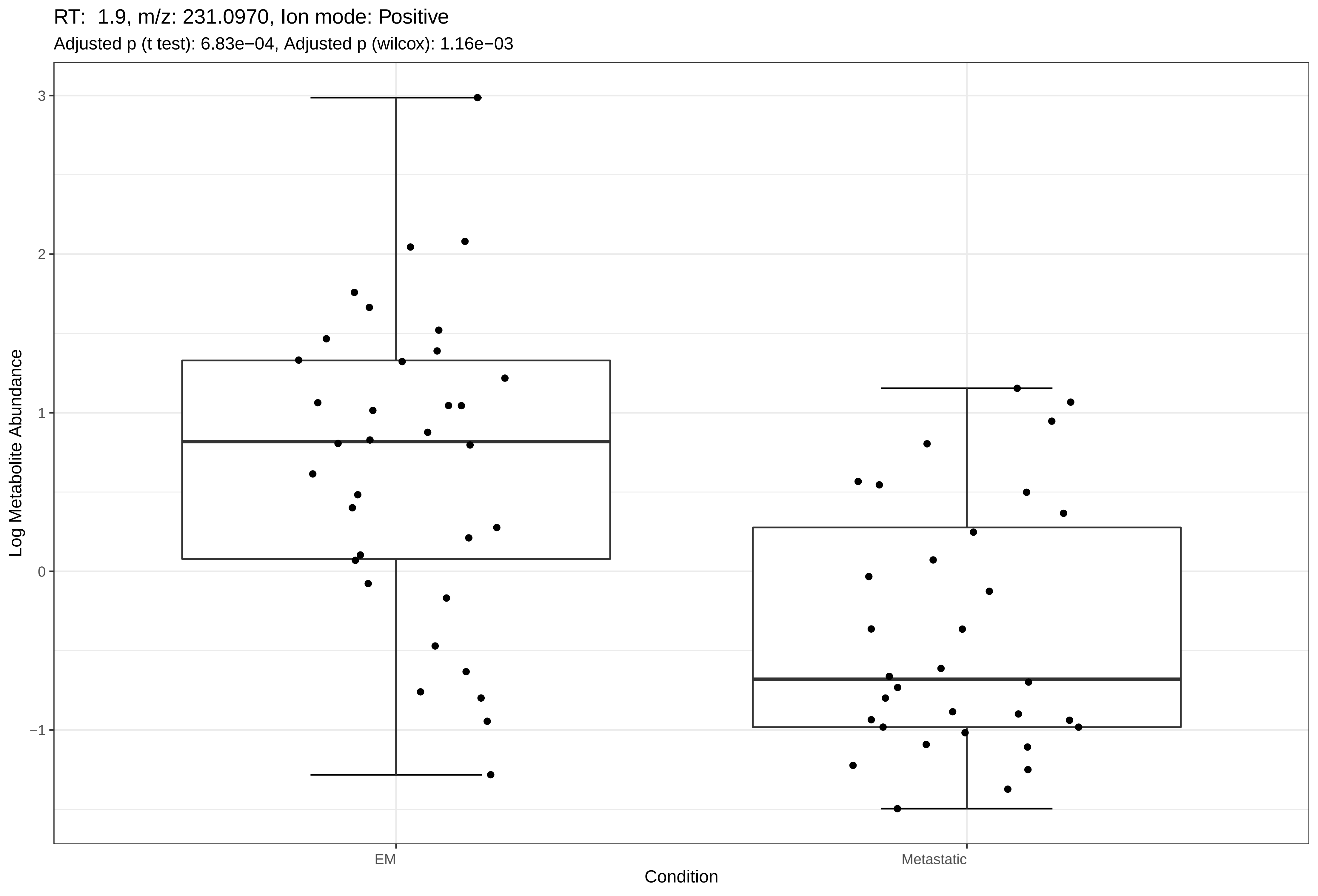
RT: 1.9, m/z: 231.0970, Ion mode: Positive
Adjusted p (t test): 6.83e−04, Adjusted p (wilcox): 1.16e−03
Log Metabolite Abundance
3
2
1
0
−1
EM
Metastatic
Condition

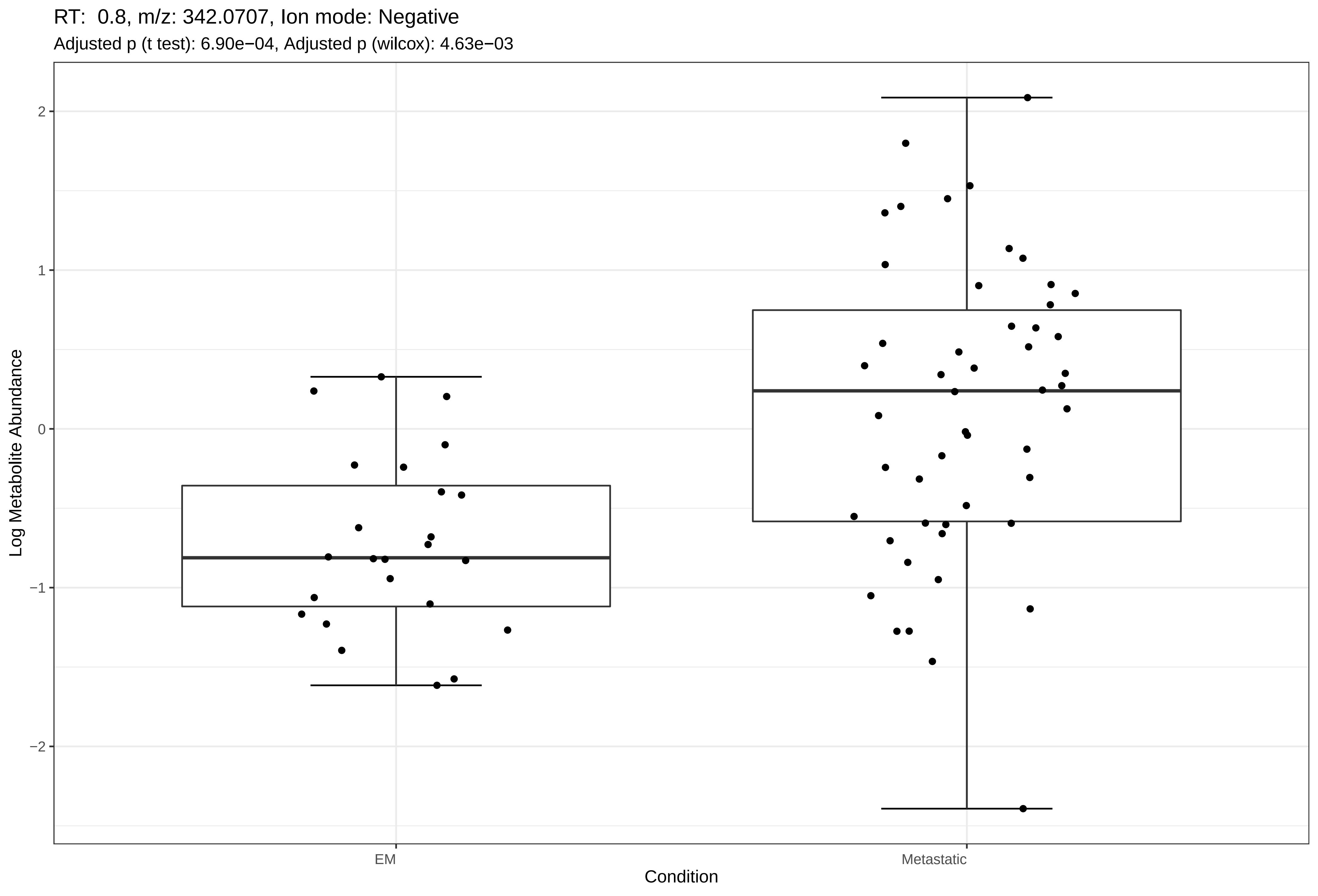

RT: 0.8, m/z: 342.0707, Ion mode: Negative
Adjusted p (t test): 6.90e−04, Adjusted p (wilcox): 4.63e−03
2
1
0
−1
−2
Log Metabolite Abundance
EM
Metastatic
Condition

RT: 13.0, m/z: 588.3308, Ion mode: Negative

Adjusted p (t test): 6.95e−04, Adjusted p (wilcox): 1.42e−03

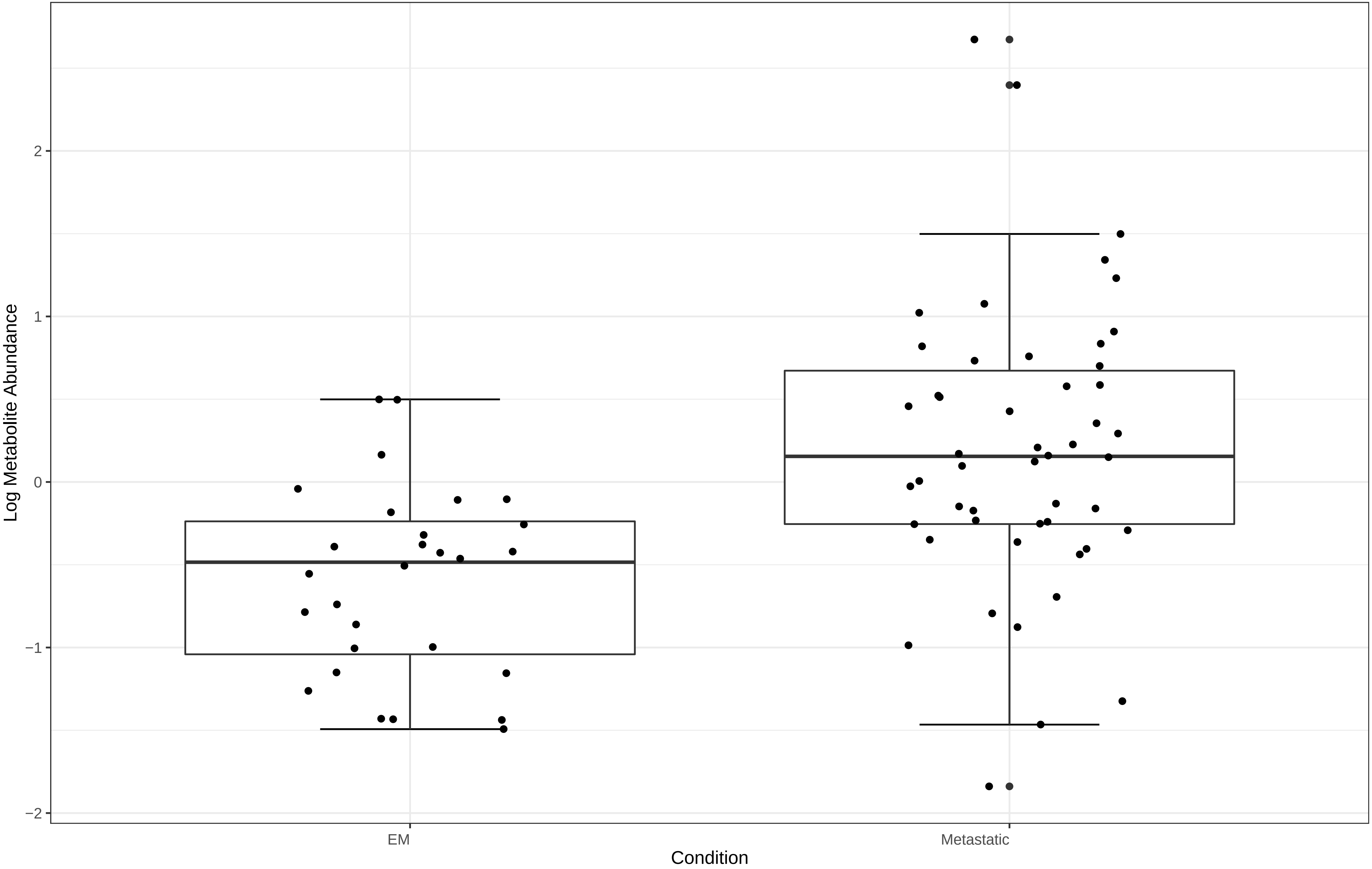

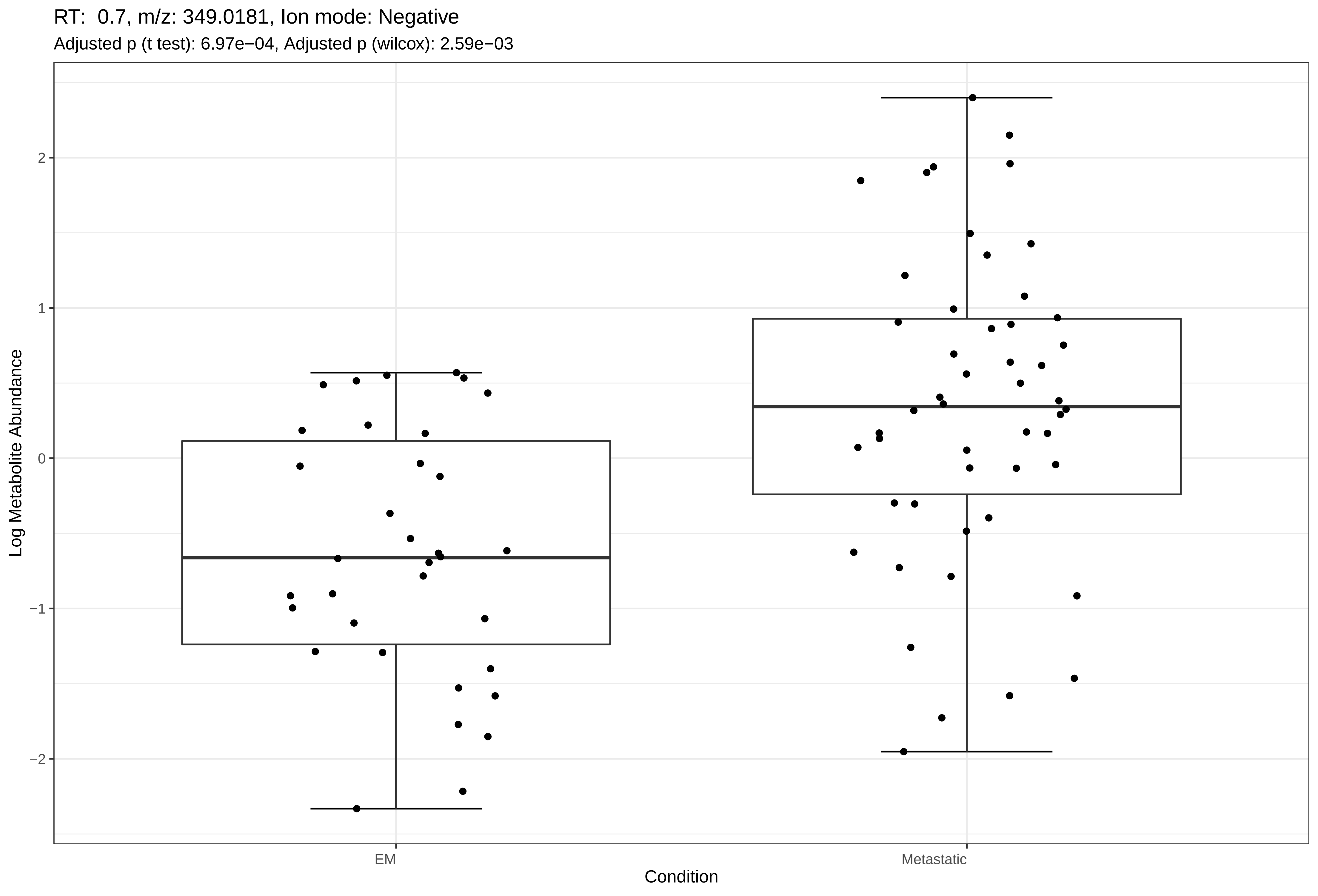
RT: 0.7, m/z: 349.0181, Ion mode: Negative
Adjusted p (t test): 6.97e−04, Adjusted p (wilcox): 2.59e−03
Log Metabolite Abundance
2
1
0
−1
−2
EM
Metastatic
Condition

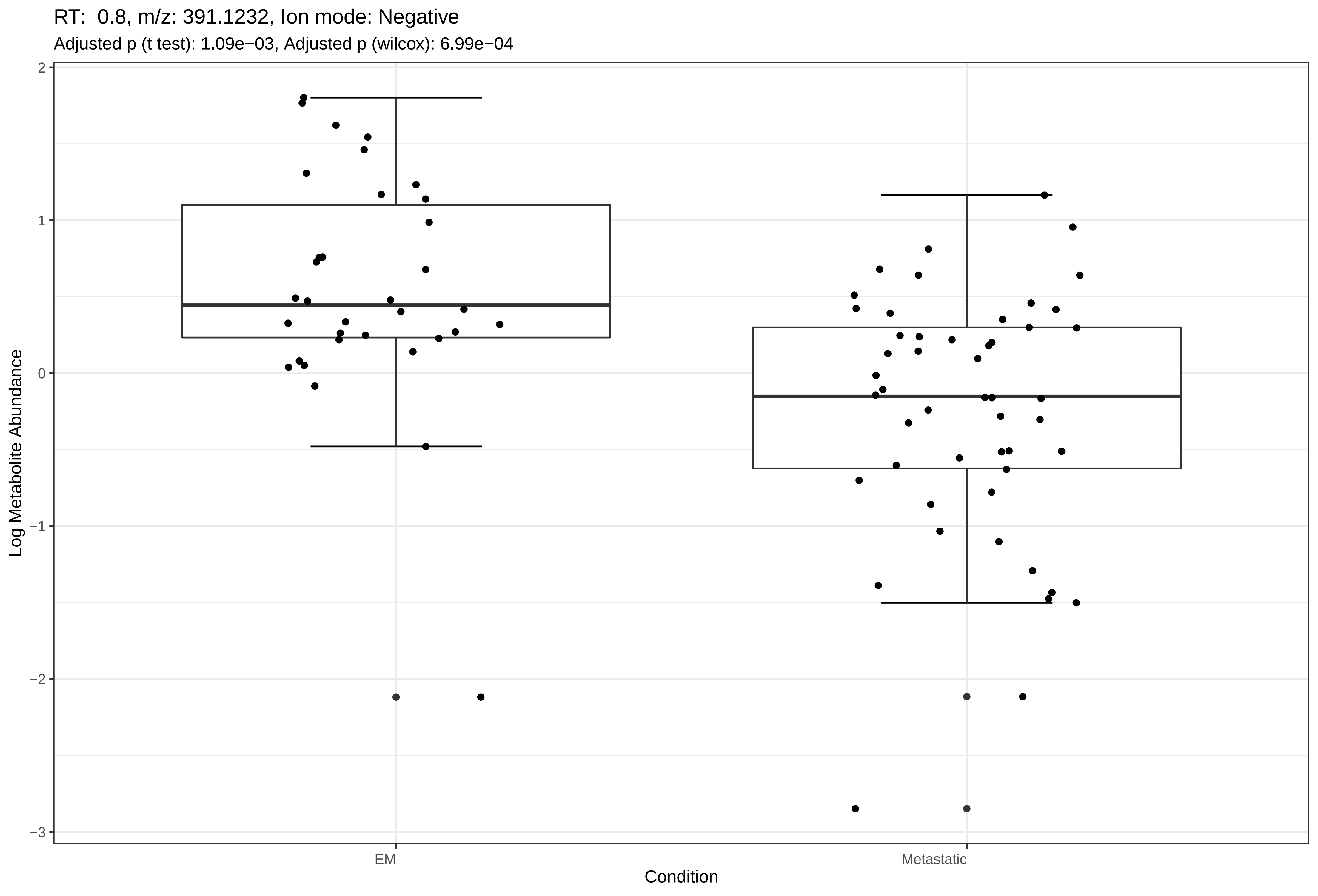
RT: 0.8, m/z: 391.1232, Ion mode: Negative
Adjusted p (t test): 1.09e−03, Adjusted p (wilcox): 6.99e−04
Log Metabolite Abundance
2
1
0
−1
−2
−3
EM
Metastatic
Condition

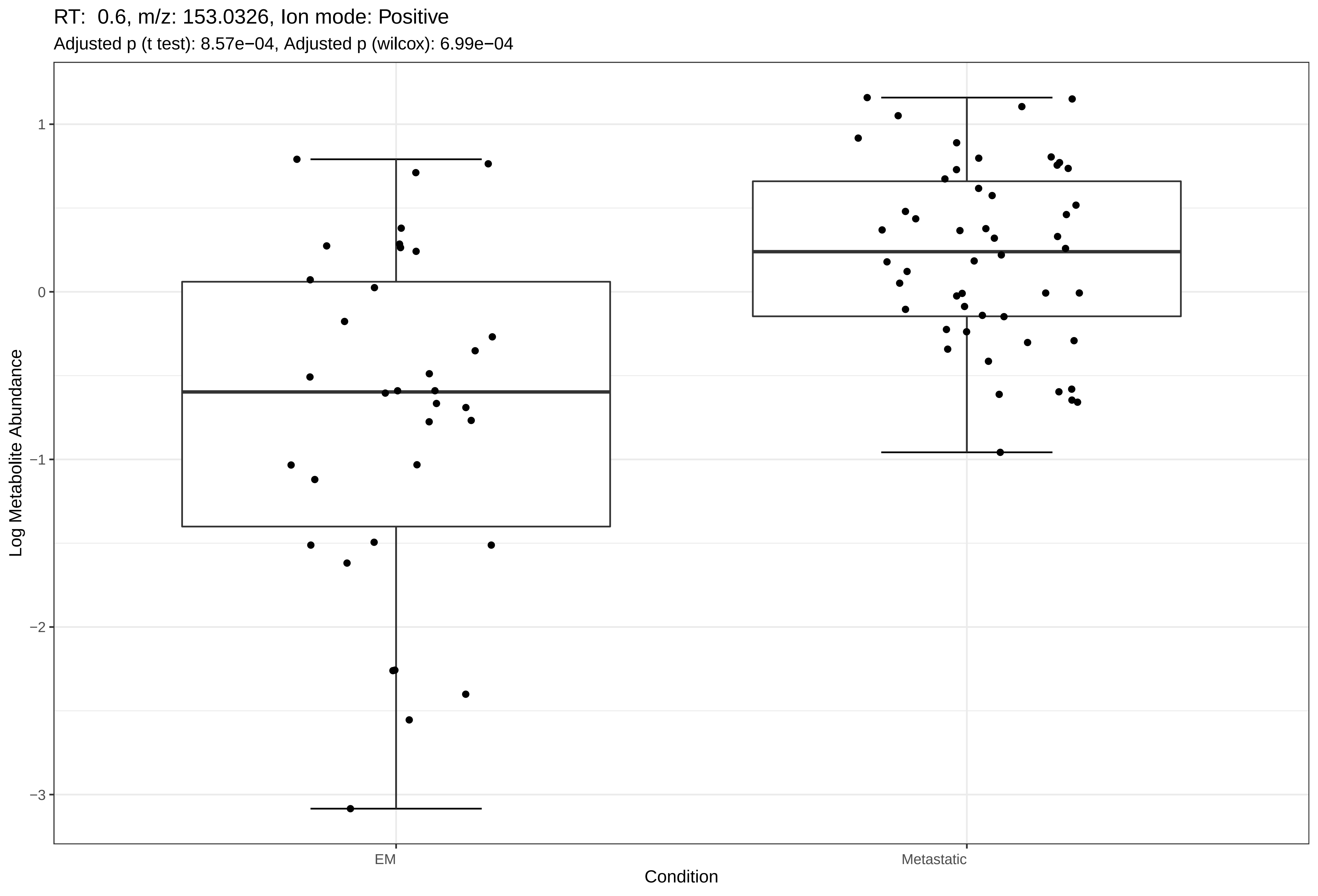
RT: 0.6, m/z: 153.0326, Ion mode: Positive
Adjusted p (t test): 8.57e−04, Adjusted p (wilcox): 6.99e−04
Log Metabolite Abundance
1
0
−1
−2
−3
EM
Metastatic
Condition

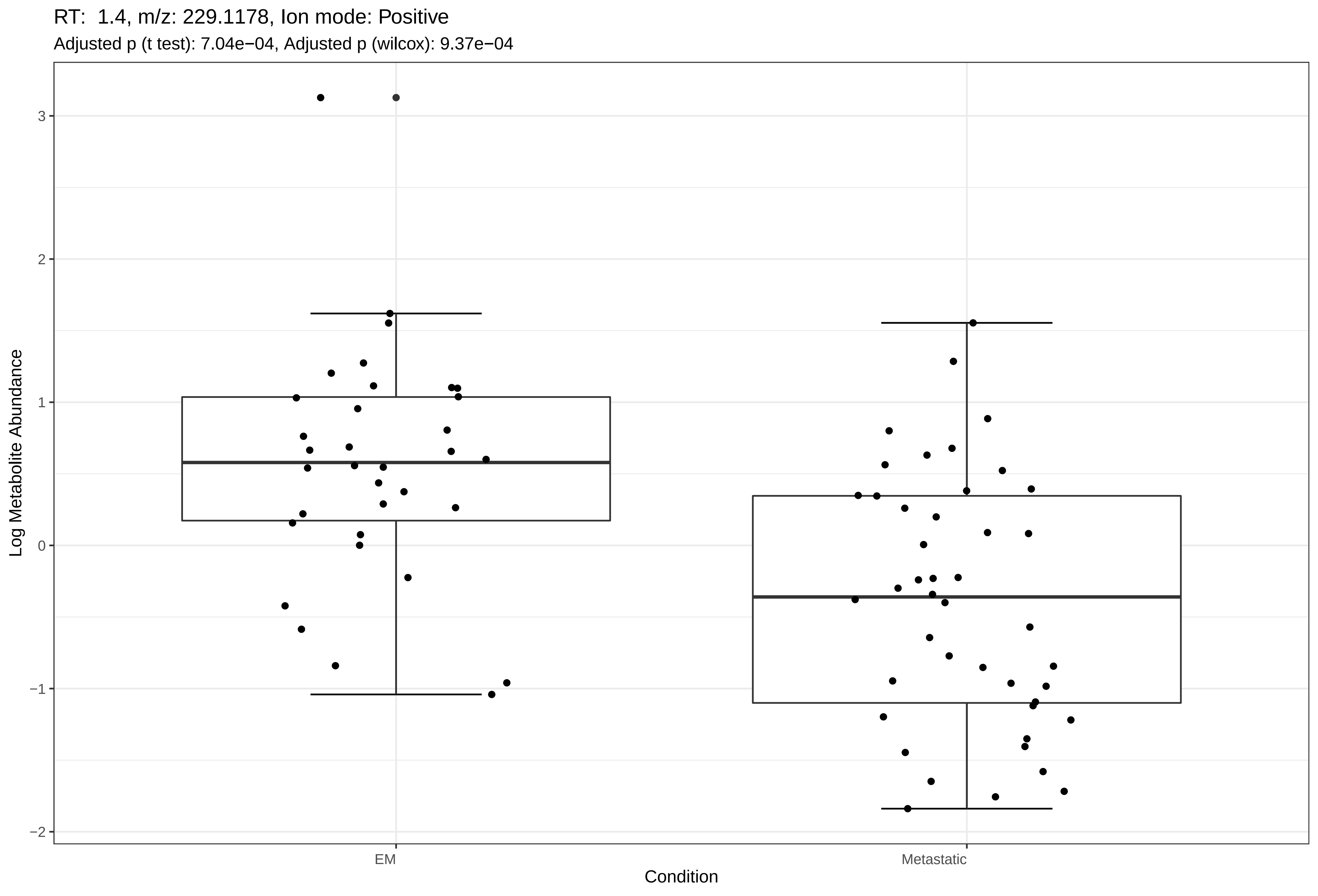

RT: 1.4, m/z: 229.1178, Ion mode: Positive
Adjusted p (t test): 7.04e−04, Adjusted p (wilcox): 9.37e−04
Log Metabolite Abundance
3
2
1
0
−1
−2
EM
Metastatic
Condition

RT: 1.4, m/z: 482.1025, Ion mode: Negative

Adjusted p (t test): 7.09e−04, Adjusted p (wilcox): 2.32e−03

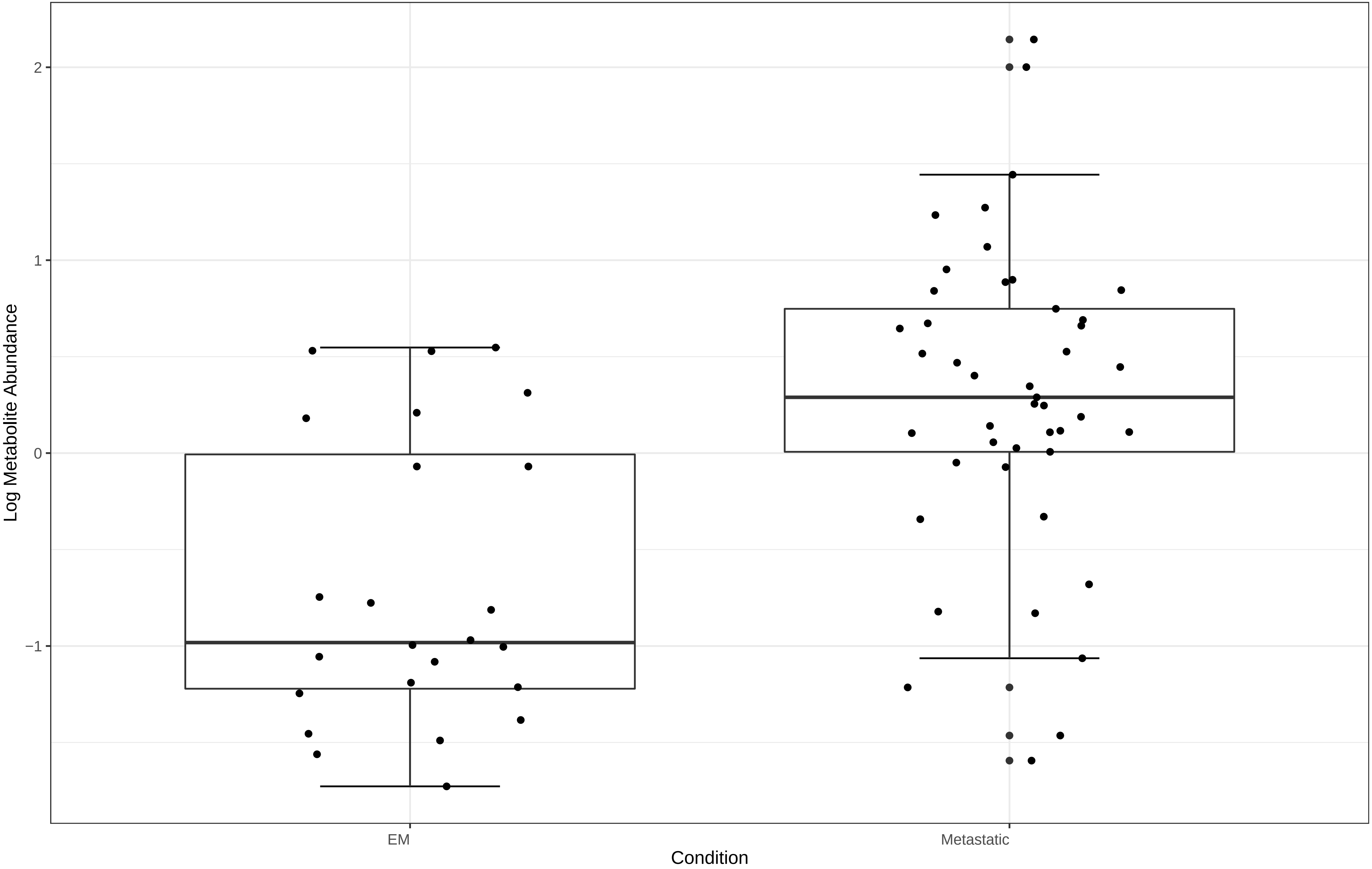

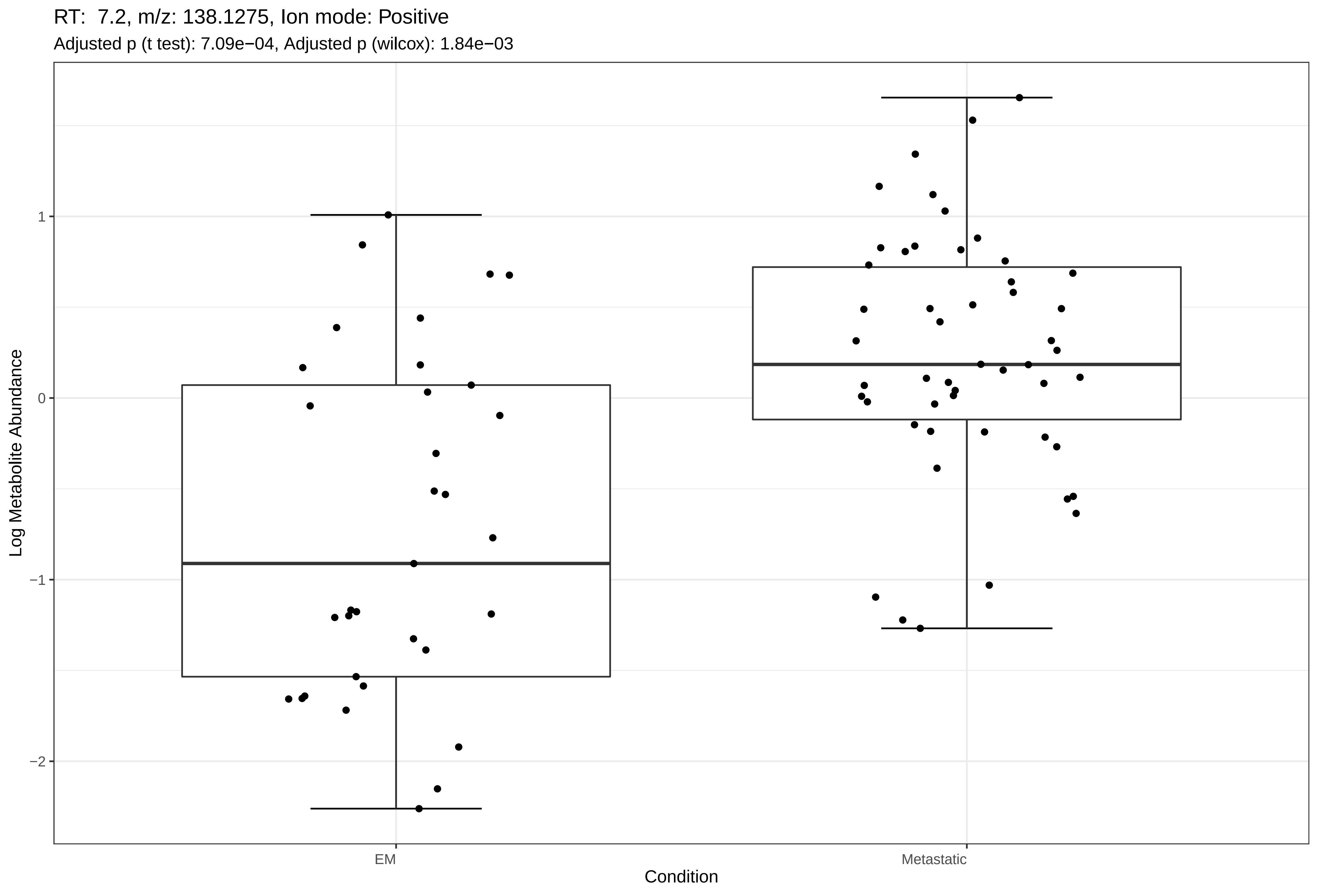

RT: 7.2, m/z: 138.1275, Ion mode: Positive
Adjusted p (t test): 7.09e−04, Adjusted p (wilcox): 1.84e−03
Log Metabolite Abundance
1
0
−1
−2
EM
Metastatic
Condition

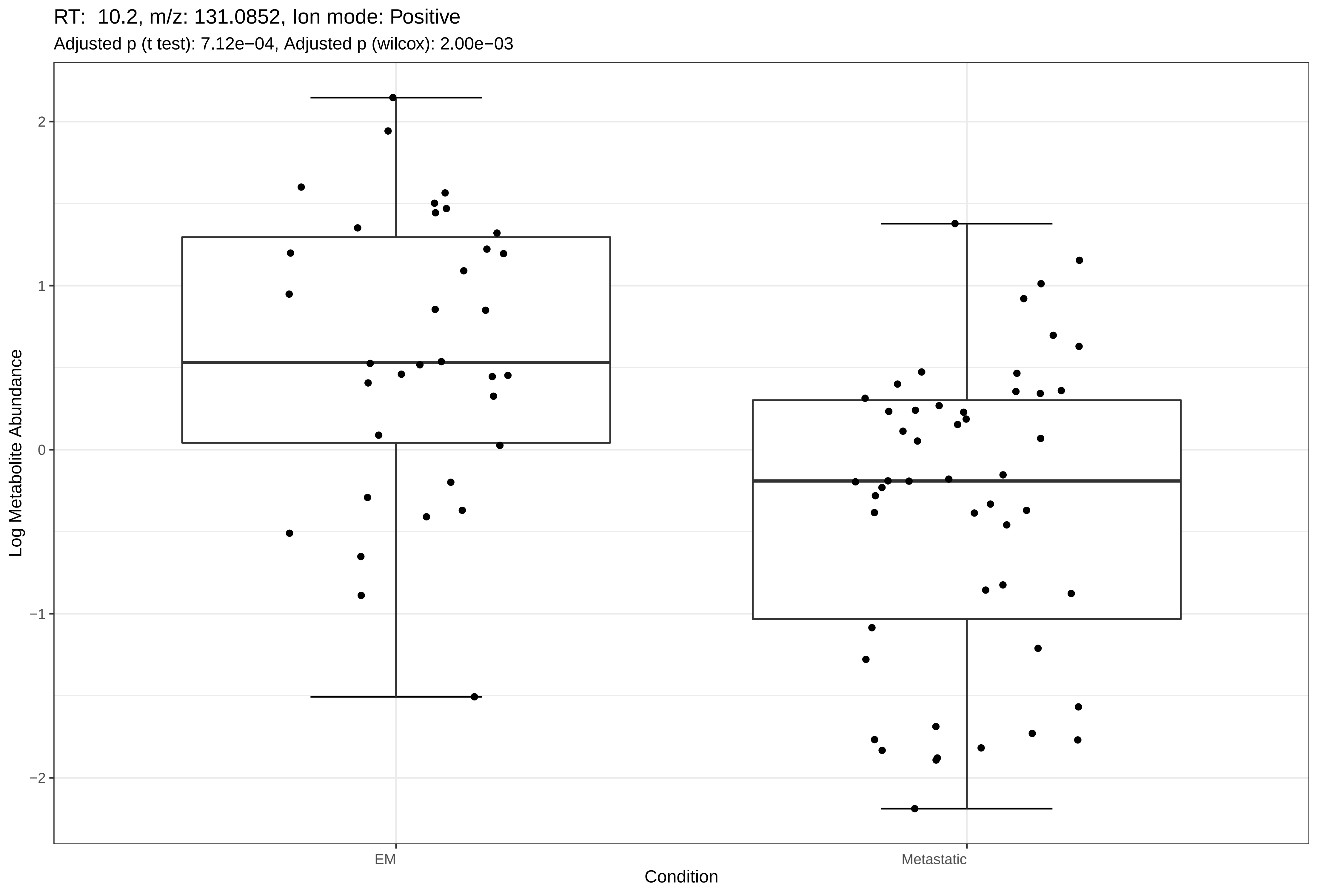
RT: 10.2, m/z: 131.0852, Ion mode: Positive
Adjusted p (t test): 7.12e−04, Adjusted p (wilcox): 2.00e−03
Log Metabolite Abundance
2
1
0
−1
−2
EM
Metastatic
Condition

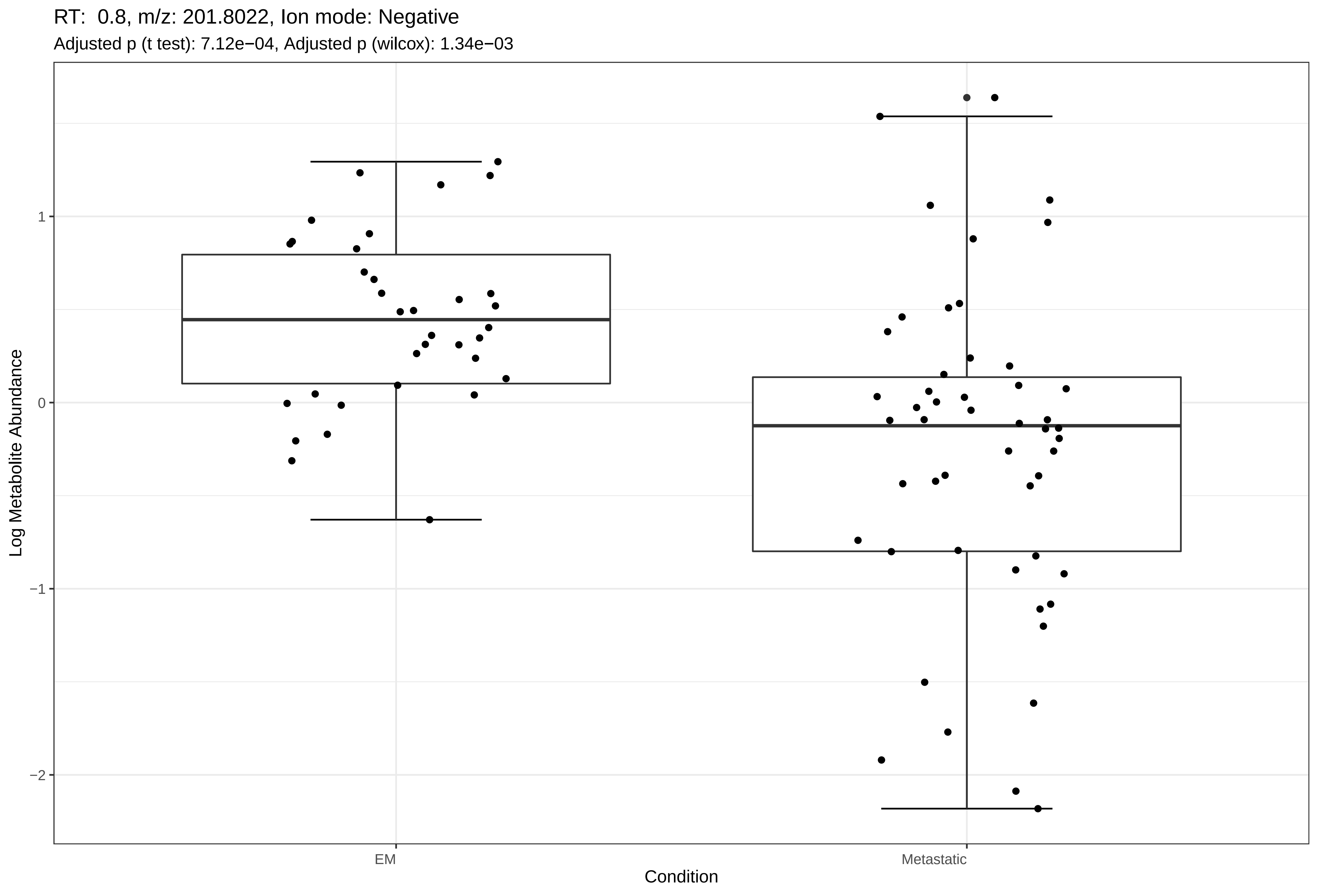

RT: 0.8, m/z: 201.8022, Ion mode: Negative
Adjusted p (t test): 7.12e−04, Adjusted p (wilcox): 1.34e−03
Log Metabolite Abundance
1
0
−1
−2
EM
Metastatic
Condition

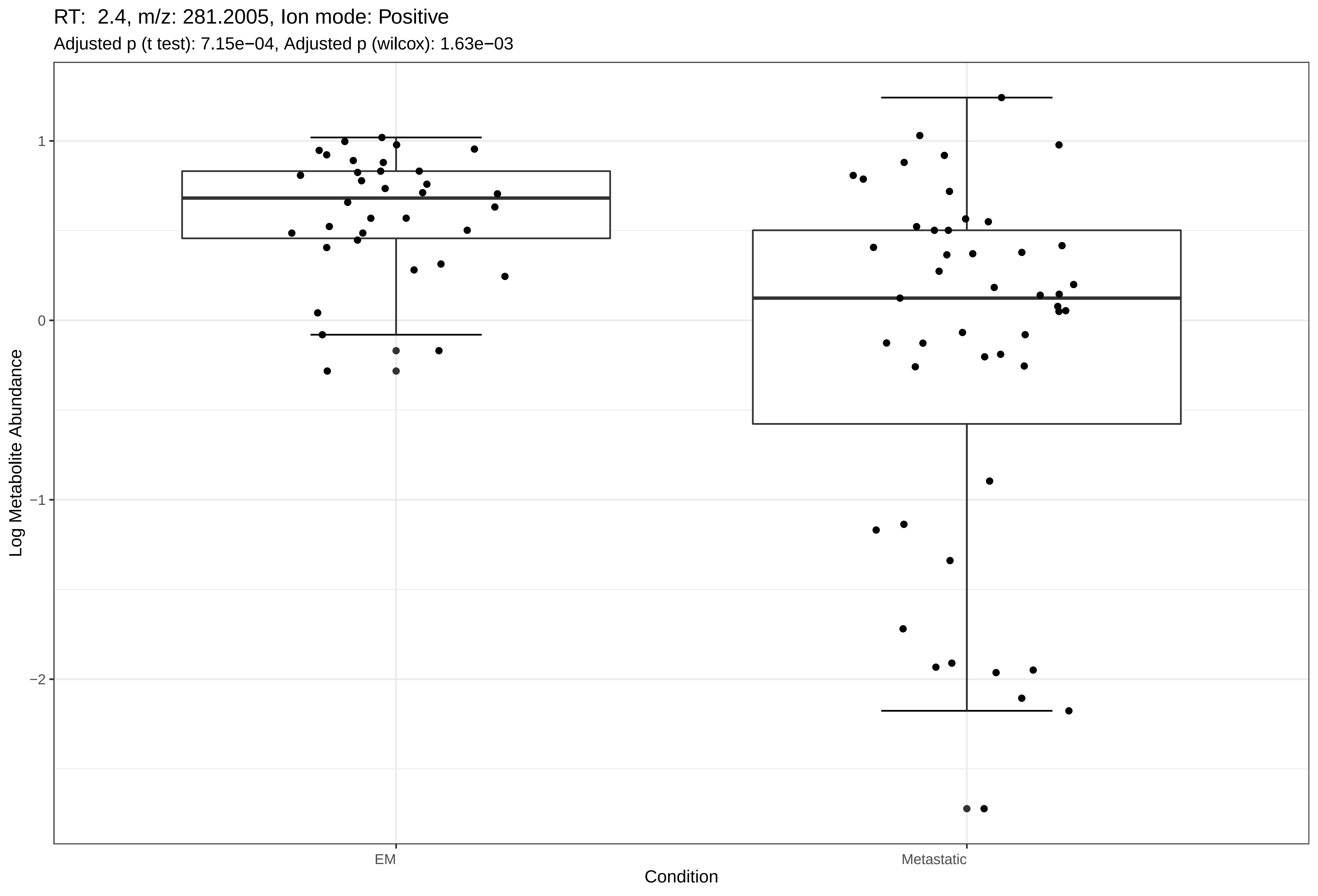
RT: 2.4, m/z: 281.2005, Ion mode: Positive
Adjusted p (t test): 7.15e−04, Adjusted p (wilcox): 1.63e−03
1
0
−1
−2
Log Metabolite Abundance
EM
Metastatic
Condition

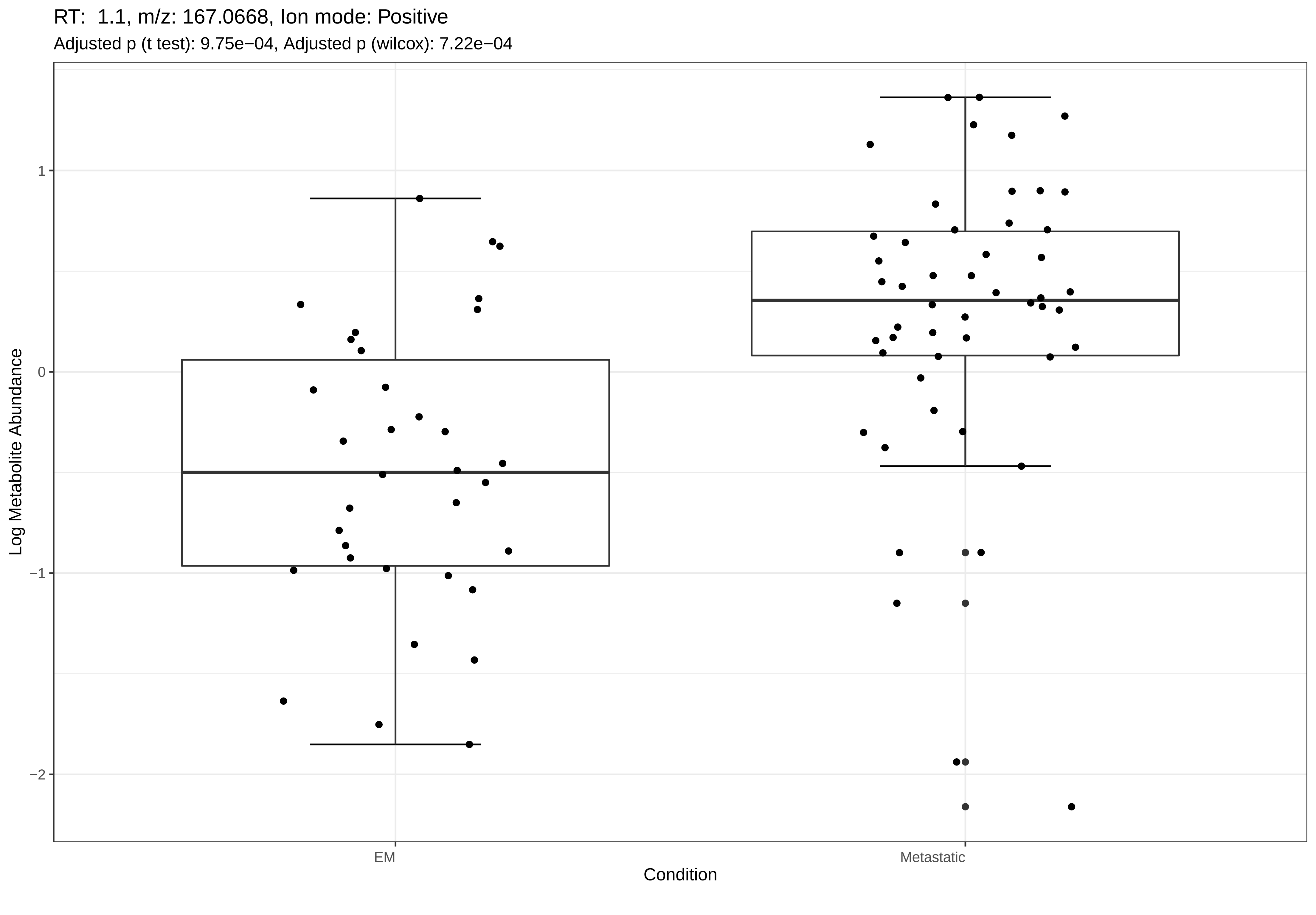
RT: 1.1, m/z: 167.0668, Ion mode: Positive
Adjusted p (t test): 9.75e−04, Adjusted p (wilcox): 7.22e−04
Log Metabolite Abundance
1
0
−1
−2
EM
Metastatic
Condition

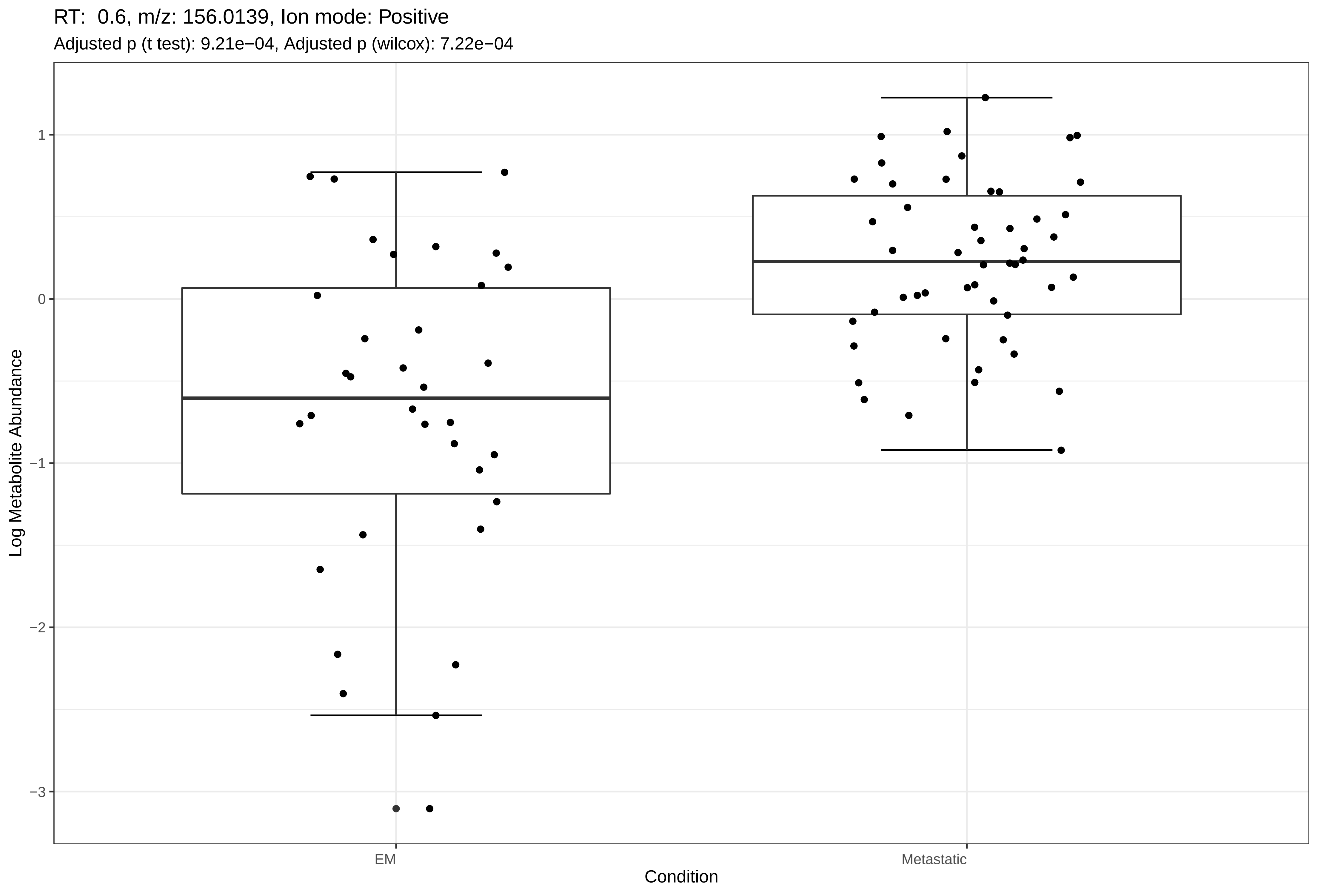

RT: 0.6, m/z: 156.0139, Ion mode: Positive
Adjusted p (t test): 9.21e−04, Adjusted p (wilcox): 7.22e−04
Log Metabolite Abundance
1
0
−1
−2
−3
EM
Metastatic
Condition

[M+Na−H] 21.9825 m/z adduct of 248.1487 m/z (RT: 5.8, m/z: 270.1309, Ion mode: Positive)

Adjusted p (t test): 7.37e−04, Adjusted p (wilcox): 2.61e−03

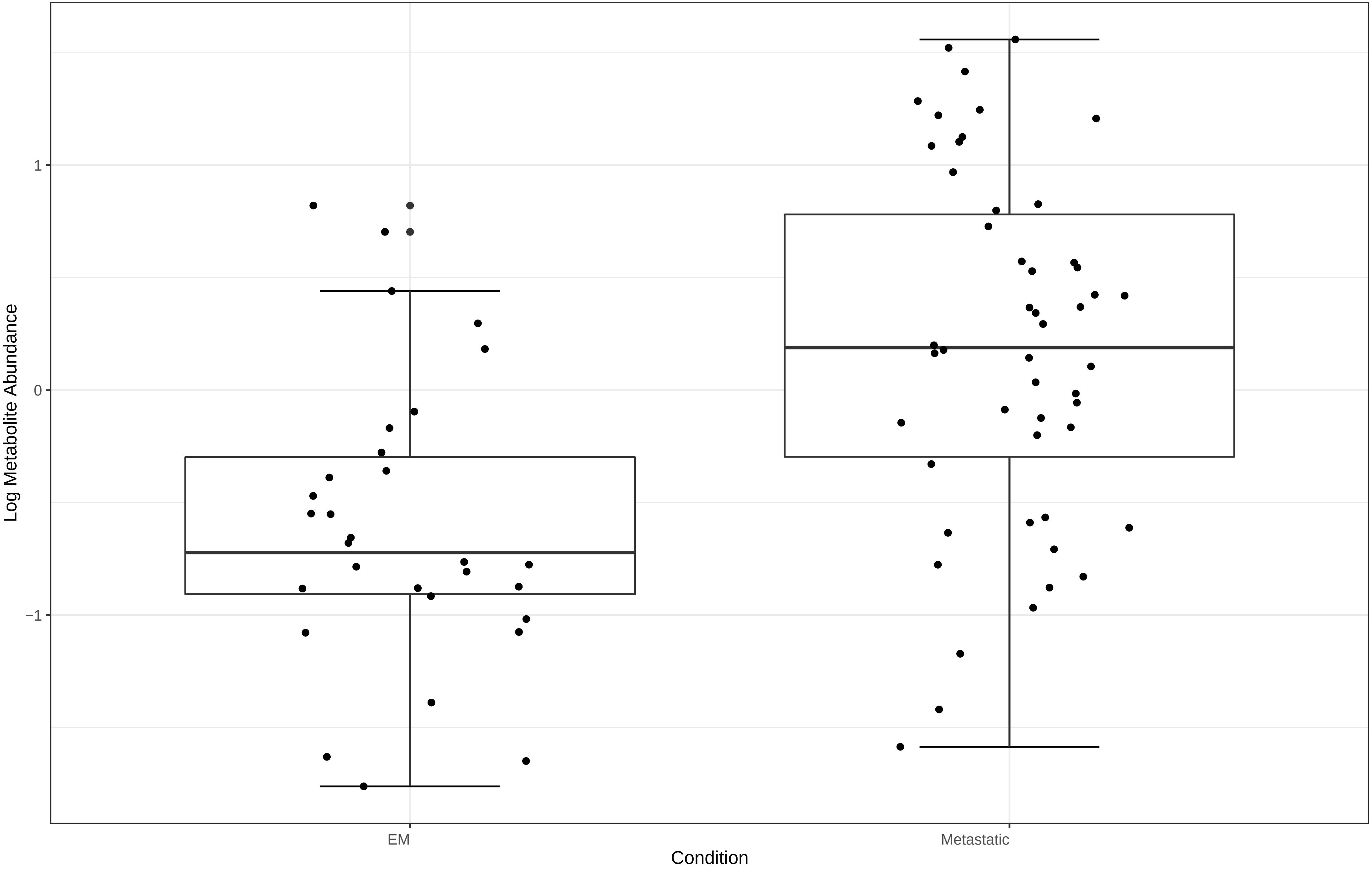

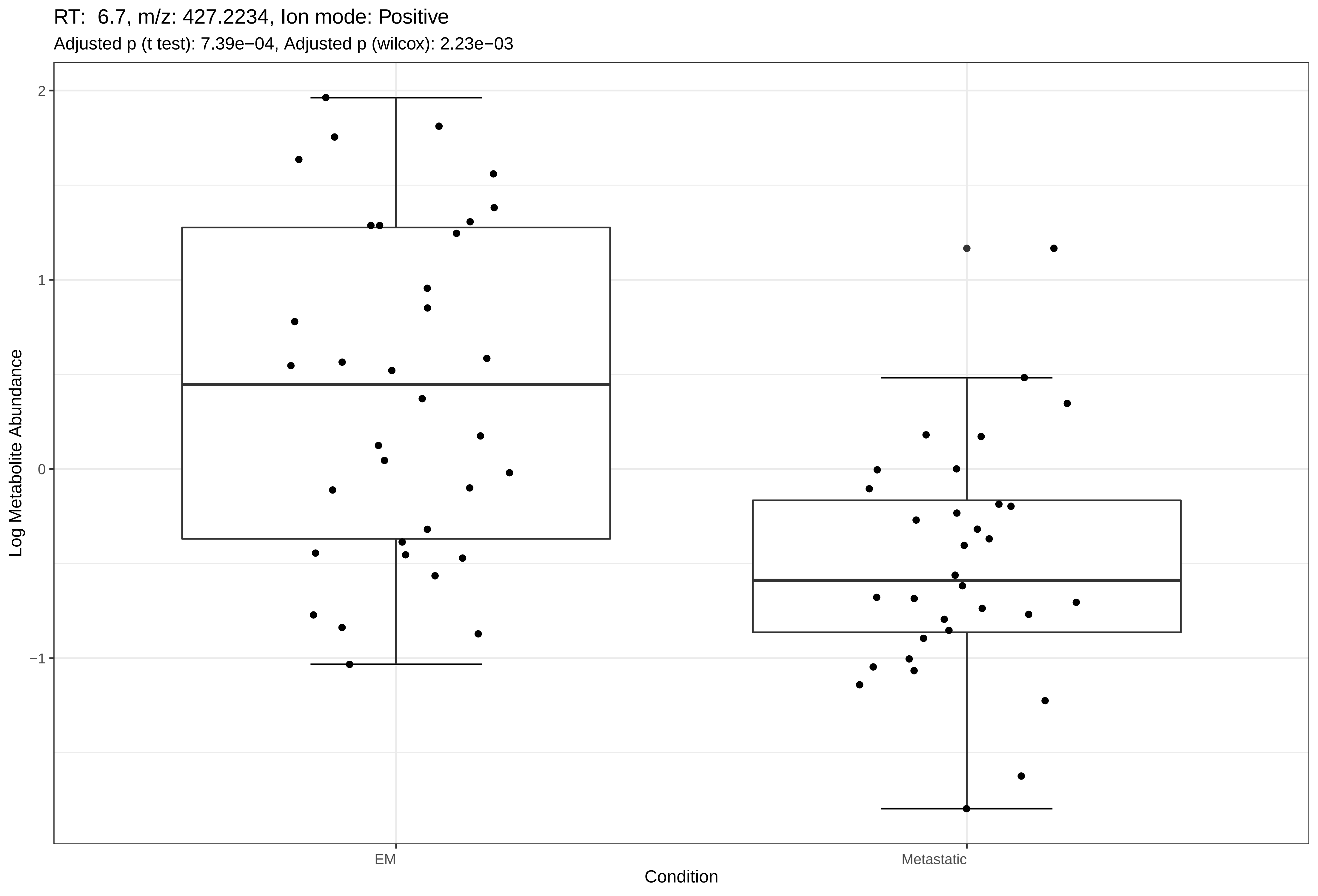

RT: 6.7, m/z: 427.2234, Ion mode: Positive
Adjusted p (t test): 7.39e−04, Adjusted p (wilcox): 2.23e−03
Log Metabolite Abundance
2
1
0
−1
EM
Metastatic
Condition

RT: 7.8, m/z: 277.1854, Ion mode: Positive

Adjusted p (t test): 7.40e−04, Adjusted p (wilcox): 9.88e−04

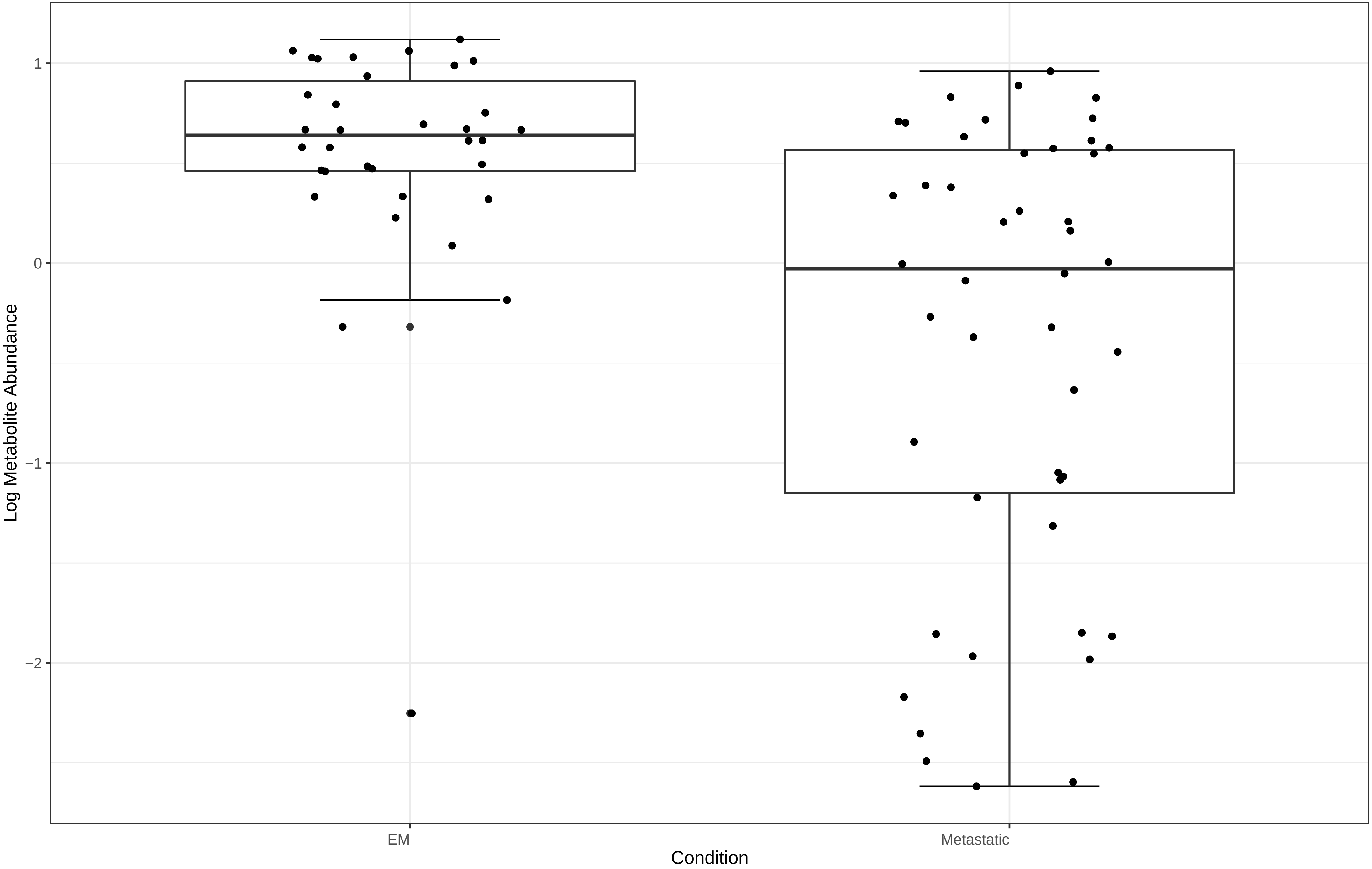

RT: 6.4, m/z: 243.1826, Ion mode: Positive

Adjusted p (t test): 7.42e−04, Adjusted p (wilcox): 2.23e−03

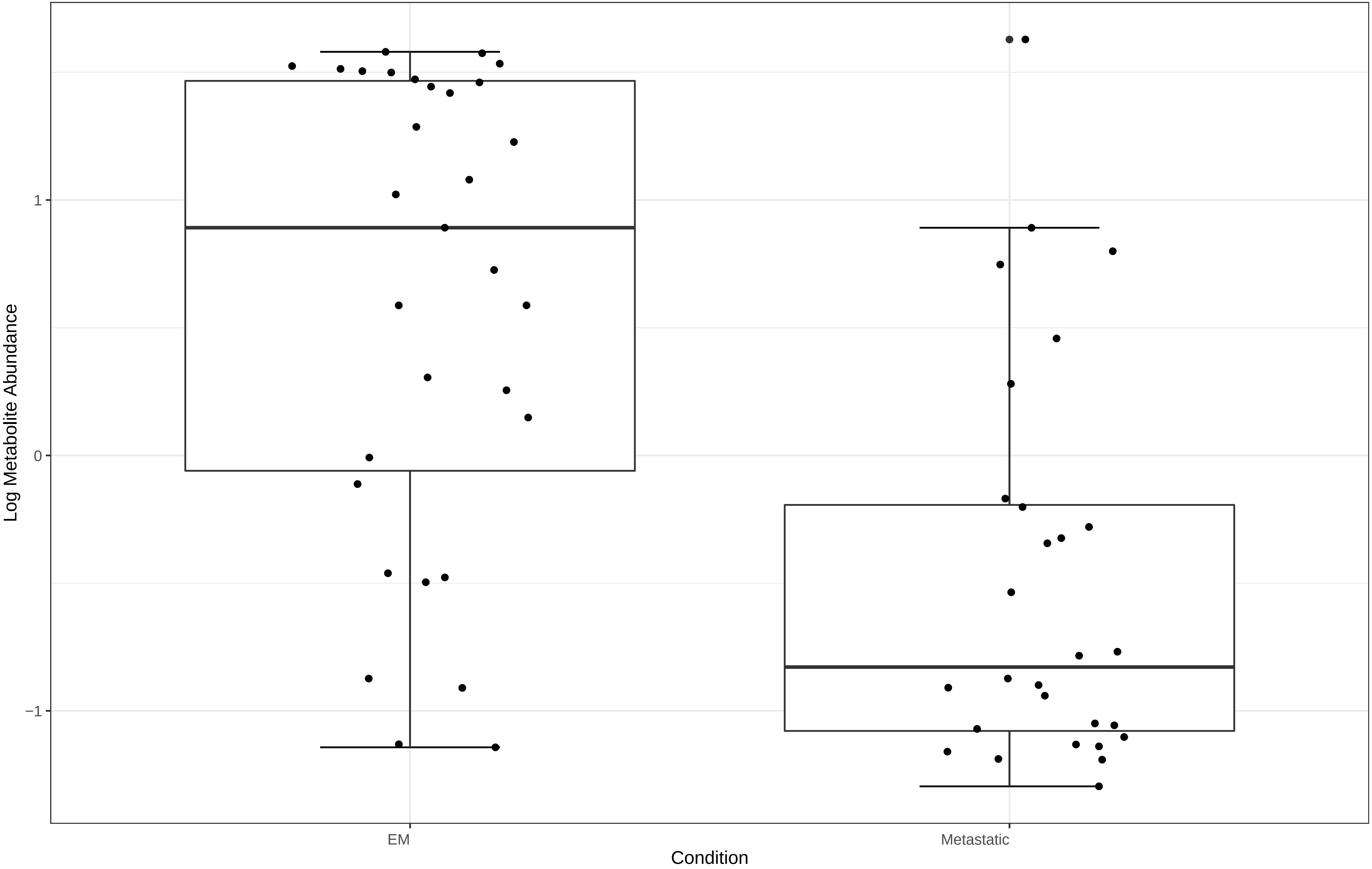

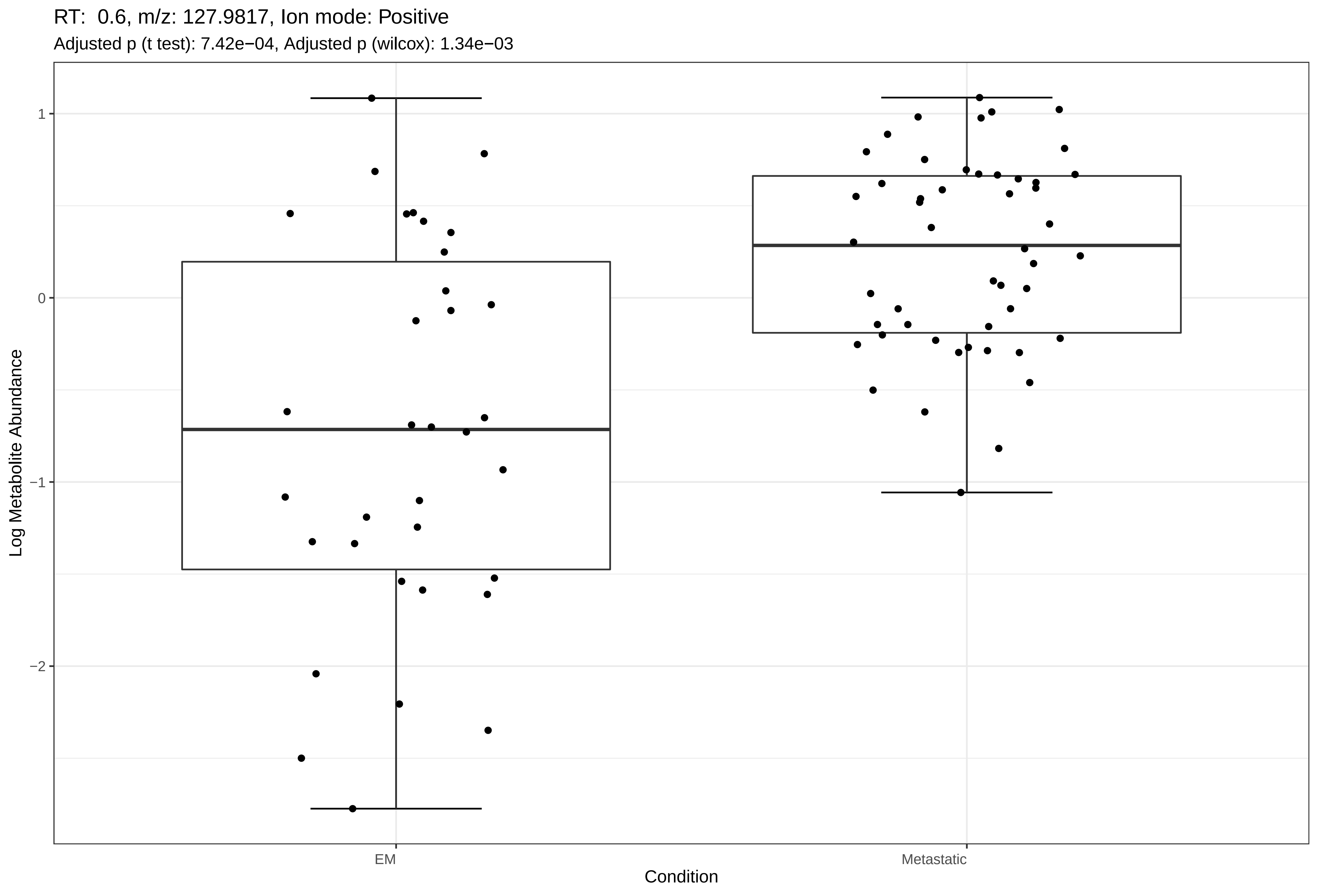

RT: 0.6, m/z: 127.9817, Ion mode: Positive
Adjusted p (t test): 7.42e−04, Adjusted p (wilcox): 1.34e−03
Log Metabolite Abundance
1
0
−1
−2
EM
Metastatic
Condition

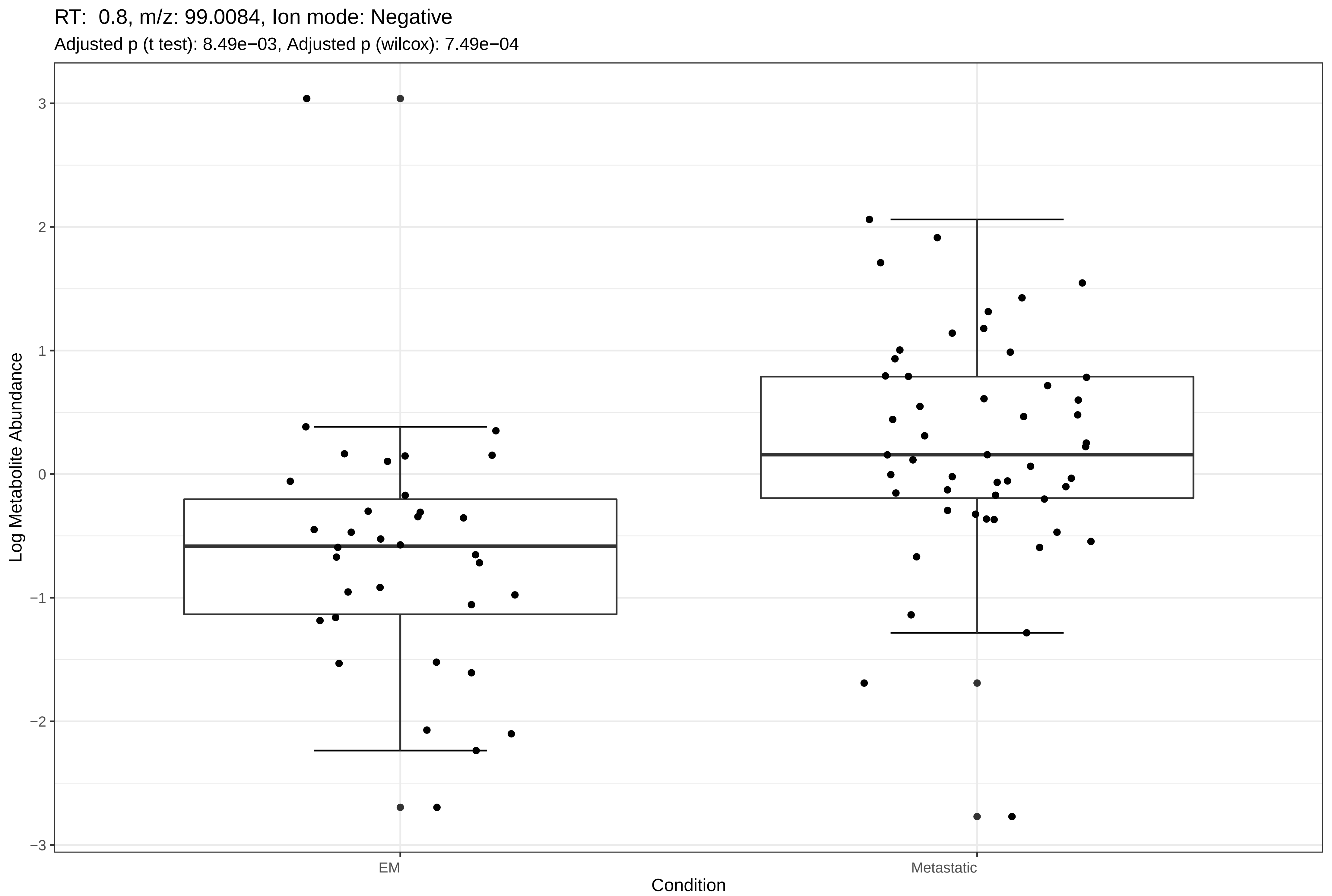
RT: 0.8, m/z: 99.0084, Ion mode: Negative
Adjusted p (t test): 8.49e−03, Adjusted p (wilcox): 7.49e−04
Log Metabolite Abundance
3
2
1
0
−1
−2
−3
EM
Metastatic
Condition

RT: 1.2, m/z: 106.0232, Ion mode: Positive

Adjusted p (t test): 1.46e−03, Adjusted p (wilcox): 7.49e−04

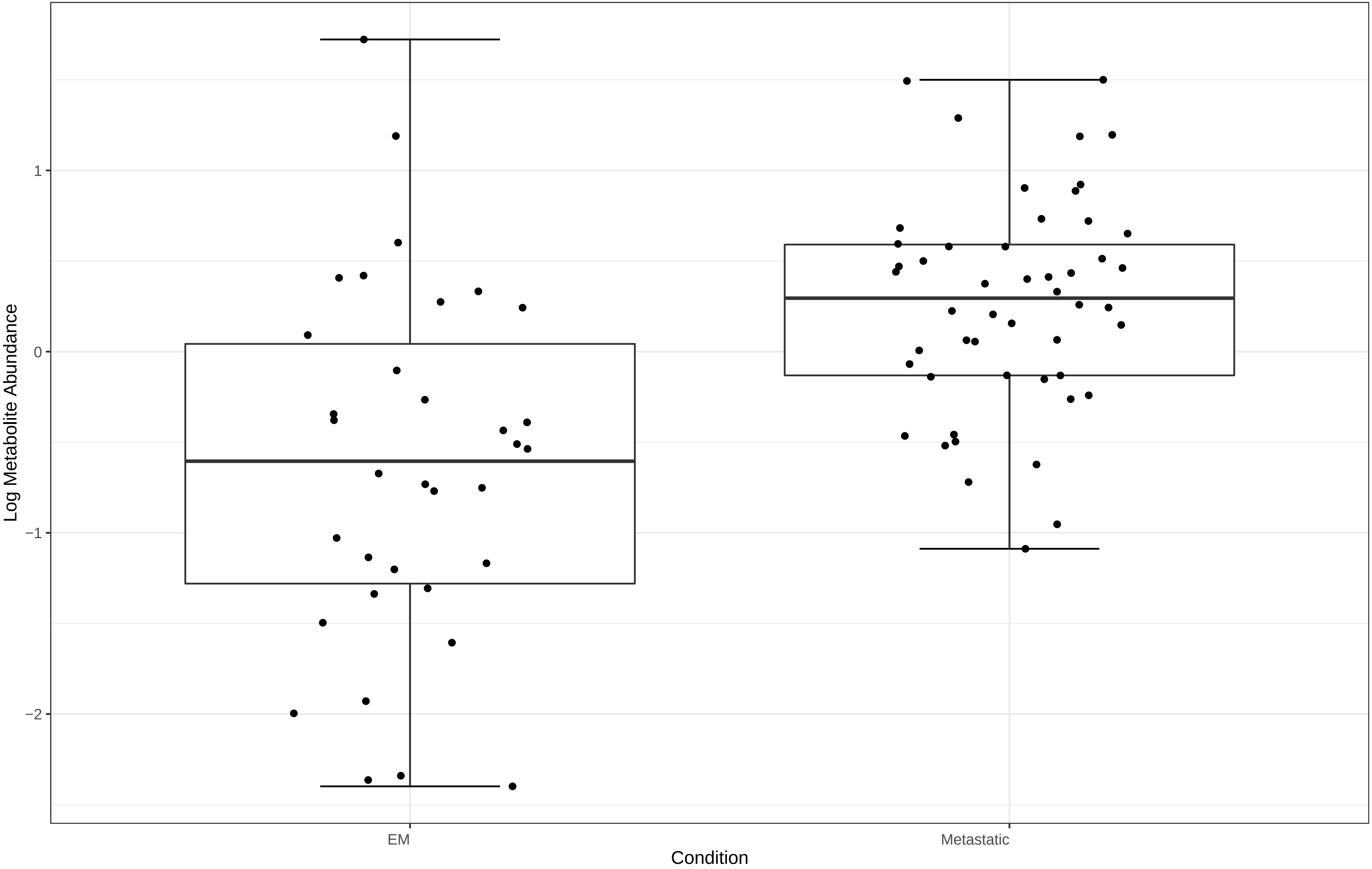

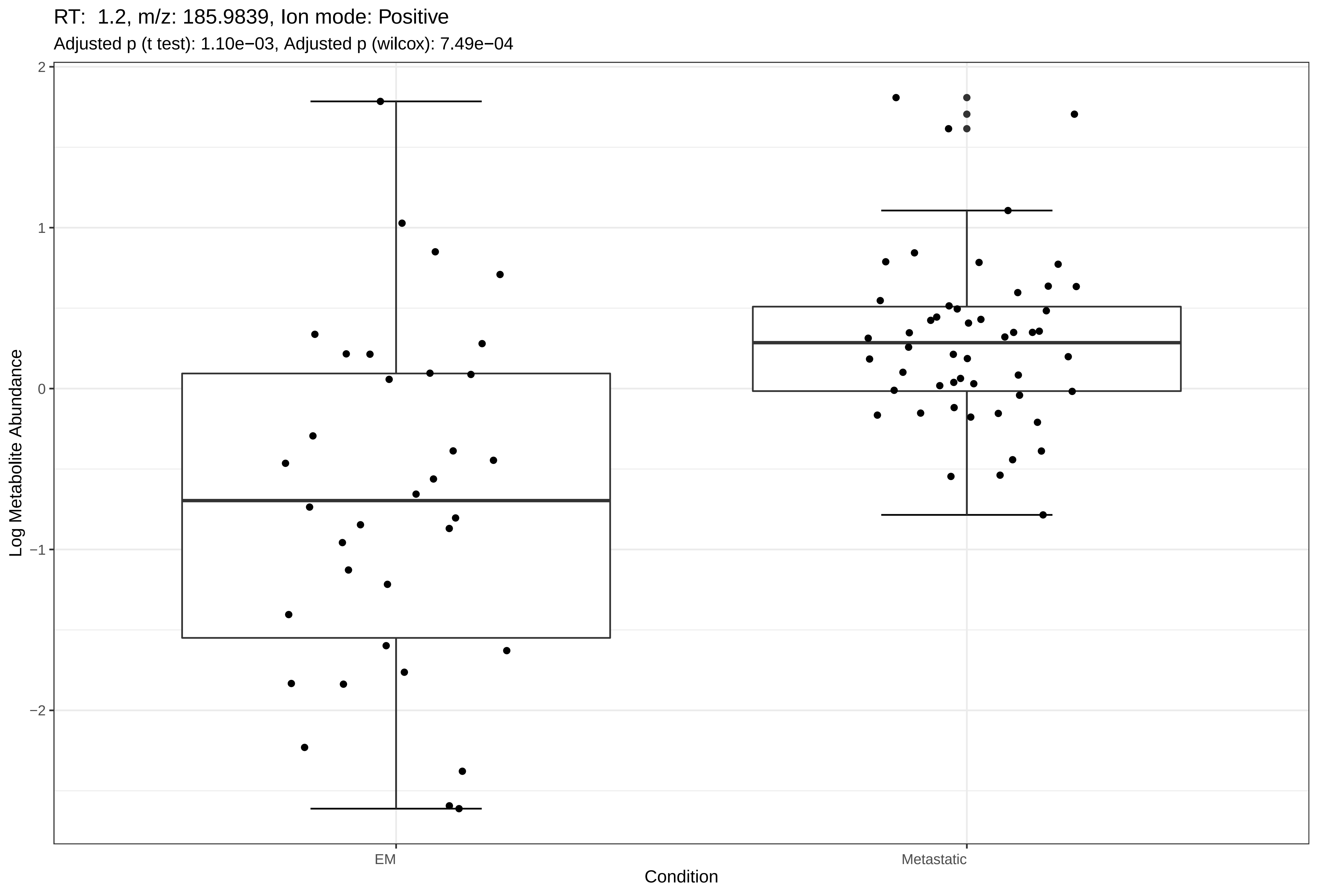
RT: 1.2, m/z: 185.9839, Ion mode: Positive
Adjusted p (t test): 1.10e−03, Adjusted p (wilcox): 7.49e−04
Log Metabolite Abundance
2
1
0
−1
−2
EM
Metastatic
Condition

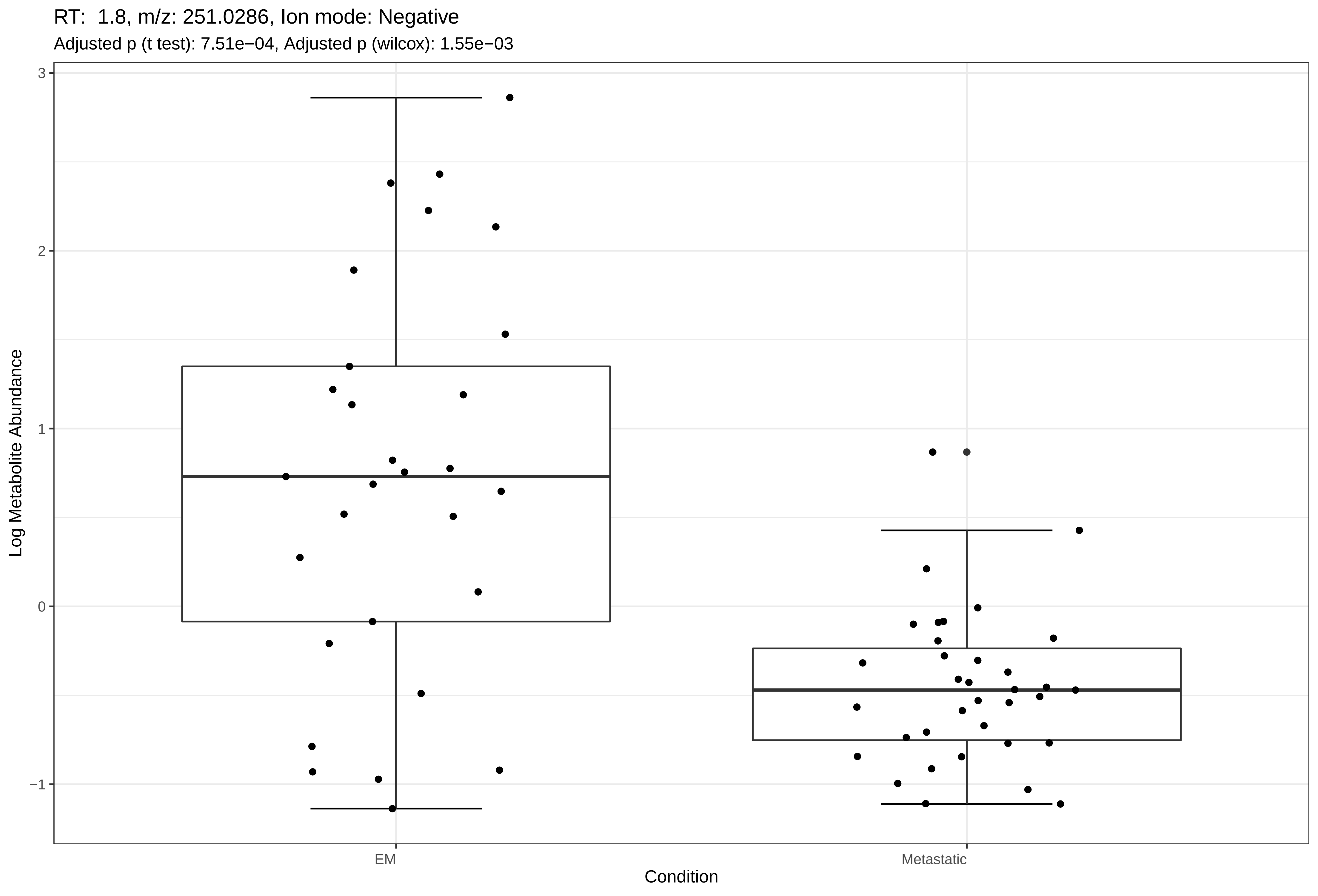
RT: 1.8, m/z: 251.0286, Ion mode: Negative
Adjusted p (t test): 7.51e−04, Adjusted p (wilcox): 1.55e−03
Log Metabolite Abundance
3
2
1
0
−1
EM
Metastatic
Condition

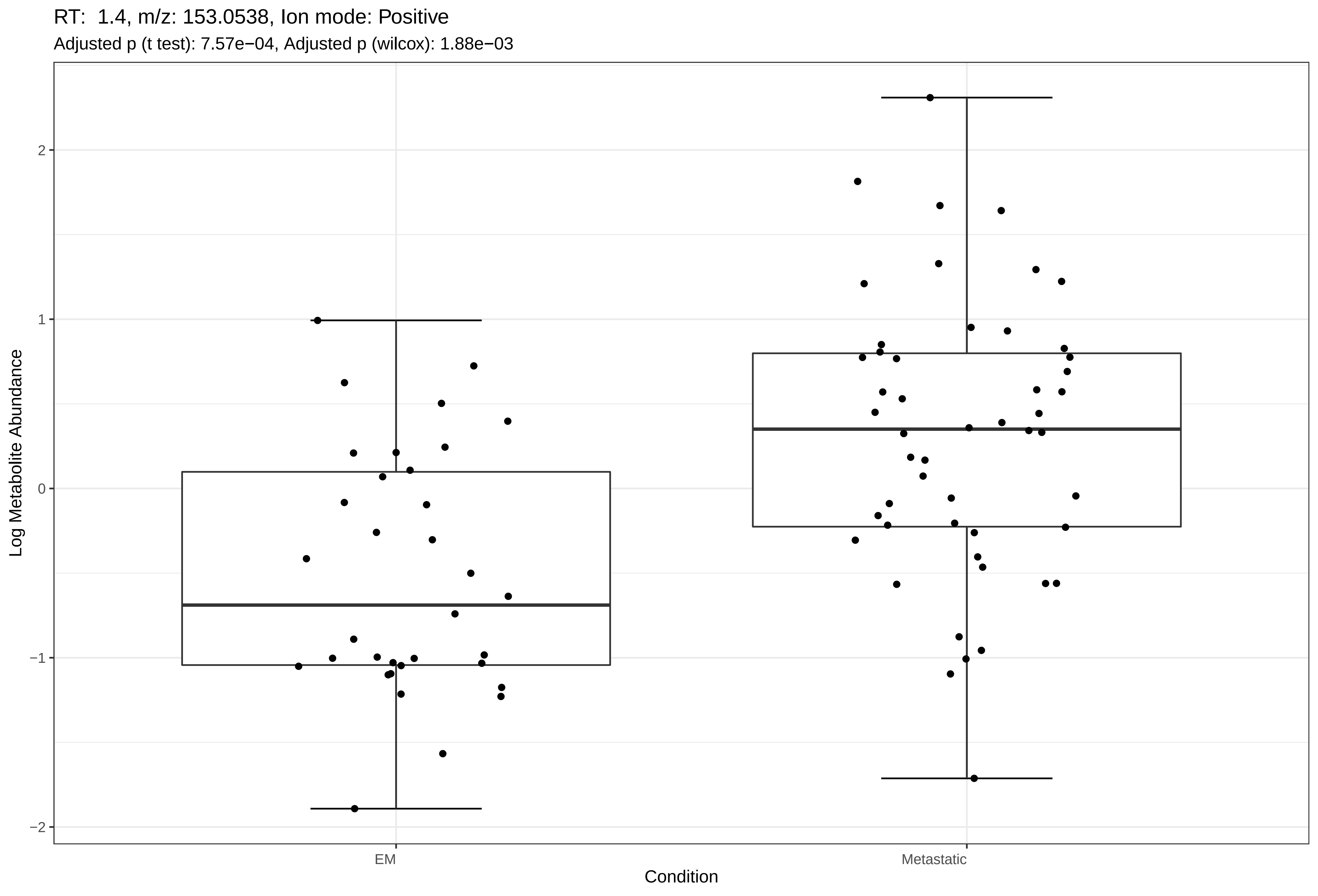

RT: 1.4, m/z: 153.0538, Ion mode: Positive
Adjusted p (t test): 7.57e−04, Adjusted p (wilcox): 1.88e−03
Log Metabolite Abundance
2
1
0
−1
−2
EM
Metastatic
Condition

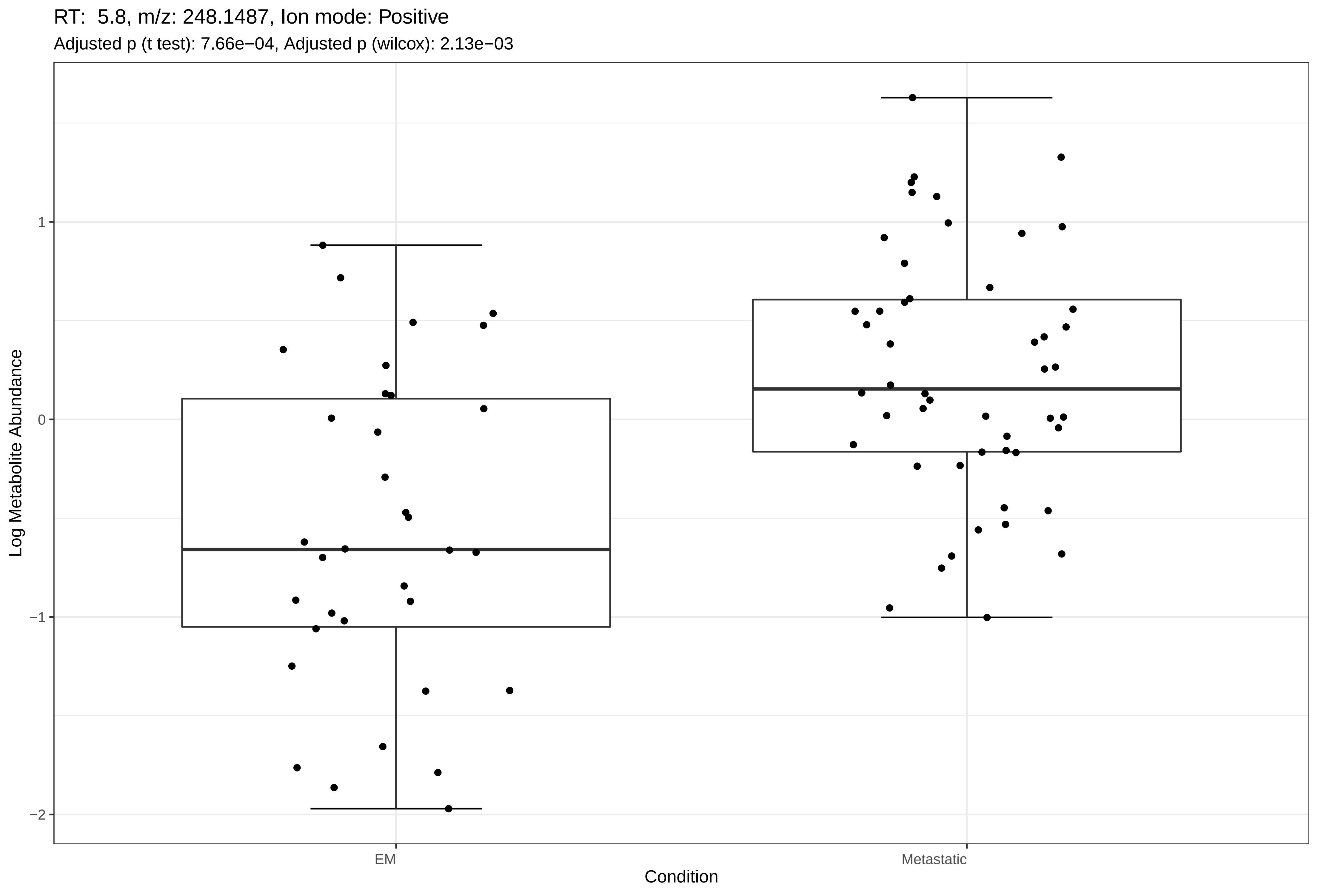
RT: 5.8, m/z: 248.1487, Ion mode: Positive
Adjusted p (t test): 7.66e−04, Adjusted p (wilcox): 2.13e−03
Log Metabolite Abundance
1
0
−1
−2
EM
Metastatic
Condition

RT: 1.2, m/z: 105.0255, Ion mode: Positive

Adjusted p (t test): 7.75e−04, Adjusted p (wilcox): 1.20e−03

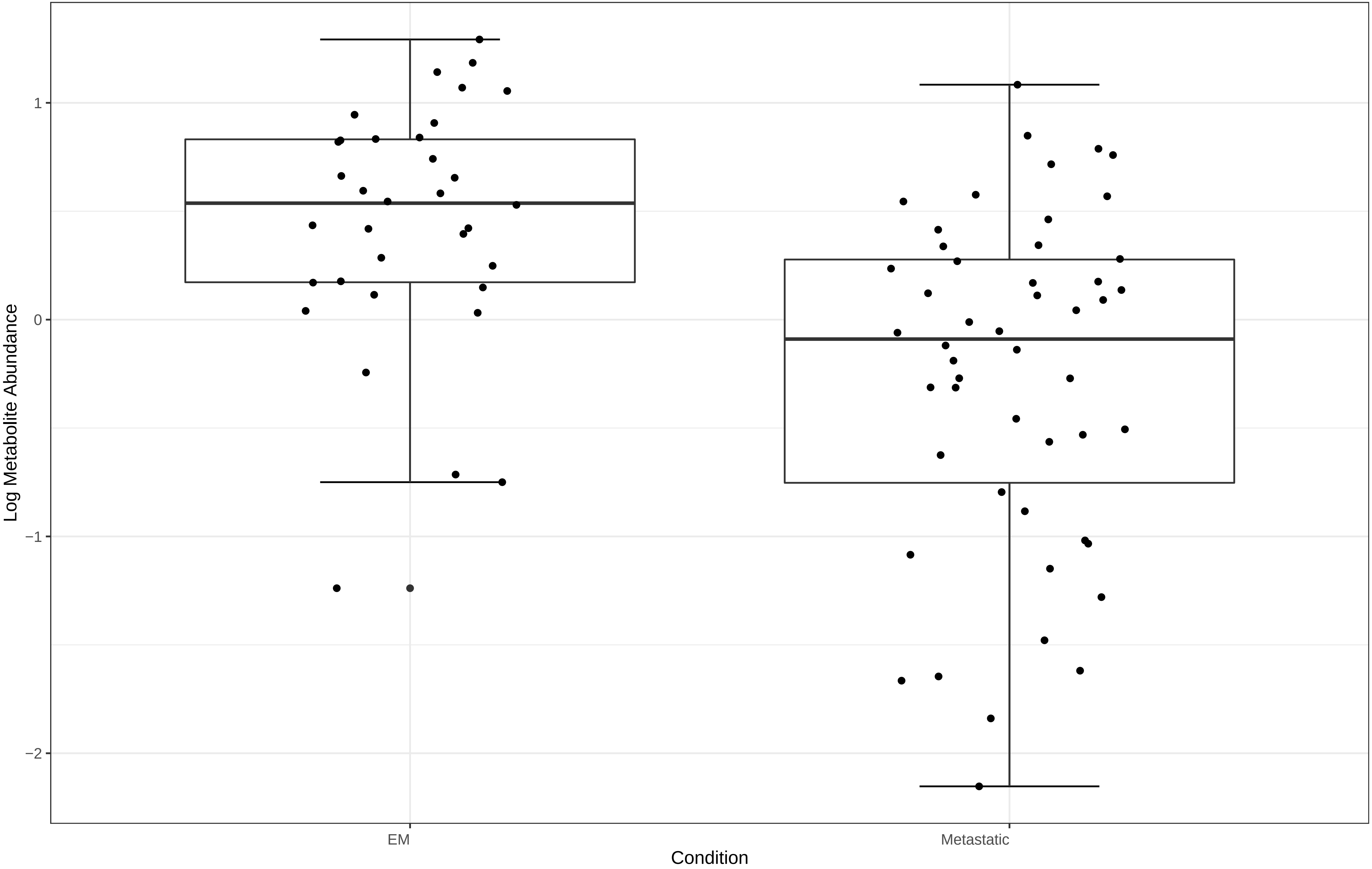

RT: 3.9, m/z: 338.0665, Ion mode: Negative

Adjusted p (t test): 1.91e−03, Adjusted p (wilcox): 7.99e−04

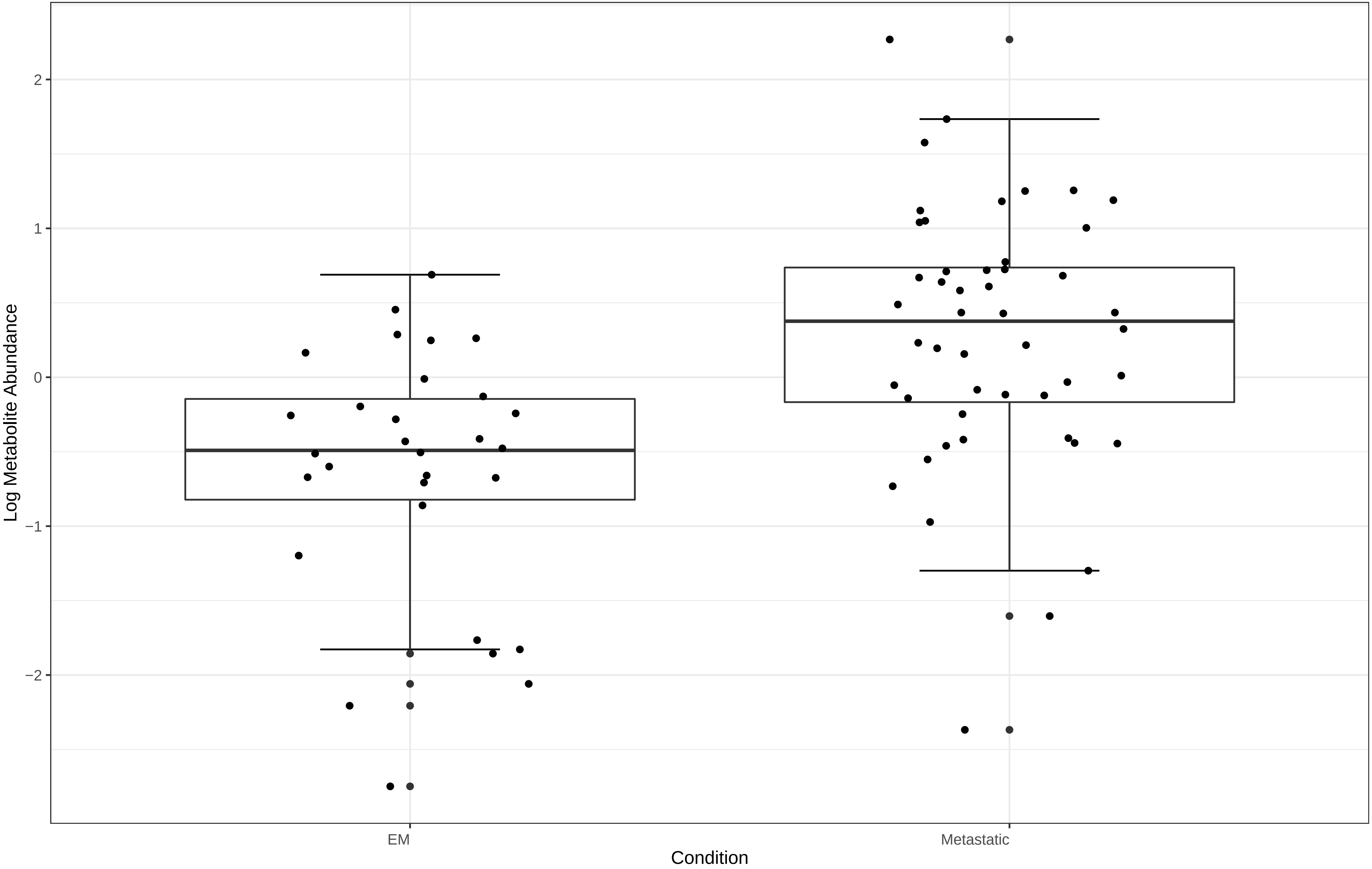

RT: 10.6, m/z: 248.9604, Ion mode: Negative

Adjusted p (t test): 8.02e−04, Adjusted p (wilcox): 1.75e−03

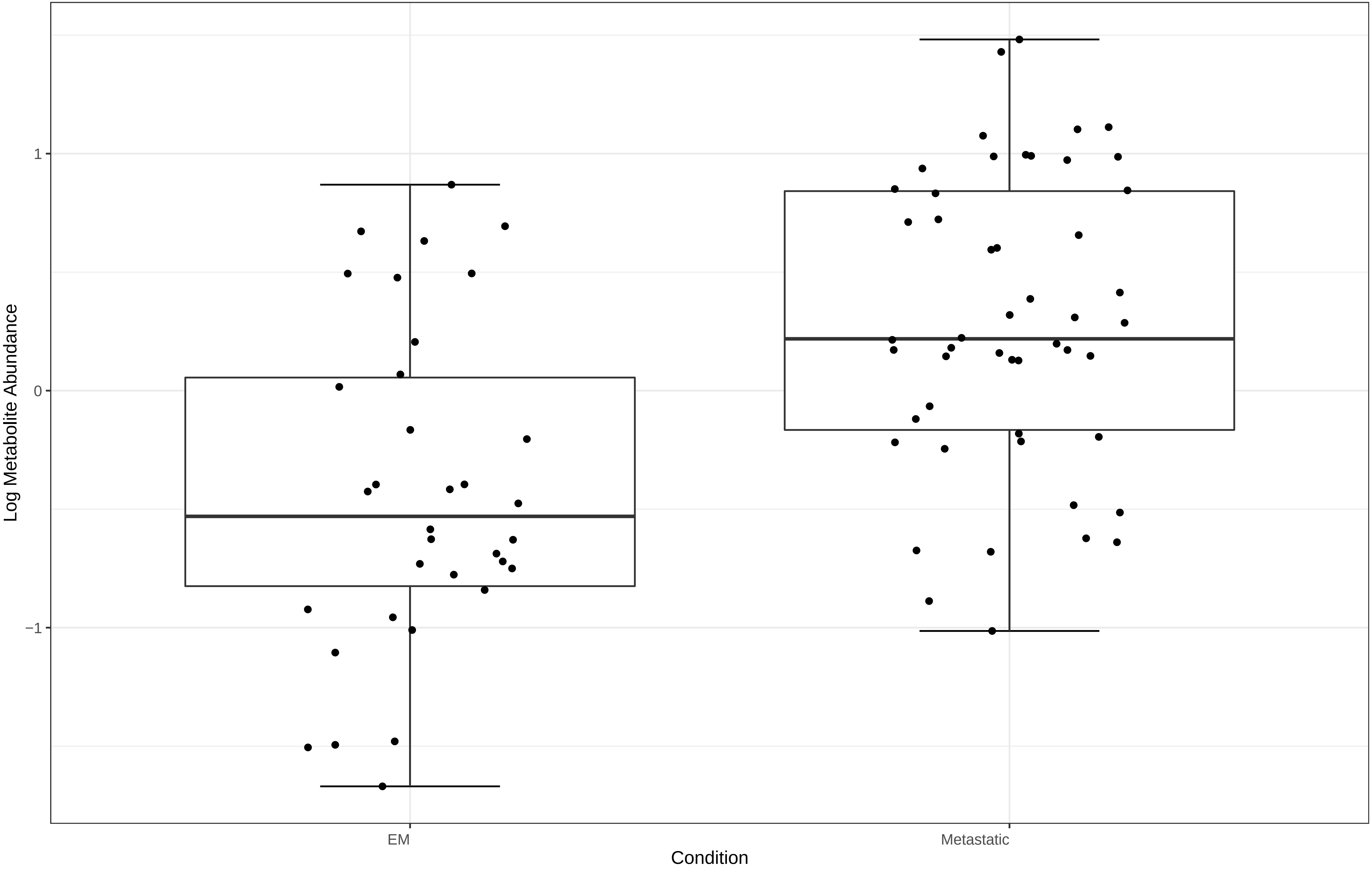

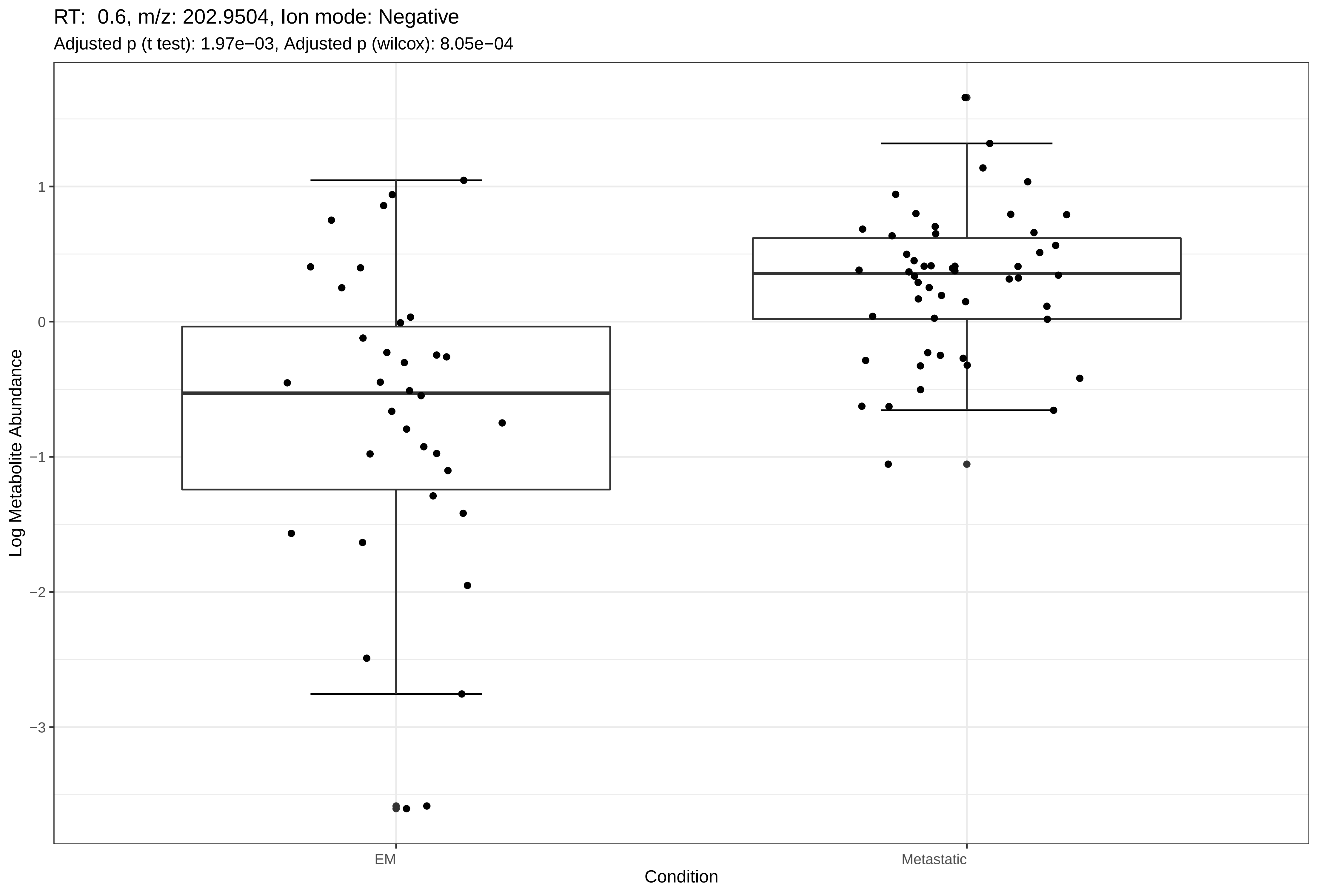
RT: 0.6, m/z: 202.9504, Ion mode: Negative
Adjusted p (t test): 1.97e−03, Adjusted p (wilcox): 8.05e−04
Log Metabolite Abundance
1
0
−1
−2
−3
EM
Metastatic
Condition

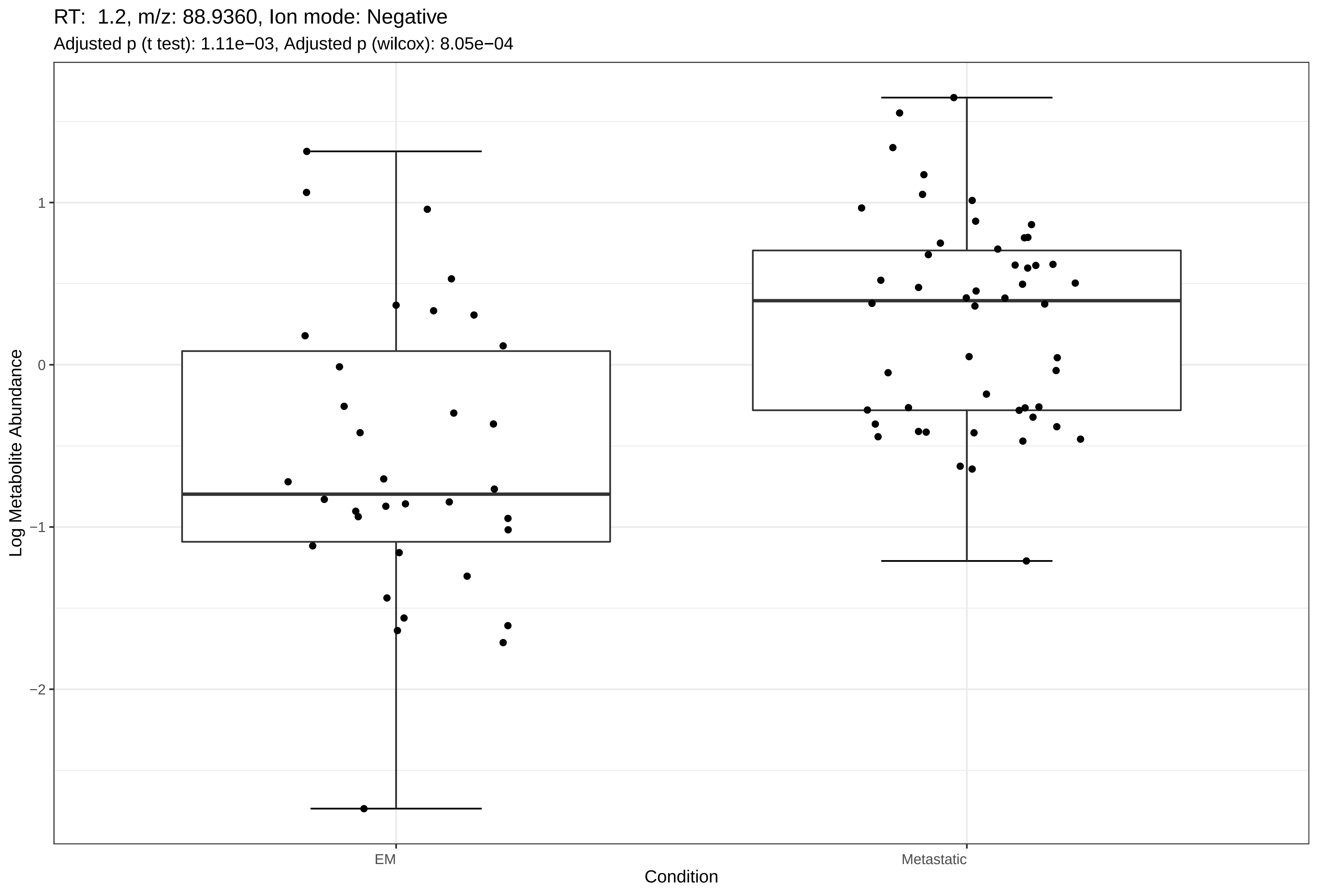
RT: 1.2, m/z: 88.9360, Ion mode: Negative
Adjusted p (t test): 1.11e−03, Adjusted p (wilcox): 8.05e−04
Log Metabolite Abundance
1
0
−1
−2
EM
Metastatic
Condition

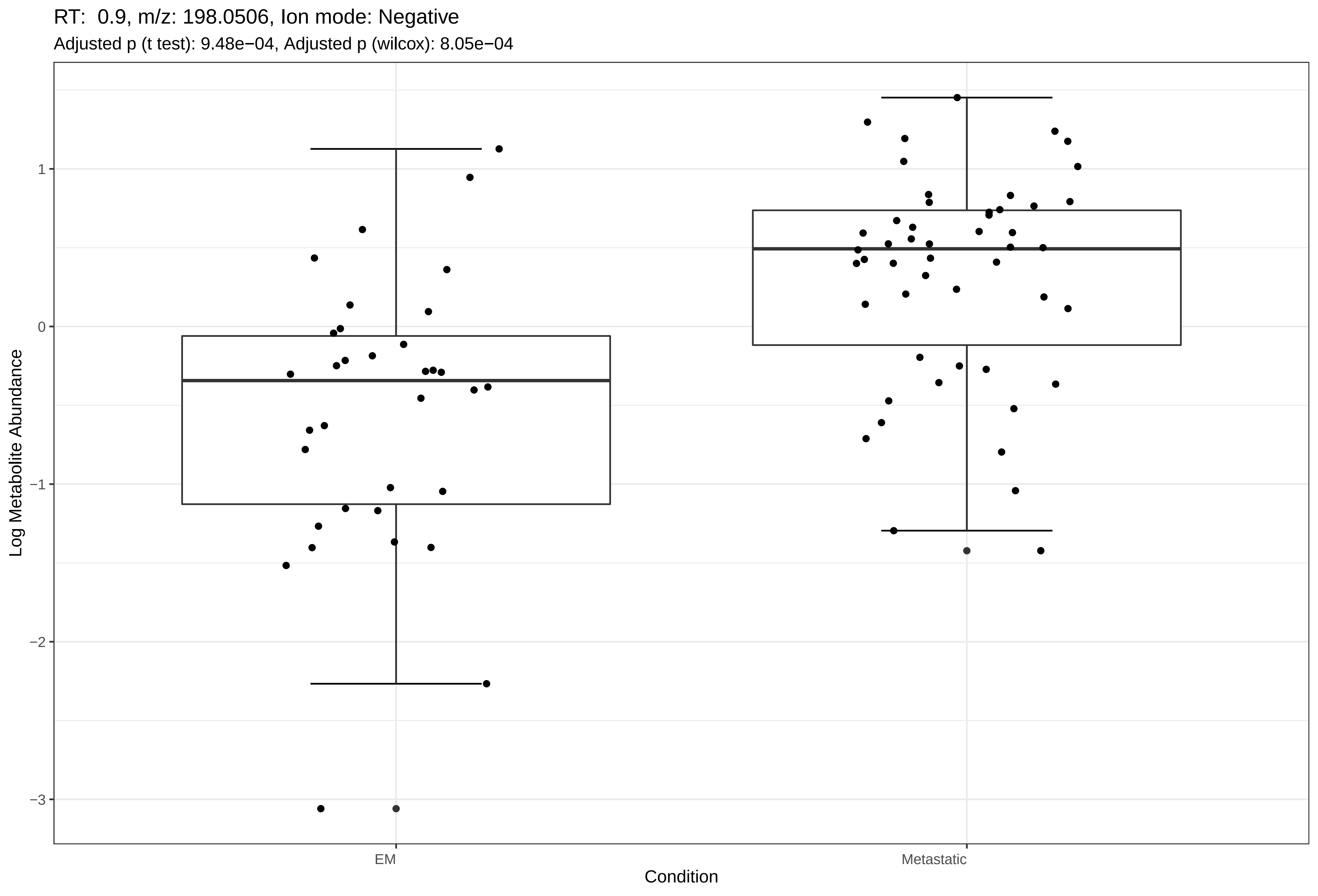

RT: 0.9, m/z: 198.0506, Ion mode: Negative
Adjusted p (t test): 9.48e−04, Adjusted p (wilcox): 8.05e−04
Log Metabolite Abundance
1
0
−1
−2
−3
EM
Metastatic
Condition

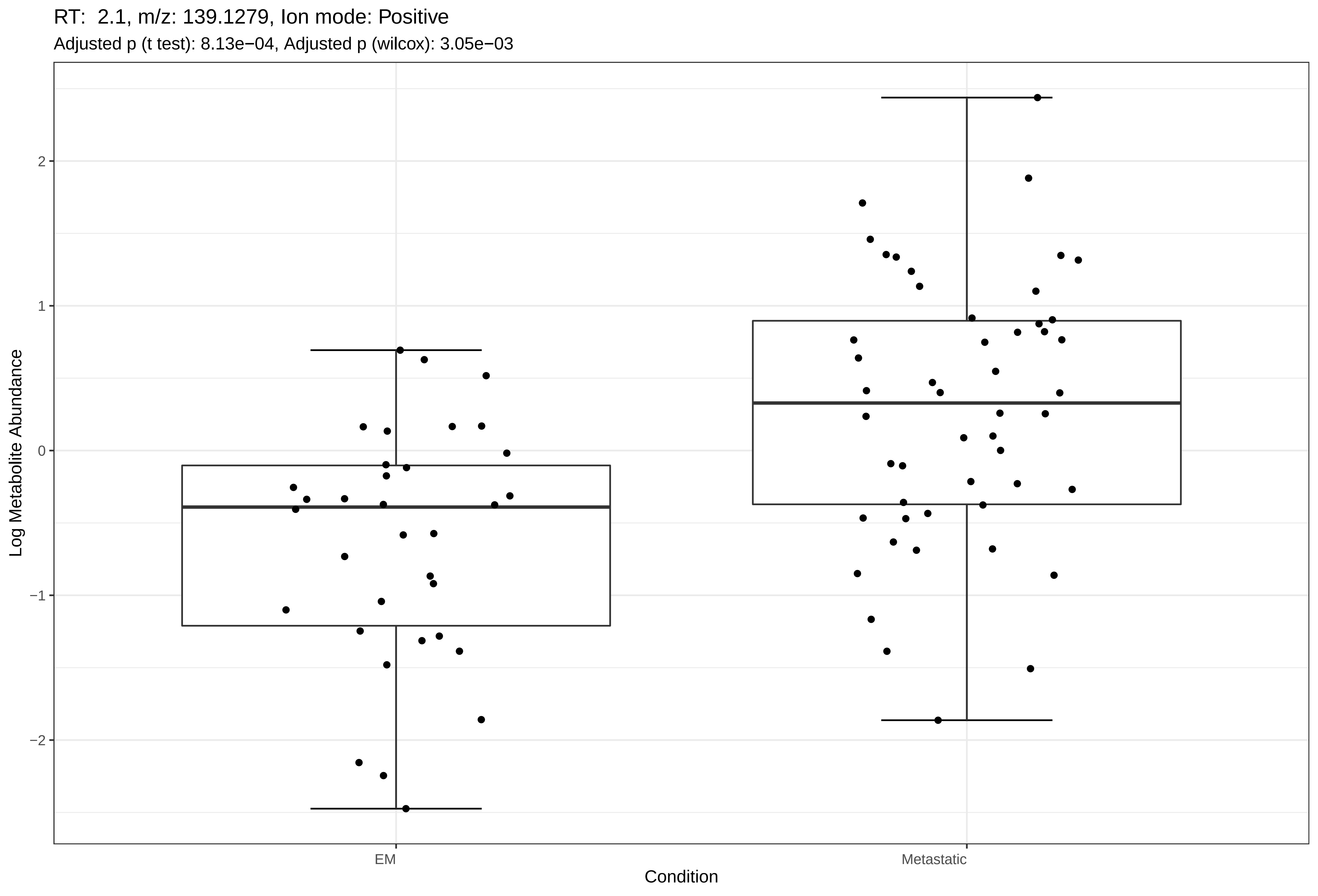

RT: 2.1, m/z: 139.1279, Ion mode: Positive
Adjusted p (t test): 8.13e−04, Adjusted p (wilcox): 3.05e−03
Log Metabolite Abundance
2
1
0
−1
−2
EM
Metastatic
Condition

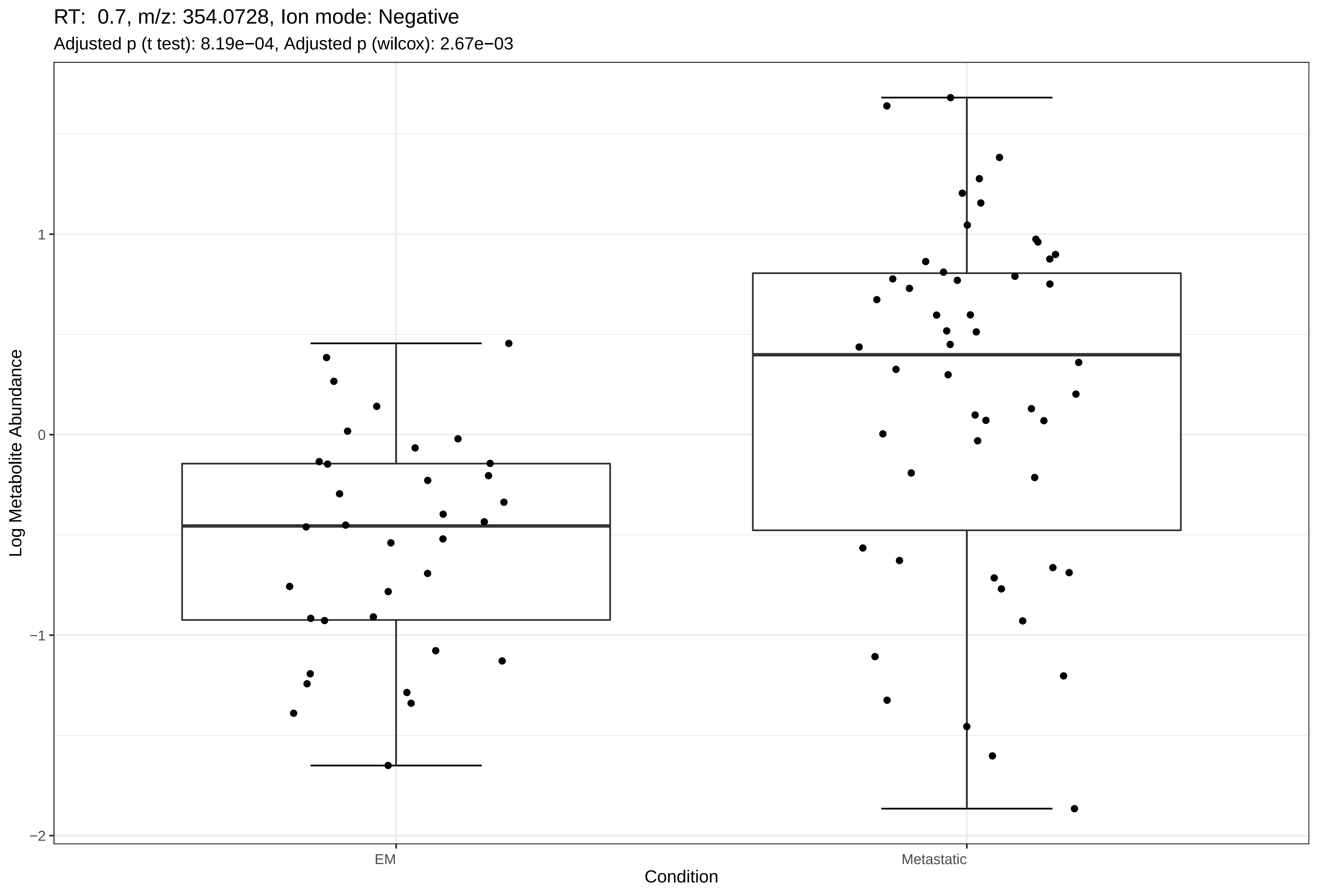

RT: 0.7, m/z: 354.0728, Ion mode: Negative
Adjusted p (t test): 8.19e−04, Adjusted p (wilcox): 2.67e−03
Log Metabolite Abundance
1
0
−1
−2
EM
Metastatic
Condition

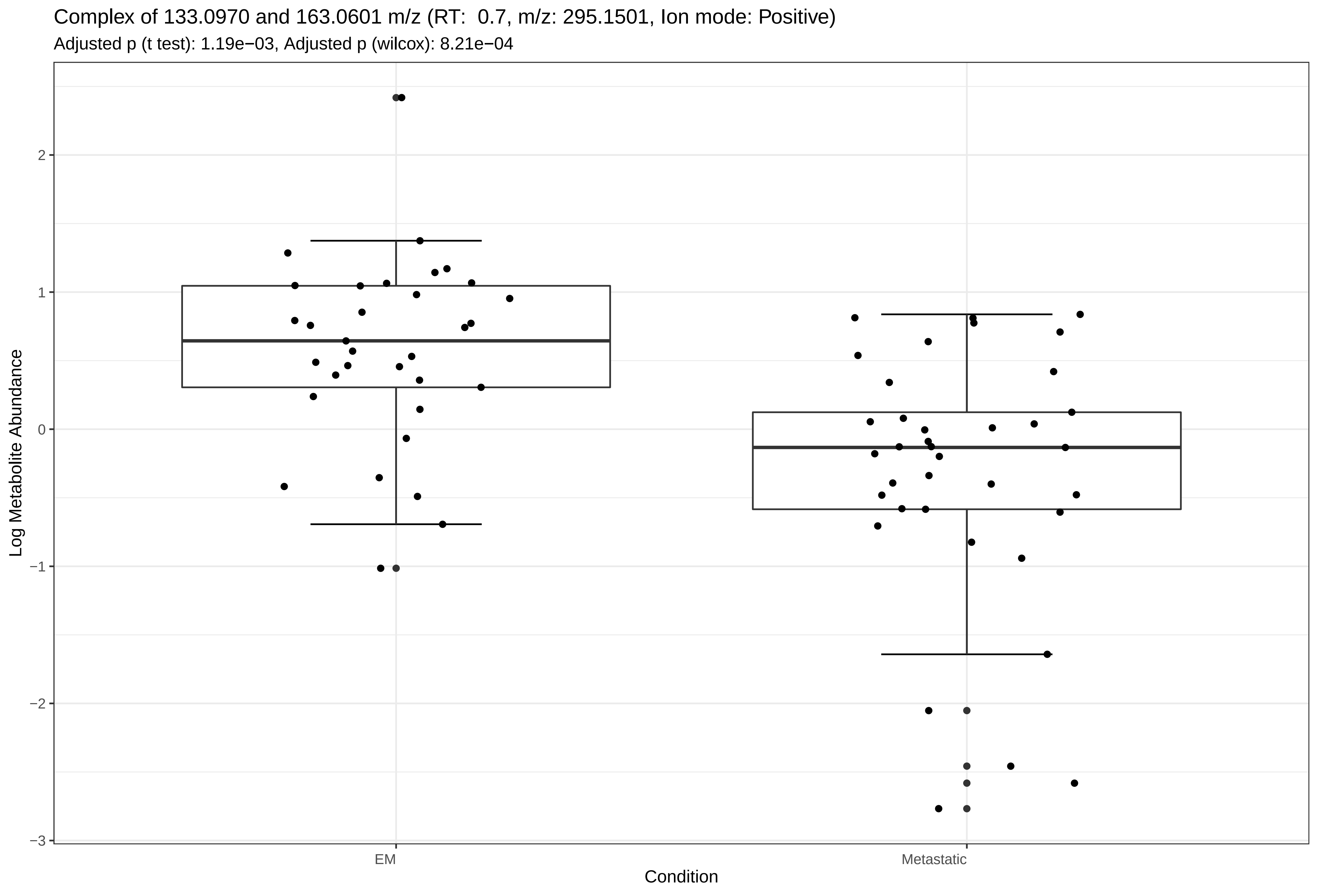

Complex of 133.0970 and 163.0601 m/z (RT: 0.7, m/z: 295.1501, Ion mode: Positive)
Adjusted p (t test): 1.19e−03, Adjusted p (wilcox): 8.21e−04
Log Metabolite Abundance
2
1
0
−1
−2
−3
EM
Metastatic
Condition

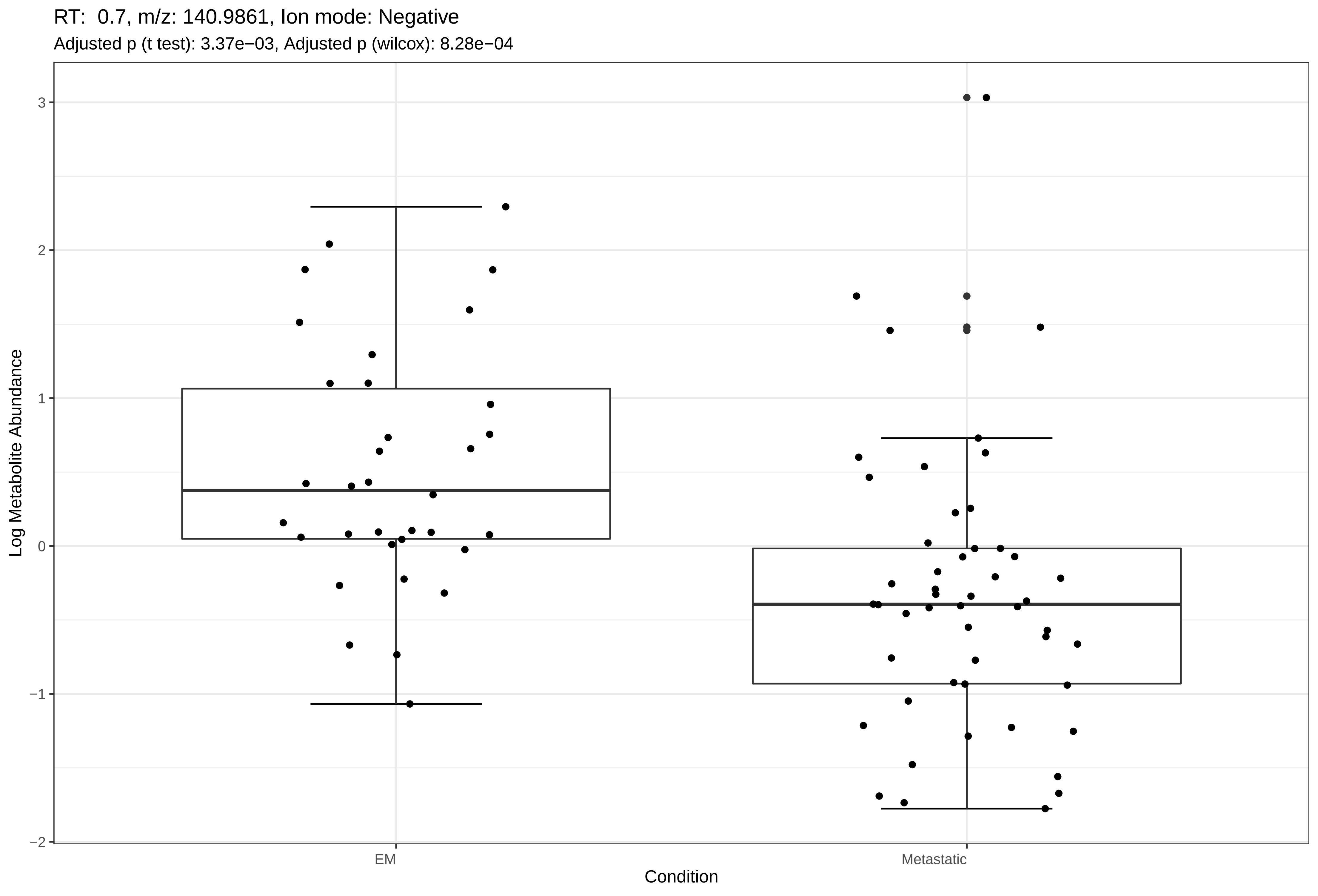
RT: 0.7, m/z: 140.9861, Ion mode: Negative
Adjusted p (t test): 3.37e−03, Adjusted p (wilcox): 8.28e−04
Log Metabolite Abundance
3
2
1
0
−1
−2
EM
Metastatic
Condition

RT: 11.6, m/z: 255.1605, Ion mode: Negative

Adjusted p (t test): 1.83e−03, Adjusted p (wilcox): 8.28e−04

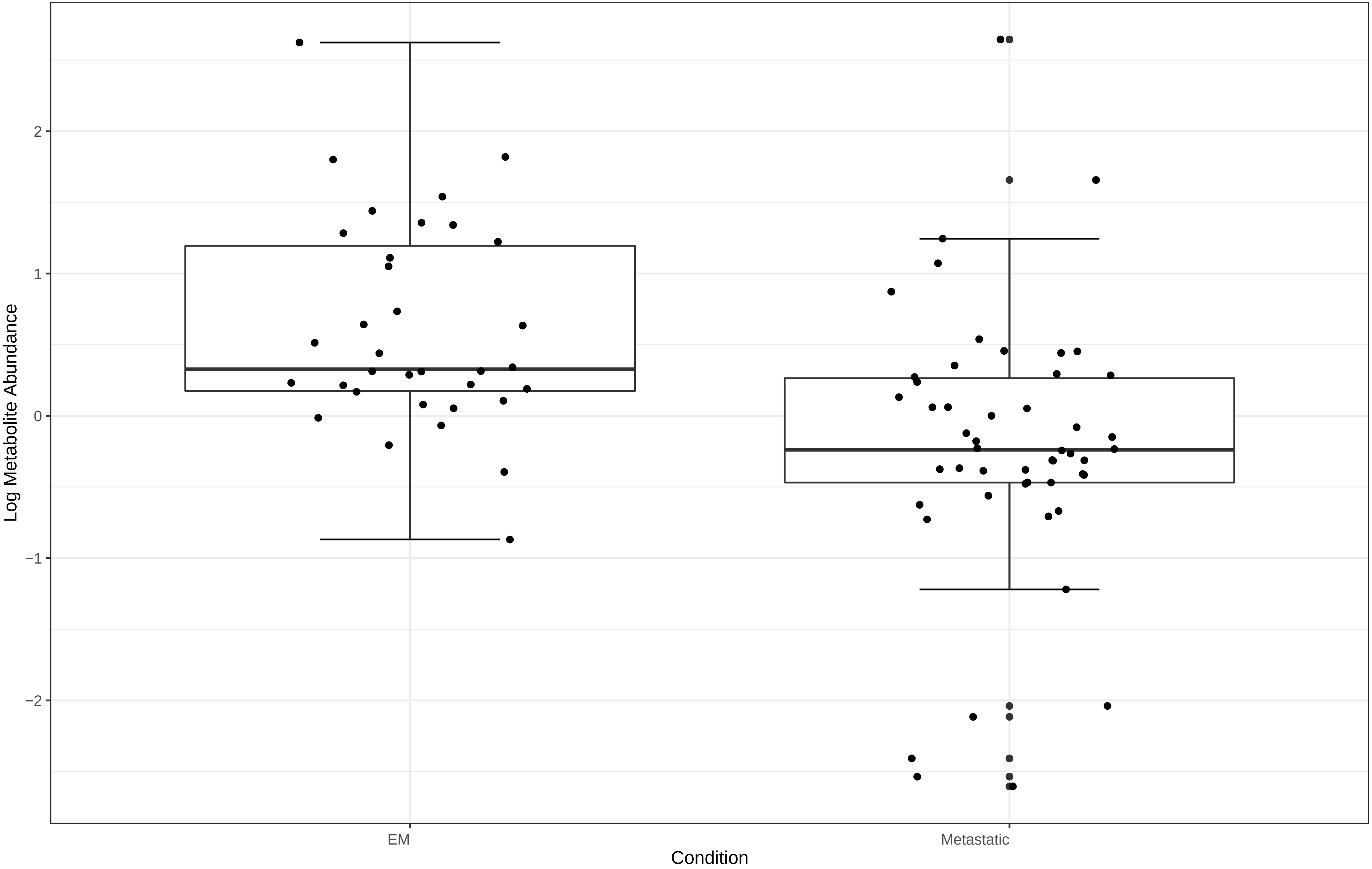

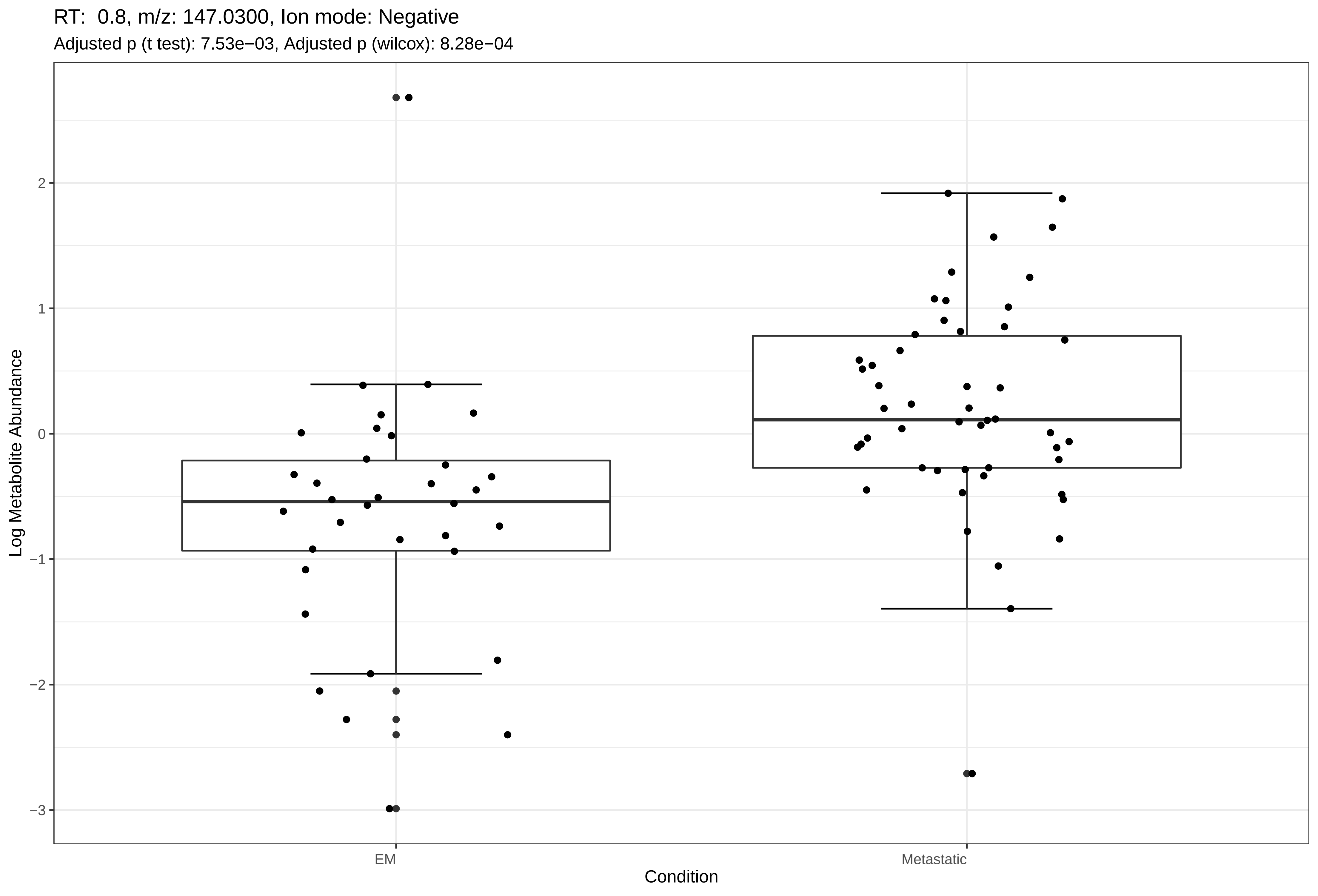
RT: 0.8, m/z: 147.0300, Ion mode: Negative
Adjusted p (t test): 7.53e−03, Adjusted p (wilcox): 8.28e−04
Log Metabolite Abundance
2
1
0
−1
−2
−3
EM
Metastatic
Condition

LysoPE (20:4) (RT: 12.8, m/z: 502.2915, Ion mode: Positive)

Adjusted p (t test): 9.48e−04, Adjusted p (wilcox): 8.28e−04

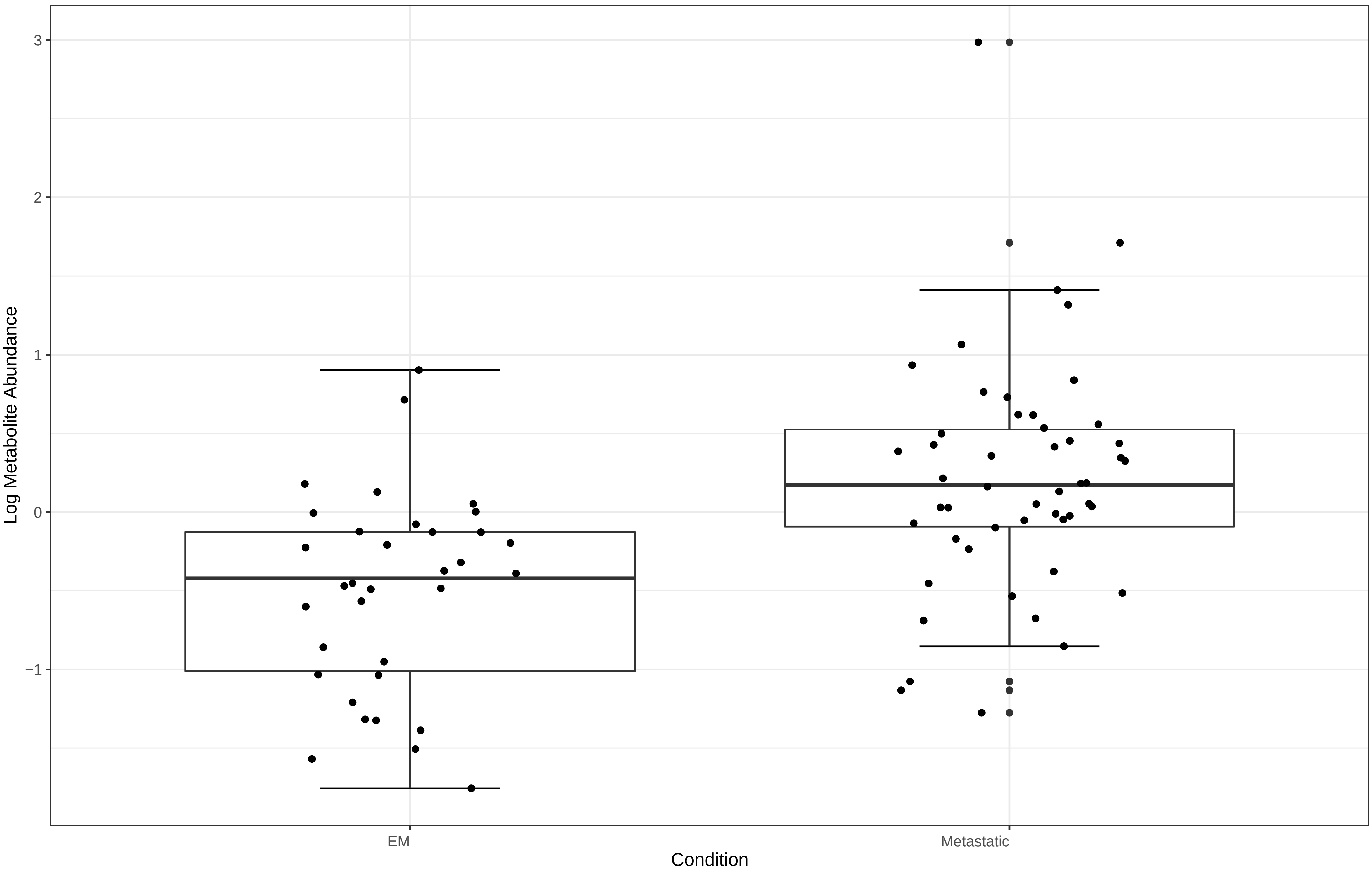

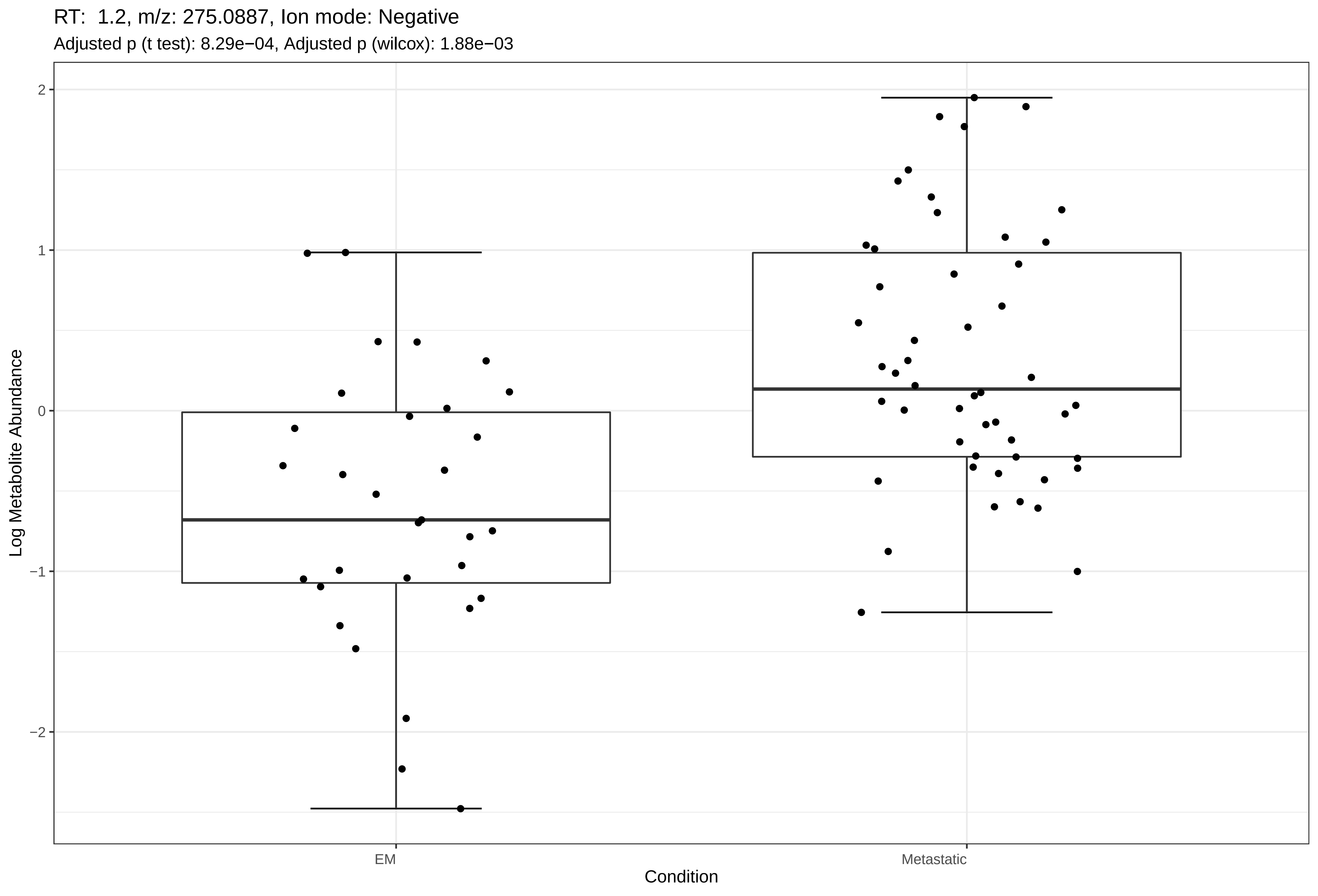
RT: 1.2, m/z: 275.0887, Ion mode: Negative
Adjusted p (t test): 8.29e−04, Adjusted p (wilcox): 1.88e−03
Log Metabolite Abundance
2
1
0
−1
−2
EM
Metastatic
Condition

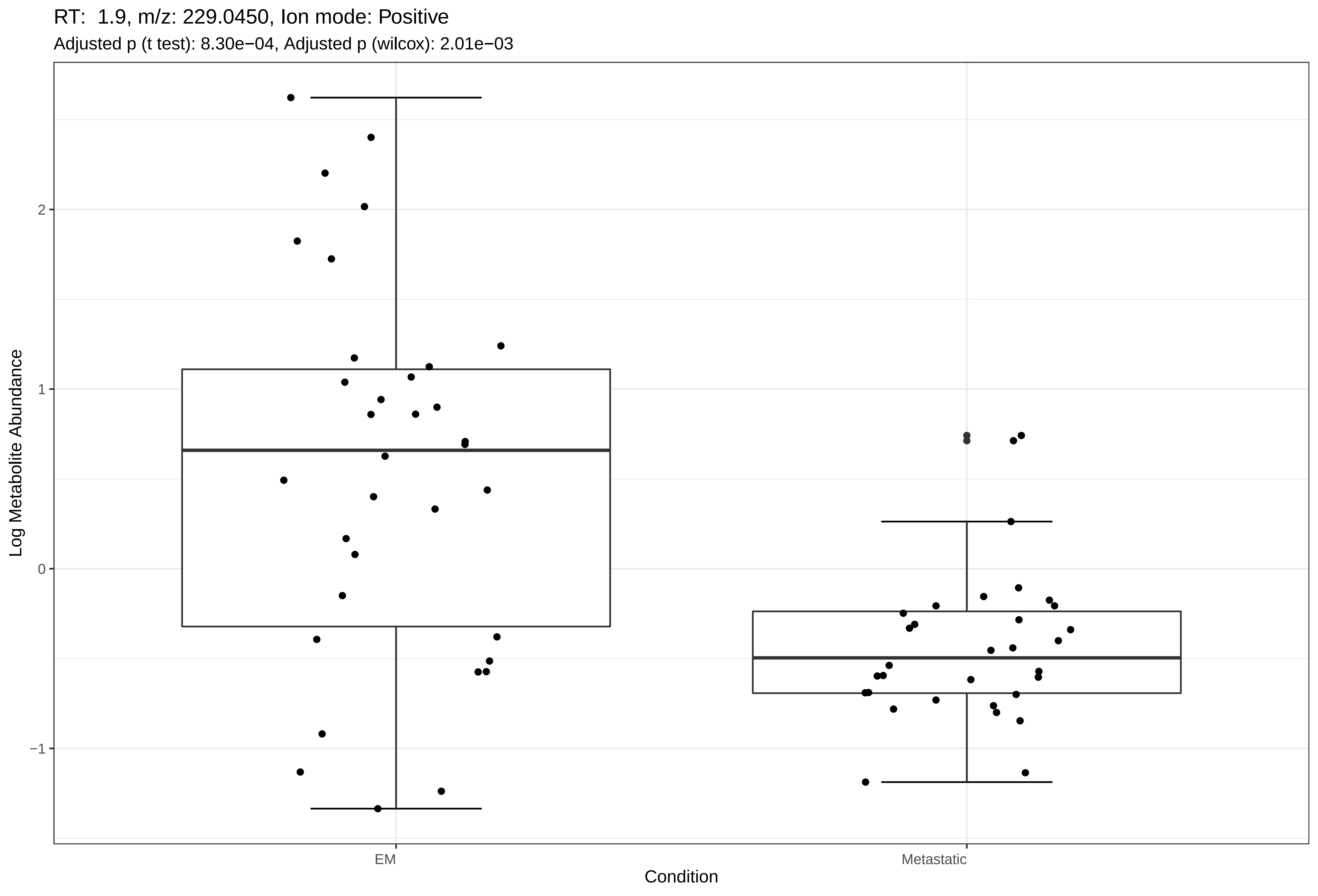

RT: 1.9, m/z: 229.0450, Ion mode: Positive
Adjusted p (t test): 8.30e−04, Adjusted p (wilcox): 2.01e−03
Log Metabolite Abundance
2
1
0
−1
EM
Metastatic
Condition

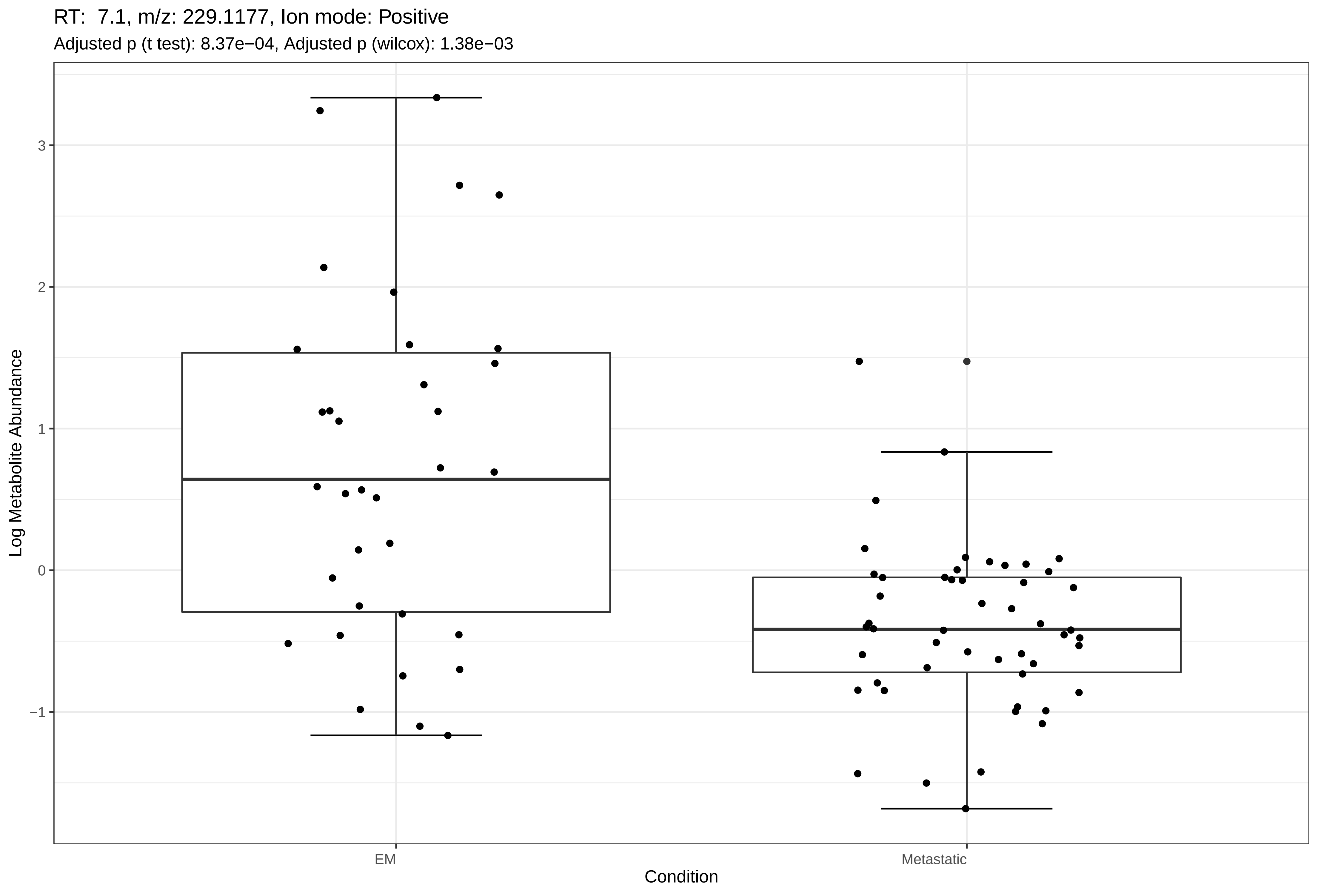

RT: 7.1, m/z: 229.1177, Ion mode: Positive
Adjusted p (t test): 8.37e−04, Adjusted p (wilcox): 1.38e−03
Log Metabolite Abundance
3
2
1
0
−1
EM
Metastatic
Condition

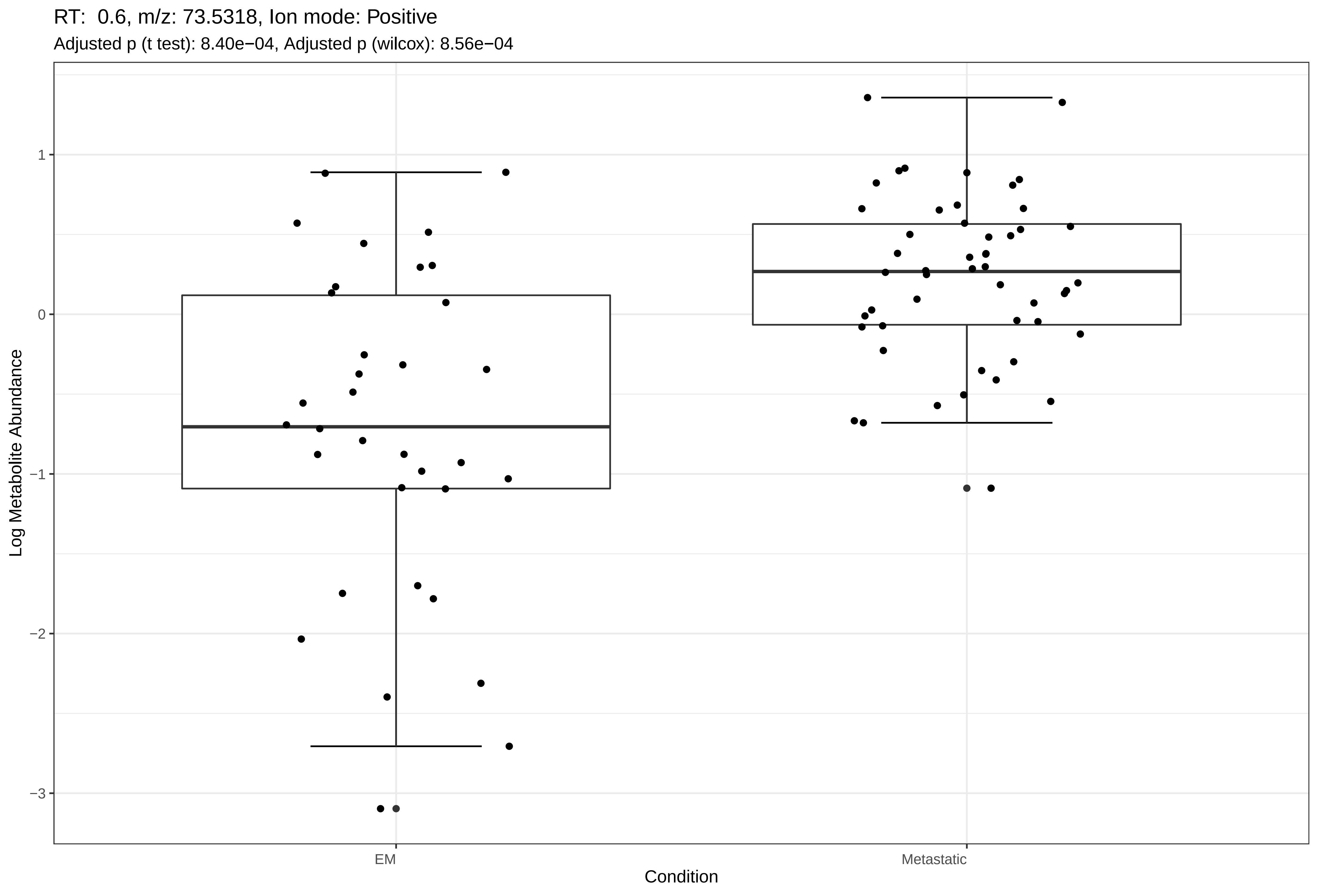

RT: 0.6, m/z: 73.5318, Ion mode: Positive
Adjusted p (t test): 8.40e−04, Adjusted p (wilcox): 8.56e−04
Log Metabolite Abundance
1
0
−1
−2
−3
EM
Metastatic
Condition

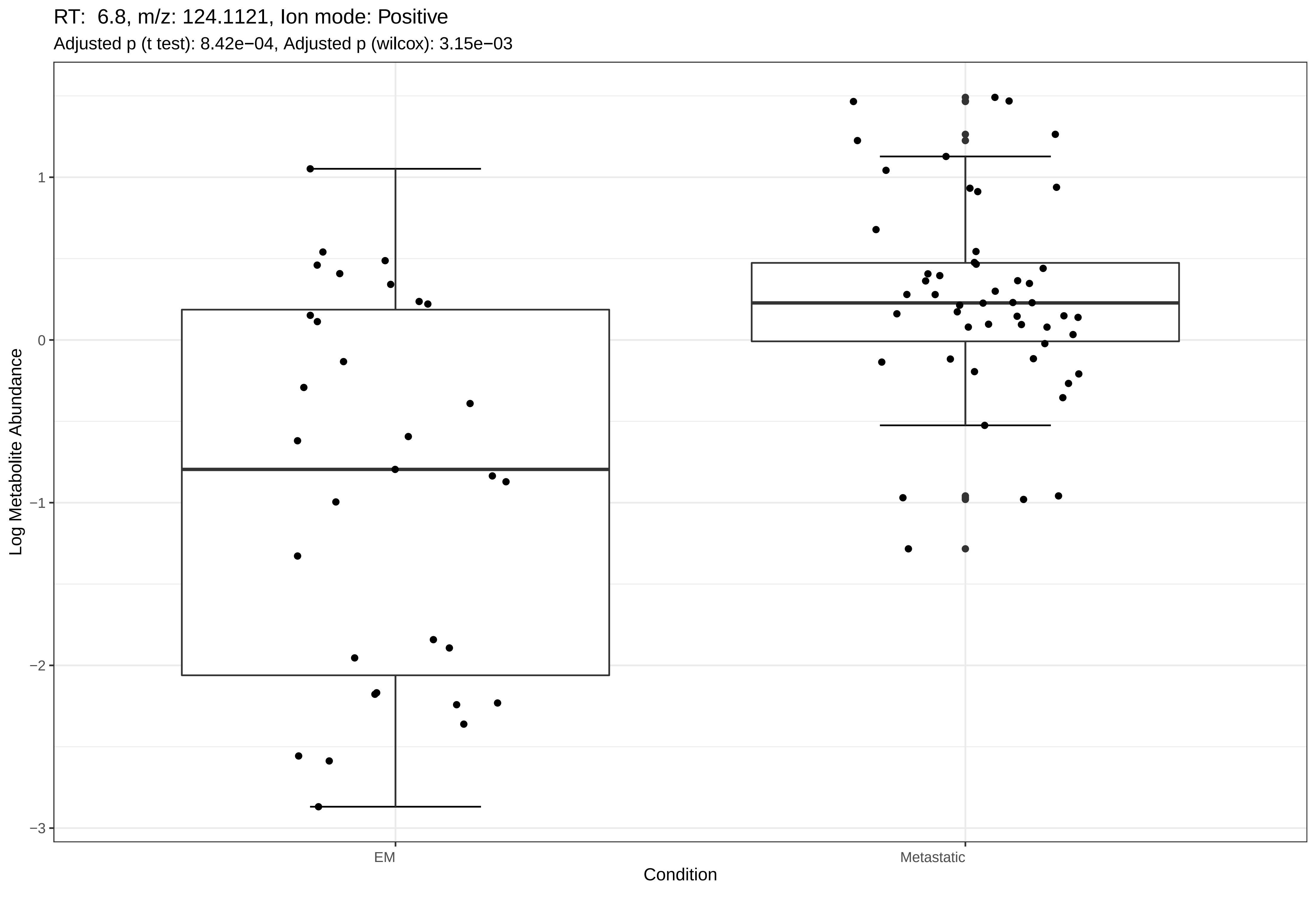

RT: 6.8, m/z: 124.1121, Ion mode: Positive
Adjusted p (t test): 8.42e−04, Adjusted p (wilcox): 3.15e−03
Log Metabolite Abundance
1
0
−1
−2
−3
EM
Metastatic
Condition

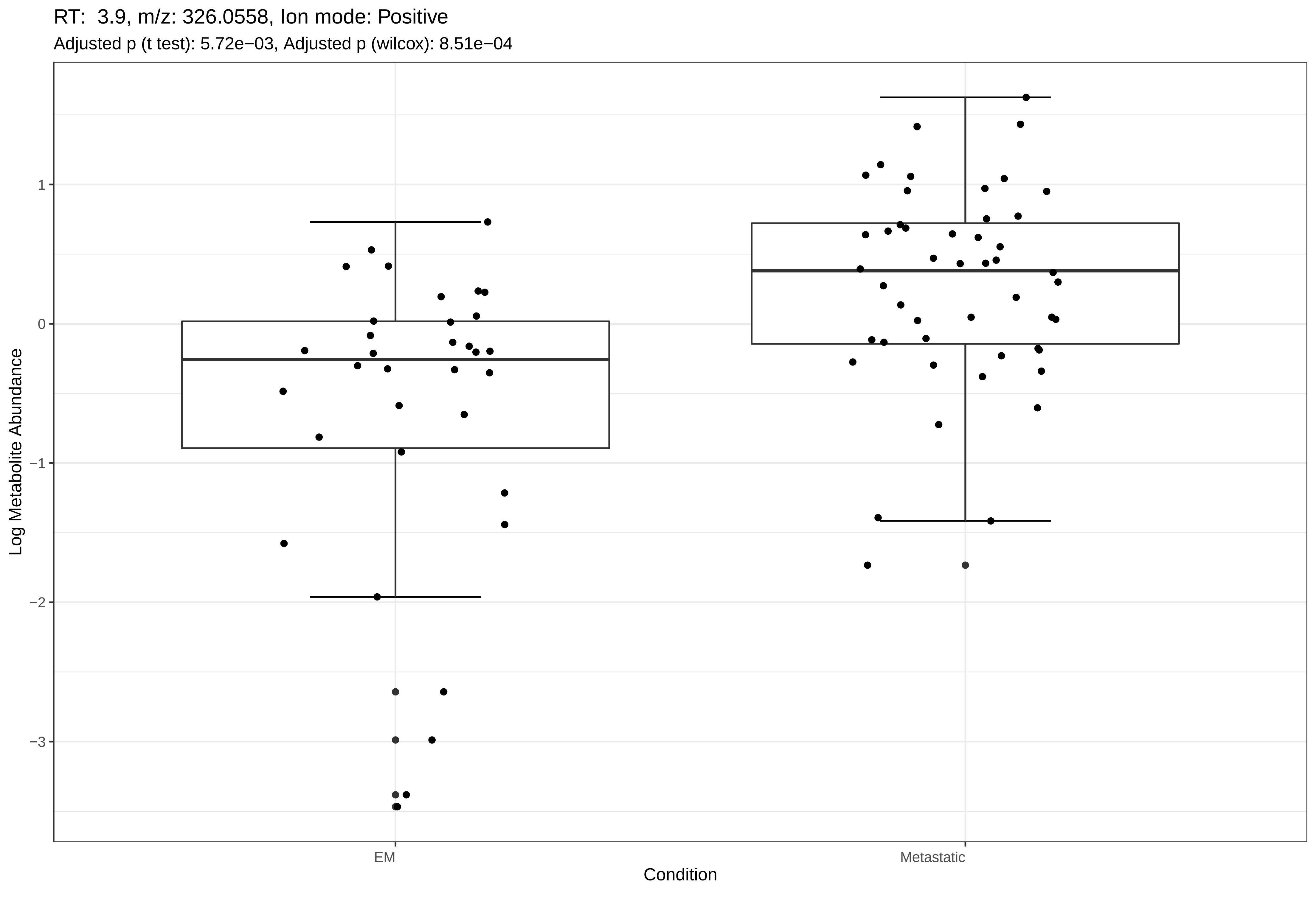
RT: 3.9, m/z: 326.0558, Ion mode: Positive
Adjusted p (t test): 5.72e−03, Adjusted p (wilcox): 8.51e−04
Log Metabolite Abundance
1
0
−1
−2
−3
EM
Metastatic
Condition

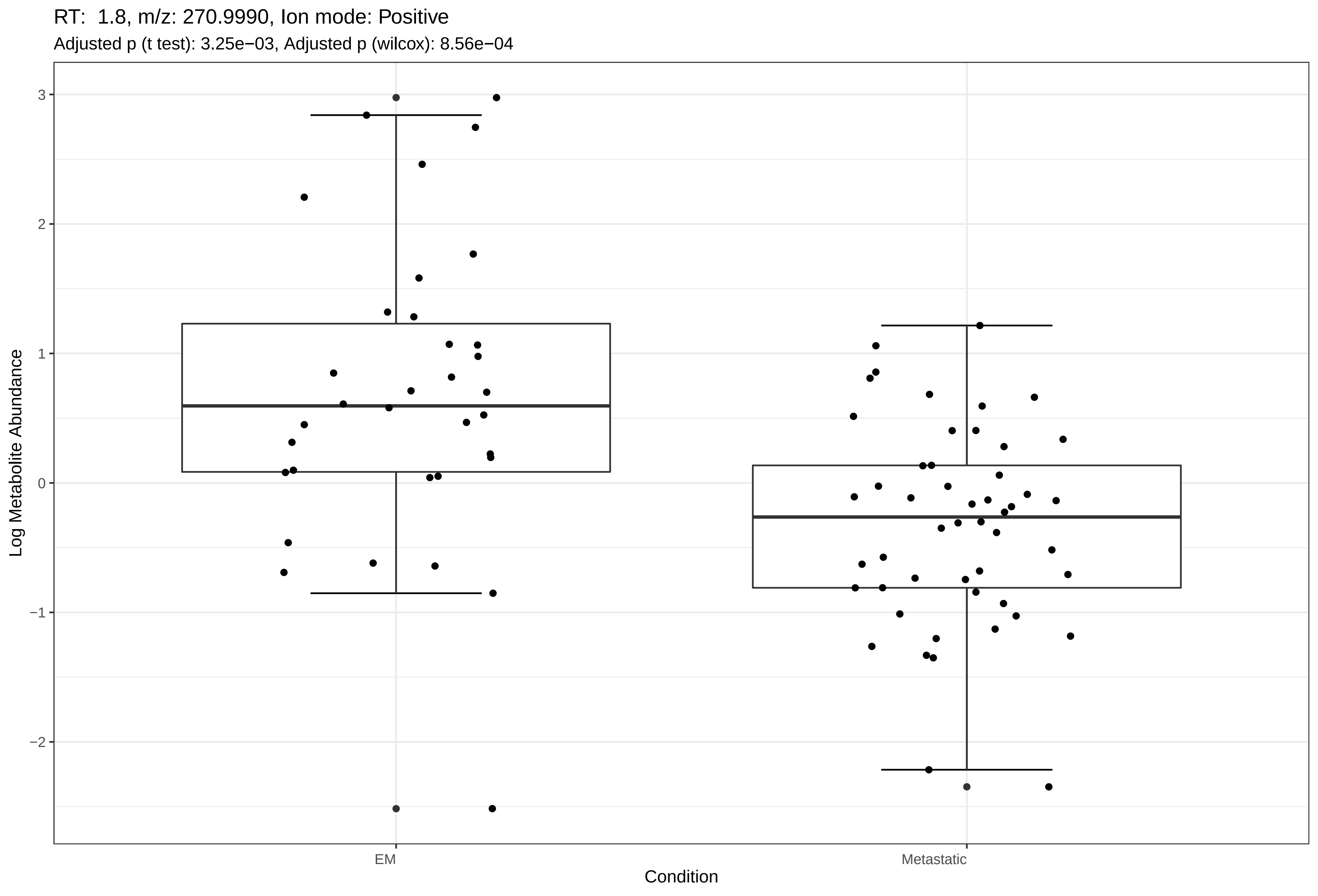

RT: 1.8, m/z: 270.9990, Ion mode: Positive
Adjusted p (t test): 3.25e−03, Adjusted p (wilcox): 8.56e−04
Log Metabolite Abundance
3
2
1
0
−1
−2
EM
Metastatic
Condition

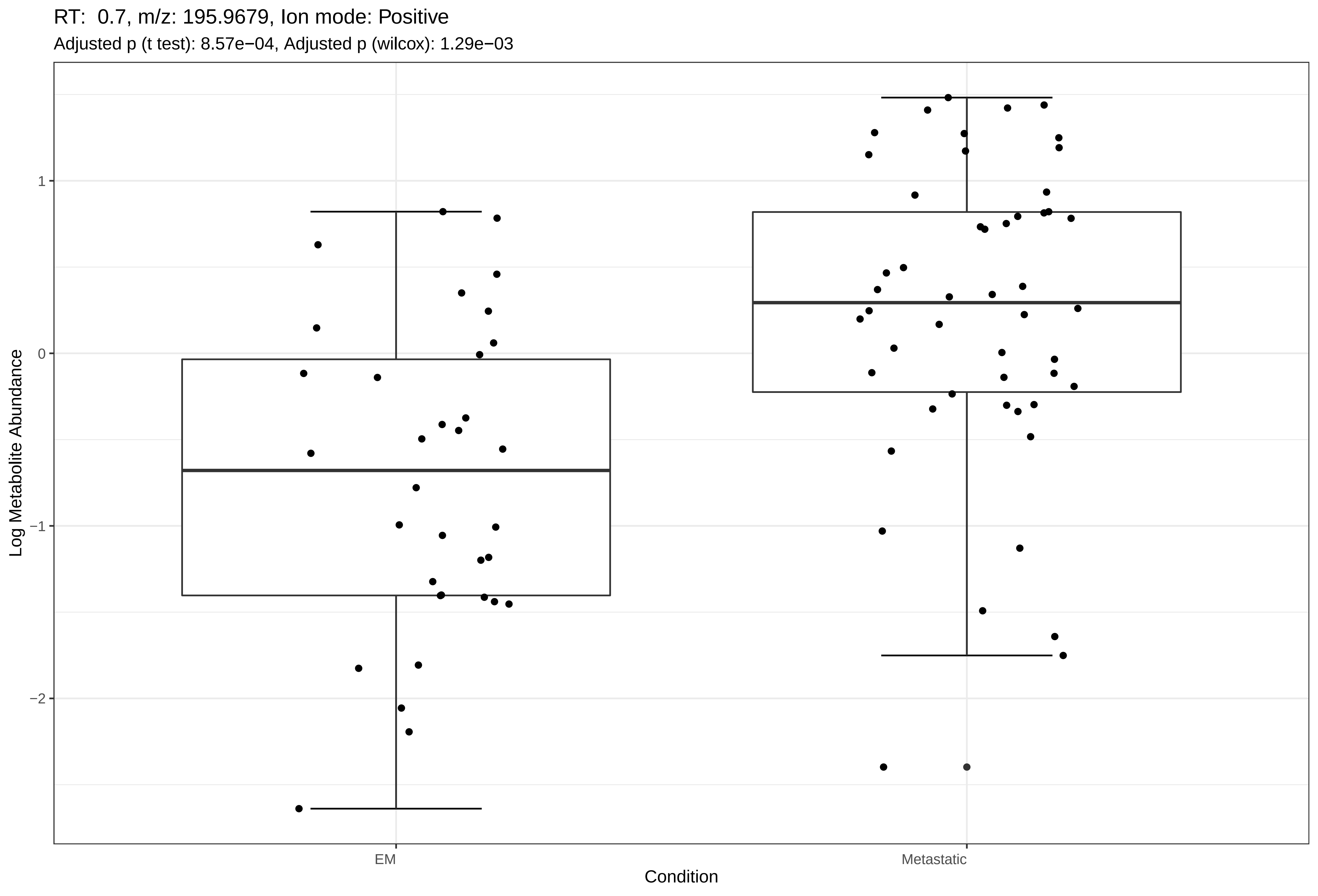

RT: 0.7, m/z: 195.9679, Ion mode: Positive
Adjusted p (t test): 8.57e−04, Adjusted p (wilcox): 1.29e−03
Log Metabolite Abundance
1
0
−1
−2
EM
Metastatic
Condition

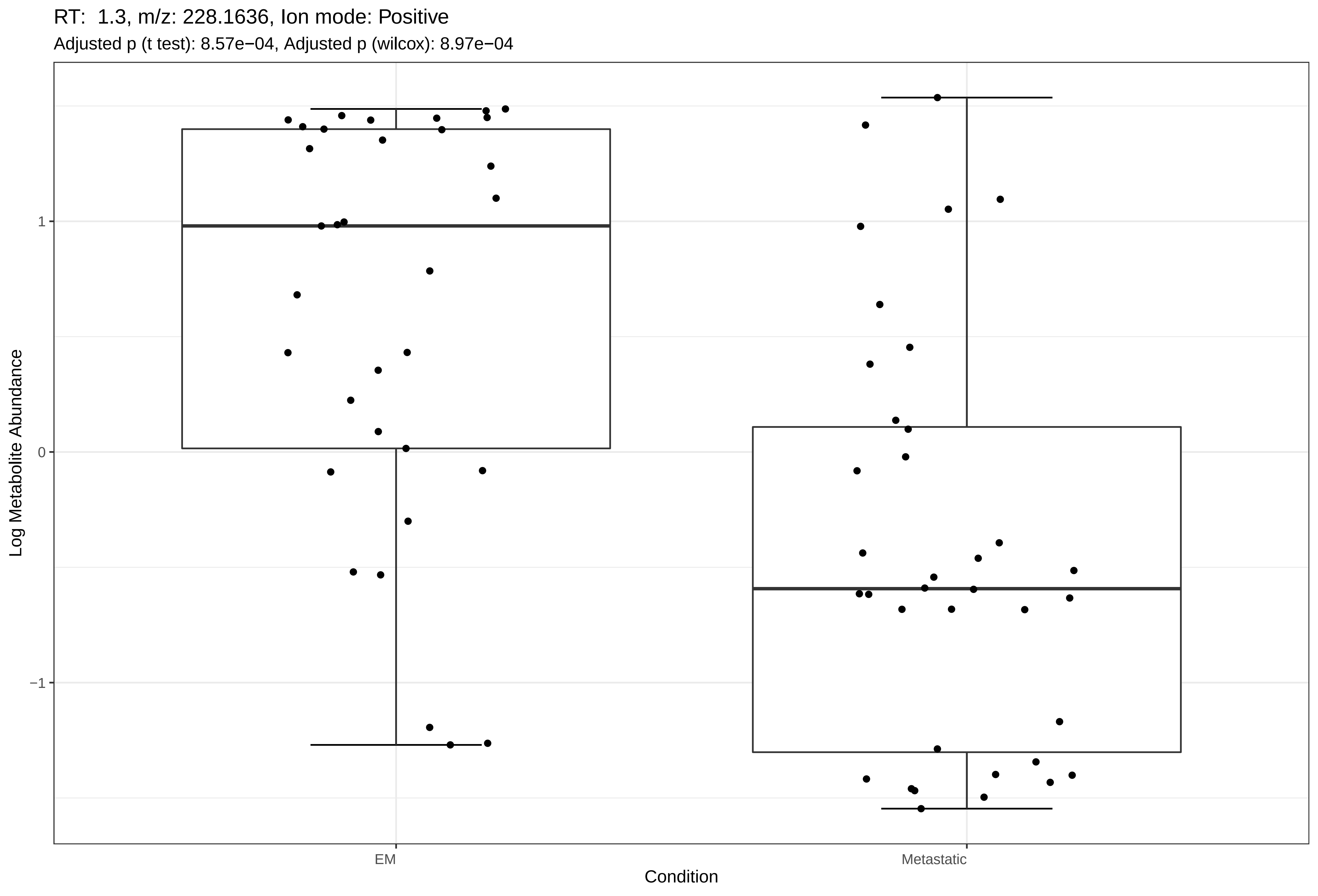
RT: 1.3, m/z: 228.1636, Ion mode: Positive
Adjusted p (t test): 8.57e−04, Adjusted p (wilcox): 8.97e−04
Log Metabolite Abundance
1
0
−1
EM
Metastatic
Condition

[M−H2O+H] −18.0106 m/z adduct of 124.0787 m/z (RT: 0.7, m/z: 106.0683, Ion mode: Positive)

Adjusted p (t test): 8.82e−04, Adjusted p (wilcox): 1.46e−03

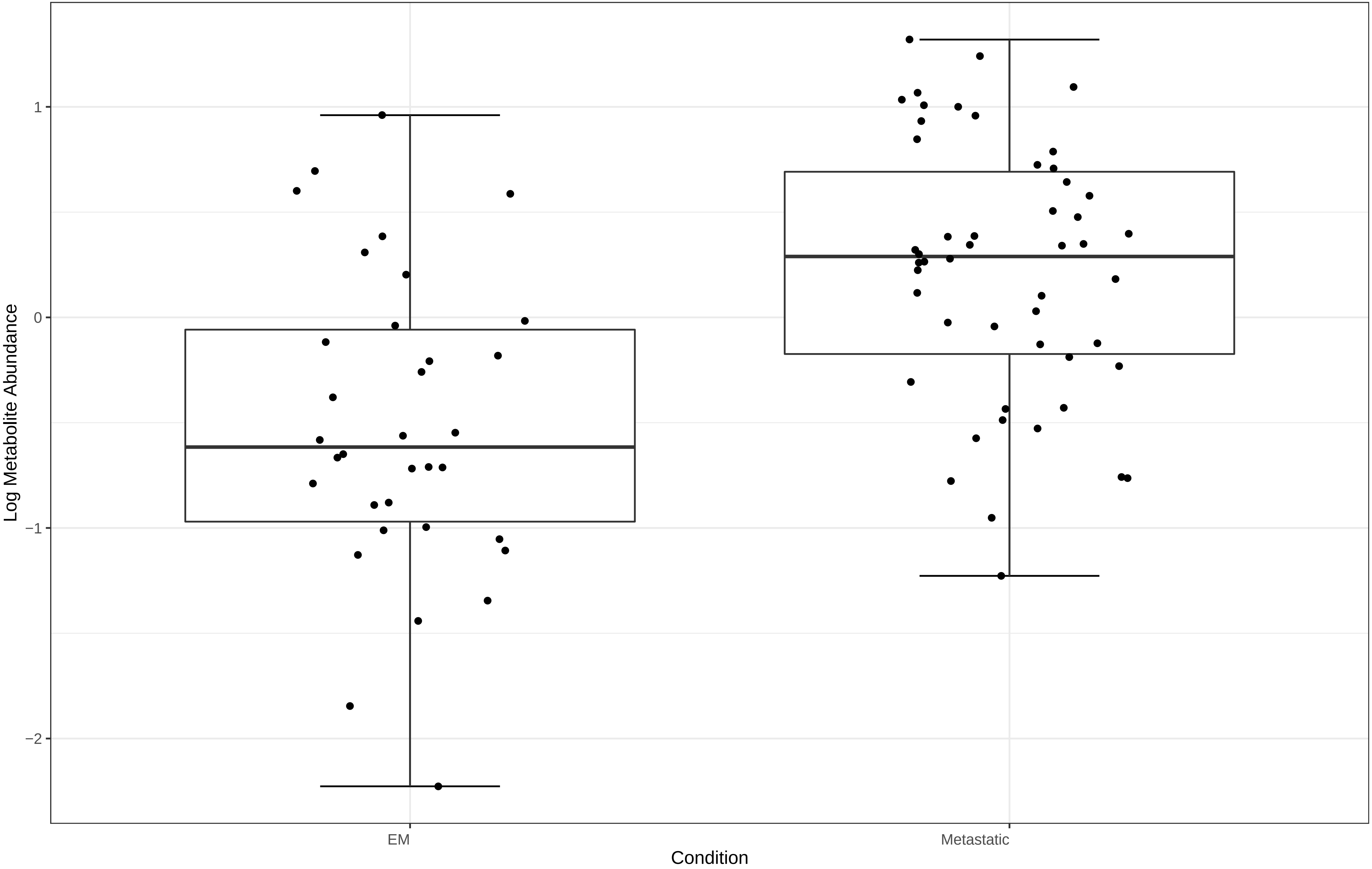

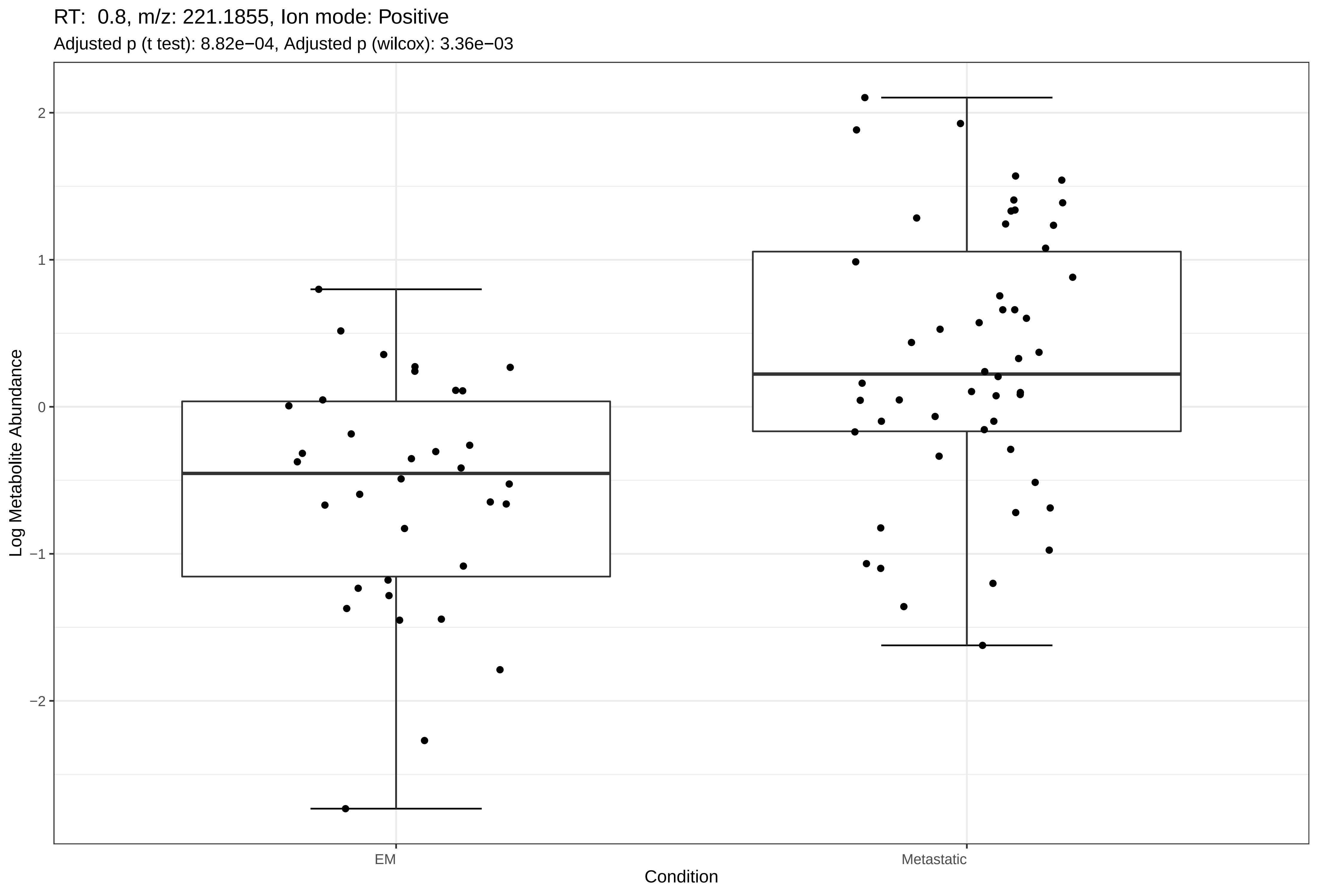

RT: 0.8, m/z: 221.1855, Ion mode: Positive
Adjusted p (t test): 8.82e−04, Adjusted p (wilcox): 3.36e−03
Log Metabolite Abundance
2
1
0
−1
−2
EM
Metastatic
Condition

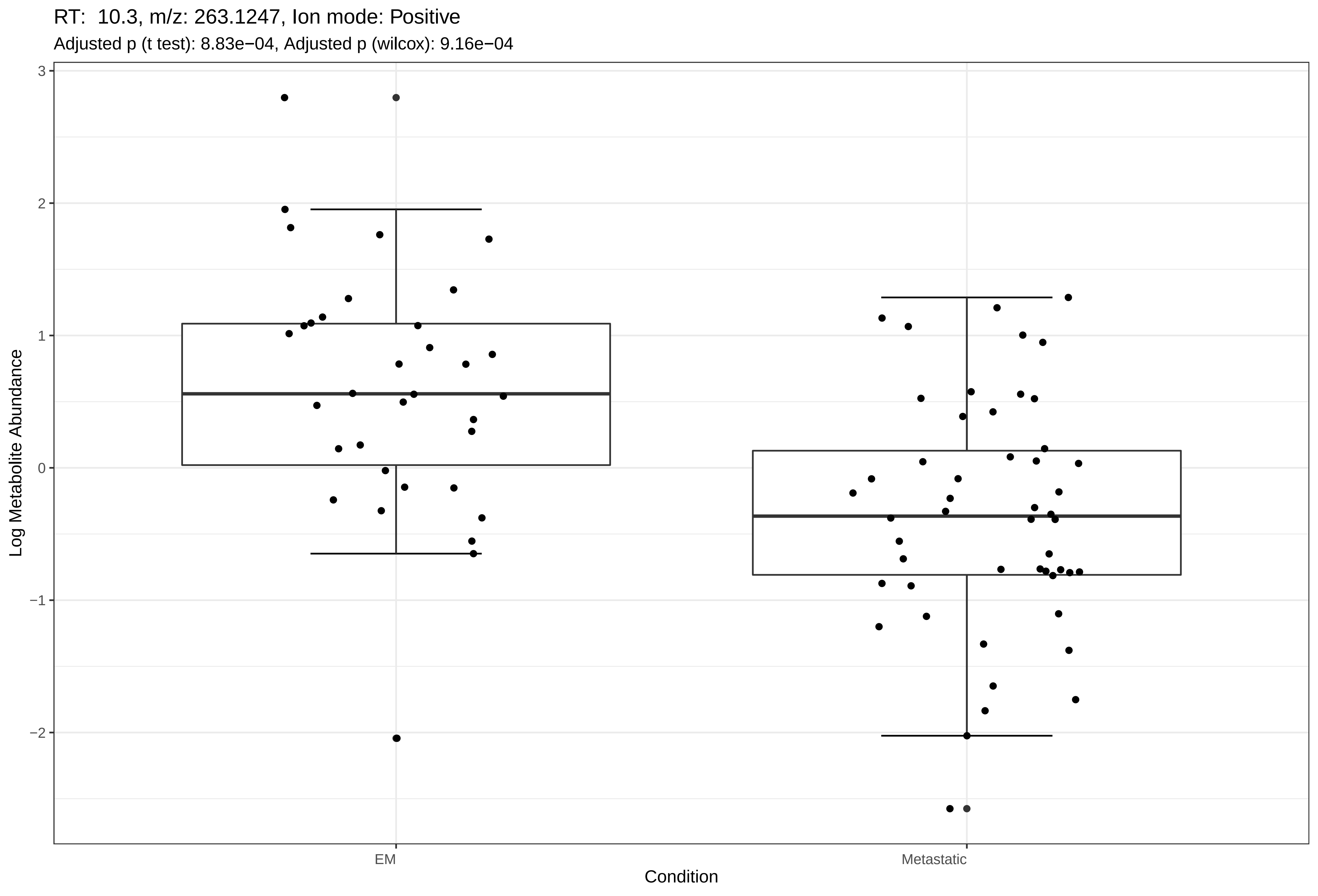
RT: 10.3, m/z: 263.1247, Ion mode: Positive
Adjusted p (t test): 8.83e−04, Adjusted p (wilcox): 9.16e−04
Log Metabolite Abundance
3
2
1
0
−1
−2
EM
Metastatic
Condition

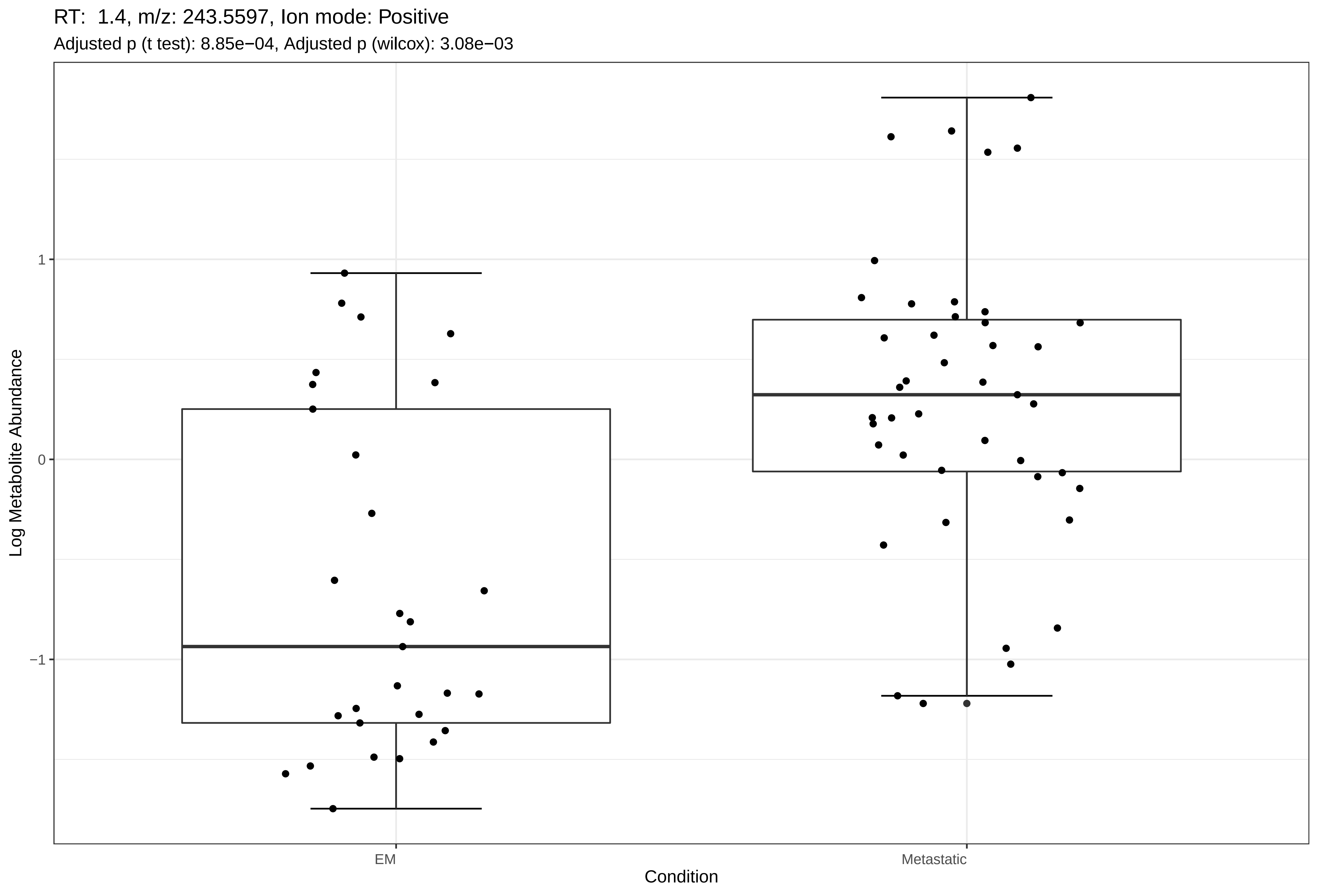
RT: 1.4, m/z: 243.5597, Ion mode: Positive
Adjusted p (t test): 8.85e−04, Adjusted p (wilcox): 3.08e−03
Log Metabolite Abundance
1
0
−1
EM
Metastatic
Condition

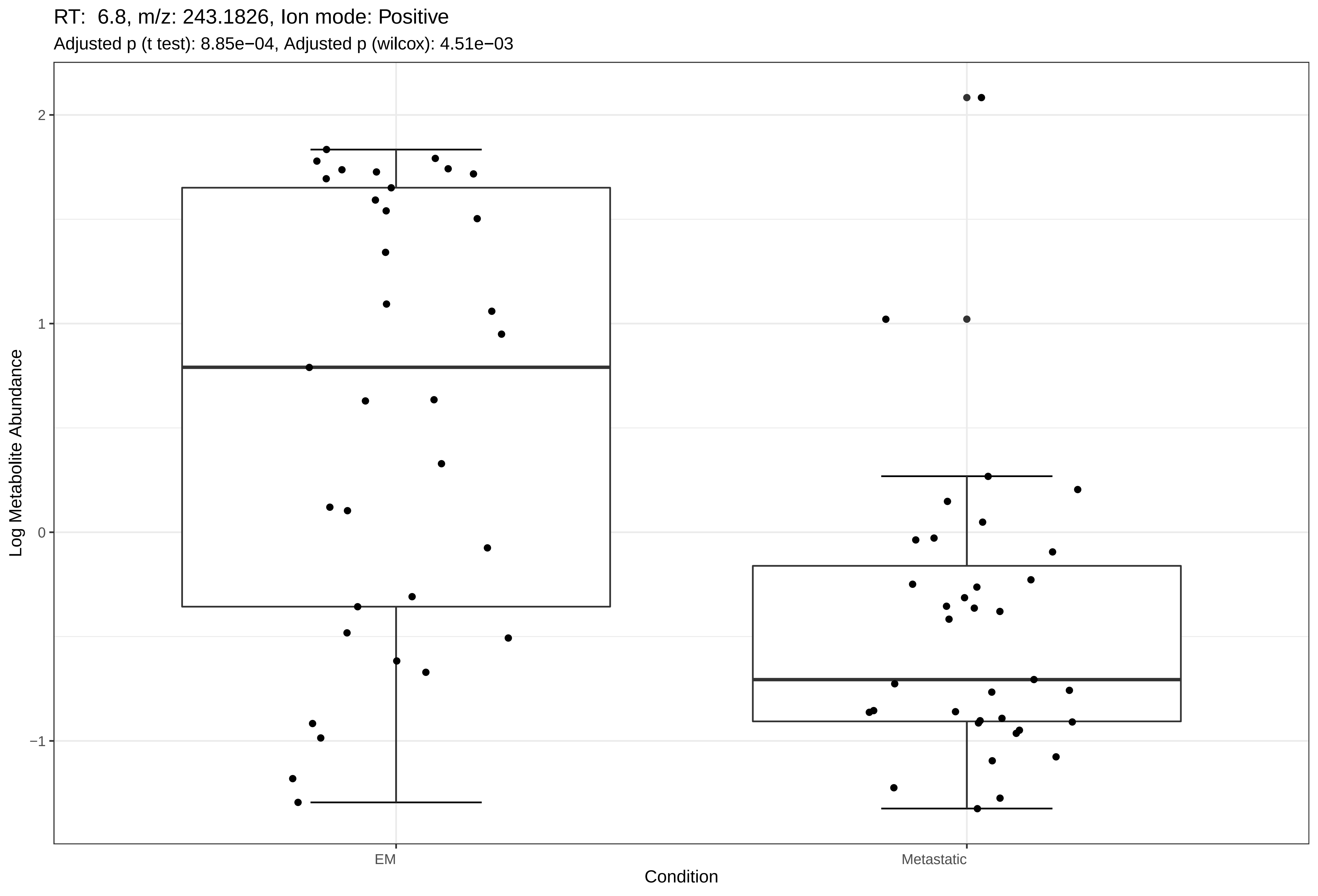
RT: 6.8, m/z: 243.1826, Ion mode: Positive
Adjusted p (t test): 8.85e−04, Adjusted p (wilcox): 4.51e−03
Log Metabolite Abundance
2
1
0
−1
EM
Metastatic
Condition

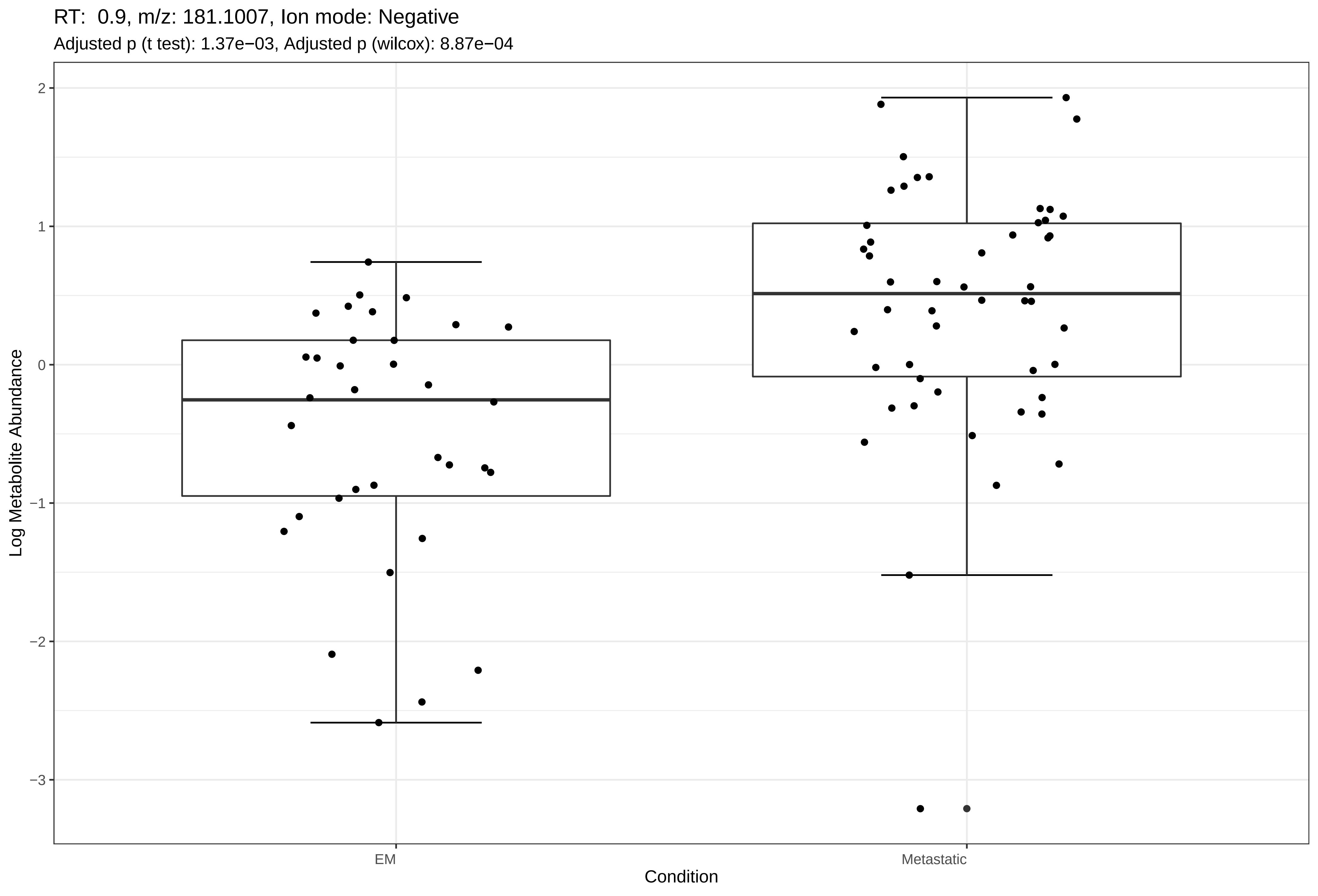

RT: 0.9, m/z: 181.1007, Ion mode: Negative
Adjusted p (t test): 1.37e−03, Adjusted p (wilcox): 8.87e−04
Log Metabolite Abundance
2
1
0
−1
−2
−3
EM
Metastatic
Condition

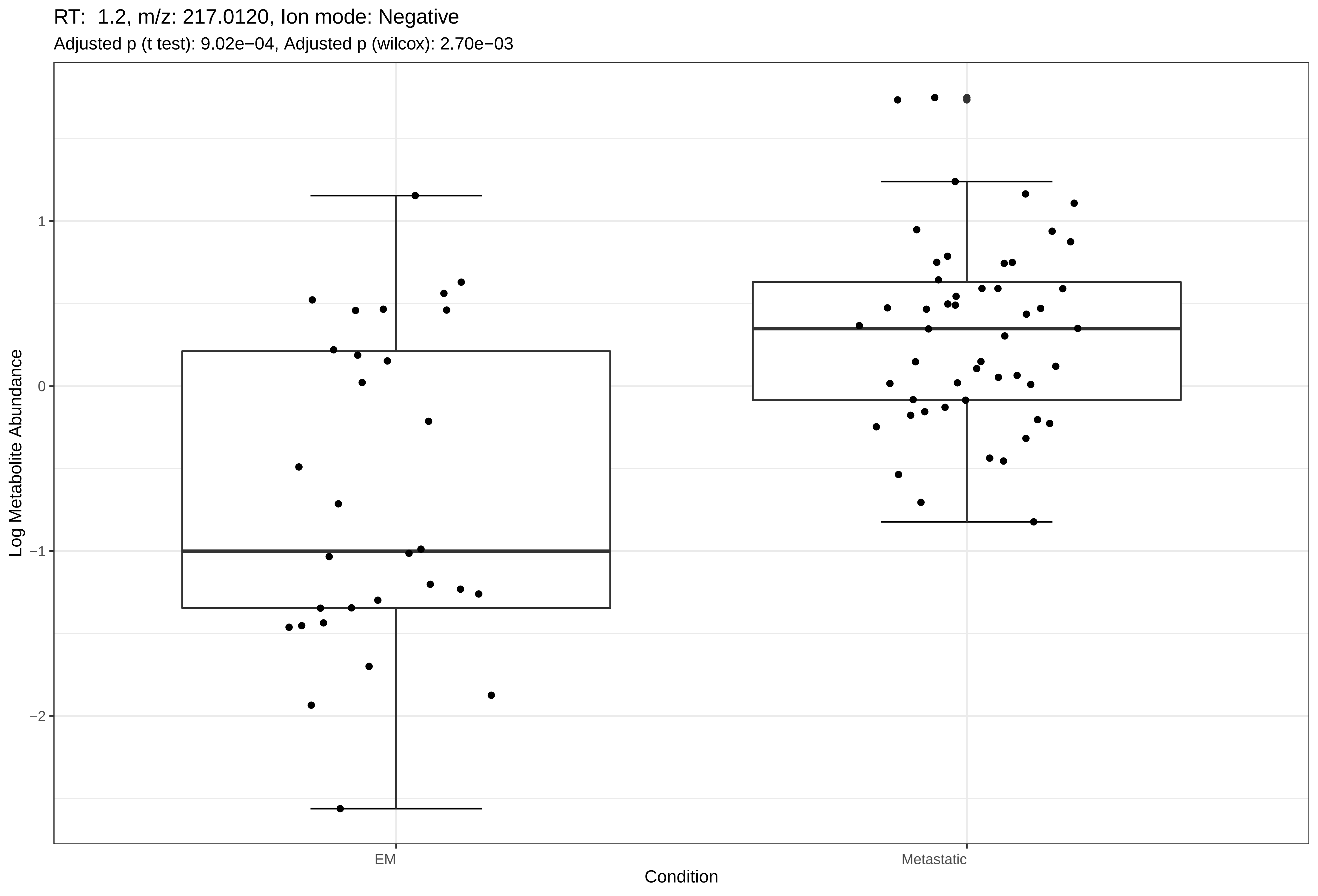
RT: 1.2, m/z: 217.0120, Ion mode: Negative
Adjusted p (t test): 9.02e−04, Adjusted p (wilcox): 2.70e−03
Log Metabolite Abundance
1
0
−1
−2
EM
Metastatic
Condition

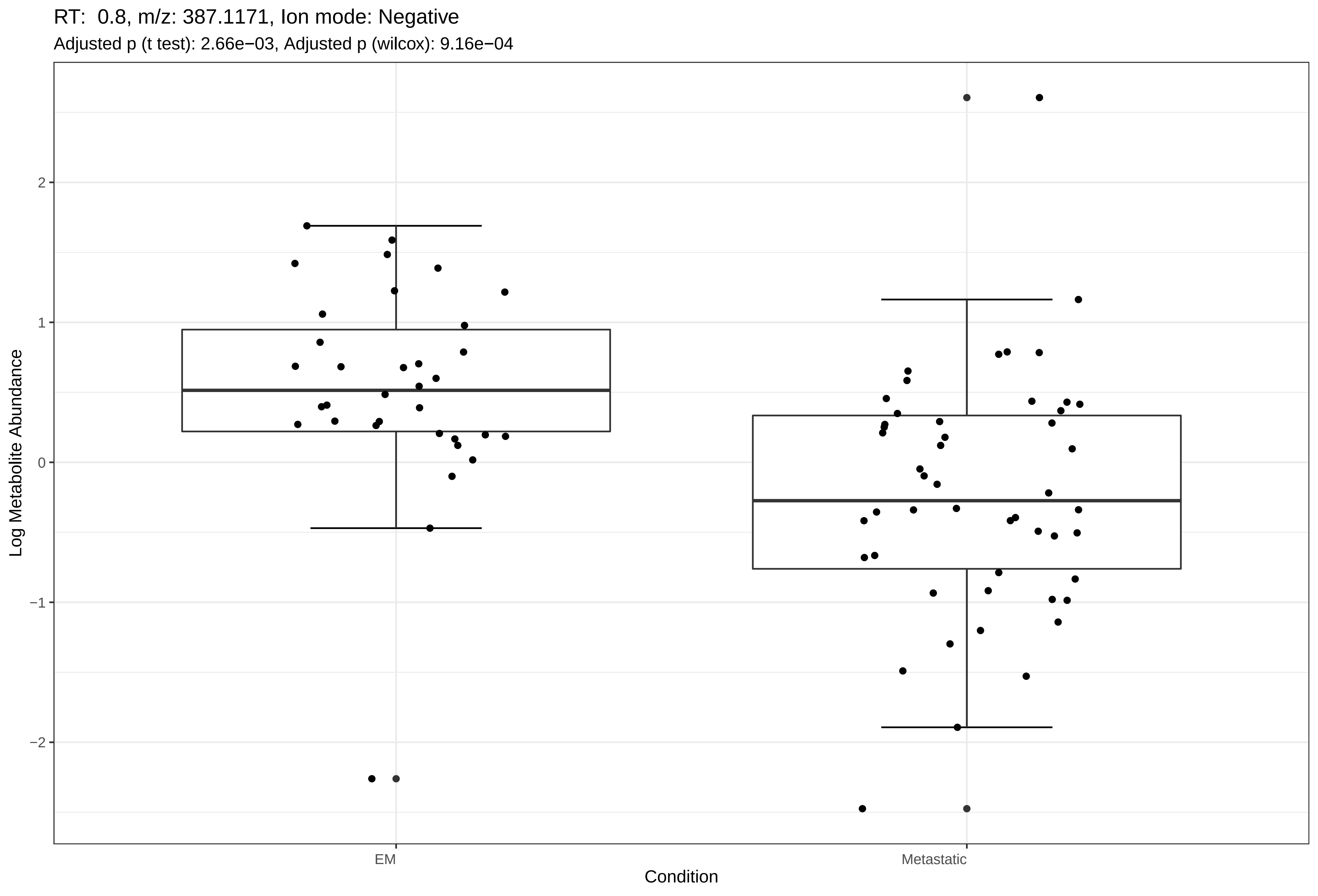

RT: 0.8, m/z: 387.1171, Ion mode: Negative
Adjusted p (t test): 2.66e−03, Adjusted p (wilcox): 9.16e−04
Log Metabolite Abundance
2
1
0
−1
−2
EM
Metastatic
Condition

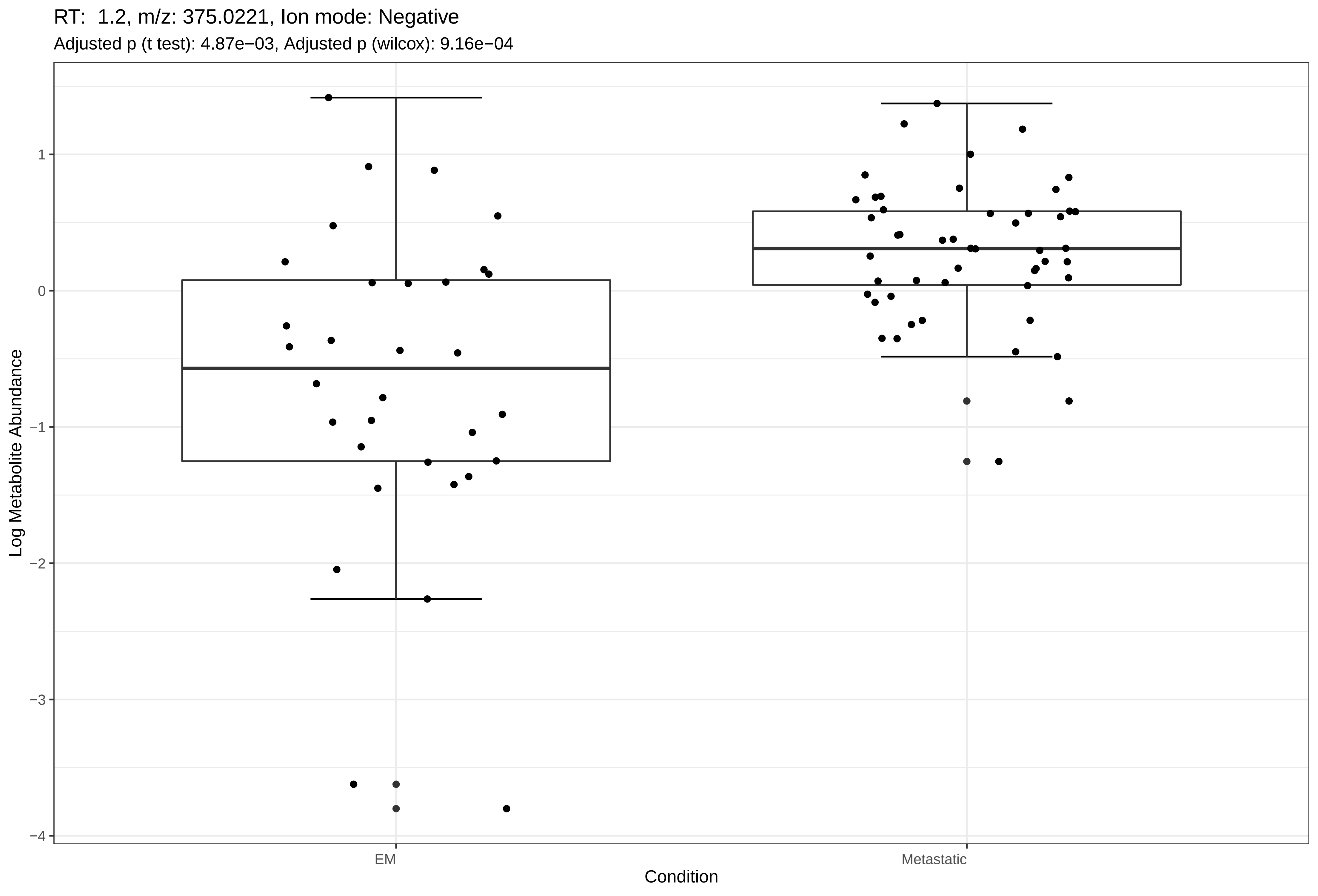

RT: 1.2, m/z: 375.0221, Ion mode: Negative
Adjusted p (t test): 4.87e−03, Adjusted p (wilcox): 9.16e−04
Log Metabolite Abundance
1
0
−1
−2
−3
−4
EM
Metastatic
Condition

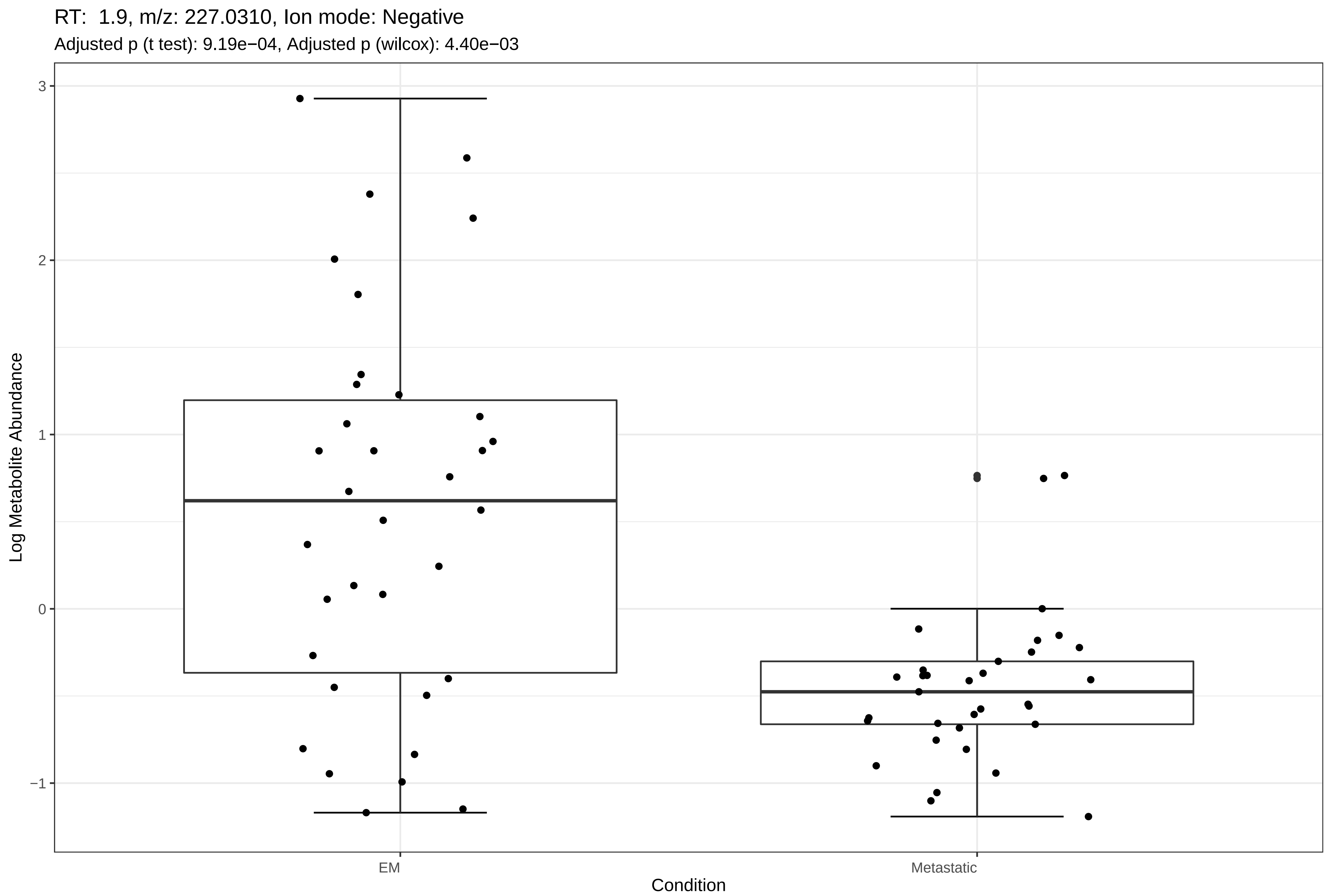
RT: 1.9, m/z: 227.0310, Ion mode: Negative
Adjusted p (t test): 9.19e−04, Adjusted p (wilcox): 4.40e−03
Log Metabolite Abundance
3
2
1
0
−1
EM
Metastatic
Condition

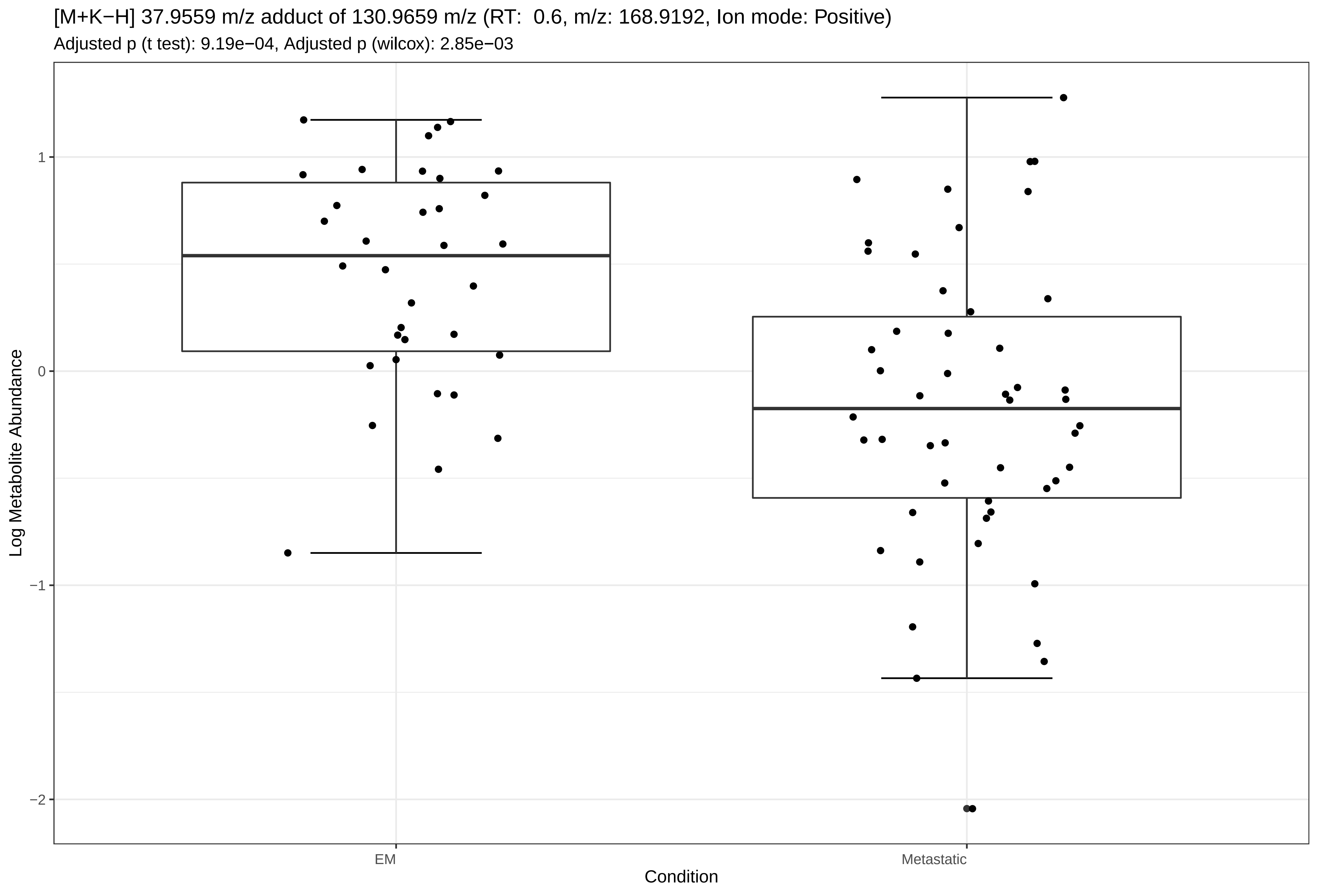

[M+K−H] 37.9559 m/z adduct of 130.9659 m/z (RT: 0.6, m/z: 168.9192, Ion mode: Positive)
Adjusted p (t test): 9.19e−04, Adjusted p (wilcox): 2.85e−03
Log Metabolite Abundance
1
0
−1
−2
EM
Metastatic
Condition

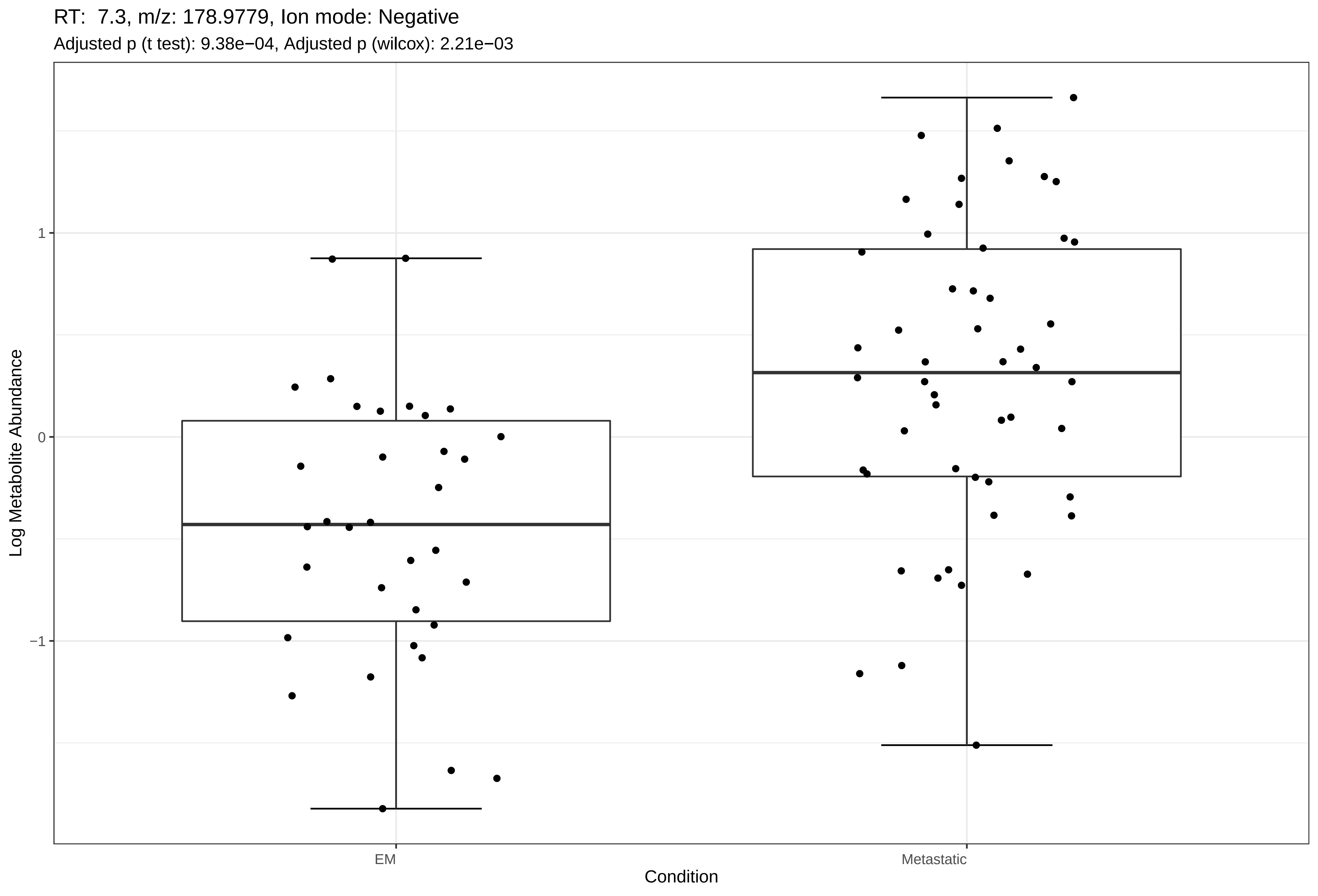

RT: 7.3, m/z: 178.9779, Ion mode: Negative
Adjusted p (t test): 9.38e−04, Adjusted p (wilcox): 2.21e−03
Log Metabolite Abundance
1
0
−1
EM
Metastatic
Condition

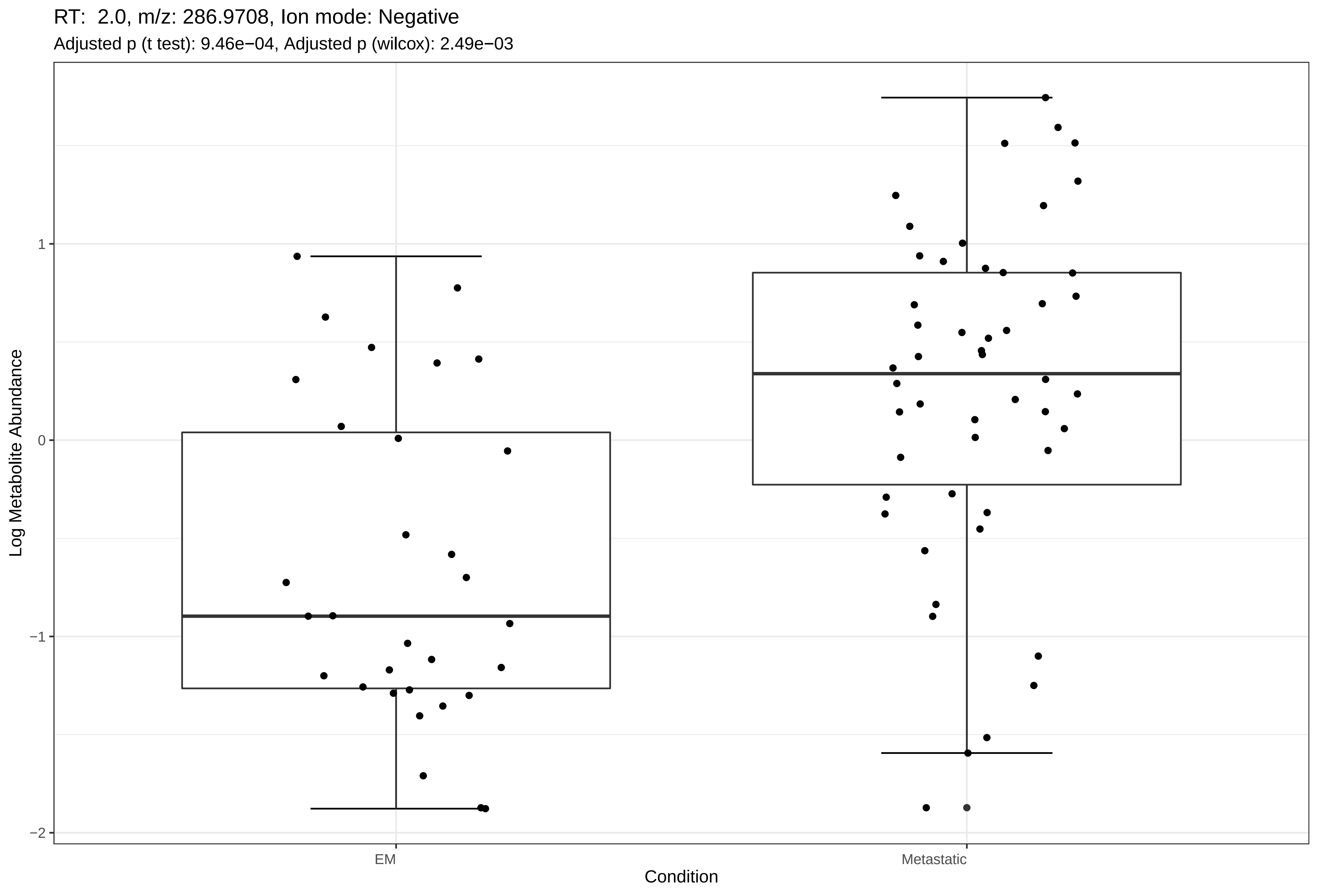
RT: 2.0, m/z: 286.9708, Ion mode: Negative
Adjusted p (t test): 9.46e−04, Adjusted p (wilcox): 2.49e−03
Log Metabolite Abundance
1
0
−1
−2
EM
Metastatic
Condition

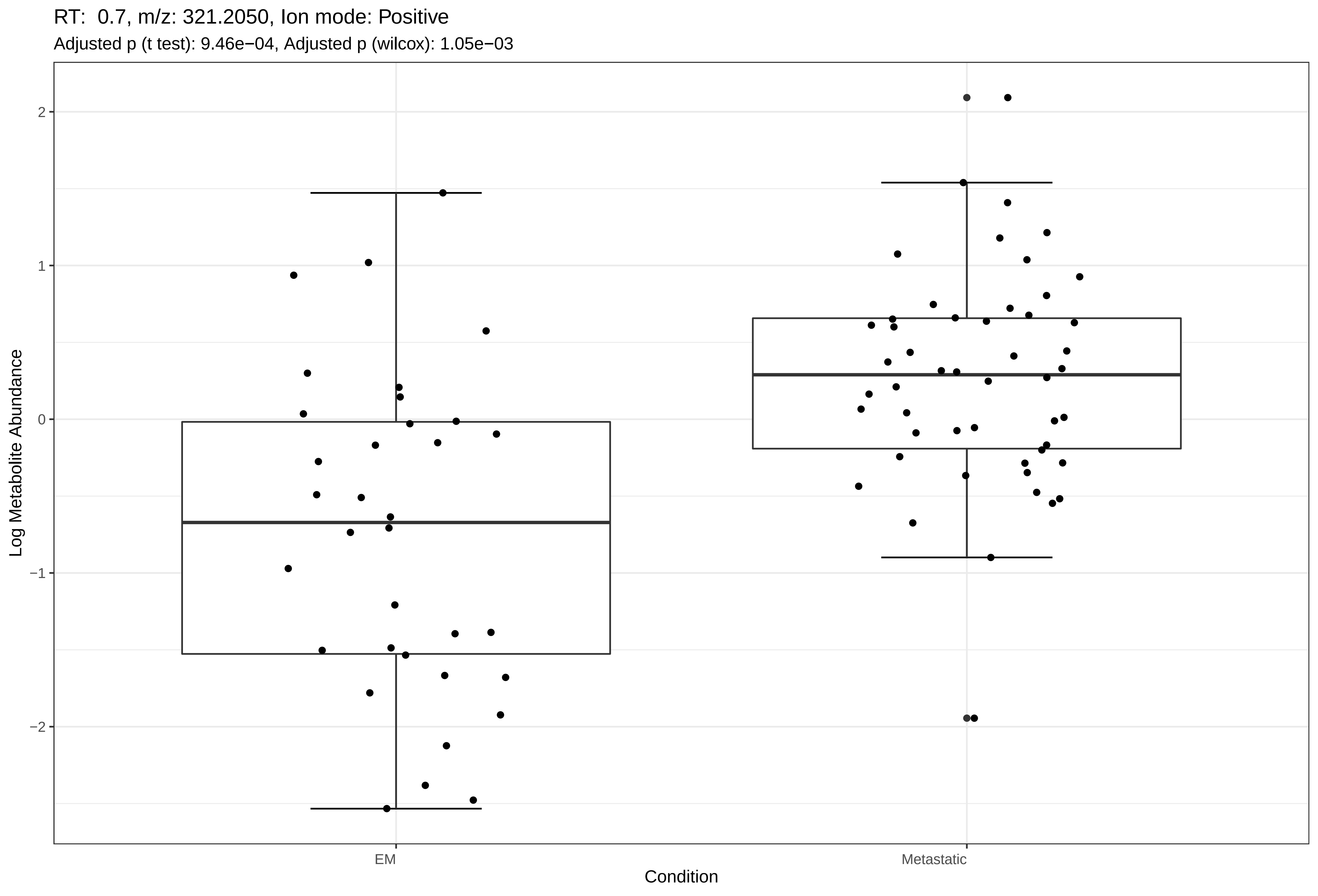

RT: 0.7, m/z: 321.2050, Ion mode: Positive
Adjusted p (t test): 9.46e−04, Adjusted p (wilcox): 1.05e−03
Log Metabolite Abundance
2
1
0
−1
−2
EM
Metastatic
Condition

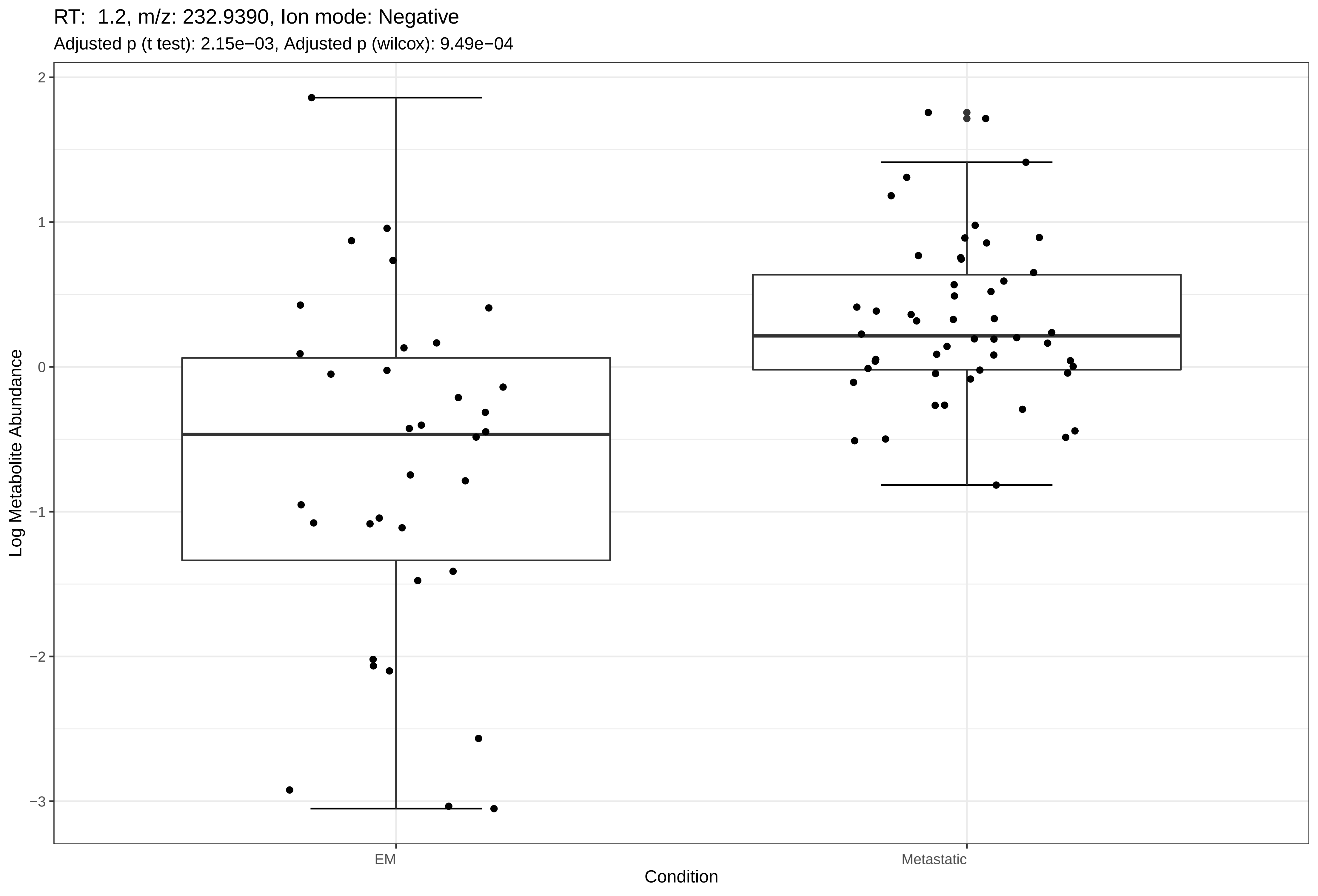
RT: 1.2, m/z: 232.9390, Ion mode: Negative
Adjusted p (t test): 2.15e−03, Adjusted p (wilcox): 9.49e−04
Log Metabolite Abundance
2
1
0
−1
−2
−3
EM
Metastatic
Condition

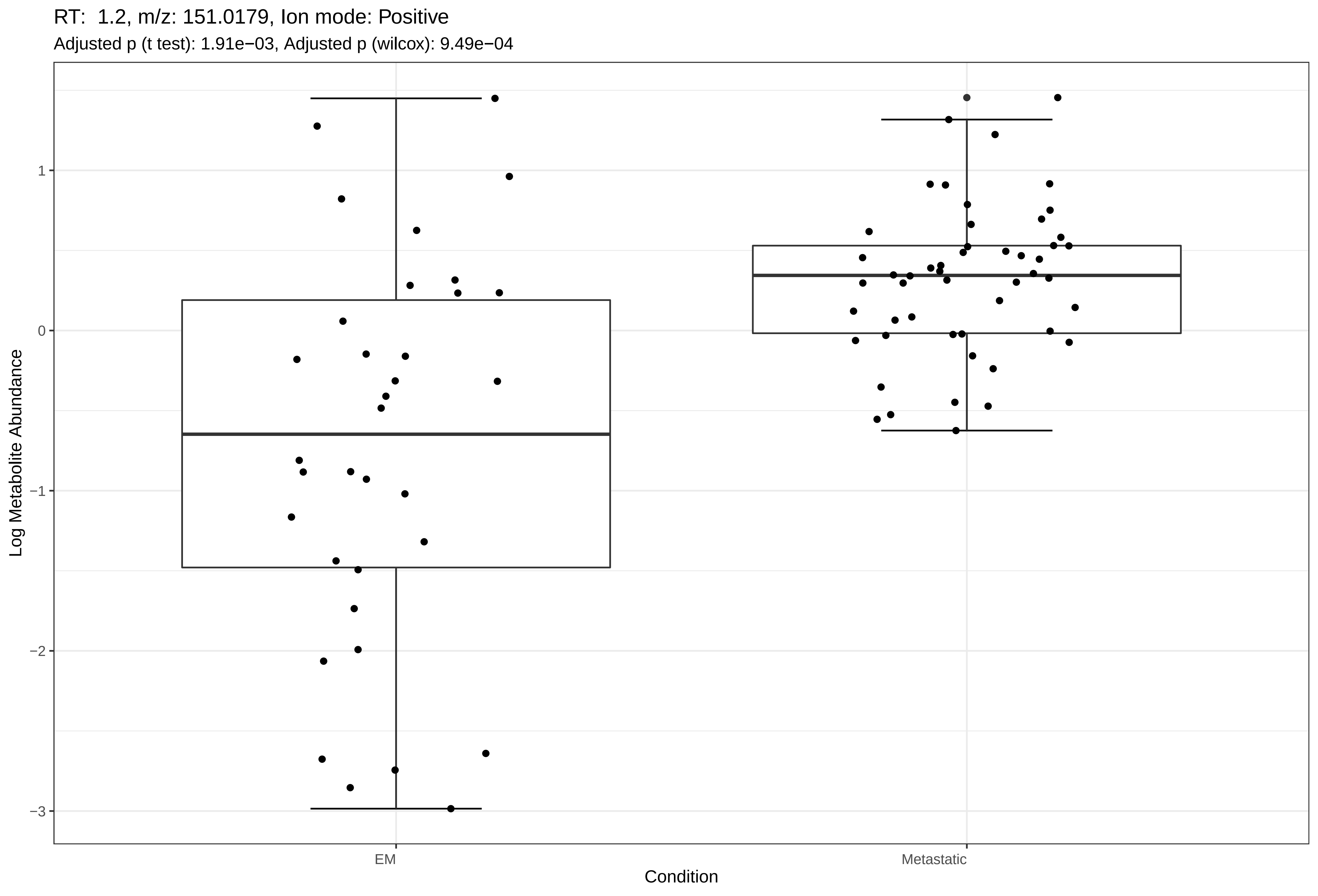

RT: 1.2, m/z: 151.0179, Ion mode: Positive
Adjusted p (t test): 1.91e−03, Adjusted p (wilcox): 9.49e−04
Log Metabolite Abundance
1
0
−1
−2
−3
EM
Metastatic
Condition

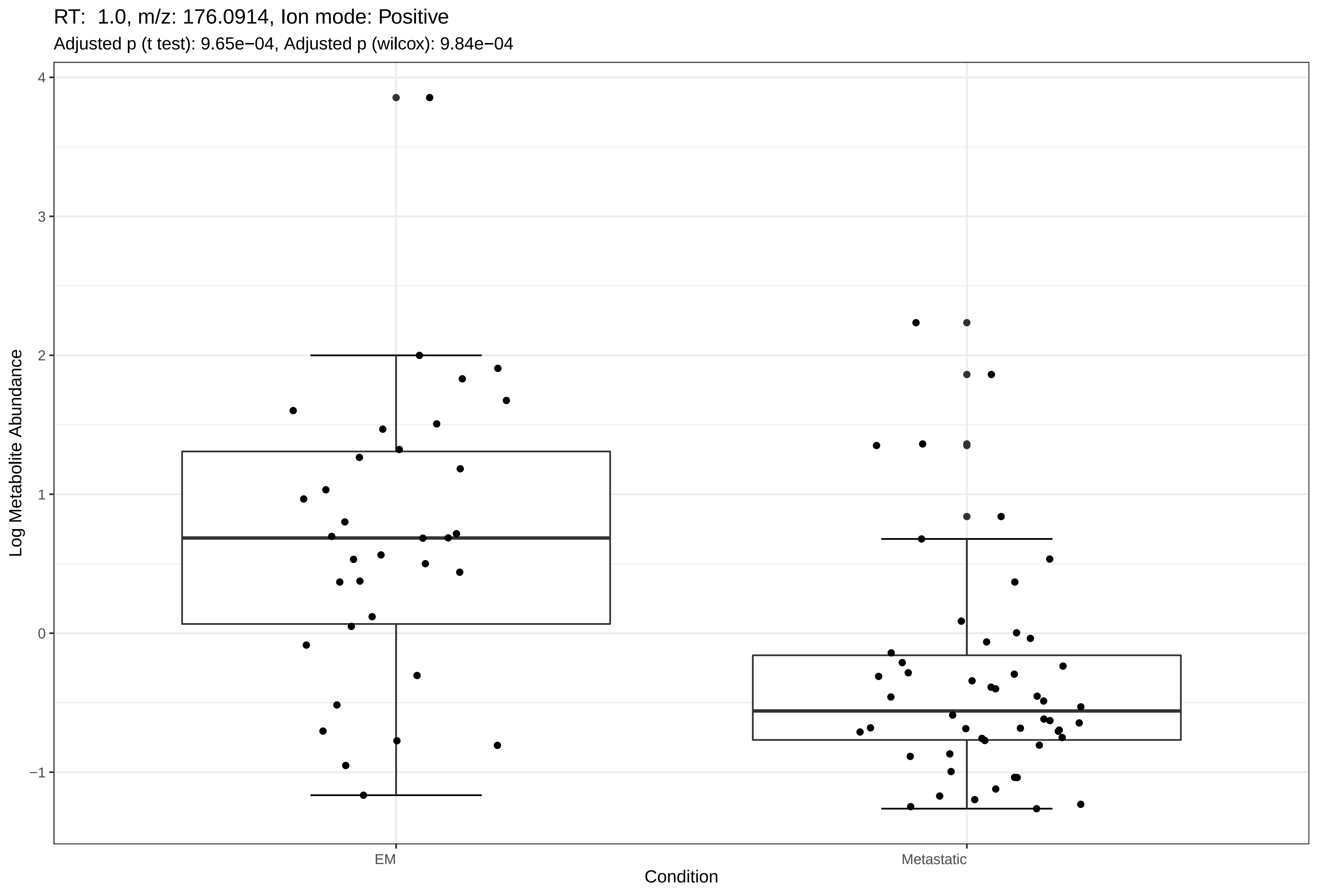
RT: 1.0, m/z: 176.0914, Ion mode: Positive
Adjusted p (t test): 9.65e−04, Adjusted p (wilcox): 9.84e−04
Log Metabolite Abundance
4
3
2
1
0
−1
EM
Metastatic
Condition

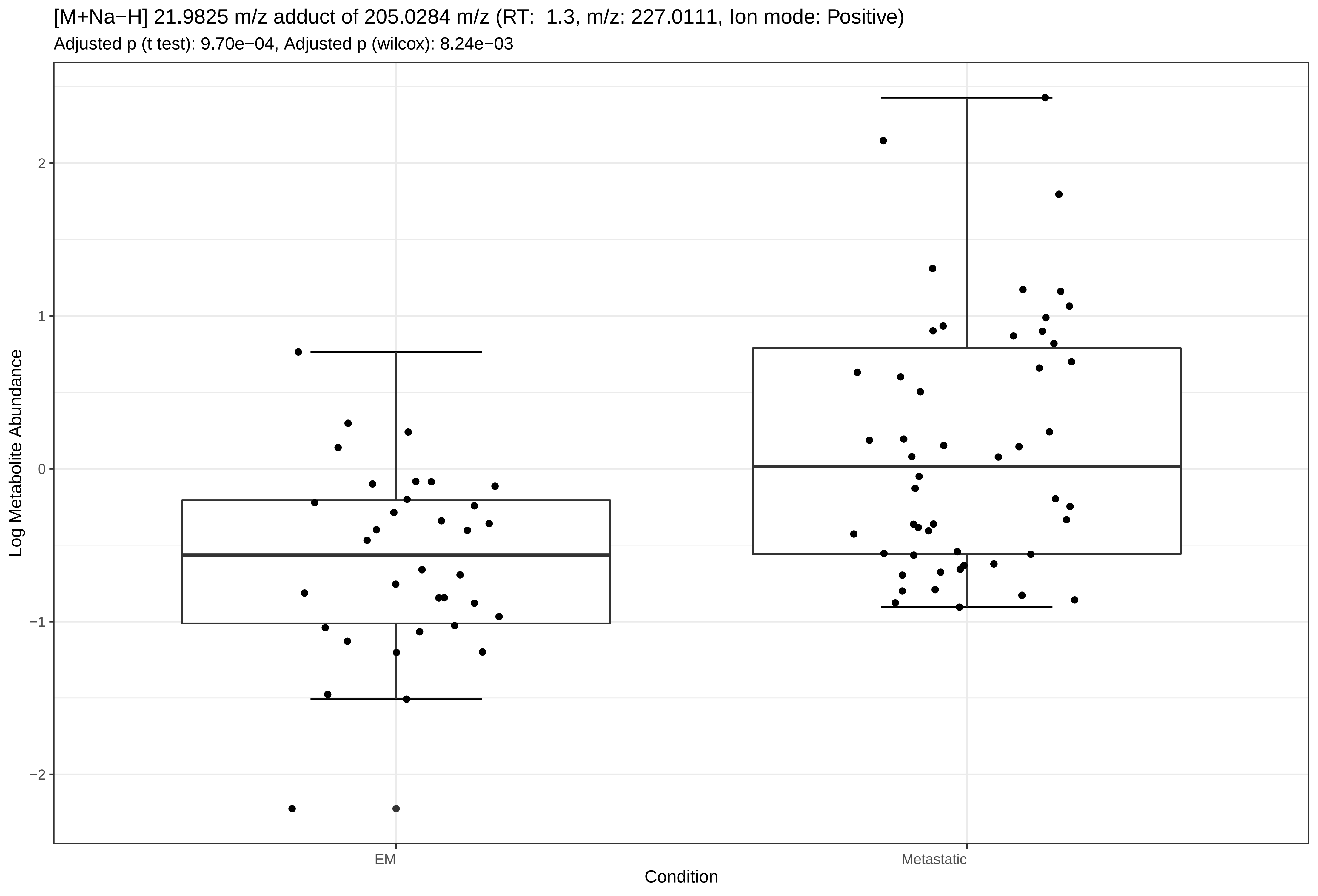
[M+Na−H] 21.9825 m/z adduct of 205.0284 m/z (RT: 1.3, m/z: 227.0111, Ion mode: Positive)
Adjusted p (t test): 9.70e−04, Adjusted p (wilcox): 8.24e−03
Log Metabolite Abundance
2
1
0
−1
−2
EM
Metastatic
Condition

RT: 6.9, m/z: 145.6180, Ion mode: Positive

Adjusted p (t test): 9.76e−04, Adjusted p (wilcox): 2.29e−03

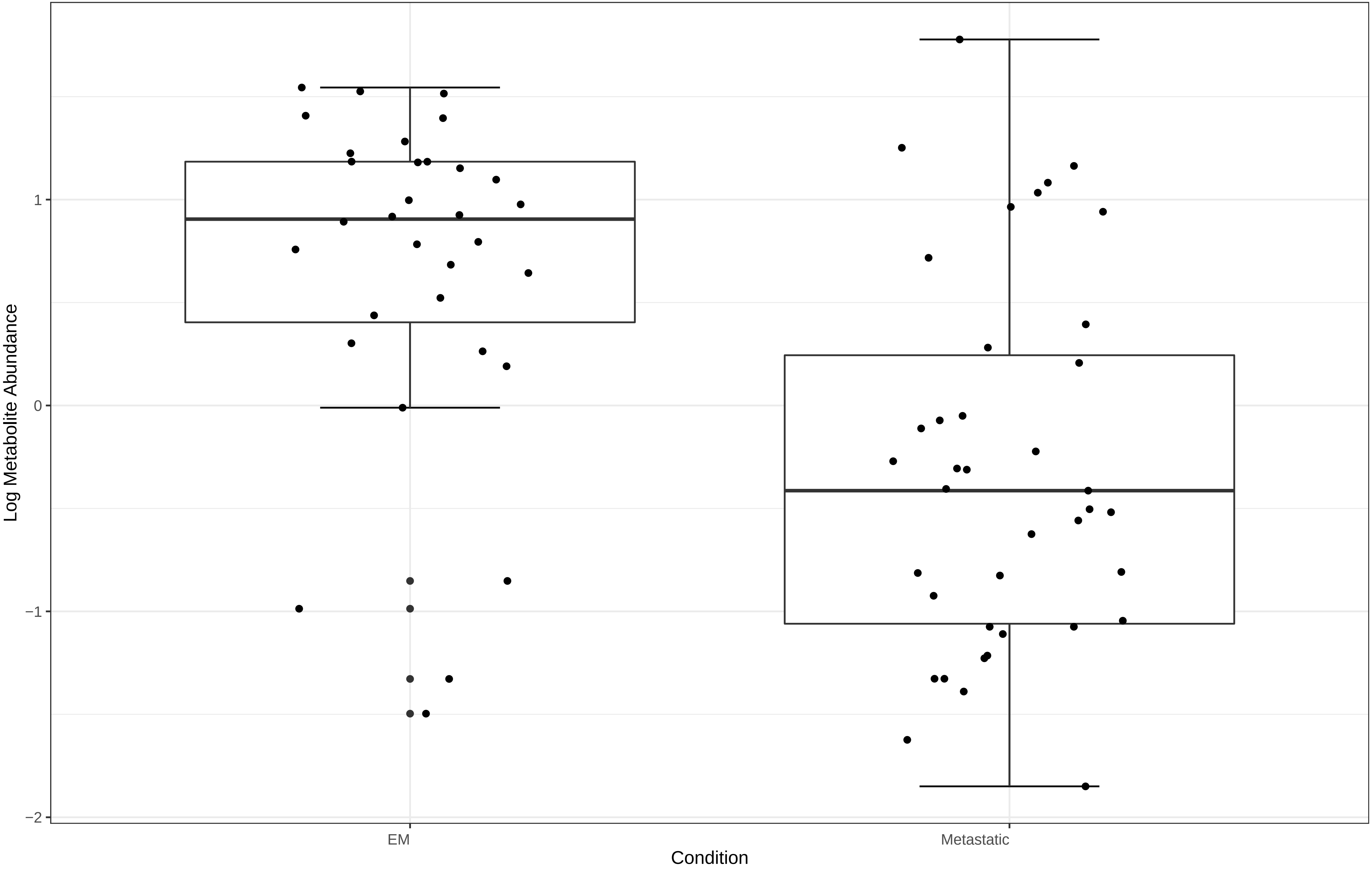

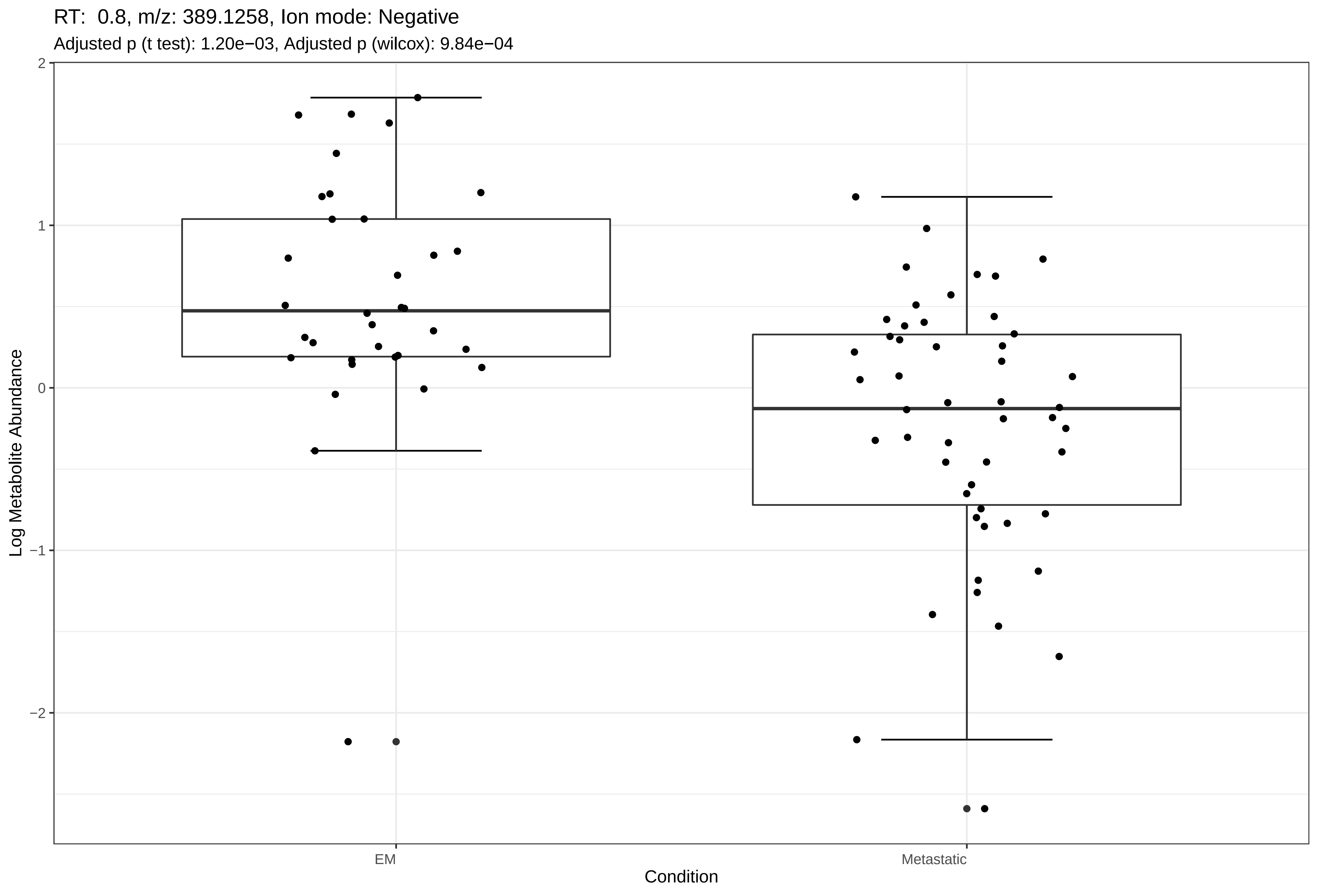

RT: 0.8, m/z: 389.1258, Ion mode: Negative
Adjusted p (t test): 1.20e−03, Adjusted p (wilcox): 9.84e−04
Log Metabolite Abundance
2
1
0
−1
−2
EM
Metastatic
Condition

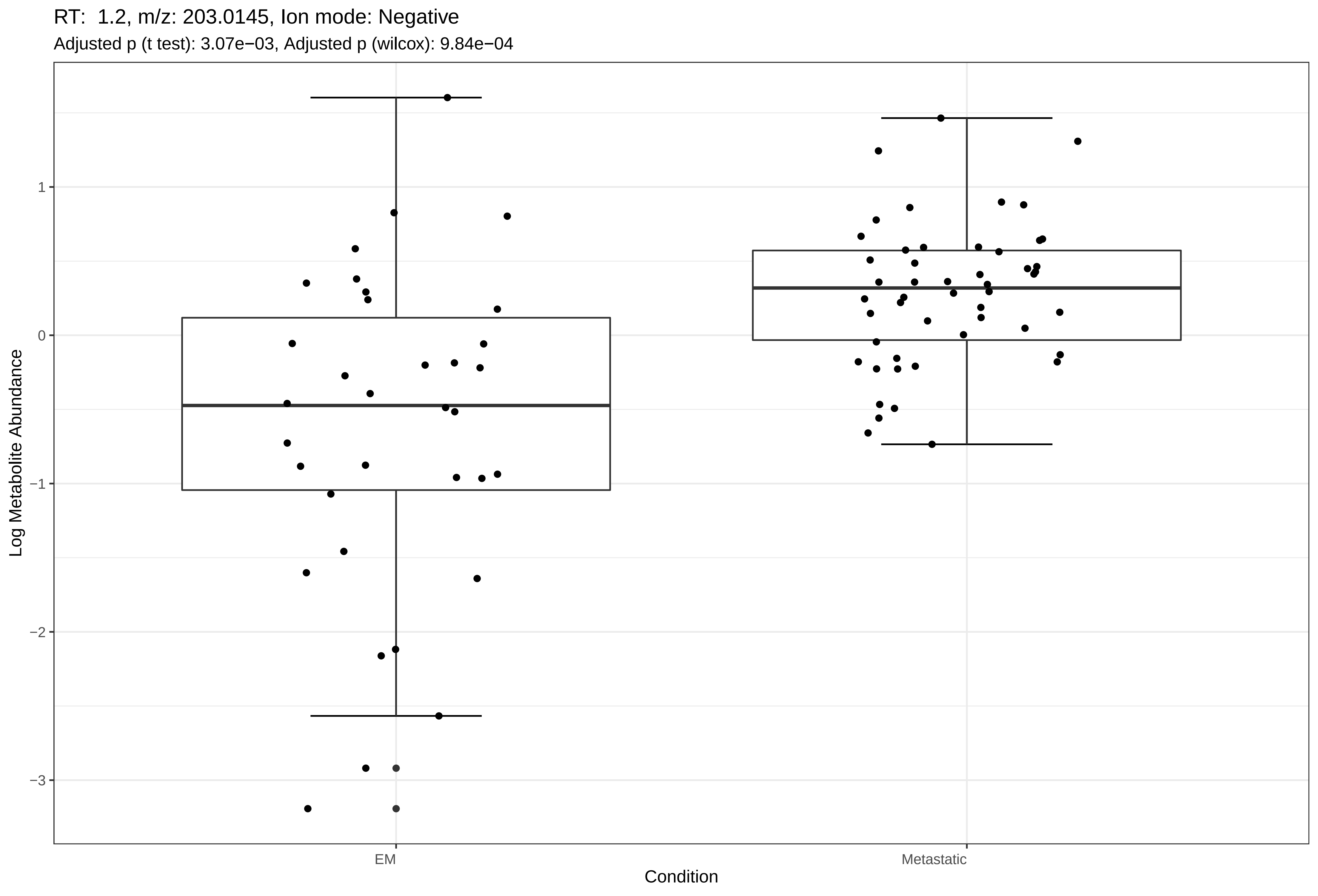

RT: 1.2, m/z: 203.0145, Ion mode: Negative
Adjusted p (t test): 3.07e−03, Adjusted p (wilcox): 9.84e−04
Log Metabolite Abundance
1
0
−1
−2
−3
EM
Metastatic
Condition

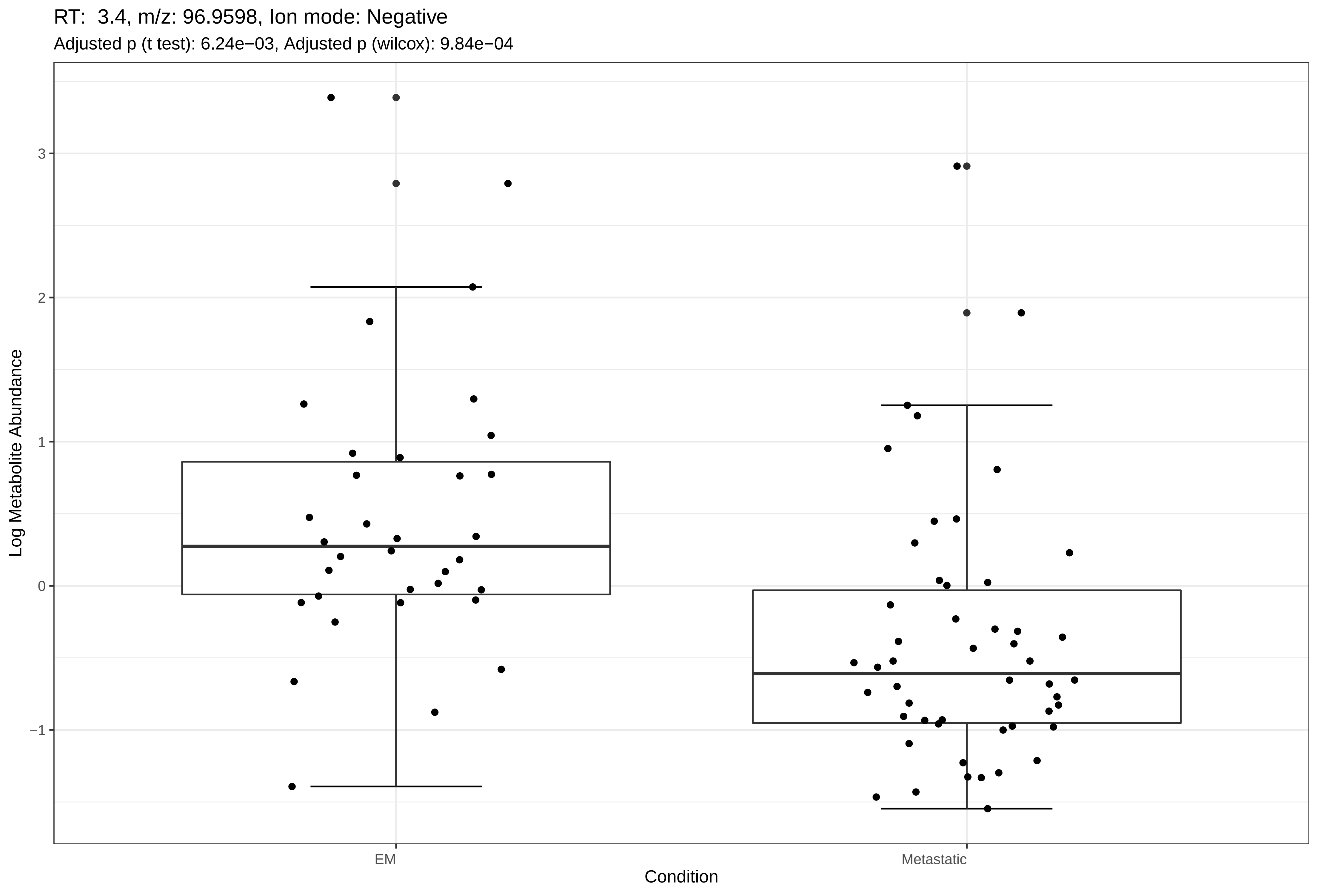
RT: 3.4, m/z: 96.9598, Ion mode: Negative
Adjusted p (t test): 6.24e−03, Adjusted p (wilcox): 9.84e−04
Log Metabolite Abundance
3
2
1
0
−1
EM
Metastatic
Condition

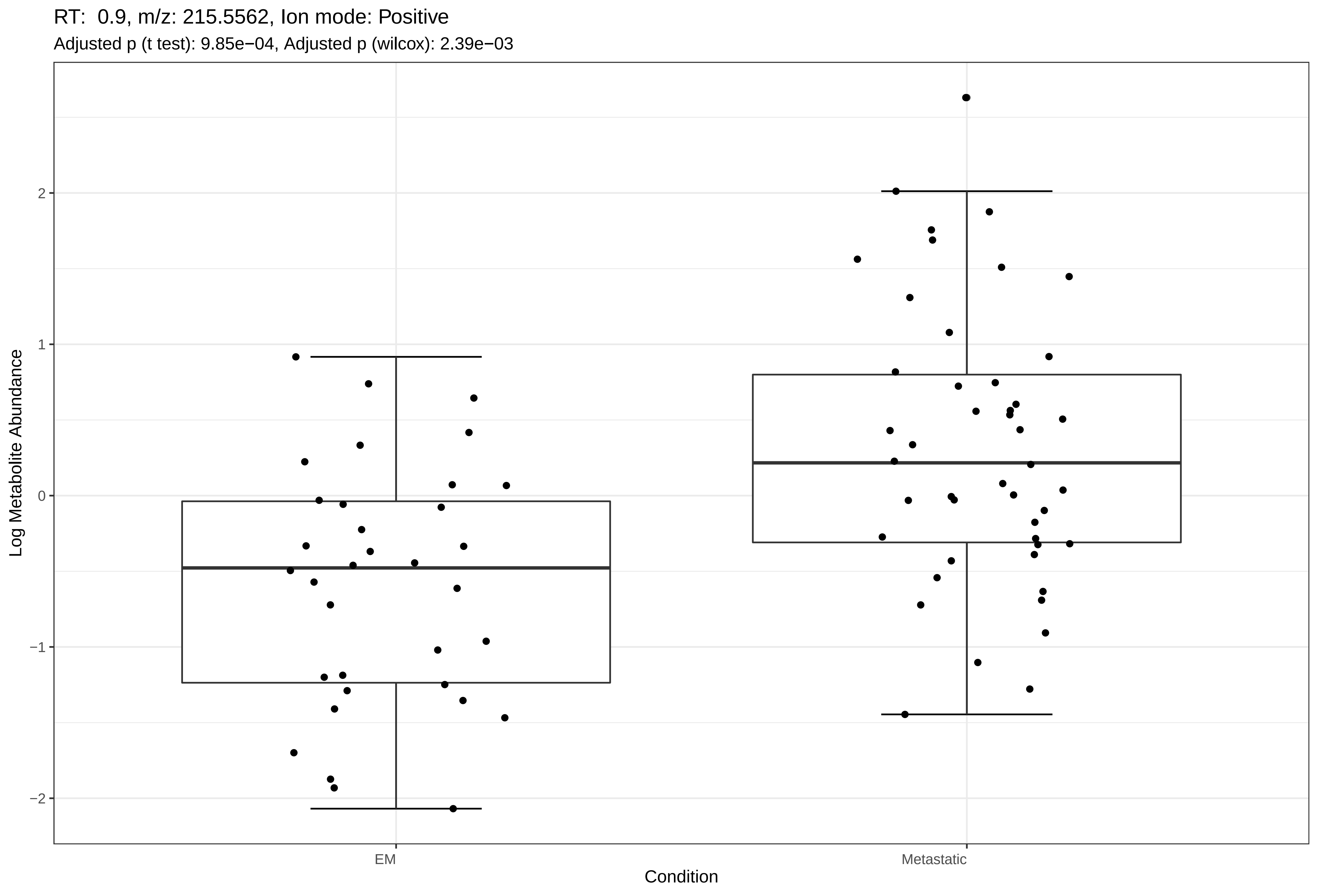
RT: 0.9, m/z: 215.5562, Ion mode: Positive
Adjusted p (t test): 9.85e−04, Adjusted p (wilcox): 2.39e−03
Log Metabolite Abundance
2
1
0
−1
−2
EM
Metastatic
Condition

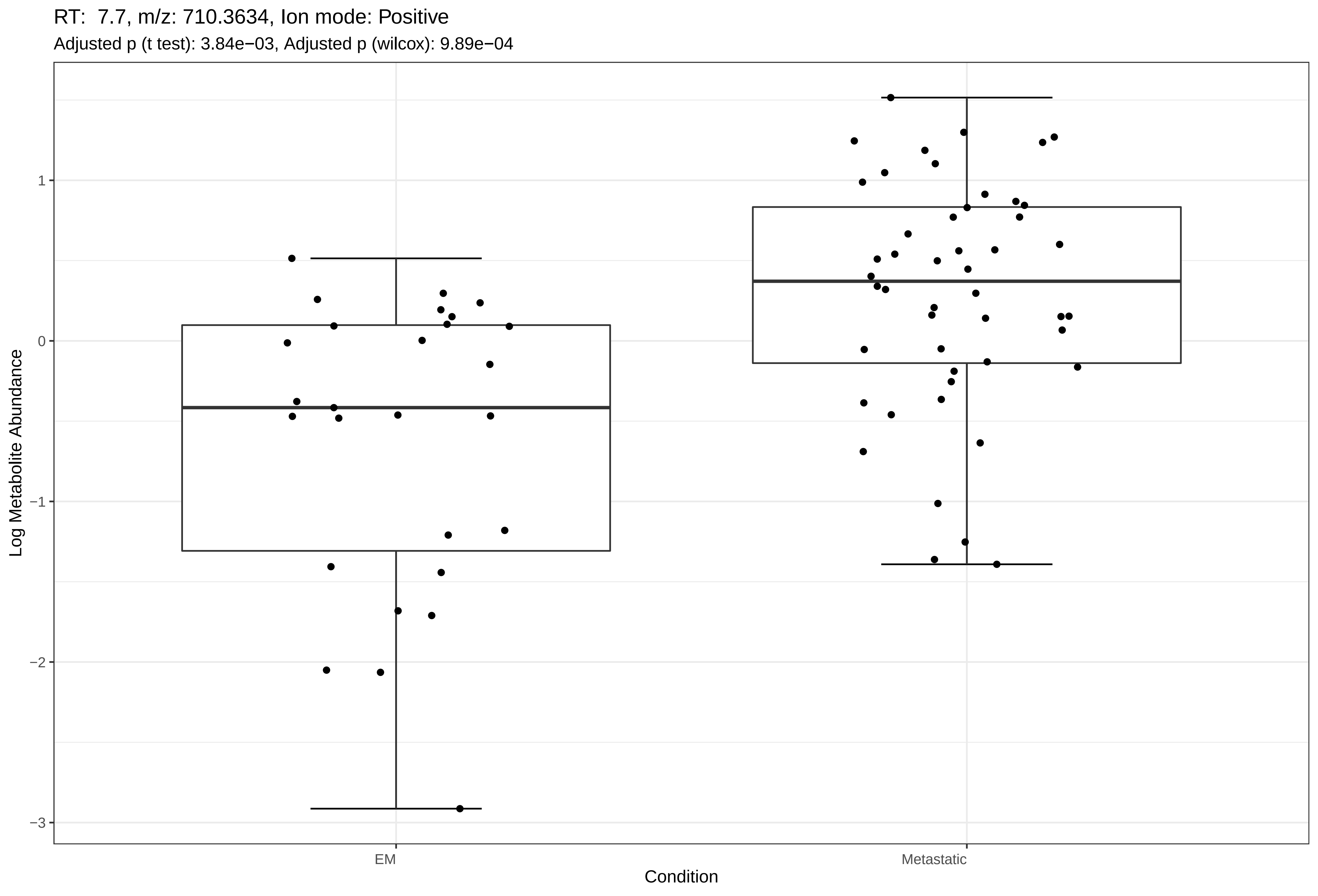

RT: 7.7, m/z: 710.3634, Ion mode: Positive
Adjusted p (t test): 3.84e−03, Adjusted p (wilcox): 9.89e−04
Log Metabolite Abundance
1
0
−1
−2
−3
EM
Metastatic
Condition

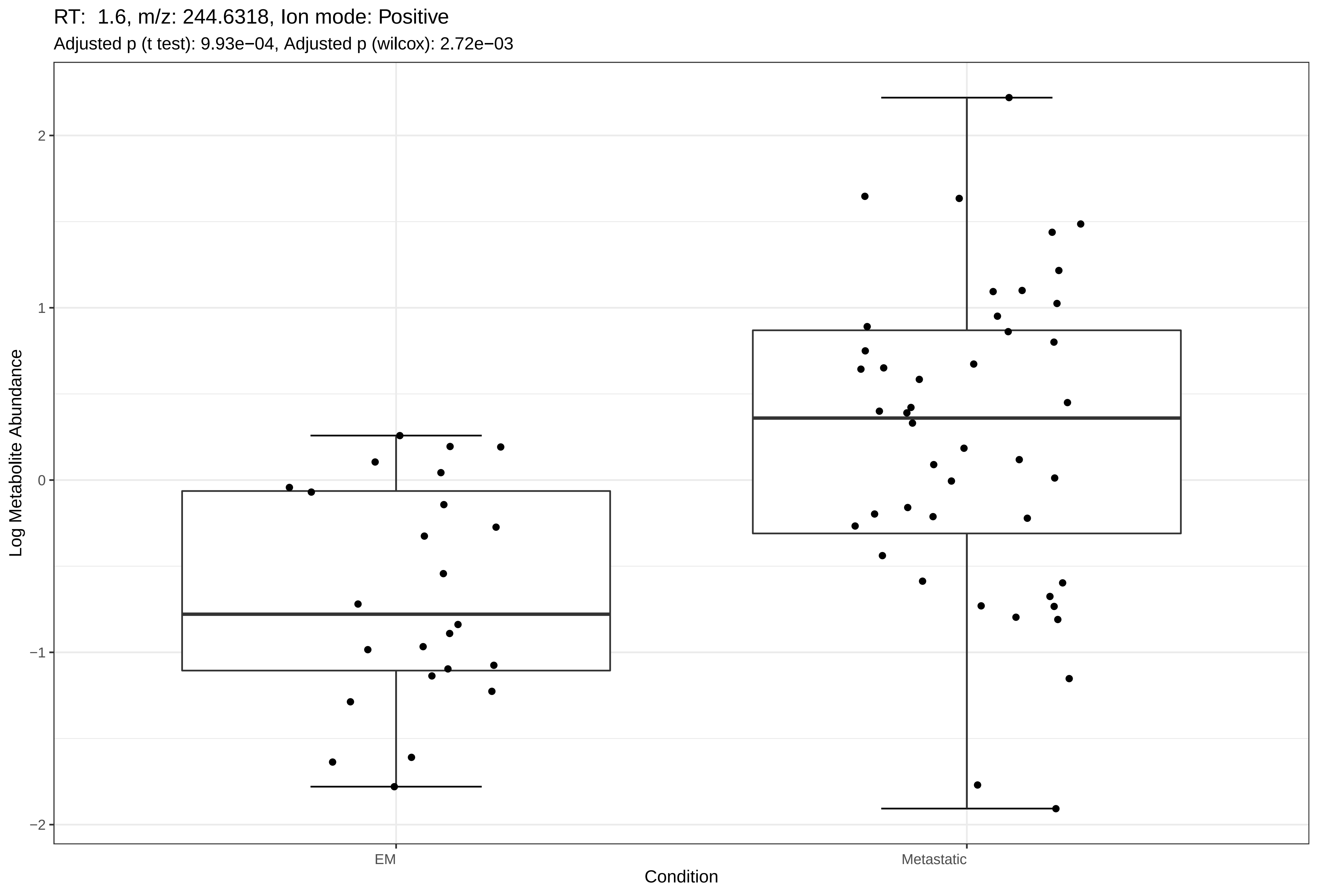

RT: 1.6, m/z: 244.6318, Ion mode: Positive
Adjusted p (t test): 9.93e−04, Adjusted p (wilcox): 2.72e−03
Log Metabolite Abundance
2
1
0
−1
−2
EM
Metastatic
Condition

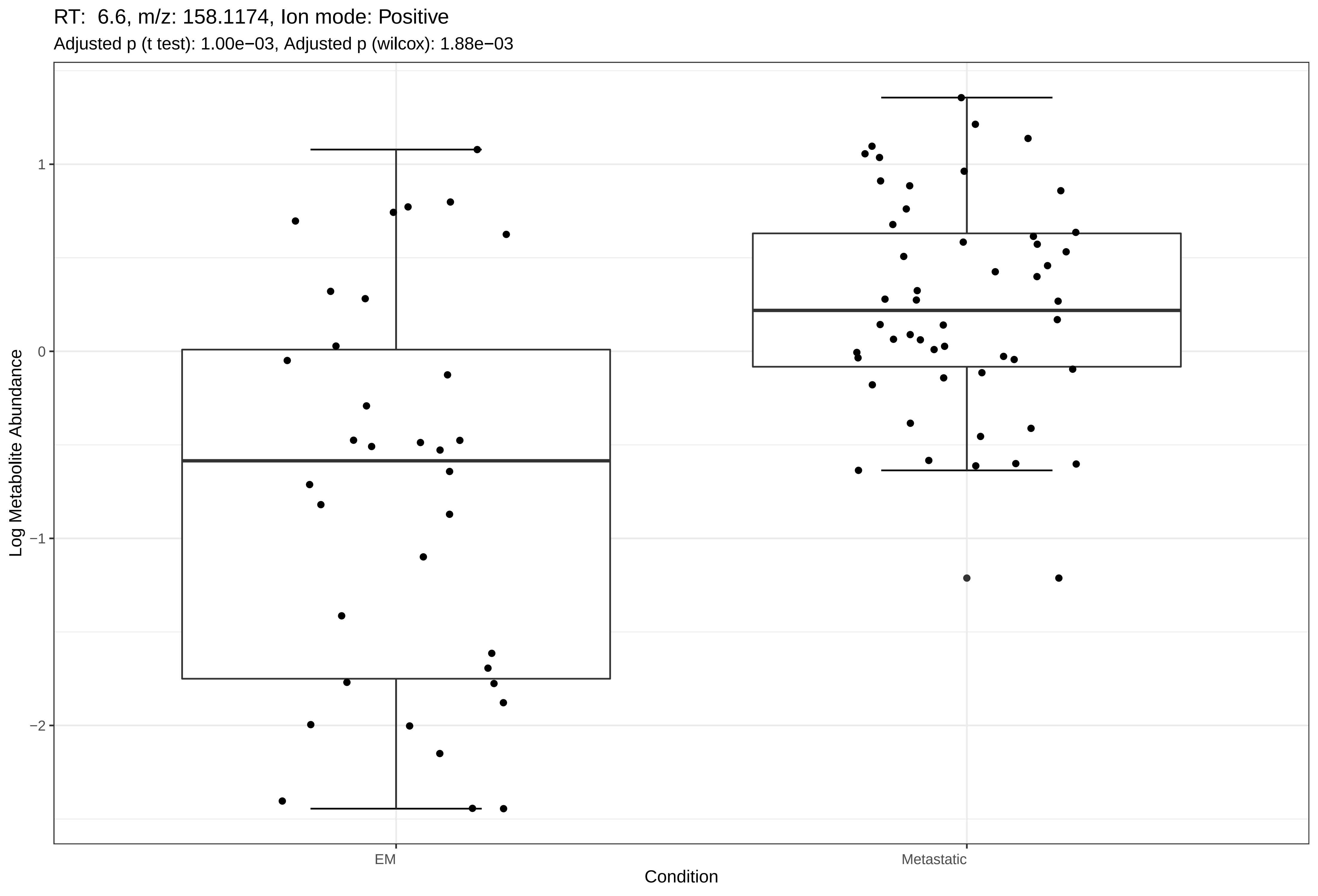
RT: 6.6, m/z: 158.1174, Ion mode: Positive
Adjusted p (t test): 1.00e−03, Adjusted p (wilcox): 1.88e−03
Log Metabolite Abundance
1
0
−1
−2
EM
Metastatic
Condition

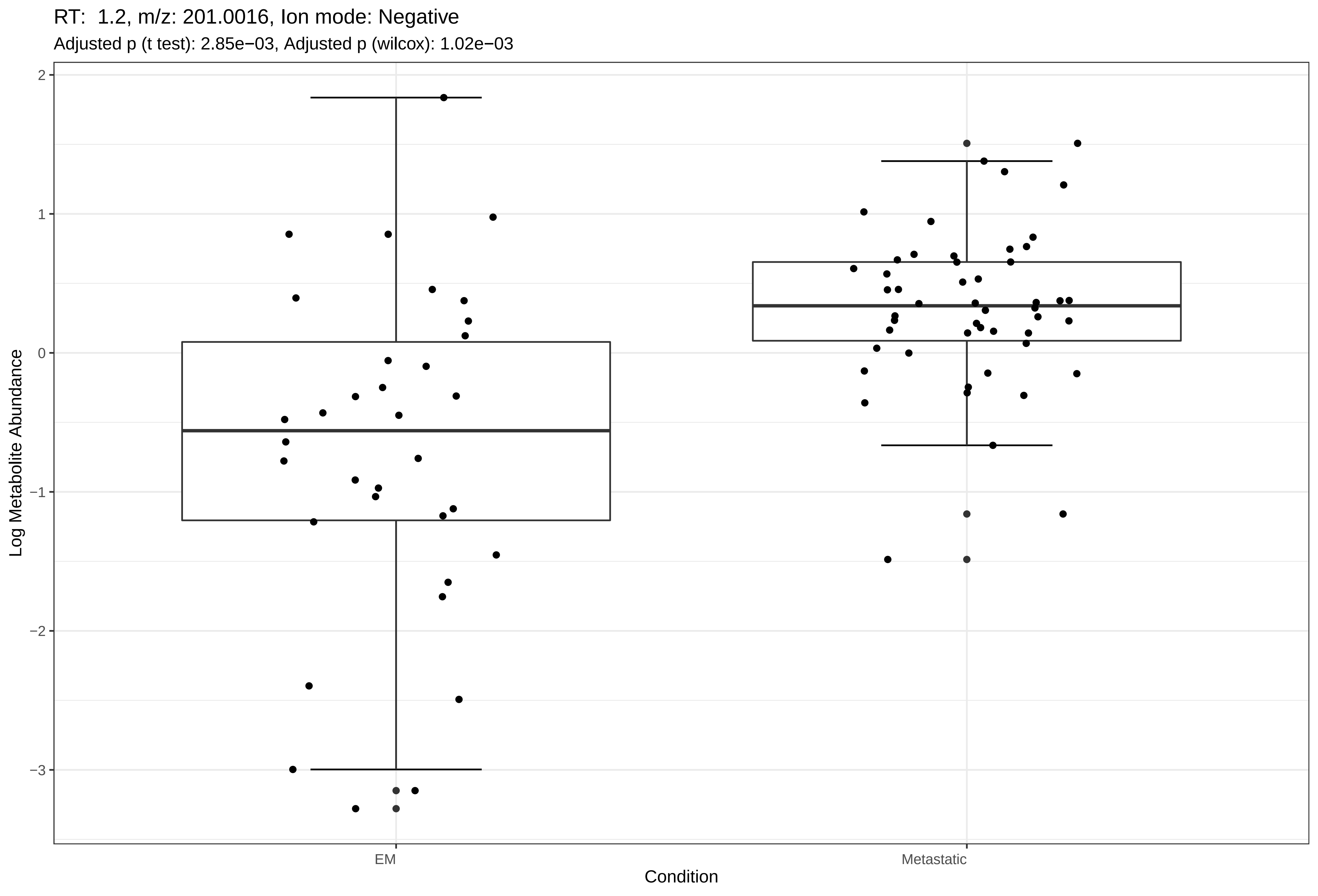
RT: 1.2, m/z: 201.0016, Ion mode: Negative
Adjusted p (t test): 2.85e−03, Adjusted p (wilcox): 1.02e−03
Log Metabolite Abundance
2
1
0
−1
−2
−3
EM
Metastatic
Condition

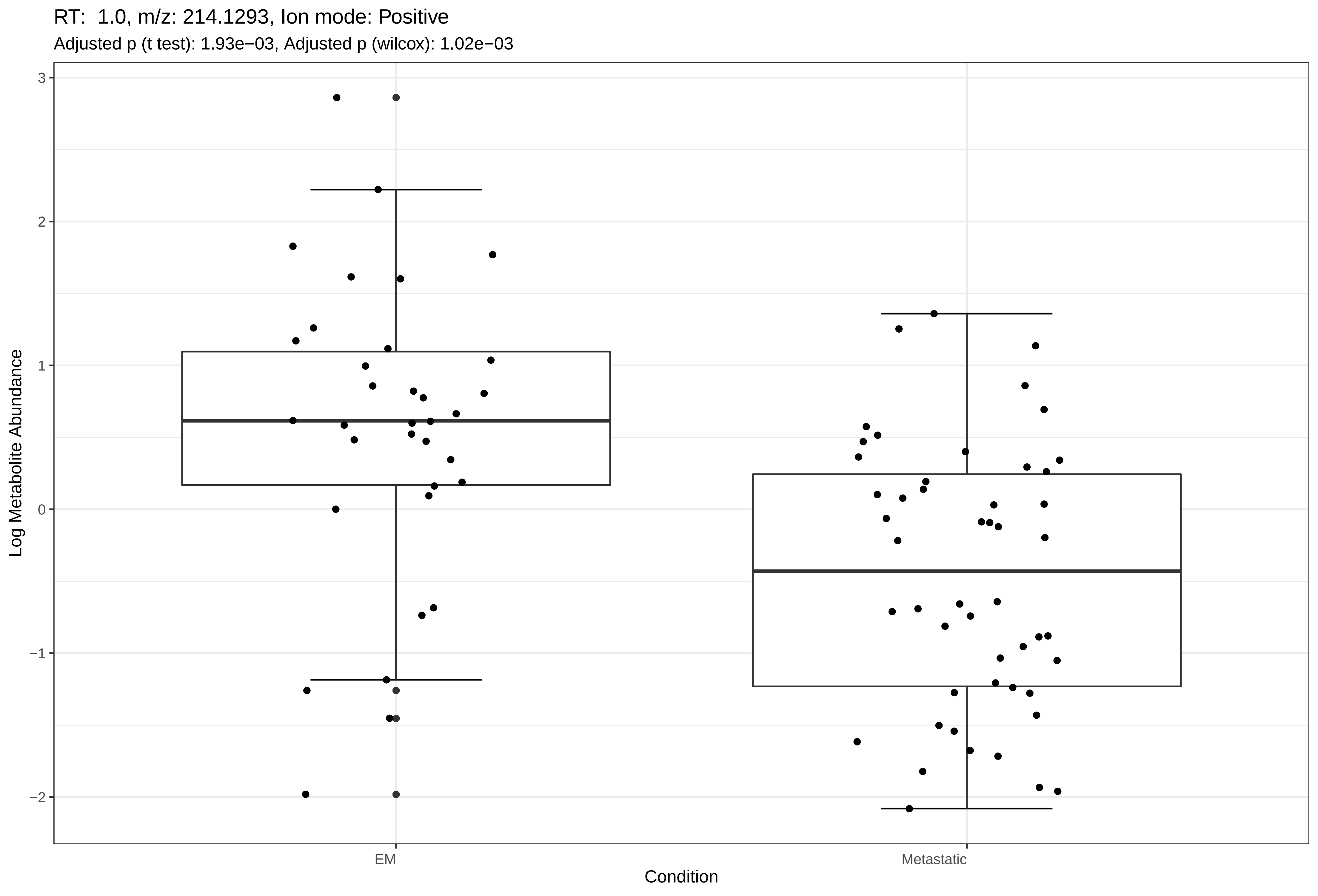
RT: 1.0, m/z: 214.1293, Ion mode: Positive
Adjusted p (t test): 1.93e−03, Adjusted p (wilcox): 1.02e−03
Log Metabolite Abundance
3
2
1
0
−1
−2
EM
Metastatic
Condition

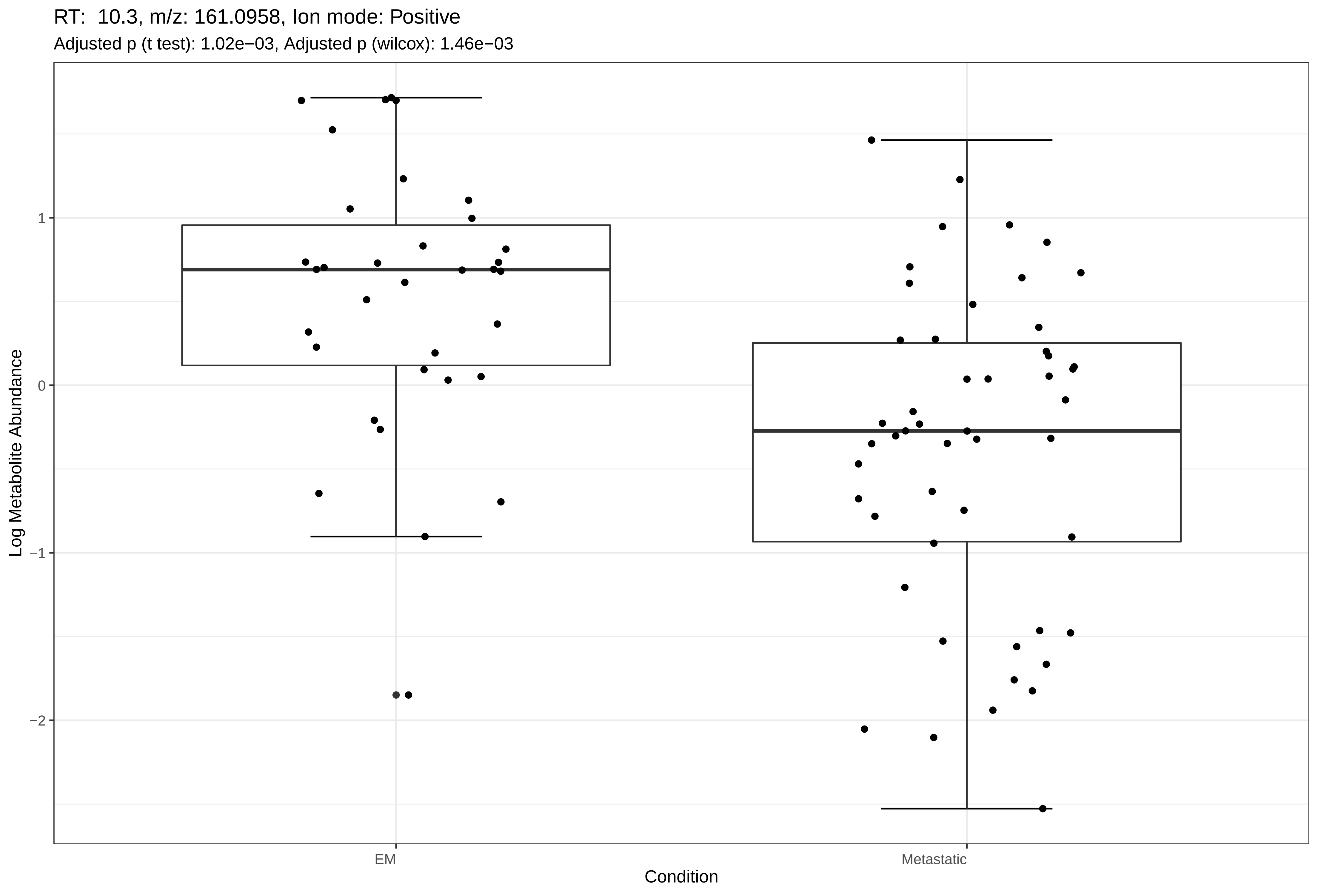

RT: 10.3, m/z: 161.0958, Ion mode: Positive
Adjusted p (t test): 1.02e−03, Adjusted p (wilcox): 1.46e−03
Log Metabolite Abundance
1
0
−1
−2
EM
Metastatic
Condition

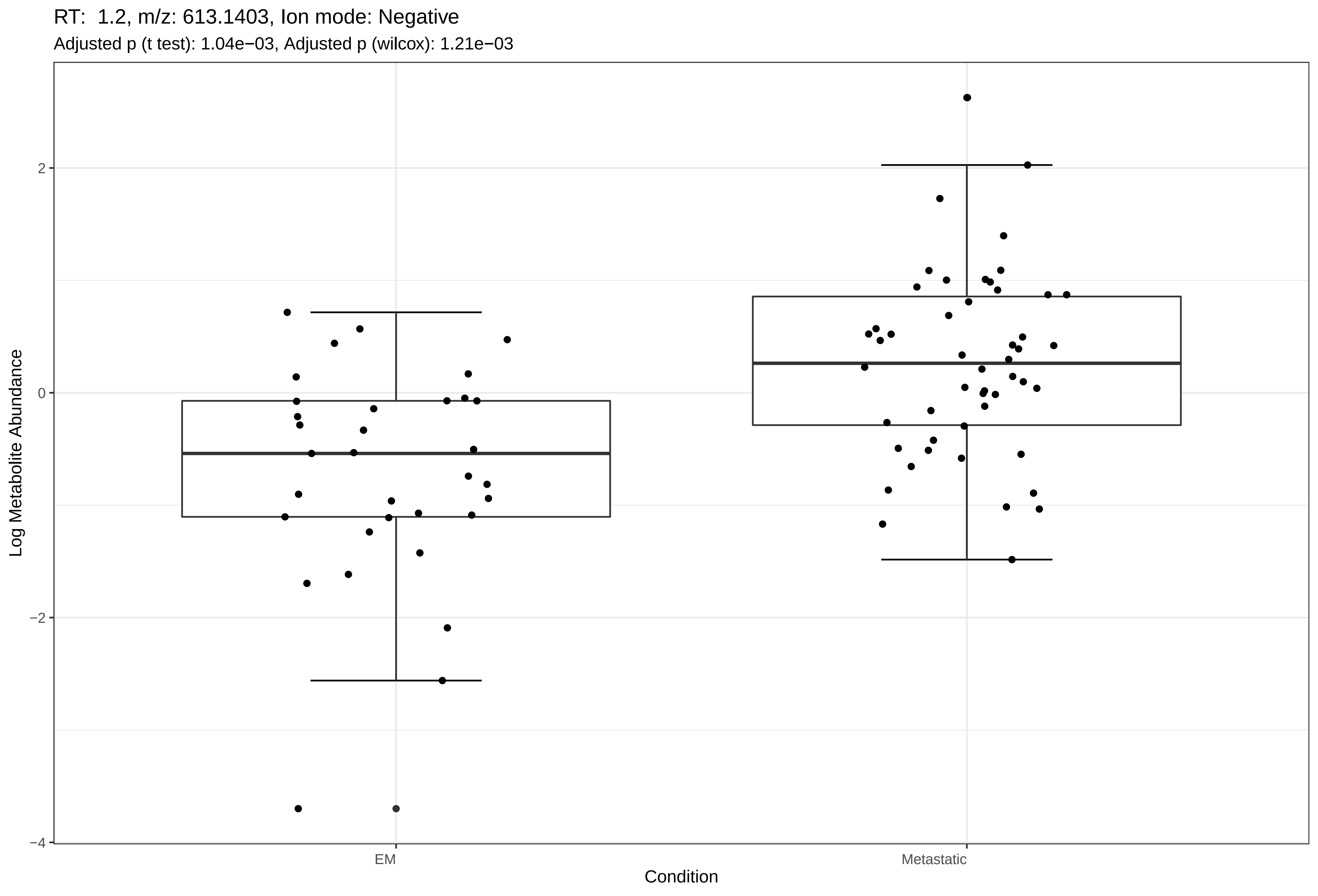
RT: 1.2, m/z: 613.1403, Ion mode: Negative
Adjusted p (t test): 1.04e−03, Adjusted p (wilcox): 1.21e−03
Log Metabolite Abundance
2
0
−2
−4
EM
Metastatic
Condition

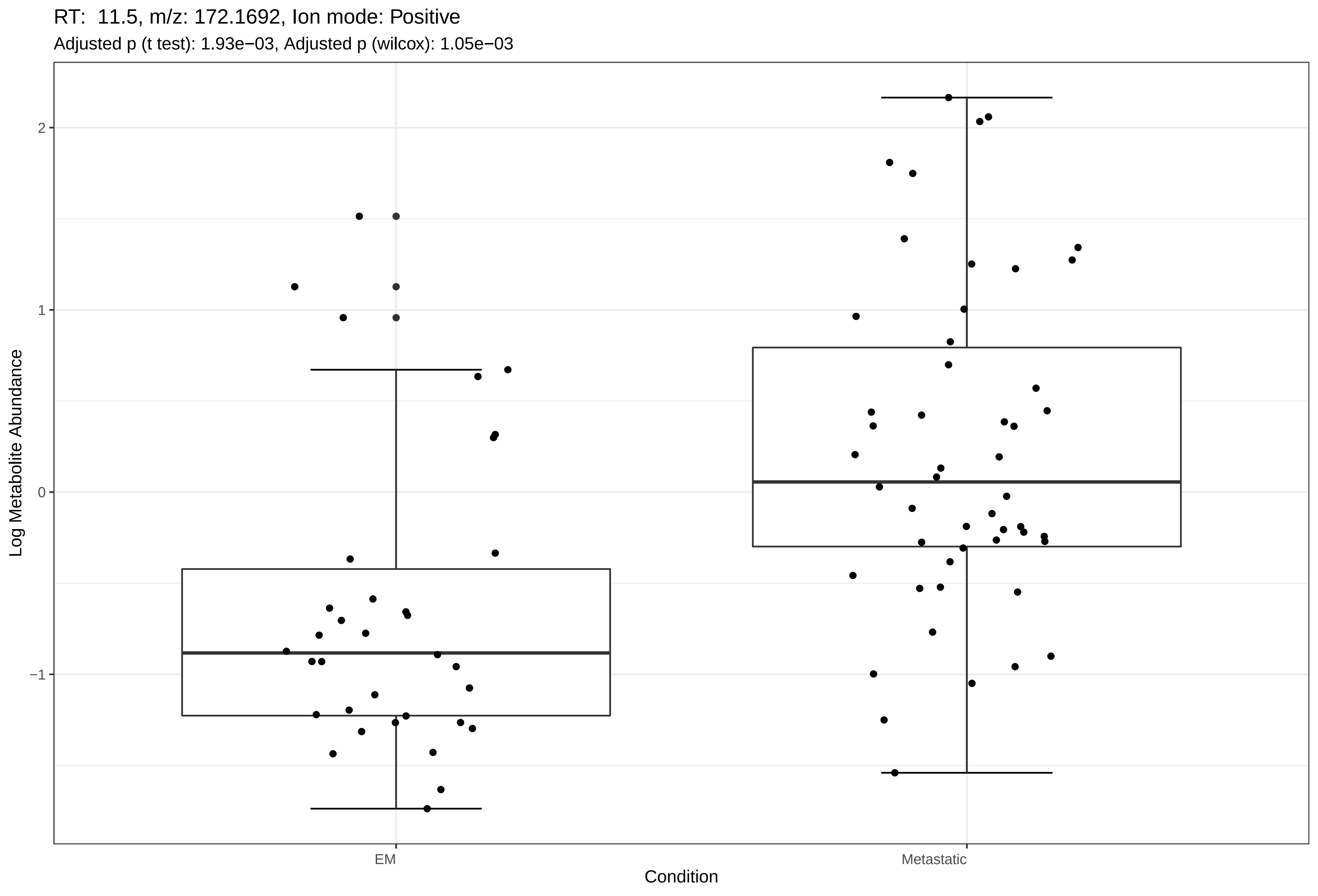
RT: 11.5, m/z: 172.1692, Ion mode: Positive
Adjusted p (t test): 1.93e−03, Adjusted p (wilcox): 1.05e−03
Log Metabolite Abundance
2
1
0
−1
EM
Metastatic
Condition

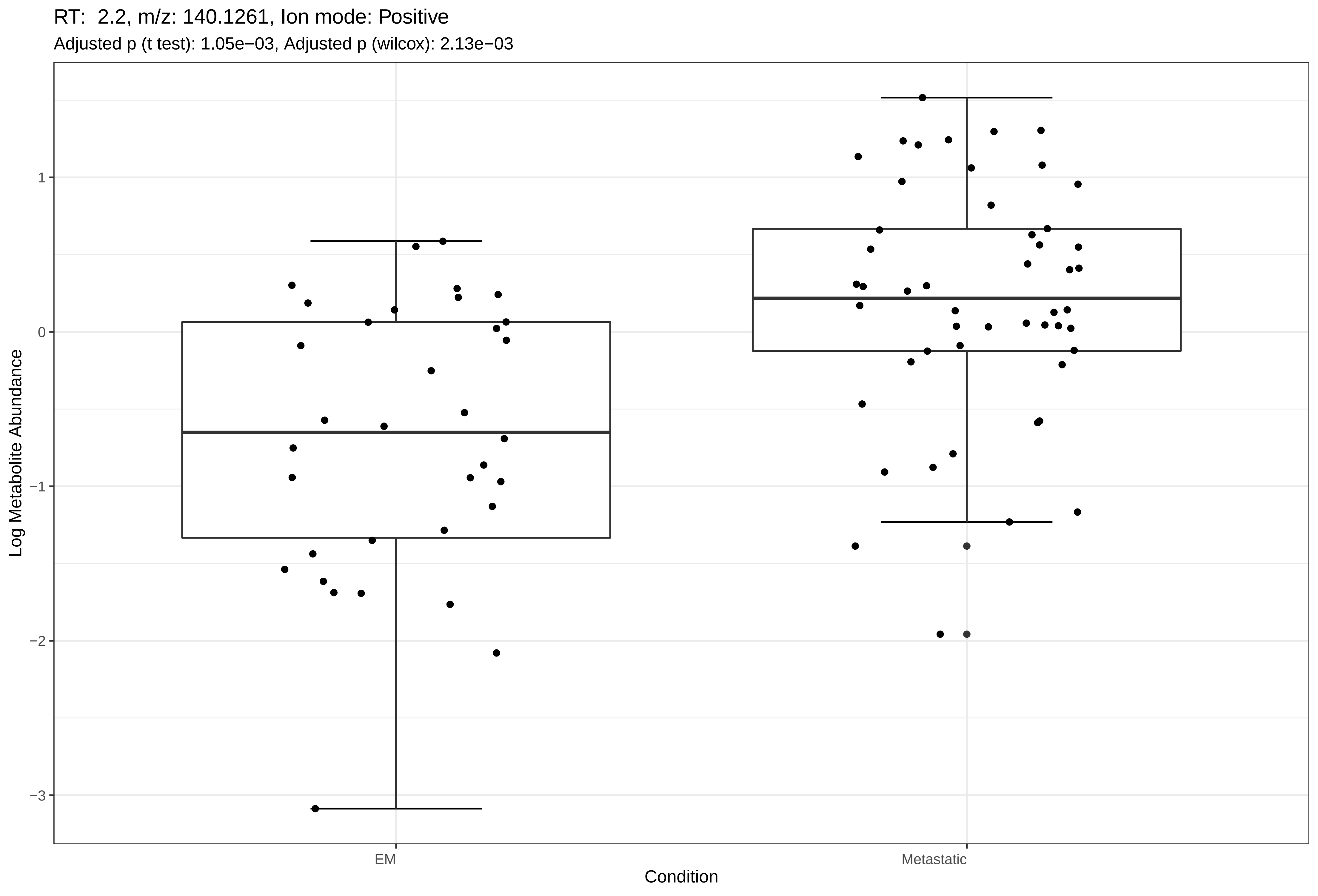

RT: 2.2, m/z: 140.1261, Ion mode: Positive
Adjusted p (t test): 1.05e−03, Adjusted p (wilcox): 2.13e−03
Log Metabolite Abundance
1
0
−1
−2
−3
EM
Metastatic
Condition

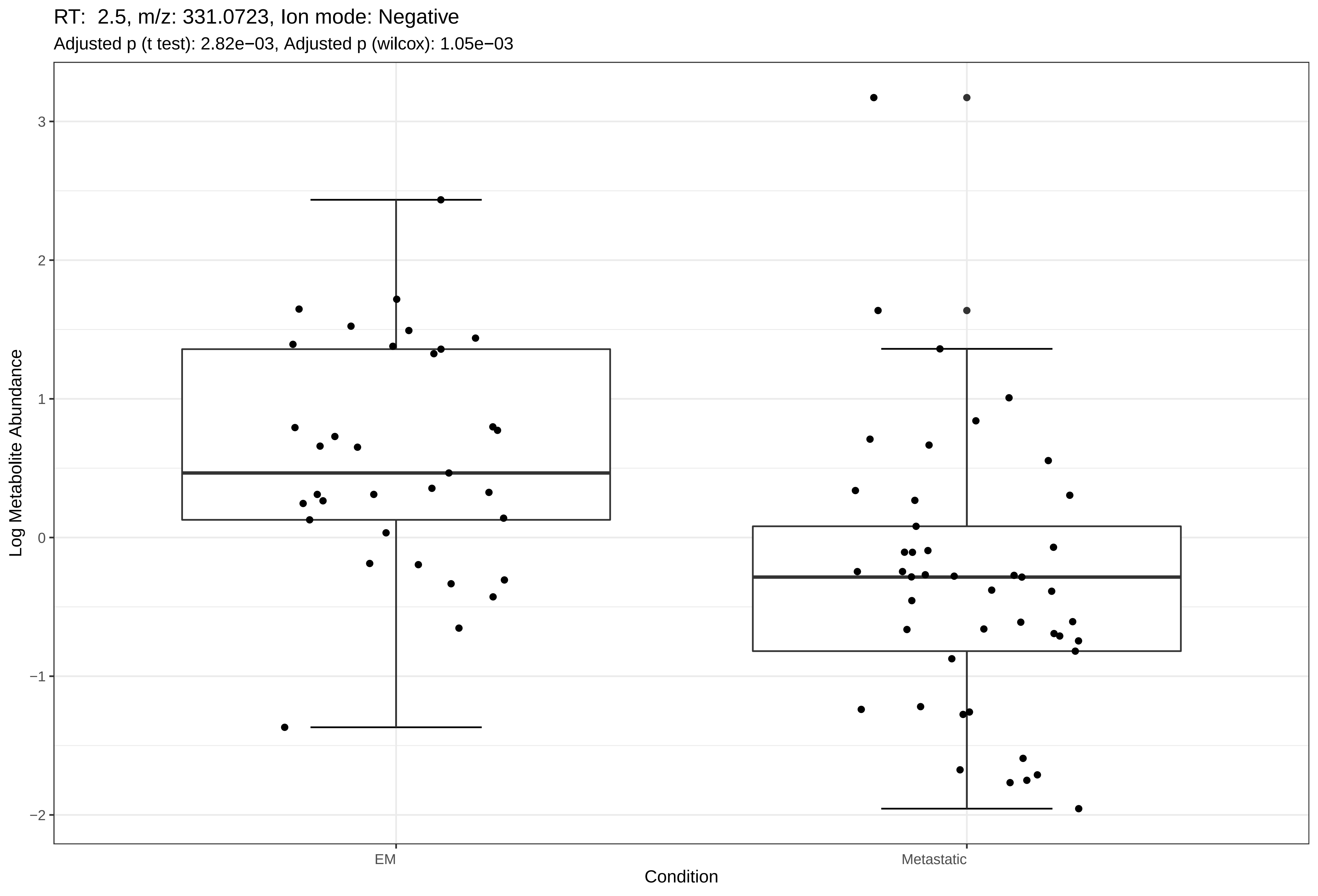

RT: 2.5, m/z: 331.0723, Ion mode: Negative
Adjusted p (t test): 2.82e−03, Adjusted p (wilcox): 1.05e−03
Log Metabolite Abundance
3
2
1
0
−1
−2
EM
Metastatic
Condition

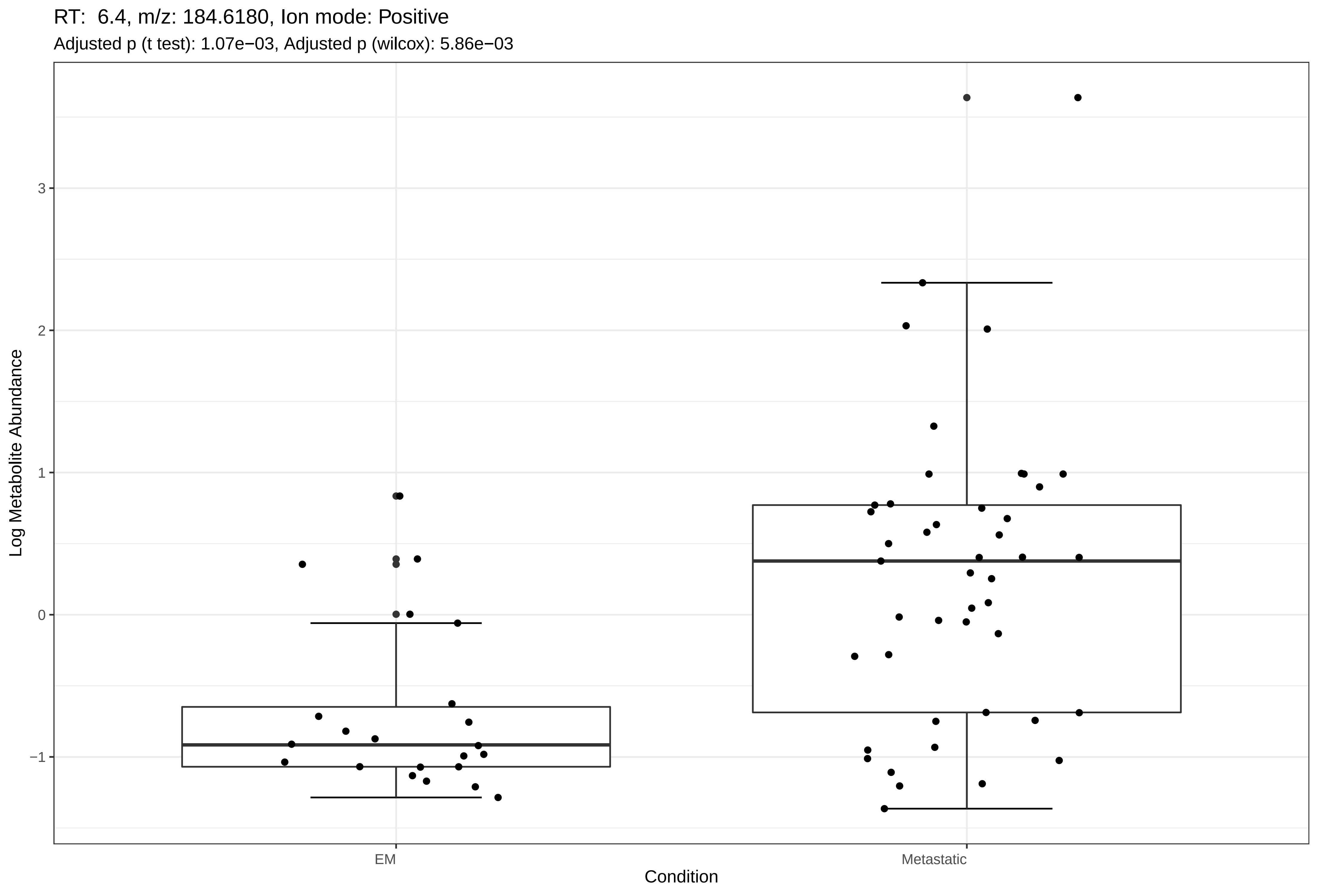
RT: 6.4, m/z: 184.6180, Ion mode: Positive
Adjusted p (t test): 1.07e−03, Adjusted p (wilcox): 5.86e−03
Log Metabolite Abundance
3
2
1
0
−1
EM
Metastatic
Condition

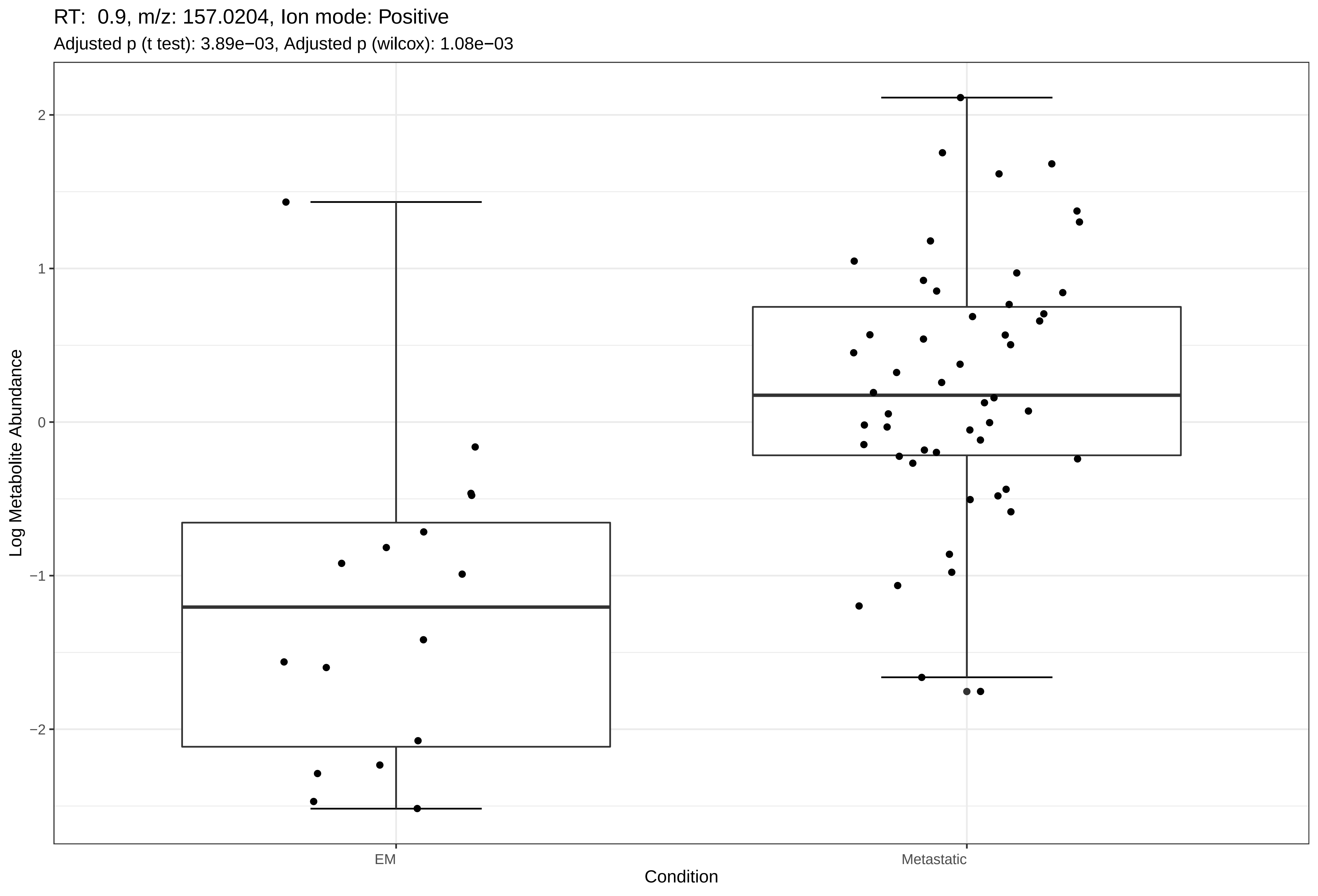

RT: 0.9, m/z: 157.0204, Ion mode: Positive
Adjusted p (t test): 3.89e−03, Adjusted p (wilcox): 1.08e−03
Log Metabolite Abundance
2
1
0
−1
−2
EM
Metastatic
Condition

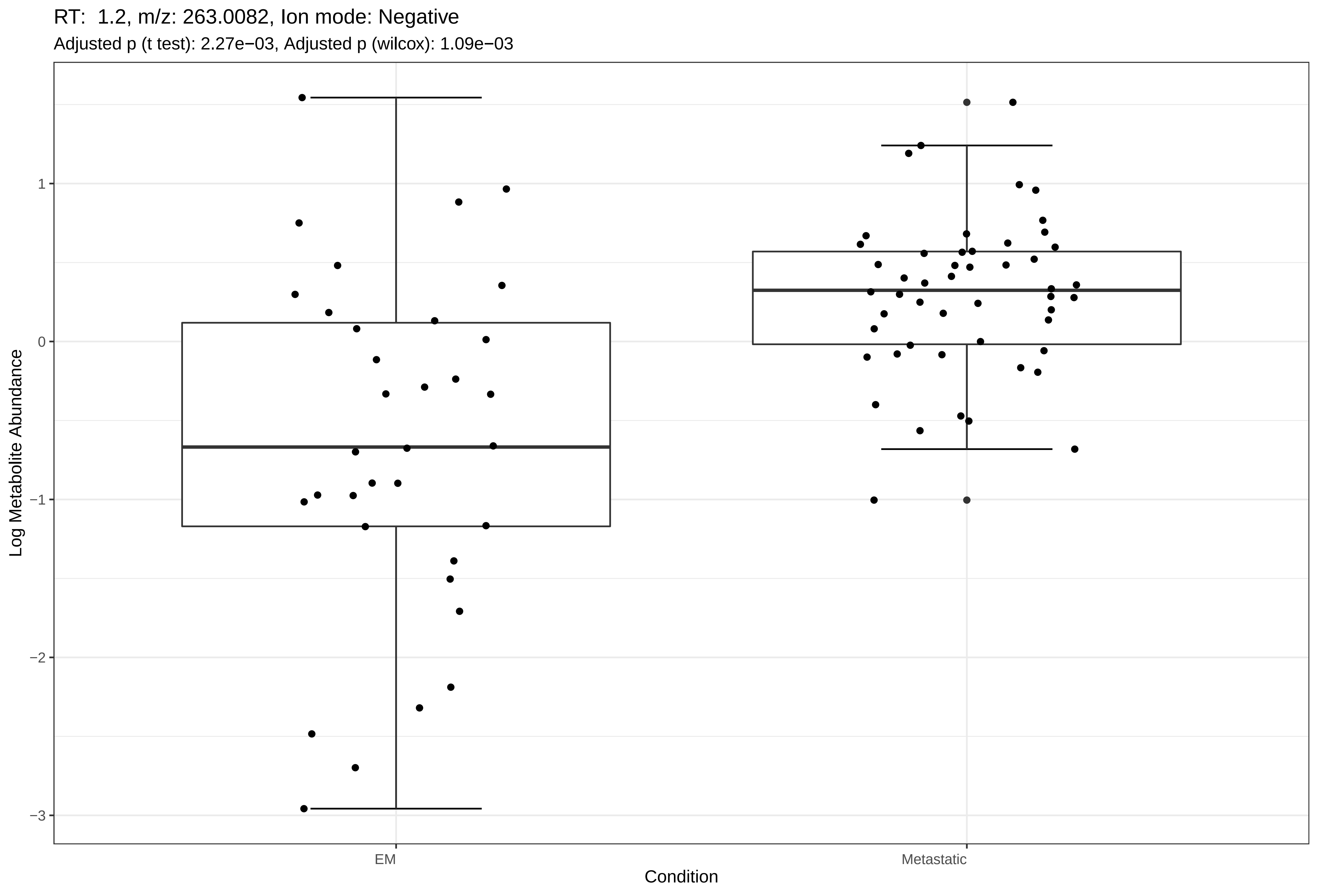

RT: 1.2, m/z: 263.0082, Ion mode: Negative
Adjusted p (t test): 2.27e−03, Adjusted p (wilcox): 1.09e−03
Log Metabolite Abundance
1
0
−1
−2
−3
EM
Metastatic
Condition

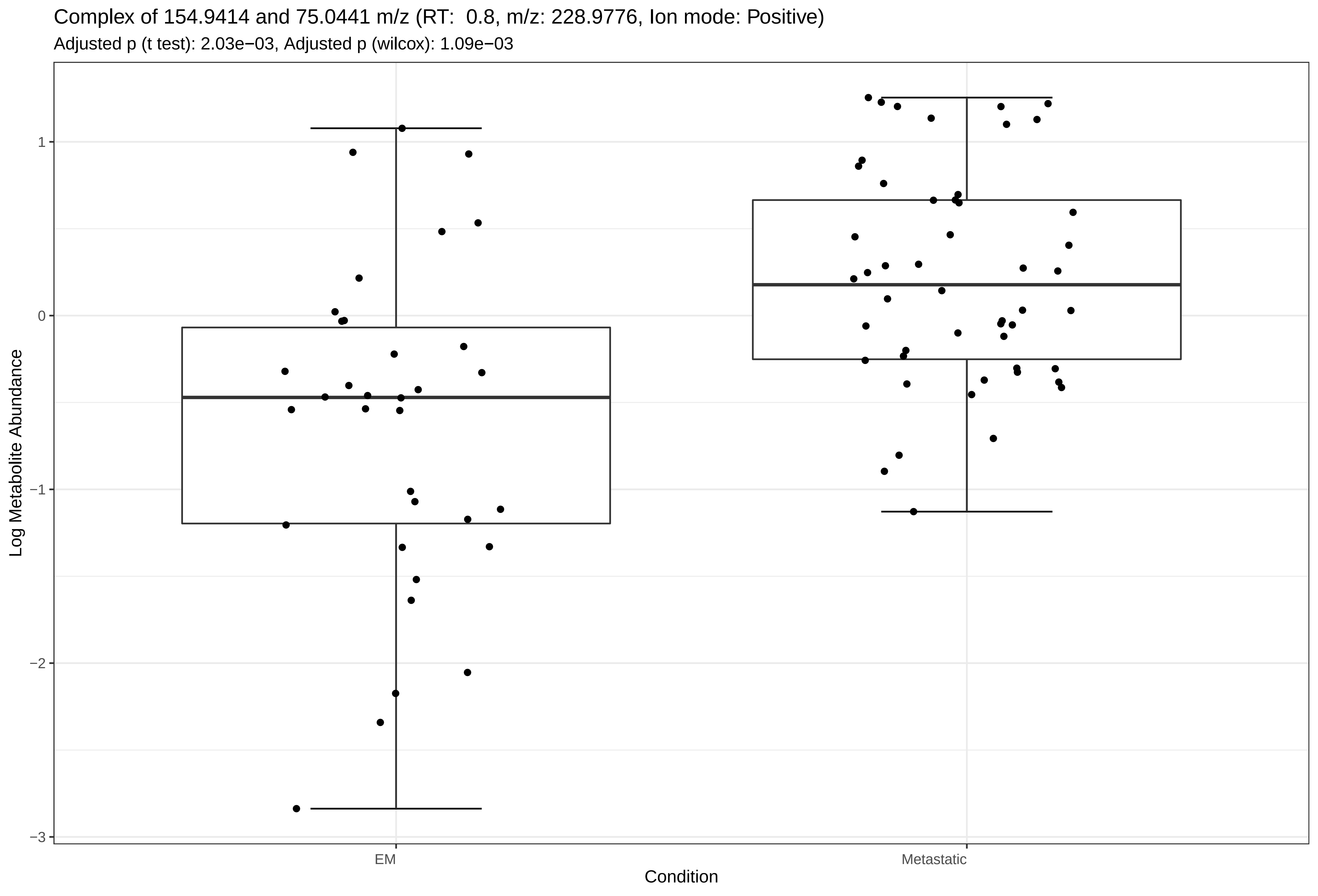
Complex of 154.9414 and 75.0441 m/z (RT: 0.8, m/z: 228.9776, Ion mode: Positive)
Adjusted p (t test): 2.03e−03, Adjusted p (wilcox): 1.09e−03
Log Metabolite Abundance
1
0
−1
−2
−3
EM
Metastatic
Condition

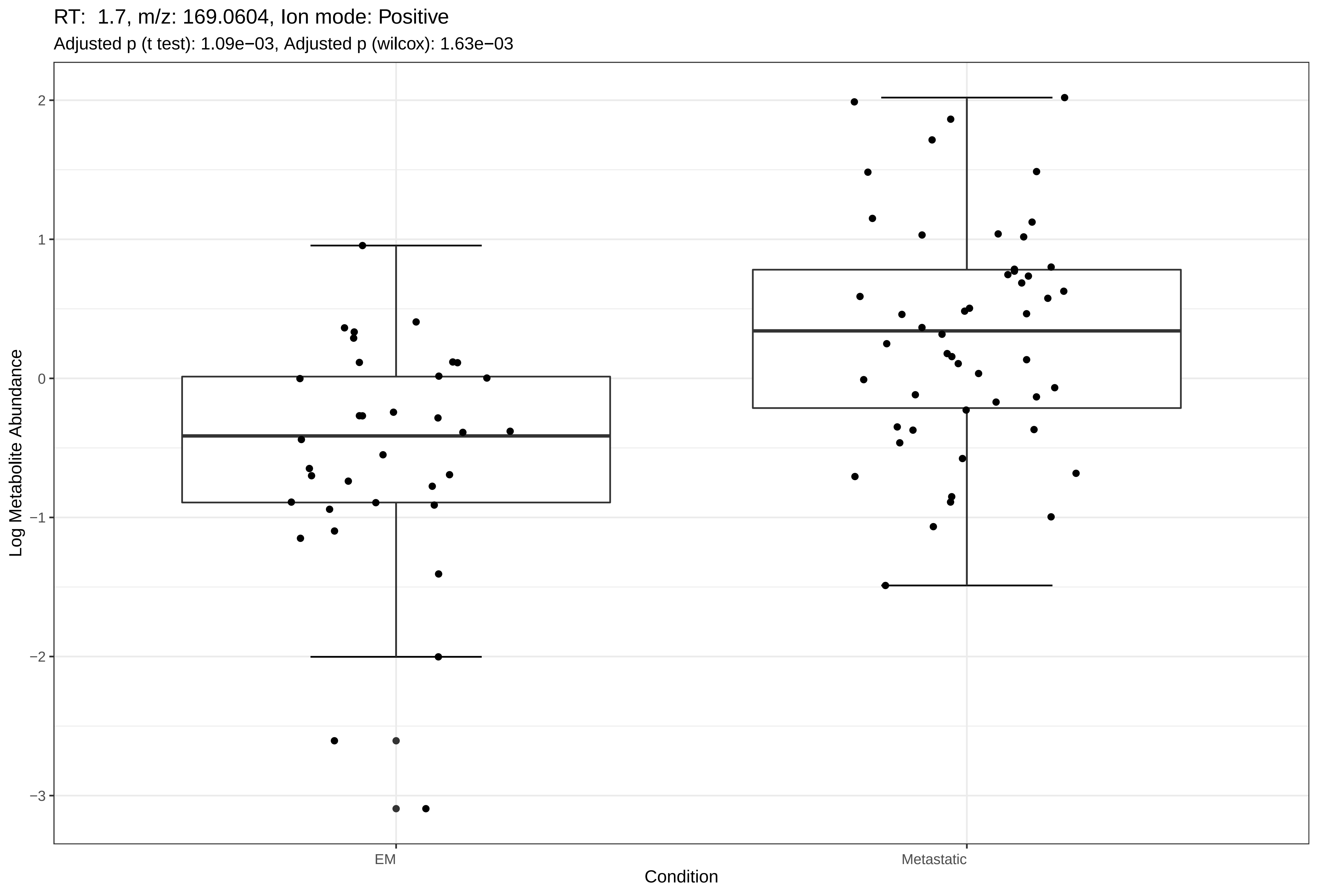

RT: 1.7, m/z: 169.0604, Ion mode: Positive
Adjusted p (t test): 1.09e−03, Adjusted p (wilcox): 1.63e−03
Log Metabolite Abundance
2
1
0
−1
−2
−3
EM
Metastatic
Condition

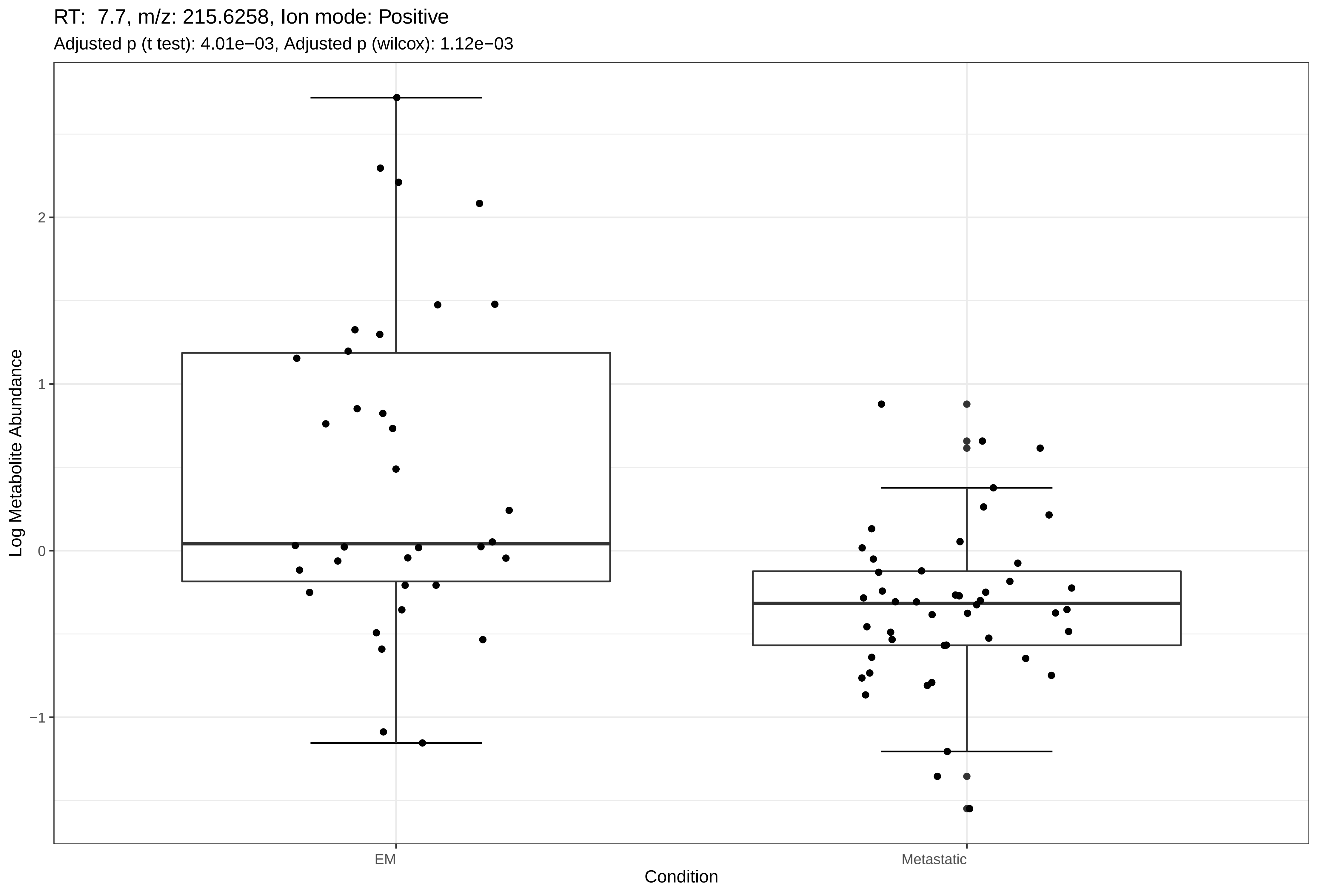
RT: 7.7, m/z: 215.6258, Ion mode: Positive
Adjusted p (t test): 4.01e−03, Adjusted p (wilcox): 1.12e−03
Log Metabolite Abundance
2
1
0
−1
EM
Metastatic
Condition

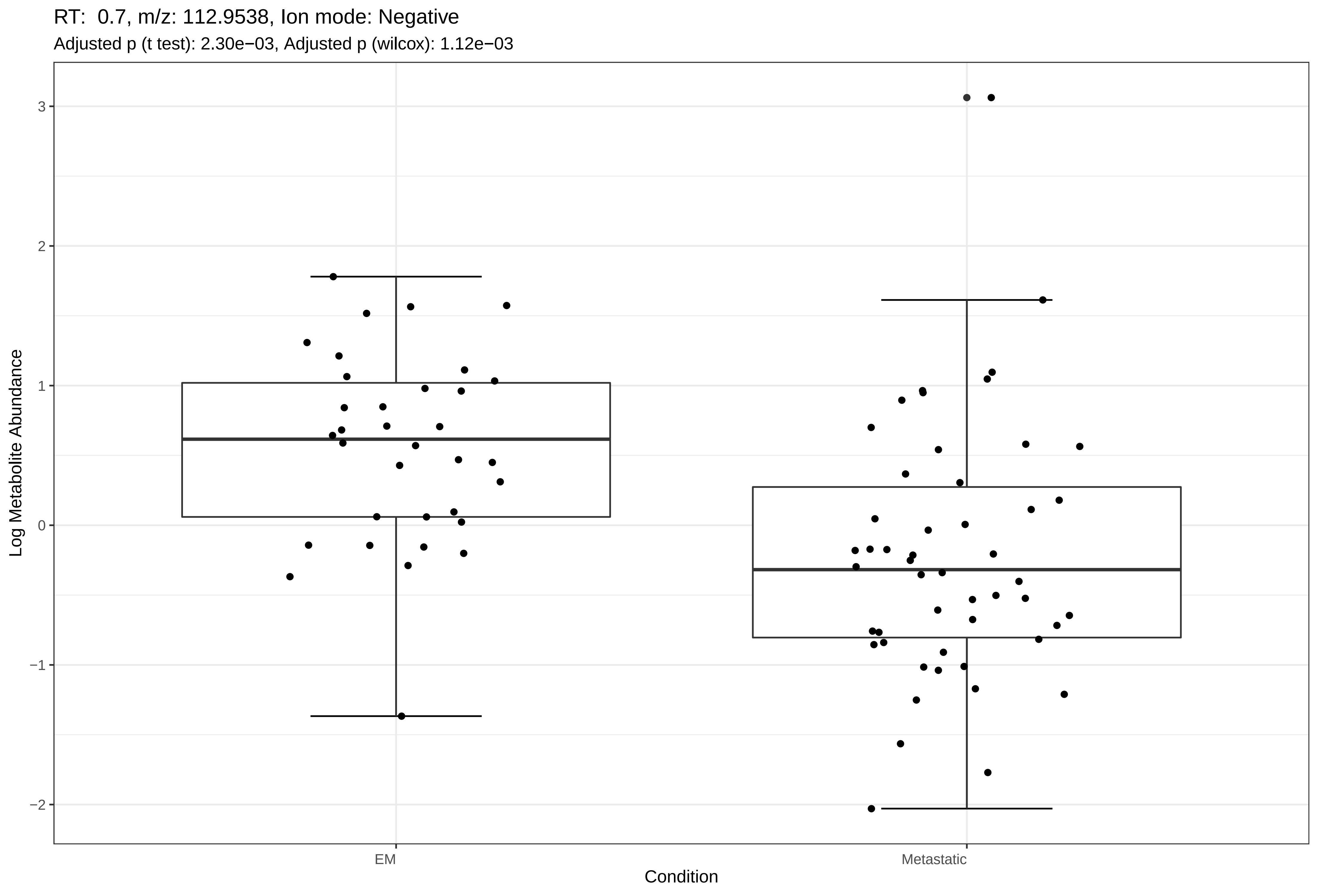
RT: 0.7, m/z: 112.9538, Ion mode: Negative
Adjusted p (t test): 2.30e−03, Adjusted p (wilcox): 1.12e−03
Log Metabolite Abundance
3
2
1
0
−1
−2
EM
Metastatic
Condition

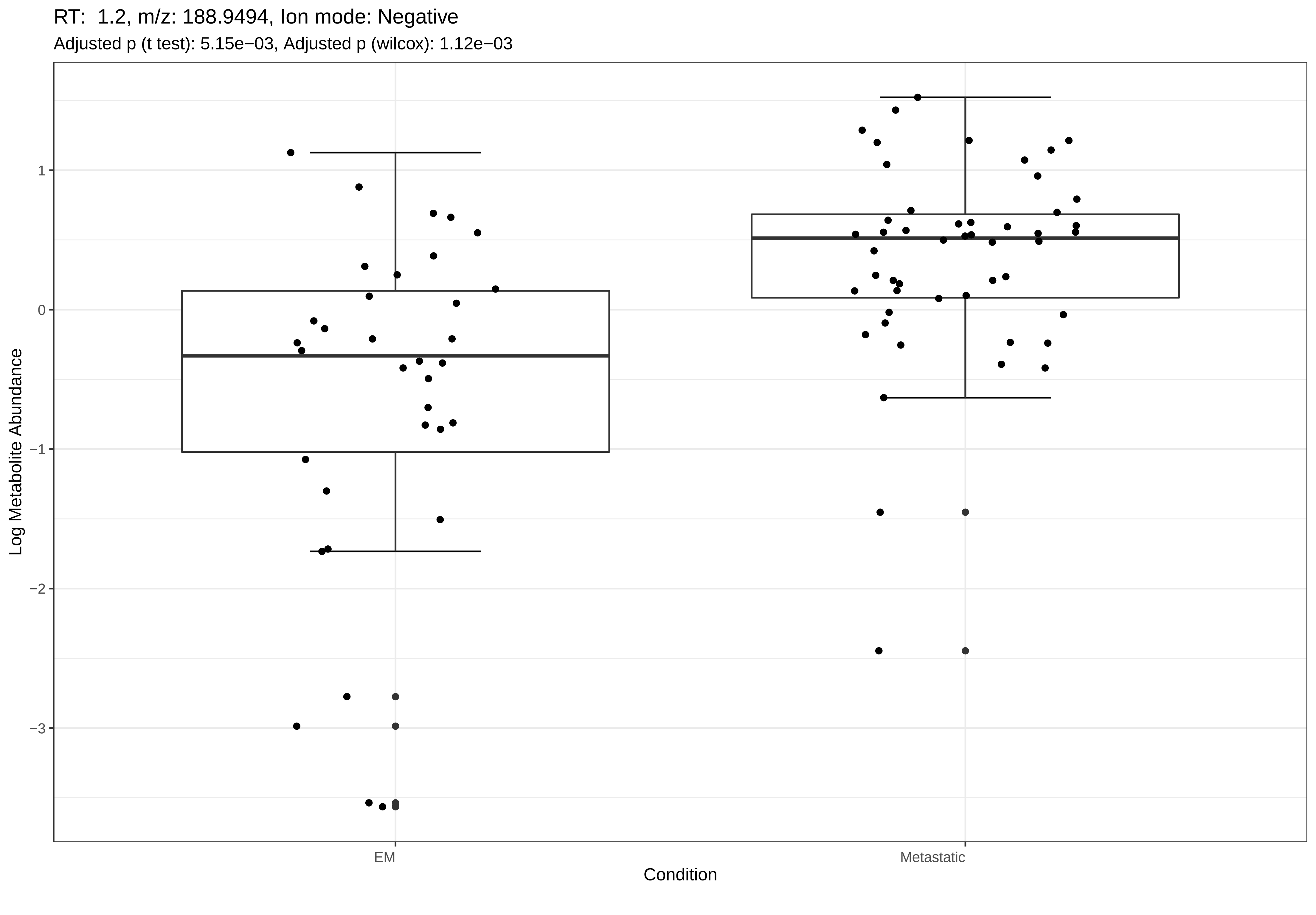

RT: 1.2, m/z: 188.9494, Ion mode: Negative
Adjusted p (t test): 5.15e−03, Adjusted p (wilcox): 1.12e−03
Log Metabolite Abundance
1
0
−1
−2
−3
EM
Metastatic
Condition

RT: 0.7, m/z: 131.0826, Ion mode: Negative

Adjusted p (t test): 3.54e−03, Adjusted p (wilcox): 1.12e−03

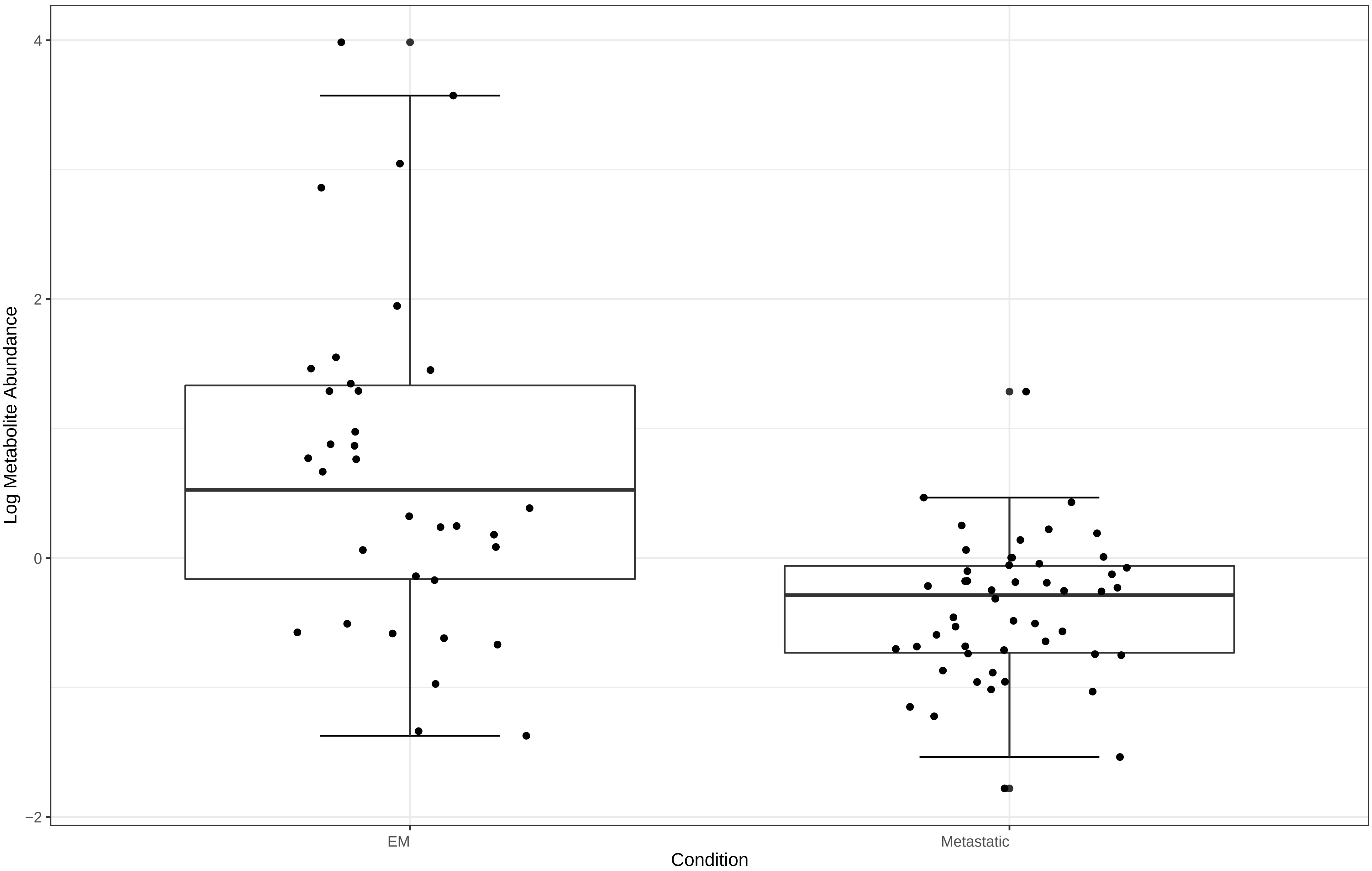

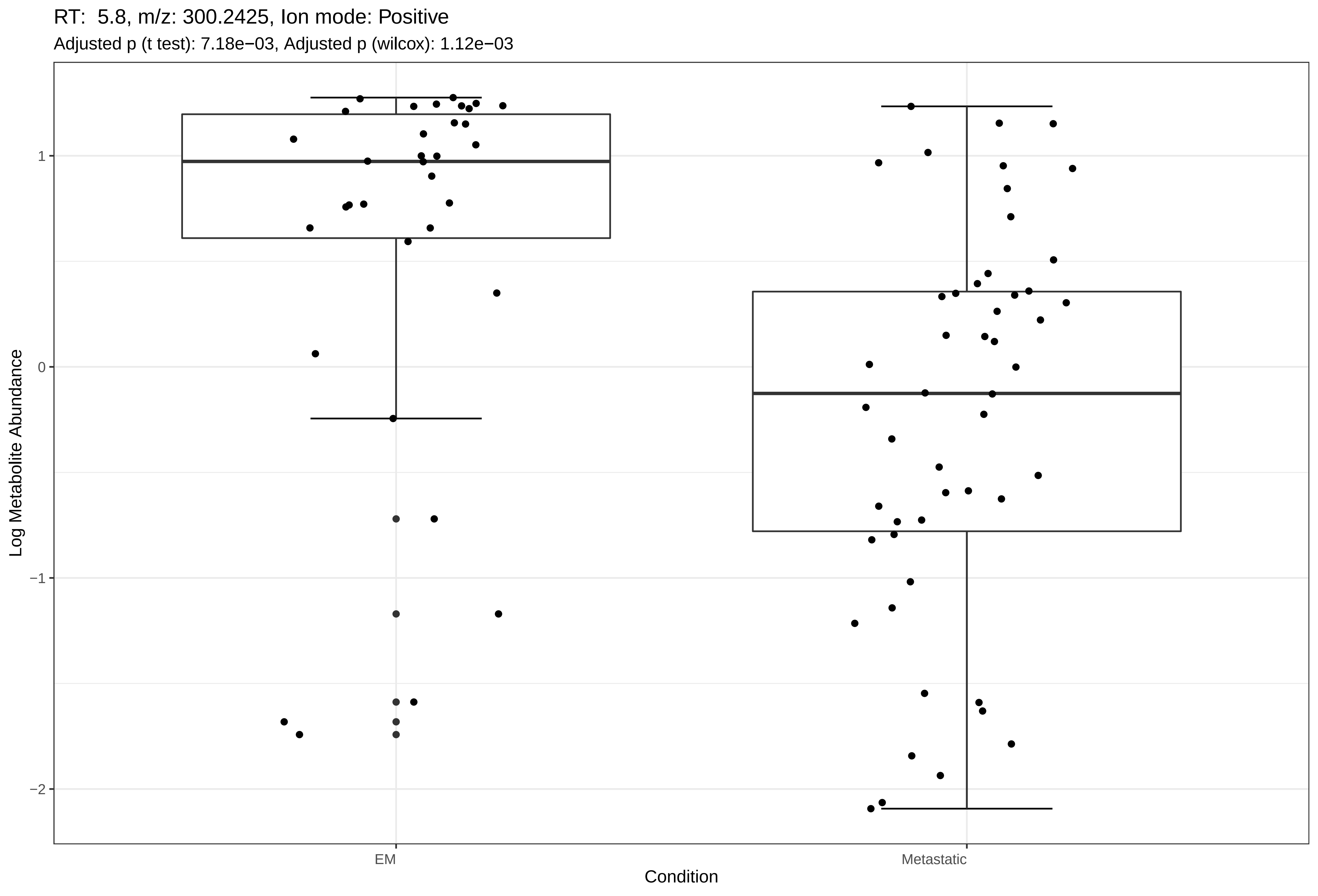
RT: 5.8, m/z: 300.2425, Ion mode: Positive
Adjusted p (t test): 7.18e−03, Adjusted p (wilcox): 1.12e−03
Log Metabolite Abundance
1
0
−1
−2
EM
Metastatic
Condition

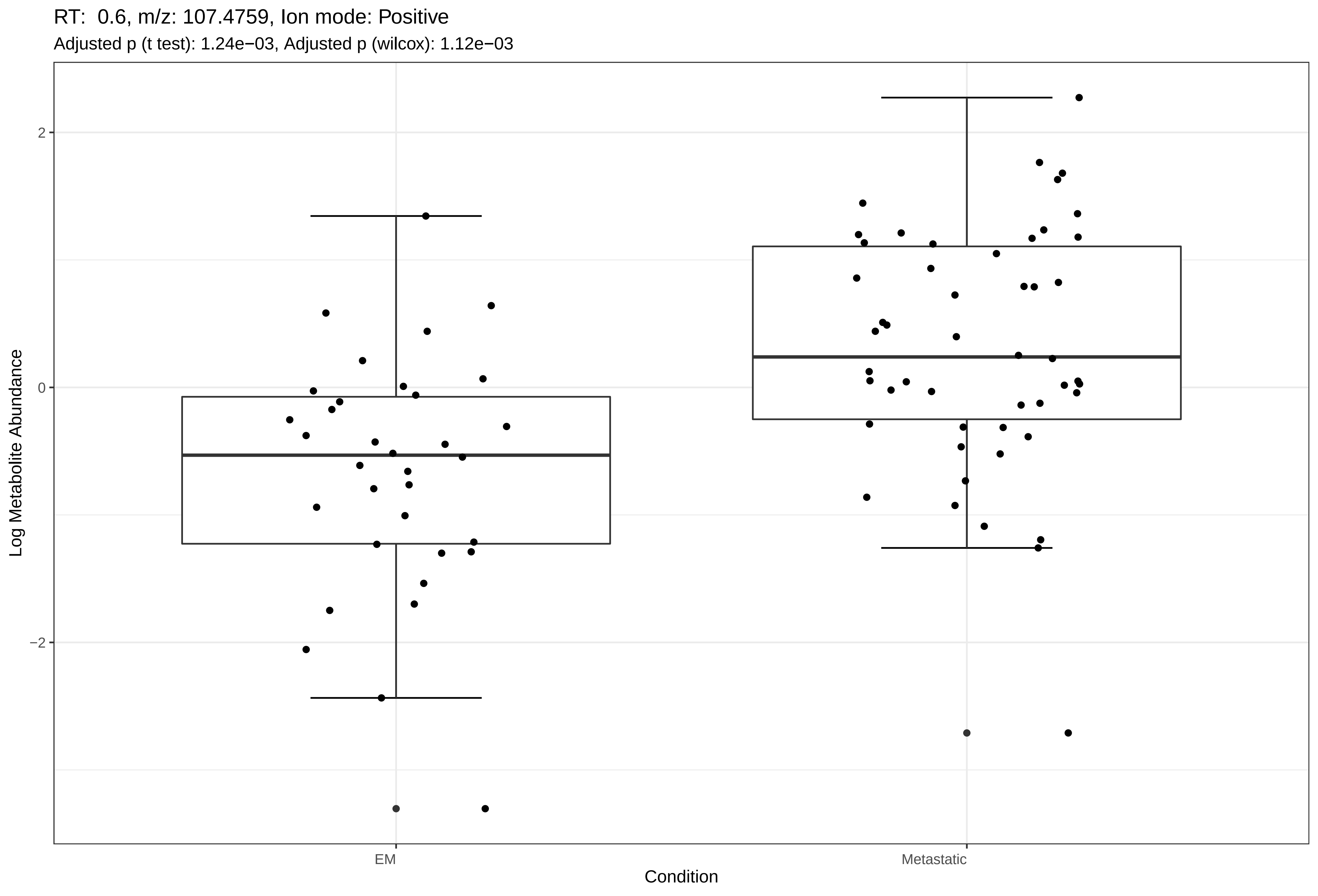

RT: 0.6, m/z: 107.4759, Ion mode: Positive
Adjusted p (t test): 1.24e−03, Adjusted p (wilcox): 1.12e−03
Log Metabolite Abundance
2
0
−2
EM
Metastatic
Condition

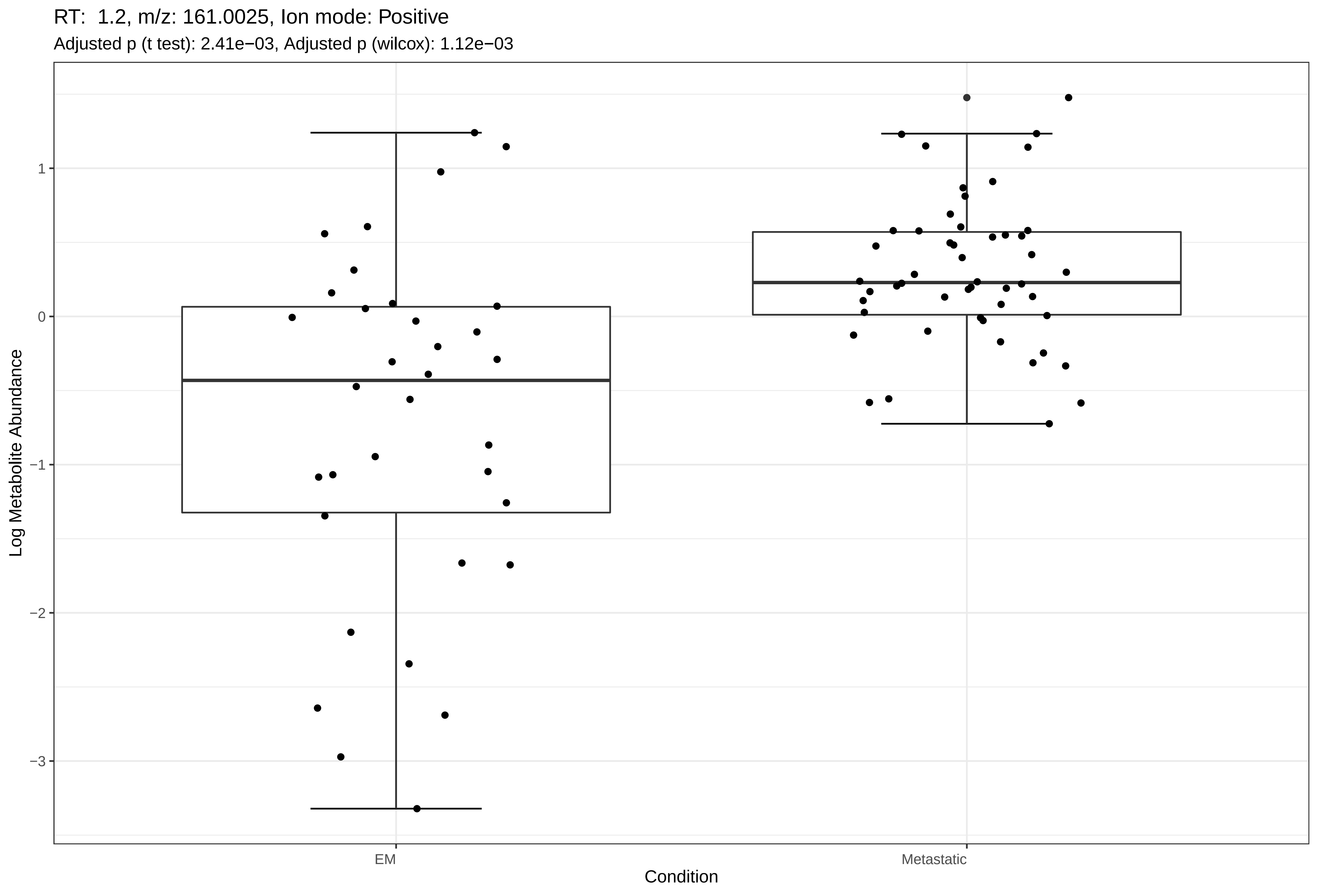
RT: 1.2, m/z: 161.0025, Ion mode: Positive
Adjusted p (t test): 2.41e−03, Adjusted p (wilcox): 1.12e−03
Log Metabolite Abundance
1
0
−1
−2
−3
EM
Metastatic
Condition

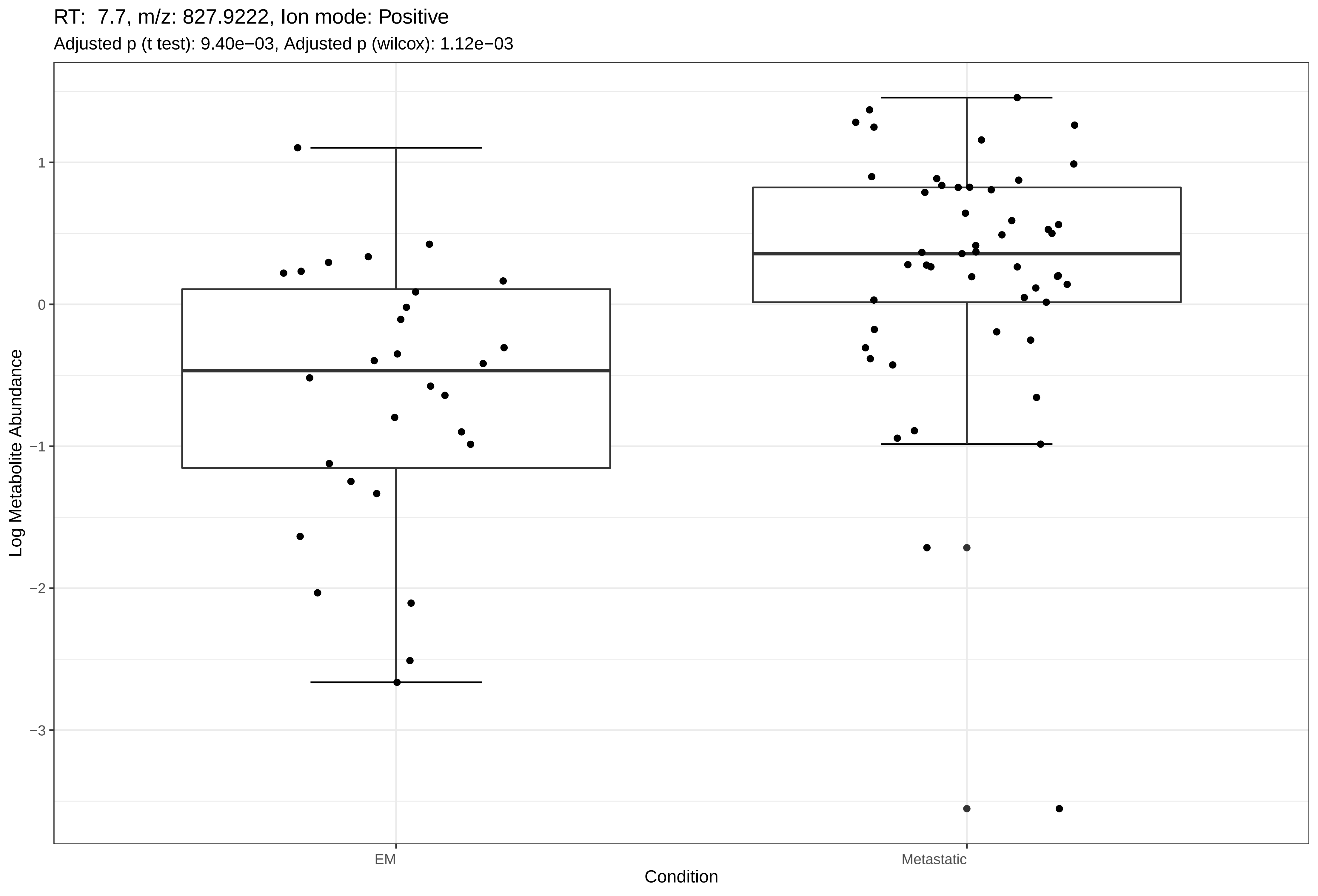

RT: 7.7, m/z: 827.9222, Ion mode: Positive
Adjusted p (t test): 9.40e−03, Adjusted p (wilcox): 1.12e−03
1
0
−1
−2
−3
Log Metabolite Abundance
EM
Metastatic
Condition

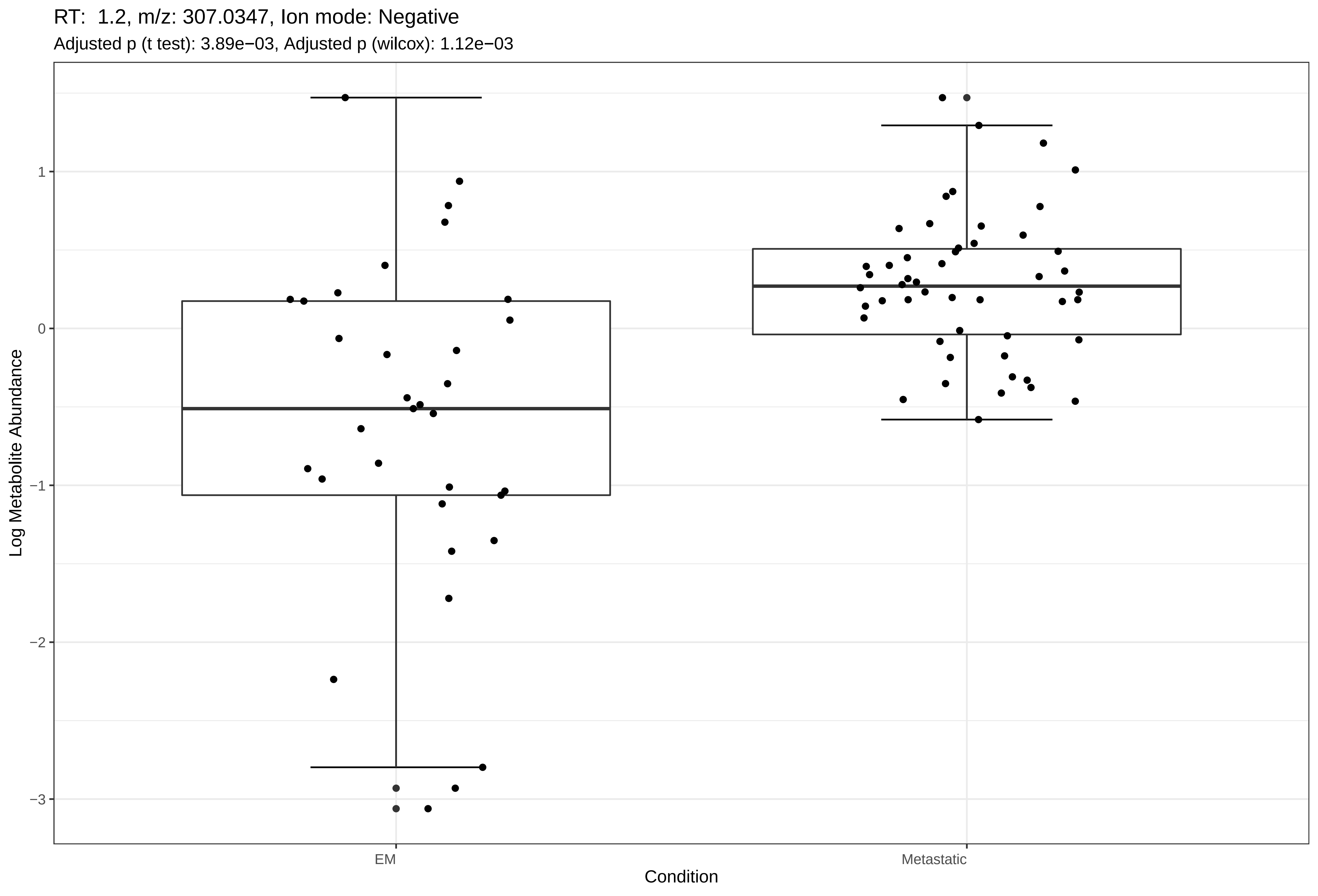
RT: 1.2, m/z: 307.0347, Ion mode: Negative
Adjusted p (t test): 3.89e−03, Adjusted p (wilcox): 1.12e−03
Log Metabolite Abundance
1
0
−1
−2
−3
EM
Metastatic
Condition

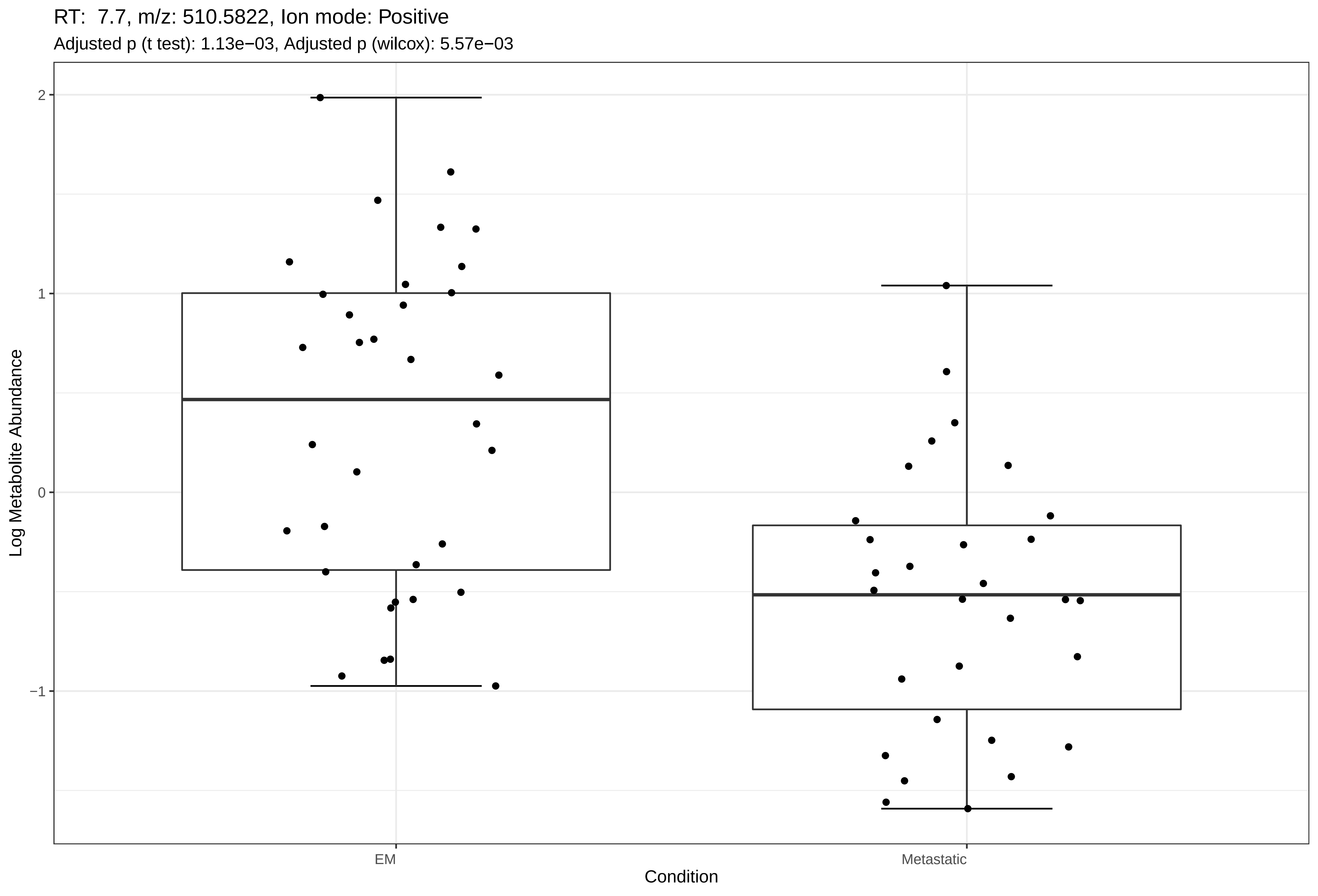

RT: 7.7, m/z: 510.5822, Ion mode: Positive
Adjusted p (t test): 1.13e−03, Adjusted p (wilcox): 5.57e−03
Log Metabolite Abundance
2
1
0
−1
EM
Metastatic
Condition

RT: 2.0, m/z: 174.0231, Ion mode: Positive

Adjusted p (t test): 1.14e−03, Adjusted p (wilcox): 3.05e−03

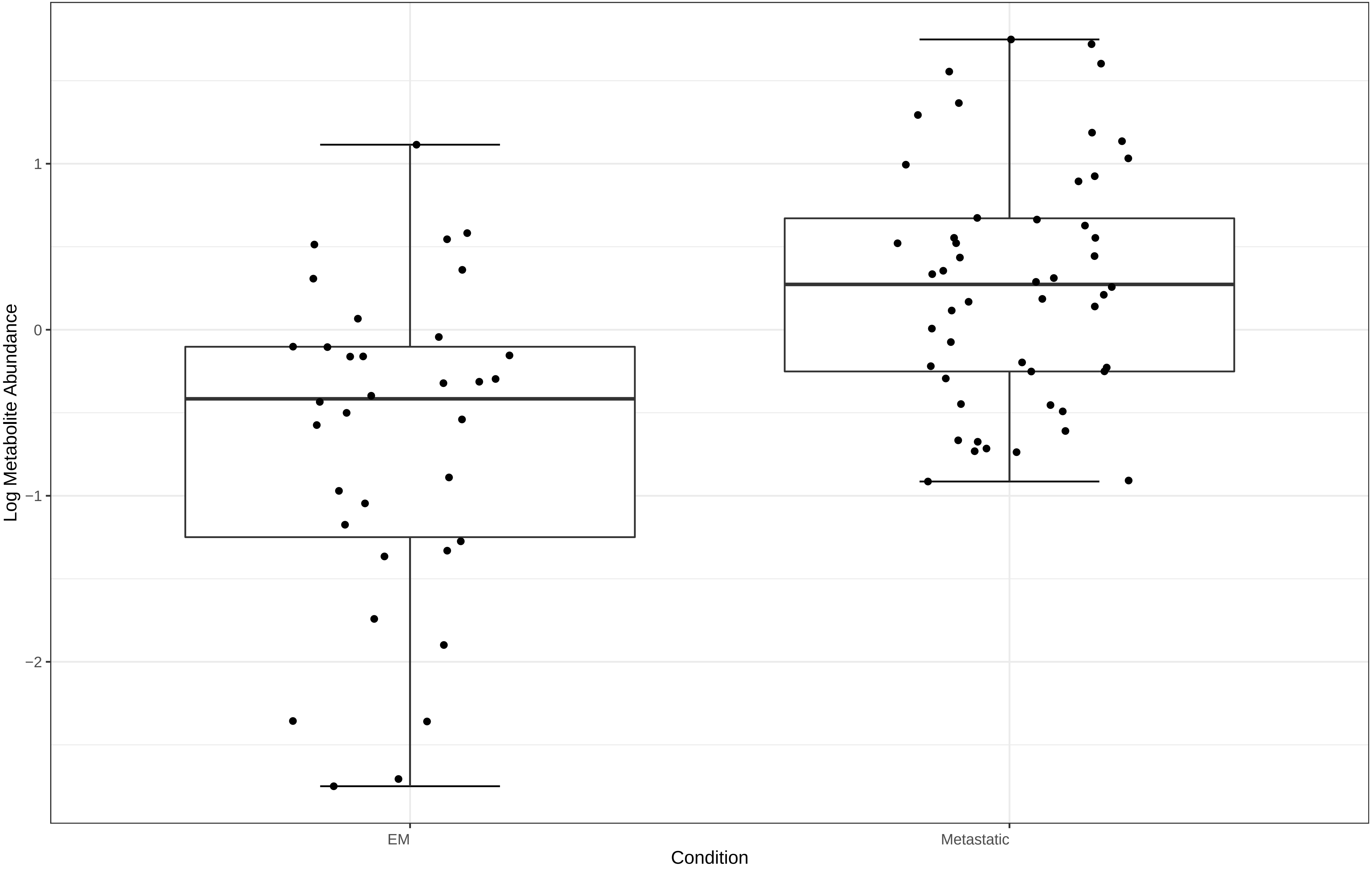

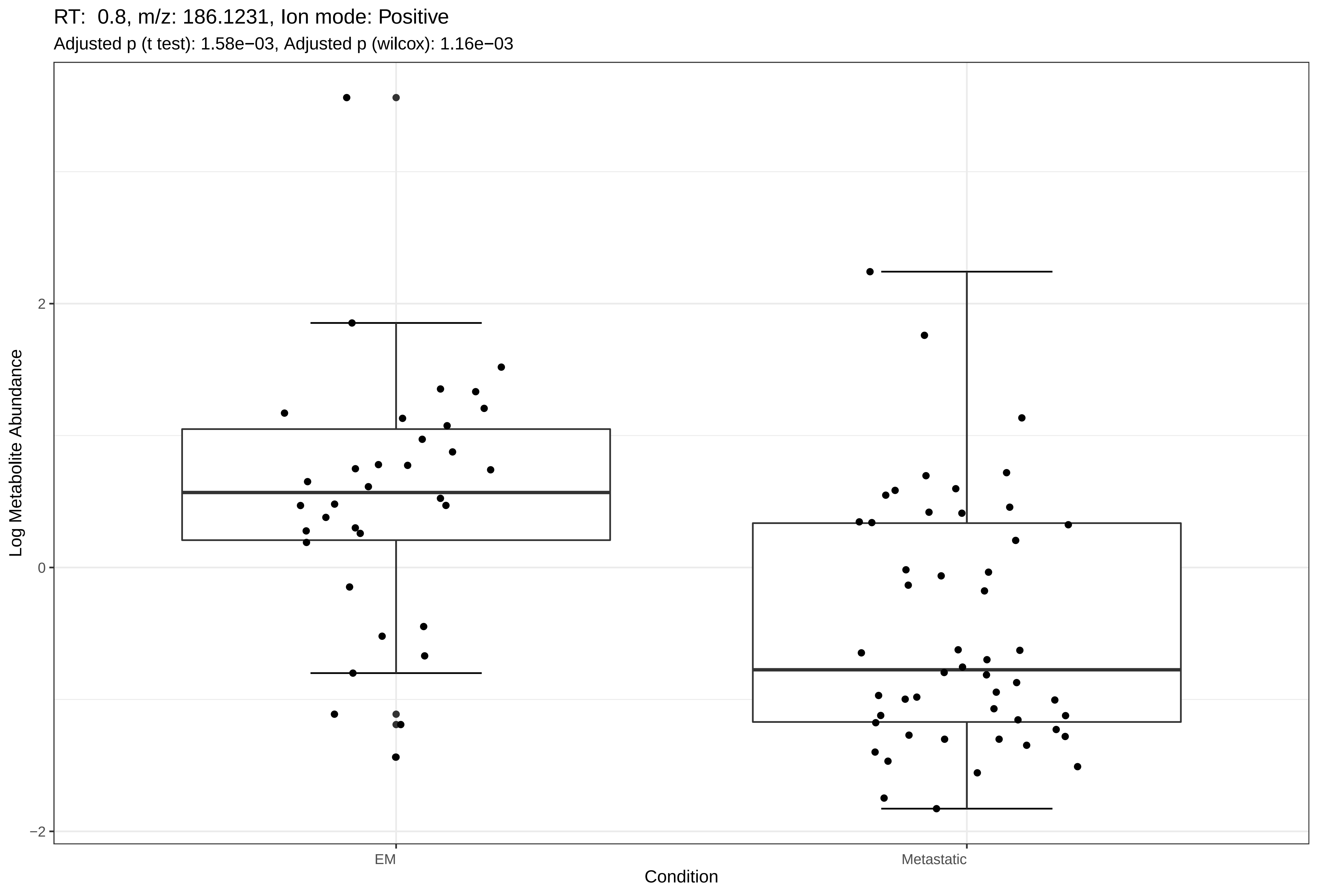

RT: 0.8, m/z: 186.1231, Ion mode: Positive
Adjusted p (t test): 1.58e−03, Adjusted p (wilcox): 1.16e−03
Log Metabolite Abundance
2
0
−2
EM
Metastatic
Condition

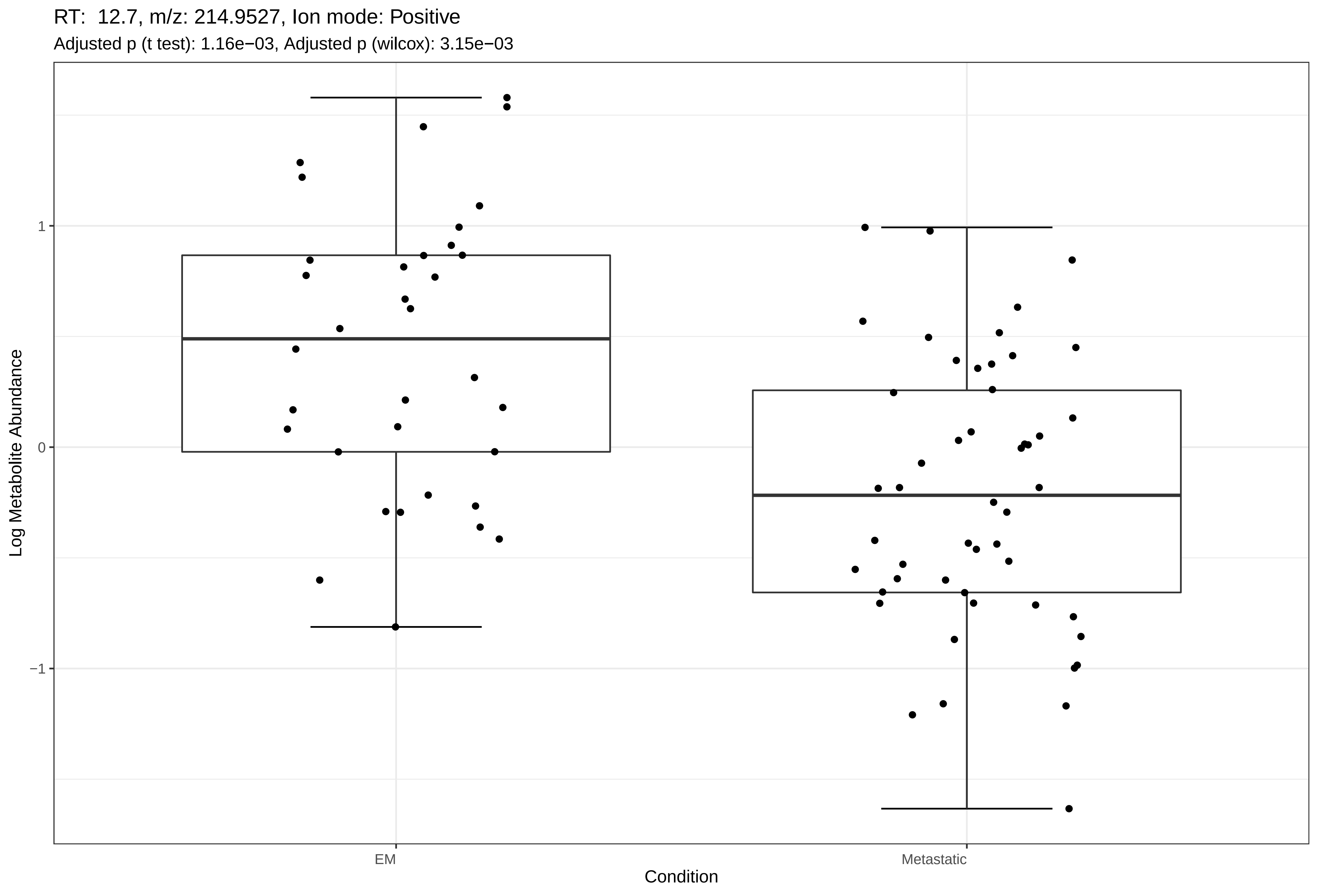
RT: 12.7, m/z: 214.9527, Ion mode: Positive
Adjusted p (t test): 1.16e−03, Adjusted p (wilcox): 3.15e−03
Log Metabolite Abundance
1
0
−1
EM
Metastatic
Condition

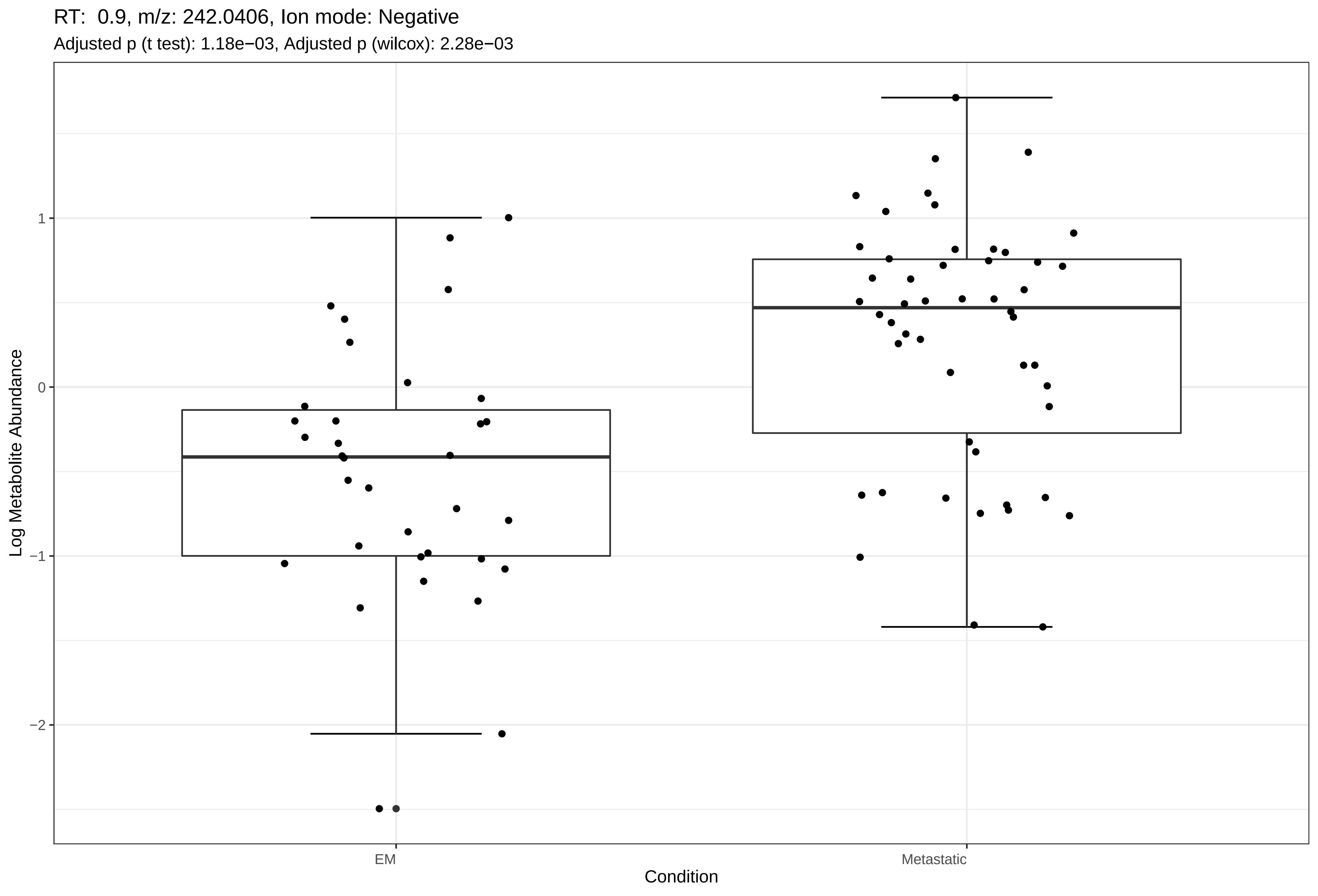
RT: 0.9, m/z: 242.0406, Ion mode: Negative
Adjusted p (t test): 1.18e−03, Adjusted p (wilcox): 2.28e−03
Log Metabolite Abundance
1
0
−1
−2
EM
Metastatic
Condition

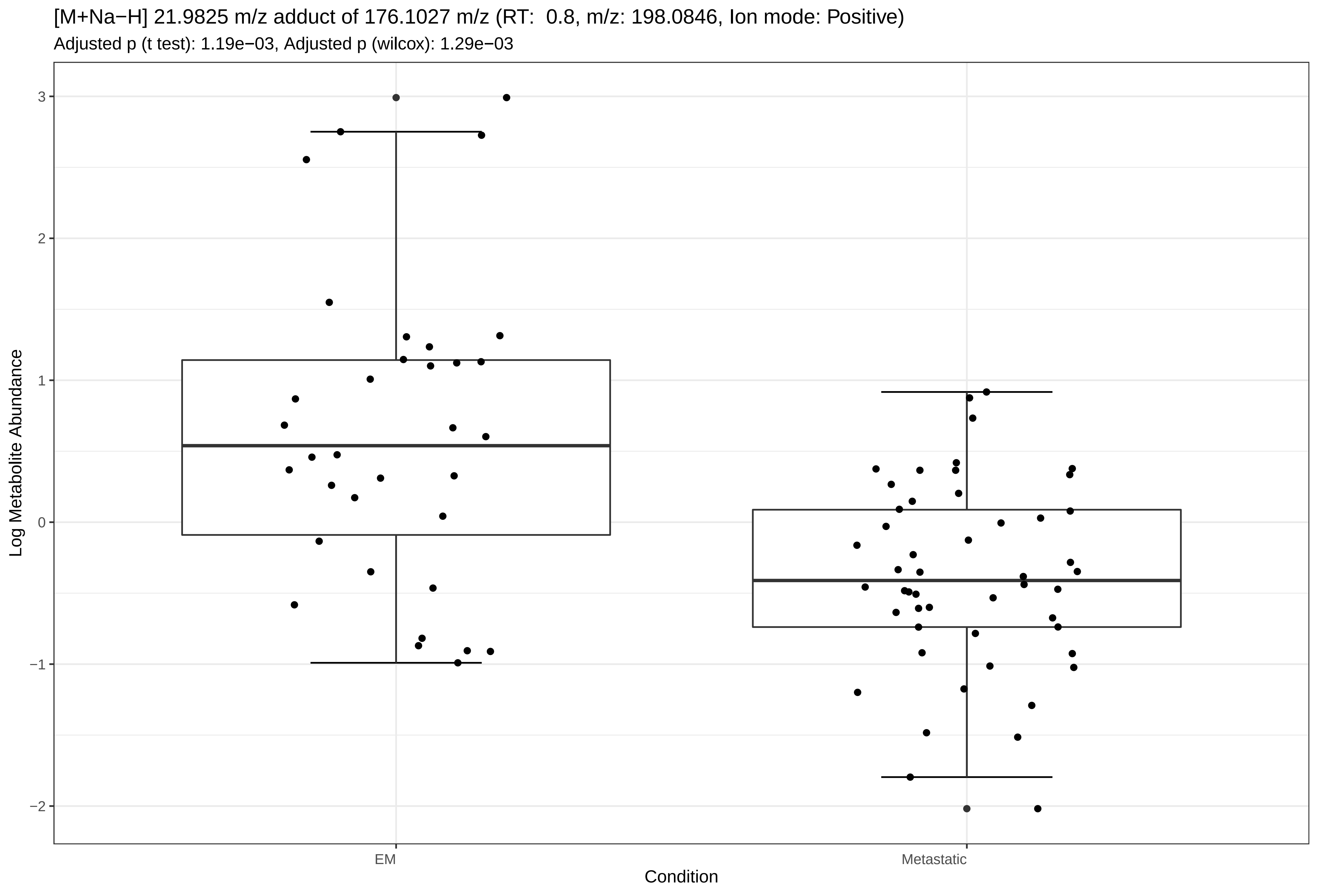

[M+Na−H] 21.9825 m/z adduct of 176.1027 m/z (RT: 0.8, m/z: 198.0846, Ion mode: Positive)
Adjusted p (t test): 1.19e−03, Adjusted p (wilcox): 1.29e−03
Log Metabolite Abundance
3
2
1
0
−1
−2
EM
Metastatic
Condition

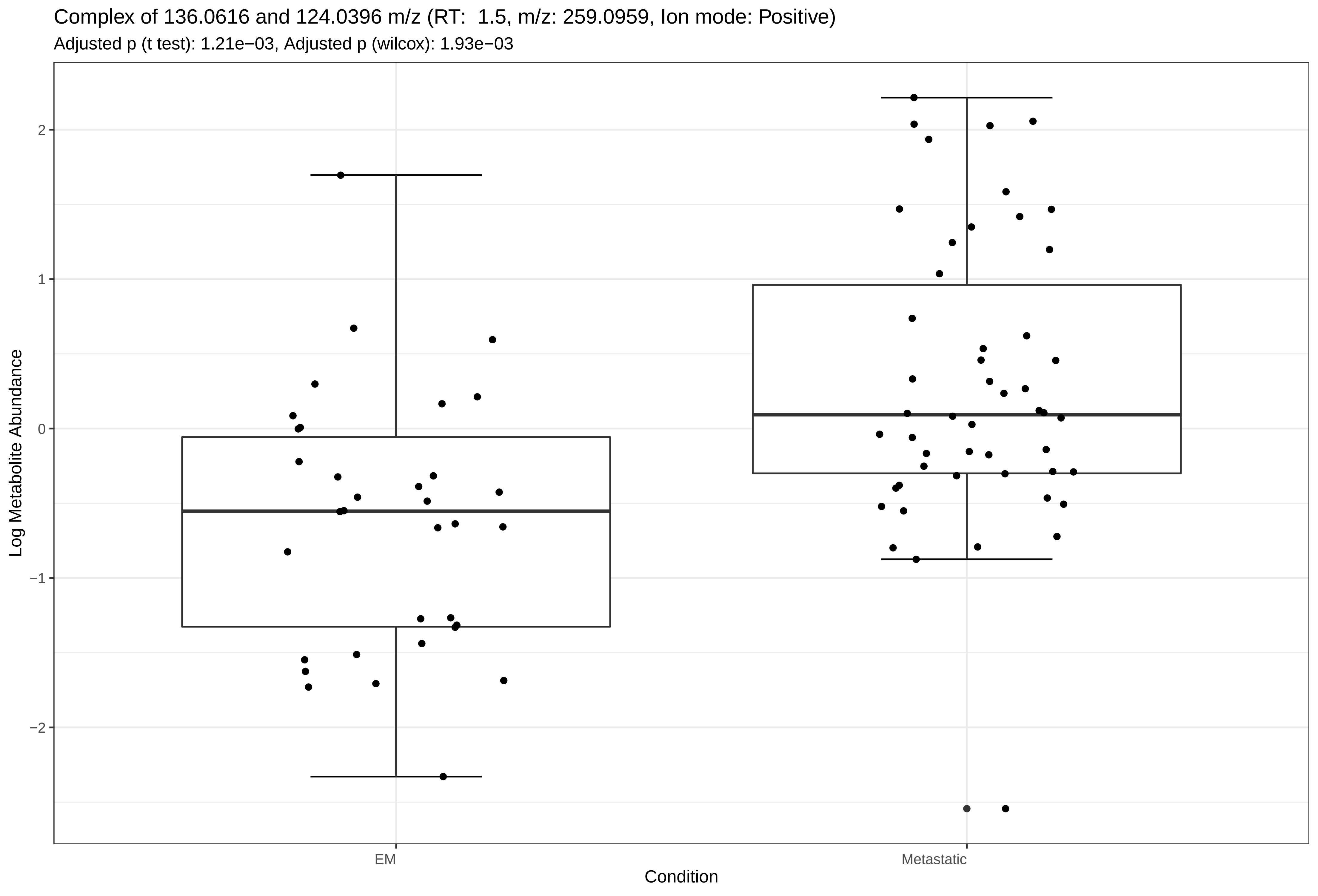

Complex of 136.0616 and 124.0396 m/z (RT: 1.5, m/z: 259.0959, Ion mode: Positive)
Adjusted p (t test): 1.21e−03, Adjusted p (wilcox): 1.93e−03
Log Metabolite Abundance
2
1
0
−1
−2
EM
Metastatic
Condition

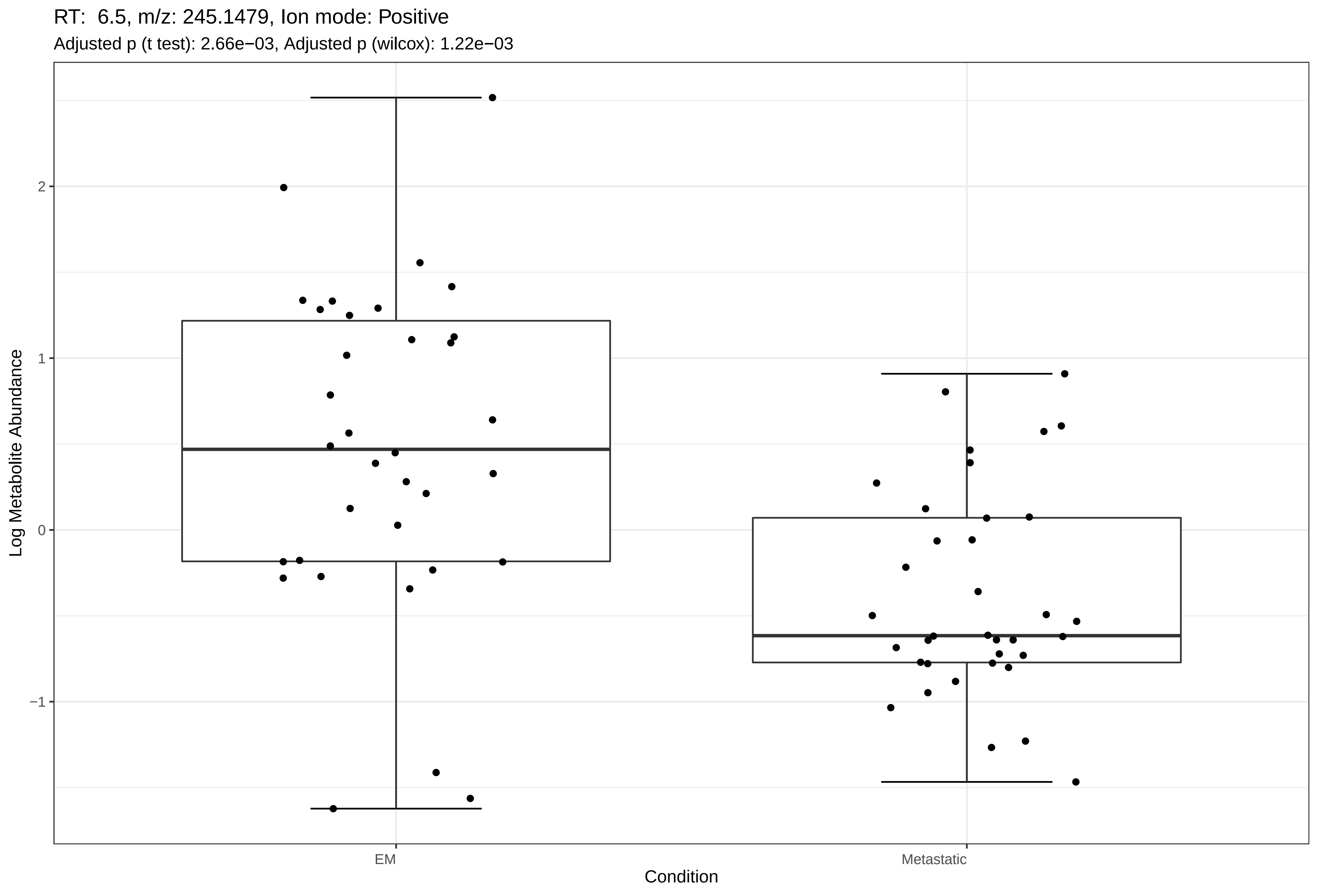
RT: 6.5, m/z: 245.1479, Ion mode: Positive
Adjusted p (t test): 2.66e−03, Adjusted p (wilcox): 1.22e−03
Log Metabolite Abundance
2
1
0
−1
EM
Metastatic
Condition

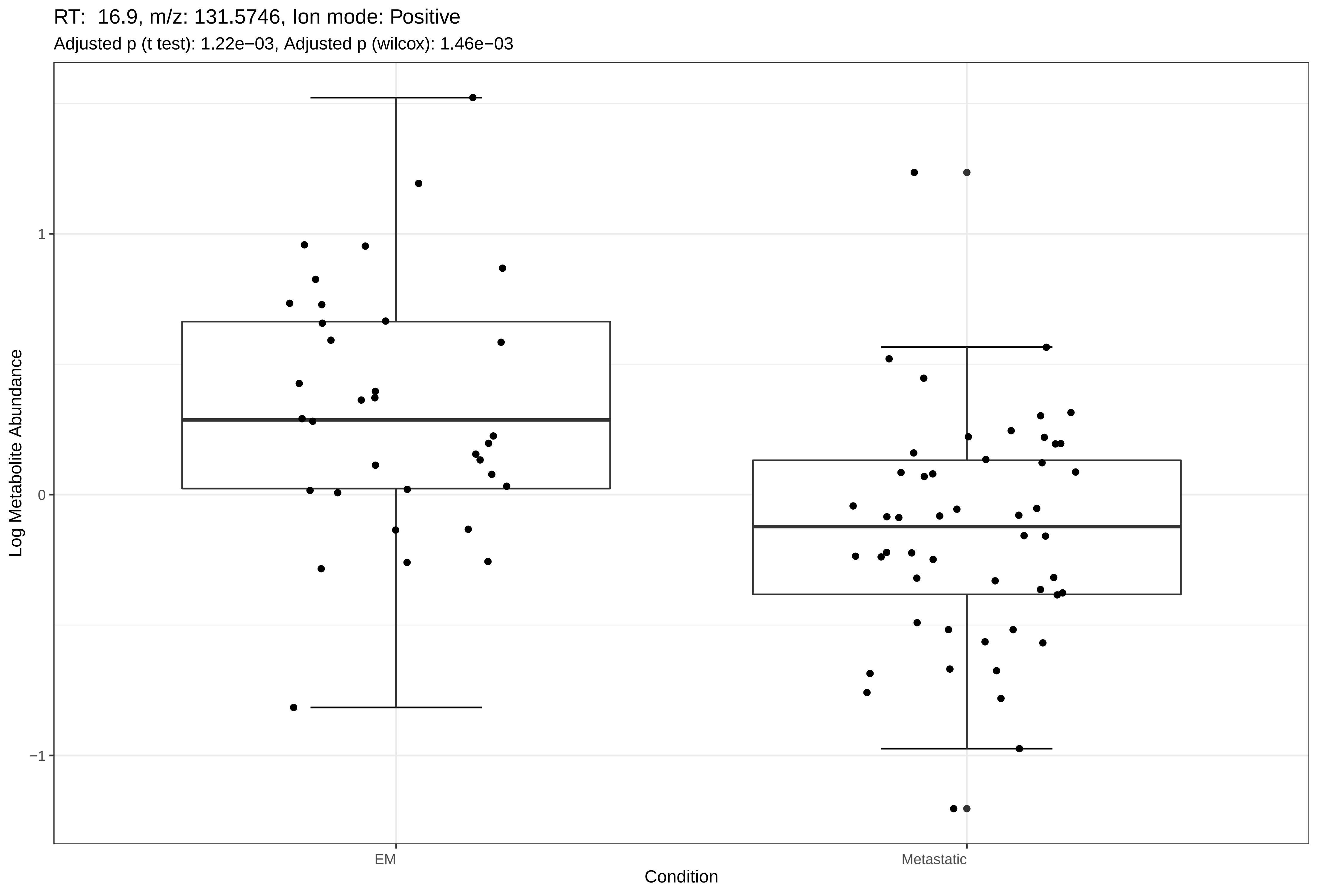
RT: 16.9, m/z: 131.5746, Ion mode: Positive
Adjusted p (t test): 1.22e−03, Adjusted p (wilcox): 1.46e−03
Log Metabolite Abundance
1
0
−1
EM
Metastatic
Condition

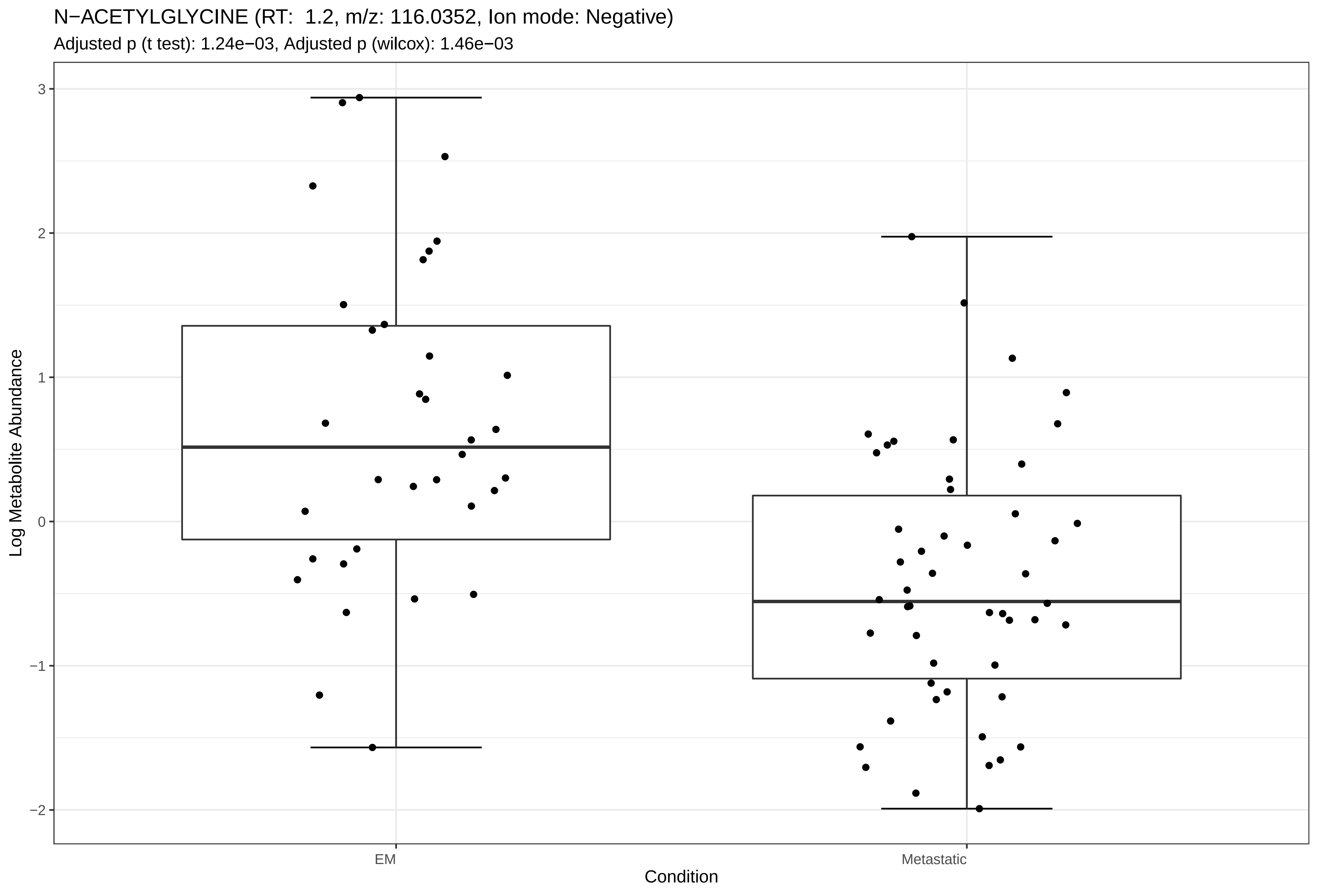
N−ACETYLGLYCINE (RT: 1.2, m/z: 116.0352, Ion mode: Negative)
Adjusted p (t test): 1.24e−03, Adjusted p (wilcox): 1.46e−03
Log Metabolite Abundance
3
2
1
0
−1
−2
EM
Metastatic
Condition

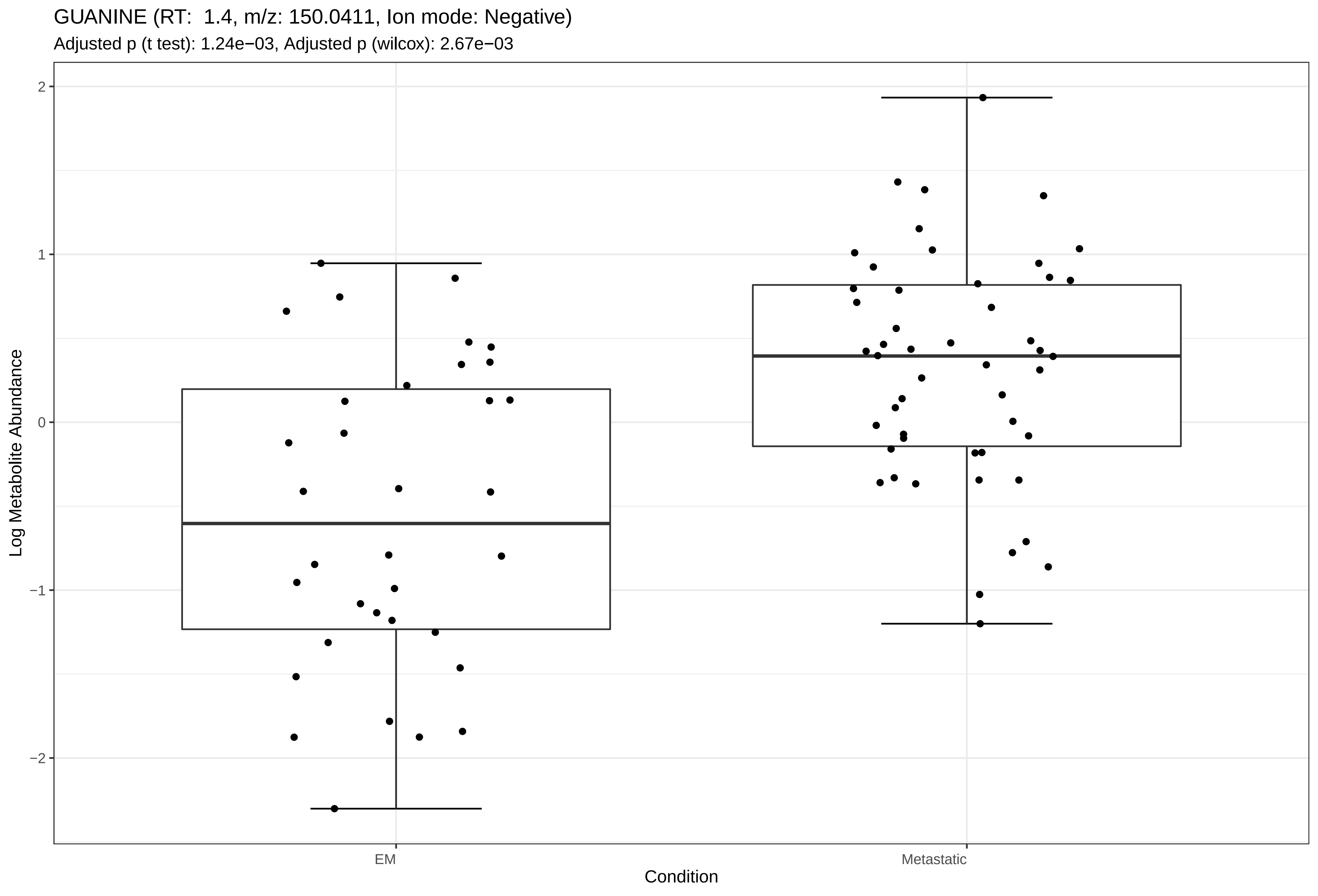

GUANINE (RT: 1.4, m/z: 150.0411, Ion mode: Negative)
Adjusted p (t test): 1.24e−03, Adjusted p (wilcox): 2.67e−03
2
1
0
−1
−2
Log Metabolite Abundance
EM
Metastatic
Condition

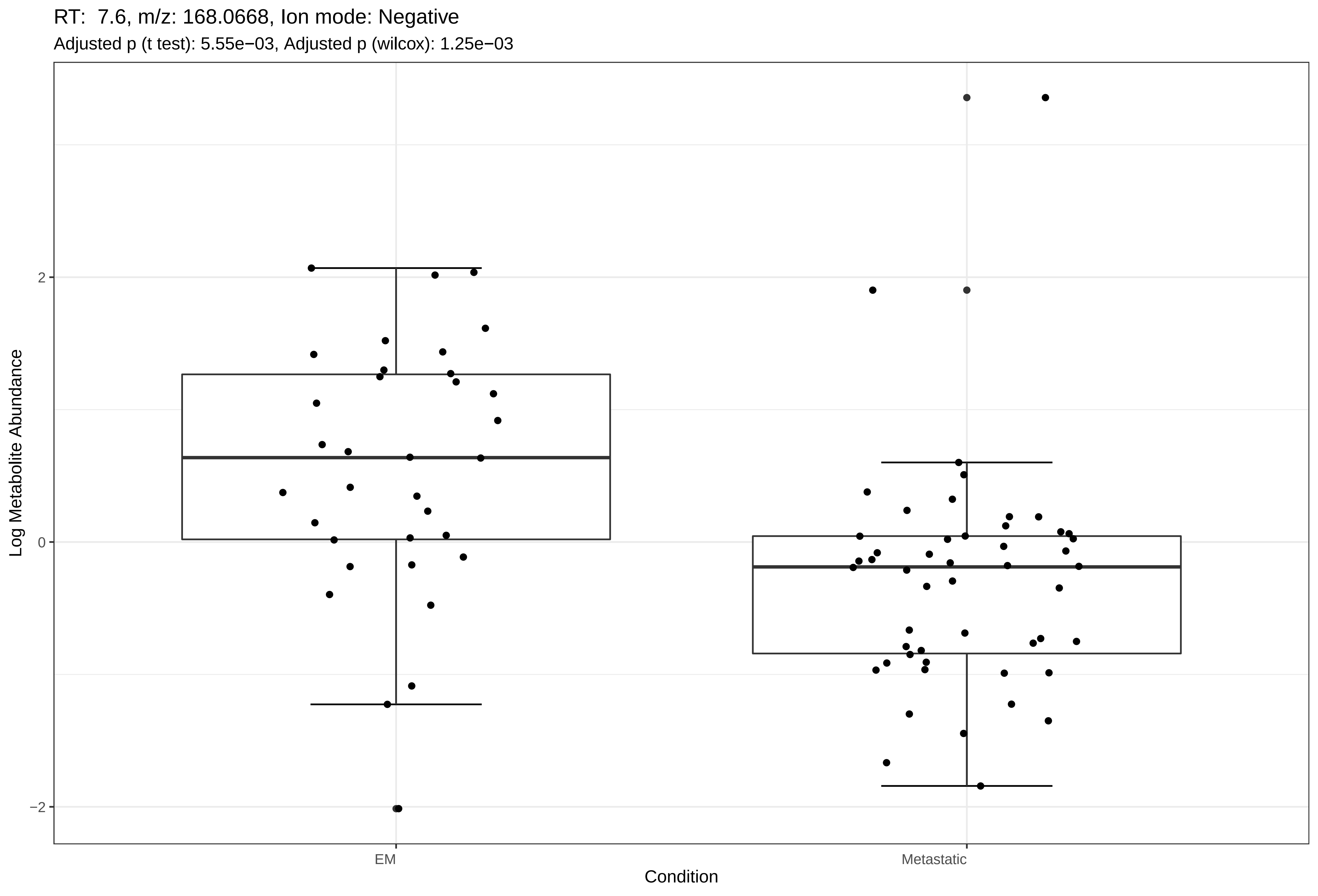
RT: 7.6, m/z: 168.0668, Ion mode: Negative
Adjusted p (t test): 5.55e−03, Adjusted p (wilcox): 1.25e−03
Log Metabolite Abundance
2
0
−2
EM
Metastatic
Condition

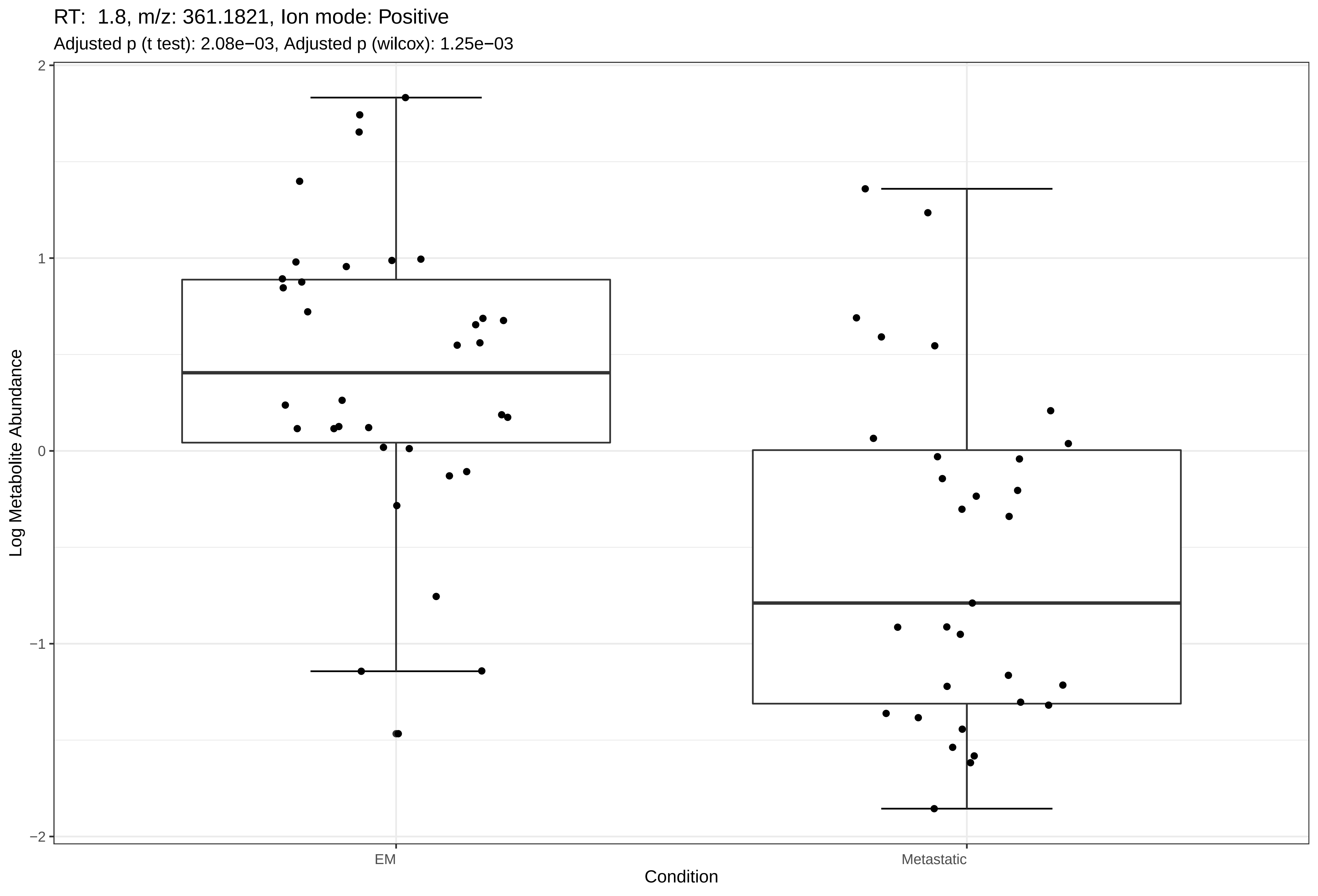
RT: 1.8, m/z: 361.1821, Ion mode: Positive
Adjusted p (t test): 2.08e−03, Adjusted p (wilcox): 1.25e−03
Log Metabolite Abundance
2
1
0
−1
−2
EM
Metastatic
Condition

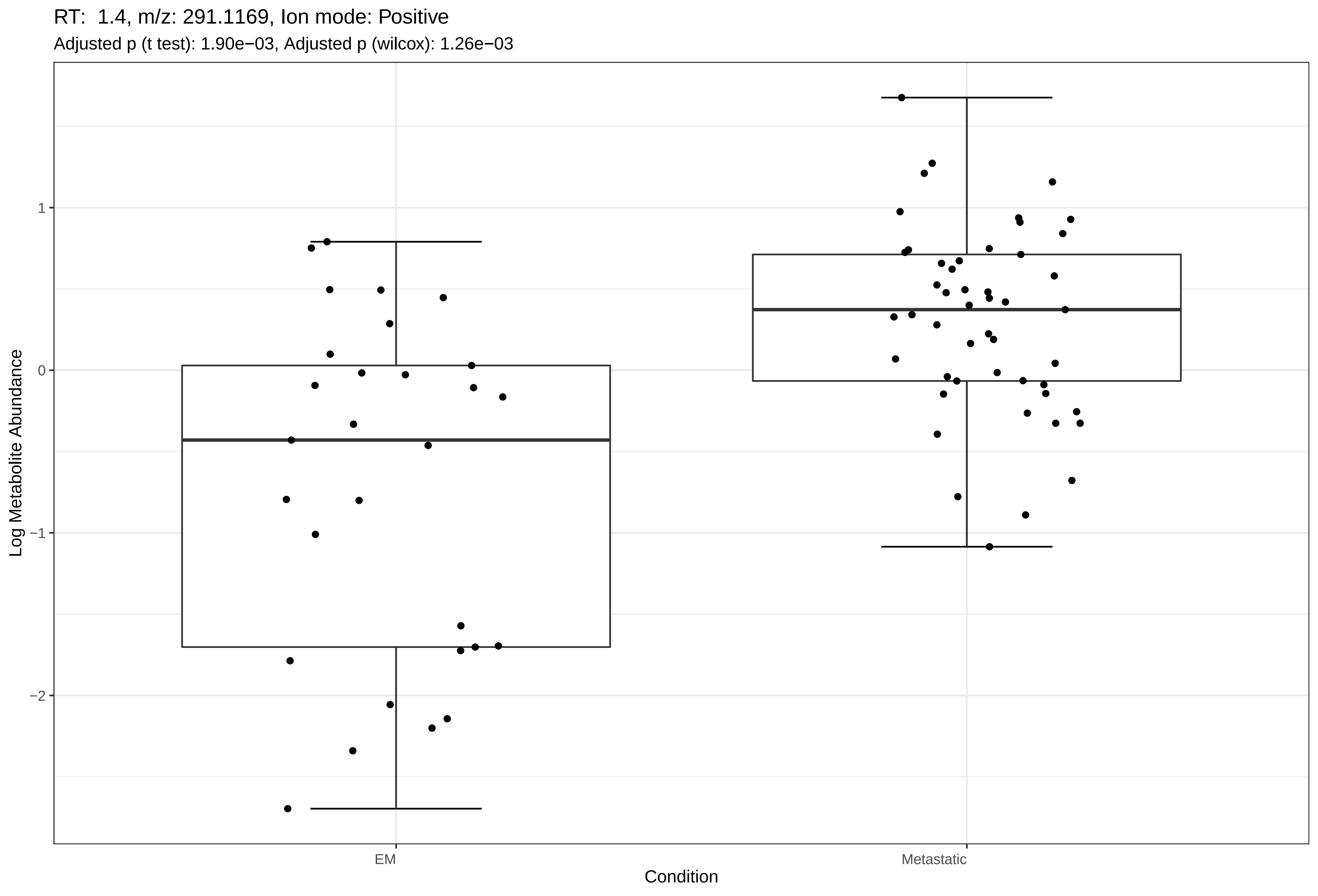

RT: 1.4, m/z: 291.1169, Ion mode: Positive
Adjusted p (t test): 1.90e−03, Adjusted p (wilcox): 1.26e−03
Log Metabolite Abundance
1
0
−1
−2
EM
Metastatic
Condition

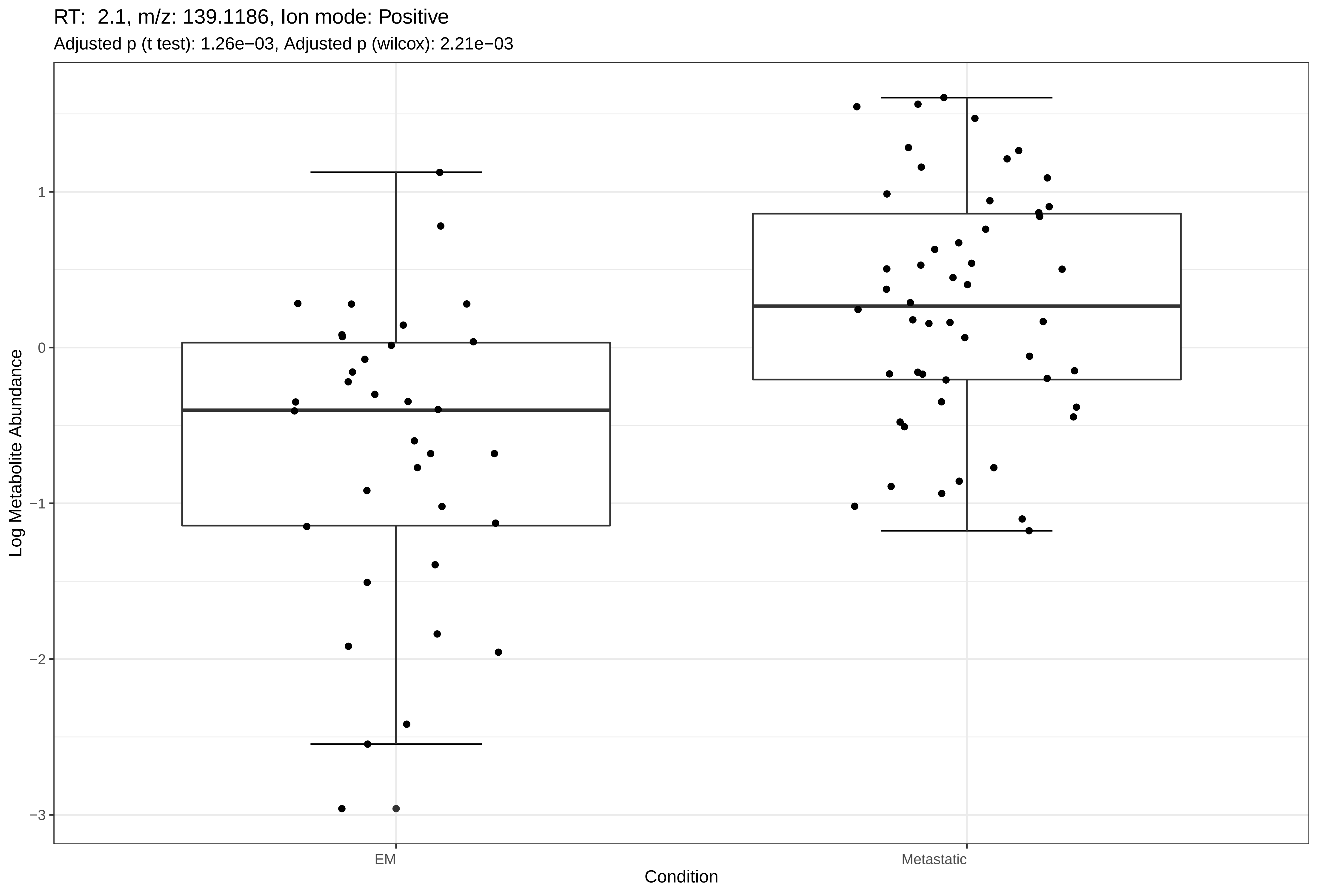
RT: 2.1, m/z: 139.1186, Ion mode: Positive
Adjusted p (t test): 1.26e−03, Adjusted p (wilcox): 2.21e−03
Log Metabolite Abundance
1
0
−1
−2
−3
EM
Metastatic
Condition

RT: 0.8, m/z: 386.1178, Ion mode: Negative

Adjusted p (t test): 1.26e−03, Adjusted p (wilcox): 4.66e−03

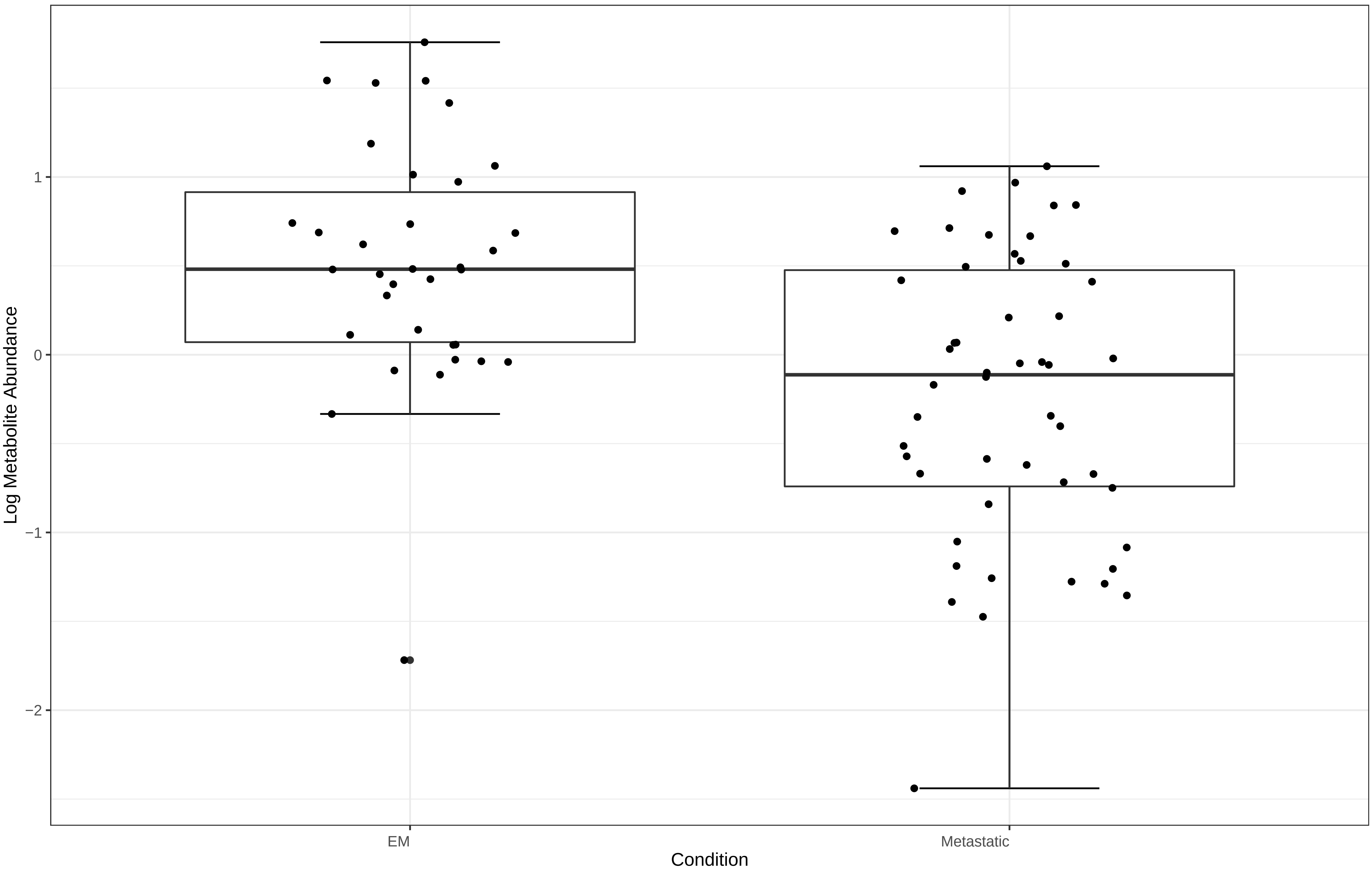

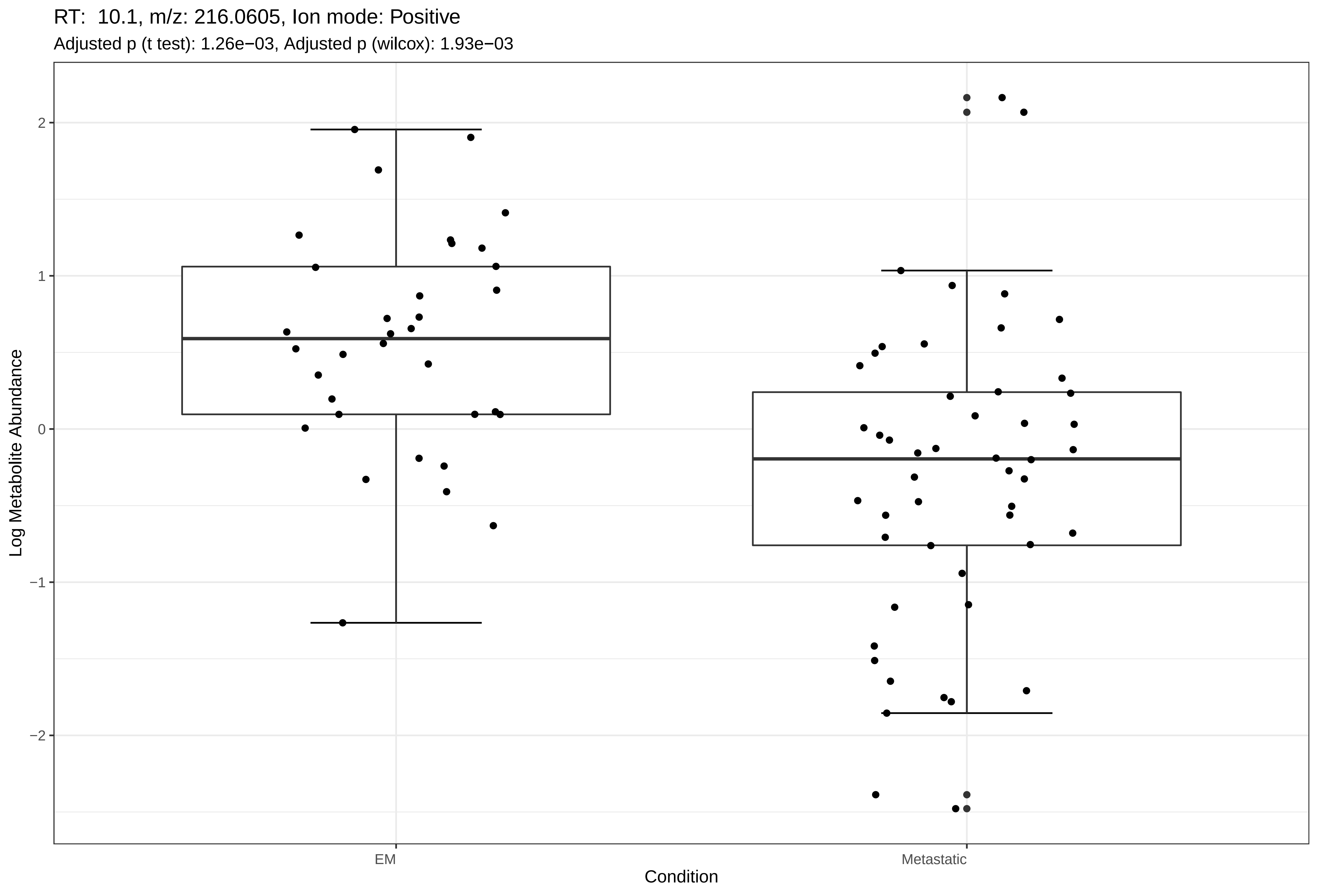
RT: 10.1, m/z: 216.0605, Ion mode: Positive
Adjusted p (t test): 1.26e−03, Adjusted p (wilcox): 1.93e−03
Log Metabolite Abundance
2
1
0
−1
−2
EM
Metastatic
Condition

RT: 0.9, m/z: 300.0392, Ion mode: Negative

Adjusted p (t test): 1.26e−03, Adjusted p (wilcox): 2.49e−03

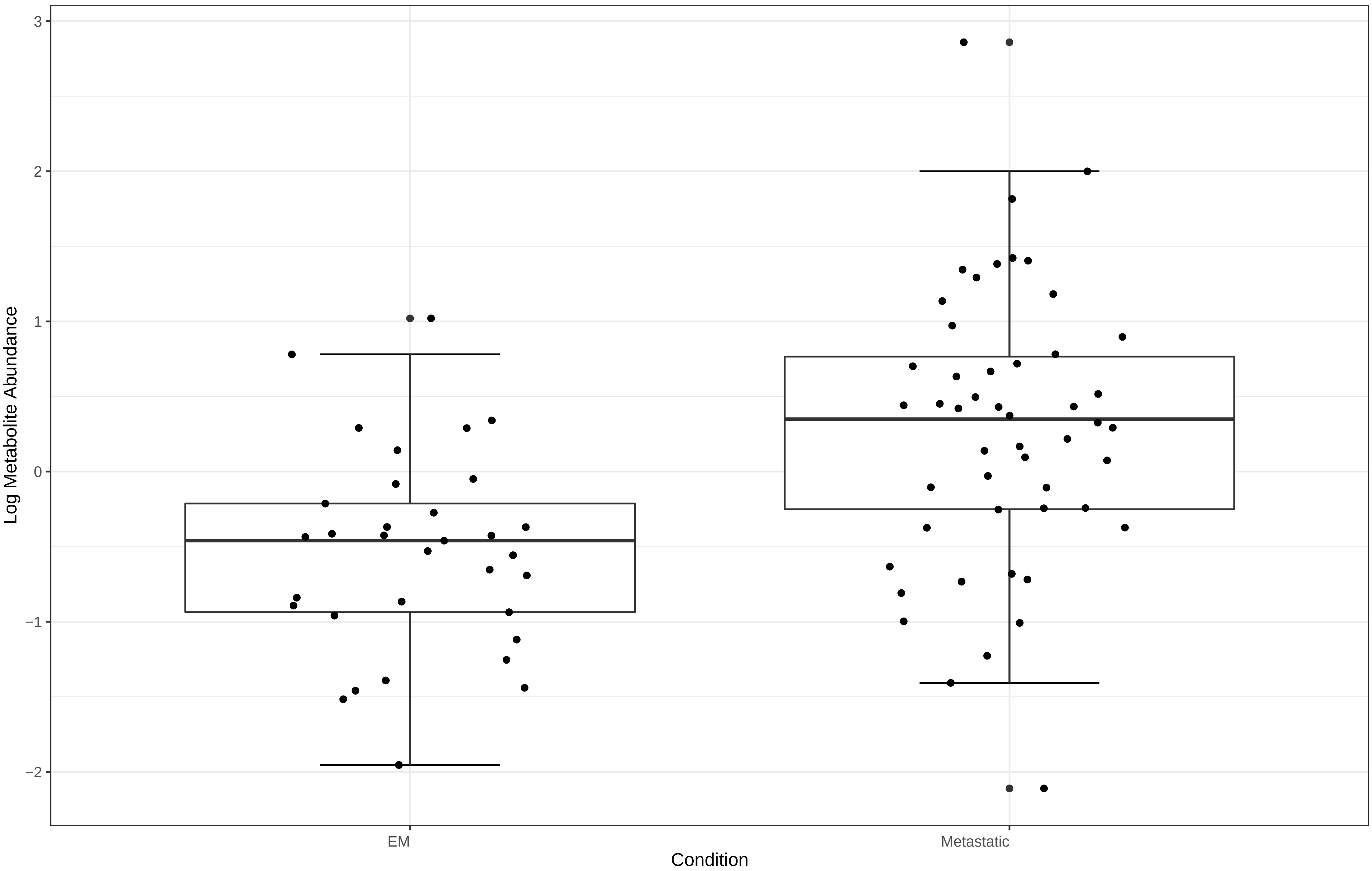

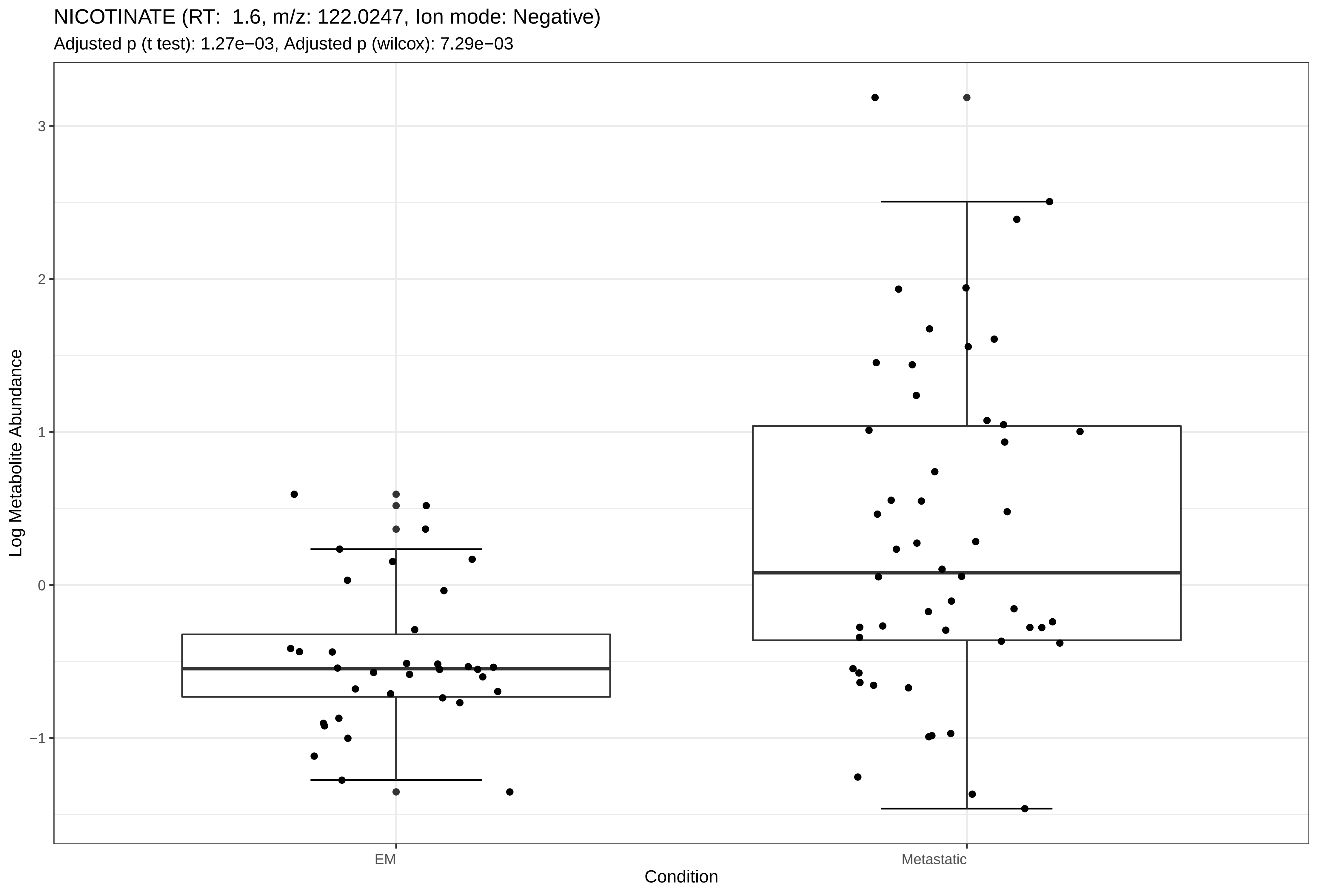

NICOTINATE (RT: 1.6, m/z: 122.0247, Ion mode: Negative)
Adjusted p (t test): 1.27e−03, Adjusted p (wilcox): 7.29e−03
Log Metabolite Abundance
3
2
1
0
−1
EM
Metastatic
Condition

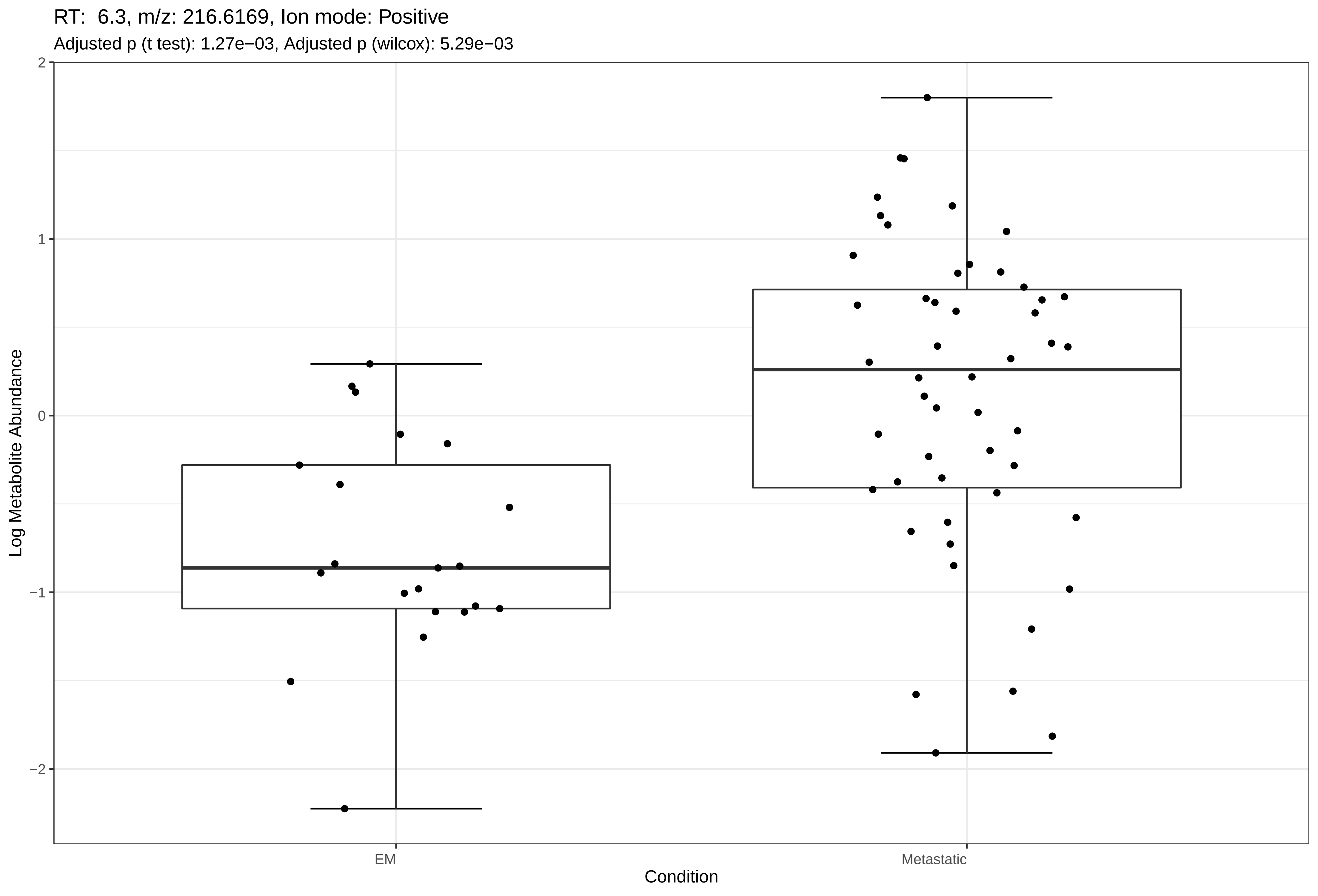
RT: 6.3, m/z: 216.6169, Ion mode: Positive
Adjusted p (t test): 1.27e−03, Adjusted p (wilcox): 5.29e−03
Log Metabolite Abundance
2
1
0
−1
−2
EM
Metastatic
Condition

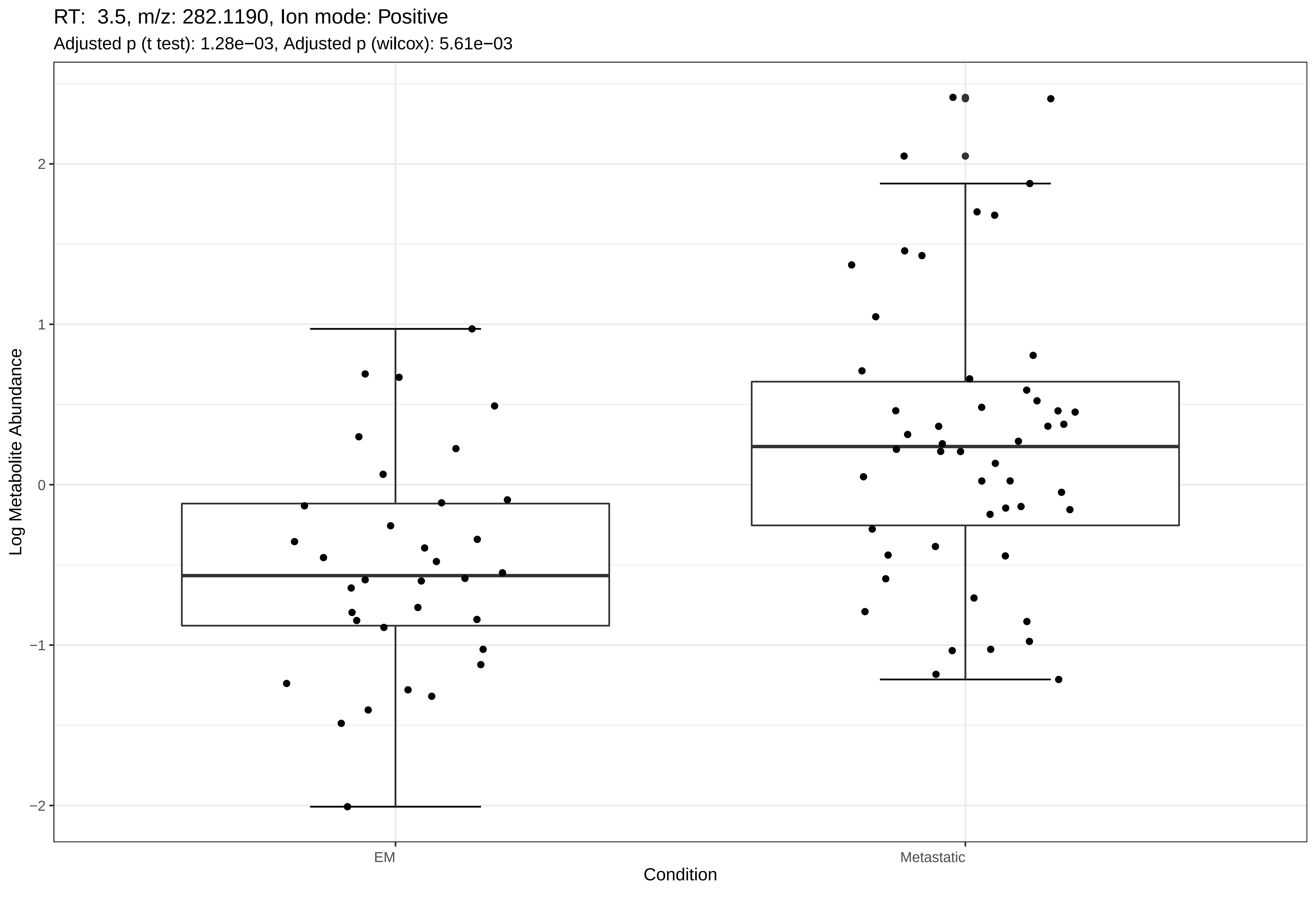

RT: 3.5, m/z: 282.1190, Ion mode: Positive
Adjusted p (t test): 1.28e−03, Adjusted p (wilcox): 5.61e−03
Log Metabolite Abundance
2
1
0
−1
−2
EM
Metastatic
Condition

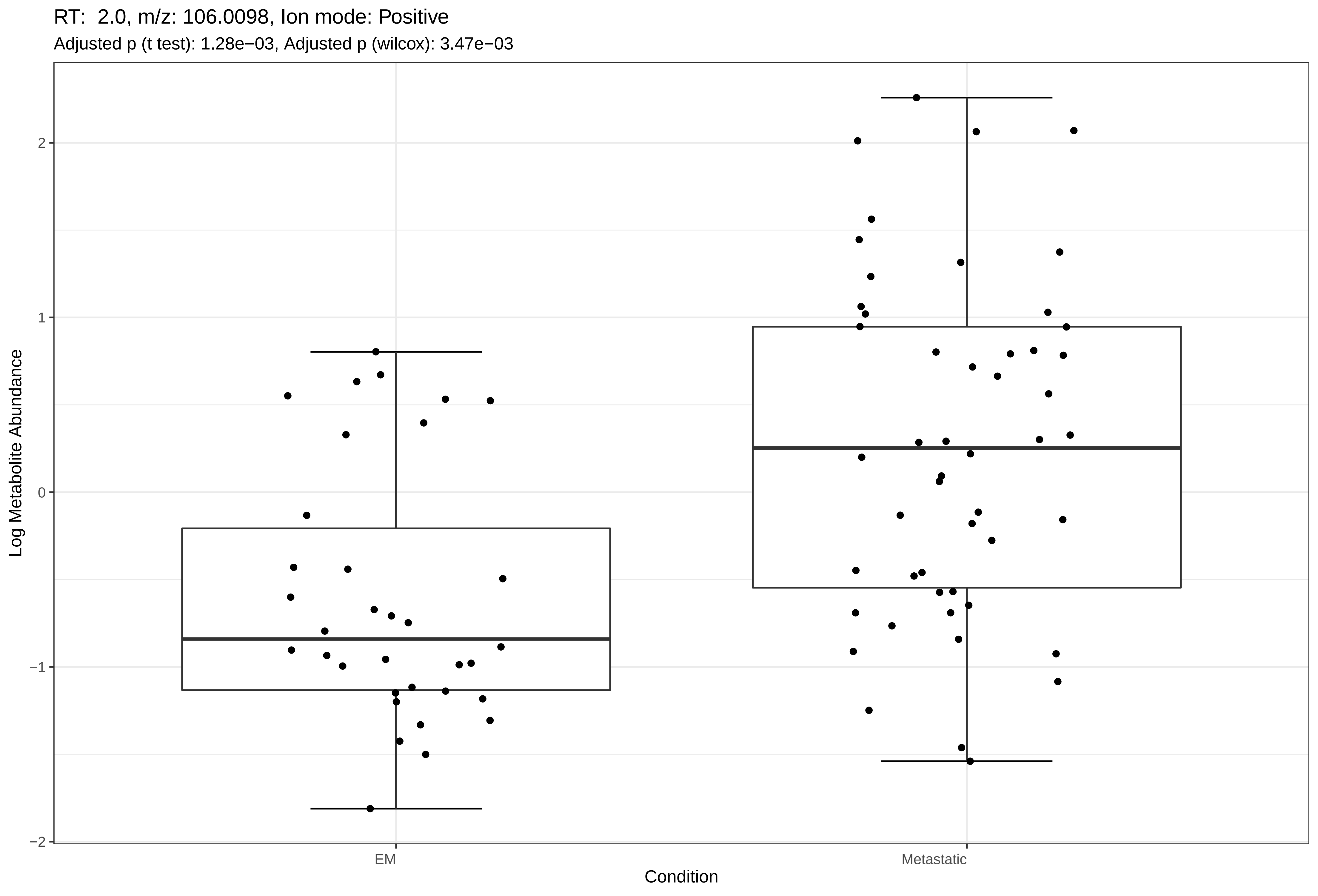
RT: 2.0, m/z: 106.0098, Ion mode: Positive
Adjusted p (t test): 1.28e−03, Adjusted p (wilcox): 3.47e−03
Log Metabolite Abundance
2
1
0
−1
−2
EM
Metastatic
Condition

RT: 3.7, m/z: 372.0842, Ion mode: Negative

Adjusted p (t test): 7.05e−03, Adjusted p (wilcox): 1.29e−03

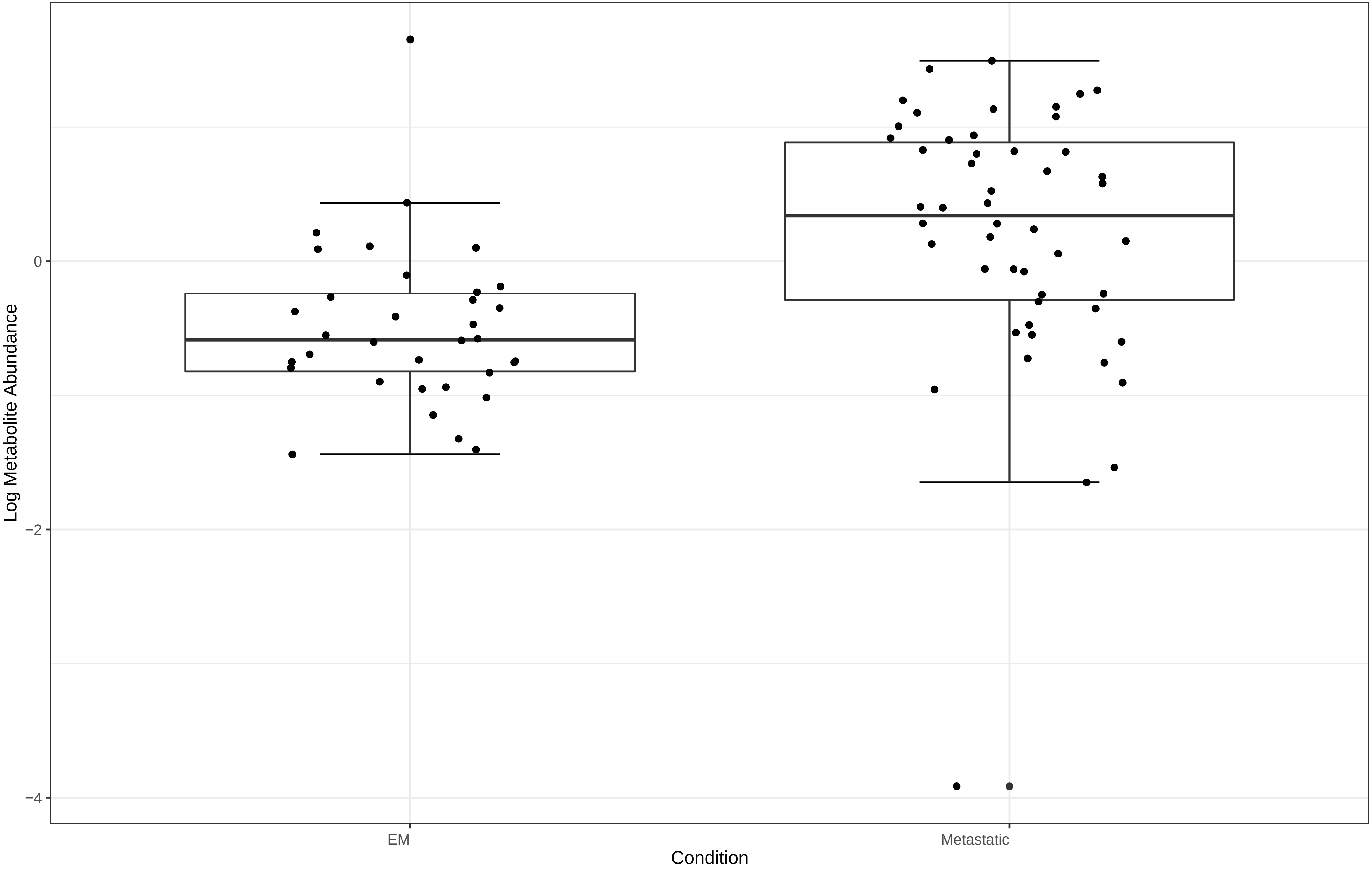

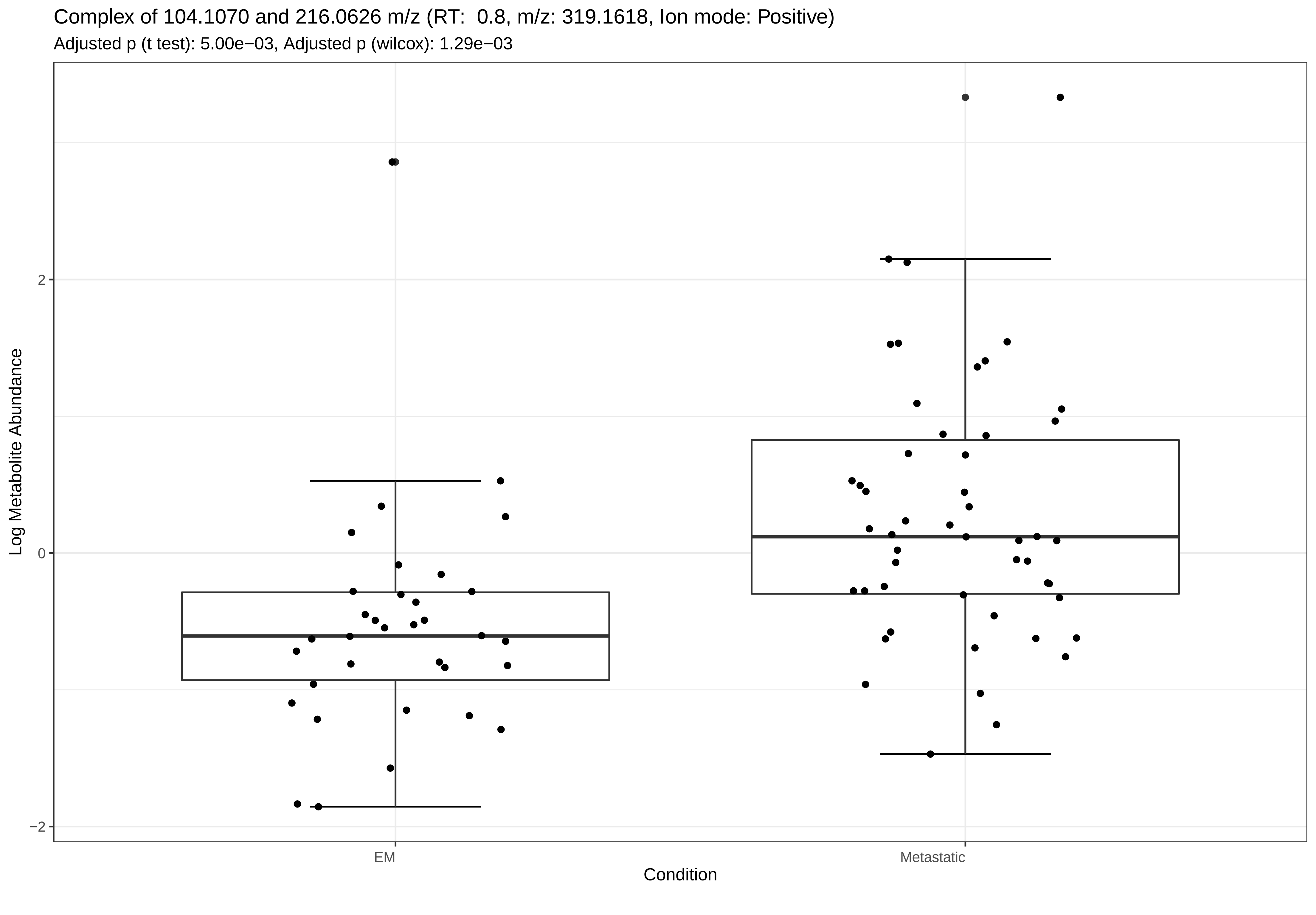

Complex of 104.1070 and 216.0626 m/z (RT: 0.8, m/z: 319.1618, Ion mode: Positive)
Adjusted p (t test): 5.00e−03, Adjusted p (wilcox): 1.29e−03
Log Metabolite Abundance
2
0
−2
EM
Metastatic
Condition

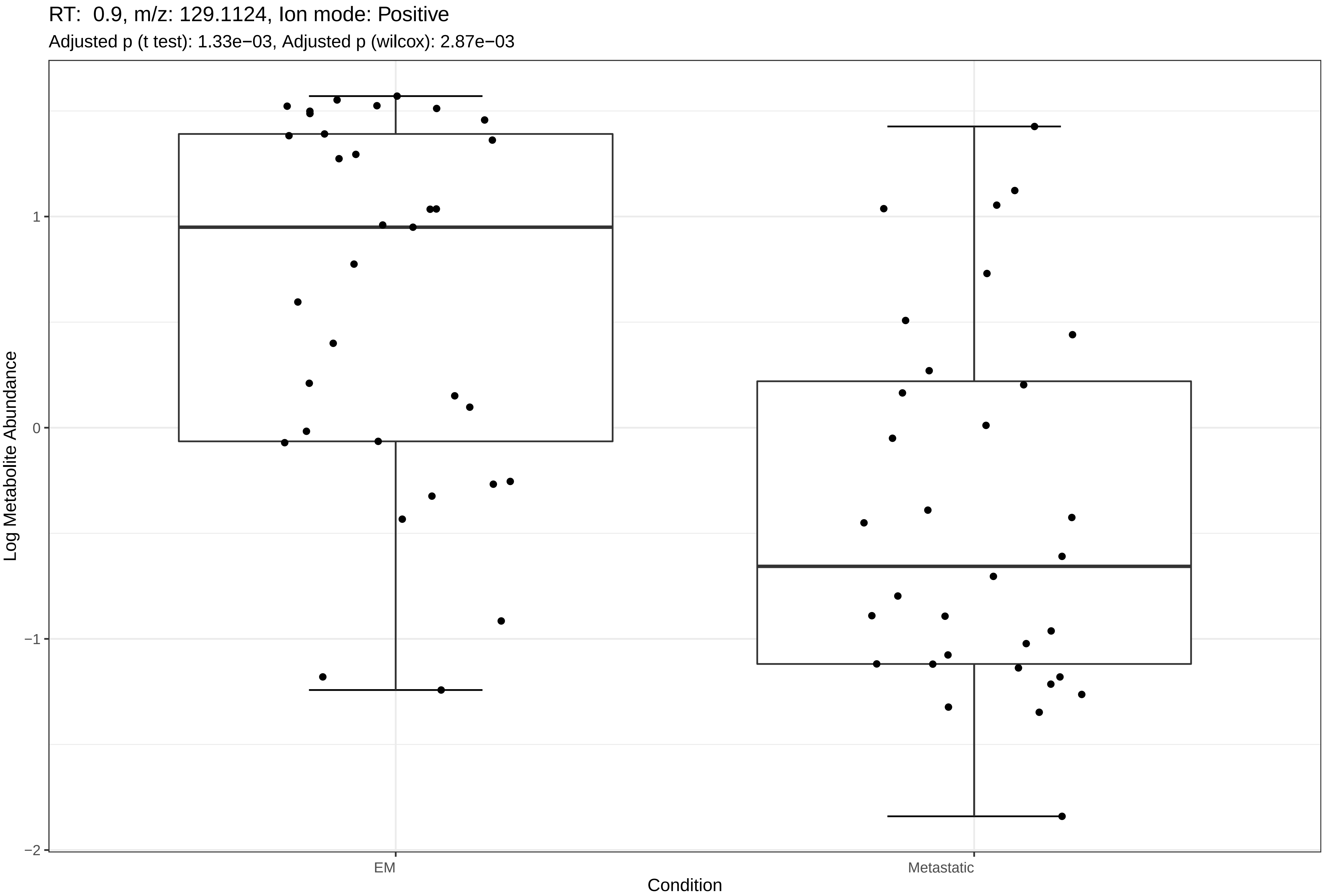
RT: 0.9, m/z: 129.1124, Ion mode: Positive
Adjusted p (t test): 1.33e−03, Adjusted p (wilcox): 2.87e−03
Log Metabolite Abundance
1
0
−1
−2
EM
Metastatic
Condition

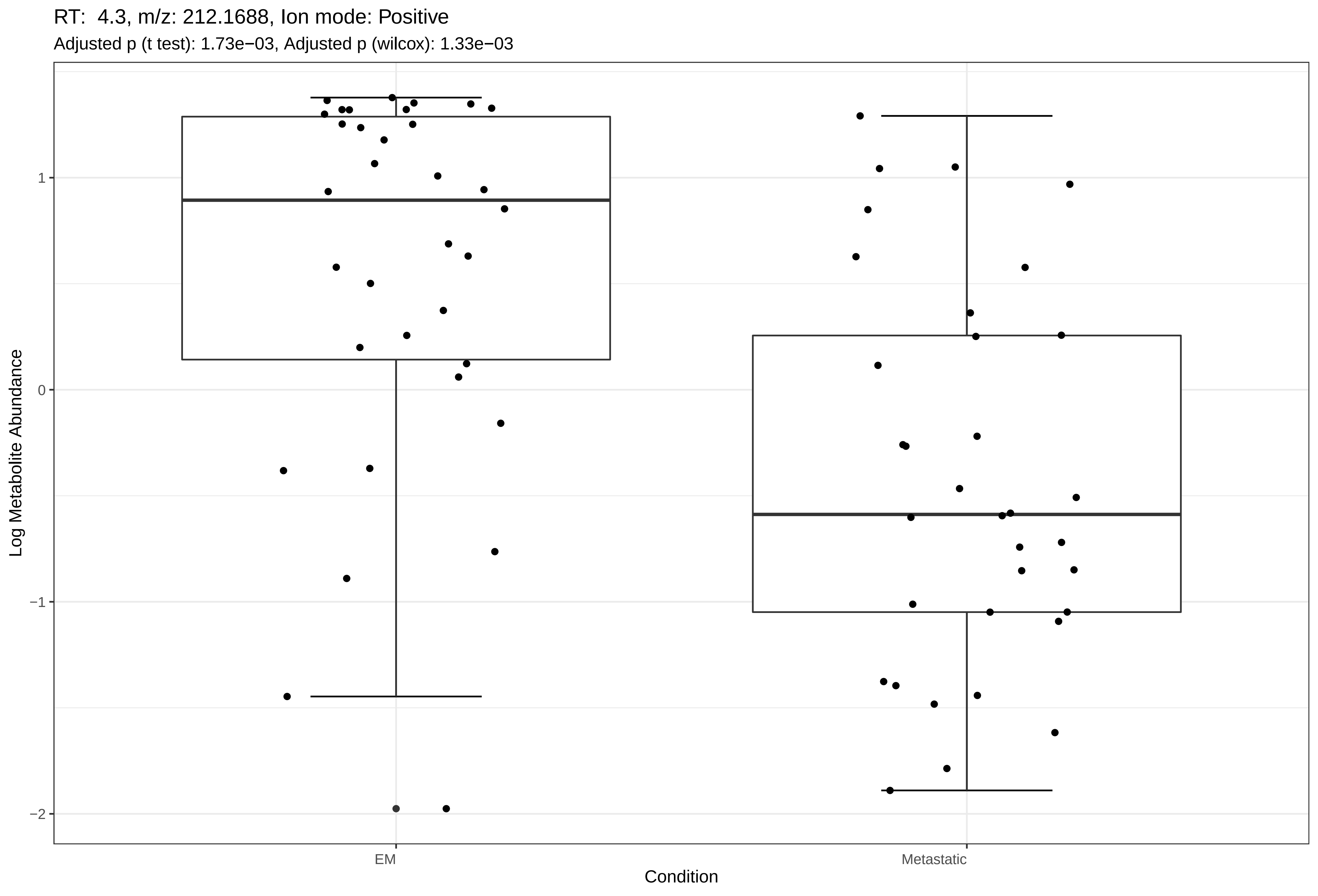
RT: 4.3, m/z: 212.1688, Ion mode: Positive
Adjusted p (t test): 1.73e−03, Adjusted p (wilcox): 1.33e−03
Log Metabolite Abundance
1
0
−1
−2
EM
Metastatic
Condition

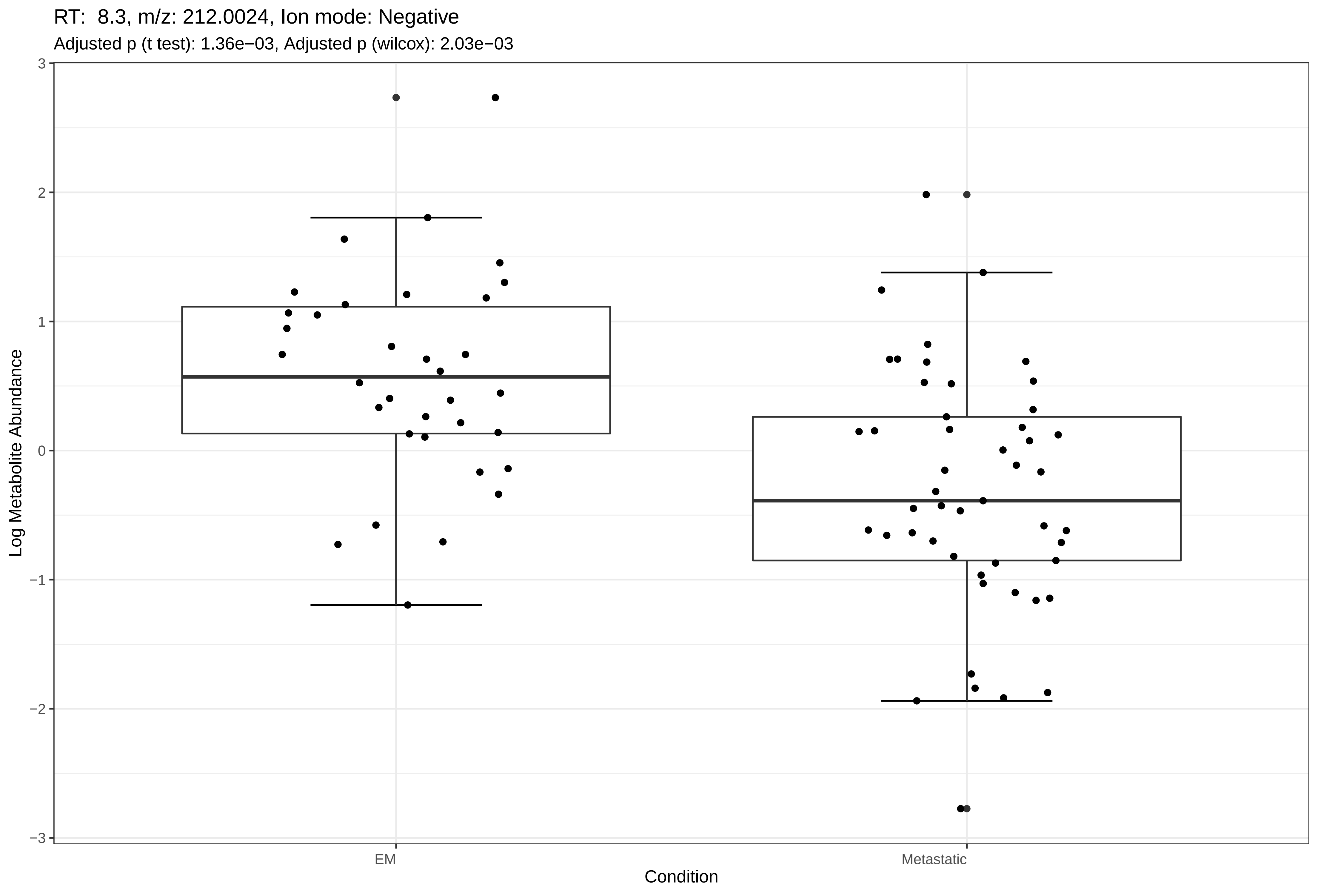

RT: 8.3, m/z: 212.0024, Ion mode: Negative
Adjusted p (t test): 1.36e−03, Adjusted p (wilcox): 2.03e−03
Log Metabolite Abundance
3
2
1
0
−1
−2
−3
EM
Metastatic
Condition

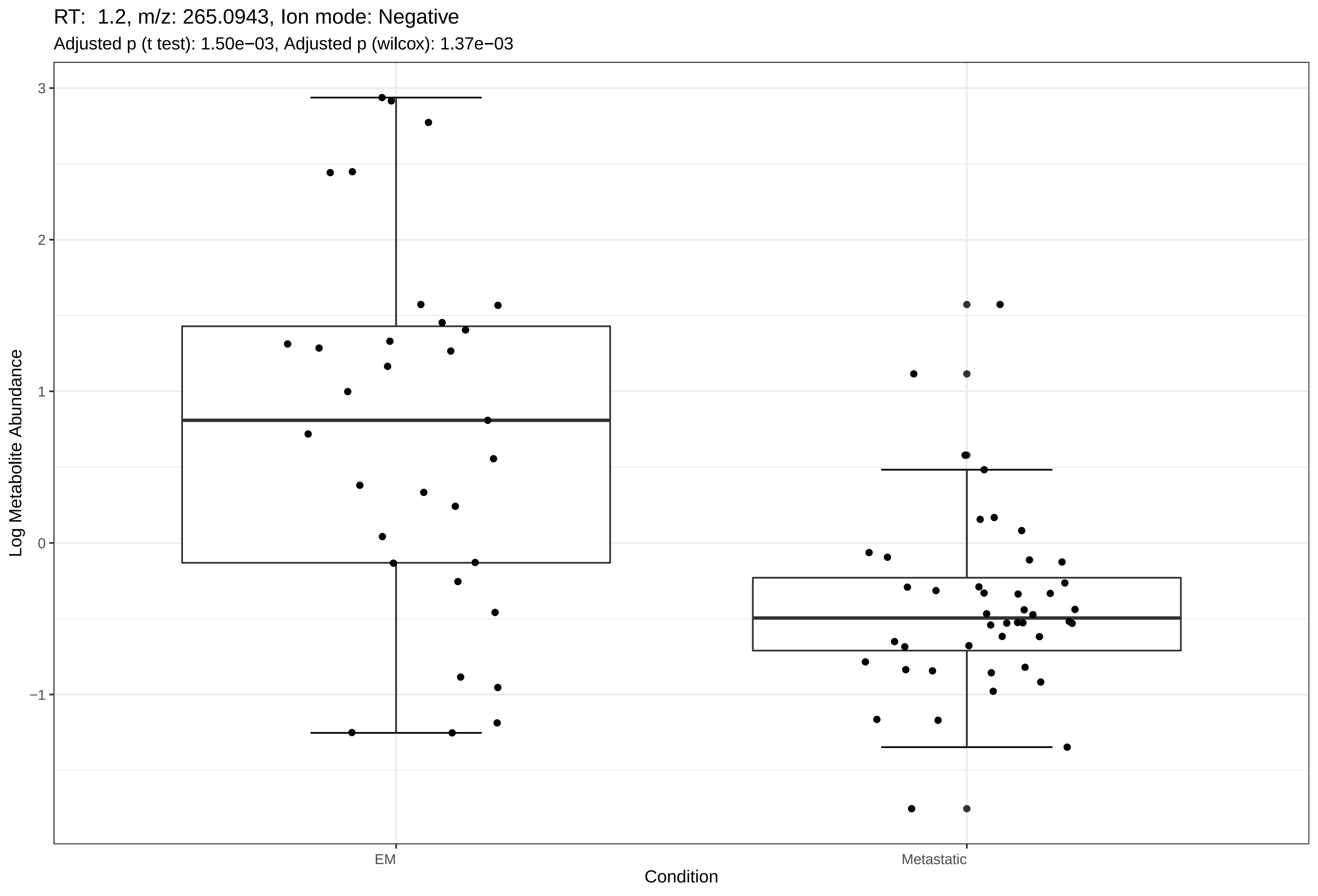
RT: 1.2, m/z: 265.0943, Ion mode: Negative
Adjusted p (t test): 1.50e−03, Adjusted p (wilcox): 1.37e−03
Log Metabolite Abundance
3
2
1
0
−1
EM
Metastatic
Condition

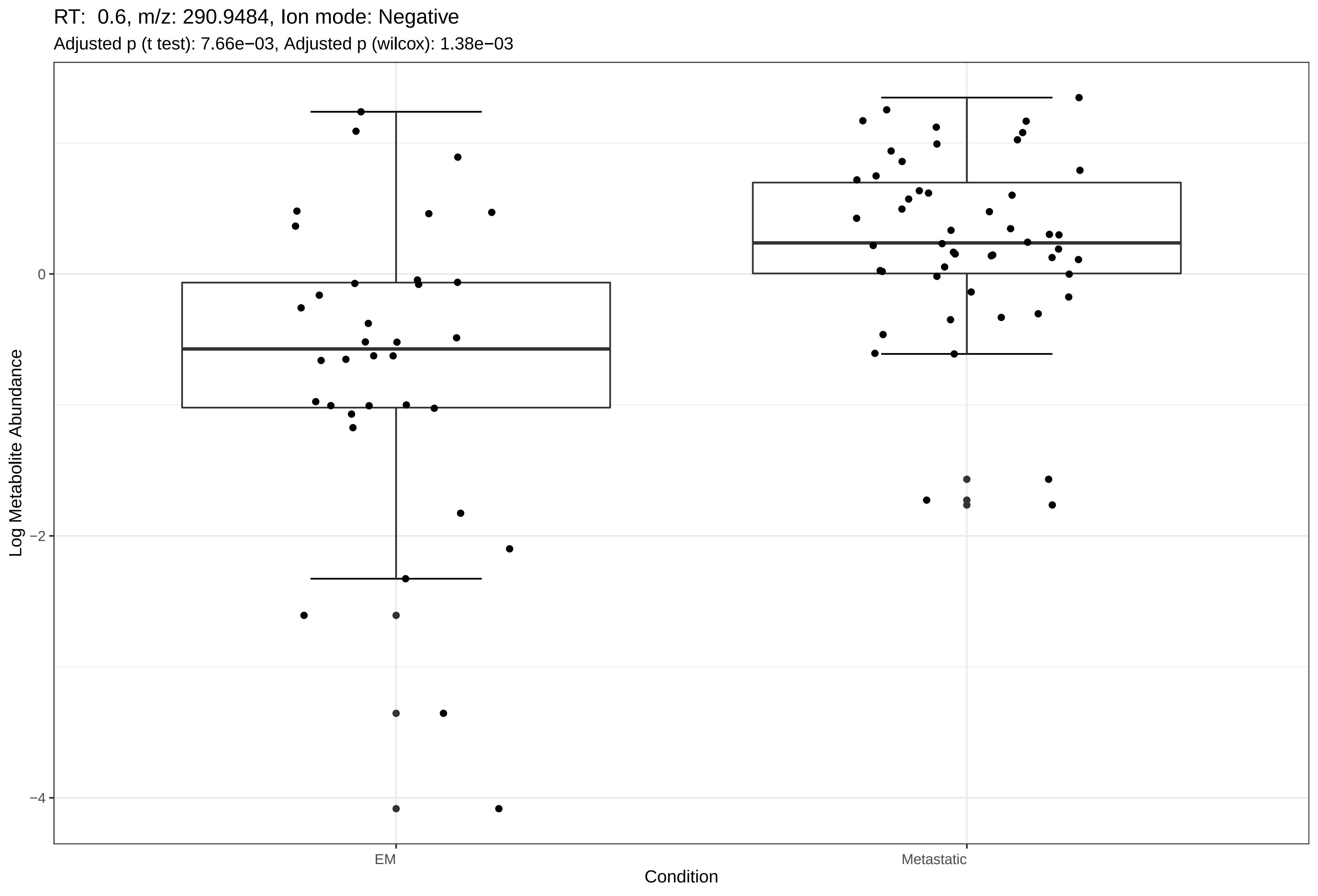

RT: 0.6, m/z: 290.9484, Ion mode: Negative
Adjusted p (t test): 7.66e−03, Adjusted p (wilcox): 1.38e−03
Log Metabolite Abundance
0
−2
−4
EM
Metastatic
Condition

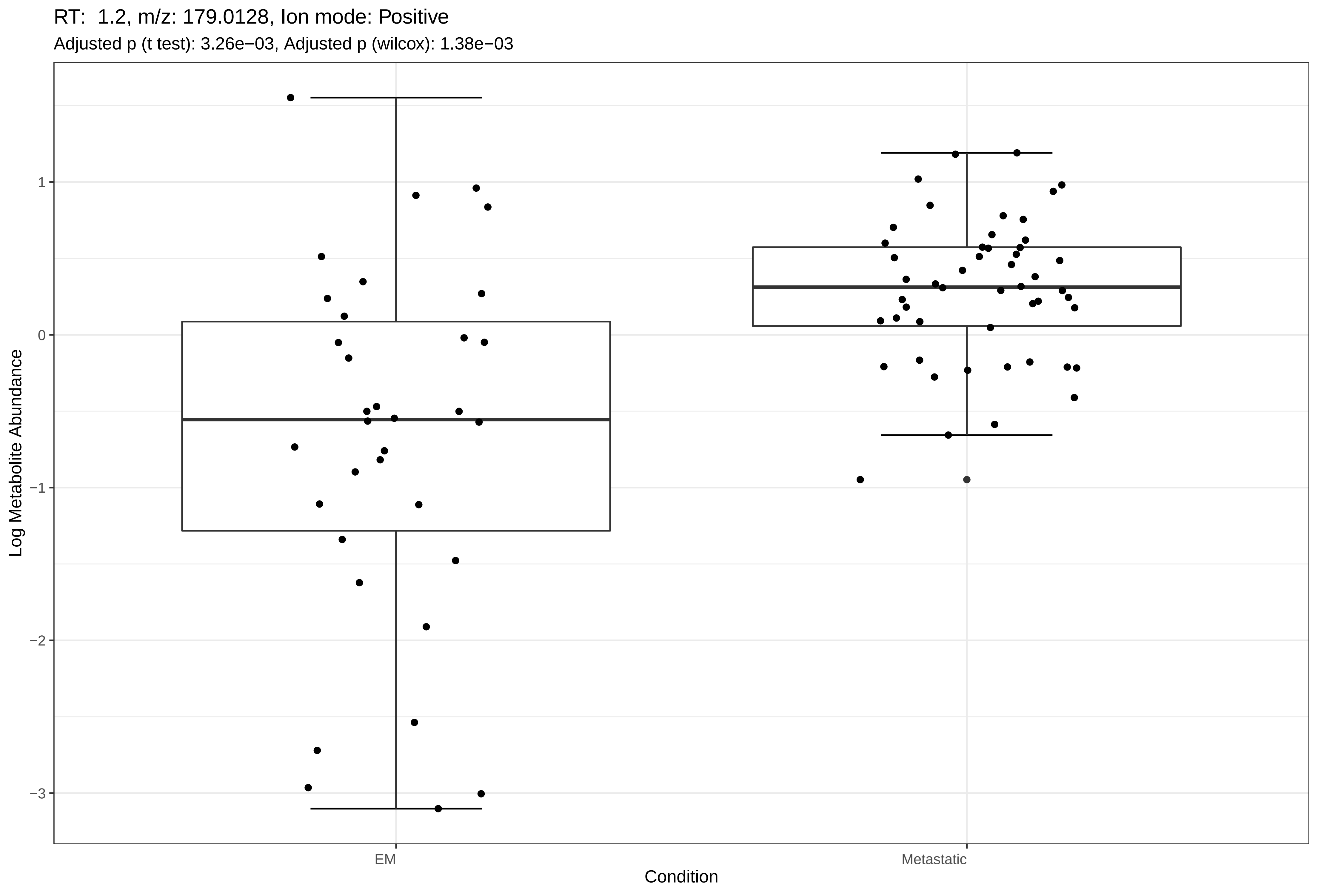
RT: 1.2, m/z: 179.0128, Ion mode: Positive
Adjusted p (t test): 3.26e−03, Adjusted p (wilcox): 1.38e−03
Log Metabolite Abundance
1
0
−1
−2
−3
EM
Metastatic
Condition

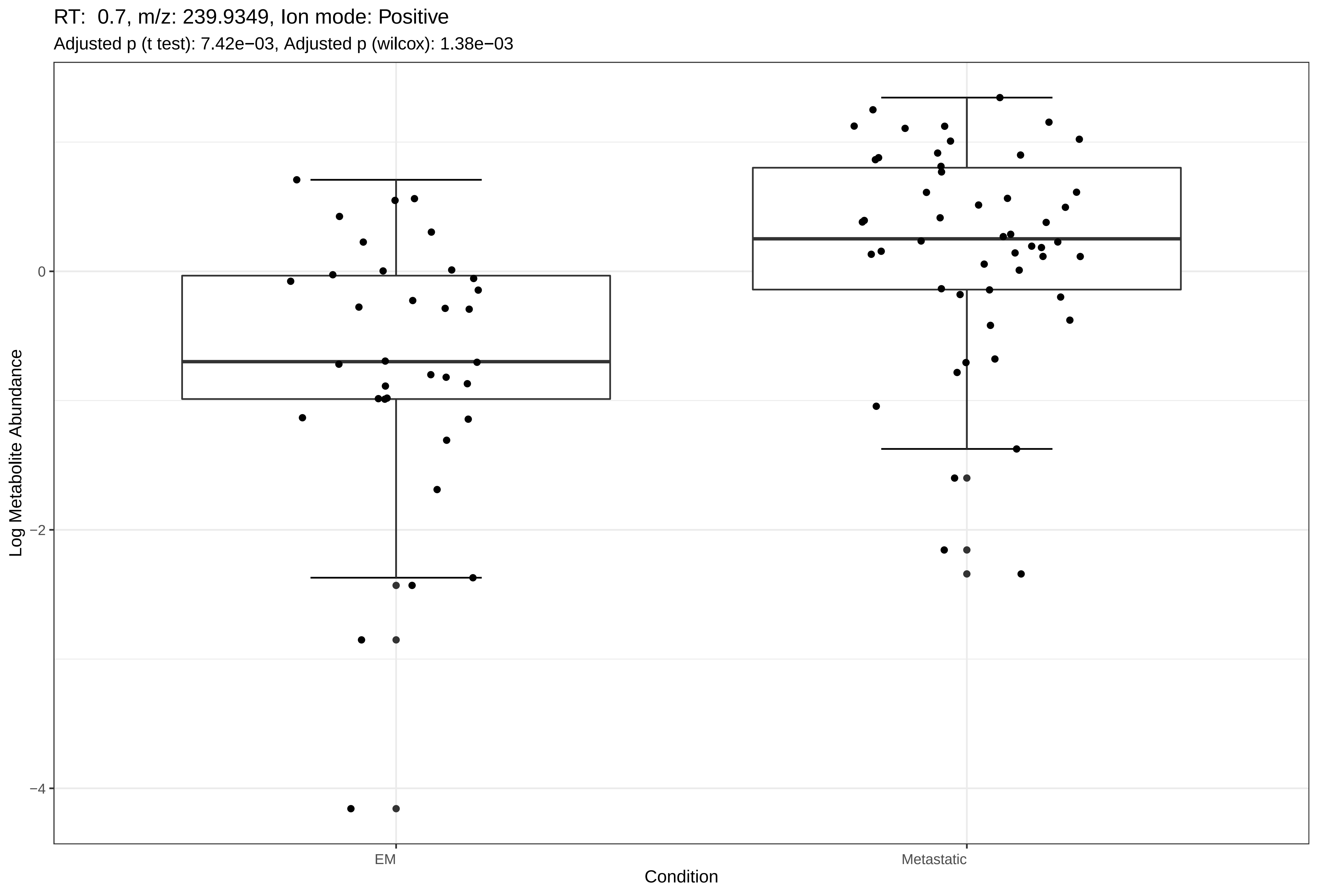
RT: 0.7, m/z: 239.9349, Ion mode: Positive
Adjusted p (t test): 7.42e−03, Adjusted p (wilcox): 1.38e−03
Log Metabolite Abundance
0
−2
−4
EM
Metastatic
Condition

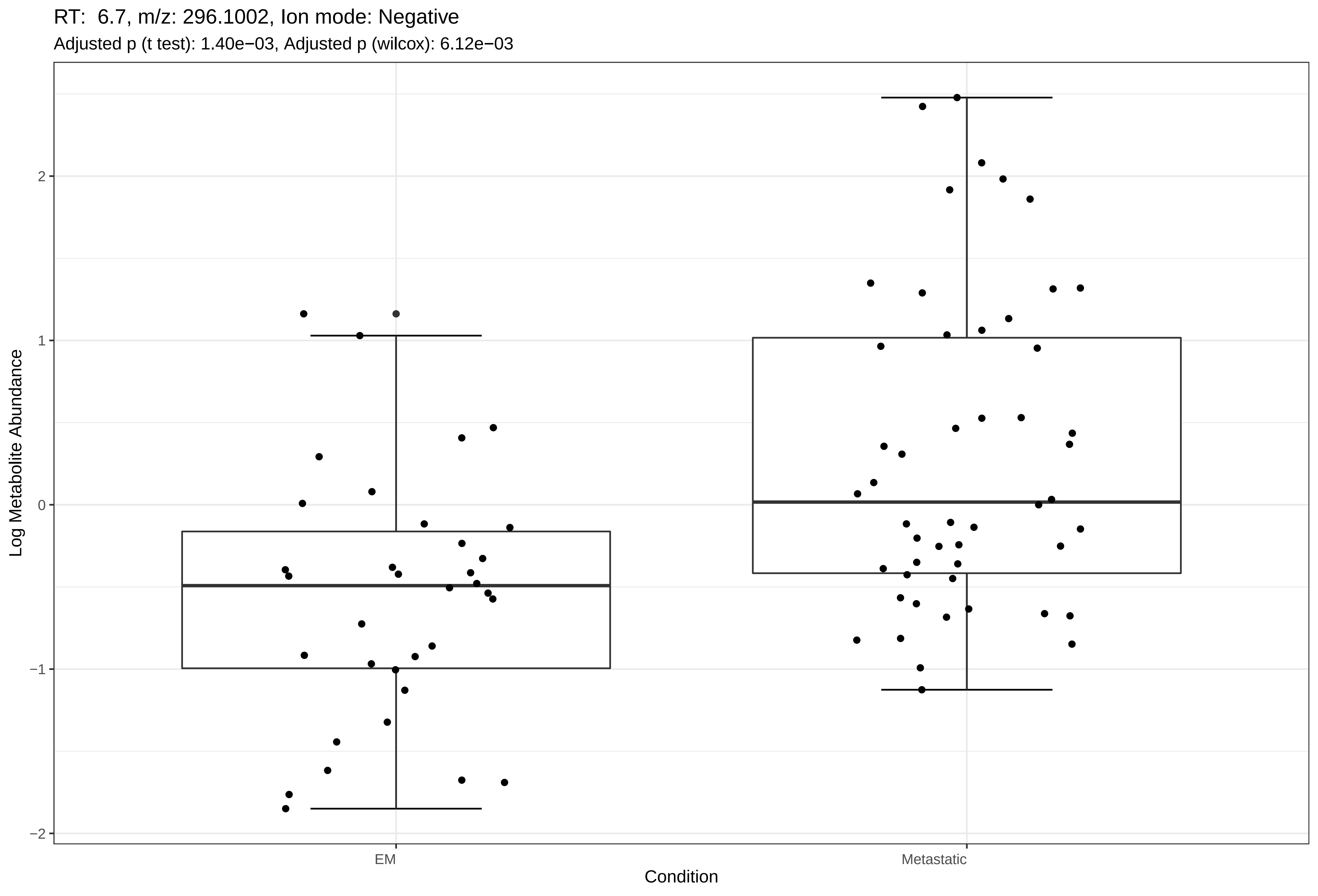

RT: 6.7, m/z: 296.1002, Ion mode: Negative
Adjusted p (t test): 1.40e−03, Adjusted p (wilcox): 6.12e−03
Log Metabolite Abundance
2
1
0
−1
−2
EM
Metastatic
Condition

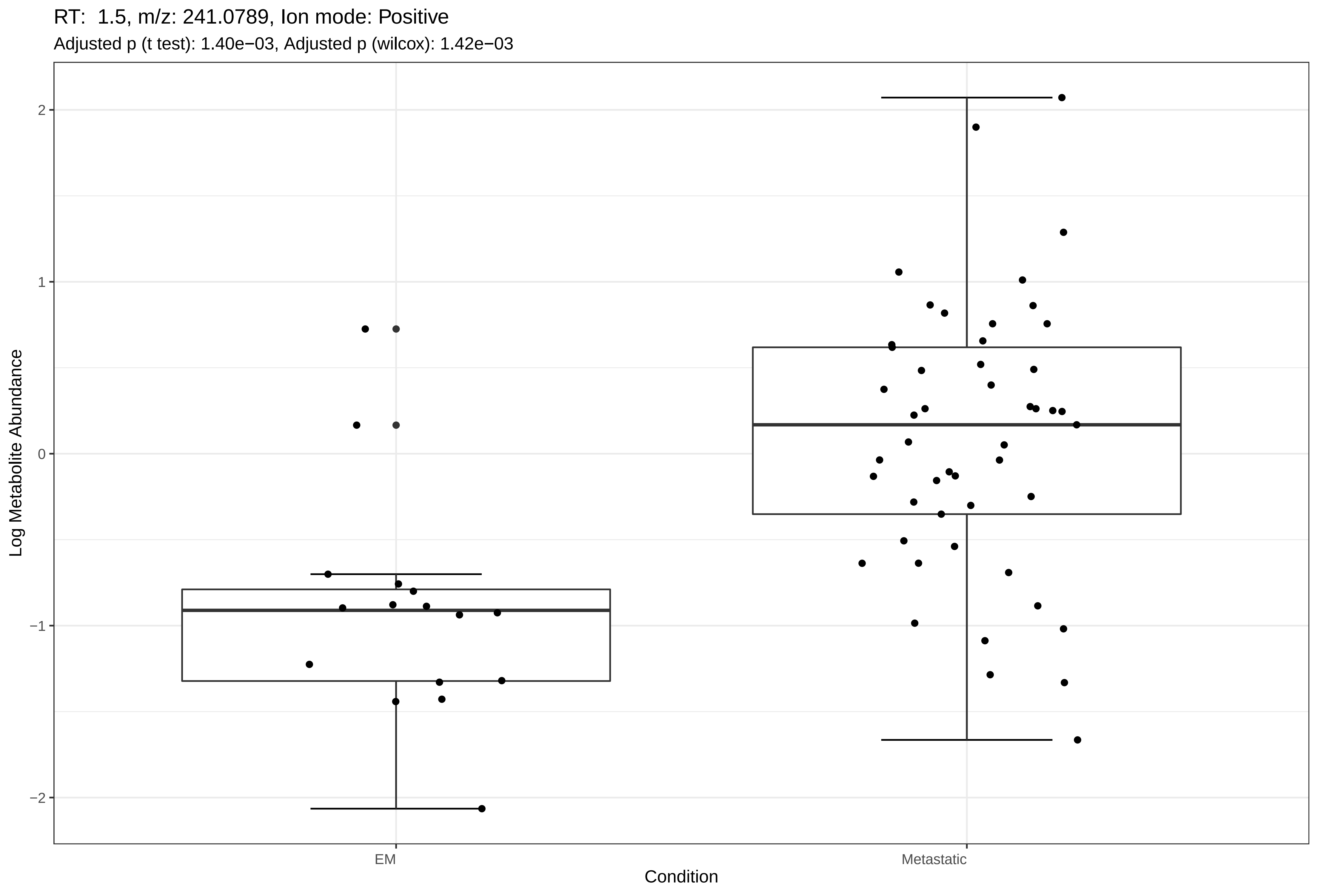

RT: 1.5, m/z: 241.0789, Ion mode: Positive
Adjusted p (t test): 1.40e−03, Adjusted p (wilcox): 1.42e−03
Log Metabolite Abundance
2
1
0
−1
−2
EM
Metastatic
Condition

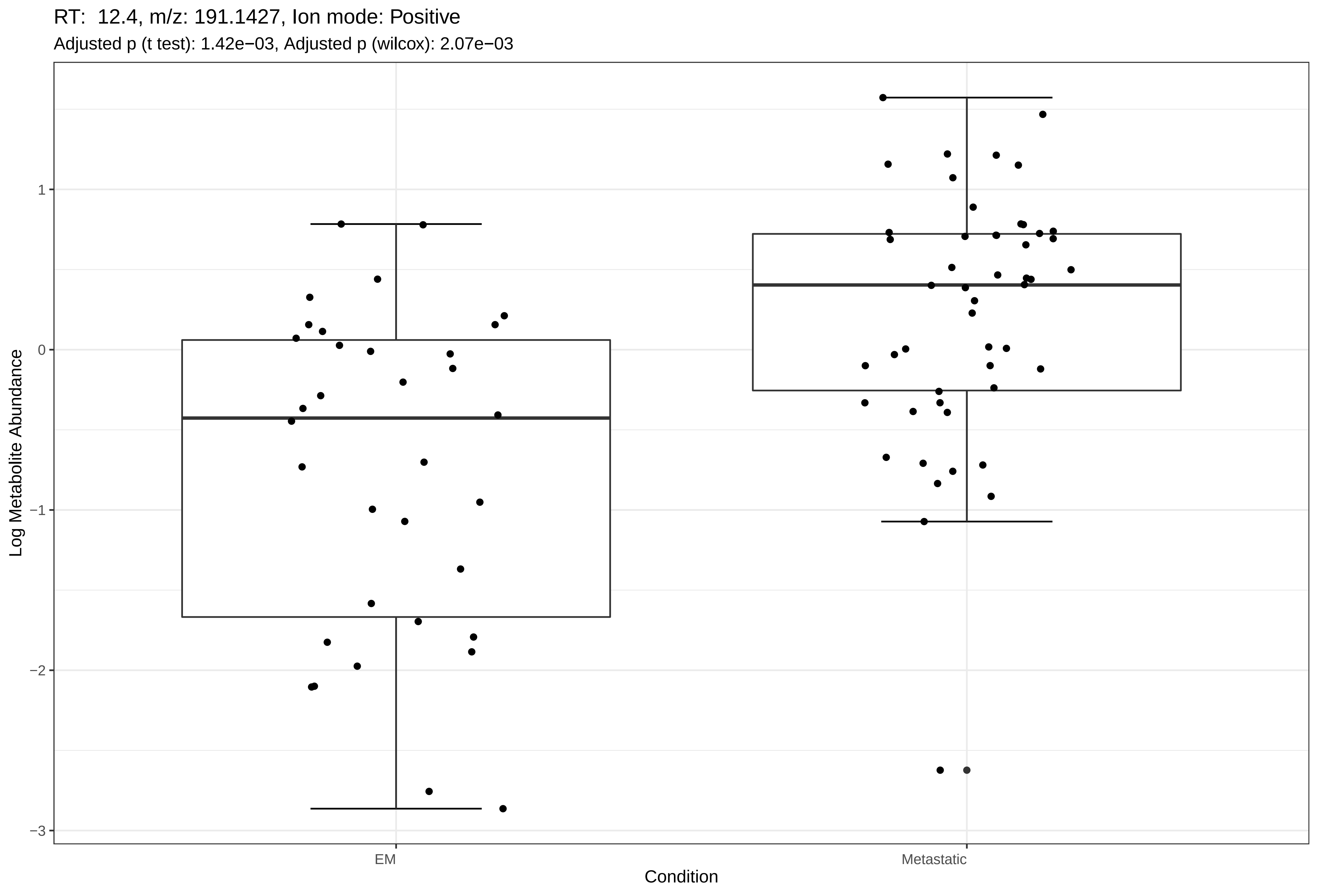
RT: 12.4, m/z: 191.1427, Ion mode: Positive
Adjusted p (t test): 1.42e−03, Adjusted p (wilcox): 2.07e−03
Log Metabolite Abundance
1
0
−1
−2
−3
EM
Metastatic
Condition

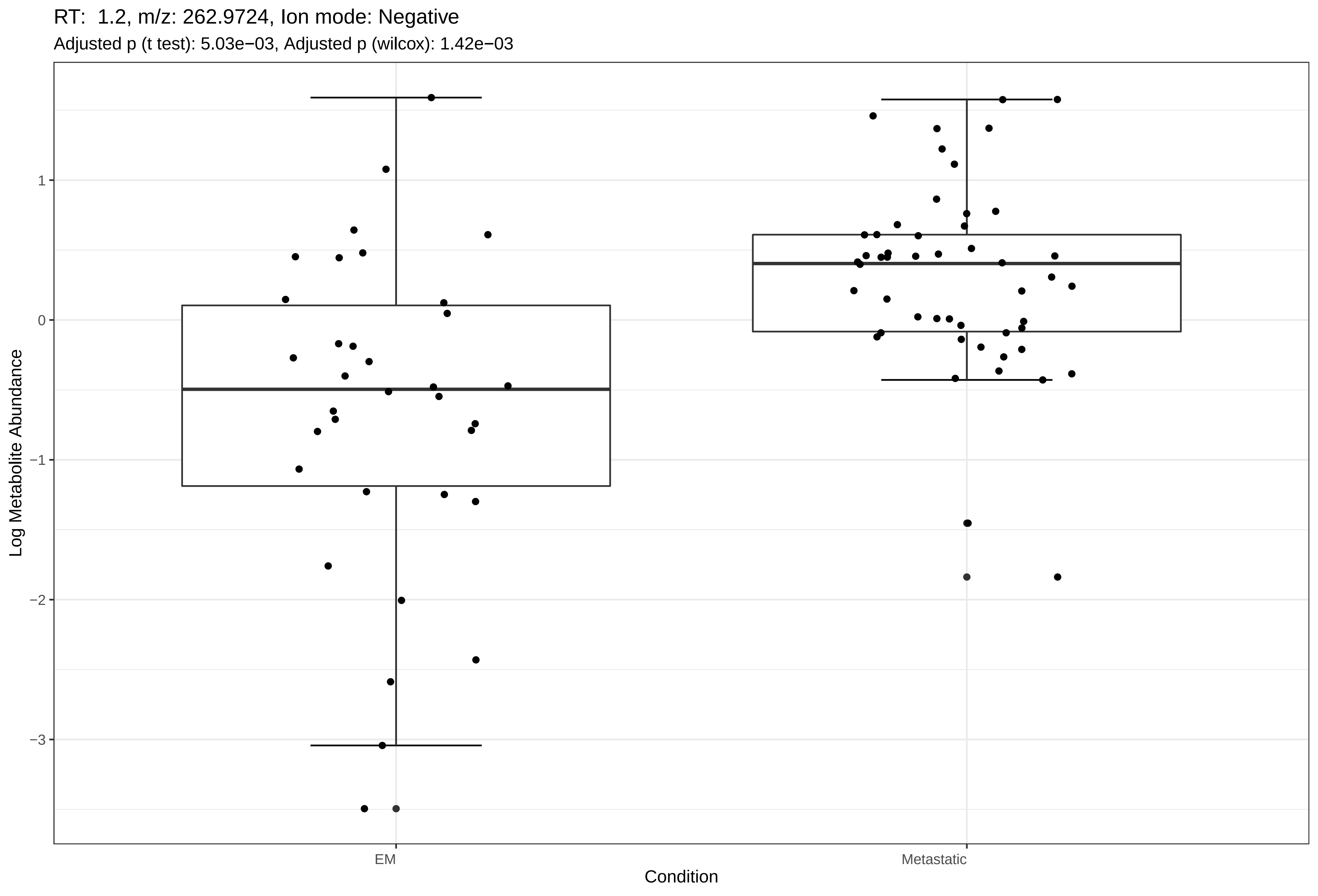

RT: 1.2, m/z: 262.9724, Ion mode: Negative
Adjusted p (t test): 5.03e−03, Adjusted p (wilcox): 1.42e−03
Log Metabolite Abundance
1
0
−1
−2
−3
EM
Metastatic
Condition

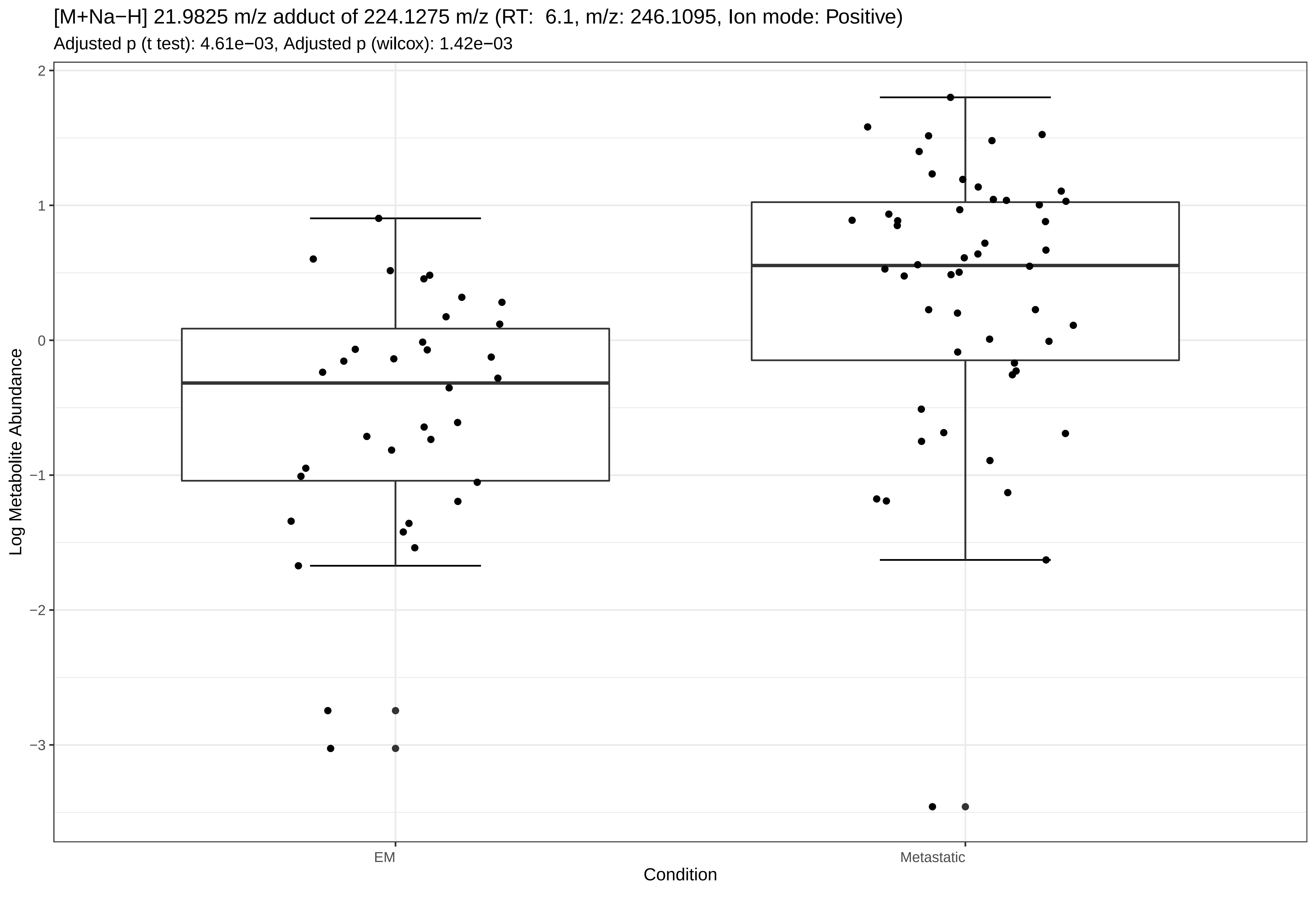

[M+Na−H] 21.9825 m/z adduct of 224.1275 m/z (RT: 6.1, m/z: 246.1095, Ion mode: Positive)
Adjusted p (t test): 4.61e−03, Adjusted p (wilcox): 1.42e−03
Log Metabolite Abundance
2
1
0
−1
−2
−3
EM
Metastatic
Condition

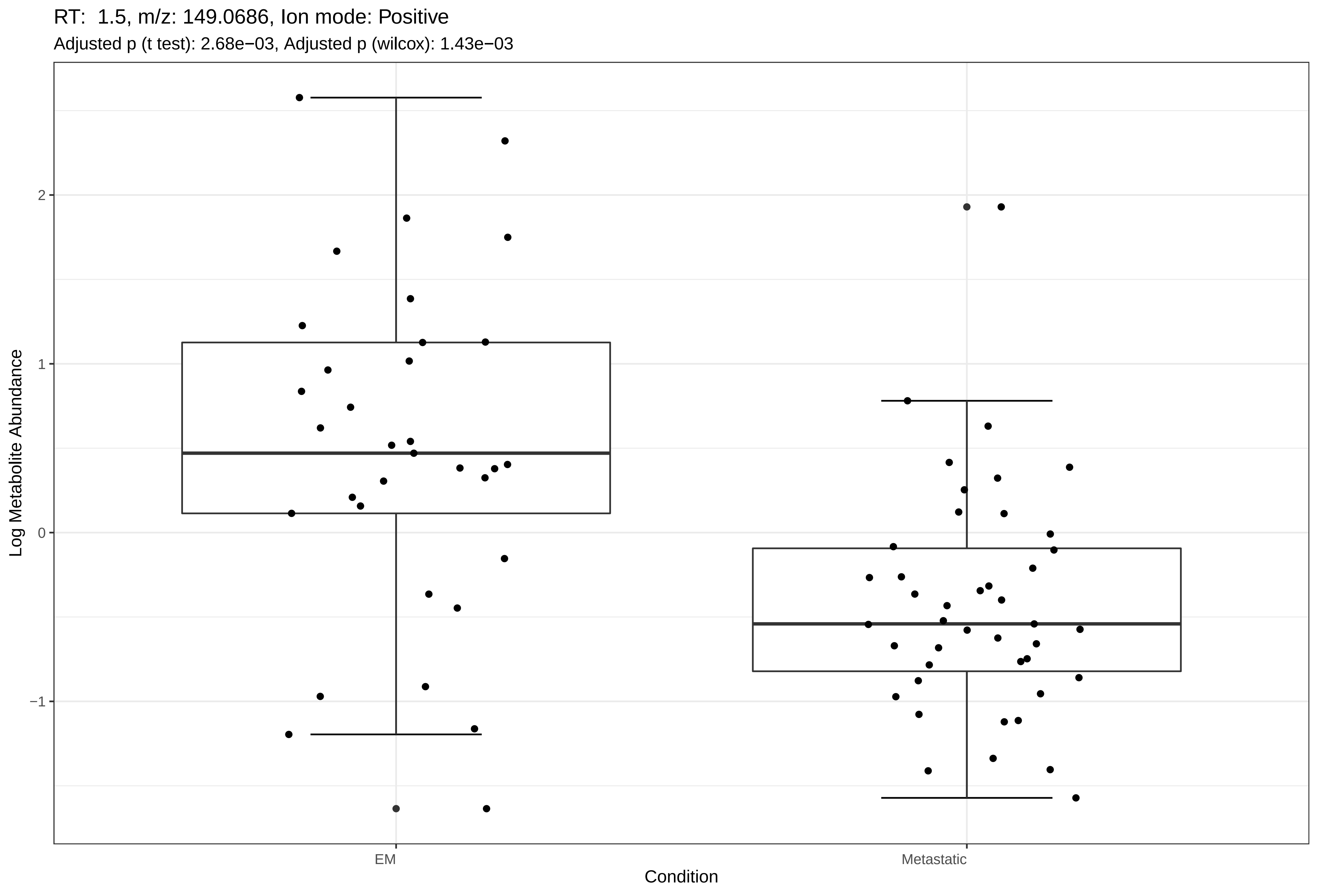

RT: 1.5, m/z: 149.0686, Ion mode: Positive
Adjusted p (t test): 2.68e−03, Adjusted p (wilcox): 1.43e−03
Log Metabolite Abundance
2
1
0
−1
EM
Metastatic
Condition

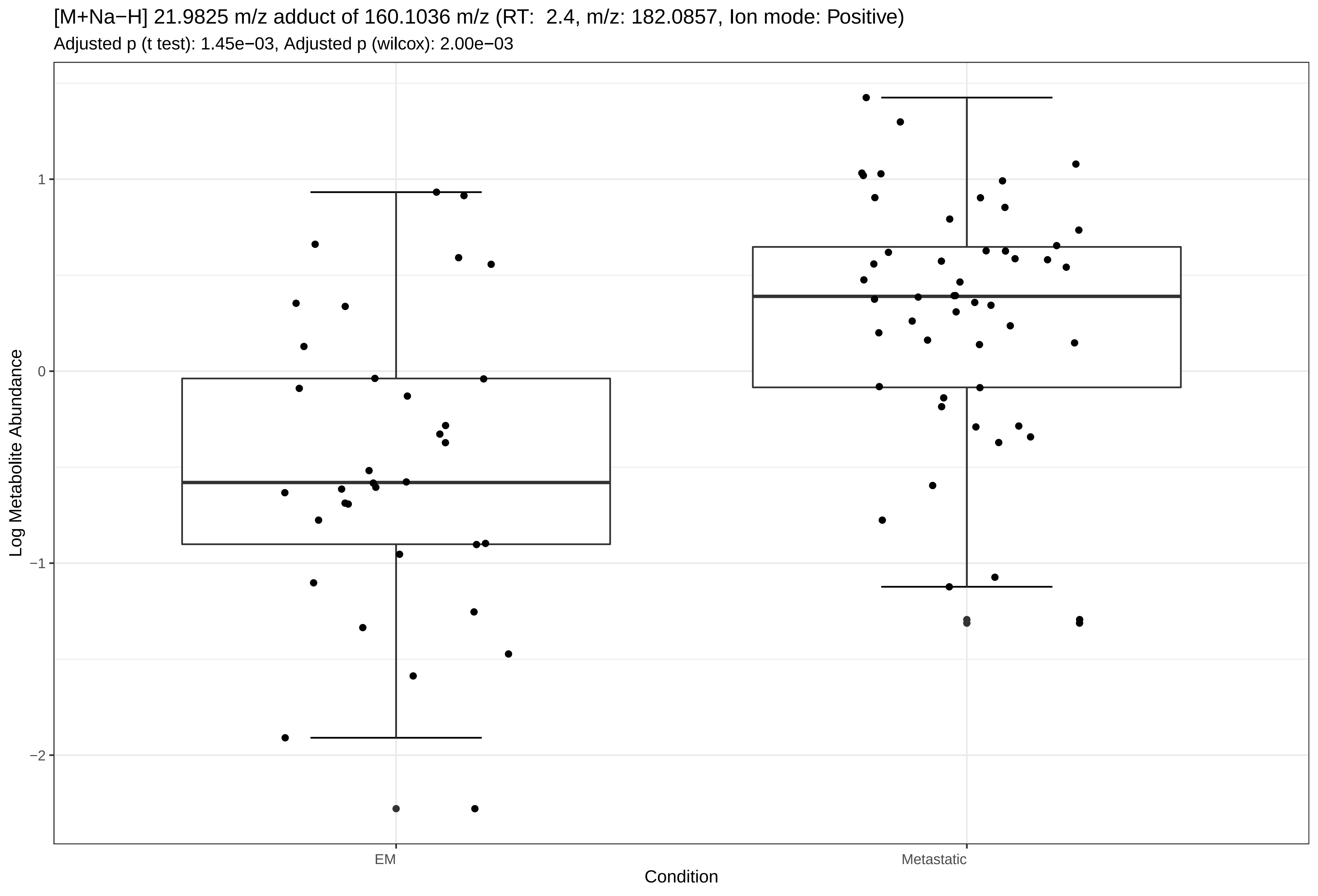
[M+Na−H] 21.9825 m/z adduct of 160.1036 m/z (RT: 2.4, m/z: 182.0857, Ion mode: Positive)
Adjusted p (t test): 1.45e−03, Adjusted p (wilcox): 2.00e−03
Log Metabolite Abundance
1
0
−1
−2
EM
Metastatic
Condition

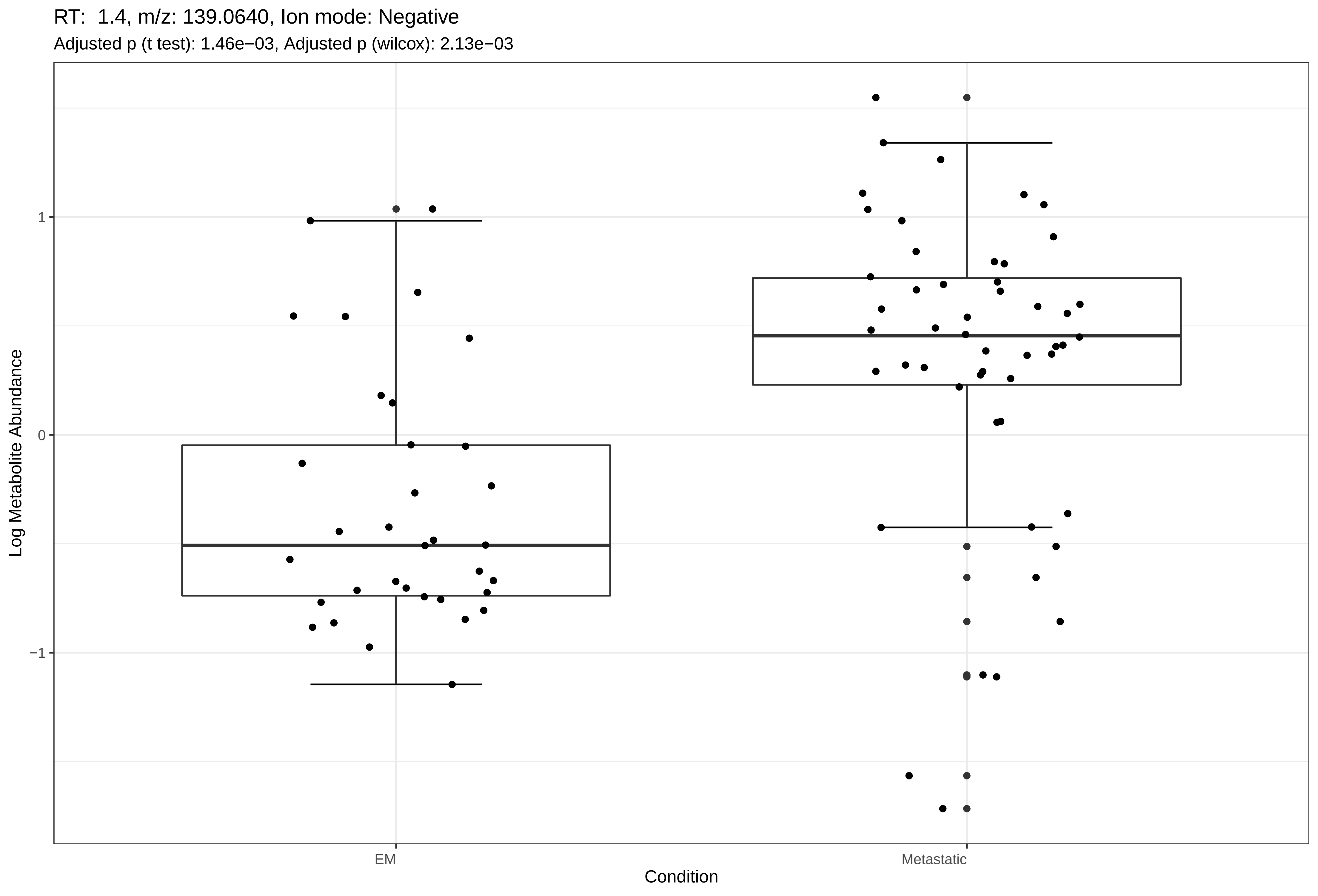
RT: 1.4, m/z: 139.0640, Ion mode: Negative
Adjusted p (t test): 1.46e−03, Adjusted p (wilcox): 2.13e−03
Log Metabolite Abundance
1
0
−1
EM
Metastatic
Condition

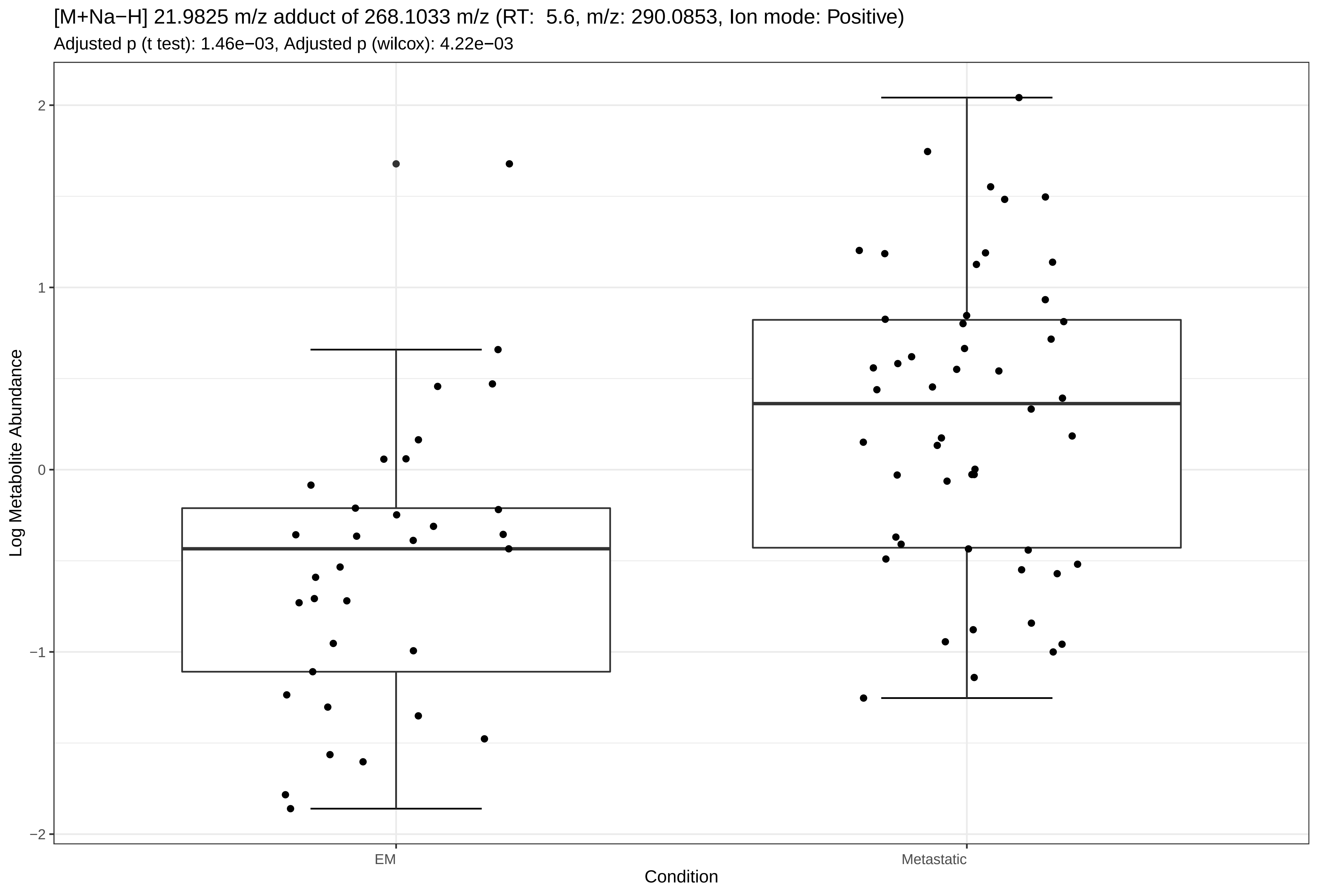
[M+Na−H] 21.9825 m/z adduct of 268.1033 m/z (RT: 5.6, m/z: 290.0853, Ion mode: Positive)
Adjusted p (t test): 1.46e−03, Adjusted p (wilcox): 4.22e−03
Log Metabolite Abundance
2
1
0
−1
−2
EM
Metastatic
Condition

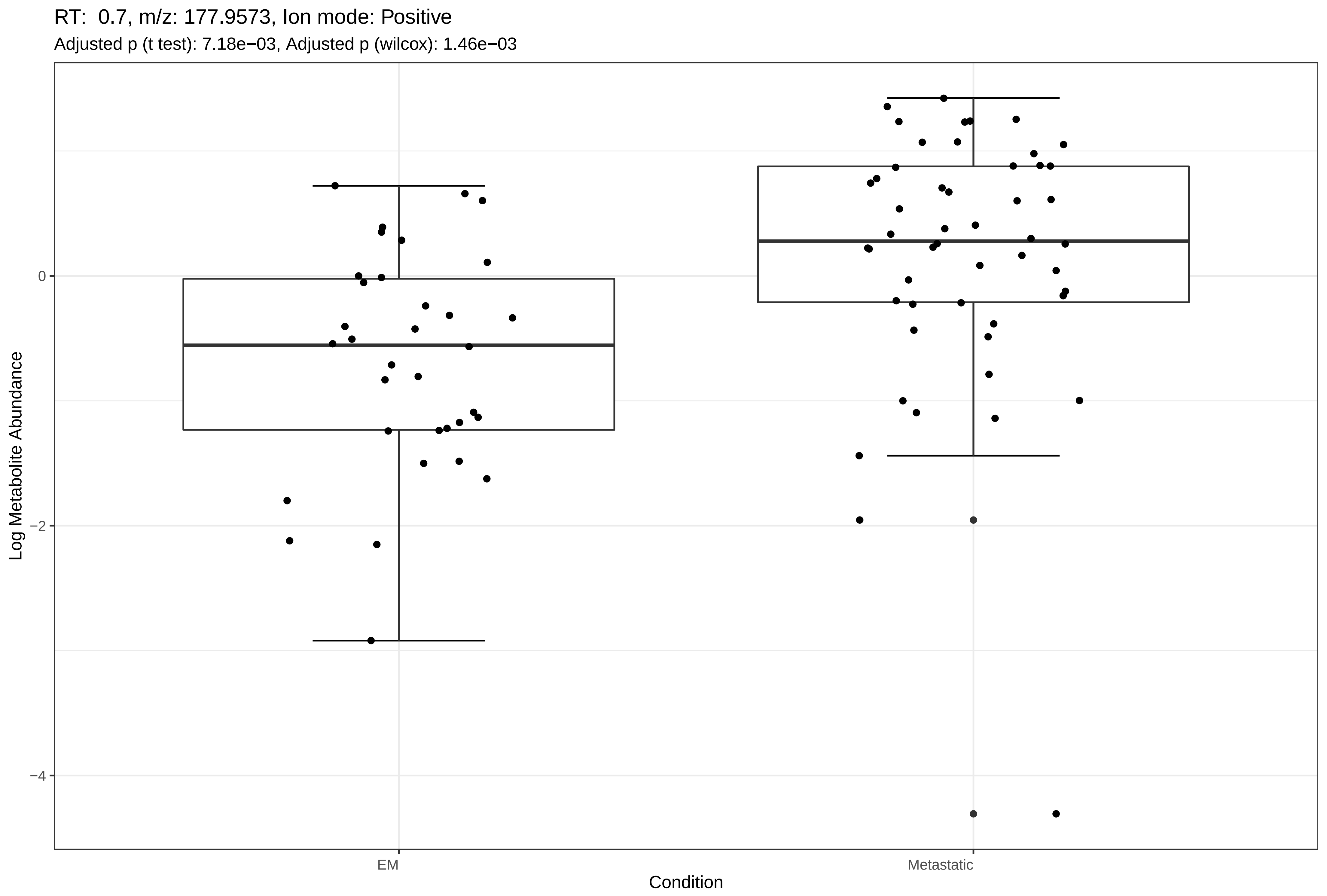
RT: 0.7, m/z: 177.9573, Ion mode: Positive
Adjusted p (t test): 7.18e−03, Adjusted p (wilcox): 1.46e−03
Log Metabolite Abundance
0
−2
−4
EM
Metastatic
Condition

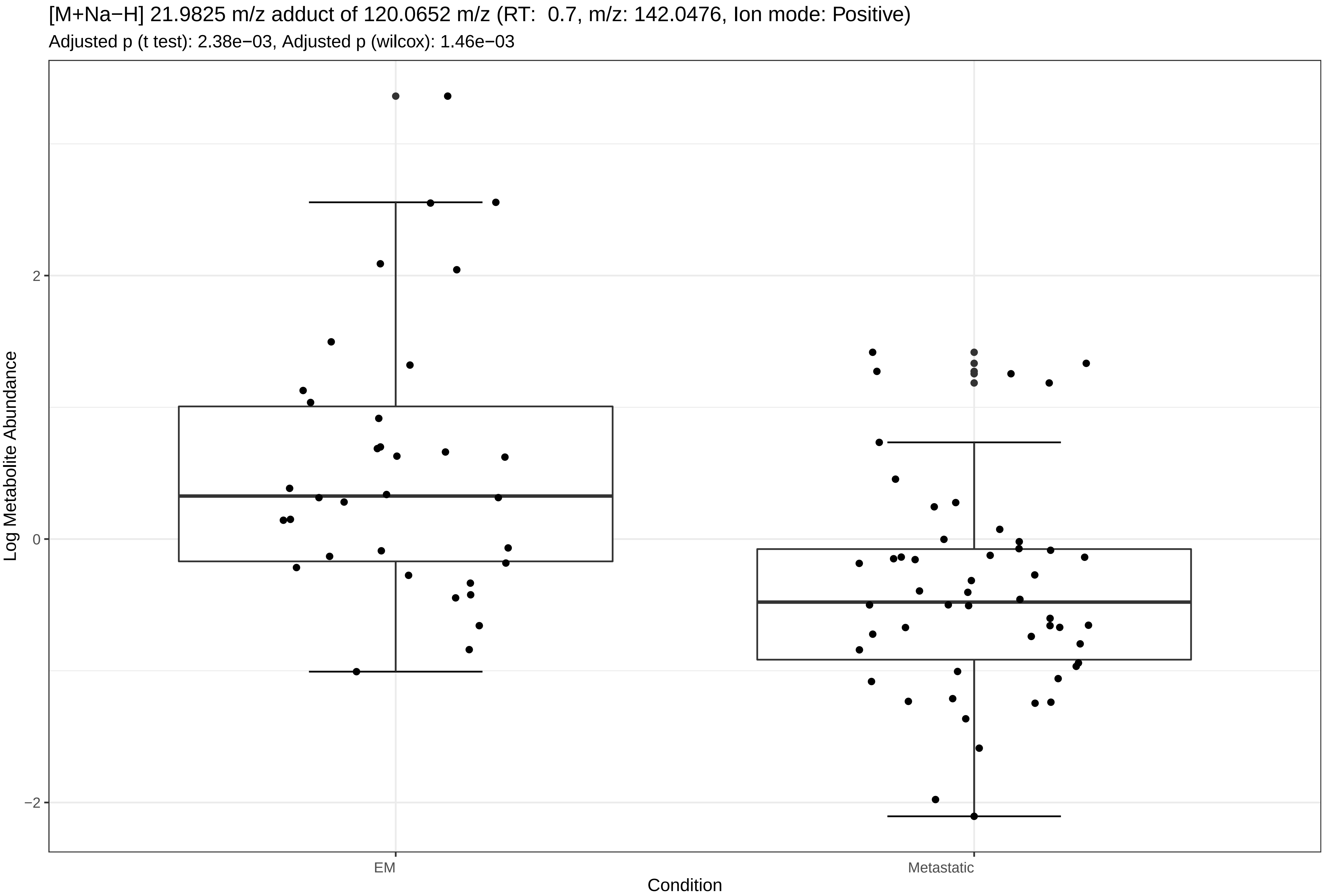

[M+Na−H] 21.9825 m/z adduct of 120.0652 m/z (RT: 0.7, m/z: 142.0476, Ion mode: Positive)
Adjusted p (t test): 2.38e−03, Adjusted p (wilcox): 1.46e−03
Log Metabolite Abundance
2
0
−2
EM
Metastatic
Condition

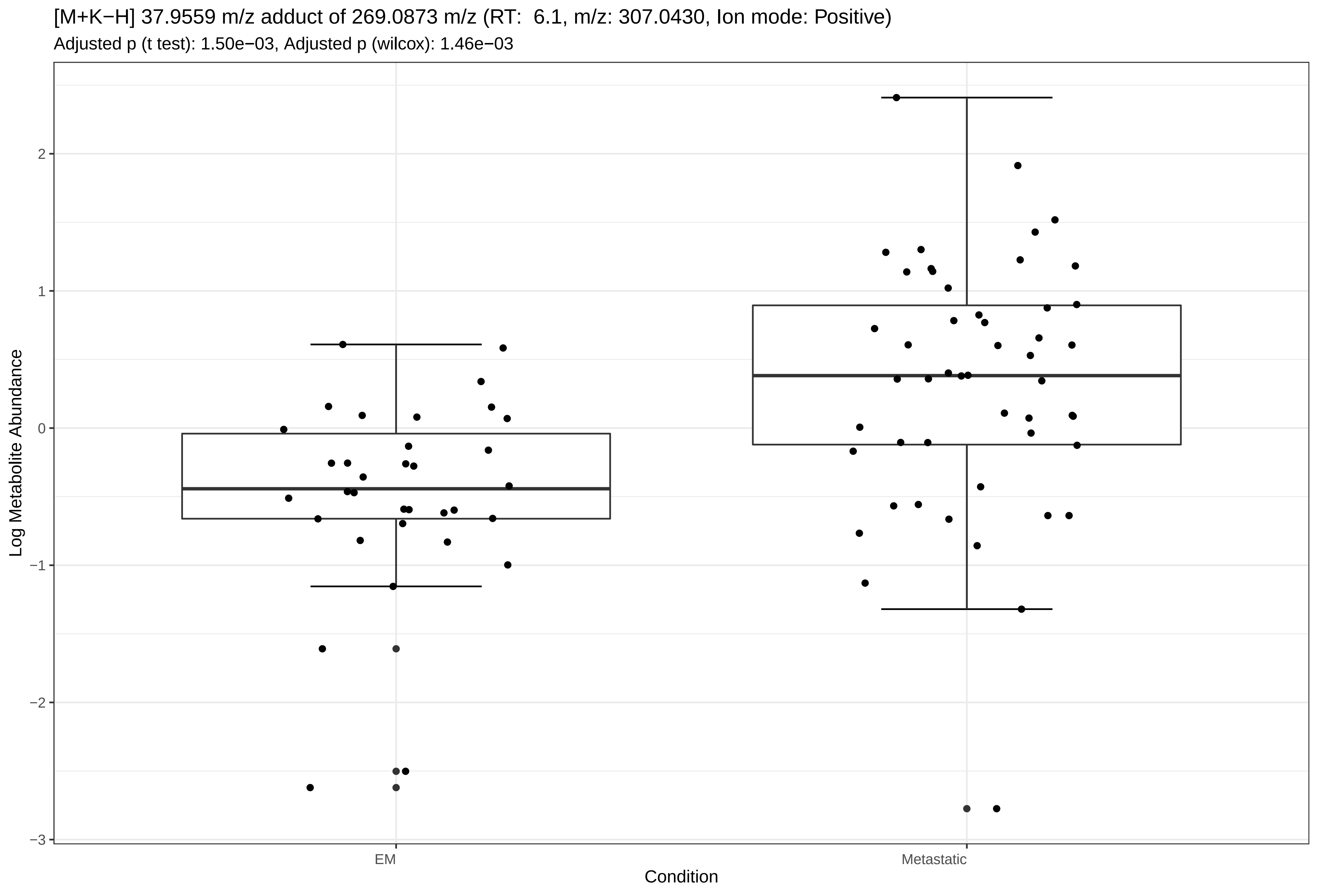

[M+K−H] 37.9559 m/z adduct of 269.0873 m/z (RT: 6.1, m/z: 307.0430, Ion mode: Positive)
Adjusted p (t test): 1.50e−03, Adjusted p (wilcox): 1.46e−03
Log Metabolite Abundance
2
1
0
−1
−2
−3
EM
Metastatic
Condition

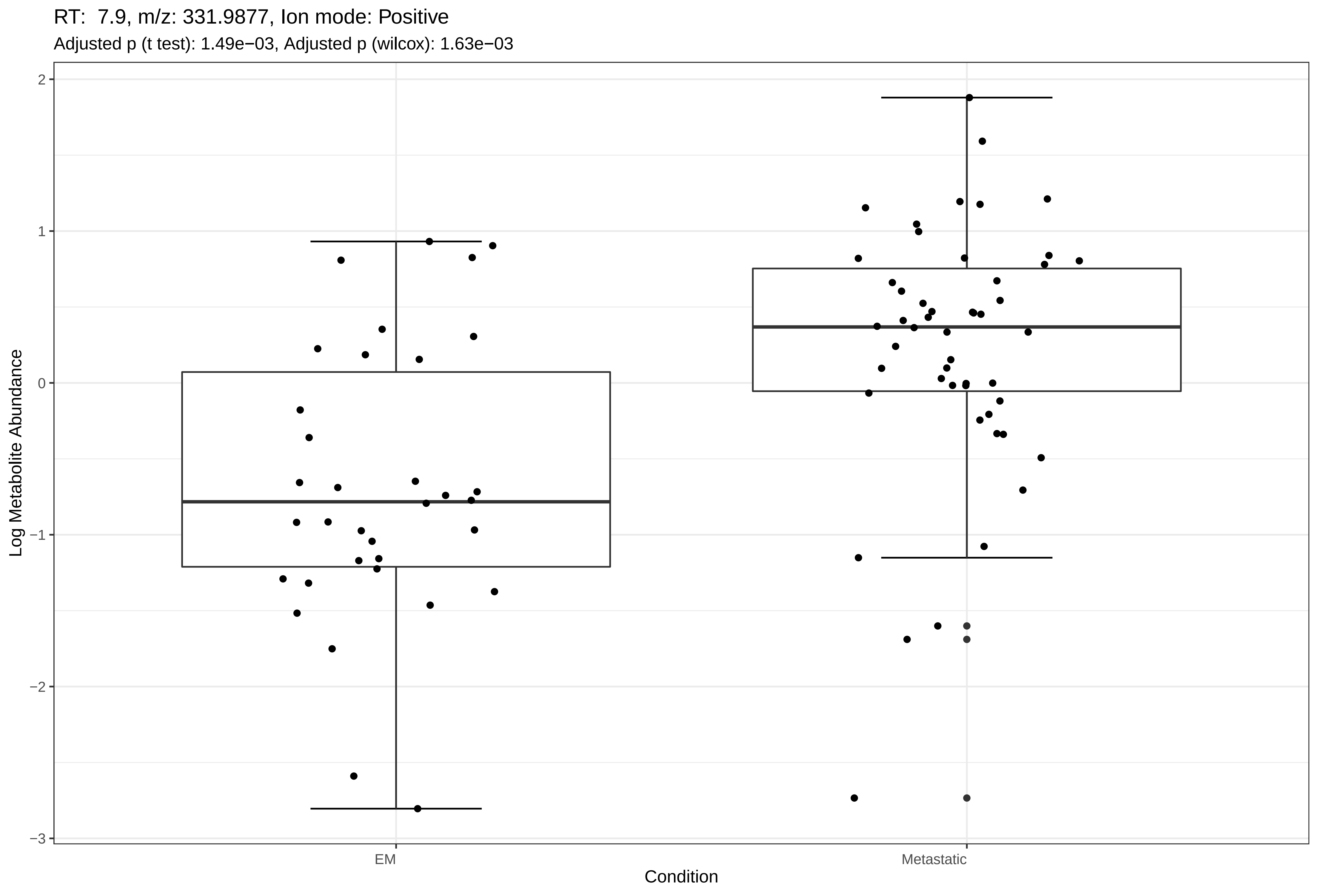
RT: 7.9, m/z: 331.9877, Ion mode: Positive
Adjusted p (t test): 1.49e−03, Adjusted p (wilcox): 1.63e−03
Log Metabolite Abundance
2
1
0
−1
−2
−3
EM
Metastatic
Condition

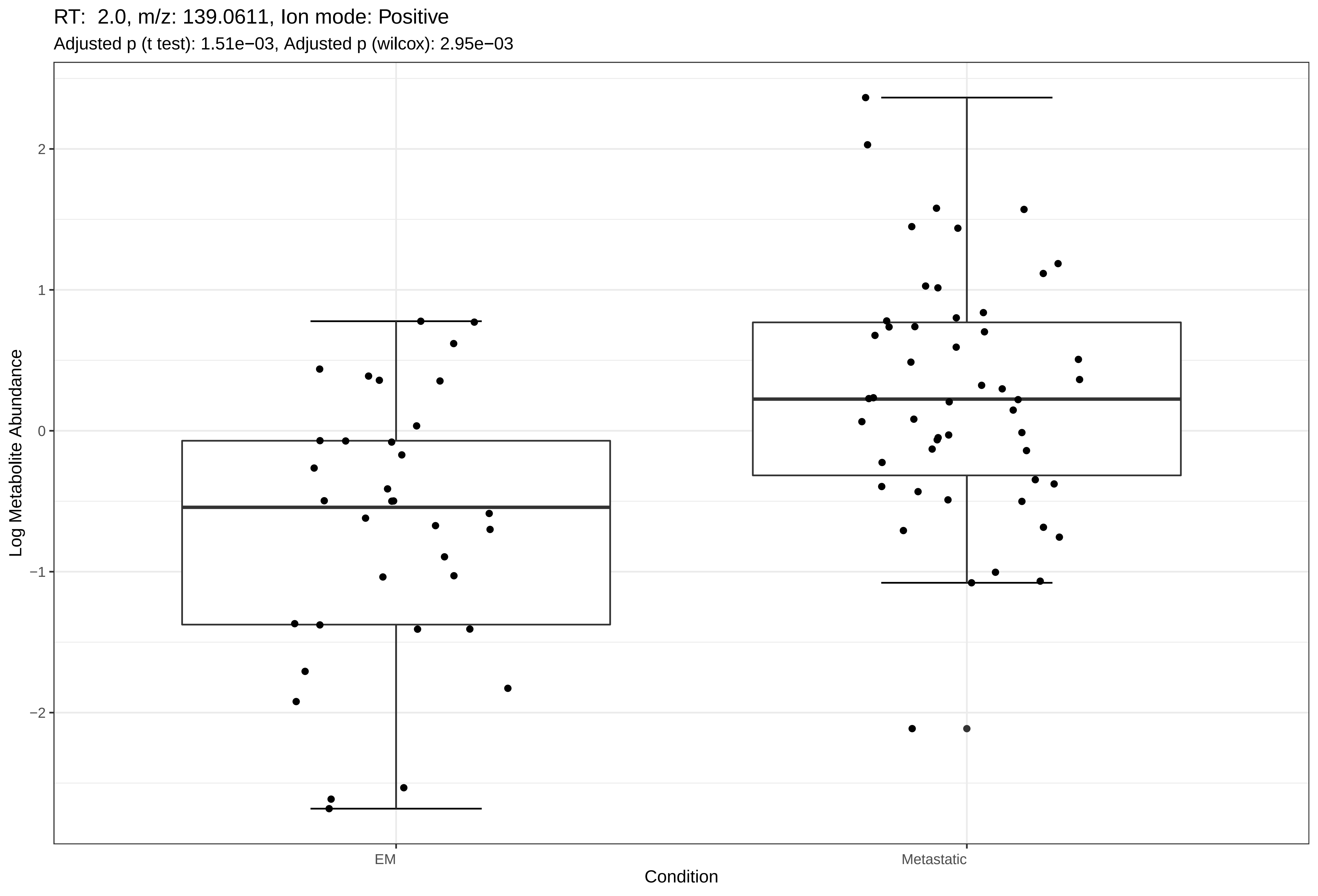
RT: 2.0, m/z: 139.0611, Ion mode: Positive
Adjusted p (t test): 1.51e−03, Adjusted p (wilcox): 2.95e−03
Log Metabolite Abundance
2
1
0
−1
−2
EM
Metastatic
Condition

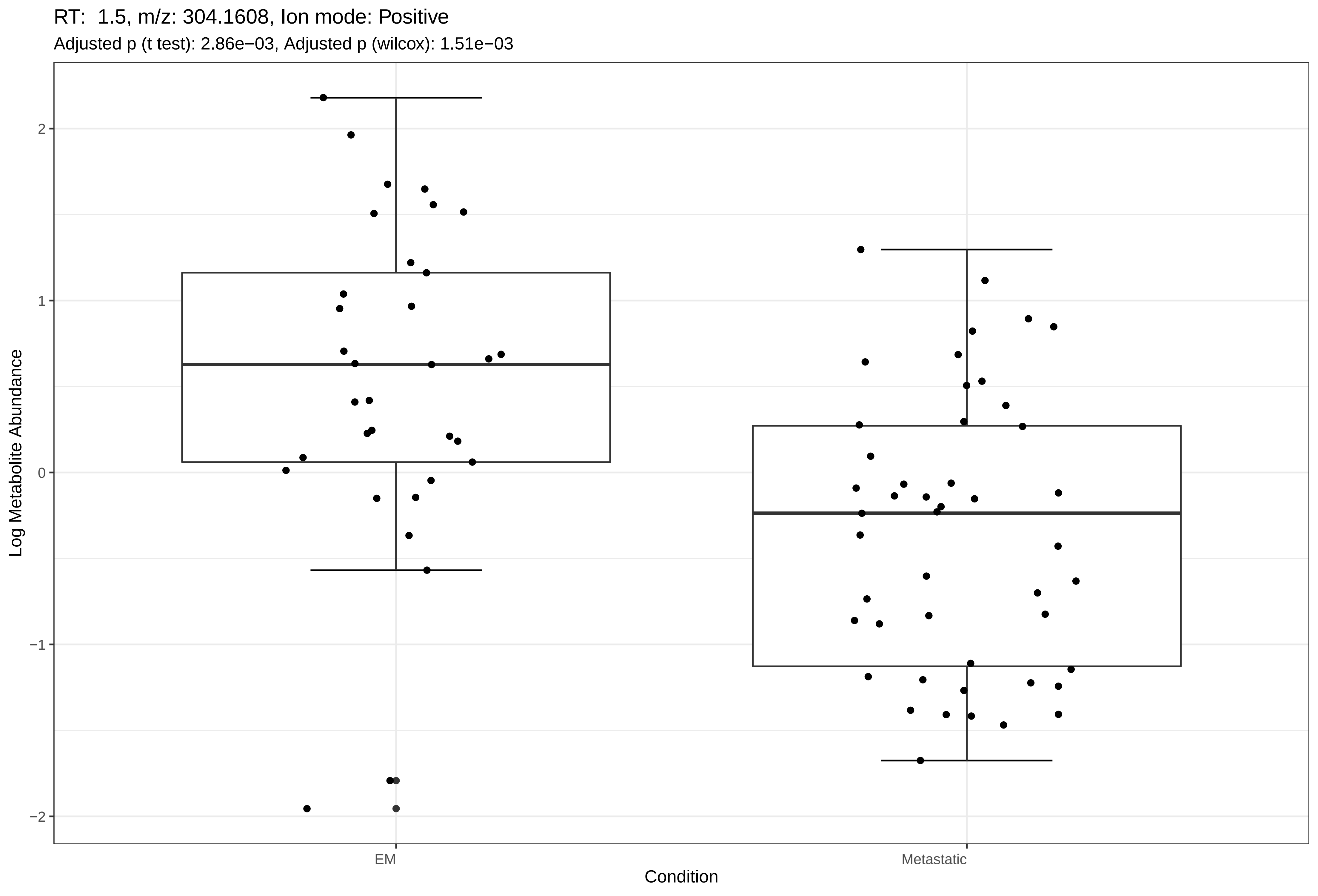
RT: 1.5, m/z: 304.1608, Ion mode: Positive
Adjusted p (t test): 2.86e−03, Adjusted p (wilcox): 1.51e−03
Log Metabolite Abundance
2
1
0
−1
−2
EM
Metastatic
Condition

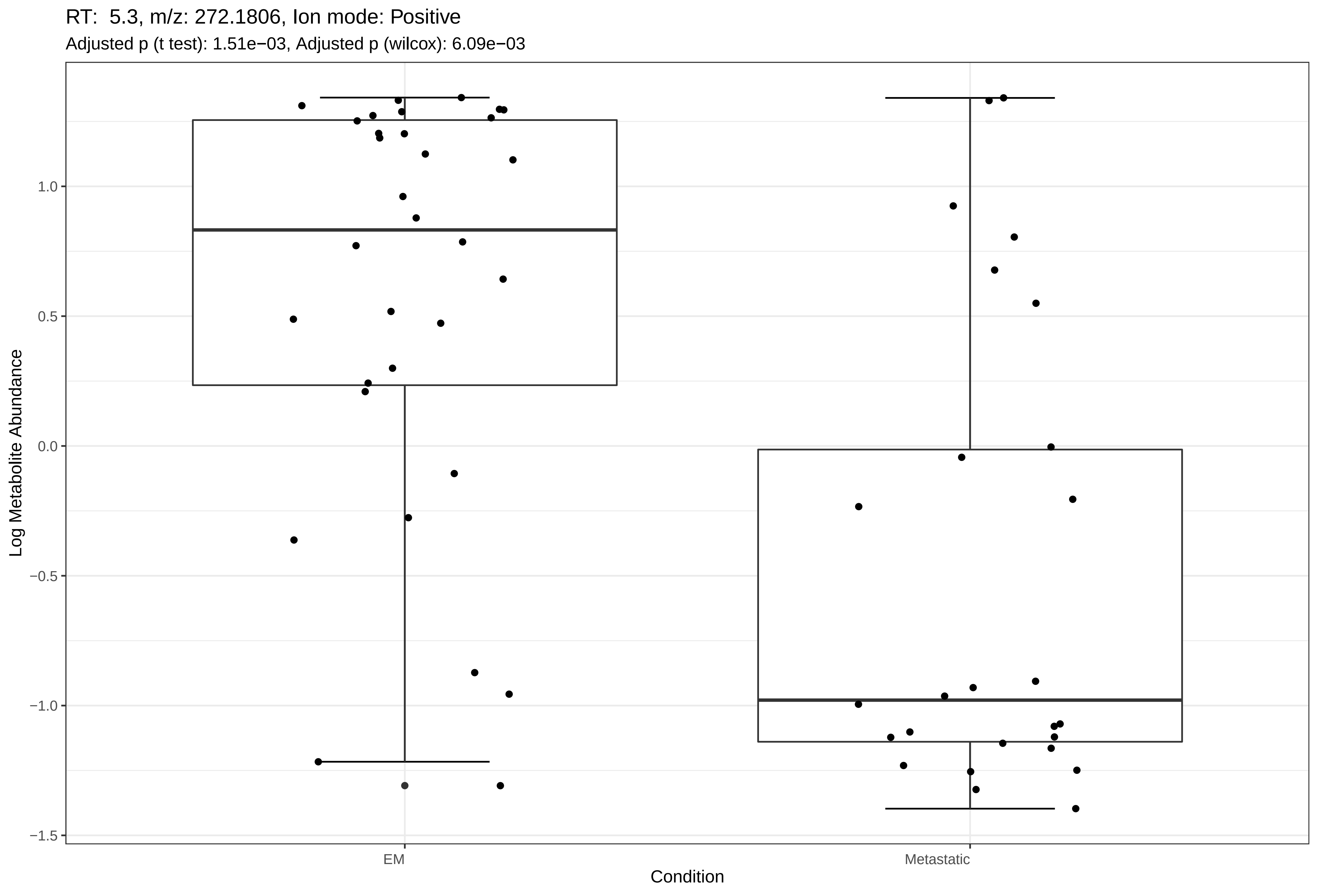

RT: 5.3, m/z: 272.1806, Ion mode: Positive
Adjusted p (t test): 1.51e−03, Adjusted p (wilcox): 6.09e−03
Log Metabolite Abundance
1.0
0.5
0.0
−0.5
−1.0
−1.5
EM
Metastatic
Condition

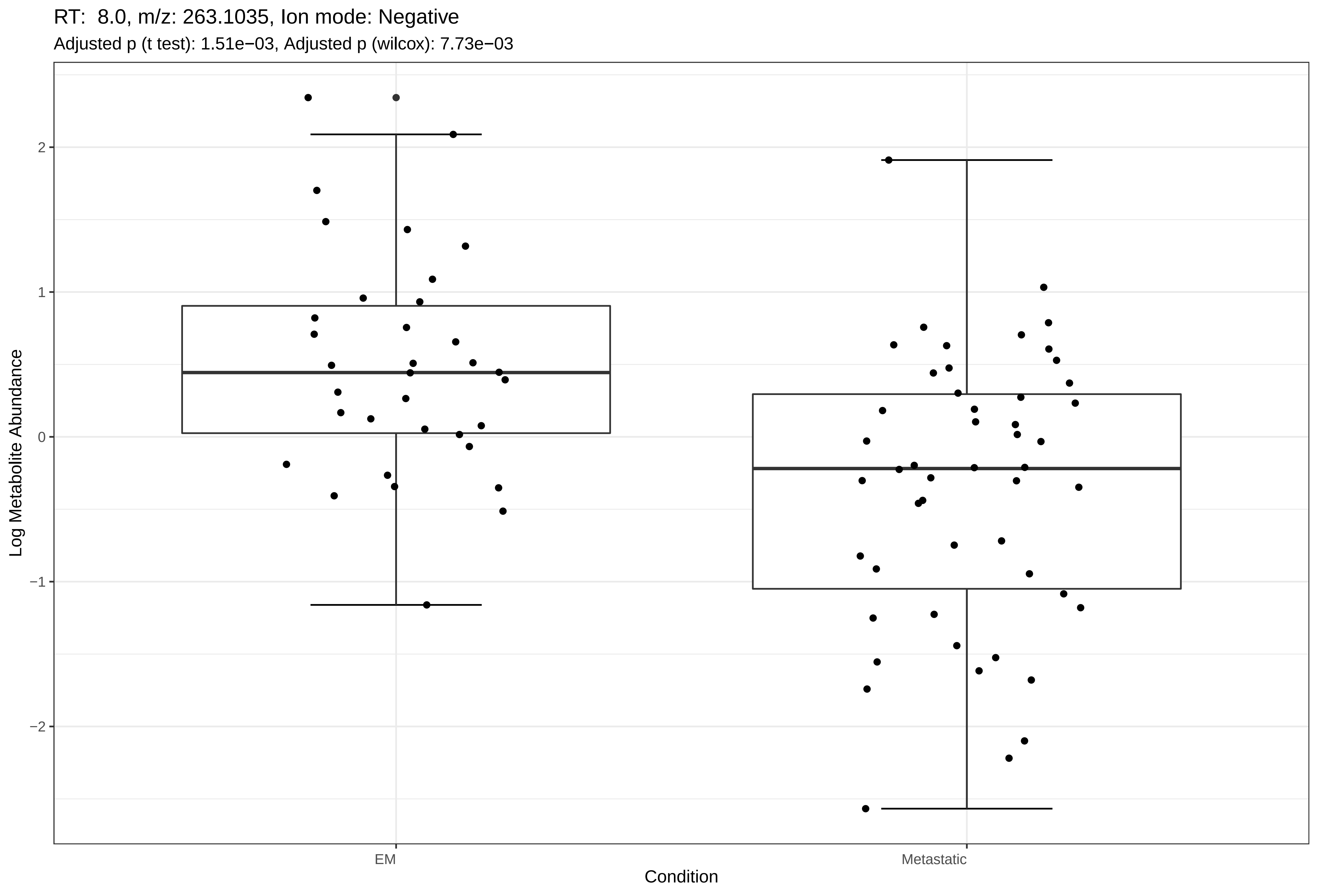

RT: 8.0, m/z: 263.1035, Ion mode: Negative
Adjusted p (t test): 1.51e−03, Adjusted p (wilcox): 7.73e−03
Log Metabolite Abundance
2
1
0
−1
−2
EM
Metastatic
Condition

RT: 6.4, m/z: 126.0913, Ion mode: Positive

Adjusted p (t test): 2.18e−03, Adjusted p (wilcox): 1.52e−03

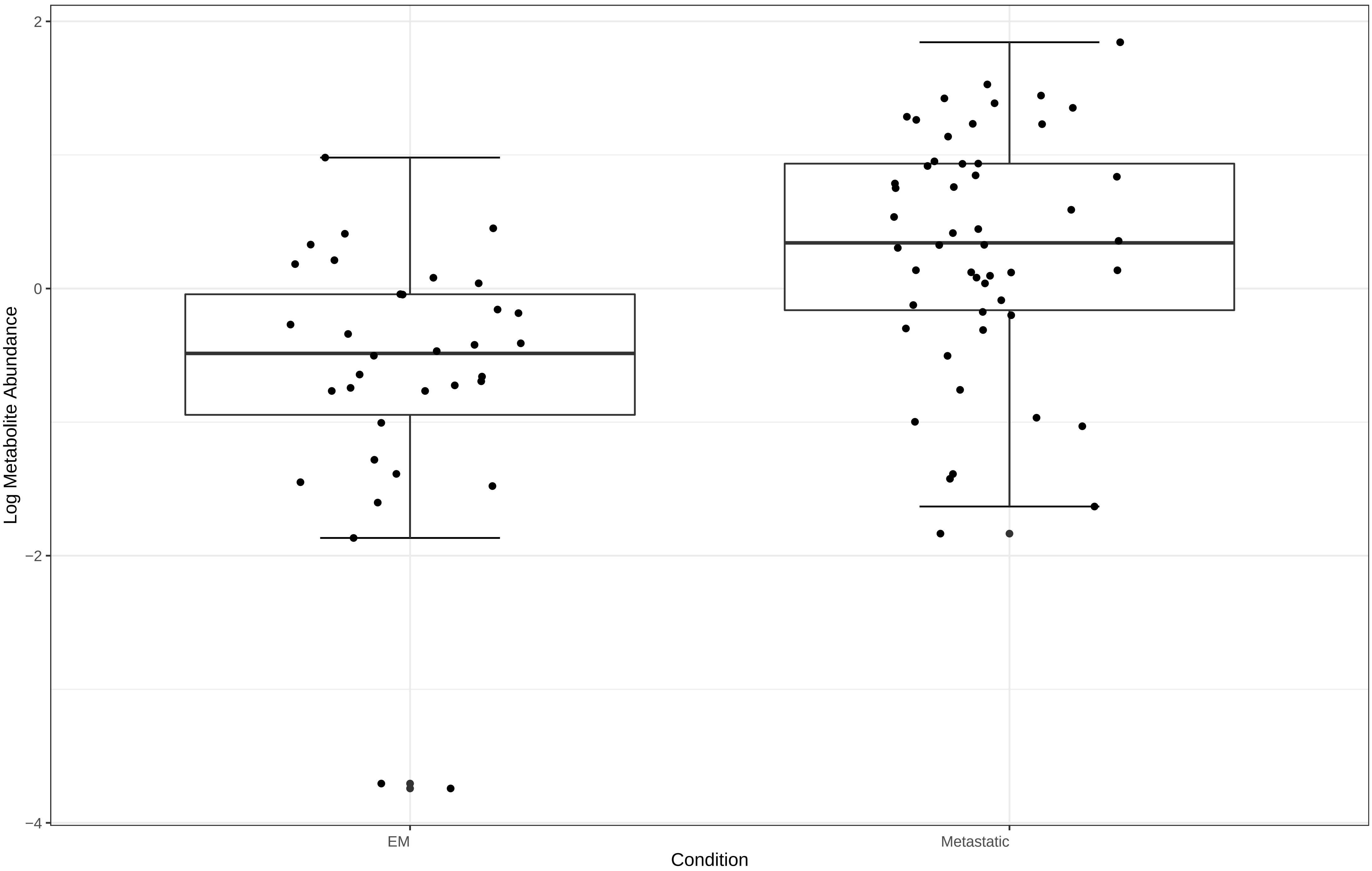

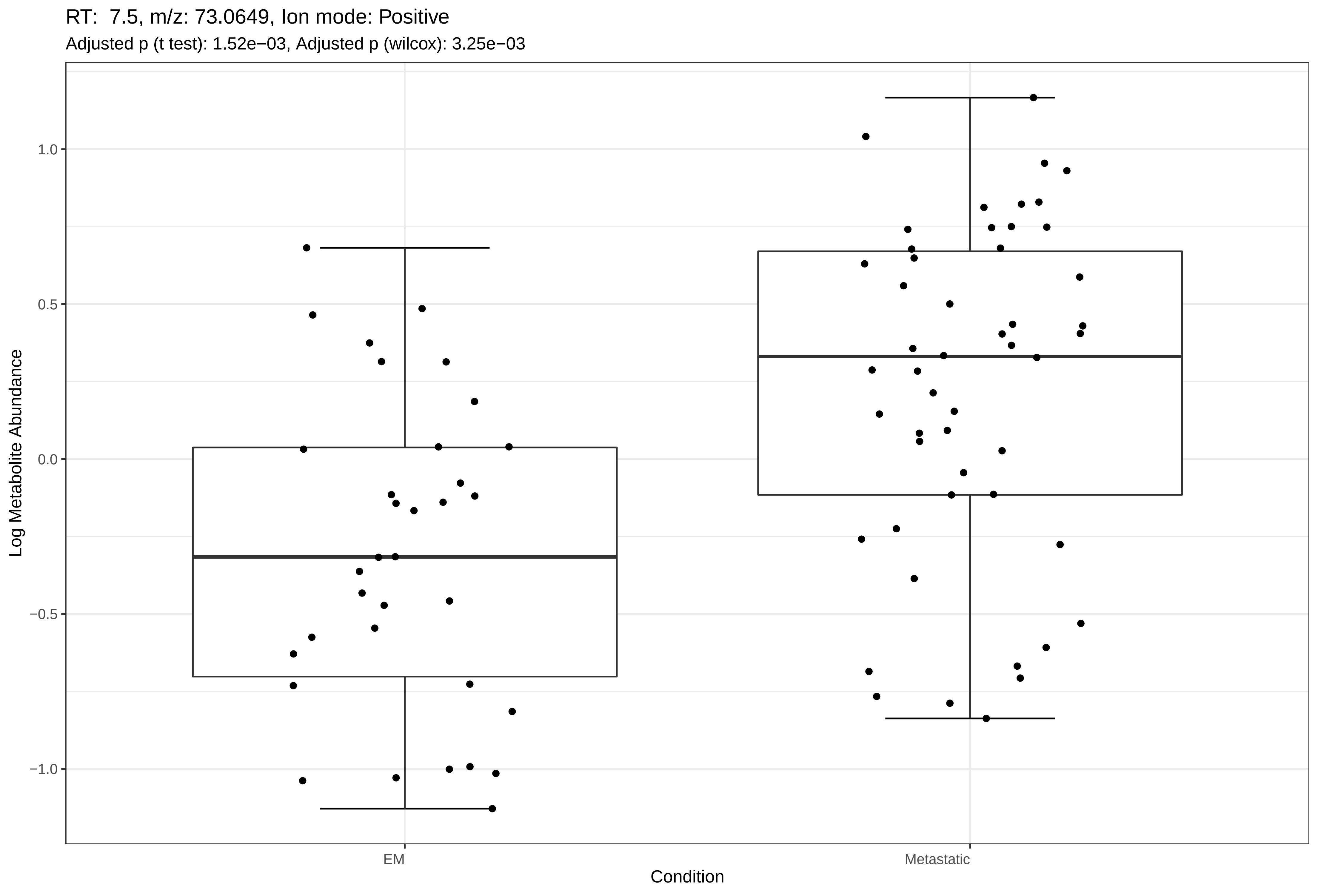

RT: 7.5, m/z: 73.0649, Ion mode: Positive
Adjusted p (t test): 1.52e−03, Adjusted p (wilcox): 3.25e−03
Log Metabolite Abundance
1.0
0.5
0.0
−0.5
−1.0
EM
Metastatic
Condition

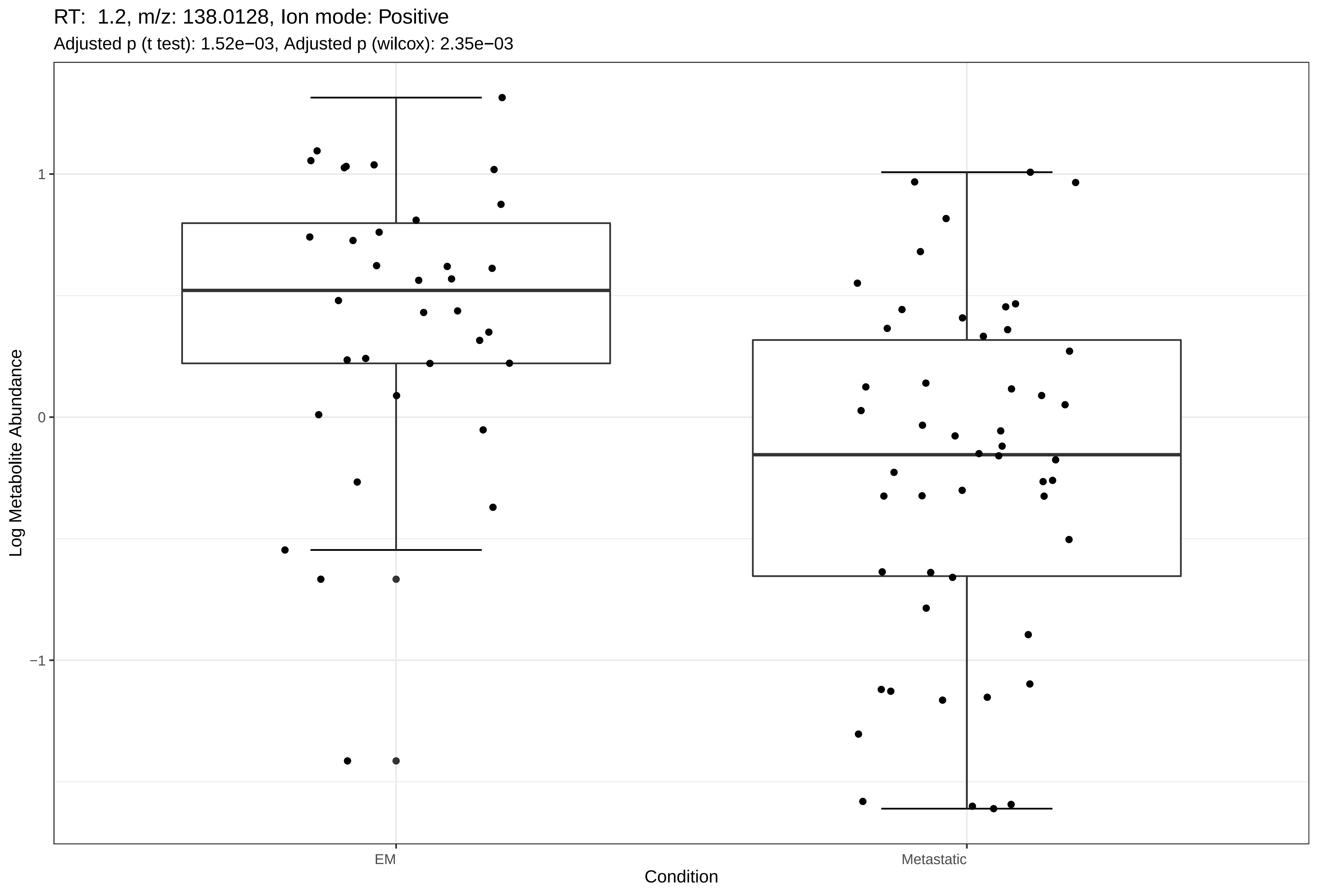

RT: 1.2, m/z: 138.0128, Ion mode: Positive
Adjusted p (t test): 1.52e−03, Adjusted p (wilcox): 2.35e−03
Log Metabolite Abundance
1
0
−1
EM
Metastatic
Condition

RT: 2.0, m/z: 188.0913, Ion mode: Positive

Adjusted p (t test): 1.56e−03, Adjusted p (wilcox): 3.36e−03

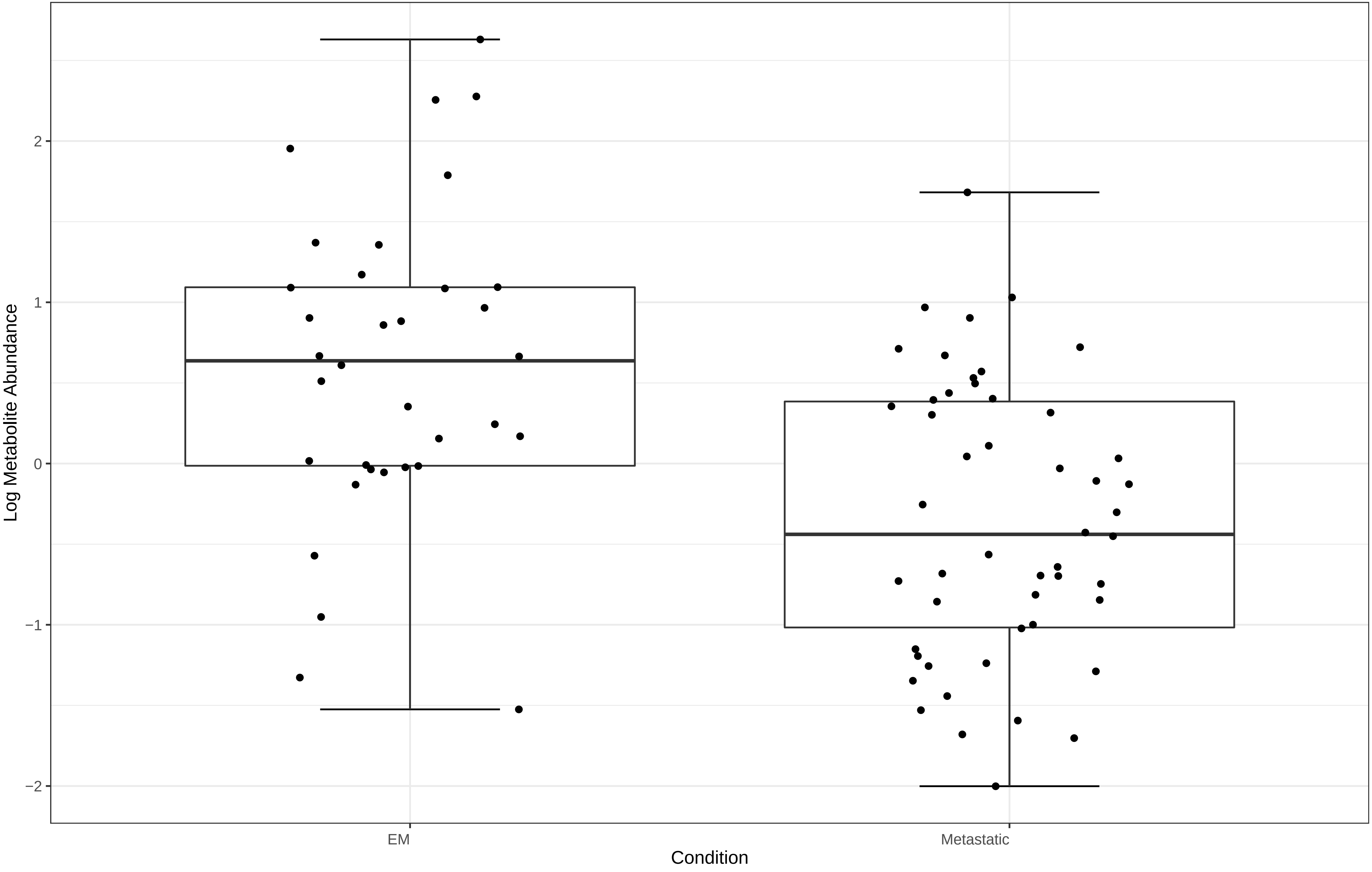

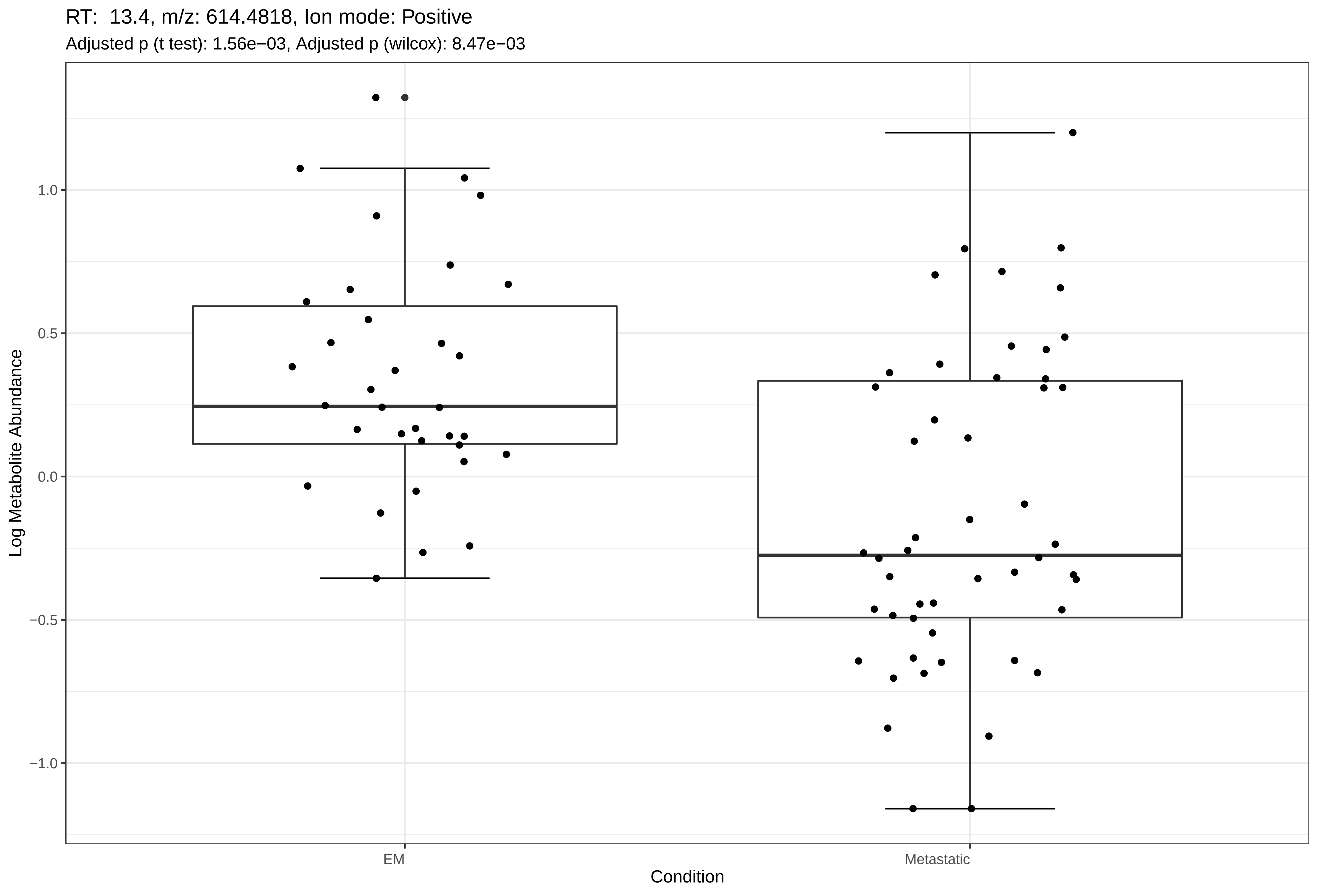
RT: 13.4, m/z: 614.4818, Ion mode: Positive
Adjusted p (t test): 1.56e−03, Adjusted p (wilcox): 8.47e−03
Log Metabolite Abundance
1.0
0.5
0.0
−0.5
−1.0
EM
Metastatic
Condition

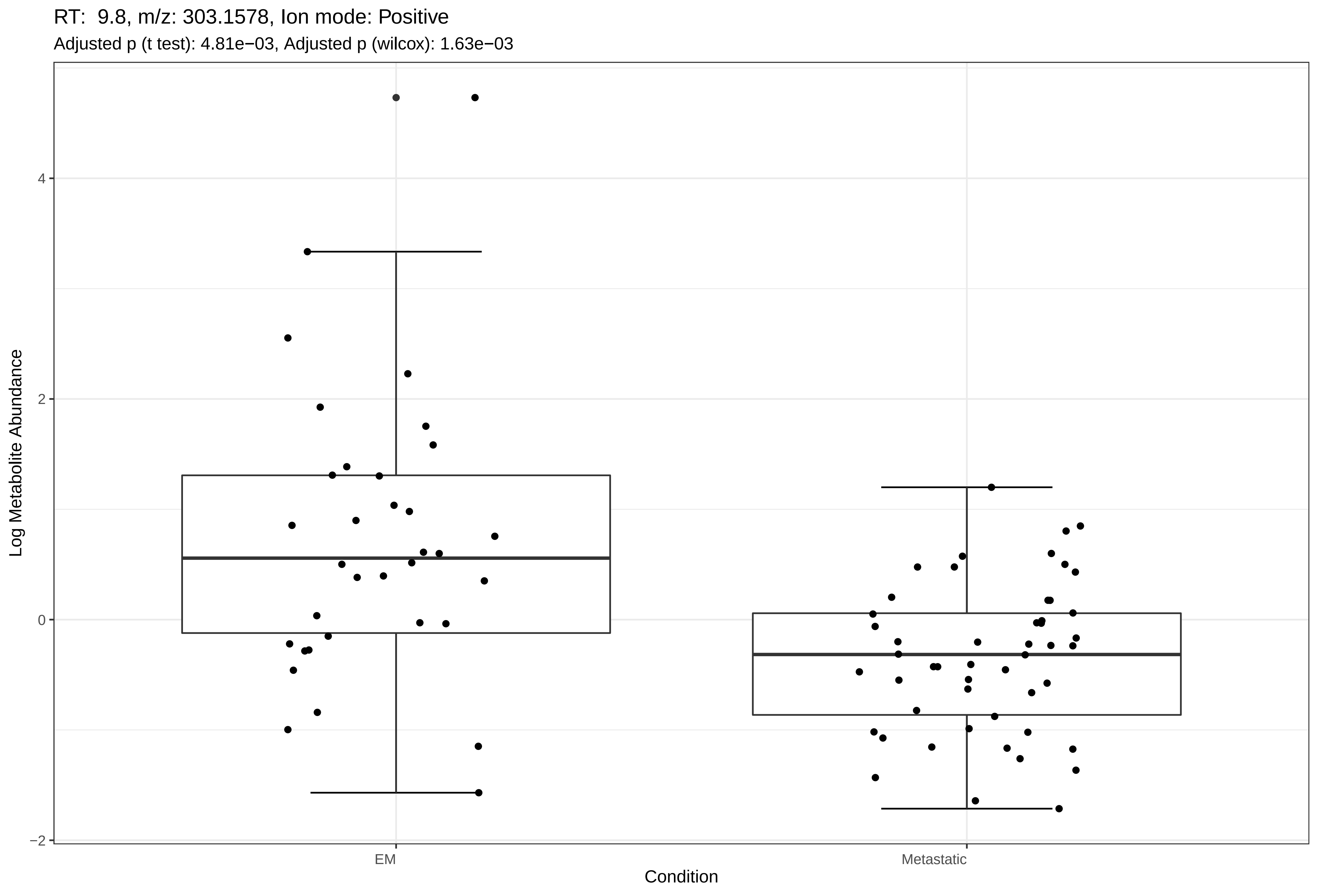
RT: 9.8, m/z: 303.1578, Ion mode: Positive
Adjusted p (t test): 4.81e−03, Adjusted p (wilcox): 1.63e−03
Log Metabolite Abundance
4
2
0
−2
EM
Metastatic
Condition

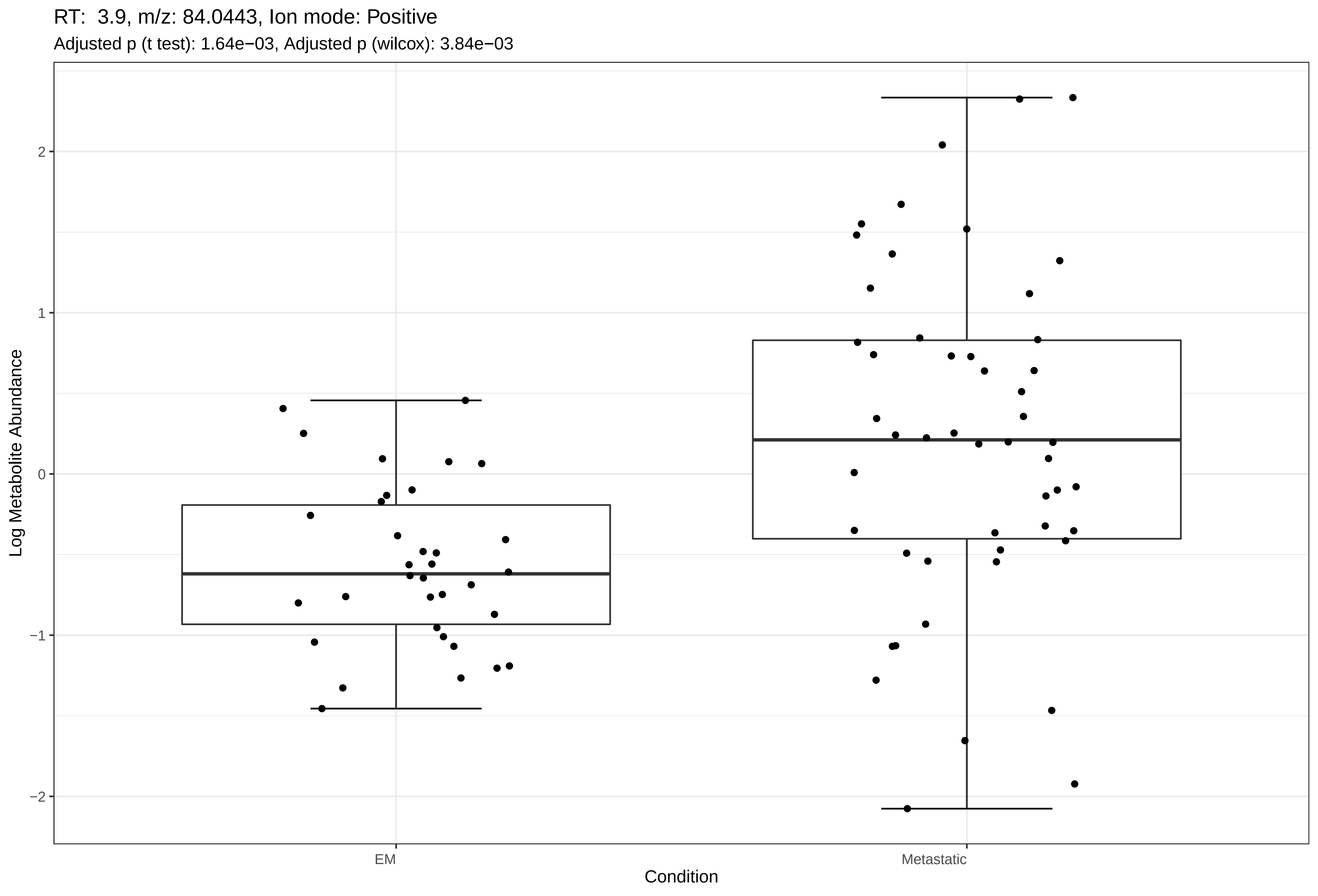
RT: 3.9, m/z: 84.0443, Ion mode: Positive
Adjusted p (t test): 1.64e−03, Adjusted p (wilcox): 3.84e−03
Log Metabolite Abundance
2
1
0
−1
−2
EM
Metastatic
Condition

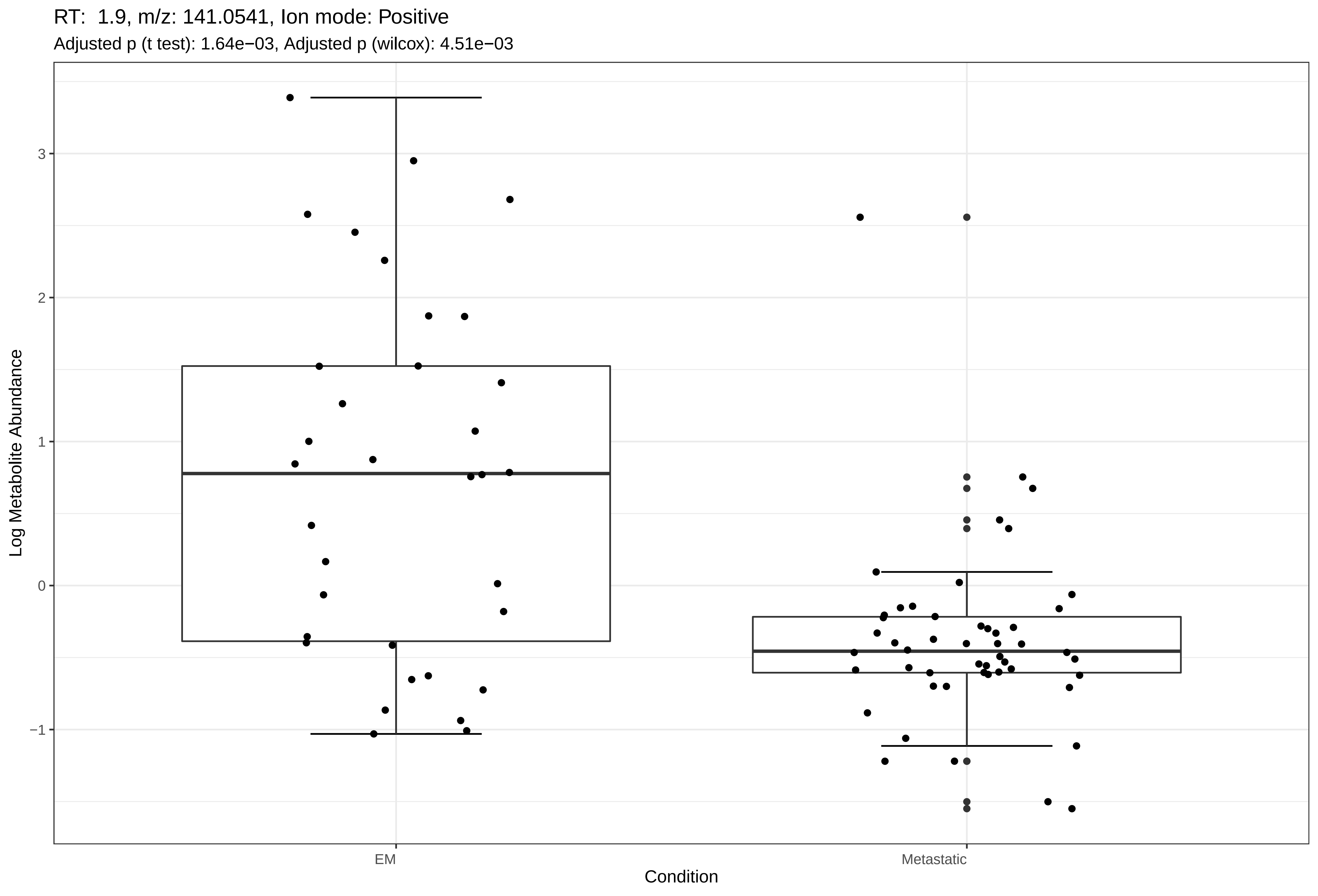
RT: 1.9, m/z: 141.0541, Ion mode: Positive
Adjusted p (t test): 1.64e−03, Adjusted p (wilcox): 4.51e−03
Log Metabolite Abundance
3
2
1
0
−1
EM
Metastatic
Condition

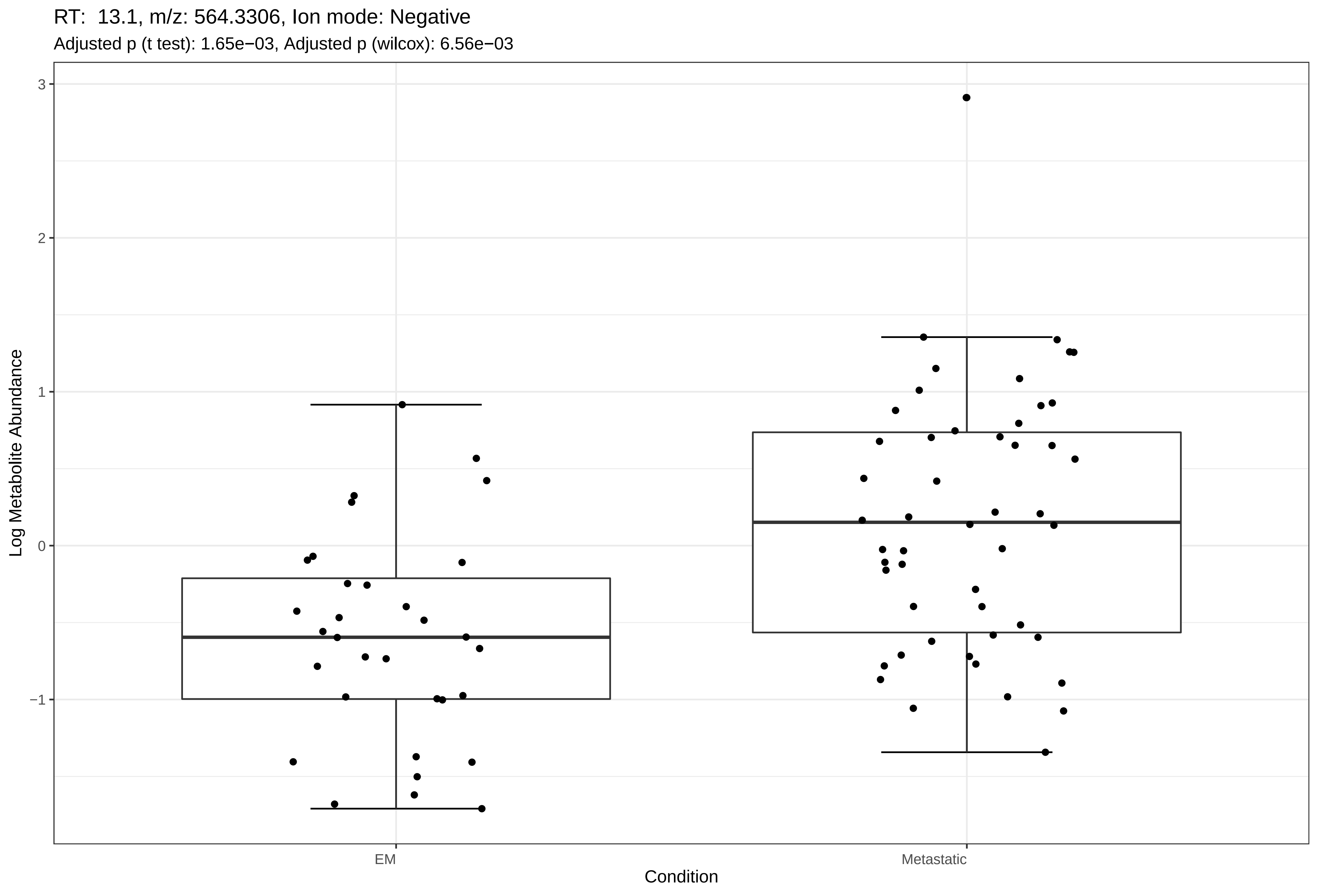

RT: 13.1, m/z: 564.3306, Ion mode: Negative
Adjusted p (t test): 1.65e−03, Adjusted p (wilcox): 6.56e−03
Log Metabolite Abundance
3
2
1
0
−1
EM
Metastatic
Condition

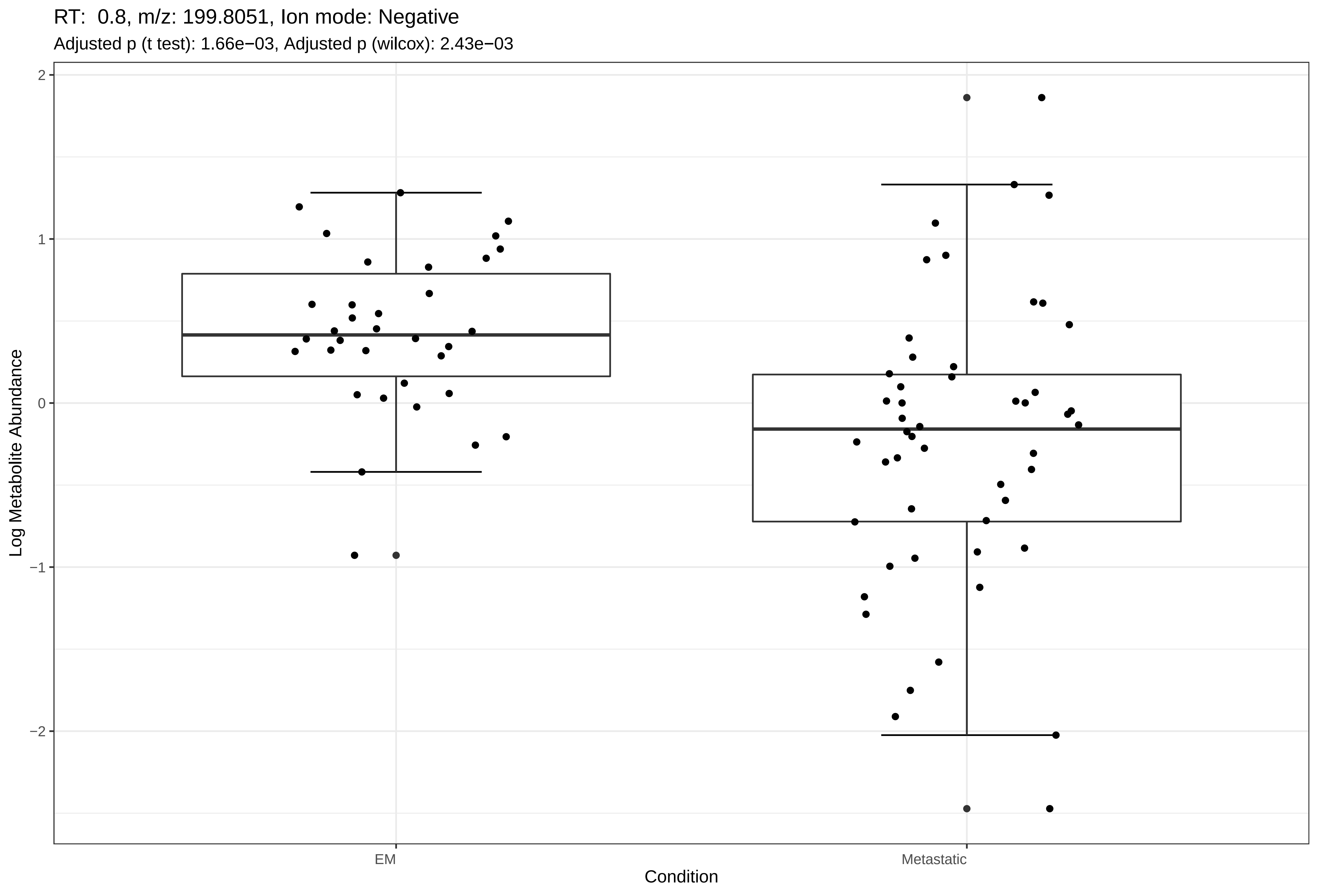

RT: 0.8, m/z: 199.8051, Ion mode: Negative
Adjusted p (t test): 1.66e−03, Adjusted p (wilcox): 2.43e−03
Log Metabolite Abundance
2
1
0
−1
−2
EM
Metastatic
Condition

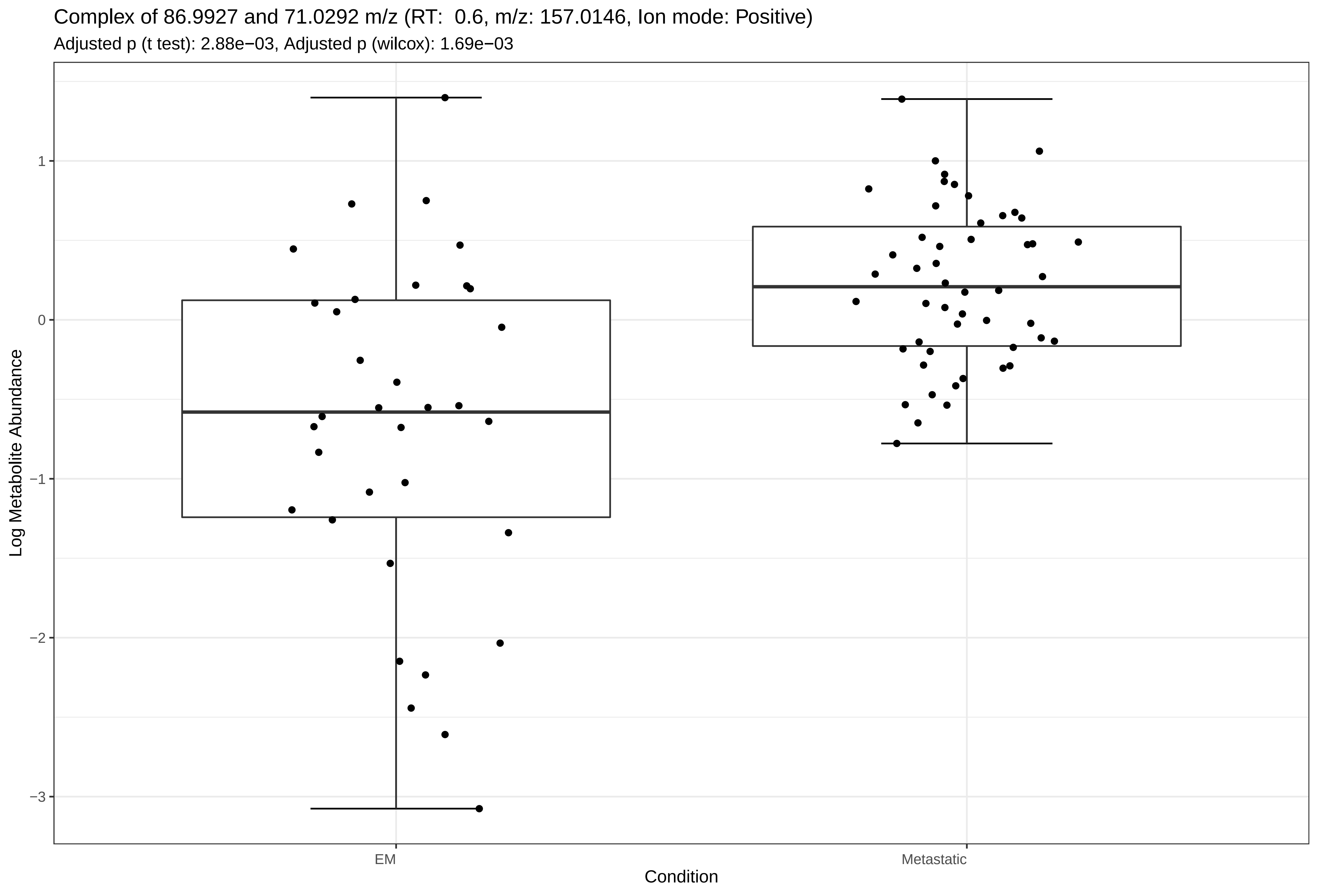

Complex of 86.9927 and 71.0292 m/z (RT: 0.6, m/z: 157.0146, Ion mode: Positive)
Adjusted p (t test): 2.88e−03, Adjusted p (wilcox): 1.69e−03
Log Metabolite Abundance
1
0
−1
−2
−3
EM
Metastatic
Condition

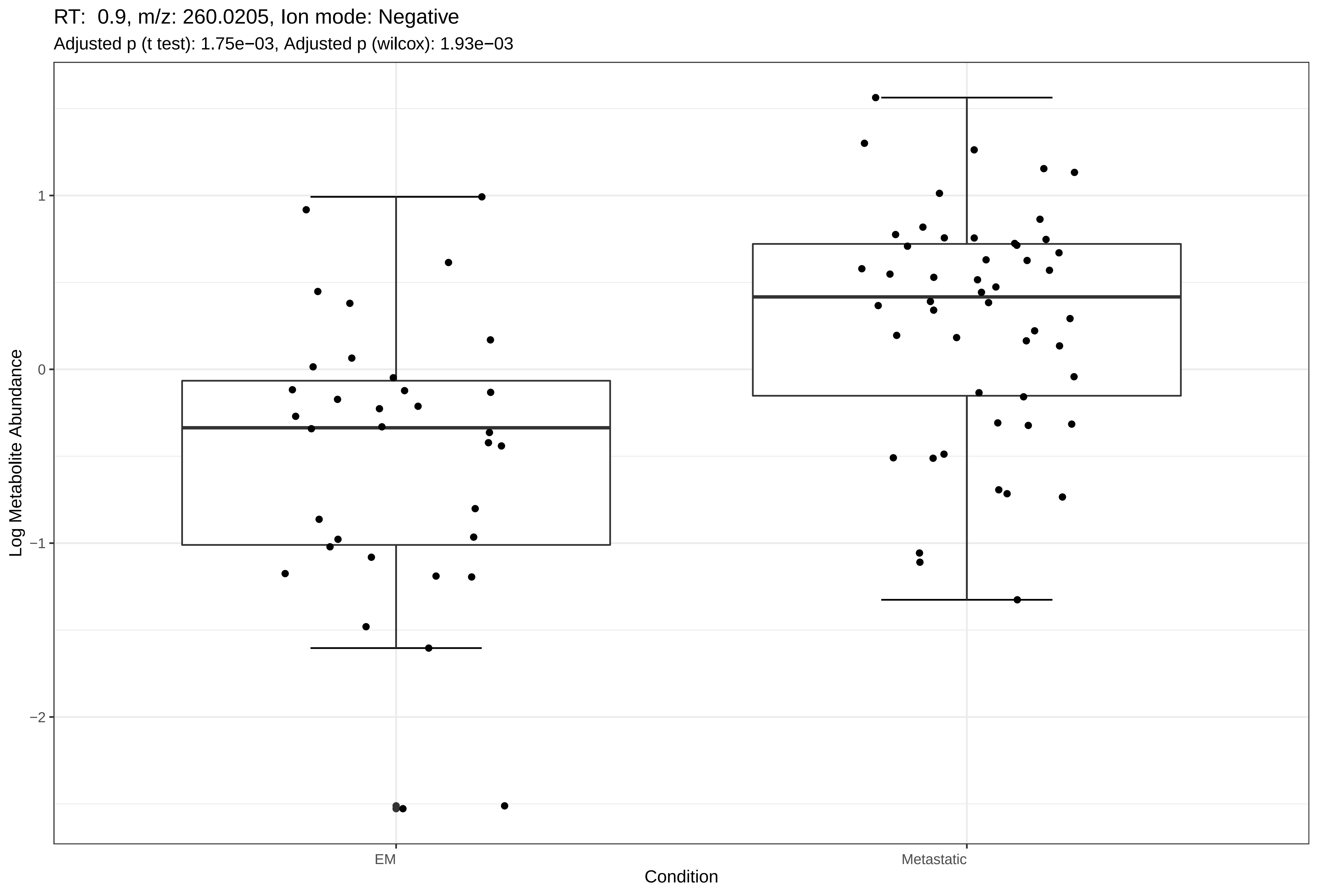
RT: 0.9, m/z: 260.0205, Ion mode: Negative
Adjusted p (t test): 1.75e−03, Adjusted p (wilcox): 1.93e−03
Log Metabolite Abundance
1
0
−1
−2
EM
Metastatic
Condition

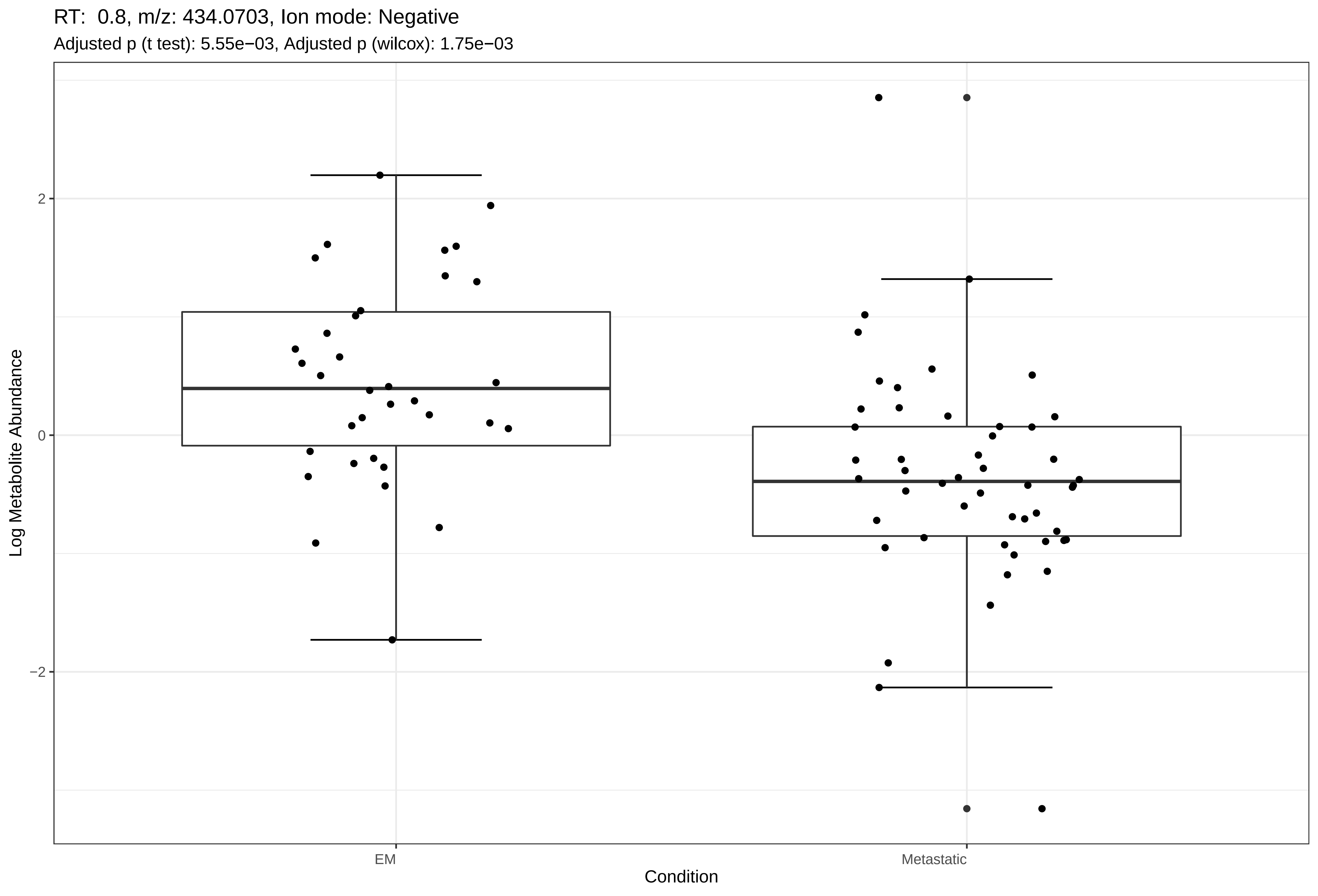
RT: 0.8, m/z: 434.0703, Ion mode: Negative
Adjusted p (t test): 5.55e−03, Adjusted p (wilcox): 1.75e−03
Log Metabolite Abundance
2
0
−2
EM
Metastatic
Condition

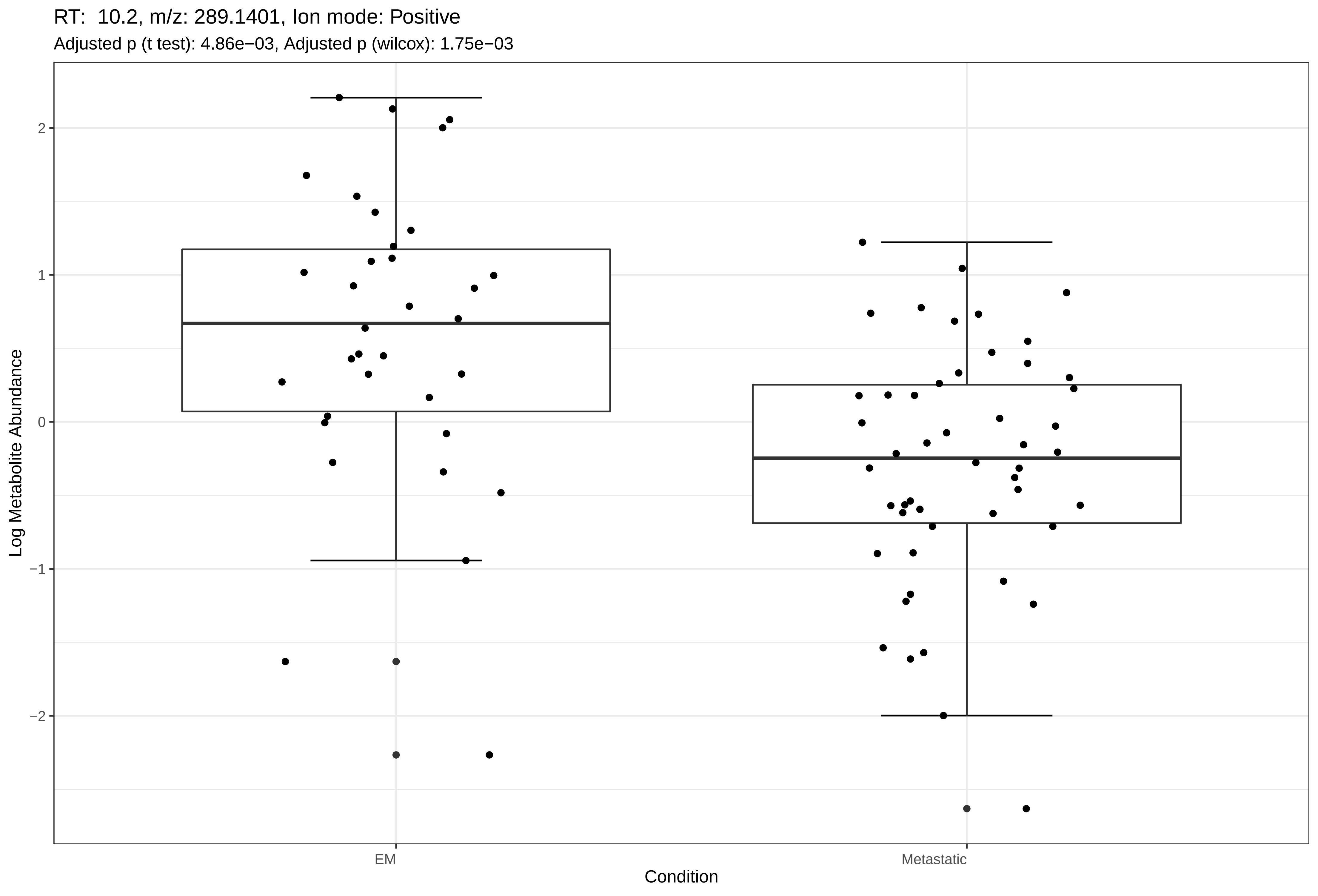
RT: 10.2, m/z: 289.1401, Ion mode: Positive
Adjusted p (t test): 4.86e−03, Adjusted p (wilcox): 1.75e−03
Log Metabolite Abundance
2
1
0
−1
−2
EM
Metastatic
Condition

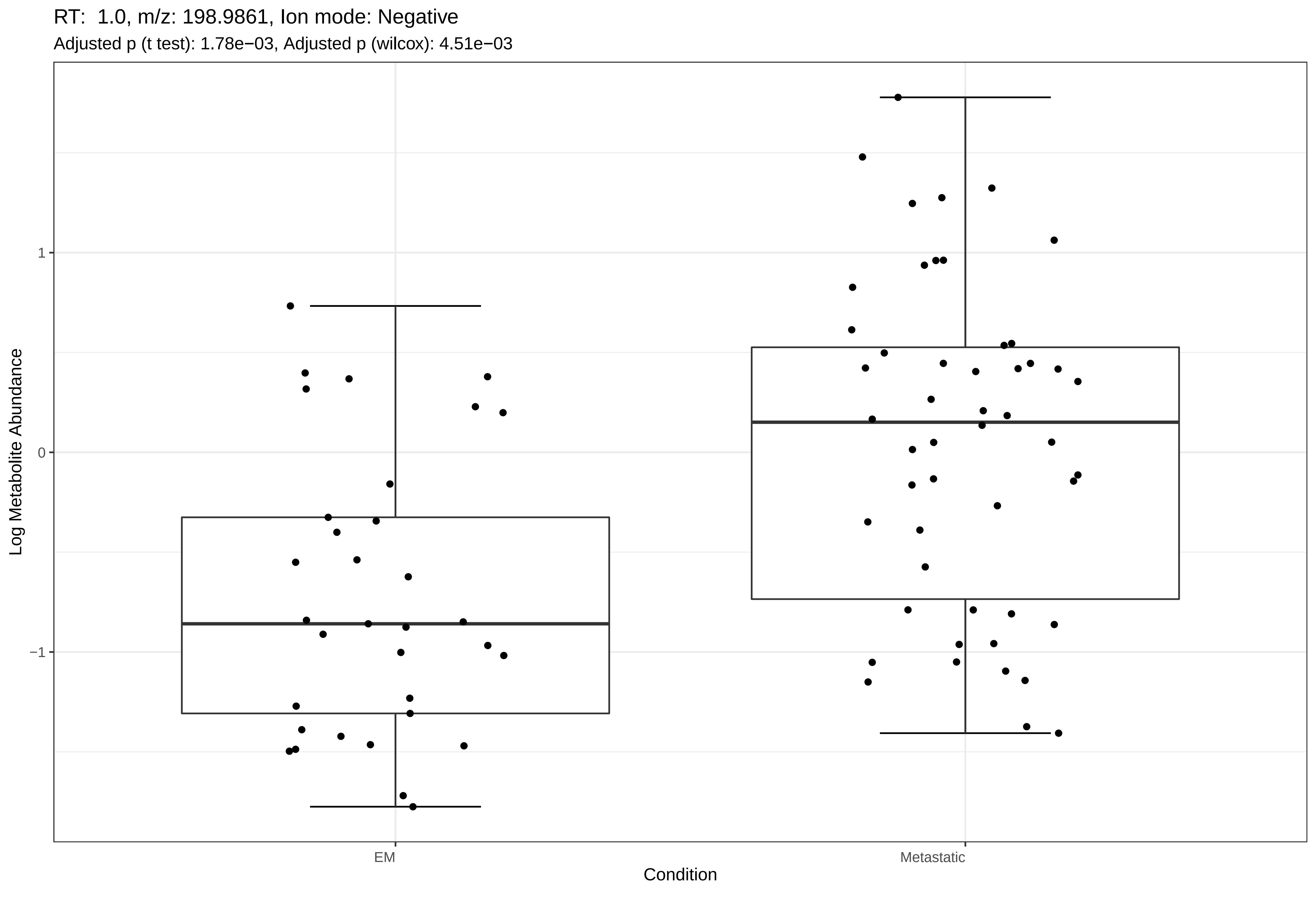

RT: 1.0, m/z: 198.9861, Ion mode: Negative
Adjusted p (t test): 1.78e−03, Adjusted p (wilcox): 4.51e−03
Log Metabolite Abundance
1
0
−1
EM
Metastatic
Condition

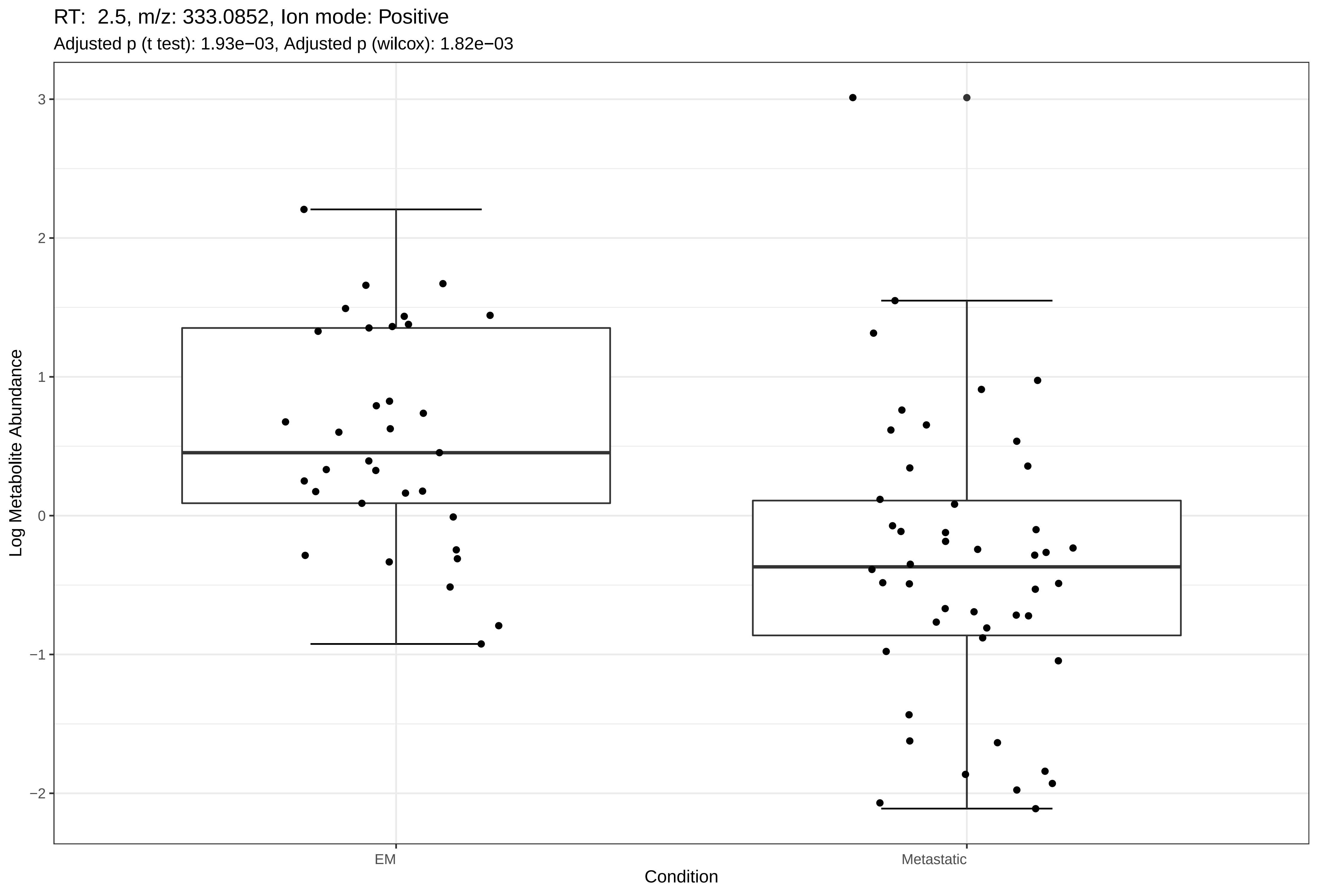
RT: 2.5, m/z: 333.0852, Ion mode: Positive
Adjusted p (t test): 1.93e−03, Adjusted p (wilcox): 1.82e−03
Log Metabolite Abundance
3
2
1
0
−1
−2
EM
Metastatic
Condition

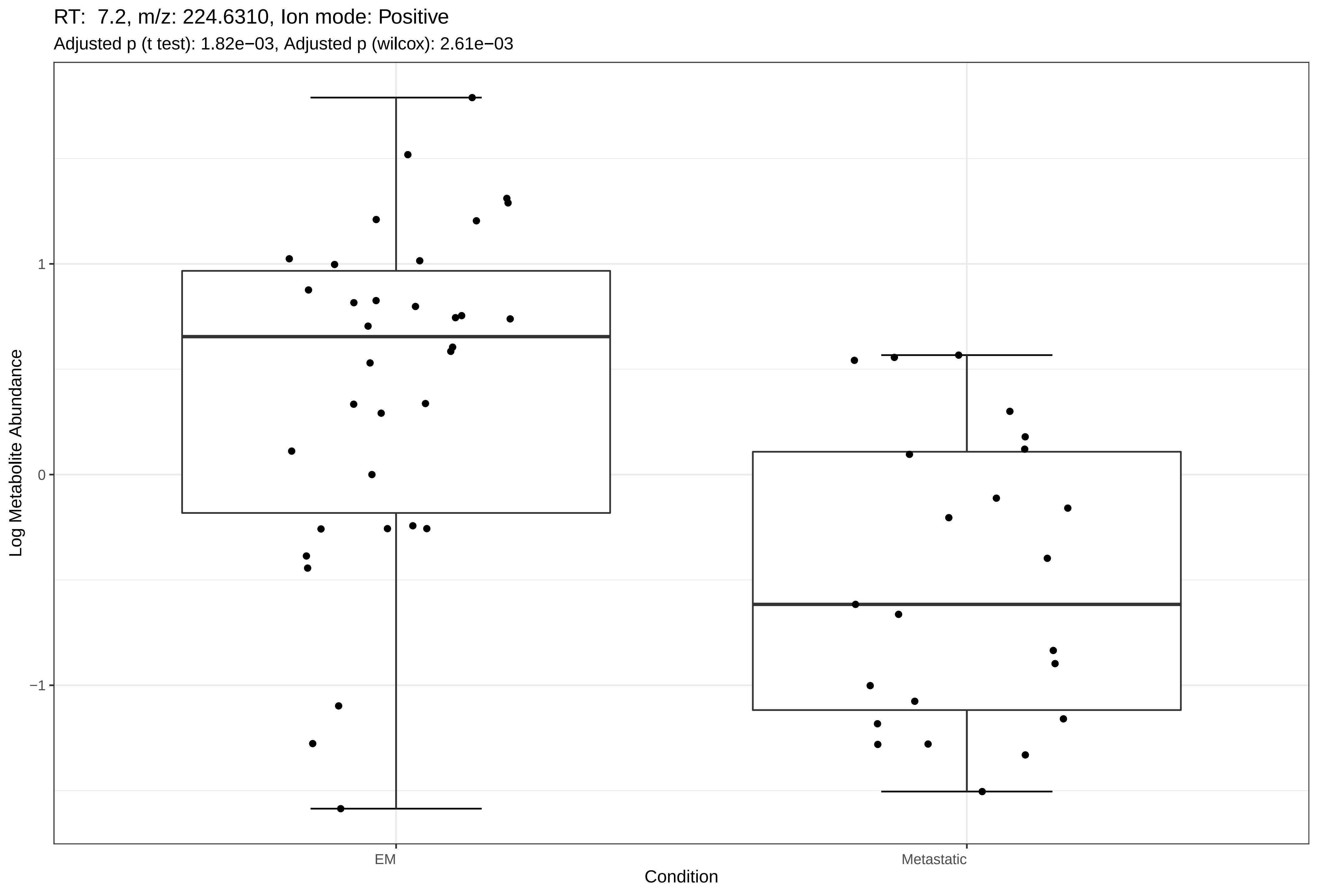

RT: 7.2, m/z: 224.6310, Ion mode: Positive
Adjusted p (t test): 1.82e−03, Adjusted p (wilcox): 2.61e−03
Log Metabolite Abundance
1
0
−1
EM
Metastatic
Condition

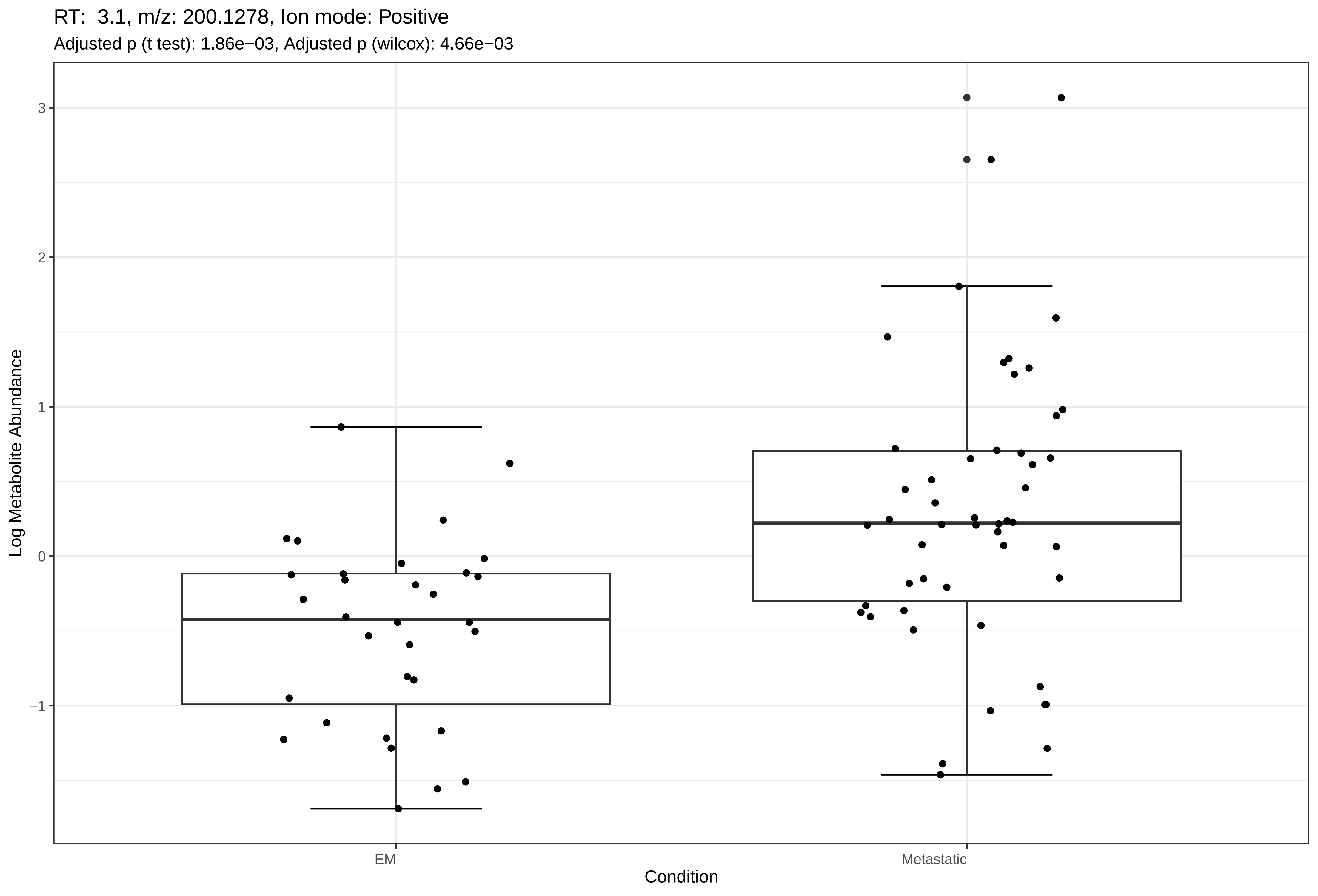

RT: 3.1, m/z: 200.1278, Ion mode: Positive
Adjusted p (t test): 1.86e−03, Adjusted p (wilcox): 4.66e−03
Log Metabolite Abundance
3
2
1
0
−1
EM
Metastatic
Condition

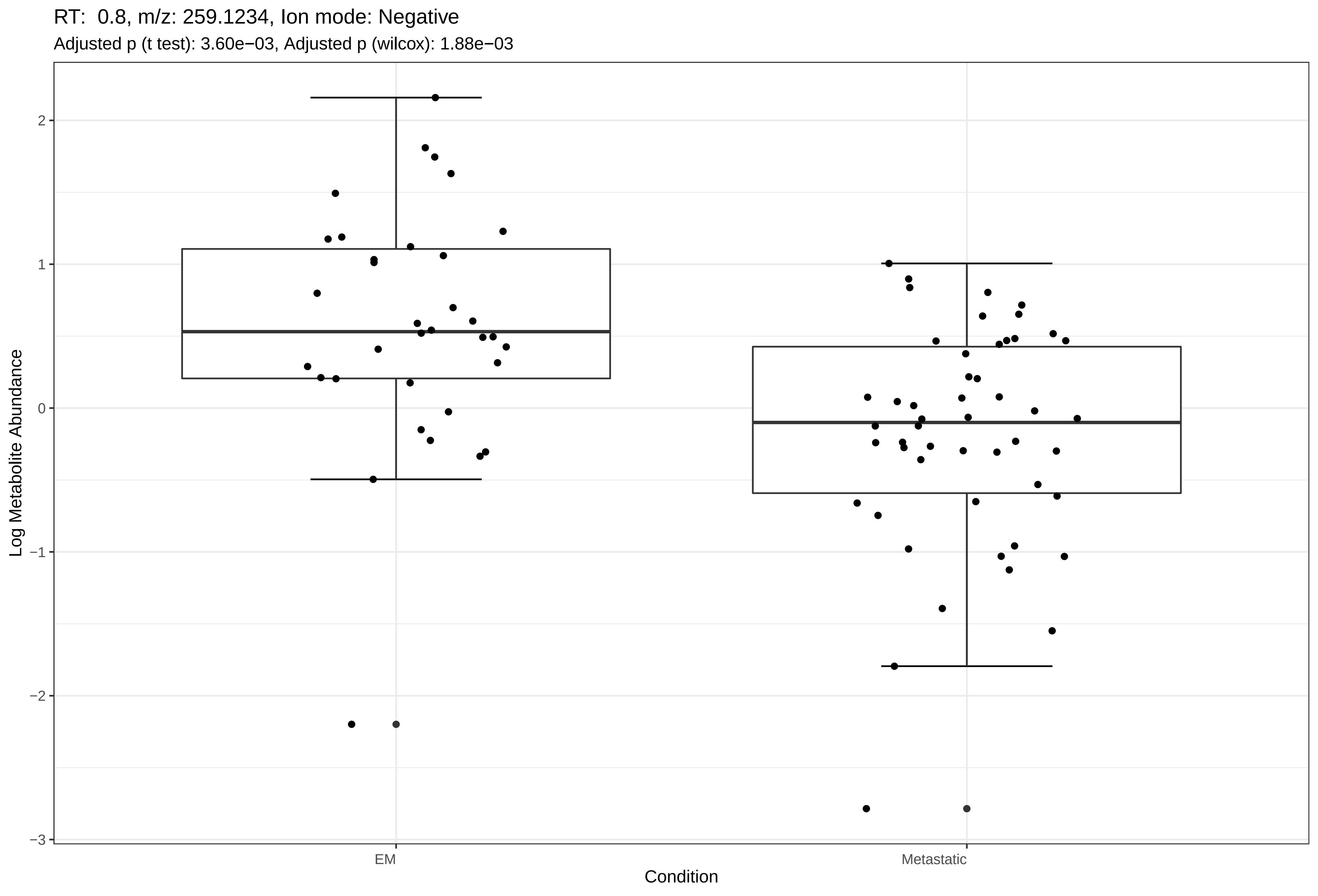
RT: 0.8, m/z: 259.1234, Ion mode: Negative
Adjusted p (t test): 3.60e−03, Adjusted p (wilcox): 1.88e−03
Log Metabolite Abundance
2
1
0
−1
−2
−3
EM
Metastatic
Condition

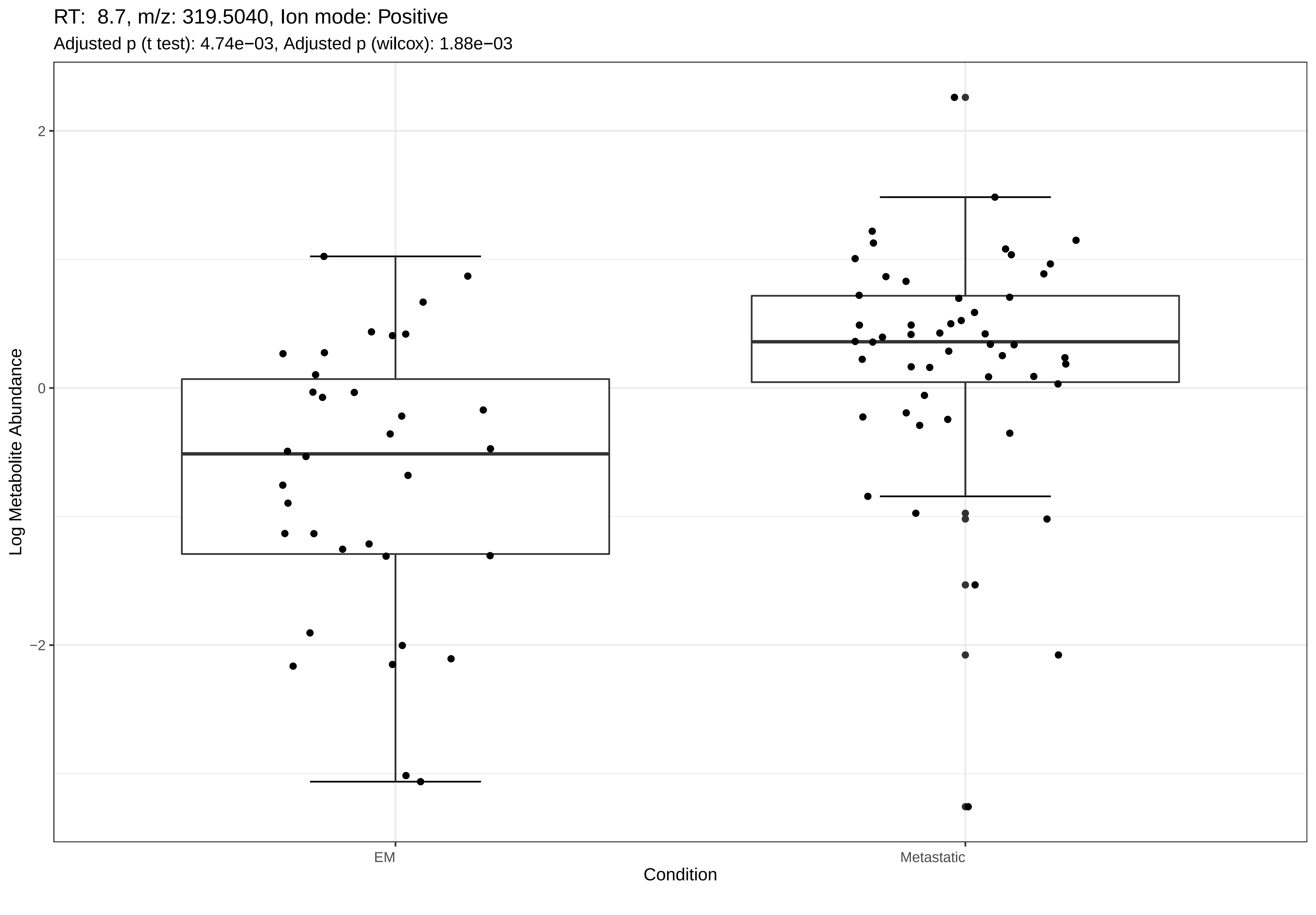
RT: 8.7, m/z: 319.5040, Ion mode: Positive
Adjusted p (t test): 4.74e−03, Adjusted p (wilcox): 1.88e−03
Log Metabolite Abundance
2
0
−2
EM
Metastatic
Condition

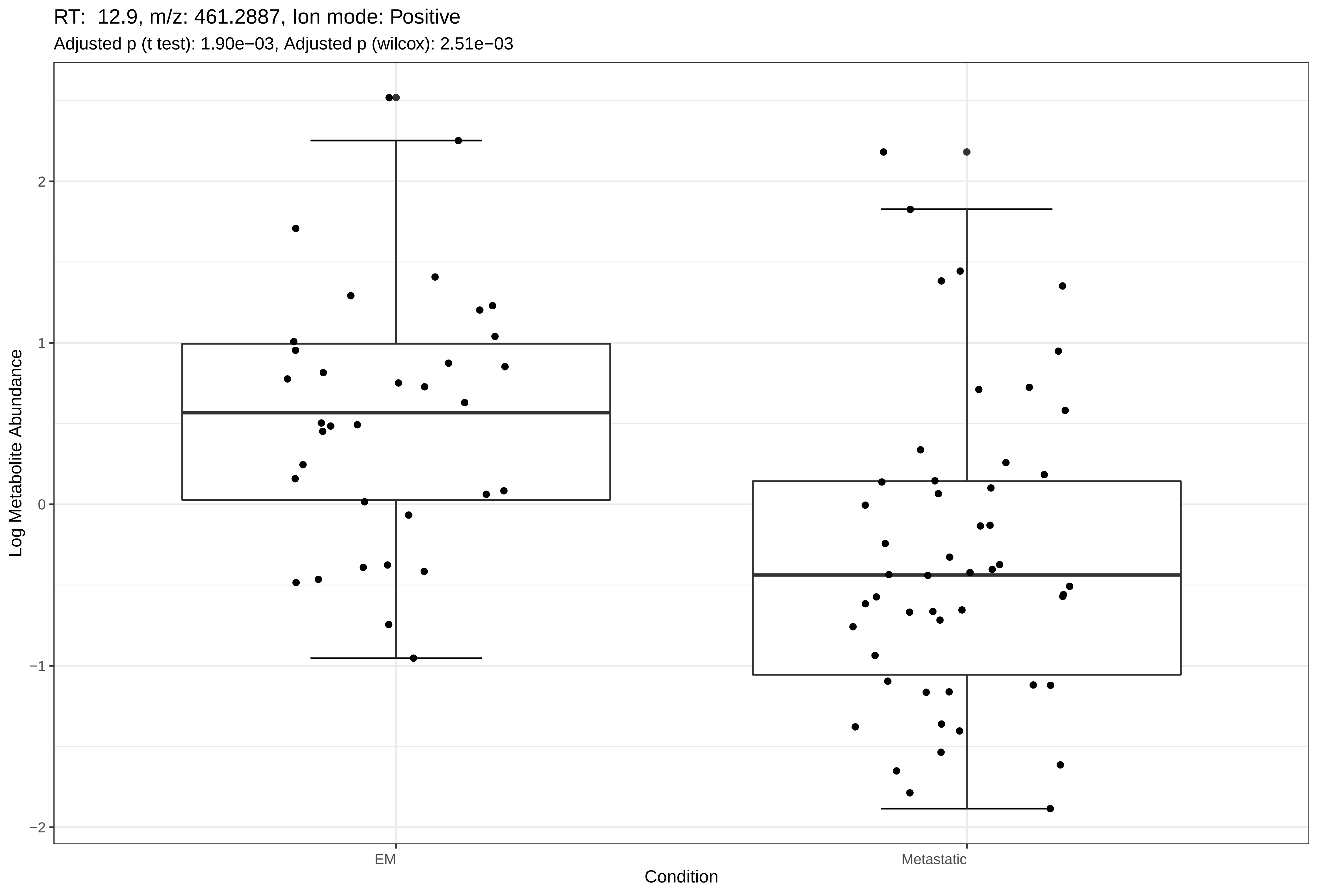
RT: 12.9, m/z: 461.2887, Ion mode: Positive
Adjusted p (t test): 1.90e−03, Adjusted p (wilcox): 2.51e−03
Log Metabolite Abundance
2
1
0
−1
−2
EM
Metastatic
Condition

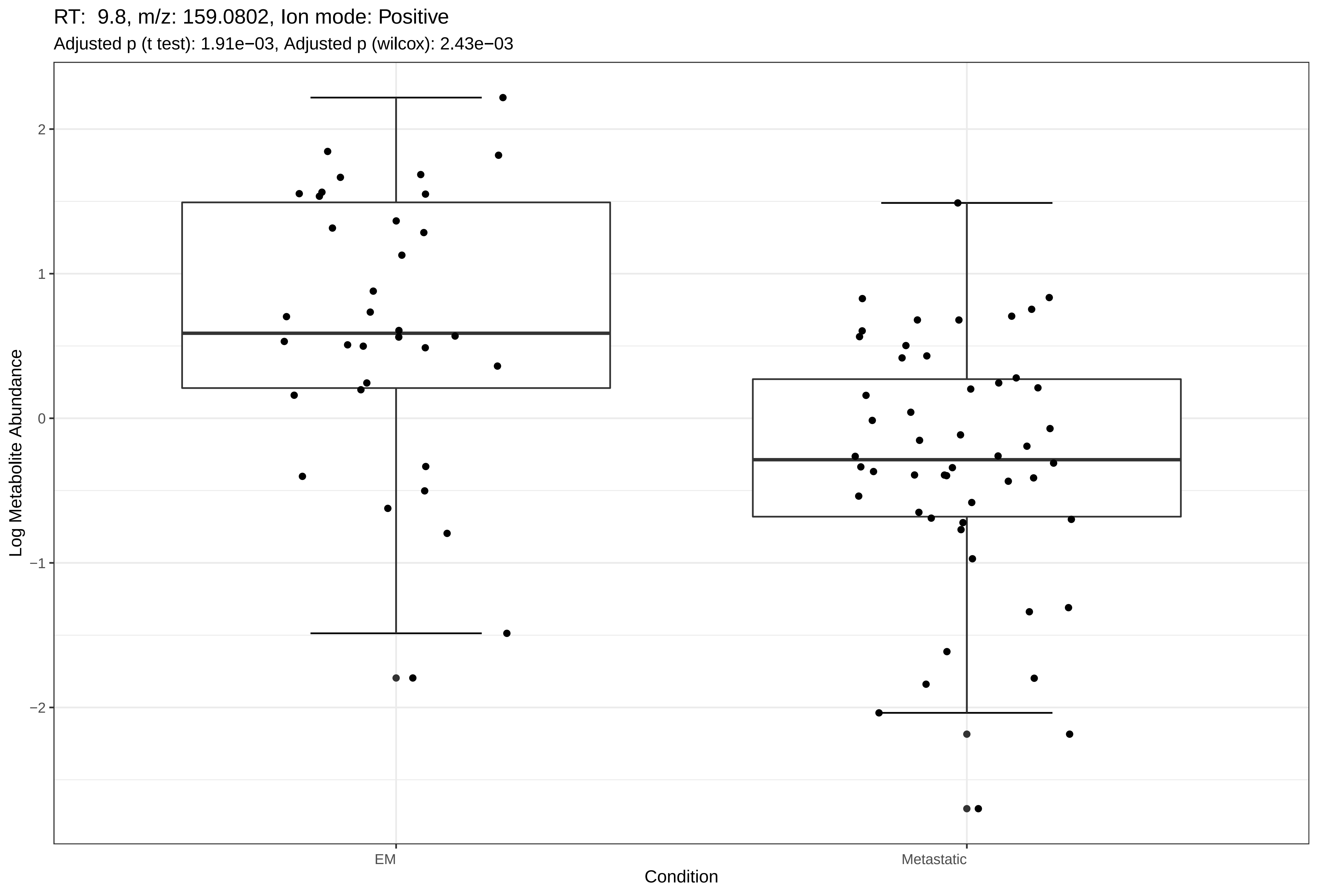
RT: 9.8, m/z: 159.0802, Ion mode: Positive
Adjusted p (t test): 1.91e−03, Adjusted p (wilcox): 2.43e−03
Log Metabolite Abundance
2
1
0
−1
−2
EM
Metastatic
Condition

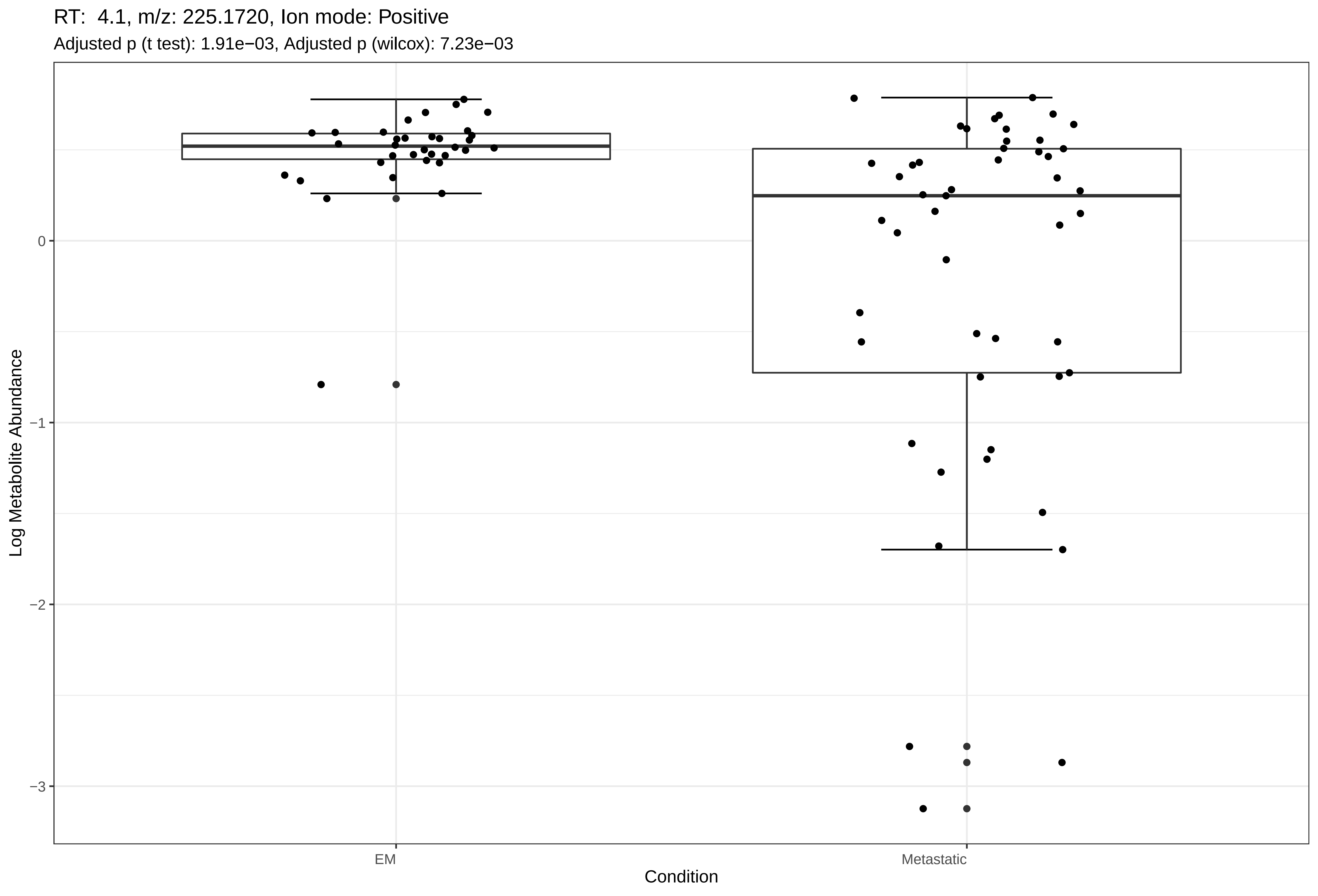
RT: 4.1, m/z: 225.1720, Ion mode: Positive
Adjusted p (t test): 1.91e−03, Adjusted p (wilcox): 7.23e−03
Log Metabolite Abundance
0
−1
−2
−3
EM
Metastatic
Condition

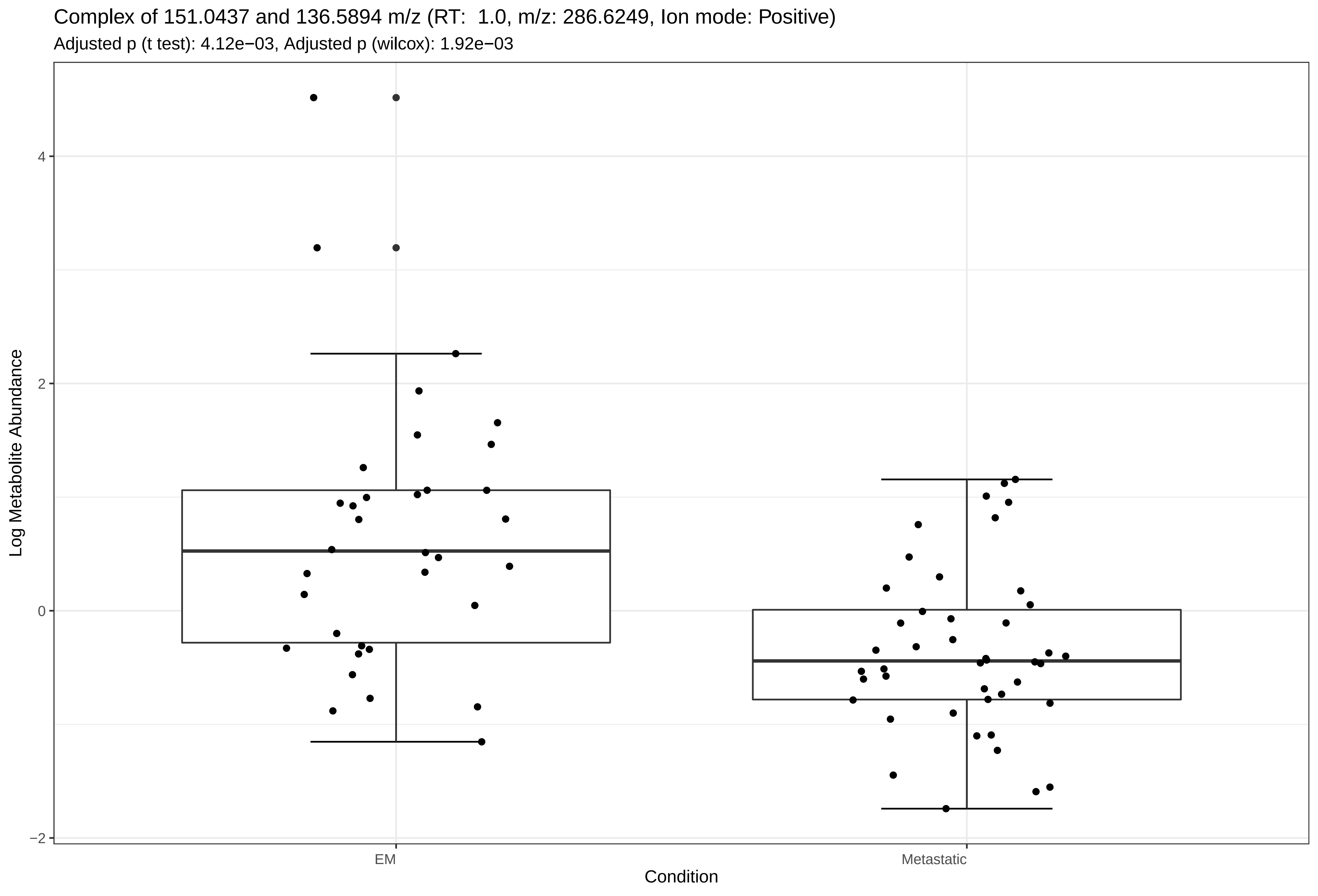

Complex of 151.0437 and 136.5894 m/z (RT: 1.0, m/z: 286.6249, Ion mode: Positive)
Adjusted p (t test): 4.12e−03, Adjusted p (wilcox): 1.92e−03
Log Metabolite Abundance
4
2
0
−2
EM
Metastatic
Condition

RT: 0.7, m/z: 137.0740, Ion mode: Positive

Adjusted p (t test): 4.25e−03, Adjusted p (wilcox): 1.93e−03

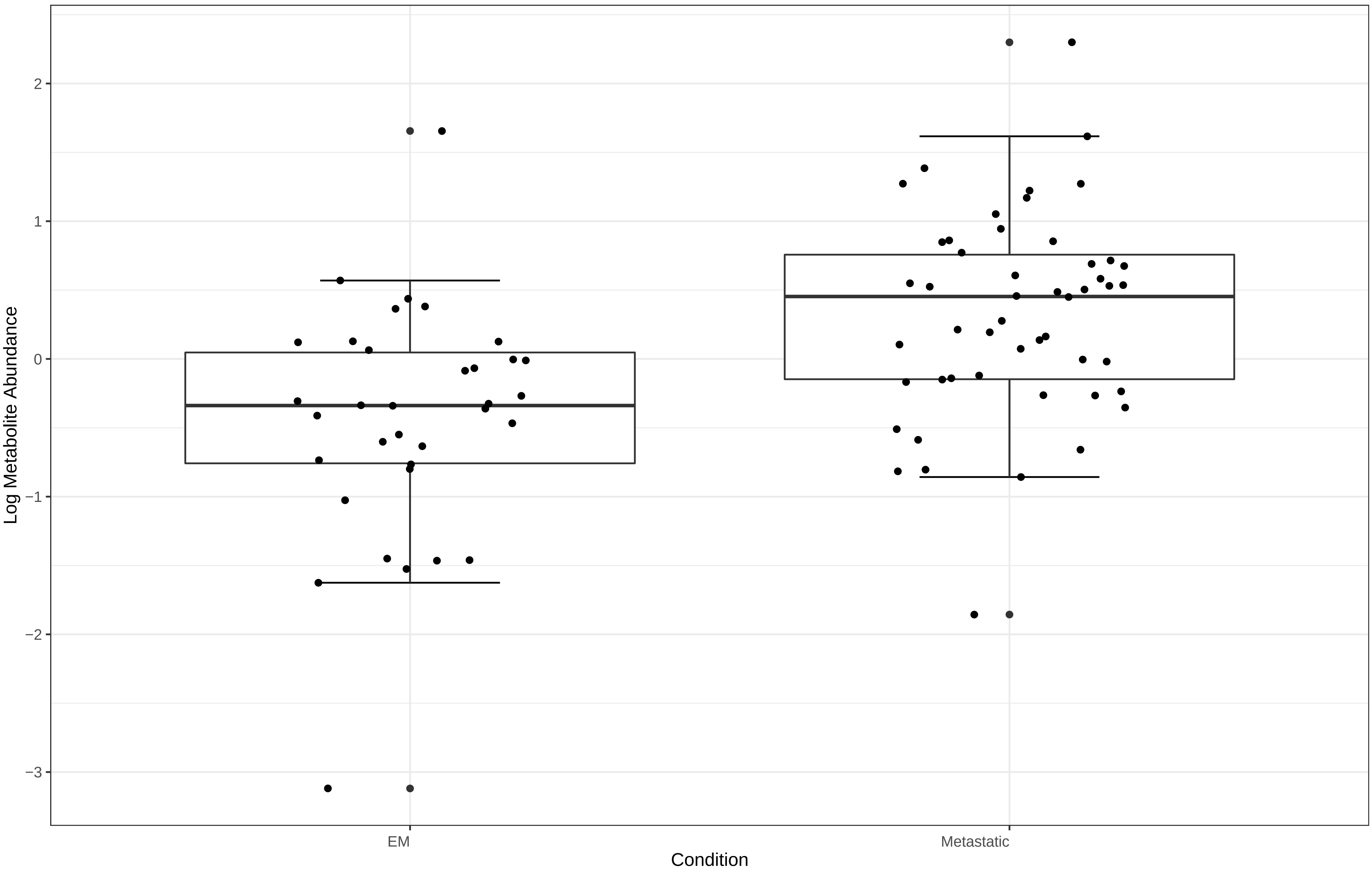

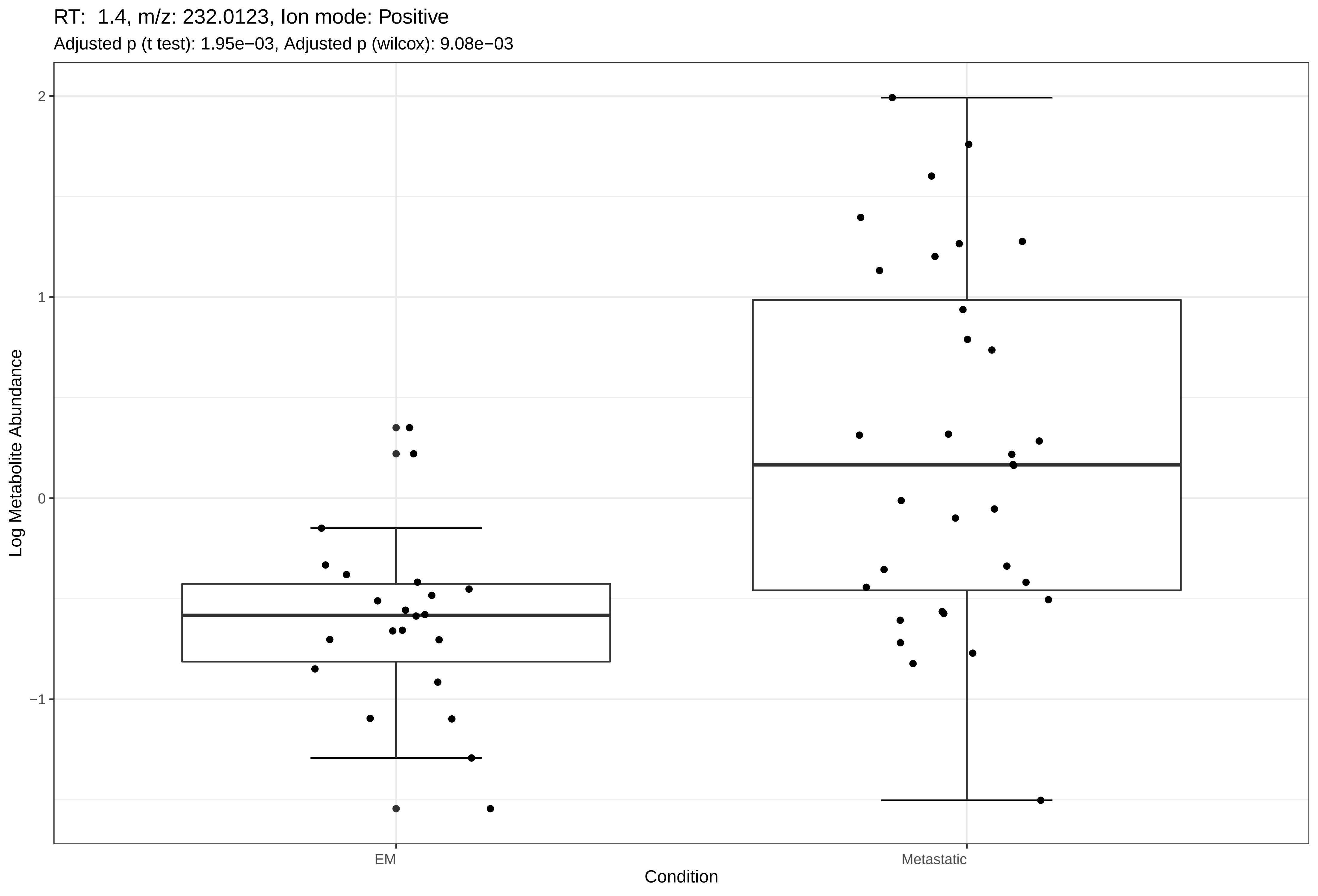

RT: 1.4, m/z: 232.0123, Ion mode: Positive
Adjusted p (t test): 1.95e−03, Adjusted p (wilcox): 9.08e−03
Log Metabolite Abundance
2
1
0
−1
EM
Metastatic
Condition

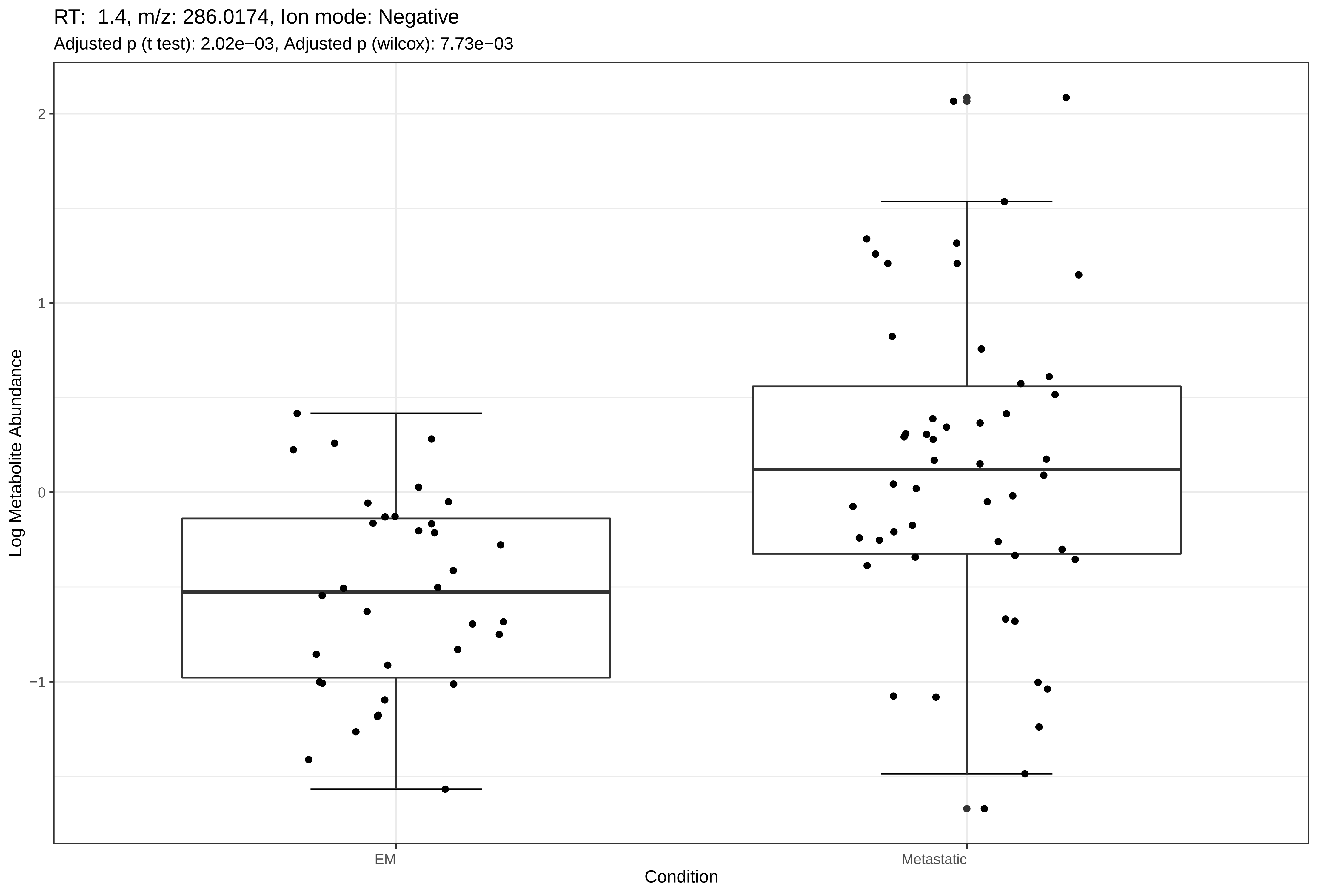
RT: 1.4, m/z: 286.0174, Ion mode: Negative
Adjusted p (t test): 2.02e−03, Adjusted p (wilcox): 7.73e−03
Log Metabolite Abundance
2
1
0
−1
EM
Metastatic
Condition

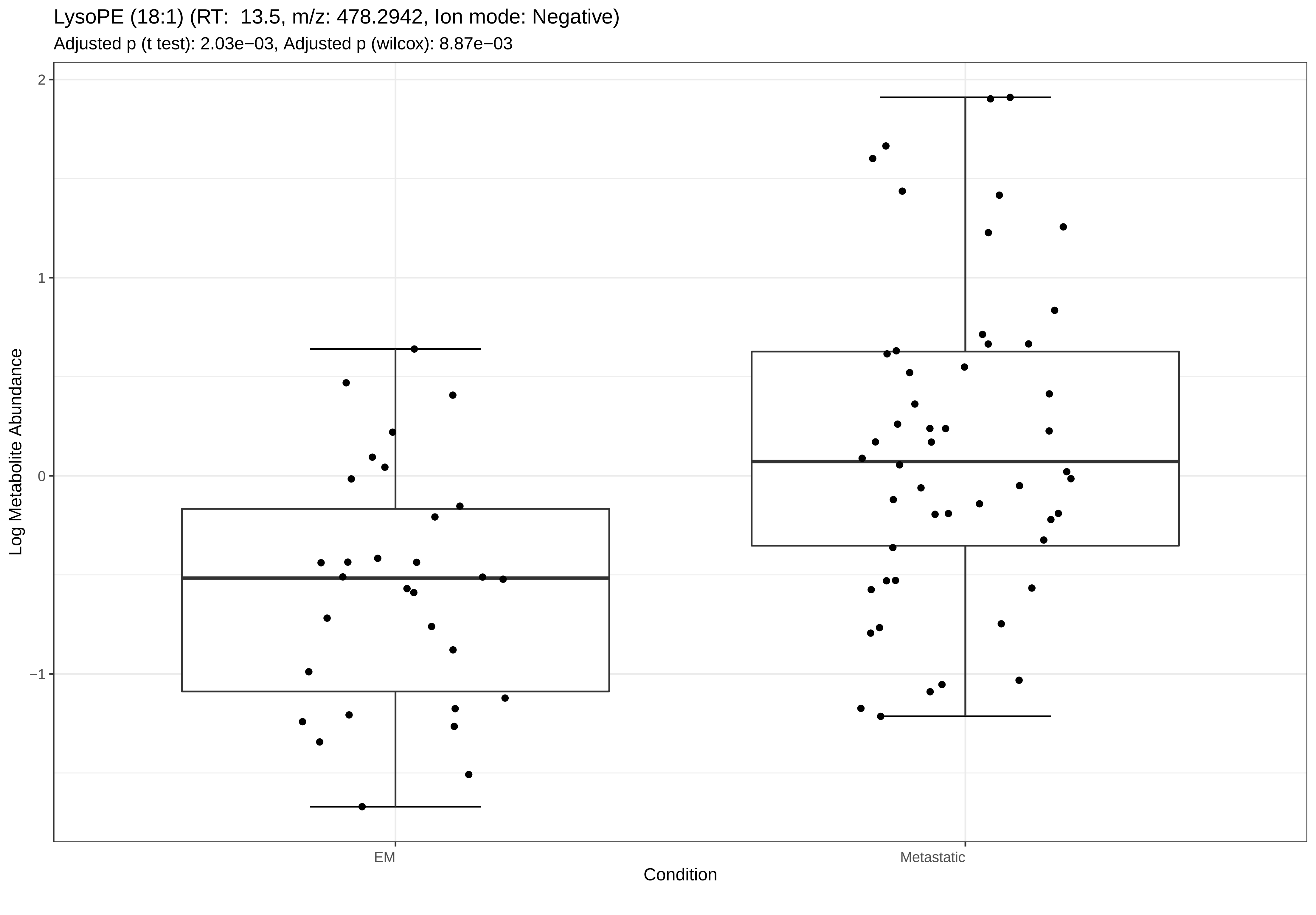

LysoPE (18:1) (RT: 13.5, m/z: 478.2942, Ion mode: Negative)
Adjusted p (t test): 2.03e−03, Adjusted p (wilcox): 8.87e−03
Log Metabolite Abundance
2
1
0
−1
EM
Metastatic
Condition

Complex of 114.0661 and 231.0259 m/z (RT: 0.8, m/z: 344.0842, Ion mode: Positive)

Adjusted p (t test): 2.03e−03, Adjusted p (wilcox): 5.12e−03

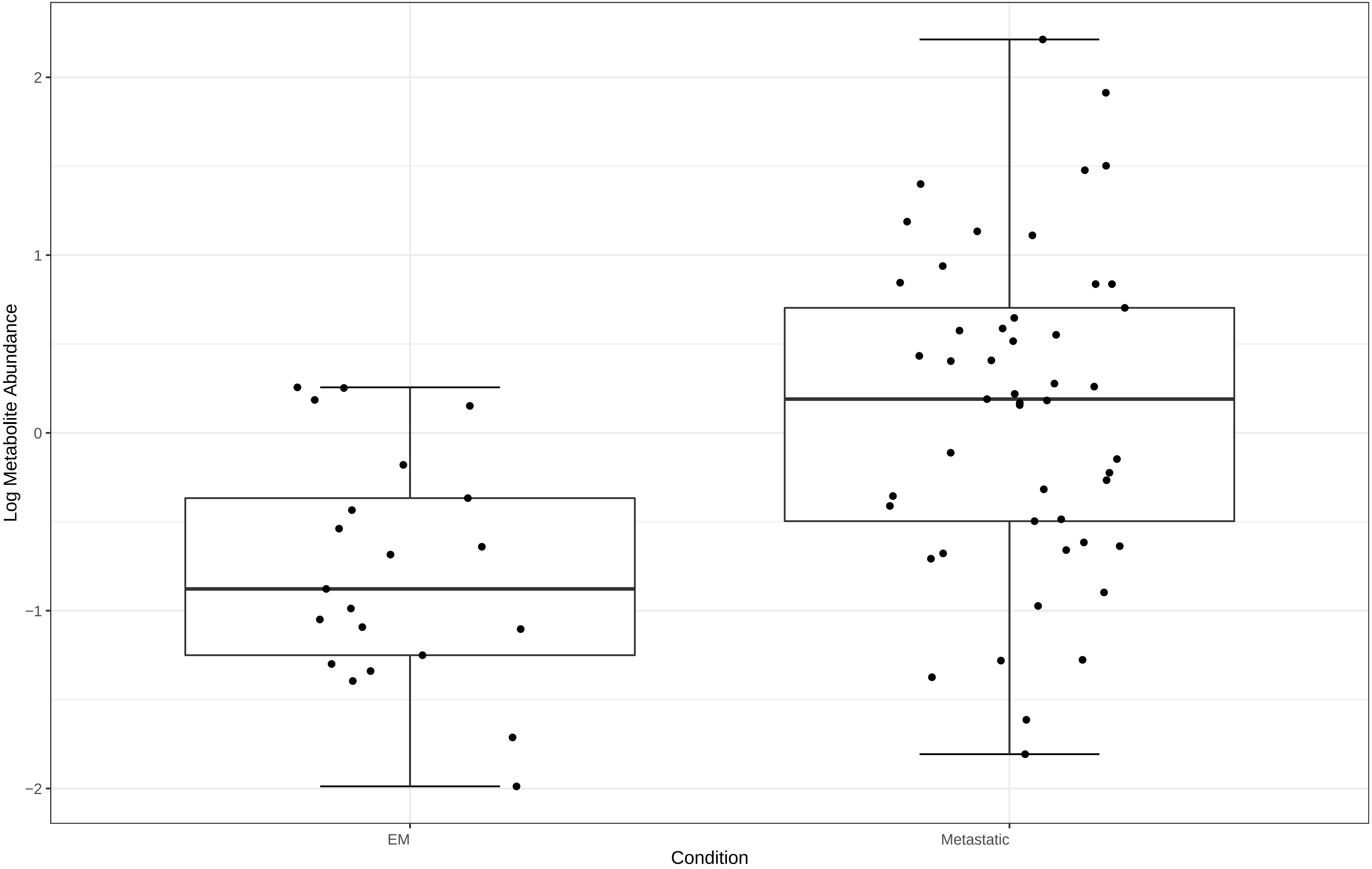

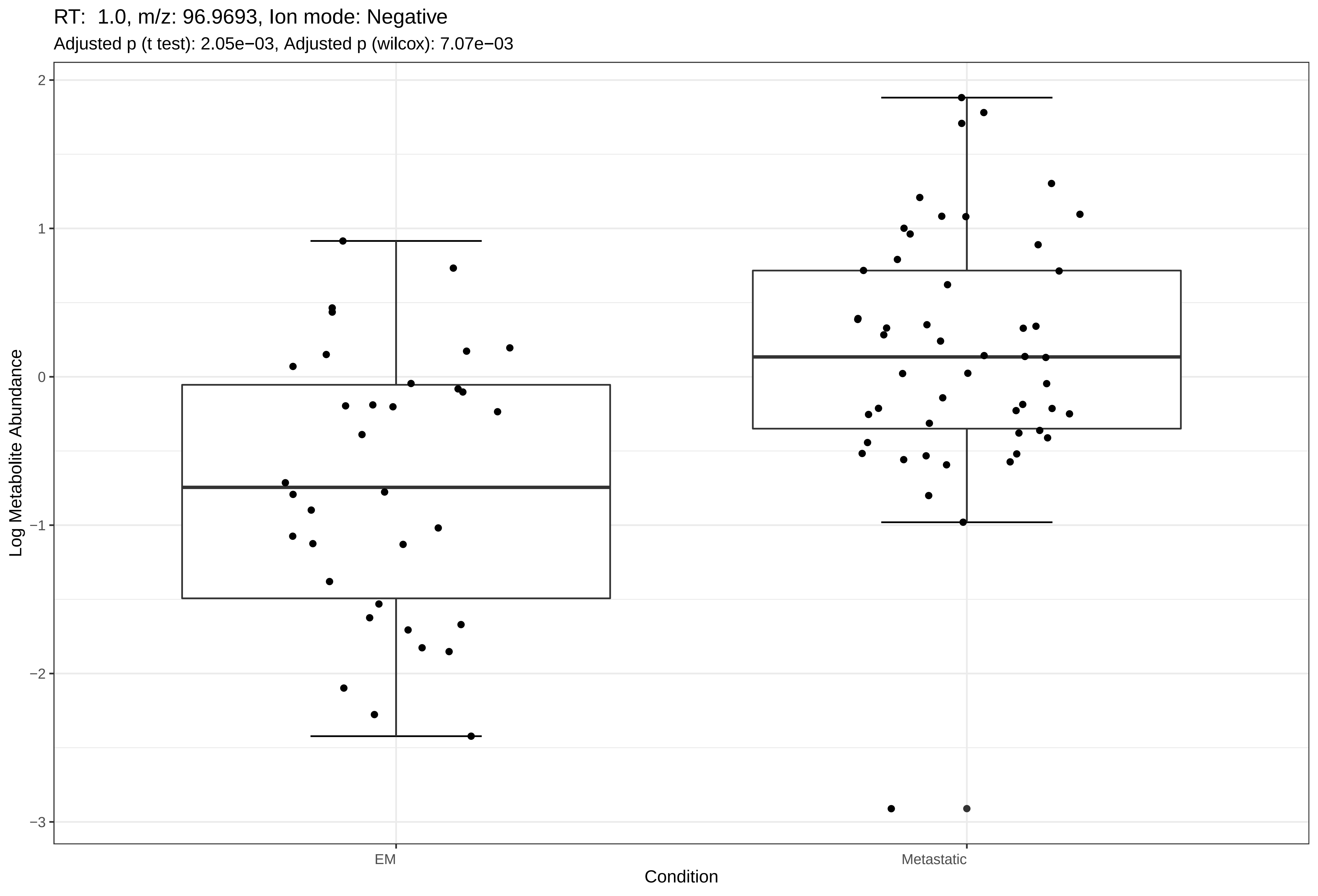
RT: 1.0, m/z: 96.9693, Ion mode: Negative
Adjusted p (t test): 2.05e−03, Adjusted p (wilcox): 7.07e−03
Log Metabolite Abundance
2
1
0
−1
−2
−3
EM
Metastatic
Condition

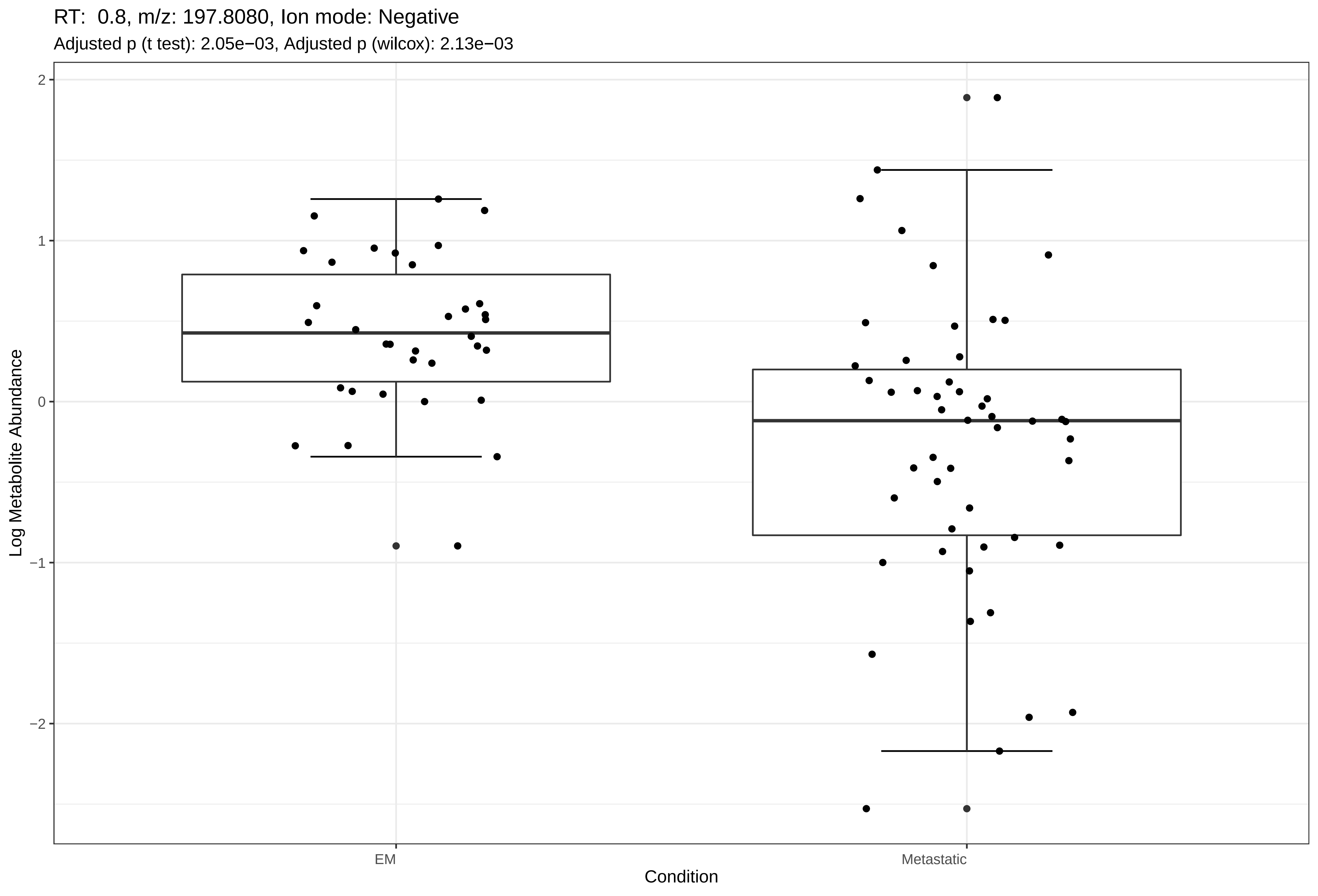
RT: 0.8, m/z: 197.8080, Ion mode: Negative
Adjusted p (t test): 2.05e−03, Adjusted p (wilcox): 2.13e−03
Log Metabolite Abundance
2
1
0
−1
−2
EM
Metastatic
Condition

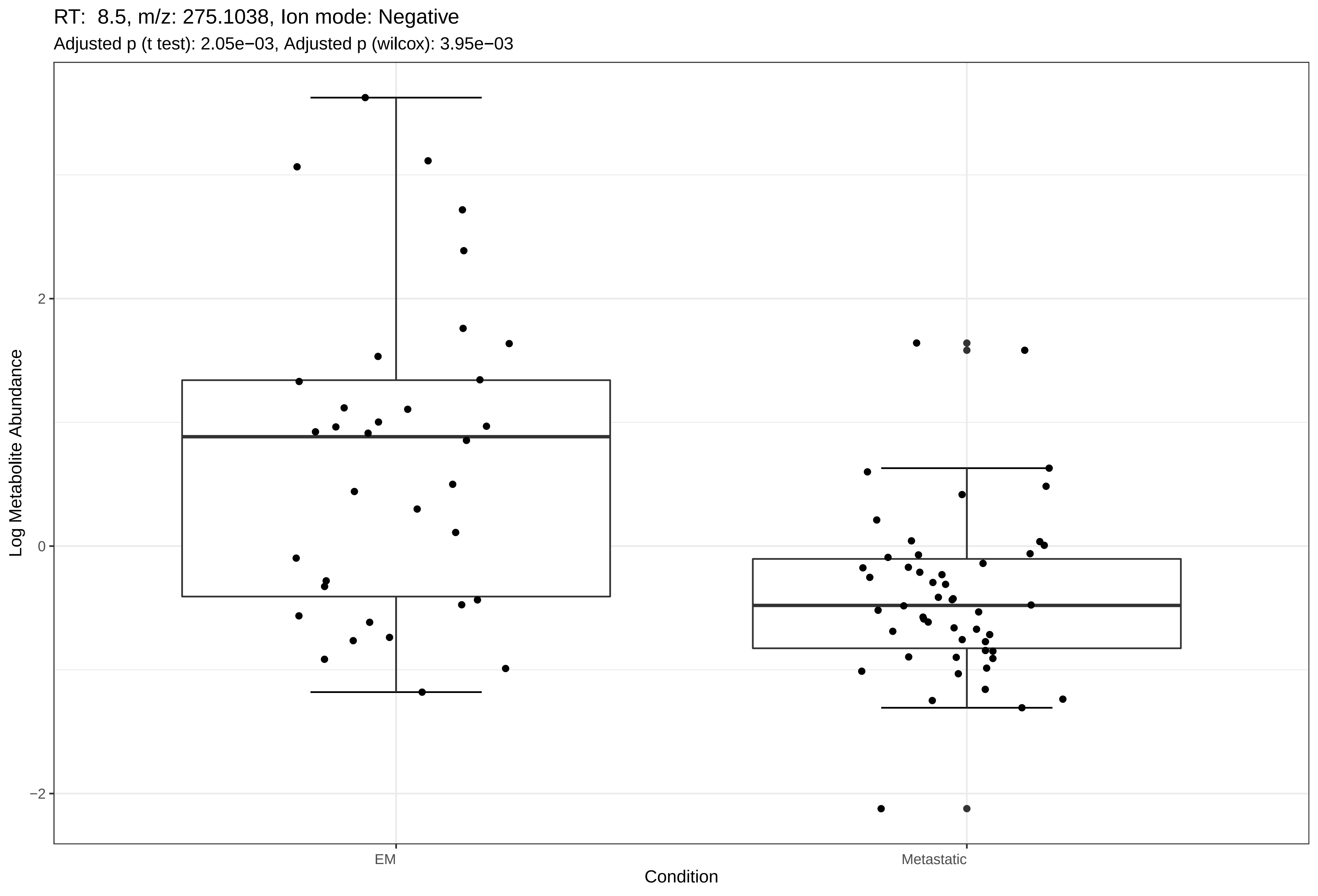
RT: 8.5, m/z: 275.1038, Ion mode: Negative
Adjusted p (t test): 2.05e−03, Adjusted p (wilcox): 3.95e−03
Log Metabolite Abundance
2
0
−2
EM
Metastatic
Condition

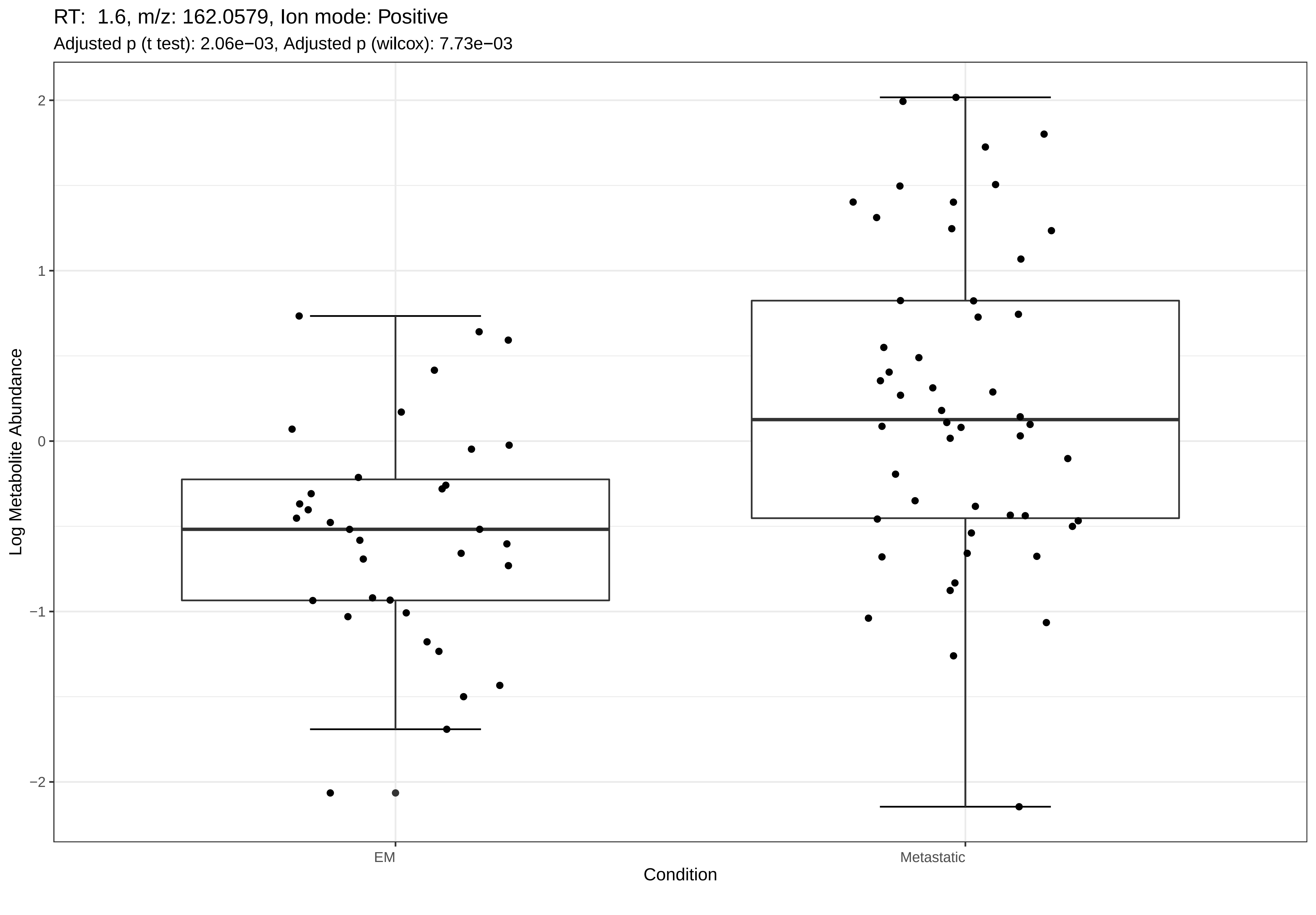
RT: 1.6, m/z: 162.0579, Ion mode: Positive
Adjusted p (t test): 2.06e−03, Adjusted p (wilcox): 7.73e−03
Log Metabolite Abundance
2
1
0
−1
−2
EM
Metastatic
Condition

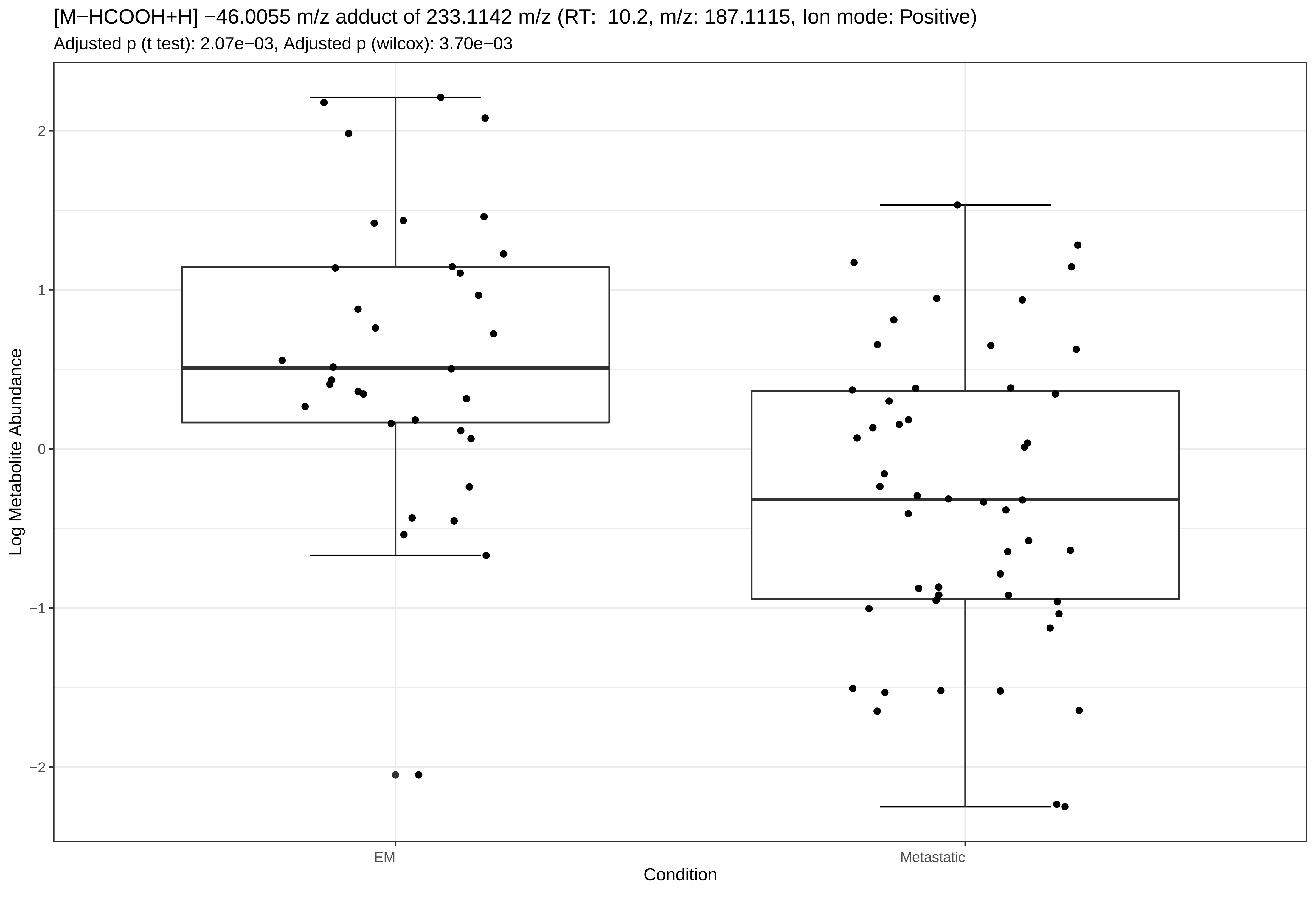
[M−HCOOH+H] −46.0055 m/z adduct of 233.1142 m/z (RT: 10.2, m/z: 187.1115, Ion mode: Positive)
Adjusted p (t test): 2.07e−03, Adjusted p (wilcox): 3.70e−03
Log Metabolite Abundance
2
1
0
−1
−2
EM
Metastatic
Condition

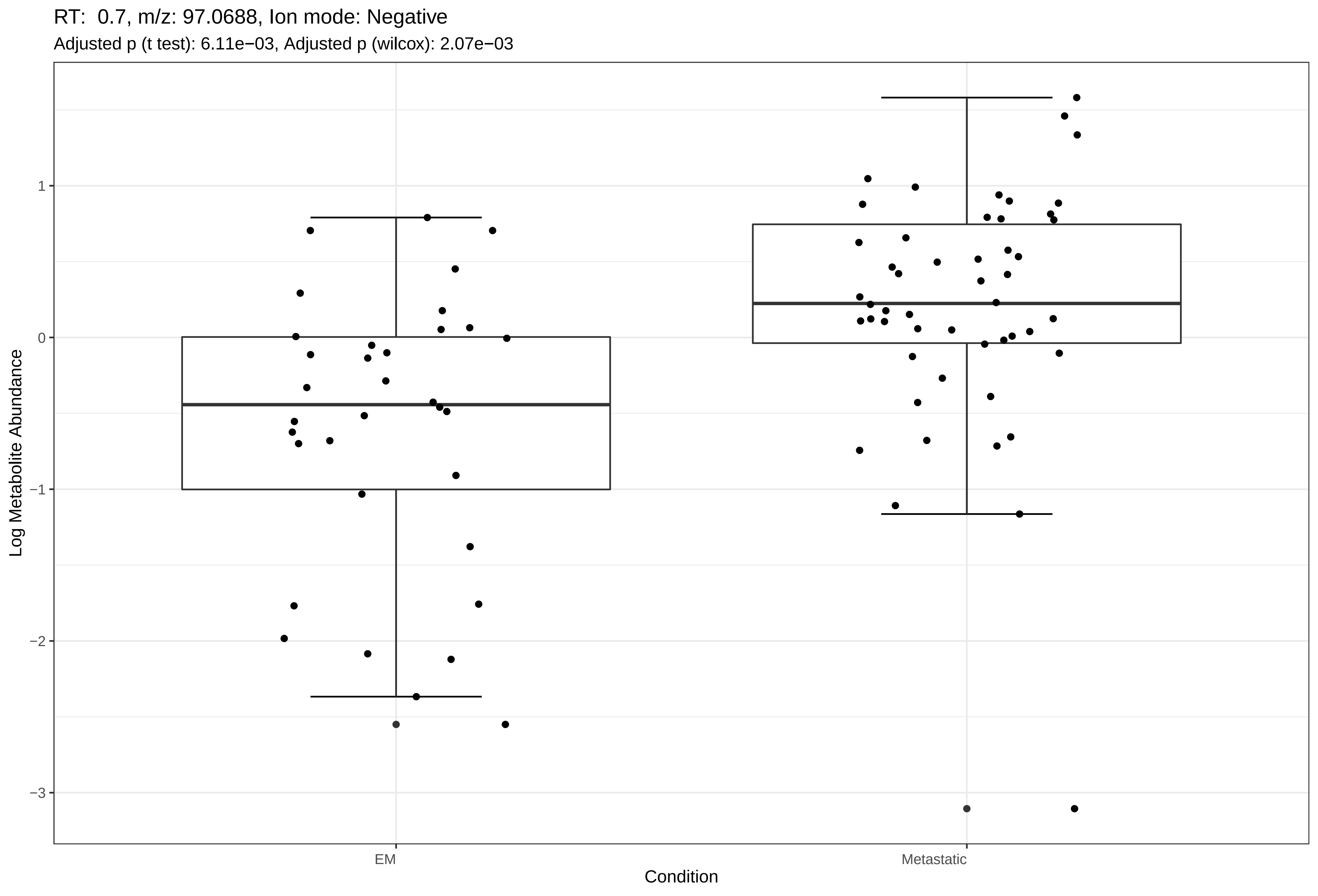

RT: 0.7, m/z: 97.0688, Ion mode: Negative
Adjusted p (t test): 6.11e−03, Adjusted p (wilcox): 2.07e−03
Log Metabolite Abundance
1
0
−1
−2
−3
EM
Metastatic
Condition

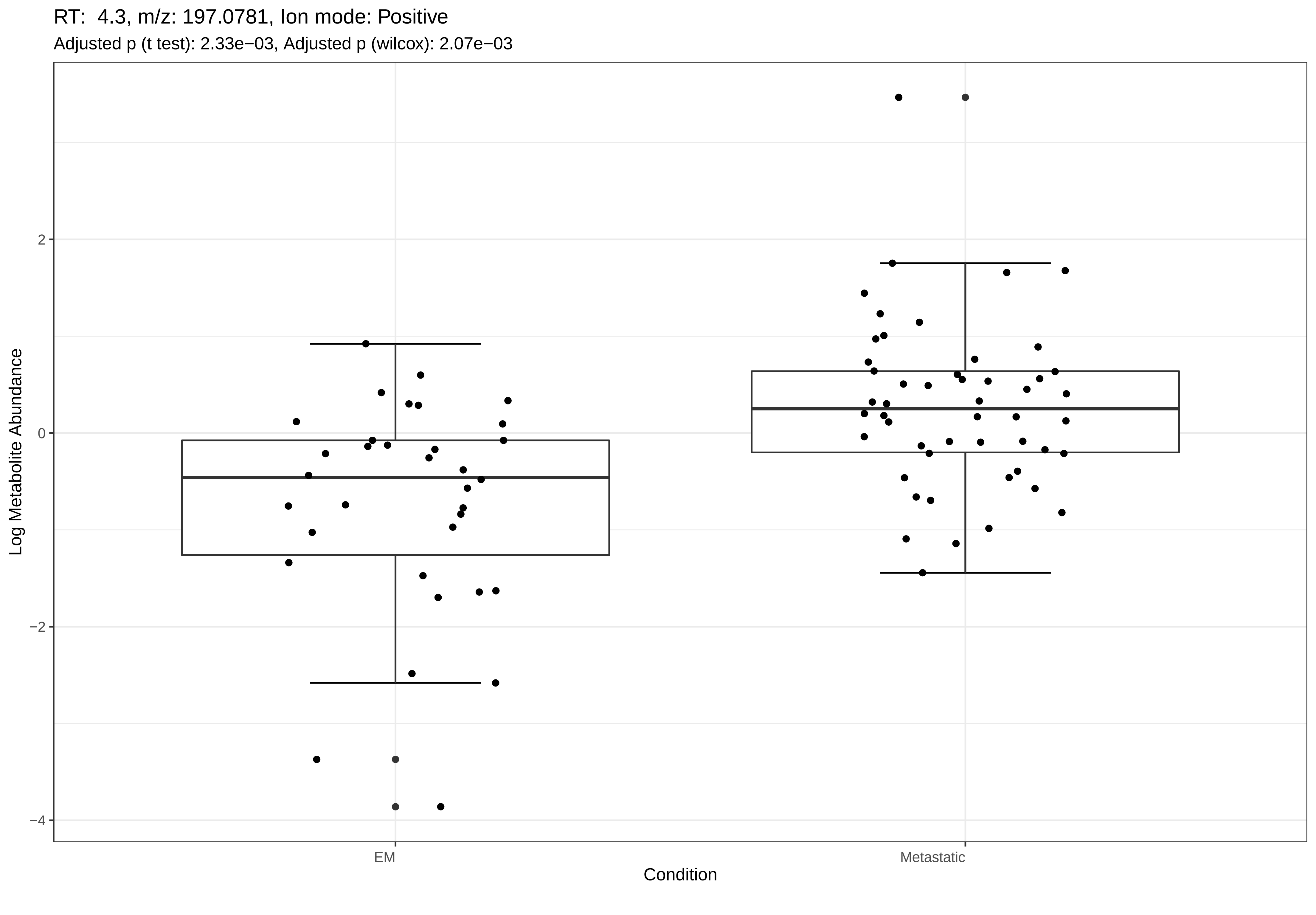

RT: 4.3, m/z: 197.0781, Ion mode: Positive
Adjusted p (t test): 2.33e−03, Adjusted p (wilcox): 2.07e−03
Log Metabolite Abundance
2
0
−2
−4
EM
Metastatic
Condition

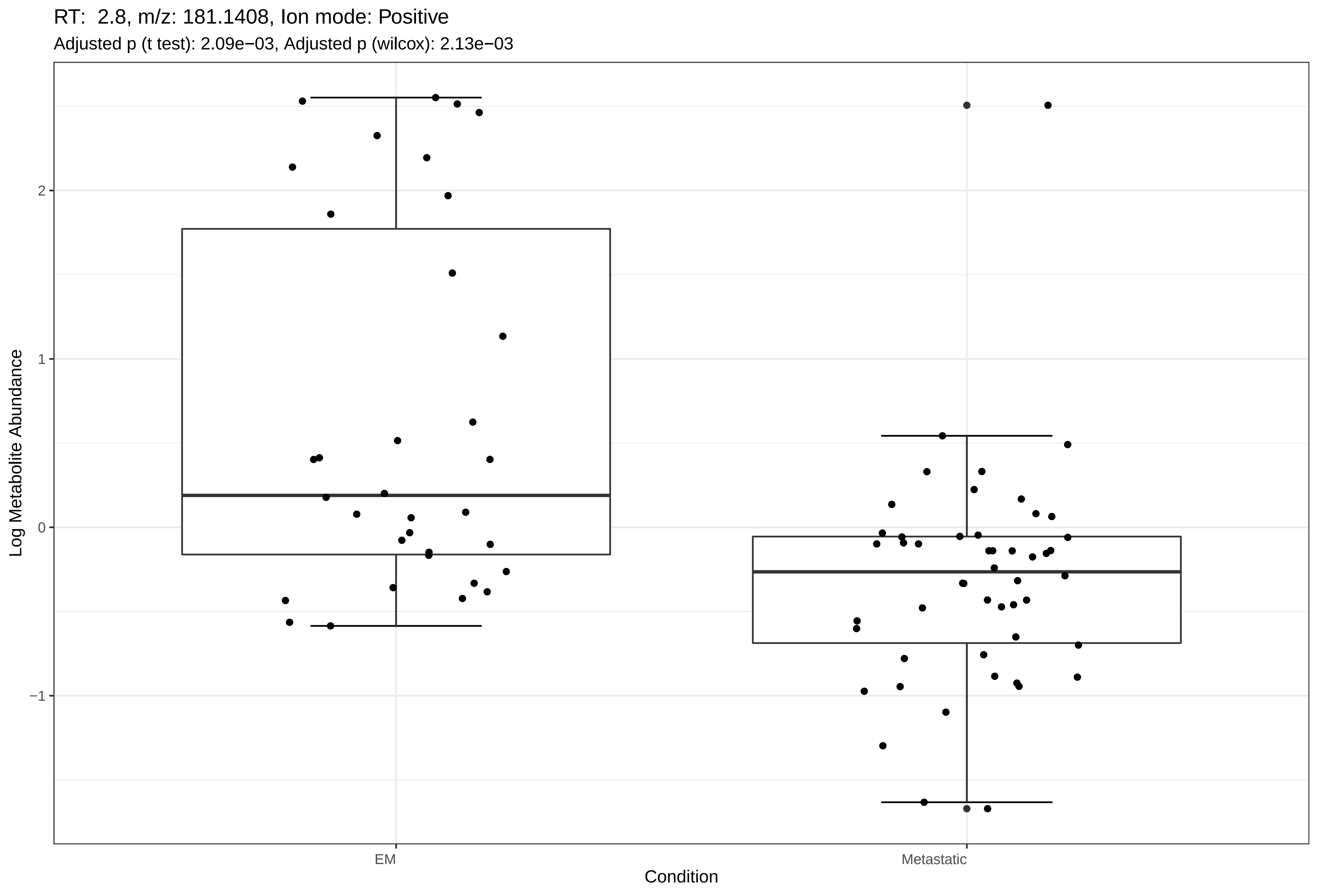
RT: 2.8, m/z: 181.1408, Ion mode: Positive
Adjusted p (t test): 2.09e−03, Adjusted p (wilcox): 2.13e−03
Log Metabolite Abundance
2
1
0
−1
EM
Metastatic
Condition

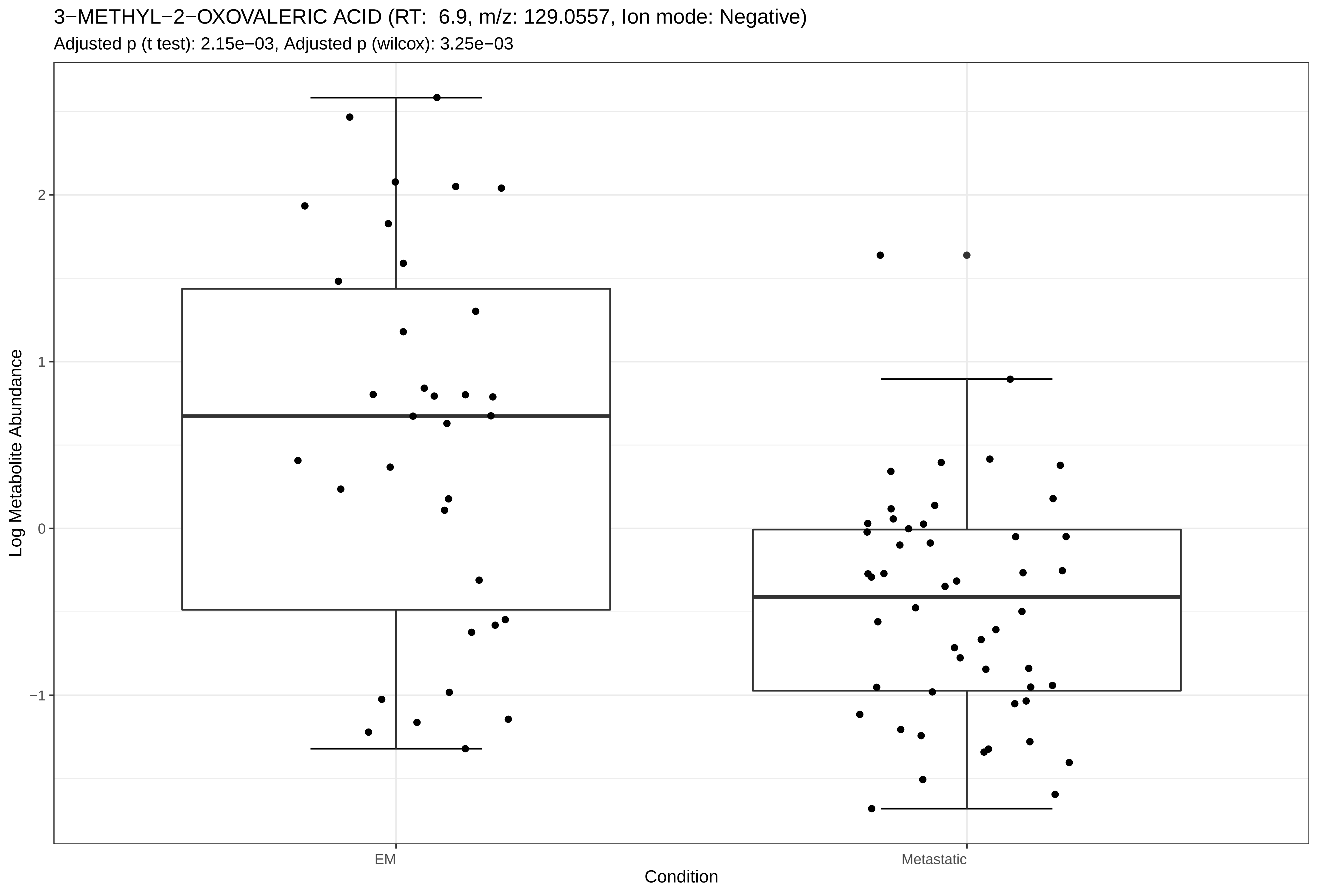

3−METHYL−2−OXOVALERIC ACID (RT: 6.9, m/z: 129.0557, Ion mode: Negative)
Adjusted p (t test): 2.15e−03, Adjusted p (wilcox): 3.25e−03
Log Metabolite Abundance
2
1
0
−1
EM
Metastatic
Condition

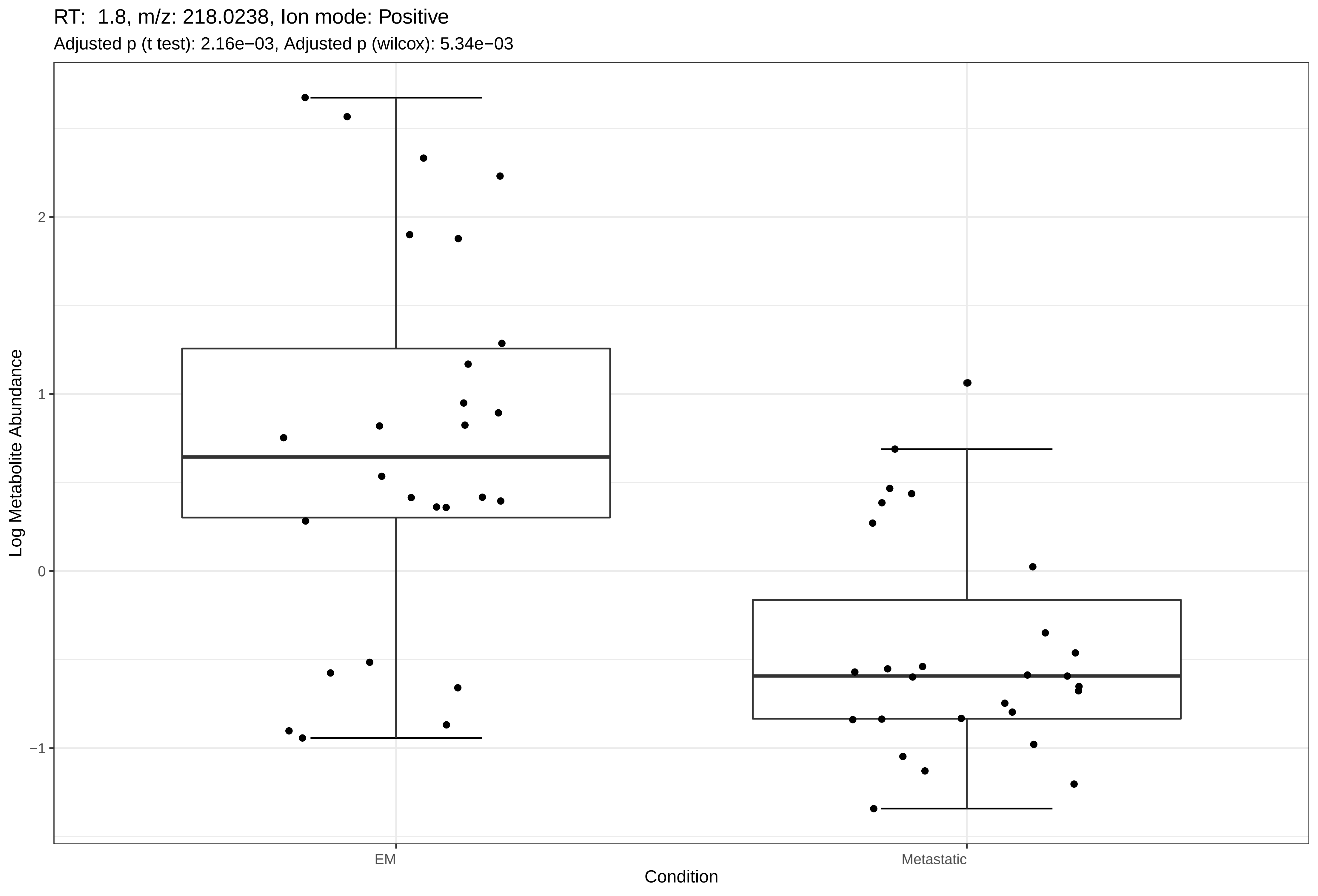
RT: 1.8, m/z: 218.0238, Ion mode: Positive
Adjusted p (t test): 2.16e−03, Adjusted p (wilcox): 5.34e−03
Log Metabolite Abundance
2
1
0
−1
EM
Metastatic
Condition

RT: 10.2, m/z: 263.0587, Ion mode: Positive

Adjusted p (t test): 2.19e−03, Adjusted p (wilcox): 2.51e−03

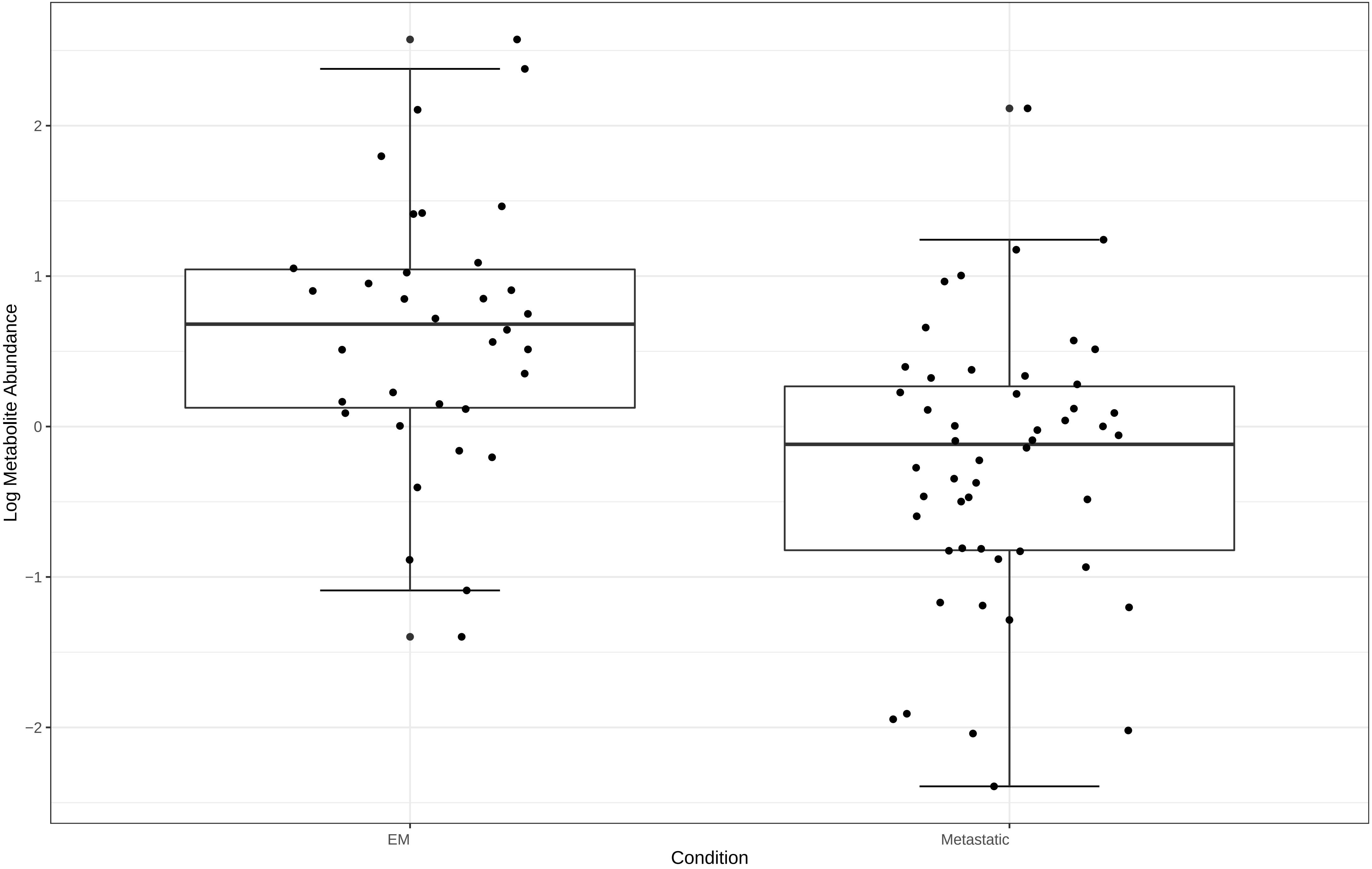

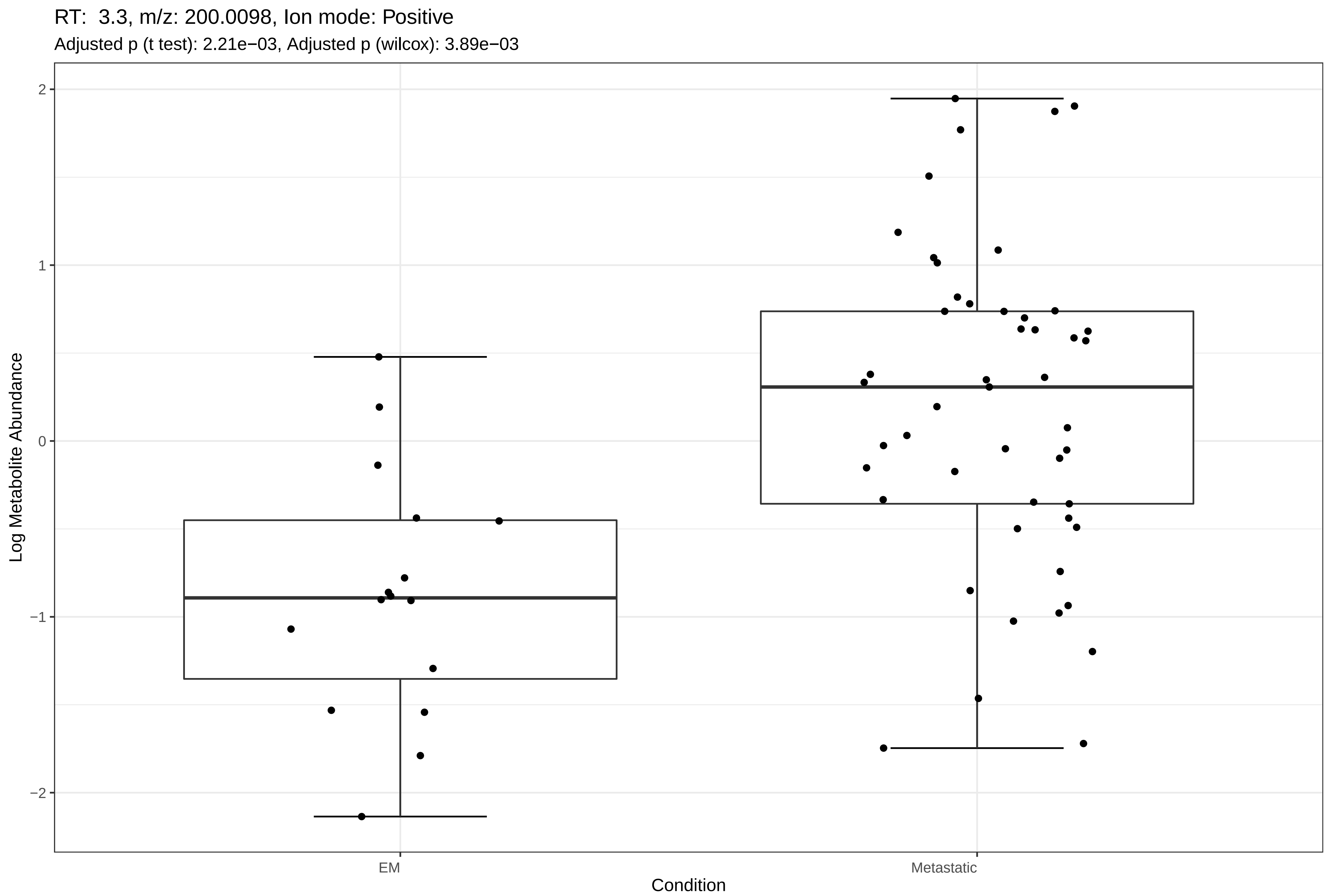
RT: 3.3, m/z: 200.0098, Ion mode: Positive
Adjusted p (t test): 2.21e−03, Adjusted p (wilcox): 3.89e−03
Log Metabolite Abundance
2
1
0
−1
−2
EM
Metastatic
Condition

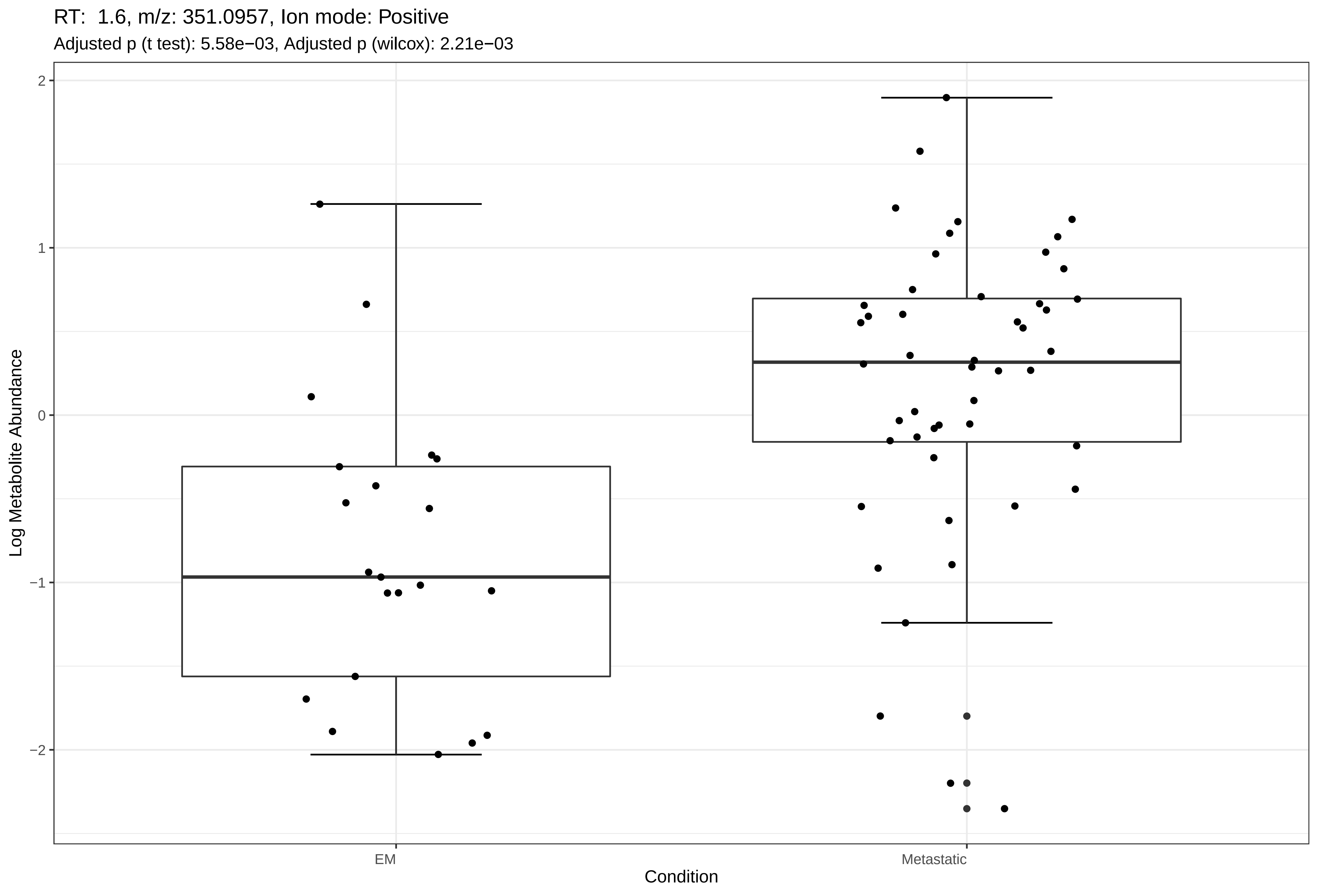
RT: 1.6, m/z: 351.0957, Ion mode: Positive
Adjusted p (t test): 5.58e−03, Adjusted p (wilcox): 2.21e−03
Log Metabolite Abundance
2
1
0
−1
−2
EM
Metastatic
Condition

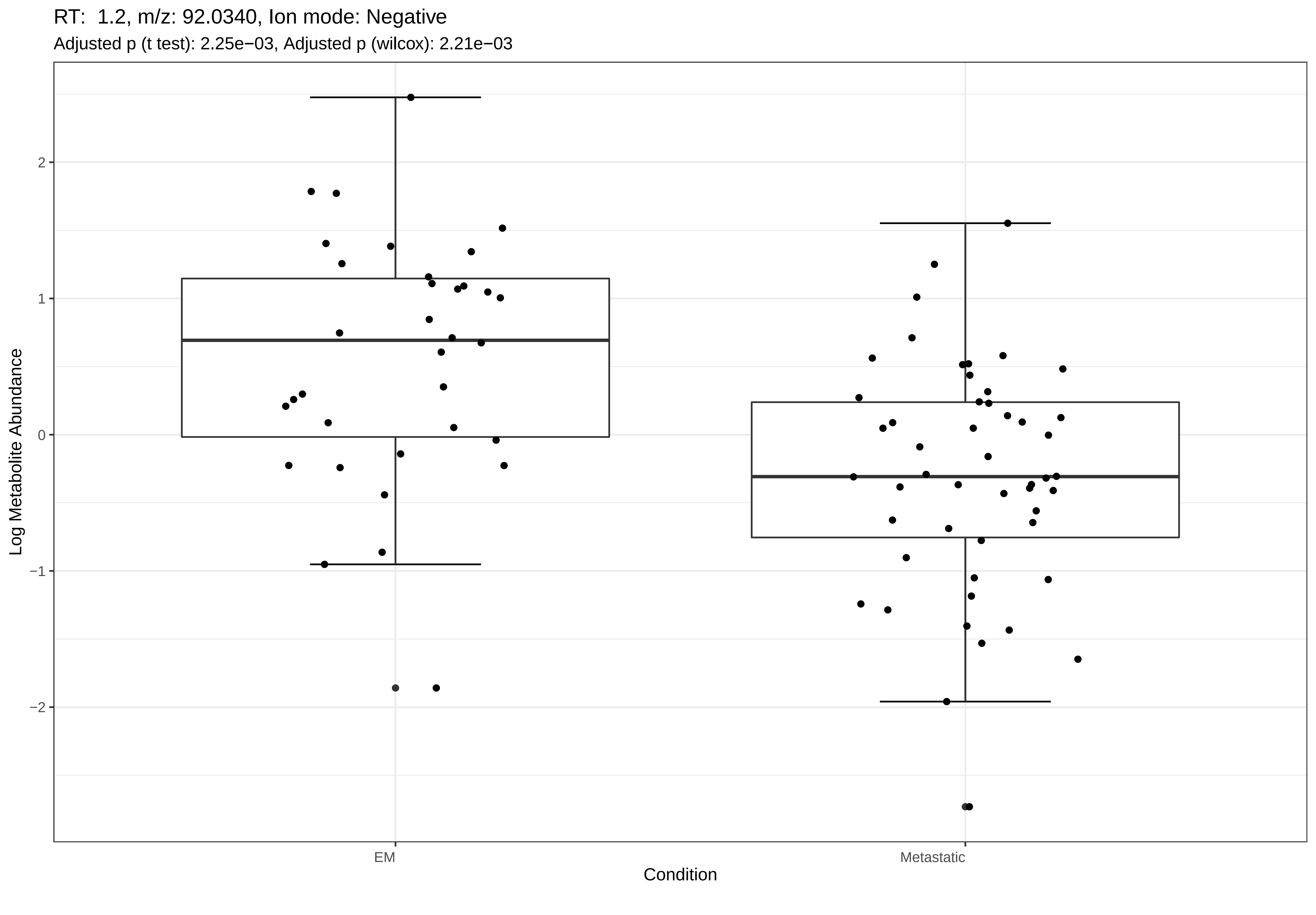
RT: 1.2, m/z: 92.0340, Ion mode: Negative
Adjusted p (t test): 2.25e−03, Adjusted p (wilcox): 2.21e−03
Log Metabolite Abundance
2
1
0
−1
−2
EM
Metastatic
Condition

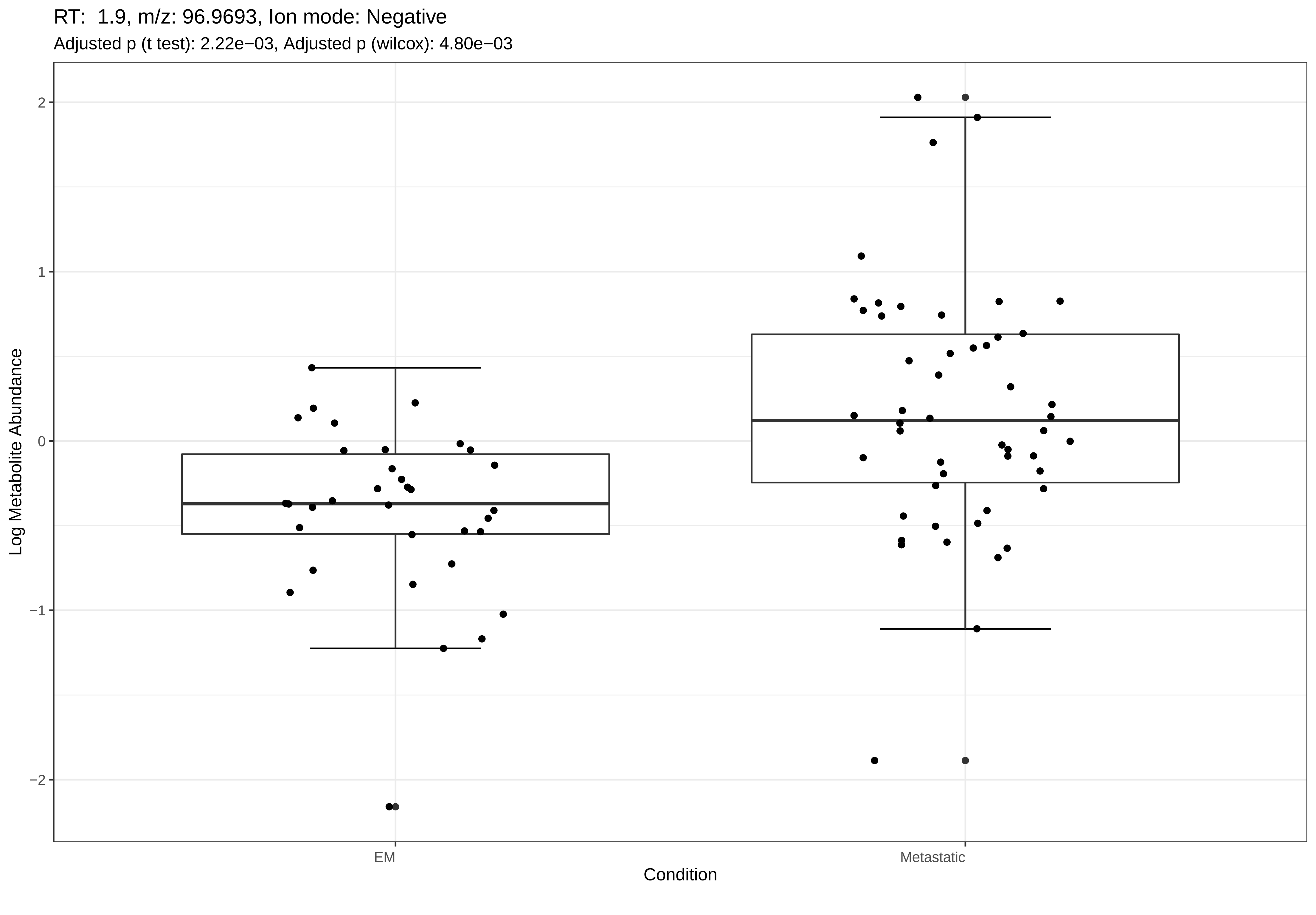

RT: 1.9, m/z: 96.9693, Ion mode: Negative
Adjusted p (t test): 2.22e−03, Adjusted p (wilcox): 4.80e−03
Log Metabolite Abundance
2
1
0
−1
−2
EM
Metastatic
Condition

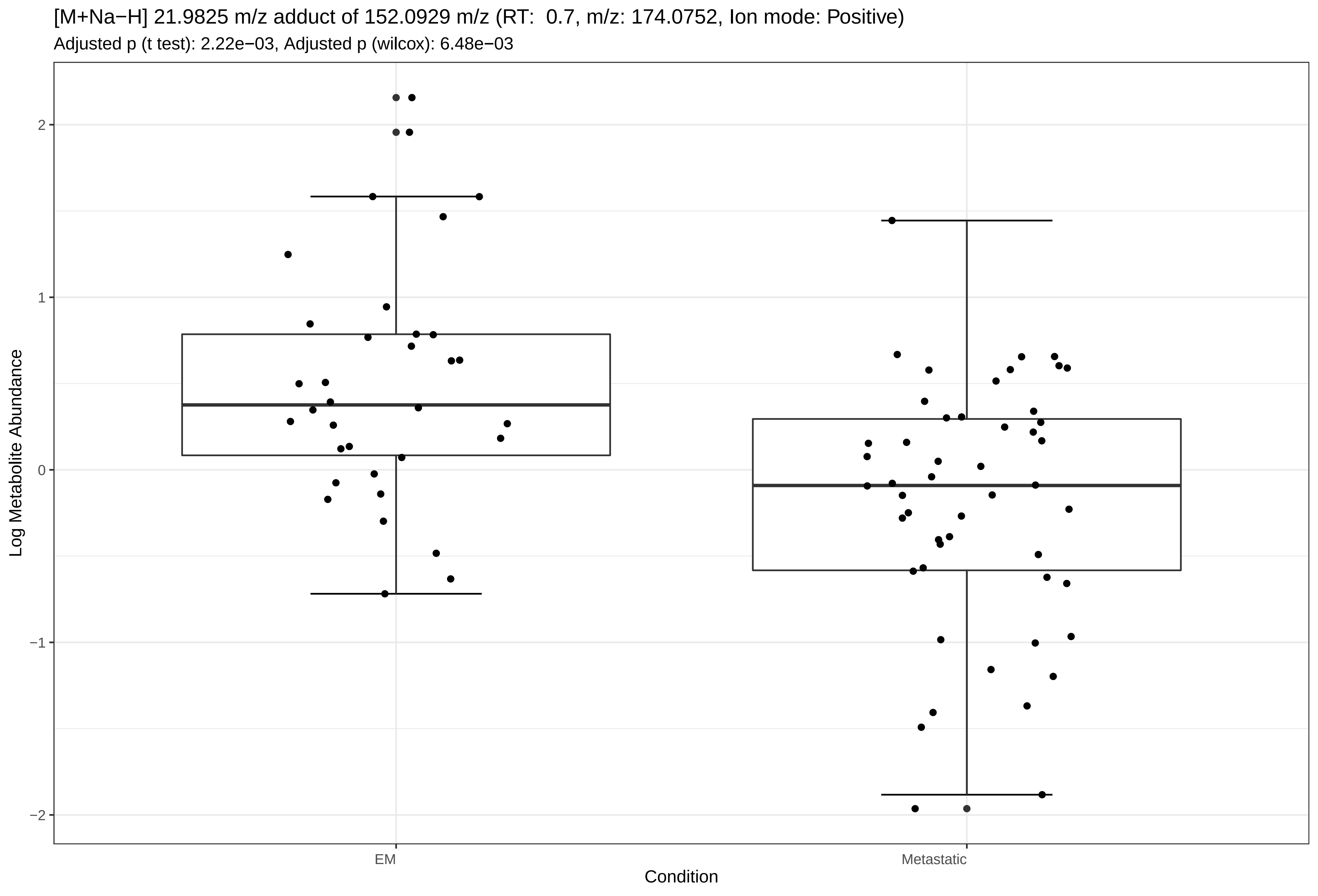

[M+Na−H] 21.9825 m/z adduct of 152.0929 m/z (RT: 0.7, m/z: 174.0752, Ion mode: Positive)
Adjusted p (t test): 2.22e−03, Adjusted p (wilcox): 6.48e−03
Log Metabolite Abundance
2
1
0
−1
−2
EM
Metastatic
Condition

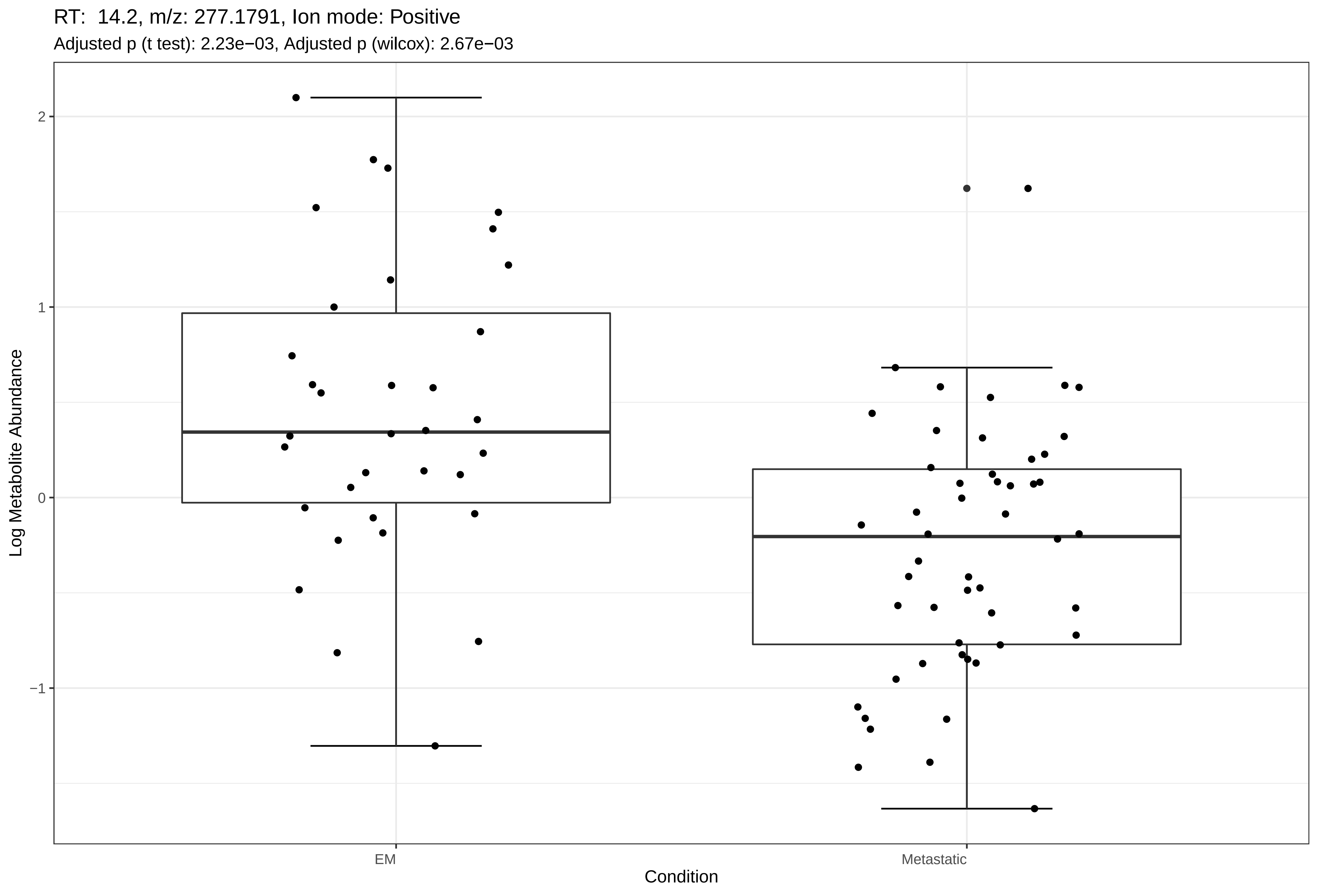
RT: 14.2, m/z: 277.1791, Ion mode: Positive
Adjusted p (t test): 2.23e−03, Adjusted p (wilcox): 2.67e−03
Log Metabolite Abundance
2
1
0
−1
EM
Metastatic
Condition

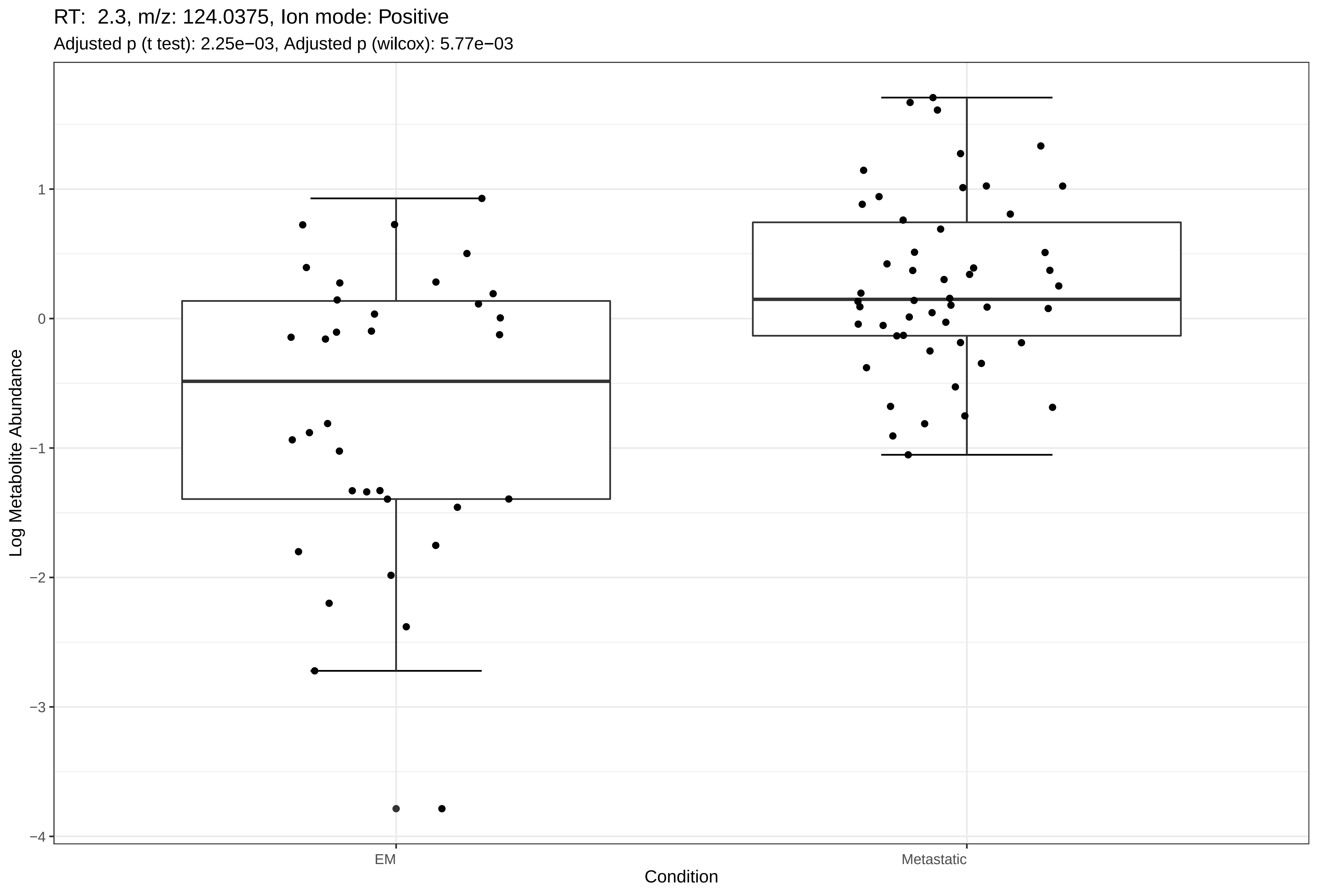

RT: 2.3, m/z: 124.0375, Ion mode: Positive
Adjusted p (t test): 2.25e−03, Adjusted p (wilcox): 5.77e−03
Log Metabolite Abundance
1
0
−1
−2
−3
−4
EM
Metastatic
Condition

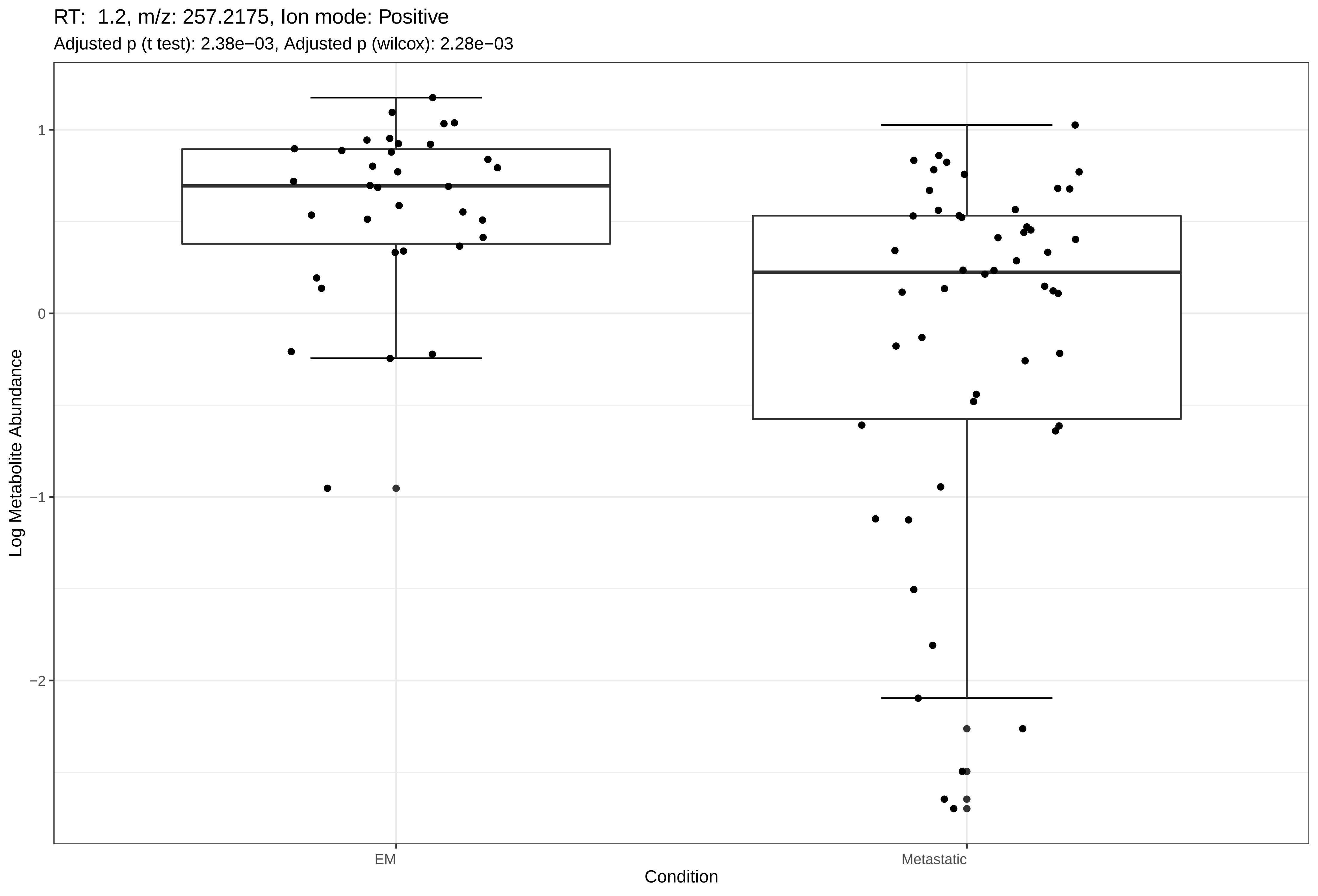

RT: 1.2, m/z: 257.2175, Ion mode: Positive
Adjusted p (t test): 2.38e−03, Adjusted p (wilcox): 2.28e−03
Log Metabolite Abundance
1
0
−1
−2
EM
Metastatic
Condition

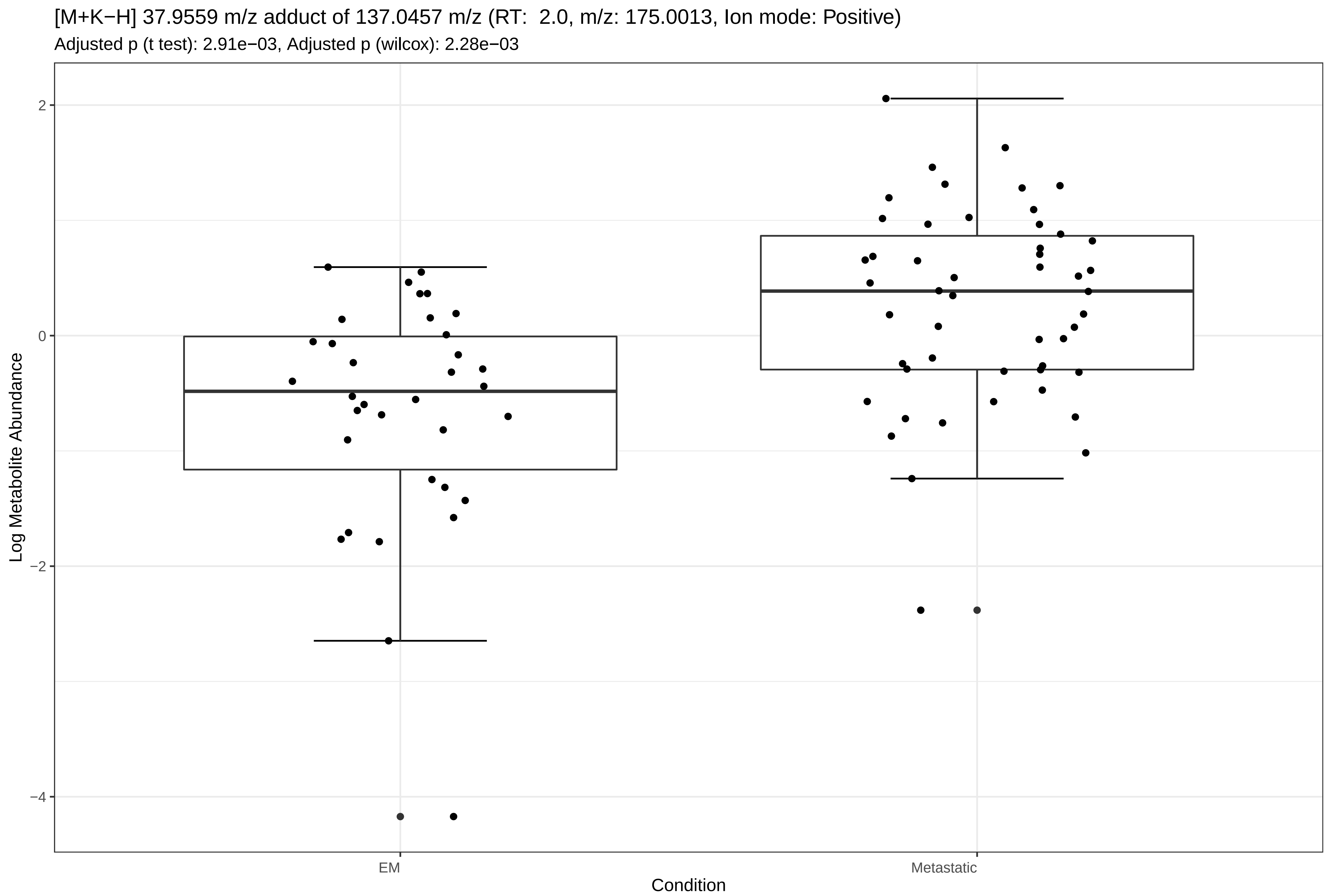
[M+K−H] 37.9559 m/z adduct of 137.0457 m/z (RT: 2.0, m/z: 175.0013, Ion mode: Positive)
Adjusted p (t test): 2.91e−03, Adjusted p (wilcox): 2.28e−03
Log Metabolite Abundance
2
0
−2
−4
EM
Metastatic
Condition

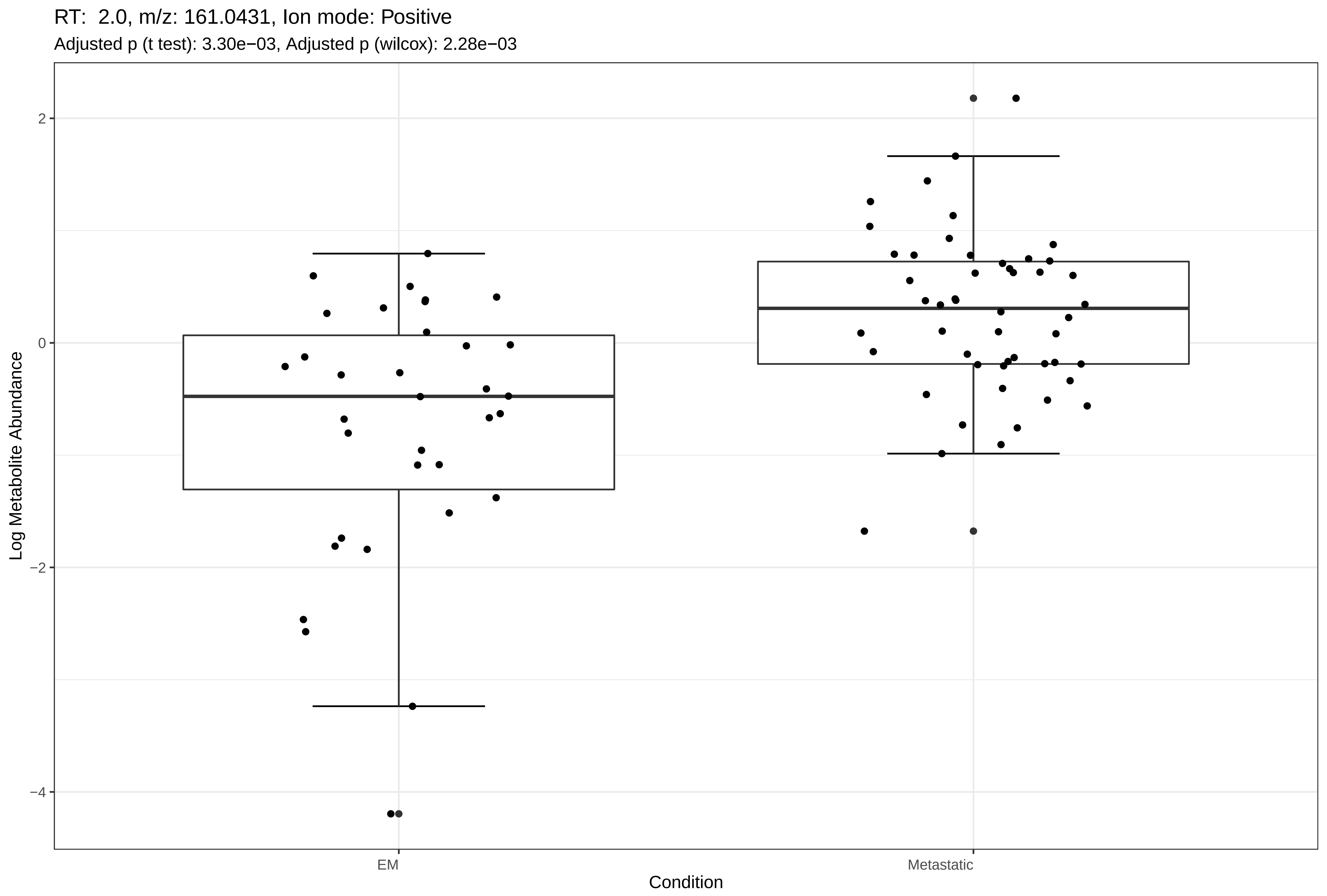
RT: 2.0, m/z: 161.0431, Ion mode: Positive
Adjusted p (t test): 3.30e−03, Adjusted p (wilcox): 2.28e−03
Log Metabolite Abundance
2
0
−2
−4
EM
Metastatic
Condition

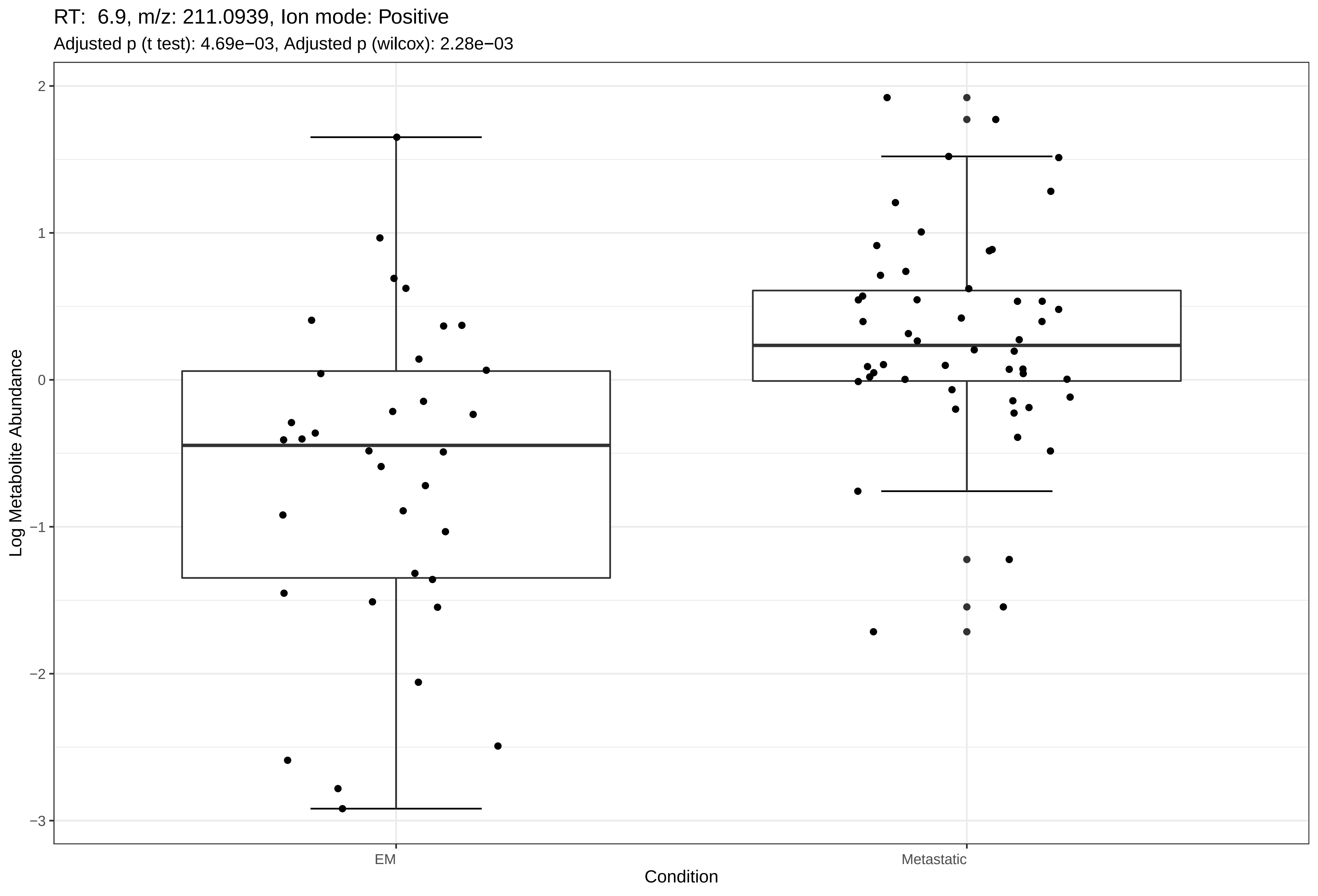
RT: 6.9, m/z: 211.0939, Ion mode: Positive
Adjusted p (t test): 4.69e−03, Adjusted p (wilcox): 2.28e−03
Log Metabolite Abundance
2
1
0
−1
−2
−3
EM
Metastatic
Condition

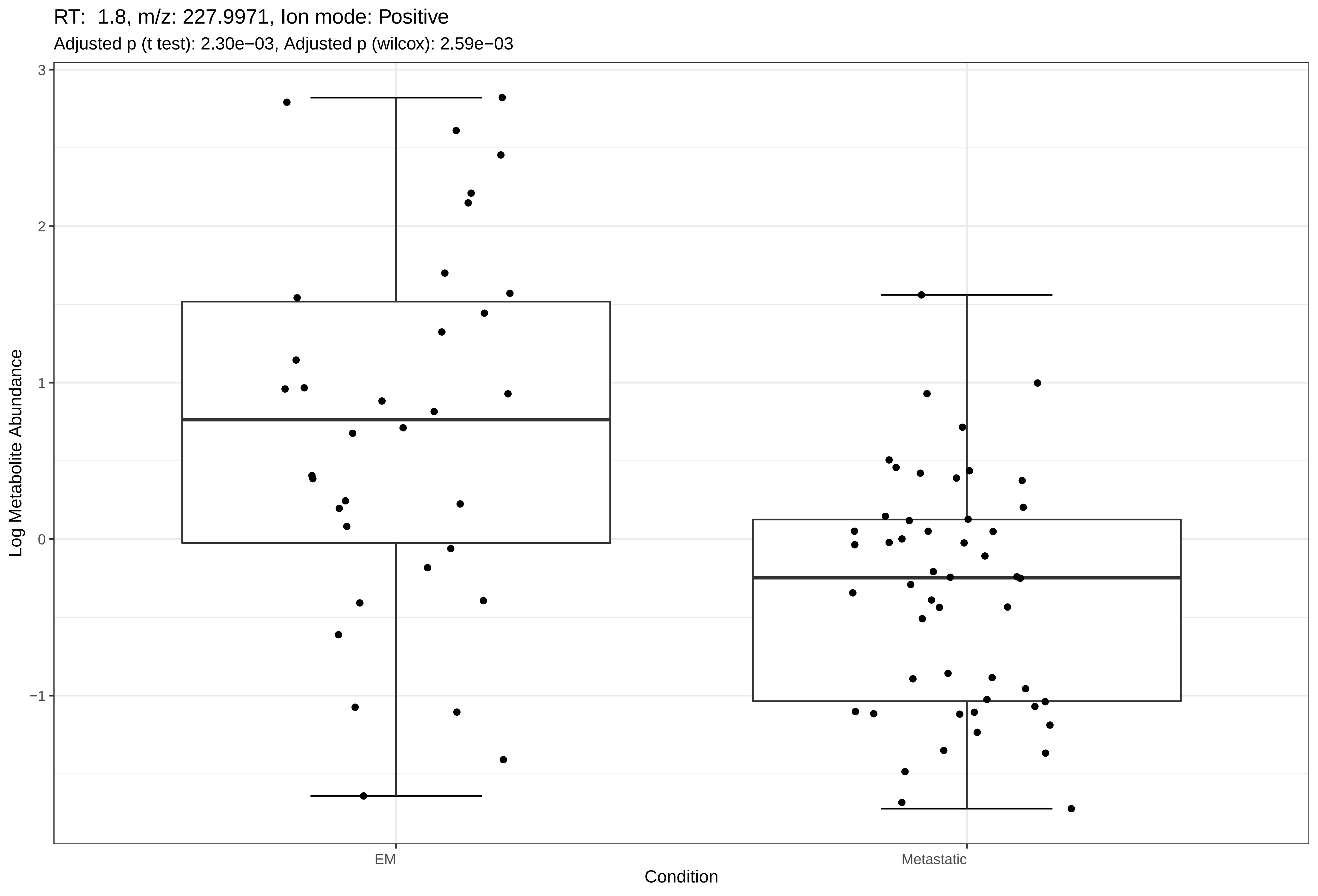

RT: 1.8, m/z: 227.9971, Ion mode: Positive
Adjusted p (t test): 2.30e−03, Adjusted p (wilcox): 2.59e−03
Log Metabolite Abundance
3
2
1
0
−1
EM
Metastatic
Condition

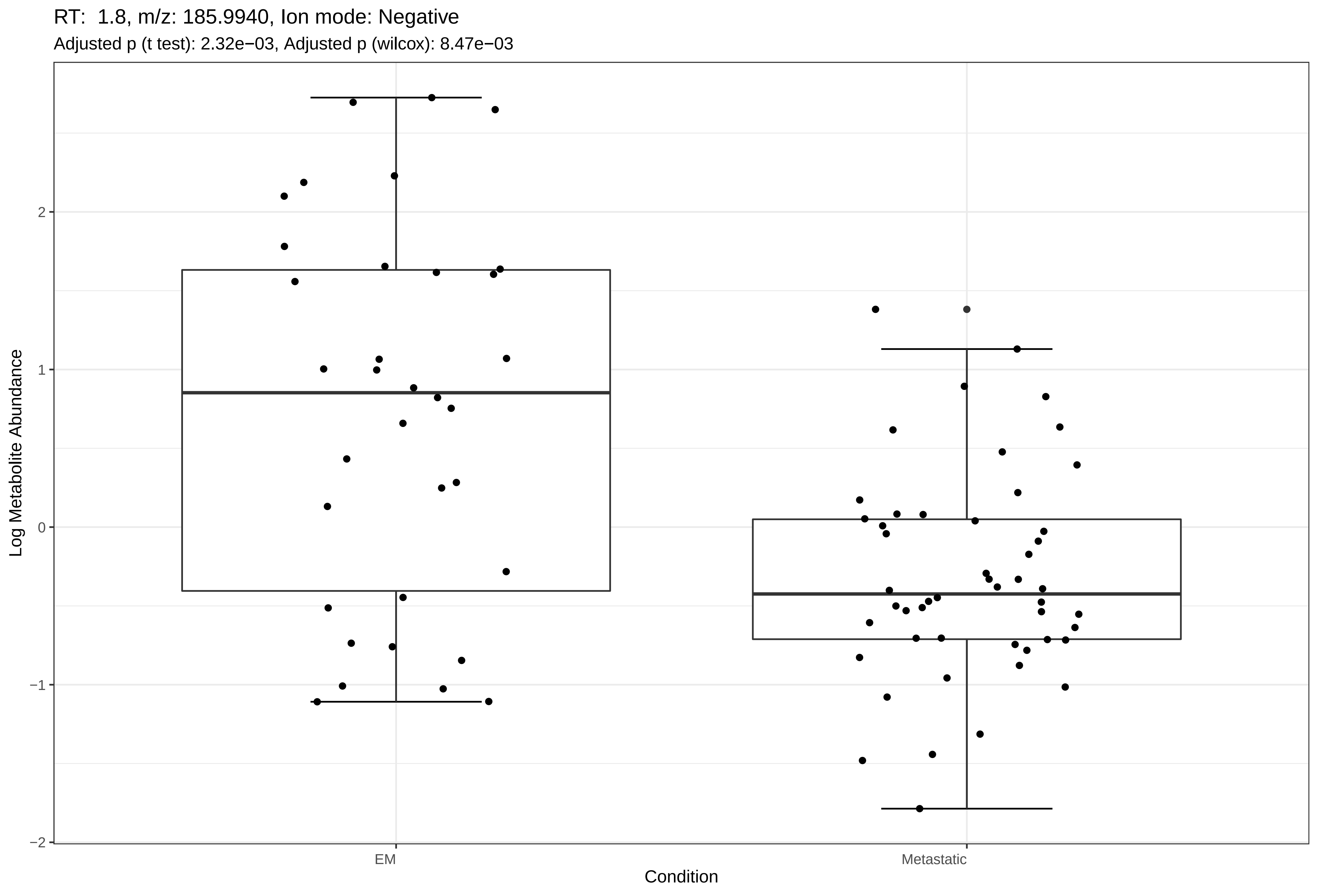
RT: 1.8, m/z: 185.9940, Ion mode: Negative
Adjusted p (t test): 2.32e−03, Adjusted p (wilcox): 8.47e−03
Log Metabolite Abundance
2
1
0
−1
−2
EM
Metastatic
Condition

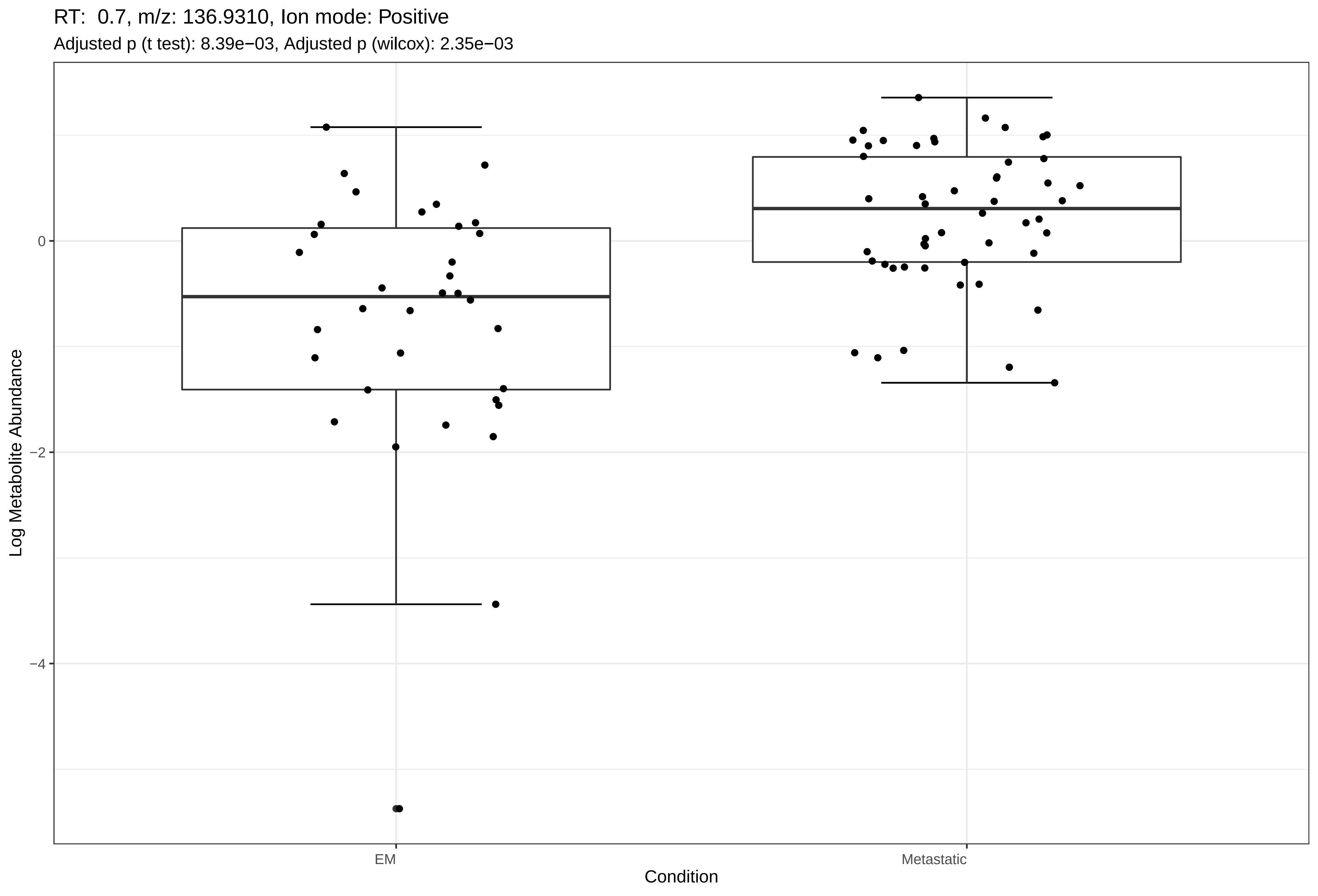
RT: 0.7, m/z: 136.9310, Ion mode: Positive
Adjusted p (t test): 8.39e−03, Adjusted p (wilcox): 2.35e−03
Log Metabolite Abundance
0
−2
−4
EM
Metastatic
Condition

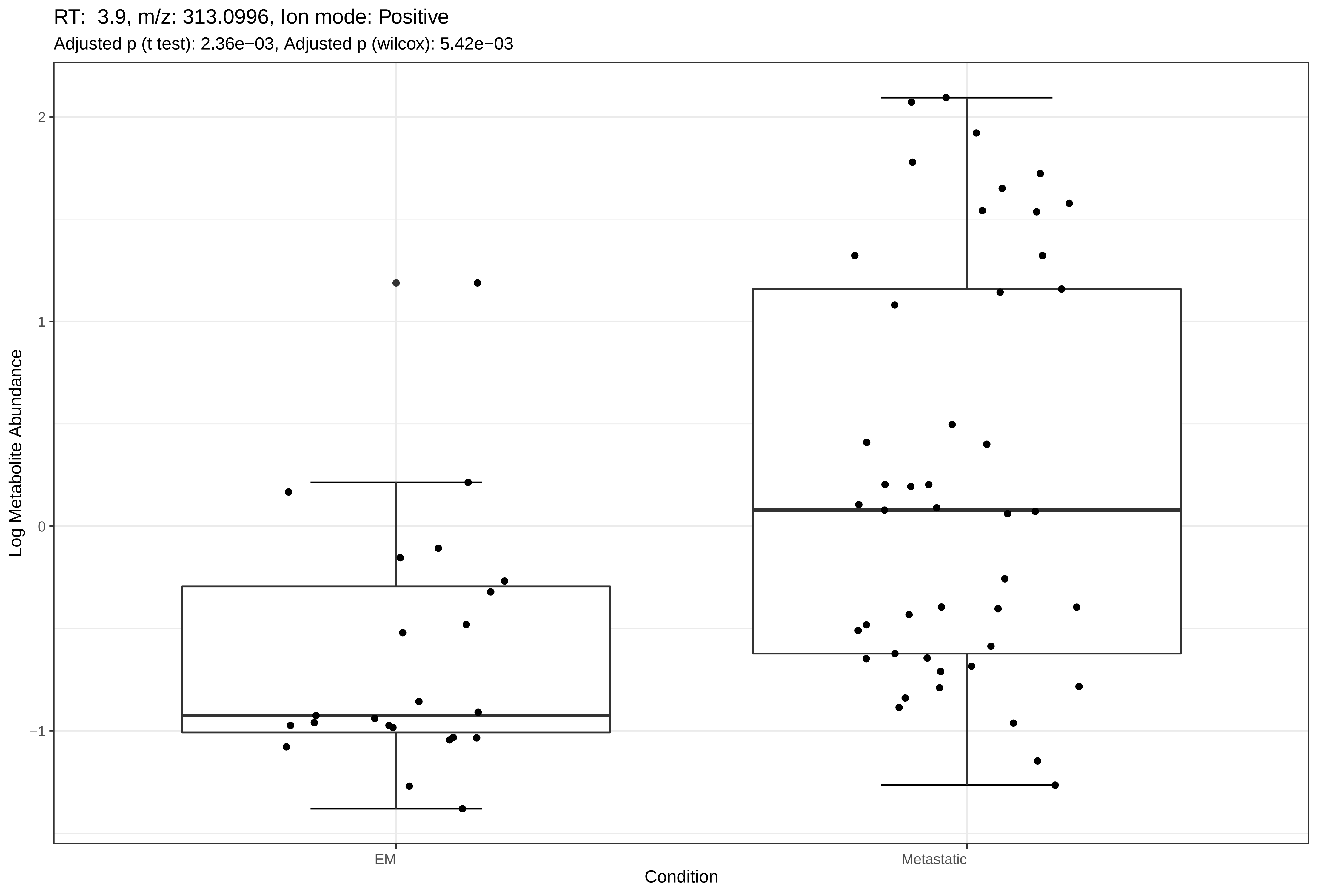
RT: 3.9, m/z: 313.0996, Ion mode: Positive
Adjusted p (t test): 2.36e−03, Adjusted p (wilcox): 5.42e−03
Log Metabolite Abundance
2
1
0
−1
EM
Metastatic
Condition

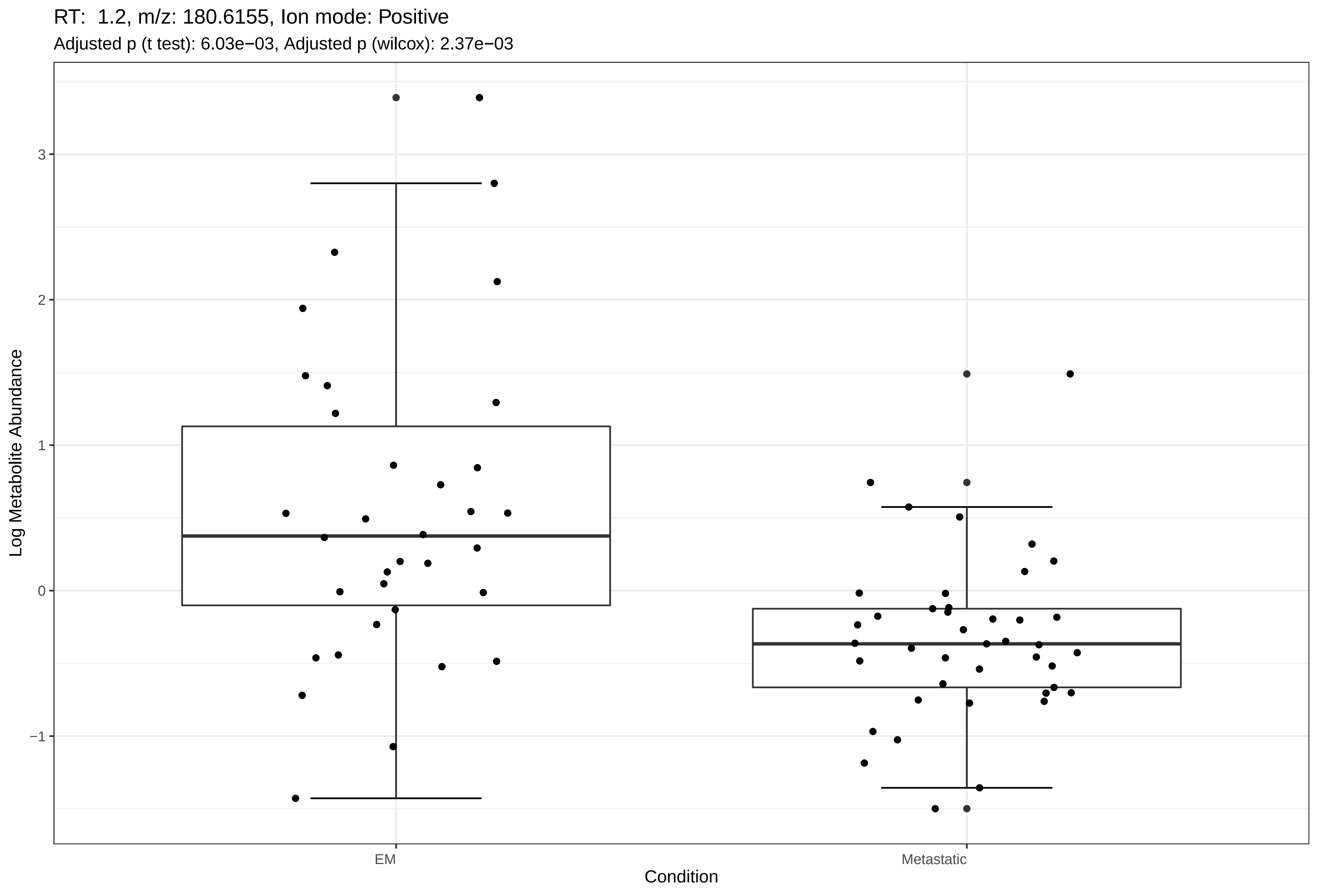

RT: 1.2, m/z: 180.6155, Ion mode: Positive
Adjusted p (t test): 6.03e−03, Adjusted p (wilcox): 2.37e−03
Log Metabolite Abundance
3
2
1
0
−1
EM
Metastatic
Condition

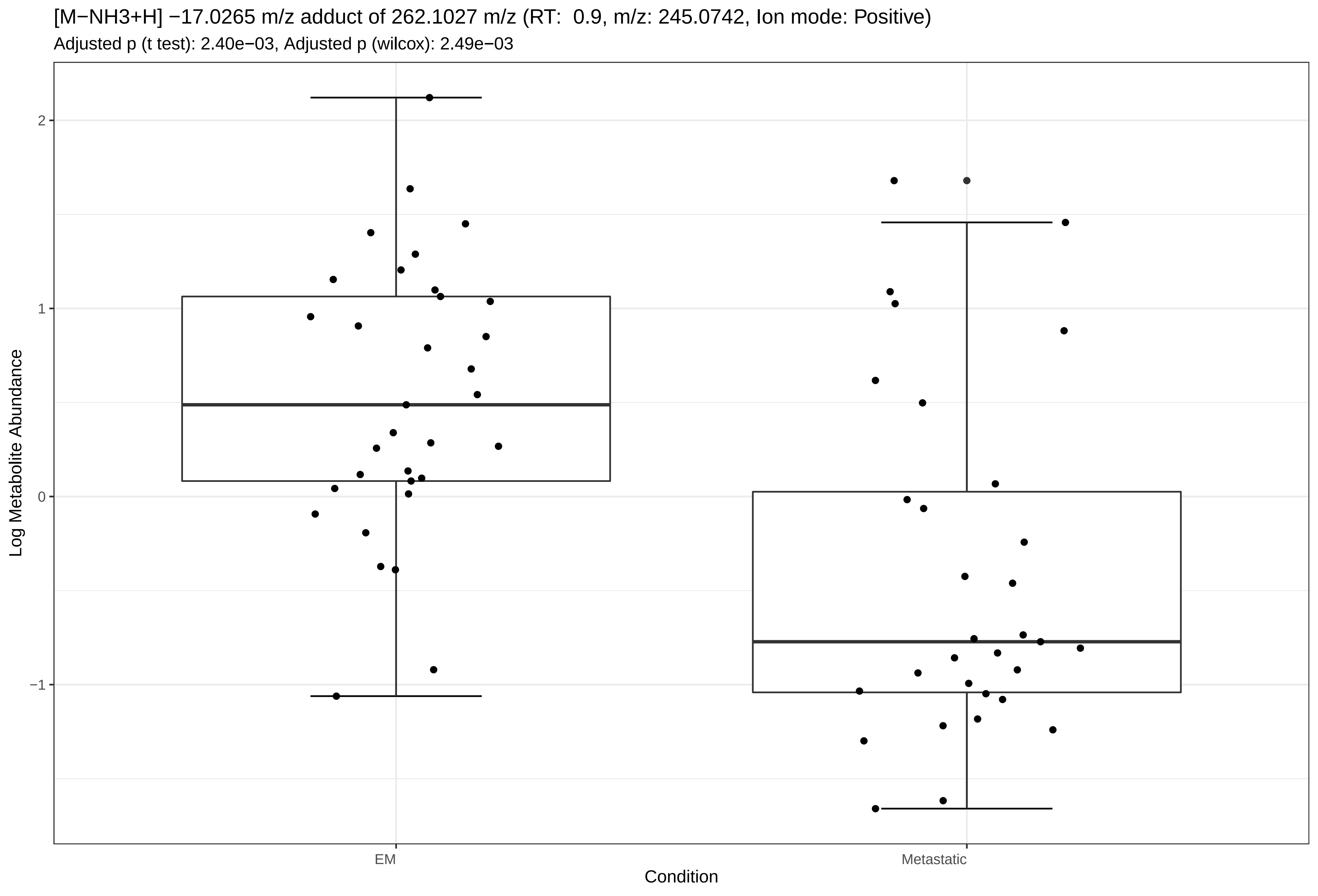
[M−NH3+H] −17.0265 m/z adduct of 262.1027 m/z (RT: 0.9, m/z: 245.0742, Ion mode: Positive)
Adjusted p (t test): 2.40e−03, Adjusted p (wilcox): 2.49e−03
Log Metabolite Abundance
2
1
0
−1
EM
Metastatic
Condition

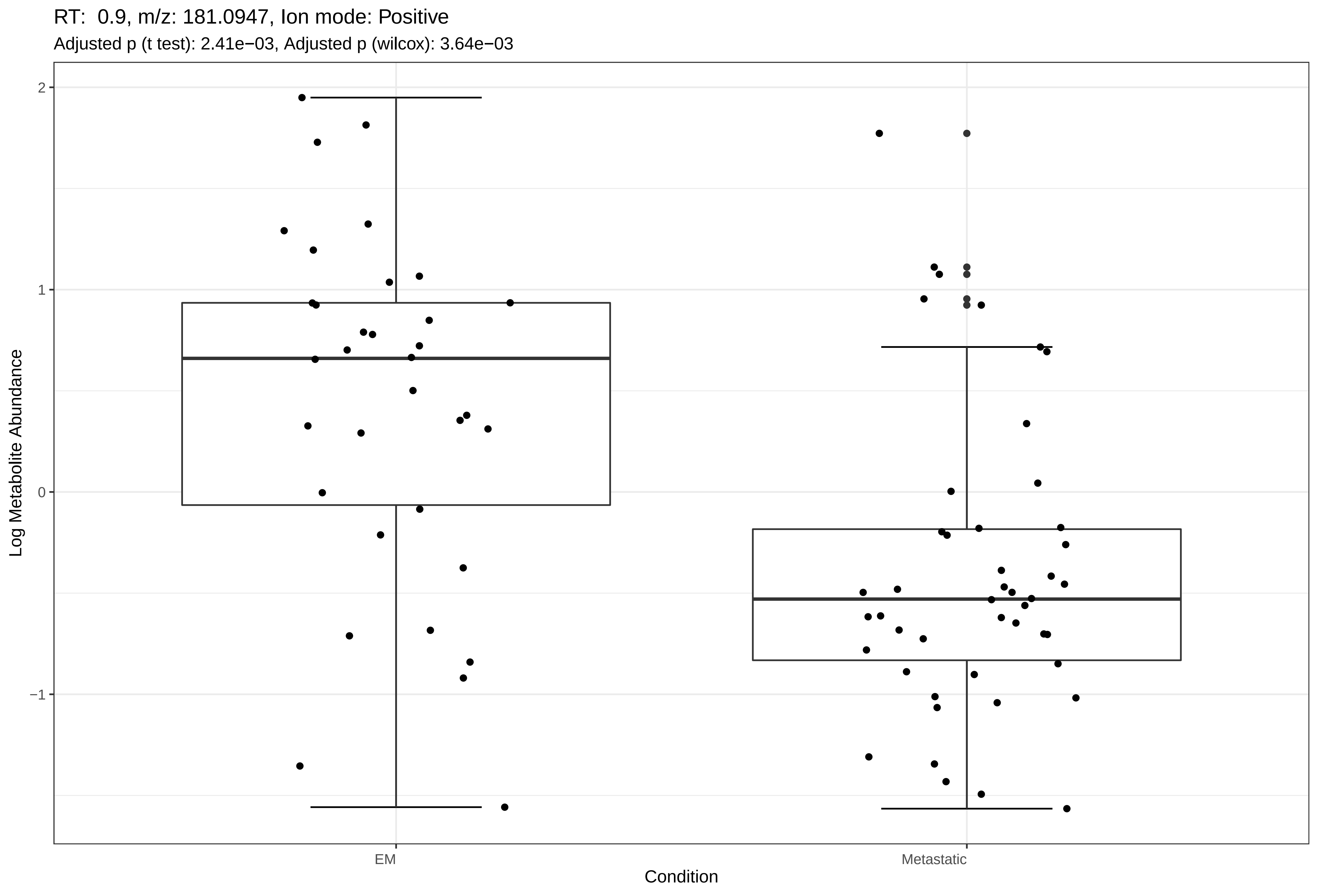

RT: 0.9, m/z: 181.0947, Ion mode: Positive
Adjusted p (t test): 2.41e−03, Adjusted p (wilcox): 3.64e−03
Log Metabolite Abundance
2
1
0
−1
EM
Metastatic
Condition

RT: 2.8, m/z: 96.9598, Ion mode: Negative

Adjusted p (t test): 5.69e−03, Adjusted p (wilcox): 2.43e−03

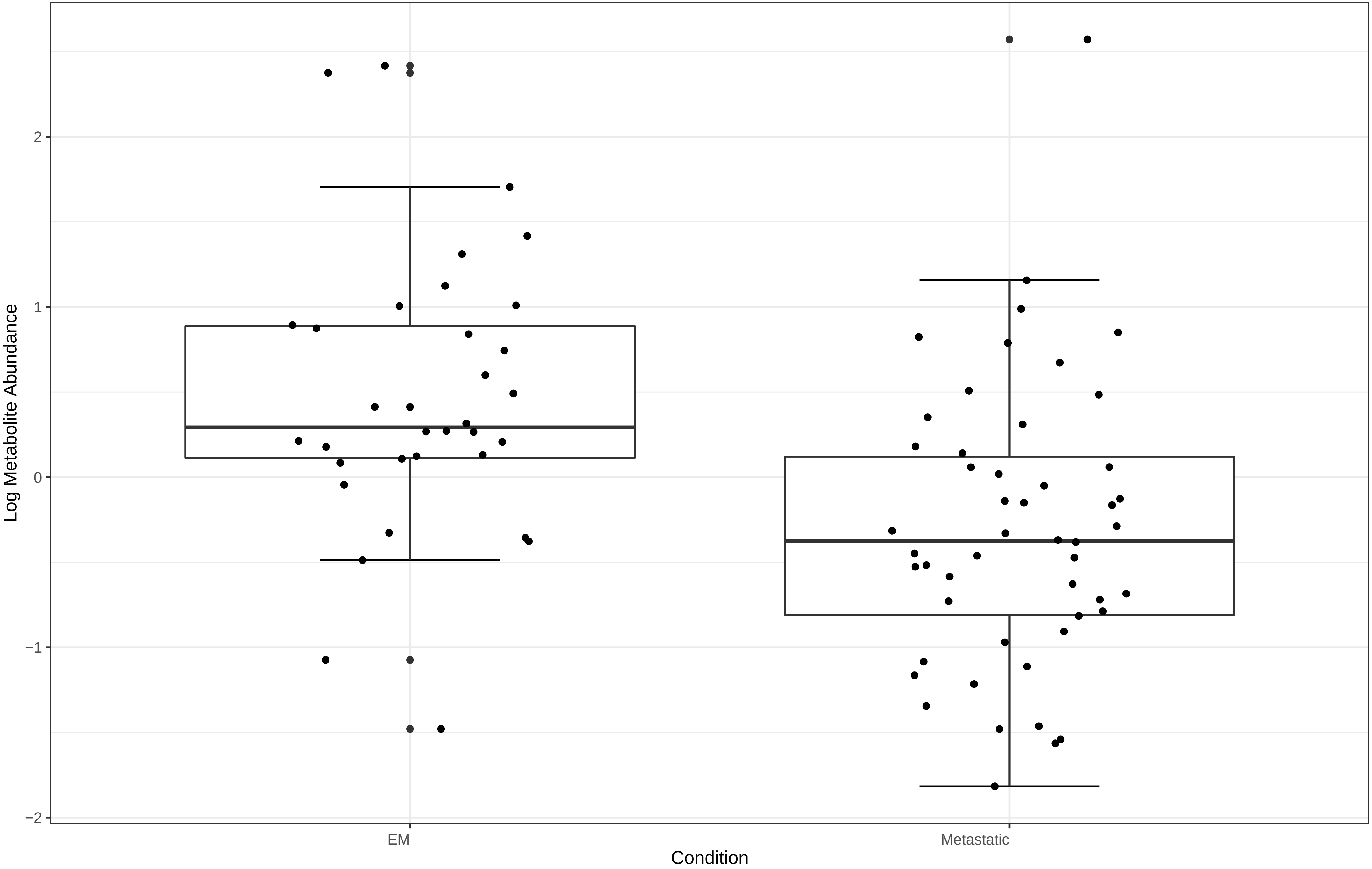

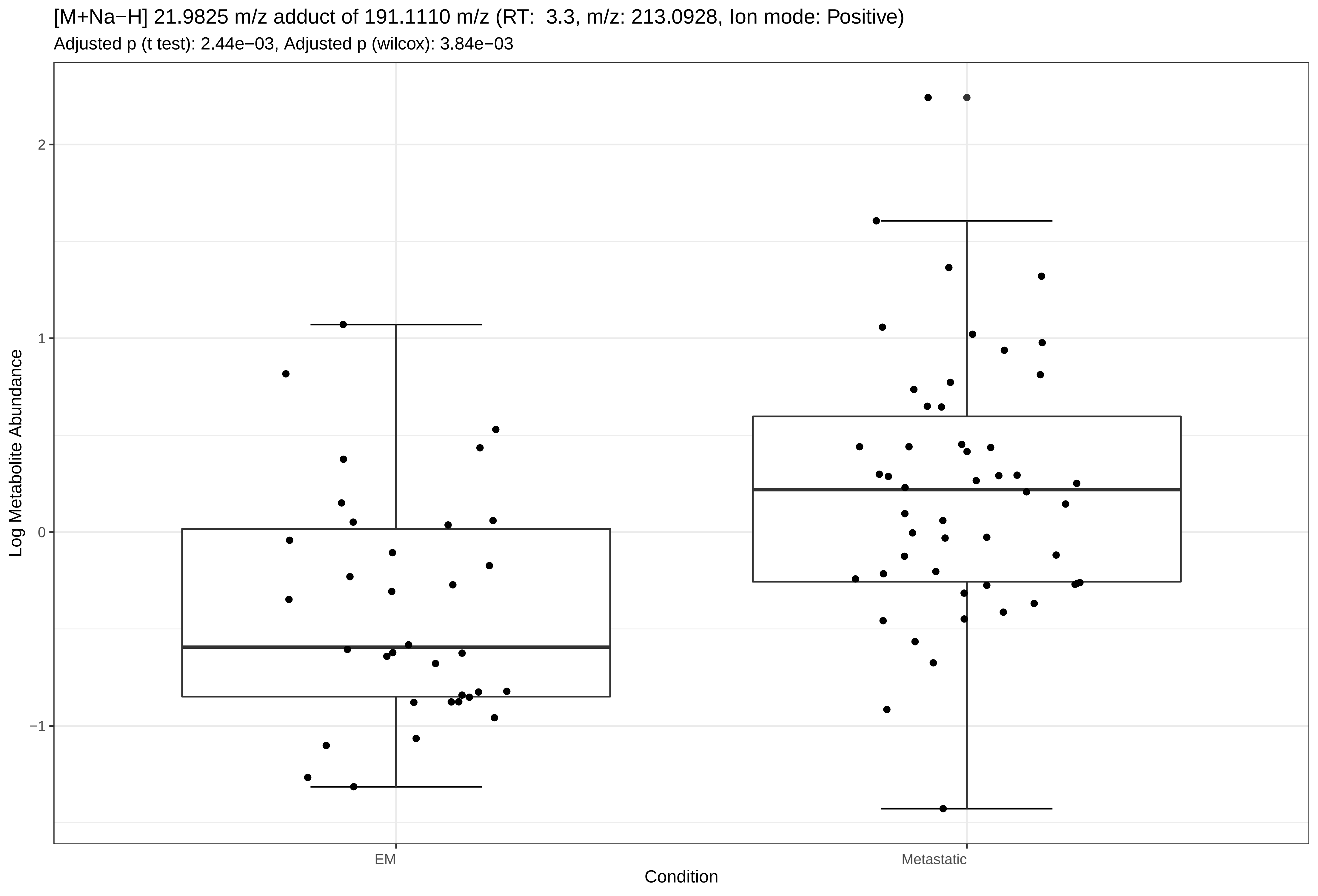

[M+Na−H] 21.9825 m/z adduct of 191.1110 m/z (RT: 3.3, m/z: 213.0928, Ion mode: Positive)
Adjusted p (t test): 2.44e−03, Adjusted p (wilcox): 3.84e−03
Log Metabolite Abundance
2
1
0
−1
EM
Metastatic
Condition

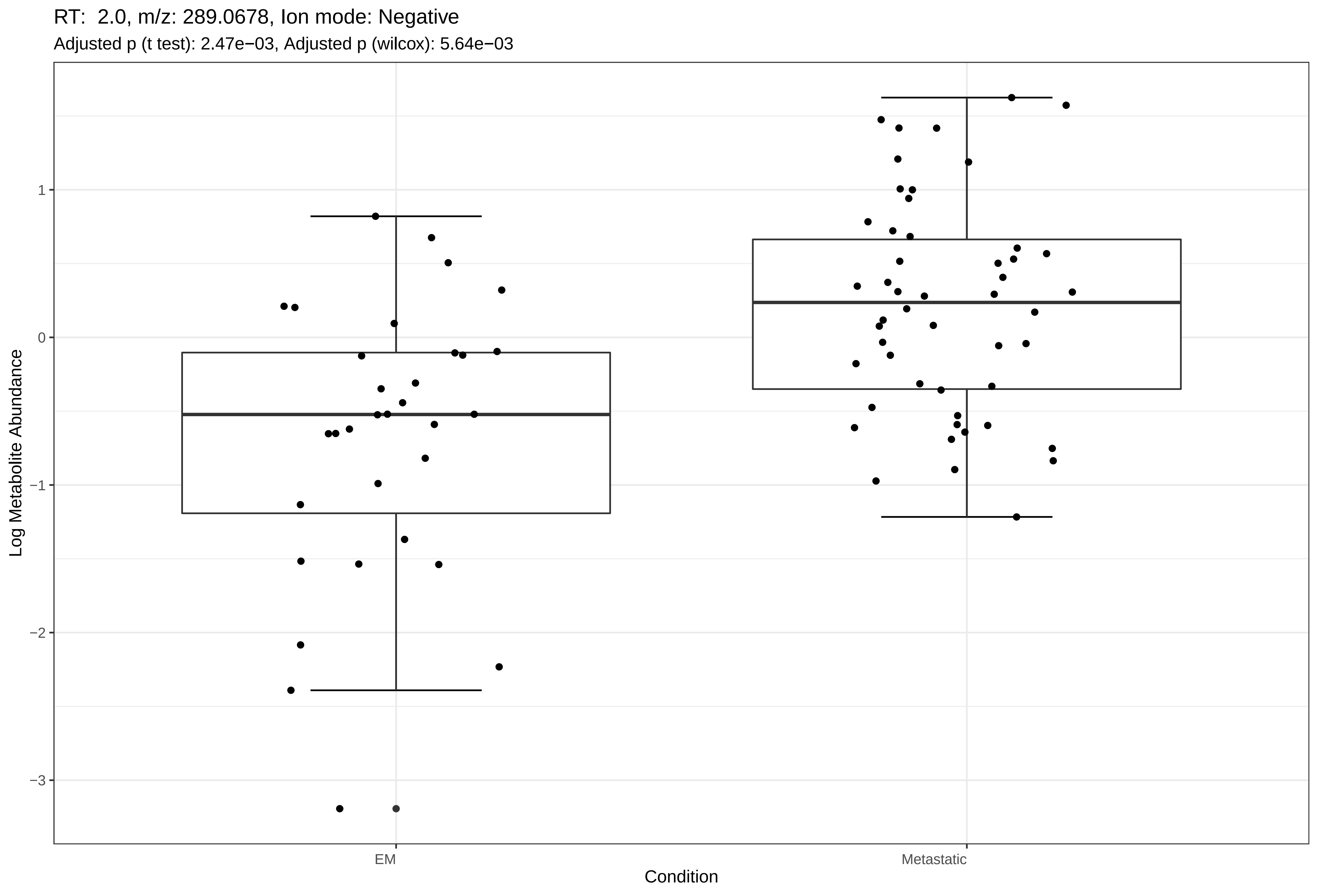
RT: 2.0, m/z: 289.0678, Ion mode: Negative
Adjusted p (t test): 2.47e−03, Adjusted p (wilcox): 5.64e−03
Log Metabolite Abundance
1
0
−1
−2
−3
EM
Metastatic
Condition

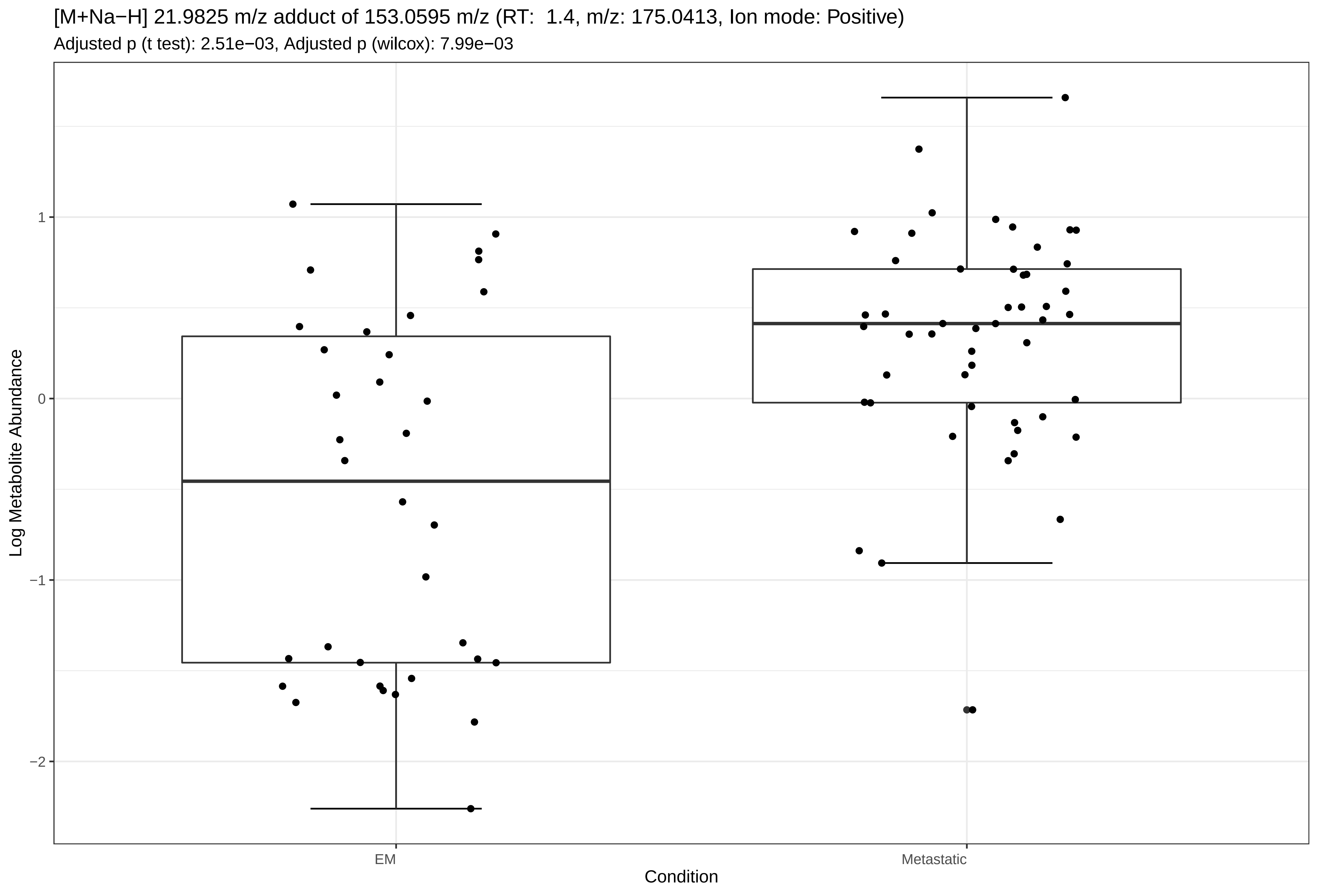
[M+Na−H] 21.9825 m/z adduct of 153.0595 m/z (RT: 1.4, m/z: 175.0413, Ion mode: Positive)
Adjusted p (t test): 2.51e−03, Adjusted p (wilcox): 7.99e−03
Log Metabolite Abundance
1
0
−1
−2
EM
Metastatic
Condition

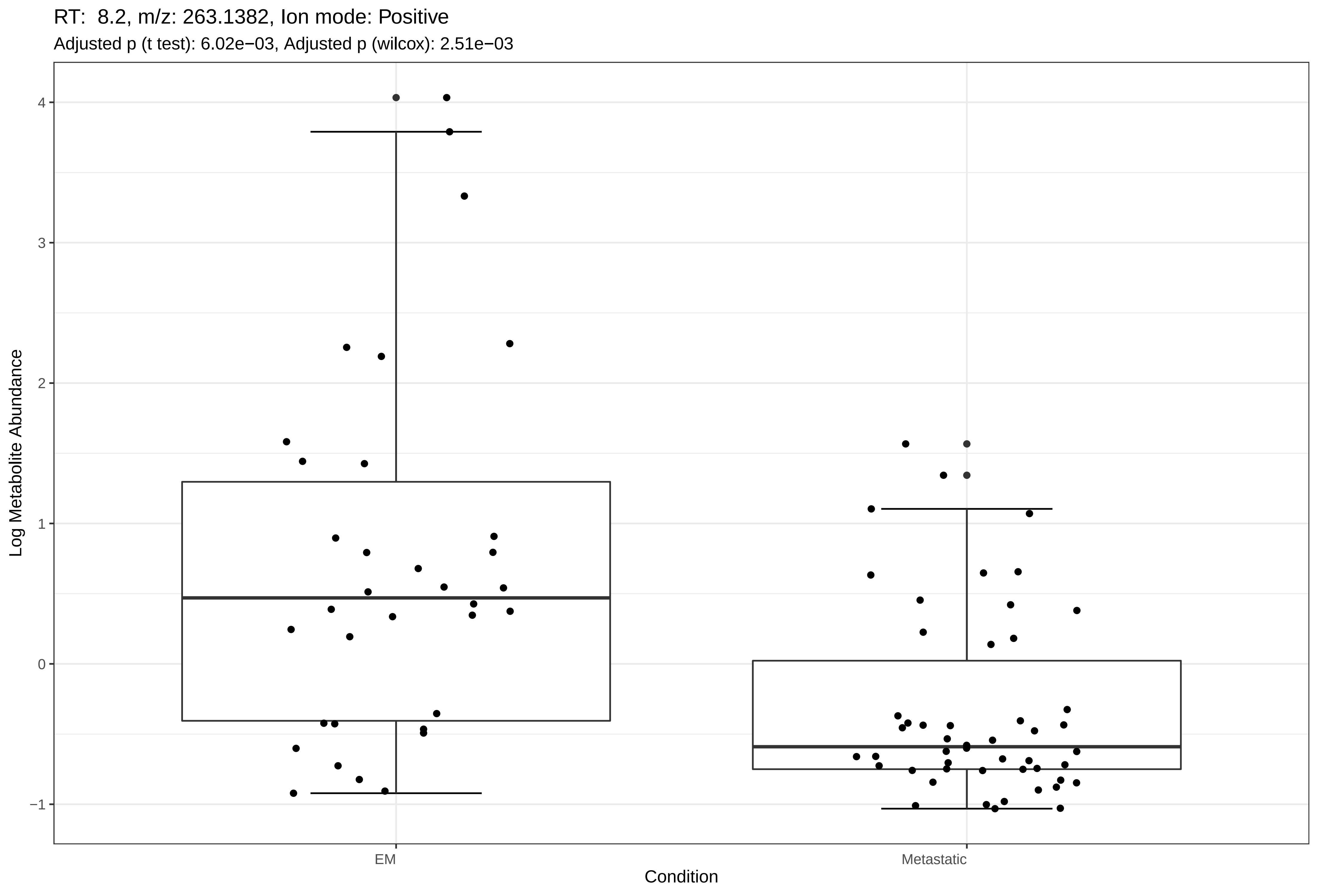

RT: 8.2, m/z: 263.1382, Ion mode: Positive
Adjusted p (t test): 6.02e−03, Adjusted p (wilcox): 2.51e−03
Log Metabolite Abundance
4
3
2
1
0
−1
EM
Metastatic
Condition

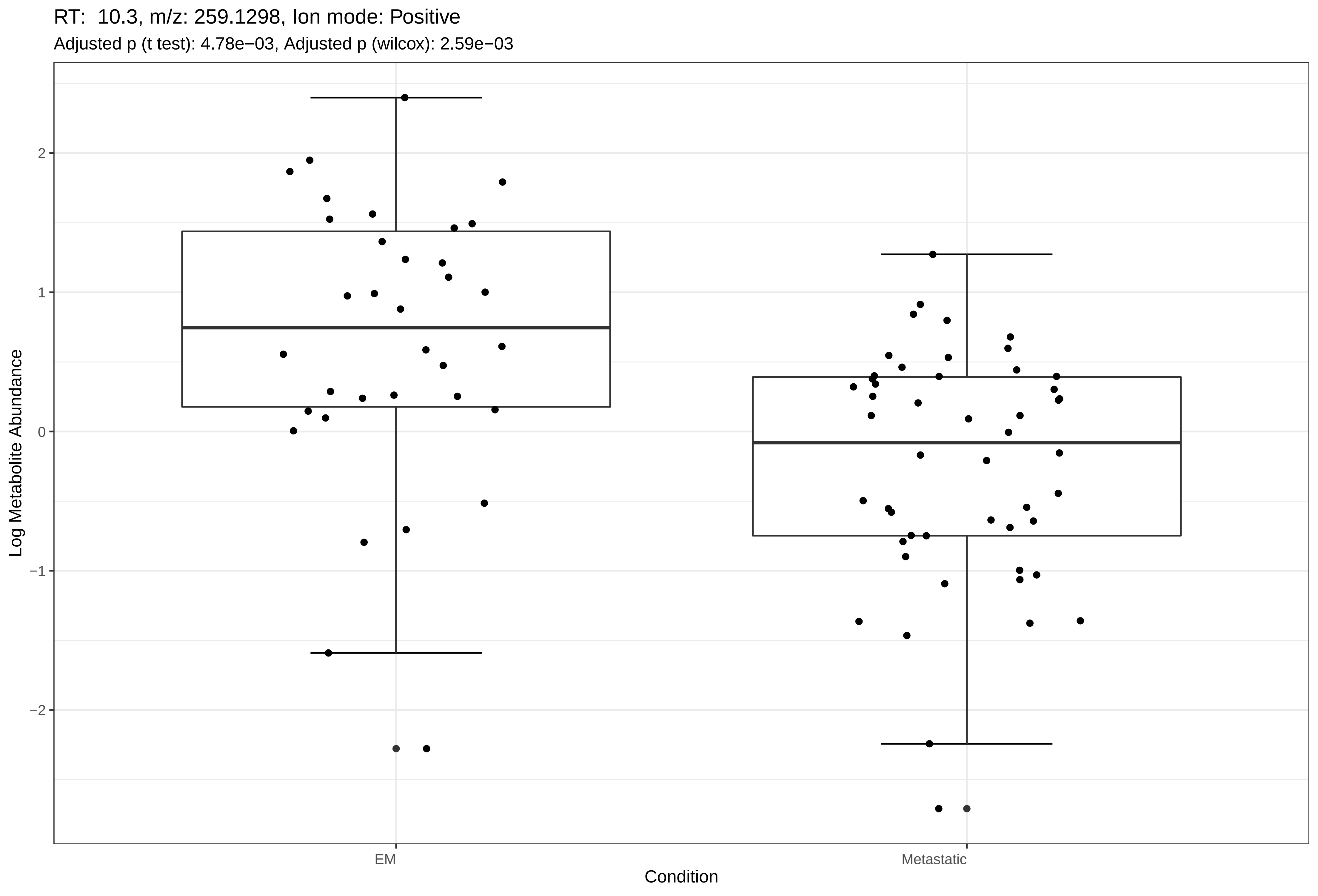
RT: 10.3, m/z: 259.1298, Ion mode: Positive
Adjusted p (t test): 4.78e−03, Adjusted p (wilcox): 2.59e−03
Log Metabolite Abundance
2
1
0
−1
−2
EM
Metastatic
Condition

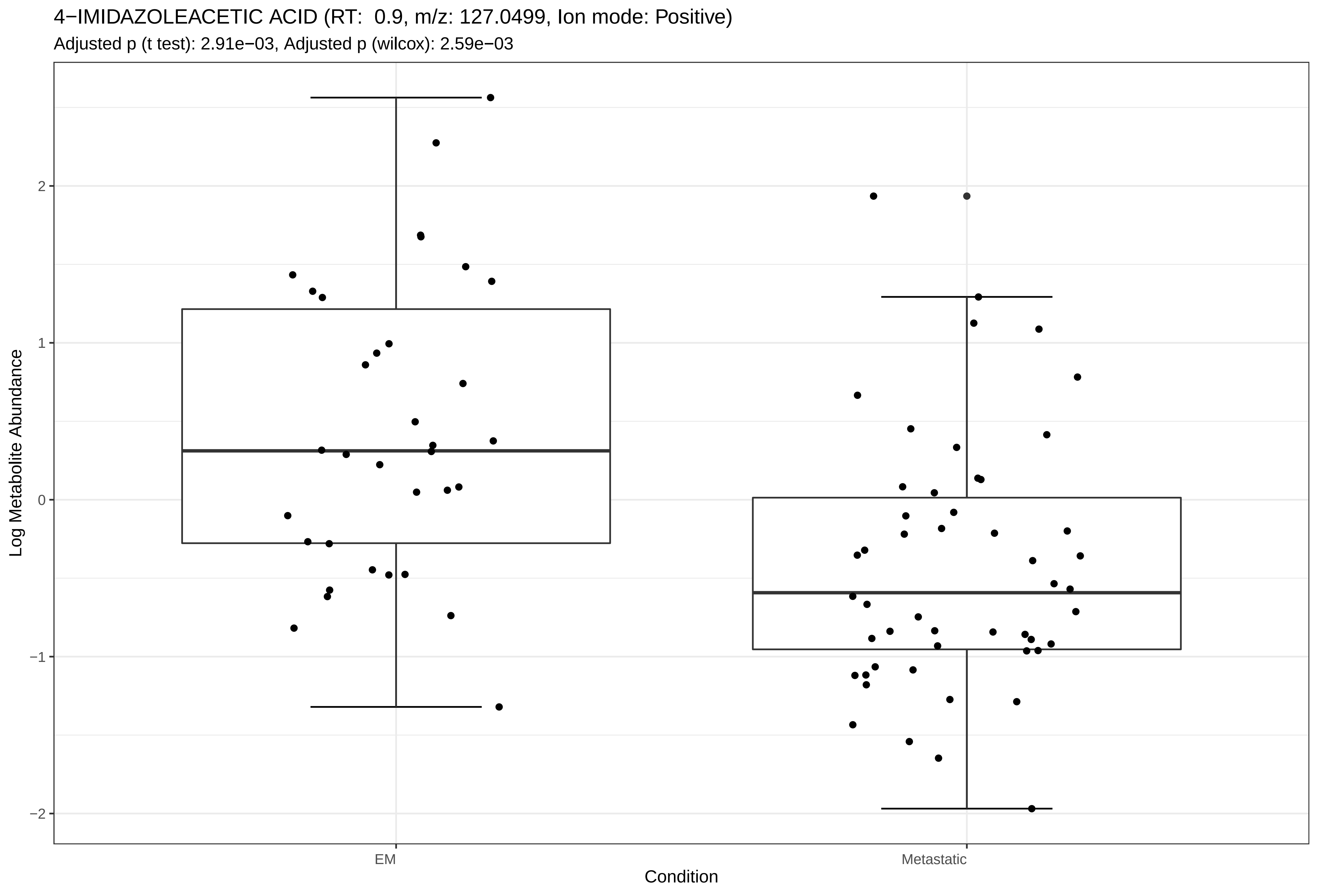
4−IMIDAZOLEACETIC ACID (RT: 0.9, m/z: 127.0499, Ion mode: Positive)
Adjusted p (t test): 2.91e−03, Adjusted p (wilcox): 2.59e−03
Log Metabolite Abundance
2
1
0
−1
−2
EM
Metastatic
Condition

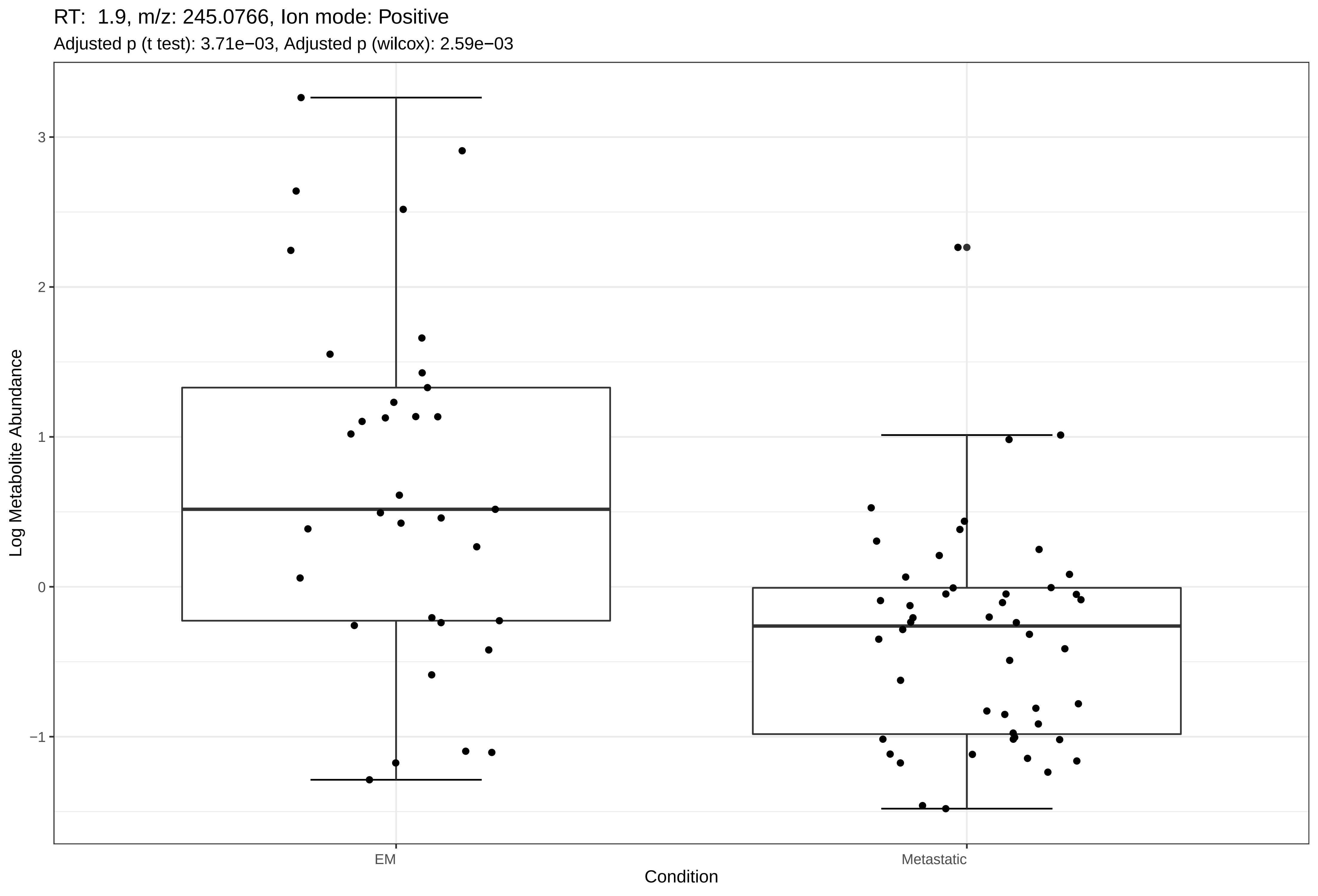

RT: 1.9, m/z: 245.0766, Ion mode: Positive
Adjusted p (t test): 3.71e−03, Adjusted p (wilcox): 2.59e−03
Log Metabolite Abundance
3
2
1
0
−1
EM
Metastatic
Condition

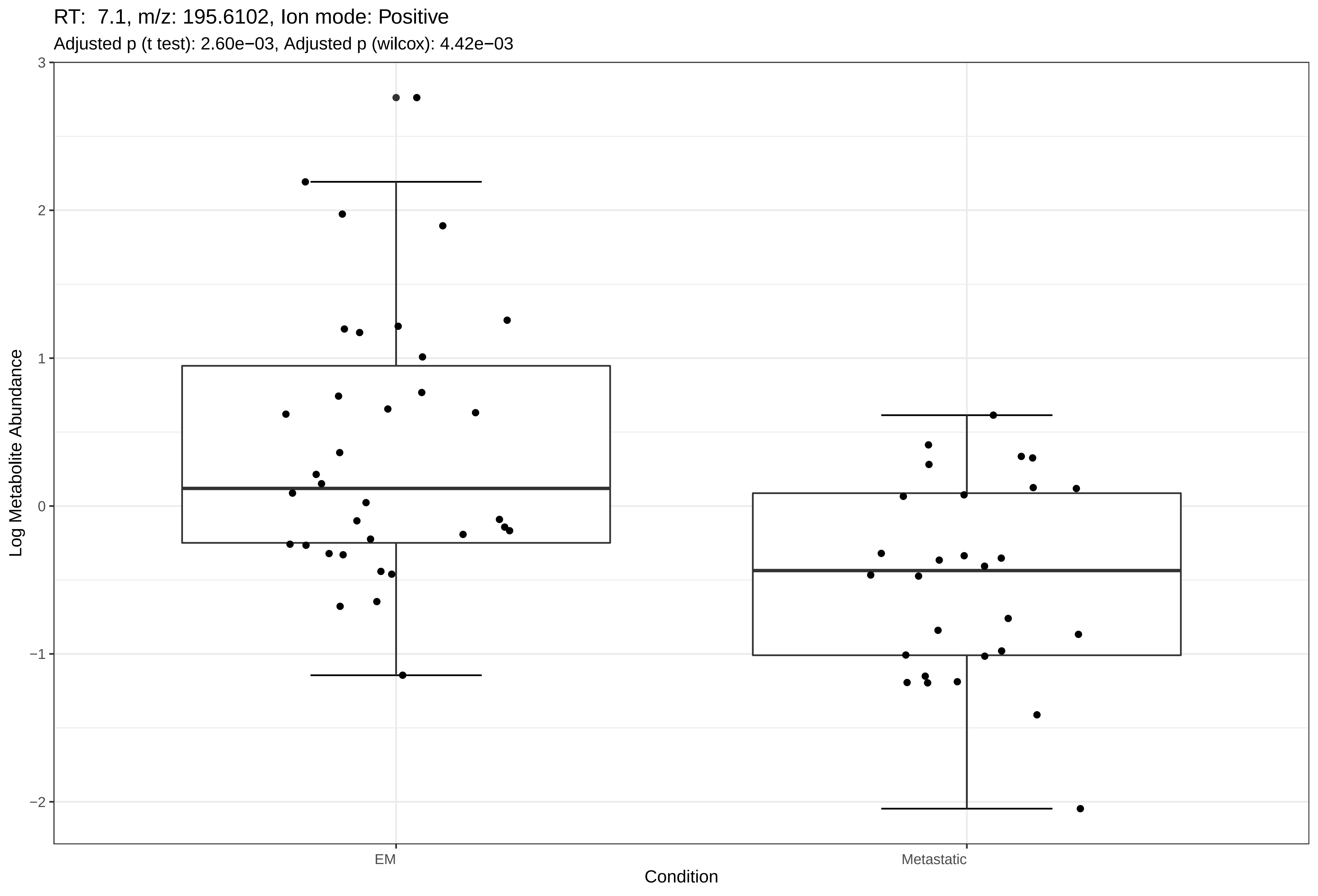
RT: 7.1, m/z: 195.6102, Ion mode: Positive
Adjusted p (t test): 2.60e−03, Adjusted p (wilcox): 4.42e−03
Log Metabolite Abundance
3
2
1
0
−1
−2
EM
Metastatic
Condition

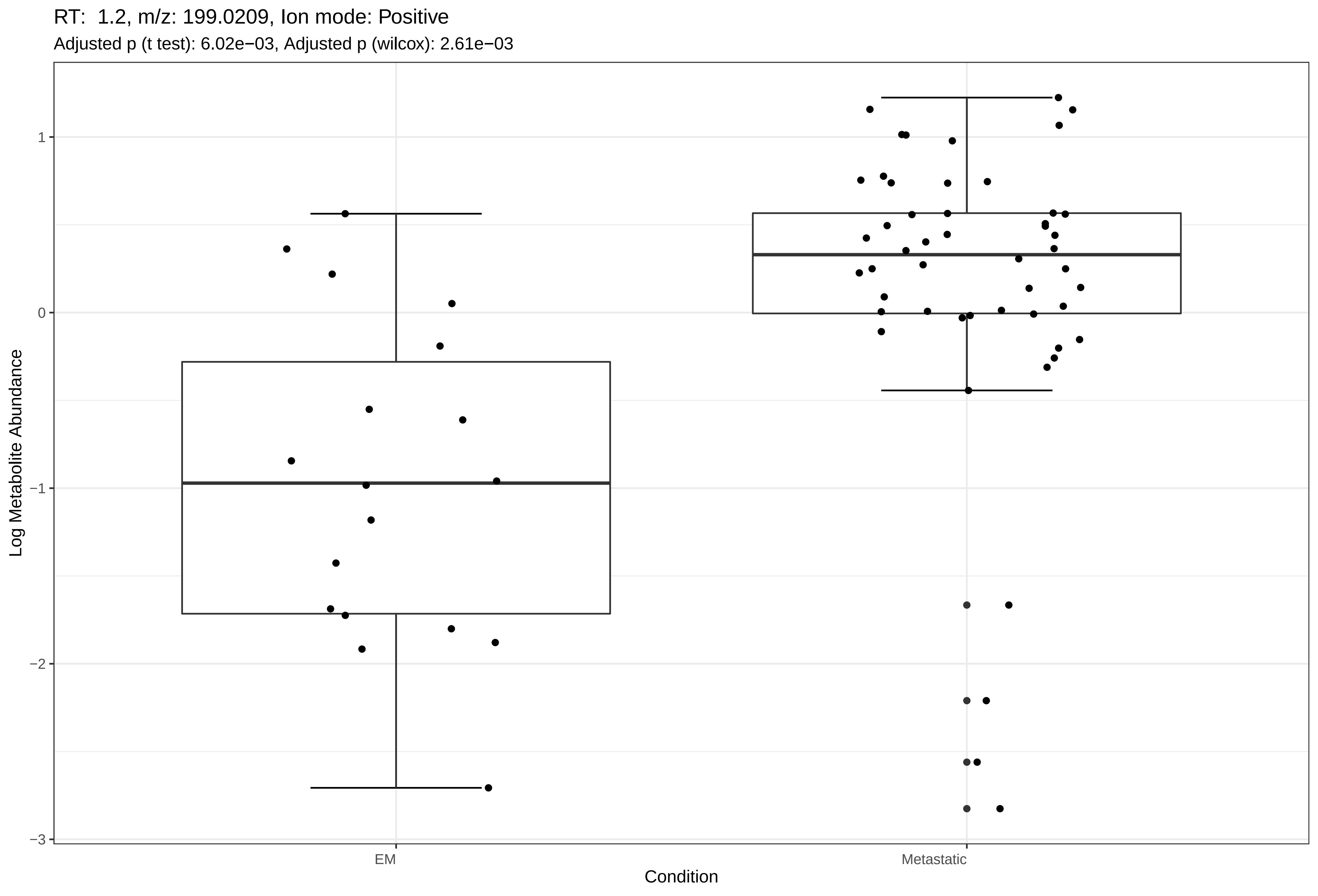

RT: 1.2, m/z: 199.0209, Ion mode: Positive
Adjusted p (t test): 6.02e−03, Adjusted p (wilcox): 2.61e−03
Log Metabolite Abundance
1
0
−1
−2
−3
EM
Metastatic
Condition

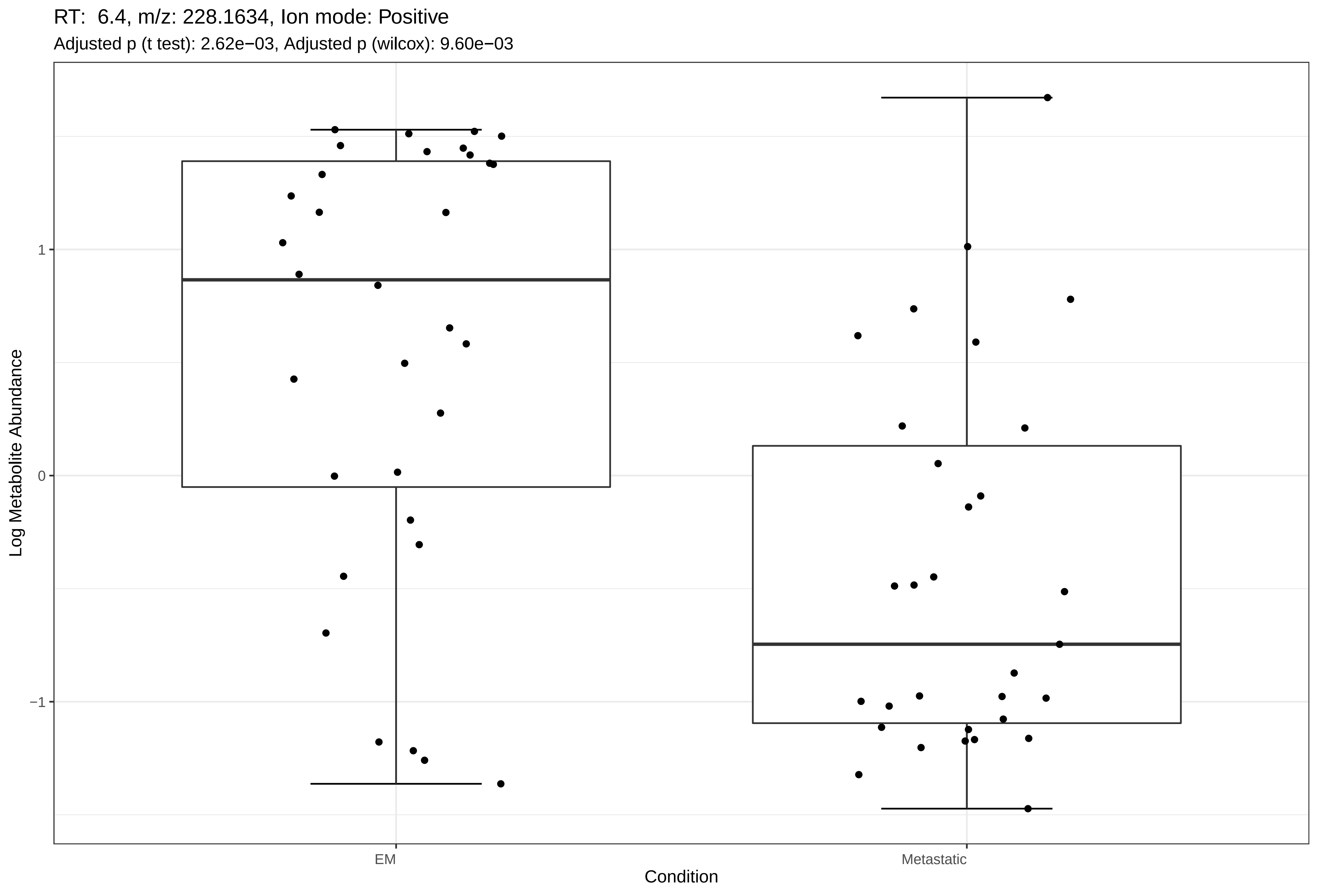

RT: 6.4, m/z: 228.1634, Ion mode: Positive
Adjusted p (t test): 2.62e−03, Adjusted p (wilcox): 9.60e−03
Log Metabolite Abundance
1
0
−1
EM
Metastatic
Condition

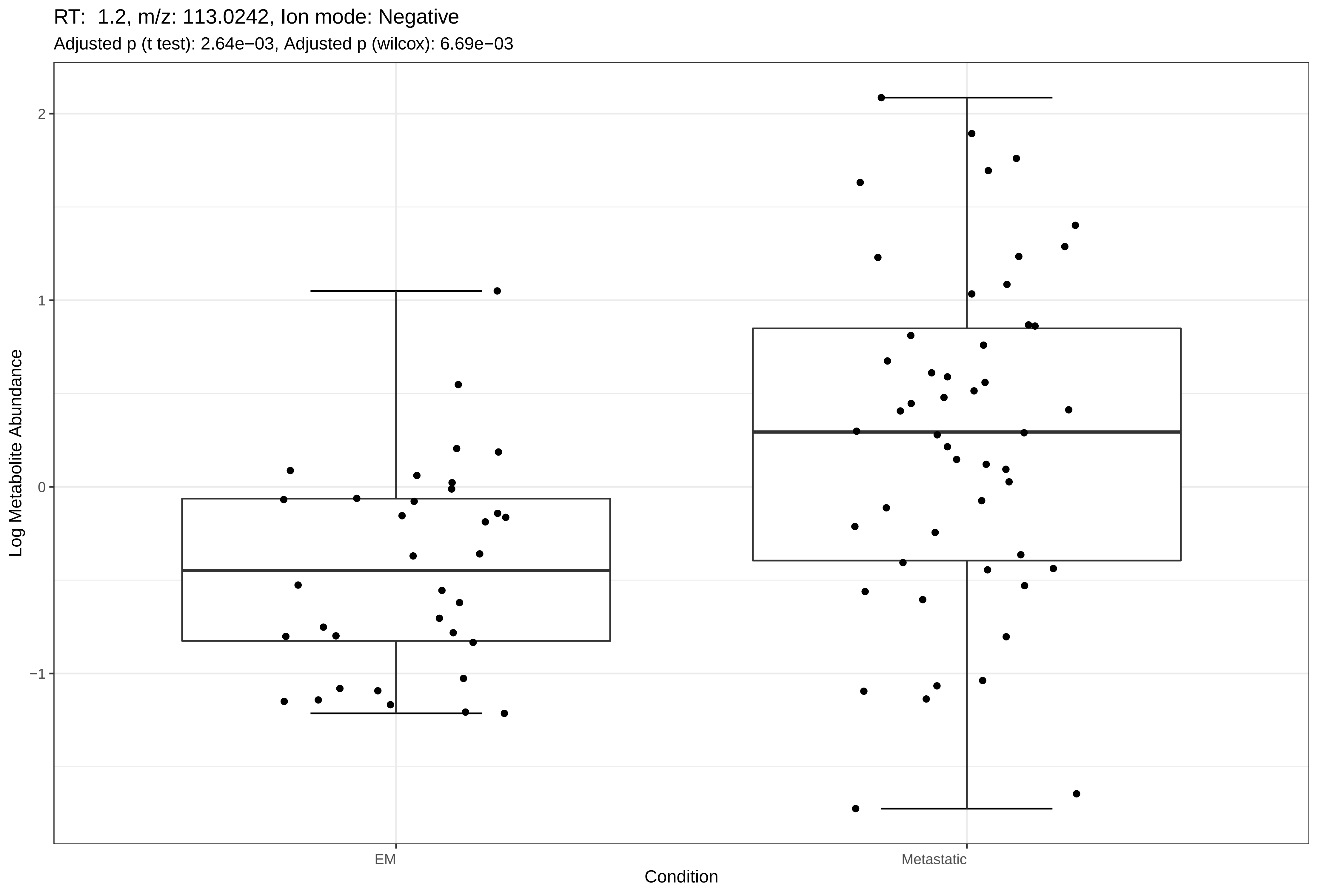

RT: 1.2, m/z: 113.0242, Ion mode: Negative
Adjusted p (t test): 2.64e−03, Adjusted p (wilcox): 6.69e−03
Log Metabolite Abundance
2
1
0
−1
EM
Metastatic
Condition

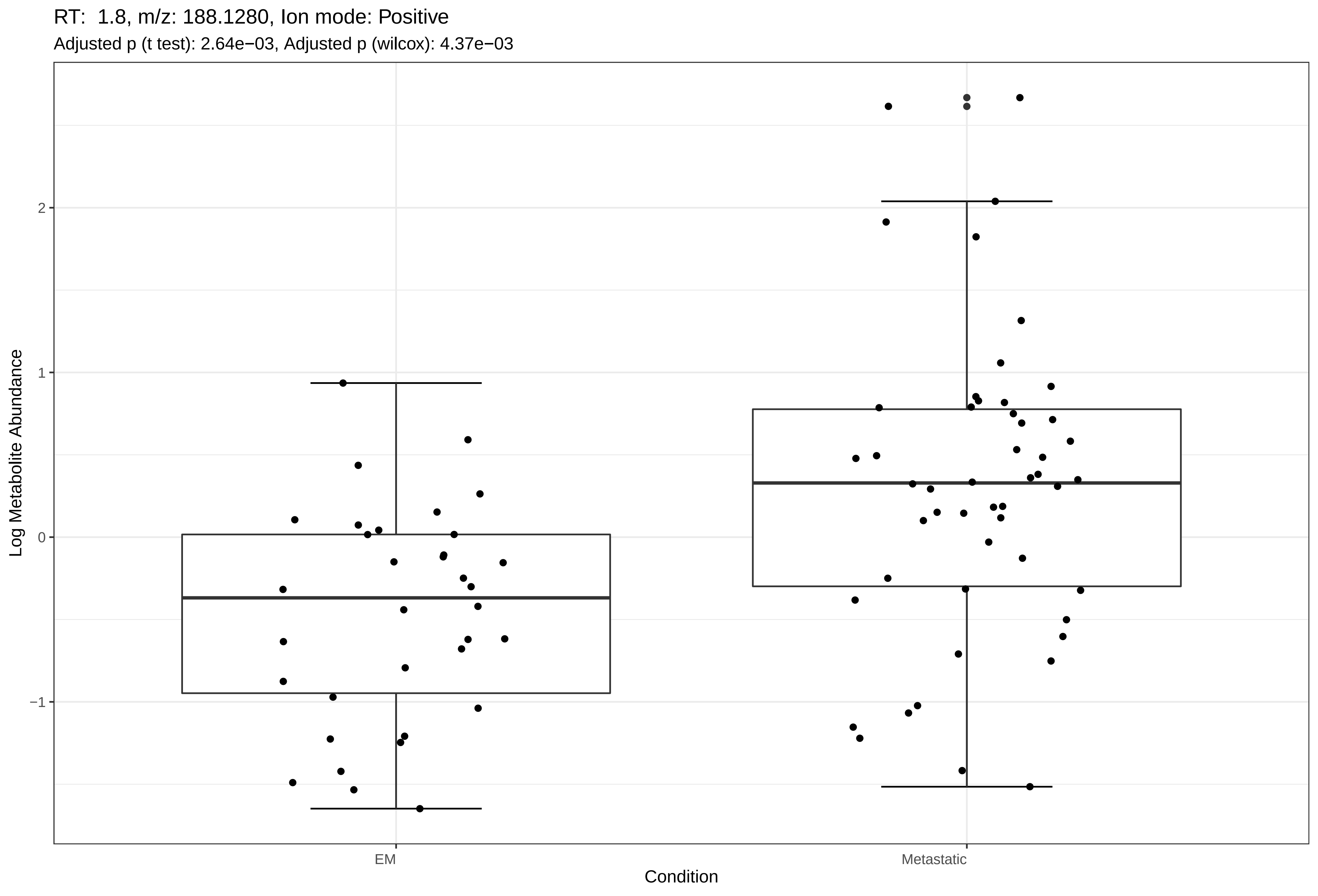
RT: 1.8, m/z: 188.1280, Ion mode: Positive
Adjusted p (t test): 2.64e−03, Adjusted p (wilcox): 4.37e−03
Log Metabolite Abundance
2
1
0
−1
EM
Metastatic
Condition

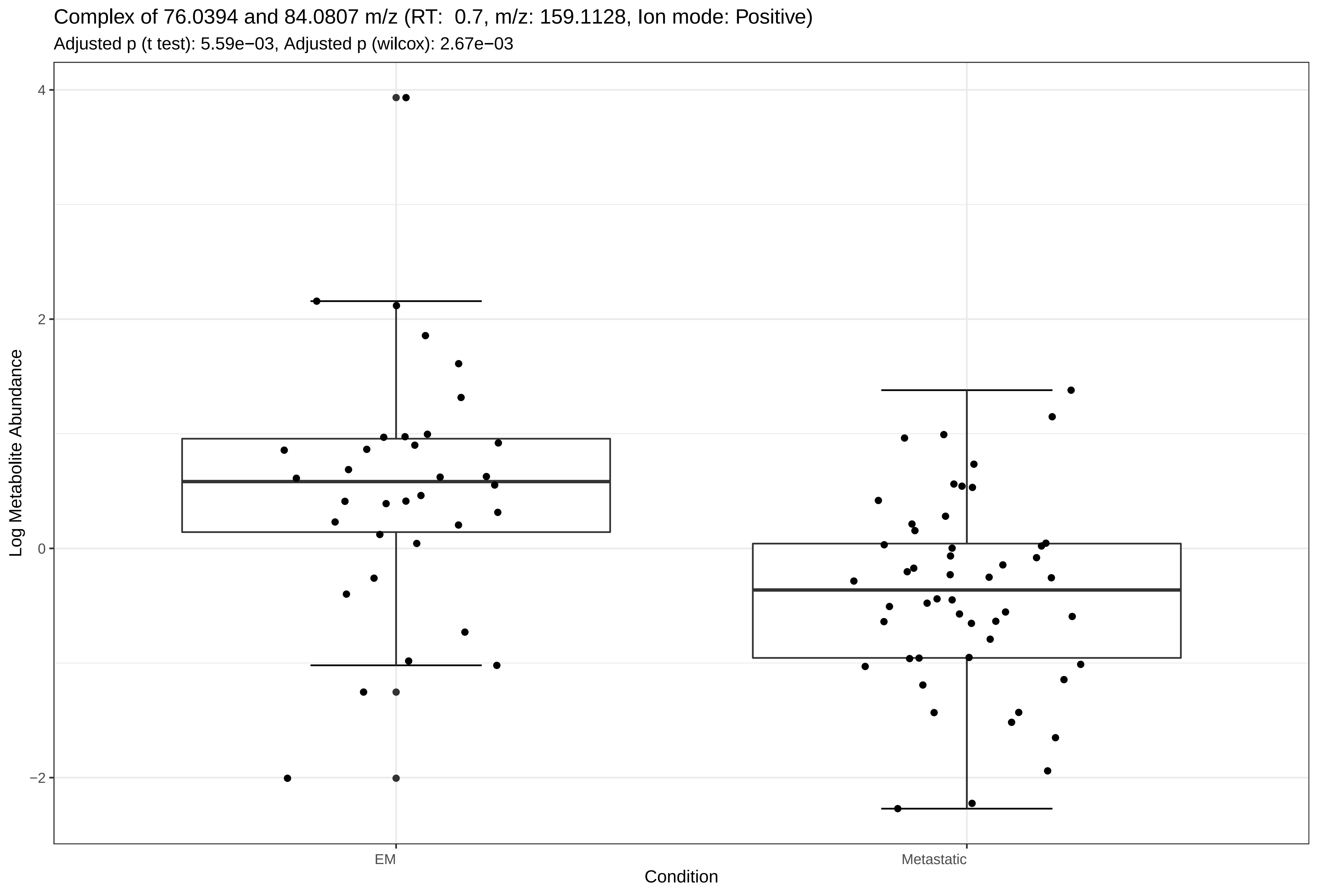

Complex of 76.0394 and 84.0807 m/z (RT: 0.7, m/z: 159.1128, Ion mode: Positive)
Adjusted p (t test): 5.59e−03, Adjusted p (wilcox): 2.67e−03
Log Metabolite Abundance
4
2
0
−2
EM
Metastatic
Condition

RT: 8.7, m/z: 266.2540, Ion mode: Positive

Adjusted p (t test): 8.02e−03, Adjusted p (wilcox): 2.67e−03

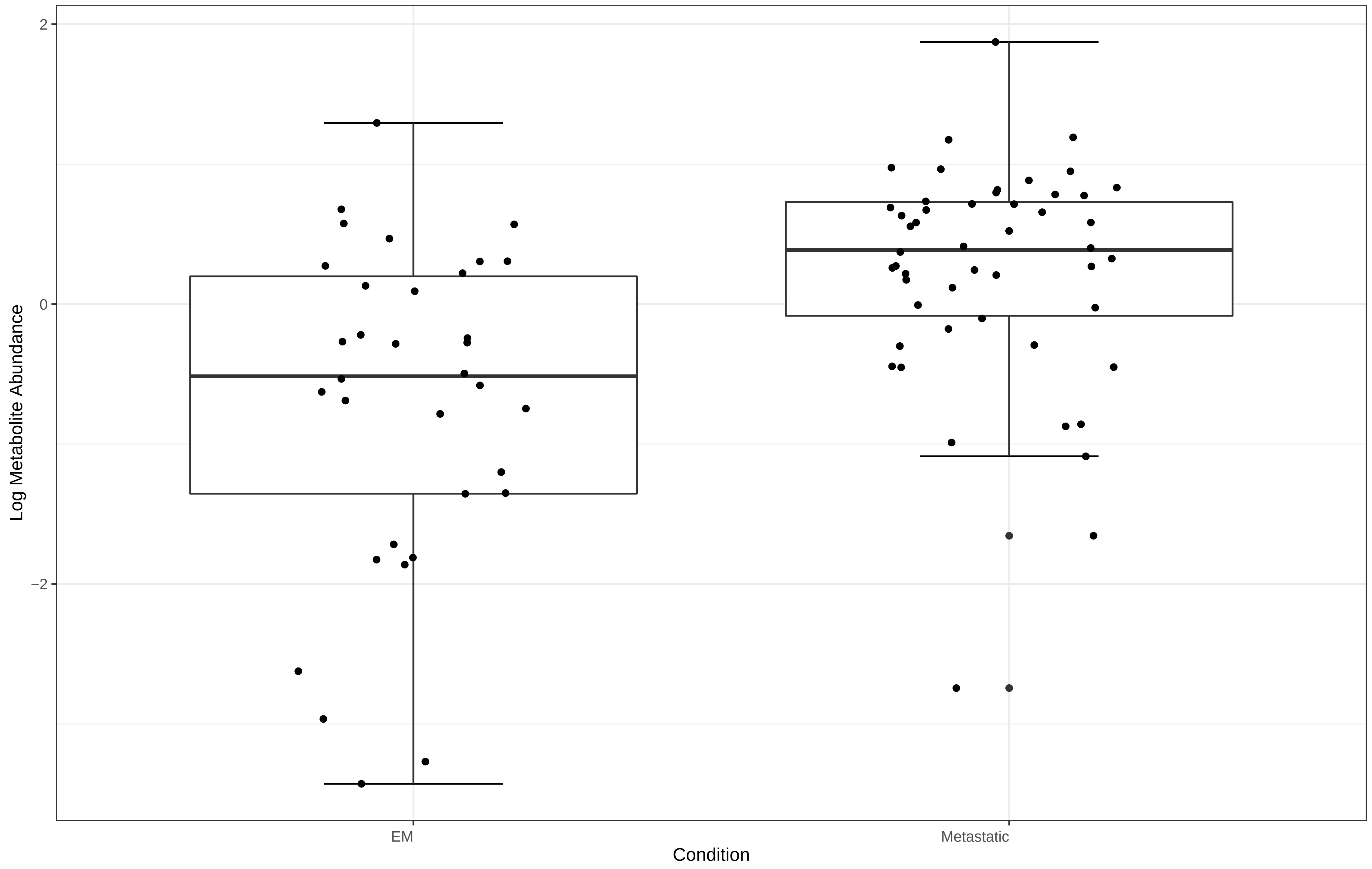

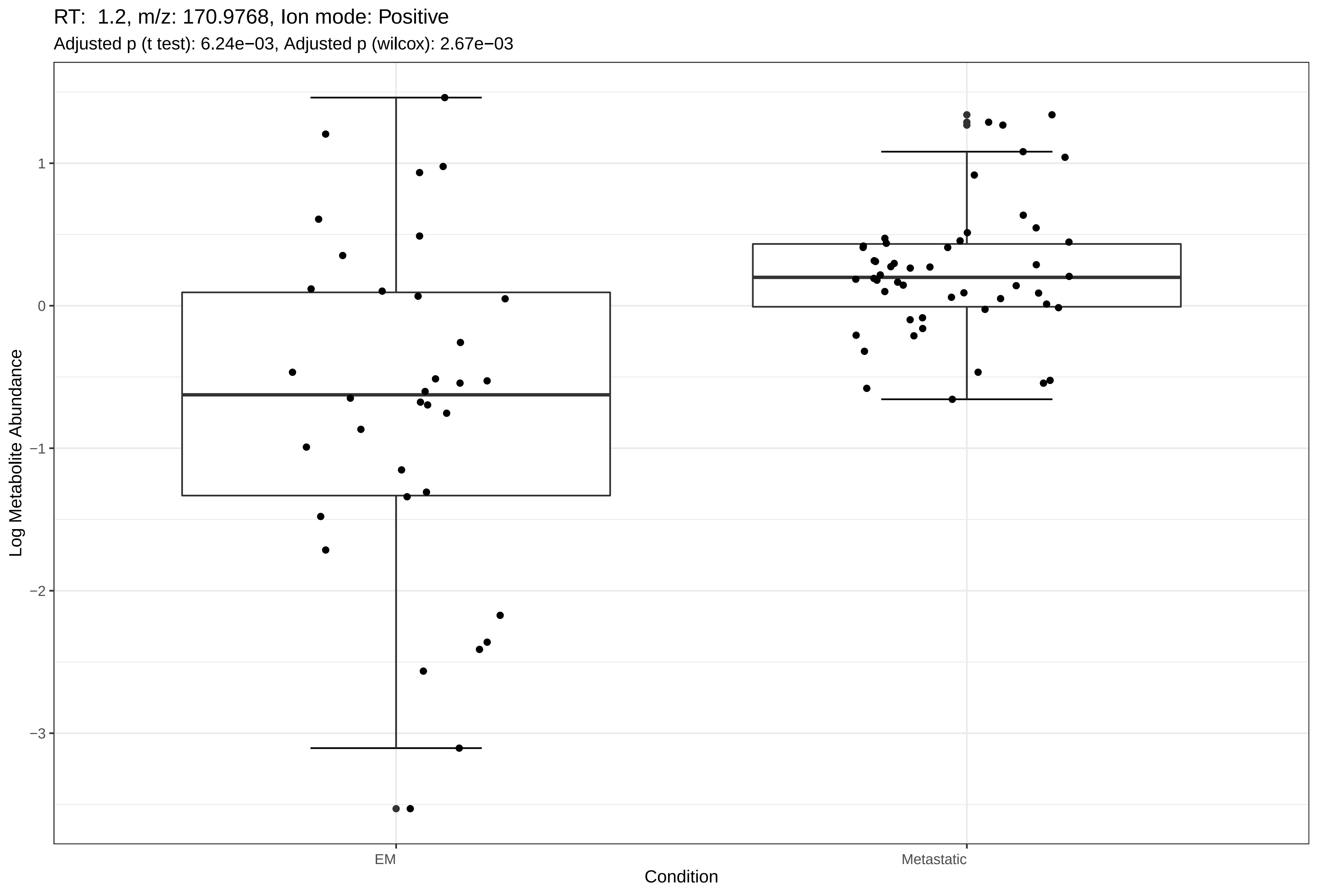

RT: 1.2, m/z: 170.9768, Ion mode: Positive
Adjusted p (t test): 6.24e−03, Adjusted p (wilcox): 2.67e−03
Log Metabolite Abundance
1
0
−1
−2
−3
EM
Metastatic
Condition

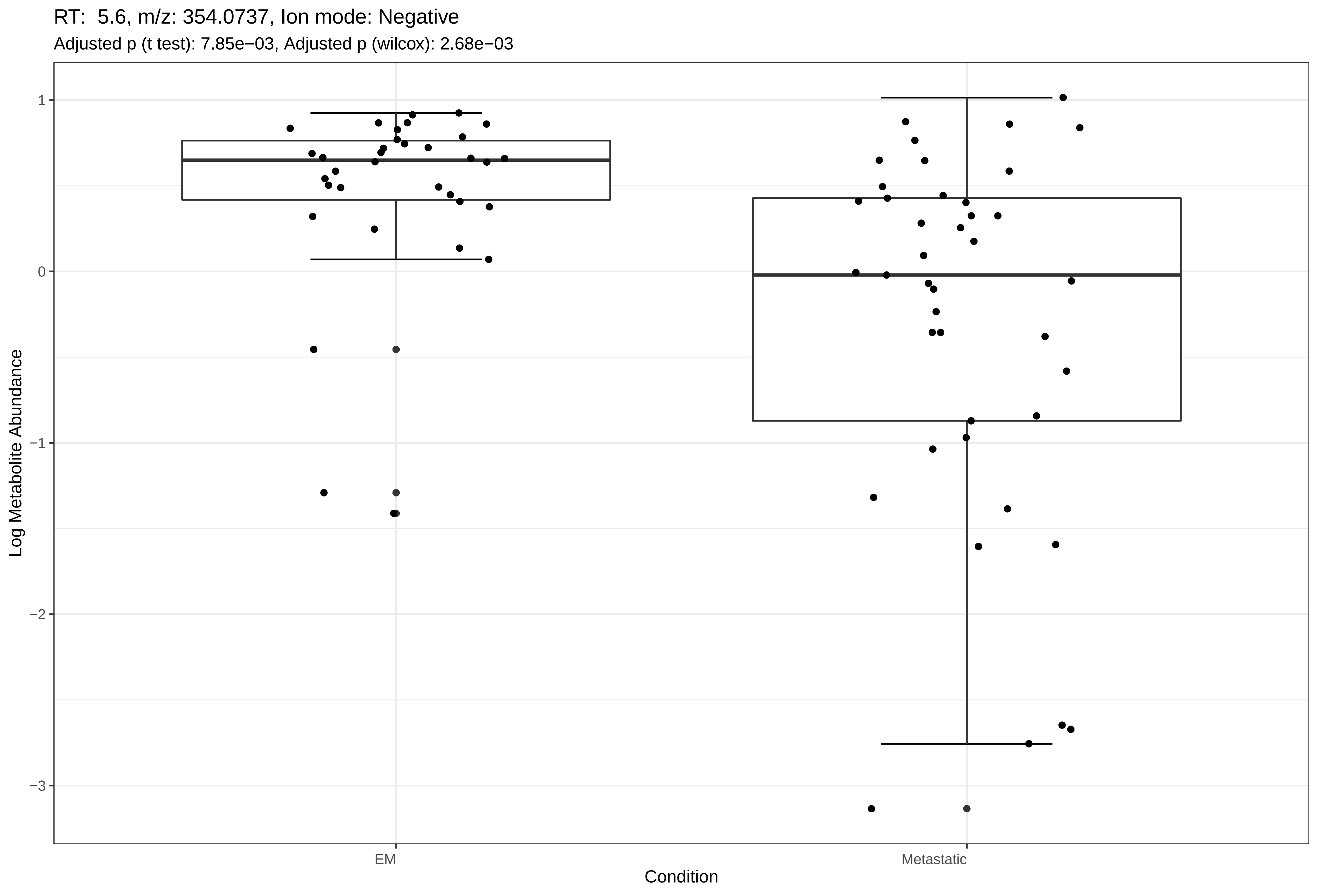

RT: 5.6, m/z: 354.0737, Ion mode: Negative
Adjusted p (t test): 7.85e−03, Adjusted p (wilcox): 2.68e−03
1
0
−1
−2
−3
Log Metabolite Abundance
EM
Metastatic
Condition

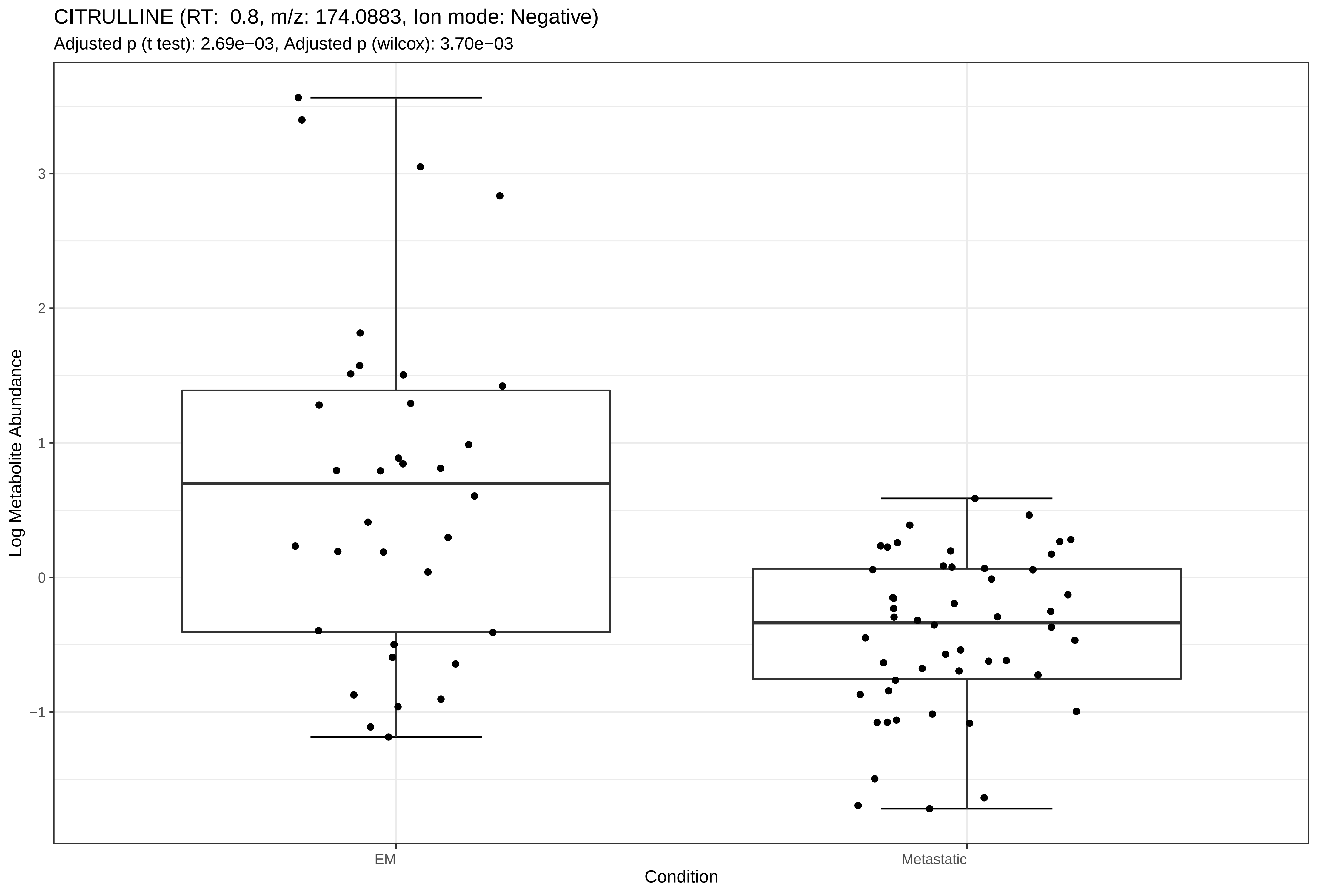
CITRULLINE (RT: 0.8, m/z: 174.0883, Ion mode: Negative)
Adjusted p (t test): 2.69e−03, Adjusted p (wilcox): 3.70e−03
Log Metabolite Abundance
3
2
1
0
−1
EM
Metastatic
Condition

[M−H2O+H] −18.0106 m/z adduct of 149.1170 m/z (RT: 7.5, m/z: 131.1066, Ion mode: Positive)

Adjusted p (t test): 2.74e−03, Adjusted p (wilcox): 4.80e−03

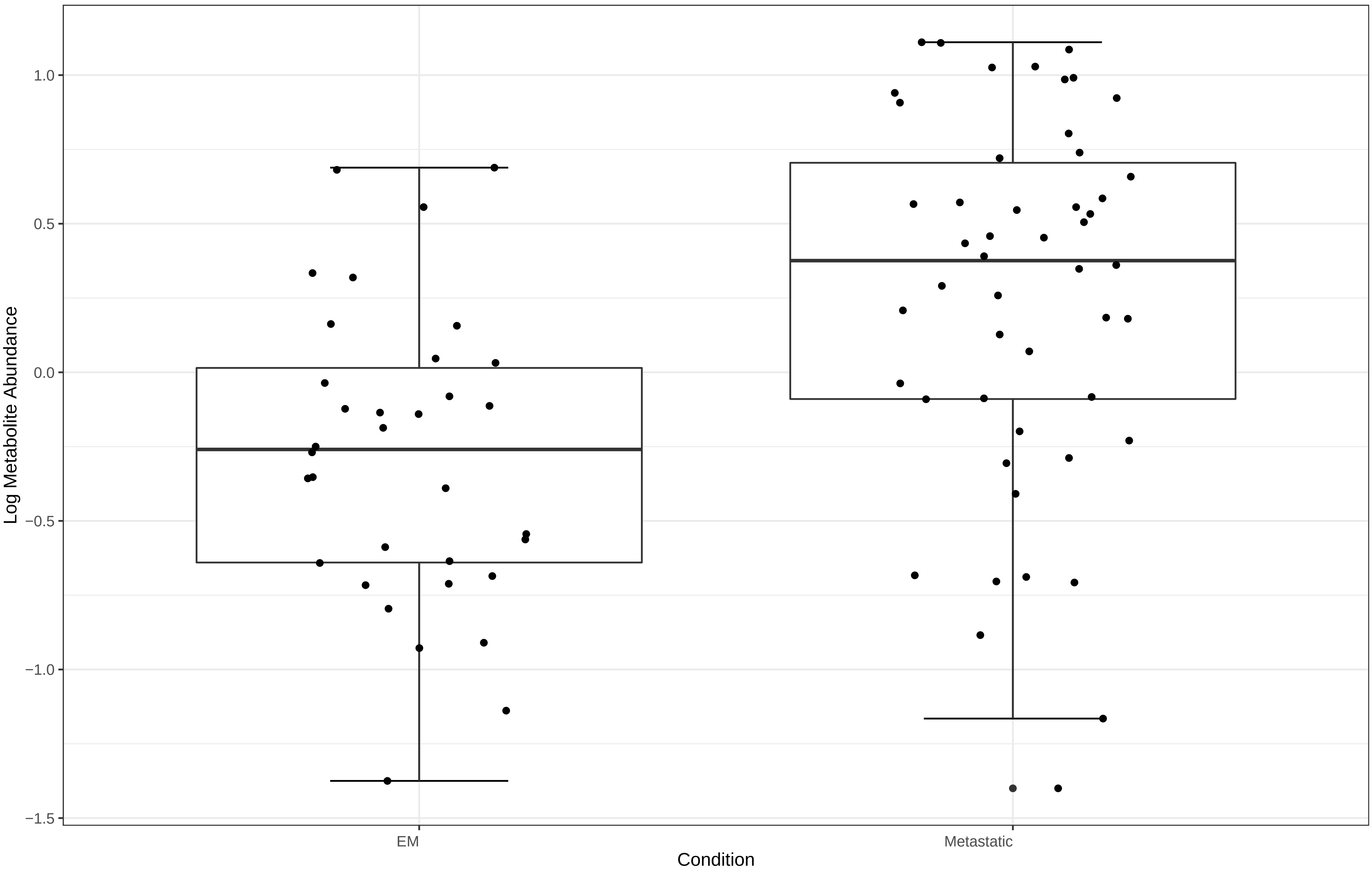

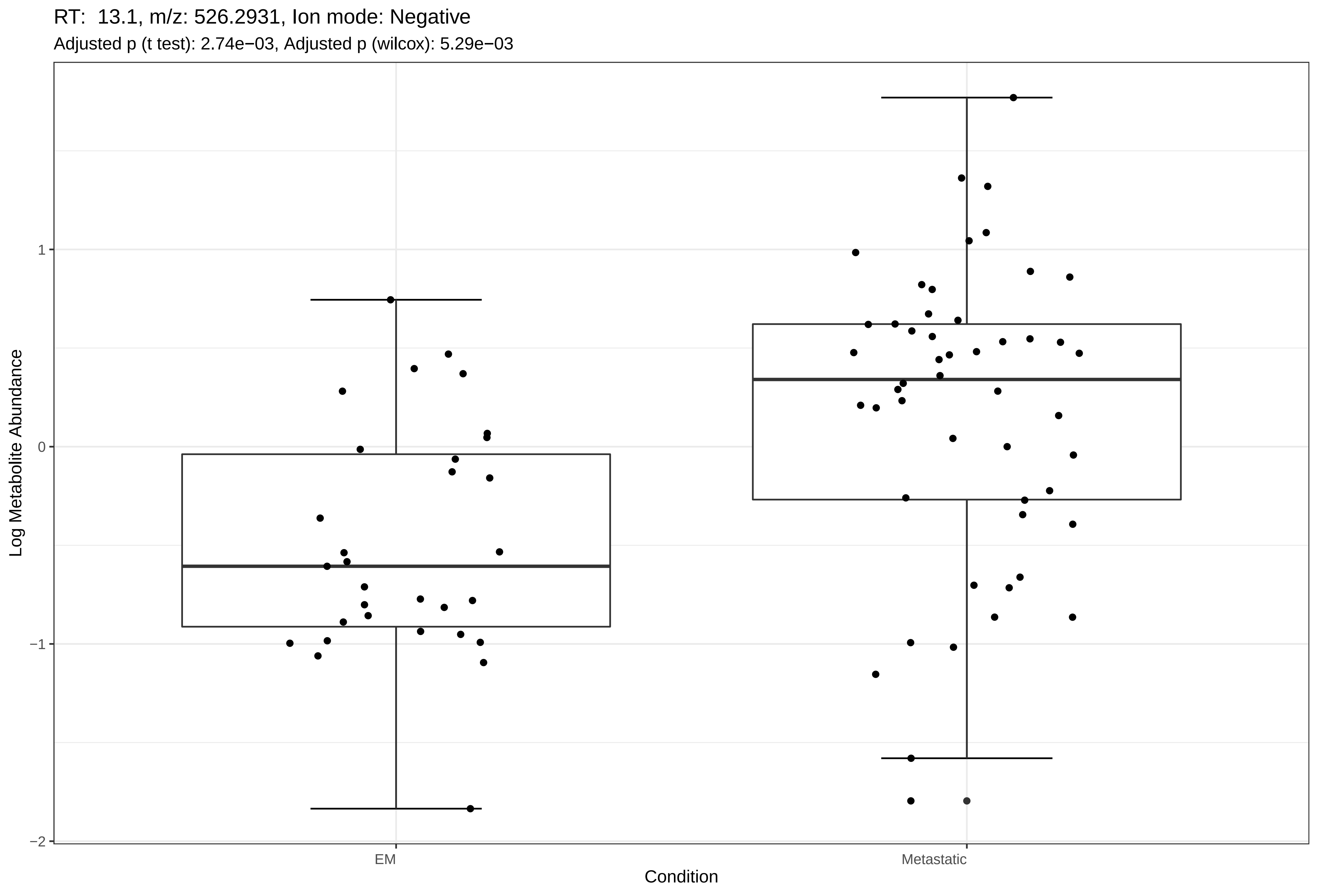
RT: 13.1, m/z: 526.2931, Ion mode: Negative
Adjusted p (t test): 2.74e−03, Adjusted p (wilcox): 5.29e−03
Log Metabolite Abundance
1
0
−1
−2
EM
Metastatic
Condition

RT: 0.7, m/z: 113.0174, Ion mode: Negative

Adjusted p (t test): 2.74e−03, Adjusted p (wilcox): 2.85e−03

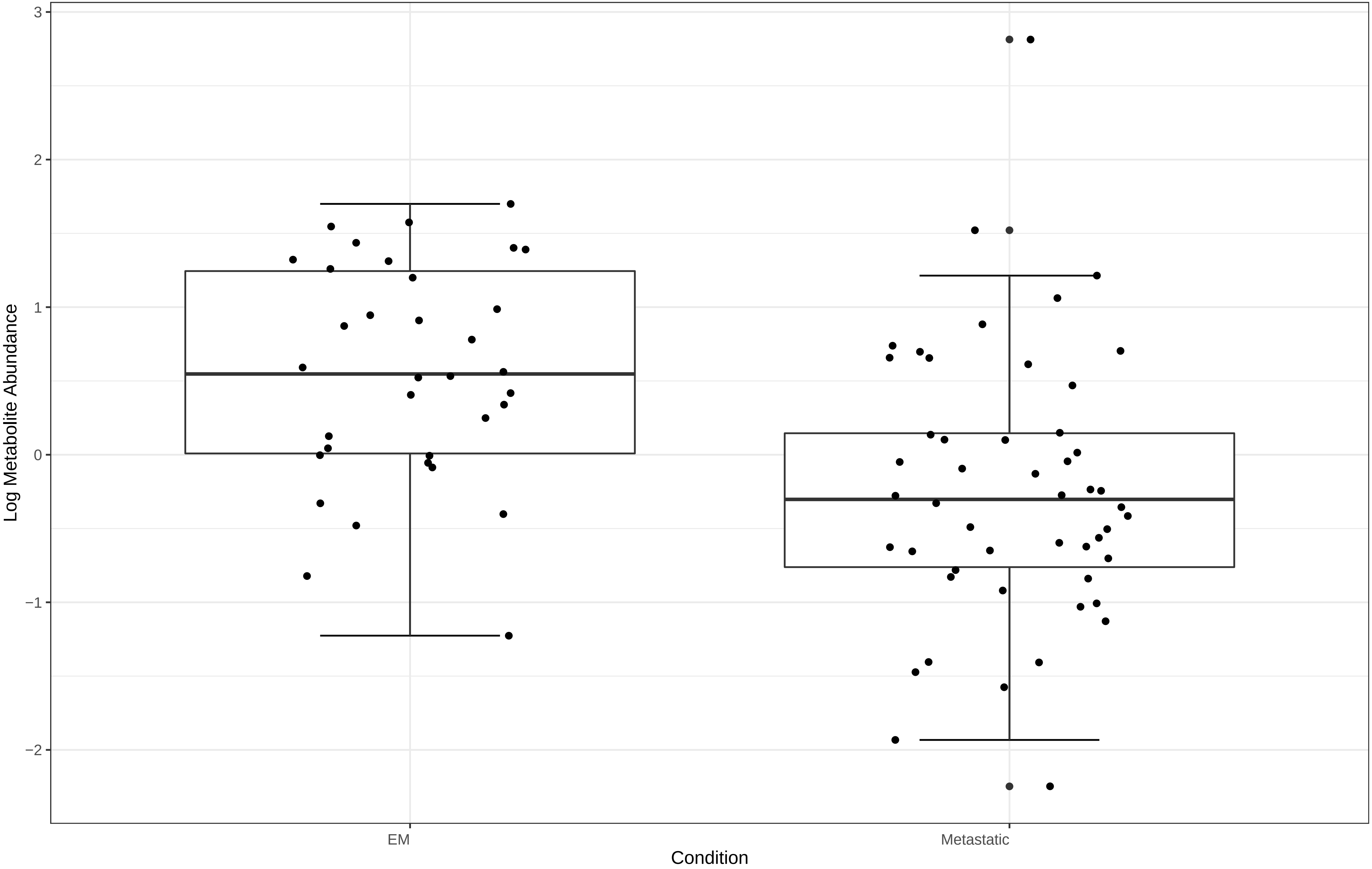

RT: 0.8, m/z: 195.8110, Ion mode: Negative

Adjusted p (t test): 2.93e−03, Adjusted p (wilcox): 2.76e−03

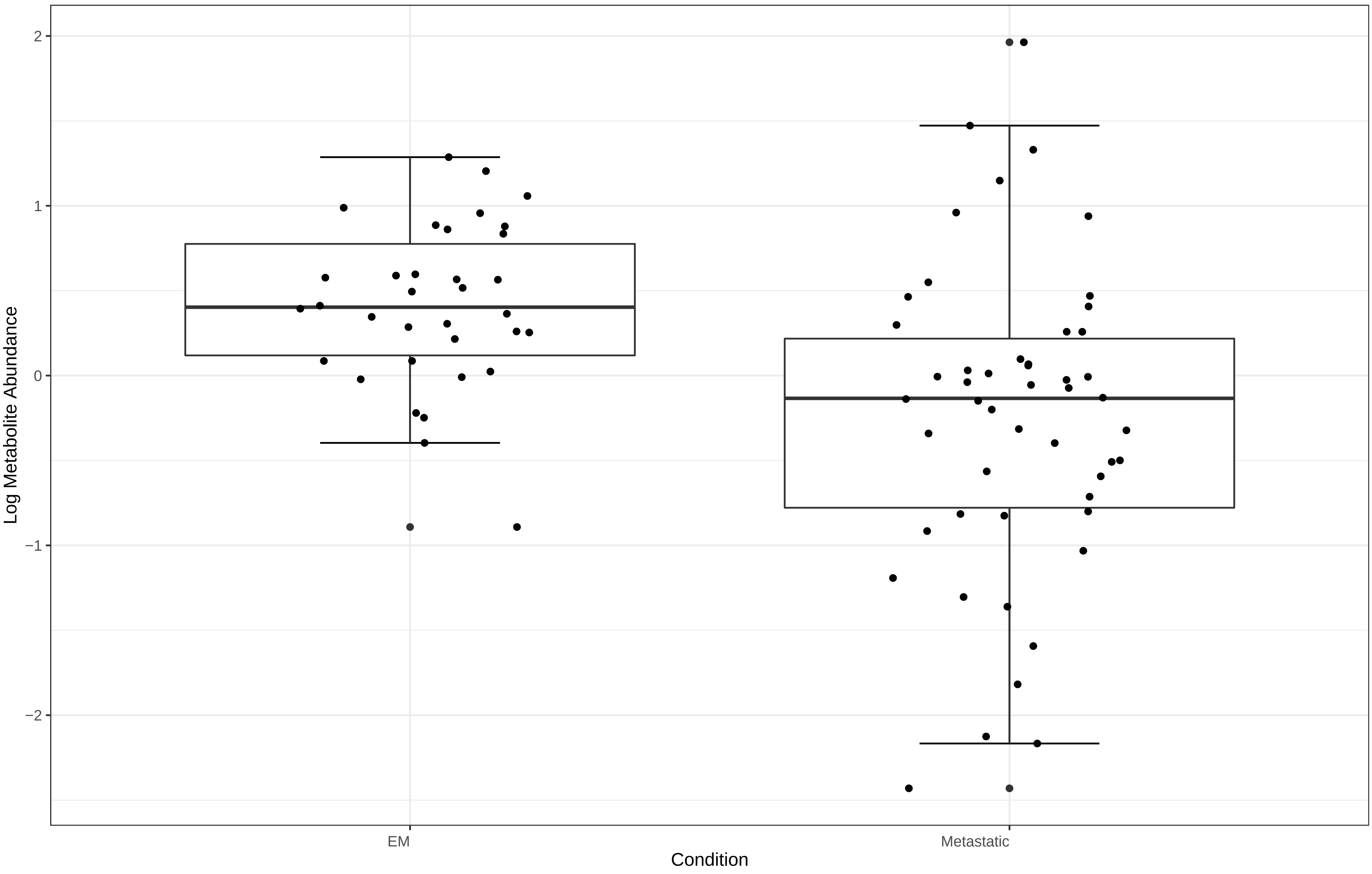

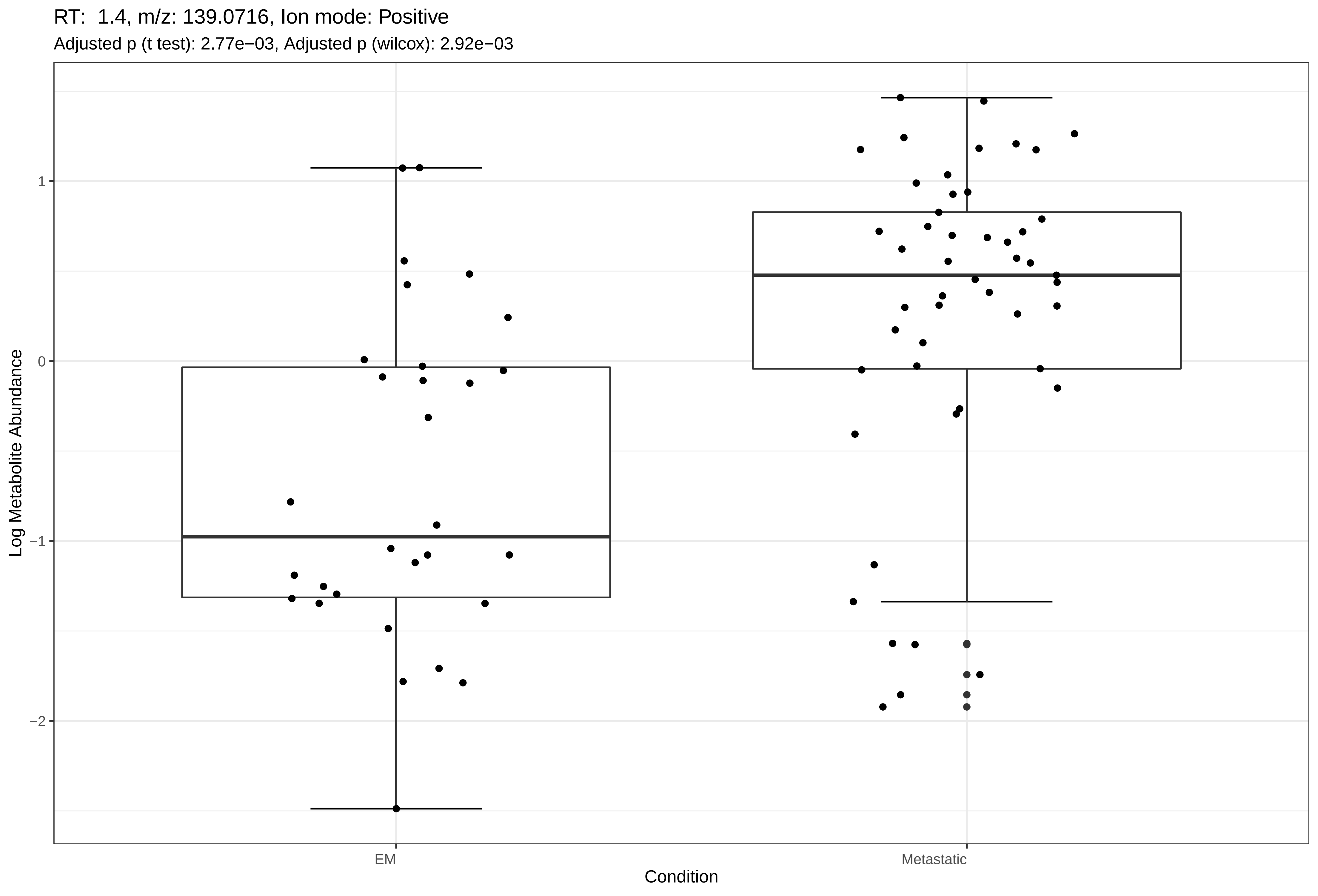
RT: 1.4, m/z: 139.0716, Ion mode: Positive
Adjusted p (t test): 2.77e−03, Adjusted p (wilcox): 2.92e−03
Log Metabolite Abundance
1
0
−1
−2
EM
Metastatic
Condition

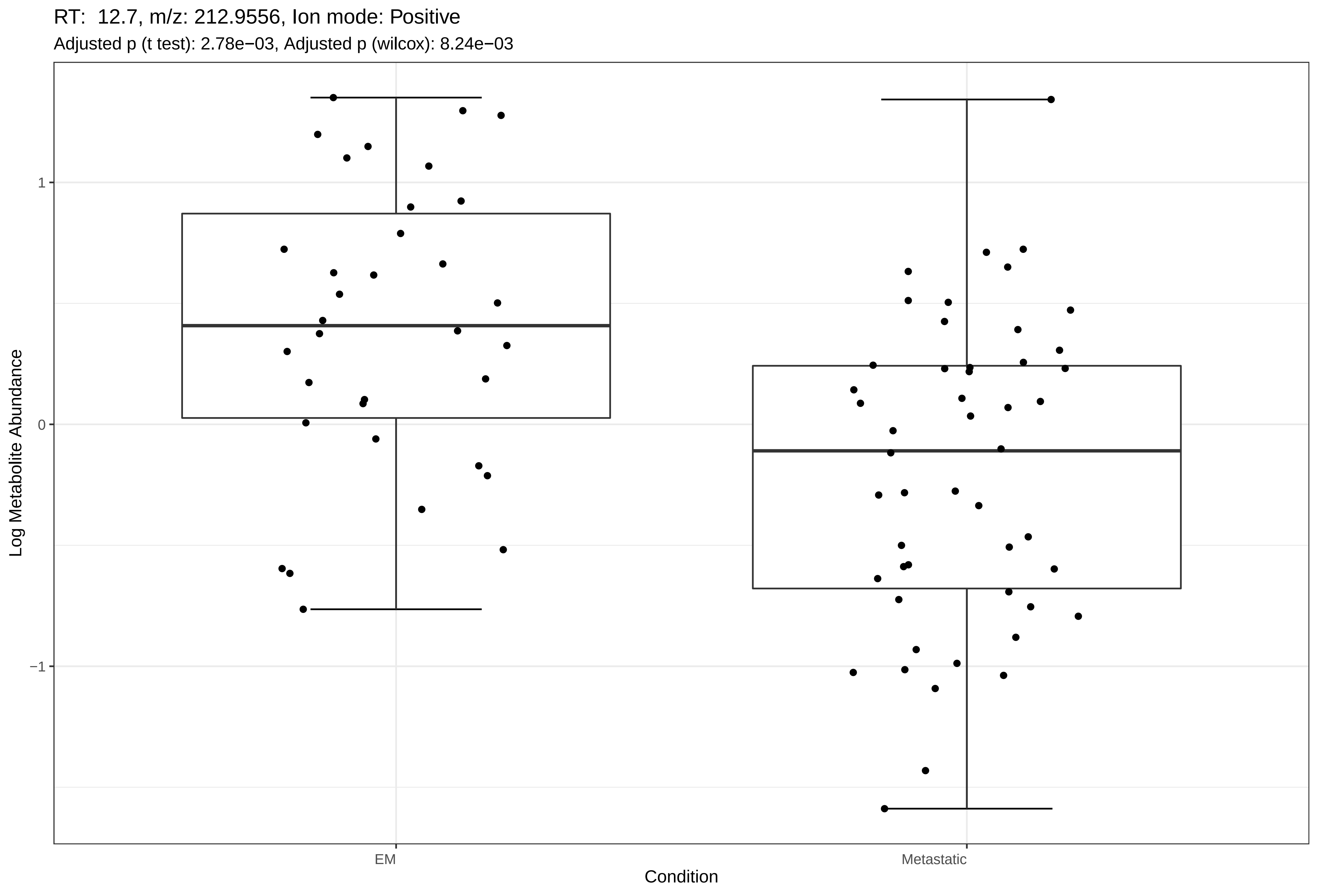

RT: 12.7, m/z: 212.9556, Ion mode: Positive
Adjusted p (t test): 2.78e−03, Adjusted p (wilcox): 8.24e−03
1
0
−1
Log Metabolite Abundance
EM
Metastatic
Condition

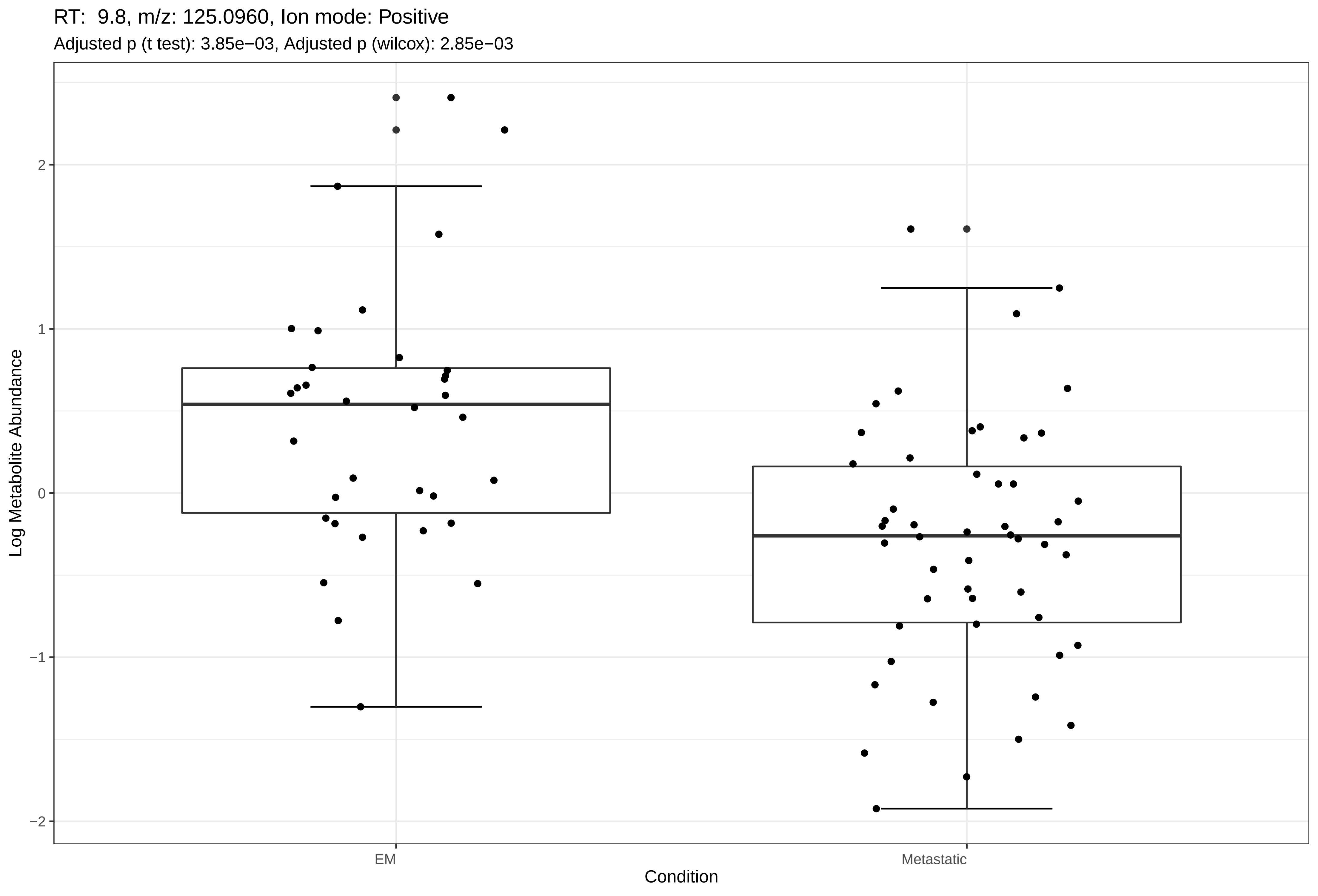
RT: 9.8, m/z: 125.0960, Ion mode: Positive
Adjusted p (t test): 3.85e−03, Adjusted p (wilcox): 2.85e−03
Log Metabolite Abundance
2
1
0
−1
−2
EM
Metastatic
Condition

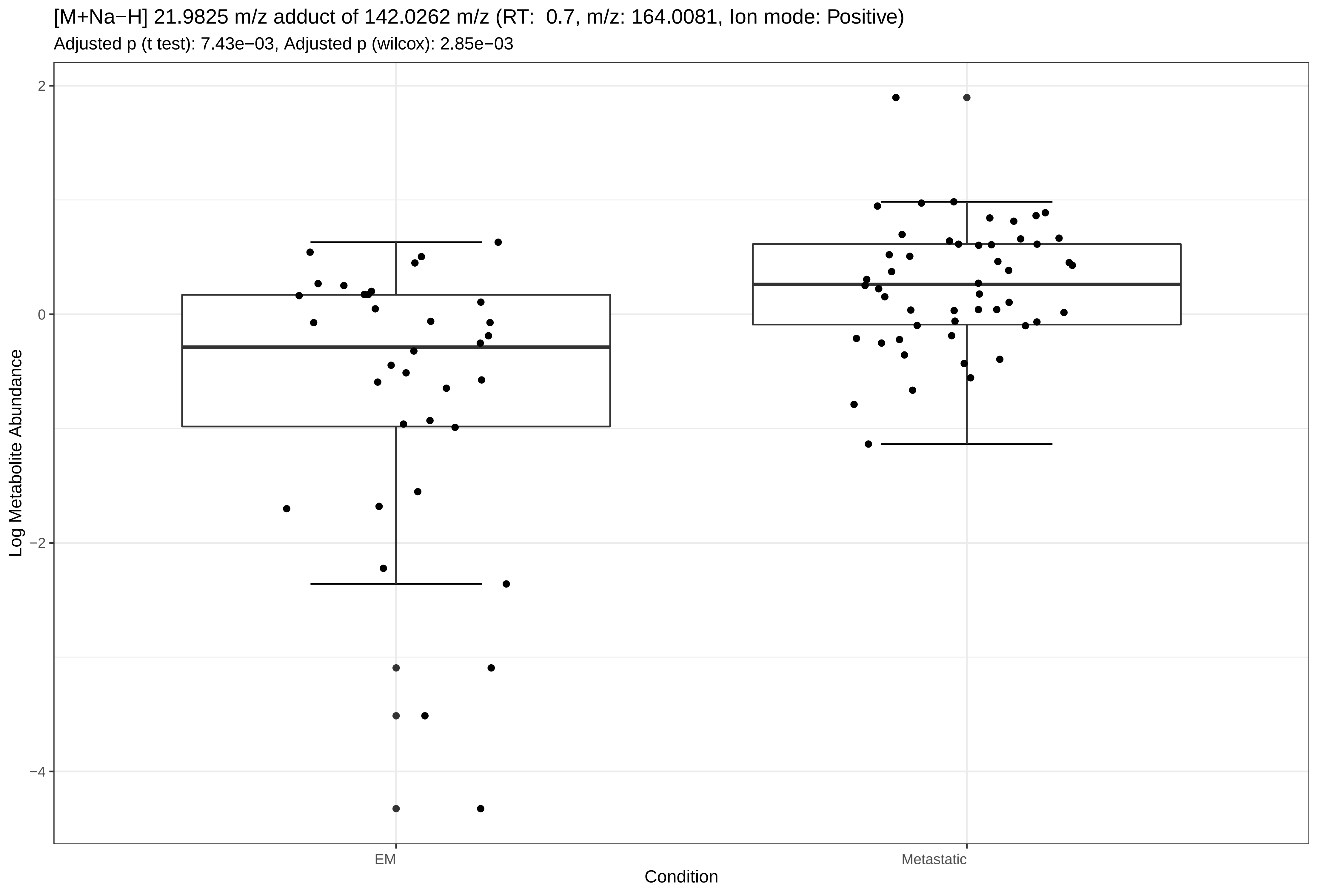

[M+Na−H] 21.9825 m/z adduct of 142.0262 m/z (RT: 0.7, m/z: 164.0081, Ion mode: Positive)
Adjusted p (t test): 7.43e−03, Adjusted p (wilcox): 2.85e−03
Log Metabolite Abundance
2
0
−2
−4
EM
Metastatic
Condition

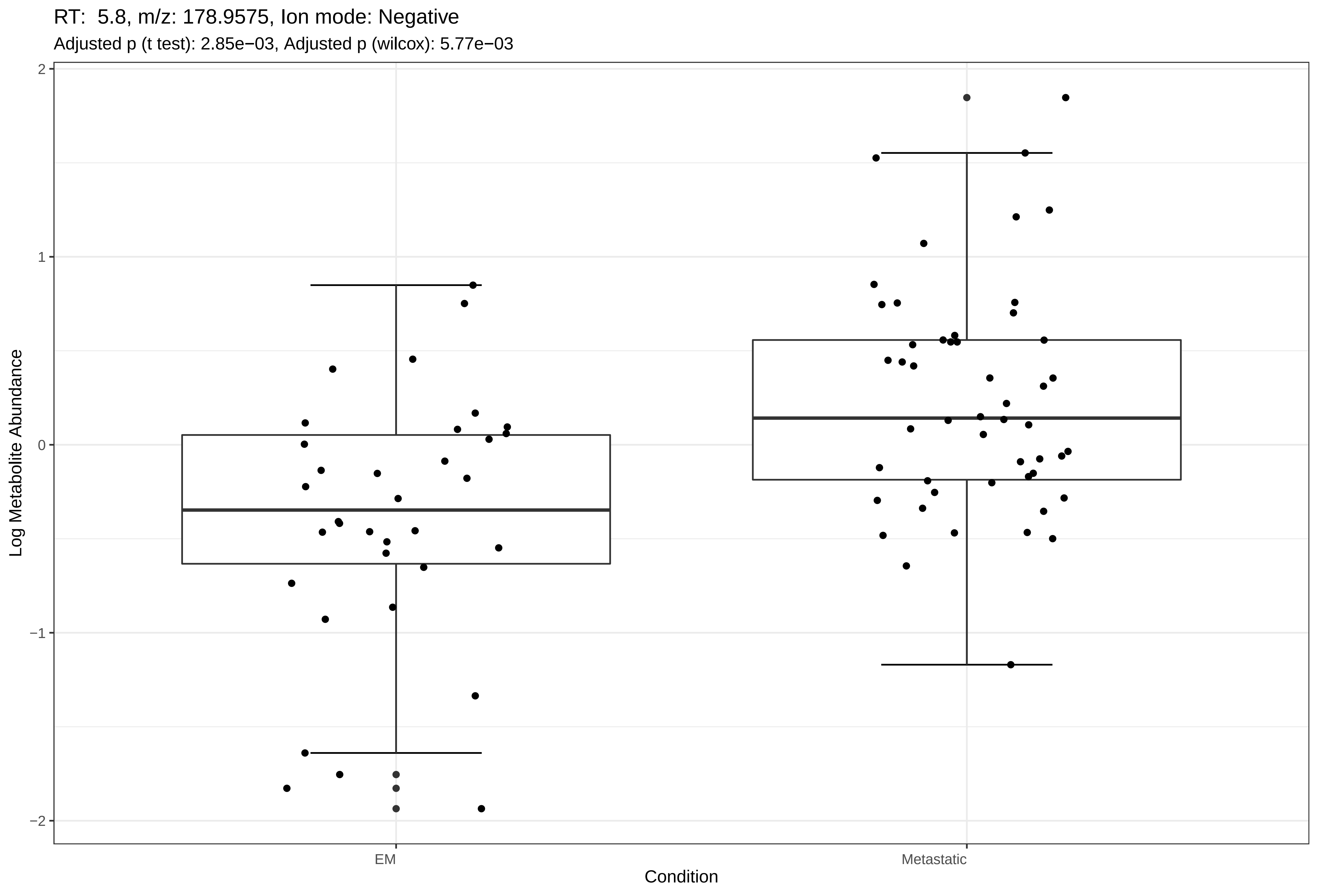

RT: 5.8, m/z: 178.9575, Ion mode: Negative
Adjusted p (t test): 2.85e−03, Adjusted p (wilcox): 5.77e−03
Log Metabolite Abundance
2
1
0
−1
−2
EM
Metastatic
Condition

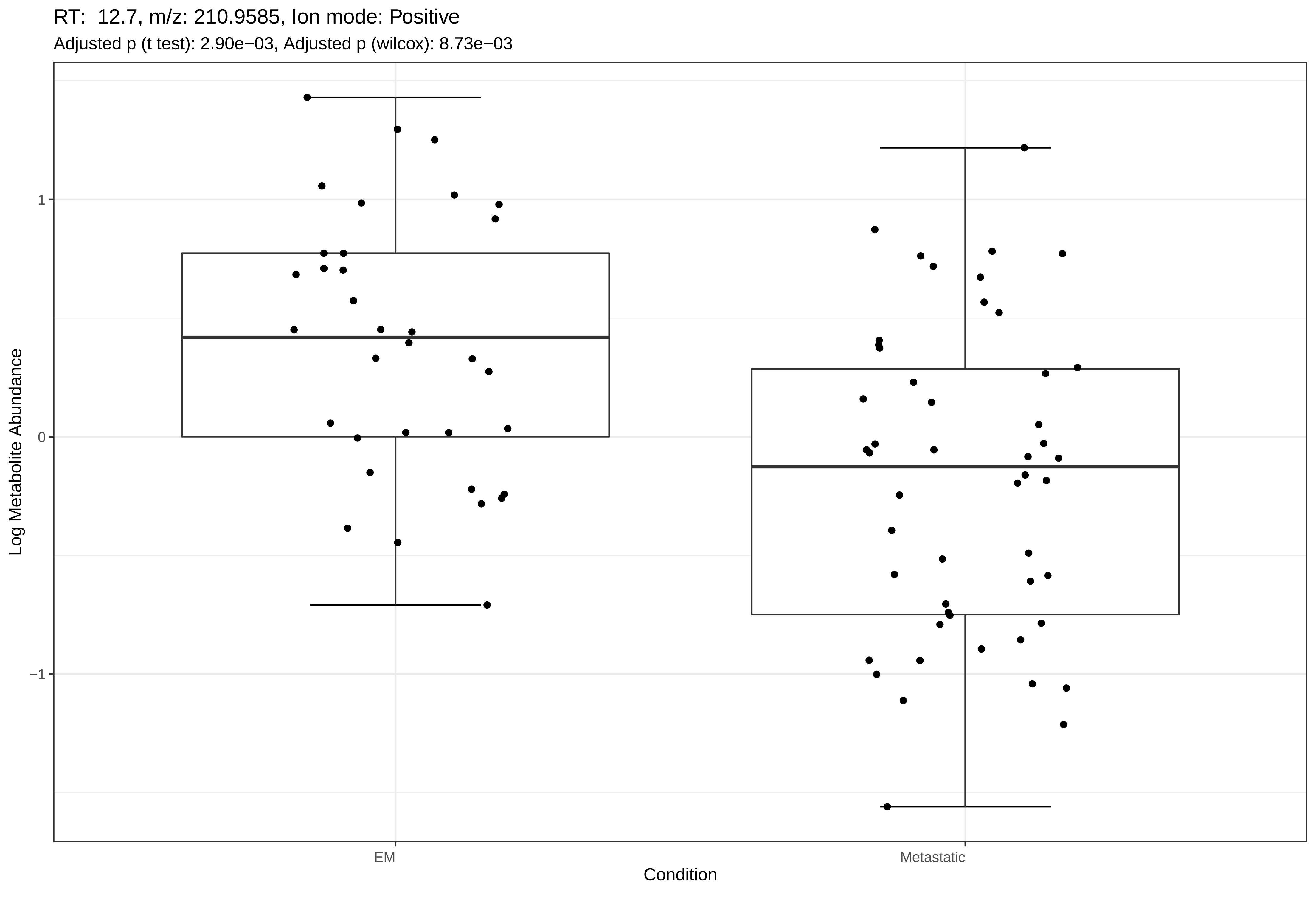
RT: 12.7, m/z: 210.9585, Ion mode: Positive
Adjusted p (t test): 2.90e−03, Adjusted p (wilcox): 8.73e−03
Log Metabolite Abundance
1
0
−1
EM
Metastatic
Condition

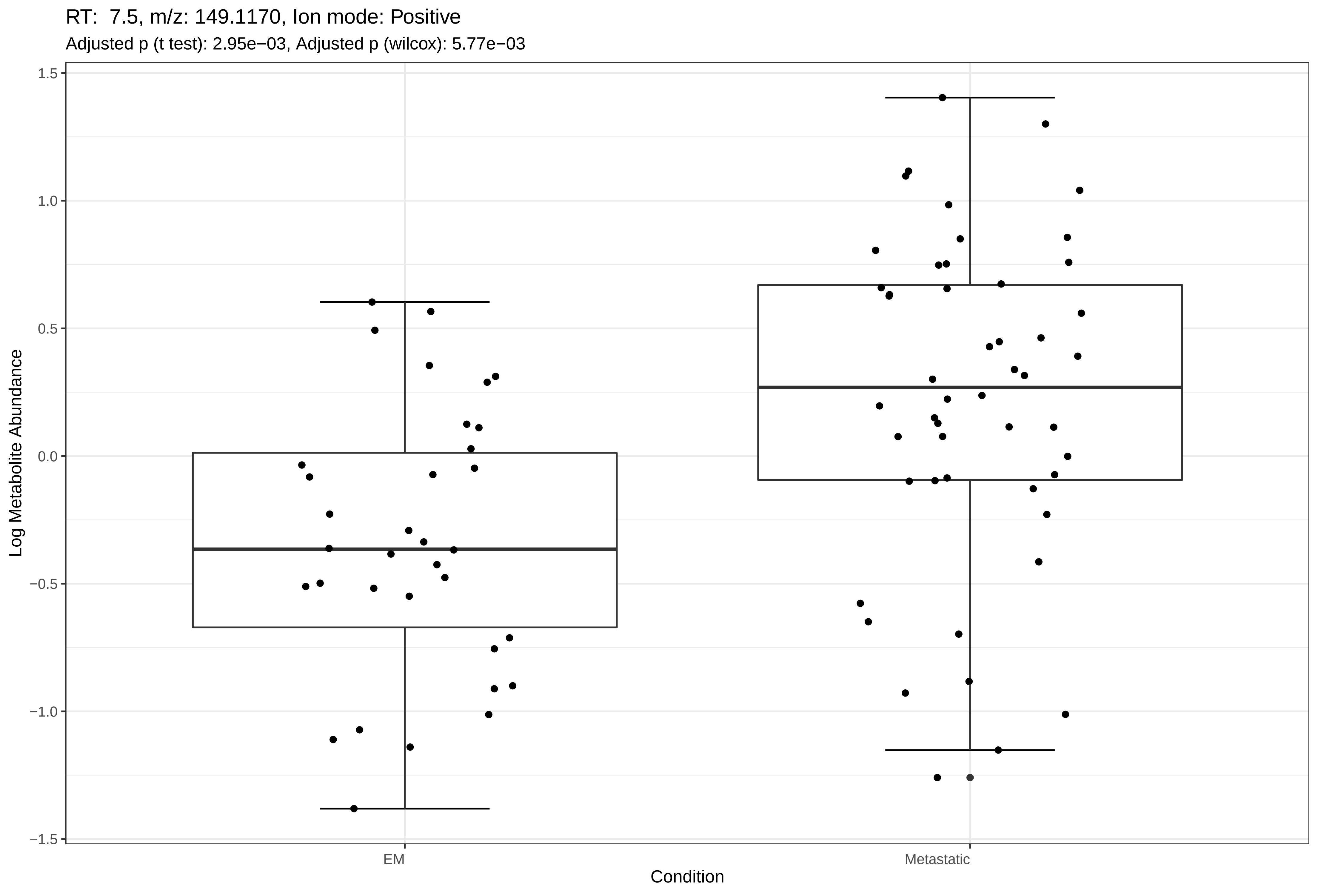
RT: 7.5, m/z: 149.1170, Ion mode: Positive
Adjusted p (t test): 2.95e−03, Adjusted p (wilcox): 5.77e−03
Log Metabolite Abundance
1.5
1.0
0.5
0.0
−0.5
−1.0
−1.5
EM
Metastatic
Condition

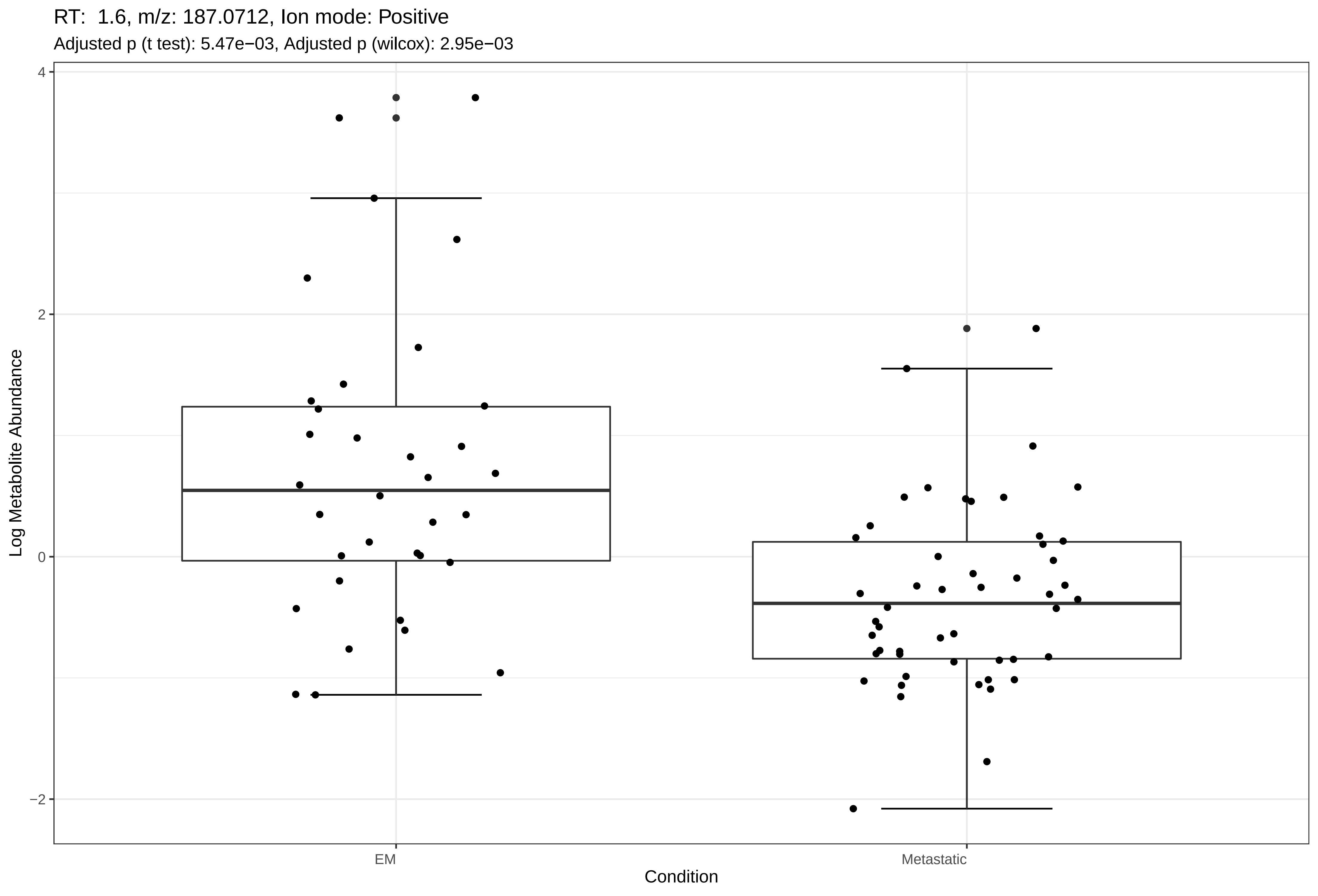
RT: 1.6, m/z: 187.0712, Ion mode: Positive
Adjusted p (t test): 5.47e−03, Adjusted p (wilcox): 2.95e−03
Log Metabolite Abundance
4
2
0
−2
EM
Metastatic
Condition

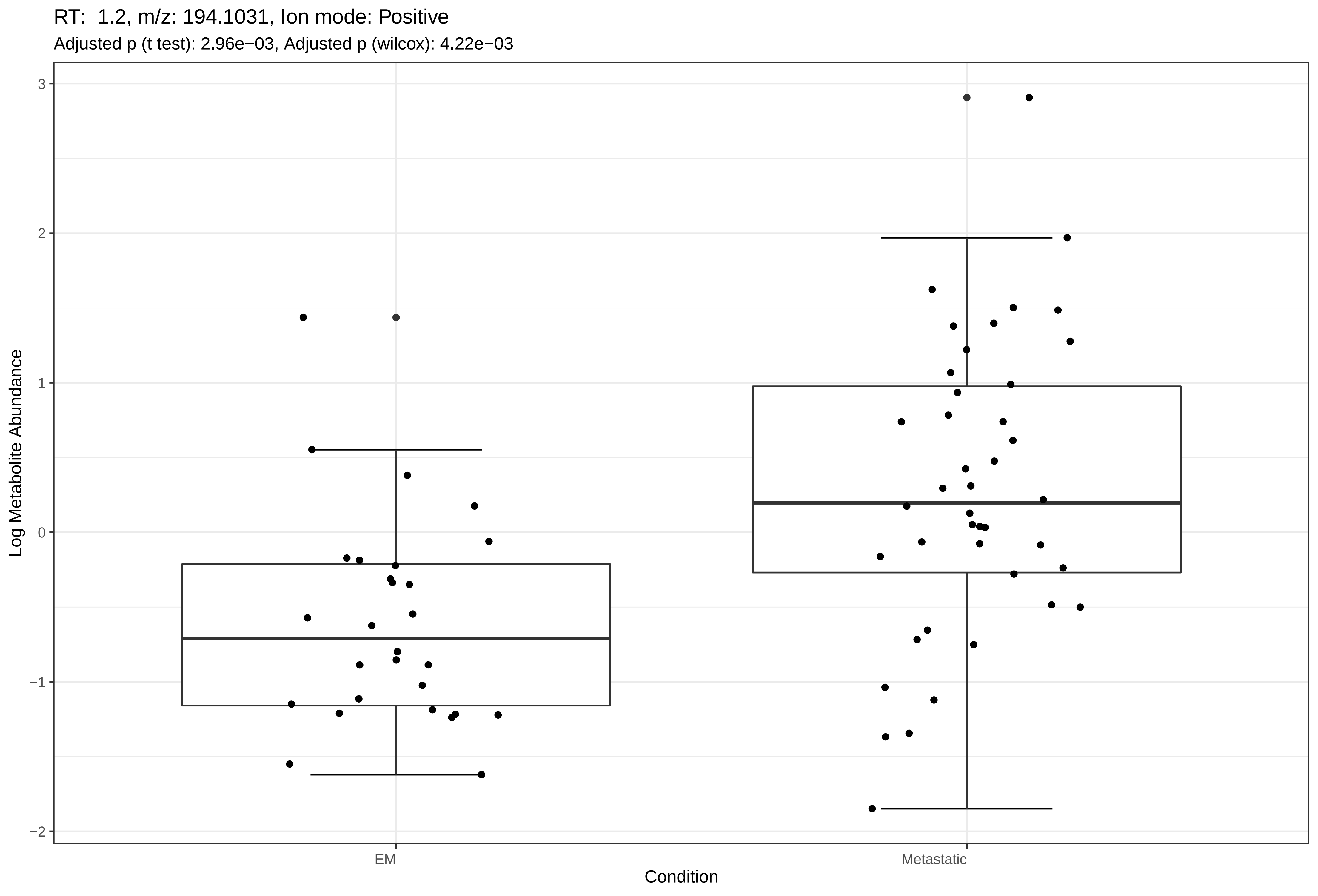
RT: 1.2, m/z: 194.1031, Ion mode: Positive
Adjusted p (t test): 2.96e−03, Adjusted p (wilcox): 4.22e−03
Log Metabolite Abundance
3
2
1
0
−1
−2
EM
Metastatic
Condition

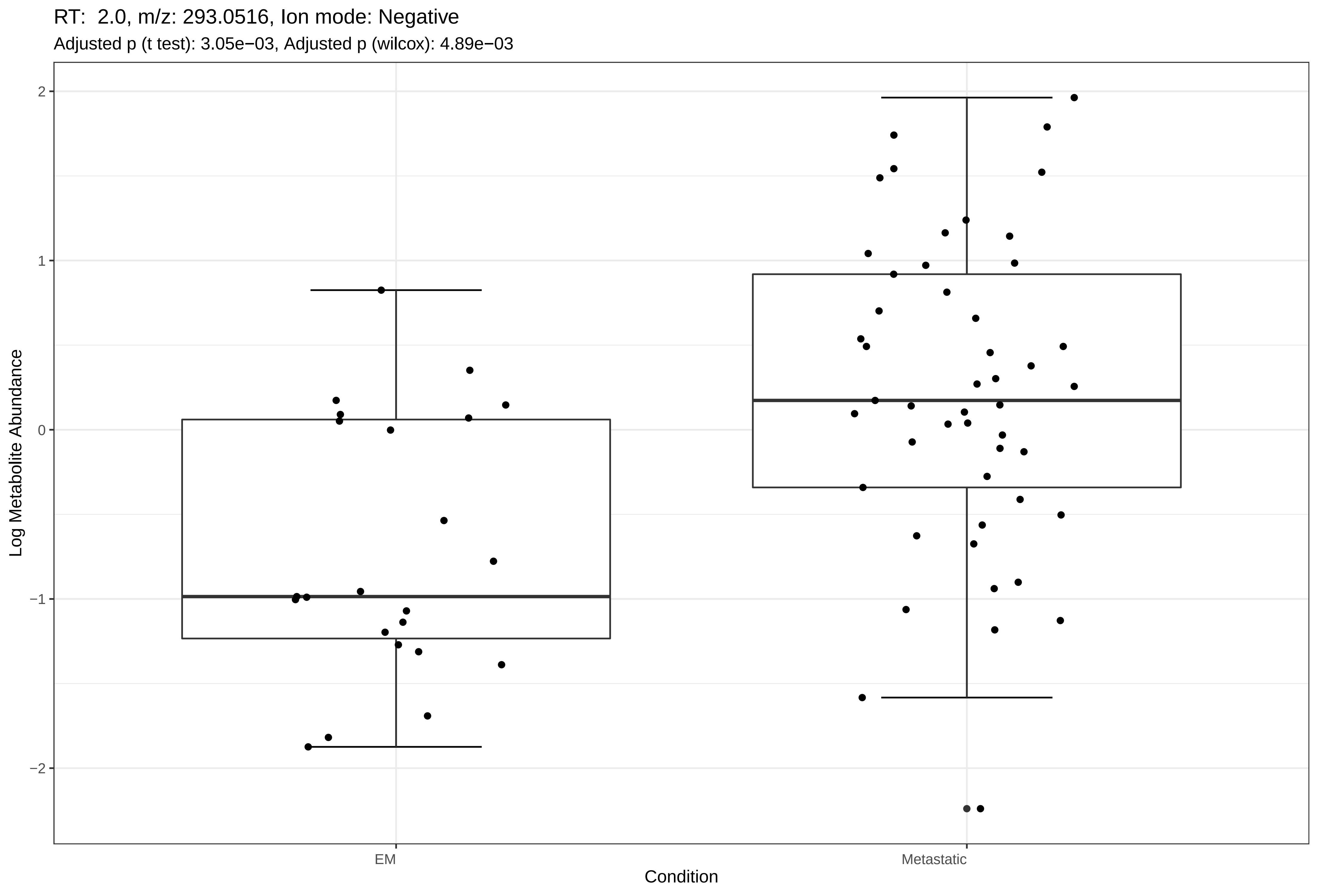

RT: 2.0, m/z: 293.0516, Ion mode: Negative
Adjusted p (t test): 3.05e−03, Adjusted p (wilcox): 4.89e−03
Log Metabolite Abundance
2
1
0
−1
−2
EM
Metastatic
Condition

RT: 3.9, m/z: 221.9407, Ion mode: Negative

Adjusted p (t test): 7.29e−03, Adjusted p (wilcox): 3.05e−03

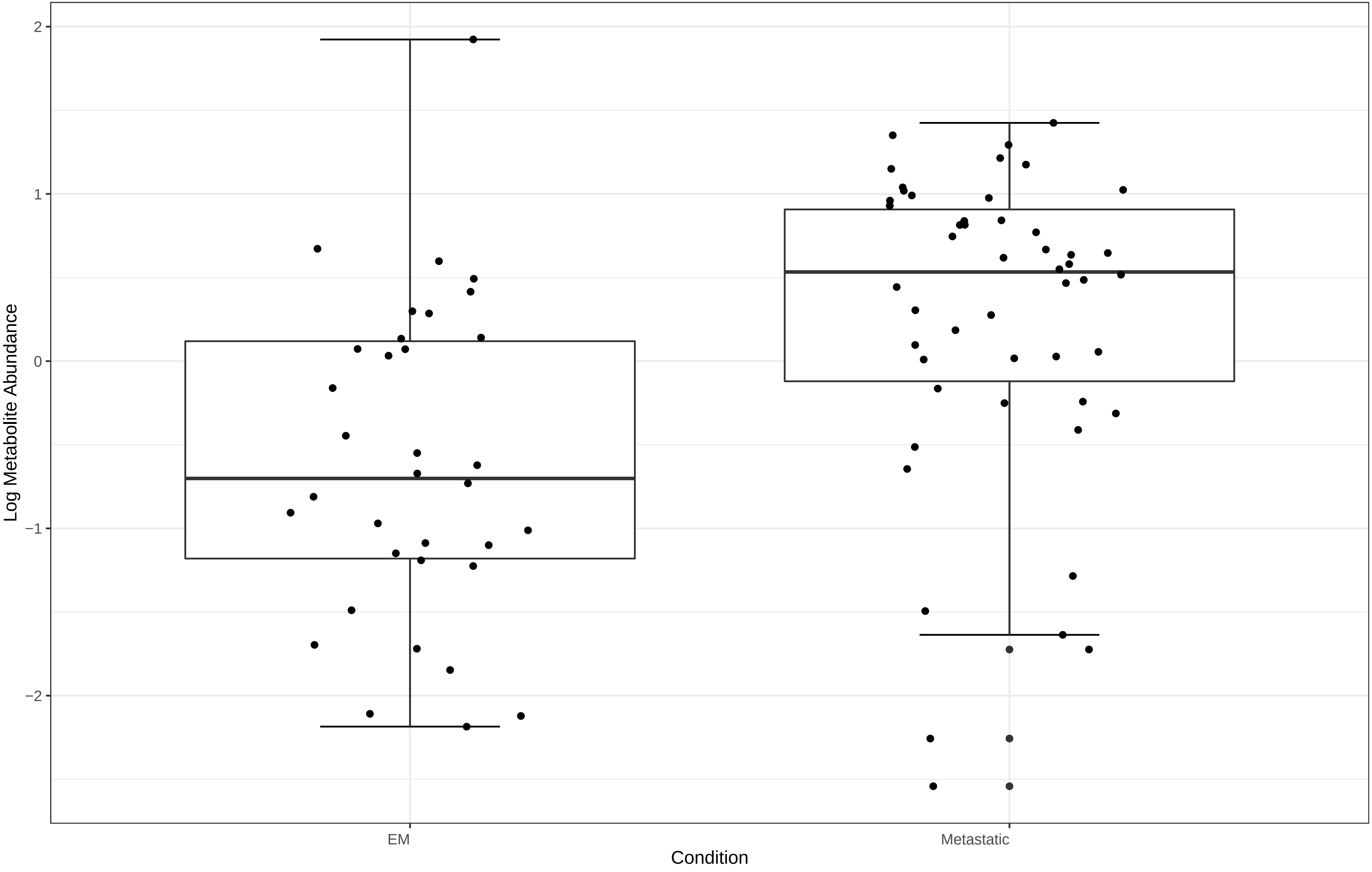

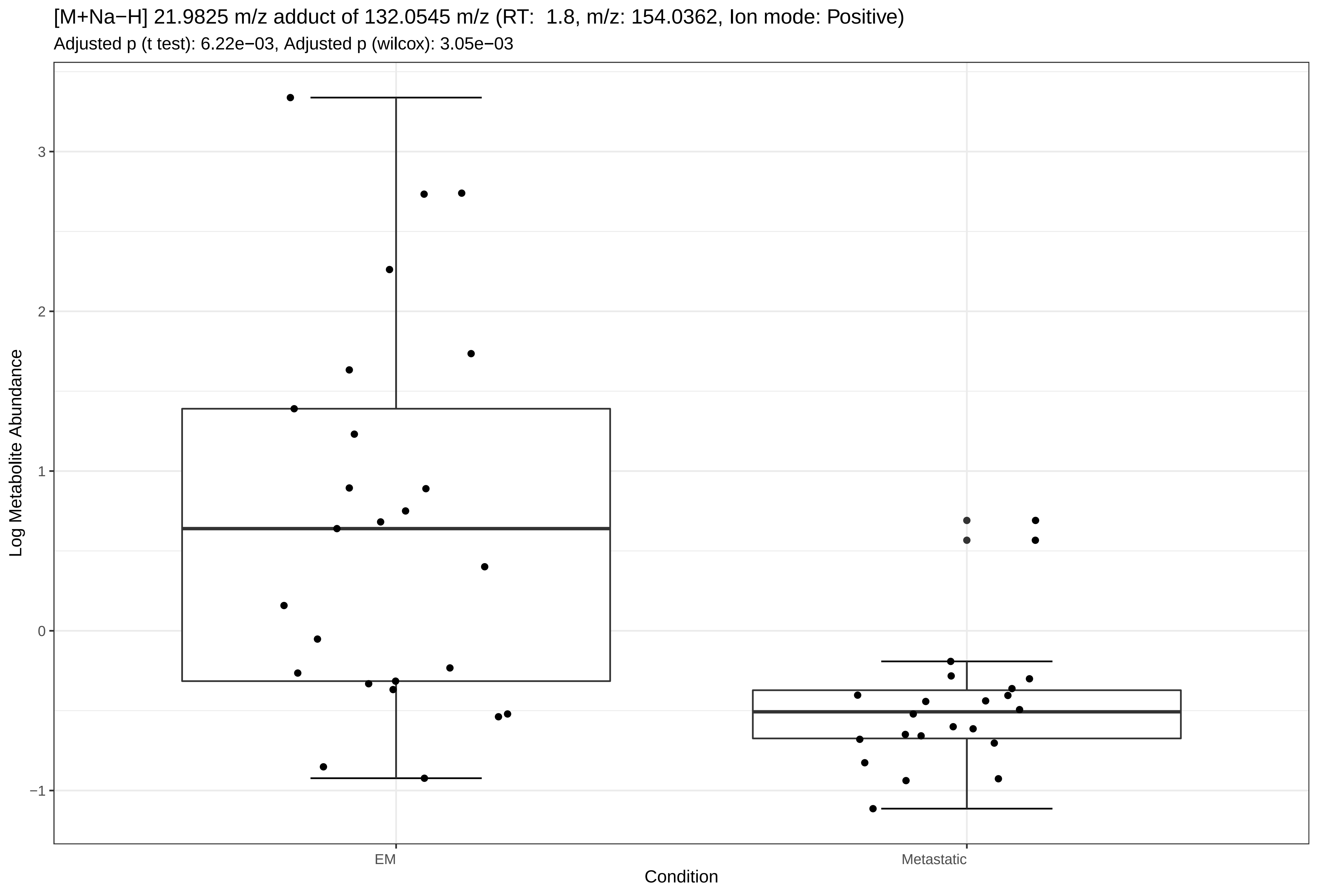
[M+Na−H] 21.9825 m/z adduct of 132.0545 m/z (RT: 1.8, m/z: 154.0362, Ion mode: Positive)
Adjusted p (t test): 6.22e−03, Adjusted p (wilcox): 3.05e−03
Log Metabolite Abundance
3
2
1
0
−1
EM
Metastatic
Condition

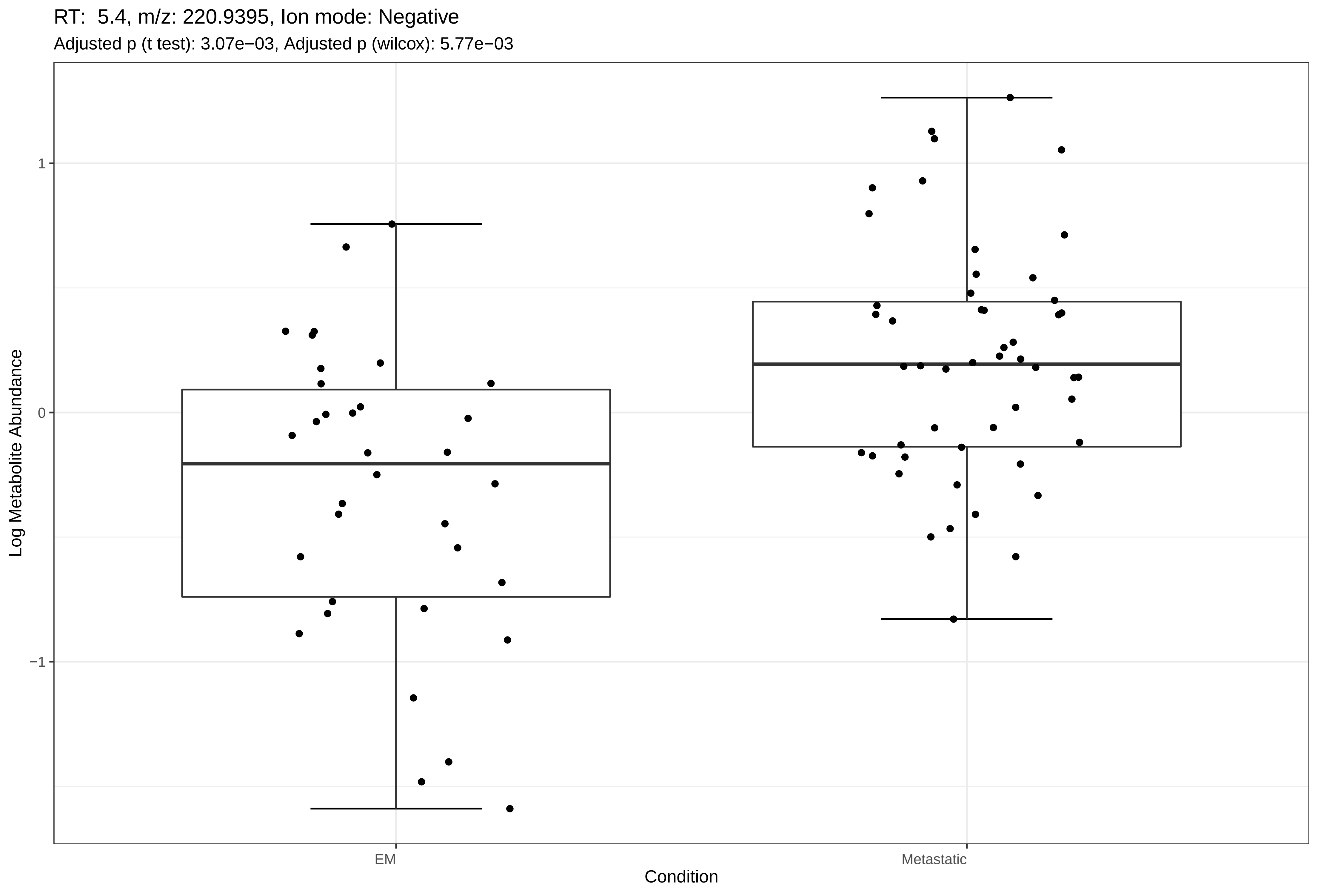

RT: 5.4, m/z: 220.9395, Ion mode: Negative
Adjusted p (t test): 3.07e−03, Adjusted p (wilcox): 5.77e−03
Log Metabolite Abundance
1
0
−1
EM
Metastatic
Condition

RT: 0.9, m/z: 157.0605, Ion mode: Positive

Adjusted p (t test): 3.09e−03, Adjusted p (wilcox): 3.59e−03

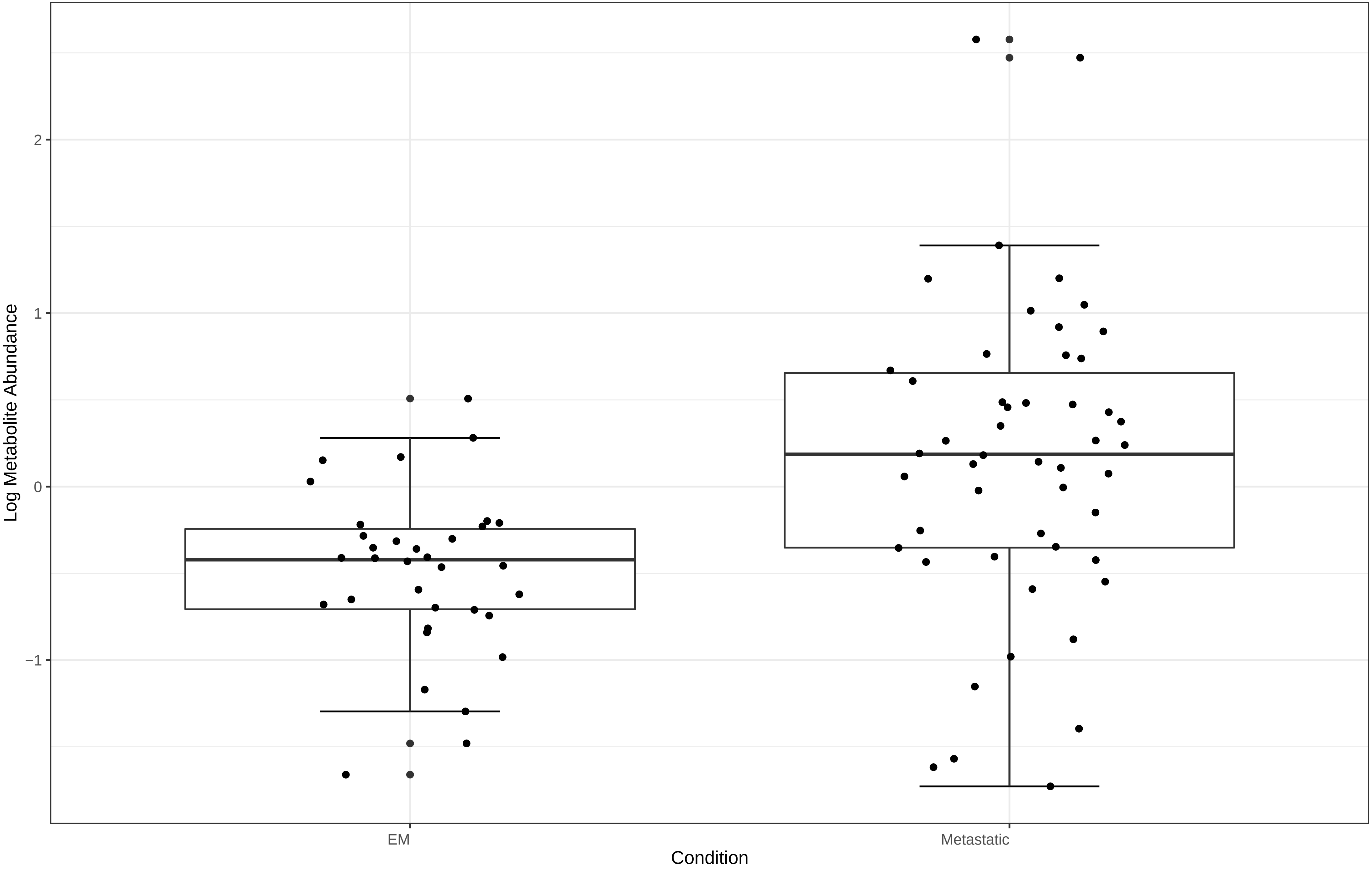

RT: 10.0, m/z: 243.0985, Ion mode: Positive

Adjusted p (t test): 8.68e−03, Adjusted p (wilcox): 3.15e−03

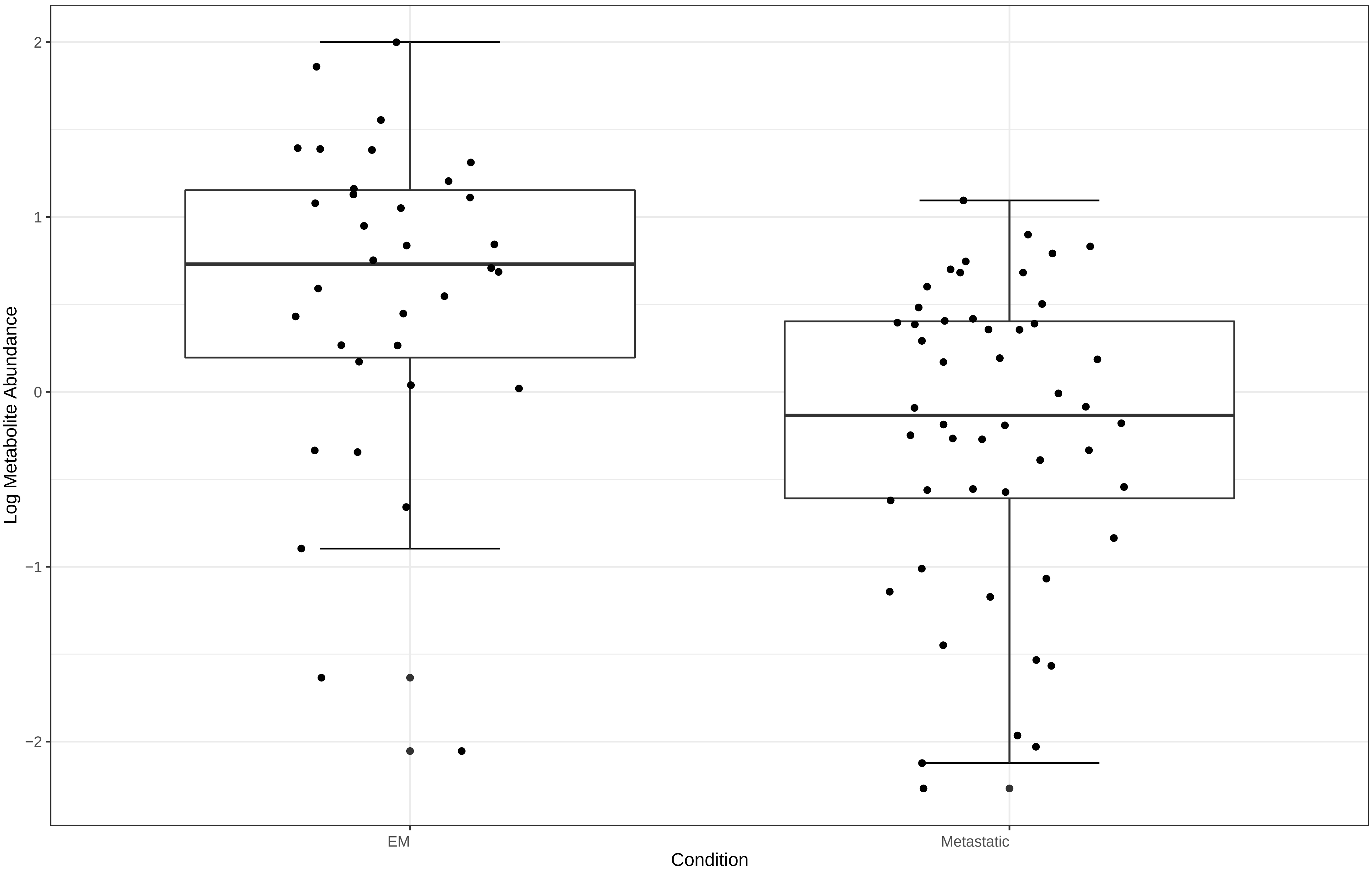

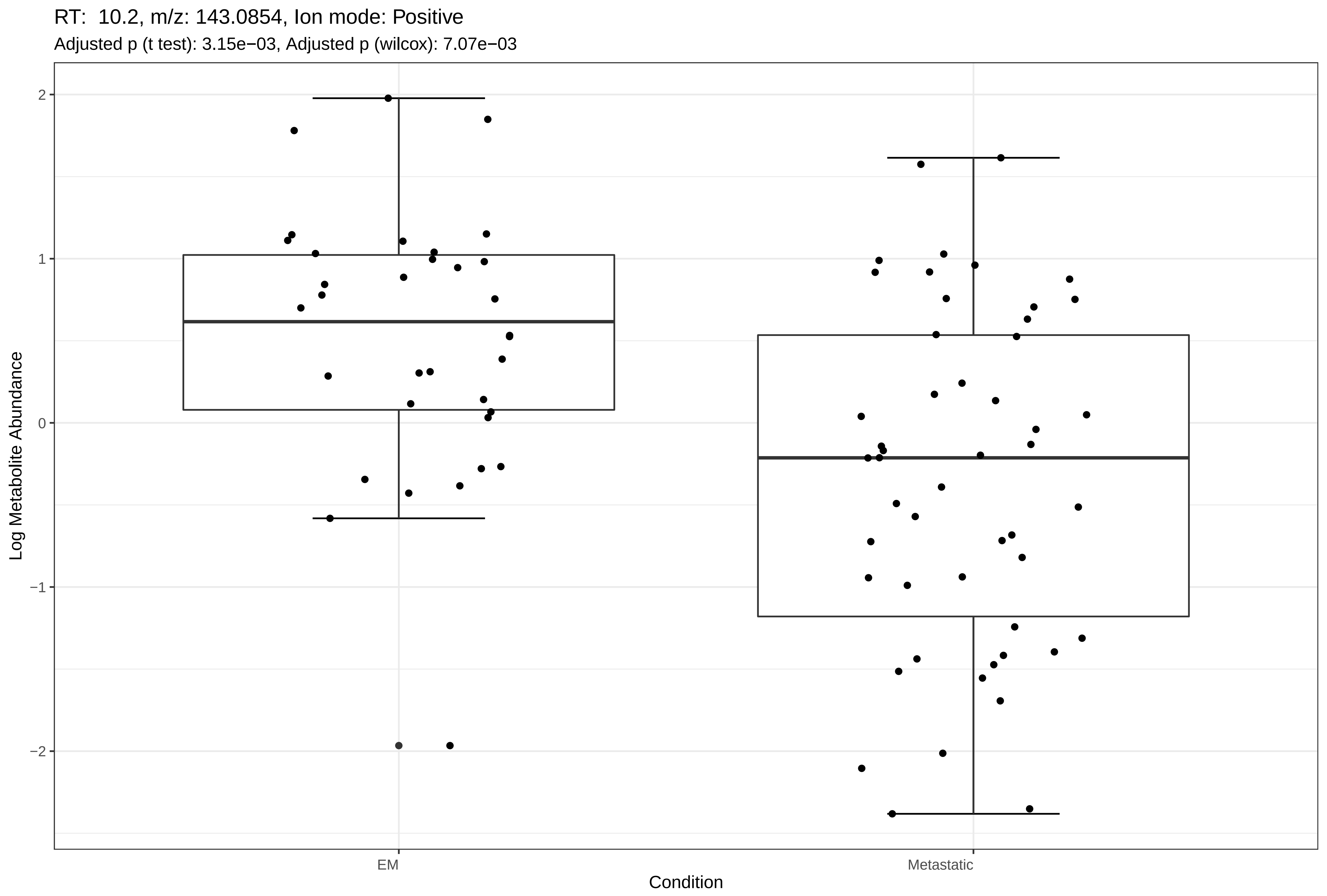
RT: 10.2, m/z: 143.0854, Ion mode: Positive
Adjusted p (t test): 3.15e−03, Adjusted p (wilcox): 7.07e−03
Log Metabolite Abundance
2
1
0
−1
−2
EM
Metastatic
Condition

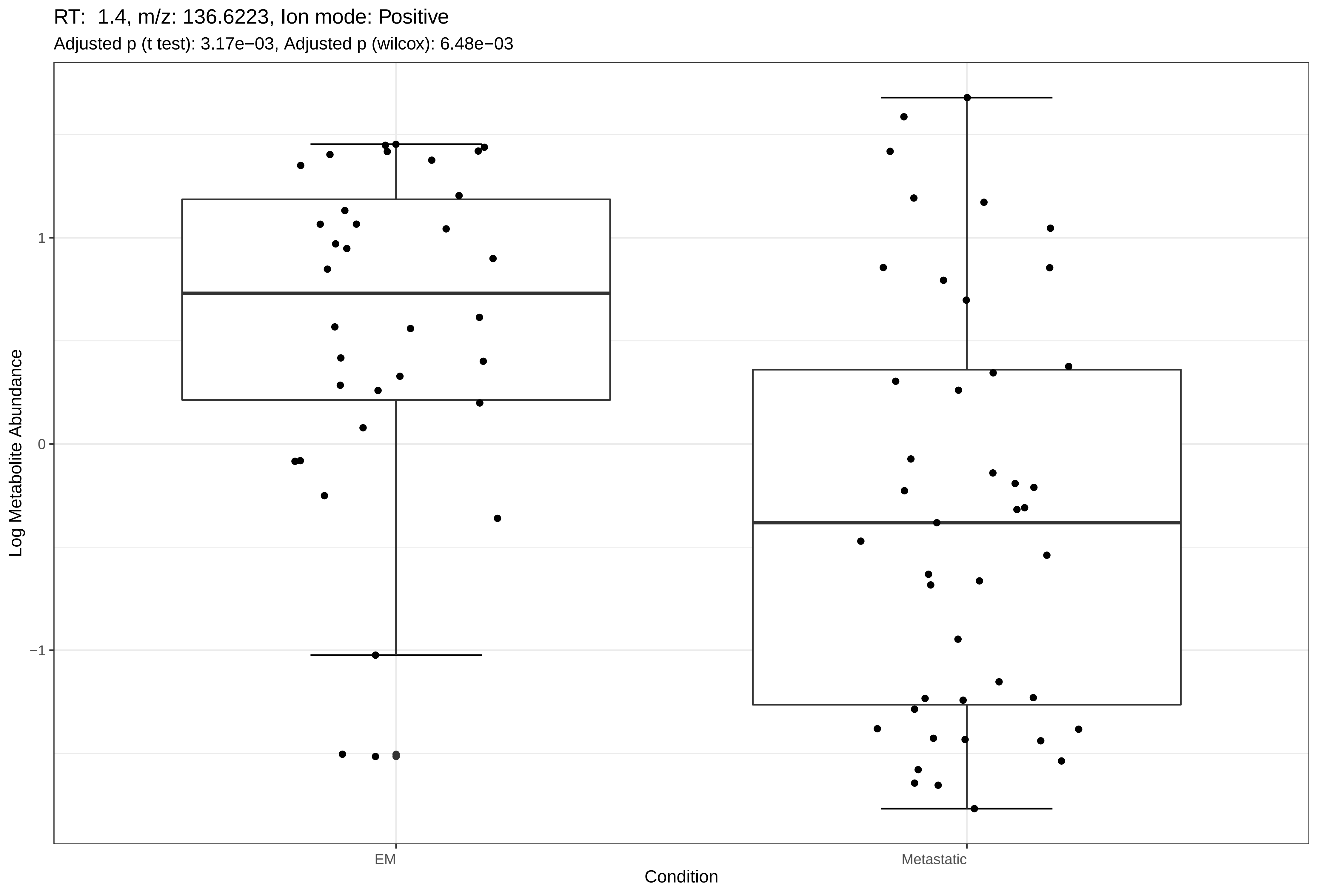
RT: 1.4, m/z: 136.6223, Ion mode: Positive
Adjusted p (t test): 3.17e−03, Adjusted p (wilcox): 6.48e−03
Log Metabolite Abundance
1
0
−1
EM
Metastatic
Condition

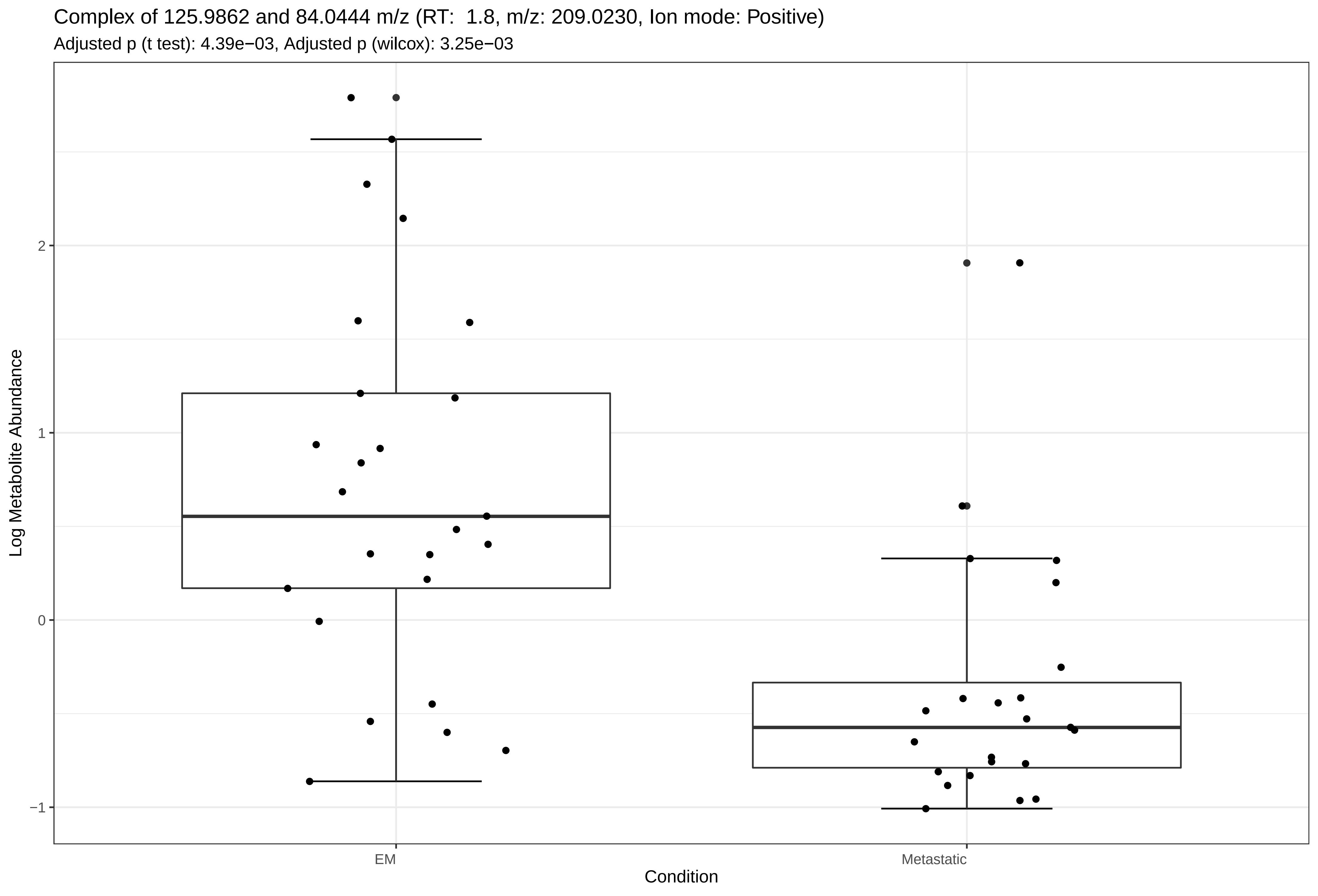

Complex of 125.9862 and 84.0444 m/z (RT: 1.8, m/z: 209.0230, Ion mode: Positive)
Adjusted p (t test): 4.39e−03, Adjusted p (wilcox): 3.25e−03
Log Metabolite Abundance
2
1
0
−1
EM
Metastatic
Condition

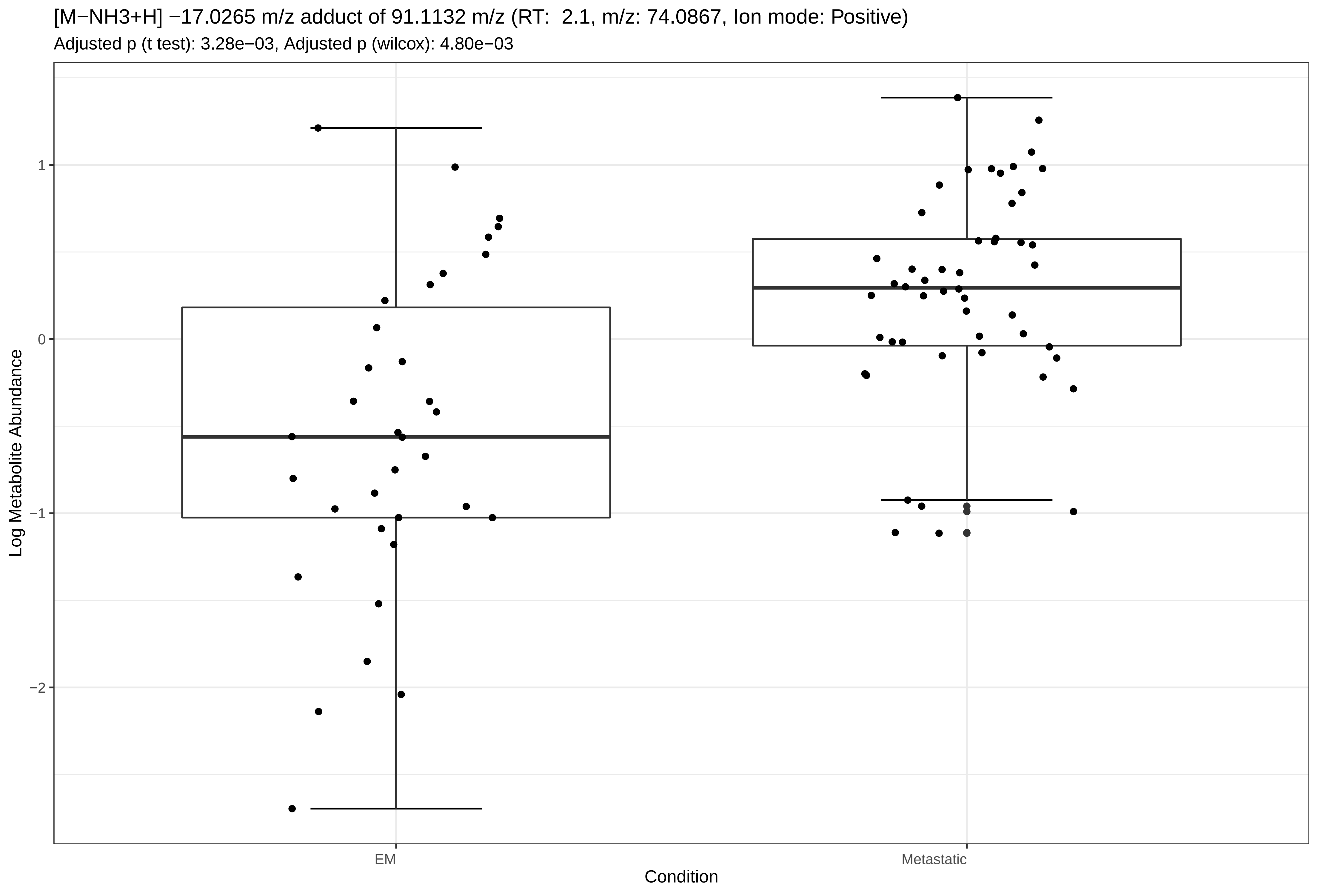

[M−NH3+H] −17.0265 m/z adduct of 91.1132 m/z (RT: 2.1, m/z: 74.0867, Ion mode: Positive)
Adjusted p (t test): 3.28e−03, Adjusted p (wilcox): 4.80e−03
Log Metabolite Abundance
1
0
−1
−2
EM
Metastatic
Condition

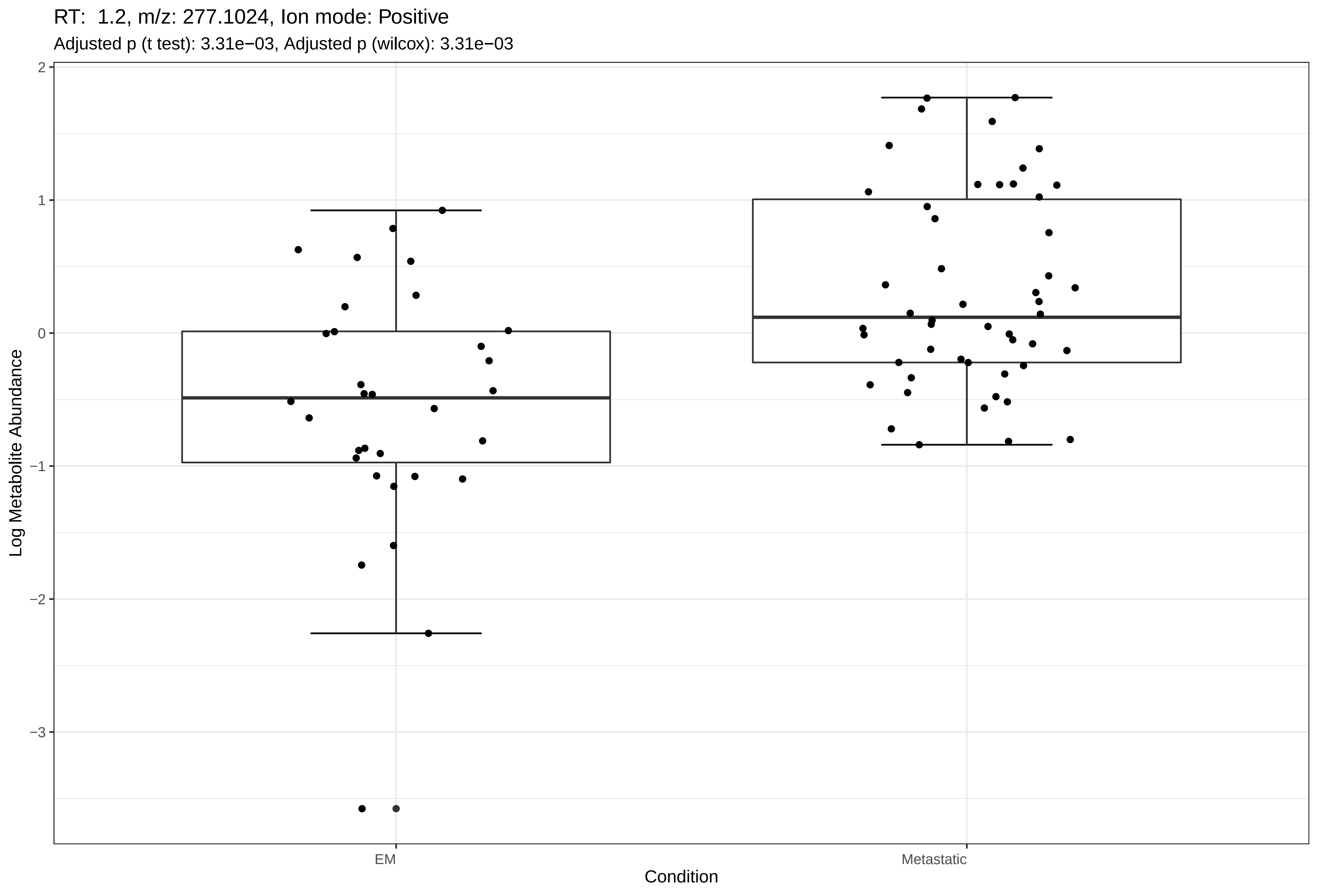

RT: 1.2, m/z: 277.1024, Ion mode: Positive
Adjusted p (t test): 3.31e−03, Adjusted p (wilcox): 3.31e−03
2
1
0
−1
−2
−3
Log Metabolite Abundance
EM
Metastatic
Condition

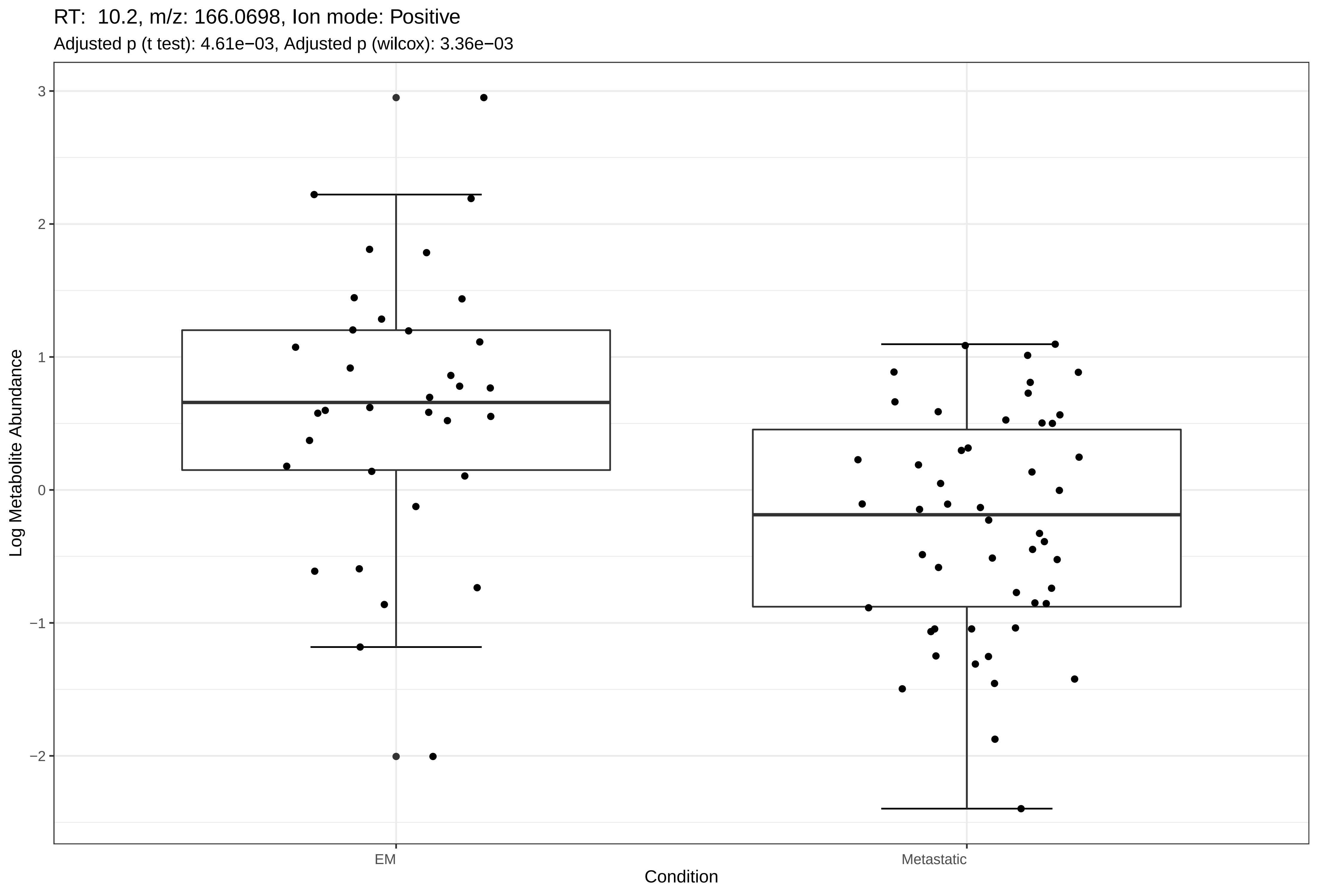

RT: 10.2, m/z: 166.0698, Ion mode: Positive
Adjusted p (t test): 4.61e−03, Adjusted p (wilcox): 3.36e−03
Log Metabolite Abundance
3
2
1
0
−1
−2
EM
Metastatic
Condition

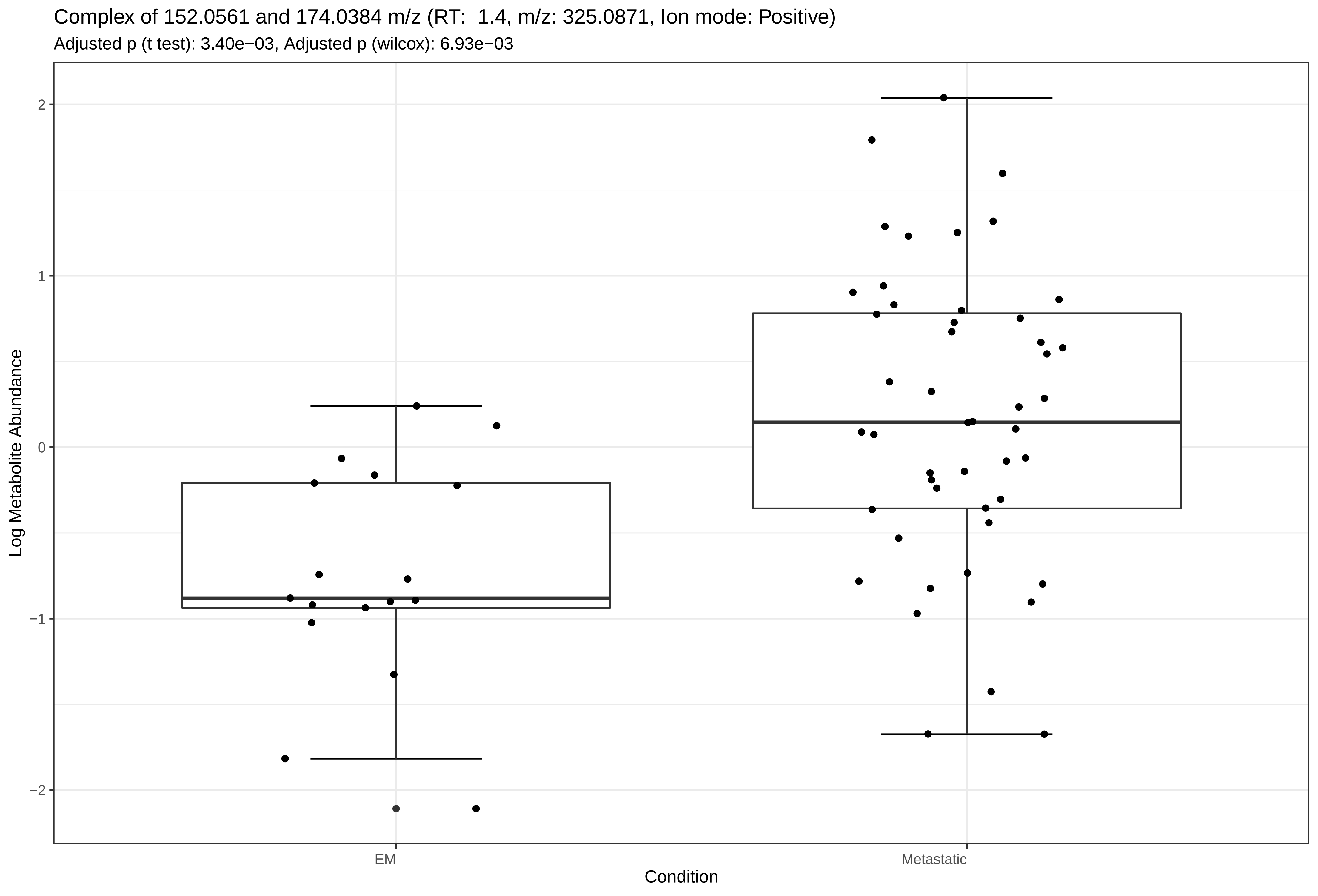
Complex of 152.0561 and 174.0384 m/z (RT: 1.4, m/z: 325.0871, Ion mode: Positive)
Adjusted p (t test): 3.40e−03, Adjusted p (wilcox): 6.93e−03
Log Metabolite Abundance
2
1
0
−1
−2
EM
Metastatic
Condition

[M+Na−H] 21.9825 m/z adduct of 141.0783 m/z (RT: 1.4, m/z: 163.0604, Ion mode: Positive)

Adjusted p (t test): 3.42e−03, Adjusted p (wilcox): 7.73e−03

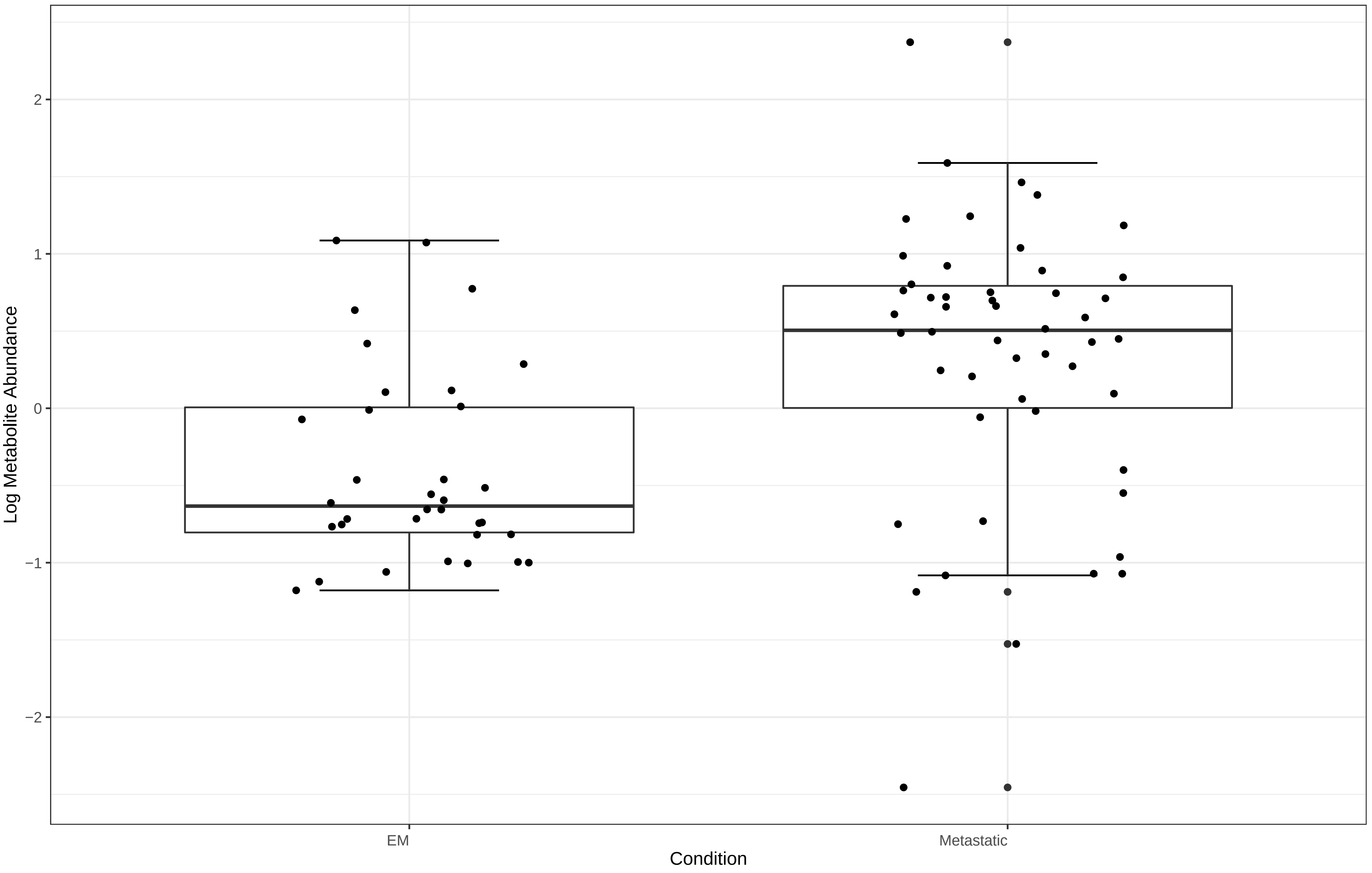

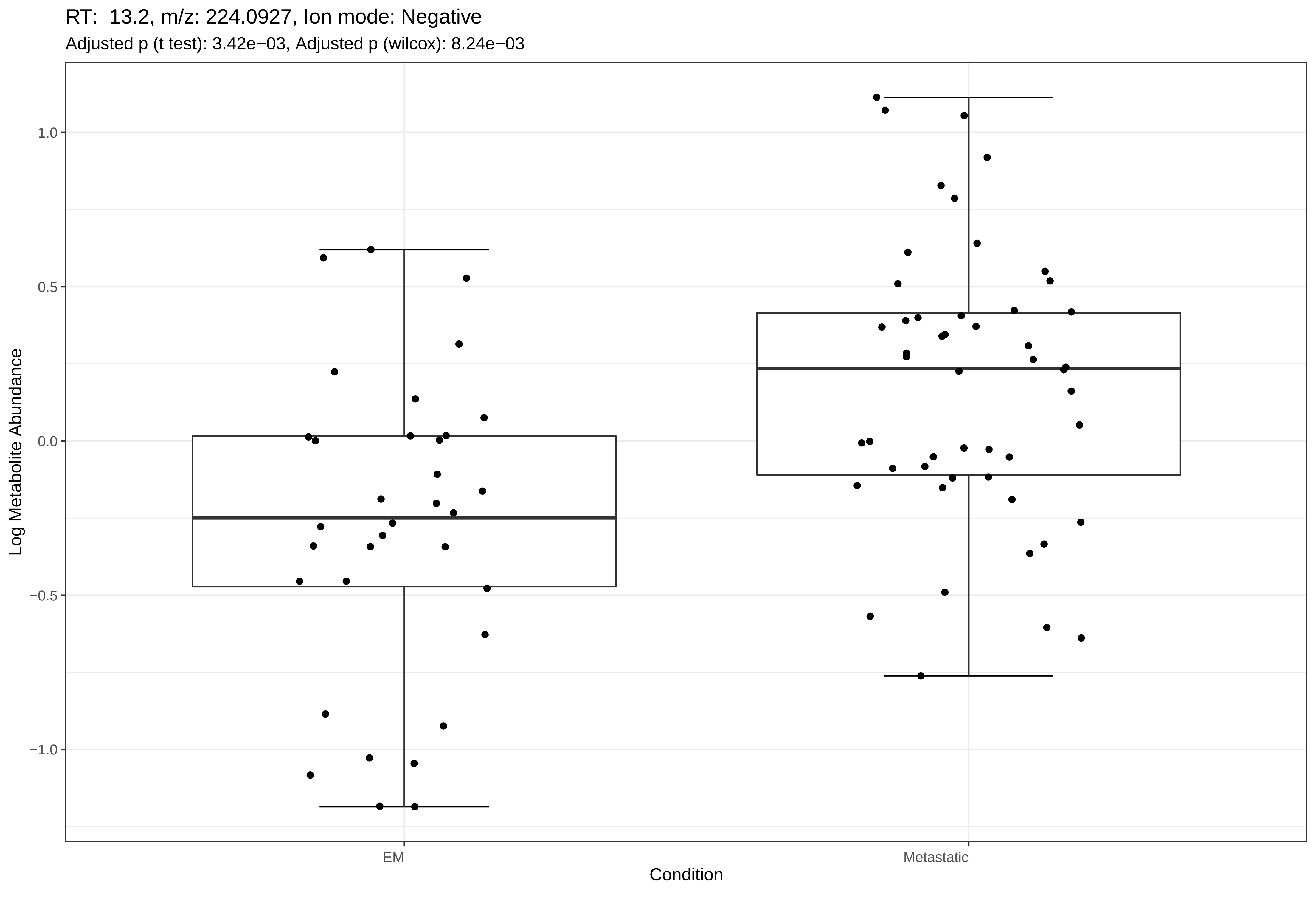
RT: 13.2, m/z: 224.0927, Ion mode: Negative
Adjusted p (t test): 3.42e−03, Adjusted p (wilcox): 8.24e−03
Log Metabolite Abundance
1.0
0.5
0.0
−0.5
−1.0
EM
Metastatic
Condition

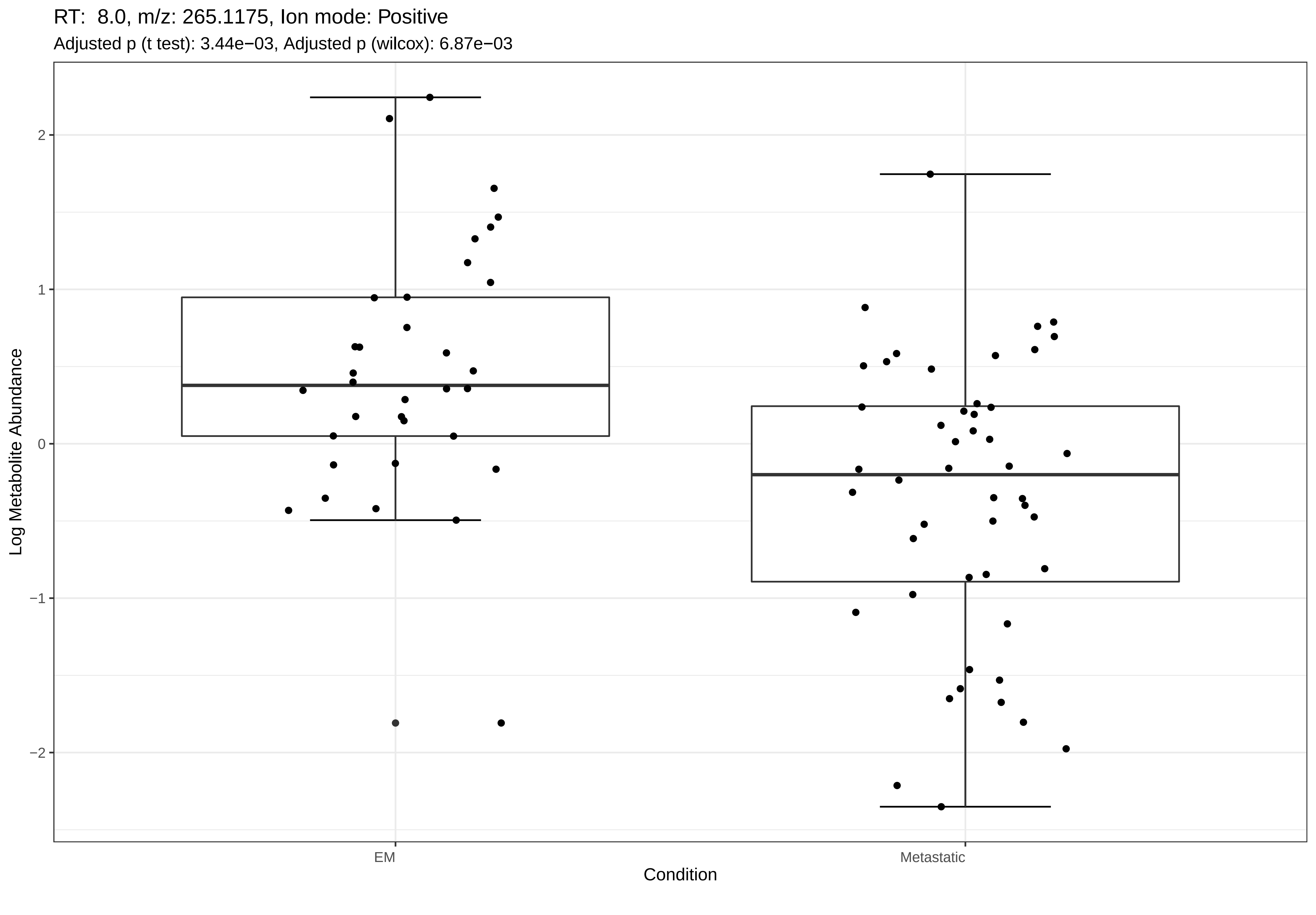
RT: 8.0, m/z: 265.1175, Ion mode: Positive
Adjusted p (t test): 3.44e−03, Adjusted p (wilcox): 6.87e−03
Log Metabolite Abundance
2
1
0
−1
−2
EM
Metastatic
Condition

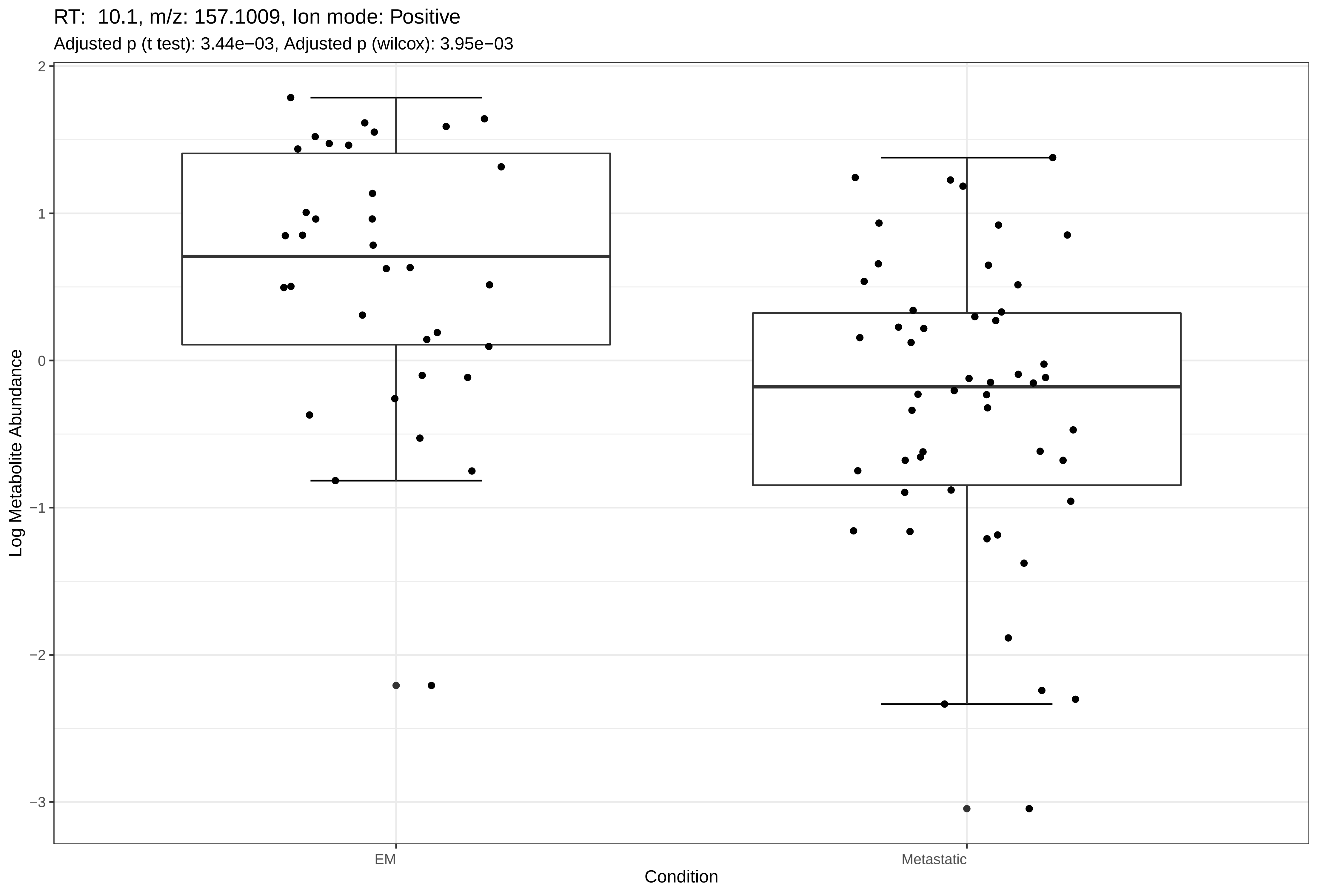
RT: 10.1, m/z: 157.1009, Ion mode: Positive
Adjusted p (t test): 3.44e−03, Adjusted p (wilcox): 3.95e−03
2
1
0
−1
−2
−3
Log Metabolite Abundance
EM
Metastatic
Condition

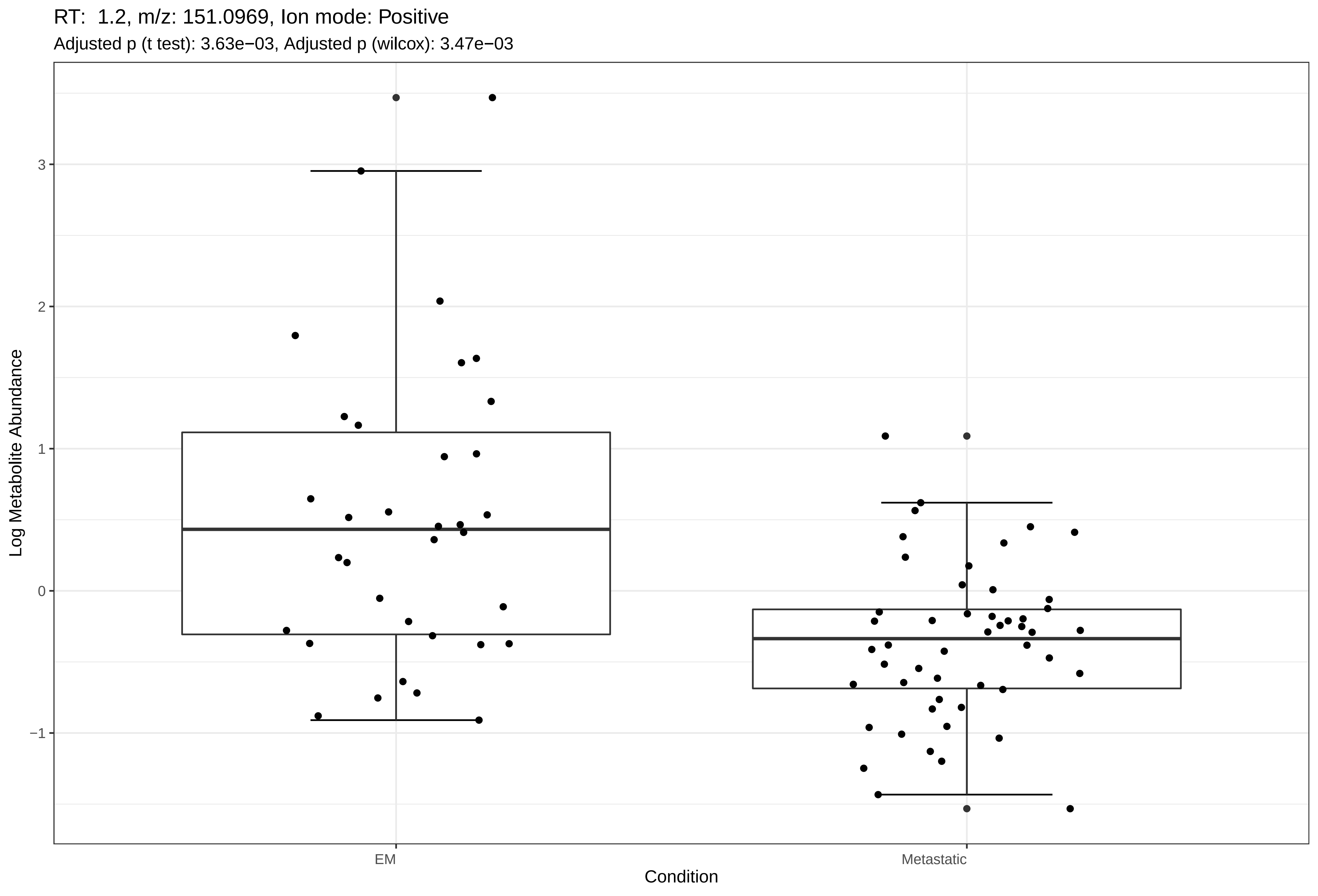
RT: 1.2, m/z: 151.0969, Ion mode: Positive
Adjusted p (t test): 3.63e−03, Adjusted p (wilcox): 3.47e−03
Log Metabolite Abundance
3
2
1
0
−1
EM
Metastatic
Condition

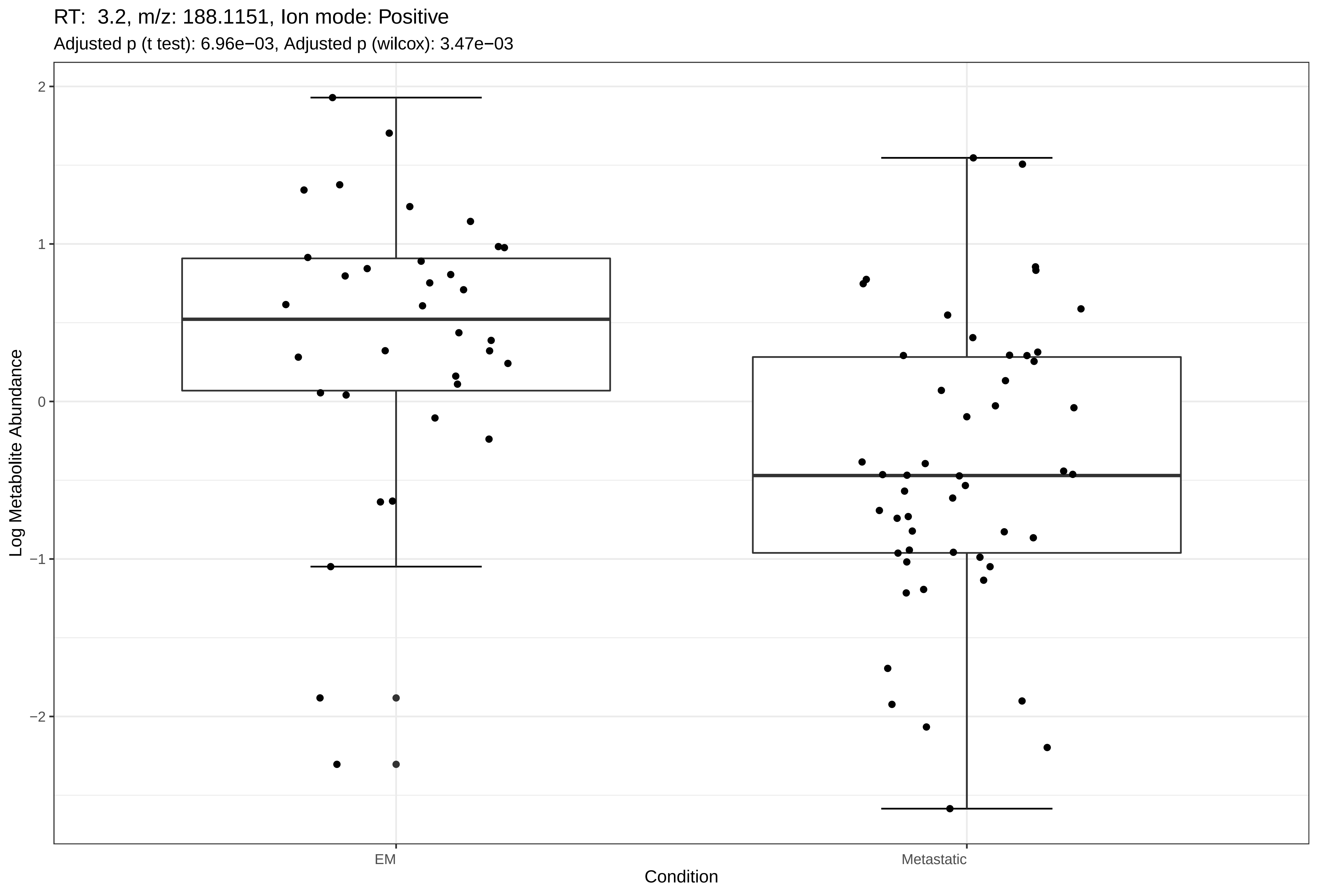
RT: 3.2, m/z: 188.1151, Ion mode: Positive
Adjusted p (t test): 6.96e−03, Adjusted p (wilcox): 3.47e−03
Log Metabolite Abundance
2
1
0
−1
−2
EM
Metastatic
Condition

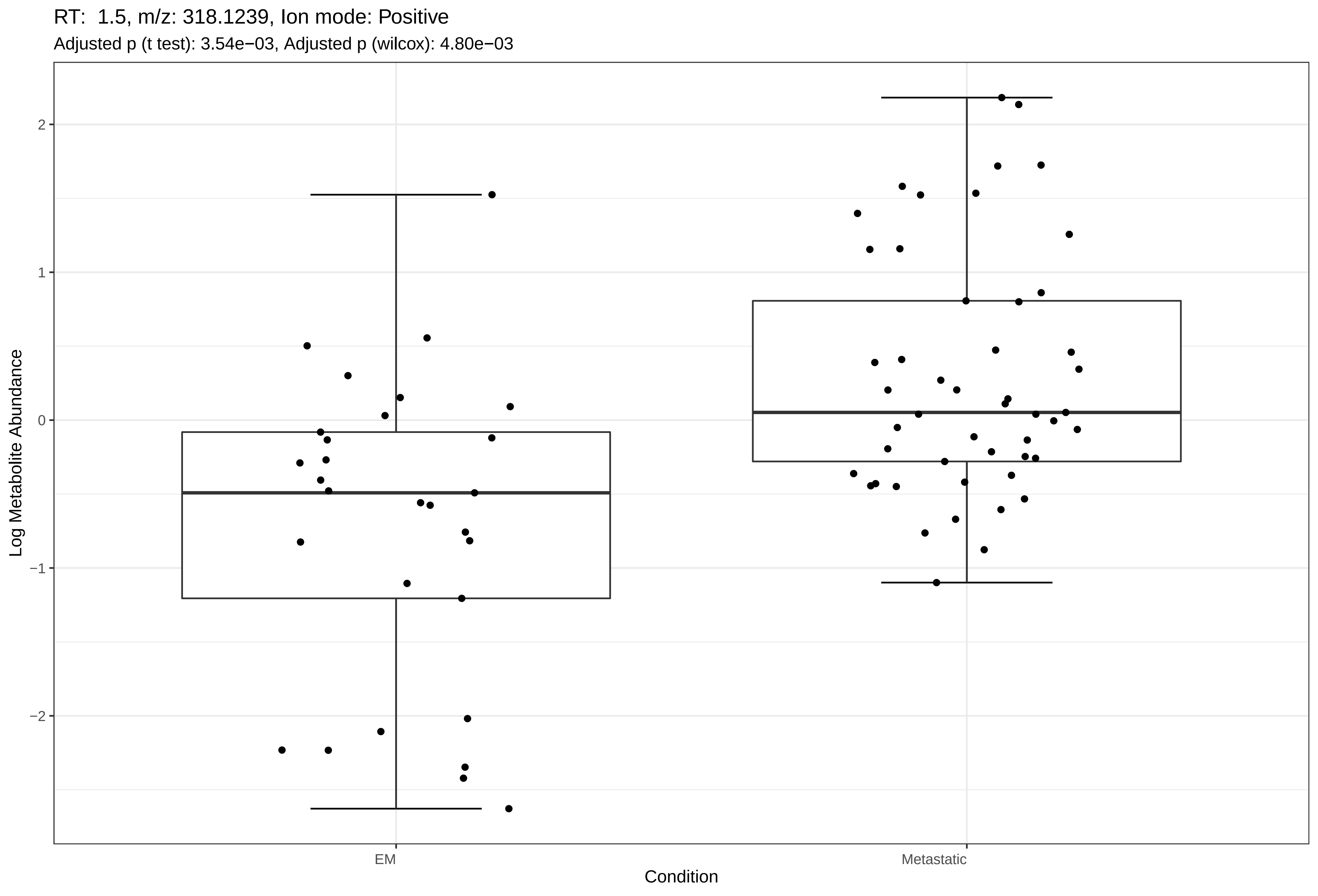

RT: 1.5, m/z: 318.1239, Ion mode: Positive
Adjusted p (t test): 3.54e−03, Adjusted p (wilcox): 4.80e−03
Log Metabolite Abundance
2
1
0
−1
−2
EM
Metastatic
Condition

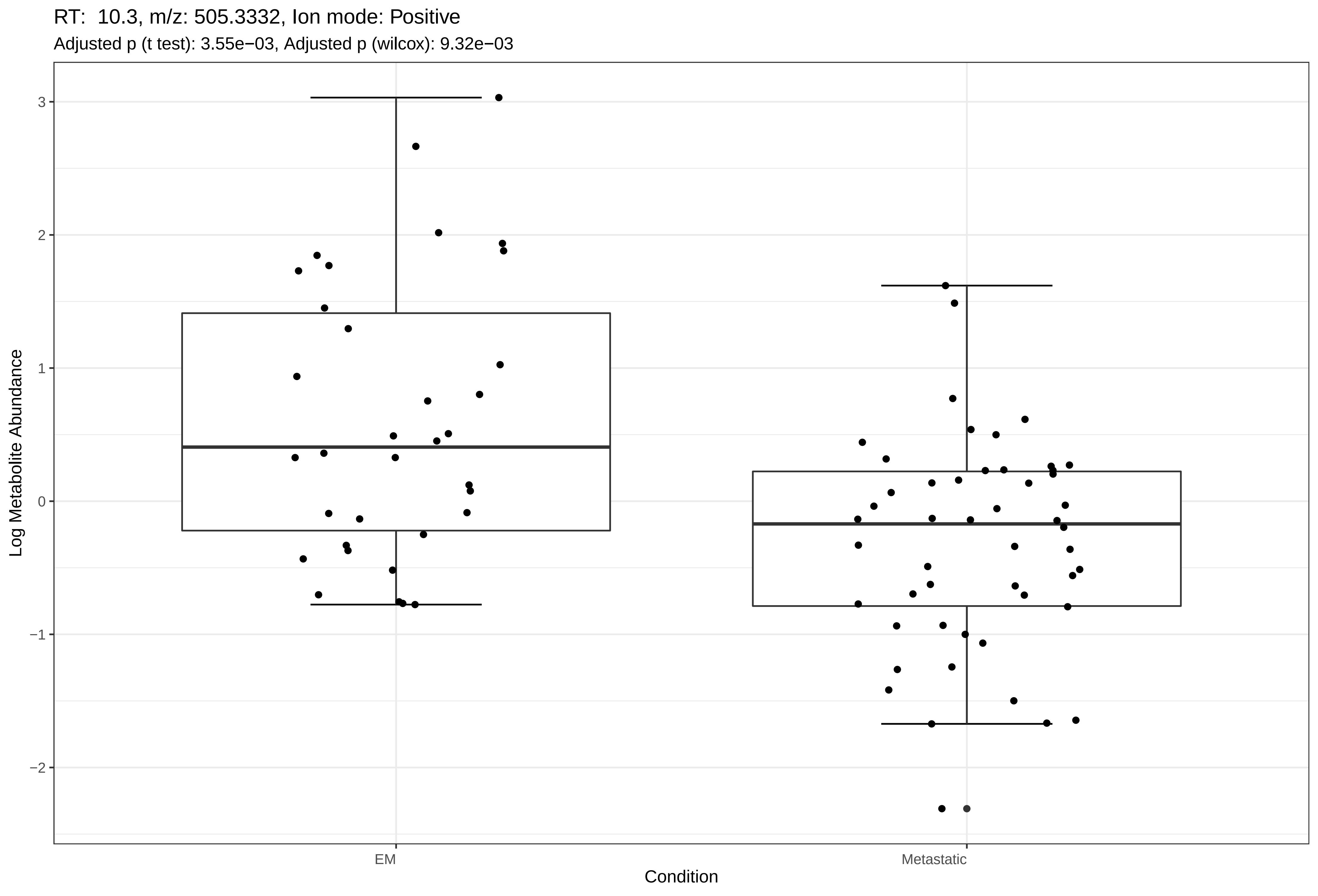
RT: 10.3, m/z: 505.3332, Ion mode: Positive
Adjusted p (t test): 3.55e−03, Adjusted p (wilcox): 9.32e−03
Log Metabolite Abundance
3
2
1
0
−1
−2
EM
Metastatic
Condition

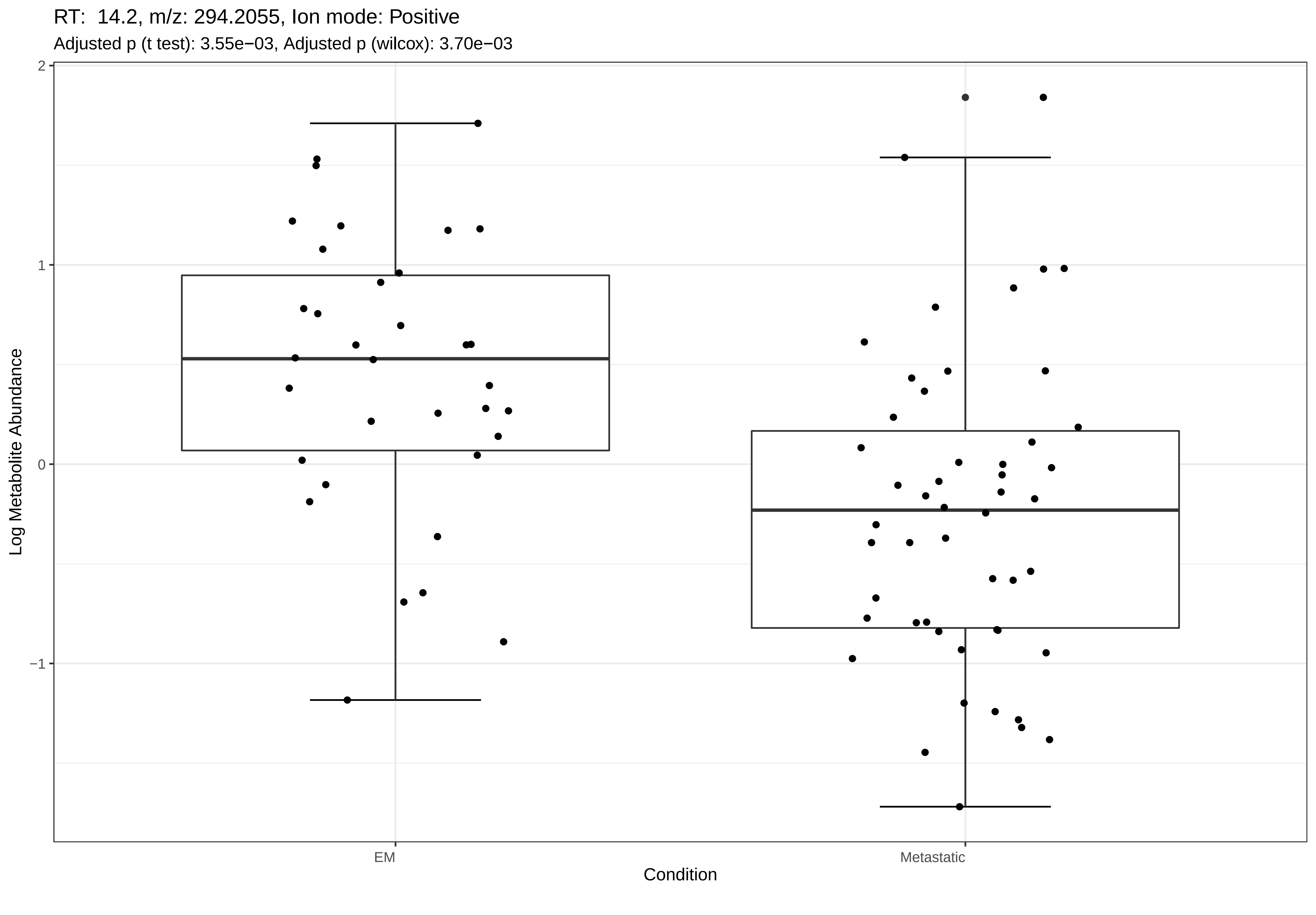

RT: 14.2, m/z: 294.2055, Ion mode: Positive
Adjusted p (t test): 3.55e−03, Adjusted p (wilcox): 3.70e−03
Log Metabolite Abundance
2
1
0
−1
EM
Metastatic
Condition

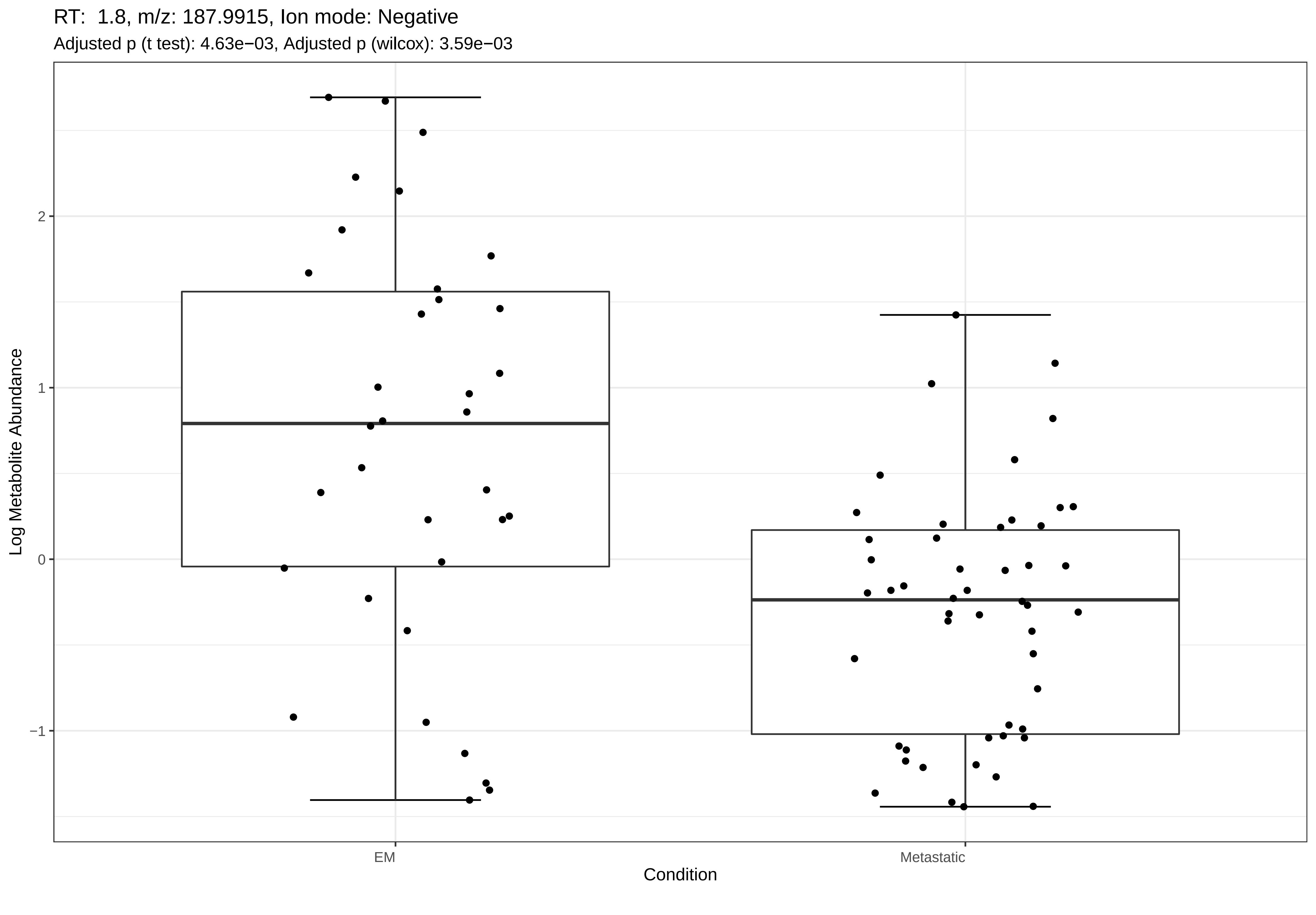
RT: 1.8, m/z: 187.9915, Ion mode: Negative
Adjusted p (t test): 4.63e−03, Adjusted p (wilcox): 3.59e−03
Log Metabolite Abundance
2
1
0
−1
EM
Metastatic
Condition

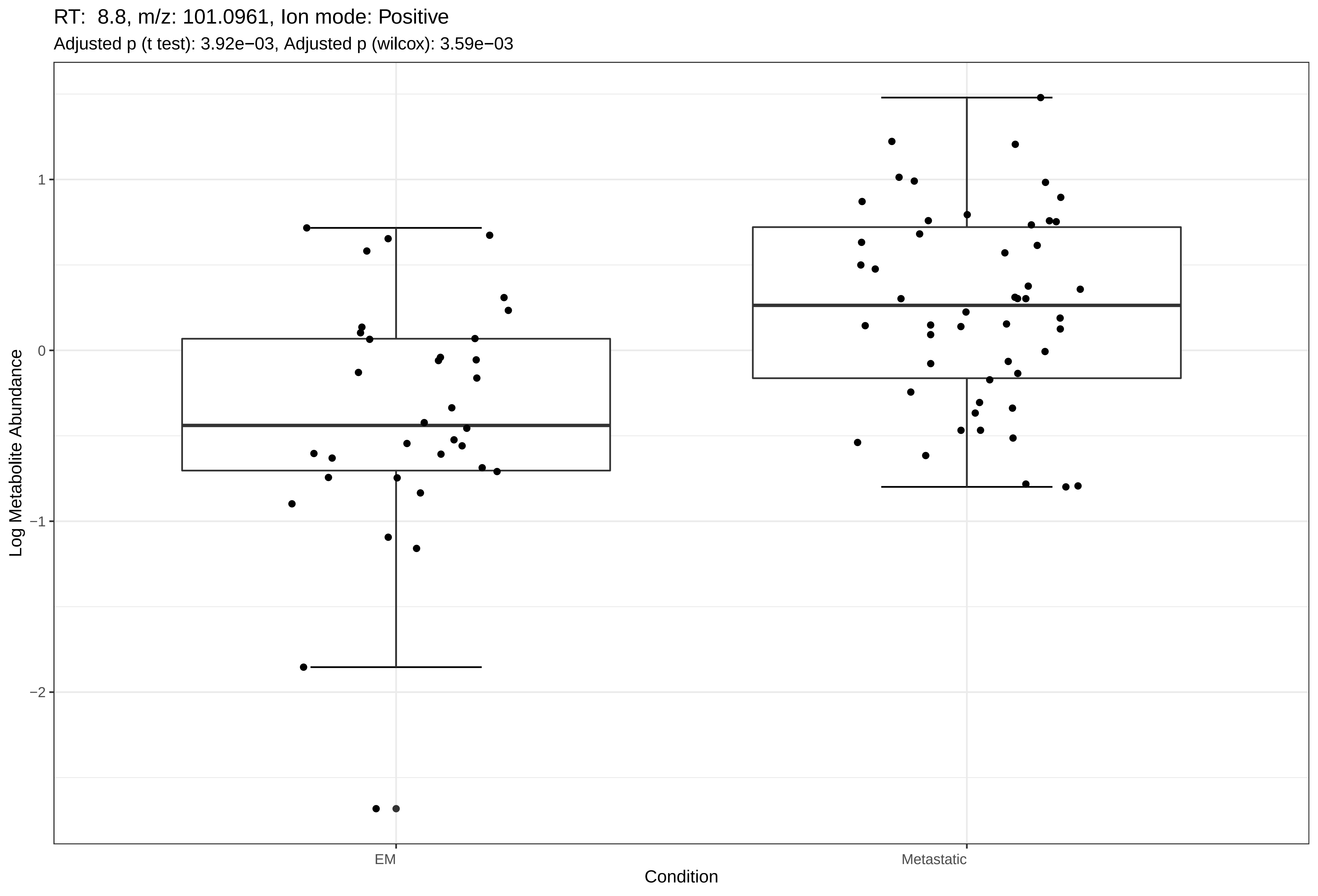

RT: 8.8, m/z: 101.0961, Ion mode: Positive
Adjusted p (t test): 3.92e−03, Adjusted p (wilcox): 3.59e−03
Log Metabolite Abundance
1
0
−1
−2
EM
Metastatic
Condition

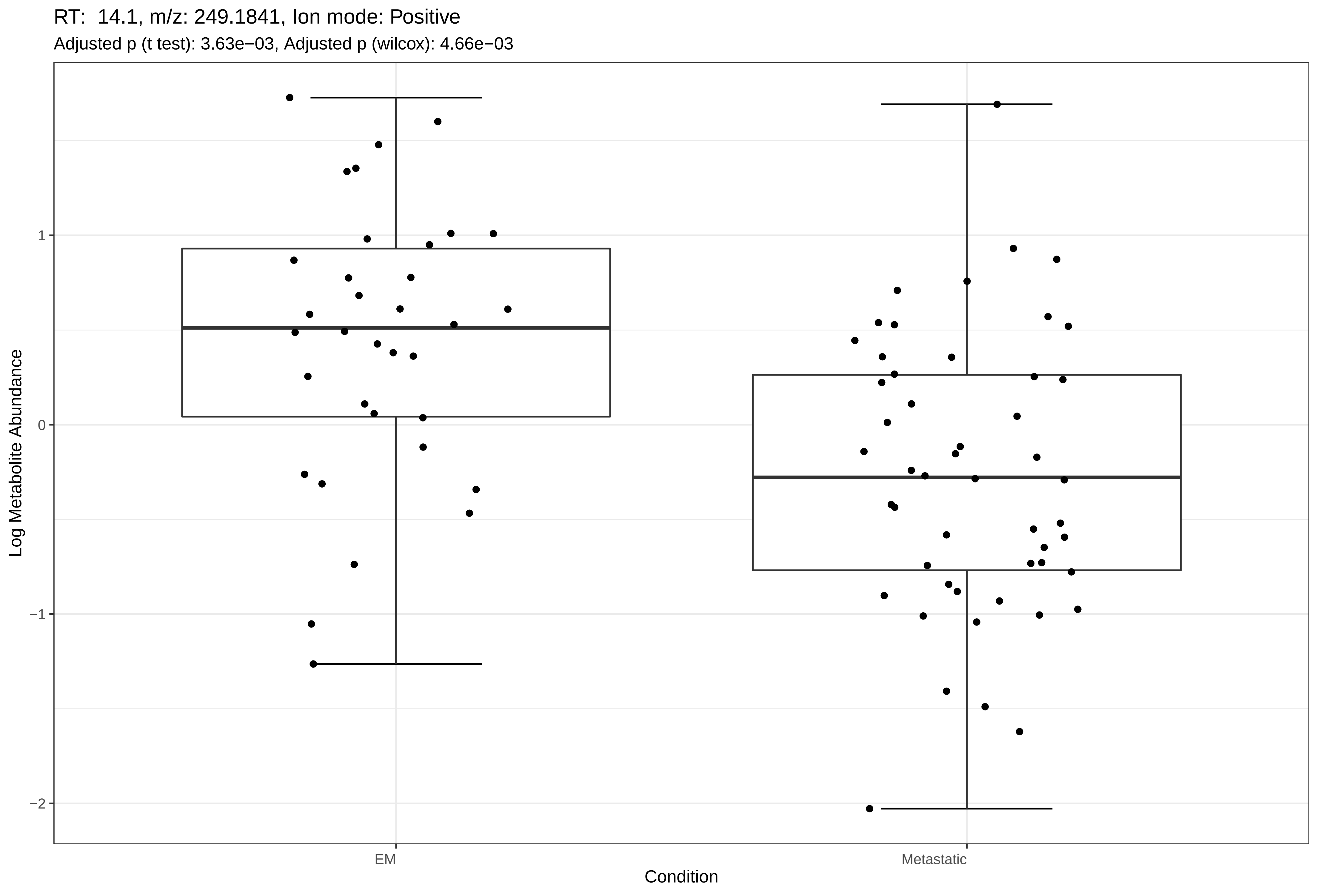

RT: 14.1, m/z: 249.1841, Ion mode: Positive
Adjusted p (t test): 3.63e−03, Adjusted p (wilcox): 4.66e−03
Log Metabolite Abundance
1
0
−1
−2
EM
Metastatic
Condition

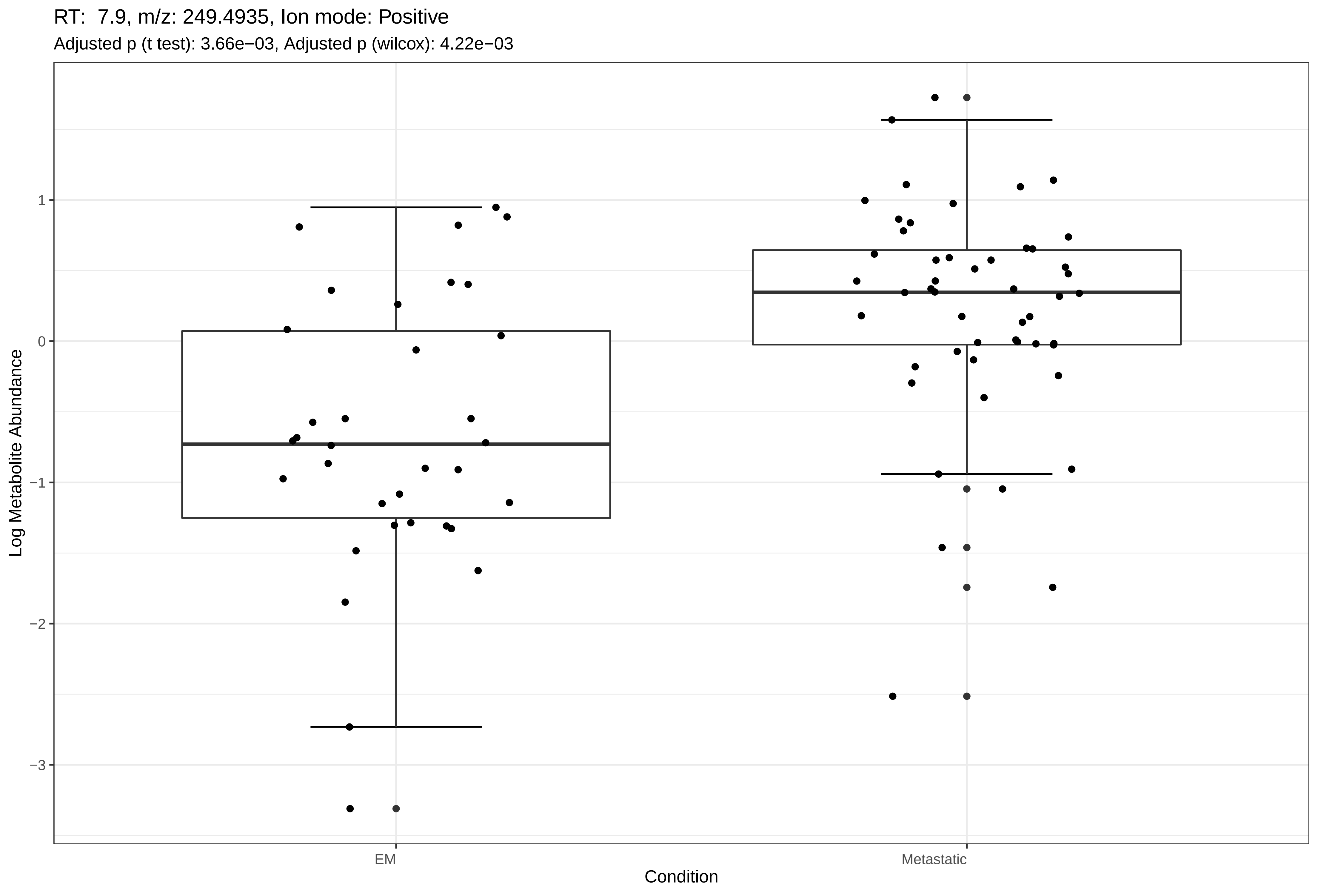

RT: 7.9, m/z: 249.4935, Ion mode: Positive
Adjusted p (t test): 3.66e−03, Adjusted p (wilcox): 4.22e−03
Log Metabolite Abundance
1
0
−1
−2
−3
EM
Metastatic
Condition

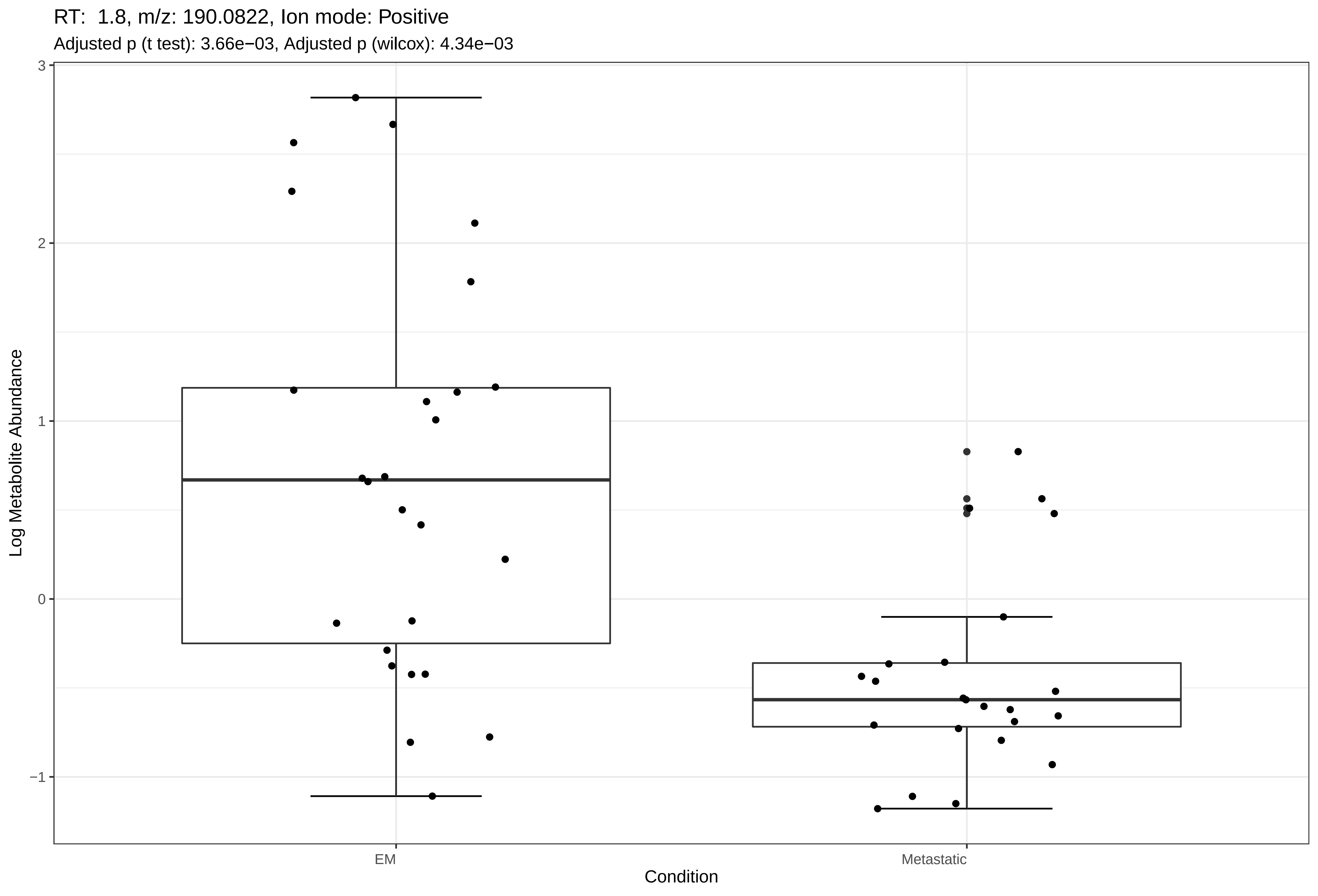
RT: 1.8, m/z: 190.0822, Ion mode: Positive
Adjusted p (t test): 3.66e−03, Adjusted p (wilcox): 4.34e−03
Log Metabolite Abundance
3
2
1
0
−1
EM
Metastatic
Condition

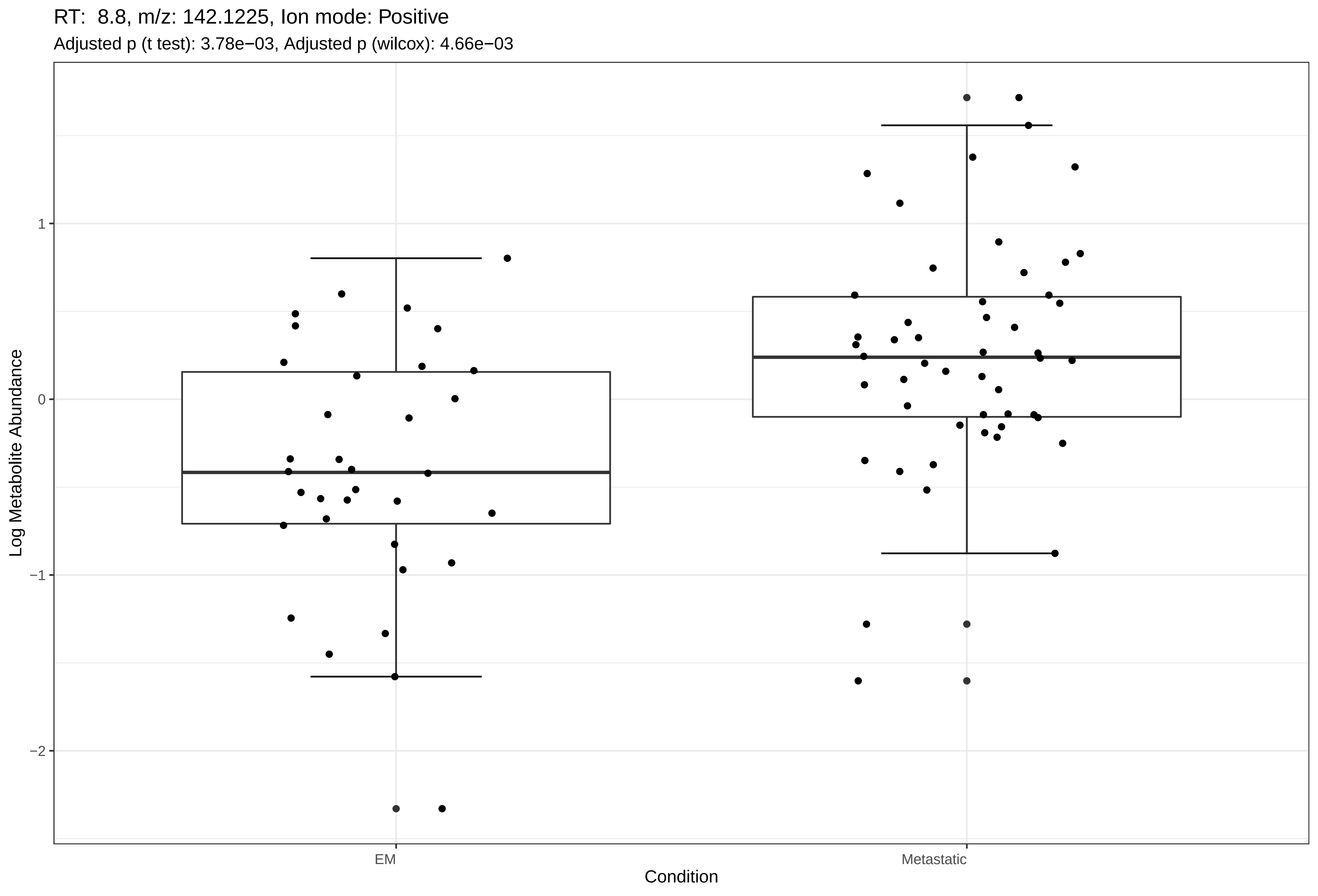

RT: 8.8, m/z: 142.1225, Ion mode: Positive
Adjusted p (t test): 3.78e−03, Adjusted p (wilcox): 4.66e−03
Log Metabolite Abundance
1
0
−1
−2
EM
Metastatic
Condition

RT: 5.0, m/z: 353.2316, Ion mode: Positive

Adjusted p (t test): 3.83e−03, Adjusted p (wilcox): 5.29e−03

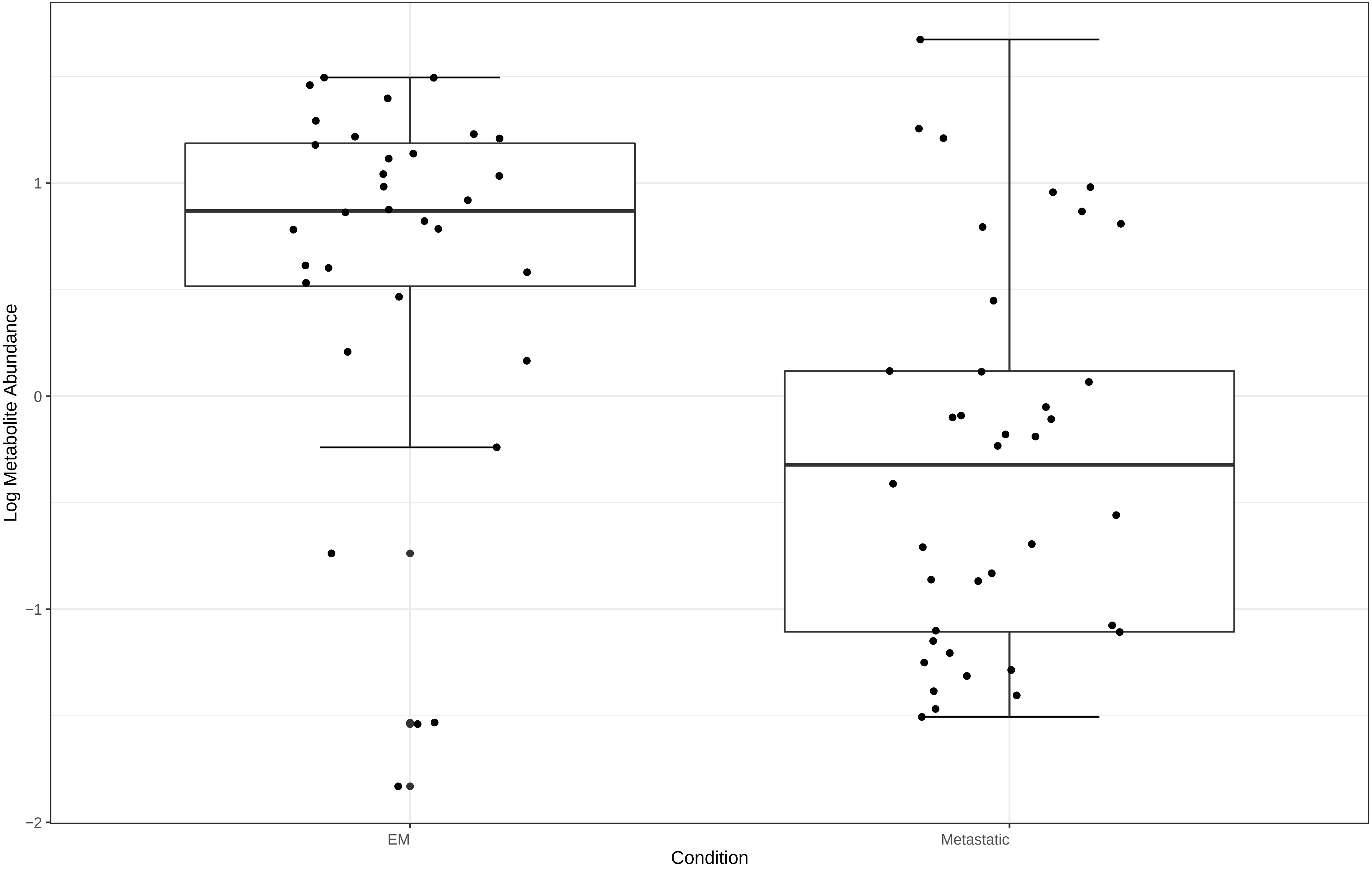

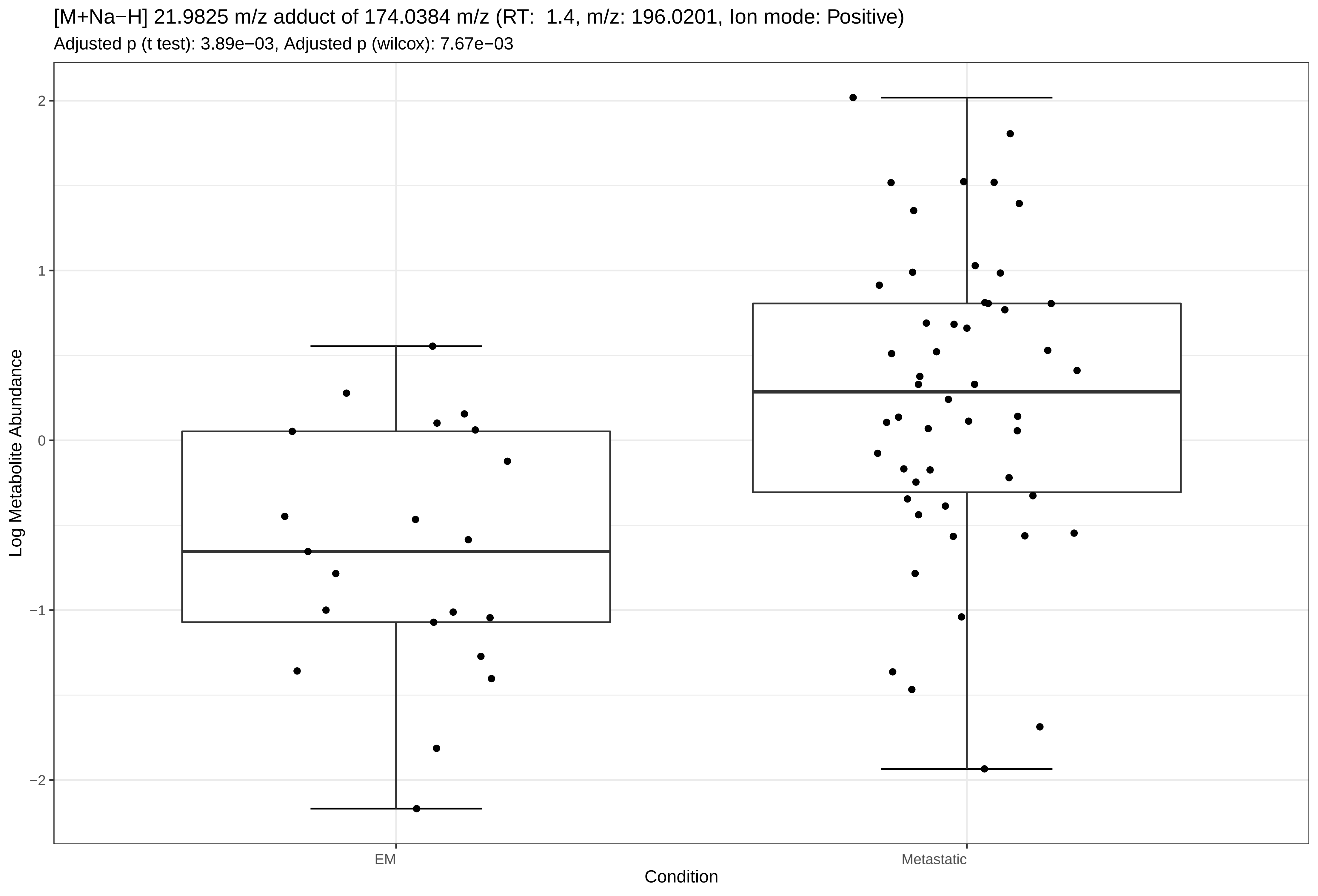

[M+Na−H] 21.9825 m/z adduct of 174.0384 m/z (RT: 1.4, m/z: 196.0201, Ion mode: Positive)
Adjusted p (t test): 3.89e−03, Adjusted p (wilcox): 7.67e−03
Log Metabolite Abundance
2
1
0
−1
−2
EM
Metastatic
Condition

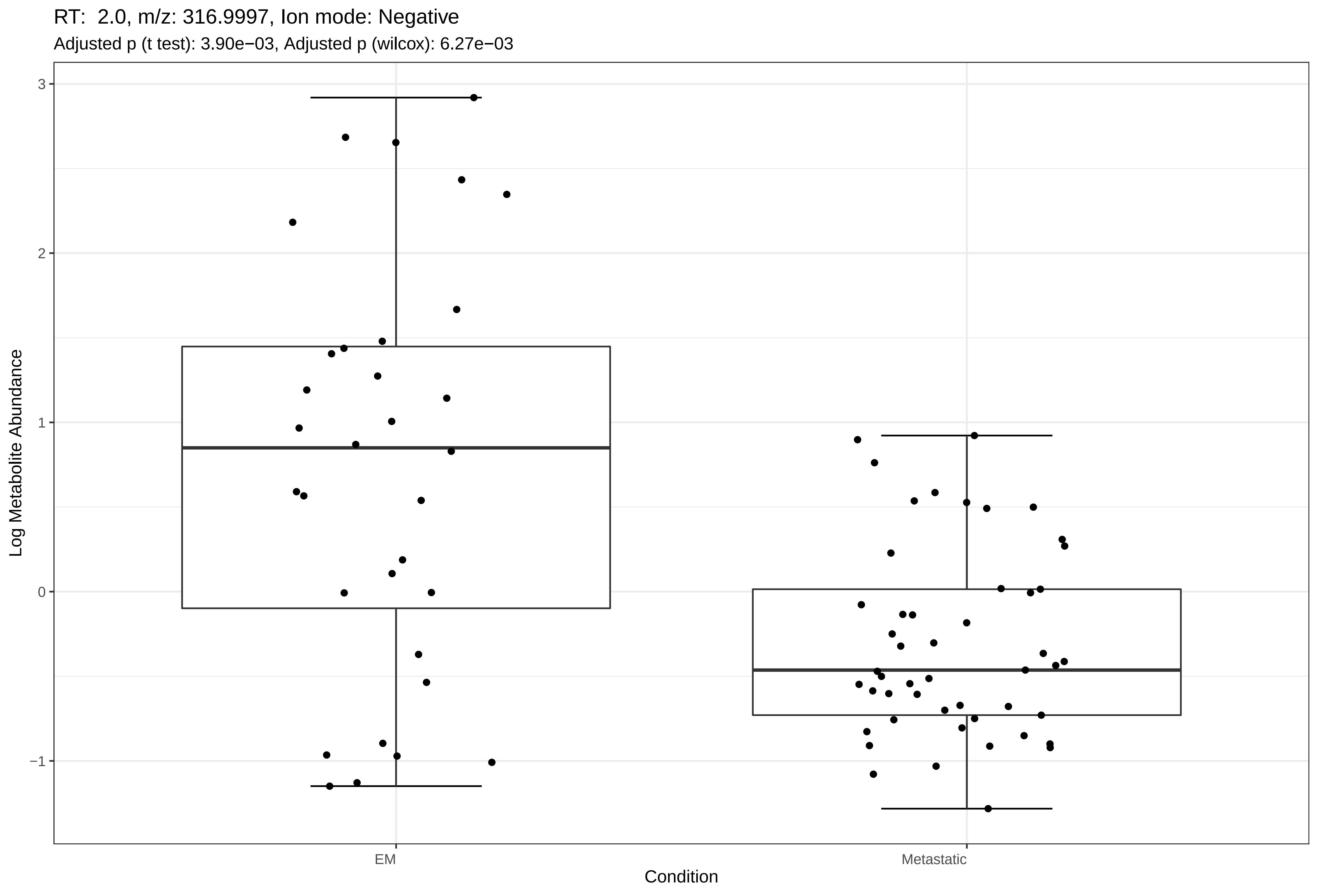
RT: 2.0, m/z: 316.9997, Ion mode: Negative
Adjusted p (t test): 3.90e−03, Adjusted p (wilcox): 6.27e−03
Log Metabolite Abundance
3
2
1
0
−1
EM
Metastatic
Condition

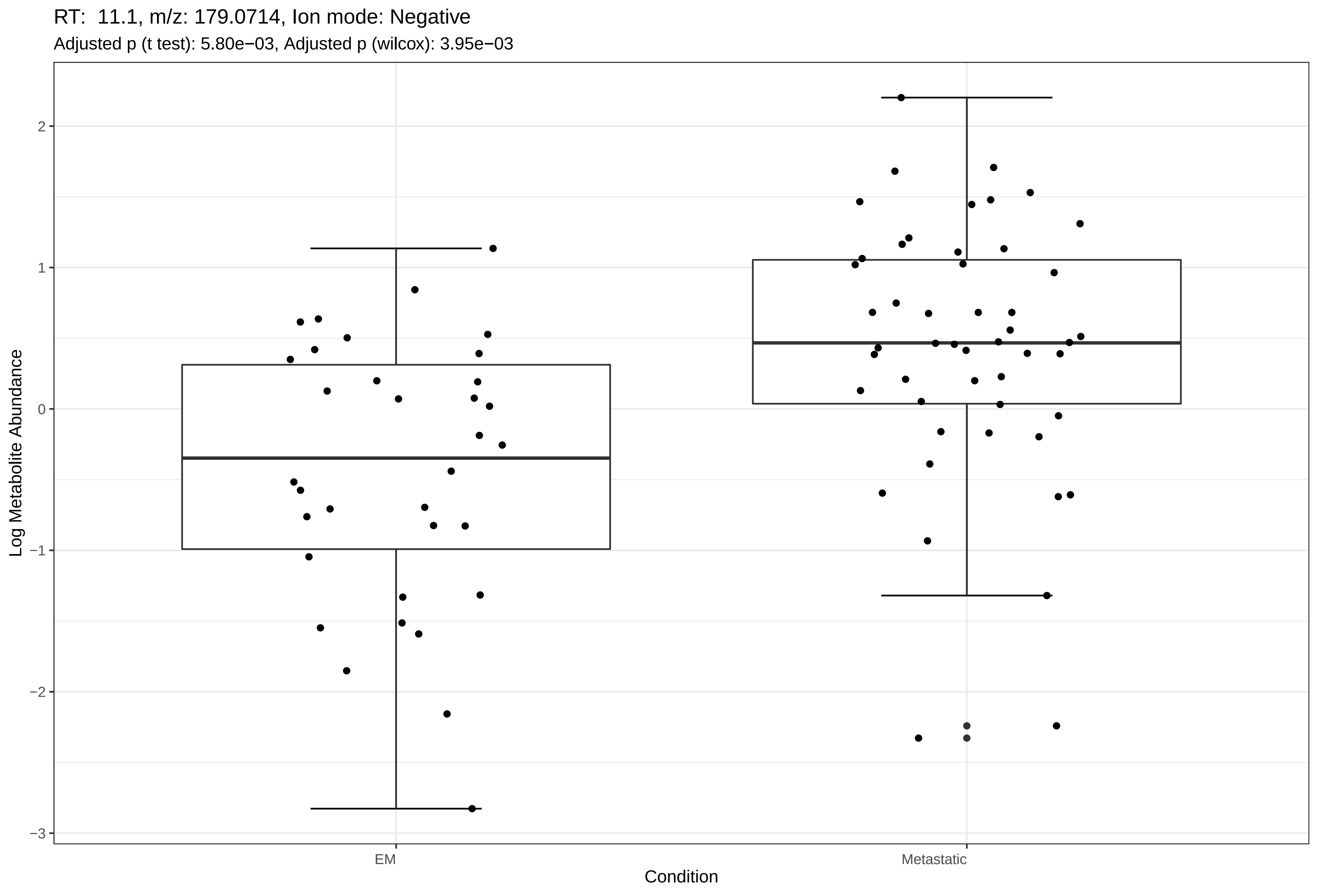
RT: 11.1, m/z: 179.0714, Ion mode: Negative
Adjusted p (t test): 5.80e−03, Adjusted p (wilcox): 3.95e−03
Log Metabolite Abundance
2
1
0
−1
−2
−3
EM
Metastatic
Condition

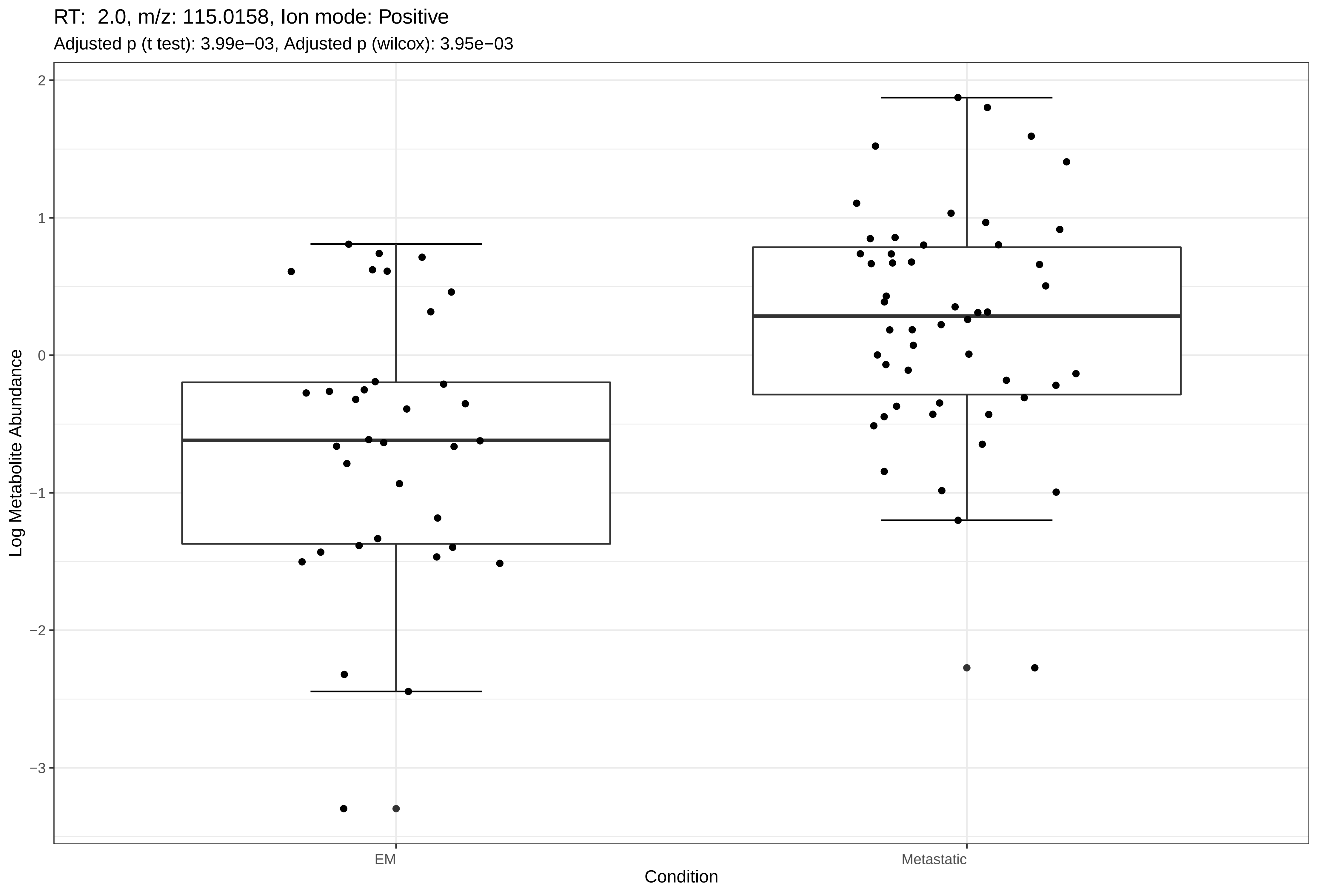
RT: 2.0, m/z: 115.0158, Ion mode: Positive
Adjusted p (t test): 3.99e−03, Adjusted p (wilcox): 3.95e−03
Log Metabolite Abundance
2
1
0
−1
−2
−3
EM
Metastatic
Condition

RT: 1.2, m/z: 174.0343, Ion mode: Positive

Adjusted p (t test): 4.01e−03, Adjusted p (wilcox): 5.12e−03

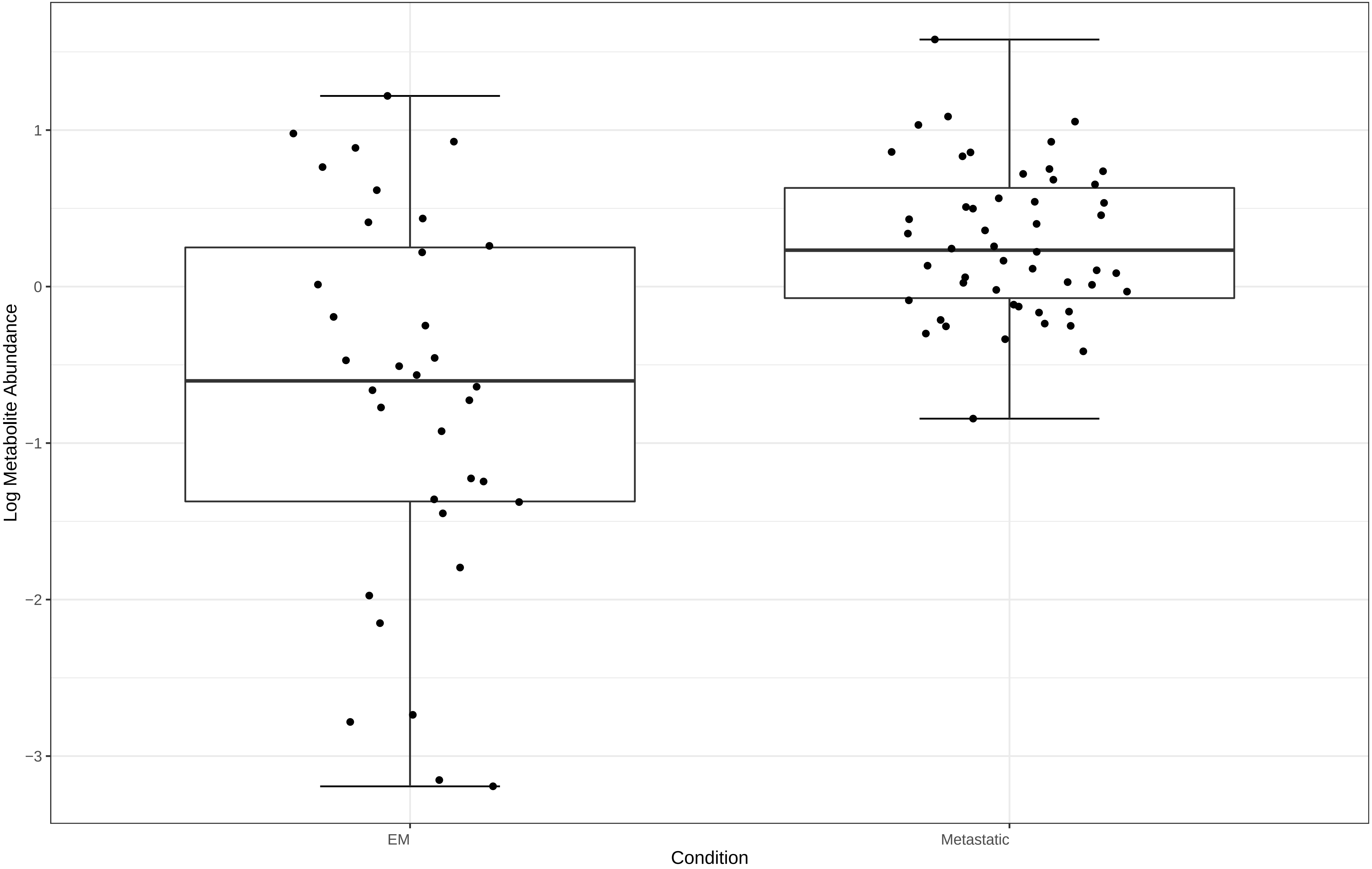

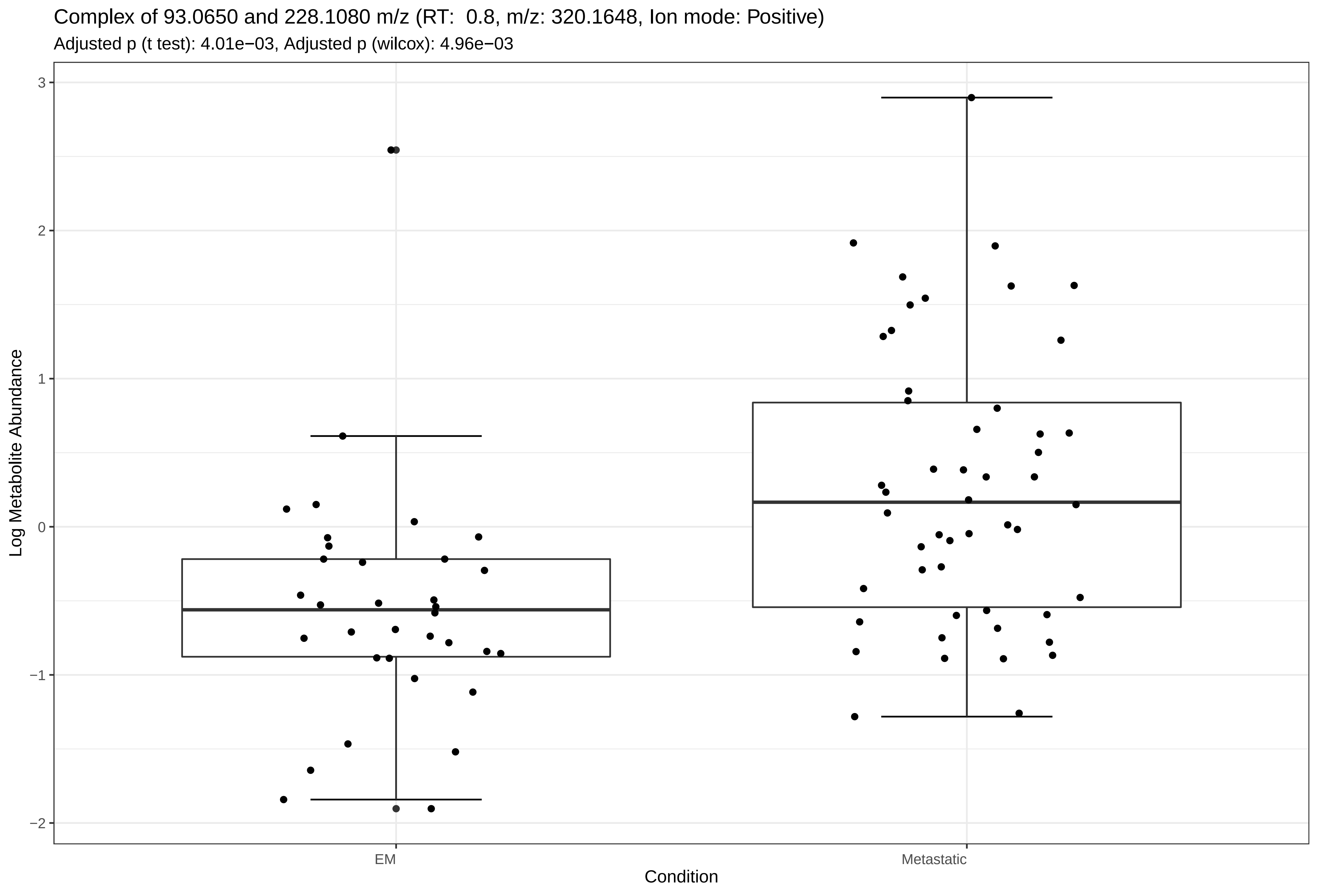

Complex of 93.0650 and 228.1080 m/z (RT: 0.8, m/z: 320.1648, Ion mode: Positive)
Adjusted p (t test): 4.01e−03, Adjusted p (wilcox): 4.96e−03
Log Metabolite Abundance
3
2
1
0
−1
−2
EM
Metastatic
Condition

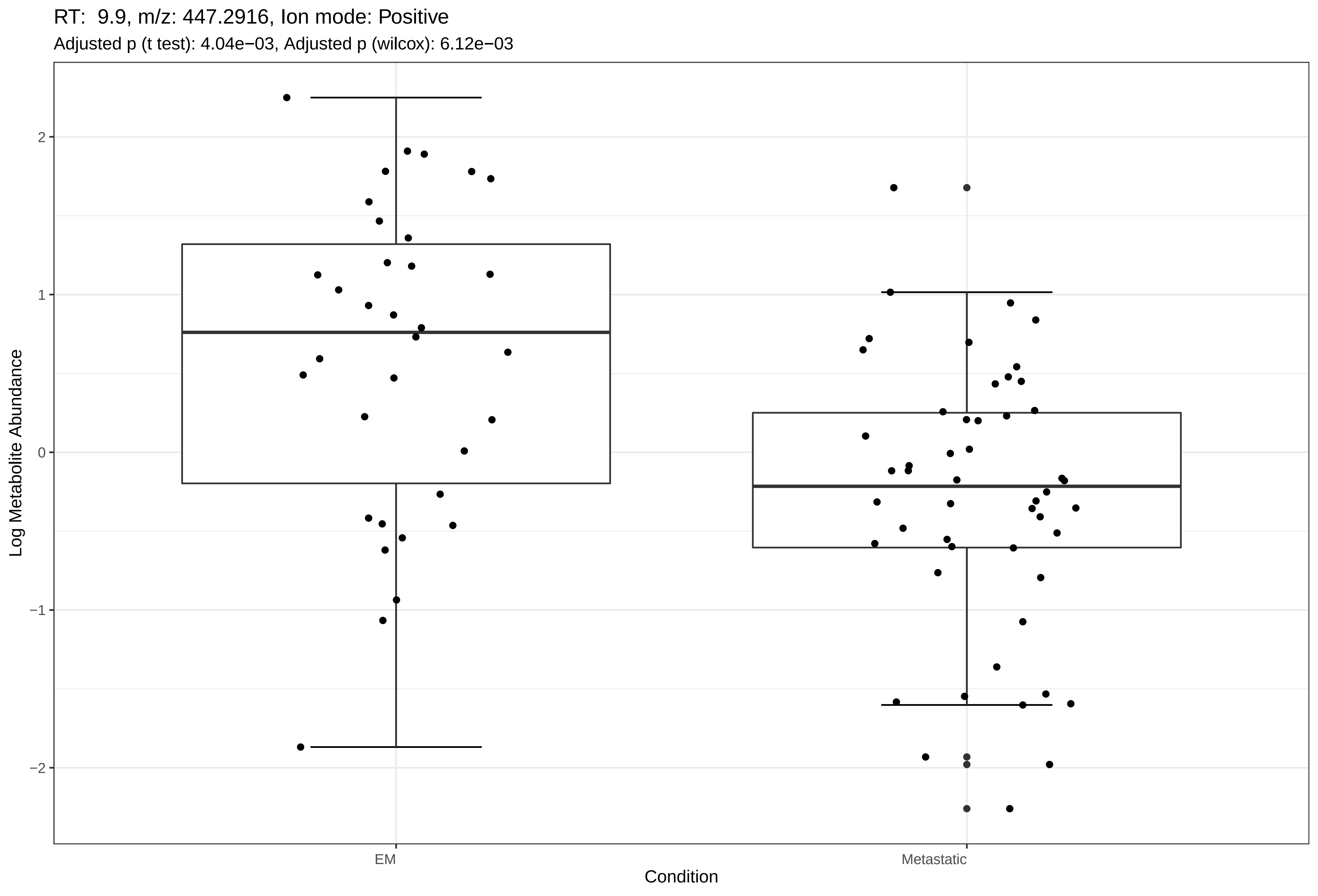

RT: 9.9, m/z: 447.2916, Ion mode: Positive
Adjusted p (t test): 4.04e−03, Adjusted p (wilcox): 6.12e−03
Log Metabolite Abundance
2
1
0
−1
−2
EM
Metastatic
Condition

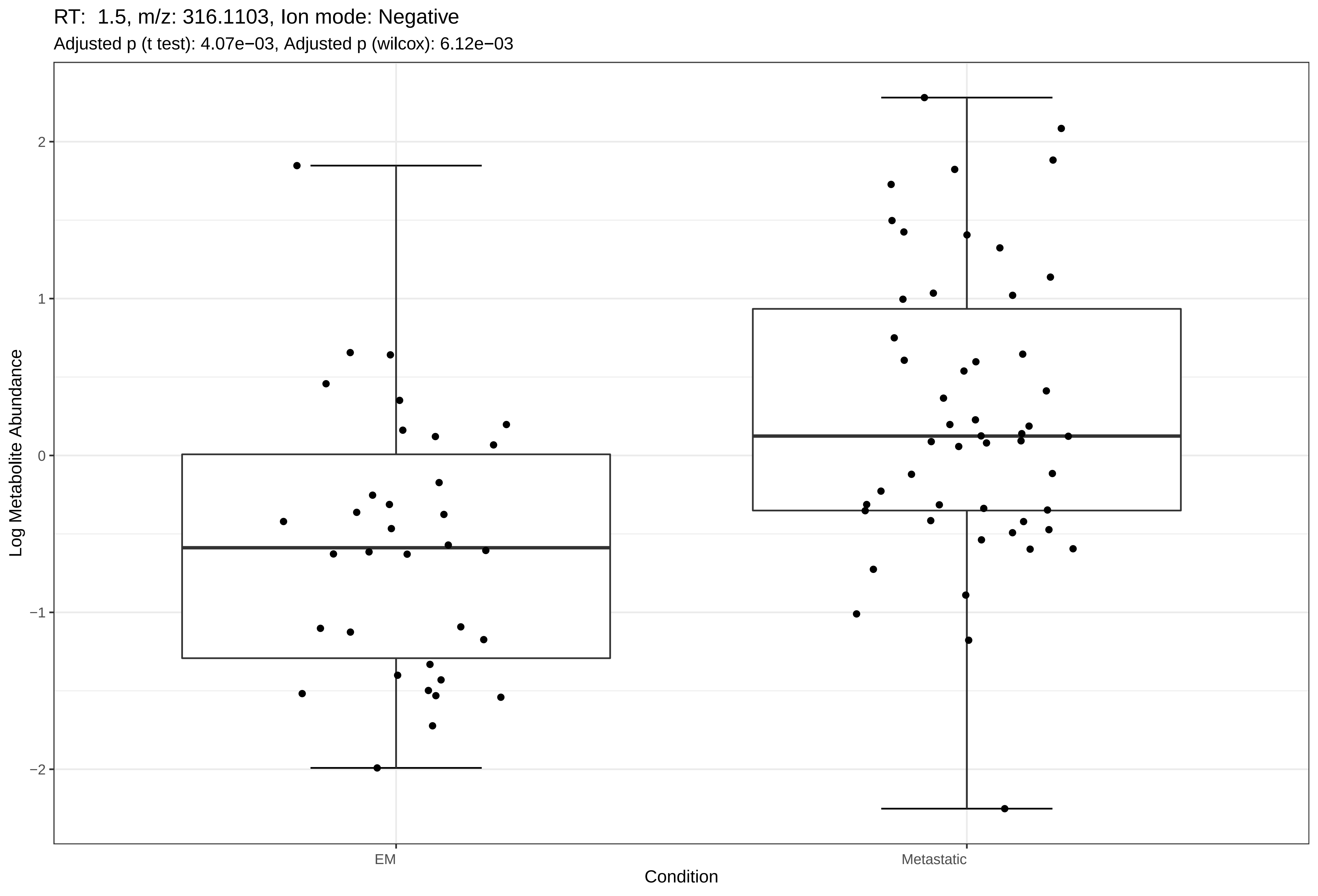
RT: 1.5, m/z: 316.1103, Ion mode: Negative
Adjusted p (t test): 4.07e−03, Adjusted p (wilcox): 6.12e−03
Log Metabolite Abundance
2
1
0
−1
−2
EM
Metastatic
Condition

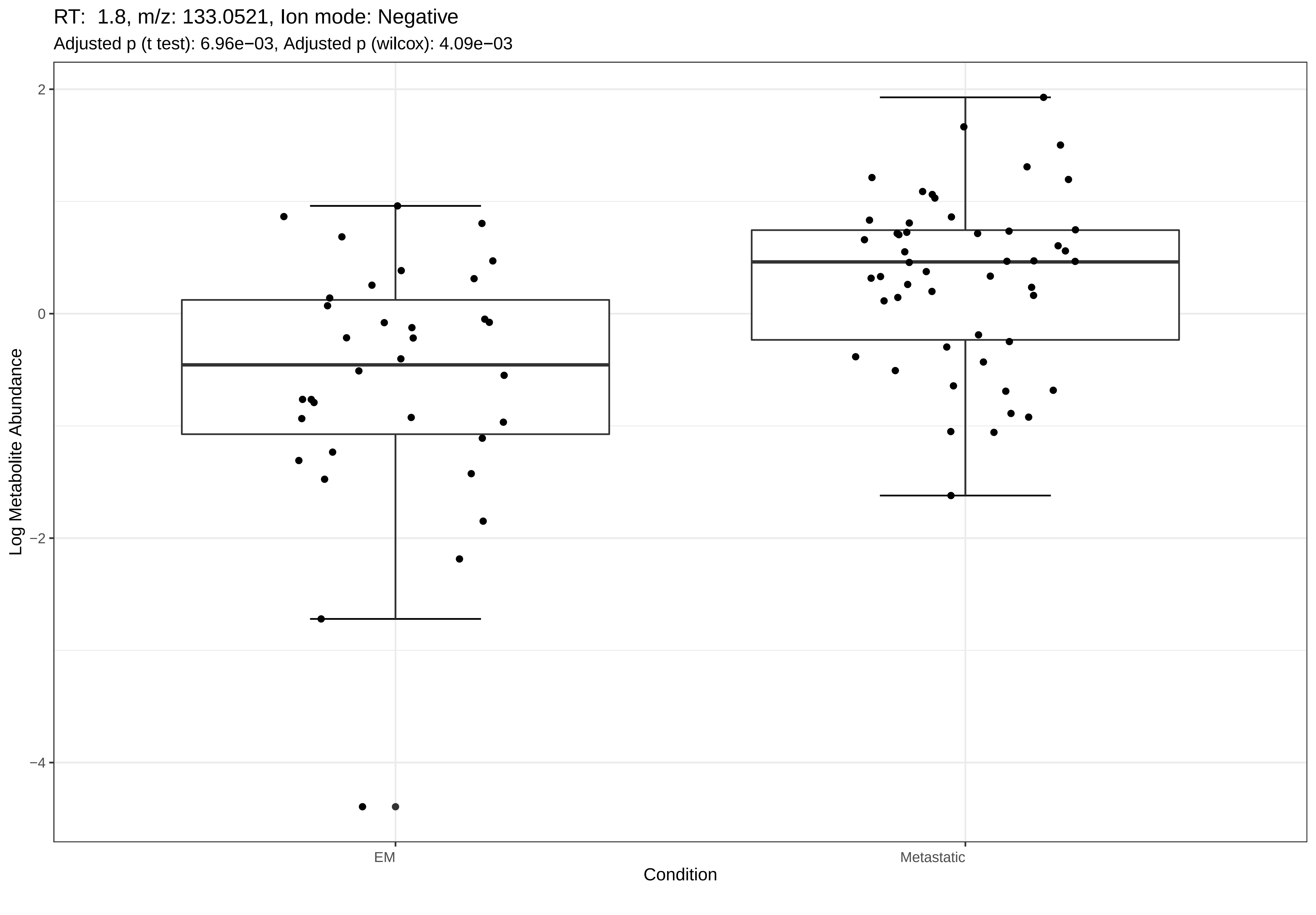

RT: 1.8, m/z: 133.0521, Ion mode: Negative
Adjusted p (t test): 6.96e−03, Adjusted p (wilcox): 4.09e−03
Log Metabolite Abundance
2
0
−2
−4
EM
Metastatic
Condition

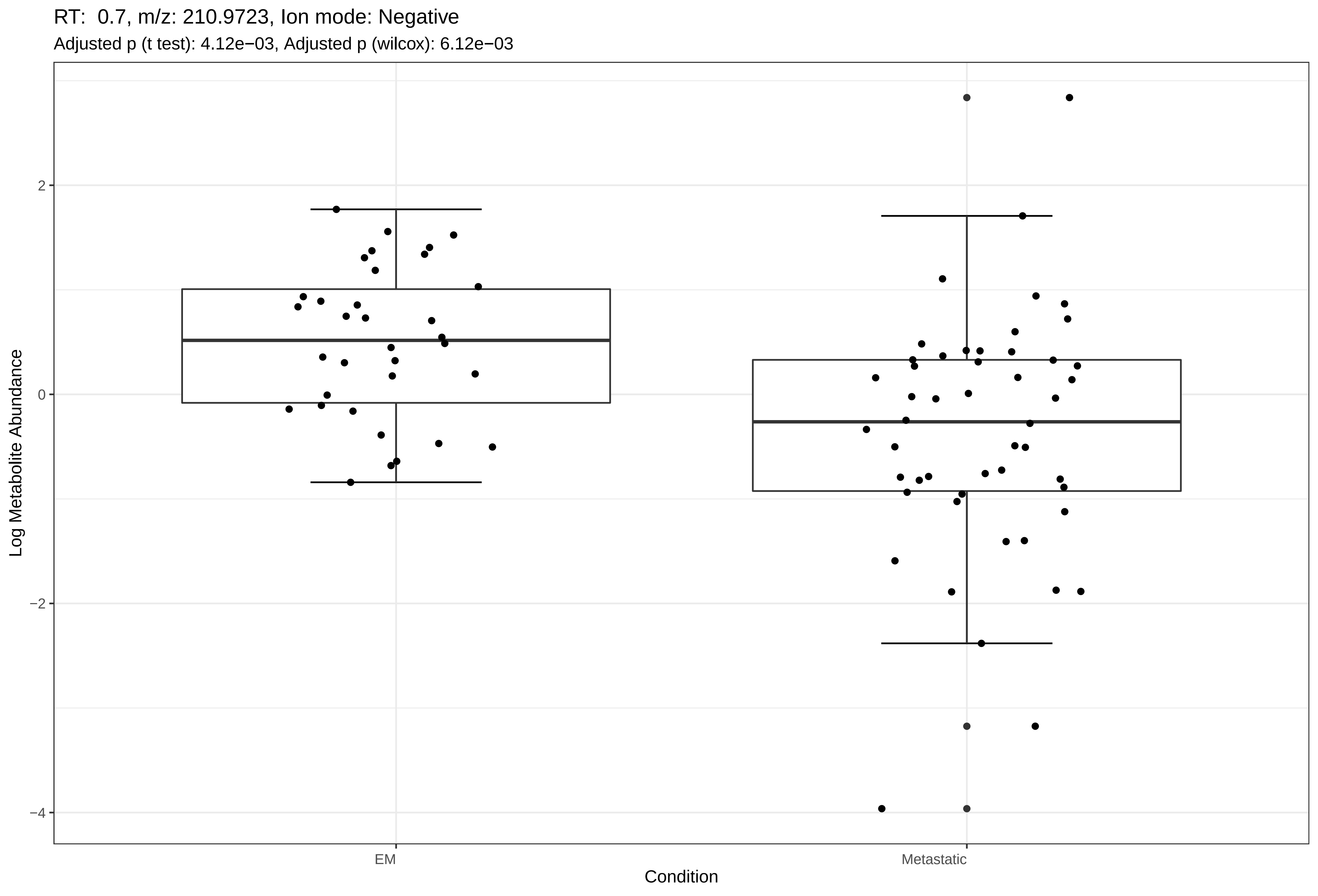
RT: 0.7, m/z: 210.9723, Ion mode: Negative
Adjusted p (t test): 4.12e−03, Adjusted p (wilcox): 6.12e−03
2
0
−2
−4
Log Metabolite Abundance
EM
Metastatic
Condition

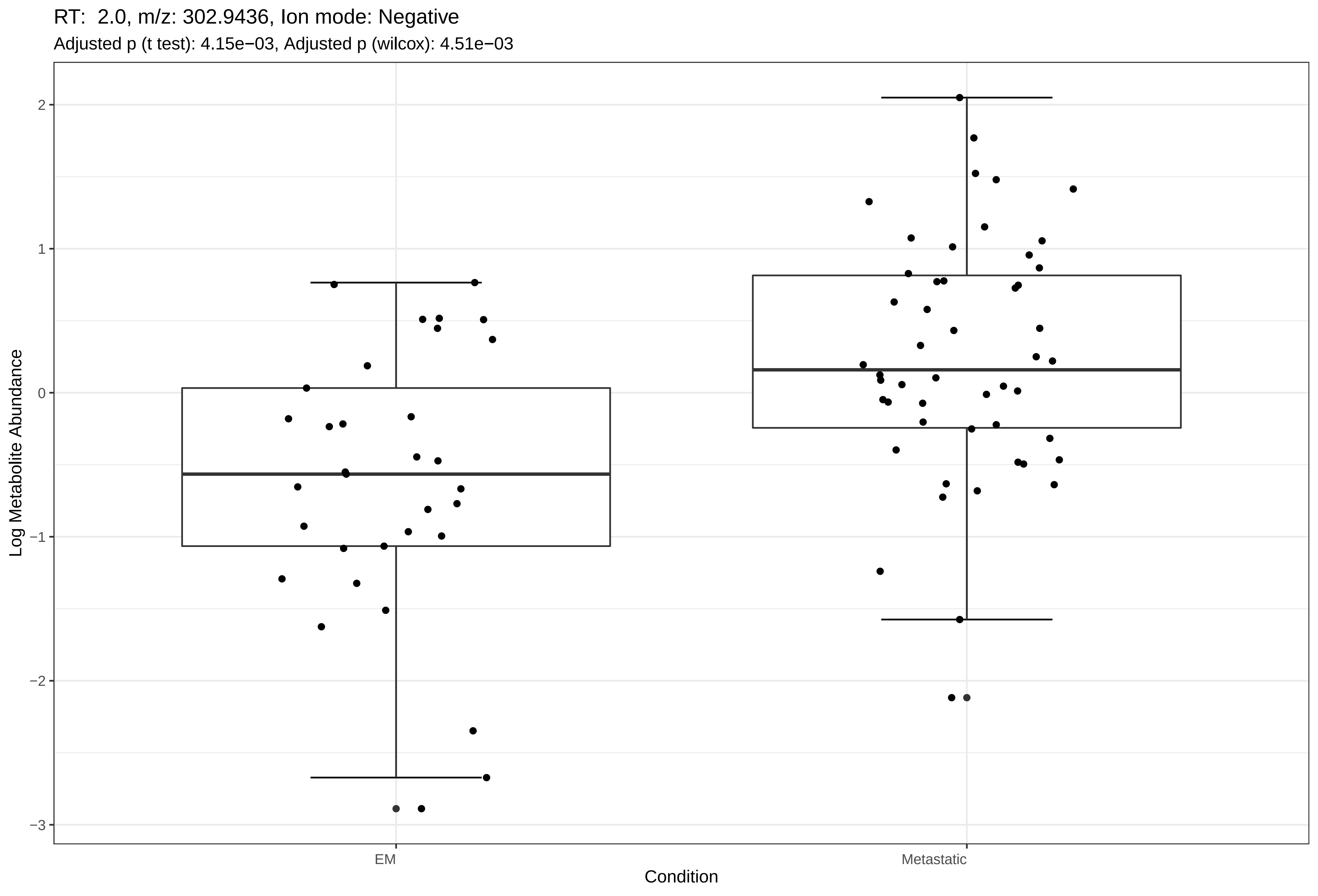
RT: 2.0, m/z: 302.9436, Ion mode: Negative
Adjusted p (t test): 4.15e−03, Adjusted p (wilcox): 4.51e−03
Log Metabolite Abundance
2
1
0
−1
−2
−3
EM
Metastatic
Condition

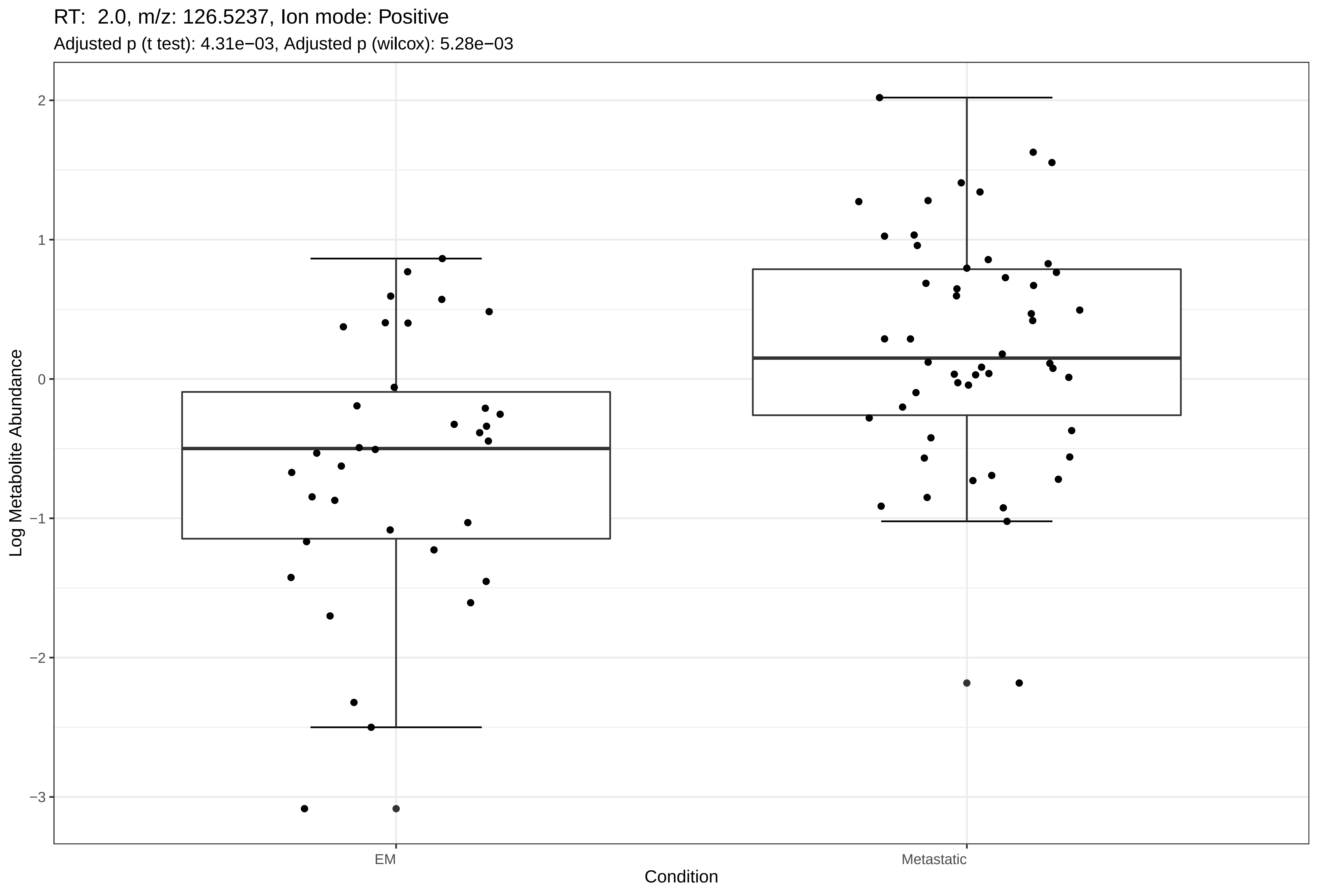

RT: 2.0, m/z: 126.5237, Ion mode: Positive
Adjusted p (t test): 4.31e−03, Adjusted p (wilcox): 5.28e−03
Log Metabolite Abundance
2
1
0
−1
−2
−3
EM
Metastatic
Condition

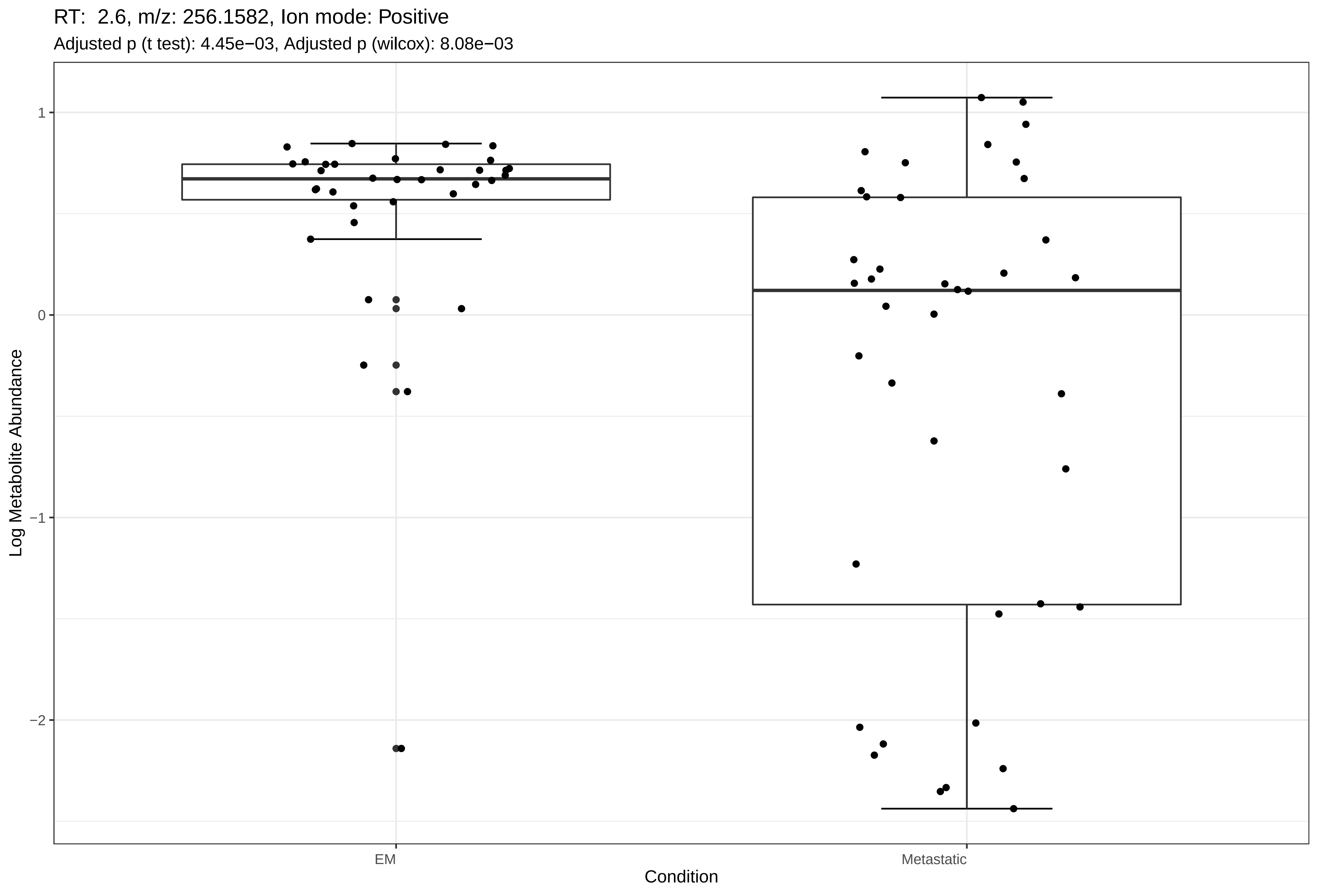
RT: 2.6, m/z: 256.1582, Ion mode: Positive
Adjusted p (t test): 4.45e−03, Adjusted p (wilcox): 8.08e−03
Log Metabolite Abundance
1
0
−1
−2
EM
Metastatic
Condition

[M−HCOOH+H] −46.0055 m/z adduct of 130.0498 m/z (RT: 0.7, m/z: 84.0444, Ion mode: Positive)

Adjusted p (t test): 4.48e−03, Adjusted p (wilcox): 7.73e−03

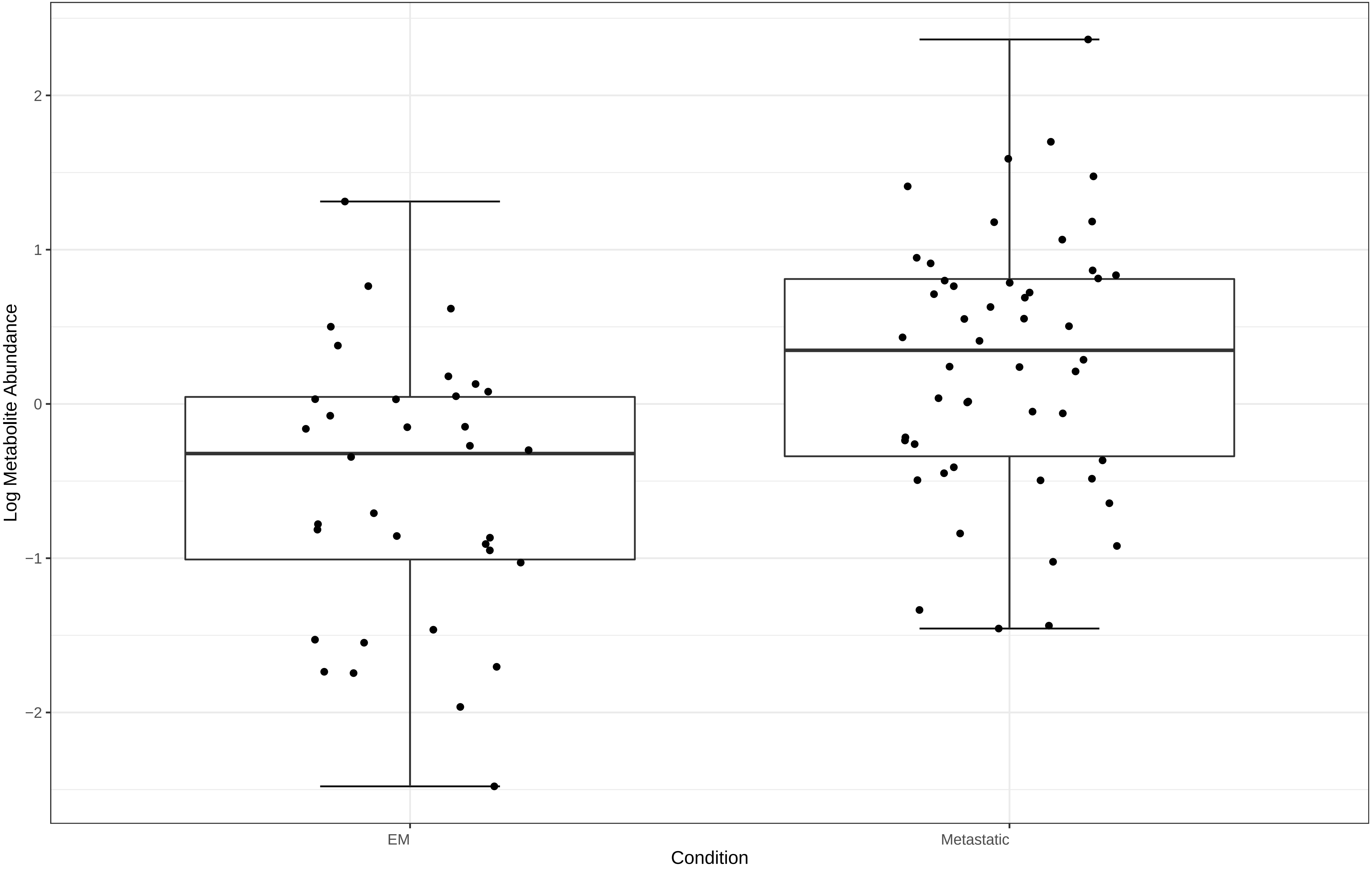

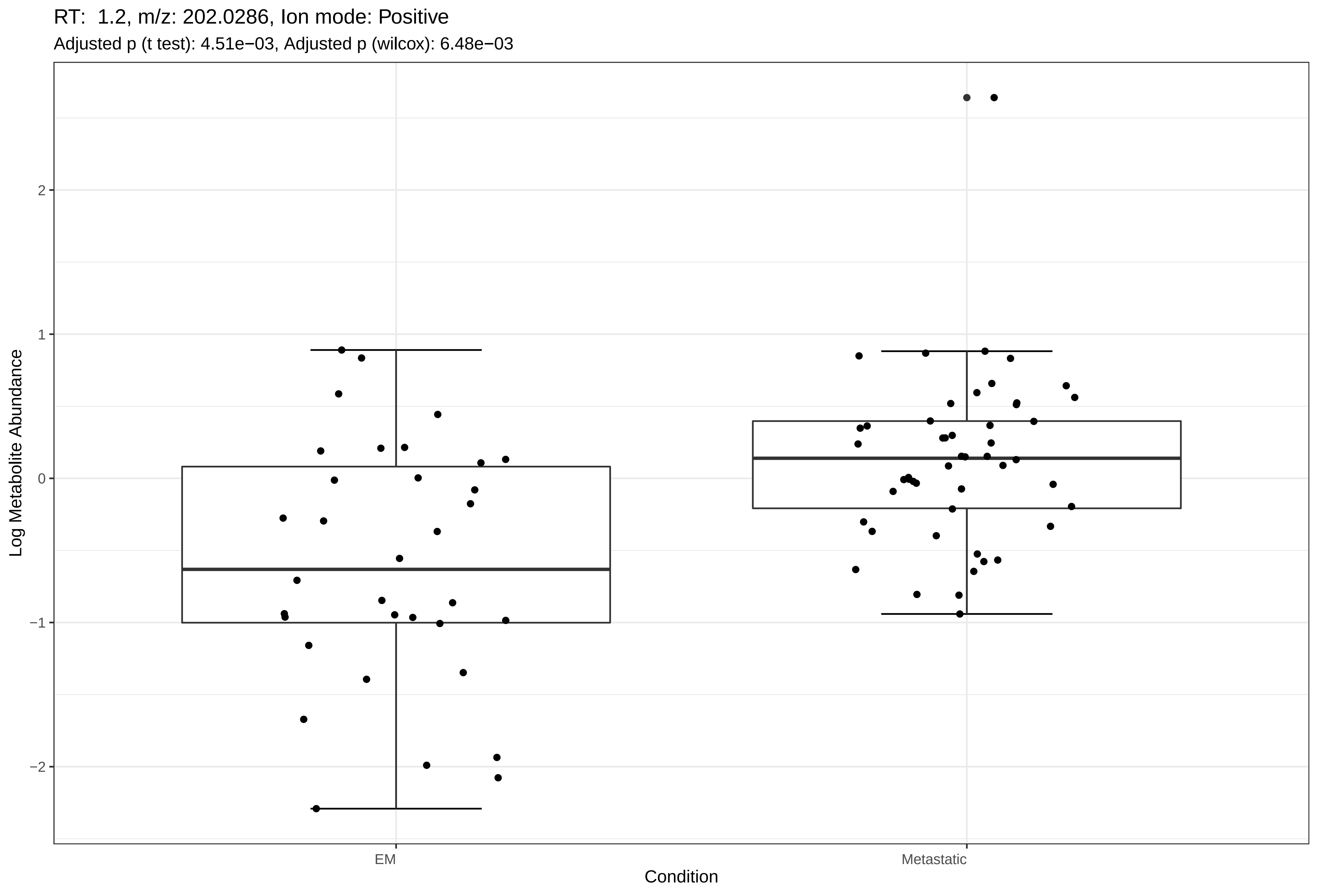
RT: 1.2, m/z: 202.0286, Ion mode: Positive
Adjusted p (t test): 4.51e−03, Adjusted p (wilcox): 6.48e−03
Log Metabolite Abundance
2
1
0
−1
−2
EM
Metastatic
Condition

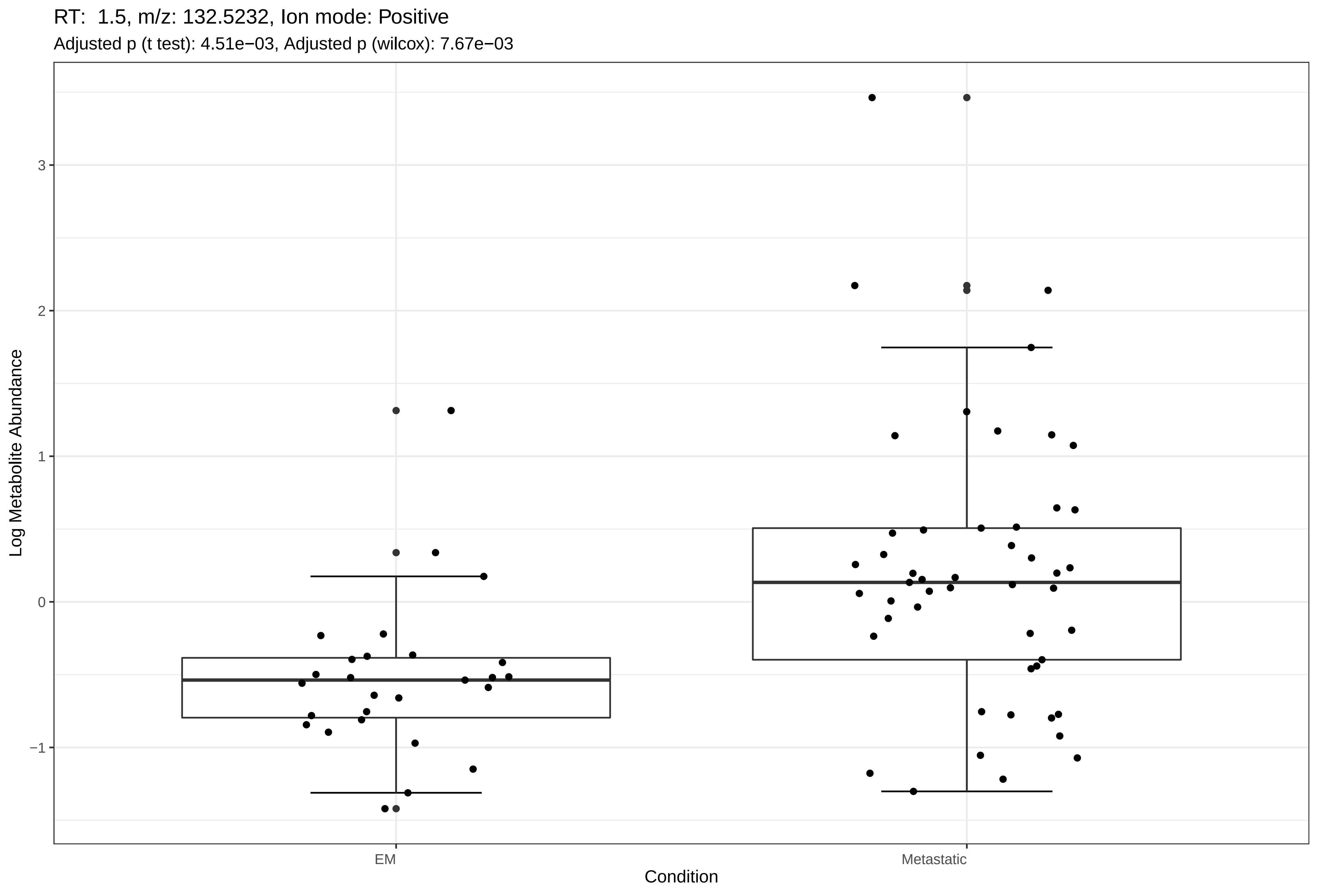

RT: 1.5, m/z: 132.5232, Ion mode: Positive
Adjusted p (t test): 4.51e−03, Adjusted p (wilcox): 7.67e−03
Log Metabolite Abundance
3
2
1
0
−1
EM
Metastatic
Condition

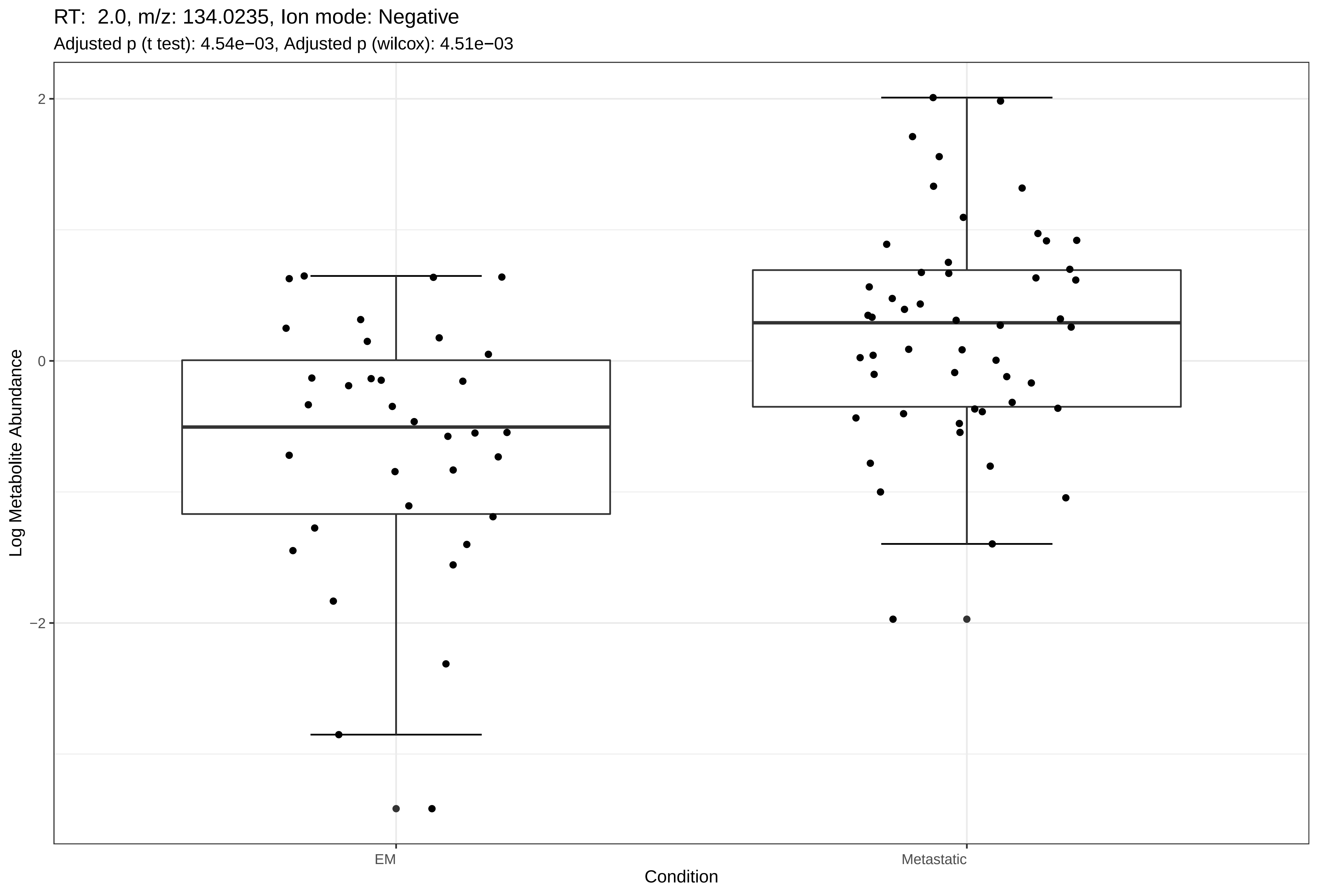
RT: 2.0, m/z: 134.0235, Ion mode: Negative
Adjusted p (t test): 4.54e−03, Adjusted p (wilcox): 4.51e−03
Log Metabolite Abundance
2
0
−2
EM
Metastatic
Condition

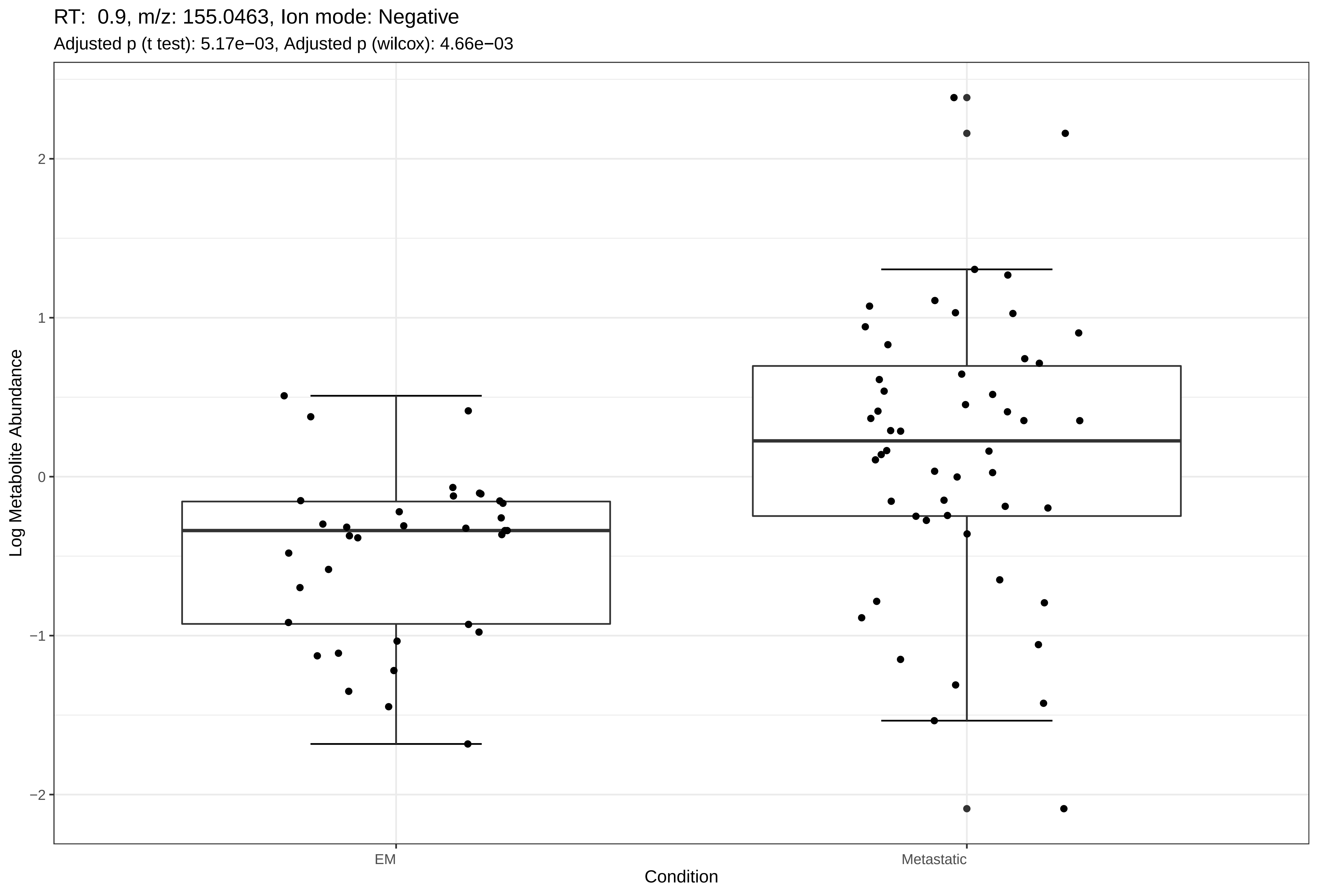
RT: 0.9, m/z: 155.0463, Ion mode: Negative
Adjusted p (t test): 5.17e−03, Adjusted p (wilcox): 4.66e−03
Log Metabolite Abundance
2
1
0
−1
−2
EM
Metastatic
Condition

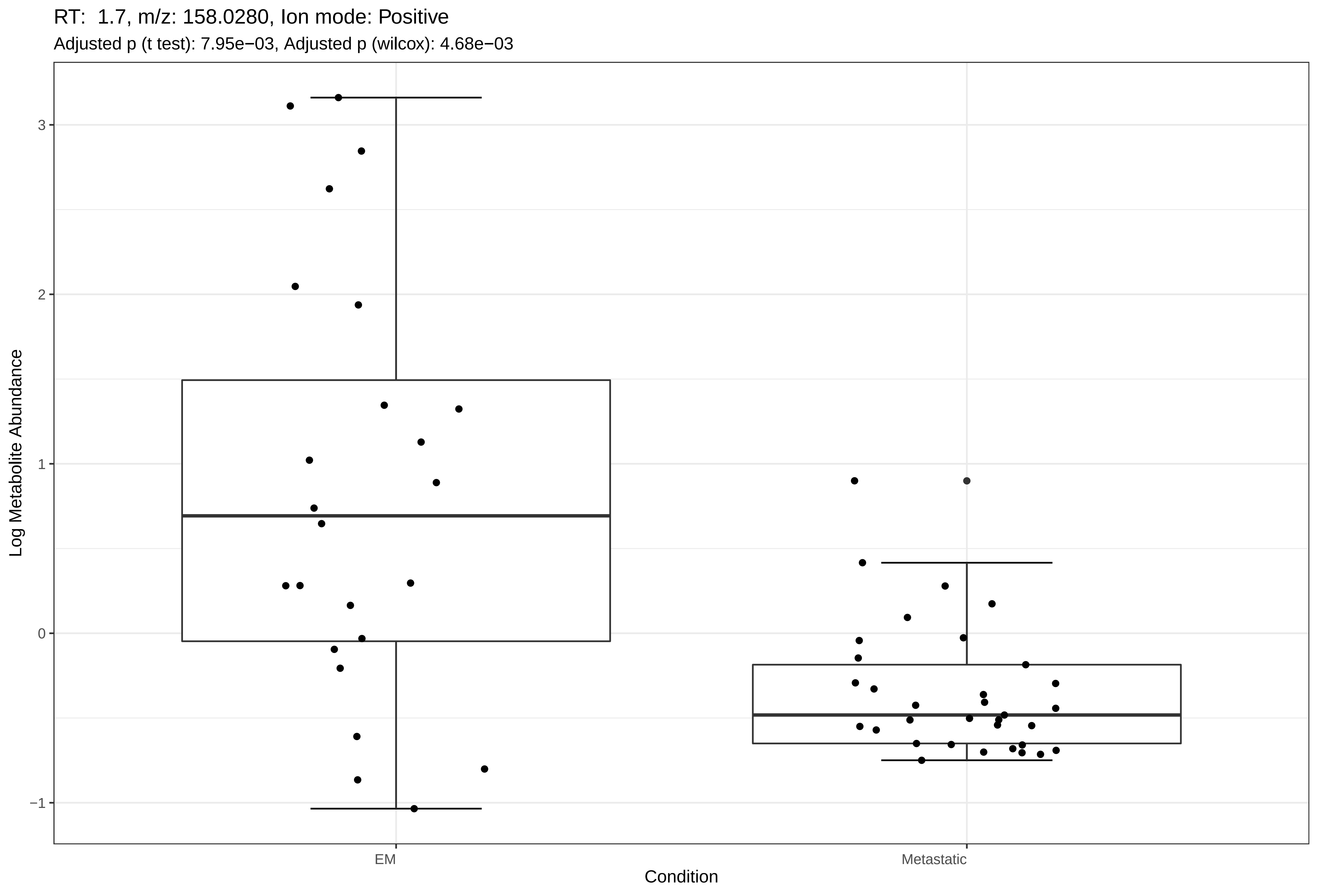

RT: 1.7, m/z: 158.0280, Ion mode: Positive
Adjusted p (t test): 7.95e−03, Adjusted p (wilcox): 4.68e−03
Log Metabolite Abundance
3
2
1
0
−1
EM
Metastatic
Condition

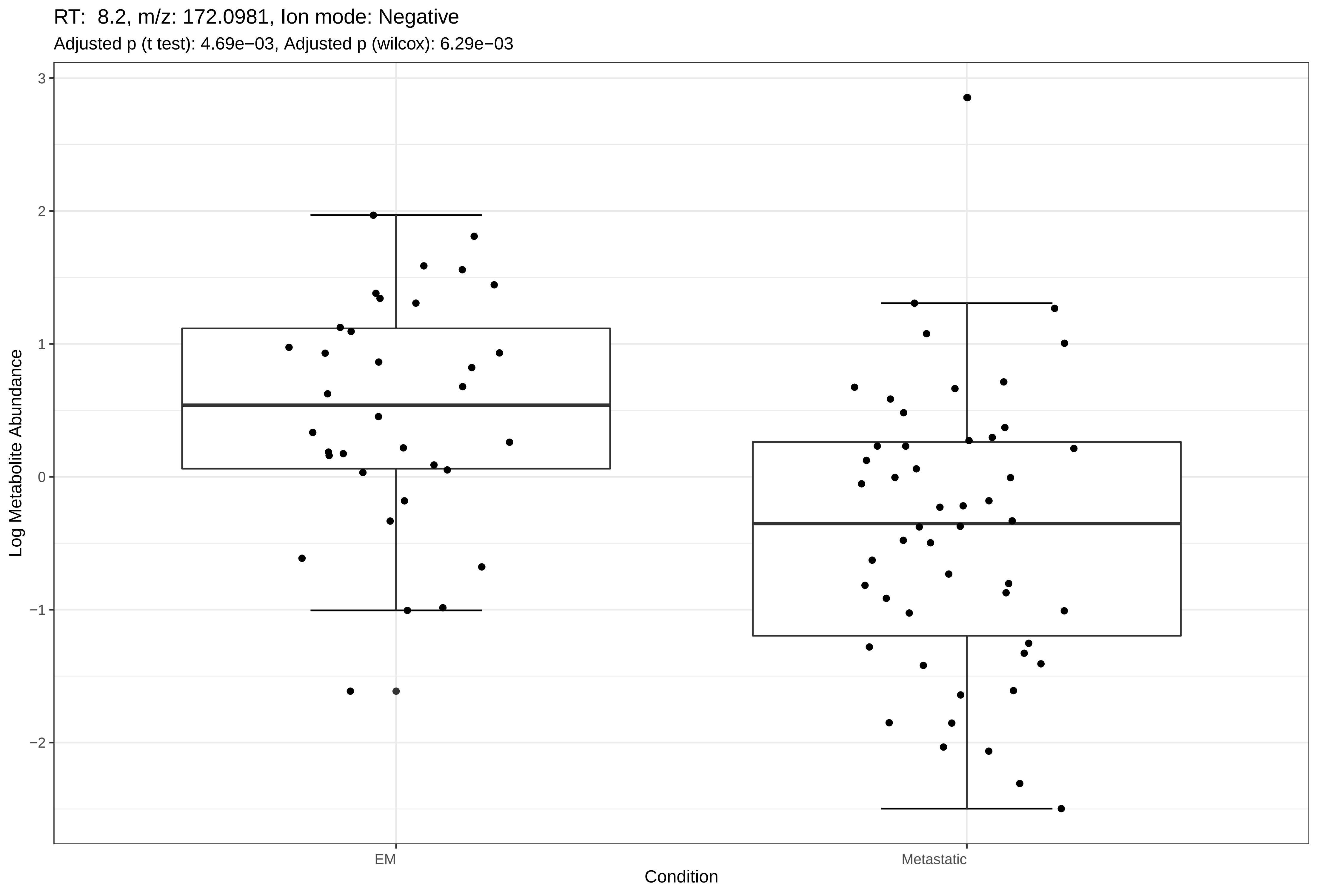
RT: 8.2, m/z: 172.0981, Ion mode: Negative
Adjusted p (t test): 4.69e−03, Adjusted p (wilcox): 6.29e−03
Log Metabolite Abundance
3
2
1
0
−1
−2
EM
Metastatic
Condition

RT: 2.0, m/z: 183.0509, Ion mode: Positive

Adjusted p (t test): 4.73e−03, Adjusted p (wilcox): 6.69e−03

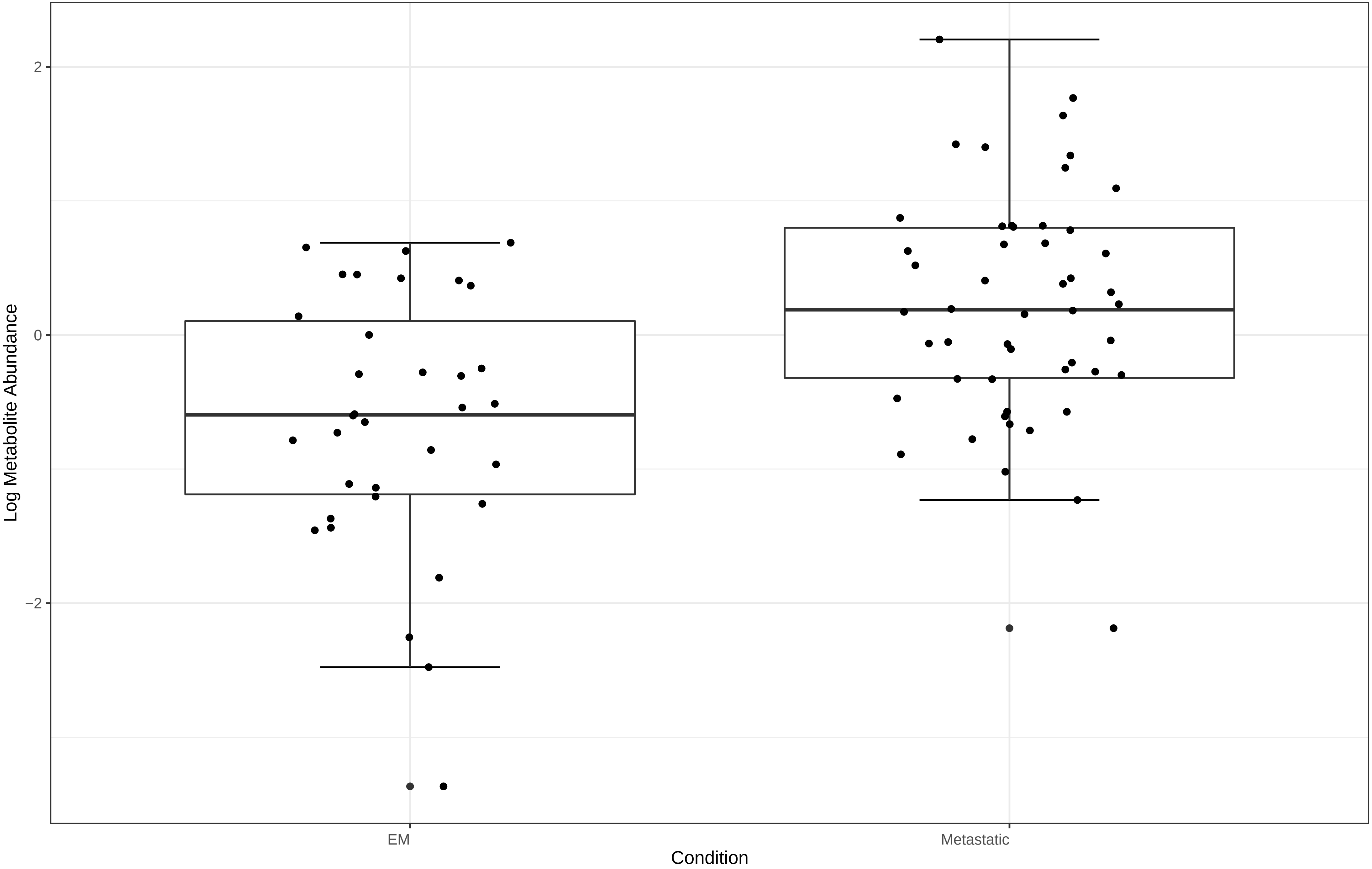

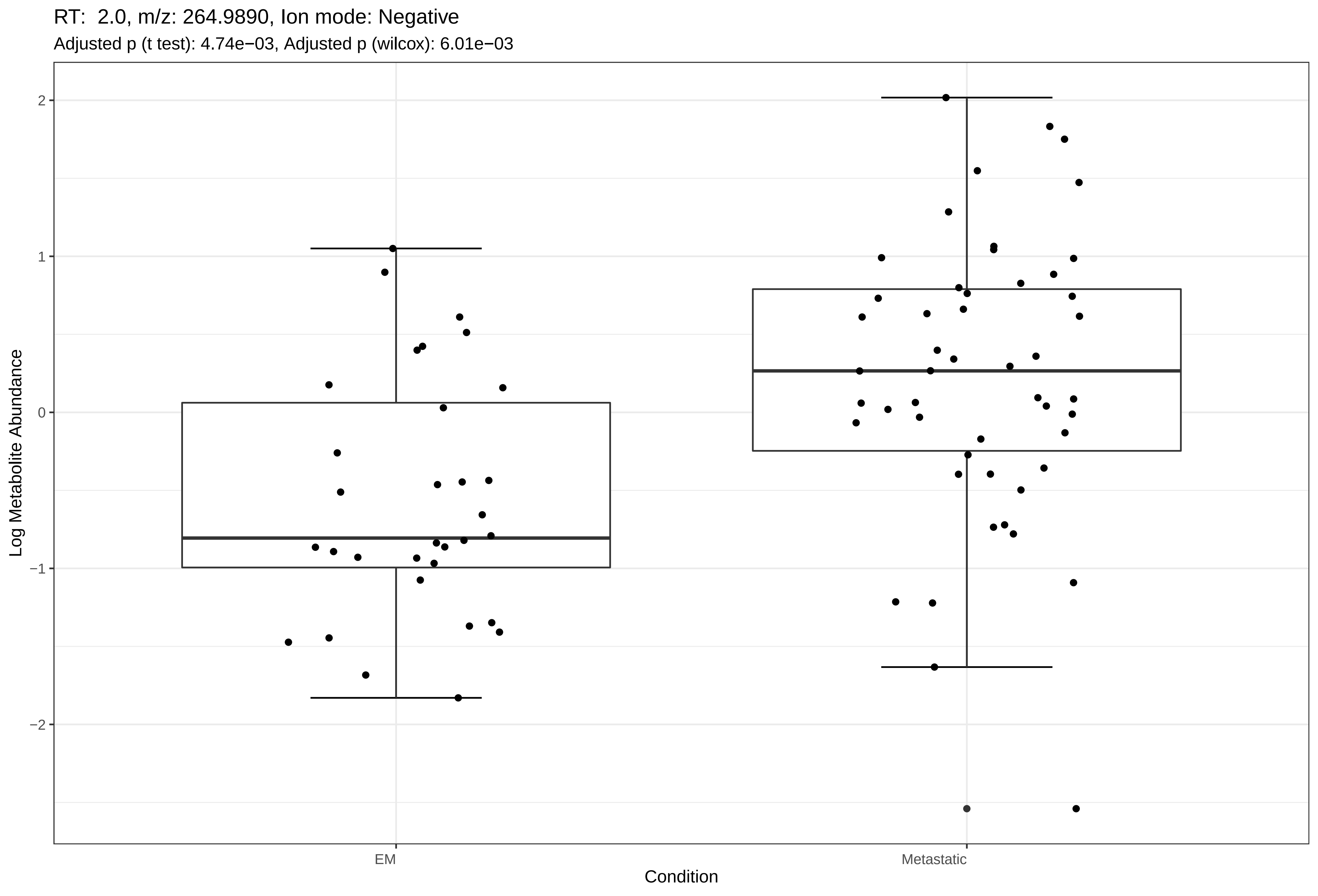

RT: 2.0, m/z: 264.9890, Ion mode: Negative
Adjusted p (t test): 4.74e−03, Adjusted p (wilcox): 6.01e−03
Log Metabolite Abundance
2
1
0
−1
−2
EM
Metastatic
Condition

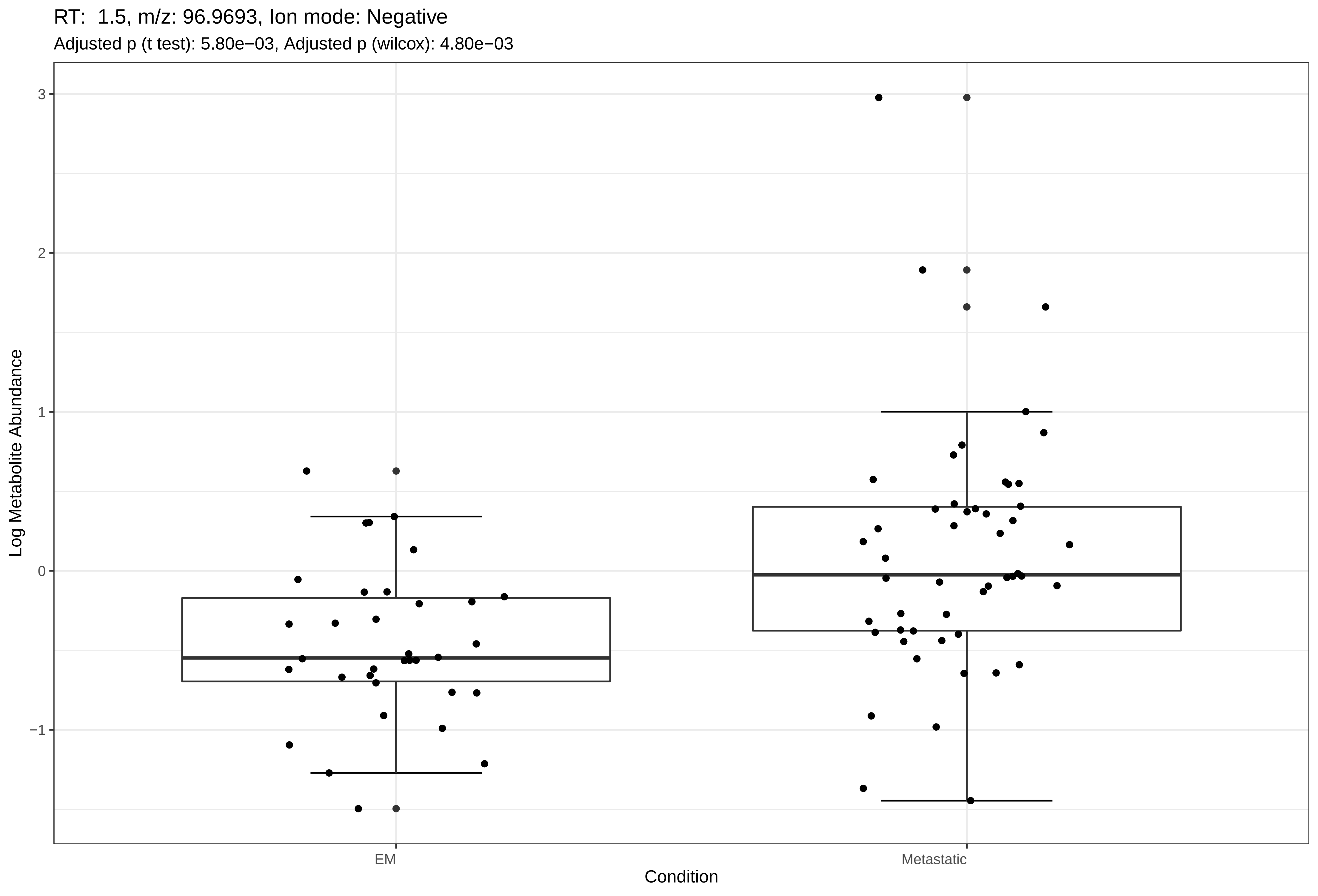

RT: 1.5, m/z: 96.9693, Ion mode: Negative
Adjusted p (t test): 5.80e−03, Adjusted p (wilcox): 4.80e−03
3
2
1
0
−1
Log Metabolite Abundance
EM
Metastatic
Condition

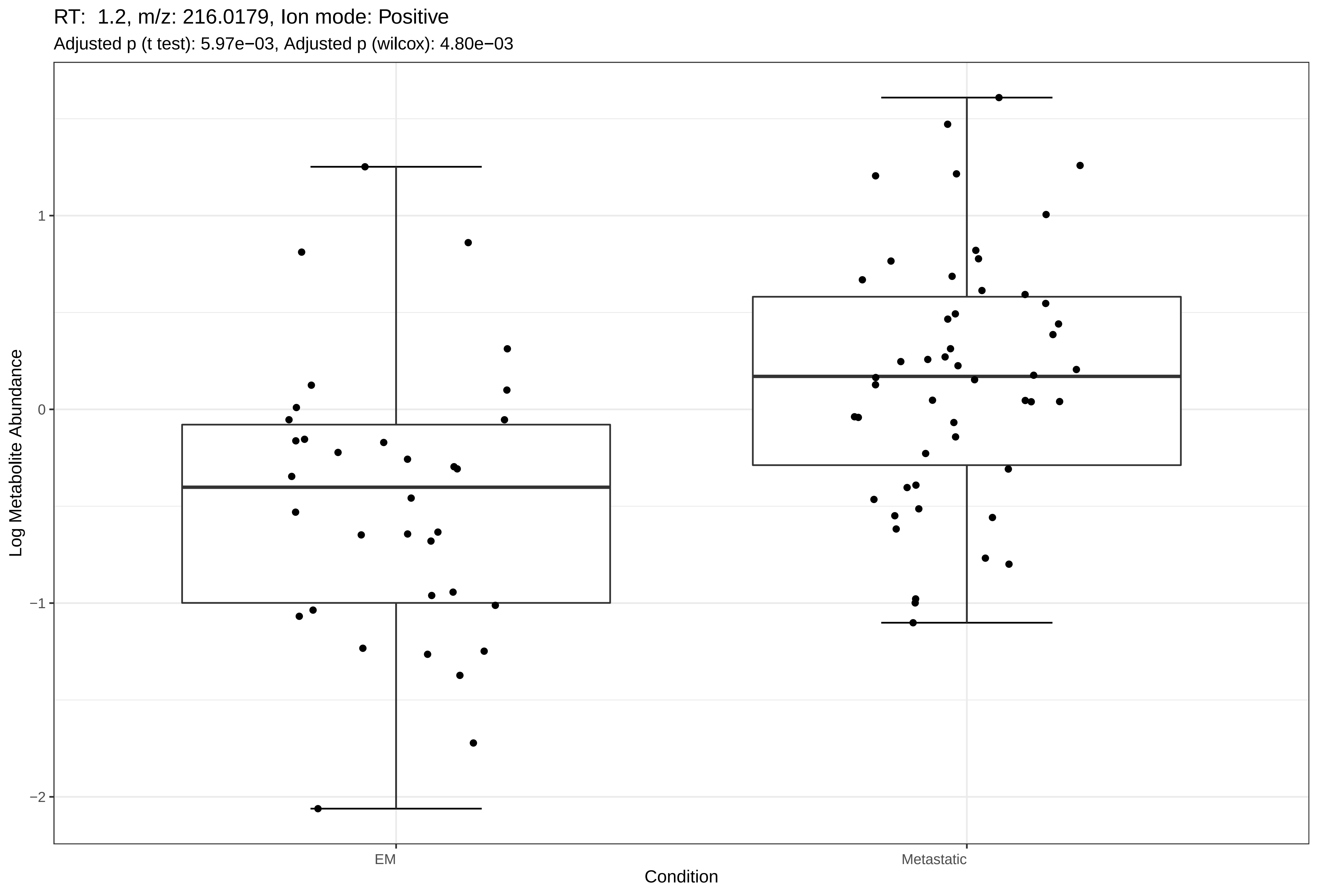

RT: 1.2, m/z: 216.0179, Ion mode: Positive
Adjusted p (t test): 5.97e−03, Adjusted p (wilcox): 4.80e−03
Log Metabolite Abundance
1
0
−1
−2
EM
Metastatic
Condition

RT: 1.7, m/z: 178.5415, Ion mode: Positive

Adjusted p (t test): 6.06e−03, Adjusted p (wilcox): 4.80e−03

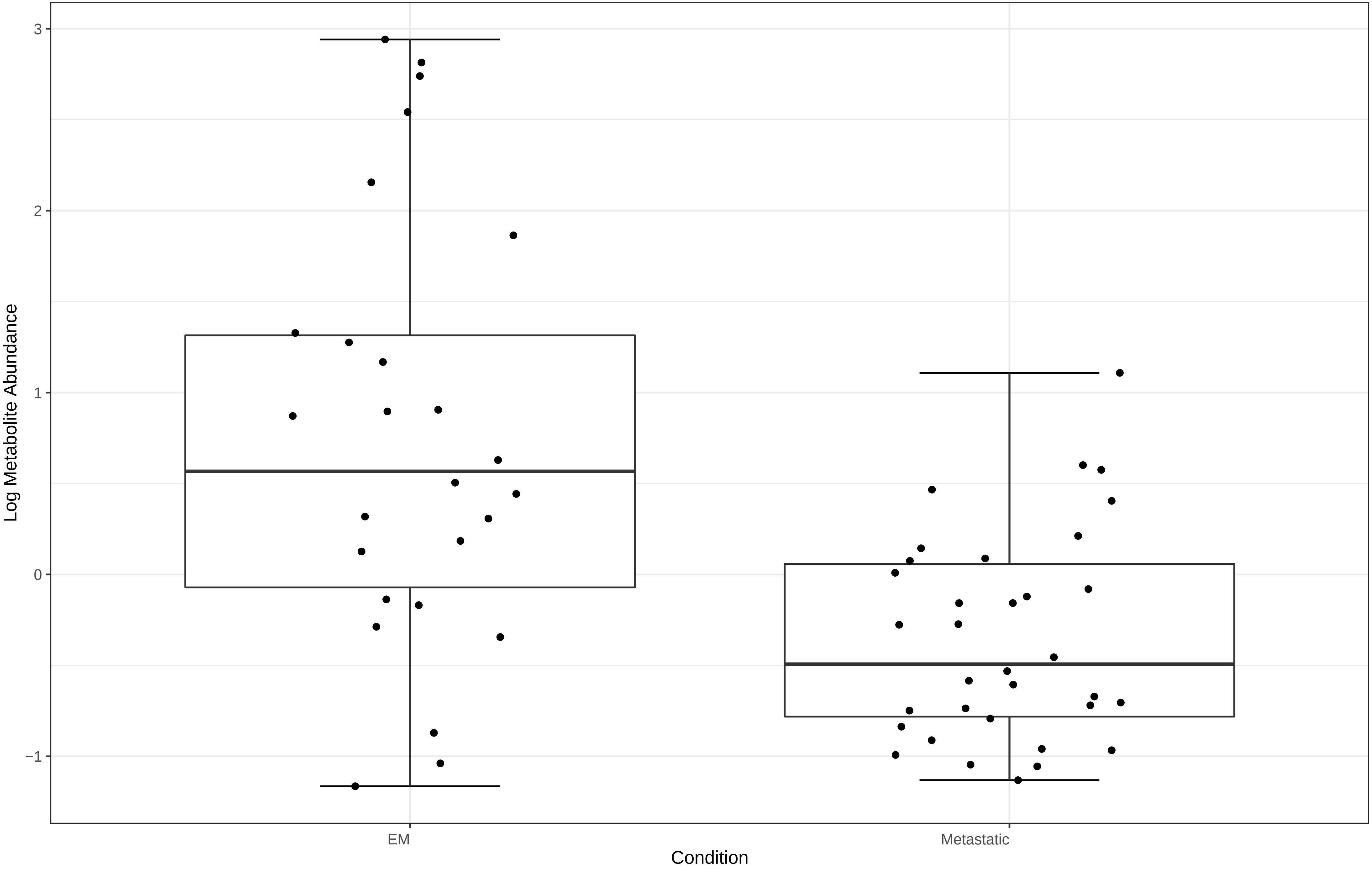

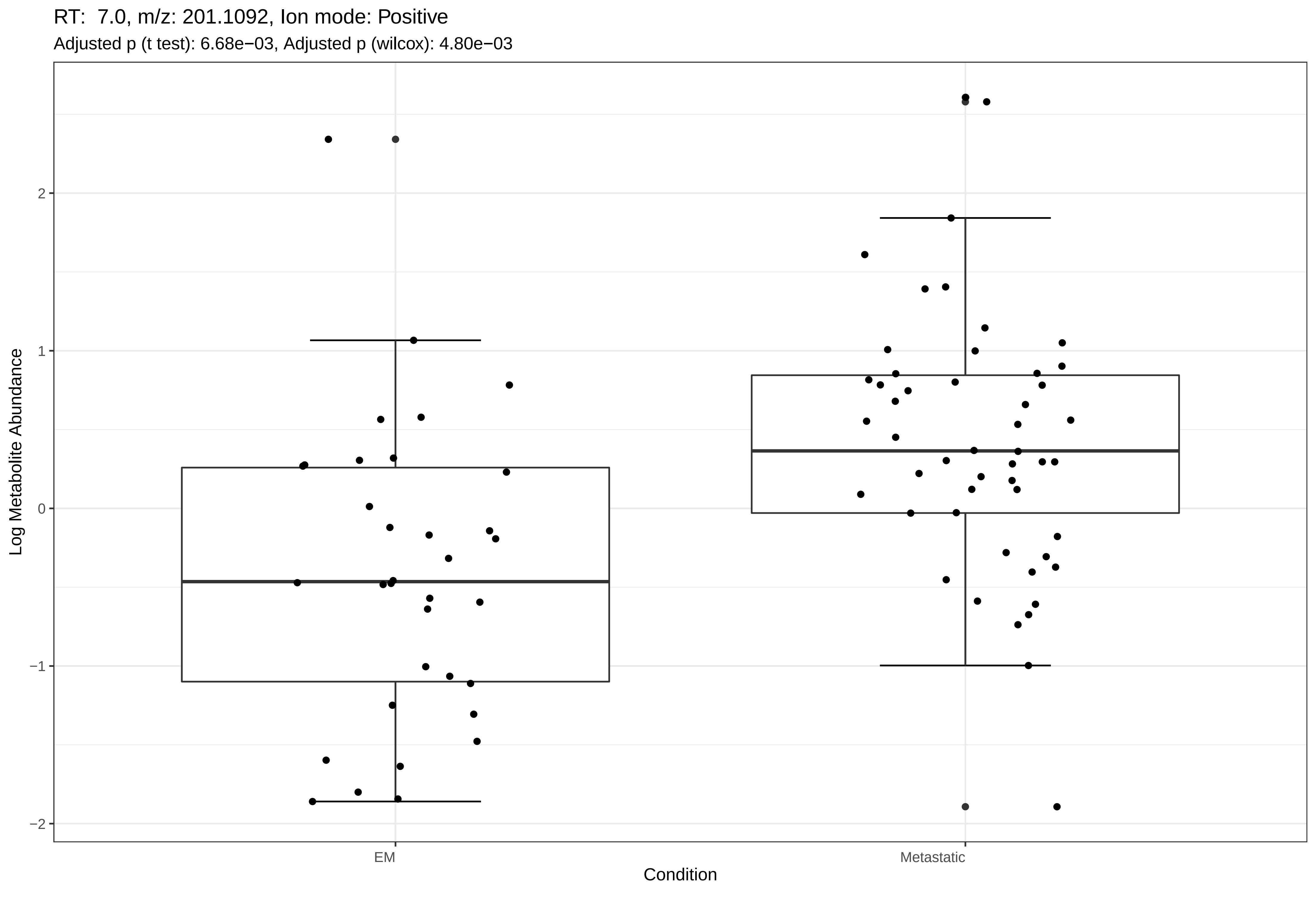

RT: 7.0, m/z: 201.1092, Ion mode: Positive
Adjusted p (t test): 6.68e−03, Adjusted p (wilcox): 4.80e−03
Log Metabolite Abundance
2
1
0
−1
−2
EM
Metastatic
Condition

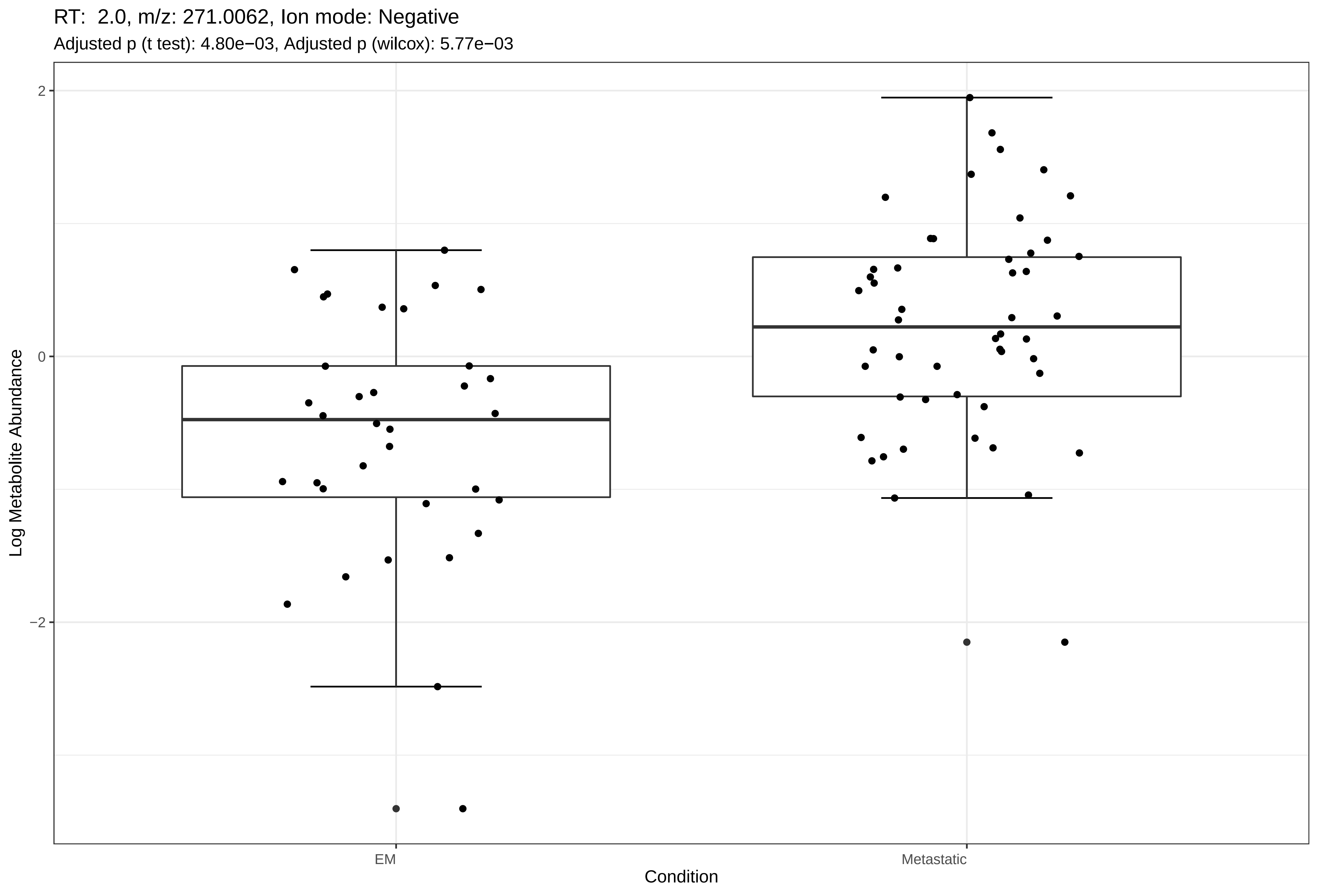
RT: 2.0, m/z: 271.0062, Ion mode: Negative
Adjusted p (t test): 4.80e−03, Adjusted p (wilcox): 5.77e−03
Log Metabolite Abundance
2
0
−2
EM
Metastatic
Condition

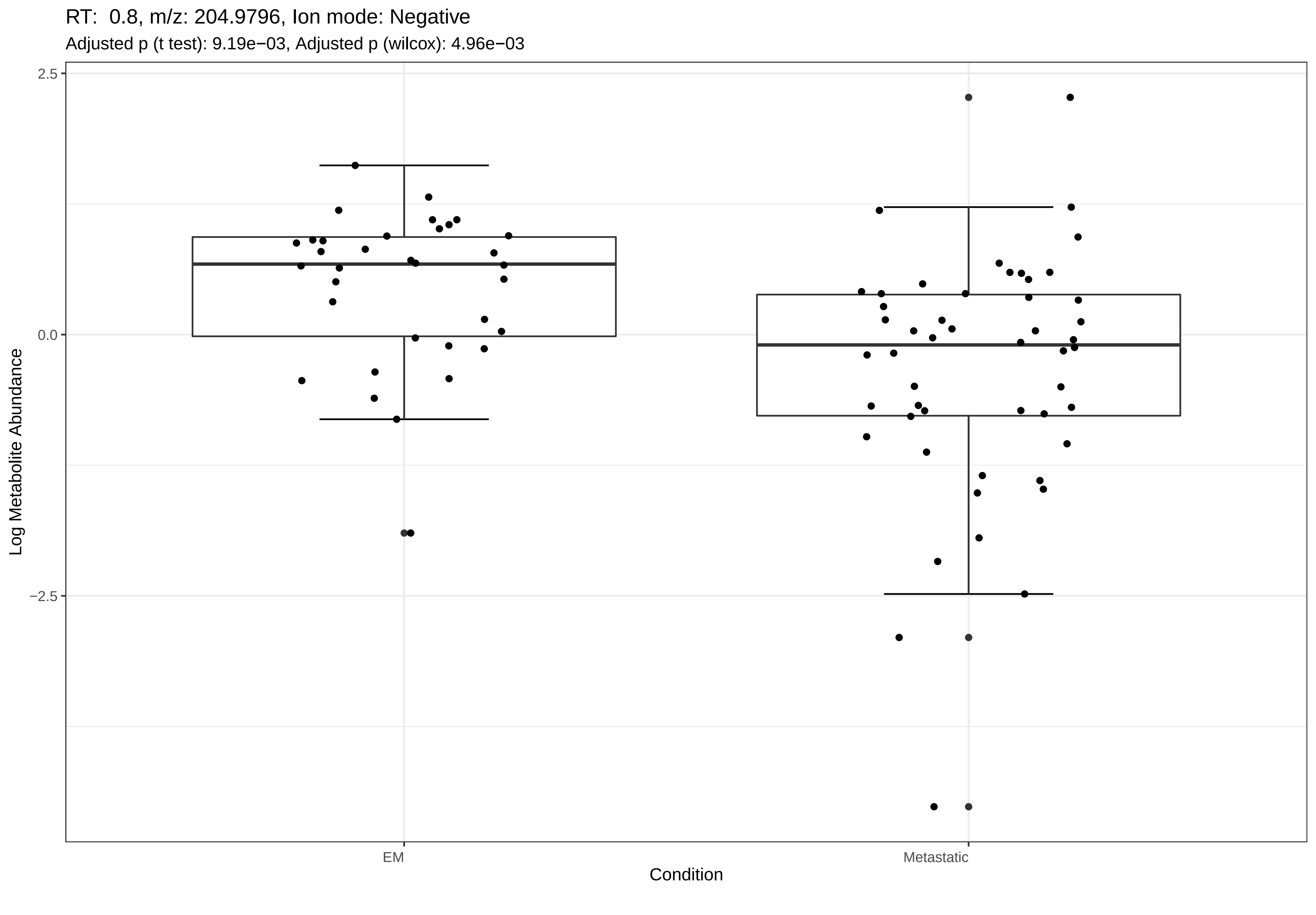

RT: 0.8, m/z: 204.9796, Ion mode: Negative
Adjusted p (t test): 9.19e−03, Adjusted p (wilcox): 4.96e−03
2.5
0.0
−2.5
Log Metabolite Abundance
EM
Metastatic
Condition

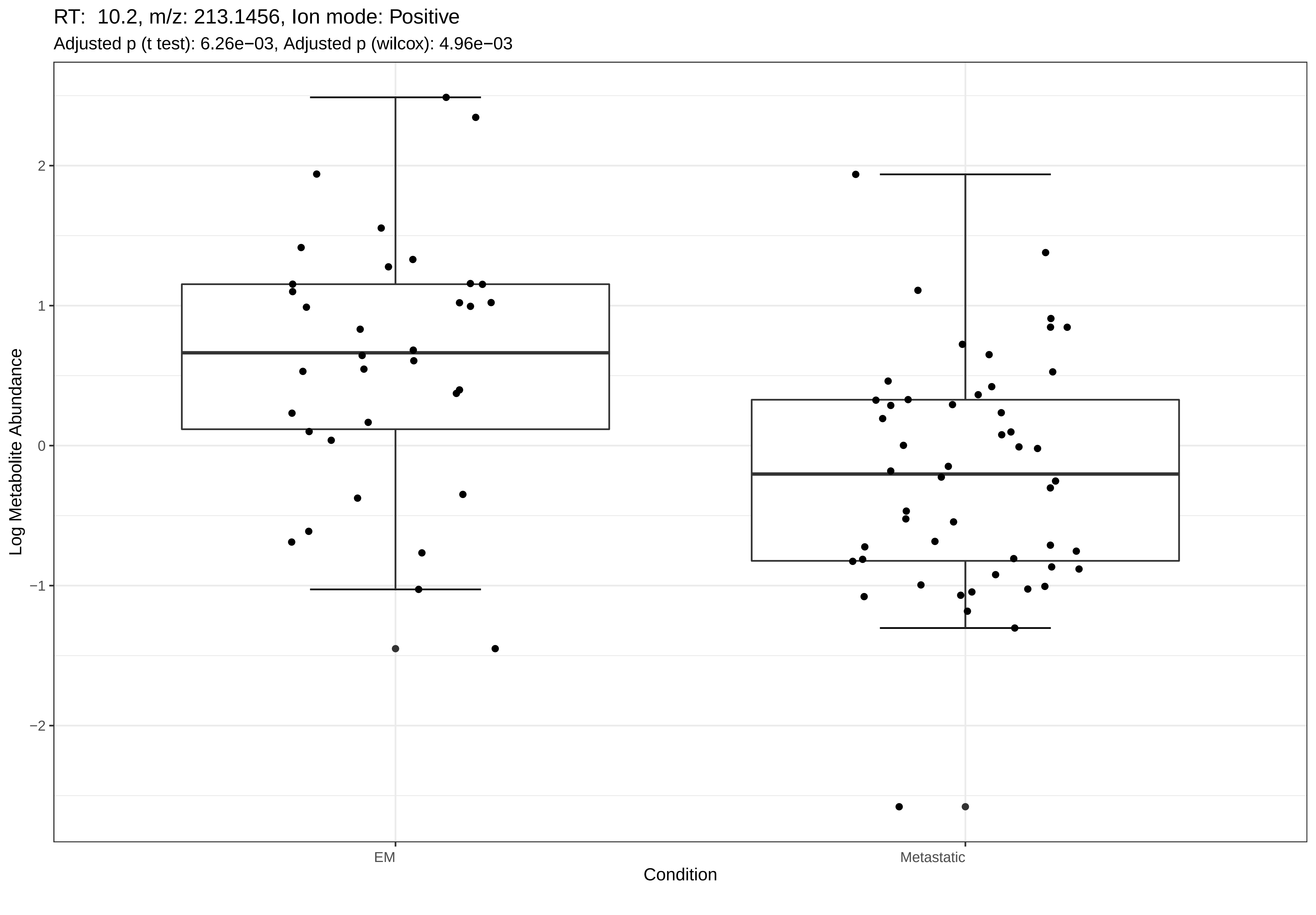

RT: 10.2, m/z: 213.1456, Ion mode: Positive
Adjusted p (t test): 6.26e−03, Adjusted p (wilcox): 4.96e−03
Log Metabolite Abundance
2
1
0
−1
−2
EM
Metastatic
Condition

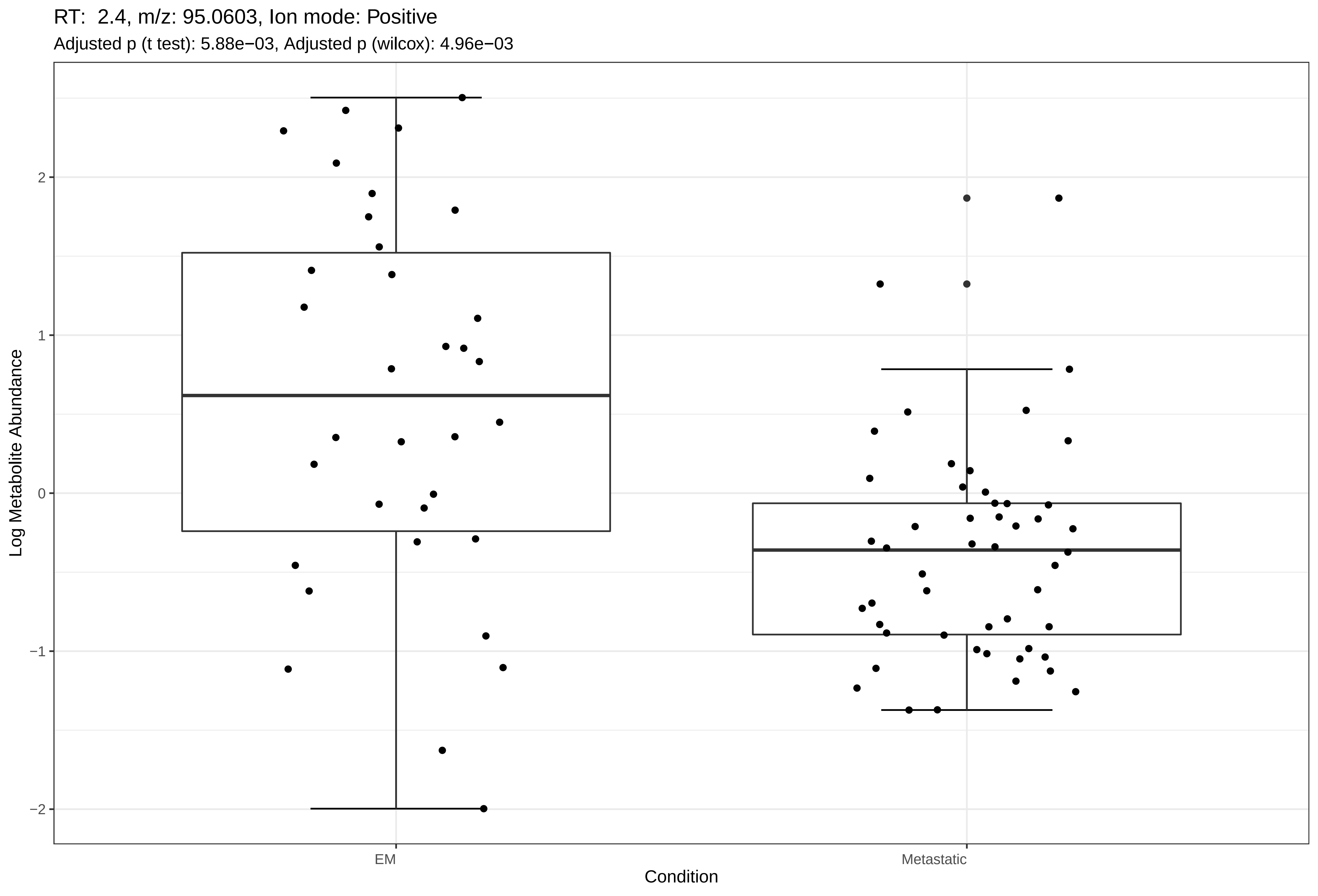
RT: 2.4, m/z: 95.0603, Ion mode: Positive
Adjusted p (t test): 5.88e−03, Adjusted p (wilcox): 4.96e−03
Log Metabolite Abundance
2
1
0
−1
−2
EM
Metastatic
Condition

RT: 10.9, m/z: 154.9737, Ion mode: Negative

Adjusted p (t test): 5.00e−03, Adjusted p (wilcox): 7.99e−03

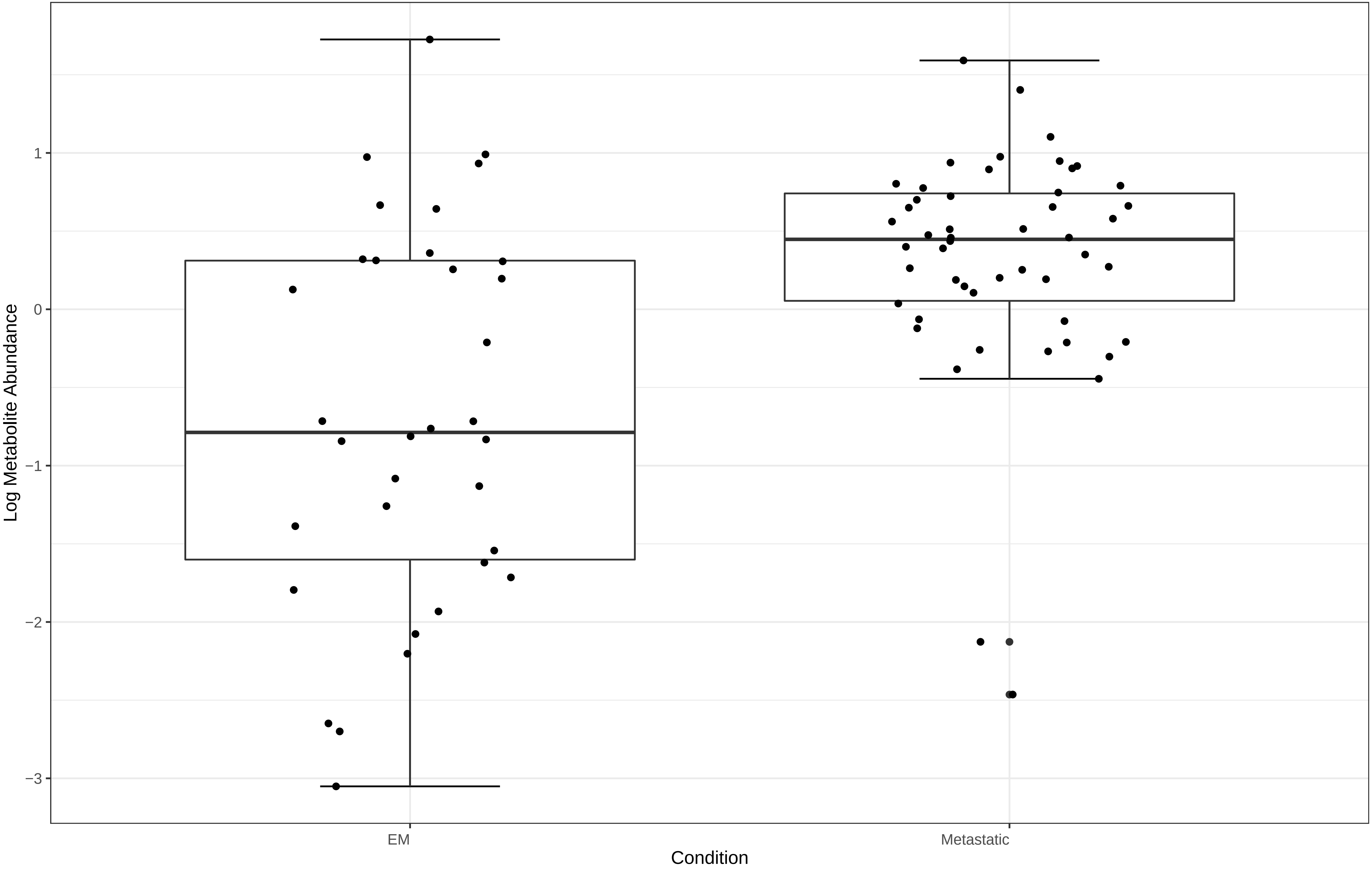

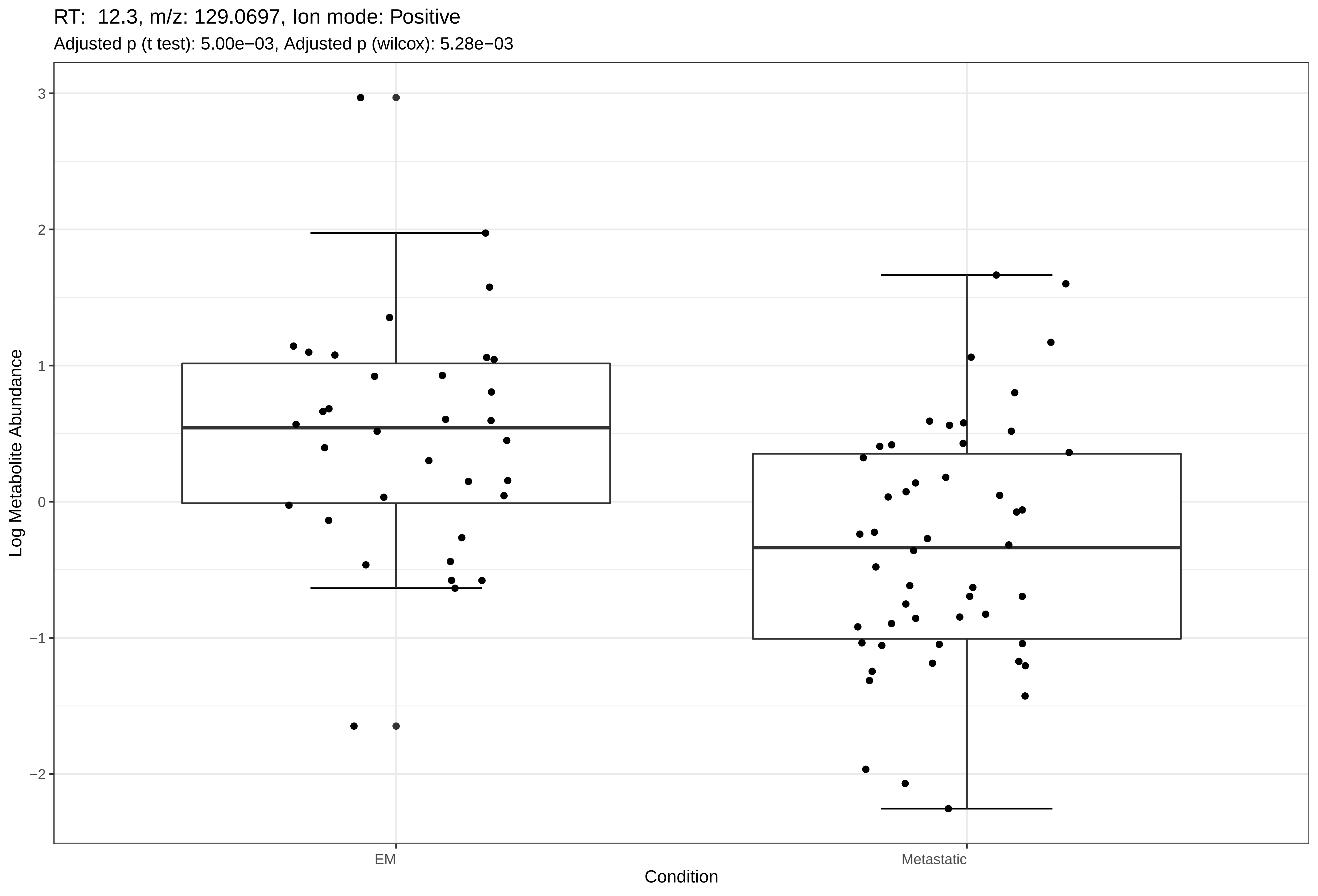
RT: 12.3, m/z: 129.0697, Ion mode: Positive
Adjusted p (t test): 5.00e−03, Adjusted p (wilcox): 5.28e−03
Log Metabolite Abundance
3
2
1
0
−1
−2
EM
Metastatic
Condition

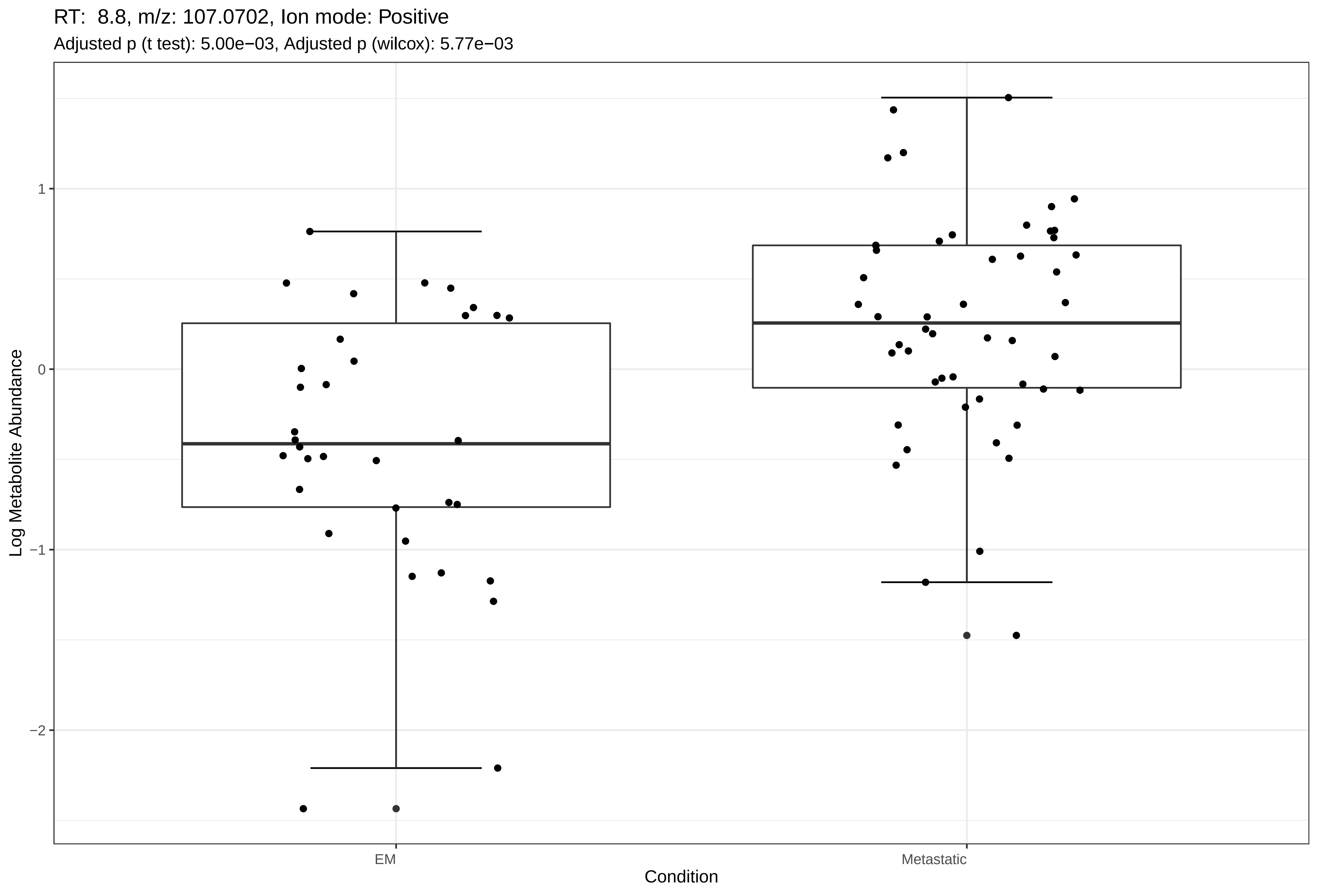
RT: 8.8, m/z: 107.0702, Ion mode: Positive
Adjusted p (t test): 5.00e−03, Adjusted p (wilcox): 5.77e−03
Log Metabolite Abundance
1
0
−1
−2
EM
Metastatic
Condition

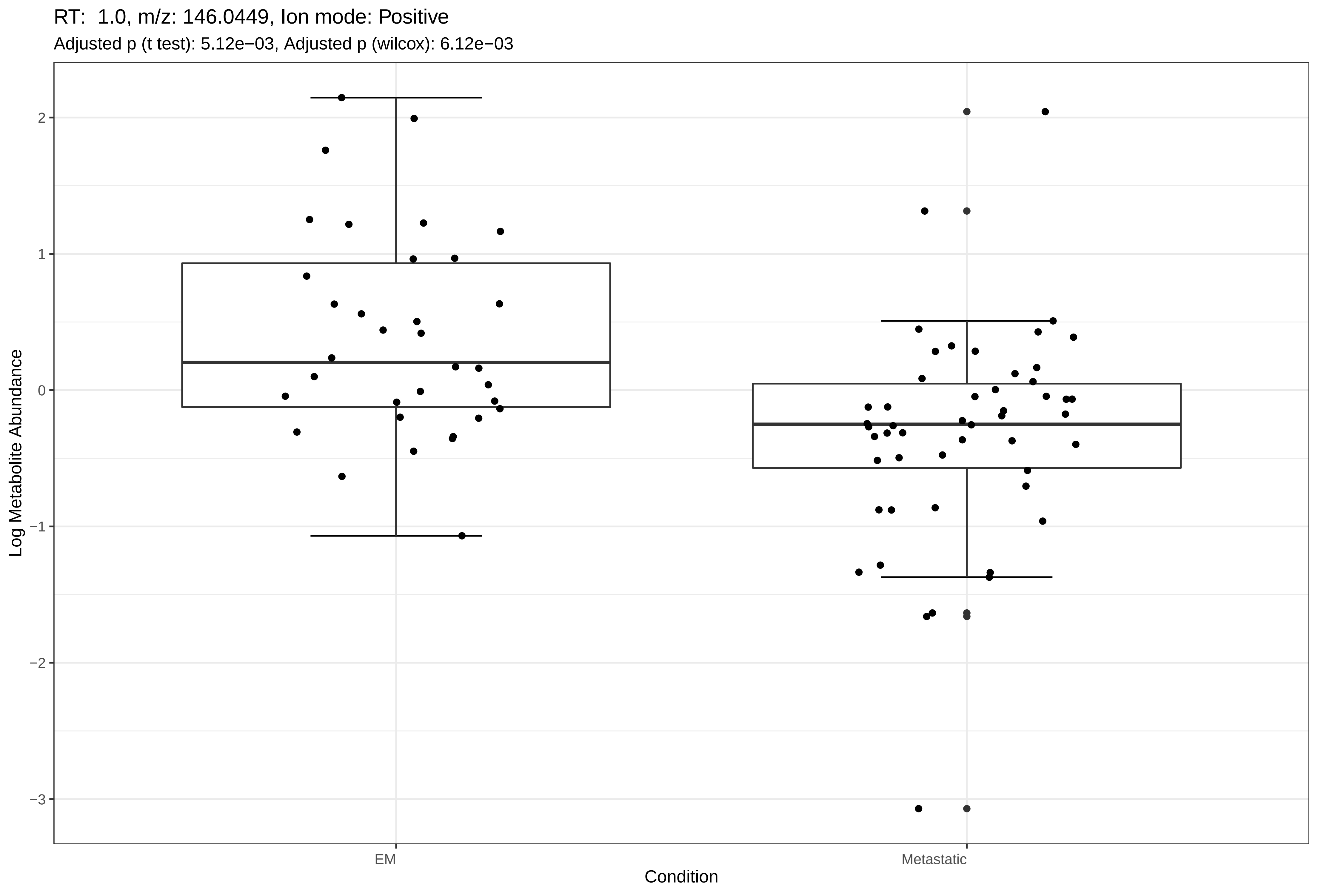
RT: 1.0, m/z: 146.0449, Ion mode: Positive
Adjusted p (t test): 5.12e−03, Adjusted p (wilcox): 6.12e−03
Log Metabolite Abundance
2
1
0
−1
−2
−3
EM
Metastatic
Condition

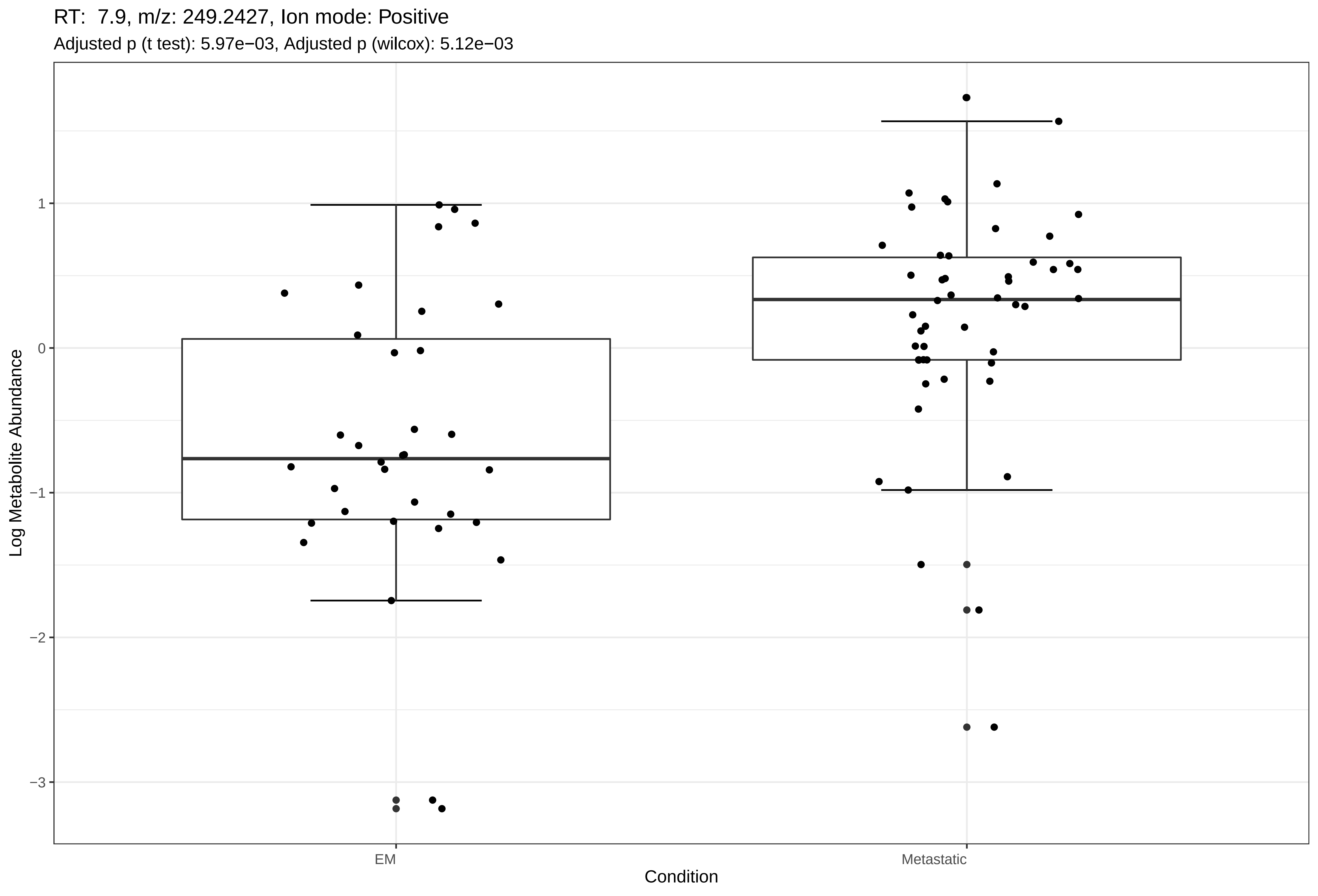
RT: 7.9, m/z: 249.2427, Ion mode: Positive
Adjusted p (t test): 5.97e−03, Adjusted p (wilcox): 5.12e−03
Log Metabolite Abundance
1
0
−1
−2
−3
EM
Metastatic
Condition

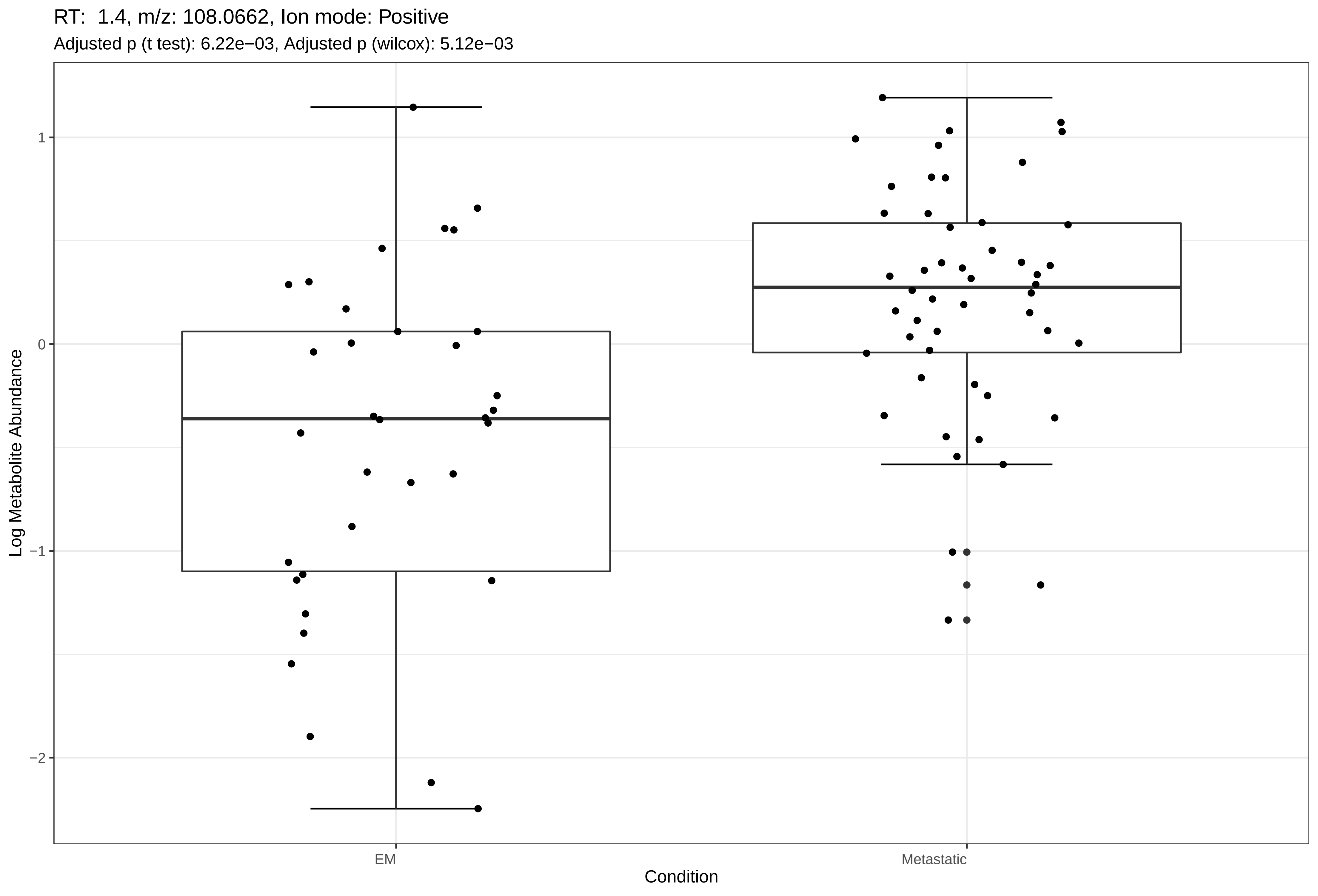
RT: 1.4, m/z: 108.0662, Ion mode: Positive
Adjusted p (t test): 6.22e−03, Adjusted p (wilcox): 5.12e−03
Log Metabolite Abundance
1
0
−1
−2
EM
Metastatic
Condition

Complex of 106.0232 and 106.0232 m/z (RT: 1.2, m/z: 211.0384, Ion mode: Positive)

Adjusted p (t test): 6.75e−03, Adjusted p (wilcox): 5.12e−03

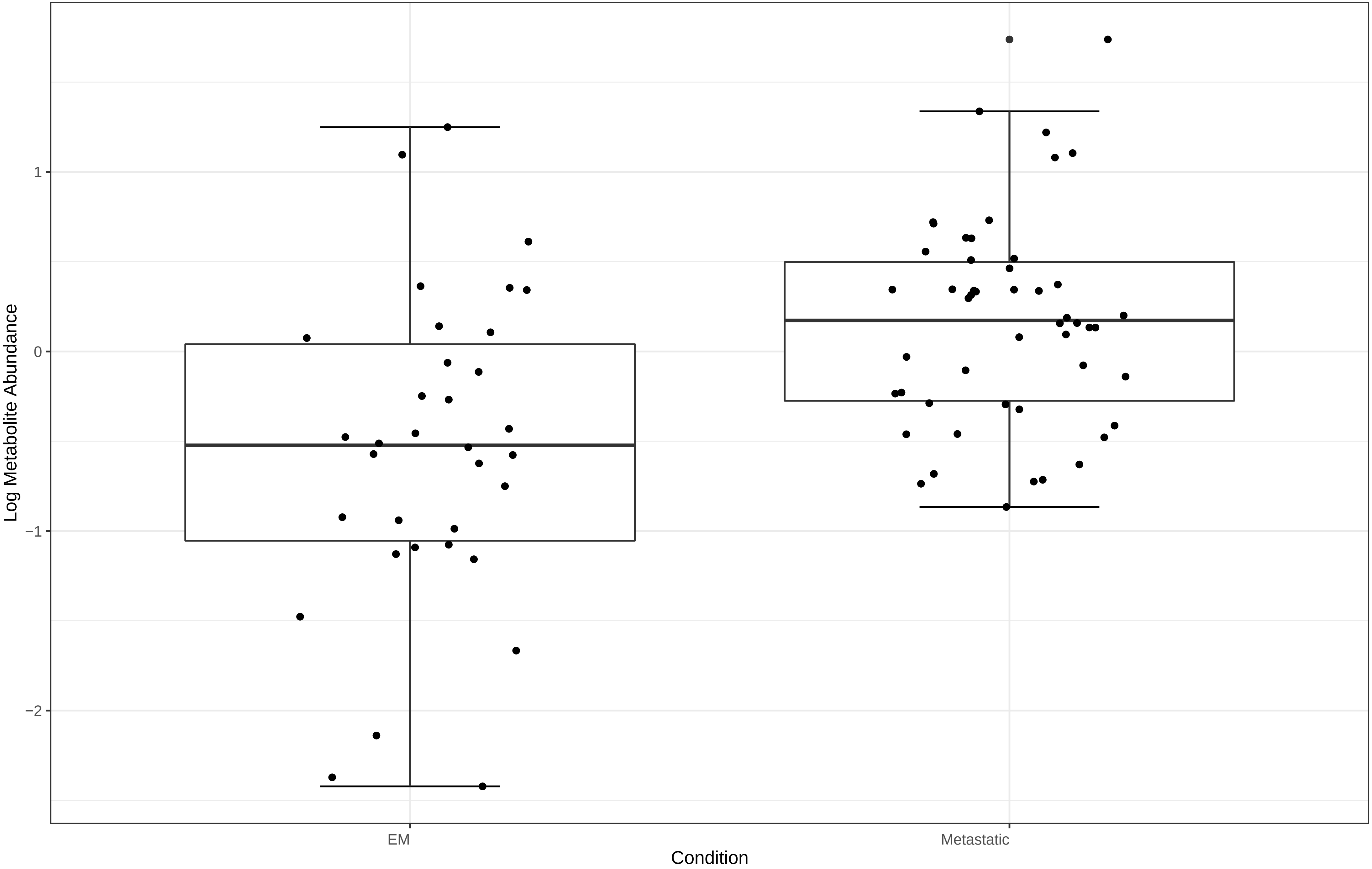

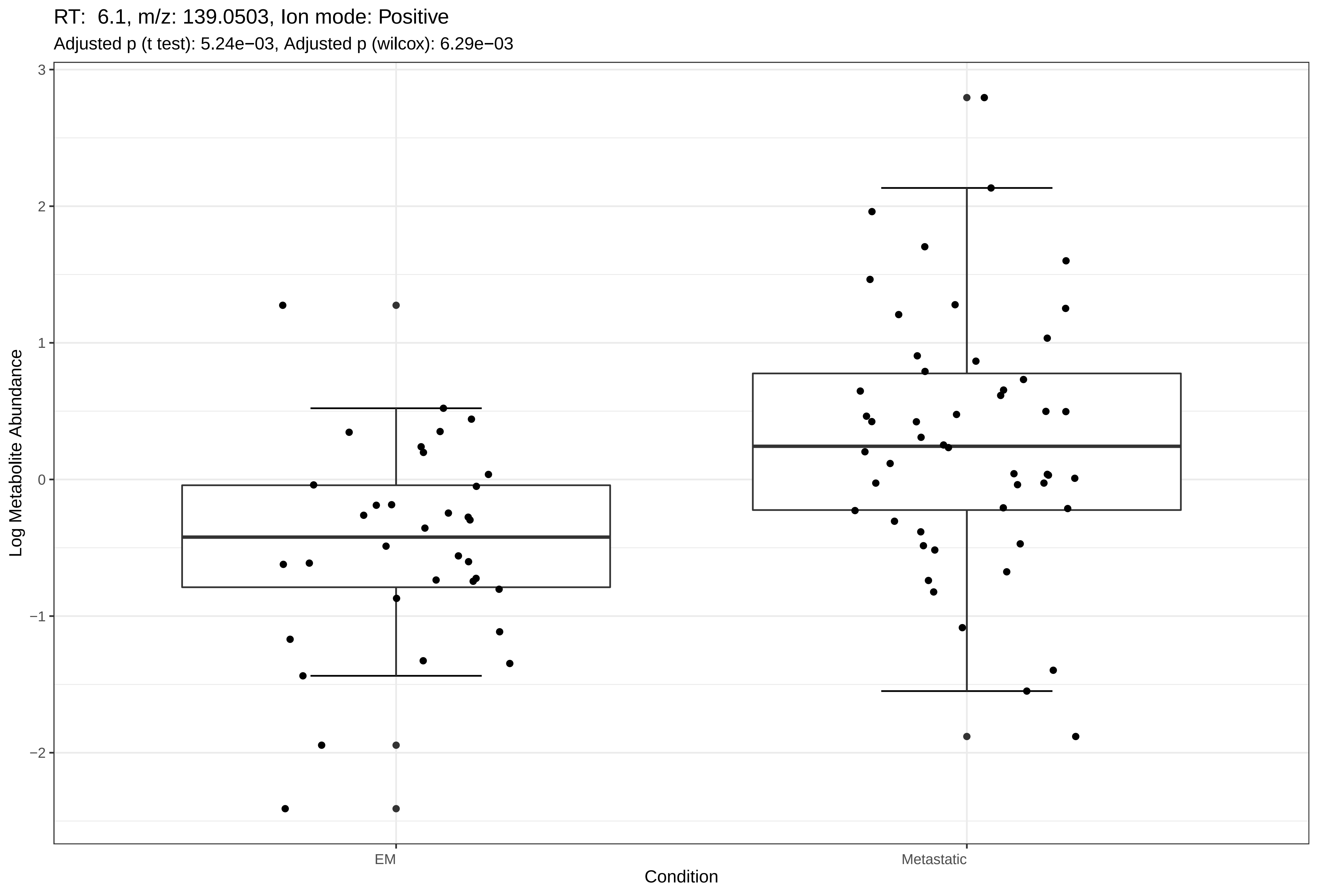

RT: 6.1, m/z: 139.0503, Ion mode: Positive
Adjusted p (t test): 5.24e−03, Adjusted p (wilcox): 6.29e−03
Log Metabolite Abundance
3
2
1
0
−1
−2
EM
Metastatic
Condition

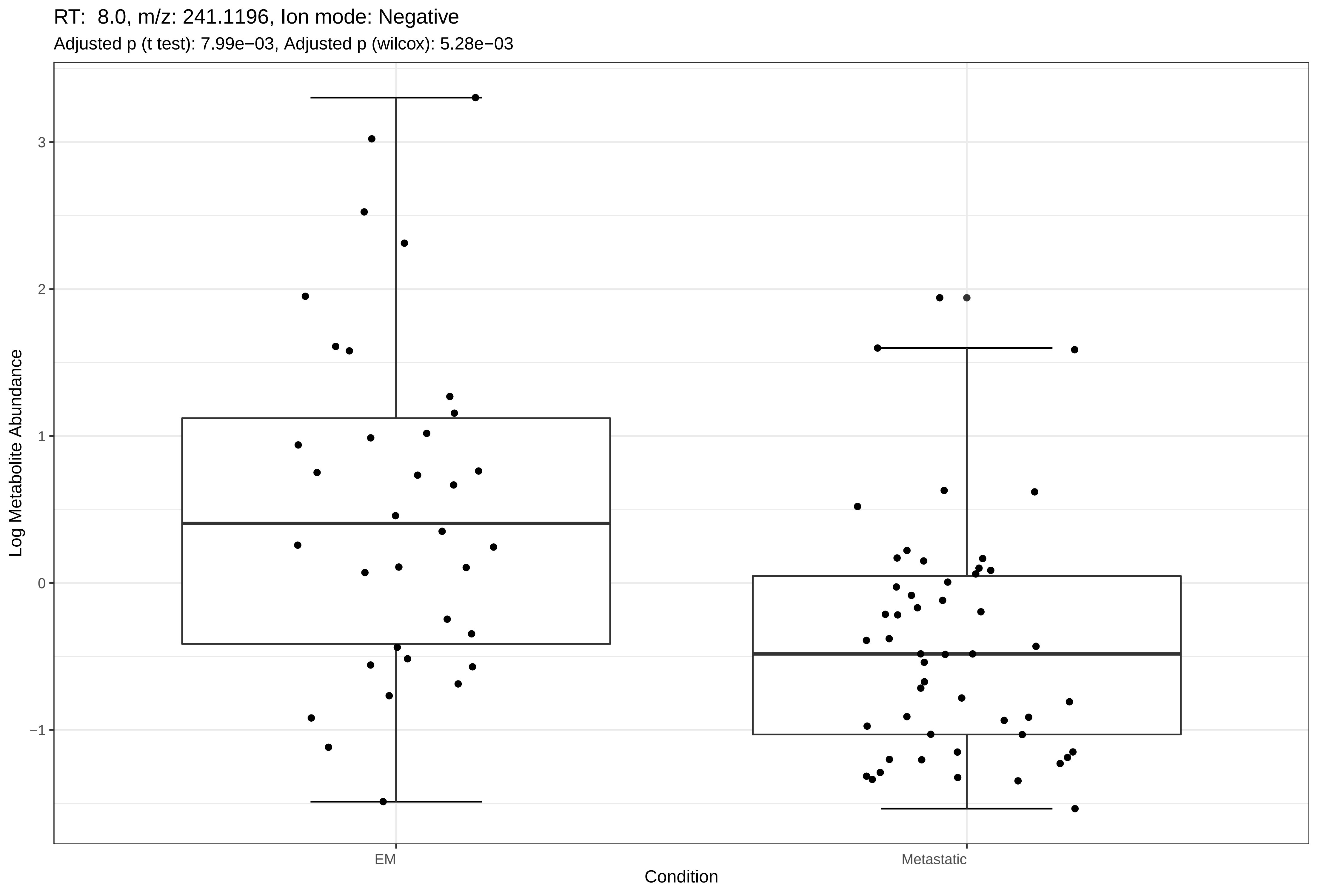
RT: 8.0, m/z: 241.1196, Ion mode: Negative
Adjusted p (t test): 7.99e−03, Adjusted p (wilcox): 5.28e−03
Log Metabolite Abundance
3
2
1
0
−1
EM
Metastatic
Condition

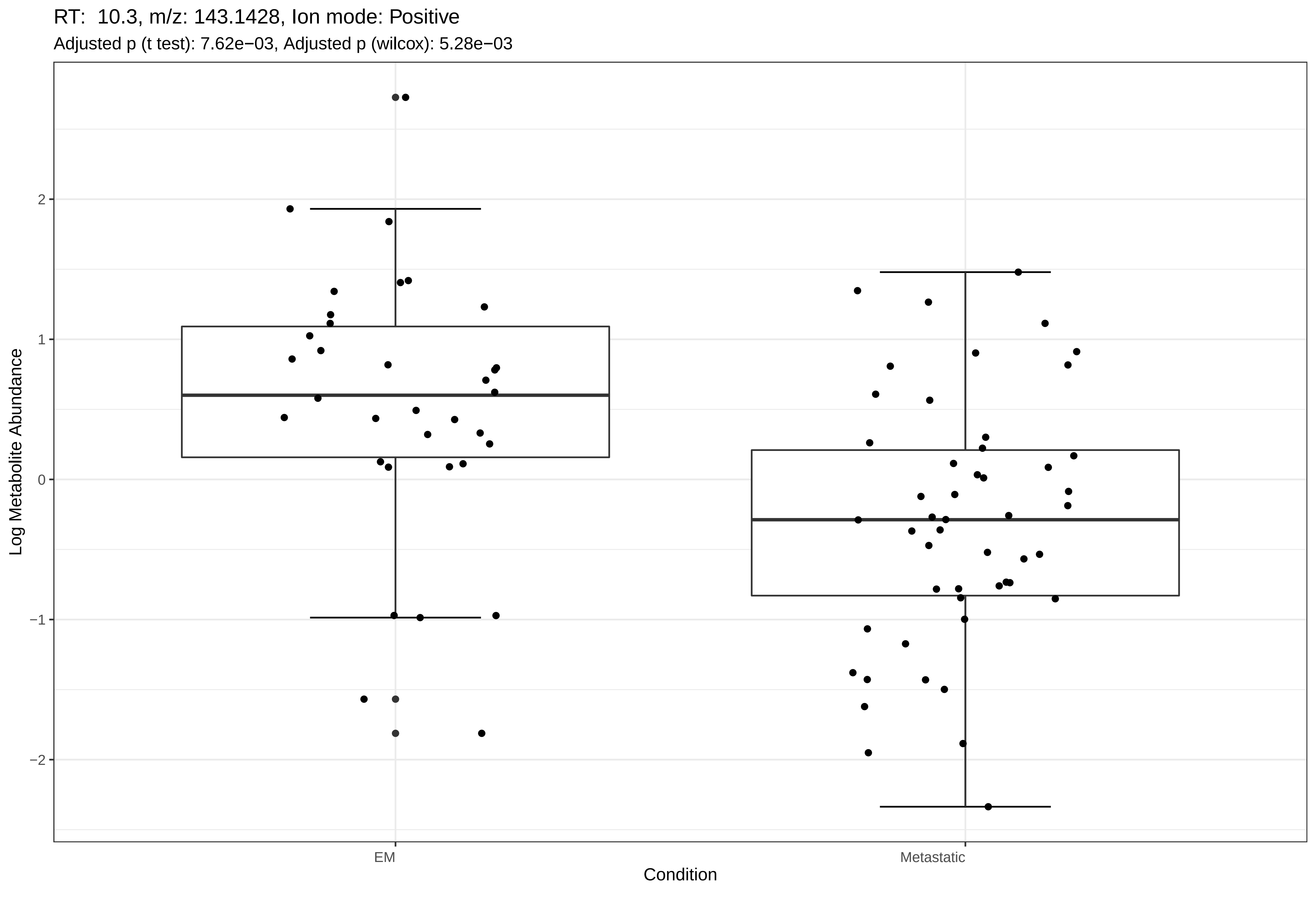
RT: 10.3, m/z: 143.1428, Ion mode: Positive
Adjusted p (t test): 7.62e−03, Adjusted p (wilcox): 5.28e−03
Log Metabolite Abundance
2
1
0
−1
−2
EM
Metastatic
Condition

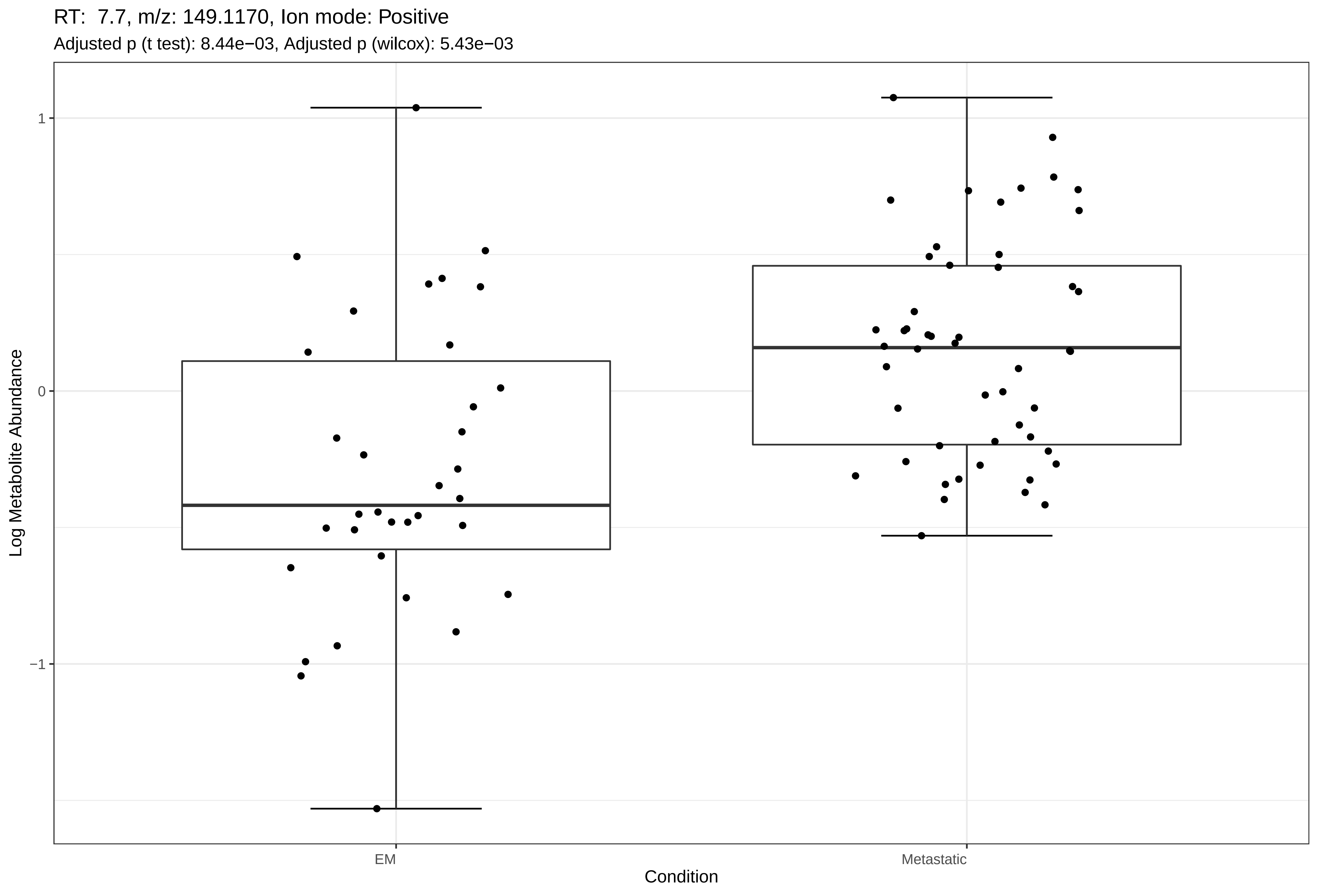

RT: 7.7, m/z: 149.1170, Ion mode: Positive
Adjusted p (t test): 8.44e−03, Adjusted p (wilcox): 5.43e−03
Log Metabolite Abundance
1
0
−1
EM
Metastatic
Condition

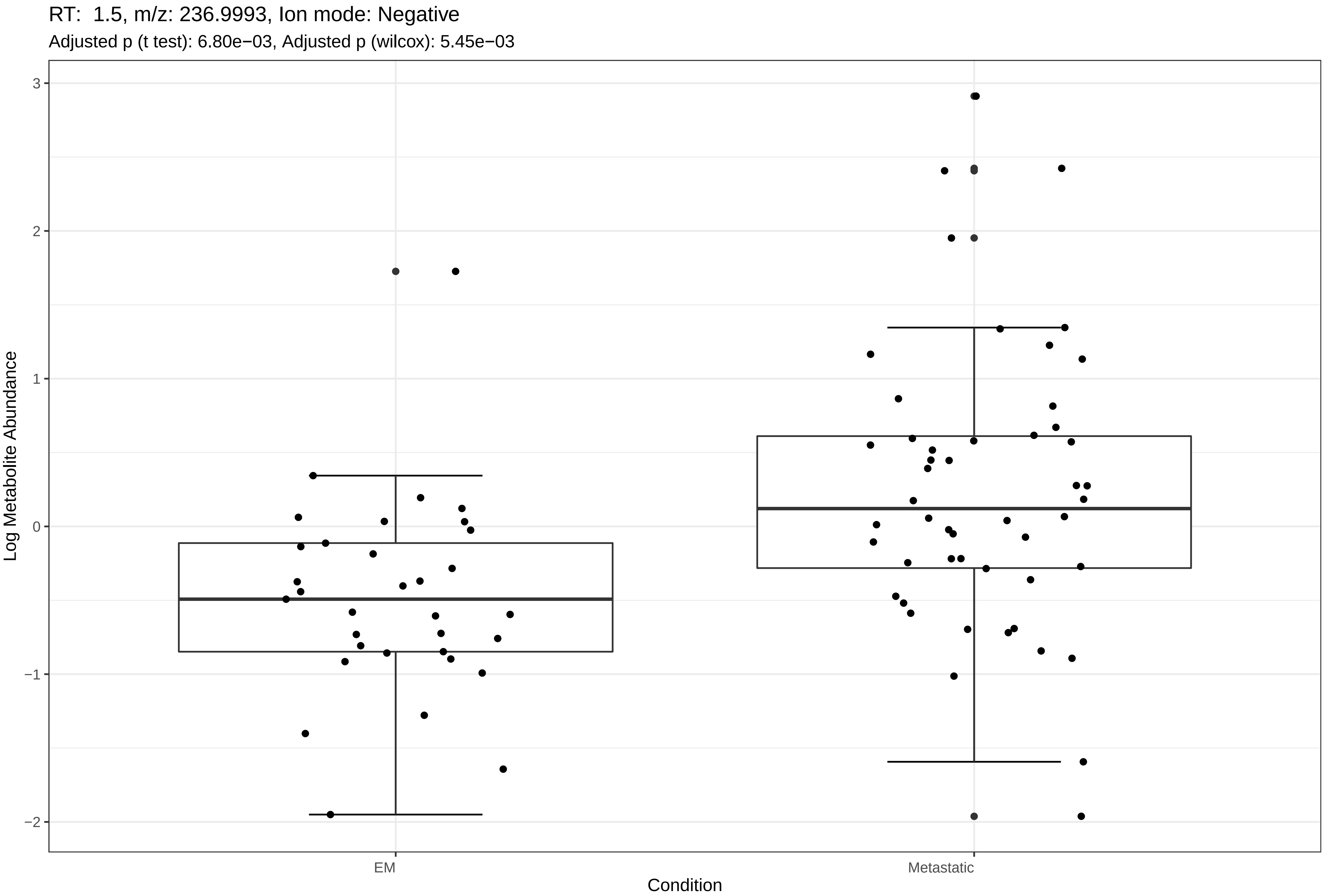

RT: 1.5, m/z: 236.9993, Ion mode: Negative
Adjusted p (t test): 6.80e−03, Adjusted p (wilcox): 5.45e−03
Log Metabolite Abundance
3
2
1
0
−1
−2
EM
Metastatic
Condition

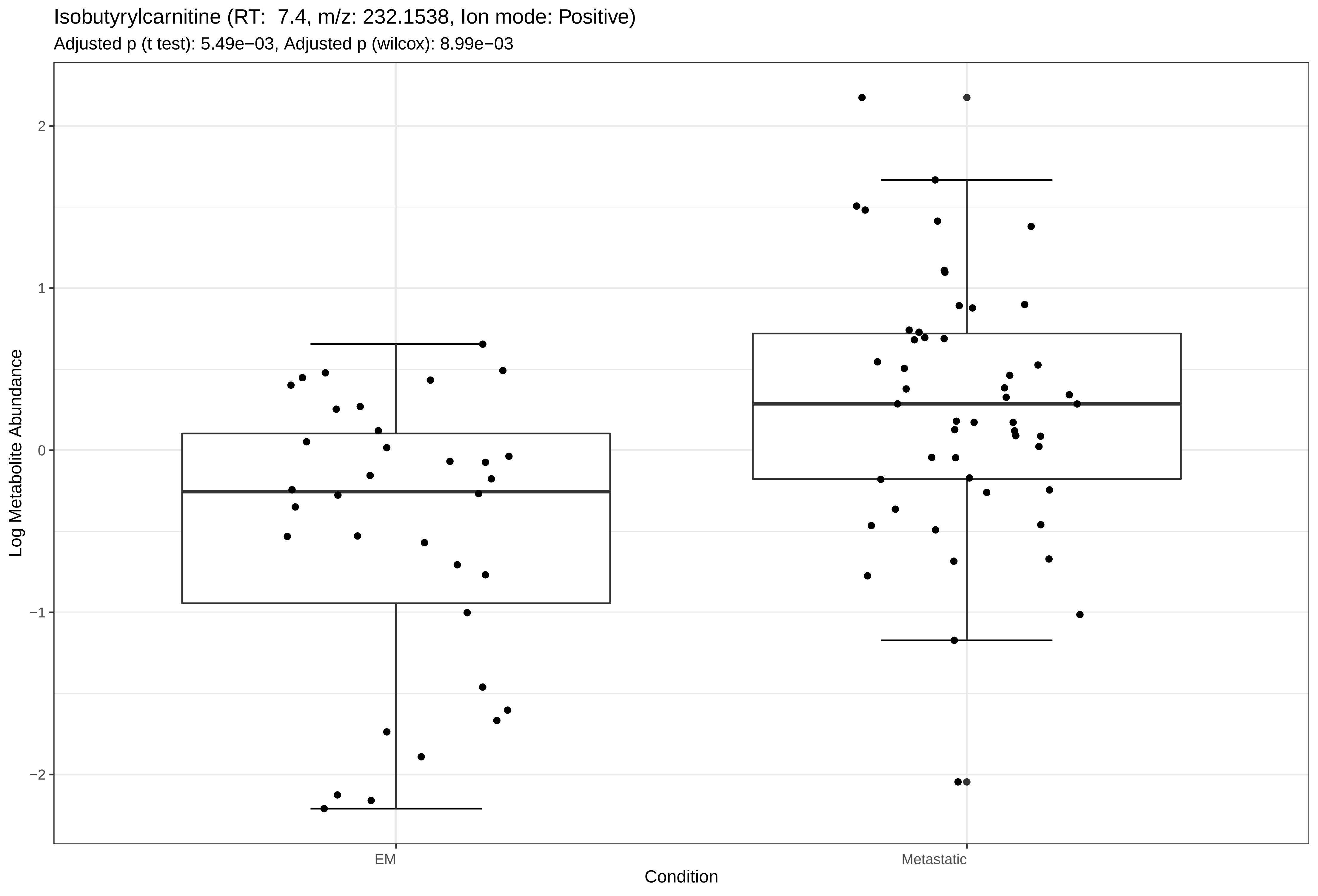
Isobutyrylcarnitine (RT: 7.4, m/z: 232.1538, Ion mode: Positive)
Adjusted p (t test): 5.49e−03, Adjusted p (wilcox): 8.99e−03
Log Metabolite Abundance
2
1
0
−1
−2
EM
Metastatic
Condition

RT: 0.8, m/z: 170.9962, Ion mode: Negative

Adjusted p (t test): 5.49e−03, Adjusted p (wilcox): 6.32e−03

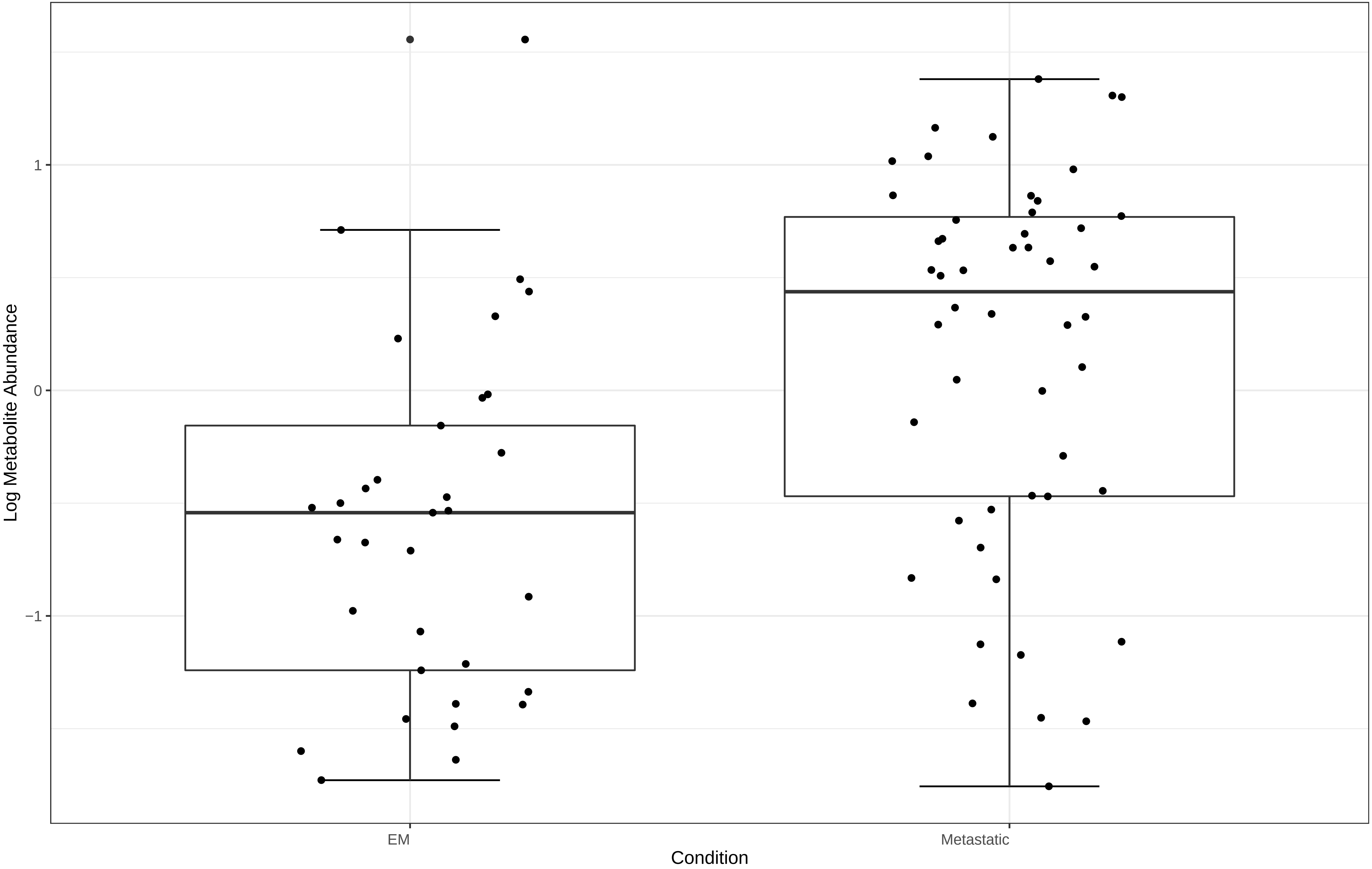

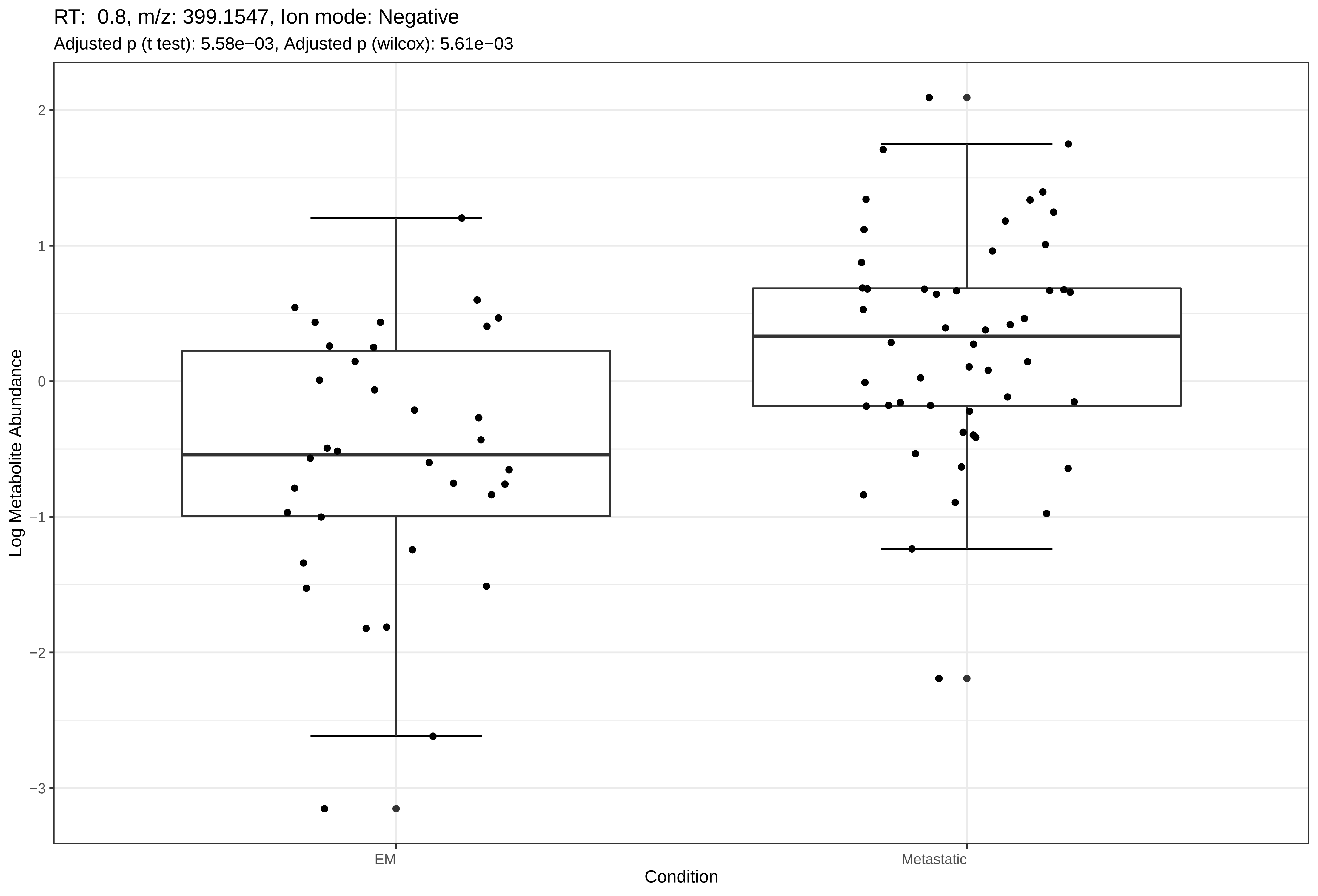
RT: 0.8, m/z: 399.1547, Ion mode: Negative
Adjusted p (t test): 5.58e−03, Adjusted p (wilcox): 5.61e−03
2
1
0
−1
−2
−3
Log Metabolite Abundance
EM
Metastatic
Condition

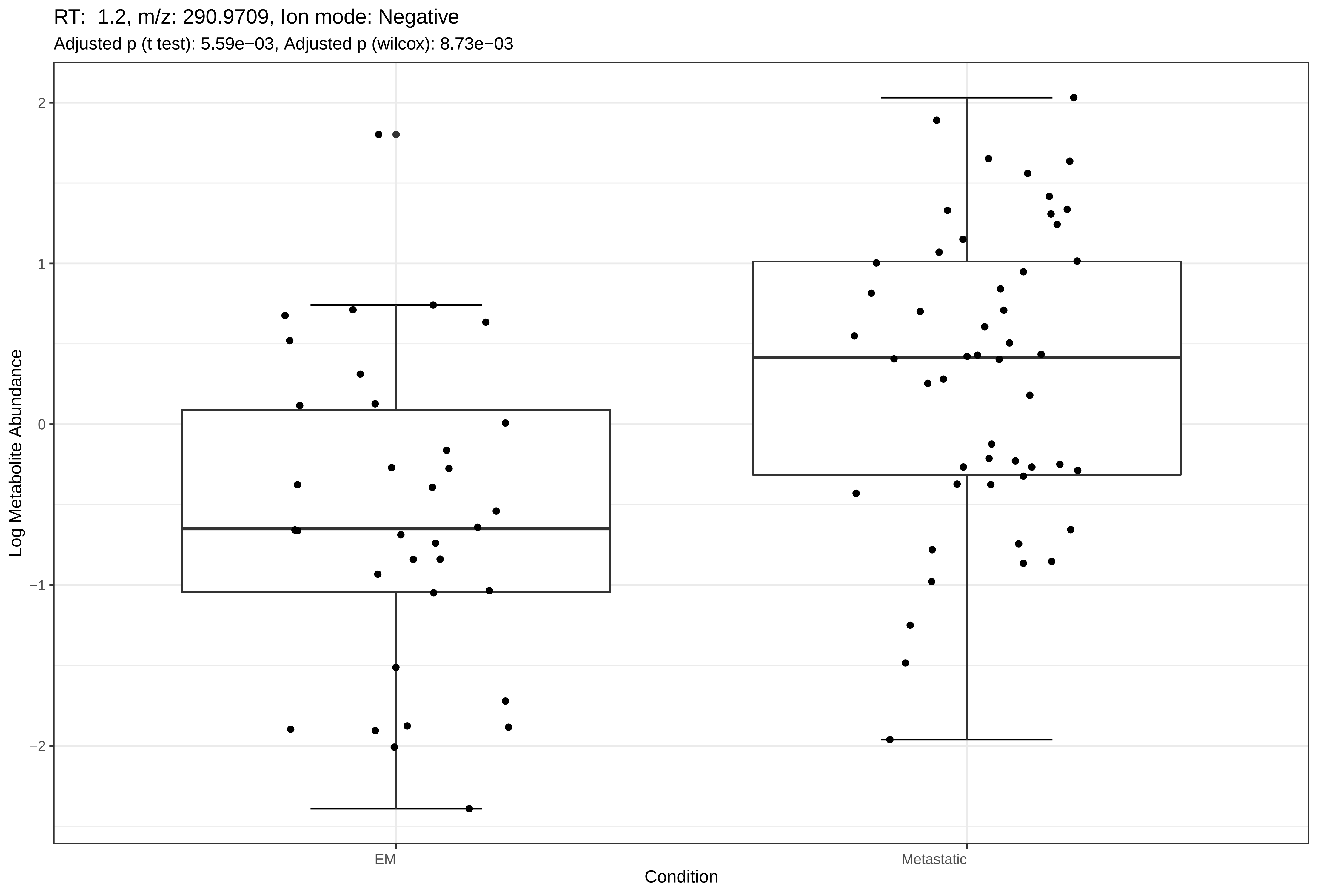
RT: 1.2, m/z: 290.9709, Ion mode: Negative
Adjusted p (t test): 5.59e−03, Adjusted p (wilcox): 8.73e−03
Log Metabolite Abundance
2
1
0
−1
−2
EM
Metastatic
Condition

L−Leucine 13C6 (RT: 2.4, m/z: 138.1220, Ion mode: Positive)

Adjusted p (t test): 6.89e−03, Adjusted p (wilcox): 5.61e−03

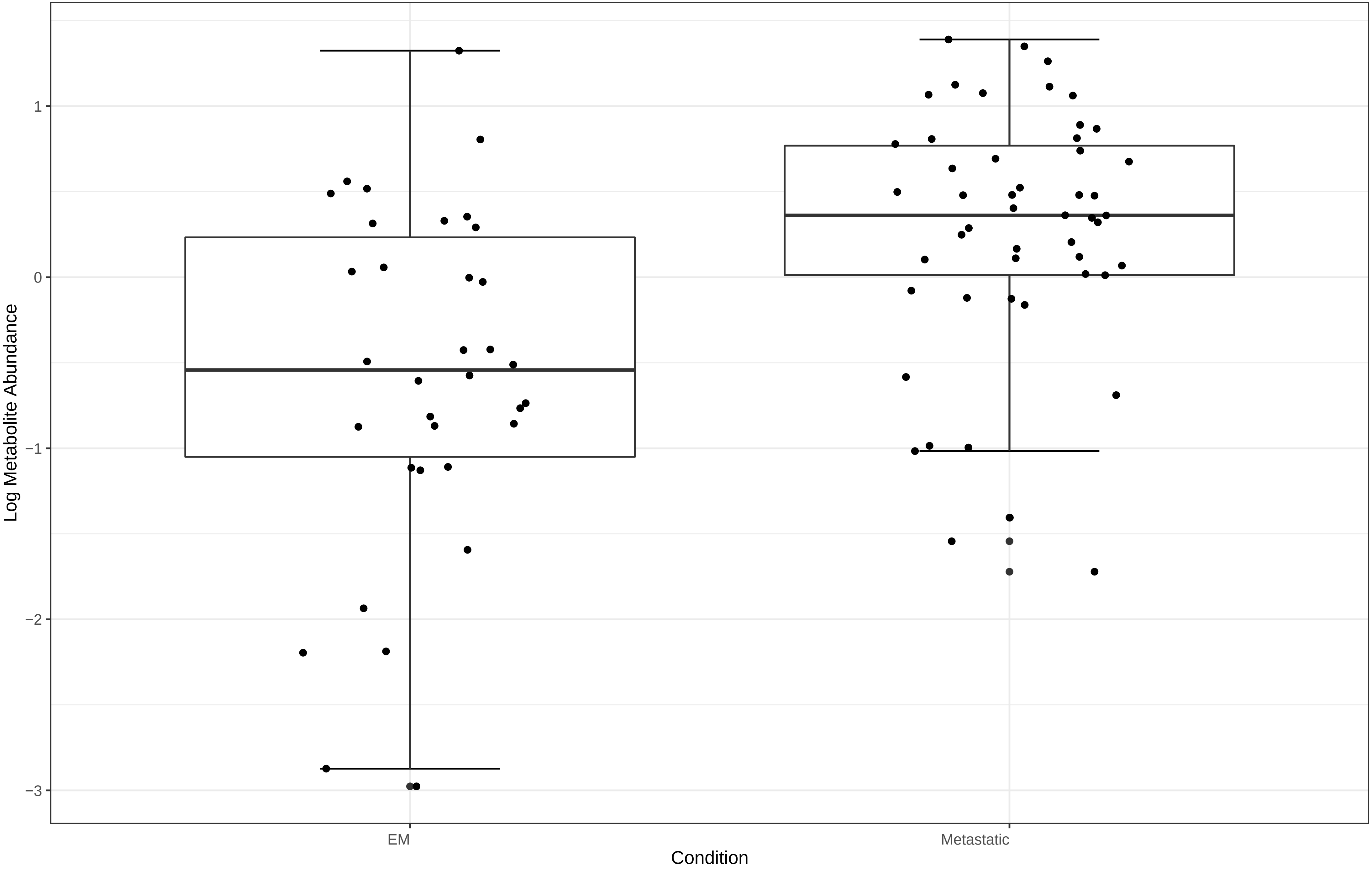

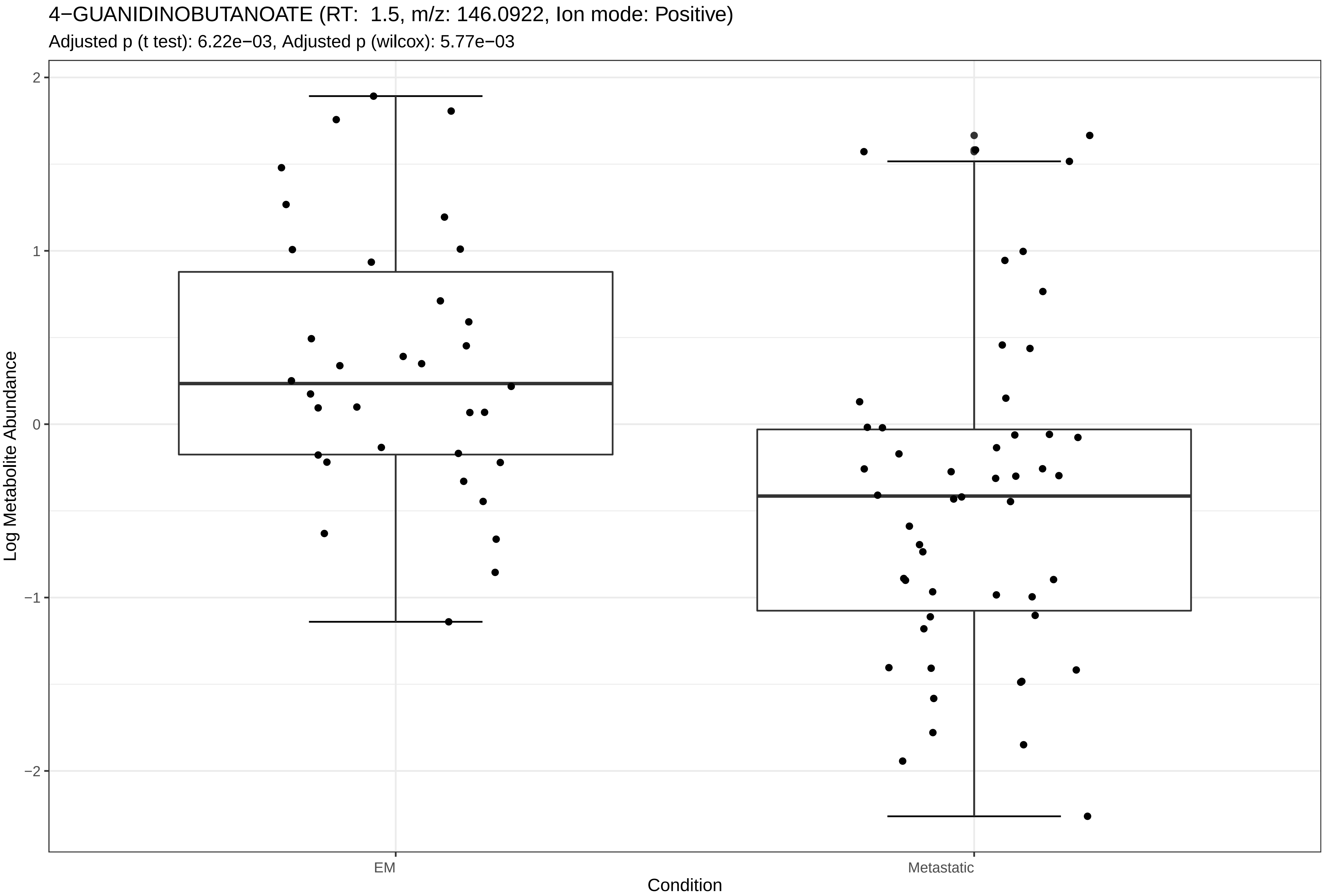
4−GUANIDINOBUTANOATE (RT: 1.5, m/z: 146.0922, Ion mode: Positive)
Adjusted p (t test): 6.22e−03, Adjusted p (wilcox): 5.77e−03
Log Metabolite Abundance
2
1
0
−1
−2
EM
Metastatic
Condition

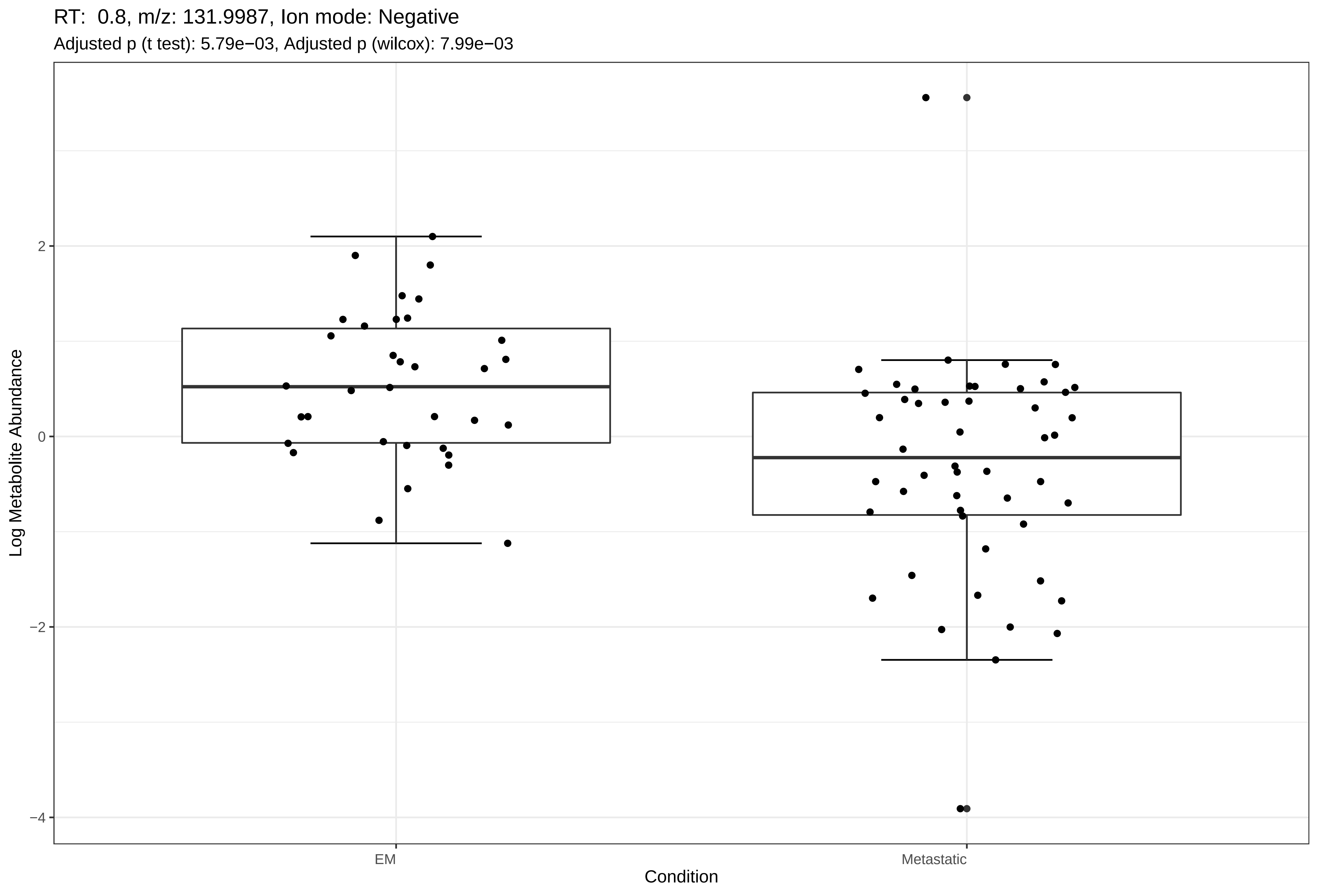
RT: 0.8, m/z: 131.9987, Ion mode: Negative
Adjusted p (t test): 5.79e−03, Adjusted p (wilcox): 7.99e−03
Log Metabolite Abundance
2
0
−2
−4
EM
Metastatic
Condition

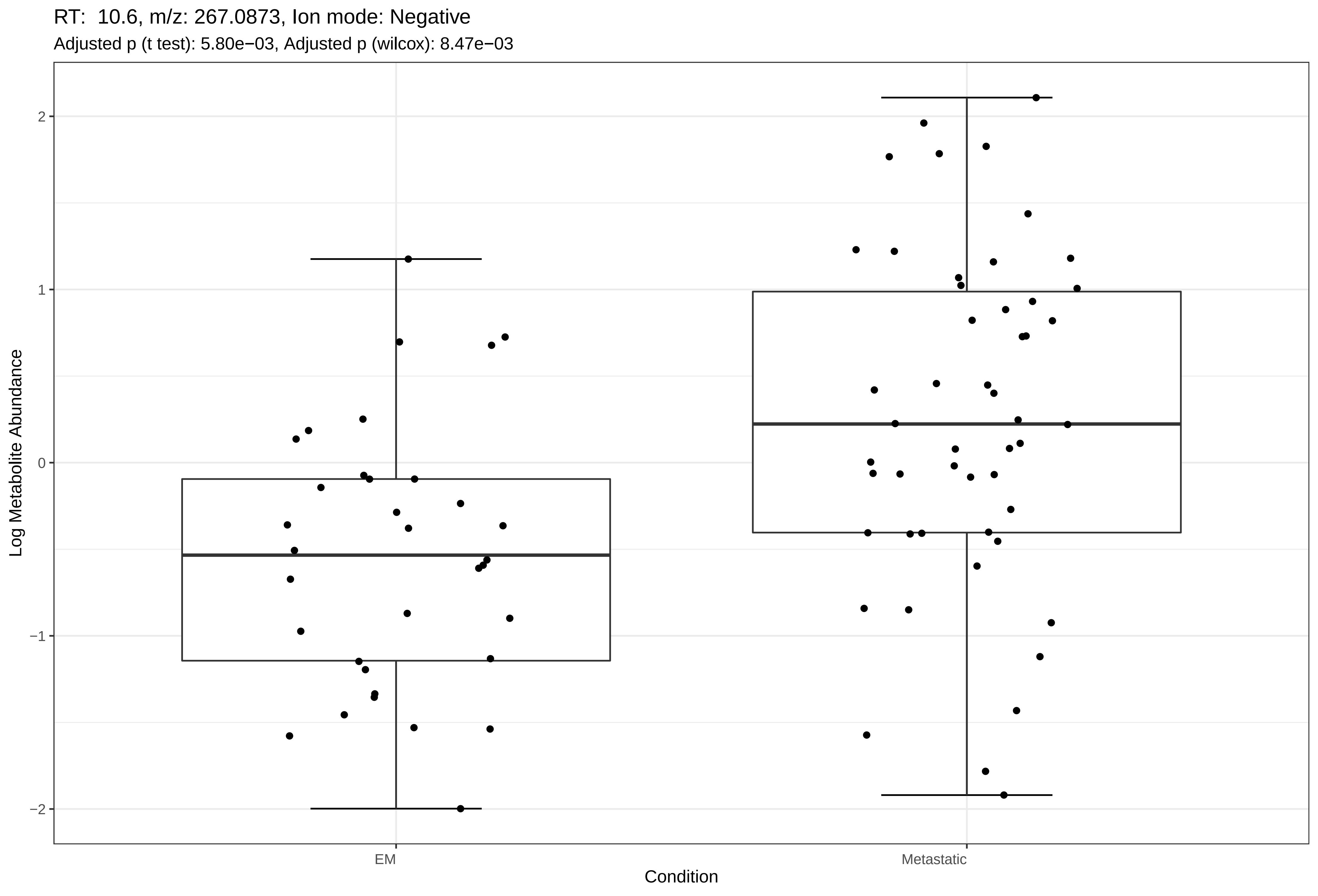
RT: 10.6, m/z: 267.0873, Ion mode: Negative
Adjusted p (t test): 5.80e−03, Adjusted p (wilcox): 8.47e−03
Log Metabolite Abundance
2
1
0
−1
−2
EM
Metastatic
Condition

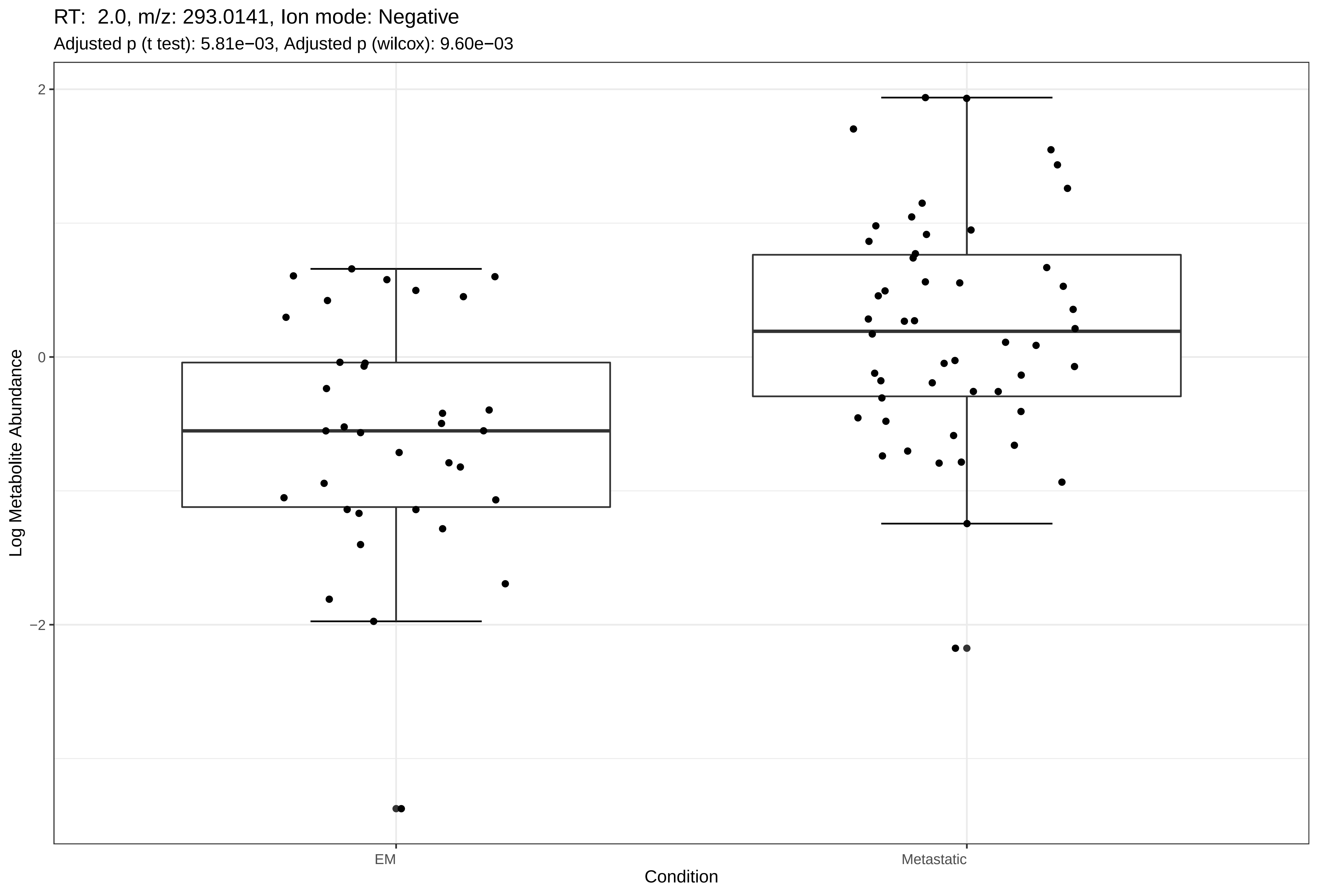
RT: 2.0, m/z: 293.0141, Ion mode: Negative
Adjusted p (t test): 5.81e−03, Adjusted p (wilcox): 9.60e−03
Log Metabolite Abundance
2
0
−2
EM
Metastatic
Condition

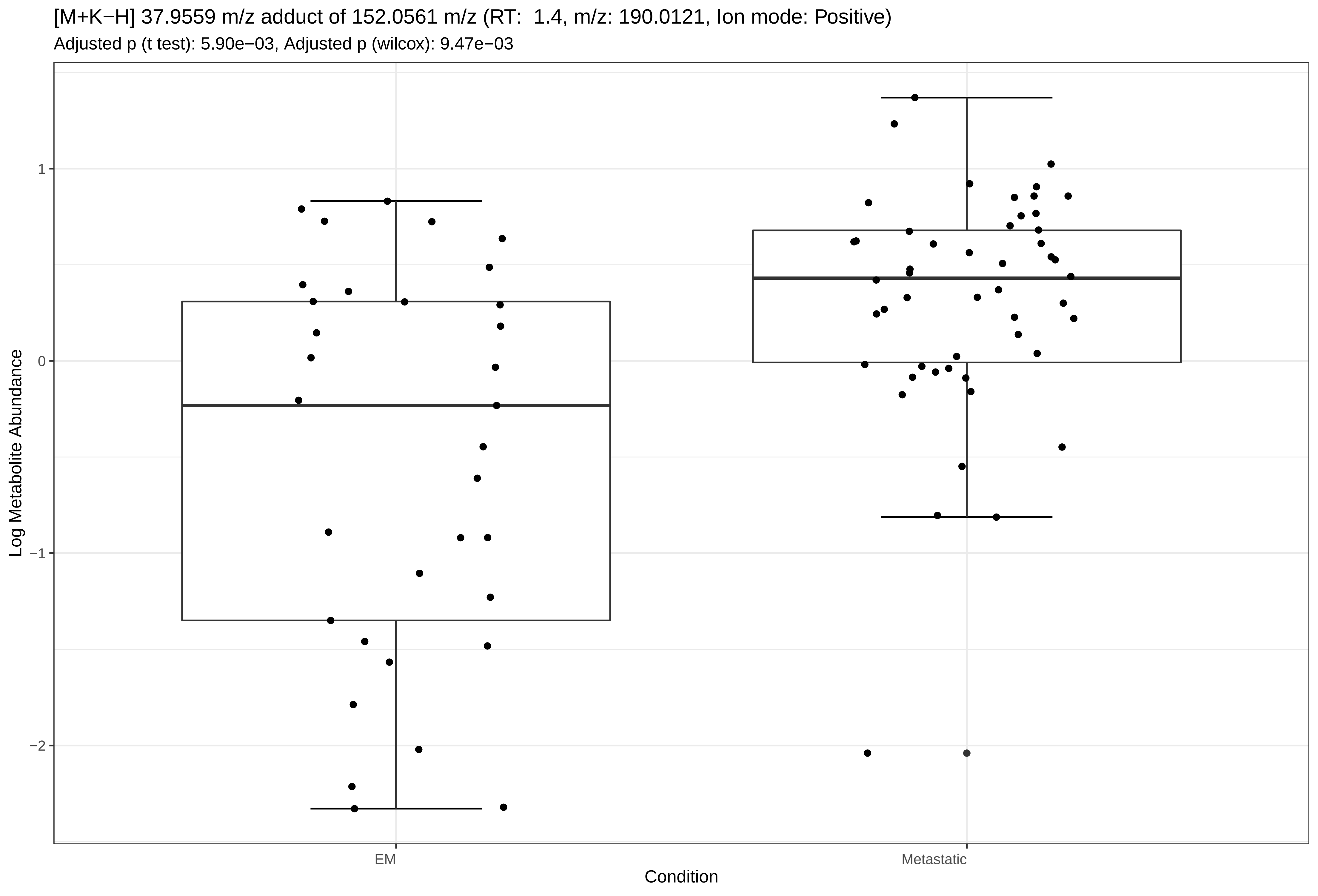

[M+K−H] 37.9559 m/z adduct of 152.0561 m/z (RT: 1.4, m/z: 190.0121, Ion mode: Positive)
Adjusted p (t test): 5.90e−03, Adjusted p (wilcox): 9.47e−03
Log Metabolite Abundance
1
0
−1
−2
EM
Metastatic
Condition

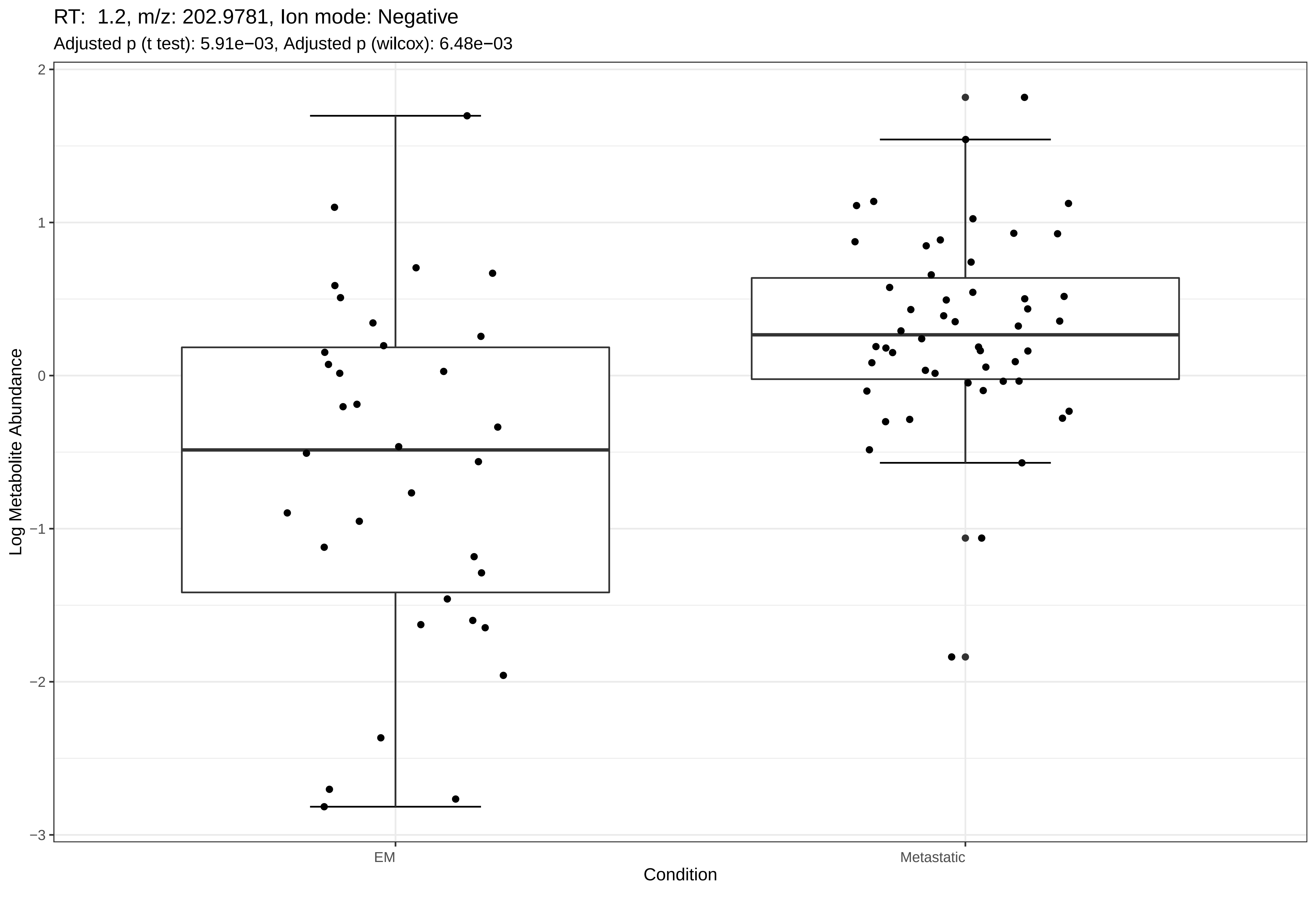

RT: 1.2, m/z: 202.9781, Ion mode: Negative
Adjusted p (t test): 5.91e−03, Adjusted p (wilcox): 6.48e−03
Log Metabolite Abundance
2
1
0
−1
−2
−3
EM
Metastatic
Condition

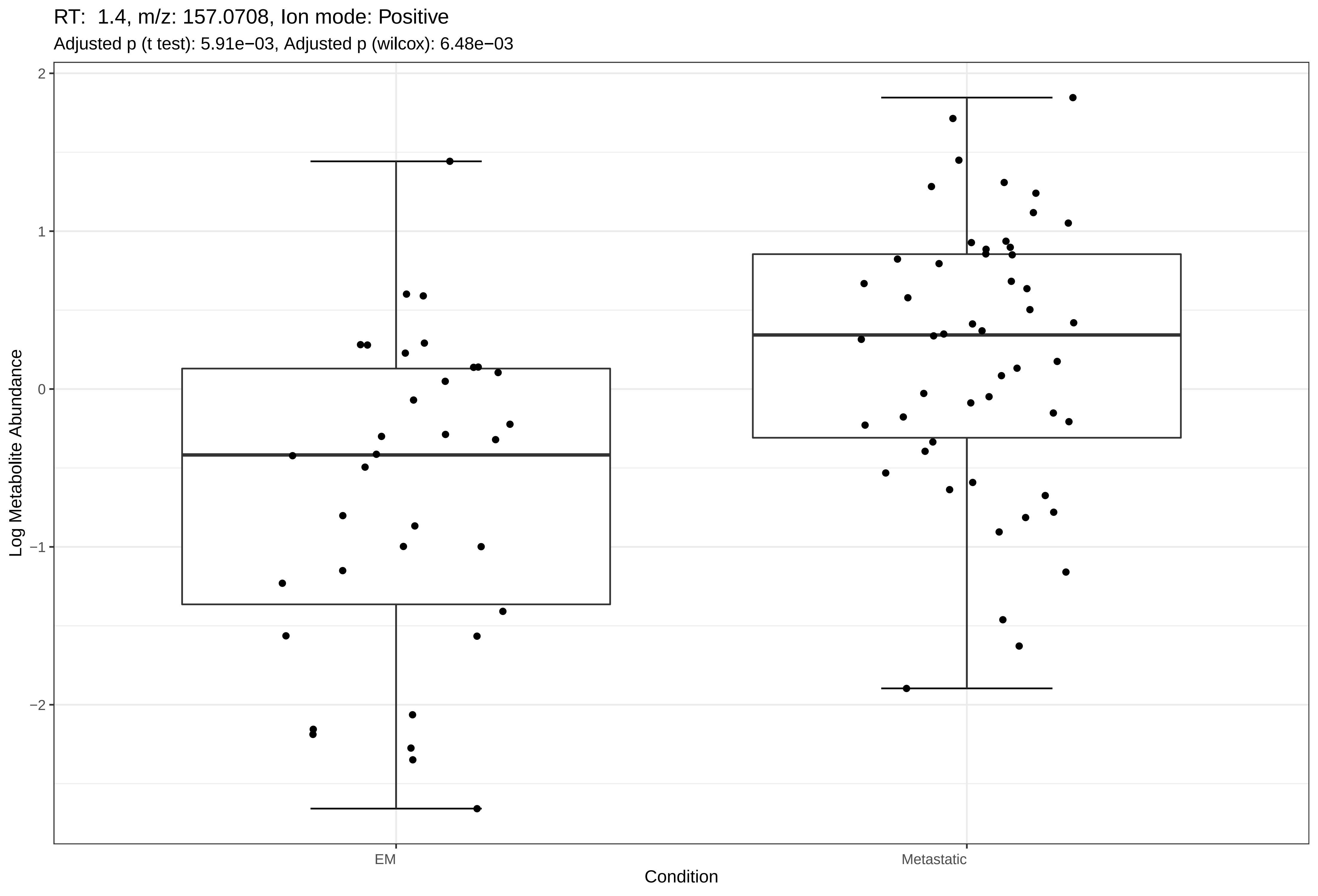
RT: 1.4, m/z: 157.0708, Ion mode: Positive
Adjusted p (t test): 5.91e−03, Adjusted p (wilcox): 6.48e−03
Log Metabolite Abundance
2
1
0
−1
−2
EM
Metastatic
Condition

RT: 2.4, m/z: 91.1132, Ion mode: Positive

Adjusted p (t test): 6.68e−03, Adjusted p (wilcox): 5.95e−03

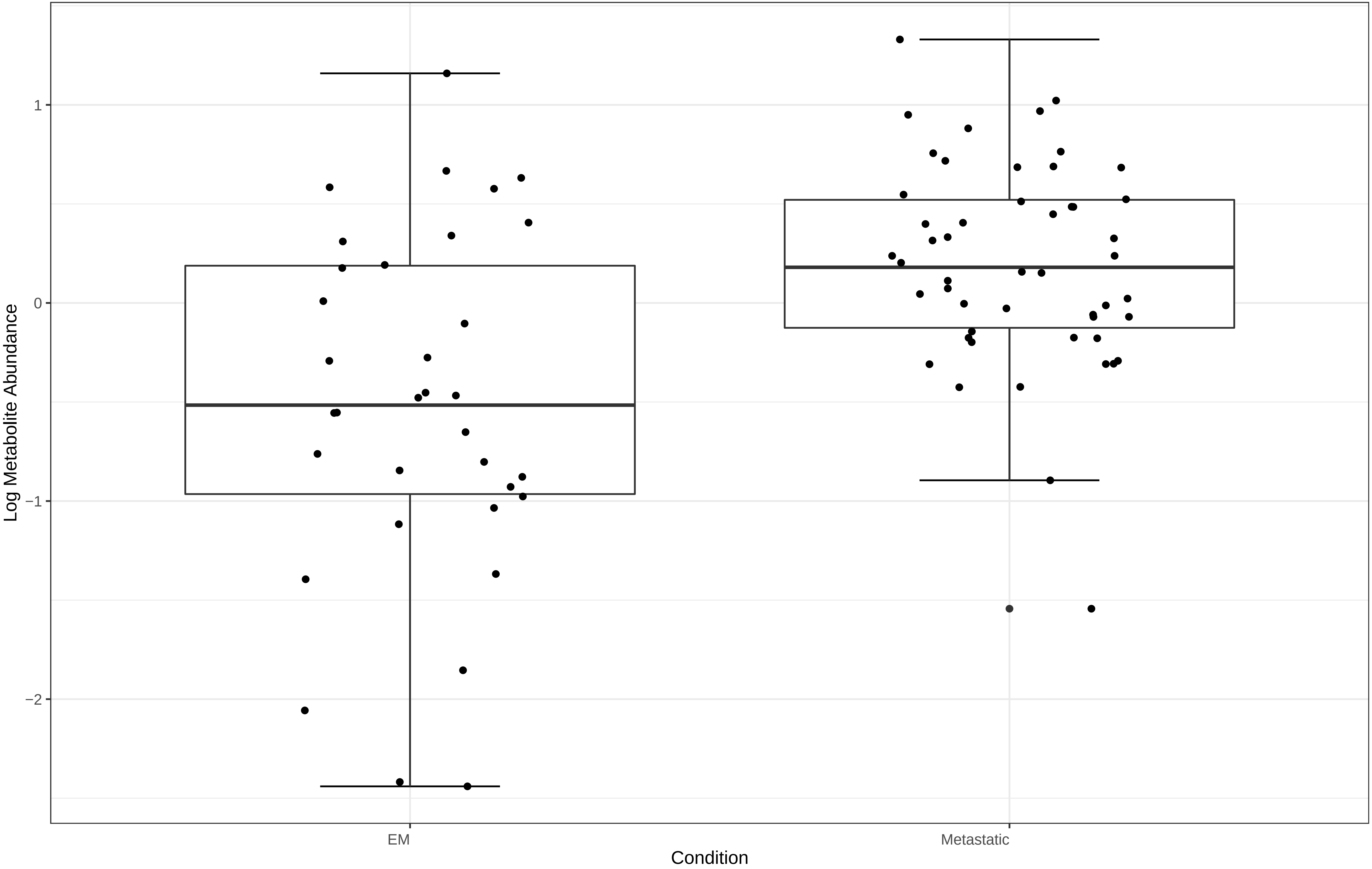

RT: 1.3, m/z: 181.1075, Ion mode: Positive

Adjusted p (t test): 7.92e−03, Adjusted p (wilcox): 5.95e−03

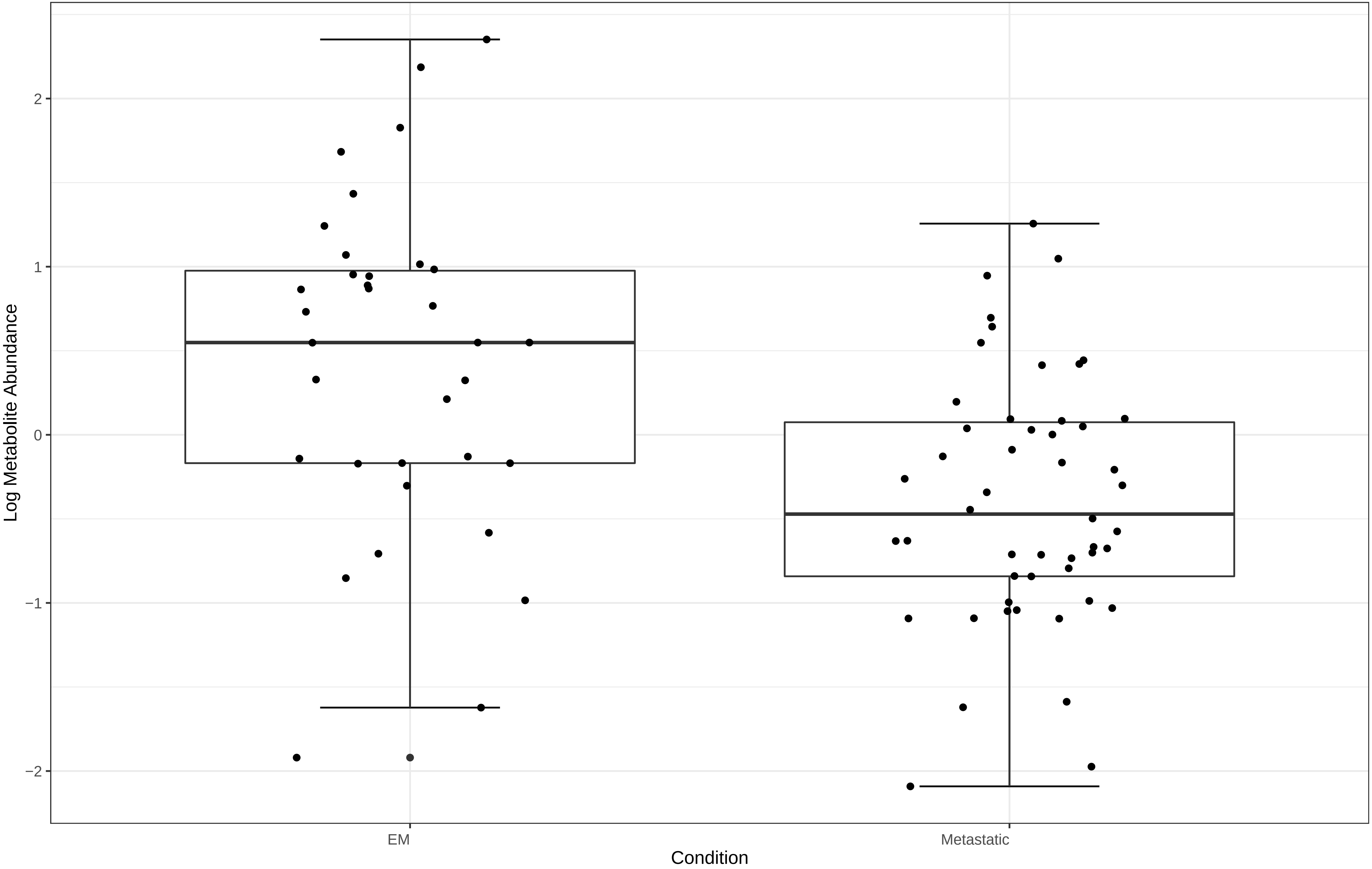

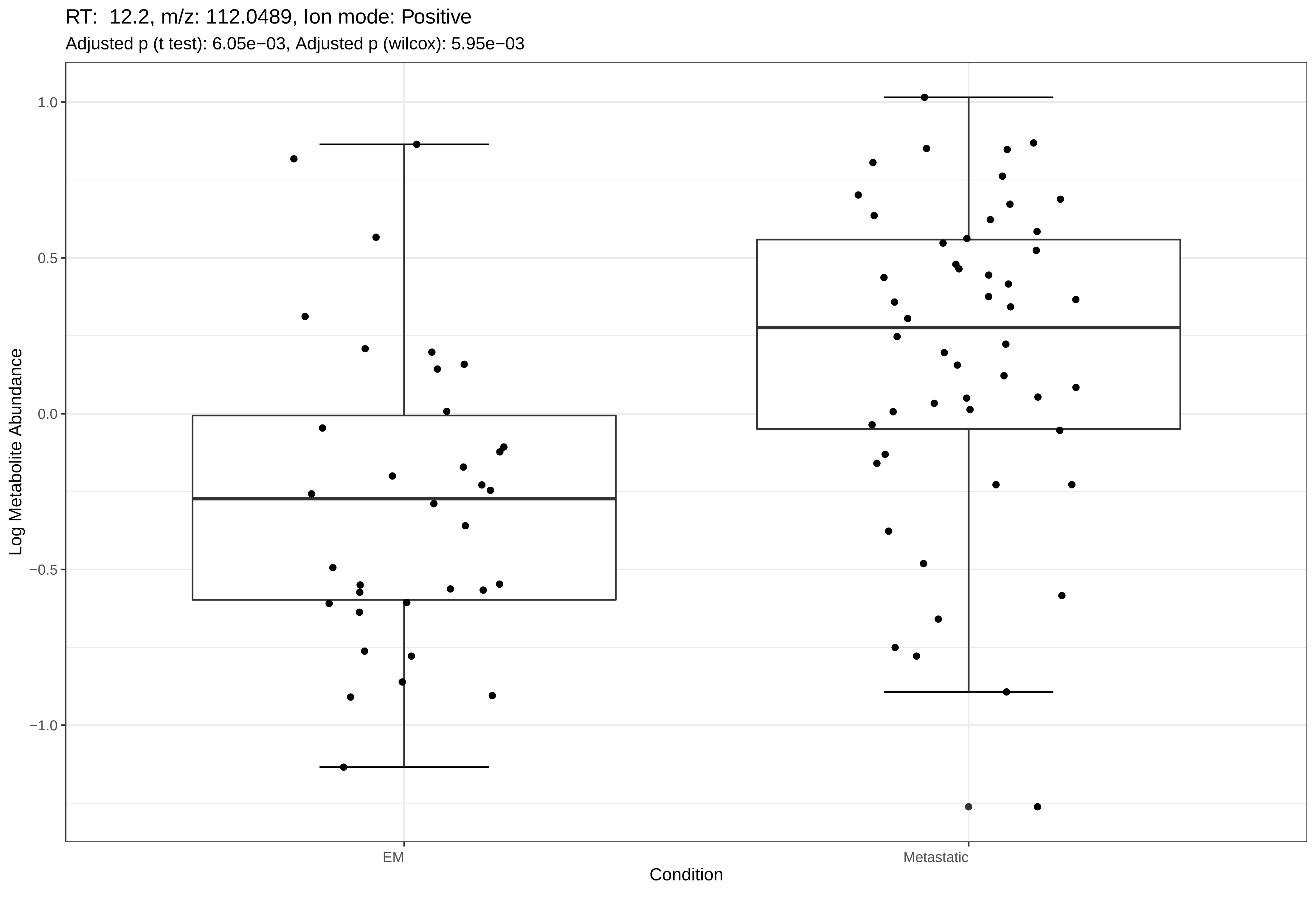
RT: 12.2, m/z: 112.0489, Ion mode: Positive
Adjusted p (t test): 6.05e−03, Adjusted p (wilcox): 5.95e−03
1.0
0.5
0.0
−0.5
−1.0
Log Metabolite Abundance
EM
Metastatic
Condition

RT: 1.5, m/z: 76.0213, Ion mode: Positive

Adjusted p (t test): 9.49e−03, Adjusted p (wilcox): 6.02e−03

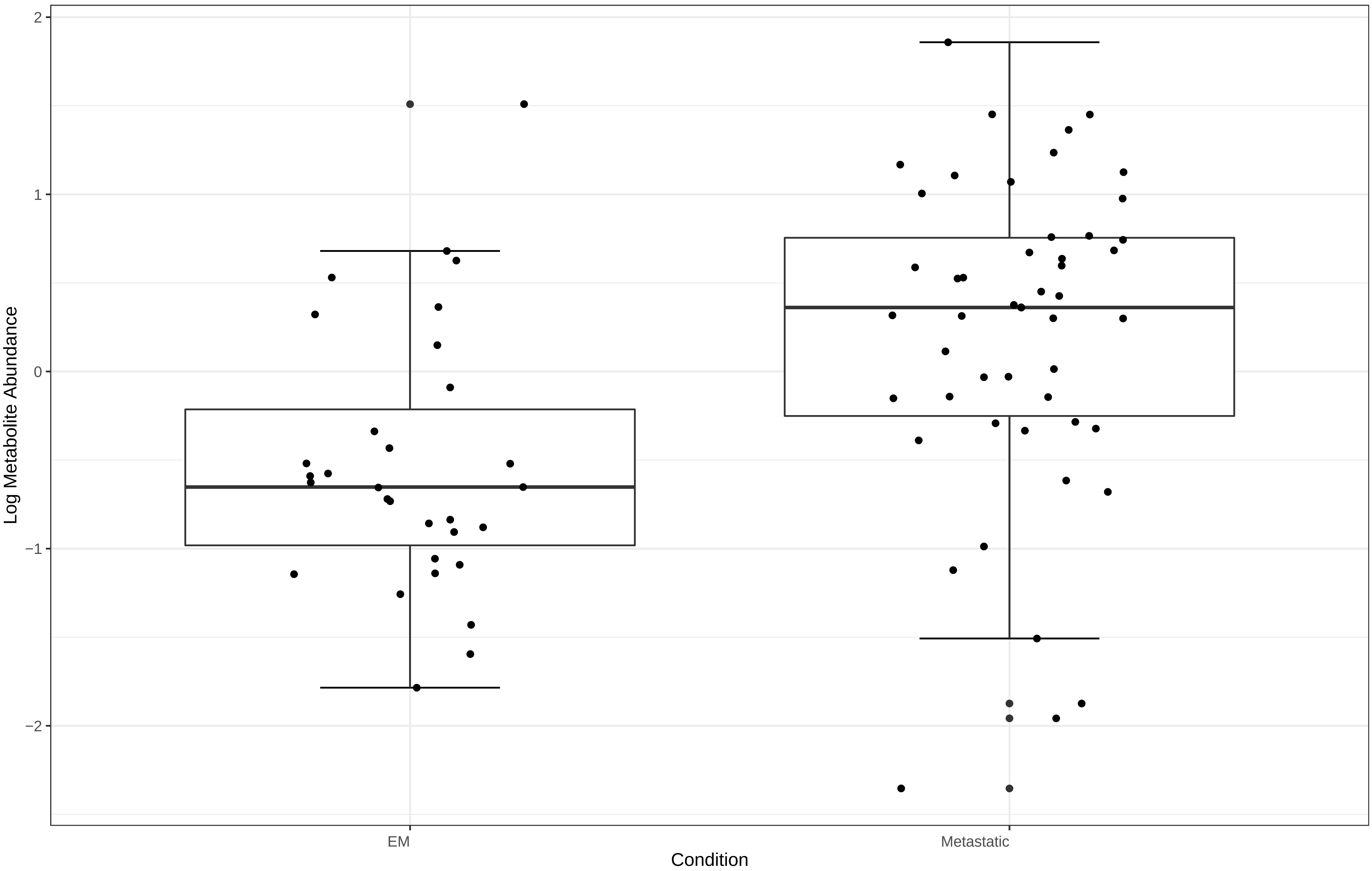

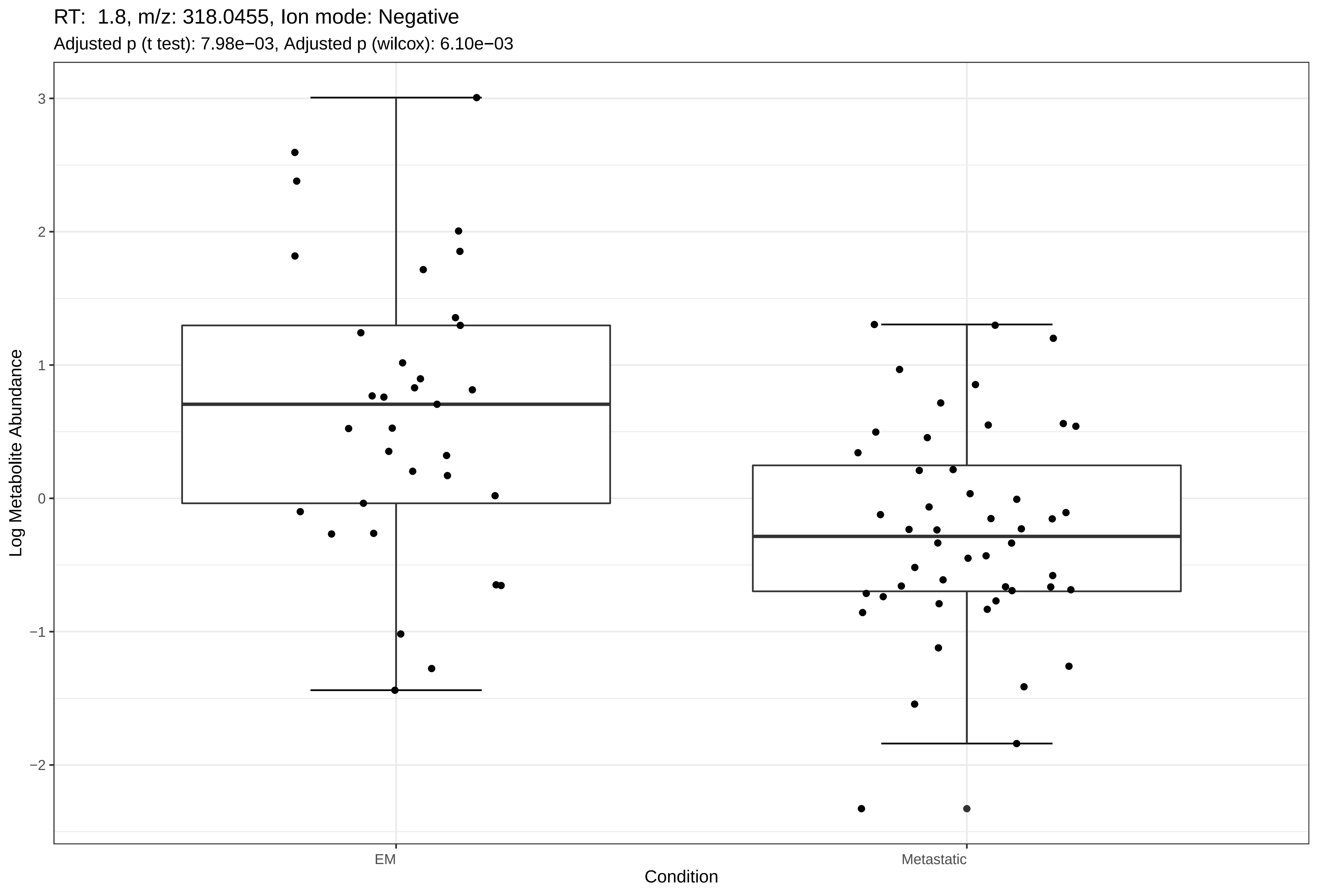

RT: 1.8, m/z: 318.0455, Ion mode: Negative
Adjusted p (t test): 7.98e−03, Adjusted p (wilcox): 6.10e−03
Log Metabolite Abundance
3
2
1
0
−1
−2
EM
Metastatic
Condition

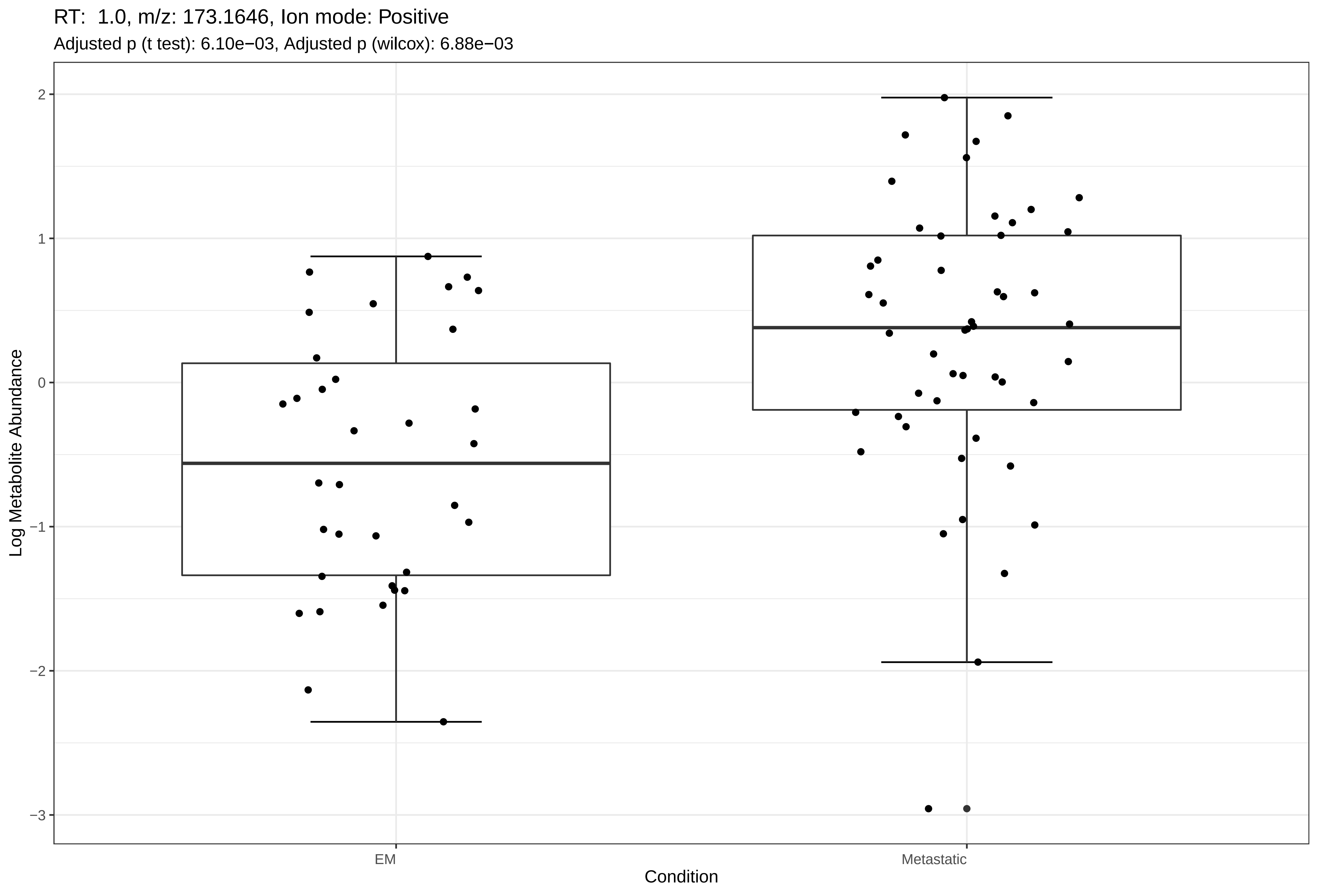
RT: 1.0, m/z: 173.1646, Ion mode: Positive
Adjusted p (t test): 6.10e−03, Adjusted p (wilcox): 6.88e−03
Log Metabolite Abundance
2
1
0
−1
−2
−3
EM
Metastatic
Condition

RT: 2.0, m/z: 137.0469, Ion mode: Negative

Adjusted p (t test): 6.11e−03, Adjusted p (wilcox): 6.12e−03

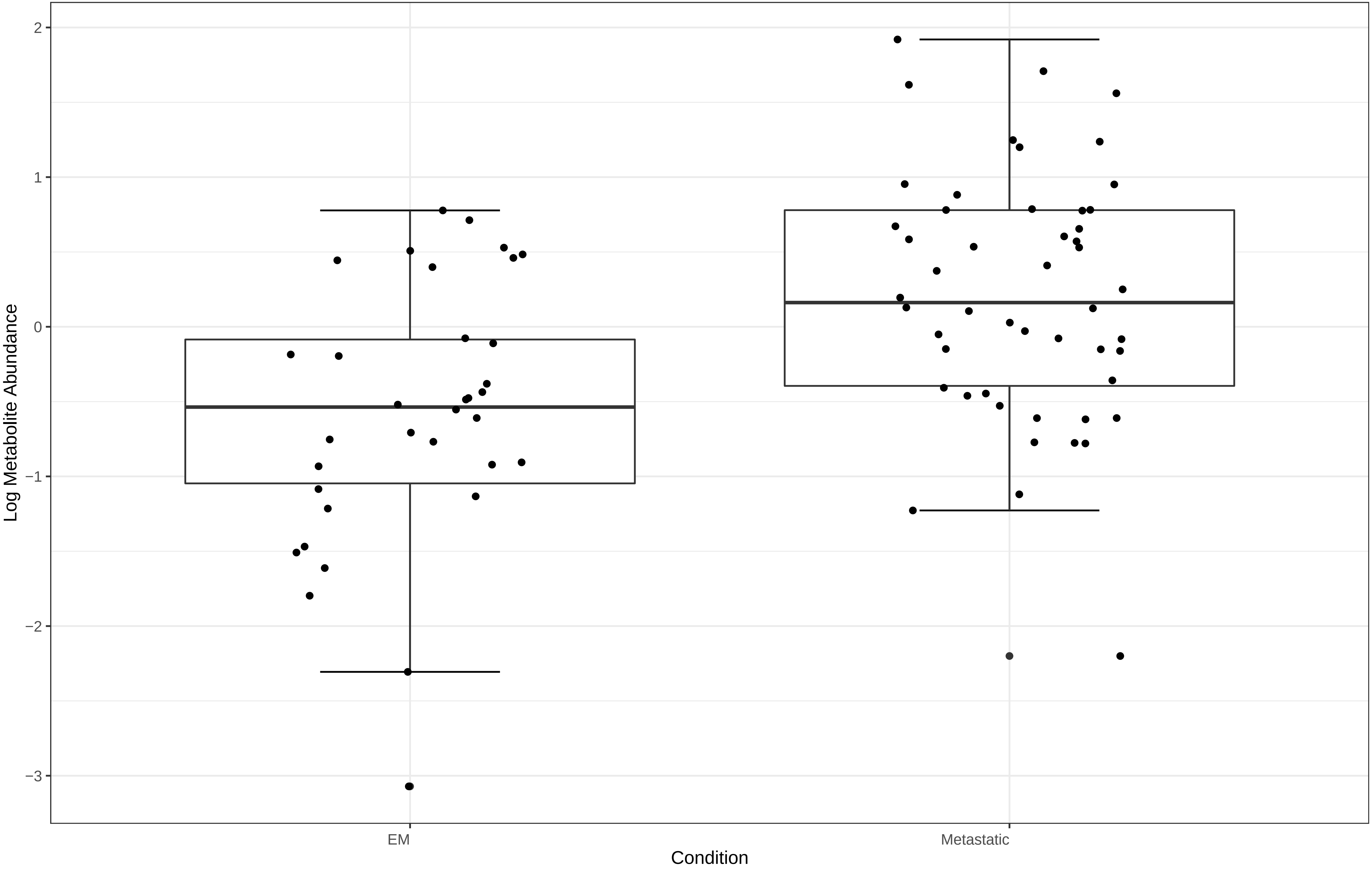

RT: 1.8, m/z: 97.5205, Ion mode: Positive

Adjusted p (t test): 6.13e−03, Adjusted p (wilcox): 8.10e−03

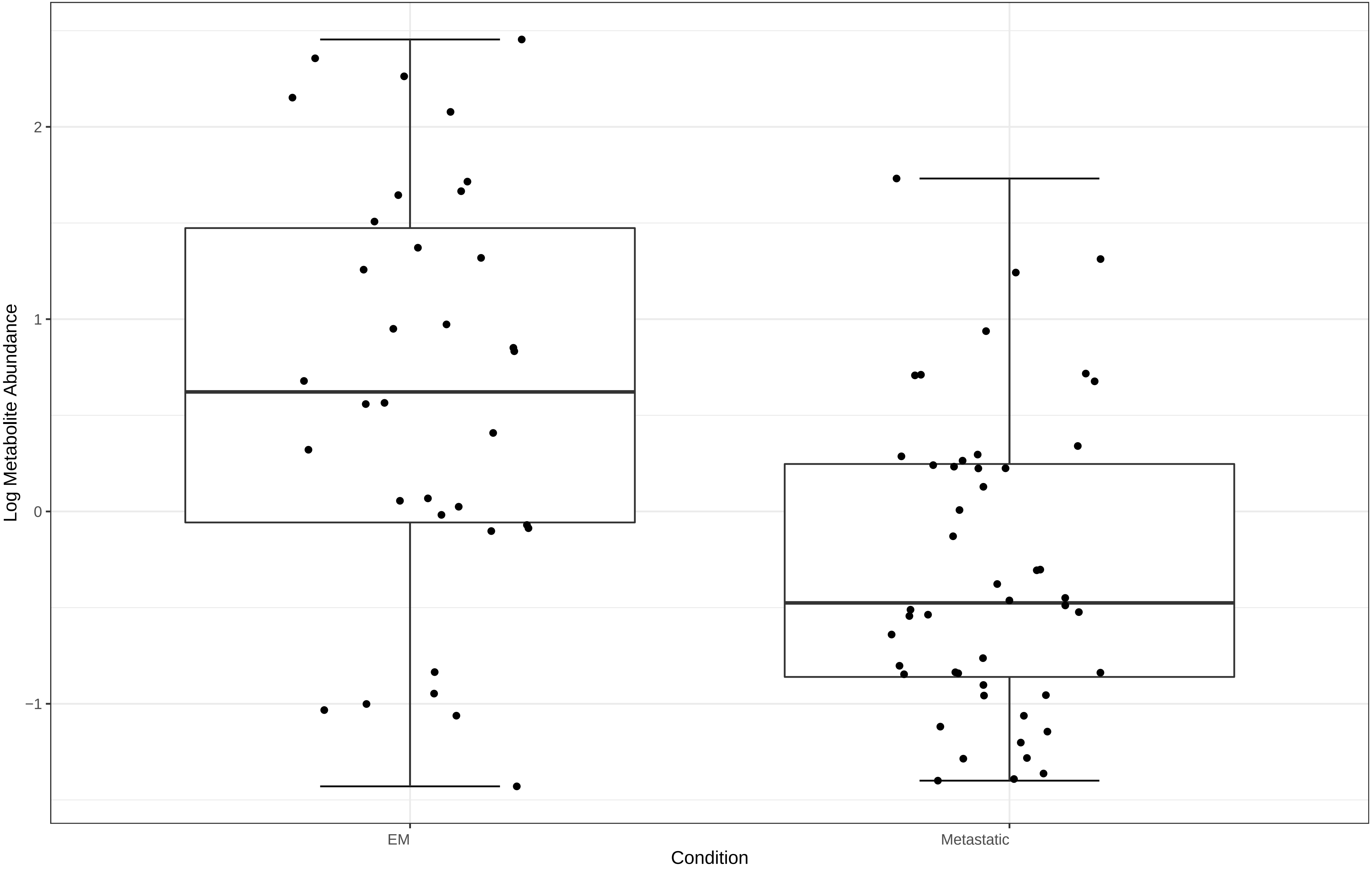

RT: 0.8, m/z: 249.0712, Ion mode: Positive

Adjusted p (t test): 6.22e−03, Adjusted p (wilcox): 9.71e−03

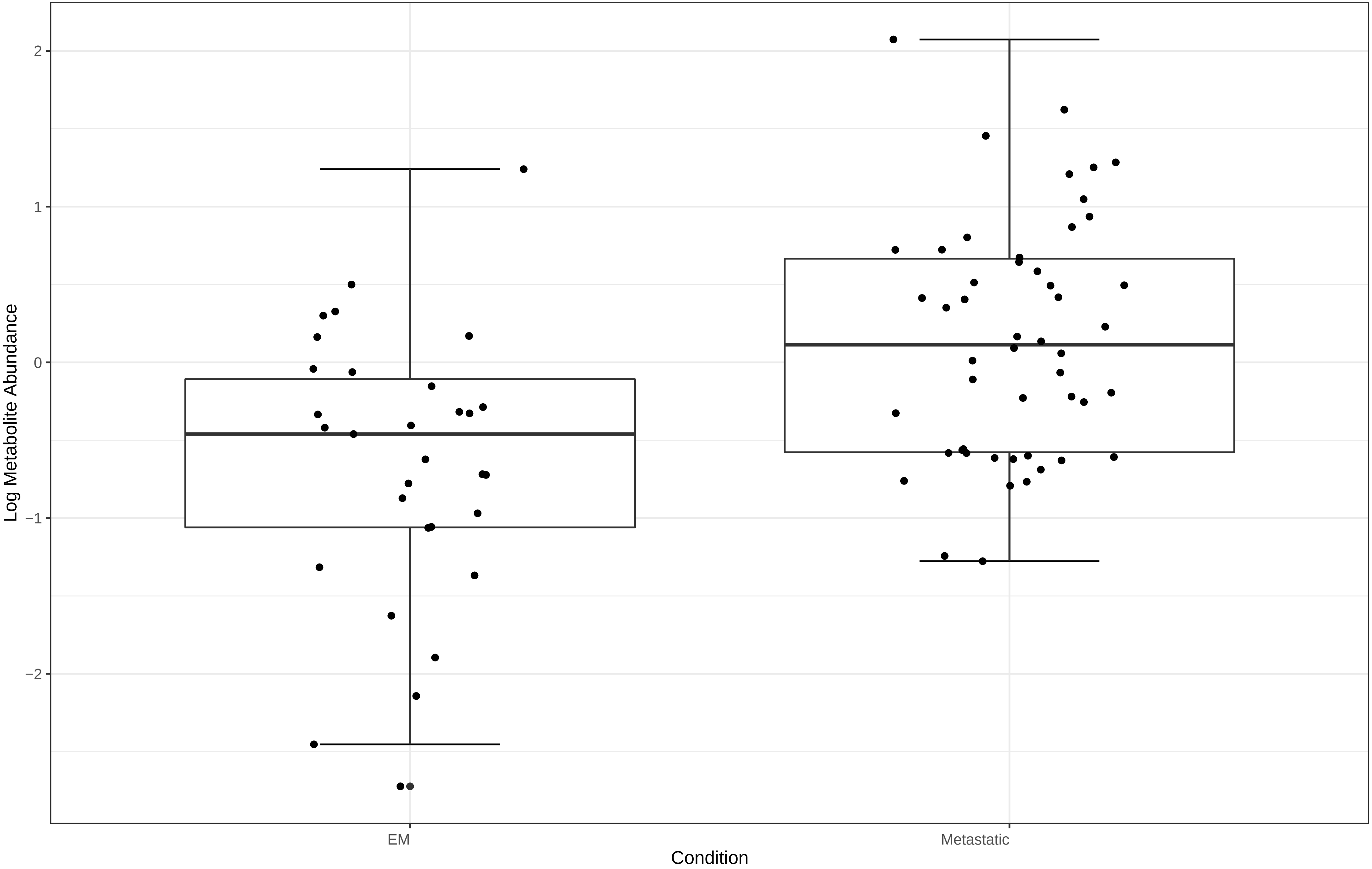

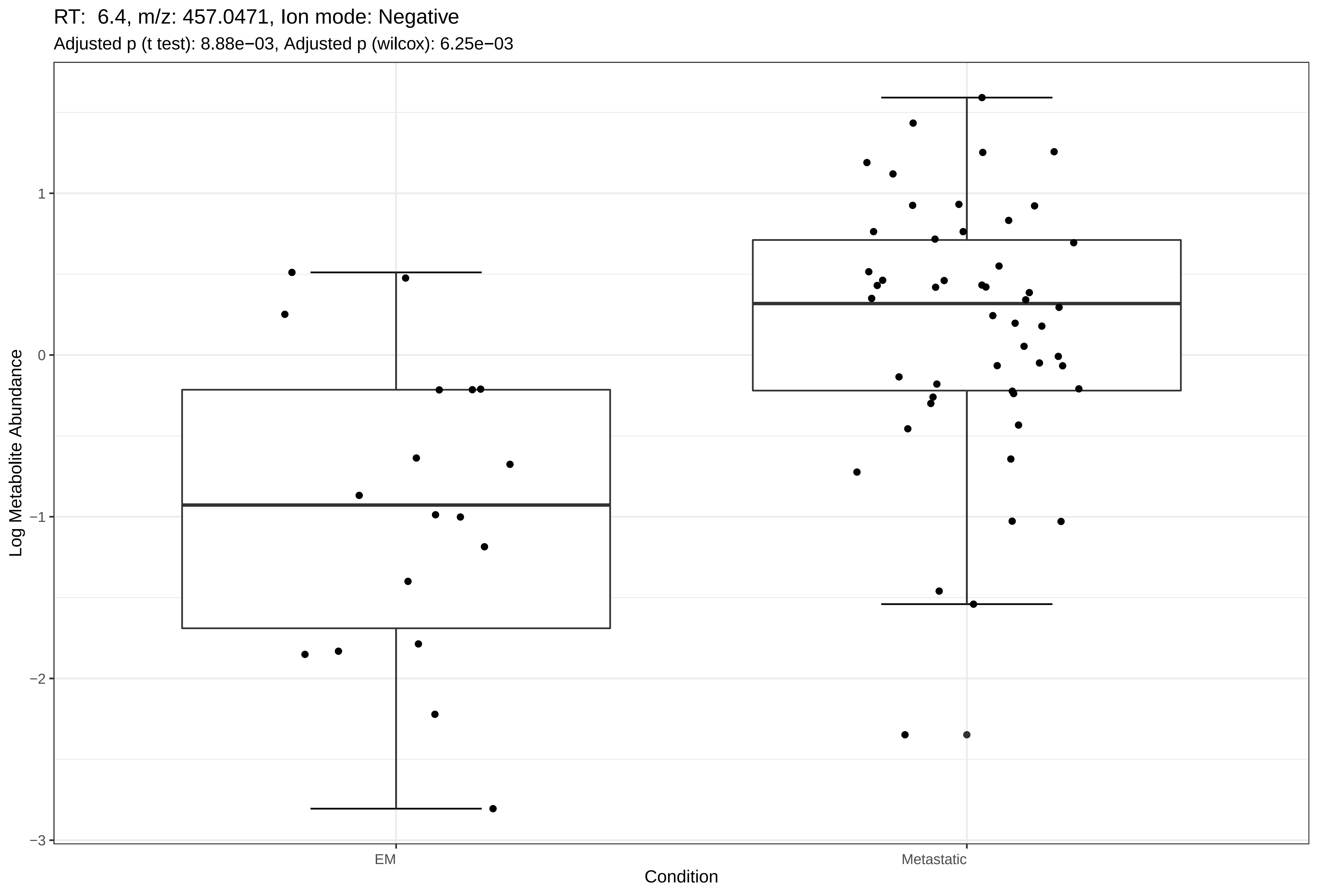
RT: 6.4, m/z: 457.0471, Ion mode: Negative
Adjusted p (t test): 8.88e−03, Adjusted p (wilcox): 6.25e−03
Log Metabolite Abundance
1
0
−1
−2
−3
EM
Metastatic
Condition

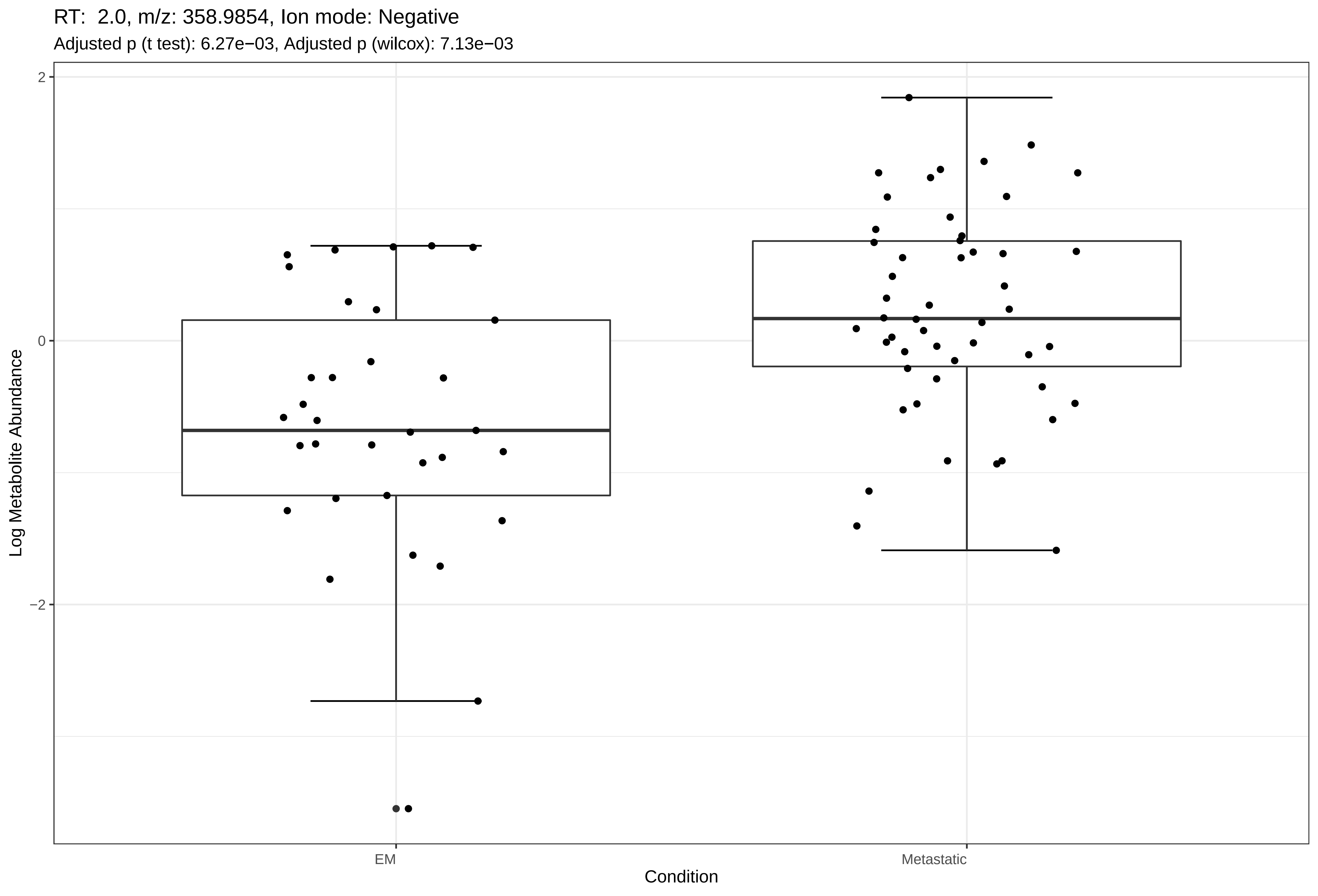
RT: 2.0, m/z: 358.9854, Ion mode: Negative
Adjusted p (t test): 6.27e−03, Adjusted p (wilcox): 7.13e−03
Log Metabolite Abundance
2
0
−2
EM
Metastatic
Condition

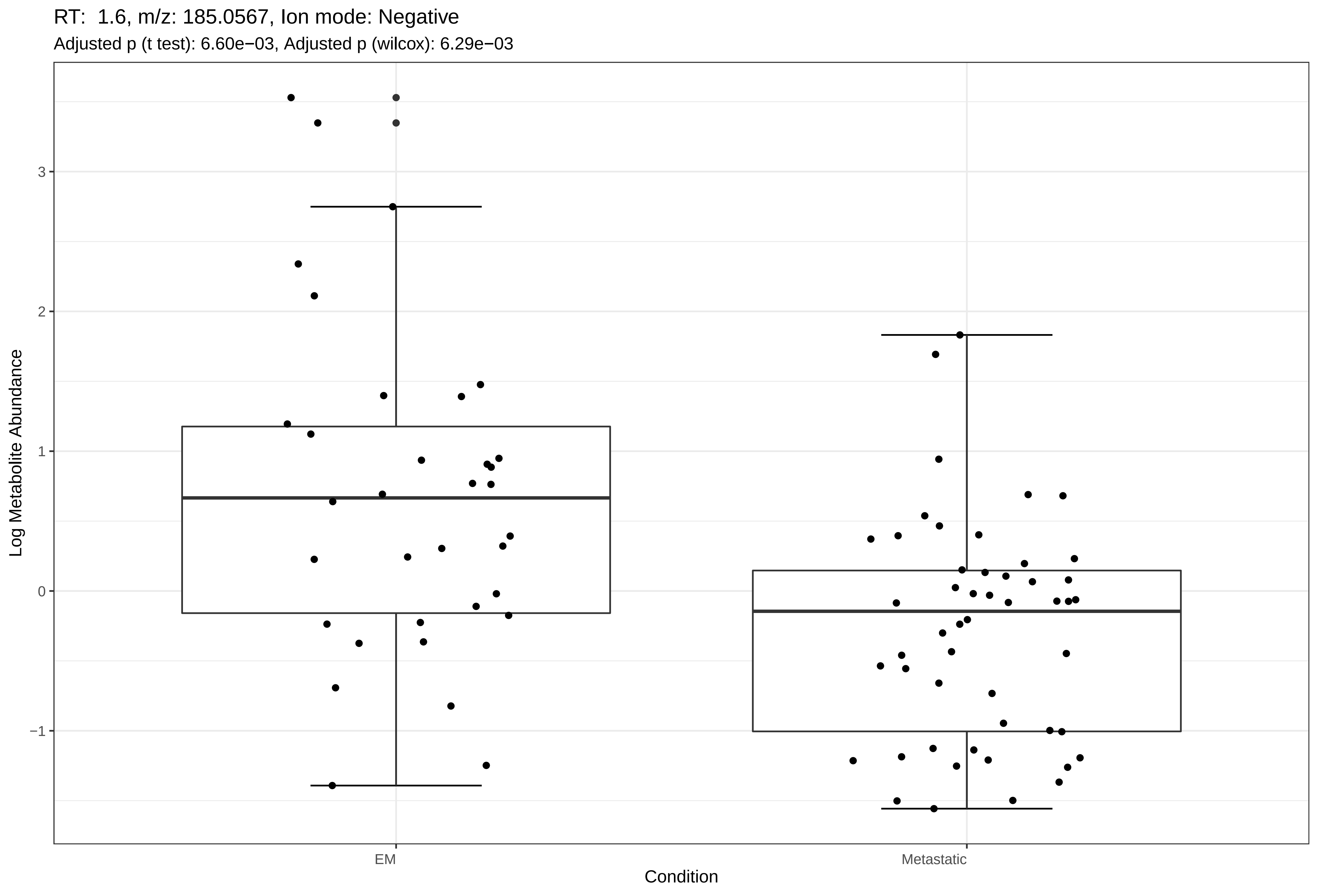

RT: 1.6, m/z: 185.0567, Ion mode: Negative
Adjusted p (t test): 6.60e−03, Adjusted p (wilcox): 6.29e−03
Log Metabolite Abundance
3
2
1
0
−1
EM
Metastatic
Condition

[M−NH3+H] −17.0265 m/z adduct of 155.0749 m/z (RT: 1.4, m/z: 138.0485, Ion mode: Positive)

Adjusted p (t test): 6.81e−03, Adjusted p (wilcox): 6.29e−03

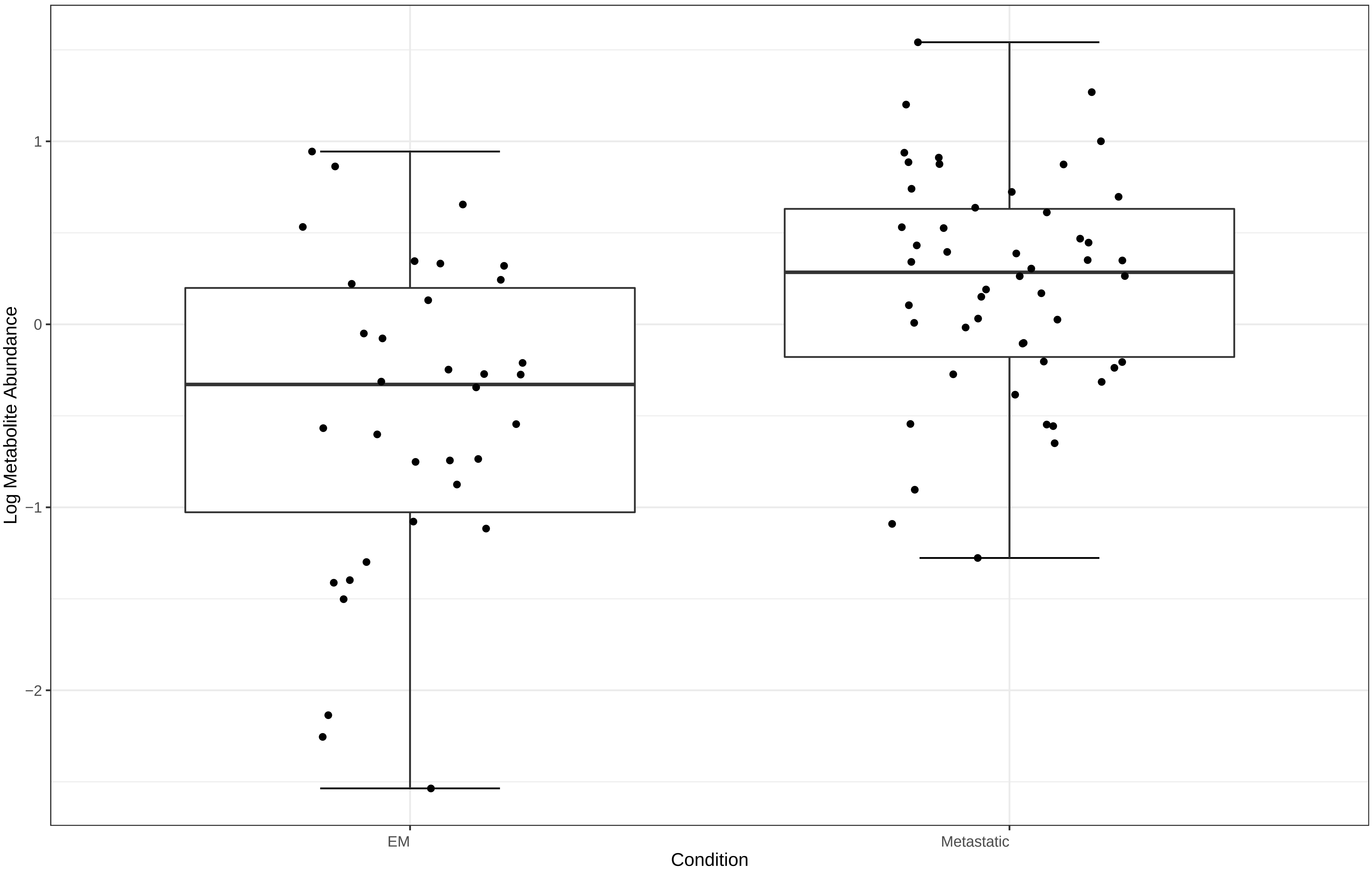

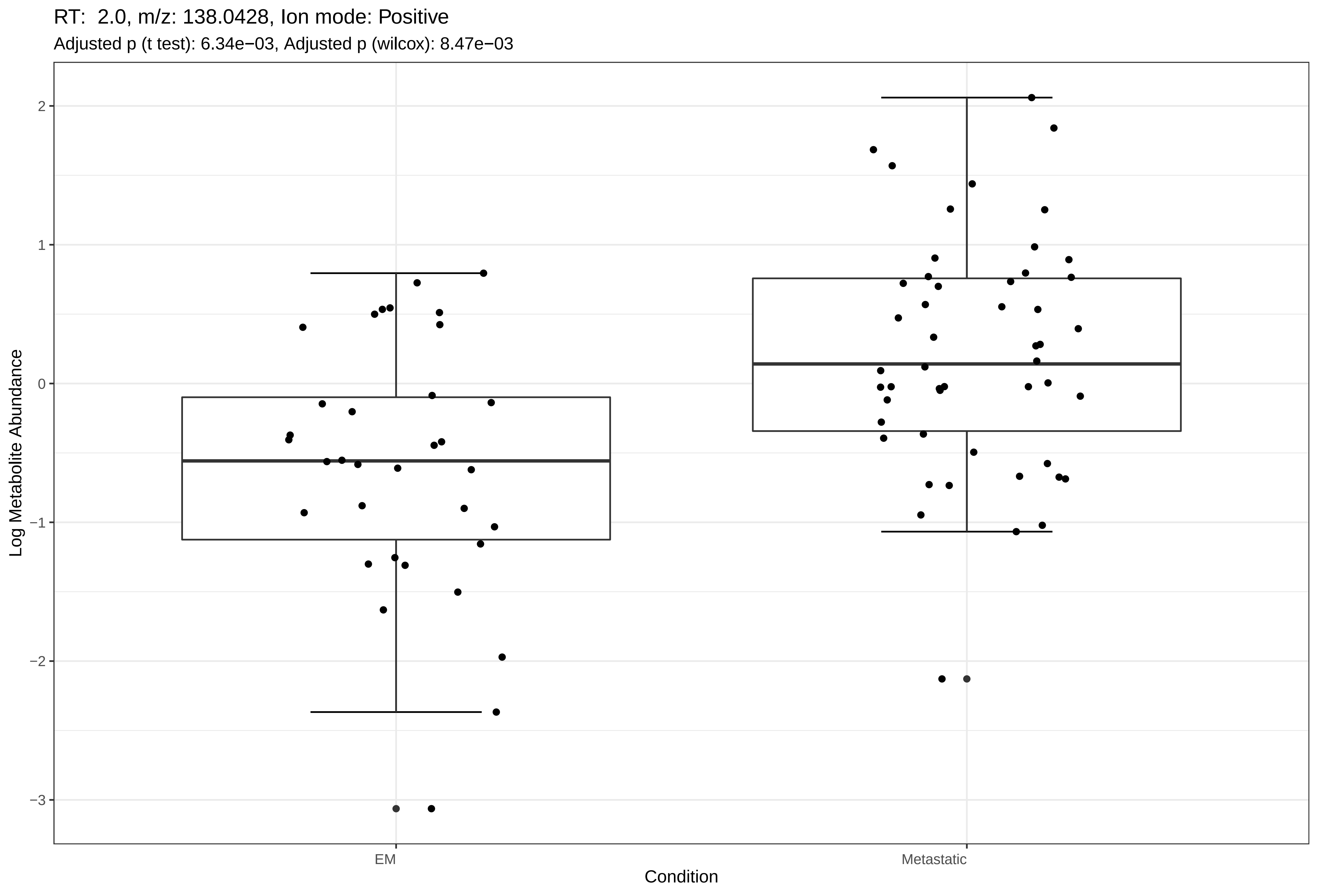
RT: 2.0, m/z: 138.0428, Ion mode: Positive
Adjusted p (t test): 6.34e−03, Adjusted p (wilcox): 8.47e−03
Log Metabolite Abundance
2
1
0
−1
−2
−3
EM
Metastatic
Condition

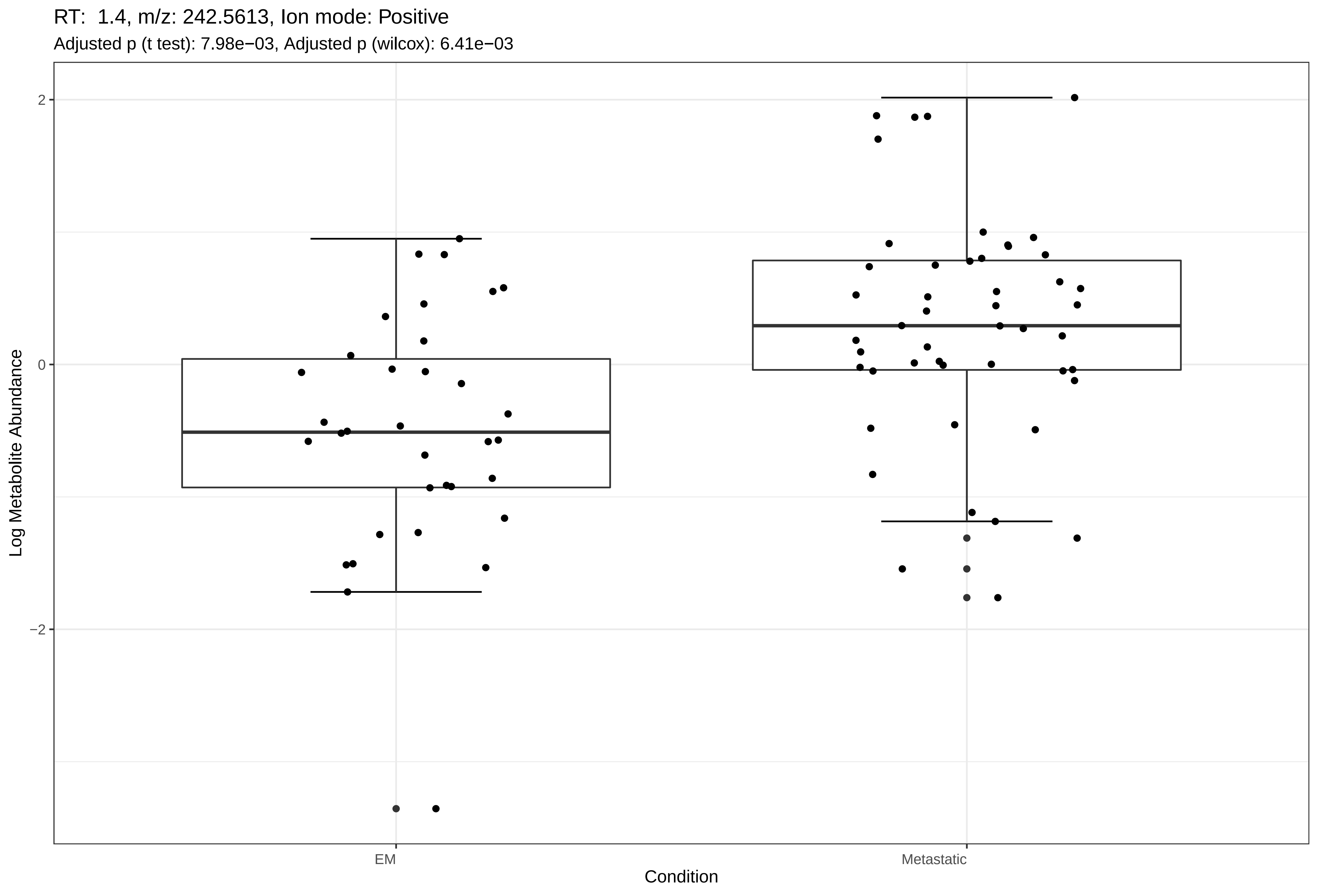
RT: 1.4, m/z: 242.5613, Ion mode: Positive
Adjusted p (t test): 7.98e−03, Adjusted p (wilcox): 6.41e−03
Log Metabolite Abundance
2
0
−2
EM
Metastatic
Condition

[M−H2O+H] −18.0106 m/z adduct of 212.1026 m/z (RT: 1.2, m/z: 194.0895, Ion mode: Positive)

Adjusted p (t test): 6.43e−03, Adjusted p (wilcox): 6.73e−03

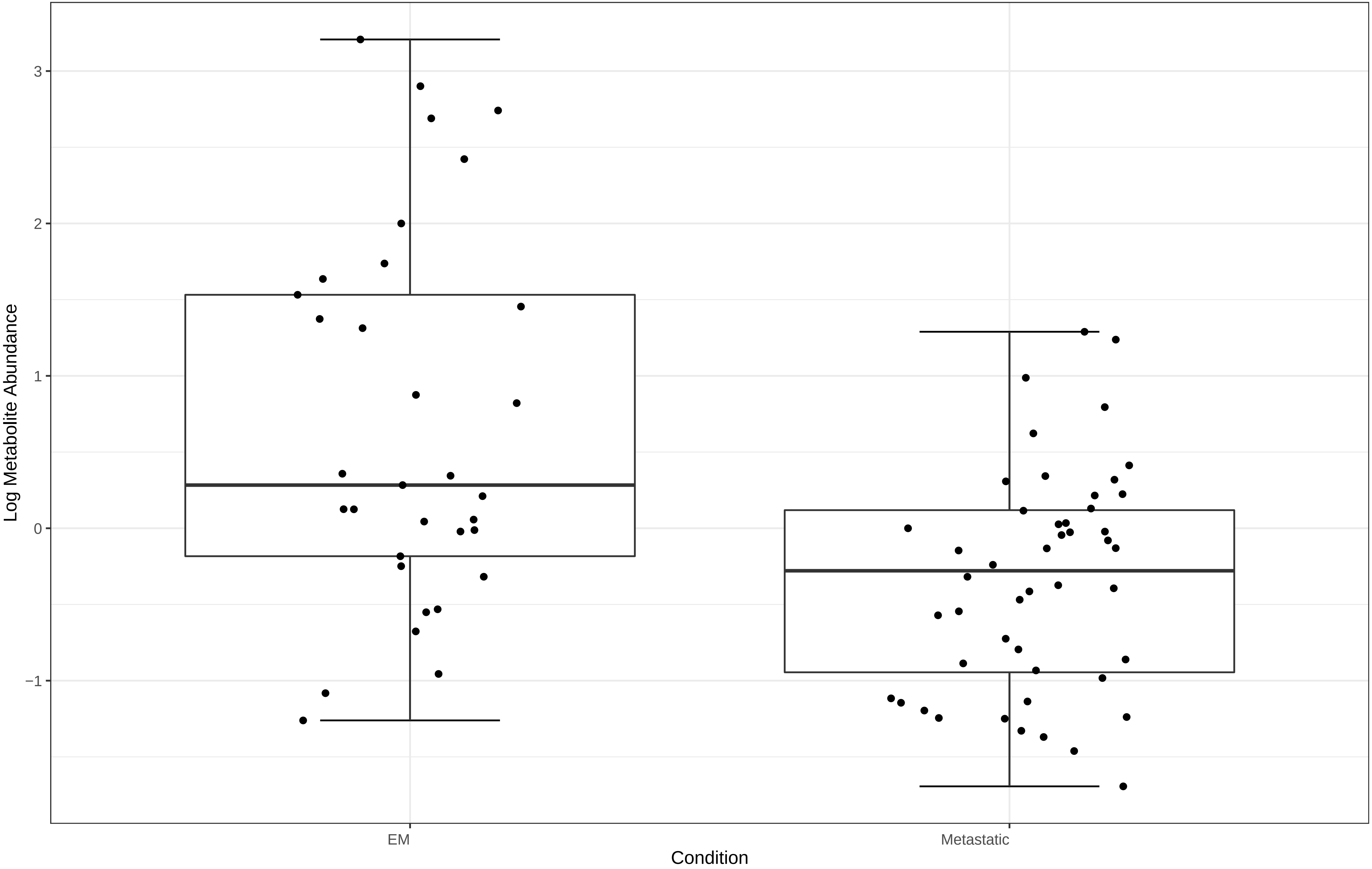

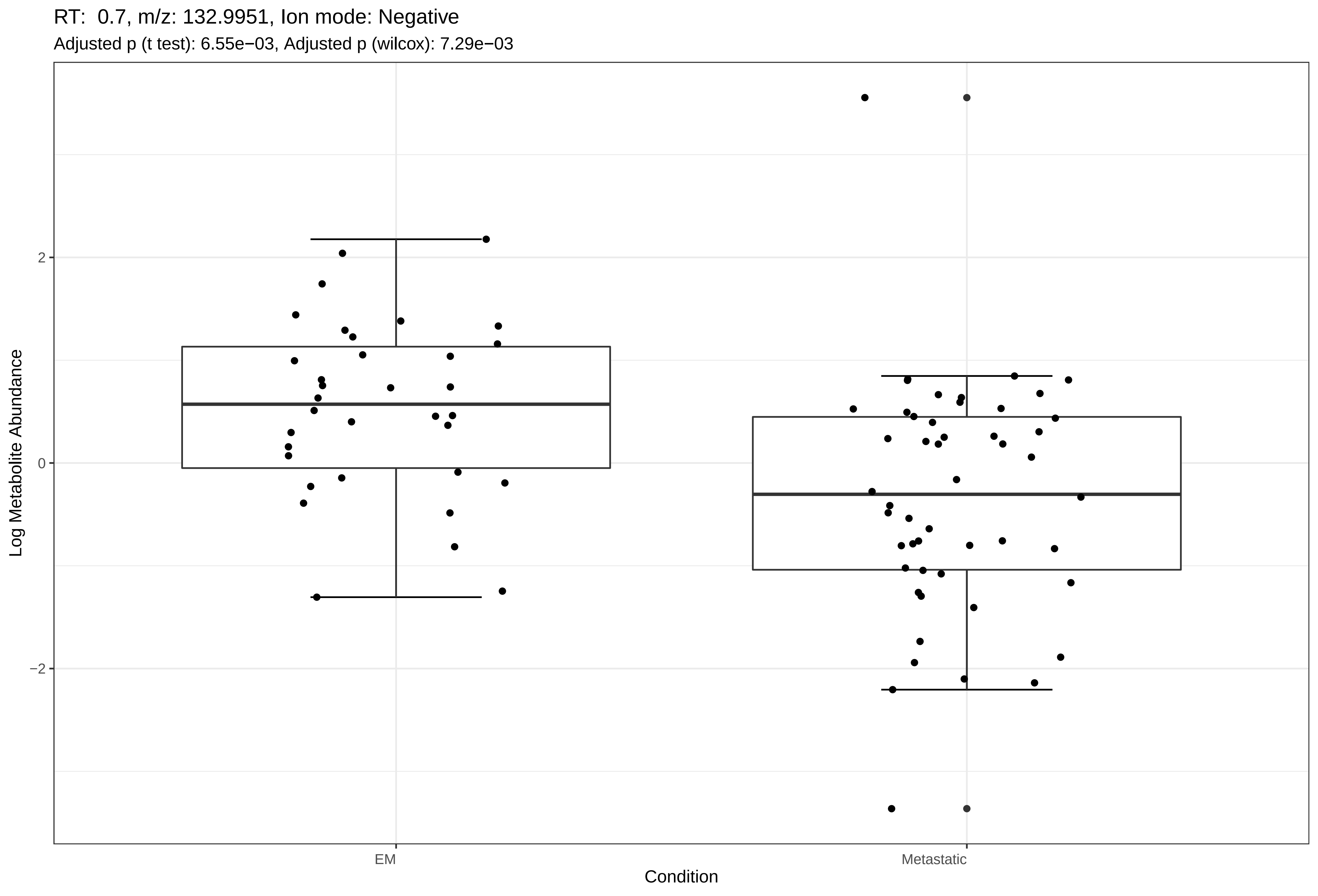
RT: 0.7, m/z: 132.9951, Ion mode: Negative
Adjusted p (t test): 6.55e−03, Adjusted p (wilcox): 7.29e−03
Log Metabolite Abundance
2
0
−2
EM
Metastatic
Condition

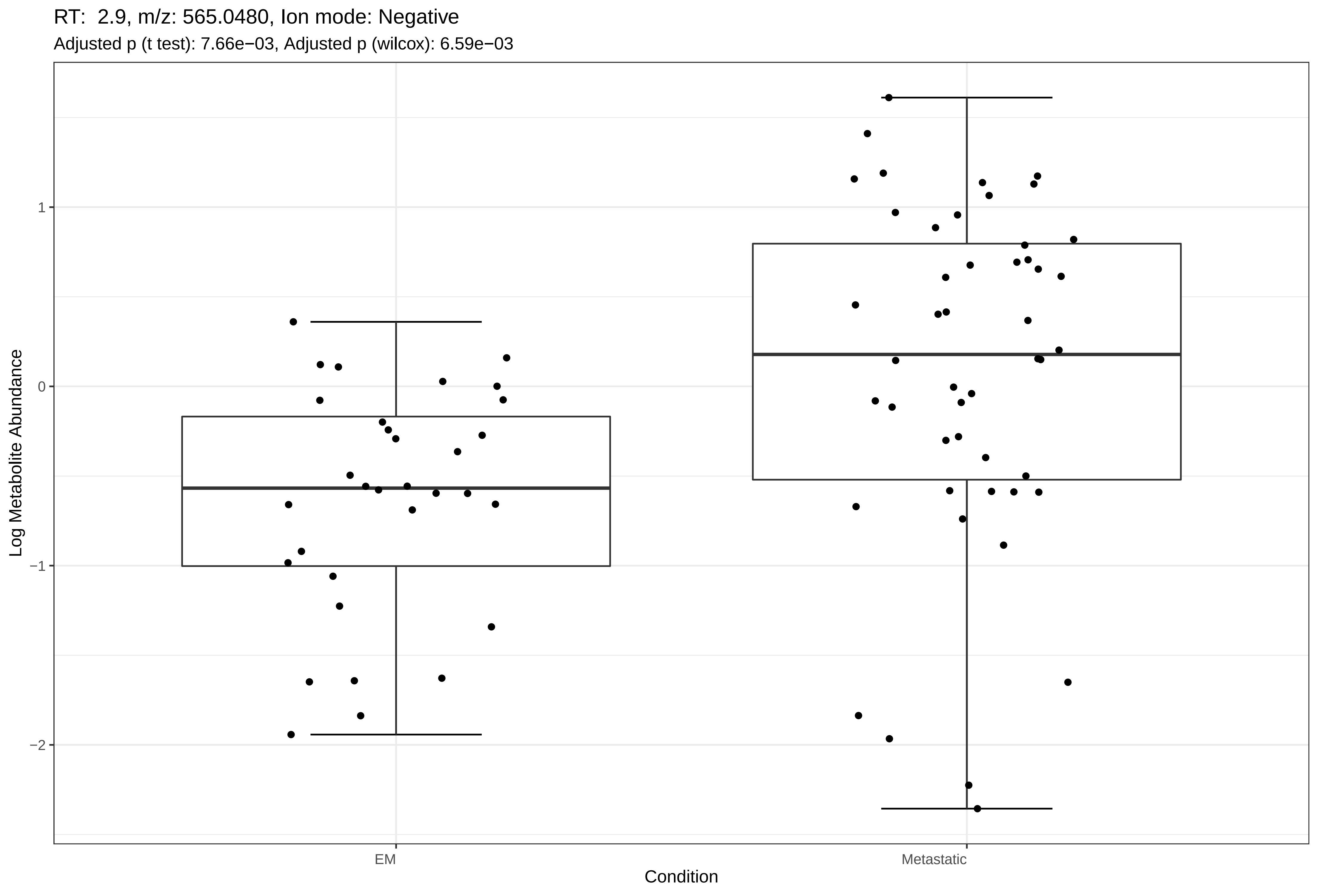
RT: 2.9, m/z: 565.0480, Ion mode: Negative
Adjusted p (t test): 7.66e−03, Adjusted p (wilcox): 6.59e−03
Log Metabolite Abundance
1
0
−1
−2
EM
Metastatic
Condition

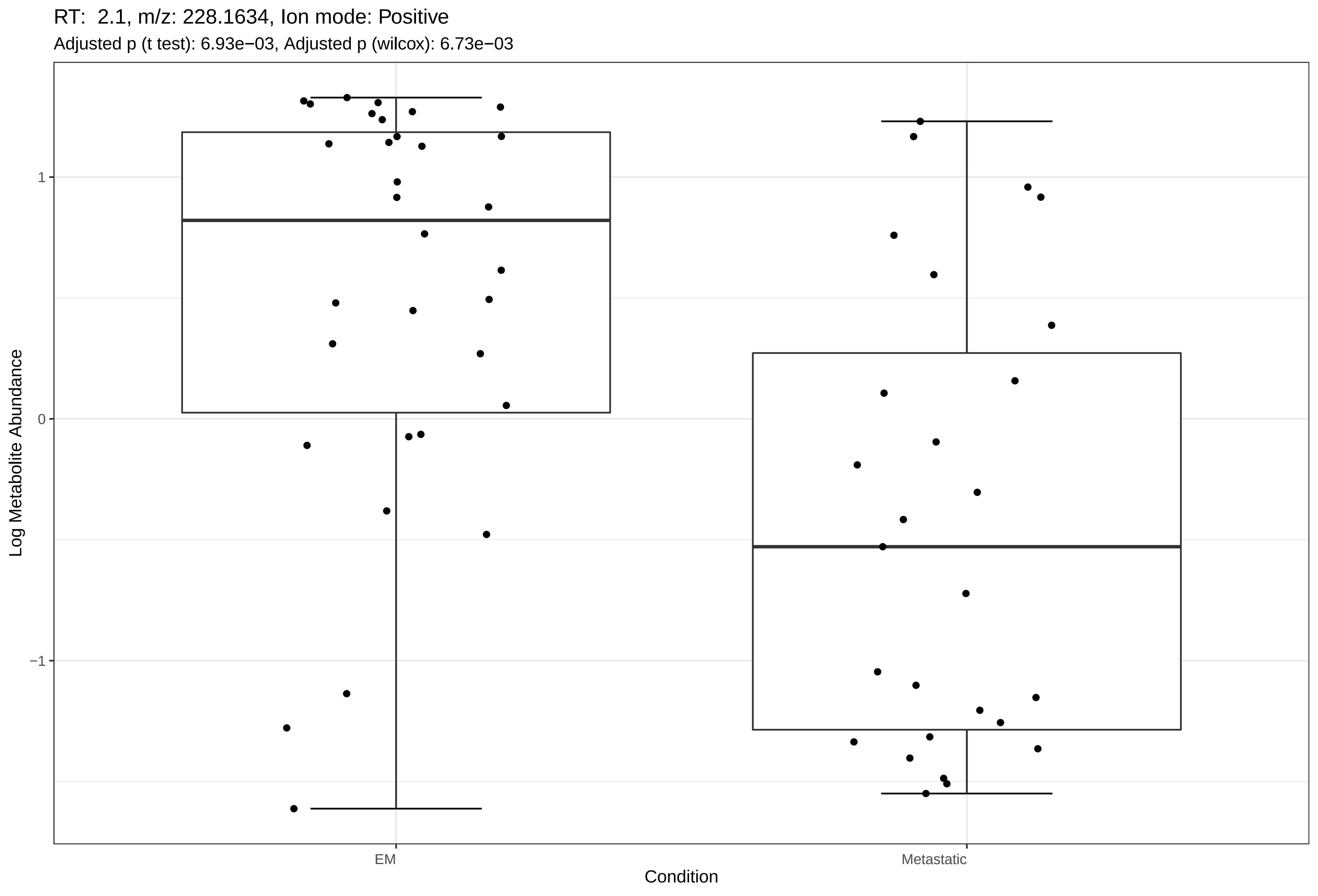
RT: 2.1, m/z: 228.1634, Ion mode: Positive
Adjusted p (t test): 6.93e−03, Adjusted p (wilcox): 6.73e−03
Log Metabolite Abundance
1
0
−1
EM
Metastatic
Condition

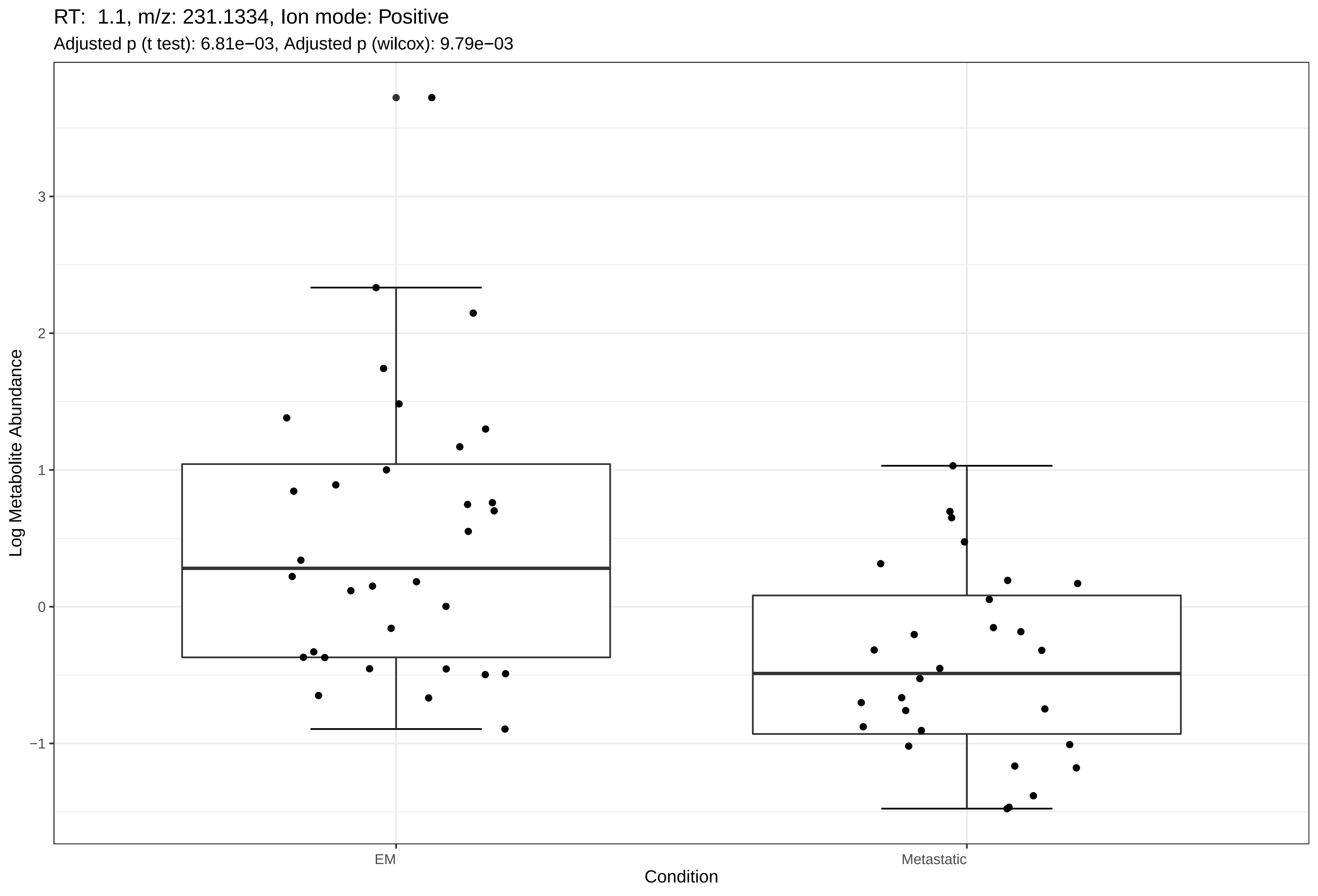

RT: 1.1, m/z: 231.1334, Ion mode: Positive
Adjusted p (t test): 6.81e−03, Adjusted p (wilcox): 9.79e−03
Log Metabolite Abundance
3
2
1
0
−1
EM
Metastatic
Condition

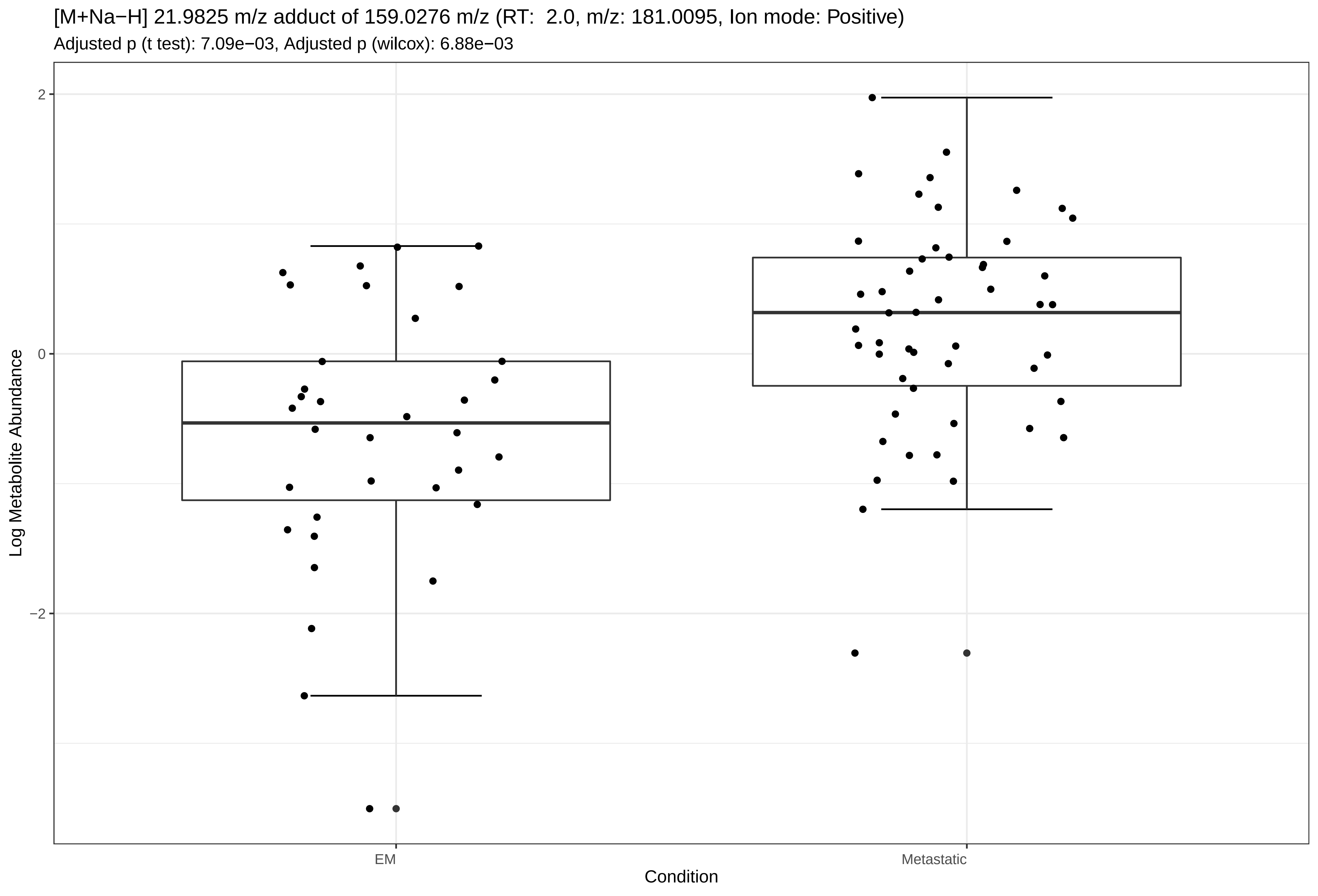
[M+Na−H] 21.9825 m/z adduct of 159.0276 m/z (RT: 2.0, m/z: 181.0095, Ion mode: Positive)
Adjusted p (t test): 7.09e−03, Adjusted p (wilcox): 6.88e−03
Log Metabolite Abundance
2
0
−2
EM
Metastatic
Condition

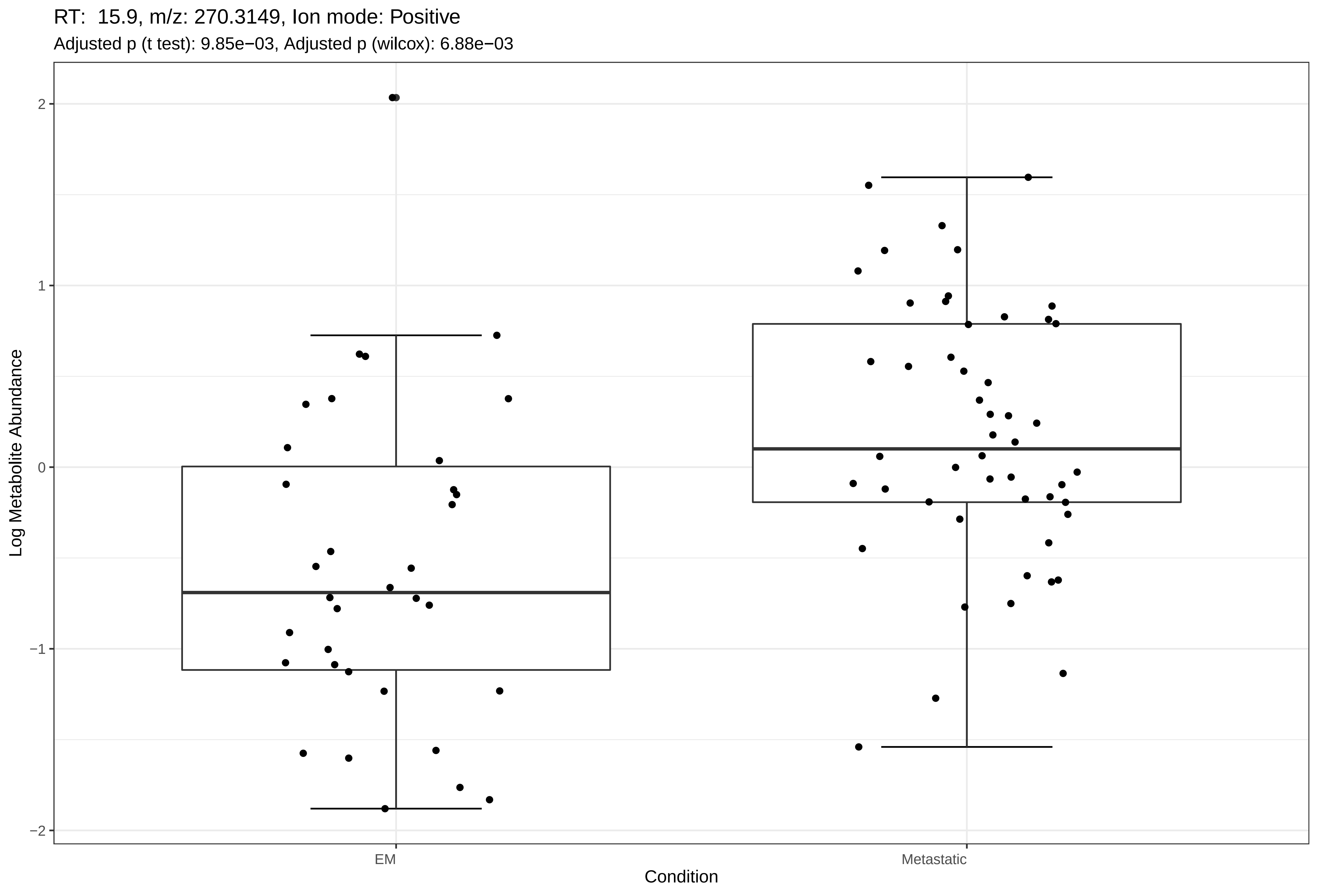
RT: 15.9, m/z: 270.3149, Ion mode: Positive
Adjusted p (t test): 9.85e−03, Adjusted p (wilcox): 6.88e−03
Log Metabolite Abundance
2
1
0
−1
−2
EM
Metastatic
Condition

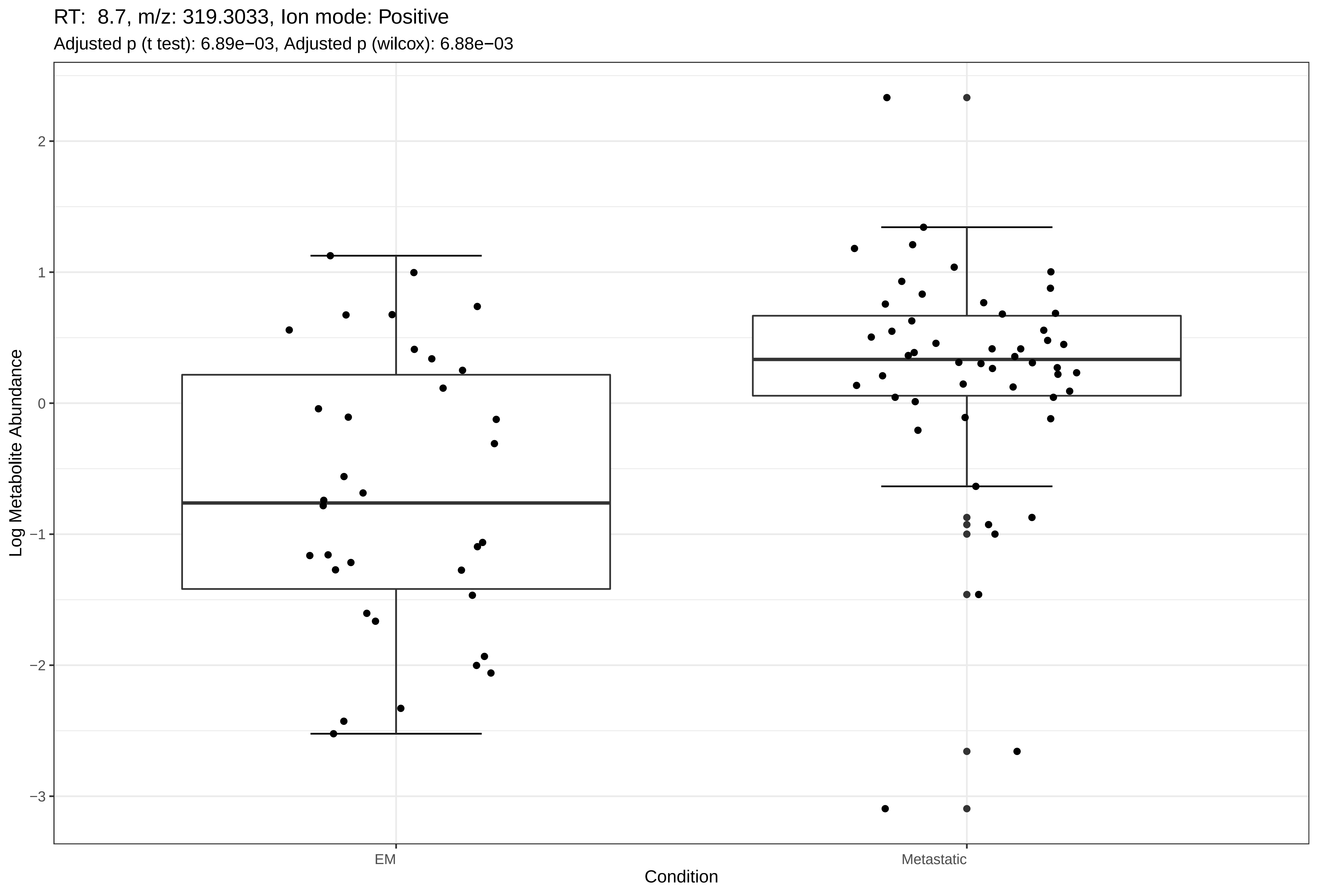
RT: 8.7, m/z: 319.3033, Ion mode: Positive
Adjusted p (t test): 6.89e−03, Adjusted p (wilcox): 6.88e−03
Log Metabolite Abundance
2
1
0
−1
−2
−3
EM
Metastatic
Condition

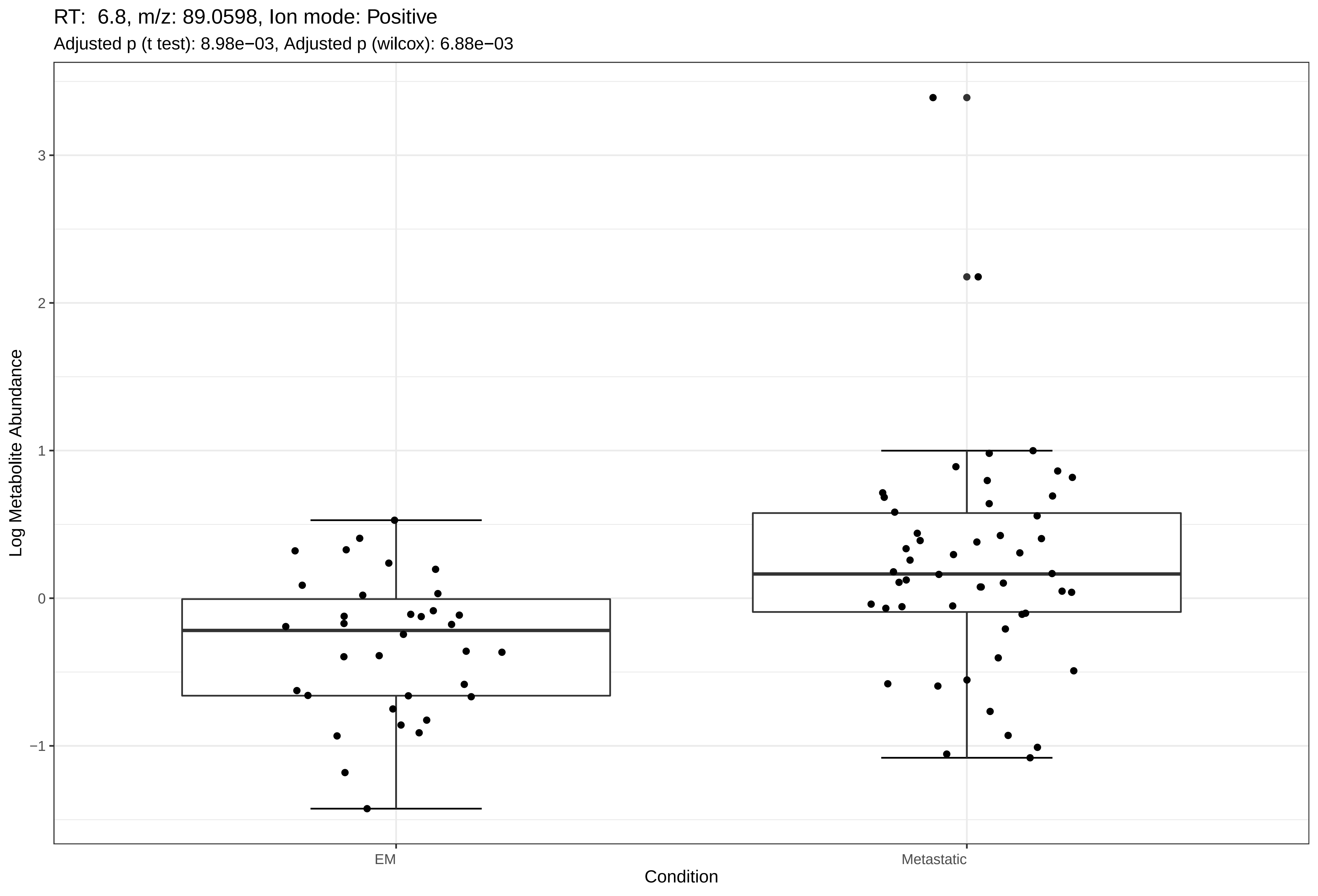
RT: 6.8, m/z: 89.0598, Ion mode: Positive
Adjusted p (t test): 8.98e−03, Adjusted p (wilcox): 6.88e−03
Log Metabolite Abundance
3
2
1
0
−1
EM
Metastatic
Condition

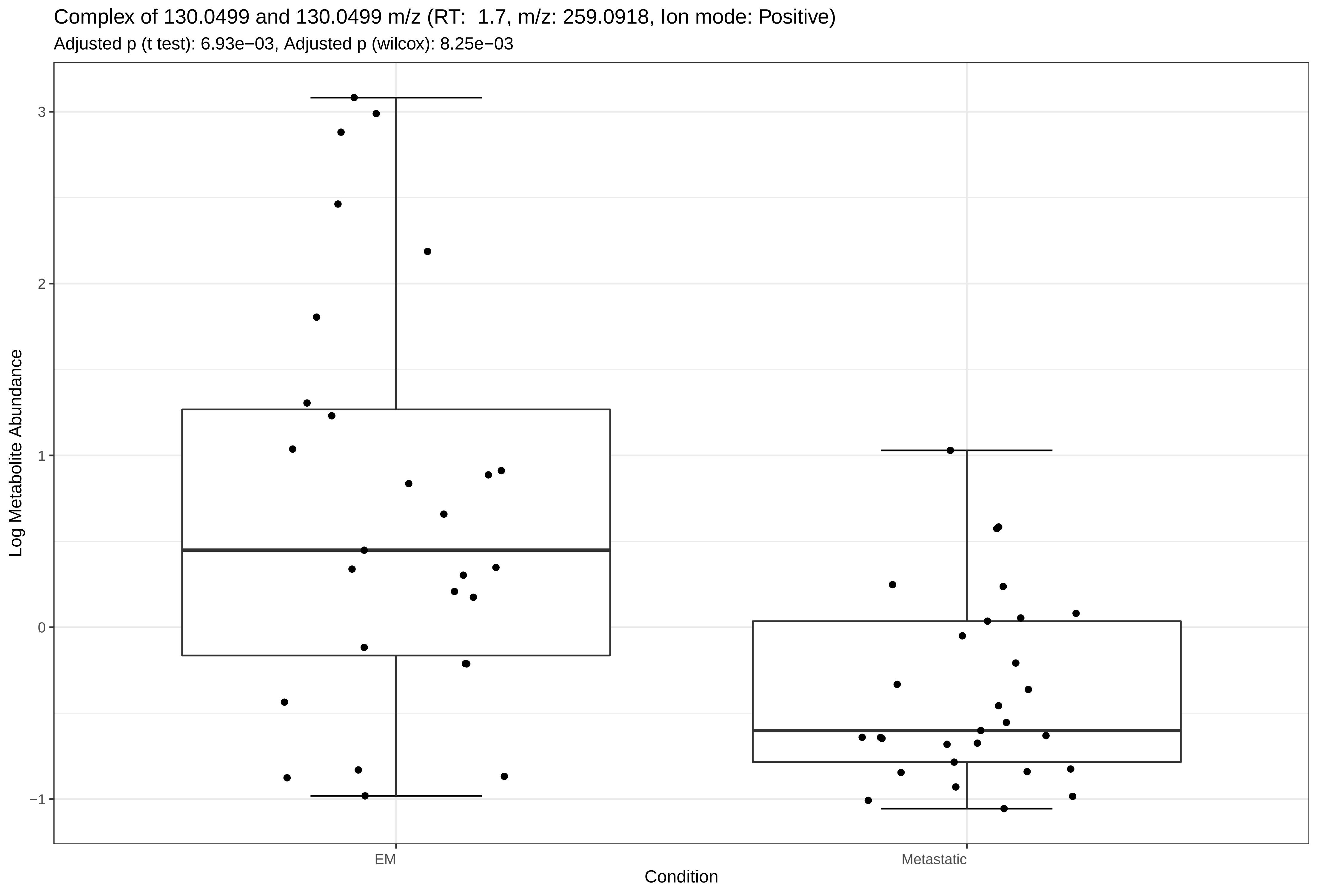
Complex of 130.0499 and 130.0499 m/z (RT: 1.7, m/z: 259.0918, Ion mode: Positive)
Adjusted p (t test): 6.93e−03, Adjusted p (wilcox): 8.25e−03
Log Metabolite Abundance
3
2
1
0
−1
EM
Metastatic
Condition

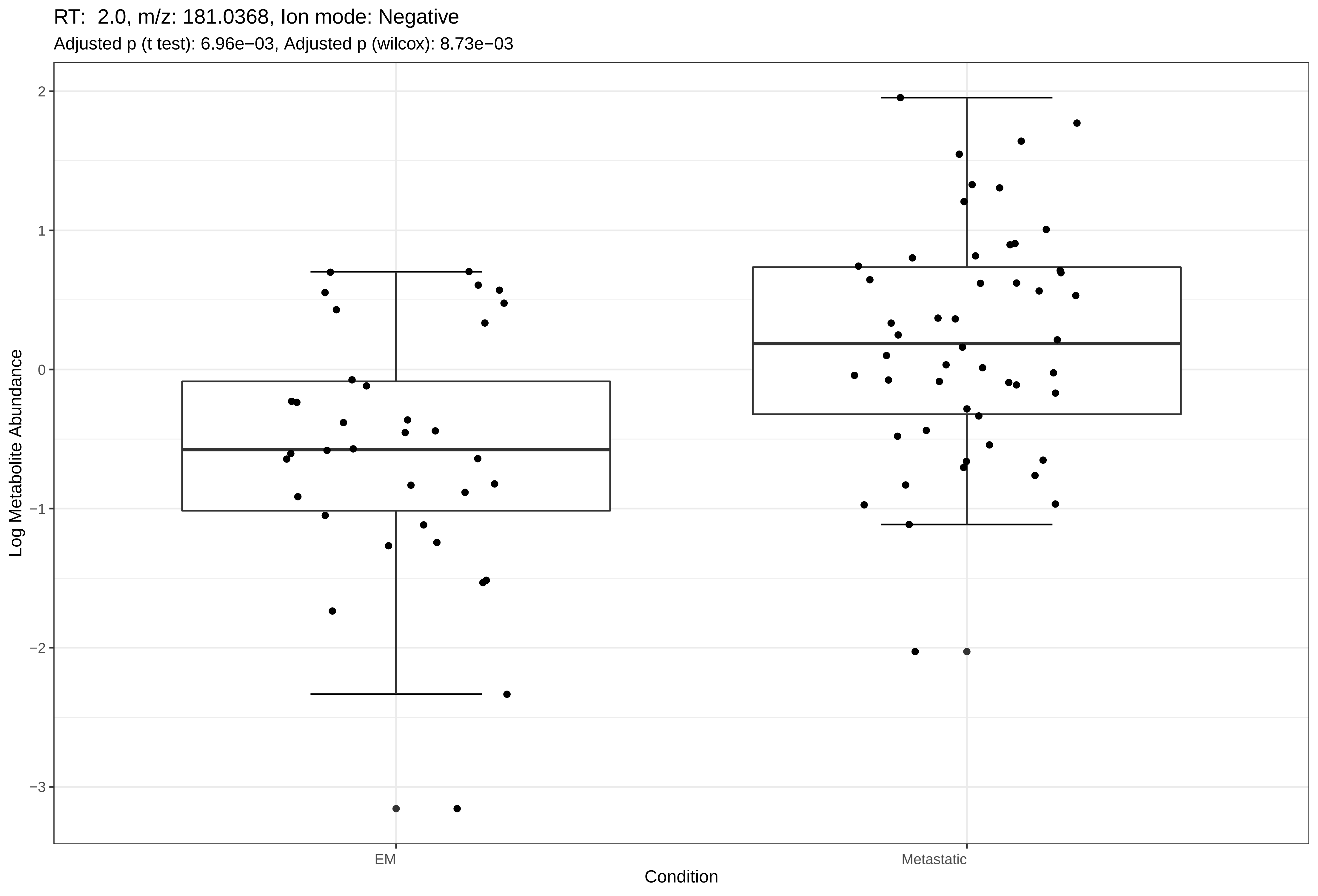
RT: 2.0, m/z: 181.0368, Ion mode: Negative
Adjusted p (t test): 6.96e−03, Adjusted p (wilcox): 8.73e−03
Log Metabolite Abundance
2
1
0
−1
−2
−3
EM
Metastatic
Condition

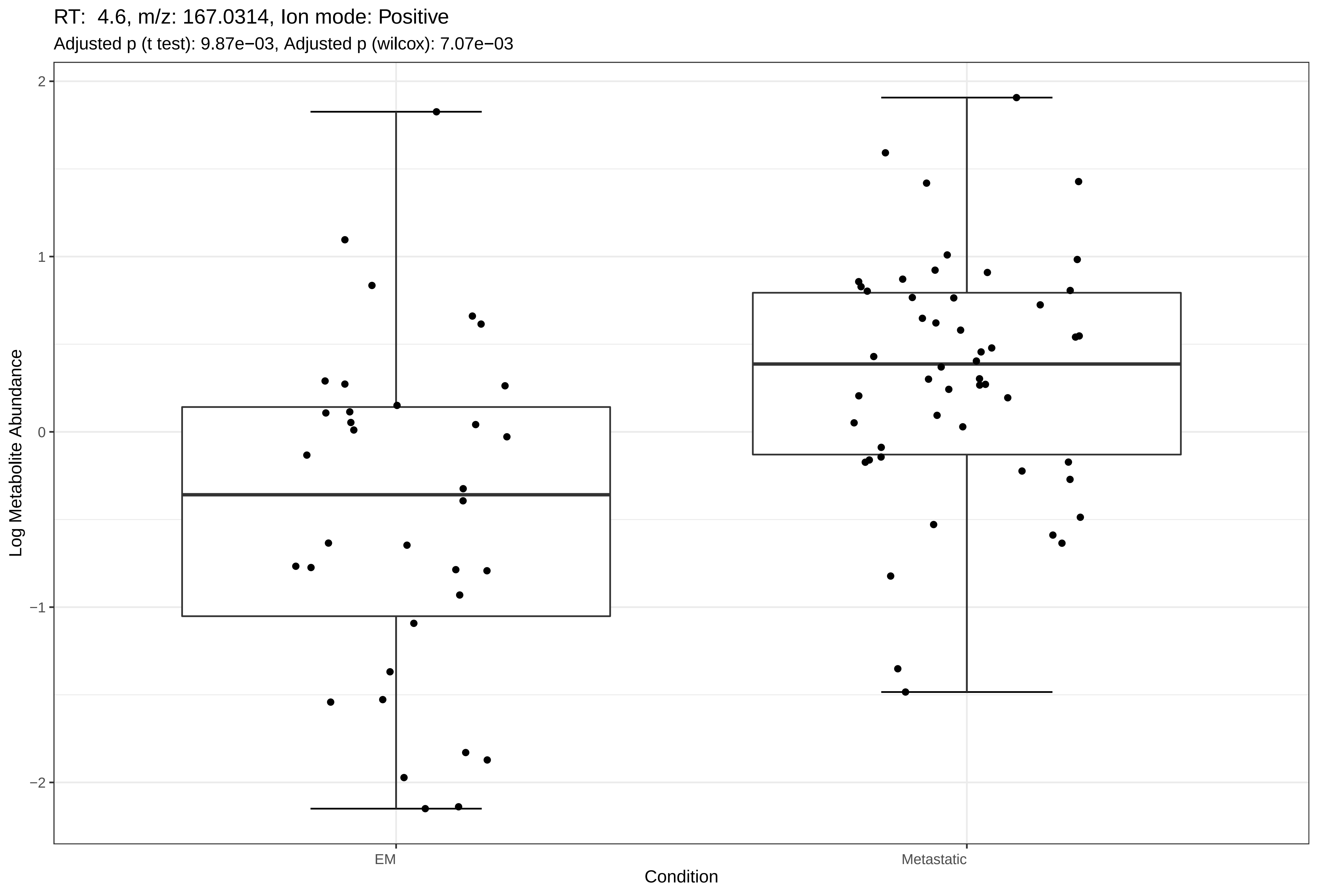
RT: 4.6, m/z: 167.0314, Ion mode: Positive
Adjusted p (t test): 9.87e−03, Adjusted p (wilcox): 7.07e−03
Log Metabolite Abundance
2
1
0
−1
−2
EM
Metastatic
Condition

RT: 6.1, m/z: 247.1130, Ion mode: Positive

Adjusted p (t test): 7.97e−03, Adjusted p (wilcox): 7.07e−03

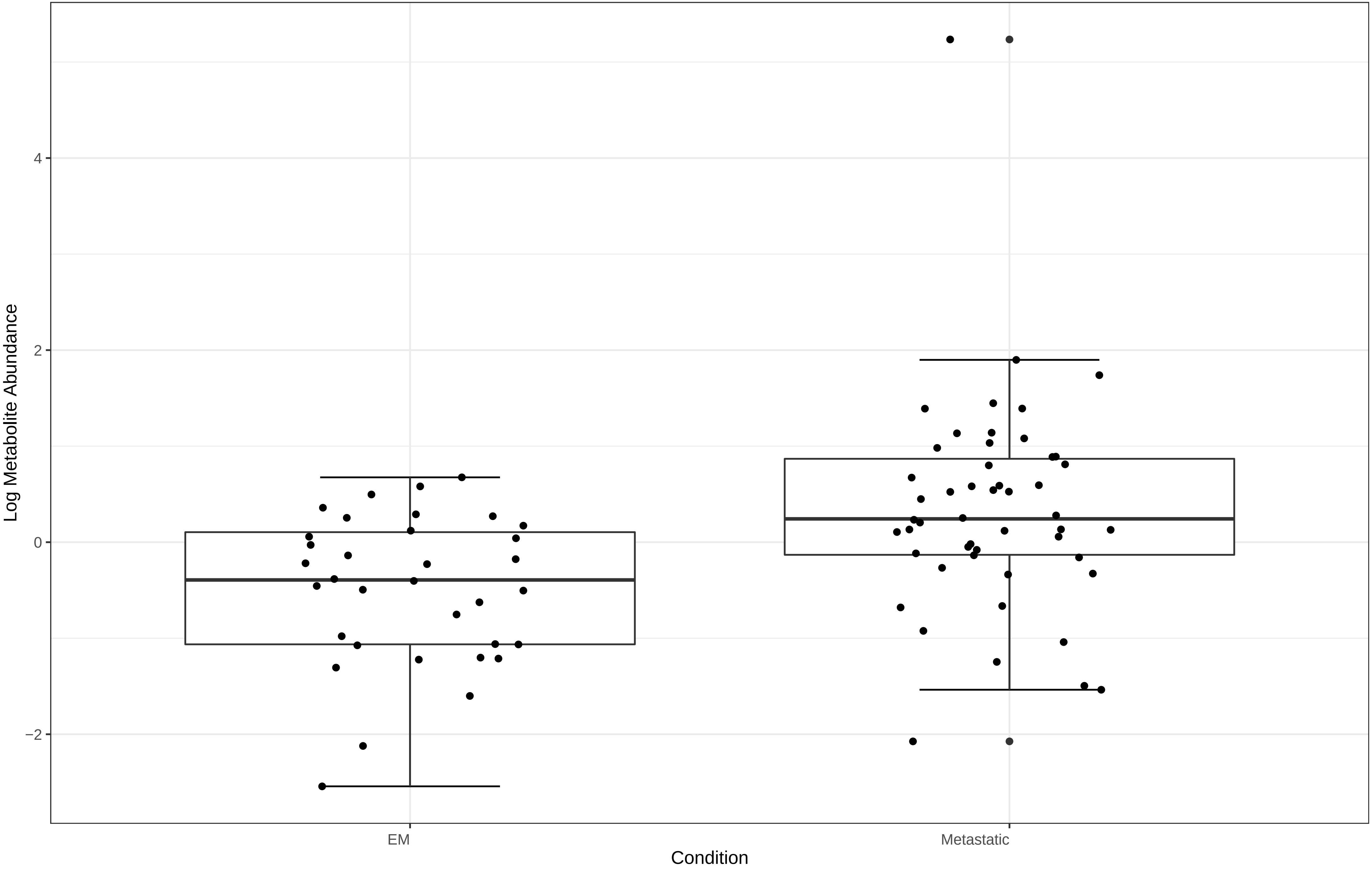

[M+K−H] 37.9559 m/z adduct of 258.1094 m/z (RT: 0.8, m/z: 296.0647, Ion mode: Positive)

Adjusted p (t test): 8.11e−03, Adjusted p (wilcox): 7.07e−03

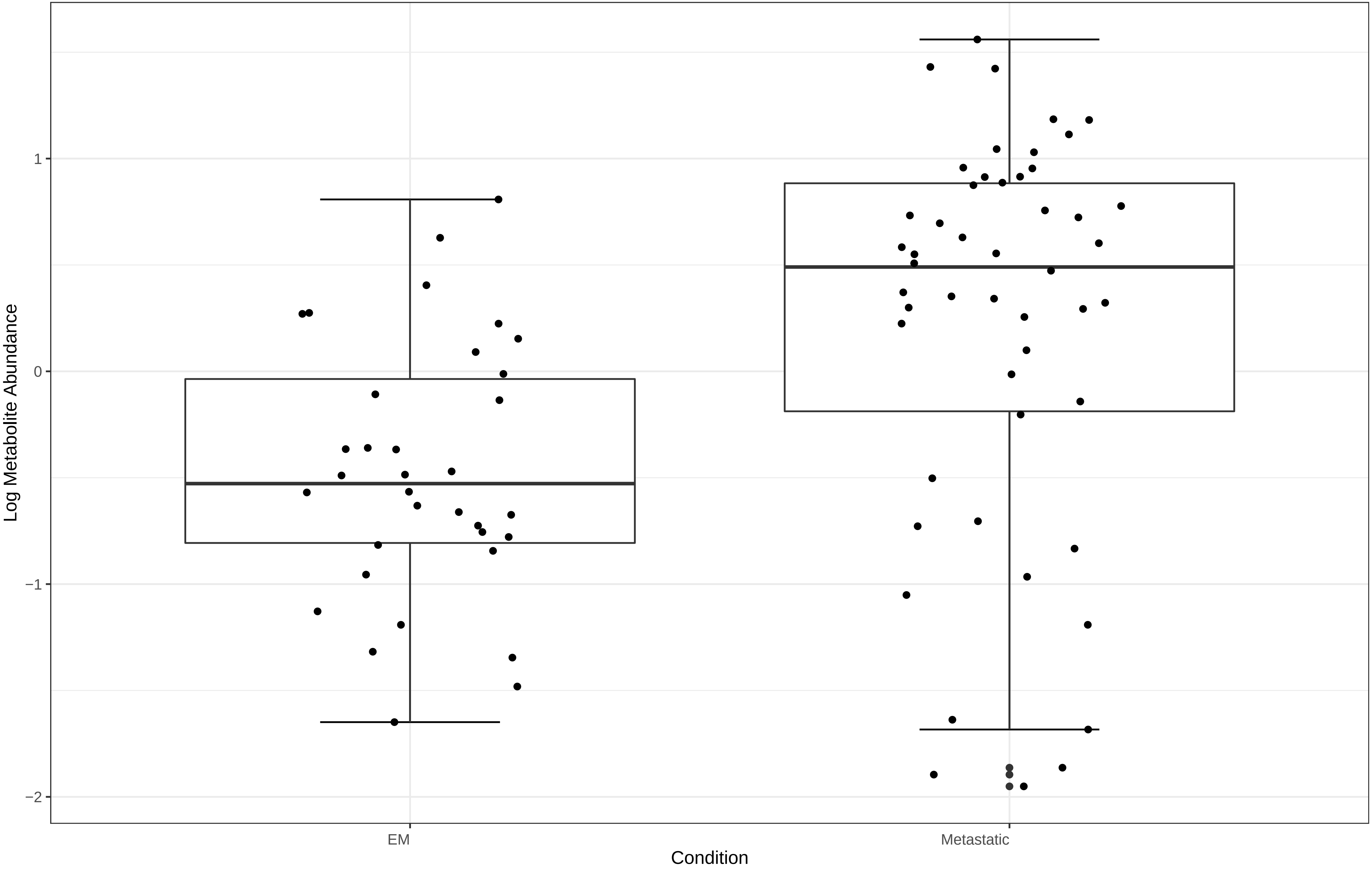

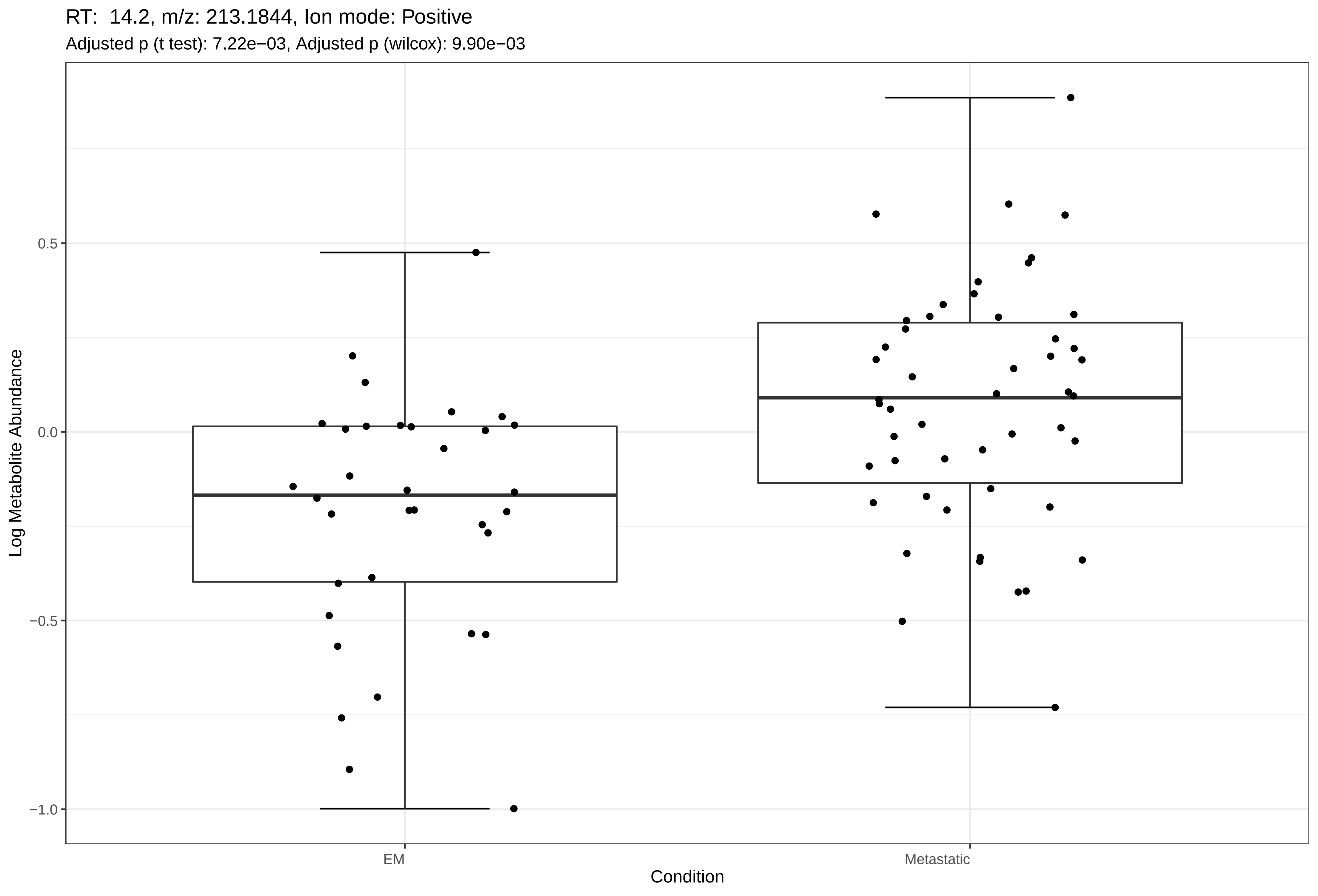

RT: 14.2, m/z: 213.1844, Ion mode: Positive
Adjusted p (t test): 7.22e−03, Adjusted p (wilcox): 9.90e−03
Log Metabolite Abundance
0.5
0.0
−0.5
−1.0
EM
Metastatic
Condition

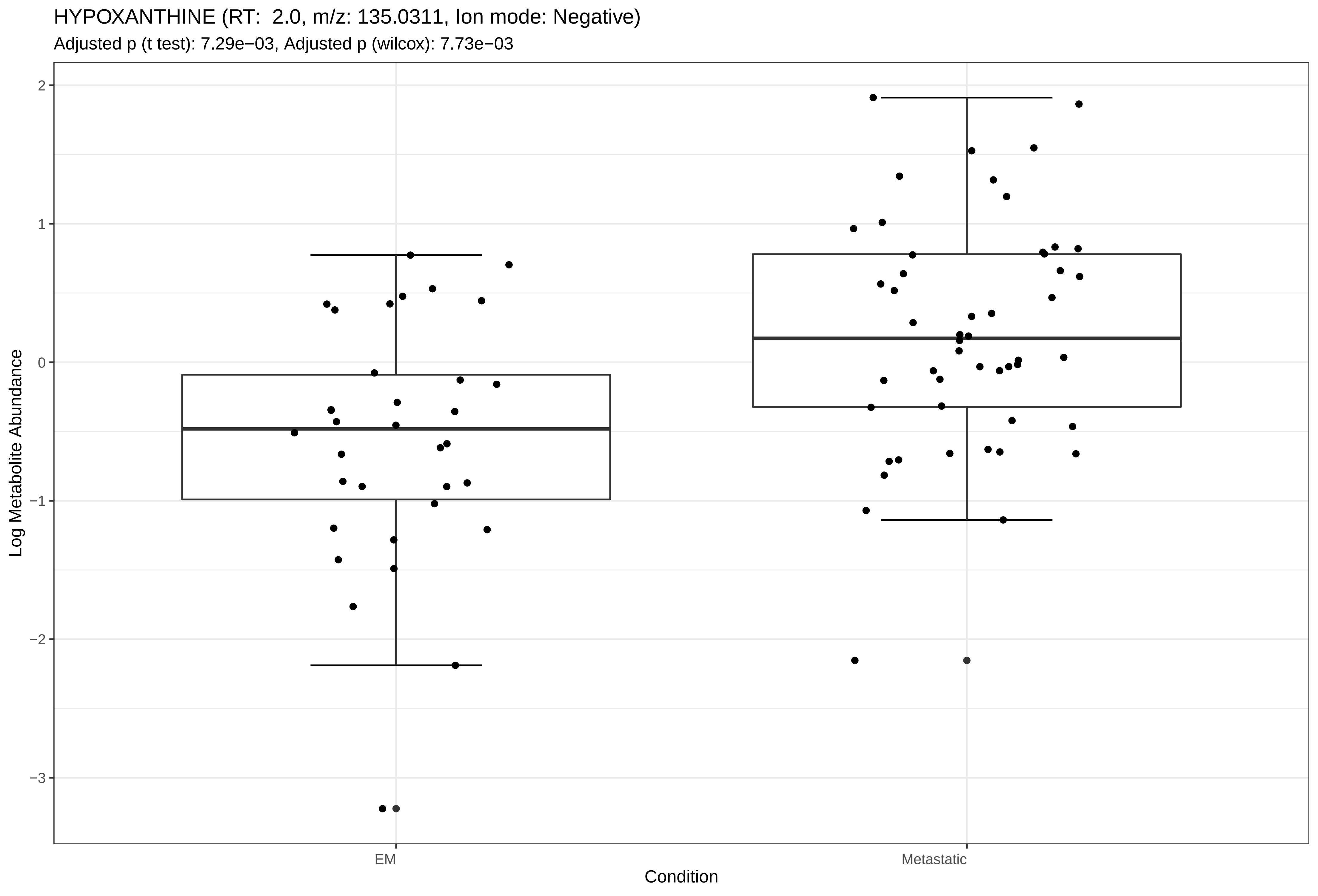

HYPOXANTHINE (RT: 2.0, m/z: 135.0311, Ion mode: Negative)
Adjusted p (t test): 7.29e−03, Adjusted p (wilcox): 7.73e−03
Log Metabolite Abundance
2
1
0
−1
−2
−3
EM
Metastatic
Condition

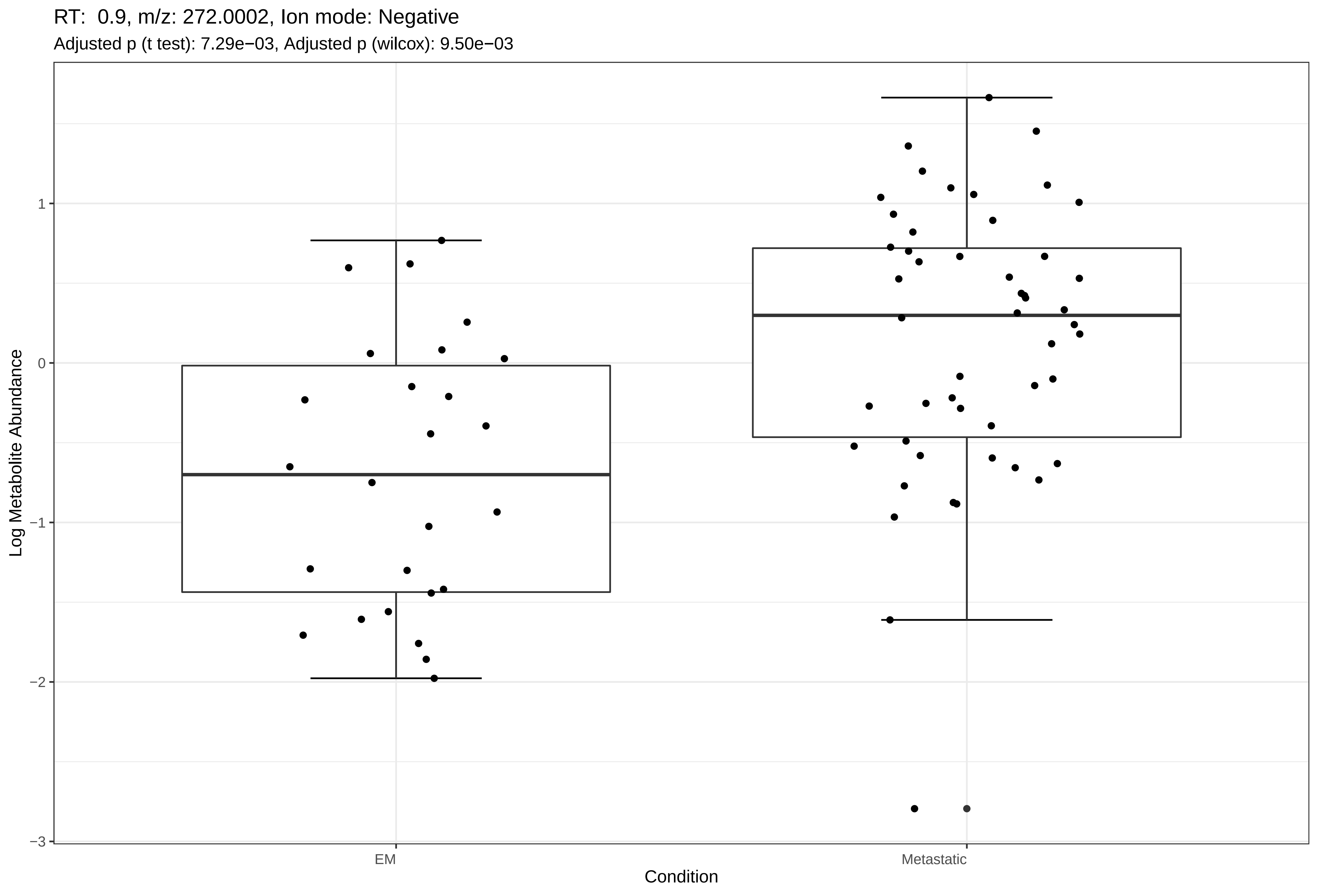
RT: 0.9, m/z: 272.0002, Ion mode: Negative
Adjusted p (t test): 7.29e−03, Adjusted p (wilcox): 9.50e−03
Log Metabolite Abundance
1
0
−1
−2
−3
EM
Metastatic
Condition

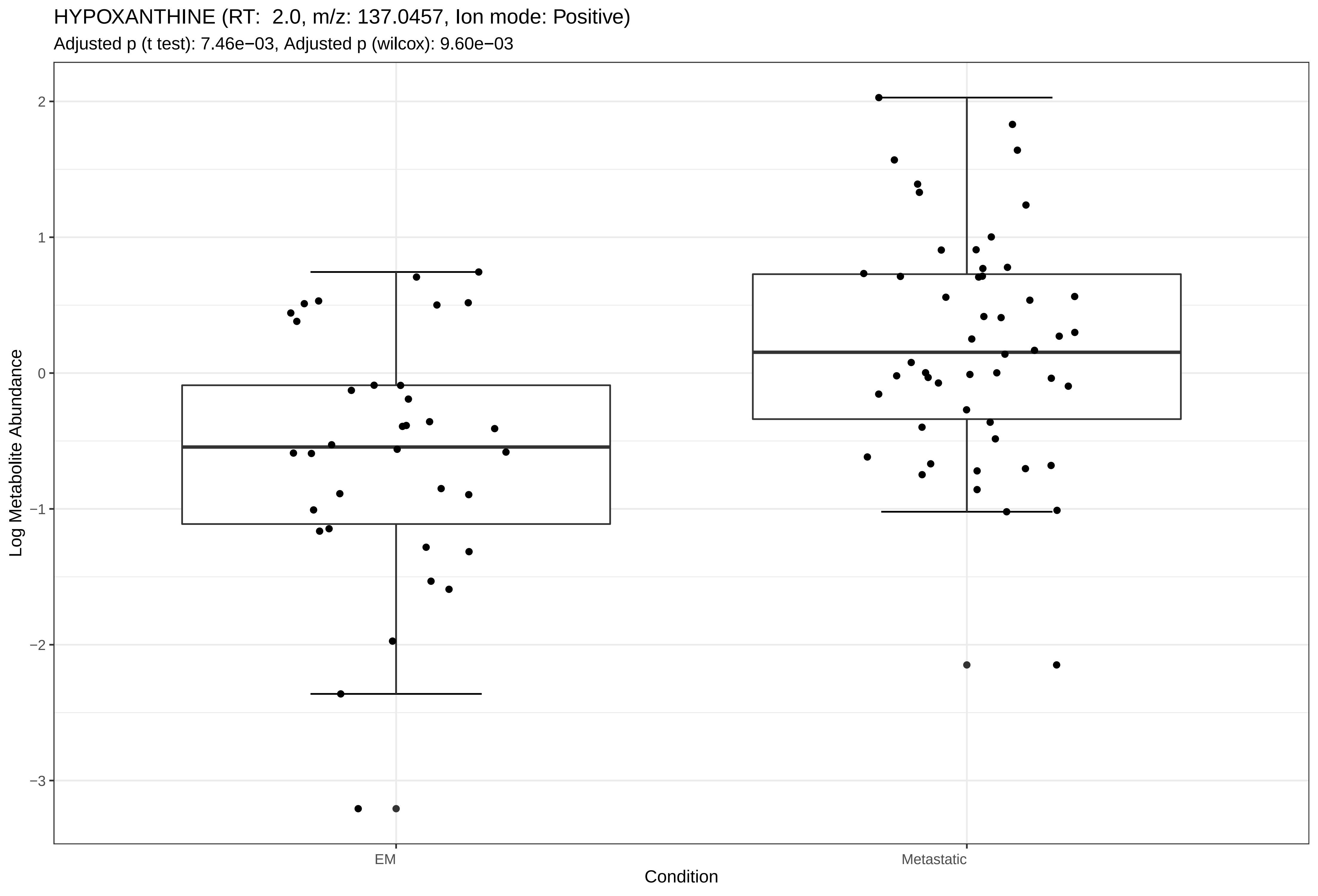
HYPOXANTHINE (RT: 2.0, m/z: 137.0457, Ion mode: Positive)
Adjusted p (t test): 7.46e−03, Adjusted p (wilcox): 9.60e−03
Log Metabolite Abundance
2
1
0
−1
−2
−3
EM
Metastatic
Condition

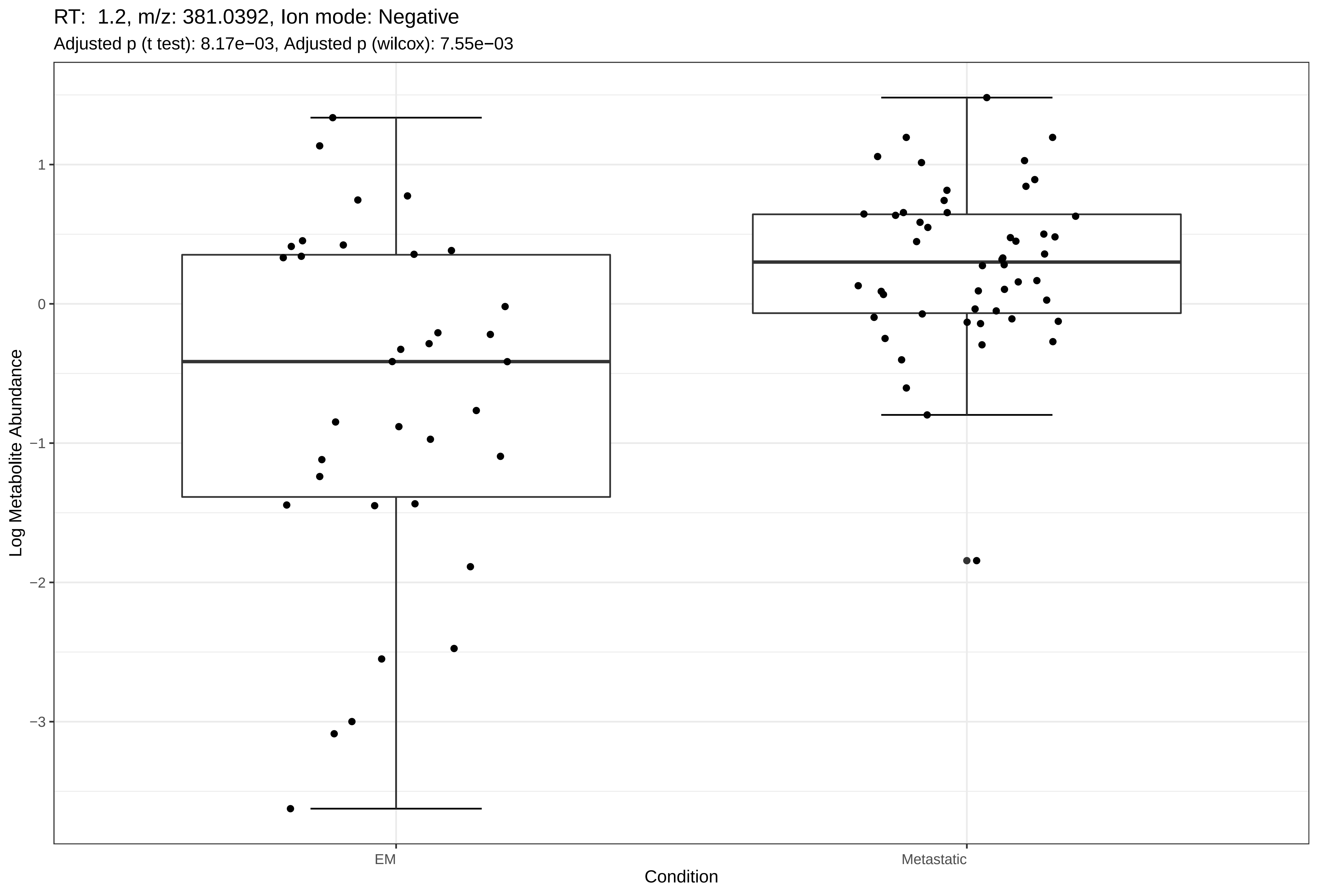
RT: 1.2, m/z: 381.0392, Ion mode: Negative
Adjusted p (t test): 8.17e−03, Adjusted p (wilcox): 7.55e−03
Log Metabolite Abundance
1
0
−1
−2
−3
EM
Metastatic
Condition

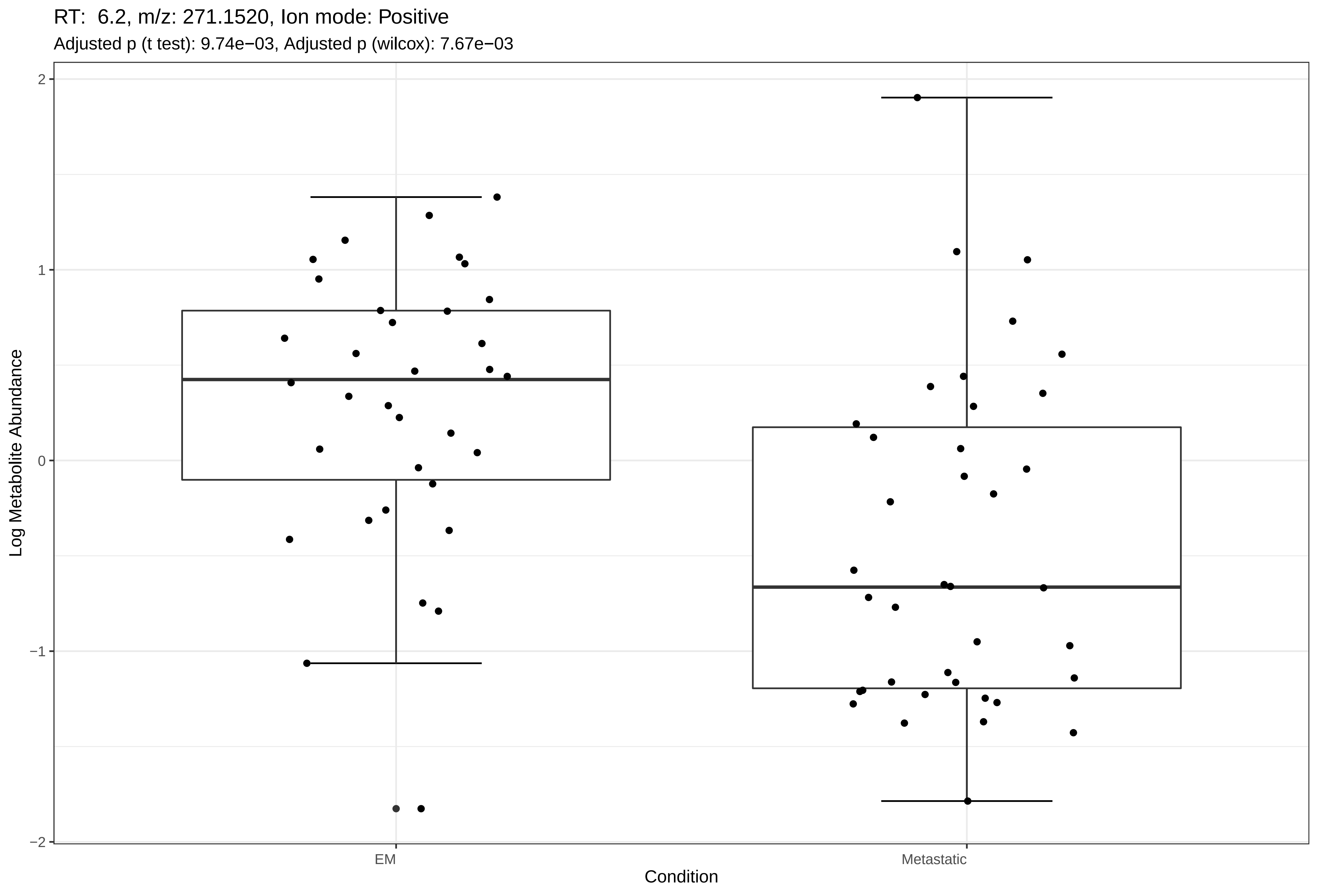
RT: 6.2, m/z: 271.1520, Ion mode: Positive
Adjusted p (t test): 9.74e−03, Adjusted p (wilcox): 7.67e−03
Log Metabolite Abundance
2
1
0
−1
−2
EM
Metastatic
Condition

RT: 1.4, m/z: 136.0616, Ion mode: Positive

Adjusted p (t test): 9.10e−03, Adjusted p (wilcox): 7.73e−03

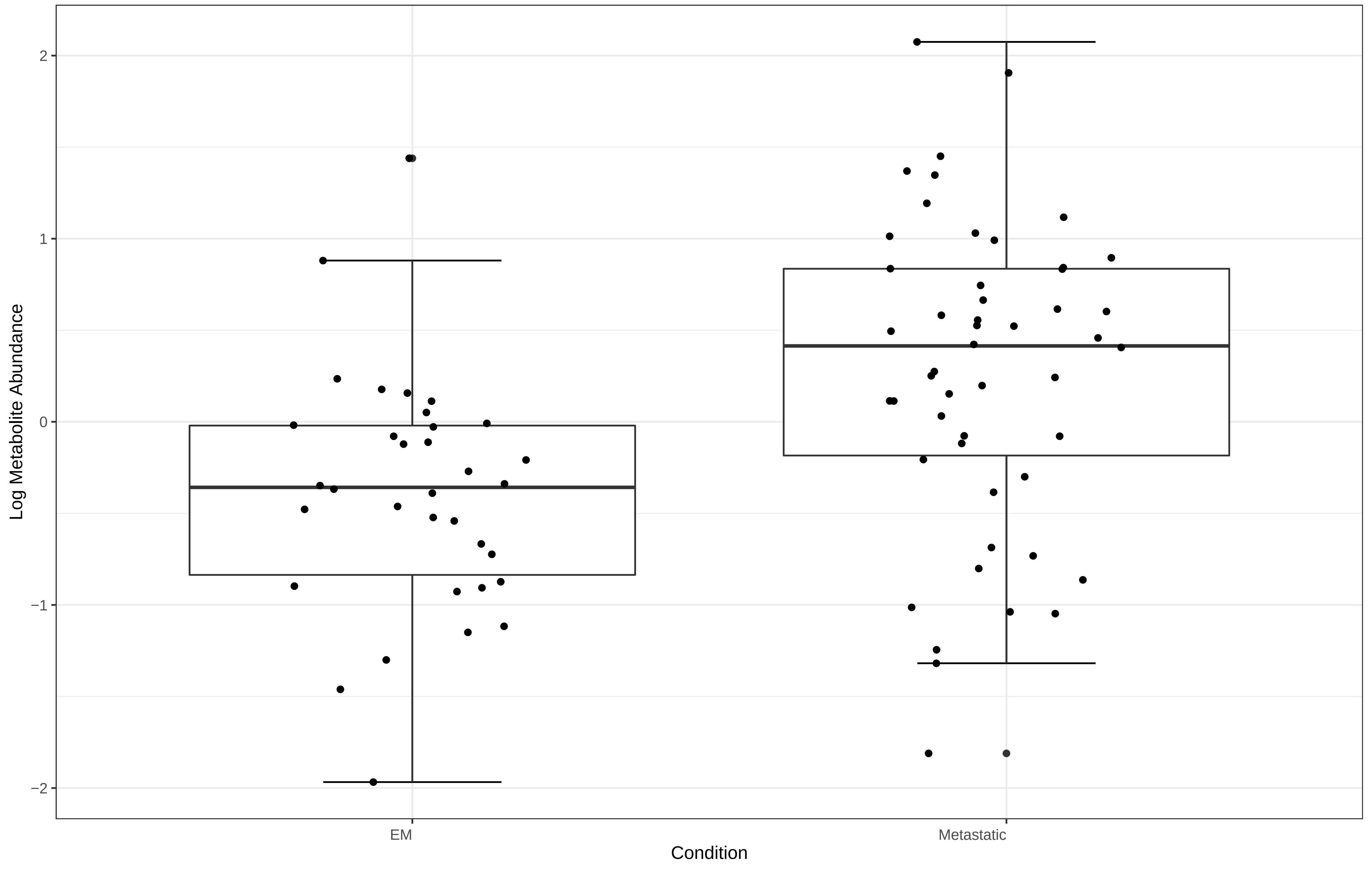

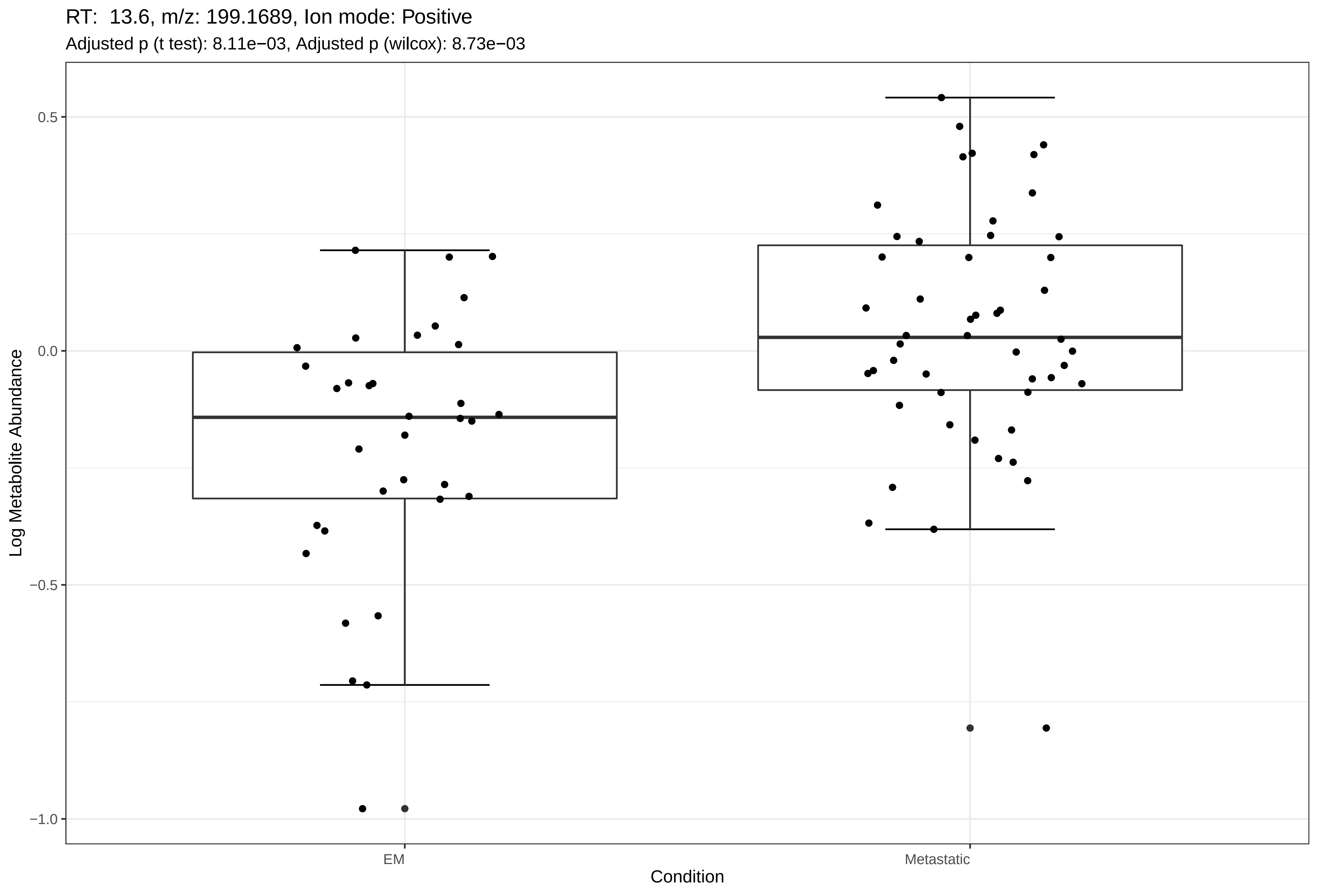

RT: 13.6, m/z: 199.1689, Ion mode: Positive
Adjusted p (t test): 8.11e−03, Adjusted p (wilcox): 8.73e−03
Log Metabolite Abundance
0.5
0.0
−0.5
−1.0
EM
Metastatic
Condition

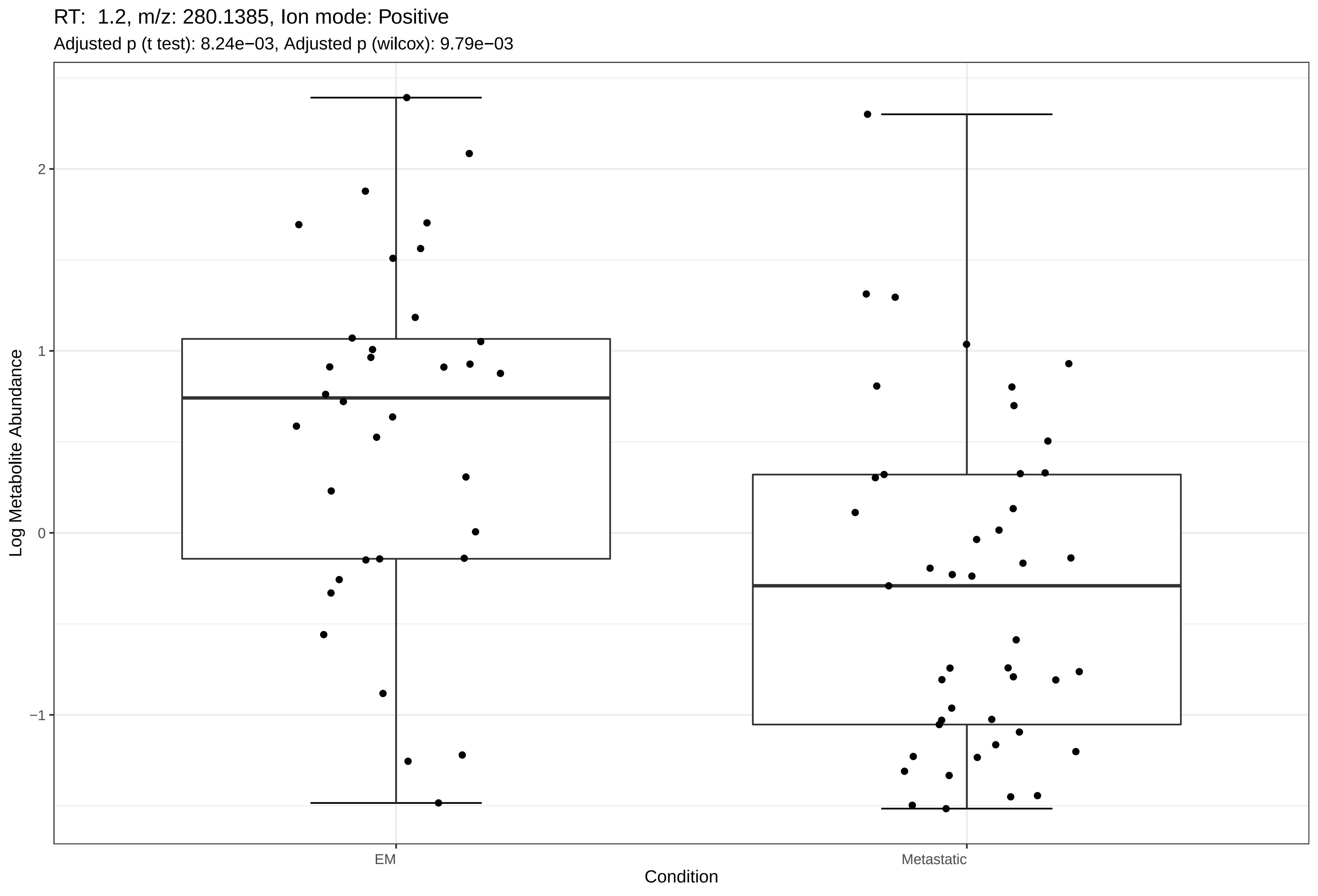

RT: 1.2, m/z: 280.1385, Ion mode: Positive
Adjusted p (t test): 8.24e−03, Adjusted p (wilcox): 9.79e−03
Log Metabolite Abundance
2
1
0
−1
EM
Metastatic
Condition

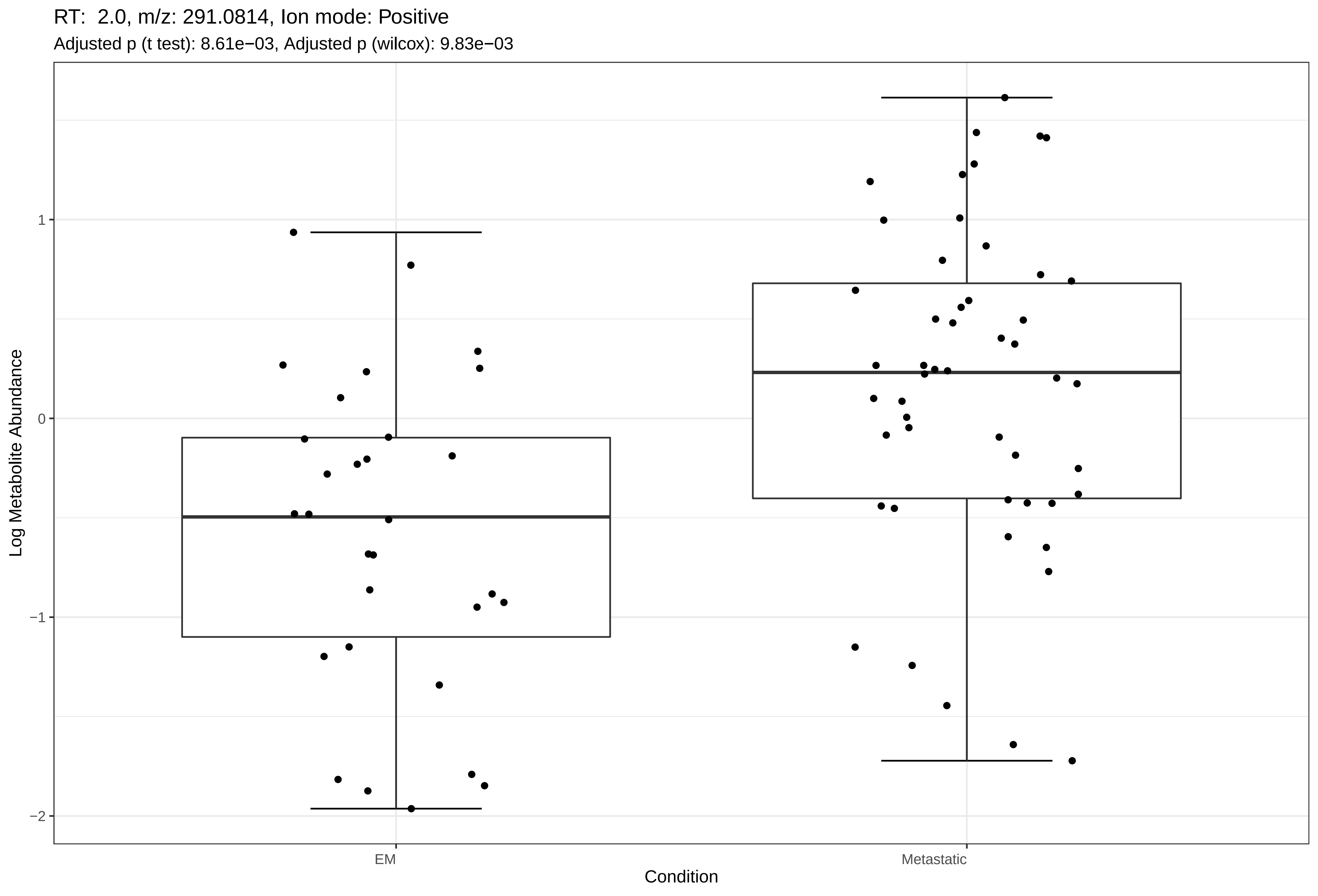

RT: 2.0, m/z: 291.0814, Ion mode: Positive
Adjusted p (t test): 8.61e−03, Adjusted p (wilcox): 9.83e−03
Log Metabolite Abundance
1
0
−1
−2
EM
Metastatic
Condition
