# Supplementary material for: Metabolomics of primary cutaneous melanoma and matched adjacent extratumoral microenvironment
Source: PLoS One. 2020 Oct 27;15(10):e0240849. doi: 10.1371/journal.pone.0240849 (PMC7591037; doi:10.1371/journal.pone.0240849)
Supplement: Attachment — Submitted filename: plosone.docx [file pone.0240849.s012.docx]

**Comments**

Article no. **Ms. Ref. No. PONE-D-20-09783,** Research Article entitled **‘Metabolomics of primary cutaneous melanoma and matched adjacent extratumoral microenvironment’.**

In the present study, authors have analysed the metabolomics of primary cutaneous melanoma and matched adjacent extratumoral microenvironment using ultra-high-performance liquid chromatography-mass spectrometry. There are few novel findings and may also hold promising translational relevance to develop the novel biomarker for melanoma patients. However, I do notice many important concerns that should be addressed to further enhance the manuscript quality:

Comment 1: First of all, authors need to provide high resolution images for all figures as it’s very hard to figure out anything out of any heatmaps.

Comment 2: Authors really need to explain their results in different subheadings and need to cite figure numbers in text rather than in headings.

Comment 3: It’s better to provide the box plot or bar diagram of potential differentially altered metabolites with their respective p-values in addition to heatmap.

Comment 4: Authors need to analyse and correlate the altered level of their identified metabolites with other pathophysiological characteristics such as TNM staging and survival if possible.

Comment 5: Authors need to provide demographic and clinical-pathological details of all the clinical bio specimens collected in their study.
